# Supplementary material for: PPARγ agonist treatment reduces fibroadipose tissue in secondary lymphedema by exhausting fibroadipogenic PDGFRα+ mesenchymal cells
Source: JCI Insight. 2023 Dec 22;8(24):e165324. doi: 10.1172/jci.insight.165324 (PMC10807713; doi:10.1172/jci.insight.165324)
Supplement: Supplemental data set 5 [file jciinsight-8-165324-s187.pdf]

"","gene","baseMean","log2FoldChange","lfcSE","stat","pvalue","padj"
"1","PLP2",640.233452733665,-1.75153887829225,0.153895268775622,-11.38
13692404409,5.17802922213276e-30,7.98193204591765e-26
"2","TNFRSF12A",110.121936055088,-2.3000091803517,0.259580314548416,-8
.86049153747642,7.96624093935279e-19,6.13998020400616e-15
"3","ANOS1",168.098137814156,2.6275811959801,0.306334689236577,8.57748
498065416,9.69700375196524e-18,4.98264376121814e-14
"4","CCN3",118.32474994201,-2.13444082876737,0.26446757136203,-8.07070
907701398,6.98910701450436e-16,2.69342711571462e-12
"5","CAV1",1821.98329407341,-1.77230739618196,0.238014887563783,-7.446
20395103232,9.60642599504762e-14,2.96166113427318e-10
"6","CD36",159.749348023261,-2.73117764576374,0.376311245263662,-7.257
76250414772,3.93545738822371e-13,1.01108459399114e-09
"7","FABP4",265.595261125657,-2.94477251342905,0.409928152144363,-7.18
36308339028,6.78840111386551e-13,1.49490290243195e-09
"8","VEGFD",794.632208681302,1.53900539590106,0.222071515075282,6.9302
2423600495,4.20174069223933e-12,8.09622909635867e-09
"9","PDGFD",699.176119500167,1.75791374299692,0.259660392469976,6.7700
49626264,1.2873822943889e-11,1.98449980680048e-08
"10","DYNLT3",91.8332152417499,-1.56421801130949,0.230748799772191,-6.
77887821238408,1.21112564274216e-11,1.98449980680048e-08
"11","CTHRC1",1623.46600024854,-1.8735508500622,0.281287661960683,-6.6
6062221500521,2.72670714320603e-11,3.8211082375019e-08
"12","ARSI",26.3102140570139,-2.98402748563309,0.4514318040619,-6.6101
4013364447,3.83956391771337e-11,4.93223981596263e-08
"13","CD9",1239.00201406808,-2.77178775329661,0.421086337097454,-6.582
46898344536,4.62699351720987e-11,5.48654654367617e-08
"14","PERP",171.254599976734,-2.58210858495597,0.393725810488684,-6.55
813897938546,5.44834211360065e-11,5.99901383436814e-08
"15","LMNA",4855.3418298381,-1.2362994509148,0.193205965282115,-6.3988
6790819104,1.56533183390319e-10,1.60863934797451e-07
"16","ANGPTL1",489.52322093608,-1.15369911815002,0.182913801683966,-6.
30733770513038,2.83875992577442e-10,2.73496776598829e-07
"17","SEMA3E",84.3002378014287,-3.05079035424434,0.486701311178682,-6.
26830107947729,3.65008516861467e-10,3.30976840436442e-07
"18","TRIL",144.462350072582,1.6025046320406,0.25748751126547,6.223620
80461611,4.85810427362251e-10,4.16042652099395e-07
"19","C1QC",19.4209726010317,-2.62252498252928,0.426001481089724,-6.15
614052754181,7.45390884710945e-10,6.04747394095748e-07
"20","TNFAIP6",835.785250372251,-2.25572096239022,0.369416647229332,-6
.10617030745201,1.02050115291798e-09,7.86551263611535e-07
"21","C1QA",46.7398565912725,-2.25781257891793,0.373325486545545,-6.04
783937954498,1.46801199412805e-09,1.04194779927264e-06
"22","SPP1",28.7519188497775,-3.37647628645835,0.558486407758652,-6.04
57626892103,1.48704843230608e-09,1.04194779927264e-06
"23","C1QB",40.5081461185447,-2.69019512651826,0.448092686432343,-6.00
365774308268,1.92921154058562e-09,1.29299112600553e-06
"24","CAPG",270.675108276406,-2.22129527034489,0.379558087598366,-5.85
231969209249,4.84763670114389e-09,3.11359665617221e-06
"25","MUC12",37.0338783765435,-2.16149506790173,0.372179983308126,-5.8

0766071482206,6.33517304025541e-09,3.90626769662149e-06
"26","TJP2",31.3936401993536,-2.20976722540196,0.385388360421075,-5.7338712123728,9.81638801060412e-09,5.47995622972045e-06
"27","ADIRF",1583.03059586396,-2.11536991977506,0.369077040301165,-5.73151317689372,9.95386146170435e-09,5.47995622972045e-06
"28","MIR497HG",75.43043467663,1.51072977618673,0.263436446192072,5.73470299202734,9.76833698851167e-09,5.47995622972045e-06
"29","NBL1",1019.44862265288,-1.31148183472325,0.230651019791441,-5.68600059045527,1.30048944995418e-08,6.64798888119972e-06
"30","CXXC5",265.028541030702,-0.880112303669745,0.155347026266974,-5.66545961528234,1.46630958132203e-08,6.64798888119972e-06
"31","MARCKS",7271.19987213329,1.52448059849426,0.268834975455749,5.67069294428617,1.42221097622406e-08,6.64798888119972e-06
"32","IL7",33.720567943225,-2.53455266671186,0.44630188257956,-5.67900957993392,1.35476892712025e-08,6.64798888119972e-06
"33","FREM1",98.4946323519748,1.51777533830322,0.267781767752295,5.66795622809989,1.44510869312958e-08,6.64798888119972e-06
"34","FXYD5",342.689772917045,-1.88338922610435,0.331162818867551,-5.68720012876088,1.29139088974176e-08,6.64798888119972e-06
"35","RNASE1",44.2420929019324,-2.57246512716116,0.457051528692156,-5.62839191135018,1.81897429116522e-08,8.01128248523198e-06
"36","RGCC",238.030822912004,-1.55903306710693,0.278774678914253,-5.59244861541558,2.23889425517363e-08,9.58682081763931e-06
"37","KCP",59.9360089557963,3.59402270884391,0.647869957463928,5.54744461822651,2.89874934765117e-08,1.20768165389305e-05
"38","GSTM1",10.7468032312369,6.4094429128127,1.16015882920957,5.52462538011242,3.30189372152856e-08,1.33943925572007e-05
"39","DBNDD2",811.665352795712,-1.74364761952678,0.315931670306185,-5.51906561895782,3.40806925099804e-08,1.34706121805474e-05
"40","ADAMTSL3",148.585877047574,1.61465587191865,0.293874647905596,5.49436939670053,3.92108979300007e-08,1.5110899789774e-05
"41","A2M",63.8002530778878,-1.85843018983322,0.346674384614991,-5.36073696906438,8.28831245772267e-08,3.11620333014134e-05
"42","FZD6",30.2978977915033,-2.06023077322312,0.388564823055715,-5.30215462383147,1.1444384233243e-07,4.20036149893907e-05
"43","AC074387.1",13.4998010565898,-3.82977610849887,0.733637773277726,-5.22025480147826,1.78677128899073e-07,6.40536730692838e-05
"44","TMCC3",65.7641531614466,1.44939861621058,0.278568348223554,5.20302692482287,1.96068449581977e-07,6.86907988705947e-05
"45","KCND2",108.832344146764,1.48827766530298,0.287898998749932,5.1694436999265,2.34791852563534e-07,7.78625705493695e-05
"46","PTGER2",26.4540471309807,-2.13610636381445,0.413383056707315,-5.16737763958929,2.37401285489482e-07,7.78625705493695e-05
"47","C16orf89",121.499388562633,1.34180691595865,0.2594750730009,5.17123629811637,2.32550272801726e-07,7.78625705493695e-05
"48","FGF12",22.2303375556067,3.55125853201795,0.68837034455107,5.15893597120887,2.48357219794375e-07,7.97588863152143e-05
"49","S100A6",33134.9246176505,-1.01474313528498,0.197371022761509,-5.14129744623727,2.72847698550176e-07,8.5835658635734e-05
"50","CSF1",801.330912069767,0.714842497836395,0.13943512433828,5.1267

0319784083,2.94859793848643e-07,9.09052744435366e-05
"51","MID1IP1",332.147299219982,-1.39399907213646,0.272385501099571,-5
.1177432958404,3.09213058468306e-07,9.34611626723322e-05
"52","KLHL13",83.0941007990936,1.76821088456386,0.346529620854717,5.10
26255135205,3.34973439939903e-07,9.93002995514154e-05
"53","F13A1",12.6631557654097,-3.30069241269399,0.64737795058507,-5.09
855550333615,3.42255182570571e-07,9.9544596968403e-05
"54","BEND6",80.2747667796532,-1.46449651458504,0.28835495226617,-5.07
879786033019,3.79830571948592e-07,0.000108427560492362
"55","ADAMTS16",148.698676194226,2.63245274532519,0.519212076226415,5.
07009152109406,3.97624447384777e-07,0.000111443288298842
"56","CRIM1",236.023107275401,1.04565434470299,0.206665740735267,5.059
64046572403,4.20047747411529e-07,0.000111682866322473
"57","IL17RD",167.262876105909,1.19332788462678,0.236105246709073,5.05
422010421136,4.32153004710742e-07,0.000111682866322473
"58","VEGFC",179.694098475054,1.82518221239556,0.360947563990861,5.056
64089325112,4.26705650775022e-07,0.000111682866322473
"59","TBC1D12",265.717237353484,1.17793563898149,0.233111655760085,5.0
5309627328889,4.3470463699957e-07,0.000111682866322473
"60","SERPINA5",117.423083488884,-1.23343367441688,0.243492013448673,-
5.06560218114456,4.07111327145151e-07,0.000111682866322473
"61","LINC00702",41.5366857574679,-2.09915213545243,0.415803125393288,
-5.04842798732439,4.45460321290265e-07,0.000112570013978515
"62","ADAMTSL1",242.555085117468,1.38851975994326,0.275560522156413,5.
0388921790296,4.68234156209873e-07,0.000116416605128632
"63","WASF1",113.873732292863,0.996042933447534,0.197936487031773,5.03
213403644802,4.85049932533628e-07,0.000118683249365173
"64","LINC00968",30.7838332297887,2.44970719669035,0.489669520539947,5
.00277655425463,5.65104282678811e-07,0.000136110664335842
"65","CRIP1",7286.074644155,-1.25912805698284,0.25203988390529,-4.9957
4923410133,5.8607777273221e-07,0.000138990597948723
"66","CRIP2",378.418553436766,-1.76201437259424,0.35298942927423,-4.99
169161019089,5.98527657381999e-07,0.000139792482402174
"67","GPX3",4646.26199242989,0.981927631830973,0.197460373303915,4.972
78322430633,6.59984472789763e-07,0.000151845681314242
"68","LUZP2",58.7591928748761,2.58360771260415,0.520205322680377,4.966
51533531418,6.81666650685812e-07,0.000154527815004732
"69","IL33",411.159839323734,0.775795686334336,0.157081038350437,4.938
82453592897,7.85948909881757e-07,0.000175585542693149
"70","CCDC34",126.368863729456,-1.08829012179664,0.220929410335299,-4.
92596309447879,8.39458420870245e-07,0.000184860736538783
"71","TUBA1C",218.018989299372,-1.38502791336113,0.282539307705854,-4.
9020715899929,9.48312621051472e-07,0.000205890690894485
"72","EMCN",61.8876818752754,-2.25376776309315,0.461050100531532,-4.88
833591077162,1.01691907459867e-06,0.000217719549096369
"73","STEAP4",378.171047245277,1.67831788925616,0.343826837425111,4.88
128821422126,1.0539507229679e-06,0.000220611150670969
"74","ABCA1",615.158056539877,1.8571157522245,0.380736805309694,4.8776
8906584671,1.07335947455872e-06,0.000220611150670969
"75","PCSK6",64.2043030394949,1.68092070333356,0.344533718381885,4.878

82785820809,1.06718152768737e-06,0.000220611150670969
"76","PXDNL",75.812602133033,2.17861114884563,0.446920291626253,4.87471969759552,1.08963060188165e-06,0.000221008628000075
"77","DTNA",9.67030791843495,-2.93660009266364,0.604675651553946,-4.85648807772716,1.19485972052517e-06,0.000239204708985657
"78","FOLR2",13.9148452189081,-2.59881574505038,0.540296861103733,-4.80997749966833,1.50947286565644e-06,0.000298314413129411
"79","S100A3",22.9979168891352,-2.31168746125782,0.482426202351585,-4.79179499369957,1.6529575711654e-06,0.000322535961512844
"80","NTM",587.853768000704,0.952373267825782,0.199118167895255,4.78295515619034,1.72736672815301e-06,0.000332841976430984
"81","KCNB2",19.2622715783645,2.7047319458758,0.566873606246107,4.77131395089427,1.83027995019633e-06,0.000348318091756499
"82","CCL18",7.46596860467055,-4.04950892254182,0.849561342218969,-4.76658802761071,1.87371886161747e-06,0.000352236295754064
"83","GPC1",72.3649767948434,-1.37932254088274,0.289677988917111,-4.76157179231669,1.9209093789926e-06,0.000356756844303264
"84","PLAUR",165.33815977764,-1.1575845765135,0.243338515544117,-4.7570955790746,1.96398193942652e-06,0.00036041406662214
"85","MAF",968.478143623124,0.891967706226398,0.188987812615041,4.71971019656855,2.36180881888467e-06,0.000428320975801261
"86","MGAT4C",16.529263426913,5.03939858188581,1.06834624930941,4.71700872740773,2.39337427694428e-06,0.000428998424175536
"87","PTGDR",9.58802018513565,-3.13857994635224,0.665732559989594,-4.71447565430974,2.42333975258372e-06,0.000429376807885954
"88","TAGLN2",1700.74164793601,-0.965587724280656,0.206213465311326,-4.68246689333737,2.83442976794268e-06,0.000496508350827687
"89","HMGA1",61.2744709897679,-1.66629829120597,0.356163893171681,-4.67845933614267,2.89038475633938e-06,0.000500621135044624
"90","CAV2",227.720454129105,-0.904546789604828,0.19363302001268,-4.67144906145447,2.99082273551154e-06,0.000512261471865671
"91","HRH2",22.1103363439358,2.41564660791992,0.519390200753785,4.65092834715426,3.3044413357628e-06,0.000541893225433868
"92","ACP5",13.5490703249553,-3.02026463695432,0.649369307417546,-4.65107389963579,3.30210964200301e-06,0.000541893225433868
"93","EYA2",25.9534888294525,2.76971471013713,0.595196710591218,4.65344424935737,3.26435893152655e-06,0.000541893225433868
"94","MT-CYB",40897.6774971142,-0.687053779634363,0.147527259944594,-4.65713102712269,3.2064638601748e-06,0.000541893225433868
"95","PHACTR2",565.12145270645,1.03050431632642,0.22322964367078,4.61634171600532,3.90563653563112e-06,0.000614340685681161
"96","PROSER2",35.0710978759136,1.39967866941833,0.303102879377416,4.61783362894248,3.87766937607251e-06,0.000614340685681161
"97","ID1",303.600542945661,-1.3515213347541,0.292763353875498,-4.61642933401035,3.90398873279051e-06,0.000614340685681161
"98","FOXRED2",72.382771024598,1.01830998347345,0.220391132694739,4.6204671259796,3.82877009902573e-06,0.000614340685681161
"99","FBN1",10811.6495938132,1.24147471616913,0.269248143205592,4.61089425311715,4.00940465709962e-06,0.000624292654436269

"100","PARP16",97.9471350997034,-1.48536369909565,0.322390437461818,-4.60734415942956,4.07844658019857e-06,0.00062869254033761
"101","ADAMTS5",3002.42371077223,1.1730905698345,0.255145849501118,4.59772546615289,4.27128296453127e-06,0.000651899276220293
"102","NID1",1270.57532449844,1.30187648017437,0.283522958701842,4.59178503968508,4.39470896454086e-06,0.000664161163611739
"103","CDA",84.351364699804,-2.09826405262104,0.45874910443321,-4.57388152335135,4.78770637595784e-06,0.000716529065877574
"104","STAC",64.5643909348906,1.32212150944006,0.289764143649354,4.56275056254019,5.04877845423899e-06,0.000748335768000904
"105","DLGAP2",16.193366524223,2.58234489049955,0.56658494569913,4.55773650553511,5.17078500840355e-06,0.00075912048480515
"106","NFATC2",56.338767089299,-2.1029961037932,0.461716641213147,-4.55473317632139,5.24521136094223e-06,0.000762782388008722
"107","C11orf96",2990.35610645884,-1.55274608756027,0.341606736432884,-4.54541998724708,5.48258217540902e-06,0.000789850506859159
"108","MAN2B1",197.739878828952,-0.728759291737188,0.160483397916604,-4.5410260575109,5.59811113982074e-06,0.000799026696484599
"109","LAMA4",1589.95109115988,0.926324655470595,0.20417756183187,4.53685824808396,5.70984569776602e-06,0.000807497903037276
"110","RDH10",168.754600382386,1.62627994270576,0.35874931458428,4.5331931702512,5.80986374450628e-06,0.000814173178377857
"111","PLEKHA2",241.269906048961,1.10823384350973,0.244694250543838,4.52905550925963,5.92479335942147e-06,0.000822799005725062
"112","SYNE2",479.46263434085,1.0201228564095,0.225364640463211,4.52654353545772,5.99562521906821e-06,0.00082520145314229
"113","TET1",159.125654000977,0.869998931636503,0.192729213942538,4.51409993243627,6.35862309004665e-06,0.000867417477283797
"114","TRIO",743.766898154242,1.08703563562548,0.240939853457034,4.51164728470016,6.43260925424873e-06,0.000869812909247756
"115","VSIG4",13.465579151281,-2.69860387389366,0.598568504136738,-4.50842945334321,6.53092774801699e-06,0.000875428271614625
"116","SSC5D",498.587796679632,-1.0481958377028,0.232614998360504,-4.50614038256602,6.60174203102017e-06,0.000877291839725654
"117","MAP4K3-DT",116.96117527313,0.969856087799973,0.21604533966235,4.48913218547426,7.15139125408472e-06,0.000938367006743659
"118","TUSC1",299.219397616594,-0.942109408920146,0.209908544003659,-4.48818990857144,7.18308834224793e-06,0.000938367006743659
"119","VGLL3",638.571664316977,1.6928384956555,0.380423259919685,4.44988168182171,8.59176097150263e-06,0.00110526974400368
"120","NAV2",48.7692453806764,-2.06009377940419,0.462986829356527,-4.4495731817412,8.60411088423238e-06,0.00110526974400368
"121","RAB13",621.588377539364,-1.14933415523537,0.259094821189101,-4.43595958406487,9.16630458476656e-06,0.00116775690226592
"122","LAMC1",2118.33251533844,1.05300210494488,0.237705597826125,4.42985825565253,9.42950450808952e-06,0.00119144108190328
"123","SLCO2B1",9.5902707102528,-2.72837280713458,0.616384562613267,-4.42641326961074,9.58128799750473e-06,0.00120077686570354
"124","VIM",57895.5874248158,-0.99124003285925,0.224227103152655,-4.42

069677983758,9.83831467927426e-06,0.00122304532887913
"125","FCGR1A",7.16399320172498,-2.89982386054232,0.658977001160856,-4.40049327280616,1.08005076148804e-05,0.00133191859906705
"126","RGS2",171.862523692025,-1.56019296547322,0.355090809969529,-4.39378581948406,1.11393566978326e-05,0.00135207231100071
"127","PIM3",140.108491122106,-0.903871837471399,0.205667840396399,-4.39481367494938,1.10867807138953e-05,0.00135207231100071
"128","RPL39L",29.6501459199545,-1.44024756160006,0.328499058249168,-4.38432782509721,1.16344457915759e-05,0.00137957678367033
"129","MS4A4A",17.1417713972449,-2.18768757008327,0.498641760808729,-4.38729312710419,1.14770068036854e-05,0.00137957678367033
"130","TBC1D2B",397.055433615311,0.795678809236307,0.181437402713529,4.38541776577679,1.15763386448389e-05,0.00137957678367033
"131","ADD3",2566.40075053702,0.687388280983194,0.156991020465359,4.37851973282044,1.19488123794293e-05,0.00140603773151834
"132","CD163",23.5891282034252,-2.35021774161624,0.537084130001569,-4.37588379609984,1.20941436018341e-05,0.00141235775471419
"133","SLITRK2",34.9730954522981,-2.17913629578685,0.498486699196111,-4.37150339076459,1.23393933661141e-05,0.00143016352435075
"134","APCDD1",40.1334567848678,2.31186201808827,0.529339629990467,4.36744556255858,1.25708114410319e-05,0.00144611237584707
"135","AGTR1",899.212626934733,0.961435770185515,0.223322082996862,4.30515315495702,1.66870248693215e-05,0.00190541102489326
"136","PLEKHG1",39.0586050054489,1.64524331842887,0.38264060669058,4.29970915177668,1.71022407516501e-05,0.0019384635381374
"137","TWIST1",743.990429115625,-1.35209910298323,0.31471785461972,-4.29622623291264,1.73730285793927e-05,0.00195478274125065
"138","SYCE1L",47.9742064270049,-1.73424325808852,0.404396780642243,-4.28846949605849,1.79908500474242e-05,0.00200963009768872
"139","LARP6",514.117691668226,-0.656869121787194,0.153271207091,-4.28566548312755,1.82192966849648e-05,0.0020205068949549
"140","PFKP",187.13008381354,-1.25041029648835,0.291959123972987,-4.28282658021689,1.84533995660964e-05,0.00202365872948233
"141","SHTN1",50.8405500027008,-1.29326637550812,0.302215856191163,-4.2792803521536,1.87498569316615e-05,0.00202365872948233
"142","LYVE1",12.2651494244078,-2.78129898315296,0.649539386716922,-4.28195585984548,1.85257736353329e-05,0.00202365872948233
"143","ABCG1",46.5744840067126,2.37998198924158,0.5561995024634,4.27900776376223,1.87728315482305e-05,0.00202365872948233
"144","EDN1",24.7085155353117,1.99770399683788,0.467654378687152,4.27175300367345,1.93942322969399e-05,0.00206180752315399
"145","AC022893.2",8.67060703287725,4.62991870682249,1.08382931039101,4.27181537021929,1.93888078124689e-05,0.00206180752315399
"146","IGFBP6",13491.9701857389,-0.88529428550138,0.207559845576844,-4.26524833375645,1.99679978974808e-05,0.00210826498349087
"147","EVA1C",33.0784112897264,-1.33835158260418,0.314804358923921,-4.25137563907627,2.12461473852806e-05,0.00222795484315714
"148","PPP1R9A",22.6489550193732,2.00157447804667,0.471928531979983,4.24126608672934,2.22262448781091e-05,0.00231498354591927
"149","APBB2",148.259450398294,1.06393463103137,0.251304739791585,4.23

364331255242,2.29935537796115e-05,0.00237882974169605
"150","ABCA6",1003.22872661286,1.05386292560014,0.24989039918864,4.21730058066211,2.47244306886622e-05,0.00254084732710485
"151","ADAMTS15",74.86135479393,2.19590310704635,0.523097724038935,4.19788312228042,2.69421610356864e-05,0.00275041994943779
"152","SH3BGRL3",4316.24412436569,-1.07725303737,0.256970381568556,-4.1921291893424,2.76348543744289e-05,0.00278425673321452
"153","RBMS3",950.992661319101,0.79535602840236,0.189676976146455,4.19321334914282,2.75030552286568e-05,0.00278425673321452
"154","MAZ",465.585531898126,-0.754732443447332,0.18025270546257,-4.18707969741989,2.82566625983803e-05,0.00282841853216904
"155","RGS22",34.2326930852895,-1.27281135044161,0.30592794531673,-4.16049390036552,3.17560098630451e-05,0.0031382171004176
"156","PLP1",175.232756050316,0.906686010212512,0.217928544304748,4.16047385212933,3.17587977726335e-05,0.0031382171004176
"157","BAALC-AS1",8.94300089871306,-2.61398282155695,0.631194892382026,-4.14132441993028,3.45306149794398e-05,0.00339037853444627
"158","CDKN2C",255.147339079625,-0.994206139936763,0.241148259803381,-4.1228003915408,3.74294025990117e-05,0.00365173570293522
"159","KANK4",10.8079066262043,3.43492840888657,0.835261758776203,4.11239754818814,3.9157125667318e-05,0.00379627101988495
"160","FAM43A",234.956495221491,1.2428842161819,0.302538832868851,4.10818077268343,3.9878792244377e-05,0.0038420723902942
"161","DACT2",28.0937982835795,2.33610895913122,0.56957929170868,4.10146399831906,4.10544389050871e-05,0.00393077127777589
"162","SLC7A4",29.4700750639392,2.21906191132916,0.542022204746852,4.09404244308691,4.23916659142481e-05,0.00403375018560576
"163","NAV2-AS3",4.09194685420289,-4.22481740829353,1.03369786432645,-4.08709116473447,4.3681550558245e-05,0.00413098835494078
"164","MS4A6A",31.2843907900949,-2.16110857714664,0.528974232304194,-4.08547041645663,4.39876051578608e-05,0.00413456666773429
"165","MYADM",2603.34076831647,-0.899986764056317,0.220899378963741,-4.07419327423299,4.61741240844279e-05,0.00431378256219065
"166","ARHGAP20",334.191162646558,1.19684526009262,0.294668006754258,4.0616735874239,4.87221696446559e-05,0.0045244111148938
"167","SPHK1",26.0150510107016,-2.4344039695432,0.600655051688853,-4.05291516769637,5.05833483583341e-05,0.00466911565834563
"168","PLEKHO1",177.736857753178,-1.0805051357791,0.266830255268409,-4.04941011914938,5.1346893375083e-05,0.00468350509690476
"169","TXN",2198.23693449783,-1.05664469543868,0.260863196641163,-4.05057021858158,5.10929743026109e-05,0.00468350509690476
"170","MAST4",194.432521807482,0.960265391495656,0.237514092905531,4.04298279629913,5.27754943073352e-05,0.00478549555733866
"171","MEG8",299.55679167622,1.74137766283345,0.431269165433148,4.03779774305099,5.39553409175297e-05,0.00486386889031416
"172","CSPG4",7.67464120906791,-3.12653317584331,0.774956178518665,-4.03446448006867,5.47269675588613e-05,0.00490474537744097
"173","IFRD2",92.3452416715141,-1.02467737040027,0.254110461405526,-4.

03240923153109,5.52079410907144e-05,0.00491925093591539
"174","MDGA2",7.84964528452806,4.53821468066699,1.12589002255457,4.030
7797295957,5.55921238104641e-05,0.00492501487665692
"175","PRELP",1687.24812487608,0.64876612189268,0.161219660418834,4.02
411294135743,5.71904682061384e-05,0.00503766324227213
"176","AFF3",145.148768077914,-1.72505313002297,0.428975930686248,-4.0
2132848634034,5.78708372131074e-05,0.00506863042977302
"177","TUBB6",613.89089820258,-0.865190092530419,0.215291363484697,-4.
01869391566149,5.85216348864288e-05,0.00509667232640847
"178","KCNIP4",4.71715477603149,-3.98105721333722,0.993705429628546,-4
.00627499320938,6.16838302609072e-05,0.00531204605291556
"179","SLC7A8",78.5083308222626,1.29908652745908,0.324243776310104,4.0
0651183576349,6.16220387829741e-05,0.00531204605291556
"180","AIF1L",130.433583074002,1.90122306600589,0.474986189496306,4.00
269125302784,6.26260058978692e-05,0.00536322156064252
"181","PCDHA10",3.71853325190884,4.87643215374306,1.21956676937419,3.9
984954298528,6.37464116959605e-05,0.00539918096864413
"182","MT-
CO2",29642.9830655043,-0.766114167293854,0.191585216993282,-3.99881671
10029,6.36599539850821e-05,0.00539918096864413
"183","SMOC2",1730.45614408706,1.17306965268553,0.293700647628566,3.99
409964587164,6.49405500339147e-05,0.00547026545777483
"184","KCNAB1",95.7666983591524,2.9611273638554,0.741801185876634,3.99
180726619633,6.55716563041033e-05,0.00549340805395518
"185","WDR81",81.2316848972317,0.79154890616101,0.198658105429943,3.98
447827964489,6.76285422096067e-05,0.00563510258465452
"186","SPI1",11.1806011329274,-2.29197617923462,0.575812324988708,-3.9
8042223094056,6.87929683341958e-05,0.00570130971436359
"187","PODN",1749.48343129128,0.613360316859051,0.154177783584729,3.97
826653489267,6.94195285487512e-05,0.0057224707624545
"188","AL355596.1",4.33604889652049,-5.39418931941958,1.35966925658582
,-3.96728049361399,7.26974157237046e-05,0.00596080140096227
"189","MS4A7",20.7992062141057,-2.29798260980505,0.579546641018909,-3.
96513834635454,7.33533903826208e-05,0.00598276461771482
"190","CYP26B1",261.560091251333,-1.91741479513459,0.485319217461121,-
3.95083220723316,7.78799130213422e-05,0.00631852031170521
"191","CALN1",8.92628026919057,-4.26941457540162,1.08119488613339,-3.9
4879279411881,7.85463025769355e-05,0.00633922122630084
"192","MAN1C1",432.158662654897,0.904816197668952,0.229325878828075,3.
94554771704284,7.96177740503249e-05,0.00637543968843007
"193","HLA-
DPA1",235.372802576186,-0.969962757431976,0.245875587501508,-3.9449331
561883,7.98222419634774e-05,0.00637543968843007
"194","APBB1IP",431.311068870854,1.1173496630533,0.283526601967147,3.9
4089886205024,8.1176849559235e-05,0.00645021204100828
"195","SEC14L2",19.3358097439833,-1.67197070034131,0.425448990311378,-
3.92989697570472,8.4982265670439e-05,0.00671795705287086
"196","IGSF10",165.613723877819,1.91680002920882,0.488504800065754,3.9
2381001978038,8.71594775417811e-05,0.0068549150321763
"197","AQP7",12.7032913481418,-2.93292653741426,0.748496930250658,-3.9

1842159784419,8.91307106496653e-05,0.00695107959307314
"198","ACTN1",127.945563996627,-2.00623431893723,0.512054787508806,-3.91800715055854,8.92840583476148e-05,0.00695107959307314
"199","PRR16",18.8212464760599,-1.73136906305002,0.442450000891147,-3.91314060246996,9.11034524812157e-05,0.00705708402009015
"200","AC025280.2",8.87104209771023,4.12220388747942,1.05537268579955,3.90592246980171,9.38666065513182e-05,0.00723476869994285
"201","KLK1",7.89971575883294,-4.54754596228768,1.16531214855177,-3.9024273178129,9.52328394416882e-05,0.00726739712868131
"202","LILRB5",11.4503303429827,-3.247007275682,0.831912425434852,-3.90306380384299,9.49826508050154e-05,0.00726739712868131
"203","FIGN",50.3729527060034,1.74111524185021,0.446486151088079,3.89959517805231,9.63536552550463e-05,0.00728686745207249
"204","PLAGL1",1128.17333027858,1.03963965896698,0.266615641691672,3.89939484559302,9.64334064367686e-05,0.00728686745207249
"205","CTNNAL1",101.502145459222,-0.941499375740364,0.241759358330067,-3.89436579515968,9.84559824341402e-05,0.0074034096059623
"206","WLS",167.625380153701,0.866302942574268,0.222804450777092,3.88817610937663,0.000101000334465575,0.00750091241244759
"207","KANK1",166.322614202968,0.843532947409821,0.216976658458395,3.88766678131679,0.000101212441244833,0.00750091241244759
"208","ATP11A",187.030877718866,0.8106426222804,0.208436476511899,3.88915911382776,0.000100592153652147,0.00750091241244759
"209","CPB1",933.432444576065,1.27507722616821,0.328297762839701,3.88390470632234,0.000102792208426656,0.0075790337709948
"210","MAN2A1",155.586828455705,1.06686219246911,0.274764430986503,3.88282496623994,0.000103249892436517,0.0075790337709948
"211","SGIP1",7.20409977259691,-3.97293078205732,1.02370606233794,-3.88092923176007,0.000104058119273473,0.00760216070426816
"212","TRIM47",242.526804103313,-1.07263367399084,0.27678661396742,-3.87530906432167,0.000106489452591767,0.00774308920614196
"213","PDCD4",594.676331010228,0.518677413894962,0.133929189880381,3.87277347349162,0.000107603830680388,0.0077873852109774
"214","LINC01615",13.4941740400965,-2.54594642467959,0.657631030043928,-3.87139035168327,0.000108216333947254,0.00779511583082672
"215","LINC00882",136.761720006002,1.16333615952573,0.301091571960725,3.86372873856952,0.00011166922614646,0.00800642381882642
"216","S100A10",25215.253541726,-0.927235740644888,0.240328182454187,-3.85820643744787,0.000114222154011418,0.00813193876568327
"217","EMILIN2",557.325026486144,0.6802567284801,0.176338936549309,3.85766604807602,0.000114474908345979,0.00813193876568327
"218","AC022424.1",15.2007553778023,3.18465903570238,0.826044816265385,3.85531023619333,0.000115582958835665,0.00817298766262281
"219","EPB41L2",1167.14504385575,0.617148257217719,0.160273233062901,3.85060091085522,0.000117828368654725,0.00828239168876242
"220","SCARA5",4120.15228710846,0.685187777896093,0.177979180771426,3.84981982120742,0.000118204746774423,0.00828239168876242
"221","SCRN1",225.80820914472,0.60016592651108,0.156321400937761,3.83930749667497,0.000123381825004187,0.00856725600197992
"222","EBF2",327.720063820624,0.995634025709284,0.259293874784061,3.83

978999325936,0.0001231395991388,0.00856725600197992
"223","MBP",541.924523824948,0.563135831524082,0.146911284788831,3.83316933299936,0.000126502809494558,0.00874457761595792
"224","TMEM37",384.590917800015,1.65973667846907,0.433180414508405,3.83151366700783,0.00012735729831726,0.00876434264982397
"225","MRC1",13.7767703298569,-2.20109332557021,0.5776446739606,-3.81046242576512,0.000138707093833034,0.0095029771174943 2
"226","VEPH1",11.9380498220471,3.05280260252412,0.802125295065093,3.80589244760868,0.000141293776953069,0.00963736093686531
"227","AIF1",48.7669495418486,-1.41063472203561,0.371110771084941,-3.80111500916996,0.000144046418294404,0.00978183056391296
"228","GALNT13",32.5355151496592,-2.23094509644368,0.587191699844236,-3.7993471246877,0.0001450777709719,0.00980865719092915
"229","LRRFIP2",230.388657847166,-0.542457024985636,0.142901783451126,-3.79601298097978,0.000147041802256283,0.00989803223484982
"230","MME",313.627721392838,1.03377285591715,0.272439849428116,3.79449943937041,0.000147941616166529,0.00989894726634595
"231","ABCA9",1008.79925888141,0.95052990273974,0.250546086717395,3.7938325646726,0.000148339722252735,0.00989894726634595
"232","CCDC71L",1003.32428621327,-1.45963640339359,0.385115476304687,-3.79012658073184,0.000150570534682364,0.0100045034143476
"233","LRRTM4",5.23723971197816,5.35329682381696,1.41438055937918,3.78490554633096,0.000153766968196522,0.0101730378315425
"234","NRN1",239.903639297398,-1.02890600253429,0.272100445912018,-3.78134625647391,0.000155982541520114,0.0102755165706519
"235","PDLIM4",304.647594802191,-0.961493849958914,0.254346908460889,-3.78024586882983,0.000156673564420259,0.0102771191299502
"236","YDJC",28.9697966651043,-1.26181277882191,0.334067900645732,-3.77711470148105,0.000158655676319142,0.0103630391968626
"237","S100A4",18918.8655103305,-0.804135591061432,0.213096598698853,-3.7735730930076,0.000160926042374895,0.0104386194665218
"238","IL6ST",1806.87185982806,0.916698155784743,0.242949807172954,3.77319976686441,0.000161167138049444,0.0104386194665218
"239","NEGR1",1410.95077460356,0.80696528357518,0.214613288828483,3.76009001110877,0.000169852231577008,0.0109551135973204
"240","FSTL3",240.301299762784,-0.810990045278144,0.215857713921798,-3.75705843698481,0.000171922360427013,0.0110424299415933
"241","SERTAD4",34.8993970788356,-3.5087694531734,0.934839789942981,-3.75333772794097,0.000174495506794405,0.0111150753604783
"242","MAP1LC3C",9.61219563766933,2.27918362036396,0.607148013811138,3.75391760908062,0.000174092107480905,0.0111150753604783
"243","RIPOR3",117.806688970488,1.02833930203492,0.27426222680711,3.74947477823171,0.000177205317228358,0.0112412344241775
"244","DPT",3182.44008060891,-1.09750079709851,0.29288851646426,-3.74716226620117,0.000178846398530128,0.0112527234013956
"245","FRMD6",752.843942558038,1.02455517418602,0.273392850290635,3.747556576907,0.000178565567861296,0.0112527234013956
"246","AC137936.1",8.73096845335487,-2.44232698456197,0.652082780798874,-3.74542474740683,0.000180088824830586,0.0112848342876564
"247","APOC1",121.692918618742,-1.65417378474613,0.441785923049655,-3.

74428812336832,0.000180905962465705,0.0112901433660277
"248","PTPRT",6.62843425808114,-3.00147570897668,0.801943530628229,-3.
74275194492232,0.000182015886845501,0.0113136084504976
"249","FZD10-
AS1",22.240476094838,-2.44833377363835,0.656344794638942,-3.7302554901
5772,0.000191285700065718,0.0118420444438275
"250","MMP23B",397.597477285171,1.60955440935159,0.43213001609204,3.72
469939465805,0.000195548098790998,0.0120094579396941
"251","ASPH",1109.14073163982,0.725703133021213,0.194824291005612,3.72
491094039351,0.000195384188642814,0.0120094579396941
"252","GAB2",68.0274747748764,0.930555402156491,0.250273417652698,3.71
815517158844,0.000200682991911463,0.0122759060329968
"253","STAB1",14.8003670955919,-2.36741945586622,0.636977872138507,-3.
71664316676834,0.0002018872616072,0.0123007594374506
"254","DYSF",5.14207091617846,-3.23418849950656,0.870900328950001,-3.7
1361497061995,0.00020431957770715,0.0123999460250225
"255","PTPRD",263.321442969243,0.893519261878431,0.24152253165313,3.69
952755862001,0.000216001185500626,0.013057483429381
"256","CYB5R2",24.2286717673353,-1.14805081487914,0.310682494148893,-3
.69525427566878,0.000219666837223214,0.0132272042804525
"257","AC009236.1",18.0444948051594,2.1277553577257,0.576481637273556,
3.69093344896261,0.000223432595984957,0.0134016088214323
"258","ENO1",1141.73530819725,-0.692937810792866,0.188247155294576,-3.
68100017080486,0.00023232084936145,0.0137824221305476
"259","GNAI1",153.572853427497,-0.921434124617645,0.25033234041285,-3.
68084332650752,0.000232463817965771,0.0137824221305476
"260","ABCA8",2168.46217245561,0.991951524939616,0.269429298982156,3.6
8167652399716,0.000231705276346907,0.0137824221305476
"261","TXNIP",9584.43596369648,1.03479259881206,0.281249807146721,3.67
926509642809,0.000233907027886832,0.0138148537734694
"262","PLXNA4",59.5162972619541,1.38985749950211,0.378012238999483,3.6
767526447841,0.000236221893469588,0.0138454771400521
"263","SREBF1",203.048844268005,0.977259611524513,0.265771357575761,3.
67706896799793,0.000235929268459904,0.0138454771400521
"264","IFI30",61.7020050654132,-1.12963378724515,0.307614318932131,-3.
67224058739079,0.000240433166880461,0.0140389290434178
"265","IRX1",114.617163737136,-0.782353846120626,0.213765194924418,-3.
6598747817541,0.000252338527423003,0.0146784845291532
"266","ASIP",23.9664969528436,-1.68140273400941,0.459539662176831,-3.6
5888490678832,0.000253315064430663,0.0146798936774386
"267","RHOBTB3",1040.57030936987,0.692950688769067,0.189477352396401,3
.65716894396626,0.000255016307646694,0.0147231325182539
"268","SOD3",3125.99419149269,-1.02485433088593,0.280750559701113,-3.6
5040886107933,0.000261823201642759,0.0150597188556833
"269","SNAI2",496.51390165035,0.911236581377125,0.249937505814313,3.64
585770514216,0.000266501421085291,0.0152718193532705
"270","PUS7",24.479585677494,-1.25442235167357,0.344326604943882,-3.64
311770761372,0.000269355586470188,0.0153782087608813
"271","SELENOM",2594.19777354981,-0.725973922691222,0.199344532586038,
-3.64180503610195,0.000270733081655684,0.0153998171724073

"272","ID4",390.636142549636,-1.09519405103141,0.301063117183881,-3.63775563501689,0.000275024171909084,0.0155863882719799
"273","IQSEC3",8.06240205375756,3.22289197016028,0.887975948926666,3.62948115211446,0.000283991464976252,0.015978512540706
"274","FLRT2",424.704841111208,1.02930450521903,0.283597264653482,3.62945850862386,0.000284016376007359,0.015978512540706
"275","EGFR",1302.13281532535,0.667233349176174,0.183992503079136,3.62641595722621,0.000287382294138893,0.0160823077783828
"276","LHFPL6",2684.67671722802,0.500524845226565,0.13804124376179,3.62590796479849,0.000287947904432933,0.0160823077783828
"277","CCL3",69.4304462159988,-2.0284365780268,0.561064057860541,-3.6153386580521,0.000299955241877603,0.0166924550669431
"278","LDHA",2573.48247197223,-0.633940139704313,0.17542887615434,-3.6136590144178,0.000301906054615559,0.0167405821291325
"279","ESR1",40.8148179115145,1.34955689360421,0.37400036186312,3.60843740065186,0.000308046820861207,0.0169590776556268
"280","VPS51",352.298316958681,-0.681057839027075,0.18872439938215,-3.60874291430645,0.000307684330826209,0.0169590776556268
"281","AL513534.2",30.1235426239589,1.34708799726695,0.373511484759993,3.60655040669645,0.000310294604405719,0.0170220331918653
"282","COL6A6",267.606577254555,1.61986078135806,0.449428900728522,3.60426483195068,0.000313037741004872,0.0171116197786883
"283","EHBP1",379.373980665159,0.73090158440143,0.203139750737448,3.59802343828854,0.000320644729107031,0.0171172685756312
"284","FGFRL1",38.7005402898477,-1.26250841456696,0.350417879030616,-3.60286529345912,0.000314728651024791,0.0171172685756312
"285","TFEC",4.75372303215035,-3.07088566966738,0.852822912990027,-3.60084798718734,0.000317180992863397,0.0171172685756312
"286","ADM",1872.34660514138,1.37559108450668,0.382118715336519,3.59990502766985,0.000318333427690526,0.0171172685756312
"287","KCNA1",20.4214362539962,-2.26195796401377,0.628141357378572,-3.60103333022621,0.000316954936242707,0.0171172685756312
"288","LASP1",463.28476315113,0.646886079865759,0.179768058998171,3.59844837548332,0.000320121376950756,0.0171172685756312
"289","TYMP",935.312072686625,-0.705310904366637,0.196039225830627,-3.59780498713052,0.000320914084875604,0.0171172685756312
"290","RGL1",209.77338497285,0.687665251517769,0.19145122257325,3.59185615153053,0.000328331076982563,0.017412949269612
"291","MMP9",8.82307797279489,-2.23467879390063,0.622204510568489,-3.59155029567187,0.000328716719912883,0.017412949269612
"292","PTMA",5661.4773932743,-0.908308915719836,0.253145808437461,-3.58808593879693,0.000333114499712254,0.0175854794967959
"293","PTGS1",78.1235539229974,1.20496650992576,0.336053034838702,3.58564388654938,0.000336247545713652,0.0176902932326824
"294","SAA1",6.7729079713768,-2.79686235146651,0.781042762925722,-3.58093370072299,0.000342368530172145,0.0179510574578354
"295","RFX2",106.683622284842,-1.10646826797868,0.309176668616931,-3.57875732644495,0.000345231843969796,0.0180398266942183
"296","STK3",203.742228543566,0.636254979811771,0.178254038284977,3.56937203742101,0.000357837951593147,0.018635378458812

"297","FCER1G",42.9334821347993,-1.46955030175981,0.412035293100208,-3
.56656414236441,0.000361692310714501,0.0187096878176646
"298","CAB39L",558.729395751602,-0.94736582388381,0.265562841165704,-3
.56738849353053,0.000360556728408285,0.0187096878176646
"299","NMB",122.892326539291,-1.12017731397058,0.31451910372307,-3.561
55572335879,0.0003686638416882,0.018979234654991
"300","C20orf194",115.871161269932,0.924044472388994,0.259486046155054
,3.5610565041206,0.000369365578754284,0.018979234654991
"301","CPNE2",58.285811260312,-0.798363630474262,0.22484525988731,-3.5
5072475565815,0.000384171958618911,0.019674454292726
"302","GALC",128.162370744342,0.724207725680004,0.204365751247818,3.54
368440532785,0.000394577261944528,0.0201404254730957
"303","ZNF185",18.7870046941855,1.38769580891954,0.392293885903554,3.5
3738831723904,0.000404105050297791,0.0205586777238959
"304","RASA3",170.239452807239,0.709664708363904,0.200753854948093,3.5
3499915878275,0.000407776456609214,0.0206772173639179
"305","TGFB1",171.854343166162,-0.895926389469101,0.253550297484263,-3
.53352529402853,0.000410056850664284,0.0207246765671801
"306","PLXNC1",176.918839352062,1.418646496276,0.401909959843512,3.529
76198158504,0.000415933676796029,0.0209529987836954
"307","PPL",808.242134287124,-1.23336429999151,0.349805691758654,-3.52
585543645887,0.000422117318957899,0.0211952393216157
"308","SCN2B",17.6946133575978,1.55442076104607,0.441380250167728,3.52
172703798002,0.000428745359701277,0.0214581484408935
"309","LGALS2",10.3752273373615,-2.44232923002107,0.695824095947075,-3
.50998081878273,0.000448139020216951,0.0223561909276514
"310","GLIS3",65.0693489170801,-1.18840188679421,0.338803654867323,-3.
50764187375605,0.000452097214599744,0.0224808985905002
"311","PTGFR",533.837333239241,-1.13848236037958,0.324746679928968,-3.
50575519549147,0.000455313786117607,0.0225680450578872
"312","ARHGAP12",375.36004669592,0.512669176251756,0.146280305757821,3
.50470402420762,0.000457115164586691,0.0225847123785379
"313","LGI2",6.25783076163339,-2.68123105961276,0.765916628850198,-3.5
0068265737728,0.000464068093880766,0.0227821963922676
"314","PRXL2C",209.277926388561,-0.608998466409081,0.173952191331875,-
3.50095311675149,0.000463597392952405,0.0227821963922676
"315","PLCB1",147.55091498228,0.810471364236774,0.232384618615303,3.48
762912565421,0.000487323544431343,0.0238479124997116
"316","STXBP6",242.982933305584,-0.843276173462172,0.242096611539106,-
3.48322171095714,0.000495417892568596,0.0241673000441295
"317","SPIN4",16.6258974591433,1.52151849194624,0.437676281989585,3.47
63558240573,0.000508277433687276,0.0247163931870327
"318","MT-
CO3",43769.9478490436,-0.530293222277869,0.152845792667779,-3.46946561
643669,0.000521494813145311,0.0252793790711791
"319","CYP11A1",19.0717625869607,1.94846642348647,0.561911269512835,3.
46756957762343,0.000525187762023621,0.0253785873090725
"320","ANTXR2",932.079129817146,0.66565557938974,0.192217676325461,3.4
6302999867015,0.000534128841547323,0.0257299877889125
"321","SUCNR1",22.3284324394086,-1.92560585420808,0.556293776509573,-3

.46149091634668,0.000537192248310301,0.025796942391599
"322","HLA-
DQB1",53.6180825414841,-1.61775041462402,0.46858498633208,-3.452416235
71255,0.000555899486285,0.0265975747146262
"323","KCNQ1OT1",1616.16717130581,1.27604122105219,0.370043226159524,3
.44835719409194,0.000564007627006619,0.0269169584220032
"324","CEBPG",142.340522720337,-0.657526913994097,0.190854279740771,-3
.44517772872155,0.000570684024888044,0.0271515254433617
"325","SLC4A4",51.5240404203985,1.70131946958357,0.494065882148899,3.4
435072953911,0.000574221101574757,0.0272357485562304
"326","LINC01137",7.02445062575159,-2.20954778323125,0.643178140059684
,-3.43535895518187,0.000591769418185844,0.027896408505611
"327","PRTG",20.2723306781224,1.30215314044531,0.379011176935977,3.435
6589453964,0.000591114603057502,0.027896408505611
"328","PLGLB1",8.07594986203602,2.44968187033021,0.713434498739738,3.4
3364650105581,0.000595520289838998,0.0279876380117932
"329","CFH",5023.01516816897,-0.801342511474098,0.233524883907852,-3.4
315080177507,0.00060023538690193,0.0280761864814832
"330","RUNX1T1",862.31605284602,0.826899586630974,0.240998397079578,3.
43114143766662,0.000601047131942229,0.0280761864814832
"331","ANKRD33B",63.8834419822549,0.862161184253835,0.252049387261614,
3.4206041665912,0.000624822021610389,0.0290985844807376
"332","GRK5",232.642686822644,0.728204212651682,0.213135937774095,3.41
661861559693,0.000634040358565253,0.0292626710397706
"333","NOVA2",14.597968375138,2.62992697072495,0.769611698968938,3.417
21282855797,0.000632658001509053,0.0292626710397706
"334","PNLIPRP3",2.84674977910268,-4.83266095179503,1.41412735305318,-
3.41741565309591,0.000632186799500712,0.0292626710397706
"335","CTXN1",40.9598702957073,-1.02148470540767,0.299150843346527,-3.
41461415913313,0.000638724211242108,0.0293908469143197
"336","HPSE2",10.1017622797908,-2.22471351310879,0.652701396017591,-3.
40847059111979,0.000653281264722201,0.0299712223086005
"337","ROR1",163.640550135705,1.101553149502,0.323867502572244,3.40124
631447479,0.000670793565412173,0.0306833317828743
"338","CTSO",417.403028869474,0.586755150238901,0.17281029624567,3.395
37147372723,0.000685355434104462,0.0312566686885215
"339","TEAD1",392.993026994155,0.843659250855074,0.248678726164898,3.3
9256704369495,0.000692409851702044,0.0314852444365398
"340","PCSK5",400.042974330135,1.22435754538689,0.361002370815175,3.39
154987437391,0.000694985130188651,0.0314976582454492
"341","SEPTIN1",158.802048340401,-0.854756503596231,0.252077567724479,
-3.3908471559456,0.000696769475296667,0.0314976582454492
"342","DIRC3",28.9177762706747,1.85636899362492,0.547980597687683,3.38
765460211229,0.000704929757814888,0.0317733690547262
"343","CXCL3",95.065775372592,-2.87036928486986,0.848801155954168,-3.3
8167457093432,0.000720454392642817,0.0323784386664403
"344","JAM2",88.2727437488381,-1.19648194058231,0.353971924783403,-3.3
8016056305719,0.000724434956504319,0.0324626885305642
"345","ANXA5",3989.45353088522,-0.667228723027277,0.197603429652507,-3
.37660497189054,0.000733863632571772,0.0327898779596924

"346","RASSF4",214.884981939645,0.851954689713895,0.252540904292931,3.
37353147641257,0.000742105613867118,0.0330623064675191
"347","PXN",555.125865726178,0.776948212114778,0.23062977409,3.3688113
9991745,0.000754930661706612,0.0335367612397909
"348","IER3",741.664690923761,-1.39782428540812,0.415059498883749,-3.3
6776845046889,0.00075779211200213,0.033567141972738
"349","CEP152",82.9586027968501,0.950084626957712,0.282429097846988,3.
36397571709286,0.000768283026971438,0.033934334844598
"350","PPP1R3C",8.1508356502584,-1.89739926540655,0.564167549705491,-3
.36318398035661,0.000770489958606068,0.0339345791768929
"351","PCDHB2",43.5490355907202,0.984328654590876,0.292958114039158,3.
35996378806326,0.000779526892658776,0.0342347779211824
"352","SNX32",2.55970464858463,4.27647995062097,1.27328361214747,3.358
62325551212,0.000783317799167669,0.0343035337334364
"353","PRUNE2",20.0963467875675,-1.94656990090254,0.579927692436024,-3
.35657345957363,0.00078914754035203,0.034363585690753
"354","PRG2",26.8301584282908,-1.25043870112954,0.372517969521648,-3.3
5672048984707,0.0007887280405783,0.034363585690753
"355","MPEG1",8.93789918540703,-1.85722607731417,0.555022598764877,-3.
34621703953525,0.000819222448662609,0.035572715622913
"356","PLEKHG3",29.2497999844915,1.06743243614616,0.319700026664527,3.
33885626248712,0.000841240811151785,0.0364261997300696
"357","GEM",372.533140123014,-1.32569963840059,0.397984368819623,-3.33
103443819281,0.00086523897442212,0.0373603887695153
"358","PLD5",6.31175671295297,3.13253671579778,0.940642155193525,3.330
2108548955,0.000867802414202042,0.0373635715809727
"359","LRRFIP1",303.838668771177,-1.04727588164726,0.314548715785875,-
3.3294552770013,0.000870160376099202,0.0373635715809727
"360","PHLDA3",353.368197183342,-0.663019887995742,0.199194644685178,-
3.32850257617933,0.000873141974710115,0.0373874542782123
"361","KCTD11",41.9198834542084,-0.832735003649815,0.250352809503634,-
3.32624588995367,0.000880242394342167,0.0375870817417853
"362","IL10",11.5807379424206,-2.21938602183787,0.669697390149495,-3.3
1401324610574,0.000919671503255474,0.0391622547587932
"363","CKB",694.397039194937,-0.944865455607021,0.28541748063291,-3.31
04680677294,0.000931400786304688,0.0395524603881178
"364","AC097518.2",5.81399059692627,3.40531511218228,1.02906222653203,
3.30914401907287,0.000935816859295313,0.0396308156209815
"365","IGSF3",61.844536787744,1.0649769579704,0.322077766721512,3.3065
8327897326,0.000944412730543645,0.0398852664146035
"366","BATF3",15.5091727681742,-1.56810144846998,0.474565531904903,-3.
30428853982638,0.000952177773620587,0.040103334372572
"367","P2RY14",4.99861857647225,-2.82144209776647,0.855955107268038,-3
.29625008812869,0.000979847482586317,0.0410444264784459
"368","HPRT1",45.7163918634766,-1.01067280950151,0.306564931650102,-3.
29676588924084,0.000978049891386016,0.0410444264784459
"369","NATD1",66.7804532262459,0.765907078892937,0.23253273384322,3.29
37602643477,0.000988567740842268,0.0412974843498199
"370","PEX11A",33.9748677612756,-0.998479486655183,0.303494367501678,-
3.2899440436886,0.00100207308093689,0.0417485311963302

"371","CMTM8",61.4998850619007,1.13228512650795,0.34482959065889,3.283
60777955401,0.00102487447597349,0.0425833963534539
"372","KCNMA1",31.4383828254458,-1.59414742403677,0.485792565451628,-3
.28153935940691,0.00103242114404879,0.0427816449879357
"373","REEP5",962.953114535427,-0.589323573661595,0.179717953926113,-3
.27915804062561,0.00104117309708616,0.0429135916887249
"374","TPP1",363.327997195597,0.670460432246017,0.204451481589872,3.27
931315064253,0.00104060094459887,0.0429135916887249
"375","CLU",4254.13640086184,-1.36841667741179,0.417580996121322,-3.27
700898777063,0.00104913029765964,0.0431262494357958
"376","AKAP12",900.143681244493,0.77596290258098,0.236920770353035,3.2
7519998109377,0.00105587200261936,0.0432006994000507
"377","ADAMTSL5",13.3078217916722,1.67824398961767,0.512437847302117,3
.27501959204085,0.00105654645954065,0.0432006994000507
"378","SMS",312.153601067032,-0.646724102942062,0.197839605220859,-3.2
6893142664782,0.00107954461088516,0.0440242861819966
"379","STRBP",22.5445455320298,1.17551958955876,0.36007773454489,3.264
62726456699,0.00109608210586691,0.0445807537254313
"380","TTC28",240.877236278024,0.577387244169331,0.176960404172191,3.2
6280473233721,0.00110315500934907,0.0447503538660946
"381","COL13A1",3.51195104949024,-4.39388468299017,1.34729844955316,-3
.2612556515948,0.00110919985583916,0.0447599889470174
"382","SNHG14",174.043745081173,0.990201235648337,0.303584013545444,3.
26170414602583,0.00110744659142904,0.0447599889470174
"383","POLG2",91.1884558777295,1.44212807110208,0.443126315729723,3.25
444014474121,0.00113616099407988,0.0457282551533719
"384","MIR4435-2HG",217.828603810226,0.813909696616381,0.2502117070673
63,3.25288415220819,0.00114240061750554,0.0458596497886666
"385","SNX18",275.344157602214,0.502755025002595,0.154661636757356,3.2
5067699749843,0.0011513057842429,0.0460970874392321
"386","KIFC3",28.650153975579,-1.20514655766616,0.370998881878857,-3.2
4838326078751,0.00116062823201804,0.0463499590584406
"387","HRCT1",225.519187782705,-1.40931282933925,0.434140837691846,-3.
24621115311796,0.00116952060730345,0.0465843931823841
"388","DOCK4",138.811809807245,1.09883968271278,0.338874032049575,3.24
261990824964,0.00118436098783955,0.04705392945244
"389","ZNF221",9.66810451434483,1.79975595665572,0.555651406672977,3.2
3900189046934,0.0011994877842533,0.0475324015276727
"390","PCYOX1",776.658825380781,0.622452663038398,0.192239965404659,3.
23789416903065,0.00120415469218055,0.0475835183887808
"391","LRRN4CL",870.969918780126,0.613872784570822,0.189642988020955,3
.23699173366215,0.00120796910832617,0.0475835183887808
"392","ITGB2",35.2020492848235,-1.16206989389497,0.359051044265806,-3.
23650331186529,0.0012100382230556,0.0475835183887808
"393","PATZ1",65.0991293833741,0.681460295933478,0.210616229912396,3.2
3555452595902,0.00121406695065714,0.0476204632172514
"394","GASAL1",36.5331054078451,-1.84522615835199,0.570562988236895,-3
.23404461276738,0.00122050388799641,0.0477514401864587
"395","NFIA-
AS2",9.59579589833233,-1.98663648811264,0.614445849466876,-3.233216547

61986,0.00122404739666224,0.047768837011515
"396","CCDC168",4.01540212938937,-2.72824783266753,0.84422329477403,-3.23166613567301,0.00123070758009664,0.047907468048459
"397","FAM171A1",127.012000090471,0.676927797211725,0.2097964892675,3.22659258777496,0.00125273694156065,0.0486421661313789
"398","FPR3",5.01444667838747,-2.83247476371752,0.878093622479166,-3.22570929933468,0.00125660919183613,0.0486699263621959
"399","SULT1A2",4.57100544846156,3.68030548817326,1.14235415215548,3.22168522014734,0.00127439061666629,0.0492349156789747
"400","CMKLR1",61.4905026255467,-1.6872142559547,0.524352192100037,-3.2177118382921,0.00129217566926827,0.0496051295451102
"401","RHOJ",79.9614597184282,1.0306304179135,0.320280597967125,3.21789838177861,0.00129133559234717,0.0496051295451102
"402","LYL1",6.0766386006339,-2.11769660050785,0.658203305344663,-3.21738979934009,0.00129362712144887,0.0496051295451102
"403","EBF1",2204.63996758919,0.532276169263458,0.165504834293508,3.21607626469395,0.00129956291428361,0.0497090876518161
"404","PTMS",1956.67845302005,-0.639127895189711,0.198920443769235,-3.21298245207595,0.00131364315346543,0.0501232901254199
"405","RBP7",7.86866635396732,-2.03419197427092,0.63503526297052,-3.20327404301224,0.00135874618288086,0.0515545914006616
"406","FCGR2A",25.5441470306221,-1.64001433013896,0.512076623840829,-3.20267368941398,0.00136158165031866,0.0515545914006616
"407","APBA1",24.8887229240597,1.15266663194819,0.359977704628154,3.20205006345839,0.00136453281164255,0.0515545914006616
"408","PTGDS",1478.74477196471,3.00677521355708,0.939007880483977,3.20207665563717,0.00136440685015714,0.0515545914006616
"409","ISYNA1",35.834810386153,-0.933348297892623,0.291687972205164,-3.19981756819282,0.00137514599682067,0.0518285465549892
"410","SMYD2",52.4975698938553,-0.825080288209015,0.258135621570089,-3.19630542731968,0.00139199675723418,0.052335682957963
"411","TGFBR3",2137.4463175047,0.629864003441913,0.197151168922454,3.19482763852981,0.0013991437304529,0.052349030594494
"412","EXOC4",155.373266723589,0.544045695834044,0.170262463827766,3.19533550497888,0.00139668374819243,0.052349030594494
"413","SCARB2",755.257169666357,0.510826417883981,0.160020341359054,3.19225926870003,0.00141164559691034,0.0526889028483605
"414","NR2F2-AS1",30.2355753943287,1.40905761156355,0.44196053215072,3.18819783455918,0.00143162550705934,0.0533055729258932
"415","RGPD2",5.77052637348862,3.86931717496874,1.21733418578847,3.17851681168599,0.00148030633302276,0.0548531781816005
"416","HAS2",395.672921388182,-1.1667864638829,0.367059084903134,-3.17874291053391,0.00147915221428679,0.0548531781816005
"417","ATM",245.314720367562,0.645703362318178,0.203264093857485,3.17667203323623,0.0014897540322924,0.0550708834719121
"418","HDGF",365.970866952468,-0.533298711104431,0.167958009765727,-3.17519070301137,0.00149738057949931,0.0552203866817747
"419","TEDC1",38.0882676627026,-1.12391399793066,0.354191832025459,-3.1731787588198,0.00150779657463128,0.0554717999950864

"420","OLFM2",22.6729722873428,-1.62913830556749,0.513858778962692,-3.1704008421461,0.00152228779275205,0.0558715864887449
"421","DDIT4L",31.0379390019192,-1.66934509138914,0.526700887732862,-3.16943663902807,0.0015273475502795,0.0559241389253169
"422","NLGN1",36.5330441332983,1.01123169437078,0.31917548605074,3.16826240913132,0.00153353036310916,0.0560174657519615
"423","CYP2U1",130.818323738752,0.577501706189871,0.182327362447405,3.16738913149396,0.00153814347385192,0.0560531481073934
"424","ZEB1",867.504208047144,0.646429055657099,0.204245161708388,3.16496630936131,0.00155100909190763,0.0563886913956511
"425","C3AR1",5.12154669298651,-2.69538124026841,0.852182888007664,-3.1629140624614,0.00156198433380223,0.0566540906013208
"426","FSTL1",9606.13315390265,0.922804338942871,0.291892243653358,3.16145549944371,0.00156982802975794,0.0568049274148327
"427","ATP6AP1L",19.3483062857865,1.45249198449542,0.459940006354139,3.15800314047272,0.00158853846602368,0.0573473546926349
"428","MT-ATP6",54678.3065933,-0.574137606179387,0.181933405802738,-3.15575693010386,0.0016008220599202,0.0576557758289182
"429","HABP4",117.270493628444,-0.663378847541003,0.210318223420559,-3.15416722693825,0.0016095682599975,0.0578356520463781
"430","PLA2G7",48.9969965422903,1.56785099987584,0.49764206258735,3.15055964466556,0.00162957979734719,0.0583106509970709
"431","CD209",3.40218058361341,-3.90751809008698,1.24031613033996,-3.15042108580493,0.00163035294062521,0.0583106509970709
"432","NOX4",198.408351465434,0.846944688337089,0.269338342073159,3.14453813674637,0.00166349227727169,0.0593581792919979
"433","ARHGEF4",40.4739839696842,0.962209212965312,0.306558190455426,3.13874899749324,0.00169670715103844,0.0602643795697177
"434","GTF3A",527.035677051651,-0.761382466821528,0.242567480626728,-3.13884806345156,0.0016961336718218,0.0602643795697177
"435","PHYHIP",34.8645105004714,-1.17863531522376,0.375740946397448,-3.13682958039137,0.00170785365631299,0.0605208370392294
"436","PLAT",229.654183812105,0.832698229654675,0.265643716715788,3.13464304727214,0.00172063340532771,0.0608338622548775
"437","DNASE1",58.5926063581836,1.0856876877477,0.346681525583852,3.13165717705688,0.00173822715351973,0.0613152667540199
"438","AVL9",75.5006453716192,0.786687150280982,0.251447162277428,3.12863801347263,0.00175618514310053,0.0618072921938235
"439","PRDM6",54.7190601653708,1.21282842892613,0.38784350128685,3.12710777646656,0.00176535198643916,0.0619883846718897
"440","AQP1",97.349325763063,-2.31918098725385,0.742329542025847,-3.12419330763089,0.00178293281391321,0.0623652627561853
"441","DHRS4",106.568950997464,-1.17784045613593,0.377031027337434,-3.12398813554882,0.00178417650830215,0.0623652627561853
"442","CLCA2",4.14365972906187,4.27695526144577,1.36988356085493,3.1221305106958,0.00179547324761893,0.0624767948353178
"443","DSTN",3856.22696839197,-0.470764781585403,0.15078217593316,-3.12215139934105,0.00179534585324558,0.0624767948353178
"444","MLIP",8.7216751440463,-2.78975489780817,0.89457378547823,-3.118

52967647246,0.00181755837133974,0.0628198706148029
"445","AL512625.1",16.9981545893195,1.27178455184354,0.407715672312475
,3.11929277731773,0.00181285727648005,0.0628198706148029
"446","GALNT16",441.752094686753,0.668425746618413,0.214333005352256,3
.11863189488644,0.00181692800401212,0.0628198706148029
"447","DDX11",23.3822415697957,1.46049827574344,0.468558381233275,3.11
700384464218,0.00182699189516711,0.0630046533870267
"448","AC108463.3",25.1484075621574,1.70324607615728,0.546922260363947
,3.11423798150739,0.00184420674916178,0.0634563549962699
"449","FMO3",22.8168462676283,1.70143374947464,0.546630290312538,3.112
58592805356,0.00185456016964386,0.0635214654038629
"450","PDE7B",45.2084820605302,1.28103178183971,0.411600656429338,3.11
231714971676,0.00185624964559992,0.0635214654038629
"451","ULBP2",8.98657291269727,2.26704012715992,0.728562004713868,3.11
16639524048,0.00186036138213014,0.0635214654038629
"452","TMOD3",610.701295908797,-0.564209362999126,0.181341316145864,-3
.11131172415943,0.00186258205400882,0.0635214654038629
"453","TMOD2",233.059998980303,0.684277190466184,0.220195631802244,3.1
0758748875059,0.00188621146299979,0.0641853194307766
"454","CACHD1",131.892560658621,0.760308903470608,0.244850948541998,3.
10519076196348,0.0019015634247211,0.0645651986609599
"455","GDF10",401.651909474479,-1.23758384513693,0.399287210766742,-3.
09948280777745,0.00193858828343697,0.0656776667894085
"456","KLHL24",278.809378114333,0.770782400179331,0.248733652983784,3.
09882635877013,0.00194288853813708,0.0656790061740857
"457","CCND1",360.153314898719,-0.902366958033733,0.291743236006662,-3
.0930175807508,0.00198132397232031,0.066831748431767
"458","SRSF8",186.922219734578,-0.471204952447493,0.152645597961601,-3
.08692133110861,0.0020224111789607,0.0680687060750391
"459","ZNF528",37.9417837378386,0.950719912283104,0.308266448015145,3.
08408494795514,0.00204179286347262,0.0685713224192384
"460","LGALSL",101.231954431017,-0.824056705943595,0.267492181083468,-
3.08067586351789,0.00206531347967717,0.0688099727264562
"461","ABRACL",108.878258268641,-1.00910676134218,0.327409537176974,-3
.08209336246895,0.00205550356979149,0.0688099727264562
"462","C6orf120",192.517612754546,0.600973073557018,0.195092798335809,
3.0804472470715,0.00206689965333368,0.0688099727264562
"463","SEMA3C",2238.49987646123,1.1982919035687,0.388947924130074,3.08
085434894354,0.00206407589852899,0.0688099727264562
"464","MEG3",3586.45396955614,1.18108673080341,0.383556049123318,3.079
30674930818,0.00207482929641401,0.0688099727264562
"465","KCNN4",3.2558575650972,-2.77973149733891,0.902749293111057,-3.0
7918435223515,0.00207568195379839,0.0688099727264562
"466","MUC20",29.6999598127674,1.85910074983342,0.604449901178461,3.07
569038593412,0.00210015801195144,0.0691791844605786
"467","NRBP2",119.594358743406,0.68407089125084,0.222413423959345,3.07
567267781409,0.00210028273289334,0.0691791844605786
"468","RNF24",211.742953249047,-0.862721378184489,0.280419691887381,-3
.07653636011755,0.00209420759673768,0.0691791844605786
"469","ERLIN2",142.623589479698,0.674186823149808,0.219458797105349,3.

0720428255431,0.00212599221240602,0.0697208532201387
"470","TMEM245",198.216353291244,0.749435679459043,0.244051798686823,3.07080580225819,0.00213481950826503,0.0697208532201387
"471","KCTD12",794.349487374744,1.12543389576258,0.36648457633439,3.07089020503745,0.00213421615037962,0.0697208532201387
"472","MT-ND4",33371.6493626764,-0.559748241861778,0.182251118642094,-3.07130209148958,0.00213127400027917,0.0697208532201387
"473","AC007563.2",42.5560103535869,1.1898314745881,0.387558989173066,3.07006548119769,0.0021401184402207б,0.0697461432473638
"474","SLC25A48",5.96553522608306,-2.52892446298377,0.824796050470583,-3.06612096595383,0.0021685556199522,0.0705238077670109
"475","KLF15",14.5779923496834,1.51173977982252,0.493436056352345,3.0636994608741,0.00218618413826846,0.070947428402965
"476","SCIN",120.26267065016,3.13101440736188,1.02259449074277,3.06183383120678,0.00219985537335782,0.0711200422084849
"477","MSANTD3",111.348094625524,-0.694067799434368,0.226692507457066,-3.06171477487328,0.00220073046600372,0.0711200422084849
"478","TNFSF14",32.0956963213764,1.44093913297595,0.470770135834733,3.06081253523227,0.00220737252171463,0.071185454858224
"479","LIFR",181.486846058441,0.931330235672695,0.304385901685797,3.05970227436507,0.00221557118943042,0.0713006886953443
"480","ALPL",467.164072465515,2.55845595774974,0.836744852931022,3.05762975271106,0.00223095033775104,0.0716432841947478
"481","TRIP10",558.493429347244,-0.500288652043492,0.163652530579636,-3.0570175130903,0.00223551214386466,0.0716432841947478
"482","LAMC2",44.6327208799169,0.962827222224913,0.315385165824208,3.05286147402881,0.00226670548792457,0.0723421637605741
"483","SIGLEC1",4.9762836308705,-2.93092204960662,0.959880332811293,-3.05342442116982,0.0022624570406728,0.0723421637605741
"484","CYP1B1-AS1",32.9880798216549,1.24331852613535,0.407384575840838,3.05195287172853,0.0022735779446477,0.0724115785469924
"485","C5AR1",29.6355526437448,-1.45200779122086,0.476331951668374,-3.04831071301251,0.00230131843523832,0.0731439663488634
"486","IGFBP5",15721.54900366,1.09671557983296,0.360137837623838,3.04526618771581,0.0023247445680886,0.0735850873040776
"487","GAS2L1",127.833453147142,-0.753676445701855,0.247458888587131,-3.04566326150165,0.00232167694467548,0.0735850873040776
"488","DMTF1",116.418392261275,0.851091990981578,0.279593737546293,3.04403095166128,0.00233431122758631,0.0737364909287767
"489","KCNIP4-IT1",3.94046965979674,-2.68329473653807,0.88317125571484,-3.03824962505851,0.00237956750823207,0.075012337708379
"490","CDH13",66.2435487716698,-2.54757722514499,0.839280600663489,-3.03542965622107,0.00240193245783789,0.0755628343623899
"491","AKR1C1",1325.71704681536,-0.951890896825596,0.313845813804681,-3.03298898680865,0.00242144437100825,0.0760215172690268
"492","DUXAP8",321.99423345181,0.850472878276482,0.280902194909714,3.02764767840222,0.0024646525453371,0.0772207702975029

"493","ENHO",3.65914849515809,-2.51321499529871,0.830281097953835,-3.0
2694473172078,0.00247039122238021,0.0772435713853771
"494","GNG4",6.69631223697876,2.21294257737478,0.732159107576845,3.022
48862914338,0.00250705501505501,0.0781976288274468
"495","NID2",286.969890812696,1.05155266885673,0.347965028389193,3.022
00676235943,0.00251104938498775,0.0781976288274468
"496","FAM117B",60.4276487560109,0.923968144138516,0.3058967838921,3.0
2052258406362,0.00252338888922621,0.0783094283234409
"497","ENPEP",28.5671151565039,1.80044653534409,0.596104607479924,3.02
035332851328,0.00252479960277328,0.0783094283234409
"498","PLPP3",3662.29339863313,0.741290642478675,0.245625098620854,3.0
179759586496,0.00254469091454993,0.0786731426497248
"499","LINC02518",5.81059973753536,2.31134342034843,0.765920505261052,
3.01773278619907,0.00254673358301737,0.0786731426497248
"500","SPATS2L",383.890986155859,-0.506370906215908,0.167867939500492,
-3.01648371763342,0.0025572495154039,0.0788400025599023
"501","EMP3",2698.94305662566,-0.638421999501647,0.211743982411394,-3.
01506561004066,0.00256923671637412,0.0790514650357426
"502","OLA1",176.033423078565,-0.620360205416201,0.205878093701227,-3.
01324047771919,0.00258474009927648,0.0792448409371741
"503","STARD10",70.363215525403,-0.857011764965584,0.28442710132172,-3
.01311570164407,0.00258580311329216,0.0792448409371741
"504","TRMT9B",24.6399021896426,1.30836830012989,0.434376274083139,3.0
1206207197097,0.00259479533036935,0.0793626389238958
"505","SERPINB8",50.2969863794525,-0.930647225465871,0.309209891146724
,-3.00975891170399,0.00261455132405439,0.0798085320005911
"506","GAB1",122.292341643237,0.60883717391767,0.202469157495825,3.007
06132947792,0.00263786538238561,0.0802592051962639
"507","PSD",3.65680117550562,-2.60857218325421,0.86754412869208,-3.006
84668016476,0.00263972864317261,0.0802592051962639
"508","TCEA3",197.707365789589,0.826689071938664,0.275021764335704,3.0
0590418338518,0.00264792422189701,0.0803465831478004
"509","BCL7A",57.9472871991186,1.13072954381817,0.376242805876921,3.00
531870950394,0.00265302697516902,0.0803465831478004
"510","DPY19L3",90.0075190148026,0.734144920072042,0.244338364150172,3
.00462402875396,0.0026590931894384,0.0803723951278293
"511","ART5",3.96938003073297,-3.16065567597706,1.05256809660056,-3.00
280398597003,0.00267504665565147,0.0806963682913256
"512","FTX",600.730805719719,0.971883742643423,0.323971601666387,2.999
90412012788,0.00270064603561385,0.0813094895292726
"513","SCARA3",381.69413342339,-0.703604719111741,0.234652316317204,-2
.99849892877514,0.00271313110123394,0.0813675407111306
"514","INO80C",57.8427290811211,-0.860095848383695,0.286811436724898,-
2.99881991529046,0.00271027451116214,0.0813675407111306
"515","MATN2",113.751011536749,-1.11509381200794,0.371965693426433,-2.
99784047753447,0.00271899953911642,0.0813851997970479
"516","SASH1",1055.03901917148,0.831457022779893,0.277409968442879,2.9
972139337563,0.00272459436797452,0.0813946166324171
"517","UBXN11",58.613985283274,-0.847734788779477,0.282899752413077,-2
.99659077658595,0.00273016938672192,0.0814034063758575

"518","CCL4L2",51.9178656484687,-1.70572807556038,0.569479558107668,-2
.99524021762671,0.00274228782660963,0.0816068858053811
"519","DPY19L4",143.326248593425,0.562469758262638,0.18808351191877,2.
99053198509798,0.00278491961777904,0.082713490615045
"520","ADGRD1",389.000374224765,0.971490669638281,0.324918393488274,2.
98995282848874,0.0027902053272671 7,0.082713490615045
"521","NHSL1",281.053343938247,0.586287763770595,0.19615855354674,2.98
884628363094,0.00280032976317169,0.0828456948299824
"522","GTF2A1",153.457295253846,0.536904467675492,0.179669344791161,2.
9882920110805,0.00280541373345772,0.0828456948299824
"523","GNS",407.517509841133,0.693224407911785,0.232614354492408,2.980
14458060631,0.0028811236365028,0.0849187779286628
"524","KCTD7",47.7198973992199,0.873212056102371,0.293404345713354,2.9
7613879569279,0.00291902718649736,0.085871763511177
"525","TTC27",80.8244089291515,1.02386291255735,0.344476424512619,2.97
222927231074,0.00295645817976494,0.0859966816210965
"526","TFPI",1180.0995042559,0.542699569439506,0.182431805839429,2.974
80785733806,0.0029317211678376,0.0859966816210965
"527","C3orf80",2.66775239613848,-4.06135618852296,1.36646229105096,-2
.97216850777444,0.0029570433993529,0.0859966816210965
"528","CDK6",134.812928636704,0.915624187906787,0.308122841309864,2.97
162061733031,0.00296232487452496,0.0859966816210965
"529","RASL12",46.4343308301217,-1.29105751877896,0.434188242337635,-2
.97349719059182,0.00294427101948174,0.0859966816210965
"530","ABCA10",402.367754863009,1.00869004554014,0.339179982114185,2.9
739079507368,0.0029403326583972,0.0859966816210965
"531","MARCH2",343.033804820968,-0.933822787759586,0.31420232539369,-2
.9720428917562,0.00295825353479251,0.0859966816210965
"532","SERGEF",79.2326523420979,-0.792353101826392,0.266712602033282,-
2.97081238676348,0.00297013165370769,0.0860612395524511
"533","CCDC18-
AS1",186.470396974694,0.693253186128093,0.233497694807223,2.9689937054
8582,0.0029877671 6609079,0.086247997875074
"534","PMS2",49.3664421309891,0.773044945351608,0.26032619688307,2.969
52421464842,0.00298261304512314,0.086247997875074
"535","CCNG2",407.717363860479,0.710817664999838,0.239543408585468,2.9
673856158151,0.00300344012353817,0.086376920717054
"536","SNHG18",84.4083273754541,-0.911545961717136,0.307144605092751,-
2.96780717161504,0.00299932426816337,0.086376920717054
"537","FMO4",45.1898411129825,0.954859311317576,0.322089196348182,2.96
458037755902,0.00303096051918714,0.0870060640656792
"538","LINC01151",7.71551739982644,-2.75685065084593,0.930537298245177
,-2.96264390051301,0.00305009203568619,0.0873925069332763
"539","CAPS",92.2983124656196,-1.19714657119159,0.404217210949595,-2.9
6164175785397,0.00306003593817611,0.0875147569331814
"540","WDR76",13.1875137794144,-1.57676743799948,0.533230367599966,-2.
95700982878451,0.00310638215713011,0.0886757054669643
"541","RAB30",275.579872650513,0.508025600909641,0.17203897814766,2.95
296802143062,0.00314734565491754,0.0896789894095266
"542","SERP2",40.3553059753146,-0.939888720004393,0.318537019691462,-2

.95064203499731,0.00317114203386968,0.0901903218673453
"543","IL6R",146.150877242151,0.781535836771988,0.265308317867668,2.94
576454689901,0.00322157527893285,0.0912878362587315
"544","NCEH1",38.7557661647392,-1.07611308870496,0.365277733052685,-2.
94601337922163,0.00321898476303993,0.0912878362587315
"545","TNFRSF19",32.7202716636111,1.19400278585738,0.405539552640382,2
.94423263546916,0.00323756542372241,0.0915726073517082
"546","AC083837.1",2.92425361273909,-3.15629591703101,1.07273711465522
,-2.94228275866586,0.00325802289986784,0.0919824597096386
"547","JAG1",311.290226687417,0.760723486526395,0.258603107507869,2.94
166413488766,0.00326453786713868,0.0919978998572992
"548","PCDHB12",25.4970325096277,1.20766736874735,0.410675413760336,2.
9406858270121,0.0032748650366593,0.0921205192337648
"549","ITGA7",11.5756020294241,-1.7182815705276,0.585248820497031,-2.9
359846792486,0.00332490743485633,0.0933578289768857
"550","ASIC1",41.2655713136441,0.906581639280467,0.309173416711621,2.9
3227551360301,0.00336488088689794,0.094308434311876
"551","SVEP1",1491.56752495212,1.13144351403924,0.385987320566229,2.93
129710162359,0.00337549786366498,0.0944343004871064
"552","ARHGEF10",430.828279291299,0.7896361290517,0.269544258584881,2.
92952308907384,0.00339482590993958,0.0948029735538381
"553","PAK3",104.869406705833,0.982560750564043,0.335505519980681,2.92
859786813827,0.00340494621524874,0.0949136454033622
"554","CYGB",813.695853541965,-0.718518553687043,0.245702012169718,-2.
92434948880569,0.00345176961610139,0.0960451780364674
"555","DDX60L",129.6800334951,0.646025496500576,0.221008016229875,2.92
308626411374,0.00346580482436453,0.0961200419507619
"556","NT5DC1",68.0524509489007,-0.697835186788641,0.238740590279277,-
2.92298509429133,0.00346693112712447,0.0961200419507619
"557","DENND2A",176.16605543644,0.501735250963194,0.17169212148102б,2.
92229629778697,0.0034746082154846,0.0961599383154311
"558","DZIP3",89.5401534602531,0.675946837744584,0.231392663134909,2.9
2121119393697,0.00348673379847144,0.0963225833394932
"559","LINC00517",5.12279868184645,-2.43008983127041,0.832637525383216
,-2.91854469344506,0.0035166945873212,0.0969764705967018
"560","FBLN5",2018.00294149311,0.650005745156791,0.222804669743822,2.9
1737936150154,0.00352986165884146,0.0971657454840019
"561","ADRA2B",8.45263891772444,1.99279251735484,0.684920534921582,2.9
095236830401,0.00361979960412055,0.0986999469320067
"562","UBE2E3",986.112977106993,-0.769040690318917,0.264218465714122,-
2.91062431325674,0.00360707443432357,0.0986999469320067
"563","SEMA3B",144.380502447423,1.09089956007292,0.374667680115762,2.9
1164575427445,0.00359530124268673,0.0986999469320067
"564","FABP5",130.801388580203,-1.37195382926259,0.471687413627655,-2.
90860809431222,0.0036304164716476,0.0986999469320067
"565","ENTPD1",66.6104471571353,2.38752123399745,0.820106011289086,2.9
1123489052912,0.00360003267496614,0.0986999469320067
"566","AC092069.1",4.52398787237473,-3.07806359788714,1.05824041382996
,-2.90866192375615,0.00362979150035189,0.0986999469320067
"567","MBNL3",88.1002309050186,0.851865357020222,0.292801739157168,2.9

0935893848282,0.00362170784234728,0.0986999469320067
"568","ATP8B4",81.7215011412302,1.32202566306201,0.454845057833006,2.9
0654067862244,0.00365449378917008,0.0991124067190055
"569","SDF2L1",137.066772058699,-0.890471733183003,0.306403891857548,-
2.90620242381581,0.00365844692981603,0.0991124067190055
"570","PINK1",418.61160880336,-0.595852164132471,0.205153057198634,-2.
90442741760144,0.00367925501106587,0.0993269982409462
"571","FBXL7",161.780054161899,0.760884478179976,0.261952381167655,2.9
0466715663483,0.00367643832183263,0.0993269982409462
"572","TGFBR2",1628.9472539152,0.524811935043013,0.180864621191056,2.9
0168376538731,0.00371163013441778,0.099677314498345
"573","POLR3G",40.015852905559,0.969297138905923,0.334015618144652,2.9
0195154433213,0.00370845897386489,0.099677314498345
"574","LGI1",15.8664379594146,1.56656800554839,0.539744104739997,2.902
42726468134,0.00370283135043192,0.099677314498345
"575","ALCAM",63.7176236823267,-0.930998652969432,0.32136427790806,-2.
89701972798539,0.00376726064665716,0.100232468642916
"576","SEPTIN11",756.660635430192,0.702189854386995,0.242290226901782,
2.89813527918996,0.00375388634268701,0.100232468642916
"577","LVRN",153.175940380323,1.00210162887698,0.345948073640314,2.896
68220531522,0.00377131571929234,0.100232468642916
"578","NOD1",38.2866219709605,0.858888806411979,0.296313254785419,2.89
858382148298,0.00374852096032235,0.100232468642916
"579","LRRC4C",33.3376784223357,-1.33460230810756,0.460565823283159,-2
.89774499243952,0.0037585605608244,0.100232468642916
"580","AC087645.2",16.1099243733995,-1.33625494958385,0.46123659843899
2,-2.89711387627579,0.00376613023516512,0.100232468642916
"581","IMPDH1",74.0230951273887,-0.734929057081442,0.253886221501683,-
2.89471816443796,0.00379499094934068,0.100688098939908
"582","SLC25A33",56.904042434547,-1.22846114402944,0.424825110776348,-
2.89168675030647,0.00383179786450308,0.101142404248827
"583","AC096642.1",14.0372659526569,1.63118855022036,0.564009318989157
,2.89213049377242,0.00382638981990862,0.101142404248827
"584","BMPR1B",2.71085511353797,-3.40547320638444,1.17728034596696,-2.
89266122385433,0.00381993074952847,0.101142404248827
"585","PRG4",944.315857717866,-2.27879482231284,0.79083069784674,-2.88
152044238735,0.00395761561674108,0.103297777092221
"586","SERTAD4-
AS1",39.756323277645,-2.5988715715198,0.901454492993505,-2.88297589253
74,0.00393937587991501,0.103297777092221
"587","NCOA1",347.679471226642,0.498894022943185,0.173342080361375,2.8
780895089243,0.00400091600169602,0.103297777092221
"588","NR4A2",132.918996812299,-1.26506013131383,0.43930699921574,-2.8
7967215084723,0.0039808889724319,0.103297777092221
"589","SLC9A9",125.152841930963,0.650789491566936,0.225940199866608,2.
8803616707038,0.00397219216858701,0.103297777092221
"590","MGST2",107.48408170534,-0.820549616177801,0.28506546491732,-2.8
7846027373324,0.00399621609132996,0.103297777092221
"591","HLA-F-
AS1",92.5801106433186,-1.01309449473393,0.351489851591732,-2.882286615

51991,0.00394800437895318,0.103297777092221
"592","ENPP5",7.07601107364998,2.12965724556488,0.739785546965524,2.87
87494623278,0.00399255374237971,0.103297777092221
"593","VPS13B",136.549244918934,0.620013098352081,0.215398369452963,2.
87844842988691,0.00399636614944228,0.103297777092221
"594","AQP3",97.0189438779322,-1.34760017798521,0.467285280626873,-2.8
8389177629857,0.00392793717214281,0.103297777092221
"595","ZDHHC12",82.2278696874255,-0.652382520622076,0.226567592607324,
-2.87941674762266,0.00398411471803632,0.103297777092221
"596","SLC38A7",52.2967996028737,0.783218772785289,0.272037241407066,2
.87908658657992,0.00398828817267324,0.103297777092221
"597","MTCL1",10.3330263822835,-1.57616924749169,0.547739555782988,-2.
87758886655277,0.00400727023685686,0.103297777092221
"598","SERTAD3",153.311344922754,-0.805574537465869,0.279489712622451,
-2.88230479006603,0.00394777664644791,0.103297777092221
"599","EFHD2",168.799481591419,-0.679730732805815,0.236314783541898,-2
.87637837387055,0.00402267188378784,0.103521681283121
"600","DMKN",4.03830625060674,-3.05043991282675,1.06363310202568,-2.86
794375524532,0.00413148972757733,0.105968243179042
"601","TOX2",8.93094200300538,-1.75481733160328,0.611811946737324,-2.8
6822992091178,0.00412775446352392,0.105968243179042
"602","H19",2092.56472627258,1.19158472865039,0.415749124614796,2.8661
1482286205,0.00415543495089314,0.106405365063152
"603","RERG",195.173792823805,-1.13146137620925,0.394955904072659,-2.8
6477899062143,0.0041730037749975,0.106501412568852
"604","SMARCE1",841.890758277207,0.486283375233102,0.169724558552996,2
.86513265598661,0.00416834583206371,0.106501412568852
"605","WNT5A",55.5728948825344,1.37380475760883,0.479898997395567,2.86
269561942103,0.00420053874693803,0.106850337927475
"606","PTPRG",352.20764191488,0.582940245780834,0.203600743706871,2.86
31538135249,0.00419446890450682,0.106850337927475
"607","CNTNAP2",2.98626447670536,4.52798943486319,1.58202495636391,2.8
6214791786232,0.00420780477709469,0.106855778239251
"608","MLH3",161.633106086954,0.630161564815409,0.220210303485496,2.86
163524068216,0.00421461648845049,0.106855778239251
"609","SLC25A3",2027.98886341398,-0.60827681747038,0.212739352833008,-
2.85925856861966,0.00424632510900309,0.107482925378132
"610","DHFR",49.7099312801716,0.828972142766525,0.29008942271947,2.857
64346385073,0.00426799650916969,0.107854370801395
"611","CIZ1",325.439346995963,-0.454648720366563,0.159267005978094,-2.
85463217930458,0.00430866980479478,0.108704001703619
"612","ELMO1",62.4197813942126,0.757871570867205,0.265655388409303,2.8
5283718657169,0.0043330815661396,0.1091412619968
"613","SLC43A1",119.527848356933,0.557201328372167,0.195507649958721,2
.85002314993716,0.00437160471208951,0.109931952099282
"614","CD1C",7.27913920991632,-1.7960483122286,0.630691022663108,-2.84
774675346534,0.0044029946196715,0.110540980557388
"615","SENCR",5.26371079558499,-1.90012464770863,0.66755692943673,-2.8
463859244363,0.00442185691926867,0.11083402343175
"616","LGALS3",7782.48185391112,-0.726390135561783,0.255247480757972,-

2.8458268555863,0.00442962731100905,0.110848547076631
"617","HS6ST1",104.109316227631,0.798564500603457,0.280683617291816,2.
84506986303094,0.00444016832827267,0.110932244376537
"618","ARVCF",19.6640031257663,1.05394399985228,0.370530468312237,2.84
44192582947,0.0044492460682328,0.110979171750499
"619","HEBP2",338.054132062984,-0.81554069281567,0.286970663636287,-2.
84189569233915,0.00448461615846182,0.111500577552724
"620","CRISPLD1",54.9627073837298,1.14594657905226,0.403176199128059,2
.8422971929657,0.00447897177093068,0.111500577552724
"621","PXDN",308.173417241265,0.762764011957266,0.268457925522586,2.84
127954305112,0.00449329066073732,0.111536353518947
"622","AC007262.2",10.8637000645702,2.32419214977354,0.818195928974317
,2.8406303031685,0.00450244747748101,0.111583967629212
"623","HIST1H2AC",78.5723618560453,1.06516407343968,0.375259927550034,
2.83847007164828,0.00453303701586167,0.111811725400893
"624","LMO7",32.9536726253758,1.21505467958938,0.42796784326166,2.8391
2611360964,0.00452372741497579,0.111811725400893
"625","APP",2267.02757056124,0.505883630934799,0.178225644831862,2.838
44466609757,0.00453339788359119,0.111811725400893
"626","WFS1",64.2011068546723,0.794183611190873,0.279947826362832,2.83
689865182791,0.004555406952396,0.112042277026496
"627","LINC01554",6.32547190734172,2.7684352841885,0.9759120372809,2.8
36767227405,0.00455728236753897,0.112042277026496
"628","CPZ",4.98582668268528,2.75116201660889,0.971299974832912,2.8324
5350344229,0.00461922836386708,0.113271660615952
"629","LMO2",35.1591082834395,-1.20783268100847,0.426455006795825,-2.8
3226286890975,0.00462198342701483,0.113271660615952
"630","AL161772.1",77.5032739807855,-0.719950888068569,0.2542640897829
93,-2.83150832932415,0.0046329026906293,0.113359039644525
"631","XG",579.706057134889,-0.726911279636726,0.256820755750003,-2.83
042263275762,0.00464865525660698,0.113564216767982
"632","SLC43A2",31.9921029103899,1.07475852360321,0.379992095848064,2.
82837073546114,0.00467855910655408,0.11411390605622
"633","PKM",836.555953995923,-0.670075374257703,0.236972448892292,-2.8
2765096697913,0.00468909002683721,0.114190083354969
"634","RARG",304.642527646215,-0.628499363454524,0.222392294776738,-2.
8260842583844,0.00471208674170333,0.114388688383239
"635","ZNF667",41.2979355915891,0.9061425746427,0.320629319887624,2.82
613759390529,0.00471130218803079,0.114388688383239
"636","ATP1B3",354.306980999414,-0.57750794033456,0.204655286671339,-2
.82185693674258,0.00477464715875778,0.11518182465141
"637","FAXDC2",268.391958135602,0.629438366079061,0.223025497976194,2.
82227086943326,0.00476848831081223,0.11518182465141
"638","GSN",60206.8620933367,-0.784188618674472,0.277805700856848,-2.8
227952711401,0.00476069613392308,0.11518182465141
"639","ENOSF1",129.09449923431,0.513348813279342,0.181913083300065,2.8
2194553556423,0.00477332830378657,0.11518182465141
"640","NEK6",111.243738260976,0.953324560479733,0.338644708058561,2.81
511725355199,0.00487594400067346,0.117258466100439
"641","CEP131",83.0601480087203,1.11829580873112,0.397218262627251,2.8

1531821154085,0.00487289571580647,0.117258466100439
"642","RXYLT1",127.118573540376,-0.549519732435565,0.195409416570046,-2.81214560731563,0.00492122201798579,0.118162986615656
"643","ZNF43",109.557346247071,0.598721936862704,0.213068625132472,2.80999577713734,0.00495421500055163,0.118585751915378
"644","DMD",88.0556825686693,0.856400011016943,0.304742440788176,2.81024201552622,0.00495042591509289,0.118585751915378
"645","SYBU",8.11869221615075,-1.71762420166324,0.611944586975035,-2.80682963494097,0.00500316942310669,0.119571870786341
"646","SOX13",60.409203935067,0.740706922870435,0.264156282453153,2.8040481036138,0.00504653752232235,0.120421634530339
"647","ARHGEF26",57.4765862539517,-1.0407795963182,0.371344158314368,-2.80273587995186,0.00506711473409169,0.120725770673916
"648","ENC1",10.468322193464,-1.73842743800418,0.620583834563064,-2.80127734752897,0.00509007522017881,0.120957666953715
"649","BZW2",202.520495528365,-0.584932374918781,0.208820813717566,-2.80112104011774,0.00509254141115544,0.120957666953715
"650","DKK3",97.0392156241448,-2.86493300428994,1.0231097388217,-2.80022063673192,0.00510676886084446,0.121108987676796
"651","TLR5",88.7563085109533,0.86814605830288,0.310089338384883,2.79966432520597,0.00511557719400084,0.121131524493891
"652","LMO3",405.283617486568,0.564644983706188,0.201764678062355,2.79853237508543,0.00513354228381648,0.121370482062931
"653","IL1RN",7.97323900596385,-2.27492455375474,0.813632388492609,-2.79601031857818,0.00517377485452577,0.122134363526056
"654","MLF1",202.135589100454,-0.626649111401163,0.224250217484541,-2.79441919134086,0.005199303332216,0.122549328541452
"655","SPRED2",133.419102455682,0.640325802187342,0.229300054782944,2.79252354646612,0.00522986611086374,0.122893881248422
"656","VSIG10L",6.34601402123513,-1.66469397243195,0.596123643703488,-2.79253136495282,0.0052297397236714,0.122893881248422
"657","AL158151.1",18.1603427555147,1.64585803855243,0.589636126739551,2.79131139344083,0.0052494941851117,0.123167355956616
"658","GPR34",4.80501239419214,-2.75510906406499,0.987231354956287,-2.79074307175645,0.00525871976304288,0.123196299615967
"659","SLC12A6",95.1548146241992,0.71842347417019,0.257621229528215,2.78868117928732,0.0052923135954756,0.123715110025042
"660","ZNF486",27.3953069011765,0.870377526688462,0.312142325805505,2.78839956882615,0.00529691680937578,0.123715110025042
"661","TMEM140",222.182984623067,0.726663470355131,0.260832913697766,2.78593471986873,0.00533736192256943,0.12428313298551
"662","ABTB2",5.85049323637606,-2.33197237803008,0.836935136438186,-2.78632390552325,0.00533095738389281,0.12428313298551
"663","RGS17",10.5265633774994,-1.68732829702927,0.605985686892254,-2.78443589267362,0.00536209196744287,0.124670660148012
"664","DPYD",232.521019268707,0.486889301438277,0.175139922590792,2.78000180790234,0.00543585958231736,0.126195444972021
"665","ZMAT3",176.125724121813,0.762081206154997,0.274329843827736,2.77797411875298,0.00546989748955986,0.126713512111915
"666","FBXO10",17.0533136970158,1.10293886587403,0.397246059371021,2.7

7646269825902,0.00549539404734215,0.126713512111915
"667","PDS5B",264.042073516649,0.52576758170037,0.189336814957066,2.77689038880048,0.00548816836056672,0.126713512111915
"668","AL161421.1",17.6670354349048,-1.01217438681008,0.364585515007847,-2.77623313364025,0.00549927600407858,0.126713512111915
"669","TYROBP",55.2483518165754,-1.2098469462844,0.435652673505958,-2.77709060419172,0.00548478873713345,0.126713512111915
"670","BOLA3-
AS1",31.8813090581946,-0.784191838786336,0.282616230412991,-2.77475868119954,0.00552426817122742,0.127086646829362
"671","ACADVL",618.38556824671,-0.553134134358822,0.199377464645421,-2.77430619023334,0.00553195848345778,0.127086646829362
"672","PPP1R16A",28.3056518764715,-0.950317877169693,0.342669025196299,-2.77328210982974,0.00554939893428182,0.127297596089218
"673","FBLN2",5625.82890597678,0.472434742775824,0.170497692791054,2.77091575282954,0.00558988876645968,0.128035862310514
"674","SHISA6",19.1553128380235,1.45771189175444,0.526707006637762,2.76759540576412,0.00564715138751357,0.128964205390402
"675","TUG1",299.61840706009,0.690553035082892,0.24949042708732,2.76785383369119,0.00564267562951065,0.128964205390402
"676","SORT1",52.7484518695797,-0.934694573741555,0.337916010343392,-2.76605589889545,0.00567388080235282,0.129191835403647
"677","EDNRA",4.72328365196211,2.61965729738413,0.947025575275537,2.76619487981825,0.00567146309311589,0.129191835403647
"678","PDZRN3",104.836956612717,1.08362966035037,0.391942480365012,2.76476706311906,0.00569634568612408,0.129213022729324
"679","AC005162.3",7.14976592735717,1.76210170445196,0.637493184949119,2.76411065412818,0.00570781794353107,0.129213022729324
"680","CCDC85B",565.455293158551,-0.81445283900233,0.294568984364786,-2.76489678897672,0.00569408089100672,0.129213022729324
"681","GPR183",57.2973962848935,-1.59773303997244,0.578034133050446,-2.76408078453908,0.00570834047866816,0.129213022729324
"682","IGF2R",275.543542751908,0.634314675461587,0.229746693862809,2.76093059184722,0.00576369240452387,0.129228193958728
"683","C8orf88",25.7247741590283,-1.01812265423554,0.368746105695823,-2.76103974661465,0.00576176638421452,0.129228193958728
"684","IL17D",172.849978097502,-0.808558553969342,0.292940338518759,-2.76014753740569,0.00577752631800132,0.129228193958728
"685","TSHR",4.14435260139691,4.21938487074971,1.52781317114506,2.76171520866479,0.00574986085464717,0.129228193958728
"686","PIF1",4.97415547213068,2.27985979255088,0.825599351999392,2.76146024948983,0.00575435209408075,0.129228193958728
"687","GJC1",3.23056926351951,-3.03497124194744,1.09972460770047,-2.75975568855697,0.00578446019017334,0.129228193958728
"688","KCNK15-
AS1",44.0878618974577,-1.05245800220887,0.38083886397372,-2.76352573691505,0.00571805827054685,0.129228193958728
"689","SDC4",112.672211375458,-0.791575807287574,0.286523132735335,-2.76269423599651,0.00573264413458571,0.129228193958728
"690","LINC01426",26.6119664190907,1.21760486570752,0.441100948249143,

2.76037689454204,0.00577347125896048,0.129228193958728
"691","MAP4K3",97.613338934618,0.602099700267915,0.218499398768805,2.7
5561261797795,0.0058582333445981,0.130498073709508
"692","AL356218.2",4.09102189347664,-2.37412613855406,0.86147849745189
5,-2.75587393716304,0.0058535552506463,0.130498073709508
"693","AL162231.1",27.90097789335,-0.999798818001064,0.362898489493645
,-2.75503714384728,0.00586854727466024,0.130539186491901
"694","HELLS",19.5625700737689,-1.17112762507324,0.425662696889265,-2.
75130433940259,0.00593584678717904,0.131656227660957
"695","ZNF528-
AS1",40.9459407962382,0.968933387917014,0.352113061743504,2.7517678075
3118,0.00592745317514388,0.131656227660957
"696","RALGPS2",193.746497681659,0.843084534724144,0.306651837074629,2
.7493216501389,0.0059718752173612,0.132265023671872
"697","AC021016.2",3.82333792575696,2.75245172844109,1.00194200459479,
2.74711681496402,0.00601217167373914,0.132775968983795
"698","HYAL3",6.57786731728598,-1.76465173793393,0.642362265023445,-2.
74712858151704,0.00601195597485423,0.132775968983795
"699","FHDC1",47.5870120931308,0.760623472805098,0.277031532598993,2.7
4562056408976,0.00603965711922258,0.13310950570907
"700","GAL",167.486844279643,2.27656373353254,0.829241978741585,2.7453
5514589763,0.00604454453430742,0.13310950570907
"701","C1orf162",21.6734431685694,-1.30505825319905,0.475831780627421,
-2.74268829097172,0.0060938501564247,0.133622617583623
"702","GPR146",32.0885721681777,1.13696484805506,0.414502694443901,2.7
4296129625989,0.00608878616035382,0.133622617583623
"703","NET1",91.9010309226597,0.822675777744102,0.299890055724831,2.74
325794416792,0.00608328791278442,0.133622617583623
"704","SESN1",188.453949411078,0.638746823054537,0.232940878981651,2.7
4209844938745,0.00610480412747027,0.133672664239992
"705","OGA",372.983707058251,0.640170605102407,0.233854211787551,2.737
47733773546,0.00619123856321123,0.135372968016881
"706","WARS2-
AS1",31.5719503074929,0.879194737893501,0.321329506618648,2.7361157932
4063,0.00621691455594742,0.13553100720342
"707","AL512308.1",23.3521654905283,1.28581749713108,0.470174265705525
,2.73476791674601,0.00624242718874009,0.13553100720342
"708","AIG1",215.771511286691,-0.543096943302716,0.198529285436693,-2.
73560115883204,0.00622664446628199,0.13553100720342
"709","NR1H3",116.842476131775,0.839454105364143,0.306936673701798,2.7
3494234247064,0.00623912035429829,0.13553100720342
"710","PRRG1",93.9046070540113,0.608282573114265,0.222400222871667,2.7
3508077132308,0.00623649708585639,0.13553100720342
"711","LRRC4B",4.95953526515164,2.64061250952741,0.965887390154649,2.7
3387201908145,0.00625943686226801,0.135709169102477
"712","SPAG17",56.6439014260524,-2.20069557454624,0.806130652063572,-2
.72994900877767,0.00633441224553994,0.136517107119473
"713","SWT1",38.8892434219914,0.752075647905428,0.27556333038623,2.729
22978123148,0.00634824525100972,0.136517107119473
"714","RASA4",10.6936149960464,-1.45326130727545,0.532117722477226,-2.

```
7310898432586,0.00631252604985762,0.136517107119473
"715","FRMD4A",85.5162390169945,0.834362412923788,0.305699762130799,2.
72935250949522,0.00634588287889674,0.136517107119473
"716","OXT",28.9670858125593,-1.23882673966653,0.453924516629163,-2.72
914701516024,0.00634983884558301,0.136517107119473
"717","ATP7A",71.7557480231732,0.598103181877258,0.219021572922061,2.7
3079575631618,0.0063181614030785,0.136517107119473
"718","CADM1",4.71403932447874,-1.95416936022106,0.716338872124029,-2.
72799569626414,0.00637204393714218,0.136711797752165
"719","AL354822.1",14.6258910015348,1.91991475615012,0.703843440268606
,2.7277582574577,0.00637663201970851,0.136711797752165
"720","VWCE",23.3446490346383,1.27798534736443,0.468860231845061,2.725
72775544494,0.00641598943413385,0.137364551565518
"721","ANXA1",7171.27324157658,-0.536375649107594,0.196974662567557,-2
.7230692623912,0.00646784963436074,0.138091277165749
"722","OLFM4",34.2170307238794,1.20383989812117,0.44208890656761,2.723
07194375859,0.00646779713851403,0.138091277165749
"723","TBCK",69.8537662986173,0.633609812405935,0.232876847408155,2.72
079349861444,0.00651254303309457,0.138853182372272
"724","PRSS12",26.0072473577377,1.47025872383177,0.541173019207527,2.7
1679975100155,0.00659164760499937,0.139959019051054
"725","EMX2",428.445355833151,-0.826415820064956,0.304165782070054,-2.
71699141974694,0.00658783155275724,0.139959019051054
"726","RBM3",1516.22954422443,-0.717929472821633,0.264180913986443,-2.
71756752593594,0.00657637345315827,0.139959019051054
"727","FOS",34957.5406591853,-0.299618522759451,0.110335639377974,-2.7
1551897871416,0.00661719835729065,0.140308270533199
"728","C3orf14",116.728700114941,-0.668259598215587,0.246219201839639,
-2.71408400816285,0.00664593103944064,0.140586892085573
"729","LAIR1",5.72278662767291,-1.98148089102854,0.730109027875881,-2.
71395204739941,0.00664857893807221,0.140586892085573
"730","CAMK2N1",740.704370821476,-0.714262139401261,0.263338499252556,
-2.71233466214997,0.00668111021487163,0.140695784101429
"731","SPRY2",571.581319886938,0.71487174072454,0.263550409257598,2.71
246682081914,0.00667844668303389,0.140695784101429
"732","HS3ST3A1",3.77971769034246,-2.35552631587394,0.868284173476774,
-2.71285183794376,0.00667069247102452,0.140695784101429
"733","AFAP1",171.08676649197,0.61926411525026,0.228367136636789,2.711
70416361257,0.00669383046141877,0.140769177336118
"734","FGFBP2",173.866667984075,-2.55545815881982,0.943330224729307,-2
.70897517309288,0.00674913898910867,0.140769177336118
"735","MSRA",49.2844863335931,-0.699378157255808,0.258049826961939,-2.
71024462790654,0.00672336002090806,0.140769177336118
"736","MEGF9",114.876528165233,0.668348839150329,0.246648152139646,2.7
0972570989272,0.00673388703592605,0.140769177336118
"737","LRRC32",252.480879641342,-0.581610951255771,0.214639637587446,-
2.70970896984868,0.00673422687887332,0.140769177336118
"738","SUPT20H",129.610092980058,0.484614335267833,0.178949358001243,2
.70810882296916,0.00676678302991007,0.140769177336118
"739","NPAS3",15.2420830481159,1.26288998720885,0.465845194282295,2.71
```

096493579702,0.00670877203380105,0.140769177336118
"740","STX16",285.692269359219,0.571579239336822,0.211055461293624,2.70819449936731,0.0067650363036522,0.140769177336118
"741","RAI2",143.919300824733,-0.816507068861043,0.301497423378387,-2.70817262619292,0.00676548220413486,0.140769177336118
"742","AC108734.4",67.9777156079756,0.880387113258439,0.325244820135412,2.70684437923377,0.00679260903325382,0.141115994942868
"743","ACTR3",519.332888122174,-0.427351599966336,0.15802151375678,-2.70438872408282,0.00684301848245535,0.14167362018881
"744","RECK",569.824697614441,0.656186415876074,0.24260287584698,2.7047759165475,0.00683504797774331,0.14167362018881
"745","NHLRC3",216.598317184059,0.510112083123054,0.188637357614335,2.70419438426383,0.0068470221888202,0.14167362018881
"746","GSTM2",59.0674031303003,0.802469857345268,0.29680295619919,2.70371248191584,0.00685695921819427,0.141689043362553
"747","ZNF674-AS1",11.2754554147533,-1.32216589351159,0.489491527301332,-2.70110067236703,0.00691104159381017,0.142615403170795
"748","CARD19",140.988481777857,-0.553605868618804,0.205045747566546,-2.69991392257055,0.00693574182913053,0.142914209158121
"749","AL161431.1",53.5405433248616,-1.1642042228628,0.431264226327257,-2.69951494186621,0.00694406374696285,0.142914209158121
"750","RNASEL",77.2573899377981,0.631751607656517,0.234087880161518,2.69877965155913,0.00695942389124627,0.143039359044748
"751","SSBP4",443.668179140171,-0.568719764662553,0.21082783291472,-2.69755542615002,0.00698506555732337,0.143375213803115
"752","MTMR11",61.1932163821938,-0.795994443488094,0.295229480037004,-2.69618888800781,0.00701378818798868,0.143732525065379
"753","RNF112",16.5232055530353,-1.0704001089222,0.397121258412366,-2.69539866287064,0.00703044592275677,0.143732525065379
"754","ZNF880",38.3977516685338,0.9933582890057,0.368537405039882,2.69540696662311,0.0070302706973338,0.143732525065379
"755","EPHB3",19.6298052297268,1.27870690251339,0.474523409957807,2.69471827033168,0.00704481686968007,0.143835565623998
"756","KRT222",570.123235916539,0.922574935395655,0.34243883753105,2.69413055495495,0.00705725155856021,0.143898852877256
"757","FAM107B",249.299715982696,0.639778947336365,0.237601985381708,2.69264983753633,0.00708866751201594,0.144348493656177
"758","MAP1A",694.673604976857,-0.57394743524969,0.21319426194729,-2.69213359687697,0.00709964992515897,0.144381403161379
"759","MINDY4",63.0293332215698,-0.774725660491893,0.287972696823178,-2.69027469978375,0.00713932242674218,0.144806125273988
"760","THOC6",64.8745668822199,-0.7371690271962,0.274000879154071,-2.69038927711501,0.0071368713791447,0.144806125273988
"761","OSMR",184.065661416225,0.505854528724426,0.18822373637345,2.6875171987914,0.00719853950706143,0.145592475197747
"762","DAB2",1504.58327846878,0.542756301740424,0.201982025944906,2.68715148885807,0.00720642611585343,0.145592475197747
"763","BAALC",2.83773834465656,-3.52102444276889,1.31005303820985,-2.68769610090005,0.00719468426930191,0.145592475197747

"764","KPNA5",99.2805676512476,0.625201534822068,0.232712949697759,2.68657818842511,0.00721880506396744,0.145651675472589
"765","ITGB1BP1",1068.26606143568,-0.683571291086883,0.254654401923033,-2.6843097387081,0.00726797376541247,0.146452046527887
"766","AC008280.3",10.6428797900154,1.3920048318766,0.518719392865271,2.68354114194097,0.00728470115360114,0.146597478175929
"767","KL",13.045442474373,1.20367760803515,0.44902628250076,2.68063954147965,0.00734816207253072,0.147681770988345
"768","AC003681.1",60.8815573538508,1.22417407215523,0.456927108272629,2.67914520717124,0.00738103777182407,0.148149345381078
"769","ARHGAP29",613.194684814537,0.646417721456579,0.241338453113564,2.67846964757166,0.00739594349928057,0.148255486399753
"770","AC068491.3",26.8645289930684,1.32015147577814,0.493447376417125,2.67536426146113,0.00746480947756018,0.148547630309788
"771","ACSL3",304.54208667174,0.661161702508061,0.247144230931263,2.67520589097605,0.00746833691145547,0.148547630309788
"772","TPBG",295.105851211368,-0.555873355353632,0.207620213982769,-2.67735662482153,0.00742056042822734,0.148547630309788
"773","ZDHHC7",235.525731462178,0.591641178996605,0.221027866977874,2.6767718798817,0.00743352276500354,0.148547630309788
"774","SEC11C",36.3059734769784,-1.0073061176605,0.376512054244067,-2.67536219971203,0.00746485538992277,0.148547630309788
"775","REM1",30.3504781500043,-1.39853592096802,0.522558196947953,-2.67632567843408,0.00744342759524373,0.148547630309788
"776","SLC38A4",2.57139203969041,3.53922166592206,1.32325914840542,2.67462474768224,0.00748129370321271,0.148613585612144
"777","TP53INP1",93.7651793900849,0.744828997897433,0.278682945835766,2.67267519963844,0.00752490692613891,0.149287567910465
"778","FAM107A",23.7958985732304,-0.868239243119007,0.324976894310729,-2.67169530609408,0.0075469140413063,0.149339768866157
"779","AC129507.1",3.91304819040655,4.16966657139289,1.5606236840446,2.67179500991972,0.00754467219089126,0.149339768866157
"780","CXCL12",4760.5458046451,0.583293325091668,0.218397970127341,2.6707818060377,0.00756748200256751,0.149554788550741
"781","LAMB1",380.639209444372,1.23691722964974,0.463350480375357,2.6695067385011,0.00759627488651048,0.149931597151804
"782","SNCG",103.254447271621,-1.41257875232822,0.529310959754957,-2.66871245776239,0.00761426051027212,0.150094406350185
"783","ARFGAP3",263.609637332013,-0.461700429587828,0.173061411221214,-2.6678415848445,0.00763402434565997,0.150291807520241
"784","ART3",5.41807927691338,2.19368051785668,0.823040984211198,2.66533570009165,0.00769115037670733,0.151223320225693
"785","LINC01220",22.6624515060336,-3.04705315394654,1.14366134391397,-2.66429670825331,0.00771494815807632,0.15149799472197
"786","POMT1",62.8240179473627,0.580562519251292,0.217945237642702,2.66379997806175,0.00772634890562001,0.151528840178286
"787","TBC1D16",204.810029772827,0.452656910819218,0.170099497242085,2.66113021001466,0.00778788335318468,0.152452638955605
"788","MAP7D3",554.280942945417,0.53666185162622,0.201684410913215,2.66089902137824,0.00779323253305332,0.152452638955605

"789","C6orf47",93.0963590816757,-0.502350605856432,0.188970932884926,-2.65834855227359,0.00785246344303165,0.15341663368103
"790","NECAB2",6.55600302852102,3.02580510325968,1.14063875377139,2.65272865160437,0.00798440291166491,0.155796925168753
"791","A4GALT",364.581605836441,-0.681797135131973,0.257295990210712,-2.64985526814319,0.00805262590911439,0.156929492274334
"792","NRK",16.973872082622,2.1988178859715,0.829986931945809,2.64921988689222,0.00806778210284081,0.157026339792034
"793","FHAD1",4.99234459171204,-2.15952633182665,0.815461840476318,-2.64822487655002,0.00809156809634188,0.157190216034899
"794","SUCLG2-AS1",56.0381575464772,0.790568424233837,0.298551353276228,2.64801487435356,0.00809659627192407,0.157190216034899
"795","PBX1",621.319366242286,0.57056998499209,0.215587696833954,2.64657952829072,0.00813103835579249,0.157640424468339
"796","SYNPO",8.47655076254635,-1.42914008455124,0.540073193550545,-2.64619703702715,0.0081402385907751,0.157640424468339
"797","SLC39A8",69.5562216861725,1.04053855917591,0.393508039526007,2.64426251730276,0.00818691335629437,0.158345381916283
"798","FKBP1B",33.6907685600728,-1.05611658268121,0.399495487293325,-2.64362581373983,0.00820232760860702,0.158444711888067
"799","RINL",41.6813806338768,-0.733031789624919,0.277496686964293,-2.64158753621171,0.0082518480254009,0.159201798888054
"800","VAMP8",31.4685195657971,-0.893079065106742,0.338300292029711,-2.63990036706296,0.00829304036843193,0.159796521599223
"801","LRRC61",7.12940509604442,-1.58617672151267,0.601375763232414,-2.63758005973988,0.00834999129449929,0.160693028470295
"802","AC007920.2",29.6473178417458,-1.03074495017117,0.390999704403869,-2.63617833610049,0.00838456526536528,0.16079887140986
"803","AC025171.2",44.1718770986732,0.762709194397311,0.289306927730471,2.63633228689179,0.00838076176940286,0.16079887140986
"804","PDE1C",27.7038248607254,1.51211054878089,0.573710833964802,2.63566671441603,0.00839721644404392,0.16079887140986
"805","PKN1",304.213359344779,-0.582785404704144,0.22109183623399,-2.63594266812891,0.0083903906503557,0.16079887140986
"806","DNASE1L3",162.768781782382,2.08701519155872,0.792630508598924,2.63302404956351,0.00846283546980884,0.161293140703632
"807","SYNJ2",80.0803070888938,-1.38315982906681,0.525138717715337,-2.63389421196055,0.00844117832974007,0.161293140703632
"808","WDR86-AS1",105.465342103896,1.21326773762682,0.46071614199319,2.63343874251481,0.0084525081450954,0.161293140703632
"809","FOLH1",21.6624632650989,1.43163670194569,0.543740323695113,2.63294193856485,0.00846488166261681,0.161293140703632
"810","C17orf49",421.53538386259,-0.712885043408536,0.271002277146881,-2.63055001202871,0.00852468258127993,0.162232076531395
"811","SLC35E4",24.6057618475208,-1.04817479825344,0.398939450414534,-2.62740322413411,0.0086039311658878,0.16353834638984
"812","ZNF561",58.5665342920323,0.625637611213358,0.238253817717053,2.62592900801429,0.00864128379955316,0.163866057467987

"813","NCF4",8.72544505582426,-1.46924904408957,0.559525549612382,-2.6
258837422302,0.00864243300171737,0.163866057467987
"814","OSBPL6",8.65118877630939,-1.38100748704437,0.526249716579785,-2
.62424366899397,0.00868416322955518,0.164455007596552
"815","SDS",4.11767452684701,-2.58723520164325,0.986282377392717,-2.62
321953727159,0.00871031261199891,0.16474781461836
"816","INKA1",17.3605547107441,-1.2726882535086,0.485330385946695,-2.6
2231315071293,0.0087335142546768,0.164782279358437
"817","FAM182A",33.0102931709863,-0.759384330774458,0.289583666054043,
-2.62233136668951,0.00873304741963971,0.164782279358437
"818","CNTNAP4",5.74271708544531,-1.80480188793614,0.688376917184839,-
2.62182220652748,0.00874610446680435,0.164818093344485
"819","ZNRF3",51.3054552684866,0.730880860185349,0.278891200764292,2.6
2066661903422,0.00877580342548869,0.165175836146408
"820","NCOA2",202.951595019679,0.491349654799825,0.187608768980565,2.6
1901220006793,0.00881847936135613,0.165776657750372
"821","TUBB3",93.115507072679,-1.41211547231342,0.539294929106193,-2.6
1844752490776,0.0088330876094463,0.165849020096973
"822","TPCN1",196.717967627739,0.630748482862381,0.240953825817313,2.6
1771516066569,0.00885206620526191,0.166003163691134
"823","FZD10",3.59256493579905,-3.69109991032388,1.41032526372035,-2.6
1719761056183,0.00886550003438285,0.166053077800743
"824","LINC01121",11.4726928695594,1.42430380878069,0.544384784919529,
2.6163549170303,0.00888741244475222,0.166059954952552
"825","DANCR",156.713872179247,-0.766938253459994,0.293115775064166,-2
.61650282483808,0.00888356292733747,0.166059954952552
"826","CLIP4",123.863305740643,0.610871432029221,0.233522920132266,2.6
1589496946693,0.00889939277704797,0.166082493532923
"827","CCDC47",336.225979132274,-0.34923678569131,0.133527267331474,-2
.61547167609106,0.00891043111594234,0.166087419168381
"828","CHSY3",14.9913134392721,1.22169829428615,0.467257427117738,2.61
461503527543,0.00893280740026194,0.166102805880625
"829","PDPR",106.369454568629,0.665757464468299,0.254613525514486,2.61
477650538412,0.00892858581079532,0.166102805880625
"830","MXRA7",1139.8847814384,-0.564501809173154,0.215986099000536,-2.
61360250398222,0.00895932040274,0.166395089166551
"831","NDFIP1",1420.55336219335,-0.517874793233695,0.198201285758028,-
2.61287302578823,0.00897846522774704,0.166549989754176
"832","LRBA",60.4497538801829,0.808097629859277,0.309392240191325,2.61
188719329081,0.0090043960011094,0.166629969216208
"833","ITPR3",102.138985983944,0.691473645288565,0.264730251772175,2.6
1199330510833,0.00900160168850773,0.166629969216208
"834","CDS1",49.8901061836094,1.12977512425673,0.43296210021459,2.6094
0882284334,0.00906988126707544,0.167439784110141
"835","LINC00294",48.101381142329,0.862363962090775,0.330449946030136,
2.60966591900163,0.00906306837854792,0.167439784110141
"836","APPBP2",214.894368634297,0.430415840156651,0.165017401408232,2.
60830576947372,0.00909916341808429,0.167779430729389
"837","RBM24",2.52010214823248,-2.57998708196597,0.989907364610642,-2.
60629143109846,0.00915285481073224,0.168567809925254

"838","TMC3-
AS1",11.2866844364957,1.364286650462,0.524708516502404,2.6000848233911
9,0.00932007199449286,0.171238271507875
"839","MAPK11",5.54345657208615,-1.98172984927084,0.762114581778996,-2
.60030433303731,0.00931411182502977,0.171238271507875
"840","CCDC110",9.34991125704734,-1.76897131849532,0.680673577170984,-
2.5988541025017,0.00935355184002944,0.171448098523557
"841","ZNF790-
AS1",18.410199714705,0.996247094152017,0.383341909672301,2.59884731884
301,0.00935373667585545,0.171448098523557
"842","CCNB1IP1",158.713375279626,-0.708830019473618,0.273056193438364
,-2.59591262350774,0.00943400525692358,0.17271400360508
"843","SNX7",383.029798737728,-0.611457576551594,0.235628415540802,-2.
59500780136474,0.00945887719997723,0.172856957980849
"844","VIT",994.246425995854,0.492114522194613,0.18965317763753,2.5948
1295449294,0.00946424083917201,0.172856957980849
"845","GLI1",25.7567348312954,-0.911630452436827,0.351529654559735,-2.
59332446242289,0.00950530483801673,0.173401507784648
"846","LINC00963",217.267245462097,-0.560048522932783,0.21599608785814
3,-2.59286419715434,0.0095180345984116,0.173428490939143
"847","PILRA",12.971250559986,1.49872472831864,0.578324810320443,2.591
4930529925,0.00955604709290501,0.173505849160342
"848","FOXP2",83.2713690206398,0.74557940019819,0.287618734001516,2.59
224908553509,0.00953507074768613,0.173505849160342
"849","NR1H2",285.415418007659,-0.392146536049141,0.151312656612117,-2
.59163076525971,0.0095522231633404,0.173505849160342
"850","LINC01894",35.3562202718702,0.998397446857364,0.385555189588627
,2.58950592241442,0.00961137695395553,0.174305147935558
"851","AC093599.2",5.76791684904622,2.02416986666337,0.781816313925806
,2.5890606663082,0.00962381381019442,0.17432560503425
"852","LAYN",16.6079172919123,-1.45081644765295,0.5605347358211,-2.588
27215324617,0.00964587369686394,0.174520120935631
"853","SLC38A5",6.32354507498291,-1.53699432200429,0.594471021111862,-
2.58548233205647,0.00972428564708254,0.175732547772306
"854","AC097534.2",32.8984408225684,-0.932221549374191,0.3608587615122
44,-2.58334187444292,0.00978483093799273,0.176013480894771
"855","HLA-
DMB",17.7077631873114,-1.24815983970568,0.483154878475503,-2.583353486
2444,0.00978450158066701,0.176013480894771
"856","KCNS2",5.41309874805671,3.3660983065806,1.303013466766,2.583318
12558702,0.00978550458169439,0.176013480894771
"857","AKT1",234.231598738935,-0.4273772396934,0.165415557796235,-2.58
365806328724,0.00977586607996964,0.176013480894771
"858","KIRREL1",244.218851586224,0.63961013967344,0.247773377973644,2.
58143205256489,0.00983913578863289,0.176771885992746
"859","NIPAL2",156.668705987208,0.69412949749517,0.268940888776745,2.5
80974208319,0.00985219420617624,0.176800435027016
"860","NMNAT3",48.5576195059705,0.684112238144231,0.265312241146836,2.
57851742982945,0.00992252938176382,0.176850160886058
"861","CDH18",2.38640641060489,3.40345498221984,1.32035838226505,2.577

67514330563,0.00994674599339686,0.176850160886058
"862","FGF1",5.48167903767628,-2.2080756559425,0.856314500476707,-2.57858024675896,0.00992072543626866,0.176850160886058
"863","MSC",38.2164026972048,-0.697165930941856,0.270332310353415,-2.57892195731404,0.00991091747823617,0.176850160886058
"864","IKBIP",338.93183910382,-0.434388953024467,0.1684837167368,-2.57822513319228,0.00993092725596168,0.176850160886058
"865","AC018926.2",53.9288806860977,-1.03577574898579,0.40169114313774,-2.57853768169996,0.00992194776665218,0.176850160886058
"866","PAQR4",7.7863296393998,-1.68387565271947,0.652895333440952,-2.57908973532548,0.00990610498727478,0.176850160886058
"867","MYO5B",13.9762639898695,1.57523706699851,0.611057916348712,2.57788504960562,0.00994070604912418,0.176850160886058
"868","GFRA2",3.67015701318385,-2.31825911120403,0.900300014304224,-2.57498508760509,0.0100244408526842,0.178026216295076
"869","ZNF706",882.585352469687,-0.554564963618947,0.21556847417906,-2.57256987938925,0.0100946575072336,0.178675511736037
"870","LINC02762",237.355832324882,-0.822608704229543,0.319766223878033,-2.57253156463238,0.0100957749414264,0.178675511736037
"871","JUNB",17734.1696737166,-0.546486116748162,0.212365721219136,-2.57332545766299,0.0100726438661577,0.178675511736037
"872","UBAC2",216.678166112629,-0.623077607347616,0.242334556298524,-2.5711463394435,0.0101362485165579,0.179186090461858
"873","AOPEP",41.1358757968253,-1.02852978662976,0.40016135347298,-2.57028765447539,0.0101614100906351,0.179219835866293
"874","MTMR3",148.34210444654,0.476039892725831,0.185199830702606,2.57041213763449,0.0101577589858764,0.179219835866293
"875","LMTK2",33.8221791493235,0.783470974752259,0.305180796249486,2.5672355023013,0.0102512963466288,0.180598552209467
"876","AGAP2-AS1",34.3535696163706,-0.849253792873802,0.330928585200335,-2.56627511449241,0.0102797259116628,0.180892665443245
"877","EGR2",84.1351676828953,-1.35028316288928,0.526360261400842,-2.56532124840821,0.010308031860638,0.181183935155911
"878","ZNF429",83.9122114921717,0.707202934251217,0.275789999167748,2.56428056269387,0.0103389932886506,0.181521163490375
"879","PREPL",169.81025653158,0.520451776612375,0.203020383419192,2.56354444734624,0.0103609433939175,0.181699593193674
"880","PTPN6",6.63505331860863,-1.83536510096028,0.716066268582619,-2.56312185266484,0.0103735634056846,0.181714181702987
"881","HIST2H3PS2",9.42976887554456,1.54979519643513,0.605033285034571,2.56150402757854,0.0104220032855998,0.182148251529287
"882","HINT3",60.6134890357721,0.595262454756551,0.23242350025729,2.56111130801146,0.0104337921570133,0.182148251529287
"883","BBOX1-AS1",11.7907916785105,-1.55083819286786,0.605369287560344,-2.56180520673221,0.0104129703558401,0.182148251529287
"884","CYP27C1",55.8051660441606,0.936532855194277,0.36586776083045,2.55975780174926,0.0104745134144666,0.182652289914031
"885","SLC37A3",192.262080695125,0.547989693107201,0.214465172763412,2

.55514536950816,0.0106143458546898,0.184464646392382
"886","AC099518.5",59.9565264029723,-1.04075502286127,0.407301914502198,-2.55524215773272,0.0106113946117874,0.184464646392382
"887","SNHG16",425.199654795416,-0.459687404834569,0.179856975623524,-2.55584974250198,0.0105928849494508,0.184464646392382
"888","WDR34",103.047817031953,-0.48583219658591,0.190213279440724,-2.55414447411022,0.010644907744938,0.184579587050866
"889","REPS2",38.7653949516035,0.87139061394194,0.341142831103158,2.55432779028102,0.0106393044213647,0.184579587050866
"890","PCNX4",197.201689434493,0.607286919695964,0.237831610700517,2.55343231249723,0.010666700902677,0.184749656645805
"891","FZD1",153.418501333,0.691329505399293,0.27093607400604,2.55163328816776,0.0107219304065249,0.185497819547229
"892","IFT57",436.131634711047,-0.451624145177817,0.177153445777722,-2.54933875655164,0.0107927407010257,0.185680912841865
"893","HLA-DQA1",56.4328968692647,-1.16608958992749,0.457140736120764,-2.55083281315678,0.0107465863767315,0.185680912841865
"894","UHRF1BP1",30.9723096322007,1.01365114165238,0.397562703561325,2.54966356897213,0.0107826916477357,0.185680912841865
"895","DHCR7",29.4695198232839,-0.904576110933833,0.354691738065703,-2.55031627143869,0.0107625234734187,0.185680912841865
"896","EXOSC6",200.899774415137,-0.444749846885420,0.174322942038,-2.54972168030329,0.0107808946756365,0.185680912841865
"897","SBF2",271.528457234146,0.504667961404513,0.198008243214682,2.54872197849535,0.0108118455098092,0.185802228019743
"898","ADAMTS19",5.0486717750185,-1.9999787607182,0.784880878248187,-2.54813031651638,0.0108302005884834,0.18591040319763
"899","LAPTM5",42.1071187032826,-1.08264633916754,0.425056735575392,-2.54706312958899,0.0108633778849913,0.186065522330156
"900","C8orf82",157.730802763097,-0.473520638884629,0.185905943006301,-2.54709790998225,0.0108622951904532,0.186065522330156
"901","DYRK3",92.8078209747229,-1.0976753540548,0.431115759697669,-2.54612671739156,0.0108925639613054,0.186358350125996
"902","IRAK1",144.408322225406,-0.705939046486846,0.277425882608495,-2.54460412939578,0.0109401687520514,0.186965300790323
"903","COL8A2",118.155408124982,-0.665278857029468,0.261548440756539,-2.54361622307945,0.0109711551535588,0.187080040588615
"904","GRIA1",33.7174456925821,1.47024768210095,0.577931231399705,2.54398378599495,0.01095961717648,0.187080040588615
"905","TRIM59",4.50417110005543,-2.03949964296809,0.802059209577786,-2.54282928069825,0.010995893952984,0.187284049314098
"906","LRRC6",31.7975869156635,0.800565586972847,0.314877940180116,2.54246323675424,0.0110074180135305,0.187284049314098
"907","AC005062.1",3.4168361837611,-2.79009933223534,1.09812029340591,-2.54079571153505,0.0110600521551629,0.187972110222531
"908","CPPED1",53.9934977327407,-0.696633572603352,0.274302885111121,-2.53965091297214,0.0110963161611948,0.188380741877553
"909","UPP1",92.647698668416,-1.01667251981918,0.400671730207021,-2.53742014514945,0.0111672841973256,0.188714340470211

"910","MPDZ",207.114096573882,0.600768129758394,0.236824722422808,2.53676273157762,0.0111882754448792,0.188714340470211
"911","SUSD1",24.2906913555512,1.09305945276401,0.430599815453637,2.53845778269198,0.0111342235519485,0.188714340470211
"912","METTL7A",1082.40881117908,0.569979588821795,0.2246532568218,2.53715257408405,0.0111758235296648,0.188714340470211
"913","LINC02254",23.306769358025,0.992599865865128,0.391170286487021,2.53751345681023,0.011164307587984,0.188714340470211
"914","MT-ND1",33375.8420267803,-0.451093468253797,0.177825001344185,-2.53672692165874,0.0111894198631056,0.188714340470211
"915","SCART1",2.80733136093225,2.94187260608842,1.16012652024849,2.53582049435288,0.0112184222318429,0.188990120808919
"916","AC110048.2",8.00656756981339,1.87653021352195,0.740117187302311,2.53545012292148,0.0112302919663295,0.188990120808919
"917","EPB41L3",446.414858339511,0.394132051782302,0.15550033933645,2.5346057343935,0.0112573947987263,0.189239630122536
"918","RTCA-AS1",50.7245264209178,0.780552269468099,0.3083481161831,2.53139950757669,0.0113608366784626,0.19011310784153
"919","HRH1",94.4479062002078,-0.725015600702102,0.286440213608358,-2.53112365602895,0.0113697757248662,0.19011310784153
"920","PRPH2",8.92748642723235,1.75862764000398,0.694811697944215,2.53108524972644,0.0113710207868888,0.19011310784153
"921","VWA2",3.16261759755061,-2.81143100575986,1.1106587053666,-2.53131856993989,0.011363458838184,0.19011310784153
"922","ITGAM",7.17155623602559,-1.6648971880793,0.657464555426078,-2.53229953514428,0.0113317144069924,0.19011310784153
"923","ZNF91",196.995498918557,0.499121553976788,0.197231412955912,2.53063924501904,0.0113854883098453,0.190148756550667
"924","AL512353.1",4.3750086828772,2.32486545649088,0.918952548323919,2.52990805752834,0.0114092419622622,0.19033924767129
"925","KIDINS220",581.621209567829,0.462474185684404,0.1828578833809,2.52914546058183,0.0114340628515629,0.19034133785836
"926","SESN3",377.363681447969,0.753931964460895,0.298072436429833,2.529358210679,0.0114271334776982,0.19034133785836
"927","ETHE1",142.722877551053,-0.720579915885961,0.285261427448946,-2.52603347858842,0.0115358487586746,0.191828596132653
"928","MLLT3",86.9943943451246,0.545335680262512,0.215943403708289,2.52536391896086,0.0115578533694773,0.19198740268372
"929","ANO1",5.00120350278022,-2.46235019856939,0.97536042543643,-2.52455413850485,0.0115845159982843,0.192223158356892
"930","YWHAQ",1516.32306137658,-0.37893959446811,0.15033621151629,-2.52061423289924,0.0117150206241524,0.193133258635451
"931","PCBP4",72.6681629809001,-0.648904238416782,0.25725042869568,-2.52246125188937,0.011653678831839,0.193133258635451
"932","EFNA5",189.629747353769,0.464300565831413,0.184130659153534,2.5215820546445,0.011682842435436,0.193133258635451
"933","KRT7",7.76974312173344,-1.58256781894694,0.627940175109659,-2.52025253627159,0.0117270664990452,0.193133258635451

"934","ALDH2",513.538875662896,-0.686837346402946,0.272421603459967,-2
.52122936536448,0.0116945595755948,0.193133258635451
"935","TBC1D4",130.416888226084,0.651561798154662,0.258498469785247,2.
52056346289384,0.0117167107961766,0.193133258635451
"936","LTBP2",643.709711003696,0.874548285018784,0.346941425728122,2.5
2073756595477,0.0117109156739156,0.193133258635451
"937","RELL1",129.384892495813,0.493200515752553,0.195896778994321,2.5
1765505428168,0.0118138957734851,0.193456863397896
"938","PDGFC",35.6660148952292,-1.43472603284448,0.569656237568173,-2.
51858215222787,0.0117828392957942,0.193456863397896
"939","AL158206.1",35.3829961495252,0.920895787999581,0.36585328113129
2,2.51711775045993,0.0118319279053975,0.193456863397896
"940","HSPA12A",112.805006291526,0.68861194065031,0.273580138458991,2.
51703922853863,0.0118345651757519,0.193456863397896
"941","AC006517.2",2.5321486944318,-2.41126992126059,0.95718578444328
1,-2.51912424991042,0.0117647133532862,0.193456863397896
"942","XYLT1",102.351943669064,-0.741894203681695,0.294647060434641,-2
.51790804424557,0.0118054137714056,0.193456863397896
"943","ZGPAT",96.7536776761097,-0.542444325633008,0.21549060432981,-2.
51725279308602,0.0118273935257475,0.193456863397896
"944","SPATA18",6.9376792017391,1.76469024596789,0.701484599238289,2.5
1565073258071,0.011881285974773,0.194014855191871
"945","TEX26-
AS1",38.5950104177631,-1.20134982438527,0.47763630630544,-2.5151978786
4918,0.011896559158618,0.194058687227616
"946","DOCK7",174.903118720921,0.582909390033714,0.231878281735458,2.5
1385936479699,0.011941804384623,0.19420696504117
"947","PM20D2",47.0536063870872,0.852873067059987,0.339220522293843,2.
51421423825651,0.0119297939066955,0.19420696504117
"948","HTRA1",3340.17730555807,-0.580650244888193,0.231018231458082,-2
.51343905294137,0.0119560434527454,0.19420696504117
"949","MEIS2",227.288826571493,0.468942125074666,0.186560292759231,2.5
1362236914941,0.0119498313284648,0.19420696504117
"950","AC079922.2",19.9979835641135,-0.945128410238567,0.3762764402817
03,-2.51179268500305,0.0120119631612954,0.194909907506703
"951","TDRD10",19.5828620634371,0.920827047545943,0.366790662059626,2.
51049752023472,0.0120561168889586,0.195420653883593
"952","ZBTB37",82.1137360398177,0.808506900370427,0.322150863037976,2.
50971514633228,0.0120828586022735,0.19564838797694
"953","HSD11B1",119.845745650309,-0.706073753067389,0.281428804714747,
-2.50888942865339,0.0121111388255104,0.195825280122077
"954","LSAMP",120.093761931953,2.65090262263759,1.05682011873931,2.508
37637894315,0.0121287399347307,0.195825280122077
"955","CISD2",161.67199694774,-0.442178739886642,0.17631330769601 2,-2.
50791472104317,0.0121445973270649,0.195825280122077
"956","PEG10",180.713849214817,0.981036218214816,0.391168551465894,2.5
0796290892611,0.0121429412730117,0.195825280122077
"957","TBC1D1",47.0736118972978,-0.726949580097124,0.290127365462712,-
2.5056224262349,0.0122236136526658,0.196893421583953
"958","SLC12A7",64.4304423657381,0.611192054335483,0.244132790012523,2

.50352299789033,0.012296368862667,0.197858586657632
"959","ITGA4",33.4567140993349,-1.08955161955297,0.435697840523168,-2.50070465863379,0.0123946494631874,0.198817400078079
"960","RBM5",342.640112491617,0.515944008897429,0.206298624655684,2.50095709439919,0.0123858182893188,0.198817400078079
"961","UBAC1",204.411240170907,-0.530177306516981,0.212004803369179,-2.50077969032497,0.0123920239837812,0.198817400078079
"962","LPP",1251.4291574983,0.506840885611665,0.202839013210376,2.49873472361054,0.0124637571569484,0.198982267566317
"963","STOX2",21.0375871343407,1.10819404636878,0.443661084798925,2.49783919378692,0.0124952860852543,0.198982267566317
"964","TPPP",30.9636208692528,-1.50877863229056,0.603908409282269,-2.4983567194961,0.012477056948969,0.198982267566317
"965","RIPOR2",55.2208689220815,0.844032228846213,0.337738744760861,2.49906841290548,0.0124520269834551,0.198982267566317
"966","GTF2I",662.534483873541,0.429257390820607,0.171845360606459,2.49792830778624,0.0124921454864803,0.198982267566317
"967","ITGBL1",468.647435288478,-0.918569499176796,0.367448312438479,-2.49986043773324,0.0124242240838587,0.198982267566317
"968","VPS18",45.2830152372013,0.665204800745187,0.266252709031307,2.4983963662393,0.0124756614182609,0.198982267566317
"969","NT5DC2",94.9129636466578,-0.671966134019655,0.269117619492624,-2.49692359529091,0.012527594569337,0.19929088780839
"970","TTC9C",90.6692503818021,0.542484395105431,0.217348405627998,2.49592074778739,0.0125630666445554,0.199597390304644
"971","Z83843.1",86.8176516386512,0.837056613856246,0.335406629746286,2.49564719245242,0.0125727580918462,0.199597390304644
"972","CFAP36",352.211830058645,-0.528782369053537,0.212039889646559,-2.49378723001103,0.0126388280393841,0.200439850027886
"973","GALNT17",49.1422576047293,1.18897767876382,0.477077657511754,2.49220993698394,0.0126950975930823,0.201125312844157
"974","SLC39A4",51.0655786629245,-0.736292614622047,0.295571650583044,-2.49107995699059,0.0127355455793929,0.201558968281664
"975","FAM53C",324.000644555056,0.72349738586179,0.290531846489472,2.49025156658001,0.0127652704883964,0.201822199567826
"976","SUSD3",3.17715975249833,-2.16623836843067,0.870049378943425,-2.48978784521554,0.012781936867958,0.201878644282349
"977","RCAN3",20.0004438625502,-0.946209764214592,0.380143830630519,-2.48908357303912,0.0128072855965722,0.202071962611219
"978","IL1B",93.4801706022745,-2.06323046652316,0.829256412014146,-2.4880488551326,0.0128446086836818,0.202453622555168
"979","PTTG1",18.0104896119237,-1.23952274929156,0.498263919788353,-2.48768313350498,0.0128578235513887,0.202454903007822
"980","MAP3K1",55.780344569056,0.744407615508116,0.299431818614743,2.48606717533213,0.0129163581817007,0.20305357179704
"981","PLEKHG2",94.2042613782913,1.22487825565334,0.492729004679914,2.48590654095762,0.0129221896810182,0.20305357179704
"982","ECHDC2",276.310779231777,0.402965263234007,0.162366441328743,2.48182604691153,0.0130711067698938,0.203965039048045
"983","NOTCH2",505.717215626042,0.405346284100582,0.163298293469585,2.

48224445882577,0.0130557673561946,0.203965039048045
"984","RREB1",140.089924468161,0.663233767153628,0.26720695851778,2.48209766254832,0.0130611472456282,0.203965039048045
"985","SMO",58.8308539561738,0.627590315697269,0.252879165127666,2.48177945138513,0.0130728159960732,0.203965039048045
"986","OMG",18.9748200200393,-1.48836847086793,0.599339550852665,-2.48334765952033,0.0130153991810906,0.203965039048045
"987","C2CD2",105.359947920337,0.658983545173202,0.26538787480094,2.48309590506909,0.013024601611863 7,0.203965039048045
"988","ARMCX2",122.145859304781,0.628874125990727,0.25337770692483,2.4819631277873,0.013066079488469,0.203965039048045
"989","KIF1C",55.1492894967107,-0.694530626472972,0.279922133786372,-2.48115651691558,0.0130956855929354,0.204115261289281
"990","ADH1B",3946.93946940839,0.788794676807993,0.318021587010417,2.48031803193962,0.0131265244889023,0.20438926767316
"991","SLC1A7",15.1057748785293,1.53116190576082,0.617608508300327,2.47917877617103,0.0131685284256401,0.204836393220224
"992","TMEM200A",9.40891460137423,-1.20780132051424,0.487255659119863,-2.4787835665078,0.013183127399957,0.204856762974131
"993","ZNF728",6.32297160980763,2.24953443685458,0.907734961808531,2.47818419637898,0.0132052953347831,0.204994589713676
"994","PHLDA2",28.3217422363473,-1.27869088178511,0.516105128526532,-2.47757832873263,0.0132277370723009,0.205136385281205
"995","DAPK3",148.811867008198,-0.442761582883983,0.178760807548954,-2.47683812215232,0.0132552005703237,0.20535569526788
"996","AL162457.2",9.01468018924299,2.81710019849311,1.13766188810019,2.4762191895146,0.0132782031893488,0.205505524260855
"997","AL020997.5",12.2875297489544,1.4057015808528,0.567880029723343,2.47534955849324,0.0133105826303379,0.205593818884428
"998","GNG10",492.52657256052,-0.507119768649037,0.204863541784619,-2.47540272042252,0.0133086012224288,0.205593818884428
"999","ST6GALNAC4",86.9030334616331,-0.663015461697224,0.267945871974973,-2.47443805276893,0.0133445961140036,0.205856107401548
"1000","KIAA1755",26.6766957010326,-0.991080590062998,0.400569458035911,-2.47417912219895,0.0133542722933213,0.205856107401548
"1001","IQCN",56.5398676839358,0.982379530681771,0.397314472080134,2.47254907564413,0.013415329307458,0.206590710563901
"1002","ACTR1B",116.771432321236,-0.480745934082884,0.194593548997391,-2.47051321361804,0.0134919334410294,0.207563027937593
"1003","SPOCK1",572.554862971946,-0.570796270837415,0.231184420523606,-2.46900837670907,0.0135488046796565,0.208230133735697
"1004","LCOR",153.067388835649,0.535580279958975,0.217069932448196,2.46731674865561,0.0136129879587168,0.209008176676912
"1005","GIPC2",5.68380280038074,-1.63651713099022,0.663609239683089,-2.46608551106333,0.0136598719768015,0.209188241725161
"1006","RAP2B",191.49490801529,-0.463467644093053,0.187949009053147,-2.46592225427481,0.0136660992928504,0.209188241725161
"1007","USP32",63.5546513331429,0.57306576969489,0.232425946803369,2.46558431868925,0.0136789975776168,0.209188241725161
"1008","SLC2A11",38.4588849932513,0.652725626774214,0.264636413820983,

2.46649966778858,0.0136440855014835,0.209188241725161
"1009","KDM3B",118.324903757336,0.523337333438564,0.212322993792062,2.46481704167705,0.0137083228438884,0.209428936212626
"1010","EEF1AKMT4",7.30741120667889,-1.53709517731604,0.623921754536297,-2.46360247281075,0.0137548570913255,0.209931804022558
"1011","FAM172A",212.442960043287,0.425062508741912,0.172680301203962,2.46155760546102,0.0138335180933686,0.209994735065999
"1012","C8orf48",27.6257093528221,-0.856664040098872,0.347840921142108,-2.46280408091746,0.0137855221288145,0.209994735065999
"1013","ANXA2",9717.63526376877,-0.510805751369782,0.207477213412199,-2.46198482700341,0.0138170511878826,0.209994735065999
"1014","BCLAF3",119.326738193218,0.503893290750025,0.204715963748529,2.46142646388341,0.0138385763132401,0.209994735065999
"1015","RTL5",46.3064936689764,0.754768075695123,0.306624850833475,2.46153589196536,0.0138343554848791,0.209994735065999
"1016","CCNQ",71.9329373760791,-0.73890164237142,0.300199217681518,-2.46137097917198,0.013840716887905,0.209994735065999
"1017","LONP2",249.419345834104,0.482780053627644,0.196329792147682,2.45902594988991,0.0139314547515664,0.210956164042628
"1018","DLGAP1-AS1",76.0958124711525,0.676457559935186,0.275078937042399,2.45913979168431,0.0139270376925952,0.210956164042628
"1019","HMCES",89.0916603484252,-0.530127971407561,0.215702305349109,-2.45768338242636,0.0139836397084544,0.211538573214744
"1020","LRATD1",3.23636264277444,-2.35613490968277,0.959019533166991,-2.45681639236487,0.0140174308206191,0.211841858921416
"1021","ELL2",104.138817155963,1.23063760721683,0.501166415214739,2.45554684004419,0.0140670419173617,0.211968182948319
"1022","BOP1",41.2005059847578,-0.662280484837367,0.269699898682171,-2.45562007280483,0.0140641759485039,0.211968182948319
"1023","CYBB",9.54147926840941,-1.58281910214184,0.644482233254067,-2.45595459497774,0.0140510909539437,0.211968182948319
"1024","GALNT2",200.741244147781,0.531024608576734,0.216329380051098,2.45470406493701,0.0141000610921877,0.212258243882884
"1025","SLC25A37",253.233117813702,0.389289847967676,0.158650831045856,2.45375234028971,0.0141374310292236,0.212613170063884
"1026","ESRRG",3.98434242044984,2.97632665985793,1.21422422297044,2.45121667279601,0.0142374222165991,0.213908248995004
"1027","ATP23",35.6185530258082,-0.97072367927232,0.396093671681704,-2.45074271232584,0.0142561813714039,0.213981534411092
"1028","ALDOA",68.3625559054473,-0.85963734479742,0.350869694461061,-2.45001879149987,0.0142848759404197,0.214203660137714
"1029","SOX9",105.055173232663,-0.869990135866798,0.355245860811531,-2.44898035934712,0.0143261259809871,0.214405079608656
"1030","ZNF626",108.849423105622,0.469312788581637,0.191615286798777,2.44924502852677,0.0143156024601084,0.214405079608656
"1031","EVA1B",570.890062169779,-0.555542835753453,0.226913878352618,-2.44825411202992,0.0143550373869657,0.214629390223159
"1032","FCGR2B",14.3178568693691,-1.86587749257326,0.762590712543496,-2.44676136475611,0.0144146242752758,0.214629637334005

"1033","PNMA2",14.4118721482183,1.67605955743218,0.685090254254117,2.4
4647993023483,0.0144258828741549,0.214629637334005
"1034","NDE1",93.2811018725886,0.499274120489203,0.204055299008957,2.4
4675890758067,0.0144147225393846,0.214629637334005
"1035","SMG6",197.575212355867,0.480719035463513,0.196465917225634,2.4
4683170624157,0.0144118115221876,0.214629637334005
"1036","ZNF681",20.7073926369071,0.950310632028593,0.388336585573164,2
.44713134773533,0.0143998351453994,0.214629637334005
"1037","KCNB1",12.436242095777,1.1554651523638,0.47235831479145,2.4461
6240718435,0.0144385944803998,0.214629637334005
"1038","AL357568.2",14.1017854896884,1.31260641414784,0.53717677100761
4,2.44352787572275,0.0145444459451703,0.214793951120817
"1039","TTC32",88.4443365241222,-1.10614679599942,0.45259382436631,-2.
44401654739362,0.0145247603291053,0.214793951120817
"1040","DTNB",11.5472899241605,1.34906478510685,0.5522238439987,2.4429
6728540036,0.0145670576959715,0.214793951120817
"1041","MMS22L",35.7216337210995,0.784218202012163,0.321022988266404,2
.44287241311632,0.0145708874920663,0.214793951120817
"1042","GARS-
DT",263.85099660649,0.73205667160519,0.299659581134405,2.4429610053978
,0.0145673111791354,0.214793951120817
"1043","VOPP1",106.331849849086,-0.537764673308373,0.220102752990393,-
2.44324373958123,0.0145559028782559,0.214793951120817
"1044","SERTM1",2.98046242999391,-3.43101186441999,1.40455834492434,-2
.44276919988312,0.0145750550030733,0.214793951120817
"1045","NQO1",150.09603144011,0.788378429449458,0.322534141027808,2.44
432551213698,0.0145123261404657,0.214793951120817
"1046","AC009271.1",11.1440006616632,1.44643533806853,0.59147452234457
8,2.44547361454374,0.0144662033240473,0.214793951120817
"1047","NCS1",66.6727777540105,-0.702237943593816,0.287546690784476,-2
.442170145231,0.0145992641976891,0.214945231716693
"1048","PARD6G",7.31210947377914,1.65974389319947,0.679888232901548,2.
44120108700251,0.0146385011600121,0.215317266585484
"1049","ELMO2",49.8038314610486,0.640554960838212,0.262458693812229,2.
44059341885045,0.0146631529115528,0.215474263233162
"1050","ANGPT1",264.399193724567,0.878934376966502,0.36021606374117,2.
4400199364736,0.0146864513789559,0.215611093339624
"1051","NEO1",74.6561959651437,0.621906939232928,0.25508014268813,2.43
808448858088,0.0147653225377587,0.216562746831161
"1052","RPS6KA1",3.83678560864842,-1.8650439869832,0.765954665113238,-
2.43492738138421,0.0148947786024969,0.218046545258774
"1053","MIR99AHG",669.944579034024,0.395281170227424,0.16232224952222,
2.4351632101631,0.0148850740811897,0.218046545258774
"1054","CRAT",190.187040771707,-0.483054495034662,0.19841531684198,-2.
43456252633647,0.0149098036302079,0.218059414572728
"1055","KLF4",4145.75396812811,0.754052378491467,0.310017043495804,2.4
3229330229282,0.0150035522757578,0.21922251974484
"1056","UGGT1",144.030269824686,0.522718472083165,0.214988270925299,2.
43138134854246,0.0150413739263424,0.219359299029866
"1057","ADAMTS1",890.404483059305,1.02566080588076,0.421798466241906,2

.43163711575123,0.0150307579743188,0.219359299029866
"1058","DCHS1",111.281932940092,-0.574618576214334,0.236376597972372,-2.43094528453064,0.0150594885617772,0.219415894309825
"1059","ZNF516",67.6479668876215,0.599469047131382,0.246702512717699,2.42992679939727,0.015101872557066,0.219825651999219
"1060","SCAP",96.2547920678798,0.494215634338081,0.203474162977585,2.42888643504347,0.0151452754841645,0.220041867661071
"1061","GM2A",89.7539671065628,0.525150758637301,0.216195778757833,2.4290518605617,0.0151383667658004,0.220041867661071
"1062","WDR19",67.2497944250184,0.692757564566624,0.285299275897234,2.42817848866941,0.0151748730504677,0.220057072505136
"1063","RANBP3L",7.99870131519749,-1.35807332499049,0.559289868165868,-2.4282101326887,0.0151735490020726,0.220057072505136
"1064","KAZN",142.489806629662,0.631348981305725,0.260144280065934,2.42691855898468,0.0152276737397831,0.22061521682214
"1065","SUOX",31.5861626715886,0.792429454150731,0.326913961412119,2.42396944666357,0.0153518970217448,0.222063659895725
"1066","MEOX1",3.68539508583492,-3.04289098296577,1.25538980524807,-2.42386147334092,0.0153564619817608,0.222063659895725
"1067","USP22",336.907578725097,0.401759251815419,0.165795807311389,2.42321719909875,0.0153837258345696,0.222249422436636
"1068","PCDHB3",12.7303651214481,1.11058268126926,0.458938878813659,2.41989234849765,0.0155251024196914,0.222864338054035
"1069","KCTD16",25.3390609053762,1.46772190844881,0.606541892893775,2.41981951394386,0.015528212186577,0.222864338054035
"1070","NOTCH4",20.8066084346531,-0.829796742200866,0.342742726334727,-2.42104843791922,0.0154758150006673,0.222864338054035
"1071","LPCAT3",86.12210417196,0.791116085453304,0.326941996502906,2.41974446206171,0.0155314171986273,0.222864338054035
"1072","RAB20",15.0420625489893,-1.09049217970086,0.450710115340919,-2.41949790471422,0.0155419502697429,0.222864338054035
"1073","NRXN3",3.98677620601188,-2.84101702672834,1.17374870145026,-2.42046446843181,0.0155006940622018,0.222864338054035
"1074","SRPX2",568.727662948591,0.47427704536846,0.195989583126052,2.4199094554093,0.0155243720971234,0.222864338054035
"1075","SLITRK4",137.904582673687,-0.689881745636228,0.285131625899687,-2.41952026001822,0.0155409949792887,0.222864338054035
"1076","FBLL1",5.96950476311941,2.46750957960964,1.0202633731365,2.41850255980866,0.0155845359095039,0.223113223996696
"1077","CAPN1",375.418178084064,-0.470641460544955,0.194607340945802,-2.41841576097599,0.015588254443363,0.223113223996696
"1078","ZNF624",42.2386062981942,0.698296555147537,0.288978502653961,2.41643080275669,0.0156735050238537,0.223894514712876
"1079","NBR2",17.0411637057681,1.0951764754855,0.453245746739509,2.416297303094,0.015679253299855,0.223894514712876
"1080","PNMA6A",29.420849632553,1.08764207316984,0.450158558636162,2.41613105494439,0.0156864142646712,0.223894514712876
"1081","C9orf16",659.226721270524,-0.736607026554993,0.305119074756204,-2.41416249424444,0.0157714269429719,0.224070549609136
"1082","CHST15",8.60395297637304,2.17891966428246,0.902344499590868,2.

41473147480857,0.0157468138664519,0.224070549609136
"1083","SULT1A1",167.324450185053,1.63307554681237,0.676107282123762,2.41540890031922,0.0157175537219639,0.224070549609136
"1084","MAP2K4",77.5253659116334,0.469182069483888,0.194329034178418,2.41436937855165,0.0157624735874133,0.224070549609136
"1085","ADGRE5",173.407556925795,-0.557031759721578,0.230689086195671,-2.41464288106418,0.0157506440503432,0.224070549609136
"1086","BMPER",96.2330766937764,1.06053730113312,0.43951995689395,2.41294458760837,0.0158242251583231,0.224588587905497
"1087","HSPA5",989.196233978944,-0.515811531888985,0.213794614737427,-2.41264978784653,0.0158370285470824,0.224588587905497
"1088","NT5E",840.132566655083,0.42926537808824,0.177969565473047,2.41201565530176,0.0158646003093309,0.224750510809436
"1089","IFIT2",138.971752011268,0.703634260117609,0.291756579447998,2.41171685467687,0.0158776066345427,0.224750510809436
"1090","AC096576.6",2.36622052383606,-3.09060764179498,1.28225766153378,-2.41028596241581,0.0159400211987813,0.225426997045151
"1091","DHRS9",3.08122104064407,-2.74226461732558,1.13852484548617,-2.40861201070634,0.0160133115819644,0.226048716150166
"1092","DIP2A",121.079493288555,0.643376236560996,0.267084189941698,2.40888925960552,0.0160011524011471,0.226048716150166
"1093","SAMD11",12.290059074901,-1.90409742469099,0.79081389387962,-2.40776931137332,0.0160503193519681,0.226156922130337
"1094","GULP1",300.643713017316,0.752355440096045,0.312440538765598,2.40799559195641,0.0160403746946463,0.226156922130337
"1095","SLC6A15",5.01488988923991,-2.91168462430253,1.21010488920367,-2.40614235202256,0.0161219812437469,0.226390146360953
"1096","ZBTB4",277.951191529393,0.461233992545039,0.191649131818389,2.40665839792123,0.016099220824473,0.226390146360953
"1097","MED29",246.503116274573,0.453247101157881,0.188377307223134,2.40605998588253,0.0161256166528917,0.226390146360953
"1098","ARHGEF6",124.676129145402,0.522519685542751,0.217106536470706,2.40674322402658,0.0160954822379929,0.226390146360953
"1099","GPAM",41.3669453505148,0.674592762672733,0.280537478741143,2.40464399159726,0.0161882274155464,0.226855932373316
"1100","AL139384.1",11.7710162080827,1.36234617090882,0.566524580896708,2.40474326595409,0.0161838308651899,0.226855932373316
"1101","HLA-
DRA",505.728056423288,-0.990616703203831,0.412017411236238,-2.40430786706692,0.01620312111309,0.226858412314516
"1102","AMPD2",52.8895316919871,-0.605082220436442,0.251928262648301,-2.40180364868849,0.0163144627570058,0.22705526629059
"1103","KCNIP3",24.3977550805828,0.811907718912052,0.337945798055728,2.40247910636298,0.0162843647897446,0.22705526629059
"1104","TTPA",5.90965085148592,1.86614077935115,0.776375892139943,2.40365626785173,0.0162320278163189,0.22705526629059
"1105","NAALADL1",89.5249862507301,-0.564044104619609,0.234809945225133,-2.40213038710439,0.0162998974016152,0.22705526629059
"1106","GLYR1",133.274254316167,0.489739313869188,0.203943924236226,2.40134299515552,0.0163350172115643,0.22705526629059

"1107","SMURF2",465.344375727308,0.603754625519111,0.251405872170444,2
.4015136174297,0.0163274013593831,0.22705526629059
"1108","DOCK11",230.487934096724,-0.452585125709171,0.188410060777682,
-2.40212822946439,0.0162999935474694,0.22705526629059
"1109","AL592183.1",23.5745643238778,-1.30147703050409,0.5416866025215
37,-2.40263839726835,0.0162772740015908,0.22705526629059
"1110","NAA10",225.580401603739,-0.554420843613869,0.230987657283614,-
2.40021847978281,0.0163852889101134,0.227548854549007
"1111","SHPRH",226.110890184047,0.432015821283998,0.180098666682429,2.
39877301282735,0.0164501084334596,0.228243403691971
"1112","WDR1",267.856298084983,-0.430682959631313,0.179620977302723,-2
.39773196927586,0.016496931701534,0.228687232175492
"1113","AZIN2",32.3523471389634,-0.790785669352771,0.329858771120905,-
2.39734619354087,0.0165143125346938,0.22871978178994
"1114","PPM1G",341.470327241539,-0.398400438035002,0.166212483736726,-
2.39693450863807,0.0165328784243418,0.22871978178994
"1115","NDST1",129.297033522034,0.568196433593771,0.237100839464994,2.
39643366457866,0.0165554898691471,0.22871978178994
"1116","AC016885.2",6.63230297485355,1.6640854750657,0.694420947863865
,2.39636416525834,0.0165586296774293,0.22871978178994
"1117","SGMS1",144.098394004801,0.471743693883251,0.196914334851846,2.
39567979770585,0.0165895756604921,0.228942084875995
"1118","ARHGAP15",16.7194487278915,1.21630795750224,0.507806076487293,
2.395221352836,0.0166103341854526,0.229023525464
"1119","HAUS4",157.806190219759,-0.528751673970615,0.22085732056633,-2
.39408715370978,0.0166617891252841,0.229527684867073
"1120","NPLOC4",145.458586814365,0.576778047841087,0.240982263665731,2
.39344605311345,0.016690935654336,0.22972390456392
"1121","HMGCLL1",35.0339350658075,-0.944158742861536,0.394644535830568
,-2.39242826680588,0.0167372995009017,0.230156531495451
"1122","SMG7",81.572311266277,0.564226594581934,0.235935988053712,2.39
143930197493,0.0167824586996261,0.230346566526313
"1123","KLF10",389.571932631365,-0.692470881642571,0.289556386196456,-
2.39148889354058,0.016780191650244,0.230346566526313
"1124","GPS2",331.818745399725,-0.407577899426926,0.170453072268331,-2
.39114434256315,0.0167959481528106,0.230346566526313
"1125","LSM10",237.52297089107,-0.578070239795298,0.24184490190788,-2.
39025191449141,0.0168368197734978,0.230701846051972
"1126","TMEM131",168.964504274723,0.460595744421532,0.192761644038286,
2.3894574396245,0.0168732787404155,0.230996085065279
"1127","FKTN",56.8033850828494,0.634994073526777,0.266079268799334,2.3
8648458556034,0.0170103204508509,0.232665563220822
"1128","DANT2",12.3549202003557,1.39259193957871,0.583869190290555,2.3
8510947783647,0.0170740394406053,0.233330069128485
"1129","CYP4B1",270.187147748334,0.794389943552286,0.333226188127713,2
.38393611263178,0.0171285756884317,0.233474942420047
"1130","TMEM64",165.572951053754,0.963834453911907,0.404264834954936,2
.38416594908471,0.017117881216345,0.233474942420047
"1131","MAOA",112.37733605918,-1.01211412791263,0.424561644196381,-2.3
8390382585874,0.017130078486998,0.233474942420047

"1132","AC008764.7",8.32221830579518,1.89153960634838,0.793587233320715,2.38353079148382,0.017147449886447,0.233505247349453
"1133","ZNF721",158.414265158254,0.50187547198456,0.210714202770846,2.38178283848457,0.0172290542847533,0.233790380105169
"1134","ANKRD50",93.1779969237252,0.609985243906507,0.256030324585048,2.38247264223533,0.0171968097168392,0.233790380105169
"1135","TGFB1I1",408.897625247934,-0.671751805812999,0.282015147894624,-2.38197065238496,0.0172202697582661,0.233790380105169
"1136","TPPP3",2107.66112335044,-0.659182601042221,0.276738949469083,-2.381965394849,0.0172205156129297,0.233790380105169
"1137","TNKS",121.344550796314,0.505806062984461,0.212450059779157,2.38082334978041,0.0172739933360548,0.234078231443806
"1138","AL035681.1",13.7304328340948,1.24595326377897,0.523359874675998,2.38068167635189,0.0172806375207948,0.234078231443806
"1139","VENTX",4.32134392468398,-2.62774531399178,1.10393157760737,-2.38035161534836,0.0172961253796062,0.234082328996162
"1140","ZNF518B",53.3008051935162,0.701128876968549,0.29481497839396,2.3781996450385,0.0173974037035133,0.235246472008471
"1141","NFKBIZ",2257.62525521219,0.674425118250108,0.283665933454823,2.37753300171139,0.0174288833051363,0.235259401180977
"1142","RBAK",41.0172584519096,0.766783831608286,0.322486239242762,2.3777257392712,0.0174197769055948,0.235259401180977
"1143","CTNND2",7.37265925948001,1.69929233562914,0.714919620990065,2.37689984403541,0.0174588279123516,0.235457421057656
"1144","QPCT",39.4911572641092,-1.45817064674601,0.613625645136087,-2.37631959860907,0.0174863096855386,0.235621908918337
"1145","PTGER3",24.7199743153315,-1.72915508054572,0.728151891283509,-2.37471755720877,0.0175623831002496,0.236233975122467
"1146","AL157392.3",28.1648515662008,0.937570873538231,0.39477862983041,2.37492813109208,0.0175523674042086,0.236233975122467
"1147","FAM149B1",66.4293444489951,0.57209794547261,0.241051467781214,2.37334354666475,0.0176278593598427,0.236907979103727
"1148","PRDM16",5.86865116216548,-1.82172591927244,0.768528032916837,-2.37040919946451,0.0177684077482432,0.237629444041217
"1149","KDM4A",92.7728930346693,0.517407135378172,0.218281025796476,2.37037155882068,0.0177702170080089,0.237629444041217
"1150","PRKACB",112.018326854293,0.475293211102851,0.200807163289939,2.36691362656516,0.0179371183971492,0.237629444041217
"1151","UHMK1",140.465371242224,0.53215079764594,0.22484170145474,2.3667798019802,0.0179436051160543,0.237629444041217
"1152","ARL13B",135.056892838459,-0.387650600615623,0.163648078158637,-2.36880631277468,0.0178455962223127,0.237629444041217
"1153","HSP90AB1",2562.7171952626,-0.341314212750915,0.14395038537649,-2.37105452589263,0.0177374141639729,0.237629444041217
"1154","PLEKHA8",46.6428735325223,0.748003434376706,0.315738157282445,2.36906251944574,0.0178332386631514,0.237629444041217
"1155","LINGO2",9.06918587615419,5.40576818935951,2.28232081664885,2.36854001853115,0.0178584482841693,0.237629444041217
"1156","SPON1",71.9065227637855,1.84732222141368,0.780372156351958,2.36723236006964,0.0179216770817766,0.237629444041217

"1157","PTHLH",43.5899400352665,-1.3312142702161,0.56235212078014,-2.3
6722548208644,0.0179220101685424,0.237629444041217
"1158","TC2N",87.2184036781282,1.23336760708851,0.520810856000825,2.36
816800740143,0.0178764160987946,0.237629444041217
"1159","IRX5",76.09976985429,-0.840921366692402,0.354723061128312,-2.3
7064194252716,0.0177572241526429,0.237629444041217
"1160","C16orf74",31.8146136481221,1.0083600415582,0.425160587040912,2
.37171570529694,0.0177057083438271,0.237629444041217
"1161","SPATA33",54.2419982521965,-0.612536746556185,0.258510591234826
,-2.36948414233352,0.0178129189418537,0.237629444041217
"1162","HOXB2",178.049513148498,-0.620418227391592,0.26184830426002,-2
.36938035228025,0.0178179191233068,0.237629444041217
"1163","AC002398.2",6.15598332297967,1.6879114284163,0.713051213163507
,2.36716717853652,0.0179248339089646,0.237629444041217
"1164","DDX17",1312.01313984623,0.454071828555529,0.191806612099792,2.
36734189496703,0.0179163732598776,0.237629444041217
"1165","LINC01871",11.4727690949648,1.84162249819971,0.778868325159756
,2.36448503387523,0.0180551568384425,0.237858785468832
"1166","EIF2A",409.131634684756,-0.44513006153656,0.188201475392387,-2
.36517838453974,0.018021388281096,0.237858785468832
"1167","PHF1",131.340676198317,-0.404301883888955,0.171055583520904,-2
.36357022417538,0.0180997960009692,0.237858785468832
"1168","F10",1757.53232231098,0.606040916846793,0.256200647324039,2.36
54933083767,0.0180060686872096,0.237858785468832
"1169","GABRB3",2.91245587769126,2.87550820425896,1.21625518089421,2.3
6423100137986,0.0180675429577549,0.237858785468832
"1170","AP3S2",105.077519286554,0.477619978398188,0.202056928761604,2.
36378916241821,0.01808910387581,0.237858785468832
"1171","PGP",114.991404731132,-0.668920459916408,0.282856035299046,-2.
36487957278055,0.0180359346504958,0.237858785468832
"1172","FBXO31",53.5217035466156,0.595884447159063,0.25187664557151,2.
36577887484168,0.0179921870568667,0.237858785468832
"1173","NFATC1",73.0025173000494,-0.853406101624612,0.360974327889015,
-2.36417394725921,0.0180703258263948,0.237858785468832
"1174","GBA",125.996729587131,0.477243348654461,0.202042777624815,2.36
209061400197,0.0181721998315412,0.238281603453002
"1175","EXOC3-
AS1",21.1331748481408,0.871567652607176,0.369050277377873,2.3616501762
2998,0.0181938013145756,0.238281603453002
"1176","AL590807.1",9.41204951148044,2.04695500267662,0.86642805034010
4,2.36252162181628,0.0181510825907478,0.238281603453002
"1177","AC104564.5",7.6052664809537,1.57767798641025,0.668016806119385
,2.36173397429209,0.018189689667068,0.238281603453002
"1178","MID1",99.8899643107288,0.785445965412021,0.332707968333446,2.3
6076691924863,0.0182371888450463,0.238647084929023
"1179","WDR48",143.70417016339,0.496861959883912,0.21053334633085,2.36
001549656224,0.0182741716751087,0.2389282072704
"1180","CAPN2",910.968045579896,-0.426279454754079,0.180759473270654,-
2.35826895841749,0.018360384944887,0.239648885626954
"1181","DUSP14",144.704628106423,-0.604466192126765,0.256287550305883,

-2.3585468408642,0.0183466442353983,0.239648885626954
"1182","PIK3AP1",5.57670454173683,-1.79081330197796,0.760037162526803,
-2.3562180775796,0.0184620756192372,0.240772331362557
"1183","ALYREF",122.500792826279,-0.684189255993843,0.290642347211567,
-2.354059078307,0.0185696594928873,0.241970668709094
"1184","JUND",11454.3013230706,-0.454744812046585,0.193218756122314,-2
.35352313187813,0.0185964507773609,0.242115108727211
"1185","TFRC",59.3934780916685,-0.771477010037379,0.32793552612014 7,-2
.35252648337567,0.0186463619119071,0.242310072579924
"1186","ZNF718",23.7837375556747,0.954553671856149,0.405781966106235,2
.35238071572221,0.0186536716220568,0.242310072579924
"1187","C5orf38",146.055610393951,-0.739648760515044,0.314480581178443
,-2.35196958026274,0.0186743020580571,0.242310072579924
"1188","PSMG1",90.8488275222816,-0.642886275618864,0.27330894589937,-2
.35223283125013,0.0186610900446647,0.242310072579924
"1189","PDXK",168.033279691084,-0.371401969111449,0.157939823370387,-2
.35154099318238,0.0186958294497499,0.242385375078129
"1190","ALDH6A1",138.227742737366,0.436646096076156,0.185741891651211,
2.35082184312032,0.018732000235146,0.242650238340148
"1191","AP000662.1",6.21419840634981,1.71803188760505,0.73101675514444
,2.35019495177726,0.0187635806481662,0.242855244073452
"1192","RPRD2",192.772563558744,0.476510179943403,0.202789828832687,2.
34977356944539,0.0187848344596135,0.242900689550127
"1193","A1BG",21.5539417259548,-1.08930051839778,0.46363063423036,-2.3
4950074040308,0.0187986067228869,0.242900689550127
"1194","NCKAP5",21.6506760167571,1.08397046405237,0.461803433344054,2.
34725509986581,0.0189123013613913,0.243798397652861
"1195","EIF1B-
AS1",17.2758793816543,1.12956966965516,0.481254960138102,2.34713356373
722,0.0189184717363523,0.243798397652861
"1196","P4HA1",282.037015676574,0.519771934249333,0.221514078904902,2.
34645101033274,0.0189531576094227,0.243798397652861
"1197","CCDC186",527.331719917122,-0.45110356085384,0.19224291753173,-
2.34652889503398,0.0189491968703462,0.243798397652861
"1198","KCTD21",24.8152654012093,0.838526970577662,0.357389073115541,2
.34625799627224,0.0189629762429958,0.243798397652861
"1199","IGF1R",178.214192882691,0.69281895939115,0.295239639673931,2.3
466325868583,0.0189439248622395,0.243798397652861
"1200","NAT14",53.8627646106733,-0.639650139431104,0.272776854655596,-
2.34495753035468,0.0190292470147937,0.244446535610871
"1201","AC106897.1",15.848302907386,-1.15300125206829,0.49207765548338
5,-2.34312864894395,0.019122788036851,0.245443611647009
"1202","HFE",40.5712297266924,0.830358956320967,0.354552819621578,2.34
198943110149,0.0191812580872902,0.245548754429445
"1203","ITGA8",4.7482194109614,-2.85314108548771,1.21832787198166,-2.3
4184996592663,0.0191884268253862,0.245548754429445
"1204","LINC01465",4.31878050467234,-1.76222572829882,0.75253219368913
9,-2.34172802582685,0.0191946966647733,0.245548754429445
"1205","RHBDL1",8.1385567996697,1.5355215108149,0.655626343547046,2.34
206804825363,0.0191772180689318,0.245548754429445

"1206","LZTS3",34.6230302914073,0.764443432075871,0.326494191478628,2.
34136916376324,0.0192131587992819,0.245581130091983
"1207","PTPN20",12.3868599603781,-1.29989828389687,0.555307978357165,-
2.34086008946335,0.0192393754262466,0.245712487320291
"1208","GBE1",135.61315297253,-0.403173484664085,0.172278729401982,-2.
34023948321183,0.0192713780820549,0.245917461204367
"1209","MGP",25088.155761124,-0.74600894586146,0.318875339944093,-2.33
950027616515,0.0193095572977367,0.246200848423996
"1210","B9D2",36.3815148066253,-0.643055345593863,0.274905539488718,-2
.33918656855678,0.0193257799119898,0.246204047391175
"1211","AC015813.1",16.7713979960931,1.03706027789379,0.44350451602493
8,2.33833081833935,0.0193700934931324,0.246564815191277
"1212","PPM1K",70.2007395204106,0.589753542020207,0.252284353657452,2.
33765405373084,0.0194052014547566,0.24680790464115
"1213","FMNL2",171.576476637802,0.678194280300482,0.290182332130556,2.
33713153837138,0.0194323455771391,0.246949387528112
"1214","MRNIP",126.969482121448,0.50401511321939,0.215713918130821,2.3
3649788380241,0.0194653077684597,0.246996302240798
"1215","VASH1",34.6430173950726,0.851746504361215,0.364548132869257,2.
33644456674995,0.0194680835045456,0.246996302240798
"1216","HDAC4",156.152944885216,0.475795772307794,0.203817043287314,2.
33442583914379,0.0195734351020693,0.2477595403822
"1217","NUTM2A-
AS1",219.44937331728,-0.432045946580002,0.185104828152871,-2.334060925
86193,0.0195925319316186,0.2477595403822
"1218","TMEM25",33.8767913360158,-0.711488347685354,0.304715877230448,
-2.3349237793319,0.0195474028710076,0.2477595403822
"1219","CMC4",130.09037781706,-0.611914477669971,0.262146827055331,-2.
33424331144322,0.0195829852022474,0.2477595403822
"1220","RFTN2",129.265997236354,0.523928541570099,0.224542398168054,2.
33331676264531,0.0196315263035785,0.247850991782939
"1221","MNT",90.6332068337563,0.599000838505293,0.25671729603265,2.333
30923845938,0.0196319209190379,0.247850991782939
"1222","RIPK2",122.950323469948,-0.695790804823333,0.298315398170086,-
2.33239989987586,0.0196796633759447,0.248047433311682
"1223","MMP14",956.446147179535,0.595544682196876,0.255306310102457,2.
33266730445432,0.0196656134806328,0.248047433311682
"1224","SLC35E2B",148.843287646991,0.625167473272719,0.268160829773376
,2.33131540427083,0.0197367345460759,0.248360622879804
"1225","HLA-
DPB1",285.992456716307,-0.501405142192678,0.215060778405169,-2.3314578
6001037,0.0197292296307056,0.248360622879804
"1226","DHX57",54.0401612697638,0.629729550458854,0.270295566289261,2.
32978128018918,0.019817714056139,0.249022087592891
"1227","MBTPS2",46.9776229518494,0.611494467108635,0.262476912896936,2
.32970763165271,0.0198216089183573,0.249022087592891
"1228","KIAA0753",31.8781043472878,0.698761963805035,0.299992247988684
,2.32926673435706,0.019844939508332,0.249112168176659
"1229","CLSTN1",337.181937125033,0.421766244919555,0.181190411915173,2
.32775145473487,0.0199253051738136,0.249563159152152

"1230","VPS45",74.8149986335173,0.473023594805635,0.203217311091809,2.32767372161485,0.0199294355443594,0.249563159152152
"1231","OPHN1",147.500605337711,0.501954235459319,0.215610068939425,2.32806490869562,0.0199086572933671,0.249563159152152
"1232","PTX3",302.25677248087,1.81170844950599,0.778703903492999,2.32656911231507,0.019988210086465,0.249690647068767
"1233","MXD4",577.509500147297,0.437714650252841,0.188115162119126,2.32684407424667,0.0199735656641564,0.249690647068767
"1234","HOXB5",11.399756630357,-1.26156614040001,0.542134161157155,-2.32703679418997,0.019963307018527,0.249690647068767
"1235","AP003068.2",7.66396173499642,-1.45727685010309,0.62648084771422,-2.3261315256805,0.0200115351970386,0.249779607337935
"1236","RABGAP1",157.674799661655,0.498535060910018,0.214389415350929,2.32537161451733,0.020052097944955,0.250083406004435
"1237","SRM",429.808819528276,-0.677894900356974,0.291849790622772,-2.32275273835362,0.0201924393364193,0.250415488633068
"1238","CDC42BPA",262.134522343795,0.494408899582873,0.212779396331718,2.32357506462751,0.0201482801597368,0.250415488633068
"1239","NECAB1",18.6124688657651,1.80295096842881,0.776212645823321,2.32275392333558,0.0201923756418083,0.250415488633068
"1240","CSAD",246.993472648904,0.64289751118658,0.276782463646828,2.32275377101532,0.0201923838292476,0.250415488633068
"1241","LYZ",110.042280809702,-1.16812219450998,0.502880984042565,-2.32286014300972,0.0201866668823637,0.250415488633068
"1242","TCFL5",53.5853065580385,-0.579455397967658,0.249434345204655,-2.32307783233391,0.0201749716048952,0.250415488633068
"1243","MIR155HG",10.2300746438047,-1.24576108405455,0.536273391567451,-2.3229962620621,0.0201793532443837,0.250415488633068
"1244","DCBLD2",182.737501336008,0.456896366204203,0.196915988078771,2.32026038445103,0.0203267957822,0.251676752596477
"1245","PSMG3-AS1",27.726146410647,0.948420023689447,0.408725377719867,2.32043341419205,0.0203174430615519,0.251676752596477
"1246","PIGW",10.5255298918215,-1.09367248124181,0.47160203622956 4,-2.31905801337431,0.0203918909484442,0.252280095481755
"1247","CHST3",73.4160974523006,0.585216295405624,0.252603361411763,2.31673993621833,0.0205179026921494,0.253432267627791
"1248","MAP2K3",123.064391649033,-0.47174813438824,0.203625639059165,-2.31674231480825,0.0205177730436358,0.253432267627791
"1249","ECHDC3",4.3131498200613,-2.26586012860703,0.978253117533149,-2.31623093041689,0.0205456632360194,0.253571976607877
"1250","DUSP10",21.2018929404726,-0.930035805879144,0.401823611140228,-2.31453747389319,0.0206382581454266,0.253618721148291
"1251","LINC01341",15.6866351985935,1.11736676815885,0.482719991893994,2.31473066564897,0.0206276764415061,0.253618721148291
"1252","TMEM161B-AS1",124.946009005892,0.50166035230422,0.216686239430962,2.31514633149583,0.0206049251881285,0.253618721148291
"1253","CRTAC1",15.9394111695219,-3.38497892003381,1.46215618822074,-2.31505973664338,0.0206096631070481,0.253618721148291

"1254","STX3",35.5601804321333,-0.940969424175608,0.406631989902604,-2
.31405656107132,0.0206646197704997,0.253618721148291
"1255","MLKL",24.513496526592,-0.862021508508711,0.372503240426342,-2.
31413156976057,0.0206605061771532,0.253618721148291
"1256","SCML1",121.925543136098,0.60337847956166,0.260713709072415,2.3
1433353354682,0.0206494337175488,0.253618721148291
"1257","EMX2OS",482.263217811611,-0.504984927422647,0.218469835715935,
-2.31146293385441,0.0208072982475205,0.254833969333258
"1258","LINC01578",835.034971229759,-0.431884542751451,0.1868116671692
99,-2.31187135844172,0.0207847735253594,0.254833969333258
"1259","AHCY",109.919465016269,-0.411964657964952,0.178235097971175,-2
.31135541009761,0.0208132317476855,0.254833969333258
"1260","SHE",60.522277759958,0.577110824936042,0.249736394325396,2.310
87994401044,0.0208394871504834,0.254939067610324
"1261","AL592295.5",92.3609771823747,-0.717150213254182,0.310412077503
499,-2.31031672163625,0.0208706258345793,0.254939067610324
"1262","KDM5B",323.580257546546,0.372893101077618,0.161404471333781,2.
31030217438328,0.0208714306405598,0.254939067610324
"1263","AC026904.2",6.54659751908643,1.46347103900765,0.63367843571098
7,2.30948531074066,0.0209166658791427,0.254966968205868
"1264","CFAP70",10.8142864815688,1.26033015555498,0.545641612577377,2.
30981312001062,0.0208985026193158,0.254966968205868
"1265","EPS8L1",3.97114360802717,-2.30489559946492,0.998064661512513,-
2.30936500243579,0.0209233353733651,0.254966968205868
"1266","KIAA1109",282.017323410509,0.532231103596997,0.230542342135786
,2.30860456550546,0.0209655343749577,0.25507791033147
"1267","AC005332.6",107.306272976973,0.536800162410697,0.2325076171508
18,2.30874226396848,0.0209578875660341,0.25507791033147
"1268","SEC63",675.244278920825,0.274385041357232,0.118899177136864,2.
30771186112911,0.0210151679292459,0.255480136931645
"1269","HES4",24.1367124398345,-1.31427806894596,0.569840211652153,-2.
30639755157931,0.0210884286795492,0.256116098208258
"1270","COL24A1",10.1147902833291,1.4038032586483,0.609053478270219,2.
30489326263313,0.0211725520390608,0.256116098208258
"1271","EMP1",2792.33581419979,-0.691657856549201,0.300108479748551,-2
.30469281350768,0.0211837836662685,0.256116098208258
"1272","WNT3",6.96022275818169,1.66209442421857,0.720740110803982,2.30
609397104944,0.0211053821205266,0.256116098208258
"1273","SMAD7",162.683459066608,-0.775072249816285,0.336148829401935,-
2.30574133247844,0.0211250901120702,0.256116098208258
"1274","SLC2A10",160.775455674072,0.461594960243936,0.200243869576522,
2.30516400437188,0.0211573899900086,0.256116098208258
"1275","CLCN5",36.0057449962981,0.819412244009796,0.355532493908485,2.
3047464241643,0.0211807792290975,0.256116098208258
"1276","NPAS2",50.6796465967282,1.10487413994537,0.479670723288031,2.3
0340124235999,0.0212562779195457,0.256791163111126
"1277","CDK14",187.544199364645,0.397191526486545,0.172521351415669,2.
30227460675033,0.0213196909111656,0.257355548469552
"1278","URGCP",91.8446844955818,0.49584763355189,0.215592325023422,2.2
9993175080802,0.0214520869417143,0.258548803914407

"1279","CRNDE",461.009648606902,-0.465074894280968,0.202203364034804,-
2.30003539506355,0.0214462148503713,0.258548803914407
"1280","LINC01816",4.53511276632342,1.82021485196104,0.791739063023071
,2.29900852057366,0.0215044554797536,0.258610350107499
"1281","USP40",82.3003038960634,0.57699606064824,0.250981940067501,2.2
9895450044357,0.0215075231163032,0.258610350107499
"1282","NF1",304.828515927058,0.453646903939318,0.197285338325323,2.29
944560396706,0.0214796488698009,0.258610350107499
"1283","NQO2",350.267761543244,-0.518563087672803,0.225637996422453,-2
.2982081736886,0.0215499437199321,0.25891845864595
"1284","CD52",49.4142822889447,-0.792594626242904,0.34495991316627,-2.
29764269989503,0.0215821332742642,0.259103258896248
"1285","ARHGAP4",12.3603815997545,-1.60164747416939,0.697373192419636,
-2.29668632459507,0.0216366701352486,0.259555852245025
"1286","BMPR1A",159.909840884552,0.450498563821114,0.196273318382928,2
.29526136070209,0.0217181505612635,0.260330708321833
"1287","SNHG9",345.192290687251,-0.923598420631756,0.403224468123864,-
2.2905316855624,0.0219905139549816,0.263390654713319
"1288","NRARP",2.44942460750161,-2.86091323508254,1.24954393974147,-2.
28956593209075,0.0220464917802629,0.263856110863938
"1289","BCL7B",170.168741770493,-0.580274317377327,0.253527660360449,-
2.28880082178146,0.0220909277158764,0.263979402187751
"1290","LILRB4",3.53141824731377,-2.41667686384519,1.05587123879781,-2
.2887988374385,0.0220910430633927,0.263979402187751
"1291","HECW1",5.25709691477845,1.83035083756844,0.800030080014304,2.2
8785252366476,0.0221461108986775,0.264432455075998
"1292","AP000525.1",30.7684203693284,1.06254504461593,0.46478883356419
,2.28608126505084,0.0222495048090623,0.265461390581808
"1293","CROT",65.7337098606042,-0.496961804078975,0.217445421713007,-2
.28545535778115,0.0222861412191928,0.265692859159982
"1294","DENND11",42.7398160032634,0.804891942119515,0.352313860450824,
2.28458778513445,0.0223370098396581,0.26609351366177
"1295","CES2",122.38538516483,0.485931024989178,0.212759156824897,2.28
394881912935,0.022374539033549,0.266334763862671
"1296","HLA-
DMA",36.3949430178902,-1.0819311065295,0.473864674886773,-2.2832069235
5475,0.0224181824870949,0.266366749665539
"1297","CDKN1C",2096.92290046423,0.647452814545975,0.283594630167249,2
.28302212268315,0.0224290652653824,0.266366749665539
"1298","MBD2",422.532568602586,-0.39322845420429,0.172199341737199,-2.
28356537392816,0.0223970867130152,0.266366749665539
"1299","TAFA2",11.9917130862151,1.26262567284646,0.553214608860946,2.2
8234333045935,0.022469078232943,0.266431416123704
"1300","CAMTA2",72.9705008014443,-0.537947289364951,0.235677049923056,
-2.2825611977941,0.0224562287855237,0.266431416123704
"1301","AADAC",930.341013826773,-1.72479373435774,0.756465327127312,-2
.28006978311573,0.02260355002769,0.267614227094349
"1302","C19orf54",16.5399636871524,0.965423626244478,0.423402104078949
,2.28015783800749,0.02259832892019,0.267614227094349
"1303","ARL3",345.648649768372,-0.509309123168293,0.223511831940364,-2

.27866739199822,0.022686844518646,0.268394250387512
"1304","PALM2-
AKAP2",131.459554781162,-0.69585200792029,0.30551425194649,-2.27764172
534304,0.0227479323575161,0.268910565407294
"1305","PCDHB13",18.6512636133069,1.00989868189084,0.443647655475302,2
.27635302345707,0.0228248889182367,0.269613534616566
"1306","GCK",3.9350386860438,-1.85732586427099,0.81609008612438,-2.275
88338083047,0.022852990438794,0.269734911388064
"1307","AL353748.3",2.4779255021153,2.74606532455053,1.20674518500256,
2.27559667001672,0.0228701608293351,0.269734911388064
"1308","CLIC1",2433.32796025825,-0.549618043030684,0.241628384765183,-
2.27464187853926,0.0229274217236657,0.270203521307575
"1309","LINC00852",4.60478600123951,-1.77392180400541,0.78001709180114
2,-2.27420888933247,0.0229534300327606,0.270303379644771
"1310","KIAA0319L",107.34433171567,0.53625567768053,0.23589885718452,2
.2732440677365,0.0230114760425962,0.270441806213095
"1311","CCNI",3651.04178046169,-0.350171701479881,0.154015427219374,-2
.27361445409692,0.0229891776399168,0.270441806213095
"1312","SYCE1",3.29723420217904,3.1068996875329,1.36678837673348,2.273
1387977985,0.0230178170451885,0.270441806213095
"1313","LINC00377",13.8871091927867,1.81381783313153,0.798142975447578
,2.2725475120725,0.0230534617308906,0.270578990420873
"1314","MMP2",8478.12150344655,-0.536817082694876,0.236237388165843,-2
.27236292638835,0.0230645989888438,0.270578990420873
"1315","AMPH",82.8937263783355,1.22788557445571,0.540425897413104,2.27
207019562408,0.0230822709259202,0.270580385036548
"1316","BIVM",82.0139017781912,0.526589936618031,0.231812775027307,2.2
7161741433793,0.0231096281531214,0.27069522642885
"1317","ARMCX4",40.5056389129205,0.784711336911594,0.345630414360768,2
.27037698161746,0.0231847198763827,0.271368608120303
"1318","ARRDC4",61.1853357639972,0.623292198487374,0.274688923031879,2
.26908384804086,0.0232632274060537,0.272080918409953
"1319","RHOB",3965.59267760426,0.550134330656806,0.242584766308424,2.2
678024635618,0.0233412491964778,0.272786471845114
"1320","AMIGO2",31.1758483743353,0.807304258266027,0.356257854212216,2
.26606725640112,0.0234472656626331,0.273817878931431
"1321","DEPTOR",116.449900962931,0.84059590906722,0.371139503496869,2.
26490551705529,0.0235184781503729,0.274441590225586
"1322","PRRX2",661.567283849302,-0.529189872193553,0.233947979514159,-
2.26199804457608,0.0236975241110632,0.276321735379757
"1323","SPPL3",251.905698078655,0.393367768418144,0.17396668314729,2.2
6116726088924,0.0237489016028944,0.276711502803187
"1324","AC009088.1",4.43372735585759,2.26876984690849,1.00353605457871
,2.2607756209227,0.0237731549761345,0.276784882142835
"1325","NCSTN",234.322570082819,0.460084632396368,0.203565516951558,2.
26013049403575,0.0238131530671152,0.27683239406454
"1326","C17orf80",46.4998457351797,0.751120121999334,0.332319388874936
,2.26023562616146,0.0238066308601136,0.27683239406454
"1327","HCFC1R1",781.944481242852,-0.611798939689526,0.271162374618548
,-2.25620881418435,0.0240575568133332,0.279252438461997

"1328","MSL1",248.011285865388,0.463017936863665,0.205212610893202,2.2
562840307345,0.0240528488472872,0.279252438461997
"1329","RHOU",226.560385114972,0.645765613517184,0.2865589569007,2.253
51746286876,0.0242265409234593,0.281002353901524
"1330","ITIH4",20.4368418058801,1.46306101983133,0.649615658366645,2.2
5219481856389,0.0243099633204217,0.281381883064617
"1331","AKR1C2",951.582631873112,-0.763463483500017,0.33899610462139,-
2.25213054985601,0.0243140232398359,0.281381883064617
"1332","SLC6A2",3.5268920677611,-2.54162106229101,1.12849768033461,-2.
2522164702522,0.0243085956936582,0.281381883064617
"1333","AC007098.1",16.1114264690205,1.00064699780088,0.44474435697223
5,2.2499374800687,0.0244529143109445,0.281930945477344
"1334","AUP1",393.445219106205,-0.412694109471321,0.183375454180003,-2
.25054171681132,0.0244145784542971,0.281930945477344
"1335","CAP2",7.90863107205962,-1.62595581228361,0.72259365429033,-2.2
5016619317047,0.0244383974556287,0.281930945477344
"1336","NUMA1",539.787738845024,0.393577138744959,0.174889567209677,2.
25043234438962,0.0244215137346817,0.281930945477344
"1337","SLC7A7",23.8337139002427,-0.837910783326315,0.372275262559663,
-2.25078286847498,0.0243992931123764,0.281930945477344
"1338","TXLNB",7.15372987951084,-1.60366213265269,0.712871890803441,-2
.24957969775647,0.0244756384226371,0.281982037582176
"1339","PTRH2",68.4058975229983,-0.617242362542249,0.274438089846173,-
2.24911331691684,0.0245052875111048,0.282112775940016
"1340","PER2",231.031222016864,-0.792804526395525,0.352676577641402,-2
.24796478319476,0.0245784356236068,0.282443840659437
"1341","HAND2",83.9611495361869,-0.885692214053058,0.394076775650485,-
2.24751182708264,0.0246073355825899,0.282443840659437
"1342","ALG10B",40.3494669957577,0.622512013752216,0.276976921686646,2
.24752304257496,0.0246066196452271,0.282443840659437
"1343","CCL3L1",23.5542550339229,-2.4411366697284,1.08604811999798,-2.
24772422582247,0.0245937802419223,0.282443840659437
"1344","METAP1D",34.059649115174,0.673351909697474,0.299717310327957,2
.24662335639099,0.0246641081965209,0.282884842149829
"1345","NGF-
AS1",10.6674883211567,1.23402143780936,0.549922901645414,2.24398990134
266,0.0248330511079997,0.284471218146417
"1346","BHMT2",244.490678796651,0.416818704119311,0.185756997743559,2.
2438923388218,0.0248393292004591,0.284471218146417
"1347","RNF19B",79.6198307887952,-0.720229449010493,0.321198988699025,
-2.24231543171319,0.0249409930955709,0.284834517014325
"1348","SAMD9L",135.050790081773,0.702879746564447,0.313468727795499,2
.24226432890936,0.0249442937344822,0.284834517014325
"1349","DCT",2.86382490255857,2.84840223807071,1.27032988712724,2.2422
5397429023,0.0249449625669373,0.284834517014325
"1350","GATD3B",209.263293334696,-0.540332396575638,0.240970767038933,
-2.24231512898964,0.024941012646831,0.284834517014325
"1351","UBE3D",42.3200398906333,0.636757831520284,0.284077123095883,2.
2414963393774,0.0249939423811558,0.285182547598457
"1352","PIK3IP1",278.396128238907,0.544028052804869,0.242783112117754,

2.2407985796846,0.0250391250023073,0.285486769164621
"1353","DDX59",99.3005694207649,-0.483655508571336,0.215922612805202,-2.23994838839633,0.0250942737183147,0.285763456525424
"1354","LINC00173",13.1340774290489,1.79188098540614,0.799999371866512,2.23985299041602,0.0251004683837447,0.285763456525424
"1355","ECSIT",152.254770763999,-0.518588845964753,0.231595266474824,-2.23920313164573,0.0251427021822419,0.286033028885061
"1356","SND1",202.917408551013,-0.383800736736062,0.171523860039964,-2.23759386388949,0.0252475520721585,0.287014023003188
"1357","TMEM161B",51.2513454348159,0.645402799951608,0.28864070077063,2.23600759777978,0.0253512734751356,0.287980752114381
"1358","SLC19A3",22.1933713832007,0.946891983477634,0.42357931938639,2.23545376306221,0.0253875738896114,0.288180744851516
"1359","IGF2BP2",63.3805149348927,-0.724689977047103,0.324302998675065,-2.23460769714684,0.0254431150766862,0.288386484490528
"1360","AC100814.1",5.57549222076976,1.67966614444718,0.751608445396981,2.23476220196013,0.0254329645510675,0.288386484490528
"1361","FLVCR2",23.4286093337167,0.984297099814608,0.440612641138421,2.23392841674142,0.0254877834562728,0.28868051578137
"1362","DENND5B",82.1555515224644,0.600977645218935,0.269173759931221,2.23267544864885,0.02557035463028,0.289403095907317
"1363","AKIRIN1",250.364786863796,-0.432833624238512,0.193960473318144,-2.23155582595716,0.0256443339633469,0.289602496736258
"1364","CD3E",11.0588555149677,1.20557522151063,0.540183791567031,2.23178710714986,0.0256290368499529,0.289602496736258
"1365","MBNL2",513.11923710281,0.450457030812313,0.201806765592441,2.23212056092329,0.0256069958588309,0.289602496736258
"1366","CDC14B",169.389302797257,0.440302396210393,0.197422882633746,2.23025006187976,0.0257308464368282,0.29036676268207
"1367","GLUL",1458.73166090881,0.489381160542855,0.21952177039074,2.22930582088408,0.0257935636151522,0.290670516276395
"1368","NR4A1",556.162337299043,-1.60510855983029,0.720262280783618,-2.22850564669858,0.0258468152126442,0.290670516276395
"1369","ZFYVE1",77.2675453258515,0.569958406399668,0.255703728898407,2.22897964317962,0.0258152592773887,0.290670516276395
"1370","ZNF99",4.90835884330413,2.10067972200329,0.942439368072285,2.22898129383128,0.0258151494448185,0.290670516276395
"1371","PFKL",223.830738081578,-0.385656728745404,0.173062301497874,-2.22842713524263,0.0258520452685656,0.290670516276395
"1372","CDO1",312.522380458317,-0.749594069410182,0.336492522827975,-2.22766931969361,0.0259025743634776,0.29102637304155
"1373","TNFSF12",523.874316330016,-0.518140762786571,0.232672701962163,-2.22690826391328,0.0259534054301231,0.291385101751892
"1374","FLYWCH1",96.5238829147142,0.427227374297351,0.191921039770214,2.22605804349991,0.0260102937915226,0.291811265499505
"1375","AGPAT2",193.445311119446,-0.615869597330571,0.276752280130996,-2.22534606413743,0.0260580153301911,0.292134040956288
"1376","FAS",139.323544842086,-0.39619533209452,0.178120353107777,-2.22431252342505,0.0261274247021473,0.292699310889245
"1377","CD58",126.143800494625,-0.433973546904316,0.195337361035171,-2

.22166176815596,0.0263061721246253,0.293490692200762
"1378","TIA1",196.940234230432,0.45248112892292,0.203668141779948,2.22
165884643756,0.0263063697253791,0.293490692200762
"1379","CAMK2A",3.16896950418093,-2.21572206563749,0.997364951443623,-
2.22157602633858,0.0263119715221877,0.293490692200762
"1380","SETX",317.347157468946,0.414338685058376,0.186515227951709,2.2
2147376173303,0.0263188899313123,0.293490692200762
"1381","RBMS2",127.135945593082,0.452300443164825,0.203504960189773,2.
22255242694352,0.0262459951257591,0.293490692200762
"1382","CPEB1",53.3138430038991,0.685125016985634,0.308440734894152,2.
22125335429754,0.0263338062889664,0.293490692200762
"1383","MN1",97.5810693517654,0.757611648170204,0.340917929243598,2.22
226988721636,0.0262650719107963,0.293490692200762
"1384","HEPH",60.5514669706174,-0.691986188145685,0.31156394271881,-2.
22100857405766,0.0263503806685602,0.293490692200762
"1385","SIGIRR",258.37121811233,-0.512355957557119,0.230844997934455,-
2.21948043986899,0.0264540564952567,0.294432693772117
"1386","DNAJC18",81.0793375597194,0.535327511893898,0.241526942871209,
2.21642979259401,0.0266620811877833,0.296300736210836
"1387","UST",191.830957741215,0.509595700594857,0.229943861898079,2.21
617440182305,0.0266795602893701,0.296300736210836
"1388","FUT11",135.693265869306,0.492917782419986,0.222377925841401,2.
21657694015697,0.0266520148096079,0.296300736210836
"1389","LPIN1",122.088547710577,0.607345701539007,0.274175768395529,2.
21516914165385,0.026748457092189,0.296852027412594
"1390","CITED4",188.678336389295,1.00831123821524,0.455469216705614,2.
21378569886303,0.0268435243182014,0.297263201314014
"1391","NUDT17",8.9821840077664,1.1536338955688,0.521449786619414,2.21
23585533477,0.0269419002762703,0.297263201314014
"1392","AC092053.4",7.97773728381756,-1.21298734057564,0.5478124772575
13,-2.21423824927859,0.0268123939646229,0.297263201314014
"1393","MYO10",8.71411888571817,2.30362245792782,1.04051440365811,2.21
392654424488,0.0268338324045547,0.297263201314014
"1394","AHSA1",298.800581585088,-0.464195356104814,0.209718423285405,-
2.21342192465891,0.0268685704928681,0.297263201314014
"1395","EHD4",164.636884186356,0.595423843129607,0.269066735257652,2.2
1292253967939,0.0269029864498843,0.297263201314014
"1396","AC015908.2",16.8434847879699,-1.04653865690703,0.4730952080111
42,-2.21211003448248,0.0269590629540702,0.297263201314014
"1397","RAD23A",575.227119745872,-0.425660293651396,0.192378052644988,
-2.21262398594347,0.0269235799570915,0.297263201314014
"1398","LSM14B",62.9043069198009,0.517758560748916,0.233988728905086,2
.21275000369328,0.0269148859012982,0.297263201314014
"1399","TNIK",15.7200330587582,0.956868364583989,0.432704086960432,2.2
1136890872835,0.0270103010497116,0.297615289979488
"1400","ANP32B",553.186971296989,-0.48509618292712,0.21946278369023,-2
.21038015999938,0.0270787895557391,0.298156815001227
"1401","LINC00622",5.2867558758931,1.66693532345263,0.754435126676906,
2.20951446255565,0.0271388775876052,0.298328786009644
"1402","TSPAN17",46.5419557840855,-0.549071426295314,0.248519327194148

,-2.20937112817133,0.0271488375188668,0.298328786009644
"1403","RGS14",14.0892264482715,-1.02213466563585,0.462821781627668,-2
.20848435879826,0.0272105269626701,0.298328786009644
"1404","ZNF727",54.4253142229987,0.695293759680053,0.314823042154587,2
.2085224604959,0.0272078738757673,0.298328786009644
"1405","SPATA20",120.33304166238,-0.420340518968349,0.19032625509734,-
2.2085261896913З,0.0272076142174537,0.298328786009644
"1406","ZNF358",369.473137513296,-0.38430498656957,0.173956375274905,-
2.20920323249004,0.0271605081621921,0.298328786009644
"1407","DDX39A",88.815140139178,-0.577123783884335,0.261390686357452,-
2.20789727410225,0.0272514349141855,0.298564939020732
"1408","MTUS1",364.3816409132,0.659193311229045,0.29860826914107,2.207
55209869163,0.0272755114378883,0.298616483533414
"1409","HSPD1",562.258549482453,-0.364799062375264,0.165312467967629,-
2.20672443440078,0.0273333170993683,0.298824881621818
"1410","WDFY1",166.156968123133,0.397596237464351,0.180173339432803,2.
20674289945454,0.0273320263126796,0.298824881621818
"1411","C9orf72",30.9067196175912,-0.726107097998371,0.329121855904765
,-2.20619531936669,0.0273703268704958,0.299017426441313
"1412","RCC1",29.5469117753671,-0.675401768769267,0.306532320945433,-2
.20336232957796,0.0275692210658172,0.300744737793846
"1413","RRS1",39.5114640895893,-0.72502717072307,0.32909251989648,-2.2
0311045341029,0.0275869645955562,0.300744737793846
"1414","GAREM1",69.3615297940744,0.712597344347392,0.323373931397292,2
.20363262204926,0.0275501911416589,0.300744737793846
"1415","TNKS2",186.197749236142,0.413626826576558,0.187770708855779,2.
20282933955504,0.0276067794175605,0.30074805987399
"1416","POPDC2",8.03710205064935,-1.24146318719244,0.56403818156569,-2
.20102685911496,0.027734122452615,0.300859604227347
"1417","BTN3A3",95.4891408533536,0.572791757845382,0.260237229104515,2
.20103695315378,0.0277334079131477,0.300859604227347
"1418","AC091182.1",4.29964854510436,2.09357809294624,0.95092270083418
3,2.20162805148061,0.0276915927642502,0.300859604227347
"1419","C8orf31",18.8403441742216,0.860256349649626,0.390720827201392,
2.20171613530655,0.0276853662443466,0.300859604227347
"1420","ZNF512B",47.6143963191396,0.673803371599992,0.305954905887825,
2.20229634705395,0.0276443820668751,0.300859604227347
"1421","LINC01278",79.1731682747373,0.5395972282161,0.245097395658777,
2.20156247179111,0.0276962292830995,0.300859604227347
"1422","DNAJC2",166.473615267235,-0.40684424686638,0.18487576203956,-2
.20063594263549,0.0277618069660195,0.300948139508573
"1423","SUSD5",4.87688106830916,2.37745511704472,1.08063576463191,2.20
005222375231,0.027803190007142,0.301184943049961
"1424","ATP1A1-
AS1",46.7583306413697,1.15652640006721,0.525978543861866,2.19880908368
564,0.0278915003349713,0.301351746161826
"1425","THNSL1",17.1296518798561,0.906497896687708,0.412436083107213,2
.19791122507597,0.0279554328259105,0.301351746161826
"1426","LUM",13068.0506249294,-0.596093857294782,0.271058267301201,-2.
19913549669526,0.0278682891893489,0.301351746161826

"1427","ZFYVE26",33.6071905910174,0.673942380220919,0.306533212962998,
2.198594970204,0.0279067349404367,0.301351746161826
"1428","TLCD2",90.0946897334861,0.582697620956026,0.265109853138428,2.
19794780940024,0.0279528253532675,0.301351746161826
"1429","HOXB7",30.0769202101403,-1.09967970905516,0.500012882825951,-2
.19930275164119,0.027856402187228,0.301351746161826
"1430","AC011043.1",15.5403754331641,1.10599418695409,0.50314321937567
2,2.19816971463209,0.0279370140053986,0.301351746161826
"1431","NLRP3",24.1661919204179,-1.20716190610093,0.549531393047797,-2
.1967114552015,0.0280410602289016,0.302063552360949
"1432","GALNT11",364.928965621602,0.328442629444897,0.149636549179604,
2.19493587125348,0.0281681982150807,0.303221211931193
"1433","CYP7B1",240.725719156747,0.524971505687864,0.239238015543712,2
.19434818707542,0.0282103877235495,0.303463452029669
"1434","GHR",174.47250524493,0.61458623343656,0.280179651866318,2.1935
4342595085,0.0282682493924566,0.303662065773323
"1435","ZFP36",16856.8464241933,-0.46675477807412,0.212761583665414,-2
.19379255424293,0.028250326352657,0.303662065773323
"1436","AASS",203.971573675722,0.684603292294337,0.312297366928925,2.1
9215198330552,0.0283685342577293,0.304527127843243
"1437","SH2D3C",7.66718275094375,-1.37258955049052,0.626335772951463,-
2.19145961282478,0.0284185492889302,0.304851730889951
"1438","CXCL14",8342.58434038536,-0.965192942573515,0.440493018122352,
-2.1911651328499,0.0284398447660252,0.304868016041918
"1439","LPP-
AS2",25.6165193498674,0.842668470102574,0.384799660349479,2.1898888095
1,0.0285323017531592,0.305052850516102
"1440","ADD1",647.057669858059,0.337269908144904,0.15396234772537,2.19
0599929968,0.0284807562299123,0.305052850516102
"1441","EPB41L4A",61.40212113368,0.537714496115054,0.245495921472224,2
.19031946799936,0.0285010759113433,0.305052850516102
"1442","ADAL",48.5219156568975,0.620689173271209,0.283441138899347,2.1
8983445974517,0.0285362445957975,0.305052850516102
"1443","DISP1",40.9990406264994,0.832726938280142,0.380427704459995,2.
18892296359481,0.0286024397047831,0.305222642664325
"1444","GUCY1A2",66.9325770869138,0.931064557833523,0.425511421442563,
2.18810708929278,0.0286618026398859,0.305222642664325
"1445","FAM20A",133.846647751792,0.57228922665823,0.261526909643802,2.
18826134349878,0.0286505709919905,0.305222642664325
"1446","GNA15",2.66940425402317,-2.49826084157734,1.14151633804338,-2.
18854584758681,0.0286298654553662,0.305222642664325
"1447","MAVS",203.305356842299,0.625071773445594,0.285719662366922,2.1
8771003811027,0.0286907304067861,0.305222642664325
"1448","AP1S2",159.916228213814,-0.63656037323593,0.290942607740916,-2
.18792420326,0.0286751239578391,0.305222642664325
"1449","RENBP",41.3461257944669,0.935542784961999,0.427436839787863,2.
1887275449311,0.0286166486954097,0.305222642664325
"1450","AC068587.4",24.4368939033476,0.847787233426194,0.3876877151006
4,2.18677868914705,0.0287586839215016,0.305734560448239
"1451","KCNE3",16.5030821545136,1.00852983912452,0.461278797901741,2.1

8637805100105,0.0287879580701905,0.305834854343203
"1452","ZNF215",13.6741406738046,-1.33000931737708,0.608392089081042,-2.18610554155302,0.0288078846667189,0.305835772821951
"1453","NDRG1",1017.82658518383,0.409860938899242,0.187625314305815,2.1844650356252,0.0289280938164717,0.306900596132768
"1454","ITM2C",318.761309008747,0.662014983554152,0.303352047070016,2.18233234272968,0.0290850136677738,0.307718246869412
"1455","GALNT8",32.3743472843316,0.900771374839723,0.412690892732494,2.18267810291516,0.0290595235823034,0.307718246869412
"1456","WASHC4",245.110331198308,0.337792557087712,0.154746172976048,2.18288149290772,0.0290445382764324,0.307718246869412
"1457","CDIP1",77.3876122498844,0.553134014540373,0.253439529030949,2.18250884798964,0.029071998967588,0.307718246869412
"1458","SYK",4.36728685520971,-1.87753794179819,0.860588120777052,-2.18169167859638,0.0291322954870406,0.308007088431229
"1459","DENND4C",175.52504834257,0.427962173465341,0.196215670045194,2.18108050884401,0.0291774622195133,0.308140289626061
"1460","GLIS2",138.876020822456,-0.550372700171129,0.252351064021517,-2.18098030339274,0.0291848733606259,0.308140289626061
"1461","TMEM123",474.242944162812,0.357221433775447,0.16383614744482,2.18035787185339,0.029230944352962,0.308415473785701
"1462","SNAPC2",164.569980141409,-0.50933825610556,0.233655213386007,-2.17987113886526,0.0292670148310722,0.308584838318042
"1463","SRPRB",162.796098520845,-0.463208275175136,0.212521187026141,-2.17958633516459,0.0292881386264891,0.30859648457097
"1464","LINC02550",10.8821714180008,1.47663491446804,0.677902838873029,2.1782397561911,0.0293881916681187,0.309338198272495
"1465","APOL1",261.044018876941,0.59898316822197,0.275002729513288,2.17809899298846,0.0293986675620632,0.309338198272495
"1466","FZD7",163.327271247032,0.493569905871513,0.226659903205323,2.17757926696194,0.0294373744997401,0.309443570749319
"1467","CELF1",303.890011763779,0.53203284977574,0.244365589734859,2.1772003593182,0.0294656215196432,0.309443570749319
"1468","RILPL2",143.436695344265,-0.49077070796505,0.225418220283669,-2.17715634231988,0.0294689044346416,0.309443570749319
"1469","ZNF317",36.1845585218135,0.610981151358806,0.280697189248807,2.17665575132367,0.0295062620996222,0.309624935511012
"1470","ZMYM2",184.738123099755,0.453466091019071,0.208406330057436,2.17587484456013,0.0295646202587997,0.310026272985984
"1471","ZNRF1",184.650567597965,-0.520469406966835,0.239276242084278,-2.17518213439476,0.0296164704331702,0.310358865892127
"1472","PNISR",1492.78724874459,0.363701380711023,0.167299591289639,2.17395259550491,0.0297086954809581,0.310902607494209
"1473","ADAM9",439.282758381553,0.447225765959398,0.205696368576775,2.17420350710991,0.0296898551153235,0.310902607494209
"1474","PTPRS",82.9332626744934,0.58897496177831,0.271089672414403,2.17262043416383,0.0298088966399721,0.311739580532679
"1475","PHC3",180.322658896222,0.453322610119792,0.208702474949855,2.17209982885307,0.0298481338924013,0.311938294204316
"1476","SLF2",105.136061625213,0.471730791439551,0.217261164167694,2.1

7126145506356,0.0299114142137358,0.312387838824348
"1477","ZBTB20",1870.67447570166,0.474117586342669,0.218398076768915,2
.17088718617394,0.0299397011847798,0.312471559758552
"1478","PLPPR4",3.10057268369902,2.43488327110948,1.12193973224б,2.170
24426636101,0.0299883462774754,0.312767495174075
"1479","TBRG1",197.962534890442,-0.344312163186747,0.158732217232317,-
2.16913849746594,0.0300721707244107,0.313429690139818
"1480","MERTK",5.4718149319369,-1.90204198305391,0.87705563922032,-2.
16866741172894,0.0301079432006537,0.313590502998701
"1481","IDH3A",77.7841852249064,-0.461084340681487,0.21269664553559,-2
.16780259754654,0.0301737091298493,0.313972685620916
"1482","KCTD2",60.2028503282659,0.550146685989028,0.253816351757265,2.
16749898964412,0.0301968266494494,0.313972685620916
"1483","NAPA-
AS1",23.3119445857517,0.834947849370967,0.385233364432942,2.1673819727
4267,0.0302057406925604,0.313972685620916
"1484","DDIT4",947.423640503744,1.48793474223182,0.686639055354069,2.1
6698239144663,0.0302361968017957,0.314077475538868
"1485","WDFY3-
AS2",180.544804626548,0.546684273588392,0.252356581459776,2.1663166873
8756,0.0302869954029707,0.314181718799996
"1486","MARCH8",70.1530147030452,0.46159668181685,0.213078582856494,2.
16632134318131,0.0302866398738006,0.314181718799996
"1487","EIF4G2",1095.62407887115,0.347654866509228,0.160505312449406,2
.16600224132029,0.0303110156673752,0.31421943948392
"1488","MRPS6",447.753565673006,-0.651341274025315,0.30091682837575,-2
.16452259430302,0.0304242644654514,0.315181476300359
"1489","TTN",8.9141742270433,1.80308607433564,0.833166177815279,2.1641
3738620989,0.0304538069743778,0.31519854914434
"1490","CALCOCO1",337.19153934225,0.497601364192039,0.229948580853468,
2.1639679720795,0.0304668075397383,0.31519854914434
"1491","SSH2",140.77276690998,0.578047528048821,0.267209740569475,2.16
32726666958,0.0305202140913786,0.31553930262817
"1492","ZNF524",164.861135986749,-0.650189798589711,0.300643414063653,
-2.1626610402051,0.0305672597075105,0.315813879618817
"1493","CABLES1",78.5308225646062,0.606057322697258,0.280319754781144,
2.16202145000601,0.0306165228673703,0.316110984595119
"1494","TRMT6",73.6817860086653,-0.54618395708218,0.252680356897346,-2
.16156081061763,0.030652044921028,0.316265911952909
"1495","TRERF1",132.35663980666,0.564051458297883,0.2611528458353,2.15
985185416517,0.0307841399219742,0.316470816178773
"1496","PPIL6",25.2994518147067,-0.743314260187019,0.344273850046609,-
2.1590784780383,0.0308440790400166,0.316470816178773
"1497","MLXIPL",115.073793539463,0.919924079463699,0.42576946962521,2.
16061541536427,0.0307250596337918,0.316470816178773
"1498","CALHM3",3.33494667802319,-1.87438025753172,0.868021830288977,-
2.15936995145354,0.0308214771652456,0.316470816178773
"1499","DDB1",303.62417635152,0.300842573293823,0.13926954691593,2.160
14613356519,0.0307613586113559,0.316470816178773
"1500","FXYD6",12.1444510707125,-1.17295015644606,0.543020944944973,-2

.16004588288011,0.0307691177797786,0.316470816178773
"1501","HELZ",226.057520360014,0.436043339668204,0.201998013432762,2.15865162363762,0.0308772045107282,0.316470816178773
"1502","ARHGAP28",107.261015126475,0.625601974478795,0.289789361361104,2.15881622272267,0.0308644274005137,0.316470816178773
"1503","TMEM221",43.3416593860569,1.14505779036415,0.530010718095414,2.16044270666619,0.0307384143832914,0.316470816178773
"1504","IFNAR2",75.8454746504685,-0.555613344215884,0.257293755676455,-2.15945133512904,0.030815168928551,0.316470816178773
"1505","PLEKHG4",68.2236835598805,-0.508788290494943,0.235880308170782,-2.15697653797608,0.0310074925720403,0.317595015281064
"1506","CMPK1",620.040541790537,-0.468230128240769,0.217269591096532,-2.15506516985497,0.0311567348183445,0.31868809174738
"1507","RBSN",117.07808141662,0.417781643910461,0.193906187559875,2.15455550525667,0.031196634077267,0.31868809174738
"1508","ANKRA2",69.6156422236344,0.51671874493808,0.239899659765201,2.15389528040104,0.0312483851757827,0.31868809174738
"1509","IQGAP2",13.4673900553054,-1.30797493220681,0.607126353333718,-2.15437021474154,0.0312111504692195,0.31868809174738
"1510","TMEM70",204.540433638568,-0.536864516243142,0.2492360592053,-2.15404030201311,0.0312370114856181,0.31868809174738
"1511","AC067930.8",4.7706592502245,1.80011142721509,0.835901345175897,2.1534974642687,0.031279603166642,0.31868809174738
"1512","PYCARD-AS1",6.80551494339031,2.08420888730252,0.967253792724823,2.15476941313526,0.0311798828932018,0.31868809174738
"1513","HSDL1",69.0373771778067,0.561653271093972,0.260791989925539,2.15364463937077,0.0312680507163444,0.31868809174738
"1514","SNTA1",142.411262200871,-0.542361990078889,0.251976389961067,-2.15243178205185,0.0313633627789001,0.319330407686093
"1515","N4BP2L2",764.278506447322,0.299867518027046,0.139359713271615,2.15175182976014,0.0314169056029501,0.31957202016981
"1516","HEXD-IT1",4.30696263717893,2.06144803753746,0.958098247202832,2.15160401718285,0.0314285554704789,0.31957202016981
"1517","SNHG15",100.850580419626,-0.612593567690819,0.285131728476947,-2.14845808624328,0.0316773826285424,0.32167776891896
"1518","IL16",74.2652769732684,-0.808347782926555,0.376227837704423,-2.14855920247353,0.0316693586481966,0.32167776891896
"1519","CSF1R",7.12665513133518,-1.61319490876932,0.751089762250201,-2.14780574819224,0.0317291901973882,0.321927100359354
"1520","SURF1",272.769716265152,-0.425642455327064,0.198192337647564,-2.14762316434233,0.0317437037006953,0.321927100359354
"1521","NECTIN3",215.727622348445,-0.345297251224298,0.160889499356443,-2.14617642919821,0.031858905373079,0.322008819401973
"1522","ANK2",1062.28184033758,-0.61461793695581,0.286241786070946,-2.14719851141326,0.0317774811776798,0.322008819401973
"1523","RANBP17",7.06207229031561,-1.39260745401743,0.64874162685372,-2.14662879083515,0.0318228459385,0.322008819401973
"1524","FGFR2",15.6163745272126,-1.48837359560299,0.693380214496025,-2

.14654754272848,0.0318293199490687,0.322008819401973
"1525","QPRT",36.657581165033,1.31952119875114,0.61476557305635,2.1463
8108668163,0.0318425870242547,0.322008819401973
"1526","LINC01915",69.2359695259361,-0.740679013228922,0.345152297746855,-2.14594837717742,0.0318770975288622,0.322008819401973
"1527","COCH",47.9006688865373,-1.00612015349442,0.4689104424541,-2.14
565525184035,0.0319004937901552,0.322034126899307
"1528","TRMT61A",61.7331853185081,-0.491664851736678,0.229220556587415
,-2.14494222968688,0.0319574662431845,0.322187274126023
"1529","ICE2",140.616005346802,0.370899401011742,0.172900448911951,2.1
4516158486448,0.0319399298725926,0.322187274126023
"1530","EVI2B",16.4481988052591,-0.90218153225315,0.420690249679931,-2
.14452684116056,0.031990697132677,0.322311500849815
"1531","C11orf80",13.7861221109448,-1.07128626625294,0.49963076033646,
-2.14415594734703,0.0320203934922556,0.322399977585317
"1532","GDPD5",97.6979161087935,0.67512374699184,0.314953572089049,2.1
4356593104763,0.0320676830260809,0.322454881831074
"1533","OGT",270.473161312337,0.509973625406741,0.237899659059081,2.14
365008938534,0.0320609341161848,0.322454881831074
"1534","SNED1",401.715812942655,0.828978796548278,0.386928689132948,2.
14245885567674,0.0321565761495183,0.32271720139637
"1535","AC005332.7",7.48116235033808,1.33316388427133,0.62220162673078
3,2.14265573569155,0.0321407521577957,0.32271720139637
"1536","CST2",3.08788087655359,2.8895991432025,1.3485907950844,2.14268
045854611,0.0321387655601257,0.32271720139637
"1537","EYA4",5.33918710796237,1.72853442613979,0.80695269387015,2.142
05174512737,0.0321893183426082,0.322835616298832
"1538","CHIC1",42.5496436708931,0.583244181086422,0.272341852617578,2.
14158850533125,0.0322266095209544,0.32299947058876
"1539","KCNA2",6.90355581314687,1.44282782365846,0.673998669169635,2.1
4069832724747,0.0322983735051974,0.323508399988705
"1540","KLF6",4686.00274702641,-0.587804102357845,0.274636057175754,-2
.14030199968127,0.0323303684927841,0.323618591114459
"1541","GOLGA8N",19.2374483932006,1.00268788497364,0.468562690352724,2
.13992258798677,0.0323610233227474,0.323715233303148
"1542","OLFML2A",290.904962681724,0.539990022408175,0.252373815759486,
2.13964361074126,0.0323835793660107,0.323730788538946
"1543","AL359258.2",10.1600756240336,-1.14604066617202,0.5360145948051
92,-2.13807735326411,0.0325104656409198,0.324788611701088
"1544","FAM89B",453.477245769145,-0.534191366882476,0.249877080743665,
-2.13781658282808,0.0325316325803421,0.32478958304791
"1545","FPR1",5.36382949266542,1.64401286221409,0.770061826769577,2.13
491021767792,0.0327683445544374,0.32694112058683
"1546","BBS2",113.194821368341,0.541315368841326,0.253639969308538,2.1
3418796066344,0.0328273978215431,0.327318458873924
"1547","SNTB1",24.5857233400203,0.906397764812982,0.424866068052805,2.
13337292141751,0.0328941465686396,0.327771990533664
"1548","YARS",92.0707920510074,-0.480766976092672,0.225570730136803,-2
.13133581560471,0.0330614864638204,0.327785407270627
"1549","CTPS1",19.5414096373357,-0.778871129398741,0.365317793327975,-

2.13203721150118,0.0330037875983258,0.327785407270627
"1550","DDR2",1626.02409281727,0.539923694997992,0.253151621750397,2.13280756909528,0.0329405150901556,0.327785407270627
"1551","CEP170",281.886822812931,0.35305324323198,0.165652699327276,2.13128578444992,0.0330656054690772,0.327785407270627
"1552","PLEC",404.775426661831,-0.589975661012856,0.276761717436135,-2.13170978442493,0.0330307119655108,0.327785407270627
"1553","AL158071.1",33.9934463084358,1.04341565549163,0.489341686240086,2.132284423812,0.0329834717496257,0.327785407270627
"1554","VWF",2.90214004316836,-2.20252725100128,1.03302156527586,-2.13212126933006,0.032996878544462,0.327785407270627
"1555","SLC23A2",68.523158478716,0.51010936460698,0.23931769228713,2.13151547523264,0.0330466989211337,0.327785407270627
"1556","RRAS2",212.394715237945,0.623825991034574,0.292781026227353,2.13069131928705,0.0331145807015596,0.328059936706003
"1557","CNIH3",35.2684217157518,-0.649524910439443,0.304980895012145,-2.12972327467783,0.0331944662840084,0.328429202675218
"1558","HNRNPAB",444.711671942042,-0.376180442310277,0.176619221842699,-2.12989525367353,0.0331802620903064,0.328429202675218
"1559","AC079848.1",5.87114480522746,-1.2878527127448,0.604985420712855,-2.12873346803518,0.0332763181970855,0.328841038681818
"1560","HMGA2",3.25562745888331,-1.93776893554729,0.91030453950639,-2.12870402315915,0.0332787557796715,0.328841038681818
"1561","ARNTL2",10.5938262735189,-1.17029344684771,0.550370847007116,-2.12637252356606,0.0334722537136857,0.330541185776083
"1562","TRIM69",144.453893978288,0.581908611999241,0.273876768753106,2.12470964459136,0.033610848199889,0.331519742181875
"1563","TPGS1",371.586971190263,-0.66516689325083,0.313068687616538,-2.12466758753453,0.0336143598462712,0.331519742181875
"1564","CYB5R3",1280.90789140626,-0.410602289299635,0.193293506678586,-2.12424253848525,0.0336498678663533,0.331657744987108
"1565","PPP1CC",711.956749519199,-0.328997384806628,0.154930797677826,-2.12351185005042,0.0337109835614305,0.332047802938946
"1566","CNST",110.350645342553,-0.530488993561775,0.250042883364902,-2.12159205022285,0.0338720105526476,0.33342084461626
"1567","CCND3",73.3493704690658,-0.58141181619632,0.274152795900359,-2.12075829570468,0.0339421479198597,0.333755724146522
"1568","AC027031.2",13.0378143433987,-1.03689078564368,0.488985047541625,-2.12049589421322,0.0339642474149223,0.333755724146522
"1569","SPG11",143.449480290474,0.4193286269745,0.197757723811714,2.12041592556833,0.0339709848320397,0.333755724146522
"1570","HLA-DOA",3.89056759995931,-1.71185354775574,0.807585649630215,-2.11971764052466,0.0340298644230051,0.334121248458996
"1571","CNBD2",24.4756236801943,-0.802154084300279,0.378675787432316,-2.11831363642085,0.0341485145203717,0.335072788880668
"1572","EDEM3",178.262933267665,0.509688711325385,0.240832098188532,2.11636536474628,0.0343137454147669,0.33647988903857
"1573","SPTSSA",182.64084265093,-0.424240162324829,0.200502285767849,-2.11588691221223,0.0343544268060856,0.336664646672479

"1574","SET",902.13614253852,-0.405522544985236,0.191696615133787,-2.1
1543925646375,0.0343925269497034,0.336823890044268
"1575","RNF126",106.584110843906,-0.457134882843009,0.21617998557122,-
2.11460317029398,0.0344637832374022,0.337307440383845
"1576","IMP4",184.495613292753,-0.436938891338639,0.206698023636164,-2
.11389970572603,0.034523834384892,0.337466649995631
"1577","SRGAP1",447.67391522949,0.514738922212207,0.243493894886972,2.
11397054719234,0.0345177829688554,0.337466649995631
"1578","TBCEL",43.2883389571284,0.613097075715505,0.290229080159992,2.
11245914908847,0.0346470862779789,0.338028376566484
"1579","ZNF676",21.6567723321122,1.01597850168778,0.480877427695006,2.
11275980774909,0.0346213313790252,0.338028376566484
"1580","SPECC1L",197.135649251629,0.441336638762523,0.208898089832459,
2.11268872356126,0.0346274190870206,0.338028376566484
"1581","PLXDC1",295.024561162615,0.56843223806878,0.269118513443796,2.
1122004235041,0.0346692622214557,0.33803078883222
"1582","AL162595.1",6.00411597300049,1.44578640856748,0.68477642327303
5,2.11132620725613,0.034744282943987,0.338139907410111
"1583","ITSN2",153.871809675561,0.517511082374456,0.245114528114962,2.
11130317878072,0.0347462610014671,0.338139907410111
"1584","MTX3",78.5459494232948,0.585262832878356,0.277193844545224,2.1
1138466598551,0.0347392619958765,0.338139907410111
"1585","NINJ1",72.7008191635632,-0.58191672779388,0.275699415299033,-2
.1106926438807,0.0347987386883979,0.338229125136803
"1586","EMP2",732.843463090254,-0.575439993271415,0.272631740958777,-2
.11068597973126,0.034799311869411,0.338229125136803
"1587","AC022034.1",26.0067140215571,0.771610135811347,0.3657087369068
43,2.1099034776626,0.0348666706576562,0.338670276110757
"1588","USP20",30.794076040825,0.674349224030021,0.319708068488836,2.1
0926557849311,0.0349216641850526,0.338990839680469
"1589","ODF3B",126.251746835643,-0.838823170558603,0.397747430918776,-
2.10893422647876,0.0349502593819064,0.339054907723151
"1590","LPL",12.4714876364037,-1.00263474534188,0.475504162825706,-2.1
0857196156533,0.0349815451909543,0.339144980577711
"1591","USP33",387.285491998181,0.426074399234194,0.202326293446438,2.
10587755044793,0.0352149902023264,0.339408599325102
"1592","NPR1",310.243086997067,-0.467714035272636,0.222080568934393,-2
.1060556424043,0.0351995192994651,0.339408599325102
"1593","KCNE4",107.14938596141,-1.05851018269453,0.502425273313994,-2.
10680122779773,0.0351348130347668,0.339408599325102
"1594","RASSF1",170.733856237152,-0.521640492201185,0.247619596107857,
-2.10662039838709,0.0351504971358787,0.339408599325102
"1595","GIMAP6",2.72426343110865,-1.99488123782065,0.94695027732517,-2
.1066377882644,0.0351489885784455,0.339408599325102
"1596","PHF19",107.821775990993,-0.421022155789413,0.199942394074052,-
2.10571728791784,0.0352289172183045,0.339408599325102
"1597","CREB3L1",220.544348686135,0.936114320026115,0.444082768219741,
2.10797262811807,0.0350333571390434,0.339408599325102
"1598","PCOTH",4.86427438912002,-1.38095561552199,0.65565383411036,-2.
10622670634691,0.0351846643850383,0.339408599325102

"1599","RBM26",248.186058537944,0.444104632774363,0.210842591458205,2.10633264229442,0.0351754677628941,0.339408599325102
"1600","DACT1",35.8081903711863,-0.847712617181696,0.402415543233509,-2.10656032411202,0.0351557089546504,0.339408599325102
"1601","SENP3",26.8612917265007,-0.79234019427453,0.376447504903232,-2.10478269600487,0.0353102281106883,0.339979491771556
"1602","TRIM7",24.4451998957565,-0.89782488837965,0.426671302426195,-2.10425421928852,0.0353562772220933,0.340049168934123
"1603","TPST2",37.9035488038851,0.651286396726531,0.309518322277832,2.104193354156,0.0353615840286344,0.340049168934123
"1604","FAM184B",4.5018869841868,2.61040998556421,1.24116193520472,2.10319855251897,0.0354484167751115,0.340671661214678
"1605","BASP1",2404.11588923246,-0.502744531989928,0.239123112784123,-2.10245060018016,0.0355138226696813,0.341087586575163
"1606","ZNF697",17.5295697066475,0.929814614865195,0.442630065204162,2.10065851364236,0.0356709535687905,0.341585600884115
"1607","TMEM173",325.370664837684,-0.478458810001777,0.227785828798015,-2.10047662985234,0.0356869343137428,0.341585600884115
"1608","MYLIP",331.001792812102,0.677573592929595,0.322622444522182,2.10020599754959,0.0357107240187394,0.341585600884115
"1609","VEGFB",1259.55017267425,-0.486454009150218,0.23154834178237,-2.10087451028879,0.0356519835159984,0.341585600884115
"1610","MRPL49",75.5870103782816,-0.461813966892249,0.21990182874747,-2.10009152503495,0.035720790699007,0.341585600884115
"1611","ZNF546",32.4136892001082,0.645638296124066,0.307247355888419,2.10136322982235,0.0356090931512735,0.341585600884115
"1612","HM13",592.040934978511,-0.42068725237833,0.200267215458004,-2.10062965831044,0.0356734884598222,0.341585600884115
"1613","POLR2J",471.933922886257,-0.477714133367703,0.227589752317286,-2.09901425043828,0.0358156445462613,0.342280322802615
"1614","IQSEC1",208.546106491144,0.677934799977153,0.323127469629529,2.09804137281927,0.0359014907692426,0.342888153784309
"1615","PTP4A2",987.851849844729,-0.298779931250289,0.142438142648095,-2.09761181728162,0.0359394503338081,0.343038159068515
"1616","DVL1",108.096259439365,-0.423954205624096,0.202288004872885,-2.09579508132726,0.0361003728809401,0.343298734089878
"1617","BCL9",42.2486216100957,0.640938704941831,0.305752582355148,2.09626587616959,0.0360586120343969,0.343298734089878
"1618","ILF2",383.363219608886,-0.533181071547095,0.254355773978545,-2.09620195841148,0.0360642793046072,0.343298734089878
"1619","LMO7DN",11.8799619270386,1.85922603916642,0.887069716659573,2.09591873586631,0.0360894003796402,0.343298734089878
"1620","RNASE6",7.54030536369906,-1.69368696378359,0.807883369423816,-2.09644984398123,0.036042304759533,0.343298734089878
"1621","STAT5B",131.995122344223,0.448817275406351,0.214098137101123,2.09631564983849,0.0360541993762008,0.343298734089878
"1622","NDRG2",162.523721358457,-0.459285016041688,0.219253709143806,-2.09476509125074,0.0361918797392743,0.343956736239774
"1623","SH3RF3-AS1",4.20072483196645,1.80330551778371,0.861161480948057,2.09403875774

662,0.0362565278119125,0.344146783387088
"1624","COBLL1",196.1973051732,-0.485790360290422,0.231966510411849,-2.09422627183518,0.0362398285062896,0.344146783387088
"1625","EFEMP1",6112.96733136435,0.607675723234432,0.290242544193524,2.093682457625,0.0362882766445394,0.344236175061892
"1626","CCL26",130.998009700818,-1.82263689809249,0.870844064566373,-2.09295437869242,0.0363532272580103,0.344640220284273
"1627","ZNF562",45.5844352448079,0.702728925367119,0.335932618958545,2.09187463707964,0.036449731466542,0.345342723144895
"1628","FAM131C",3.7088865230727,-2.40188662889282,1.14864999443243,-2.09105179169886,0.0365234215225119,0.345828343224521
"1629","XPO1",261.533075745331,0.338800295448241,0.162182880051877,2.08900159708515,0.0367075794050134,0.347331511837553
"1630","IL17RE",20.1152534306964,-0.834678397601078,0.399671686358752,-2.08841012783641,0.0367608546778837,0.347331511837553
"1631","PLSCR4",710.770104401072,0.46076797622384,0.220660817568071,2.08812774874134,0.0367863125638496,0.347331511837553
"1632","MDGA1",43.2268627093937,0.599294550418052,0.286944070590788,2.08854132857378,0.0367490313831817,0.347331511837553
"1633","ACIN1",249.819683003165,0.413154334571137,0.197867696960088,2.088033271315,0.0367948335277797,0.347331511837553
"1634","KCTD10",149.581822947537,0.431732902022408,0.206801452597984,2.08766861450284,0.0368277378675398,0.347429363052708
"1635","ZNF135",21.7615241224118,0.89872851247862,0.430647566548582,2.08692346663297,0.0368950532668421,0.347851526671787
"1636","TRIB2",10.6603152381428,-1.0778185455512,0.516527198106748,-2.08666368296147,0.0369185463066023,0.347860263640754
"1637","GPRC5B",290.490083085636,0.539671825088117,0.258675077070747,2.08629231389199,0.0369521524810111,0.347964221438477
"1638","EIF3G",1277.7836292564,-0.490514579394489,0.235168616373266,-2.08579948701969,0.0369967899167105,0.348171866035466
"1639","USP46",101.189296021315,0.419132873756692,0.200998807041315,2.08525055410175,0.0370465631635803,0.348215104369872
"1640","PCDHGA6",21.5978906988875,0.888961467580118,0.426286207896645,2.08536295829597,0.0370363665285455,0.348215104369872
"1641","CD8B",5.36224212339518,-1.44382321309155,0.692543431972623,-2.08481251346071,0.0370863223977192,0.348376392297892
"1642","MBTPS1",381.36799186639,0.31375637673346,0.15056207711035,2.08390042668913,0.0371692255383456,0.348942516244578
"1643","IRF4",113.890378759749,0.9766109642864,0.46879047746227,2.08325683058483,0.0372278194102603,0.349279875964189
"1644","EPB41L4A-AS1",585.291641782332,-0.528847449893448,0.253910386942922,-2.08281140547563,0.0372684175495095,0.349448087911003
"1645","PHF21A",141.353883325993,0.480227806487909,0.230730941626538,2.08133249534086,0.037403482779655,0.349879812037772
"1646","STRAP",394.243204397386,-0.473184998272653,0.227279593524375,-2.08195109351911,0.0373469371263126,0.349879812037772
"1647","TM6SF1",4.27742850260471,-1.7206018860901,0.826690527653562,-2.08131317407709,0.0374052500965455,0.349879812037772

"1648","REEP6",8.53753157036763,1.49138896578071,0.716518652721853,2.0
8143774082551,0.0373938572181717,0.349879812037772
"1649","PARVA",577.891183476419,0.351215814760885,0.168813328950049,2.
08049812740088,0.037479867215607,0.350152820077928
"1650","FXYD7",6.04035876977208,-1.33889078223537,0.64347009390506,-2.
08073505655868,0.0374581634204352,0.350152820077928
"1651","GFRA1",352.185304802318,0.645998763725417,0.310543242204435,2.
08022161145644,0.0375052108743195,0.350177362584879
"1652","DBT",108.115261017481,0.5460912672462,0.262644889130496,2.079
20005241333,0.0375989668527461,0.350627993971616
"1653","TLR3",116.471256327417,0.407920083028163,0.196190728568951,2.0
7920163202208,0.0375988217266422,0.350627993971616
"1654","USP10",105.952570540068,0.437912543789797,0.21067728899355,2.0
7859397603699,0.0376546851345401,0.350821879948847
"1655","TMEM35A",49.2375701775618,0.653790934907093,0.314552600666929,
2.07847887291631,0.0376652748177322,0.350821879948847
"1656","LBX2",15.1224878652798,1.03379095370943,0.497652054433465,2.07
73368551373,0.0377704797559419,0.351165226440195
"1657","CCDC36",32.803705053196,-0.73970299293136,0.356079106113387,-2
.07735579041758,0.0377687333644776,0.351165226440195
"1658","CDKL5",70.4202617240673,0.5372606222614,0.258614665915572,2.07
745612708908,0.0377594805096284,0.351165226440195
"1659","CCL28",17.6952920717975,-0.910772110791201,0.438728741788273,-
2.07593445343668,0.0379000136587633,0.351310108568753
"1660","SNX13",154.317717324945,0.370480088094901,0.17844114126774,2.
07616872043029,0.0378783491577637,0.351310108568753
"1661","RMRP",7.92019772798005,1.33582406332224,0.643413232080286,2.07
615261346622,0.0378798383574004,0.351310108568753
"1662","DDX21",550.718171718474,-0.392428038195562,0.189006283306917,-
2.07626980082096,0.0378690047170807,0.351310108568753
"1663","KCNMB4",47.2174950927157,0.647583383647264,0.311836389983271,2
.07667675886706,0.0378314030597051,0.351310108568753
"1664","GZMH",13.7224210639829,1.03911121339252,0.500724482806824,2.07
521551086888,0.037966565714999,0.351715511115811
"1665","HCST",31.1779004096032,-0.945886822765303,0.456029574085611,-2
.07417868602472,0.0380627189443319,0.352394481998124
"1666","AC087071.1",4.72143443643784,1.68535932335932,0.81264885821579
,2.07390843698421,0.0380878153394741,0.352415170142853
"1667","LINC01985",18.9248606956573,-0.821165542706931,0.3961161416872
96,-2.07304236381051,0.038168337145788,0.352674639243947
"1668","TFEB",56.4925309034388,-0.56319920841983,0.27166196218858,-2.0
7316182170867,0.0381572221352172,0.352674639243947
"1669","LRRC8A",139.646678405023,0.476905656477846,0.230070355943651,2
.07286877321409,0.0381844938630002,0.352674639243947
"1670","MFSD13A",4.52208249720904,-1.54559661740293,0.745887659175216,
-2.07215737972125,0.0382507666395841,0.353075190268975
"1671","AC010864.1",2.87906218163939,-1.86048756464393,0.8981294676747
96,-2.07151377569275,0.0383108084432921,0.353349703518755
"1672","ZSWIM8",119.88386428133,0.468946592741486,0.226396891966829,2.
07134730811673,0.0383263512347298,0.353349703518755

"1673","STK38",236.082021467737,0.420283161281531,0.202970692601717,2.07065934443175,0.038390642088405,0.353730871364473
"1674","SGCD",231.956145236042,0.595344310178442,0.287560529832575,2.07032693438514,0.0384217389539979,0.353805917548314
"1675","MAN2B2",219.386444577648,0.408175431593508,0.197217660439482,2.06966977847686,0.0384832787098327,0.354161039589296
"1676","GREM2",7.74853483650988,1.47700140330847,0.713818168870545,2.06915635902836,0.0385314164557775,0.354392472950961
"1677","NAP1L5",46.6041532594249,0.549220788428675,0.265506120384193,2.06858051947707,0.0385854675316118,0.354677985688608
"1678","DNAJC13",157.829827870532,0.357202936831658,0.172758762783759,2.06764004948778,0.038673883132318,0.355043969681313
"1679","DYNLT1",620.752115300771,-0.515442860329949,0.249270777441773,-2.06780299568147,0.0386585518927316,0.355043969681313
"1680","NUDT2",175.478432274918,-0.535882869051603,0.259203391935427,-2.06742228583607,0.0386943800885245,0.355043969681313
"1681","TKT",391.674455856655,-0.390868566951374,0.18924225108716,-2.06544027407151,0.03888136122098,0.356396693915375
"1682","PARD6A",22.7062786716271,-0.960900833221734,0.465244018649736,-2.06536955813108,0.038888046653627,0.356396693915375
"1683","GJA1",643.638297371755,0.568197362156189,0.275175051622532,2.06485783796856,0.0389364534056253,0.35641652568154
"1684","LAMA2",1033.3338760006,0.569601409866334,0.275828376802175,2.06505732466698,0.0389175766499645,0.35641652568154
"1685","LNP1",9.25550985769551,1.03335476779529,0.500877726423621,2.06308788209384,0.0391042792808866,0.357607970254918
"1686","SMAD5",397.287377265996,0.329099668203842,0.159525112972791,2.06299598897619,0.0391130092669343,0.357607970254918
"1687","LIMD1",44.3865194591929,0.629503750296259,0.305239914266197,2.06232448927788,0.0391768530138778,0.357641768397511
"1688","FAM184A",61.251106094694,-0.809502425686257,0.392538340958336,-2.06222511592104,0.0391863085840673,0.357641768397511
"1689","SMIM25",4.01220633189933,-1.70027109882655,0.824464054688299,-2.06227438195515,0.0391816205819006,0.357641768397511
"1690","ADAMTS3",20.6278777575587,-1.10730575937655,0.537037071669095,-2.06187955690857,0.0392192042915034,0.357730197723979
"1691","ZNF593",163.484735690477,-0.500222707884123,0.242885046078736,-2.05950393389787,0.0394459885535986,0.357863350298482
"1692","AL359541.1",13.9524229781541,0.879966457589974,0.427288073200539,2.05942199836919,0.0394538301896789,0.357863350298482
"1693","SLC30A6",65.2728904013075,0.462835531717445,0.224777975364916,2.05907865735535,0.0394867040186619,0.357863350298482
"1694","PCBP1",1178.6555093175,-0.240843102866374,0.116915531716698,-2.05997526017304,0.0394009059854447,0.357863350298482
"1695","PPP1R11",121.770895957481,-0.423022453799964,0.205455000404718,-2.05895428666457,0.0394986178590409,0.357863350298482
"1696","ADGRA2",185.030303685253,0.384512046513415,0.186776788841806,2.05867147035643,0.0395257210379201,0.357863350298482
"1697","EIF4EBP2",516.018610114844,0.362028057102266,0.175713382518887,2.06033286658375,0.0393667299646296,0.357863350298482

"1698","NEK9",98.5141560819944,0.52133191551018,0.253189029072933,2.05906202736772,0.0394882968782107,0.357863350298482
"1699","SLC30A4",70.8806240325296,0.466217044420622,0.226251864488127,2.06061083949691,0.0393401818109639,0.357863350298482
"1700","MFAP4",4909.57103990524,-0.423495798786745,0.205671111702094,-2.05909228224604,0.0394853990350551,0.357863350298482
"1701","LDLR",103.146910623946,-0.997258753221014,0.484442850808189,-2.058568418457,0.0395356007498096,0.357863350298482
"1702","TSPAN6",125.471656256765,0.514193133362402,0.249598259298948,2.06008301022061,0.039390605790719,0.357863350298482
"1703","STK26",13.7496516754441,-1.10411500464989,0.535973332549189,-2.06001854494982,0.0393967679711826,0.357863350298482
"1704","ZNF445",67.2602592095001,0.617995949231244,0.300321871164182,2.05777869868623,0.039611381756875,0.358226463978706
"1705","PPP1R1A",5.55397864840002,-1.46278246230463,0.7108939796723,-2.05766612762557,0.0396221940372166,0.358226463978706
"1706","AC099063.4",5.47468647695681,1.63846108411101,0.796625187806659,2.05675279816619,0.0397100105926853,0.358800409375094
"1707","PRRC1",216.780666169508,0.391611375154643,0.190424112070405,2.05652199659387,0.0397322282713776,0.358800409375094
"1708","KAT14",32.1371067362573,0.631520149354418,0.307184584739337,2.05583281430055,0.0397986338961001,0.359189661304674
"1709","KCND3",13.6420564713606,1.19102310029984,0.579796952790366,2.05420724370463,0.0399556376180115,0.359196497268971
"1710","PIK3R4",56.8535062238302,0.504565106722518,0.245540686155121,2.05491446091243,0.0398872674622676,0.359196497268971
"1711","WDR46",182.758330248505,-0.48947283459131,0.238162030845868,-2.05520935832162,0.039858787622418,0.359196497268971
"1712","SQLE",61.9640525435641,-0.648181781027074,0.315393491433159,-2.05515268587729,0.0398642594476046,0.359196497268971
"1713","BDKRB1",22.0348698643799,-0.933244444942214,0.454321841522679,-2.05414831436324,0.039961339091206,0.359196497268971
"1714","FBXL20",157.548208344364,0.493275099492378,0.240131142070609,2.05419045292898,0.0399572620721757,0.359196497268971
"1715","UXT",688.7532431144,-0.499958795323074,0.243391249344614,-2.05413627921844,0.0399625035884713,0.359196497268971
"1716","WNT2",97.4544746599652,-0.978779835908963,0.476636687179892,-2.05351342486893,0.0400228090782375,0.359528905560042
"1717","AVEN",106.931642574176,-0.471731353346454,0.229780796708168,-2.05296247599652,0.0400762169241182,0.359799000515598
"1718","KIAA0040",51.1145085619268,1.00555696404041,0.489879983732062,2.0526598298215,0.0401055805363575,0.359853040726397
"1719","POC1B-AS1",22.7235900742769,0.735750844674004,0.358498108773942,2.0523144381159,0.0401391137398601,0.35994440855145
"1720","C19orf18",3.47517389182437,2.10997696528595,1.028708532954,2.05109309167196,0.0402578819582457,0.360799564178115
"1721","PSMC5",762.712533992668,-0.503379038479122,0.24549372963831,-2.05047615358958,0.0403179884937908,0.361128293220096
"1722","KANSL1L",92.692992921124,0.476013092266303,0.232176845871871,2

.05021775741151,0.040343185906485,0.361130165697708
"1723","SLC25A6",2442.22127736023,-0.525108661470441,0.256151361879311,-2.04999363508305,0.0403650519297536,0.361130165697708
"1724","TNFRSF11B",51.845291265471,0.871643168481958,0.425458629353456,2.0487142775939,0.0404900622524772,0.361308531534289
"1725","PTPRD-AS1",41.4901100357824,0.882420968735415,0.430649745992143,2.04904560364356,0.0404576558149796,0.361308531534289
"1726","CLCF1",20.4891846956637,-1.08589905000882,0.529956185970767,-2.04903552171899,0.0404586415863841,0.361308531534289
"1727","ZSWIM1",8.71490424889744,1.1868140097083,0.579332012682191,2.04859041745957,0.0405021824515895,0.361308531534289
"1728","MT-ND3",40921.3987507276,-0.785568306577897,0.383373720157541,-2.04909273972947,0.0404530473018404,0.361308531534289
"1729","CAMKK1",29.0479373982943,-1.16523020276866,0.568996365001866,-2.04786932648478,0.0405728051231816,0.361729202414022
"1730","FNDC10",24.9533219201428,1.0965161244485,0.535539199304833,2.04749927899182,0.0406090875865049,0.361843401818482
"1731","RPUSD1",41.6383582532214,-0.640243324783593,0.312789675826134,-2.04688125684646,0.0406697448259359,0.362174532924207
"1732","ITGB3BP",91.1193797047118,-0.583943475517713,0.285468639682642,-2.04556085798737,0.0407995958347141,0.363121114198682
"1733","TBC1D5",264.982520671054,0.39895975010153,0.195144402791371,2.04443347795149,0.040910742848636,0.363900231397418
"1734","ZGRF1",7.91224697589131,1.2069707583128,0.590851670571418,2.04276439998135,0.0410757662588015,0.365048370202802
"1735","CDC25B",62.4831384288123,0.551274129837632,0.269881948214672,2.04264914153922,0.0410871827636628,0.365048370202802
"1736","CHIC2",254.412383429999,-0.430297297614836,0.210716588027537,-2.04206655794276,0.0411449296012431,0.365318105632605
"1737","MRC2",1568.42900864125,0.509687805845785,0.249618777406065,2.04186484343145,0.0411649399600282,0.365318105632605
"1738","ZNF704",321.5230379751,0.460174512993631,0.22542762548453,2.04134037256765,0.0412170067832233,0.365569712061788
"1739","LIMCH1",167.475718710474,0.45985321562814,0.225379994309985,2.04034620302485,0.0413158560244258,0.365605006094445
"1740","ENPP6",4.33866085650693,2.53493080447455,1.24198445465822,2.04103263528538,0.0412475833501002,0.365605006094445
"1741","TCF7",83.6650256286933,0.881948717792691,0.432230398281165,2.04045972078758,0.0413045589267475,0.365605006094445
"1742","ZNF462",124.196703111177,0.583625707838514,0.286028855596951,2.04044346022526,0.041306176990123,0.365605006094445
"1743","NBPF11",50.2636234907771,0.61126076246111,0.29968457561576,2.03968042467704,0.0413821658675416,0.365981690675935
"1744","SOCS7",39.6562421367714,0.601330744593024,0.294876512773976,2.03926294073461,0.0414237921759199,0.366139768573283
"1745","ZNF610",3.04252546264499,2.24948803065188,1.10341551224002,2.03865905970927,0.0414840664219844,0.366462397647501
"1746","IQCE",55.0674848220836,-0.51184402544711,0.251107304955343,-2.

03834781125996,0.0415151615763077,0.366476756746056
"1747","CREBL2",285.664330247869,0.315404549628796,0.154753160567373,2
.03811378373421,0.0415385550013658,0.366476756746056
"1748","TBCD",124.780875899054,0.374090295914865,0.183585338374122,2.0
3769156746341,0.0415807880343076,0.366476756746056
"1749","RALGAPA2",44.0203597220007,0.671830445746722,0.329687383216275
,2.03778027291387,0.0415719120774556,0.366476756746056
"1750","UBE3B",76.3756884625833,0.485317007574431,0.238297463335857,2.
03660165232403,0.041689977082454,0.367019415605956
"1751","PLCXD1",8.38738334514442,1.29870066967385,0.637647725785363,2.
03670556195319,0.0416795568300188,0.367019415605956
"1752","LY86",3.48579082062961,-1.74862981842048,0.859258809123626,-2.
03504438924977,0.0418464067341432,0.367837504025439
"1753","AGTPBP1",15.886100349947,-0.895384412665687,0.440000115093122,
-2.03496404194391,0.0418544912137931,0.367837504025439
"1754","MYH10",213.630969968524,0.520617960276385,0.255791192373269,2.
03532402912709,0.0418182799049089,0.367837504025439
"1755","MYO9A",355.054207418078,0.397000995640376,0.195122461753718,2.
034624779086,0.041888642140256,0.367927873841622
"1756","CLIC3",40.0770026848416,-1.08348361780373,0.53270811425211,-2.
03391611431501,0.0419600539446345,0.368345234371606
"1757","ZMYM4",202.137524890927,0.339405521206295,0.166983176297931,2.
03257315336204,0.0420956661164287,0.369115297602246
"1758","AL591846.2",12.9665440116493,-1.24360514890041,0.6118252799341
76,-2.03261484885719,0.0420914501343776,0.369115297602246
"1759","PRRT2",52.5673191859433,-0.612979707739899,0.301686679049606,-
2.03184214056434,0.0421696395278539,0.369291016189409
"1760","HSF4",30.9983747964837,0.692406247891068,0.340767205968272,2.0
3190399711038,0.0421633758188989,0.369291016189409
"1761","TLE2",126.510112047622,-0.518296838977647,0.255109392774266,-2
.03166505686547,0.0421875757061011,0.369291016189409
"1762","CCT3",415.559515502034,-0.517855729963205,0.255349071133649,-2
.02803060008846,0.0425571250218928,0.371537810521445
"1763","GPR1",43.4162799791012,1.21214233210222,0.597855510156149,2.02
748375069025,0.0426129645800787,0.371537810521445
"1764","ANXA3",25.3119439826204,1.21308502859647,0.598173816682359,2.0
2798082223756,0.0425622053466571,0.371537810521445
"1765","NAV3",135.949300850038,0.523479985959327,0.258125401684112,2.0
2800647493016,0.0425595871701943,0.371537810521445
"1766","UNC119B",38.0830896304376,0.748799292610267,0.369259726202946,
2.0278390506056,0.0425766773623463,0.371537810521445
"1767","GANC",55.8429066411621,0.537228234111613,0.26490791651501,2.02
798104782638,0.0425621823219178,0.371537810521445
"1768","MED13",289.928002815681,0.444514725326156,0.219243043017736,2.
02749751694605,0.0426115581285609,0.371537810521445
"1769","TMTC1",81.7286079245632,0.555224399135141,0.274088569321581,2.
02571161763302,0.0427943454699773,0.372731848311712
"1770","KDM6A",82.9587308837023,0.501244598567856,0.247445920217951,2.
02567331935139,0.0427982725599566,0.372731848311712
"1771","CCN4",2.48762494865831,-2.01845395459778,0.99701688101711,-2.0

2449325886904,0.0429194248846627,0.373547350198373
"1772","MXRA5",339.587801994727,0.713881774741524,0.352657950209534,2.02428946892411,0.042940376552158,0.373547350198373
"1773","ZNF529",34.9781896635557,0.703539151068758,0.347633334645009,2.02379657229128,0.04299108705706,0.37377556110874
"1774","IL7R",27.7132394734464,0.935320873500885,0.462676300003555,2.02154481112108,0.0432233984931067,0.375585506071725
"1775","DDI2",143.346899785035,0.582568153372314,0.28821965278386,2.02126450346254,0.0432523915981227,0.375625699428204
"1776","AFAP1L2",35.6125730131141,-0.786064200397036,0.388959604754324,-2.0209404544555,0.0432859294868415,0.375705294504314
"1777","CNN2",223.225957541501,0.49773636770288,0.24632199366036,2.02067367313201,0.0433135568685454,0.375733527928321
"1778","CXCL8",298.494388253913,-1.97846324481415,0.979751221804333,-2.01935266910978,0.0434505772404683,0.375880839778945
"1779","CLTB",835.407319243914,-0.503889149932598,0.24962612532616,-2.01857537657254,0.0435313724468942,0.375880839778945
"1780","CBWD1",100.201530937098,-0.532447217072068,0.263797151385159,-2.01839638630011,0.0435499954489261,0.375880839778945
"1781","RNF38",109.723287081243,0.44822448334293,0.222065708533749,2.01843177995584,0.0435463123903957,0.375880839778945
"1782","KLHL33",11.2514785538679,1.23642147380622,0.612541840361878,2.01850941819054,0.0435382342888351,0.375880839778945
"1783","AL121839.2",22.0853658581022,1.13915769922554,0.56399484679573,2.01980160935429,0.0434039701560503,0.375880839778945
"1784","HSD3B7",429.785994825978,-0.647408129911054,0.320625073558182,-2.01920617974868,0.0434657942942541,0.375880839778945
"1785","TAF9B",43.66670707484,0.595263075764743,0.294682507889317,2.02001496467623,0.0433818353154878,0.375880839778945
"1786","AMOT",94.378418901475,0.487870168962503,0.241693784467886,2.01854660862132,0.0435343651480305,0.375880839778945
"1787","SFMBT2",33.6438397608982,1.04970064670542,0.520159255480766,2.0180370447032,0.0435874033809457,0.375993185851862
"1788","KHDRBS3",350.12852440928,-0.54065897768009,0.268125604788173,-2.01643919127838,0.04375407083924,0.376377791287324
"1789","MPP7",17.1337102665311,0.905521355010943,0.449041471682115,2.01656508833995,0.0437409193788223,0.376377791287324
"1790","SLC3A2",407.196510242183,-0.589280165655225,0.292115824095775,-2.01728258809431,0.043666031620726,0.376377791287324
"1791","BRCA1",43.1598432406233,0.580104581758181,0.287624159993448,2.01688405372969,0.0437076145544199,0.376377791287324
"1792","ADGRG2",17.8025415465514,1.06526511693952,0.528260451913296,2.01655284449414,0.0437421982490355,0.376377791287324
"1793","C15orf61",547.064040000482,-0.433385520827988,0.214951903694875,-2.01619763946441,0.0437793131776255,0.376384892712269
"1794","RNF213",452.191406675621,0.493011629383064,0.244567473434924,2.01585117783149,0.0438155401356877,0.376486371901687
"1795","SLC30A1",110.309780362006,0.437927688645727,0.217331012694974,2.01502621837207,0.0439019020184136,0.377018283907435
"1796","IFFO2",22.630374528956,0.838859803661864,0.416434513257629,2.0

1438588050674,0.0439690356019411,0.377036986481915
"1797","TCF4",2689.22579996989,0.460275418846839,0.228490892462715,2.01441472737014,0.0439660094082528,0.377036986481915
"1798","AF165147.1",10.6563258916465,1.07070823468505,0.531552028800772,2.01430561200315,0.0439774571323051,0.377036986481915
"1799","S100A8",25.6755942200715,1.00191952687094,0.497651888575333,2.01329393070166,0.0440837164923938,0.377711812062052
"1800","TARBP1",65.3164900296008,0.576952403652358,0.286633580312613,2.01285698285285,0.04412967716664,0.377711812062052
"1801","AC079781.5",18.4338723580802,1.13530866388731,0.56397563227038,2.01304559794,0.0441098325937128,0.377711812062052
"1802","ZSCAN30",61.8345560234234,0.582376894488503,0.289431524044375,2.01214051030327,0.0442051273823977,0.378147635182942
"1803","KIF22",110.742610315395,-0.457573459806261,0.227451651726433,-2.01173944586961,0.0442474101461596,0.378299405104298
"1804","ACE",389.522227504168,0.525650728506944,0.261553918157146,2.00972224851598,0.0444605938539032,0.379911338280442
"1805","FASN",44.6439216236636,0.579914700042563,0.288824851735398,2.00784211108621,0.0446600723268653,0.380844522231312
"1806","KLHL22",72.015634635379,0.432881052475246,0.215603807438422,2.00776163286857,0.0446686277128909,0.380844522231312
"1807","LGALS1",11826.3766300394,-0.557892613037017,0.277839468635237,-2.00796746328885,0.0446467492820531,0.380844522231312
"1808","ALG13",173.116861287107,-0.346206590229245,0.172389883527468,-2.00827672218992,0.0446138940736514,0.380844522231312
"1809","TTN-AS1",62.4807818460807,0.610012180825713,0.303978148456468,2.00676326217268,0.0447748763682897,0.381458372585414
"1810","E2F4",111.49516928565,-0.474085452465936,0.236260677843822,-2.00662021624829,0.0447901170534934,0.381458372585414
"1811","CKAP2",43.2192914680236,0.591510112304487,0.294859257813485,2.00607610793977,0.0448481285110128,0.381741524570548
"1812","ATP9B",79.1776086690659,0.482443687723189,0.240549736638674,2.00558809360852,0.0449002132632148,0.381973944510186
"1813","DYNC1I1",4.73509822756045,-1.33017382975387,0.663375738004804,-2.00515899745527,0.0449460519383123,0.382133322473801
"1814","PRSS23",1117.9717474547,-0.650659288388371,0.324557784939391,-2.00475637492374,0.0449890984191994,0.382133322473801
"1815","APAF1",64.5760315913277,0.524800664575563,0.261813185008113,2.00448523843175,0.0450181066242247,0.382133322473801
"1816","CD93",7.03934322573158,-1.46335246454097,0.729973395511172,-2.00466547622087,0.0449988216838556,0.382133322473801
"1817","NCF2",9.21457734261307,-1.3591336603794,0.678214249630257,-2.00398865273084,0.0450712759629275,0.382374088590274
"1818","RPL22L1",518.29199066487,-0.469473740864619,0.234495764527366,-2.00205637748238,0.0452786684333893,0.38249721770777
"1819","FAM198B-AS1",12.584956805631,1.13462469550637,0.566785030220304,2.00186073204043,0.0452997119744708,0.38249721770777
"1820","ASL",72.0135083872157,-0.495564728961451,0.247489492250081,-2.

0023667447695,0.0452453023644021,0.38249721770777
"1821","MINDY3",91.4649423365329,0.433083333080934,0.21629662323606,2.00226580795152,0.0452561513129791,0.38249721770777
"1822","NPM3",81.6601856582018,-0.543954276107478,0.271597909999534,-2.00279256975288,0.0451995577323346,0.38249721770777
"1823","PPP1R14B",1412.95294721979,-0.530833002289693,0.265181366362808,-2.00177338841913,0.0453091092789094,0.38249721770777
"1824","HOXC9",324.535110918127,-0.430633528913825,0.215006052288913,-2.00289026438737,0.0451890682988374,0.38249721770777
"1825","PYGL",101.179690638711,-0.407382652222862,0.20346295124888,-2.00224487909124,0.0452584010751253,0.38249721770777
"1826","AC006942.1",11.0745128681252,-1.18279042190007,0.590748969567116,-2.00218787138434,0.0452645296357175,0.38249721770777
"1827","SH3GL2",3.03314442113434,-1.90938113810295,0.954086141664119,-2.00126702896303,0.0453636209000681,0.382747792104296
"1828","NUP133",73.9418710937474,0.443651004528117,0.221731910537886,2.00084418815448,0.0454091837488407,0.382786345791886
"1829","SYS1",241.285927096707,-0.332843767771258,0.166358354861987,-2.00076376114316,0.045417854456916,0.382786345791886
"1830","ARL2",868.928141709033,-0.503316926620314,0.251649686266243,-2.00006975604892,0.045492732028767,0.383207903947237
"1831","GREB1L",9.34094444137943,1.2032773947669,0.601698181557636,1.99980227902956,0.0455216184107645,0.383241806554852
"1832","AL513218.2",2.57123078277527,-2.00603941364137,1.00376069458226,-1.99852357685339,0.0456599264772489,0.384196379174013
"1833","WDR86",167.524606908087,0.751777930769795,0.376497814205029,1.99676572454256,0.0458506385888971,0.38540489235618
"1834","AKR1C8P",5.10566085243141,1.65850557066528,0.830607141087155,1.99673887765341,0.0458535564438037,0.38540489235618
"1835","ZBTB40",50.905964038656,0.617073950270839,0.309151126363569,1.99602685433902,0.0459309998240904,0.385671464952452
"1836","KLF12",126.175992161438,0.548166418627685,0.274648114852964,1.99588633230253,0.0459462967457344,0.385671464952452
"1837","LDLRAD4",56.4801973453314,-0.682043415811565,0.341746645356381,-1.99575745681516,0.0459603296216448,0.385671464952452
"1838","CPXM2",156.550030735711,0.930349926602182,0.466290135992773,1.99521682915592,0.0460192363319512,0.385745801009803
"1839","HDAC7",537.967036763428,0.440451534419802,0.220746259980157,1.99528424381638,0.0460118873719645,0.385745801009803
"1840","FAM160B1",167.26649536609,0.443646167239882,0.222528062435993,1.99366391089434,0.0461887958392883,0.386956678186212
"1841","SAP25",10.6066693800868,-1.15433303750393,0.579166681831208,-1.99309296220936,0.0462512685122811,0.387269583985233
"1842","SGSM2",75.7329512976604,0.492768489412343,0.247389159564495,1.99187583756627,0.0463846826749584,0.388175832483433
"1843","ODF2L",497.498781859628,0.354003405393758,0.177826098283976,1.99072806978219,0.0465107910116462,0.388387780847522
"1844","TTC14",250.005256809198,0.413077324250968,0.207498304355624,1.99075036074998,0.0465083390977537,0.388387780847522
"1845","PDLIM3",326.343276994664,-1.39695335153848,0.701672220006982,-

1.99089163245565,0.0464928023264545,0.388387780847522
"1846","AL353746.1",7.21342877913298,5.81113898716073,2.91824860421009
,1.99131046572836,0.0464467655806815,0.388387780847522
"1847","ATG101",188.467166271466,-0.448745655340087,0.225614570491751,
-1.98899235258608,0.046702047072955,0.389773717178993
"1848","CCZ1",212.586339380706,-0.633162319039882,0.318393369386477,-1
.98861653513685,0.046743544881717,0.389868160496088
"1849","TSC22D1",570.492702693063,-0.589328504898423,0.296378523862336
,-1.98843187832381,0.0467639460757228,0.389868160496088
"1850","KCNS3",37.3087415905736,-0.724890375250809,0.365058169447433,-
1.98568457281214,0.0470683590583835,0.391770386007009
"1851","SCG5",3.76003967297483,-1.53588083712687,0.77336201254332,-1.9
8597915622451,0.047035638395926,0.391770386007009
"1852","ANPEP",469.532619315692,0.487809670212234,0.245653382971999,1.
98576410514093,0.0470595231699126,0.391770386007009
"1853","DIPK2A",129.726224927659,0.469648250687951,0.236648057007773,1
.98458528088623,0.047190631294744,0.39257613675579
"1854","ACVR2B",38.0023058978499,0.56210730993871,0.283454421498301,1.
98306065210586,0.0473606550879298,0.392890605555364
"1855","CPED1",320.150017401326,0.349996772250828,0.176502426126133,1.
98295728808119,0.0473722006755368,0.392890605555364
"1856","BAG4",111.884768470894,0.360248268904367,0.181576837321724,1.9
8399902883024,0.0472559481967009,0.392890605555364
"1857","PNP",22.4171293970929,-1.02805582543934,0.518467205408802,-1.9
8287531923786,0.047381358139956,0.392890605555364
"1858","AC022467.1",14.0782608863921,1.27702310840612,0.64397772508429
3,1.98302372685168,0.0473647793049853,0.392890605555364
"1859","KCTD15",7.85063717477148,1.30775598775232,0.659331151051732,1.
98345851802429,0.0473162362154059,0.392890605555364
"1860","RNF130",533.507731529005,-0.324427345386803,0.163642895515396,
-1.98253241831864,0.047419682774395,0.392996994606074
"1861","FOXJ3",117.625588537668,0.516653456962759,0.260891054436571,1.
98034178702884,0.0476651363377308,0.393115384145299
"1862","GSTM3",663.562194517887,0.669570484178495,0.338184277428908,1.
97989832427751,0.0477149547561849,0.393115384145299
"1863","SMPD4",75.2631633728487,0.464689333715614,0.234751924740051,1.
97949104881751,0.0477607464585651,0.393115384145299
"1864","GTDC1",75.775461489411,0.458127166559895,0.231266003509238,1.9
8095335937084,0.0475965042937586,0.393115384145299
"1865","ACVR2A",135.806961384022,0.611070887438926,0.308703836249526,1
.97947293063438,0.0477627844203845,0.393115384145299
"1866","EGFLAM",16.188858107876,-0.833510969090428,0.42050912677431,-1
.98214715453198,0.0474627732250858,0.393115384145299
"1867","PANK3",136.50442405821,0.461555037745474,0.232908242137088,1.9
8170332449552,0.0475124549189367,0.393115384145299
"1868","ENTPD7",14.6296638668846,-1.03839646557456,0.524524064476624,-
1.97969270792309,0.0477380684577949,0.393115384145299
"1869","DIXDC1",282.428857328581,-0.482830355106165,0.243837912656539,
-1.98012831493625,0.0476891122419372,0.393115384145299
"1870","NCOA6",106.408377382661,0.430234286213025,0.217092838456523,1.

98179861331164,0.0475017847420152,0.393115384145299
"1871","CHAF1B",4.87785897610471,-1.31480452896081,0.663762225569256,-1.98083662840742,0.0476095976898733,0.393115384145299
"1872","MAGT1",293.248138478506,0.329156366844555,0.166243266146621,1.97996811825299,0.047707111229524З,0.393115384145299
"1873","LINC00892",2.66043187010479,2.28672600242002,1.15523370424659,1.97944882841811,0.0477654955889812,0.393115384145299
"1874","ANGPT2",44.133048851984,-0.826582609118825,0.417661634552694,-1.97907238955303,0.047807856585377,0.39325406043948
"1875","MYLK",26.1871443640361,-2.11809129539023,1.07040812673919,-1.97876981917413,0.0478419279811203,0.393324437242117
"1876","AC021546.1",14.1350152002092,-1.03675579362772,0.524246242330452,-1.97761225530008,0.0479724656567586,0.394187397707321
"1877","TSPAN33",4.6338524068055,-1.40963943914626,0.713223906447209,-1.97643324403989,0.0481057294791696,0.394861458957082
"1878","LINC00654",12.9290127884339,0.955429257708529,0.483385447117341,1.97653707492894,0.0480939809816159,0.394861458957082
"1879","TSPAN9",72.3304422811906,0.478146160387602,0.242041284104338,1.97547357326647,0.0482144307085067,0.395541121313475
"1880","CCL2",1031.4863466281,-1.30628936469013,0.661328821863424,-1.97524940922641,0.0482398513181533,0.395541121313475
"1881","SRGAP2",142.088034741409,0.489941948996791,0.248174120573369,1.9741863005895,0.0483605631381689,0.39575903052744
"1882","LYPLA1",136.215451319367,-0.395404973984635,0.200289848669606,-1.97416382613023,0.0483631177609614,0.39575903052744
"1883","DIO3OS",202.151994441403,0.787556803563,0.398969277095157,1.97397857122508,0.0483841796003898,0.39575903052744
"1884","IRX3",160.301390661818,0.68441286052394,0.346590355719002,1.97470255369376,0.0483019129702033,0.39575903052744
"1885","TMSB4X",29548.264439055,-0.537634016355767,0.27237349484897,-1.97388522203265,0.0483947954942734,0.39575903052744
"1886","AL356215.1",3.50948213634276,-2.36626490299972,1.19906598222734,-1.97342342962998,0.0484473404140925,0.395825243763767
"1887","WDR7",55.1283544103386,0.548177156074253,0.277820082201987,1.97313726109873,0.0484799260607195,0.395825243763767
"1888","LAGE3",123.866892008705,-0.500337904669902,0.253565636230988,-1.97320864178186,0.048471796310594,0.395825243763767
"1889","ETNK2",39.4741565621461,-0.564090424461323,0.286119775652091,-1.97151847744782,0.0486646016407805,0.396548190411251
"1890","NR2F1-AS1",20.2267192657779,0.934307131202298,0.473797935500811,1.97195272751605,0.0486150032400811,0.396548190411251
"1891","ZFAND1",171.836517136938,-0.451161802079671,0.228808097379986,-1.97179124010816,0.0486334427614222,0.396548190411251
"1892","STARD13",38.2464459687721,0.863638023786583,0.438120457659023,1.97123418614414,0.0486970953258838,0.396548190411251
"1893","STON2",71.3671687860237,0.637817522757889,0.323551322435819,1.9713024751565,0.0486892884246769,0.396548190411251
"1894","KLHL8",91.0482502897117,0.474638517470681,0.240883292114908,1.97040862943814,0.0487915573764444,0.397107633029509

"1895","STAT4",4.88788390568868,1.56908809186484,0.797230405343828,1.9
6817391979439,0.0490480299592847,0.397374873962836
"1896","SLC9B2",78.3506181877532,-0.613655437261367,0.311928273913037,
-1.9672966145815,0.0491490251248178,0.397374873962836
"1897","PCDHB5",20.062583429534,1.08811995813043,0.553129757798956,1.9
6720560191941,0.0491595124649451,0.397374873962836
"1898","TREM2",5.94698164324107,-1.68989089331909,0.858819513345827,-1
.96769037854711,0.0491036735458138,0.397374873962836
"1899","PRRC2B",352.218327964907,0.439832691366577,0.223576595911196,1
.96725730425414,0.0491535546023462,0.397374873962836
"1900","KLC2",32.0185310885821,-0.555276124702238,0.282153176490215,-1
.96799529819043,0.0490685787020769,0.397374873962836
"1901","KDM5A",359.897392652346,0.450274026271592,0.228825708564098,1.
96775978143847,0.0490956837421908,0.397374873962836
"1902","AC005906.2",8.80714977875973,-1.83744241143506,0.9337531702791
76,-1.96780313033443,0.0490906938816913,0.397374873962836
"1903","LRRC23",51.8909028058323,-0.561302526943864,0.285169568957267,
-1.96831144710246,0.0490322136519008,0.397374873962836
"1904","MON2",185.017606641127,0.449563502392816,0.22848895384852,1.96
755026805742,0.0491198066787674,0.397374873962836
"1905","SERPINF2",9.43340775460879,1.23925187939659,0.629182263544079,
1.96962303485175,0.048881589667721,0.397374873962836
"1906","RAB34",692.007698357936,-0.414160375360113,0.21035119316117,-1
.96889957758778,0.0489646240810183,0.397374873962836
"1907","CXorf40A",27.0276013823671,-0.724607215631552,0.36798826702577
3,-1.96910412793352,0.048941134930396,0.397374873962836
"1908","FAM168B",145.862244918676,0.386462589233701,0.196563387450712,
1.96609650579311,0.0492874639632312,0.398200344336063
"1909","FAM174C",232.558540442173,-0.520181326993171,0.264656943476687
,-1.96549283823719,0.049357223768412,0.39855505730229
"1910","ASAP3",175.519293907917,0.377837168833221,0.192334892423489,1.
96447542134626,0.049474983890283,0.398731206509643
"1911","SERINC2",6.4023695448577,-1.37586030995119,0.700753800423988,-
1.96340042553994,0.0495996644246525,0.398731206509643
"1912","LINC01358",30.0297779347171,-0.972063040275017,0.4948821159267
39,-1.96423149875741,0.0495032515196854,0.398731206509643
"1913","EXOC1",243.267917069309,0.43204966177023,0.220017034286555,1.9
6371005168409,0.0495637262657057,0.398731206509643
"1914","NNT-
AS1",174.056661833506,0.35608716652794,0.18137216121669,1.963295602474
04,0.0496118361391823,0.398731206509643
"1915","HIST1H2BK",3.47970577175994,1.77854101151657,0.905427835588265
,1.9643100660375,0.0494941450590303,0.398731206509643
"1916","SDK1",239.859751364708,0.556331199537664,0.283338892407814,1.9
634833566156,0.0495900365064736,0.398731206509643
"1917","AL360020.1",4.96087520895967,1.59659698552686,0.81316674887194
1,1.96343122458183,0.0495960886158869,0.398731206509643
"1918","UBXN1",866.28527986315,-0.474355924191807,0.241535489943094,-1
.96391811531948,0.0495395886908851,0.398731206509643
"1919","ANO8",29.4570687204382,0.661947291405777,0.337282778847046,1.9

6258846558532,0.0496940120545383,0.399183530912302
"1920","SERPINE1",156.899859731269,-1.64758488649067,0.83968664459576,-1.96214254102356,0.0497458913241214,0.39939214310486
"1921","ARHGAP25",4.11372016803943,1.90380495282149,0.971381943283211,1.95989329015809,0.0500082640486843,0.400969282623344
"1922","IL15",7.60027123147409,-1.24238308669552,0.634006946283695,-1.95957330432716,0.0500456841518484,0.400969282623344
"1923","CNOT2",339.268702082119,0.318330903880613,0.162425314167708,1.95986017026835,0.0500121360993292,0.400969282623344
"1924","SPART",568.463677367134,0.302847434522836,0.154583176271215,1.9591228607665,0.0500984001513176,0.400969282623344
"1925","KIF23",6.41058995245653,1.28654588030448,0.656657340076723,1.9592347511933,0.0500853011248472,0.400969282623344
"1926","GATA6",32.2179286177121,0.767086200017207,0.391463427715473,1.95953477568471,0.050050191389872,0.400969282623344
"1927","VASH2",108.60636882726,0.56189443844134,0.286914161931387,1.95840607747941,0.0501823822051129,0.40097984564448
"1928","RICTOR",211.209623817753,0.457844322637097,0.233772575693812,1.9585031361282,0.0501710034065638,0.40097984564448
"1929","ARHGAP23",100.35387391017,0.487870732209291,0.249139427705032,1.95822370109521,0.0502037691919459,0.40097984564448
"1930","PCMTD2",231.660792956389,0.415946164786501,0.212345187474113,1.95882077542826,0.0501337796642016,0.40097984564448
"1931","HIVEP3",13.137592801836,-0.944202546973159,0.482560689989392,-1.95665035830812,0.0503885900230763,0.40114689294859
"1932","RBM43",263.84943794261,0.364159870202622,0.186077581200428,1.95703247996532,0.0503436497866804,0.40114689294859
"1933","GTF2H2",43.5830507172032,0.509557962619061,0.260444267753403,1.95649521110415,0.0504068460357716,0.40114689294859
"1934","BBS9",71.1359815182882,0.435592653397314,0.222585794954955,1.95696519396248,0.0503515606637562,0.40114689294859
"1935","C12orf76",214.027635954773,-0.44666185490568,0.228250285631687,-1.95689505346964,0.0503598082548127,0.40114689294859
"1936","KANSL1",274.12727119949,0.421877336292631,0.215586958824992,1.9568778120531,0.0503618357899146,0.40114689294859
"1937","AC245060.5",49.9446209717367,0.877386039539571,0.448433292025387,1.95655865686685,0.0503993797680673,0.40114689294859
"1938","ALMS1",51.9679183149013,0.58170101164194,0.297415126996962,1.95585549906438,0.0504821788502311,0.401416531182622
"1939","ARHGEF3",116.604543700064,0.509299872403516,0.260409505354899,1.95576529247431,0.0504928092094132,0.401416531182622
"1940","POLR3GL",329.588979580601,-0.452772678919476,0.231599367200873,-1.9549823662807,0.0505851516404439,0.401736276423206
"1941","CSGALNACT1",21.5653486771724,-1.28196619864563,0.655712264861967,-1.95507430216438,0.0505743009124699,0.401736276423206
"1942","ANKIB1",260.408253840541,0.433593585267471,0.221867810348144,1.95428793652895,0.0506671747001916,0.401797272062245
"1943","NOC4L",78.7258316723224,-0.628619192289853,0.321666867457711,-1.95425533645456,0.0506710280174508,0.401797272062245
"1944","MT-

ND2",22898.6593506799,-0.416247437127037,0.212992623834343,-1.95428099
63728,0.0506679950036253,0.401797272062245
"1945","F2R",88.189280974225,-0.868731340724393,0.444695240192408,-1.9
5354315091953,0.0507552693798091,0.402051632831325
"1946","Z95114.1",18.5246070724943,-1.1831133465531,0.60560608327252,-
1.95360215036133,0.0507482861357064,0.402051632831325
"1947","BRD7",369.404656644329,-0.352168906905015,0.180312462983909,-1
.9531035241665,0.0508073294744697,0.402257310656883
"1948","TOGARAM1",115.919817773423,0.423518924157068,0.216875616910625
,1.95281945564033,0.0508409923247819,0.402317195424288
"1949","CCDC136",33.895226545415,0.699803360635453,0.358409848392737,1
.95252268812833,0.0508761799880061,0.402389078766092
"1950","PGPEP1",74.3206039506057,0.483066630043596,0.247457943087412,1
.95211608088473,0.0509244244427331,0.4025641039921[?]7
"1951","OMA1",68.9339537814537,0.448761600692394,0.229923583551086,1.9
5178586624928,0.0509636330075053,0.402666631270551
"1952","APOBR",6.22423105641241,1.74090032712645,0.892052566819653,1.9
5156697248583,0.050989637641266,0.402666631270551
"1953","H2AFY",322.202471480872,-0.35955104857029,0.184277266610208,-1
.95114164207151,0.0510401987745683,0.402859531034291
"1954","NES",66.2801483432612,-0.584314527733108,0.299520650119267,-1.
95083219637924,0.0510770104950766,0.402922189569264
"1955","SPINT2",4.97867081977691,-1.48147567273756,0.759483577021807,-
1.95063556021439,0.051100413922018[?]2,0.402922189569264
"1956","TMEM255A",35.3592874985603,0.782345718577324,0.401162080269364
,1.95019857822058,0.0511524552039993,0.403126327694095
"1957","NOCT",16.8048256560502,-1.57236898232352,0.806733005097043,-1.
94905746063331,0.0512885630413367,0.403875058367847
"1958","EML2",16.2089801503318,0.742815135827154,0.381133553143864,1.9
4896284963599,0.051299861452108,0.403875058367847
"1959","WDYHV1",54.8198499773489,-0.52638275960414,0.2702443545354,-1.
94780298189425,0.0514385418614788,0.404553634078926
"1960","ABCC3",4.12023910656729,-1.63612147979061,0.839912534522364,-1
.94796649953679,0.0514189717777686,0.404553634078926
"1961","TASOR",239.747815927631,0.36509699865155,0.187534003370142,1.9
4683093247333,0.0515550072039406,0.404754850442607
"1962","FAM114A1",880.067381908708,-0.28367364872115,0.145740644808418
,-1.94642784169132,0.0516033679031,0.404754850442607
"1963","PXDC1",621.253237585486,-0.494346684369235,0.25395833572455,-1
.94656608911399,0.0515867774331833,0.404754850442607
"1964","RELA",188.617088975436,-0.298820264862122,0.153528642939772,-1
.94634863658208,0.0516128749743767,0.404754850442607
"1965","PDE1B",6.45943073385715,-1.31415612019783,0.675291787267953,-1
.94605671944353,0.0516479267480122,0.404754850442607
"1966","FGF14",70.8750402615883,0.58022765590814,0.29799542502977,1.94
710256323624,0.0515224397790573,0.404754850442607
"1967","NIN",179.609229419601,0.452525719387993,0.23251629453273,1.946
21078190412,0.0516294253097904,0.404754850442607
"1968","ASB7",29.5578377707382,0.601264581537347,0.309030449572115,1.9
4564834102873,0.0516969959635722,0.404933532915887

"1969","STMN1",62.2813649158279,-0.765972578283255,0.39453916147646,-1
.94143611857643,0.0522054008684229,0.405291469603775
"1970","CDC14A",48.2196464971728,0.692500400758128,0.356481373769549,1
.94259911376408,0.0520646140799542,0.405291469603775
"1971","SERPINE2",63.608554284476,-0.903347053070177,0.465019229611222
,-1.94260150021197,0.0520643255144898,0.405291469603775
"1972","MFSD4B",39.8032357890323,0.729282239207134,0.374979909979665,1
.94485683045442,0.0517922119781428,0.405291469603775
"1973","AC006001.2",6.66024983277528,1.47779131690457,0.76094502090090
5,1.94204742302534,0.0521313595530647,0.405291469603775
"1974","RHOBTB2",36.7308404989846,0.601773975781564,0.309812055222327,
1.9423839893826,0.0520906320724594,0.405291469603775
"1975","ZEB1-
AS1",83.489423988611,0.500142020715637,0.257416485689306,1.94292925480
808,0.0520247066591657,0.405291469603775
"1976","ANXA11",981.373855079866,-0.33220149460517,0.170919507621299,-
1.94361368827026,0.0519420539064797,0.405291469603775
"1977","STAMBPL1",55.298439022075,0.606968983762847,0.312653189403478,
1.94134908689371,0.0522159493112615,0.405291469603775
"1978","DGKZ",78.5307039067228,-0.387763319061124,0.199636524967629,-1
.9423465677135,0.0520951591071619,0.405291469603775
"1979","UCP2",56.3835600357885,-0.872251775252092,0.449047133366594,-1
.94245038090467,0.0520826012595563,0.405291469603775
"1980","ZNF740",57.2415143377211,0.454137356019874,0.233889071469324,1
.941678391243,0.0521760462414987,0.405291469603775
"1981","SLC39A9",104.937953332612,0.479145315156795,0.246748388216305,
1.94183766962144,0.0521567550227538,0.405291469603775
"1982","SNX33",166.7617064852,0.397980923595864,0.204961274675939,1.94
173716095933,0.0521689275722996,0.405291469603775
"1983","PYCARD",282.120503516072,-0.518146590360073,0.266706034463753,
-1.94276290524087,0.052044811781115,0.405291469603775
"1984","RBL2",254.737624637592,0.393118027330737,0.202275768233964,1.9
434756360734,0.0519587163546413,0.405291469603775
"1985","ZNF415",19.5806331240138,0.786706462598258,0.404596244394005,1
.9444235419846,0.0518443972162706,0.405291469603775
"1986","LINC01260",8.151713215257,-1.27299625870226,0.654821437511224,
-1.94403571077412,0.0518911448886195,0.405291469603775
"1987","SLC6A6",48.5437870708334,-0.861739640666621,0.444183236125279,
-1.94005439778365,0.0523730792487021,0.406306500563031
"1988","RXRA",258.810388809347,0.365966575635958,0.188689991552001,1.9
395123855051,0.0524389778708623,0.406408669622595
"1989","RNF185",78.8677224557951,0.430113057030141,0.221752537462926,1
.93960827664509,0.0524273142449933,0.406408669622595
"1990","PYGO1",61.142745917375,0.559729112008981,0.288707343036946,1.9
3874220905199,0.0525327362488937,0.406930718229496
"1991","MRPL30",84.2018872788725,0.388665124201056,0.200553039578994,1
.93796675940166,0.0526272781106789,0.407450538434854
"1992","COL5A1",1206.67247624926,0.727672722367923,0.375564899243331,1
.93754188379692,0.0526791387026056,0.407450538434854
"1993","PLEKHM1",118.259249607147,0.363169165954086,0.18743298710924,1

.93759471881235,0.0526726873015282,0.407450538434854
"1994","FUCA1",121.668254904415,-0.367174258864838,0.189534970277564,-1.93723753630863,0.0527163138075704,0.407454806069119
"1995","APOL6",487.248855268197,0.516983992679148,0.26688490933756,1.93710462671855,0.0527325551805315,0.407454806069119
"1996","EML2-AS1",3.50636990283211,-1.80210134785891,0.930550153825048,-1.93659776472158,0.0527945314258028,0.407729309583542
"1997","UBXN7",208.626752206318,0.406267167491034,0.209840789900883,1.93607338059932,0.0528587142364159,0.408020570833426
"1998","DCUN1D3",221.227154427758,0.607377302098142,0.313867909975571,1.93513666989854,0.0529735266746437,0.40870215900382
"1999","ANKMY2",110.698444484733,0.361102477770055,0.186797229743589,1.93312544444973,0.0532207463035635,0.409584525346695
"2000","KRIT1",208.568057939166,0.332635354369188,0.172069142363315,1.93314937123848,0.0532177995701835,0.409584525346695
"2001","AL731577.1",2.79669871834867,2.69541412430149,1.39386803714003,1.93376564529881,0.0531419485246234,0.409584525346695
"2002","AC010542.2",7.39737346435269,1.31128185831036,0.678041349460146,1.9339260936732,0.0531222153548884,0.409584525346695
"2003","PCBP3",10.9529715934782,1.39192034404764,0.71998602213233,1.93326023181018,0.0532041481774616,0.409584525346695
"2004","PRIMA1",22.5140900538286,0.872279616809527,0.451418477771564,1.93230817913248,0.0533214794971677,0.410154993237944
"2005","MRPS30-DT",30.2520644444285,0.59271801399198,0.306786191276468,1.93202311853025,0.0533566524853358,0.410220846913442
"2006","SAMD5",3.56823191921618,-1.70702586785842,0.883697755234192,-1.93168519185163,0.0533983736007992,0.410265609659157
"2007","SDSL",19.2683433078983,1.00292034957363,0.519232225469988,1.93154488565465,0.0534157040925026,0.410265609659157
"2008","TNPO1",296.74958145381,0.303751271482897,0.157369524989655,1.93017848597348,0.0535847262852643,0.41135884247378
"2009","TCF7L2",1033.46925714399,0.373942569483706,0.193759423374125,1.92993229940445,0.0536152267342384,0.411388113543198
"2010","ECM1",1494.82288964292,-0.620720869058938,0.321870463937917,-1.92848036276688,0.0537954044923919,0.411745859111332
"2011","LYPLAL1-AS1",17.9526668595994,1.51549514619609,0.785765415119438,1.9286864973126,0.0537697935238335,0.411745859111332
"2012","TRMT112",971.354878929007,-0.447494590721276,0.231979964071552,-1.92902258827513,0.0537280580866009,0.411745859111332
"2013","DICER1",269.589056571425,0.46382234044507,0.240466436723107,1.92884440242758,0.0537501816560066,0.411745859111332
"2014","ORAI3",335.981137996962,0.529717093472166,0.274562233918413,1.92931520811261,0.0536917428780541,0.411745859111332
"2015","HSPB7",4.35102742734033,-1.65927386568742,0.860547344051073,-1.92816104443051,0.0538350979673637,0.411845178742884
"2016","WDR13",353.775813224729,-0.40713808853592,0.211195838589384,-1.92777514583275,0.0538831004538392,0.412007933281712

"2017","LUC7L3",637.330300014183,0.463773739172324,0.240696751476413,1
.92679683596715,0.0540049539614657,0.412734935704508
"2018","PDGFB",2.57701915993458,-2.02336702237351,1.05027667323997,-1.
92650858000272,0.0540409015563043,0.412805003711809
"2019","SMIM26",608.987195929898,-0.454769467370721,0.236180160954869,
-1.92551933884752,0.0541644189531367,0.413543594929471
"2020","CISD1",155.618954631647,-0.557690697925819,0.289683873375092,-
1.92516998419067,0.0542080958917922,0.413643786059308
"2021","CMIP",103.58785044761,0.375155749074363,0.194887601365025,1.92
498520401867,0.0542312093172794,0.413643786059308
"2022","GLUD1",477.598283316326,-0.324913157151405,0.168817903001082,-
1.92463685056746,0.0542748058465973,0.413771578696982
"2023","HSPA4L",10.981443300776,1.04931133220922,0.545324471433623,1.9
241963036257,0.0543299822889118,0.413987482443685
"2024","AC003991.1",3.44626513100432,2.41064253722858,1.25331989980816
,1.92340561862742,0.054429129259918,0.414538057085789
"2025","FASTKD3",28.0977116736005,-0.73209669318662,0.380717897201075,
-1.92293742576532,0.0544879088466171,0.414780797466964
"2026","SATB1",114.955209469787,0.533538842312871,0.277613036209396,1.
9218796408049,0.054620904151949,0.41558797507517
"2027","AC009404.1",18.18617282863,0.758751064925443,0.394895029395585
,1.92139938070825,0.0546813765659276,0.41584283165455
"2028","CSRNP1",473.878099931504,-0.632541253248258,0.329355311326289,
-1.92054365451422,0.0547892645389438,0.416186556534542
"2029","SLC37A4",27.9097387407974,-0.579820367231004,0.301921516791516
,-1.92043406973006,0.0548030935474019,0.416186556534542
"2030","CENPB",133.925405581148,-0.520751195247471,0.271168286223904,-
1.92039859269341,0.0548075711816491,0.416186556534542
"2031","CRABP2",826.552933212987,-0.75990713504584,0.395855632445666,-
1.91965725067747,0.0549012073925841,0.416395991620877
"2032","CACNA2D1",164.315075232058,0.42826801834291,0.223197206556697,
1.91878753748704,0.0550112276958699,0.416395991620877
"2033","MAB21L1",23.764189984129,0.955513181317192,0.498033481083677,1
.91857217960142,0.0550384992362428,0.416395991620877
"2034","CYP46A1",26.3933690364566,0.838765562648716,0.436918863467084,
1.91972842736259,0.0548922115398984,0.416395991620877
"2035","MSI2",50.9844857840605,0.568070588020098,0.296105823815675,1.9
1847151366304,0.0550512507897079,0.416395991620877
"2036","ANKRD29",17.2424070405636,-0.752678451890778,0.392264025652677
,-1.91880570908438,0.0550089270769184,0.416395991620877
"2037","HSBP1L1",45.6212129927865,-0.842395564074782,0.438947668484759
,-1.91912527291173,0.0549684817379485,0.416395991620877
"2038","POLR2I",428.001011582647,-0.481187033740891,0.250707293094328,
-1.91931805334377,0.0549440946297816,0.416395991620877
"2039","RNPEPL1",181.068390195463,-0.342845524918075,0.178782446069298
,-1.91766883413813,0.0551530159348956,0.416961128316045
"2040","KREMEN1",254.754238858552,0.499573637927203,0.260540540807111,
1.91745068302847,0.0551807006075158,0.416965931306302
"2041","CHL1",940.972992530658,0.420659735669869,0.219501020699007,1.9
1643635337215,0.0553095772609754,0.417468224051147

"2042","TNIP2",194.613328058898,-0.359661078497036,0.187663145065936,-1.91652483693944,0.0552983249158011,0.417468224051147
"2043","PSMD7",487.169293444596,-0.336990350140671,0.175855774841491,-1.9162882222345,0.0553284191849817,0.417468224051147
"2044","FAM177A1",299.800428168567,-0.322535115355763,0.168342823100207,-1.91594217927408,0.0553724558621984,0.417596089586981
"2045","MAP4",595.169441593609,-0.409483629481357,0.213882593970764,-1.91452526303908,0.055553074329467,0.418434382655329
"2046","ECPAS",159.035640935493,0.371141717761804,0.193865239216728,1.91443148478461,0.0555650458187129,0.418434382655329
"2047","EHD1",153.341677943479,0.456341982025528,0.238342144954653,1.91465081474513,0.0555370500883757,0.418434382655329
"2048","ZNF148",392.778333071688,0.293747093834521,0.153484819020946,1.91385112682991,0.0556391806360758,0.418583684482727
"2049","DTX4",29.1463834392849,0.751677234197205,0.392729121851224,1.91398394561114,0.055622207122,0.418583684482727
"2050","HEBP1",644.841211950233,-0.488252386807234,0.255229775571413,-1.91299148273012,0.0557491428339061,0.419206359407152
"2051","SOS1",197.388428338075,0.39010101275579,0.203999401528664,1.91226547643071,0.0558421515813202,0.419340332456852
"2052","FGD2",3.71676423544241,-1.64610487920694,0.86083652837556,-1.9122154148282,0.0558485697394692,0.419340332456852
"2053","ZNF483",42.5589040053764,0.594589526398926,0.310930620694904,1.91229003135834,0.0558390037363081,0.419340332456852
"2054","PLOD1",126.675635000975,0.443553733544771,0.23201284662556,1.91176368031298,0.0559065122491462,0.419460402007547
"2055","ARID5A",70.3053450139055,-0.745798366623831,0.390157923572049,-1.91152946426348,0.0559365740810061,0.419460402007547
"2056","N4BP2L1",174.428950344851,0.400445432252956,0.209497754353396,1.91145453319493,0.0559461943903676,0.419460402007547
"2057","CASP9",27.4423881134615,-0.590201583581365,0.308840727167535,-1.91102251634449,0.0560016873917638,0.419468421352788
"2058","TMEM120B",38.7084375952848,0.642630877931377,0.336258848556921,1.91111960529596,0.0559892122259599,0.419468421352788
"2059","AL139339.1",17.3612094539683,-0.893443621510109,0.467681651534741,-1.91036705968299,0.05608596899354,0.419496485637278
"2060","HERPUD1",892.959928217291,-0.366747013411141,0.191965536512337,-1.91048362156178,0.0560709732193879,0.419496485637278
"2061","KCNJ12",70.266618509521,0.562344935075184,0.294385960348793,1.9102301427993,0.0561035877198245,0.419496485637278
"2062","AL022323.4",36.313013436245,0.915563155142849,0.479315546167043,1.91014700537957,0.0561142882506693,0.419496485637278
"2063","FAM43B",39.9405421774015,-0.896699251836585,0.469609832204206,-1.90945587239465,0.0562033090631209,0.419818189169675
"2064","TMEM192",94.4044154575906,0.453925296255791,0.237767692469758,1.90911259448559,0.0562475683785691,0.419818189169675
"2065","ALDH5A1",17.2027448779608,0.901352083544866,0.472211994007614,1.9087869325283,0.0562895832636331,0.419818189169675
"2066","CCT6A",303.338087533437,-0.376884772393622,0.197450405737893,-1.908756636813,0.0562934931569067,0.419818189169675

"2067","C1RL",223.589718396232,0.453704475375288,0.237695248376767,1.9
0876544009045,0.056292357003461,0.419818189169675
"2068","C1QTNF6",92.312587921808,0.859255932666859,0.45037674818446,1.
9078603327784,0.0564092703713082,0.420478192830617
"2069","BMP8B",4.39380129359362,2.28616680321772,1.198799694816,1.9070
4653421572,0.0565145618286573,0.420652810520885
"2070","GPATCH4",97.0338783712236,-0.516014933948887,0.270577886894021
,-1.90708464713156,0.0565096270279578,0.420652810520885
"2071","AL118506.1",6.69814828894673,1.43623570622111,0.75301630631834
1,1.90731023242136,0.056480425941136,0.420652810520885
"2072","MGST3",2241.88401234623,-0.473019753302586,0.24810529640107,-1
.90652823685769,0.0565817057729998,0.42094932166544
"2073","MPHOSPH10",242.066775345709,-0.390058793298275,0.2046449167743
,-1.90602727615495,0.0566466668901225,0.421229315056072
"2074","OSBPL10",101.89575095196,0.656489747653987,0.344679417047815,1
.90463867345731,0.0568270558221341,0.421276997532558
"2075","PHLDB2",268.394837167028,0.489706754908537,0.257130660740192,1
.9045054895392,0.0568443824111399,0.421276997532558
"2076","ANKRD37",35.0731903019001,-0.648593033751037,0.340529269539697
,-1.90466163048996,0.0568240696663472,0.421276997532558
"2077","WAC-
AS1",184.325202583329,0.487515089241894,0.255834364017915,1.9055887629
2223,0.0567035811858943,0.421276997532558
"2078","RTN1",5.85937515405218,-1.1738339755457,0.61616464563325,-1.90
50654461671,0.0567715643386429,0.421276997532558
"2079","MEX3B",101.789602995937,0.550482159130352,0.289038664772323,1.
90452775431955,0.0568414855640381,0.421276997532558
"2080","ZNF787",103.728945190453,-0.521717533637044,0.273908505119618,
-1.90471461778526,0.0568171777967618,0.421276997532558
"2081","SCO1",112.4343044896,0.370450435110088,0.194541997438319,1.904
21831783413,0.0568817570301757,0.421351410197097
"2082","NINL",37.4007268856711,0.654555564595766,0.343895559518135,1.9
0335567435918,0.0569941507146935,0.421981187928434
"2083","WDFY3",161.709918391215,0.447652738439747,0.2352786971665,1.90
264883234608,0.0570863827102505,0.422461156734763
"2084","SPSB1",332.28368882586,-0.560241907844838,0.294498122272886,-1
.90236156183607,0.0571239025539861,0.422535968267609
"2085","TNFAIP8",39.8892602017881,-0.607428335792239,0.319424326471481
,-1.90163455145133,0.0572189476204617,0.423036008426579
"2086","INSYN1",23.5979318017735,-0.790043207175499,0.415543886809826,
-1.90122687940555,0.0572723018004245,0.423227484301795
"2087","TMEM97",28.3881872602593,-0.57623748810389,0.303211843723239,-
1.90044518389545,0.0573747221271129,0.42378118907017
"2088","SLC2A5",11.4912640303861,1.14269538496108,0.601527554608787,1.
89965592798861,0.0574782875241975,0.424091382186703
"2089","FAM131B",7.56050938040595,1.13808187991065,0.599149302192613,1
.89949629540715,0.0574992532449049,0.424091382186703
"2090","ARC",45.957048935609,-0.942372740339815,0.496078251711742,-1.8
9964534241949,0.0574796776080204,0.424091382186703
"2091","SEMA3G",2.93838769287022,-2.13909777892798,1.12658888816326,-1

.89873857394019,0.05759885711843,0.424582784679396
"2092","PLPBP",255.241931382714,-0.325668896575244,0.171533735873466,-1.89857053434362,0.0576209656535386,0.424582784679396
"2093","SOCS5",189.883593383377,-0.458455312890226,0.241546364158169,-1.89800129879008,0.0576959109373831,0.424728971871901
"2094","FZD2",21.9477644074092,0.919682099753875,0.484513684500167,1.8981550556258,0.057675659401592 8,0.424728971871901
"2095","CARF",55.4878860420485,0.487021698068803,0.256840094638448,1.89620588154034,0.057932825766456,0.425457126817493
"2096","LRAT",4.95569139819788,1.89373770707864,0.998434213718453,1.89670754573386,0.0578665472638826,0.425457126817493
"2097","PAM",1433.30539704944,0.463259545677149,0.244241417615681,1.89672804145814,0.0578638407653482,0.425457126817493
"2098","AC009403.1",10.9486613143056,0.930704745093433,0.490751293518928,1.89648964227852,0.0578953283269465,0.425457126817493
"2099","SWAP70",412.771467131606,0.424069347318736,0.223634904605947,1.8962574203967,0.0579260136870042,0.425457126817493
"2100","AC114760.2",3.36548609387724,2.00637279556872,1.05869923821397,1.89513010225027,0.0580751675089548,0.425916658492218
"2101","DHTKD1",51.9351439472758,0.53730502215256,0.283551685424308,1.89491034535215,0.0581042803617969,0.425916658492218
"2102","SF3B2",571.941785568008,-0.291724351246406,0.1539256588925,-1.89522886142162,0.0580620880852187,0.425916658492218
"2103","IQCD",12.3817103118631,-0.835022858599868,0.440669031288365,-1.894897983093,0.0581059184436675,0.425916658492218
"2104","TMEM265",108.237910302085,-0.560533495552063,0.295867142837894,-1.89454459246656,0.0581527612906191,0.426057421718105
"2105","AC082651.1",7.38540979306901,1.19778818225643,0.632341910023138,1.89420970407071,0.0581971805701618,0.426180303320211
"2106","NANOS1",10.6273485255502,1.1581716441282,0.61160330942252,1.89366477631024,0.0582695195251621,0.426305004024857
"2107","GLCE",124.265630172254,0.385966741448412,0.203817099961635,1.893691655514,0.0582659495703145,0.426305004024857
"2108","MYOM1",27.5518281280133,0.658724857412208,0.347911021444054,1.89337162898168,0.0583084656148915,0.426387569949503
"2109","ZNF514",51.9873624979031,0.504806219116401,0.266672289433844,1.89298340741786,0.0583600760727322,0.426562623357595
"2110","PTPN23",125.775290429899,0.331825787071183,0.175390734463183,1.89192312858943,0.0585012236909857,0.4266843721812
"2111","ABCB5",73.3598085739149,0.893763015001236,0.472275736573301,1.892460157886,0.0584296972832832,0.4266843721812
"2112","VIM-AS1",26.1643956847051,-0.713732297899129,0.377273082949048,-1.89181876512383,0.0585151321953329,0.4266843721812
"2113","RECQL5",24.9678200439403,0.632513859865229,0.334307064337477,1.89201463965092,0.0584890302842445,0.4266843721812
"2114","CBX7",143.855652419886,0.481192273219821,0.254275415712215,1.8924058067982,0.0584369329461799,0.4266843721812
"2115","RAB3GAP1",210.096678228492,0.327866035596486,0.173356601151035,1.89128094009433,0.0585868516137238,0.427005351123193

"2116","GLIDR",22.7153309876266,0.638362332202531,0.337615961673112,1.
89079428898746,0.0586518098468649,0.427096138987305
"2117","NUP50",110.92778319678,0.426006238200197,0.22530803717202,1.89
077248884355,0.0586547211311142,0.427096138987305
"2118","CERS6",18.045401054866,-0.75416136404571,0.399001992058481,-1.
89011929528205,0.0587420070681347,0.427529763434984
"2119","AC024560.1",2.6976092415675,-1.71634577278925,0.90820963649792
6,-1.88981233386547,0.0587830634105301,0.427612581968553
"2120","ZNF629",41.5381346176991,0.625634218016335,0.331090050429652,1
.88961950745562,0.0588088662843551,0.427612581968553
"2121","LINC01359",16.2523998376939,0.983805011777796,0.52081931779944
8,1.88895645410109,0.0588976638840002,0.427678431800733
"2122","CCDC9B",9.70624241581943,1.11373212829977,0.589594501607938,1.
88897984167493,0.0588945298745348,0.427678431800733
"2123","AC022467.2",4.00763972362919,2.19769178959625,1.16354617100844
,1.88878778028338,0.0589202708138569,0.427678431800733
"2124","CHMP1B",284.805498750048,-0.365649212932205,0.193595955046971,
-1.88872341285999,0.0589288997174672,0.427678431800733
"2125","PTPMT1",237.094813287233,-0.45912831827808,0.243212877742834,-
1.88776319140284,0.0590577487339833,0.428309921813188
"2126","MRGPRF",283.340864945001,-0.410874428821829,0.217663256095831,
-1.88766095018321,0.0590714819185753,0.428309921813188
"2127","NAF1",50.1146218671135,-0.502389402134271,0.266219802666361,-1
.88712258480594,0.0591438396497871,0.428632951669707
"2128","LRFN4",52.9568774144937,-0.534849403591613,0.283487053936937,-
1.88668017168288,0.059203356196847,0.42886265778872
"2129","FBXL19",17.1662120744,0.821334712487464,0.435453255042395,1.88
616046148857,0.059273334777763,0.429167898355667
"2130","ATP11C",120.425131776312,0.419826763010876,0.222608471284828,1
.88594243780466,0.0593027119323015,0.429179016167337
"2131","ITGAX",12.2054483703396,-0.984731798211863,0.522277290933228,-
1.88545781198394,0.059368055125884,0.429450290833178
"2132","TTC17",261.893343140839,0.301734144069413,0.160068558723764,1.
88503067982343,0.0594256958427583,0.429565788498246
"2133","RABEP1",243.103120388752,0.362293520149744,0.192226685808382,1
.88472021262902,0.0594676219692025,0.429565788498246
"2134","PPM1N",6.09241432453567,-1.32132202000417,0.701020197172812,-1
.88485585056324,0.0594493021264731,0.429565788498246
"2135","GPRASP1",40.788485520539,0.592168582726144,0.31423604233551,1.
88447059835957,0.05950134818787,0.4296080947616
"2136","POMT2",32.4338210240042,0.584902651393898,0.31044526135231,1.8
8407659645389,0.0595546153962771,0.429791384051317
"2137","RFTN1",455.433661620407,0.502558829840869,0.266821575511165,1.
88350147051673,0.0596324407420377,0.430151649058732
"2138","ZNF770",121.762001005549,0.402442486313962,0.213709161678412,1
.88313165029188,0.0596825289212955,0.430311591824963
"2139","MT-
ND5",7548.95367510035,-0.380821714545509,0.202269072599596,-1.88274811
196356,0.0597345119307677,0.430485040398684
"2140","UCHL5",119.789361786567,-0.337052497860267,0.17904353313906,-1

.88251701667708,0.0597658516440395,0.43050962761349
"2141","TMEM167A",469.755443329586,-0.326807351247872,0.173753118487692,-1.88087186055899,0.0599893518905549,0.431468705041339
"2142","PCYOX1L",24.6003875458119,0.62561649255996,0.332695882547382,1.88044555216538,0.0600473803224013,0.431468705041339
"2143","LRTOMT",34.9452481573014,-0.637677401237241,0.339062699819632,-1.88070643446318,0.0600118639082327,0.431468705041339
"2144","GCHFR",50.3763760812437,-0.696360497556667,0.370178280821267,-1.88114898586633,0.0599516549730882,0.431468705041339
"2145","GABPB1-AS1",297.151060395826,0.754916719055804,0.401484918744432,1.88031152307455,0.0600656337668485,0.431468705041339
"2146","CRKL",94.6328172906305,0.458514290184042,0.24385141426893,1.88030195173842,0.0600669374647236,0.431468705041339
"2147","FBXO4",72.3237833368093,-0.453892137265174,0.24147796939872,-1.87964201618626,0.0601568829299835,0.431511563688086
"2148","CX3CL1",3.13134961539694,-1.68588602614084,0.896852475709622,-1.879780757484,0.0601379640146629,0.431511563688086
"2149","HPS4",120.213565187246,0.384690460136472,0.204650462826326,1.87974390491819,0.0601429887896864,0.431511563688086
"2150","REEP2",7.00593417772038,-1.13132980128916,0.602125380048843,-1.8788940622257,0.0602589597789453,0.432042727903462
"2151","SMAD1",65.088764750986,0.45269668281101,0.24099777397438,1.87842682256117,0.0603227990457229,0.432299371124974
"2152","GSTM5",319.941661736041,0.61874379272662,0.329446853849999,1.87812931128595,0.0603634774169828,0.432389871925088
"2153","BTNL9",3.01992454635575,-2.12083971182133,1.12948191002759,-1.87771020765577,0.0604208195449453,0.432533934964827
"2154","BEGAIN",4.77774979381244,2.26417691725732,1.20590669298395,1.8775722287889,0.0604397078114977,0.432533934964827
"2155","R3HDM4",82.8357396908001,-0.447993833857958,0.238698622858094,-1.87681784039571,0.0605430644434568,0.433072546819437
"2156","CTSC",93.6284966523693,0.52528204107606,0.27996405971829,1.8762481212932,0.0606212171042935,0.433430455316644
"2157","PGBD2",19.2840731617864,-0.653966108011155,0.348762646839945,-1.87510363835286,0.0607784671231073,0.433820377081887
"2158","COX17",337.42682521576,-0.412031592470932,0.219746434579444,-1.87503198065301,0.0607883240023922,0.433820377081887
"2159","LINC01277",6.1482329535586,-1.23902850117388,0.660764351414843,-1.87514429088198,0.0607728757507607,0.433820377081887
"2160","DOCK9-DT",4.12646899277483,-1.31713418688512,0.702427063444896,-1.87511879229927,0.0607763827907334,0.433820377081887
"2161","EFHD1",313.363425992794,0.530426833639069,0.283164861971265,1.87320852575591,0.0610395954726234,0.433880996562517
"2162","SUN1",477.4181659874,0.266358054508057,0.142121932849144,1.8741516468875,0.0609095264913003,0.433880996562517
"2163","KDM7A-DT",78.1320579425458,-0.638972422919178,0.341011454786196,-1.8737564792942,0.0609639973965314,0.433880996562517

"2164","FICD",35.8949164929628,-0.579227519348796,0.309142629302916,-1
.87365786677461,0.0609775966865974,0.433880996562517
"2165","PCNX1",128.565600065545,0.442558916403399,0.236252386518005,1.
87324633171344,0.0610343771039176,0.433880996562517
"2166","CPSF2",96.5352464853292,0.420617371639091,0.224552961111948,1.
87313215357422,0.0610501382772688,0.433880996562517
"2167","ZNF90",17.3686871152885,0.745218920442353,0.397517401082676,1.
87468251304893,0.0608364140226871,0.433880996562517
"2168","SLC5A3",179.459189283135,-0.685663569154995,0.365963562136339,
-1.87358425836819,0.0609877493892548,0.433880996562517
"2169","MED14",55.5307379850691,0.458048079075407,0.244392762596388,1.
8742293110859,0.060898825803109,0.433880996562517
"2170","MAP3K13",127.261281334459,-0.341867440716372,0.182624385248478
,-1.8719703847393,0.0612107008360359,0.434821637505758
"2171","C21orf62-
AS1",29.0496354822232,0.818103628376115,0.43722896655234,1.87111031281
177,0.0613297924555017,0.435466951037107
"2172","DENND3",15.1307272020456,-0.940212237756818,0.502601185827459,
-1.87069243819808,0.0613877235400928,0.435477109236323
"2173","RWDD2B",84.7438426871295,0.394273803686337,0.210745040402681,1
.87085685590977,0.0613649244649259,0.435477109236323
"2174","TRNAU1AP",143.668041398728,-0.327812337529711,0.17529558459180
6,-1.87005473237136,0.061476217748292,0.435557925285147
"2175","SLC40A1",239.698467028793,-0.553635066404347,0.296067199542632
,-1.86996420832706,0.0614887882940216,0.435557925285147
"2176","GPR150",7.47499959195599,-1.24514994848384,0.665951788522793,-
1.86972986625026,0.0615213398964164,0.435557925285147
"2177","GNAL",52.5623918818907,0.642855230104653,0.343781805995457,1.8
6995128565108,0.061490582964192,0.435557925285147
"2178","ZNF177",15.0934062584719,0.862717332466405,0.461446661962551,1
.86959274729008,0.0615403932060364,0.435557925285147
"2179","RBBP4",304.146143718646,0.257166995484863,0.137622915432037,1.
86863499205451,0.0616736139743016,0.436300486192684
"2180","DAAM2",21.2600408689651,0.787850798689951,0.421765902940865,1.
86798125025392,0.061764684439263,0.436744316803321
"2181","DHRS11",17.1111320745854,-0.75860845865807,0.406204340863222,-
1.86755379582098,0.0618242917720193,0.436965363441393
"2182","MDM1",63.8276667650329,0.424810521426126,0.227598632468883,1.8
664897791643,0.0619728726145268,0.43781477147247
"2183","TWF2",347.008818023668,-0.446102360299676,0.239069732510947,-1
.86599263576475,0.0620423958192436,0.437968579720448
"2184","AC010624.5",2.40090978135704,2.22003360758254,1.18977465840791
,1.8659277972463,0.0620514679279571,0.437968579720448
"2185","OBSL1",116.583232439652,0.425166759199144,0.227909131282183,1.
86550998113686,0.0621099544645945,0.43798030561378
"2186","ROBO3",216.330327362143,-0.456608563430208,0.244755567191829,-
1.86556967291509,0.0621015959267117,0.43798030561378
"2187","PNO1",62.372488479295,-0.438436745490415,0.235143179274831,-1.
86455225638494,0.0622441903547323,0.43872619767636
"2188","PLD1",99.820318574796,0.599036218739764,0.321354221912616,1.86

409942018019,0.0623077439604326,0.438954473985423
"2189","AP000350.5",3.03686307908232,1.95117026638742,1.04681244688466
,1.86391580668929,0.0623335286120072,0.438954473985423
"2190","ST8SIA4",5.06110600623208,-1.54432157654996,0.828746234919321,
-1.86344324894617,0.0623999299977776,0.439221425075681
"2191","EGR3",125.561836486164,-0.818464511698842,0.439301536382758,-1
.86310414126511,0.0624476157151997,0.439256221217273
"2192","HMCN2",223.122350317309,-0.856907671233454,0.460010471505791,-
1.8628003584972,0.0624903595932196,0.439256221217273
"2193","AL359532.1",3.26156807494087,1.72432202180987,0.92557131252809
2,1.86298127272342,0.0624649010700397,0.439256221217273
"2194","LRRC59",215.757621157596,-0.381369066957476,0.204763998499303,
-1.86248105014795,0.0625353140861158,0.439371862642422
"2195","SRPK2",371.208138154918,0.327799219035516,0.17602157815889,1.8
6226724282417,0.0625654303445908,0.439383193057798
"2196","UBFD1",101.426703599724,0.367885379776814,0.19757850481881,1.8
6197066383402,0.0626072254296284,0.439476493623735
"2197","MAT2B",160.21773056144,0.440825529812153,0.236795026517642,1.8
6163339785902,0.06265478234064,0.439570636538856
"2198","LRRK2",150.554553094859,0.52600878340179,0.282576916711411,1.8
6147116871188,0.0626776684471233,0.439570636538856
"2199","ZKSCAN3",17.9099041584047,0.713004917826044,0.38329457376783,1
.8602009175792,0.0628571052953146,0.439828542045971
"2200","NELFE",313.796818893437,-0.360249907474212,0.193651725161776,-
1.86029795073223,0.0628433833366264,0.439828542045971
"2201","VEZT",158.989035964351,0.338820836314018,0.18212963980693,1.86
032782293531,0.0628391594529206,0.439828542045971
"2202","BTBD6",300.455556243743,-0.460912296356358,0.247712696457975,-
1.86067288010226,0.0627903859113363,0.439828542045971
"2203","AL683813.1",20.3839974455874,0.947415160274791,0.5092886798423
84,1.86027138982944,0.0628471392046936,0.439828542045971
"2204","LINC01625",9.95580884057773,1.15170109533927,0.619332009147798
,1.85958593828214,0.0629441303018147,0.440037990295906
"2205","HELLPAR",217.22913495078,-0.646010924730674,0.347393805911908,
-1.85959252507366,0.0629431976851722,0.440037990295906
"2206","TRANK1",83.5851070065207,0.452839239545434,0.243609174466723,1
.85887596613194,0.0630447214814594,0.440516477467714
"2207","AK1",268.324545115382,-0.596647194984676,0.321002485426149,-1.
85869961160143,0.0630697285612225,0.440516477467714
"2208","FBLIM1",24.4333017310828,-0.652689154546717,0.351191878661879,
-1.85849728938383,0.0630984279582947,0.440517331058475
"2209","FAM3B",6.86169635839308,-1.3129497785375,0.706537706694999,-1.
85828691957453,0.063128280351874,0.440526229798161
"2210","HIF1AN",58.1962951128535,0.426337359181448,0.229578295529612,1
.85704558088966,0.0633046694812686,0.441557230793554
"2211","SDC2",1983.82051644936,-0.371527609581613,0.200091065369207,-1
.85679260038959,0.0633406668847471,0.441608493907
"2212","TES",85.1570712517735,-0.891416378540939,0.48026791409426,-1.8
5608147532001,0.063441945752813,0.441715263676429
"2213","RGS3",9.23038059460776,-1.08598918684126,0.585032545088503,-1.

85628850216696,0.0634124470651672,0.441715263676429
"2214","RNASEH2A",20.3135835506574,-0.740211949770842,0.398801724122629,-1.85609014454319,0.0634407102715662,0.441715263676429
"2215","BTN3A1",81.470753470397,0.554792132935223,0.298979355798676,1.85562020311798,0.063507711939689,0.441964728374556
"2216","ZNF277",144.248824167845,-0.405306536767474,0.218443676973776,-1.85542810111244,0.063535117617776,0.441964728374556
"2217","SLC2A13",70.2231191624727,0.401437859233022,0.216432768179444,1.8547924263491 9,0.0636258739908299,0.442396412976384
"2218","AGPS",233.166344601326,-0.398833534487486,0.215130628658349,-1.85391330362761,0.0637515642921007,0.442937414832253
"2219","BRMS1",130.396168098053,-0.390655719991177,0.210727133175507,-1.85384631824235,0.0637611497575588,0.442937414832253
"2220","LRMDA",105.404020803416,0.479886232813787,0.25898462531716,1.85295259217069,0.0638891539835322,0.443626715610878
"2221","TOP1MT",30.1311783518363,-0.552374600028276,0.298218696909384,-1.85224670938764,0.0639904042318853,0.443929829538484
"2222","CHMP4B",419.898880777856,-0.288980901183143,0.156000945097411,-1.8524304516405,0.0639640359058557,0.443929829538484
"2223","NPEPPS",442.241690256274,0.434877772789909,0.234824016297446,1.8519305633503,0.0640357944557873,0.444044881482664
"2224","LNX1",58.289398937788,-0.531595816973303,0.287089657070265,-1.85167178225162,0.0640729683738428,0.444102881062404
"2225","STX6",63.0189927612376,0.414014895542605,0.223676856670564,1.85095097322642,0.0641766065770357,0.444196731153956
"2226","CRPPA",13.7585737965856,0.947924009429968,0.512160045925212,1.8508355287995,0.0641932180732266,0.444196731153956
"2227","AC007036.3",10.6473497514308,0.914921900741396,0.49445193710718,1.85037580415642,0.0642594038581461,0.444196731153956
"2228","VDAC2",849.759115143755,-0.432036549635914,0.233485773440598,-1.85037633458138,0.0642593274613041,0.444196731153956
"2229","ZNF319",21.6272118053933,0.665952130934023,0.359845771201874,1.85065987772974,0.0642184996158499,0.444196731153956
"2230","PLXNB2",118.522537306508,0.497997783909381,0.269108072284487,1.8505494082055,0.0642344037575639,0.444196731153956
"2231","RPS15",12089.8522808584,-0.418937185924451,0.226436849101057,-1.85012813765785,0.064295083331699,0.44424415488935
"2232","POLR2A",334.695590348505,0.455755655647543,0.246427012429701,1.84945494064925,0.0643921484758902,0.444715487793838
"2233","AC116407.2",19.3543443259699,-0.896253624419785,0.484763743383183,-1.84884624036608,0.0644800182371223,0.445122920342696
"2234","COX15",86.3629968449528,0.419895456360075,0.227162693430498,1.84843492573108,0.0645394501370863,0.445333761800889
"2235","MTR",235.035240796807,0.484896114702955,0.26245174832134,1.84756290557934,0.0646656000848714,0.445392069319477
"2236","MYO3B",2.66816896521653,2.62001145683187,1.41825902568792,1.84734340439754,0.0646973860450612,0.445392069319477
"2237","SKIL",270.852815328156,-0.687689273234281,0.372331898189121,-1.84697920478728,0.0647501542228315,0.445392069319477
"2238","RSPH9",2.83507829579933,-2.07752227396234,1.12475793607206,-1.

84708389897437,0.0647349816486439,0.445392069319477
"2239","PRKCSH",420.749631387236,0.255362369139886,0.138226543564984,1
.84741918993173,0.0646864100869782,0.445392069319477
"2240","IER2",3067.22669683744,-0.466110236615726,0.252330110381033,-1
.84722400315948,0.0647146819484926,0.445392069319477
"2241","TNMD",94.0334995951753,-1.51076698382364,0.817734097237426,-1.
84750396116232,0.0646741345583909,0.445392069319477
"2242","ACVR1B",73.3399800867582,0.508462523886602,0.275396586091614,1
.84629203688624,0.0648498133661224,0.445857316207273
"2243","PCBP2-
OT1",14.1530679069012,0.909420598996227,0.492613426470167,1.8461141132
7682,0.0648756380313275,0.445857316207273
"2244","EBNA1BP2",212.989864323728,-0.437148702630816,0.23723136601247
1,-1.8427103885067,0.065371306435287,0.446191448217459
"2245","VCAM1",55.0812638253754,0.998470664590117,0.541816942268812,1.
84281920090779,0.065355412415685,0.446191448217459
"2246","PLGLB2",9.68582380905134,1.01668814062047,0.551333216645125,1.
84405384969737,0.0651752927362006,0.446191448217459
"2247","CLK1",522.896463974791,0.328636259157864,0.178354604806964,1.8
4260036074511,0.065387381221099,0.446191448217459
"2248","PCDH7",87.7622080368701,0.993021691843958,0.538115053701973,1.
84537058573709,0.0649836489259615,0.446191448217459
"2249","PCDHGA5",38.8332874892674,0.577092071279014,0.312858514115283,
1.84457844438386,0.0650988850276396,0.446191448217459
"2250","BTN3A2",186.516113181336,0.61797388163519,0.335271300723974,1.
84320542885942,0.0652990225639085,0.446191448217459
"2251","ZFAND5",1204.74811453045,0.552564592201611,0.299802392716319,1
.84309600465551,0.0653149945844301,0.446191448217459
"2252","ASB6",34.1272488169873,0.51315861468899,0.278297526003853,1.84
392086432664,0.0651946739277942,0.446191448217459
"2253","TPI1",985.145742708936,-0.338126999729299,0.183309790112969,-1
.84456596410329,0.065100701933874,0.446191448217459
"2254","NDUFA4L2",15.8506502239406,-1.24684987766016,0.676375025967757
,-1.84342979824864,0.0652662827301983,0.446191448217459
"2255","IL4R",210.841478355618,0.340908897226494,0.184886021286512,1.8
4388681661443,0.0651996367809205,0.446191448217459
"2256","MBTD1",79.1309134267636,0.460251017973396,0.249427700494122,1.
84522816456082,0.0650043551111104,0.446191448217459
"2257","PARD6G-
AS1",10.493982668811,-0.902401449818798,0.489398354733587,-1.843899639
40528,0.0651977676722839,0.446191448217459
"2258","CDC37",486.127738559597,-0.32353890678373,0.175566595395962,-1
.84282725340797,0.065354236329012,0.446191448217459
"2259","ZNF284",18.2618056212015,0.783577369699097,0.425094098629441,1
.8433033350156,0.0652847344968158,0.446191448217459
"2260","ZDBF2",42.2931485644742,0.620534067771888,0.336858929062185,1.
84211850788534,0.0654578171386234,0.446276979739885
"2261","INTS6",722.600742941827,0.410976725868909,0.223084647971273,1.
84224566596726,0.0654392234485433,0.446276979739885
"2262","NDUFB2",997.477700980919,-0.449017183825434,0.243890727827167,

-1.84105885379796,0.0656129345194127,0.447136775250551
"2263","ZCWPW1",49.5108956937185,0.460688343841848,0.250300217997231,1
.84054311869175,0.0656885399271817,0.447383279213905
"2264","PAPPA",15.5966626508742,-0.940813245809297,0.511195906617776,-
1.8404162350086,0.065707151744423,0.447383279213905
"2265","ESRG",24.8634852783615,0.626674366117932,0.340807049260343,1.8
3879519944793,0.0659453146150415,0.448662911533949
"2266","CDK9",133.904221511254,-0.369080285983167,0.200746054777866,-1
.8385431603703,0.0659824080731406,0.448662911533949
"2267","MED21",229.053702598617,-0.475171963758675,0.258446045214576,-
1.83857316665133,0.065977991044069,0.448662911533949
"2268","AC245297.3",413.765066644592,-0.380231784709001,0.206858991485
89,-1.83812065396701,0.0660446283640528,0.448690148185048
"2269","XPO7",95.0129571709431,0.427999389895026,0.232834494770239,1.8
3821297749449,0.066031028238313,0.448690148185048
"2270","ZNF587",43.9983715111758,0.703614142626611,0.382921087445253,1
.83749123695677,0.0661374089440291,0.44912253694811
"2271","RAD51B",46.0338403620121,0.604711571254985,0.329236396804521,1
.83670935875909,0.0662528129337715,0.449708107166045
"2272","MTMR12",65.3880948974176,0.436546891060428,0.237740250035514,1
.83623467627049,0.0663229562181417,0.449948139992076
"2273","TAF6",96.5692573222474,-0.335860050651958,0.182922830630342,-1
.83607507873458,0.0663465534999668,0.449948139992076
"2274","HOXB6",9.3554706499059,-1.11194295019129,0.605675374104914,-1.
83587280865522,0.0663764700643983,0.44995307213839
"2275","FAM124A",9.0217139523172,0.955901622165,0.520820231469202,1.83
537728453532,0.0664498070489838,0.450252209081356
"2276","GDPGP1",11.2534231069748,0.855121441981781,0.466046130685528,1
.83484291721068,0.0665289675627317,0.450590525034934
"2277","TUFM",590.886748934313,-0.495416686678473,0.270101881393752,-1
.83418450890485,0.0666266101431288,0.45105366506646
"2278","NBPF14",90.2578800903913,0.843800096270981,0.460279655494543,1
.83323352704861,0.066767849969087,0.451194121841831
"2279","MAMDC2",134.360881305506,-1.26312556497426,0.68887007265076,-1
.83361945179847,0.0667105027290314,0.451194121841831
"2280","EIF5A",658.949090090581,-0.421659244142813,0.229977147823147,-
1.83348323141685,0.0667307400286068,0.451194121841831
"2281","UNC119",39.1370637666918,-0.597847429304904,0.326147292474716,
-1.83305961171286,0.066793706522417,0.451194121841831
"2282","AGPAT3",75.4133731289127,0.510758603311488,0.278581407850002,1
.83342674320354,0.0667391335660245,0.451194121841831
"2283","RASA1",124.108553883398,0.371976572534176,0.203114731090762,1.
83136186398986,0.0670465494912015,0.451755594876089
"2284","MBLAC1",18.733128889479,-0.776558684599487,0.42404293727885,-1
.83132087892511,0.0670526630604276,0.451755594876089
"2285","MOSPD3",130.510601581187,-0.582168770517914,0.317768532722582,
-1.83205292711018,0.0669435355975754,0.451755594876089
"2286","RPL41",26729.6055681284,-0.447101333733838,0.244065505528878,-
1.83189071624436,0.0669677039832483,0.451755594876089
"2287","ANKRD10-

IT1",12.6907201409869,0.981120210796308,0.535740273330937,1.8313355549999,0.0670504738395196,0.451755594876089
"2288","DNASE1L1",13.8231015788598,-0.789258196681043,0.43088068481449,-1.83173259906243,0.0669912693492346,0.451755594876089
"2289","EN1",100.989230462913,0.563629030533431,0.30788339047695,1.83065747606683,0.0671516839111657,0.452223201313368
"2290","ZKSCAN1",400.47860630202,0.458837105856457,0.25069413240029,1.83026663393868,0.0672100781192946,0.452223201313368
"2291","AL441992.1",9.13648339161956,-0.956341015825649,0.522509537997225,-1.83028432263896,0.0672074344164744,0.452223201313368
"2292","HNRNPA0",1048.69400397671,-0.290420339010882,0.158741583850943,-1.8295164503561,0.0673222772368089,0.45278049895524
"2293","GLIPR1L2",14.7032682635413,0.933534062942981,0.510368521342233,1.82913722909057,0.0673790530361472,0.452964719822159
"2294","AREG",71.7134828562453,-0.866100191940034,0.473650510295257,-1.82856383158996,0.0674649751078838,0.453147098600448
"2295","SUGCT",9.71086430094319,-0.954761869368972,0.522095289106698,-1.8287119023858,0.0674427784676885,0.453147098600448
"2296","MAD2L2",117.556034666499,-0.43378059950001,0.237333958276164,-1.82772243235103,0.0675912197120011,0.453205155224227
"2297","TMEM135",117.891563039658,0.360458675744001,0.197210042709735,1.82779066821939,0.067580974279467,0.453205155224227
"2298","NR2C2AP",80.9758335252781,-0.471118509374435,0.257680407261107,-1.82830551372519,0.0675037127905402,0.453205155224227
"2299","ZFP82",38.1779916778947,0.543443166256377,0.297299735048222,1.82793020709632,0.0675600268694979,0.453205155224227
"2300","FAM225A",12.7697367914755,1.00065984820676,0.547578581126016,1.82742693505113,0.0676356025890519,0.453305571265319
"2301","LATS2",298.357805250677,0.370946506476419,0.203056540747693,1.82681387711286,0.067727758667291,0.45352884442063
"2302","AC003102.1",5.0528378732583,1.33552969099917,0.731010095494351,1.82696477002278,0.0677050665699607,0.45352884442063
"2303","UIMC1",80.1530978154445,-0.391863010832499,0.214535966220592,-1.82656091533656,0.0677658144719953,0.453586639203564
"2304","AL139220.2",3.51008226166168,-1.59462827277058,0.873576093817862,-1.82540282873521,0.0679402627540675,0.453704411262789
"2305","IVNS1ABP",131.585542971048,-0.550301289615572,0.301348572614999,-1.8261287413451,0.0678308718236941,0.453704411262789
"2306","HLX",43.7484062740953,0.641768674860135,0.351647930042547,1.82503185723995,0.0679962220581437,0.453704411262789
"2307","BOLA3",169.568556151552,-0.4443631835271,0.243502805871202,-1.82487910945117,0.0680192743573575,0.453704411262789
"2308","RBM12B",87.0982718319015,0.426578196283643,0.233609751884474,1.82602906275333,0.0678458842382754,0.453704411262789
"2309","RPL6",10057.3695924944,-0.480610250254921,0.263283336652097,-1.825448797354,0.067933331242481,0.453704411262789
"2310","PFN1",1396.5557344754,-0.47414815889633,0.259782244432981,-1.82517538845367,0.067974566556038,0.453704411262789
"2311","MORC2",47.3258204365298,0.472869878533475,0.259151254727148,1.82468681863549,0.0680483035251098,0.453704411262789

"2312","GPR82",9.72499053663343,-0.914511767856025,0.500948851723902,-1.82555916578896,0.0679166913922817,0.453704411262789
"2313","HS3ST1",3.89128927494098,1.68617126836983,0.924393377146645,1.82408410754153,0.0681393577758679,0.454115088679206
"2314","SIRT7",70.2966719255157,-0.391275216756235,0.214566240677577,-1.82356374199702,0.0682180522826982,0.454443075167586
"2315","AC006059.1",11.4020522367251,-0.934948316539284,0.512782365876184,-1.82328484510529,0.0682602604072547,0.454463217822172
"2316","SYNRG",186.409931160865,0.398764160199385,0.218722124793046,1.82315419885709,0.0682800397324781,0.454463217822172
"2317","PYROXD2",58.0714999847469,0.515551112038217,0.282923289591331,1.82222931446508,0.0684201983632788,0.454611446009742
"2318","AC074032.1",44.2692258230531,-0.504653699297664,0.276859688180015,-1.8227778215568,0.0683370481095008,0.454611446009742
"2319","RAB37",5.41430394899223,1.59467458826375,0.875031399745533,1.82241984542211,0.0683913056363809,0.454611446009742
"2320","KIAA1671",94.0608840122363,0.598204716738721,0.328281890791489,1.8222288024979,0.0684202760131432,0.454611446009742
"2321","ZNF714",40.7237303035245,0.745116100333897,0.408950183178532,1.82202168132691,0.068451695944307,0.454624253761953
"2322","NFIC",1945.37099036594,-0.298555548839551,0.163929854872583,-1.82123963369338,0.0685704382006899,0.4552167548939
"2323","JTB",1022.25710865502,-0.402419877998109,0.221037999798949,-1.82059138412463,0.0686689934996858,0.455312721061959
"2324","MKLN1",311.08211065316,0.400972586428466,0.220235381845436,1.82065471528037,0.0686593599486694,0.455312721061959
"2325","ADAM33",349.75446749734,0.455516510206491,0.250206572446158,1.82056172926677,0.0686735047985115,0.455312721061959
"2326","AC069243.1",2.40530668515813,-1.93080043849656,1.06102393804213,-1.81975200489765,0.0687967797335163,0.455933946514254
"2327","POLR1A",49.4068795817673,0.517244392185084,0.284582661516871,1.81755413147129,0.0691323076521915,0.456913840441873
"2328","ST3GAL6",7.2443852382894,-1.33574878550166,0.734642786424486,-1.81822895451375,0.0690291462929244,0.456913840441873
"2329","SPEF2",46.8864454854009,0.494611602488056,0.272135944815438,1.81751661958327,0.0691380458725678,0.456913840441873
"2330","BTRC",50.7931969930327,0.428615975738821,0.23580250509967,1.81769050993607,0.069111449037264,0.456913840441873
"2331","KLF5",35.103129927666,-0.844614967029688,0.464584657853901,-1.81800012710557,0.0690641133521722,0.456913840441873
"2332","FN3K",26.9922461394998,0.609433493741247,0.335174578130853,1.81825691297901,0.0690248749640532,0.456913840441873
"2333","AP5S1",47.1671161440244,0.534580661674337,0.294141865244772,1.81742459962129,0.0691521238891268,0.456913840441873
"2334","INPP4A",42.3322217242149,0.572731722809807,0.315257765399648,1.81670932699711,0.0692616328394938,0.457441332571036
"2335","KIAA0825",27.1917490899227,0.562288264360502,0.309546289525606,1.81649169570805,0.0692949806540361,0.457465578921613
"2336","EXOC6B",76.4086350905956,0.466175694417446,0.256802281017263,1.81530978841308,0.069476315589387,0.457878326126721

"2337","JCHAIN",53.6825195764216,-0.868905452373246,0.478589866239566,-1.81555338645282,0.0694389095483121,0.457878326126721
"2338","AC021321.1",6.32602412151021,-1.23893684300299,0.682447652080235,-1.815431321694,0.0694576513069936,0.457878326126721
"2339","PGM2L1",57.6917094308133,-0.727543398903312,0.400760251217913,-1.81540808174539,0.0694612200271128,0.457878326126721
"2340","RCOR3",141.296006915763,0.33679987497679,0.185661642285313,1.81405200789519,0.0696697192229007,0.458398512537266
"2341","AC016575.1",5.81364999053364,-1.26723741043812,0.698578494410735,-1.8140229345409,0.0696741949318725,0.458398512537266
"2342","LIMK1",55.2769233557046,-0.492320358563418,0.271375341920465,-1.81416762141826,0.0696519233891702,0.458398512537266
"2343","PLD6",13.7725090332949,0.803181896773605,0.442681468956916,1.81435626538904,0.0696228943425248,0.458398512537266
"2344","AL590399.1",8.32380245029761,1.05182831578085,0.57990591371207,1.81379132529953,0.069709858539036,0.458437486936536
"2345","LINC01232",12.1152661289913,0.926953362083796,0.511215342194125,1.81323463045012,0.069795640716204,0.458805885560889
"2346","GLB1",167.606223821092,0.396632385560116,0.218792988211923,1.81282036870367,0.0698595313208506,0.459030125878479
"2347","BDKRB2",285.259131623651,0.762151686484292,0.420497553736856,1.81249969164206,0.0699090215392918,0.459159593961731
"2348","PRKG1",73.1240127996717,0.443269660879847,0.244596472295024,1.81224878969305,0.0699477633997348,0.459218387055755
"2349","AGA",70.7876250353433,-0.600173486013164,0.331351074047237,-1.81129180805856,0.0700956930947281,0.45922444374632
"2350","TSC1",125.853349670743,0.478498602768866,0.264177160464799,1.81127922613365,0.070097639710353,0.45922444374632
"2351","ARHGDIB",96.2843249532053,-0.584210406172038,0.322447120820574,-1.81180221018975,0.0700167634962569,0.45922444374632
"2352","PRMT7",58.5182470231984,0.403131224370954,0.222557890765338,1.81135444348729,0.0700860030989813,0.45922444374632
"2353","SETBP1",254.085207238428,-0.568512892603654,0.313755174202347,-1.81196340123784,0.0699918517533612,0.45922444374632
"2354","SHKBP1",69.9713258950147,-0.519559237261637,0.286891075033554,-1.81099825848842,0.0701411212470431,0.459314096866257
"2355","RNF165",7.10865789558202,1.11636161713796,0.616608741403391,1.810486200045,0.0702204224840547,0.459638136981615
"2356","POM121",112.544729139451,0.445923475683629,0.246363663758658,1.81002128674488,0.070292486173019,0.459677734201348
"2357","YTHDF3",176.731480533625,0.353507368095679,0.195289630463224,1.81016968108939,0.0702694777805532,0.459677734201348
"2358","MOSPD2",100.117756587212,0.363143485356465,0.200646158739468,1.80987010983845,0.0703159323546402,0.459677734201348
"2359","USP24",79.1896909104695,0.484884017110895,0.268060959025118,1.80885727960654,0.0704731787787927,0.459968368301616
"2360","GMPPB",60.6246066513849,-0.653215446638521,0.361111258324097,-1.80890357633841,0.0704659847178336,0.459968368301616
"2361","SEL1L3",18.7408640633267,0.97958816117306,0.541412308902291,1.80932007836905,0.0704012914464506,0.459968368301616

"2362","EEF2K",269.095816667561,0.495636447417273,0.274011682801739,1.
80881501967159,0.0704797460868257,0.459968368301616
"2363","PARP15",11.755904963511,1.07982715779362,0.597112412688768,1.8
084151909206,0.0705419054024342,0.460179209385748
"2364","PPP4R1",110.551607370605,-0.33063665334735,0.18295326013224,-1
.8072192488309,0.0707281008183965,0.461198677713868
"2365","AF117829.1",113.383759344277,0.608923696022848,0.3371054605625
84,1.80632996868884,0.0708668133654222,0.461907791978005
"2366","MALL",34.8121997456212,0.726215762026455,0.402135994143609,1.8
0589594714845,0.0709345942910087,0.46191050856319
"2367","AC105446.1",11.0292700166744,-1.10607174455221,0.6125271196227
23,-1.80575146653666,0.0709571695448427,0.46191050856319
"2368","ALOX5",7.14336263051774,-1.48367930396551,0.821803323073999,-1
.80539462704499,0.0710129513488543,0.46191050856319
"2369","JRKL",69.1290216494039,0.438790509922381,0.243073426356835,1.8
0517679986224,0.0710470201623954,0.46191050856319
"2370","PDZRN4",17.0224979965235,1.42547463664743,0.789577428935339,1.
80536396356914,0.0710177463966785,0.46191050856319
"2371","BCORL1",64.3139437141692,0.481147356817409,0.266410262516309,1
.80603912279075,0.0709122287500339,0.46191050856319
"2372","KCNJ8",94.232307876068,-0.561226509738297,0.310996685619581,-1
.80460608003007,0.0711363459794226,0.462296278782799
"2373","NOSTRIN",48.2567050152409,0.72218435457589,0.400256118617524,1
.80430559580276,0.0711834130437297,0.462407211154274
"2374","NMI",181.998573367106,-0.375587944471834,0.208207144993603,-1.
8039147719132,0.0712446688678225,0.46261018138O575
"2375","MELTF",86.4594836163673,0.896972912175402,0.497313119266672,1.
80363814551698,0.0712880520450183,0.462696977799561
"2376","CCDC8",103.848515727883,0.464352479481984,0.257521690041435,1.
80315871415441,0.0713632923050817,0.462990383368196
"2377","ALG1L9P",5.59289340573491,1.33681304259888,0.741650926839713,1
.80248280453885,0.0714694777140475,0.463289318318773
"2378","BCL2L1",120.760023405385,0.420948580740252,0.233514325087164,1
.80266705515015,0.0714405191115369,0.463289318318773
"2379","NPR2",81.9293162098581,0.449798261752127,0.249637010233464,1.8
0180920021222,0.0715754297466385,0.46378110531502
"2380","HMG20B",278.496162693372,-0.419548599742015,0.23289034242233,-
1.80148560639407,0.0716263739537589,0.463916199368569
"2381","SLC25A5",553.887655305782,-0.42745808593299,0.237349235306339,
-1.80096677110117,0.0717081175324922,0.464250580329008
"2382","CLCN4",40.0145570346426,0.840226697667045,0.466592663320887,1.
80077134451041,0.0717389272080876,0.464255064195076
"2383","SARDH",5.6846690879842,1.38454909586191,0.769020658744459,1.80
040559394386,0.0717966181878299,0.464433432381619
"2384","GTPBP6",237.195267458772,-0.471348463290608,0.261831012162307,
-1.80020105104442,0.0718288979461203,0.464447341375606
"2385","PGD",270.647609053373,0.4342866959861,0.241302213058566,1.7997
6259016197,0.0718981333250531,0.464638322058703
"2386","SRPRA",266.996014220613,-0.323459130374072,0.179736233315908,-
1.7996322967643,0.0719187179002313,0.464638322058703

"2387","FRMPD1",14.0882706973778,1.13332604778843,0.629877722619001,1.
79927945867987,0.0719744857312658,0.464803811289259
"2388","KLHL36",155.202629351324,0.328139212136146,0.182424340634124,1
.79876879913889,0.0720552607603124,0.46513058819942
"2389","PRF1",9.48186298923476,1.30419550403769,0.725481064590275,1.79
769751092574,0.0722249560907115,0.466030848948647
"2390","TRAK1",100.991312002401,0.506926444234125,0.282203317410966,1.
79631638949127,0.0724442127309192,0.467250016421389
"2391","TRIML2",4.94049069736132,-4.178945798486,2.32757836888407,-1.7
954049815687,0.0725891993556588,0.467989338380376
"2392","MFSD6",17.9386978803763,-0.945405719517371,0.526955677665497,-
1.79408963521501,0.0727988631259511,0.468557192102938
"2393","AC107294.2",4.32035343514575,1.69976149112189,0.94738436844218
6,1.79416248329794,0.0727872383264205,0.468557192102938
"2394","VPS35L",116.29755898593,0.325082120613492,0.181143740015785,1.
79460863833972,0.0727160758676974,0.468557192102938
"2395","BRWD1",221.351140699268,0.375235738736946,0.209112664789962,1.
79441899950843,0.0727463166101418,0.468557192102938
"2396","PPP1CB",636.215535349079,-0.384134956697637,0.214158528060404,
-1.79369441962774,0.0728619565005334,0.468701784451419
"2397","EBP",125.484020874835,-0.452450150151994,0.252262605322164,-1.
79356805410842,0.0728821393013333,0.468701784451419
"2398","SPATA6",234.515570548812,0.377446432487768,0.210997218919085,1
.7888692297528,0.073635878325839,0.469173307626942
"2399","ACKR1",3.68821706996496,-1.93087789044469,1.07993933174119,-1.
78795033544295,0.0737840208867585,0.469173307626942
"2400","LHX4",2.67799588826126,2.10587893301877,1.17521135583326,1.791
9150649507,0.0731465724011143,0.469173307626942
"2401","B3GALNT2",78.0422937749235,-0.35556100119081,0.198444904587238
,-1.79173661289198,0.0731751667388894,0.469173307626942
"2402","TMEM178A",2.40515003977305,2.18914897798196,1.22448101096965,1
.78781782516039,0.0738054040565295,0.469173307626942
"2403","KCMF1",221.839207059057,-0.311962986312615,0.174350753089131,-
1.78928384756178,0.0735691140338729,0.469173307626942
"2404","ST3GAL5",567.184381128716,0.433321384795319,0.24218523364671,1
.78921471912458,0.0735802420770187,0.469173307626942
"2405","LRP1B",82.5687440549901,0.635630782623966,0.355138519845081,1.
78981086844998,0.0734843213737932,0.469173307626942
"2406","FAP",273.464452088097,-0.848962613058446,0.474725905327447,-1.
78832164735747,0.0737241293055725,0.469173307626942
"2407","KLF7",294.227724738298,0.388946382113124,0.217577855474159,1.7
8761933867538,0.0738374432772269,0.469173307626942
"2408","RPL14",10843.3423930164,-0.423028432890073,0.236644009700646,-
1.7876152175802,0.0738381086151775,0.469173307626942
"2409","PDS5A",202.293533763565,0.355493893915115,0.198455027723192,1.
79130706837503,0.0732440324777886,0.469173307626942
"2410","AP002026.1",7.36264583362014,1.07115925000316,0.59764672366795
6,1.79229502577894,0.0730857196775145,0.469173307626942
"2411","TMEM131L",29.1742155192174,0.564685126637538,0.315297282940058
,1.79096096665347,0.073299559003583,0.469173307626942

"2412","MTREX",200.120631671916,0.344747871565922,0.192638685633842,1.78960871972103,0.073516835732106,0.469173307626942
"2413","TUBB2B",34.2508234117213,-0.932449286784754,0.520846524005855,-1.79025729040725,0.0734125588591051,0.469173307626942
"2414","AC002429.2",3.73253649498,1.66206171004041,0.928581055959473,1.78989405326933,0.0734709450320697,0.469173307626942
"2415","TMEM176B",2037.30788829426,0.57598215859746,0.321752088903293,1.79014271689959,0.073430971127947,0.469173307626942
"2416","SLC52A2",122.561234218138,-0.464764982882284,0.259816286261283,-1.78882159224959,0.0736435523798419,0.469173307626942
"2417","ZNF37A",215.428444820624,0.440599523718339,0.245874053761933,1.79197242237259,0.073137383652982,0.469173307626942
"2418","BHLHE41",111.545796022065,-0.94489840712926,0.52763178170742,-1.79082921061268,0.073320706215583,0.469173307626942
"2419","AC022506.2",2.9588328425487,-1.61368006299937,0.90122431553391,-1.79054208279254,0.0733668083198762,0.469173307626942
"2420","MIA2",82.9253176344918,-0.377454913479954,0.210858129123861,-1.79008945516267,0.0734395317173109,0.469173307626942
"2421","EPG5",68.9624361570873,0.422696115265541,0.236437062258878,1.78777434987213,0.0738124207627813,0.469173307626942
"2422","AC007193.1",3.27991964435348,2.24329720900562,1.25446117172879,1.78825559496123,0.0737347804661532,0.469173307626942
"2423","AC008440.2",7.51715234687589,-1.10112519391296,0.615366558906775,-1.78938094372427,0.0735534861727545,0.469173307626942
"2424","ZNF8",26.7026122360786,0.67595503562335,0.37758935097127,1.79018564449605,0.0734240721060743,0.469173307626942
"2425","AL359555.4",5.44094288273318,-1.29763779000609,0.723856673011111,-1.79267227669279,0.0730253419455712,0.469173307626942
"2426","AC009509.1",7.01891864882607,5.77478563846671,3.23033710374857,1.78767275767148,0.0738288193906188,0.469173307626942
"2427","HGF",53.5103561161235,-0.781348743732615,0.437177863376019,-1.78725596419454,0.0738961277932634,0.469348500178474
"2428","FAM72D",3.62882536831731,1.85361190781605,1.03820524260636,1.78540025781671,0.0741964168703155,0.469708986176861
"2429","CCL20",12.9452383817078,-1.19435453561916,0.668484040729441,-1.78666125569115,0.0739922547244589,0.469708986176861
"2430","RING1",160.860211835778,-0.327099310691963,0.183154158033287,-1.78592347672781,0.0741116491795755,0.469708986176861
"2431","PHKG1",62.5457471362651,-0.78624531231203,0.440234645057223,-1.7859687353998,0.0741043204572092,0.469708986176861
"2432","CSRP2",46.0160923188268,-0.874707687487794,0.489974439846361,-1.78521085255401,0.0742271223046923,0.469708986176861
"2433","TM9SF1",320.190162042866,-0.386384647107035,0.216416107623782,-1.78537841452507,0.0741999574659166,0.469708986176861
"2434","ARL6IP1",590.854100219659,0.279000222637302,0.15627507788956,1.7853148845299,0.0742102558755368,0.469708986176861
"2435","KSR1",41.5989356068484,0.655627489118851,0.367012879010476,1.7863882348939,0.074036419224244,0.469708986176861
"2436","KIAA0355",128.526822666605,0.350189091625092,0.196102778195874,1.78574263376989,0.0741409389352323,0.469708986176861

"2437","B3GNT9",62.5527897034134,0.654230001820671,0.366602063988831,1
.78457806457032,0.0743297820418575,0.470165609427671
"2438","CHRAC1",187.029644595738,-0.313444669739179,0.175755272716397,
-1.78341545544958,0.0745186991902727,0.470395474208867
"2439","KIF5B",658.826419351353,-0.319051864484452,0.17888751044237,-1
.78353348255265,0.074499502606342,0.470395474208867
"2440","DRC3",23.7950271234269,-0.646223152327171,0.362309555660615,-1
.78362160818222,0.0744851719990064,0.470395474208867
"2441","PNPLA6",47.3973185500111,-0.430819969073413,0.241564224285241,
-1.78345932783779,0.0745115630693932,0.470395474208867
"2442","RPA4",11.6915233260544,-0.787742825637169,0.441571990629221,-1
.78395107106922,0.0744316161231277,0.470395474208867
"2443","SELENBP1",579.44971448065,0.413378848390343,0.231916967693596,
1.78244331366255,0.0746769675075401,0.470623652546497
"2444","ANAPC15",58.0614308829357,-0.587920102305696,0.329803437596957
,-1.78263788452131,0.0746452686840536,0.470623652546497
"2445","SENP8",8.89865537926132,1.02391698472595,0.574401757030962,1.7
8257982708565,0.0746547260534865,0.470623652546497
"2446","BTG3",210.877656127984,-0.406642980974068,0.228100771262833,-1
.782733915027,0.0746296277720115,0.470623652546497
"2447","VWA1",60.5478942994361,0.65902076648819,0.369915743985869,1.78
154289781558,0.0748238034190675,0.470738962553177
"2448","VDAC1",539.237719689719,-0.383188646010733,0.215151116734135,-
1.7810209485654,0.0749090285528993,0.470738962553177
"2449","ZNF397",143.916908276186,0.456476522251172,0.256280086823129,1
.78116266429318,0.0748858810300189,0.470738962553177
"2450","PBX4",18.2151720086064,-0.703773180521126,0.395116622209478,-1
.78117836851725,0.0748833162975608,0.470738962553177
"2451","TCEANC",10.9939320422682,0.841870033770199,0.472444962077004,1
.78194308617261,0.0747585133498096,0.470738962553177
"2452","GEMIN8",76.8818300282422,-0.496182213830449,0.278453829592407,
-1.78191915893829,0.0747624157301324,0.470738962553177
"2453","CA5B",119.428834330508,-0.628667965575794,0.352976881172573,-1
.78104572596196,0.0749049810481421,0.470738962553177
"2454","ADCY6",64.0873364343291,0.394756594605989,0.221729542738679,1.
7803518183918,0.0750184016897771,0.470850432429932
"2455","ZNF664",102.327197746638,0.382025571605045,0.21455063783211,1.
78058464642733,0.0749803298374471,0.470850432429932
"2456","FAM110A",7.89655831065341,-0.976508883717144,0.548457951933628
,-1.78046262302222,0.0750002810378732,0.470850432429932
"2457","ZNF500",43.1013669712787,0.581052826116187,0.326628782201769,1
.77893944985305,0.0752496895134224,0.472109875396584
"2458","LINC02289",5.13637041605978,1.300323573885,0.731149738138531,1
.77846411761776,0.0753276601773345,0.472406786669492
"2459","AC093627.6",13.3536291002258,-2.69246504230131,1.5144018672009
6,-1.77790657857398,0.0754191995922678,0.472788516354131
"2460","NNMT",5170.43906940938,-0.46362546858986,0.260800112935386,-1.
77770424779196,0.0754524416708409,0.472804629413014
"2461","TRIM61",9.19600236752518,1.29753796115354,0.730439696832619,1.
77637930520482,0.0756704197436989,0.472825099452419

"2462","ERGIC1",537.768884238599,0.281111739464971,0.158211539202636,1.77680933313547,0.0755996157690589,0.472825099452419
"2463","AC004854.2",5.92722999537616,-1.03019853838553,0.579903950664409,-1.77649856878058,0.0756507776094321,0.472825099452419
"2464","UBE2H",415.000999354817,0.339753357598991,0.191178862576018,1.77714917340245,0.0755436994243875,0.472825099452419
"2465","AC139019.1",3.66914822780212,1.5507422564667,0.872543417539845,1.77726658100184,0.0755243893612674,0.472825099452419
"2466","POLR3K",79.8946041815234,-0.528598087890501,0.297524425415048,-1.77665442813008,0.0756251146004242,0.472825099452419
"2467","MORC2-AS1",14.892574268056,-0.686297991346774,0.386317308082235,-1.77651370256671,0.0756482854455597,0.472825099452419
"2468","ADORA2B",6.02780050734155,-1.27572592692179,0.718482961519188,-1.77558271420153,0.075801721191471,0.473453619192272
"2469","SLC36A4",79.5271265530613,-0.395510323663161,0.222788864903963,-1.77526971033158,0.0758533642138969,0.473584288925565
"2470","IQCH-AS1",59.803248263203,0.656190444142393,0.369768369071271,1.77459863803525,0.0759641822898852,0.474084157894162
"2471","VAV3",2.8968406439667,-1.61498417423245,0.910718242217641,-1.77330825206695,0.0761776424126356,0.474135865796456
"2472","DEGS1",355.964721298495,-0.368344329594998,0.207699394084006,-1.7734492255958,0.0761542983025499,0.474135865796456
"2473","C2orf68",114.593336476931,0.432220602256166,0.243745278187236,1.77324707773067,0.0761877742184768,0.474135865796456
"2474","AC116351.2",3.39398198897427,1.71545069211165,0.967155153013708,1.77370785521455,0.0761114864399119,0.474135865796456
"2475","RNF170",157.127551218031,0.326610951118654,0.184171193850692,1.7734095343023,0.0761608702790288,0.474135865796456
"2476","AL138756.1",5.02545442651037,1.34055620990555,0.755950608655183,1.77333835644417,0.0761726568739424,0.474135865796456
"2477","YES1",202.956619706414,0.302430077421781,0.170506085706341,1.77372013537775,0.0761094541500034,0.474135865796456
"2478","WDR82",303.723348639126,0.294544633115472,0.166177818666261,1.77246659920968,0.0763171349974392,0.474557739405214
"2479","TLE1",36.0149672208805,0.731250550404135,0.412528436123205,1.77260641054509,0.0762939487405407,0.474557739405214
"2480","STAT6",288.73140977215,0.360055378050594,0.203242738095677,1.77155347061452,0.0764687092558664,0.475308529507734
"2481","AC010883.3",7.76075690444303,1.5075190422195,0.851069972494008,1.77132208977108,0.0765071561445203,0.475355829088182
"2482","OVGP1",2.75973285465796,-1.65082893626438,0.93210423871856,-1.77107759807413,0.0765477986949594,0.47541672718888
"2483","DNAJC4",230.702714148215,-0.515109454383597,0.290932848594921,-1.77054415433442,0.0766365356740514,0.475734671161622
"2484","RHNO1",28.2496403764538,0.595982912197599,0.336637645154045,1.77039888668683,0.0766607150934459,0.475734671161622
"2485","TRMT10A",53.0290315827196,0.400917444121936,0.226513130838852,1.76995233184588,0.0767350819256047,0.475842604916135

"2486","CACNB1",17.5916765890286,-0.701113000025834,0.3961675707374,-1
.76973849404389,0.0767707141372966,0.475842604916135
"2487","CALR",1580.69125696324,-0.385320830725939,0.217715216318519,-1
.76983877030539,0.0767540032283097,0.475842604916135
"2488","MBD5",133.369883426431,0.400873237831194,0.226604093477326,1.7
6904676204053,0.0768860711936288,0.475889406068652
"2489","CLOCK",138.818199680975,0.397375433933785,0.224573801530581,1.
76946478719012,0.076816342126553,0.475889406068652
"2490","SGPL1",58.3461124062262,0.42193099579496,0.238519134757103,1.7
6896078473803,0.0769004191111317,0.475889406068652
"2491","SMIM10L2A",2.93428135830948,1.82354576595762,1.03086174435006,
1.7689527969702,0.0769017522229654,0.475889406068652
"2492","BICC1",588.311178850137,0.506869159971286,0.286602638109268,1.
76854324620013,0.0769701291030153,0.476121404543732
"2493","UBE2QL1",4.40168348734898,1.63888611095967,0.927267980914425,1
.76743524492616,0.0771553647509221,0.476502382866773
"2494","GALNT10",141.938028769559,0.580989255681548,0.328634843104987,
1.76788696594762,0.0770798022187823,0.476502382866773
"2495","QKI",829.568507428826,0.288145286230474,0.163018898118142,1.76
755756269221,0.0771348978461895,0.476502382866773
"2496","AL138820.1",3.39265721730191,1.78657997338404,1.01069160673172
,1.76768062728978,0.0771143104425038,0.476502382866773
"2497","CENPK",5.27849743562959,-1.11538973856558,0.631274912796453,-1
.76688430976065,0.0772476052208102,0.476880990980693
"2498","AC073349.1",14.6590307304833,-0.771027342871559,0.436478749337
386,-1.76647166452444,0.0773167512987857,0.476925858851853
"2499","EXD3",35.6860762503177,0.549398372759222,0.311001277065774,1.7
6654699923637,0.0773041238610012,0.476925858851853
"2500","HS2ST1",93.2187503041676,0.369641686768004,0.209285677828652,1
.76620631952962,0.0773612412248407,0.47693627805283
"2501","KMT2C",449.050092565083,0.496907873357894,0.281448178152921,1.
76553949156461,0.0774731391660256,0.47693627805283
"2502","PPP2R2D",144.33085248574,-0.307852288109672,0.174327207276268,
-1.76594516093979,0.0774050495880462,0.47693627805283
"2503","RELB",50.7275557908653,-0.476266364524675,0.269727727349177,-1
.76573009087836,0.0774411419489293,0.47693627805283
"2504","MRTFA",108.829013120469,0.394140035910894,0.223227066549533,1.
76564626325651,0.077455213341605,0.47693627805283
"2505","LINC02384",91.2827600856479,0.673923295945254,0.38186799907175
,1.76480694266981,0.0775962174417166,0.477503270205214
"2506","CFLAR",646.491154862516,0.433831913884411,0.245883841187399,1.
76437748731023,0.0776684458634492,0.477639904369255
"2507","CRYGS",3.80845911478988,1.48541362252139,0.841924932356047,1.7
6430649032403,0.0776803918426027,0.477639904369255
"2508","VRK3",89.1770818141316,0.366608517955336,0.207815851323564,1.7
6410276511841,0.0777146790293891,0.477660198260779
"2509","MBOAT1",123.584261693577,-0.451971093411406,0.256253905956897,
-1.76376274821516,0.0777719317180193,0.477821573309393
"2510","LINC01303",6.8125058819726,-1.34141648607163,0.760839494399276
,-1.76307420414703,0.0778879752794374,0.4783438800528

"2511","CSF2",3.42847361724786,-2.51388526016811,1.42651032751153,-1.76226222249189,0.078025003492286,0.478803912752225
"2512","CKAP5",120.187757712792,0.315880682064319,0.179229085156516,1.76244096647857,0.0779948222197033,0.478803912752225
"2513","CEP63",186.381713384618,-0.344018776660981,0.195292979830593,-1.7615521917859,0.0781449874928495,0.47896818377824
"2514","PIM1",339.31858070288,-1.30751309256369,0.742174537591957,-1.76173261993872,0.0781144837521986,0.47896818377824
"2515","TBXAS1",4.44140325357043,-1.26297276780256,0.716964202930816,-1.76155624317053,0.0781443024468401,0.47896818377824
"2516","SIPA1L2",175.461907662294,0.532144784640968,0.302184606215143,1.76099236591192,0.0782396951118224,0.479108701477686
"2517","AC007364.1",11.7123353668426,0.8896591695234,0.505339614828654,1.76051736973967,0.0783201250570884,0.479108701477686
"2518","TCTA",82.0272847452272,0.519029429692218,0.294827501832203,1.76045120101318,0.0783313345850773,0.479108701477686
"2519","PCNP",558.186188215597,-0.253514176899163,0.144011260041799,-1.76037746510641,0.0783438275942065,0.479108701477686
"2520","TM4SF1",8.50349586711716,-1.4940821740503,0.848880226847932,-1.76006240550347,0.078397226133008,0.479108701477686
"2521","THAP6",67.2443577573366,0.395959055000643,0.224966617639422,1.76007915821221,0.0783943860194684,0.479108701477686
"2522","R3HCC1",212.463354176652,-0.43863455783101,0.249063696443242,-1.76113405564495,0.0782157161580054,0.479108701477686
"2523","AC011462.5",7.13383842982936,1.41337302858665,0.803076409702028,1.7599483828831,0.0784165587952125,0.479108701477686
"2524","GMPPA",115.528895069606,-0.411695403579365,0.23396550240284,-1.75964148283071,0.0784686133180336,0.47915684141953
"2525","MAOB",42.3927621788842,-0.47423560650472,0.269523190441502,-1.75953544378828,0.0784866055520152,0.47915684141953
"2526","TBC1D9",106.612902871093,0.389966313625834,0.221753549382819,1.75855725742015,0.0786527382594583,0.4799809818961
"2527","TNFRSF25",2.66193795559974,-1.61452740647283,0.918709078690142,-1.75738701611042,0.0788518648994074,0.480815465753309
"2528","AL353150.1",5.40998979821467,1.39683579066626,0.794769734544662,1.7575352079386,0.0788266261259835,0.480815465753309
"2529","MCC",122.80250223406,0.528041456185476,0.300555490968251,1.75688507464751,0.0789374001546893,0.48095653098203
"2530","TRIR",1297.27163391692,-0.394474227107866,0.224520871534608,-1.75696016326509,0.0789245995925501,0.48095653098203
"2531","CD164",1136.63583512112,0.246135038622136,0.140156484827041,1.75614449039498,0.0790637400692909,0.481535975175077
"2532","SEPSECS",29.3286520450156,0.531674720531749,0.302804714141158,1.75583369644602,0.0791168089102482,0.481668882050346
"2533","SLC39A1",527.915912316161,-0.384849140822466,0.219271765744143,-1.75512401022723,0.0792380982120382,0.481924959538081
"2534","ATF2",165.609700880097,0.31246856546556,0.178096443767789,1.7544907627295,0.0793464513336134,0.481924959538081
"2535","JRK",23.0078716725383,0.784518432112006,0.447080468840631,1.75475890088963,0.0793005563017044,0.481924959538081

"2536","MFAP5",16526.9573531606,0.548417616199929,0.312577568779269,1.
75450086946962,0.0793447210533399,0.481924959538081
"2537","ABAT",17.2214106545981,0.811995015529446,0.462717003519664,1.7
5484153241181,0.0792864172869534,0.481924959538081
"2538","CD37",16.9472076633113,-0.708351951402382,0.403644285600836,-1
.75489156336743,0.0792778575259163,0.481924959538081
"2539","RUNX3",13.476863798532,1.16765890674987,0.665783323738456,1.75
381218651335,0.0794626942132989,0.482250957203938
"2540","ENTR1",93.8275048402677,-0.345431491653786,0.196958445240917,-
1.7538292975011,0.0794597613303959,0.482250957203938
"2541","AL356055.1",5.73738104374508,-1.2372794671613,0.70595936367111
,-1.75262137005625,0.0796670206067798,0.483300717297722
"2542","SVBP",568.565198633127,0.470370683170117,0.268458760715497,1.7
5211522960354,0.0797539960263997,0.483447836707413
"2543","BMP5",30.5929434349083,1.25447730776681,0.715972014972818,1.75
213176148293,0.0797511539614685,0.483447836707413
"2544","PRIMPOL",57.2164064982437,0.395070835139048,0.225592843401642,
1.75125606460693,0.079901812046236,0.484153471970412
"2545","HLTF",163.941069079928,0.302502848365933,0.172856698023697,1.7
5002098168312,0.0801146933073743,0.48483304497996
"2546","KCNJ5",3.63507720184745,-1.43074853666664,0.817531032339148,-1
.75008468189022,0.0801037025574348,0.48483304497996
"2547","PHKG2",47.6037593732654,-0.492754016598557,0.281560396391552,-
1.75008283449533,0.0801040212872668,0.48483304497996
"2548","LMNB2",48.3270682681292,-0.500763953610054,0.286200965194294,-
1.74969344799413,0.0801712248883502,0.48483304497996
"2549","YIPF2",213.632055220104,-0.381256064465512,0.217889461088474,-
1.74976826580292,0.0801583086468035,0.48483304497996
"2550","SETD7",481.585701142907,0.315721298586955,0.180501010705066,1.
74913867436917,0.0802670515109244,0.485222195702313
"2551","MMADHC",258.896403758328,-0.318678255532804,0.182229588746309,
-1.74877338924608,0.0803301983798476,0.485413566454469
"2552","OSR2",1298.18337281448,0.644317664984429,0.368493077036529,1.7
4852040685843,0.0803739551145968,0.485487663828962
"2553","TOE1",35.1765924325803,-0.543709701624324,0.311143743648151,-1
.74745503557084,0.0805584380285778,0.485587455108655
"2554","RPL5",10445.0483393804,-0.460204781828986,0.263484980382056,-1
.74660726832203,0.080705485565253,0.485587455108655
"2555","RBM47",32.8310863961485,-0.657280238685431,0.376217570471369,-
1.7470748053099,0.0806243630678315,0.485587455108655
"2556","CDK19",84.8898939359072,0.409906427869366,0.234583364567334,1.
74738063214925,0.0805713347875149,0.485587455108655
"2557","FAM66C",24.0391043237165,0.603367905172397,0.345446109692569,1
.74663395604416,0.0807008531866173,0.485587455108655
"2558","AC093525.6",9.824310250673,0.93040074849642,0.532619355863255,
1.74683991156959,0.0806651112768786,0.485587455108655
"2559","CBX1",349.547793415064,0.258636572609477,0.147973723424008,1.7
4785473139966,0.0804891852204358,0.485587455108655
"2560","HSPA12B",143.52661244931,0.456700706254268,0.261345778402287,1
.74749601484388,0.0805513355792863,0.485587455108655

"2561","TRIOBP",1074.06861514881,0.330803196063508,0.189393054937143,1.74664903194733,0.080698236449817,0.485587455108655
"2562","RAB33A",10.2976341058278,-1.06066218741513,0.607004168030643,-1.74737216526261,0.0805728025063386,0.485587455108655
"2563","STXBP3",207.959451444006,-0.352175165194197,0.201684904727797,-1.74616521583263,0.0807822471902003,0.48585967242955
"2564","CCDC167",119.094278439359,-0.522991689219311,0.299571374237713,-1.74579994684109,0.0808457202218253,0.486051785186988
"2565","FBXW5",329.345952637053,-0.297129306993529,0.170264999227072,-1.74509915920691,0.080967609918766,0.486594817504007
"2566","TNRC6A",535.206486172786,0.372043260247118,0.213216399214088,1.74490921720122,0.0810006726807782,0.48660380723858
"2567","MYLPF",4.87405514692746,-1.43176203278388,0.820782048674501,-1.74438760581578,0.0810915247407428,0.486959818417822
"2568","GALNS",70.357146473201,0.50991536917663,0.292604248102572,1.74267930996641,0.0813896476978857,0.488559742703625
"2569","MAP3K2-DT",13.3491782477924,-0.791878608181536,0.454496171976186,-1.74232184341264,0.0814521433088805,0.488690280778266
"2570","AC022966.1",8.21363388807799,1.10326346435022,0.63332745730099,1.7420111059955,0.0815065009329174,0.488690280778266
"2571","ZBTB33",46.9716988098724,0.424251217710025,0.243522261853766,1.74214552082629,0.0814829839933538,0.488690280778266
"2572","DNMT3A",69.8518183010329,0.444265758582622,0.255251422868699,1.74050257424481,0.0817708084478706,0.488754173022073
"2573","SMC2",116.032768239666,-0.288523749616428,0.165723273828832,-1.74099716322544,0.0816840757725494,0.488754173022073
"2574","VPS26B",156.610388158515,0.320959878233723,0.184312873609089,1.74138611128407,0.081615921059642,0.488754173022073
"2575","TMED2",855.003710938762,-0.286061578189882,0.164305309239846,-1.74103672920454,0.0816771405837378,0.488754173022073
"2576","FAM161B",30.7325511463988,0.624720295306318,0.358859865905055,1.74084748577486,0.0817103158004835,0.488754173022073
"2577","MEX3D",133.283384391963,-0.39190116188695,0.22503093559004,-1.74154349427277,0.0815883562189383,0.488754173022073
"2578","ARSA",169.484098867709,0.496497668392435,0.285069401039376,1.74167296308261,0.0815656860698905,0.488754173022073
"2579","BGN",377.738971301561,-1.26467036052667,0.726585897825896,-1.74056551924671,0.0817597660675858,0.488754173022073
"2580","ACOT7",21.8331844921937,-0.850708017951082,0.48930766478858,-1.73859532390251,0.0821059696289844,0.489040454712012
"2581","AKR1A1",525.650084665755,-0.457373363647298,0.263093092613326,-1.73844687104541,0.0821321039202409,0.489040454712012
"2582","FLAD1",42.5399371492829,-0.441663786691739,0.253867879855738,-1.73973874498309,0.0819049027133588,0.489040454712012
"2583","SPSB4",68.5039987165889,0.733007068851712,0.421693795756648,1.73824485023896,0.0821676793839838,0.489040454712012
"2584","DRAP1",676.816654177887,-0.446681597588238,0.256714127711618,-1.73999616448854,0.0818596914455312,0.489040454712012
"2585","IGDCC4",103.16675771258,0.621595891574579,0.357570873284838,1.

73838513708979,0.0821429738219628,0.489040454712012
"2586","STAP2",13.2724401654802,-0.879202510798135,0.505571688993703,-1.73902639316713,0.0820301205285354,0.489040454712012
"2587","SLC25A23",77.2580812028077,0.366945151005454,0.211000011875563,1.73907644717034,0.0820213169242702,0.489040454712012
"2588","CDS2",119.081198955522,0.340408686418498,0.195698703870628,1.73945294315048,0.0819551225691205,0.489040454712012
"2589","PQBP1",207.8492230931,-0.406370508406383,0.233699565514854,-1.73885863891584,0.0820596310904785,0.489040454712012
"2590","RTL8B",72.6683335728656,-0.375646845629276,0.216053310605269,-1.73867664687437,0.0820916560361094,0.489040454712012
"2591","BTG2",1710.30862641533,0.302760053645696,0.174215760260977,1.73784537743404,0.0822380625377909,0.489081687507734
"2592","CDK12",231.236026826681,0.353580265746769,0.203450269168497,1.73791987197586,0.0822249336300372,0.489081687507734
"2593","FAM126B",103.319280669322,0.414894513235099,0.238796812066103,1.73743740398112,0.0823099938715324,0.489132056873428
"2594","ZNF776",13.5732512875229,0.788516541606909,0.453793481282094,1.73761099295461,0.0822793815141585,0.489132056873428
"2595","MAFIP",3.39071673853996,1.71943881343524,0.989806276728802,1.73714680727,0.0823612611733696,0.489248108280344
"2596","PARP8",42.4653985284198,0.585864766834829,0.337322377046703,1.73680967140142,0.0824207713881563,0.489312494923129
"2597","OLR1",6.92836541853721,-1.47969233553228,0.852001135882973,-1.73672577795192,0.0824355854242858,0.489312494923129
"2598","UBXN8",44.2656707779217,0.476307406548793,0.274319367375038,1.73632438389815,0.0825064940867775,0.489544883120737
"2599","ZDHHC11",10.0816459896601,-1.52736478122593,0.879952656524925,-1.73573517836487,0.0826106703238017,0.489785955015924
"2600","PPP1R15A",2039.83912725432,-0.259050226626949,0.149232335254393,-1.73588536415618,0.0825841061585439,0.489785955015924
"2601","NUDC",648.528769214456,-0.375723265171546,0.216562289550254,-1.73494317016979,0.0827508716472305,0.489919314555978
"2602","LRRC8C",57.0882287291282,0.4703726768151,0.271125361346092,1.73488999509223,0.0827602916058233,0.489919314555978
"2603","AC005828.5",5.86838575347614,1.44155087354095,0.830822023688223,1.73508986574712,0.0827248890486814,0.489919314555978
"2604","APCDD1L",25.4051617280676,1.06780816736239,0.615467937758724,1.73495336126022,0.0827490663960208,0.489919314555978
"2605","KIT",9.11416063676156,1.08546101734689,0.625991373857556,1.73398718045896,0.0829203577869497,0.49044297046152
"2606","PTPN12",404.955272993229,0.289267937902355,0.166841630731718,1.73378752433497,0.0829557900390608,0.49044297046152
"2607","AC005670.3",92.6664382827161,0.443045331272166,0.25555290993357,1.73367359184967,0.0829760147235578,0.49044297046152
"2608","PPP1R9B",93.616711367415,-0.351279758178734,0.202597072715461,-1.73388368089648,0.0829387239497763,0.49044297046152
"2609","ARL9",4.93186824762916,-1.30144311788125,0.751228475970846,-1.7324198423114,0.0831988376240567,0.491194975861675
"2610","FILIP1",488.295384556676,0.327663702030566,0.189115958680369,1

.73260736067421,0.083165480087311,0.491194975861675
"2611","TMUB1",90.7242153301469,-0.518898266977247,0.299520005178993,-1.73243275242051,0.0831965407045836,0.491194975861675
"2612","PLA2G4A",40.2353284129313,0.586918648730894,0.338922186942943,1.73172094168536,0.0833232601614777,0.491378996042415
"2613","SLC26A2",133.722208370279,0.423359052674754,0.244474906197949,1.73170759837398,0.0833256370843252,0.491378996042415
"2614","RAB4B",57.0057168440701,-0.440267423683363,0.254187454604871,-1.73205803711969,0.0832632295794247,0.491378996042415
"2615","ZC3H12C",45.0385884442407,0.566706943743748,0.32745317932055,1.73065030218866,0.0835141541658985,0.492302365761883
"2616","FIBIN",281.697041454193,-0.656478853054538,0.379376712815543,-1.73041420540149,0.0835562976273766,0.492315952770894
"2617","HECTD4",102.307257845706,0.503510546611214,0.290999529224036,1.73027959170192,0.0835803339864697,0.492315952770894
"2618","KIF13B",106.853959828639,-0.640092473438481,0.370103407081022,-1.72949630074157,0.0837203079973597,0.492952080893545
"2619","RNF5",202.365113961094,-0.348761177349338,0.201711914093939,-1.72900633517818,0.0838079612497087,0.493279771922207
"2620","ERMP1",19.5634256128523,0.627589721909548,0.363110842679487,1.72836954489821,0.0839219919889476,0.493285261827195
"2621","TNFAIP2",1146.37422213862,0.515009921470159,0.298033450655188,1.72802724102941,0.0839833406080367,0.493285261827195
"2622","HSD11B1L",47.9795500387817,-0.576529498630394,0.333616450971765,-1.72812071152686,0.0839665849722658,0.493285261827195
"2623","ICAM1",193.335342452794,-0.684897552938324,0.3963689619513,-1.7279293251586,0.0840008960296066,0.493285261827195
"2624","PSMC4",272.714486695289,-0.484560985829982,0.280387730658828,-1.72818184551587,0.0839556274824553,0.493285261827195
"2625","XBP1",550.087762253212,-0.438123288110219,0.253476148807898,-1.72845962103622,0.0839058543324589,0.493285261827195
"2626","AL109615.2",3.3651315070259,-1.57285213853786,0.910446068951866,-1.72756211726916,0.0840667595128299,0.493393166479662
"2627","EEF1D",3846.71085930625,-0.398720449050069,0.230883276087346,-1.72693516744465,0.0841793076770361,0.493393166479662
"2628","AP003120.1",3.21848427122535,1.97551676601848,1.14385437347475,1.72707016892136,0.0841550623170598,0.493393166479662
"2629","CORO6",15.9563703414977,0.801157996085068,0.463892447535797,1.72703392853414,0.0841615702925535,0.493393166479662
"2630","AC008969.1",21.0905989336181,0.580335189195481,0.336030707333187,1.72703022828225,0.0841622347992284,0.493393166479662
"2631","UPF1",147.863447269101,0.360919330629021,0.209024226222873,1.72668660064403,0.0842239633950708,0.493467273179406
"2632","PRELID1",662.727317713243,-0.431047537061153,0.249682421743657,-1.72638319530439,0.0842784969906325,0.493599175953875
"2633","TUBE1",91.8599603375278,0.336512726288411,0.194987227290744,1.72581933167673,0.0843799208451362,0.493630542629137
"2634","MESP2",8.85715411255047,1.17920980854913,0.683145509174117,1.7261473474,0.084320907656443,0.493630542629137
"2635","ZNF75A",52.7722487359034,0.488200404988466,0.282851348985058,1

.7259963819874,0.0843480636387026,0.493630542629137
"2636","GNB4",252.208905315832,0.423685641677298,0.245684569972203,1.7
2451058576953,0.0846157096137324,0.494159944999686
"2637","WDR37",35.2610588816314,0.538467813408863,0.312200760351663,1.
72474856500136,0.0845727947399304,0.494159944999686
"2638","RTRAF",844.501422284657,-0.429147591071834,0.248889454345593,-
1.72424979676296,0.0846627580112988,0.494159944999686
"2639","PRPSAP2",58.1531788775492,-0.45323283886195,0.262804599962643,
-1.72460009804385,0.084599565769038,0.494159944999686
"2640","ZNF708",107.660988649828,0.406724006768525,0.235806738611044,1
.72481927006931,0.0845600478652369,0.494159944999686
"2641","MSN",532.38872790084,-0.353306567769848,0.204902112212897,-1.7
2427001339428,0.0846591100147162,0.494159944999686
"2642","DCLK1",1550.56697097932,0.3664925150728,0.212626601446418,1.72
364376131533,0.0847721733303254,0.494611298973113
"2643","CMSS1",136.19264058396,-0.43159393497529,0.250455492868863,-1.
72323605296718,0.0848458464635305,0.494853849124224
"2644","TBX15",252.055493823292,-0.343983428667052,0.19969380791686,-1
.72255430579131,0.0849691542631166,0.495052358004322
"2645","ARL6IP5",2413.44174107042,-0.306695865211106,0.178051178045584
,-1.7225152261143,0.0849762270048288,0.495052358004322
"2646","CIB2",34.9126628337173,-0.529449245932282,0.30734628817935,-1.
72264727538641,0.084952330297219,0.495052358004322
"2647","MAPK14",188.377348773678,0.36504764606646,0.211987758720441,1.
72202229161669,0.0850654804595886,0.495385108154348
"2648","AMMECR1L",54.5700628335691,0.488898889812059,0.284037198400209
,1.72124951437945,0.0852055563615453,0.495952455219616
"2649","AC011379.2",22.5513433280849,0.943726166465136,0.5483430003539
92,1.72105081282317,0.0852416037237131,0.495952455219616
"2650","SLC8B1",102.852627338326,0.411964518202663,0.239381674400013,1
.72095261358336,0.085259423051053,0.495952455219616
"2651","PRICKLE1",219.263920650159,0.424437165409528,0.246700147778962
,1.72045768610489,0.0853492789086979,0.496287866607913
"2652","TRIB3",6.59248398393275,1.43182087978807,0.832440027806902,1.7
2002886929916,0.0854271940261695,0.496553618368553
"2653","EGOT",3.33913946161649,-1.84566870459247,1.07322112737638,-1.7
1974689792441,0.0854784589640081,0.496664321496489
"2654","CPLANE1",130.786841910254,0.498944569198085,0.290235688877869,
1.71910136595242,0.0855959161330363,0.496972145834559
"2655","UBTD2",227.991790934206,0.333448632270539,0.193948573877437,1.
71926313044847,0.085566470182497,0.496972145834559
"2656","DDX19A",92.7536994449209,0.370404375369212,0.21559237759312,1.
71807732492409,0.0857825121485529,0.497868006313984
"2657","GABPB2",140.244763486396,0.478464770522926,0.278523491960663,1
.71786145274418,0.0858218892991303,0.497881627529378
"2658","CEP70",28.5398697417315,-0.482416322356416,0.280848465219775,-
1.71771037445017,0.0858494561124285,0.497881627529378
"2659","GSAP",17.8943585091662,-0.789467844342799,0.459657194044725,-1
.71751438805064,0.0858852278372211,0.497901762734397
"2660","TRPV2",107.987630576161,-0.34391434413816,0.200261149992877,-1

.71732931799499,0.085919018150502,0.497910400296988
"2661","CLEC7A",14.4369388484015,-1.15413502687284,0.672501994294739,-1.71618082424156,0.0861289517631501,0.498751987764447
"2662","LRRC37A3",13.4708241047395,0.852589932537278,0.496767211346011,1.71627658401035,0.0861114319781735,0.498751987764447
"2663","TRBC2",22.6520663820303,0.909227007928546,0.529922110600092,1.71577480867693,0.0862032665704677,0.498774724586898
"2664","CTNND1",235.694344122921,0.281975189053072,0.164373653773263,1.71545246199873,0.0862623039733162,0.498774724586898
"2665","COX5A",430.078966357234,-0.464249158386679,0.270601211762745,-1.71562113621915,0.086231407422308,0.498774724586898
"2666","APOE",848.778972382636,-1.42480528241954,0.83053600528541,-1.71552500235064,0.0862490154488894,0.498774724586898
"2667","NSMCE4A",182.575326889583,-0.416112486495833,0.242637866134808,-1.71495279415556,0.0863538820773237,0.499117019955735
"2668","MRPL3",286.571255814628,-0.396624887873527,0.231347400114329,-1.71441255738132,0.0864529839012354,0.499502528799679
"2669","ZNF618",122.627383328442,0.424201890910556,0.247504957877793,1.7139127011751,0.0865447600436451,0.499658230738872
"2670","GANAB",367.917100850027,0.234989718123407,0.137098647198519,1.71401923305006,0.0865251936552591,0.499658230738872
"2671","SMC5-AS1",5.13247399653083,-1.14164223924239,0.666477132004863,-1.71295035406265,0.0867216734054604,0.500370530218849
"2672","NME1",209.521350038412,-0.580076129959521,0.338688636639569,-1.71271211138045,0.0867655158790128,0.500370530218849
"2673","ATRX",903.112805545022,0.339649324963547,0.198299943919971,1.71280595571233,0.0867482440937533,0.500370530218849
"2674","ZNF471",33.1377417247488,0.582653183567976,0.3402677580704,1.71233732773303,0.086834521441726,0.500581207189307
"2675","ZNF669",37.4739248503449,-0.504132455245797,0.294544887849997,-1.71156409783808,0.0869770293485236,0.500840832053602
"2676","ACSM5",7.20076935289758,-1.1307163040952,0.660621707302315,-1.71159423251855,0.0869714719299676,0.500840832053602
"2677","ACTG1",11991.0351741497,-0.440283092314344,0.257190872356669,-1.71189237114047,0.0869165048400286,0.500840832053602
"2678","MAPKAPK3",39.1534139965011,-0.469108099588475,0.274157217647315,-1.71109155401464,0.0870642130751326,0.501033113758782
"2679","LATS1",131.203139630551,0.354220400222701,0.207021638826307,1.71103079963489,0.0870754272954769,0.501033113758782
"2680","EML1",227.619545981073,-0.401842299575614,0.234899382508838,-1.71069968462133,0.0871365659619886,0.501168766479934
"2681","ZNF426",110.723229716914,0.357859866828275,0.209207361393951,1.71055102671269,0.0871640261390012,0.501168766479934
"2682","DENND2D",6.80525467693977,1.07406102859083,0.628037121080575,1.71018717292196,0.0872312668943636,0.501368374040498
"2683","SPRYD7",79.5878720331116,-0.40195158972123,0.235077811218244,-1.70986614022904,0.0872906289980427,0.501522566531803
"2684","AC073316.1",14.3447054884112,0.977283093712532,0.571668686386034,1.70952706871301,0.0873533620506628,0.501696004475025

"2685","AC245407.2",49.071373346154,-0.533599810827989,0.312280809628446,-1.70871790508956,0.0875032158297701,0.501879589834276
"2686","AMOTL2",563.045077394581,0.349622564200667,0.204640060011314,1.70847567275605,0.0875481165789405,0.501879589834276
"2687","GTF2IRD2B",89.5950842638555,0.353345914742363,0.206806094881321,1.70858559533816,0.087527738770165,0.501879589834276
"2688","AC130895.1",9.60035160242447,0.895770792770147,0.52428077896289,1.7085707291084,0.0875304944968036,0.501879589834276
"2689","AC026401.1",3.31929816733376,-1.62934662594991,0.953464521349431,-1.70886969516591,0.0874750891379267,0.501879589834276
"2690","CDC26",435.88615188685,-0.425430371110616,0.249191954603555,-1.70723959281688,0.0877775287442029,0.502822771563074
"2691","RAB8B",362.170797421529,0.371421248904316,0.217556848017644,1.70723768196069,0.0877778837675142,0.502822771563074
"2692","BBS10",55.8753499925946,0.434748562790068,0.254704296273293,1.7068756560101,0.0878451664791242,0.502834475037393
"2693","RCVRN",7.18084846446115,1.13473770694915,0.664787282616386,1.70691849351148,0.0878372029380864,0.502834475037393
"2694","PANTR1",2.5681827721079,-1.76800371284729,1.03629309461378,-1.70608462223346,0.0879923252580544,0.503489864087939
"2695","LARP1B",122.646379591256,-0.374574015129267,0.219636023073311,-1.70543069341699,0.0881141279662722,0.503918996309955
"2696","PLEKHO2",80.9925380971038,0.436971810465662,0.256238701958391,1.7053310336259,0.0881327028252766,0.503918996309955
"2697","FRY",8.56956684874961,-0.968915561928948,0.568519109763521,-1.70427967202716,0.0883288508207853,0.504563901392321
"2698","MGA",117.295345912321,0.416846211734148,0.244579721827182,1.70433676438919,0.0883181903163372,0.504563901392321
"2699","PBDC1",279.792931754114,-0.428947906873303,0.251700418383743,-1.70420021399936,0.0883436892544842,0.504563901392321
"2700","KAT6A",238.003009871052,0.349518298990122,0.205164762554336,1.70359809666417,0.0884561973124685,0.504863905818836
"2701","CCDC189",10.36564586009,-0.802113462361936,0.470843077411007,-1.70356855785682,0.0884617197286199,0.504863905818836
"2702","NADK",116.806940708058,0.342740937116241,0.201221437863405,1.70330229599543,0.0885115111541928,0.504961119334523
"2703","DYNLL1",2750.47386041827,-0.46517705882083,0.273135124611733,-1.70310229957457,0.0885489256825467,0.504987676432282
"2704","SENP7",153.429154220539,0.327882221706216,0.192568672100428,1.70267685875312,0.0886285578448742,0.505254888749532
"2705","FNDC3B",734.758025208565,0.42029396070802,0.246874817362261,1.702457809179,0.088669581094001,0.505300955875925
"2706","MOK",74.3070147194044,-0.398905030887314,0.234335222812601,-1.70228370323278,0.0887021982873989,0.505300955875925
"2707","PCID2",248.591064082749,-0.279595702414937,0.164305150381778,-1.70168556350954,0.0888143280961719,0.5057528140386
"2708","LINC00886",9.24594897196904,0.93345201717588,0.548848365085355,1.70074664799396,0.0889905715148405,0.506569298338725
"2709","C7",44.2516892120089,-0.78049878191828,0.459313102059054,-1.69927393409721,0.0892675814823292,0.507711789687633

"2710","NDUFAF4",74.6686398459872,-0.445713088879315,0.262314639655281
,-1.69915445613347,0.0892900851017303,0.507711789687633
"2711","ZNF433-
AS1",22.4165510658334,-0.596265012125614,0.350860894618105,-1.69943422
385279,0.0892373981562208,0.507711789687633
"2712","GPD1L",41.740278204434,0.560177251954183,0.329866683700464,1.6
9819287498234,0.0894713648794267,0.508180209880753
"2713","MFHAS1",69.6943256337654,-0.396375632830406,0.233380740973005,
-1.69840763714198,0.0894308516751433,0.508180209880753
"2714","AC120053.1",15.2873330510825,0.818168784629642,0.4817594852734
47,1.69829304796199,0.0894524661903596,0.508180209880753
"2715","HEATR5A",131.094995850322,0.418775388858537,0.246635855400775,
1.69795015480633,0.0895171699345049,0.508253102961471
"2716","HPF1",307.756595691816,-0.39552367549758,0.233003550697544,-1.
69750063599245,0.0896020509630842,0.508547722973469
"2717","CENPA",26.3681177589301,2.36667628559739,1.39453290594386,1.69
711039123567,0.089675792023988,0.508591734381815
"2718","TNKS1BP1",323.06099243762,0.348833482351827,0.20554031522735,1
.69715358257566,0.0896676281389025,0.508591734381815
"2719","INHBB",110.501860092033,0.819165341007604,0.482883713573672,1.
69640291851058,0.0898096013961471,0.508976104971179
"2720","JAGN1",269.005852293253,-0.40983053172956,0.241569756608397,-1
.69653079708122,0.089785402914348,0.508976104971179
"2721","HECW2",101.928071644438,0.462972028097744,0.273080708432349,1.
69536702447964,0.0900058173668263,0.509491010947208
"2722","SLC25A25",73.3323813766,-0.793695763250928,0.468081061283742,-
1.69563742030957,0.0899545665435315,0.509491010947208
"2723","WDR11",192.019912569761,0.451514701986473,0.266340856272925,1.
6952513718879,0.0900277453328228,0.509491010947208
"2724","AR",438.748936313901,0.396225167645158,0.233730074954438,1.695
22543353651,0.0900326638871356,0.509491010947208
"2725","EREG",19.1891539235914,-1.15053119028959,0.678999721253139,-1.
69445016585015,0.0901797737217577,0.509575224311179
"2726","SLC27A4",26.1388123573467,-0.690532337180413,0.407509757735801
,-1.69451730681773,0.0901670258353731,0.509575224311179
"2727","LENG8",268.442925521453,0.426491457485765,0.251684446024557,1.
69454832915719,0.0901611361917843,0.509575224311179
"2728","RPRD1B",72.7589411327277,0.392643428731714,0.231656003574856,1
.69494173547217,0.0900864741922276,0.509575224311179
"2729","PRDX6",1411.82553013574,-0.410295772375848,0.242171231227909,-
1.69423828873264,0.0902200118070642,0.509615786737228
"2730","PSMD6-
AS1",5.29647985704913,1.22995440561824,0.726225193047703,1.69362673918
895,0.0903362336690045,0.509711948026246
"2731","AC135279.4",3.06054322691445,-2.08274113262585,1.2295138772066
8,-1.69395496158011,0.09027384171444,0.509711948026246
"2732","CSF2RA",3.05630093100804,-1.81861153841169,1.07377360077253,-1
.69366385716997,0.0903291761542959,0.509711948026246
"2733","APPL2",284.224558025792,0.299639635790406,0.176951967954359,1.
69333881535407,0.0903909938156434,0.509745428811736

"2734","ACOT4",13.3267105391365,0.865405588269925,0.5110920992743,1.69
324783047657,0.0904083037542191,0.509745428811736
"2735","SEC14L5",11.3999737161126,0.885776897977467,0.523195234969417,
1.6930140772961,0.090452787693654,0.509809770492752
"2736","PTPRH",10.6995082952638,1.05269480938977,0.621909093330597,1.6
9268277418509,0.0905158658452544,0.50997882748706
"2737","C1QTNF12",3.37787950343229,-1.62351101665029,0.961080004407081
,-1.68925688725767,0.091170211506944,0.510331911659202
"2738","EIF4G3",225.989746238488,0.340611038037086,0.201570757118392,1
.68978398903879,0.0910692880198414,0.510331911659202
"2739","PALMD",35.8383063637694,-0.927448132761657,0.548325384514373,-
1.6914192903597,0.090756750582038,0.510331911659202
"2740","TMEM17",20.9681044158954,0.659479309990959,0.390188057400225,1
.69015759832576,0.0909978079562085,0.510331911659202
"2741","LOXL3",27.2001528524146,-0.652889960423131,0.38633399738791,-1
.68996248023075,0.0910351329125158,0.510331911659202
"2742","NR2C2",103.747210606847,0.385243974050286,0.227736795610797,1.
69161936707263,0.0907185713888195,0.510331911659202
"2743","GNL3",240.150782686998,-0.345197088525136,0.204291425570724,-1
.68972871749643,0.0910798665718117,0.510331911659202
"2744","POLK",242.59562751677,0.300767056002495,0.177869093727542,1.69
09461317839,0.0908470914349987,0.510331911659202
"2745","AC087854.1",16.4686612103355,0.889089761643978,0.5263269328972
15,1.68923478179219,0.0911744459753124,0.510331911659202
"2746","NOL8",164.364752068119,0.274252205182676,0.162200407651803,1.6
9082315607625,0.0908705832026274,0.510331911659202
"2747","IRF7",104.572430882775,-0.563582321251121,0.333548122108468,-1
.68965820490468,0.0910933635787365,0.510331911659202
"2748","EGLN3",18.5939331660882,-1.06842447112313,0.632392556307433,-1
.68949564707356,0.091124485342604,0.510331911659202
"2749","MYZAP",2.99740611048572,-1.63822480712012,0.969383178472956,-1
.68996620067286,0.0910344210984752,0.510331911659202
"2750","ZNF48",17.0785651296981,-0.639971896269698,0.378585717307272,-
1.69042799823924,0.0909461023710804,0.510331911659202
"2751","TSR1",73.3493952369118,-0.362109009720026,0.21407064484386,-1.
69153977176153,0.0907337584391583,0.510331911659202
"2752","FAM117A",74.244802255115,0.458845621563848,0.271435443636584,1
.6904410692149,0.0909436035517188,0.510331911659202
"2753","EPS15L1",98.2717267251966,0.356877516419366,0.211225080835232,
1.68956032592432,0.091121015260247,0.510331911659202
"2754","LILRA6",3.02079346636312,-1.83812111687557,1.08788615726054,-1
.68962634978667,0.0910994615808483,0.510331911659202
"2755","PPP1R32",7.38916754786649,0.985419132358795,0.583531386946491,
1.6887165873207,0.0912737553362585,0.510346046625653
"2756","DIP2B",56.2530663696861,0.495676088184769,0.293554678268078,1.
68853070613343,0.0913093997141454,0.510346046625653
"2757","N4BP2L2-
IT2",6.28348152104962,1.22905213335982,0.727869703795792,1.68856064066
192,0.0913036587451254,0.510346046625653
"2758","JMJD6",70.7931274525636,-0.380943571142126,0.225583635071655,-

1.68870215705639,0.0912765220677734,0.510346046625653
"2759","AC097448.1",51.5298177624433,-0.621009119395267,0.367862381154256,-1.68815609099985,0.0913812695128691,0.51056262034827
"2760","MRTO4",138.654455000199,-0.409398493001305,0.242566597175761,-1.68777769803424,0.0914539102431072,0.510604774120825
"2761","UTP3",105.705218174455,-0.334894222573698,0.198423887865544,-1.6877717001525,0.0914550620400647,0.510604774120825
"2762","NPFF",10.3746387634696,0.907180614851828,0.537563340332667,1.6875790196006,0.0914920694527748,0.510626448448415
"2763","SLC27A3",383.85416970957,-0.435912405126037,0.258755578246913,-1.68464930526087,0.0920562526544284,0.510951647592535
"2764","TPM3",801.758814212272,-0.300645098376831,0.178267856274235,-1.68647957439023,0.0917034660111668,0.510951647592535
"2765","UAP1",1010.89336562383,-0.458295784108511,0.271827711149945,-1.68597889512342,0.0917998644359067,0.510951647592535
"2766","RMDN2",43.4430432710668,0.526149556752912,0.312474551412075,1.68381570395169,0.092217291301042,0.510951647592535
"2767","SLC3A1",3.29331816550029,1.97429856780593,1.17068461351575,1.68644786564402,0.091709568649898,0.510951647592535
"2768","BHLHE40",206.773821826316,-0.725298852606762,0.4303055254608,-1.68554389774583,0.0918836828667327,0.510951647592535
"2769","CD47",828.418078307727,-0.313553640507115,0.186245389900613,-1.68355115084694,0.0922684460696567,0.510951647592535
"2770","NOP14",68.3553341631393,-0.412227651159678,0.244409044754371,-1.68663009821901,0.0916745007725223,0.510951647592535
"2771","RGS12",88.6255181289163,0.426794789045001,0.253232113676997,1.68538967213845,0.0919134149295881,0.510951647592535
"2772","MARCH3",168.614428789652,0.447545164949289,0.265297705002617,1.68695452885608,0.0916120957188148,0.510951647592535
"2773","AC114296.1",3.67918039503283,-1.41584212787357,0.840604375142829,-1.68431448817168,0.0921209068762649,0.510951647592535
"2774","PDAP1",693.816931599657,-0.335618572123814,0.199116249671477,-1.68554084700547,0.09188427092244,0.510951647592535
"2775","FBXL13",3.35225027470802,1.53599109699865,0.91105120005467,1.68595474865351,0.0918045155406976,0.510951647592535
"2776","RC3H2",136.872055456691,0.343329426951232,0.203619850680382,1.68612945056202,0.0917708686446212,0.510951647592535
"2777","ACAD10",30.0523212492305,0.573017477176772,0.34030209531727,1.68384939458729,0.092210778415902,0.510951647592535
"2778","SECISBP2L",246.841384098846,0.363737615474025,0.215943362608054,1.68441211195837,0.0921020516531352,0.510951647592535
"2779","AC009560.3",4.76257997583133,1.55294287191272,0.921531572139756,1.68517598187858,0.091954623536086,0.510951647592535
"2780","BOLA2B",18.6726010566205,-0.851948557006177,0.506059842814943,-1.68349369961315,0.0922795580212532,0.510951647592535
"2781","DHODH",22.3707239293092,-0.563219457050048,0.334220747423839,-1.68517203492399,0.0919553848170541,0.510951647592535
"2782","SLC13A3",7.7842106192106,-0.889458767789234,0.528255845537404,-1.68376512120632,0.0922270703681169,0.510951647592535
"2783","ICOSLG",14.3482620287908,-0.893856416113103,0.530468024699894,

-1.68503354489423,0.0919820997121411,0.510951647592535
"2784","GPM6B",8.26493259611756,-1.0100486631437,0.599897222336825,-1.68370285031356,0.0922391102266001,0.510951647592535
"2785","TIMM23B",31.8044839852857,-0.554879824482018,0.329711305854988,-1.68292628923699,0.0923893616457683,0.511375946057278
"2786","LRRC8D",22.9033019042835,0.59456508506984,0.353393404260195,1.6824453368464,0.0924825163108752,0.511524215619714
"2787","NUDT14",42.4960979080448,0.658581400340915,0.391438349977698,1.68246519631619,0.0924786682792684,0.511524215619714
"2788","AKT3-IT1",2.86301910090853,1.83255842013382,1.08934878711291,1.68225130629707,0.0925201190286914,0.511548649507632
"2789","TMEM9B-AS1",15.3808258728534,0.791608191873567,0.470680010167356,1.68183941270865,0.0925999838442653,0.511623208229157
"2790","PTPN9",107.690876603191,0.369219950120368,0.219532404149166,1.68184715851558,0.0925984814471884,0.511623208229157
"2791","FRS2",143.11017617668,0.375475422245387,0.223358834572761,1.68104128481685,0.0927548957681881,0.512295492033901
"2792","MT1F",101.603198984828,-1.33727113396944,0.795861978264272,-1.68028021251367,0.0929028091403898,0.51274500640856
"2793","AC015912.3",39.2587656136359,-0.791895596837024,0.471275882314033,-1.68032277176736,0.092894532812283,0.51274500640856
"2794","AL590617.2",4.97995368140095,-1.35378812516552,0.805924680760308,-1.67979484619872,0.092997238261283,0.513082472368532
"2795","PELI1",53.2548170125762,-0.701040975785122,0.417387634502248,-1.67959210536063,0.0930367047556469,0.513116566657709
"2796","IGF1",1084.96237187884,-0.58766386926196,0.349979954712115,-1.67913579434959,0.0931255816003351,0.513382828533595
"2797","TMSB15B.1",18.8247312277803,0.746981401810872,0.44489599028256,1.67900232442296,0.0931515907498193,0.513382828533595
"2798","DACT3",20.9742420635035,-0.708833758281461,0.422283433143481,-1.67857344770761,0.0932352049846681,0.513660001729328
"2799","RPS6KC1",85.5799198776128,0.420332684764508,0.250576347854698,1.67746352903286,0.0934518754795028,0.514669760813339
"2800","RHBDD1",46.9858841059363,0.434603144291972,0.259302070682506,1.67604964799571,0.0937284682219533,0.514721887296548
"2801","PRDM5",83.1495787182644,0.36436680999015,0.217262718907355,1.67707930666892,0.0935269747174619,0.514721887296548
"2802","ACSL1",102.868034162805,0.425172879923394,0.253631284666852,1.67634241368081,0.0936711416261352,0.514721887296548
"2803","AC106739.1",32.3557265622736,-0.695269972102857,0.414781607142292,-1.67623144356144,0.093692867430275,0.514721887296548
"2804","VMO1",9.41090784277804,-0.889794837675418,0.530813849597076,-1.67628414057175,0.0936825498704762,0.514721887296548
"2805","RPL28",14122.9353253087,-0.366616360887461,0.21870288971128,-1.67632152172954,0.0936752315575146,0.514721887296548
"2806","ASPHD2",12.3967179197033,-0.685188699078963,0.408789679754946,-1.67613991500399,0.093710789993943,0.514721887296548
"2807","TSIX",40.2461137896334,0.948425874949318,0.565668624233942,1.6

76645715031,0.0936117817004613,0.514721887296548
"2808","ZNF180",32.9738693764304,0.455368635812355,0.271733891273962,1
.67578888918664,0.0937795512255867,0.51481901073448
"2809","CCRL2",3.90764558394616,-1.52905884809822,0.912882488125567,-1
.67497883680281,0.0939383840244639,0.515285056254778
"2810","NSD1",198.268615345938,0.321253134193574,0.191808499862237,1.6
7486391074592,0.0939609358672404,0.515285056254778
"2811","PHIP",509.222166578807,0.326876150002825,0.195187896352132,1.6
7467428109947,0.0939981562237065,0.515285056254778
"2812","PEG3",62.2572684626238,0.629851674801743,0.376082210134308,1.6
747712543404,0.093979120916145,0.515285056254778
"2813","AC104984.3",5.1031474983168,1.28793807761777,0.769375461579597
,1.67400462054446,0.0941296912706064,0.51582267719033
"2814","ZNF736",32.132345970054,0.514759454394259,0.30759994597804,1.6
7347056176339,0.0942346970947397,0.516214589806472
"2815","WTAP",491.860826228535,-0.364327680615375,0.217766180142719,-1
.67302232319363,0.0943229015474731,0.516332274296497
"2816","HK1",116.219028045335,-0.360957822679562,0.215756506251466,-1.
67298696549555,0.0943298620558437,0.516332274296497
"2817","KRT27",37.7004310606152,1.01568507808355,0.607158189757104,1.6
7285082408239,0.09435666666839,0.516332274296497
"2818","RABAC1",1823.39536177637,-0.410919056309329,0.245691830281602,
-1.67249784349097,0.0944261927541513,0.516529368809525
"2819","SLC4A11",5.07710313247278,1.87101398589093,1.1188513449587,1.6
7226324955618,0.0944724230985555,0.516598936525092
"2820","DCTN5",103.990137550743,0.314196494603467,0.187956201792877,1.
6716473923521,0.0945938735658661,0.517076608266241
"2821","ZC3H4",85.2485722090041,0.427472742155646,0.255745021274316,1.
67148021113237,0.0946268642179089,0.517076608266241
"2822","WDR26",185.286686307366,0.349509360800493,0.209159605249383,1.
6710175006487,0.0947182211209986,0.517209131625998
"2823","ZBTB7C",21.044923175348,0.703572436641006,0.421013343848829,1.
67114046839721,0.0946939356498614,0.517209131625998
"2824","ADSS",221.347452395527,-0.346826670660217,0.207577514419229,-1
.67082967358284,0.0947553255933187,0.517228521253898
"2825","NBEA",22.6025836504803,0.782785252899386,0.468596212255215,1.6
7048992805996,0.094822470525243,0.517356589183421
"2826","ADRM1",286.503943340465,-0.419014233607922,0.250850943708295,-
1.67037136641262,0.0948459111924974,0.517356589183421
"2827","EAF1",48.2388562932385,-0.407052035781448,0.243749635782101,-1
.66995956517174,0.0949273639448446,0.517617727346933
"2828","MYCBP",25.2855608254822,-0.593739280520295,0.35584595479637,-1
.66852895899873,0.0952107681391479,0.517924717245849
"2829","RGS16",366.284850063736,-1.03873708250379,0.62293316310809,-1.
66749363177434,0.0954162896493028,0.517924717245849
"2830","EIF5A2",69.1360266938774,-0.384277719465811,0.230402457031573,
-1.66785425996196,0.0953446615146752,0.517924717245849
"2831","DNAJB11",219.479554500037,-0.356772028773598,0.21393312562637,
-1.66768015812892,0.0953792363215973,0.517924717245849
"2832","FAM8A1",213.989532908822,0.353932744575098,0.212250656323864,1

.66752249771632,0.0954105546966407,0.517924717245849
"2833","AC004839.2",3.1894600973693,-1.39036084371396,0.833710607744955,-1.66767800577067,0.0953796638203058,0.517924717245849
"2834","PDE7A",137.028626341896,0.454955474796536,0.272814217008414,1.66763843829482,0.095387522936357,0.517924717245849
"2835","OSGIN2",106.211213720043,-0.480509903363197,0.288147405897089,-1.66758365173279,0.0953984058096325,0.517924717245849
"2836","MRPL41",491.098601787771,-0.379777935262369,0.227714631559608,-1.66778011874461,0.0953593839593257,0.517924717245849
"2837","LARP4B",93.954944657111,0.42393609695295,0.254238702459126,1.66747270518778,0.0954204474199294,0.517924717245849
"2838","SFXN4",60.999083458542,-0.49224032221567,0.295163070947184,-1.66768939161685,0.0953774023951629,0.517924717245849
"2839","NSMCE1-DT",11.4266929876983,0.97916815756263,0.586757682055594,1.66877773825185,0.0951614361556778,0.517924717245849
"2840","CLTC",569.734942139145,0.251935445047069,0.150970970825952,1.66876747012189,0.095163471881785,0.517924717245849
"2841","STK17A",214.910617024667,0.428837609472146,0.257349061213915,1.66636554821424,0.0956406283844471,0.518327705993765
"2842","FJX1",26.578330906414,-0.71091098760264З,0.426538338849339,-1.66669891743014,0.095574288290725,0.518327705993765
"2843","PCED1B",11.5427975877078,0.922790642273416,0.553730753797892,1.6664970040841,0.0956144644149464,0.518327705993765
"2844","IKZF3",6.20221886110327,1.3382401384343,0.802960534131974,1.66663251996685,0.0955874983709424,0.518327705993765
"2845","LGI4",98.3876429359706,0.730871341354112,0.438631386658733,1.66625408847623,0.0956628169673864,0.518327705993765
"2846","TAOK1",374.202444218563,0.380810215392796,0.228708458951954,1.66504648379794,0.0959034823197654,0.519449114532391
"2847","HARS",157.510261984117,-0.352427386712757,0.211721930066812,-1.66457667659436,0.0959972417519986,0.519477104610884
"2848","IL10RA",13.3786356752363,-0.919303599437545,0.552275403908151,-1.66457458168902,0.0959976599965366,0.519477104610884
"2849","ADAM10",166.540665505002,0.473855314616763,0.284680876343756,1.66451403656836,0.0960097483643469,0.519477104610884
"2850","AL021707.2",7.20889764892519,0.962331400933081,0.57845217118841,1.6636317553377,0.09618604187215,0.520248363319015
"2851","AL158151.3",3.79320022566261,1.5208746498378,0.914422143984655,1.66320846432096,0.0962707139048765,0.520523695139835
"2852","RABGGTB",140.082404384021,-0.359477570697898,0.216472744674095,-1.66061353931231,0.0967910879910932,0.520643950233382
"2853","DUSP19",11.405198076931,0.805102278665579,0.484648263554359,1.6612094568565,0.096671386929661,0.520643950233382
"2854","CLASP2",115.817219572789,0.410738066754801,0.247308392336113,1.66083351589854,0.0967468878293779,0.520643950233382
"2855","HIST1H2BC",9.61935239148272,0.883647696771676,0.532136074424041,1.66056717302636,0.0968004064855911,0.520643950233382
"2856","ANKS1A",95.188056260177,0.45341078883395,0.272955438445331,1.66111652296227,0.0966900466243613,0.520643950233382

"2857","BICRAL",137.426358697253,0.335416979246594,0.202009349198511,1
.66040324656947,0.096833357464749,0.520643950233382
"2858","FOXO3",551.702501060317,0.522802405423878,0.314583289470884,1.
66188867280017,0.0965350982688046,0.520643950233382
"2859","AC144652.1",11.3017536216579,-0.855022594334361,0.514388857028
859,-1.66221056823241,0.0964705617702608,0.520643950233382
"2860","FAM92A",221.169686987927,-0.404050830117404,0.243141841374091,
-1.6617906150334,0.0965547646356283,0.520643950233382
"2861","FBP1",21.0797386997702,0.772337459158451,0.464523554288993,1.6
6264434177208,0.096383649541939,0.520643950233382
"2862","ZER1",88.0234368935072,0.328763204512699,0.197871632899816,1.6
6149740462875,0.0966135897395856,0.520643950233382
"2863","PLCE1",73.1556038786762,0.476639116737999,0.28700642992703,1.6
6072626616478,0.0967684356310413,0.520643950233382
"2864","SLC25A45",43.3548689264534,0.563506468900141,0.338931560567577
,1.66259662557388,0.0963932070414597,0.520643950233382
"2865","VPS11",94.1048731790635,0.336554749533606,0.20252511236286,1.6
6179268144588,0.0965543501649672,0.520643950233382
"2866","TRAFD1",80.180207020262,0.348837621138439,0.209934109916613,1.
66165289326732,0.0965823913859172,0.520643950233382
"2867","SLC46A3",30.2079328777836,0.555160059508205,0.334055016176793,
1.66188212307639,0.0965364117749215,0.520643950233382
"2868","UBR2",180.162132487842,0.309742176966321,0.18661244910108,1.65
981518627703,0.0969516377194374,0.520916519848424
"2869","LINC01422",5.57901313964283,1.19139139155715,0.717772243547472
,1.65984600584287,0.0969454359193504,0.520916519848424
"2870","MRPL28",193.360792884085,-0.441799013351447,0.266263359445874,
-1.65925576193016,0.0970642654520226,0.52133994841217
"2871","AC004951.1",22.1713391074167,0.848114795372749,0.5112505394171
64,1.65890249492865,0.0971354418325008,0.521540521019854
"2872","NOTCH2NLA",33.3904825244242,0.668043438588288,0.40305406029941
6,1.65745368770636,0.0974277847244059,0.522502024703074
"2873","ZNF169",8.47437133410628,0.884970322445601,0.533944626008916,1
.65741966364659,0.0974346586021653,0.522502024703074
"2874","ZCCHC8",100.340950038667,0.431916639865553,0.260564787844544,1
.65761706882374,0.0973947822513665,0.522502024703074
"2875","ADGRG4",2.61552844838306,2.08022895125043,1.25515880699821,1.6
57343229918,0.0974501019151045,0.522502024703074
"2876","ARHGDIA",618.967848054073,-0.296345598556001,0.178879469491244
,-1.6566775348722,0.0975846872761561,0.523041708748938
"2877","RUFY3",440.478796611403,0.328349970404988,0.198409265659503,1.
65491248260795,0.0979422518236817,0.52459340231482
"2878","PIP4K2B",143.059434171467,0.343170303596848,0.207360505382689,
1.65494534729995,0.097935584539013,0.52459340231482
"2879","TRIM52",63.5009492102906,0.508109931573905,0.307161830851273,1
.65420921657395,0.0980850108570572,0.524993209153312
"2880","FAM199X",62.7230334354289,0.368780504547873,0.222934469549694,
1.65421033944537,0.0980847827880821,0.524993209153312
"2881","AC147067.1",39.7942945107077,-0.612220701368877,0.370237624907
445,-1.65358856091929,0.0982111384875769,0.525292643913144

"2882","JMY",113.83604931478,0.418218075299136,0.252921669646135,1.653
54781930812,0.0982194223946451,0.525292643913144
"2883","CREB5",749.531810189938,0.444815260024181,0.269025601162145,1.
65343096754604,0.0982431847162889,0.525292643913144
"2884","RARB",3.07223030856176,1.56125644371823,0.94469467030378,1.652
65719474863,0.0984006507239445,0.525587675297853
"2885","MEST",769.10863892814,0.763689153417479,0.462071935899873,1.65
274948354138,0.0983818589879879,0.525587675297853
"2886","PODXL",8.52758535334849,-1.145578939509,0.693079386315887,-1.6
5288271751726,0.0983547350972292,0.525587675297853
"2887","GPR157",16.1328763179761,-0.677556961148445,0.410256072243072,
-1.65154645352087,0.098627043482766,0.525811177995159
"2888","AC118549.1",116.355107785331,0.311595653182194,0.1888165712525
9,1.65025586003972,0.0988906160972581,0.525811177995159
"2889","PDK1",28.6418076022999,0.60391249056347,0.365532658450403,1.65
214373217328,0.0985052537170253,0.525811177995159
"2890","SLC12A8",4.68470229975853,-1.59107471328597,0.963966863780703,
-1.65054917660316,0.0988306639400449,0.525811177995159
"2891","SERPINI1",36.6976150736337,-0.746425167447079,0.45198798889492
6,-1.65142699758909,0.0986514158981966,0.525811177995159
"2892","AL355312.2",11.1722692667854,0.883706997367918,0.5349495175480
89,1.65194465716754,0.0985458333101006,0.525811177995159
"2893","TRA2A",482.285282121412,0.302140529725569,0.183020526853669,1.
65085597183938,0.0987679878709342,0.525811177995159
"2894","ZNF189",44.6699947972006,0.50827613043114,0.307896863409756,1.
65079996204675,0.0987794279020189,0.525811177995159
"2895","MDM2",159.317941296139,0.310316981799824,0.188058137992023,1.6
501119553411,0.0989200399731405,0.525811177995159
"2896","PGAM5",30.6877872101275,-0.510667097585868,0.309364036025072,-
1.65069962283684,0.0987999248860408,0.525811177995159
"2897","ADAMTS17",6.47013749088193,1.20500141064006,0.730011316628823,
1.65066127495768,0.0988077593691323,0.525811177995159
"2898","FLCN",155.82658316697,0.425393223115611,0.257628278722692,1.65
118994399485,0.0986997958287705,0.525811177995159
"2899","SOGA1",121.834699448585,0.381059122307633,0.230897833408958,1.
65033650026813,0.0988741308225017,0.525811177995159
"2900","SMARCA1",429.755282700909,0.352429741149951,0.21343972782174,1
.65119092282713,0.0986995960213437,0.525811177995159
"2901","ELP5",174.006017028143,-0.50022224937576,0.303313779815765,-1.
64919064896952,0.09910858307368,0.526631784929603
"2902","YBX1",3897.25124717551,-0.337089837242319,0.204510552431092,-1
.64827600940494,0.0992960454153943,0.526938702554842
"2903","ZNF660",31.4000063723823,0.611064418541166,0.370737136290535,1
.64824172904625,0.0993030769329754,0.526938702554842
"2904","VPS36",303.971757439436,0.370053090975281,0.224486217713881,1.
64844458935529,0.099261472418832,0.526938702554842
"2905","SCO2",134.553013221947,-0.442831014564223,0.268615547343417,-1
.64856807040315,0.0992361545664594,0.526938702554842
"2906","ZNF304",15.9435207395342,0.719570379137302,0.436657466947119,1
.64790581543965,0.0993719998737219,0.527122979371446

"2907","ZC3H12A",44.5607737537776,-0.64389657363429,0.390817664123485,
-1.6475626174124,0.099442456849527,0.527315263961286
"2908","CHMP3",682.238456140211,-0.361773252324923,0.21976245876031,-1
.64620133195498,0.0997223151434396,0.527452451441018
"2909","HIST1H2BD",8.59783859567037,1.21394877392869,0.737276065902527
,1.64653218797039,0.0996542387720943,0.527452451441018
"2910","CCDC6",228.354553817746,0.370916837995074,0.225385091337798,1.
64570263185314,0.0998249969159639,0.527452451441018
"2911","EBF3",347.846074712217,0.407136476928804,0.247312158191431,1.6
4624529544424,0.0997132671542133,0.527452451441018
"2912","RASSF3",20.6951186405092,-0.655302139886666,0.398190454167093,
-1.64570027490333,0.0998254824093282,0.527452451441018
"2913","STX4",389.99091896047,-0.311907276092722,0.189512408628202,-1.
64584091538113,0.0997965160522996,0.527452451441018
"2914","CDRT4",15.6848476570412,0.658360092685223,0.399995727442077,1.
64591781241103,0.0997806811507854,0.527452451441018
"2915","ACACA",45.4039576897838,0.47456605900907,0.288383606520167,1.6
4560692175088,0.0998447131563342,0.527452451441018
"2916","SGSH",67.4864990421991,0.417858152736341,0.253903134342272,1.6
4573845777363,0.0998176175903147,0.527452451441018
"2917","ZNF737",66.730516458312,0.49081900060323,0.298070127538624,1.6
4665612302806,0.0996287476511844,0.527452451441018
"2918","ZMYM3",44.6056171401814,0.464553107414909,0.282104873801974,1.
64673903415439,0.0996116972888086,0.527452451441018
"2919","LRCH3",198.906149764501,0.359073457165454,0.218307189891638,1.
64480820509708,0.100009369574083,0.527812470411869
"2920","RWDD1",1091.06015675885,-0.353384505635267,0.214870959359609,-
1.64463595587081,0.100044907331002,0.527812470411869
"2921","INTS10",208.485072864409,-0.263534416611018,0.160206084753191,
-1.64497133187551,0.0999757232047825,0.527812470411869
"2922","CC2D2B",2.61668685534902,2.19392501427722,1.33400756101891,1.6
4461212843615,0.100049824102723,0.527812470411869
"2923","RASSF5",82.6693507648448,0.470178788231412,0.28607747625063,1.
64353654958663,0.100271969647387,0.528261248159425
"2924","FAM71A",21.6652283618368,1.016441007564,0.618350642744894,1.64
379388861304,0.100218784170924,0.528261248159425
"2925","TNNI2",2.44789024044533,-1.54343189186696,0.938928899573033,-1
.64382190447947,0.100212995356807,0.528261248159425
"2926","COTL1",507.679233514771,-0.413652652041245,0.251679282183555,-
1.64357053330898,0.100264944781102,0.528261248159425
"2927","TPM2",389.278325555195,-0.502398311893151,0.305731515054013,-1
.64326635350102,0.100327836566912,0.528374991690792
"2928","STXBP5",87.450400197301,0.482971672513744,0.2939690137962,1.64
293394829896,0.10039660015178,0.528556554419291
"2929","NCK1",283.590206576922,0.267485234054023,0.162873079932067,1.6
4229247807918,0.100529405213621,0.529075036315453
"2930","ADCY3",366.440224484364,0.402039479070628,0.244879639665288,1.
64178401936622,0.100634772043432,0.529123778073707
"2931","MFSD1",151.755089348938,-0.334241417034719,0.203572769752251,-
1.64187684552061,0.100615529310111,0.529123778073707

"2932","CTTN",602.185584795715,-0.249994704223181,0.152288582242978,-1
.64158534107509,0.100675967634783,0.529123778073707
"2933","NAALAD2",47.8551101937311,0.510989279214081,0.311271306424848,
1.64162024788961,0.100668728795681,0.529123778073707
"2934","C16orf70",30.4368759154771,0.480009429303283,0.29246298530964,
1.64126557347106,0.100742299100672,0.529220957720922
"2935","TNRC6B",936.996426936013,0.341238285739418,0.207924393822242,1
.64116523062296,0.100763121045145,0.529220957720922
"2936","PYGB",117.757527846878,-0.393877908277785,0.240038915861654,-1
.64089188148473,0.100819860576194,0.529338607214585
"2937","AC023157.3",18.7608990996534,-0.852419775714867,0.519582453739
798,-1.64058614677883,0.100883352591159,0.529491617362178
"2938","BAZ2B",334.219679398604,0.454341877952081,0.277093063051905,1.
63967250911285,0.101073277858706,0.529521956345729
"2939","PTPRG-
AS1",8.5161409265168,1.03874292681842,0.63328969710652,1.6402334217095
3,0.100956642683873,0.529521956345729
"2940","ACTRT3",9.21542438320597,-0.876271044569071,0.534504564320484,
-1.63940797340632,0.10112832218479,0.529521956345729
"2941","NCOA4",292.786466185825,0.231780012301608,0.141387091244933,1.
6393293776734,0.101144680907104,0.529521956345729
"2942","ATXN3",288.706561154253,0.32836444087158,0.20024576115294,1.63
980719981776,0.101045260722546,0.529521956345729
"2943","RPLP1",28712.1657246786,-0.389437133647831,0.237477736303958,-
1.63988902584693,0.101028243037006,0.529521956345729
"2944","C17orf53",2.84510083175656,-1.55346014704535,0.947768170484423
,-1.63907186949668,0.101198292792379,0.529521956345729
"2945","PIAS2",104.730216721989,0.410522251236867,0.25039376447377,1.6
3950668699608,0.101107779167257,0.529521956345729
"2946","ARHGAP35",168.734047769019,0.300260751095672,0.183183920806416
,1.63912176229146,0.10118790360011,0.529521956345729
"2947","CDHR4",7.77565287592732,-1.01068976227077,0.616748440342045,-1
.63873906468292,0.101267614580756,0.529583764573203
"2948","CCND2-
AS1",12.586927580346,-0.823567934601206,0.502578457457672,-1.638685308
49349,0.101278815307285,0.529583764573203
"2949","RGS10",338.886474158842,-0.471504965276507,0.287815716248301,-
1.63821827182549,0.10137616935414,0.529913072429323
"2950","TRMT1L",73.3781914752071,0.36539632678351,0.223127420083339,1.
63761283416908,0.101502484076508,0.530393488826906
"2951","EPB41L5",36.7005831696704,0.470035168573681,0.287072093065924,
1.63734190792883,0.101559049006956,0.530509230919088
"2952","FAM222B",63.3454207339168,0.446455048338287,0.272835096591383,
1.6363549041747,0.101765331428107,0.531406701884915
"2953","LURAP1L",207.735119663003,0.460708475388587,0.28167399883131,1
.63560881480046,0.101921484344682,0.532041883228335
"2954","TPMT",46.6461309891919,-0.456205707883907,0.279110899141278,-1
.6344962138257,0.10215470059711,0.533078777828183
"2955","ASB16-
AS1",26.3517906792192,0.587535642634528,0.359710720175747,1.6333559432

0756,0.102394157252368,0.53414752421159
"2956","FOXF2",46.0336369544003,0.581393657994779,0.356030881568509,1.63298659777327,0.102471815425482,0.534191083795674
"2957","CUL9",31.9698038080676,0.463029500680574,0.283519741915014,1.633147298855,0.102438020832309,0.534191083795674
"2958","CAMK2D",469.520619065404,0.380529266083893,0.233102752796984,1.63245290550175,0.102584111754903,0.534545114605944
"2959","TIGD7",13.2839411599395,0.728783030755738,0.446466775558144,1.63233429821213,0.102609081681413,0.534545114605944
"2960","AC004846.1",18.5929059445621,-0.578947948092238,0.354776532699256,-1.63186652647911,0.102707606965996,0.534877622088118
"2961","SULF2",124.574070539947,0.566717265254921,0.347365231686487,1.63147377330616,0.102790389431934,0.535127947684316
"2962","KIAA1549",7.09095284184361,1.03661586830996,0.635663480986057,1.63076202946555,0.102940542308253,0.535407833733371
"2963","ANK1",18.1502843930285,-0.856541385659412,0.525247550571331,-1.63073846746686,0.10294551604118,0.535407833733371
"2964","LTBP4",2643.3309548071,0.486136684117675,0.298110761642695,1.63072504138693,0.102948350255317,0.535407833733371
"2965","ARID1A",236.027183000432,0.347273864499179,0.21320814701498,1.62880203857679,0.103354933019962,0.53615136355628
"2966","ZRANB2-AS2",5.87572618874887,1.40105222261094,0.859727550768616,1.62964676583688,0.103176174396009,0.53615136355628
"2967","ANKRD36B",83.4311274729237,-0.679251359403241,0.416813317319794,-1.62962969554568,0.103179784324132,0.53615136355628
"2968","RSRC1",353.8293147858,-0.261170322591672,0.160407839654677,-1.62816432883776,0.103490046249047,0.53615136355628
"2969","BASP1-AS1",3.81075050396438,-1.66361316140595,1.0218274624614,-1.62807638522319,0.103508690103373,0.53615136355628
"2970","SH3BGRL2",28.1798796571208,0.567794383229254,0.348585752343125,1.62885137849913,0.103344485089036,0.53615136355628
"2971","CCDC170",12.2362641772013,1.01825072849533,0.62525097228323,1.62854721325259,0.103408906695657,0.53615136355628
"2972","IQUB",6.09207161736831,-1.08670522134402,0.66733183408594,-1.62843306109097,0.103433092137957,0.53615136355628
"2973","RIN1",22.4446279597645,-0.749500961470906,0.460284789790534,-1.62834179641693,0.103452431637827,0.53615136355628
"2974","ITGAE",246.600620256158,-0.423730596168521,0.260242199514641,-1.62821631910117,0.103479025682195,0.53615136355628
"2975","OCEL1",124.326555634469,-0.479801029410661,0.294456315277047,-1.62944723722168,0.103218375846642,0.53615136355628
"2976","AURKC",7.07405645341378,1.01329466082162,0.622127039285655,1.62875843169446,0.103364167655151,0.53615136355628
"2977","AC132192.2",8.0927906038346,-0.900966027114689,0.55376511843084,-1.62698226581639,0.103740864314729,0.537173471082148
"2978","RFC2",82.5371738173645,-0.447523471050181,0.275094938486726,-1.62679645620516,0.103780334544348,0.537197399933219
"2979","PAFAH1B3",26.5781604533692,-0.764850203662241,0.47030808528831

,-1.62627483470408,0.103891202709509,0.537590765279317
"2980","MIR1915HG",3.27560032070981,1.60662210134569,0.98816891480436,1.6258577630564,0.103979916990455,0.537869268593241
"2981","ECM2",1118.03707768032,0.334971102866278,0.20608334127127,1.62541572161988,0.104074008223621,0.538175389723959
"2982","PLEK",20.7817427429927,-1.02312197483233,0.629699071627712,-1.62477923333706,0.104209607464664,0.53843907351948
"2983","JADE2",97.093545723245,0.352257030005792,0.216774247245818,1.62499482517561,0.104163661468732,0.53843907351948
"2984","NUFIP1",56.4221764843399,0.38273867375055,0.235577221027127,1.62468456025499,0.104229788866826,0.53843907351948
"2985","UFC1",560.575471279299,-0.400677479128938,0.246725399693984,-1.62398147748834,0.104379761738373,0.538481540911955
"2986","DISC1",23.3311382659359,0.741328037031424,0.456446326162323,1.62412970494101,0.104348129452508,0.538481540911955
"2987","CYTIP",18.4565336304476,-0.642967146374552,0.396037631790505,-1.62350012918638,0.104482535768255,0.538481540911955
"2988","SGMS2",198.679634684769,0.350867925981782,0.215996123092027,1.62441770231354,0.104286691517537,0.538481540911955
"2989","CSNK2B",623.153614202761,-0.356640946462295,0.219661960671232,-1.62358992595936,0.104463356911555,0.538481540911955
"2990","GOLGA8B",23.9827145713802,-1.01879067349589,0.62750263926162,-1.62356396571448,0.104468901231238,0.538481540911955
"2991","THRA",293.237175218285,0.320205080061352,0.197154595694323,1.62413196067624,0.104347648129102,0.538481540911955
"2992","SOCS3",6721.00469746152,-0.41012964248538,0.25271891267549,-1.6228688155683,0.104617451114305,0.538996660737639
"2993","MANBA",120.135187982789,0.43050879226292,0.26535267040032,1.62240233577993,0.104717229366898,0.539315884245516
"2994","AL442128.2",6.79272672405887,-0.940168345437032,0.57954516191974,-1.62225208182695,0.104749384199226,0.539315884245516
"2995","ANKFY1",178.05797340552,0.290708679343542,0.179220250009061,1.62207495709243,0.104787299532673,0.539330959030437
"2996","NBEAL1",316.456417902721,0.355407228177644,0.219245544976991,1.62104652213089,0.105007661736571,0.539744283317521
"2997","CHL1-AS2",30.023010984496,0.80867034802374,0.49885544192204,1.62105147115969,0.105006600430728,0.539744283317521
"2998","C5orf49",4.73700505018496,-1.16253331813611,0.717140871033918,-1.62106688531092,0.105003294962234,0.539744283317521
"2999","TMEM106C",302.943978436213,0.574874444021859,0.354587775569761,1.62124721614595,0.104964630275566,0.539744283317521
"3000","ROR1-AS1",3.77331484471177,-2.01401661415762,1.24337956165625,-1.6197922792577,0.105276905355968,0.539803550493732
"3001","CPOX",53.864729187266,0.410460855214764,0.25324487055171,1.62080619568147,0.105059209340342,0.539803550493732
"3002","AFF1",353.235794698019,0.428734084165837,0.264664585956447,1.61991481639477,0.105250576644515,0.539803550493732
"3003","STK32A",11.1844980952819,-1.22210937365618,0.754443571013718,-

1.61988175207601,0.105257680432349,0.539803550493732
"3004","SERINC1",1013.47582047407,0.264303244919932,0.163152702017347,
1.61997467189866,0.105237717804036,0.539803550493732
"3005","SCAI",57.7963361556057,0.454378989607959,0.280563123995774,1.6
1952498652247,0.105334354841722,0.539803550493732
"3006","CCL13",10.7472423049333,-1.03703930995881,0.640191093875663,-1
.61989024820801,0.105255855022893,0.539803550493732
"3007","CXXC1",95.3782447978255,-0.324537550114468,0.200376031025621,-
1.61964257128624,0.105309079170858,0.539803550493732
"3008","ZNF431",112.932825964723,0.414921359884183,0.256032018397725,1
.62058387259845,0.105106913285714,0.539803550493732
"3009","SLC19A2",178.650476762499,0.672383229398929,0.415585783243171,
1.61791682129198,0.105680524618596,0.539859603032118
"3010","HPS3",80.7512820175392,0.35193874991212,0.217353965852095,1.61
919635803474,0.105405021372093,0.539859603032118
"3011","RNF139",288.021606283475,0.277597416736937,0.171568454083067,1
.61799800680453,0.105663027217426,0.539859603032118
"3012","RAD23B",459.720834846745,0.305476658265923,0.18877331498494,1.
61821949405451,0.105615303155567,0.539859603032118
"3013","PAXX",187.629131539732,-0.367174562588074,0.226826508079793,-1
.61874626425457,0.105501868193476,0.539859603032118
"3014","HERC4",191.231704306783,0.280581246990302,0.173364347855764,1.
61844837453973,0.105566004034227,0.539859603032118
"3015","ATF7IP",234.215268317593,0.349431005107367,0.215827895439202,1
.61902614301218,0.105441638294887,0.539859603032118
"3016","PIGL",50.4963451761558,0.392710194884465,0.242628576940513,1.6
1856529777508,0.105540826702117,0.539859603032118
"3017","RASD1",1641.0403299635,0.565464589643031,0.349516662827847,1.6
178473010929,0.105695509695163,0.539859603032118
"3018","SLC39A6",280.362817770181,0.334175750773886,0.206520760870254,
1.61812182642417,0.105636345581714,0.539859603032118
"3019","NPC1",48.8895059387043,0.462937772333982,0.28626651875015,1.61
715653774387,0.105844495307363,0.540441502207024
"3020","UQCRH",1199.74795659966,-0.396959053364887,0.245524481707546,-
1.61677992599419,0.105925794032915,0.540443036409975
"3021","BPNT1",40.0155958175974,0.429710265635817,0.26579995235353,1.6
1666795584777,0.105949974442487,0.540443036409975
"3022","POLR1D",586.413665487867,-0.307827289832546,0.190382035675833,
-1.61689252213212,0.105901482851704,0.540443036409975
"3023","MEG9",11.2944935780168,1.26205196889511,0.780764246354541,1.61
64315602152,0.106001039420561,0.54052465189148
"3024","SOX4",1569.13534812553,0.540319194916441,0.334354955340313,1.6
1600474671145,0.106093286927569,0.540802749632799
"3025","FBXL6",35.0935983347183,-0.509030611990001,0.315022524838707,-
1.61585465118924,0.106125742305496,0.540802749632799
"3026","C2orf69",116.522987268159,-0.398992154936351,0.246960288683911
,-1.61561260339726,0.106178097243954,0.540890736621135
"3027","AL031118.1",3.97434038623712,-1.23995746558132,0.7678020190561
7,-1.61494426272225,0.106322765684213,0.54144877205885
"3028","LRIG2",67.1226886768249,0.43414914971869,0.269088514146523,1.6

1340646997028,0.106656228425301,0.541561054728419
"3029","ZNF692",30.1139836601616,-0.531121329552352,0.32918898584358,-1.61342375472041,0.106652475712086,0.541561054728419
"3030","SAP130",38.3688772619389,0.481361830840576,0.29837564099584,1.61327455965914,0.106684871081173,0.541561054728419
"3031","RNF175",2.62831995950816,-1.54618666555601,0.95820681803962,-1.6136251970314,0.106608748042391,0.541561054728419
"3032","FER",183.839703861819,0.380485083356099,0.235668486662733,1.61449283586488,0.106420569542285,0.541561054728419
"3033","TPST1",522.281459945749,0.420918368077225,0.2606921772643 7,1.61461833068496,0.106393373314858,0.541561054728419
"3034","AC083862.2",18.7181235892038,0.637278404368397,0.394945121120426,1.61358723095627,0.106616988362092,0.541561054728419
"3035","RPS13",11763.5028048877,-0.411223373833384,0.254809870788111,-1.61384397143445,0.106561274143062,0.541561054728419
"3036","RFX7",47.8869838557038,0.449722153162633,0.278772518743448,1.61322269207069,0.106696135141759,0.541561054728419
"3037","ADAMTS7",55.9701756943117,-0.376635260639517,0.233388273974335,-1.6137711386517,0.106577076947206,0.541561054728419
"3038","KMT2A",608.638890540993,0.338481356224185,0.209969543738071,1.61204977730688,0.106951108256517,0.542497970968811
"3039","ATRN",104.037125168036,0.320451962471253,0.198768910653895,1.61218352214667,0.106922009879314,0.542497970968811
"3040","DYRK2",183.775515906934,-0.49902496798192,0.309594132246307,-1.61186830112434,0.1069906014009,0.542519776511472
"3041","LINC00342",362.116201005388,-0.569113186864852,0.353116319282534,-1.61168758221422,0.10702994122825,0.542540790540436
"3042","RSRP1",1004.58901696467,0.287072111654787,0.178237662578729,1.61061420746575,0.107263835236289,0.542797735546003
"3043","FAM76A",175.64783234847,-0.34964781185115,0.217215870713662,-1.60967893691369,0.107467965545663,0.542797735546003
"3044","AC104506.1",12.1029414775775,-0.797256905353486,0.495257643724009,-1.60978213149552,0.107445427409799,0.542797735546003
"3045","CREG1",553.250465804889,0.252696152511241,0.156916452700272,1.61038659849084,0.107313484423059,0.542797735546003
"3046","CSRP1",314.089550296332,-0.415816762485776,0.258139400127873,-1.61082253340558,0.10721840827965,0.542797735546003
"3047","PTH2R",2.4290009337589,1.73070451390307,1.07448369185178,1.61073130009106,0.10723830047838,0.542797735546003
"3048","SLC35A5",96.0859400486565,0.309776177990688,0.192407390018775,1.61000145555979,0.107397538534035,0.542797735546003
"3049","GTF3C6",364.320152454569,-0.416426930314529,0.258664401573131,-1.60991202415921,0.107417063621068,0.542797735546003
"3050","FAN1",55.1360750505405,0.41547608358883,0.25805896577638,1.61000445126506,0.107396884546131,0.542797735546003
"3051","DLX3",3.85848759156721,-1.39224054541611,0.864315854865935,-1.61080065531375,0.107223178237242,0.542797735546003
"3052","SNRPB",553.096734432075,-0.397649031570521,0.247002227515195,-1.60990058903845,0.107419560393517,0.542797735546003
"3053","S100A2",7.20387523709683,-0.931905552589701,0.579186046891645,

-1.60899171793074,0.107618152604265,0.543022527788785
"3054","RAB4A",406.008463757689,-0.292583440254114,0.181815859788955,-1.60922947312591,0.107566174022251,0.543022527788785
"3055","ZNF418",2.36232904756024,1.79719563942483,1.11687302343823,1.60913156796666,0.107587575862718,0.543022527788785
"3056","MRPS18C",338.857258510997,-0.406018177439816,0.252539477516991,-1.60774141703251,0.1078918236338,0.543180883256939
"3057","MED10",428.633204636199,-0.285068789156862,0.177312299495685,-1.60772146076533,0.10789619620712,0.543180883256939
"3058","AC008883.3",3.35191827041346,1.41412778183273,0.879467293676647,1.60793674989426,0.107849032091322,0.543180883256939
"3059","PTAR1",126.862851241927,0.369068832478347,0.229434211452065,1.60860418392946,0.107702918799652,0.543180883256939
"3060","CDT1",8.39859764044978,0.946400725923204,0.588479836142458,1.60821266558078,0.107788610177246,0.543180883256939
"3061","ZADH2",143.908932762324,0.296550011157871,0.184406909867595,1.60812852062211,0.10780703397766,0.543180883256939
"3062","C19orf44",26.4826428803617,0.654065451613087,0.40676757058721,1.60795869412323,0.107844225611856,0.543180883256939
"3063","ABCD3",95.6164569642727,-0.304414319526298,0.189433517394088,-1.60697179524471,0.108060555399076,0.543676983980753
"3064","PBLD",34.0297721350015,0.482212975344675,0.300109609958747,1.6067895173732,0.108100548550179,0.543676983980753
"3065","POLI",71.8308820818252,0.399068012970716,0.24836092160216,1.60680678101996,0.108096760272611,0.543676983980753
"3066","PTCD3",144.487477807314,0.317411191305504,0.19758269421064,1.60647263452697,0.108170102943362,0.543849359710346
"3067","GLMP",210.53190205451,0.388276703964627,0.241742563519824,1.60615779989768,0.108239242835879,0.544019539717991
"3068","CPE",3920.20001186186,0.43800786741508,0.272741880234737,1.60594283150833,0.10828647149217,0.544079516966034
"3069","ZNF589",41.0662327665487,0.457335947847741,0.284930548713562,1.60507867588286,0.108476491463896,0.54449753085592
"3070","TLR2",5.15558910476305,-0.996809659762872,0.621067748382235,-1.60499343647996,0.108495249121589,0.54449753085592
"3071","CACNB2",3.19968678543811,1.60484506821402,0.999952026919217,1.60492206126971,0.108510957819616,0.54449753085592
"3072","NUDT11",20.4879157084183,-0.774587634098978,0.482537097560348,-1.60523955156029,0.108441096390682,0.54449753085592
"3073","SEC61G",963.751776928103,-0.402973169649653,0.251204619935908,-1.60416305142982,0.108678116746721,0.544981512573424
"3074","AC073578.1",4.6612425935089,1.20900353312586,0.753613855471852,1.60427455565938,0.10865354709069,0.544981512573424
"3075","ST5",239.43008031789,0.279832528042644,0.174515892076433,1.60347877040382,0.108828992490751,0.545206018604137
"3076","VPS13C",446.021393692021,0.297835890142516,0.185710822123332,1.60376162647495,0.108766606049267,0.545206018604137
"3077","AC009120.2",31.8623587298048,0.656885459357516,0.409662345822882,1.60348019791285,0.108828677569793,0.545206018604137
"3078","C1QBP",483.548024658986,-0.368751606668326,0.229996773840318,-

1.60329034408258,0.108870567310613,0.545237100420112
"3079","ELL3",5.24212356027831,1.21470976023519,0.757907241189113,1.60
271560188472,0.108997457364233,0.545518118594043
"3080","AC007216.3",6.91136095530143,1.17075897912702,0.73043381370649
1,1.60282691895951,0.108972871947524,0.545518118594043
"3081","BCAP29",341.90432329193,-0.290979006947,0.181580970607235,-1.6
0247522619755,0.109050561546286,0.545606753078868
"3082","LNPEP",285.382404384997,0.335652910508342,0.209491841459267,1.
60222425928508,0.109106027390406,0.545650737316779
"3083","CD6",5.56698034620795,1.23590056744993,0.771418061961864,1.602
11515440383,0.109130147463356,0.545650737316779
"3084","PELI2",88.9783684133792,-0.501983135687314,0.313400937684359,-
1.60172825070768,0.109215715165578,0.545838511490134
"3085","NUMB",218.289774357141,0.336200097684087,0.209911847013454,1.6
0162517012458,0.109238521436722,0.545838511490134
"3086","LINGO1",2.4588195264981,-2.0359847532636,1.27163727766175,-1.6
0107350502285,0.109360639704003,0.546113860649519
"3087","SNHG30",98.9754595154056,-0.483124880363837,0.301753863231599,
-1.6010561561329,0.109364481856962,0.546113860649519
"3088","TINF2",183.617402612227,-0.299853666144548,0.187343555510077,-
1.60055500883466,0.10947551397083,0.5464912719755
"3089","C11orf53",8.70059786997808,-1.22731953855752,0.7670624318473,-
1.60002561408436,0.109592901244317,0.546602717141023
"3090","ZMYND15",5.45645795748334,-1.03813924096473,0.648816721726105,
-1.60005006992248,0.109587476248847,0.546602717141023
"3091","PRR5",88.828676239235,-0.446103751088319,0.278819269346532,-1.
59997460768709,0.10960421658663,0.546602717141023
"3092","SCAANT1",3.89601008834952,-1.51472377735958,0.947081143333764,
-1.59936008442496,0.109740616027546,0.546990175699883
"3093","GARS",220.455621149626,-0.315086053789968,0.197014377727297,-1
.59930487015574,0.109752877939652,0.546990175699883
"3094","COL9A2",2.59109888773182,-1.58412820183152,0.991449707126132,-
1.59778977233587,0.110089771592519,0.547191524194872
"3095","MAN1A2",311.245410480655,0.24874537956074,0.155773870507386,1.
59683635484261,0.110302189816388,0.547191524194872
"3096","AC092803.1",26.2361479261002,-0.715300095325888,0.447744012621
69,-1.59756484768511,0.110139854897456,0.547191524194872
"3097","TMEM63A",58.781422407394,0.531068538776318,0.332502642728225,1
.59718591833988,0.110224270678473,0.547191524194872
"3098","AC007389.1",2.80600131003394,1.48435660272921,0.92868641891599
9,1.59833994822688,0.109967341395531,0.547191524194872
"3099","RPS14",17047.694279757,-0.407449250117388,0.255130636493078,-1
.59702204218992,0.110260793928565,0.547191524194872
"3100","MICU3",121.198699168176,0.469703874230347,0.293951906397728,1.
59789361459293,0.110066655417308,0.547191524194872
"3101","TOX",3.33917826984551,1.77080841505372,1.10874599160951,1.5971
2723063209,0.110237349371029,0.547191524194872
"3102","CPT1A",167.652781957177,0.401456002692349,0.251355829350093,1.
5971620937949,0.110229579886069,0.547191524194872
"3103","KITLG",220.063338140324,-0.40395651170415,0.252823543842598,-1

.59778043438725,0.110091850487769,0.547191524194872
"3104","NSRP1",357.693690999818,-0.288582209108445,0.180600717069244,-1.59790179015623,0.110064835629812,0.547191524194872
"3105","PEX12",20.0392549086763,0.608599725688016,0.380827163621942,1.59809956805547,0.110020819760074,0.547191524194872
"3106","AL118522.1",10.3034642704163,-1.12669670877664,0.705670568412013,-1.59663270541675,0.110347604164146,0.547191524194872
"3107","GABPA",180.187287058132,0.296533933509614,0.185493992064965,1.59861745498344,0.109905628873939,0.547191524194872
"3108","DDX3X",1371.11072982594,0.348707763999183,0.218284397158914,1.59749285124268,0.110155889934484,0.547191524194872
"3109","HAUS7",40.3040434120699,-0.575384480080653,0.36038752244993,-1.59657159096176,0.110361235726361,0.547191524194872
"3110","SLX4IP",29.0221590470401,0.510285263679578,0.319709788258991,1.5960889607365,0.11046893299994,0.547549389773014
"3111","INAFM1",157.190484737533,-0.348068007394218,0.218159001282678,-1.5954785516423,0.110605262554758,0.548048898194019
"3112","EFCAB1",3.00523407370136,-1.37101295560064,0.860089304096816,-1.59403558336346,0.110928065256093,0.549118858677802
"3113","AC068888.1",162.156545904165,-0.375059014044637,0.235258862550387,-1.59423968125455,0.110882361933866,0.549118858677802
"3114","PGK1",571.653678159345,-0.353320220650067,0.221631449261881,-1.59417908345932,0.11089592995152,0.549118858677802
"3115","RPS7",8060.10887308954,-0.443939476354071,0.278685612003963,-1.59297594576845,0.111165587349892,0.549412481243536
"3116","AC044849.1",28.6415072057744,-0.524191069987183,0.329012063952252,-1.59322750567364,0.111109162861819,0.549412481243536
"3117","PLAAT3",554.704996069626,-0.405755299351969,0.254678543351947,-1.59320567022109,0.111114059623044,0.549412481243536
"3118","C1RL-AS1",49.2315089950538,0.69468518460172,0.436056535106117,1.59310806896327,0.111135949507283,0.549412481243536
"3119","SLC38A10",277.552964491093,0.278905980032033,0.175078357402995,1.59303516533485,0.111152302459903,0.549412481243536
"3120","AC159540.2",14.5267575591667,-0.736967896848206,0.462736452630966,-1.5926298709731,0.111243248336295,0.549620087533329
"3121","SYT11",48.9122640120194,0.477918506976825,0.300220504909694,1.59189162352712,0.111409058012162,0.549899612923874
"3122","SPAST",77.3413464093149,0.384605298696798,0.24158736564515,1.59199260139173,0.111386366972141,0.549899612923874
"3123","DCUN1D4",89.5830517855427,0.346344711202594,0.217628783448075,1.5914471685025,0.111508976207022,0.549899612923874
"3124","COL28A1",3.8193235468479,-1.18866812433611,0.746735831494725,-1.59181878544221,0.111425427942148,0.549899612923874
"3125","RECQL",328.871250111344,-0.330002709709398,0.207324199879215,-1.59172305935175,0.111446944702919,0.549899612923874
"3126","CAND1",243.942256864548,0.276511427075037,0.173750789612069,1.59142544153267,0.111513862471621,0.549899612923874
"3127","C1QTNF3",161.029313341807,0.489380371424907,0.307558647313382,1.59117740860091,0.111569655563779,0.549997028767272

"3128","MPP5",65.8475731724386,0.412608131085697,0.259335531457467,1.5
9102043891493,0.111604976061241,0.549997028767272
"3129","ZFHX2-
AS1",16.2892329624929,0.857731144281707,0.539271475145703,1.5905368331
4134,0.111713849967065,0.550357621362196
"3130","YIF1A",446.015125059776,-0.405471091742925,0.254967987923289,-
1.59028235287685,0.111771174610089,0.550464107544578
"3131","NICN1",22.4292954994998,0.652385927463861,0.410281763355328,1.
59009243337695,0.111813971309142,0.550498999594512
"3132","CLEC4A",3.67112991402798,-1.25604171109288,0.79023602636024,-1
.58945134009911,0.111958531550108,0.550683077168128
"3133","TIMP2",6595.61151507241,0.21049141516052,0.132424522103605,1.5
895199153208,0.111943061473185,0.550683077168128
"3134","ARHGEF1",113.916642096395,-0.305950256451923,0.192479821137348
,-1.58951860327013,0.111943357446558,0.550683077168128
"3135","THRB-
AS1",5.01733411515106,-1.07859182614388,0.678727860788036,-1.589137397
26491,0.112029376329007,0.550855769094625
"3136","SLC1A4",10.92186088403,-0.774147590720941,0.487263623138782,-1
.58876541149153,0.112113364921279,0.551092959267067
"3137","EIF3H",1087.31732860502,-0.363665156981228,0.228921652726339,-
1.58860095866932,0.11215051163293,0.551099820472303
"3138","FGL2",1105.04731468964,-0.43190613906153,0.271943964783769,-1.
58821740870385,0.112237185870027,0.551349974565479
"3139","MYO1B",199.958303704363,0.400299684233955,0.25220495807072,1.5
8719990001826,0.112467377418298,0.551426406775782
"3140","MSL2",67.1365714217834,0.410929955016408,0.258867111242013,1.5
8741662100224,0.11241831734016,0.551426406775782
"3141","KIAA1958",5.02423272494037,1.35795994631419,0.855326378810662,
1.58765119369105,0.112365235110683,0.551426406775782
"3142","ABI1",247.646493441807,0.315185449677226,0.198559841714562,1.5
8735747850927,0.112431704010914,0.551426406775782
"3143","PLBD1-
AS1",9.45403806928852,0.874193888319997,0.550663131131801,1.5875293603
2458,0.112392802718867,0.551426406775782
"3144","CCDC117",91.2818900124515,0.332485046099968,0.209393595267988,
1.58784725805221,0.112320882222628,0.551426406775782
"3145","DNAH5",7.68228954286581,-0.900153116270183,0.567369382985326,-
1.58653805310017,0.112617307098918,0.551985942426018
"3146","PARD3B",174.365493519296,0.393348190099657,0.248023860581314,1
.58592882627395,0.112755455857239,0.552136388830793
"3147","HMBOX1",111.158763554344,0.454577127450324,0.286628831371314,1
.58594348403647,0.112752130483856,0.552136388830793
"3148","ZNF623",52.0828205224292,0.429010533443096,0.270475216252208,1
.58613620644289,0.112708415177565,0.552136388830793
"3149","ADCY9",85.6181702402667,0.360157970138134,0.227160575063054,1.
58547745372701,0.112857895572428,0.55228713023777
"3150","CD320",118.301611121128,-0.50754694754975,0.320097110296159,-1
.5856030286564,0.112829388781204,0.55228713023777
"3151","SCAF11",832.445326205352,0.269799933610452,0.170212960456124,1

.58507279873085,0.112949795007991,0.552561437654136
"3152","METTL14",108.134643174214,0.361819682372633,0.228418467630226,
1.58402114385236,0.113188907577235,0.55262911727399
"3153","MRPL22",283.357810844643,-0.436710771252698,0.275667049119673,
-1.58419648865292,0.113149012114448,0.55262911727399
"3154","USP47",267.12016880348,0.291191398430359,0.183782351335844,1.5
8443613499229,0.113094504314291,0.55262911727399
"3155","TPT1-
AS1",196.648810877385,0.356515593148824,0.225036942184622,1.5842536327
0505,0.113136012763409,0.55262911727399
"3156","AC027807.2",6.32269461374099,1.18893020056273,0.75063068579373
6,1.58390833610209,0.11321458010712,0.55262911727399
"3157","LINC00205",34.92324604353,0.620627128177714,0.39183201494538,1
.58391122855091,0.113213921792928,0.55262911727399
"3158","UFD1",305.576192865253,-0.375052896536593,0.236786363638224,-1
.58392945765078,0.113209772964317,0.55262911727399
"3159","ELOVL4",35.3178934584151,-0.508190632827939,0.320934117403084,
-1.58347338369659,0.113313608529319,0.552762428949192
"3160","ROMO1",624.936088789268,-0.383264724696569,0.24203872961334,-1
.58348511128297,0.113310937539129,0.552762428949192
"3161","PGM1",179.32918981568,-0.331302996371877,0.209304899842871,-1.
58287262563176,0.113450499200653,0.553069134992938
"3162","ZNF382",3.58812168040059,1.32873723939795,0.839524928505554,1.
58272517501445,0.113484117676462,0.553069134992938
"3163","NBDY",316.022049635793,-0.374710428026606,0.236712741506685,-1
.5829753212335,0.113427089422968,0.553069134992938
"3164","ST7-
AS1",31.0104254801329,-0.601106121040237,0.379835364338038,-1.58254385
314496,0.113525469494324,0.553095800333441
"3165","AC092164.1",18.634668009259,0.648144087959328,0.40969240888914
1,1.58202611006812,0.11364361003178,0.553321619911527
"3166","SOX15",22.8776030396342,-0.532739563728456,0.336745028788556,-
1.58202651319009,0.113643518008262,0.553321619911527
"3167","BDNF",10.4153103731146,-0.997505357072579,0.630610378424782,-1
.58180929334572,0.113693112832209,0.55338785421803
"3168","UBE4A",152.321870189101,0.282994558487105,0.1789314796431,1.58
158060868647,0.113745343676766,0.553467320952446
"3169","EXOC2",47.4032079464536,0.410674938699964,0.259738511809304,1.
58110915412296,0.113853082023901,0.553816743262366
"3170","HSPA8",1505.20421018181,-0.405124262394792,0.256261088718712,-
1.58090432074719,0.113899916253888,0.553869782035861
"3171","TIMM10",228.780564208943,-0.397118848230408,0.251266298190024,
-1.58047000768118,0.113999270164271,0.55389253733908
"3172","STAT2",253.283473764563,0.458933812102136,0.290362102997208,1.
58055685423435,0.113979397600016,0.55389253733908
"3173","GPCPD1",135.775235317487,0.415503825404581,0.262908437329123,1
.58041266999899,0.114012391889517,0.55389253733908
"3174","EFCC1",3.49529794129096,-1.46864987859247,0.929374877834701,-1
.58025562517269,0.114048337677794,0.553892604065279
"3175","MYOSLID",2.36088440415957,-1.6754061127791,1.0605831017382,-1.

57970281634061,0.114174940366427,0.553934912411227
"3176","SPSB3",275.699388625419,-0.289484592803737,0.183283844789469,-1.57943321811181,0.114236723097975,0.553934912411227
"3177","ZNF559",79.2664215479471,0.359423542508324,0.227537880885705,1.57962068166077,0.114193760050819,0.553934912411227
"3178","BTBD3",37.306199416141,0.453438288675774,0.287008732883039,1.57987627805234,0.114135202758753,0.553934912411227
"3179","PLA2G6",20.8195914078374,0.611098848497624,0.386909815642541,1.57943485481952,0.114236347940934,0.553934912411227
"3180","MICOS10",989.557800256018,-0.379147213449989,0.240261920368305,-1.57805786646832,0.114552316517938,0.553945142551517
"3181","AGO3",246.941828725763,0.413468370602813,0.261909867753361,1.57866663883078,0.114412540764276,0.553945142551517
"3182","FNDC4",145.769328443523,-0.527402412374004,0.334202192849812,-1.57809381164358,0.114544059678838,0.553945142551517
"3183","AC011346.1",12.8034349565577,0.824369024765528,0.522295590746507,1.57835723557856,0.114483563790312,0.553945142551517
"3184","BEND7",70.4094933728368,-0.458228907598363,0.290383183322624,-1.57801461625709,0.114562251991842,0.553945142551517
"3185","RASSF8",292.19492581537,-0.322985194936728,0.204676295788346,-1.57802931547444,0.114558875199539,0.553945142551517
"3186","C16orf91",79.2835147111992,-0.455761045861018,0.288675270471674,-1.57880183195581,0.11438151828538,0.553945142551517
"3187","AC087393.2",2.96206280025146,1.65809503665843,1.05016964840215,1.57888303016684,0.114362889087062,0.553945142551517
"3188","KCNK6",26.776804331882,-0.748308195161269,0.473995404064227,-1.57872457991148,0.114399244336935,0.553945142551517
"3189","TENT5A",944.581475336706,-0.337216074537033,0.213740573393848,-1.57768863993671,0.114637157356757,0.554133515413739
"3190","ABHD1",5.06005040383731,-1.00416989229264,0.637056390150629,-1.57626531625435,0.11496467129547,0.554393089237149
"3191","MSX1",811.894139056118,-0.3653975794847,0.231807583105857,-1.57629692087268,0.114957390931735,0.554393089237149
"3192","AC058791.1",100.276331723526,-0.720399303119183,0.456919052789279,-1.57664535703092,0.11487715005643,0.554393089237149
"3193","PPP3CB-AS1",2.56787357591307,1.67115006108995,1.05989116559134,1.57671854935934,0.114860300298856,0.554393089237149
"3194","WBP1L",232.387209753869,0.378427638306976,0.239945469551653,1.57714016861448,0.114763276313027,0.554393089237149
"3195","FXYD2",3.04527817084231,-1.28937619023216,0.817495158693033,-1.57722792180634,0.114743090457701,0.554393089237149
"3196","AP000866.1",9.79420925637482,0.848639236154739,0.538406648640339,1.5762049712756,0.114978573226803,0.554393089237149
"3197","AL627171.2",137.139471866005,0.557369264973123,0.353597236787306,1.5762828636254,0.114960629080568,0.554393089237149
"3198","TGFBI",116.297024312783,-0.571913252421969,0.362968198622416,-1.57565664042351,0.115104955163167,0.55469980403453
"3199","SCAPER",121.633210322014,0.447202204395781,0.283826764390657,1.57561675113998,0.115114153299154,0.55469980403453

"3200","IFI44L",112.012161076294,-0.607371341028624,0.385568603940037,-1.57526140567991,0.115196118514447,0.554747943423992
"3201","ZFHX4",1056.97112016891,0.416530008001454,0.264409109708538,1.57532396845404,0.115181684239813,0.554747943423992
"3202","AC008875.3",139.966065388947,-0.26271330044539,0.166815171481841,-1.57487654217344,0.115284944235804,0.555002315863496
"3203","KLF3-AS1",69.8051076778743,0.38295638910278,0.243267691481178,1.57421804256489,0.115437049531042,0.555049720203976
"3204","NUP43",41.1280805977215,0.481967752091085,0.306180842898878,1.57412771983995,0.115457925268702,0.555049720203976
"3205","ME3",57.2889061927379,-0.402713481242453,0.255790302829722,-1.57438916482513,0.115397507230289,0.555049720203976
"3206","PHB2",704.470136637254,-0.38896128057458,0.247076822648558,-1.57425239812088,0.115429109921206,0.555049720203976
"3207","AC134312.5",9.75758902240426,-0.79050436410563,0.502208984955849,-1.57405460233876,0.115474826642501,0.555049720203976
"3208","CLIC4",438.792439850621,0.23555341360428,0.149718641059336,1.57330718431331,0.115647706612559,0.555469844354155
"3209","SEC61B",1431.8042617373,-0.387948096071326,0.246621001596281,-1.57305376898273,0.115706368486616,0.555469844354155
"3210","LNX2",14.6115615743057,-0.707937767743275,0.449942975674382,-1.57339442110904,0.115627518004559,0.555469844354155
"3211","TMTC4",34.110902099732,0.542500299498711,0.344858322671422,1.57311064815333,0.11569319977039,0.555469844354155
"3212","SLC35B1",143.667081993018,-0.4137339086325,0.263076453337764,-1.5726755602156,0.115793961686387,0.555717284992422
"3213","AC103591.3",232.418629368124,-0.587635026199992,0.373778239689205,-1.5721488406832,0.115916036832956,0.555825250676849
"3214","CDC42SE1",121.211578078876,-0.361295463556533,0.22981537874904,-1.57211177738923,0.11592463061473,0.555825250676849
"3215","RPS5",7924.83138138872,-0.365820336622974,0.23267015290071,-1.57227015181051,0.115887912199051,0.555825250676849
"3216","DPM2",180.710086849931,-0.411081457838264,0.261587656397686,-1.57148645123111,0.116069699080695,0.55600199233341
"3217","TFCP2",88.0725884149552,0.316156198056736,0.201180162120668,1.57150782027457,0.11606473935552,0.55600199233341
"3218","AL121601.1",5.52380908933059,1.06913068221504,0.680201010973465,1.57178637633155,0.116000102118305,0.55600199233341
"3219","IPO9",131.424715162011,0.347021556767868,0.221299243616719,1.56810999936762,0.11685546332312,0.55677394078925
"3220","PDCL3",83.8888950675662,-0.426203082347093,0.27147417676504,-1.56995809849595,0.116424859775502,0.55677394078925
"3221","PHF7",29.6863092823437,0.531344189450581,0.338528862156802,1.56956835545818,0.116515565276584,0.55677394078925
"3222","TMEM108",44.047985762486,0.800573435566745,0.510200176488598,1.56913594400655,0.116616266016066,0.55677394078925
"3223","STARD4-AS1",15.7011288622831,0.795201948020185,0.506649145852955,1.56953180426554,0.11652407473872,0.55677394078925

"3224","AL662884.5",33.3414579751666,-0.559827642086314,0.356663503716031,-1.56962413101857,0.11650258114127,0.55677394078925
"3225","STMP1",498.435416686303,-0.292519347817592,0.186485985652645,-1.56858622267975,0.116744384768552,0.55677394078925
"3226","XRCC2",2.4907748615734,-1.62987068739,1.03780686163512,-1.57049519293218,0.116299951865791,0.55677394078925
"3227","HSPA14",41.2232179452319,0.395479495160586,0.251874577289027,1.57014455137634,0.116381485929917,0.55677394078925
"3228","IFIT5",117.841427407175,0.322639767167424,0.205627914602719,1.56904653626807,0.116637095973806,0.55677394078925
"3229","SSSCA1-AS1",7.98230300133993,0.924151953785785,0.589035486258944,1.56892407222393,0.116665632037369,0.55677394078925
"3230","VWA5A",79.4158128645844,-0.38884650706043,0.247970345342808,-1.56811697190189,0.116853836388812,0.55677394078925
"3231","LINC00648",22.5575087099824,0.598136967526631,0.380939831930808,1.57016126272475,0.116377599050593,0.55677394078925
"3232","BTBD1",121.737474264092,-0.327562804241138,0.208791583011766,-1.56885061895756,0.116682750443532,0.55677394078925
"3233","AC024337.2",3.95237858416729,1.49289527378468,0.951914951963127,1.56830741097815,0.116809407218416,0.55677394078925
"3234","SHPK",52.10299970759,0.496325403775438,0.316420500832014,1.56856272735291,0.116749863081783,0.55677394078925
"3235","QTRT1",122.627863833733,-0.361360957076856,0.23011480398278,-1.57035075893638,0.116333531411189,0.55677394078925
"3236","WFDC3",28.532945235673,-0.972126005370005,0.619978079848273,-1.56800060674389,0.116880990748882,0.55677394078925
"3237","IBA57",40.3171978593635,0.480380034483352,0.306505491746788,1.56728035033123,0.117049176263054,0.557402858231382
"3238","LINC01089",24.2059356278192,0.550284067105483,0.351164741010841,1.56702539532149,0.117108755770794,0.557514351515377
"3239","CDC37L1",131.968275182178,-0.349586801204517,0.223130152831242,-1.56673939747138,0.117175617899848,0.55752653901187
"3240","PTDSS2",76.9369012121963,-0.404933754234434,0.258462022541245,-1.56670504336789,0.117183651404376,0.55752653901187
"3241","SIKE1",168.633445489238,0.26033946735376,0.16618888257043,1.56652757589504,0.117225158013023,0.557551931740434
"3242","CHST2",7.71807016284072,-1.05738803029438,0.675121388162833,-1.56621912567722,0.117297326708982,0.557618551092457
"3243","LINC02381",268.512397741522,0.366819143810082,0.234215847586228,1.56615851399652,0.117311512240859,0.557618551092457
"3244","AL357497.1",2.94948469513545,1.66055891391396,1.06053950236435,1.56576809276027,0.117402918537946,0.557764119435259
"3245","OXA1L",272.433490775816,-0.277979025326488,0.177540855869542,-1.56571862834067,0.117414503247967,0.557764119435259
"3246","SESN2",80.8156006409322,0.518441973278901,0.331236365600591,1.56517226705731,0.117542522337349,0.557903464090623
"3247","USP49",21.3801746097553,0.774309563062553,0.494725375291814,1.56513007364101,0.1175524133225,0.557903464090623
"3248","TBX3",146.3282013513,-0.396203510686674,0.253137605396446,-1.5

6517049320336,0.117542938151228,0.557903464090623
"3249","GAP43",16.0221796972177,-1.16364478583822,0.743586456035981,-1
.56490852730367,0.117604358958513,0.557978206631418
"3250","AAK1",283.916679512559,0.325325442666919,0.207995706010834,1.5
6409691770259,0.117794809729362,0.558537985843777
"3251","RBPJ",1891.08273527587,0.375430649009208,0.240011243919639,1.5
6422108763751,0.117765656577699,0.558537985843777
"3252","STARD5",35.637253496315,0.600639315412778,0.384128906587498,1.
56363997895577,0.117902140539309,0.558874998897124
"3253","A1BG-
AS1",3.44720018492121,-1.44429544851768,0.923870129858913,-1.563310038
75863,0.117979688222915,0.559070671366809
"3254","C1QTNF2",179.53314564108,-0.408676942008893,0.261446563526625,
-1.56313755475036,0.11802024402505,0.559090983910923
"3255","ZNF678",45.8264295978606,0.476016892081203,0.304596790580644,1
.56277710994192,0.11810502996203,0.559320748652746
"3256","PDGFA",25.0106245669802,-0.747607515097645,0.478492680102915,-
1.5624220519671 2,0.11818859548373,0.559544594404696
"3257","XIST",2377.974989463,0.473951919138913,0.30342303216447,1.5620
1694959665,0.118283995960368,0.559824316158758
"3258","AC009061.2",16.6692232937481,0.619514942169833,0.3967409854590
63,1.56150981339375,0.118403510238342,0.560217958969932
"3259","NCAPG",4.14941642767504,1.27856312780677,0.818953035447662,1.5
612166623762,0.118472637957194,0.560373032865955
"3260","CCT5",386.367208814247,-0.35465786812661,0.227384988667148,-1.
5597241937803,0.118825072245991,0.561564377473554
"3261","LINC00621",2.70441924755138,-1.50947226815062,0.96780218479529
3,-1.55969090777564,0.118832941803641,0.561564377473554
"3262","NDUFV2",745.500566558676,-0.398452413894981,0.255469424920753,
-1.55968728554731,0.118833798204264,0.561564377473554
"3263","LINC01686",14.5214920794328,-0.720724914517551,0.4624131344509
68,-1.5586168748716,0.119087086607681,0.561783283805972
"3264","ZNF124",17.2711262078048,-0.7221405075254,0.463610484845209,-1
.55764490047396,0.119317448665634,0.561783283805972
"3265","CHAC2",5.79647089797236,-1.15791277822518,0.743177851343022,-1
.55805609132817,0.119219952110464,0.561783283805972
"3266","C2orf27A",87.9823326401437,0.296275312807848,0.190150225687939
,1.55811181257326,0.119206744973785,0.561783283805972
"3267","KIF2A",167.454349296266,-0.257510932609107,0.165192048629718,-
1.5588579156514,0.119030012894441,0.561783283805972
"3268","ELP3",60.1380457295867,0.401646448419266,0.257676090298757,1.5
5872610436453,0.119061220556326,0.561783283805972
"3269","SURF2",117.088203857218,-0.424919078308179,0.272681891004805,-
1.55829592035758,0.119163115611302,0.561783283805972
"3270","C12orf10",293.393091280166,-0.426746858664814,0.27392249690813
5,-1.55791095467391,0.119254358010466,0.561783283805972
"3271","MAP3K12",144.133765542576,0.318490394121507,0.204288744388291,
1.55902076286765,0.118991466014754,0.561783283805972
"3272","STARD9",163.724363411269,0.550467338989696,0.353177344243136,1
.55861452599502,0.119087642880935,0.561783283805972

"3273","RBPMS2",9.11822787115705,0.831623948288773,0.533890647235799,1
.55766719757029,0.119312160248809,0.561783283805972
"3274","SCML2",9.00112267372217,0.92853404700264,0.595990521000923,1.5
579678103659,0.119240878949715,0.561783283805972
"3275","MBD3",138.314429418,-0.323252383816987,0.207548888956154,-1.55
747585758108,0.119357548169773,0.561800490087651
"3276","C6orf62",352.879270438723,0.31305867180719,0.201027058483053,1
.55729618773475,0.11940018012169,0.561805883076427
"3277","RTL10",33.7477860819809,0.455749000432224,0.292678895333145,1.
55716386695208,0.119431584744823,0.561805883076427
"3278","THAP7",145.364133295293,-0.373034137699986,0.239632314154717,-
1.55669379989853,0.119543201404152,0.562159380611654
"3279","HYAL1",15.9878265403477,-0.745464302620163,0.478943293887751,-
1.55647716991498,0.119594667343524,0.562174900223147
"3280","GFPT2",614.91462472414,0.476191526162685,0.305962356144362,1.5
563729217002,0.119619440332918,0.562174900223147
"3281","ZNF703",302.002433056152,0.452742864937863,0.290978918849757,1
.55593012279914,0.119724709503362,0.562326751064692
"3282","ZFAS1",2207.12063393312,-0.342799090960095,0.22030789621712,-1
.55600002018201,0.11970808756845,0.562326751064692
"3283","TRMT61B",60.1607489330178,-0.393893023824325,0.253230054516746
,-1.55547501885593,0.119832979627581,0.56266383824525
"3284","S100A13",4870.40691428396,-0.38497767767006,0.247562477475639,
-1.55507281069258,0.119928729563214,0.562705843923196
"3285","ATP6V1C1",152.875390295336,0.274382525279177,0.176425557564892
,1.55523116415974,0.119891024685624,0.562705843923196
"3286","COPE",830.751504948193,-0.435904824742541,0.280328709188239,-1
.55497746201169,0.119951437115253,0.562705843923196
"3287","MZB1",4.26333637292949,1.40384433508788,0.903374761082324,1.55
399995169884,0.120184428150132,0.563284572798504
"3288","PEX16",113.235305789812,-0.413338593544401,0.265935027733248,-
1.55428413123152,0.120116657032853,0.563284572798504
"3289","CFL1",2668.73072555241,-0.400075128704068,0.257438894775136,-1
.55405860118192,0.120170438973778,0.563284572798504
"3290","LINC00853",4.59591481946548,1.22751035371584,0.790666461219608
,1.55250085076632,0.120542429302118,0.563369468432547
"3291","ACKR4",588.408734024987,0.551914074126718,0.355271991559318,1.
55349728444486,0.120304377516458,0.563369468432547
"3292","LMNB1",9.02156920123419,0.926919094815169,0.596801367773289,1.
5531450577494,0.120388483718799,0.563369468432547
"3293","SCX",4.63402523364985,-1.16994111490706,0.753167958283969,-1.5
5336017954438,0.12033711054337,0.563369468432547
"3294","RHOBTB1",18.8151488194041,0.777612428133719,0.500911814260321,
1.55239386653715,0.1205680101433,0.563369468432547
"3295","GRID1",22.6854854758644,0.552309384477012,0.355765260230465,1.
5524545148653,0.120553508089467,0.563369468432547
"3296","LRP6",304.413165324733,0.430998627925873,0.277559033950579,1.5
52817870099,0.120466652222526,0.563369468432547
"3297","ZNF268",69.8000524842768,0.357359395951134,0.230186038413681,1
.55248076040521,0.120547232754713,0.563369468432547

"3298","CPD",136.475319727682,0.288972553957043,0.185999306116897,1.55
362167735954,0.120274685436545,0.563369468432547
"3299","FAM171A2",16.031692308781,0.830948005265662,0.535061108807374,
1.55299645514847,0.120423981489467,0.563369468432547
"3300","TMEM43",555.195188360165,-0.290597112315826,0.18721166289308,-
1.5522382944795,0.120605216330105,0.563372548402596
"3301","PUSL1",47.8395858697957,-0.490649097600618,0.316190993510361,-
1.55174912527842,0.120722263248665,0.563419959213159
"3302","SORBS2",44.0183033436488,0.776027943028234,0.500208194571385,1
.55140989581986,0.120803485229677,0.563419959213159
"3303","ENPP2",228.086210685557,-0.565858462300241,0.364803650510216,-
1.55113157861449,0.120870154870654,0.563419959213159
"3304","AC121761.2",8.05914032996315,0.888310027081184,0.5727318633303
38,1.55100507577108,0.120900467576393,0.563419959213159
"3305","MED1",160.402685966888,0.400252653078901,0.258025665403219,1.5
512125603994б,0.120850753077245,0.563419959213159
"3306","ERBB2",106.094313475056,0.33839698333887,0.218150035577338,1.5
5121213912845,0.12085085399997,0.563419959213159
"3307","AC008622.2",3.55667488687218,1.31246808056634,0.846221508319O2
9,1.55097461795019,0.120907766790602,0.563419959213159
"3308","DYRK1A",126.490412875176,0.375578433472652,0.242019713679928,1
.55185058176441,0.120697979745145,0.563419959213159
"3309","DMXL1",142.393769744648,0.288542708284811,0.186108705457376,1.
55039877138308,0.121045833269862,0.563892873936213
"3310","MZT2A",581.059247120233,-0.38239021265281,0.246926651444257,-1
.54859838100195,0.121478295380264,0.563906568469583
"3311","TLR1",10.7523140207068,0.763946317827021,0.493251354972663,1.5
4879720070786,0.121430478695149,0.563906568469583
"3312","CENPH",26.0739181276278,-0.539102799801348,0.348062474397524,-
1.54886791727406,0.121413474716379,0.563906568469583
"3313","KLHL3",8.5460276662595,1.02563798778146,0.662071399428141,1.54
913501575109,0.121349267006303,0.563906568469583
"3314","EPM2A",13.7251934181076,-0.697812922585135,0.450301755706709,-
1.54965623327402,0.121224048191216,0.563906568469583
"3315","C1GALT1",269.113831068975,0.297751969994126,0.192222017795494,
1.54900033517963,0.121381639501681,0.563906568469583
"3316","LINC00174",25.7615150461956,0.557741711005054,0.36000225453181
4,1.5492728281116,0.121316148729276,0.563906568469583
"3317","RBP4",3.94579680756329,-1.18222983237484,0.762881690789203,-1.
5496896132765,0.121216032327399,0.563906568469583
"3318","KAT8",151.046382398167,-0.300395685456693,0.193900315025307,-1
.54922742347008,0.121327059331604,0.563906568469583
"3319","WSB1",1628.1152071293,0.457130861033685,0.294991832323552,1.54
963904401359,0.12122817617682,0.563906568469583
"3320","RBM42",233.104946842528,-0.339189274463766,0.219035413280499,-
1.54855906350357,0.121487753090333,0.563906568469583
"3321","PRPF31",259.145200659084,-0.349674253642723,0.225562900884083,
-1.55022945826726,0.121086451682567,0.563906568469583
"3322","MUL1",87.4115685892504,0.327580085925463,0.211723165978242,1.5
4720946294147,0.121812744859009,0.564056311805833

"3323","CLHC1",16.0713112826809,0.906306365068213,0.585713444039358,1.547354554162,0.121777773462199,0.564056311805833
"3324","STK16",131.315144960682,-0.421671408364884,0.272496004244963,-1.54744070296832,0.121757012694355,0.564056311805833
"3325","SMC4",110.022090090383,-0.425644736049689,0.275070938531506,-1.54739987554497,0.121766851239689,0.564056311805833
"3326","GGCT",162.551378285132,-0.406276323104097,0.262420315415313,-1.54818929495269,0.121576728018815,0.564056311805833
"3327","PLPP7",28.753549688856,0.447914943745794,0.289358791075915,1.547956922547,0.121632668302396,0.564056311805833
"3328","AGAP4",24.603641658936,0.516495845758688,0.333632901697352,1.54809625528844,0.121599123551859,0.564056311805833
"3329","FIBP",341.656824077209,-0.426429759119519,0.275605693580879,-1.54724582637978,0.121803979427193,0.564056311805833
"3330","QARS",252.49925009923,-0.28022192763217,0.181144151746885,-1.54695542157898,0.121873995462832,0.564112537358927
"3331","AC011446.2",6.26515535611559,-0.983186755529632,0.635603462986596,-1.54685556763615,0.121898077323554,0.564112537358927
"3332","PCDHB16",43.604446341255,0.569874699448072,0.36847725992217,1.5465668073206,0.121967738829938,0.564251097741796
"3333","NXF1",119.000707132542,0.393285126978432,0.254318414348058,1.54642803977295,0.122001226647642,0.564251097741796
"3334","EPHA3",3.91951286034472,1.48643593453789,0.961371106818802,1.5461624798113,0.122065332444579,0.564378254239106
"3335","COQ10B",404.845490401806,-0.333840408836462,0.215938913291554,-1.5459946692689,0.122105855244993,0.564396329415763
"3336","FAM50A",377.804058351359,-0.338927127763875,0.219257442113236,-1.54579531940739,0.122154007807787,0.564449649387601
"3337","MKKS",81.920374992855,-0.357207482103132,0.231168264443778,-1.54522716585956,0.122291325598294,0.564914828917499
"3338","RANBP9",143.972333066185,0.348722240787516,0.225716956036545,1.54495367521728,0.122357468915091,0.565051043536888
"3339","ADI1",703.713223934997,0.387790479040075,0.251230128252626,1.5435667757576,0.122693318993909,0.56542635683049
"3340","DYNC2LI1",183.917387990037,-0.255888983816236,0.165772649584288,-1.54361400664063,0.122681869781073,0.56542635683049
"3341","GPAT2",30.6830951006301,-0.871088530808933,0.564552384375179,-1.5429720162691 6,0.122837565770061,0.56542635683049
"3342","PIK3CA",198.010552699477,0.297576002084929,0.192900613061699,1.54263896501847,0.122918398394097,0.56542635683049
"3343","TSPYL4",83.1474433984372,-0.368826381266661,0.239132982005178,-1.54234843798617,0.122988944174676,0.56542635683049
"3344","VLDLR",15.758536560672,1.8335692300497,1.18874777509663,1.54243757040946,0.122967297675855,0.56542635683049
"3345","BANF1",525.987559583051,-0.443595324742025,0.287349763605302,-1.54374696250434,0.122649644503884,0.56542635683049
"3346","SERPINA1",9.25953354723292,-0.878104629090513,0.568655707088415,-1.54417623554068,0.122545644273623,0.56542635683049
"3347","NFATC3",94.5351347897844,0.35203636468852,0.228232407326182,1.54244688040906,0.122965036842047,0.56542635683049

"3348","VAT1",1330.63967398843,0.226843223699407,0.147035243277672,1.5
4278129952164,0.122883848263993,0.56542635683049
"3349","DBF4B",9.62573071919554,0.826980751199504,0.536051681668941,1.
54272578461984,0.122897322991402,0.56542635683049
"3350","ALPK2",39.8397533459342,1.33301082099763,0.864004481947609,1.5
428285950471,0.122872369476265,0.56542635683049
"3351","AC036176.1",75.9920411708001,0.513062522652074,0.3326083200842
16,1.54254265955273,0.122941779786637,0.56542635683049
"3352","NDUFS7",465.929989300205,-0.375463218782426,0.243269846533311,
-1.54340221006804,0.122733217795101,0.56542635683049
"3353","MAP3K7CL",14.2763481647042,-0.616265485283665,0.399105553517974,-1.54411653722055,0.12256010329444,0.56542635683049
"3354","ARF1",1088.18386951651,-0.244554575242837,0.158579953091474,-1
.5421531566589,0.123036380159371,0.565475790148093
"3355","ARRDC1-
AS1",38.2383205085281,0.479284736390085,0.310909660805722,1.5415562679
7835,0.123181459580168,0.565973829933916
"3356","GATD1",74.2725595660204,0.34175041939779,0.221715579882592,1.5
4139109023715,0.123221631177455,0.56598970339704
"3357","PSMD2",415.886909587555,-0.260833640174497,0.169240401907583,-
1.54120196616485,0.123267639137332,0.566032367382181
"3358","MCOLN3",98.8178445029711,0.669255573750128,0.434437796929683,1
.5405095470053,0.123436197480084,0.566228465574237
"3359","MPC2",516.158603551418,-0.367320917340006,0.238436222131469,-1
.54054159244929,0.123428392561272,0.566228465574237
"3360","MANF",342.023492652856,-0.331807575390018,0.215343224196334,-1
.54083127819941,0.123357854811708,0.566228465574237
"3361","PI16",1516.41028208443,0.649333919490339,0.421529612875834,1.5
4042302048565,0.123457273616284,0.566228465574237
"3362","HOMER3",361.225490353229,-0.37957077401433,0.246464980980585,-
1.54005965676816,0.123545812451652,0.566466002064907
"3363","TMEM160",395.530696933469,-0.324337766319218,0.210622694713694
,-1.53989942422919,0.123584871176733,0.566476595060759
"3364","PARP11",36.6373566249208,0.407284523791292,0.264658802665883,1
.53890412746054,0.123827703394451,0.567420941684144
"3365","MPZL1",700.48752368654,0.288236305231411,0.18738809354508,1.53
817833234997,0.124005017342192,0.567703203855114
"3366","PTK2B",24.1622187368122,0.563274121497891,0.36630267326319,1.5
3772866706101,0.124114971296276,0.567703203855114
"3367","POGLUT3",418.822420038693,0.281639193063471,0.183182850040438,
1.53747576807163,0.124176844582235,0.567703203855114
"3368","GLIPR1",207.936682794332,0.315784441183552,0.205415461721697,1
.53729635800924,0.124220752942154,0.567703203855114
"3369","VASN",673.760324237992,-0.444597039274063,0.289182160645651,-1
.53742899728469,0.124188289977974,0.567703203855114
"3370","ZNF423",141.992301282073,0.318302759372164,0.207046408098235,1
.53734982555767,0.124207666164231,0.567703203855114
"3371","CTU2",41.0342066045744,-0.436255470434103,0.283716825483497,-1
.53764398600843,0.124135686354881,0.567703203855114
"3372","EXOC7",272.177675718614,0.248490128174911,0.161567118988346,1.

53799937593017,0.124048767372212,0.567703203855114
"3373","RPS6KA6",9.94211714369465,-0.868438003455018,0.564552724993747
,-1.53827616980263,0.123981103808422,0.567703203855114
"3374","EEF1AKMT2",146.573353816418,-0.330251368440934,0.2148748501869
33,-1.53694752156256,0.124306160953288,0.567783836324395
"3375","RSL1D1",588.489847168207,-0.320453970542729,0.20850353743721,-
1.53692342336989,0.124312062769694,0.567783836324395
"3376","SUMO4",13.6480337027851,0.72737485199916,0.473338166003009,1.5
36691744385,0.124368813731432,0.56787478189278
"3377","RSF1",625.696328563498,0.240984026065637,0.156856691108655,1.5
3633245966349,0.124456862346431,0.568108538072324
"3378","AC016405.2",3.2770397945149,-1.24219483182139,0.80872062730627
8,-1.53599993604584,0.124538396031455,0.568266612079332
"3379","ARFIP2",111.22055221708,-0.358630808311509,0.2335002343517,-1.
53589057119033,0.124565221032505,0.568266612079332
"3380","POLR2H",219.436242343391,-0.323671097409367,0.21082636932151,-
1.53524959164747,0.124722531047339,0.568815921921517
"3381","EEF1E1",101.818229998348,-0.415933586275145,0.27099282977685,-
1.53485089113851,0.12482045875852,0.568859555848434
"3382","SELENOS",797.526897447245,-0.302942333297152,0.19738743912957,
-1.53475993524742,0.124842807488501,0.568859555848434
"3383","RAP2C",121.881681108404,0.289096831946954,0.188357305997742,1.
53483205982156,0.124825085536611,0.568859555848434
"3384","TIAM1",41.7727718757665,0.56988561563595,0.371397697665372,1.5
3443497150974,0.124922679666439,0.569055291683851
"3385","VPS53",128.479196023693,0.459877337508962,0.299742086989782,1.
53424346286292,0.124969768843746,0.569101620894046
"3386","DYNLRB2",9.86310528953448,-0.739092790243956,0.481889700481644
,-1.53373850801385,0.125093996212744,0.569499099710411
"3387","NPM1",2381.03133646691,-0.391358353518861,0.255192772826372,-1
.53357929844327,0.125133184394728,0.569509311321148
"3388","CYP20A1",183.685671882984,-0.333710598573081,0.217757673469313
,-1.53248605781099,0.125402535496622,0.569954748450076
"3389","SLC4A7",192.190478268179,-0.445944837697702,0.291033167751108,
-1.53228183970795,0.125452900518398,0.569954748450076
"3390","MBNL1-
AS1",17.0162871393438,-0.602327214631993,0.393036496133778,-1.53249690
692077,0.12539986029004,0.569954748450076
"3391","REEP3",252.199917483972,-0.259311864367549,0.169187613366679,-
1.53268823412943,0.12535268955158,0.569954748450076
"3392","DNMT1",176.279174956051,-0.363994040165564,0.237546441108374,-
1.53230685531299,0.125446730230516,0.569954748450076
"3393","ATP6AP2",1161.80891276339,-0.271364131112217,0.177080646155159
,-1.53243246511788,0.125415751198908,0.569954748450076
"3394","NRM",50.8574728085074,-0.430237256242995,0.280997066403775,-1.
53110942313103,0.125742351313899,0.570455194946508
"3395","GIMAP2",17.155593390986,0.593700349036236,0.387716685732114,1.
53127366163045,0.12570177215873,0.570455194946508
"3396","PDGFRL",2053.71997311269,-0.242485268735845,0.15836589232975,-
1.53117104427349,0.125727125101032,0.570455194946508

"3397","RPL30",17267.1925932793,-0.411022806733941,0.268447927278532,-1.53110814041592,0.125742668280335,0.570455194946508
"3398","NPC2",1363.41487934204,-0.358122282125967,0.233900794592969,-1.53108621434641,0.125748086437122,0.570455194946508
"3399","ZIC1",41.3785809415118,-0.626213007947715,0.409179344242535,-1.53041207177002,0.125914762774031,0.571043268067574
"3400","ZNF213-AS1",21.3691710768296,-0.571540499994887,0.373547726867143,-1.530033403732,0.126008460861135,0.571300124757177
"3401","GLDN",7.80110020896274,-1.11310251449594,0.727981716735903,-1.52902537097611,0.126258154463258,0.571591615580361
"3402","ALDH3A2",467.913402828461,0.210213482195978,0.137459909513062,1.52927121035244,0.126197223602923,0.571591615580361
"3403","POLDIP2",147.802627576364,-0.367752583864345,0.240502743684144,-1.52909932847718,0.126239821816774,0.571591615580361
"3404","CCDC9",46.59848411206,-0.476257119750802,0.311422780834939,-1.52929441601521,0.126191473302965,0.571591615580361
"3405","AC244090.1",38.0644063693711,0.596784577920332,0.390236571682213,1.52928920871701,0.126192763639502,0.571591615580361
"3406","EPHA2",28.5541371560016,0.629055429579775,0.411515565966466,1.52863094765906,0.126355959456067,0.571866445982169
"3407","CREBBP",349.757136697097,0.339582991006094,0.222285428374909,1.52768894249491,0.126589786811213,0.572756549367434
"3408","HDAC9",10.7940145986088,-0.934610022050644,0.611931818895152,-1.52731071206281,0.126683767038559,0.573013576554986
"3409","TMEM39B",76.5880319520098,-0.334102505371051,0.218787770857581,-1.5270620659531,0.126745578583402,0.573124990866279
"3410","DPCD",149.704701418479,-0.381896969566516,0.250240153471025,-1.52612186441428,0.126979517826688,0.573846164555377
"3411","ADAP2",5.32018451639321,-1.21776564203451,0.797917981852826,-1.52617896792696,0.1269652998556,0.573846164555377
"3412","CRAMP1",50.2808171193974,0.521927190069848,0.342034655565718,1.52594826745433,0.127022748636365,0.573873291392017
"3413","LINC00623",178.137871578967,-0.335232023195454,0.219970051452779,-1.52398938392492,0.127511363149406,0.57404819583176
"3414","RABIF",61.6384060057468,-0.382129395940465,0.250911457668732,-1.5229651108439,0.12776743439124,0.57404819583176
"3415","SPTBN1",2758.4416379306,0.300766409701627,0.197451723375135,1.52324023594469,0.127698613064343,0.57404819583176
"3416","FAIM",141.911670321072,-0.34129593177415,0.223837936985356,-1.52474569936944,0.127322538458795,0.57404819583176
"3417","LRRC1",7.5298256863967,-1.06954185508898,0.701856743483922,-1.52387487192888,0.127539971643477,0.57404819583176
"3418","LRRC4",4.31062392300954,1.2320002106073,0.808076421925544,1.52460853599911,0.127356767046453,0.57404819583176
"3419","SEMA4D",10.3117387891105,0.871005980404386,0.57170977565832,1.52351073479796,0.127630977085313,0.57404819583176
"3420","IER5L",416.46914277891,-0.404041070421869,0.265054993383767,-1.52436694462446,0.127417072647145,0.57404819583176
"3421","ERCC6",76.6771948913735,0.406422765193423,0.266798642934413,1.

52333145597496,0.127675801142968,0.57404819583176
"3422","BUD13",50.1591163921677,-0.428973863826824,0.281671352640597,-1.52295879508265,0.127769014589606,0.57404819583176
"3423","DENND4A",84.086173003202,0.451524298269087,0.296354442141128,1.52359551288273,0.127609784769333,0.57404819583176
"3424","AC068338.2",6.20966222566757,-0.894142340245386,0.586407858535599,-1.52477891834239,0.1273142498695,0.57404819583176
"3425","AP000919.4",3.77337966145953,1.5764306906744,1.0335911447991,1.52519755863505,0.12720982935508,0.57404819583176
"3426","MKNK2",331.269714209151,0.333308497984923,0.218787430606788,1.52343531372218,0.127649832693296,0.57404819583176
"3427","FBL",395.218941303158,-0.326514069040721,0.214276898381553,-1.52379501246706,0.127559925861381,0.57404819583176
"3428","SPHK2",36.3343227562781,0.507146791033587,0.332692786432754,1.52436966389139,0.127416393745071,0.57404819583176
"3429","HAS1",276.617072701589,-1.28959258468024,0.84640646501082,-1.52360909089199,0.127606390874507,0.57404819583176
"3430","APMAP",196.917109835179,0.308186916542711,0.202136824535362,1.52464508755947,0.127347645046563,0.57404819583176
"3431","TAF1",126.257021419159,0.394635895724176,0.258806142186216,1.52483203215567,0.127300998116967,0.57404819583176
"3432","AL031280.1",5.97217207788665,1.16363103087808,0.764357486528662,1.52236492922013,0.127917667267894,0.574447070856748
"3433","AC027702.1",3.05957144857442,-1.32396887638225,0.869712481444901,-1.52230639967668,0.127932325283893,0.574447070856748
"3434","C1orf167",27.7566029439661,0.517259503952378,0.339958140703742,1.52153880734142,0.128124680405027,0.574475999937103
"3435","STT3B",232.030532648362,0.293648285846715,0.19302421463387,1.52130283966553,0.12818385798884,0.574475999937103
"3436","ZKSCAN8",58.0830009910477,0.443075380377107,0.291277370711188,1.52114590740533,0.12822322629577,0.574475999937103
"3437","RRP36",152.034669155354,-0.367594255911888,0.241636780382641,-1.52126781084315,0.128192644566983,0.574475999937103
"3438","AC007566.1",15.7835352427029,0.74806454478425,0.4917946109238,1.52109138280118,0.128236906635327,0.574475999937103
"3439","AP003119.3",3.95322445210596,1.20350097212304,0.790737111309093,1.52199884754442,0.128009369458723,0.574475999937103
"3440","GABPB1",43.0870728156773,0.380015598681629,0.249705307537,1.52185631306744,0.128045087673724,0.574475999937103
"3441","ZNF329",27.3404514801914,0.482917855312463,0.317403237695416,1.52146480552248,0.128143236799539,0.574475999937103
"3442","IGSF6",10.4929499047498,-0.768255074841691,0.505203060048054,-1.52068571154065,0.128338726049854,0.574765096472545
"3443","CTCF",226.22002691464,0.314922428257035,0.207195084053382,1.51993195058575,0.128528079332256,0.575445931718478
"3444","HNRNPR",597.68362061547,-0.264872596899914,0.174309379290334,-1.51955447250337,0.128622987659344,0.575703645403247
"3445","CD83",59.8156623288691,-0.812857356735852,0.5353523110918,-1.51835966688573,0.128923753559978,0.576714933583011
"3446","GMFG",26.7355740485434,-0.664286459194911,0.437494354440305,-1

.5183886430826,0.128916452983568,0.576714933583011
"3447","BMPR2",378.690099729637,0.304569789480442,0.20061947307444,1.5
1814669240722,0.128977422510428,0.576787632143387
"3448","UBAP2L",173.455584082936,0.272011504310008,0.17927442964215,1.
5172911432655,0.129193193338679,0.576864435706209
"3449","ZC2HC1B",6.49061021958914,1.40077227736833,0.923355443352138,1
.51704556187267,0.129255181112979,0.576864435706209
"3450","PAPSS2",271.843675927694,0.333727306088669,0.219952672768189,1
.51726870098273,0.129198857088976,0.576864435706209
"3451","MAPK8IP1",56.4135307831597,-0.357229421372066,0.23555585877414
6,-1.51653804422917,0.129383358026976,0.576864435706209
"3452","AMBRA1",38.9968561647141,0.456695622913622,0.30110496796537,1.
516732274461,0.129334292251062,0.576864435706209
"3453","USP12",128.504149742589,-0.314330050602373,0.207214465146325,-
1.51693102303648,0.129284100027262,0.576864435706209
"3454","FNDC3A",351.443830971498,0.23130593712123,0.152546349118119,1.
5162993965993,0.129443664165279,0.576864435706209
"3455","ABHD2",226.521189280362,-0.443337935424243,0.292104446993602,-
1.51773771329799,0.129080532725762,0.576864435706209
"3456","ECI1",309.393634919508,-0.361417240706953,0.238130078488652,-1
.51773032201883,0.129082396775102,0.576864435706209
"3457","DSEL",246.200501314943,0.309819913604309,0.204164610321215,1.5
1750057523126,0.12914034837074,0.576864435706209
"3458","USF2",292.812080701619,-0.275625684097251,0.181723970622827,-1
.51672717227448,0.129335580963098,0.576864435706209
"3459","EIF3K",1668.34418216277,-0.407109815490992,0.268474516029789,-
1.51638159744674,0.129422889587693,0.576864435706209
"3460","LINC01914",29.3756632295561,-0.544983722509978,0.3596020738476
74,-1.5155188530443,0.129641059597164,0.57691020025701
"3461","ABHD18",38.033833324322,0.459137415539413,0.302951559934752,1.
51554728960068,0.129633864038983,0.57691020025701
"3462","PABPC1",2169.42626277833,-0.265028365386598,0.174831811656981,
-1.51590470220936,0.129543451145168,0.57691020025701
"3463","EVI2A",9.26182406706803,-0.812998994393174,0.536355223732188,-
1.51578461142968,0.12957382444995,0.57691020025701
"3464","RBL1",37.3527254010557,0.502424409854921,0.331475867546633,1.5
15719420462,0.129590314835012,0.57691020025701
"3465","TBCE.1",19.7657901205535,0.588333602522603,0.38830648100265,1.
51512691985841,0.129740265578843,0.577137542585685
"3466","TAX1BP3",887.600044209788,-0.358375396832404,0.236571185520135
,-1.51487340288068,0.1298044670869,0.577137542585685
"3467","AL121894.2",2.43552297294814,1.64382070896441,1.08506473629661
,1.51495173880121,0.129784626397447,0.577137542585685
"3468","PPP1R8",106.919264035575,0.287419456897803,0.189958421141795,1
.51306509693117,0.130263124302951,0.578350545827047
"3469","MLLT11",18.3100295141709,-0.569961611016889,0.376650368793058,
-1.51323789445177,0.130219241810629,0.578350545827047
"3470","MTX1",222.374296696161,-0.374489982195183,0.247458243698297,-1
.51334615730872,0.13019175395205,0.578350545827047
"3471","UBR3",128.448506287704,0.337367324858851,0.222947249406022,1.5

1321590985162,0.13022482423431,0.578350545827047
"3472","KLHDC8B",144.672485765116,-0.380868933376789,0.251824829587679
,-1.51243598179099,0.130422987089645,0.578350545827047
"3473","ENPP4",11.5837475382235,0.961141553054173,0.635313951538675,1.
51286076864261,0.130315028942991,0.578350545827047
"3474","SRSF5",1739.32432544085,0.260272043256871,0.172117960601797,1.
51217247954165,0.130489990164545,0.578350545827047
"3475","HMG20A",88.6507573163689,0.278733062229987,0.184301441493522,1
.51237592050947,0.130438257059829,0.578350545827047
"3476","CLEC10A",16.7420712355391,-0.926387153826038,0.612429616474159
,-1.51264264318139,0.130370456128994,0.578350545827047
"3477","TOMM40",145.216700278062,-0.321374052310258,0.212418797565142,
-1.51292661475359,0.130298300585143,0.578350545827047
"3478","ZNF134",35.0385459395522,0.51860558625043,0.342931387420147,1.
51227214910793,0.130464643152341,0.578350545827047
"3479","ATP13A2",24.212096688198,-0.58259114050821,0.385491252269927,-
1.51129535904558,0.130713215859772,0.578674388994369
"3480","GORAB",43.2111335158534,0.417144033480052,0.275978939738023,1.
51150676162475,0.130659387183522,0.578674388994369
"3481","LCORL",62.0300958216115,0.379441005300689,0.251053697484104,1.
51139381376653,0.130688144549505,0.578674388994369
"3482","ANGPTL5",422.680962146045,-0.603817527627304,0.39948195181757,
-1.51150139544489,0.130660753341807,0.578674388994369
"3483","RNF217",97.6879662969463,0.397777119359616,0.263273428555672,1
.51088972989731,0.13081654791412,0.578915706130699
"3484","NME2",3634.70844465362,-0.392320220321643,0.259679445658812,-1
.51078657506495,0.130842836208846,0.578915706130699
"3485","FAM66B",11.033983889353,0.779434091807329,0.515974111635529,1.
51060697471175,0.130888615892242,0.578952084355496
"3486","BSDC1",215.722802349547,0.27572053258318,0.182708291989318,1.5
0907509222022,0.131279593951509,0.579741665982606
"3487","FNDC5",114.815248562516,0.641917428170963,0.425433504361201,1.
50885490115504,0.131335867051138,0.579741665982606
"3488","PLK3",303.151577232709,-0.458042326483168,0.303442880914305,-1
.50948450365037,0.131175012470081,0.579741665982606
"3489","DPH3",150.005647364291,-0.410370652626221,0.271865765861389,-1
.5094605653124,0.131181125583803,0.579741665982606
"3490","NPHP3",212.301145815966,0.44012477987409,0.291747311007796,1.5
0858213004174,0.131405603694014,0.579741665982606
"3491","SLC39A7",375.813905242652,-0.290077909771931,0.192275409754075
,-1.50865838820964,0.131386104646201,0.579741665982606
"3492","PHTF2",123.75449254492,0.312571156386598,0.207189430311347,1.5
0862501005429,0.131394639091935,0.579741665982606
"3493","WRN",81.8477833887315,0.431039146023235,0.285651372431319,1.50
896928082105,0.131306633293724,0.579741665982606
"3494","AXIN2",14.1329122524793,0.705099776032576,0.467355887243582,1.
50869989076458,0.131375493475724,0.579741665982606
"3495","TRPM4",15.9892340268562,-0.570789229542991,0.378442519729026,-
1.50825871773523,0.131488324451276,0.579940635598404
"3496","STBD1",31.2969838431501,-0.679737192187621,0.450722938674295,-

1.50810427839977,0.131527840397865,0.579948987337842
"3497","FEN1",20.0770754278426,-0.55926795178954,0.371022609519269,-1.50736892426631,0.131716119612254,0.579949724028248
"3498","WNT10B",65.998749493623,0.502538856430839,0.333381937214691,1.50739677329073,0.131708985377041,0.579949724028248
"3499","SALL2",22.7765370234782,0.643425973160098,0.426852143979143,1.50737435019546,0.131714729599146,0.579949724028248
"3500","ARID3B",12.1827468632466,0.680625672532009,0.451421529931733,1.50773861546865,0.131621438215304,0.579949724028248
"3501","COQ8B",61.3876354923446,-0.338278945595831,0.22434155858576,-1.5078746342333,0.131586615807187,0.579949724028248
"3502","GNG12-AS1",5.87354060407943,-0.975899108207389,0.647666379010872,-1.50679291041446,0.131863747365996,0.580102644305602
"3503","ZNF26",46.1344566679622,0.485655046555182,0.322289389953021,1.50689120304573,0.131838546679941,0.580102644305602
"3504","SLC44A2",146.520275911071,0.490176424050218,0.325299815058123,1.50684507448194,0.131850372854014,0.580102644305602
"3505","NT5DC3",18.7340661599237,0.809192683608627,0.537119006503868,1.50654263545001,0.131927930786707,0.580219415999168
"3506","IGFBP7",2295.01109029659,-0.560672657798239,0.372219823598996,-1.50629445894926,0.131991599954322,0.580249403854181
"3507","ZNF160",94.828120711102,0.359197201053202,0.238475504814976,1.50622262580758,0.132010033040325,0.580249403854181
"3508","TMEM242",214.151546104833,-0.34114657166637,0.22651510744654,-1.50606542544578,0.132050379147646,0.580261286933001
"3509","AL359851.1",6.17323837302459,-1.27772385542727,0.848643607100959,-1.50560711791854,0.132168060154903,0.580612894638881
"3510","ANXA7",648.534494457412,-0.247223664688084,0.164266314037481,-1.50501742330247,0.132319597326614,0.581112989398792
"3511","HDC",3.43797520124326,-1.76684850899263,1.17409174185587,-1.50486409707626,0.132359020474427,0.581120564116575
"3512","SYTL2",61.2808568656472,-0.374288908049599,0.248808425164714,-1.50432569878538,0.132497525188691,0.581232029249762
"3513","DHRS1",111.046944101939,-0.361818849844893,0.240511361512435,-1.5043732136795,0.132485297313141,0.581232029249762
"3514","AC060766.4",2.59892706446557,1.82401491244095,1.21250550250786,1.5043353689268,0.132495036523736,0.581232029249762
"3515","CFAP69",32.6907977537992,-0.671377074500473,0.446379444899127,-1.50405015771323,0.132568452437661,0.581377722425758
"3516","SNRNP40",154.562686473436,-0.468149814104706,0.311816540641045,-1.50136299101473,0.133261700678647,0.582137250831178
"3517","NEXN-AS1",7.90378369100555,0.913122791561514,0.608306831271057,1.50108916195063,0.133332501658687,0.582137250831178
"3518","NBPF9",43.5490765896773,0.759944888493174,0.505800665794219,1.50245924904012,0.132978544654321,0.582137250831178
"3519","BMP2K",92.0708112285346,0.351566871426795,0.234187378615001,1.50122040524124,0.133298563887546,0.582137250831178
"3520","TENM3",17.4014467309078,-0.564967658617095,0.376270023457529,-

1.50149526509083,0.133227510444944,0.582137250831178
"3521","TSLP",10.0124324222642,1.13609165764902,0.756047392570175,1.50267254250675,0.132923506563318,0.582137250831178
"3522","RPS10",6885.08090362858,-0.503454610096778,0.335199181153867,-1.50195656315066,0.133108327140361,0.582137250831178
"3523","GALT",94.7993105564075,0.387202884580559,0.257738852369292,1.50230700967726,0.133017839177615,0.582137250831178
"3524","PTGES2",88.0337074633683,-0.420951903856402,0.28032849013428,-1.50163796642561,0.133190632584765,0.582137250831178
"3525","FADS3",75.9094089944601,-0.386475193621721,0.257322215521809,-1.50191149581862,0.133119967325785,0.582137250831178
"3526","SPA17",37.3658889044279,-0.462821173368224,0.307930725385143,-1.50300419936774,0.132837961101504,0.582137250831178
"3527","AC127502.2",49.5393006085879,-0.550825791532667,0.366735557552602,-1.50196996224905,0.133104866515229,0.582137250831178
"3528","TRADD",235.574020205435,-0.344173892153858,0.229048804189947,-1.50262252348822,0.13293641185128,0.582137250831178
"3529","BCL2",85.0580271572774,0.487068070487732,0.324487618453391,1.50103745963945,0.133345873025293,0.582137250831178
"3530","SERTAD1",1185.78210663493,-0.505911675578442,0.336898054286478,-1.50167586052083,0.133180841059862,0.582137250831178
"3531","C20orf27",102.409779005033,-0.40629092020212,0.270477721879203,-1.50212341844395,0.133065237891856,0.582137250831178
"3532","TAB2",164.583242187969,0.340018612500026,0.226608370922716,1.50046801499662,0.133493212731907,0.582615479689225
"3533","EEPD1",24.2807559468867,-0.633101476880605,0.422004026905051,-1.50022615074014,0.133555831472952,0.582723787759849
"3534","HIGD2A",939.534974446978,-0.46144380589784,0.307802586685359,-1.49915506190835,0.133833409968373,0.583439483784637
"3535","AC008033.3",2.74747708311416,1.53416573756804,1.02324460636997,1.49931475623468,0.13379199602917,0.583439483784637
"3536","USP36",95.6896469867524,0.427084589812704,0.284871693342189,1.49921736625369,0.133817251243118,0.583439483784637
"3537","SRGAP2C",213.540666158102,0.287369703017171,0.191726216130838,1.4988545062667,0.133911380728098,0.583449387768125
"3538","AC026124.2",17.6064553838565,-0.648877035992225,0.432880650616741,-1.49897445189048,0.133880259976668,0.583449387768125
"3539","LINC00891",49.0226353958087,0.541178700872118,0.361190845558075,1.49831787689952,0.134050681586114,0.583891284727309
"3540","AC021092.1",3.5888929344032,1.42331586514258,0.950238523145949,1.49785115049895,0.134171927796348,0.584254312706414
"3541","BCL3",111.634652877576,-0.486987736660721,0.325197982737772,-1.49751155453326,0.134260201345953,0.584473596088073
"3542","ANKRD28",484.613643742525,-0.472018810408914,0.315314337329879,-1.49697858462773,0.134398830473897,0.58452555419224
"3543","PPP3CA",393.565899798603,-0.377414958741685,0.252133977560061,-1.49688257962686,0.134423813792507,0.58452555419224
"3544","HMBS",28.3092306353579,-0.590174424733848,0.394256556238535,-1.49692989347976,0.134411500890788,0.58452555419224
"3545","SPIRE2",7.19365259130753,-0.909243097074658,0.607365398379286,

-1.49702814730788,0.134385934215239,0.58452555419224
"3546","CWC22",232.033394588256,0.263673877378885,0.176235994996877,1.49614088417953,0.134616945756106,0.584572515834804
"3547","SETD2",230.6059285641,0.330897243881364,0.221171299304952,1.49611294467787,0.134624225184142,0.584572515834804
"3548","COA4",294.717052842438,-0.333946794524525,0.223191565407688,-1.49623393659401,0.134592703840244,0.584572515834804
"3549","PXN-AS1",34.1322347351118,0.566035335138671,0.378247990530602,1.49646620552998,0.134532208109905,0.584572515834804
"3550","HEXIM1",234.910470637937,0.356852553888789,0.238495586832487,1.49626480987857,0.134584661510692,0.584572515834804
"3551","NOC2L",105.74006474498,-0.282491275835736,0.188878996082366,-1.49562037968768,0.134752609282118,0.584639795544602
"3552","ARNT",230.002355861811,0.285691158099051,0.190985194156871,1.49588118262398,0.134684620784376,0.584639795544602
"3553","ZNF658",17.9817946686548,0.542485089882525,0.362787143550804,1.49532611484772,0.134829352783721,0.584639795544602
"3554","NOC3L",141.333434499781,-0.292650714834689,0.195706937112122,-1.4953517701165,0.134822660612746,0.584639795544602
"3555","ABHD12",173.569530534537,-0.364258492064384,0.243576399534936,-1.49545888994118,0.134794721204836,0.584639795544602
"3556","KLHL9",135.795452785971,0.282572834622785,0.189031866489565,1.49484232405007,0.134955597576852,0.585022648101006
"3557","HNRNPLL",118.636447872626,-0.322225280476314,0.215704154182346,-1.49382973961605,0.135220126289317,0.585483722820261
"3558","NISCH",152.634859882441,0.339845941080454,0.227545629780835,1.49352875468443,0.135298833124893,0.585483722820261
"3559","FAM20C",192.373463488846,-0.220120105563084,0.147359480267457,-1.4937627709026,0.135237635386212,0.585483722820261
"3560","SFRP4",1944.21515610344,-1.00806520725216,0.674643809047099,-1.49421842123772,0.13511853932389,0.585483722820261
"3561","LINC01257",3.05342163233508,1.45208906591756,0.972232695357996,1.49356123575218,0.135290337700517,0.585483722820261
"3562","NEK3",48.2451921406076,0.400537934671557,0.26816577709878,1.49362062156058,0.13527480640586,0.585483722820261
"3563","GTF2F1",187.738322872463,-0.290816914117699,0.194732441704664,-1.49341789982154,0.135327830321673,0.585483722820261
"3564","ADGRA3",61.0200711297559,0.44987954926046,0.301338911049981,1.49293547153571,0.135454078877776,0.585865495482859
"3565","USP30",36.5777140370919,0.506930727753888,0.339625432643309,1.49261709822035,0.135537445063159,0.586061631317978
"3566","ATPSCKMT",39.9858661556842,0.43440312050548,0.291071575098988,1.49242714737002,0.135587202645553,0.586112374868537
"3567","ARHGAP30",9.12549723214677,-0.848809089124419,0.569033866474394,-1.49166708544686,0.135786441880009,0.586315966829227
"3568","MMAA",30.8484086694979,0.457469330354169,0.306680473678195,1.49168065663743,0.135782882407422,0.586315966829227
"3569","MLF2",412.790899902593,-0.311615431233653,0.208867082046059,-1.49193174999657,0.135717038256818,0.586315966829227

"3570","FAM217B",108.447837212665,0.349633409717319,0.234382114466375,
1.49172393342956,0.135771532185552,0.586315966829227
"3571","KBTBD11",11.2001726901879,0.981078131853539,0.657866020558441,
1.49130385396822,0.13588173756335,0.586342603584071
"3572","SLC25A32",77.8974416305478,-0.314139763842936,0.21067565145457
8,-1.49110617042837,0.135933622573343,0.586342603584071
"3573","ZNF518A",133.598176675351,0.325933155701145,0.218530698649956,
1.49147537492308,0.135836731707885,0.586342603584071
"3574","SUN2",205.452356371486,-0.291911536362408,0.195774015047694,-1
.49106374659218,0.135944759338921,0.586342603584071
"3575","TET2",76.0027284400309,0.406718817497751,0.272839259234722,1.4
9069022778666,0.136042842912977,0.586437478608375
"3576","RAMP2",2060.52721753359,-0.462037911515071,0.309936965451215,-
1.4907480004601,0.136027668619235,0.586437478608375
"3577","TSPAN15",53.1256140173653,-0.80200612500971,0.538149984291977,
-1.49030223621557,0.136144784784275,0.586712848042941
"3578","NUFIP2",462.767951806891,0.377169890326867,0.253113141473322,1
.49012369777182,0.13619171422698,0.586751055005282
"3579","DPYSL3",770.375330504592,-0.376750812783719,0.2528850134053,-1
.48981075513517,0.13627400239975,0.586941533107613
"3580","TPM4",1159.1292916263,-0.437575436828034,0.293761424836475,-1.
48956057478144,0.136339814849941,0.587060962545207
"3581","IGBP1",593.745590715469,-0.373086398196288,0.250491929798772,-
1.4894148426099,0.136378162464606,0.587062098406002
"3582","SERTAD2",174.693484685676,-0.290088068227827,0.194894440959273
,-1.48843685227762,0.136635723929709,0.587490012440369
"3583","CBWD2",46.9101222286842,-0.709758530243898,0.476887653843095,-
1.48831391319143,0.136668127448016,0.587490012440369
"3584","BOC",447.841135350966,0.489143603594524,0.3285350554142,1.4888
6274244871,0.136523516293954,0.587490012440369
"3585","LRRC10B",15.4512885395935,0.916663593960154,0.615793883032519,
1.48858833973144,0.136595803964388,0.587490012440369
"3586","ADCY4",2.99560334043274,-1.3552606546045,0.910443478741328,-1.
48857198304954,0.136600113842795,0.587490012440369
"3587","ABHD17A",423.893126297767,-0.306541018901428,0.206013907756874
,-1.48796274115236,0.136760719847232,0.587687799100719
"3588","SLC27A1",155.731206708442,0.302391449756014,0.20324051269433,1
.48785026049804,0.136790387490975,0.587687799100719
"3589","H6PD",357.727550908954,0.375033633063314,0.252230352328842,1.4
8686956030719,0.137049265188902,0.587817034748727
"3590","ZBTB9",6.98833818370964,0.92284889666284,0.620627290046757,1.4
8696151694089,0.137024975138176,0.587817034748727
"3591","ORC5",32.813580423441,0.415494271520182,0.279427003307102,1.48
695103409006,0.137027743981977,0.587817034748727
"3592","TMEM68",56.5963497910029,0.334354285866712,0.224829680958957,1
.48714477750716,0.136976577349837,0.587817034748727
"3593","FZD4",222.837819235641,0.420176818452092,0.28246252462496,1.48
754890231893,0.136869897518875,0.587817034748727
"3594","PPTC7",140.212095896611,-0.378738268304346,0.254627533331415,-
1.48742071742647,0.136903728494195,0.587817034748727

"3595","AC107027.3",3.01337084554186,1.40971762623085,0.948458224833159,1.48632547994281,0.137193050247157,0.588106470956599
"3596","GPR108",214.770482335287,-0.296173620750995,0.199265060670957,-1.48632991530844,0.137191877635214,0.588106470956599
"3597","IDS",460.673654542415,-0.337533015172864,0.227150774120666,-1.48594261445731,0.137294300496264,0.588376881331639
"3598","FLG-AS1",5.53243540965871,1.05366317631537,0.710349239369354,1.48330302605914,0.137993918734206,0.58842352187715
"3599","MNDA",11.2863293622032,-0.765633026403194,0.515605145023516,-1.48492123050532,0.137564691346024,0.58842352187715
"3600","MCFD2",377.56985703559,0.270039035681837,0.182205931480419,1.48205403352007,0.138325918230085,0.58842352187715
"3601","PSMD14",235.118561087888,-0.371245142422402,0.250314624094024,-1.48311407599963,0.138044104808865,0.58842352187715
"3602","AOX1",402.326603773104,0.268836565480827,0.180962640376222,1.48559152829509,0.137387197224597,0.58842352187715
"3603","RPSA",5467.27667271199,-0.337113982605491,0.227430565658092,-1.4822721019491,0.138267908318916,0.58842352187715
"3604","ALPK1",83.5697515301415,0.388503529891206,0.262001040720181,1.4828320102214,0.138119049053176,0.58842352187715
"3605","DDX60",57.1476302449917,0.408355067602982,0.275422836173573,1.4826478198984,0.138168004929457,0.58842352187715
"3606","PLK2",144.763217968178,-0.559900470623188,0.377737246836898,-1.48224850822017,0.138274183747572,0.58842352187715
"3607","CYSTM1",723.137071637893,-0.364097190485486,0.245244523696079,-1.48462923859901,0.137642065746821,0.58842352187715
"3608","PHYKPL",323.403907513987,0.239948864650939,0.161964164169345,1.48149355063541,0.138475102251025,0.58842352187715
"3609","RACK1",9301.40718571991,-0.374171154691061,0.252250470161092,-1.48333184256151,0.137986266164015,0.58842352187715
"3610","TREM1",6.93531690062072,-0.938134000023519,0.632064162401658,-1.48423855650807,0.137745644354562,0.58842352187715
"3611","DDHD2",96.3281511683564,0.315501214972298,0.212777714018578,1.48277377838899,0.138134525024591,0.58842352187715
"3612","THEM6",20.0190682165515,-0.636441079933351,0.428808229348763,-1.48420910881287,0.137753454035425,0.58842352187715
"3613","C9orf40",45.8490340762199,0.474187317101663,0.320021842154691,1.48173422760454,0.138411025958163,0.58842352187715
"3614","GDI2",840.042526289943,-0.235416614171829,0.158464624308583,-1.48560989683978,0.137382335741366,0.58842352187715
"3615","RPLP2",10259.5835369392,-0.24419406229425,0.164907837026268,-1.48079112974693,0.13866224096663,0.58842352187715
"3616","IGF2-AS",3.94385188164333,1.32459391320081,0.894018250430268,1.48161842620474,0.138441853328181,0.58842352187715
"3617","C11orf91",3.66853462801378,-1.62738203578075,1.09610047586238,-1.48470151379177,0.137622910551732,0.58842352187715
"3618","HSPB2",603.446056524107,-0.309957896032288,0.209228427515917,-1.48143299508719,0.138491227769718,0.58842352187715

"3619","GLT8D2",400.059637060605,0.386764073936654,0.260884422403305,1.48251118397077,0.138204329979404,0.58842352187715
"3620","PSME2",368.894999463896,-0.436237024057775,0.294067183019703,-1.48346041057069,0.137952127361786,0.58842352187715
"3621","TRMT5",73.8479986848284,0.34239258871219,0.231219655446764,1.48081091138475,0.138656968085376,0.58842352187715
"3622","SMAD6",66.009850933014,0.555748874792178,0.374514956417499,1.48391637041231,0.137831108335324,0.58842352187715
"3623","NR2F2",398.611579041288,0.336859927198002,0.227490953808906,1.48076185693505,0.138670044044845,0.58842352187715
"3624","UNKL",90.8080252926461,0.4041844318469,0.272388911358428,1.48385053499864,0.137848577049021,0.58842352187715
"3625","NPIPB5",47.8278483828935,0.64858008133663,0.437408309713582,1.48277951500584,0.138133000374411,0.58842352187715
"3626","ZNF668",53.6565249829788,-0.457280524961716,0.308573239780364,-1.48191892883258,0.138361867782142,0.58842352187715
"3627","LPCAT2",54.0602163701632,-0.378867707847714,0.255778934206756,-1.48123108348501,0.138545005870345,0.58842352187715
"3628","ZNF532",170.740151045112,0.280446396054647,0.189224101200739,1.48208602537968,0.138317406677702,0.58842352187715
"3629","PHLPP1",60.5505485411177,0.412073572241439,0.277671982365127,1.48403007293541,0.137800942621425,0.58842352187715
"3630","PAF1",186.380108971981,0.266450837073181,0.17994597738147,1.48072683229994,0.138679380796606,0.58842352187715
"3631","ZBED1",177.709522191106,0.339339609701675,0.228471754900029,1.48525847254142,0.137475367936334,0.58842352187715
"3632","MSL3",88.7208045853128,-0.300546042415122,0.202651371211181,-1.48306937485228,0.138055979713077,0.58842352187715
"3633","BEX3",876.283239419175,0.427317749276257,0.28800680874372,1.48370710796807,0.137886639766567,0.58842352187715
"3634","AL034417.3",31.5855533080407,1.59295333776572,1.07598103950574,1.48046599268836,0.138748929792829,0.588491359918137
"3635","PAM16",138.148273138807,-0.36576666692832,0.247076112203147,-1.48038053402583,0.138771721913878,0.588491359918137
"3636","C2orf49",156.686020672003,0.28973605699447,0.195806494570142,1.47970606200031,0.138951707143221,0.588524869078248
"3637","PLPP5",143.335875386676,-0.362894525136394,0.245238431875869,-1.47976205181445,0.138936759225731,0.588524869078248
"3638","ZNRD2",163.46864362452,-0.400318824576511,0.270475005934096,-1.48005847414254,0.138857642311931,0.588524869078248
"3639","AC004803.1",3.27861587121943,-1.15397067501752,0.779708732843934,-1.48000224495176,0.138872647556017,0.588524869078248
"3640","SLC46A1",21.587205058832,0.597615016603405,0.403893371872872,1.47963561232074,0.13897051725234,0.588524869078248
"3641","PLSCR3",142.34378124619,-0.278898821093595,0.188535482959763,-1.47929088315496,0.139062588433353,0.588753035072821
"3642","REL",99.917021516993,-0.408596020802152,0.276303167948168,-1.47879600453514,0.139194844034836,0.589151158922845
"3643","TRRAP",92.6837484183857,0.3121143451302,0.211098782181443,1.47852271768168,0.139267921049962,0.589298655774133

"3644","TBC1D24",57.08487025278,0.384465395401272,0.260087053669559,1.47821811957521,0.139349405510074,0.589481637194784
"3645","RSPH3",20.0947323520506,-0.793109333893349,0.536597643098172,-1.47803357710285,0.13939879117887,0.589528769827787
"3646","SH3D19",837.619530643682,0.268430287453424,0.181636082251882,1.4778467148459,0.139448811375833,0.589578559341322
"3647","TSEN15",59.9662912358118,-0.468828016378553,0.317374912830015,-1.47720565623174,0.139620518068782,0.590142661373805
"3648","FABP3",59.1406185108668,-0.566109498644022,0.383269158636267,-1.47705466481657,0.139660984585112,0.590151885246575
"3649","TRIP12",274.721994820899,0.354059423484289,0.239775743198615,1.47662736339016,0.139775552598119,0.5903123680274
"3650","LRRC20",18.4603905165068,0.576812650015412,0.390615847887909,1.47667498165854,0.139762781613365,0.5903123680274
"3651","FHIT",46.3516985311564,0.559379097037928,0.378904098673936,1.4763078546672,0.139861266491376,0.590512578188049
"3652","MYSM1",139.454705560466,0.388011000043441,0.263015148171488,1.4752420259477,0.140147486501661,0.591293730058535
"3653","ZNF507",82.5744948088656,0.365218632956721,0.247577782901245,1.47516723300814,0.140167588471009,0.591293730058535
"3654","ZFP30",30.3413012515926,0.65078273908605,0.441194361912996,1.47504772333057,0.140199713484525,0.591293730058535
"3655","RBBP9",50.6580936817552,0.353805437507889,0.239801274476882,1.47541099720885,0.140102080552331,0.591293730058535
"3656","MAGOH",351.042213531977,-0.369173790383654,0.250348472423925,-1.4746396764847,0.140309441933948,0.591419242868403
"3657","ZP3",2.35794100725346,-1.39158322486965,0.943638748832585,-1.47469911191251,0.140293454960792,0.591419242868403
"3658","C9orf92",6.82709895320236,-1.20457176547163,0.816930716786462,-1.47450908714759,0.14034457284545,0.591419242868403
"3659","AHI1",410.14330677315,-0.339787177528346,0.230491671030365,-1.47418419073192,0.14043200525715,0.591625952729973
"3660","SNAPC5",126.525944718943,-0.358252438880887,0.243085414969116,-1.4737718382915,0.14054303315299,0.591931927883427
"3661","C4orf46",49.7474175350806,0.530339271048197,0.359972330667011,1.47327787684543,0.140676123523642,0.592167001504178
"3662","AP4M1",83.3069375453269,-0.326073815568557,0.221331537783403,-1.4732370218638,0.140687135612481,0.592167001504178
"3663","PRKX",29.1417167424989,0.56756418335374,0.385275893593513,1.47313702412057,0.140714091891651,0.592167001504178
"3664","EFNA3",4.73465744439309,-0.976133002825129,0.66275852514896,-1.472833567257,0.140795918729566,0.592349641707495
"3665","TEX10",39.7620839270669,0.382067246237294,0.259456752238431,1.47256620974809,0.140868041723989,0.592491367851376
"3666","AL035071.1",29.9670155387731,0.623287502921777,0.423319904108756,1.47237939173691,0.140918455048498,0.592541730652646
"3667","LINC00242",4.80454497029079,-1.09672649575999,0.745021864098029,-1.47207289961585,0.14100119278375,0.592727948394195
"3668","ZNF385B",67.3296645162835,0.70340778670518,0.477928138664814,1.47178567194283,0.14107876396071,0.592730756733263

"3669","AKR1B1",310.318099248542,-0.254565874012682,0.17296317904432,-1.47179229370809,0.141076975260471,0.592730756733263
"3670","FIS1",1048.9376296685,-0.376811710379815,0.256109617783966,-1.47129074511237,0.141212505114549,0.592969426951994
"3671","ZNF888",6.31215627788267,-0.880858480763097,0.598645755643061,-1.47141856842681,0.141177954845426,0.592969426951994
"3672","GTF2E1",29.5641273687663,0.459983413454089,0.312796311121693,1.47055255160964,0.141412163815787,0.593484482771672
"3673","EYA1",3.70121956204609,1.90465340553517,1.29507317363713,1.47069172947662,0.14137450388664,0.593484482771672
"3674","UNC93B1",72.3122509496254,-0.368544295477848,0.250744368638852,-1.46980088716833,0.14161568882328,0.594072140722509
"3675","COX6A1",1171.36496699667,-0.351100023074207,0.238884053310598,-1.4697507774523,0.141629264817076,0.594072140722509
"3676","YWHAB",1802.21502370308,-0.267066205755636,0.181742916107941,-1.46947243653238,0.141704692639634,0.594226832709455
"3677","CCNL2",229.619332133215,0.351810965516995,0.239588751763337,1.46839516850319,0.141996913228361,0.595290295734344
"3678","AL117339.4",4.96884464717095,1.30265091152668,0.887450380794068,1.46785774136589,0.142142869082888,0.595416393182806
"3679","AL731569.1",2.82685160132481,1.39490977368299,0.950219649924309,1.46798666370887,0.142107845519225,0.595416393182806
"3680","DEF8",145.699726753011,-0.368616500955859,0.251081599406921,-1.46811435735063,0.142073162282846,0.595416393182806
"3681","AFF4",529.159197659803,0.378296567861441,0.257914407388801,1.46675236832026,0.14244343130633,0.595469949107624
"3682","TUBB2A",425.528606560776,-0.504094265884791,0.343684376445955,-1.46673605328714,0.142447871181751,0.595469949107624
"3683","FKBP9",970.910798305006,0.312994634331646,0.213396457827636,1.46672834927962,0.142449967741156,0.595469949107624
"3684","EXT2",264.000615423328,0.273942818181661,0.186686560613393,1.46739442454546,0.142268790318266,0.595469949107624
"3685","CIB1",578.824173447332,-0.311183097965413,0.212169195737224,-1.46667425911733,0.14246468844041,0.595469949107624
"3686","AC005632.6",2.42733790389181,1.70849242885419,1.16474135613648,1.46684276286141,0.142418833802225,0.595469949107624
"3687","CCDC124",307.086861254679,-0.370228843376633,0.252274870707222,-1.4675613244345,0.142223420043106,0.595469949107624
"3688","CFAP410",151.169145295926,-0.362775575198273,0.247331786739844,-1.46675678035617,0.142442230659547,0.595469949107624
"3689","SOAT1",125.643903531668,0.37314908324314,0.254446759135504,1.466511440393,0.142509006790582,0.595483190095074
"3690","PIR",71.6978321086367,0.404043167405744,0.275538056967714,1.46637880753107,0.142545116539139,0.595483190095074
"3691","SRP19",289.778459338581,-0.322855038143799,0.22021480292396,-1.46609144279588,0.142623376662525,0.595648699878846
"3692","HLA-DOB",3.01682564678551,1.50992683656111,1.03006862796349,1.46585071671033,0.142688960727823,0.595761194371451
"3693","AL354743.1",3.21055205882377,-1.4966241425063,1.02125371607344

,-1.46547730397553,0.142790740104195,0.59587138710016
"3694","GAPLINC",13.2308691803517,0.912087366642689,0.622385453746976,
1.46547025023096,0.142792663246707,0.59587138710016
"3695","MREG",15.8798235072205,-0.593529588664307,0.40518449165944,-1.
46483787233192,0.142965156411583,0.596107083063173
"3696","PDZD2",12.1281863605523,0.753968554020782,0.514677701360153,1.
46493339817958,0.142939089661669,0.596107083063173
"3697","ZNF616",32.3494015454121,0.502038005254898,0.342685638886003,1
.46501034267708,0.142918095976057,0.596107083063173
"3698","FAHD2B",42.9280018778865,-0.640848948757293,0.437874332475856,
-1.46354536273859,0.143318210478478,0.596132251203034
"3699","STAM2",161.9809481215,0.233748856514887,0.159631986884409,1.46
429835947696,0.14311244522703,0.596132251203034
"3700","WDR17",7.33478016567112,-1.13679023864162,0.776557637738673,-1
.46388391974604,0.143225667712971,0.596132251203034
"3701","CDKN2AIP",170.894833711736,0.285826894968116,0.195294324535451
,1.46356989967843,0.143311501894426,0.596132251203034
"3702","CITED2",1024.149250192,0.470661613828565,0.321554546452101,1.4
637069169808,0.143274044761181,0.596132251203034
"3703","WEE2-
AS1",4.99941082219798,1.15880118663305,0.791778789191715,1.46354158819
538,0.143319242488384,0.596132251203034
"3704","FOSL1",33.1467959097955,-1.4146471871681,0.966517700524057,-1.
46365367794202,0.143288598102046,0.596132251203034
"3705","DEXI",271.03195205777,-0.338159512402963,0.231001133294314,-1.
46388681120504,0.14322487754546,0.596132251203034
"3706","CLDN7",5.70692271777411,-1.09081344675945,0.744785918217164,-1
.46459998783354,0.143030085331575,0.596132251203034
"3707","ATG2B",78.4467598164982,0.346319958564944,0.236707291697516,1.
46307262476519,0.143447507638326,0.596183157251223
"3708","USB1",56.5297667968944,-0.41640389651418,0.284603803543846,-1.
46310025139924,0.143439949098748,0.596183157251223
"3709","NDC80",9.73087966856038,-0.746342408518437,0.510085949050304,-
1.46316990285265,0.143420894088196,0.596183157251223
"3710","RASSF8-
AS1",172.453760322201,-0.234433229366969,0.160253249365163,-1.46289220
527925,0.143496877220671,0.596227590931712
"3711","TEX41",7.14176903635064,-0.850537436497784,0.581468647515878,-
1.46273997769511,0.143538542570949,0.596239998310746
"3712","RERE",346.568757642276,0.372735810963505,0.255104297901477,1.4
6111145139334,0.143984858093298,0.596326326573936
"3713","NOTCH2NLC",25.3641766963684,0.554906936524095,0.37947879505172
4,1.46228707311158,0.143662559393485,0.596326326573936
"3714","NDUFS6",666.445483305534,-0.400547771955342,0.273985449682815,
-1.46193081573873,0.14376016947651,0.596326326573936
"3715","DUSP22",226.059244765388,0.258565563190729,0.176885023171795,1
.46177193837154,0.143803716290946,0.596326326573936
"3716","AL159169.2",3.68083985782864,1.34809660828141,0.92217869747818
5,1.46186049620096,0.14377944216276,0.596326326573936
"3717","ADAMTSL2",5.23228282713433,-1.20320346943986,0.823273069355928

,-1.46148770587281,0.143881647020033,0.596326326573936
"3718","ZDHHC16",59.8010606093195,-0.429790403664919,0.294129092398612,-1.46123050990976,0.143952192790515,0.596326326573936
"3719","PITPNM2",39.4874783401709,0.37654376589322,0.25755205595835,1.462010328328,0.143738379609719,0.596326326573936
"3720","FLT3LG",108.291280454927,-0.375712172847619,0.257025843235254,-1.46176807794278,0.14380477452457,0.596326326573936
"3721","HCK",5.46505211876951,-1.11648791285938,0.76409739686827,-1.46118533767477,0.143964585730781,0.596326326573936
"3722","CHADL",35.8578924946797,0.546526017367248,0.373948005761441,1.46150269274575,0.14387753711789,0.596326326573936
"3723","HMOX2",198.104026204929,-0.328597660432011,0.224946797170213,-1.46077945792385,0.144075974978976,0.596543420440752
"3724","AL513166.1",9.14165037395868,0.739194433920735,0.507199235790737,1.4574044709833,0.145004763653484,0.596753721267641
"3725","TIMM17A",134.7135606675,-0.293703824426095,0.201505195028374,-1.4575496397735,0.144964719363123,0.596753721267641
"3726","TAF1A-AS1",2.99600950036464,1.47264619232473,1.01106139154355,1.45653488961387,0.145244811981394,0.596753721267641
"3727","AC007389.5",4.31508308010042,1.29498945403357,0.888636565048204,1.4572768046781,0.14503998694836,0.596753721267641
"3728","TMEM237",195.941718974529,-0.316306836216204,0.21705289089089,-1.45727999713769,0.145039106064836,0.596753721267641
"3729","ATP6V1A",133.304262520214,0.290170925096322,0.198995663049532,1.45817713134831,0.14479172550621,0.596753721267641
"3730","OPA1",120.316399457304,0.252832160829308,0.173230169182373,1.45951575307377,0.144423208057509,0.596753721267641
"3731","TMEM170B",18.69754448828,0.601452928197936,0.412716542083032,1.45730269293866,0.145032843814849,0.596753721267641
"3732","MYO6",115.840274854737,-0.365745427318461,0.251109415863953,-1.45651817181008,0.145249429918663,0.596753721267641
"3733","FAM162B",9.78755330479166,0.998940092117001,0.68471899047705,1.45890519470043,0.14459120318332,0.596753721267641
"3734","DOK2",10.4529501906961,-0.873766745286662,0.599058949583351,-1.4585655483394,0.144684721673476,0.596753721267641
"3735","UBAP1",131.090065923641,0.326984772264235,0.224393899217467,1.45719100833193,0.145063661950719,0.596753721267641
"3736","GARNL3",34.4638669622627,-0.458175173478151,0.314207916536154,-1.45819105555678,0.144787888520209,0.596753721267641
"3737","ADIRF-AS1",10.8079105254446,-0.770212124562562,0.527394886475755,-1.46040878346281,0.144177760236447,0.596753721267641
"3738","SBF2-AS1",30.3809669188916,0.554725789568643,0.380654189302926,1.45729590047199,0.145034717977893,0.596753721267641
"3739","NDUFV1",395.403579430902,-0.338892440342578,0.232186298547295,-1.45957122561884,0.144407952204033,0.596753721267641
"3740","METTL1",27.0655767697731,-0.518210365111341,0.35493083962741,-1.46003194778829,0.14428129382755,0.596753721267641

"3741","GPC6",143.577648358986,0.554052658826145,0.380322135976765,1.45679834649434,0.145172052476744,0.596753721267641
"3742","SNF8",522.274644812271,-0.394576011205292,0.27054500299975,-1.45844871215625,0.144716902149473,0.596753721267641
"3743","LSM7",580.80380756716,-0.383874159893623,0.263134909444558,-1.45884922948433,0.144606609500689,0.596753721267641
"3744","ANGPTL4",821.502203375162,-0.580687898519557,0.398633084286447,-1.45669770375178,0.14519984392307,0.596753721267641
"3745","AC020916.1",1027.55073331363,-0.478273171871967,0.327685251300648,-1.45955049845425,0.144413652367379,0.596753721267641
"3746","ZNF780A",45.3674279611211,0.41522723858715,0.284466672349482,1.45966919483989,0.144381012089373,0.596753721267641
"3747","UBOX5",26.8815935616364,0.500718662388866,0.343714532364628,1.4567864179152,0.145175346216929,0.596753721267641
"3748","ACSS1",24.7827329902611,0.672182869208574,0.461092059274987,1.45780621393806,0.144893965018365,0.596753721267641
"3749","MATN4",47.2464736886864,0.699548503810669,0.479252531681682,1.45966574523034,0.144381960616524,0.596753721267641
"3750","AP001627.1",6.61814114508844,1.01204373793422,0.694711216532895,1.45678335666586,0.145176191503694,0.596753721267641
"3751","CARD10",3.47199650920272,-1.2174239734735,0.835488650642336,-1.45714004916467,0.145077725237517,0.596753721267641
"3752","PHF5A",194.433264898579,-0.378705019498503,0.259787145980156,-1.45775118345321,0.144909138307407,0.596753721267641
"3753","SH3BP5",1511.8255555436,0.224165923329292,0.153937790171211,1.45621112970359,0.14533426376094,0.596906387087457
"3754","ESR2",4.4324045145123,1.35276586100816,0.929031445635403,1.45610341540479,0.145364033546955,0.596906387087457
"3755","ST8SIA1",127.312427324,0.559952674670097,0.384640131003903,1.45578328815725,0.145452537011236,0.597110747810442
"3756","HIPK1",175.551197593378,0.333991789886702,0.229488398602711,1.4553754870411,0.14556533884249,0.597371140914487
"3757","CMYA5",87.7241848872732,0.651678067013966,0.447854725070087,1.45511039748879,0.145638701154416,0.597371140914487
"3758","FBXO38",52.9362839581625,0.382374923215148,0.262827037032232,1.45485383670118,0.145709730122509,0.597371140914487
"3759","AC092111.1",4.28395687619418,1.18283201825087,0.813011220324702,1.45487785245876,0.145703080224518,0.597371140914487
"3760","LAMP2",900.769637107291,0.287354298024941,0.197484197962448,1.45507489201532,0.145648529279796,0.597371140914487
"3761","ZNF333",56.2781998713219,0.42657137876587,0.293307100005269,1.45435067462808,0.145849107768839,0.597783567204641
"3762","DLD",130.069981442605,0.310143717749975,0.21327560932696,1.45419215412726,0.145893039633484,0.597804679944218
"3763","IFNGR2",563.917772487894,-0.258348648713611,0.17769988692943,-1.45384813225125,0.145988415598991,0.598036520451356
"3764","WDR6",111.348642143438,0.302173223934458,0.207923284446199,1.45329189436042,0.146142726874798,0.598350633406377
"3765","PSMC3",541.137062629731,-0.320348483981948,0.220423641473854,-1.4533308761254,0.146131908504654,0.598350633406377

"3766","SPRYD3",344.257699785027,-0.259316041585799,0.178547610113545,
-1.45236355401727,0.146400544615472,0.599111887823325
"3767","METRN",148.051171342829,-0.494344761396774,0.340377529910134,-
1.45234252545193,0.146406388675346,0.599111887823325
"3768","NCKAP1",391.122384565674,0.2500599631273,0.172227969084664,1.4
5191262752669,0.146525900964483,0.599158688422716
"3769","NBR1",262.719928215345,0.255581578923177,0.176006649380701,1.4
5211320039595,0.146470132111878,0.599158688422716
"3770","PTTG1IP",632.663077613393,0.289788549585677,0.199595117758612,
1.45188195402727,0.146534431096571,0.599158688422716
"3771","AC073140.1",3.42868918161343,1.27010107971889,0.87504736180563
6,1.4514655265037,0.146650274900362,0.599473345953083
"3772","TUBA4A",23.0098343988446,0.657387797727391,0.452997906626101,1
.45119389761329,0.14672587566863,0.59962337577729
"3773","ANAPC7",83.916322474084,-0.291849472124883,0.201177712279954,-
1.45070479635812,0.14686207931812,0.599963233303138
"3774","LINC00685",27.5548977243513,0.701873503692774,0.48384521531482
4,1.45061577851108,0.146886879175222,0.599963233303138
"3775","CCN2",2350.35084256542,-0.483087297592255,0.33323364799636,-1.
44969543290998,0.147143469898423,0.600156416014425
"3776","DEFB1",67.5882509045584,2.34012271851765,1.6136196653212,1.450
23190334746,0.146993861261229,0.600156416014425
"3777","MFSD14C",92.7895374356713,0.337554690265903,0.232904067869205,
1.44932930263574,0.147245641606653,0.600156416014425
"3778","RPS25",9426.93613909327,-0.311324525902427,0.214800020785811,-
1.44936916096887,0.147234516175915,0.600156416014425
"3779","CFL2",316.484689305523,0.299951383535414,0.206905154727102,1.4
4970473998598,0.14714087338787,0.600156416014425
"3780","CTSH",774.739226621737,0.562251931068695,0.387904241235303,1.4
494606433747,0.147208983640536,0.600156416014425
"3781","CRLS1",135.07680153126,-0.278472852530349,0.192050529582946,-1
.44999783721022,0.147059122338252,0.600156416014425
"3782","VAMP7",112.40247397978,0.2578950642665,0.177843126162902,1.450
12669216279,0.147023192944228,0.600156416014425
"3783","FTSJ3",133.593730581162,-0.356680522490574,0.246139572788983,-
1.44909864939254,0.14731003516141,0.600260161779841
"3784","PRXL2B",12.2510028518926,0.715641421559353,0.493969217588462,1
.44875712104711,0.147405422258886,0.600331462119083
"3785","ATG14",122.335853806496,0.411919884160327,0.284299297375023,1.
44889518885078,0.147366854952606,0.600331462119083
"3786","AL590822.3",3.67938540082745,-1.12118770161951,0.7741247199936
35,-1.44832954259455,0.147524909255432,0.600337402293834
"3787","CHTOP",178.660187916984,0.249967591195208,0.172620215589055,1.
44807831656455,0.147595149005514,0.600337402293834
"3788","AC008906.1",4.84874074192771,1.16339797463867,0.80341809737554
3,1.44806045375259,0.147600144204172,0.600337402293834
"3789","SORBS3",436.475957061141,-0.211035578927315,0.145716168772061,
-1.44826466894989,0.147543044691968,0.600337402293834
"3790","RAB31",194.078430890294,-0.319312468903026,0.220511250521138,-
1.44805522688021,0.147601605883466,0.600337402293834

"3791","TOLLIP",138.535548742163,0.275263015554216,0.190150359063607,1
.44760713000883,0.147726955972603,0.600688743423284
"3792","BTBD2",204.984315255177,-0.240368318816064,0.166062895036327,-
1.44745350105743,0.147769950675937,0.600705113309487
"3793","PTDSS1",78.5556420638746,-0.292526233618719,0.202140339762646,
-1.44714426601936,0.147856522400091,0.60074019314639
"3794","ZNF264",165.347056428064,0.322239739166749,0.222670775743734,1
.44715775157494,0.147852746250811,0.60074019314639
"3795","PAAF1",85.1534990296848,0.322409002955682,0.222820307744638,1.
44694622415286,0.147911985455667,0.600807182028748
"3796","SH3BP4",69.4548372388299,-0.448004732254988,0.309722731901647,
-1.44647029781867,0.148045337068277,0.601184289823502
"3797","AL137026.1",52.5150103568548,0.49275248251285,0.34072282314177
9,1.44619746329059,0.148121825024305,0.601184289823502
"3798","CNIH1",376.641898958534,-0.271485936275583,0.187721925206428,-
1.44621325387029,0.148117397382057,0.601184289823502
"3799","DERL1",263.317953296571,-0.273987956655335,0.189483000133351,-
1.44597645415426,0.148183806089914,0.601277539056598
"3800","TSTD2",75.8551172719098,0.345324184187759,0.23886545170741,1.4
4568493149336,0.148265592671982,0.601451081852265
"3801","SYNPO2",88.3921320019105,0.560710359271006,0.387987016108594,1
.44517815285362,0.14840785130891,0.601869778460102
"3802","AL391001.1",4.14579575605964,1.15172434149136,0.79706451813933
7,1.4449574849724,0.148469827910506,0.601899295490608
"3803","AC021739.2",3.97369540555297,1.23513898095233,0.85484187784098
8,1.44487420769772,0.148493222234887,0.601899295490608
"3804","CCDC80",14946.9866343561,-0.386557054880517,0.267634963041703,
-1.44434438044735,0.148642127703317,0.602163318876837
"3805","ARFGEF1",154.707696246267,0.290305241593179,0.201039644392626,
1.44401987215128,0.148733385528724,0.602163318876837
"3806","UBTD1",45.8805375479732,-0.560889162031065,0.388432224100284,-
1.44398205717932,0.148744022591197,0.602163318876837
"3807","TPP2",154.39614935931,0.279309406125537,0.193432415909354,1.44
396379899647,0.148749158686352,0.602163318876837
"3808","SPATA5L1",33.8621870046468,-0.433617963606916,0.30030031663448
,-1.44394774027064,0.148753676177943,0.602163318876837
"3809","CTNNBIP1",98.3491680519176,-0.336215159426366,0.23340258035605
,-1.44049461198534,0.149727513307985,0.602472621745024
"3810","BCL10",170.338302615477,-0.310164528122834,0.215295907971341,-
1.44064293207152,0.149685584960978,0.602472621745024
"3811","SNRPG",470.571384426197,-0.317873627158356,0.220322491383257,-
1.44276521730778,0.14908662050405,0.602472621745024
"3812","AC007038.2",5.89812468843002,1.02242617488366,0.70851164544657
8,1.44306191924231,0.149003029415678,0.602472621745024
"3813","RBMS3-
AS3",12.8116218899425,0.64126512761254,0.445113472755009,1.44067786500
251,0.149675711134944,0.602472621745024
"3814","FBXL18",30.9137573739786,0.457232646017808,0.317070853866363,1
.44205195918298,0.149287716177837,0.602472621745024
"3815","COQ4",174.822391802696,-0.240799346437062,0.167048604624753,-1

.44149271391987,0.149445534270334,0.602472621745024
"3816","WASHC2C",148.027120324347,0.323664390493408,0.224663911379148,
1.44066035575774,0.149680660077451,0.602472621745024
"3817","SSRP1",208.61378344912,-0.267658281428741,0.185551168974851,-1
.44250388131491,0.149160277441803,0.602472621745024
"3818","FAM168A",109.683287116431,0.334915025847317,0.23208772675789,1
.44305358377134,0.149005377314545,0.602472621745024
"3819","COMMD6",1964.96557876918,-0.383983848197446,0.266422537124451,
-1.44125888275766,0.149511558769968,0.602472621745024
"3820","COPS3",174.642004948478,-0.328301371625041,0.227474830924988,-
1.44324262288737,0.148952136548516,0.602472621745024
"3821","AOC3",13.1555412376247,0.81470024649678,0.565122200488004,1.44
16355361606,0.149405217969671,0.602472621745024
"3822","SNHG25",122.912968653671,-0.699194090974122,0.484950261558292,
-1.44178516107487,0.149362990292016,0.602472621745024
"3823","PTPRM",92.6143272498108,0.36219504109661,0.251019754599526,1.4
4289457088528,0.149050172774677,0.602472621745024
"3824","SMAD2",213.40613597367,0.246879943873585,0.171191156156096,1.4
4213024444133,0.149265634359095,0.602472621745024
"3825","C19orf53",873.088718772836,-0.351652345489173,0.24410917767612
9,-1.44055356229058,0.149710847676072,0.602472621745024
"3826","RPL18A",13648.1153632338,-0.313327151416555,0.217512500086097,
-1.44050181618312,0.149725476558848,0.602472621745024
"3827","ZNF302",219.906045519719,0.330579579128946,0.229295070535058,1
.44172126490788,0.149381022182052,0.602472621745024
"3828","BCKDHA",98.7211158391649,-0.389476332545932,0.27025539999954,-
1.44114172203995,0.149544648605115,0.602472621745024
"3829","ISOC2",218.466991912184,-0.446200988780389,0.309682126183022,-
1.44083546015402,0.149631172893407,0.602472621745024
"3830","DGCR6",24.5474327264944,0.707940498259547,0.490478006216279,1.
44336848806096,0.148916696075661,0.602472621745024
"3831","FAM120C",36.5868636752562,0.397512750725116,0.275956746761555,
1.44048933533991,0.149729005118728,0.602472621745024
"3832","NKD2",3.30159513756279,1.2716139732638,0.882967727421266,1.440
15906105378,0.149822402679932,0.602533873548434
"3833","SETD9",35.0834123898225,-0.446180095673416,0.309784301826945,-
1.4402927877303,0.149784581045404,0.602533873548434
"3834","ZNF253",38.4315205351154,0.54110221114613,0.375811137259917,1.
43982484151845,0.149916961144031,0.602756900374346
"3835","MPG",561.666590684456,-0.340738527272636,0.236678627239647,-1.
43966749869486,0.149961492837383,0.602778725446744
"3836","TOMM70",158.358221060211,0.234748646438018,0.163089580125277,1
.43938470046766,0.150041556701729,0.60285021135
"3837","AL445250.1",16.6412903285128,0.636984810917668,0.4425569596491
69,1.43932842322179,0.150057493412257,0.60285021135
"3838","SERBP1",1297.18415959882,-0.202925590754649,0.141068748913338,
-1.43848720795923,0.150295864443723,0.603264052810918
"3839","PFDN2",393.024896426646,-0.361613426070765,0.251466033391537,-
1.43802095731842,0.15042810790128,0.603264052810918
"3840","LIMA1",2173.56851001161,-0.253011521323346,0.175934762532299,-

1.43809851834652,0.150406102986476,0.603264052810918
"3841","AC073896.2",22.8695270690739,-0.630238234586718,0.438103413097588,-1.43856043058567,0.150275104242814,0.603264052810918
"3842","RPL13",29376.3260727188,-0.347434603455505,0.241604507464057,-1.43803030457614,0.150425455851622,0.603264052810918
"3843","TRIM37",71.6715198236913,0.346103782509494,0.240683737833972,1.43800235788361,0.150433385129901,0.603264052810918
"3844","AC005306.1",5.00220240952856,1.10064845699142,0.765403004168984,1.43799861118447,0.150434448200141,0.603264052810918
"3845","TUT4",283.648192574928,0.295218507550802,0.205364116476627,1.43753695930808,0.150565478900211,0.603376438479813
"3846","NTMT1",157.697987097137,-0.29185229342719,0.203029635843001,-1.43748616902842,0.150579900021527,0.603376438479813
"3847","EIF3J",297.502801539031,-0.269066099730033,0.187157062798133,-1.43764865566547,0.150533768135543,0.603376438479813
"3848","CXCL9",49.0586239864872,-1.76490036507503,1.22813861107736,-1.43705307296446,0.150702913783377,0.603712426187828
"3849","MDN1",81.3718598224564,0.406847881493959,0.283142403372914,1.4369019851757,0.150745845784262,0.603727516956197
"3850","WNK2",2.89099202402557,-1.40111818152942,0.975303630466336,-1.43659690968185,0.150832562219559,0.603824556809351
"3851","ZNF98",3.88910599218742,1.52856689483601,1.06406064615103,1.43654114111373,0.150848418311567,0.603824556809351
"3852","AL358472.2",4.69788173387944,1.05276247870308,0.73303152677414,1.43617626289005,0.150952191657725,0.603926300130764
"3853","C17orf75",55.4879611954107,-0.333885554644727,0.232480743686328,-1.4361858507095,0.150949464134051,0.603926300130764
"3854","DNAJC16",71.3461066461938,0.337187369296968,0.234825429506776,1.43590653706114,0.151028938112534,0.604076564868892
"3855","PGGT1B",128.505105520253,0.28331671379118,0.197350613255663,1.43560087864612,0.151115944582587,0.604267778402226
"3856","RUSC1",149.834087598489,-0.326204937922852,0.22732083725132,-1.43499796088736,0.151287678665663,0.604330150093384
"3857","MGAT4B",180.986369097001,-0.339731393340666,0.236753117095591,-1.43496059316295,0.151298327316827,0.604330150093384
"3858","CCDC180",25.8673296267968,0.658005111642565,0.458680154105548,1.43456198344947,0.151411954350859,0.604330150093384
"3859","CNTN1",63.601691600579,1.46154889801287,1.01867552011057,1.43475411861692,0.151357176501211,0.604330150093384
"3860","HOTAIR",10.5971817396664,0.844902240359687,0.589009654130397,1.43444548732751,0.151445174817434,0.604330150093384
"3861","C12orf66",17.9493787569143,-0.500475958501038,0.348806947144786,-1.43482222070909,0.151337764178876,0.604330150093384
"3862","TCF20",61.1321873512598,0.387251441746018,0.26983910356455,1.43511980521156,0.151252960750256,0.604330150093384
"3863","SYTL4",67.5403240149837,0.445562621085003,0.310600731755142,1.43451890331107,0.151424238596148,0.604330150093384
"3864","CABIN1",133.611921902731,-0.378745469394099,0.264094377580889,-1.43412924146064,0.151535384656413,0.604533632111443
"3865","AC099791.2",8.75780341695659,-0.77876641935902,0.5434002854469

3,-1.43313583046521,0.151819023581226,0.605011277942075
"3866","TRNT1",67.135278579394,-0.346477403595233,0.241705144019475,-1
.4334713685999,0.151723175479632,0.605011277942075
"3867","COQ2",46.5537818718366,-0.398891275136774,0.278463277838084,-1
.43247353199912,0.152008347678862,0.605011277942075
"3868","PCDHGB4",2.56105339280397,1.53458079398377,1.07094857101708,1.
43291735524367,0.1518814568725,0.605011277942075
"3869","TRIM4",108.259382485689,0.302768195219049,0.211336839700464,1.
43263330542926,0.151962658533524,0.605011277942075
"3870","DNAJC24",71.1028824797216,-0.298060462784394,0.20802034131192,
-1.43284286961852,0.151902746970778,0.605011277942075
"3871","ACER3",77.8130857422229,0.415960386039416,0.290374251948228,1.
43249748642858,0.15200149695354,0.605011277942075
"3872","NCAM1",3.89058920708764,-1.1718628345617,0.81801649684787,-1.4
3256626128853,0.151981829343283,0.605011277942075
"3873","EIF6",368.858494836249,-0.398728250483638,0.278200867491348,-1
.43323870295278,0.151789632654452,0.605011277942075
"3874","ETS2",52.2583741123195,-0.653022762324988,0.455924944745232,-1
.43230321098112,0.152057064551108,0.605048954583205
"3875","RC3H1",150.626631492345,0.372891258107721,0.260460170514589,1.
43166326494758,0.152240214373336,0.605308977189831
"3876","PELO",68.8634362262109,-0.375314409351974,0.262136056952302,-1
.43175423371942,0.152214169265351,0.605308977189831
"3877","GPD1",13.5174582567485,-1.25113397930158,0.873825190901418,-1.
43178978167354,0.1522039925153,0.605308977189831
"3878","ERI3",220.443082179407,-0.345793735576075,0.242262270068206,-1
.42735282501366,0.153478221339997,0.605640416455023
"3879","ENAH",467.311431831477,-0.337185116573639,0.235865421088932,-1
.42956570325968,0.152841704900306,0.605640416455023
"3880","PAPOLG",57.1316933463676,0.396650010184554,0.277271698301824,1
.43054632915611,0.152560278875955,0.605640416455023
"3881","ZIC4",18.0462111589128,-0.67694404033135,0.473177027571478,-1.
43063589499617,0.152534594389217,0.605640416455023
"3882","LEKR1",2.85139091444201,1.34574232539917,0.942018954649049,1.4
2857244937341,0.153127157441598,0.605640416455023
"3883","CNOT6L",127.417861605096,0.325008202247336,0.227306025250392,1
.42982660441719,0.152766791353986,0.605640416455023
"3884","NEK1",128.745275904304,0.268383394620001,0.188029723230228,1.4
2734558137591,0.15348030821828,0.605640416455023
"3885","AC008914.1",4.86693555137519,1.29736377602935,0.90724079986353
5,1.43001039660529,0.152714035175321,0.605640416455023
"3886","TRIM23",144.175090750782,0.333066034684287,0.233559553729227,1
.42604329117027,0.153855846308003,0.605640416455023
"3887","HAVCR2",4.11203456988608,-1.11840079903059,0.78369882651622,-1
.42707984392707,0.15355688158886,0.605640416455023
"3888","ZNF12",137.558224183738,0.258124200149525,0.180860068916493,1.
4272039245363,0.153521123621839,0.605640416455023
"3889","AC093726.2",3.34707881431443,1.49565444594742,1.04741579411631
,1.42794719570682,0.153307057782856,0.605640416455023
"3890","E2F5",8.40136665838655,-0.689133586759042,0.482979412242085,-1

.4268384309798,0.153626470929313,0.605640416455023
"3891","PPRC1",60.9944750503951,-0.383840417024424,0.268596778836788,-1.4290581543335,0.152987519430964,0.605640416455023
"3892","MTMR2",75.0137955981218,-0.324441572928817,0.227184009664574,-1.42810039055054,0.153262965125396,0.605640416455023
"3893","RPUSD4",74.004836532873,-0.325800824730066,0.228046404028373,-1.42866021553021,0.153101917889203,0.605640416455023
"3894","AP001783.1",3.22952372029134,-1.31774606433348,0.922714995185617,-1.42811818514817,0.153257844095936,0.605640416455023
"3895","CACNB3",25.5274800800168,-0.504262267777739,0.352471008322649,-1.43064892110543,0.152530859210955,0.605640416455023
"3896","ATP2B1",583.290492551393,0.367981924737862,0.257936445043597,1.42663796376531,0.15368427553304,0.605640416455023
"3897","MMAB",87.4639262105945,-0.384366699580059,0.268858329412224,-1.42962541060327,0.152824558436843,0.605640416455023
"3898","KBTBD6",49.5602122317879,0.401681820810081,0.281096991946923,1.42897943527594,0.153010144229165,0.605640416455023
"3899","DLEU2",76.4963436670249,0.48455763470322,0.339756241711211,1.42619200242711,0.15381292753071,0.605640416455023
"3900","RABGGTA",44.1354551686324,-0.451774910712491,0.316745345829952,-1.42630323273962,0.153780831882416,0.605640416455023
"3901","BAHD1",29.5705202179136,0.545082874891207,0.382095856801402,1.42656054806299,0.153706602730704,0.605640416455023
"3902","CNOT1",176.795722370254,0.287129524837823,0.201066258005034,1.42803435885615,0.153281969235539,0.605640416455023
"3903","NDEL1",144.307957837632,-0.267595838574578,0.187642319046929,-1.42609534956585,0.153840820971527,0.605640416455023
"3904","AC015726.1",5.85809420025784,0.895507201815554,0.626047387757495,1.43041440524697,0.152598116233828,0.605640416455023
"3905","C17orf58",1539.0552793875,0.503789364581663,0.352296713430508,1.43001437531445,0.152712893269806,0.605640416455023
"3906","ENGASE",33.9038778100258,0.547521526007953,0.383736176609459,1.42681758818164,0.153632480167838,0.605640416455023
"3907","MRPL4",214.696569304011,-0.354901185087777,0.248282071418685,-1.42942735679572,0.152881440187169,0.605640416455023
"3908","PLA2G4C",57.5551916549562,0.621231868835588,0.434186080134528,1.43079637339619,0.152488582782974,0.605640416455023
"3909","RPL18",10452.5109271342,-0.374461660752281,0.262322967651818,-1.42748331991007,0.153440629703601,0.605640416455023
"3910","OSCAR",8.78381276225186,-0.827921257608249,0.580087534264271,-1.42723504420468,0.15351215644438,0.605640416455023
"3911","PPP1R12C",173.871087780373,-0.220004490906141,0.154066368075696,-1.42798518361936,0.153296123173316,0.605640416455023
"3912","FAM227A",7.1896622402582,-0.904626958663396,0.634245239143912,-1.42630468915216,0.153780411666494,0.605640416455023
"3913","UBQLN2",162.487607800538,0.31314404256276,0.218953844136967,1.43018289446826,0.152664533560648,0.605640416455023
"3914","EFNB1",61.509109108993,0.541440702690553,0.379324574918967,1.4273810306285,0.153470095561488,0.605640416455023
"3915","MORF4L2",849.534474351122,-0.252609667962065,0.176677261544371

,-1.42978029970554,0.152780084963223,0.605640416455023
"3916","RPL39",13839.9920945031,-0.317132351203894,0.222001057931144,-1.42851729698629,0.153143019634123,0.605640416455023
"3917","GBP4",73.4295438210391,0.382925873935203,0.268963599757715,1.42370891183843,0.154530752868881,0.606006592088343
"3918","PACC1",20.3196864977395,-0.518927998325428,0.364284583695434,-1.42451265178789,0.154298125316612,0.606006592088343
"3919","LTBP1",245.518654693139,0.460144136838067,0.323307502651065,1.42323989720302,0.154666623465879,0.606006592088343
"3920","LIMD1-AS1",8.07891801377484,1.02960056456143,0.722959781900662,1.42414639145571,0.154404099523228,0.606006592088343
"3921","RPL35A",12361.3492499274,-0.360006346913625,0.252970749612334,-1.42311452001988,0.154702959816092,0.606006592088343
"3922","LETM2",6.09284990022965,0.880309319193008,0.618114211811177,1.42418553460137,0.154392771159664,0.606006592088343
"3923","RPL8",12370.1541751858,-0.336357102909334,0.236040331685757,-1.42499843356062,0.15415765390738,0.606006592088343
"3924","TUBB4B",1255.54190816331,-0.320298139775733,0.224963010984193,-1.42378135131841,0.154509775692616,0.606006592088343
"3925","AL450306.1",9.62917817127085,-0.915793803105795,0.643015462898535,-1.42421738814437,0.154383552935962,0.606006592088343
"3926","RBP5",7.90256295376556,-1.18626595791793,0.833190331280881,-1.42376347081975,0.154514953363747,0.606006592088343
"3927","HOMER2",5.72798073754185,0.929521244926092,0.653017582081635,1.4234245301069,0.154613125645956,0.606006592088343
"3928","PAGR1",170.157905037056,-0.290335571034883,0.203951887247951,-1.42354932309066,0.154576974546298,0.606006592088343
"3929","CD2BP2-DT",3.05898106932051,-1.11201079297115,0.781602224425726,-1.42273238000083,0.154813750220168,0.606006592088343
"3930","RIPOR1",104.676758645638,0.265900915061117,0.18682662155243,1.4232496035716,0.154663810672534,0.606006592088343
"3931","SHMT1",46.7619340570414,-0.397442076322536,0.278955966654797,-1.42474843283909,0.154229933389711,0.606006592088343
"3932","AC006141.1",16.0394677757143,0.611926566507539,0.430091432647366,1.42278250636362,0.154799214103613,0.606006592088343
"3933","MIB1",193.190711583571,0.344186301669694,0.241576498149142,1.42475076965974,0.154229257655701,0.606006592088343
"3934","CA11",34.5931164078312,-0.52970125028722,0.37221356866835,-1.42311107083577,0.154703959537475,0.606006592088343
"3935","AC010642.2",194.716567453878,-0.306203824831143,0.214983214755916,-1.42431503398437,0.154355297423129,0.606006592088343
"3936","TP53TG5",3.16829198700015,-1.24674284734629,0.874719570188321,-1.42530576637022,0.154068834015893,0.606006592088343
"3937","TOB2",294.923409873879,0.314500984198939,0.220628649119029,1.42547663440239,0.154019469585693,0.606006592088343
"3938","PARVG",7.87035304222926,-0.90310114096575,0.634725448686935,-1.42282169847453,0.154787849526917,0.606006592088343
"3939","ARID3A",40.3956901150894,0.523860481904799,0.368273210153577,1

.42247784378978,0.15488757903549,0.606141668147264
"3940","SPAG16",173.785498996532,-0.309452642543753,0.217601232346903,-1.42210886954178,0.154994648337798,0.606406726935826
"3941","IL6R-AS1",4.09721988279236,-1.30300101064735,0.91640071422615,-1.42186817450013,0.155064523742014,0.606526169368977
"3942","ADAMTSL4",668.480701197085,0.304970495873761,0.214557452134908,1.42139316457768,0.155202492527832,0.606841584047559
"3943","CDR1",10.4454383372849,0.698032343281031,0.491115714969595,1.42131950170694,0.155223896587708,0.606841584047559
"3944","GLRX3",235.408614610064,-0.336954296275113,0.237116967442275,-1.4210467513555,0.155303168575611,0.606843686588856
"3945","ASPA",165.799791298454,0.376414225920479,0.264873229543195,1.42111087092361,0.155284530142487,0.606843686588856
"3946","UBA6",111.77105577222,0.283517080808056,0.199576351525665,1.42059456764644,0.155434658669356,0.606934585905234
"3947","MRPS36",352.081782181792,-0.369012470842732,0.259767798640043,-1.4205473995415,0.155448379506121,0.606934585905234
"3948","TGFBR1",166.426056517358,0.336870071236677,0.237184019995922,1.42028991347085,0.155523296420738,0.606934585905234
"3949","DENND5A",170.771702427031,0.258186941058573,0.181775159238423,1.42036426836477,0.155501659662934,0.606934585905234
"3950","PHLDB3",12.5901167049718,-0.815460076857745,0.57397248745565,-1.42073025219829,0.155395194209099,0.606934585905234
"3951","AC007405.3",35.5295417477929,-0.560224649748884,0.394561768696677,-1.41986551712658,0.155646836556484,0.607262967734296
"3952","NDUFAB1",550.538272759128,-0.361153416456945,0.254413899405198,-1.41955065073605,0.155738541062942,0.607331842905119
"3953","SYNGR1",64.7243214373648,0.393128716778047,0.276942022943145,1.41953435813082,0.155743287382675,0.607331842905119
"3954","ASCC1",68.7167437543468,0.357784241179465,0.252094237971414,1.41924799257028,0.155826728581226,0.607503546049469
"3955","INKA2",5.48812752086002,-0.978634784092924,0.690212219728842,-1.4178751927594,0.156227206175969,0.607528350959274
"3956","CALM2",2350.84082289877,-0.27272771903515,0.192226932229744,-1.41877995903922,0.155963177219272,0.607528350959274
"3957","TET3",10.2725586175214,0.740523540369566,0.521997722574178,1.41863366130747,0.156005846871439,0.607528350959274
"3958","LINC01088",57.0599986660673,-1.12960271508415,0.796647760834829,-1.41794500733976,0.156206820815944,0.607528350959274
"3959","CLPTM1L",233.863048538877,-0.277880934016825,0.195979099805214,-1.41791106446052,0.156216731642781,0.607528350959274
"3960","ANKHD1",340.182489395825,0.312807013061982,0.220533424477474,1.41841089985855,0.156070835179794,0.607528350959274
"3961","ASS1",107.021493908447,-0.53050766611329,0.374153385481692,-1.41788818890494,0.156223411241208,0.607528350959274
"3962","SYNGR2",6.61780110148427,-0.98031535850285,0.691298079137441,-1.41807910087936,0.156167672117818,0.607528350959274
"3963","AC245060.6",7.38295898352419,1.00121391308112,0.705719874108177,1.41871293386254,0.155982724891818,0.607528350959274

"3964","TMLHE",51.571162294172,0.393935024038872,0.27781342604455,1.41
798411130678,0.156195403617869,0.607528350959274
"3965","ARL2BP",409.875669034244,-0.243806980272054,0.171989066231255,
-1.41757255629397,0.156315597202983,0.607565539809377
"3966","ALDH3A1",5.11860753546916,0.977216380087466,0.689349294539573,
1.41759248588216,0.156309775201987,0.607565539809377
"3967","MINK1",82.9244368446214,0.306435659465486,0.216198401339021,1.
41738170850284,0.156371357614341,0.607629059144205
"3968","MDH2",446.007350256495,-0.352908929463036,0.249017503731721,-1
.41720531358006,0.156422908714983,0.607676194012467
"3969","ISY1",164.95899374832,-0.272492723729772,0.192300038492649,-1.
41701856050428,0.156477501013591,0.607735116685439
"3970","ELOVL2",13.1672201953894,0.755205869521912,0.533057871962163,1
.41674273891131,0.156558156555456,0.607895209899837
"3971","ZFAND3",206.683653430109,0.236032252912783,0.166639509824153,1
.4164243111484,0.156651310165844,0.608103738656883
"3972","AC021097.2",9.65182634819693,-0.790600604557319,0.558356874108
256,-1.41594138304463,0.156792667294854,0.608499236241231
"3973","LOX",920.755849787971,0.577517899612551,0.408070045617831,1.41
524207869306,0.156997530968433,0.609140936289554
"3974","GLRX5",462.545673378532,-0.373664141478641,0.264157680985984,-
1.41454959811851,0.157200595483566,0.609621931919287
"3975","KXD1",273.322376161247,-0.264969791954654,0.187310021610571,-1
.41460552765053,0.157184187199251,0.609621931919287
"3976","PITHD1",336.06526556894,-0.275414457714836,0.19477558673031,-1
.41400912885545,0.157359222139992,0.609768153718839
"3977","SUPT7L",89.3340105357242,0.319645526694132,0.226276395649069,1
.41263310199562,0.157763630830554,0.609768153718839
"3978","AC093495.1",9.00243773236006,0.853239119156309,0.6038176094607
36,1.41307425584744,0.157633891851139,0.609768153718839
"3979","TMEM243",361.605977102664,-0.349761831703398,0.247659759888735
,-1.41226750708526,0.157871209957307,0.609768153718839
"3980","LCNL1",2.37134507703582,1.71290821430579,1.212647415381,1.4125
3606990752,0.157792177856778,0.609768153718839
"3981","CD82",17.0409111317263,-0.538101075434748,0.380925270328213,-1
.41261585237206,0.157768705416886,0.609768153718839
"3982","AP001160.3",14.2382351768419,-0.539336530450881,0.381838394846
716,-1.41247328118323,0.157810652505004,0.609768153718839
"3983","TSKU",210.736155907673,0.487056963102522,0.344803634005177,1.4
1256331160132,0.157784162903211,0.609768153718839
"3984","MDP1",179.437768277289,-0.377567344354658,0.267248479779452,-1
.41279510613586,0.157715977621676,0.609768153718839
"3985","KLF13",418.321007962209,-0.302666470524528,0.214257544052857,-
1.41262923488874,0.157764768465944,0.609768153718839
"3986","DNAJA2",346.0212229013,-0.224840470139175,0.159140161041749,-1
.41284556121688,0.157701138583012,0.609768153718839
"3987","NUDT21",224.471664270954,0.195340231661733,0.138308559933647,1
.41235099082404,0.157846639327779,0.609768153718839
"3988","EMC8",114.628287635416,-0.329871959840964,0.233427578060786,-1
.4131661844817,0.15760686673041,0.609768153718839

"3989","SGTA",112.493973518423,-0.28924084256681,0.204630922467694,-1.41347573025027,0.157515892476053,0.609768153718839
"3990","COL6A2",13972.6266453564,-0.295155322238623,0.208948434303206,-1.41257494090777,0.157780741464256,0.609768153718839
"3991","CERK",98.0829268897309,0.339546362342807,0.240120179427462,1.41406841837456,0.157341814862462,0.609768153718839
"3992","RBM8A",567.073971335645,-0.317236281618907,0.224806696625838,-1.41115138641491,0.158199980866155,0.610030608829882
"3993","HIST2H2BE",9.79733513320635,0.788349758985434,0.558625151979973,1.41123212263399,0.158176181359548,0.610030608829882
"3994","SEPSECS-AS1",25.3453516163022,0.49187042989834,0.348556306699423,1.41116491207977,0.158195993567521,0.610030608829882
"3995","PTPN13",189.260040799223,0.300272225048924,0.212759497393531,1.41132230865128,0.158149599437723,0.610030608829882
"3996","EMB",16.3498920705941,-0.700704684526949,0.49656753495992,-1.41109644750236,0.158216177366323,0.610030608829882
"3997","PRKAG2",193.570136829932,0.408011958527071,0.289139552170356,1.41112468171313,0.158207853502699,0.610030608829882
"3998","LINC00667",218.115975899777,-0.26053046195246,0.184529590022376,-1.41186279079072,0.157990365649537,0.610030608829882
"3999","UTP11",154.706905608191,-0.307543865040713,0.218082949749911,-1.41021508280859,0.158476183528167,0.610075042372593
"4000","ETAA1",72.04974573808,0.338029912292064,0.23982609092631,1.40947930638593,0.158693488205673,0.610075042372593
"4001","PAIP2B",31.467307402851,0.451260305494181,0.320035402118247,1.41003246049463,0.158530098288392,0.610075042372593
"4002","AP2M1",1041.65259721904,-0.253172966122402,0.179594046948068,-1.4096957578756,0.158629537881316,0.610075042372593
"4003","ALDH7A1",180.022695338857,0.296911605473331,0.21058409779362,1.40994314662978,0.158556471072723,0.610075042372593
"4004","RNF144B",7.54624253085549,-1.02924417518168,0.730174561159502,-1.40958646045852,0.158661827229004,0.610075042372593
"4005","MAP3K5",34.042738887362,0.496349690944904,0.351842729106001,1.41071464573414,0.158328770751211,0.610075042372593
"4006","FAM3C",709.456151156292,-0.279117441141984,0.197983240295807,-1.4098033789373,0.158597748634113,0.610075042372593
"4007","FAU",8759.29751267489,-0.352172694847775,0.249830079773845,-1.40964889082441,0.158643383038527,0.610075042372593
"4008","RAB27A",46.5270900526811,-0.411351967209281,0.29179320967757,-1.40973797047513,0.158617068494667,0.610075042372593
"4009","TK2",304.346238893938,0.308968431084367,0.219084856310345,1.41026831469673,0.158460470731715,0.610075042372593
"4010","LRRC75A",150.874097541146,-0.579296494995907,0.411009368258348,-1.40944839639708,0.158702622115738,0.610075042372593
"4011","LINC02427",4.86754916734479,1.28335989975582,0.910717794610967,1.40917406835564,0.158783703565122,0.6100824502633
"4012","AC093627.4",2.38678473161985,-1.36796883643167,0.970700957975174,-1.40925876830819,0.15875866597671,0.6100824502633
"4013","COLEC11",6.46250703303766,1.4198697781104,1.00794561474936,1.4

086769735721,0.158930706685697,0.610495101809124
"4014","TRIM5",69.8541719801239,0.344021157253264,0.244254410800987,1.4084542265792,0.158996611842533,0.610596106515357
"4015","PATL2",9.79585783228762,0.692420467940188,0.491681323358987,1.40827083528377,0.159050888173385,0.610652413746632
"4016","DSTYK",168.255039352756,0.356532809866,0.253301090260624,1.40754550049177,0.159265695032484,0.610764860324422
"4017","NFIB",2579.82866857258,0.270215172307067,0.191936308198457,1.40783770847396,0.159179131671675,0.610764860324422
"4018","HAPLN3",17.1399435459867,-0.85773577388075,0.609460334234783,-1.40736931626189,0.159317904856601,0.610764860324422
"4019","WDR59",67.4477053177006,0.378036592509681,0.268524859573416,1.40782716769776,0.159182253640213,0.610764860324422
"4020","RPS16",11228.5105701009,-0.303629372013003,0.215730008528882,-1.40745079501702,0.159293758115468,0.610764860324422
"4021","ZNF865",44.570882459695,-0.356489776817375,0.253288243632499,-1.40744699282061,0.159294884858676,0.610764860324422
"4022","DUSP2",63.0652476984206,-0.757865796799056,0.538555160487844,-1.40722037852641,0.159362050647689,0.610782200580341
"4023","TTC37",335.959252363584,0.237176078226649,0.168579491346556,1.40690944273332,0.159454243191515,0.610983633804922
"4024","KHNYN",92.6327941071328,0.354097522572485,0.251735933372245,1.40662287592005,0.159539246047121,0.611056331452879
"4025","TAB1",53.2005887124701,0.363308163201797,0.258292186816234,1.4065782154699,0.159552496535701,0.611056331452879
"4026","AC110772.2",8.49376109251659,-0.850236274742319,0.60479550768443,-1.40582438847405,0.15977627812152,0.611477055313719
"4027","CAPZA2",481.790161618614,-0.284580223535107,0.202429812803491,-1.40582169984697,0.159777076693732,0.611477055313719
"4028","TLCD3A",51.2729744392932,0.389236240606393,0.276877379453221,1.40580729771084,0.159781354447205,0.611477055313719
"4029","DARS2",18.5007553902856,0.51194213464711,0.364297480739311,1.40528595917866,0.159936261859848,0.611766123218251
"4030","LINC00603",4.9439028282358,-1.59153375666961,1.13245380612104,-1.40538514513104,0.159906781595452,0.611766123218251
"4031","HCG11",190.197054591991,0.25623821991935,0.182422651757841,1.40464036373891,0.160128247576165,0.611893142386363
"4032","PWP1",249.528288598253,-0.254911579248372,0.181443306738447,-1.40491034819946,0.160047939021125,0.611893142386363
"4033","GAA",330.899865200717,0.353689574925986,0.251779183474859,1.40476098954902,0.16009236291538,0.611893142386363
"4034","HIF3A",40.1100459934059,-0.78275901561447,0.557200868438794,-1.40480580693936,0.160079031854848,0.611893142386363
"4035","MGME1",48.9802317111344,0.373313802642191,0.26584433755423,1.40425711556124,0.160242299439327,0.612082739031488
"4036","PCSK1N",2.49791089290577,2.01764427215457,1.43685693061512,1.40420679969217,0.160257277634193,0.612082739031488
"4037","NBPF12",79.0933925172087,0.617277352146292,0.439825299760981,1.40346031135941,0.160479619099391,0.612677971218191
"4038","ZNF891",39.266680578465,0.453508698763277,0.323146145308094,1.

40341670587125,0.160492614192608,0.612677971218191
"4039","KTN1",854.345827491869,-0.276792090456141,0.197246618912435,-1
.40327926522796,0.160533578781281,0.612682623647797
"4040","AURKAIP1",632.870970844603,-0.363489375519598,0.25961132096352
2,-1.40012913986394,0.161474650394187,0.613420911344429
"4041","CZIB",571.902006446054,-0.376524479938048,0.268992616848695,-1
.39975767494703,0.161585896209401,0.613420911344429
"4042","ACOT11",5.09704456729245,-1.13824382669531,0.812168376513235,-
1.40148749891244,0.161068343238064,0.613420911344429
"4043","AL078459.1",10.4854635146199,0.819789157961872,0.5849193517349
23,1.40154220497288,0.161051995963261,0.613420911344429
"4044","LRIF1",59.5936762873566,-0.320414339038688,0.228878940191732,-
1.39992931971057,0.161534485075963,0.613420911344429
"4045","RPL29",12446.1448381173,-0.384119133743124,0.274078498582625,-
1.40149313327958,0.161066659517569,0.613420911344429
"4046","SIAH2",76.3371820673945,-0.326866992174949,0.233480290111019,-
1.39997681183077,0.161520262391875,0.613420911344429
"4047","LMBRD2",54.1813928674088,0.373825646421688,0.266855589025715,1
.40085372686597,0.161257818498062,0.613420911344429
"4048","CEP41",38.346080155763,0.41625857844903,0.297390794895733,1.39
97022960814,0.161602485953275,0.613420911344429
"4049","TMEM86A",11.5876046856191,0.713455716524054,0.50970277741414,1
.3997485360853,0.161588633832632,0.613420911344429
"4050","ESAM",2.57090961741947,-1.4611170214438,1.04233141054381,-1.40
177779031095,0.160981612629933,0.613420911344429
"4051","AC010168.2",5.60564557445128,1.15709007447459,0.82568220804462
3,1.40137460054372,0.161102083511338,0.613420911344429
"4052","MAPKBP1",35.2040894253239,0.403195629238109,0.287688065496626,
1.40150279971498,0.161063770923248,0.613420911344429
"4053","AC027279.1",34.8393787669305,0.491599143980721,0.3507803878554
83,1.40144421125178,0.161081279352871,0.613420911344429
"4054","TNFSF13",56.8427435796258,-0.535209055949381,0.381784938652603
,-1.40186005723076,0.16095704008941,0.613420911344429
"4055","AC020910.5",37.1464924763367,-0.599332311357807,0.428136371297
391,-1.39986310796636,0.16155431539032,0.613420911344429
"4056","TMEM147",450.939762314278,-0.308032726098085,0.219988677282503
,-1.40022082001302,0.16144720304651,0.613420911344429
"4057","LIG1",19.2951032478829,-0.499991959626216,0.357116651411879,-1
.40008021930501,0.161489297749971,0.613420911344429
"4058","ZHX3",205.354632659599,0.372868765411929,0.266370728524969,1.3
9981133616555,0.161569822244609,0.613420911344429
"4059","ATP6V1E1",310.017448068932,0.216574629550319,0.154643539407079
,1.40047641421485,0.161370701453602,0.613420911344429
"4060","RRP7A",146.486331154947,-0.456760018991503,0.326187370929639,-
1.4002995201492,0.161423644485919,0.613420911344429
"4061","PHF6",104.859558686451,0.31848741520433,0.227475707577974,1.40
009418410165,0.161485116433265,0.613420911344429
"4062","ADIPOR2",97.4152090226881,0.28211103234716,0.201590364265889,1
.39942716694072,0.161684924944664,0.613582746928113
"4063","ELOVL6",64.5606695406784,0.409827784574623,0.292951961977108,1

.39895900272772,0.161825277446539,0.613878014356383
"4064","RBM7",325.417417426965,-0.302985267469255,0.216587917485285,-1.39890198394765,0.161842377576668,0.613878014356383
"4065","TRAF2",31.1914450192372,0.431840609272283,0.308827432425885,1.3983233480268,0.162015989666023,0.614385358106209
"4066","RPL36AL",2296.31091902122,-0.351297905053702,0.251312891327049,-1.3978507158892,0.162157900984663,0.614470020569955
"4067","AC015712.2",25.5663837269486,0.501268606247137,0.358570857543624,1.39796248273231,0.162124333691972,0.614470020569955
"4068","ZNF850",9.18181899677985,0.745464447469488,0.533195703891277,1.39810662769611,0.162081049918175,0.614470020569955
"4069","LINC00640",15.8652944074175,0.814837570737643,0.583149001944802,1.39730595100079,0.162321587051093,0.614637009185115
"4070","MARK3",257.250296498747,0.278889652333276,0.199567278011713,1.39747184564449,0.162271727319632,0.614637009185115
"4071","SMCR8",49.1165910862501,0.449681444585869,0.321799723955381,1.39739537081834,0.162294710437016,0.614637009185115
"4072","FOXP4",31.8326773433388,-0.484691945724904,0.346962565915933,-1.39695746267435,0.16242636306619,0.614882707923702
"4073","AC107223.1",5.94518260399672,0.987810715278159,0.707191981482599,1.39680700735217,0.162471614521742,0.614903004628689
"4074","CCDC40",9.26827089124294,-0.877794234413179,0.628572398315303,-1.39648867300861,0.162567389195276,0.6151144586267
"4075","LINC02591",37.3497377258917,0.448412237455109,0.321196143410122,1.39606980549125,0.16269347535487,0.615188674965128
"4076","PLA2R1",178.360674978218,0.426126470524173,0.305231310101495,1.39607719267882,0.162691251048485,0.615188674965128
"4077","GPAA1",370.818023204178,-0.313662628184798,0.224682545563907,-1.39602579006558,0.16270672901932,0.615188674965128
"4078","MDFI",3.83294564761746,1.34696036583478,0.965063645199527,1.39572179776422,0.162798287619219,0.615297837737502
"4079","DCAF10",181.051747113647,0.305059870758857,0.218576732046232,1.3956648903248,0.162815431730864,0.615297837737502
"4080","AC073195.1",3.52876529020608,-1.04007728634243,0.745303653090462,-1.39550810200603,0.162862673310141,0.615323226666127
"4081","PITPNM1",14.1686150241528,-0.657547742975086,0.471277541820074,-1.39524523157975,0.162941901475909,0.615323226666127
"4082","FOPNL",114.138564650043,0.242082488473302,0.173490690326338,1.39536299047483,0.162906405790941,0.615323226666127
"4083","CHAMP1",69.7670432439651,0.292055204553628,0.209344489454012,1.39509382508865,0.162987548017996,0.615344857383642
"4084","TOR1AIP1",311.339340328094,0.239851251310991,0.171999719084923,1.39448629676289,0.163170804700328,0.615568064589326
"4085","FAM110B",126.929479255376,0.362761860188293,0.260260890150499,1.39383931246267,0.163366133781053,0.615568064589326
"4086","EXPH5",14.3955178205578,-0.679089008455045,0.487150197567218,-1.39400335224403,0.16331659235172,0.615568064589326
"4087","AP000821.2",12.5984059320281,-0.67028188823522,0.480694316205396,-1.39440360669631,0.163195759589626,0.615568064589326
"4088","AC004494.1",4.29122142359151,1.27710280306611,0.91565434148292

1,1.39474334932744,0.163093247634943,0.615568064589326
"4089","SF3B3",169.613952802551,0.256833968443641,0.184214740606167,1.39420964684214,0.163254305682974,0.615568064589326
"4090","KIF3B",148.958619932339,-0.33205471609981,0.238164008811732,-1.39422710323246,0.163249035885154,0.615568064589326
"4091","FAM3A",170.005368326957,-0.367739445490687,0.263807809505112,-1.39396724524776,0.163327496003839,0.615568064589326
"4092","CCDC12",256.45293591996,-0.297065346759146,0.213163604897932,-1.39360256597925,0.163437653235276,0.61568705391539
"4093","SFRP1",1255.98308597973,1.10296939075287,0.791613045422513,1.39331886599238,0.163523388099721,0.615709093199122
"4094","PICALM",287.273545846941,0.236705032272281,0.169873440797587,1.39341989637054,0.163492852574199,0.615709093199122
"4095","POGZ",232.182581211285,0.264643062306726,0.190065899849129,1.39237528939592,0.163808783256839,0.616156359033465
"4096","SRRT",86.3621247801303,-0.362347290605869,0.260257332464075,-1.39226544426327,0.163842031506855,0.616156359033465
"4097","KLRD1",18.5367396438902,0.75570246523584,0.542780138170315,1.39228098467876,0.163837327379063,0.616156359033465
"4098","ZFP1",34.5058626121742,0.387457562200701,0.278217161292034,1.39264436601019,0.163727359833261,0.616156359033465
"4099","CENPX",279.135902277797,-0.396040860802368,0.284403280307611,-1.39253267534049,0.163761154075044,0.616156359033465
"4100","BANP",57.1576511388966,0.327973890349488,0.235640777408807,1.39183843287232,0.16397132888965,0.616492203618036
"4101","SEMA6D",9.37398104263903,1.00592270013364,0.722840534102108,1.39162464288637,0.164036092538502,0.61658531248013
"4102","ENTPD3-AS1",62.4241053182117,0.35013005424475,0.25169357181633,1.39109652947455,0.164196157152884,0.617036509632302
"4103","ERCC5",167.019156193718,0.310954812348463,0.223553692885806,1.39096253939899,0.164236786587701,0.61703876803544
"4104","RTN4",3853.06921238222,-0.218952497770709,0.157442303298534,-1.39068403588801,0.164321260656377,0.617109927956819
"4105","SELENOW",1525.07289869226,-0.338818641481448,0.243642915097805,-1.39063613380851,0.164335793335241,0.617109927956819
"4106","DNAJC11",48.1458808841177,0.351889523425266,0.253091658142227,1.39036397330614,0.164418380594741,0.61726968749828
"4107","MCU",84.5546588380087,0.349461244304515,0.251390013586152,1.39011585750503,0.164493698712661,0.61740208562349
"4108","YRDC",49.6327386824291,-0.414034118398695,0.29797243481389,-1.3895047662959,0.16467931258055,0.617919149969862
"4109","CLDN11",102.560245475352,1.10690228088913,0.797601867713651,1.38778797504838,0.165201617557183,0.617919149969862
"4110","FRAS1",17.0344074670817,1.05988633625691,0.764216445206442,1.38689286641901,0.165474433411487,0.617919149969862
"4111","TRIM52-AS1",117.834107090129,-0.360683935538964,0.26018869822779,-1.3862398251564,0.165673684516733,0.617919149969862
"4112","AL590004.3",5.311379896964,-1.12860705309737,0.814125205112568

,-1.38628192077817,0.165660835172434,0.617919149969862
"4113","HSPA1L",4.77288777997936,1.26098159195845,0.908864042585589,1.38742598768803,0.16531190514404,0.617919149969862
"4114","DINOL",16.7471181377786,-0.749247149501109,0.539755712580445,-1.3881226859446,0.16509968964554,0.617919149969862
"4115","GSTA4",200.900719704985,0.369219935837156,0.265923153861846,1.38844598702742,0.165001281263286,0.617919149969862
"4116","YWHAZ",1223.37735413432,-0.217450639541544,0.156566895433442,-1.38886728857688,0.164873109165843,0.617919149969862
"4117","TSSC4",197.421578087829,-0.329247310439548,0.237228472802579,-1.38789120272905,0.165170177074304,0.617919149969862
"4118","P4HA3",15.4329827438069,-0.885984921944028,0.639055628713133,-1.38639718067758,0.165625656867827,0.617919149969862
"4119","MORN3",3.89050330982145,-1.07622066040672,0.776132282373183,-1.38664591700265,0.165549759572659,0.617919149969862
"4120","AL139385.1",2.36690593063533,-1.76677745660437,1.27196250726081,-1.38901692975932,0.164827602042613,0.617919149969862
"4121","PLEKHH1",2.36515013401008,1.62584392524454,1.1713007265882,1.38806703380126,0.165116633845146,0.617919149969862
"4122","RDH11",244.422778268226,-0.2365761550204,0.17064029310429,-1.38640265271821,0.165623986894111,0.617919149969862
"4123","OTUB2",3.3474579319068,1.45488186962362,1.04871316886284,1.38730199335744,0.165349695542838,0.617919149969862
"4124","NEDD4",86.7594995415588,0.311586636073742,0.224649504786291,1.3869900864912,0.165444785771924,0.617919149969862
"4125","TSR3",335.954861987537,-0.323992268199206,0.233601660424682,-1.38694334453872,0.165459039413366,0.617919149969862
"4126","CARHSP1",901.711607702911,0.33073132196742,0.238074168785092,1.38919448361477,0.164773618712878,0.617919149969862
"4127","C16orf86",51.2435792952801,0.481408389502121,0.347254846185524,1.38632590672305,0.165647409623544,0.617919149969862
"4128","KRT25",9.9229472289944,1.21109826250806,0.872515877255146,1.38805297883869,0.16512091331398,0.617919149969862
"4129","CEBPA",120.017336993203,-0.410238107768274,0.295305823455896,-1.38919748675238,0.164772705755863,0.617919149969862
"4130","Z95331.1",29.2660836099373,0.792781947854574,0.571893120231015,1.38624144933643,0.165673188735155,0.617919149969862
"4131","SH3KBP1",323.687407094298,0.238676047546297,0.17198366061241,1.38778327369243,0.165203049575909,0.617919149969862
"4132","FAM104B",132.492815602152,-0.355469694987415,0.256062329138461,-1.38821550277784,0.165071432963939,0.617919149969862
"4133","LINC00629",4.25832010251725,1.06964226151907,0.771039047840443,1.38727378920038,0.165358292378432,0.617919149969862
"4134","LMO4",656.271152456785,-0.302981561915635,0.219156767011377,-1.38248782388684,0.166821966565854,0.618250705587772
"4135","COX20",307.020459786012,-0.32950007189759,0.238454325970574,-1.38181628937296,0.167028116125582,0.618250705587772
"4136","CRIM1-DT",42.9227974133344,0.601517823818044,0.434446007398094,1.38456289981935,0.16618616238519,0.618250705587772

"4137","HAAO",35.2463253924365,0.483511759901225,0.349190570162566,1.3
8466442457517,0.166155101981354,0.618250705587772
"4138","LBX2-
AS1",31.1600549500958,0.418062436714796,0.302094363230937,1.3838802956
916,0.166395111140676,0.618250705587772
"4139","PAX8-
AS1",124.560997241671,-0.712774285497131,0.516108197567744,-1.38105592
752879,0.167261765183965,0.618250705587772
"4140","PDCD6IP",351.791869261115,0.181817828233668,0.13156215444686,1
.38199187295239,0.166974196508265,0.618250705587772
"4141","SELENOK",707.109783158378,-0.267524531282195,0.19330492256382,
-1.38395095031204,0.166373474224724,0.618250705587772
"4142","ZBED3-
AS1",7.05741677438642,0.896950447462487,0.648430992616514,1.3832627645
4363,0.166584310842703,0.618250705587772
"4143","SRFBP1",152.439060862816,-0.312132711798675,0.225289219892165,
-1.38547557645269,0.165907095587409,0.618250705587772
"4144","PCDHGA8",13.6559520495174,0.813575537065987,0.589242549635153,
1.38071416867254,0.167366863082567,0.618250705587772
"4145","DCTN4",125.072284532773,0.294076121000774,0.212546507053123,1.
38358482140228,0.166485618627949,0.618250705587772
"4146","FBXL4",98.5291156833784,0.342442179608987,0.247733630858236,1.
38229992602396,0.166879628703717,0.618250705587772
"4147","BX322234.1",65.7540753593627,-0.622602095308684,0.450079341741
767,-1.38331631240676,0.166567898402999,0.618250705587772
"4148","ADCY1",31.0242109237537,0.510070120211507,0.368360891317102,1.
38470215550764,0.166143559722438,0.618250705587772
"4149","PEBP4",10.8362929046125,0.689694987106093,0.499469747991418,1.
38085437582487,0.167323740490686,0.618250705587772
"4150","AC090578.1",6.98280911743483,0.993355108749397,0.7193119534299
79,1.38097956528133,0.167285243847415,0.618250705587772
"4151","SIT1",4.25220255848096,1.13842664807577,0.821837319692992,1.38
52214067147,0.165984776978509,0.618250705587772
"4152","SEC16A",104.979437341347,0.29472296196375,0.213321452990439,1
.38158770280636,0.167098331930868,0.618250705587772
"4153","CREM",130.860750210514,-0.332025258563648,0.240150369728566,-1
.38257233973416,0.166796035207253,0.618250705587772
"4154","INPP5F",34.7792956942609,-0.438836859630245,0.31720450514072,-
1.38345090475801,0.166526651165799,0.618250705587772
"4155","UEVLD",72.1288842021221,0.341542843790707,0.247281647156964,1.
38118961806296,0.167220666081893,0.618250705587772
"4156","PTPRJ",17.2588364424303,0.610348481065155,0.441924039796889,1.
38111626908931,0.167243214060756,0.618250705587772
"4157","EMSY",72.8080400367369,0.323131598900557,0.233253959306965,1.3
8532096029852,0.165954347356206,0.618250705587772
"4158","KATNAL1",149.596281341907,0.219650121643054,0.158749287985877,
1.38362901925327,0.166472077917181,0.618250705587772
"4159","COL4A2",637.720903440083,-0.385224820486066,0.278713954553428,
-1.38215117755154,0.166925287290291,0.618250705587772
"4160","F7",3.35091106673128,1.21740592763607,0.880843155916732,1.3820

9160105134,0.166943577027319,0.618250705587772
"4161","ZNF219",57.736430792644,-0.367281555206075,0.265904578591292,-1.38125321930091,0.167201116473042,0.618250705587772
"4162","HOMEZ",22.4751804149845,0.491544061378336,0.355922321120271,1.38104308780409,0.16726571276752,0.618250705587772
"4163","ADSSL1",11.3883232788946,1.05364677392703,0.762816135624902,1.38125915895038,0.167199290844313,0.618250705587772
"4164","TUBGCP4",96.8993102299089,0.290093442692333,0.209406139441207,1.38531488840985,0.165956203174033,0.618250705587772
"4165","RPL4",4007.86229292524,-0.23540286612836,0.169977684693482,-1.38490453351484,0.166081660614462,0.618250705587772
"4166","CDR2",102.505912416228,-0.33098464717612,0.23942193508204,-1.38243242860227,0.166838964726166,0.618250705587772
"4167","FBRS",93.1970722974177,-0.264590971479822,0.191389222143311,-1.38247581821352,0.166825650421634,0.618250705587772
"4168","SLFN13",12.8696784864037,0.692010369944094,0.50026180049774,1.38329644449281,0.166573987784065,0.618250705587772
"4169","ZNF260",60.6797799169346,0.323917651104324,0.234522465703757,1.38117962444369,0.167223738054765,0.618250705587772
"4170","EIF3D",677.345633778582,-0.336775739113797,0.243908295369831,-1.38074737721878,0.167356648593864,0.618250705587772
"4171","DMC1",4.86472675822018,-0.941446829405354,0.680441789046615,-1.38358173257471,0.166486564970091,0.618250705587772
"4172","ZRSR2",70.4722241783245,-0.303495023732674,0.219715818996668,-1.38130711351865,0.167184551930939,0.618250705587772
"4173","G6PD",142.403186601716,-0.292933598543879,0.211983708441808,-1.38186844968935,0.16701209695024,0.618250705587772
"4174","LRIG3",243.035874279973,0.280868016404942,0.203458557913518,1.38046794042612,0.167442614090961,0.618382342168704
"4175","AADAT",24.5941765143865,0.521944820695325,0.378145743577473,1.38027421849954,0.167502229867907,0.618454340937433
"4176","HTRA2",184.739651697203,-0.330555229225327,0.239558311517878,-1.37985289314689,0.167631943123671,0.618488847116178
"4177","PLIN1",4.35621981983016,-1.17065167912962,0.848381094811519,-1.37986535330528,0.167628105937719,0.618488847116178
"4178","ZNF630",4.32491131193227,1.00783147900527,0.730273933777926,1.38007319224918,0.167564110325387,0.618488847116178
"4179","MESD",566.722514337269,0.226461310716351,0.164144206905608,1.37964851142495,0.167694893206736,0.618573050677634
"4180","MRM3",56.4562098753793,-0.393495082727822,0.28524624042188,-1.37949261713614,0.167742920973557,0.618602183446741
"4181","LGALS9",48.0713972575617,-0.412743418612901,0.299376577568684,-1.37867638799567,0.167994552737518,0.619381973319502
"4182","NBN",267.415663087498,-0.239119778860722,0.173483089539804,-1.37834632467655,0.168096386976551,0.619609231287309
"4183","HTR2A",11.8331447325361,-0.802377157212206,0.582261461974847,-1.37803583031375,0.168192225916523,0.619666147825812
"4184","ZNF738",36.9148696820978,0.409594731890869,0.297215860609867,1.3781052298165,0.168170801115934,0.619666147825812
"4185","H2AFY2",25.5081549562974,0.568001249088194,0.412225934869995,1

.37788819441292,0.168237810432984,0.619685985143238
"4186","GKAP1",30.0525284871976,-0.467125186560275,0.339057136248827,-1.37771819737621,0.168290310730202,0.619731280436232
"4187","EFHB",5.43099436527548,-1.52145498949539,1.10495081256728,-1.37694363603425,0.168529674705209,0.619777667615749
"4188","ZKSCAN7",13.7351439133167,0.608377834832477,0.441695055675766,1.37737071541744,0.168397662073086,0.619777667615749
"4189","GOLT1B",154.277476843654,-0.299708546899108,0.217650500751742,-1.37701749301723,0.168506839544813,0.619777667615749
"4190","SEL1L",322.012174927236,0.222220946053705,0.161374349864999,1.37705246366357,0.168496028102839,0.619777667615749
"4191","ALKBH5",232.711349790705,-0.231212554002505,0.16787234709489,-1.37731173718452,0.168415887954927,0.619777667615749
"4192","LRP5L",8.9253330840238,-0.694988748024593,0.504750049228016,-1.37689684050063,0.16854414418717,0.619777667615749
"4193","PCDH18",790.88147181688,0.213060229177341,0.154773498241683,1.3765937424548,0.168637886642837,0.619974486668096
"4194","HIST1H1C",66.5854303524127,0.465587158094079,0.338322586741398,1.37616339062209,0.168771053481055,0.620002000305419
"4195","MYH11",7.82666449327031,0.862531655346829,0.626817235371609,1.37604967871612,0.168806253342967,0.620002000305419
"4196","ZNF625",16.4069464200963,0.576230103302975,0.418696196020892,1.37624871871113,0.168744643532128,0.620002000305419
"4197","USP25",121.911470350622,0.259736971541111,0.188714172328262,1.37635116820642,0.168712938423324,0.620002000305419
"4198","SH3RF3",35.5985934523876,0.510016146242668,0.370728914435459,1.37571181093148,0.168910873829547,0.620126240611335
"4199","FBLN7",112.422239449305,0.366514975800469,0.266424468702264,1.37568061066515,0.168920537419851,0.620126240611335
"4200","FLNB",223.086706747636,0.413348742038079,0.300516491240238,1.37546109477114,0.168988539323777,0.620228174684766
"4201","HACE1",31.0182512135514,0.438729191859192,0.319096608143986,1.37491023302016,0.169159276386041,0.620707032966156
"4202","AC092802.1",15.0462639123536,0.623565934016314,0.453660817453621,1.37452014815025,0.16928025962996,0.620855389530297
"4203","STEAP1",164.336882354095,-0.417346517752048,0.303605330504167,-1.3746350140131,0.169244627707932,0.620855389530297
"4204","AC123768.3",7.12644518697845,0.925523468245284,0.673427597943113,1.37434739988703,0.169333857533688,0.620904237364845
"4205","ADAM15",141.644359589704,-0.248915255760441,0.181221351114614,-1.37354265504297,0.169583710213978,0.621322576525242
"4206","ACOX3",40.5348473810248,0.348039075526541,0.253366090542923,1.37366083512102,0.169547001017196,0.621322576525242
"4207","PIGN",60.243162717547,0.424675115658116,0.309200778735576,1.37346069241725,0.169609173014481,0.621322576525242
"4208","ELOF1",225.623973514417,-0.317576663457445,0.231211311381407,-1.37353428584456,0.169586310087487,0.621322576525242
"4209","CLASP1",104.766571356188,0.314739557690372,0.229199716081996,1.37321094053089,0.169686779499049,0.621459184123982
"4210","LINC02478",8.91270976361216,-1.07488530973456,0.78290100368368

5,-1.37295175849442,0.169767344402368,0.621606559135985
"4211","RPL19",16154.8681471438,-0.361780291037883,0.263538528205453,-1.37277950780632,0.169820903170982,0.62165500412745
"4212","NDUFS5",1385.06614348644,-0.321483140964411,0.234240998799546,-1.37244608165082,0.169924613024209,0.621886968131096
"4213","KLHL21",98.226139200466,0.452068371727105,0.329504841101878,1.37196276150411,0.170075030625174,0.622100541251823
"4214","AC007878.1",12.2479961699622,0.857375666652068,0.625025975461153,1.37174405594821,0.170143128369739,0.622100541251823
"4215","GPC2",7.01596224115463,-0.890708780773639,0.649327709794869,-1.37173998173438,0.170144397140298,0.622100541251823
"4216","AC005332.1",2.87191115714868,-1.2328529585826,0.898638877534575,-1.37191144229698,0.170091007910265,0.622100541251823
"4217","XAF1",227.98126264912,0.366692759759939,0.267356439190021,1.37155013311393,0.170203526677737,0.622169163798273
"4218","RPS27A",15388.1246478371,-0.345893881179671,0.252247378573952,-1.37124866523941,0.17029745236917,0.622217404188374
"4219","ARHGAP21",806.027686530916,0.267420792715522,0.195006695778476,1.37134159238976,0.170268495734946,0.622217404188374
"4220","SP140L",59.6131321870906,0.338578721009178,0.246998419789397,1.37077282234383,0.17044578558099,0.622381374698249
"4221","ZNF292",488.778339717326,0.298447962240741,0.217746439918257,1.37062154656939,0.170492962640841,0.622381374698249
"4222","TRIM34",57.2602188227072,0.347929141249179,0.253811234397932,1.37081852217655,0.170431535495623,0.622381374698249
"4223","HAGH",143.136749078275,-0.324285552123592,0.236603457052335,-1.37058670301619,0.17050383038279,0.622381374698249
"4224","SCYL1",164.530000307749,-0.284554746058346,0.207639944760446,-1.37042391523768,0.170554610943965,0.622419348414115
"4225","INTS11",203.075769784626,-0.24591693900684,0.179556582528314,-1.36957907944178,0.170818333837497,0.622644742517146
"4226","CEP85",8.72504365851124,-0.771559379020775,0.563221260865898,-1.36990456971489,0.170716693050998,0.622644742517146
"4227","TMSB10",20719.4558506164,-0.316586834830322,0.23110374203331,-1.36989056103077,0.170721066607001,0.622644742517146
"4228","GPR137",81.1686759340857,-0.376960001828756,0.275198365950943,-1.36977558179234,0.170756966664701,0.622644742517146
"4229","NKG7",42.6775488073661,0.578008035020613,0.421998557359284,1.36969196918013,0.170783076647195,0.622644742517146
"4230","LST1",23.0095277929909,-0.563469629271526,0.411525522304033,-1.36922158829128,0.170930019754883,0.622904551896339
"4231","UBR4",94.1274644105333,0.410000258000756,0.299548740501742,1.36872636257461,0.171084826484855,0.623321342534636
"4232","ZMYND12",3.08015795028097,1.29624270094741,0.947219217622323,1.36847170837728,0.171164471824617,0.623349540134085
"4233","ENSA",573.192654477371,-0.329909632732345,0.24112948320018,-1.36818454696584,0.171254317383578,0.623349540134085
"4234","ZBTB24",47.4599509949404,-0.403808928322092,0.29512263359184,-1.36827502319109,0.171226005846643,0.623349540134085
"4235","NLRC3",3.0255750273736,1.6071751531612,1.17452386961013,1.3683

6312547202,0.171198440524853,0.623349540134085
"4236","GXYLT2",295.828875796515,0.318559585711008,0.232935347393329,1
.36758799931337,0.171441074953319,0.623882004344997
"4237","S100PBP",102.909355614984,0.28855469724165,0.211110819332934,1
.36683992868496,0.171675484328281,0.624587583412898
"4238","AGBL5",22.215070920077,0.46688971148419,0.342055372372421,1.36
495359872861,0.172267634451577,0.624725467644042
"4239","PPM1B",127.958026850426,0.27231458439132,0.199330590355597,1.3
6614547674555,0.171893306819269,0.624725467644042
"4240","ROBO2",48.0486985118353,0.499206617244369,0.365513945277605,1.
36576626882245,0.172012336823029,0.624725467644042
"4241","SNHG8",632.239652943079,-0.347457162993083,0.254598657457676,-
1.3647250400401,0.172339486454256,0.624725467644042
"4242","LUC7L2",313.369409650941,-0.180800456986832,0.132317800423918,
-1.36641069007787,0.171810095342601,0.624725467644042
"4243","LSM1",219.894483016919,-0.323232417694464,0.236870242716236,-1
.36459697929084,0.172379754714323,0.624725467644042
"4244","LINC00824",12.9490087359864,-0.722684456415447,0.5293254354280
73,-1.3652932733735,0.17216089232578,0.624725467644042
"4245","SCRIB",51.3821085123105,0.330254519788693,0.241741433650879,1.
36614776706274,0.171892588096327,0.624725467644042
"4246","PPIF",72.9467223992335,0.387995568299315,0.284243909888695,1.3
6500925719481,0.172250140491249,0.624725467644042
"4247","SEC31B",18.2809382376141,0.608936035035369,0.446201451261988,1
.36471101407922,0.172343896526016,0.624725467644042
"4248","CPSF7",149.627089576034,0.242261353782305,0.177626020936916,1.
36388437068206,0.172603960583773,0.624725467644042
"4249","CEP295",56.7556990587392,0.369453731418757,0.270707676988921,1
.36477005575973,0.172325333088386,0.624725467644042
"4250","AC092794.1",2.90470185322729,1.40181614490557,1.02723659372816
,1.36464778753446,0.172363777395544,0.624725467644042
"4251","TUBA1A",3374.97863115074,-0.382502868179716,0.280366113053896,
-1.36429778910615,0.172473861462685,0.624725467644042
"4252","MRPL57",394.881819564151,-0.303484602654576,0.222500105276,-1.
36397509690217,0.172575403560935,0.624725467644042
"4253","MZT1",105.86456780498,-0.306673244177391,0.224790471413771,-1.
36426264978509,0.172484916643282,0.624725467644042
"4254","ITPRIPL2",424.271113198062,-0.375872391642798,0.27540776024828
3,-1.36478504201895,0.172320621460377,0.624725467644042
"4255","ZNF844",61.4614693585049,0.605467677820041,0.443423544802719,1
.3654387208722,0.172115200853291,0.624725467644042
"4256","PLEKHA4",178.904690160936,-0.512865289950739,0.375482793475446
,-1.36588226907467,0.171975918827088,0.624725467644042
"4257","SMIM17",3.96854904253846,1.10248674265528,0.807996668345657,1.
36446941657888,0.172419873364824,0.624725467644042
"4258","GSTT2B",6.79246762961403,-1.18026295179861,0.864094030823201,-
1.36589642989919,0.171971473465978,0.624725467644042
"4259","GABRE",121.777267130843,0.550800356284968,0.403847771838084,1.
36388113218513,0.172604979999739,0.624725467644042
"4260","SLC2A1",4.35293841993702,1.07011883656402,0.785039085651438,1.

36314083734571,0.17283812861936,0.625040073795598
"4261","AC104794.5",7.91762185948587,-0.754609005236631,0.553601675326
8,-1.36309017632791,0.17285409241587,0.625040073795598
"4262","TMEM158",7.94980590893558,-0.885842946221844,0.649788222568796
,-1.36327947391822,0.172794448476297,0.625040073795598
"4263","CD99L2",125.793924901338,0.258503461513744,0.18963089568272,1.
36319274653566,0.172821772654717,0.625040073795598
"4264","ZDHHC13",21.0499451897669,-0.486544895677972,0.357119162413331
,-1.36241609772495,0.173066606302071,0.625661758008075
"4265","SRRM3",33.8079155114474,-0.61332012069263,0.450292349226119,-1
.36204872622574,0.173182508147816,0.625933965556526
"4266","SF3A3",197.912194489934,-0.25294859927821,0.185838937247963,-1
.3611173364638,0.173476611822738,0.626047719516421
"4267","PAIP1",353.838459357161,-0.301419994184284,0.221465909758014,-
1.36102208467945,0.173506710373292,0.626047719516421
"4268","HOMER1",14.6978493409798,-0.672960892336936,0.494248631084336,
-1.36158372530142,0.173329294234165,0.626047719516421
"4269","PRR3",30.2055330906219,0.434652509877567,0.319281049130493,1.3
6134766238481,0.173403847406756,0.626047719516421
"4270","HAS2-
AS1",56.421624448867,-0.340489066155575,0.250190309965438,-1.360920277
85813,0.173538884560082,0.626047719516421
"4271","RSL24D1",797.391979901404,-0.323163209820326,0.237450074337922
,-1.36097329394988,0.173522129236126,0.626047719516421
"4272","NORAD",1096.26309858396,0.193400615854573,0.142078963309956,1.
36121922168489,0.173444421495792,0.626047719516421
"4273","USP51",58.9407702369928,0.328007237605647,0.240891778615525,1.
3616373273127,0.173312369034325,0.626047719516421
"4274","LHCGR",13.33092255715,-0.615076021985668,0.452182568689314,-1.
3602382413115,0.173754544744134,0.62665529002847
"4275","TMEM126A",181.521847021798,-0.392004455163691,0.28821089791898
5,-1.36013057796962,0.173788606219378,0.62665529002847
"4276","ZNF879",9.9542030672644,0.698764706621535,0.513953145014845,1.
35958834652399,0.173960227932257,0.627120629053206
"4277","DCP1B",43.1359331707685,0.342864406768846,0.252205240172044,1.
35946583241077,0.173999022410675,0.627120629053206
"4278","CHST12",263.672844042668,-0.23509736040455,0.172968878137075,-
1.35918879128209,0.174086772181779,0.627290227485304
"4279","DGKD",34.2642786527482,0.419080239481782,0.308401408382718,1.3
5887913638097,0.174184891124946,0.627497101353362
"4280","APOL3",310.762458759656,0.260554432459049,0.19178446605389,1.3
5857943982718,0.174279893936525,0.627692655381201
"4281","CAPZA1",358.960053861217,0.250156090172343,0.18427858352559,1.
35748867495286,0.174625989484581,0.627873643727822
"4282","TRAPPC11",95.2404641107867,0.337658704240037,0.248837803835939
,1.3569429525373,0.174799337586961,0.627873643727822
"4283","AGGF1",181.839830786363,0.219973352883413,0.161987044282672,1.
3579688045888,0.174473583009102,0.627873643727822
"4284","NRG2",4.65005410091052,-1.10562449022584,0.814462736930532,-1.
35748934861356,0.17462577557637,0.627873643727822

"4285","NOP16",40.2158255853758,-0.45934973228677,0.338584265516344,-1
.35667772861878,0.17488363203004,0.627873643727822
"4286","SLC25A27",26.8442476001155,0.564449111044752,0.416028638717912
,1.35675542141578,0.174858936277536,0.627873643727822
"4287","RHBDD2",99.2173644367412,-0.422716356080054,0.311431711414393,
-1.35733241216912,0.174675613061285,0.627873643727822
"4288","SNAPC4",56.6488823143932,0.373020502772214,0.274748870019074,1
.35767802337574,0.174565873173796,0.627873643727822
"4289","APLP2",1496.59359822002,0.246147697794665,0.181244465098157,1.
35809773645423,0.17443267337631,0.627873643727822
"4290","LRP1",3719.96732751655,0.24251435809334,0.178638770147152,1.35
756844885112,0.174600660152099,0.627873643727822
"4291","BAZ1A",192.230801049617,-0.323164171400053,0.237967611359036,-
1.35801746109254,0.174458143628044,0.627873643727822
"4292","PPP2R3C",158.408682865333,-0.29862967724742,0.220036564736233,
-1.35718205565243,0.174723370951844,0.627873643727822
"4293","CTNS",45.5053453393929,0.370764529658879,0.273299195724366,1.3
5662503022076,0.1749003844416,0.627873643727822
"4294","MICOS13",633.074944937921,-0.345064420335417,0.254312057341685
,-1.35685434635842,0.174827495355037,0.627873643727822
"4295","PRPS2",78.5745217177309,-0.39288231232017,0.289641627455455,-1
.35644284204484,0.17495830987632,0.627935354305814
"4296","CASTOR2",51.2846466924749,0.382921931898304,0.282444669043518,
1.3557414030686,0.175181461093924,0.628589902877755
"4297","FGF7",1629.75061047363,0.240215151269405,0.177206536351261,1.3
5556597524849,0.175237303741046,0.628623319663178
"4298","ACLY",218.566283156472,0.236930694553472,0.17479778262328 5,1.3
5545595028566,0.175272333954741,0.628623319663178
"4299","FAM66D",11.4976573773217,0.767342544131935,0.566242216289252,1
.35514894873178,0.175370106038572,0.628827677270199
"4300","SETD5",321.8035914236,0.270281866231438,0.199600764219141,1.35
411238172764,0.175700526556356,0.629440080613154
"4301","POU5F1",2.6197375236684,1.31848371771148,0.97356546928215,1.35
428356829834,0.175645926408591,0.629440080613154
"4302","ACBD5",130.96837083696,0.276002939369385,0.20382747245219,1.35
41007796882,0.175704227497788,0.629440080613154
"4303","PPIL2",78.2061025163106,0.310323248831586,0.229117177083927,1.
35443030846139,0.175599133544516,0.629440080613154
"4304","TADA1",19.1995023264691,0.50790390677392,0.375220862413078,1.3
5361318533182,0.175859818084671,0.629457436765961
"4305","TPRKB",230.182462869415,-0.32391563582447,0.239289285019304,-1
.35365708413704,0.175845805837961,0.629457436765961
"4306","IGFBP3",756.396965111686,-0.524455964698404,0.387496825431909,
-1.35344583562417,0.175913242788697,0.629457436765961
"4307","RPP38",77.9906715022406,-0.306157651570664,0.226203449931039,-
1.35346145986721,0.175908254394728,0.629457436765961
"4308","SCGB1B2P",2.42964772425544,1.63211293953687,1.20565526487936,1
.35371443818162,0.175827500010621,0.629457436765961
"4309","MIGA1",217.298547060113,0.300422479229791,0.222001526980187,1.
35324510293392,0.175977340636825,0.629540660458728

"4310","PUM2",199.428588013435,0.257471225523571,0.19035488174643,1.35258535615885,0.176188133285059,0.629877114390102
"4311","CENPP",20.8387696993431,0.532379267749763,0.393643753002397,1.35243926440901,0.176234835832923,0.629877114390102
"4312","TRUB2",92.9293255510673,-0.279593086994632,0.206698181386988,-1.35266350733473,0.176163153739209,0.629877114390102
"4313","AC103703.1",6.60589088809072,0.880392205239493,0.650943409605073,1.35248654836774,0.17621971910727,0.629877114390102
"4314","SPAG1",20.9197579931715,-0.542004692210844,0.400885802287983,-1.35201767964206,0.176369659613711,0.630212865773146
"4315","DAZAP1",173.109761888018,-0.215752614972588,0.15963348822734,-1.35154983686961,0.176519366796805,0.630601631326245
"4316","HMOX1",83.6585998156862,0.405817523709862,0.300299539265864,1.35137577867071,0.176575088655589,0.630654539301646
"4317","SAP30",133.553829610269,-0.357757223910612,0.264787681553195,-1.35110977146699,0.176660271795107,0.630682357386488
"4318","FAM204A",463.250128982235,-0.233339773302313,0.172715668695259,-1.35100523921787,0.176693754401243,0.630682357386488
"4319","ZNF480",93.0272252804768,-0.304510501141183,0.225401678641585,-1.35096820474612,0.176705618005335,0.630682357386488
"4320","RPL32",17708.9105109309,-0.346525846821399,0.256541889794656,-1.35075736402647,0.176773169925037,0.630777410739456
"4321","ATP5MG",1674.04422823778,-0.323541598826649,0.239579234264844,-1.35045760463943,0.17686924390043,0.630974171424469
"4322","MELTF-AS1",16.830166702109,0.593842817297923,0.440250169294289,1.3488758408653,0.177376849322673,0.632412398602537
"4323","ISOC1",57.6792603902236,-0.385732422009629,0.28594168243972,-1.34898983148757,0.17734023223771,0.632412398602537
"4324","KLHDC10",148.795271123017,0.315607191347208,0.233987996341682,1.34881787220547,0.177395472692661,0.632412398602537
"4325","PACSIN3",18.1791127030704,0.610913748074569,0.45300015413659,1.34859501149389,0.177467083863597,0.632521409886093
"4326","KLHL35",15.1437008832905,0.597517581703054,0.443217291598941,1.34813689138224,0.17761435784738,0.632899982944373
"4327","SEC24B",100.936153841359,0.306161036230719,0.227144146611626,1.34787112412013,0.177699836974477,0.633058235951367
"4328","FH",144.60971032259,-0.388703545870333,0.288473028283163,-1.34745195481078,0.177834717279914,0.633102089494832
"4329","ANKRD36C",237.459630633759,-0.616793670280314,0.457883459570111,-1.34705383518199,0.177962894777877,0.633102089494832
"4330","NSUN3",63.3824409334133,0.302357164589044,0.224380032127464,1.34752260137517,0.177811979288762,0.633102089494832
"4331","MAATS1",8.72416108001498,-0.966710436191285,0.717708747851453,-1.34693974273722,0.177999640341914,0.633102089494832
"4332","ERBIN",222.627286619249,0.290510859922695,0.215622491994662,1.34731241270455,0.177879636046252,0.633102089494832
"4333","PDK4",2781.90246754961,0.428751912759204,0.318265341361993,1.34715238211108,0.177931160477485,0.633102089494832
"4334","EXOC3L4",13.3802742589159,0.644531238827838,0.478423853713735,

1.34719712201786,0.177916754624034,0.633102089494832
"4335","CCL8",53.1173784140192,-1.64786709607533,1.2240046673637,-1.34629151343399,0.178208521680644,0.633698814695994
"4336","AP003307.1",27.9595179763256,-0.58298542736539,0.433249388815903,-1.34561165558415,0.178427790740824,0.634332194250415
"4337","UQCRC1",282.760884283544,-0.231268881651941,0.171929622921952,-1.34513690963498,0.178581025710358,0.634584258027932
"4338","NUP85",86.4003765599981,0.288324988062717,0.214341688311932,1.34516523749275,0.178571879509045,0.634584258027932
"4339","PTPDC1",39.1739786687537,0.376016718165946,0.279602011665379,1.34482837203601,0.178680665662602,0.634654867509108
"4340","TRIM29",11.2333692616348,-0.586072736604495,0.435800002673391,-1.34482040617087,0.178683238727832,0.634654867509108
"4341","CASZ1",3.31594668161646,1.32450434599972,0.98548795242673,1.34400866366573,0.178945585353118,0.634709203455665
"4342","BTBD8",69.0163165240117,0.426955304093342,0.317625279892951,1.34421071344585,0.17888025822916,0.634709203455665
"4343","PRKCD",29.4513527183634,-0.431557616319233,0.321091600054929,-1.34403271915368,0.178937806756039,0.634709203455665
"4344","NIM1K",3.22884243581637,-1.20157012673589,0.893953853921178,-1.34410755260509,0.178913610174153,0.634709203455665
"4345","DVL2",95.6948320473033,0.26354760007867,0.196051064387182,1.34428038379934,0.178857736389662,0.634709203455665
"4346","UTP6",121.995258938143,-0.29096821814844,0.216482156949274,-1.34407482930162,0.178924190598682,0.634709203455665
"4347","EIF2B1",215.096616479557,-0.239259090361721,0.178076976731701,-1.34357116092666,0.179087100386737,0.635065022420417
"4348","AC090229.1",2.89612551421639,1.31108506032557,0.976187051535503,1.34306745645036,0.179250132144194,0.635496961132186
"4349","C8orf89",4.17514921013062,1.09961865002338,0.818816689696846,1.3429362931409,0.179292603285365,0.635501374946863
"4350","AC008011.2",3.27440569205147,-1.23871980782732,0.922498363464013,-1.34278808167842,0.179340603682064,0.635525380634257
"4351","AC068790.9",2.48559988117905,1.39767069901759,1.0410513540175,1.34255691962156,0.179415487883275,0.635600588818022
"4352","TTC9",4.49394767345408,1.14187122983758,0.850576111246982,1.34246802224853,0.179444292087968,0.635600588818022
"4353","USP9X",260.329513725453,0.249093835575855,0.1856018037963,1.34208736381268,0.179567670534008,0.635891486625712
"4354","ZNF451",224.894462352165,0.336132346101326,0.250517131007757,1.34175393414879,0.179675793059267,0.636128238403446
"4355","MED20",30.0970030267819,0.50822198559851,0.37890839754302,1.3412792878015,0.179829791925585,0.636381139240792
"4356","NIP7",83.7040084004337,-0.260695266255609,0.194347006974636,-1.34139069242076,0.179793637923072,0.636381139240792
"4357","PKIA",38.7608387737059,-0.411282994654984,0.306694196503733,-1.3410198149934,0.179914019259668,0.636533074796368
"4358","GGPS1",175.610461076786,-0.274998664996099,0.205135640205516,-1.34056990155679,0.180060134909349,0.636781107139018
"4359","TFAM",192.533231530701,-0.240777424540245,0.179610983220883,-1

.34054956006861,0.180066743173466,0.636781107139018
"4360","SUMF1",164.971387976319,0.215771537100485,0.160983245663035,1.34033536354542,0.180136339346345,0.63688111720732
"4361","AC012020.1",3.46592009342714,1.29306509026214,0.965205315642694,1.33967879093282,0.180349795719667,0.636905406877127
"4362","AC004540.1",37.0775884677813,0.606200124379967,0.45243062963168,1.33987419214625,0.18028624978185,0.636905406877127
"4363","DCUN1D5",165.437284284855,-0.269716993683575,0.201300728556437,-1.33987092653744,0.180287311645601,0.636905406877127
"4364","UBE2N",298.767374634448,-0.279469772073035,0.208590577304568,-1.33980055899157,0.18031019388184,0.636905406877127
"4365","ZXDB",34.0230749156992,0.466892586560788,0.348476010841736,1.33981270456184,0.180306244210941,0.636905406877127
"4366","WDR55",92.1823113564424,-0.261390208253596,0.195171854716878,-1.33928228859011,0.18047879238371,0.637069059902655
"4367","RPS21",6960.20157797836,-0.37270498363119,0.278279826287996,-1.33931729296637,0.180467401426969,0.637069059902655
"4368","SNX27",284.988859166691,0.19729845464838,0.147427836211405,1.33827138563889,0.180807986038128,0.637812615025204
"4369","ZBTB10",165.322952464506,0.313202708764773,0.23400447386515,1.33844752449161,0.180750595568635,0.637812615025204
"4370","SLC2A6",5.6269120881912,-0.8460418558733,0.632198141067029,-1.33825426067426,0.180813566504064,0.637812615025204
"4371","OTUD5",132.379558564932,0.253085417704974,0.189159855465872,1.33794465576041,0.180914478679021,0.638022578091308
"4372","PRDX1",1982.09790390218,-0.352502766214126,0.263614934324586,-1.33718814951543,0.181161229131909,0.638042995171808
"4373","ARL15",37.0355890303761,0.384798567574217,0.287808605303273,1.33699465715677,0.181224380843988,0.638042995171808
"4374","KIAA1324L",24.1652953261151,-0.61517256740915,0.460144432883405,-1.33691189862777,0.181251396422792,0.638042995171808
"4375","FUS",1336.56035014272,0.341585143178404,0.255454382715059,1.33716689276542,0.181168166075539,0.638042995171808
"4376","SPG7",243.786832149327,0.334521778731433,0.2502068225803,1.33698104344886,0.181228824678404,0.638042995171808
"4377","NXPH3",118.504835718216,0.407646950773816,0.304895516137582,1.33700539757976,0.181220874974417,0.638042995171808
"4378","SKA2",138.075079448269,0.337883915675641,0.252623998498191,1.33749729908602,0.181060363316181,0.638042995171808
"4379","AC090360.1",3.45498729875605,1.20587029815395,0.901932008877221,1.33698581077646,0.18122726850136,0.638042995171808
"4380","MALINC1",24.2215992022722,-0.425319661939262,0.318270460484952,-1.33634664458397,0.18143599710508,0.63823924684402
"4381","SPAAR",4.84041503757321,1.13453770488124,0.848899407427153,1.33648073606247,0.181392192887064,0.63823924684402
"4382","GALK2",120.227568044735,0.288752696037601,0.216094388984225,1.33623412155639,0.181472761525614,0.63823924684402
"4383","RBFADN",3.56052853981436,1.11851442817444,0.83689303219569,1.33650823360291,0.181383211121664,0.63823924684402
"4384","SMYD3",64.5946051307335,-0.333146319747837,0.249415230054296,-

1.3357096103366,0.181644206962215,0.638410511423487
"4385","SLC5A6",18.5287787280214,-0.519112120345185,0.388679634381122,
-1.33557839008402,0.181687117328241,0.638410511423487
"4386","DLG1",161.212171546862,0.302871105322525,0.226761651491656,1.3
3563635354662,0.181668161755232,0.638410511423487
"4387","NUCB2",860.771223888264,-0.282470328138973,0.211441052766808,-
1.33592944436623,0.181572335836471,0.638410511423487
"4388","ZNF596",13.3954091030856,0.595307191113782,0.445997767562019,1
.33477616797936,0.181949615729908,0.638689535310406
"4389","NIBAN2",63.4917781779763,-0.458273100231388,0.343306707005325,
-1.33487954321929,0.181915774109433,0.638689535310406
"4390","THOP1",42.8407995654635,-0.339797068654713,0.25458634716883,-1
.33470263599554,0.181973690501674,0.638689535310406
"4391","ZNF529-
AS1",14.9155849161926,0.551465352596265,0.413154925903287,1.3347664956
2047,0.181952782377898,0.638689535310406
"4392","RNF215",51.5879600923838,-0.353995134100991,0.265204342844135,
-1.33480142257338,0.181941347783467,0.638689535310406
"4393","MOB1A",430.182208029271,0.164532318569129,0.123308082927578,1.
33431900539532,0.182099331572533,0.638839598586844
"4394","TBL1XR1",537.884241177547,0.226161834244056,0.169481760150751,
1.33443170547019,0.182062415023325,0.638839598586844
"4395","FBXO6",59.8904300549439,0.360114814106965,0.270134456987663,1.
33309470447679,0.182500726977938,0.638939065719945
"4396","PSMA5",351.161595103018,-0.337980117386723,0.253460035638558,-
1.33346512216503,0.182379213857483,0.638939065719945
"4397","DVL3",174.003506800005,0.300520292733134,0.225310852838841,1.3
3380300569937,0.182268425686786,0.638939065719945
"4398","MTHFD2L",29.6951093541673,-0.450756983415187,0.338122348825126
,-1.33311798223759,0.182493089092277,0.638939065719945
"4399","DNAJB9",181.646969855547,-0.296939609421712,0.222688923448158,
-1.33342783657059,0.182391442438702,0.638939065719945
"4400","ZNF33B",32.1173356123196,-0.38037889878194,0.285255217299548,-
1.33346868247637,0.182378046211602,0.638939065719945
"4401","MRPL23",101.005671951645,-0.440768088434158,0.330589240665702,
-1.33328019855271,0.182439869347817,0.638939065719945
"4402","BAD",577.828979559439,-0.33467866877867,0.250922841906885,-1.3
3379116159886,0.182272308388438,0.638939065719945
"4403","RPS4X",19677.3276976014,-0.350771963247302,0.263031785919561,-
1.33357252630514,0.182343991848222,0.638939065719945
"4404","USO1",333.322619610233,0.2211031430612,0.165936457418391,1.332
45669156183,0.182710163701015,0.639236762017963
"4405","RPS6KA2",41.5475408119194,0.545707590703029,0.409537351497374,
1.33249772873654,0.182696687311282,0.639236762017963
"4406","C15orf48",9.11065059387824,-0.885773350048843,0.66470005394298
1,-1.33259106087695,0.182666040276946,0.639236762017963
"4407","RAB9B",41.7640887794878,0.402897685869758,0.302413792080668,1.
33227285401813,0.182770544016998,0.639302912643981
"4408","ACAT2",80.878207573562,-0.409658313264853,0.307607607938401,-1
.33175611621052,0.182940342606261,0.639461537704196

"4409","CCDC122",49.2286433281797,-0.427822531630518,0.321224045458347
,-1.33185089248244,0.182909190637611,0.639461537704196
"4410","ADPRHL1",9.69057191417014,0.684463349226752,0.513873334878778,
1.33196899463214,0.182870377199708,0.639461537704196
"4411","GSDME",64.2683523051355,0.518869977505934,0.389712453350133,1.
33141749268084,0.183051676740775,0.639705644289061
"4412","AC002451.1",43.8827082850005,0.409769675889893,0.3078594350039
78,1.33102848020428,0.183179639944148,0.639937056269662
"4413","SYMPK",98.4391226466352,0.289382169965108,0.217423021076902,1.
33096379827578,0.183200923082583,0.639937056269662
"4414","ZMPSTE24",200.643071165984,-0.203894174080667,0.15323107124109
3,-1.33063204759471,0.183310112136694,0.639962208394874
"4415","NCL",1320.21238181235,-0.279678180188304,0.210195285294898,-1.
33056352713108,0.183332670273874,0.639962208394874
"4416","XPO5",27.3338736302786,0.430368927802812,0.323432034924282,1.3
3063172886868,0.183310217062127,0.639962208394874
"4417","NDUFS3",374.686998355626,-0.370921834826056,0.27880477812451,-
1.33039984938998,0.183386564178684,0.640005407927193
"4418","BCL2L12",54.4025491065813,-0.340238701922701,0.255868770333502
,-1.32973907475786,0.183604255718582,0.640620100023074
"4419","PPP3CB",251.300800713301,0.20468945668917,0.153962672013615,1.
32947456686819,0.183691451206025,0.640634325868978
"4420","ZNF112",8.65253006247327,0.67169648440926,0.50522984358851,1.3
2948695120301,0.183687368004144,0.640634325868978
"4421","TWIST2",582.64057508732,-0.290448959500266,0.218598093013457,-
1.32868935632657,0.183950477900651,0.641102558634078
"4422","GNA12",189.707126624626,0.255447779664138,0.192253756057067,1.
32870111306597,0.183946597572301,0.641102558634078
"4423","LINC00641",21.3381984043264,0.699020518746921,0.52607997234145
2,1.32873432842492,0.183935635123967,0.641102558634078
"4424","PEX11B",49.7724778708162,0.337390083643918,0.253990353241361,1
.32835786610881,0.184059911663224,0.641338955309359
"4425","SRGN",191.410662696553,-0.42472517175946,0.319834201671931,-1.
32795420108048,0.184193237338855,0.641390163696861
"4426","RIMKLB",116.044613753118,0.307343979315555,0.231403858021128,1
.32817137079665,0.184121499925387,0.641390163696861
"4427","BAZ2A",178.4404054675,0.349424867556956,0.263139233847791,1.32
790866054994,0.184208283312379,0.641390163696861
"4428","TM9SF4",142.468551466914,0.263097880340384,0.198144294194487,1
.32780952088452,0.184241040859533,0.641390163696861
"4429","TRA2B",803.340902178767,0.266304897647142,0.200678947170696,1.
32701960719689,0.184502196783317,0.642009337113959
"4430","NDUFC1",688.6003735982,-0.31190546618942,0.235023890858783,-1.
32712238338711,0.184468202116704,0.642009337113959
"4431","LINC00345",6.38209265861244,-1.21223437276329,0.91391524954167
4,-1.32641880455679,0.184701013470035,0.642467872489987
"4432","NUP62",130.642260613953,0.235382656476582,0.177463860577127,1.
32636952510274,0.184717327984147,0.642467872489987
"4433","NABP2",131.739364369054,-0.275901803821761,0.208041597330768,-
1.3261857597791,0.184778174951772,0.642534528960425

"4434","CDK11B",89.507443050149,-0.329802213610257,0.248801467339733,-1.32556378037723,0.184984230067036,0.642746429454909
"4435","MEX3A",7.48227378585747,-0.822612718076587,0.620670531571514,-1.32536132494282,0.18505133804575,0.642746429454909
"4436","ABCB8",60.9927021211415,-0.344060365631888,0.259564116049225,-1.3255313210037,0.184994988175596,0.642746429454909
"4437","ABCF2",72.2542270393286,-0.321676132772269,0.242752398991073,-1.32512030410088,0.18513125279657,0.642746429454909
"4438","C11orf98",10.5466854060936,0.878185203516826,0.662817502711487,1.32492760062054,0.185195165429968,0.642746429454909
"4439","ATP5MC2",1679.21818825883,-0.352616407728367,0.26615176767345,-1.32486968172612,0.185214378179611,0.642746429454909
"4440","PSMC6",424.693786929538,-0.233862793072651,0.176421542810107,-1.32559090770661,0.184975239529099,0.642746429454909
"4441","BAIAP2",59.0650245542658,-0.513112747313121,0.387120623159487,-1.3254596025532,0.18501875968029,0.642746429454909
"4442","COL5A3",186.935851980537,0.520363968093275,0.392662240982757,1.32522028802898,0.185098098247141,0.642746429454909
"4443","TRAF5",92.6819719096871,0.268590943009235,0.202801359211929,1.32440405751204,0.185368887765228,0.642848460045216
"4444","ROR2",13.6478786260191,1.00128045216401,0.755961295487632,1.32451285289432,0.1853327773101,0.642848460045216
"4445","MPRIP-AS1",3.43731614268384,-1.07983191265727,0.815218203581893,-1.32459249304385,0.185306347114941,0.642848460045216
"4446","AC145124.1",50.5286299543029,-0.348533017409817,0.263232905792176,-1.32404805683749,0.185487084910351,0.643053970179427
"4447","PTPRA",907.848213904656,0.253605489899235,0.191548649993006,1.323974300568,0.185511579979754,0.643053970179427
"4448","AL157700.1",7.62585340504277,0.730723144917079,0.552094361861313,1.32354755888748,0.1856533513966,0.643400722072525
"4449","NUDT8",69.9137970479828,-0.338838997670667,0.256043382070674,-1.32336557551462,0.185713833973035,0.643465666597962
"4450","LARP1",323.837740025129,0.231965114920357,0.175319942653253,1.32309600043126,0.18580345462145,0.643631517525765
"4451","KLRC3",3.60440894055229,1.35724824337094,1.02613713990959,1.32267724320994,0.185942734506992,0.643969277111948
"4452","TIFA",89.081737778355,-0.492359085242452,0.372366848791831,-1.32224199560177,0.18608758092778,0.644036834306629
"4453","MSMO1",77.4195086599956,-0.350515371679536,0.265070474459454,-1.32234784879126,0.186052346282256,0.644036834306629
"4454","NCOA5",47.50806124443,0.316660484313857,0.239447348580705,1.3224639411997,0.186013709046412,0.644036834306629
"4455","GLG1",932.418118506442,0.279841712532083,0.211662026360904,1.32211581521442,0.186129588195639,0.644037621107919
"4456","CYCS",417.987716953146,-0.305990076249394,0.231463929343182,-1.32197736864527,0.186175687115587,0.644052562137965
"4457","LINC01715",3.50773865223161,-1.09391470589486,0.828263450919742,-1.32073279906306,0.186590473685556,0.644330679180745
"4458","HIST2H4B",2.44444636872386,1.45682445543117,1.10236564609112,1

.32154377324523,0.186320117119913,0.644330679180745
"4459","PTH1R",89.0650128357434,0.475200036058045,0.359761468201581,1.32087529671683,0.186542947895308,0.644330679180745
"4460","TNRC18",238.744202365639,0.264272092279466,0.200065722246247,1.32092639014989,0.186525909401843,0.644330679180745
"4461","TOLLIP-AS1",14.7811361479785,-0.724471959437254,0.548477352839231,-1.32087852978245,0.186541869707653,0.644330679180745
"4462","INPPL1",73.800003887584,0.277744809840985,0.210243135065266,1.32106482218677,0.186479751256203,0.644330679180745
"4463","AC009041.2",14.7204635639329,0.596369696589801,0.451535981860371,1.32075785883708,0.186582115106301,0.644330679180745
"4464","UBN1",176.517912672829,0.291034374904871,0.220270944926581,1.32125630550991,0.186415917844343,0.644330679180745
"4465","HERC6",16.8971927071059,-0.558645964532017,0.423127722005087,-1.32027739020442,0.186742421541227,0.644422303124696
"4466","TMPO-AS1",4.13356905435515,1.03859086167215,0.786522044657478,1.32048538083181,0.18667301381674,0.644422303124696
"4467","HORMAD2-AS1",4.41405469094681,1.38810626747771,1.05132814054672,1.32033588176938,0.186722900627376,0.644422303124696
"4468","HES1",240.568444618746,-0.327690587909528,0.248233437114458,-1.3200904427651,0.186804823294103,0.644493364162624
"4469","DUSP7",38.2683927986622,-0.497130636125616,0.37664574398857,-1.31988916391606,0.186872025989947,0.644580953375482
"4470","RHBDL2",3.33830856858727,1.44405719267698,1.09632169418806,1.31718381596603,0.187777017398282,0.644660305212947
"4471","ADGRL4",6.20438512112132,-1.20880964155251,0.916353700526558,-1.31915180880254,0.1871183656018,0.644660305212947
"4472","PRPF38B",294.982238952998,-0.203865799291658,0.154960416197976,-1.31559919812812,0.18830860285539,0.644660305212947
"4473","MRPL24",204.434989256629,-0.269721721604514,0.204782845125985,-1.317110920295,0.187801447091019,0.644660305212947
"4474","RXRG",10.4046682984934,-0.712929099999785,0.540669456075253,-1.31860435611616,0.18730141663772,0.644660305212947
"4475","YIPF4",389.375655759774,0.196173702401927,0.148866146670545,1.3177858552091,0.187575344283402,0.644660305212947
"4476","NIFK",275.06948069175,-0.25873720220291,0.196676596840709,-1.31554646744505,0.188326311275092,0.644660305212947
"4477","DENND6A",134.869799585645,0.326727993240715,0.24826716255255,1.31603386400953,0.188162676843025,0.644660305212947
"4478","FAM200B",420.24569931413,-0.328154548606922,0.249188134337716,-1.3168947609768,0.187873902848047,0.644660305212947
"4479","BTF3",4152.90443951176,-0.336965465344566,0.255395024003332,-1.3193893133179,0.187038992632138,0.644660305212947
"4480","UBE2D2",697.851932324178,-0.290329253953362,0.220837812201196,-1.31467184473308,0.188620212937991,0.644660305212947
"4481","NOL7",659.469861429426,-0.327020996451432,0.247953747205678,-1.31887902536987,0.187209559319317,0.644660305212947

"4482","MRPL2",180.889914002043,-0.36221242608181,0.27528749409034,-1.31576055526498,0.188254422303175,0.644660305212947
"4483","SGCE",1008.1117191406,0.329515321480879,0.250568538999015,1.31507061021007,0.188486172846305,0.644660305212947
"4484","FGFR1",1985.97592736019,0.235333073919196,0.178425486963079,1.31894315058191,0.187188118789903,0.644660305212947
"4485","AC040970.1",4.75413596115519,-0.943601874975759,0.717284724329621,-1.31551926727236,0.188335446324579,0.644660305212947
"4486","GPR107",120.218509232173,0.297149634179567,0.225987288726286,1.3148953459036,0.188545077122514,0.644660305212947
"4487","PLXDC2",747.696107727177,0.45181586033111,0.342726946030655,1.31829687033332,0.187404288225725,0.644660305212947
"4488","ITGB1",2154.34269662502,0.227355280090774,0.172625523345449,1.3170432487889,0.187824128096942,0.644660305212947
"4489","SCD",4.20412961018182,1.17017494356188,0.890088454899693,1.31467264530888,0.188619943763907,0.644660305212947
"4490","EDRF1-DT",32.8089982138613,0.50784020837459,0.385686625946753,1.31671718491141,0.187933441083431,0.644660305212947
"4491","STK32C",52.3655866284676,-0.337815302565952,0.256951365529815,-1.31470522396097,0.188608990227322,0.644660305212947
"4492","CXCR5",2.8236251218493,1.53974833952353,1.16818735276501,1.31806626383949,0.187481466661996,0.644660305212947
"4493","ZBTB44",305.402124117639,0.2433792744318,0.184964130204245,1.31581877071544,0.188234877523624,0.644660305212947
"4494","SP1",214.531566740694,0.284241158365971,0.216144959687802,1.31504874680643,0.18849352013853,0.644660305212947
"4495","HOXC10",214.777473445,-0.218406730764573,0.165687523439637,-1.31818453333417,0.187441881781556,0.644660305212947
"4496","ORAI1",65.0739568096621,0.423728615699282,0.32233031759551,1.31457884216469,0.188651484710711,0.644660305212947
"4497","CEP128",18.4196486566305,0.571359981192642,0.434350897789537,1.31543409740917,0.188364052334977,0.644660305212947
"4498","CHP1",261.920766105087,-0.292565839604611,0.221917211925862,-1.31835578261658,0.187384575466367,0.644660305212947
"4499","HACD3",276.214843559927,0.269402218268245,0.204371954697576,1.31819563338277,0.187438166904494,0.644660305212947
"4500","NOL3",38.8344940969078,0.422214514169892,0.321079258364441,1.31498532892043,0.188514833191014,0.644660305212947
"4501","CISD3",209.658815887441,-0.312455647652249,0.237519223816849,-1.31549624755082,0.188343177644678,0.644660305212947
"4502","TOB1",263.53230225194,0.350635674889939,0.266190231376707,1.3172371994137,0.187759128369356,0.644660305212947
"4503","ME2",100.033123936057,-0.343402836397087,0.260551129123711,-1.31798636817282,0.187508211288628,0.644660305212947
"4504","SH3GL1",331.233939533548,-0.252915533227808,0.191912180059502,-1.31787119061121,0.187546771288468,0.644660305212947
"4505","ZNF490",24.9919754332429,0.513719912796722,0.390155010776957,1.31670720253905,0.187936788416467,0.644660305212947
"4506","DMAC2",83.2902325069618,-0.360151841243505,0.273283762656667,-

1.31786769086597,0.187547943050813,0.644660305212947
"4507","CARD8",62.0353907261,0.299247321286052,0.227339928566626,1.316
29900287556,0.188073705411799,0.644660305212947
"4508","AC010332.3",7.24889606414297,-0.807420315449058,0.612701515441
71,-1.31780368597093,0.187569373714138,0.644660305212947
"4509","CABLES2",7.17057550790237,0.727715815417429,0.55246059975846,1
.31722663251568,0.187762669283668,0.644660305212947
"4510","AL031595.3",8.50614257979122,0.79794984970388,0.60664741588467
5,1.31534368862386,0.18839442144952,0.644660305212947
"4511","CCDC120",13.8677371961122,0.558352219827862,0.424493917985098,
1.31533620664846,0.188396934873367,0.644660305212947
"4512","FAM136A",168.670233345653,-0.25647398706342,0.195304699849821,
-1.31319925870005,0.189115813724273,0.645120416922998
"4513","AC092683.1",86.5129117925988,-0.516119525735805,0.393000279638
323,-1.31328030150714,0.189088513722009,0.645120416922998
"4514","MFAP3",14.7101972540933,-0.532502094046086,0.405389631327521,-
1.31355627498985,0.188995571351078,0.645120416922998
"4515","RPA1",123.498192808264,0.283172861843752,0.215502432067076,1.3
1401237158945,0.188842040941116,0.645120416922998
"4516","RPS6KB1",146.801881921676,0.25703357599351,0.195708494773796,1
.31334910265696,0.189065339712949,0.645120416922998
"4517","ATP8B1",34.2848218359699,-0.514146934866927,0.391393931580541,
-1.31363031815716,0.188970640819047,0.645120416922998
"4518","PAK4",41.0395465911465,0.363364298755019,0.276704772883947,1.3
1318406606386,0.189120931824523,0.645120416922998
"4519","MPP1",47.5244662651768,-0.386151781955854,0.294039982004194,-1
.31326284039273,0.189094395410696,0.645120416922998
"4520","SEC22C",199.04461420353,-0.22022815297181,0.167798517690032,-1
.31245589057366,0.189366359571126,0.645296714069699
"4521","AL391684.1",2.37108257346609,1.39851670073588,1.06558995902836
,1.31243419561788,0.189373675342153,0.645296714069699
"4522","SF1",667.627258871529,-0.169904888633022,0.129460246685192,-1.
31240973954096,0.189381922442512,0.645296714069699
"4523","AC125494.2",3.63981448369582,-0.994081936384776,0.757204198109
112,-1.31283204565848,0.189239549168777,0.645296714069699
"4524","LRP3",74.4089002799232,-0.417126865017539,0.317801695606599,-1
.31253819845534,0.189338606367696,0.645296714069699
"4525","ALDH9A1",468.711509879609,0.303467947295382,0.231385295088766,
1.31152650465089,0.189679945146953,0.645380052601259
"4526","LAMB3",18.0303411402706,-0.734996817015361,0.560187619560989,-
1.31205473193315,0.189501668241074,0.645380052601259
"4527","FAM13B",110.497175509949,0.306019312216911,0.233341285005633,1
.31146664513107,0.189700155550422,0.645380052601259
"4528","ADAMTS2",494.394130096645,0.279445025056028,0.213080998660516,
1.31144976235654,0.189705855977972,0.645380052601259
"4529","NHLRC1",7.37108010840792,0.804245559869791,0.613427059050363,1
.31106958521659,0.189834255284134,0.645380052601259
"4530","TMEM225B",25.4900210441042,-0.592127811284213,0.45158706618940
9,-1.311215168939,0.189785078934256,0.645380052601259
"4531","TRPS1",75.5243303118158,0.368942979948738,0.281319084010633,1.

31147512173327,0.189697293494003,0.645380052601259
"4532","DLG5",87.0080472848653,0.303540888060703,0.231417174780314,1.3
1166102234571,0.189634533650533,0.645380052601259
"4533","RPS6KA5",196.04813029141,-0.449970851431063,0.343093160218961,
-1.31151215939103,0.18968478840058,0.645380052601259
"4534","PICK1",17.8227360717026,-0.512700356311462,0.391033756527856,-
1.31114091239573,0.18981016068749,0.645380052601259
"4535","F8A1",116.623202987894,-0.386082436342115,0.294500698587415,-1
.31097290496755,0.189866917842797,0.645380052601259
"4536","AC004889.1",104.385567440996,0.359671838250138,0.2743926452413
2,1.31079256127225,0.189927856413975,0.645423979265472
"4537","LSM12",183.892047278103,-0.240747814021309,0.183680650011049,-
1.31068685790706,0.18996358053373,0.645423979265472
"4538","ZNF449",29.0964752764092,0.454184289290712,0.346565250922057,1
.3105303779947,0.190016474476008,0.645461426630159
"4539","CAMTA1",291.173492043085,-0.298948921333501,0.228177937747665,
-1.31015699538883,0.190142730528788,0.645464314905061
"4540","NABP1",618.713484946139,0.79434341747975,0.60641123469668,1.30
99088078028,0.190226687162743,0.645464314905061
"4541","ABCF3",78.045142993088,-0.267150390292192,0.203934290557454,-1
.3099826888452,0.190201691908405,0.645464314905061
"4542","RSPO3",120.346652706647,0.633080844427249,0.483254319706978,1.
31003659690227,0.190183455392294,0.645464314905061
"4543","NUTF2",221.769648723355,-0.308785052500784,0.235666197179605,-
1.31026450206371,0.190106371757265,0.645464314905061
"4544","ZNF217",200.916323044491,0.238161670709901,0.181835753783627,1
.30976260583657,0.190276156986108,0.645490088015152
"4545","SPATA9",3.77486422176342,1.06737116964257,0.815301304791908,1.
30917387641738,0.190475459094939,0.645967146985603
"4546","DISP2",3.45021867502719,1.74547202414713,1.3333377696052,1.309
09966246884,0.190500593590435,0.645967146985603
"4547","TNFRSF4",2.91981296499042,1.20046908774068,0.917424049835482,1
.30852149336608,0.190696489311851,0.646489197876001
"4548","TRAPPC3",259.02173605226,-0.281155768879529,0.215013133714687,
-1.3076213718768,0.191001764301937,0.646807892428187
"4549","CCDC17",7.82134673790084,0.778117742680719,0.59528660923304,1.
30713127191495,0.191168132202583,0.646807892428187
"4550","GNG5",963.912665007174,-0.310626704808453,0.237624548576089,-1
.30721639102447,0.191139230269001,0.646807892428187
"4551","PTRHD1",208.358528641192,-0.364162253536492,0.278488974606769,
-1.30763616064404,0.190996745807003,0.646807892428187
"4552","NKTR",559.98409652461,0.365468333765006,0.279562044679339,1.30
728881377371,0.191114641877772,0.646807892428187
"4553","HSD17B13",3.04425248051538,1.25765227001833,0.961535380180646,
1.30796255233172,0.190886011109462,0.646807892428187
"4554","CXCL16",62.9307998280106,-0.529636651506637,0.405090098357908,
-1.30745395568442,0.191058582925928,0.646807892428187
"4555","ZNF549",9.44292975339819,0.665798322229701,0.509255132180926,1
.3073963916247,0.191078122205272,0.646807892428187
"4556","UBE2M",432.888004145632,-0.374035760249074,0.286023456784734,-

1.30771008942312,0.190971659897067,0.646807892428187
"4557","PITPNA",151.404411445094,-0.264141406075717,0.202153662118031,
-1.30663675991926,0.191336105856976,0.647234161023761
"4558","DYNC1H1",903.151901507257,0.226265344780211,0.173241839867815,
1.30606639223442,0.191529980668775,0.647747839405257
"4559","HRAS",153.055175073378,-0.330940869560244,0.253439198753467,-1
.3057998572753,0.191620628625797,0.647912259325875
"4560","PIP5K1A",83.3315680918591,0.267759383183072,0.205215755413831,
1.30477010716413,0.191971140883293,0.648213029990461
"4561","MRPL9",201.371625902768,-0.262721236953366,0.201385900461491,-
1.30456619034064,0.192040607155454,0.648213029990461
"4562","FDPS",360.330351507537,-0.321552278550148,0.24646372529674,-1.
30466371131493,0.19200738337034,0.648213029990461
"4563","TCOF1",69.9467576910303,-0.313438097858245,0.240107809485597,-
1.30540567809831,0.191754745944323,0.648213029990461
"4564","AC026979.4",12.7300308886101,0.663316363319206,0.5081839737023
51,1.30526816594913,0.191801549944764,0.648213029990461
"4565","CD44",2941.81749956303,-0.328002797420221,0.251315472758806,-1
.30514366592547,0.191843932350293,0.648213029990461
"4566","HDAC5",126.936992044736,-0.275567004210832,0.21123523085908,-1
.30455039668392,0.192045988191141,0.648213029990461
"4567","AC132938.6",3.70603239259527,-1.00479921122826,0.7700432119118
3,-1.30486081259464,0.191940247124458,0.648213029990461
"4568","ZNF280D",303.086931933525,0.23847964348326,0.18284867952287,1.
30424591583354,0.192149749128249,0.648238354258258
"4569","N6AMT1",54.3873244148762,0.378715680778322,0.290390905472531,1
.30415820069181,0.19217964832697,0.648238354258258
"4570","Z82195.3",3.08916862939232,1.69795172471037,1.30186309711882,1
.30424752684683,0.192149200018943,0.648238354258258
"4571","TAF6L",28.6908753491818,-0.485948901461374,0.372660829327132,-
1.30399779965818,0.192234332595483,0.648280953174222
"4572","ZNF862",20.2327097933044,0.477279481373768,0.366059992684492,1
.30382858250543,0.19229203487395,0.648333709007422
"4573","LNPK",285.379949720971,0.218976823403053,0.167975344975206,1.3
0362478752686,0.192361544934307,0.648426244295286
"4574","SLC7A5",4.23881345722997,1.09418056515041,0.839681046536318,1.
30309070290904,0.192543797279061,0.648898695902213
"4575","IFT22",217.970468541765,-0.297287195637934,0.228261411840723,-
1.30239795347177,0.192780381826949,0.649554007838781
"4576","GLB1L2",2.42124908262361,-1.75028855162728,1.34416686286146,-1
.3021363641574,0.192869774126565,0.649713192342876
"4577","HDAC1",42.0029007419218,-0.362734594638787,0.278740829274709,-
1.30133283876149,0.193144551431952,0.649903453818532
"4578","NRBP1",380.591086931234,-0.19907891579566,0.152965335751086,-1
.30146424886494,0.193099594158638,0.649903453818532
"4579","FARP1",226.261568145571,0.290653150256685,0.2233149419742,1.30
153919700664,0.193073956771772,0.649903453818532
"4580","VCPKMT",26.2926669365257,-0.417199685205429,0.32058079535512,-
1.30138701771976,0.193126015105007,0.649903453818532
"4581","FGF11",2.38091334141867,1.36044810700955,1.04550810383843,1.30

123152753658,0.193179216697795,0.649903453818532
"4582","SNRPD1",389.324508836075,-0.363784787534163,0.279512375176096,-1.30149796517944,0.193088060565245,0.649903453818532
"4583","SLC29A1",311.211267980521,-0.392295274684819,0.301538378330819,-1.30097958626822,0.193265442271497,0.650051667600945
"4584","LINC02604",8.99904897270357,0.653911874728894,0.502784161529783,1.30058169044006,0.193401677600418,0.650367988702103
"4585","EIF3I",779.715295347656,-0.293088964970377,0.225423785268058,-1.30016876711504,0.193543132750527,0.650434870360247
"4586","HLA-C",3157.32930799296,-0.387166052686208,0.297894261915523,-1.29967610049501,0.193712004948789,0.650434870360247
"4587","ORC3",89.2173075389091,0.267680374563092,0.205913554787231,1.29996480726917,0.193613031304606,0.650434870360247
"4588","RELA-DT",9.51928257803259,-0.622020350361202,0.478553386802016,-1.29979301686259,0.193671919533551,0.650434870360247
"4589","CRTC1",56.333770045053,0.321502280281192,0.247318253809469,1.29995370470663,0.193616836767185,0.650434870360247
"4590","TBC1D20",146.446874206383,0.273876098294655,0.210676513718796,1.29998400609674,0.193606450933575,0.650434870360247
"4591","Z83745.1",4.83100373563589,0.997829934983416,0.76776126330026,1.2996617342925,0.193716930900026,0.650434870360247
"4592","TMCO4",61.2241171550827,-0.272618777176684,0.209902895311202,-1.29878521576608,0.194017649724827,0.650503943536765
"4593","AK2",178.730478998391,-0.246367236357883,0.189688284611334,-1.29880048661246,0.194012407621807,0.650503943536765
"4594","ANXA4",1105.55813601942,-0.321125132373174,0.247131205160374,-1.29941150962616,0.193802743865934,0.650503943536765
"4595","SCN7A",3.15972519121397,3.19375409745261,2.45911586674862,1.29874079568089,0.194032898630039,0.650503943536765
"4596","NCBP2",217.814842856415,-0.2295205059049,0.176664427226592,-1.29918914355359,0.193879026322043,0.650503943536765
"4597","DAP",220.726440848105,-0.273862693299213,0.210829031955181,-1.29897998752578,0.193950797201297,0.650503943536765
"4598","KDELR3",345.420041735847,-0.378465626941289,0.291296865585106,-1.2992437326131,0.19386029755927,0.650503943536765
"4599","TEAD3",57.0494838652107,0.393645113119838,0.303160155236866,1.29847246189814,0.194125033246026,0.650671317131439
"4600","AC072022.2",28.4971540808402,0.591357871355721,0.455555434964549,1.29810298806278,0.194251947674751,0.6509551681318
"4601","ATP6V0B",438.916966084742,-0.312701013247415,0.240937846991268,-1.29784928832185,0.194339128905756,0.650978884928571
"4602","AC074117.1",7.95314124145863,0.790479108719916,0.609189768520089,1.29759091430612,0.194427945910551,0.650978884928571
"4603","LRWD1",31.5600745317687,-0.493545453446342,0.380300676327284,-1.29777695431075,0.194363990982829,0.650978884928571
"4604","HTATIP2",145.704695631375,0.508771558663078,0.392054425564713,1.29770645473584,0.194388224787894,0.650978884928571
"4605","AC004263.2",3.71357826936207,1.3688690218165,1.05536733300491,

1.29705456953928,0.194612411452808,0.651455010324654
"4606","SRP72",524.263778004783,-0.196548115800041,0.151575891954995,-1.29669773514114,0.194735208942972,0.65159673821667
"4607","DOP1A",55.0674593341555,0.308467653213309,0.23818220466995,1.29509109902125,0.195288805096401,0.65159673821667
"4608","HOXA7",82.0683690148298,0.503134620870398,0.388267874888704,1.29584406388146,0.195029213634094,0.65159673821667
"4609","SMIM30",196.451998033212,0.33418256239779,0.257855398863664,1.29600762237475,0.194972858833114,0.65159673821667
"4610","FAM160B2",74.265454171396,0.294166085543372,0.227098078412996,1.29532617624535,0.195207732904581,0.65159673821667
"4611","CYC1",376.704357960441,-0.339550690519507,0.262177388669803,-1.29511813449004,0.195279479990461,0.65159673821667
"4612","DNLZ",19.8711853792884,-0.616222479659967,0.475787108044542,-1.29516430613811,0.19526355516584,0.65159673821667
"4613","PLAU",382.835931400932,0.457648868698596,0.353033264028142,1.29633356210342,0.19486059047823,0.65159673821667
"4614","RCBTB1",29.3962158238015,0.375645793819626,0.28977744705857,1.29632515446828,0.194863485851336,0.65159673821667
"4615","YLPM1",146.866396473274,0.304186902693988,0.234589352383659,1.29667821494518,0.194741928071181,0.65159673821667
"4616","SMG1",257.619200515005,0.305959707612491,0.236035427823571,1.29624484948584,0.19489114241023,0.65159673821667
"4617","MTHFSD",31.0477221424878,0.381908117752727,0.294723370535013,1.29581891337442,0.195037880411968,0.65159673821667
"4618","PSMB6",755.980026679272,-0.365469856266738,0.281990003315428,-1.29603834167813,0.194962275693905,0.65159673821667
"4619","PRCD",35.7007015297086,-0.494763862912811,0.382015891542688,-1.2951394794463,0.195272117893504,0.65159673821667
"4620","BEX5",6.18310628793004,-1.2612623903501З,0.973803192172054,-1.29519229397564,0.195253902488948,0.65159673821667
"4621","HERC3",86.673477446847,-0.30932559057685,0.238920461262161,-1.29468020002454,0.195430573060244,0.651646611231593
"4622","LINC00683",5.78770097569766,1.04157090373228,0.804432141607397,1.29479026242168,0.195392592044854,0.651646611231593
"4623","PITPNB",294.584904747562,-0.216384701040247,0.167105837680846,-1.29489612118469,0.195356066751717,0.651646611231593
"4624","STARD7",129.096715690342,0.274139243118875,0.211838302358535,1.29409667688375,0.195632029273066,0.651754426463002
"4625","PAX8",6.39888856218437,-1.04322307519268,0.80592834879767,-1.29443650511738,0.195514688071782,0.651754426463002
"4626","ZNF655",232.981479552622,0.284979900716403,0.220199440394152,1.29418994074778,0.195599820530654,0.651754426463002
"4627","GON7",70.6921971514604,-0.368243280724552,0.284549835208316,-1.29412579155059,0.195621974084601,0.651754426463002
"4628","IL27RA",6.26114253471912,-1.07705414126783,0.832413164770372,-1.29389368987809,0.195702144419992,0.651847138339277
"4629","TDRD6",10.0385165672298,0.641360404336991,0.495773516712652,1.29365603993874,0.195784256134839,0.651903775451086
"4630","TMEM176A",1387.37336098917,0.407859677235328,0.315345149030221

,1.29337546015728,0.195881233273047,0.651903775451086
"4631","IRF9",315.069738201824,-0.363659518740085,0.281155362184499,-1.29344685413272,0.195856553946163,0.651903775451086
"4632","BX537318.1",9.78538834662518,0.686788387188202,0.531013056561689,1.29335499137283,0.195888309301942,0.651903775451086
"4633","TBX18",95.8607114268562,0.266881561664568,0.206392081810423,1.2930804288786,0.195983243265296,0.652078932642895
"4634","ZBTB42",6.97047215845107,-0.752600896310543,0.582129112752404,-1.29284187961691,0.196065752499159,0.652212683378192
"4635","SLC30A7",167.382694909762,0.283685442231653,0.21949679225375,1.2924354808051,0.196206376018778,0.652258202788325
"4636","GLIPR2",539.454506735318,-0.267145689745645,0.206689163584518,-1.29249973783171,0.196184136663754,0.652258202788325
"4637","DOK4",41.1754524515339,-0.350799694886562,0.271402766781262,-1.29254281025547,0.196169230333115,0.652258202788325
"4638","LINC01266",5.09610875893552,0.908063168638364,0.702975933613471,1.2917414739516,0.196446689484891,0.652775537488597
"4639","DBN1",1121.94501875945,-0.265583315330756,0.205590467184772,-1.29180753839168,0.196423804097751,0.652775537488597
"4640","SH2D1A",5.33767809539444,1.01587604378602,0.78659845000029,1.29147984436741,0.196537339790132,0.652936011393294
"4641","EXOSC8",194.669128594942,-0.307354236791234,0.238028452651248,-1.29124998867913,0.19661700624819,0.653032855342148
"4642","CANT1",92.9761306562682,0.247094402225938,0.191375249083299,1.29115130305271,0.196651217288242,0.653032855342148
"4643","AC005089.1",3.30643883826531,1.30865566323517,1.013826735678,1.29080800217802,0.196770262299475,0.653146768593111
"4644","BCL2A1",29.5439764715918,-1.28574537740959,0.995986607858355,-1.29092637116306,0.196729210021034,0.653146768593111
"4645","STARD4",6.13568443240288,-0.970896746718212,0.75236193191441,-1.29046500830755,0.196889253546137,0.653232085869735
"4646","RAPGEF1",145.00974004281,0.316965885279982,0.245608147968467,1.29053489430927,0.196865004459479,0.653232085869735
"4647","AC005291.1",2.66177148683847,-1.9876640391198,1.54038601902511,-1.29036748877905,0.196923094585576,0.653232085869735
"4648","FAM72B",5.09662803639272,1.09925407724404,0.852431742885266,1.28955084840382,0.197206650734081,0.653437618457051
"4649","AZI2",390.333173030839,0.220219237988602,0.170819275726542,1.28919430814788,0.197330543324189,0.653437618457051
"4650","PEX6",44.7290241627657,-0.411439248476508,0.319112166861125,-1.28932485565667,0.197285173340888,0.653437618457051
"4651","CASTOR3",27.3834175659359,0.49149357164017,0.381172832956909,1.28942445301639,0.197250564789435,0.653437618457051
"4652","CCDC7",37.1150747034742,0.409049290665843,0.317222792757364,1.28947005071831,0.197234721772068,0.653437618457051
"4653","CALHM2",624.863643974531,-0.214405665952134,0.166303500380106,-1.28924325382259,0.197313532036279,0.653437618457051
"4654","PTGES3",1141.66288323345,-0.27668021135705,0.214632077311502,-1.28909068403366,0.197366561890109,0.653437618457051
"4655","MEGF8",62.3374059493208,0.364466623085993,0.28265066602419,1.2

8945962948766,0.197238342568442,0.653437618457051
"4656","SHOX",4.81168033180741,1.56714026892889,1.21478140221934,1.29005948400741,0.197030005777193,0.653437618457051
"4657","POU2F1",143.447407762415,0.323861040230435,0.251321959940854,1.28863009148366,0.197526716861289,0.653827429765251
"4658","WASF3-AS1",4.79293257662982,1.19507212068031,0.92780145622397,1.28806881328264,0.197722010288969,0.653843685548472
"4659","ISLR",2579.72246644364,0.254813825824255,0.197897724430652,1.28760361725911,0.197883979533292,0.653843685548472
"4660","DDX28",14.0421299488198,-0.578989987548012,0.449573438895771,-1.28786520166785,0.197792890655148,0.653843685548472
"4661","CDYL2",9.19086494224698,0.660124409234981,0.512405244643612,1.2882858170082,0.197646488381986,0.653843685548472
"4662","MEX3C",87.8879633626289,0.34043219543854,0.264409407120582,1.28751922689078,0.197913372479349,0.653843685548472
"4663","ADNP2",86.8598437590008,0.365010446360812,0.283398246467196,1.28797708140746,0.197753941279009,0.653843685548472
"4664","AP3D1",178.201137033631,0.258344544672916,0.20060629782642,1.28781871492617,0.19780907601854,0.653843685548472
"4665","FBXO46",22.7941992024147,0.413689621845844,0.321296839751857,1.28756206306089,0.197898452353453,0.653843685548472
"4666","CBFA2T2",92.1749496860159,0.359606708948533,0.279162876868058,1.28816092233673,0.197689951802496,0.653843685548472
"4667","AQP5",3.64246283474977,-1.29760055254615,1.00794588463473,-1.28737124911858,0.197964920463354,0.653873848069981
"4668","FADS2",18.636637280775,0.929988704413354,0.722562768966837,1.28706978044704,0.198069967311673,0.654080665404765
"4669","AC103691.1",4.92972587572578,0.900204918662399,0.699601591919536,1.28673938004123,0.198185142255248,0.654320832697505
"4670","HPSE",17.2306840448801,0.573626134325493,0.445844991354968,1.28660441509545,0.198232204046499,0.654336065391172
"4671","NAGLU",336.202588683754,0.355311158696545,0.276195072087293,1.28645002972481,0.198286047691536,0.654373672696431
"4672","KAT2B",86.2858489520893,0.3705564883023,0.288175515771063,1.28587082532259,0.198488147115752,0.654393919325752
"4673","FAM120AOS",288.070513471981,-0.204777162857584,0.159283827026968,-1.2856117703834,0.198578586828052,0.654393919325752
"4674","ZNF239",10.1455081602732,-0.771758399240562,0.600116284995519,-1.28601475836691,0.198437911088811,0.654393919325752
"4675","ATXN2",255.775760110785,0.248579317585936,0.193267446386083,1.28619341867517,0.198375567361123,0.654393919325752
"4676","RUVBL2",135.494944281358,-0.351180681759162,0.273085331187238,-1.28597416870546,0.198452076896583,0.654393919325752
"4677","C22orf39",165.8721517803,-0.253967639782788,0.197527571470308,-1.2857326088321,0.198536396682462,0.654393919325752
"4678","NUP50-DT",11.5764035081758,-0.61164503631191,0.475773253679423,-1.28558096021942,0.198589345092823,0.654393919325752
"4679","AL450384.2",6.45878403135044,0.910352056836859,0.7082681200950

07,1.28532123783115,0.198680051655316,0.654552895120047
"4680","CCL19",5.27746717305317,-1.53110899611372,1.19157318422277,-1.28494751005362,0.198810627094325,0.654582438068418
"4681","EDF1",1933.96485612184,-0.343485742989322,0.267416819514832,-1.28445826112396,0.198981658862881,0.654582438068418
"4682","BNIP2",306.250829661754,0.205101473058059,0.159680949681857,1.28444547371929,0.198986130528784,0.654582438068418
"4683","KIF7",41.3962888638355,-0.442239895887358,0.344304245869571,-1.28444508365106,0.198986266934065,0.654582438068418
"4684","SRRM2-AS1",3.25827920179762,1.06363906753755,0.827742168459581,1.28498837931257,0.198796344872947,0.654582438068418
"4685","GSDMB",3.77576300241495,1.19845036105827,0.932807922821179,1.28477721054693,0.198870148243436,0.654582438068418
"4686","PROCR",1499.29135122788,0.46963280320028,0.3656201643869,1.28448277459695,0.198973086878323,0.654582438068418
"4687","STX12",326.126447431734,0.250099750491386,0.195021983842628,1.28241824620758,0.199695966892157,0.654658156664985
"4688","TMEM127",126.033652350636,0.263019810616492,0.205114112866638,1.28230967114147,0.199734036709551,0.654658156664985
"4689","SLC22A4",24.8174766575154,0.487336810565026,0.380070356869492,1.2822278869077,0.199762716322509,0.654658156664985
"4690","HDAC3",162.347647005144,-0.318914653040706,0.248481708382062,-1.28345323733185,0.199333332643265,0.654658156664985
"4691","FAM193B",54.0634024194203,0.366947059540518,0.286070023287442,1.28271762040517,0.199591024381165,0.654658156664985
"4692","ECI2",420.700332939771,-0.272438008596282,0.212449741333752,-1.28236451071169,0.199714807576839,0.654658156664985
"4693","PDCD2",254.317238443222,-0.279988732293583,0.218366477390391,-1.28219649664003,0.199773724875257,0.654658156664985
"4694","RGP1",43.0679619711154,0.385829604998722,0.300547217910856,1.28375703385537,0.199226981560308,0.654658156664985
"4695","SLC35D2",122.310910953751,-0.271888291792797,0.211937094268615,-1.28287260298189,0.19953671267966,0.654658156664985
"4696","FBH1",199.976357399536,-0.214213305035801,0.166887178076942,-1.28358156392961,0.199288403855232,0.654658156664985
"4697","FAM53B",47.363061544201,0.405666629384217,0.3161144432343,1.28329039709059,0.199390355754314,0.654658156664985
"4698","AP001107.2",3.0544607874672,1.25086971476121,0.975249779497726,1.28261471169513,0.199627093414174,0.654658156664985
"4699","WBP11",249.368995121808,-0.185673839857591,0.144749393820019,-1.28272619979644,0.199588017558702,0.654658156664985
"4700","ZC3H10",25.8397812535417,-0.413888044403881,0.322339579022698,-1.28401248664141,0.199137586429769,0.654658156664985
"4701","PPAN",56.4425124810303,-0.407013418474268,0.317295408773249,-1.28275861301585,0.199576657984245,0.654658156664985
"4702","ZNF420",53.5110196523355,0.290332859706557,0.226154717673775,1.28377980655419,0.199219011114973,0.654658156664985
"4703","ZNF224",100.997212061427,0.269811994631291,0.210215568960437,1.28350148357503,0.199316440146528,0.654658156664985

"4704","DUSP15",2.87250986087811,1.36964177780707,1.06650937443839,1.28422854091489,0.199062001604137,0.654658156664985
"4705","ALG3",125.213422977027,-0.342303186356888,0.267139014886899,-1.28136725555349,0.20006469983247,0.654915554877367
"4706","TMEM179B",547.326878440533,-0.292757639797523,0.2284340915729,-1.28158471348002,0.19998836543238,0.654915554877367
"4707","MPHOSPH9",33.8439401937512,0.363915221338169,0.283968797966461,1.28153242167525,0.200006719516702,0.654915554877367
"4708","SMAD9-IT1",4.25644304692833,1.19636275069219,0.933358900688694,1.28178212026417,0.19991908801734,0.654915554877367
"4709","CIC",102.824801381209,-0.249711716901326,0.194874682866303,-1.28139639910354,0.200054468318373,0.654915554877367
"4710","LRIG2-DT",12.7941056170104,0.555790521644241,0.433956480091889,1.28075175078974,0.200280876205731,0.654920313989142
"4711","SLC25A46",132.850987714747,0.21284536741832,0.166202684014908,1.28063736563501,0.200321069118314,0.654920313989142
"4712","CEP170B",36.6754987428663,-0.354924140254779,0.27710156470387,-1.28084495168432,0.200248131419201,0.654920313989142
"4713","CCNDBP1",417.186148199646,-0.26930479232118,0.210266566252467,-1.28077800061578,0.200271653313532,0.654920313989142
"4714","AC005899.6",11.7851622605841,0.693027611760903,0.540910828249225,1.2812234023934,0.200115208393415,0.654920313989142
"4715","CD99",5140.91883636223,-0.305132714100909,0.238183715332356,-1.28108134376497,0.200165096009385,0.654920313989142
"4716","ANAPC5",443.339002222786,-0.174953829689353,0.136650919755105,-1.28029749088327,0.200440529813361,0.655171918378489
"4717","FAF1",118.176432627387,-0.284514379728383,0.222384007411123,-1.27938327508596,0.200762120511211,0.65599901359016
"4718","LRPPRC",177.708339499138,0.240410660706602,0.187923562543441,1.27930025087209,0.200791344306158,0.65599901359016
"4719","GAS2",3.84091626008136,-0.947763864918462,0.740894699650302,-1.27921533973154,0.200821235493478,0.65599901359016
"4720","FRK",16.0115908759036,-0.530746134696136,0.414998933284578,-1.27890963597294,0.20092887898365,0.656072584099337
"4721","MAGEL2",3.06963825877166,1.58006728057955,1.23547652173436,1.27891323937217,0.200927609920381,0.656072584099337
"4722","C20orf96",42.9384252130762,-0.386576038265132,0.302334170001797,-1.2786382639542,0.2010244689523,0.656245698623402
"4723","CAPN7",128.802080840215,0.240890583902261,0.18841643245338,1.27850092885006,0.201072857467498,0.656264683011113
"4724","SLC33A1",111.103469924936,0.269923693082528,0.211159537211591,1.27829269114211,0.201146243945882,0.656365230826792
"4725","ACOXL-AS1",2.42257427019444,1.2836103013787,1.0043134876016,1.27809724475978,0.201215140319421,0.656451087412461
"4726","ADAM17",194.270586496873,-0.347118075888099,0.271660501979102,-1.27776424382372,0.201332565392899,0.656695195838243
"4727","ILKAP",107.561447082242,-0.262597089159863,0.205597238056171,-

1.27724035421195,0.20151740409979,0.657020047419261
"4728","EIF4E",195.017017804148,-0.268628983512565,0.210310261767562,-1.27729850771361,0.201496880280204,0.657020047419261
"4729","ZBTB18",11.4635467641227,0.609679207300663,0.477758474408574,1.27612431795249,0.201911576516478,0.657331985639179
"4730","MVP",431.313748628205,-0.197723067596248,0.154876741938643,-1.2766479015589,0.201726582287826,0.657331985639179
"4731","SP2",59.9695010737999,0.289998922590629,0.227242078106654,1.27616735864614,0.201896364577489,0.657331985639179
"4732","FBXO27",14.9483176635265,0.632909762937458,0.495912464790324,1.27625298389113,0.201866104399065,0.657331985639179
"4733","NAA20",284.550579663283,-0.307359508031972,0.240851743622722,-1.27613569828844,0.201907554265469,0.657331985639179
"4734","AP001442.1",3.68131078541976,1.11304792506902,0.872106750569707,1.27627486467903,0.201858372203671,0.657331985639179
"4735","LINC00630",18.6937461777525,0.602396748000984,0.471897455616341,1.27654163172844,0.201764119883366,0.657331985639179
"4736","CNNM3",80.1203319799864,0.344902991451188,0.270421150615554,1.27542905081977,0.202157421630813,0.657498690209128
"4737","CCDC14",167.791323752621,0.26623076561033,0.208773894209134,1.27521099617771,0.202234570255369,0.657498690209128
"4738","AL021368.2",51.5893868039827,0.453799102131753,0.355883097534089,1.27513530503731,0.202261355106824,0.657498690209128
"4739","UAP1L1",36.829863884806,0.437577911455579,0.343095712749912,1.27538146119167,0.20217425720577,0.657498690209128
"4740","STXBP4",62.124540958401,0.352174240114527,0.276048722542129,1.27576841099411,0.202037397286227,0.657498690209128
"4741","KIAA1328",96.3994733359858,0.253408400228618,0.198726275353393,1.27516303406776,0.202251542324641,0.657498690209128
"4742","KCNK15",17.8002677407358,-0.581126299215512,0.455657211381395,-1.27535850349814,0.202182379214156,0.657498690209128
"4743","AC084018.2",2.65688751802067,-1.28698009127554,1.00946032675394,-1.27491894150412,0.202337934006914,0.65760895060438
"4744","AC034236.2",12.7294497126277,0.687475509496241,0.539298330100873,1.27475920306976,0.202394484787738,0.65765408579321
"4745","ST6GALNAC6",350.364600478748,-0.230833578883401,0.181142826988417,-1.27431807663111,0.202550712685377,0.657773101866237
"4746","CALM1",3622.34882606535,-0.230023607600484,0.180527501309257,-1.27417488156797,0.202601445066202,0.657773101866237
"4747","AC145343.1",3.50581091066295,1.06011532771151,0.832080719407702,1.27405346979573,0.202644467127003,0.657773101866237
"4748","ZNF582-AS1",18.2776096718565,0.606001640977913,0.475623249279304,1.2741211492419,0.202620484206206,0.657773101866237
"4749","TSPYL2",118.556313238052,0.442099035772555,0.346991485105421,1.27409188625548,0.202630853598244,0.657773101866237
"4750","AC004148.1",49.044533871468,-0.492790218689748,0.386865578649225,-1.27380218320371,0.202733531258075,0.657785178771463
"4751","MAST3",14.5342113233533,0.585682144125993,0.459757652375904,1.27389319372792,0.202701270878736,0.657785178771463

"4752","AC109630.1",2.78227427858222,-1.13897489217715,0.894669314498416,-1.27306801934489,0.202993906135114,0.658491385326764
"4753","AUXG01000058.1",117.46881541203,1.03502546234231,0.813217710384662,1.2727532235528,0.203105624608275,0.658527661793236
"4754","MYBBP1A",32.2114766322228,-0.376788979531482,0.29603462735092,-1.27278684559032,0.203093690284522,0.658527661793236
"4755","PTGIR",26.8311429385833,-0.855771615593689,0.672419387258712,-1.27267540438187,0.203133248902163,0.658527661793236
"4756","KRT24",18.3749377222286,0.777315046619711,0.610874383799518,1.27246299277597,0.203208664822328,0.658633635037047
"4757","ZYG11B",235.003488072428,0.275348874522967,0.216779112386751,1.27018176009376,0.204019893281082,0.658707565962923
"4758","HDAC4-AS1",17.7733685868998,-0.461089695889341,0.36248951054322,-1.27200838225183,0.203370141043075,0.658707565962923
"4759","MUSTN1",122.4078656562,-0.595361684272901,0.468613388720773,-1.27047519042964,0.203915414741585,0.658707565962923
"4760","FILIP1L",1589.12403149818,-0.317688889940566,0.249810811345905,-1.27171793818272,0.203473354756367,0.658707565962923
"4761","ATP2C1",164.793442305219,0.249068675482679,0.196134325221039,1.26988825235962,0.204124438341786,0.658707565962923
"4762","RAET1G",8.31248740008247,0.887000232197512,0.697433995612879,1.27180527157706,0.203442315498957,0.658707565962923
"4763","ZNF3",63.2810471045106,0.337706789028829,0.265722717695677,1.27089919882422,0.20376451148717,0.658707565962923
"4764","LERFS",21.1950315619023,2.05203383055517,1.61380142499105,1.27155286813962,0.203532031897722,0.658707565962923
"4765","C9orf116",20.8721009213571,-0.526493419318001,0.414466791371201,-1.27029096245848,0.203981006236191,0.658707565962923
"4766","LINC02664",4.99581079848671,0.901905534271,0.710107534177158,1.27009712031304,0.204050037281919,0.658707565962923
"4767","AC022387.2",4.70692491200438,-0.857556649672344,0.674440928808335,-1.27150742643623,0.203548187137636,0.658707565962923
"4768","KDM2A",217.573043268423,0.315484861741892,0.248460483173282,1.26975870654597,0.204170593900626,0.658707565962923
"4769","ZCRB1",554.512254132692,-0.25257175711985,0.198531252451279,-1.27220149977059,0.203301534900893,0.658707565962923
"4770","LIN52",32.4441388890585,0.379276887124905,0.29848568905424,1.27067025667681,0.203845981179416,0.658707565962923
"4771","RPP25",68.4318724691804,-0.41744477990079,0.328383906923157,-1.27120961502682,0.203654086881859,0.658707565962923
"4772","MCRIP2",50.0027336622647,-0.394056267543232,0.309760551805102,-1.27213186200407,0.203326272186845,0.658707565962923
"4773","LINC02175",13.6313897951239,0.621385541998427,0.4892678303279,1.27003147045654,0.204073420355399,0.658707565962923
"4774","AXL",1061.97012461478,-0.220689030277987,0.1737953202685,-1.26982147699397,0.204148228624157,0.658707565962923
"4775","EMC10",232.018556998069,0.213634909118854,0.168183035639958,1.27025242650631,0.203994728302091,0.658707565962923
"4776","Z82217.1",16.4473325222565,-0.699539919105546,0.55083280638476

6,-1.26996778513752,0.204096105565746,0.658707565962923
"4777","PUDP",30.8051055677527,0.379062799364594,0.298527241958661,1.2
6977624178461,0.204164345869332,0.658707565962923
"4778","RPL36A",5292.77892839205,-0.313142295396223,0.246616124230902,
-1.26975596738774,0.204171569910532,0.658707565962923
"4779","PAXIP1-
AS1",66.7375238458334,-0.295070071307616,0.232431087657912,-1.26949486
095378,0.204264622277475,0.658869879139417
"4780","AC254633.1",15.9774754280778,-0.660533096933096,0.520660273676
988,-1.26864508457368,0.204567676760248,0.65926387754896
"4781","UBE2E2",235.180022440115,-0.279993626864442,0.220781497916732,
-1.26819334729779,0.204728912217118,0.65926387754896
"4782","TMEM181",115.605732313111,0.290468852238445,0.229039859090491,
1.26820219586183,0.204725753073311,0.65926387754896
"4783","CTBP2",247.901679007529,0.278746944323071,0.219695244294414,1.
26878915935714,0.204516272576015,0.65926387754896
"4784","AAGAB",47.1724180993786,-0.343162478504313,0.270579238621602,-
1.26825132723585,0.204708212671396,0.65926387754896
"4785","AC027237.3",4.03411532581985,1.01465384390872,0.79984495369542
,1.26856316242398,0.20459690980792,0.65926387754896
"4786","AC093525.7",4.62134395786284,1.00784430574321,0.79465123316401
2,1.26828508367166,0.204696161912743,0.65926387754896
"4787","CCDC102A",101.495903866923,-0.273007231966367,0.21527058746164
2,-1.26820498418073,0.204724757585757,0.65926387754896
"4788","KATNB1",40.5317193192084,-0.335668694725622,0.264737887613467,
-1.26792843197313,0.204823509588079,0.659430743588188
"4789","HERC5",28.1910511056424,0.4641268156991,0.366124728475515,1.26
767404548622,0.204914377176185,0.659447833856136
"4790","ABCC9",305.835990586502,0.391158414441246,0.308545138856589,1.
26775101980478,0.204886878634991,0.659447833856136
"4791","AKAP9",868.162331055564,0.26813499386697,0.211616935463324,1.2
6707719909044,0.205127687486008,0.659996514839662
"4792","CDKN1B",215.465874007154,0.267655940709259,0.211279995279137,1
.26683049360939,0.205215906089545,0.660142569359421
"4793","CDKN2B",40.5214733979117,0.576929372181774,0.455472401006054,1
.26666153845424,0.205276338118139,0.660199197181537
"4794","DLGAP1",3.26241437769975,1.14261376428817,0.902248425547081,1.
26640704703401,0.205367389247949,0.660348164322403
"4795","AP001469.3",2.37472831985076,1.4915674528558,1.17790107216485,
1.26629263535219,0.205408332658185,0.660348164322403
"4796","FBXL15",165.26289826823,-0.301055637771368,0.237840009369483,-
1.26579055630493,0.205588077008345,0.660788199975737
"4797","CCT4",479.37591284521,-0.237637102856413,0.187772482459377,-1.
26555872161845,0.205671112411611,0.660917281180944
"4798","AC010896.1",7.93549784713681,0.875248769549235,0.6916577068970
23,1.26543630009684,0.20571496952197,0.660920436678026
"4799","CKAP4",428.46714501855,0.291177335055357,0.230140619353319,1.2
6521487546852,0.205794311431316,0.660959583934626
"4800","BMF",19.3642386217611,0.530137720511064,0.419027215025759,1.26
516298106908,0.205812909690964,0.660959583934626

"4801","PUF60",310.244563839266,-0.3046168567976,0.240883068392968,-1.26458392791916,0.206020517444122,0.661374344123181
"4802","DUSP6",110.993233082635,-0.40354676417437,0.319119432276776,-1.26456343098646,0.206027868989913,0.661374344123181
"4803","CA12",247.877733641376,-0.461370951679803,0.364904657500003,-1.264360271093,0.206100745770915,0.661470538425702
"4804","PCGF1",62.1797655530225,-0.261121269611963,0.206638140879085,-1.26366443533171,0.206350495342515,0.661858694487071
"4805","CD2AP",111.444820566864,0.26548859631216,0.210081992849415,1.26373799444325,0.206324083101201,0.661858694487071
"4806","SFXN3",221.802952525965,-0.269009162790414,0.21287156960128,-1.26371578551207,0.206332057213517,0.661858694487071
"4807","ZNF691",20.2750368832498,0.487426212739136,0.385863028312619,1.26321045804946,0.206513555422101,0.661946804578585
"4808","MICA",117.780679458883,-0.353743575631093,0.280084164101335,-1.26299027567695,0.20659267446173,0.661946804578585
"4809","PDE3A",31.5128776870666,0.547572125113228,0.433449739848964,1.26328862327621,0.206485473281053,0.661946804578585
"4810","SLC38A1",24.5594563449039,-0.593356961183699,0.469765829081543,-1.26309093691168,0.20655650071495,0.661946804578585
"4811","HIC2",61.0355720242838,0.4474296356817,0.35414566381859,1.26340565872605,0.206443431558161,0.661946804578585
"4812","DUSP23",112.282813039055,-0.367811662704982,0.291345594860867,-1.26245829418025,0.206783924381382,0.662284270587783
"4813","NDUFS1",283.523664605483,0.198373963929415,0.157131737230947,1.262469106657,0.206780035964625,0.662284270587783
"4814","ADGRL2",90.9056608759257,0.338508888488783,0.268243554935802,1.26194602725794,0.206968208249155,0.662578116797664
"4815","CAST",2067.35300861996,-0.265044689046455,0.210065266409952,-1.26172543217691,0.207047602245498,0.662578116797664
"4816","PRKAR1B",7.30386927613406,-0.718347323123759,0.569308912578626,-1.26178829674399,0.207024974515651,0.662578116797664
"4817","PNKP",90.3920272653222,-0.312172239710353,0.247391775778462,-1.26185374888897,0.207001417309549,0.662578116797664
"4818","ANTXR1",684.938040107722,0.301826230692266,0.239247566310901,1.2615644762716,0.20710554555442,0.662625982715107
"4819","SYAP1",370.285316790232,-0.193959752583407,0.153812580883457,-1.26101357554341,0.207303956086334,0.663123154818603
"4820","TTLL3",210.731799322222,0.300773327634873,0.238665953124337,1.26022720751535,0.207587410606808,0.66389210259418
"4821","CERS2",441.171427147816,-0.264721657522805,0.210149273678822,-1.25968390415371,0.207783414102498,0.664105604061788
"4822","VPS54",71.6240531374401,0.262174178155858,0.208112981871588,1.2597684959299,0.207752887740083,0.664105604061788
"4823","NDUFA5",529.837332120655,-0.286557556854342,0.227464792652225,-1.25978861833121,0.207745626712725,0.664105604061788
"4824","CTDSP1",435.637500424527,-0.204456791831394,0.162400367029429,-1.25896754774171,0.20804205376877,0.664106079694676
"4825","SEPTIN2",1055.4283874773,0.219541015803558,0.174367171481867,1.259073103829,0.208003928205772,0.664106079694676

"4826","AC097376.3",58.813861471819,0.324290899268019,0.25748212092366,1.25946958221681,0.207860770333145,0.664106079694676
"4827","TMEM250",109.091756092862,-0.263103926711748,0.208933596474375,-1.25927055845237,0.20793262362292,0.664106079694676
"4828","SOS2",149.64902607091,0.271486695851453,0.21560412986683,1.25919061021298,0.207961492301835,0.664106079694676
"4829","KRT10",1385.91287165737,-0.325847093764522,0.258768563322045,-1.25922210017063,0.207950121179478,0.664106079694676
"4830","TIAL1",341.764337743678,-0.168097015704999,0.133552747511407,-1.25865636489913,0.208154478679286,0.664327388994036
"4831","CCDC152",76.0106051040898,0.439184653541049,0.349276797162975,1.2574114773966,0.208604675315643,0.664925723421579
"4832","JAZF1",262.336494269607,0.230952365314136,0.183612141847794,1.25782730373891,0.208454218950054,0.664925723421579
"4833","SBDS",795.672923574503,-0.228575147906703,0.181774849550518,-1.25746300146533,0.208586028348852,0.664925723421579
"4834","SMNDC1",149.699217081096,-0.222944524910947,0.177255289956599,-1.25775950024134,0.208478746581754,0.664925723421579
"4835","TLN2",280.653139765972,0.316681742220114,0.251873825141663,1.25730310421101,0.208643900369132,0.664925723421579
"4836","ZNF682",17.7425330683771,0.483733870400399,0.384707687310722,1.25740630186497,0.208606548448126,0.664925723421579
"4837","ZNF254",113.215621403352,0.266283294340652,0.211788240436874,1.25730915839032,0.208641708952669,0.664925723421579
"4838","TRIM35",35.0885901715338,-0.375154836828697,0.298442843533561,-1.25704082023502,0.208738854673796,0.665082324270319
"4839","C12orf57",1943.65282159133,-0.334566201439198,0.266177476385384,-1.25692904592272,0.20877932968823,0.665082324270319
"4840","IER5",383.426280093438,0.282226478520014,0.224661871066447,1.25622775765337,0.20903340558986,0.665174710718392
"4841","FN1",5208.945882213,-0.281980842231166,0.224455766985389,-1.25628691130721,0.209011965645027,0.665174710718392
"4842","TMEM144",15.001185951236,0.643986782740811,0.512819220933173,1.25577738987426,0.209196691376112,0.665174710718392
"4843","LMX1B",22.1654187429236,0.485379782416314,0.386332019510283,1.25637989579943,0.208978267103237,0.665174710718392
"4844","AGAP9",23.9372039209769,0.559414234129222,0.445383365363718,1.25602857590422,0.209105609729706,0.665174710718392
"4845","EIF3F",1530.47384468239,-0.303182115087751,0.241345764071906,-1.2562147765619,0.20903811073447,0.665174710718392
"4846","IRAK4",55.3784752358457,-0.306475844618175,0.243966675847941,-1.25622011101711,0.209036177191071,0.665174710718392
"4847","TTBK2",106.665618911189,0.304114449134743,0.242136252733031,1.25596413466449,0.209128973791521,0.665174710718392
"4848","KANTR",44.1104355874812,0.459160885101178,0.365631869881983,1.25580104723744,0.209188111840626,0.665174710718392
"4849","BNIP3L",831.395432049422,0.229633991293838,0.182919119007761,1.25538539951143,0.209338886977817,0.665253782060522
"4850","AL133453.1",41.4001275805008,-0.504092848408966,0.401572191976184,-1.25529819664122,0.209370529579849,0.665253782060522

"4851","AC051619.6",2.96066912922812,-1.15300959886036,0.918534605742044,-1.25527072322865,0.209380499353005,0.665253782060522
"4852","NFAT5",319.256831514042,0.304330443444431,0.242495301706521,1.25499521558874,0.209480496796742,0.665253782060522
"4853","FLOT2",104.488024486738,0.326378724555696,0.260063149198884,1.25499797092012,0.209479496558571,0.665253782060522
"4854","MYL9",1495.53830412318,0.414543698873319,0.330282877560156,1.25511713454724,0.209436241184406,0.665253782060522
"4855","AL136441.1",5.27105571890793,1.04789031236936,0.835292252605709,1.25451937223223,0.209653288948379,0.665665386022504
"4856","AL080250.1",8.65446076679789,0.752259196927501,0.599753293169122,1.25428106105517,0.209739865237746,0.665803134810515
"4857","PXYLP1",75.6363666263578,0.339576607558907,0.270841164169753,1.25378506845464,0.209920137846416,0.665987757900654
"4858","ARHGAP32",126.844295377818,-0.37419984677123,0.298544552567317,-1.25341374864595,0.210055170499161,0.665987757900654
"4859","AC022364.1",5.74937261802626,0.846913842484032,0.675567252647831,1.25363365255587,0.209975193547228,0.665987757900654
"4860","NCLN",71.9080733219325,0.347419584177541,0.277179955706526,1.25340803699884,0.210057248064417,0.665987757900654
"4861","PRNP",598.026903242511,0.267369370581803,0.213252171324985,1.25377091787894,0.209925282635263,0.665987757900654
"4862","EIF1AX",1362.11670058953,-0.163050045743323,0.130059693492247,-1.2536554666957,0.209967261154809,0.665987757900654
"4863","BRPF3",54.1457325696283,0.327409266075946,0.261251366641564,1.25323465398422,0.210120321806755,0.666050742474013
"4864","WIPF2",243.811936866332,0.244923225556672,0.195479502342951,1.25293558977338,0.210229148406822,0.666258701211175
"4865","AL353622.1",3.08156111287382,1.19742033035758,0.955919112351603,1.25263771263226,0.210337583586931,0.666424703882124
"4866","FCGBP",2.41086707301462,-1.17475300725271,0.937885969480609,-1.2525541968639,0.210367992805087,0.666424703882124
"4867","ZC3H7B",130.628362874751,0.242148811116568,0.193367269303065,1.25227403784167,0.21047002572108,0.666610940310346
"4868","IRX2",94.1568774406856,-0.447491638221256,0.357513174107304,-1.25167873698256,0.210686951039972,0.666934089176569
"4869","C6orf132",154.659043079893,-0.31928407983264,0.255117205668336,-1.25151919485871,0.210745115042431,0.666934089176569
"4870","SIRT4",6.20985600760238,0.806213462415052,0.644155918032087,1.25158123964467,0.210722494099561,0.666934089176569
"4871","ALKBH7",524.044312450865,-0.318825416355633,0.254679607711239,-1.25186864869497,0.21061773038836,0.666934089176569
"4872","CISH",20.2944746858529,-0.825491039350946,0.659845989202291,-1.25103592786691,0.210921369752311,0.667354867555803
"4873","GPD2",69.5407724020875,0.36747197267898,0.293775572759304,1.25085952255144,0.210985733967803,0.667421524546212
"4874","ZNF345",35.9194976130488,0.369622744213546,0.295524544283956,1.25073450365731,0.211031357650665,0.667428883501231
"4875","SPATA17",9.25582641969863,-0.613698441713549,0.491169108916663,-1.24946465600644,0.211495172953027,0.667508478300003

"4876","LINC01354",36.173765795953,0.575604750678289,0.461003400001467,1.24859111814893,0.211814662578602,0.667508478300003
"4877","N4BP2",76.2676324760657,0.302408339856684,0.242218748458892,1.24849270248792,0.211850679185852,0.667508478300003
"4878","AC098487.1",60.3700683373426,0.333656645825023,0.267214912688568,1.24864530376675,0.211794834472158,0.667508478300003
"4879","NUDT12",58.5029849458684,0.326471619229444,0.261383322777125,1.24901472580872,0.211659687862956,0.667508478300003
"4880","N4BP3",3.47345803324436,1.13062813096454,0.905737000454544,1.2482962829134,0.211922574946494,0.667508478300003
"4881","PPP1R35",116.36062585669,-0.3246952621377,0.259956495694536,-1.24903692546785,0.211651568490846,0.667508478300003
"4882","ENTPD1-AS1",66.4099282858281,0.260752579507936,0.208757523054267,1.24906913864921,0.211639787142446,0.667508478300003
"4883","INPP5A",46.991255313287,-0.293907382989865,0.235437019989232,-1.2483482122026,0.211903565474127,0.667508478300003
"4884","BATF2",4.41111366243724,0.978847254513549,0.783845584223087,1.24877561884046,0.211747153858084,0.667508478300003
"4885","PPP1CA",317.70944641798,-0.271962063586046,0.217769614592322,-1.24885220601219,0.211719135214094,0.667508478300003
"4886","LPAR6",92.8781339485856,0.244276609160503,0.195638502308922,1.24861214064489,0.211806969671616,0.667508478300003
"4887","MAP3K3",158.189830394338,0.271614376445592,0.217244415882536,1.25027092338453,0.211200596274173,0.667508478300003
"4888","SLC16A3",6.76737597032225,-0.993828830313641,0.795827964646024,-1.24879857766205,0.211738754323609,0.667508478300003
"4889","C3",10035.2262827036,0.301972872436593,0.241864931578733,1.24851862758902,0.211841191097984,0.667508478300003
"4890","ZNF324",52.2555950996801,-0.407566290705479,0.325936549711108,-1.25044672365441,0.211136405559133,0.667508478300003
"4891","PLCG1",41.7856666995739,0.40414348729458,0.32326538688964,1.25019103091464,0.211229772421514,0.667508478300003
"4892","MRPL39",81.8890306329862,-0.376383706727236,0.301119511739497,-1.24994791786476,0.211318573461755,0.667508478300003
"4893","SPIN3",34.6454872714403,0.37506563367619,0.300104106198016,1.24978507767806,0.211378068611184,0.667508478300003
"4894","HDX",31.4622128766413,0.375162794792569,0.300138288666666,1.24996646199054,0.211311798964022,0.667508478300003
"4895","PAXIP1-AS2",74.2436776214996,0.36552890956309,0.29293001453706,1.24783699663131,0.212090757027414,0.667565078426522
"4896","CD4",6.55696070901885,-0.751405664068665,0.602196839987896,-1.2477741731155,0.212113769324236,0.667565078426522
"4897","PPM1A",212.024616345937,0.214249235861796,0.17169446682388,1.24785172070553,0.212085363849898,0.667565078426522
"4898","TRIM28",274.633528294117,-0.194451324171601,0.155811492764826,-1.24799089413189,0.212034391889767,0.667565078426522
"4899","FAM162A",361.568892371286,-0.370568623639187,0.297087091558742,-1.24734003653577,0.212272843160153,0.66774201912078

"4900","STEAP2",106.274559713574,-0.436114612551844,0.349631025810045,-1.24735672854384,0.212266725377619,0.66774201912078
"4901","PRDM11",27.7000157023231,0.360697603180616,0.289190577535292,1.24726609786101,0.212299943931946,0.66774201912078
"4902","CAPRIN1",391.767313612868,0.195334953049266,0.156625433314003,1.24714708790403,0.212343569947819,0.667742988728198
"4903","C1orf21",978.899281185694,0.213230971458812,0.171000242423134,1.24696297757976,0.212411072856406,0.667819026734957
"4904","FAM13A-AS1",15.8699025059566,0.627956252091015,0.503785629904654,1.24647511722369,0.212590018782364,0.668121960526814
"4905","GIPC1",220.661253621575,-0.304527684856285,0.24431326811701,-1.24646396490606,0.212594110696336,0.668121960526814
"4906","AL354953.1",6.74008881322425,-0.74803006527028,0.600313451991577,-1.24606580576971,0.212740237145093,0.668142987577971
"4907","ARIH2OS",29.0779668477122,0.480669149938867,0.385851141246427,1.24573727678023,0.212860863574143,0.668142987577971
"4908","AC009570.1",5.10571228804697,-0.80266470741666,0.644093275762185,-1.24619327296474,0.212693448138619,0.668142987577971
"4909","PCDHGB7",34.9793337046495,0.442371139913917,0.354975066491675,1.24620341447078,0.212689725845148,0.668142987577971
"4910","LYRM4",200.042465737511,-0.266525241630944,0.213944119886274,-1.2457703524295,0.212848716908441,0.668142987577971
"4911","PLGRKT",154.866711647434,-0.311780575877948,0.250249195372748,-1.24588043295623,0.2128082946594,0.668142987577971
"4912","ERICH6-AS1",14.611888549422,-0.646511114193626,0.51927873477795,-1.24501750388466,0.213125316160212,0.668428636543166
"4913","ARHGEF17",39.4004045134508,0.404372392666259,0.324703719879336,1.24535805384838,0.213000164760904,0.668428636543166
"4914","SINHCAF",22.2376190595913,-0.514526614283638,0.413212713668599,-1.24518582624322,0.21306345141262,0.668428636543166
"4915","AP1G1",128.315727119667,0.247138122028273,0.198490794015005,1.24508606686107,0.213100115142983,0.668428636543166
"4916","MRPL47",199.546432390034,-0.348010926152997,0.279712659255879,-1.24417295620017,0.213435914703205,0.669198274740231
"4917","ESRRA",143.944713143009,-0.306085642233548,0.246029214634213,-1.24410283018066,0.213461719567199,0.669198274740231
"4918","DTX3",59.4384054592631,0.362101415370857,0.291079188820295,1.24399623634519,0.213500948113685,0.669198274740231
"4919","ENDOG",147.394623098978,-0.357353352480008,0.287398436316058,-1.24340743485124,0.213717731932864,0.669605454826241
"4920","FERMT2",819.118281461954,0.256586854386022,0.206353468655671,1.24343368714665,0.213708063031107,0.669605454826241
"4921","FMO2",39.1355791278605,0.654989789590534,0.526870927501451,1.24316935211562,0.213805433860226,0.669608037983621
"4922","LACTB",145.606836656864,-0.485588062844836,0.390594200776659,-1.2432034625176,0.213792867105579,0.669608037983621
"4923","MTRNR2L12",50.136228835692,-0.481420379916602,0.387474881699808,-1.24245571172166,0.2140684712924,0.669628732402229

"4924","ANAPC4",72.2812862858076,-0.251697095019957,0.202565648959131,-1.24254579349107,0.214035255601085,0.669628732402229
"4925","CAMLG",876.27653889406,-0.24434552400825,0.196683821979611,-1.24232649919513,0.214116122089561,0.669628732402229
"4926","KMT5B",239.072480592212,0.23003603554574,0.185094736406471,1.24280160533889,0.213940950819728,0.669628732402229
"4927","ZDHHC17",155.767052381347,0.267680395720989,0.215442040833815,1.24247057206197,0.214062991609372,0.669628732402229
"4928","CDK2AP1",1063.36177039359,-0.276891123330221,0.222777811779074,-1.24290260829391,0.213903724436296,0.669628732402229
"4929","PKIG",372.839807524286,-0.310960185335944,0.250302337875824,-1.24233831763175,0.214111763386815,0.669628732402229
"4930","DNAH1",5.61374916632032,0.832516524331417,0.670191887527713,1.24220620963126,0.214160489165811,0.669631630931231
"4931","P2RY1",12.4640735144311,0.701153384453346,0.564615595338294,1.24182433188592,0.214301383217752,0.669800450588329
"4932","FIGNL1",29.3169314163658,0.474774531086959,0.382307696353386,1.24186495750821,0.214286391190472,0.669800450588329
"4933","RNF4",113.012690571521,0.247262443487655,0.199250009009851,1.24096578322077,0.214618389370977,0.670655275117295
"4934","C11orf1",48.9593027672303,-0.474574700222228,0.382476176567707,-1.24079545157819,0.21468132193055,0.670715966266603
"4935","LINC02062",3.41924142392015,1.04278230439568,0.84065928775936,1.24043393034418,0.214814937530857,0.670997418852718
"4936","PBXIP1",205.169861390816,0.226523979948072,0.182636229138298,1.24030145068611,0.214863916046886,0.671014437978675
"4937","ZNF786",11.9737997461917,0.654930146705322,0.528278051569404,1.23974513943871,0.215069675483555,0.671249047900183
"4938","DBX2",20.9464431762845,0.591753252912468,0.47728210005987,1.23983961023939,0.215034724141038,0.671249047900183
"4939","TBX21",2.5274917016935,1.37372066131166,1.10787050236305,1.23996501250062,0.2149883354159,0.671249047900183
"4940","UACA",507.864823629613,0.289676364232713,0.233736580617848,1.2393283219383,0.215223934247088,0.67159452356657
"4941","STAT1",292.358025460355,0.359490125513138,0.290127751223069,1.23907528320771,0.215317619487515,0.671648128833878
"4942","NHP2",439.793733122661,-0.273758265287491,0.220984641277739,-1.23881127532037,0.215415397272442,0.671648128833878
"4943","YME1L1",467.259496493382,0.185314463357246,0.149587438215305,1.23883706792624,0.215405843330483,0.671648128833878
"4944","EFCAB13",34.7035062079273,0.486353277667581,0.392522916003073,1.23904428974479,0.215329096548872,0.671648128833878
"4945","EIF4A1",2439.12827178467,-0.213784857192754,0.172653496278831,-1.23823068631925,0.215630536341602,0.672182956057795
"4946","UBXN10-AS1",3.68801451857461,-1.26889965787609,1.02522159957802,-1.23768330514922,0.215833511843093,0.672213150841646
"4947","CPS1",8.48047300674587,-0.698404060639049,0.564242086810774,-1.2377737800216,0.215799953187066,0.672213150841646
"4948","PSMG3",174.409578230189,-0.316587163265966,0.255803911038893,-

1.23761658678405,0.215858261217395,0.672213150841646
"4949","DLEU1",13.6247859288022,-0.56439520039504,0.455920980541865,-1.23792329040057,0.215744505502003,0.672213150841646
"4950","PDHA1",178.232798202979,-0.293461122329893,0.237042549260158,-1.23801032028142,0.215712234170721,0.672213150841646
"4951","SMURF1",53.1917033515032,0.333646594772636,0.269681334480457,1.23718831121705,0.216017179969339,0.672490014336848
"4952","LGMN",582.745735302432,-0.189643725867272,0.153307962756569,-1.23701158411712,0.216082782038808,0.672490014336848
"4953","DUS1L",151.751705335741,-0.312850334028913,0.252929584596109,-1.23690684317728,0.216121669219899,0.672490014336848
"4954","HSD17B14",36.264006128469,-0.714609869415144,0.577682249392239,-1.23702930143165,0.216076204627545,0.672490014336848
"4955","TSC22D4",187.076825999157,0.215889884811633,0.174611795730949,1.23639920148515,0.216310212799651,0.672940853745029
"4956","MATN1-AS1",5.28574577028626,-0.746016133654627,0.603652318720918,-1.23583743575336,0.216518996610994,0.673318606568182
"4957","RPL36",7862.84215215789,-0.291948997874605,0.236229524433073,-1.23587006567131,0.216506865528053,0.673318606568182
"4958","ERMAP",37.6643261011939,0.382597713044498,0.309866278308042,1.23471877977039,0.216935184119998,0.673436445597939
"4959","AL356488.3",11.936698679626,-0.674131028762491,0.546579317908026,-1.23336358818452,0.217440143495049,0.673436445597939
"4960","PEAR1",47.410214297994,0.715643340941078,0.57942046698104,1.23510193671584,0.216792568713999,0.673436445597939
"4961","LINC00467",86.2601446401013,0.346781610553643,0.280898462631102,1.23454435209588,0.217000130452771,0.673436445597939
"4962","AL390728.6",59.8981594358095,0.530542230230657,0.429588899648122,1.2349998583884,0.21683055684324,0.673436445597939
"4963","AC010207.1",4.24664504571587,1.18931919076866,0.962923310813973,1.23511309510548,0.216788416444682,0.673436445597939
"4964","C4orf48",86.359010655289,0.58192799728844,0.471975634028784,1.2329619483131,0.217589961205174,0.673436445597939
"4965","RASGEF1B",25.6884521142912,-0.579840421509942,0.470240347053373,-1.23307245995234,0.217548731302645,0.673436445597939
"4966","ATXN1",187.218379767428,0.294677663984343,0.238974785900303,1.23309102621093,0.217541805117107,0.673436445597939
"4967","ARMC10",236.307202439822,-0.283837085803284,0.230200624239191,-1.23299876679901,0.217576224267025,0.673436445597939
"4968","AK8",5.79607893937178,0.82375570912683,0.668126133622526,1.23293442311632,0.217600231234347,0.673436445597939
"4969","SFR1",71.5287110820262,-0.284384080665285,0.230613670117828,-1.23316228617316,0.217515222896276,0.673436445597939
"4970","FTH1",37796.4072488613,-0.329896602930227,0.267043351713915,-1.23536721964023,0.216693866901203,0.673436445597939
"4971","RELT",8.46845293106438,-0.820689554421306,0.664223422471617,-1.23556250300158,0.216621229963566,0.673436445597939
"4972","CD27-AS1",152.548544194149,0.284667670087963,0.230570707490511,1.2346220089

5436,0.216971214000846,0.673436445597939
"4973","PA2G4",643.395060292752,-0.23544647261378,0.190857347358329,-1
.23362540595168,0.217342521480364,0.673436445597939
"4974","AL136295.7",5.67940585327133,0.798061931155087,0.6459921144787
23,1.23540506651397,0.216679788137149,0.673436445597939
"4975","STUB1",772.605474283244,-0.280282971447519,0.227161381311688,-
1.23384956469755,0.217258966150556,0.673436445597939
"4976","ZNF205",75.0349501125057,-0.287689831203665,0.233234245700847,
-1.23348023074049,0.217396647971509,0.673436445597939
"4977","AC009152.1",3.57334455787734,-1.02609103565588,0.8322441981084
59,-1.23292062352372,0.217605380183155,0.673436445597939
"4978","TMEM220",129.333806901277,-0.279382032417359,0.226348513509906
,-1.2343002747625,0.217091033592394,0.673436445597939
"4979","MED16",85.3115967451199,0.310101169545501,0.251177290729979,1.
23459078901709,0.216982838781384,0.673436445597939
"4980","ZNF44",70.852967355356,0.316540098909937,0.256537598340957,1.2
3389359281844,0.217242557352597,0.673436445597939
"4981","MTMR1",26.113155052092,0.437834541370665,0.354746164326541,1.2
3421924012021,0.217121219851333,0.673436445597939
"4982","AC244453.2",2.95805834983032,1.38898464492185,1.12834546782361
,1.23099235520568,0.218325723528092,0.67350230702132
"4983","SLC25A12",91.9435427334224,-0.305108630813827,0.2477331812396,
-1.23160179547662,0.218097869918678,0.67350230702132
"4984","WDR12",51.5052545677597,0.324659869553456,0.263713431228922,1.
23110858647024,0.218282254534926,0.67350230702132
"4985","RCAN2",81.1222151439906,-0.487077521288622,0.395474374768127,-
1.23162852605609,0.21808787997551,0.67350230702132
"4986","VCP",573.068732589221,0.158287503518516,0.128563063887251,1.23
120512791553,0.218246154008593,0.67350230702132
"4987","AL596244.1",9.07839814108146,0.668771517746873,0.5425643238379
77,1.23261240808488,0.217720405358838,0.67350230702132
"4988","BMI1",260.606672322109,0.201120215625214,0.163169210954977,1.2
3258680021876,0.217729964112323,0.67350230702132
"4989","RAN",851.752437051661,-0.3224773154911,0.261823256136808,-1.23
1660320207,0.218075998066476,0.67350230702132
"4990","METTL3",67.3248357003791,0.269442566110072,0.218751547464219,1
.23172873167512,0.218050433342847,0.67350230702132
"4991","PTGR2",34.3267255826767,-0.390975445169891,0.31731950975385,-1
.23211915168146,0.217904578273849,0.67350230702132
"4992","CES1",171.647693079429,0.874307008555035,0.709797833300607,1.2
317690580844,0.218035364751281,0.67350230702132
"4993","POLR2C",230.550535348514,-0.281282474632862,0.228477349477626,
-1.23111754962129,0.218278902690399,0.67350230702132
"4994","UTP18",97.396033821769,-0.288867111465104,0.23462834629891,-1.
23116885074532,0.218259718922099,0.67350230702132
"4995","TEN1",109.372845731913,-0.388754349750966,0.315435814896593,-1
.23243566960971,0.21778638340108,0.67350230702132
"4996","UBXN6",357.478706588948,-0.22811649107827,0.185139818502618,-1
.23213089935617,0.217900190605613,0.67350230702132
"4997","ZNF561-

AS1",32.6272653369674,0.346341440679167,0.281304039317698,1.2311996710
7197,0.218248194415991,0.67350230702132
"4998","SLC25A30",16.1700289727111,0.513392475471056,0.41711243646314,
1.23082514591105,0.218388268557065,0.673560456143887
"4999","BRD2",869.782750210108,0.30526798037507,0.24806660833665,1.230
5887617119,0.218476710585307,0.673589003880315
"5000","ATP5F1D",1216.53456666583,-0.307433785654539,0.249831036262889
,-1.23056682729778,0.218484918546972,0.673589003880315
"5001","DESI2",326.305496617043,-0.199039252010955,0.161900958231166,-
1.22938896832693,0.218926004384907,0.673946022625884
"5002","AC025171.3",3.6393684975541,-1.20498301340615,0.98001094502743
3,-1.22956077125487,0.218861627639456,0.673946022625884
"5003","AC011379.1",18.807661204604,-0.562563446933792,0.4576338638069
53,-1.22928719097392,0.218964148069568,0.673946022625884
"5004","DEK",1340.26970067887,-0.232143409049863,0.188898192119524,-1.
2289339905539,0.219096556045884,0.673946022625884
"5005","POLH",30.875929146553,0.441898666976145,0.359601325698833,1.22
885716874758,0.219125362659807,0.673946022625884
"5006","AL139407.1",2.36082629334323,1.43120016662527,1.16432858205384
,1.22920641877629,0.218994422927943,0.673946022625884
"5007","LINC01001",4.66895255684695,0.984700459459182,0.80094695214739
1,1.22942032155705,0.218914254915852,0.673946022625884
"5008","RPS6KA4",68.4304671428992,-0.323880763505352,0.263384173438248
,-1.22968954162042,0.21881338465868,0.673946022625884
"5009","AC008149.2",6.12434374005804,-0.779535101186725,0.634274791825
548,-1.22901794495583,0.219065077963693,0.673946022625884
"5010","APEX1",478.398819818602,-0.327445696073436,0.266212830502054,-
1.23001470461023,0.21869159845349,0.673946022625884
"5011","METTL9",588.939405821396,-0.265476183406464,0.215809870803328,
-1.23013920734144,0.218644980224626,0.673946022625884
"5012","NDUFB11",828.359841375501,-0.309628645608032,0.251937217390245
,-1.22899128924022,0.219075071975256,0.673946022625884
"5013","DCP1A",204.896716534134,0.248983550437153,0.202673872535213,1.
2284935760227,0.219261739429174,0.674230942210395
"5014","AC112715.1",6.93781795330423,-0.836643179954792,0.681557022919
57,-1.22754685495116,0.219617122525529,0.674724863355328
"5015","SYPL1",627.739299762092,-0.291083741347305,0.237145770451342,-
1.22744648067435,0.219654825559964,0.674724863355328
"5016","OLFM1",134.874696869782,0.42363243764185,0.345142365193921,1.2
2741361352099,0.219667172276597,0.674724863355328
"5017","AL450326.1",37.6388311444092,-0.377028278165478,0.307148123554
394,-1.22751288141504,0.21962988329693,0.674724863355328
"5018","EMID1",11.2722987994769,0.861044759086104,0.701485953961839,1.
22745830365257,0.219650384318683,0.674724863355328
"5019","COX7B",710.326443817402,-0.325479217988709,0.265185093552022,-
1.22736619026763,0.219684987945533,0.674724863355328
"5020","RADIL",66.2916302951958,0.281190438728985,0.229170592520583,1.
22699180394941,0.21982567144822,0.675022455253846
"5021","PNPT1",112.372991317057,-0.278426581145282,0.226955268687206,-
1.2267905599011,0.219901319826608,0.67512026391698

"5022","AL451064.1",2.86637057844804,1.16824627980604,0.952682568254218,1.22627023809918,0.220096997290862,0.675586462213985
"5023","LINC02649",2.64712049569519,1.42244465608547,1.16026421668999,1.22596615117843,0.220211413106704,0.675803092383008
"5024","CD86",5.98674837852949,-1.28371169278741,1.04791221025112,-1.22501835576454,0.220568304567906,0.676188162929621
"5025","KLHL2",39.7737769199404,0.355646229232566,0.290247840100269,1.22531912419987,0.220455005597649,0.676188162929621
"5026","DNAJC3",463.654851001466,-0.188182007198107,0.153593340820794,-1.22519639323211,0.220501233107209,0.676188162929621
"5027","KLHDC1",59.2815457753157,0.328280131813265,0.267883236663263,1.22545977830603,0.220402035756173,0.676188162929621
"5028","WHAMM",77.0926317003754,0.268846515082281,0.219458813007185,1.22504314772485,0.220558963898735,0.676188162929621
"5029","ANKRD54",55.2527200340981,-0.279395108717172,0.22808992559991,-1.22493401662665,0.220600082476358,0.676188162929621
"5030","PDPN",284.354488268194,0.459856482325328,0.375502717672941,1.22464222143356,0.220710052502994,0.676357292891917
"5031","AP005205.2",4.69337652407809,-1.00939263075145,0.824293566229431,-1.22455478497632,0.220743012684997,0.676357292891917
"5032","MAST2",77.3566108773078,-0.283479242720246,0.231587958101408,-1.2240672833089,0.220926846764493,0.676786037932167
"5033","BLOC1S2",215.694205379332,-0.321428485109024,0.262668372665332,-1.22370455889853,0.221063699253339,0.677070718058857
"5034","SDHB",261.1543223729,-0.266859022398989,0.218156903201681,-1.22324353931759,0.221237725267439,0.677230951825496
"5035","UBLCP1",160.882366512545,-0.250727988046682,0.20499377835857,-1.22310047677698,0.221291748578983,0.677230951825496
"5036","ZNF487",43.7443667308499,-0.389385064025588,0.318299184771645,-1.22333038428905,0.221204935459157,0.677230951825496
"5037","MAGEH1",320.524466050607,-0.327318368608106,0.267598937078068,-1.2231676709262,0.221266373530586,0.677230951825496
"5038","POU5F2",32.6202276997206,0.51701320165479,0.422767049170501,1.22292691133145,0.221357303097225,0.677268014499559
"5039","DST",1584.43057319044,0.323823773508549,0.26482025029881,1.22280593399924,0.221403003663764,0.677268014499559
"5040","PRR5L",22.7134551433686,0.642994078568754,0.525872113873367,1.22271948180084,0.221435666109489,0.677268014499559
"5041","LMCD1-AS1",10.8568323285112,0.615850089080261,0.503793946242967,1.22242455208712,0.221547119284787,0.677299274191813
"5042","AC004540.2",7.31533663564076,1.06538218110533,0.872004199092663,1.22176267294799,0.221797388006505,0.677299274191813
"5043","FUOM",122.143099491484,0.419686563284778,0.343499516573314,1.221796663563,0.221784530596159,0.677299274191813
"5044","AC132192.1",5.41447711338746,-0.990097934757413,0.810280274241214,-1.22192032341475,0.221737759087703,0.677299274191813
"5045","ERN1",63.693407196096,-0.31716819952921,0.259536548287139,-1.22205601339165,0.221686445591173,0.677299274191813
"5046","AC087741.1",11.1888962439836,0.651988870545838,0.5333940683510

69,1.22233993445295,0.221579103488911,0.677299274191813
"5047","PPFIA3",3.27464876208687,-0.994269410534223,0.813527824101203,-1.22217013490928,0.221643295165198,0.677299274191813
"5048","LRCH2",39.8239059284155,0.361385138837539,0.295692913980257,1.22216367640676,0.221645737025475,0.677299274191813
"5049","MED19",155.474208732059,-0.228975995737141,0.187445920917886,-1.22155763441471,0.221874957933346,0.677401956138349
"5050","SLC66A1L",199.673163361113,0.555482864516859,0.455107930229382,1.22055193421237,0.222255714626903,0.677893122472045
"5051","CUTA",738.550030704895,-0.383168169187544,0.313927824640435,-1.22056134917768,0.22225214796589,0.677893122472045
"5052","ZHX1",235.567306081114,0.218651906897855,0.179101698892568,1.22082542069582,0.22215212673751,0.677893122472045
"5053","AC068790.7",4.92010603225827,0.909306274538394,0.744755711202921,1.22094568844553,0.222106584126937,0.677893122472045
"5054","ZNF213",39.046243386461,-0.327260590188911,0.268116252939623,-1.2205921371824,0.222240484865897,0.677893122472045
"5055","STAG2",500.652046831106,0.253248942038676,0.20751585318762,1.22038359069226,0.22231949493694,0.677953514234013
"5056","NPIPB13",4.49771018391116,1.00838793512335,0.826422800160947,1.2201840691314,0.222395104641367,0.67804994818961
"5057","TRNP1",3.45281746386885,-1.03218683719348,0.846732090630003,-1.21902411472971,0.222835039900869,0.678166232791736
"5058","RAB3GAP2",167.587988737921,0.238068325843832,0.195363490803842,1.21859168703567,0.222999205789479,0.678166232791736
"5059","AL591848.4",2.99732105169641,1.24383529721699,1.01995686955524,1.21949793598565,0.222655258786199,0.678166232791736
"5060","ORC4",140.778831205352,0.239170815168722,0.196553640123895,1.21682211033062,0.223671905686589,0.678166232791736
"5061","PDE6D",61.5716131113461,-0.368944553747062,0.302817825813893,-1.21837131864823,0.223082899237599,0.678166232791736
"5062","PLXNB1",38.492425223326,0.428678050108748,0.351897725362239,1.21818931812495,0.223152037945291,0.678166232791736
"5063","NSA2",1064.65187281397,-0.295621386293051,0.242734481620837,-1.21787965318758,0.223269709288438,0.678166232791736
"5064","DMGDH",27.0918604260656,0.393308817291332,0.322616677238188,1.21912115845441,0.222798210323768,0.678166232791736
"5065","RBM22",199.722102042437,-0.1940476241758,0.159444932092863,-1.21701970472654,0.22359671880079,0.678166232791736
"5066","GNL1",253.426513650293,-0.199854677031561,0.163852615042502,-1.21972223012565,0.222570191496525,0.678166232791736
"5067","BCCIP",117.066665692583,-0.222628781993054,0.182926080237039,-1.21704232499033,0.223588112689775,0.678166232791736
"5068","TMX2",187.843946801079,-0.26895693304974,0.220708318179327,-1.2186080491593,0.222992992534207,0.678166232791736
"5069","AC009318.2",3.19786011293176,0.997520686172911,0.819031102042721,1.21792772421587,0.223251439594405,0.678166232791736
"5070","SHMT2",137.28107686589,-0.285418097101296,0.234502199651951,-1.2171233256017,0.223557297120811,0.678166232791736
"5071","MAPKAPK5-

AS1",123.281285423632,-0.311504877532569,0.255786023125914,-1.21783385083253,0.223287117754154,0.678166232791736
"5072","HMGB1",3757.90247591074,-0.258458056363895,0.212284708828508,-1.21750670498217,0.22341148692088,0.678166232791736
"5073","MEDAG",1516.12886383085,0.293244279411216,0.240802814502553,1.21777762447252,0.223308489482136,0.678166232791736
"5074","CARS2",165.827149617096,-0.251728683021216,0.206667000566732,-1.2180400466979,0.223208754904198,0.678166232791736
"5075","OSGEP",126.590580211,-0.2518202298836,0.20693515513969,-1.21690405728002,0.223640721758256,0.678166232791736
"5076","ZC2HC1C",5.53099049469181,-0.786158279565316,0.645883163200322,-1.21718342319056,0.223534435782488,0.678166232791736
"5077","SETD3",116.851777326676,-0.257560149818185,0.21151261064473,-1.2177058806711,0.223335761538976,0.678166232791736
"5078","DNAJC17",97.4212246248501,-0.304383368260229,0.249981450541612,-1.2176238180903,0.223366959007094,0.678166232791736
"5079","ARMC7",22.8743548585943,0.413376293752475,0.339513412572583,1.21755511990002,0.223393078174981,0.678166232791736
"5080","SOCS6",65.3284758049006,0.253812659766808,0.208088240414167,1.21973572010429,0.222565075939226,0.678166232791736
"5081","HPN",2.54728664669621,1.91050862765438,1.56716098958384,1.2190889387578,0.222810437708044,0.678166232791736
"5082","CSNK2A1",291.471745189694,0.266991516373056,0.218989775888072,1.21919626288635,0.222769710034487,0.678166232791736
"5083","TRMT2A",60.8940969141905,-0.311507693850658,0.25557472234087,-1.21885173540436,0.222900471234016,0.678166232791736
"5084","TOMM22",313.682170252622,-0.27846202605781,0.22862026511131,-1.21672446190747,0.22370906868284,0.678166232791736
"5085","AC245140.2",3.7420542547074,1.24123471509314,1.01857905175301,1.21859438691276,0.222998180545717,0.678166232791736
"5086","AC099343.3",64.4514238281726,-0.412751822634547,0.339357303612455,-1.2162750535816,0.223880161244671,0.678293983002295
"5087","PTCH1",47.6270212958025,0.475303979361426,0.390783521966911,1.21628459912819,0.22387652622305,0.678293983002295
"5088","ZNF688",168.142400978463,-0.271649461069012,0.223346892054751,-1.2162670300441,0.223883216705525,0.678293983002295
"5089","ZNF778",14.0720198424626,0.592425966625751,0.487218283677024,1.2159354163697,0.224009525317926,0.678543295888353
"5090","MRPS15",397.415605971867,-0.317924514212253,0.261599485569708,-1.21531016591979,0.22424781606615,0.678598367620673
"5091","LINC02580",8.47797431800689,0.841288349521206,0.692029081858722,1.21568351905326,0.224105504743135,0.678598367620673
"5092","AC093388.1",2.75434332043145,-1.23167924231629,1.01323564214466,-1.21559012640857,0.224141097237977,0.678598367620673
"5093","SCAMP1",243.19757217891,0.195447417356521,0.160817778395543,1.21533464338628,0.224238483991693,0.678598367620673
"5094","LRRC8E",8.30034337313472,-0.741944962516676,0.610489419582791,-1.21532812644603,0.224240968558792,0.678598367620673
"5095","FRS3",29.0398905023358,-0.390741963861617,0.321573064767342,-1.21509543762416,0.224329693482912,0.678712899909537

"5096","PLA2G15",64.5601896706633,0.290227152072974,0.239058283059217,1.21404348913977,0.22473111763391,0.679793991037427
"5097","SNRPF",512.91971921511,-0.277024187091547,0.228424084992194,-1.21276259944749,0.225220598510147,0.681140970381384
"5098","CLCN6",63.4068992114541,0.303223506631771,0.250112129538716,1.21235026542299,0.225378330268724,0.681184928121818
"5099","LSM2",416.108004877604,-0.313613500889893,0.258725615295942,-1.21214708690969,0.225456081951185,0.681184928121818
"5100","HPS5",49.4796138400789,0.344450496572386,0.284103405250658,1.21241241817741,0.225354549682943,0.681184928121818
"5101","HIPK3",352.331674569897,-0.24398837794625,0.201227677209936,-1.21249910215732,0.22532138607087,0.681184928121818
"5102","PRMT2",437.225074480713,0.174319597320041,0.143804028311349,1.21220246308138,0.225434888882231,0.681184928121818
"5103","MRPL13",234.875646926686,-0.337892550258224,0.278832753615961,-1.21181082880818,0.225584802166673,0.681440275406481
"5104","MBLAC2",41.1603251192812,0.309881396429586,0.255802037724421,1.21141097696581,0.225737934465333,0.681602038501158
"5105","PTPA",94.4698534131883,-0.240034866229484,0.198177867947713,-1.21120924710329,0.225815219631879,0.681602038501158
"5106","VAMP1",18.6064438608791,0.495388470460949,0.408910575219757,1.21148363598744,0.225710102535383,0.681602038501158
"5107","SMIM10L1",195.012863660899,-0.277791067035633,0.229331379876251,-1.21130857532681,0.225777163419949,0.681602038501158
"5108","TMEM220-AS1",20.4546681507161,-0.413719882370544,0.34169461783942,-1.21078840804268,0.225976508997273,0.681955341854535
"5109","RPL15",12561.7226804778,-0.292431033196946,0.241573520328538,-1.21052602453796,0.226077110821703,0.682125398965854
"5110","CEP350",303.657989313187,0.290037693745885,0.239934131269505,1.20882215552777,0.226731177848612,0.682128694709167
"5111","ADIPOR1",163.462199820746,-0.259582119351694,0.214744038555887,-1.2087977906038,0.226740540630227,0.682128694709167
"5112","LINC02613",5.32294543814255,0.879345779434885,0.726798453669518,1.20988944733602,0.226321317027748,0.682128694709167
"5113","NAALADL2",125.768473675375,0.266631307325664,0.220576285397492,1.20879407704767,0.22674196767368,0.682128694709167
"5114","PGBD1",23.2397546344625,0.427703334015462,0.353711716409768,1.2091862219231,0.226591309817731,0.682128694709167
"5115","CCM2",205.715787337707,-0.262423864914295,0.217012812965339,-1.20925516483771,0.226564830071637,0.682128694709167
"5116","BAG1",528.824089304421,-0.279874306843861,0.231385777890503,-1.20955708425738,0.226448894198373,0.682128694709167
"5117","NANS",254.665844956692,-0.285661410760854,0.236169138366762,-1.20956282745645,0.226446689242736,0.682128694709167
"5118","MRE11",133.030831536502,0.271703545458677,0.22459162900881,1.20976701873434,0.226368305132888,0.682128694709167
"5119","GDE1",181.160803350742,-0.234046585788175,0.193555907377965,-1.20919371027588,0.22658843356788,0.682128694709167
"5120","PDP2",13.4498391666537,0.59837615420764,0.494851762264996,1.20

920283575186,0.226584928539993,0.682128694709167
"5121","PHF12",76.3804127085269,0.27698587930046,0.229036038766516,1.2093550027855,0.226526488006338,0.682128694709167
"5122","SLC66A2",176.928544105577,-0.217622728627992,0.179846912649918,-1.21004428389386,0.226261900713759,0.682128694709167
"5123","ZNF442",5.50902244072715,0.792802887449631,0.655810436500389,1.20889031848941,0.226704986129945,0.682128694709167
"5124","FTSJ1",66.5006393237163,-0.31346356688081,0.25926516272719 8,-1.20904622735851,0.226645086030248,0.682128694709167
"5125","BRK1",1308.91255452416,-0.255214110376602,0.211244907004532,-1.20814325891003,0.226992162680091,0.682614941028796
"5126","PFDN6",165.569927821997,-0.325127758200802,0.269111153483984,-1.20815415485985,0.226987972313834,0.682614941028796
"5127","CUX1",209.483687747268,0.206535741341366,0.170994317476584,1.20785149114473,0.227104391293436,0.682819229917751
"5128","SENP6",343.779441410479,0.230606762936399,0.19099789511814,1.20737855667864,0.227286389795754,0.683099960752887
"5129","YIF1B",102.768644934667,-0.347563473814466,0.287850344064981,-1.20744505254441,0.227260794033747,0.683099960752887
"5130","AL645728.1",8.43301002667912,-0.680376994079441,0.563636156562944,-1.20712091684888,0.227385580597542,0.683215311987691
"5131","SIL1",283.459774923102,0.283801375631905,0.235120082704573,1.20704863815695,0.227413413286334,0.683215311987691
"5132","SNX15",134.90190834209,-0.305628799499774,0.253284426720995,-1.20666242080663,0.22756217693715,0.683529025231133
"5133","ZNF638",417.350402141943,0.264268105108223,0.219032604935565,1.20652404780541,0.227615492484161,0.683555974409378
"5134","NOL9",85.6345821214834,0.239352223221921,0.198443339262642,1.20614893959799,0.227760067616508,0.683575219483606
"5135","BORCS7",469.116176888705,-0.234778851828784,0.194641989109294,-1.20620865468526,0.227737047701562,0.683575219483606
"5136","TSG101",260.603294689666,-0.291558550315188,0.241747207804233,-1.20604722992827,0.227799280083508,0.683575219483606
"5137","LINC00324",22.7154924326409,-0.443223978762794,0.367485871097725,-1.2060980125272,0.227779701098084,0.683575219483606
"5138","P2RX4",74.0924370578514,0.292405281377909,0.242495864702316,1.20581553725405,0.227888623290416,0.683710223437477
"5139","CAVIN1",3281.89105379417,-0.248895609078282,0.206454085719793,-1.2055736664669,0.227981917921184,0.683723981469854
"5140","STXBP2",6.54793939746247,-0.823565263772203,0.683095052971238,-1.20563786868309,0.227957151128674,0.683723981469854
"5141","NEDD4L",10.8764028215264,0.560126330433067,0.464738616942955,1.20525024177584,0.228106712117669,0.683965175509409
"5142","SIX5",95.6008148029424,0.298713652001128,0.247886258782808,1.20504320597639,0.228186622921269,0.684071721573581
"5143","MIIP",54.5839178492503,-0.363500935634103,0.302601128131639,-1.20125439676474,0.229652534333597,0.68416563407269
"5144","CEPT1",91.5017509654408,0.254765446113684,0.211874831436568,1.20243374064916,0.229195523259376,0.68416563407269
"5145","AL358472.4",10.5525631665052,-0.639172981234599,0.530757477840

83,-1.20426561644466,0.228486931734989,0.68416563407269
"5146","XCL2",5.36587245929376,1.07229413367981,0.891094417664337,1.20334513652371,0.22884278915969,0.68416563407269
"5147","PAPPA2",3.60999722926239,1.25614845714923,1.04471734961286,1.20238115851595,0.229215885723239,0.68416563407269
"5148","ACKR2",45.5435593453181,0.486441203874174,0.404092787669821,1.20378590936802,0.228672337230378,0.68416563407269
"5149","KCNMB2",8.0319263974253,0.848490255410075,0.706745873175958,1.20055919335921,0.229922238199366,0.68416563407269
"5150","UCHL1",80.01992947679,-0.296450908775597,0.247009212467164,-1.20016134545996,0.230076684432724,0.68416563407269
"5151","FAM241A",55.183679350377,-0.436469695318154,0.362829494237893,-1.20296090105613,0.228991451211061,0.68416563407269
"5152","SH3RF1",106.319331733,0.262616539744986,0.218260469496828,1.20322539555796,0.228889109988338,0.68416563407269
"5153","MXD3",8.79722763126057,0.641326269844086,0.532977059064993,1.2032905712099,0.228863896484369,0.68416563407269
"5154","GOPC",425.556264625738,0.182883886647338,0.152019161608387,1.20303180673013,0.228964012385756,0.68416563407269
"5155","Z94721.3",3.30957625499403,1.09862139132121,0.912228125403885,1.20432747108602,0.228463032876326,0.68416563407269
"5156","SEM1",1152.430092415,-0.294905757726183,0.245071510745142,-1.20334573704434,0.228842556869937,0.68416563407269
"5157","LINC01004",11.711271302181,-0.630985793855275,0.525159683314185,-1.2015122521844,0.229552556675564,0.68416563407269
"5158","FLNC",222.822667829659,0.375318867476683,0.312522159552387,1.20093521692746,0.229776332058007,0.68416563407269
"5159","ADAM28",2.36954108851682,-1.15321519601615,0.960047190971896,-1.20120678114657,0.229670999609754,0.68416563407269
"5160","AC113139.1",4.4591162622882,-1.19009274919876,0.988254348452899,-1.20423730091533,0.228497872637096,0.68416563407269
"5161","ANKRD46",63.5807400751862,0.33653171096127,0.279568162591659,1.20375549147495,0.228684097274813,0.68416563407269
"5162","RNF121",77.6461803286015,-0.247249619754585,0.205251831485725,-1.20461590020833,0.22835161564366,0.68416563407269
"5163","SLC35E3",158.639354849255,0.259738070207609,0.215997298525445,1.20250610531136,0.229167502103869,0.68416563407269
"5164","ELK3",225.50053884305,-0.287550317072248,0.239499169343933,-1.20063179283645,0.229894062733352,0.68416563407269
"5165","SH2B3",32.7229166243839,-0.500921714310773,0.416299215828757,-1.20327325938761,0.228870593450455,0.68416563407269
"5166","SPART-AS1",5.0388208568739,0.833212768852561,0.693380586214454,1.2016672883813,0.229492459774464,0.68416563407269
"5167","GNPNAT1",115.200439301773,-0.284068114804331,0.236321158538933,-1.20204266330021,0.229346999027666,0.68416563407269
"5168","TLNRD1",248.209480931898,0.327121219570577,0.272359056392954,1.20106606294968,0.229725576120407,0.68416563407269
"5169","ELOB",1801.21169824949,-0.281399044653326,0.23445807864786,-1.20021048656621,0.230057603655315,0.68416563407269

"5170","AC009152.6",4.16622259695924,-1.30166929582901,1.0820195541164
,-1.2029997894927,0.228976402012504,0.68416563407269
"5171","NUPR1",3760.24820700557,0.268524084806603,0.223654461753142,1.
20062029034317,0.229898526625303,0.68416563407269
"5172","CDH11",374.298478433961,0.372619149802474,0.310477781549543,1.
20014755304806,0.230082040028078,0.68416563407269
"5173","MIEF2",36.7121682395617,0.339916738897316,0.283208607197529,1.
2002344923798,0.23004828295591,0.68416563407269
"5174","ERAL1",81.5790669963559,-0.28762362802411,0.239413789541864,-1
.20136617266073,0.229609191932951,0.68416563407269
"5175","RETREG3",144.002110658088,0.230895405531648,0.191970282908522,
1.20276639713906,0.229066731660911,0.68416563407269
"5176","DCAF7",220.542509191374,0.251173890177403,0.208817770774675,1.
20283771465232,0.229039127012486,0.68416563407269
"5177","SCAMP4",118.874330647299,0.266422741900766,0.221338588164699,1
.20368862975904,0.228709948598154,0.68416563407269
"5178","MAP1S",88.5488544058705,0.261576657054735,0.217584491374158,1.
20218428897549,0.229292135009306,0.68416563407269
"5179","SIPA1L3",31.2652085128569,0.392490869388782,0.32664406460028,1
.20158579911464,0.229524046178827,0.68416563407269
"5180","LYPD3",3.0658727483447,-1.18252160980027,0.984598182463666,-1.
2010194928873,0.229743640005869,0.68416563407269
"5181","KDELR1",1265.8716695088,-0.253930616769418,0.211378809512093,-
1.20130592728544,0.229632552053201,0.68416563407269
"5182","TMEM86B",7.52058221068381,-0.67759733064065,0.562826170563825,
-1.20391937347521,0.228620742959428,0.68416563407269
"5183","TGIF2",20.8039864968595,0.469484417879511,0.390234848797612,1.
20308173225964,0.228944693786239,0.68416563407269
"5184","CHM",172.290119082699,0.201197664371856,0.167488477649616,1.20
126272084674,0.229649306373646,0.68416563407269
"5185","TSPAN5",32.8710201258697,-0.717664267884446,0.598368264734368,
-1.19936886726945,0.230384547620617,0.684642246913471
"5186","AC011978.2",3.74831497848461,0.96922919807059,0.80840846099634
4,1.19893499985891,0.230553221130576,0.684642246913471
"5187","GRINA",628.651605971019,0.165615620504519,0.138133866667868,1.
19895015248309,0.23054732880516,0.684642246913471
"5188","CD81",7919.1025289026,-0.265134993260712,0.221094522737529,-1.
19919295140326,0.230452927399183,0.684642246913471
"5189","ARRB2",44.0830279911345,-0.373476630902201,0.311483346901581,-
1.19902599807433,0.230517836717145,0.684642246913471
"5190","AP001033.3",2.45612099103062,-1.11128704571392,0.9265443095292
06,-1.19938899228531,0.230376725799945,0.684642246913471
"5191","UQCRFS1",459.059013323211,-0.281171004485829,0.234477015021117
,-1.19914100945249,0.230473120372486,0.684642246913471
"5192","MYL3",18.1203634532626,0.494136903002563,0.41238428345236,1.19
824378093606,0.230822125815743,0.685308757598167
"5193","SP110",207.745082936213,0.218660800791338,0.182533491748286,1.
19792153591666,0.230947564843639,0.68531787548737
"5194","MTDH",1442.52244847369,-0.149520151544781,0.124819260104524,-1
.19789326919236,0.230958570428601,0.68531787548737

"5195","B4GALNT1",3.22544900682472,-1.01079706708328,0.843741042917803
,-1.19799442680632,0.230919186660638,0.68531787548737
"5196","PSMA7",1607.88824867342,-0.255785246549245,0.213554417162889,-
1.19775207625017,0.231013549160045,0.685349087817956
"5197","SRRD",85.4520789643772,-0.26354099562211,0.220088171904431,-1.
19743370732594,0.231137552082237,0.685585023157147
"5198","ANKRD13C",99.9042142202964,0.233345745844435,0.194921630400184
,1.19712596988525,0.231257459040857,0.685676809216158
"5199","ATF3",3206.56965870544,-0.328452229647531,0.274363076248195,-1
.19714443407977,0.231250263397692,0.685676809216158
"5200","PRUNE1",45.1755021511837,0.349980543699247,0.292472898594476,1
.19662555190971,0.231452536471244,0.685793446846619
"5201","FAM89A",28.4341894426152,0.437342389668721,0.365549629143261,1
.19639675382443,0.231541767520938,0.685793446846619
"5202","RBM34",15.6677067314718,-0.475682880474395,0.397484433756707,-
1.19673335626912,0.231410501333262,0.685793446846619
"5203","EFHC1",205.730642205477,-0.219057094073293,0.183123439404909,-
1.19622640763605,0.231608218247389,0.685793446846619
"5204","PSMD8",863.711775748657,-0.305007113672883,0.254934030478736,-
1.19641584569983,0.231534320772372,0.685793446846619
"5205","RGS19",31.036309278419,-0.383674025925648,0.320715407325384,-1
.1963068102194,0.231576852136013,0.685793446846619
"5206","THAP7-
AS1",41.0122301214924,-0.402167199654703,0.336130322963946,-1.19646212
251383,0.231516271297628,0.685793446846619
"5207","S100A1",4.53303382677645,-1.15175825548758,0.963039379095992,-
1.19596174412799,0.231711488293858,0.685936831454473
"5208","CD74",400.204945008275,-0.466057980941813,0.389870200465987,-1
.19541832226409,0.231923630589732,0.685936831454473
"5209","NUDT3",189.147049773204,0.243765300049084,0.203902402973089,1.
19549988864651,0.231891779722571,0.685936831454473
"5210","METTL23",181.283074638909,-0.296106601159946,0.247699880086506
,-1.1954248869904,0.231921067013989,0.685936831454473
"5211","KLF16",26.3768190720748,-0.375848501762402,0.314387777910059,-
1.19549336256299,0.231894327979462,0.685936831454473
"5212","AC005261.1",269.03500558262,0.477007998452968,0.39900579601190
4,1.19549140193126,0.231895093556342,0.685936831454473
"5213","MTURN",624.936967512609,0.265129558896999,0.221878799511446,1.
19492966196314,0.23211451265593,0.686369693572062
"5214","CHMP2B",397.255970995774,-0.216015115800323,0.180913555482941,
-1.19402393714324,0.232468604660302,0.686713936340303
"5215","SEPTIN7",1632.99590946915,-0.172148150171516,0.1441449613214,-
1.19427102129278,0.232371969416981,0.686713936340303
"5216","REEP4",28.2900868071433,0.370904623733293,0.310635643053487,1.
19401823978528,0.232470833247897,0.686713936340303
"5217","TNFRSF10D",27.6810658818207,0.591433391472499,0.49520649028048
,1.19431672056139,0.232354099440447,0.686713936340303
"5218","RCL1",40.7302997604452,-0.408286638466268,0.341826781507222,-1
.19442554110595,0.232311550813144,0.686713936340303
"5219","CPAMD8",2.84455350907328,1.33087454061722,1.11468362124577,1.1

9394823360716,0.232498218213431,0.686713936340303
"5220","SMIM13",66.0436431678777,-0.323026125828306,0.270640699200639,-1.19356078661632,0.232649820836619,0.686885884531718
"5221","ATP6V1H",124.847592392033,0.20433590552265,0.171202087478891,1.19353629696743,0.232659405652052,0.686885884531718
"5222","HYKK",40.601450261516,-0.315291674740894,0.264183336212301,-1.1934578435618,0.232690112813794,0.686885884531718
"5223","AC018638.6",4.40107246072225,1.24147381662854,1.04033320700195,1.19334248707319,0.232735269298924,0.686887646226866
"5224","FSD1L",25.089277420043,-0.386469723446182,0.323946144702832,-1.19300608994963,0.232866987998576,0.68703894823152
"5225","LINC01550",29.668399571782,0.501127750561915,0.420062454351235,1.19298391315615,0.232875673338287,0.68703894823152
"5226","ERICH2",10.5615383079022,-0.618803039719613,0.518990866434908,-1.1923197106923,0.233135908711043,0.687193054137891
"5227","TNFRSF21",38.795391811632,0.645257869304901,0.541195815548674,1.19228170426766,0.233150805912499,0.687193054137891
"5228","CDH5",6.61549665138935,1.04978616645263,0.880159611973378,1.19272249279757,0.232978073567156,0.687193054137891
"5229","RAB8A",270.892997950085,-0.225037378496504,0.188717759764142,-1.19245469413029,0.233083005337079,0.687193054137891
"5230","HSD17B10",239.719931094974,-0.364253976769967,0.305475932179307,-1.19241465005551,0.233098698719804,0.687193054137891
"5231","CBLB",484.237547302927,0.309700666632577,0.259801151718674,1.19206810510193,0.233234541942248,0.687252923392567
"5232","AL121832.2",10.1996913657046,-0.630218469047798,0.528750953008928,-1.19190039367581,0.233300303814626,0.687252923392567
"5233","MCAT",73.0007636669779,-0.298132777371113,0.250134734855818,-1.19188875364696,0.233304868512053,0.687252923392567
"5234","ADAR",333.602257769059,0.223336504893205,0.18749674260207,1.19114871967242,0.233595206669468,0.687319748507469
"5235","SP5",66.3137425062245,-0.600499734301162,0.504152342687197,-1.19110769395699,0.233611309813275,0.687319748507469
"5236","CCR1",15.1575353252497,0.742896044288725,0.623423467660477,1.19163952405673,0.233402620378022,0.687319748507469
"5237","CD200",47.1829202209439,0.356359974319558,0.299249419677236,1.19084599964779,0.233714046860827,0.687319748507469
"5238","CCDC112",92.1299138632545,-0.267020479051633,0.224360023008645,-1.19014285821028,0.233990247685349,0.687319748507469
"5239","CUL7",44.3135794319448,0.328423331966294,0.275839848991903,1.19063048057256,0.233798680286549,0.687319748507469
"5240","CDK5",24.9425810089718,-0.427188831360762,0.358868833342938,-1.1903759582057,0.233898658111252,0.687319748507469
"5241","AJM1",3.52449879572521,-0.888764316443004,0.746186089475861,-1.19107596480027,0.233623764472686,0.687319748507469
"5242","JCAD",17.6385767846574,-0.705810438677038,0.593054584031478,-1.19012727948086,0.233996369780551,0.687319748507469
"5243","METTL15",88.3959321205559,0.263870900898362,0.221521080938548,1.19117738041176,0.233583957411582,0.687319748507469
"5244","TCIRG1",152.458391387335,-0.215667747626839,0.181061726287625,

-1.19112830772551,0.233603218534163,0.687319748507469
"5245","TRHDE-
AS1",2.92769614474174,-1.16340837586114,0.977171893577175,-1.190587227
80309,0.23381566808233,0.687319748507469
"5246","SERPINA3",198.243259036852,-0.771731530005101,0.64844340959100
1,-1.19012934450496,0.233995558265346,0.687319748507469
"5247","MAPK8IP3",35.1598955135528,0.494511663960586,0.415380699024086
,1.19050226725126,0.233849039417473,0.687319748507469
"5248","STAT3",1025.49920984426,-0.18213320837816,0.15297462001023,-1.
19061062786742,0.233806477452067,0.687319748507469
"5249","DCTN3",697.785798973335,-0.322082278768178,0.270716740246547,-
1.18973905520158,0.234148969974622,0.687375047068901
"5250","HPS6",32.6440586662616,-0.367505944434056,0.308892730846122,-1
.18975264787676,0.234143625880175,0.687375047068901
"5251","TRIP4",80.6189689916307,-0.295856783304124,0.248670217023893,-
1.18975560018793,0.234142465161314,0.687375047068901
"5252","RP2",62.0630059748937,-0.280827957710345,0.236127501747613,-1.
18930643670008,0.234319103360086,0.687743522143133
"5253","GMIP",10.1831314875391,-0.549982732940042,0.46254513297391,-1.
18903582317245,0.234425570497242,0.687925027453832
"5254","HEMK1",58.4676607030333,0.330943646897536,0.278388277840114,1.
1887844181701,0.234524511150153,0.688084381305599
"5255","FBXL22",3.48665691677772,-0.99242274462003,0.835437383788727,-
1.1879079915234,0.234869660879588,0.688965676309486
"5256","YPEL1",35.6589217398019,0.394878845569016,0.332447033523215,1.
18779476352717,0.234914277955411,0.688965676309486
"5257","MTRF1L",108.109130324442,-0.239505540854882,0.201667271433874,
-1.18762721958786,0.234980309038641,0.689028241169993
"5258","MOV10",44.5622184504767,0.326808495158746,0.275425832310616,1.
18655716646862,0.235402339839791,0.689099994463742
"5259","NPR3",44.6322762172271,1.21623744464934,1.02498407983395,1.186
5915467159,0.235388771874946,0.689099994463742
"5260","SRA1",177.643550869949,-0.373266136717852,0.314395214908026,-1
.18725132895883,0.235128499871672,0.689099994463742
"5261","TRBC1",17.0660928573254,0.716468290779296,0.60376563552266,1.1
866662304473,0.235359300281349,0.689099994463742
"5262","SARAF",873.563457377728,-0.219662628337054,0.185124390983304,-
1.18656772978589,0.235398171029096,0.689099994463742
"5263","CARS",92.1827721215128,-0.227054294319784,0.191357494370743,-1
.1865450844579,0.235407108066563,0.689099994463742
"5264","RPS2",16590.7264112148,-0.272113467868365,0.22928586443143,-1.
18678693317242,0.23531167425599,0.689099994463742
"5265","LINC01431",4.7037124228019,0.84416920947129,0.711365133359689,
1.18668904319837,0.235350298464965,0.689099994463742
"5266","FANCB",6.81577604543039,-0.666389718691065,0.561566227510431,-
1.18666274082283,0.235360677294095,0.689099994463742
"5267","FSBP",12.9584575198908,0.554656780229906,0.467686932529359,1.1
8595740366359,0.235639121803272,0.689255609601031
"5268","EIF2S1",222.037323313843,-0.22370582320774,0.188615723702271,-
1.18604016047389,0.23560644000149,0.689255609601031

"5269","ODF3L1",6.35979735893477,-0.8041958502378,0.677998869796749,-1
.18613154986362,0.235570352802452,0.689255609601031
"5270","VASP",85.7346215555842,-0.30759107069968,0.259349801634282,-1.
18600850573784,0.235618940512994,0.689255609601031
"5271","AC127070.1",3.79534472233989,-1.05503383072258,0.8897572228427
63,-1.18575472458854,0.235719176103929,0.689292406593316
"5272","IMPA2",253.620837727498,-0.452585874245129,0.381703804843554,-
1.1856991429012,0.235741133153419,0.689292406593316
"5273","RFWD3",30.1558350516794,0.370244047562762,0.312317804545843,1.
18547211261668,0.235830834468376,0.68929319554987
"5274","MRPL54",390.738273959108,-0.339407542713108,0.286302122567118,
-1.18548734347417,0.235824815890352,0.68929319554987
"5275","COG8",58.2742114266967,0.317893075762465,0.268284601880155,1.1
8490988127776,0.236053080096527,0.689801711224922
"5276","ULK2",95.706505105206,0.300757582591893,0.253845507774814,1.18
480561357302,0.236094312580129,0.689801711224922
"5277","TPRG1-
AS1",2.96338022330282,-1.02038143654266,0.86153580206976,-1.1843749663
0005,0.236264665270268,0.690168621402535
"5278","GPN2",67.3599361406995,0.271230001338976,0.229275776710088,1.1
8298585760299,0.236814752362992,0.691104837105737
"5279","TATDN2",51.8042997238148,0.322020116068908,0.272185969564615,1
.18308859411088,0.23677403772664,0.691104837105737
"5280","BCL6",428.239529144238,0.351735881884896,0.29735388926993,1.18
288643457291,0.23685415857474,0.691104837105737
"5281","SLC2A8",37.5602093954744,-0.402888505607378,0.340586293029212,
-1.18292636507489,0.236838331606042,0.691104837105737
"5282","ARHGAP19",38.5379829405005,0.336998796836455,0.284860652452236
,1.18303034812069,0.236797120094813,0.691104837105737
"5283","ST20-
AS1",12.3468124520832,-0.562004273916036,0.475033518860947,-1.18308340
696385,0.236776093282383,0.691104837105737
"5284","LUZP1",204.421764664103,-0.260272209367718,0.220096542764381,-
1.18253656372216,0.236992866365114,0.691378697013291
"5285","SSH1",68.6679737320578,0.393795231245618,0.333137516671862,1.1
820801066771,0.237173917001361,0.691618304485191
"5286","SACS",122.670129685797,0.281598863787313,0.238241289844436,1.1
8199017463005,0.237209599468907,0.691618304485191
"5287","ERVK3-1",183.870384124838,-0.246932516706656,0.208885908720676
,-1.1821406155111,0.237149910958958,0.691618304485191
"5288","PCDHGA10",42.4013287002369,0.422693281055251,0.357765339325667
,1.181481922905,0.237411330534869,0.692075578705562
"5289","GAS6-
DT",7.71360722746169,0.758530814780889,0.642165884422947,1.18120696408
92,0.237520515418696,0.692262950497107
"5290","HPGDS",3.31577697618562,-1.31014192160277,1.10981247539179,-1.
18050747369754,0.237798439733493,0.692680073411149
"5291","CLDN23",19.2845449354043,-0.730119402238322,0.618425412104198,
-1.18061028532784,0.237757575814589,0.692680073411149
"5292","AC105339.6",3.17886963350121,1.15600706808971,0.97920220420145

8,1.18056011631677,0.237777515569355,0.692680073411149
"5293","RCSD1",5.42208081747678,-0.885333909541024,0.751071706357327,-1.17876083208468,0.2384934248808,0.693405481397708
"5294","LIN9",16.1713784493488,0.435213729266517,0.369288519044304,1.17851952287286,0.238589553921422,0.693405481397708
"5295","MTHFD2",46.6364508774079,-0.573850218805312,0.486753183535122,-1.17893470082236,0.23842417868l972,0.693405481397708
"5296","AC009948.3",4.7605577148584,-0.783779994276853,0.665227373481433,-1.17821368380405,0.238711428657965,0.693405481397708
"5297","PIKFYVE",83.1123420381034,0.28125179769597,0.238586717040034,1.17882420775662,0.238468182791556,0.693405481397708
"5298","TUSC2",127.796348486826,-0.26008561552727,0.220587689213452,-1.1790577092251,0.238375197053933,0.693405481397708
"5299","PPM1M",98.6719850860531,0.311974250754716,0.264632416564475,1.17889657965885,0.238439359872626,0.693405481397708
"5300","RAB6B",23.0645764946646,0.537280791090479,0.455988691644279,1.1782765689935,0.238686365761264,0.693405481397708
"5301","SPRY1",1522.5400911294,0.320248065468885,0.27169375611998,1.17870969889887,0.238513792247523,0.693405481397708
"5302","AC095055.1",2.68989399842517,-1.02564210009044,0.869500787394631,-1.17957581517973,0.238168966568244,0.693405481397708
"5303","CARMIL1",194.232292024637,0.243516804534587,0.206467542734498,1.17944351596091,0.23822161588643,0.693405481397708
"5304","RPS12",20653.9561358699,-0.280829819373222,0.238357669479924,-1.17818663014271,0.238722211467897,0.693405481397708
"5305","FMNL3",27.6920715218283,-0.474582886351423,0.40235203440459,-1.17952152784246,0.238190569566508,0.693405481397708
"5306","MX1",103.436682204088,-0.476109752919421,0.404051472241277,-1.17833935928617,0.238661342538514,0.693405481397708
"5307","RPL10",37007.2604544737,-0.305862230094889,0.259468738338147,-1.17880185510549,0.238477085481738,0.693405481397708
"5308","ARHGAP39",5.70700120317949,0.836301849842543,0.709950440318168,1.17797215460244,0.238807707402065,0.693523136699856
"5309","CD84",6.91220739895257,-0.850083019252419,0.721724849899938,-1.1778491753059,0.238856740125512,0.693534874559195
"5310","C12orf65",104.425833930922,0.242846197205929,0.206198118942438,1.17773235978802,0.238903321884275,0.693539492814707
"5311","DAP3",188.413757981467,-0.205926033184817,0.175406417785788,-1.17399372146292,0.240397540471387,0.694067716543761
"5312","CNIH4",427.179251380482,-0.28447160233001,0.242597429704758,-1.17260765159884,0.240953179587885,0.694067716543761
"5313","CMPK2",12.5401347851081,-0.650414982392778,0.55255741588585,-1.17709936324001,0.239155849236146,0.694067716543761
"5314","HADHA",758.172342699873,-0.207351613214809,0.176536182795552,-1.17455588951385,0.240172439554502,0.694067716543761
"5315","EDEM1",81.18525245426,-0.315173912477209,0.268918712978776,-1.17200439116368,0.24119529316712,0.694067716543761
"5316","AC018809.2",7.35592053866749,0.751128602770103,0.639772934226063,1.17405498511554,0.240373002327977,0.694067716543761
"5317","RBM5-

AS1",7.40847694370854,0.845542905761938,0.721529120836922,1.1718763405
9893,0.241246707231443,0.694067716543761
"5318","PRICKLE2",126.675120092024,0.347120731234387,0.295789201349123
,1.17354091917871,0.240578957673145,0.694067716543761
"5319","PARP14",257.867619430899,0.229158902480777,0.194810580088267,1
.17631651410794,0.23946841905632,0.694067716543761
"5320","CLCN3",189.037436327891,0.231912292202536,0.197834869281867,1.
17225185349968,0.241095955495254,0.694067716543761
"5321","HMGB2",267.715983615339,0.297996140214334,0.253843507840503,1.
17393642543568,0.240420491045208,0.694067716543761
"5322","CFAP97",359.979076287548,-0.190435034515865,0.161890162398059,
-1.17632246268071,0.239466042871469,0.694067716543761
"5323","SLC1A3",66.7033766956739,0.438719887193022,0.373528712926394,1
.17452787967996,0.240183651614526,0.694067716543761
"5324","HOTAIRM1",52.517845584893,-0.546996384414551,0.466500364423274
,-1.17255296272017,0.240975121458959,0.694067716543761
"5325","LAMTOR4",1177.48098428154,-0.279916883689248,0.237959867835504
,-1.17631971405677,0.239467140819922,0.694067716543761
"5326","IFRD1",563.127906773474,0.358115018324491,0.304972878205217,1.
17425202015346,0.240294095166163,0.694067716543761
"5327","AC124067.2",4.0056273402288,-1.07333053976263,0.91523848963290
3,-1.17273317492705,0.240902823359062,0.694067716543761
"5328","SLC45A4",6.99507337123662,0.803275769232037,0.684695094767038,
1.17318756242199,0.240720598578245,0.694067716543761
"5329","ZNF251",45.5297522969689,0.31780454570575,0.27041800647071,1.1
7523440784692,0.239900948049876,0.694067716543761
"5330","ACER2",4.35962935429188,0.869901705527276,0.741634934180655,1.
17295136115497,0.240815311168017,0.694067716543761
"5331","TRMT10B",82.9627350201288,0.291058601213732,0.24745436507499,1
.17621122232185,0.239510481098158,0.694067716543761
"5332","IDNK",52.9736797719147,-0.350228374550222,0.29879602231785,-1.
17213198433297,0.241144070427504,0.694067716543761
"5333","PTGES",904.65920486297,-0.309829136691974,0.264162154335577,-1
.1728748104408,0.240846012300588,0.694067716543761
"5334","NTNG2",12.7787161458058,0.579622430655611,0.494633188579379,1.
17182276490651,0.241268220898836,0.694067716543761
"5335","NACC2",49.0177736301187,0.366258180348221,0.311949737272878,1.
17409356888747,0.240357549141675,0.694067716543761
"5336","CELF2",1552.62154738651,0.272963219313367,0.232138754459115,1.
17586234125092,0.239649889600158,0.694067716543761
"5337","LHPP",34.809452499233,0.432031501003861,0.36835077137157,1.172
88067402485,0.240843660576822,0.694067716543761
"5338","PSMA1",606.849582454434,-0.271811081187797,0.231225833254187,-
1.17552211776007,0.239785893732159,0.694067716543761
"5339","RCE1",54.4261208049456,-0.357700875149408,0.304241098730829,-1
.1757151701121,0.239708714496235,0.694067716543761
"5340","ST3GAL4",243.525396763178,-0.353387228677666,0.300295832487624
,-1.17679697966581,0.239276548433044,0.694067716543761
"5341","MTERF2",100.844781773116,0.325958130477649,0.277157643724658,1
.1760748363176,0.239564972426007,0.694067716543761

"5342","ACACB",104.589474958272,0.27814848793062,0.237134026662468,1.17295898798417,0.240812252532473,0.694067716543761
"5343","POSTN",1517.47832027358,-0.788677963895911,0.671452608672425,-1.17458470442949,0.240160905613114,0.694067716543761
"5344","NAXD",70.2635815080482,0.27937218498087,0.238161351250012,1.17303745345145,0.240780786618771,0.694067716543761
"5345","EVL",271.881178304746,-0.169717239184509,0.144729577411622,-1.17265069255208,0.240935912001523,0.694067716543761
"5346","BCKDK",140.166455707208,-0.272872128995823,0.232237907584649,-1.17496808266051,0.240007485359364,0.694067716543761
"5347","TXNDC17",302.728545522083,-0.254783938323623,0.217004429090074,-1.17409556750506,0.240356748694421,0.694067716543761
"5348","PSMD11",347.413445903938,-0.209594076754505,0.178462118697719,-1.17444574951795,0.240216529634182,0.694067716543761
"5349","NAT9",73.1573403084687,-0.281814929091046,0.240001082131145,-1.17422357677976,0.240305484834597,0.694067716543761
"5350","CYBC1",170.080492542452,-0.241377420021307,0.205890180077747,-1.17236004131018,0.241052535210032,0.694067716543761
"5351","SIN3B",99.5852407345181,0.280084557010472,0.238552649662608,1.17409954325221,0.240355156411472,0.694067716543761
"5352","MRPS12",198.923686661008,-0.314773851671637,0.267569401427072,-1.17641946348425,0.239427297798891,0.694067716543761
"5353","CYTH2",218.569513918149,-0.254668127627105,0.217337253298087,-1.17176472860738,0.241291527275901,0.694067716543761
"5354","CNOT3",88.9743013877175,0.239031509011024,0.203919196077108,1.17218738406873,0.241121832388109,0.694067716543761
"5355","PRELID3B",98.5896513342387,-0.237709168259259,0.202609127166324,-1.1732401772015,0.240699504542915,0.694067716543761
"5356","TFIP11",53.9373587114326,0.322483733550937,0.274629370061703,1.17425071280061,0.240294618665182,0.694067716543761
"5357","POLDIP3",154.916090488137,0.201577016732486,0.171964643146837,1.1722003607472,0.241116623620979,0.694067716543761
"5358","PPARA",104.586856424635,0.309760004160642,0.263508616780292,1.17552134706439,0.239786201879041,0.694067716543761
"5359","LMF2",328.356626485581,-0.243245974881313,0.207045978633955,-1.17484037355469,0.240058584298774,0.694067716543761
"5360","STIM2-AS1",2.45986620587675,1.20939965678329,1.03225822912984,1.17160573067339,0.241355386224143,0.694121880344246
"5361","C11orf24",112.037887585834,-0.239978037930334,0.204872437246481,-1.17135345855049,0.24145673165466,0.69425151645309
"5362","FMN1",7.37368144474219,-0.718079256812872,0.613077830440981,-1.17126932529327,0.241490537218389,0.69425151645309
"5363","ZXDC",50.8670094323421,0.342887254978127,0.292869113740272,1.17078667189949,0.241684536440446,0.694420713742679
"5364","HINT1",1559.94750742944,-0.276716745646588,0.236334902401475,-1.1708670316351,0.241652228787388,0.694420713742679
"5365","IPO8",79.5593340226604,0.315654400651902,0.26956849515358,1.17096176417821,0.241614146625931,0.694420713742679
"5366","INPP5B",41.8975144291164,0.419497301328864,0.358519464497159,1

.17008236056926,0.241967826315091,0.694514333809666
"5367","FGGY",32.9115833366739,0.379884508447582,0.324774724818624,1.16968618373777,0.24212728019088,0.694514333809666
"5368","NBAS",129.962424558266,0.198247602195474,0.16945912169278 8,1.1698845138291,0.242047446745049,0.694514333809666
"5369","MRPS9",116.074326923708,-0.283111362888868,0.242036863191385,-1.16970348712958,0.242120314351015,0.694514333809666
"5370","BCHE",26.6704004474377,0.630760175990687,0.538907546471133,1.17044227738324,0.241823030491895,0.694514333809666
"5371","TADA2B",55.7959709145632,0.2687720830893,0.229817746792085,1.16950099303017,0.242201841387021,0.694514333809666
"5372","KRBA1",13.4473926246671,0.552161835843202,0.4719826978295,1.16987728232925,0.242050357301994,0.694514333809666
"5373","SGCA",30.7176440623817,1.41690535742535,1.21046016198943,1.1705510035924,0.241779301542752,0.694514333809666
"5374","NXT1",180.337495279355,-0.321682277941616,0.274947298126018,-1.16997795626338,0.242009839953116,0.694514333809666
"5375","TGM2",3.08868134807669,-1.43717578177728,1.2289078734316,-1.16947397998526,0.24221271868704 3,0.694514333809666
"5376","PPP2R3B",45.5214800519541,-0.303331877468875,0.259211571033743,-1.17020963323195,0.241916617097038,0.694514333809666
"5377","SIPA1",78.3766010488213,0.299015754122965,0.255848467100353,1.1687220858184,0.242515620628997,0.694865853530852
"5378","KMT2D",57.3063447535531,0.35120174442005,0.30049933162581,1.16872720654626,0.242513556833862,0.694865853530852
"5379","FANCM",35.6209172187332,0.374469181015948,0.320375129529407,1.16884597617174,0.242465692851097,0.694865853530852
"5380","BCL2L13",108.294148672924,0.213476509275522,0.182631526955736,1.16889188210786,0.242447194607323,0.694865853530852
"5381","MAPKAPK2",123.38331741488,-0.265793698240159,0.227498186770474,-1.1683332602045,0.242672364195897,0.694927455708667
"5382","CTNNBL1",214.270747534883,-0.259227172482967,0.221837226139174,-1.16854676284284,0.242586288094238,0.694927455708667
"5383","WAS",12.0864043859939,-0.630887283942664,0.53997634368837,-1.16836096861821,0.242661192010209,0.694927455708667
"5384","FAM180A",138.850527457548,0.317859187627298,0.272088194301477,1.1682211660940 6,0.242717564852547,0.694927797585812
"5385","SRSF7",1181.57149430752,0.183194923873774,0.156899273851969,1.16759574073373,0.242969869069685,0.695202061945033
"5386","ADCK2",37.6830826333005,-0.30762148343015,0.263440501909205,-1.16770762734188,0.24292471911162,0.695202061945033
"5387","LSM4",258.421716562101,-0.321564110497294,0.275401500097719,-1.16761931355928,0.242960356162121,0.695202061945033
"5388","RBX1",916.44291981731,-0.302253311967052,0.258881239975338,-1.16753655844605,0.242993753471284,0.695202061945033
"5389","APC",258.102812004286,0.242636499973393,0.207842179238128,1.16740740913518,0.24304588043824,0.695222164957408
"5390","DNER",23.4859442371635,0.566699385857505,0.485567069856104,1.16708776405584,0.243174928697591,0.695333245385526
"5391","APRT",1115.24825166956,-0.325076227433334,0.278524160304315,-1

.16713834476031,0.243154504867199,0.695333245385526
"5392","ENTPD4",92.836157928405,0.348792399434858,0.298904733409282,1.16690155909062,0.24325012622937,0.695419268513675
"5393","ACTN4",408.622337389349,-0.189931689731125,0.162783370705222,-1.16677575177544,0.243300941855872,0.695435568089797
"5394","DENND6B",25.8575328924194,-0.419248974019859,0.359396592268532,-1.16653575197677,0.24339790237836,0.69558373473534
"5395","FHL3",114.866264685897,-0.349664568731074,0.29989010673493,-1.16597567201551,0.243624281703176,0.696101631594895
"5396","RADX",3.893675363067,0.94516829647576,0.810709154890904,1.1658537353053,0.243673587035743,0.696113481126014
"5397","FAM86C1",6.46413370253565,-0.705976504299102,0.605720197614926,-1.16551587198007,0.243810239311864,0.696374808040092
"5398","DUSP11",143.006011699818,-0.292546693205372,0.251054084864223,-1.1652735838319,0.243908268367916,0.696397652622819
"5399","FGF18",187.895340099929,-0.402227938606498,0.345179208721492,-1.1652727871308,0.243908590756445,0.696397652622819
"5400","EPHA4",20.9755246619963,0.519194110136187,0.445648732334637,1.1650299270824,0.244006879071581,0.696549266831189
"5401","AL606760.1",3.98077014562856,-0.98574323932943,0.846196263205851,-1.16491088674263,0.244055066253404,0.696557831197226
"5402","LINC01119",10.3923511135858,-0.580097805121625,0.498123992583825,-1.16456507568044,0.244195087465986,0.696570553902327
"5403","CRIP3",2.90097375308747,1.35946385815344,1.16734227077411,1.16458033962218,0.244188905804095,0.696570553902327
"5404","CCDC43",124.661944086397,0.218627899357768,0.187717118231257,1.16466682110702,0.244153884206393,0.696570553902327
"5405","AL356966.1",5.02289609053309,0.81546559010372,0.70037377848561,1.16432912703695,0.244290657019483,0.696585364031692
"5406","AMMECR1",87.0341691791425,0.326583142309884,0.28048920585173,1.1643340830824,0.244288649333638,0.696585364031692
"5407","ZNF346",64.8571525137518,0.304882597686556,0.262033184103484,1.16352666830988,0.244615884541332,0.697086254182997
"5408","UBN2",139.739649449413,0.304269009673712,0.26156788412532,1.16325064405817,0.244727824293126,0.697086254182997
"5409","HSPA14.1",63.5467864481974,-0.309038293649326,0.265576386791616,-1.16365124694543,0.244565374298513,0.697086254182997
"5410","GLTP",262.901192230773,-0.196294378739249,0.168718324963241,-1.16344433114788,0.244649272053478,0.697086254182997
"5411","SMG8",27.573180397905,0.352788586895492,0.303284534843364,1.16322643051251,0.244737645646343,0.697086254182997
"5412","FKBP8",1080.81741829714,-0.267316543250926,0.229702354019861,-1.1637518665908,0.244524583541497,0.697086254182997
"5413","MRPL20",710.354035516495,-0.269492040856792,0.232056678167026,-1.16131991109009,0.245511825182408,0.697687962178846
"5414","ZNF644",312.369606277101,0.164123939191754,0.141332967433701,1.16125729312762,0.245537281532289,0.697687962178846
"5415","MZT2B",1739.60576888437,-0.299611491942182,0.257888897845082,-1.1617851502943,0.24532274745234,0.697687962178846
"5416","GK5",127.262970962263,0.371721838104697,0.319920793645977,1.16

191834193823,0.245268635887519,0.697687962178846
"5417","UTRN",596.803471017761,0.339771156730919,0.292422029709899,1.16192051969543,0.245267751203019,0.697687962178846
"5418","SLC26A7",2.87042899739397,-1.07005930982091,0.920987110187444,-1.1618613311571,0.24529179654507,0.697687962178846
"5419","FKBP15",114.493884245462,0.250820984269933,0.215831571666323,1.16211443179269,0.245188986001801,0.697687962178846
"5420","DCTN2",602.839404442622,-0.23842258306463,0.205123874918982,-1.16233462905671,0.245099565541562,0.697687962178846
"5421","LIG4",30.5915877552399,0.458525586183977,0.394804354546829,1.16139951574316,0.245479465832513,0.697687962178846
"5422","ZDHHC1",124.723529041483,-0.274700204380838,0.236554037492205,-1.16125772907127,0.245537104299842,0.697687962178846
"5423","VPS4A",194.702473361731,-0.187956947179927,0.161709122265589,-1.16231505400932,0.245107513894995,0.697687962178846
"5424","KRBA2",28.1627627096739,0.450335603001377,0.387498965900922,1.16215949623081,0.245170683808421,0.697687962178846
"5425","ZNF846",46.5086781363694,-0.342382987378824,0.294727345202706,-1.1616939959994,0.245359785394416,0.697687962178846
"5426","ZNF24",225.857101591096,-0.194383037209871,0.167407330234588,-1.16113814692273,0.245585723664078,0.697697001526311
"5427","AC105942.1",15.8937566640575,-0.446143182623467,0.384525157490692,-1.16024445717642,0.245949291240511,0.69826321928839
"5428","SDHAF3",124.921142543587,-0.349137667030627,0.300928119507024,-1.16020286705868,0.245966219963409,0.69826321928839
"5429","NAGPA",56.1022667908066,0.330264664915373,0.284641400272905,1.16028330593767,0.245933479089424,0.69826321928839
"5430","FCGRT",2886.12888350603,0.313207748507969,0.269901243941522,1.16045314921124,0.245864358162993,0.69826321928839
"5431","MAGIX",3.38187864980799,-0.925212539731458,0.797732307435037,-1.15980327123307,0.246128911938517,0.69859642377688
"5432","ZNF672",49.2599820327181,-0.291801508167142,0.251719320612573,-1.15923365539454,0.246360956466625,0.698611873424028
"5433","THUMPD3-AS1",175.397260994156,-0.353026906956425,0.30446289670234,-1.15950715433666,0.246249522015636,0.698611873424028
"5434","ATF6B",129.980178420062,0.221544117461192,0.191079185096822,1.15943616437831,0.246278442779743,0.698611873424028
"5435","PARG",52.4520324192693,0.27657397963593,0.23853160576335,1.1594856738202,0.246258272761982,0.698611873424028
"5436","ZNF792",7.32932540665974,-0.654621973390632,0.564686717882778,-1.15926575331018,0.246347876656837,0.698611873424028
"5437","SLC23A3",3.21519118146883,1.04688400734846,0.903718008990333,1.15841888391499,0.246693136528168,0.699167990362515
"5438","CUL1",135.099414740863,-0.196502874412216,0.16960809816011,-1.15857011866684,0.246631454835566,0.699167990362515
"5439","SS18",211.76123140712,0.170769455481933,0.147410585406521,1.15846128017872,0.246675843953934,0.699167990362515
"5440","EMC1-AS1",25.2050908396733,-0.44914584096174,0.387971159376905,-1.157678425

58989,0.246995291387285,0.69927991862676
"5441","SKP2",32.3026375709061,0.319810752330337,0.276158888827689,1.1
5806792853184,0.246836316711004,0.69927991862676
"5442","ZFP62",44.9788965537539,0.347326177447241,0.30003496111041,1.1
5761901933631,0.247019544206455,0.69927991862676
"5443","ADRB1",4.46481589279489,-0.950124854268399,0.820811006635094,-
1.15754399805556,0.247050174300443,0.69927991862676
"5444","TBC1D15",209.940257777653,0.205100427202595,0.177144589256619,
1.15781367109937,0.246940083136829,0.69927991862676
"5445","MRPS23",173.806503006471,-0.287198554550997,0.248078654524034,
-1.15769151965944,0.246989945910082,0.69927991862676
"5446","TRABD",86.1028966064497,-0.245475483314314,0.211999009733498,-
1.15790863185115,0.246901324597877,0.69927991862676
"5447","PYURF",657.011300368215,-0.257847491839625,0.222809676949078,-
1.15725445757257,0.247168414398575,0.699486158978158
"5448","LLPH",231.188959313062,-0.249161447134993,0.21533085677669,-1.
15710981168568,0.247227498507126,0.699524943004284
"5449","MCM6",77.8307548880693,0.26977871972488,0.233219338239049,1.15
675964850032,0.247370572074331,0.699544554857057
"5450","MARF1",168.419945712284,0.300676612611682,0.259928374241509,1.
15676718053225,0.247367493943009,0.699544554857057
"5451","SAMD4B",547.177146990545,0.235216754981121,0.203340788912202,1
.15676129830834,0.247369897841526,0.699544554857057
"5452","SNAP47",85.1191948772474,-0.307116167241269,0.265555338204085,
-1.15650534204379,0.247474515767974,0.699655563135511
"5453","OXR1",323.16775108247,0.200277792428857,0.173200527097035,1.15
633477441239,0.247544249687897,0.699655563135511
"5454","RAB3IL1",276.24872075531,0.224244358933297,0.193927562748285,1
.15633051720638,0.247545990356216,0.699655563135511
"5455","PPIH",115.545365764312,-0.364561086411528,0.315424631206091,-1
.15577875138525,0.247771666552499,0.700095704667408
"5456","DDAH2",2202.91565530243,-0.269119870457157,0.232857504666954,-
1.15572770927897,0.247792550416178,0.700095704667408
"5457","AC124016.2",10.4885880262836,-0.605694712666024,0.524253539606
684,-1.15534692072931,0.247948388826556,0.700331750329202
"5458","TEX9",11.4315623638851,-0.607330308081815,0.525689857011397,-1
.15530155277211,0.247966960317664,0.700331750329202
"5459","PRRG3",270.90081546918,0.324364035428718,0.280827907787235,1.1
5502778190573,0.248079049793807,0.700519976657179
"5460","BTBD7",269.105859950673,0.266775035019727,0.231178319048422,1.
15397947401741,0.248508584247956,0.701604363769642
"5461","ALG6",59.0643280206191,0.34601632154084,0.300296986098624,1.15
224706726561,0.249219561537568,0.701829745757099
"5462","CD46",463.153438324885,0.203519543688228,0.176518541253659,1.1
5296411494682,0.248925113904919,0.701829745757099
"5463","COX5B",1395.62499726096,-0.276850444520899,0.24017153255455,-1
.15271964822899,0.249025474095698,0.701829745757099
"5464","STK39",51.604428218254,0.316649209260458,0.274661024056149,1.1
5287274686533,0.248962619660107,0.701829745757099
"5465","STK32B",31.0573221927279,0.460603632337458,0.399702283496264,1

.15236677736359,0.249170386998264,0.701829745757099
"5466","THBS2",1202.19588406443,0.268106919261419,0.23269211324452,1.1
5219598775006,0.249240546056455,0.701829745757099
"5467","ERICH1",265.622249000321,0.241869338528437,0.209864620367251,1
.15250173233192,0.249114958301789,0.701829745757099
"5468","GTF3C4",78.399382265369,0.310113146465498,0.269041976687515,1.
15265710683388,0.24905115357132,0.701829745757099
"5469","BBS1",76.9908923381258,0.253220888563912,0.219699414234938,1.1
5257880611883,0.249083306428136,0.701829745757099
"5470","TCP11L2",123.839386478235,-0.33203347046822,0.288039344281851,
-1.1527365169368,0.249018548126992,0.701829745757099
"5471","KNSTRN",16.5178571803952,-0.426585445080308,0.370120892541608,
-1.15255705278067,0.249092239581525,0.701829745757099
"5472","AC139530.1",31.8756847455705,-0.450811970151561,0.390912431497
283,-1.15323006849603,0.248815964911236,0.701829745757099
"5473","ZNF550",15.9011678037448,0.514149986692647,0.446263874464943,1
.15212101205615,0.249271349855343,0.701829745757099
"5474","IFNAR1",396.343056423936,0.188465048016248,0.163563996541883,1
.15224041965732,0.249222292442302,0.701829745757099
"5475","DNAL4",88.5554488849974,-0.313345763481563,0.271749677143838,-
1.15306765687787,0.248882615672177,0.701829745757099
"5476","LRRC2",69.6447070000384,-0.514102712485841,0.446313219174392,-
1.15188771113893,0.249367218669144,0.701954113930257
"5477","KIAA0513",31.6325066758695,0.410426456981697,0.35633733509059,
1.15179190212374,0.249406596302045,0.701954113930257
"5478","AC009060.1",2.91427203337973,1.04736109597318,0.90963515645225
4,1.15140788979406,0.249564469497405,0.702142050976912
"5479","FKBP1A",1470.449707656,-0.294884574719405,0.256099688711152,-1
.15144448711923,0.249549420781458,0.702142050976912
"5480","BECN1",181.309839659671,-0.212700024428296,0.18475689732197,-1
.15124267354214,0.24963241384342,0.702205047335095
"5481","MAU2",115.945540404584,0.309595309519519,0.268959825144767,1.1
5108384441014,0.249697743703437,0.702260667613297
"5482","RPL7A",15122.2362684408,-0.293323941642975,0.254919923127,-1.1
5065130274986,0.249875718012724,0.702504868350563
"5483","ARMCX3",486.167221626978,0.133315041839327,0.115853226265303,1
.15072360206903,0.24984596344809,0.702504868350563
"5484","OIP5-
AS1",342.169470887223,0.199978531827619,0.173849501456235,1.1502968380
8418,0.250021632686255,0.702658790858455
"5485","CPNE1",430.531695825011,0.244959262489772,0.212936024720484,1.
15038900914641,0.249983684927319,0.702658790858455
"5486","ARHGAP42",89.5497979935357,0.28215732501495,0.245335453832258,
1.15008785158246,0.250107689447904,0.702673906166044
"5487","MT-
ATP8",447.710491369215,-0.426211036592295,0.370598189382556,-1.1500623
8239424,0.250118178600914,0.702673906166044
"5488","PRDM2",395.838493494236,0.318328686770687,0.277074264712624,1.
1488930128565,0.2506000991203,0.702718474370203
"5489","TSNAX",260.19801468511,-0.197045502439189,0.171365476084669,-1

.14985530890616,0.25020347052042,0.702718474370203
"5490","PID1",380.726747081363,0.385909427116893,0.335878062641442,1.14895692824351,0.25057374157761,0.702718474370203
"5491","KIF9",49.233885844016,-0.304195942452523,0.264651947545843,-1.14941886985294,0.250383302806173,0.702718474370203
"5492","ZBTB49",11.7183522927575,0.512904073011986,0.446179646659327,1.14954610066202,0.250330868740577,0.702718474370203
"5493","GRPEL2",29.7683820947559,0.337230248130603,0.293494326265385,1.14901794668995,0.250548580486426,0.702718474370203
"5494","HIF1A-AS3",4.1628329309631,1.21233699540902,1.05534273770114,1.1487613948525,0.250654382087244,0.702718474370203
"5495","MT1M",2043.69635326364,-0.541636179587354,0.471118505238488,-1.14968139345995,0.250275120599421,0.702718474370203
"5496","PRAM1",2.66259046480432,-1.19720431103173,1.04159738594621,-1.14939258410692,0.250394136582597,0.702718474370203
"5497","CHRDL1",1776.03504539763,0.239377361960465,0.208294674891747,1.14922458812195,0.250463384537657,0.702718474370203
"5498","UBE2A",329.703632617093,-0.22287518253896,0.194023054401183,-1.14870463835766,0.250677792595025,0.702718474370203
"5499","DCAF12L2",32.3631332072111,-0.378482796252941,0.329488897090008,-1.14869666199875,0.250681082748086,0.702718474370203
"5500","UBA52",7757.77420267614,-0.259736348604869,0.226164028483387,-1.14844235109629,0.250785998769749,0.702884758370123
"5501","STAU2",90.1638267106731,0.256404753646631,0.223308726597304,1.14820749530765,0.250882915798994,0.703000575748314
"5502","OGN",2745.04465406049,0.342408509455143,0.298245447029887,1.14807623340124,0.250937094528674,0.703000575748314
"5503","PSMF1",328.568324766649,-0.172693059478632,0.150428089099343,-1.14801072401169,0.250964136772168,0.703000575748314
"5504","QRSL1",56.523352864805,-0.262614609718141,0.228848393957689,-1.14754840607138,0.251155039214059,0.703279732876424
"5505","FKRP",34.9795638968814,0.324922115602821,0.283139938794175,1.14756723119524,0.251147263879189,0.703279732876424
"5506","ARHGAP5",263.49350065549,0.274862170420579,0.239545036022346,1.14743421523015,0.251202207020271,0.703284057613054
"5507","DIAPH1",273.422692421747,-0.421335354900652,0.367264343138516,-1.14722641272513,0.251288058030684,0.703333240837445
"5508","AL110292.1",24.5419447403093,-0.485122823854132,0.422897983856699,-1.14713912662804,0.251324125296494,0.703333240837445
"5509","LINC00653",4.65517799070178,-0.747483892882957,0.651651722541117,-1.1470604113009,0.251356654153324,0.703333240837445
"5510","YBEY",177.2956160959,0.358508670883259,0.312672617514192,1.14659439554852,0.251549293840942,0.703744530772799
"5511","TICAM2",71.4642506063228,0.243840418827347,0.212780482282287,1.14597173674912,0.251806846683741,0.704337242175625
"5512","ATP2A2",232.486513542354,0.261365905721607,0.228115377827364,1.14576188686151,0.251893689148463,0.704452325512257
"5513","FBXO28",72.5137299601864,0.248562424771916,0.217047777979134,1.14519681835127,0.252127636046642,0.704467556581293

"5514","WNT9A",27.296440007414,-0.449805898662659,0.392759370139597,-1
.14524549345006,0.25210747786021,0.704467556581293
"5515","HEATR1",80.5246287516837,0.246122031896427,0.214845032284661,1
.14557934749138,0.251969246647476,0.704467556581293
"5516","LINC00944",3.58893273730037,-0.970224664253762,0.8470634794149
93,-1.14539782180649,0.252044400227786,0.704467556581293
"5517","ELMO3",2.87832755555283,-1.05586816554523,0.921739455057461,-1
.14551694597842,0.251995079780416,0.704467556581293
"5518","FGD4",162.502367043679,0.30878960909305,0.269674390648997,1.14
504609929745,0.252190061593914,0.704480812050799
"5519","CSNK1G2",157.165579071553,-0.206239807176514,0.180127656268138
,-1.14496469586828,0.252223782141314,0.704480812050799
"5520","AP000254.2",8.50410339591233,0.599106302955994,0.5233418482746
44,1.14477048783913,0.252304243547738,0.704577883023257
"5521","LRRC58",364.61133725748,0.242261162209483,0.211723498601477,1.
14423370013116,0.252526730579608,0.704816141930953
"5522","DCAF13",145.679079627181,-0.233287706946877,0.203854174703133,
-1.14438523168146,0.252463910144095,0.704816141930953
"5523","FEM1B",160.618358959374,0.21919396709219,0.191555250369089,1.1
4428587402249,0.252505099619627,0.704816141930953
"5524","RPS23",18047.5509107255,-0.28551149013068,0.249563888711222,-1
.144041678486,0.252606352642199,0.704888608144312
"5525","ZNF836",40.22975070582,0.349220452025631,0.305275831093247,1.1
4395054064716,0.252644149205146,0.704888608144312
"5526","EPB41",8.632454516463,-0.650696326698996,0.569033973490347,-1.
14351050554635,0.252826695423716,0.705085203324963
"5527","SNRPE",673.601710356335,-0.237817323707669,0.207991498290519,-
1.14339925267277,0.252872862636581,0.705085203324963
"5528","ADAMTS12",52.9899050314114,-0.325613905810529,0.28479191484130
4,-1.14333971170485,0.252897573090089,0.705085203324963
"5529","SLC20A2",64.4455313718576,0.283647623440634,0.24802507120783,1
.14362480397377,0.252779270497058,0.705085203324963
"5530","PIGC",211.191983327153,-0.247625232885425,0.216686476445844,-1
.14278120604039,0.253129443770853,0.705153158884231
"5531","LYST",199.321327631608,0.251420003569019,0.219982111790696,1.1
4291112819317,0.25307549173575,0.705153158884231
"5532","AC064836.4",30.1182045560554,-0.399772250079889,0.349919116300
151,-1.1424704494766,0.253258522382327,0.705153158884231
"5533","QRICH1",134.902717083769,0.205867880490988,0.180189706106946,1
.14250633367925,0.25324361485991,0.705153158884231
"5534","CREB3",174.397180528831,-0.241758365822118,0.211623269657711,-
1.1423997285986,0.253287904037755,0.705153158884231
"5535","TMEM72-
AS1",11.7209045203733,0.582325433516852,0.509652215966588,1.1425937438
7774,0.253207304237917,0.705153158884231
"5536","SLC22A17",98.9984833948178,0.290424088169502,0.254160601439384
,1.14267941815037,0.253171718246991,0.705153158884231
"5537","PIAS1",227.634179039967,0.229435307484954,0.200762563782473,1.
14281917486144,0.253113675845528,0.705153158884231
"5538","WWC2-

AS2",8.08864404229663,0.701764145352357,0.61443289628583,1.14213309475198,0.253398700930143,0.70533423164286
"5539","AGO4",62.7926200213957,0.325871711329854,0.28541320929956,1.14175413299751,0.253556232570438,0.705417141253294
"5540","TAF1A",27.2505571056852,0.38017766709115,0.333138067899664,1.14120151289841,0.253786074917138,0.705417141253294
"5541","AC023481.1",4.29531846612122,0.868324455423062,0.76067931823141,1.14151184948991,0.25365698378583,0.705417141253294
"5542","RFC1",458.710721000916,0.177953085251326,0.155854134244234,1.14179252359428,0.253540270775751,0.705417141253294
"5543","AC093827.4",44.4981444956421,-0.377999881518063,0.331235675098893,-1.14118106814795,0.253794580953018,0.705417141253294
"5544","TAF2",99.1970433809532,0.244588424005384,0.214305691288796,1.14130624592596,0.253742503866381,0.705417141253294
"5545","AC083805.1",8.77471403618639,-0.549813228964502,0.48162135553302,-1.14158814315036,0.253625254810931,0.705417141253294
"5546","AC100830.1",2.50893110637595,1.29976007526604,1.13864339440366,1.14149880608296,0.25366240854894,0.705417141253294
"5547","NKAPL",8.08762506858158,0.788212619031266,0.690836904826125,1.14095326049446,0.253889373724782,0.705553397506311
"5548","ORAI2",91.0779569338038,-0.330352615964251,0.289601407149239,-1.14071481632688,0.25398861883799,0.705701975376283
"5549","ZNF85",32.1536766946646,0.385608608297477,0.338089794356847,1.14055086765049,0.254056873138119,0.705764407897659
"5550","RHOC",747.372955196134,-0.283252316156976,0.248478837027181,-1.13994543577967,0.254309034136916,0.705956016787423
"5551","COMTD1",95.6103284508287,-0.33508998007958,0.293893473893194,-1.14017496081372,0.254213416993873,0.705956016787423
"5552","RPL23A",11624.5564792032,-0.272792277596618,0.239301107952024,-1.13995409353186,0.254305426975901,0.705956016787423
"5553","THAP8",27.608972975376,0.45236806882417,0.396799500969093,1.14004192978913,0.254268832939671,0.705956016787423
"5554","PKN2",325.530490298192,0.199857686991653,0.175494212619752,1.13882779385261,0.254774986804086,0.706105073999459
"5555","AC009126.1",4.56147603927862,-0.794388959557337,0.697503003881715,-1.13890399774114,0.254743198024794,0.706105073999459
"5556","FAM50B",73.9632265579701,-0.34242680542725,0.300651188746399,-1.13895044571432,0.254723823406598,0.706105073999459
"5557","POLD2",240.564151618777,-0.327540885134212,0.28741708566882,-1.13960130230958,0.254452442544826,0.706105073999459
"5558","LRATD2",4.12593283650952,0.867012489664533,0.760989816552082,1.13932206556038,0.254568848322354,0.706105073999459
"5559","RMI1",17.4161418464962,0.462712183263819,0.406052614621354,1.13953750475243,0.254479034648971,0.706105073999459
"5560","SUFU",40.5240485029862,0.326113193514032,0.286249346304514,1.13926266635771,0.254593614920664,0.706105073999459
"5561","KNTC1",13.1976235071739,0.516837808933066,0.453814321564186,1.1388750516988,0.254755272665701,0.706105073999459
"5562","ZNF519",54.9648363803246,0.333021537256959,0.292389319851327,1.13896614769067,0.254717273949024,0.706105073999459

"5563","XPO6",90.3550156880113,0.272052800844887,0.238931457365716,1.13862278263542,0.25486052180867,0.706215161546044
"5564","OAZ3",6.2391568881694,-0.689676433326594,0.605986009459883,-1.13810619809739,0.255076140330605,0.70643167502628
"5565","POLR3H",76.628636184616,-0.315258790194252,0.276966610313282,-1.13825558191891,0.255013775609064,0.70643167502628
"5566","LINC00893",13.3316638824779,0.557549002028205,0.489882028318429,1.13812912047835,0.255066570011854,0.70643167502628
"5567","ADH5",1225.70640624625,0.239375106918292,0.210355841257579,1.1379532200638,0.255140016552512,0.7064816517257
"5568","RPAP2",125.301889715481,0.240753230724979,0.211800857145199,1.13669620590785,0.255665305971792,0.70651156247698
"5569","LIX1L",165.214633159046,0.189446085686738,0.166599902317681,1.13713203339995,0.25548309443601,0.70651156247698
"5570","OPN3",31.4246679306801,-0.342995316733531,0.301534498748136,-1.13749941767037,0.255329567891904,0.70651156247698
"5571","DGUOK",358.220912466216,-0.266346363089019,0.234457989566139,-1.13600890113358,0.255952839051842,0.70651156247698
"5572","FRZB",25.8956797796197,0.727204116756596,0.640478996464499,1.13540665778398,0.256204971407481,0.70651156247698
"5573","NDUFB4",990.279761665153,-0.26883679942929,0.236734366436171,-1.13560529244821,0.256121792897797,0.70651156247698
"5574","TXNDC15",444.269644770514,-0.207544834653795,0.182734447856751,-1.13577290482468,0.256051619603614,0.70651156247698
"5575","ZNF76",51.8348974653126,0.272731980039449,0.239915880122967,1.13678169156482,0.255629558857895,0.70651156247698
"5576","AMD1",541.270432445731,0.316076438268335,0.278179666069954,1.13623128078977,0.255859782229301,0.70651156247698
"5577","SULF1",175.786909682863,0.407623483095779,0.358985810352857,1.13548633773328,0.256171603077375,0.70651156247698
"5578","FZD8",60.4258091888846,0.415566668631443,0.36578754350043,1.13608753500638,0.255919931294062,0.70651156247698
"5579","NFKB2",79.5702016386484,-0.340470597925037,0.299308816517704,-1.13752278294448,0.255319805926012,0.70651156247698
"5580","ARAP1",254.774970978257,0.249037605889651,0.219192541969774,1.13615912134453,0.255889975439398,0.70651156247698
"5581","CCDC153",3.91055874173466,-0.923328860941878,0.812886106489083,-1.1358649798184,0.256013076788406,0.70651156247698
"5582","A2M-AS1",15.6696345605279,0.438705742562292,0.386321652428267,1.13559708549795,0.256125229196747,0.70651156247698
"5583","DECR2",35.7357181764694,0.429794702956498,0.378368824680178,1.13591468144816,0.255992273241049,0.70651156247698
"5584","NHLRC4",16.7935373686227,0.550891148587786,0.484897893305197,1.13609722004103,0.255915878374193,0.70651156247698
"5585","MIR193BHG",64.5387792512442,-0.301963126274161,0.265660129246853,-1.13665203404902,0.255683778460497,0.70651156247698
"5586","AC127024.5",3.78089262050467,0.893138203382128,0.786146998822116,1.1360956725909,0.255916525936398,0.70651156247698
"5587","IGFBP4",7195.32984741065,-0.342179164539948,0.301214208859264,

-1.13599941329403,0.255956809848948,0.70651156247698
"5588","IGFLR1",14.5828652901822,-0.474913236220157,0.417428661525064,
-1.13771113484415,0.25524112234586,0.70651156247698
"5589","TBCB",518.991842174635,-0.309847654434419,0.272627205811995,-1
.13652507097216,0.255736879058474,0.70651156247698
"5590","ZFP14",75.1315920089112,0.318447827289602,0.280281762982553,1.
13617034480201,0.255885279118084,0.70651156247698
"5591","HOXD4",9.72662673678972,0.651378833017789,0.573784312693499,1.
13523290652552,0.256277745343633,0.706585842330907
"5592","ANAPC1",65.295476920864,0.273338191913715,0.240914324052162,1.
13458671662268,0.256548521351531,0.706851505445565
"5593","CCDC74A",56.6383056852832,-0.387405773576003,0.341425078790085
,-1.13467286863889,0.256512409192266,0.706851505445565
"5594","INIP",104.367854778079,0.209154108011804,0.184359533180078,1.1
3449033203782,0.2565889268589,0.706851505445565
"5595","ZFPL1",148.701190357996,-0.261140003736078,0.230108734930065,-
1.1348548059918,0.256436158493652,0.706851505445565
"5596","AC006305.1",3.74884033205472,1.13019234985786,0.99624179311529
,1.13445586971783,0.256603374925292,0.706851505445565
"5597","SMAGP",9.02510065113886,-0.599350634350612,0.528420405212172,-
1.1342306777687,0.256697798848266,0.706858979858168
"5598","UBR7",79.3059198638379,-0.240991275400591,0.212457131911113,-1
.13430541602819,0.256666458110337,0.706858979858168
"5599","TMEM54",265.140042956415,-0.288662004573821,0.254602354326916,
-1.13377586525838,0.256888577184098,0.707257977726893
"5600","FAM87B",10.6519958106733,-0.698077655424093,0.615809656408586,
-1.13359322667218,0.256965215532564,0.707300949742555
"5601","TP53",86.9751419977928,-0.259809725924126,0.229206127146567,-1
.13351998551936,0.256995953260334,0.707300949742555
"5602","CCT7",507.867367395769,-0.246969182759372,0.217970363870862,-1
.13304019121467,0.257197375671484,0.707629451631363
"5603","RBM6",273.581050867493,0.254721296480036,0.224898225157403,1.1
3260696611438,0.257379341965897,0.707629451631363
"5604","EBLN2",11.1680016990258,0.651662513608632,0.575286319036716,1.
13276205611808,0.257314189694689,0.707629451631363
"5605","NSUN7",9.80470507301603,0.662151620190182,0.584640133750335,1.
13257982469084,0.25739074507279,0.707629451631363
"5606","SRD5A1",26.6683981332067,-0.396209060098314,0.349804781315851,
-1.13265764581006,0.257358050506442,0.707629451631363
"5607","TAP2",72.4642926960544,-0.241541564542721,0.213254242107113,-1
.13264600111167,0.257362942547318,0.707629451631363
"5608","DCAF8",226.935257220838,0.231315658368578,0.204302615583867,1.
13222073886578,0.257541643188202,0.707665673751538
"5609","PTPN11",170.152360355673,-0.228621886719934,0.201908901195354,
-1.13230216878222,0.257507418632931,0.707665673751538
"5610","NCBP3",205.030870963735,0.220338870163417,0.194595382812229,1.
13229238525165,0.257511530431346,0.707665673751538
"5611","ADAM23",12.9899120948551,0.739249943631898,0.653140992813331,1
.13183822752828,0.257702452870306,0.70798134218424
"5612","PRKCE",80.4311646940989,0.325140131369943,0.28734488076395,1.1

3153270907596,0.257830944379523,0.70819936169532
"5613","RAB11FIP4",3.57555451377278,-1.02934691558461,0.909774295873439,-1.13143108159192,0.25787369556898,0.70819936169532
"5614","AC068193.1",3.52943733188564,0.993790117916034,0.878669032546775,1.13101757442798,0.258047694516184,0.708294083094113
"5615","MCRIP1",667.622522795883,-0.27587695587305,0.243941940224528,-1.13091236225771,0.258091979548468,0.708294083094113
"5616","L3MBTL1",14.2340499608877,0.567874683860219,0.502071989674715,1.13106226903464,0.258028883628525,0.708294083094113
"5617","C21orf58",15.2914500537616,0.491960374014133,0.434968982313745,1.1310240362364,0.258044974837244,0.708294083094113
"5618","GASK1B",191.286962063086,0.893746918740508,0.790601045711575,1.1304651360992,0.258280281074525,0.708566943270738
"5619","MOCS2",212.109375208973,-0.254497896450827,0.225128155110569,-1.13045787776226,0.25828333793307,0.708566943270738
"5620","PEF1",175.660287810837,-0.324724209848119,0.287408473169251,-1.12983520028964,0.258545672781787,0.708727224711279
"5621","AC083798.2",16.7452806031602,-0.616526352761851,0.545517092233896,-1.1301687179721,0.258405138378837,0.708727224711279
"5622","SMAP1",304.031396579121,-0.21505067853297,0.190357017123103,-1.12972288483538,0.258593011085578,0.708727224711279
"5623","SART1",127.215665278412,-0.269418288518553,0.238434176534145,-1.12994828356736,0.258498016927081,0.708727224711279
"5624","APBA2",4.51788298221956,0.891814755933793,0.789168574037004,1.13006876512036,0.258447249971153,0.708727224711279
"5625","MPHOSPH6",84.0489792748419,-0.310493644469811,0.274854742060313,-1.12966449893623,0.25861762173214,0.708727224711279
"5626","SLC50A1",86.0688761562741,-0.273282375286619,0.242072173363034,-1.12892932504382,0.258927648907285,0.709154243106184
"5627","NAIP",56.1181021073616,0.346815694074632,0.3071726023667,1.12905803252793,0.258873353613707,0.709154243106184
"5628","MCTP1",2.47531382829626,-1.11536250177587,0.988044415206392,-1.12885866729269,0.258957459256874,0.709154243106184
"5629","LFNG",13.1168086571926,0.744903171218898,0.659728726351618,1.12910525412211,0.258853435147324,0.709154243106184
"5630","C11orf65",7.19121410816488,0.669413044162134,0.593093114295375,1.12868119360555,0.259032345501683,0.709233322541463
"5631","NIPBL",423.84838390733,0.262445688160399,0.232672896225218,1.12795986304465,0.259336870309273,0.709530366018265
"5632","AC004130.2",37.5723391489731,-0.425451307718057,0.377187798028456,-1.12795617976476,0.259338425919085,0.709530366018265
"5633","TKFC",34.5308453640517,0.427625992169347,0.379141690102443,1.12787911045552,0.259370977133501,0.709530366018265
"5634","AC084824.4",3.58476163753386,0.882596483041242,0.782383079095246,1.12808738663147,0.259283015519803,0.709530366018265
"5635","PFDN5",4064.42821850604,-0.290642573515945,0.257599326918059,-1.12827380798397,0.259204301414716,0.709530366018265
"5636","TRIM33",152.118306174654,0.239214245227402,0.212197986018438,1.12731628473899,0.259608779606867,0.709622273528802
"5637","ECRG4",5.08696072272922,-0.820776059471109,0.727954707160984,-

1.12750979064635,0.25952700337724,0.709622273528802
"5638","PIK3R1",2002.47061326751,0.297141026577923,0.263622495776372,1
.12714594292433,0.259680781380212,0.709622273528802
"5639","BCL2L2",55.3113409392392,-0.309588429233867,0.274554212010746,
-1.127604005659,0.259487194262208,0.709622273528802
"5640","AC022613.2",3.78081434502149,-0.907615054647084,0.805012835746
886,-1.1274541402871,0.259550519573575,0.709622273528802
"5641","QPCTL",3.91973171499144,0.923013583393639,0.818850450127722,1.
12720654088872,0.259655165645271,0.709622273528802
"5642","TMEM14C",1092.09636429754,-0.277530295129041,0.246299603608979
,-1.1267996012273,0.259827219168597,0.709896594024092
"5643","PHF8",42.3657864100374,0.361595188789403,0.320945575642684,1.1
2665578288568,0.259888044235479,0.709936948766598
"5644","NRAS",93.5302940964615,0.225058336533389,0.199805752911942,1.1
2638566834748,0.260002310372188,0.710123248474003
"5645","STRIP1",59.0816609395537,0.263334138600045,0.233889936391498,1
.12588913684282,0.260212448036508,0.710190652455185
"5646","TMEM45A",134.467360540684,0.508440651240324,0.451642741903845,
1.12575849021077,0.260267758672975,0.710190652455185
"5647","ARHGAP26",79.1627852524198,0.320685694789357,0.284788651784975
,1.12604801061907,0.260145198100966,0.710190652455185
"5648","AL365272.1",5.26513475597437,-0.783109719068713,0.695413682513
136,-1.12610628574154,0.260120533746504,0.710190652455185
"5649","COX7A2",1093.3585057353,-0.251161603526115,0.223090705170949,-
1.1258272877557,0.260238631489064,0.710190652455185
"5650","AC023794.2",23.4381064837142,-0.378639972460864,0.336367261865
152,-1.12567427151296,0.260303417863886,0.710190652455185
"5651","LINC-
PINT",411.006123332081,-0.392863722988664,0.349143174508058,-1.1252224
06373,0.260494800631234,0.710587037998667
"5652","CLTCL1",5.12272919333416,-0.713394747064725,0.634251871163053,
-1.12478146222343,0.260681651759799,0.710891475927022
"5653","HS6ST3",3.94203694312402,-2.72605730982556,2.42371921463771,-1
.12474138644523,0.260698638560847,0.710891475927022
"5654","CHN1",3.16523680394567,0.998950231126807,0.888342893519293,1.1
2450973426413,0.260796843287758,0.711033487669047
"5655","PRAC2",9.09072078369933,-1.02632142363594,0.912855422547467,-1
.12429788801805,0.260886674046338,0.711152622533032
"5656","PLK1",3.2613788441422,0.962079143629122,0.856115203858197,1.12
377299140745,0.261109342102817,0.711507956251532
"5657","ZNF417",12.3281462630735,0.545110764922547,0.485044735845844,1
.12383606013569,0.261082580573111,0.711507956251532
"5658","FOXO3B",7.85690938674057,0.647738770142713,0.57649649751877,1.
12357797997138,0.261192101995202,0.711607679790747
"5659","GATAD1",390.708547526307,0.239290784561689,0.213029566739436,1
.12327499052924,0.261320722092213,0.711832290343074
"5660","EIF3L",783.24848490214,-0.261834572782864,0.233166860814447,-1
.12294934137845,0.261459010132328,0.712083152153682
"5661","PCDH10",2.61692046743016,1.08301767901613,0.964683084525238,1.
12266680777255,0.261579029984577,0.712158379938583

"5662","AC007686.3",10.4267620379702,0.537603903486343,0.478835604564148,1.12273168152499,0.261551468344284,0.712158379938583
"5663","EHHADH",5.19459972337526,0.86819372523404,0.773483974499438,1.12244565350678,0.261673002551234,0.712288422095581
"5664","CFI",40.7918739974455,0.727507565435037,0.648301230007963,1.12217520461299,0.261787953033496,0.712475511301437
"5665","ITPK1",53.8522549329469,-0.410555926993085,0.365992886536525,-1.12175930761461,0.261964792263874,0.712830939584751
"5666","CLEC12A",11.2774010716184,0.934742015030951,0.833390846344448,1.12161300922738,0.262027017896368,0.712842023776554
"5667","LINC01697",86.1088189031768,0.52440847294985,0.467582141872065,1.12153229558825,0.262061352497031,0.712842023776554
"5668","ZNF853",45.2287717760771,0.349545468758974,0.311861885545735,1.12083420565259,0.262358441169265,0.713524236172233
"5669","HP1BP3",985.916356068362,0.179244615704579,0.160065868074413,1.11981784662081,0.2627913925337,0.713793146041251
"5670","AL109936.2",2.62394034152073,1.12058733287373,1.00105003005589,1.11941191671625,0.262964449426975,0.713793146041251
"5671","RPL11",18249.5396249062,-0.297455513626423,0.265877565738725,-1.118768756589,0.263238803850117,0.713793146041251
"5672","AC239799.2",15.9197458456174,-0.465082637328523,0.415161466413028,-1.12024519362746,0.262609290038062,0.713793146041251
"5673","ARPC5",827.876817275925,-0.203076761970421,0.181585065065734,-1.11835608229623,0.263414943359941,0.713793146041251
"5674","NENF",1409.13258174019,-0.268483799067444,0.239903510534629,-1.11913243148932,0.263083645878386,0.713793146041251
"5675","LGALS8",144.648243968249,0.290997469856861,0.259975442055715,1.11932676238895,0.263000762579849,0.713793146041251
"5676","WBP1",207.835205404635,0.229689066288312,0.205413620372482,1.11817836554271,0.263490822276467,0.713793146041251
"5677","ATOH8",146.366069723306,0.418521640296245,0.374112548028748,1.11870516640378,0.26326594040183,0.713793146041251
"5678","DAPP1",4.85247570292902,-1.27177943541669,1.13744230185677,-1.11810456964773,0.263522335006212,0.713793146041251
"5679","SELENOP",5066.88012030509,0.288624783934194,0.257658380302921,1.12018395673708,0.262635379150831,0.713793146041251
"5680","EDIL3",135.838367470309,-0.523698659073136,0.468260185941337,-1.1183924552978,0.263399415203369,0.713793146041251
"5681","OSTF1",185.439283880792,-0.258251499238269,0.230838095689478,-1.1187559768543,0.263244257334389,0.713793146041251
"5682","GCNT1",5.09044255770819,-0.985532190136658,0.881346913778215,-1.1182114269985,0.263476705034845,0.713793146041251
"5683","NYNRIN",99.8430484353304,0.309466587199586,0.276549075434832,1.11902954914239,0.263127533117127,0.713793146041251
"5684","TCF12",217.823109049881,0.214103332589406,0.191204189248352,1.11976277000558,0.262814868300758,0.713793146041251
"5685","ISG20",48.6521339276989,-0.308265628266523,0.275422685316361,-1.11924559849686,0.263035377276138,0.713793146041251
"5686","ZNF598",65.6837220334069,-0.252779490647078,0.226007830107645,-1.11845457091766,0.263372898613895,0.713793146041251

"5687","AC092140.2",3.22223566723975,1.15803647765,1.03529021335268,1.
11856217968083,0.26332696577796,0.713793146041251
"5688","SMAD4",212.838106141718,0.183570596493643,0.163868944198399,1.
12022810296129,0.262616571095102,0.713793146041251
"5689","HMGN1",514.938423370344,0.218456481630903,0.195179216338379,1.
11926098346541,0.26302881566845,0.713793146041251
"5690","MIF",2061.86101962518,-0.311190594677714,0.277927575675015,-1.
11968232703039,0.262849158785583,0.713793146041251
"5691","PPP6R2",108.56117562407,0.286938204062694,0.25620342254436,1.1
1996241585342,0.262729778466644,0.713793146041251
"5692","ZFYVE9",92.7203711202051,0.276799214223805,0.247677270304213,1
.11758020380241,0.263746327483609,0.714148891298057
"5693","FUT8-
AS1",2.76362627865042,1.08909935728487,0.974456629621109,1.11764785027
769,0.263717423675883,0.714148891298057
"5694","PXK",343.547836872696,0.273491668269715,0.244938734404503,1.11
657173755972,0.264177481416732,0.71481409093188
"5695","AC108863.2",41.0895850027542,0.462147950719605,0.4138252642732
02,1.11677074992333,0.264092358331898,0.71481409093188
"5696","ANKRD16",65.9522871463662,0.255498577116986,0.228789759932817,
1.11673956558201,0.264105695486164,0.71481409093188
"5697","CD151",1225.66567311381,-0.277359916175604,0.248389562466383,-
1.1166327337653,0.264151389644365,0.71481409093188
"5698","CYP19A1",16.9949808682145,-0.639661723989719,0.573116840074364
,-1.11611050184238,0.264374837402334,0.715222555029304
"5699","HNRNPUL1",529.980365423696,-0.216710337405885,0.19420347088108
9,-1.11589322488771,0.264467842229507,0.71534862045409
"5700","LEO1",147.170783081159,-0.227598870662023,0.203997837472349,-1
.11569256557866,0.264553753948474,0.715455459143111
"5701","TMEM81",3.71346629834077,-0.91750070940054,0.823858988251978,-
1.11366231659042,0.265424081388765,0.715605537099114
"5702","SPRTN",68.7940543873164,-0.241095427663054,0.216417039471524,-
1.11403163194448,0.265265616665124,0.715605537099114
"5703","ZNF141",90.843914245847,0.285722832431013,0.256447092221093,1.
1141589867772,0.265210986750619,0.715605537099114
"5704","MRFAP1L1",172.449107903129,0.195072894102483,0.175173470580515
,1.11359838596576,0.265451519168153,0.715605537099114
"5705","ZSCAN16",9.12885088899525,-0.625988628946392,0.561984914830514
,-1.1138886692985,0.265326950915349,0.715605537099114
"5706","CNKSR3",9.99769714487306,-0.573826025664746,0.515297887173624,
-1.11358117304176,0.265458906953783,0.715605537099114
"5707","AGMO",6.01228976340879,0.80405491278285,0.72137295397895,1.114
61749203078,0.265014371293808,0.715605537099114
"5708","TBRG4",78.6095251524526,-0.278753295649644,0.250148209534768,-
1.11435255190544,0.265127970222341,0.715605537099114
"5709","LY6E-
DT",11.28615484968,0.628801658072076,0.564083099322711,1.1147323130706
7,0.264965149655999,0.715605537099114
"5710","SSNA1",340.916310496888,-0.270956874238182,0.243292211761031,-
1.11370960984285,0.265403785302643,0.715605537099114

"5711","ZNF511",222.571726789201,-0.289837794191696,0.259856278283449,-1.11537730050741,0.264688772655032,0.715605537099114
"5712","STRN3",214.951301389107,-0.188990354069431,0.169689311853637,-1.11374341733699,0.265389277338184,0.715605537099114
"5713","DCAF5",243.004101809413,0.231706917063138,0.207777560683399,1.11516814568923,0.264778373679967,0.715605537099114
"5714","RNF40",104.927651402013,0.249741231796339,0.224232954157792,1.11375793417321,0.265383047829553,0.715605537099114
"5715","NTN1",30.8767379764626,-0.633115064059264,0.567722175799246,-1.11518466434389,0.264771296399157,0.715605537099114
"5716","FHOD3",9.42370224206603,0.626767683841929,0.562659786439286,1.11393722982824,0.265306116240193,0.715605537099114
"5717","WDR83",52.9726367695874,-0.262945287270325,0.236141831013298,-1.11350575263185,0.265491279057401,0.715605537099114
"5718","ATF5",107.311278992192,-0.375109950397269,0.336825968808778,-1.11366101528302,0.265424639865092,0.715605537099114
"5719","RHBDD3",56.7258350356078,0.333635399252267,0.29927099724778,1.11482703743602,0.264924547829944,0.715605537099114
"5720","RPL22",7198.66658666863,-0.286934374654768,0.25775970284448,-1.11318554253568,0.265628750569893,0.715711475588494
"5721","FAM131A",37.1269503936066,-0.331810942804893,0.29827575040042,-1.11243016691586,0.265953240053451,0.715711475588494
"5722","AC098818.2",53.6023613958684,-0.420300313427622,0.377554482937138,-1.11321764784237,0.265614965017918,0.715711475588494
"5723","GPSM3",16.325385081188,-0.467329832601701,0.420017494274074,-1.11264373263642,0.265861470183894,0.715711475588494
"5724","VPS52",56.825803244002,0.300179884254447,0.269885493853125,1.11224905039842,0.266031083484265,0.715711475588494
"5725","SMARCD3",84.0623851452713,0.231686519633448,0.208223185874001,1.11268357873289,0.265844350603051,0.715711475588494
"5726","GSTO1",723.085416654387,-0.274747868901832,0.246859993592081,-1.11297041251583,0.265721136984949,0.715711475588494
"5727","PSMD9",449.235101854898,-0.256728764525296,0.230795901739002,-1.11236275250511,0.265982212768017,0.715711475588494
"5728","RBBP6",573.87355061346,0.27580845256868,0.24798630318653,1.11219228249563,0.266055485416429,0.715711475588494
"5729","NUP93",50.9795321069004,-0.284259289736382,0.255379800995196,-1.113084467247,0.265672154046739,0.715711475588494
"5730","ATMIN",188.053419521786,0.164365978521192,0.147795546936919,1.11211725879228,0.266087737048178,0.715711475588494
"5731","LILRB2",6.740200804499,-1.05491278013493,0.948435263352297,-1.11226650979454,0.266023578796009,0.715711475588494
"5732","NSD2",42.3877429026401,0.368919601779995,0.331847443293212,1.11171446167818,0.266260939855583,0.715927505298067
"5733","STK38L",61.3699871862773,-0.284494640185247,0.255904996914447,-1.11171975387553,0.266258663707175,0.715927505298067
"5734","GRHL1",38.9366180030725,-0.563530424877122,0.507320041213658,-1.11079866572784,0.266655020929151,0.715987586609569
"5735","CFAP44",31.6239457422325,0.512675861237637,0.461488098136925,1.1109189236025,0.266603249242543,0.715987586609569

"5736","HIST1H4H",15.3124968604547,0.507604686064658,0.456850986294417
,1.11109464856782,0.266527611100221,0.715987586609569
"5737","FAM86B1",9.56126505042823,0.555003048440226,0.49970209710658,1
.11066783920631,0.266711350331944,0.715987586609569
"5738","ASAP1-
IT2",11.5703659541996,0.674654025381083,0.607536198117349,1.1104754374
6648,0.266794206778162,0.715987586609569
"5739","MAF1",525.725995409664,-0.209540536718958,0.18866656002992,-1.
11063951495023,0.266723546860845,0.715987586609569
"5740","PI4K2A",89.2109655506152,0.269513676172829,0.242621236671264,1
.11084124320907,0.266636690294849,0.715987586609569
"5741","LIN7A",4.07849626493991,-1.05770095190831,0.952430088267135,-1
.11052870435111,0.266771265999393,0.715987586609569
"5742","SDR39U1",95.1648375532267,-0.254580060961854,0.229111383548984
,-1.11116286331283,0.266498253078094,0.715987586609569
"5743","GOLGA6L4",49.8540186646202,0.367596359009903,0.330801804227452
,1.11122839812914,0.266470050530031,0.715987586609569
"5744","CSTF2",14.2780234631472,-0.450988754016467,0.405811960729483,-
1.11132444988998,0.26642871889353,0.715987586609569
"5745","SLC1A1",5.4232909043758,-0.795117749008458,0.71630997448155,-1
.11001909415536,0.26699079858946,0.716039068739922
"5746","PYGM",15.1508453327297,0.580187688636305,0.522678156275595,1.1
1002857431522,0.266986713541903,0.716039068739922
"5747","PI4KA",97.3066739386485,0.225086134046049,0.202800065066795,1.
10989182361413,0.267045644254675,0.716039068739922
"5748","CRELD2",226.272323808697,-0.289669271962664,0.260921713911006,
-1.1101769477931,0.26692278428112,0.716039068739922
"5749","SPIN2A",33.5689700229423,0.359617043060114,0.324005310035332,1
.10991095491892,0.267037399360771,0.716039068739922
"5750","DIRAS3",5.92773599971706,-0.795299942695498,0.717052017411863,
-1.10912447546841,0.267376487634352,0.716207593691543
"5751","TIPRL",289.768840044282,-0.187355613803074,0.16890346968521,-1
.10924668482094,0.267323778018634,0.716207593691543
"5752","AC092903.2",18.8263742910979,0.505187479565334,0.4554933845293
77,1.10909948799213,0.267387265760287,0.716207593691543
"5753","CEP164",143.563102661608,-0.238758680802344,0.215182671345025,
-1.10956276966893,0.267187481893722,0.716207593691543
"5754","ZMYM5",123.577753193831,0.201870208131217,0.182006898839202,1.
10913492520722,0.267371980320919,0.716207593691543
"5755","SMCHD1",278.822995708671,0.242299122417364,0.218388151274665,1
.10948840861163,0.267219542161909,0.716207593691543
"5756","PCNX2",162.347672464843,0.330829816737931,0.298390616950983,1.
10871387350721,0.267553635018023,0.716404252875251
"5757","AP001830.1",3.40431994273818,-0.949825433330701,0.856684810577
761,-1.10872215965884,0.267550059298323,0.716404252875251
"5758","FRYL",114.566928775183,0.252158367212308,0.22746215354477,1.10
857284731843,0.267614497030204,0.716405760594738
"5759","SWI5",269.237279499849,-0.247987435911524,0.223714986096966,-1
.10849720100574,0.267647147276361,0.716405760594738
"5760","TIGD1",7.34788889235965,0.742601628045066,0.670045174345621,1.

10828591336443,0.267738357150457,0.716525481853176
"5761","MED8",183.400101998182,-0.24758329535523,0.223551663217436,-1.1074992321324,0.268078144099698,0.717061355421974
"5762","EIF3M",578.283216095507,-0.282870639946735,0.255403988093511,-1.1075419849872,0.26805967048151,0.717061355421974
"5763","SNRNP25",128.31240758955,-0.36284933099812,0.327620346438847,-1.10752990448305,0.268064890409669,0.717061355421974
"5764","AL034417.4",7.09397334830903,0.813767470186336,0.735520068696381,1.10638377499154,0.268560445149898,0.717074905619092
"5765","CYP1B1",300.313866207558,0.497662350184831,0.449748837861794,1.10653393247402,0.268495485410986,0.717074905619092
"5766","CPEB2",56.4924458204783,-0.285707913456096,0.258175659878837,-1.10664155401086,0.268448933818426,0.717074905619092
"5767","USP46-AS1",17.0187678060873,0.441139503336298,0.398750663771959,1.10630412289064,0.268594907884829,0.717074905619092
"5768","LYRM7",130.089840044802,0.254993219098381,0.230421845969724,1.10663647374774,0.268451131156693,0.717074905619092
"5769","DNAJB5",7.1417753748417,-0.618801421704012,0.559220242981634,-1.10654331539342,0.268491426617592,0.717074905619092
"5770","COX8A",949.081082131862,-0.263313324806531,0.23786782978375,-1.10697325084234,0.268305493545948,0.717074905619092
"5771","C12orf81",31.6883263487041,0.540055094559979,0.488138006030256,1.10635739870357,0.268571856929647,0.717074905619092
"5772","GAS2L3",5.39256986086753,-0.835604421548763,0.754821696615574,-1.10702226141008,0.268284303689998,0.717074905619092
"5773","PIP4P1",101.331549432875,-0.248902866345222,0.224984227778965,-1.1063125126698,0.268591277771751,0.717074905619092
"5774","SEC23B",48.1811587111301,-0.265270316918185,0.239635876365539,-1.10697246564844,0.268305833036083,0.717074905619092
"5775","CCDC24",4.98333322404898,-0.786270700370635,0.710943435945597,-1.10595394881851,0.268746452232543,0.717136351541708
"5776","HOXB-AS1",104.126781145579,0.310706022980642,0.280907773307049,1.1060784090194,0.26869258304005,0.717136351541708
"5777","MANBAL",233.517584411647,-0.264739586133994,0.239382200675414,-1.10592844993084,0.268757489643623,0.717136351541708
"5778","C3orf70",12.535331488449,-0.691188405036807,0.625052304600459,-1.10580890583009,0.268809239477829,0.717150298814595
"5779","BIN1",868.978837858486,0.314673550872297,0.284614152905034,1.10561455802689,0.26889338593215,0.717199569859021
"5780","SPTY2D1OS",12.9333073114194,0.507260548805943,0.458919571927589,1.10533648995467,0.269013812061295,0.717199569859021
"5781","NADSYN1",69.016875526812,-0.235012694752193,0.212579837470887,-1.10552674020356,0.268931414204661,0.717199569859021
"5782","JMJD8",196.626862696564,-0.285134626613698,0.257941342514116,-1.1054242946653,0.268975781469121,0.717199569859021
"5783","FOXK1",108.500471372465,0.265450548389475,0.240179752643882,1.10521617858047,0.269065928161553,0.717214470449651
"5784","RRN3",91.0470847381948,0.216988262665561,0.196442699527099,1.1

0458807167648,0.269338122061342,0.71781589757531
"5785","USP53",960.307044881699,0.319509988078583,0.289300793456919,1.10442140258479,0.269410380827477,0.717884359629311
"5786","AC239868.1",4.5864330823226,0.824738023608646,0.747241170595648,1.10371063060032,0.269718682712427,0.71796123191367
"5787","HAX1",336.627477407258,-0.244428613412056,0.221353981196616,-1.10424313170561,0.269487684228798,0.71796123191367
"5788","IKBKE",22.2979767314957,-0.388601593675646,0.351984428969307,-1.10403063798465,0.269579847524508,0.71796123191367
"5789","PSMD6",184.074102519494,-0.249047057599614,0.225640399001909,-1.10373434323482,0.269708393304696,0.71796123191367
"5790","AL133346.1",2.75024722320793,0.990439225095372,0.897282164995381,1.10382136604763,0.269670634594918,0.71796123191367
"5791","NUCB1",1081.97208488247,0.210752585362836,0.19090899046734,1.10394269461548,0.269617996863953,0.71796123191367
"5792","ARPC1B",1363.03649886793,-0.29593628659896,0.268265793700126,-1.1031458111643,0.269963849275479,0.718117814768163
"5793","FCN1",10.6232674993633,-0.565557200127103,0.512601412230126,-1.10330792431216,0.269893466510201,0.718117814768163
"5794","BACE1-AS",22.3331542327458,0.481844127819456,0.436782141569439,1.10316810593057,0.269954169071585,0.718117814768163
"5795","ZNF133",28.5976440576869,0.338135988476896,0.306514302258598,1.10316545096033,0.269955321825311,0.718117814768163
"5796","P4HA2",459.165269225464,-0.22543212217897,0.204395965703879,-1.10291864813794,0.270062495162158,0.718256273796527
"5797","SCLY",18.6214582882975,0.391881420331145,0.355723026550578,1.10164760524837,0.270614903564207,0.71836361759829
"5798","PSMD6-AS2",10.58711095201,0.574345537337277,0.521101407210394,1.10217613959616,0.270385103060616,0.71836361759829
"5799","B3GNT5",6.25771923503384,-0.704933099163213,0.640513215624367,-1.10057541666186,0.271081489689864,0.71836361759829
"5800","CLCN2",10.3345850962567,0.565275617854841,0.513518391380174,1.10078943099888,0.27098831270414,0.71836361759829
"5801","CEP44",98.6619196158435,0.222769353825221,0.202112073391012,1.10220705813178,0.270371664189336,0.71836361759829
"5802","ZBTB22",51.8001185903736,-0.285056319377564,0.258655332590121,-1.10207014300873,0.270431178398537,0.71836361759829
"5803","AC004160.1",22.4976391761186,0.52175390374895,0.474007632765018,1.10072890747648,0.271014661046449,0.71836361759829
"5804","POM121C",106.878726481964,0.245853676184202,0.223145973438047,1.10176165133654,0.270565306345764,0.71836361759829
"5805","UPK3BL1",164.928572556677,-0.34519998359009,0.313363214884727,-1.10159701966638,0.270636904596672,0.71836361759829
"5806","NMRK1",73.4399687993519,-0.483211764832182,0.438773304853909,-1.10127885969058,0.27077530904802,0.71836361759829
"5807","ANAPC16",618.800889069974,-0.231825707830434,0.210626756610665,-1.10064700022398,0.2710503213884,0.71836361759829
"5808","FRMD8",49.4655996479405,-0.257540322451329,0.233557268336897,-

1.10268596770809,0.270163562615531,0.71836361759829
"5809","SPCS2",1217.40108220962,-0.25879976682462,0.234776585950737,-1.10232358042264,0.270321021401772,0.71836361759829
"5810","HCFC2",137.073707772529,0.20656643233753,0.187682609129491,1.10061573256907,0.271063935380825,0.71836361759829
"5811","AKAP11",180.601324924725,0.226743754887976,0.205860695117531,1.10144267587614,0.27070404044667,0.71836361759829
"5812","LTB4R",18.8984751460846,-0.456924845956855,0.414816287207675,-1.10151134381109,0.270674170131131,0.71836361759829
"5813","AC092376.1",2.35927029377238,-1.00218894095261,0.910201515300581,-1.10106270326484,0.270869368066517,0.71836361759829
"5814","SPIRE1",54.4945327753044,-0.284146688674874,0.257709177084086,-1.10258661290188,0.270206726433504,0.71836361759829
"5815","CHERP",91.2935745616611,0.225327585250417,0.204722745578942,1.10064753485601,0.27105008861274,0.71836361759829
"5816","KIAA0930",316.712473722813,-0.271572083067711,0.246567427340284,-1.1014110257674 8,0.270717808895739,0.71836361759829
"5817","UXT-AS1",2.47026344627027,1.08393057159447,0.984056852198417,1.10149181845839,0.270682663362533,0.71836361759829
"5818","SLC20A1",306.674379047364,0.394278570155513,0.358485961532205,1.0998438222527,0.271400175062445,0.718978040724373
"5819","AC018553.1",2.76169639786743,1.03920365109469,0.944877471079263,1.0998290073608,0.271406631136888,0.718978040724373
"5820","EXOSC10",108.534244982032,0.237744574989818,0.216301644733937,1.09913438375495,0.271709454227812,0.71918852625834
"5821","C1orf54",100.167496559287,0.448167200376921,0.407873093147316,1.09879079524143,0.27185932809,0.71918852625834
"5822","PRKRA",211.373798054944,-0.252085254195357,0.229323810550643,-1.09925460243339,0.271657028011091,0.71918852625834
"5823","ERCC6L2",128.142935113396,0.240413777770582,0.218736381950724,1.09910283614704,0.271723212985654,0.71918852625834
"5824","NUBP2",202.472847385806,-0.267781511098723,0.243569741110741,-1.09940384990997,0.27159195225181,0.71918852625834
"5825","AC002546.1",2.93002264960077,1.45092696839201,1.32040635305448,1.09884882410297,0.271834011835497,0.71918852625834
"5826","C18orf21",89.8442449149561,-0.301090478438329,0.273888322050354,-1.09931842359812,0.2716291990262,0.71918852625834
"5827","CEP250",43.0035938624249,0.371535334956867,0.33807536155493,1.0989719370499,0.271780306688887,0.71918852625834
"5828","AL590064.1",3.73355267710285,1.07084934323299,0.974844576902011,1.09848212587496,0.271994018421734,0.719288467641191
"5829","CCNF",5.6136305401291,0.675929902922779,0.615320183733023,1.09850110689048,0.27198573458671,0.719288467641191
"5830","MRTFA-AS1",3.34865608497505,1.14673754206021,1.0440229732252,1.09838343740436,0.272037091556805,0.719288467641191
"5831","QSER1",170.654025461331,0.240289390762252,0.218827230386592,1.09807810635698,0.272170384571542,0.719517488967642
"5832","AIMP2",71.6201754548597,-0.292170229744487,0.266142108985174,-

1.09779783010873,0.272292779198861,0.719594238187973
"5833","ALDH1B1",27.3837707879217,0.36363609076172,0.331231865510926,1.09782943196244,0.272278977012057,0.719594238187973
"5834","MAD2L1",11.5672870894757,-0.524330575854154,0.477730514300276,-1.09754466201962,0.272403368264223,0.719763099381727
"5835","KLHL5",51.0534183357664,-0.326433016944996,0.297647913294927,-1.09670856862869,0.272768809528543,0.720234874765756
"5836","ZNF354B",40.2365072803227,0.316000247268129,0.288091948356333,1.0968728875313,0.272696962247853,0.720234874765756
"5837","CBWD3",15.2634374726889,-0.430241091043459,0.392263428813649,-1.09681672937156,0.272721515550475,0.720234874765756
"5838","TARBP2",30.3453016102956,0.367709552988942,0.335263110968696,1.09677903997996,0.272737994841649,0.720234874765756
"5839","RPUSD3",86.073630814992,-0.278825072600347,0.254336151143115,-1.09628564931555,0.272953787566752,0.720476478654364
"5840","PPA2",86.0810906228871,-0.291906403058943,0.266258834332702,-1.09632570048058,0.272936266160575,0.720476478654364
"5841","MAP7D1",213.164563042736,-0.20009860416983,0.182651344863948,-1.09552220553798,0.273287922675153,0.72049483975329
"5842","NEB",2.65270487853163,1.43616752725954,1.31080941856245,1.09563412264353,0.273238922623173,0.72049483975329
"5843","FBXO25",118.469407541833,0.22355487739739,0.204050149771353,1.09558791134382,0.273259154334635,0.72049483975329
"5844","GTF2F2",162.395196336792,-0.186572122326106,0.17022508139198,-1.09603191727359,0.273064807011738,0.72049483975329
"5845","MAP2K5",62.6712727761532,0.264903040307146,0.241754913052977,1.09575039018585,0.273188024206224,0.72049483975329
"5846","GNAS",5543.14846436805,-0.213855134397188,0.195179046347957,-1.09568695205087,0.273215794701139,0.72049483975329
"5847","DGCR6L",337.333406982767,-0.278254995006942,0.253911488119858,-1.09587398769288,0.273133924062315,0.72049483975329
"5848","UBR5",217.139299166385,0.237533996272147,0.216872477151169,1.09527036068563,0.273398208496526,0.72053913215489
"5849","ABITRAM",47.1000051672356,0.329780468855643,0.301071943410551,1.09535436985553,0.273361416511731,0.72053913215489
"5850","KCNK1",52.2826542895886,0.44605286785893,0.40732051028707,1.09509061437776,0.27347694012439,0.720623424276491
"5851","CCNC",176.611157172612,-0.221470588460062,0.20229565360437,-1.09478668727699,0.273610100095549,0.720727903788942
"5852","CCNY",432.46025818755,-0.172005556805055,0.157107520893397,-1.09482700654265,0.273592432414483,0.720727903788942
"5853","NCKAP5L",166.314101231419,0.349524906122933,0.3193260591898,1.09457056843326,0.273704815481501,0.72080902705647
"5854","PCBP2",1668.33363252272,-0.190147259238037,0.173734031329269,-1.09447330372287,0.273747449638859,0.72080902705647
"5855","URI1",406.703686000423,-0.164745743644682,0.150535719017998,-1.09439636465938,0.273781177646165,0.72080902705647
"5856","SH3BP2",46.3649803422865,0.291705537134559,0.266581321670616,1.09424597082232,0.273847114443835,0.720859506344214
"5857","AC103706.1",11.1973446556614,-0.678119531168129,0.619869444246

402,-1.09397154104368,0.273967459980318,0.7210531663986
"5858","RBM17",357.234701343574,-0.197826465771997,0.180853016591435,-1.0938521762062,0.274019816252073,0.721067850379944
"5859","ITK",7.14423354978233,0.755003362092978,0.690504027614964,1.09340906337766,0.274214235930283,0.721390335644127
"5860","DYNC2H1",198.975639927457,0.271019070889989,0.247877351025542,1.09335955773572,0.274235962820278,0.721390335644127
"5861","AC119674.1",14.9354539923558,0.470732565326516,0.430738492784549,1.09285000809522,0.274459660812694,0.721526354798623
"5862","ANKS6",46.7593429497134,0.303920887047235,0.2781353144544,1.09270873295402,0.274521704242227,0.721526354798623
"5863","TTC25",7.77676233486486,-0.668307675772499,0.611592139971958,-1.09273424573956,0.274510499151088,0.721526354798623
"5864","RPL17",12545.6754801423,-0.270433299219829,0.247441479080541,-1.09291821332754,0.274429710729872,0.721526354798623
"5865","OSER1",251.237214705941,-0.282019230608707,0.258010945376372,-1.09305142150971,0.274371223179008,0.721526354798623
"5866","CATSPERE",4.04362015497102,1.06998167898544,0.979373604682734,1.09251635317664,0.274606206562138,0.721625413255259
"5867","AC092645.2",3.87075180454289,0.89867889782833,0.822680741572457,1.09237867427237,0.274666692569543,0.721661337303478
"5868","SNX3",1513.2460994408,-0.245839471681776,0.225095861968967,-1.09215455820183,0.274765172183023,0.72179705678277
"5869","ARHGEF9",69.4979588515736,0.250139870332469,0.229089701273354,1.09188614303529,0.274883149141183,0.721983940025786
"5870","PPCS",393.502730547376,-0.266939303147457,0.244561224427211,-1.09150297138337,0.275051625125194,0.722094366890072
"5871","WDR36",89.5130330571311,0.262574651449045,0.240569535892864,1.09147091494569,0.275065723151379,0.722094366890072
"5872","SLC12A2",36.1136805328773,-0.302063660473581,0.276743686842377,-1.09149250673106,0.27505622729607,0.722094366890072
"5873","FAM193A",100.959442188444,0.283385212161496,0.259714774434549,1.09114012777472,0.275211228079798,0.722353325532111
"5874","ABCA7",3.39278946192043,0.900546349122158,0.825937198507807,1.0903327162757,0.275566608610088,0.723162967607168
"5875","MARC2",68.4588409970575,0.312253809602593,0.286485126995078,1.08994771518438,0.275736176261814,0.723238583814168
"5876","RAB3IP",10.1918728858732,0.7072892613607,0.648883706539365,1.09000927937122,0.275709056507492,0.723238583814168
"5877","POLR2E",576.055105653781,-0.242118410025946,0.222118454557867,-1.09004184505015,0.275694711675683,0.723238583814168
"5878","ADAMTS14",9.163475747977,0.942736680083598,0.865399132486202,1.0893663336306,0.275992371472324,0.723664297711494
"5879","RTEL1",10.8103594659041,0.505436810563768,0.46395657527981,1.0894054260551,0.275975139671683,0.723664297711494
"5880","UBAP1L",9.89001753863685,0.611074766305893,0.561021260536553,1.08921855425135,0.276057518723744,0.723712015497706
"5881","RNASEH2B",200.544743501822,-0.20497966709372,0.188217859349003,-1.0890553521472,0.27612947713507,0.723777570147441
"5882","SCRN3",46.5716967232845,0.326139049279533,0.299720150996027,1.

08814521878396,0.276531004024981,0.724337370780812
"5883","EAF2",11.998457761974,-0.520638809835724,0.478395848536108,-1.0883012706504,0.276462129789896,0.724337370780812
"5884","FBXO9",221.151249449244,-0.188278056093195,0.173014198584631,-1.08822314950699,0.276496607466891,0.724337370780812
"5885","ZNF578",8.03751173078344,0.623642973274228,0.573060632507731,1.08826699636502,0.27647725590679,0.724337370780812
"5886","FCER1A",11.6605489982124,-0.868067468533548,0.798339676001279,-1.08734100863122,0.276886131471957,0.724354344721775
"5887","C1orf112",15.3399301471285,-0.420574456485548,0.386662570233758,-1.08770408325608,0.276725764559028,0.724354344721775
"5888","PARP3",37.8349067925609,0.346355635100561,0.318493702527938,1.08748032489019,0.276824589201605,0.724354344721775
"5889","INPP4B",2.42569258966564,1.20446381214511,1.10748361634846,1.08756806363999,0.276785835826263,0.724354344721775
"5890","RASGRP2",6.57534167891077,-0.734892362037108,0.675900277408444,-1.08727927269812,0.276913405997108,0.724354344721775
"5891","DNAJC7",333.712035217335,-0.182698007376781,0.168026400641415,-1.08731727085363,0.276896618447771,0.724354344721775
"5892","PLD3",974.033005894126,0.2110578731632,0.193989588296557,1.08798557188827,0.27660147704697,0.724354344721775
"5893","MT-ND4L",2131.05602849901,-0.355256062977596,0.326600379792805,-1.08773928310485,0.276710220458123,0.724354344721775
"5894","RMND5A",116.548632750869,0.257715599228712,0.237053784504273,1.0871608726587,0.276965719470724,0.724368266990364
"5895","EMILIN1",515.237389599012,0.225626740337173,0.207623802536152,1.0867094118358,0.27716525319086,0.724767154866345
"5896","RER1",735.842417242062,-0.213788114573189,0.196789204535862,-1.08638131383995,0.277310325248322,0.72502351826711
"5897","FAM41C",3.19248022540613,-0.941975903713254,0.867443850995278,-1.08592147218804,0.277513736219261,0.725432218181922
"5898","AASDH",95.0200532700928,0.223035317706246,0.205428296134993,1.08570884295162,0.277607827120023,0.725432218181922
"5899","DYNLRB1",1125.78051712341,-0.294860750536641,0.271560728839003,-1.08580040934951,0.277567305264848,0.725432218181922
"5900","NUP153",118.907700106212,0.230783929452475,0.212606710121069,1.08549692209176,0.277701626171226,0.725554333462618
"5901","HPGD",149.510212209909,0.357979834688614,0.329868948789048,1.08521834505115,0.277824960982427,0.725630595314151
"5902","ERAP1",87.7272232788372,0.289559232333504,0.266810220876831,1.08526289353501,0.27780523546364,0.725630595314151
"5903","NCAM2",52.8321327377763,0.492905200938286,0.454495278924745,1.08451115730929,0.278138222720412,0.726325716285812
"5904","AGT",10.9108196663174,0.987503358118997,0.910762771350532,1.08425968779408,0.27824967366618,0.726370655302992
"5905","GGA3",78.774618045287,0.269711935007438,0.248731776890193,1.08434852345588,0.2782102983504,0.726370655302992
"5906","ISG15",282.02231989241,-0.435630642784596,0.401882898041501,-1.08397407530293,0.278376293594316,0.726455149103839

"5907","BAK1",31.5152620071988,-0.334990464834759,0.309016403622063,-1.08405398842342,0.278340861944351,0.726455149103839
"5908","THBS3",314.559163813536,-0.267936025510585,0.247237804907738,-1.08371786268921,0.278489913103544,0.72662864090913
"5909","SRSF11",1000.28168043795,0.19724994657794,0.182104627216213,1.08316822912877,0.278733758897889,0.727141799528001
"5910","NDUFA7",195.095948906707,-0.328961625355534,0.303780765875728,-1.08289155308176,0.278856561608861,0.727339068900268
"5911","RSAD2",15.3005674873011,-0.745353627866361,0.688565493837985,-1.0824731046452,0.27904235993329,0.727454418801229
"5912","DEF6",5.39716229783537,0.824932989624444,0.762074279499207,1.08248370508783,0.279037652114697,0.727454418801229
"5913","GCN1",75.3240769394015,0.248563806192644,0.229604193537612,1.0825752019722,0.278997019195345,0.727454418801229
"5914","LPIN2",252.5795726674,0.172330450799749,0.159241024806946,1.0821988304124,0.279164188093638,0.727648961694866
"5915","SAC3D1",68.5537632754678,-0.309997715263937,0.286561069743745,-1.08178586693981,0.279347687984406,0.727881103833607
"5916","ZPR1",105.962370099516,-0.225049793749312,0.2080167572608,-1.08188300170047,0.279304518879498,0.727881103833607
"5917","ATL2",78.0844298784084,0.237876410632077,0.219924944386949,1.08162542132348,0.279419003944268,0.727943881325146
"5918","LINC01138",14.7284047418418,0.446742021440169,0.413175935098626,1.08123920947509,0.279590720772218,0.728100025277669
"5919","ASH1L",553.943901213487,0.232183693807108,0.214751868783424,1.08117193634885,0.279620638964891,0.728100025277669
"5920","TRPC4AP",148.535324740587,0.189668981080177,0.175416876273945,1.0812470562067,0.279587231258536,0.728100025277669
"5921","KPNA3",155.364873999294,-0.260561405806521,0.241045765160374,-1.08096238750829,0.279713844991677,0.728219712978669
"5922","WNT2B",29.8989799695775,0.436098594120993,0.40388327107923,1.07976394505194,0.280247310497085,0.728255612156534
"5923","CHD1L",10.9714778367075,-0.528543473113229,0.489034229413464,-1.08079034415884,0.27979038459254,0.728255612156534
"5924","MARS2",4.034898916008,-0.746526400791426,0.69130921591299,-1.07987335277386,0.280198580930535,0.728255612156534
"5925","ZNF197",62.1322308587169,0.264251335659749,0.244519219876787,1.08069760648224,0.279831648149117,0.728255612156534
"5926","CASC1",4.1353048460758,-0.770034609649398,0.713085542155657,-1.07986288338084,0.280203243688545,0.728255612156534
"5927","ARPC3",1247.36747044591,-0.276326525884907,0.255873577174946,-1.0799338053416,0.280171658175781,0.728255612156534
"5928","CSNK1G1",125.940958221986,0.235246942362821,0.217715903456579,1.08052254625367,0.279909552332779,0.728255612156534
"5929","SNHG22",2.72570150457806,1.08900724849057,1.00826033965789,1.08008537642181,0.280104163212264,0.728255612156534
"5930","CDKN2D",66.7469574108725,-0.289457331614884,0.268017282261133,-1.07999502559265,0.280144395321891,0.728255612156534
"5931","SHANK3",8.3702683616238,-0.580412695208343,0.537347463670434,-1.08014410497771,0.280078014216124,0.728255612156534

"5932","TMSB15B",9.28334066078915,0.553066239112493,0.51201728562415,1
.08017103062898,0.280066026075581,0.728255612156534
"5933","FAM71F2",12.2873742280398,0.701482056538854,0.649736141501512,
1.0796414293928,0.280301885095971,0.728274660164233
"5934","RNF7",751.438718459356,-0.260782034569477,0.241612237974758,-1
.07934116564377,0.280435668107314,0.728499464758045
"5935","XIAP",293.645459402852,0.251713429201215,0.233286527106695,1.0
7898828244844,0.280592951229631,0.728785230531551
"5936","SLC35B2",176.514856118694,0.23091625634809,0.214044557200339,1
.07882330374773,0.28066650422496,0.728853464054541
"5937","ICK",216.908986549489,0.235547196571968,0.218370460782911,1.07
865869645315,0.280739904683238,0.728921278540022
"5938","RNF11",284.507387384581,0.170241631041858,0.157935092443867,1.
07792149551794,0.281068791733609,0.729371893226201
"5939","DPP4",448.520921541107,0.376239038222992,0.349135302096335,1.0
7763103863722,0.281198444900872,0.729371893226201
"5940","NMUR1",4.44795055314429,0.837715618141948,0.777146277167226,1.
07793814723723,0.281061360025182,0.729371893226201
"5941","NAT1",12.1878951058513,-0.506158936526665,0.469617540910276,-1
.07781096835855,0.281118123692165,0.729371893226201
"5942","CCT2",347.182335130853,-0.203857202091088,0.189156298698545,-1
.07771828637846,0.281159495282254,0.729371893226201
"5943","NUBP1",128.802400840196,-0.260793220410634,0.242053095268114,-
1.07742154720956,0.281291982175139,0.729371893226201
"5944","COLGALT1",189.932846475887,0.179780646566636,0.166851884259606
,1.07748646270553,0.281262995349367,0.729371893226201
"5945","ZNRF3-
AS1",3.06799242432334,1.01792469122611,0.944636394941974,1.07758360431
226,0.281219622331034,0.729371893226201
"5946","SLC25A24",173.210112446736,-0.187943789578773,0.17450949458821
5,-1.07698317516911,0.281487782255869,0.729479526099122
"5947","FAM178B",4.59078264832935,-0.726301706233189,0.674328338419197
,-1.07707427502725,0.281447084642257,0.729479526099122
"5948","AC078785.1",4.6848874216045,0.725289253264732,0.67348974855860
5,1.07691209081799,0.281519540989457,0.729479526099122
"5949","CHCHD2",2854.80036186037,-0.298674444556958,0.277345253710509,
-1.0769048345378,0.281522783053109,0.729479526099122
"5950","OXCT1-
AS1",2.54521753834106,-1.09233622251573,1.01477358327172,-1.0764334434
0315,0.281733452144528,0.729902716774437
"5951","GTF3C3",65.1152241950798,0.293295330077713,0.272522878921059,1
.07622277894206,0.281827634625006,0.729951027980088
"5952","TRMT1",51.5870575348644,-0.301728182282289,0.280369649764799,-
1.0761799022662,0.281846806262568,0.729951027980088
"5953","ACADL",35.530300131034,0.418133206439256,0.388591376431092,1.0
7602286566285,0.2819170302865,0.729982832734417
"5954","REV3L",690.238753407396,-0.256442399713167,0.23834251273242,-1
.07594065688595,0.281953797346787,0.729982832734417
"5955","NEK10",3.88618865539504,-0.820339501969268,0.762668023889129,-
1.07561806221539,0.282098106035685,0.730111199553405

"5956","ZNF410",103.705634512754,-0.203335771237244,0.189027572666555,
-1.07569371160433,0.282064260733155,0.730111199553405
"5957","GGCX",175.945564144696,0.262840322745639,0.244507428615558,1.0
7497888401136,0.282384182330957,0.730728919024962
"5958","NFKBID",9.28812858491353,-0.611000764020402,0.568579212717178,
-1.07460974716345,0.282549486078319,0.730911281741448
"5959","ZNF571",31.0053979197689,0.350600372438182,0.326253429288078,1
.07462586126139,0.282542268630002,0.730911281741448
"5960","TAF15",182.579447638206,-0.227628408995805,0.211972283322921,-
1.07385930569532,0.282885744642278,0.731658347929649
"5961","PRKD3",174.650446354711,0.226068454527164,0.210578115393631,1.
07356101133576,0.283019479969725,0.731751104901775
"5962","AC009831.1",3.73826244017315,-0.889118564281568,0.828272281795
055,-1.07346169106933,0.283064018068718,0.731751104901775
"5963","PMAIP1",12.3591870622151,-0.669543886013449,0.623652825622597,
-1.07358430605207,0.283009034628243,0.731751104901775
"5964","AL110114.1",2.90628038750818,1.02379383678015,0.95389746366888
4,1.07327451405776,0.283147966597698,0.731845389856391
"5965","PAQR3",84.7538729012869,-0.402379673002735,0.375029921477847,-
1.07292685185522,0.283303937183397,0.731999095009243
"5966","CEP72",3.95585118332714,0.802221442887819,0.747839573881269,1.
07271862964447,0.283397379112239,0.731999095009243
"5967","AKAP10",72.3615048656511,0.24928038765501,0.232333615290017,1.
07294154289227,0.283297345212782,0.731999095009243
"5968","SUGP2",168.176099108376,0.294916824042311,0.274921669913383,1.
07273036765427,0.283392111000472,0.731999095009243
"5969","NPEPL1",179.948680815353,-0.203917179528982,0.19013413599121,-
1.07249115718184,0.283499483653902,0.732066661175364
"5970","CU638689.5",3.91171072549743,-0.788546785792222,0.735276889745
88,-1.07244875609344,0.283518518794481,0.732066661175364
"5971","AC073352.2",2.94155867035183,-1.19607007275333,1.1155986931863
9,-1.07213290949373,0.283660339196798,0.732187563415713
"5972","NAE1",202.184348699031,-0.233943668780453,0.218194598652352,-1
.07217901004596,0.283639636284625,0.732187563415713
"5973","AC026471.2",3.3105775574902,-0.885344826103792,0.8258924062550
66,-1.07198567198155,0.283726467715267,0.732235643701798
"5974","EIF3A",657.513814864123,-0.226824318349625,0.211626559348486,-
1.07181404379453,0.283803563948857,0.732312008415071
"5975","AC005072.1",4.62534793382523,0.983540882139354,0.9180610814850
68,1.07132401315647,0.28402376620569,0.732634932406412
"5976","ZNF202",12.6333490787547,0.452983300993989,0.422816936853249,1
.07134615837589,0.284013812440807,0.732634932406412
"5977","AC046134.2",18.438964458433,0.488789212268754,0.45652854870207
2,1.07066516137578,0.284320012902018,0.732841238011691
"5978","SNX8",55.4560665446551,0.311072308808888,0.290447551983689,1.0
7101026221202,0.284164815386579,0.732841238011691
"5979","EI24",409.829678985775,-0.209779287422124,0.195942329131992,-1
.07061750440258,0.284341449532788,0.732841238011691
"5980","IFT20",323.868726762429,-0.244370129695279,0.228226194801413,-
1.07073655549449,0.284287901088712,0.732841238011691

"5981","ADAT3",6.332207593647,-0.956140146212155,0.893003700032356,-1.07070121453865,0.284303796519489,0.732841238011691
"5982","CCND2",211.807691788755,-0.289468651291315,0.270407446783002,-1.07049067891836,0.284398502359176,0.732865749559795
"5983","AC005070.3",8.33694122970248,0.62176677785813,0.581184023701222,1.06982771807535,0.284696863377177,0.733144361670429
"5984","AC090517.5",26.3025618326082,0.385769764291196,0.360557316082928,1.06992632539585,0.284652472422295,0.733144361670429
"5985","RPS15A",11828.8443692059,-0.256284508331867,0.239533136937943,-1.06993341968491,0.284649278902207,0.733144361670429
"5986","SCAF4",87.4883427063655,0.268532493553351,0.250958660927241,1.07002680266614,0.284607244472262,0.733144361670429
"5987","CD68",382.09356324146,-0.277755375533528,0.259734432175732,-1.06938218859486,0.284897489820724,0.733538467610901
"5988","SEC14L1",161.564054273846,-0.190005792633906,0.177739358071799,-1.06901360900129,0.285063537215197,0.733843424544466
"5989","ZNF280C",19.5498802322841,0.382214595296868,0.357888057425545,1.0679724773336,0.285532926992359,0.734929048186211
"5990","ANKRD35",330.340634175784,-0.309379934545112,0.289781111612282,-1.06763319673869,0.285686003014883,0.735200289895563
"5991","SEPHS2",244.515911408043,-0.352533153251106,0.330263981378649,-1.06742840009224,0.285778429646963,0.735315388584199
"5992","TNS2",412.743565970509,0.260823413065765,0.244390093073055,1.06724216921427,0.285862494917389,0.735408938443183
"5993","AP003696.1",2.68695942162112,1.29288418991142,1.21209484153475,1.06665266248834,0.286128710484894,0.735970978161963
"5994","FNTB",22.2743269459757,0.388412680851075,0.364305085869005,1.06617419277736,0.286344905912979,0.73640419163306
"5995","BLOC1S3",26.56772560516,-0.354696021035464,0.332761589313791,-1.06591635701376,0.286461454144374,0.736581036803256
"5996","GVQW3",38.2624004179396,0.338396535096547,0.317556702665312,1.06562554736311,0.286592945842596,0.736796240854505
"5997","LAMTOR5-AS1",2.65268070175074,1.01099981139633,0.949635965550958,1.06461828329108,0.287048702672943,0.736870313152177
"5998","AL512343.2",3.56739971662301,0.927351447627524,0.870791775539766,1.06495200537775,0.2868976492578,0.736870313152177
"5999","CXCL1",36.7918203407565,-0.603900200004808,0.567197629963697,-1.06470860966655,0.287007812734657,0.736870313152177
"6000","SNHG6",1294.47502802886,-0.266749048599481,0.250520234061499,-1.06478045415684,0.28697529218735,0.736870313152177
"6001","PRXL2A",153.033776607317,0.343426147977839,0.322464159551266,1.06500563800871,0.286873378399798,0.736870313152177
"6002","MS4A4E",2.54724738161592,-1.07099568581444,1.0054221369304,-1.06521991756044,0.286776422376085,0.736870313152177
"6003","SLIRP",494.393266032544,-0.269981855940712,0.253596706479295,-1.06461104991817,0.287051977325905,0.736870313152177
"6004","TRAPPC8",93.1045168778042,0.236057956774499,0.221677426724912,1.06487142268858,0.286934118674837,0.736870313152177
"6005","BRWD3",55.4837376290025,0.348270111887383,0.327119455590486,1.

06465728630759,0.287031045875555,0.736870313152177
"6006","AC019171.1",8.20117440480945,0.592504360026666,0.5566272418019
84,1.06445447784506,0.287122865957737,0.736929566889529
"6007","ELOC",465.532318060338,-0.276546336624064,0.259931303977003,-1
.06392086059989,0.287364552054627,0.737427096707522
"6008","AL357873.1",7.3091364382632,0.599077118091978,0.56324825138678
2,1.06361114591476,0.287504891078755,0.737541670157932
"6009","CELF2-
AS2",4.77378530529433,1.04257626779865,0.980139765259206,1.06370163190
24,0.287463884963972,0.737541670157932
"6010","PI4KB",150.400035845682,0.224167296264513,0.210806160515257,1.
06338114463353,0.287609139998826,0.737623656683146
"6011","AC091607.2",26.6849962337592,-0.55527968297268,0.5225850107242
92,-1.06256335634861,0.287980012051277,0.737623656683146
"6012","CERCAM",585.783665887343,0.311809772727345,0.293386416260481,1
.06279553328232,0.287874685601486,0.737623656683146
"6013","DIP2C",158.603133482703,0.275210762164212,0.258976917246141,1.
06268452451553,0.287925041191755,0.737623656683146
"6014","ABLIM1",1532.7154946395,0.301524860961688,0.283622257837267,1.
06312129118827,0.287726950200785,0.737623656683146
"6015","CDADC1",115.463959972461,-0.233566402790637,0.219791160326235,
-1.06267423332201,0.287929709764707,0.737623656683146
"6016","ARL17B",24.2367674676574,0.539384158325652,0.507533225202983,1
.06275635079837,0.287892458808593,0.737623656683146
"6017","RTCB",260.446589128851,-0.261495370621277,0.246036511375119,-1
.06283156576948,0.287858341888779,0.737623656683146
"6018","TIMP3",7786.61233695968,-0.205592693764161,0.193501656172413,-
1.06248544757143,0.288015360984486,0.737623656683146
"6019","FAM118A",57.9802346550598,0.347599511973201,0.327015067125293,
1.06294647224931,0.287806226478239,0.737623656683146
"6020","ALG14",79.7407120223956,-0.286109015294144,0.269353562087484,-
1.06220616901,0.288142099882801,0.737744352922809
"6021","SNX1",229.267185263099,0.183800873160804,0.173058896821891,1.0
6207121700289,0.288203355683987,0.737744352922809
"6022","PCTP",46.0969335898961,0.339125618402893,0.319307706297238,1.0
6206524839462,0.28820606508603,0.737744352922809
"6023","CEP120",74.2735570005697,0.316824980379721,0.298448871802768,1
.06157204906138,0.288430008330453,0.737865404873726
"6024","LY6G5C",4.44368804941659,-0.819878480579858,0.772441681192589,
-1.06141149622329,0.288502934631281,0.737865404873726
"6025","ALDH1A1",587.440938047813,0.638577136146564,0.601735209496604,
1.06122614410545,0.288587140716044,0.737865404873726
"6026","AP004609.3",13.0161931786004,-0.477444809518238,0.449874728422
206,-1.06128390717284,0.288560896985302,0.737865404873726
"6027","DHRS7",596.180167353142,-0.207839305947758,0.195868235515239,-
1.06111797760892,0.288636288769936,0.737865404873726
"6028","MLX",158.414875175613,-0.213663546905654,0.201237175045982,-1.
06174988223141,0.288349247465114,0.737865404873726
"6029","GPX4",7125.86508163967,-0.311790719395562,0.293823950667623,-1
.06114807416861,0.288622613106424,0.737865404873726

"6030","SBF1",74.9249278741751,0.24685001723227,0.232581143128877,1.06135009017256,0.288530829769873,0.737865404873726
"6031","C3orf18",30.5380963731485,0.31402277715711,0.296048413039197,1.06071427282244,0.288819771586454,0.737967309631225
"6032","THG1L",78.3872428019798,-0.231446050912376,0.218180088910326,-1.06080280775531,0.288779525953386,0.737967309631225
"6033","CD248",3135.61965461353,0.308053847685509,0.29037519334701,1.06088210957253,0.2887434806588,0.737967309631225
"6034","AC113383.1",5.92950635927054,-0.826410683602536,0.779356663156874,-1.06037546436706,0.288973819835388,0.73811622746686
"6035","GRK6",49.7825215559033,-0.297266276644657,0.280323049133927,-1.06044179229313,0.288943657727404,0.73811622746686
"6036","IRF2BP1",94.60992205234,-0.234310597684921,0.221084723673188,-1.05982264985112,0.289225290458131,0.738636158451306
"6037","GRPEL1",101.004535215666,-0.263014136989772,0.248285623430486,-1.05932084732006,0.289453683766919,0.739050556412208
"6038","GRB10",57.3164780184512,0.253696374780962,0.239524370365376,1.05916727552177,0.289523605593552,0.739050556412208
"6039","ATP5F1B",817.62776942798,-0.230113831746044,0.217262701786456,-1.05915018939707,0.289531385674559,0.739050556412208
"6040","PSMG4",177.26898020236,-0.285259487524444,0.269433108439327,-1.05873954829378,0.289718411392327,0.739405515167668
"6041","FZD3",15.0461622001065,0.451067448781125,0.426127950125018,1.05852584569679,0.289815774013303,0.739418116428529
"6042","TVP23A",2.98147552565725,1.32143047273856,1.24837772646815,1.0585181429639,0.289819283779512,0.739418116428529
"6043","MAFG-DT",9.42409783436728,-0.494193593922429,0.46695497617269,-1.05833242847736,0.289903913632839,0.73951163803578
"6044","CCDC39",17.2089393877401,0.488153762786194,0.461308774823618,1.05819310064683,0.289967416068199,0.739551243992602
"6045","LINC02482",38.4942663303051,0.453565160327703,0.428791124428692,1.0577764661804,0.29015736438253,0.7396836110566
"6046","LRRC17",437.77329731806,0.4575100772997,0.432498519934646,1.05783038834175,0.290132775948963,0.7396836110566
"6047","CTDNEP1",555.482953798182,-0.182852739948769,0.17286731577244,-1.05776351724853,0.29016326928701,0.7396836110566
"6048","EDRF1",67.7405027403084,0.337692979549276,0.319313373449214,1.05755977553187,0.290256189171547,0.739798140886144
"6049","ITPRID2",110.842270532544,0.234651344457705,0.221905351439117,1.05743887173485,0.290311338876795,0.739816381019309
"6050","SLC45A1",10.1508462899557,-0.526607331328545,0.498436010168369,-1.05651943396036,0.290730966856125,0.739832440221344
"6051","FAM49A",84.306217951841,0.262400347478797,0.248226415493156,1.05710082046458,0.290465576813768,0.739832440221344
"6052","SEC13",304.073233210599,-0.225305208876488,0.213199565430899,-1.0567808073207,0.290611635540015,0.739832440221344
"6053","AL356599.1",55.2904128648274,0.314464557413252,0.297606088883892,1.05664692074206,0.290672758010587,0.739832440221344
"6054","PLAG1",4.88249860262999,-0.838422125980991,0.793423990407291,-

1.05671385806043,0.290642198420234,0.739832440221344
"6055","TOMM5",682.484072856203,-0.26930712790044,0.254847293580867,-1
.05673921082856,0.290630624421619,0.739832440221344
"6056","MTG1",138.707095480352,-0.252406388437576,0.23893265825861,-1.
05639132916013,0.290789465778992,0.739832440221344
"6057","CEP57",266.613605089808,0.174712767895562,0.165405645806484,1.
05626846679687,0.290845578186269,0.739832440221344
"6058","ARID4A",280.602095502343,0.208491758688135,0.197307365695575,1
.05668512654422,0.290655315253467,0.739832440221344
"6059","UBE2O",51.0624553968543,0.277128656777148,0.26211583983758,1.0
5727550440626,0.290385869325616,0.739832440221344
"6060","TXNL4A",384.931193039198,-0.242289761397809,0.229372866276738,
-1.05631396307132,0.290824798756908,0.739832440221344
"6061","CRYBG1",42.4491289837941,-0.498106149698846,0.471656088570477,
-1.05607912580655,0.290932066272638,0.739930341790582
"6062","MIR210HG",4.63884808215763,-0.754632549499359,0.71463523796823
4,-1.05596884873021,0.290982447135159,0.739936394356397
"6063","TPD52L1",16.1939419678468,-0.614074081179939,0.581607585580632
,-1.05582199476799,0.291049547515708,0.739984953810761
"6064","MEIS1",71.6577797449973,0.277438505617127,0.262869845016715,1.
05542157412344,0.29123256022098,0.740084016795182
"6065","SPEGNB",4.06171126675723,-0.900694393982434,0.853737453652411,
-1.05500161686609,0.291424585270629,0.740084016795182
"6066","MEF2C",59.5801640357911,-0.35542613203142,0.336842797103326,-1
.05516916225581,0.291347965100799,0.740084016795182
"6067","SEMA6A",62.0012027551218,0.46837184296964,0.443787779433186,1.
0553959903264,0.291244255953364,0.740084016795182
"6068","PHF3",561.753289891675,0.228032932040611,0.216126038687911,1.0
5509235918534,0.291383086225953,0.740084016795182
"6069","DRD4",3.09100511777881,0.896736769374125,0.849566302307175,1.0
5552299677947,0.291186197566576,0.740084016795182
"6070","MYEF2",3.54390280191591,0.902708955647806,0.855598233503214,1.
05506173376691,0.291397091657084,0.740084016795182
"6071","TMEM222",167.681654762683,-0.267376404656767,0.253899768903162
,-1.05307856644306,0.292304985291467,0.740158573068232
"6072","NDC1",29.178785230501,0.377340297417938,0.358158505494573,1.05
355671198392,0.292085916720818,0.740158573068232
"6073","AC253572.2",36.4876459624895,-0.42112754471583,0.3992952001994
54,-1.05467720249447,0.291572981686877,0.740158573068232
"6074","NBPF15",30.9315138580664,0.344742553924962,0.327246644291218,1
.0534639848535,0.292128392232711,0.740158573068232
"6075","MRPL53",26.0363778176794,-0.465353225882154,0.442056467700982,
-1.05270086489704,0.292478112146917,0.740158573068232
"6076","AC007405.1",4.20019909166752,0.92908007519358,0.88170974995298
1,1.05372553183531,0.292008596074074,0.740158573068232
"6077","FOXP1-
IT1",6.02291666992022,0.707288494259419,0.671404635887621,1.0534459496
6098,0.292136654094436,0.740158573068232
"6078","CCDC58",63.9030029597887,-0.301464101223444,0.286152660854217,
-1.05350794335974,0.292108255644727,0.740158573068232

"6079","KPNA4",347.445517838274,-0.185581762080892,0.176240925528242,-1.05300038299647,0.292340816545292,0.740158573068232
"6080","UTP15",26.933020698221,-0.393829056713236,0.373463074403669,-1.05453278705555,0.291639057775664,0.740158573068232
"6081","HULC",10.6473285598944,0.540639342723801,0.513095345723065,1.05368202465746,0.292028521343638,0.740158573068232
"6082","IPCEF1",2.38227364472994,1.08743923444817,1.03296520115818,1.05273559383115,0.292462190589749,0.740158573068232
"6083","HOXA-AS3",3.79069474144731,0.848555271375365,0.806237603576955,1.05248783684941,0.292575788065936,0.740158573068232
"6084","AC100810.1",370.22619161185,0.263784240114456,0.250282018166335,1.05394803049393,0.291906711174572,0.740158573068232
"6085","SH3GLB2",222.920088994357,-0.186645681495578,0.177137605882345,-1.05367621158631,0.292031183663405,0.740158573068232
"6086","ATP5F1C",868.902686392376,-0.283320497183869,0.268614297913357,-1.05474838601203,0.291540415960248,0.740158573068232
"6087","HPS1",174.995611411666,-0.198749721353894,0.188787037038382,-1.05277207837891,0.292445464794564,0.740158573068232
"6088","MCRS1",120.225292802438,-0.250837798758519,0.238119522858299,-1.05341131104059,0.292152522371509,0.740158573068232
"6089","EID3",50.1616905301383,-0.321137193645595,0.305123038049188,-1.05248425585558,0.292577430181809,0.740158573068232
"6090","POP5",149.648929488631,-0.300153722975781,0.285069522966006,-1.05291410969798,0.292380358779175,0.740158573068232
"6091","AL139383.1",3.89670405550252,0.90343518964798,0.857361559319425,1.05373885711079,0.292002493592383,0.740158573068232
"6092","AC000403.1",6.00343289988953,0.679116512356234,0.644895183694373,1.05306494687372,0.292311226889632,0.740158573068232
"6093","ZNF652",232.386537314924,0.200616813617284,0.190315891069807,1.05412539378385,0.291825511432823,0.740158573068232
"6094","TSPO",1933.12368901257,-0.290183595978515,0.275729505513098,-1.05242126858538,0.292606314906118,0.740158573068232
"6095","TMEM119",296.97375479799,-0.232064982306819,0.220560613499859,-1.05215966996287,0.292726299384278,0.740340591469834
"6096","GNG12",671.696611155301,0.204557682756409,0.194548260297321,1.05144956034966,0.293052163841095,0.740729954368613
"6097","CP",397.727739560523,-0.480231557736059,0.456797532701762,-1.05130068215495,0.293120513892218,0.740729954368613
"6098","OTUD4",90.5182071696833,0.239464349490572,0.22768909049268,1.05171639524938,0.292929686580848,0.740729954368613
"6099","PHACTR1",72.5707741454085,0.396734093256199,0.377306373186367,1.051490569602,0.293033338352899,0.740729954368613
"6100","AP001527.2",6.24369960289816,-0.704984675176828,0.670524032404788,-1.0513935983002,0.29307785479011,0.740729954368613
"6101","VAMP4",118.297671243776,0.192827306869263,0.183612886698524,1.05018395133599,0.293633546665657,0.74081196756974
"6102","FAM228B",90.5161351770244,-0.275944467247993,0.262630898050232,-1.05069308027578,0.293399575141801,0.74081196756974
"6103","MPV17",215.702653009439,0.223343594960327,0.212640222662056,1.

0503355957978,0.293563844975578,0.74081196756974
"6104","MED22",54.7468984889752,0.285838277881831,0.272083775932008,1.05055245173185,0.293464188845713,0.74081196756974
"6105","LINC02328",28.1311224801118,0.354812730779191,0.337602341472811,1.0509782877432,0.29326856199157,0.74081196756974
"6106","NAA60",116.301035920441,0.197677997796992,0.188216487869045,1.05026929380666,0.293594318582542,0.74081196756974
"6107","EZH1",248.926909746482,0.209113536200632,0.199055236664917,1.0505301930471,0.293474416777321,0.74081196756974
"6108","AC008735.2",4.41456157703938,1.2689836515743,1.20810940429201,1.05038802534441,0.293539748900198,0.74081196756974
"6109","RTF2",495.711664227914,-0.212470269345929,0.202173368026278,-1.05093104705221,0.293290259803209,0.74081196756974
"6110","HMGXB4",197.772191421166,0.159760356137179,0.152103284937472,1.05034126122164,0.293561241141641,0.74081196756974
"6111","ERRFI1",1506.6273384961,0.369350317798442,0.351843710640097,1.04975677162595,0.293829954750338,0.740822497951662
"6112","COL25A1",4.81939607280542,0.883934488129665,0.841819169795355,1.05002893714638,0.293704808687599,0.740822497951662
"6113","ABCD2",3.20405452469126,1.05779780706112,1.00764641775504,1.0497708208181,0.293823493831585,0.740822497951662
"6114","NACA2",3.90539196643401,-0.956013659655781,0.910662388570711,-1.04980031200833,0.293809931782792,0.740822497951662
"6115","MROH8",9.55238439498262,-0.660094343530551,0.628889201941947,-1.04961945839147,0.293893107145557,0.74086054728516
"6116","SASS6",56.9074694429513,-0.28858688215026,0.27511016801907,-1.04898660863111,0.294184281798637,0.74111573838214
"6117","TASOR2",160.515609420393,0.256776849251939,0.24474417193739,1.04916430581083,0.294102503668181,0.74111573838214
"6118","TUB",50.8124673812354,0.33650815942855,0.320795143603966,1.0489814641458,0.294186649572515,0.74111573838214
"6119","UCKL1-AS1",2.92256014897525,1.08286001783708,1.0320089075732,1.04927390634975,0.294052071936551,0.74111573838214
"6120","POC5",33.1065998787109,0.325672793643741,0.310498747388896,1.04886991133603,0.294237995427236,0.741123970508308
"6121","COX7A2L",731.879408505833,-0.253013089973345,0.241252621546738,-1.04874752593861,0.294294334248712,0.741144774129047
"6122","ARPC4",433.213143858099,-0.202845477076473,0.19359964451016,-1.04775748731206,0.29475035392195,0.741399325139288
"6123","HMGCR",42.9907357982581,0.260578748110347,0.248699503258012,1.04776545468211,0.294746682198874,0.741399325139288
"6124","SEMA3A",57.3977975541165,0.631341831022442,0.602451990251414,1.04795376434722,0.294659909544572,0.741399325139288
"6125","HNRNPC",877.276800296329,0.155429662485812,0.148354237808538,1.04769277090962,0.294780179291514,0.741399325139288
"6126","BTG3-AS1",4.77186646556756,-0.860848796272278,0.821548114869724,-1.04783734597064,0.294713552840256,0.741399325139288
"6127","TBC1D22A",104.322321767603,0.227970192792876,0.217442953056334

,1.04841380044086,0.294447997992959,0.741399325139288
"6128","NAP1L2",3.11765599719721,0.965722295286234,0.921455770105559,1
.04803977208326,0.294620283076506,0.741399325139288
"6129","KLHL4",25.4480487060502,-0.559634990423805,0.533957185280722,-
1.04808963312214,0.294597312163794,0.741399325139288
"6130","RMND5B",122.210677643745,-0.231285235794869,0.220781178402585,
-1.04757677927206,0.294833640559828,0.741412817166353
"6131","PUS3",58.7691116805614,-0.314866844630131,0.300636607379753,-1
.04733368093262,0.294945707174526,0.741573654558036
"6132","NR4A3",38.3043395625946,-0.635216598123475,0.606773526121339,-
1.04687592780119,0.29515680558751,0.74174146692703
"6133","FAM111A",127.669352089661,0.23923932072693,0.228515933567498,1
.04692621206768,0.295133611440634,0.74174146692703
"6134","BRMS1L",76.6179061021925,-0.231733928024111,0.221318227843197,
-1.04706209823934,0.295070938622616,0.74174146692703
"6135","TMEM165",569.039663419812,-0.153687017408964,0.146848524296631
,-1.04656834752061,0.295298706804316,0.741977109272784
"6136","HMGCL",175.915164700188,0.267990116033417,0.256195568943009,1.
04603727979789,0.295543820804874,0.742074557152055
"6137","ACY1",42.81909973377,0.398254999557879,0.380614264378689,1.046
34806635003,0.295400360920867,0.742074557152055
"6138","SNHG7",700.207350219501,-0.317847415874378,0.303826303895491,-
1.04614844665889,0.295492500533226,0.742074557152055
"6139","SNHG21",24.3795586756508,-0.418159869348089,0.39969233568197,-
1.04620437275738,0.295466684460888,0.742074557152055
"6140","H3F3B",9505.2069908554,-0.212983857134385,0.203624687961999,-1
.04596284107815,0.295578188836433,0.742074557152055
"6141","GPC3",5239.29229375523,0.261011153330291,0.249573645958279,1.0
458281856167,0.295640365473158,0.742109792178591
"6142","INPP5D",5.26998768730766,-0.72250794606415,0.690975828742834,-
1.04563418286091,0.295729960889048,0.742150969674742
"6143","CASS4",3.55278362773427,-0.832207734706878,0.795926100740607,-
1.04558417412435,0.295753059144466,0.742150969674742
"6144","TYW3",200.303561028818,-0.204910687916633,0.196072173523896,-1
.0450778620642,0.295986984805854,0.742375426420801
"6145","ALAD",63.8363418828469,0.251415855549747,0.24052762839445,1.04
526809343266,0.29589907982827,0.742375426420801
"6146","EXOC5",178.960189958588,0.178346768443494,0.170642013391026,1.
04515157140588,0.295952922008372,0.742375426420801
"6147","ESD",1108.72205841157,-0.252435533592835,0.241578486969028,-1.
04494210871185,0.296049726447365,0.74241199498717
"6148","AC008494.3",2.40905708447998,-0.958456731210115,0.917658039020
536,-1.04445958129798,0.296272810017661,0.742850580094704
"6149","SLC25A22",13.978781488487,0.451580200326644,0.432442988962536,
1.04425372095873,0.296368018231147,0.742968450322513
"6150","NDUFS8",391.831750798176,-0.298213485813241,0.285706688293384,
-1.04377495533816,0.29658952138918,0.74328588064004
"6151","NDUFA11",1281.16199461918,-0.249669424213828,0.239223169226537
,-1.04366740488,0.296639295342039,0.74328588064004
"6152","ZNF66",25.617635344094,0.463876868343421,0.444448726572605,1.0

4371289781982,0.296618240696498,0.74328588064004
"6153","LINC02447",8.5791845662311,0.535991439560178,0.513795841433766
,1.04319925607898,0.296856017961999,0.743356152476101
"6154","SETDB2",54.7586349039756,0.298717350820567,0.286320072406245,1
.04329867029627,0.296809986754871,0.743356152476101
"6155","GRN",1107.67509969222,0.280550815552749,0.268935451525709,1.04
319015570891,0.296860231893797,0.743356152476101
"6156","STARD8",6.4266450868892,0.759057021433032,0.727470930740947,1.
04341904172022,0.296754258239684,0.743356152476101
"6157","LMX1A",17.908620805406,0.384240816637723,0.368404648360221,1.0
4298579930517,0.296954869784301,0.743472359546045
"6158","MCM2",17.9420177409032,0.42154179106128,0.404402670353067,1.04
238132427081,0.297234921563902,0.743847399644305
"6159","NDUFA8",263.431372388471,-0.296169446909944,0.284130825049652,
-1.04236999578693,0.297240171707298,0.743847399644305
"6160","PIP5K1C",105.553628069116,-0.254265734380357,0.243935079732411
,-1.04235001648503,0.297249431190977,0.743847399644305
"6161","ZSWIM5",5.93631617787894,0.740956292593562,0.711459222053404,1
.04145995951114,0.297662127141463,0.74387525049038
"6162","HSPE1",677.346910530779,-0.279179628423352,0.267977274711155,-
1.04180337203694,0.29750285046272,0.74387525049038
"6163","AC012467.2",7.52501195994786,0.661229024745338,0.6350701851247
66,1.04119047033429,0.29778715764995,0.74387525049038
"6164","ATXN7",174.462558157302,0.350622255295001,0.336859628233393,1.
04085567372316,0.297942536658079,0.74387525049038
"6165","HEG1",428.395609285021,0.254216969327781,0.244120520895304,1.0
4135845850012,0.297709214795121,0.74387525049038
"6166","MRPS22",204.2193659818,-0.270913971619738,0.260302613257375,-1
.04076546996426,0.297984409456899,0.74387525049038
"6167","EIF4A2",1681.28767116733,0.202063218264743,0.194001199366032,1
.04155654153199,0.29761732609338,0.74387525049038
"6168","UBE2D3-
AS1",36.6910830591198,-0.279638895169911,0.268547491724521,-1.04130145
984297,0.297735659403997,0.74387525049038
"6169","RPP21",299.617313917332,-0.266448306980709,0.255825939235446,-
1.04152185574695,0.297633415106856,0.74387525049038
"6170","BLVRA",170.919772982581,-0.256485227513963,0.246375530212433,-
1.0410336906949,0.297859912292124,0.74387525049038
"6171","USP54",39.344376580923,0.343463970443701,0.329995518353429,1.0
4081404546787,0.297961860104426,0.74387525049038
"6172","FAM24B",21.7582945651818,-0.384159067995027,0.368644353110296,
-1.042085860678,0.297371873336757,0.74387525049038
"6173","SNHG1",70.1306934304703,-0.27452540938061,0.26366271991817,-1.
04119918608824,0.297783113394367,0.74387525049038
"6174","SLX4",22.2104651517154,0.434636200541034,0.417519358217435,1.0
4099652384186,0.297877161560635,0.74387525049038
"6175","WDR45",370.981798556987,-0.258961857911394,0.248699848033636,-
1.04126263027057,0.297753675338748,0.74387525049038
"6176","AP003469.4",11.0918168789622,0.558936146392907,0.5371837534998
38,1.04049339309193,0.298110731997978,0.743949641209136

"6177","LONRF3",32.6871860136938,-0.40474087025315,0.388988167564547,-1.04049661147081,0.298109237528171,0.743949641209136
"6178","MAP4K2",13.1412378643766,-0.459687382079003,0.441920798498083,-1.04020309440357,0.298245554183548,0.743955282924308
"6179","POLE2",5.66106877173692,-0.677061778414427,0.650910299568583,-1.04017677837204,0.298257778039068,0.743955282924308
"6180","USP8",398.0786423921,0.173466900292853,0.166747668239561,1.04029580817669,0.298202491094577,0.743955282924308
"6181","ITGB5",846.213056366721,-0.220396675707865,0.211965242548684,-1.03977743264792,0.298443316087916,0.74407974842877
"6182","FOXC1",82.2814672783381,-0.274193837672362,0.263758430837942,-1.03956425886091,0.298542389255956,0.74407974842877
"6183","ZBTB6",37.5724268112165,0.364509430591518,0.350604226413044,1.03966068612673,0.298497571670136,0.74407974842877
"6184","OIP5",3.03803701132675,-0.939538147965483,0.903793147728993,-1.03954997924725,0.29854902653467,0.74407974842877
"6185","SLC7A6",73.5042932162125,0.31997544500946,0.307739740419276,1.03975991067489,0.298451458650119,0.74407974842877
"6186","TTC30B",16.5762153090521,-0.401341156073003,0.386331571813416,-1.038851560045,0.298873777629858,0.744523283116759
"6187","ASNSD1",143.850612960669,-0.230691569614067,0.222042139221019,-1.03895400406154,0.298826128440316,0.744523283116759
"6188","DAGLB",27.788497177054,0.36678077099975,0.353065408058859,1.03884652143153,0.298876121341678,0.744523283116759
"6189","GOLGA2",532.449067003352,-0.193977047804718,0.186756730313718,-1.03866161866762,0.298962137376037,0.744523283116759
"6190","MED13L",557.386478794797,0.262347247298612,0.252585333056869,1.03864798531095,0.298968480213606,0.744523283116759
"6191","FLVCR1-DT",15.8435869255701,-0.515024497710666,0.495937821185632,-1.03848602729956,0.299043837079943,0.744590655562481
"6192","INTS3",94.0756044105767,0.239531126153987,0.230748306651197,1.03806233566891,0.299241035003877,0.744841039009326
"6193","HCG27",6.41723237486096,0.734785415367264,0.707842370794205,1.03806362219152,0.299240436089001,0.744841039009326
"6194","GAPDH",3571.639141429,-0.313243510021153,0.301900407927086,-1.03757233112718,0.299469204988846,0.744927835227216
"6195","GPR162",4.06100247939968,-0.834865042982385,0.804560630973105,-1.03766579029927,0.299425676890497,0.744927835227216
"6196","TMBIM4",1565.35375980049,-0.2421741939317,0.233392008010677,-1.03762847749534,0.299443054618678,0.744927835227216
"6197","AC004687.1",7.25963577798016,-0.855414785076123,0.824230409969165,-1.03783453598627,0.299347095200719,0.744927835227216
"6198","LIME1",17.8569049473663,0.408773014505193,0.394023903959018,1.03743201972769,0.29953456218166,0.744970196197206
"6199","RALB",188.942252623076,-0.207857814896155,0.200616556188439,-1.03609502049728,0.300157815069619,0.745317770505506
"6200","CEP57L1",116.263379726557,-0.191005496923159,0.184347011764072,-1.03611930074358,0.30014648893636,0.745317770505506
"6201","SLC12A9",54.4943031208386,-0.235321952561457,0.226962323114484

,-1.03683267483456,0.299813844764098,0.745317770505506
"6202","STX17",197.916782607726,0.239793410927547,0.231390260699645,1.
03631592013637,0.300054781357611,0.745317770505506
"6203","PPP6R3",157.799331237162,0.172411367842454,0.166363338008162,1
.03635434289011,0.300036862328545,0.745317770505506
"6204","NACA",9193.6499564735,-0.285090384091303,0.275110439676266,-1.
03627613850925,0.300073334869524,0.745317770505506
"6205","AC022558.1",3.34296815184816,-0.808558588427534,0.780048007304
852,-1.03654977752099,0.29994572949372,0.745317770505506
"6206","CEP192",66.9904632946349,0.239868340868504,0.231490537171146,1
.03619069617159,0.300113186389021,0.745317770505506
"6207","ZNF558",29.6949364184354,0.321572148960962,0.310356459151076,1
.03613809050588,0.300137724173406,0.745317770505506
"6208","POLR2F",454.709583654834,-0.253804369374365,0.244840324872256,
-1.03661179794131,0.299916812701457,0.745317770505506
"6209","FAAP20",313.495852701095,-0.233872437289053,0.225822138853988,
-1.03564884504203,0.300365995589949,0.74547445210418
"6210","EFR3A",193.184705057757,-0.16265904028607,0.157045226987102,-
1.03574647511851,0.300320434255302,0.74547445210418
"6211","PRR14L",132.906799012616,0.262354922071423,0.253284014410017,1
.03581318656266,0.300289304465469,0.74547445210418
"6212","AL445423.1",7.30481982051801,0.609614011621495,0.5889347866615
48,1.03511292833825,0.300616175576132,0.74572033971842
"6213","IL1R2",4.81155934096406,-0.727169983784942,0.702439610512922,-
1.03520640479537,0.300572528316979,0.74572033971842
"6214","TAOK2",51.6719820638489,0.326321608057693,0.315279834792413,1.
03502213604162,0.300658573554977,0.74572033971842
"6215","NOL12",51.1986516133877,-0.36741244514683,0.354973365764378,-1
.03504228931561,0.300649162081169,0.74572033971842
"6216","SLC6A8",35.8549703277642,-0.291565516169541,0.281906893330567,
-1.03426174764605,0.301013814410146,0.746481330298005
"6217","PDSS2",68.7958333634628,0.218859067391635,0.211652199707658,1.
03405052106206,0.301112545570807,0.746606062405338
"6218","ROM1",21.4543208281598,-0.350758251529326,0.339275174721681,-1
.03384590934797,0.301208205385117,0.746723140239882
"6219","FKBP14",217.162561660977,0.30571975447373,0.295829340513519,1.
03343283645578,0.301401386379274,0.747081905617706
"6220","EPS8L2",109.219384040384,-0.191999564160677,0.185839541547459,
-1.03314699639229,0.301535112931208,0.747293209941249
"6221","CETN3",155.609350530596,0.266752564639382,0.258317707789507,1.
03265303382433,0.30176629999167,0.747345525650693
"6222","STOML2",416.453503245134,-0.244262409885039,0.236509419647443,
-1.03278089409358,0.301706446815185,0.747345525650693
"6223","UHRF1BP1L",136.509595317452,-0.218031975485679,0.2111729869410
79,-1.03248042585349,0.301847112727941,0.747345525650693
"6224","AC233723.1",16.0452827019058,-0.732694617544393,0.709420361793
531,-1.03280742561719,0.301694028028822,0.747345525650693
"6225","MAP2K6",53.7319052279198,0.291574018660753,0.2823819874966,1.0
3255176169572,0.301813712497395,0.747345525650693
"6226","RRBP1",1389.95559594026,0.222119765481792,0.215031533238869,1.

03296368740044,0.301620892442919,0.747345525650693
"6227","ZRANB3",14.8596910473143,0.417423669938409,0.404430193898463,1
.0321278584932,0.302012224792706,0.747634245251256
"6228","CUEDC2",331.38024756367,-0.260057561638072,0.252029877764624,-
1.03185211191883,0.302141402523973,0.747833930620912
"6229","XRN2",357.131380299847,-0.14220284753012,0.137893648731057,-1.
03125016154636,0.302423523163338,0.748412041991147
"6230","HDLBP",1089.58815308603,0.184044111977653,0.178489052684412,1.
03112268909322,0.302483289120042,0.748439791618851
"6231","PPP1R3F",28.194713469584,-0.395861071103797,0.383965361330405,
-1.03098120552379,0.30254963344211,0.748483806693969
"6232","PHTF1",43.2066678499084,-0.261063817596477,0.253341096870895,-
1.03048348973368,0.302783098699005,0.748744264834081
"6233","NUP54",198.584741589293,-0.193278148962618,0.187587305419894,-
1.03033703975856,0.302851817298249,0.748744264834081
"6234","MIOS",43.847123193317,-0.285458928105007,0.277057807689607,-1.
03032262647805,0.30285858099031,0.748744264834081
"6235","POT1",40.034414240381,0.272171140515575,0.264187085237834,1.03
022121717476,0.302906171975963,0.748744264834081
"6236","PPFIA1",235.479456249317,0.20222028219175,0.196207998280303,1
.03065129857897,0.302704370643671,0.748744264834081
"6237","AC243829.1",4.19261867318752,-0.839343747831168,0.814789564992
625,-1.03013561279318,0.302946349644513,0.748744264834081
"6238","RAC1",2538.32479585717,-0.199889044259675,0.194121271427334,-1
.02971221437987,0.303145120091295,0.748995356019764
"6239","AC142472.1",20.5936311969758,0.414388060334152,0.4023925149534
08,1.02981055793777,0.303098943571834,0.748995356019764
"6240","TMEM214",165.804246171953,-0.224842288085833,0.218401736508847
,-1.02948947055064,0.303249725165744,0.749013701879497
"6241","BBS7",92.9827478252434,-0.213854201154388,0.207718516134711,-1
.02953845970909,0.303226716793447,0.749013701879497
"6242","GID4",72.2849333405651,0.224879950652531,0.218487932450267,1.0
2925570364724,0.303359532702284,0.749044881724445
"6243","ZNF75D",32.0881352287813,0.321811778725242,0.312647885924331,1
.02931058616891,0.30333375031276,0.749044881724445
"6244","SSR2",1941.42292942114,-0.249217101463771,0.24236260011377,-1.
02828200946344,0.303817191189203,0.749076603494942
"6245","GNAI2",1440.88390193851,-0.173171656801127,0.168327678787067,-
1.02877707367538,0.303584442423221,0.749076603494942
"6246","SLC25A36",521.473745288401,0.138199172830485,0.134364044507729
,1.02854281691807,0.303694560781667,0.749076603494942
"6247","SMARCAD1",105.533489184241,0.21634822781236,0.210415009072182,
1.0281976973332,0.303856841381493,0.749076603494942
"6248","ADTRP",5.99736705810355,-0.77248143519269,0.750947504475897,-1
.02867568050822,0.303632101608247,0.749076603494942
"6249","DDX43",2.7252399066647,1.10866597307227,1.0773771359147,1.0290
4167548627,0.303460091512692,0.749076603494942
"6250","COPS7A",187.536024517252,0.182810327495055,0.177788581424085,1
.02824560514936,0.303834310938125,0.749076603494942
"6251","LDHB",724.267362345116,-0.250959919734957,0.244078242664053,-1

.0281945534997,0.303858319925648,0.749076603494942
"6252","C19orf12",168.383101222692,0.174159171317011,0.169254111826683
,1.02898044506801,0.303488864035505,0.749076603494942
"6253","AC010331.1",2.89059456251314,-1.13860097897481,1.1071468557289
3,-1.02841007322843,0.303756972114307,0.749076603494942
"6254","SP2-
AS1",9.94084145361019,0.523152922727139,0.508867602544699,1.0280727641
3551,0.303915601113881,0.749098015857128
"6255","RPS6",14001.485272871,-0.253532505253163,0.246655979331224,-1.
02787901570675,0.304006741588272,0.749202865161185
"6256","ATP10A",30.4474633493031,-0.386120657036896,0.375687796186947,
-1.02777002861375,0.304058017776921,0.749209453969189
"6257","RANBP1",492.148948664379,-0.262857848036905,0.2558096683193,-1
.02755243679378,0.30416040744042,0.749341965909233
"6258","HIGD1A",265.770197838396,-0.279325840381226,0.271867042334836,
-1.02743546250525,0.304215460139611,0.749357832862274
"6259","AL024507.2",3.6513406449058,0.936938466725149,0.91205670965728
2,1.02728093199075,0.304288198419825,0.749417251740151
"6260","MARCH7",291.21737512937,0.167636718031713,0.163258918979376,1.
02681506823459,0.304507552685258,0.749497188815536
"6261","CCDC146",240.092918015315,-0.471551498666689,0.459177253464192
,-1.0269487329983,0.30444460523028,0.749497188815536
"6262","ARHGEF35",31.6573465068808,-0.306253206227308,0.29819417246996
6,-1.02702612760869,0.304408161326289,0.749497188815536
"6263","LCP1",34.515085044964,-0.518978407114011,0.505433321333893,-1.
02679895687204,0.304515140678022,0.749497188815536
"6264","CXCL2",352.823735301596,-0.886221636975873,0.863223161318797,-
1.0266425609131 5,0.30458880524269,0.749528012393762
"6265","ZNF184",21.3385807768733,-0.445484343711519,0.433955907811101,
-1.02656591532206,0.304624910648519,0.749528012393762
"6266","TENT2",110.609141875362,0.208230047077564,0.2029194968904,1.02
617072419627,0.304811118251146,0.749627215673488
"6267","ITM2B",10570.9640022272,-0.216707136335154,0.211155041918903,-
1.02629392301408,0.304753060878117,0.749627215673488
"6268","ERCC2",25.2726391195209,0.342278614909568,0.333531867102901,1.
02622462399963,0.304785717096187,0.749627215673488
"6269","PNRC2",868.388560568193,0.163749552559552,0.159671716701637,1.
02553887402322,0.305108992648383,0.749642193445143
"6270","NPL",14.0073012451911,-0.522128158387108,0.509007449913363,-1.
02577704604516,0.304996688060377,0.749642193445143
"6271","LINC02767",22.3326156232579,0.431474341188824,0.42072491412711
5,1.02554977540137,0.305103851752897,0.749642193445143
"6272","SMIM27",68.7965052435818,0.282380307844532,0.275213633337435,1
.02604040512161,0.304872539030029,0.749642193445143
"6273","FANCG",40.9354332512157,0.348587792041424,0.339879395401756,1.
02562201992084,0.305069783978686,0.749642193445143
"6274","TCF4-
AS2",4.65204106131168,0.765670390305662,0.746440202369033,1.0257625297
7211,0.305003532092969,0.749642193445143
"6275","ZFYVE28",30.0595410941373,-0.312772110668863,0.305105048734996

,-1.02512925291029,0.305302204312748,0.749925391340842
"6276","LARP4",118.602303444571,0.303209498569044,0.295788681247935,1.02508823965069,0.305321554074286,0.749925391340842
"6277","EPAS1",94.0530924483966,0.27911103479955,0.272368532653666,1.02475507019916,0.305478771177354,0.750072516358539
"6278","IKZF2",23.5502070655634,0.410065857278563,0.400143362845931,1.02479734853544,0.30545881776213,0.750072516358539
"6279","LINC02511",307.235247403091,-0.213361305815796,0.208270468417869,-1.02444339534356,0.305625893941512,0.750314246712599
"6280","DAPL1",3.40522512890239,-0.811234429162201,0.791991526625907,-1.02429685405635,0.305695083418468,0.750364603645809
"6281","MTMR10",46.7724504173258,-0.24310968858537,0.237391214279232,-1.02408882031924,0.305793324397274,0.750366777393183
"6282","AC007114.2",3.49378023077459,-0.900170699904972,0.878955834708551,-1.02413644048845,0.305770834601123,0.750366777393183
"6283","ACAP3",125.353233742982,-0.19257911769804,0.188205849324183,-1.02323662303568,0.306195980968648,0.750731393609889
"6284","RPL24",10124.318669475,-0.255194564736564,0.249349330101131,-1.02344195042779,0.306098933256107,0.750731393609889
"6285","ARID5B",2200.39243348044,-0.261886024756086,0.255857236020449,-1.02356309647289,0.306041683307672,0.750731393609889
"6286","SOCS2",36.8098140043415,0.414780036972537,0.405336350685339,1.02329839470659,0.306166782527869,0.750731393609889
"6287","ZNF136",75.8821959592276,0.223799106434249,0.218676584730939,1.02342510383365,0.306106895008003,0.750731393609889
"6288","TIMP1",5159.00996091724,-0.296790982770356,0.290074046491365,-1.02315593676938,0.306234122803696,0.750731393609889
"6289","CACNB4",141.758161547724,0.421088960344879,0.411864751642097,1.02239620813873,0.306593414472248,0.75086305855332
"6290","MASP1",119.749766464314,-0.701331347405292,0.685872275069439,-1.02253928741221,0.306525727932615,0.75086305855332
"6291","GPAT3",8.49307661849677,-0.807426591703672,0.78967338166763,-1.02248171262725,0.30655296365669,0.75086305855332
"6292","AC008264.2",3.95163685211533,-0.91015834632252,0.889838403436139,-1.02283554273216,0.306385609848792,0.75086305855332
"6293","AC048341.1",20.4051044134342,0.426270253865103,0.417005039138921,1.02221847185663,0.30667751000011,0.75086305855332
"6294","AL357093.2",3.69724685099566,-0.802686996304088,0.785194352998306,-1.02227810635543,0.306649292365858,0.75086305855332
"6295","AC108134.3",2.60932380278621,-1.11877874334325,1.09420852312413,-1.0224547878214,0.306565700973268,0.75086305855332
"6296","FAM215B",33.3461263738142,-0.313838611041958,0.306957442714908,-1.02241733663855,0.306583418585582,0.75086305855332
"6297","P3H1",198.735088453489,0.286439221784111,0.280556369822222,1.02096852039259,0.307269353366048,0.750946363134815
"6298","ALKAL2",12.4893740291989,0.96335541426056,0.943884682782432,1.02062829478356,0.307430578688774,0.750946363134815
"6299","CEP97",59.5054192462929,0.266235718656676,0.260541774534404,1.02185424633898,0.306849890237218,0.750946363134815
"6300","LHFPL2",139.22685883977,0.280180947198741,0.274466878695618,1.

02081879070538,0.307340300024307,0.750946363134815
"6301","AC004918.1",10.6748608167449,0.551222270873449,0.5398946925217
9,1.02098108855034,0.307263398669023,0.750946363134815
"6302","SAXO1",4.27605330587063,0.735037844943832,0.719267404853408,1.
02192569826466,0.306816068492007,0.750946363134815
"6303","PANX1",59.2572749966678,-0.411637099466013,0.403327092956183,-
1.02060364070492,0.307442263882181,0.750946363134815
"6304","NCKAP1L",7.52470767356428,-0.632012356153166,0.618828323858636
,-1.02130483008975,0.30711003873701,0.750946363134815
"6305","LINC01481",2.57499383182425,-1.04615577153577,1.02466025125583
,-1.02097819277521,0.307264770658357,0.750946363134815
"6306","ZNF84",104.609760008515,0.214021421424423,0.209649272368262,1.
02085458731515,0.307323337472758,0.750946363134815
"6307","ALG5",276.127410786412,-0.259586660665587,0.254327855594665,-1
.02067726737453,0.307407368221068,0.750946363134815
"6308","AC005899.5",4.55988110440077,0.828391634309506,0.8107842675098
26,1.02171646331244,0.306915116759401,0.750946363134815
"6309","GALNT1",472.275295103079,-0.13824571273576,0.135441998615681,-
1.02070047805508,0.307396367967755,0.750946363134815
"6310","DHPS",216.469698524334,-0.237957395585271,0.233150965377547,-1
.02061509889072,0.30743683305588,0.750946363134815
"6311","MORC4",233.553905173597,-0.246568647557451,0.241565381149411,-
1.02071185193935,0.307390977630334,0.750946363134815
"6312","ATP11B",122.773463578615,0.216976735368543,0.212621201306736,1
.02048494710329,0.307498524721184,0.75096479064909
"6313","TFB2M",49.7101195830199,-0.308299787803336,0.302142989871692,-
1.02037710004213,0.307549650202744,0.750970672877443
"6314","SHARPIN",278.572458063052,-0.24988632515498,0.244936740528109,
-1.02020760387437,0.307630012146131,0.751027515277219
"6315","SLC2A4RG",568.305352649322,-0.279263853704029,0.27375521627260
8,-1.02012249303018,0.307670370351971,0.751027515277219
"6316","EFS",187.671748820984,0.213023144388751,0.208911795603453,1.01
967983077941,0.307880330244246,0.751421040328539
"6317","RPL27",6238.7033030382,-0.227583212961101,0.22326165370684,-1.
0193564778479,0.308033760239795,0.751676494237208
"6318","C19orf25",109.230527548651,-0.259858839321846,0.25502306321227
5,-1.01896211287191,0.308220953699968,0.752014245217633
"6319","RNF212",110.122587170707,-0.21333956852164,0.209585981715355,-
1.01790953180916,0.308720951385157,0.75287667546309
"6320","AC024075.2",41.0658707152827,0.405174940148111,0.3980272259725
02,1.01795785240104,0.308697986370654,0.75287667546309
"6321","AL022328.4",4.25031651315673,0.809001746792653,0.7947099695689
17,1.01798363902681,0.308685731390415,0.75287667546309
"6322","AP003117.1",11.9209172641589,-0.54787788475166,0.5384983916309
79,-1.01741786654603,0.308954686169154,0.753089261115987
"6323","BAMBI",19.5662816694469,0.569296969017413,0.559508950140287,1.
01749394513649,0.308918511205127,0.753089261115987
"6324","DAD1",1811.38683996539,-0.27842214488837,0.273602527287032,-1.
01761539869214,0.30886076649048,0.753089261115987
"6325","DYNC1LI2",583.11494668413,-0.241055021455277,0.23699243943426,

-1.01714224314799,0.309085767073965,0.753170581638504
"6326","BAIAP2L2",2.55638605374949,1.00689287724659,0.989897180009442,
1.01716915411052,0.309072967146268,0.753170581638504
"6327","MXD1",68.3920338283648,0.417471466613317,0.410670882274247,1.0
1655969447215,0.309362936356297,0.753268986880107
"6328","TNS1",416.782387600492,-0.258602525512951,0.254306209546592,-1
.01689426292035,0.309203732856001,0.753268986880107
"6329","PLD2",50.5746277276253,-0.287804386343389,0.283050879762063,-1
.01679382372993,0.30925152088731,0.753268986880107
"6330","BPTF",808.865231430146,0.234023972374907,0.23019749938295,1.01
662256541541,0.30933301525411,0.753268986880107
"6331","CTIF",78.9155264100047,0.236979095765602,0.233122356037257,1.0
1654384330145,0.309370480437104,0.753268986880107
"6332","AL352979.4",6.29857825109776,-0.666338497764828,0.655783457520
098,-1.01609531335945,0.309584000616347,0.753431854989105
"6333","TSHZ2",1557.90978305227,-0.267401209354495,0.26312409348642,-1
.01625512818459,0.309507910497674,0.753431854989105
"6334","FOXO4",16.3147520073242,0.46528456128183,0.457891339348248,1.0
1614623666852,0.309559753960859,0.753431854989105
"6335","ZBTB39",18.1621441635308,0.49867535639255,0.490856646233405,1.
01592870386729,0.309663338929227,0.753505977836469
"6336","TMEM143",19.8621412210245,0.453859142013118,0.446915790279722,
1.01553615218887,0.309850322481683,0.753841969863502
"6337","CD109",407.523546384762,-0.278977607785802,0.274758126818768,-
1.01535707429618,0.309935647149457,0.753930566641768
"6338","TMEM35B",732.095885167725,0.23795170168861,0.234411807223046,1
.01510117816803,0.310057600081919,0.753986806034344
"6339","PRKAB2",91.2333951880345,0.222308004519585,0.219105961929809,1
.01461412807563,0.310289802061957,0.753986806034344
"6340","PPOX",50.9454975936453,-0.303428640787148,0.299065183439207,-1
.01459032207548,0.310301154556074,0.753986806034344
"6341","TMCO6",21.4588413665788,0.349480000432459,0.344330813567472,1.
01495418551607,0.310127666992347,0.753986806034344
"6342","MACORIS",4.35256766705651,-0.708469644233119,0.698238487928168
,-1.0146528105824,0.310271355913644,0.753986806034344
"6343","AP001372.2",22.4419243754797,-0.375364859684001,0.369870823750
563,-1.01485393164491,0.310175460949102,0.753986806034344
"6344","FDXR",27.2146544068192,0.365461982647669,0.360048674053332,1.0
1503493550857,0.310089174641678,0.753986806034344
"6345","ZNF493",107.115449059579,0.242829956763667,0.239380241858035,1
.01441102606822,0.310386665207857,0.754075720122792
"6346","AL390198.1",6.71059887839181,-0.868520103583485,0.856382512438
459,-1.01417309551367,0.310500164125264,0.754232592182626
"6347","CLDND1",263.290514248557,-0.203265280880333,0.200509426785904,
-1.01374426199608,0.310704797777706,0.754254245313911
"6348","TCAF1",356.423896653457,0.194188809618367,0.191524003324643,1.
01391369357087,0.310623936650363,0.754254245313911
"6349","TLCD1",5.34705240337777,-0.822425095451736,0.81106460780642,-1
.01400688371305,0.310579467626249,0.754254245313911
"6350","STS",57.5630897458585,0.256778970988551,0.253286650858555,1.01

378801495522,0.310683915371344,0.754254245313911
"6351","FGF10",52.6188820415589,-0.460374920251619,0.454244138193021,-1.01349666741543,0.310822987136909,0.754422350293725
"6352","TCP1",479.220972843509,-0.190484719831348,0.187990963046004,-1.01326530140033,0.310933456593866,0.754452893655665
"6353","DZANK1",11.5788400638596,-0.488382401666015,0.481978293587097,-1.01328712965735,0.310923033231932,0.754452893655665
"6354","PPP2R5A",125.477307896079,-0.194285076467363,0.191782898389004,-1.01304693014538,0.311037745251505,0.754492379453494
"6355","LSM11",23.1071856705564,0.37390582511009,0.369097862398209,1.01302625455656,0.311047620592083,0.754492379453494
"6356","ACAP2",348.615259097416,0.154016835012215,0.152052495730284,1.01291882301897,0.311098936754031,0.754498129336593
"6357","NOP53",2265.57824403966,-0.241918464913687,0.238867943084736,-1.01277074600114,0.311169676946015,0.754550978468275
"6358","IL17RA",109.713251764511,-0.252683957564911,0.249567900487035,-1.01248580875904,0.311305828629809,0.754762401435751
"6359","IGSF8",116.44071245673,-0.258502231313637,0.25545394687154,-1.01193281403333,0.311570178440493,0.754928371685035
"6360","ZDHHC21",95.275600277552,0.267252676239959,0.26407490680758,1.01203359103974,0.311521992664921,0.754928371685035
"6361","ITIH5",1275.66478846774,-0.335746877270107,0.331691903325016,-1.012225121881,0.311430427163661,0.754928371685035
"6362","CTDP1",34.8846167181799,0.346657798781672,0.342509374340348,1.01211185664426,0.311484573938839,0.754928371685035
"6363","RPARP-AS1",12.9644794926265,-0.41545036146834,0.41059436284468,-1.01182675424479,0.311620895446394,0.754932595207633
"6364","CCDC121",44.3444097566752,0.295703672667446,0.292359972277797,1.01143692949345,0.311807353525769,0.755211929076538
"6365","LCMT2",11.0297772674802,0.454939911577943,0.449820570892656,1.01138085053587,0.31183418284607,0.755211929076538
"6366","SPRY3",4.11858557487037,0.811750336364003,0.802948544170544,1.01096183841089,0.312034694979537,0.755578828638009
"6367","HSCB",118.67992993023,-0.280499696060125,0.277495437320009,-1.01082633562963,0.312099556035789,0.755617191187638
"6368","TRAM1L1",22.2854606650981,-0.355650604300463,0.351883715303352,-1.01070492561403,0.31215767886076,0.755639230470888
"6369","CNTN4",152.629868078819,0.457616797369868,0.452925826008372,1.01035704102554,0.312324261746171,0.755805101227194
"6370","DHRS4-AS1",143.41640230744,0.196231161551414,0.194215439724276,1.01037879290132,0.312313844247256,0.755805101227194
"6371","AL591623.1",2.75241128254307,0.938513205498426,0.929881424736293,1.0092826682333,0.312839089764612,0.755943840658311
"6372","GCA",42.238297169186,-0.330493524117361,0.327426428159305,-1.00936728282838,0.312798523087666,0.755943840658311
"6373","RAB43",30.1718750909103,-0.28974536639092,0.287005933824595,-1.00954486386334,0.31271339688394,0.755943840658311
"6374","RWDD4",202.568030033834,-0.171308704548159,0.169691576128452,-

1.00952980964997,0.312720612760962,0.755943840658311
"6375","BIN3",92.9189357483429,-0.209157286781293,0.207295374993262,-1
.00898192633623,0.312983302142973,0.755943840658311
"6376","TRIM22",308.832486719551,0.235812809354712,0.233692944448608,1
.00907115493412,0.312940510494267,0.755943840658311
"6377","AMPD3",4.26775266989044,0.799279586380603,0.791680679673288,1.
00959844910002,0.312687712972606,0.755943840658311
"6378","CCNK",185.839175203071,0.181858982047301,0.180212958753085,1.0
0913376765802,0.312910485407033,0.755943840658311
"6379","GNB5",80.2592668258047,-0.308980074168739,0.306252202315493,-1
.00890727260938,0.313019107033539,0.755943840658311
"6380","UQCRC2",402.700784617199,-0.203139767462749,0.201288070485741,
-1.00919923854672,0.312879091754358,0.755943840658311
"6381","ZNF771",47.7299344675308,-0.305102430229125,0.302239577764814,
-1.00947212964458,0.312748261306665,0.755943840658311
"6382","NR1D1",58.9933801745404,-1.02969097784185,1.01994610125491,-1.
00955430544315,0.312708871344493,0.755943840658311
"6383","RGN",28.0612927360778,-0.386810622561583,0.383018106622492,-1.
0099016622805,0.312542406199519,0.755943840658311
"6384","PPWD1",130.04111756919,0.204200681477994,0.202446416018507,1.0
0866533225921,0.313135163279696,0.755987242279799
"6385","AL021368.3",3.05559732903649,1.03859826961816,1.02964869436417
,1.0086918725804,0.313122430783046,0.755987242279799
"6386","TBC1D19",40.5980091478629,0.264484397778569,0.262247163032763,
1.0085310160077,0.31319960560318,0.756024415968215
"6387","PPFIBP2",42.4406939921853,-0.354295794335036,0.351356204758341
,-1.00836640861008,0.31327859293437,0.756096682336514
"6388","OGFOD1",62.386983203667,0.282924062373135,0.280629310745565,1.
00817716303929,0.313369419165989,0.756197494746981
"6389","AC010680.2",2.79181061806344,0.949177374944403,0.9424116839705
27,1.00717912467444,0.313848702888436,0.756288534473229
"6390","POMGNT2",98.7863324512487,0.20163439059317,0.200127995016927,1
.00752716068591,0.313681512297284,0.756288534473229
"6391","CCDC69",163.954107917618,0.356936032902268,0.354243342575045,1
.00760124469143,0.313645931165364,0.756288534473229
"6392","SLC16A9",2.49891755868677,1.20614912243002,1.19753013326816,1.
00719730462092,0.313839968099832,0.756288534473229
"6393","HNRNPUL2",293.13183073775,0.224853102848431,0.223074942497217,
1.00797113441478,0.313468319935154,0.756288534473229
"6394","PKNOX2",17.839240573119,0.453025247373587,0.449670000433494,1.
00746157612662,0.313713013523978,0.756288534473229
"6395","C2CD5",73.0828421057823,0.244897381421003,0.243063556468046,1.
00754463145197,0.313673121182942,0.756288534473229
"6396","ACSF2",38.4154899330613,0.342411185401349,0.339922382941614,1.
00732167866734,0.313780215282956,0.756288534473229
"6397","MTG2",68.5110700200384,-0.230528931256275,0.22888520021976,-1.
00718146492188,0.313847578477554,0.756288534473229
"6398","ITGAL",8.33937788117797,0.77417803540841,0.768807126488608,1.0
069860290504,0.313941488030198,0.756393879022429
"6399","NVL",46.079704606023,0.302113701347784,0.300171244531492,1.006

47116221717,0.314188976923597,0.756607695021643
"6400","AC106845.1",10.509887584944,0.634650988248966,0.630643562316518,1.00635450224486,0.314245071477916,0.756607695021643
"6401","PGM5",25.0382070845956,0.446149476957951,0.443209981866571,1.0066322853989,0.314111513595589,0.756607695021643
"6402","SLC5A12",4.65357666517697,-0.914264832057467,0.908641373846448,-1.00618886435604,0.314324727792319,0.756607695021643
"6403","ISM1",327.826824040939,0.361725081650469,0.359469884097987,1.00627367591069,0.314283939717533,0.756607695021643
"6404","CTSZ",1016.08574886361,-0.190195863305286,0.188984490089116,-1.00640990811256,0.314218429407015,0.756607695021643
"6405","WIPF1",304.362784333674,0.181688819436722,0.180616438462845,1.00593733872179,0.314445713458231,0.756780745192603
"6406","TBC1D31",12.400809338758,0.440287370317076,0.438031736160419,1.00514947655718,0.314824879051749,0.757024034955962
"6407","SMARCA2",397.6379365777,0.185261754236857,0.18430963072944,1.00516589124317,0.314816976275795,0.757024034955962
"6408","HEXA",694.78295564119,0.307846743647784,0.306189372123833,1.00541289696783,0.314698072236236,0.757024034955962
"6409","SPATA22",7.49053590771905,-0.770896658284883,0.766605741636328,-1.00559729260493,0.314609326795913,0.757024034955962
"6410","AC040904.1",2.42072896430687,-0.957839567717421,0.953158548238428,-1.00491106069147,0.314939677987193,0.757024034955962
"6411","AMZ2",102.875103570993,0.206882935791929,0.205837560181885,1.00507864361159,0.314858982746744,0.757024034955962
"6412","ARFGEF2",74.8639385632385,0.243447084851434,0.242205069264524,1.00512795042103,0.31483524290976,0.757024034955962
"6413","CLDN5",3.81944348377236,-1.05606381197687,1.05085148723369,-1.00496009646129,0.3149160646682,0.757024034955962
"6414","LINC00957",3.88527848121649,-0.927973695918252,0.923606721718093,-1.00472817498776,0.31502775739799,0.757117692592768
"6415","C6orf89",341.426810556936,0.162393011012031,0.161646134212207,1.00462044331258,0.315079649523806,0.757124364366247
"6416","CD302",630.380997954468,0.193122468907418,0.192391231945607,1.00380078111885,0.315474647826241,0.757129331502648
"6417","STX18-
AS1",19.1412948633612,0.449538168939531,0.447646386810992,1.00422606366158,0.315269661981705,0.757129331502648
"6418","UTP23",171.745923877317,0.175328449962939,0.174662715366168,1.00381154383965,0.315469459123943,0.757129331502648
"6419","ATP5MD",1071.57296939638,-0.267886921664032,0.266749678762804,-1.00426333372359,0.315251702013055,0.757129331502648
"6420","COG4",116.528318614414,0.236596282731769,0.235696533231064,1.0038174065964,0.315466632715443,0.757129331502648
"6421","LDHD",13.4128488505582,0.517314891600015,0.515096363962451,1.00430701475059,0.315230653537553,0.757129331502648
"6422","OSBPL2",88.0893190328102,0.230844530214172,0.22990560682677,1.004083951672,0.315338150109504,0.757129331502648
"6423","AL022311.1",29.6287204629692,-0.314030877467892,0.312720743123867,-1.00418947055106,0.315287296390049,0.757129331502648

"6424","ZNF436",128.003200582337,0.220352469481494,0.219742222127055,1
.0027771055946,0.315968417509498,0.757340535733592
"6425","LRRC41",212.968061585975,0.180977718522874,0.180793261334619,1
.00102026583786,0.316817010812472,0.757340535733592
"6426","NSUN4",45.629970222898,0.273170952507608,0.273506606748843,0.9
98772774649852,0.317904777504837,0.757340535733592
"6427","LINC01750",2.77179307639558,0.955742193251474,0.95734339630248
2,0.99832745171984,0.318120600194085,0.757340535733592
"6428","LHX9",2.95941089042905,1.5154914896093,1.51825283062632,0.9981
81237695515,0.318191482741631,0.757340535733592
"6429","FAM72A",15.0891288727095,0.420712999239443,0.42144920470097,0.
998253157312161,0.318156615807969,0.757340535733592
"6430","KCTD3",190.492443252903,0.183441289099424,0.183644822527872,0.
998891700698955,0.317847157051826,0.757340535733592
"6431","ACBD3",400.072338275129,-0.200364096799239,0.200026157018414,-
1.00168947794559,0.316493590123161,0.757340535733592
"6432","SIX2",35.0672692172665,0.329280948107925,0.329105829620499,1.0
0053210387561,0.317053069252578,0.757340535733592
"6433","PEX13",77.4142154715539,-0.217601379786441,0.217836159163935,-
0.998922220358663,0.317832371179593,0.757340535733592
"6434","ALMS1-
IT1",5.05129504686421,0.758576227688468,0.757794804015181,1.0010311810
917,0.316811733886938,0.757340535733592
"6435","CHST10",20.9162886877138,0.366141789358391,0.366671575279942,0
.99855514864727,0.318010236457002,0.757340535733592
"6436","FZD5",11.2590543968702,-0.606566847620176,0.605710860582492,-1
.00141319413831,0.316627087887699,0.757340535733592
"6437","HTR2B",11.7161909174675,0.71889694722146,0.720075921578227,0.9
98362708262507,0.31810350985535,0.757340535733592
"6438","DUSP28",24.522019607511,-0.317176028377108,0.317590093085977,-
0.998696229139754,0.317941867928422,0.757340535733592
"6439","VHL",84.8763912836215,0.279322275649654,0.278493712580011,1.00
297515897923,0.315872846933263,0.757340535733592
"6440","GSK3B",149.50677482363,0.174159773105379,0.173911736703961,1.0
014262200247,0.316620793070859,0.757340535733592
"6441","WDR5B",20.3667552723427,0.399532015047696,0.398676743493948,1.
00214527575964,0.31627343360198,0.757340535733592
"6442","LINC02035",16.5105691564128,0.429040756354052,0.42962731057184
2,0.99863473712365,0.317971666177044,0.757340535733592
"6443","LAP3",248.932560498244,-0.165817645952258,0.166050240591871,-0
.998599251414612,0.317988862932845,0.757340535733592
"6444","SIRT5",34.4462722391903,0.289966496647779,0.289805736321591,1.
00055471754365,0.317042131508217,0.757340535733592
"6445","TNF",9.14034186820554,-0.657267325557953,0.656122837056232,-1.
00174432047946,0.316467095115299,0.757340535733592
"6446","ZNF117",58.0931660014851,0.372170265420628,0.371522670824319,1
.00174308231278,0.316467693270655,0.757340535733592
"6447","POLR2J2",9.62557364010356,-0.618485462834276,0.619318704679875
,-0.998654583109952,0.317962048864599,0.757340535733592
"6448","WDR60",199.579603834988,-0.238501112735116,0.238878759908296,-

0.998419084336651,0.318076183235651,0.757340535733592
"6449","HOOK3",512.269526547425,0.214789775446214,0.215191896583717,0.998131336988581,0.318215676285856,0.757340535733592
"6450","VCPIP1",119.293416556255,0.257991758854552,0.258074620011033,0.999678925589516,0.317465914022839,0.757340535733592
"6451","GDAP1",10.4075778764956,0.486664374498633,0.485908784693876,1.0015550033845,0.316558562365332,0.757340535733592
"6452","MINCR",35.961789139646,0.346489796077,0.345937152517674,1.00159752589539,0.316538016396779,0.757340535733592
"6453","RPL35",8098.06497416317,-0.221269209284907,0.220830703044082,-1.00198571228901,0.316350493483232,0.757340535733592
"6454","AKR1C3",730.125907064593,-0.327856230713634,0.328345293618641,-0.998510522567214,0.318031864552311,0.757340535733592
"6455","SLC25A28",131.013480175734,-0.196725955750275,0.196669673759908,-1.00028617523633,0.317172035620866,0.757340535733592
"6456","ACADSB",68.8267753658035,0.21632009582933,0.216379781189214,0.999724163877252,0.317444014806333,0.757340535733592
"6457","MADD",58.1648481470464,-0.246225149076694,0.246638758664858,-0.998323014637265,0.31812275107846,0.757340535733592
"6458","MTCH2",113.931086150093,-0.313499790497712,0.312982389146262,-1.00165313247452,0.316511149806406,0.757340535733592
"6459","TMEM258",1328.40037709173,-0.241263014267721,0.240910206840641,-1.00146447687587,0.31660230575414,0.757340535733592
"6460","STX5",216.958141625005,-0.241972211365185,0.241281143193225,-1.00286416154538,0.315926406359458,0.757340535733592
"6461","AP002433.1",7.92180929770673,-0.558557610567938,0.557818957170982,-1.00132418123741,0.316670105960976,0.757340535733592
"6462","SIAE",37.0764442540271,0.332533390699438,0.332131735222729,1.00120932580092,0.31672561885703,0.757340535733592
"6463","RNF41",122.366838686539,-0.175951622260259,0.175391125265999,-1.00319569757826,0.315766448463914,0.757340535733592
"6464","ING1",132.547907286059,-0.305711279948519,0.30469100491454,-1.00334855646383,0.315692715767246,0.757340535733592
"6465","GPR176",30.9542430397963,-0.432437280799973,0.431924619799246,-1.00118692238698,0.316736447807651,0.757340535733592
"6466","CAPN3",20.5648678196947,0.461992672129845,0.461415690200893,1.00125046014084,0.316705736727955,0.757340535733592
"6467","AC104041.1",11.3125878708852,0.578671171870239,0.578492583017991,1.00030871416072,0.317161131345519,0.757340535733592
"6468","ASGR1",3.88918783153465,-0.755612583776467,0.755232459234691,-1.00050332124517,0.317066991149185,0.757340535733592
"6469","PSME3",89.6801841520014,-0.204249634561958,0.204064254344141,-1.00090844042438,0.316871075577278,0.757340535733592
"6470","NMT1",384.091495915842,0.196024026275386,0.195539200697132,1.00247942906857,0.316112096896764,0.757340535733592
"6471","TEX2",37.6040349167162,0.263799537003506,0.263853519243546,0.99979540830005,0.317409528395002,0.757340535733592
"6472","SAMD1",154.622823074619,-0.219864575523271,0.219450401564384,-1.00188732376853,0.316398015560599,0.757340535733592
"6473","ANKRD27",62.8632769809151,0.212052419285318,0.212185434723367,

0.999373117018034,0.317613977611783,0.757340535733592
"6474","C19orf48",76.779267635837,-0.332260412580007,0.33223609807615,
-1.00007318441312,0.317275092187965,0.757340535733592
"6475","AC011468.5",4.69242038887,0.785505781645105,0.784464687658866,
1.00132713938896,0.316668676286605,0.757340535733592
"6476","HSPBP1",62.8506469831908,-0.298607449322301,0.299023278939218,
-0.998609373763832,0.317983957471552,0.757340535733592
"6477","HAR1B",2.36733857209009,-1.04745571156999,1.04903639168681,-0.
998493207548044,0.318040256561323,0.757340535733592
"6478","AL021155.5",44.0115788197518,0.45036730153597,0.45136142644018
6,0.997797496981398,0.318377564169013,0.75734457192394
"6479","DNTTIP2",184.720486243257,-0.189673116057575,0.190122700829233
,-0.997635291473893,0.318456241395458,0.75734457192394
"6480","ISG20L2",111.856741566326,-0.199593368640234,0.20002903066958,
-0.997822005996389,0.318365677262444,0.75734457192394
"6481","AC005082.1",4.38302973751295,0.691609865443056,0.6933292985360
23,0.997520033991643,0.318512154380986,0.75734457192394
"6482","NINJ2",175.270604962607,0.317770889310505,0.318536474716496,0.
997596553403585,0.318475033047452,0.75734457192394
"6483","DCTPP1",160.857177148568,-0.25236444100822,0.252895003287582,-
0.997902045226418,0.318326860148667,0.75734457192394
"6484","PTPN4",67.1864342365062,0.22794960880527,0.228690569655051,0.9
96759985114828,0.31888102549212,0.757520574504705
"6485","SH3TC1",2.69367521267507,-0.877192214823762,0.879797497510696,
-0.997038770064355,0.31874569157871,0.757520574504705
"6486","KAT6B",358.521749735586,0.20116220422277,0.201717958842979,0.9
97244892703671,0.318645655184736,0.757520574504705
"6487","DAPK2",16.1653258632573,0.466136272918611,0.467646027338623,0.
996771587201107,0.318875392602499,0.757520574504705
"6488","AC020978.5",2.84770053252054,-1.13927448377545,1.1428539438061
2,-0.996867963706063,0.318828603690003,0.757520574504705
"6489","IL1RAPL1",3.20753282465748,1.35296956711868,1.35698744560312,0
.997039118897184,0.318745522264178,0.757520574504705
"6490","DEDD",85.4798364875678,-0.190985281438767,0.191877467360346,-0
.995350230885093,0.319565955339644,0.75754632558929
"6491","ALS2",50.4002364205425,0.31896041198172,0.320489030237705,0.99
5230357011435,0.319624240492907,0.75754632558929
"6492","CTDSPL",291.440468805887,-0.188066907588216,0.188806299636637,
-0.996083859225862,0.319209401595694,0.75754632558929
"6493","AC008522.1",4.88048450729145,0.948250107998961,0.9525344140938
38,0.995502203351936,0.319492073183764,0.75754632558929
"6494","HILPDA",278.498656762879,0.399572227849915,0.401563902767394,0
.995040204301846,0.319716710932175,0.75754632558929
"6495","CLEC2L",3.4347583833581,-0.957174502100452,0.96097164546365,-0
.996048641621089,0.319226511897945,0.75754632558929
"6496","PTK2",209.805191810339,0.19137785565478,0.192374795664206,0.99
4817720242492,0.319824926180428,0.75754632558929
"6497","AP001462.1",4.99517972571818,0.692425451271138,0.6956608588133
18,0.995349159721738,0.319566476130599,0.75754632558929
"6498","ACVRL1",507.578689232512,0.238192711573147,0.239142581219486,0

.996028019596111,0.319236531287265,0.75754632558929
"6499","LINC00327",2.51708243125973,1.0225728692995,1.0272495058934,0.99544741898919,0.319518705580561,0.75754632558929
"6500","AC009118.3",7.12185619209879,0.718820700147291,0.72164540996257 5,0.996085737155273,0.319208489229666,0.75754632558929
"6501","CCL5",42.3177832263984,0.561078723328152,0.563600873554752,0.99552493556177,0.319481022770044,0.75754632558929
"6502","ATP5F1A",685.905397683475,-0.170283962385624,0.171171253682585,-0.994816353342799,0.319825591108343,0.75754632558929
"6503","PDCD2L",28.5755310451553,-0.289156722351269,0.290654093719741,-0.99484827015746,0.31981006542008,0.75754632558929
"6504","NUMBL",264.483430038733,-0.24405156308708,0.244933925534934,-0.996397549069907,0.319057023391343,0.75754632558929
"6505","CCN5",12241.8208232845,-0.254504847149599,0.255515361462135,-0.996045191542483,0.319228188134162,0.75754632558929
"6506","TNNC2",66.5562023641623,0.367333703538854,0.36886593498271,0.99584610206977,0.319324926407325,0.75754632558929
"6507","PCIF1",82.5859959348301,-0.264482067593002,0.265681373106558,-0.995485925492132,0.319499986210232,0.75754632558929
"6508","RAB22A",341.943547293601,-0.156802182607797,0.157567301721246,-0.995144175821435,0.319666147864319,0.75754632558929
"6509","EDC3",45.7199227990117,0.246817994312223,0.248130883880134,0.994708882879149,0.319877872910908,0.757553758015309
"6510","TSC22D2",454.20065882938,-0.418363324567444,0.420690456936607,-0.994468302451857,0.3199949297784,0.757714568745627
"6511","TUT7",127.736227933719,0.222671511379114,0.223997815304811,0.994078942583026,0.320184436151996,0.757930448907096
"6512","DNMBP",150.547019829402,0.218732065136579,0.220017063167657,0.994159552842956,0.320145196096701,0.757930448907096
"6513","PRPF4",86.4857788781607,-0.223831329935711,0.225225164039227,-0.993811374899151,0.320314707258837,0.758006019710619
"6514","TMEM208",354.74493960836,-0.273721527457331,0.275404379013867,-0.993889525059253,0.320276654583444,0.758006019710619
"6515","RGS1",82.4346631098372,-0.501476645914557,0.504809112114645,-0.993398561713499,0.320515762235498,0.758016335510923
"6516","RCHY1",96.8903812518704,0.252022204395244,0.253679678459723,0.993466271817505,0.320482779307983,0.758016335510923
"6517","LRCH4",37.4584796843855,-0.296563628539436,0.298497842079573,-0.993520175802071,0.320456523199796,0.758016335510923
"6518","C11orf54",148.272738803085,0.242575074164691,0.244174811655441,0.993448392649905,0.320491488387688,0.758016335510923
"6519","SELENOI",24.6419877453682,-0.331762825892823,0.334234887877512,-0.992603818229787,0.320903063311966,0.758036402570571
"6520","LARP7",440.489918942996,-0.195414912469026,0.196887094817142,-0.992522707750434,0.320942607941663,0.758036402570571
"6521","MPC1",413.627485894511,-0.236690129073641,0.238485197591848,-0.992473040103397,0.320966824494202,0.758036402570571
"6522","NCALD",294.820439097828,-0.25587338399491,0.257657582762111,-0.993075310464087,0.320673254875101,0.758036402570571
"6523","ZFP41",13.9641796267368,0.401222419882925,0.404147470916913,0.

992762416581867,0.320825749418,0.758036402570571
"6524","DEPP1",2114.77268772483,0.390499366163993,0.393346233577511,0.992762438863018,0.320825738557188,0.758036402570571
"6525","DAAM1",357.48646700874,-0.222949428893,0.224485906450133,-0.99315557229659,0.320634145441593,0.758036402570571
"6526","NFE2L1",567.001654975124,0.252005739094084,0.253882340582596,0.992608381172926,0.32090083878776,0.758036402570571
"6527","PDXP",13.7526501438384,-0.420797905176822,0.423832704690674,-0.992839628749113,0.320788114256358,0.758036402570571
"6528","TNNI3K",8.12676369954093,0.581614883854613,0.586299647359085,0.992009608865409,0.32119283811,0.758266148354295
"6529","LINC02739",4.51334267005925,0.736975736968622,0.743086876086658,0.991776009892383,0.32130680281889,0.758266148354295
"6530","PRDM4",72.1503481185526,0.28870712934349,0.291056368249841,0.991928577545041,0.321232367445121,0.758266148354295
"6531","PHETA1",36.2596588701664,0.262934781642928,0.265197727945751,0.991466946868842,0.321457624359087,0.758266148354295
"6532","IGHG2",43.2872255528878,1.00813267846856,1.01630322479532,0.991960523072816,0.321216783147459,0.758266148354295
"6533","UBE2S",329.423700663909,0.307354844036332,0.309965440504696,0.991577782142056,0.32140353186571,0.758266148354295
"6534","POFUT1",93.497107217441,0.191410153119853,0.193004503143889,0.991739311787727,0.321324708931263,0.758266148354295
"6535","XPNPEP2",105.41968088471,0.335572979734724,0.338458766366097,0.991473742393036,0.321454307673013,0.758266148354295
"6536","HTATSF1",278.664815504723,-0.200326815986546,0.202071917961559,-0.991363955998354,0.321507893766871,0.758268693760146
"6537","SLFN12",46.4314053434407,0.300986356846164,0.303661550203413,0.991190213724927,0.32159270826798,0.758352699701837
"6538","MKNK1",120.926574148015,0.218499003364604,0.220607847962106,0.990440754411128,0.321958733772994,0.758596161731341
"6539","DENND1B",74.0731241370865,0.247657093263648,0.249945371324927,0.990844887228161,0.321761327159989,0.758596161731341
"6540","ACP1",328.539277535355,-0.22577289062789,0.227913452239973,-0.990608006719015,0.321877026512061,0.758596161731341
"6541","ROCK2",541.893804300301,0.188931221612279,0.190846536317764,0.989964110732949,0.322191661314435,0.758596161731341
"6542","SOS1-IT1",2.97250542244621,1.00876377996028,1.01866344698785,0.990281709767005,0.322036443925038,0.758596161731341
"6543","CHPF",154.456419432923,-0.296502928971223,0.299537002981818,-0.989870787313782,0.322237279728629,0.758596161731341
"6544","GPX8",601.658468056977,0.176427630608825,0.178168177431711,0.990230877096145,0.322061283649365,0.758596161731341
"6545","AOAH",5.4093101328422,-0.921674072440853,0.931053239971057,-0.98992628227093,0.322210152139822,0.758596161731341
"6546","WNT11",1025.26012733636,-0.394180312013964,0.398036865007303,-0.99031106580224,0.322022099471787,0.758596161731341
"6547","EIF1",14997.6774741508,-0.203843733584026,0.205881180137262,-0.990103774653525,0.322123398579248,0.758596161731341

"6548","AC011815.2",2.66001804236913,0.956957545374215,0.966622742884537,0.990001065481369,0.322173598235205,0.758596161731341
"6549","AKR1C4",4.86341236189521,0.930389265917074,0.940465670785707,0.989285728143363,0.322523364757709,0.758914941650197
"6550","RAD9A",57.5844319800271,-0.263171011870454,0.266046666447651,-0.98919116478476,0.322569620349795,0.758914941650197
"6551","NARS2",45.377976375746,0.287308305998302,0.290365836578228,0.989470074661824,0.32243320424373,0.758914941650197
"6552","DIO3",13.9611393675366,0.575046208290714,0.581233353187265,0.989355144775118,0.322489412421196,0.758914941650197
"6553","CD5",2.93986296082636,-0.862324255373364,0.872141908249319,-0.988743055707915,0.322788871384926,0.759314886677648
"6554","SERP1",798.469059626981,-0.16830299032489,0.17026149052276,-0.98849710411992,0.322909252002449,0.759482166557485
"6555","NDUFA13",1852.98659664167,-0.243372877073689,0.246265908103967,-0.988252409549695,0.323029046421376,0.759648016870406
"6556","NAXE",253.228293495453,-0.278993202051259,0.283082692998578,-0.985553723175371,0.324352156300714,0.759839613701686
"6557","RNF144A",62.9057575903988,0.372602531107881,0.377591809965216,0.986786580837667,0.323747274591101,0.759839613701686
"6558","ATAD2B",102.884257869656,0.297470203677985,0.301985417720499,0.985048238167932,0.324600376937457,0.759839613701686
"6559","KHK",17.5470081735046,-0.451151278329118,0.457496144755853,-0.986131322636345,0.324068674886883,0.759839613701686
"6560","RBKS",18.5475120571272,0.445880145429673,0.45292765389113,0.984440101192959,0.324899169108446,0.759839613701686
"6561","MAP3K20",740.721150712454,-0.180028964308861,0.18283833679475,-0.984634663959764,0.324803556324159,0.759839613701686
"6562","DTYMK",176.701537974678,-0.285643797080234,0.289258567515696,-0.987503324563533,0.323395953913027,0.759839613701686
"6563","XRN1",238.485293210156,0.211906200990047,0.214539331233752,0.987726585010947,0.323286570855209,0.759839613701686
"6564","COMMD2",249.861930356973,-0.181913220691968,0.184653144965131,-0.985161778459392,0.324544611712113,0.759839613701686
"6565","AC104825.1",20.8086491879968,0.421344139028379,0.427421815004573,0.985780613523132,0.324240780996728,0.759839613701686
"6566","COX18",36.0993835454595,0.28151988277505,0.28538203180712,0.986466740713795,0.323904128337786,0.759839613701686
"6567","LAMTOR3",194.975397578953,0.174468574680532,0.176713513896267,0.987296165605915,0.323497469863005,0.759839613701686
"6568","LINC02569",2.68179254530538,-0.844855764442174,0.856726129190573,-0.986144504826048,0.324062207052676,0.759839613701686
"6569","YIPF3",600.018233039815,-0.22833624776653,0.23115649980363,-0.987799382498457,0.323250910048526,0.759839613701686
"6570","AFG1L",11.2713954738828,0.472879471745541,0.48015158625678,0.984854544440992,0.324695523856207,0.759839613701686
"6571","TCF21",10.9621298444123,-0.713384585852168,0.724529223604971,-0.984618097669889,0.324811696680802,0.759839613701686
"6572","AL080276.2",15.5876568936086,-0.463180897955048,0.470021173543323,-0.985446877772106,0.324404612902055,0.759839613701686

"6573","NT5C3A",168.541644032651,-0.190622483302055,0.193159473079115,
-0.986865827822893,0.32370841849263,0.759839613701686
"6574","TNS3",49.1867943767366,-0.485573244720391,0.493334244152826,-0
.984268273438502,0.324983624595214,0.759839613701686
"6575","ATXN7L1",42.8102052633227,0.275735860867902,0.280017320969209,
0.984710016914356,0.324766531005222,0.759839613701686
"6576","LSM8",459.205099580629,-0.21429703738894,0.217079064406417,-0.
987184268436551,0.323552312467335,0.759839613701686
"6577","PTPRZ1",9.03709070219336,0.725806367660244,0.735014955681605,0
.987471563741419,0.323411516603951,0.759839613701686
"6578","PCM1",559.927807658367,0.21124456506658,0.214611568033597,0.98
431117670931,0.32496253576384,0.759839613701686
"6579","TSPYL5",87.6399900985035,0.228061430012907,0.231378746693802,0
.985662828897225,0.324298595677966,0.759839613701686
"6580","LINC01230",7.53926978618674,0.599725745318763,0.60744820685064
1,0.987287045307261,0.323501939641443,0.759839613701686
"6581","MARK2",88.5031277130871,0.265454223183869,0.269157640586255,0.
986240712341214,0.324015005433736,0.759839613701686
"6582","GSTP1",3203.20014436961,-0.267693210239521,0.27129851119101,-0
.986710944576652,0.323784363126325,0.759839613701686
"6583","UPF3A",410.614863671596,-0.234369921345483,0.237571450013159,-
0.986523933462973,0.323876076637399,0.759839613701686
"6584","ZSCAN29",40.3631327301416,0.308334067696972,0.31317700246626,0
.984536109831981,0.32485198588426,0.759839613701686
"6585","TP53BP1",174.127222773841,0.181481452593035,0.184038039030763,
0.986108380358802,0.324079931703379,0.759839613701686
"6586","RAB40C",32.5161867976282,0.300231233318567,0.304005225420223,0
.987585765683997,0.323355560332267,0.759839613701686
"6587","TRIM16",36.5899309846485,-0.317372329876104,0.321501766672564,
-0.987155788165028,0.323566272074198,0.759839613701686
"6588","MAP2K2",905.707407273534,-0.237205866165704,0.240604842481658,
-0.985873200718255,0.324195339207356,0.759839613701686
"6589","DOP1B",32.6566622793271,0.318174039645899,0.322817404687095,0.
985616125482153,0.324321521946432,0.759839613701686
"6590","TRO",15.8629657707945,0.414491344425809,0.421066462534336,0.98
4384607434767,0.324926443421762,0.759839613701686
"6591","TMSB15A",5.60522802633087,-0.85028310630026,0.862831438748877,
-0.985456797370745,0.324399742563535,0.759839613701686
"6592","APLN",8.58458105899463,0.667953473021904,0.677109831483482,0.9
86477292108554,0.323898953007381,0.759839613701686
"6593","AC145212.1",3.75905708857203,0.911579088173093,0.9260485699435
4,0.984375029301834,0.324931151074915,0.759839613701686
"6594","PKP4",28.1966365188341,-0.400478031438088,0.406972899651456,-0
.984041030203901,0.325095339420322,0.759870304346364
"6595","TMEM128",140.268582489089,0.228249040054129,0.231950559481702,
0.984041774092528,0.3250949736772,0.759870304346364
"6596","AL450998.2",24.4694267857142,-0.342787442698637,0.348402388699
669,-0.983883732766621,0.325172682892584,0.759935856092964
"6597","PROSER3",25.0310447911567,-0.333906393698074,0.339410534535605
,-0.983783235116546,0.325222104060373,0.759936142805921

"6598","PPP1R7",330.804204250138,-0.236689494685823,0.240659242984636,-0.983504692154849,0.325359107116374,0.760019394201448
"6599","SMARCC1",145.944251106748,0.210359133154166,0.213907844018405,0.983410094751207,0.325405643965589,0.760019394201448
"6600","ZNF665",25.9004207232669,0.359709376013664,0.365774650167785,0.983418002993539,0.325401753368651,0.760019394201448
"6601","NDUFAF2",308.163522474313,-0.272051477411233,0.276711378884461,-0.983159704194263,0.325528843586877,0.76011582078687
"6602","AC234772.2",11.8773474850595,-0.734100144497692,0.746700145351351,-0.983125755456055,0.325545549713585,0.76011582078687
"6603","MCUB",454.767429406883,-0.200640533663906,0.20412879996642,-0.982911444621789,0.325651024646398,0.760246940015785
"6604","GBP3",147.262229743708,-0.206921728726077,0.210697013560081,-0.982081925271676,0.326059489231349,0.760770044187056
"6605","KRTCAP2",1565.25175430652,-0.25242111570186,0.257155002456831,-0.981591309872475,0.326301230398134,0.760770044187056
"6606","DPY30",313.946017894717,-0.23778306636717,0.242096900037179,-0.982181375848486,0.326010501094528,0.760770044187056
"6607","FAT1",196.90188035514,-0.361140602391422,0.367766516319455,-0.98198336815884,0.326108041979303,0.760770044187056
"6608","IRF1",2303.18193045467,-0.443411657965824,0.451732884590287,-0.981579320637658,0.326307139317287,0.760770044187056
"6609","DCUN1D2",22.2316378201076,0.394831084349794,0.40225071552843,0.981554709806077,0.32631926903437,0.760770044187056
"6610","LINC02076",2.42561490846051,-0.923200868888557,0.94014541950992,-0.981976670555715,0.326111341627936,0.760770044187056
"6611","MYDGF",952.285406023728,-0.24719508575243,0.251774282120777,-0.981812295005769,0.32619232987193,0.760770044187056
"6612","LARGE1",62.9967777623141,0.23840226362177,0.242808207208039,0.981854222981455,0.32617167061173,0.760770044187056
"6613","HFM1",3.99241060796269,0.786158235464989,0.801355781813408,0.981035207215917,0.326575379932254,0.760848552590179
"6614","ZBTB20-AS2",17.6923958067946,0.349250114098487,0.355969077471565,0.981124867865484,0.326531168579276,0.760848552590179
"6615","PRR7",4.65785753726303,0.710702852132605,0.72419861897269,0.981364550433375,0.326413001036465,0.760848552590179
"6616","ABCC4",7.01955412523445,-0.554238277908244,0.56498092459507,-0.980985824088618,0.326599732240624,0.760848552590179
"6617","PDCD5",699.645360180582,-0.242975253846738,0.247671043154657,-0.98104021669991,0.32657290967063,0.760848552590179
"6618","SNX16",60.1194719501724,0.223927720708243,0.228293649495812,0.980875820255135,0.326653982687157,0.760859949096786
"6619","SPRR2F",3.73022339481175,1.16720489928861,1.19132673271316,0.979752126127809,0.327208488764624,0.760963973821545
"6620","ACYP2",85.0893339743949,0.254603857372518,0.259785110378206,0.980055619823074,0.327058664403239,0.760963973821545
"6621","METTL8",101.243455483714,-0.217306310101315,0.221653556046546,-0.9803872041452,0.326895023603279,0.760963973821545
"6622","MCM9",21.6598034761641,0.340909493460233,0.347791009740363,0.9

80213645300184,0.326980670320156,0.760963973821545
"6623","DLL1",16.4451912507643,0.411064907408943,0.419451627725566,0.980005512525726,0.327083397575402,0.760963973821545
"6624","ACSS3",67.5718002448866,0.234911881636676,0.239779193542126,0.979700857970419,0.327233802483417,0.760963973821545
"6625","AL606834.2",2.70698740513306,0.915405727492483,0.934217758804642,0.979863333644792,0.327153584280024,0.760963973821545
"6626","MTSS2",13.109454528583,0.434125092343888,0.443114398347994,0.979713351591328,0.327227633624819,0.760963973821545
"6627","CRLF1",76.246546005011,-0.721569006388218,0.73653168782351,-0.979684945423723,0.327241659582421,0.760963973821545
"6628","RBM10",104.473132025858,0.203424588143072,0.207592326412947,0.979923447355256,0.327123907911337,0.760963973821545
"6629","TIMM8A",30.6627161680733,-0.372685631089569,0.380213495888655,-0.980200953200013,0.326986934108938,0.760963973821545
"6630","POLE4",356.590846503325,-0.229489214841798,0.234315132658712,-0.979404156436009,0.327380324219832,0.761065893288014
"6631","NPAT",75.2370541563186,0.307011859793809,0.31347053003994,0.979396244216932,0.327384232137063,0.761065893288014
"6632","AC012510.1",9.59456609589741,-0.479503002384303,0.489644588595379,-0.979287862161067,0.327437766072377,0.761075567552125
"6633","CORT",3.67810834594655,-0.742641743592597,0.758946790690361,-0.978516218399274,0.327819073916674,0.761394047577226
"6634","RPL37",16592.7218316418,-0.253741349903887,0.259296919778586,-0.978574485653577,0.327790271093645,0.761394047577226
"6635","ARPC5L",151.899536706828,-0.246813338828496,0.252228263122457,-0.978531651342612,0.327811444903482,0.761394047577226
"6636","RCCD1",35.620236041386,0.305607841348236,0.312230569526408,0.978788982167194,0.327684254760997,0.761394047577226
"6637","AL021707.6",5.71918960186772,-0.662211820266027,0.676754736365638,-0.978510802631822,0.327821751136558,0.761394047577226
"6638","BAG2",166.778544937933,-0.252580120198665,0.258205097432377,-0.978215080609764,0.327967959320409,0.761618875101553
"6639","UGDH",732.331204671953,-0.244008974317022,0.249576099301339,-0.977693677399794,0.328225849780398,0.762047363635276
"6640","AL606834.1",29.7713293557713,0.329277997999833,0.33680830965846,0.977642144084084,0.328251345737154,0.762047363635276
"6641","CTC1",18.9874215648958,0.381870729286423,0.390728398800264,0.97733036671755,0.328405624069147,0.762290723539513
"6642","ATAD3A",46.0951185769665,-0.250896834285561,0.256845209024924,-0.976840624117752,0.328648060748637,0.76262379293094
"6643","RETN",3.10388437005106,-3.04978545279435,3.12184836495084,-0.976916587952977,0.328610448862668,0.76262379293094
"6644","ARHGAP31",19.277067764008,-0.382678753229927,0.39180929841742,-0.976696456096439,0.328719450169479,0.762674642438669
"6645","DDX18",448.117043054902,-0.198476317810649,0.203385194484078,-0.97586413954132,0.329131794964059,0.762838389091642
"6646","GLS",392.055806764732,0.269933692755825,0.276527400851591,0.976155317427999,0.328987502025755,0.762838389091642
"6647","TMEM120A",144.518156344814,-0.191165927601309,0.19587365133359

7,-0.975965507865729,0.329081557329633,0.762838389091642
"6648","SRI",1278.2136768771,-0.243667994199849,0.249658308230292,-0.9
7600594960005,0.32906151599534,0.762838389091642
"6649","ASAH1",671.936631301133,0.222177456476521,0.22756759746812,0.9
76314110393707,0.328908829594663,0.762838389091642
"6650","COG7",65.2655378148558,0.251334124183361,0.257510431376766,0.9
76015312620991,0.32905687616287,0.762838389091642
"6651","TXLNG",177.274455329285,0.216042686991454,0.22138815262730l,0.
97585478006655,0.329136433723549,0.762838389091642
"6652","PIFO",34.4866442840151,0.35877921599487,0.368046943333892,0.97
4819170470289,0.329649966086551,0.762868038423861
"6653","BRINP3",2.54113371473128,1.28395476619479,1.31723980761217,0.9
7473122112995,0.3296936018151,0.762868038423861
"6654","CLSTN3",57.0165041729373,-0.397082006693025,0.40733065515051,-
0.974839486476417,0.329639886909756,0.762868038423861
"6655","RB1",93.5577781006578,0.234110611462173,0.240022477872927,0.97
5369530124242,0.329376992201477,0.762868038423861
"6656","NIPA2",130.850638909484,-0.181570351287918,0.186090931209843,-
0.975707682837983,0.329209343878456,0.762868038423861
"6657","SH2B1",83.9360417544302,0.199703661505554,0.204753211428896,0.
975338360321176,0.329392448255362,0.762868038423861
"6658","RABEP2",71.2092377907834,-0.213703218524676,0.219111084816483,
-0.97531906568608,0.329402016049498,0.762868038423861
"6659","AC018557.1",5.22489239286321,0.933183956815751,0.9572706706308
21,0.974838136637784,0.329640556585497,0.762868038423861
"6660","ERCC1",313.169324501491,-0.256760903968017,0.263301355451235,-
0.975159826002379,0.329480986455188,0.762868038423861
"6661","GYG2",2.86146775249136,0.975701811782343,1.00044027866576,0.97
5272420142452,0.329425147314118,0.762868038423861
"6662","SLC25A14",22.8714138306953,-0.345284999992301,0.35423423046556
3,-0.974736404041192,0.329691030229795,0.762868038423861
"6663","CCDC93",133.49998089152,0.19939883202177,0.204595824577704,0.9
74598735987595,0.32975934087337,0.762905634033168
"6664","YEATS2",61.7326323733948,0.260082500353253,0.26701116110904,0.
974051044431966,0.330031195217862,0.763419999142158
"6665","DCBLD1",9.48903080557409,0.478542029236264,0.491426060838833,0
.973782359892397,0.330164613577422,0.763614031252208
"6666","PCDHGA7",4.77597649378398,0.679612634063524,0.698027435407004,
0.973618800050828,0.330245848169271,0.763687331162513
"6667","RAD54L2",65.1038190340768,0.285526769575712,0.293476051017852,
0.972913355571708,0.330596366599629,0.764039428955515
"6668","VPS50",67.0927991369869,0.210256271128814,0.216101422249489,0.
972951815588116,0.330577250537973,0.764039428955515
"6669","VPS28",1094.44650139327,-0.237408720956077,0.24397534833755,-0
.973084873425868,0.330511121351349,0.764039428955515
"6670","S1PR3",164.854132345446,-0.443452106029341,0.455740176232886,-
0.973037114469219,0.33053485636724,0.764039428955515
"6671","MCTP2",3.46060234531071,-0.811511124672763,0.834243226134008,-
0.972751230397652,0.330676956716973,0.764111120940209
"6672","POMC",10.3187211333423,-0.669348570234686,0.688364156900047,-0

.972375687381814,0.330863682628344,0.76412921563535
"6673","IWS1",193.354957573896,-0.213168640460624,0.219326345671889,-0
.971924461731207,0.331088129339334,0.76412921563535
"6674","PMPCB",216.385911712954,-0.199093657727343,0.204780213091458,-
0.97223093345657,0.330935674773433,0.76412921563535
"6675","ELP1",58.7661108446341,0.217020141951216,0.223148652331714,0.9
72536198106241,0.330783865818959,0.76412921563535
"6676","SFXN2",2.47309619891431,0.970145506197596,0.998112185134715,0.
971980425293231,0.331060286838175,0.76412921563535
"6677","CACUL1",217.283549956545,0.168693133265581,0.173565982278717,0
.971925091834463,0.331087815847452,0.76412921563535
"6678","PYCR1",65.1261191405838,0.414339177748242,0.426261780014467,0.
972029858586382,0.331035694478878,0.76412921563535
"6679","DDX49",134.612934890795,-0.237266174338089,0.244141578510919,-
0.97183845449528,0.331130921858199,0.76412921563535
"6680","RBM41",75.1886644184067,0.309613161190531,0.318557123371836,0.
971923521638332,0.331088597059041,0.76412921563535
"6681","NELFA",93.8050119978961,0.216411188910424,0.222725115531403,0.
97165148346241,0.331223960806584,0.764229509928678
"6682","ARSK",74.7190605534004,-0.197495149564355,0.203288111843198,-0
.971503683976802,0.331297519502757,0.764284834351241
"6683","HBP1",445.498891053913,0.207757429632942,0.213995333774664,0.9
7085027962203,0.331622840521657,0.764806416313784
"6684","IREB2",200.179842500767,0.184089745493248,0.189615622672188,0.
970857479457303,0.331619254698114,0.764806416313784
"6685","PPIL3",172.008138333908,-0.258165923946127,0.266157699872629,-
0.969973530991866,0.332059685913953,0.765333464082942
"6686","RBM27",288.650600611408,0.182407457931962,0.187996417688373,0.
970270924174335,0.331911466279704,0.765333464082942
"6687","ZNF800",153.360114639719,-0.171357602152829,0.176678921923614,
-0.969881411359953,0.332105606663597,0.765333464082942
"6688","RBIS",837.888899564989,-0.227904313771585,0.234942540350851,-0
.970042774847181,0.332025171219891,0.765333464082942
"6689","MAN1B1",243.386079768261,0.167937775306837,0.173168531007125,0
.969793843778273,0.332149262063891,0.765333464082942
"6690","TTC7B",47.0947177710393,0.270596130613155,0.278985964610568,0.
969927397569538,0.332082682467971,0.765333464082942
"6691","YTHDC2",96.0722576388499,0.267548198925759,0.275927798067493,0
.969631189026904,0.332230360796199,0.765405920142492
"6692","USP3",142.256012055633,0.188451832169448,0.194433423595839,0.9
69235786133025,0.332427559768977,0.765745791069752
"6693","PTGS2",81.6931492385312,-0.493522834503258,0.509243769198397,-
0.969128861959595,0.332480898961611,0.765754229417784
"6694","ZNF649",23.9769402185594,-0.326663421853051,0.337161324580923,
-0.968863858448412,0.332613119959594,0.765944314935336
"6695","CMTR1",58.8234791727626,0.292262543461514,0.301815867649365,0.
968347177163828,0.332871010835978,0.766068872010944
"6696","PPIA",3153.11306172699,-0.234113426793843,0.241742266064332,-0
.96844226127814,0.33282354185836,0.766068872010944
"6697","ABCB4",11.5625358851748,-0.557644594303508,0.575975192880191,-

0.968174673487203,0.33295714124981,0.766068872010944
"6698","AL132656.3",3.79724491699859,-0.741006225367182,0.765074295643753,-0.968541525426207,0.332773990739904,0.766068872010944
"6699","PCLAF",3.92592275447446,-0.782837474200592,0.808584285645866,-0.968158160005906,0.332965387121202,0.766068872010944
"6700","ZNF227",45.2315252411451,0.250081599496057,0.258269053619655,0.968298741142813,0.332895193300004,0.766068872010944
"6701","MFSD10",445.037161327675,-0.216232051134625,0.22340203633693б,-0.967905461741195,0.333091586370443,0.766157425051241
"6702","TMEM246",4.189051899328,0.955006640245714,0.986869434436496,0.967713262688113,0.333187592566943,0.766157425051241
"6703","TTI1",45.9707294913677,0.239846677388792,0.247856647465743,0.967683053253363,0.333202684238957,0.766157425051241
"6704","LINC01560",19.1816690111858,-0.40666151842331,0.420231640288624,-0.967707995866295,0.333190223671723,0.766157425051241
"6705","HIST2H2AB",2.67959288770099,0.990217394782862,1.02429607425727,0.966729659196322,0.333679196229454,0.766567035749185
"6706","TLL1",3.79919018553954,-1.15804493534476,1.19749299105726,-0.967057798244261,0.333515140800698,0.766567035749185
"6707","AL135905.2",2.52598987304816,-0.841264728680593,0.87021044307673,-0.966737109826221,0.333675470657041,0.766567035749185
"6708","TTC23",64.2964370541608,0.27285003048604,0.282221535946864,0.966793797541416,0.333647125719332,0.766567035749185
"6709","PSMG2",740.220290975034,-0.243062089087984,0.251425863163456,-0.966734631154334,0.333676710075658,0.766567035749185
"6710","ADA",278.791869496715,-0.260153421294048,0.269004681752902,-0.967096258692685,0.333495915636477,0.766567035749185
"6711","POLR3F",54.4503941174969,-0.254643408562635,0.263441064531756,-0.966604842017483,0.333741613122338,0.766596180342846
"6712","GABARAPL2",1139.96937789482,-0.223300173962222,0.231062250781813,-0.966406988621776,0.333840568412717,0.766709231537848
"6713","LINC02802",895.492937913406,0.236503756456598,0.244803810317072,0.966095078954351,0.33399660675101,0.766953328328142
"6714","TRAT1",3.20093167121855,0.880362758965555,0.9115298110691,0.965807972789184,0.334140278263829,0.767168958808003
"6715","UBE3A",309.025449253827,0.156290750228141,0.16189035548997,0.965411125048915,0.334338931431432,0.767510741327704
"6716","PSME4",76.5031340087148,0.266031187642072,0.275629760880827,0.965175846004142,0.334456742841346,0.76766686880574
"6717","WDR74",214.174586721082,-0.274357068780749,0.284353670124466,-0.964844479273499,0.334622713671286,0.767819162137968
"6718","SGPP1",76.6093325264449,0.225578424902409,0.2337976491778,0.964844709498599,0.334622598340589,0.767819162137968
"6719","ADAMTSL4-AS1",32.3444313212191,0.407735574207099,0.422684904650598,0.964632447766602,0.334728941209116,0.767948597817908
"6720","PAIP2",619.678365124315,0.177847422361561,0.1844075742728,0.964425800094659,0.334832492360508,0.767957576214438
"6721","ATP5PO",1097.72892723867,-0.263940399378389,0.273648010677879,-0.964525189584086,0.334782685709716,0.767957576214438

"6722","C1R",8096.34506063734,0.233975234780455,0.24263313835653,0.964316895726941,0.334887072645441,0.767968495214143
"6723","CDC123",232.066757507489,-0.243058805127222,0.25208214819107,-0.964204751789848,0.334943282516213,0.767983147402563
"6724","CAPZB",865.761406003421,-0.23648207940849,0.245416730726803,-0.963593959988577,0.335249536206723,0.768146842308967
"6725","SMU1",215.935725274221,-0.155730433124954,0.161603976854039,-0.963654707987849,0.335219068818691,0.768146842308967
"6726","AC007541.1",36.6670238964614,0.31677089104197,0.328748698913781,0.963565459235619,0.335263831012308,0.768146842308967
"6727","AC011921.1",2.60289748276013,-1.06606511721243,1.10604324299377,-0.963854825718087,0.335118714925916,0.768146842308967
"6728","PCDH19",2.44611489516342,1.02652345337508,1.06501399186533,0.963859124120198,0.335116559600182,0.768146842308967
"6729","AP000866.6",8.07422680861477,0.594712271742922,0.617371963181704,0.963296533062496,0.33539873264924,0.768239253412232
"6730","CLBA1",18.3106778222236,-0.341079763130983,0.354079304934496,-0.963286355281571,0.335403838823505,0.768239253412232
"6731","AP001062.1",4.45366969028733,-0.734819487269022,0.763012889939281,-0.963049899887665,0.33552248215843,0.7683968299617
"6732","SLC29A3",34.1347790369601,0.325421519423124,0.337999340510228,0.96278743896921,0.335654205609084,0.768581770002329
"6733","B4GAT1",178.041217993476,0.23828683067876,0.247521789149373,0.962690321113349,0.335702955395763,0.768581770002329
"6734","PAQR7",31.0317467619697,-0.323798686297088,0.336446847470926,-0.962406658677545,0.335845370174134,0.768657522832858
"6735","STUM",3.71384486430542,0.819537646918692,0.851709103433939,0.962227177817475,0.335935499923773,0.768657522832858
"6736","LINC01011",14.0358749096168,0.451076745617368,0.468701629151523,0.962396368098698,0.33585053736461,0.768657522832858
"6737","COX6B1",1435.16833406138,-0.248984312062113,0.258737601213348,-0.962304322582041,0.335896758295946,0.768657522832858
"6738","OSBPL3",11.0565145656656,-0.548697530767725,0.570312014055082,-0.962100599751228,0.335999072883294,0.768688885202728
"6739","DCAF16",94.6395054368191,0.24041542405003,0.249921969373656,0.961961946172837,0.336068719570995,0.768734131501244
"6740","AHCYL1",698.07615578956,0.149180543615975,0.155259718563093,0.960845124521798,0.336630045874735,0.768852867540145
"6741","FCGR3A",12.2462519297821,-0.525040340240233,0.546297689978604,-0.961088340426254,0.336507751669346,0.768852867540145
"6742","TMCC2",3.69332245438148,0.760508881620669,0.79115856653482,0.961259744619346,0.336421583112022,0.768852867540145
"6743","PSD4",4.72371760950969,-0.814516350220675,0.847329611645639,-0.961274501712225,0.336414165069238,0.768852867540145
"6744","CARMN",4.64494422052504,-0.844980075752268,0.879749279556252,-0.960478281014879,0.336814556774451,0.768852867540145
"6745","HIST1H1D",5.61105511967637,-1.02184947096034,1.06412573531959,-0.960271363659339,0.336918658464722,0.768852867540145
"6746","AL645933.2",8.70065021316616,-0.533517671664035,0.555193376765543,-0.96095827866718,0.336573145972962,0.768852867540145

"6747","MAD2L1BP",58.5803404380182,-0.26775611883442,0.278746135757998
,-0.960573383757618,0.336766716802675,0.768852867540145
"6748","GLI3",389.862613187171,0.186673951622571,0.194385844348668,0.9
60326881044566,0.336890725216714,0.768852867540145
"6749","MRPS33",243.901912640843,-0.254713936208946,0.265203651869925,
-0.960446564038554,0.336830512483242,0.768852867540145
"6750","BNC2",279.693794576992,0.320287285701072,0.3331000180365,0.961
534879490687,0.336283296605114,0.768852867540145
"6751","TIGAR",14.4283167339952,-0.442572789987551,0.460287926442313,-
0.961512923895946,0.336294330440912,0.768852867540145
"6752","AL139317.5",51.1479645909525,-0.354624662420026,0.368986739266
267,-0.96107698375611,0.336513461412009,0.768852867540145
"6753","AC005225.2",3.56312527953984,-0.736825533046619,0.767241614930
753,-0.96035657960644,0.336875783170227,0.768852867540145
"6754","FAHD1",115.168043904676,-0.224264169728061,0.233452465076121,-
0.9606416863276,0.33673236094192,0.768852867540145
"6755","LINC00323",6.31453019852469,-0.634618283330249,0.6602945859938
53,-0.961113867645974,0.336494917689819,0.768852867540145
"6756","NMNAT1",39.9436054476906,0.267663384680062,0.278874882085289,0
.959797392574788,0.337157194874403,0.769072419173232
"6757","YWHAG",399.978893580443,-0.175197095807706,0.182538274450118,-
0.959782798076036,0.337164541600564,0.769072419173232
"6758","SIDT2",163.379303992477,0.179957637956189,0.187463724767515,0.
959959790510753,0.337075452305115,0.769072419173232
"6759","RAB32",714.559969374617,0.266471704063895,0.277804258190981,0.
959206693947452,0.33745462892186,0.76962022560025
"6760","MRPL18",504.206466265184,-0.23561776521258,0.245703410126758,-
0.958951953865945,0.337582950033811,0.769685131603491
"6761","AL049840.4",9.99241439762919,0.598471635395216,0.6240860501271
58,0.958956918318037,0.337580448973468,0.769685131603491
"6762","BIRC6",238.199876915681,0.217303458371089,0.226823326474553,0.
958029589586623,0.338047838076012,0.769743610111203
"6763","PTPN18",157.450777604879,0.19325712219384,0.201717887465807,0
.958056445203174,0.338034296557417,0.769743610111203
"6764","ROBO1",199.215334396711,0.186049056591042,0.194203787240812,0.
958009414926299,0.338058011057596,0.769743610111203
"6765","CNBP",1374.08142854383,-0.144709950349855,0.151023089006271,-0
.958197526630158,0.337963164208808,0.769743610111203
"6766","SAMD8",156.256814488925,0.253247235683945,0.264241363676413,0.
95839361468808,0.337864313840029,0.769743610111203
"6767","ANGEL1",13.9954906647771,0.427555328245536,0.44621182558341,0.
958189146346624,0.337967389225286,0.769743610111203
"6768","ZWILCH",32.0219777079188,0.312898688490911,0.326476207648108,0
.958411918421233,0.337855087653964,0.769743610111203
"6769","ALDH16A1",142.641491898686,-0.252558659972395,0.26357658478217
5,-0.958198393006399,0.337962727417181,0.769743610111203
"6770","TASP1",142.64952752188,0.258704926511252,0.270023694408532,0.9
58082315990555,0.338021251952785,0.769743610111203
"6771","SCOC",262.389749273655,-0.209291678354619,0.218507982329873,-0
.957821659982469,0.338152695071774,0.769845487303411

"6772","TMEM255B",4.00251049092312,-0.703184926902115,0.734275641748138,-0.957657978723082,0.338235252723894,0.769919731355408
"6773","RAB2A",1136.50631748893,-0.148391020270988,0.155007110568333,-0.95731750451262,0.338407022770126,0.770196996309094
"6774","AC083855.2",7.23300418633295,-0.625868138123945,0.653988319191508,-0.957002013273989,0.33856623883157,0.770233951360804
"6775","CTBP1-DT",50.6351148783994,0.273944877736967,0.286257289886947,0.956988301835588,0.338573159547247,0.770233951360804
"6776","SLC35A2",61.8794604825738,-0.297182101062471,0.310535167789842,-0.956999824456572,0.338567343609862,0.770233951360804
"6777","NRDC",243.745691472058,0.170558490277274,0.178285073089102,0.956661639261487,0.338738066169753,0.770417418318334
"6778","WWC2",139.757382092421,0.253021574731124,0.264492467519418,0.956630550216136,0.338753763305979,0.770417418318334
"6779","PPIL4",309.603244279777,-0.164206513975149,0.171705275016951,-0.956327718871416,0.338906689973933,0.770570744368284
"6780","PSMB3",574.400467086819,-0.272716282181864,0.285208378782414,-0.95620010655409,0.338971146127884,0.770570744368284
"6781","SAMM50",174.058847883851,-0.192619580863495,0.201425499582519,-0.956282006313625,0.338929778186295,0.770570744368284
"6782","AC009812.4",2.53589978812969,0.933690870044415,0.977036925876527,0.955635191788452,0.339256575390777,0.771105884642999
"6783","TEX261",145.424959481443,0.176600902909156,0.184955656853253,0.954828340553403,0.339664512936925,0.771578023419201
"6784","GOLGA8R",9.06110362163029,0.626544585418365,0.656073881448411,0.95499089833472,0.339582299728753,0.771578023419201
"6785","CFD",145924.408534051,0.24372095149344,0.255198381539845,0.955025459106947,0.339564822346106,0.771578023419201
"6786","TBXA2R",23.7388976399758,-0.427467883623685,0.447678868452992,-0.954853833286462,0.339651619204559,0.771578023419201
"6787","MRPS17",139.703282133784,-0.234455036639012,0.245698334520307,-0.954239421674362,0.339962464111256,0.771967874581531
"6788","CASD1",124.00858007275,0.17085723375522,0.179036953272542,0.954312674742233,0.339925394134562,0.771967874581531
"6789","AC069544.1",4.42036844487859,-0.66488741063374,0.696806599364071,-0.954192183656898,0.339986370453066,0.771967874581531
"6790","PLA2G2A",18624.8688527499,-0.361964145397498,0.379611878221396,-0.95351111533553,0.340331167090724,0.772213501661466
"6791","TGFBRAP1",69.1673461553439,-0.240765763043866,0.252451043002345,-0.95371268892571,0.340229095407436,0.772213501661466
"6792","AC080013.1",8.30608699040529,0.56249952828014,0.589999629975158,0.953389628911842,0.340392694167986,0.772213501661466
"6793","BMS1",107.427087538397,-0.195606443233836,0.205191784415322,-0.953285940717367,0.340445212928402,0.772213501661466
"6794","CCDC86",31.4132606112968,-0.287645238651536,0.301706930924939,-0.953392876224972,0.340391049466118,0.772213501661466
"6795","STX1B",4.30282734818024,-0.707739148754383,0.742330167181296,-0.953402111410535,0.340386372049449,0.772213501661466
"6796","TERF2IP",626.54945238971,-0.145427608172246,0.15255200114347 7,

-0.953298593805199,0.340438803777121,0.772213501661466
"6797","ARPC2",1650.75318271116,-0.185062479839367,0.194261499303552,-0.952646203714249,0.340769358800715,0.772392697721258
"6798","FYCO1",109.159608974511,0.217556652048536,0.228420833261574,0.952437870670939,0.340874960921033,0.772392697721258
"6799","SLC49A3",80.7529149169061,-0.30484101117786,0.320057845496594,-0.952455987150937,0.340865777010551,0.772392697721258
"6800","AL117336.2",9.60793894432789,0.610179444668106,0.640576721928391,0.952547015494448,0.340819633791494,0.772392697721258
"6801","ABCB9",4.6514178748682,-0.64185087514774,0.67382166686913,-0.952553036963118,0.340816581587148,0.772392697721258
"6802","AC006504.5",14.3669114152884,-0.392678447989214,0.412249706666334,-0.952525718367676,0.340830429169201,0.772392697721258
"6803","MT-CO1",33028.0656638579,-0.226470840837685,0.23766901287874,-0.952883331716583,0.340649186279704,0.772392697721258
"6804","EIF5B",615.038592449327,-0.268590761553169,0.282050759539501,-0.952278100550737,0.34095596113646,0.772444961272009
"6805","FST",624.702184664345,-0.361992745692847,0.380241407015063,-0.952007695675571,0.341093079013375,0.772444961272009
"6806","PRKRIP1",115.011822798477,0.175024416804888,0.183812434908971,0.95219029600236,0.341000481512204,0.772444961272009
"6807","DCAF12",79.0490260017677,0.200122207006995,0.210213048439549,0.951997073885469,0.341098465869514,0.772444961272009
"6808","PUM1",443.076481612829,0.15482437997878,0.162674920436437,0.951740929477057,0.341228386370136,0.772625672135084
"6809","SPATA1",7.22499067549198,0.565140437092623,0.594175850081622,0.951133300040702,0.341536712341131,0.772756263134968
"6810","AC239809.3",17.8758827976764,-0.416461979673779,0.437747274228404,-0.951375380709923,0.341413853354004,0.772756263134968
"6811","ARHGEF33",5.88399039180246,0.698298498698459,0.734104345757641,0.951225125874676,0.341490106243939,0.772756263134968
"6812","PLCB3",50.1318548568759,-0.25223076632218,0.265142909318119,-0.951301194404385,0.34145150083139,0.772756263134968
"6813","AC007114.1",10.8761496331227,0.519066194842623,0.545670772956926,0.951244267729173,0.341480391330277,0.772756263134968
"6814","RBM12",107.270896163388,0.178268406535165,0.187467783819427,0.950928222989378,0.341640813661704,0.772764951224528
"6815","BCAP31",437.063591709724,-0.163280797805902,0.171695494901113,-0.95099057724224,0.341609159214955,0.772764951224528
"6816","SNRPD2",1017.40876159928,-0.202390577882993,0.212857341902195,-0.950827329113173,0.341692036915703,0.772767422103222
"6817","MINDY1",58.9932189804773,0.22655784575756,0.238340728789299,0.950562863965413,0.341826327707922,0.772844359286833
"6818","ZNF701",42.8867567753685,0.264697747347009,0.278462546380417,0.950568580183118,0.341823424755205,0.772844359286833
"6819","CENPF",24.6727955247167,-0.413991618656215,0.435728965177001,-0.950112688717041,0.342054996503643,0.772998424468241
"6820","PBX3",155.081401362066,0.197172929443611,0.207538519286134,0.950054621772488,0.342084499025098,0.772998424468241

"6821","DMXL2",54.4748076933087,0.307603746291477,0.323781880648575,0.950033848945805,0.342095053631031,0.772998424468241
"6822","CMTM3",726.813764651756,0.243769312802,0.256576395954439,0.950084718024046,0.342069207586706,0.772998424468241
"6823","ARIH1",202.392890906198,0.250339884051535,0.263566536732047,0.949816646511696,0.342205425959421,0.773134492329543
"6824","PDIK1L",34.6409329169987,-0.331114258434342,0.34865058319432,-0.949702293341055,0.342263544153709,0.773152481408182
"6825","AC022382.1",2.93565332842886,0.924296392400161,0.973479368701382,0.949477125163083,0.342378000777516,0.773297711646215
"6826","ABI2",221.039095055186,0.177800084335388,0.18736713728274,0.948939536110246,0.342651364942695,0.773501650555982
"6827","LRP11",210.2891079309,0.186941782164504,0.197010091296087,0.948894449693692,0.342674297736585,0.773501650555982
"6828","GNG11",1192.43314126272,-0.272844621437658,0.287590538164208,-0.948726001833446,0.342759985882227,0.773501650555982
"6829","HOXC8",154.019181526869,-0.192464223089315,0.202914255467136,-0.948500255175452,0.34287484296552,0.773501650555982
"6830","CPM",23.128090956038,-0.440168620020302,0.464111330886447,-0.94841170798305,0.342919901388231,0.773501650555982
"6831","KDM2B",36.8405656038474,0.264980167381605,0.279385275575772,0.948439987882395,0.342905510372063,0.773501650555982
"6832","AC009996.1",8.54863625178442,0.682851436100585,0.719988578336568,0.948419817545185,0.342915774571701,0.773501650555982
"6833","SRP68",163.623466416136,-0.175749448231883,0.185211601418851,-0.94891166042256,0.342665543540796,0.773501650555982
"6834","SLC25A5-AS1",6.30991062440398,0.62220280731923,0.655827155976594,0.948729862203869,0.342758021987816,0.773501650555982
"6835","AC136469.1",8.33259518001059,-0.505456825575555,0.533264325214244,-0.94785419101959,0.343203688267597,0.774028508360645
"6836","HMGN2",1239.50726443138,0.191887622344423,0.202499008888411,0.947597834664786,0.34333422893905,0.774209645859487
"6837","LRP8",4.45752603844565,0.684969198016339,0.723296042234105,0.947010847592388,0.343633251398245,0.774315991813393
"6838","CYP4V2",135.539052679139,0.19946832125915,0.210604407205437,0.947123205567945,0.343576001225748,0.774315991813393
"6839","MT2A",11828.9681543743,-0.567576620336438,0.599396381485766,-0.946913658253234,0.343682777553502,0.774315991813393
"6840","UBL5",1298.28174298505,-0.22546641158485,0.23805924100798,-0.947102118910362,0.343586745123563,0.774315991813393
"6841","AC092296.2",5.71135951839124,0.729274684892188,0.770122883386692,0.946958856338784,0.343659744754572,0.774315991813393
"6842","ZC3H12B",8.89967607368147,0.469766272518943,0.495919915734044,0.947262365584999,0.343505102955684,0.774315991813393
"6843","OAF",1386.97950913425,0.300821773696467,0.3177200649123,0.946813899775271,0.343733617641824,0.774317363137326
"6844","C2orf88",50.8376287876475,0.389770211151322,0.411766541812936,0.946580578002361,0.343852544577251,0.774362007733433
"6845","ZNF607",9.8711276706628,0.485407961156801,0.51280296763356,0.9

46577909634223,0.343853904828761,0.774362007733433
"6846","AF001548.2",8.55513110563983,0.561260447073704,0.593195180719331,0.946164880154788,0.344064495901104,0.774508888134885
"6847","C16orf54",2.98500775405739,0.830065299457631,0.877259779136715,0.946202389757882,0.344045367505832,0.774508888134885
"6848","PLLP",4.17195212511875,-0.657142582345682,0.69454055980199,-0.946154365028055,0.344069858316425,0.774508888134885
"6849","CLEC3B",2550.89690014599,0.352237549961262,0.372347972095532,0.945990246647267,0.34415356093354,0.77458419357432
"6850","GATAD2B",158.254331216558,0.224296051397783,0.237162742178043,0.945747419421383,0.344277429977235,0.774615866511803
"6851","AC107068.1",5.69554279633922,0.765915285844586,0.810089309292975,0.945470180952093,0.344418887387084,0.774615866511803
"6852","SORBS1",15.3340627635481,-0.498092637516958,0.526806789735641,-0.945493959496815,0.344406753232531,0.774615866511803
"6853","DNAAF4",39.3421698067339,-0.281354346287962,0.297526176949287,-0.945645687962167,0.34432933285569,0.774615866511803
"6854","AC011477.2",6.36712297758619,0.649088929061647,0.686420785347609,0.94561374439287,0.344345631334092,0.774615866511803
"6855","CCNT2",108.7359680002,0.206808661492,0.218808413380066,0.945158635800613,0.344577893597055,0.774860427395858
"6856","CCDC85C",12.3195458066262,0.645559944200041,0.683254377839897,0.944831039708774,0.344745142384231,0.775010408320392
"6857","LINC02246",7.59929133159302,0.587350126904265,0.621624670601069,0.944862961015265,0.344728843210386,0.775010408320392
"6858","AC012485.3",11.9480096807642,-0.54259806584544,0.57444995449171,-0.9445523697979,0.344887453469782,0.775104256485885
"6859","BYSL",32.8926771399915,-0.281346078807457,0.297847549765545,-0.944597593731837,0.344864355975064,0.775104256485885
"6860","SDCCAG8",220.66374730178,0.161344142603897,0.170854728408341,0.94433524964136,0.344998358306791,0.775226043068439
"6861","IMPDH2",508.012890445787,-0.219491448246609,0.232458451465456,-0.944217974708596,0.345058271726667,0.775226043068439
"6862","MIEF1",71.66096862048,0.218865163384138,0.231811621297717,0.944150953946558,0.345092514274124,0.775226043068439
"6863","ZNF554",13.0028138917619,0.434784070110259,0.460665089052701,0.943818145638813,0.345262586278673,0.775495084873341
"6864","BCL7C",680.808865459125,-0.250615168240698,0.265610013261715,-0.943545633551693,0.345401885440281,0.775694939402962
"6865","C1orf220",10.5505140311365,0.456358761416167,0.483941669677657,0.943003651080794,0.345679035413709,0.775696729494942
"6866","ATG7",72.9313201557491,0.216117164683166,0.229191713926468,0.942953656485604,0.345704607955255,0.775696729494942
"6867","SCRG1",4.99180544087558,0.632591460520865,0.670726071584508,0.943144283964458,0.345607107298087,0.775696729494942
"6868","RAB24",55.01324395135,-0.292227418395481,0.309873141705014,-0.943055008858008,0.345652766851559,0.775696729494942
"6869","UNC13B",32.7994666732273,-0.30125513985195,0.319347336365001,-0.943346336565737,0.345503782143618,0.775696729494942
"6870","MFGE8",891.003341200154,0.159327053944754,0.168879688949341,0.

943435264098263,0.345458312844979,0.775696729494942
"6871","KLRG1",9.06749402089664,0.488035060109593,0.517765593898082,0.942579162967051,0.345896202020611,0.775860901164758
"6872","ACBD4",49.6182824427144,0.303357745076602,0.321859681463477,0.9425155201088,0.3459287689721 3,0.775860901164758
"6873","CNFN",7.9465860206406,0.732751671393908,0.777319051994982,0.942665266615179,0.34585214467438,0.775860901164758
"6874","FRRS1",34.7967071233634,-0.269096183392159,0.285612218656882,-0.942173218840596,0.346103962886391,0.775963447315202
"6875","USF1",25.4821800061157,-0.353777881097934,0.376008386196677,-0.940877634875104,0.346767570071549,0.775963447315202
"6876","CDC42EP3",6.30292190704027,0.618710461968261,0.657085198547867,0.941598537504098,0.346398218796665,0.775963447315202
"6877","AC010680.3",4.68769298374968,0.704980638733139,0.748844735433224,0.941424310508494,0.346487460261848,0.775963447315202
"6878","POLR1C",52.1291214558309,-0.228069992091741,0.242212840966721,-0.941609830351961,0.346392434949732,0.775963447315202
"6879","DPY19L1",77.0925999125428,0.206217725731853,0.219126529020104,0.941089728633149,0.346658878751964,0.775963447315202
"6880","WASHC1",91.9625076971756,0.289933641254382,0.30768267322211,0.942313839834212,0.346031984528018,0.775963447315202
"6881","FAM225B",10.5633165281148,0.503490397731538,0.534472748629758,0.942031935252732,0.34617629000522,0.775963447315202
"6882","ZRANB1",147.359931638113,0.186614341127786,0.198291458434055,0.941111344893597,0.346647802322629,0.775963447315202
"6883","AP000941.1",2.55263308726377,0.974307390387671,1.03478778364706,0.941552853430269,0.346421617392077,0.775963447315202
"6884","MARS",51.519438687063,-0.315616104819746,0.335429906077192,-0.940930129071777,0.346740666441764,0.775963447315202
"6885","TMED2-DT",2.69696983119038,1.07168292106149,1.1387821902659,0.941078048306375,0.346664863984306,0.775963447315202
"6886","DGLUCY",105.134735631389,-0.218978609604168,0.232719590625149,-0.940954773149659,0.346728036644017,0.775963447315202
"6887","TTC39C",120.299403721942,-0.22661648406415,0.240539746563637,-0.942116582816789,0.3461329553345,0.775963447315202
"6888","LTN1",227.935210913415,0.158906483279288,0.168708148055333,0.941901651526458,0.346242994511389,0.775963447315202
"6889","CD40LG",3.831060008625,0.856521398534777,0.910366312393827,0.940853573857029,0.346779901949687,0.775963447315202
"6890","CCDC74B",27.3657687640405,-0.3241279704166,0.344619364786044,-0.940539051303266,0.346941128360796,0.776211537544509
"6891","TSPAN14",255.873060461891,0.22959219088625,0.244201977462854,0.940173348601052,0.347128650041585,0.776485190036912
"6892","NAT10",56.0566617996001,0.251184222507946,0.267196900815601,0.94007161662887,0.347180826694736,0.776485190036912
"6893","RASSF9",40.9757723384734,-0.267995025355797,0.28509931547877,-0.94000585341901,0.347214558217608,0.776485190036912
"6894","MTRF1",21.3506184068198,-0.319168671001963,0.339643891405166,-0.939715622976485,0.347363449260125,0.776686667732423

"6895","MRPL34",430.39760263131,-0.242264907463031,0.25782906271168,-0
.939633821397186,0.347405421603312,0.776686667732423
"6896","KDM4A-
AS1",12.6727498614596,0.48945199588566,0.521000571578077,0.93944617834
7064,0.347501713332576,0.776769016163945
"6897","GUSB",487.937653086602,-0.237710121478827,0.253071059357369,-0
.939301878620382,0.347575774359111,0.776769016163945
"6898","ANKFN1",3.56806879553388,0.772171388296442,0.822099559883708,0
.939267487754975,0.347593426759578,0.776769016163945
"6899","RAB5IF",314.925059663737,-0.200718465820325,0.213736415842672,
-0.939093439126772,0.347682772485779,0.776856057090635
"6900","MACF1",639.004827353625,0.239345759314714,0.254955926012153,0.
938773077599715,0.347847264322661,0.776998345099813
"6901","KLHDC9",14.417571880894,-0.465327662541723,0.495636420560711,-
0.938848807792009,0.347808375667126,0.776998345099813
"6902","FBXO44",36.8657590010025,0.248096238074801,0.264335115084608,0
.938567083663444,0.347953059466591,0.777009475833333
"6903","TADA3",294.201078456447,-0.237265618646752,0.252772680471254,-
0.938652144703328,0.347909371016881,0.777009475833333
"6904","SYDE2",100.985368449059,0.297418423499943,0.317156013153927,0.
937766938555868,0.348364195059351,0.777589641882406
"6905","GTPBP4",133.945115422961,-0.210669153764026,0.224645898885827,
-0.937783217093564,0.348355827643198,0.777589641882406
"6906","IDH3B",253.648476442746,-0.178220801648086,0.190020865565729,-
0.937901220044902,0.348295176149133,0.777589641882406
"6907","WDR73",114.899754808115,0.20106087756997,0.214442386347446,0.9
37598582978855,0.3484507398802,0.777670212140333
"6908","SDF2",409.256010285089,-0.224004405952151,0.23898889111165,-0.
937300495057326,0.348604008383905,0.777899651018803
"6909","STX17-
AS1",2.82875325142499,-0.798133633447589,0.851705575449344,-0.93710039
76656,0.348706916911852,0.778016662931856
"6910","CBR4",108.090542622152,0.24339770719347,0.259834299324272,0.93
6742022998707,0.348891274440776,0.778165600482235
"6911","MAPK8",88.2236935599726,0.193760288546775,0.206897577883187,0.
936503416469049,0.349014054358187,0.778165600482235
"6912","AL353719.1",5.09843098102064,-0.664245245807512,0.709241997739
834,-0.93655656027743,0.348986705738955,0.778165600482235
"6913","DNAAF2",56.7982965532547,-0.262803230399295,0.280628752828122,
-0.936480056839563,0.349026076012596,0.778165600482235
"6914","YJU2",77.763935831693,-0.257946773940045,0.275330671780894,-0.
936861746174495,0.348829678746423,0.778165600482235
"6915","IFITM1",411.928073205907,-0.686115605582723,0.732780815946322,
-0.936317641854017,0.349109667521241,0.778239410678225
"6916","PIGM",87.4349314924108,0.256838349622744,0.274530166835869,0.9
35556017697312,0.349501828717821,0.77828645939633
"6917","TOR1AIP2",281.675165284435,0.195023721790596,0.208552732468068
,0.935129065357396,0.349721789265664,0.77828645939633
"6918","MTA3",91.6799975771134,0.18892799898166,0.202063578551299,0.93
4992839066723,0.34979198984475,0.77828645939633

"6919","KDM3A",88.0616502074398,0.257730681321324,0.275450275197535,0.
935670444099199,0.349442892581264,0.77828645939633
"6920","SLC16A14",3.41980964505555,0.771943472306224,0.826809245680776
,0.933641558000023,0.350488820684535,0.77828645939633
"6921","AC116351.1",2.4539071777286,1.011876500509,1.08312223543403,0.
934221888726641,0.350189447000351,0.77828645939633
"6922","CDKN2AIPNL",95.7496820962966,-0.281620800709277,0.301336758344
408,-0.934571680722082,0.350009079101027,0.77828645939633
"6923","LY6G5B",15.6807472744963,-0.470776941429329,0.504156839727023,
-0.933790646744437,0.350411895178762,0.77828645939633
"6924","STK19",166.485308523349,-0.199473431029583,0.213362813929282,-
0.934902513498426,0.349838541643178,0.77828645939633
"6925","COL21A1",24.6486508698059,1.10797184476493,1.18618522596723,0.
934063096142074,0.350271346788174,0.77828645939633
"6926","COQ3",35.3539926860792,-0.296963487274419,0.317509902137444,-0
.935288900520238,0.34963943388625,0.77828645939633
"6927","TFB1M",38.2993226720973,-0.257419646061331,0.275781454600715,-
0.933418987270304,0.350603680707985,0.77828645939633
"6928","REPIN1",125.682079260533,-0.196311473155419,0.210362336020734,
-0.933206375575096,0.350713423551342,0.77828645939633
"6929","AL512625.3",4.25652063061524,-0.766788174453075,0.820622835468
933,-0.934397802877257,0.350098730678186,0.77828645939633
"6930","VSIR",892.672431688699,0.28123935445456,0.300621864606781,0.93
5525281311212,0.349517660788327,0.77828645939633
"6931","AP003119.2",5.46462031133787,0.621108103794716,0.6643953667362
97,0.934847133034324,0.349867085452544,0.77828645939633
"6932","DDN-
AS1",4.55956661580672,-0.616781355894939,0.659080283663385,-0.93582128
1842426,0.3493652121638,0.77828645939633
"6933","NR2C1",86.8528817367737,0.183719718854425,0.196593552030522,0.
934515486173735,0.3500380514688,0.77828645939633
"6934","AC023355.1",2.84447442310636,-0.805955278228993,0.861982978281
006,-0.935001384640165,0.349787585848926,0.77828645939633
"6935","TRPM7",166.84485108562,0.206196756158328,0.220317107187256,0.9
35908966810612,0.349320060037805,0.77828645939633
"6936","AC092756.1",2.78530929565847,1.00581267089169,1.07630361551459
,0.934506450032506,0.350042710397368,0.77828645939633
"6937","REXO5",18.9359942029805,0.381460941592752,0.408650515023588,0.
933464972069674,0.350579947800749,0.77828645939633
"6938","AC002553.1",2.52723643526555,0.921235334252264,0.9840445267313
58,0.936172408084294,0.349184426993472,0.77828645939633
"6939","PIGS",76.9307118692729,0.211560676266897,0.226458192947184,0.9
34215156950577,0.350192918773768,0.77828645939633
"6940","AATF",157.468036581901,-0.192795852194054,0.206375367510263,-0
.934199921821898,0.350200776054963,0.77828645939633
"6941","VPS25",235.986261580053,-0.228934643928744,0.245303016923224,-
0.933272842707832,0.350679113164071,0.77828645939633
"6942","BST2",502.872963303138,0.434686421681734,0.465008003227381,0.9
34793420037503,0.349894771239392,0.77828645939633
"6943","AC093227.1",10.3386435573285,0.509183159721876,0.5454132309064

02,0.933573171438623,0.3505241097666,0.77828645939633
"6944","SLC17A7",115.643968543872,0.233295104662989,0.250010169143697,
0.933142461612829,0.350746417997165,0.77828645939633
"6945","ZNF444",59.6300983277799,0.236468999699048,0.25320972508172,0.
933885930418869,0.350362737151176,0.77828645939633
"6946","MZF1",51.624508285553,0.249074162548402,0.266556827428119,0.93
4412991599582,0.350090898778285,0.77828645939633
"6947","IGLC3",37.8424271587237,0.917359811668032,0.982771107147204,0.
933441983587564,0.350591812105154,0.77828645939633
"6948","MTF2",122.296160679184,0.218139855015856,0.23396441915098,0.93
2363373060961,0.351148766789416,0.77834323456679
"6949","GEMIN6",92.792865947549,-0.279574699358186,0.299827250801034,-
0.93245259932598,0.351102672375564,0.77834323456679
"6950","AL357054.4",9.62502981974271,-0.716511303490984,0.768336552767
711,-0.932548765134183,0.351052997277799,0.77834323456679
"6951","AL356417.2",16.19794006039,-0.407873802469,0.437213887341501,-
0.932893062818968,0.350875184508125,0.77834323456679
"6952","UHRF2",90.0794893880811,0.234253201478925,0.251200835753319,0.
932533527511684,0.35106086807762,0.77834323456679
"6953","FAM214B",43.2425137301794,-0.302999810467256,0.32495941325344,
-0.932423552325108,0.351117677676021,0.77834323456679
"6954","NPDC1",501.49842878778,0.232351603427854,0.249210339554796,0.9
3235137772751,0.351154963887954,0.77834323456679
"6955","RAB5C",619.573495272191,-0.21584941896211,0.231520890616369,-0
.932310766373876,0.351175945274864,0.77834323456679
"6956","EFCAB14",352.798858615388,0.181485670146642,0.194967261306129,
0.930852025775144,0.351930113479137,0.778389266560005
"6957","AL451085.2",21.2931923806142,-0.400754217846845,0.430474513956
902,-0.930959220240777,0.35187465910314,0.778389266560005
"6958","INTU",84.649220953383,0.23700361425488,0.254539396382831,0.931
107787725021,0.351797810580272,0.778389266560005
"6959","ZNF622",159.896910688507,-0.210686998284831,0.226297996518314,
-0.931015747051833,0.351845418599545,0.778389266560005
"6960","ANXA6",634.628490377871,0.172069069076014,0.184641995813228,0.
931906462114218,0.351384867723628,0.778389266560005
"6961","SERAC1",16.378256777514,-0.373501017252291,0.401124051350313,-
0.931135931627551,0.351783253966963,0.778389266560005
"6962","PVT1",14.0011608112118,0.429599985324978,0.461079392198361,0.9
31726710397327,0.35147777891636,0.778389266560005
"6963","DMAC1",372.280470221456,0.232301112349994,0.249457138468104,0.
931226557702602,0.351736382862885,0.778389266560005
"6964","EGFL7",19.3501755299653,-0.432911593005675,0.46452261311722,-0
.931949448274615,0.35136265107373,0.778389266560005
"6965","CUL2",138.76993887861,-0.194419853552368,0.208814698475405,-0.
931064024572328,0.351820446553252,0.778389266560005
"6966","SIRT1",154.137857332273,0.271674004666847,0.291869766720033,0.
930805570305752,0.351954147773158,0.778389266560005
"6967","NLRC5",29.1025247580037,0.367227735875842,0.394403933439464,0.
931095520963426,0.351804155324548,0.778389266560005
"6968","SCPEP1",1073.29283485329,0.192421872045638,0.206531675625282,0

.931682132840273,0.351500822851334,0.778389266560005
"6969","CCDC130",95.3100741762216,-0.190710338914158,0.204651769973093
,-0.931877300348937,0.351399939981865,0.778389266560005
"6970","AP001347.1",28.2714316305603,0.391247344963627,0.4202894303063
56,0.930899796072531,0.351905400026925,0.778389266560005
"6971","AC005332.4",21.1105733870604,0.399997724641488,0.4298322413344
24,0.930590323796293,0.352065521698834,0.778523887101926
"6972","ZDHHC2",106.430294936848,-0.263593400803157,0.283346729373732,
-0.930285665854571,0.352223197472686,0.778662898715633
"6973","MUS81",75.3036052593144,-0.253473463129281,0.272471934064619,-
0.930273659191512,0.3522294124388,0.778662898715633
"6974","SPIN1",229.602783796684,0.174828821286189,0.188021277836316,0.
929835300015292,0.352456366265486,0.779052894462642
"6975","ERCC3",77.9944120852822,0.227006471664164,0.244249294395227,0.
92940482070273,0.352679330457863,0.779284476204006
"6976","CLN8",114.356029214332,0.217610022187647,0.234155391007745,0.9
29340218267491,0.352712798603656,0.779284476204006
"6977","SNAPC3",103.877770939702,0.196359711311287,0.211272997986851,0
.929412244737054,0.352675484467444,0.779284476204006
"6978","FAM20B",115.816497509231,0.206017431060863,0.221760644096905,0
.929008083917894,0.352884896916482,0.779329611170139
"6979","SEMA4C",57.1109788529547,-0.250542708514132,0.269661974765388,
-0.929099138772197,0.352837710764451,0.779329611170139
"6980","ZNF763",12.0321529150035,0.427307540314066,0.459878661835524,0
.929174531839646,0.352798643833824,0.779329611170139
"6981","KBTBD4",51.1024123968189,-0.260151589256322,0.280117792005776,
-0.928722118625574,0.353033114878488,0.779433610119148
"6982","AC103810.2",3.63350352495947,0.787270537121909,0.8476364681280
59,0.928783230457907,0.353001436848359,0.779433610119148
"6983","COA5",218.982987637405,0.217653348696454,0.234485557658714,0.9
28216436311359,0.353295310187654,0.779565875542898
"6984","CRYM",11.8092781799837,-0.446236061808711,0.480716832889214,-0
.928272178710144,0.3532664018239,0.779565875542898
"6985","KATNAL2",14.9835856032763,0.516020879876904,0.555763272567624,
0.928490429914323,0.353153229750251,0.779565875542898
"6986","MIDN",620.804126457783,-0.334171405022454,0.35999882533217,-0.
928256931711137,0.353274308866276,0.779565875542898
"6987","CYSLTR1",4.77271946731515,0.711414441076092,0.766567543332192,
0.928051868702457,0.35338066472648,0.779642614392256
"6988","CLIC6",47.1819556882595,0.35302362312533,0.380614222654421,0.9
27510329654336,0.353661631083576,0.780150836169622
"6989","AC016727.1",12.2065072535851,0.456621438792639,0.4926237949219
34,0.92691713940655,0.353969557596673,0.780160241686118
"6990","PTPRE",53.804298977261,0.422601508135197,0.455684328030521,0.9
27399697860339,0.353719047471249,0.780160241686118
"6991","RPLP0",10104.8793069224,-0.157294171407174,0.169688947442277,-
0.926955902420692,0.353949430448911,0.780160241686118
"6992","PLCB2",6.08486949440554,-0.699605412730065,0.75470519115472,-0
.926991653071379,0.35393086806941,0.780160241686118
"6993","SHLD1",20.0651040874107,-0.410516933724671,0.442729060143876,-

0.927241897315851,0.353800953989473,0.780160241686118
"6994","IL13RA1",1036.47131983596,-0.285450568534329,0.307930378906731
,-0.926997100928418,0.353928039498267,0.780160241686118
"6995","MAPK3",381.595301460855,-0.16802493069568,0.181300684668277,-0
.926774937464314,0.354043400140011,0.780211438621626
"6996","SRGAP2B",165.508265975551,0.24219354500176,0.26136784459809,0.
926638643610449,0.35411418386876,0.780255881123071
"6997","PRADC1",112.450494746333,-0.276447528611701,0.298372226799092,
-0.926518971210567,0.354176342675714,0.78028130946779
"6998","MAP11",11.5953640554684,-0.436234631313162,0.470937539707351,-
0.92631101692213,0.354284372200185,0.780327753982268
"6999","SART3",101.149896807528,0.224672005233054,0.242552096102433,0.
926283502980623,0.354298666890814,0.780327753982268
"7000","OAS3",43.0093527688526,-0.407654254561085,0.440223424856143,-0
.926016725925703,0.354437288077751,0.780521542245503
"7001","GRK2",97.4361917013103,-0.213641537033489,0.230795438453369,-0
.925674868035373,0.354614972408316,0.780578294969897
"7002","GART",78.3699617031957,0.208423892417423,0.225140652961284,0.9
25749702135157,0.354576071768799,0.780578294969897
"7003","AL672277.1",3.84642513662984,-0.787156372467114,0.850333901320
44,-0.925702681317045,0.354600514053171,0.780578294969897
"7004","CDC42EP1",165.221607353139,-0.222566391322314,0.24051234002158
3,-0.925384499200091,0.354765938916165,0.780799107423284
"7005","AC069113.2",3.34913556764845,-0.752484731768784,0.813248750028
892,-0.925282371158949,0.354819046248044,0.780804510765681
"7006","ZFP69",12.4602448115854,-0.424557865846216,0.459315015708811,-
0.924328296106414,0.355315414715608,0.78098587034874
"7007","RANBP2",188.010045549229,0.196414622962347,0.212426446860988,0
.924624150451854,0.355161446258091,0.78098587034874
"7008","DIRC3-
AS1",2.94134684713595,0.825772062802862,0.893453330029439,0.9242475628
53286,0.3553574372122,0.78098587034874
"7009","AMACR",59.8248842371769,0.276430810430566,0.299001377278733,0.
924513502066158,0.355219024938486,0.78098587034874
"7010","ACO1",174.29516775951,0.212721095751142,0.230115829823364,0.92
4408789757862,0.355273520056005,0.78098587034874
"7011","PSMD13",470.31828462381,-0.214561586043583,0.23201193812706,-0
.924786835434649,0.355076799718151,0.78098587034874
"7012","ZFP36L1",6478.56118865971,0.206064967215622,0.222892525596357,
0.924503711662327,0.355224119905896,0.78098587034874
"7013","AC009113.1",25.2754983431726,0.297073497165099,0.3211689782333
18,0.924975689742009,0.354978553001738,0.78098587034874
"7014","SPC24",4.94866177539757,0.744087778664958,0.804922032387459,0.
924422178453654,0.355266551922444,0.78098587034874
"7015","TTC30A",14.5791914329306,0.416230862974188,0.450397151651304,0
.924141863349155,0.35541245964191,0.780995447666434
"7016","PTER",95.09372729466,0.21539897580168,0.233104588432658,0.9240
44340997206,0.355463230179571,0.780995680333251
"7017","AGO1",86.6511290240604,0.225742013512677,0.24435590868806,0.92
3824656930459,0.355577615370639,0.781024357500485

"7018","ING2",134.797121224353,0.20907974590142,0.226299042747466,0.92390910435595,0.355533642503054,0.781024357500485
"7019","CDC42",1650.02428377961,-0.206556641085648,0.223648070185835,-0.92357891089342,0.355705598030781,0.781187979702752
"7020","ZNF791",221.768770616342,0.224208297393706,0.242784460162991,0.923487019075215,0.355753462050816,0.781187979702752
"7021","AIMP1",291.290014523745,-0.136623099436059,0.147996193106508,-0.923152795813712,0.355927584370374,0.781290338232713
"7022","ZNF608",11.4617378847677,0.61718218052107,0.668461811234084,0.923287119995166,0.355857598228815,0.781290338232713
"7023","LINC00472",25.3604649773498,0.493889898138739,0.535030713425329,0.923105694207344,0.355952127499731,0.781290338232713
"7024","LINC01637",18.2402363140248,-0.445345367503238,0.482508330106787,-0.922979645563167,0.356017812635729,0.781323260503953
"7025","APLF",19.1495337477769,0.336722333755386,0.365193546548014,0.922038017753185,0.356508745536394,0.781346166486234
"7026","CARD6",183.434426898081,0.195597865293858,0.211961090941761,0.92280080473638,0.356111021389115,0.781346166486234
"7027","AL355347.1",2.82824720906723,-0.79115946512713,0.857856836227227,-0.922251163267025,0.356397581332764,0.781346166486234
"7028","NDUFB9",675.595347200816,-0.229986888722132,0.249347447903741,-0.92235509388858,0.356343385138313,0.781346166486234
"7029","STK33",3.30273331996788,-0.987654621014929,1.0706738581383,-0.92246076011632,0.356288289211675,0.781346166486234
"7030","PRDX5",1828.2153452772,-0.231837642441162,0.251454229014672,-0.921987446183038,0.35653512390945,0.781346166486234
"7031","AC026356.1",2.71353545503674,0.890189100144482,0.96479906046647,0.922667876266469,0.356180311381033,0.781346166486234
"7032","YEATS4",103.661260475601,-0.253552250030957,0.274995687000915,-0.922022642595532,0.356516765162094,0.781346166486234
"7033","RNF152",10.5244408497612,-0.569250047811007,0.616991470200633,-0.922622232728596,0.356204105387931,0.781346166486234
"7034","UBE2V1",614.358982233046,-0.159498090433178,0.172969627696311,-0.922116168933513,0.356467983925688,0.781346166486234
"7035","SMKR1",2.51088809255438,1.09943414609607,1.19283241323504,0.921700428238976,0.356684857142016,0.781460484145209
"7036","NME3",516.106163012171,-0.224952175015668,0.244064074693854,-0.921693105787244,0.356688677680551,0.781460484145209
"7037","ELOVL1",220.966564361618,-0.198914176724265,0.216012534156509,-0.920845531028972,0.357131079742057,0.781652079259379
"7038","PLPP1",1045.48002573398,0.295669983900029,0.320972220594452,0.92117001076429,0.356961672746929,0.781652079259379
"7039","SLC22A23",24.3871659689378,0.346232604147458,0.375992453191423,0.92084987666278,0.357128810604067,0.781652079259379
"7040","CASC15",10.5531235930183,-0.478242546987281,0.519244807561962,-0.92103481830237,0.357032248964436,0.781652079259379
"7041","AC104051.2",16.9832422568861,0.607227581213208,0.65921230987928,0.921141143927375,0.35697674172613,0.781652079259379
"7042","BBIP1",168.188236383045,-0.195799973306383,0.212576524373393,-0.921079944662462,0.357008690107179,0.781652079259379

"7043","AC022150.4",6.51065427022449,0.675181727444582,0.732796310338826,0.921377083807089,0.356853588848008,0.781652079259379
"7044","ATP5PD",1047.07802239072,-0.227394479001,0.246985093081764,-0.920680985899509,0.357217006135016,0.781729152409322
"7045","ANKRD53",8.88721542285209,-0.460960120400667,0.500822632471804,-0.920405929192144,0.357360671365286,0.781737807623851
"7046","VPS4B",449.098596503838,0.162417796940463,0.17643102689166 8,0.92057389112285,0.357272938624166,0.781737807623851
"7047","ZNF182",24.3416535582564,0.295908921085967,0.321506588339939,0.920382137777821,0.357373099599434,0.781737807623851
"7048","FBXO42",92.3419650270146,0.222126635305293,0.241575376082304,0.919492039741728,0.357838267365714,0.781802984589317
"7049","THEMIS2",11.5583793032628,-0.46853627976055,0.509673469951685,-0.919287165967193,0.357945388906143,0.781802984589317
"7050","SOWAHC",116.275484661742,0.216730664129892,0.23559698152835,0.919921226171618,0.357613925764898,0.781802984589317
"7051","RGPD5",42.6112535583017,0.41792096042843,0.454594493480688,0.919326930752151,0.357924595672184,0.781802984589317
"7052","DLC1",821.342208518209,0.207446558728001,0.225498950728726,0.919944674055525,0.357601671787335,0.781802984589317
"7053","ZBED5",143.677453527674,0.189283015864887,0.205805374628019,0.91971852633589,0.357719868698591,0.781802984589317
"7054","GIT2",122.398664081741,0.239078498560595,0.259972421638413,0.919630232521825,0.357766022448594,0.781802984589317
"7055","GNG2",172.743889308926,-0.260793015066103,0.283699562300709,-0.91925772796813,0.35796078269422,0.781802984589317
"7056","NEK8",13.6851461472243,-0.405052381594926,0.440623636079294,-0.919270661917087,0.357954019191742,0.781802984589317
"7057","TTPAL",57.2156713301712,-0.216617189585365,0.235581090230082,-0.919501600802525,0.357833268704451,0.781802984589317
"7058","URB1-AS1",26.329547435172,0.440574873583706,0.47905459878146,0.919675700232015,0.357742254683236,0.781802984589317
"7059","TFAP2E",3.35924837990059,-0.782209038174658,0.851672203395329,-0.918439083788639,0.358389036908856,0.781984610816457
"7060","CCN1",2416.71644530304,0.299267138547436,0.326365230398066,0.916970040535326,0.359158339532778,0.781984610816457
"7061","NEK7",217.646358531307,0.163523959894356,0.178357204380579,0.916834060402899,0.359229601499007,0.781984610816457
"7062","MIR29B2CHG",20.0849487213878,0.463727819779515,0.505887169665859,0.916662543716635,0.359319499479776,0.781984610816457
"7063","ARL6IP6",109.946328271803,-0.219001732721615,0.238597222094186,-0.917872097585295,0.358685831050173,0.781984610816457
"7064","BZW1",1067.32763901072,0.1433357814167,0.15611712090926 7,0.918129802688359,0.358550913791139,0.781984610816457
"7065","AC008875.2",4.63906000846303,-0.615773559188495,0.670993362602581,-0.917704396955724,0.358773645093685,0.781984610816457
"7066","NR2F1",15.6693516281241,0.479860972514887,0.522867823290337,0.917748140429042,0.358750738144656,0.781984610816457
"7067","RNF44",68.0685286212388,0.270910792958389,0.295148952473602,0.

917878212637803,0.358682629245526,0.781984610816457
"7068","RAB23",165.051197522481,0.184319446388727,0.200584865207419,0.918910039389702,0.358142628342977,0.781984610816457
"7069","ZC2HC1A",286.934400951952,-0.197402358912534,0.214884123392074,-0.918645620702078,0.358280961655518,0.781984610816457
"7070","GSDMD",239.032120111081,-0.198985000875712,0.216564640263482,-0.918824978231061,0.358187125282371,0.781984610816457
"7071","AL731563.3",3.28142496124934,0.823691862182272,0.898015839302094,0.917235338323671,0.359019332726204,0.781984610816457
"7072","ANO4",2.64408169693733,1.04069847745502,1.13526308135179,0.916702475884119,0.359298568351604,0.781984610816457
"7073","POGLUT2",54.2355784247286,0.257153678466866,0.280323898282121,0.917344828759708,0.358961973408462,0.781984610816457
"7074","SPRED1",174.178099957748,0.206547035672018,0.224999136615613,0.917990347780229,0.358623919146981,0.781984610816457
"7075","CMTM4",17.623266706719,0.433010454672684,0.47232834660752,0.916757289251777,0.359269838235675,0.781984610816457
"7076","RTN4RL1",6.3772812227076,0.998743243161599,1.08879918541959,0.917288749418666,0.358991351261543,0.781984610816457
"7077","CNP",205.311375650118,0.159603160884069,0.174045094006629,0.917021889039803,0.359131170062175,0.781984610816457
"7078","ARSG",59.4404074702026,-0.261641141149501,0.284992677281666,-0.918062680224284,0.3585860515768,0.781984610816457
"7079","CASKIN2",29.3852821565536,0.327515698437385,0.356480253425894,0.918748500905308,0.358227134841845,0.781984610816457
"7080","AC092279.1",24.7940018017859,0.454184864290112,0.495521374131632,0.91657976426555,0.359362892184481,0.781984610816457
"7081","CHD6",225.524712351552,0.188352681057038,0.205406962468763,0.916973206717285,0.359156680364232,0.781984610816457
"7082","PHKA1",3.7173436919633,-0.694359700982433,0.757322026206202,-0.916861885637767,0.359215018638772,0.781984610816457
"7083","TCEAL3",132.318203548813,-0.250147377062324,0.272315388527887,-0.918594349054594,0.358307788826802,0.781984610816457
"7084","NKAP",240.483132074519,-0.181349507138669,0.197835394282243,-0.916668666881447,0.359316289868537,0.781984610816457
"7085","TBL2",107.117907982986,-0.243705800116625,0.265944531051294,-0.916378310744882,0.359468507186115,0.782104027985033
"7086","MRPL20-
AS1",39.420600555319,-0.342427248783058,0.373772650372755,-0.916137787078758,0.359594630830372,0.782157645583489
"7087","TUBG1",45.87122239173,-0.332029521249252,0.362402311278208,-0.916190407500908,0.359567035829764,0.782157645583489
"7088","PPP1R3E",20.1864995086316,0.34468810905427,0.376316637425565,0.915952351754445,0.35969188671858,0.782258808375692
"7089","MTERF4",118.815114741936,-0.184503526505471,0.201690162710057,-0.914786938670416,0.360303492988831,0.782350176362809
"7090","NR3C2",41.629966026908,0.330771189353757,0.361612451430599,0.914711835959107,0.360342929106451,0.782350176362809
"7091","AC002454.1",3.03985247279344,0.938985883565617,1.02558535289038,0.915560933977163,0.359897229154673,0.782350176362809

"7092","ARHGEF5",32.7821195590783,-0.295520736296403,0.322921485317654
,-0.915147333741832,0.360114288748809,0.782350176362809
"7093","PSMB7",662.35353293673,-0.222706925449462,0.243291567093297,-0
.915391059830935,0.359986370061745,0.782350176362809
"7094","CNPY2",588.209692815919,0.213534734609857,0.233415719636313,0.
914825852100137,0.360283060776222,0.782350176362809
"7095","KBTBD7",87.2169187615019,0.190475642836946,0.208075855788771,0
.915414439195234,0.359974100993525,0.782350176362809
"7096","TMEM219",650.436861521181,-0.223833711326453,0.244611540663201
,-0.91505785344218,0.360161259296214,0.782350176362809
"7097","HOXB3",60.2723713426644,0.283601690713122,0.309901494341601,0.
915134957047063,0.360120785370094,0.782350176362809
"7098","SF3A2",119.186819460729,-0.276950240724539,0.302597980206472,-
0.915241537751068,0.360064842758884,0.782350176362809
"7099","GRWD1",78.0094413786571,-0.213519025545452,0.233402956695642,-
0.914808572129105,0.360292133853328,0.782350176362809
"7100","GGT5",380.545876854365,-0.301592265919943,0.329507930968093,-0
.915280749188239,0.360044262637351,0.782350176362809
"7101","RPS8",17812.6172524939,-0.20410962050722,0.223351365495307,-0.
913849888737343,0.360795727933201,0.78248555485739
"7102","TTLL7",61.5045920873805,0.263327543577512,0.288037538369245,0.
914212588638163,0.360605150636993,0.78248555485739
"7103","ADCYAP1R1",3.84088392736449,0.826168289602146,0.90402403227588
8,0.913878680329174,0.360780597349951,0.78248555485739
"7104","CTR9",220.890923977062,0.17472820386035,0.191083482748285,0.91
4407678504163,0.360502668609609,0.78248555485739
"7105","BIRC3",33.8227351141205,-0.463449748241651,0.50695847153724,-0
.914176947938836,0.360623874912446,0.78248555485739
"7106","RAP1B",1681.05372565248,0.190127592784769,0.208079999902425,0.
913723533611716,0.36086213489985,0.78248555485739
"7107","CLN6",28.7423341169824,-0.321179315707544,0.351333961745472,-0
.914170990222193,0.360627004930956,0.78248555485739
"7108","LCA5L",4.95082421745798,-0.717637345961865,0.785389450739723,-
0.913734383987402,0.360856432095488,0.78248555485739
"7109","WWC3",141.111897652355,-0.232171551123515,0.254036613410536,-0
.913929484441338,0.360753899698234,0.78248555485739
"7110","TSBP1-
AS1",6.85433397523608,-0.615306602074308,0.673769166451386,-0.91323057
318727,0.361121287577755,0.78289800375653
"7111","EBLN3P",253.13104285002,0.160466996102882,0.175725502997859,0.
913168512056203,0.361153921810748,0.78289800375653
"7112","GBP1",216.033911183669,0.310844694993615,0.340650935797825,0.9
12502102087575,0.361504463466133,0.783048535351666
"7113","C2CD6",13.6125140379108,0.457781230951079,0.502199356400749,0.
911552802918718,0.362004177298512,0.783048535351666
"7114","HUS1B",2.6280534422681,-0.9040264241794,0.991710769941012,-0.9
11582743256054,0.361988410010878,0.783048535351666
"7115","OSTM1",134.018529123072,0.168727069609139,0.185014101036261,0.
911968702191359,0.361785193464662,0.783048535351666
"7116","AL356124.1",4.19013042725785,0.881488405383808,0.9656272422518

9,0.912866131788218,0.361312951947322,0.783048535351666
"7117","MAFK",80.0411269128007,-0.271033132729389,0.297090342069592,-0
.912291967626135,0.361615041569693,0.783048535351666
"7118","LAMTOR1",795.216613948616,-0.22479975337787,0.2463307261719,-0
.912593231349449,0.361456515519177,0.783048535351666
"7119","CDCA4",44.4722961506121,-0.278622957498238,0.305618713855734,-
0.911668510030312,0.36194324558717,0.783048535351666
"7120","ZNF263",59.6269674585047,-0.203277771078488,0.22296335728752,-
0.911709320991043,0.361921755946307,0.783048535351666
"7121","LINC02185",7.16210261897619,-0.778812801242198,0.8536295586547
67,-0.912354537569585,0.361582113455917,0.783048535351666
"7122","MYO15A",3.55258507867761,0.844555598775428,0.925805640237533,0
.912238554259337,0.36164315242252,0.783048535351666
"7123","RFFL",42.9554667902704,0.287995231572641,0.31579121476366,0.91
1979871853553,0.361779313436992,0.783048535351666
"7124","PSMD3",291.46333797094,-0.198953476031997,0.218054675154848,-0
.912401790471648,0.361557247347673,0.783048535351666
"7125","SUPT5H",196.6130031616,0.208522231554244,0.228794402597303,0.9
11395686201556,0.362086925720835,0.783048535351666
"7126","BCR",45.1265619697486,-0.282871857764022,0.310299432191208,-0.
91160933091787,0.361974408681977,0.783048535351666
"7127","CTPS2",43.871906856835,0.258613316394015,0.283332247473189,0.9
12756379481608,0.361370684577138,0.783048535351666
"7128","ZDHHC9",192.917003925368,-0.238491798692247,0.261673666347521,
-0.911409245038485,0.362079784241628,0.783048535351666
"7129","CALHM5",62.9567838397302,0.217434782153431,0.238640975446314,0
.911137669240485,0.362222840829693,0.783232584007535
"7130","AC002558.3",44.4901152430141,0.310024159677858,0.3403478331103
58,0.910903873970992,0.362346024320572,0.783389055385922
"7131","TOMM20",1122.66987479985,-0.179621023169428,0.19731601571711,-
0.910321559639379,0.362652951732242,0.783704438688522
"7132","ZCCHC4",27.0919275091322,0.324352988532886,0.356238259260181,0
.910494535894283,0.36256176206703,0.783704438688522
"7133","CALU",1255.14012956206,-0.18290637961807,0.200908117016629,-0.
910398157795341,0.36261256892569,0.783704438688522
"7134","ZC3HAV1L",4.01849994460238,0.699649436923041,0.768807376086895
,0.910045166949546,0.362798690304131,0.783704438688522
"7135","TMC5",2.83904533070762,1.27560573949284,1.40174917024382,0.910
009983648472,0.362817244670074,0.783704438688522
"7136","MATK",5.37376621005814,-0.814922756671973,0.89556667475736,-0.
909952077987675,0.362847783257865,0.783704438688522
"7137","PARD6B",3.44196928870088,0.701800237661178,0.771118379068689,0
.910107003945063,0.362766081209529,0.783704438688522
"7138","CD72",11.581446076936,0.527453099194927,0.579786543872392,0.90
973670356692,0.362961382660148,0.783839971099213
"7139","SMIM12",129.111200977493,-0.241918003950537,0.266077814628334,
-0.909200206294747,0.363244455341036,0.783935145648484
"7140","IPO9-
AS1",9.60155882141786,0.484432615596668,0.532633675750587,0.9095042946
99365,0.363083991865047,0.783935145648484

"7141","PLEKHM3",70.1702780641447,0.254623176874922,0.279990787911676,
0.909398408333503,0.363139861680807,0.783935145648484
"7142","RPS27L",1459.16672024208,-0.214856989340386,0.236273468240295,
-0.909357241592068,0.363161584326934,0.783935145648484
"7143","AC027277.1",5.25821532133528,-0.695307538907823,0.764770694979
982,-0.909171263323608,0.363259730481162,0.783935145648484
"7144","PRKAA2",27.1808664867572,0.390391810353414,0.429490105014433,0
.908965784765391,0.363368186804848,0.784059434434033
"7145","EPHX2",46.918337664162,0.277405022524438,0.305280832975812,0.9
08687976969775,0.363514852378963,0.784266123082116
"7146","WNT4",3.1170073536088,1.03723143275622,1.1443815051101,0.90636
8573875569,0.364740800704828,0.784507674118327
"7147","GPATCH3",30.0434213750238,0.279081462364667,0.307257714993026,
0.908297656158127,0.363720980524752,0.784507674118327
"7148","ABHD10",69.1962802555054,0.218217020899653,0.240652083222732,0
.906773870300078,0.364526390362278,0.784507674118327
"7149","MSH3",100.340262037942,0.243097171047113,0.268155614856979,0.9
06552604452339,0.364643434890269,0.784507674118327
"7150","FUT10",53.5502666400769,-0.245107242138435,0.270047204216303,-
0.907645916386192,0.364065326750483,0.784507674118327
"7151","TCEA1",703.985871828925,-0.143838885292049,0.158468228095978,-
0.907682801911132,0.364045832870896,0.784507674118327
"7152","PPA1",952.079556525688,-0.195579436770615,0.215650934889633,-0
.906925986064978,0.36444593825287,0.784507674118327
"7153","REXO2",943.722068428972,-0.243710148744891,0.268441285927306,-
0.907871335450569,0.363946203703906,0.784507674118327
"7154","SNX19",71.5773582296708,0.202685821202072,0.223227962913026,0.
907976843748029,0.363890456058768,0.784507674118327
"7155","BCDIN3D-
AS1",2.39211441402363,0.969527161691442,1.06857622393301,0.90730744328
467,0.364244239152122,0.784507674118327
"7156","DIS3",240.704947713038,0.171632276137586,0.189300664743222,0.9
06664941564773,0.364584008215451,0.784507674118327
"7157","IPO4",24.1078758629577,-0.305792047868966,0.337092968466968,-0
.907144546086643,0.364330364042743,0.784507674118327
"7158","NEDD8",1247.21569740835,-0.230772956252128,0.254599981300778,-
0.906413877460182,0.364716830243837,0.784507674118327
"7159","DIO2",18.1338630549921,-1.00109979743517,1.10317973546838,-0.9
07467537019367,0.364159608873656,0.784507674118327
"7160","AP4E1",34.8226317318646,0.299620629658454,0.330426094094731,0.
906770485180127,0.364528180835757,0.784507674118327
"7161","SNN",41.0593996829243,0.305173659251101,0.336059851906293,0.90
8093178997758,0.363828993944118,0.784507674118327
"7162","ABR",103.196190949099,0.236664810485453,0.260890073191982,0.90
7143792747137,0.364330762368189,0.784507674118327
"7163","GNA13",182.92705751411,0.174625064075239,0.192625453071864,0.9
06552385940869,0.3646435504894,0.784507674118327
"7164","POP4",133.500924322431,-0.206889244100271,0.228264401977564,-0
.90635790034666,0.364746448291018,0.784507674118327
"7165","TMEM238",21.5157035377507,-0.497106151994297,0.547856843946677

,-0.907365048893468,0.364213785709269,0.784507674118327
"7166","NOL4L",23.132593055771,0.355247528368799,0.391173511926191,0.908158445134774,0.363794515451466,0.784507674118327
"7167","GPC4",147.200275105846,0.19443545218625,0.214402083534245,0.906872960286295,0.364473981658352,0.784507674118327
"7168","AC087623.3",7.66591972350941,-0.50779325932552,0.56054585776001,-0.905890664065748,0.364993726269528,0.784601630239127
"7169","IPPK",22.3192100342049,-0.294458916016941,0.325031381052846,-0.90593995897604,0.364967632718214,0.784601630239127
"7170","BRF1",56.6660288255313,0.215255500671876,0.237575137255228,0.906052304794106,0.364908168430413,0.784601630239127
"7171","TLK2",142.932596472014,0.192406634053633,0.212345145892843,0.90610328408792,0.364881187247088,0.784601630239127
"7172","AC093297.2",17.8935216681984,0.329812765149536,0.364121695992301,0.905776197297812,0.365054322100186,0.784604035410021
"7173","PJA2",641.775575842873,0.122024245455377,0.134729766306841,0.905696260004437,0.36509664262057,0.784604035410021
"7174","DTX2",58.4756151585051,0.229332493624344,0.253300835848842,0.905375984472466,0.365266234098657,0.784640328683222
"7175","SEC11A",1844.54706746062,-0.230745122194822,0.25481058008481,-0.905555499767795,0.365171171561862,0.784640328683222
"7176","TCF3",130.277889502078,0.156469614682187,0.172809305968333,0.905446693425528,0.365228788244501,0.784640328683222
"7177","ADCY2",11.6184662481277,-0.517941705366404,0.572319367091904,-0.904987206702777,0.365472164793617,0.784863948215882
"7178","DOCK1",275.42077109394,0.213948302863075,0.23638972287904,0.905066008189163,0.36543041878158,0.784863948215882
"7179","AJUBA",5.70598382756002,0.708492476561072,0.783125215928227,0.904698842727604,0.365624954373806,0.785082695594404
"7180","ABCC10",19.5994447902672,-0.341202515446913,0.37728070734455,-0.904373080321096,0.365797607459285,0.785234663554502
"7181","ATP5F1E",3312.61168191208,-0.233032938558653,0.257658083093004,-0.90442704440418,0.365769003143496,0.785234663554502
"7182","COL15A1",122.860113725736,-0.390695778888146,0.432065830645503,-0.904250582149599,0.365862544279889,0.785264706220341
"7183","TXNDC9",155.392083967906,-0.226934786067497,0.251166175473463,-0.903524471954521,0.366247606179554,0.785484642138322
"7184","TMEM115",236.097251614273,-0.206892690676,0.229006124766243,-0.903437368267702,0.366293814920231,0.785484642138322
"7185","ACOX2",76.0741433339363,0.263116546566648,0.291103460525537,0.903859219301744,0.36607005565204,0.785484642138322
"7186","BCLAF1",1105.88562171855,0.198253536853101,0.21943183007256,0.903485774090042,0.366268135052072,0.785484642138322
"7187","CLPB",39.806096858278,-0.253726915517123,0.280862503143042,-0.903384797464048,0.366321705632981,0.785484642138322
"7188","GOLGA6L9",13.7415187996613,-0.544266298054164,0.602473281028055,-0.903386615129939,0.366320741273375,0.785484642138322
"7189","MED26",42.4172519959431,0.341622426129774,0.378107262868119,0.903506649246592,0.366257060878245,0.785484642138322
"7190","PTBP3",176.918263174197,0.197551934197462,0.218710912817613,0.

903255953955093,0.36639006737601,0.785521959471654
"7191","COL5A2",1665.44507984707,0.311013393968268,0.344484494407019,0.902837134959102,0.366612339174691,0.785889195991916
"7192","BBS5",53.1066516744165,0.241726536293452,0.267921622812909,0.902228546376975,0.366935473581447,0.786361991868373
"7193","AP2A2",255.895604252481,0.20248100203738,0.224403421579846,0.902307997854368,0.366893278185347,0.786361991868373
"7194","IFT52",204.431006818915,-0.205705633089844,0.228021267277415,-0.902133540199909,0.366985933798318,0.786361991868373
"7195","CAPN10",37.2505135700415,0.25600923470685,0.283897292167992,0.901767088906786,0.367180605990159,0.786451025076217
"7196","SLAIN2",221.595519466833,0.167686589469347,0.185973102330289,0.901671195286801,0.367231558773832,0.786451025076217
"7197","PDCD6",636.429215779525,-0.206584398593279,0.229080632797595,-0.901797747240409,0.367164316707201,0.786451025076217
"7198","DOK6",31.4788171143739,0.500700061948805,0.555190947537428,0.901851991949221,0.367135496693848,0.786451025076217
"7199","ATG5",156.277632060476,-0.189411531564791,0.210144713735108,-0.901338549983933,0.367408342994135,0.786571245041424
"7200","NUDT4",323.131225316609,0.345311003594128,0.383135038394688,0.901277536612051,0.367440774281109,0.786571245041424
"7201","HDDC3",111.096483938239,0.227632940316981,0.252557215074072,0.901312363023243,0.367422262295459,0.786571245041424
"7202","KIF1B",322.023590762186,0.258239812343512,0.286634230200165,0.900938496296049,0.367621021589672,0.78681351541376
"7203","GPRC5A",601.159422889681,-0.566893045304081,0.62933806493713,-0.900776668197741,0.367707075254021,0.78681351541376
"7204","DDX54",138.645802220385,-0.14785216069892,0.164128672051149,-0.900830786304322,0.36767829602861,0.78681351541376
"7205","HOXB-AS3",2.75407155631776,-0.766076049887794,0.850562166773769,-0.900670262343744,0.367763664437423,0.786825383386936
"7206","GRAMD4",36.6993161628217,0.324681651588903,0.360563429349854,0.900484145533973,0.367862658857242,0.786927960905409
"7207","ID3",1728.21442420172,-0.32798212949773,0.36437725984169,-0.900116899831313,0.368058043324476,0.787236677930734
"7208","TMA7",2234.82336849698,-0.217979206781931,0.242237181612376,-0.89985858211783,0.368195513907805,0.787317151970566
"7209","ZNF195",65.8772868620677,-0.21503813242721,0.23896995678063,-0.899854255004159,0.368197816967616,0.787317151970566
"7210","POU3F1",7.54684630566849,-0.707145893781341,0.785989072707421,-0.899689217491922,0.368285663105708,0.787395769316851
"7211","DUSP5",81.4068030349024,-0.500899392031078,0.556824423028842,-0.89956433539039,0.368352143998721,0.787428692239674
"7212","ZNHIT6",134.914935067836,0.228615111712215,0.254241529340074,0.899204438809127,0.368543776450619,0.787729106209968
"7213","CYP4X1",43.9146556758669,0.43838300959475,0.487677772260058,0.898919398280425,0.368695594666373,0.787816098917378
"7214","APEH",93.4065537106086,-0.1774774431881,0.197497668350949,-0.898630574578361,0.368849467558081,0.787816098917378

"7215","CRYBG3",228.045135740885,0.23131165288503,0.257391415232561,0.898676642637957,0.36882492179263,0.787816098917378
"7216","PDE8B",6.93836573479667,-0.487573425594988,0.542529506143278,-0.898703978445411,0.368810357337483,0.787816098917378
"7217","TEFM",64.3889580005099,0.223843768877038,0.249169173635314,0.898360602201368,0.368993333388483,0.787816098917378
"7218","AC144831.1",30.9460952670478,0.365791659495141,0.407135937464191,0.898450924704512,0.368945197461405,0.787816098917378
"7219","PEX26",96.1879467572798,0.238107838318156,0.264890688783905,0.898890932751516,0.368710758105993,0.787816098917378
"7220","SMDT1",722.159720583684,-0.250884505578695,0.279253805580431,-0.898410336995158,0.368966827545841,0.787816098917378
"7221","AKR1E2",5.11163501568198,0.660515541551651,0.735588954926453,0.897941081262826,0.36921696153594,0.788184387491555
"7222","MRPL15",189.501280417844,-0.277492305888594,0.309115942935005,-0.897696518833191,0.369347365879737,0.788353592500159
"7223","LIMS2",202.764273864075,0.19593460076386,0.218373956949555,0.897243441941759,0.36958902890838,0.788438620990724
"7224","DDX56",112.421155292092,-0.190397250516969,0.212220005880232,-0.897169188773002,0.3696286435788,0.788438620990724
"7225","DDX19B",136.73843975378,-0.1940157573061,0.216259713798987,-0.897142393735141,0.369642939597792,0.788438620990724
"7226","STX8",351.327219451588,-0.242438019714155,0.270211333915202,-0.897216323983792,0.369603496253947,0.788438620990724
"7227","EBF4",4.13062362944939,0.655652876581025,0.730718612789935,0.897271350565022,0.369574140112907,0.788438620990724
"7228","CNNM1",4.36759569198544,-0.688451095642751,0.767704383325014,-0.89676587837234,0.369843859058702,0.788648926184796
"7229","ZFP64",43.7564328590662,0.235688340788528,0.262798369348215,0.896840955950661,0.369803790080686,0.788648926184796
"7230","LOXL1",709.421418293534,0.320279121194281,0.357188108657609,0.896667927714504,0.369896139477823,0.78865131259345
"7231","SKI",375.19867748376,-0.170682728218706,0.190444966715555,-0.896231237623804,0.370129275364357,0.788703635963305
"7232","SLC25A51",28.3473101211276,-0.315003666496817,0.351438632391725,-0.896326235829713,0.370078550872574,0.788703635963305
"7233","RAB11FIP3",47.4080585307184,-0.251200217342886,0.280251207821215,-0.896339463782574,0.370071488121896,0.788703635963305
"7234","FTO",133.291836769363,0.190741761925975,0.21284203150556,0.896165858673415,0.370164187101329,0.788703635963305
"7235","ZNF829",30.5976668299419,0.31991134128127,0.356987015192012,0.896142794183144,0.370176503807623,0.788703635963305
"7236","IFI44",107.462981644866,-0.18820898411837,0.210357572179637,-0.894709813239557,0.370942231510433,0.788805616806641
"7237","BHLHE40-
AS1",7.50317243728911,-0.58590166511027,0.654608033260802,-0.895041972203909,0.370764651569787,0.788805616806641
"7238","VPS8",72.1654139122654,0.213350615249877,0.23831981700704,0.895228176696586,0.370665125388397,0.788805616806641
"7239","ETV1",43.7666816581439,0.400163042972142,0.447187417327106,0.8

94844146921589,0.370870407229445,0.788805616806641
"7240","TRIM73",33.2535364946381,0.340669694710046,0.380792708525041,0
.894632925167063,0.370983345193122,0.788805616806641
"7241","PLAC9",9745.68467668227,-0.216901317241772,0.242154375645325,-
0.895715044024064,0.370404973511936,0.788805616806641
"7242","ITPRIP",41.7249640658852,-0.312451258142111,0.349132602439721,
-0.894935780728346,0.370821418272874,0.788805616806641
"7243","TMEM19",84.3773042653277,-0.187130055374353,0.209131279002743,
-0.894797068457168,0.370895577721364,0.788805616806641
"7244","TSPOAP1",4.01469355438325,0.786558017016087,0.878338449350315,
0.895506757785548,0.370516254951559,0.788805616806641
"7245","AP000894.4",2.43319078166773,0.946596449822736,1.0568784092334
9,0.895653124855926,0.370438053001924,0.788805616806641
"7246","AC006504.7",3.25927808948901,0.852854025100987,0.9533176342114
24,0.894616856433648,0.37099193784289,0.788805616806641
"7247","APLP1",2.67475892333242,-0.973517646712339,1.08819475851667,-0
.894617106995948,0.370991803855396,0.788805616806641
"7248","FOSB",9694.21930788645,0.285812105269869,0.319055335120702,0.8
95807321829435,0.37035567872915,0.788805616806641
"7249","DDRGK1",283.688338367091,-0.190890998821526,0.213350727264039,
-0.894728606128828,0.37093218298417,0.788805616806641
"7250","HSPA13",93.2788072863007,-0.173274146762814,0.193607582779497,
-0.894976034901272,0.370799898992765,0.788805616806641
"7251","RPS24",15077.6497799792,-0.229025894739041,0.256135855532777,-
0.894157884543942,0.371237422200087,0.78904043907463
"7252","CRYAB",1401.62939049329,-0.255815764343798,0.286107936678237,-
0.894123271496288,0.371255939319383,0.78904043907463
"7253","CBARP",2.52698961284888,-0.825379249766901,0.922967911902722,-
0.894266462704387,0.371179339304297,0.78904043907463
"7254","TAS2R14",13.5855337486719,0.355539874081677,0.397766109728231,
0.893841545034078,0.371406677281868,0.789251989288668
"7255","AP002813.1",3.769058337544,0.74529032649876,0.833937736949365,
0.893700205035825,0.371482315658483,0.789303913973192
"7256","GPBP1L1",154.329982884786,0.159423573267015,0.178414077287121,
0.893559385510007,0.371557685005452,0.789355252805822
"7257","WDR83OS",1183.72358681047,-0.222955845327957,0.249635741410052
,-0.893124694679554,0.371790399810055,0.789740803785587
"7258","TIGD2",5.31573758149533,-0.613921809956981,0.687748385710241,-
0.892654672424976,0.372042131222565,0.789842784502372
"7259","MARCH6",341.347816957893,0.181283365776677,0.203056664704697,0
.892772300974782,0.371979122590177,0.789842784502372
"7260","HOXC5",55.4945225753067,-0.266538152910357,0.298584515354328,-
0.892672389906282,0.372032640294953,0.789842784502372
"7261","LINC00632",335.371523233754,0.187059226683017,0.20955439406314
3,0.892652370852468,0.372043364143478,0.789842784502372
"7262","AC006449.6",11.0078393213003,-0.398748549626372,0.446806009574
324,-0.892442225668054,0.372155946718587,0.789972998990225
"7263","TRDC",6.30000587656594,-0.713575405704615,0.79970221293668,-0.
892301401898354,0.372231403055395,0.790007671399886
"7264","AC090527.3",2.59342732633539,0.861326658563786,0.9653742547710

09,0.892220456788644,0.372274779438779,0.790007671399886
"7265","VAMP5",843.214495775554,-0.221557363215362,0.248499488959338,-0.891580759957276,0.372617686571147,0.790626515966172
"7266","TIAF1",3.11699972006305,0.737122848291758,0.827069483534029,0.891246579600624,0.372796900516628,0.790897911018968
"7267","SHISA4",125.899893954305,-0.260643442597635,0.292616918001871,-0.890732649285744,0.373072614787956,0.791254226838199
"7268","RAD50",368.093005341993,0.147009301372915,0.165071694326633,0.890578496650203,0.37315533948766,0.791254226838199
"7269","NAPEPLD",41.3055357292524,0.311968527315689,0.350278690403657,0.890629478362444,0.373127979328748,0.791254226838199
"7270","AC137932.3",12.3606852067692,0.415048472017362,0.466058136054984,0.890550856016805,0.373170173799138,0.791254226838199
"7271","SLC35F5",194.217114299211,0.165175373427129,0.18555277950112,0.890180000920614,0.373369241457372,0.79141871265346
"7272","AC004816.2",6.01208760675709,-0.743822525521893,0.835636660596343,-0.890126726837322,0.37339784332772,0.79141871265346
"7273","CDK10",77.7143763869443,0.207832259734215,0.233488065771032,0.890119411662023,0.373401770815999,0.79141871265346
"7274","UBXN2A",212.229497535049,0.135596110870005,0.152776104032282,0.887547903704583,0.37478398740528,0.791420036013944
"7275","MRPS5",216.1994456762,-0.164631321787944,0.185131881443819,-0.889265103903265,0.373860621103741,0.791420036013944
"7276","AC009237.14",4.58659730261823,0.63470069548431,0.713817133728559,0.889164276807155,0.373914798533393,0.791420036013944
"7277","RIF1",348.111377347799,0.215209514563927,0.242635300317058,0.886967041822469,0.37509664502873,0.791420036013944
"7278","AC092944.1",65.810367045549,0.252590423622681,0.284415060872813,0.888104950727755,0.374484299858624,0.791420036013944
"7279","TPRG1",43.5489402410236,-0.328680604008751,0.370095686092567,-0.888096285257816,0.374488960688184,0.791420036013944
"7280","ZSCAN9",23.4746725780208,0.304446504769754,0.343178283333943,0.887138025786731,0.375004593609638,0.791420036013944
"7281","EIF3B",305.481817375743,-0.155095499018962,0.174462721941574,-0.888989334185109,0.37400881198838,0.791420036013944
"7282","PIP4P2",370.799510360386,-0.178299483898635,0.20036919258844,-0.889854780544348,0.373543867397611,0.791420036013944
"7283","SIGMAR1",160.442054708176,-0.170705590495385,0.192290480800097,-0.887748523926404,0.374676037999929,0.791420036013944
"7284","PDCD4-AS1",4.34914361142257,0.692635063721168,0.779890674201095,0.888118151214835,0.37447719988327,0.791420036013944
"7285","OTUB1",186.870469077348,-0.161610327496702,0.181858215311761,-0.888661132078371,0.374185225916242,0.791420036013944
"7286","STIP1",134.258439027783,-0.221143495387983,0.248871895228153,-0.888583643344903,0.374226884870118,0.791420036013944
"7287","AP002495.1",19.9922994880897,0.324958521318332,0.366316285041771,0.887098211539455,0.375026026875382,0.791420036013944
"7288","INTS4",40.4897911890165,0.23531123095849,0.264719262627055,0.888908606888967,0.37405219944023,0.791420036013944

"7289","CLEC2D",44.1023151261826,0.303048641116419,0.341380185227104,0
.887715966627693,0.374693555071994,0.791420036013944
"7290","NELL2",3.36287097422906,0.804759885642794,0.907160073707318,0.
887120045257236,0.375014273002343,0.791420036013944
"7291","GTF2H3",98.7390816865237,-0.171124209324263,0.192698550512544,
-0.888040978352476,0.374518709032321,0.791420036013944
"7292","DCAF4",23.768638385743,0.304944466671332,0.343119611213377,0.8
88741000821707,0.374142290440118,0.791420036013944
"7293","SLX1A",3.30358994329222,0.865118912927985,0.974497106414319,0.
887759345034083,0.374670215942178,0.791420036013944
"7294","MT1G",180.975946708252,-0.675362482568221,0.760700363295697,-0
.887816695186323,0.374639360894372,0.791420036013944
"7295","NPIPB15",43.576124093225,0.316070563221322,0.355737708661432,0
.888493278968456,0.3742754695607,0.791420036013944
"7296","MEAK7",51.6877744873682,0.217229633919077,0.244408472630094,0.
888797477360156,0.374111931878606,0.791420036013944
"7297","TOM1L2",73.8123246186047,0.199334773076644,0.224211841759131,0
.889046588764869,0.373978041999915,0.791420036013944
"7298","COPRS",568.689874816869,-0.23080924069446,0.26021332730653,-0.
887000074452634,0.375078860398805,0.791420036013944
"7299","MIF4GD",96.5698780671381,-0.255093258372662,0.286873968239177,
-0.889217170656563,0.373886376472388,0.791420036013944
"7300","FAAP100",60.3577787920865,0.202504006858694,0.228212565821214,
0.887348188431216,0.374891468970746,0.791420036013944
"7301","SLC39A3",143.330492361064,-0.26953804329342,0.303546256387948,
-0.887963655031661,0.37456030196197,0.791420036013944
"7302","ZNF628",22.0866150468621,0.318874571514705,0.359486129493485,0
.887028859678113,0.375063362978683,0.791420036013944
"7303","NRSN2",58.8766469048506,0.268940127697878,0.302822609175521,0.
888111123638048,0.374480979704795,0.791420036013944
"7304","CEBPB",2034.76686442172,-0.284753882002324,0.320765999451806,-
0.887730876991242,0.374685532663226,0.791420036013944
"7305","TTC28-
AS1",63.6342879322605,-0.202307780136072,0.227585691034491,-0.88893013
97486,0.374040626149215,0.791420036013944
"7306","TCEAL7",131.083528922701,-0.260904660925138,0.293508579843797,
-0.888916641087593,0.374047881264279,0.791420036013944
"7307","STK10",37.24442872771,-0.254647563845687,0.287252824095356,-0.
88649281220349,0.375352025709127,0.791534816180296
"7308","SVIP",7.53457123164886,-0.558321604073226,0.629634243826376,-0
.886739578648434,0.375219124398146,0.791534816180296
"7309","TMEM116",36.9549857960264,0.299249824452245,0.337567168553848,
0.8864897191698,0.375353691712638,0.791534816180296
"7310","MED4",385.553417924866,-0.169844513317248,0.191607979988201,-0
.886416700012739,0.375393023413567,0.791534816180296
"7311","WDR90",43.1534766564985,0.280501555842086,0.316454134009416,0.
886389292148544,0.375407787291219,0.791534816180296
"7312","ENY2",781.40865543342,-0.189826606744264,0.214250011426635,-0.
886005118414036,0.37561476907464,0.791754637670666
"7313","SLFN5",574.504644025181,0.228913775929797,0.258356541745077,0.

886038241507616,0.375596920526486,0.791754637670666
"7314","USP37",47.8935358172971,0.268811995351324,0.303567795861134,0.885508934137041,0.375882203199626,0.791810596295166
"7315","SMPD2",25.6475263464318,-0.320884447383111,0.362336198506322,-0.885598647625907,0.375833840588185,0.791810596295166
"7316","IKZF1",11.6668494554057,0.550300333603389,0.621471667484648,0.885479358746443,0.375898147498412,0.791810596295166
"7317","REXO1",65.2411500265671,-0.237245835590733,0.26787804161815,-0.885648686087224,0.375806867599565,0.791810596295166
"7318","CHMP2A",839.503368641449,-0.22901967433131,0.258624993027492,-0.885528005821794,0.375871921743172,0.791810596295166
"7319","DCP2",151.788786418738,0.171633602836676,0.194011577775858,0.884656497330095,0.376341925224748,0.792420921656363
"7320","DMRT3",14.9539344105796,0.511502628722793,0.578222499160529,0.884612116383225,0.376365869516894,0.792420921656363
"7321","BRD3",220.178263808259,0.163593076542699,0.184940424911265,0.88457175666808,0.376387645100801,0.792420921656363
"7322","ALG10",15.5371198936918,-0.357716786380732,0.404400413028429,-0.884560882868298,0.376393512057599,0.792420921656363
"7323","SMIM4",115.145582768,-0.225292095877987,0.254804165363799,-0.884177444887231,0.376600432033058,0.792748280730518
"7324","ZNF667-AS1",149.197446107898,-0.185305757572565,0.209608464413214,-0.884056653395734,0.376665630960422,0.792777266692368
"7325","FARP2",69.224878728807,0.226151448225985,0.25591157152844,0.883709348800791,0.37685313235002,0.792777971500271
"7326","AC117453.1",2.65927441662064,1.12912196042491,1.27789478199565,0.883579756591218,0.376923110809308,0.792777971500271
"7327","DIMT1",128.217973493318,-0.199168292655198,0.225404741022884,-0.883602943537813,0.376910589508184,0.792777971500271
"7328","OR2A1-AS1",17.3043564080611,-0.45699997227477,0.517049832677471,-0.883860594071288,0.376771471594632,0.792777971500271
"7329","CSTB",1597.93094699634,-0.201015709114315,0.227447840558325,-0.883788162687646,0.376810577581128,0.792777971500271
"7330","ZSCAN31",16.2207387729828,0.367271013103103,0.415767147674642,0.883357463804499,0.377043165287698,0.79292229098361
"7331","AC002070.1",2.92589802888264,-0.84346690491357,0.95509402351483,-0.883124471671949,0.377169023507085,0.793078774704912
"7332","FPGT",47.3224933680341,0.278408861383538,0.315675192472358,0.881947229375385,0.377805344929206,0.793471440366053
"7333","OLFML3",1596.45388768395,0.207806193441615,0.23568499183424,0.881711609315231,0.377932781362459,0.793471440366053
"7334","NCK2",126.824901683427,-0.173899013076545,0.197225509964822,-0.881726776153664,0.377924577495344,0.793471440366053
"7335","ROPN1L",5.20125939772114,0.621388255240444,0.704258244692953,0.882330111039539,0.377598317705558,0.793471440366053
"7336","PRKAR2B",55.2064554667869,-0.303639299898183,0.344113368015505,-0.882381587350892,0.377570489378714,0.793471440366053
"7337","GIMAP4",6.86102950763216,-0.641202073029335,0.7266837679906,-0

.882367408318978,0.377578154501397,0.793471440366053
"7338","PCGF5",124.140989812605,-0.169292812280888,0.19195925001484,-0.881920575683643,0.377819759399896,0.793471440366053
"7339","C12orf60",5.37675941458908,0.607633375358201,0.689008460593263,0.881895375907293,0.377833387896349,0.793471440366053
"7340","LRRC49",43.7387224834283,-0.253939004555781,0.288031436572966,-0.881636419889365,0.377973453558737,0.793471440366053
"7341","ZNF606",16.6908846306021,0.383934913360017,0.435307945247788,0.881984621579724,0.377785123579892,0.793471440366053
"7342","SDCBP2",16.6426522843574,-0.410714944411387,0.465512287474139,-0.882285936295944,0.377622199779588,0.793471440366053
"7343","LAMA5",7.5609093623213,-0.496524454242127,0.562845332632867,-0.882168555825975,0.377685663393305,0.793471440366053
"7344","LDB2",177.498401387804,0.195345601099729,0.221600404565518,0.881521861310379,0.378035426884057,0.793472921471208
"7345","AC009812.1",15.7826077704724,-0.326149201699997,0.370016584026322,-0.881444821069955,0.378077107246579,0.793472921471208
"7346","PHF10",202.240207389464,0.173191667958469,0.196562993130504,0.881100074842076,0.378263656753681,0.793756366574733
"7347","AP001160.2",4.52678144716563,0.8034152019765,0.912025793686754,0.880912807003836,0.378365015113558,0.793860991966176
"7348","RBM15B",144.980014205881,-0.170522851079566,0.193623040462827,-0.880695038524118,0.378482902916608,0.793892223222141
"7349","XPOT",109.899849169336,-0.174191998289111,0.197788608707977,-0.880697828995273,0.378481392166938,0.793892223222141
"7350","PRKAR1A",610.288645500352,0.137291010489477,0.155922624288724,0.880507310056903,0.378584546867427,0.793997386389305
"7351","SCLT1",74.1487775156914,-0.194452501177598,0.220932090622787,-0.880146024189849,0.378780209251977,0.794299676998943
"7352","NBPF3",9.91888321832203,0.501546945697054,0.570146481416097,0.87968085754268,0.379032222217331,0.794456144298492
"7353","GAS5",1551.30835916316,-0.194831347904232,0.221510239462922,-0.879559104701544,0.37909820118959,0.794456144298492
"7354","LMCD1",364.871189510598,-0.357158885320738,0.405976774382966,-0.87975201503479,0.378993664596694,0.794456144298492
"7355","PC",9.05323251941419,0.434101026675604,0.493558714756583,0.879532695293805,0.379112513620481,0.794456144298492
"7356","AC008870.2",2.57447446371524,0.825697025186293,0.938702264316782,0.879615461231748,0.379067660194776,0.794456144298492
"7357","CCNA2",3.15260882212536,-0.671671437381834,0.763816471943477,-0.87936233644296,0.379204846636424,0.794541621707283
"7358","PAK1IP1",120.344758096622,-0.211046517600366,0.240126771497593,-0.878896244196917,0.379457535276241,0.794929580077713
"7359","SLC14A2-AS1",3.53337702564704,-0.798608231467905,0.908716933103231,-0.878830582303216,0.379493141731553,0.794929580077713
"7360","COL1A1",26082.8984474973,0.355401549146387,0.404484290166649,0.87865352941139,0.379589162389811,0.795022681825942
"7361","DCLK2",10.2691055796916,0.452498525765199,0.515090966570087,0.878482744083669,0.379681798130525,0.795108669770688

"7362","SMCR5",11.068686253049,0.440614635711694,0.501735849416427,0.878180493230005,0.379845776207871,0.795344015246446
"7363","ZFAND2B",123.467165168241,-0.198077456561744,0.225592510982623,-0.878032057442728,0.37992632199344,0.795404624952992
"7364","USP44",34.1544392385742,0.39064182531538,0.444988770442205,0.877868951450577,0.380014840385566,0.795481907189503
"7365","ZNF717",30.2791606110079,0.298437395503763,0.340046833263537,0.877636155701158,0.380141201694084,0.795629419440253
"7366","NOA1",152.196017843991,-0.206724969753961,0.235570867117763,-0.877548961309453,0.380188537372488,0.795629419440253
"7367","LINC02588",4.04931980109044,0.756257206124294,0.861893629115301,0.877436821177761,0.380249420800837,0.795648815209027
"7368","TRAF3IP1",80.7116835376256,-0.251729963570302,0.286957108121801,-0.877238989540742,0.380356842703616,0.795751158176712
"7369","RIPK3",8.37920057166158,-0.476105756720271,0.542799265042183,-0.877130437314191,0.380415794113564,0.795751158176712
"7370","TMEM229B",4.74192002923523,0.612089159875825,0.697886193452487,0.877061569090199,0.380453197259966,0.795751158176712
"7371","KPNA6",219.247686298691,0.19865046003198,0.226636349352735,0.876516324937806,0.380749405551977,0.796154650920201
"7372","DMPK",34.3879709276077,0.25839361802002,0.29478439382654,0.876551213128555,0.380730448019946,0.796154650920201
"7373","RTTN",26.1912851161033,0.316482863879464,0.361164362687886,0.87628486244908,0.380875192233848,0.796309655267159
"7374","SMOC1",29.5568100610033,-0.599681206074603,0.684503105642902,-0.876082520489615,0.380985174468668,0.796431579120492
"7375","LYPLAL1",188.261775987344,0.225197835746186,0.257142567466229,0.875770347808173,0.381154893037581,0.796431660449095
"7376","PPP2CA",208.473829680184,-0.167756934253166,0.191535096694535,-0.875854802322256,0.381108973168154,0.796431660449095
"7377","POLR3D",92.1678711577241,-0.203990029388863,0.232937568818966,-0.875728335378133,0.381177737430951,0.796431660449095
"7378","AC092868.2",11.1978294992874,0.650588351993244,0.742940685976206,0.87569353014822,0.381196663522756,0.796431660449095
"7379","HOXB4",51.750083155208,-0.235767694303913,0.26927602628908,-0.875561399033741,0.381268517907708,0.796431660449095
"7380","TMEM205",250.281224830345,-0.189141230076245,0.216034915381198,-0.875512320508475,0.38129520947871,0.796431660449095
"7381","CYHR1",221.111104163445,-0.18360653634773,0.20976579848799,-0.87529300615821,0.381414498562815,0.796466481402463
"7382","AC138331.1",6.11233057497009,-0.552765276789857,0.631521223911575,-0.875291685948555,0.381415216718325,0.796466481402463
"7383","ARL16",243.541211270197,-0.222702759100029,0.254480324345868,-0.875127614177944,0.381504473431822,0.796544962474811
"7384","OAS1",90.0994386612445,-0.322392629675894,0.368460148022322,-0.874972860447211,0.381588672782336,0.796612864428455
"7385","LINC01285",2.58108470785919,0.808427223371713,0.924219062170907,0.874713860015818,0.381729616820838,0.796799193404634
"7386","TMEM187",73.709620615798,0.263620939704197,0.301432709401278,0.874559832036195,0.381813451613874,0.796866281698872

"7387","SDC3",82.1750621509767,0.269149215474216,0.308122623197548,0.8
7351331973328,0.382383349368368,0.797028160710837
"7388","CCDC30",41.9425649784781,-0.266407284272218,0.304814346463177,
-0.87399850880838,0.382119065811255,0.797028160710837
"7389","DPH2",23.3139888335489,-0.307820474649211,0.353102203727445,-0
.871760276202676,0.383339169590215,0.797028160710837
"7390","RETREG2",268.992933970616,-0.145593011679652,0.16705137655186,
-0.871546315180791,0.383455928682371,0.797028160710837
"7391","COPS9",885.484591185989,-0.202742575019344,0.232032019559572,-
0.873769815925305,0.382243621369052,0.797028160710837
"7392","AC116036.2",2.71450438693107,0.807531465840081,0.9238332525936
25,0.87410954690467,0.382058598870755,0.797028160710837
"7393","PPP4R2",244.937346971641,0.13158563474605,0.150657934308512,0.
873406603840683,0.382441492780906,0.797028160710837
"7394","PPARGC1A",8.32199771294102,0.669455102295053,0.768184640743581
,0.871476812719192,0.383493861043936,0.797028160710837
"7395","AC008467.1",10.2774632231152,0.476852849083281,0.5461027336353
28,0.873192569297245,0.382558124349844,0.797028160710837
"7396","CLK4",139.419906135072,0.167597423981217,0.192248769066615,0.8
71773716913341,0.383331835685996,0.797028160710837
"7397","RNF8",139.873289144432,-0.173211832864247,0.198716667822238,-0
.871652261294932,0.383398110887658,0.797028160710837
"7398","MAP3K7",112.991014157261,0.182339961839955,0.209181565631459,0
.871682747423385,0.383381474733247,0.797028160710837
"7399","AL360178.1",8.3290141322269,0.540028123936855,0.61935831468839
5,0.871915515025495,0.383254468987924,0.797028160710837
"7400","MYO1G",6.32545044712243,0.681187223681771,0.780640931418379,0.
872599932012396,0.382881177629535,0.797028160710837
"7401","HIPK2",80.0657718016353,0.265481656582639,0.304357191284833,0.
872270030689658,0.383061082971693,0.797028160710837
"7402","GIMAP7",4.4557007914997,-0.685970332627437,0.78665408487074,-0
.872010132306316,0.383202850020214,0.797028160710837
"7403","TMEFF1",10.6235946028363,-0.469105418857067,0.537932046073333,
-0.872053305396711,0.383179298122921,0.797028160710837
"7404","FNBP1",258.521521004489,0.184762270573378,0.211589926239273,0.
873209201672687,0.382549060265057,0.797028160710837
"7405","ZNF32",238.786594622719,-0.226214919966927,0.259530747148054,-
0.871630519515589,0.383409975556933,0.797028160710837
"7406","PKP3",5.32161090380084,-0.657059180498944,0.753359890042407,-0
.872171705958433,0.38311471248889,0.797028160710837
"7407","LINC01068",3.12614810958466,0.91124347810656,1.04388924769653,
0.872931185101607,0.382700587228467,0.797028160710837
"7408","AL160153.1",3.15289410877102,-0.745642858685103,0.854137011091
899,-0.872978045678994,0.382675044299602,0.797028160710837
"7409","CHD8",180.427445922426,0.231970249202,0.265794331276742,0.8727
43403095663,0.382802954589635,0.797028160710837
"7410","MRPL52",249.309470452215,-0.246211378838513,0.281729686068142,
-0.873927708061841,0.382157624151865,0.797028160710837
"7411","MTA1",143.965787511172,-0.168725219118686,0.193408187214843,-0
.872378887100894,0.383001714497255,0.797028160710837

"7412","MYO1E",170.082437094146,-0.2961306515726,0.339212531198218,-0.87299443368605,0.382666111713498,0.797028160710837
"7413","FANCI",14.8129065493274,0.361250321998589,0.413340508897478,0.873977542056471,0.38213048413092,0.797028160710837
"7414","NOB1",206.819245413521,-0.205658763338211,0.235533625123118,-0.873160947744552,0.382575357394764,0.797028160710837
"7415","RPL26",15410.6345428062,-0.239244018636095,0.274342077368705,-0.872064617031241,0.383173127517035,0.797028160710837
"7416","AC068234.2",3.97247609828815,-0.693535784083464,0.795257013843753,-0.872090119307927,0.383159216002107,0.797028160710837
"7417","AL022322.1",7.8587353990937,0.514175406176424,0.589025768988018,0.872925147332363,0.382703878393018,0.797028160710837
"7418","JHY",39.1351947502678,0.282120257930372,0.323989461757271,0.870769858995393,0.38387982574553,0.797516083751568
"7419","AC124068.2",3.32133670867909,-0.708835102448572,0.813997118268358,-0.870807877006371,0.383859063586725,0.797516083751568
"7420","MLLT1",139.418383186982,0.175271949248946,0.201285593120054,0.870762514753888,0.383883836616064,0.797516083751568
"7421","MAPRE3",47.1237925244117,-0.219964196577997,0.252732632354474,-0.870343471394242,0.384112728961767,0.797630482812001
"7422","ZNF354A",55.8983128534383,-0.214912134230946,0.246921686150667,-0.870365570482173,0.384100655781461,0.797630482812001
"7423","FAM135A",43.2943933890816,0.243128122897458,0.279549931912437,0.86971268865704,0.384457436279132,0.797630482812001
"7424","EZR",30.4277862633789,-0.406882752659712,0.467634227910383,-0.870087620570166,0.384252522366835,0.797630482812001
"7425","MROH1",42.7806507677884,0.34904884056925,0.401317796346036,0.869756695933523,0.384433381241857,0.797630482812001
"7426","CCDC59",367.315331020641,-0.194053838205929,0.223147846181621,-0.869620036789368,0.384508084189165,0.797630482812001
"7427","SLC25A39",141.241774862664,-0.238357493468138,0.27391392030399,-0.870191238194861,0.38419590335812,0.797630482812001
"7428","EID2",90.8200529279749,-0.218287511830342,0.250860231221592,-0.870155906208675,0.384215208979223,0.797630482812001
"7429","THBD",35.3889621707276,-0.554151922958588,0.637214947868161,-0.869646772745263,0.384493468627329,0.797630482812001
"7430","DSN1",51.8612383753075,-0.239607829833338,0.275456388255355,-0.869857589257342,0.384378234913194,0.797630482812001
"7431","LINC00899",43.1385966739427,0.31287055471363,0.359458682852419,0.870393649225281,0.384085316129126,0.797630482812001
"7432","PPP1R12B",292.120911988014,0.259819128051429,0.298866999896239,0.869346994287203,0.384657365918771,0.797725453469374
"7433","TAF9",285.745704318028,-0.230832700123768,0.265505634756357,-0.869407914207144,0.384624055831166,0.797725453469374
"7434","INTS7",24.2134950777854,0.344571827911028,0.39641426559851,0.869221563938396,0.384725954885241,0.797760370534838
"7435","RASA2",127.068329970652,0.178588694063229,0.205607679910633,0.868589607843698,0.385071640628547,0.798043865711091
"7436","ETFDH",103.576536647958,0.178815659465994,0.205916739684973,0.868388163777066,0.385181872264424,0.798043865711091

"7437","MAP1B",1711.95300327223,-0.251869693089657,0.289990168022387,-0.868545629692566,0.385095704142237,0.798043865711091
"7438","ZNF510",38.659300842221,0.258802620277854,0.297906301568368,0.868738321127659,0.384990276028547,0.798043865711091
"7439","GAPVD1",132.972206441016,0.191639451248747,0.220651307621851,0.868517179046935,0.385111271964382,0.798043865711091
"7440","CCNJ",18.1440906152913,-0.304351489796859,0.350488045642093,-0.868364823226107,0.38519464562775,0.798043865711091
"7441","ZNF17",19.6686923269182,0.329500242759933,0.379473381955322,0.868309237032933,0.385225066802221,0.798043865711091
"7442","AC026329.1",7.14534947646677,0.551041062169739,0.63506365305211,0.867694221707448,0.385561749968716,0.79823972392198
"7443","CDC5L",348.57544914699,-0.163382070905632,0.188305269787282,-0.867644708457687,0.385588863252999,0.79823972392198
"7444","TRMT11",138.984948712805,0.193745784191985,0.223309274201561,0.867611902303297,0.385606828431217,0.79823972392198
"7445","BSG",1078.36694591421,-0.205239819195027,0.236568864685397,-0.867569024638835,0.385630309701394,0.79823972392198
"7446","ZNF808",27.9285106359976,0.276391172689825,0.318472673732487,0.867864641102584,0.385468437801831,0.79823972392198
"7447","DEPDC5",30.1705353311675,0.290952163580757,0.335272410758022,0.867808248590867,0.385499313672011,0.79823972392198
"7448","PPT2",23.4068097327399,0.304604335776641,0.351226998301822,0.867257748548372,0.385800801283515,0.798378218792507
"7449","AC005736.1",17.5499901778082,0.427462938602292,0.492869052465435,0.867295149622465,0.385780313601037,0.798378218792507
"7450","NDUFB6",450.98582206124,-0.22877294375598,0.263855230471323,-0.867039638923678,0.385920291323477,0.798518294060591
"7451","AQR",115.397664666396,0.187646678648857,0.216487263470641,0.866779299809962,0.386062946119402,0.798599076010544
"7452","PEAK1",187.318448728471,0.281853776616732,0.325143957193251,0.866858418805582,0.386019588858992,0.798599076010544
"7453","KIAA1614",37.4162531535171,0.260158651815568,0.300371190974092,0.866123848202231,0.386422248368351,0.798763410961996
"7454","HSD17B11",465.995029771932,-0.21081690043532,0.243307343479607,-0.866463368595324,0.38623610627005,0.798763410961996
"7455","LTC4S",43.2087434751518,-0.336375111704469,0.388393763481219,-0.866067232103572,0.386453293477429,0.798763410961996
"7456","ZNF438",93.7501278535768,0.192738506117942,0.222489583266372,0.866281033423436,0.386336064744961,0.798763410961996
"7457","DEGS2",3.10729459762887,0.93850419936259,1.08360130745253,0.866097330178523,0.386436789183204,0.798763410961996
"7458","THBS1",573.388866902692,-0.664868657038626,0.767419075998939,-0.866369729177211,0.386287438625182,0.798763410961996
"7459","GATA2",2.94616873889161,-1.05799249864329,1.22236299098061,-0.865530539168678,0.386747661541893,0.798914711361424
"7460","AIP",464.149520134673,-0.206191272170651,0.23814313107663,-0.865829181125124,0.386583844006059,0.798914711361424
"7461","AC004825.2",7.9262403304717,-0.44273970303333,0.511442209048479,-0.865669073065033,0.386671664670794,0.798914711361424

"7462","LITAF",425.345307435054,-0.150372362918867,0.173748216426381,-0.865461332563267,0.386785630288051,0.798914711361424
"7463","RPS9",13041.0413339318,-0.236223019135404,0.272902648950833,-0.865594452980052,0.386712598595713,0.798914711361424
"7464","CYTH1",90.6994730865705,0.269509070564817,0.311450880759942,0.86533410953025,0.386855434458119,0.798951838447469
"7465","AC105345.1",5.11426009151409,0.621545163690452,0.718591560501214,0.864949155897305,0.38706669591882,0.79908979175807
"7466","RBM4B",54.4408887732801,0.26791709202314,0.309883525090501,0.864573526278607,0.387272908206029,0.79908979175807
"7467","SAP18",1430.04113153898,-0.228444463699005,0.264166949554034,-0.864773069018153,0.38716335534983,0.79908979175807
"7468","TP53I13",362.628604304945,-0.215385786258136,0.249082427433067,-0.864716907080945,0.387194187437456,0.79908979175807
"7469","SHC2",166.921693614986,0.238787824362814,0.276089878910764,0.864891626251872,0.387098274067803,0.79908979175807
"7470","GDF15",97.1707485278692,0.439363102869157,0.508197827928806,0.864551319827183,0.387285101149824,0.79908979175807
"7471","MIR503HG",13.2873921895292,0.578764997711407,0.669104118609599,0.864984957668595,0.387047045040957,0.79908979175807
"7472","DUSP16",68.1689620279524,0.224423708345292,0.259640679218,0.86436266081734,0.387388697998147,0.799196571151155
"7473","SIVA1",627.397548095592,-0.226264304499296,0.261860551987335,-0.864064108862949,0.387552674023193,0.799356982937254
"7474","MRPS34",384.609406882478,-0.21998156563231,0.254598785040999,-0.864032267855816,0.387570164805257,0.799356982937254
"7475","CXCL10",11.7564672764413,-0.722839672317927,0.836702389968797,-0.863914912857944,0.387634633965626,0.799374625239971
"7476","SPCS3",244.516658786879,-0.146814084845634,0.169994432843168,-0.86364054628236,0.387785383197894,0.799374625239971
"7477","LOXL2",120.537880113585,-0.341682711985126,0.395631256216408,-0.863639327319042,0.387786053030415,0.799374625239971
"7478","AC105020.1",5.17509970835033,-0.677039557672,0.783938004593706,-0.863639157311796,0.387786146451152,0.799374625239971
"7479","RPIA",127.970439480366,0.173890994091817,0.201423419617656,0.863310703501605,0.387966660821421,0.799532897936123
"7480","AC079915.1",4.47558724400326,0.734583927178827,0.850849841251379,0.863353192965805,0.387943306226136,0.799532897936123
"7481","DDIAS",2.6839130818441,-0.829891571734157,0.9617660637507,-0.862882984764239,0.388201806772107,0.799910553588026
"7482","AC020659.1",3.09938748119332,0.800590895536195,0.927981754202931,0.862722668748853,0.388289965705924,0.799985274172256
"7483","TBC1D7",38.872908171917,-0.279537351446994,0.324133661063601,-0.862413828078607,0.38845983379146,0.800121370643419
"7484","COA8",304.463931116015,-0.198699063807323,0.230392623372433,-0.862436743411366,0.388447228380035,0.800121370643419
"7485","TMEM42",137.657443362208,0.247394693619133,0.287140214782557,0.861581488355706,0.388917861342231,0.800850765774845
"7486","AC106791.1",17.7596292677275,0.409287554734093,0.474995568425337,0.86166048992681,0.388871313541269,0.800850765774845

"7487","TNPO2",124.120007692264,0.153556964568126,0.178252487723314,0.
861457624122919,0.388986050579643,0.800884195229758
"7488","CYP4Z1",4.42750767237682,0.870051413021102,1.01022434480528,0.
861245739617181,0.389102713252073,0.801017404484602
"7489","NDUFAF3",534.978807484447,-0.21477571658059,0.249463543835465,
-0.860950314737158,0.389265408422784,0.801245329261212
"7490","HSPA9",358.714031318039,-0.147711941546554,0.171695064801072,-
0.860315593332254,0.389615099550426,0.801430045312225
"7491","IGF2",188.783973393976,0.740705249501894,0.860781764549043,0.8
6050295209256,0.389511856918079,0.801430045312225
"7492","CCDC84-
DT",3.06252808683679,0.784433779149674,0.911772256762556,0.86033960052
149,0.389601869640135,0.801430045312225
"7493","ZNF609",114.244259459995,0.231889338020687,0.269490443643358,0
.860473324714877,0.389528181753354,0.801430045312225
"7494","CFAP53",5.40158651990181,0.643520375761219,0.747991871555565,0
.860330707101026,0.389606770604959,0.801430045312225
"7495","PGAM1",596.394951265972,-0.200499401686866,0.233146036665453,-
0.85997345077998,0.389803677569661,0.801497090801163
"7496","NDUFB10",1091.87953628138,-0.208662642931136,0.242611974325836
,-0.86006737099833,0.389751906226538,0.801497090801163
"7497","ARAF",83.0351757106766,-0.170908353574531,0.198725356576503,-0
.860022880415548,0.389776430107651,0.801497090801163
"7498","AC091057.6",3.06705827500665,-0.706147587784766,0.821266658503
064,-0.859827414730159,0.389884184862759,0.801555709477117
"7499","ZSCAN16-
AS1",203.042397409586,0.231112572408143,0.2688521396987,0.859627052502
345,0.389994657742242,0.801569019879555
"7500","WDR4",14.0407161620588,-0.37821883830212,0.439977995696051,-0.
859631258840053,0.389992338315942,0.801569019879555
"7501","MED25",91.1089624908443,-0.183851829100634,0.213916412746104,-
0.859456395797204,0.390088767018985,0.801655558405234
"7502","AC100786.1",3.44304113167213,0.835246024183862,0.9720004261420
78,0.859306232507529,0.390171586542545,0.801718875840221
"7503","CPSF4",73.4500931054224,0.240001490667056,0.279359863713086,0.
859112284338554,0.390278570546251,0.801831822600353
"7504","UBE2J2",189.504378359028,-0.205133033167268,0.23886863750173,-
0.858769218565926,0.390467853162933,0.801917647096677
"7505","GSC",170.526835952121,-0.281427343444167,0.327706457089365,-0.
858778755669811,0.390462590421214,0.801917647096677
"7506","LIPE",8.90991323123438,0.490312052323494,0.570957709235406,0.8
58753712214678,0.390476409932382,0.801917647096677
"7507","IRAK3",147.498362296684,0.222366585970491,0.258969608497478,0.
858659003504871,0.390528674899995,0.801918146207995
"7508","FBXL2",34.5430402429078,0.253243887477118,0.295026185795657,0.
858377661610425,0.390683958390716,0.802004472189746
"7509","SLC25A25-
AS1",2.90129564655644,-0.71269857446797,0.830360627607293,-0.858300057
556475,0.390726797674018,0.802004472189746
"7510","ARHGAP6",233.816423007799,0.20689295733918,0.241017202062973,0

.858415729534204,0.39066294503471,0.802004472189746
"7511","MECR",93.5802379766971,-0.214974176534885,0.250655028633499,-0
.857649566046466,0.391085996655262,0.802053324886362
"7512","CEBPD",7074.1138439165,-0.231681903600108,0.27002064668444,-0.
858015512683604,0.39088389762306,0.802053324886362
"7513","MACROD1",104.959782387569,-0.208958668981085,0.243508578026814
,-0.858116254771425,0.390828272565138,0.802053324886362
"7514","CLEC2B",973.138933747222,-0.226991703715335,0.264578131790851,
-0.857938266397511,0.390926552657764,0.802053324886362
"7515","AC092828.1",12.6002142708136,0.372990450822683,0.4349248869608
49,0.857597396711535,0.391114813050327,0.802053324886362
"7516","AC068338.3",5.62218335472394,-0.554845734115928,0.646860214631
438,-0.857752141136185,0.391029341760738,0.802053324886362
"7517","KARS",177.4540238979,0.134409153653908,0.156701975756157,0.857
737453566388,0.391037453782444,0.802053324886362
"7518","BCL2L10",2.65853207830821,-0.762435729176386,0.889137421318009
,-0.857500439072953,0.391168372258748,0.802056458947672
"7519","GALNT7",40.6551112178664,-0.257985406964394,0.301063486355572,
-0.856913636679606,0.391492615771337,0.802104230808699
"7520","CFB",828.622937802686,-0.563416266873523,0.657416346408396,-0.
857015907729683,0.391436093147534,0.802104230808699
"7521","COX6C",1819.40551067074,-0.224530861684003,0.262042254653312,-
0.856849831265048,0.391527881920221,0.802104230808699
"7522","CCIN",3.62212175157021,-0.703127752739684,0.820644461673921,-0
.856799485742547,0.391555709958604,0.802104230808699
"7523","OASL",11.9233405409685,-0.653983292557667,0.76315349571414,-0.
856948564385053,0.39147331155455,0.802104230808699
"7524","OXLD1",107.817178040256,0.200730483345202,0.234130106074812,0.
857345886483572,0.391253756011153,0.802104230808699
"7525","CYB5A",1252.29617116974,-0.243311007791433,0.283976722773946,-
0.856799125698466,0.391555908974081,0.802104230808699
"7526","PSTPIP1",4.82829281838021,-0.572714734318099,0.66865777649047,
-0.856513981373342,0.391713542656753,0.802320523525624
"7527","AL139420.1",5.14530688220473,-0.643494621708551,0.751577746125
761,-0.856191691445951,0.391891757550798,0.802546400474438
"7528","RPL10A",9725.17152478097,-0.219119865053877,0.255943400279915,
-0.856126256095037,0.391927946984857,0.802546400474438
"7529","COL1A2",29478.5533295244,0.280574731420177,0.327874781462678,0
.855737456136482,0.392143017067438,0.80266692445818
"7530","MT1H",5.09076810773639,-0.742020233087252,0.867024987024655,-0
.855823354795831,0.392095494874682,0.80266692445818
"7531","BCAT2",67.6039710679593,-0.217290749775688,0.253888612574728,-
0.855850711743648,0.392080360771839,0.80266692445818
"7532","TRIM2",139.83359001072,0.211563776046279,0.247400939726744,0.8
55145401953415,0.392470657505058,0.803230906192309
"7533","SCML4",2.86247355630174,0.901630273722581,1.05466341965475,0.8
54898593162296,0.392607289514624,0.803237608063261
"7534","RPF2",172.625177200943,-0.19608718379344,0.229430581544423,-0.
854668904526457,0.392734469800376,0.803237608063261
"7535","AGPAT5",34.7341344723376,-0.256288619673131,0.299862833549541,

-0.854686179808908,0.39272490348114,0.803237608063261
"7536","NDUFA12",556.880435644684,-0.225780287704384,0.264131585367257
,-0.854802303898838,0.392660602547717,0.803237608063261
"7537","AC006504.1",2.81181119650779,0.915275007563358,1.0705883483169
,0.854927114611589,0.392591498721375,0.803237608063261
"7538","CDC42EP4",437.194307204798,-0.239063150089456,0.27985285616128
7,-0.854245882527912,0.392968765527922,0.803610177847297
"7539","MRPL11",399.747534633789,-0.216639357469332,0.253672486630296,
-0.854012038700371,0.39309831903721,0.803768482286589
"7540","AUH",93.3412879258091,0.161703222501214,0.189366540569936,0.85
3916547318951,0.393151230348335,0.803770055148485
"7541","CDC7",10.1092649440054,-0.399142008778899,0.46772193148511,-0.
853374584149909,0.393451611241559,0.803839371322471
"7542","APH1A",444.475887944995,-0.149720664572608,0.175459762985268,-
0.853304837674825,0.3934902780357,0.803839371322471
"7543","BMP1",241.058408515252,0.233417740776135,0.273554720405412,0.8
53276230913533,0.393506138022407,0.803839371322471
"7544","ADAM32",19.6388939085396,0.331606681646754,0.388663752762063,0
.85319682962502,0.393550161230664,0.803839371322471
"7545","TMEM109",649.577599546207,-0.152510212922997,0.178652795519594
,-0.85366821425568,0.39328885069381,0.803839371322471
"7546","RAD52",16.9145495568221,-0.373636422946198,0.437886546386679,-
0.853272214068563,0.393508365048176,0.803839371322471
"7547","KANK2",599.011109683355,0.196496031015406,0.230204668549634,0.
853571008152859,0.39334272797677,0.803839371322471
"7548","PIGO",31.4822280625679,-0.271525810704549,0.318412337068314,-0
.852749027266157,0.393798496371587,0.803950375788481
"7549","TRIM21",39.7207961971973,0.2287525792714,0.268261406124872,0.8
52722658006641,0.393813122775143,0.803950375788481
"7550","ANKRD13D",109.935506286324,-0.180259399101422,0.21139132054990
2,-0.852728478314551,0.393809894359945,0.803950375788481
"7551","ATL1",165.835786531552,-0.185581613387373,0.217568039528027,-0
.852981962745804,0.393669306864406,0.803950375788481
"7552","PPIC",1680.44295853094,0.289662443098398,0.339841272937464,0.8
52346275055592,0.394021929330175,0.804270132497967
"7553","GNLY",35.8557987763852,0.528901290130512,0.620616657297467,0.8
52218972712822,0.394092568211551,0.804307816626646
"7554","MFSD9",36.4639869476876,0.266380325703113,0.312678678127951,0.
851929934263404,0.394252981402757,0.804422198322104
"7555","LPAR2",9.96737537751483,-0.488741152590304,0.573636424475719,-
0.85200508847916,0.394211267830919,0.804422198322104
"7556","HSPA6",5.73897637858055,-0.781944657499031,0.918027917467226,-
0.8517656627005,0.394344167937508,0.804445003394069
"7557","CYB561",216.218652957924,-0.186285538458802,0.218740561088255,
-0.851627780106325,0.394420715903495,0.804445003394069
"7558","LILRB3",4.71937280822266,-0.607541564860842,0.713339098809601,
-0.851686898804074,0.394387894009763,0.804445003394069
"7559","AL645568.1",17.6578738873158,0.360132606337554,0.4229558022006
74,0.85146628669888,0.394510383260789,0.80452143907462
"7560","AP1S3",4.49819047544955,0.778266887903102,0.914703831845697,0.

850840305689666,0.39485806854329,0.805123958544289
"7561","PSMD4",506.790317205079,-0.199853164738519,0.234947915474158,-0.850627528808616,0.394976292251005,0.805152016007569
"7562","ADSL",174.59217730317,-0.199395118492314,0.234384372523546,-0.850718485816641,0.394925751835654,0.805152016007569
"7563","SLC47A1",6.24085202680921,0.670563202115193,0.788403512910076,0.850532996282674,0.395028823553025,0.805152626612439
"7564","HIPK1-AS1",3.91462758648666,0.67522975897615,0.794744336367984,0.849618837250202,0.395537035540148,0.805152751739022
"7565","NBPF10",32.7959990431284,0.44626879520266,0.524842473435724,0.850290930688774,0.395163357576738,0.805152751739022
"7566","AC092910.3",3.80173085275054,0.675335185518593,0.794664000682371,0.849837396608741,0.395415495063081,0.805152751739022
"7567","TSPAN18",49.4111813807772,0.301892023090127,0.355290420780555,0.849704932733299,0.395489155298062,0.805152751739022
"7568","EED",67.1802798872633,-0.188566142511328,0.2217751766134,-0.850258109995952,0.395181600631791,0.805152751739022
"7569","BCO2",18.6442057879737,-0.34914901632136,0.410794913648967,-0.849935100753742,0.395361169259761,0.805152751739022
"7570","RMDN3",83.9778354497354,-0.164025943328711,0.192978696933786,-0.849969172426274,0.395342225669081,0.805152751739022
"7571","CIAO3",49.3159434192945,-0.266605936251401,0.31380416509506,-0.849593363971568,0.395551202654532,0.805152751739022
"7572","SARM1",19.5732327994259,0.311959949176935,0.366962408276579,0.850114186469509,0.395261605100817,0.805152751739022
"7573","ARL4D",288.448384097243,0.263395634752086,0.310014278000463,0.849624205862196,0.395534049793995,0.805152751739022
"7574","CCR7",5.38454387466725,-0.762086653185435,0.897252783202245,-0.849355574541201,0.395683465440379,0.805315635036102
"7575","SLC35A3",163.749997516402,0.161344430321061,0.190123120590055,0.848631296500511,0.396086486600568,0.805862481692112
"7576","SEC24A",99.1400622815082,0.198742594858954,0.234203077985745,0.848590874928851,0.396108986297836,0.805862481692112
"7577","CCDC144NL-AS1",22.2346043640105,-0.39606496371107,0.466665491209341,-0.848712774292968,0.39604113628961,0.805862481692112
"7578","ARNTL",68.5507834972101,0.789123564917525,0.930416091080348,0.848140495937939,0.396359731152592,0.806053463814934
"7579","HELB",35.6982873850584,0.486021646932055,0.572999124236876,0.848206613892023,0.396322914517349,0.806053463814934
"7580","AARSD1",130.014918968975,-0.212619145095809,0.250642161382626,-0.848297604532813,0.396272251334024,0.806053463814934
"7581","HSPA1A",978.75932473403,-0.278181728547528,0.328057973901082,-0.847965148475274,0.396457380342238,0.806145695551457
"7582","CASP8",53.6297924940357,0.208240788213,0.24566116554877,0.847674835979151,0.396619084291942,0.806261794060436
"7583","ABCF1",244.324074743967,-0.17640862512648,0.208087031957412,-0.847763666322967,0.396569601598345,0.806261794060436
"7584","UBE2T",7.7024519392569,0.477744709023025,0.564329303372593,0.8

46570798588494,0.397234396130488,0.806264433061585
"7585","SLC39A10",170.876236168656,0.157511031606758,0.186042280498618
,0.846641049467939,0.39719522614885,0.806264433061585
"7586","SF3B1",796.389301745505,0.143247595408015,0.169092114480844,0.
847157159562537,0.396907528614028,0.806264433061585
"7587","KLF7-
IT1",3.66184044462407,0.751131016424838,0.887427253300083,0.8464141862
12561,0.397321727321693,0.806264433061585
"7588","CNOT9",78.3390491480215,-0.192277988130288,0.226981136718282,-
0.847109988566733,0.396933818129519,0.806264433061585
"7589","TIGIT",2.4202941428105,0.827140562186257,0.977293082728779,0.8
46358760543697,0.397352636910079,0.806264433061585
"7590","ZCCHC24",651.499784177646,0.149308528451299,0.176396219687423,
0.84643836878067,0.397308241728115,0.806264433061585
"7591","GSEC",26.9743289377783,0.41144925257985,0.486114681297272,0.84
6403674708681,0.397327589228086,0.806264433061585
"7592","OTUD7A",10.7442922401082,-0.391548773378867,0.462428979405309,
-0.846721963408101,0.39715011362118,0.806264433061585
"7593","SCAMP2",262.310004925657,-0.188167330524914,0.222244809216086,
-0.846666930888638,0.397180795959111,0.806264433061585
"7594","PER1",362.200634862642,0.220090901474539,0.259955306529519,0.8
46649004449338,0.397190790814198,0.806264433061585
"7595","ANKRD40",127.665580870852,-0.179413139645126,0.21190758239287,
-0.846657479733311,0.397186065415438,0.806264433061585
"7596","MAFG",245.006450788955,-0.170078770122639,0.200751911825696,-0
.847208719338681,0.396878794328793,0.806264433061585
"7597","RLIM",198.565267699004,0.199113672776373,0.235222878481597,0.8
46489397892267,0.39727978586057,0.806264433061585
"7598","ZBTB5",17.8450390159324,0.352613009209007,0.416669928964371,0.
846264596260697,0.397405153434296,0.806264864462974
"7599","ICA1L",67.0187006830874,0.253140738636588,0.299453409725139,0.
845342649024901,0.397919555272892,0.807099432822313
"7600","PAK6",3.8950394044688,-0.907926186406142,1.07403713881155,-0.8
45339656886337,0.397921225394069,0.807099432822313
"7601","SMIM10",214.84202054467,-0.21356234904082,0.252681489039747,-0
.845183989743019,0.398008119908316,0.807169473541205
"7602","APOBEC3C",271.24339845697,0.151941087451811,0.179814526277769,
0.844987836061142,0.398117630578598,0.807285355875965
"7603","THRB-
IT1",9.83292751866817,0.496801538040999,0.588211615736075,0.8445966124
27166,0.39833610108971,0.807339064627714
"7604","B4GALT1",498.01205209021,-0.249988092355,0.295941543279236,-0.
844721189140799,0.398266526040289,0.807339064627714
"7605","ZNF576",85.793102923237,-0.219006248933294,0.259299329645255,-
0.8446078485159,0.398329825528042,0.807339064627714
"7606","STRN4",81.0216504393492,-0.196312894113128,0.232442539599134,-
0.844565260953032,0.398353611778034,0.807339064627714
"7607","NUDT15",68.9537967090259,-0.200725719689995,0.237715070380081,
-0.844396273947022,0.398448003802988,0.807424211729074
"7608","E2F6",53.5502344975593,-0.245849078931268,0.291209596739949,-0

.844234124436538,0.398538589232966,0.807501623689033
"7609","SRP9",779.028416361562,-0.21180010159202,0.250906306881487,-0.84414020605732,0.398591062758199,0.807501804759843
"7610","ZNF467",3.01535450131777,0.756119755955923,0.896077995939945,0.843810203332566,0.398775472932402,0.807663108034816
"7611","AC087392.1",11.7396463673063,-0.721985132532607,0.855619846429216,-0.843815317685406,0.398772614568128,0.807663108034816
"7612","Z99572.1",11.481865825301,0.444413792081127,0.526734641652722,0.843714760598812,0.398828817252402,0.807665031259297
"7613","FAM78B",15.8760474054182,-0.312318789528173,0.370318844181963,-0.843378063079904,0.399017036657136,0.807843622808539
"7614","WASHC5",141.215368013102,0.169603514039341,0.201143636849782,0.843196019996416,0.399118823974373,0.807843622808539
"7615","ENG",302.699555449932,0.147946045566342,0.175461567711433,0.843182056880151,0.399126631937064,0.807843622808539
"7616","APCDD1L-DT",14.6726344359209,0.526381383868563,0.62425042887002,0.843221501379482,0.39910457540866,0.807843622808539
"7617","LINC02716",4.36671200421596,0.619412979382111,0.734807339448576,0.842959706753799,0.399250979139946,0.807883150885045
"7618","C12orf29",116.349148781709,-0.194605053738286,0.23085653601005,-0.842969651636002,0.399245417060777,0.807883150885045
"7619","AC253572.1",4.88938779860128,-0.570156272446733,0.67648823286052,-0.842817723577891,0.399330394081005,0.807937790360769
"7620","GMFB",157.991906805948,-0.155783063088801,0.184882111462125,-0.842607550599692,0.399447966826174,0.808069607431164
"7621","CACNA2D3-AS1",2.56575765260148,0.993941778298961,1.18004239024683,0.842293282439671,0.399623810244116,0.808087821627284
"7622","TAF7",585.681556929057,0.116818031404339,0.13869882407153,0.842242406785612,0.399652281231612,0.808087821627284
"7623","SLC13A4",4.2864517474691,0.68052064307166,0.807967215692499,0.842262693156918,0.399640928444609,0.808087821627284
"7624","MEN1",43.3001981429922,0.235502521545918,0.279622236550175,0.842216715134742,0.39966665923363,0.808087821627284
"7625","MYO5A",86.6152568872924,-0.250502622025777,0.297543415472277,-0.841902757714081,0.399842386596615,0.808337100247451
"7626","SWSAP1",9.81791249164534,-0.421982831112394,0.501290480043159,-0.841793028018533,0.399903815148304,0.808355272818135
"7627","ZBTB2",41.5466543176876,-0.265763902561623,0.315789805948954,-0.841584805953436,0.400020397005272,0.808378922369726
"7628","PPM1F",76.5798306584171,0.216660063419759,0.25743723579735,0.841603440732678,0.400009962710039,0.808378922369726
"7629","PLEKHA3",276.662553853668,-0.121766106320297,0.144791955575886,-0.840972869217719,0.400363133702452,0.808541557188955
"7630","AC079610.1",2.47705667887957,-0.796334312725636,0.946792139083907,-0.841086738950061,0.400299343591488,0.808541557188955
"7631","HDAC11",26.4531221593843,-0.295194440603577,0.350907892725777,-0.84123055286836,0.400218787402306,0.808541557188955
"7632","AC004908.2",4.41998813360526,-0.607451851627072,0.722067472965

182,-0.841267436036914,0.400198129167451,0.808541557188955
"7633","HIF1A",228.950383399343,0.191285161439351,0.227440800791355,0.841032746867734,0.400329589330456,0.808541557188955
"7634","SLC25A20",52.2100787173227,0.271591399505746,0.323167348180546,0.840404827513745,0.400681443340008,0.808680177837164
"7635","ZYX",589.506166847839,0.194856736935975,0.231866782347297,0.840382287464154,0.400694077088567,0.808680177837164
"7636","CD44-AS1",4.32173226548683,2.22307587296113,2.64524973654153,0.840403022161394,0.40068245523525,0.808680177837164
"7637","SULT1A3",8.57246806472074,-0.428686856980959,0.50992842642176,-0.840680446056157,0.400526977881574,0.808680177837164
"7638","GPS1",160.132226905623,-0.134408242053417,0.15991561249454,-0.840494808210213,0.400631011341597,0.808680177837164
"7639","NCDN",39.2932084482193,0.232453641275982,0.276825314685913,0.839712370740823,0.401069676167625,0.808697064502805
"7640","AC108488.3",5.39860626722269,0.582798249600232,0.693926881226179,0.839855416136091,0.400989457826378,0.808697064502805
"7641","AC026748.3",9.28423523710531,0.526740728395177,0.627250101350268,0.839761886464862,0.401041907183991,0.808697064502805
"7642","TNFAIP3",245.479008589301,0.313808694424501,0.373567111152416,0.840032982176706,0.400889894078856,0.808697064502805
"7643","RCC1L",143.272934766189,-0.181195957174643,0.215740437175744,-0.839879438211386,0.400975987446238,0.808697064502805
"7644","MAN2A2",60.99292949106,0.223058479996041,0.265629957835439,0.83973389829105,0.401057603129687,0.808697064502805
"7645","PHF20",298.466460231737,-0.150926891614314,0.179707849417559,-0.839845850381462,0.400994821899509,0.808697064502805
"7646","AC023794.1",3.20390776849136,0.712175433748025,0.848292902927298,0.839539540281951,0.401166610511481,0.808786725220309
"7647","DAG1",172.846920610397,0.167782641821823,0.199906625971318,0.839305055580778,0.401298146974054,0.80880410254617
"7648","AC108519.1",12.129460574317,-0.493454884677116,0.587975762444655,-0.839243581445356,0.401332635768774,0.80880410254617
"7649","COPS6",565.391956383319,-0.191317609883832,0.22793100187016,-0.839366335926587,0.401263768672013,0.80880410254617
"7650","AC011389.1",3.67621715711118,0.86809210680922,1.03465328847308,0.83901739498681,0.40145954833006,0.808913797188699
"7651","ADNP",222.309208867218,0.136195644576798,0.162338753178163,0.838959533139487,0.40149201831273,0.808913797188699
"7652","SMAP2",91.8943399133678,-0.208917986887564,0.249137624213771,-0.838564578701704,0.401713694602395,0.809254652678505
"7653","APOO",20.0924770381293,-0.408967133898336,0.487801326011313,-0.838388729367355,0.401812417278801,0.80934776066284
"7654","AC104695.4",58.1880136214623,0.27116762841856,0.32361342031105,0.837936906812825,0.402066139414504,0.809546055673665
"7655","TTC12",18.2969611652582,0.339244277436822,0.404858545168367,0.837932857007482,0.402068414027738,0.809546055673665
"7656","PIAS4",57.5386998255218,0.191462963009479,0.228457968340386,0.838066469733342,0.401993373192197,0.809546055673665

"7657","PCDHB9",17.6532770667481,0.424470691196604,0.506794374187374,0
.837559990434437,0.40227787129696,0.80986200679674
"7658","ZNF564",25.2771754088837,0.267276495729862,0.319334556085208,0
.836979558386861,0.402604058363793,0.810412844042553
"7659","TEKT4",2.43620663681537,-0.752742313436464,0.900730421811835,-
0.835702109319579,0.403322508342447,0.810441536359688
"7660","PNKD",379.504280941546,-0.221014629386049,0.264345761407235,-0
.836081608456613,0.403108994136564,0.810441536359688
"7661","ARMC9",33.4307021817837,0.291754690146019,0.349099932489336,0.
835734020529872,0.403304551815389,0.810441536359688
"7662","BLOC1S5",125.265239075944,0.196929696075292,0.235544549337137,
0.836061359218399,0.403120385073779,0.810441536359688
"7663","NDUFA4",1816.90422088786,-0.235518441575469,0.281788713110928,
-0.835797995510046,0.403268554357947,0.810441536359688
"7664","PREX2",31.6654212432271,0.423409678443989,0.506558937957757,0.
835854718408498,0.403236639113442,0.810441536359688
"7665","C9orf78",329.914497769648,-0.142972267261106,0.170988293306031
,-0.836152373339489,0.403069187815948,0.810441536359688
"7666","AC008014.1",9.89048338991276,0.443197872255939,0.5302255170250
16,0.835866735993072,0.403229877591337,0.810441536359688
"7667","PLCG2",4.10996768124834,0.772984321864772,0.924676950150287,0.
835950676329868,0.403182651655253,0.810441536359688
"7668","MED9",30.4029208063827,0.272849932746982,0.326484407828395,0.8
35721174440882,0.403311780287915,0.810441536359688
"7669","VPS16",99.6416330785862,-0.165484689219484,0.197965167360587,-
0.835928317217842,0.403195230861604,0.810441536359688
"7670","RALY",480.72050350218,-0.177887676360127,0.21277057693471,-0.8
36053926829896,0.403124566112572,0.810441536359688
"7671","IL10RB-
DT",4.28536182614195,-0.599184127409178,0.716626822624176,-0.836117360
518351,0.403088882764853,0.810441536359688
"7672","JPX",488.697545503341,0.147568949357582,0.176592763721933,0.83
5645505780444,0.403354360489882,0.810441536359688
"7673","HHLA3",51.6915258811047,0.253283219307022,0.303371847912779,0.
834893616693932,0.403777609144367,0.810458334716123
"7674","LANCL1",80.4416268410549,0.166628491150654,0.199605256990088,0
.834790093524084,0.403835904570519,0.810458334716123
"7675","RAD18",39.5762654763193,-0.220446655767431,0.264048700849656,-
0.834871200116029,0.40379023182315,0.810458334716123
"7676","RABL3",90.4739013388863,0.161975944694814,0.194025283710241,0.
834818749378614,0.403819767531254,0.810458334716123
"7677","ACTL6A",141.880232137949,-0.204187513581792,0.244542200074617,
-0.834978639758242,0.403729735169113,0.810458334716123
"7678","NIPSNAP3A",71.4587897327513,-0.227948344459564,0.2728915844243
97,-0.835307343538532,0.403544683751579,0.810458334716123
"7679","CBLN3",18.7111240873146,0.354972926919313,0.425008153608562,0.
835214392724916,0.403597007416649,0.810458334716123
"7680","BMERB1",305.501262017067,-0.1628299653557,0.194948982699345,-0
.835243985893583,0.403580348455239,0.810458334716123
"7681","LAMA3",10.5913021114706,-0.487413331861905,0.58345263295311,-0

.835394862124269,0.40349542169733,0.810458334716123
"7682","SMARCA4",128.193733205032,0.235743917174468,0.282460786988886,0.834607591685794,0.403938686321107,0.810559079619873
"7683","CIT",5.30167426337114,0.733430519374856,0.879339711771572,0.834069597399669,0.40424176612137,0.811061671841848
"7684","BDP1",329.087861773002,0.188859687395157,0.226468789297376,0.833932516622258,0.404319012507545,0.811111085086389
"7685","NFIA",3094.79033736634,0.17334515092894,0.207929079229884,0.833674402690406,0.404464486252839,0.811191784489658
"7686","L2HGDH",14.5037942120218,0.358533268655619,0.430060482162204,0.833681036799825,0.404460746857923,0.811191784489658
"7687","SMARCA5",362.024212997655,0.153412698571704,0.184134592895657,0.833155227158418,0.404757189871004,0.811462099344717
"7688","TXNL4B",44.9485684099961,0.296047439763491,0.355307997348622,0.8332135554861,0.404724298892758,0.811462099344717
"7689","MLLT6",228.656341120225,0.219031527590462,0.262853155765889,0.833284755331389,0.404684151912931,0.811462099344717
"7690","METTL26",527.341199265459,-0.204955851232364,0.246033575468761,-0.833040168773173,0.404822075259351,0.811486643709089
"7691","AC008269.1",6.43425843010495,0.622321154847363,0.747375942755732,0.832674855110715,0.405028129443037,0.811567428290519
"7692","NFKBIE",22.6845213902982,-0.299611819438219,0.359832074126408,-0.832643449491294,0.405045846624179,0.811567428290519
"7693","AP3M2",46.1685166282995,0.243397736436907,0.292336161492007,0.832595376482573,0.405072967451654,0.811567428290519
"7694","SLC26A11",62.1830564290915,0.195865833925642,0.235207908799334,0.832734897927023,0.404994258149596,0.811567428290519
"7695","EPSTI1",171.698601337365,0.330739084915509,0.397396601194804,0.832264503322666,0.405259662017311,0.811768280930272
"7696","BRICD5",4.34037211434033,0.589470746841313,0.708381217826804,0.832137741666432,0.405331200669498,0.811768280930272
"7697","DDX27",256.607619382833,-0.170154424833517,0.204456049337835,-0.832229838073219,0.405279224795703,0.811768280930272
"7698","AC092807.3",9.01428920234054,-0.463361266451219,0.556996419774755,-0.831892719595215,0.405469501641612,0.811911972781505
"7699","PIP4K2A",170.196298468517,0.158594359723034,0.190679936266313,0.831730714979544,0.405560959482166,0.811911972781505
"7700","TMBIM6",2215.52630383493,0.111066351437207,0.133521523229238,0.831823579832304,0.405508532191191,0.811911972781505
"7701","AL031846.2",13.6539612877692,0.47956193691619,0.576682664276582,0.831587225736662,0.405641974974154,0.811968711105907
"7702","FMOD",266.502649856482,-0.291228232799146,0.350346133585831,-0.831258589379574,0.405827562844083,0.812089652082873
"7703","USP13",56.7289641125017,-0.213957991522109,0.257369570519475,-0.831325906517449,0.405789543309343,0.812089652082873
"7704","CCHCR1",50.1864016201084,0.224637216103229,0.270256392230542,0.831200380679998,0.405860439808398,0.812089652082873
"7705","EPHB2",48.941369128253,-0.328817877478807,0.396652763918002,-0.828981687234083,0.407114769733784,0.812184282711028
"7706","CAP1",669.052218149722,-0.145433117747387,0.175072559489171,-0

.830701956787136,0.406142020661068,0.812184282711028
"7707","AK4",53.3614212616741,-0.294850470676823,0.355137146108487,-0.830243960418469,0.406400865093062,0.812184282711028
"7708","NBPF19",101.418767508051,0.366661856584561,0.441742952928941,0.830034421949325,0.406519322143881,0.812184282711028
"7709","HK2",4.38099742869305,-0.571471660739793,0.687997136271951,-0.830630871280112,0.406182189388902,0.812184282711028
"7710","GCC2",601.702787703507,0.143308522913847,0.172862275221775,0.829032955455363,0.407085759369075,0.812184282711028
"7711","THRB",163.421294685888,0.212251559160718,0.255554006153339,0.830554614876047,0.4062252827045,0.812184282711028
"7712","AC139887.2",28.5519681701056,0.402134425221746,0.484049047804416,0.830772061314397,0.406102408584602,0.812184282711028
"7713","GNPDA2",146.525755467032,-0.173122918268752,0.208852381922792,-0.828924796906322,0.407146962837156,0.812184282711028
"7714","PCDHGA12",28.1435942238541,0.367843772150353,0.443431068980266,0.82953991698476,0.406798959179659,0.812184282711028
"7715","TBPL1",103.81304083184,-0.207531377693058,0.250291133268496,-0.82915992661407,0.407013917448568,0.812184282711028
"7716","RAET1E-AS1",3.65152946430924,0.653607460254277,0.787719435161624,0.829746520244445,0.406682113387931,0.812184282711028
"7717","ATP6V1F",1022.18383335556,-0.213197420631694,0.25697128779413,-0.829654637534815,0.406734075767675,0.812184282711028
"7718","UBE2R2",604.249659176419,-0.142075206669216,0.17113067638326,-0.83021472053922,0.406417393851075,0.812184282711028
"7719","PPP1R26-AS1",4.32874569567946,-0.652414522089311,0.78646892177516,-0.829549018436391,0.406793811373908,0.812184282711028
"7720","NAP1L4",335.199130777678,0.136418263124059,0.164349903327194,0.8300477235601,0.406511801816325,0.812184282711028
"7721","PSME1",972.773194502587,-0.191473105570544,0.230950880494749,-0.829064193911556,0.407068083545894,0.812184282711028
"7722","ZCCHC14",150.421356637037,0.164637160637235,0.198626020790951,0.828880123468372,0.407172243710076,0.812184282711028
"7723","CBX8",9.21990649809738,0.482527982566174,0.581778377885309,0.829401711902911,0.406877133125094,0.812184282711028
"7724","ZBTB14",53.2783431311061,-0.186016364016276,0.224377416637903,-0.829033361750782,0.407085529469822,0.812184282711028
"7725","ESCO1",107.157257830456,0.174544156869929,0.210467757483642,0.829315420835876,0.406925947113214,0.812184282711028
"7726","TIMM29",77.3889543720476,0.201403053961669,0.242689035865247,0.82988114087485,0.406605988608555,0.812184282711028
"7727","AL109809.5",3.54244069496467,0.747619601234979,0.900907919784387,0.829851292032049,0.406622866688759,0.812184282711028
"7728","TMEM184B",93.8361662769203,0.174298576032,0.210140767413794,0.829437229991573,0.406857041914724,0.812184282711028
"7729","AC087190.1",17.6330539325562,-0.467720213926934,0.564369384872348,-0.828748380872441,0.407246802810323,0.812227903392565
"7730","COPS7B",57.2554722299922,0.229152632942731,0.27655059762177,0.

828610152765374,0.407325041106905,0.812278849762346
"7731","NOMO3",4.5684504603839,0.645539018598841,0.779217041814939,0.82844571404042,0.407418126510215,0.812359386904017
"7732","SMIM1",10.7464263894126,0.474015306337382,0.572438683693491,0.828063022014758,0.407634809747598,0.812383119487503
"7733","BACH2",40.3587023638306,0.334991758646575,0.404487663335446,0.828187826259519,0.407564137054931,0.812383119487503
"7734","GDF11",73.7274717459995,0.27770497939225,0.335371268403232,0.82805238717812,0.407640832256623,0.812383119487503
"7735","SNAI3-AS1",9.35275916453343,0.432063629226022,0.521761532517743,0.828086400201089,0.407621570864838,0.812383119487503
"7736","PSMA2",797.975379677413,-0.214024156141187,0.258545115616245,-0.827801970387483,0.407782658636427,0.812410825693463
"7737","TMEM41B",119.069818474149,-0.189250453176157,0.228610895358141,-0.82782779394516,0.407768031812895,0.812410825693463
"7738","IRGQ",65.4283760083483,0.213705850890376,0.258177216601569,0.827748682488033,0.407812842634837,0.812410825693463
"7739","BCAR3",11.763741046783,0.360110819877791,0.435220781969054,0.827421012040267,0.40799847509109,0.812413070332018
"7740","SF3B5",914.486251572636,-0.210787498121551,0.254711133484811,-0.827555102274793,0.407922503963341,0.812413070332018
"7741","ATP2B1-AS1",102.806486639732,-0.253633861109055,0.30645322245006,-0.827642989299576,0.407872714631683,0.812413070332018
"7742","SNPH",7.79017639559792,0.459862600387713,0.555809433943913,0.827374586150185,0.408024780441809,0.812413070332018
"7743","COX7C",2524.00828725417,-0.192024991102903,0.232282252318558,-0.826688174349002,0.408413825796588,0.812977676220868
"7744","CYREN",71.4087735022826,-0.228090848465426,0.275897920371555,-0.82672188379765,0.408394714755831,0.812977676220868
"7745","CSTF2T",112.71045149793,-0.212786837195337,0.257519328275191,-0.82629462658409,0.408636980902757,0.813316857406843
"7746","UNC50",315.951944600348,-0.212588890399339,0.257493748638712,-0.82560796727388,0.409026514114164,0.813380023879004
"7747","AC021148.2",3.29347522448608,0.755391434640469,0.914650662943616,0.82587972134563,0.408872325023988,0.813380023879004
"7748","TRPM3",7.14307023767386,0.582055084713584,0.705019177231654,0.825587591814305,0.409038076231595,0.813380023879004
"7749","NEAT1",12131.6967253021,0.312208429168459,0.378045828177746,0.825848100674365,0.408890264328856,0.813380023879004
"7750","ZBTB25",73.1914739448745,0.191346669965032,0.23165526157415,0.825997513135633,0.408805502519198,0.813380023879004
"7751","ARNT2",3.91530758575675,0.676747322836139,0.81954792150376,0.825756865558757,0.40894202722692,0.813380023879004
"7752","TSC22D3",3867.07438884282,0.257400912436743,0.311733732030142,0.825707602319581,0.40896997870088,0.813380023879004
"7753","MAPK10",72.4934286976988,0.363797683464226,0.440907339480148,0.825111425664091,0.409308333563694,0.813497674301746
"7754","ZNF503",892.734503110358,0.14401508609578,0.174513475409768,0.

825237625676893,0.409236695954811,0.813497674301746
"7755","PRDM10",15.0935624917945,0.455300399436454,0.55178980070663,0.825133771688765,0.409295648268876,0.813497674301746
"7756","TFE3",120.274939244458,0.17998387426342,0.218125749807906,0.825138134410653,0.409293171685039,0.813497674301746
"7757","ZBTB7B",40.3963099851064,-0.2667952241202,0.323494505698184,-0.824728764849924,0.409525597097397,0.813551422162899
"7758","ARRDC3",451.31072246577,-0.267318951604468,0.324135574594496,-0.824713399443711,0.409534322554428,0.813551422162899
"7759","PCMT1",512.436994067823,-0.216783481788906,0.262944106103319,-0.824447008915746,0.409685613650897,0.813551422162899
"7760","BUD23",198.247465775287,-0.179168435647992,0.217328439210122,-0.824413207489907,0.409704812860888,0.813551422162899
"7761","TMEM170A",124.119785293416,-0.174858270542047,0.212098738210137,-0.824419192766745,0.409701413184642,0.813551422162899
"7762","IZUMO4",5.96687735238448,-0.518051529345407,0.62823550717589,-0.824613577914764,0.409591010273556,0.813551422162899
"7763","MAPK1",292.118424558881,0.174806537139635,0.211945588126376,0.824770822950105,0.409501714392894,0.813551422162899
"7764","ZNF766",105.828514119722,0.182034538776974,0.220867535929879,0.824179696715436,0.409837461596071,0.813710003928829
"7765","CCDC191",50.5569828079543,0.216796571437382,0.263095509361585,0.824022317839825,0.409926877050054,0.813759587364832
"7766","ADAM8",8.30717601555258,-0.485918135226318,0.589810456037298,-0.823854732062583,0.410022104346079,0.813759587364832
"7767","AL133467.1",56.3632467543028,0.303224324465922,0.368096054769756,0.823764124979793,0.410073595501136,0.813759587364832
"7768","LRRC3",7.68653291338341,0.599093006355366,0.727164870069697,0.823875067421705,0.410010548480385,0.813759587364832
"7769","KLF11",156.729340834723,0.205215249550511,0.249236894418978,0.823374284248365,0.410295182171812,0.813906893964885
"7770","ZFP2",7.72391050172487,0.448525879369941,0.544882673725856,0.823160472883023,0.410416743417361,0.813906893964885
"7771","PAXIP1",37.5278310672393,0.24710026965975,0.300369514679696,0.822654289411657,0.410704616460392,0.813906893964885
"7772","NFRKB",66.419898274246,0.169474060146887,0.205989873128785,0.822730057418562,0.410661518590551,0.813906893964885
"7773","GOLGA5",144.581962670267,-0.170438204014349,0.20710557733324,-0.822953230950937,0.410534590069466,0.813906893964885
"7774","CDIPT",473.330688924233,-0.124167691556271,0.150888322335163,-0.822911207670944,0.410558488760257,0.813906893964885
"7775","CCL22",2.89469543345783,-0.948011621430383,1.15190663445743,-0.822993455434793,0.410511715130412,0.813906893964885
"7776","ZNF594",14.8611807794825,0.411780467009033,0.500229070833185,0.823183799220562,0.410403480319881,0.813906893964885
"7777","TSEN54",24.4477902688759,0.25561630102295,0.310737351498253,0.822612086350319,0.410728623298919,0.813906893964885
"7778","AAR2",71.7588433816465,0.181870346445789,0.221038673348487,0.822798760464209,0.410622441688901,0.813906893964885
"7779","PIM2",9.78385977605771,0.50446303748625,0.612642370556211,0.82

3421724860874,0.410268212979846,0.813906893964885
"7780","ADPGK",165.271307869652,0.143783216040413,0.174825998921045,0.822436118928446,0.4108287297942,0.814000625935423
"7781","IL6",239.054232490556,0.734112245137483,0.892915173078525,0.822152279713724,0.41099023424536,0.814215969784375
"7782","PSMD12",287.68749067507,-0.125702723544008,0.152940508700735,-0.821906011768114,0.411130391191881,0.814388972015272
"7783","DNM1",502.810822185652,-0.185931296373135,0.226315742824763,-0.821557060292983,0.411329036360293,0.8145731109319
"7784","SAFB",207.607718641272,0.139302218327508,0.169546771771614,0.821615279795206,0.411295890192157,0.8145731109319
"7785","LMBR1L",98.4776666319832,0.190400544356168,0.232029975253429,0.820585978808159,0.411882136902703,0.815533558554557
"7786","C16orf46",8.5871189851684,0.442454215793699,0.53923647759397,0.820519816774812,0.411919836970858,0.815533558554557
"7787","AC097500.1",2.55272219319036,0.948545757628047,1.1563211535782,0.820313417853513,0.412037459164738,0.815661671121668
"7788","TMEM14B",579.355615104161,-0.187014413936522,0.228072889431156,-0.81997651892323,0.412229493221727,0.815832281167406
"7789","POC1B",47.908719302137,0.250132656432262,0.305014334405945,0.820068528646129,0.412177041957783,0.815832281167406
"7790","GDAP2",53.4426593284699,0.231698367496685,0.282858483794277,0.819131759417897,0.41271124361414,0.815859622094042
"7791","ZBED6",4.56383367776636,0.630145318820865,0.769298841612529,0.819116427499118,0.412719990199303,0.815859622094042
"7792","TTL",99.1497792486416,-0.194980167621229,0.237865020964916,-0.81970929071571,0.412381852355355,0.815859622094042
"7793","NIF3L1",69.665178208185,-0.220117343592013,0.268715320434295,-0.819146981408655,0.412702559849613,0.815859622094042
"7794","HTRA3",1245.85722395246,-0.262396817138913,0.320421597395323,-0.818911144791461,0.4128371108899,0.815859622094042
"7795","DHX15",185.89378632794,0.154812863584513,0.18897894244821,0.819206952790204,0.412668348731925,0.815859622094042
"7796","PDGFRA",2430.84059462111,0.195968854906899,0.23929595721009,0.818939263294149,0.412821067174312,0.815859622094042
"7797","DNAJC21",293.151851718568,-0.126991279494616,0.15504126319467,-0.819080526550963,0.412740471477209,0.815859622094042
"7798","GTF2H2C",52.583391187231,-0.2410235419188,0.294306752989841,-0.818953488053737,0.412812951022367,0.815859622094042
"7799","NFYA",39.1844216424378,-0.24906826566942,0.304065520001145,-0.819126962072129,0.412713980402202,0.815859622094042
"7800","ZNF574",22.6944376166144,0.303318426827359,0.370425105264168,0.818838740994749,0.412878424388947,0.815859622094042
"7801","AC022137.4",12.7457575342907,-0.445328103119812,0.543374605004471,-0.819560021794077,0.412466971985845,0.815859622094042
"7802","RND3",1357.86252138199,-0.320837610105523,0.391950922263716,-0.818565774134484,0.413034200898645,0.816062830921893
"7803","ST3GAL3",52.9039689724467,0.202600075287636,0.247587315637406,0.818297475240395,0.413187347420103,0.816156196883763
"7804","MTIF3",354.1967573172,-0.170176899566002,0.207951362078315,-0.

818349530703785,0.413157631238773,0.816156196883763
"7805","TRMT12",33.0564829304734,-0.226764411883492,0.277219714078872,-0.817995259236774,0.41335989429058,0.816392411337512
"7806","SLC30A9",119.704582126411,0.171757097507799,0.210130559536135,0.817382763777696,0.413709722607498,0.816581161868348
"7807","SRPK1",116.018642100775,-0.142899076805669,0.174831670310821,-0.817352351273766,0.413727097350891,0.816581161868348
"7808","AC246817.1",7.03320670028582,0.665770722725744,0.814678419507679,0.817219048379946,0.413803258739688,0.816581161868348
"7809","TMEM216",94.8930389787732,-0.248784423306153,0.304506635610907,-0.81700821660926,0.413923732481953,0.816581161868348
"7810","ENDOD1",81.861788501173,-0.207969186388513,0.25442534130712,-0.817407516562867,0.413695581595121,0.816581161868348
"7811","CALML4",18.6795046599369,-0.371877967508012,0.455141474975959,-0.817060162508053,0.413894047567586,0.816581161868348
"7812","ICAM2",56.0829021708375,-0.305364397316805,0.373747384794444,-0.817034204760392,0.413908881180128,0.816581161868348
"7813","SNRNP70",404.187408268756,0.132779028605877,0.162392021632201,0.817645025114631,0.413559909938773,0.816581161868348
"7814","BRCC3",70.9535539953161,0.182100839918022,0.222891456036747,0.816993361504178,0.413932221786524,0.816581161868348
"7815","RPL34",17371.943221236,-0.20420003607474,0.249998231047311,-0.8168059238631,0.414039346354385,0.816649029079605
"7816","ASMTL",161.00938301107,-0.220467382389488,0.269933249207623,-0.81674778130023,0.414072579389309,0.816649029079605
"7817","ZNF287",32.248578195578,-0.287155414131586,0.351703586231308,-0.816469963268245,0.41423139597056,0.816857741958064
"7818","SYF2",657.249273382599,-0.179462843263384,0.219917948958835,-0.816044548037215,0.414474657324814,0.816919427523591
"7819","CYB5RL",25.8752441415686,0.289371580631907,0.354558917361799,0.816145262358827,0.414417059130975,0.816919427523591
"7820","CCSAP",13.1266409152972,0.415380745553416,0.509013322362242,0.816050832669187,0.414471063025686,0.816919427523591
"7821","PDGFRB",1568.01052039281,-0.150248211594546,0.184106180592462,-0.816095424450394,0.414445560677442,0.816919427523591
"7822","SRSF3",1867.51598145707,0.169895894869648,0.208305990335168,0.815607340894434,0.414724749550503,0.817247228123886
"7823","PARVB",116.956928200182,-0.254720583521619,0.312322755359902,-0.815568443702074,0.414747003932089,0.817247228123886
"7824","RFX1",90.2864282157554,-0.199124620573792,0.244191420812874,-0.81544478471413,0.414817758054927,0.817282175411133
"7825","KANSL3",71.2837252184566,0.219214107625638,0.268938671376816,0.815108167610793,0.415010396843202,0.817557222662998
"7826","AP1G2",19.8269510125581,0.37447062784625,0.459836991418018,0.814355162448934,0.415441517018073,0.818301940305851
"7827","AC092835.1",6.02073724401828,0.511016943182317,0.627692185809962,0.814120288151925,0.41557604437487,0.818462338576545
"7828","ARRB1",128.585685733212,0.134443697315068,0.165191176772783,0.813867301762688,0.415720974450219,0.818490905143294
"7829","UFM1",417.661109607325,-0.103350533249546,0.126988263728898,-0

.81385893636742,0.415725767302237,0.818490905143294
"7830","RBCK1",353.344217542877,-0.165502122601641,0.203365300497296,-0.813816920570683,0.415749840238209,0.818490905143294
"7831","FCMR",2.80031272638908,-0.740940060749181,0.910583434794455,-0.813698154871917,0.415817891455449,0.818520341819148
"7832","RPH3AL",11.8870923189013,0.430909864546372,0.5296432013078,0.813585189958761,0.415882624999936,0.818543241110063
"7833","CD38",13.4530736933124,-0.462317212551011,0.568738462188837,-0.812881918996202,0.416285762013565,0.819022976571679
"7834","NECTIN1",39.6411655259247,-0.279529495935881,0.343857892768797,-0.812921563861938,0.416263030198754,0.819022976571679
"7835","FBXO34",77.9209700368483,-0.191778790032468,0.235920634818108,-0.812895362799979,0.416278053441304,0.819022976571679
"7836","ATP10D",104.719792653424,0.176692268626017,0.217424321460774,0.812661009766079,0.416412441708528,0.819130075258902
"7837","PTP4A3",15.8033870477762,0.343991424561703,0.423321090232764,0.81260166927322,0.416446474200714,0.819130075258902
"7838","UBXN10",5.39686550223477,-0.818568852934476,1.0076011088242,-0.812393759559956,0.416565725889739,0.819225082947726
"7839","MLH1",79.6118638278523,0.174889383870994,0.215298543488038,0.812311040463269,0.416613177047622,0.819225082947726
"7840","ARSJ",19.2806393177925,0.474742310306208,0.584685640439401,0.811961637965713,0.416813644214013,0.819225082947726
"7841","CHD1",275.386562393869,-0.246208853031302,0.303215771589265,-0.811992238203279,0.416796085275349,0.819225082947726
"7842","YAE1",74.843738913588,0.190676986544065,0.234764722129214,0.812204597073648,0.416674242150545,0.819225082947726
"7843","JDP2",943.312227043954,-0.155664870478827,0.191672610401232,-0.812139356546407,0.416711672349849,0.819225082947726
"7844","MIPOL1",49.818296945483,-0.249815299489853,0.307711791019167,-0.811848316447167,0.416878673835194,0.819248439210799
"7845","GMNN",85.1119553427215,-0.193074959407696,0.23791433763782,-0.811531416410963,0.417060558869389,0.819501404075414
"7846","AL732509.1",9.67435587660868,0.399782603338424,0.492881206095986,0.811113506447167,0.417300490158832,0.819868347667396
"7847","NOP56",266.513437923518,0.17058092827486,0.210339688542806,0.810978324902035,0.417378118265599,0.819916362057373
"7848","TRIT1",38.8158366778437,-0.271900228339891,0.335344016016207,-0.810809841099863,0.417474882133165,0.819927415648916
"7849","TTF2",54.9491178374368,0.222572542448659,0.274575740116505,0.810605271806675,0.417592388443344,0.819927415648916
"7850","ASAP1",248.154337841135,0.225006804411878,0.277581212302861,0.810598104047401,0.417596506017492,0.819927415648916
"7851","UNC45A",139.670783199122,-0.136582295595732,0.16846918696833,-0.810725676627188,0.417523224549502,0.819927415648916
"7852","HMCN1",365.459214910153,0.283911242218774,0.3505056796669,0.810004683771705,0.417937483807692,0.82007886846279
"7853","TRIM56",372.987130222824,0.238895322358647,0.294881622265759,0.810139745308863,0.417859863378441,0.82007886846279
"7854","MAPRE2",148.1120123854,-0.134797741489109,0.166359327246896,-0

.810280636017804,0.417778901950219,0.82007886846279
"7855","HNRNPM",623.130481741245,-0.148379997718429,0.183184972580271,-0.810000927632914,0.417939642597709,0.82007886846279
"7856","TSR2",189.080857822103,-0.186584147565309,0.230309078906826,-0.810146731735196,0.417855848481072,0.82007886846279
"7857","EGFL6",3.53492807413514,1.17058978520253,1.44547592720745,0.809830010427098,0.418037881901365,0.820167232978178
"7858","SLC66A3",492.509631012557,-0.184169172520554,0.22757228163183,-0.80927769937512,0.418355430638238,0.820581367004509
"7859","YJEFN3",15.0435187177509,0.372368566388519,0.460097424776494,0.809325473989358,0.418327957230822,0.820581367004509
"7860","ZNF347",57.8077673497638,0.260834212337293,0.322349894001263,0.809164877021088,0.418420314802642,0.820604217898567
"7861","TSHZ3",66.8293797135853,0.192457585249254,0.237879960995202,0.809053374836967,0.418484445557099,0.820625585582328
"7862","D2HGDH",89.1413380450949,0.192534431349706,0.23814661621682,0.808470153421847,0.418819981051699,0.820779690707645
"7863","ELF2",411.082508452041,-0.157295259638476,0.194506630510287,-0.808688419648281,0.418694390885643,0.820779690707645
"7864","SGK3",77.1383933382618,0.195399573672745,0.241672825064083,0.808529356252331,0.418785913612242,0.820779690707645
"7865","MRPL43",481.517678328398,-0.198200286898852,0.245085783837357,-0.808697606999439,0.418689104979204,0.820779690707645
"7866","PDCD7",322.826762716143,-0.114106170221565,0.141141198161217,-0.808454028364052,0.418829260272873,0.820779690707645
"7867","UTP20",65.530553377847,0.203009946573844,0.251139913210797,0.808353972804974,0.41888684029996,0.820788183961343
"7868","POGLUT1",82.5008675583144,-0.169772452815548,0.210067152769851,-0.808181815086292,0.418985924617352,0.820877990337631
"7869","BBS12",17.6188007021919,-0.331142224873362,0.409925908783679,-0.807809942669685,0.419200000555781,0.821088692321138
"7870","ZNF441",42.4473549471132,0.270056533429298,0.334271741897122,0.807895192984672,0.419150918791188,0.821088692321138
"7871","PIK3CB",26.7180618034848,-0.278024293516842,0.344311532192811,-0.807478889092659,0.419390632413364,0.821357718034812
"7872","CENPW",60.7367852897584,0.298338154657854,0.369571917700507,0.807253312194627,0.419520556424318,0.821507796910678
"7873","SHOX2",226.382193252241,-0.174876671419416,0.216795727100624,-0.806642611264421,0.41987241646994,0.821586330038842
"7874","LXN",202.453038872404,0.279109273704699,0.346098884648976,0.806443724855629,0.419987043834322,0.821586330038842
"7875","FUCA2",412.881043865185,-0.203472838128601,0.252275213909742,-0.806551047862351,0.419925186376442,0.821586330038842
"7876","UBR5-AS1",9.06815661615448,0.422767464790513,0.523955517414201,0.806876635018435,0.419737561389331,0.821586330038842
"7877","KCTD21-AS1",12.8210003268096,0.422188677861058,0.523490094241079,0.80648837963805,0.41996130563325,0.821586330038842
"7878","RPS17",3924.68149974993,0.184114006641185,0.228257363690747,0.

806607084495337,0.419892890828387,0.821586330038842
"7879","MIEN1",237.23025826692,-0.227273608807389,0.281632509817822,-0
.806986412734823,0.419674311273036,0.821586330038842
"7880","H1F0",360.002178734828,-0.154743740334546,0.191739270838631,-0
.807052930042582,0.419635989031249,0.821586330038842
"7881","FARP1-
AS1",2.95531043883475,0.973445737596161,1.20726386700687,0.80632392321
1249,0.420056099893248,0.82161715262713
"7882","RAP1GDS1",57.987709466443,-0.183879239119621,0.228238641758757
,-0.805644643267625,0.420447776547931,0.822103046413316
"7883","B4GALT1-
AS1",28.1101081977114,-0.33731949672831,0.418659205193424,-0.805713794
283982,0.420407893877625,0.822103046413316
"7884","CHD2",347.379958391694,0.164036252674807,0.203616025175563,0.8
0561563135009,0.420464509758195,0.822103046413316
"7885","IPO11",34.0160936185526,0.227982678046345,0.2830803931868,0.80
536371834097,0.420609822122839,0.822178595995887
"7886","DMWD",120.494084267304,0.175682528852828,0.218136987917396,0.8
05377073049874,0.42060211791263,0.822178595995887
"7887","LINC02018",7.644856550187,-0.442290087326785,0.549451913363398
,-0.80496596075053,0.420839322847279,0.822333740820431
"7888","ZFYVE16",249.173501688879,0.152566266661177,0.189557137222818,
0.804856355695227,0.420902576391385,0.822333740820431
"7889","DELE1",79.5529290527919,0.199220666827405,0.247498817210724,0.
804935833926775,0.420856708609417,0.822333740820431
"7890","ALOX12-
AS1",44.9186453365382,0.227864581100327,0.28306306892707,0.80499579815
9158,0.420822104518062,0.822333740820431
"7891","YIPF1",57.4828492283563,0.195816917613881,0.243392708756929,0.
804530746274078,0.42109051988631,0.822356993893131
"7892","KDM6B",172.739083070808,-0.295732617740513,0.367655777555041,-
0.804373644573664,0.42118121743667,0.822356993893131
"7893","SNHG11",52.6640027071351,-0.254993221052697,0.316983889435022,
-0.804435902112205,0.421145273703197,0.822356993893131
"7894","B4GALT5",154.409303235456,-0.163168609457853,0.202766132340077
,-0.804713329463663,0.42098512583056,0.822356993893131
"7895","SCARF2",28.5070562688245,-0.446134278943443,0.554609434236258,
-0.804411629884743,0.421159286803719,0.822356993893131
"7896","VPS72",144.040310633133,-0.191403185403523,0.238018310246327,-
0.80415319815286,0.421308504324353,0.822397188066342
"7897","SLC37A1",17.3601374213138,0.30735584797912,0.382209910268996,0
.804154575067939,0.421307709216526,0.822397188066342
"7898","ALKBH1",18.8397487154862,0.29824194056183,0.370975571084644,0.
803939568553911,0.421431876674901,0.822533854006534
"7899","CRYZ",71.6904099303957,0.192363271217068,0.239431777859446,0.8
03415791073444,0.421734450916861,0.822989909530451
"7900","DCTN6",292.541582495335,-0.21625924968836,0.269196714854743,-0
.803350255611598,0.421772318215411,0.822989909530451
"7901","LYSMD1",21.9688431591139,0.299428929613822,0.372845500771869,0
.803091170455165,0.421922040731235,0.823177858229589

"7902","ERVK13-1",24.2107184666853,0.340650541949813,0.424284394115876,0.802882563379831,0.422042615150874,0.8233088980702
"7903","RPTOR",31.4064322861672,-0.245560338948739,0.305923677244808,-0.802684974109523,0.422156839928527,0.823427519612583
"7904","GGT7",76.8926996017729,0.177850847677303,0.221650046907377,0.802394811816231,0.422324613269014,0.823650545741631
"7905","ERI2",26.9713949399842,0.312435239669503,0.389433111676466,0.802282164257949,0.422389757197987,0.823673384845917
"7906","ZC3H7A",172.375138128004,0.160694124459893,0.200322683485157,0.802176376954333,0.422450939211806,0.823688493289905
"7907","UBIAD1",54.1046556410453,-0.191654896206785,0.238979054621252,-0.801973614426296,0.422568221292477,0.823812967146015
"7908","ARL1",635.022166748594,-0.163102947069314,0.203417033938875,-0.801815580097016,0.422659644874828,0.823887003761441
"7909","AC015917.2",2.87424079072375,-0.719326570832576,0.897549039085222,-0.801434283262907,0.422880274533573,0.824212850162476
"7910","PHACTR4",168.395070669986,-0.16007681894759,0.19986784043131,-0.800913336543527,0.423181818755034,0.824405414111661
"7911","TBC1D8",25.0427753937282,0.347537247339669,0.434086800379464,0.80061694351421,0.423353438735512,0.824405414111661
"7912","AP1AR",69.7387612576167,-0.237981451070785,0.297054610388522,-0.801137039278822,0.423052315493462,0.824405414111661
"7913","FBXW11",176.637263715261,0.167173398705677,0.208724193558655,0.800929666347942,0.423172364518501,0.824405414111661
"7914","PDLIM2",1183.97812191983,-0.208466810081512,0.26035205286973,-0.800711220763144,0.423298845113721,0.824405414111661
"7915","AL627171.1",5.70350615376184,-0.538642925208764,0.672569253305544,-0.800873549543697,0.423204854190789,0.824405414111661
"7916","ARSD",79.5182994704689,0.246378938331187,0.307724778568429,0.800647057014291,0.423336000304905,0.824405414111661
"7917","AC005726.5",3.34981171614808,0.768677601112939,0.960438222835994,0.800340493366849,0.423513547932352,0.824613027835949
"7918","LINC01117",19.5740112229945,-0.396226440562196,0.495147490647188,-0.800219021698573,0.423583910812582,0.824645868297039
"7919","ZBTB43",127.826611617272,0.245997182649703,0.307493356942846,0.800008120810968,0.423706092121399,0.824779569396561
"7920","SPARCL1",343.508802417655,-0.765819510950648,0.957500957798863,-0.799810699627017,0.423820482909325,0.824858729616318
"7921","AL158212.3",13.4637657751276,0.340876599840581,0.426255561787808,0.799700063527313,0.423884596139819,0.824858729616318
"7922","USP3-
AS1",4.6977792189665,0.547008098063422,0.684050068578233,0.799660906694086,0.423907288746057,0.824858729616318
"7923","AC092958.1",7.07210774529498,0.605842862329962,0.757969600960464,0.799297045108757,0.424118191862478,0.82516495362364
"7924","TMCC1",82.5894768548445,0.217073678200882,0.271665715951423,0.79904701055358,0.424263153597385,0.825238676681853
"7925","HMGCS1",120.204764933815,0.247701306313337,0.309975112857416,0.799100624659749,0.424232067478776,0.825238676681853
"7926","SSB",724.920139875508,-0.144788136070348,0.181233038434346,-0.

798905858011089,0.424345001947239,0.825293742747501
"7927","AC069185.1",5.55343842563645,0.540866761406851,0.677114749255666,0.798781538877142,0.424417096965001,0.825313173954326
"7928","AC037459.2",8.16007621197946,0.423930338873296,0.530834131896849,0.798611681879705,0.424515611825096,0.825313173954326
"7929","REXO4",140.648218875614,0.161968779502076,0.20281146690364 8,0.798617464657584,0.42451225766901 5,0.825313173954326
"7930","STRN",202.176956521682,0.135309793614963,0.169485854403926,0.798354494484751,0.424664802622718,0.82540469202319
"7931","AC090198.1",4.49933679822544,-0.56231436082334,0.704349263429753,-0.798345920155025,0.424669776998762,0.82540469202319
"7932","C17orf100",8.15849360519622,0.424829732415498,0.532212501933313,0.798233282518285,0.424735126605019,0.825427631948609
"7933","SLC7A2",36.4434666744851,-0.361800741795644,0.45362954604117,-0.797568731915906,0.425120802226892,0.825440005073638
"7934","TRIM68",18.5133038473166,0.308940644205458,0.38721886880414,0.797845015041671,0.424960434948252,0.825440005073638
"7935","TSPAN31",239.293051780331,0.145290750142482,0.182101515923914,0.797855797110374,0.424954177262297,0.825440005073638
"7936","ERH",923.915029614942,-0.206297036543844,0.258535156828446,-0.79794577679327,0.424901957045568,0.825440005073638
"7937","PDPK1",165.104485945372,0.139040164679375,0.17431697000265,0.797628393134992,0.425086169152713,0.825440005073638
"7938","SIAH1",184.090724940752,-0.174843265989492,0.219209773753543,-0.79760707287654,0.425098545279343,0.825440005073638
"7939","AC022098.1",15.2114384360098,-0.318854287000222,0.399653433746473,-0.797826967258067,0.424970909622824,0.825440005073638
"7940","MACROD2",6.80074682115302,0.60252333120721,0.755530118423807,0.797484198861853,0.425169876113181,0.825440005073638
"7941","AC025181.2",50.8249941367983,-0.282387672111475,0.354248046437154,-0.797146730805112,0.425365819060066,0.825592948577315
"7942","ILK",556.75801194208,-0.176709423568778,0.221723901175012,-0.796979588724164,0.42546288572807,0.825592948577315
"7943","NSMCE1",317.858180973175,0.186235977453095,0.233660226208894,0.797037563794013,0.425429215621444,0.825592948577315
"7944","UBE2Z",217.180906994639,0.164698597475512,0.206596831400131,0.797198080722391,0.425336000528928,0.825592948577315
"7945","CHI3L1",335.365763336686,-1.01249861737601,1.27064180972966,-0.796840313000109,0.425543779068878,0.825645456865103
"7946","CCNT1",113.163447857076,0.171451204392875,0.215188592957535,0.796748573130492,0.425597067807338,0.825645456865103
"7947","DNAJB14",305.279554229913,0.125865359151399,0.158064293645637,0.79629216851198 2,0.425862236455304,0.825951984770825
"7948","RNFT1-
DT",14.2693146444709,-0.43999592301237,0.552516493653019,-0.796348938116384,0.425829248368703,0.825951984770825
"7949","CARM1",160.053901863521,0.16666255557452,0.209386361139204,0.79595707508246,0.426056985314309,0.82622574268714
"7950","NSFL1C",287.012682276918,-0.139392569889213,0.175155068439105,-0.795823787067145,0.426134463790675,0.826244786239205

"7951","RBM39",1463.36898053557,0.132671494030838,0.166723886118694,0.795755767931098,0.426174005539275,0.826244786239205
"7952","HECTD3",80.8904712921535,0.175291324565208,0.220394551541954,0.795352350313614,0.426408569384387,0.826283858838506
"7953","VPS33A",56.9617604519881,0.186537376616106,0.234517406563847,0.795409515008948,0.426375326866139,0.826283858838506
"7954","ARF6",470.007283449243,-0.154065620054642,0.193693330486313,-0.795410041573574,0.42637502066434,0.826283858838506
"7955","F8A3",12.1440752150335,-0.454028090007274,0.570816950966333,-0.795400503153686,0.426380567356346,0.826283858838506
"7956","GINM1",794.103778613789,-0.103164089596906,0.129769846583353,-0.794977356551334,0.426626673919133,0.826498702835672
"7957","LRRC28",65.0705903526558,-0.220321432039828,0.27712845806364,-0.795015544701775,0.426604459885414,0.826498702835672
"7958","SUGT1",374.631377436044,-0.132634234262009,0.166878750806847,-0.79479402632589,0.426733326436955,0.826601435916771
"7959","KCTD18",78.3998327505579,-0.187057004371466,0.235464767754576,-0.794416108003196,0.426953229787369,0.826715743898039
"7960","ARL4A",276.824505657205,-0.274988073303668,0.346086171259647,-0.794565331237584,0.426866391788806,0.826715743898039
"7961","AC078846.1",16.9724252954777,0.334052165472677,0.420489863461837,0.794435715340365,0.426941819033594,0.826715743898039
"7962","LSMEM1",25.7509078954873,-0.372634752449848,0.469220243822822,-0.794157450270955,0.427103775773496,0.826799535796615
"7963","TEPSIN",29.9601741178876,0.262457667861426,0.330458257586932,0.794223360547685,0.427065411224847,0.826799535796615
"7964","ZNF551",14.4880303666355,0.361756635124139,0.455601022110833,0.794020683816937,0.427183390149517,0.826849819080212
"7965","DNAH14",16.3458365500687,-0.368908320450184,0.464871940313079,-0.79356977364935,0.427445934847707,0.827150274375772
"7966","NDUFB3",414.285215574266,-0.219184026611799,0.276185533353142,-0.793611540585442,0.427421611891464,0.827150274375772
"7967","ARID2",113.075277445079,0.209561493331798,0.264132369458586,0.793395727155114,0.427547299423713,0.827242578212192
"7968","KLHL15",55.7391816297419,0.283141748113518,0.356931304469136,0.793266784303592,0.427622404691593,0.827284057269191
"7969","CCBE1",81.4282802635914,-0.729833328842356,0.920353203962786,-0.792992652929218,0.427782103362207,0.827347116010367
"7970","PNMA8B",28.3025486207007,0.328064376239861,0.413651216351295,0.79309418967416,0.42772294780925,0.827347116010367
"7971","JOSD1",180.221320516464,0.153985927690308,0.194197550115721,0.792934450504393,0.427816014383304,0.827347116010367
"7972","AL353801.3",3.06867625641294,0.820375846466598,1.03491557117638,0.792698331453343,0.427953602679205,0.827405592035613
"7973","AL049838.1",11.014969442471,-0.365882479569139,0.461557607081316,-0.792712489092783,0.427945352193419,0.827405592035613
"7974","NASP",427.07359895379,0.209083765179105,0.263813743138753,0.792543112771562,0.428044063769361,0.827461489876124
"7975","AL161729.1",7.67736262909742,-0.471922067550125,0.595511920433353,-0.792464518941465,0.42808987231671,0.827461489876124

"7976","HERPUD2",151.499073496557,-0.15287082202397,0.193010944977792,
-0.792031882137873,0.428342086490882,0.827845193487581
"7977","MRPS27",101.769815882602,-0.178209861770237,0.225040848011016,
-0.791900063234357,0.428418950107119,0.827889948088409
"7978","ZNF175",25.5109842923962,0.284408481139252,0.359294639321697,0
.791574518551625,0.428608809621975,0.828153020847674
"7979","ZNF362",85.2474472641922,0.178207890231364,0.225282951440179,0
.791040285525935,0.428920483401424,0.828367418162767
"7980","NUDCD2",303.196660024559,-0.192355557511409,0.243177801643191,
-0.791007880701415,0.428939392749632,0.828367418162767
"7981","HIST1H3A",3.28445074515923,0.707960103576335,0.895148542438403
,0.790885612847939,0.429010744682064,0.828367418162767
"7982","PPP2R5D",101.205624813611,0.166411622453472,0.21047509195384,0
.790647581662388,0.429149672490941,0.828367418162767
"7983","TP53TG1",222.953289314092,-0.230048397209056,0.290928688168574
,-0.790738096876022,0.42909683986015,0.828367418162767
"7984","PPDPFL",35.2031180323063,0.449343602347441,0.568199102156348,0
.790820683528286,0.429048638333304,0.828367418162767
"7985","DNAJC3-
DT",13.501511245603,0.359510342684174,0.454339230389304,0.791281753011
124,0.428779593943806,0.828367418162767
"7986","AC093462.1",3.25448256499078,-0.660009798528411,0.834258170955
112,-0.791133753922709,0.428865944038984,0.828367418162767
"7987","RAMAC",97.0552761407701,-0.258229831039282,0.326673684700184,-
0.790482500224286,0.429246038257642,0.828449690715106
"7988","HSPG2",1886.35804237388,0.138377425991125,0.175098485950514,0.
790283395312923,0.429362281849978,0.828476289757066
"7989","ABCD4",156.210422443069,0.154610640561811,0.195641621940671,0.
79027478421078,0.429367309689861,0.828476289757066
"7990","AC083799.1",17.3334653898521,-0.34685696315864,0.4391070061728
85,-0.789914436077286,0.429577739993772,0.828674866975847
"7991","CACFD1",14.6025421570329,-0.341616496685119,0.432469709263918,
-0.789920055364258,0.429574458073163,0.828674866975847
"7992","EXOC8",41.2326470579224,0.211045915713934,0.267330829800192,0.
789455955647441,0.429845562614268,0.82896635524637
"7993","PRKN",22.9826408602512,0.312257890550865,0.395804502940127,0.7
88919499983809,0.430159057775914,0.82896635524637
"7994","IDE",59.9711306427525,0.216456121276042,0.274317999177216,0.78
9070064397073,0.430071057209447,0.82896635524637
"7995","ITFG2",69.9822443850402,0.210328647536147,0.266505877620565,0.
789208288440076,0.429990278438444,0.82896635524637
"7996","NFYB",312.551795522971,-0.167944447754029,0.212875520440989,-0
.788932646675947,0.430151373496162,0.82896635524637
"7997","NXN",37.6976183371245,0.275460661749502,0.349071171295777,0.78
9124638184736,0.430039162982485,0.82896635524637
"7998","ZNF544",21.2065312327756,0.356924807870996,0.452307825043985,0
.789119241605617,0.430042316811219,0.82896635524637
"7999","PRAF2",145.47817479748,-0.215900203234954,0.273537760417045,-0
.789288480339187,0.429943417960038,0.82896635524637
"8000","RABEPK",99.7510475315502,-0.174583113290865,0.221333692469424,

-0.788777846440993,0.430241859738964,0.829022283484516
"8001","TTC21A",13.8869502503931,-0.372245122533275,0.472091649645108,-0.788501814876642,0.430403237422411,0.829023665006137
"8002","DCAKD",62.9008474214473,-0.206819647537188,0.262294834073508,-0.788500651443355,0.430403917680449,0.829023665006137
"8003","DHRSX",142.74307393053,-0.212647077964305,0.269654717490743,-0.78859023844671,0.430351538093914,0.829023665006137
"8004","PPM1D",48.2577835261282,-0.229174968396063,0.290685886130338,-0.788393862003005,0.430466359994626,0.829040347241024
"8005","PTPN22",3.0225690887201,0.761449501372024,0.966120044211373,0.788152058260608,0.430607767797071,0.829119958292027
"8006","SRGAP3",12.2242025201068,0.458326971081719,0.581530461888507,0.788139231078821,0.430615269937462,0.829119958292027
"8007","RCC2",75.9267888203743,0.189146441819546,0.240103521166897,0.787770378794527,0.430831030332171,0.829141205204921
"8008","IAH1",512.150283577847,-0.200804802232021,0.255070022456253,-0.787253634505251,0.431133405707467,0.829141205204921
"8009","SLC7A14-AS1",3.1439740448086,0.956569570895176,1.21514938433535,0.787203271652389,0.431162882350424,0.829141205204921
"8010","HLA-B",5107.63763964928,0.328484726722075,0.417116666790252,0.787512830042953,0.430981720826443,0.829141205204921
"8011","ATP6V1G2",5.04097921452636,0.591050236468508,0.750322118228295,0.787728659611062,0.430855437944223,0.829141205204921
"8012","TMEM60",153.540649089227,0.183803253732371,0.233329625440734,0.787740748244834,0.430848365463356,0.829141205204921
"8013","PRPF39",60.9360894109409,0.201921001278481,0.256504995753664,0.787201047235731,0.431164184295988,0.829141205204921
"8014","RGMA",189.199583361342,-0.172606988796374,0.219046522061469,-0.787992373364128,0.430701166944284,0.829141205204921
"8015","AC087477.2",23.6165052554373,0.281972402968199,0.358170372369118,0.78725775418858,0.431130994568631,0.829141205204921
"8016","IER3IP1",470.340329868651,-0.18369784509773,0.233291106427077,-0.787418980136522,0.431036639538942,0.829141205204921
"8017","AC007529.1",6.58796369303338,0.53698025281003,0.682353711485729,0.786952930380977,0.431309420785636,0.829317041463213
"8018","PSPC1",110.804798507395,0.150571202834204,0.191395042585312,0.786703776651316,0.431455292754527,0.82949405560127 6
"8019","STK40",108.080877582035,-0.171154728360928,0.218030009166308,-0.785005371578805,0.432450419182709,0.829497039365858
"8020","NAV1",772.974936069177,0.195201387107334,0.249015872454051,0.783891344690702,0.433103868889876,0.829497039365858
"8021","LINC01703",3.5298446179728,0.673434199213764,0.860960518201514,0.782189409359352,0.434103267589118,0.829497039365858
"8022","RNASEH1",175.411767892578,-0.171932959032593,0.219494384822133,-0.783313701495914,0.43344291921664,0.829497039365858
"8023","HS1BP3",108.378223949959,0.159125150889404,0.202361530006912,0.786340916101835,0.431667787772622,0.829497039365858
"8024","IL1RL1",2.58915466987375,-0.988972971536522,1.25789871331937,-

0.786210337179531,0.431744271023886,0.829497039365858
"8025","CHCHD5",254.334119764514,-0.19110510558731,0.244159905602965,-0.782704699673629,0.433800541801202,0.829497039365858
"8026","ORC2",48.2477065460267,0.240294212933331,0.307146416478312,0.782344185188624,0.434012326149088,0.829497039365858
"8027","AC012076.1",3.12494652151182,-0.941971947481999,1.20453267480064,-0.782022743914277,0.43420120730737,0.829497039365858
"8028","UBE2E1",303.577583522517,-0.140455880189283,0.179516818221606,-0.782410704360283,0.433973244945843,0.829497039365858
"8029","NDUFB5",551.036662628909,-0.204601474185653,0.260482154193677,-0.785472136542319,0.432176800755527,0.829497039365858
"8030","PGRMC2",611.856001166705,0.118403405057689,0.150869179473995,0.784808437816806,0.432565892173992,0.829497039365858
"8031","C5orf34",8.56817614088917,0.501521490700271,0.641240547844127,0.782111319046189,0.43414915519559,0.829497039365858
"8032","PCBD2",40.2614106442822,0.236455078112125,0.30194563289122,0.78310481210805,0.433565565582964,0.829497039365858
"8033","TRIM41",36.6173365500448,0.23318132225403,0.296682085808483,0.785963606864202,0.431888808407172,0.829497039365858
"8034","MCUR1",171.420346917142,-0.194135073790278,0.247889647919447,-0.783151194169122,0.433538331297288,0.829497039365858
"8035","ZNF391",14.3254737275073,0.344458509604347,0.439662100814814,0.783461910785511,0.43335591245694,0.829497039365858
"8036","FLOT1",474.260679906712,-0.138799632274033,0.177230858541883,-0.783157252726572,0.433534773950062,0.829497039365858
"8037","TBC1D32",29.355867649856,0.298231568468901,0.379464487994201,0.785927479130692,0.431909974791128,0.829497039365858
"8038","TOMM7",3146.54848929419,-0.174978912546029,0.223351372639848,-0.783424388567251,0.433377939045784,0.829497039365858
"8039","ZNF107",55.0485060713919,0.213825246057567,0.272347599185222,0.785118894740633,0.43238386248006,0.829497039365858
"8040","EIF4H",603.154417448207,-0.100669024686153,0.128527613215553,-0.783248223222832,0.433481361673385,0.829497039365858
"8041","MRPS28",148.094614832426,-0.208747209963228,0.265621025944615,-0.785883606995608,0.431935679244721,0.829497039365858
"8042","EIF3E",2224.97291831145,-0.199239172263931,0.253893802020142,-0.784734289213272,0.432609374164288,0.829497039365858
"8043","LINC01235",17.0665962877412,0.495622912913465,0.63340697415413,0.782471512214298,0.433937521023364,0.829497039365858
"8044","ZCCHC7",193.733722795005,-0.133846424712481,0.170372584468287,-0.785610109338893,0.432095940068669,0.829497039365858
"8045","GNAQ",308.628257716116,0.162311156742927,0.207170182697215,0.783467749218281,0.433352485192249,0.829497039365858
"8046","BICD2",151.168752439033,0.151680011523873,0.193721115995263,0.782981301468354,0.433638092498117,0.829497039365858
"8047","SEC24C",75.1904872853264,0.165962158134438,0.211346486447276,0.785261022902504,0.432300543513733,0.829497039365858
"8048","AP002748.4",13.8979249279116,-0.398596315256535,0.507917223856691,-0.784766289731099,0.432590608211169,0.829497039365858
"8049","CD3D",14.9946957340581,0.439314651851417,0.561748964925537,0.7

82047995245796,0.43418636772826,0.829497039365858
"8050","AACS",71.042496944388,0.227114242836766,0.290406988825258,0.782055017875014,0.434182240755921,0.829497039365858
"8051","HNRNPA1L2",20.3479070518814,0.31682711077618,0.40298637387311,0.786198073476155,0.431751454576659,0.829497039365858
"8052","OBI1",82.85500191467,0.22979900999195,0.293372841910757,0.783300214482204,0.433450837314654,0.829497039365858
"8053","ABHD4",63.2244995430805,0.245804666027327,0.313076552523793,0.785126398147131,0.432379463569196,0.829497039365858
"8054","KLHL28",114.436624377766,0.152923011075961,0.195164927421024,0.783557850771334,0.433299595953345,0.829497039365858
"8055","CBFB",112.71461888545,0.154698802896932,0.197562698011493,0.783036496535053,0.433605680432235,0.829497039365858
"8056","CYB5B",169.490202180705,0.165076973699826,0.21101828479677,0.782287534271308,0.434045611180046,0.829497039365858
"8057","SPATA2L",36.1398847129702,-0.251257355553972,0.320415650711127,-0.784160683151194,0.432945831909747,0.829497039365858
"8058","ZNF276",66.0638260720519,0.225813109918294,0.288260721567973,0.783364131921965,0.433413312732311,0.829497039365858
"8059","COX10-AS1",25.2309155920061,0.317994058643805,0.405057635359197,0.785058793822796,0.432419097889038,0.829497039365858
"8060","TTYH2",10.1740947442295,0.378712379537729,0.481714638521879,0.786175775558308,0.431764515920437,0.829497039365858
"8061","AC027307.2",19.1342317968291,0.296665530183577,0.377514585897736,0.785838590787431,0.431962054925964,0.829497039365858
"8062","CLPP",238.988221897302,-0.1801486957032,0.229750261316159,-0.784106597621222,0.432977564446364,0.829497039365858
"8063","HAUS8",23.5794818410292,-0.268076118872687,0.341641779268527,-0.784670187137685,0.432646966740504,0.829497039365858
"8064","MEF2B",23.9924396108099,-0.283311930136227,0.360388211532364,-0.786129848508612,0.431791419108283,0.829497039365858
"8065","PLCG1-AS1",9.93845777261187,0.577884077617744,0.736777691902456,0.784339813717178,0.432840743774687,0.829497039365858
"8066","RPL3",14623.0012439845,-0.19868300230858,0.253919718146377,-0.7824638581004,0.433942017634696,0.829497039365858
"8067","PMM1",249.817880207365,-0.191444876310132,0.24388505827748,-0.784979931375358,0.432465335155699,0.829497039365858
"8068","PRPS1",91.9252839448036,-0.164496656942525,0.210009151874218,-0.783283278249929,0.433460780534695,0.829497039365858
"8069","FAM122B",48.6303421305542,0.219654091891011,0.279883981596144,0.784804084315048,0.432568445070849,0.829497039365858
"8070","TMED9",739.641048185952,-0.184463511676843,0.235911908065559,-0.781916916315989,0.434263402740727,0.829513054925441
"8071","AC096751.2",2.5099765131011,-0.877462893487501,1.12268816660138,-0.781573120293744,0.434465488979625,0.829796247382099
"8072","ZC3H11A",7.69272515932468,0.5330103700682,0.682256902142259,0.781245845068873,0.434657914609155,0.829876998587774
"8073","BICD1",53.8069038435098,0.226678924322448,0.290126736169794,0.

781310014082212,0.434620181743543,0.829876998587774
"8074","FARSA",72.4002567293936,-0.201544485463246,0.257984693419015,-0.78122652469114,0.434669275809127,0.829876998587774
"8075","DXO",33.0437164689773,-0.24593959752695,0.314988093194389,-0.78079014045516,0.434925933888062,0.830073037583508
"8076","Z97989.1",20.9747137462744,0.317927423094306,0.407123931836561,0.780910671745863,0.434855035027533,0.830073037583508
"8077","AP002387.2",10.4376061414139,-0.383393470276635,0.491040764226354,-0.780777275957281,0.434933501431203,0.830073037583508
"8078","RWDD3",142.727867958199,-0.184420640724329,0.236302890468633,-0.780441747278621,0.435130903078873,0.830346975855513
"8079","ALPK3",7.76768030705184,-0.611935275210082,0.784630144364122,-0.779902836521792,0.435448068739325,0.83084103644209
"8080","CD3EAP",26.0943847299639,0.27131434826043,0.347919782263107,0.779818688364361,0.435497604570359,0.83084103644209
"8081","RPS27",22755.187890988,-0.222533039779933,0.285732260632806,-0.778816642149871,0.43608773290056,0.831079178784918
"8082","AC004067.1",2.87653149608761,0.680538402422908,0.873031077564921,0.779512230333291,0.435678035915835,0.831079178784918
"8083","ABLIM3",40.8469336940176,0.233567557260111,0.29997973177645,0.778611127748356,0.436208822056618,0.831079178784918
"8084","POR",180.366078644895,-0.163650060731186,0.210185071912052,-0.77859982748757,0.436215480736216,0.831079178784918
"8085","NDUFA9",219.764204996162,-0.213178397890584,0.273557256515872,-0.779282555344005,0.435813288458599,0.831079178784918
"8086","REC8",20.1618499672449,-0.428104481933315,0.549601751525301,-0.778935803507182,0.436017531863748,0.831079178784918
"8087","MNS1",95.1937701365631,0.262797524846456,0.337463024385823,0.778744650098315,0.436130148436879,0.831079178784918
"8088","UBL7",86.1368986078487,-0.19629321356865,0.252027674146934,-0.77885579126604,0.436064668425265,0.831079178784918
"8089","LINC01569",4.83629376010857,0.529276615302216,0.679556591321105,0.77885583343878,0.436064643579818,0.831079178784918
"8090","MCM3AP-AS1",5.63588530036309,0.499554343452434,0.641508704916563,0.778717949770313,0.436145880065987,0.831079178784918
"8091","Z93943.1",3.43893161100229,0.734789726974357,0.943206624255695,0.779033679448759,0.435959875489542,0.831079178784918
"8092","Z84485.1",8.95271843267482,-0.375847602221561,0.48281045291276,-0.778457881253607,0.436299127556145,0.831135819485661
"8093","TAPBP",469.026400789756,0.120715616719197,0.15510178066593,0.778299360593442,0.436392552366089,0.831179037745956
"8094","WWP1",165.555502213558,-0.164952620297407,0.211981872514089,-0.778144934475208,0.436483575125106,0.831179037745956
"8095","PTEN",547.335286528661,0.146401628534361,0.188139131897635,0.778156181851719,0.436476945260357,0.831179037745956
"8096","B4GALT2",177.426569871848,-0.231888655120539,0.298274300220821,-0.777434244079577,0.436902615469336,0.83137857190487
"8097","CAMSAP2",154.963789852144,0.169910942786605,0.218601763124788,0.777262453686669,0.437003942023104,0.83137857190487

"8098","AC012459.1",3.12708546901638,0.83751075773634,1.07836491684447
,0.776648743532089,0.437366035135667,0.83137857190487
"8099","NXPE3",34.971346927647,-0.268069289467314,0.344824862378384,-0
.777407080273568,0.436918636505679,0.83137857190487
"8100","GTPBP8",89.5500584153588,-0.182786824165953,0.23538911148075,-
0.776530498866775,0.437435820118432,0.83137857190487
"8101","SLC9A3-
AS1",5.46877004064621,0.584454058824081,0.751610446914989,0.7776023619
98815,0.436803468139454,0.83137857190487
"8102","TRAPPC13",48.4731117904467,-0.214609132355526,0.27637855258805
9,-0.77650429219593,0.437451287494025,0.83137857190487
"8103","JAK2",59.6107953453192,-0.222617066101203,0.286294118939752,-0
.777581694397471,0.436815656132467,0.83137857190487
"8104","DNA2",6.16009578747957,0.472702904004149,0.608539869197232,0.7
7678214350643,0.43728731356949,0.83137857190487
"8105","IFITM10",7.08657029919121,-0.532999880531353,0.686349518967086
,-0.776572090169867,0.437411273262553,0.83137857190487
"8106","TM7SF3",220.366746557097,-0.135257042026068,0.173981225974211,
-0.777423203386997,0.436909127157295,0.83137857190487
"8107","ESYT1",93.0694323058141,0.187935048652643,0.242006314141189,0.
776570848242412,0.437412006226763,0.83137857190487
"8108","LGR5",2.64026085932813,-0.782747391770943,1.00733661820526,-0.
777046498285287,0.437131337471815,0.83137857190487
"8109","VSIG10",91.5547502588178,0.225071714348246,0.289632182617446,0
.777094977202608,0.437102737143691,0.83137857190487
"8110","ERG28",99.4932743795106,-0.211027121224996,0.271599189452111,-
0.776979937424317,0.437170607068907,0.83137857190487
"8111","AJ011932.1",9.52848525532671,-0.419103932556005,0.539314037053
601,-0.777105552167098,0.437096498544905,0.83137857190487
"8112","SEC61A1",321.872511662147,0.131778995832014,0.16986575144757,0
.775783197666471,0.437877006415542,0.831535385332502
"8113","PCDHGC3",68.0264344629339,-0.212972063818474,0.274558500651568
,-0.7756892003455,0.437932517993095,0.831535385332502
"8114","LRRN3",38.6725011887415,-0.694251898374361,0.89518251983823,-0
.775542286616387,0.438019288284133,0.831535385332502
"8115","STXBP1",162.104845211598,0.150312853926225,0.193660696606219,0
.776166029351142,0.437650961036464,0.831535385332502
"8116","FAM102A",146.415710170515,-0.145170576329235,0.187141594066902
,-0.775725872450022,0.437910860230759,0.831535385332502
"8117","LRP5",111.898831913555,-0.240631898463918,0.31007079311014,-0.
776054706895413,0.437716685157154,0.831535385332502
"8118","CLMN",8.6056713169635,-0.469639338481575,0.605198456238476,-0.
776008817670407,0.437743779537511,0.831535385332502
"8119","POLR2M",158.219398057869,0.149955319256273,0.193289043456984,0
.775808688243859,0.437861953256919,0.831535385332502
"8120","AC026471.4",5.27926340854634,-0.539980088457081,0.696227180179
484,-0.77558030457512,0.437996833142094,0.831535385332502
"8121","HEXB",669.387531623571,-0.138474009353366,0.178610603987312,-0
.77528436868845,0.438171643868169,0.83161978456388
"8122","ZNF230",25.384247343473,-0.235225614173745,0.303376683769323,-

0.775358248535021,0.43812799893661,0.83161978456388
"8123","OTUD6B",43.5474223613322,0.235053699276924,0.303219684851606,0.775192743149107,0.438225775639534,0.831620131907351
"8124","SV2A",3.01740202847175,-1.08938317953561,1.40628631739253,-0.774652477281794,0.438545039363411,0.831765550503143
"8125","HHAT",11.8957020224902,0.42863763034917,0.554449246812832,0.773087226311748,0.439470760264969,0.831765550503143
"8126","EML4",101.805378186899,0.184241781481695,0.237937573667972,0.77432823509747,0.438736710621815,0.831765550503143
"8127","ATP5MC3",1141.45305446808,-0.234556713465872,0.302819175398367,-0.774576818516551,0.438589759685007,0.831765550503143
"8128","COL4A4",14.8140236407815,-1.04701144617314,1.35436764772363,-0.773062947813997,0.439485127886852,0.831765550503143
"8129","NIT2",189.2811831265,-0.220568751910022,0.28505944363939,-0.773764058099576,0.439070330732276,0.831765550503143
"8130","FRG1",391.073163539059,-0.184064912886312,0.237573770130236,-0.774769507531952,0.43847587038066,0.831765550503143
"8131","MICALL2",39.9223597496023,-0.301469789439293,0.389885875415924,-0.773225726932759,0.439388802997106,0.831765550503143
"8132","UFSP1",5.17344511743072,0.591993443893768,0.76509094306127,0.773755655144855,0.439075300831906,0.831765550503143
"8133","CHCHD3",242.514144818325,-0.176174311446644,0.227807812229971,-0.773346224267308,0.439317506251704,0.831765550503143
"8134","ACTR3B",14.857290584517,0.325708179840728,0.420700516935841,0.774204372775705,0.438809942815799,0.831765550503143
"8135","DCLRE1A",9.32148445019682,0.368134328983106,0.475500416506184,0.774204009510721,0.438810157602433,0.831765550503143
"8136","MYCBP2",810.123965419531,0.150506753080849,0.194260716704728,0.774766796055922,0.438477472887135,0.831765550503143
"8137","GEMIN2",75.5783224118408,-0.192701362531295,0.248891477455723,-0.774238493423608,0.438789768668561,0.831765550503143
"8138","DICER1-AS1",11.3679249375727,0.352021845219751,0.455332718449034,0.773109049617205,0.439457845816276,0.831765550503143
"8139","ZNF207",390.148181619473,0.155322342512411,0.200882443569371,0.773200184907018,0.439403916743299,0.831765550503143
"8140","PRDX2",1023.37139287713,-0.204132605503933,0.264049759922094,-0.773083851938213,0.439472757148458,0.831765550503143
"8141","GATAD2A",108.516004011451,-0.154978841388212,0.200392380834828,-0.773376915542276,0.439299347674912,0.831765550503143
"8142","ESS2",42.4130878816596,-0.211479436467411,0.272993296176725,-0.774668973301482,0.438535289257491,0.831765550503143
"8143","RPS19BP1",674.718730079719,-0.175579370728674,0.226810316779056,-0.77412426922234,0.438857306873466,0.831765550503143
"8144","SREBF2-AS1",6.75133137525483,0.428902546759533,0.554805103547206,0.773068856103339,0.439481631432052,0.831765550503143
"8145","ZMAT1",43.8551958087242,0.316908662820114,0.40994291842294,0.773055585492901,0.439489484842562,0.831765550503143
"8146","SDHD",623.61246456424,-0.217667211342149,0.281656560158607,-0.

772810728142017,0.439634403356786,0.831915841856553
"8147","STK11",109.484756311973,-0.148189031335784,0.191771118039643,-
0.772739048771412,0.439676831891361,0.831915841856553
"8148","CHI3L2",188.584371171242,-0.662855278116342,0.85823044084066,-
0.772351161847693,0.439906471127372,0.831954611711218
"8149","ZSCAN26",63.9975381477188,0.184710946580898,0.239148750170449,
0.772368437841504,0.439896241821617,0.831954611711218
"8150","BBS4",103.749843005272,0.139642913854279,0.180805023239339,0.7
72339790966026,0.439913204025828,0.831954611711218
"8151","CALCOCO2",467.233886421944,0.113376484617905,0.146779666274466
,0.772426368689928,0.439861941325995,0.831954611711218
"8152","C1orf43",427.105252939588,-0.175292880380209,0.227027569427725
,-0.772121556963654,0.440042435663129,0.832032903470396
"8153","TMEM217",9.41926069531526,-0.818577923929429,1.06044280699255,
-0.771920860353556,0.440161301405239,0.832032903470396
"8154","POMZP3",15.7940826261616,0.358416490512731,0.464367739721457,0
.771837618883088,0.440210607886283,0.832032903470396
"8155","MRPL48",81.9246349678622,-0.202291100510211,0.262098184346853,
-0.771814200141521,0.440224480097603,0.832032903470396
"8156","LCMT1",152.442536279835,-0.176445117010316,0.228549058896718,-
0.772022942741814,0.440100839194497,0.832032903470396
"8157","KIAA2026",253.15157226979,0.173018042722535,0.224247086926702,
0.771550904378474,0.440380461945383,0.832225673763404
"8158","BICRA",27.704526992213,0.271722631321742,0.352248810584644,0.7
71394035002564,0.440473409668647,0.832299290272395
"8159","LAMA1",4.90326891991752,-0.525839954562032,0.681929047362327,-
0.771106549275118,0.44064377895517,0.832519163205533
"8160","ZNF595",58.408346634424,0.239240329059882,0.310441858912981,0.
770644557720365,0.440917642698454,0.832934492916258
"8161","CEP104",69.2803634051599,0.208289392032458,0.270395643901045,0
.770313415657996,0.441114000239373,0.833018437241173
"8162","DNAAF5",65.5758063735526,0.171625551642353,0.222776065936535,0
.770394929638654,0.441065660188923,0.833018437241173
"8163","VPS39",92.2533690332536,0.17579005707632,0.228248894304688,0.7
7016827446997,0.441200080501924,0.833018437241173
"8164","MIR762HG",20.3390542787276,-0.280528506837726,0.36423164481645
8,-0.770192570662246,0.441185670257856,0.833018437241173
"8165","ZNF875",41.0783794316044,0.238432542142358,0.309606817162121,0
.770113992733906,0.441232276359013,0.833018437241173
"8166","AVPI1",72.7345149578681,-0.205284295437936,0.266627306762832,-
0.769929749245595,0.441341565822951,0.833081779760398
"8167","PRIM1",34.5548092310809,-0.237140900455295,0.308025111658069,-
0.769875219519568,0.441373914713147,0.833081779760398
"8168","CKS2",36.5343493784826,-0.291929052229168,0.379425292415625,-0
.769397976530746,0.441657089454779,0.833208276507544
"8169","HNRNPF",845.553749146406,-0.152120856497066,0.197714158711566,
-0.76939788980407,0.44165714092398,0.833208276507544
"8170","AC010186.3",3.68377192037933,0.593484247819444,0.7713218588478
01,0.769437869563283,0.441633414721746,0.833208276507544
"8171","AC116158.3",3.92521372009836,0.702556789857017,0.9129003817507

41,0.769587573739063,0.441544578460151,0.833208276507544
"8172","ADAMTS9-
AS2",10.7419112470547,0.465809592153594,0.60641771389596,0.76813322150
6638,0.44240804170831,0.833606496615883
"8173","HOXA6",18.4918594521175,0.314895846885617,0.409901045825897,0.
768224063081232,0.442354079903133,0.833606496615883
"8174","AC091563.1",14.8783983040513,0.364269809462025,0.4741371576951
05,0.768279396689405,0.442321212421995,0.833606496615883
"8175","EMG1",150.077785775124,-0.210492872691368,0.273920353209198,-0
.768445536176021,0.442222536010758,0.833606496615883
"8176","BTG1",2567.85680737685,-0.162557384243415,0.21155761698415,-0.
768383509706455,0.442259374361055,0.833606496615883
"8177","NPIPA5",7.08404537359929,0.833820757381273,1.08471783651442,0.
768698300436021,0.442072434091489,0.833606496615883
"8178","MC1R",7.7926187510964,-0.397874243971148,0.517791867206984,-0.
768405742093474,0.442246170047017,0.833606496615883
"8179","LINC02193",8.39936200936381,0.452797694281424,0.58947931611874
8,0.768131606826744,0.442409000896175,0.833606496615883
"8180","GEMIN4",44.3402674664581,0.219284430798501,0.285470634495827,0
.768150570673518,0.442397735647526,0.833606496615883
"8181","AC067852.3",9.09794356956935,0.497440523347861,0.6472986901643
37,0.768486837539514,0.44219800755211,0.833606496615883
"8182","KDM4B",96.5375512439932,0.216532584563218,0.282002505966387,0.
767839221219642,0.442582709872862,0.833831883731382
"8183","TMEM184A",5.15415911646655,0.627736642837209,0.817772907623549
,0.767617314030877,0.442714573007502,0.833978387255364
"8184","AC092155.1",5.52251878668448,0.636650491675856,0.8296952638205
25,0.767330512101818,0.44288503159475,0.834197551568068
"8185","C7orf50",222.271743333282,0.206148560570256,0.268824467352836,
0.766851926092289,0.443169559153785,0.834223806857443
"8186","STARD3NL",117.276313704006,-0.158402507872467,0.20650966057557
6,-0.767046478266312,0.443053881962123,0.834223806857443
"8187","SKIDA1",9.61744486668084,-0.38730407514244,0.505049059882295,-
0.766864263112783,0.443162223272759,0.834223806857443
"8188","NRL",3.32224044565906,-0.615577771020538,0.802549338525292,-0.
767027946408479,0.443064899924682,0.834223806857443
"8189","SGSM3",130.311922184898,-0.1373787462624,0.179070306359808,-0.
767177702741867,0.442975868018278,0.834223806857443
"8190","TNFRSF14-
AS1",4.36433849639749,0.554366857295753,0.72385101010415,0.76585768280
6837,0.443760987607003,0.834387542250605
"8191","SAPCD1",3.13035388701244,0.726572396581318,0.948291214700909,0
.766191213540325,0.443562535671291,0.834387542250605
"8192","RXRB",99.539833035208,0.158995472677303,0.207464085651979,0.76
6375887072896,0.443452676093538,0.834387542250605
"8193","CLDN15",25.4054225936831,-0.263312683860195,0.343648864678351,
-0.766225967621488,0.44354185979025,0.834387542250605
"8194","RND1",20.0577011370344,0.259707483384757,0.339045701343514,0.7
65995505489766,0.443678976488805,0.834387542250605
"8195","ZDHHC20",100.345550503492,0.231732543516456,0.302432609954182,

0.766228693233719,0.443540238293632,0.834387542250605
"8196","HHIPL1",17.5805535240703,0.300009228501967,0.391807935243107,0
.765704830137804,0.443851952413556,0.834387542250605
"8197","HIC1",683.353121662288,0.109811295695676,0.143381158497028,0.7
6586977568571,0.443753791416581,0.834387542250605
"8198","ANAPC11",840.659128543586,-0.17630667334005,0.230235757465312,
-0.765765818832953,0.443815655893324,0.834387542250605
"8199","KLHL26",25.1021437203201,-0.243176209405751,0.317393852425013,
-0.766165467754936,0.443577852696401,0.834387542250605
"8200","TAB3",41.2748809878339,0.244931415695261,0.319772308635708,0.7
65955678714797,0.443702674444042,0.834387542250605
"8201","MEA1",233.214132335194,-0.172628558784126,0.225527928481015,-0
.76544204501332,0.444008364481057,0.834478046631979
"8202","PRR11",6.41185160996103,0.476447107723423,0.622376777610117,0.
765528414400272,0.443956953174299,0.834478046631979
"8203","TRAPPC3L",4.10486630127131,0.555791933134952,0.726619369397208
,0.764901070000417,0.444330456725196,0.834772674094839
"8204","WDR61",259.454424449568,-0.16554997253203,0.216457552231356,-0
.764814952518197,0.444381742693627,0.834772674094839
"8205","GHDC",94.7874543835554,0.177337884674098,0.231795379993241,0.7
65062205637011,0.444234503899909,0.834772674094839
"8206","SAP30BP",201.567962027749,-0.155441220565568,0.203201405372624
,-0.76496134601296,0.444294562264212,0.834772674094839
"8207","DENND2C",45.7255861708911,0.256513992681087,0.335940382014363,
0.763569985671207,0.44512354126206,0.834837929251672
"8208","VANGL1",95.4387409902127,0.199079360893668,0.260530841576401,0
.764129727172005,0.444789938679555,0.834837929251672
"8209","PDE1A",76.1215199906772,-0.376408841198139,0.493131926334583,-
0.763302518244885,0.445283000603779,0.834837929251672
"8210","DCK",51.1344513178229,-0.242123700221429,0.317176273778426,-0.
763372673930119,0.445241171876803,0.834837929251672
"8211","ZNF300",15.1789448600345,-0.309215142635288,0.40460166642541,-
0.76424584546859,0.444720750735378,0.834837929251672
"8212","TMEM248",406.845461579055,0.100346344398228,0.131264809741751,
0.764457317963955,0.444594762703062,0.834837929251672
"8213","FRMPD2",2.7878628988975,0.866658154164617,1.13525611740707,0.7
63403201159635,0.445222971412005,0.834837929251672
"8214","CNNM2",29.6505687627134,0.337675071457059,0.441690729544012,0.
764505679812807,0.444565953239695,0.834837929251672
"8215","PELI3",44.7465760273288,-0.210550865374644,0.275611090671071,-
0.763941918527246,0.444901855606781,0.834837929251672
"8216","FADD",78.3439458420537,-0.208985119193415,0.273717306318373,-0
.763507145398893,0.445161002592909,0.834837929251672
"8217","ITFG2-
AS1",5.18037328483753,-0.593782147440617,0.77748736542632,-0.763719352
680448,0.445034505466763,0.834837929251672
"8218","CHD4",370.533970038095,0.182312952978576,0.238627539616606,0.7
6400633921588,0.444863464906653,0.834837929251672
"8219","OAS2",30.0317498309215,0.3584879767443,0.469608509900661,0.763
376236133654,0.445239048053962,0.834837929251672

"8220","AC009283.1",11.4305482251271,0.360215422120591,0.471180159169108,0.76449615950681,0.444571624462803,0.834837929251672
"8221","MPPE1",139.778186095493,-0.121473491956355,0.159136177388155,-0.763330462940962,0.445266338947774,0.834837929251672
"8222","TP53RK",75.1159763771951,-0.219974562317808,0.288113228847227,-0.763500389058672,0.445165030395652,0.834837929251672
"8223","GUK1",2149.56279139619,-0.20756418382889,0.271979119913321,-0.76316220118309,0.445366668171387,0.834893249405562
"8224","CSE1L",108.964830631566,0.160206836036018,0.209982000450502,0.762955089923444,0.44549017982754,0.835023239547852
"8225","MAIP1",81.4495512129969,-0.202744782482059,0.265873911738463,-0.762559895991209,0.445725909515886,0.835186873814117
"8226","ZNF398",47.8084607722404,0.193972132537266,0.254395605711945,0.762482244905218,0.445772236054775,0.835186873814117
"8227","WWP2",51.3102044910556,0.194131101621882,0.254616388005487,0.762445430722622,0.445794200307659,0.835186873814117
"8228","CACTIN",38.1501206434081,-0.211648069675102,0.277577276222071,-0.762483415630093,0.445771537581441,0.835186873814117
"8229","ABCA5",56.1100445578287,0.250290702377093,0.3283774787822,0.76204226992988,0.445938123677141,0.835354985597658
"8230","USP38",59.5303425632756,0.174476241606851,0.228940334713985,0.762103549052741,0.445998204823289,0.835366017904131
"8231","VILL",30.8365991882772,-0.26944791877459,0.353606199301632,-0.761999985596254,0.446060012756795,0.835380281453772
"8232","COL3A1",20214.8773699007,0.326956189212479,0.429354321101347,0.761506693059001,0.446354482703112,0.835627198308048
"8233","BUD31",556.657887848176,-0.198184349202062,0.260217611233813,-0.761610054993501,0.446292771849514,0.835627198308048
"8234","LINC00598",3.81118340909041,-0.550091698665006,0.722306050608115,-0.761577032619177,0.446312486886155,0.835627198308048
"8235","S1PR1",7.96748820566688,0.410324331626981,0.538927823335018,0.761371586064704,0.446435153842288,0.835676733027186
"8236","PPP1R15B",336.792785973527,-0.311621934392574,0.40940571504811,-0.761156776612058,0.446563431669657,0.835763409038052
"8237","PCDHB15",11.4913906862913,-0.464367050438215,0.610116203385134,-0.761112469824187,0.446589892977388,0.835763409038052
"8238","MAGEE1",22.9176726806113,0.260825160604831,0.342872365341172,0.760706277233219,0.446832524643535,0.836115970791465
"8239","MANEAL",6.92516562323024,0.498821308536226,0.656129572223868,0.760248173002679,0.447106254740826,0.836377617687288
"8240","RFK",157.522512718023,-0.130643654137791,0.171824526460461,-0.760331815422487,0.447056268938334,0.836377617687288
"8241","PAFAH1B2",270.962993233145,0.121856506814694,0.16029535410819,0.760199866631494,0.447135124707164,0.836377617687288
"8242","ZHX2",133.824204873802,0.210874286239277,0.277522366420692,0.759846094421147,0.447346586516594,0.836671636878584
"8243","PON3",27.0252012081438,0.348630157595337,0.458924074857598,0.759668487000852,0.447452770012084,0.836768706749517
"8244","AC012184.3",3.70380376210004,0.640907438103443,0.843804051762621,0.759545343216417,0.447526400558035,0.836804884109911

"8245","AC112220.2",6.38570974720246,-0.482703847297408,0.635755560306478,-0.759260126745429,0.447696964598185,0.836857765494603
"8246","C9orf85",122.390099645521,0.147954014015082,0.194874872604269,0.759225712570605,0.44771754732624,0.836857765494603
"8247","AC109460.2",2.84314092421502,0.757956929541427,0.998307232715275,0.759242149813817,0.447707716330216,0.836857765494603
"8248","NIT1",122.442267047734,-0.179189737908088,0.236217452323453,-0.758579589042065,0.448104086311138,0.837478720960984
"8249","XRCC6",639.61013634481,-0.127595540624221,0.168227536089305,-0.758470007885548,0.448169661285996,0.837499736783081
"8250","CRCP",167.564576406249,-0.162931795812329,0.214879916447879,-0.758245807731639,0.448303842936861,0.837648938045055
"8251","TLCD4",58.2766771824547,-0.292872302756401,0.386505533322057,-0.75774413949299,0.448604169214237,0.837754076926953
"8252","SNRNP27",250.041497921261,0.108508960516508,0.143173835540686,0.757882612467084,0.448521260243793,0.837754076926953
"8253","LINC01473",26.6183394125414,0.2697637044286,0.356201326292257,0.757334924146981,0.448849232652592,0.837754076926953
"8254","DNPEP",218.3293415703,-0.122766637929094,0.162057585055395,-0.75754947160992,0.448720738899214,0.837754076926953
"8255","WASHC3",414.709947844078,-0.204556346119043,0.269916904592751,-0.757849333029644,0.448541185098705,0.837754076926953
"8256","ZCCHC2",82.0098006935761,-0.248238926761762,0.327768374234677,-0.757360826349973,0.448833718560657,0.837754076926953
"8257","AL117332.1",11.5321593765067,-0.347611469429428,0.458918342443933,-0.757458217028874,0.448775389259356,0.837754076926953
"8258","SLA2",2.97547354502596,0.703271172338873,0.928609565075467,0.757337850899392,0.448847479662661,0.837754076926953
"8259","AC006946.2",6.59028423923494,0.529700389774498,0.699396198058917,0.757368128743926,0.448829344855969,0.837754076926953
"8260","STN1",75.8166788333266,-0.234318249260453,0.309455572557837,-0.757195119556811,0.448932973690375,0.8378089333459
"8261","XAB2",122.362453488292,-0.154427457198849,0.203981419908658,-0.757066291959342,0.449010147508114,0.837851522072095
"8262","KLC4",28.9708989606762,0.233082421275513,0.308010535060872,0.756735224103451,0.44920850732207,0.837999367846103
"8263","MVK",24.7344784976746,-0.270625586651395,0.357657229597427,-0.756661865764623,0.449252466810262,0.837999367846103
"8264","MMD",52.8301845086536,0.322621747035399,0.426316050557231,0.756766597489597,0.449189707777672,0.837999367846103
"8265","LCK",8.02445590670053,0.564812448406261,0.746756494353725,0.756354250250042,0.449436829894775,0.83824183095317
"8266","MCEE",149.878504578182,-0.201054688834545,0.265913103144996,-0.756091694830528,0.449594221064843,0.838423398507907
"8267","COLCA1",35.6665419974132,0.296764726926909,0.39254053519169,0.756010399746334,0.449642960458311,0.838423398507907
"8268","TMEM63B",53.6156971974986,0.198521466496218,0.262658704637177,0.755815295634102,0.449759944772026,0.838540100225057
"8269","ANKS1B",15.0337693727009,-0.373907534452258,0.494874264390562,-0.755560677443443,0.449912639641244,0.838723345032021

"8270","LURAP1",28.2000601110879,-0.229907363858118,0.304562088377412,-0.754878471851094,0.450321904041892,0.838755338044119
"8271","NRP2",22.1197651875143,-0.520851157397456,0.690481439089179,-0.754330424992329,0.450650837507186,0.838755338044119
"8272","DHFR2",55.51622674479,0.183219148815769,0.242868006471867,0.75439804310739,0.450610246283824,0.838755338044119
"8273","KLKB1",2.49517171850203,0.970732533801184,1.2854856169564 5,0.755148498743622,0.450159886028869,0.838755338044119
"8274","TTC1",319.672365927008,-0.146851835903432,0.194457838331618,-0.75518599385538,0.450137391312946,0.838755338044119
"8275","GTF2IRD1",13.3724696510881,0.320085567337693,0.424196710651831,0.754568716117203,0.450507800311091,0.838755338044119
"8276","AC007032.1",32.1872570445463,-0.277482530654083,0.367985152498951,-0.754059039528433,0.45081377130749,0.838755338044119
"8277","UBQLN1",190.139408653628,0.125400643103674,0.166229061664556,0.754384593451701,0.450618319960877,0.838755338044119
"8278","STIM1",179.343925477408,0.162989163403944,0.215938727186495,0.754793572822994,0.450372850874068,0.838755338044119
"8279","LRP4",22.0381865301483,-0.37377805883492,0.495816777997405,-0.753863272527005,0.450931326167477,0.838755338044119
"8280","USP5",94.7109685234024,-0.171267406295597,0.227118029952526,-0.754089872703618,0.450795258075274,0.838755338044119
"8281","EFNB2",7.30219036212275,-0.602269954219574,0.798339734179971,-0.754403079834435,0.450607222814306,0.838755338044119
"8282","KIAA0586",101.9555188163,0.165314666741848,0.219291372483483,0.753858507380628,0.4509341877757,0.838755338044119
"8283","ZNHIT3",216.39198290783,-0.212716890153097,0.281919993369484,-0.754529281909815,0.450531469406329,0.838755338044119
"8284","RAD51C",92.2850704817881,0.183724676558405,0.243387391399715,0.754865219195656,0.45032985657763,0.838755338044119
"8285","RAC3",5.05084840777653,-0.520801647801714,0.690302141990172,-0.754454630982629,0.450576278116117,0.838755338044119
"8286","ROCK1",588.983221940153,-0.159012994857005,0.210859067273547,-0.754119786799195,0.450777297099469,0.838755338044119
"8287","SEPTIN5",17.2743168381638,0.400524032257856,0.53133322895335,0.753809493614451,0.450963622556578,0.838755338044119
"8288","NDP",3.2670418425242,-0.726084675805959,0.962632697866649,-0.754269699559429,0.450687292811069,0.838755338044119
"8289","SRD5A3",99.0831931743358,0.169195406168803,0.224516787354553,0.753598018938387,0.451090634258945,0.838789158878364
"8290","PFAS",22.7169633487815,0.359797278139749,0.477395770802522,0.753666664316935,0.451049403621595,0.838789158878364
"8291","AL691432.2",20.48099835033,-0.290065206447425,0.385035188868262,-0.753347264960425,0.451241263344232,0.838940799612863
"8292","TMED8",51.1374881387554,0.205838832053487,0.273256345398252,0.753281069295909,0.451281032136871,0.838940799612863
"8293","AC009163.7",4.50722555516042,0.589004650721867,0.782052575762436,0.753152241903477,0.451358434278291,0.838983511925703
"8294","ERO1A",126.162223556976,0.130516207170329,0.173329228717072,0.752995949594701,0.451452347987939,0.839056901884987

"8295","DNM3OS",82.8109648417624,-0.18717952201993,0.248706538522028,-0.752611986529466,0.451683112578221,0.839182256284594
"8296","INO80",135.770433514829,-0.162370078221101,0.215713196795006,-0.752712771557514,0.451622533588293,0.839182256284594
"8297","RRP1B",136.111212839084,-0.159153310931032,0.211443274505273,-0.752699802362657,0.451630328741753,0.839182256284594
"8298","ASTN2",11.1799829511271,0.345767782369547,0.459506538499431,0.752476305339832,0.45176467390729,0.839232640188103
"8299","PLRG1",191.219845102876,-0.165104190799267,0.219535154312086,-0.75206265400465,0.452013381378815,0.839504186666318
"8300","TTYH3",57.6405663912126,0.270442989136442,0.359650061716669,0.751961470117851,0.45207422987461,0.839504186666318
"8301","ZNF383",45.2265850673738,0.206552767737756,0.274675594685124,0.751988060586668,0.452058238836296,0.839504186666318
"8302","FALEC",6.74806863362902,0.639032236299594,0.850800395585143,0.751095368097585,0.452595263124625,0.839561489899651
"8303","MR1",149.09498784302,0.157946144293121,0.210278776824959,0.751127368524687,0.45257600614153,0.839561489899651
"8304","HSPB1",1570.06826301638,-0.163171156394856,0.217087165170768,-0.751638892453636,0.452268247850829,0.839561489899651
"8305","TRPV3",9.59899696686892,0.404793898105762,0.538690056262873,0.751441192202419,0.452387180151572,0.839561489899651
"8306","TRAF4",23.9035547563146,-0.316369339174684,0.421143109434311,-0.751215755612476,0.452522819610795,0.839561489899651
"8307","CFAP298",233.439798559569,-0.166755817309431,0.221904488091602,-0.751475640459306,0.452366455535636,0.839561489899651
"8308","CRYBB2",2.93109061756088,-1.13657670688955,1.51276817992452,-0.751322457712098,0.452458616809234,0.839561489899651
"8309","ACOT9",283.123948884027,-0.130174294527619,0.173207510152422,-0.751551098523762,0.452321060645835,0.839561489899651
"8310","RBMX",821.445419893724,0.116278723229004,0.154720263909199,0.751541655185162,0.452326741532093,0.839561489899651
"8311","P2RX7",25.6035134416796,0.30530773534623,0.406646374199672,0.750794190522691,0.452776526240676,0.839695639076037
"8312","AC130371.2",12.4204409084447,0.336755460069538,0.448530407099224,0.750797392416342,0.452774598971843,0.839695639076037
"8313","ZNF10",21.389886499618,0.258994599511613,0.345067877476789,0.750561313923041,0.452916710654942,0.83985457653626
"8314","CKS1B",530.811792915852,-0.20145474378641,0.268589582621319,-0.750046750958464,0.453226548320212,0.839937693874673
"8315","PRKG2",26.3127484052997,0.444692225099313,0.59301375632522,0.749885176112905,0.453323863272455,0.839937693874673
"8316","CDK5RAP2",112.889571462917,0.167398143191534,0.223216463904708,0.74993636339924,0.453293032393431,0.839937693874673
"8317","TMEM132C",37.7749664022585,-0.407183493467467,0.543017405619833,-0.749853483983046,0.453342952516204,0.839937693874673
"8318","ARGLU1",1135.26699912647,0.132470505396044,0.176629682481701,0.749990055662179,0.453260694000783,0.839937693874673
"8319","PPP2R5E",326.910793249023,0.126384009496048,0.16842691443938,0.750378939831114,0.453026511252221,0.839937693874673

"8320","RHBDF1",164.431221684003,0.177069797670783,0.236117605497389,0
.749922045405213,0.453301656219204,0.839937693874673
"8321","ARL5A",464.857729731143,-0.119912844726497,0.159961730361108,-
0.749634581070098,0.453474817546401,0.839980090420305
"8322","RNF168",151.685321059259,-0.180026935888705,0.240149884414379,
-0.749644066361773,0.453469103248467,0.839980090420305
"8323","FZD4-
DT",4.41270465517299,0.599283544433019,0.799559809940292,0.74951684287
1043,0.453545750866388,0.840010543025997
"8324","SMAD9",87.3747991236321,-0.294417197913616,0.393000896103269,-
0.749151467166763,0.453765917339896,0.840317349326585
"8325","BVES",9.21192737539132,-0.376327322455174,0.502524855985335,-0
.748873051696684,0.453933724163494,0.840527130087719
"8326","CNTNAP1",40.479374733155,0.209014749784068,0.279168385588223,0
.748704941441211,0.454035064671495,0.840613802775773
"8327","AL691447.2",8.74507829690744,0.468631555430713,0.6261395044788
32,0.748445916730296,0.454191235353678,0.840801956644282
"8328","MUTYH",40.571572406328,-0.224711929696186,0.300340889490965,-0
.748189599082032,0.454345803704954,0.840987099437064
"8329","WEE1",156.203048672168,-0.245632429219021,0.328393824742038,-0
.747981267345607,0.454471456742046,0.841118682396283
"8330","ZCCHC12",9.41555723518126,-0.637333809161971,0.852197097096351
,-0.747871368411753,0.454537748986843,0.841140384229553
"8331","ARHGEF7",143.115544417188,0.189547825727324,0.25352503159813,0
.74764935254118,0.454671688022211,0.841287248933187
"8332","TXNDC12",303.657706836827,0.167828676565458,0.224911711474204,
0.746198032398625,0.4555477965485,0.841356388794157
"8333","PYGO2",38.6397568519251,0.203520650211877,0.272278849246258,0.
747471391095113,0.454779065700543,0.841356388794157
"8334","FOSL2",292.595942795841,0.21994194651658,0.294612636050987,0.7
46546208827635,0.455337528675174,0.841356388794157
"8335","AC064836.3",16.0417812051322,-0.297670599793497,0.398420962894
659,-0.747125848074916,0.454987598884233,0.841356388794157
"8336","DCUN1D1",149.463558722628,0.130301937421307,0.174432504613058,
0.747004910067386,0.455060596982278,0.841356388794157
"8337","CSNK1A1",787.825512145557,0.126373634104447,0.169430228962893,
0.745874185958424,0.455743420296401,0.841356388794157
"8338","MAPK9",131.88323113735,-0.138188219602989,0.185137887831849,-0
.746407022470178,0.455421578410803,0.841356388794157
"8339","AC002467.1",16.0368322992529,-0.353172619725895,0.473266718481
554,-0.746244360598663,0.455519815247713,0.841356388794157
"8340","RBM33",219.5542507985,0.167377301887563,0.22413649259198,0.746
765062449061,0.455205388348552,0.841356388794157
"8341","VPS37A",132.619337897561,0.130782098465085,0.175078524308458,0
.746991094319882,0.455068936577701,0.841356388794157
"8342","AC022893.1",3.51280719792975,0.714022424875527,0.9567067570775
39,0.746333627930734,0.455465902302803,0.841356388794157
"8343","SPOUT1",47.5925286006117,-0.211940973086739,0.284051975909287,
-0.74613447911527,0.45558618302675,0.841356388794157
"8344","SELENOH",743.355668572945,-0.184690509873007,0.247369497452944

,-0.746617961287406,0.45529420330503,0.841356388794157
"8345","VDR",35.5211385257505,0.418355376739088,0.560001635928914,0.747060990357882,0.455026746137496,0.841356388794157
"8346","CFAP54",3.38209356965009,0.667759066844034,0.894187009317754,0.746777866246927,0.45519765828818,0.841356388794157
"8347","SELPLG",6.17073594497106,0.51818454632121,0.694133060978195,0.746520480656789,0.455353064352833,0.841356388794157
"8348","AC005520.3",3.04024971132922,-0.639091762790592,0.856936263993274,-0.745786810109378,0.455796208935506,0.841356388794157
"8349","HIRIP3",79.255820863798,-0.198210691951605,0.26580875417383,-0.745689104813992,0.455855242245138,0.841356388794157
"8350","MT1X",2256.87471160536,-0.317029480373582,0.42502638045896,-0.745905418932447,0.455724551543936,0.841356388794157
"8351","DOHH",63.4321258232272,-0.20370245504287,0.273146467313258,-0.745762729595379,0.455810757926,0.841356388794157
"8352","NDUFA1",956.57593004633,-0.184786899208731,0.247444317088078,-0.746781746225987,0.455195315835898,0.841356388794157
"8353","METTL6",57.3503639068178,0.204846942485306,0.274764257698939,0.74553708040788,0.455947103586356,0.841425188768548
"8354","PPARD",41.7713321916634,-0.199924327643066,0.268258172201441,-0.745268358471252,0.456109505334335,0.841443723386195
"8355","METTL27",67.8925044336911,0.363732437746478,0.48806104923927,0.745260123325595,0.456114482747464,0.841443723386195
"8356","NRBF2",277.126012925763,-0.205232222217592,0.275442204451015,-0.745100855646437,0.456210751902751,0.841443723386195
"8357","ITGA5",260.413309823776,-0.206741620920582,0.277426102900613,-0.745213297375433,0.456142785450393,0.841443723386195
"8358","GALK1",185.786675837802,-0.197440219774948,0.264995926476788,-0.745068886152266,0.456230077201545,0.841443723386195
"8359","RIC8B",50.3026916056885,0.19349170360201,0.259818858706697,0.744717702807087,0.456442394972712,0.84173459965359
"8360","CEP295NL",6.78040160234233,0.621568978067202,0.834856040859762,0.744522345944925,0.456560527499746,0.841851738206769
"8361","EIF2S3",622.707805026044,-0.148904713383721,0.200076877321398,-0.744237491994262,0.456732809816735,0.842068683569546
"8362","AC005632.3",9.4644346509533,0.378248805917834,0.508349424887643,0.74407245764355,0.456832640838261,0.842152016087274
"8363","PRPF38A",171.618537487229,-0.146784419297941,0.19731305473101,-0.74391640987996,0.456927047054919,0.842180894085894
"8364","GSK3A",127.872592925561,-0.145251716795047,0.195265982799502,-0.743865955106939,0.456957573670738,0.842180894085894
"8365","SEMA6C",52.4824259100553,0.203254738426313,0.273277196062536,0.743767651874621,0.457017053296954,0.842189823858046
"8366","ST6GAL1",6.62028033755701,-0.559047987678217,0.752321672223282,-0.743097013310946,0.457422947751246,0.842559165024472
"8367","AGPAT1",155.452030540665,0.148228447030215,0.199503959716669,0.742984987569802,0.457490769461877,0.842559165024472
"8368","KIF21A",58.3482977775317,-0.179212058480311,0.241202750323896,-0.74299342872193,0.457485658891729,0.842559165024472
"8369","VMP1",1769.32355204019,-0.225136564261434,0.302955707089062,-0

.743133596738115,0.457400800933954,0.842559165024472
"8370","RAVER1",43.5799606364662,-0.191134207792011,0.25720325925381,-0.74312513903021,0.457405920994284,0.842559165024472
"8371","ZNF852",7.38426454044002,-0.408637201079522,0.550089328482557,-0.742856077951492,0.457568819857328,0.842602240843473
"8372","PFN4",11.0426706612365,0.34938460994574,0.470513621167107,0.742560032755467,0.457748093460808,0.842605107561998
"8373","ATG2A",55.8078628044575,0.21121623223532,0.284408044799851,0.74265210178552,0.457692335768247,0.842605107561998
"8374","NEDD1",89.4999214917053,0.146574837388355,0.197406572947983,0.742502314889878,0.457783049775483,0.842605107561998
"8375","FBXW9",35.0301539699063,-0.252234563238596,0.339713301991696,-0.742492453959786,0.457789022110395,0.842605107561998
"8376","SUPT16H",375.22401892187,-0.142579710308123,0.192094364104516,-0.74223786300439,0.457943231876418,0.842788314156517
"8377","AC245014.3",9.60797156148756,-0.393396119562644,0.530446037135383,-0.741632686497457,0.458309913817598,0.842965032789359
"8378","COP1",103.432038812269,-0.145044151793871,0.195576610856528,-0.741623199004469,0.458315663686462,0.842965032789359
"8379","ANO10",78.8290888415555,-0.149256833795057,0.201247131087209,-0.741659436279752,0.458293702403923,0.842965032789359
"8380","NUDCD3",127.734944706924,0.174759493866656,0.235712580132734,0.741409278063337,0.458445320620499,0.842965032789359
"8381","LTBR",323.245984589385,-0.121596690146632,0.16398074716113,-0.741530284815385,0.458371976211743,0.842965032789359
"8382","RAB5B",208.106823130116,0.131032059757434,0.176729211845981,0.74142841689142,0.458433719788157,0.842965032789359
"8383","SRRM2",1640.63726098796,0.143303564314293,0.193209300199501,0.741701171560183,0.458268409834971,0.842965032789359
"8384","MIS12",45.0292497825019,-0.18068158180219,0.24371722473989,-0.741357456351413,0.458476732721764,0.842965032789359
"8385","CYBRD1",3077.20459636952,0.160664929729336,0.216792869891216,0.741098772344107,0.458633553929967,0.843086394050919
"8386","SLC39A11",50.4658659834542,0.221986280173263,0.299549079844881,0.741068142450034,0.458652124587156,0.843086394050919
"8387","AP001107.9",6.58155120457467,-0.459633468359578,0.620494888923744,-0.740753028855432,0.458843199886761,0.84314583654129
"8388","SLFN12L",7.46169394547515,0.432545587443587,0.583833581792399,0.740871373167076,0.458771434264739,0.84314583654129
"8389","ZNF813",21.5553031215928,0.268012116904203,0.361814666141,0.740744204105197,0.458848551589029,0.84314583654129
"8390","AC092040.1",66.0409421114945,-0.25506303554119,0.344456118151887,-0.740480491128105,0.459008494448805,0.843245082612194
"8391","AC005838.2",22.0651140579167,0.362215238898,0.489166216498022,0.740474764367675,0.45901196809594,0.843245082612194
"8392","FAM174A",116.604838452353,-0.184360564094402,0.249029108944318,-0.740317326259334,0.459107470165024,0.843320025332918
"8393","ZBTB17",55.4557356065295,-0.167953249573127,0.226919874570091,-0.740143409171722,0.459212981335206,0.843413333406673
"8394","AC233266.2",6.28934507898981,-0.522523631270025,0.706269619241

434,-0.73983591681494,0.459399562533859,0.843555003747401
"8395","AC084757.3",2.73637024392934,0.698914439125285,0.9446164079829
,0.739892334305014,0.459365326168173,0.843555003747401
"8396","SEPTIN8",193.606430597482,0.146984682371849,0.198718908560237,
0.73966128053332,0.459505547798591,0.843646810688363
"8397","TCERG1",161.968092284755,0.165445856409763,0.223704509768465,0
.739573183307749,0.459559018446331,0.843646810688363
"8398","HINT2",283.66162770016,-0.216588411186639,0.29301855903839,-0.
739162774867998,0.459808161937007,0.844003669475943
"8399","PRMT1",381.235929408212,-0.21373753202701,0.289246801696476,-0
.738945187201403,0.459940281869179,0.844145665557019
"8400","RPS18",25355.5375165398,-0.190857632559664,0.258391782603935,-
0.738636618534466,0.460127682183733,0.844389073912172
"8401","CASP1",63.2522040808019,0.254347425956065,0.34450421578047,0.7
38299893891991,0.460332230962537,0.84439741471138
"8402","IL18",38.1464270835563,-0.322234999778029,0.436391886787223,-0
.738407402920268,0.460266917353657,0.84439741471138
"8403","ZNF232",18.2354798677058,0.317121518080117,0.429547707682352,0
.73826844471168,0.460351337867949,0.84439741471138
"8404","UPF3B",104.253145254103,-0.154872707060732,0.209749479003288,-
0.738369924906009,0.460289685312017,0.84439741471138
"8405","NPHP4",25.4243753896116,0.237008084184542,0.321183225712252,0.
737921738157263,0.460562008350933,0.844599665241204
"8406","LINC01948",2.85026001840117,0.651297388394828,0.88312582108719
9,0.737491049229009,0.460823784401004,0.844599665241204
"8407","CCNB1",17.4839852437674,-0.263867532032737,0.357725260381639,-
0.737626221171047,0.460741616897483,0.844599665241204
"8408","AP002840.2",5.18852195151434,0.499365538727554,0.6767490826286
26,0.737888756033358,0.460582052199164,0.844599665241204
"8409","R3HDM2",294.977308844861,-0.135534682447585,0.183767185221763,
-0.737534736052179,0.460797227422918,0.844599665241204
"8410","ANKRD10",251.094960515183,-0.184830129051057,0.250524284767502
,-0.737773302985727,0.46065221899928,0.844599665241204
"8411","MTHFD1",60.37786495687,-0.179491221606694,0.243422222274559,-0
.737365799759416,0.460899927603569,0.844599665241204
"8412","RIBC1",5.28524809234479,-0.494097779687127,0.670010641146948,-
0.737447660295832,0.460850161140323,0.844599665241204
"8413","CHEK1",13.022370380689,-0.340253874678398,0.461721991057018,-0
.736923692760348,0.461168754963059,0.844991840931363
"8414","STX10",283.649337722139,-0.193846671348485,0.263286045288936,-
0.736258813625134,0.461573205955947,0.845632394795688
"8415","FUNDC2",499.107405595059,-0.151094990463,0.205269629518965,-0.
736080592229255,0.461681653035233,0.845730562274286
"8416","PSMB9",386.831869315025,0.223322765277832,0.303504939689494,0.
735812621390302,0.461844739106604,0.845928784853648
"8417","CENPL",53.0744503366549,-0.197635851957365,0.268631094110848,-
0.735714726590113,0.461904325544127,0.845937409797162
"8418","UQCRHL",2.99563565047547,0.61653640844064,0.839922438761931,0.
734039692223762,0.462924547308199,0.846148252608864
"8419","BX470102.2",6.1796481287249,0.432694387250822,0.58980412492980

```
1,0.733623874370701,0.46317800614037,0.846148252608864
"8420","ZBTB41",128.967717951099,0.131672912636812,0.179169195983742,0
.734908207372656,0.46239539957084,0.846148252608864
"8421","WDR43",155.19076868983,-0.166782412535932,0.22746423072628,-0
.733224788821007,0.463421338640993,0.846148252608864
"8422","AC010913.1",6.6741221135147,-0.421266333075898,0.5734615983327
58,-0.734602516194036,0.462581605144269,0.846148252608864
"8423","LONRF2",14.6463888446411,0.433930137142695,0.59175224500322,0.
7332969850251,0.463377313522198,0.846148252608864
"8424","PMS1",76.1297546372342,0.162865517247791,0.221829665994395,0.7
34191779614843,0.462831862832811,0.846148252608864
"8425","MATR3.1",42.32034541988,0.266027156411581,0.362339661352274,0.
734192761064993,0.462831264755083,0.846148252608864
"8426","AC008429.1",2.55303572760019,0.732534944724219,0.9991531575420
34,0.733155812194289,0.463463402644143,0.846148252608864
"8427","HOXA10",319.313965845368,-0.176369278108841,0.240521133305138,
-0.733279756690193,0.463387819118923,0.846148252608864
"8428","PAG1",8.73210547915426,0.454127349727903,0.618318103871675,0.7
34455851905886,0.462670957562575,0.846148252608864
"8429","FNBP4",308.967418958138,0.192850426500556,0.262622238621089,0.
734326336996923,0.462749870051936,0.846148252608864
"8430","MAP3K11",36.8277514790457,-0.212935577430361,0.290164243265251
,-0.733844994249371,0.463043214453262,0.846148252608864
"8431","SIK2",154.897216370325,0.184983339764345,0.251717351395118,0.7
34885135009938,0.46240945217279,0.846148252608864
"8432","VPS29",587.844478604481,-0.195114213439945,0.265551768447342,-
0.734750194211702,0.462491644856945,0.846148252608864
"8433","AC079949.2",2.8289051158316,-0.596755656327107,0.8116661669763
74,-0.73522302715924,0.462203677210877,0.846148252608864
"8434","RCBTB2",60.7004286155312,-0.188629131762311,0.257094098160199,
-0.733696856956916,0.463133514525174,0.846148252608864
"8435","AL122035.2",4.7055035069572,-0.508758465942238,0.6922221532632
48,-0.734964149216935,0.462361328259847,0.846148252608864
"8436","IGHG4",127.561788507293,0.661713092307563,0.902631458746193,0.
733093319422659,0.46350151443589,0.846148252608864
"8437","AC103858.3",3.88767942389676,0.571385721352661,0.7789756494128
88,0.733509092079211,0.463247984494753,0.846148252608864
"8438","CSNK1D",207.118373434301,0.145553298247337,0.198477532579187,0
.733348990970883,0.463345601831679,0.846148252608864
"8439","PEPD",397.237239248973,-0.197757110199669,0.269631930386111,-0
.733433573377166,0.463294028560274,0.846148252608864
"8440","LMTK3",5.54412903541223,-0.547117219230786,0.745149567319228,-
0.734238122420322,0.462803622847046,0.846148252608864
"8441","ZNF584",29.769903503324,-0.222334927167972,0.302819235269167,-
0.734216658893367,0.46281670198961,0.846148252608864
"8442","KIZ",149.753280111198,-0.15590686033373,0.212145775174723,-0.7
34904384522038,0.462397727924409,0.846148252608864
"8443","NANP",30.438754409477,-0.216499051811021,0.295264445590641,-0.
733237797656065,0.463413405704022,0.846148252608864
"8444","SPATC1L",145.498773695953,0.223829174354363,0.305276612503734,
```

0.733201186028048,0.463435732088311,0.846148252608864
"8445","DDHD1",146.188543624208,0.173709693257987,0.237139402283877,0.732521426574405,0.463850369645412,0.846684836954888
"8446","SPARC",6208.55835471621,0.25967309069976,0.354578493385595,0.732343036996811,0.463959217377966,0.846738234717956
"8447","UBAP2",95.2423077861177,-0.159086413508015,0.217276308122549,-0.73218481519065,0.464055771268459,0.846738234717956
"8448","PIP5KL1",4.51267939222921,-0.516979132879153,0.706146228493084,-0.732113423564388,0.464099341234642,0.846738234717956
"8449","CETN2",247.17007150866,0.158257879171254,0.216150460248314,0.732165358285367,0.464067645498427,0.846738234717956
"8450","LINC01091",44.4208562756437,0.251360436909819,0.343432895906994,0.731905533527838,0.464226228494894,0.846869504408141
"8451","ZBTB8OS",174.213875588523,-0.178557905486949,0.244136989685869,-0.731384071363783,0.464544591833024,0.846949128693799
"8452","ATP5PB",548.137004347504,-0.14332320330781,0.195906584143804,-0.731589517188482,0.464419148446444,0.846949128693799
"8453","RN7SL832P",43.0821243079997,-0.302304267255568,0.413331062359691,-0.731385310191121,0.464543835359617,0.846949128693799
"8454","KCNK5",2.97986067444404,-0.672871170633436,0.919895301626617,-0.731464949808552,0.464495205926743,0.846949128693799
"8455","DNAJB1",1738.74344640304,0.164829496240538,0.225265789050311,0.731711179648876,0.464344871322018,0.846949128693799
"8456","TSSK3",4.64171343932863,0.58151451119038,0.795563941873347,0.73094628927131,0.464811960548874,0.847040697050457
"8457","GNA14",7.83596665593384,0.385713427986107,0.527646198726452,0.731007688328051,0.464774456854653,0.847040697050457
"8458","GNB3",4.59521470481382,0.559532293997347,0.7654948468956,0.730941947246913,0.464814612802453,0.847040697050457
"8459","ATG4A",32.7952937037924,-0.203530077085321,0.278391830761175,-0.731092132009879,0.464722879821134,0.847040697050457
"8460","PAQR8",26.4275985110869,0.247282769150293,0.338477769047848,0.73057314767203,0.465039918624206,0.847351104679922
"8461","EMC1",108.267228773687,0.146134218309579,0.200358267867319,0.729364552134937,0.465778695249667,0.847473399993347
"8462","AL596325.2",30.9220048032711,0.435731985027053,0.597630676779688,0.72909909406756,0.465941048742083,0.847473399993347
"8463","CD247",11.4842768255186,0.468740923986166,0.6432368615937,0.728722111517057,0.466171664332219,0.847473399993347
"8464","SFT2D2",460.288767924315,0.198814281060196,0.272336368634398,0.73003206313255,0.465370586086844,0.847473399993347
"8465","TOR3A",134.138467694491,-0.178795332902936,0.24521707825617,-0.72913083450148,0.465921634717571,0.847473399993347
"8466","RBMS1",1098.30811229278,0.130563063096508,0.179203278224988,0.728575193432494,0.466261557271721,0.847473399993347
"8467","CDYL",162.931107744551,-0.173149274373558,0.23749905677429,-0.729052471724604,0.46596956609532,0.847473399993347
"8468","FRAT2",43.9481433281094,-0.235597856273144,0.323132952367811,-0.729105015588046,0.465937426812205,0.847473399993347
"8469","AL139243.1",6.08469552359114,-0.547158663408446,0.750568765582

637,-0.728992050426862,0.466006525257311,0.847473399993347
"8470","CCDC90B",339.944029055009,0.156169668294417,0.214256165993063,
0.728892293813721,0.466067549039049,0.847473399993347
"8471","PTS",110.297545727807,-0.206682842703634,0.283584908876593,-0.
728821725818902,0.466110720043965,0.847473399993347
"8472","ETNK1",178.938617348209,0.135292149276703,0.185467001769427,0.
729467495489567,0.465715743808054,0.847473399993347
"8473","TMEM263",375.777124059818,0.140588182654124,0.192930141388812,
0.72869994103615,0.466185228891859,0.847473399993347
"8474","GMPR2",171.04655450082,-0.1657236940676,0.227436334906862,-0.7
28659711015242,0.466209843374835,0.847473399993347
"8475","CSK",84.6489612501773,0.152995175234568,0.209542374719273,0.73
0139550243897,0.465304888187567,0.847473399993347
"8476","AC009090.6",2.48380261454338,0.839048615941622,1.1504796164415
2,0.729303330498658,0.465816135461492,0.847473399993347
"8477","CNTROB",26.8007058362685,0.226765046176686,0.311213722658985,0
.728647323900834,0.466217422497464,0.847473399993347
"8478","AC005921.2",57.1008146862944,-0.335126250006816,0.459696894465
516,-0.729015692821818,0.465992063224019,0.847473399993347
"8479","CDK3",15.1440555426256,-0.301972444428223,0.413877482744225,-0
.729617959464687,0.465623741281532,0.847473399993347
"8480","ZNF77",23.2190609254151,-0.266910252538452,0.36624789763759,-0
.728769377954397,0.466142746051652,0.847473399993347
"8481","DGCR2",95.1567200612474,0.150077053170988,0.205530208125989,0.
730194624621757,0.465271227815045,0.847473399993347
"8482","TTLL4",18.6666253893043,-0.322151934689324,0.442488234023183,-
0.728046329639685,0.466585226235706,0.847570440271152
"8483","NDUFA10",486.581130030307,-0.159252792792479,0.218924962242569
,-0.727430947851558,0.466962001820975,0.847570440271152
"8484","ATG4B",109.639464752481,-0.132640176316722,0.182423189882056,-
0.727101507228765,0.467163775505586,0.847570440271152
"8485","NEK4",39.5110280367902,-0.226460696590631,0.311516855045453,-0
.726961295746223,0.467249666002222,0.847570440271152
"8486","C4orf3",1234.71033508708,-0.177500533793173,0.243958032144466,
-0.727586348491538,0.466866839812976,0.847570440271152
"8487","WDR70",139.101986389919,0.195532878289393,0.268887481057809,0.
727192197718403,0.467108225152561,0.847570440271152
"8488","DSE",662.158474184123,0.167472346358525,0.230320702024906,0.72
7126762319505,0.46714830571831,0.847570440271152
"8489","AL021408.1",2.85546685259751,0.690100744223993,0.9490144683767
08,0.727176209867919,0.467118017873059,0.847570440271152
"8490","ZSCAN21",41.9608239395929,0.215879355441625,0.296854836698592,
0.727221957514593,0.467089997278907,0.847570440271152
"8491","GTF2E2",115.534037726083,-0.156431365020564,0.215178538476933,
-0.726984048352636,0.467235727656585,0.847570440271152
"8492","CH25H",51.091208075481,-0.676880151449018,0.930240099976099,-0
.727640263482954,0.466833826640132,0.847570440271152
"8493","CYB561A3",152.302477996578,-0.148586523611518,0.20399667749491
3,-0.728377174746991,0.466382731724851,0.847570440271152
"8494","STT3A",195.899452633983,0.144876819388767,0.199020246870422,0.

727950154152371,0.466644099816265,0.847570440271152
"8495","AC015961.2",3.84068697320899,0.576425117245098,0.792466234292009,0.727381296895354,0.466992408626918,0.847570440271152
"8496","TEAD2",86.6569704829802,-0.174206465563814,0.239551069593503,-0.72722057079280,0.467090846637169,0.847570440271152
"8497","CLDND2",11.6487125165516,0.381673024082738,0.524127783145463,0.728206052715223,0.466487461267992,0.847570440271152
"8498","PKNOX1",82.9819707074029,-0.161723084200324,0.222093036982253,-0.728177192755697,0.466505125331732,0.847570440271152
"8499","SMPDL3A",108.062767821127,0.220815273161571,0.303847179292698,0.726731357768698,0.467390539944567,0.847726223466937
"8500","PLEKHA6",33.8382897182438,-0.491596333812057,0.676598368678958,-0.72657037996099,0.467489178717546,0.847741517896937
"8501","ZBED8",20.4458997578396,0.276900419347018,0.381123055527065,0.726538096636753,0.467508961637487,0.847741517896937
"8502","RRP9",45.8194641270725,-0.226755374163043,0.312196327487322,-0.726323003182192,0.467640780744405,0.847880808653846
"8503","AC034243.1",2.50277984688912,-0.977886646575909,1.34670006990918,-0.726135439082482,0.467755745413736,0.847989511413941
"8504","MMACHC",13.808903821479,0.301168092252744,0.415131959567141,0.725475563401026,0.468160330992862,0.848359984375218
"8505","IDUA",92.9874590282847,-0.240046739842718,0.330789402643548,-0.72567844654137,0.468035917852901,0.848359984375218
"8506","MIR4458HG",112.302679075733,0.226784676364629,0.312560554909758,0.72557036645301,0.468102193052819,0.848359984375218
"8507","SNX9",1275.75449094666,-0.146003624413134,0.201286202130172,-0.725353366837897,0.468235273893244,0.848359984375218
"8508","AC023794.3",6.24060383646935,-0.579872347134101,0.799368927146115,-0.725412669221889,0.468198903033655,0.848359984375218
"8509","SOD2",2670.4786374745,-0.256840809412011,0.354192619851478,-0.725144441235706,0.468363422922575,0.848492439105828
"8510","RASAL2",48.7534319133362,-0.223419022282059,0.308623133805799,-0.723921825065278,0.469113730128842,0.848570982054957
"8511","HACL1",69.9645410652357,-0.192106127184732,0.265076665060169,-0.724719119056092,0.468624363459178,0.848570982054957
"8512","B3GALNT1",53.0997995497178,-0.202586455310671,0.279785442928504,-0.724077897656882,0.469017912951121,0.848570982054957
"8513","PDCD10",283.369901262811,-0.151869821800718,0.209707149253345,-0.724199543703899,0.468943238666986,0.848570982054957
"8514","KCNMB3",8.49180821684295,0.439005827472349,0.606604369987957,0.723710294868242,0.469243611508017,0.848570982054957
"8515","TRMT44",31.7781362172822,-0.20411787784623,0.282026735892547,-0.723753644136773,0.469216993060396,0.848570982054957
"8516","THAP9-AS1",55.3286237822694,-0.227727613505763,0.314578975282801,-0.723912376219802,0.469119531377762,0.848570982054957
"8517","GFOD1",6.01873046896378,-0.418278124712876,0.577213265009857,-0.724650922056917,0.46866621066471,0.848570982054957
"8518","IKBKB",65.1505397523429,0.237178304377686,0.327757887886861,0.723638738053979,0.469287552514986,0.848570982054957

"8519","AC048341.2",8.26327003002281,-0.369838297918296,0.510695845308
327,-0.724185053228719,0.468952133521404,0.848570982054957
"8520","TMTC3",145.738915860848,0.16135871501882,0.222653082748293,0.7
24709099138027,0.468630511775625,0.848570982054957
"8521","NME4",334.194769339557,-0.213082611645118,0.2941779654018,-0.7
24332331804936,0.468861732172411,0.848570982054957
"8522","MYO1D",104.566119962271,-0.253631316324765,0.350072043174572,-
0.724511771990559,0.468751602675641,0.848570982054957
"8523","AC006449.2",5.77006897737831,0.514055294371462,0.7102008998333
34,0.723816731986819,0.469178255706623,0.848570982054957
"8524","MED24",84.5228579809984,-0.17877380651719,0.24673546289062,-0.
72455659361963,0.468724096114285,0.848570982054957
"8525","SOX9-
AS1",2.37135422843151,0.727041621565739,1.00394225601527,0.72418669222
2145,0.468951127434607,0.848570982054957
"8526","EIF4E3",83.9037722352375,-0.17230689159928,0.238153583842496,-
0.7235116466407 5,0.46936560135106,0.84861256683399
"8527","PAFAH2",30.3282817675671,0.227650142225096,0.315196164919411,0
.722249086638796,0.470141349013263,0.848929155460513
"8528","CERT1",327.881462251788,0.153951421975342,0.213158048670504,0.
722240717324813,0.470146493685787,0.848929155460513
"8529","C6orf136",34.3856129249944,-0.227674838265273,0.31520339302014
8,-0.722310873889354,0.470103368944956,0.848929155460513
"8530","CYP51A1",161.508886914871,-0.12976157350919,0.179494708503101,
-0.72292701323253,0.469724726358909,0.848929155460513
"8531","BRCA2",18.7426501733759,0.327076824762128,0.452863729378384,0.
722241158970899,0.470146222202246,0.848929155460513
"8532","GPR180",55.7529206370148,0.229635722234933,0.317635274587504,0
.722954094230083,0.469708087856866,0.848929155460513
"8533","TRDV3",2.50670434555578,0.702202276046399,0.971530721248055,0.
722779280869607,0.469815498515118,0.848929155460513
"8534","C14orf93",34.7454224688425,-0.220412093244136,0.30512655180086
4,-0.722362875152159,0.47007140554905,0.848929155460513
"8535","FUT8",167.872811233257,0.15529571513356,0.214985281072207,0.72
2355104307821,0.470076181944715,0.848929155460513
"8536","ACTL10",8.23998168568657,-0.421083244931257,0.58250823410151,-
0.722879472391936,0.469753936128889,0.848929155460513
"8537","DIDO1",164.497297034747,0.157780789166849,0.218325351524341,0.
72268652295864,0.469872497315103,0.848929155460513
"8538","AL135999.1",10.1107737292329,0.396711036187927,0.5493553296359
88,0.722139232636179,0.470208879468194,0.848942360857602
"8539","AL031848.2",3.36878427611846,0.750312502266417,1.0436513840287
8,0.718930203848346,0.47218392535913,0.84898466369217
"8540","KTI12",44.5743567155914,-0.215200731427297,0.298248884554243,-
0.721547481221705,0.470572738443872,0.84898466369217
"8541","AL357078.2",2.77711894477524,0.683804260284236,0.9553274079831
69,0.715780008581396,0.474127198452339,0.84898466369217
"8542","PRMT6",33.566764337273,0.230732343844943,0.321356815709556,0.7
17994243674234,0.472760836107739,0.84898466369217
"8543","SLC16A1-

AS1",101.023896909464,0.222917932403845,0.309380557858515,0.7205298676
389,0.471198816956022,0.84898466369217
"8544","NMNAT2",3.07199051147635,0.615802548449759,0.85481326072739,0.
720394239001103,0.471282296064966,0.84898466369217
"8545","LDAH",29.9862118456112,0.211589279694304,0.294681850562237,0.7
18026167171828,0.472741152589261,0.84898466369217
"8546","CAD",45.6449980921597,-0.215357654185574,0.2996135175484,-0.71
8784839708659,0.472273500020355,0.84898466369217
"8547","ZNF513",23.8110930137562,-0.278868265659237,0.388542544917985,
-0.717729034585135,0.472924377236345,0.84898466369217
"8548","SNRNP200",266.03217605721,0.127666171153409,0.177327505834316,
0.719945676519538,0.471558443316468,0.84898466369217
"8549","TMEM87B",87.0722137999006,0.170726678873165,0.238634677497555,
0.715431137936415,0.474342677609291,0.84898466369217
"8550","CCDC173",3.94958688649835,-0.559382582238231,0.777047471046165
,-0.7198821218543,0.471597576509816,0.84898466369217
"8551","STK36",26.0660801702094,0.291717021070633,0.40417362284649,0.7
2176165039209,0.47044103112114,0.84898466369217
"8552","MFF",353.448625867729,-0.14416860124971,0.200842194391319,-0.7
17820285157876,0.472868104076604,0.84898466369217
"8553","CAPN10-
DT",5.63726271751534,-0.468098348380911,0.652971010053432,-0.716874625
632472,0.473451460020193,0.84898466369217
"8554","SACM1L",94.351537186323,0.176537844504324,0.246024251257238,0.
717562775223078,0.473026916913252,0.84898466369217
"8555","DALRD3",202.45817600567,-0.159837725504264,0.223567906944088,-
0.71494038517898,0.474645880976005,0.84898466369217
"8556","STAG1",319.59243907555,0.146842513473654,0.203788148208809,0.7
20564540991823,0.47117747689778,0.84898466369217
"8557","ZBTB38",533.819014634321,0.135660257274426,0.18861553912673,0.
719242210384782,0.47199169579651,0.84898466369217
"8558","RARRES1",296.466226344594,-0.447191416621498,0.625769211371257
,-0.714626748160972,0.474839712082612,0.84898466369217
"8559","PRKCI",115.762686008526,0.167760761363179,0.234416773112011,0.
715651696489393,0.474206443868536,0.84898466369217
"8560","LSM6",341.256683432971,-0.167306333787519,0.233253296158336,-0
.717273181314226,0.47320555180202,0.84898466369217
"8561","AC025171.4",3.72590821829193,0.623000984880065,0.8693774068617
89,0.716605906667077,0.473617298854072,0.84898466369217
"8562","PCDHGB2",3.86630210450111,0.608059048907309,0.845152546628462,
0.719466623313173,0.471853459979878,0.84898466369217
"8563","ADRB2",3.49386101250724,-0.607465822297404,0.846429242030755,-
0.717680571668307,0.472954265248212,0.84898466369217
"8564","TNIP1",146.927684965794,-0.141148734869195,0.197596295230283,-
0.714328852697857,0.475023854968859,0.84898466369217
"8565","RPL26L1",190.922010276591,-0.181191723784295,0.252280890013749
,-0.718214224527351,0.472625208586739,0.84898466369217
"8566","FAM153CP",23.6633084076402,0.35283406056909,0.493470740932161,
0.715005027253673,0.474605936868212,0.84898466369217
"8567","HMGN4",231.262389085142,-0.150351619314389,0.210345578825905,-

0.714783834077299,0.474742625836979,0.84898466369217
"8568","PSORS1C1",44.8273049029255,0.207288092427829,0.29017846710042,
0.714346913811817,0.475012689447626,0.84898466369217
"8569","LHFPL5",3.72587585156886,0.955034282858323,1.33506319855986,0.
715347620913022,0.474394269688095,0.84898466369217
"8570","HDAC2",245.638049871483,0.121063305763935,0.168915067461294,0.
716711111586754,0.473552368254411,0.84898466369217
"8571","ENPP1",115.199788550662,0.335785837247672,0.466446242579267,0.
719881106536321,0.471598201696939,0.84898466369217
"8572","AL358852.1",7.85775056840222,-0.400842832256291,0.556637810466
667,-0.720114273085829,0.471454640225974,0.84898466369217
"8573","TBP",41.2017724802907,0.19716649006316,0.273596466488902,0.720
647063148963,0.471126689963875,0.84898466369217
"8574","AC073332.1",54.9903536791602,-0.255292674732156,0.356634850440
59,-0.715837710242745,0.474091564337833,0.84898466369217
"8575","HOXA1",3.79362415475692,0.61743813690523,0.856043106099128,0.7
21269913286039,0.470743464303162,0.84898466369217
"8576","PTCD1",26.9810273309319,0.229571837786712,0.31859319945986,0.7
20579843436476,0.471168059026332,0.84898466369217
"8577","PCOLCE",3480.17281289322,0.263435000394649,0.367663823363327,0
.716510528517027,0.473676168777638,0.84898466369217
"8578","SH2B2",3.8210105296225,0.670254301117732,0.936523984971083,0.7
156830063871,0.47418710623664,0.84898466369217
"8579","CHMP7",70.7537886325929,0.149692215823681,0.207670324218742,0.
720816594218864,0.471022364270201,0.84898466369217
"8580","EXT1",269.737415930578,0.157888714244686,0.221005719388621,0.7
14410082605379,0.474973639140767,0.84898466369217
"8581","GAS1RR",59.0036646264301,0.2203636372579,0.306650128850896,0.7
186158312852,0.472377656264292,0.84898466369217
"8582","GSN-
AS1",4.26871124681455,0.566943175924561,0.790977878541714,0.7167623663
12704,0.473520736525439,0.84898466369217
"8583","RSU1",288.672278937708,0.137594717302162,0.191154826727656,0.7
19807705918918,0.471643399704697,0.84898466369217
"8584","MALRD1",4.12302664137119,0.555644136448729,0.769674611056841,0
.721920833124238,0.470343151921771,0.84898466369217
"8585","AL137026.3",27.0097895200212,0.406937329209013,0.5647014687446
4,0.720623819367169,0.471140994669878,0.84898466369217
"8586","FAM13C",53.1262324138918,-0.328880993131716,0.458801554862275,
-0.716826239245072,0.473481319127573,0.84898466369217
"8587","ACCS",67.654414647905,0.206813348688853,0.289340693274001,0.71
4774497664607,0.474748395860274,0.84898466369217
"8588","DLG2",27.9418415636196,0.265674669494092,0.371608398275816,0.7
14931822657309,0.474651172131042,0.84898466369217
"8589","HIKESHI",357.979830515725,-0.193747809746936,0.270220313287349
,-0.716999426837712,0.473374450318455,0.84898466369217
"8590","IGSF9B",4.36942292888878,0.696097982074363,0.971171339645821,0
.716761248667228,0.473521426265222,0.84898466369217
"8591","HIST4H4",7.45073947179633,0.430475294099795,0.596658172271744,
0.721477244601852,0.470615936194612,0.84898466369217

"8592","AC079630.1",14.5371010797494,-0.317452126690899,0.443765367588
664,-0.715360300457589,0.474386436786357,0.84898466369217
"8593","RPAP3",108.576675251747,0.142034682290408,0.198409081045526,0.
715867850110235,0.474072951810945,0.84898466369217
"8594","FKBP11",245.86604476236,0.200901119769168,0.279064732256407,0.
719908668303445,0.471581230563189,0.84898466369217
"8595","CDK2",38.7532489101053,-0.217967487629016,0.304451080003123,-0
.715935997424547,0.474030869704979,0.84898466369217
"8596","HSPB8",298.131505741424,-0.170056243817295,0.237577326011819,-
0.715793239498094,0.474119027465686,0.84898466369217
"8597","NRAV",13.3906562233079,-0.355503448798516,0.497069025579736,-0
.715199359654103,0.474485864675217,0.84898466369217
"8598","MICU2",174.151202783267,-0.154839214871591,0.215470448027474,-
0.71860998243178,0.472381261012913,0.84898466369217
"8599","RPL21",14624.3934014997,-0.167879562447402,0.233282333002564,-
0.719641133070961,0.471745979374783,0.84898466369217
"8600","NOVA1",5200.3182906315,-0.162830383869273,0.227032420180074,-0
.717212034035145,0.473243275011213,0.84898466369217
"8601","ARHGAP5-
AS1",10.0125693005986,-0.404035247096294,0.56115873418093,-0.720001708
047947,0.471523943909722,0.84898466369217
"8602","AC090825.1",13.195005436251,-0.375713466693566,0.5227587096149
3,-0.718712973659149,0.472317787969629,0.84898466369217
"8603","ANKRD11",689.692966901167,0.163431212270988,0.226496374905551,
0.721562154533994,0.470563714167479,0.84898466369217
"8604","TCF25",875.98520007993,-0.102411365103532,0.143033678591003,-0
.715994765095653,0.473994581334179,0.84898466369217
"8605","NT5M",7.88054203680196,-0.482917394665991,0.671913580622485,-0
.718719502913751,0.472313764176916,0.84898466369217
"8606","AC073508.3",21.0637088774366,0.256024971076583,0.3560739668241
29,0.719021874472048,0.472127441927454,0.84898466369217
"8607","ZNF521",292.101349027472,-0.121525978395204,0.169989109208662,
-0.714904495711135,0.474668058857808,0.84898466369217
"8608","MCOLN1",145.04800429436,-0.143184834034631,0.198941434176464,-
0.719733597112927,0.471689036217541,0.84898466369217
"8609","NUDT19",37.7959479815579,0.21029217696316,0.294346614472404,0.
714437220010477,0.47495686361359,0.84898466369217
"8610","MYPOP",45.6340053454455,-0.212301975222217,0.296717180528009,-
0.715502805885475,0.474298407673937,0.84898466369217
"8611","BBC3",44.5881196155573,0.234340294350198,0.324574540503614,0.7
21992223994503,0.470299258343594,0.84898466369217
"8612","MAMSTR",13.1651688026658,-0.341509173449088,0.476897308979434,
-0.716106312656537,0.473925706179383,0.84898466369217
"8613","PTOV1",304.429096662887,-0.13154354888423,0.183327338057724,-0
.717533731072945,0.473044831011066,0.84898466369217
"8614","ZNF83",213.562138477168,0.165049568885666,0.229326512463583,0.
719714293443835,0.471700923897365,0.84898466369217
"8615","EPN1",310.705711523772,-0.130370953418062,0.182421637564253,-0
.71466825514129,0.474814057820166,0.84898466369217
"8616","DTD1",289.048168503954,-0.202110062882919,0.282303574143212,-0

.715931647327956,0.474033555901009,0.84898466369217
"8617","EDEM2",91.5917690863717,0.218015554590397,0.303135366346236,0.719201976391576,0.472016481838654,0.84898466369217
"8618","AURKA",5.02807149278301,-0.535183572993152,0.744661614331224,-0.718693649160092,0.472329697215884,0.84898466369217
"8619","AP000692.2",52.8773683991296,-0.318690157018855,0.445036638245488,-0.716098697570741,0.473930407946882,0.84898466369217
"8620","IL2RB",8.40496571879842,0.495183792599678,0.69053993895149,0.717096527901863,0.473314538003206,0.84898466369217
"8621","AL592156.2",2.38981488142034,-0.738852341498729,1.02571175510076,-0.72033135802967,0.471321001923176,0.84898466369217
"8622","PRICKLE3",17.6901228108148,-0.262056435239437,0.363274640275124,-0.721372774716589,0.470680192539719,0.84898466369217
"8623","ZDHHC15",25.7235679469686,-0.243098103585573,0.339191943006894,-0.716697753580286,0.473560612306373,0.84898466369217
"8624","HMGN5",36.6942347685689,-0.218801278789626,0.30368512905124,-0.720487300360063,0.471225016138136,0.84898466369217
"8625","WDR44",57.7692093502204,0.180824097604894,0.252004484415163,0.717543173981763,0.473039006692535,0.84898466369217
"8626","LSM14A",355.13420060822,0.0983100270256469,0.137646545931232,0.714220806345279,0.475090653073511,0.849005612929303
"8627","MTERF1",32.1366053008728,0.219721542826268,0.307815184542603,0.713809954348946,0.475344703481917,0.849361145725484
"8628","NPIPA1",74.2123277552236,0.265708621686369,0.372387205688771,0.713527794798728,0.475519220054039,0.849574498972301
"8629","PABPC4L",9.07789823198288,0.433873154330384,0.608234602476097,0.713331915948394,0.475640392405565,0.849594049702408
"8630","DUSP1",8435.50030591292,-0.207387567756382,0.290723744026585,-0.713349260311598,0.475629662349129,0.849594049702408
"8631","SCARB1",22.8704769120769,0.266693652188868,0.373982718853647,0.713117582027191,0.475773000623801,0.849634013509718
"8632","OFD1",143.939269493444,0.145382204408641,0.203860696649088,0.71314484252397,0.475756133452027,0.849634013509718
"8633","ZSCAN25",27.3866447724553,0.239246420906243,0.335658605185828,0.712767130679673,0.475989868245935,0.849824394140732
"8634","CNKSR2",72.3180473449627,-0.406209143148961,0.569883383271507,-0.712793450507456,0.475973579029194,0.849824394140732
"8635","TRAM2",459.135647155398,0.219324255886347,0.307814113750186,0.71252176586141,0.4761417380723,0.849898667483153
"8636","GCSH",323.051454331762,-0.203789983598744,0.285994547260463,-0.712565975648294,0.476114372234204,0.849898667483153
"8637","MFAP3L",2.59983130532575,-0.706327458167207,0.991894946722011,-0.712099058979441,0.47640343696289,0.849972104257286
"8638","TEP1",26.4119061493646,0.256967696078584,0.360836209438861,0.712144982562022,0.476375001742599,0.849972104257286
"8639","AC026401.3",13.2383280973659,0.28889604507003,0.405642988467217,0.712192872263532,0.476345350120321,0.849972104257286
"8640","NPIPA9",2.90052195215411,-0.64069288912941,0.899397887896984,-0.712357564711998,0.476243386056139,0.849972104257286
"8641","RRAGB",81.0333620362259,0.163536298378302,0.229724940341582,0.

711878728252741,0.476539875505336,0.850117137011314
"8642","ATE1",119.899483215201,0.151582827988587,0.212979366103202,0.7117254162318,0.47663482572485,0.850188132208813
"8643","VANGL2",19.4675543962612,0.278896429978251,0.392253742387008,0.711010246278504,0.477077886412244,0.850191401045635
"8644","FRMD4B",38.6074804637715,-0.217039117983842,0.305182422857554,-0.711178304279818,0.476973751188973,0.850191401045635
"8645","ARF5",456.3661036825,-0.304100874899121,0.427680838549837,-0.71104629314293,0.477055549335577,0.850191401045635
"8646","FNTA",404.339153874099,-0.129878716468893,0.182581626743027,-0.711346036212556,0.476869830419592,0.850191401045635
"8647","JMJD1C-AS1",6.2465568654151,-0.423578886883464,0.595632883330439,-0.71114087005246,0.47699694580361,0.850191401045635
"8648","LGR4",200.561620463093,-0.274332720768915,0.385731685351898,-0.711200897376747,0.476959752583198,0.850191401045635
"8649","C11orf52",12.3719183224221,-0.363904716479462,0.51170090429574,-0.711166842631065,0.476980852873433,0.850191401045635
"8650","AP2S1",797.122240366614,-0.173453756378461,0.243914491224735,-0.711125261592788,0.477006617140804,0.850191401045635
"8651","SSR1",451.248330035939,0.101657059238549,0.14300654921603,0.710855969854795,0.477173492993314,0.850263483353594
"8652","SLC44A3",6.89402274541198,-0.41083373034637,0.578287214968408,-0.710431978629881,0.477436298429923,0.85033859506612
"8653","TNFAIP8L3",8.17987618285347,-0.457867212749013,0.644417085817558,-0.710513769460545,0.477385595285057,0.85033859506612
"8654","RMI2",8.45617564889125,-0.432241343756329,0.608344222411683,-0.710520997541125,0.477381114650439,0.85033859506612
"8655","TANGO2",43.6542392148773,-0.181546275403086,0.255506330213906,-0.710535332925406,0.477372228317988,0.85033859506612
"8656","AL592435.1",6.73688483876295,0.417016309405241,0.587176463507451,0.710206105527848,0.477576335254635,0.850476774856275
"8657","ZSCAN22",12.1771653741456,0.329345790387189,0.46378313139694,0.710128868626984,0.477624225749645,0.850476774856275
"8658","SZT2",57.5880553840596,0.237888839164303,0.335498663853298,0.709060466685862,0.478286954396497,0.850534954700246
"8659","CNRIP1",300.128227743848,0.143540488594649,0.2023982150124,0.709198391823046,0.478201371331306,0.850534954700246
"8660","IL1RAP",16.7771545506649,0.279494665430199,0.393717607341662,0.709886121977922,0.477774757138678,0.850534954700246
"8661","CLEC4E",4.3223804619301,-0.563849028196429,0.795131448720131,-0.709126810546883,0.478245786736959,0.850534954700246
"8662","PPFIA2",2.3807394435011,0.828512335328589,1.16809544112132,0.709284794856535,0.478147762189299,0.850534954700246
"8663","RAB35",102.927840533905,0.126263927346457,0.178106253578298,0.708924727850442,0.478371189025476,0.850534954700246
"8664","SEMA4B",9.79036452782436,0.370277102093427,0.522114377997018,0.709187713837564,0.478207996762427,0.850534954700246
"8665","RPGRIP1L",39.2055802605994,0.20493891293343,0.288753043009328,0.70973767340284,0.477866825447098,0.850534954700246

"8666","CENPT",100.19273100067,0.15918731627918,0.22453650620998,0.708959620714474,0.478349534997574,0.850534954700246
"8667","AP2A1",279.797505271035,-0.121458239496365,0.171225181304999,-0.709347997593967,0.478108549853489,0.850534954700246
"8668","SDCBP2-AS1",14.5577990081436,0.30777684306848,0.434148967673114,0.708919900738348,0.478374184706528,0.850534954700246
"8669","LINC00310",11.0025863828945,-0.508662547739975,0.717322944458679,-0.709112334506229,0.478254769237295,0.850534954700246
"8670","THOC2",385.413537134377,0.145536162687578,0.205137116694594,0.709457971490604,0.478040323876664,0.850534954700246
"8671","H2AFJ",1309.6068999795,0.171698811410353,0.242285625875339,0.708662805686603,0.478533751391501,0.850720537158343
"8672","RAB11FIP1",14.7595194157609,-0.392417588996472,0.554018865221341,-0.708310878258077,0.478752223165966,0.850821768409695
"8673","ACTR6",113.438683816211,-0.170343136143109,0.240436727393603,-0.708473859171488,0.478651040053396,0.850821768409695
"8674","MLST8",83.311722808541,-0.177671701809405,0.250840902389147,-0.708304347963834,0.478756277598812,0.850821768409695
"8675","CACNA2D3",13.9277653174348,-0.325228542672281,0.459707643049384,-0.70746820852257,0.479275562427921,0.850850809265934
"8676","B4GALT4",107.765496470113,0.127731749664468,0.180531977802094,0.707529775165331,0.479237315951138,0.850850809265934
"8677","ATG12",372.670560869115,0.101388424550522,0.14322261179036l,0.707907943327602,0.479002426584521,0.850850809265934
"8678","DDX58",73.4958574751568,-0.198979556148065,0.281287199160836,-0.707389304389539,0.479324581749294,0.850850809265934
"8679","HSDL2",166.418277490886,-0.149182132194648,0.210804309211148,-0.707680657728981,0.479143591614413,0.850850809265934
"8680","ZNF488",3.27468619655959,0.621473916289748,0.878516993943975,0.707412515152075,0.479310161741141,0.850850809265934
"8681","CYFIP1",298.748162624932,0.098620349962192,0.139343481812948,0.707750005088709,0.479100518189033,0.850850809265934
"8682","GFER",132.911649706998,-0.182455112203539,0.257829127623327,-0.707659037151516,0.479157021142383,0.850850809265934
"8683","EEF2",4094.53131919667,-0.100510986378388,0.142016152293856,-0.70774334295731,0.479104656118084,0.850850809265934
"8684","MX2",289.470886651795,0.244429008870845,0.345321639401255,0.707829979304671,0.479050846775215,0.850850809265934
"8685","ELF3-AS1",4.46557563394125,0.647990457625784,0.916197614854076,0.707260581254614,0.479404557079656,0.850872621578736
"8686","LAG3",9.32214647144568,0.374797735848747,0.52998028463303,0.707191846029263,0.479447265068628,0.850872621578736
"8687","LRRC42",96.5904051930574,-0.183450803931858,0.259887035129146,-0.705886708972212,0.480258593607326,0.85100250933687
"8688","S100A16",800.673617553571,-0.16916814947542,0.239502081179059,-0.706332690900269,0.479981268186749,0.85100250933687
"8689","GALNT5",31.8115584951126,-0.883924960141973,1.25166192548331,-0.706201045302752,0.480063120424825,0.85100250933687

"8690","AL121933.2",16.3746417699216,-0.380883997561007,0.539604452076636,-0.70585777432932,0.480276589083989,0.85100250933687
"8691","COG5",92.0198428624778,0.134454020426848,0.190472024468587,0.705899046340122,0.480250920674119,0.85100250933687
"8692","HACD1",8.66731757604813,-0.485069632054487,0.687148516753916,-0.705916727210519,0.480239924592844,0.85100250933687
"8693","WAC",574.233546831757,0.131229610606904,0.185732817745121,0.706550475032305,0.479845874985961,0.85100250933687
"8694","TM9SF3",681.09775462672,0.0891468731051422,0.126136395135977,0.706749808483431,0.479721970562319,0.85100250933687
"8695","AL132800.1",3.89695368533361,0.675569122882636,0.956076758388879,0.706605528222507,0.479811652523568,0.85100250933687
"8696","PNN",556.341224870193,0.103828753998583,0.147101469423868,0.705830841834786,0.480293339684124,0.85100250933687
"8697","ATXN1L",91.8815743353123,0.150047924861992,0.212317756518347,0.706713971184158,0.479744245516034,0.85100250933687
"8698","ASPSCR1",80.5681093234132,-0.159491323656327,0.22586097020923,-0.706148226975999,0.480095962995721,0.85100250933687
"8699","NCOA3",201.475636647581,0.138679698367737,0.196355219209298,0.70626947898908,0.48002057000515,0.85100250933687
"8700","USP16",430.054000635049,-0.118157148713002,0.167257584626222,-0.70643821012394,0.479915665831665,0.85100250933687
"8701","KHDC4",66.3615417479662,0.182404344134428,0.258479240079302,0.705682762292499,0.480385443087197,0.851067877851872
"8702","IFT74",130.663467049787,-0.147718641606931,0.209363178057016,-0.705561708500157,0.480460744002423,0.851103466880872
"8703","GOLGA6L10",13.728917706806,-0.370784516251126,0.525597080644063,-0.705453911191381,0.480527804201215,0.851124451541046
"8704","USP6NL",33.3947851711946,-0.213540072479962,0.302806038166589,-0.705204142469851,0.480683203745252,0.851301882552051
"8705","INTS12",53.970537107472,-0.194542856310781,0.275973610513474,-0.704932822920338,0.480852042667714,0.851405265072688
"8706","POLR2K",424.110964296198,-0.190868182870341,0.270754713227469,-0.704948699120101,0.480842162208137,0.851405265072688
"8707","TMEM175",88.594789407281,-0.192340857644663,0.272910219295504,-0.704777044044653,0.480948996600538,0.851479129734386
"8708","MANEA",58.2945343264404,0.170578777943814,0.242162045185005,0.704399311681962,0.481184134536575,0.851558187396424
"8709","MAD1L1",81.5050350254022,-0.175678137265996,0.249512970675,-0.704084187650605,0.4813803467384,0.851558187396424
"8710","TOR1A",145.64188851883,-0.150326251105872,0.213468755805633,-0.704207276322661,0.481303700306954,0.851558187396424
"8711","SLC25A15",9.09899757351615,-0.436601807553352,0.619851829489315,-0.704364796201473,0.481205623501447,0.851558187396424
"8712","UBALD1",45.3807081831312,-0.211094579900627,0.299701034815009,-0.704350520614404,0.481214511480292,0.851558187396424
"8713","AC005562.1",66.8487648474131,0.163276208575279,0.231822632943545,0.704315219364458,0.481236490418135,0.851558187396424
"8714","PTGIS",1293.86995055069,-0.233630229587253,0.331798900384601,-0.704132018871801,0.481350561791564,0.851558187396424

"8715","PSAP",3680.81356718292,0.0978372602478759,0.138994295438357,0.703894069460324,0.481498745022851,0.851630053427177
"8716","CRY1",64.0258158572296,-0.241861718498679,0.343630927328098,-0.703841532481591,0.481531465823631,0.851630053427177
"8717","RPA2",223.244192567645,-0.170159803186044,0.242094417292422,-0.702865456746616,0.482139600119426,0.851630793610742
"8718","DYNC1LI1",236.386920513395,-0.135265392170175,0.192302977328651,-0.703397285102887,0.481808197974363,0.851630793610742
"8719","RBP1",13.569232788916,-0.47520276305924,0.675939696738727,-0.703025381335048,0.482039932096003,0.851630793610742
"8720","NNT",116.606275748517,0.153559771453803,0.218380960950681,0.703173805927533,0.481947441112681,0.851630793610742
"8721","SERF1A",4.61378953739502,-0.546946197816107,0.777419685339042,-0.703540453284994,0.481719005696945,0.851630793610742
"8722","NEU1",588.574898160214,0.203951152082553,0.289983216623868,0.703320538536872,0.48185601397911,0.851630793610742
"8723","RAB21",310.059722345854,0.110400544735783,0.157066635453061,0.702889855743908,0.482124393479276,0.851630793610742
"8724","DCN",80454.3198075831,0.129976655994076,0.184826312661575,0.703236753048624,0.481908218444841,0.851630793610742
"8725","FBXL3",349.063962899853,0.119616896171219,0.170094917453061,0.703236157566131,0.481908589484843,0.851630793610742
"8726","PSKH1",61.7268291017566,0.170901198438505,0.243139235779929,0.702894363759503,0.482121583893551,0.851630793610742
"8727","CXorf56",53.6099119435762,0.207234791940201,0.294593165927267,0.703460962130283,0.481768526744301,0.851630793610742
"8728","TAP1",131.782120661537,0.199525360039386,0.283932349360528,0.702721477453191,0.482229340317864,0.85169171413839
"8729","ZNF512",67.5109506005566,0.154622057563571,0.220128000404711,0.70241885302776,0.482417991317166,0.851892816592774
"8730","WAKMAR2",8.37359934390104,0.401144592431298,0.571136911236378,0.702361525825592,0.482453732653579,0.851892816592774
"8731","RAE1",76.1919442138758,-0.182900457590688,0.260507927695624,-0.702091714477065,0.482621969121758,0.852092275113034
"8732","RRP12",23.5047679164799,-0.257375724226082,0.366695949581073,-0.701877739637206,0.482755412306558,0.852230265770224
"8733","RPS19",9669.98186149051,-0.156236793428639,0.222644365672748,-0.701732527371845,0.482845983842894,0.852292550204765
"8734","CD34",2459.8592083425,-0.148813025043432,0.212092326960914,-0.701642662777032,0.482902038651769,0.852293900368333
"8735","FBXL14",31.4358050859668,-0.196680369486689,0.280409267685128,-0.701404668648619,0.48305050924633,0.852425880793393
"8736","THAP2",65.7758760960454,0.207607076235148,0.296012551989599,0.701345516734854,0.483087414506071,0.852425880793393
"8737","CBWD5",34.4587442979813,-0.239676391915836,0.34195980754295,-0.700890533416658,0.483371332702351,0.852577344307495
"8738","LINC00937",4.81852657919729,-0.495133963328232,0.706472903993906,-0.700853437589876,0.483394485192832,0.852577344307495
"8739","AC087500.1",11.5965836558463,0.327609950132445,0.467278856301256,0.701101592153431,0.483239616796546,0.852577344307495

"8740","NPB",23.9878295481705,-0.350493097904032,0.50004552681082,-0.700922374287397,0.483351460449558,0.852577344307495
"8741","LINC01551",4.41853668662939,0.72519805913731,1.03499529369181,0.700677639364469,0.48350421371857,0.852673315921721
"8742","POLR3B",26.0782158369725,0.253612979435618,0.36200925344461,0.700570432999782,0.483571135684034,0.852676328167498
"8743","NTAN1",765.709512432821,-0.16966061383821,0.242200106858657,-0.70049768366626,0.483616551227274,0.852676328167498
"8744","SETDB1",47.3653518432417,0.182223049910263,0.260257142906923,0.700165412848755,0.483824008801676,0.852719271806619
"8745","AL590399.3",2.50374569079408,0.682651200178495,0.975025654814864,0.700136654668964,0.483841966612317,0.852719271806619
"8746","KEAP1",156.94013986966,0.1565794601756,0.223641740321734,0.70013522498234,0.483842859377895,0.852719271806619
"8747","RTN2",154.807369059262,0.199310868253915,0.284687398010892,0.700104288586351,0.483862177780895,0.852719271806619
"8748","QDPR",205.808459616843,-0.183403728751325,0.262003448815563,-0.700005017416513,0.483924171035565,0.852731035266717
"8749","ZBTB7A",627.18461330944,-0.135353391020067,0.193426952949359,-0.69976489292836,0.484074142743179,0.8528978066506
"8750","MSTO1",23.8498271598156,-0.295378154523588,0.422908615808639,-0.698444400237149,0.484899317469698,0.853236178973264
"8751","AL358115.1",2.93270268525022,-0.60764214889337,0.870386601079937,-0.698129024664941,0.485096508055201,0.853236178973264
"8752","IK",412.260138202374,-0.148779449302159,0.213078983404868,-0.6982361513311,0.485029521554687,0.853236178973264
"8753","CD24",34.7604199600857,-0.398034980106863,0.570169931119337,-0.698098862080389,0.485115369678463,0.853236178973264
"8754","HIBADH",311.891600003939,-0.150005191699243,0.214810093508279,-0.69831537824577,0.484979984042815,0.853236178973264
"8755","TAS2R4",2.94065883139593,0.653606982776671,0.934847122305211,0.699159217782007,0.484452534039754,0.853236178973264
"8756","COPS5",322.094180706757,-0.17774187387832,0.254593613528674,-0.698139562162666,0.485089918716405,0.853236178973264
"8757","AC067930.3",2.52340055250959,0.885011228348179,1.26627929378009,0.698906815183127,0.484610268106673,0.853236178973264
"8758","POLR2L",1760.70703660093,-0.205013557410739,0.293504127199359,-0.698503150081723,0.484862588553671,0.853236178973264
"8759","TAS2R30",3.44562635089654,0.623567170371646,0.893310754564034,0.698040594704323,0.485151807246231,0.853236178973264
"8760","TUBA1B",2278.82347617494,-0.225014286900585,0.322032585904368,-0.698731422687162,0.484719892603727,0.853236178973264
"8761","CLPTM1",252.136190086538,-0.11819307203322,0.169273123694188,-0.698238854779744,0.485027831148114,0.853236178973264
"8762","DHX34",15.4136651753265,0.280925183816366,0.402414872692423,0.698098412558139,0.485115650782024,0.853236178973264
"8763","ZFP28",14.6361223453303,0.339357840842452,0.485915381566251,0.698388760093579,0.484934103668951,0.853236178973264
"8764","KDM5C",128.468657939955,0.139300151780969,0.199231829426488,0.699186230342614,0.484435654717057,0.853236178973264

"8765","ZXDA",15.0608420128818,-0.295936991113049,0.423460088437324,-0.698854506466317,0.484642960897345,0.853236178973264
"8766","MTOR",40.566512444449,0.205589109222208,0.294895330960103,0.697159594059569,0.485702922545973,0.853359226517876
"8767","PUS10",14.3206652621975,0.279735601440818,0.401204529544516,0.697239389989936,0.48565299176895,0.853359226517876
"8768","SLMAP",129.410326815404,0.188885539561408,0.270899383993616,0.69725348495388,0.485644172403557,0.853359226517876
"8769","RETREG1",91.2807427938682,-0.321110123528348,0.460462830197624,-0.697363831496521,0.48557513042075,0.853359226517876
"8770","PCDHGB5",2.74564738110579,0.717445228877828,1.02894338283623,0.697264048581784,0.485637562688638,0.853359226517876
"8771","EEF1A1",42441.8065138766,-0.136925296162307,0.19641625161265,-0.697117957593124,0.485728976871238,0.853359226517876
"8772","AL359715.1",5.15839018208306,0.571298688316041,0.819602241520418,0.69704383342857,0.485775362484227,0.853359226517876
"8773","COMP",50.919089559254,-0.986232568927706,1.41426971446729,-0.697344048903137,0.485587507667128,0.853359226517876
"8774","ZNF506",142.307974259406,-0.134800347617829,0.193234364302244,-0.697600285045489,0.485427203286895,0.853359226517876
"8775","AC008079.1",3.76771846430949,0.666548007392248,0.955925326195586,0.697280414198242,0.485627322736541,0.853359226517876
"8776","CACYBP",449.87513445091,-0.148688314437277,0.214095210042571,-0.694496221600251,0.487371069526453,0.853600631302829
"8777","ZNF281",84.776777745561,-0.172153360643629,0.247203243130864,-0.69640413476491,0.486175774361699,0.853600631302829
"8778","RNASEH1-AS1",45.8390294433178,-0.247194469390986,0.355956976392297,-0.694450413351514,0.487399787587548,0.853600631302829
"8779","AC008074.2",2.54940615210248,-0.647286656513319,0.931578732583161,-0.694827644592602,0.487163321376948,0.853600631302829
"8780","ZC3H6",250.369301613387,0.131091692331521,0.18824336166338,0.696394768841525,0.486181638177075,0.853600631302829
"8781","PBRM1",308.854775015683,0.130613578713266,0.187822254330491,0.695410558130341,0.486798045744098,0.853600631302829
"8782","SLC25A26",115.652876798114,-0.15030031586121,0.215872786139104,-0.696244851189162,0.486275503795393,0.853600631302829
"8783","PACRGL",60.611357269381,-0.146413933533067,0.210847326508678,-0.694407351316549,0.487426784828145,0.853600631302829
"8784","LIAS",86.9694658921613,-0.165573950726178,0.237912523731292,-0.695944661211629,0.486463486549151,0.853600631302829
"8785","FGF10-AS1",10.3788064651339,0.454631633940593,0.653000365902918,0.696219569972161,0.486291333697831,0.853600631302829
"8786","CCDC125",32.7080758292417,0.20984825979257,0.301661301611783,0.695641962264785,0.486653080221556,0.853600631302829
"8787","SOBP",328.621252046194,-0.121999363686393,0.175751444847312,-0.694158524798375,0.487582799520794,0.853600631302829
"8788","AC073957.3",2.69461120574082,0.872337890514897,1.25638093369037,0.694325954113756,0.487477818060316,0.853600631302829

"8789","PLOD3",172.401632376955,0.113965738212529,0.1642846798835б,0.6
93708861308943,0.487864807723908,0.853600631302829
"8790","LINC02680",2.87830248572388,0.792995570302318,1.14031727661022
,0.695416606034094,0.48679425667508,0.853600631302829
"8791","PGGHG",18.9347768192001,0.330049933828763,0.475821204715659,0.
693642760259065,0.487906270672023,0.853600631302829
"8792","PCNX3",46.1552251483395,-0.193826349567806,0.278432071201624,-
0.69613514251901,0.486344200195009,0.853600631302829
"8793","NDUFC2",1111.24451774242,-0.190887170848737,0.275168745248152,
-0.693709493338685,0.487864411282249,0.853600631302829
"8794","FBXW8",38.5213352946511,0.251982478831159,0.36323380548118,0.6
93719788821293,0.487857953448924,0.853600631302829
"8795","AL355001.2",39.9888566804053,-0.235490186682398,0.339366755987
209,-0.693910592383639,0.48773828041381,0.853600631302829
"8796","RASL11A",84.3228475510124,0.251071155763258,0.3608665753953,0.
695745111578233,0.486588468781736,0.853600631302829
"8797","IRS2",192.297899468835,0.214213630829407,0.308762338093984,0.6
93781606110918,0.487819179572769,0.853600631302829
"8798","ZNF710",18.6661969821105,0.278917401160461,0.401707102160769,0
.694330271135793,0.487475111364311,0.853600631302829
"8799","HNRNPA1P48",87.0604659632446,-0.223323326690851,0.321605048205
82,-0.694402429118367,0.487429870794302,0.853600631302829
"8800","CHMP1A",182.180292119168,-0.135684492104043,0.195555008845583,
-0.693843092565242,0.487780614857166,0.853600631302829
"8801","FANCA",5.44545415970011,-0.467719253651572,0.673033937919544,-
0.694941558366829,0.487091927069527,0.853600631302829
"8802","SLC25A35",17.4845385991749,-0.319366092795444,0.45874773561129
9,-0.696169306143553,0.486322807357807,0.853600631302829
"8803","TNFAIP1",56.0133993698933,0.179591703063825,0.258526396056692,
0.694674531510673,0.487259292357096,0.853600631302829
"8804","TMEM94",49.2414444810026,0.1802936442214,0.259143668106318,0.6
95728533669713,0.486598852663579,0.853600631302829
"8805","FAAP24",27.8100318555999,0.264395726118823,0.380615002592028,0
.694653979265821,0.487272175241075,0.853600631302829
"8806","TSKS",35.0886209671136,0.246922564851363,0.354976143956342,0.6
95603265333042,0.486677320691561,0.853600631302829
"8807","NDUFAF5",44.0808763647272,-0.181504316578225,0.261350786568885
,-0.694485442194702,0.48737782725831,0.853600631302829
"8808","SUSD2",8.30602584583464,-0.454016226649399,0.65351628131028,-0
.694728256408716,0.487225616533517,0.853600631302829
"8809","NDUFA6",411.220306540099,-0.182383875335999,0.262613995070622,
-0.694494119732471,0.487372387207283,0.853600631302829
"8810","KCND1",4.59335551214675,-0.491959814591199,0.707188395251184,-
0.695655949524541,0.486644318505256,0.853600631302829
"8811","PIN4",292.896124348174,-0.180033562403121,0.258765703832387,-0
.695739658450783,0.486591884436486,0.853600631302829
"8812","RAB40B",142.226082737348,0.15253881063539,0.220055723277122,0.
693182655573533,0.488194931412992,0.85400872307436
"8813","ZNF322",198.709289659025,0.142010897511581,0.204968913560565,0
.692841148663351,0.488409245751794,0.854274265170304

"8814","RECQL4",3.83933328777363,-0.521684050680927,0.753047109879093,-0.692764162875131,0.488457565566725,0.854274265170304
"8815","LYSMD3",123.387655142294,0.148319584393223,0.214177811064255,0.692506771155327,0.488619135137207,0.854459894287017
"8816","PEA15",339.061588623812,-0.119225028162152,0.172408245255459,-0.691527414976562,0.489234158461833,0.854913486113957
"8817","LAMB2",1389.29323015847,0.119437905552317,0.172744203582858,0.691414838096306,0.489304882028873,0.854913486113957
"8818","AC095057.3",3.33849484618723,0.668317091346105,0.967609308703958,0.690688985042183,0.489761012939578,0.854913486113957
"8819","PLA2G12A",174.437503974142,0.118108264149283,0.170741626187719,0.691736788423407,0.489102639551138,0.854913486113957
"8820","CNOT6",53.6819727347268,0.217519850433638,0.314935220170285,0.690681246498964,0.489765877124382,0.854913486113957
"8821","THSD7A",11.2223258836932,0.384366707247312,0.556496648758666,0.690690066336768,0.489760333276686,0.854913486113957
"8822","IMMP2L",100.796446835073,-0.177551408491763,0.256764461563659,-0.691495261495693,0.489254357508253,0.854913486113957
"8823","AC107375.1",22.5715348285264,0.32088825233424,0.464257438077506,0.691186023131996,0.489448646186742,0.854913486113957
"8824","SEPHS1",139.032583688416,-0.141735663735869,0.205147163506421,-0.690897506518207,0.489629953228298,0.854913486113957
"8825","CS",97.3983585562556,0.187088815252062,0.270704619522869,0.691117926180262,0.489491435807682,0.854913486113957
"8826","ZNF605",64.997277518716,0.177416092586544,0.256840406762768,0.69076394490535,0.489713897067312,0.854913486113957
"8827","TENT4B",144.20776455107,-0.132966140762295,0.192360208877244,-0.691235165205855,0.48941776836529,0.854913486113957
"8828","ATP5MC1",556.263051205423,-0.197593754029761,0.285680348611794,-0.691660294416216,0.489150687411812,0.854913486113957
"8829","RELCH",56.1250556880389,0.178073583058619,0.257540180822092,0.691440001673494,0.489289073172576,0.854913486113957
"8830","PALM",690.836337444468,-0.107494265036914,0.155580477170709,-0.69092386777402,0.489613386022155,0.854913486113957
"8831","GCDH",64.866786899799,-0.194033530543794,0.280436514202747,-0.691898239768853,0.48900123613898,0.854913486113957
"8832","WNK1",423.185285893361,0.153123226412934,0.221755558742966,0.690504568548005,0.489876937941575,0.854969138212732
"8833","DDX51",59.0854405621971,-0.204878997259332,0.296769150769948,-0.690364873598847,0.489964760614896,0.854969138212732
"8834","VIPAS39",63.5004465776375,0.178383954319608,0.258514543954947,0.690034500924235,0.490172491241317,0.854969138212732
"8835","COQ9",115.338870281336,-0.147906522505667,0.214353236161616,-0.690013013818694,0.490186003472211,0.854969138212732
"8836","AC010538.1",3.27899980454816,-0.540325151942468,0.782949524422012,-0.690114924511062,0.490121918405312,0.854969138212732
"8837","AC008736.1",9.03061536228544,0.35497151729721,0.514119187482845,0.690445962608728,0.489913780978568,0.854969138212732
"8838","ENTPD6",75.5596799126976,-0.159946208983352,0.231789940538122,-0.690048103951459,0.49016393704033,0.854969138212732

"8839","ZC3H3",40.29879665711,0.224733701703217,0.325808684497066,0.68
9771980909983,0.490337591468809,0.855136777066601
"8840","TRAF3IP3",6.09955623655564,0.539503341132129,0.782350860598447
,0.689592570677873,0.49045044071337,0.85514009089431
"8841","ITGB1-
DT",5.15661021426096,0.516186785812924,0.748518066106833,0.68961165960
589,0.490438433091659,0.85514009089431
"8842","EGFL8",35.3780838048485,-0.219146344763647,0.317894732305004,-
0.689367650651686,0.490591935395548,0.855193337568968
"8843","CLN3",206.404965152635,-0.14370621683226,0.208439885090981,-0.
689437229201762,0.49054816196099,0.855193337568968
"8844","YAP1",240.256783148568,0.154842943177979,0.224649078601143,0.6
89265872542915,0.490655970080419,0.855208251785353
"8845","FNBP1L",69.9078787031919,0.187024029847838,0.271431885087069,0
.689027487643337,0.490805969836094,0.855237647191239
"8846","AP4B1",27.180599217762,0.203835579917828,0.295853565629697,0.6
88974559032211,0.490839277632235,0.855237647191239
"8847","AC023632.2",9.73759128037264,-0.334549366448215,0.485544402837
264,-0.689019097930664,0.490811249372922,0.855237647191239
"8848","ZC3H11B",2.62956957357997,0.644224176976921,0.935495149711468,
0.688645127850869,0.491046614866578,0.85526231477256
"8849","MSRB3",136.072944189831,-0.156098668439603,0.226634917668555,-
0.688767071047239,0.490969860833441,0.85526231477256
"8850","USP14",253.548065094871,-0.121417891901682,0.176325861427554,-
0.688599453980656,0.491075364777939,0.85526231477256
"8851","SMOX",22.6363734100141,-0.256205706489439,0.372057421093318,-0
.688618723788817,0.491063235078604,0.85526231477256
"8852","CCNH",164.135381229909,-0.13865796157436,0.201393975245292,-0.
688491110051719,0.491143566651692,0.855284464520542
"8853","HACD2",77.5462773736037,0.147329106977349,0.214079392284043,0.
688198454813771,0.491327816854104,0.855400010881884
"8854","OTUD6B-
AS1",324.701388521229,-0.128078380137271,0.186151457539956,-0.68803318
4536198,0.49143188429257,0.855400010881884
"8855","TMEM254",54.6368973956959,0.2303861755047,0.3347948635969,0.68
8141308470282,0.491363799442347,0.855400010881884
"8856","TACC2",109.429467340186,-0.183694788742333,0.266971223554342,-
0.688069621499646,0.491408939637394,0.855400010881884
"8857","WHRN",12.0630232761354,0.304256498483802,0.442412893074589,0.6
87720686369115,0.49162869057556,0.855645959717992
"8858","CCDC137",90.479097356842,0.181190471856182,0.263590725449902,0
.687393198478143,0.491834982536339,0.855908360329382
"8859","COX14",427.686433347969,-0.17808749023114,0.259166316600784,-0
.687155231308331,0.491984912525329,0.85607262970741
"8860","TRAK2",132.804803131034,-0.189549958618873,0.276045964170303,-
0.686660858051641,0.492296468221851,0.856409926550971
"8861","AC135507.1",10.2893284223022,0.342243583220466,0.4985375833498
94,0.686495050023671,0.492400984691616,0.856409926550971
"8862","ADAMTS13",7.53819773169753,0.366771902022586,0.534092722499532
,0.686719527474759,0.492259489020568,0.856409926550971

"8863","REX1BD",759.297563719615,-0.146386088976514,0.213209884038581,-0.686582095556249,0.49234611438243,0.856409926550971
"8864","RRAS",1235.11613849076,0.185794113966893,0.270836527849988,0.686000944709354,0.492712512674339,0.856855074782823
"8865","B4GALT7",123.825970657882,-0.210630866368746,0.307243673052199,-0.685549890340494,0.492996989719442,0.857253084774416
"8866","PRPF8",282.465674436824,0.148829864467163,0.217163354079282,0.685336000165243,0.493131919609991,0.857390992644711
"8867","STPG1",13.7399983932607,-0.314348219732294,0.459176406821224,-0.68459140117511,0.493601794658282,0.857596303679816
"8868","FOXD2-AS1",2.75427569918956,-0.588706135415295,0.859770348293746,-0.684724864707895,0.493517555612084,0.857596303679816
"8869","DCAF6",230.766670472055,0.127901943211636,0.186942152307642,0.684179258839141,0.49386197779862,0.857596303679816
"8870","PTPN14",106.842561490225,-0.216875555610806,0.316783746461688,-0.68461705511471,0.493585601896753,0.857596303679816
"8871","OST4",2221.45679725824,-0.162211125152264,0.236897874506054,-0.684730183799599,0.493514198485877,0.857596303679816
"8872","ZNF316",48.7247140284438,0.208264900133482,0.304341345531179,0.684313528843704,0.493777205839676,0.857596303679816
"8873","ANKRD26",154.018036273967,0.166708807731724,0.243647839606366,0.684220340311889,0.493836040002889,0.857596303679816
"8874","IMPACT",117.674592466437,0.141410994238125,0.206457280161853,0.68494070118169,0.49338134099193,0.857596303679816
"8875","SIRT2",243.078435539268,-0.144263780898446,0.210689712873964,-0.684721522140693,0.49351966526799,0.857596303679816
"8876","HAR1A",4.6214007734146,0.523822505199999,0.765428510087679,0.684351965332458,0.493752940227062,0.857596303679816
"8877","NHS",15.8009446054169,-0.309149477194388,0.451841745683865,-0.684198572060862,0.49384978383144,0.857596303679816
"8878","ACSS2",50.7539828422476,-0.177371839944913,0.259310294383404,-0.684013877531058,0.493966402719767,0.857681020266412
"8879","ATG9A",86.637149170593,0.158199580571214,0.231355822496048,0.683793383129211,0.494105645476978,0.857694864252425
"8880","COL6A3",8969.50789800004,0.140288416420494,0.205275206662617,0.683416271752033,0.494343840899109,0.857694864252425
"8881","NKIRAS1",69.601472076812,0.190732466076,0.279014758495685,0.683592750090852,0.49423236397832,0.857694864252425
"8882","ZFR",364.605197716613,0.102594282174196,0.150051619773583,0.683726589083164,0.49414783022496,0.857694864252425
"8883","IGIP",50.8015986157147,0.229444829710024,0.335724366169847,0.683432162900996,0.494333802309752,0.857694864252425
"8884","RPS20",8572.95667523714,-0.123541289200714,0.180778571625148,-0.683384585297432,0.494363857858112,0.857694864252425
"8885","MFSD12",76.1371510063815,-0.181695362771683,0.26570802776051,-0.683815857213956,0.494091452076176,0.857694864252425
"8886","GOLM1",268.60661141335,0.112509271950453,0.164723279537211,0.683019863777284,0.494594290849066,0.857998086139811
"8887","IL18BP",11.2285788971524,-0.39461003627013,0.577849455140139,-

0.682894191142622,0.494673704786292,0.858039288767941
"8888","RNF227",10.8791538830809,-0.310760503741033,0.455126490424702,
-0.682800298991709,0.494733040730252,0.858045659637357
"8889","ABCE1",192.7689611269,-0.11535400358744,0.169030927272839,-0.6
82443180360967,0.49495875963715,0.858049175759052
"8890","MIR3945HG",3.0601846125314,0.644412638127045,0.94463630962731,
0.682180677959846,0.495124710890481,0.858049175759052
"8891","PCDHB7",15.7630814878531,-0.345844640411219,0.506791023253289,
-0.682420612328742,0.494973025741557,0.858049175759052
"8892","HSD17B8",36.0647139157106,0.251894892882098,0.369061830511213,
0.682527620190853,0.494905383984732,0.858049175759052
"8893","AL162231.2",58.2062922585941,0.164122571797199,0.2405800999342
73,0.682195126870581,0.495115575668311,0.858049175759052
"8894","ZDHHC24",128.739945519842,0.139474509503504,0.204369613699083,
0.682462069478043,0.494946819282683,0.858049175759052
"8895","BCAS3",52.3630386703508,0.182750685267868,0.267859258238532,0.
682263836873341,0.495072135484652,0.858049175759052
"8896","DLST",184.342085923204,0.125774663043304,0.184493175559772,0.6
81730707174888,0.495409246814944,0.858234867634483
"8897","CD226",4.02510885923816,0.592725556798906,0.869533752163601,0.
681659056159772,0.495454562898428,0.858234867634483
"8898","SMG9",84.285869659788,-0.141682870542081,0.207811138112917,-0.
681786702236794,0.495373833967085,0.858234867634483
"8899","CSF2RB",3.08165588252097,-0.758075631737915,1.11196128461161,-
0.681746426093155,0.495399305593619,0.858234867634483
"8900","NPIPB2",17.0830075333935,-0.277989888818849,0.408003352171781,
-0.681342168732494,0.495655006655601,0.858292735070331
"8901","C17orf97",39.6511570777488,-0.373858894595384,0.54864120546069
6,-0.681426934168121,0.495601384943923,0.858292735070331
"8902","ZNF446",34.3391834790133,-0.190086564031118,0.27897943296337,-
0.681364077674054,0.495641146995746,0.858292735070331
"8903","KALRN",6.42211974662059,-0.494547518093087,0.726017993597993,-
0.681178045797753,0.495758837879001,0.858369252411038
"8904","GRK4",49.2217415006168,0.166711506378161,0.24489156885469,0.68
0756414595396,0.496025633116375,0.858369252411038
"8905","AC108471.3",5.69853997420678,0.475182578275612,0.6977004214924
75,0.681069644847186,0.495827423345852,0.858369252411038
"8906","TSPAN12",23.9601625725641,0.30473288966487,0.447546161871739,0
.68089711325064,0.495936594825466,0.858369252411038
"8907","VCL",431.862292192683,-0.131203317130532,0.19273082844563,-0.6
80759368849726,0.496023763488766,0.858369252411038
"8908","DOK5",72.4713947086956,-0.209286795207754,0.307438188777416,-0
.680744301936014,0.496033298765976,0.858369252411038
"8909","ASTE1",49.890061736564,-0.164496386904212,0.241688745868894,-0
.680612522162883,0.496116701345678,0.858417213070336
"8910","AL359504.2",9.40080794173443,0.338754775517617,0.4984887100030
41,0.679563586335889,0.496780832846359,0.858517154802083
"8911","AC009315.1",2.54143949518457,-0.628919662777228,0.924704251386
568,-0.680130605903651,0.496421766773945,0.858517154802083
"8912","TOMM6",769.904581238979,-0.174055760612283,0.256039644531221,-

0.679800040071758,0.49663108130478,0.858517154802083
"8913","AL954642.1",5.63782686097526,1.48265334174009,2.18161299458201
,0.679613361958436,0.496749306809944,0.858517154802083
"8914","CPNE8",109.996704883696,0.199292750272567,0.292934383314899,0.
680332393955719,0.496294017557052,0.858517154802083
"8915","AL049775.3",5.70684633338565,0.484357664403726,0.7120546191019
02,0.680225436939985,0.496361728376202,0.858517154802083
"8916","CORO7",44.1764692925216,0.180857745676403,0.266070111530676,0.
679737173919856,0.496670893523215,0.858517154802083
"8917","MILR1",18.2530323210182,0.372578985509571,0.548270111863833,0.
6795537043648,0.496787091847848,0.858517154802083
"8918","AC027601.6",5.16963378728653,-0.440457274444587,0.647973295841
688,-0.679746028534175,0.496665285921983,0.858517154802083
"8919","GZF1",32.6923194141238,-0.220995339751073,0.325055463457044,-0
.679869636402153,0.496587008946686,0.858517154802083
"8920","ACOT8",69.3528427053864,0.181381615669626,0.266630786873127,0.
680272588911251,0.496331877472799,0.858517154802083
"8921","KLHDC4",52.2676793624334,-0.155642144367967,0.229077457425302,
-0.679430207220275,0.49686531549715,0.858556085459989
"8922","DENND4B",48.7682962477758,-0.166910464216735,0.24581538846842,
-0.679007385406948,0.497133182447124,0.85892266391195
"8923","PCYT1A",121.439528425495,0.135547439257224,0.199810972623243,0
.67837835669219,0.497531828341054,0.85951508840943
"8924","ARID1B",539.426958750261,0.156346630135364,0.230541851908707,0
.678170270781361,0.49766373989489,0.859646632729687
"8925","VTA1",135.04344966046,0.119935515728057,0.17702475538453,0.677
507027011758,0.498084313127263,0.859987644137181
"8926","STK24-
AS1",3.16315447393656,-0.666317466891051,0.983399659944928,-0.67756528
0964573,0.498047365812339,0.859987644137181
"8927","GOLGA8O",6.31838617698074,0.425902690909369,0.628430704534362,
0.677724191126121,0.497946585152206,0.859987644137181
"8928","ATP6V0D1",349.20682110196,-0.150284258638591,0.221803127170879
,-0.677556987385532,0.498052625889562,0.859987644137181
"8929","AK9",33.2569950403658,-0.232113079697892,0.342817546997107,-0.
677074676401704,0.498358575181228,0.859997428624548
"8930","NOP2",23.4979622279256,-0.262754211844846,0.388039522638892,-0
.677132602519367,0.49832182502991,0.859997428624548
"8931","DERA",299.308846174963,-0.169922940359494,0.250977247253413,-0
.677045199192587,0.498377277003374,0.859997428624548
"8932","C15orf41",36.7840610430344,0.209465035496253,0.30933454383525,
0.677147249379989,0.498312532829077,0.859997428624548
"8933","TMEM202-
AS1",9.5276695991832,-0.318840986246515,0.470982148649724,-0.676970426
927245,0.498424717958593,0.859997428624548
"8934","PKD1",221.992464555415,0.148365346940721,0.219097201328705,0.6
77166782783925,0.498300140671024,0.859997428624548
"8935","MDM4",305.252628751142,0.161273258623023,0.238477424510985,0.6
76262161727574,0.498874211924815,0.860086249981113
"8936","RAB11FIP5",63.687345639428,-0.1602556773288,0.23691218793158,-

0.676434921849954,0.498764551446895,0.860086249981113
"8937","IBTK",184.088850888519,0.123215098598097,0.182223607385355,0.676175279186131,0.498929365958117,0.860086249981113
"8938","TSTD3",86.6144437827352,0.148256366109091,0.219214669054501,0.676306776131989,0.498845891448671,0.860086249981113
"8939","NEIL2",40.4315464672193,-0.189933054119857,0.280925283430965,-0.67609811334953,0.498978354432766,0.860086249981113
"8940","AGAP5",13.8834151248501,0.355221259930734,0.525060853810597,0.676533505312265,0.49870198077151,0.860086249981113
"8941","RHOG",226.675716055273,-0.149987483458118,0.221731995119602,-0.67643590803039,0.498763925499992,0.860086249981113
"8942","ADAT1",31.467857923034,0.20311721854604,0.300140595793532,0.676740239050387,0.498570780939774,0.860086249981113
"8943","AC011511.5",3.52201334731219,0.656283911689095,0.970086923757519,0.676520727799376,0.498710090391402,0.860086249981113
"8944","AL390728.5",3.47878307282397,0.683527229652217,1.01120410444364,0.675953772980671,0.499069995297349,0.86014802968567
"8945","ITCH",136.386609285797,0.138505816399535,0.204964573181666,0.675754908516673,0.499196267840111,0.860269476663534
"8946","AC067817.2",2.59540969446956,0.645068291463106,0.954763162154339,0.675631734688596,0.499274487772791,0.860308096246096
"8947","PIGV",75.4045334148876,-0.176236924375686,0.261329578706075,-0.674385675162721,0.500066147536357,0.860577644231137
"8948","ZMYM6",55.3039740236436,0.223143813713767,0.330703453078263,0.674755015820649,0.499831424681034,0.860577644231137
"8949","PRRC2C",1237.75235277852,0.117420343252073,0.174157524137404,0.674219180788494,0.500171976928033,0.860577644231137
"8950","THADA",83.2729860719546,0.144948503434708,0.214966876143723,0.674282968776071,0.500131429746123,0.860577644231137
"8951","RPL9",14286.0841162473,-0.146737591052157,0.217519350786733,-0.674595572860211,0.499932746473269,0.860577644231137
"8952","OCIAD2",5.2168344636447,-0.430705518690811,0.638836135555857,-0.674203437656278,0.500181984404127,0.860577644231137
"8953","LPCAT1",98.5134645805043,0.176766898032662,0.262239119819932,0.674067614908257,0.500268327600079,0.860577644231137
"8954","EGR1",8896.99502560217,0.143099911755171,0.212160389759464,0.674489295185638,0.500000289183363,0.860577644231137
"8955","PCDHGA1",4.22111320872057,-0.518534181122676,0.768745782201296,-0.674519708762314,0.499980959931372,0.860577644231137
"8956","GLO1",453.796856732708,0.13804102072837,0.20477659074093,0.674105473818592,0.500244259707921,0.860577644231137
"8957","PURB",169.827460925313,0.16303860822427,0.241620632793595,0.674771050548263,0.499821235638129,0.860577644231137
"8958","PSPH",22.2608673860813,0.250135629949166,0.370681983619007,0.674798455287914,0.499803821935663,0.860577644231137
"8959","ODF2",49.3474853717988,-0.192209880637703,0.285082112409417,-0.674226379947904,0.500167400655821,0.860577644231137
"8960","PCDH9",142.397996656063,-0.249731572381511,0.370238337884083,-0.67451570199005,0.499983506400072,0.860577644231137
"8961","NLE1",48.9957429672291,0.2054231661397,0.30458038534853,0.6744

46471346589,0.500027506410384,0.860577644231137
"8962","SRSF9",803.731025017531,-0.156518888982618,0.232310306095876,-0.673749226252673,0.500470759835222,0.860829810629317
"8963","KIFAP3",317.884405921608,-0.112237327153776,0.166706594336906,-0.673262672062927,0.50078019599209,0.861077011942724
"8964","IQCA1",9.81508974523477,0.42626828859758,0.633182532644297,0.673215489406187,0.500810208361204,0.861077011942724
"8965","CEP162",117.033650612855,0.175034998458159,0.26001529575579,0.673171929941207,0.500837916904214,0.861077011942724
"8966","MICU1",275.642651498912,0.128348942339899,0.19065788896042,0.673189780080611,0.50082656218175,0.861077011942724
"8967","EXOC3L1",3.6372624556057,-0.557554238824515,0.828407164929917,-0.67304371863042,0.5009194779154,0.861121194609779
"8968","RPP40",21.8102699512281,-0.333773576741447,0.496010984758604,-0.67291569541325,0.501000926294603,0.861165173821511
"8969","LINC01719",13.30801458903,0.360173384612484,0.535412706249799,0.672702347942493,0.501136673559586,0.861206446256524
"8970","PIN1",377.46192078088,-0.164829454242436,0.244998672513207,-0.672776927938459,0.501089218096229,0.861206446256524
"8971","RMDN1",124.542996188327,-0.12130449837131,0.180357053269919,-0.672579730994875,0.501214700246807,0.861244521714918
"8972","GTPBP2",59.3283071509937,-0.21620292650074,0.321602464537704,-0.672267629576554,0.501413333472253,0.861297808722396
"8973","AL138762.1",15.8144291688994,0.322407874480095,0.479548645494469,0.67231526459147,0.501383014039537,0.861297808722396
"8974","PGAP2",42.993837620538,0.196486131750918,0.292261600545958,0.672295407210092,0.501395653038769,0.861297808722396
"8975","ZNF468",27.6687665893331,0.20985471992801,0.312200788203194,0.67217869991868,0.501469939323106,0.861299065700911
"8976","KDM4C",47.8713799107746,0.189978324903889,0.282872230731199,0.671604718543112,0.501835373566514,0.861400871370334
"8977","GPRC5D-AS1",2.54906635010598,-0.810274715687296,1.20629644236532,-0.671704472657237,0.50177185342794,0.861400871370334
"8978","TBX5-AS1",10.3088782529784,-0.482175216788989,0.71772525163578,-0.671810300236831,0.501704470558963,0.861400871370334
"8979","SNHG19",172.305611476686,-0.193044080927493,0.287441486681201,-0.671594358755864,0.501841970582199,0.861400871370334
"8980","AC124283.1",9.4192033503015,0.350770975904404,0.522157255179502,0.671772674658747,0.501728427086454,0.861400871370334
"8981","ZNF577",28.1953068182305,0.211886767437149,0.315514753745855,0.67155898391941,0.501864497293349,0.861400871370334
"8982","KANSL1-AS1",37.8036129286205,0.204688214834866,0.304846402123363,0.671447041556469,0.501935785764631,0.861427314357804
"8983","NHLRC2",129.200271117025,0.160181410842311,0.238626064596978,0.671265358680937,0.502051498626999,0.86143408852796
"8984","C17orf82",4.53159055744587,0.50430827865876,0.751264899287576,0.671278904600754,0.502042870815407,0.86143408852796

"8985","LRP10",832.436193060051,-0.0875073829040835,0.130382173351128,-0.671160639947458,0.502118199846028,0.861452648928939
"8986","SNX11",41.9215206634474,-0.21570098567275,0.321526137222522,-0.67086609983271,0.502305834056955,0.861678659246379
"8987","RAD51-AS1",53.3079162282165,0.227624608421106,0.339354447496284,0.670757699215357,0.502374899059634,0.861701242795622
"8988","PIK3CD",8.42490042566916,-0.416978689801786,0.622203848619899,-0.670164112174298,0.502753178652835,0.86172775792959
"8989","TMEM18",237.12681866549,-0.1360515780606,0.203105607382842,-0.669856336384406,0.50294937645751,0.86172775792959
"8990","ABCB6",103.689101738819,-0.144761571893558,0.216068154957275,-0.669981061865329,0.502869862840918,0.86172775792959
"8991","LARS2",21.9552995310649,0.226550439762344,0.338144353709206,0.669981436263079,0.502869624168985,0.86172775792959
"8992","PIGG",70.4922464821722,0.165775160174776,0.247235842774357,0.670514268135764,0.502530013969538,0.86172775792959
"8993","MRPL1",167.004168730497,0.138278962835939,0.206277109488119,0.67035534470636,0.502631294048911,0.86172775792959
"8994","PIWIL4",16.8292112111732,0.308563828948803,0.460587519848197,0.669935279728163,0.502899048593614,0.86172775792959
"8995","CRTC3",131.492952990938,0.118317668702777,0.176532348806062,0.670232223742517,0.502709765045507,0.86172775792959
"8996","PCGF2",157.992097825821,-0.144226595431091,0.215220509683726,-0.670134066883478,0.502772329839555,0.86172775792959
"8997","PAXBP1-AS1",9.01853455017844,0.363996844786842,0.54286306707948,0.670513186216717,0.502530703427658,0.86172775792959
"8998","AC096921.2",16.9987502721278,0.270213288569279,0.403464685114182,0.669732193519757,0.503028525248002,0.861767583540559
"8999","HIST2H2BF",4.41653824935045,0.490842565108831,0.733155606305197,0.66949302561086,0.503181028203403,0.861770458667653
"9000","WDR75",74.428367856336,0.148555939953725,0.221885459772822,0.669516335616694,0.503166163743952,0.861770458667653
"9001","MMP11",47.6859690189485,0.280232818324157,0.418591223418153,0.669466540735893,0.503197917513302,0.861770458667653
"9002","PDE4B",51.4329279324086,0.274523348804041,0.410706636533356,0.668417124011449,0.503867367742912,0.862184009445849
"9003","AC010969.2",26.2833322608443,0.23651636207042,0.353728671198365,0.668637804419835,0.503726550950626,0.862184009445849
"9004","ANKAR",53.590650133181,0.202815939121556,0.303409737400688,0.66845560349869,0.503842812377973,0.862184009445849
"9005","MYNN",64.7202262695012,-0.16204986927901,0.242315730927145,-0.668755052175019,0.503651743278217,0.862184009445849
"9006","AC006160.1",3.4805329727474,-0.597005203520327,0.892845081879123,-0.668654860330128,0.503715668390963,0.862184009445849
"9007","BCKDHB",64.7727754118983,-0.143126360703568,0.214137088686078,-0.668386600293186,0.503886846649215,0.862184009445849
"9008","SUV39H2",11.1323797266551,-0.344525843062513,0.51511486954679,-0.668833037892373,0.503601989241073,0.862184009445849

"9009","RTKN2",5.25122030673304,-0.447553807192996,0.669429846985579,-0.668559684346787,0.503776397214578,0.862184009445849
"9010","HSD17B12",344.163325485908,-0.0882626025108974,0.132093603409125,-0.668182260404595,0.504017257692597,0.862311434775959
"9011","HELQ",76.5877604051612,-0.152872604743539,0.228833657581237,-0.668051222706468,0.504100896173374,0.862351333948746
"9012","AC015712.1",9.37816400367186,-0.389965344931405,0.583806293030381,-0.667970437432594,0.504152463285507,0.862351333948746
"9013","JADE1",187.841289089845,0.200302777329508,0.30006075829072,0.667540728986097,0.504426802906344,0.862724860401786
"9014","MLLT10",155.058671094563,0.124341045004314,0.186310730975959,0.66738531029841,0.504526046551771,0.862798869269531
"9015","ATP13A1",62.9585664023261,0.139839700652275,0.209591163765363,0.667202271985212,0.504642940095436,0.862903041771619
"9016","UNG",60.700616083181,0.153247479913623,0.229733237201847,0.667067080846368,0.504729286220521,0.862918869438288
"9017","SNX5",361.008198133278,-0.112319885443148,0.168392476835831,-0.667012491019124,0.504764154766464,0.862918869438288
"9018","AC011445.2",2.77401890957985,0.58671619194507,0.879765809085865,0.666900424960487,0.504835739490183,0.862945544936923
"9019","ASB3",86.9780832537708,0.154558303071552,0.231815121036179,0.66673089477813,0.504944040888186,0.862996702388077
"9020","SSTR2",2.53494166714232,-0.637651532246056,0.956513731776995,-0.666641273472821,0.505001298841424,0.862996702388077
"9021","TRAPPC2B",90.1326230343846,-0.20175999530321,0.302674489017954,-0.666590686112438,0.505033619996292,0.862996702388077
"9022","THAP11",124.575703435013,-0.175897271060748,0.263940542970807,-0.666427632075469,0.50513780550817,0.863079059178502
"9023","FAM111A-DT",25.8513389984545,-0.213123851666421,0.319846226979241,-0.666332236210038,0.505198765195183,0.863087550203229
"9024","ME1",29.8387446681711,-0.205033649079133,0.307764431148175,-0.666203200656472,0.505281227418235,0.863132770462334
"9025","TSNARE1",7.9069336736997,-0.339962688400387,0.510520882837678,-0.665913383426627,0.505466465569916,0.863353525402798
"9026","RBBP7",473.34934576064,-0.117228218344937,0.176071316586583,-0.665799635156875,0.505539178123656,0.863382055259933
"9027","AFDN-DT",6.11969835852827,0.45833621472068,0.688928686323475,0.665288329285037,0.505866093788287,0.863507518450824
"9028","STYXL1",51.4199383494981,0.213839740430744,0.321444280269862,0.665246680548241,0.505892727805993,0.863507518450824
"9029","ARHGAP27",19.1491130213801,0.331276308757041,0.497834590324562,0.665434494097861,0.505772628456663,0.863507518450824
"9030","TOB1-AS1",5.57422765569007,0.450323352767803,0.676817084928829,0.665354588108776,0.505823723347407,0.863507518450824
"9031","BAHCC1",35.6642141344578,-0.202923495601386,0.304898559456603,-0.665544291068612,0.505702424580805,0.863507518450824
"9032","AC005520.2",21.5792428720532,0.226241249520957,0.3401327878002

4,0.665155661658319,0.505950936190886,0.863511257903289
"9033","MAT2A",469.100367734885,0.154407488279865,0.232180165923545,0.665033068891467,0.506029342266032,0.863549464300995
"9034","BRF2",71.4798943311232,0.188769171254446,0.283914615402772,0.664880076661961,0.506127199748291,0.863620852791665
"9035","SSU72",515.528021088952,-0.155427202593429,0.23389512102662,-0.664516651357337,0.506359695156045,0.86369138162497
"9036","PIK3R3",2.8724985245229,0.698515270414415,1.0512976563273,0.66443149208063,0.506414182535098,0.86369138162497
"9037","JAK1",856.496063342969,-0.107042309747848,0.161148472698608,-0.664246504824443,0.506532553377823,0.86369138162497
"9038","SHROOM1",24.8340782022567,0.24965026911527,0.375852002847021,0.66422492689731,0.50654636174885,0.86369138162497
"9039","ZFAND2A",84.2255578701754,-0.216452375944524,0.32588312830695,-0.664202461382558,0.506560738324447,0.86369138162497
"9040","NLRX1",31.6234446319762,0.192980889481539,0.290436247348073,0.664451807388426,0.506401183930996,0.86369138162497
"9041","ZNF772",6.26943010702018,0.389294353410877,0.585750831437671,0.664607427795518,0.506301617149028,0.86369138162497
"9042","STK35",76.4967879425748,-0.223355797267896,0.336335301643108,-0.664086690206856,0.506634828301294,0.863722171893877
"9043","WDSUB1",51.0927510058661,0.180883247448942,0.27243419061053,0.663952079742926,0.506720982003004,0.863773519581589
"9044","TENT4A",63.6176382196876,-0.162423730947055,0.244731030091554,-0.66368261877659,0.506893466170838,0.863972001440012
"9045","LSM5",404.136219748705,-0.175572437649894,0.264617644209946,-0.663494825426668,0.507013692471565,0.863989696506207
"9046","PORCN",39.2348931843695,-0.196579307768453,0.296280155756358,-0.663491306957821,0.507015945157,0.863989696506207
"9047","STK25",262.698033633211,-0.11806734260498,0.17813732539221,-0.662788342336605,0.507466120873387,0.864291392174859
"9048","MRPS25",196.076753890424,0.171631537210901,0.25895816349213,0.662777086832858,0.507473330559497,0.864291392174859
"9049","CD96",4.22442040390887,0.559132221713112,0.843318147315554,0.663014573435825,0.50732122048721,0.864291392174859
"9050","BRD8",118.666805888955,0.186140562162093,0.280846246797221,0.662784581545401,0.50746852983325,0.864291392174859
"9051","RNF169",145.726964219973,0.144595480477237,0.218159309986376,0.662797661425806,0.507460151593702,0.864291392174859
"9052","TMEM50A",1307.70103790201,-0.149159587687928,0.225274737418605,-0.662123012092388,0.507892388934623,0.864468915182715
"9053","SPICE1",46.9679745484303,0.164184935911204,0.247966076240305,0.662126603770151,0.507890087291076,0.864468915182715
"9054","TNFSF13B",200.3484476493,-0.185611040087846,0.280244443977872,-0.662318358406069,0.507767213719165,0.864468915182715
"9055","GZMM",8.09320807549087,0.358347767400979,0.540968047612107,0.662419470027418,0.507702429151199,0.864468915182715
"9056","SETD4",54.1912392045416,0.16651843769766,0.251468402348358,0.662184338639025,0.507853089988098,0.864468915182715
"9057","CBX6",324.340781863325,-0.126432456704962,0.190959847113952,-0

.662089222502967,0.507914042478744,0.864468915182715
"9058","POLR2B",163.590954508125,0.109445758742942,0.16544858611456,0.661509181270123,0.508285828597666,0.864664489262536
"9059","ABHD11",55.6992426358587,0.18018488674618,0.272401283788407,0.661468566668511,0.508311866481022,0.864664489262536
"9060","IGHG1",86.6223528323129,0.671820748553952,1.0157787785921,0.661384902611487,0.508365505428892,0.864664489262536
"9061","PPP4C",313.707075939321,-0.177688111428944,0.268483186903507,-0.661822118093397,0.508085229323608,0.864664489262536
"9062","ZNF805",59.4809408617812,-0.183018798493757,0.276716539305536,-0.661394504835421,0.508359349071878,0.864664489262536
"9063","GMEB2",28.7627039418983,0.234074631281047,0.353754960801616,0.661685791629972,0.508172612430073,0.864664489262536
"9064","KLF9",1634.77191132219,-0.195989000874795,0.296479552870543,-0.661054022030226,0.508577669646188,0.864834503871592
"9065","POLD1",18.1582754098731,0.267338615270808,0.404389747657307,0.661091476278867,0.508553651246041,0.864834503871592
"9066","ZFC3H1",199.370535009475,0.14771773301985,0.223586965225269,0.660672382538138,0.508822438664362,0.865137552395561
"9067","CHFR",42.3551806062866,0.208906104741077,0.316236359745752,0.660601155759046,0.508868127639997,0.865137552395561
"9068","HOXA13",12.4171286915711,0.345708289527307,0.523613911129815,0.660235112511361,0.509102962860243,0.865250515158837
"9069","IFIT3",392.853159026691,0.210814406856059,0.319223045917536,0.660398456665682,0.508998162325969,0.865250515158837
"9070","SH3BGRL",1044.06334141655,-0.138655663595183,0.209984743286218,-0.660313037153323,0.509052965512882,0.865250515158837
"9071","L3HYPDH",82.0202671343832,-0.18186735864869,0.275555883860462,-0.660001724879826,0.509252722440298,0.865409625886583
"9072","AKNAD1",18.6915961498091,0.256824149467492,0.389390599793067,0.659554056014644,0.509540046018021,0.865802448122551
"9073","THOC7",530.135848139119,-0.167447620815191,0.253987146559081,-0.659275963700155,0.509718574418161,0.865967528562757
"9074","TCEAL1",149.273240946818,-0.133817533216279,0.202991364451731,-0.659227714330179,0.509749552655106,0.865967528562757
"9075","SARNP",293.157756863975,-0.14818636552633,0.22484918397643,-0.659047824438022,0.509865058634765,0.866068306209906
"9076","ZNF492",4.61055191963,-0.538111556495629,0.817012576980214,-0.658633136939654,0.510131378603107,0.866425209471891
"9077","SNX4",153.73037853332,0.123587624142606,0.187742722173535,0.658281837569026,0.510357046465055,0.86662602828825
"9078","KLF3",444.453812050012,-0.131520882639739,0.199823113018102,-0.658186536348399,0.510418275110543,0.86662602828825
"9079","RITA1",39.6800406925558,-0.204516380374682,0.310721464599826,-0.658198430668687,0.510410633098124,0.86662602828825
"9080","BX284668.5",112.674597720173,0.205125981545357,0.312140572995353,0.657158983136776,0.51107869618648,0.866845490915078
"9081","ABCB10",42.1714140677243,-0.167888147173757,0.255482275856836,-0.657142052655879,0.511089581351409,0.866845490915078
"9082","CCDC115",251.17750017498,-0.17692698721866,0.269338347912036,-

0.656894900374315,0.511248497497158,0.866845490915078
"9083","ATIC",105.497663019562,-0.157764890767675,0.239814565506178,-0
.65786200448117,0.510626807507458,0.866845490915078
"9084","ACKR3",2862.4362280836,0.237159410791069,0.36066126024271,0.65
7568297275593,0.51081557147907,0.866845490915078
"9085","RNF13",555.288185915625,-0.0898887914243699,0.136824546399238,
-0.656963927818075,0.511204111027708,0.866845490915078
"9086","SSBP1",693.716906974284,-0.178943623331462,0.272302799539592,-
0.65714940732897,0.511084852774474,0.866845490915078
"9087","AL512598.1",4.02622039760884,0.602805964374574,0.9181435892070
67,0.656548683082538,0.511471154721319,0.866845490915078
"9088","LIPA",206.651618323163,-0.111387682893249,0.169449267231498,-0
.65735122206857,0.510955107927763,0.866845490915078
"9089","PCF11",369.917430342162,0.0960950023352249,0.146349788167221,0
.656611830728622,0.511430539804702,0.866845490915078
"9090","ALG9",55.8333212178134,0.152883516099669,0.232739476203672,0.6
56886913184761,0.511253633600914,0.866845490915078
"9091","TAGLN",3020.60813355664,0.272559987040916,0.415119110786783,0.
656582604747654,0.51144933698298,0.866845490915078
"9092","LETMD1",270.782194214213,-0.155962639338051,0.237438501749287,
-0.656854883218278,0.511274230507033,0.866845490915078
"9093","HERC2",211.649601645884,0.180609779868832,0.274897619418374,0.
657007435171552,0.511176135684466,0.866845490915078
"9094","SBNO2",68.5929605015477,-0.245579448348338,0.373536260596789,-
0.657444736304801,0.510894994317301,0.866845490915078
"9095","ICAM3",55.4491956398612,-0.189452592053247,0.288494793891064,-
0.656693278578832,0.511378157161537,0.866845490915078
"9096","ZBTB45",35.3167998401287,-0.257094368408545,0.391614626434656,
-0.656498381455227,0.511503508619108,0.866845490915078
"9097","MRPL33",539.948517654992,-0.153275184008586,0.233515121608066,
-0.656382263183127,0.511578199720273,0.866876766921844
"9098","INTS6L",17.149672440291,0.321896574865331,0.490694249765185,0.
65600233754394,0.511822620200954,0.867195613365322
"9099","BPI",2.4245310005974,-0.933442585414389,1.42395486748443,-0.65
5528210008099,0.51212772976648,0.867617205665489
"9100","BRD9",174.770742282832,-0.164218692912974,0.250697615652542,-0
.655046887803574,0.512437566243771,0.868046712488761
"9101","GADD45A",352.181687981794,0.264241205047486,0.403492745054622,
0.654884649813753,0.512542024016157,0.868051934630449
"9102","BCAS2",263.345533582556,-0.15272365019161,0.233360994004597,-0
.654452346858798,0.512820418704381,0.868051934630449
"9103","MICB",6.71762143175507,-0.40462676705344,0.618463553473525,-0.
654245128562392,0.512953891185235,0.868051934630449
"9104","HOXA5",31.108320790557,0.296049642496949,0.452265267622462,0.6
54592920772511,0.512729883195735,0.868051934630449
"9105","AL162390.1",36.6546891784455,-0.294854707048312,0.450625430213
965,-0.654323274450601,0.51290355409527,0.868051934630449
"9106","ASPN",1900.11421748249,0.1968752415705,0.300955309248568,0.654
167697064599,0.513003770644398,0.868051934630449
"9107","TMTC2",15.2243636051385,0.340142447383353,0.51980887596131,0.6

54360598891872,0.512879512743544,0.868051934630449
"9108","PSMC3IP",19.9833507391929,0.239364514140387,0.365767664468965,0.654416826287545,0.512843296763493,0.868051934630449
"9109","ELP2",184.336959076592,0.110456877615958,0.168792670980315,0.654393801427787,0.512858126879539,0.868051934630449
"9110","LZTR1",92.6526583404737,0.152316065621901,0.232695139661464,0.654573472585195,0.51274240814924６,0.868051934630449
"9111","SSBP2",342.810681668195,-0.148001335079468,0.22629942597023,-0.654006674762568,0.513107505325965,0.868132169311794
"9112","MAP3K8",219.673314938103,-0.209166305529274,0.319948018019075,-0.653750902488176,0.513272302819154,0.86816298135968
"9113","AC140912.1",4.71071460149178,0.467186197625071,0.714547334382365,0.653821202802322,0.513227004641043,0.86816298135968
"9114","DDTL",7.29777868860124,-0.480570234664846,0.735135899555576,-0.653716183572824,0.513294674804549,0.86816298135968
"9115","CSDE1",1086.99808298898,-0.101774807457422,0.155794301589894,-0.653263992449027,0.513586101623869,0.868281610315827
"9116","RAB33B",97.1240004263801,-0.137056849814817,0.209801897655531,-0.653267922484892,0.513583568434843,0.868281610315827
"9117","LINC00535",5.12388002253462,0.564494359892315,0.864104909704063,0.653270631323738,0.513581822398371,0.868281610315827
"9118","AC110285.2",4.65971233428089,0.495465623589147,0.758453488020271,0.653257755966579,0.513590121495927,0.868281610315827
"9119","XRCC4",64.0831769319171,-0.166332862376255,0.254662308868378,-0.653150688515213,0.513659136904447,0.868303059039593
"9120","SPESP1",21.2084946904615,0.252320797390523,0.386435804276372,0.652943631512124,0.513792619017244,0.868433467341099
"9121","FAM114A2",94.0565024368135,0.123834615502522,0.189817280117696,0.652388525563839,0.514150564656167,0.86875659141336
"9122","PROSER1",40.6618796834489,0.175371773099826,0.268834681646862,0.652340583534504,0.514181484910529,0.86875659141336
"9123","AC005899.7",6.28001079313091,0.415966012781027,0.637439145941416,0.65255799777828,0.514041271152802,0.86875659141336
"9124","UQCR11",1136.77143926466,-0.144669345195451,0.221784273969668,-0.652297580013434,0.514209220892345,0.86875659141336
"9125","TPH1",4.67746609424383,0.530350639353247,0.813197948345066,0.652179017953206,0.514285693896078,0.868790572209101
"9126","PRELID2",55.2591071492546,-0.189047439677468,0.289939471806092,-0.652023812073095,0.514385811247621,0.868864483934044
"9127","GPR161",62.2616799479084,0.179733514571764,0.275744940862747,0.651810742236708,0.514523270917001,0.868876918291407
"9128","ASXL2",129.33155371354,0.140740878287428,0.215971892408619,0.651662939643768,0.514618635355209,0.868876918291407
"9129","GTPBP10",56.9302466918193,0.151648130012677,0.232616609002622,0.651923053400577,0.514450812221031,0.868876918291407
"9130","MCM3AP",91.3408709694937,0.186700501980572,0.286495905182372,0.651669006793399,0.514614720558942,0.868876918291407
"9131","CCDC96",10.2813396997936,-0.31257662810529,0.479730016096644,-0.651567793586467,0.514680029870911,0.868885408001324
"9132","NEMP1",32.3401511414478,0.233942424397741,0.359289797223835,0.

651124596928081,0.514966059757884,0.869177905525871
"9133","SYNJ1",47.1976956338888,0.17251468137953,0.264944355989833,0.65113552140039,0.514959008338632,0.869177905525871
"9134","SOX2-OT",3.40925431435821,0.596015733267511,0.915661994691163,0.650912385490605,0.515103045826615,0.869218768628054
"9135","MIR100HG",261.170018495955,-0.128597721694915,0.197561126576617,-0.650926242035993,0.515094100614919,0.869218768628054
"9136","EIF1B",939.723581399186,-0.136973568352015,0.210616468129131,-0.650345956176776,0.51546877817465,0.869264983651924
"9137","POLN",9.10848333167148,0.418481486825348,0.643313621377633,0.650509289589089,0.515363303150562,0.869264983651924
"9138","PACS1",188.059013718432,-0.105025728449256,0.161477074192948,-0.650406436790904,0.515429720595929,0.869264983651924
"9139","KLRC1",2.57160184263538,0.72769737848115,1.11882977808082,0.650409376598288,0.515427822146451,0.869264983651924
"9140","PMM2",91.5837640668175,-0.175504113395559,0.269818417383792,-0.650452682575484,0.515399856717916,0.869264983651924
"9141","SNHG29",2892.94573285832,-0.182081383873515,0.279858440888213,-0.650619589302456,0.515292081779431,0.869264983651924
"9142","TPRG1L",192.411246028999,-0.112589515832247,0.173196717993342,-0.650067259568827,0.515648776598141,0.869315222650797
"9143","INO80B",56.8692904861373,-0.148160145244659,0.227925401330854,-0.650037882480642,0.515667751924677,0.869315222650797
"9144","HEXIM2",51.0953474893766,0.231242418132988,0.355723061758037,0.650063049019518,0.515651496265046,0.869315222650797
"9145","LYPLA2",70.1086335780272,-0.16479200669178,0.253670126979841,-0.649631111992445,0.515930531436322,0.869546550180082
"9146","SMIM20",337.642785305599,-0.171863816267237,0.264516816445316,-0.649727373014738,0.515868339175245,0.869546550180082
"9147","YY2",9.77794957255531,0.345832456025896,0.532407443660665,0.649563525348297,0.515974200097127,0.869546550180082
"9148","RPS3A",14515.8379622142,-0.171242098282727,0.263721415218829,-0.649329513648467,0.516125413086495,0.869626992877086
"9149","AC021097.1",4.47701170455561,0.508210613682957,0.782792498149558,0.649227751778811,0.516191176440177,0.869626992877086
"9150","EFTUD2",69.1842047639931,-0.158800568277603,0.244567229386723,-0.649312537398455,0.516136383643621,0.869626992877086
"9151","SMIM19",683.363965677143,-0.194102900335369,0.29908440932705,-0.648990366205003,0.516344603162439,0.869741145611322
"9152","SOX12",25.776672585458,0.225257624812977,0.347111806231012,0.648948323765924,0.516371778438847,0.869741145611322
"9153","ECE1",280.258461549527,-0.170299124867991,0.263199459176194,-0.647034478722039,0.517609629005124,0.87020277599098
"9154","GLRX2",130.314856501075,-0.146640592650422,0.226427705685793,-0.647626544668126,0.517226524325309,0.87020277599098
"9155","AL353593.1",6.70073576009332,-0.368875423369808,0.570310137242821,-0.646797942525002,0.517762724152051,0.87020277599098
"9156","CAMKMT",24.2940108655905,-0.207730771043532,0.32054658681501,-0.648051732846606,0.516951490879346,0.87020277599098

"9157","CEP68",154.536513026113,0.136407981511563,0.210454170021522,0.648160031695324,0.516881449755489,0.87020277599098
"9158","SFXN5",37.8219018644863,0.180132178846721,0.278516317585934,0.646756284902921,0.517789688962114,0.87020277599098
"9159","INO80D",173.355892526655,0.170326165547108,0.263055217445322,0.647492063458166,0.517313529413958,0.87020277599098
"9160","BAP1",86.1928903406407,-0.134842713784364,0.208478112277496,-0.646795542761251,0.517764277489688,0.87020277599098
"9161","TMED7",323.813233848087,-0.106300991668468,0.164143633780401,-0.647609591796187,0.517237491879791,0.87020277599098
"9162","CD14",124.499910841813,-0.327997257856818,0.50716300339441,-0.646729465007409,0.517807049756298,0.87020277599098
"9163","PCDHGB6",28.0760280687331,-0.218034947528173,0.336666772873032,-0.647628352710653,0.517225354630609,0.87020277599098
"9164","SAP30L-AS1",5.54318811384113,-0.434425675895428,0.671516028783554,-0.64693269747021,0.517675502798243,0.87020277599098
"9165","AL023806.1",5.82447961891284,-0.416479984250549,0.643701545457008,-0.647007898598194,0.517626831495294,0.87020277599098
"9166","GAS1",2553.21629505685,-0.156306427285553,0.241734118883277,-0.646604740810404,0.517887788967969,0.87020277599098
"9167","TMEM38B",54.5071087868652,-0.239217466843196,0.369201575609094,-0.647931868786162,0.517029017411726,0.87020277599098
"9168","ZNF25",87.4586833854901,0.154385732093841,0.23840484160312 4,0.647578006619722,0.517257926033334,0.87020277599098
"9169","POLE",34.2103238959134,0.204226006504729,0.315821029886712,0.64665106873341,0.51785779819725,0.87020277599098
"9170","ACOT2",90.2957851187486,-0.268872477553284,0.414704383831799,-0.648347324108195,0.51676033199109,0.87020277599098
"9171","MORF4L1",2161.43608761079,-0.0930652554180957,0.143540744715765,-0.648354274616457,0.516755837537702,0.87020277599098
"9172","ZNF790",30.3106909303599,0.196906241144075,0.304507945891102,0.646637448385313,0.517866615350939,0.87020277599098
"9173","ZNF234",44.9099317621551,0.213130169523188,0.329238991206644,0.647341825286478,0.517410737718256,0.87020277599098
"9174","NR0B1",25.0376202456422,-0.471858236522221,0.729219426757396,-0.647073047162804,0.517584668277204,0.87020277599098
"9175","MTFR1L",154.603179056698,-0.142349185902818,0.220418261220205,-0.645813940799609,0.518399858530975,0.870356717605285
"9176","GPAT4",115.053251871154,-0.13501098867579,0.208997742500935,-0.645992569394313,0.518284167640226,0.870356717605285
"9177","NSMAF",90.0267658296633,0.123648694719039,0.191430599319851,0.645919174668836,0.518331700973875,0.870356717605285
"9178","ADHFE1",10.6637918468241,0.301448850814362,0.466727427269437,0.645877729059061,0.518358543789269,0.870356717605285
"9179","FLI1",22.4209088156057,-0.27941812969243,0.432660090278106,-0.645814430244363,0.51839954151814,0.870356717605285
"9180","CACNA2D4",16.7723954092785,0.285209396675524,0.441253620181062,0.646361601653245,0.518045201876437,0.870356717605285
"9181","PITPNC1",63.3755668369861,-0.227297550196639,0.351902752608381

,-0.645910122929871,0.518337563394949,0.870356717605285
"9182","ZNF585A",107.215556696445,-0.13769322785076,0.213224743609144,
-0.645765709551805,0.518431098349123,0.870356717605285
"9183","GALNT4",65.7643511132425,0.156617741474331,0.242605936866442,0
.64556433983951,0.51856153785561,0.870480900146382
"9184","GALM",103.327179360886,0.129237027612347,0.200331475399908,0.6
45115937744482,0.518852056297781,0.870541540031446
"9185","HVCN1",31.4920975650033,-0.213286844526042,0.330468487459284,-
0.64540751272788,0.518663136135523,0.870541540031446
"9186","LIPC",29.5274366479216,-0.309020673375087,0.478963548895447,-0
.645186202765803,0.518806526235944,0.870541540031446
"9187","MINDY2",350.984889470576,0.124201321690937,0.192498461826347,0
.645206826654957,0.518793162838338,0.870541540031446
"9188","GADD45B",4665.35784815234,-0.193151881689033,0.299426500943701
,-0.645072767708526,0.518880030477387,0.870541540031446
"9189","SLC4A2",96.6321358398704,-0.135568002258337,0.210314359444509,
-0.644596986227688,0.519188388434796,0.870693326933894
"9190","ZFP37",34.3993915326508,0.195471854167035,0.303304308039317,0.
644474374368914,0.519267869535912,0.870693326933894
"9191","NRDE2",101.223297815517,0.151518468250597,0.235086466284342,0.
644522292777722,0.519236806475523,0.870693326933894
"9192","AC092376.2",29.2202720013282,0.236496040921947,0.3668173563971
08,0.644724238909573,0.519105905639875,0.870693326933894
"9193","CCDC22",66.0564630770197,-0.1797895388125,0.278947683026028,-0
.644527808448312,0.51923323100917,0.870693326933894
"9194","EDA2R",21.9463924510476,0.297148846551352,0.46111746445554,0.6
44410306389518,0.519309403037965,0.870693326933894
"9195","NIBAN1",691.509186377297,0.143911238436225,0.223386865184268,0
.644224262324087,0.519430019987958,0.870706150295168
"9196","BUB3",272.801648663868,-0.0988416168383184,0.153411569383798,-
0.644290500614337,0.519387074415431,0.870706150295168
"9197","IGKC",811.421682197829,0.468735343024092,0.728085727939091,0.6
43791417737149,0.519710699736846,0.870987218573982
"9198","CLTA",1427.33648812171,-0.158590969215398,0.246318897104766,-0
.643844102419575,0.51967653196861,0.870987218573982
"9199","ZBTB48",35.5422343572943,-0.192106346437761,0.298955914238825,
-0.642590888114341,0.520489597092772,0.87119207110078
"9200","IPP",73.948843639418,0.156924592608829,0.244083444716383,0.642
913708429389,0.520280093842302,0.87119207110078
"9201","AC046143.1",7.40902376267528,-0.45468091450437,0.7067905679613
1,-0.643303596730028,0.520027122916686,0.87119207110078
"9202","ZNF282",49.6362555142036,0.170258594280828,0.264847944413423,0
.642854127706792,0.520318757143343,0.87119207110078
"9203","MSR1",33.5306191988305,-0.284652033591191,0.442507641605918,-0
.64327032310256,0.520048709343009,0.87119207110078
"9204","AP001160.4",6.50071379030469,-0.414031461337042,0.644314332801
712,-0.642592350129296,0.520488648179389,0.87119207110078
"9205","AC023509.3",7.86183043559273,-0.432284167289217,0.672067186055
78,-0.643215702623722,0.520084145643885,0.87119207110078
"9206","DHRS12",141.378729157223,-0.170030312434267,0.264614845827141,

-0.64255772159261,0.52051112389479,0.87119207110078
"9207","PABPN1",375.268637111645,-0.102218379705119,0.158951530958138,
-0.643078925311135,0.520172888555935,0.87119207110078
"9208","INTS14",56.7032516795667,0.154532782153563,0.240204730870587,0
.643337796027087,0.520004936439263,0.87119207110078
"9209","FBXO17",358.001440471959,0.120106629996023,0.186840113635269,0
.642831069084465,0.520333720811113,0.87119207110078
"9210","SAE1",126.570278006984,-0.147920925213612,0.230122299247444,-0
.642792661542794,0.520358645502602,0.87119207110078
"9211","TECTA",2.94742606437648,0.580756369675347,0.903943351839351,0.
642469872137031,0.520568144989102,0.871192916622192
"9212","RUNDC1",52.4965132009827,-0.209381407697925,0.3259561083635,-0
.642360742215106,0.520638983234942,0.871216883040233
"9213","PTPN21",117.131857230086,0.126889088463304,0.197623618586482,0
.642074511998552,0.52082480414209,0.871433230853177
"9214","VPS9D1",24.7657821386394,-0.218348150369004,0.340161591874807,
-0.641895368508755,0.520941121630155,0.871533252651274
"9215","EIF2B4",93.2937543310065,-0.137953942022416,0.21504550160772,-
0.641510475648396,0.521191076988275,0.871667619808427
"9216","PPP1R21",75.5881719458025,0.13933579890166,0.217148265252253,0
.641662040172408,0.521092641280811,0.871667619808427
"9217","U47924.2",5.35032208116986,-0.452152559326796,0.70473691701759
4,-0.64159056863415,0.521139058281863,0.871667619808427
"9218","CCNYL1",43.2972428817742,-0.243774761570074,0.380240729550112,
-0.641106390308319,0.521453562656608,0.871917417111575
"9219","RIOK2",91.0302510289299,-0.179857916824572,0.28052592398819,-0
.641145439492943,0.521428194126755,0.871917417111575
"9220","KBTBD3",56.6690482960635,-0.190620762995125,0.29743935030154,-
0.64087271170367,0.521605386592142,0.872076684850095
"9221","HOXD3",5.3697991550572,-0.53229129075798,0.831045618463545,-0.
640507908268732,0.521842449438748,0.872283816753232
"9222","DHRS7B",170.012195878019,-0.171413524891382,0.26760031944034,-
0.640557998024357,0.521809895970191,0.872283816753232
"9223","ATP5MGL",2.47085520761215,0.653852235700119,1.02116016632343,0
.640303311138977,0.521975428515569,0.872316915716338
"9224","BCOR",24.960642696393,-0.252780382274667,0.394770257595661,-0.
640322763457967,0.521962784619864,0.872316915716338
"9225","CHTF8",7.23349598900074,0.357834529806095,0.559024175184694,0.
64010564424673,0.522103919791612,0.872437064887555
"9226","C1S",8829.45978973422,0.129654281666725,0.202584324640198,0.64
000154946341,0.522171592042054,0.87245557027187
"9227","SARS",466.74179324733,-0.121817911408711,0.190446450728759,-0.
639643904848661,0.522404131955316,0.872503673424293
"9228","CASP10",18.1997092960008,0.24699132547241,0.386220311485705,0.
639508897194683,0.522491927533192,0.872503673424293
"9229","FAM76B",92.431613744081,-0.159316978874208,0.249145121080051,-
0.639454540323986,0.522527277987159,0.872503673424293
"9230","MYO19",50.422725392775,0.190135372180961,0.297223549117836,0.6
39704938405068,0.522364444276494,0.872503673424293
"9231","PNPO",44.9107475318475,0.158173287005924,0.247364138127061,0.6

39434997342569,0.522539987872402,0.872503673424293
"9232","ZNF674",14.3420994919167,-0.249192241773406,0.389636522343805,-0.639550523329857,0.522464857218152,0.872503673424293
"9233","OAZ2",263.693330822757,0.113750966079915,0.177930707121514,0.639299241373951,0.522628281895418,0.872507903699167
"9234","ZNF430",133.602492045006,0.121721664995975,0.190411141939016,0.639257050592971,0.522655723824723,0.872507903699167
"9235","PARS2",10.9175580117179,-0.321463439867907,0.503036226996778,-0.639046300476419,0.52279281200412,0.872547769277123
"9236","ABHD8",65.7981762222846,-0.201785740875805,0.315729682936452,-0.63910918669125 8,0.522751904012184,0.872547769277123
"9237","DHX9",237.721006470962,-0.0929156392891517,0.145479300680093,-0.638686320698447,0.523027013402102,0.87257288594554
"9238","PLCL1",22.5645112064271,0.25899747662834,0.405578265152923,0.638588156420771,0.523090888032613,0.87257288594554
"9239","ZNF22",484.020727385774,0.131415620901444,0.205726900679615,0.638786762777812,0.522961660771138,0.87257288594554
"9240","SNHG10",24.5155793258431,-0.23569860649289,0.368984608880414,-0.638776254673751,0.522968497671833,0.87257288594554
"9241","RHOT2",113.839142167213,-0.13664357055104,0.21396688158113,-0.638620189915833,0.523070043679485,0.87257288594554
"9242","GALE",9.62426910772934,-0.341843883928072,0.535403103639695,-0.63847945894262,0.523161621195589,0.872586777986437
"9243","DAGLA",13.1770364469648,0.281205963795282,0.440484575709518,0.638401386342131,0.523212428733613,0.872586777986437
"9244","PBX2",76.8108258176731,0.145906034269995,0.228593127476955,0.638278306440792,0.52329253094937,0.872625958955489
"9245","DHCR24",29.0288301336702,-0.257154777816483,0.403157660940366,-0.637851646466718,0.523570256269494,0.872873188202832
"9246","ERV3-1",49.6853669030234,0.163148857515743,0.255741191648166,0.637945168176872,0.523509373813054,0.872873188202832
"9247","MAP2K7",127.00613612545,0.169940513802677,0.266452320621017,0.637789580539585,0.523610663075679,0.872873188202832
"9248","ZNF653",8.79965434659318,-0.312180319898482,0.489635014112462,-0.637577605564742,0.523748677290849,0.873008851691008
"9249","STRADB",63.2988300942297,0.153814130053515,0.241301243574189,0.637436126624122,0.52384080281326,0.873027841612683
"9250","DCAF1",45.9706426693769,0.212545735711584,0.333464622308956,0.637386161805975,0.523873339923277,0.873027841612683
"9251","AC106707.1",34.4188878555165,-0.275382132873902,0.432410951944361,-0.636852817061248,0.524220718795153,0.873229131211075
"9252","ISCU",1071.2037393318,-0.158598363026739,0.248991419471118,-0.636963166697137,0.524148836026054,0.873229131211075
"9253","LCAT",46.6024836154935,0.209889869189152,0.329553637732814,0.636891374142015,0.52419560178342,0.873229131211075
"9254","LYRM9",92.9913766086612,-0.168976218739124,0.265234382222732,-0.637082633567563,0.524071019902187,0.873229131211075
"9255","AL133330.1",109.414871767494,0.697670795612992,1.0956726806785,0.636751109994779,0.524286976188798,0.873245136461408
"9256","RFC4",49.1863324938236,-0.20272341054355,0.318455131694687,-0.

636583902619935,0.524395913293812,0.873332217310297
"9257","SERINC3",790.1548821909,0.107129934853604,0.168323661709389,0.636452022048831,0.524481842849219,0.873380966568079
"9258","ZNF670",21.2328954774763,0.225984743577967,0.355183410347267,0.636248025652492,0.524614775192091,0.873413625616815
"9259","AP000926.2",2.95692231783674,-0.559238081105106,0.878863996493474,-0.636319252280645,0.524568359065624,0.873413625616815
"9260","SLC12A4",136.816774049265,0.13355415628134,0.209989522030548,0.6360039062421,0.524773876000431,0.873584157510437
"9261","SEMA6A-AS1",3.09953239973512,-0.545048455871055,0.857661629485533,-0.635505235553095,0.525098953183335,0.873595194184839
"9262","DDX46",409.742440950007,-0.132138871482767,0.207923968135877,-0.635515340859668,0.525092364637424,0.873595194184839
"9263","HUS1",46.3674485238601,0.154854152614743,0.243634242100954,0.635600937205602,0.525036558482441,0.873595194184839
"9264","TNPO3",143.348236977779,0.1163696182847,0.183038778805285,0.635764831060702,0.524929713259211,0.873595194184839
"9265","DENR",281.153999016855,0.103092185555941,0.162203186128545,0.635574355945398,0.525053888312248,0.873595194184839
"9266","SRP14",3976.41730523558,-0.13498821140375,0.21242192151102,-0.635472132271182,0.525120536446105,0.873595194184839
"9267","XKR8",34.2083655224563,0.191341033184035,0.301176180060841,0.635312637092951,0.5252245332947,0.873673916125801
"9268","AC010654.1",3.96519639683696,0.486044423600885,0.765193889243597,0.63519119850963,0.525303722880311,0.873711360401381
"9269","AC092490.1",5.60466267324278,0.601833362964476,0.947630147435615,0.635093094698495,0.525367700420628,0.873723497894485
"9270","APIP",115.327452092621,-0.174276641894856,0.27450372941007,-0.63487895872814,0.525507361162292,0.873861485686811
"9271","FGF2",165.980562651275,-0.127410047063217,0.200746610286479,-0.63468093872865,0.525636527871289,0.873981995160816
"9272","AL133245.1",4.24267739361749,0.469307868396867,0.739704004346231,0.634453599871549,0.525784839031917,0.874040040297315
"9273","TCF19",28.1228310373297,-0.194420418667975,0.306397889453499,-0.634535763332931,0.525731234822913,0.874040040297315
"9274","TIMELESS",5.39664812978259,0.394686677235619,0.622459860627913,0.634075708652883,0.526031414767706,0.874280310888743
"9275","AVP",7.40858110175518,-0.380245095656975,0.599700572472065,-0.634058250252358,0.526042807881485,0.874280310888743
"9276","MYD88",81.9842724973677,-0.133169201977598,0.210106908049928,-0.633816390015853,0.526200655544088,0.874309871551534
"9277","GGACT",26.8542995463685,0.305707339301365,0.482231703863316,0.633942847084179,0.52611812158522,0.874309871551534
"9278","MEIS3",105.525854509963,-0.140009740724021,0.220915596895633,-0.633770284631217,0.52623074850828,0.874309871551534
"9279","CATSPERG",4.87228795373164,0.440571570314354,0.695460729498447,0.633495971270852,0.526409810895835,0.874513119405032
"9280","RBBP5",79.3568049575538,0.152806148345759,0.241410992129229,0.632970963741207,0.526752604638502,0.874769085505239

"9281","ZNF860",3.56517938759848,-0.537743038187858,0.849841976746847,-0.632756504034211,0.526892664829781,0.874769085505239
"9282","MAGEF1",172.502265159388,-0.122381779264234,0.193416024396038,-0.632738572961491,0.526904376186581,0.874769085505239
"9283","AC100793.4",72.3034888666384,-0.246398781400867,0.38932328521741,-0.632889916315359,0.526805533176489,0.874769085505239
"9284","MKS1",25.2751488792077,0.208731556395113,0.329743225185055,0.633012418308129,0.526725533518923,0.874769085505239
"9285","HDHD5-AS1",2.46297539815057,0.632265829670067,0.998786164323872,0.633034229201683,0.52671129061329,0.874769085505239
"9286","PPIL1",21.7486190872129,-0.264668129972045,0.418471161985675,-0.632464442032697,0.527083436401667,0.874877912364779
"9287","CADPS2",68.6202731898697,-0.142121398074802,0.224704739848333,-0.632480641800114,0.527072853974888,0.874877912364779
"9288","OTUD3",38.4921807770794,-0.184095116009024,0.291372053577228,-0.631821459020709,0.527503549764358,0.874915775728167
"9289","AC004522.5",4.35125756372948,0.459218021606824,0.726690292216127,0.631930860403246,0.527432056843644,0.874915775728167
"9290","LINC01252",9.55879092908578,0.448263320642512,0.709262093040371,0.632013644943234,0.527377961099735,0.874915775728167
"9291","FAIM2",21.0198009669947,0.302062748957689,0.477781315410824,0.632219677108883,0.527243341205322,0.874915775728167
"9292","INTS6-AS1",20.4082691933349,-0.211775826046134,0.335037456888669,-0.632095969247121,0.52732416890485,0.874915775728167
"9293","NLRP1",53.2148972609534,0.199936866554925,0.316428596144011,0.631854607931611,0.527481886696696,0.874915775728167
"9294","ZNF414",82.4304465774585,-0.180415078441836,0.285537210003107,-0.631844369565259,0.52748857749925,0.874915775728167
"9295","NUP42",110.029567547955,-0.158266205400742,0.250563829062394,-0.631640273031313,0.527621964215953,0.875018028874548
"9296","BTN2A1",77.9805564744275,-0.140681975375511,0.222794110924548,-0.631443868923244,0.527750339806092,0.87513677798095
"9297","Z97200.1",101.741184201929,0.179187010500289,0.283979921779438,0.630984787154989,0.52805047145757,0.875196299379304
"9298","SLC26A6",9.91951892710022,-0.347174348976228,0.549982980983287,-0.631245622101856,0.527879935995344,0.875196299379304
"9299","ZFPM2",153.076921447075,0.142886392473166,0.226460602216698,0.630954749190497,0.52807011226253,0.875196299379304
"9300","AC092068.2",14.0005814118083,-0.315339432271238,0.49973012665786,-0.631019455201138,0.528027803662193,0.875196299379304
"9301","ARMCX6",207.38773335963,0.17747845418003,0.281242745181201,0.631050781650145,0.528007321205598,0.875196299379304
"9302","CNTFR",53.8338622872074,0.664552858942314,1.05363333018578,0.630724977943836,0.528220364191775,0.875351205548936
"9303","MTLN",307.199619886278,-0.213927167120925,0.339489477309744,-0.630143734692967,0.528600547773357,0.875571879425613
"9304","PPIP5K2",172.093176451986,0.113155763639787,0.179510275262132,0.630358142309958,0.528460290349078,0.875571879425613

"9305","PRPF4B",491.829523180679,0.117819875977021,0.186989791395583,0
.630087210096774,0.528637527201698,0.875571879425613
"9306","PPP2R2A",264.573518158187,0.108248631051418,0.171763838218348,
0.630217816359058,0.528552084181978,0.875571879425613
"9307","AC062004.1",105.830960467153,0.266571466872829,0.4230404841725
6,0.630132284843202,0.528608038368859,0.875571879425613
"9308","UTP25",64.5838739394025,0.17021191963751,0.27044990205819,0.62
936580247268,0.529109600957087,0.875894069600413
"9309","AL392172.1",32.561450459963,-0.204558064957697,0.3250176957154
7,-0.629375162196624,0.529103474780449,0.875894069600413
"9310","HLA-
A",4351.68330207713,0.164546416299337,0.261559512775381,0.629097426254
36,0.52928527534785,0.875894069600413
"9311","FAM126A",311.178728447245,-0.176608188216909,0.280675727122081
,-0.629225013604731,0.529201755194719,0.875894069600413
"9312","ZNF273",53.1431295272151,0.168399936430029,0.26758908708921,0.
62932288555507,0.529137691629924,0.875894069600413
"9313","LIG3",38.2937172772471,0.196136453900161,0.311476436933716,0.6
29699170283947,0.52889142552192,0.875894069600413
"9314","NDUFB7",1174.00468492291,-0.15348257506212,0.243973461111505,-
0.629095371123063,0.529286620715397,0.875894069600413
"9315","RALGAPB",86.5884451788644,0.17116598105308,0.272006624472813,0
.629271369345595,0.529171411851872,0.875894069600413
"9316","BRWD1-
AS2",2.48359021216187,-0.619382983288566,0.984812448801064,-0.62893496
5274473,0.529391633884581,0.875973812401333
"9317","FDFT1",145.699225318871,-0.125239236190817,0.199237069206905,-
0.628594049738596,0.529614856822282,0.876249116444723
"9318","CYB561D2",113.173452382872,-0.151801327772069,0.24178603306998
1,-0.627833319586879,0.530113136297077,0.876327923902185
"9319","AC099343.2",2.69826450686447,0.628102440267911,1.0004380297732
9,0.627827433159696,0.53011699284468,0.876327923902185
"9320","MAML1",97.4620858536791,0.135313559238835,0.215457903034589,0.
62802782972,0.529985709188572,0.876327923902185
"9321","MRPS10",249.514250124505,-0.129579524162428,0.206260610330932,
-0.628232040788232,0.529851943571023,0.876327923902185
"9322","KDELR2",1220.84832898211,-0.145804141598378,0.232022986380857,
-0.628403865809424,0.529739405276246,0.876327923902185
"9323","MED30",193.133789793522,-0.152581202875521,0.242914222109496,-
0.62812791095757,0.529920150223133,0.876327923902185
"9324","VMAC",40.4776116641354,-0.187868409781828,0.299235954949047,-0
.627826992962184,0.530117281244754,0.876327923902185
"9325","CASK",113.366783766711,-0.12211287799776,0.194358509243391,-0.
62828675972628,0.529816103607283,0.876327923902185
"9326","DOCK2",8.95259260145421,-0.462981750918007,0.737774892856517,-
0.62753795961453,0.530306661716771,0.876528817752334
"9327","RBM4",545.216075160473,-0.138751612281848,0.221159981100288,-0
.627381190717904,0.530409394225999,0.876528817752334
"9328","HECTD1",329.764634985659,0.110353112101121,0.175880879194327,0
.627430978322516,0.530376766725722,0.876528817752334

"9329","CTSK",3821.19983569933,-0.144716990110615,0.231211913990086,-0
.625906284901997,0.531376411944405,0.876571680850281
"9330","CADM3",240.710771425427,-0.273824600314833,0.437023132649029,-
0.62656774861101,0.530942614652308,0.876571680850281
"9331","BDH2",603.070990517506,0.147822518818823,0.236229570260582,0.6
25757895828884,0.531473752278908,0.876571680850281
"9332","UQCRQ",885.788601787861,-0.145124668693414,0.231714877295527,-
0.626307082165997,0.531113541953944,0.876571680850281
"9333","BOD1",394.404132561301,-0.149128358285148,0.237964339289962,-0
.626683639784503,0.530866630077402,0.876571680850281
"9334","MAP7",2.48510751468616,-0.606235716082111,0.967044314516369,-0
.626895486568572,0.530727746034021,0.876571680850281
"9335","RNASET2",232.258077040924,-0.163772393575766,0.261371393333925
,-0.626588822467395,0.530928797072069,0.876571680850281
"9336","ZC3HAV1",596.749977874942,0.273219106201463,0.436665796804582,
0.625693856035476,0.53151576392524,0.876571680850281
"9337","HGSNAT",360.8009698832,0.122503700708277,0.195313759616722,0.6
27214902568437,0.530518375857608,0.876571680850281
"9338","URM1",215.698256671854,-0.172654287308173,0.275619640673598,-0
.62642293005738,0.531037991089936,0.876571680850281
"9339","CAPS2",28.803281883013,0.206918787183469,0.330258810368,0.6265
35252618706,0.530963921787962,0.876571680850281
"9340","DRAM1",162.535898878207,0.147291092986233,0.234944386315594,0.
626918971319371,0.530712350867177,0.876571680850281
"9341","AC106886.3",3.24671423221911,0.570334313703747,0.9098416407160
59,0.626850089269255,0.530757506370552,0.876571680850281
"9342","MIR22HG",403.42222381865,-0.209571590672505,0.3349278519372,-0
.625721597831765,0.531497564432272,0.876571680850281
"9343","AC132938.3",12.6240642751778,-0.304108897642282,0.486021447984
697,-0.625710858858758,0.531504609499651,0.876571680850281
"9344","SPPL2B",116.431467199182,0.118999443688444,0.190106372494336,0
.625962413185227,0.531339595242971,0.876571680850281
"9345","KLF2",4768.8839245096,-0.166103052517409,0.265421013725573,-0.
62580972842319,0.531439750081825,0.876571680850281
"9346","LINC00649",11.8628870338086,-0.30024034342802,0.47914853791749
2,-0.626612249998601,0.530913436462547,0.876571680850281
"9347","TCEAL4",749.486079552523,-0.118902249508645,0.189896514113293,
-0.626142349499411,0.531221576808968,0.876571680850281
"9348","SNAPIN",167.336262924637,-0.154081306704942,0.246661209658381,
-0.624667765630193,0.532189133439058,0.876650512071284
"9349","AP3S1",1134.4720027785,0.128614227856398,0.205693869334956,0.6
25270107817167,0.531793795376703,0.876650512071284
"9350","AC087623.2",4.47677332072683,-0.48458761401429,0.7753663054182
05,-0.6249789430209,0.531984878345654,0.876650512071284
"9351","GPSM1",48.0147653044189,-0.20598172771154,0.32964409164905,-0.
62486097257534,0.532062308810197,0.876650512071284
"9352","TGDS",54.2994731710742,-0.187956848009079,0.300704026090032,-0
.625055974318095,0.531934321567139,0.876650512071284
"9353","AC093249.6",5.8203222057445,-0.413412637333646,0.6615930943118
53,-0.624874474791263,0.532053446277106,0.876650512071284

"9354","AKTIP",118.343821881297,-0.140933750226456,0.225374092479,-0.625332524587263,0.531752837597113,0.876650512071284
"9355","AC010618.3",15.9572465578744,0.286018813261079,0.457835162191787,0.6247200671347,0.532154800248528,0.876650512071284
"9356","PRX",11.31019582277,0.282017881040903,0.451334279094929,0.624853670779095,0.532067101566574,0.876650512071284
"9357","LENG8-AS1",14.1140995809859,-0.278131756987092,0.445151640526679,-0.624802273351214,0.532100838452611,0.876650512071284
"9358","TCEA2",267.363893624871,-0.136238775900902,0.218044567068115,-0.624820777388792,0.532088692416362,0.876650512071284
"9359","PARK7",1933.73787665389,-0.162904127032076,0.26223548720292,-0.62121312706247,0.534459403476365,0.876742061079599
"9360","KHDRBS1",708.425416527587,0.081161304408836,0.130588924919384,0.621502202112,0.534269246045438,0.876742061079599
"9361","TTC4",92.692480480383,-0.128402983714949,0.206343632654644,-0.622277421711657,0.533759464900252,0.876742061079599
"9362","WDR78",11.2125789955168,-0.269429224874633,0.434334761012884,-0.620326183992998,0.53504305957759,0.876742061079599
"9363","TAF13",89.0504535416607,-0.14395388543339,0.231010295790209,-0.623149219133167,0.533186468307486,0.876742061079599
"9364","ARV1",72.6584907691857,-0.19325001081311,0.311025642130464,-0.621331442286835,0.534381570011628,0.876742061079599
"9365","PGM5P4-AS1",2.59303766861587,-0.555205410699222,0.89501536732146,-0.620330589809651,0.535040159519906,0.876742061079599
"9366","ITGAV",455.638154414871,0.103358216134903,0.165755144888957,0.623559625881567,0.532916832547207,0.876742061079599
"9367","TYW5",43.8055608468128,0.196933838650448,0.317443240717562,0.620374962797415,0.535010952158969,0.876742061079599
"9368","UBA7",92.3449001483443,0.140489206918133,0.226163499039705,0.621184264988175,0.53447839120981,0.876742061079599
"9369","DNAJC19",424.588174289532,-0.133668542225447,0.214968553790733,-0.621805095993576,0.534070035051583,0.876742061079599
"9370","LETM1",64.0561560835109,-0.185566377062987,0.298330217627297,-0.622016698606157,0.533930887887034,0.876742061079599
"9371","PI4K2B",51.0988524181172,-0.150779553687887,0.242452639897007,-0.621892810702897,0.53401235276151,0.876742061079599
"9372","C5orf56",146.734747781173,0.16310758844288,0.2620832364581,0.622350329029746,0.533711534000424,0.876742061079599
"9373","SAR1B",215.564580761897,-0.113661807112103,0.182130992986569,-0.624066257193716,0.532584072752694,0.876742061079599
"9374","ARHGAP18",203.717282481616,-0.185904461083619,0.298305122482438,-0.623202375931589,0.533151540593305,0.876742061079599
"9375","MOXD1",64.4542096842771,-0.581547835863803,0.935776197324407,-0.621460385000792,0.534296751786891,0.876742061079599
"9376","MALSU1",241.090232749146,-0.157474626648889,0.25334061893276,-0.621592492006521,0.534209859146887,0.876742061079599
"9377","DBF4",29.3424846395938,-0.215546985959119,0.345287260136784,-0.624254094615975,0.532460726249078,0.876742061079599

"9378","ST7",86.4380206269375,-0.167727170768901,0.270051448722391,-0.621093393730769,0.5345381756647,0.876742061079599
"9379","FASTK",186.502551279478,-0.158951727366431,0.255734262621254,-0.621550377087486,0.534237559223319,0.876742061079599
"9380","LYN",23.1025592070589,-0.285225299686086,0.459027956183636,-0.621368036181178,0.534357497940278,0.876742061079599
"9381","CPNE3",619.228545618163,0.080551307610865,0.129736239500394,0.620885173803886,0.534675176861679,0.876742061079599
"9382","AC027373.1",3.49019720750117,0.54056801401662,0.870030593232853,0.621320696330897,0.534388638983846,0.876742061079599
"9383","RNF139-AS1",9.05777814852816,0.355658943821444,0.573217337235806,0.620460898019098,0.534954389828786,0.876742061079599
"9384","RAB11FIP2",215.716158771087,-0.128401726113855,0.206236653319306,-0.622594112381457,0.533551281172738,0.876742061079599
"9385","RNH1",1967.39962855456,-0.149846253529581,0.240053798346705,-0.62421946480997,0.532483465388087,0.876742061079599
"9386","BTBD10",91.1877473709421,-0.120522991257654,0.194127511863102,-0.62084446506813,0.534701963810711,0.876742061079599
"9387","LIPT2",3.59832793820737,0.545553403941916,0.87586796298127,0.622871742088804,0.533368808808284,0.876742061079599
"9388","THY1",2891.81457020556,-0.232286417577378,0.373623488907043,-0.621712564852074,0.534130888097739,0.876742061079599
"9389","ARHGEF12",294.625068229712,0.164568611134391,0.26504109823051,0.620917330304989,0.534654017887919,0.876742061079599
"9390","ETV6",82.2804843353345,0.158614324322097,0.255045084585955,0.621907003538589,0.534003019670961,0.876742061079599
"9391","ERGIC2",421.511580424023,-0.111830632612675,0.179388021805445,-0.623400779423059,0.533021185917509,0.876742061079599
"9392","RESF1",172.23987587981,0.118246117271911,0.189809256701079,0.622973396171771,0.533302004465531,0.876742061079599
"9393","AC008035.1",2.79235923399709,0.571982009999896,0.922179807649643,0.620249982980765,0.535093218917696,0.876742061079599
"9394","MMP17",64.6809996398058,-0.165063261304505,0.266112751242904,-0.620275655839723,0.535076319485441,0.876742061079599
"9395","CDAN1",16.4191080582074,-0.223389978544976,0.357788714848241,-0.624362841180525,0.532389322655762,0.876742061079599
"9396","EID1",3661.37693658705,-0.118571429765073,0.190615222582041,-0.622045963375458,0.53391164519145,0.876742061079599
"9397","C15orf65",16.5997301767894,-0.254023159868765,0.407103722933041,-0.623976509078858,0.532643012419644,0.876742061079599
"9398","SYNM",24.0095499802797,-0.305278437447612,0.492030145552593,-0.620446613295935,0.534963791785906,0.876742061079599
"9399","LUC7L",162.146232022982,-0.152983884441849,0.246602058750723,-0.620367426033909,0.535015912980134,0.876742061079599
"9400","MYH3",2.5782871441534,0.603702740481325,0.967874741770596,0.623740567273129,0.532797976615666,0.876742061079599
"9401","ZNF18",19.2876032565961,-0.220335785031863,0.354614117519948,-0.621339574895714,0.53437622019916,0.876742061079599
"9402","VTN",3.25008914618159,-0.566437623663121,0.908902366043783,-0.

623210638265448,0.533146111769124,0.876742061079599
"9403","PDE4A",150.876933874611,-0.121675142352059,0.196113089127692,-0.62043356153976,0.534972382298382,0.876742061079599
"9404","KPTN",28.3198638557983,0.189972876348738,0.30595306807707,0.620921625472583,0.534651191700155,0.876742061079599
"9405","ZNF419",13.3830843871855,0.2750900398517,0.442105056481989,0.622227761972922,0.533792113567066,0.876742061079599
"9406","LPIN3",88.877677525314,-0.152498130081628,0.245897007959287,-0.620170742813093,0.535145381297304,0.876742061079599
"9407","CD40",178.957032077593,-0.136611790333812,0.21970326578883,-0.621801363959325,0.53407248935377,0.876742061079599
"9408","AP001505.1",11.0064117274837,-0.311612345183737,0.50170334167438,-0.621108769464769,0.534528059689038,0.876742061079599
"9409","MFNG",2.87221457651323,-0.507984460706185,0.818341278930675,-0.620748914645938,0.534764840058343,0.876742061079599
"9410","PRPF3",68.4697029769799,-0.137354826321025,0.221566344622006,-0.619926399721732,0.535306244105745,0.87680761312196
"9411","DCTN1",222.949449021693,0.131185307246855,0.211745947401399,0.619541053119527,0.535559985824017,0.87680761312196
"9412","LCA5",153.686254410665,0.128235694473667,0.206900404534053,0.619794314865928,0.535393212066069,0.87680761312196
"9413","AK3",574.741710123572,0.12018084573479,0.194130180193791,0.619073477471762,0.535867954798702,0.87680761312196
"9414","RPP25L",89.5749821765558,0.174309955938405,0.28154457635121,0.619120276431692,0.535837126620305,0.87680761312196
"9415","ZBTB26",14.750136265647,0.270846170435647,0.437438539514485,0.619163941833429,0.535808363436251,0.87680761312196
"9416","LSP1",1534.5532726461,0.144555189482421,0.233309482250627,0.619585574010814,0.535530666763219,0.87680761312196
"9417","PITPNA-AS1",126.263617310009,-0.168981930002738,0.272598029618476,-0.619894172526644,0.535327462637304,0.87680761312196
"9418","ZNF443",9.50923510268469,-0.296394778719604,0.478432079278661,-0.619512761699597,0.535578617449448,0.87680761312196
"9419","ALKBH6",36.2011210718919,0.215458208913355,0.348031147985936,0.619077373275976,0.535865388456576,0.87680761312196
"9420","E2F1",6.44237023906573,0.41674726573154,0.672873928397525,0.619354158548005,0.535683073344069,0.87680761312196
"9421","SON",1498.2291703077,0.121823304857094,0.196746001542776,0.619190753061415,0.535790702784358,0.87680761312196
"9422","GPR89A",39.5427461446518,0.179895409264759,0.29076571100165,0.618695404781542,0.536117037791975,0.876846278072111
"9423","XYLB",17.3049192038274,0.259263186953001,0.418909815627388,0.618899766205553,0.53598239254319,0.876846278072111
"9424","PSMB1",1566.77754497141,-0.160050181699642,0.258675183391968,-0.618730330451219,0.536094025513488,0.876846278072111
"9425","AKR1B10",5.82238209899689,-0.459338315161872,0.742540533034852,-0.618603691955376,0.536177469096391,0.876846278072111
"9426","ARHGEF40",151.033471455211,-0.160418202462877,0.259358316515251,-0.618519601061046,0.536232881179747,0.876846278072111

"9427","PAFAH1B1",442.234821723741,0.0854158781365093,0.138086963636677,0.618565836245399,0.536202413934682,0.876846278072111
"9428","IP6K1",101.95520503711,0.118948605964787,0.192905609448773,0.6166155888607,0.537488309783021,0.877038456155951
"9429","AC104162.2",11.4425468028649,-0.326623874532238,0.528554502586004,-0.617956848223218,0.536603783934084,0.877038456155951
"9430","MITF",319.1446319448,-0.180229129813625,0.292207097897351,-0.616785598674736,0.537376152185781,0.877038456155951
"9431","HMGXB3",96.9960580917562,0.129323142777849,0.20971824411605,0.616651847925482,0.537464388238698,0.877038456155951
"9432","CDKN1A",1732.23261175168,-0.236061144551191,0.382442250962597,-0.617246509654808,0.537072142622559,0.877038456155951
"9433","MICAL1",52.1448703530732,-0.209962500404663,0.339681495423485,-0.618115803284782,0.536499005734886,0.877038456155951
"9434","LINC01013",2.49235791539157,0.602956002929827,0.975860448931051,0.617871134740935,0.53666028784918,0.877038456155951
"9435","AP5Z1",82.482932816054,-0.119625728431123,0.193775704262893,-0.61734121357561,0.537009688128718,0.877038456155951
"9436","ZNF395",144.562416346703,0.144024055986518,0.233039946976335,0.618023037917803,0.536560152509317,0.877038456155951
"9437","PTRH1",86.9303991125299,-0.170921428447794,0.276991207772626,-0.617064454219421,0.537192213166087,0.877038456155951
"9438","ANKRD49",92.5830683686889,-0.164024882053534,0.265801920646631,-0.617094419989523,0.537172448992872,0.877038456155951
"9439","TPTE2",2.58799548586557,0.736475346584097,1.19300358847389,0.617328693475441,0.537017944562851,0.877038456155951
"9440","TRAP1",120.229724641208,-0.113338717871804,0.183760706058737,-0.616773413112463,0.537384190762147,0.877038456155951
"9441","FCSK",21.7946401942213,0.222378477922778,0.360186719070456,0.617397772179598,0.536972391108326,0.877038456155951
"9442","AC130343.2",2.71581298390205,-0.575621037885854,0.931909036825883,-0.617679424857216,0.536786677386666,0.877038456155951
"9443","ADPRM",32.4400760409294,0.210945373540949,0.341422601167443,0.617842441653404,0.536679203532498,0.877038456155951
"9444","AC010327.7",4.19561395365221,0.445370683594264,0.722039617373322,0.616823056350369,0.537351442470343,0.877038456155951
"9445","AC245452.1",45.4868159301244,-0.200199068567154,0.324260922228097,-0.617401157042062,0.536970159035052,0.877038456155951
"9446","CXorf40B",38.8234462939329,-0.189853929069936,0.307406873024671,-0.617598192265206,0.53684023652026,0.877038456155951
"9447","FLNA",995.732123522695,-0.126862655231374,0.205600408022909,-0.617035036317817,0.53721161634357,0.877038456155951
"9448","ACD",65.3676815942282,-0.140521417348203,0.228023292163775,-0.61625904974337,0.537723561274783,0.877329455657364
"9449","Z95115.1",5.07584835050541,0.437193899572147,0.709732980336255,0.615997722643542,0.537896022844974,0.877517958742224
"9450","MAP2K1",123.264461612514,0.120883012240718,0.196382970923486,0.615547324048867,0.53819332640323,0.877910066296909
"9451","FAM185A",21.6745761860993,-0.197682023160005,0.321390058454771,-0.615084436993638,0.538498959364347,0.877944099270377

"9452","EIF5AL1",16.4923521235114,-0.334859990405229,0.544323601562127
,-0.615185506276471,0.538432218369419,0.877944099270377
"9453","MTRNR2L8",4.83447042091845,0.564399321975843,0.917261567309137
,0.615309026444393,0.538350657590711,0.877944099270377
"9454","RASSF2",116.796579380715,-0.251560762615019,0.408856358486557,
-0.615279076363662,0.538370433159625,0.877944099270377
"9455","EMD",208.034454400965,-0.111251981982031,0.180855785276202,-0.
61514195861707,0.538460974511879,0.877944099270377
"9456","CDCA7L",33.3329507378855,0.228343364477757,0.371353486046705,0
.614894899489481,0.538624131442577,0.878001348756365
"9457","FAM78A",5.69346109268198,0.403106543611248,0.655608343437961,0
.614858776045142,0.538647989308397,0.878001348756365
"9458","ANGEL2",120.823324321861,0.124803255227916,0.203141398769014,0
.614366426460545,0.538973216281788,0.87806722986512
"9459","IKZF4",50.903989266786,0.196041408529986,0.319038239625939,0.6
14476210625525,0.538900688609886,0.87806722986512
"9460","AC073548.2",3.46543274905778,0.849840383281218,1.3831055128931
3,0.614443638145549,0.53892220674143,0.87806722986512
"9461","ELK1",54.4474710723187,-0.14421612221904,0.234712131417715,-0.
614438296597375,0.538925735532458,0.87806722986512
"9462","GRIA3",51.2065126340186,-0.262513945312289,0.427097145911342,-
0.614646920086846,0.538787921046097,0.87806722986512
"9463","FNIP2",177.645724278894,-0.154773157280299,0.252001473398424,-
0.614175604583061,0.53909929224658,0.878179815067213
"9464","AC026304.1",17.6878165784953,0.239359421522349,0.3898116752596
09,0.61403861585967,0.539189809772351,0.878234458753254
"9465","AC012313.1",14.734943407561,0.291015404661868,0.47405125586471
,0.613890167068603,0.53928790832055,0.878301437587033
"9466","TTC39B",59.7228405630246,0.152407910404228,0.248349771669366,0
.613682506650871,0.539425150342532,0.878432145840918
"9467","TMEM30A",339.518349018625,0.0972091207812056,0.158470392941222
,0.613421339955037,0.53959777930214,0.878527647649186
"9468","RTL8A",316.270160990582,-0.140711488243136,0.229367120928734,-
0.613477152581326,0.539560885307912,0.878527647649186
"9469","GPX7",163.84541461614,0.145904783619569,0.238229955056185,0.61
2453558097506,0.540237715943048,0.878563240398904
"9470","RIT1",94.2723727717901,-0.140148893525461,0.229309552227044,-0
.611177738407936,0.541081919423401,0.878563240398904
"9471","RRNAD1",64.5213982122129,-0.195723434744649,0.319670393595099,
-0.612266380203342,0.540361529362535,0.878563240398904
"9472","COPA",503.045476095826,0.113681222920329,0.185668521492601,0.6
1228054172263,0.540352161381249,0.878563240398904
"9473","AL513217.1",2.86587064833598,-0.527637421156,0.862243485211154
,-0.611935526572042,0.540580415337508,0.878563240398904
"9474","HIST3H2A",106.394559097535,0.279334562056788,0.456139947332362
,0.612387850900621,0.540281177967197,0.878563240398904
"9475","TMEM198",8.58142616771744,-0.360740182603478,0.588433640892759
,-0.613051595853988,0.53984222429575,0.878563240398904
"9476","U73166.1",4.63607030906327,0.443932111832006,0.726343920369016
,0.611187206752509,0.541075651840836,0.878563240398904

"9477","AP001816.1",57.9911005428141,-0.181284872642991,0.296768811396
405,-0.610862279597306,0.541290758511042,0.878563240398904
"9478","ZCCHC9",180.046433047351,-0.137907185085953,0.225714362461712,
-0.610980992001986,0.541212164160165,0.878563240398904
"9479","PDLIM7",261.378456443487,-0.16267264117518,0.265512412304583,-
0.612674337004516,0.540091694551102,0.878563240398904
"9480","HIST1H2BJ",3.3301212107137,0.538026135941249,0.879723582647333
,0.611585441784087,0.54081207251037,0.878563240398904
"9481","UQCC2",175.679260854881,-0.201983542521643,0.32974179509651,-0
.612550624534951,0.540173514550109,0.878563240398904
"9482","LEMD2",74.9609351398795,0.178373961739183,0.291536045177382,0.
61184187921138,0.540642378558564,0.878563240398904
"9483","PEX2",232.618990188986,0.144846707502931,0.237230432300707,0.6
10573888426453,0.541481713577612,0.878563240398904
"9484","RRM2B",70.1504766348325,0.14718510390852,0.241028264875203,0.6
1065495362018,0.54142803374613,0.878563240398904
"9485","NELFB",182.54397308679,-0.106736037937593,0.174409915785528,-0
.611983770858802,0.540548495146609,0.878563240398904
"9486","RNF141",94.8486112341828,0.122874149996135,0.201131618881025,0
.610914140102547,0.541256423211461,0.878563240398904
"9487","BDNF-
AS",40.3151446569285,0.152306677951779,0.248996606986869,0.61168174054
6009,0.54074834500375,0.878563240398904
"9488","TRAF6",47.2665397520109,-0.163532736470449,0.266984708247827,-
0.612517239446727,0.540195595584734,0.878563240398904
"9489","JAML",7.38377586684826,-0.434035129337447,0.710421976145024,-0
.610953973711033,0.54123005130151,0.878563240398904
"9490","CERS5",126.525998508082,-0.110036203369139,0.179868764428101,-
0.611758265638855,0.540697705769098,0.878563240398904
"9491","SLC11A2",85.1119022305991,0.140601324525538,0.230120771599851,
0.610989279881367,0.541206677326401,0.878563240398904
"9492","COG3",61.5146926823066,0.138687391954669,0.226411374052754,0.6
12546046040755,0.54017654275828,0.878563240398904
"9493","BLOC1S6",523.753169569708,0.0987447618320162,0.161016734772692
,0.613257758402657,0.53970591940149,0.878563240398904
"9494","AMDHD2",110.067925799574,0.15596071776485,0.255252873291965,0.
611004748951281,0.541196436395617,0.878563240398904
"9495","EVI5L",15.0772338482918,0.296831555317211,0.485575540321519,0.
611298409142822,0.541002043993674,0.878563240398904
"9496","TUBA8",2.91774103328988,-0.696588454679346,1.13900364361465,-0
.611577020481434,0.540817645643361,0.878563240398904
"9497","DRG1",150.485138096281,0.126884109827365,0.207820855926208,0.6
10545603143978,0.541500444179694,0.878563240398904
"9498","CDPF1",28.161901995383,-0.205789289237185,0.336154373530432,-0
.612186856520418,0.540414136552765,0.878563240398904
"9499","RPGR",58.191069823366,-0.215063291566249,0.351073285973788,-0.
612588026940638,0.540148776981524,0.878563240398904
"9500","HMGB3",10.2837692682968,-0.315109042908047,0.515959335975923,-
0.610724568656224,0.541381938112322,0.878563240398904
"9501","UBL4A",109.661943037718,-0.181835066263425,0.29768672492342,-0

.610826923203252,0.54131416755258,0.878563240398904
"9502","KAT2A",23.1464661093657,0.248567449202324,0.407283201469712,0.610306166091187,0.541659013098894,0.878635555816001
"9503","EIF2S2",725.19668760859,-0.0976327361429876,0.15997050788012,-0.610317098050051,0.541651772825203,0.878635555816001
"9504","BAIAP2-DT",23.7943739432483,0.207739283711457,0.340513312036561,0.610076835084649,0.541810910942839,0.878728472045731
"9505","DLGAP4",556.620324007339,0.105587846778034,0.1730813363326,0.610047559230375,0.541830303392454,0.878728472045731
"9506","CORO1B",315.082372371084,-0.13943346148807,0.228712700004854,-0.609644595502776,0.542097263456941,0.87879691832236
"9507","C12orf4",51.0172825495704,0.14215665988898,0.233189324283833,0.609619073795804,0.542114173581084,0.87879691832236
"9508","ACTR10",415.817691193108,-0.133173540217705,0.21849304659846,-0.609509283205919,0.542186921421912,0.87879691832236
"9509","BCL11B",11.4964538159196,0.477775999228933,0.783796954557659,0.609566031675345,0.542149318970143,0.87879691832236
"9510","RGS9",189.128962063518,-0.18919420863544,0.310425391634296,-0.609467568485265,0.542214563098538,0.87879691832236
"9511","GRK3",21.5678247104821,-0.211348964495977,0.346658242681083,-0.609675289591809,0.542076926574207,0.87879691832236
"9512","EPS15",380.572586332294,0.104940688916509,0.172276535443343,0.609140929415902,0.542431030206267,0.878803273474277
"9513","HNRNPH1",752.921384772591,0.1283388858592,0.210691344756419,0.609132216644083,0.542436804840623,0.878803273474277
"9514","ITGB4",2.47425155695754,0.648928866376494,1.06535899617009,0.609117554466955,0.542446522679711,0.878803273474277
"9515","SMARCB1",252.316912390675,-0.161787040833824,0.265545076844145,-0.609263944022508,0.542349502090155,0.878803273474277
"9516","TRAF3IP2",102.144072921306,-0.140395271607036,0.230546791756421,-0.608966494555982,0.542546647646379,0.878819865589622
"9517","SLC48A1",102.489620115796,0.137698371528217,0.226131663076211,0.608930079295484,0.542570785651407,0.878819865589622
"9518","ASB9",3.21970502324746,-0.502176055157083,0.825271422910543,-0.608498054356497,0.542857195995076,0.879191392757312
"9519","GREB1",9.94616587065742,0.441839391326322,0.726486227185043,0.608186879245243,0.543063535765028,0.87922025188551
"9520","FOXO1",390.266721354834,0.157591689974004,0.259103530205886,0.608218999751877,0.543042234895354,0.87922025188551
"9521","STAT5A",99.2667934442316,-0.158772078064705,0.260988844387252,-0.608348140080351,0.542956599060506,0.87922025188551
"9522","RAMP2-AS1",172.553084242892,0.189132428594808,0.311008044448096,0.608127127163013,0.543103161755032,0.87922025188551
"9523","EFEMP2",1065.60599323618,0.166245251216589,0.273480683869152,0.607886629741389,0.543262667801805,0.879342366450324
"9524","EEF2KMT",37.3320136935759,-0.242743244500181,0.399409454013835,-0.607755380001029,0.543349726917894,0.879342366450324
"9525","CFAP20",206.290894260785,-0.158100965128324,0.260124014742016,

-0.607790731221507,0.543326277456624,0.879342366450324
"9526","AC005041.4",8.51341555150908,-0.325108867386218,0.535262238102097,-0.607382408553555,0.543597160089402,0.879468455073856
"9527","SIX4",6.06501892163594,-0.381662962192225,0.628458732894673,-0.607299958160006,0.543651866112391,0.879468455073856
"9528","C1QTNF1-AS1",2.77479510712941,0.570446582314637,0.939083548442876,0.607450299028784,0.543552116661806,0.879468455073856
"9529","CHAF1A",29.2671080009089,-0.194148182103238,0.319693914656827,-0.607293955881657,0.54365584874465,0.879468455073856
"9530","EGLN1",159.001686837333,0.120660242679868,0.198822175144381,0.606875176736432,0.543933752955401,0.879723242957307
"9531","LINC02308",9.26901692459862,0.424952067377731,0.700318106033053,0.606798630103781,0.543984557370681,0.879723242957307
"9532","SPECC1",16.7604194283014,-0.329202141003775,0.542387656599475,-0.606949913034016,0.543884152346287,0.879723242957307
"9533","MIB2",95.4063117309369,-0.121418235650507,0.200333734503125,-0.606079829498774,0.544461744329174,0.879834612438308
"9534","AKR7A2",523.414454578314,-0.180290120145078,0.297230830384473,-0.606566014406613,0.544138960151722,0.879834612438308
"9535","HES6",16.4196835580241,-0.249477298817069,0.411514134762356,-0.606242356562402,0.544353830012878,0.879834612438308
"9536","FBXL5",224.84216167454,-0.0849388515079550,0.14015924384285,-0.60601676478214,0.544503620746046,0.879834612438308
"9537","DNAJC15",586.123142978908,-0.16180784148652,0.267041179194286,-0.605928426375007,0.544562282163594,0.879834612438308
"9538","LMO7-AS1",7.75927360029814,0.607412802714291,1.00153168982624,0.60648385755989,0.544193498417701,0.879834612438308
"9539","AC011939.2",38.1150631298769,0.355541274915786,0.586311903611012,0.606402961846856,0.544247202159262,0.879834612438308
"9540","VPS33B",25.2025973544742,0.228251952045344,0.376590845221397,0.606100639305649,0.544447926491795,0.879834612438308
"9541","AC100793.2",6.57089182669515,0.353306767892271,0.583090334916063,0.605921152754368,0.544567112375861,0.879834612438308
"9542","ZCWPW2",40.9483948094726,0.171040791486674,0.282410978891619,0.605644979377074,0.544750527299919,0.880038710786863
"9543","PLCD4",4.96239619338627,-0.405257868108291,0.669642616913296,-0.605185300147591,0.545055881927658,0.88035776595532
"9544","EVC2",100.665780894898,0.227140591559253,0.375433036641453,0.60500960062334,0.545172617635439,0.88035776595532
"9545","AC136475.1",19.073862852391,0.257882803927368,0.426170064881582,0.605117123838871,0.545101177159861,0.88035776595532
"9546","CLUH",34.1639907951353,-0.163710338140117,0.270632344686988,-0.604917857580783,0.545233577137557,0.88035776595532
"9547","ZZEF1",81.6259383244965,0.144056373293531,0.238108657291335,0.605002669505092,0.545177222962081,0.88035776595532
"9548","PRR13",341.271629704226,-0.119692830317782,0.197977407679146,-0.604578227995406,0.545459276557916,0.880445523365474
"9549","MAX",232.545154665624,0.0940490233868996,0.155538524976138,0.6

04667064968812,0.545400235842048,0.880445523365474
"9550","AL391097.1",3.36608413103931,0.699249139539107,1.1564877662970
1,0.604631678705995,0.5454237530382,0.880445523365474
"9551","LINC01060",12.11565241555,-0.84732019206931,1.40344238426244,-
0.603744194682141,0.546013726534869,0.880606547257596
"9552","ZUP1",27.7059980767058,0.19537470032869,0.323355853277968,0.60
420956771962,0.545704320661757,0.880606547257596
"9553","AC124242.3",3.1206428094439,0.516765943712367,0.85560150519547
9,0.603979703839232,0.545857136082204,0.880606547257596
"9554","RNF208",20.4046402719571,0.244728677325598,0.405038459784475,0
.604210961733907,0.545703393974196,0.880606547257596
"9555","NUP160",96.2541729637582,-0.157819064415813,0.261343523310322,
-0.603875934696176,0.545926129623451,0.880606547257596
"9556","ADGRL1",28.8988605861626,-0.177887581527748,0.294683020803925,
-0.603657384271591,0.546071452546335,0.880606547257596
"9557","SAMHD1",747.625708850422,-0.14256180308115,0.236164450800208,-
0.603654794775848,0.54607317452062,0.880606547257596
"9558","BCAS4",6.59021846109718,0.349175768840314,0.578331380391,0.603
764175141667,0.546000440627705,0.880606547257596
"9559","TBC1D10A",67.5624146596457,-0.172231975827647,0.28509328476321
5,-0.604124983058423,0.545760550780795,0.880606547257596
"9560","COG2",77.2683085489634,-0.134609393473528,0.223119027310583,-0
.603307548872335,0.546304112010291,0.880787700583299
"9561","PDIA6",979.884859169712,-0.12742071731141 8,0.211351762132819,-
0.602884575106326,0.546585478026587,0.880787700583299
"9562","UBE2B",999.988492155475,-0.101604734974127,0.168406678226202,-
0.603329606903431,0.546289440775618,0.880787700583299
"9563","RNF217-
AS1",3.89029372376635,0.488480877336057,0.810024608986622,0.6030444901
50946,0.54647909263866,0.880787700583299
"9564","PANK1",2.7357724243762,0.533405184303005,0.884637305641536,0.6
02964831916263,0.546532084945732,0.880787700583299
"9565","SCUBE2",9.47534073525639,0.348839287993829,0.578561817130506,0
.602942119001161,0.546547195083751,0.880787700583299
"9566","CYP2R1",16.0317013771862,0.231839742018219,0.384547756398116,0
.602889337308208,0.546582309766883,0.880787700583299
"9567","ZNF501",21.2995740319282,0.20341561719633,0.337916420390528,0.
601970206009058,0.547193969902289,0.880810549752853
"9568","LIN54",36.0000755924466,0.192625861346566,0.32004233422837,0.6
01876191820033,0.547256553210239,0.880810549752853
"9569","TUBB",2662.4981965976,0.143328370288124,0.237943683523851,0.60
2362576578997,0.546932815141036,0.880810549752853
"9570","KIAA0895",3.15877531800786,-0.599897780960788,0.99599374199215
7,-0.602310793400057,0.546967277554726,0.880810549752853
"9571","ZNF782",15.9152873470257,0.251524341948334,0.417848090557005,0
.601951636569748,0.547206330914633,0.880810549752853
"9572","TSPAN4",1704.99506436795,0.126969948380442,0.210998555538331,0
.601757429364846,0.547335615991267,0.880810549752853
"9573","IVD",101.510520134764,0.147570615796421,0.245148258635605,0.60
1964772736866,0.547197586623181,0.880810549752853

"9574","CIAO2B",553.598716449317,-0.155899857833023,0.259115648038489,
-0.601661300709502,0.547399615091296,0.880810549752853
"9575","MPP3",12.4063615051857,0.294776446385256,0.489261151593861,0.6
02493055957879,0.546845984096988,0.880810549752853
"9576","SKAP1",4.52871796409382,0.525922420974394,0.872893605599169,0.
602504609497502,0.546838295809414,0.880810549752853
"9577","DHX40",198.837572200205,0.124090145251199,0.205877525129956,0.
602737696467205,0.546683199827512,0.880810549752853
"9578","FUZ",81.4128267397674,0.162308054145922,0.269705969274113,0.60
1796299068792,0.547309738951541,0.880810549752853
"9579","ZNF614",25.7407353867304,0.198023584221999,0.329124517897814,0
.60166767728766,0.547395369673722,0.880810549752853
"9580","ATP5PF",1405.67146897988,-0.144685255590764,0.240171815183196,
-0.602423958366648,0.546891966114288,0.880810549752853
"9581","BTBD19",27.1336983473447,-0.240269825808789,0.399648511374686,
-0.601202854434073,0.54770488351888,0.881025856145626
"9582","PPP2R1A",397.464544075234,0.0943976106842415,0.156995245842456
,0.601276874198912,0.547655589827452,0.881025856145626
"9583","USP11",172.572341496884,0.12221630254361,0.203230692134674,0.6
01367348897391,0.547595340901661,0.881025856145626
"9584","SLCO3A1",273.646860383881,-0.135211836839369,0.224983249087854
,-0.600986239586975,0.547849151449097,0.881165971367678
"9585","TOPORS",314.834658774302,-0.149363043813077,0.248695163315498,
-0.600586846249169,0.54811520115307,0.88150191192223
"9586","AL513548.1",6.44734076118171,-0.447523000723671,0.745882909705
268,-0.599990956892293,0.548512262288447,0.881550145318543
"9587","AL157786.1",3.45328557184241,-0.47198090662231,0.7867715627155
75,-0.599895737198798,0.548575723534558,0.881550145318543
"9588","OSBPL5",71.9288722961445,0.124458975690284,0.207310009345459,0
.600351985334614,0.548271679927198,0.881550145318543
"9589","PCK2",47.6864595844064,0.220250384189427,0.367192434114506,0.5
99822773365596,0.548624354329301,0.881550145318543
"9590","CYB5D2",214.411720771765,0.155123814837802,0.258402453032618,0
.600318661906128,0.548293883830652,0.881550145318543
"9591","TMEM100",180.130640232054,-0.253332122811055,0.422276529118282
,-0.599919970309541,0.548559572504289,0.881550145318543
"9592","PCNA",152.67015726819,-0.137355858319259,0.229014421800626,-0.
599769469709805,0.548659882853461,0.881550145318543
"9593","ZMYND8",144.25500436945,0.149132574082451,0.248505637504293,0.
600117468481475,0.548427951482947,0.881550145318543
"9594","GAB3",17.575108994973,-0.276499339508065,0.460887150692317,-0.
599928505476284,0.54855388399063,0.881550145318543
"9595","BRAF",124.652619085416,0.146631871563518,0.244579571750291,0.5
99526242172119,0.548822015895365,0.881626862758133
"9596","MCM5",56.6848242880378,-0.161755987949459,0.269779438080063,-0
.599586051111333,0.548782145662749,0.881626862758133
"9597","FASTKD2",98.9819554397345,0.114607334579813,0.191314578402391,
0.599051758297062,0.54913837014312,0.881968871547957
"9598","TULP3",295.501751665828,0.082613483203042,0.137910878058152,0.
599035292692479,0.549149349926519,0.881968871547957

"9599","BSCL2",283.183692032527,-0.153037276689102,0.255524256676836,-0.598914868902835,0.549229655592211,0.882005952802785
"9600","NFIL3",681.45932763804,-0.233687035254155,0.390248461815913,-0.598816031629586,0.549295570426985,0.882019918555413
"9601","CD53",28.2754366493576,-0.253705971593,0.424083191167761,-0.598245761390333,0.549675961313995,0.882248542043908
"9602","DUSP4",4.93643332798323,-0.470482287200756,0.786618982294569,-0.598106958757031,0.549768567337462,0.882248542043908
"9603","SAMD12",3.77566462469461,0.579940067920771,0.97026241199223,0.597714660232984,0.550030341737973,0.882248542043908
"9604","TRIB1",87.3599334608714,-0.529305697251847,0.885396400921881,-0.597817764676624,0.549961535878019,0.882248542043908
"9605","CDC42EP2",208.906621931459,0.218408736883384,0.365189572774906,0.598069477241087,0.549793575487977,0.882248542043908
"9606","RALGAPA1",185.390552431219,0.147292804317215,0.246317926395938,0.597978419485606,0.549854332731286,0.882248542043908
"9607","FCF1",115.948630155418,0.1307513494217,0.218481567683656,0.598454829887605,0.549536489986223,0.882248542043908
"9608","JMJD7",20.7133959459405,-0.212100749727652,0.354463108657058,-0.598371860279707,0.549591837611375,0.882248542043908
"9609","DRG2",135.403500862807,-0.124329691281044,0.208017585089655,-0.59768836960326,0.550047887241158,0.882248542043908
"9610","CIRBP",2593.24585747557,-0.130629648809958,0.218363527115972,-0.598221005747818,0.54969247716357,0.882248542043908
"9611","HDHD5",62.1965310155351,-0.184467414659774,0.308649960963791,-0.59765895995501,0.550067514601622,0.882248542043908
"9612","USP18",59.0442173697246,0.246059279615678,0.411987200799783,0.597249815377779,0.550340604585825,0.882594716988191
"9613","AC105285.1",3.94760869039436,0.439821750295578,0.736639018710131,0.597065508511502,0.550463644908688,0.882662408239883
"9614","ARL6IP4",1413.0929253016,-0.13570003724272,0.227309663964753,-0.596983141305255,0.55051863631918,0.882662408239883
"9615","AREL1",24.6234833889188,0.2248296512339,0.376643693528615,0.596929286476473,0.550554593268016,0.882662408239883
"9616","NOSIP",239.777927209144,-0.135532224535683,0.227103605399004,-0.596785877958929,0.550650347658783,0.882724117009166
"9617","ZNF354C",50.8861691955497,0.173170004377042,0.290367927798959,0.596381307294169,0.550920525136862,0.883016731056232
"9618","AC008267.5",23.2550321842057,0.273189591600367,0.458175586221911,0.59625523449062,0.551004731497236,0.883016731056232
"9619","ZBTB34",34.9615736193528,0.182198031752197,0.305555983356197,0.596283632710942,0.550985763247274,0.883016731056232
"9620","SLC19A1",9.86422743309762,0.343846693431201,0.576910157176279,0.596014282560354,0.551165685362447,0.88318285237652
"9621","G0S2",270.947762146513,-0.226587736520328,0.380509258262352,-0.595485475320818,0.551519005185002,0.883421860577006
"9622","ZNF2",13.5254979309299,0.259727985287677,0.436145023781685,0.595508308304545,0.551503747145898,0.883421860577006
"9623","CXCR4",117.609633647629,0.241956246319148,0.406449473705861,0.595292310537587,0.551648094987194,0.883421860577006

"9624","WDR31",18.1745001038507,0.239077888109568,0.401537513729584,0.
595406107611095,0.551572043915399,0.883421860577006
"9625","MIPEP",38.0373822199077,-0.17127116857998,0.287799936869364,-0
.595104955348625,0.551773316486242,0.883421860577006
"9626","AC008915.2",24.7869054377664,0.224671062424553,0.3774447089490
53,0.595242315225775,0.551681508697289,0.883421860577006
"9627","HOOK2",62.1147159720179,0.153030373365991,0.257004866216842,0.
595437649172272,0.551550965466921,0.883421860577006
"9628","CRYZL1",114.895857155774,-0.114535323268164,0.192453960177847,
-0.595131028544809,0.551755889259201,0.883421860577006
"9629","ZCCHC18",7.06679256631137,-0.355959930918647,0.598406871382648
,-0.594845995160758,0.551946419188418,0.883607233543408
"9630","RPS6KB2",82.0773648278833,-0.13361501678644,0.224729052162986,
-0.594560496297271,0.552137292652776,0.883710070854392
"9631","DNMT3B",3.55721398319472,0.483087561736795,0.812604734198797,0
.594492674489651,0.552182640445638,0.883710070854392
"9632","STK4",172.207363660108,0.120591551485181,0.202789844997055,0.5
94662673995993,0.552068976883645,0.883710070854392
"9633","C5orf51",66.9792596072331,0.138488776284313,0.233045487400584,
0.594256416758089,0.552340624070515,0.883871142951
"9634","CSMD2",9.86220276546968,-0.301822906257259,0.507997577500159,-
0.594142412533778,0.552416865703482,0.883901389331448
"9635","USP2",3.28747238898726,-0.579624289538912,0.975706622380935,-0
.594055914189147,0.55247471590955,0.883902205059233
"9636","ADAMTS4",67.2206176712752,0.652309487140272,1.10056304658972,0
.59270524224992,0.553378432516328,0.884215153827136
"9637","CIR1",549.819894652225,0.120373198444838,0.203539969489528,0.5
91398331967573,0.554253557757721,0.884215153827136
"9638","ACAD11",60.1331914141136,0.188312831929902,0.317345275053621,0
.593400459162606,0.552913181679638,0.884215153827136
"9639","AL391840.3",21.3209089316388,0.220640735109605,0.3720438181827
87,0.593050399781683,0.553147423321211,0.884215153827136
"9640","AL009176.1",13.010486437855,-0.384874655906921,0.6501150928776
27,-0.592010030413749,0.553843871703785,0.884215153827136
"9641","INTS1",131.789137548072,0.117022654615381,0.197835373645404,0.
591515321345562,0.554175192459679,0.884215153827136
"9642","AUTS2",279.569851053331,0.13834985581817,0.233657370467465,0.5
92105678247519,0.553779824815547,0.884215153827136
"9643","RBPMS",304.771924626425,0.165461506051268,0.279796284002367,0.
59136420142688,0.55427642111136,0.884215153827136
"9644","ZNF707",8.99482284551686,0.294678878192594,0.496929371106411,0
.592999519300887,0.553181473957338,0.884215153827136
"9645","PHF2",99.7840035256541,0.143762836225261,0.24275399141871,0.59
2216158362949,0.553705850575558,0.884215153827136
"9646","AL022345.4",7.25369882846655,-0.34765998952705,0.5878484692963
93,-0.59141089529955,0.554245141953866,0.884215153827136
"9647","AL133482.1",39.4778614111693,0.312417296817605,0.5264356369911
96,0.593457727526204,0.552874865281461,0.884215153827136
"9648","RIC8A",212.032914748253,-0.110959973125865,0.187165260170883,-
0.592844916971014,0.553284944449762,0.884215153827136

"9649","KLRK1",6.9298454449358,0.46163805835932,0.779567410645444,0.59
2172084229517,0.553735360740418,0.884215153827136
"9650","TRIAP1",121.657523071491,-0.18031769831262,0.304031399598017,-
0.593089064323725,0.553121548618878,0.884215153827136
"9651","UBC",8606.31039632748,-0.119534826935584,0.201753611704172,-0.
592479242011568,0.55352971697807,0.884215153827136
"9652","IGHA1",125.547205851782,0.431267915237864,0.728067784618064,0.
592345828711679,0.553619033292955,0.884215153827136
"9653","MPI",71.4893987352524,0.195113751187464,0.329881125726641,0.59
1466852666034,0.554207658531521,0.884215153827136
"9654","AC015871.1",24.3413128333186,-0.232639109368538,0.393155485576
093,-0.591722913461709,0.554036150294944,0.884215153827136
"9655","MMP25-
AS1",11.066274786359,0.289143056032706,0.488526773244164,0.59186736913
6375,0.553939406072583,0.884215153827136
"9656","GTF3C1",127.645814574464,0.125274535136101,0.211640879288428,0
.591920311223874,0.553903952000465,0.884215153827136
"9657","SCARF1",8.24222223198798,-0.352132379748696,0.594047950218232,
-0.592767603388472,0.553336691564066,0.884215153827136
"9658","RARA",159.186700777184,-0.139312479142773,0.234675631304544,-0
.593638454782652,0.552753955095819,0.884215153827136
"9659","MYL12B",2981.70718120926,-0.16629157152747,0.28106253489618,-0
.591653283099062,0.554082785790647,0.884215153827136
"9660","ATP9A",99.4230895564862,0.147248690753281,0.248707190122143,0.
592056428609745,0.55381280248579,0.884215153827136
"9661","Z82215.1",4.21239949072397,-0.451798873837257,0.76354310070593
7,-0.591713648410345,0.554042355526832,0.884215153827136
"9662","MAPK8IP2",5.18492788477997,-0.476478138433428,0.80368584210323
3,-0.592866159227706,0.553270727111263,0.884215153827136
"9663","ZNF81",7.89457236903985,-0.316851840174037,0.535464266250686,-
0.591732931858607,0.554029440551115,0.884215153827136
"9664","AL109615.4",3.4931108422938,0.489908852744521,0.82876325048717
9,0.591132452430213,0.554431677245114,0.884354877151324
"9665","AL118516.1",108.292134517365,-0.215401087184552,0.364430491277
772,-0.591062197977205,0.554478747172724,0.884354877151324
"9666","GNAI3",594.954575862734,0.11139193121015,0.188558912342773,0.5
90753997390777,0.554685262220527,0.884422413718913
"9667","NOLC1",191.274080406257,-0.113713079869751,0.192491907836882,-
0.590742131176298,0.55469321413133,0.884422413718913
"9668","AP003555.1",10.3601353782639,-0.308676151340872,0.522438033259
228,-0.590837825139178,0.554629088287623,0.884422413718913
"9669","MANEA-
DT",4.31452330693438,0.413428079990737,0.700135353808714,0.59049736274
8077,0.55485725332345,0.884592466644015
"9670","CFAP57",3.44232493478553,-0.594326606256215,1.00775805914447,-
0.58975128093817,0.55535740956543,0.884882522527986
"9671","AC093702.1",7.23568239486139,0.369545817956698,0.6267673107760
39,0.589606081241124,0.55545477372674,0.884882522527986
"9672","FAHD2A",138.936445885214,0.144762412320503,0.245523857383125,0
.589606296770624,0.555454629196508,0.884882522527986

"9673","SLBP",220.354048038317,-0.12043810312853,0.204187434515832,-0.589840914619023,0.555297309551076,0.884882522527986
"9674","NSUN5",76.4784304779698,-0.140228346667348,0.237850561072567,-0.589564918556426,0.555482377024543,0.884882522527986
"9675","AL162426.1",3.27025306754726,0.485157599018272,0.822976058505227,0.589516054573282,0.555515145608123,0.884882522527986
"9676","MTFMT",39.2586185221913,-0.164072612874375,0.278322661501841,-0.589505044213905,0.555522529374774,0.884882522527986
"9677","AC067852.2",20.6162737226454,0.206725063425377,0.350734405493844,0.589406286316001,0.555588760534027,0.884882522527986
"9678","ZNF611",47.4552919722095,-0.168035271398979,0.285110080197457,-0.589369801595943,0.55561322968202З,0.884882522527986
"9679","RNF114",225.653709052322,-0.122612468069818,0.207996738633608,-0.5894922625965,0.555531101042503,0.884882522527986
"9680","HPCA",2.86230810904187,-0.596591112676246,1.0128945431231,-0.588996274811347,0.555863772549051,0.885008207585442
"9681","OPTN",802.089193346293,-0.0958326317379217,0.162728884172206,-0.588909782214865,0.555921795267586,0.885008207585442
"9682","UBE2G1",113.476369783112,0.119450547669257,0.202778570114487,0.589068892249396,0.555815060128413,0.885008207585442
"9683","SPATA2",34.447636817488,-0.166304775733393,0.282385879627872,-0.588927378212218,0.555909990924129,0.885008207585442
"9684","GSTK1",591.317569622507,-0.149530788112663,0.254000407596551,-0.58870294550934,0.556060561647471,0.885137707331243
"9685","RAPGEF4",9.67159425413219,0.322100658343468,0.547295090326193,0.588531971210436,0.556175280759537,0.885217602102157
"9686","C5orf24",313.653699537872,0.107924294601602,0.183402184201967,0.588456975423766,0.556225604538533,0.885217602102157
"9687","AL445183.2",2.61703910876267,0.557443884669811,0.947852228794519,0.588112648507214,0.556456683777847,0.885273708935766
"9688","MAP3K2",366.347996147149,-0.133980207456122,0.227833413846152,-0.58806215117592,0.556490576684241,0.885273708935766
"9689","RAD21",397.778124997314,0.11320128071502,0.192455095256752,0.588195810373323,0.556400869213168,0.885273708935766
"9690","MAPT",10.5529055144176,0.356967185372817,0.606774551250853,0.588302829505517,0.556329046724485,0.885273708935766
"9691","NAA40",23.5703017643648,0.202786665101214,0.344937537481722,0.587893873718977,0.556603528771425,0.885362026211074
"9692","ST6GAL2",2.92498855588609,-0.563454203351072,0.958634078551895,-0.587767758269371,0.556688187978322,0.885405325803325
"9693","FCRLB",4.76166586987533,-0.409324854005258,0.696639559605027,-0.587570499495223,0.556820617101763,0.885439050307255
"9694","FSIP2",12.8312575675771,0.363191061748649,0.618239530773104,0.587460108373337,0.556894734570041,0.885439050307255
"9695","SEC22A",57.7646100961981,-0.147135099225574,0.250559281897707,-0.587226695858918,0.557051465395464,0.885439050307255
"9696","RUVBL1",85.096234342516,-0.146352150774926,0.249408068639944,-0.586797979604284,0.557339393896494,0.885439050307255
"9697","HDDC2",342.151005436764,-0.163353471558667,0.278382406570553,-0.586795241736182,0.557341232898559,0.885439050307255

"9698","AC084824.5",3.72810741003296,0.480808773390636,0.819094833360372,0.587000129665203,0.557203619631089,0.885439050307255
"9699","MAPK7",72.0388594001469,0.134698648404192,0.229505039436403,0.586909327720961,0.557264604843671,0.885439050307255
"9700","DUSP3",120.228090815505,0.124079120899019,0.211353178646315,0.58707004878624,0.557156662141786,0.885439050307255
"9701","RAB11B",379.945786246997,-0.106982029142905,0.18228288009602,-0.586901134580223,0.55727010775388,0.885439050307255
"9702","ZNF547",3.76066135764154,0.512064413527586,0.872243428290275,0.587065946178932,0.557159417388337,0.885439050307255
"9703","TMEM189",110.989367956501,0.130579110009901,0.222286025034193,0.587437334352507,0.556910025825039,0.885439050307255
"9704","ZNF619",17.2837164427051,-0.219666980732426,0.374453738687277,-0.586633161956169,0.557450105733859,0.885520752255507
"9705","ZBTB21",97.0999059689167,-0.172598189596494,0.294329852320156,-0.586410750509775,0.557599521630773,0.885666834202819
"9706","COA7",32.1947476185935,0.18652364820234,0.318323306605101,0.585956618105043,0.557904668067481,0.885766565198481
"9707","ANKRD17",355.921171291889,0.132218988849302,0.22571427519659,0.585780357640842,0.558023125125492,0.885766565198481
"9708","TCAF2",7.93404942165052,0.345246294424546,0.589125094947292,0.586032232178864,0.557853854858426,0.885766565198481
"9709","PRKAG2-AS1",95.170378055937,-0.18103749104537,0.309081718360037,-0.585726946277964,0.558059023014373,0.885766565198481
"9710","LPAR1",489.881025948838,-0.142211923251504,0.242792422862377,-0.58573460231959,0.558053877303803,0.885766565198481
"9711","MASTL",25.4427728785211,-0.219332789774751,0.37468650951558,-0.585376799549893,0.558294385174728,0.885766565198481
"9712","ROBO4",40.4922939694902,0.289862512803278,0.49515147470163,0.585401695466916,0.558277649005879,0.885766565198481
"9713","FGFR1OP2",374.660507149743,-0.112158149913836,0.191589643832281,-0.585408207199448,0.558273271563056,0.885766565198481
"9714","PSMA3",472.935484380194,-0.14030298622562 9,0.239656837407081,-0.585432853673649,0.558256703384446,0.885766565198481
"9715","MRPL12",140.117964278993,-0.17646680069572,0.301383954032463,-0.58552155260632 2,0.55819707899402,0.885766565198481
"9716","CCDC57",38.4391478165767,0.227839359046795,0.389044160489423,0.585638809640967,0.558118262288356,0.885766565198481
"9717","SH3BP5L",34.5385084044791,0.205739257872888,0.35156490797436,0.585209880753777,0.558406601888146,0.885853428846946
"9718","LRRC8C-DT",16.2952859467214,0.24560004381959,0.420028744656834,0.584721990920517,0.558734664899145,0.885861527037581
"9719","CCDC88A",280.498946770134,0.137959135500557,0.235920087661302,0.58477061817058,0.558701963148873,0.885861527037581
"9720","RNF181",408.447690497454,-0.165561169023667,0.283095131106328,-0.58482520832011,0.558665252465876,0.885861527037581
"9721","RBM18",85.4256699466862,0.133013721358073,0.227495359671727,0.584687624178402,0.558757777040727,0.885861527037581

"9722","DOCK9",156.508972557966,0.152085734698273,0.26015038055311,0.584607004513778,0.558811996778289,0.885861527037581
"9723","ZNF69",11.9311849255694,0.24936220154308,0.426548872825391,0.58460405695447,0.558813979170512,0.885861527037581
"9724","ZNF566",74.7291025401225,0.13266036695604,0.226883979278764,0.584705748628664,0.558745588022395,0.885861527037581
"9725","NAB1",96.8654848793741,-0.161623734580054,0.276601166542408,-0.584320509563988,0.559004696014577,0.885997566240704
"9726","LINC02019",4.77644526716625,-0.412111536834872,0.705301403868368,-0.584305567200863,0.559014747275841,0.885997566240704
"9727","UBE2D3",1169.9763184708,-0.0898524732149757,0.153837632113062,-0.584073428463453,0.559170911029587,0.886153962529154
"9728","WDR89",28.6429044091803,0.190895189779041,0.326905004611533,0.58394697874352,0.559255984857759,0.886197677485851
"9729","TMEM256",368.121151603578,0.159018302229076,0.272476840214097,0.583603003118096,0.55948743924497,0.886473314416816
"9730","PTPRU",37.5272851733232,-0.181326095008873,0.310958685497479,-0.583119570108755,0.55981281028647,0.886501872330591
"9731","TRAPPC9",40.7431769037508,0.176482207161366,0.302642021961484,0.583138475012653,0.559800084755694,0.886501872330591
"9732","AC090559.2",5.52438385044031,0.423093869633542,0.725745704373044,0.582978124547143,0.559908026533288,0.886501872330591
"9733","UBL3",798.63385992497,-0.120902270255686,0.207298404299409,-0.583228176137149,0.559739705804341,0.886501872330591
"9734","MT1A",802.758569227203,-0.478774605574203,0.821255120735958,-0.582979141908005,0.559907341653201,0.886501872330591
"9735","ZNF155",15.4571805019142,-0.238466984394916,0.408812284793629,-0.583316581387215,0.559680202212905,0.886501872330591
"9736","AP000692.1",3.34331880498657,0.476742723768087,0.817595162225342,0.583103650553021,0.559823526387141,0.886501872330591
"9737","GNB1",695.024838269971,0.0938239791994712,0.161199350117424,0.582036956917793,0.560541787822925,0.88652401424898
"9738","METTL5",327.164300609663,-0.147462385362403,0.253068226285534,-0.582698142421178,0.560096523943066,0.88652401424898
"9739","SP100",581.067125359168,0.0818718038935154,0.140629034856971,0.582182790181164,0.560443564211201,0.88652401424898
"9740","CAVIN3",1420.33705347192,-0.147620415858409,0.253571139579303,-0.582165683773497,0.560455085520889,0.88652401424898
"9741","AC025031.4",2.57772064460919,0.542146115038264,0.93149389144016,0.582017896220517,0.560554626460253,0.88652401424898
"9742","FIGNL2",3.3413281023172,-0.473271861819145,0.812522395785704,-0.582472390021316,0.560248533694446,0.88652401424898
"9743","PITPNM3",2.73068613315608,-0.541295125866028,0.929595788576424,-0.58229085428083,0.560370784781594,0.88652401424898
"9744","PDK2",127.096308438656,0.143720983656776,0.24674285578313,0.582472725301911,0.560248307919366,0.88652401424898
"9745","CST7",17.6495501665219,0.261023610880272,0.448144865786799,0.582453645702263,0.56026115602847,0.88652401424898
"9746","APOBEC3F",30.7072738920666,0.17474617492319,0.299970970417346,0.582543619737831,0.56020056920387,0.88652401424898

"9747","AC005052.2",2.89039026847569,0.508527129798391,0.8731558697095
14,0.582401318526978,0.560296393625194,0.88652401424898
"9748","SUCLG1",383.021124672987,-0.135941488575848,0.233862285367339,
-0.581288634729274,0.561045939135535,0.886627178804293
"9749","GAK",101.493267115981,0.14471921187168,0.248927034952589,0.581
372014892812,0.560989754315869,0.886627178804293
"9750","RB1CC1",639.06502973159,0.161767861671395,0.278242425872361,0.
581391788704442,0.560976430346695,0.886627178804293
"9751","MTFR1",63.8608796843072,-0.145357542285026,0.249980508378685,-
0.581475504741475,0.560920022590802,0.886627178804293
"9752","EXOSC4",68.2758978009722,-0.181565871329812,0.312257865958729,
-0.58146132131027,0.560929579175114,0.886627178804293
"9753","SENP1",46.5328212644573,0.152821419934903,0.262923956595681,0.
581238096039719,0.561079995409399,0.886627178804293
"9754","MAPKAPK5",80.7470919397123,-0.12524827169361 8,0.21534250365035
2,-0.58162355118236,0.560820275814814,0.886627178804293
"9755","TUBG2",48.0246619502438,0.14579140099701,0.25062378253896,0.58
1714151466639,0.560759237811728,0.886627178804293
"9756","ELOA-
AS1",24.2228339911254,-0.223042669484123,0.384190035697277,-0.58055297
8369979,0.561541771178953,0.886761619845903
"9757","PDE12",82.5806391328996,-0.144425715580291,0.248898937510954,-
0.580258465643047,0.561740332000988,0.886761619845903
"9758","WDR27",75.7222648118391,0.14472603488701,0.249318013067744,0.5
80487679595318,0.561585792760017,0.886761619845903
"9759","CYP3A5",41.6258801789973,-0.24296496227303,0.418242812819215,-
0.580918439782135,0.561295423871322,0.886761619845903
"9760","IL11RA",392.061139781032,-0.172018283886348,0.296318578630878,
-0.580518051487518,0.561565317149624,0.886761619845903
"9761","PDCL",187.939006668029,0.101274126845019,0.174487695969924,0.5
80408413797124,0.561639232540361,0.886761619845903
"9762","ACTR1A",180.623393453741,-0.125197423514084,0.215540614497787,
-0.580853050854458,0.561339496876398,0.886761619845903
"9763","TNFRSF1A",825.195800656226,-0.1404208470854,0.241986759879172,
-0.580283182251436,0.561723666730295,0.886761619845903
"9764","TSNAXIP1",3.17539901668572,-0.490506905615871,0.84513454916511
9,-0.580389129873376,0.561652233836593,0.886761619845903
"9765","NUDT7",56.379921624387,0.185115646997777,0.318919034207011,0.5
80447157875244,0.561613111572414,0.886761619845903
"9766","AL512329.2",5.40091260576392,0.444246550645938,0.7660909766999
08,0.579887460050268,0.561990512701749,0.886813187115936
"9767","GABARAPL1",618.659139914683,-0.144732617331724,0.2495498099420
72,-0.579974864999177,0.56193156795701,0.886813187115936
"9768","NLK",15.0732569827661,0.229769044382269,0.396183469602202,0.57
9956161757518,0.561944180925231,0.886813187115936
"9769","COMMD7",235.035858540488,-0.122389521803833,0.211064170941788,
-0.579868772884186,0.562003115467764,0.886813187115936
"9770","TRAF7",165.947788452075,-0.0974123807379773,0.16802882983056,-
0.579736113357497,0.562092585980699,0.886863583714685
"9771","MTF1",78.2169005190811,0.141969958189146,0.244941748752835,0.5

79607024576298,0.56217965485612,0.886873360739332
"9772","PRRX1",3757.72140506375,0.0889845712179434,0.153606937684276,0.579300470144405,0.562386448344273,0.886873360739332
"9773","SDCBP",2866.38851706041,0.153967074096249,0.265734509434282,0.579401879056007,0.562318036516644,0.886873360739332
"9774","TMOD1",13.4395570854141,-0.323822185131613,0.558797674730258,-0.579498089872919,0.56225313510996,0.886873360739332
"9775","WDR20",94.5353677323577,-0.111852640417962,0.193081805244164,-0.579301816017917,0.562385540373425,0.886873360739332
"9776","RNF166",64.0925548168718,-0.132252357410794,0.228342942135202,-0.579183031339273,0.562465679177747,0.88690757411262
"9777","FGD5",142.760123990168,0.135088684770613,0.233518485705189,0.578492466507164,0.562931682108994,0.88712619951696
"9778","AC012557.2",3.06648199409298,0.492489973987153,0.850995442505761,0.578722222691362,0.562776618704547,0.88712619951696
"9779","MRPS30",138.684502199762,-0.12248221211607,0.211688988280172,-0.578595103652551,0.562862409325155,0.88712619951696
"9780","ASCC3",115.687886409058,-0.103913318214947,0.179691356948203,-0.578287792912044,0.563069834528718,0.88712619951696
"9781","SMCO4",82.2438085638844,-0.175036020362187,0.302710087030366,-0.578229890121333,0.563108921241614,0.88712619951696
"9782","ZBTB16",1114.51823987814,-0.239443612076159,0.413640572000702,-0.578868777107657,0.562677719288593,0.88712619951696
"9783","ING4",197.79281267755,0.146072973275201,0.252629573553088,0.578210109057186,0.563122274555527,0.88712619951696
"9784","ANO6",386.52346794484,0.11548890936959,0.199583261290133,0.578650276696822,0.562825173138647,0.88712619951696
"9785","MORF4L2-AS1",2.75321514419634,0.553761721332613,0.957706255597613,0.578216669355536,0.563117845973858,0.88712619951696
"9786","PLA2G5",125.909272481192,-0.442286275945555,0.765124348952905,-0.578058032724794,0.563224939603362,0.887167947842907
"9787","AC093157.1",32.9155452877552,0.220920413663987,0.382321625611164,0.577839177448655,0.5633727022168,0.887167947842907
"9788","RNASE4",1632.26813539909,-0.125227957552848,0.216721154901516,-0.577829873644567,0.56337898419943,0.887167947842907
"9789","AC022819.1",5.61768349534752,0.398919308618652,0.690291104142352,0.577900115219197,0.563331557514749,0.887167947842907
"9790","CPEB4",73.9865607083349,0.17293299957073,0.299397313044221,0.577603712646506,0.563531699808248,0.887216001343732
"9791","SLC25A40",29.8344962221102,0.169267050591116,0.293088408855155,0.577528982644854,0.563582165757887,0.887216001343732
"9792","RNF214",91.4077042892233,-0.122543306893079,0.212177689515893,-0.577550387944535,0.56356771031313,0.887216001343732
"9793","KIAA1522",30.1080944730021,-0.294061701438476,0.509468449062713,-0.577193154903844,0.563808980619088,0.88733071865576
"9794","PYCR2",110.195263184206,-0.147832593473226,0.256331090114941,-0.576725177608915,0.564125121354964,0.88733071865576
"9795","MORN2",166.605530796363,-0.160844468260223,0.279092005650817,-0.57631341996037 5,0.564403353643836,0.88733071865576

"9796","ATP6V1E2",46.0333129806821,-0.171426130863833,0.297267206747325,-0.576673534694811,0.564160013808303,0.88733071865576
"9797","FXR1",509.368708733196,0.0883662683563991,0.153308390961605,0.576395511048901,0.564347877899921,0.88733071865576
"9798","ZNF454",8.85973029014557,0.301628480829324,0.523075722069101,0.576644007938638,0.564179963981388,0.88733071865576
"9799","AL049775.1",11.1441641677231,0.295610519874451,0.512845738286619,0.576412160237628,0.564336626985057,0.88733071865576
"9800","IGHA2",13.0168383787482,-0.462766189636064,0.802795485034369,-0.576443438288959,0.564315490710038,0.88733071865576
"9801","DTWD1",415.567839943073,0.107966475643639,0.187131695931142,0.576954508462142,0.563970186881076,0.88733071865576
"9802","CCT6B",16.1200079225196,0.242220615660972,0.419784798654424,0.577011403074587,0.563931752414132,0.88733071865576
"9803","PPP5D1",22.6860078141767,0.189204374743138,0.327871366265625,0.577068918515603,0.563892899836426,0.88733071865576
"9804","SPIN2B",71.9668940610153,-0.188056105984422,0.325987153232321,-0.576881954149894,0.564019201899042,0.88733071865576
"9805","HNRNPH2",252.438942314558,-0.128332426423143,0.222577287399536,-0.576574671757861,0.564226813294208,0.88733071865576
"9806","CYP27A1",258.881705239539,0.110731022119812,0.19221452148298,0.576080419239376,0.564560825814545,0.887400599570974
"9807","FGFBP3",25.2602431553986,-0.179748214666876,0.312047341315907,-0.576028668947716,0.564595803814686,0.887400599570974
"9808","FBXL12",128.609580178016,-0.124125708653768,0.215498968387257,-0.575992124615239,0.564620504741622,0.887400599570974
"9809","CCDC142",28.0741082468874,0.235230875543354,0.408818575858087,0.575391847226163,0.565026316689406,0.887405060801548
"9810","NUS1",101.733609605343,0.115034900421097,0.199902853152731,0.575454019824358,0.564984278972779,0.887405060801548
"9811","TMEM209",45.4025787743098,-0.167942359349483,0.291813200361676,-0.575513236348916,0.564944241390173,0.887405060801548
"9812","CAT",516.952449913574,0.141024619547195,0.244903465158411,0.5758375834167,0.564724967476645,0.887405060801548
"9813","MANSC1",83.6558216500806,0.140567755878048,0.244297119166934,0.575396698730592,0.565023036312877,0.887405060801548
"9814","COG6",87.3745222375408,0.142485024366492,0.247566183620118,0.575543162975482,0.564924007864858,0.887405060801548
"9815","RPL23",8760.85903759881,-0.0983244240077746,0.170838494595623,-0.575540215573251,0.564926000601143,0.887405060801548
"9816","TIRAP",13.540148125196,0.237951666803724,0.413685898712152,0.575198882883108,0.565156798219985,0.887429158048392
"9817","MPDU1",100.933424497092,0.181519740927635,0.315544652928908,0.575258491128769,0.565116489874133,0.887429158048392
"9818","DDOST",679.419197320091,-0.142939994315094,0.248775158486825,-0.574575030660323,0.565578743082447,0.887639617655866
"9819","CCDC50",540.820342843179,0.0937772079186843,0.163190128457277,0.574650003681045,0.565528026790464,0.887639617655866
"9820","ZNF581",156.682021764686,-0.156545620672514,0.272431783316534,-0.574623191048991,0.565546164227733,0.887639617655866

"9821","BMP2",6.54729603049602,0.457840703432531,0.796600209008731,0.574743388533951,0.565464858675835,0.887639617655866
"9822","AL031663.3",3.40731309103908,-0.492531768284046,0.856879664847621,-0.574796892130277,0.565428668881202,0.887639617655866
"9823","ZNF843",8.22963901746454,0.313083015814941,0.545127427738886,0.574329963754651,0.56574453642154,0.887809429801286
"9824","CDK11A",109.725854221396,-0.158623175210717,0.277534806934995,-0.571543356894582,0.567631379112219,0.888135671298445
"9825","SLC6A9",3.91288481709665,-0.42326449871874,0.739405355477277,-0.572439049275655,0.567024567316813,0.888135671298445
"9826","POGK",69.3547400214286,0.154018829050832,0.268395132910931,0.573851050801074,0.566068599291891,0.888135671298445
"9827","BCS1L",64.7674546175617,-0.136253800255006,0.23829905959557,-0.571776491633032,0.567473405552676,0.888135671298445
"9828","AC007370.2",4.14281400440061,0.599803933731723,1.05004040986972,0.57121985791589,0.567850618917201,0.888135671298445
"9829","LINC01023",18.5761040716222,-0.230606200900921,0.403686384497651,-0.571250876315505,0.567829595555331,0.888135671298445
"9830","AC116366.1",27.5234854254328,0.320444139334183,0.560633127805778,0.571575462528136,0.567609622883754,0.888135671298445
"9831","PCDHGA2",7.00650277975303,0.359099918103922,0.626270934504484,0.573393875269093,0.566378036345289,0.888135671298445
"9832","AL451165.2",44.7907784114235,0.162485784440481,0.284332603177462,0.571463780884345,0.567685305134581,0.888135671298445
"9833","TMEM106B",359.011134165938,0.0962287298183617,0.168145801786447,0.572293383456441,0.567123231519503,0.888135671298445
"9834","PCOLCE-AS1",3.1001731549342,0.530784044421304,0.926875305063237,0.572659603208536,0.566875194614726,0.888135671298445
"9835","CTTNBP2",10.8004718129544,-0.320976581850157,0.559761401079469,-0.573416782992131,0.566362529432423,0.888135671298445
"9836","CHD7",13.026408915375,-0.316703900440727,0.554369034522237,-0.571287140367909,0.567805017316132,0.888135671298445
"9837","PPP6C",160.720378875094,0.116059556348755,0.202959698173804,0.571835479620037,0.56743343826075,0.888135671298445
"9838","SURF6",86.3221998888526,0.156318129105627,0.273179243512398,0.572218178422962,0.56717417355722,0.888135671298445
"9839","DDX50",219.308440690167,-0.116984387333811,0.203936551934059,-0.573631289851545,0.566217333301954,0.888135671298445
"9840","ABCC2",182.799246192678,0.414715604259802,0.723855162548667,0.572926222974778,0.566694648481355,0.888135671298445
"9841","UBQLNL",3.12732543156798,0.539651681000697,0.944441743250445,0.571397531777239,0.567730201827464,0.888135671298445
"9842","C2CD2L",14.0517757565277,-0.233509083506727,0.408201961340311,-0.572043021890465,0.567292828777618,0.888135671298445
"9843","SPPL2A",644.414585706227,0.0939631563903356,0.163914499207006,0.573244934675793,0.566478863589646,0.888135671298445
"9844","UBE2Q2",248.601244180546,-0.0946443200091711,0.165035862948045,-0.573477293471457,0.566321569085255,0.888135671298445
"9845","NUP88",51.5123931837188,0.157661361742147,0.276010409279003,0.

571215274648486,0.567853725353064,0.888135671298445
"9846","RPAIN",195.101793608909,-0.129774040865759,0.22685355319093,-0
.572060869403862,0.567280737901527,0.888135671298445
"9847","AC005332.3",15.3277611828602,0.275625138940045,0.4805569994229
55,0.573553479131531,0.5662700000214,0.888135671298445
"9848","NDUFAF8",404.111246339642,-0.158860990917052,0.277489203223528
,-0.572494313550222,0.566987137176641,0.888135671298445
"9849","VAPA",801.870422116139,-0.114432133240188,0.199728728387958,-0
.572937774970021,0.566686826473505,0.888135671298445
"9850","ATP8B3",5.51790366579126,-0.354892269625526,0.618732033984929,
-0.573579918498563,0.566252104090853,0.888135671298445
"9851","KIF16B",77.9027653977222,0.154784129217011,0.270706764557958,0
.571777840386667,0.567472491690018,0.888135671298445
"9852","PXMP4",84.6482401673578,-0.120787574447131,0.210735358457395,-
0.573171846107408,0.56652834498211,0.888135671298445
"9853","AL031651.2",2.97063393484317,-0.612671827875924,1.070171276022
05,-0.572498852850264,0.566984062790344,0.888135671298445
"9854","HELZ2",150.328176099905,-0.229512949657044,0.401504044402719,-
0.571632970717566,0.567570653733282,0.888135671298445
"9855","ASCC2",167.424275803361,-0.126804306689801,0.221820630016561,-
0.571652450361959,0.56755745407428,0.888135671298445
"9856","PWP2",4.45330397930285,1.78844729212153,3.12847667338485,0.571
667133508311,0.567547504681542,0.888135671298445
"9857","NUP155",45.6995316365915,-0.158775131638677,0.278023705953073,
-0.571084868804232,0.5679421149173,0.888183798463039
"9858","FMO1",36.2321666715394,-0.544729877633135,0.954182233891554,-0
.570886627611477,0.568076496141644,0.888213732429601
"9859","ZNF530",9.06273075264787,-0.272895697300215,0.47798403700758,-
0.570930567072239,0.568046709705445,0.888213732429601
"9860","LINC02777",3.614203378354,-0.433739655011041,0.760109716226699
,-0.570627694596763,0.568252041274093,0.888324000332839
"9861","S1PR4",2.67520984341245,-0.502946634647545,0.881415225997055,-
0.570612600977721,0.568262274880449,0.888324000332839
"9862","DNM3",8.96583404399829,-0.342070480265754,0.599614134089647,-0
.570484351215464,0.568349232902357,0.888369846399294
"9863","AL139011.1",2.47872393136403,0.539795696117199,0.9466323453888
83,0.570227394771143,0.56852347789764,0.888411211915781
"9864","LINC00504",3.74239942982755,0.574160881175832,1.00711245575144
,0.570106027283151,0.568605787406627,0.888411211915781
"9865","BRI3BP",38.2057138212333,0.179508071057817,0.314868230443167,0
.570105376478169,0.568606228787615,0.888411211915781
"9866","ULK3",45.2148204367854,-0.194317536875611,0.3407151796388,-0.5
70322511258853,0.568458975382791,0.888411211915781
"9867","HNRNPU",1353.34681391872,0.10692423839966,0.187595953243379,0.
569970921819089,0.568697420492351,0.888463640102321
"9868","OSR1",155.392166212135,-0.198629865980738,0.348650320099131,-0
.569710837851124,0.56887383808389,0.888469119965873
"9869","AP000787.1",15.3882720397071,0.224592024720088,0.3942015607542
31,0.569739055041723,0.568854696813107,0.888469119965873
"9870","SCYL2",75.7930317088056,-0.126566016916142,0.222137435871714,-

0.569764463245337,0.56883746129583,0.888469119965873
"9871","MSRB1",79.1867032411323,-0.144835455732158,0.254264848250296,-0.569624376821382,0.568932491266902,0.888470707413564
"9872","PHKB",218.771590274144,0.111080661732303,0.195061113482091,0.569465947104321,0.569039973855764,0.888548541023765
"9873","AL590560.3",6.03017148503919,0.376820927790182,0.66206243458249,0.569162224145541,0.56924605404204,0.888692761400193
"9874","EIPR1",50.7602313307928,-0.136936190858506,0.24104836423581,-0.568085957739773,0.569976602259688,0.888692761400193
"9875","AC093635.1",2.71536435946504,-0.516018240420324,0.908824993934941,-0.567786145697995,0.570180188299645,0.888692761400193
"9876","NME6",110.317997170072,0.11651781298478,0.205101444963885,0.568098450039184,0.569968120171411,0.888692761400193
"9877","BST1",231.021547389043,-0.185193304140689,0.325696678932755,-0.568606670315252,0.569623097098899,0.888692761400193
"9878","FAT4",173.68078962998,0.130406959886803,0.229650624017421,0.567849360064922,0.570137259979683,0.888692761400193
"9879","CDK7",44.606623054894,0.165198485524717,0.290952617712016,0.567784840101455,0.570181074935,0.888692761400193
"9880","ATG10",85.6995563764007,-0.12231284964341,0.215502799123269,-0.567569656361849,0.570327215975021,0.888692761400193
"9881","MRPS18B",294.423901625,-0.131418886576918,0.231625108398637,-0.56737755023838,0.570457699039838,0.888692761400193
"9882","CENPQ",61.6538901706598,-0.145867853683544,0.256867894269593,-0.567871100038878,0.570122496910886,0.888692761400193
"9883","PILRB",69.7622590800071,0.140098530660791,0.246508498033247,0.568331444062003,0.569809931675682,0.888692761400193
"9884","AC105046.1",4.33343588957348,0.534774402282824,0.942336960296333,0.567498065781751,0.570375840343191,0.888692761400193
"9885","UPF2",223.322128805628,-0.115177271936987,0.202652616535715,-0.568348309071491,0.569798482192743,0.888692761400193
"9886","AP006623.1",20.4519840716691,0.285827818393771,0.5039216085421,0.567206909861838,0.570573613984931,0.888692761400193
"9887","OSBP",153.551879987831,0.117310221389412,0.206679696305592,0.567594318582506,0.570310465836373,0.888692761400193
"9888","RSRC2",775.837221612815,0.0997865932022447,0.17590540354568,0.567274178000631,0.570527917817358,0.888692761400193
"9889","TSC22D1-AS1",5.82608911285587,0.3837455527768,0.674907260404498,0.568590049760032,0.56963437896645,0.888692761400193
"9890","EFCAB11",48.062540017799,0.165637171722986,0.29122905795096,0.568752214797457,0.569524307507195,0.888692761400193
"9891","TMEM106A",32.3301040165148,-0.272818367007623,0.480502685369093,-0.567776987131843,0.570186407929067,0.888692761400193
"9892","C17orf67",19.3030661636734,-0.217448462737067,0.382611347982963,-0.568327269652101,0.569812765656934,0.888692761400193
"9893","TNFAIP8L1",3.18053458427549,-0.489250253606204,0.862526039639472,-0.567229545685028,0.570558236965708,0.888692761400193
"9894","GRAMD1A",123.919490561048,-0.106913416934532,0.187996513758931,-0.568698933809099,0.569560471495814,0.888692761400193

"9895","AL121894.1",4.10602084738596,-0.433411264446797,0.763753914537382,-0.567475015443058,0.570391496568538,0.888692761400193
"9896","PDE9A",19.5641568512871,0.224364927078514,0.394681758239054,0.568470476263104,0.569715547409264,0.888692761400193
"9897","CFP",6.34535639171296,-0.380841872909857,0.671225789514362,-0.567382658502141,0.570454229201066,0.888692761400193
"9898","RNF207",15.4793997927613,0.261791404390076,0.461639527583069,0.567090530052082,0.570652676500532,0.888726107118175
"9899","C11orf58",1202.59517099571,-0.09483790662431,0.167269432031564,-0.566976915461839,0.570729865500459,0.888756528607898
"9900","UCN",3.71766975540664,0.448260372807441,0.790976777421402,0.566717488557348,0.570906137059482,0.888874890957066
"9901","TRIM66",24.844006815577,0.235509934520251,0.415709243650592,0.566525614037583,0.571036525796161,0.888874890957066
"9902","PMP22",4276.30528419978,0.0946338342493411,0.167010443057861,0.566634232666248,0.570962712035746,0.888874890957066
"9903","PCED1A",90.5820929510317,-0.113346392079983,0.200071629909361,-0.566529058274442,0.571034185132758,0.888874890957066
"9904","TTLL5",46.1075957021247,-0.180186628009222,0.318169641537115,-0.566322503739576,0.571174565273285,0.888999992294799
"9905","RNASEH2C",592.692999374797,0.135852712455947,0.239946265778406,0.566179732013039,0.571271606462494,0.889061263363891
"9906","ZMIZ1-AS1",7.51378531111465,-0.308042903481892,0.544296106587984,-0.565947284489896,0.571429616599744,0.889127641050273
"9907","KCTD17",61.1988598551235,-0.135801589463921,0.239952223030966,-0.565952620686476,0.571425988996666,0.889127641050273
"9908","BTF3L4",335.826309606287,-0.129975131274647,0.229698729223101,-0.565850458617059,0.571495441758727,0.889140314363219
"9909","ETV5",43.6460560301402,0.187869514735499,0.332204394580749,0.565523869642349,0.57171749342432,0.889313462459251
"9910","CNTD1",5.5882480231202,0.429813646903783,0.760036554498089,0.565517071988231,0.571722115664689,0.889313462459251
"9911","XRCC5",808.698305501023,-0.102901160967744,0.182083261290171,-0.565132457748318,0.571983672984577,0.889451056093742
"9912","HOPX",9.56556341909289,0.380424716976542,0.673121991154869,0.565164594197630,0.571961816381032,0.889451056093742
"9913","CIPC",21.4939908231538,0.191580571029739,0.338982263065387,0.565163997954621,0.571962221893501,0.889451056093742
"9914","LIX1L-AS1",4.36831655927097,-0.404370963745665,0.717376511326515,-0.563680239541067,0.572971767927148,0.889641398327657
"9915","DUSP12",87.7244224982385,-0.168839655817217,0.299295942393277,-0.564122769146535,0.572670583269949,0.889641398327657
"9916","MARC1",8.41145438655415,0.387212045226761,0.685728683762757,0.564672376109508,0.572296626673361,0.889641398327657
"9917","WDR54",59.3056915016593,-0.138096477569601,0.244672214428309,-0.564414222073688,0.572472262120185,0.889641398327657
"9918","RPL37A",14406.0238961658,-0.151864869299341,0.26934673291849,-0.563826661841481,0.572872104918121,0.889641398327657

"9919","EXOG",79.1830421448193,-0.132085250887915,0.234302702241124,-0
.563737633516428,0.572932701474754,0.889641398327657
"9920","SLC10A7",44.8785594456211,0.15225852125921,0.269776903194643,0
.564386793147209,0.572490924931579,0.889641398327657
"9921","LUCAT1",14.306621684873,-0.448439229680399,0.795511882551002,-
0.563711541608114,0.572950461340568,0.889641398327657
"9922","TBCC",87.1737358456375,-0.169875734880514,0.300989914906735,-0
.564390122284168,0.572488659750775,0.889641398327657
"9923","PRRT4",2.43248988733129,0.54602319320258,0.96679124127369,0.56
4778795971741,0.572224231228011,0.889641398327657
"9924","BMP4",247.482562550404,0.159994038251303,0.283831857749408,0.5
63693024172644,0.572963065681355,0.889641398327657
"9925","PARP6",60.3891714403848,-0.149277017832956,0.264596561917091,-
0.564168395656367,0.572639534244177,0.889641398327657
"9926","CIITA",9.85776891939472,-0.367444731967073,0.65137380748959,-0
.5641073186274,0.572681097592285,0.889641398327657
"9927","ZNF570",51.0648072054117,0.13799665240469,0.244651700510296,0.
564053518192826,0.572717710352989,0.889641398327657
"9928","YIPF6",266.718518673244,0.107417862857021,0.190280272757413,0.
564524431778416,0.572397277667538,0.889641398327657
"9929","LDLRAD2",12.4452651320136,0.286217016854133,0.507920498470529,
0.563507512919839,0.573089345667914,0.889734340162242
"9930","TRIM24",55.5709661076791,-0.15148995416951,0.268942387790654,-
0.563280319677352,0.573244017125254,0.889884846322838
"9931","SRSF10",624.293793320784,-0.088599776805203,0.157517549653053,
-0.562475590817354,0.573792029700094,0.889916081680517
"9932","EVI5",258.358765210671,0.0947180753936991,0.168462153021738,0.
562251364444313,0.573944769900406,0.889916081680517
"9933","PARP1",203.450158557009,0.107356736909342,0.190774781872686,0.
562740713712293,0.573611456154098,0.889916081680517
"9934","ODC1",86.2062994910871,-0.182896084638498,0.32501249978501,-0.
562735540200701,0.573614979542815,0.889916081680517
"9935","FBXL17",103.505084911106,-0.118633976882248,0.210900723018309,
-0.562511001311027,0.573767910275854,0.889916081680517
"9936","GNPDA1",150.411765244859,0.0935217121848974,0.166306802012189,
0.562344480522468,0.573881338054458,0.889916081680517
"9937","CYTH3",136.599480962143,0.145957861679323,0.259893309088953,0.
561606846251538,0.574383914843687,0.889916081680517
"9938","PDIA4",303.823463222722,0.136810422326843,0.243159861392349,0.
562635714395698,0.573682967310808,0.889916081680517
"9939","LINC00865",3.38375658449161,0.499726471347125,0.88879738972570
4,0.562250156361675,0.573945592883348,0.889916081680517
"9940","BTAF1",77.9125453627468,0.188427168357451,0.334665608487933,0.
563031167764123,0.573413660638342,0.889916081680517
"9941","USP35",10.2650600679419,-0.269375274264324,0.479588984805089,-
0.561679443855037,0.574334442240389,0.889916081680517
"9942","UBASH3B",3.05093187206591,0.519541736964627,0.924143447987128,
0.562187329354808,0.573988393337627,0.889916081680517
"9943","ATP6V1D",244.895423929326,-0.124784258516906,0.222171641664698
,-0.561657003485759,0.574349734313355,0.889916081680517

"9944","MESP1",19.7493918711661,0.255934405651805,0.454843263064084,0.
562687031852872,0.573648016358119,0.889916081680517
"9945","NFATC2IP",170.197575930328,0.126898057525279,0.225975892292264
,0.561555731622721,0.574418748797999,0.889916081680517
"9946","EPN2",293.630180525606,0.147180454996402,0.26196526806304 7,0.5
61831940870038,0.574230527689034,0.889916081680517
"9947","TUBD1",47.2763792764398,-0.170115970540584,0.302929882509703,-
0.561568799786977,0.574409842918992,0.889916081680517
"9948","STAU1",467.113140464541,-0.0747957729655774,0.132999054767819,
-0.562378229650961,0.573858348542846,0.889916081680517
"9949","URB1",46.4181331144622,0.164801587130792,0.293356030103499,0.5
61780124555982,0.574265835375281,0.889916081680517
"9950","TTC38",38.5440012180162,-0.158321223336425,0.281822800961409,-
0.56177577824196,0.574268797004559,0.889916081680517
"9951","TCP11L1",35.5506755346673,0.173762144855262,0.309661737517584,
0.561135341577016,0.574705277065769,0.890270510096355
"9952","PURA",621.318677079365,-0.108290408274611,0.19310172617803,-0.
560794615449336,0.574937557822987,0.890306816783361
"9953","ACTB",11430.2616127701,-0.166243332413013,0.296459652562358,-0
.560762083393606,0.574959737987568,0.890306816783361
"9954","ITGB8",39.2839919166004,0.227072760265858,0.404759572998752,0.
561006521929893,0.574793091131825,0.890306816783361
"9955","FAM122A",88.8404896614645,0.124939417863426,0.222769492024037,
0.560846176593808,0.574902404566349,0.890306816783361
"9956","AL445223.1",2.47806712583973,-0.494590280956077,0.882383963818
026,-0.560515944573621,0.575127567100917,0.890477244562137
"9957","SDE2",87.6568806105505,-0.114244299331636,0.20409340901417,-0.
55976476596412,0.575639899419798,0.890695836047845
"9958","ALAS1",58.4774224743371,0.145619000707805,0.260061461387638,0.
559940715286337,0.57551987597314,0.890695836047845
"9959","HIST1H4C",843.556245070579,-0.203027377162362,0.36259583425399
,-0.559927494975426,0.575528893768603,0.890695836047845
"9960","AC015987.1",3.06121988106608,-0.513354907966795,0.916991555520
285,-0.559825120391132,0.575598727446771,0.890695836047845
"9961","AC104211.1",5.8087408608438,0.375992322187538,0.67170366398596
,0.559759224709837,0.575643679566358,0.890695836047845
"9962","MRPS2",143.087703730818,-0.138501042955507,0.247336707957401,-
0.559969622379552,0.575500158193338,0.890695836047845
"9963","HAUS1",142.877747723773,-0.128066854102842,0.228806877561539,-
0.559715929292367,0.575673215345097,0.890695836047845
"9964","SCMH1",60.8959074109839,0.142045708952759,0.254219932897478,0.
558751264441733,0.576331487364172,0.890992662259179
"9965","OLFML2B",415.215773183943,-0.126082797326591,0.225566830264933
,-0.558959831011077,0.576189134753665,0.890992662259179
"9966","SESTD1",1125.31685320297,0.130665441810179,0.233883645099423,0
.55867712235558,0.576382095442161,0.890992662259179
"9967","ANKH",168.929032055588,-0.143297135758853,0.256249415022586,-0
.55920961125403,0.576018674477371,0.890992662259179
"9968","EXTL3",27.3565478383905,0.202606673064597,0.362598435867544,0.
558763229576116,0.576323320371317,0.890992662259179

"9969","SOX5",118.1471109363,0.171614596890543,0.307275977194502,0.558503136032378,0.576500863663513,0.890992662259179
"9970","PARP2",66.5775919282514,-0.184913296934329,0.33104156395259,-0.558580302504888,0.576448186117658,0.890992662259179
"9971","YWHAE",1259.82763171391,-0.0941907319976553,0.168599598181201,-0.558665222300379,0.576390218404818,0.890992662259179
"9972","ANKRD13B",71.4284685850204,-0.156969288765336,0.28076350627536,-0.559080098577298,0.576107056265224,0.890992662259179
"9973","RHOT1",100.975048162963,-0.110537952728652,0.197795331326407,-0.558850160857636,0.576263985681421,0.890992662259179
"9974","CCDC97",95.2270615076852,-0.13787340946029,0.246585466341613,-0.559130314960591,0.576072786939337,0.890992662259179
"9975","WASF3",128.156230975337,-0.126796157087489,0.227116921177093,-0.558285822255492,0.576649224699331,0.891132611402526
"9976","PPP2R5B",28.6637904813889,-0.173239164815944,0.310388118252519,-0.558137230868496,0.576750679030919,0.891200051850604
"9977","METAP2",488.580975774852,-0.112342351474812,0.201365060212785,-0.557903895323738,0.576910011448314,0.891356903525685
"9978","GLRB",2.87535797693127,-0.530827840647432,0.951837883913263,-0.557687238151371,0.577057973665559,0.891496157952956
"9979","EBPL",312.589252802711,-0.183147817233587,0.328574662529076,-0.557400914068898,0.577253541103566,0.891530241069178
"9980","IFI27L1",170.796926104105,0.168200197048311,0.30167975493995,0.557545524000416,0.577154764532483,0.891530241069178
"9981","ZNF267",68.2931519034717,-0.164408982002512,0.294912116551482,-0.557484663312611,0.577196334773589,0.891530241069178
"9982","CANX",887.072228390475,-0.0750641171834509,0.134751221083303,-0.557057046162471,0.577488453853071,0.8915454043293
"9983","AC016405.3",5.81605872125162,-0.396191834640024,0.711128096782315,-0.5571314597647,0.577437614516614,0.8915454043293
"9984","KHSRP",310.622182231575,0.107605310868419,0.193170661357376,0.557047898020828,0.577494704004415,0.8915454043293
"9985","SAT1",3157.83957658968,-0.159901463658469,0.287026224592437,-0.557097052318204,0.577461121405189,0.8915454043293
"9986","TULP4",94.8909714054444,0.133146122943238,0.239139487799937,0.556771799455503,0.577683353784006,0.891747336128626
"9987","MTSS1",258.132350346228,0.19674475201807,0.353508512894538,0.556548837840194,0.577835717863823,0.891893220273439
"9988","HPCAL1",69.5063273204542,-0.140778415271356,0.25311519501988,-0.556183184736498,0.578085633187224,0.89199704971215
"9989","TTC21B",75.7187676665176,0.126020267866709,0.22656332796674,0.556225356493747,0.578056807186308,0.89199704971215
"9990","SLC9B1",11.7094917098014,0.241400716971672,0.434080119474464,0.556120186439161,0.578128696180267,0.89199704971215
"9991","AC027796.1",6.76493955235497,0.356513213616205,0.64108194069864,0.556111771340312,0.578134448503022,0.89199704971215
"9992","SLC16A11",3.42935267824464,0.444137466774925,0.798784472974025,0.55601665004994,0.578199472589464,0.892008093471436
"9993","FOXD2",3.73761682729714,-0.471121216799483,0.847549525109441,-0.55586275827203,0.578304678952529,0.892081119388895

"9994","NKX3-2",2.64382096073619,-0.597039349936738,1.07440950190422,-0.555690683001761,0.578422326911161,0.89217332092611
"9995","DESI1",96.7076705815616,0.124106961520903,0.22338815328621,0.555566442065949,0.578507277502427,0.892215075807895
"9996","AC012146.1",32.4179009849933,-0.19571942261925,0.352472442064085,-0.55527581524704,0.578706018481617,0.892343030398532
"9997","MFSD11",131.538031234772,-0.113138958330601,0.203746175216862,-0.5552936550106,0.578693818091201,0.892343030398532
"9998","TF",6.2167212748806,0.382922175891794,0.68987937465585,0.555056709852816,0.578855871767697,0.892374405466012
"9999","CREBRF",641.452061372225,0.126037835699488,0.227095208335478,0.554999978305564,0.578894675283687,0.892374405466012
"10000","SLC25A21",5.60725209258718,-0.355840626693485,0.641219746100073,-0.554943338625679,0.578933417182615,0.892374405466012
"10001","SNU13",747.847821951104,-0.128291823557548,0.231194967882502,-0.554907508292952,0.578957925985442,0.892374405466012
"10002","GPATCH2L",182.920545949468,0.125609338949764,0.226472789984872,0.554633247367837,0.579145543143607,0.892447716436133
"10003","MHENCR",14.3609909227843,0.251035851282018,0.452656063134611,0.554584090940069,0.579179173222645,0.892447716436133
"10004","TRMT2B",20.8742130291811,-0.212023953808218,0.382281210300556,-0.554628237264188,0.579148970734395,0.892447716436133
"10005","AL354733.3",10.2034637266607,0.408417720589055,0.736796474314816,0.554315519721865,0.57936293081854,0.892478898894383
"10006","CEP126",546.795861562401,0.119060742430657,0.214827309616713,0.554216047499177,0.579430997089522,0.892478898894383
"10007","TJP1",240.874375409635,-0.134501141431795,0.242668631726441,-0.554258457201085,0.579401976767731,0.892478898894383
"10008","TRIM25",206.695851336097,0.207293380158362,0.373952448432565,0.554330854169399,0.579352438186294,0.892478898894383
"10009","LRRC47",118.266577827144,-0.10255222262179,0.185481170878648,-0.55289829224167,0.580333056905326,0.89268216924069
"10010","TAF5L",63.011390532823,-0.150055733334229,0.271649879498798,-0.552386526403347,0.580683559811127,0.89268216924069
"10011","ERO1B",58.7170066203439,0.182480420231472,0.330022501785757,0.552933267410759,0.580309106408388,0.89268216924069
"10012","CENPO",7.71865250704079,0.303295059658637,0.548970434794506,0.552479770194125,0.580619690759121,0.89268216924069
"10013","SEMA4F",15.746995847039,-0.233527489518614,0.42293250017128,-0.552162554128707,0.580836987186775,0.89268216924069
"10014","KYNU",3.07832264131597,-0.653684570202901,1.1830818883579,-0.552526901675594,0.580587408434558,0.89268216924069
"10015","NDNF",56.6836466155968,-0.19952044756413,0.360872716137426,-0.552883159745858,0.580343419566169,0.89268216924069
"10016","PTGER4",235.409742986189,0.170624070591412,0.308249988143627,0.553524986712768,0.579903975690948,0.89268216924069
"10017","SNX2",635.837279806993,0.107950611429789,0.195459643650742,0.552291047980632,0.580748962924405,0.89268216924069
"10018","AC244517.1",5.04173773930409,0.379444882195207,0.686037315984104,0.553096563925101,0.580197289414389,0.89268216924069

"10019","SPACA9",35.4044003188942,-0.165268601988268,0.299270703835404
,-0.552237823048541,0.58078542371959,0.892682169240692
"10020","BRD3OS",60.8131715694711,-0.122731867953714,0.221930337050704
,-0.553019788032284,0.580249860310958,0.892682169240692
"10021","GOT1",47.8168400059522,-0.177549158013886,0.320771609924233,-
0.553506459177679,0.579916658872496,0.892682169240692
"10022","ECHS1",517.975877171158,-0.145599430064112,0.263243310826224,
-0.553098308964162,0.580196094556568,0.892682169240692
"10023","TMEM132A",19.9748508289331,-0.266643815019365,0.4817592975039
48,-0.553479333768707,0.579935228036267,0.892682169240692
"10024","TRPT1",93.199104279878,-0.129006613764319,0.233083854963269,-
0.5534772615831 7,0.579936646597866,0.892682169240692
"10025","LINC02356",4.81366671821267,0.433107269595014,0.7837110633324
69,0.552636411375093,0.580512403903285,0.892682169240692
"10026","PDIA3",1391.3188050865,-0.129640459938652,0.234212979348372,-
0.553515267596775,0.579910628978152,0.892682169240692
"10027","EMC6",189.930099572635,-0.155806871976066,0.281816439552609,-
0.552866512058037,0.580354819923417,0.892682169240692
"10028","SMARCD2",281.503918540289,0.110235867127448,0.199604253018302
,0.552272135791317,0.580761918261324,0.892682169240692
"10029","B3GNTL1",11.3211678965337,0.239064802972909,0.432626781968885
,0.552589004973119,0.580544872570123,0.892682169240692
"10030","CCDC159",22.270404208032,-0.184144259921294,0.332868783576332
,-0.553203751769253,0.580123898221058,0.892682169240692
"10031","AC006480.2",6.89041474495185,0.320340720954609,0.580267838864
42,0.552056652978585,0.580909539092945,0.892704670034667
"10032","ACAT1",380.942156442458,0.109270846393576,0.197972094084425,0
.551950752952979,0.580982094470409,0.892727171676771
"10033","GLT8D1",601.207469906186,-0.104667098471638,0.189726217906778
,-0.551674405500807,0.581171448614979,0.892770912526196
"10034","KIAA1549L",9.4119853008896,-0.396499638317085,0.7185641567247
8,-0.551794345162348,0.581089262034657,0.892770912526196
"10035","ZNF74",19.9729612884632,0.199205925792119,0.36110557277024,0.
551655639828267,0.581184307959804,0.892770912526196
"10036","AL669970.3",13.5505536940362,-0.264912447981438,0.48050104742
0379,-0.551325432907272,0.581410606981507,0.892851614526791
"10037","ADK",327.807575100798,0.0946911309811609,0.171700680539134,0.
55148954962691,0.581298128557611,0.892851614526791
"10038","RALY-
AS1",11.8939506294371,-0.28290024671009,0.513109785499703,-0.551344477
740921,0.581397553963183,0.892851614526791
"10039","PDIA5",75.6992689481213,0.179074896234133,0.325171729026137,0
.550708687899923,0.581833388016043,0.892861548687703
"10040","LINC00887",3.85246115129032,-0.442800814152625,0.804013445784
38,-0.550738070954319,0.581813242562061,0.892861548687703
"10041","INMT",188.227291259304,0.23024276349,0.417900833684479,0.5509
50715891218,0.581667459785062,0.892861548687703
"10042","FUBP3",133.501262446555,0.106536267695295,0.193477150444498,0
.550640049486653,0.58188044879122,0.892861548687703
"10043","SLC43A3",419.748285137699,0.119964012348916,0.217738414632148

,0.550954743340012,0.581664698855794,0.892861548687703
"10044","MAFTRR",5.68590528850745,0.378034907950905,0.68627471286115,0.550850702883746,0.581736023475692,0.892861548687703
"10045","SEPTIN9",163.48834471256,-0.102831830928251,0.186669660545231,-0.550875973245444,0.581718699079834,0.892861548687703
"10046","CC2D1A",57.9500396746047,-0.14352340019173,0.260458496935606,-0.551041343939007,0.581605333195039,0.892861548687703
"10047","NCAPG2",26.8032952079881,-0.19474916928197,0.353818162750909,-0.550421628352288,0.582030217408539,0.892951094469404
"10048","AMN1",25.1271570501603,0.19233302729141,0.349569573500957,0.550199565039902,0.582182501881088,0.892951094469404
"10049","SLC7A1",49.523173260257,-0.211882639084629,0.385066231483101,-0.550249857715523,0.582148011016881,0.892951094469404
"10050","PPP2R5C",267.113625916888,-0.102108627483801,0.185607309079627,-0.550132578238046,0.58222844310814,0.892951094469404
"10051","CPT1C",7.87014897516896,-0.333045637523169,0.605182572100267,-0.550322585079316,0.582098136065943,0.892951094469404
"10052","AC093425.1",84.765751599444,0.283655485206839,0.515898731353167,0.549827840170938,0.582437461477685,0.892969845994722
"10053","AC015982.2",6.69820084290487,0.345665807849515,0.628782886832467,0.549737938306222,0.582499131423961,0.892969845994722
"10054","ZEB2-AS1",6.26077903787324,0.343473040531144,0.624861187956419,0.549678948142799,0.582539598538976,0.892969845994722
"10055","KLHL7",97.9070255603914,0.11052558273654,0.201183991366233,0.549375633647413,0.582747692295102,0.892969845994722
"10056","TUSC3",162.426305776009,-0.166536354815159,0.303149026266058,-0.549354741021003,0.582762027292047,0.892969845994722
"10057","IFIT1",375.641394799972,0.163981741362397,0.29841373564603,0.549511372214139,0.58265456239465,0.892969845994722
"10058","AL049840.5",23.2085412504139,0.176664464476346,0.321490139598632,0.549517520807652,0.582650344024039,0.892969845994722
"10059","TLE5",1391.10676410226,-0.107109075206744,0.1947962289507,-0.549851892840553,0.582420962594365,0.892969845994722
"10060","EWSR1",324.470123649701,0.115827872650378,0.210592871580623,0.550008515392861,0.58231353298706,0.892969845994722
"10061","DNALI1",44.7433044201937,-0.171807126190989,0.312977756750925,-0.548943567027089,0.583044178383717,0.893142113694126
"10062","GMDS",45.529045603232,-0.191600793242454,0.34894650708542,-0.549083568260368,0.582948101193824,0.893142113694126
"10063","MRPL38",194.638657144243,-0.121210008902733,0.220817503645123,-0.548914859111578,0.583063880378593,0.893142113694126
"10064","EID2B",39.1442842242972,-0.180144849603759,0.328220470824436,-0.548853181373072,0.58310621032875,0.893142113694126
"10065","LAS1L",74.3010227473137,-0.146149856389495,0.266484162682419,-0.548437306436361,0.58339166619202,0.893490564764032
"10066","C1orf216",81.4043350142629,-0.147232250822619,0.268677565932467,-0.547988628345794,0.583699711105639,0.893561420046755
"10067","OSBPL11",121.546448020487,0.126777303404191,0.231403144879916,0.547863355400723,0.583785732162885,0.893561420046755

"10068","ADH1C",268.005995723234,0.186313172700778,0.340044296859598,0
.54790853550973,0.583754707698516,0.893561420046755
"10069","AP1S1",209.868889937781,-0.156374543481242,0.285414231087887,
-0.547886287537953,0.583769984931943,0.893561420046755
"10070","TMPO",133.74391349768,0.136449306457557,0.248921801212015,0.5
48161333371273,0.583581129587795,0.893561420046755
"10071","PPP6R1",72.8406394583667,0.121981194762887,0.222511245100374,
0.548202382795807,0.583552946169827,0.893561420046755
"10072","MTHFR",115.986222012666,-0.110476808374387,0.201715093028435,
-0.547687367939358,0.58390658728294,0.893576944030192
"10073","LINC01309",3.89149092071366,-0.504339328568636,0.920865372608
29,-0.547679762504402,0.583911810393521,0.893576944030192
"10074","C17orf113",4.9911068930123,0.385216848430733,0.70353876434037
5,0.547541753142636,0.584006593519508,0.893633277655669
"10075","ICMT",146.288152770904,0.132946344101736,0.242899227969446,0.
54733127483822,0.584151161212076,0.893699865820509
"10076","TMEM107",110.047767799388,0.117983842048785,0.215570576488921
,0.547309581717655,0.584166062147742,0.893699865820509
"10077","MRPL46",108.571585676743,-0.160798571320453,0.294022414598439
,-0.546892220921535,0.584452780393192,0.894045072519161
"10078","ZNF266",28.8712514523832,0.196687238139737,0.359697914492941,
0.546812283904962,0.584507702941817,0.894045072519161
"10079","YTHDF2",347.283709806288,-0.109669994827919,0.200836377014601
,-0.546066387265819,0.585020303905815,0.894052395630858
"10080","AL603839.3",2.57354218377802,-0.573814354281055,1.05388247327
238,-0.5444766080029,0.586113541155558,0.894052395630858
"10081","KLHL20",152.999418152233,0.0949777575706873,0.174409730068953
,0.544566851477481,0.586051458417462,0.894052395630858
"10082","AL078645.1",2.39406873041794,-0.575586158236741,1.05408615141
663,-0.546052291326649,0.585029993039319,0.894052395630858
"10083","GEN1",17.4690798719378,-0.273556938903296,0.50140926102846,-0
.545576159367685,0.585357315923444,0.894052395630858
"10084","PPP1R1C",12.395712186357,0.374591316523113,0.685788378054628,
0.54621998346737,0.584914731234003,0.894052395630858
"10085","AC096992.2",2.65067268642684,0.497047598668117,0.910332929649
965,0.546006392253918,0.58506154322711,0.894052395630858
"10086","CPA3",7.73784937942739,0.487447367321128,0.893299256019991,0.
545670853340797,0.585292210588358,0.894052395630858
"10087","ARHGAP10",357.592035811592,0.0810871658723483,0.1485331855309
95,0.545919523522422,0.585121257377501,0.894052395630858
"10088","AC107214.1",4.00702181263039,0.411255467826986,0.754597497245
665,0.544999777136948,0.585753670889673,0.894052395630858
"10089","MAN1A1",637.408677865296,-0.108708613829511,0.199077681449648
,-0.546061281394851,0.585023813522552,0.894052395630858
"10090","IL20RA",7.91473429997284,-0.301189276754684,0.553720330132177
,-0.543937544577401,0.58648445180793,0.894052395630858
"10091","AC002456.1",9.05912526219941,-0.299345159859979,0.54804755312
1136,-0.546202894539362,0.584926476684199,0.894052395630858
"10092","CCDC25",342.488704327811,-0.105237849071168,0.193054897336189
,-0.545118774624531,0.585671830881626,0.894052395630858

"10093","PLEKHF2",58.7006631044579,-0.13465899775837,0.247380237936235,-0.544340157814381,0.586207417462474,0.894052395630858
"10094","TLR4",149.062766652796,0.21489764704344,0.394594935173257,0.544603156016394,0.586026483675153,0.894052395630858
"10095","ZNF485",10.1659497352284,-0.245331264140876,0.449997074832023,-0.545184130880084,0.585626884657608,0.894052395630858
"10096","WASHC2A",157.216192811874,0.104926919507623,0.192575052436569,0.544862474032998,0.585848107101605,0.894052395630858
"10097","TALDO1",635.811434001477,-0.142381987691509,0.261649680380651,-0.544170310028164,0.586324280710097,0.894052395630858
"10098","AL109628.2",6.22197589979886,0.329570927380644,0.605859050474544,0.543972937472017,0.586460095860237,0.894052395630858
"10099","C14orf28",67.9412599099591,0.125623639765441,0.230237088062342,0.545627295856981,0.585322157425781,0.894052395630858
"10100","TMEM251",101.186586500496,-0.150966440081055,0.277044227605733,-0.54491819369685,0.585809782615133,0.894052395630858
"10101","ZNF839",22.9934966304482,0.23066400791935,0.423899673442923,0.544147642402958,0.586339877917035,0.894052395630858
"10102","AL928654.1",8.12284588224065,0.316868832544554,0.582387953204453,0.544085485973839,0.586382647684585,0.894052395630858
"10103","SAXO2",6.76895252762951,0.317873549003863,0.58396206031917,0.544339385387685,0.586207948903837,0.894052395630858
"10104","CYB5D1",30.6419644582384,-0.273168037967446,0.501723624698206,-0.544459189323128,0.586125524637318,0.894052395630858
"10105","CHD3",286.272389509061,0.134319451972528,0.24669748087797,0.544470302228055,0.586117879309207,0.894052395630858
"10106","PEMT",112.203855149256,-0.152170076458955,0.278990587101542,-0.545430862165867,0.585457219243514,0.894052395630858
"10107","NARS",356.084004290016,-0.11708124089433,0.214732939435859,-0.545241178190559,0.585587653896554,0.894052395630858
"10108","GADD45GIP1",668.990987644655,-0.138310331317936,0.253834497567933,-0.544883901294468,0.585833369100552,0.894052395630858
"10109","ZNF181",70.5569822461351,0.131023097483512,0.240324841903678,0.545191651623037,0.585621712661756,0.894052395630858
"10110","HUNK",86.7569023536494,0.192799119669441,0.353710957004979,0.545075338637947,0.585701703178597,0.894052395630858
"10111","PES1",73.6649918786337,-0.127146302559693,0.232814231569586,-0.546127707496651,0.584978155052761,0.894052395630858
"10112","NONO",412.377975224617,0.0826458826542344,0.151833319870043,0.544319802300131,0.586221422441758,0.894052395630858
"10113","ATF1",151.052008568719,0.095455705951757,0.17558829283889,0.543633658078455,0.586693593406462,0.894108524924643
"10114","ARHGEF25",80.3387200810285,-0.152822856126632,0.28115852587104,-0.543546939055755,0.586753281747499,0.894108524924643
"10115","AL132639.2",4.21825906450132,-0.413076080989968,0.75990669984426,-0.543587891874919,0.586725093742531,0.894108524924643
"10116","TMEM104",22.1040396896367,0.205810432438908,0.378472084371859,0.543792900288768,0.586583994986275,0.894108524924643
"10117","METTL21A",74.6764129961624,0.12867265129062,0.236812647209382,0.543352108964231,0.586887392746375,0.894224489392644

"10118","OLFML1",532.254598423149,0.0866195802103813,0.159453474024176
,0.543227927396854,0.586972880349493,0.894266352103917
"10119","MYL5",66.3621869487681,0.195846542157103,0.360645163100548,0.
543044971055112,0.587098839493417,0.894369059786066
"10120","AC073389.1",5.30175580713319,-0.355920983552796,0.65561969027
8112,-0.542877202790867,0.587214353168652,0.894369059786066
"10121","SHFL",186.598247000175,-0.0966941942987661,0.178089998476017,
-0.542951289382978,0.587163340945144,0.894369059786066
"10122","AC018362.1",10.548284328542,0.313195161995653,0.5771563079090
52,0.542652237710631,0.587369265145809,0.894428254689583
"10123","MIR222HG",164.955278421988,-0.358609691613637,0.6607845271049
3,-0.542702918884617,0.58733436420835,0.894428254689583
"10124","CTH",5.70737273969445,-0.405908277862734,0.749951089082619,-0
.541246334289965,0.588337804827234,0.894502089070145
"10125","CCDC18",47.8771968816825,-0.144880812522791,0.26731189940174,
-0.541991631674621,0.587824270753821,0.894502089070145
"10126","CSTA",27.7565698321697,-0.243583380098788,0.450050763844002,-
0.541235344249343,0.588345378872372,0.894502089070145
"10127","UBA5",131.579760023991,-0.113942595003513,0.210556416003582,-
0.541149954801539,0.588404228554737,0.894502089070145
"10128","AC124852.1",61.817247979889,0.160167695797865,0.2957796731308
39,0.541510152142925,0.588156002068114,0.894502089070145
"10129","HMGN3",714.181221281632,-0.128850422923329,0.237957431360976,
-0.541485181556974,0.58817320874253,0.894502089070145
"10130","BHLHE22",45.0665799527724,0.268543219851833,0.49537405827307,
0.542101903333422,0.587748307612148,0.894502089070145
"10131","C11orf68",189.356359718401,-0.109031834057287,0.2014694966226
8,-0.541182838519154,0.588381565053786,0.894502089070145
"10132","CDON",332.554371005392,0.130895235502591,0.241848354403909,0.
541228555493845,0.588350057525501,0.894502089070145
"10133","MAGOHB",115.951910031867,-0.169840321576004,0.313423640207909
,-0.541887400271851,0.587896077101621,0.894502089070145
"10134","ANP32A",308.231280933443,-0.107210529500915,0.198013493784211
,-0.541430421998156,0.588210943148952,0.894502089070145
"10135","GGA2",112.384361109962,0.101562112107216,0.187536039506253,0.
541560504181543,0.588121306307787,0.894502089070145
"10136","COX4I1",2522.37533593577,-0.134788456067094,0.248775042194091
,-0.541808594939094,0.58795036979676,0.894502089070145
"10137","RAB11B-
AS1",86.9581989802743,0.158838197778968,0.292963809124487,0.5421768588
19697,0.587696675411886,0.894502089070145
"10138","ZNF331",124.459872062602,-0.134213957782234,0.247424353320189
,-0.542444411721062,0.587512391873397,0.894502089070145
"10139","SS18L1",35.4573120524742,0.170109341605538,0.313947995485173,
0.541839234688064,0.587929260359122,0.894502089070145
"10140","OPRL1",3.66033065237137,0.462275856788258,0.853722897792113,0
.541482321703904,0.58817517941841,0.894502089070145
"10141","AC092821.3",32.0366060734936,-0.197148395327508,0.36451920681
5983,-0.540845013489323,0.588614413712277,0.894733378106178
"10142","CDK2AP2",79.0886180232656,-0.13678925785509,0.252958376942555

,-0.540757967806512,0.588674417554163,0.894736358370876
"10143","ENKD1",67.9499042649753,0.12549114663592,0.23211533695257,0.540641339273338,0.588754818387673,0.894770336729367
"10144","ODR4",172.653426966403,0.095314313415606,0.176366619919581,0.540432840744282,0.588898564762327,0.894771694696502
"10145","SLC25A4",197.821315411686,0.0961288832202087,0.177895471116519,0.540367231480815,0.588943801495838,0.894771694696502
"10146","CDKL3",32.2511071647186,0.171879332832656,0.317984673985179,0.540527097355225,0.588833578862858,0.894771694696502
"10147","SRSF12",5.19611478472845,-0.440836908771717,0.815906402640254,-0.54030328398598,0.588987894004892,0.894771694696502
"10148","NAPRT",150.71898862459,0.160386488701915,0.296986536718285,0.540046328275325,0.589165083184435,0.894952676122199
"10149","NPTN-IT1",10.4409704080783,0.327279391276879,0.606206113837354,0.539881376657789,0.589278841978968,0.895037279446821
"10150","SNX21",538.437833766525,-0.122619268179885,0.227305549136652,-0.539446875123004,0.58957854421476,0.895404261977391
"10151","TATDN1",252.57966533211,-0.13684792962325,0.253741520866166,-0.539320207257011,0.589665928003068,0.89544875186359
"10152","MYBL1",9.40269102853425,0.35246040876674,0.653705680584671,0.539172932460218,0.589767535307116,0.895514830256027
"10153","RAD54B",3.72558376873635,0.404405077636117,0.75069568483969,0.538707076386721,0.590088990192367,0.895594041054748
"10154","SYCP3",2.44771578649761,0.554674675412815,1.02940863490548,0.538828465785839,0.590005220033534,0.895594041054748
"10155","DIABLO",2.94063613368035,-0.438663911271935,0.814336683851627,-0.538676348457194,0.590110196236982,0.895594041054748
"10156","MRPL58",105.547363606949,0.139396031937655,0.25870491657871,0.538822507825218,0.590009331461641,0.895594041054748
"10157","ZCCHC3",80.1304722314988,0.141202848716076,0.262048751832842,0.53884190528848,0.58999594584331,0.895594041054748
"10158","RTL8C",607.615508737595,-0.0851164087398264,0.158078140794984,-0.53844515321202,0.590269760600737,0.895748017292809
"10159","PPT1",245.121669583053,0.100958720453198,0.187551589371975,0.538298399876338,0.590371055874128,0.895767212778113
"10160","IMPA1",63.0356412042123,0.118179472365746,0.219632318547323,0.538078699653132,0.590522717088998,0.895767212778113
"10161","NCBP1",42.9564486542547,0.15527449366636,0.288493294847311,0.538225658757654,0.590421267794682,0.895767212778113
"10162","FBXO3",145.441619836337,-0.0933244776818461,0.173463662583794,-0.538005921769146,0.590572960328509,0.895767212778113
"10163","CENPV",47.7393084165212,-0.208512498054269,0.387552524819213,-0.538023841159433,0.59056058924191,0.895767212778113
"10164","ZNHIT1",779.608197233284,-0.127797856306379,0.237620128798559,-0.537824202657172,0.590698421270808,0.895781226157354
"10165","AC104389.5",4.48899939616389,0.451141191926928,0.838700743667707,0.537904843095822,0.59064274468096,0.895781226157354
"10166","VARS2",25.9959450104316,0.181810318310832,0.33817111568824,0.53762817069365,0.59083377771365,0.895898355641936

"10167","OXSR1",91.2478176301497,-0.12195969116552,0.226908004450251,-
0.537485186831562,0.590932514429001,0.895959939994398
"10168","SNX24",85.9673318261928,0.125799518348766,0.234092028955181,0
.537393429884158,0.59099588067658,0.895967889519029
"10169","PLCD1",59.3064907253966,0.114883917752687,0.213864112003768,0
.537181842602289,0.591142012225727,0.896024834326913
"10170","DGCR8",76.2425141814882,-0.130577229208418,0.243083297212887,
-0.537170717632901,0.591149696082044,0.896024834326913
"10171","LINC01873",2.50495159665981,0.535318367930234,0.9971221111000
44,0.536863401153206,0.591361973305576,0.896148490736473
"10172","SNHG32",740.656600696797,0.14098402586183,0.262561519285988,0
.536956162674651,0.591297895086873,0.896148490736473
"10173","TENM4",7.6363971915749,0.523530309052795,0.975432687553741,0.
536715978183734,0.591463817369632,0.896148490736473
"10174","SORD",43.5539484534149,-0.142322171357789,0.265136173949249,-
0.536788961075645,0.591413397663332,0.896148490736473
"10175","GMCL1",98.3165376742444,0.116128343203391,0.216612904061735,0
.536109996338418,0.591882531833613,0.896216326007116
"10176","UXS1",175.609095187844,-0.11236782332983,0.209686317780485,-0
.535885338248276,0.59203779810815,0.896216326007116
"10177","RYBP",217.0526195161,-0.123248273290221,0.229994204849844,-0.
53587555986763,0.592044556592198,0.896216326007116
"10178","LRPAP1",878.960828216547,-0.107961527753355,0.201524017450712
,-0.535725364743485,0.592148370801796,0.896216326007116
"10179","AL031963.3",16.9586078810252,-0.259436785075863,0.48431329718
7556,-0.535679665585133,0.592179959516774,0.896216326007116
"10180","NUP214",109.443501163346,0.112139885311147,0.209223951157951,
0.535980152800424,0.591972267351842,0.896216326007116
"10181","VSTM4",151.97943087435,0.143396381575736,0.267706327860683,0.
535648083934576,0.592201790212217,0.896216326007116
"10182","MXI1",248.083757196864,-0.165824127805592,0.309250906370048,-
0.536212261273594,0.591811860422752,0.896216326007116
"10183","SIK3",140.787002537605,-0.115661406705512,0.215797375520305,-
0.535972258358762,0.591977723441349,0.896216326007116
"10184","SUMO2",3149.87722072549,-0.128175723104184,0.239293809210829,
-0.535641617837489,0.592206259922704,0.896216326007116
"10185","TNRC6C",259.687462476529,0.144017949318468,0.268547277485275,
0.536285270389176,0.591761408967989,0.896216326007116
"10186","MPND",52.6731968282177,0.133595874484007,0.249029972267324,0.
536465041808691,0.591637190029129,0.896216326007116
"10187","UFSP2",229.521573605367,-0.121204616782648,0.226395407700447,
-0.535366940583083,0.592396145756476,0.896330141859976
"10188","AC015922.3",78.8973486175811,0.109502933847075,0.204571124141
851,0.53528050112852,0.592455907584255,0.896330141859976
"10189","MPRIP",213.079217348205,0.111898288844356,0.209041054521308,0
.535293361873801,0.59244701584859,0.896330141859976
"10190","AC108673.3",13.5070032938563,0.252074198197025,0.471041594098
507,0.535142121959424,0.592551584838482,0.896386916612876
"10191","LY75",7.53661636622192,-0.40637403976157,0.759595625716105,-0
.534987335371321,0.592658614802409,0.896432253128274

"10192","RAB28",136.722308700761,-0.132621461428489,0.247922751310856,
-0.534930581107508,0.592697860777384,0.896432253128274
"10193","BEX4",275.154553338449,0.148507086523997,0.277711206069436,0.
534753669561558,0.5928020400847,0.896529328440161
"10194","ARL4C",382.681692776584,0.332531853232627,0.622070780586516,0
.534556297466828,0.59295671039802,0.896647801725082
"10195","AC121247.1",12.1021343861506,-0.264337213013145,0.49502575045
7969,-0.533986793148833,0.593350671414152,0.896657799942188
"10196","SYCP2L",2.63205060117165,0.583115578248333,1.09117684006314,0
.534391454106182,0.593070730318956,0.896657799942188
"10197","SYNGAP1",67.6388602748031,-0.152744227960202,0.28603391465866
,-0.534007403081836,0.593336412170965,0.896657799942188
"10198","USP15",220.272396005878,-0.0963233518972147,0.180319372833416
,-0.534181937213152,0.593215664813788,0.896657799942188
"10199","RNASEK",1704.46963735691,-0.132386666491217,0.247934542375863
,-0.533958137589889,0.593370497386329,0.896657799942188
"10200","TMEM11",176.087030134056,-0.134543854711589,0.251921189108399
,-0.534071211666504,0.593292266388568,0.896657799942188
"10201","PSPN",9.10727844641461,0.288047144343293,0.539258245014938,0.
534154362971889,0.593234740659083,0.896657799942188
"10202","TCF7L1",386.374238619962,0.0955794677016756,0.179118738979993
,0.533609538823024,0.593611707319536,0.896758571964979
"10203","DCAF17",26.2595489312695,0.183758510341348,0.344360508079214,
0.533622485825458,0.593602747949536,0.896758571964979
"10204","C1GALT1C1",233.697660633242,-0.147860103773841,0.277008663661
849,-0.533774293624036,0.593497701051463,0.896758571964979
"10205","PLEKHB2",126.223618772573,0.137991782468024,0.258731330079523
,0.533340057524579,0.593798203350929,0.896916644821396
"10206","AC022075.1",6.1478074063366,0.361173374164015,0.6772548549236
5,0.533290195763502,0.593832713399103,0.896916644821396
"10207","ETFA",269.512219777079,-0.114444545938161,0.214648649527831,-
0.533171516289099,0.593914856876345,0.896952828328486
"10208","GLYCTK",10.0132195787878,-0.285598982961004,0.535872260853538
,-0.532960938314108,0.594060620290554,0.896976250506972
"10209","TM7SF2",5.12784330890954,-0.363895227146549,0.68286230894267,
-0.532896928679512,0.594104931409419,0.896976250506972
"10210","FOXJ2",81.865707067622,-0.111550192796099,0.209309900750527,-
0.532942743731236,0.594073215464497,0.896976250506972
"10211","FGR",8.33812267055816,-0.296944660879484,0.557372124552538,-0
.532758363396578,0.594200859376613,0.897013326931615
"10212","SS18L2",235.45962888196,-0.147742610638303,0.277350206775198,
-0.532693349524177,0.594245870556319,0.897013326931615
"10213","EFCAB7",64.4002053560324,0.163416286655688,0.306880063138925,
0.532508645182693,0.594373755774374,0.897118520049151
"10214","SLC22A5",27.2897633136366,-0.163324220287041,0.30680707643993
9,-0.532335245269396,0.59449382550312,0.897211897408518
"10215","TAF12",246.670973358235,-0.144889467332535,0.272763788200583,
-0.531190259118956,0.595286942395543,0.897357365423626
"10216","RAVER2",21.1711310101864,-0.220646660742572,0.415827744072942
,-0.530620344331491,0.59568189480368,0.897357365423626

"10217","SSX2IP",16.7154716807802,-0.20830433590886,0.391481502277651,
-0.532092409722909,0.594661994148479,0.897357365423626
"10218","NDUFS2",299.948317493958,-0.111437665750448,0.20984630474345,
-0.531044212985724,0.595388141351517,0.897357365423626
"10219","AC007365.1",3.90040272778857,0.410314839855943,0.773064105776
467,0.530764314097628,0.595582112106559,0.897357365423626
"10220","LINC01886",28.0989341633,-0.24407564705667,0.460294983110336,
-0.530259194674218,0.595932234280601,0.897357365423626
"10221","EPM2AIP1",167.141946538367,0.0946532963649334,0.1779733573975
24,0.531839696396323,0.594837026435546,0.897357365423626
"10222","CGGBP1",322.11838336546,0.0820014621741089,0.154562968637872,
0.530537572464926,0.595739265855905,0.897357365423626
"10223","NPY1R",27.2886915716563,0.552641237884165,1.04004683995723,0.
531361873958378,0.59516803628708,0.897357365423626
"10224","SNHG4",3.05762080786293,-0.472171500095233,0.890134240336489,
-0.53044976667423,0.595800128788676,0.897357365423626
"10225","SPRY4",20.7538798035283,0.291694005840407,0.548407892522242,0
.531892428642568,0.594800501505417,0.897357365423626
"10226","AL138724.1",16.44889534747,-0.244451302468151,0.4607584774666
66,-0.530541084804753,0.595736831322567,0.897357365423626
"10227","PRRC2A",241.866299714194,0.0962828845479612,0.181703772715452
,0.529889297888935,0.596188686738471,0.897357365423626
"10228","SDHAF4",43.7084066349614,-0.171798791658927,0.324208950357789
,-0.52990144617949,0.596180263431024,0.897357365423626
"10229","MAPKAP1",156.058868869663,-0.10066670243284,0.189771276167247
,-0.530463326515872,0.595790729545455,0.897357365423626
"10230","GBGT1",17.5024079823837,-0.224675768323016,0.422335557200972,
-0.531984021928094,0.594737061955909,0.897357365423626
"10231","CLNS1A",297.494051333927,-0.130843323176318,0.246407037468571
,-0.531004814312606,0.595415443005035,0.897357365423626
"10232","DAZAP2",498.668667011438,0.0713197768958676,0.134494560897165
,0.530280008500855,0.595917805379297,0.897357365423626
"10233","GPR137C",2.47250978806504,0.553892892328562,1.04445618648986,
0.530317019989175,0.595892148063433,0.897357365423626
"10234","SPG21",262.177736444053,-0.107339114293645,0.201955791733402,
-0.531498073773202,0.595073675766416,0.897357365423626
"10235","EME2",10.3580134166665,0.305842914354663,0.576683980434719,0.
530347512209567,0.595871010450161,0.897357365423626
"10236","ZKSCAN2-
DT",9.67511910845482,-0.300491483320503,0.566199997252077,-0.530716151
146008,0.595615492080479,0.897357365423626
"10237","CRK",336.813790102489,0.0845837208647628,0.159630444372204,0.
529872113038426,0.596200602357675,0.897357365423626
"10238","AP2B1",490.019942154204,0.112110924597466,0.211250193337668,0
.530702115942045,0.595625219525728,0.897357365423626
"10239","LIN37",73.3942432655638,0.135568304159272,0.25577390855561,0.
530031796147015,0.596089885579844,0.897357365423626
"10240","ZNF565",37.898782103563,-0.149752601731352,0.281829281708293,
-0.53135927120005,0.595169839569185,0.897357365423626
"10241","ZIM2",2.7841216229684,-0.54296441399932,1.02204014877044,-0.5

31255464526055,0.595241762500894,0.897357365423626
"10242","PLTP",2744.7665110438,-0.153227080364191,0.28919288931405,-0.529843872467394,0.596220184020031,0.897357365423626
"10243","C7orf31",12.8777449011501,-0.304748512641838,0.57577306674641,-0.529285807625418,0.596607199362938,0.897501705687488
"10244","MYORG",7.60969718478915,0.374712128567472,0.707908072268651,0.529323146954127,0.596581301144177,0.897501705687488
"10245","KLRB1",18.9720699761507,0.25933527789672,0.489817885615081,0.529452446537478,0.596491624084571,0.897501705687488
"10246","AC138150.2",7.05454560014081,-0.294487832402298,0.556069809943975,-0.529587881118682,0.596397698616674,0.897501705687488
"10247","MORC3",235.281518645221,0.111849938159898,0.21125330320433,0.529458884019031,0.596487159463264,0.897501705687488
"10248","AL139246.5",2.78382945586081,-0.55991925688103,1.05828535999972,-0.529081548365348,0.596748880785378,0.897627244077537
"10249","EIF4EBP3",248.276069180008,0.167143952635924,0.316168571307364,0.528654546354117,0.597045113889844,0.897631867657895
"10250","RIOK1",110.013427878493,-0.111391478273491,0.210627648061787,-0.528854968939379,0.596906062175017,0.897631867657895
"10251","AL513550.1",11.0192781149123,0.236332355979807,0.446961509923712,0.528753261148023,0.596976624450655,0.897631867657895
"10252","HTRA4",7.89323028606833,0.38274389710058,0.72410722509694,0.528573509329837,0.597101340964259,0.897631867657895
"10253","RFX3",39.6479245804102,0.169900110609977,0.321335329825074,0.528731498968589,0.59699172298944,0.897631867657895
"10254","FKBP2",87.8282894600497,-0.179507653868605,0.339501588756605,-0.528738774171973,0.596986675454495,0.897631867657895
"10255","SETMAR",63.3647495248706,-0.163053046043008,0.308583314030263,-0.528392296762414,0.597227082978863,0.897720433017878
"10256","RHEB",647.533478018617,-0.13162095872817,0.249164966828892,-0.528248254171935,0.597327041620125,0.897720433017878
"10257","AIFM1",67.076082961051,-0.135278933031332,0.256095227540601,-0.528236837251819,0.597334964739823,0.897720433017878
"10258","AC022509.1",2.77604359971252,-0.519691360479443,0.984257328244981,-0.528003547005436,0.597496874079672,0.897756334958144
"10259","Z93241.1",23.0616304005135,0.22311138750032,0.422598924165726,0.527950675550763,0.597533570980899,0.897756334958144
"10260","IDH3G",390.62836303559,-0.140691940878057,0.266441897026224,-0.528039855774672,0.597471673563493,0.897756334958144
"10261","LCLAT1",22.5113093154237,0.211193917373323,0.400136705979303,0.527804408386985,0.597635097103962,0.897821364570469
"10262","B4GALT3",57.0965643301676,0.14120276336204,0.267683384069441,0.527499171653517,0.597846991515039,0.897964666686576
"10263","Z69706.1",5.38858427696414,0.326645511574237,0.6191918317869,0.527535239978189,0.597821951215882,0.897964666686576
"10264","GLCCI1",27.8307076216102,0.176414686617218,0.334667274820787,0.52713455987499,0.598100148621956,0.898108305208256
"10265","CENPJ",38.918862543951,-0.159418919510437,0.302439736248838,-0.527109702870762,0.598117409099446,0.898108305208256
"10266","CUL4B",131.692936094467,0.105061819713153,0.199287554571932,0

.527187058613996,0.598063694718689,0.898108305208256
"10267","RAPH1",76.8896828924238,-0.134937442736539,0.256104796745028,
-0.52688369937436,0.598274354245213,0.898168988185621
"10268","AKT1S1",129.980814168545,-0.115542516021278,0.219274340729532
,-0.526931311875636,0.598241288817844,0.898168988185621
"10269","SSR3",899.926528883689,0.132635161296493,0.252015896217541,0.
526296806222103,0.598682001720461,0.89821939155974
"10270","NACAD",23.6305451237245,-0.192447177009103,0.365605794083361,
-0.526378903517113,0.598624970551239,0.89821939155974
"10271","RFC5",37.9019912285896,-0.145656729959544,0.276573939709762,-
0.526646617943819,0.598439012402432,0.89821939155974
"10272","COX16",520.365394632784,-0.119265952182737,0.226575343755282,
-0.526385396601462,0.598620460054778,0.89821939155974
"10273","B2M",22941.5180244448,0.142220322129453,0.270053119386445,0.5
26638323795608,0.598444773239178,0.89821939155974
"10274","RANGRF",135.567278103431,-0.132596218651585,0.251847811951242
,-0.526493431188739,0.598545414836129,0.89821939155974
"10275","PLEKHH3",142.575558526983,-0.10397893743793,0.197585378749167
,-0.526248136862043,0.598715812408455,0.89821939155974
"10276","PRKCH",10.1628726982584,0.286718065545914,0.544928082624824,0
.526157624626066,0.59877869371693,0.898226310203043
"10277","GPANK1",118.118876494942,-0.127448751343692,0.242336796721251
,-0.525915804236242,0.598946707596573,0.898390921241722
"10278","IFIH1",50.2470068637969,-0.166289043831132,0.31638094661719,-
0.525597529210051,0.599167873801916,0.898601309276275
"10279","ANKZF1",55.7745232336418,-0.135759507286515,0.258369081483723
,-0.525447961911292,0.599271819436606,0.898601309276275
"10280","AC002074.1",17.5690985999342,-0.264325361987257,0.50304186642
6083,-0.525454002199034,0.599267621425234,0.898601309276275
"10281","FGF16",49.7601644587467,0.151503160947366,0.288369588276183,0
.525378427916141,0.599320146653869,0.898601309276275
"10282","GNPAT",113.376748887139,-0.105588216771231,0.201140291415577,
-0.524948114712007,0.599619260344174,0.898874929320766
"10283","GCAT",17.036217332606,0.213472717220137,0.406632877577894,0.5
24976530406704,0.599599506313604,0.898874929320766
"10284","STAM-
AS1",4.93387550789955,0.421787247022018,0.804019766996998,0.5245981060
8561,0.5998626036034,0.899107378614844
"10285","ZNF100",47.6493437309701,0.184983692304858,0.352647254443986,
0.524557301875268,0.599890975611656,0.899107378614844
"10286","AC017083.1",5.38084842780455,-0.362737771009923,0.69205321779
0824,-0.524147221174488,0.600176146887231,0.899178288743625
"10287","LRR1",31.3583370314157,-0.188404187468386,0.359478780217086,-
0.524103779796433,0.600206359735566,0.899178288743625
"10288","ST3GAL2",91.455541046307,0.128297751314434,0.244849577523112,
0.523986002394976,0.600288275670493,0.899178288743625
"10289","IST1",238.485970570491,0.0918719688759408,0.175266296924085,0
.524185028658043,0.600149852890689,0.899178288743625
"10290","KPNB1",542.020516712969,0.0983992733472495,0.187734916817293,
0.524139435622481,0.600181561575129,0.899178288743625

"10291","SCAND1",597.111354846787,-0.147822211738333,0.282090525814331,-0.524024021408034,0.600261832328681,0.899178288743625
"10292","SNCAIP",48.2599483731548,0.151058089152826,0.28851731432848,0.523566807435499,0.600579873013889,0.899206983855411
"10293","NUDT1",311.077461279393,-0.150224441445085,0.286928959866405,-0.523559704517209,0.600584814445714,0.899206983855411
"10294","AGBL3",8.50394562299287,-0.255018866714359,0.487033467728295,-0.523616719614489,0.6005451501101,0.899206983855411
"10295","CA13",5.11682410270582,-0.350637952818973,0.669756989133749,-0.523530113918604,0.600605400537824,0.899206983855411
"10296","TUT1",23.770039344511,0.191456846618942,0.36562023368307,0.52364948375615,0.600522357245974,0.899206983855411
"10297","BCAR1",65.1444143671184,-0.130561276809203,0.249501929562024,-0.523287643660275,0.600774098392921,0.899206983855411
"10298","PECAM1",6.99399604172374,-0.346756585007903,0.662588757968503,-0.523336052472516,0.600740416416488,0.899206983855411
"10299","SNHG20",22.4669870147003,-0.17048062065011З,0.325699848415694,-0.523428615270729,0.60067601527054,0.899206983855411
"10300","B3GALT6",64.090825365111,0.140905085414103,0.269467611911006,0.522901748432157,0.601042627822472,0.899224872036054
"10301","ABCG2",2.55565786322078,0.48145750479683,0.921080505289763,0.522709472225089,0.601176445560469,0.899224872036054
"10302","DNAJA1",1678.49087773876,0.123916922632509,0.236884741476286,0.523110614302333,0.600897279484888,0.899224872036054
"10303","FAM219A",44.4351726410413,-0.137087440435174,0.262360237100018,-0.522516071606205,0.601311059419244,0.899224872036054
"10304","TRUB1",59.5503187965818,-0.137860744175362,0.263679834782345,-0.522833853749792,0.601089878689063,0.899224872036054
"10305","ANKDD1A",33.6597701793658,0.177927071145521,0.340253320573867,0.522925304139373,0.601026234771808,0.899224872036054
"10306","DPEP2",3.09418200987411,-0.570868845452766,1.09240501296977,-0.522579847835763,0.601266667342331,0.899224872036054
"10307","U2AF1L4",56.2962381036478,0.136256046556368,0.260696209561238,0.522662169832433,0.601209368487735,0.899224872036054
"10308","THOC5",80.4004129153461,0.109745854767109,0.209814650760029,0.523060970097023,0.60093182511103,0.899224872036054
"10309","COA6-AS1",22.5942248414316,-0.226920410539033,0.43512934929388,-0.521501045395525,0.602017777521826,0.89925285927401
"10310","HNRNPA3",851.558744653238,-0.10948893438141,0.209768481446658,-0.521951313306579,0.60170422957099,0.89925285927401
"10311","ATP13A3",154.343061366369,0.144933932690517,0.277936893096204,0.5214634555202 8,0.602043956887147,0.89925285927401
"10312","JARID2",54.7904621819423,0.148787332518268,0.285482260118269,0.521178907777418,0.602242146023804,0.89925285927401
"10313","CBLL1",66.8523389091649,0.130792676194604,0.250967018614812,0.521154839056149,0.602258911374822,0.89925285927401
"10314","AGAP3",144.742526521784,-0.104108191803844,0.199659897990338,-0.521427651980881,0.602068892640942,0.89925285927401
"10315","HNRNPH3",617.658971527099,0.0876309442765347,0.16814958218172

,0.521148748272427,0.602263154015237,0.89925285927401
"10316","CDK4",436.590679517116,-0.127505603590927,0.244635694880655,-0.521206047437724,0.602223241826331,0.89925285927401
"10317","UBAC2-AS1",6.11128514418188,-0.291153674040626,0.558496290001937,-0.521317113923202,0.602145880977814,0.89925285927401
"10318","GSKIP",48.088719099882,0.151700414215306,0.290439467235131,0.522313360713108,0.601452168169327,0.89925285927401
"10319","DUT",735.21260850444,-0.125197958083864,0.240064115151803,-0.521518836768653,0.602005386947828,0.89925285927401
"10320","BABAM1",259.548479883869,-0.12422973250334,0.238077161514771,-0.521804492765816,0.601806461229883,0.89925285927401
"10321","EXOSC5",54.7099304011031,-0.177329656348053,0.339697871401593,-0.522021688320834,0.601655229978442,0.89925285927401
"10322","SRC",62.944244174099,0.166555036429911,0.319371172542203,0.521509299365151,0.602012029136076,0.89925285927401
"10323","NDUFV3",231.467092939151,-0.137765899504011,0.264314138421585,-0.521220318847537,0.602213301147826,0.89925285927401
"10324","TMEM191B",5.87465324612059,0.45320337494002,0.868348101739679,0.521914395888073,0.60172993456213,0.89925285927401
"10325","POLG",63.8549374799128,-0.124456365269924,0.238858608598432,-0.521046178742337,0.602334602612714,0.899272435765132
"10326","PARP9",125.808924354817,0.105354838842702,0.202615353670738,0.51997460673148,0.60308127393869,0.899690103335421
"10327","NAA15",127.012962170254,-0.111668351206085,0.214750908249854,-0.51999012305067,0.603070459197391,0.899690103335421
"10328","BAG6",214.041199333388,-0.0925878372265407,0.177978705558673,-0.520218623547736,0.602911206417272,0.899690103335421
"10329","PRCP",290.59615034435,-0.0969843785211795,0.186466999752486,-0.520115509178114,0.60298306934446,0.899690103335421
"10330","FAM214A",124.190022835034,0.126920589514211,0.243865993847164,0.520452185694062,0.602748445497854,0.899690103335421
"10331","COLEC12",775.138275005354,0.155339463132669,0.298583645755595,0.520254425655389,0.60288625595397,0.899690103335421
"10332","ETV2",8.36905865354163,-0.313273874047912,0.602442089314397,-0.520006619066788,0.603058961711437,0.899690103335421
"10333","Z68871.1",5.25770982558354,-0.369250838426732,0.709902196543746,-0.520143253851699,0.602963733023556,0.899690103335421
"10334","CLPX",139.728520745407,0.100679130814571,0.193762145229796,0.519601652299881,0.60334124622604,0.899990837098355
"10335","AC006213.4",3.4271780024663,0.419485324258416,0.807556291134266,0.519450258593393,0.603446791374497,0.900061179394085
"10336","IFT27",129.547144829212,-0.135436140110187,0.260822294764979,-0.519265963180891,0.603575285372557,0.900165733747869
"10337","LZIC",317.701229553744,0.0809699609977908,0.156133814123831,0.518593371027066,0.60404433274359,0.900186650686287
"10338","SPON2",2471.6294595529,-0.129863611264762,0.250751363232883,-0.517897927215465,0.604529488300691,0.900186650686287
"10339","AC109347.1",4.64449180840233,-0.364394322678332,0.703294901162316,-0.51812450520558,0.604371403745613,0.900186650686287

"10340","RNF150",7.80900093771364,0.401377355646139,0.775340231469051,
0.517678999947728,0.604682252532338,0.900186650686287
"10341","FANCE",10.1847397836409,-0.271336750178784,0.523021040587174,
-0.518787446627703,0.603908972925324,0.900186650686287
"10342","MTHFD1L",38.2942991978046,0.177886322402412,0.34329671700411,
0.518170764797272,0.604339130486637,0.900186650686287
"10343","DBNL",261.09542263382,-0.104299024308319,0.200954153737168,-0
.519019001939787,0.603747490366535,0.900186650686287
"10344","CARNMT1",37.5604157259995,0.165590428017706,0.319937278016875
,0.517571534783678,0.604757246481167,0.900186650686287
"10345","FANCC",10.4904902895178,0.253311854311051,0.48803794999889,0.
519041304701053,0.60373193784174,0.900186650686287
"10346","DPP7",817.91738443843,0.130925623611551,0.252302361912577,0.5
1892349567824,0.603814092384697,0.900186650686287
"10347","IMMP1L",62.2099602396813,-0.160288586071392,0.309492852515514
,-0.517907230388647,0.604522997066693,0.900186650686287
"10348","POLR2G",319.995947467096,-0.123978988606784,0.239023138299095
,-0.518690322154697,0.603976711581872,0.900186650686287
"10349","PRR4",11.364419271826,0.32391816879883,0.625208385991704,0.51
809632765087,0.604391062350973,0.900186650686287
"10350","ZSCAN32",26.812118162678,0.189373993727785,0.365805830716569,
0.517689926803039,0.604674627522638,0.900186650686287
"10351","GIT1",97.3397949902709,0.0973547963620538,0.188078901087633,0
.517627420189427,0.604718246650433,0.900186650686287
"10352","TMEM241",22.7610900309791,0.168708076270954,0.325181011900744
,0.518812815314228,0.603891280307882,0.900186650686287
"10353","ZNF580",190.906150726082,0.132564440342397,0.255640273811054,
0.518558513359975,0.604068645988479,0.900186650686287
"10354","GUCD1",132.457136041798,0.0979534402490436,0.189223486328811,
0.517660054517922,0.604695473189051,0.900186650686287
"10355","TRMU",58.6734100870541,-0.131058845220966,0.252791224057151,-
0.518446974216699,0.604146447564583,0.900186650686287
"10356","NEXMIF",4.78857880042736,0.38560826001446,0.744573716396395,0
.517891313543453,0.604534102970194,0.900186650686287
"10357","CHDH",9.17464477345079,0.27943902000582,0.540479170755027,0.5
17020886513453,0.60514157865343,0.900634692280451
"10358","FP565260.1",16.4754569310494,-0.230491144668432,0.44584766162
8685,-0.516972868774159,0.605175098452216,0.900634692280451
"10359","ANKRD42",47.6939258365681,-0.153953838221098,0.29804212262285
1,-0.516550603204214,0.605469905693912,0.900986446208287
"10360","USP42",74.8910507937018,0.131968537407761,0.255530874573953,0
.516448502075502,0.605541197866011,0.901005556477275
"10361","ZNF28",28.5305451285269,0.18858648617939,0.365352815121316,0.
51617635987496,0.605731239677364,0.901201337672673
"10362","AL603840.1",11.562451495878,-0.358147355364582,0.694368396485
831,-0.515788675258193,0.606002012947529,0.901379457402544
"10363","CIP2A",17.9278593124197,-0.183770377741523,0.356314161008017,
-0.515753786550707,0.606026383167043,0.901379457402544
"10364","ZBED3",95.1119409009961,-0.0998745114919038,0.193599532838116
,-0.51588198601397,0.605936836316443,0.901379457402544

"10365","NAMPT",1435.48324291942,-0.211999620639104,0.411116991626821,
-0.515667376821878,0.606086743383304,0.901382262349603
"10366","KATNA1",144.952841820724,-0.119937679815923,0.232766370522221
,-0.515270653345831,0.606363903116436,0.901489027121198
"10367","LPXN",20.824094084905,-0.222328872167849,0.431514011828875,-0
.515229786457125,0.606392456842018,0.901489027121198
"10368","RFLNB",230.293627727137,-0.326230028985418,0.63300153211148,-
0.51537004641556 7,0.606294459614109,0.901489027121198
"10369","OSBPL1A",731.408820585849,0.0834032618869636,0.16183093157184
8,0.515372809616034,0.606292529084469,0.901489027121198
"10370","ZC3H15",488.93172894108,-0.0849544950628895,0.165017037774287
,-0.5148225674678,0.606677013891491,0.901663363956227
"10371","KDM1B",51.2598529271826,0.135133811427887,0.262492159392829,0
.514810848981033,0.606685203435225,0.901663363956227
"10372","WDR18",108.091281930481,-0.129753313768975,0.251994789015946,
-0.5149047497199,0.606619581657055,0.901663363956227
"10373","NGLY1",102.736177933119,-0.108492245093945,0.211048811419581,
-0.51406233640546,0.607208408840352,0.902282438814799
"10374","ZNF16",17.4637201758007,0.226255528275784,0.4401452178695,0.5
14047453181389,0.607218814159243,0.902282438814799
"10375","SCNM1",109.159759146289,-0.146628724579638,0.286149469820573,
-0.51242004631908,0.608357064154576,0.902336171802458
"10376","CREB3L4",27.6526220501987,-0.184588718693086,0.35991944036567
,-0.512861207234453,0.608048411011644,0.902336171802458
"10377","WDR92",74.5191146387347,0.143984521740957,0.280719067150015,0
.512913223895875,0.608012022755886,0.902336171802458
"10378","AC009961.4",2.62041453272811,0.558700917517915,1.091068247035
51,0.51206780055779,0.608603558940903,0.902336171802458
"10379","NEPRO",109.365984325289,-0.0995633649122147,0.194016388958923
,-0.513169869032528,0.607832500862247,0.902336171802458
"10380","CTBP1",213.482682615525,-0.0984300946421152,0.192040173872327
,-0.512549497625189,0.608266487806345,0.902336171802458
"10381","MIR3936HG",13.0359100545593,-0.215118607163169,0.420423629399
432,-0.511671067276741,0.608881238477463,0.902336171802458
"10382","NFKBIL1",188.633330421968,-0.127691760731782,0.24872154187430
3,-0.513392445903675,0.607676828679626,0.902336171802458
"10383","CLIP2",30.5822633918145,-0.162433128509426,0.316214963890614,
-0.513679449292652,0.607476122291593,0.902336171802458
"10384","RALGDS",190.81734174621,-0.105243654565988,0.205626790721071,
-0.511818786827001,0.608777840773449,0.902336171802458
"10385","RET",3.82747232619401,0.445302426793882,0.868147638770299,0.5
12933983699636,0.607997500506103,0.902336171802458
"10386","DENND10",242.665237720645,-0.102674888320584,0.20007583845528
1,-0.513179847768237,0.607825521267684,0.902336171802458
"10387","UVRAG",149.218637170911,0.106783918763308,0.207941483007673,0
.5135286967217,0.607581542459548,0.902336171802458
"10388","PPHLN1",243.015551121576,-0.0884515162587631,0.17213166964411
1,-0.513859631069868,0.607350133204744,0.902336171802458
"10389","FGD6",19.6468136553524,0.234256011640522,0.4570350927237,0.51
2555852646836,0.608262041388311,0.902336171802458

"10390","PSEN1",155.747439880256,0.103309954215733,0.201161679851188,0
.51356677023257,0.607554917162049,0.902336171802458
"10391","LOXL1-
AS1",25.0269004136395,0.232439925570502,0.45413791243215,0.51182673634
461,0.608772276654643,0.902336171802458
"10392","MTHFS",114.72365913258,-0.14980747482688,0.291970450462268,-0
.513091220668716,0.607887512458007,0.902336171802458
"10393","BFAR",291.763121714323,-0.098232574414548,0.191784008508494,-
0.512204198767684,0.608508104829123,0.902336171802458
"10394","AC012645.1",10.9051873877305,-0.244857564599747,0.47837180994
6472,-0.51185617444127,0.608751672196854,0.902336171802458
"10395","CES4A",8.29386490913191,0.386576909017662,0.754868593878992,0
.51211152795639,0.608572956933412,0.902336171802458
"10396","CENPBD1",17.3827471435378,-0.201065200345583,0.39207347886660
6,-0.51282530235612,0.60807352883576,0.902336171802458
"10397","TRIM65",65.0573864784836,-0.119550287071876,0.233483204884831
,-0.512029493217066,0.608630368359891,0.902336171802458
"10398","PIK3R2",99.2728638865571,0.128629134149156,0.251362130396748,
0.511728373506894,0.60884112550873,0.902336171802458
"10399","LINC00662",95.3127319682576,0.102031537992403,0.1994155567499
53,0.511652850235452,0.608893990210131,0.902336171802458
"10400","RCN3",557.388788101575,-0.142952199352596,0.279277281386208,-
0.511864762658263,0.608745661148163,0.902336171802458
"10401","TXN2",517.715335592005,-0.120182499921782,0.233984444145171,-
0.513634572421477,0.607507503505426,0.902336171802458
"10402","FGD1",37.7248918002346,0.151401431527458,0.295258117058234,0.
512776525962866,0.608107651872128,0.902336171802458
"10403","CNOT11",62.224787509341,-0.136859565278562,0.267565697926651,
-0.511498919103148,0.609001745089626,0.902350576480124
"10404","CD2BP2",233.840113337977,-0.113997941349604,0.222882197676392
,-0.51147172155544,0.609020784800467,0.902350576480124
"10405","NLGN3",5.25028400903768,0.339186406766067,0.663339959609196,0
.511331183735558,0.609119172870487,0.902409615550077
"10406","POFUT2",112.166368226303,0.130192835858626,0.254685592149185,
0.51119042408321,0.609217723328841,0.902468883827992
"10407","ARMC5",26.69022521784,-0.1833945013368,0.358953702641585,-0.5
10914081641106,0.609411220379704,0.902668776991749
"10408","GTF2H5",590.132425751409,0.129152355140443,0.252960697221428,
0.510562931550547,0.609657137722111,0.902946269983315
"10409","MRI1",84.697893680252,-0.12258484865382,0.240195694281356,-0.
510354063675384,0.609803432934506,0.903054549940805
"10410","ZNF428",508.52800575744,-0.13898816031503,0.272370245010786,-
0.510291277630292,0.609847412577605,0.903054549940805
"10411","PEX1",78.0837560943353,0.124777405739582,0.244576539825412,0.
510177328654062,0.609927233838496,0.903085996505659
"10412","LDB1",51.9035413394853,0.144197802753859,0.282718954132512,0.
510039389457677,0.61002386645797,0.903142326301346
"10413","SMARCAL1",73.6242232570597,0.107614495991329,0.21114396186645
9,0.509673566035439,0.610280175162064,0.903405435181191
"10414","DNAJC1",400.533329272638,-0.0782309684400079,0.15355930138642

2,-0.509451187480625,0.61043600472574,0.903405435181191
"10415","KLHL42",116.13187450314,0.138600482987019,0.27201477496002,0.509532921538509,0.610378728344126,0.903405435181191
"10416","ATF4",1093.71941069451,-0.0790068644590898,0.155034565650141,-0.509608061452444,0.610326075013358,0.903405435181191
"10417","ZNF496",50.4856358994667,0.138138571342688,0.271309722146245,0.509154519970451,0.610643919023246,0.903626381083165
"10418","RNLS",30.2851578907113,-0.176227304563367,0.346188461305965,-0.509050197394116,0.610717039162788,0.903647836311613
"10419","CCDC3",4.3536016260561,-0.358747025912343,0.705302841790804,-0.508642535738923,0.611002808188954,0.903897148582796
"10420","ELANE",107.677515921798,-0.18800329740482,0.369558849868498,-0.508723569931333,0.610945998856435,0.903897148582796
"10421","PEX5",49.8350411745396,-0.127170656386338,0.250100868477585,-0.508477468152956,0.611118536698225,0.903931425340272
"10422","CHCHD10",139.496239258052,0.197919268668621,0.389266006771964,0.508442209762651,0.611143257534629,0.903931425340272
"10423","SHQ1",32.2208864731393,0.161316470594725,0.317628265648286,0.50787819612173,0.611538766588371,0.90408267833123
"10424","HMGN3-AS1",35.8740842454428,0.144328301601092,0.284132012946968,0.507962126844293,0.611479903810921,0.90408267833123
"10425","AXIN1",45.0459267878334,-0.127543497237672,0.251110325944012,-0.507918170064057,0.61151073152009,0.90408267833123
"10426","CWC25",175.769588876507,-0.086463066674619,0.170175370163158,-0.508082142508176,0.61139573809459,0.90408267833123
"10427","TAF4B",28.4274535311816,0.187379461931707,0.368858825966529,0.507997772428821,0.611454905403598,0.90408267833123
"10428","AC048382.5",11.1065964816365,0.262426662820354,0.516831958081218,0.507760131154883,0.611621572837814,0.904118387542664
"10429","PSMB2",507.532829441818,-0.128108834974026,0.253678206595768,-0.505005284818043,0.613555124118072,0.904133561211736
"10430","ASAP2",331.009337950794,-0.138870753737908,0.274001155331768,-0.506825431337177,0.612277310670997,0.904133561211736
"10431","LBH",63.1287088389508,-0.225962131440645,0.446829671942838,-0.505700819863081,0.613066692647144,0.904133561211736
"10432","HEATR5B",57.1491529097934,0.142425466712369,0.281234448266539,0.506429662476433,0.612555055625491,0.904133561211736
"10433","AC007319.1",3.07278726823643,0.479397000410454,0.948795966112186,0.505268801231149,0.613370052553842,0.904133561211736
"10434","CUL3",236.342982327411,0.0907476914106633,0.178823319380785,0.507471238789758,0.611824211819954,0.904133561211736
"10435","NCK1-DT",19.1146405642902,-0.177146845834578,0.34939021070158,-0.507017198561074,0.61214275119562,0.904133561211736
"10436","EXOSC9",88.490819532707,-0.135427371949459,0.267644481261674,-0.505997251693946,0.61285857900221,0.904133561211736
"10437","ZNF131",187.103770943341,-0.113943357294315,0.224800427724141,-0.506864503986347,0.612249893062601,0.904133561211736
"10438","HIST1H1E",34.2202784661282,0.18603651858472,0.367160691834912

,0.506689639500868,0.612372601173305,0.904133561211736
"10439","SLC2A12",9.79948840244705,0.267776759287197,0.528914323747499,0.506276247899521,0.612662734767611,0.904133561211736
"10440","AC006483.2",2.94143860666322,-0.490522666879852,0.969446154430684,-0.505982374202016,0.612869023186164,0.904133561211736
"10441","RALA",416.57404419057,-0.0939837526372127,0.185900335940628,-0.50555988595539,0.613165647989451,0.904133561211736
"10442","THAP5",124.574494695269,-0.136176513405105,0.26830458970655,-0.507544479779654,0.611772835273735,0.904133561211736
"10443","CREB3L2",255.208905385054,-0.116244480300651,0.229096341787189,-0.507404349601673,0.611871134419719,0.904133561211736
"10444","AC093673.1",67.1692846069797,-0.185781587479775,0.367098874492954,-0.506080515055735,0.612800128540369,0.904133561211736
"10445","PUM3",174.0913198157,-0.116034429374645,0.22906654061575,-0.506553375550769,0.612468229567387,0.904133561211736
"10446","RIC1",86.2099222243568,0.10681448933264,0.21165584237687,0.50466119022809,0.613796824032869,0.904133561211736
"10447","MRPL50",140.803263179976,-0.115095597537973,0.227351534035256,-0.506245088806503,0.612684605839133,0.904133561211736
"10448","VAV2",25.487731227345,0.162464908835349,0.320232001515667,0.507335019818126,0.61191977077195,0.904133561211736
"10449","AL731559.1",2.46108188192321,0.553290545486979,1.0963995118653,0.504643188453875,0.61380947004668,0.904133561211736
"10450","SPOCK2",8.27836062116747,0.286460706909855,0.567688360793218,0.504609089588503,0.613833424373963,0.904133561211736
"10451","PDLIM1",1042.06040008864,0.119097456489273,0.235706204723383,0.505279259105809,0.613362708338499,0.904133561211736
"10452","NUDT22",118.98001005804,-0.13408284526796,0.265628897723407,-0.504775069343461,0.613716828087861,0.904133561211736
"10453","FAM118B",28.6015117371372,-0.206999792784715,0.408224133189475,-0.507073874264648,0.612102985418551,0.904133561211736
"10454","AC010809.2",3.03848029816502,0.472205223678065,0.935903269005891,0.504544902572718,0.613878516634211,0.904133561211736
"10455","PPCDC",45.3112442056394,-0.175021667992876,0.346576083937308,-0.505002151344452,0.613557324952188,0.904133561211736
"10456","ZSCAN2",9.53774612589218,0.234421033767562,0.463141180245223,0.506154589068156,0.612748131025828,0.904133561211736
"10457","CLEC16A",35.7598763253669,0.133781148761974,0.265182626529112,0.504486853128319,0.613919298423824,0.904133561211736
"10458","DHX33",58.6402604916537,0.125740573557771,0.249213951197048,0.504548693818311,0.613875853191523,0.904133561211736
"10459","DDX42",299.936346017977,0.0936474062361431,0.185158218416294,0.505769644129942,0.613018370935141,0.904133561211736
"10460","NOL11",132.599355239579,-0.0825003921633484,0.163373155207295,-0.504981323637096,0.613571953639783,0.904133561211736
"10461","GRB2",234.905938660908,0.0931287413393014,0.184272623331772,0.50538565987433,0.613287988846175,0.904133561211736
"10462","GATA6-AS1",3.55414648174196,-0.412600224722301,0.816867366468921,-0.50510063403053,0.613488156079188,0.904133561211736

"10463","HDGFL2",201.662774942417,-0.101212365064318,0.19972025423207,
-0.506770660058902,0.612315745054212,0.904133561211736
"10464","GEMIN7",46.83463632072,-0.168449463844485,0.332642190665355,-
0.506398372099313,0.61257701714215,0.904133561211736
"10465","ZDHHC8",109.580058419132,-0.111063904363674,0.219485223511353
,-0.506019961557592,0.612842636547724,0.904133561211736
"10466","TMEM191C",3.29816493193051,-0.468900373532757,0.9256829543557
45,-0.506545325617562,0.612473879119778,0.904133561211736
"10467","MCTS1",323.806993726692,-0.13446543226027,0.26613218842163,-0
.505258056373241,0.613377598348334,0.904133561211736
"10468","DLEU2L",17.9416874054016,0.201589996406776,0.399711832638341,
0.504338325628639,0.614023649687209,0.904188907108944
"10469","PRIM2",48.2970283711738,0.137627148192625,0.273061091216228,0
.504015960602908,0.614250161222502,0.904188907108944
"10470","AL133338.1",5.18035522678173,-0.315332189532679,0.62551169693
1101,-0.504118773605303,0.61417791511001,0.904188907108944
"10471","AC016831.6",7.98082540846088,0.369317941091416,0.732601355607
77,0.504118560885037,0.614178064583466,0.904188907108944
"10472","FAM27C",4.62190756402271,0.411422737596464,0.816287826286217,
0.504016750400741,0.614249606221782,0.904188907108944
"10473","PRKACA",210.931195122577,0.0906651089971964,0.17991816650248,
0.503924149293434,0.614314679672273,0.90419753529534
"10474","CEP95",152.294337928837,-0.126161233873123,0.250468018672659,
-0.503701967786972,0.614470825363782,0.904254679998348
"10475","OGFR",154.697041800764,0.0921362399566914,0.182913926518931,0
.503713641219962,0.614462621022437,0.904254679998348
"10476","PPARG",149.548603702319,-0.17636029907837,0.350217775291442,-
0.50357323791349,0.614561302703925,0.90430149686722
"10477","AARS",115.846622699655,0.10008562668736,0.198785074453573,0.5
03486627265544,0.614622179994859,0.904304753710104
"10478","CCDC51",131.91205502358,0.165202390835695,0.328682430687452,0
.50262008373909,0.615231406193525,0.904380500184037
"10479","PSMC2",294.612638777907,-0.10219643297727,0.203344048477892,-
0.502578923466162,0.615260350660396,0.904380500184037
"10480","GCC1",28.1275482318197,0.1603023243849,0.318856548857314,0.50
2741201205918,0.6151462382079,0.904380500184037
"10481","POLD4",463.930411600646,-0.117256766525041,0.2331472956097,-0
.502929987750468,0.615013496683521,0.904380500184037
"10482","YARS2",80.1980892321794,-0.141463170499999,0.281094475129646,
-0.503258452286383,0.614782573383384,0.904380500184037
"10483","BAG5",202.474812985479,-0.0858318150501417,0.170757572831495,
-0.502653051497994,0.615208223246446,0.904380500184037
"10484","AC008105.3",48.4997744932893,0.17784491689469,0.3536555894078
,0.502876024644467,0.615051438487868,0.904380500184037
"10485","ZNF799",20.6772626290282,0.170135544878357,0.338312515878227,
0.502894622259839,0.615038362269485,0.904380500184037
"10486","AL050403.2",23.1265832859593,0.204417269864758,0.406513529198
863,0.502854776488284,0.615066378484669,0.904380500184037
"10487","ACSL4",215.926072056535,0.116524463315341,0.23168613110809,0.
502941038196963,0.615005727171058,0.904380500184037

"10488","FOXO6",4.90993969820933,0.369936397743961,0.736378631805894,0.50237253196325,0.615405497015984,0.904495291994632
"10489","CDC6",16.0636903630762,-0.205258504574182,0.4086364543553,-0.502301012027953,0.615455797452591,0.904495291994632
"10490","MOB1B",44.6305993692362,0.141602972793334,0.282006522081137,0.502126588237534,0.615578478418303,0.904503121229448
"10491","AC004812.2",20.9952618351234,0.258151579005797,0.514114084648273,0.502128976260998,0.615576798729544,0.904503121229448
"10492","TMEM88",7.90367071000483,-0.37359667233798,0.744392659351219,-0.501881188166997,0.615751098740501,0.904619708191995
"10493","GRIN2D",11.5431017946276,-0.26648780354568,0.531014106892279,-0.501846937937827,0.615775192867895,0.904619708191995
"10494","ANKRD65",170.179230762422,0.138060236209229,0.275189752876606,0.501691050506283,0.615884860771261,0.904624791006182
"10495","AC009093.2",7.6632076158835,0.325952051930632,0.649727276004359,0.501675185833579,0.615896022160875,0.904624791006182
"10496","TMEM268",19.5949303285385,0.195279014423533,0.389437995741448,0.501438012107018,0.616062893323049,0.904783679551715
"10497","AC012615.2",2.91549733906188,0.613355806033881,1.22359482719244,0.501273618033542,0.616178569678493,0.904867357492043
"10498","DIS3L",104.186018480486,0.105086704102765,0.209701510032652,0.501125166368149,0.61628303630439,0.904887013482929
"10499","MED11",152.981180985413,-0.144056832943064,0.287488227351316,-0.5010877637331,0.616309358064046,0.904887013482929
"10500","HDHD2",92.1015571450528,0.11073980036786,0.221058638866084,0.500952149782057,0.616404799268933,0.904940950545771
"10501","LANCL3",7.76672471872357,-0.380145396411265,0.759080706759055,-0.500797073389365,0.616513945528091,0.905014995744741
"10502","MYH7B",3.83578341904386,0.397867198935133,0.794749055609411,0.500619907789699,0.616638649042677,0.905111862025601
"10503","GINS3",10.9352308152838,-0.262642038904659,0.524859134818466,-0.500404816228453,0.616790062789873,0.905247911825753
"10504","HCLS1",24.2449707333961,-0.226925785129625,0.454510788635194,-0.499274804479423,0.617585802391316,0.905445264815188
"10505","DKK2",67.7768174662207,0.221285678199768,0.442765864354097,0.499780348971973,0.617229748965927,0.905445264815188
"10506","AC016596.1",8.60319307905951,0.354262397037489,0.709653576078187,0.499204695050332,0.61763518734406,0.905445264815188
"10507","SMIM15",276.339372790974,-0.111932176980003,0.224216314161525,-0.499215132487491,0.617627835123035,0.905445264815188
"10508","FCHSD1",30.1390102109145,-0.167290457043155,0.335107963753908,-0.499213612142048,0.617628906065133,0.905445264815188
"10509","MMUT",132.452060782136,0.0869690666442865,0.17409942246337,0.499536790034926,0.617401275548833,0.905445264815188
"10510","CHN2",7.91763641146848,0.331378217130048,0.662779791622533,0.499982379243654,0.617087484821311,0.905445264815188
"10511","PARP10",87.6264139639412,0.109767012480613,0.219916878862552,0.499129548620129,0.61768812227029,0.905445264815188
"10512","MIGA2",28.7230150892774,0.170751664481809,0.342031488263537,0.499227908368028,0.61761883573441,0.905445264815188

"10513","ANKLE2",176.71615226712,-0.0912302104284592,0.182633298698049
,-0.499526707773547,0.617408376439653,0.905445264815188
"10514","TOP3A",53.2244030206882,0.132319136463057,0.264964059312137,0
.499385225326655,0.617508025655208,0.905445264815188
"10515","BLVRB",1006.95672260653,-0.154558143387156,0.309232996315721,
-0.499811291901576,0.617207958876707,0.905445264815188
"10516","RANGAP1",56.7374126580598,-0.118198825954536,0.23650094981809
3,-0.49978161206308,0.61722885948738,0.905445264815188
"10517","HGH1",36.7095374808401,0.155205374664601,0.311231921809584,0.
498680770796893,0.618004293331917,0.905815229751003
"10518","THYN1",251.376013623862,-0.150154628071468,0.301149764416113,
-0.498604501194269,0.618058033507691,0.905815229751003
"10519","TBX2",6.80046609803414,0.318609650378804,0.639136870685601,0.
498499875366338,0.618131757032629,0.905837155115313
"10520","SKP1",2103.25363038258,-0.0958477989611508,0.192610488301414,
-0.497625024506243,0.618748362231903,0.906230818235791
"10521","AL136366.1",2.83734524026599,0.411359564554853,0.826395501985
952,0.497775657740506,0.618642175007725,0.906230818235791
"10522","TRIM44",302.607271923698,0.0964128235817834,0.193846008904573
,0.497368112589027,0.618929487796718,0.906230818235791
"10523","PAK1",80.3841544197961,-0.111632376494952,0.224145807159749,-
0.498034640529284,0.618459626582161,0.906230818235791
"10524","PAN3-
AS1",5.99460894093222,-0.314188720900711,0.631518252285186,-0.49751328
6059113,0.618827136150639,0.906230818235791
"10525","HERC1",186.758660714558,0.102226856462087,0.205532245655567,0
.497376244472118,0.618923754380082,0.906230818235791
"10526","TMC6",7.59508239080361,0.31382807932775,0.630649444984982,0.4
97626822354888,0.618747094811545,0.906230818235791
"10527","AC004832.6",3.20741790586927,0.522218286659258,1.049803993048
17,0.497443608633039,0.618876259901252,0.906230818235791
"10528","EIF4ENIF1",62.6051274195664,-0.114755227993102,0.230502427294
725,-0.497848241078924,0.618591011032484,0.906230818235791
"10529","GATB",36.6406189508601,0.137604230347659,0.276797092168235,0.
497130332077422,0.619097146146924,0.90639020874298
"10530","VPS26C",91.1632412694664,-0.109452509917041,0.220227460102096
,-0.496997558189606,0.619190773254634,0.90644119370562
"10531","ZNF724",11.6977524807461,0.235655885789094,0.474399514648883,
0.4967456300277,0.619368440417776,0.906615184601654
"10532","GABBR1",67.9074331802454,-0.179376698506082,0.361287227458758
,-0.496493329608665,0.619546392392328,0.906693845262601
"10533","AC092171.5",2.58939839731474,-0.525759297199724,1.05903060557
786,-0.496453355012195,0.619574589232516,0.906693845262601
"10534","PSMB5",704.912543355298,-0.133492412543749,0.268918124081526,
-0.496405413356516,0.61960840652709,0.906693845262601
"10535","GABPB1-
IT1",223.296765436687,0.116552966845807,0.234826799986251,0.4963358818
18561,0.619657454417224,0.906693845262601
"10536","NUP107",111.472413877178,-0.126565250890597,0.255208698519984
,-0.495928436705248,0.619944902227638,0.906862893086897

"10537","SUGP1",48.9563665205943,-0.130315648683205,0.26277451006783,-0.495921954719149,0.619949475663297,0.906862893086897
"10538","APOOL",143.532137777017,0.10650708156323,0.21469594555273,0.496083339110248,0.619835613528188,0.906862893086897
"10539","ZNF451-AS1",3.15106280296856,0.497705798327948,1.0047939318979,0.495331214220072,0.620366340740632,0.906889601350563
"10540","KHDC1",9.26852477172563,-0.243244963364515,0.490772486904043,-0.495636919051811,0.620150600207842,0.906889601350563
"10541","CHST1",2.76985223278577,0.569956671667731,1.15050634912873,0.49539637230108,0.620320354955208,0.906889601350563
"10542","PPP1R14B-AS1",9.03532461162522,-0.309240199404372,0.624034310473921,-0.495549994950631,0.620211940538391,0.906889601350563
"10543","SLC25A29",68.2381356643599,0.151109262716508,0.30488865190528,0.495621144874401,0.620161731484251,0.906889601350563
"10544","AC092338.3",24.8489653449014,0.168323848863206,0.339724495552846,0.495471627941595,0.620267244591597,0.906889601350563
"10545","CMTR2",75.8170080999156,0.111594149489802,0.225300495384258,0.495312490545013,0.620379555383827,0.906889601350563
"10546","SNX17",413.221513002856,-0.114307906174625,0.230822005798087,-0.495221007110631,0.620444123580741,0.906897986440083
"10547","CCNL1",2855.61156313213,0.14057074640182,0.284124560574264,0.494750422553059,0.620776304205967,0.906957958928063
"10548","PAPSS1",182.906691256752,0.0876739720686568,0.1772476130829,0.494641200204209,0.620853414159887,0.906957958928063
"10549","DROSHA",68.3263816436545,0.103913378986647,0.209975323596142,0.494883766372993,0.620682170368435,0.906957958928063
"10550","AL391834.1",3.57119157723023,-0.450369104190214,0.910610210501039,-0.494579457814789,0.620897005550947,0.906957958928063
"10551","MRPL17",185.557352354974,-0.129468743907577,0.261688946114047,-0.494742883985454,0.620781626230132,0.906957958928063
"10552","SNUPN",171.391902956035,0.10583696456396,0.213869004977846,0.494868176783836,0.620693175491124,0.906957958928063
"10553","AC023043.1",4.51778408728205,0.384536161947154,0.777058193018545,0.494861472927005,0.620697907955408,0.906957958928063
"10554","CNOT10",63.2550907356723,-0.113613441750658,0.229865113234058,-0.494261352460965,0.621121615537469,0.907114135813367
"10555","LINC01290",2.93058036631734,0.500770224331505,1.0130266260713,0.494330762335028,0.621072603129693,0.907114135813367
"10556","LY96",302.409777903991,-0.177069949968259,0.358317082490261,-0.494171108833673,0.621185341801691,0.907121262208514
"10557","BEST1",21.4010751443465,0.198072663670331,0.401053373462435,0.493881056180379,0.621390184063669,0.907248502305499
"10558","C14orf132",212.391452525671,-0.12072987464992,0.244433279718962,-0.493917500876844,0.621364444317394,0.907248502305499
"10559","CPT1B",4.34813755311712,-0.378364199985986,0.766793374251067,-0.493436971016523,0.621703864699445,0.907620520346808
"10560","C8orf37",18.8946588445689,0.199501686842603,0.40453716205235,0.493160346086538,0.62189929414206,0.907739863559895

"10561","HECTD2",55.6224645868616,0.131284561909803,0.266213828474858,
0.493154554224075,0.621903386250798,0.907739863559895
"10562","ZNFX1",172.505193656348,-0.118288928915647,0.239903233376703,
-0.493069339877994,0.621963593860531,0.907741791266814
"10563","PHF13",71.2789663086278,0.127199079248578,0.258482668140929,0
.492099064759062,0.622649313378723,0.907824441767728
"10564","CTBS",183.720143616952,0.0924700941218897,0.188033929429098,0
.491773449624991,0.62287950776669,0.907824441767728
"10565","IRS1",119.901938269234,0.143207491944204,0.290803126789808,0.
492455131157218,0.622397633615656,0.907824441767728
"10566","AC007620.2",8.34951013350402,0.304913743664075,0.619583951561
041,0.492126600270787,0.622629848774765,0.907824441767728
"10567","AC016949.1",16.887838429715,0.238122101340487,0.4850311148458
56,0.490941908780765,0.623467534763395,0.907824441767728
"10568","GZMA",21.9122569640635,0.220480079004056,0.447495127620395,0.
492698278473965,0.622225794229036,0.907824441767728
"10569","AC011416.3",3.41890620383761,-0.382165587894806,0.77820253642
3275,-0.491087563979541,0.623364516829619,0.907824441767728
"10570","ATP6V0E1",1852.08139049559,-0.116402673454184,0.2365070318064
51,-0.492174260380737,0.622596158897887,0.907824441767728
"10571","CDKAL1",60.9236395190983,0.108958637543193,0.221545330727068,
0.491811933862981,0.622852299322423,0.907824441767728
"10572","ATAT1",41.3902593593867,0.132001290903558,0.268035245030717,0
.492477363894552,0.622381920229002,0.907824441767728
"10573","CALD1",3931.24000185586,-0.086149242118111,0.175374362495628,
-0.491230536163793,0.623263403684746,0.907824441767728
"10574","RRM1",114.53180036169,-0.0893552360295594,0.181516212530039,-
0.492271377768927,0.622527511211533,0.907824441767728
"10575","PUS1",17.9768867573742,-0.209689107655764,0.425709068471055,-
0.49256434308263,0.622320447777948,0.907824441767728
"10576","BORA",8.39550207051758,0.251157969878214,0.511124302724179,0.
491383345576796,0.623155341281316,0.907824441767728
"10577","GRTP1",22.3096653193214,0.17356945782735,0.352453696866678,0.
492460312859213,0.622393971339275,0.907824441767728
"10578","EAPP",434.155063750454,-0.107727963112397,0.219456656388748,-
0.490884919533114,0.623507843702948,0.907824441767728
"10579","GPR135",13.3591604688237,0.215373550324471,0.438130742902705,
0.491573699890533,0.623020739557812,0.907824441767728
"10580","MRTFB",81.1001817932781,0.120737178352717,0.245688584217222,0
.491423639960286,0.623126847608884,0.907824441767728
"10581","LAT",11.2577973377021,0.226146515123524,0.460240920009282,0.4
91365511608492,0.623167952530439,0.907824441767728
"10582","AC007906.2",10.9907819337391,-0.321106047881625,0.65421923248
8521,-0.490823308052563,0.623551423252462,0.907824441767728
"10583","RPS28",12716.8868427141,-0.137104984299704,0.278773909991435,
-0.491814260179213,0.622850654628032,0.907824441767728
"10584","ZNF93",18.2581923033854,0.208605331376496,0.424522789936111,0
.491387827277518,0.623152172074471,0.907824441767728
"10585","ZNF461",6.36336304455837,0.346869936605621,0.705195729659442,
0.491877534160812,0.622805920948929,0.907824441767728

"10586","SNRPA",157.321583908202,0.113938533881382,0.231956193085763,0
.491207121334564,0.623279962692483,0.907824441767728
"10587","LINC01684",13.1539719961214,0.246073935853846,0.5006163942933
77,0.491541904457965,0.623043221597636,0.907824441767728
"10588","AF129075.2",3.4923976974888,0.393492732483873,0.8014290564470
13,0.490988852124167,0.623434332217388,0.907824441767728
"10589","POMP",962.850542543791,-0.11950741479824,0.243542530447939,-0
.490704496575748,0.623635465704405,0.9078610542859
"10590","FEZ2",393.615737493014,0.0785732069389325,0.160217393960227,0
.490416208857061,0.623839409189081,0.908003834050821
"10591","CMBL",611.957065010034,-0.0970163347292686,0.197856909403782,
-0.490335844331217,0.623896266634712,0.908003834050821
"10592","ELAC1",53.7135215580974,-0.150977585812896,0.307918901545217,
-0.49031607041741,0.623910256909912,0.908003834050821
"10593","ZNF526",28.6074552397824,0.189714910800168,0.387067579922133,
0.490133818074697,0.624039208959098,0.908031582557581
"10594","COMT",1050.1266615819,0.13555039129775,0.276564244813061,0.49
0122616498648,0.624047134973403,0.908031582557581
"10595","FUBP1",161.724068427125,0.105568710593897,0.216000072409018,0
.488743866687194,0.625023043219602,0.908071086505458
"10596","DHX30",138.147906841309,-0.0887365977374056,0.18154567012463,
-0.48878388383754,0.624994708958692,0.908071086505458
"10597","SLC38A9",44.3177389027695,0.122941214013589,0.2511438282139,0
.489525125454724,0.624469970897798,0.908071086505458
"10598","GGH",41.0955039330741,0.182573677846337,0.372721841472073,0.4
89838956378994,0.624247861781319,0.908071086505458
"10599","EBAG9",180.753034804133,-0.0923182071396725,0.188760933906507
,-0.48907475307045,0.624788774799901,0.908071086505458
"10600","AC067750.1",9.42702166686153,-0.240396960550136,0.49097989419
7623,-0.48962689387312,0.624397942090775,0.908071086505458
"10601","USPL1",48.5826198680477,0.142674389045431,0.291257749726894,0
.489856112598595,0.624235720709006,0.908071086505458
"10602","G2E3",70.0042551867744,0.107860229292369,0.220697164349724,0.
488725034642719,0.625036377495801,0.908071086505458
"10603","MGAT2",147.616350930125,-0.0947489586906279,0.193727762294537
,-0.489083018192172,0.624782923557424,0.908071086505458
"10604","TTC8",61.1321221022854,-0.139586262404314,0.285189809153109,-
0.48945038681019,0.624522871081084,0.908071086505458
"10605","ZNF747",38.0082119010964,0.147615578419437,0.301672365245555,
0.489324165636722,0.624612215109602,0.908071086505458
"10606","ZNF23",42.169977916503,-0.129288998725428,0.264473987866212,-
0.488853364251576,0.624945514463838,0.908071086505458
"10607","BRIP1",2.50189691593219,0.472753519333438,0.9674300380604,0.4
88669465216587,0.625075724872489,0.908071086505458
"10608","AFG3L2",151.906951351275,-0.134046373463404,0.273986253876482
,-0.489244885708154,0.624668335205422,0.908071086505458
"10609","CCDC157",3.97963531050919,-0.361568667588651,0.73833393678302
7,-0.489708856082155,0.624339934167532,0.908071086505458
"10610","RTL6",46.1391587610651,0.150909914124499,0.30838058015431,0.4
89362572860411,0.624585028467878,0.908071086505458

"10611","TMEM47",235.316946552563,0.101536278948311,0.207657634012212,
0.488960010698861,0.62487000840482,0.908071086505458
"10612","TTLL12",31.5331123174661,0.145884316535548,0.298612285572827,
0.488540905996879,0.625166758665109,0.908117752056413
"10613","SUMO1",812.454326992158,-0.124235965042901,0.254379593363448,
-0.488388095130719,0.625274972676171,0.908189362461432
"10614","MAN1B1-
DT",12.5717348939352,-0.226159984583769,0.463212966017673,-0.488241912
8465,0.625378500172259,0.908254153020103
"10615","CCDC183",11.1710884095132,-0.278137120668155,0.56976977972159
2,-0.488157025112953,0.62543862175483,0.908255897724984
"10616","CRTAP",967.265783365206,-0.0707745948487921,0.14520764292945,
-0.48740268363958,0.625972992129499,0.908775068155606
"10617","TRAPPC2",87.5834343867923,-0.118171021336093,0.24242915060464
4,-0.487445594068871,0.625942589399556,0.908775068155606
"10618","TBC1D25",44.1713232680609,-0.124045998418573,0.25442194449142
5,-0.487560138204799,0.625861436149752,0.908775068155606
"10619","AC016394.1",13.951308795058,-0.2099175973708,0.43086907645999
8,-0.487195783683234,0.626119593003053,0.908827138654845
"10620","MAP6",6.63989671229901,0.306639496295296,0.629409950935394,0.
487185650369184,0.626126773435903,0.908827138654845
"10621","SERPINH1",915.523502579916,0.148995523167628,0.30592969820630
1,0.487025365766073,0.626240355290524,0.908906419056909
"10622","PFDN4",266.128203171596,-0.110900105565702,0.227761550666886,
-0.486913200410633,0.626319843863564,0.908936207226214
"10623","AC108075.1",2.54765739956831,-0.523551268262248,1.07557452022
309,-0.486764290542745,0.626425378992587,0.909003785858112
"10624","SAP30L",184.808824345453,0.0800484020449655,0.164526899806226
,0.486536865030849,0.626586574348119,0.909010103199578
"10625","H2AFV",557.941879497685,0.103986498757218,0.213740328563231,0
.486508556696896,0.626606640064789,0.909010103199578
"10626","UBE3C",122.465285132106,0.099270985606084,0.20403890279782,0.
486529697253129,0.626591655037695,0.909010103199578
"10627","STIM2",51.7734783458134,-0.162884584879042,0.33488182574827,-
0.486394221349839,0.626687686879011,0.909042127904391
"10628","IQCG",33.2706644033505,0.130989758144338,0.269376154897335,0.
486270799263064,0.626775179903692,0.909083496256625
"10629","ELN",1213.6952526104,-0.145771476106674,0.299871817461122,-0.
486112624190077,0.626887316746104,0.909160597200225
"10630","STON1",18.7876506324238,0.233794230847512,0.481218396486663,0
.485838098780979,0.627081959615543,0.909186268573514
"10631","MTERF3",47.3569431163598,-0.12479348877437,0.256845042632676,
-0.485870731610117,0.62705882106222,0.909186268573514
"10632","COQ7",88.2251001284619,-0.123887135828718,0.254971645664027,-
0.485885932555664,0.627048042842519,0.909186268573514
"10633","GBP5",7.53419843763344,0.410446509962539,0.845459681252909,0.
48547141757758,0.627341982900874,0.909273242036022
"10634","CAPRIN2",70.8632873525888,0.113431188642466,0.233676002909898
,0.485420784461994,0.627377891812853,0.909273242036022
"10635","MED31",141.347858591572,-0.145854969222472,0.300469435092333,

-0.485423647758552,0.627375861144682,0.909273242036022
"10636","GABARAP",3267.89021142328,-0.117141498335894,0.24131346773925
8,-0.485432907799689,0.627369293883804,0.909273242036022
"10637","SLU7",305.719555100572,-0.0761579913024162,0.156923177538619,
-0.485320221633123,0.627449213402343,0.909291118228553
"10638","PLXND1",168.097286191814,0.108596399311589,0.223837378691007,
0.485157572638925,0.627564575369062,0.909372807794142
"10639","PPM1L",69.6595139674432,-0.204666182389118,0.4221129759657 8,-
0.484861148655402,0.627774843264613,0.909506504598121
"10640","PIH1D2",13.6573680585065,0.235288205991698,0.485214976371728,
0.484915382767249,0.62773637012165,0.909506504598121
"10641","MIAT",9.37569516033524,0.272823832862327,0.562927148336029,0.
484652114698632,0.627923139351353,0.9096358606429
"10642","DDX31",16.4884895350246,0.244875403658798,0.505382590116129,0
.484534703901314,0.628006441316013,0.90967104800661
"10643","ASPRV1",6.33747295499405,-0.286538166513933,0.591781807587105
,-0.484195632309561,0.6282470363362,0.909682776448589
"10644","LINC02268",2.87540187211016,0.527742223142402,1.0906964649690
9,0.483858011914761,0.628486640880796,0.909682776448589
"10645","GBA2",48.1795025680245,0.133003221319735,0.27464054259209,0.4
84281089981961,0.628186394413621,0.909682776448589
"10646","TMEM9B",274.281052750031,-0.0913295029384232,0.18870372102826
1,-0.483983582521647,0.628397520577189,0.909682776448589
"10647","AICDA",6.60659016297454,-0.275488374619462,0.569226615748237,
-0.483969594881535,0.628407447654133,0.909682776448589
"10648","ALOX5AP",17.8219092767611,-0.197855563060364,0.40886744081413
9,-0.483911271257974,0.628448840857358,0.909682776448589
"10649","TMEM161A",85.7215307852352,-0.131138506360403,0.2709428047751
99,-0.484008078639359,0.628380135758963,0.909682776448589
"10650","HSPB6",591.005296685191,0.142752000740238,0.294760984866316,0
.484297475138989,0.628174767570831,0.909682776448589
"10651","AL139260.1",16.3868499173404,-0.214756989075133,0.44414342232
9321,-0.483530720659635,0.62871895234441,0.909687177995434
"10652","PHF20L1",289.373314013613,-0.0815071197977694,0.1686496984546
85,-0.483292413473658,0.628888126013119,0.909687177995434
"10653","OAT",439.664823917303,0.0864270016669247,0.178802671298528,0.
483365270995458,0.628836402574337,0.909687177995434
"10654","FEZ1",430.225392476877,0.131389299332177,0.271640778467691,0.
483687685160291,0.628607534289706,0.909687177995434
"10655","SRP54",198.091619203417,-0.0790613933840345,0.163624273513568
,-0.483188659520487,0.628961786771024,0.909687177995434
"10656","AL133445.2",2.48811089229777,0.498033633360599,1.030225394920
69,0.483422012130597,0.628796121836875,0.909687177995434
"10657","CDK5RAP3",211.915131565788,-0.0837842243786442,0.173368642714
153,-0.483272078889064,0.628902562385671,0.909687177995434
"10658","EHD2",551.165375849947,0.11540664530088,0.238822832526527,0.4
83231205659793,0.628931580430737,0.909687177995434
"10659","AZIN1-
AS1",47.1091134925534,-0.134441205965545,0.278295406188286,-0.48308812
4978198,0.629033165411159,0.909705060963788

"10660","KIAA1191",100.735987166964,0.099601036275416,0.206216387885684,0.482992827566303,0.629100828935369,0.909717568296314
"10661","FHOD1",36.7264855985227,-0.165295534355322,0.34229105344545,-0.482909303913971,0.62916013535959,0.909717989547705
"10662","CEMIP2",98.1702710561206,-0.352668503010962,0.730524258572338,-0.48276083767592,0.629265560525469,0.909754037626454
"10663","ZNF101",14.2115631710873,0.211688036777722,0.438542656187227,0.482707973309092,0.629303101084066,0.909754037626454
"10664","RBM26-AS1",18.8106962763831,0.203033311302609,0.420725259435591,0.482579324034361,0.629394462761112,0.909800791772557
"10665","STMN3",230.338468145833,-0.105913514937113,0.21951429191961,-0.482490292595164,0.62945769272388,0.909806876074882
"10666","RABGEF1",141.71770820404,0.138350766711088,0.286927183452123,0.482180757663116,0.629677544992983,0.909954003568655
"10667","APEX2",35.2164932871256,-0.132011086436664,0.273755458491628,-0.482222663847637,0.629647778516194,0.909954003568655
"10668","GTF2B",257.598095209293,-0.122852686267465,0.255032078040985,-0.481714642374213,0.630008672941546,0.910133748665185
"10669","SFT2D3",55.2792472396591,-0.13119281911835,0.272305888199976,-0.481784731081558,0.629958877225636,0.910133748665185
"10670","CHPF2",87.8946794635507,0.12788359996397,0.265636142603597,0.481423945968105,0.63021522109972,0.910133748665185
"10671","IDI1",163.763292300254,0.0908365394577764,0.188678774108152,0.481434861378251,0.630207464864397,0.910133748665185
"10672","PDHX",66.2215884587518,0.105254565517554,0.218532371585479,0.481642901479168,0.630059644223531,0.910133748665185
"10673","PRC1",15.5638887507939,-0.195025948356056,0.405009603537191,-0.481534133148395,0.630136926535912,0.910133748665185
"10674","ZNF470",14.2125803780656,0.192528575883672,0.399604348444107,0.481798000030025,0.629949450264487,0.910133748665185
"10675","LSR",11.8607161562556,-0.229247489234654,0.47635270177129,-0.481255776197364,0.630334723759034,0.910221055432834
"10676","C1orf174",128.405691529685,-0.11817763962644,0.246019954071622,-0.480357945242262,0.630972892547747,0.910323439303263
"10677","MRPL37",180.120988046808,-0.12802955968507,0.266427156289527,-0.480542454711111,0.630841722673546,0.910323439303263
"10678","PTGFRN",289.46261142114,-0.16637887761193,0.346592960858488,-0.480041132975754,0.631198145099438,0.910323439303263
"10679","UBE2Q1",269.835926721264,0.170557765750333,0.355364625156777,0.47995144613814,0.631261918389303,0.910323439303263
"10680","CSNK1G3",302.103976823156,0.142221897413921,0.296238670532871,0.480092275455105,0.631161780633863,0.910323439303263
"10681","PHAX",301.09433995421,-0.0848000431992669,0.176415796802439,-0.480682822832646,0.63074194117272,0.910323439303263
"10682","AL662844.4",63.7190799060517,-0.230888440635397,0.481053104466773,-0.479964557949018,0.631252594850811,0.910323439303263
"10683","AL035530.2",9.17200683962248,-0.223542806386164,0.465586594411688,-0.4801315352918,0.631133865835494,0.910323439303263
"10684","AC104232.3",3.86002603852555,0.355426774459372,0.738965557483

795,0.480978809986237,0.630531559039123,0.910323439303263
"10685","AL133415.1",2.44333820144174,0.48742282630473,1.0139735383425
9,0.480705667232162,0.630725702734551,0.910323439303263
"10686","ADO",56.5620710230356,0.12055825425371,0.250909556448405,0.48
0484904442052,0.630882634564759,0.910323439303263
"10687","CCDC91",145.006640182541,0.0908553874694933,0.188986983044879
,0.480749446367518,0.630694583798893,0.910323439303263
"10688","SHF",32.2780164233674,-0.150133709374412,0.312690516894929,-0
.480135153650535,0.631131293111729,0.910323439303263
"10689","CELF6",14.218249114692,-0.239479920055189,0.498591881656476,-
0.480312513833083,0.631005192035753,0.910323439303263
"10690","AC008764.8",6.7129994934931,0.281367481601829,0.5862922706261
59,0.479909928372975,0.631291441203496,0.910323439303263
"10691","RNF2",72.6509013664928,-0.12136276736414,0.253389429676966,-0
.47895749841996,0.631968864844658,0.910439924895199
"10692","AC009948.2",3.34501909469841,0.44743699758761,0.9342610882203
85,0.478920724869217,0.63199502653573,0.910439924895199
"10693","CAVIN2",52.3522878777699,-0.167772986349257,0.350522074967121
,-0.478637433505419,0.632196583060806,0.910439924895199
"10694","AC007383.2",4.43540258049006,0.343398939137682,0.717343757394
455,0.47870903677336,0.632145636088723,0.910439924895199
"10695","TFG",563.7441802851,-0.0940860071717775,0.19641849644911,-0.4
79007877937577,0.631933024251596,0.910439924895199
"10696","AK6",258.84299883014,-0.131807091879487,0.275363096613178,-0.
478666507969459,0.632175895862756,0.910439924895199
"10697","AL499604.1",18.0035436901452,0.204076690138715,0.426373236742
614,0.478633911682192,0.632199088944418,0.910439924895199
"10698","RNF31",43.7789408706411,-0.12627237610954,0.263697462710319,-
0.478853208565962,0.632043060645665,0.910439924895199
"10699","AC020663.3",4.08559763471641,0.449790526480486,0.937782592601
694,0.479631985098626,0.631489098677463,0.910439924895199
"10700","SAT2",807.129170755505,-0.127075747315627,0.265255193858494,-
0.479069779811431,0.63188898770036,0.910439924895199
"10701","ZNF236",70.8889111135598,0.121502519679832,0.253497913306763,
0.47930382579836,0.631722500859739,0.910439924895199
"10702","ZNF440",12.5757282640719,-0.19639826369098,0.410139556175067,
-0.478857161505163,0.632040248305581,0.910439924895199
"10703","NFKBIB",55.660892280675,-0.133655688241084,0.279040632301868,
-0.47898288911736,0.631950801489902,0.910439924895199
"10704","ZNF525",14.7167110065458,0.238033596481781,0.496577746376589,
0.479348094469912,0.63169101277545,0.910439924895199
"10705","NMRAL1",109.809780971474,0.137214413435939,0.286748943678364,
0.478517589902084,0.632281857778413,0.910474062368448
"10706","BID",69.3022017747904,-0.140689079609078,0.294081279246999,-0
.478402025349302,0.63236409236916,0.910507424235998
"10707","AL513477.2",6.30060990723577,0.315385783172645,0.659371247289
336,0.478312914718667,0.632427505715613,0.910513682694142
"10708","BOLA1",116.539352815692,0.13690724624575,0.28652429378375,0.4
7782072660505,0.632777807731604,0.91052027243756
"10709","ARL8B",226.924242264514,0.0765484327407302,0.160169679577593,

0.477920870807805,0.632706526035935,0.91052027243756
"10710","OMD",653.723105944966,-0.0620294094133458,0.129751778627714,-0.478062112669159,0.632605997214859,0.91052027243756
"10711","SLC15A4",159.70434659332,0.0951416218405271,0.198971945217801,0.478166013486885,0.63253205019516З,0.91052027243756
"10712","AC007952.4",181.698872652379,-0.173650816095396,0.363431800310536,-0.477808535045694,0.632786485801076,0.91052027243756
"10713","SRXN1",30.8302501513378,-0.135179133943331,0.282819320143223,-0.477969941639329,0.632671599127857,0.91052027243756
"10714","CENPS",41.9226988280906,-0.157795128887248,0.330673686256232,-0.477192880612144,0.633224780272559,0.910562482162359
"10715","SPEN",233.14536219777,0.13199503012407,0.278006948845688,0.474790398844801,0.634936377034283,0.910562482162359
"10716","ZSCAN20",9.57961468881851,0.248418940319692,0.521173935010279,0.476652655921469,0.633609481161839,0.910562482162359
"10717","SFPQ",1080.07411400614,0.127508713684104,0.268773270950818,0.47440994870147,0.635207599968876,0.910562482162359
"10718","MIER1",462.406514466008,-0.0696376684494758,0.147653549480572,-0.471628814169743,0.637191756056501,0.910562482162359
"10719","AGL",216.989164549543,-0.1068008227172,0.225804955911878,-0.472978204955253,0.636228728732311,0.910562482162359
"10720","AL445426.1",71.0156833477941,0.142235007483918,0.298848772536503,0.475943087457537,0.634114924422325,0.910562482162359
"10721","KCNT2",51.4445729987471,0.209369847971043,0.441521507183829,0.47420079104747,0.635356729333312,0.910562482162359
"10722","KIF21B",2.81526429627762,0.417506766197694,0.874134523322509,0.477623014602818,0.632918547277631,0.910562482162359
"10723","EIF2D",164.277334574811,-0.0888029124111139,0.186119959296997,-0.477127293314138,0.633271480536765,0.910562482162359
"10724","MTIF2",84.0517347682608,-0.124483802726743,0.262226872544652,-0.474717947549583,0.63498802379258,0.910562482162359
"10725","MDH1",526.517501560656,-0.131906466755144,0.279787471180288,-0.471452371325616,0.637317724367544,0.910562482162359
"10726","STEAP3",5.7328846950801,0.381520438021831,0.802007675076154,0.475706716878494,0.634283334981106,0.910562482162359
"10727","AC108047.1",6.05532720879146,-0.346323648671645,0.73166071796076,-0.473339131335215,0.63597124846524,0.910562482162359
"10728","MLPH",7.59343002536814,0.289849725308021,0.611970559019937,0.473633446962239,0.635761320013987,0.910562482162359
"10729","RYK",242.713534300707,-0.0963992193192846,0.203084054668593,-0.474676455897022,0.635017601832593,0.910562482162359
"10730","NAAA",184.240937249935,-0.122443233930272,0.256734348965754,-0.476925796737099,0.633414961716878,0.910562482162359
"10731","FBXW7",126.142189136381,-0.106059648588953,0.222481423438128,-0.476712378723378,0.633566946920714,0.910562482162359
"10732","OXCT1",59.0640879135928,-0.134099441609549,0.284270056483602,-0.471732560468552,0.637117693077712,0.910562482162359
"10733","RAD17",90.2963917653133,0.0993332772825429,0.210533754626981,0.471816395705949,0.637057846964896,0.910562482162359
"10734","HSPA4",167.459279603412,0.0820066923149617,0.172982862539679,

0.474074085206855,0.635447077766561,0.910562482162359
"10735","LMAN2",665.468810962629,-0.101930123525332,0.214092717640381,
-0.476102712174206,0.634001204877369,0.910562482162359
"10736","AL031775.1",4.28164773934444,0.409505231709626,0.861308367086
325,0.47544551626207,0.634469433400987,0.910562482162359
"10737","LINC00240",3.00637829841157,0.399930020143998,0.8431996138490
77,0.474300525730056,0.635285616679751,0.910562482162359
"10738","HLA-
DRB5",47.7477629561933,0.785225154811772,1.65912965343269,0.4732753423
98448,0.636016751478415,0.910562482162359
"10739","AL390208.1",4.48155371393531,0.362415441181973,0.767622594424
388,0.472127115348571,0.636836059177921,0.910562482162359
"10740","SAMD3",4.68612025138013,-0.375804792655661,0.796069014433202,
-0.472075643998319,0.636872796534876,0.910562482162359
"10741","RBAKDN",3.39809152056276,0.391838510948438,0.828508231850374,
0.472944620083393,0.636252689986614,0.910562482162359
"10742","MPP6",37.3045685332905,0.167637458891696,0.355092509992029,0.
472095170059935,0.636858859824431,0.910562482162359
"10743","ZNF394",102.825701897595,-0.0937217516955595,0.19728112067213
5,-0.475067007812253,0.634739213235876,0.910562482162359
"10744","LONRF1",101.699410987954,0.109580380557127,0.231869153280681,
0.472595767943649,0.636501602374662,0.910562482162359
"10745","EIF4EBP1",154.425185503628,0.146322994312608,0.30936406879238
,0.472979925832333,0.636227500976277,0.910562482162359
"10746","AC064807.1",22.2243829204287,-0.152056336068145,0.32016316276
5858,-0.474933889191204,0.634834095476665,0.910562482162359
"10747","YTHDF3-
AS1",16.0008155288796,0.222998807334261,0.470549968730859,0.4739110023
44173,0.635563373062651,0.910562482162359
"10748","AC016877.3",6.13071916194148,-0.303708606777409,0.64329296647
3828,-0.472115540827641,0.636844320342634,0.910562482162359
"10749","TONSL",5.36123179410022,-0.328530321384939,0.691471824774353,
-0.475117437347715,0.634703270415178,0.910562482162359
"10750","TBC1D2",12.6563783831248,-0.212899402905982,0.451019862244947
,-0.472039971468833,0.636898258105238,0.910562482162359
"10751","TMEM141",200.175505949719,-0.133876434925419,0.28057756227709
8,-0.477145905178272,0.63325822814278,0.910562482162359
"10752","CDNF",10.7174330694622,-0.230634838155981,0.484316648722578,-
0.47620671055661,0.633927119191709,0.910562482162359
"10753","SGMS1-
AS1",20.2266344683722,0.171637502545353,0.360390029962169,0.4762548580
03064,0.633892821472006,0.910562482162359
"10754","TCTN3",139.552532239342,0.088569612538066,0.186240855169661,0
.475564893950799,0.634384390820405,0.910562482162359
"10755","R3HCC1L",64.9967042333909,0.132819086357973,0.279289954725773
,0.475559840626506,0.634387991689269,0.910562482162359
"10756","ENO4",7.22696446296819,-0.268859745112915,0.56414544944654,-0
.476578771266669,0.633662103069904,0.910562482162359
"10757","LINC00900",10.1967105252324,-0.244429262656511,0.513146708929
969,-0.476334074452516,0.633836393532891,0.910562482162359

"10758","CCDC15",22.1434036887905,-0.168816538164208,0.357269744596065,-0.472518428211923,0.636556791247784,0.910562482162359
"10759","AC007406.5",14.621835639868,0.199832158634787,0.422206036412107,0.47330483555601,0.635995712745096,0.910562482162359
"10760","WNT5B",33.9656490698229,0.186270529136946,0.391771525107994,0.475457038603303,0.634461247644358,0.910562482162359
"10761","PHC1",91.4027056986763,0.123986820832874,0.261562685705883,0.474023351221786,0.635483255490066,0.910562482162359
"10762","PPP1R12A",573.442354766075,-0.0838360485643822,0.177813933059724,-0.471481886271664,0.637296651953342,0.910562482162359
"10763","MYCBP2-AS1",3.40437586656806,-0.365209406214685,0.770899796701775,-0.473744328091926,0.635682238686117,0.910562482162359
"10764","TRAC",238.58862679099,0.337716972656115,0.711578917978839,0.47460227407434,0.635070485075879,0.910562482162359
"10765","SEC23A",326.981887453324,-0.0720086453791426,0.15132882666911,-0.475842223614106,0.634186786109478,0.910562482162359
"10766","RCOR1",120.297748825147,0.116726867548593,0.246062137825546,0.474379636705222,0.635229211499287,0.910562482162359
"10767","MAPK6",184.970266402503,-0.113043759194883,0.239275567868935,-0.472441713133052,0.636611536367279,0.910562482162359
"10768","AC103740.1",8.24325060205261,0.294280356909309,0.622849488234013,0.472474269415702,0.636588303435719,0.910562482162359
"10769","IQCH",2.97913672591785,-0.377060189757583,0.797263356543826,-0.472943082938312,0.636253786677715,0.910562482162359
"10770","AC026471.1",12.5239211823365,0.260449753147869,0.550568931291374,0.473055667229492,0.63617346443012,0.910562482162359
"10771","ACSF3",65.9504203631192,0.115799828049426,0.245525211442029,0.471641292433088,0.637182847813448,0.910562482162359
"10772","SUZ12",165.880601101651,-0.0843231765554462,0.17793518206767,-0.473898279000143,0.635572446528033,0.910562482162359
"10773","MRPL45",132.704334477331,-0.108852824189403,0.22882106432958,-0.475711554390021,0.634279888135493,0.910562482162359
"10774","TMEM99",116.984514422933,0.164990492738799,0.348817393954408,0.472999614120058,0.636213454481069,0.910562482162359
"10775","KLHL11",10.5970708843143,-0.245772984734126,0.514696058119275,-0.477510913202235,0.632998351576097,0.910562482162359
"10776","WIPI1",228.559977136004,-0.0909035635068603,0.192621298635849,-0.471928930760216,0.636977517096782,0.910562482162359
"10777","AC145207.5",9.84710195869794,-0.248136591007876,0.524964710007255,-0.472672898344818,0.636446564886726,0.910562482162359
"10778","PRELID3A",9.53174284756708,-0.239338870832589,0.50646122071959,-0.472570970967,0.636519297034026,0.910562482162359
"10779","LMAN1",732.273079269222,-0.0713967886858308,0.149501728332022,-0.477564971872892,0.63295986700715,0.910562482162359
"10780","SAFB2",204.645955001297,0.123069329336006,0.258459214287053,0.476165377487069,0.633956563333445,0.910562482162359
"10781","SCN1B",140.103872495845,0.128759866742971,0.271567633080367,0.47413554142097,0.635403255330903,0.910562482162359
"10782","AC010632.2",5.58564183525899,0.355677697912505,0.753532127734

49,0.472013978994974,0.636916810723844,0.910562482162359
"10783","AC008532.1",2.8592558295727,0.418437349815083,0.885347157274593,0.472625168982503,0.636480622611193,0.910562482162359
"10784","CU638689.4",13.5153113478775,0.263214685092392,0.553028860458516,0.47595108304865,0.63410922801255,0.910562482162359
"10785","DNAJC28",3.18838737312966,0.39380275672802,0.82961014147882 5,0.47468411611512 5,0.635012141070177,0.910562482162359
"10786","HIRA",41.6853080642592,-0.125775463199695,0.265541531856797,-0.47365464660015,0.635744903262202,0.910562482162359
"10787","APOL2",142.151896497796,0.131288148703916,0.277168358935629,0.473676538000526,0.635730586639884,0.910562482162359
"10788","PNPLA4",56.8276202833425,-0.129485793855425,0.274103953883819,-0.472396665647183,0.636643683905528,0.910562482162359
"10789","HUWE1",247.909530629055,0.0872516049133875,0.185095376648243,0.471387273379612,0.637364202564505,0.910562482162359
"10790","SMIM10L2B",2.63494160886598,-0.424997184830833,0.894113372783284,-0.475328071101162,0.634553153988258,0.910562482162359
"10791","VAPB",157.752729770929,0.0831185351280739,0.176361257411128,0.471297020378521,0.637428643159213,0.910570154230309
"10792","MAP3K21",5.95784279901215,-0.277266880005836,0.588799413647397,-0.470902099389449,0.63771064876851,0.910596394274317
"10793","NSUN2",50.4838388839892,-0.105296539580713,0.223643395297618,-0.470823381305707,0.637766866133613,0.910596394274317
"10794","C5orf22",31.8936204248423,-0.163515903529622,0.347735485218906,-0.470230708340524,0.638190196778783,0.910596394274317
"10795","CAMK4",2.64896945846178,0.414752115379809,0.882083350482793,0.470196059309816,0.638214949318178,0.910596394274317
"10796","C5orf63",13.7565446842122,-0.219613481014034,0.466878727705855,-0.470386565036212,0.638078861018015,0.910596394274317
"10797","DGAT1",79.459446600227,-0.11307067033505,0.240140419370359,-0.47085230646102,0.637746208681069,0.910596394274317
"10798","PAOX",30.6583374476007,-0.134936880677194,0.286471230038738,-0.471031177053791,0.637618471078076,0.910596394274317
"10799","MAML2",255.376055065653,-0.122864625074106,0.261031177933218,-0.470689463407853,0.637862509826266,0.910596394274317
"10800","ADGRG1",3.18743302519966,0.45011274906942,0.9566511744401,0.470508750833717,0.63799158366932,0.910596394274317
"10801","RHBDF2",3.10955488353905,-0.455847148620288,0.968329554116155,-0.470756207618142,0.637814840591824,0.910596394274317
"10802","GNG7",3.20376138548038,0.379947948133671,0.807783475049694,0.470358654106284,0.638098798506784,0.910596394274317
"10803","PLIN3",372.176273533416,-0.0950164826708889,0.202057311304518,-0.470245209428185,0.638179837626204,0.910596394274317
"10804","CEP89",122.324042273886,0.0883474440850043,0.187711231856247,0.470656141411199,0.637886309216096,0.910596394274317
"10805","RNPS1",365.80360467417,-0.0741119676427804,0.157744119056292,-0.469823966092411,0.638480790307132,0.910891382006889
"10806","GAL3ST4",53.3798243367781,0.130195369294224,0.277459515099003,0.46924096024520 1,0.638897410702835,0.911232752219116
"10807","AP000873.2",47.5703001191272,-0.168707980056139,0.35947841087

904,-0.469313246499542,0.638845748204964,0.911232752219116
"10808","CDK5RAP1",59.697696398206,-0.122603062745545,0.261218604623759,-0.469350423650467,0.6388191786287,0.911232752219116
"10809","ACTR8",52.03805520445,-0.130570289109569,0.278353063790306,-0.469081558979829,0.63901133990336,0.911247460225674
"10810","AC008124.1",98.3942865935867,-0.0955909987513007,0.203792204175438,-0.46906111614068 1,0.639025951673016,0.911247460225674
"10811","TGOLN2",829.021705382312,0.0750193676404969,0.160018826997774,0.46881588278072,0.639201246141018,0.911311556891839
"10812","TWISTNB",179.6123708892,-0.116714675481973,0.248940977862701,-0.468844769888967,0.63918059639285,0.911311556891839
"10813","AL391121.1",38.7844947541372,0.21124635277858,0.450658768741272,0.46875012189069,0.639248255898246,0.911311556891839
"10814","ABCC5",84.1568227495577,0.114948610259984,0.245364212855475,0.468481564292717,0.639440251727536,0.911431076438024
"10815","MAPRE1",211.679188090674,-0.0842532159913641,0.179848603995029,-0.468467444949936,0.639450346524634,0.911431076438024
"10816","LDOC1",46.8366873418791,-0.146427059724315,0.312654637684573,-0.468334840028953,0.639545157295562,0.911481934144886
"10817","MOB3C",66.5754089325981,0.119685499981737,0.255672502352851,0.468120344895598,0.639698530954065,0.911531970295518
"10818","FGD5-
AS1",525.383930693278,0.0636413622244306,0.135947489718732,0.468131940914108,0.639690238885928,0.911531970295518
"10819","ZNF654",64.875356200745,0.107869734122783,0.230661440453856,0.467653951655447,0.640032076221231,0.911689158370289
"10820","PLOD2",107.666825469183,0.122468619501421,0.262194492777661,0.467090739412569,0.640434959433871,0.911689158370289
"10821","LMLN",45.618222305218,0.1320818947031,0.282532508396665,0.467492733677436,0.640147389614544,0.911689158370289
"10822","WRNIP1",81.6561309676907,-0.0931793781962366,0.199476982141341,-0.467118447431763,0.640415136547062,0.911689158370289
"10823","TECPR1",30.376443487229,0.164890393635859,0.352828914010288,0.467338098121546,0.64025800300548,0.911689158370289
"10824","AC083862.3",29.2686486954383,-0.188945185366018,0.404554977706917,-0.467044519973503,0.640468026342088,0.911689158370289
"10825","MRPL16",213.071278094314,-0.126442400738135,0.270365253540948,-0.46767252478686,0.64001879208998,0.911689158370289
"10826","JAM3",209.257844113734,-0.0914390546965372,0.195650099759272,-0.467360123041306,0.640242247724956,0.911689158370289
"10827","NUP58",98.7088245215471,0.109172645191562,0.233588970813435,0.467370718794581,0.64023466822889,0.911689158370289
"10828","KLHDC2",340.015895933822,-0.0842987074539089,0.18043163825048,-0.467205797560201,0.640352646148014,0.911689158370289
"10829","KATNBL1",227.316144247577,-0.07341525335062,0.157214899925141,-0.466973889787655,0.640518558880975,0.911689158370289
"10830","DGKE",17.8460850339876,-0.223054863080257,0.477489170119937,-0.467141198247971,0.640398860337043,0.911689158370289
"10831","SLC16A8",17.4054754199066,-0.202842172088402,0.434475811740474,-0.466866432163007,0.640595442897636,0.911714407927897

"10832","C1orf109",50.8297946488045,0.117314066692368,0.251440768592404,0.466567404121085,0.640809412435463,0.911934738985659
"10833","FBXO30",62.939796698867,0.119937359849359,0.257111737270824,0.466479520236858,0.640872303432524,0.911940049608821
"10834","AC104794.3",66.6567106034951,0.128060644718007,0.27463893838193,0.466287284215749,0.64100987933749,0.911967447160813
"10835","SNRPC",294.313965203278,-0.130050870607542,0.27890506335176,-0.466290819695587,0.641007349019616,0.911967447160813
"10836","POMGNT1",62.3591350759259,-0.0959971680190795,0.205974313221585,-0.466063784933254,0.641169844656403,0.911999451220559
"10837","TOMM40L",19.5840668798269,0.185446320838203,0.397831664692877,0.466142686207158,0.641113370681685,0.911999451220559
"10838","AC093772.1",9.96905649143449,-0.285077330051084,0.611743589567767,-0.466007874724945,0.641209863920105,0.911999451220559
"10839","TRAPPC1",531.288225115329,-0.130639903437902,0.28043839289382,-0.465841720492832,0.64132879951819,0.912084458397722
"10840","TMCC1-AS1",4.35382896864473,0.364868680755783,0.7834903786076,0.465696440847454,0.641432800329723,0.912148211907997
"10841","GYPC",1768.6349082164,-0.106221917024126,0.228166423316008,-0.465545786625274,0.641540656051814,0.91221743501879
"10842","LINC02609",113.032629260161,0.119355050346644,0.256923648521088,0.464554551648629,0.642250485269213,0.912248073333716
"10843","RNF187",405.284102911209,-0.102747728307148,0.221090466077066,-0.464731610232949,0.642123668572274,0.912248073333716
"10844","USP39",71.8832184277337,0.107903339011459,0.231880365826408,0.46534055881315,0.64168759403479,0.912248073333716
"10845","EIF2AK3-DT",7.14139566176937,0.267006065921654,0.573956355483379,0.465202734268504,0.641786280840308,0.912248073333716
"10846","PPIG",795.714273046763,0.0814731380675053,0.175402437447334,0.464492621956683,0.642294844344055,0.912248073333716
"10847","NMD3",166.759803481305,-0.0827201724547125,0.178161392304785,-0.464299090754754,0.642433475332024,0.912248073333716
"10848","MARCH1",5.47990325509235,-0.443647857282311,0.95390490075336,-0.465086044669583,0.641869839291737,0.912248073333716
"10849","ANXA2R",49.9425871364144,-0.16436173888148,0.353479060600093,-0.464982957130321,0.641943661427299,0.912248073333716
"10850","AL354740.1",37.0229010422092,-0.165384034924588,0.356421527193524,-0.464012474854783,0.642638807980822,0.912248073333716
"10851","ZDHHC14",136.540301368615,0.138137747290191,0.297635978558269,0.464116428260193,0.642564332234977,0.912248073333716
"10852","NOXA1",4.81343383916898,-0.304763379492547,0.656258989150495,-0.464394979011339,0.642364786743312,0.912248073333716
"10853","SMARCC2",328.513581057183,0.0878663310415608,0.189054387035619,0.46476747997922,0.642097978444227,0.912248073333716
"10854","GIHCG",105.319940347072,0.122578924934277,0.263837892367752,0.464599394098477,0.642218366261436,0.912248073333716
"10855","RILP",104.654644942134,-0.153359975377783,0.33055583139341,-0.463945756852378,0.642686608913666,0.912248073333716

"10856","METRNL",1600.41270929718,0.123180197431856,0.265441287870947,
0.464058166760191,0.642606072307339,0.912248073333716
"10857","ZNF555",21.7260574496391,-0.175087620221723,0.376356708418603
,-0.465217216287751,0.641775910951344,0.912248073333716
"10858","PSENEN",328.528561034163,-0.136091923825028,0.292738356265834
,-0.464892696539717,0.642008300947621,0.912248073333716
"10859","ITPA",284.421541769675,-0.122445099172458,0.263275399189372,-
0.465083709110188,0.641871511772661,0.912248073333716
"10860","TMEM185A",124.13793574049,0.100464817746365,0.216514653750307
,0.464009322261509,0.642641066661713,0.912248073333716
"10861","SNAPC1",110.004842427138,-0.105007804243057,0.22644407119343,
-0.463725120687124,0.642844696942504,0.912304456211443
"10862","F8",24.2369304658086,0.175570236869649,0.37856027041986,0.463
784106755114,0.64280243124782,0.912304456211443
"10863","LINC01366",4.42455122538371,0.382706002151835,0.8254514249715
2,0.463632372026057,0.642911157119267,0.912314782932293
"10864","SPAG8",11.9576639356838,0.219279350612095,0.473154608589703,0
.463441223294187,0.643048136072867,0.912341188915163
"10865","TMEM91",141.084661538665,0.107750709472731,0.232462234251211,
0.463519202677412,0.642992253858133,0.912341188915163
"10866","AL109627.1",2.66039083520287,0.440153067702489,0.953734060880
993,0.461505031387792,0.64443631246399,0.912420533909187
"10867","C1orf50",114.376821613167,-0.112512319643629,0.24404431159718
2,-0.461032338378538,0.644775404101815,0.912420533909187
"10868","PTCH2",57.6734079379548,-0.130458869010631,0.282234098938204,
-0.462236382851796,0.643911815049741,0.912420533909187
"10869","MSH6",102.998946478648,0.0980826829010561,0.212659277724422,0
.461219862827514,0.644640872485452,0.912420533909187
"10870","AC012306.3",5.87099257364206,-0.272118209171539,0.58951570114
0125,-0.461596202858145,0.644370918093014,0.912420533909187
"10871","MTX2",193.902013146263,0.133937855776253,0.291283112290854,0.
459820189103555,0.645645290607727,0.912420533909187
"10872","AC009950.1",23.0078988714372,0.229135874711342,0.496267742048
992,0.461718252661931,0.644283379992058,0.912420533909187
"10873","NR1D2",224.52815876399,-0.373273244561358,0.80715945203638,-0
.462452918837323,0.643756557692974,0.912420533909187
"10874","AC092053.3",8.08613124737415,0.304839079586296,0.660096301327
138,0.461810010105208,0.644217571808562,0.912420533909187
"10875","ARMC8",116.532967766957,-0.0979568267898321,0.213111881331958
,-0.459649767894675,0.645767630551361,0.912420533909187
"10876","SMIM14",1199.34119386716,0.0989677053128286,0.214944046893135
,0.460434735194285,0.645204207228689,0.912420533909187
"10877","HHIP",5.57419264735209,-0.424225563483011,0.922243024876685,-
0.459993246942405,0.645521067721835,0.912420533909187
"10878","BNIP1",56.6074008955697,-0.148087933714122,0.320186038317119,
-0.4625059059179,0.64371856806536,0.912420533909187
"10879","RUFY1",170.296660516095,-0.0800716031034783,0.172998247852281
,-0.462846324153813,0.643474523948165,0.912420533909187
"10880","MGAT1",534.345467222993,0.0893915879906589,0.194461327536724,
0.459688253304645,0.645740002266679,0.912420533909187

"10881","SERPINB1",518.485455306882,-0.0908136887148549,0.196715130658374,-0.461650755643027,0.644331790446393,0.912420533909187
"10882","SERPINB9",39.58257611364,0.187773258615866,0.40563140486754,0.46291597830593,0.643424593975373,0.912420533909187
"10883","PPP1R3G",32.3611504953764,-0.161115307998686,0.349605949734434,-0.460848301126086,0.644907445283073,0.912420533909187
"10884","IER3-AS1",8.0552611425874,-0.26299132523238,0.570143949660687,-0.461271798795542,0.644603615242604,0.912420533909187
"10885","RBM48",65.1978392671791,0.111975237545729,0.243531776565434,0.459797235190137,0.645661767930575,0.912420533909187
"10886","DOCK5",8.30003491128651,0.322510515646522,0.701075500274838,0.460022516148533,0.645500058930193,0.912420533909187
"10887","AL162253.2",33.4911777043717,0.173764944071318,0.377660191186143,0.460109241393865,0.64543781111967,0.912420533909187
"10888","PDCD1LG2",51.7087273494824,0.147819241154985,0.320685708777502,0.460947392131979,0.644836349081344,0.912420533909187
"10889","RANBP6",54.8920381722674,0.123235328512073,0.266643791760601,0.462172127460281,0.643957889468823,0.912420533909187
"10890","SLK",421.652625909982,-0.0938371887440994,0.202599416159281,-0.463166136028377,0.643245287455348,0.912420533909187
"10891","C11orf49",96.5096368985963,-0.0963890803038645,0.208228562056056,-0.46290037904558,0.643435775805558,0.912420533909187
"10892","ZBTB3",11.8523534555863,0.243951126533192,0.529004627958637,0.461151214261714,0.644690120193883,0.912420533909187
"10893","AP003486.1",17.4960608142858,0.169830410686714,0.368211663286442,0.461230394417458,0.644633317379314,0.912420533909187
"10894","IFNG",5.27526802409655,-0.366214334580557,0.79612324778566,-0.459997036387452,0.645518347725685,0.912420533909187
"10895","CEP83",71.4611593575234,0.117957633933846,0.255620553207485,0.461455984089434,0.644471493656036,0.912420533909187
"10896","FAM155A",11.0922145856563,-0.261222999018693,0.568001146751006,-0.459898717657352,0.645588920692963,0.912420533909187
"10897","LINC00645",22.1265634142067,-0.268976127288272,0.583387522221073,-0.461059102300005,0.644756202727876,0.912420533909187
"10898","TUBGCP5",58.5320921526175,-0.12023045745213,0.260615508386666,-0.461332705012123,0.644559924160456,0.912420533909187
"10899","AC022306.2",8.55527030126075,0.234434304047407,0.508777285503345,0.460779816094729,0.644956584088143,0.912420533909187
"10900","AQP9",2.60992446574485,-0.469455179113611,1.02113515422099,-0.459738534290059,0.645703906794715,0.912420533909187
"10901","FBXL8",41.088483027342,-0.155368208039366,0.33757715297781,-0.460245033376353,0.645340350214778,0.912420533909187
"10902","ATXN7L3",54.6132290048784,0.109970846379988,0.237783355823805,0.462483364317034,0.643734729382129,0.912420533909187
"10903","STRADA",70.0565103219751,0.109146900359065,0.236449286458445,0.461608076699556,0.64436240157001,0.912420533909187
"10904","RNF213-AS1",15.3306633153828,0.252485849400258,0.546146539258658,0.462304219199089,0.643863174386238,0.912420533909187

"10905","MALT1",118.457675917625,0.120491340051634,0.260163256250956,0.463137422970318,0.643265867170018,0.912420533909187
"10906","ARHGEF18",45.4171058226978,0.136084580194569,0.294664671182276,0.461828625904016,0.644204220949599,0.912420533909187
"10907","ZNF567",52.4566868479428,-0.101862542343952,0.221165354891701,-0.460571875707348,0.645105793231423,0.912420533909187
"10908","LENG9",53.3451434146716,-0.0997035462208051,0.216783423063266,-0.459922372347205,0.645571941117297,0.912420533909187
"10909","ESF1",249.784040396228,-0.10174018023437,0.220362610675742,-0.46169438600492,0.644300497549118,0.912420533909187
"10910","NXT2",38.1482387170972,-0.124365882025576,0.269334763942624,-0.461752059797487,0.644259133283918,0.912420533909187
"10911","TMEM240",50.0957274971893,0.137633903781539,0.299597788663253,0.459395592990304,0.645950112403591,0.912438280160315
"10912","MSH5",4.07816738158561,0.336217455099389,0.731886115765744,0.459384934154158,0.645957765253942,0.912438280160315
"10913","FKBPL",27.832504345615,-0.197173854353095,0.42912872725066,-0.459474842470575,0.645893213894294,0.912438280160315
"10914","DFFB",17.815507326088,0.19185077901781,0.41877974617759,0.458118571322818,0.646867257222727,0.912461556119008
"10915","PATJ",16.1832120745524,-0.214528413263738,0.469195987229613,-0.457225592508644,0.647508905058049,0.912461556119008
"10916","KCNC4",11.033044236652,0.235705543307338,0.515549978503201,0.457192421948428,0.647532744736426,0.912461556119008
"10917","FOXN2",85.5958195431365,-0.109479324365661,0.239275664807994,-0.457544750543322,0.647279544688439,0.912461556119008
"10918","SMYD5",34.9328590108151,0.135357852886843,0.295754084592192,0.457670273847562,0.647189347544609,0.912461556119008
"10919","EEFSEC",58.9861140679775,-0.101070838742333,0.220184093220332,-0.459028793879285,0.646213489028165,0.912461556119008
"10920","SMIM3",105.74032921113,-0.137038004239696,0.298503206866515,-0.459083859360268,0.646173946951691,0.912461556119008
"10921","CPQ",1307.24047387999,-0.0925268175936513,0.20206089124158,-0.457915517570533,0.647013137982345,0.912461556119008
"10922","RNF20",122.360352984311,0.115587697743925,0.25294037568986,0.456976065717763,0.647688248806921,0.912461556119008
"10923","TAF3",157.49438326286,-0.1104312059305,0.241253895826745,-0.457738539525205,0.647140296126259,0.912461556119008
"10924","CDH23",19.2155617979013,-0.1696013677395,0.370621821336423,-0.457613011365426,0.647230493941295,0.912461556119008
"10925","CREBZF",218.434967676053,0.0954227505890151,0.20874264632354,0.457131076325988,0.647576834781846,0.912461556119008
"10926","MMP27",39.2972463441333,0.265361679534826,0.580572066224723,0.457069320024969,0.647621221237175,0.912461556119008
"10927","VSIG10L2",6.45800898264466,0.35877524946529,0.781969731753909,0.458809637887875,0.646370873076836,0.912461556119008
"10928","PIP4K2C",19.6458149965079,-0.212983641376532,0.464056098719637,-0.458960978994066,0.646262187722101,0.912461556119008
"10929","EP400",121.894740830919,0.112349569745116,0.245985837278712,0.45673186305364,0.647863785718682,0.912461556119008

"10930","LINC00346",12.4288699820486,-0.283539312965111,0.620076546895764,-0.457265017334666,0.647480570909696,0.912461556119008
"10931","MBIP",179.269978464164,0.101000072753521,0.221139594799214,0.456725412946626,0.647868422427671,0.912461556119008
"10932","CGRRF1",110.347482306208,-0.115145849553494,0.251038138826677,-0.458678709504749,0.646464905158502,0.912461556119008
"10933","PGF",12.0483480881105,-0.205869810048212,0.448995202441597,-0.458512271241897,0.646584448402638,0.912461556119008
"10934","CDC42BPB",289.71275972459,0.117801505647023,0.256861437145479,0.45861888400283,0.64650787340165,0.912461556119008
"10935","INF2",105.600785081803,0.12420654668262,0.271381232479657,0.457682889666773,0.647180282495695,0.912461556119008
"10936","AC027020.2",6.13852895703851,-0.329239634214587,0.719148112658606,-0.457818950532216,0.647082519804859,0.912461556119008
"10937","ABI3",2.35701295330119,-0.418524358586373,0.914035455950262,-0.457886349880444,0.647034094162694,0.912461556119008
"10938","AC068473.5",10.0691256156495,0.275681016481092,0.603561510115538,0.456757118969232,0.647845630441152,0.912461556119008
"10939","COX7A1",747.746245838939,-0.123883011699715,0.270817600289437,-0.457440770346222,0.647354265548371,0.912461556119008
"10940","ZSWIM9",30.8678688393573,-0.16360751845827,0.356748280665959,-0.458607727983597,0.646515886077419,0.912461556119008
"10941","SOD1",1768.63845025363,-0.104646662352845,0.228295573735457,-0.45838235337014,0.646677767443842,0.912461556119008
"10942","Z98885.3",5.01865343389884,-0.36562802928189,0.798013605001672,-0.458172676493559,0.646828388507173,0.912461556119008
"10943","SMC1A",206.717753788291,-0.122252407440883,0.266374578475857,-0.458949229090807,0.646270625624619,0.912461556119008
"10944","RBMX2",197.041301922019,-0.0873069729833792,0.191110356605486,-0.456840615726595,0.647785610080933,0.912461556119008
"10945","CCL17",2.6470056516925,-0.73917778073113,1.61125208076617,-0.458759861076265,0.64640662185779,0.912461556119008
"10946","CSF3R",14.6545594899168,0.327267688744291,0.717070573866534,0.456395368421859,0.648105695698291,0.912545606429408
"10947","KLC1",285.369165032029,-0.0742795473591215,0.162736227764475,-0.456441373746385,0.64807261973206,0.912545606429408
"10948","MPP2",11.1082161987815,-0.22316752195615,0.488970347333943,-0.456402976525972,0.648100225731448,0.912545606429408
"10949","CAND2",6.07233488266448,0.317980131304828,0.697206889564447,0.456077150217885,0.648334500719554,0.912784393879983
"10950","G3BP2",286.862709136978,0.0795071738243021,0.174469524473215,0.455708090363415,0.648599903458089,0.912907917440325
"10951","METTL16",89.1527174505699,0.0992722767074544,0.217763837535892,0.45587126784121,0.648482551810755,0.912907917440325
"10952","ZNF569",28.574228485671,-0.141886300162337,0.311325216516943,-0.455749462731411,0.648570149043614,0.912907917440325
"10953","AC011603.2",11.2688529622588,0.228097138253197,0.500756271991247,0.455505304698778,0.648745752116317,0.913029833732588
"10954","THAP9",10.9494266665643,-0.209046225378146,0.45904121145304,-0.45539751151413,0.648823285227694,0.913055590814762

"10955","IFNGR1",580.39572547148,-0.101586972570725,0.223356569631366,-0.454819720496186,0.649238941625765,0.913062624369372
"10956","LINC01239",49.4001876492267,0.190225189743504,0.41811527439156,0.454958719267837,0.64913893748012,0.913062624369372
"10957","RORB",2.93402491886513,0.508643732607004,1.11825293507,0.454855709880308,0.649213048073687,0.913062624369372
"10958","ECD",74.7008144490486,0.0946869788727831,0.208085551528975,0.455038700078118,0.64908139730114,0.913062624369372
"10959","PCF11-AS1",9.55195582575039,-0.260237389870006,0.572033102107706,-0.454934144389789,0.649156617677533,0.913062624369372
"10960","MRPL42",211.653772120037,-0.111364298195374,0.244856682766035,-0.454814207794299,0.649242907928166,0.913062624369372
"10961","TPD52L2",212.524818333505,0.0954782402785728,0.209743809483037,0.455213627109671,0.648955557754211,0.913062624369372
"10962","AL136171.2",2.75651141798207,-0.441996864380438,0.974090215771728,-0.45375352018115,0.650006240922716,0.913213829497367
"10963","TMEM184C",73.7291305882928,0.107350034207807,0.236379145343395,0.454143423066599,0.649725601197142,0.913213829497367
"10964","ADRA1B",36.8654595116157,-0.198436025105858,0.43753246084701,-0.453534406845403,0.65016397354472,0.913213829497367
"10965","UFL1",325.02877288986,0.0711068438837629,0.156791396642482,0.453512408247129,0.650179810491963,0.913213829497367
"10966","HOXA9",40.4643951690422,-0.155620673261427,0.342930153308458,-0.453796995569095,0.649974946257628,0.913213829497367
"10967","CCAR2",51.8842066582039,-0.122741192208458,0.270580680726365,-0.453621418495081,0.650101334788236,0.913213829497367
"10968","ZFHX4-AS1",23.3610816572181,0.198292681619795,0.436709893942571,0.454060428605429,0.649785333815376,0.913213829497367
"10969","TOR2A",51.1717063891022,0.143645177079128,0.316172286021348,0.454325642790301,0.649594462257015,0.913213829497367
"10970","ZNF408",19.5172565829059,0.182371905545971,0.401499264219487,0.454227247216757,0.649665273721938,0.913213829497367
"10971","CHORDC1",138.733844413312,-0.0949954596094147,0.209250834404342,-0.453978880800312,0.649844027441549,0.913213829497367
"10972","CASP4",501.14890113541,-0.0823369165883335,0.181210710408532,-0.45437113735004,0.649561722663048,0.913213829497367
"10973","MFAP1",155.557191387992,-0.120247600239524,0.264797244478164,-0.454111977171425,0.64974823311306,0.913213829497367
"10974","IDH2",84.8934238036727,0.142731248280937,0.314700768962054,0.453545915225099,0.650155688643337,0.913213829497367
"10975","TSEN34",208.353696567277,-0.103410999019099,0.227818878201899,-0.453917602594169,0.649888133554832,0.913213829497367
"10976","TMED10",793.78726341309,-0.0763289946326435,0.168384768221172,-0.453301064217317,0.650331966610653,0.913344320818441
"10977","IMMT",161.476800591464,-0.0921228062730039,0.20329646664885,-0.45314514212451,0.650444231317121,0.913394221480299
"10978","ELMSAN1",328.810363135228,0.129424487559581,0.285650330674783,0.453087126676329,0.650486004762291,0.913394221480299

"10979","AC093323.1",60.7029368271739,-0.118807878507735,0.262431150713974,-0.452720182739375,0.650750244518565,0.913608924857723
"10980","TAGAP",13.6981791240252,-0.238766056380454,0.52756857799547,-0.452578235966327,0.650852473492814,0.913608924857723
"10981","PSD3",170.292600705633,-0.130091722794466,0.287466551141345,-0.452545599750496,0.650875978773111,0.913608924857723
"10982","CYP4F12",30.292835939835,0.215390514956228,0.475830534513249,0.452662238619388,0.650791974634748,0.913608924857723
"10983","AC007390.2",11.3162167180972,0.221565477062906,0.48986521042784,0.452298861699925,0.651053695872694,0.913691981234302
"10984","PPP1R18",210.327324151693,-0.0882191229754914,0.195038821205904,-0.452315710431607,0.651041559668229,0.913691981234302
"10985","SLC35D1",46.0319089106204,-0.121805859910776,0.270205510446218,-0.450789695997042,0.652141128399143,0.913941975476053
"10986","GPR137B",116.50876918667,0.153329460406131,0.339403792235942,0.451761188041001,0.651441032773974,0.913941975476053
"10987","ELMOD3",72.8278931421262,0.103654874067592,0.229486231809917,0.451682322072589,0.651497855263197,0.913941975476053
"10988","AAMP",250.23415701755,-0.0813118435590043,0.180713266953024,-0.449949496957195,0.652746856406858,0.913941975476053
"10989","TCAIM",38.3658385530749,-0.120780936607845,0.268865866206598,-0.449223764667236,0.653270245818926,0.913941975476053
"10990","CCDC71",41.0653311371591,0.1347760438902,0.298712836565608,0.451189327649126,0.651853100852041,0.913941975476053
"10991","HESX1",4.63934234983379,-0.347071940573524,0.77168882480858,-0.449756338845021,0.652886143038744,0.913941975476053
"10992","QTRT2",41.054250946409,0.123624339765911,0.274309789971229,0.450674180381521,0.652224393934888,0.913941975476053
"10993","REST",278.604351012469,0.0763315630030257,0.170139672672176,0.448640589253401,0.65369094860319,0.913941975476053
"10994","PRMT9",28.4057483440553,0.14666366410224,0.32486091838473,0.451466014537789,0.65165371402099,0.913941975476053
"10995","MRPL36",298.190527049333,-0.129777931285458,0.288617649370569,-0.449653482967807,0.652960317518437,0.913941975476053
"10996","MDC1",48.2043095940638,0.140442514954711,0.311446455972202,0.450936307868106,0.652035454420066,0.913941975476053
"10997","AGER",6.29859557993355,0.303633176969459,0.675671407701092,0.449379940469203,0.653157599287284,0.913941975476053
"10998","KCTD20",255.164146081948,0.0857782202365563,0.191060911492222,0.448957453236311,0.653462349239218,0.913941975476053
"10999","B3GAT2",12.4254384193588,-0.259495554593919,0.578820203115043,-0.448318066987622,0.65392366347197,0.913941975476053
"11000","AC018647.2",29.8688291757365,-0.146172182093787,0.324092018715399,-0.451020616530974,0.651974690117814,0.913941975476053
"11001","COA1",149.655147412966,0.0870303172479842,0.193248949012036,0.45035337937369,0.652455655255366,0.913941975476053
"11002","NDUFB2-AS1",3.91613386935326,0.353226509945628,0.784530729853425,0.450239227737608,0.652537953762816,0.913941975476053
"11003","PIWIL2",8.51662758735317,0.256514173740528,0.572300216816076,

0.448216104420882,0.653997241224904,0.913941975476053
"11004","AC138696.2",5.17452879395666,-0.274985499009753,0.609285995066667,-0.4513241749134,0.651755923752608,0.913941975476053
"11005","RFX3-AS1",5.8467612013506,0.287401454712249,0.639697785872846,0.44927692585351,0.653231900814384,0.913941975476053
"11006","PLPP6",18.2461267968996,0.191713067938748,0.427638738380991,0.448306130227022,0.653932277047796,0.913941975476053
"11007","AL358781.1",5.86906443285005,0.385056492151346,0.859773334633915,0.447858146607094,0.654255575697583,0.913941975476053
"11008","CAMK2G",130.668846411546,0.0815408453199635,0.181320187907721,0.449706379972769,0.652922170433389,0.913941975476053
"11009","NUTM2B-AS1",149.262729402069,0.109750229088872,0.244208022507095,0.449412873345238,0.653133846461294,0.913941975476053
"11010","MMS19",63.9885633655237,0.0932884723748438,0.208208508586879,0.448053122362755,0.654114858562428,0.913941975476053
"11011","CATSPERZ",8.72946439962061,-0.349628398515483,0.778009592250775,-0.449388287751069,0.65315157877973,0.913941975476053
"11012","AP003396.5",7.92646783397611,0.298362356810586,0.665128057677884,0.448578816314317,0.653735518055749,0.913941975476053
"11013","HYLS1",17.7791623956195,-0.173405591953053,0.385075169213521,-0.450316213084362,0.652482450115485,0.913941975476053
"11014","DYRK4",18.6772452607111,-0.338026308232334,0.753811006112228,-0.448423153139806,0.653847835211262,0.913941975476053
"11015","SPSB2",45.1156375500435,0.140297072655297,0.311536216777453,0.450339527476252,0.652465641662739,0.913941975476053
"11016","AC079601.1",3.99755384887487,-0.321350854211047,0.717373043118928,-0.447955017676588,0.654185660754265,0.913941975476053
"11017","PAN2",37.9107342916227,-0.147788403509646,0.32967974645287,-0.448278685905787,0.653952081066375,0.913941975476053
"11018","SETD1B",56.2605312219666,0.1275694601847,0.283315810118606,0.450273001465379,0.652513603891169,0.913941975476053
"11019","EIF5",1175.76483957056,-0.101957512576966,0.226870113079785,-0.449409184810122,0.65313650679855,0.913941975476053
"11020","RPUSD2",28.6152918062997,0.160312365417489,0.355492675437448,0.45095827985828,0.652019618189565,0.913941975476053
"11021","MAN2C1",110.859857938889,0.110684033525239,0.245671207781291,0.450537262892342,0.652323091903439,0.913941975476053
"11022","KLHL25",4.72818652588389,-0.379515675226982,0.846507312693856,-0.448331242431021,0.653914156115181,0.913941975476053
"11023","PRSS27",2.58624608165436,-0.443576676012896,0.988075379135378,-0.448929996010072,0.653482156781681,0.913941975476053
"11024","AC007611.1",6.85060732311196,0.300806735036747,0.666496110268523,0.451325567249832,0.651754920402208,0.913941975476053
"11025","AKAP1",48.6206786214732,0.136546177229435,0.304613661750158,0.448260187822531,0.653965429553071,0.913941975476053
"11026","B4GALT6",15.5258500979385,-0.21171257330964,0.469608373970439,-0.450827934603583,0.652113566344335,0.913941975476053
"11027","TYK2",65.9230079980244,0.124233167090654,0.277140356640124,0.

448268049434514,0.653959756485668,0.913941975476053
"11028","AC011472.4",3.5403409020541,-0.401479563595216,0.891511652545038,-0.45033574429352,0.652468369126909,0.913941975476053
"11029","ZNF20",13.9486968568774,-0.180884485526169,0.400558237422319,-0.451580990295446,0.651570867213173,0.913941975476053
"11030","PLEKHF1",236.257977260137,0.144650164196185,0.322797621027121,0.448114096181742,0.654070855300676,0.913941975476053
"11031","CCNE1",3.15670934035548,-0.433353245165605,0.967153359669353,-0.44807086780297,0.65410205200338,0.913941975476053
"11032","PIH1D1",679.888453052651,0.113027183969964,0.25136525269968,0.449653175035309,0.652960539589001,0.913941975476053
"11033","PREX1",9.80876120653512,0.279106170970249,0.623116162074286,0.447919967347878,0.654210957345186,0.913941975476053
"11034","FAM210B",384.221275189801,0.0969068717397613,0.214738448117038,0.451278625646696,0.651788747973845,0.913941975476053
"11035","HLCS",24.7139877137052,-0.16536922455506,0.368035791229302,-0.449329191605791,0.653194202576517,0.913941975476053
"11036","ZNF140",43.2605815260923,0.121400225466869,0.271122029265888,0.447769684357934,0.654319424376605,0.913948344215781
"11037","C2orf73",3.12120161243056,-0.437588361771181,0.977566232735695,-0.447630397938972,0.654419961129835,0.914001858561355
"11038","SYNCRIP",407.471670313336,-0.0753412691305628,0.168349925002401,-0.447527785530573,0.654494030640848,0.914001858561355
"11039","DERL2",265.445264310277,-0.105948849585258,0.236772984880223,-0.447470177557859,0.654535615741732,0.914001858561355
"11040","MRPL27",376.419938722986,-0.112236124000402,0.250978274608967,-0.447194579591681,0.654734574722336,0.914196872223262
"11041","YPEL3",820.39288796232,-0.107384698559874,0.240179624641883,-0.447101617050109,0.654801691534696,0.914207777828761
"11042","CDKN2A",31.0960685451345,0.297687321978447,0.666365379254536,0.44673287545561,0.655067941941024,0.914417377440912
"11043","MCM8",19.390223694395,0.168957931454362,0.378210913427808,0.446729392134825,0.655070457287057,0.914417377440912
"11044","APH1B",162.129241238281,0.092443469724277,0.207000767155298,0.446585155188933,0.655174615882263,0.914479962316651
"11045","GMDS-
DT",71.9175444241503,0.103949316011987,0.232893375520285,0.446338655102422,0.655352637835129,0.914619197364271
"11046","DGCR11",3.01752454140505,-0.386590141149678,0.866244923201593,-0.446282720735451,0.655393036268942,0.914619197364271
"11047","NCBP2AS2",192.3878042438,-0.116843242883094,0.261977799387202,-0.446004368142662,0.655594090613627,0.914696132006879
"11048","CHMP5",611.739719974044,-0.097332295820882,0.218253448386211,-0.44595994491985,0.655626179860137,0.914696132006879
"11049","WDR93",2.47909330332845,-0.529062922957083,1.18615539530135,-0.446031713089895,0.655574338226165,0.914696132006879
"11050","TMEM183A",185.031239957239,-0.0831518783745056,0.186492402792685,-0.445872738670978,0.65568917540106,0.914701234281207
"11051","CTSW",8.81007559268297,0.337709967463038,0.758196368305847,0.445412272572123,0.656021844817589,0.915082502747546

"11052","SUCLG2",351.684302779087,-0.0745735684327378,0.167536732947444,-0.445117719086304,0.656234684408673,0.915256314434076
"11053","CIAO2A",130.796208660805,0.0970281829259676,0.218003877081996,0.445075492347657,0.656265199055455,0.915256314434076
"11054","ING3",127.527248268319,-0.0864778482421014,0.19452225059498 3,-0.444565328529731,0.65663390813311,0.915687687160475
"11055","RRAGA",633.689890876974,-0.103831862001716,0.233613287137299,-0.44446042977295,0.656709731647516,0.915710584653683
"11056","RGS5",7.6001155240246,0.245582304364063,0.553313381809664,0.443839445127575,0.657158667678209,0.915713023523493
"11057","TP53BP2",125.398888976887,0.121460681549137,0.273568741438403,0.443985964589764,0.657052731410081,0.915713023523493
"11058","C1orf35",166.05622901593,-0.0898054603921175,0.202392868385173,-0.443718502082836,0.657246116916246,0.915713023523493
"11059","OTULINL",19.6749107162423,0.188742788987309,0.424770289039272,0.444340844587317,0.656796175202001,0.915713023523493
"11060","AC055822.1",4.00238890370181,0.393261575552565,0.885951694354148,0.44388602455267,0.657124989149444,0.915713023523493
"11061","NTRK2",2148.95296022091,0.131982745112665,0.297165823110582,0.444138372748037,0.65694254481853,0.915713023523493
"11062","TECPR2",71.6122004599579,-0.0966053184239155,0.217713160465213,-0.44372750924881,0.657239604021138,0.915713023523493
"11063","AC006441.4",4.08030939068294,0.334818147494552,0.754039075301416,0.444032886970365,0.657018807121942,0.915713023523493
"11064","SLC25A18",8.99025394045485,0.261090377653333,0.58793589164996,0.444079671544833,0.656984983169592,0.915713023523493
"11065","NFU1",211.444302119643,-0.117274751378007,0.264471729155265,-0.443430198579591,0.657454596954819,0.915837937109934
"11066","NUDT16L1",101.766010172417,-0.11339326349899,0.255717108440534,-0.443432448421258,0.657452969930425,0.915837937109934
"11067","RAB7A",780.53147415921,-0.0802745447973385,0.18109398168129,-0.443275607792504,0.657566396701021,0.91591090676301
"11068","GCLM",123.630385901451,0.141921520315314,0.321664767000019,0.441209404557838,0.659061405397808,0.916690184586995
"11069","MSH2",63.3364763459198,0.128804135034696,0.291933636164698,0.441210326863568,0.659060737755651,0.916690184586995
"11070","IL18R1",8.48753982786975,-0.229841606810045,0.520294397032471,-0.441752992384619,0.658667958110055,0.916690184586995
"11071","LINC01124",4.11671197179047,0.372055197252342,0.84172679177496,0.442014203287726,0.658478928016612,0.916690184586995
"11072","LSM3",621.793599368193,-0.118469488087034,0.268411871278768,-0.441372013550002,0.658943699585027,0.916690184586995
"11073","NUDT9",156.737814347395,-0.0753201021780673,0.170281836858856,-0.442326108101,0.658253240927957,0.916690184586995
"11074","LRRCC1",94.8266617308815,-0.109806180933287,0.248615642412872,-0.441670443048526,0.65872770099804,0.916690184586995
"11075","CDC16",175.344443918938,0.0791739515102608,0.179359354777658,0.441426384525126,0.658904344606469,0.916690184586995
"11076","NAA38",684.483497117234,-0.119626898541349,0.27060701581169,-0.442068725315663,0.65843947489701,0.916690184586995

"11077","AC091271.1",44.9666151652731,-0.174343576950252,0.395169001524613,-0.441187381291579,0.659077347763715,0.916690184586995
"11078","TYMSOS",2.8945308509891,0.421934793669972,0.955734167192724,0.441477147258762,0.658867602209759,0.916690184586995
"11079","DOT1L",30.512179697145,-0.158215436785081,0.357882440172376,-0.442087733359804,0.658425720558087,0.916690184586995
"11080","CTU1",75.4822786247763,0.142679221643893,0.323059889356657,0.441649447500355,0.658742896315478,0.916690184586995
"11081","LINC01597",3.86793326851735,0.361075994693079,0.817681738898073,0.441585004918508,0.658789536867762,0.916690184586995
"11082","ZNF70",15.4191868356944,0.203018638806138,0.460106967287777,0.44124226156123,0.659037620913585,0.916690184586995
"11083","LPAR4",19.6413291056922,0.198442896588443,0.449554427551871,0.441421292787836,0.658908030084465,0.916690184586995
"11084","AKIRIN2",245.119798061703,-0.0906895229503837,0.205614838660736,-0.441065068752267,0.659165891094025,0.916730621726308
"11085","DZIP1",123.001842710462,0.109774835121144,0.24903936754692,0.44079310111668,0.659362788436417,0.916921730604183
"11086","SREK1",429.699188848079,0.0807514600965515,0.183291622843651,0.440562742823402,0.659529580185671,0.916948892937354
"11087","SYVN1",120.314338960996,-0.0890453349678272,0.202137026530739,-0.440519663794926,0.65956077358997,0.916948892937354
"11088","AC008735.1",4.07287526700113,-0.299498413905093,0.679807471969551,-0.4405635805052,0.659528973628486,0.916948892937354
"11089","LINC01116",191.514968801339,0.188004125727565,0.427057323823573,0.44023159243426,0.659769380470674,0.916997024653439
"11090","ADAP1",17.3067634366133,-0.339934272529974,0.772326410932693,-0.440143270666421,0.659833343980275,0.916997024653439
"11091","IL32",345.07324674228,0.127183520906701,0.28893868752921,0.440174772005371,0.659810530109539,0.916997024653439
"11092","AC087294.1",2.54856790942585,0.4005284186775,0.909864714290209,0.440206563005309,0.659787506781504,0.916997024653439
"11093","SRRM1",722.949404487368,-0.069508520281048,0.157989337194248,-0.4399570345408,0.659968226198355,0.917101794541391
"11094","MEGF6",180.795274632792,0.161334067562605,0.366848704447406,0.439783664509941,0.660093800031958,0.91717663067395
"11095","MYLK-AS1",8.56470524742502,-0.237147689158101,0.539518748724643,-0.439554120628226,0.660260075938296,0.91717663067395
"11096","PODXL2",46.2951555520064,0.144081333936494,0.32774808295699,0.439609997521792,0.660219598554409,0.91717663067395
"11097","PRPF18",184.551191519354,-0.0814911313790538,0.185326188958678,-0.439717299734813,0.660141871307882,0.91717663067395
"11098","RBMXL1",77.1017570442671,-0.108951464136038,0.24800000827618,-0.439320405242512,0.660429390816534,0.917246514049632
"11099","STOM",807.972634259242,0.0877177362192851,0.199649082010375,0.439359576993831,0.66040101166738,0.917246514049632
"11100","ATAD3C",2.36341483679798,-0.485891486799267,1.10646123351367,-0.43914009102359,0.660560031153825,0.917251654563691
"11101","IL22RA1",4.18984992579931,-0.408464426593741,0.93081912246735

4,-0.438822556106293,0.660790115078157,0.917251654563691
"11102","SLC25A38",59.3324901859928,-0.0962537367161526,0.219257102215779,-0.438999401813793,0.660661969766678,0.917251654563691
"11103","STX1A",7.014573197302,0.322886110691675,0.735417940152973,0.439051174933959,0.660624455993727,0.917251654563691
"11104","TMX1",205.193301647757,-0.0777305359244477,0.177112738728968,-0.43887603162976,0.660751364784276,0.917251654563691
"11105","CCDC32",186.122736066565,-0.104883756077219,0.238982833183051,-0.438875691112431,0.660751611532542,0.917251654563691
"11106","PRPSAP1",148.569129370316,-0.106084209999049,0.241793030712203,-0.438739734088188,0.66085013269575,0.917252367684584
"11107","SNHG5",1390.84973275762,0.166391007997283,0.379443098353968,0.438513728986218,0.661013920139899,0.917397099032731
"11108","FAM49B",65.465545909239,-0.116305892410607,0.265289020439198,-0.438412008978197,0.661087642623558,0.91741681770275
"11109","HSD17B7",53.9443128000437,-0.123003018839776,0.280848860115936,-0.437968730900313,0.661408950740628,0.917480497206926
"11110","AC009951.6",53.0786147607693,-0.157691260601363,0.359962779089632,-0.438076572806151,0.661330776276775,0.917480497206926
"11111","IP6K2",335.718272682571,-0.0944994718287882,0.215852586850034,-0.437796336878941,0.661533926621845,0.917480497206926
"11112","HNRNPDL",978.874290880002,0.101525848304117,0.231954511227407,0.437697235405705,0.661605773835376,0.917480497206926
"11113","LYNX1",9.41679852951916,0.235436028143256,0.537973159867746,0.437635268274602,0.661650700742102,0.917480497206926
"11114","NEK5",9.30035438581996,-0.221373054077095,0.505514492923094,-0.43791633509264,0.661446933729493,0.917480497206926
"11115","KMT5C",31.8585444172324,0.135554568550536,0.30946128010823,0.438034019968918,0.661361622334655,0.917480497206926
"11116","ZMAT5",112.442811487227,0.143391537534897,0.327669883196404,0.437609755697165,0.661669198018125,0.917480497206926
"11117","SRPX",1583.14067978033,0.143804717896142,0.328189934756648,0.438175284085945,0.661259223829725,0.917480497206926
"11118","MYL12A",1783.47892823534,-0.113652955524571,0.259806011460216,-0.437453140078612,0.661782752901782,0.917555417879202
"11119","TJAP1",58.1309867242785,0.103020105246081,0.235643281864895,0.437186685021417,0.661975965258996,0.91774075946285
"11120","RASIP1",2.96423956277703,0.432797815452853,0.990282103447267,0.437044973292198,0.662078732685593,0.917800689239966
"11121","LEPR",414.488581442863,0.187028517339174,0.428313920069277,0.43666224368548,0.662356314816593,0.917874964067048
"11122","ARFIP1",184.740070429853,0.0754512143363288,0.172753330639597,0.436756929993654,0.662287637399731,0.917874964067048
"11123","PALLD",632.074707676675,-0.0963584335957702,0.220763287370745,-0.436478522961782,0.662489578346414,0.917874964067048
"11124","RNMT",309.031991411479,-0.0754886592993425,0.172930744543772,-0.436525381871787,0.662455587786305,0.917874964067048
"11125","NNAT",5.66697972350578,-0.400517415175803,0.917291678773191,-0.436630380983577,0.662379425956601,0.917874964067048
"11126","NF2",93.7491629342668,0.0861938467732523,0.197335882678868,0.

436787499582722,0.662265465419121,0.917874964067048
"11127","KIN",152.804097496959,0.0975255174829603,0.22361260736165,0.436136041852201,0.662738028720253,0.917889192517762
"11128","CHID1",433.399151955245,0.126317778049999,0.289623068630278,0.436145430843605,0.66273121705138,0.917889192517762
"11129","MTA2",33.5469363738585,-0.130915661597608,0.300169151461458,-0.436139626474632,0.662735428090335,0.917889192517762
"11130","INPP5K",57.4140460234269,-0.109328618108722,0.250670159096516,-0.436145325405993,0.662731293545714,0.917889192517762
"11131","AIFM2",90.0836822831298,0.102872398909046,0.23597164391079,0.435952376328475,0.662871282798377,0.917991269817355
"11132","FBXO22",92.8528957712086,0.0883283405919246,0.202685150040758,0.435790883417767,0.662988458889158,0.918071064837978
"11133","LINC01128",57.0483534437731,-0.12255270894339,0.281449944860663,-0.435433408963936,0.663247864674354,0.918100380204308
"11134","AAMDC",448.789216848624,-0.101821435339677,0.233782403861406,-0.435539346237712,0.663170985762052,0.918100380204308
"11135","SCRN2",142.07651856351,0.108006217682634,0.248028211820844,0.43545940556411,0.663228998556116,0.918100380204308
"11136","ZNF613",4.87439646491105,0.320936606887726,0.73687324955231,0.435538414622477,0.663171661821816,0.918100380204308
"11137","ATG16L1",29.8075137515523,0.131200275509063,0.301484735762396,0.435180491567122,0.663431421723798,0.918147404399246
"11138","PFKFB3",99.1893666427814,0.197489044099832,0.453861337441677,0.435130793940363,0.663467492593683,0.918147404399246
"11139","SLC26A10",2.43487783161825,0.46640818200482,1.0715790864124,0.435253158557186,0.663378680940689,0.918147404399246
"11140","RNFT1",108.366159239679,-0.101166341979311,0.232535118303483,-0.435058337499277,0.663520083360856,0.918147404399246
"11141","TMEM39A",91.2921753528663,0.0875550734997946,0.201615706753706,0.434267125858166,0.664094473169221,0.918808989330975
"11142","TMEM203",208.276975306649,-0.0945512117996773,0.217741759044434,-0.434235546799189,0.664117402473287,0.918808989330975
"11143","SLC25A21-
AS1",31.6681326201317,0.162253343204289,0.373972881187626,0.433863928018045,0.664387255536994,0.919029247913174
"11144","JUP",96.3221296609475,-0.102959363873609,0.237323280071379,-0.433835921375444,0.664407594481292,0.919029247913174
"11145","SECTM1",46.8206697549209,-0.14740494155967,0.339951476505275,-0.433605828322921,0.6645747016859,0.919029247913174
"11146","TMEM259",305.529098828731,-0.0716841612248268,0.165308754267535,-0.433638022030057,0.664551319705362,0.919029247913174
"11147","ZNF793",9.00584136575408,-0.340856464679468,0.785988477895991,-0.433665981455485,0.664531013307837,0.919029247913174
"11148","HARBI1",7.67629088436086,-0.240352720955087,0.554721285407078,-0.43328555668944,0.664807329671683,0.919186024476544
"11149","AP000842.1",2.42557767040607,0.55088897063305,1.27139497204894,0.433294910507042,0.664800535106915,0.919186024476544
"11150","RNF123",34.0788131855428,-0.125661641587323,0.290198818768352,-0.433019135366058,0.66500086830457,0.919371155597753

"11151","LRRC40",81.2777725481727,0.0864666823922413,0.199931157751832
,0.43248227722249,0.66539093040005,0.919415667991822
"11152","RAB7B",113.255863871366,0.106302836386727,0.245738617921102,0
.432584985160359,0.665316299443992,0.919415667991822
"11153","MARK1",33.528527957011,-0.135271112595849,0.312698090450003,-
0.432593343954166,0.665310225816175,0.919415667991822
"11154","RRAGD",56.8873870056245,-0.180822065097046,0.418037096130188,
-0.432550285060667,0.6653415133036,0.919415667991822
"11155","LRP12",80.3451042559189,-0.116802488170877,0.270064648443118,
-0.432498251230678,0.665379322944098,0.919415667991822
"11156","DOLPP1",16.144543318597,-0.191302114949395,0.442119729826862,
-0.432693006087538,0.665237811473161,0.919415667991822
"11157","GATC",112.792041731962,0.102534095041419,0.237219330449826,0.
432233304288437,0.665571856056241,0.919583235736037
"11158","ERMARD",39.3572748090723,-0.129335497741562,0.299352772582372
,-0.432050442111649,0.66570475221788,0.91968441973818
"11159","SELENOT",264.869712740114,-0.087814344658325,0.20333370199882
7,-0.431873043155589,0.665833687985887,0.919697697159717
"11160","WAPL",183.359307495665,0.0856871013116042,0.198395444497034,0
.431900548567713,0.665813696063028,0.919697697159717
"11161","ATP5IF1",856.679928818014,0.118524830867781,0.27495483525432,
0.431070181974254,0.666417340953828,0.92008270618305
"11162","ARF4",952.127607241482,-0.101609719134247,0.236262737055237,-
0.430070862636672,0.667144094566868,0.92008270618305
"11163","TOPBP1",99.2272009529879,0.095406652196694,0.221600148493176,
0.430535145600915,0.666806406528648,0.92008270618305
"11164","NLN",39.7220880016113,-0.121341696897369,0.281570178117586,-0
.430946550194303,0.666507235041377,0.92008270618305
"11165","LINC01184",102.384454934346,0.081075843387894,0.1884998440713
89,0.430110930793072,0.667114949039333,0.92008270618305
"11166","FAM133B",720.35393857205,-0.112213925804422,0.260372264669002
,-0.430974957901426,0.666486579047298,0.92008270618305
"11167","WDR91",31.1857774106385,-0.118600364774411,0.275645866785797,
-0.430263533995141,0.667003950258124,0.92008270618305
"11168","ATP6V1G1",988.556848756524,-0.0878947959707169,0.203976085839
158,-0.430907356659568,0.666535734110679,0.92008270618305
"11169","MVB12B",21.9155548021287,0.153260901549158,0.356438587732496,
0.429978422157196,0.667211337573522,0.92008270618305
"11170","CHUK",33.1006032292513,0.136099028706995,0.315896756986796,0.
430833890177238,0.66658915560496,0.92008270618305
"11171","GRAMD1B",3.26568251243251,0.411871471221116,0.957522190355947
,0.430143003858749,0.66709161949287,0.92008270618305
"11172","ULK1",110.038726486744,0.123101707605857,0.286329582745047,0.
429930105110615,0.667246485398658,0.92008270618305
"11173","IRF2BPL",226.863484774396,0.0928129701331982,0.21522762190178
3,0.431231685380757,0.666299917188418,0.92008270618305
"11174","AC023449.2",2.50272998971145,-0.399024800209726,0.92674711358
7795,-0.43056492365533,0.666784750287788,0.92008270618305
"11175","IRX6",15.6220075067406,-0.25371459297303,0.589494633252853,-0
.430393388949147,0.666909503578021,0.92008270618305

"11176","PGLS",899.356684741015,-0.112279577476312,0.261086534178847,-0.430047370422402,0.667161183008107,0.92008270618305
"11177","NDRG3",97.2139916404274,0.0949542599272635,0.220793076861581,0.430059951502882,0.667152031399134,0.92008270618305
"11178","BACE2",250.590365494802,-0.085055129745353,0.197594008658646,-0.43045399161009,0.666865427588771,0.92008270618305
"11179","UTP14A",67.1555605478543,-0.113965872348164,0.26472789394121,-0.430501941640775,0.666830554604191,0.92008270618305
"11180","LAPTM4A",6903.71283802825,-0.10501789037181,0.244492472653266,-0.429534247955127,0.667534475813555,0.920120231190947
"11181","HIVEP1",194.920583521964,0.112692422124983,0.262391203198323,0.429482470263331,0.66757214827373,0.920120231190947
"11182","SBNO1",199.230464276387,0.0935939146428223,0.217882441294043,0.429561529083993,0.667514626921972,0.920120231190947
"11183","GPI",257.974078472366,-0.0832937552548789,0.193855952138897,-0.429668288932389,0.667436954049372,0.920120231190947
"11184","UBA2",326.205394529003,-0.0749252307983697,0.174328182721192,-0.429794136718557,0.66734539836866,0.920120231190947
"11185","DYNC1I2",843.44304608852,0.0747495975760224,0.174112792150147,0.429317091828391,0.667692480076868,0.920150142643191
"11186","AL157938.3",14.1799159027997,-0.172881848578669,0.402717101063848,-0.429288570368557,0.667713233578121,0.920150142643191
"11187","BGLAP",31.0874316382096,0.150217664308163,0.350200034565033,0.428948170992448,0.667960943135258,0.920150654897099
"11188","NFXL1",46.4396144061242,0.135330279068825,0.315364333660613,0.429123602843637,0.667833276378606,0.920150654897099
"11189","SCAF8",124.639938498936,0.0856371206790108,0.199798759127069,0.428616879570043,0.668202059542598,0.920150654897099
"11190","SLC29A4",2.67904575985411,-0.395189176496073,0.921864329936734,-0.428684746402102,0.668152662758616,0.920150654897099
"11191","TMEM74",4.60464350206468,-0.326799057368767,0.762409775272255,-0.428639647559697,0.668185487731254,0.920150654897099
"11192","GRHPR",506.619907532683,-0.0983705492787334,0.229492827522504,-0.428643240578345,0.668182872546879,0.920150654897099
"11193","CCDC61",54.406466720031,-0.117676885054204,0.274593210206661,-0.428549871883719,0.66825083240824,0.920150654897099
"11194","PMEPA1",128.508260720949,-0.103907697138086,0.24219490223484,-0.429025120592067,0.667904943536081,0.920150654897099
"11195","MED14OS",17.5055590302509,-0.200422093190525,0.467138630453848,-0.429042001933784,0.667892658490446,0.920150654897099
"11196","FMO5",20.1021924825676,0.151270425574332,0.353529096605364,0.427886776581763,0.668733554876759,0.920155850698123
"11197","ATF6",183.022791141962,0.0854280862988043,0.199597369757273,0.428002064369345,0.668649617421905,0.920155850698123
"11198","NOL10",84.3669755483496,0.0851203664044854,0.198975520859474,0.427793157855842,0.66880171880783,0.920155850698123
"11199","EIF2AK2",478.752346113409,-0.0724646555942736,0.169377212212342,-0.427830017082979,0.668774881224864,0.920155850698123
"11200","PLXNA1",174.973446800904,0.105455186152057,0.246573073093779,0.427683302271813,0.668881708300224,0.920155850698123

"11201","RELN",7.7289094398104,-0.695487570004601,1.62632836323717,-0.427642772349028,0.668911220429657,0.920155850698123
"11202","AL589843.1",4.77162657110717,0.321291571364233,0.750134505615793,0.428311948002554,0.668424021310369,0.920155850698123
"11203","JMJD1C",676.773366458082,-0.117107491254229,0.27361594276337,-0.427999516663787,0.668651472282388,0.920155850698123
"11204","ZNF557",39.677103092783,-0.141843783548982,0.331445308106363,-0.427955321978681,0.668683648604598,0.920155850698123
"11205","AC008569.2",3.47396837456859,0.399076241378341,0.933042170730207,0.427715117169913,0.66885854243163,0.920155850698123
"11206","B3GNT8",42.0862876916746,-0.15352794809007,0.358358118832925,-0.428420454349044,0.668345035477149,0.920155850698123
"11207","CNDP2",141.17789185981,0.0685707859360592,0.160404334257909,0.427487114068915,0.669024568782888,0.920229653590454
"11208","AL391988.1",7.64283903362936,0.241311211617822,0.564647400434177,0.427366196022986,0.669112624950913,0.920268657531971
"11209","ZWINT",10.345050980105,0.229156712284302,0.536407258251671,0.427206583727445,0.669228866406192,0.920346415884686
"11210","PLEKHH2",321.874050682935,-0.173669029110237,0.406616731176863,-0.427107435071817,0.669301077767155,0.920363614074994
"11211","C15orf39",32.2001654149714,0.140928851095122,0.330067200786924,0.426970176858317,0.669401049902216,0.92041897995207
"11212","CDHR3",18.4823068373035,0.162865919072423,0.381592964744012,0.426805350517087,0.669521109048529,0.920501952906089
"11213","UBR1",114.976462125722,0.114138202089885,0.267618768029855,0.426495506761886,0.669746821421984,0.920710534071249
"11214","PLXNA3",54.0340340464956,0.123337282725214,0.289230077079664,0.426433115015361,0.669792275645474,0.920710534071249
"11215","ZNF200",29.9055869594454,0.137507685547377,0.322574397907985,0.426282080782499,0.669902313535884,0.920779684632693
"11216","BCDIN3D",43.0426723291559,0.13126722168274,0.308293188493974,0.425786967023134,0.670263084563398,0.92094709408546
"11217","C18orf54",17.8908612947554,0.201221233305263,0.472513686903843,0.425852708360195,0.670215176909211,0.92094709408546
"11218","ZNF814",10.0284151411019,0.20253297127532,0.475572775616805,0.42587166814299,0.670201360604626,0.92094709408546
"11219","CDK16",127.765235910369,0.0758563115307291,0.178106843323412,0.425903407838109,0.67017823161809,0.92094709408546
"11220","SEMA5A",22.7002595405808,0.45363959613917,1.06562700006315,0.425702047820001,0.67032496967995,0.920950036329449
"11221","GPR155",31.1333504979337,-0.141891810451992,0.33370267837852,-0.425204290062795,0.670687757072341,0.921120079763911
"11222","DDX39B",567.739435961121,-0.0786432582499789,0.184858619323056,-0.425423810574627,0.6705277515545,0.921120079763911
"11223","AHNAK",6515.66691746003,0.0757192023281645,0.178015066300701,0.425352774355966,0.670579527253943,0.921120079763911
"11224","ZNF543",13.6920349368893,0.194792618443016,0.458040957440078,0.42527336317626,0.670637409007897,0.921120079763911
"11225","AL360012.1",48.7557104568872,0.174143007427387,0.410597757322519,0.424120697986668,0.671477791313671,0.921128275112573

"11226","TESK2",13.220244528491,-0.210211341678588,0.495657497758719,-0.424106046270115,0.67148847619808,0.921128275112573
"11227","ZNF326",175.195951957412,-0.0858431116782978,0.202181619579844,-0.424584152885358,0.671139847306046,0.921128275112573
"11228","ZEB2",834.245715546577,0.0742674872116107,0.175065958331196,0.42422574850965,0.671401184303358,0.921128275112573
"11229","IMPG2",3.29604729576005,-0.460024504496742,1.08349779259546,-0.424573550255951,0.671147577833341,0.921128275112573
"11230","CASP6",88.0037354933457,0.0916310577017677,0.215696913753941,0.424813948920461,0.670972308313547,0.921128275112573
"11231","BRIX1",154.056949409038,-0.0955623475516285,0.22531658656222,-0.424124779314635,0.671474814983617,0.921128275112573
"11232","ZNF680",99.4211696411488,-0.115946547243729,0.273003665292546,-0.424706925159713,0.671050334836395,0.921128275112573
"11233","ZC3HC1",34.5443684484427,0.121011965294042,0.284796538855097,0.424906727379899,0.670904670311313,0.921128275112573
"11234","RNF34",79.7578817367383,-0.0964986350653571,0.227190214972302,-0.424748200872656,0.671020242029832,0.921128275112573
"11235","AC016355.1",3.43622473748074,-0.385257675164074,0.908873471322149,-0.42388482810884,0.671649809423634,0.921128275112573
"11236","DLGAP1-AS2",10.1563430887561,-0.269229637692964,0.633697508073498,-0.424855130820142,0.670942285267573,0.921128275112573
"11237","AC027575.2",6.99020595653479,-0.270621848487417,0.638208796444091,-0.42403340410731,0.671541452071394,0.921128275112573
"11238","ZNF121",46.8022103795369,0.122256920470999,0.288398191344249,0.42391708457376,0.671626284013419,0.921128275112573
"11239","AL021707.1",2.55245185971648,0.46590133977235,1.09840122378336,0.424163165229902,0.671446822103534,0.921128275112573
"11240","MIRLET7BHG",37.8179215556475,-0.189900704315557,0.447657613246622,-0.424209705579023,0.671412883224186,0.921128275112573
"11241","AC068870.2",6.78310542179513,-0.236868883564636,0.55909784653515,-0.423662664831503,0.671811847143467,0.921194963720026
"11242","XYLT2",49.3199658753956,-0.121408892425174,0.286575387409208,-0.423654290491497,0.671817955377264,0.921194963720026
"11243","WRAP53",25.9906278775564,-0.162648429250709,0.384110041436031,-0.423442273580333,0.671972607482716,0.921325068428895
"11244","FBXO48",26.0579620167123,0.146700979740765,0.347031311748223,0.422731248663807,0.672491353762425,0.921342679635737
"11245","PLCL2",90.135733414211,0.121091838396433,0.286458990729712,0.422719629389079,0.672499832194896,0.921342679635737
"11246","SEMA3D",359.474969140049,0.162221199478791,0.383979789699505,0.422473275496459,0.672679603201332,0.921342679635737
"11247","AC090136.3",5.2959190560596,0.315413602163413,0.745819089857886,0.42290899556287,0.672361659371808,0.921342679635737
"11248","TAF10",488.864965065604,-0.110121849052643,0.260180388585717,-0.423251920143715,0.672111469366983,0.921342679635737
"11249","C1QTNF4",249.30580100973,-0.299662617895418,0.708682715261231,-0.422844541629547,0.672408687553058,0.921342679635737
"11250","AL354811.1",3.75385847769623,0.315237905312162,0.745925206031

389,0.42261328986233,0.672577428688188,0.921342679635737
"11251","AL008727.1",2.85161648248734,0.377419046410247,0.89228662144708,0.422979609173296,0.672310138313984,0.921342679635737
"11252","CD70",120.358200495418,-0.315086104915618,0.744834277521133,-0.423028470123917,0.672274489318826,0.921342679635737
"11253","S1PR2",219.81603616001,-0.0905568242643634,0.214389707037118,-0.422393525864024,0.672737802644715,0.921342679635737
"11254","ZNF473",16.5922555897327,0.161049793605161,0.381248707551218,0.422427120185123,0.672713286044581,0.921342679635737
"11255","DDT",464.979207552167,-0.0914280873427913,0.216469697699468,-0.422359749722217,0.672762452285427,0.921342679635737
"11256","EP300",262.074127932286,0.0942382374913387,0.223029350985712,0.422537379384546,0.672632823083032,0.921342679635737
"11257","NBEAL2",15.3496357773141,-0.232828613916935,0.551554295723843,-0.422131811359347,0.672928809629548,0.921406786324346
"11258","TPSB2",24.538250436229,-0.306081446819116,0.725038222688123,-0.422159049331634,0.672908929567848,0.921406786324346
"11259","CCNT2-AS1",2.74014548380383,0.381787252080091,0.904863512509239,0.421927999971369,0.67307757182432,0.921504081346233
"11260","SHLD3",9.46892215853789,-0.226574958743426,0.537072093291705,-0.421870660519247,0.673119426270424,0.921504081346233
"11261","ELOA",130.602753445775,-0.08652431784421,0.205755780886263,-0.42051949875488,0.67410598810551,0.921519611306852
"11262","EXTL2",148.98346980626,0.0847481137563545,0.201333616707234,0.420933747390977,0.673803461398979,0.921519611306852
"11263","NTPCR",271.98020612489,0.1166528778262,0.277344500162276,0.420606421825368,0.674042503621768,0.921519611306852
"11264","AC007038.1",3.45454973298561,-0.366348289199287,0.870945987533602,-0.420632616078449,0.674023373038052,0.921519611306852
"11265","SNX25",11.5315734107257,-0.21276719996108,0.506089793663004,-0.420413931727613,0.674183092370632,0.921519611306852
"11266","FAM149A",83.2318369988128,0.105646446218928,0.250672602704978,0.421451906107447,0.673425123186616,0.921519611306852
"11267","MRS2",58.7689189747964,0.105133176451405,0.250138655866528,0.420299597785891,0.674266603693155,0.921519611306852
"11268","SAYSD1",138.101166113474,0.105953256586502,0.251543015921636,0.42121327121048,0.673599354329743,0.921519611306852
"11269","CBX3",596.512737942495,-0.0601497572334246,0.142775036968346,-0.421290433612425,0.673543014913417,0.921519611306852
"11270","TMEM67",48.9192263013608,0.123528322529269,0.293650009240917,0.420665140956708,0.673999619269608,0.921519611306852
"11271","UQCRB",2286.63889269394,-0.100172730939338,0.238182356211197,-0.420571584448155,0.674067946909396,0.921519611306852
"11272","CNTRL",93.5960125165046,0.106691397552652,0.253379672480535,0.421073231755987,0.673701607523401,0.921519611306852
"11273","RCN1",685.191128633196,-0.0790700935006021,0.188107722797316,-0.420344748874554,0.674233624143092,0.921519611306852
"11274","NEU3",12.4903857516715,0.217073871541109,0.515531358574042,0.421068220062374,0.673705267044435,0.921519611306852

"11275","TLCD5",52.8262697178139,0.117294279837899,0.27860603959166,0.421004081640915,0.673752101375799,0.921519611306852
"11276","MYL6",6073.91082007654,-0.111100650345827,0.264255742737896,-0.420428518202621,0.674172438466883,0.921519611306852
"11277","PLEKHJ1",345.198519810686,0.099316038181165,0.235927291328432,0.420960362923458,0.67378402581894,0.921519611306852
"11278","DEDD2",47.7448801153122,-0.104606324660986,0.24880887324296,-0.420428432867179,0.674172500795363,0.921519611306852
"11279","TST",61.0128188724165,0.141631087264659,0.33660117426495,0.420768250657307,0.673924317709456,0.921519611306852
"11280","KCTD6",63.4739716906834,-0.104753938649726,0.24944842705586,-0.419942269775338,0.674527627600064,0.921635510643929
"11281","SMPD1",132.316612392168,0.122298062948759,0.291228877769332,0.419937967297407,0.674530770748285,0.921635510643929
"11282","PRR34-AS1",20.0702434283236,-0.157417984990284,0.374726678336296,-0.420087477329304,0.674421550464315,0.921635510643929
"11283","SLC25A13",59.7525688021507,0.116278972580527,0.277017891725785,0.419752572139381,0.674666215410145,0.921738873575059
"11284","DDX5",5662.69995646481,0.0878579793333787,0.209431452295719,0.419507091080678,0.6748455734154,0.921902207922579
"11285","AL359921.2",2.95700795319072,0.347779811970204,0.829621339960837,0.419203069181683,0.675067729237619,0.922123973965254
"11286","PSIP1",580.132288474634,-0.0749838756975088,0.178988754070756,-0.418930653418968,0.675266813779155,0.922164340511352
"11287","RAB1B",316.798113501241,-0.0826953867710559,0.197373691597157,-0.418978771192256,0.675231647109906,0.922164340511352
"11288","TSPAN3",520.104596720448,0.0671459930258442,0.160284694467457,0.418917060352741,0.675276748342014,0.922164340511352
"11289","VDAC3",472.658801028753,-0.102328474226059,0.244557346491671,-0.41842322749255,0.675637706994063,0.922248849438191
"11290","RABL6",176.297942644281,-0.0779953282312529,0.186402999892527,-0.418423138448533,0.675637772085987,0.922248849438191
"11291","NPTN",457.65105995544,-0.0758499071063814,0.181142069321669,-0.418731592227141,0.675412304333418,0.922248849438191
"11292","TFAP4",43.7476561645092,-0.12804768917156,0.305971853337714,-0.418494994800152,0.675585245278311,0.922248849438191
"11293","ETFB",753.021126864858,-0.113719360069425,0.271723741595709,-0.418510945718631,0.675573585412274,0.922248849438191
"11294","SEC22B",336.041965468312,0.0745038505126168,0.1781395516913,0.418233063939249,0.675776723675475,0.922285012741432
"11295","TSHZ1",151.863450436434,0.0754293443921612,0.180356666473519,0.418223212188478,0.675783925975639,0.922285012741432
"11296","LINC01504",36.2621949129547,0.134630152644992,0.322055460355024,0.418034063128693,0.67592221256243,0.922392077430052
"11297","ZNF436-AS1",10.924457829535,-0.196859899246281,0.471097176239363,-0.41787535390842,0.676038253066353,0.922428397855006
"11298","MAGI2-AS3",538.01308178989,0.0785915065819229,0.188139497510742,0.4177299696

32961,0.676144557769346,0.922428397855006
"11299","AL355377.3",2.51779505335734,0.455195640408969,1.089695284423
22,0.417727457313816,0.676146394829032,0.922428397855006
"11300","HOXC-
AS2",17.5949679899817,0.159919218807447,0.382958891931589,0.4175884727
49224,0.676248026218581,0.922428397855006
"11301","PRR34",3.76829711106571,0.378160749042687,0.90550131569532,0.
417625841606098,0.676220699950725,0.922428397855006
"11302","SPATA7",96.909762750725,-0.0903209887439095,0.216377684406922
,-0.417422845574275,0.676369147514261,0.922511980970831
"11303","DTX3L",109.161486794432,0.0914313600404274,0.219090091491014,
0.417323117710128,0.676442081422315,0.922529831471732
"11304","LINC01006",12.1874992660892,0.238717439508515,0.5723436336621
6,0.417087612176401,0.67661432556389,0.922683105853446
"11305","ZNF710-
AS1",5.19016426519832,0.302114328749293,0.72475423771123,0.41685072405
1464,0.676787597974669,0.922837755221541
"11306","NUDCD1",56.578782387251,-0.0986527070298129,0.236805127836459
,-0.416598694171622,0.676971964646226,0.923007503539853
"11307","MRFAP1",1485.00366567958,0.0876241170824155,0.210539121474213
,0.41618924059749,0.677271532275202,0.923170985058116
"11308","PINX1",52.0921788705323,-0.113754197737039,0.273277640255791,
-0.41625870902048,0.677220703646526,0.923170985058116
"11309","DPM1",206.589243376909,0.0943560335854757,0.226639642958159,0
.41632625410946,0.677171283691434,0.923170985058116
"11310","KAT7",87.1815791399142,-0.0945960170449805,0.227371943102395,
-0.416040852509141,0.677380109735812,0.923237346735415
"11311","CDC42EP5",226.695379441603,-0.100437055197125,0.2415737856239
99,-0.41576139951481,0.677584607260685,0.923434419673191
"11312","TMEM150C",71.2444821814593,-0.167708805797218,0.4038363193619
45,-0.415289060830872,0.677930308285088,0.923578939656618
"11313","ELMOD2",84.4655482363223,0.0884751387409233,0.213029874310959
,0.415317987803797,0.677909134907514,0.923578939656618
"11314","POLE3",183.448501961418,-0.0901426494387262,0.217021538492472
,-0.415362687339225,0.677876417150524,0.923578939656618
"11315","PWWP3A",277.840346781103,0.089257347085928,0.214842659244984,
0.415454488413069,0.677809225398999,0.923578939656618
"11316","Z92544.2",2.40921204227671,-0.409114824597855,0.9858455567596
06,-0.414988759438732,0.678150131817198,0.923796772884597
"11317","KRBOX1",23.366231023718,-0.178990106838045,0.431526529694937,
-0.414783552159772,0.678300361293928,0.923826639384639
"11318","AC127070.2",3.1585501000706,0.353161532944638,0.8515801019342
91,0.414713227965827,0.678351847628591,0.923826639384639
"11319","NSMCE3",186.758227129136,-0.0960462466924497,0.23154948907888
6,-0.414797921060098,0.678289841599699,0.923826639384639
"11320","C2orf92",2.60526291826002,0.360127011786417,0.869153511212089
,0.414342238903457,0.678623484081582,0.923909079823062
"11321","GET4",3.42469854194282,-0.323095325644087,0.779895156510234,-
0.414280461863398,0.678668720997396,0.923909079823062
"11322","AP3B2",2.88894585873333,0.367804539487856,0.887915943862028,0

.414233511663362,0.678703101571136,0.923909079823062
"11323","GPT2",4.69181152014748,0.307490511950106,0.742355615055917,0.414209181844667,0.678720918012268,0.923909079823062
"11324","IRF8",31.4357932075357,-0.19118095605546,0.461634275121073,-0.414139431057885,0.678771996691286,0.923909079823062
"11325","DNAJC5",50.0977506001216,0.0975733569589472,0.23541369209903,0.414476133860139,0.678525442004251,0.923909079823062
"11326","EFNA4",55.8338877283554,0.150717461675535,0.364096984301479,0.413948668003078,0.678911700498099,0.924017646404574
"11327","PAMR1",449.012215387913,0.125172230866762,0.302493452366392,0.413801455494475,0.679019517950647,0.924050336652451
"11328","AL008721.2",3.69747387403217,0.346259694666482,0.836877029502354,0.413752179185016,0.679055609056047,0.924050336652451
"11329","PMVK",321.039157756615,-0.109167442021412,0.264393290957726,-0.412897928029749,0.679681399189956,0.924204030486805
"11330","CCDC138",17.7103929816587,0.179499747428386,0.434433447317338,0.413181233021562,0.679473836830097,0.924204030486805
"11331","HBEGF",30.3209470320913,0.19263600195518,0.466519190468961,0.41292192452263,0.679663817306307,0.924204030486805
"11332","SMIM29",95.0024233059953,0.101644168823806,0.245933518133451,0.413299372916917,0.679387289247634,0.924204030486805
"11333","BPGM",83.1809373697127,-0.0906498845259801,0.219456483109516,-0.413065420722808,0.679558683349619,0.924204030486805
"11334","AP006621.3",44.7263091990981,0.113630594716763,0.27525765831501,0.412815379642307,0.679741882531133,0.924204030486805
"11335","CSRNP2",50.0597461804549,0.11216326968934,0.271726778501976,0.412779595399812,0.679768102345728,0.924204030486805
"11336","TXNRD1",289.74988775421,0.0887570615996985,0.214730732676915,0.4133412134035,0.679356638524229,0.924204030486805
"11337","ZNF433",15.1667832556501,0.163164259181364,0.394812612657782,0.413270128537641,0.679408712864351,0.924204030486805
"11338","ZNF275",64.9849806943496,0.111657946799329,0.270351027846197,0.413010994220638,0.679598558748898,0.924204030486805
"11339","TMEM14A",209.2856258046,-0.0905145669201163,0.219631052725107,-0.412120990165291,0.680250744418227,0.924277819947989
"11340","AL359546.1",2.3971564021885,0.444239166850054,1.07786763602101,0.412146308140374,0.680232188362389,0.924277819947989
"11341","MRGPRF-AS1",2.92057018645809,-0.346544356892576,0.840457047691634,-0.41232845609943,0.6800986941485,0.924277819947989
"11342","PFKM",79.0257938618736,-0.0888082713424926,0.215527384608771,-0.412050986020635,0.680302052879006,0.924277819947989
"11343","EMC7",584.438733854267,-0.0981077285661216,0.238029443251994,-0.412166357345373,0.680217494032051,0.924277819947989
"11344","AC080038.1",38.8620380541502,-0.115914285935596,0.280989970701654,-0.412521079119476,0.67995753378606,0.924277819947989
"11345","HEXD",83.6636248231975,0.0820399772794751,0.198925397278907,0.412415802113239,0.68003468278249,0.924277819947989
"11346","CR381653.1",3.17480457334893,0.370930660153159,0.899528764175539,0.412361088300645,0.680074779429911,0.924277819947989

"11347","IARS",125.000739421154,-0.0911104439406001,0.221430903518817,
-0.411462187493887,0.68073366225514,0.924675844510996
"11348","TMEM260",75.4175657684145,0.118707742367139,0.288547248365741
,0.411397935830163,0.680780767244064,0.924675844510996
"11349","CILP",3788.72034403592,-0.119377926620702,0.290200467815802,-
0.411363660159481,0.680805896364735,0.924675844510996
"11350","AP005329.3",19.443798445248,-0.153130897264906,0.372287753625
504,-0.411324024960932,0.680834955251366,0.924675844510996
"11351","DR1",185.685311901456,0.0629189865015427,0.153447433434241,0.
410036095706392,0.681779469254186,0.924701311224567
"11352","ACBD6",278.205571965314,-0.076044565630545,0.185532961169669,
-0.409870920784812,0.681900637828248,0.924701311224567
"11353","ASB1",87.7836879983432,0.0907305068053584,0.220731381141686,0
.411044892375856,0.681039617120686,0.924701311224567
"11354","ARF4-
AS1",10.6826351983621,0.211457021303605,0.515120619618733,0.4105000134
92209,0.68143919359468,0.924701311224567
"11355","TMEM44-
AS1",56.2662833449063,0.118217571368771,0.287921026546872,0.4105902677
08446,0.681373001217746,0.924701311224567
"11356","MFSD8",60.2257467182141,0.114650862473907,0.279302394964413,0
.410490080074378,0.681446478905955,0.924701311224567
"11357","HCG18",139.902132297321,0.110292081821238,0.26911931811092,0.
409825955993914,0.681933624399111,0.924701311224567
"11358","ANKRD6",37.9438438824328,0.113796121311972,0.277383352664674,
0.410248561129544,0.681623621508486,0.924701311224567
"11359","C9orf170",2.70383475362959,0.411957589787735,1.00540140869011
,0.409744392863399,0.681993461389043,0.924701311224567
"11360","TOR1B",58.5524966756852,0.0888738208956195,0.216302348375058,
0.410877743876902,0.681162182754958,0.924701311224567
"11361","EXOC6",21.4993844046418,0.175194149721734,0.426895803012309,0
.410390892778777,0.681519225921961,0.924701311224567
"11362","C11orf95",113.355072794782,0.0840875396392018,0.2047004693536
65,0.410783326021212,0.681231420626348,0.924701311224567
"11363","AHNAK2",846.068545403276,-0.104633411200705,0.255322259776436
,-0.409809200703158,0.681945916385021,0.924701311224567
"11364","RRAD",33.8080594546872,-0.373350270626563,0.909771274496063,-
0.41037816986843,0.681528557510373,0.924701311224567
"11365","WDR45B",241.951411664226,0.0967993884976552,0.235813651366363
,0.410491029407227,0.681445782650328,0.924701311224567
"11366","CCDC102B",80.2850964785421,-0.151780187651794,0.3701741562642
93,-0.410023728245977,0.681788541458303,0.924701311224567
"11367","LINC00909",66.5004357625143,-0.113390435591662,0.275753966001
962,-0.411201467872471,0.680924812031701,0.924701311224567
"11368","DUS3L",50.7939488172337,-0.11037642243697,0.269065646379081,-
0.410221163208117,0.681643717683045,0.924701311224567
"11369","PRKD2",53.0843154989871,-0.100738188593181,0.245322536105069,
-0.41063568880617,0.681339690345318,0.924701311224567
"11370","PMF1",286.484558204781,0.120534413530509,0.29443978815308,0.4
09368632841981,0.682269155256037,0.924912411245433

"11371","LSS",83.7031447294198,0.107239649755292,0.261929154490179,0.409422349199821,0.682229741145659,0.924912411245433
"11372","TIGD6",25.7831288074528,-0.12499047063469,0.305584746030946,-0.409020647326528,0.682524508887133,0.925088056929327
"11373","C6orf163",4.13168903118689,0.32971008481342,0.806214528941561,0.408960733126802,0.682568477915667,0.925088056929327
"11374","SLC35B4",98.2096976176985,0.0873309528103069,0.213593638497864,0.408864952273286,0.682638770520344,0.925088056929327
"11375","AL512506.1",2.5114712988343,-0.359540549884669,0.879277761477951,-0.408904404997493,0.682609816229559,0.925088056929327
"11376","MYO9B",128.183006895088,-0.100855916300785,0.246774344032636,-0.408696927940964,0.682762088537255,0.925173839205501
"11377","AC009228.1",2.72001642988224,-0.35215494226807,0.861830247284057,-0.408612883311811,0.682823774548825,0.925176099557892
"11378","TMEM154",11.7361547091196,0.193989875136405,0.47496585042139,0.408429100669653,0.682958672383594,0.925277547441826
"11379","ZNF768",51.8990760684411,-0.0940650376729925,0.230498010772352,-0.408094791611431,0.683204083694834,0.925528688826422
"11380","LBR",91.6366480922832,-0.0846569712763569,0.207629403997415,-0.407731128859817,0.683471081138163,0.925583119624633
"11381","AL022069.3",12.1157480622936,-0.210560384979891,0.516333006789744,-0.407799583236082,0.683420819614974,0.925583119624633
"11382","ZFAND4",13.508511948557,0.171099018773576,0.419557974696617,0.407807809867749,0.683414779437614,0.925583119624633
"11383","AL162458.1",13.402161356269,0.175871739624882,0.431361688288169,0.407712934180171,0.683484440524632,0.925583119624633
"11384","ZC3H8",73.5393358918222,-0.0863393872974846,0.211831302954838,-0.407585593314752,0.683577942929489,0.925594315350883
"11385","ACAA2",581.380592511822,-0.092474896061073,0.226911030316667,-0.407538125987173,0.683612797941603,0.925594315350883
"11386","WASL",395.900888865061,0.0700167590520877,0.171841680090407,0.407449223117764,0.683678080670876,0.925601406423815
"11387","CNTLN",237.807041750121,-0.0820353651959389,0.201540563346861,-0.407041460208446,0.683977537468348,0.925681627750182
"11388","AC023509.2",4.2662424384426,0.342375720343035,0.840714187763537,0.40724389492441,0.683828865323674,0.925681627750182
"11389","AL163051.1",3.781451967462,-0.370181694821919,0.909169458553317,-0.407164683480412,0.683887038348939,0.925681627750182
"11390","MPV17L",27.433236902411,-0.145057424941213,0.35631012727022,-0.407110025338132,0.683927180479799,0.925681627750182
"11391","C16orf87",86.2887709161469,-0.0942351670841493,0.231783204994332,-0.406565985169001,0.684326784113568,0.925991693917719
"11392","ARHGAP8",4.2288998518793,-0.329555811485623,0.810455739266218,-0.40663023965257,0.684279583865927,0.925991693917719
"11393","YAF2",128.84078369804,-0.0828712774657428,0.203901004715624,-0.40642898048159,0.684427429537395,0.926046592321509
"11394","RTF1",604.36446729292,-0.0826697649988905,0.203594988924213,-0.40605009698772,0.684705791451059,0.926260620905491
"11395","CHMP6",117.590658029906,-0.0977204227173643,0.240649930412494,-0.406068776125816,0.684692067071165,0.926260620905491

"11396","ZNF662",45.4140260378622,0.104390133585074,0.257257924993024,
0.405780049683232,0.684904218648017,0.926447747495541
"11397","ITM2A",4429.07291224777,-0.119013274735165,0.293451070227454,
-0.405564289279701,0.685062772174099,0.926580910157387
"11398","AC004837.4",4.12991954175794,-0.32359289120417,0.798208567583
844,-0.405398919963584,0.685184304736812,0.926663981182484
"11399","GUF1",49.4251512623744,0.114884722970595,0.283645258363047,0.
405029590953183,0.685455759964914,0.926929841481271
"11400","PPID",135.118547807025,-0.0836250924900996,0.206539826029692,
-0.404886041097361,0.685561279450404,0.926929841481271
"11401","MED7",76.4579359494825,-0.11308975720098,0.279287657888763,-0
.404922143913793,0.685534740702102,0.926929841481271
"11402","NAA35",82.5572616634656,0.102918418279692,0.254283749511724,0
.404738480053548,0.685669753840589,0.926995198689061
"11403","SNHG3",104.191144795052,-0.0991797770740188,0.245322674445664
,-0.404282960383207,0.686004654208567,0.927122720026745
"11404","AC036214.2",3.23808416599584,0.370944860755313,0.916996930262
193,0.404521376804665,0.685829361551513,0.927122720026745
"11405","TM2D3",287.285015949687,0.0794546055963209,0.196456406432279,
0.404438862744392,0.685890027057724,0.927122720026745
"11406","SSR4",1556.06391585422,0.10042853452734,0.248410213605331,0.4
04285045569418,0.686003121028535,0.927122720026745
"11407","XRRA1",156.309353031159,-0.141516952421945,0.350155062492083,
-0.404155094645088,0.686098672836126,0.927168496692284
"11408","HOXD-
AS2",139.971352910421,-0.153896991713466,0.381122301602502,-0.40379949
1833401,0.686360170206287,0.927265591605151
"11409","FKBP7",281.319444169855,-0.104081206443363,0.257779546150509,
-0.40376053103373,0.686388822843908,0.927265591605151
"11410","AC019131.2",6.75993841540702,0.29503042737529,0.7304395450854
82,0.403908070640894,0.68628032131782,0.927265591605151
"11411","TMEM87A",422.014428183661,-0.0560249772730311,0.1387683621289
1,-0.403730190466514,0.686411136283255,0.927265591605151
"11412","CCDC85A",30.9284426923226,0.213974031192267,0.530223096487301
,0.4035547161371,0.68654019116997,0.927277407069578
"11413","OGDH",185.526449949068,0.0846646214099313,0.209783504095288,0
.40358092870579,0.686520912213352,0.927277407069578
"11414","AL162258.2",3.8108072287486,0.379002809873346,0.9395254536919
04,0.403398128687242,0.686655363171188,0.927351710468185
"11415","TNC",77.0932570280383,0.442236541107759,1.09661459311867,0.40
3274353526592,0.686746406477267,0.927393417069388
"11416","DHX37",28.8458340177461,0.123717952810571,0.307115067945441,0
.402839084510661,0.687066606396633,0.927744546041003
"11417","FZR1",75.0343504582578,0.0927575550246073,0.230358952493792,0
.402665292668003,0.687194469765118,0.927835924623745
"11418","MIR34AHG",10.7302469226663,0.279150137633008,0.69371933401811
1,0.402396363982153,0.687392345522752,0.927836865346859
"11419","CCDC66",226.934083497826,-0.0893343586509741,0.22223564611919
1,-0.401980331287905,0.687698501516926,0.927836865346859
"11420","MAML3",31.2392460140353,-0.147650936123721,0.367063148618663,

-0.402249413157827,0.6875004799117,0.927836865346859
"11421","ABHD16A",146.49618610527,-0.0801208773387556,0.199337924152652,-0.401934943786207,0.687731905009087,0.927836865346859
"11422","DPH6",67.4814988015743,0.0830389999427387,0.206552631625589,0.402023442108745,0.687666774140495,0.927836865346859
"11423","VPS35",423.152707150697,0.0585356083695147,0.145444909656471,0.402458968882247,0.68734627938297,0.927836865346859
"11424","RSAD1",106.973326247122,-0.0858296938984916,0.213534803202175,-0.401947095327724,0.687722961872366,0.927836865346859
"11425","ZNF57",9.63726243811543,-0.210055687396559,0.522548629168751,-0.401983041713663,0.687696506764914,0.927836865346859
"11426","ZFP92",2.61909279033059,-0.381788033513338,0.949891175159778,-0.401928182403757,0.68773688118412,0.927836865346859
"11427","TRAF3",53.9244273520798,-0.123827499316255,0.308214920755261,-0.401756991559083,0.687862877025172,0.927844439039467
"11428","AC018521.5",66.7883551898978,-0.122091582501253,0.303887046576159,-0.401766326919286,0.687856006010479,0.927844439039467
"11429","IFI6",876.798094229187,0.137658804915544,0.343008089209768,0.401328158856796,0.688178534376043,0.927987019162665
"11430","SUMF2",389.086052323631,-0.0755783213178779,0.188317743276933,-0.401334043211932,0.688174202622315,0.927987019162665
"11431","MYC",2418.56531729315,-0.143444821590837,0.357377802383536,-0.401381453000521,0.688139302394948,0.927987019162665
"11432","INTS13",43.5020764730363,-0.102242901572175,0.254883315874953,-0.401136108972845,0.688319917045777,0.927987019162665
"11433","RAP2A",143.098798593538,-0.0749161041310653,0.186766049875972,-0.401122710368484,0.688329781194026,0.927987019162665
"11434","SUSD6",55.8993110653496,-0.122927239813539,0.306419177119987,-0.401173454510659,0.688292423273873,0.927987019162665
"11435","SUPT4H1",374.144630245939,-0.0919877997031906,0.229393912775838,-0.40100366478809,0.688417425728655,0.928024015531895
"11436","PHF23",59.1028153422713,-0.11764573949177,0.293492104204605,-0.400848056238522,0.688531995206198,0.928097298540009
"11437","ACADM",180.481256230784,-0.0836283250818789,0.209297319499247,-0.399567109994353,0.689475385301827,0.92830843833044
"11438","PEX19",123.165544471213,0.0976934414932496,0.244536971322618,0.399503768141311,0.689522047793222,0.92830843833044
"11439","RNF149",239.37631631568,0.0719529490664746,0.179980474654174,0.399781971931841,0.68931711023525б,0.92830843833044
"11440","SLC11A1",10.4349538125425,-0.18466502995,0.461035874075221,-0.400543732785251,0.688756079248642,0.92830843833044
"11441","ARAP2",6.82008353050988,-0.27348483241296,0.684499649599318,-0.399539769776432,0.689495526068902,0.92830843833044
"11442","TXNDC5",482.155944625626,0.0824841992672318,0.206063733905129,0.400284891009533,0.688946695033579,0.92830843833044
"11443","GNRH1",9.51487143556879,0.198348305545847,0.495402127246588,0.400378388862102,0.688877839237559,0.92830843833044
"11444","DOCK8-
AS1",6.53691621188708,-0.287099037199232,0.717817284413348,-0.399961164816295,0.689185120678615,0.92830843833044

"11445","ALKBH8",32.0370935213068,-0.112658310428029,0.281784708433204
,-0.399802782253298,0.689301781322581,0.92830843833044
"11446","SMARCD1",114.336688097903,-0.0819089988257354,0.2049623144635
4,-0.399629556487594,0.689429383557534,0.92830843833044
"11447","ZC3H14",191.244028011168,-0.0657114191390983,0.16413180900203
2,-0.40035761220596,0.688893139828251,0.92830843833044
"11448","TIPIN",32.9526639592194,-0.123208997495441,0.307937951972465,
-0.400109816624546,0.689075634188519,0.92830843833044
"11449","IFT140",38.4041803851334,0.104853963461707,0.2622511803582,0.
399822655968565,0.689287142434425,0.92830843833044
"11450","AC008760.2",53.8756702912027,0.191517873189134,0.479405175286
691,0.399490625178594,0.689531730060561,0.92830843833044
"11451","DHDDS",73.5445689702067,-0.101600525023181,0.257070490760063,
-0.395224378818379,0.69267730248522,0.928603479093633
"11452","SRSF4",447.262199415737,-0.0751297558079776,0.190346344739534
,-0.39470028127298,0.693064095165245,0.928603479093633
"11453","NFYC-
AS1",2.74962469328653,-0.345135413341524,0.874264576105572,-0.39477227
2346818,0.693010959815876,0.928603479093633
"11454","GSTM4",81.0446706607427,0.0948397077255507,0.238583005454312,
0.397512419398674,0.690989630055545,0.928603479093633
"11455","RRP15",140.443435054333,-0.103227675103741,0.259531336826072,
-0.397746477809423,0.690817073548651,0.928603479093633
"11456","JMJD4",56.0444779713389,0.121113218529627,0.305714566987456,0
.396164369016138,0.691983774879856,0.928603479093633
"11457","CEBPZOS",201.83196433616,0.0743726015208766,0.187822040438128
,0.395973770423266,0.692124378243228,0.928603479093633
"11458","OGG1",82.0152078018603,0.0829273057740849,0.209528470186427,0
.395780610149546,0.692266882169087,0.928603479093633
"11459","RAB5A",289.0825224679,-0.06472374812347,0.162491871966506,-0.
398319911883416,0.690394384668766,0.928603479093633
"11460","RPP14",77.6590587474737,0.0996213353042353,0.25193257685034,0
.395428556916699,0.692526637341698,0.928603479093633
"11461","U2SURP",526.838349312137,0.056611654073036,0.14278720727325,0
.396475672814995,0.691754150889858,0.928603479093633
"11462","AC069224.1",6.9789152520631,0.26674940574769,0.67469740917136
3,0.395361538552965,0.692576089548931,0.928603479093633
"11463","ZNF639",103.46685574836,0.0800285366666471,0.2026593632085,0.
394891878665937,0.692922683827484,0.928603479093633
"11464","C6",13.536058633278,0.214339928985532,0.540410181444749,0.396
624520308831,0.691644367975776,0.928603479093633
"11465","AC109454.2",5.8667104294049,0.283053464393512,0.7127881439819
35,0.397107424952744,0.691288244843367,0.928603479093633
"11466","LRRTM2",4.04007669743023,-0.329852902514127,0.828248557345159
,-0.398253518933286,0.690443319203131,0.928603479093633
"11467","LINC00847",42.9319456920326,0.112423329153455,0.2822983858026
22,0.398242904697511,0.690451142483304,0.928603479093633
"11468","TRIM38",275.075593260219,-0.0859489354047607,0.21730425528849
4,-0.395523480617784,0.692456596296599,0.928603479093633
"11469","HIST1H2BN",4.66241601220888,0.311467985026747,0.7861933726514

86,0.396172234289259,0.691977972946219,0.928603479093633
"11470","CDC40",286.669666744807,0.0602637046100278,0.152508072669487,
0.3951509159822,0.69273151445111,0.928603479093633
"11471","AC104248.1",8.90444082580836,0.267144633078585,0.672009647256
431,0.39753094939818,0.690975968470046,0.928603479093633
"11472","SHB",12.0092256830986,-0.193168701527678,0.487073774838322,-0
.396590232335539,0.691669656598545,0.928603479093633
"11473","ARRDC1",56.5722328410182,0.12004984992979,0.302151112224016,0
.397317253099449,0.691133526136878,0.928603479093633
"11474","AL022344.1",6.68324237381166,-0.219774917449698,0.55627426219
0804,-0.39508374265627,0.692781086444675,0.928603479093633
"11475","NT5C2",83.4670020518089,0.0872758543908596,0.221076525648702,
0.394776669005301,0.693007714768263,0.928603479093633
"11476","VTI1A",75.796861002422,-0.0928015577610103,0.234727460981335,
-0.395358759358751,0.692578140317958,0.928603479093633
"11477","UROS",223.830634694324,-0.0948161969907344,0.239746473378411,
-0.395485262638581,0.692484795757421,0.928603479093633
"11478","PIK3C2A",158.293048009808,0.078504853208712,0.198635230742859
,0.395221194725217,0.692679652156996,0.928603479093633
"11479","SLC39A13",213.090226538178,-0.0824785284256967,0.206808663250
423,-0.3988156353287,0.690029055527223,0.928603479093633
"11480","SSH3",60.8535969223892,-0.109221439985938,0.274070402573815,-
0.398515997934222,0.690249868151087,0.928603479093633
"11481","TSFM",97.609495229674,-0.0980550619297433,0.24725713737277,-0
.39657120911302,0.691683687062804,0.928603479093633
"11482","PHLDA1",289.008793471612,-0.183416219082686,0.463458281689275
,-0.39575561885343,0.692285320285313,0.928603479093633
"11483","AC024941.2",6.13962706711356,0.286365716810076,0.721209180330
921,0.397063327284158,0.691320762302977,0.928603479093633
"11484","CHPT1",598.338275040096,-0.115915208408919,0.293160925513475,
-0.395397879870558,0.692549273476918,0.928603479093633
"11485","ENOX1",5.24807665111571,0.256156517127754,0.648977359634699,0
.394707940615897,0.693058441866753,0.928603479093633
"11486","HSP90AA1",4728.65573896535,0.0772475769683816,0.1953395656148
73,0.395452793832259,0.692508753477037,0.928603479093633
"11487","ATP5MPL",975.063554321845,-0.0885238813644754,0.2224210765889
95,-0.398001316790925,0.690629215103739,0.928603479093633
"11488","FLYWCH2",243.807335529885,-0.114938658532122,0.28989339868263
,-0.396485946401128,0.691746573367509,0.928603479093633
"11489","ALG1",47.9630946816762,-0.12155564566586,0.307010602073956,-0
.395933055225819,0.692154414959802,0.928603479093633
"11490","USP7",225.919931826986,0.0773545863963581,0.194261647444194,0
.398197932603139,0.690484289776786,0.928603479093633
"11491","KNOP1",190.901326535398,-0.103219240475137,0.259457174292841,
-0.39782765983043,0.690757226915386,0.928603479093633
"11492","AC135050.6",19.7734350989998,0.16045474715382,0.4047979112499
82,0.396382349549059,0.691822985048273,0.928603479093633
"11493","ITFG1",285.515236075948,-0.0631691857953091,0.159624288330958
,-0.395736679272374,0.692299293679525,0.928603479093633
"11494","NKIRAS2",104.581423739314,-0.0770117970818217,0.1929880792347

22,-0.39904950288746,0.689856729185453,0.928603479093633
"11495","GPATCH8",272.682178283459,0.0791423086447314,0.19857918921959
7,0.398542812848391,0.690230106286779,0.928603479093633
"11496","DYNLL2",184.685577708887,-0.0713751608980426,0.1806581212776,
-0.3950841533903,0.692780783330709,0.928603479093633
"11497","APBA3",41.5821160091138,0.100060536525592,0.252120093921044,0
.396876484414318,0.691458545868533,0.928603479093633
"11498","PODNL1",6.71146375409273,-0.25190010479024,0.632724212577015,
-0.398119907193498,0.690541800873911,0.928603479093633
"11499","BX322562.1",4.83585753068812,0.29869514767439,0.7522218308357
54,0.397083859348413,0.691305621963016,0.928603479093633
"11500","PCNT",60.8079421037563,0.138885379468985,0.348456702031039,0.
398572846093843,0.690207972850357,0.928603479093633
"11501","MICAL3",40.3277001367347,-0.171441478479283,0.432447194528954
,-0.396444885406244,0.691776859043936,0.928603479093633
"11502","AL022322.2",3.88886921936051,0.335699534423869,0.849010755772
252,0.395400802806696,0.692547116674062,0.928603479093633
"11503","CHKB-
DT",13.0997778164731,0.21273141009208,0.537857630885112,0.395516207034
199,0.692461963139233,0.928603479093633
"11504","UBA1",321.198716947943,0.0667877840702015,0.168004832307127,0
.397534899163541,0.690973056445355,0.928603479093633
"11505","GPR173",16.5882498358999,0.170488644437078,0.429616063024736,
0.396839548402224,0.691485784807177,0.928603479093633
"11506","GPATCH2",166.664177092573,-0.0659920916810397,0.1672362139787
76,-0.394604075941438,0.693135104990657,0.928617907477054
"11507","MTCH1",1162.46839902892,-0.0721782760427969,0.182976225987356
,-0.394468055362474,0.693235507327605,0.928671708130271
"11508","HYI",336.444423779251,-0.105589695337069,0.267831970668609,-0
.394238578290252,0.693404905924196,0.928737216510685
"11509","SOCS1",571.571183691223,0.155726732219271,0.3949892489673,0.3
94255622466735,0.693392323488495,0.928737216510685
"11510","WDR77",44.2376910868752,-0.116409006804183,0.295791773483888,
-0.393550521818431,0.693912916917704,0.928795457495577
"11511","YOD1",17.8323251186202,-0.172704087832718,0.438811998893358,-
0.393571935745288,0.693897104351947,0.928795457495577
"11512","MTMR14",99.8411061212219,0.0781242648573039,0.198723596003237
,0.393130289651316,0.69422325355689,0.928795457495577
"11513","SLC49A4",43.6303950649899,0.105095264550918,0.266977129026036
,0.393648942642831,0.693840241682403,0.928795457495577
"11514","TCIM",60.1064816800784,-0.306495588783256,0.77843927259561,-0
.393730891507161,0.693779731707932,0.928795457495577
"11515","AC009318.3",3.50373986661596,0.310856398402023,0.789224581874
746,0.393875717433441,0.693672798903831,0.928795457495577
"11516","AC011611.4",5.17790560662354,0.280359677133073,0.713167659581
425,0.393118887776856,0.694231674425173,0.928795457495577
"11517","XRCC3",6.56016550633072,0.251691614649752,0.639934784120075,0
.393308225924668,0.69409184339676,0.928795457495577
"11518","CTF1",36.588221567987,0.164981751496904,0.419470437453756,0.3
93309603647794,0.694090825951413,0.928795457495577

"11519","CSNK2A2",283.891226659433,-0.0856060824680052,0.217533931803299,-0.393529789850959,0.693928226034983,0.928795457495577
"11520","DHX58",37.587712994677,0.112751551217705,0.286643942249301,0.393350546091925,0.694060590302611,0.928795457495577
"11521","ZNF257",11.0774437512832,-0.256990842818736,0.652939959534641,-0.393590312655848,0.693883534499336,0.928795457495577
"11522","CBR1",477.817230247814,-0.0891538271021179,0.226739660314585,-0.393199085587512,0.694172445035335,0.928795457495577
"11523","SMG7-AS1",3.2310530831838,-0.375566371128717,0.956095197208031,-0.392812736875405,0.694457796656946,0.928936735115136
"11524","AC022007.1",3.38582185243157,0.32451148046156,0.826062030592677,0.392841540276015,0.694436521383574,0.928936735115136
"11525","SLC1A2",24.274635903504,0.189470348282061,0.482500552810069,0.39268420974565,0.69455273455505,0.928983115242178
"11526","MMP16",50.9421491982775,-0.174966195709829,0.446003963974639,-0.392297400567,0.694838484073946,0.929262416313863
"11527","CCDC88C",4.33903323880354,-0.316168763061391,0.806062876836796,-0.392238337909967,0.694882119549134,0.929262416313863
"11528","GHRL",2.643826856128,-0.338038263131563,0.86211231625655,-0.392104667521031,0.694980878929331,0.929313866125575
"11529","VPS13D",133.80716952272,0.0924943225636425,0.236064172859541,0.39181855274022,0.695192285838195,0.929357054421012
"11530","AC139795.2",2.7427881955704,-0.391767023961598,1.00093076483894,-0.391402720071888,0.695499582048164,0.929357054421012
"11531","MAK",2.67194822421693,-0.376812438449008,0.962044544827881,-0.391678785015535,0.695295567214483,0.929357054421012
"11532","DAXX",176.831651899436,-0.0739719205806703,0.189035195095791,-0.391312953882403,0.695565924936047,0.929357054421012
"11533","TRAM2-AS1",57.849334340842,-0.0963493417017227,0.246048030267835,-0.391587535152555,0.695362999362106,0.929357054421012
"11534","USP45",33.4656884032421,0.120080363414877,0.306748752756981,0.39146161911214,0.695456053200999,0.929357054421012
"11535","ZNF696",25.5928814754704,0.14329859276168,0.36568669398289,0.39186165403214,0.695160437274483,0.929357054421012
"11536","NTN4",194.172839454386,-0.0909745036579923,0.232719668413321,-0.390918843595194,0.695857224919061,0.929357054421012
"11537","AC009133.1",14.281788462949,-0.188718542304537,0.482693256149612,-0.390969917023335,0.69581947232245,0.929357054421012
"11538","AC009107.2",87.8425851379858,-0.119304962800565,0.304814745551393,-0.391401546486042,0.695500449387247,0.929357054421012
"11539","COG1",58.3162710361936,0.107691702250136,0.275287472798613,0.39119724975251,0.695651441039094,0.929357054421012
"11540","SLC25A10",11.2913492794559,0.179905807265151,0.46000998516794,0.391091091641133,0.695729905152433,0.929357054421012
"11541","C19orf47",17.1940319588264,0.156152311490976,0.399367316675581,0.390999225451952,0.695797808378258,0.929357054421012
"11542","LINC01204",2.41398982907076,0.396148278097163,1.01164776915231,0.391587161240032,0.695363275682266,0.929357054421012

"11543","AMIGO1",6.94040390603487,0.283812256115683,0.727263554153408,
0.390246774356874,0.696354077087729,0.92944512539693
"11544","AC021851.1",2.97146680864002,-0.348663198235613,0.89350952862
3114,-0.390217660882586,0.696375603252395,0.92944512539693
"11545","PCDHB4",64.4458228851183,-0.108774126415138,0.278549463867589
,-0.390502013196638,0.696165366927405,0.92944512539693
"11546","ZNRD1",164.077386037765,-0.0798433310815718,0.204633517852609
,-0.390177190518126,0.696405526975968,0.92944512539693
"11547","AC124242.1",6.9184744148723,0.2342169568801,0.600122541285912
,0.390281885393327,0.696328116720605,0.92944512539693
"11548","AL133215.2",2.88939641164436,-0.348290508590055,0.89161447318
9767,-0.390629043227662,0.696071454619321,0.92944512539693
"11549","CEP290",175.346033226798,0.074986557039639,0.192098552761499,
0.390354617261167,0.696274341428121,0.92944512539693
"11550","RFNG",308.752060519752,-0.0800824788626668,0.204971249462033,
-0.390701032817292,0.696018235347592,0.92944512539693
"11551","AC011484.1",12.6724226045762,0.211820434265987,0.543012945967
228,0.390083580583309,0.696474743824386,0.929457031949867
"11552","PPDPF",1379.42801133832,-0.125191951677572,0.321075649866652,
-0.389914189162481,0.696600001274195,0.929543717074249
"11553","YY1AP1",124.111600282398,0.0671445624511368,0.172414953945895
,0.389435840189403,0.696953763803217,0.929675386117392
"11554","CYFIP2",5.40029787513002,0.248876599738968,0.639086859605235,
0.389425312065874,0.696961550608481,0.929675386117392
"11555","SLC44A1",566.720474510147,-0.0744574078475979,0.1912638993453
17,-0.389291487324375,0.69706053277618,0.929675386117392
"11556","TANC2",94.9737341877647,0.158461001670398,0.406996871783486,0
.389342062940218,0.697023124407856,0.929675386117392
"11557","CASTOR1",32.1125355003778,-0.125925787523155,0.32345086723076
2,-0.389319678136199,0.697039681287776,0.929675386117392
"11558","HDAC8",101.027725501095,0.0868644900035925,0.222988371085376,
0.389547174952611,0.696871420397502,0.929675386117392
"11559","Z95118.2",3.18294195620413,0.457541720383865,1.17595244964253
,0.38908182088736,0.69721562088395,0.929801781808642
"11560","EIF2B3",138.830042709122,-0.101838193013246,0.261876934381374
,-0.38887805546455,0.697366356195308,0.929834262176687
"11561","PSMB8-
AS1",45.5440937667164,0.104079785324014,0.267667844498874,0.3888393300
24389,0.697395004660146,0.929834262176687
"11562","SEC61A2",21.2463447850105,-0.138825151209402,0.35705664629333
4,-0.388804276997978,0.697420936703655,0.929834262176687
"11563","RLF",143.429906831208,-0.0848948251672255,0.218586578385769,-
0.388380777054848,0.697734267642565,0.929921193876454
"11564","MOB3B",33.5105710524003,-0.221950965915601,0.571106219682734,
-0.388633424512346,0.697547337527688,0.929921193876454
"11565","HMGA2-
AS1",18.0258086750289,-0.210271549516169,0.541433602554326,-0.38836073
0704871,0.69774910042584,0.929921193876454
"11566","TEX22",2.84988059640013,-0.357239842244939,0.919841612528037,
-0.388371038425977,0.697741473477166,0.929921193876454

"11567","STOML1",92.4002300530688,0.0926878437727046,0.238696424184694
,0.388308472107594,0.697787768379432,0.929921193876454
"11568","SIM1",53.3486241236057,0.211936858719814,0.54597612440145,0.3
88179719309445,0.697883040400571,0.929967761737102
"11569","KAZALD1",170.042291413288,-0.135806686914557,0.35009297479086
2,-0.387916058571825,0.698078153864331,0.930009403426807
"11570","NCOR1",533.10924515942,-0.0575317739004662,0.148318704054227,
-0.387892911196366,0.698095284271916,0.930009403426807
"11571","RBFA",159.325161010918,0.0807542531723358,0.208148949693466,0
.387963779261244,0.698042838256966,0.930009403426807
"11572","NDUFS4",822.23247990987,-0.102587954894716,0.264719831293193,
-0.387534074774676,0.698360863817156,0.930282813320208
"11573","SPCS1",903.827651222335,-0.0961425542977187,0.2484201783644,-
0.387015881442168,0.698744450675977,0.930582511138676
"11574","DTNBP1",147.815831952067,-0.0916719710473318,0.23686979572031
3,-0.387014185445469,0.698745706245057,0.930582511138676
"11575","TBX1",27.0594185419992,-0.26642580535508,0.688464591535928,-0
.386985487170368,0.698766952087588,0.930582511138676
"11576","GUCY1B1",25.6297612864052,0.187347219902301,0.484247556725385
,0.386883149538626,0.698842716373966,0.930603012517682
"11577","SAMD9",116.755513208272,-0.0819321070653956,0.211854219820332
,-0.386738140665218,0.698950076875582,0.930665581328246
"11578","TRIM45",8.84537050402124,-0.19069297059523,0.493212660531589,
-0.3866343787479,0.699026902983827,0.930687485705276
"11579","PHC2",218.314020769364,-0.0702338657119043,0.181694187384586,
-0.386549876597003,0.699089471292516,0.93069040504138
"11580","DEAF1",135.379009911096,-0.0948829654814381,0.245563913414618
,-0.386388065583703,0.69920928745434,0.930769530752042
"11581","VPS37B",49.2204229170288,0.117253870053721,0.303711826403577,
0.386069490418565,0.699445204620777,0.930922796514357
"11582","PRSS36",17.585401505158,-0.200402087245987,0.518989286996501,
-0.386139159838453,0.699393609255168,0.930922796514357
"11583","AL365295.1",33.6672418717282,0.154109642974233,0.399333706016
112,0.385916942778718,0.69955818229259,0.930992780802924
"11584","ACP6",6.02365133947235,0.30946054530102,0.802453107220716,0.3
85643151626432,0.699760970296997,0.931101886674856
"11585","GOSR2",115.898596747575,-0.0822118745160308,0.21314996810774,
-0.385699680116656,0.699719099769034,0.931101886674856
"11586","DNPH1",285.179830812993,0.125922707620842,0.326634884027426,0
.385515184625133,0.69985575854427,0.931147636626956
"11587","PTPRF",32.258322216372,0.226139437180214,0.586880317462031,0.
385324623184086,0.699996920670699,0.931177581910494
"11588","RTN3",793.519219025698,0.0688312250182809,0.178633135763754,0
.385321708226137,0.699999080063497,0.931177581910494
"11589","BRAP",63.1006569877762,0.110681334577991,0.287332152384789,0.
385203443677854,0.700086692159473,0.931186851372857
"11590","CTSA",631.175809890731,0.0866211861181288,0.224902925530147,0
.385149218997232,0.700126863925489,0.931186851372857
"11591","EYA3",63.4351014398638,-0.104035828148081,0.271039361246232,-
0.383840294154054,0.701096821008712,0.931202944014725

"11592","SLC16A4",75.3094752267777,0.104226464649489,0.271390298449572
,0.384046390917159,0.700944064045448,0.931202944014725
"11593","UCK2",20.6646145825699,0.12892415818773,0.335117098629546,0.3
84713757414833,0.70044950132574,0.931202944014725
"11594","MRPL44",105.33397597234,-0.101061520573232,0.262824537984561,
-0.384520872168976,0.700592429015683,0.931202944014725
"11595","DZIP1L",27.0730291121534,-0.169383428673564,0.441165037593466
,-0.38394572153211,0.701018677727323,0.931202944014725
"11596","MED28",236.680073776264,0.0600270557864229,0.155949741973948,
0.384912825289898,0.700302003437502,0.931202944014725
"11597","G3BP1",388.800413725573,0.0604270312316207,0.157243970071998,
0.384288384501819,0.700764716131896,0.931202944014725
"11598","CPSF1",73.7546450760326,-0.0868247330477675,0.225915619673928
,-0.384323727474375,0.700738523905838,0.931202944014725
"11599","PLAA",85.6539917872984,-0.0728196421350136,0.189437713166655,
-0.384398866085084,0.700682840822278,0.931202944014725
"11600","STAM",164.267837947787,0.079455573528168,0.206964661521413,0.
383908890262153,0.701045976882905,0.931202944014725
"11601","ERLIN1",132.217665382568,0.0717523000284592,0.186941882905641
,0.383821425745862,0.701110806697565,0.931202944014725
"11602","CBX5",367.790799805799,-0.092456219794019,0.24074816368348,-0
.384037071682816,0.70095097111248,0.931202944014725
"11603","JPT2",37.616715953258,-0.146952756365198,0.381782524475607,-0
.384912212959573,0.700302457121858,0.931202944014725
"11604","DHRS13",7.54928374724677,-0.197328303647088,0.513150688471418
,-0.384542607230819,0.700576322836031,0.931202944014725
"11605","LINC00672",8.87801454601488,-0.220207263659765,0.572356839596
001,-0.384737716797791,0.700431748197826,0.931202944014725
"11606","ASXL3",8.89700650021723,-0.251604848378266,0.655652802355511,
-0.383747079970291,0.701165914445599,0.931202944014725
"11607","GAMT",179.378009346186,0.122892464947702,0.320224634739244,0.
383769553044449,0.701149256433412,0.931202944014725
"11608","CPSF3",55.2457070386536,-0.0951871600558729,0.248916875530161
,-0.382405410855076,0.702160677531959,0.931606472297451
"11609","HOXD8",100.676235074697,-0.137087040488418,0.357906435894182,
-0.383024798494959,0.70170137710524,0.931606472297451
"11610","SLC35B3",132.615055921603,-0.0751944096387124,0.1966418426417
83,-0.382392722873799,0.702170087309508,0.931606472297451
"11611","TIAM2",28.3977526649007,0.131403104020879,0.343609417208217,0
.382419972911431,0.702149877941857,0.931606472297451
"11612","ZFYVE27",44.4000765156507,-0.122052816277871,0.31897381232849
3,-0.382642121580112,0.7019851343163,0.931606472297451
"11613","CTSF",1039.8854461709,-0.0921508284821367,0.240461379348768,-
0.383225068124058,0.701552892558158,0.931606472297451
"11614","MED17",92.144561283725,0.0723216529515226,0.189088219722538,0
.382475719839369,0.702108535162891,0.931606472297451
"11615","NIPA1",24.7115940948948,0.12937124880578,0.338215230657421,0.
382511599357336,0.702081926826895,0.931606472297451
"11616","PGBD4",9.42309481155771,-0.199479894592509,0.521078044866115,
-0.382821530398125,0.701852096433317,0.931606472297451

"11617","PRKCA",53.6099366676062,-0.150963738752721,0.394821818586254,-0.382359159616051,0.702194979021997,0.931606472297451
"11618","DPP9",80.5151404879198,-0.0934089502767818,0.244276498328714,-0.38239024595434,0.702171924270659,0.931606472297451
"11619","UBE2L3",628.26099429554,-0.0781923306168521,0.204399152400894,-0.382547235144553,0.70205549960367,0.931606472297451
"11620","DFFA",190.743634395409,0.0740255098521708,0.193878748432342,0.381813429531207,0.702599756780445,0.931822700745983
"11621","TSEN2",25.7487645707814,-0.139418132321832,0.365059749484647,-0.381904969032188,0.702531854405786,0.931822700745983
"11622","SLC26A1",3.29451347224074,0.337549808641081,0.883973940692291,0.381854931579461,0.702568971004661,0.931822700745983
"11623","CNPY3",185.2977662433,-0.0811124238983366,0.212313361491968,-0.382041070464636,0.702430901171033,0.931822700745983
"11624","SMC6",131.378087900921,-0.0748331703592454,0.19614349303402,-0.38152257412009,0.702815523902451,0.932028673516542
"11625","GIGYF1",143.442730706928,0.0817742289269669,0.214476994713857,0.381272728275895,0.703000887755252,0.932114113602892
"11626","AC125437.1",2.67335973887828,-0.393661061380613,1.0323728495015,-0.381316751569647,0.702968225024185,0.932114113602892
"11627","AC024560.5",24.3903160334014,-0.155478759376136,0.408268612516134,-0.380824669371299,0.703333352655718,0.932158312679187
"11628","PPP1R10",383.798258564501,-0.0708802799487969,0.186165090330435,-0.380738836819447,0.703397047880915,0.932158312679187
"11629","XPNPEP1",123.956432879483,-0.082176904434845,0.215626849664853,-0.38110701224162,0.703123844358554,0.932158312679187
"11630","GSTZ1",104.185200496221,-0.12942634342633,0.339825093011156,-0.380861643498707,0.703305915276158,0.932158312679187
"11631","LMF1",88.6866737749769,-0.0945819319505847,0.248291852231842,-0.380930469930479,0.703254842295758,0.932158312679187
"11632","LIF",10.4357518437258,-0.229299282125642,0.602179396503299,-0.380782344027584,0.703364761485001,0.932158312679187
"11633","C5orf58",16.3862211594159,-0.197135096549403,0.518012386729238,-0.380560584263488,0.703529333444122,0.93217334322169
"11634","COQ6",62.2088973998878,-0.0917128718390149,0.24095400342472,-0.380623980243052,0.703482284715201,0.93217334322169
"11635","HSPB11",204.192056849534,-0.0936146763644611,0.246606187126186,-0.379612034294011,0.704233426327908,0.932590590740235
"11636","DIPK1A",84.3517200816554,0.0892793241782281,0.235210763130926,0.379571593535167,0.704263450471169,0.932590590740235
"11637","XPR1",64.3290033554968,0.104666151580374,0.275867120805525,0.379407851413216,0.7043850210737,0.932590590740235
"11638","COPS8",341.111324147803,0.0950474929081855,0.250402300462106,0.379579152159464,0.704257838740788,0.932590590740235
"11639","AC069287.2",2.50196179216737,-0.350371942146207,0.922344306137843,-0.379871095657682,0.704041104188425,0.932590590740235
"11640","POU6F1",25.3006562856491,-0.138513374925,0.365124633556938,-0.3793591617625,0.704421172240375,0.932590590740235
"11641","CTDSP2",585.226148334434,-0.0592470664568253,0.15616614965631,-0.379384819227573,0.704402121963054,0.932590590740235

"11642","GAS7",1091.81720811603,0.0992249943443528,0.261098161055948,0
.380029464562529,0.703923543500749,0.932590590740235
"11643","RPL38",6299.3833242631,-0.092123845404196,0.242864892466035,-
0.37932137687245,0.704449227283769,0.932590590740235
"11644","CALM3",832.222532584818,0.0753339507106984,0.198435124789972,
0.379640201251837,0.704212514807237,0.932590590740235
"11645","AC018638.7",6.05221776073796,0.266857644031242,0.703693555307
461,0.379224226253777,0.704521362853539,0.932605994709086
"11646","ZFP91",383.608001711309,0.0751746383589129,0.198340109165042,
0.37901884129931,0.704673872541564,0.932727781661361
"11647","TTC7A",110.381902712312,0.104899280971484,0.276852771990466,0
.378899153572846,0.704762752773731,0.932746157594867
"11648","HACD4",72.0766454303331,-0.139690836966839,0.368735813447776,
-0.378837183350033,0.704808773510542,0.932746157594867
"11649","AL157893.2",7.47402859240736,0.251982637256646,0.665364766293
506,0.378713526807777,0.704900607378726,0.93278760947232
"11650","NCAPD2",34.5943449381206,0.118754969989078,0.313983517027873,
0.378220395494634,0.705266875454282,0.932843290676633
"11651","ZGLP1",3.51354559852833,-0.297451156752302,0.786097758745507,
-0.378389524003973,0.705141249338415,0.932843290676633
"11652","ZNF709",18.7925335762956,-0.153827261287861,0.406575744754394
,-0.378348347811022,0.705171833655678,0.932843290676633
"11653","JAK3",4.91551536253995,-0.274587058339219,0.726039994016097,-
0.378198254369347,0.70528332214447,0.932843290676633
"11654","YWHAH",812.656482889767,-0.0885678873453643,0.234128443273228
,-0.378287601912621,0.70521695457428,0.932843290676633
"11655","FBXO7",263.120836024713,-0.0703832791161269,0.186116420332284
,-0.378168025102071,0.705305777024726,0.932843290676633
"11656","ACOT13",316.611565380687,0.119023578856788,0.314864105284899,
0.378015711727736,0.705418922229048,0.93291289345923
"11657","RNF180",7.24960540683016,0.286995291950748,0.759497996935648,
0.377874982039044,0.70552346834269,0.932933942942836
"11658","C16orf58",122.446853944721,-0.064838719801207,0.1716075686514
06,-0.377831352723823,0.705555881078663,0.932933942942836
"11659","DDX41",103.629503789093,0.0761344098003419,0.201562418325286,
0.377721255941048,0.705637675660216,0.932962069671689
"11660","CDK15",13.5998194479894,-0.22504010082239,0.596169827008221,-
0.377476501874837,0.705819523825742,0.932962438670367
"11661","TCTEX1D2",93.6409910958184,0.125347235169494,0.33201620001361
1,0.377533491330709,0.705777180115057,0.932962438670367
"11662","COX11",255.250919493812,-0.0783571621875437,0.207515777731784
,-0.377596166633755,0.705730612817464,0.932962438670367
"11663","AL139424.2",3.95636925677275,0.348095728242121,0.923714150271
849,0.376843559384336,0.706289867361294,0.933000662332307
"11664","ANP32E",192.868036430815,-0.0610543639625475,0.16189888503929
8,-0.377114171896414,0.706088759765938,0.933000662332307
"11665","TNFSF4",13.9161577175509,0.170092227432499,0.451620711537395,
0.376626277509451,0.706451356763501,0.933000662332307
"11666","SCCPDH",319.002286392062,-0.0799838508006647,0.21280763602920
4,-0.37585047366293,0.707028061654343,0.933000662332307

"11667","AC019080.1",16.131837541863,0.153147357328989,0.407339639431075,0.375969688446937,0.706939430694881,0.933000662332307
"11668","TRAIP",10.8556684428821,-0.182929805572505,0.486691413324537,-0.375864049712591,0.707017968256888,0.933000662332307
"11669","CCDC126",74.7371411008402,-0.0965074356284583,0.25603377092511,-0.376932446371252,0.706223808107988,0.933000662332307
"11670","NIPSNAP3B",11.6613112436105,0.223430239630497,0.592330761850522,0.377205193484246,0.706021121043158,0.933000662332307
"11671","ZSWIM8-AS1",7.12177379197311,0.248090578201546,0.659578911127443,0.376134794512268,0.706816688142742,0.933000662332307
"11672","ZW10",38.3847881855056,0.101404558586867,0.269670991137106,0.376030651866855,0.706894108602956,0.933000662332307
"11673","RNF6",97.5987095516429,-0.0704714974800439,0.187192495468653,-0.376465398912559,0.706570934333996,0.933000662332307
"11674","CHMP4A",344.057906661742,-0.075252786368272,0.200175857234389,-0.375933378819791,0.706966424888351,0.933000662332307
"11675","RAB15",3.86281299900668,0.337037420765756,0.893530769872964,0.377197330108367,0.706026964276317,0.933000662332307
"11676","AC005480.1",3.47936087449253,-0.316353252139254,0.84006144022437,-0.376583469959959,0.706483173978926,0.933000662332307
"11677","CERNA1",2.69700723035025,0.328190624826957,0.871965889470126,0.376380118523203,0.706634324334388,0.933000662332307
"11678","UBE2Q2L",4.56431427407585,0.328837655107101,0.875001754637845,0.375813709360164,0.707055395101084,0.933000662332307
"11679","CBX4",84.785108859457,0.104548036630917,0.277691592832299,0.376489743764244,0.706552838868825,0.933000662332307
"11680","ZNF350",43.5459206960226,0.112309365244565,0.298027481747774,0.376842311943615,0.706290794453155,0.933000662332307
"11681","ZNF337",37.0869968310114,0.126070390082926,0.335164576093209,0.376144733290268,0.706809299735656,0.933000662332307
"11682","ATP6AP1",279.250719777483,0.0801392045984985,0.21324454814122,0.375808925935245,0.707058951499579,0.933000662332307
"11683","VPS41",117.451577845989,-0.0757766780279162,0.201755113560892,-0.375587397466612,0.707223661309708,0.933020905609156
"11684","CHKA",21.3551289214873,-0.129040985391362,0.343610759936495,-0.375544076137812,0.707255872983652,0.933020905609156
"11685","DCAF11",59.2070235587783,-0.0925943722443263,0.246463819253259,-0.375691541764104,0.707146226721661,0.933020905609156
"11686","BBX",546.941856089989,0.0582977818016601,0.155315581046574,0.375350505138171,0.707399809548463,0.933051087891636
"11687","NDST1-AS1",2.74972664324753,0.481484840216855,1.28271601850575,0.375363551456808,0.707390108167961,0.933051087891636
"11688","AL391069.2",2.68779907119123,-0.35431397328538,0.944226361986798,-0.375242619301423,0.70748003641821,0.933077067195988
"11689","TSN",188.566667178113,-0.0697380378497513,0.186097845623165,-0.374738555496048,0.707854915125328,0.933353378134958
"11690","PFN2",163.694974540381,-0.0755482090184698,0.201736355043845,-0.374489808750884,0.708039937367091,0.933353378134958

"11691","RBM28",131.560896374054,-0.098553803815147,0.263128689383677,
-0.374546021743157,0.70799812363844,0.933353378134958
"11692","PPP4R3A",180.036673630608,0.0801184400727294,0.21378168267293
9,0.374767562267256,0.707833340496944,0.933353378134958
"11693","AC027097.1",26.2141089608756,0.141092067610547,0.376775537348
911,0.374472473991562,0.708052831911138,0.933353378134958
"11694","C22orf31",4.85433990883375,0.343818185533488,0.91795901937365
2,0.374546334070648,0.707997891317793,0.933353378134958
"11695","AMY2B",11.5858403418558,-0.181135428160192,0.484375588545643,
-0.373956558595488,0.70843663636059,0.933485990270459
"11696","PGM2",55.4195287769461,-0.101629142627003,0.271635957913282,-
0.374137295399776,0.708302172570055,0.933485990270459
"11697","SURF4",413.174981526987,-0.0639833950398719,0.171013116772331
,-0.374143201688165,0.708297778588411,0.933485990270459
"11698","SLC1A5",148.108462037086,-0.110665201880155,0.295951464277761
,-0.373930239372939,0.708456217980804,0.933485990270459
"11699","TP53INP2",66.9179900323876,-0.183304221301412,0.4902007162843
55,-0.373937073553931,0.708451133300816,0.933485990270459
"11700","FBXO2",2.59908491358242,0.436201689671119,1.17713105398661,0.
370563403449284,0.710962744156342,0.933660998085701
"11701","AHDC1",175.364558286011,0.100385050020798,0.269773060790622,0
.372109245180376,0.709811513161233,0.933660998085701
"11702","CD2",9.52587745881363,0.211831873271643,0.567889873948965,0.3
73015760606219,0.709136713900212,0.933660998085701
"11703","SMG5",47.932600649767,0.0957188869004795,0.256630269913381,0.
372983619324357,0.709160635588476,0.933660998085701
"11704","TBC1D8-
AS1",2.99654423564163,-0.324638005925443,0.872905790837606,-0.37190497
4549353,0.709963601227786,0.933660998085701
"11705","PHOSPHO2",26.3401505390895,0.127091447585367,0.34308090932284
1,0.370441619255979,0.711053468268084,0.933660998085701
"11706","CREB1",245.239457923808,0.0727657146722327,0.195476283201039,
0.372248302866472,0.709707985484845,0.933660998085701
"11707","GASK1A",59.0659556767363,-0.0832642051928861,0.22482919930897
6,-0.370344267776618,0.71112599397729,0.933660998085701
"11708","NAA50",234.837848566034,-0.0541342885121092,0.146313843269519
,-0.369987468734524,0.711391827424337,0.933660998085701
"11709","DHX36",524.344142869588,-0.0588903801110436,0.158823786918288
,-0.370790681003858,0.710793442869741,0.933660998085701
"11710","PARL",206.417074437142,-0.0926052320290046,0.249319420021346,
-0.371432085078155,0.710315731633007,0.933660998085701
"11711","EIF2B5",84.6090079407136,-0.0786406168694505,0.21064928526742
7,-0.373324869199596,0.708906669288979,0.933660998085701
"11712","SLC30A5",118.981633696711,0.0848283212003912,0.22784910663730
9,0.372300433617329,0.709669175810791,0.933660998085701
"11713","AC008393.1",12.5608376475073,0.161871088028827,0.433888311218
495,0.373070866035184,0.709095701427578,0.933660998085701
"11714","OGFRL1",330.067794419188,0.0755808039089703,0.202489557158994
,0.373257786571307,0.708956591227559,0.933660998085701
"11715","POP7",176.604422393103,-0.0835566568068597,0.224833156449207,

-0.371638499082924,0.710162020774868,0.933660998085701
"11716","RINT1",47.3738261588601,-0.0959519568793547,0.258833818829655
,-0.370708732395217,0.710854485550343,0.933660998085701
"11717","FSCN3",2.64941504390268,0.367449918128133,0.993109349873457,0
.369999454918992,0.711382896536975,0.933660998085701
"11718","PTPRN2",85.3299905124277,-0.0968315314214816,0.26056299008548
1,-0.371624271696125,0.710172615142019,0.933660998085701
"11719","AC034111.1",3.28324594709262,-0.346482167124699,0.93243567075
5633,-0.371588280019269,0.710199416451525,0.933660998085701
"11720","SNX30",41.4359566945317,0.139303855648242,0.376569655266987,0
.369928521058012,0.711435749822552,0.933660998085701
"11721","DAB2IP",42.848427346628,-0.138839131500073,0.372725868944005,
-0.372496633768479,0.70952311783614,0.933660998085701
"11722","PNPLA7",24.7217072037063,-0.192772509037872,0.520936488306171
,-0.370049926171756,0.711345290919752,0.933660998085701
"11723","NRP1",234.07915349135,0.105895638234198,0.284417781110664,0.3
72324254203345,0.709651442398288,0.933660998085701
"11724","ANKRD2",8.06423542045294,0.229266132482663,0.614219219977708,
0.373264341176076,0.70895171332874,0.933660998085701
"11725","FAM180B",326.648032022422,-0.122855084830371,0.33081590571708
7,-0.371369945359992,0.710362007688095,0.933660998085701
"11726","UBE2L6",356.289879085624,0.110878613936833,0.297059281957531,
0.373254163970828,0.708959287150926,0.933660998085701
"11727","ZDHHC5",105.276105851271,0.0766351535188594,0.206925526522835
,0.370351376201052,0.711120698196037,0.933660998085701
"11728","DPF2",157.949797092428,0.0739393466128586,0.198683337826506,0
.3721466904156,0.709783634864071,0.933660998085701
"11729","AP000766.1",17.6789871828027,0.145237117396316,0.389561126216
282,0.372822408660152,0.709280623655996,0.933660998085701
"11730","CRTAM",3.12876948723145,0.4383679587635,1.17477052205879,0.37
315199056515,0.70903532565192,0.933660998085701
"11731","GRASP",136.585944352408,0.0974800239730401,0.262722082638508,
0.37103856285719,0.710608809504062,0.933660998085701
"11732","GPN3",127.744084336899,-0.0949655828102021,0.255581205145046,
-0.371567161037166,0.710215142931401,0.933660998085701
"11733","ANKRD9",107.919944123154,-0.115774049099377,0.312205312053529
,-0.370826647176096,0.710766652621458,0.933660998085701
"11734","DNAJA4",44.3013649482215,0.134922180988617,0.362937539708747,
0.371750414952641,0.710078685019931,0.933660998085701
"11735","AC145285.2",2.65453281445256,0.378889384354295,1.016209179559
39,0.372845858879739,0.709263169359069,0.933660998085701
"11736","ZNF720",133.526466353069,0.0618530305534628,0.167187344815826
,0.369962395309287,0.711410509722476,0.933660998085701
"11737","IL34",60.4732573213407,0.142494868170144,0.383237949477282,0.
371818261642668,0.710028166123579,0.933660998085701
"11738","AC009063.3",5.23743423266865,0.328830902843238,0.885486155129
959,0.371356345819972,0.710372135540826,0.933660998085701
"11739","NT5C3B",206.722950656098,0.0900933813217104,0.24311744974344,
0.370575544522967,0.710953699790515,0.933660998085701
"11740","LINC00526",23.3900945192376,-0.166938135647496,0.449255254132

77,-0.371588610509963,0.710199170349013,0.933660998085701
"11741","RAB3A",10.4399433925171,0.204703888099701,0.551312021794573,0
.37130314596328,0.710411755042349,0.933660998085701
"11742","TBC1D17",142.191292068181,0.0654166464302378,0.17662364533149
4,0.370373096464299,0.711104516670876,0.933660998085701
"11743","ZNF548",38.5526160681828,0.0988100251027928,0.266866925314319
,0.370259540354853,0.711189117040035,0.933660998085701
"11744","PANK2",126.518127630001,0.0762785004732672,0.2052367227831,0.
371661072340746,0.710145211802907,0.933660998085701
"11745","MRGBP",70.2379441180062,0.0894190432629389,0.241289761717003,
0.37058780541138,0.710944566211254,0.933660998085701
"11746","SNAP29",196.757994427903,0.0727328955277026,0.19541259916208,
0.372201668876919,0.709742703634397,0.933660998085701
"11747","CAMKK2",256.670489792939,-0.0943689397621757,0.25533693035974
7,-0.36958594132552,0.71169102775029,0.93391650572663
"11748","AHCYL2",29.6297204095578,0.134492032915409,0.363979426142429,
0.36950449189065,0.711751725656913,0.933916653983769
"11749","NEIL1",23.9382574708644,-0.129338792726305,0.350226701413621,
-0.369300205279193,0.711903972559819,0.93395742442635
"11750","EPOR",28.2744656887154,-0.121118108349096,0.32789663450058,-0
.369378931057255,0.711845299924532,0.93395742442635
"11751","ZNF212",25.5953080712038,-0.136161984289858,0.368998641444739
,-0.369004026022272,0.712124723891211,0.934167527766404
"11752","SSBP3",250.40282591326,-0.0720465919520799,0.195680653498677,
-0.3681845428453,0.712735635095115,0.934173949068208
"11753","ATP8A1",18.4623379464771,0.21848251280354,0.592268167238978,0
.368891196401888,0.712208825556967,0.934173949068208
"11754","AC096733.2",12.1535107397725,-0.171412637172551,0.46555605979
3235,-0.368189036673004,0.712732284517594,0.934173949068208
"11755","TAF11",208.608661593333,-0.0731883273436505,0.198727880606174
,-0.368284143726619,0.712661374430812,0.934173949068208
"11756","TGS1",200.151359070413,0.0743829631417809,0.201947198107321,0
.368328770287032,0.712628102536336,0.934173949068208
"11757","TNFSF15",5.28671909661333,-0.43263733439765,1.17437196699669,
-0.368398894520674,0.712575821635166,0.934173949068208
"11758","TMEM233",36.0453677596405,-0.208175783766787,0.56467266669515
8,-0.368666301815476,0.712376469366993,0.934173949068208
"11759","VWA8",33.5554682666664,-0.106188716054187,0.287947485435213,-
0.368778063450319,0.712293156836335,0.934173949068208
"11760","MOCOS",5.16247643908362,-0.298428239262024,0.809729395448888,
-0.368553051104913,0.712460895428429,0.934173949068208
"11761","ACTR5",22.4393168933719,-0.129165131321132,0.350666975634731,
-0.368341304701802,0.712618757443826,0.934173949068208
"11762","LSG1",101.173231537416,-0.0906834877711667,0.246769887841025,
-0.367481983172871,0.713259528540658,0.934385955390823
"11763","HYMAI",460.51117687416,0.094786419389505,0.257713727527719,0.
367797324181383,0.713024364384701,0.934385955390823
"11764","AKNA",70.0182163615469,-0.0969727978467176,0.263845966554619,
-0.367535646320533,0.713219507561906,0.934385955390823
"11765","POLA2",14.6629882006658,0.161324588785446,0.439002479037461,0

.367479903847378,0.713261079278872,0.934385955390823
"11766","MPV17L2",14.1409781335514,-0.164014368206948,0.445962884359471,-0.367775826103822,0.713040395614468,0.934385955390823
"11767","TOP3B",40.4721852881849,0.11127031947897,0.302594804542819,0.36772052199338,0.713081636763595,0.934385955390823
"11768","SLC4A5",7.27543905715192,0.209619380207117,0.57114515094628 5,0.367015950078216,0.713607120544063,0.934443050068068
"11769","RARS",165.365972300303,-0.0689140126393285,0.18797698021822,-0.366608786668065,0.713910853107469,0.934443050068068
"11770","LINC00513",40.0389128668963,-0.185274204720506,0.505218041468225,-0.366721275792283,0.713826934819118,0.934443050068068
"11771","AC087672.2",10.9246558299642,0.205747184352336,0.560526401150684,0.367060648579558,0.713573779470917,0.934443050068068
"11772","PHPT1",1072.11261230483,-0.101123230813055,0.275652993849325,-0.366849746128024,0.71373109858227,0.934443050068068
"11773","GOLGA3",253.483996885175,0.0899321962209404,0.245034925958726,0.367017868449042,0.713605689600244,0.934443050068068
"11774","LRRC57",74.7436752340479,0.0860171225106526,0.234611917375411,0.366635776532244,0.71389071801688,0.934443050068068
"11775","WTIP",188.694029583725,0.0851534615860389,0.232022469006763,0.367005238546776,0.713615110467081,0.934443050068068
"11776","ZNF497",3.58150632784423,0.274979725847705,0.749635855793927,0.366817733866904,0.713754978650372,0.934443050068068
"11777","SMTN",87.5014287056506,0.110017146045544,0.299698389686138,0.367092883484494,0.713549735462364,0.934443050068068
"11778","PPP4R3B",259.273458242002,0.073027958336868,0.199322847653865,0.366380267974523,0.71408134155585,0.934458753255731
"11779","FAM151B",6.7141809795027,0.222881008566038,0.608384467538923,0.366348946197872,0.714104710564548,0.934458753255731
"11780","ZNF563",26.2938541114076,0.1327649876986,0.362269651842293,0.366481119860398,0.7140060983295,0.934458753255731
"11781","KCTD5",41.3965414799818,-0.104477085454276,0.285301333080019,-0.366199079150372,0.714216529271449,0.934525744734691
"11782","HADHB",487.85295037232,-0.0881140659150575,0.240731835497877,-0.366025813465101,0.714345813803992,0.934615576284886
"11783","ZNF165",3.61282495691016,-0.282557106056857,0.772755524982831,-0.365648768493936,0.714627179335462,0.934904351137753
"11784","SLC24A1",10.4472572360961,0.179169426627872,0.490115470483505,0.36556574402991,0.71468914061249,0.934906067764896
"11785","STAG3",5.67973728091436,0.236271591105897,0.646616878376123,0.365396572541157,0.714815399357679,0.934991886389361
"11786","SLC4A1AP",111.892128340448,-0.0751989370954205,0.206257569377458,-0.364587526762734,0.71541932708103,0.935147030183505
"11787","ZNF789",15.5786720657457,0.160684196599313,0.44064204336604,0.364659248971922,0.715365781461562,0.935147030183505
"11788","GZMB",16.7264955245047,0.213949255353557,0.586480801330225,0.364801805733944,0.715259357078134,0.935147030183505
"11789","AL136040.1",4.38221126939836,0.254956510746928,0.699059245041735,0.364713738578346,0.715325102115188,0.935147030183505
"11790","AC016876.1",84.996734322371,-0.115982337090401,0.317729753831

901,-0.36503454804476,0.715085617432906,0.935147030183505
"11791","ARRDC2",50.7802402556846,0.101664473770274,0.278833709930256,
0.364606108048065,0.715405454724282,0.935147030183505
"11792","MAP4K1",2.39744338695323,-0.363702824171985,0.997529778891898
,-0.364603475372939,0.715407420212767,0.935147030183505
"11793","CLEC11A",346.81445874612,0.0961961160720584,0.263479345578587
,0.365099267499761,0.715037307674605,0.935147030183505
"11794","AC112206.2",5.91950860643433,-0.273040842477696,0.74956318976
447,-0.364266610482156,0.71565893055865,0.935380906779853
"11795","SNIP1",97.20930791686,0.0943571146459488,0.259112288754479,0.
364155305406439,0.715742040032197,0.935410220186207
"11796","NPHP1",64.0757232273748,-0.101547117195194,0.279066846540035,
-0.363880978533315,0.715946889294692,0.935598618046598
"11797","GRIP2",6.8180777344451,-0.230310022178226,0.63328640890555,-0
.363674348508836,0.7161012005073,0.935634779705085
"11798","PCDHGA9",6.99436611320681,0.230090034674883,0.632894869330595
,0.363551745834574,0.716192765613181,0.935634779705085
"11799","PTK7",88.3045511933314,-0.103486889754516,0.284744614141056,-
0.363437566911276,0.716278043159242,0.935634779705085
"11800","KMT2E",607.150991116084,0.0747985232824759,0.205700698152622,
0.363627950484535,0.716135852126221,0.935634779705085
"11801","C16orf95",25.6755490521003,0.15017910415009,0.413191053943178
,0.363461654643526,0.716260052305322,0.935634779705085
"11802","TENT5C",18.2688973565888,-0.17236910248062,0.474866564287554,
-0.362984289574539,0.71661662021701,0.935926654266376
"11803","ZNF225",17.5627024335544,0.150084106197327,0.413574943929549,
0.362894581502727,0.716683634574135,0.935926654266376
"11804","PJA1",87.0265387894417,0.10763401108455,0.296551819456181,0.3
62951781182561,0.71664090461341,0.935926654266376
"11805","PPIP5K1",6.69765558083937,-0.235485596147719,0.64944061275826
3,-0.362597582475755,0.716905516449381,0.936137105977739
"11806","FCHO2",196.676848415374,0.0675492475756237,0.186391844587988,
0.362404523250139,0.717049759849267,0.936147644289144
"11807","E2F3",46.4054550303376,0.105892230989016,0.29271541042728,0.3
617583059069,0.717532651860142,0.936147644289144
"11808","LINC00271",18.1201650156055,-0.14508087286768,0.4012064283523
57,-0.36161153614484,0.7176423426899,0.936147644289144
"11809","AC007285.1",4.92083698282612,0.28570078955999,0.7896229325446
94,0.361819265607283,0.717487094322945,0.936147644289144
"11810","TRAF1",10.9131878404474,-0.208088617887635,0.575241733668124,
-0.361741170204609,0.717545458212824,0.936147644289144
"11811","TRAPPC4",219.170766381938,-0.101369943586996,0.27992531344565
,-0.362132107093907,0.717253311617481,0.936147644289144
"11812","FAM71D",3.58509056882862,0.328420049185987,0.906734891540692,
0.362200740536131,0.717202026207811,0.936147644289144
"11813","ABHD17C",9.82456388396168,-0.169388409915882,0.46798089703513
9,-0.361955821250463,0.717385044666416,0.936147644289144
"11814","ZNF174",31.3142240357696,0.107153827985229,0.296313438842226,
0.361623247342095,0.717633589917527,0.936147644289144
"11815","SH2D3A",6.75780260118069,-0.219027404209948,0.604425816867362

,-0.362372681804246,0.717073551023203,0.936147644289144
"11816","DONSON",25.2270504484905,-0.134048336170758,0.370334973503034
,-0.361965101223851,0.717378109814927,0.936147644289144
"11817","FUNDC1",108.214532616368,0.101831372935189,0.281229559333532,
0.362093419968061,0.717282220609804,0.936147644289144
"11818","AC027097.2",23.2070788544929,0.145181570076687,0.401672075062
884,0.361443025517663,0.717768289097006,0.936153496609725
"11819","AC027307.3",7.58994578384612,-0.209916087992746,0.58067759242
7642,-0.361501960347993,0.717724239785237,0.936153496609725
"11820","AC084036.1",9.83114094977197,-0.238431040950385,0.66063036752
7492,-0.360914442735578,0.71816340653307,0.936272742871049
"11821","GLE1",63.4290091131234,0.0836064241645161,0.231641982494109,0
.360929496735949,0.718152152572983,0.936272742871049
"11822","MPHOSPH8",822.846102477517,0.0590554646886086,0.1635594214537
59,0.361064279658782,0.71805139526084,0.936272742871049
"11823","AL356019.2",5.41275769017302,0.222385313547855,0.615726258610
575,0.361175620558528,0.717968165790036,0.936272742871049
"11824","ZNF426-
DT",3.49930802264442,0.281029043683513,0.778142580067875,0.36115366371
4174,0.71798457868993,0.936272742871049
"11825","MALAT1",195116.05869378,-0.0539092260308352,0.149412907524144
,-0.360807020786501,0.718243714027087,0.936282284061087
"11826","TAPBPL",112.558326423205,0.126290257616949,0.350084548608122,
0.360742163911713,0.718292201836291,0.936282284061087
"11827","KRR1",355.159380200657,-0.0608851006100801,0.168938264705672,
-0.360398520229596,0.718549133068475,0.936458816896393
"11828","ZNF341",8.93158350136218,-0.195321652402332,0.541878269736977
,-0.360453008195253,0.718508392060784,0.936458816896393
"11829","TERC",2.82248373339458,-0.326245992207398,0.905973733140386,-
0.360105354353407,0.718768348838107,0.936478611871269
"11830","COL4A1",760.966903474822,0.126764150708676,0.352013933040139,
0.360111174048959,0.718763996915979,0.936478611871269
"11831","NPIPB4",13.8331354908289,0.221281176331704,0.614579059460374,
0.36005323143616,0.718807326348418,0.936478611871269
"11832","ICAM4",22.3234195391166,0.183342500164619,0.50911192963312,0.
360122184323476,0.718755763545061,0.936478611871269
"11833","SNRPD3",577.148016730467,-0.0774304151513561,0.21512547027141
5,-0.359931416087854,0.718898422701363,0.936518142985
"11834","AC016394.2",3.40630516505836,0.284878916056653,0.792274094195
805,0.359571161222706,0.719167853043853,0.936729035835913
"11835","C12orf73",43.6463665611182,0.116402933965898,0.32374395631917
5,0.359552454011336,0.719181844898996,0.936729035835913
"11836","WDR53",18.4873380743597,0.147057143820036,0.409142901642301,0
.359427337562861,0.719275426804324,0.93675417636879
"11837","PAN3",93.8085038311374,0.0842705765820247,0.234499114387527,0
.359364157097675,0.719322684766615,0.93675417636879
"11838","SH3BP5-
AS1",14.0433033904484,0.152201597250583,0.423644406344207,0.3592673359
33903,0.719395107507345,0.936769351429779
"11839","SFT2D1",410.382595069111,0.082982524603527,0.23103432758326,0

.359178332811265,0.719461684534447,0.93677691250093
"11840","TARS2",54.3898916446372,0.0926113978613233,0.257962815865688,0.359010648687998,0.719587123111215,0.936781986551759
"11841","ALG1L",6.35227046193575,0.237397391916972,0.661211007183155,0.359034240715859,0.719569474290928,0.936781986551759
"11842","UBE2D4",147.42708488543,-0.107824772426174,0.300848106487417,-0.358402695915535,0.720041974431815,0.936828757482013
"11843","CPEB3",21.9486673161585,0.154433969090225,0.430641813493798,0.358613502570273,0.719884244228064,0.936828757482013
"11844","GTF2H1",123.473929152789,0.0696196397736089,0.194271269793156,0.358363024279062,0.720071658960664,0.936828757482013
"11845","AC012085.2",2.90006828183148,0.331307088926064,0.92415171655262,0.358498591726848,0.719970221585893,0.936828757482013
"11846","C12orf43",93.4971401623068,-0.0858053236876625,0.239308073405425,-0.358555908568513,0.719927336151052,0.936828757482013
"11847","VAMP2",736.826476057672,0.0934407173763288,0.260779736606018,0.358312799117125,0.720109240830644,0.936828757482013
"11848","AC060780.1",59.6795160359146,0.0975619489244321,0.271870883532568,0.35885398118679,0.719704327365517,0.936828757482013
"11849","TUBGCP6",47.4779082272377,0.113230731166929,0.315871447227601,0.358470929109782,0.719990919541302,0.936828757482013
"11850","RAB10",366.820052132005,-0.0494637455816673,0.138106433587968,-0.358156707813044,0.720226043239714,0.936901641902126
"11851","NME7",102.472124568506,-0.0860932280093898,0.240787025563948,-0.357549281601659,0.720680639623258,0.937136203456226
"11852","AC099661.1",2.54394330968901,0.345170424973254,0.965139290570868,0.357637937182197,0.720614283832486,0.937136203456226
"11853","AL137186.2",26.0005540218161,0.125207252116494,0.35024896158996,0.357480723277847,0.720731954719581,0.937136203456226
"11854","COLCA2",32.3719758180477,0.159373436177144,0.445832050078488,0.357474156802066,0.720736869715214,0.937136203456226
"11855","MT1E",3079.84127297098,-0.2185800151135,0.611724241859438,-0.357317889592032,0.720853838852315,0.937136203456226
"11856","WWOX",67.8644061552614,0.095972852969087,0.268611070128631,0.357292992143355,0.720872475694678,0.937136203456226
"11857","LINC01415",5.14038890633975,0.229443591902274,0.642220686278882,0.357265962938227,0.720892708438789,0.937136203456226
"11858","SF3A1",165.486707950803,0.0612103806152708,0.171308414194988,0.357310999012572,0.720858996739443,0.937136203456226
"11859","THUMPD3",130.432315595775,0.0632595571932216,0.177267284863278,0.356859740036139,0.721196810608727,0.937203117400196
"11860","SNRK",108.615134690989,0.0788248320610621,0.221027713548309,0.356628726758438,0.721369768923342,0.937203117400196
"11861","SPATA5",33.5746431200317,-0.109477269306787,0.306667398863632,-0.356990243216132,0.721099109929017,0.937203117400196
"11862","AL359091.5",9.46765772939268,0.227762086294909,0.638194442169007,0.356885098404842,0.721177825811984,0.937203117400196
"11863","CORO1C",153.348814001721,0.0927361117723523,0.260030219693697,0.356635901325587,0.721364397151662,0.937203117400196
"11864","AC012615.1",11.9942939833707,-0.176558939742188,0.49498979373

7022,-0.356692081283578,0.72132233433567,0.937203117400196
"11865","INE2",4.05731551765951,0.308383516634131,0.86391819930168,0.356959162202397,0.721122378191808,0.937203117400196
"11866","WDCP",17.3542683069889,0.134162033879492,0.376327353537304,0.356503540384271,0.721463501196737,0.93724590181592
"11867","RIN3",41.1515977416509,-0.118275582969018,0.331977302066851,-0.3562761135555,0.721633795876359,0.937388132083431
"11868","GNA11",355.70829450148,0.113903425754742,0.319783547279346,0.356189137070402,0.721698926549074,0.937393743912536
"11869","FTL",35485.6620873256,0.102046128469624,0.286589978284577,0.356070121783165,0.721788052133796,0.937430518463431
"11870","COMMD5",170.361177323153,-0.0947637487216494,0.266269919641493,-0.355893556618185,0.721920281379511,0.937523263476425
"11871","TMEM230",915.048662338982,-0.098201617052402,0.276042674091488,-0.35574795591154,0.722029327665084,0.93758588880105
"11872","MFSD14B",146.558467118306,0.0676011416347985,0.190120958938801,0.355569117745503,0.722163274564088,0.937609830045195
"11873","ERP29",910.900200894006,-0.0865602403885794,0.243447002125354,-0.355560921403372,0.722169413696179,0.937609830045195
"11874","OSBPL10-
AS1",4.94375870864519,0.241687001971149,0.681618697268939,0.354578011048586,0.722905751782523,0.937635481755405
"11875","PCGF3",104.93637339574,0.0914412159432148,0.257444526336767,0.355188036989371,0.722448726306494,0.937635481755405
"11876","SGCB",359.601087487528,0.0667720232667888,0.188078603086734,0.355021901327055,0.722573183717145,0.937635481755405
"11877","UNC5C",3.44772110759208,0.354544949997525,0.998245453142154,0.355168109087332,0.722463654532411,0.937635481755405
"11878","SGK1",288.43891565274,-0.283081902498187,0.798258163215846,-0.354624500622417,0.722870918765894,0.937635481755405
"11879","GPNMB",3925.19926191132,-0.0692467586567591,0.194908497496165,-0.355278294924631,0.722381114348242,0.937635481755405
"11880","CBL",42.8095836909252,-0.140087615575398,0.394731430225448,-0.354893491748018,0.722669384371065,0.937635481755405
"11881","MGST1",3193.71773923014,-0.0937082750596725,0.264294387535588,-0.354560215725559,0.722919085349529,0.937635481755405
"11882","AC004241.1",9.5029489922191,0.195787734669818,0.551628792566329,0.354926605188536,0.722644576342481,0.937635481755405
"11883","NOP9",26.1128078060581,-0.127982158857269,0.360801744565699,-0.354716020043978,0.722802348132562,0.937635481755405
"11884","CINP",185.302463554,-0.0804904139848423,0.22668577532991,-0.355074833732727,0.722533529602751,0.937635481755405
"11885","DUSP18",60.5865889126706,-0.0982740529293377,0.27703737906116,-0.354732106051445,0.722790295973013,0.937635481755405
"11886","GORAB-
AS1",4.18691134987526,0.249048198313017,0.702999159577762,0.354265285982137,0.723140080689521,0.937664703551174
"11887","ERLEC1",537.005484387508,0.0504467791551111,0.142382304443898,0.354305117845515,0.723110232719709,0.937664703551174
"11888","FGF9",5.23888408021379,0.378129509921783,1.06696886978201,0.3

54396009697112,0.723042124572511,0.937664703551174
"11889","TRAPPC6A",151.121389256014,0.103768265679945,0.29296067827012
2,0.354205439080348,0.723184927701583,0.937664703551174
"11890","THORLNC",9.54320408640155,0.19344095653822,0.54656382051723,0
.35392199277874,0.723397344586036,0.937703503766713
"11891","MB21D2",36.1985965719706,0.151964855906381,0.429370553675318,
0.353924726802049,0.723395295585689,0.937703503766713
"11892","SEC31A",929.029392997744,0.0670450912918441,0.189368914957737
,0.354044861622653,0.723305263091949,0.937703503766713
"11893","SLC9A1",58.8913498257315,0.0927563626622585,0.262343014770148
,0.353569020099609,0.723661894874266,0.937809845270013
"11894","CAHM",12.6001743389416,0.182177237288534,0.515030435164842,0.
353721304315202,0.723547754983232,0.937809845270013
"11895","BRD4",481.913333814622,0.0779654264578143,0.220488331361771,0
.35360341282592,0.723636116340046,0.937809845270013
"11896","GJA9",3.77620298578703,0.369591452290549,1.04636266544797,0.3
53215442881192,0.723926931362854,0.937916763065927
"11897","H3F3A",5663.65176724677,-0.0775447522451869,0.219479427480685
,-0.353312167501489,0.723854424663391,0.937916763065927
"11898","MTPAP",68.1625445490658,-0.0953479104737339,0.269911924532284
,-0.35325564307304,0.7238967961985,0.937916763065927
"11899","ELK4",168.308192595682,0.0872165633148941,0.247342129062746,0
.352615074695863,0.724377034681542,0.938189942840395
"11900","FBXO36",27.1254486560044,-0.120595335827633,0.34317104808246,
-0.351414656048301,0.725277288739342,0.938189942840395
"11901","NME5",12.3044043431389,-0.241187077880108,0.6858234439752,-0.
351675172376914,0.725081882186892,0.938189942840395
"11902","GPER1",19.2501705051039,-0.145687999206351,0.413205029017824,
-0.352580411600138,0.724403024936547,0.938189942840395
"11903","PNPLA8",273.833768960874,-0.0588978618454978,0.16751435295533
,-0.351598897684927,0.725139092005483,0.938189942840395
"11904","ZNF746",31.7635538915433,-0.100018275921958,0.28465879961731,
-0.351361967578099,0.725316811171928,0.938189942840395
"11905","AL391834.2",11.4056040955941,0.173670346737101,0.494148447831
88,0.351453793893506,0.725247931312832,0.938189942840395
"11906","CD59",2417.41638387996,0.0799658342667041,0.226936425644695,0
.352371083837829,0.724559984832616,0.938189942840395
"11907","DPAGT1",65.2900662818762,-0.0916617111104796,0.26088707607870
4,-0.35134630848033,0.725328557443268,0.938189942840395
"11908","ATXN7L3B",328.055489829346,-0.0597701072207332,0.170054385043
78,-0.351476424470593,0.725230956226723,0.938189942840395
"11909","ZC3H13",474.177251553204,-0.0754126934473313,0.21404601089863
1,-0.352320013490211,0.724598280585595,0.938189942840395
"11910","LINC01588",7.48053261507868,0.19271407005194,0.54783785972880
8,0.351772092106482,0.725009189775546,0.938189942840395
"11911","IGHG3",75.7038756258072,0.474991978607826,1.3478279195003,0.3
52412924332303,0.724528610716579,0.938189942840395
"11912","GLIS2-
AS1",3.54454735842629,0.283516916338247,0.805871265515443,0.3518141525
45701,0.72497764408275,0.938189942840395

"11913","RARA-
AS1",70.3394497550721,-0.106044451502161,0.301047989838341,-0.352250986824745,0.724650042208097,0.938189942840395
"11914","NT5C",438.572987646849,-0.0917518647071531,0.261230230478989,-0.351229888435645,0.725415888985707,0.938189942840395
"11915","CAPNS1",818.654574626171,-0.071439343127682,0.203387848864826,-0.351246859271134,0.725403158230179,0.938189942840395
"11916","ZNF765",49.7736065009607,0.0993407620448618,0.281746096595774,0.352589665820243,0.72439608612705,0.938189942840395
"11917","NECAB3",79.2160303949336,-0.128727840546595,0.36607669418567,-0.351641725876452,0.725106968539464,0.938189942840395
"11918","TAF4",33.7356620758793,0.115996789105946,0.32878094558541,0.352808733789021,0.724231835689019,0.938189942840395
"11919","PLS3",705.83507574604,0.0677886541860658,0.192855516725686,0.351499689181757,0.725213505620467,0.938189942840395
"11920","LRRC37B",19.5490202992869,0.133670438441014,0.38071970241946,0.351099345769456,0.725513818744031,0.938237878853963
"11921","LONP1",87.5167393727208,-0.0695808033386234,0.198232529774563,-0.351005979784212,0.725583862263547,0.938249747235347
"11922","ABCA3",17.8470705189015,-0.136518723995749,0.389114361809326,-0.350844732024171,0.725704836355679,0.93832746623241
"11923","CYBA",1364.58899794486,0.0976475816048797,0.278462789287583,0.350666535570877,0.725838533951327,0.938421622147086
"11924","CTSS",222.807091790552,-0.0918834648699778,0.262285699031617,-0.350318241555754,0.726099876809946,0.938440790200412
"11925","ZNF687-
AS1",4.05654270148537,-0.263539331664972,0.753968608798374,-0.349536212236984,0.726686789649841,0.938440790200412
"11926","LINC01133",654.453918194596,-0.0997627501876016,0.285634000349327,-0.349267769472797,0.72688829289607,0.938440790200412
"11927","TRABD2A",19.367877755432,-0.200722859699502,0.573180672788416,-0.350191255966505,0.726195168543138,0.938440790200412
"11928","BARD1",28.983634512842,0.137181504840669,0.392522191248686,0.34948725931716,0.726723534145515,0.938440790200412
"11929","TEX264",323.1319720186,-0.100112026684199,0.286152357203993,-0.349855677102917,0.726447011931942,0.938440790200412
"11930","C2",467.916358294634,0.109081043495955,0.311806728680671,0.349835437989114,0.726462201816995,0.938440790200412
"11931","HLA-
DQA2",8.90992939049569,-0.707646399264514,2.02279894421405,-0.349835262317416,0.726462333662809,0.938440790200412
"11932","AL139393.3",53.3335988808518,-0.077447807511671,0.221702595594847,-0.34933198370489,0.726840089555979,0.938440790200412
"11933","FAM120B",63.3183228553448,0.0864330853192646,0.246706084629588,0.350348413372284,0.726077236086926,0.938440790200412
"11934","DUS4L",19.5769337102028,0.127228134053677,0.363614637250419,0.349898274216217,0.726415042244504,0.938440790200412
"11935","GLI4",81.9876105538212,-0.102302292763917,0.292267246497055,-0.35002996055854,0.726316212945894,0.938440790200412
"11936","SH3PXD2A",312.105620412331,0.0937889589932151,0.2684157839694

8,0.349416705702669,0.726776493420048,0.938440790200412
"11937","EIF3J-
DT",142.98046884182,0.0775470166731325,0.221752782027434,0.34970031024
7015,0.726563620815198,0.938440790200412
"11938","TERF2",100.815264733195,0.0757969584396264,0.216226486630244,
0.350544281696822,0.725930263667742,0.938440790200412
"11939","TMEM92",3.45639452138045,-0.365271790942784,1.04374909385291,
-0.349961301134562,0.726367740596827,0.938440790200412
"11940","MRO",2.55824566374551,0.391176766301585,1.11954659921132,0.34
9406417363205,0.726784216209223,0.938440790200412
"11941","TCP10L",5.50436602812286,-0.235241357502765,0.673784543798117
,-0.349134392689853,0.726988417557772,0.938491454371749
"11942","RHOH",7.89116324851872,0.245072258817454,0.703602100178016,0.
348310868821241,0.72760673217636,0.938590647803382
"11943","FAM91A1",102.235548856262,0.0745633248436522,0.21407653193285
9,0.348302189737629,0.727613249513487,0.938590647803382
"11944","AL359644.1",8.24948736445874,0.227287188616484,0.652126993927
691,0.348532096865915,0.72744061325484,0.938590647803382
"11945","TMEM80",121.305169156098,0.0959384930936445,0.275174804389464
,0.348645630207698,0.727355366665896,0.938590647803382
"11946","AP000487.1",8.22168454063883,0.202406057781156,0.580886663907
335,0.348443285682738,0.727507299545225,0.938590647803382
"11947","PHF11",212.299778840172,0.0660287472803849,0.189297611757279,
0.348809193456959,0.727232561018447,0.938590647803382
"11948","THUMPD1",300.403468284593,0.0538450190460544,0.15446031081891
,0.34860100151671,0.72738887574724,0.938590647803382
"11949","TRAPPC2L",511.662820679767,0.108715495137276,0.31156398351204
3,0.348934732159353,0.727138309501586,0.938590647803382
"11950","GLOD4",236.375994510254,-0.0640017860880602,0.183613890246018
,-0.348567235312679,0.727414229167059,0.938590647803382
"11951","LTB",15.5915617686761,0.193234072556631,0.555098951925619,0.3
48107435415449,0.727759500499521,0.938674295134506
"11952","AL136038.5",24.6311119665429,-0.116574047194746,0.33500928945
503,-0.347972581251047,0.727860775202254,0.938674295134506
"11953","ZNF830",118.111034814834,-0.0877810953113216,0.25226305753094
,-0.347974436568284,0.727859381837421,0.938674295134506
"11954","MED15",126.464192501512,0.0726187609753537,0.208772029104604,
0.3478375972433,0.72796215217594,0.93872649956434
"11955","SPDYE16",3.4243977003886,0.280271873033901,0.806439534086632,
0.347542327958085,0.728183924715562,0.93879517929104
"11956","MRPL51",839.703041346914,-0.0911948873812275,0.26241363404223
7,-0.347523434573331,0.72819811604171,0.93879517929104
"11957","ITGB2-
AS1",4.95503131913466,-0.248270259282743,0.714103219643682,-0.34766718
9354816,0.728090140327029,0.93879517929104
"11958","PLEKHA1",183.175077729389,-0.0787429323313615,0.2267215617237
26,-0.347311176461084,0.728357555184312,0.938922203810517
"11959","LINC01772",3.37229455723885,0.285198255750381,0.8271858943870
04,0.344781333537766,0.73025876726976,0.939624420243833
"11960","TMEM200B",59.5987294196438,-0.0871595160307483,0.254490849645

534,-0.342485854214986,0.731985287762416,0.939624420243833
"11961","LINC01135",4.13411722003901,0.260592179877726,0.760192686215972,0.342797536207408,0.731750779600922,0.939624420243833
"11962","TMEM9",326.788788512902,0.0571400896429156,0.166599563954228,0.342978626634428,0.731614539465375,0.939624420243833
"11963","PFKFB2",11.7552117598641,0.146846722124216,0.42887375728845,0.342400810561721,0.732049278581523,0.939624420243833
"11964","EIF2AK3",66.0847282975757,0.0799443984112827,0.231741607269977,0.344972140967971,0.730115314856585,0.939624420243833
"11965","LIPT1",57.4593569800039,0.0968629787999185,0.282168956447522,0.343280068861625,0.731387773578654,0.939624420243833
"11966","TMEM185B",98.6678094774493,-0.0855600750947414,0.249528171897634,-0.342887436092151,0.731683143977271,0.939624420243833
"11967","LINC01963",6.5954058641512,0.201998603323318,0.586179109671671,0.344602187267406,0.730393461206095,0.939624420243833
"11968","UBE2F",150.610089587799,0.0734780141712982,0.212136736291654,0.346371003230094,0.72906391403166,0.939624420243833
"11969","SLC41A3",141.344422582366,0.0682383592111156,0.197458971023652,0.345582471423605,0.729656521013467,0.939624420243833
"11970","MRAS",64.9259445270073,-0.0797111849370899,0.231273735094422,-0.344661640477879,0.730348759454171,0.939624420243833
"11971","DGKQ",22.6031311124165,-0.111823432810217,0.323691443933514,-0.345463047930254,0.729746285695441,0.939624420243833
"11972","SLC35A4",102.543178592074,0.0651044492120268,0.190050068629997,0.342564723503346,0.731925944489728,0.939624420243833
"11973","RWDD2A",35.2790163551896,-0.0984271649636656,0.28659956328415,-0.34343096631337,0.731274266793142,0.939624420243833
"11974","FAM221A",18.3809295689209,-0.163764667255387,0.474099236810769,-0.345422760764222,0.729776568383702,0.939624420243833
"11975","METTL2B",105.539805510186,0.0825721281996969,0.240418595011889,0.343451504637624,0.731258818085952,0.939624420243833
"11976","AC090114.2",57.4993524683367,-0.115214889733148,0.334178082125604,-0.344770934707331,0.730266585566305,0.939624420243833
"11977","CSPP1",101.123590132891,-0.0810527381242124,0.236165542139266,-0.343203065908811,0.731445698344602,0.939624420243833
"11978","LY6E",1294.98090627805,-0.098506818515143,0.284580283861448,-0.346147727377708,0.729231696596582,0.939624420243833
"11979","TSTA3",124.387888431745,-0.0876403862533187,0.255083482445555,-0.343575308809047,0.731165696224739,0.939624420243833
"11980","HSF1",161.303797356014,-0.05437550048205,0.158579954838493,-0.342890124653076,0.731681121287152,0.939624420243833
"11981","ZNF503-AS2",17.378266634767,-0.153406840470632,0.446593748329025,-0.343504227375818,0.73121916110677,0.939624420243833
"11982","TSPAN32",4.71258406839664,-0.245598543324604,0.715385262365914,-0.343309481260997,0.731365648774726,0.939624420243833
"11983","TMEM223",156.531458138878,-0.0924172484564432,0.267733515382632,-0.345183711215105,0.729956263628544,0.939624420243833
"11984","CRADD",37.8907652169107,0.0954941727440645,0.277257064200447,0.344424669645299,0.730526938820106,0.939624420243833

"11985","GPALPP1",119.717830203218,0.0734371775539265,0.212191329203388,0.346089436498775,0.729275501915278,0.939624420243833
"11986","LAMP1",1494.53700494286,0.0601999292609762,0.175832614523398,0.342370665556846,0.732071961539308,0.939624420243833
"11987","NFATC4",136.731890294142,0.0942368238828313,0.274256409186234,0.343608465386271,0.731140757493665,0.939624420243833
"11988","AP4S1",44.0905382615774,-0.0852917484141206,0.249071925624995,-0.342438226227618,0.732021125056348,0.939624420243833
"11989","TRIP11",448.238342244318,0.0623616265901476,0.18158254250034,0.343434042344852,0.73127195302832,0.939624420243833
"11990","AC123768.4",7.02350779241359,0.194356373075944,0.567291580041442,0.342604015137587,0.73189638105604,0.939624420243833
"11991","NOP10",634.444477238628,-0.0846238461480357,0.24642657421453,-0.343403897967454,0.731294627479024,0.939624420243833
"11992","AC090517.2",6.62140504465069,0.220710962582078,0.637824170658697,0.346037313628526,0.729314672753154,0.939624420243833
"11993","AC009269.4",2.71261073776533,-0.356628772806165,1.03103166582626,-0.345895072505232,0.729421571918708,0.939624420243833
"11994","PDE8A",115.933686198332,0.0747400841451002,0.216775770201694,0.344780618588323,0.730259304799193,0.939624420243833
"11995","ARPIN",135.15357367675,0.0680418087342384,0.196540826333829,0.346196818256314,0.729194805720058,0.939624420243833
"11996","SNX29",250.418511612765,0.0971568894175812,0.28224692892109,0.344226560015979,0.73067590944562,0.939624420243833
"11997","EIF3CL",2.54960892333259,0.377499608453846,1.09864813862599,0.343603739160712,0.731144312308499,0.939624420243833
"11998","RSPRY1",149.00620752996,0.0571282202287638,0.16536531818123,0.345466757220246,0.729743497551475,0.939624420243833
"11999","HYDIN",7.77449056071065,0.246720514825363,0.72061794681838,0.342373536372034,0.73206980135111,0.939624420243833
"12000","ATP2A3",6.25219671367679,0.234885283889856,0.683118135234783,0.343842846170564,0.730964476009697,0.939624420243833
"12001","AC015802.6",14.8923295682586,-0.190053375035307,0.551854537348274,-0.34439034595699,0.730552748151763,0.939624420243833
"12002","NAPG",130.945943370844,0.0680272582077309,0.198361295487123,0.342946228701895,0.731638912847589,0.939624420243833
"12003","KCTD1",39.9821532644919,0.124566619720715,0.360053453334963,0.345967018416093,0.729367501399725,0.939624420243833
"12004","ZNF781",13.2362000986575,0.193241140963115,0.559783988948549,0.345206623944502,0.729939039324358,0.939624420243833
"12005","DYRK1B",47.9062993397475,-0.0869469331547196,0.252369197190563,-0.344522763168543,0.730453180122732,0.939624420243833
"12006","IRF3",187.650479899524,0.0633425348262871,0.183961342670462,0.344325247395891,0.730601699159076,0.939624420243833
"12007","RDH13",49.2322412308787,0.0859903350614495,0.250995402851064,0.342597251123657,0.731901470342016,0.939624420243833
"12008","SLC27A5",41.3568574533284,-0.126137473755994,0.364917827781258,-0.345659938082292,0.729598295171756,0.939624420243833
"12009","GPKOW",70.5344829394373,-0.0784671272088629,0.227487170301522,-0.344929901342828,0.73014707054299,0.939624420243833

"12010","OCRL",64.0916142482483,0.0880227037776184,0.255790566263211,0
.344120211560274,0.730755883479689,0.939624420243833
"12011","PGAP1",50.995994924697,0.0979336747878758,0.286413861499533,0
.341930639373176,0.732403090973642,0.939712829112978
"12012","CAAP1",67.6288636183942,0.0761067852641185,0.222616415511909,
0.341874093557341,0.732445646564543,0.939712829112978
"12013","THSD1",65.3613215852351,-0.150756570743184,0.440880437592913,
-0.341944341114961,0.732392779358061,0.939712829112978
"12014","DNAL1",62.2752844714896,-0.0843086274892671,0.246502672837908
,-0.342019121004443,0.732336502585382,0.939712829112978
"12015","KCNJ14",6.3734842518307,0.212385702701047,0.620691120480407,0
.342176157662226,0.732218326883478,0.939712829112978
"12016","LINC00960",4.60253436985373,-0.287032184842423,0.839952705153
538,-0.341724222186957,0.73255844164327,0.939779325726615
"12017","SLIT2",220.666125214106,0.102943268618954,0.301381424636566,0
.341571378339234,0.732673479795733,0.939783412290944
"12018","FOCAD",60.1345717858837,0.0877521352886524,0.256978179013386,
0.341476990869646,0.732744523666873,0.939783412290944
"12019","JADE3",11.5201182984582,-0.209541065945422,0.613507623713787,
-0.341545985487504,0.73269259234573,0.939783412290944
"12020","MOSMO",209.046964748141,-0.0592411910444786,0.173578563123913
,-0.341293244847221,0.732882832794876,0.939882601292264
"12021","GUCA1B",8.03899489291681,0.1882510027174,0.551942571551966,0.
341069909117666,0.733050953526898,0.939941810731753
"12022","SLC16A7",122.172125414763,0.0926337521293321,0.27155257969333
,0.341126393400295,0.733008432568688,0.939941810731753
"12023","SIRT3",47.3315961633935,0.084803107697922,0.248787617293493,0
.340865468388165,0.733204861872317,0.940060961969705
"12024","LINC01960",6.67398654535897,0.21521274165365,0.63180177588982
9,0.340633328151926,0.733379636136928,0.940113875964641
"12025","ZDHHC4",163.400254754504,-0.0790810904091221,0.23216025678807
,-0.340631473720811,0.733381032360863,0.940113875964641
"12026","SGF29",102.578587534211,-0.0833161299990952,0.24463900834531,
-0.340567641124075,0.733429093243644,0.940113875964641
"12027","ID2",1038.6206819098,-0.0647678228314199,0.190275768809847,-0
.340389232094739,0.733563426633631,0.940120559029673
"12028","ATP6V0A2",46.9452348309566,-0.104344458789635,0.3064959501447
63,-0.340443189348345,0.73352279856464,0.940120559029673
"12029","PVR",28.0126445015354,0.130543386952433,0.383592674695568,0.3
40317726494743,0.733617269190265,0.940120559029673
"12030","BLZF1",115.067532803304,0.0646954052577337,0.190232926460366,
0.340085212699562,0.733792357361521,0.940247766072681
"12031","DCXR",74.2076094000186,0.081880882481981,0.240809193877274,0.
340023905082756,0.733838525697076,0.940247766072681
"12032","DHX35",27.3868954695138,0.112533187732201,0.331347870254895,0
.339622486921775,0.734140841573321,0.940556937570872
"12033","DDO",2.99097831844486,0.327586885981913,0.965090343050658,0.3
39436497671719,0.734280927694485,0.940633488836621
"12034","HSPA2",28.5125763515939,0.12127097782993,0.357329763169125,0.
33938112726572,0.734322634100544,0.940633488836621

"12035","ID2-
AS1",4.13076973345837,-0.243119801956329,0.716624566962382,-0.339256862190563,0.734416236586599,0.94064579871355
"12036","SLC25A42",27.2299127602512,0.114286988995852,0.336924675623979,0.339206348671834,0.734454286948835,0.94064579871355
"12037","KPNA1",95.3747356163551,0.0788116309346778,0.232510156604096,0.338959949473835,0.734639901632334,0.940727204158699
"12038","INO80E",113.426467091813,-0.0940064906801857,0.277328155153988,-0.338972040642567,0.734630792886763,0.940727204158699
"12039","AC093110.1",4.86191278313573,0.28836338897479,0.851905555113561,0.338492204028826,0.734992301031388,0.940735114491073
"12040","AC006994.2",2.83321379444069,0.310291294201527,0.917632859308875,0.338143181179482,0.735255291187766,0.940735114491073
"12041","TOP2B",208.956232917954,0.0619328118993943,0.183034796499477,0.338366327517245,0.735087145921575,0.940735114491073
"12042","HSPBAP1",27.2551593544856,0.125171946904878,0.36998218947895,0.338318844702118,0.735122924117421,0.940735114491073
"12043","PDP1",188.403004546521,0.08394666867307,0.248377764875587,0.337979805539836,0.735378406073139,0.940735114491073
"12044","LTBP3",848.691949673,-0.0626375148803914,0.185272609319024,-0.3380829746535,0.735300660193188,0.940735114491073
"12045","TBX5",27.9293873388956,-0.193194376006256,0.571123470230311,-0.338270769941127,0.735159148928711,0.940735114491073
"12046","SFSWAP",231.040329055716,-0.0638541265390594,0.188604927475981,-0.338560224240119,0.734941051138181,0.940735114491073
"12047","SCFD1",310.646390940932,-0.0579040412956048,0.171295276389469,-0.338036415925154,0.735335745448281,0.940735114491073
"12048","PIGQ",54.204162210454,0.0865004836369494,0.255447850686959,0.338622867267544,0.734893853733248,0.940735114491073
"12049","DLG4",38.5139596249245,-0.105786235643046,0.312460611663864,-0.338558626892941,0.734942254647407,0.940735114491073
"12050","ZNF835",14.6687214619423,0.143446777823275,0.424240104869291,0.33812639629502,0.735267939449921,0.940735114491073
"12051","RNF146",362.369934629553,0.0572913398337536,0.169556474330214,0.337889426281521,0.735446516038889,0.94074417432076
"12052","BANK1",6.46922702244865,-0.251129670864469,0.743701819021782,-0.337675213965173,0.735607955118169,0.940872604393178
"12053","CDK5R1",3.81267982605225,0.302517167061332,0.896111347055827,0.33758881421963,0.735673072771783,0.940877824340582
"12054","TPM1",1282.48102218404,-0.088315845907696,0.261773887522197,-0.337374543899866,0.735834571957098,0.941006298881589
"12055","HNRNPD",704.496925072745,-0.0590471965716882,0.17506655284792,-0.337284281955231,0.735902607415722,0.941015237935575
"12056","HNMT",531.061048398073,-0.0740076184520976,0.219619969993799,-0.33698036865312,0.736131699035949,0.941152039532152
"12057","HOXC4",65.1055844286576,-0.106139822044919,0.314936310263965,-0.337019957958985,0.736101855056675,0.941152039532152
"12058","TMED5",315.964134942356,-0.0806113776757374,0.2396516393967,-0.336368980736659,0.736592637873512,0.941351391896209
"12059","AC132217.1",35.9846717096795,0.400102609937554,1.188646443675

44,0.336603547729792,0.736415781425665,0.941351391896209
"12060","ZNF214",21.1192490882615,-0.118293843874894,0.351679260625706
,-0.336368552596551,0.73659296069102,0.941351391896209
"12061","SLC35C1",73.5141692331443,0.0724851512235966,0.21539496744536
1,0.336522027804498,0.736477243411338,0.941351391896209
"12062","PYM1",96.7580545917523,-0.0845856541139635,0.25133858185302,-
0.336540667534394,0.73646318982959,0.941351391896209
"12063","NACC1",28.8809380402355,0.113417991537876,0.337325562332327,0
.336227088020499,0.736699627482203,0.941409662408866
"12064","MYOM2",15.5886255081496,0.154317801140278,0.459157358323044,0
.336089138816995,0.736803648506445,0.941464542583459
"12065","SLIT3",2186.11502468442,-0.118594037186837,0.353017729287498,
-0.33594357265341,0.736913418352658,0.941526758715808
"12066","TCTN2",30.7235604136491,0.11508867983883,0.342679903617093,0.
335848932557855,0.736984788289647,0.941539906471482
"12067","MCM3",100.958980131938,-0.0750122093664338,0.223452584269648,
-0.335696316118296,0.73709988409386,0.941608909696433
"12068","DLAT",61.3893312826903,-0.0737986784178165,0.219985901352527,
-0.335470036780013,0.737270543681604,0.941670845211031
"12069","AC010198.2",6.42839154219605,-0.236801426145007,0.70578867348
2843,-0.335513213858289,0.737237978583944,0.941670845211031
"12070","MEAF6",434.987446921236,-0.066623281026707,0.198735449329807,
-0.335236019801096,0.737447052615265,0.941818253195055
"12071","TRIM27",84.7073806096737,0.0748404259480135,0.223381157674338
,0.33503464091238,0.737598955128252,0.941934213677575
"12072","TMEM234",52.9852275660279,-0.0972393440784768,0.2909452922369
96,-0.334218654410357,0.738214568386745,0.94194003573228
"12073","ETV3",60.9029853007333,-0.105139978520822,0.314295484593019,-
0.334525895772787,0.737982753333322,0.94194003573228
"12074","TAF1B",122.90191725018,-0.0821650282113973,0.245797560654477,
-0.334279266208417,0.738168834614441,0.94194003573228
"12075","KIAA1841",44.0524048071988,0.100017010086684,0.29900676540101
3,0.334497481863148,0.738004190762337,0.94194003573228
"12076","SLC35A1",128.439007933013,-0.0769858575673455,0.2303364599200
08,-0.334232181887667,0.738204361340343,0.94194003573228
"12077","CALHM6",15.6339610923194,-0.140360223303314,0.419780987913455
,-0.334365365141909,0.738103871478723,0.94194003573228
"12078","APTR",54.1434996793946,0.100337330707483,0.299562236268148,0.
334946527163952,0.737665423610021,0.94194003573228
"12079","RNF26",66.9336980225586,0.0793931989774994,0.237382767660104,
0.334452242511463,0.738038322895126,0.94194003573228
"12080","DGKH",43.3392736712324,-0.137670890145629,0.411580244938244,-
0.334493435578487,0.738007243577391,0.94194003573228
"12081","NECTIN2",158.652236858639,-0.104718000820199,0.31287378727912
3,-0.334697264768867,0.73785346496862,0.94194003573228
"12082","KBTBD2",110.847410670647,0.0839170023905242,0.251233909363443
,0.334019410847631,0.738364911317769,0.941975925512158
"12083","RUSC2",75.6643587449263,-0.076480174388694,0.228939893539675,
-0.334062243177553,0.738332590543178,0.941975925512158
"12084","AC090617.10",6.33185569281485,0.243350162218657,0.72908872204

0761,0.333773044160533,0.738550825712389,0.942135135580642
"12085","ZNF713",17.1804584012628,-0.123600147818789,0.370451015089304
,-0.333647750402284,0.738645381346153,0.942177786797762
"12086","ILVBL",146.122245871893,-0.0900499919056843,0.270022029918711
,-0.333491278221979,0.73876347199836,0.942250448523475
"12087","NDUFAF7",61.6425122059105,0.0863253863722233,0.25913648143188
8,0.33312710697939,0.739038338446668,0.942290334976199
"12088","ZNF311",2.48618941221772,0.295521740901103,0.887117302988691,
0.33312588978424,0.73903925720806,0.942290334976199
"12089","GSE1",128.031869080462,0.0916479661402044,0.275105208549396,0
.333137880680106,0.73903020627453,0.942290334976199
"12090","ZNF837",9.14732896923139,-0.177920064555359,0.533706280937975
,-0.333367005242414,0.738857266078694,0.942290334976199
"12091","AP001885.2",2.89409523756321,-0.312197106241132,0.93755792593
9558,-0.33298967200162,0.73914207926089,0.942339101389169
"12092","ABCC1",67.1911648401071,0.0791168676619797,0.237650101532687,
0.332913249991176,0.739199767369305,0.942339101389169
"12093","TAF8",56.3545615578273,0.0865220500096741,0.260073489133929,0
.332683082377221,0.73937352113122,0.94248266172478
"12094","CCDC77",26.6629417629549,-0.101248291585902,0.304509924826753
,-0.332495867395804,0.739514859720819,0.942584881974237
"12095","NCOA7",726.314898706344,0.0573304674427361,0.172804986012875,
0.331763965644282,0.740067495839938,0.942899450233296
"12096","TAX1BP1",600.362726090757,-0.0504063743149506,0.1518166607650
55,-0.332021360902921,0.739873129418143,0.942899450233296
"12097","MDK",357.276324962104,-0.117646718540249,0.354522924341092,-0
.331845165609261,0.740006177662704,0.942899450233296
"12098","CDK17",65.0005419147466,0.0929077366408368,0.279962425022857,
0.331857879260945,0.739996577094517,0.942899450233296
"12099","CYTH4",5.65868668481772,-0.273635703755641,0.824708646806149,
-0.331796816748983,0.740042688119659,0.942899450233296
"12100","ATP6V1B2",195.491887763663,0.0641978183013555,0.1936549470747
82,0.331506213866898,0.74026214811212,0.94306950521887
"12101","EPB41L1",100.232490557916,-0.0636385635112591,0.1921596883129
21,-0.331175409733323,0.740511993364388,0.943309840319976
"12102","DBR1",70.9120785310833,0.0693371075123873,0.209722083954504,0
.330614240546213,0.74093588819539,0.943382069590479
"12103","CCDC127",173.02716648698,-0.0707190543577239,0.21364120361451
4,-0.331017861541946,0.740630993846424,0.943382069590479
"12104","HOXC-
AS1",41.569222805085,0.119592645302952,0.361605912177952,0.33072646567
8189,0.740851109509435,0.943382069590479
"12105","TSC2",131.949263100413,0.0722473363413379,0.218441221861479,0
.330740396550027,0.740840585874023,0.943382069590479
"12106","TADA2A",43.1532415833662,-0.103165719071689,0.312032464130877
,-0.330624953909981,0.740927794819782,0.943382069590479
"12107","RNF125",19.1963318047473,0.144004851330106,0.435459493117316,
0.330696318730408,0.740873883231231,0.943382069590479
"12108","ABHD3",32.6820186896146,0.0962509651406874,0.291340244562417,
0.330373049851911,0.741118102502092,0.943536137270379

"12109","PDE5A",69.9412875127828,-0.129759150126895,0.392900325768713,
-0.330259716311051,0.741203728534212,0.943567220691625
"12110","AC118553.1",5.02087499899351,-0.248765018130047,0.75404985799
4367,-0.329905264874284,0.741471545226691,0.94359498405193
"12111","ITGA11",847.36440961686,-0.103406314926529,0.31348369462717,-
0.329861861075458,0.74150434245577,0.94359498405193
"12112","LINC00921",26.3936795881596,-0.111386119334812,0.337489935284
345,-0.33004278850854,0.741367631292014,0.94359498405193
"12113","GAN",11.6125462181109,0.17612253440715,0.533700200509331,0.33
0002751055872,0.741397883418098,0.94359498405193
"12114","PET117",62.4526809819536,-0.0891810767650971,0.27045485470842
1,-0.329744780737043,0.741592814258134,0.94359498405193
"12115","RRP1",84.5948640725786,-0.0790302603550695,0.23962518677461,-
0.329807819531946,0.741545178544225,0.94359498405193
"12116","AIDA",604.613100691892,0.0608839271337887,0.185008824387506,0
.329086611600027,0.742090223557057,0.943682626310182
"12117","AC006460.2",5.39967762774194,0.238035595363313,0.723266525299
596,0.329111865456116,0.742071136044023,0.943682626310182
"12118","ARF3",346.749144971602,0.047300441487595,0.143697104649791,0.
329167672535034,0.742028956183129,0.943682626310182
"12119","LEMD3",93.1150919736531,0.072693033453064,0.220764962059492,0
.329277946893922,0.741945611380613,0.943682626310182
"12120","TBC1D10B",99.9075196670712,-0.0729187156840848,0.221537871227
357,-0.329147857565404,0.7420439325576,0.943682626310182
"12121","ZNF396",17.6280473571334,0.139281379103367,0.423089553736784,
0.329200704373849,0.742003990569049,0.943682626310182
"12122","TOP1",459.596039646691,-0.0608946568918028,0.185040162136914,
-0.329088864755457,0.742088520557869,0.943682626310182
"12123","MRPS21",723.877789043903,-0.0807269479274963,0.24553348529606
1,-0.328781827171788,0.742320599962723,0.943732208673336
"12124","PON2",125.279952532954,0.0803022202425906,0.244294251824343,0
.328711050886007,0.742374100705344,0.943732208673336
"12125","AC093799.1",4.01566148224622,-0.319236912200896,0.97071367752
4974,-0.328868253937509,0.742255270500985,0.943732208673336
"12126","NGFR",8.14443254886224,0.289696585650195,0.8808531758753,0.32
8881808665019,0.742245024734089,0.943732208673336
"12127","H2AFZ",898.437419154272,-0.0804977065666979,0.245085893705251
,-0.328446918546472,0.742573772867787,0.943896170222484
"12128","ERI1",44.6460746718087,0.0845914986019177,0.257603693364014,0
.3283784385901,0.742625543461453,0.943896170222484
"12129","ICE1",157.89437951916,0.0751657864696886,0.229030389173443,0.
328191323173128,0.7427670079603,0.943926186954231
"12130","MTAP",150.880062771218,0.0568979273020218,0.173371392673418,0
.328185212246643,0.74277162813849,0.943926186954231
"12131","ATP5ME",1264.49667916916,-0.0912119331515904,0.27799903189951
2,-0.328101621535721,0.742834827992844,0.943928684651693
"12132","AFTPH",111.757231275698,-0.0832454431167525,0.253905640726287
,-0.32785976270016,0.743017698302245,0.944005424819015
"12133","PACS2",167.840422616537,-0.0539630743465987,0.164560324748359
,-0.327922750694115,0.742970071463326,0.944005424819015

"12134","IFT122",62.0455610429611,-0.0740030517478499,0.225940518980062,-0.327533335242008,0.743264534234264,0.9441292223971
"12135","FPGS",66.0789557237604,-0.0766416377844063,0.234021261408842,-0.327498609840031,0.743290794231293,0.9441292223971
"12136","RDH5",56.4855362906631,0.079454263760122,0.242677406029971,0.327406926998013,0.743360127942498,0.9441292223971
"12137","P4HB",1480.52540580727,-0.0717846415466342,0.219236514076605,-0.327430135664133,0.743342576556017,0.9441292223971
"12138","SYNC",134.881904783737,-0.104500973373558,0.321708895475457,-0.324830848146475,0.745309096043782,0.944258405380543
"12139","ATRAID",1154.13406593064,-0.0854792495133621,0.261497096845673,-0.326884124315189,0.743755529026825,0.944258405380543
"12140","RFX8",12.1938633468307,-0.164438314245672,0.502848278038873,-0.327013776177156,0.743657465675485,0.944258405380543
"12141","AC008063.1",12.0812270095308,0.19241280799926,0.590057215814222,0.326091780326335,0.744354915782917,0.944258405380543
"12142","IDH1",155.625305885756,0.0520053317135582,0.159269042489476,0.326525047810183,0.744027141459666,0.944258405380543
"12143","AC097634.1",4.22825780157973,-0.231352070752227,0.712679495232137,-0.324622880691789,0.745466508028851,0.944258405380543
"12144","XXYLT1",76.7402532595175,0.0685823039805989,0.211197803081059,0.324730195958888,0.745385279043704,0.944258405380543
"12145","SFRP2",1416.0077667693,0.2063242218265,0.633907000790239,0.325480270085821,0.744817612623584,0.944258405380543
"12146","PTCD2",34.7986611965876,0.106055340884606,0.32660706918702,0.324718448833933,0.745394170530067,0.944258405380543
"12147","AC008771.1",35.8940617713203,0.102763819440956,0.314300204230353,0.326960714812771,0.743697598612948,0.944258405380543
"12148","EPB41L4A-DT",9.79941450110771,0.236533814337508,0.728697170227981,0.324598233671616,0.745485164231045,0.944258405380543
"12149","GRAMD2B",83.3409010801408,0.0958166089634145,0.294036909084989,0.325865923640625,0.744525798627718,0.944258405380543
"12150","HEIH",113.196174406417,0.0808706792302803,0.247841071761405,0.326300554849658,0.744196968470817,0.944258405380543
"12151","ABT1",112.689787055081,-0.0779903166712028,0.239049544335258,-0.32625168513948,0.744233939639157,0.944258405380543
"12152","RSBN1L",349.767122236882,-0.0669837040481094,0.206292422783558,-0.324702687303201,0.745406100603343,0.944258405380543
"12153","ATP5MF",815.07510639449,-0.0896836032019436,0.275350674884252,-0.325706858135153,0.744646154909841,0.944258405380543
"12154","COPG2",37.5523219930418,-0.0915289139983887,0.281611815660381,-0.325018017385929,0.745167435461047,0.944258405380543
"12155","ST3GAL1",109.071852826886,-0.0799987074068408,0.244797524403023,-0.326795410214749,0.743822631129155,0.944258405380543
"12156","ZNF7",73.5170685875173,-0.0996172963656127,0.306962267254424,-0.324526194234308,0.745539694287337,0.944258405380543
"12157","APTX",99.3185796354031,-0.0817226739360032,0.251714673592583,-0.32466392510862,0.745435440387843,0.944258405380543
"12158","RUFY2",167.652579315209,0.0573613519185864,0.176187276178693,

0.32557034289133,0.744749453598581,0.944258405380543
"12159","SUPV3L1",48.1693175276238,-0.0772283319512194,0.237158410284339,-0.325640283465499,0.744696530220114,0.944258405380543
"12160","ACTA2",302.951288028256,0.0701742223528153,0.21628191366804,0.324457191832148,0.745591926663501,0.944258405380543
"12161","NAP1L1",1197.28426114755,-0.0499938352421898,0.153869759561305,-0.32491007579869,0.745249130890156,0.944258405380543
"12162","ALDH1L2",154.648561531685,0.089622662698439,0.275777769519862,0.3249814619012 8,0.745195102104101,0.944258405380543
"12163","WDFY2",187.820316529017,0.102536445008058,0.316107229155326,0.324372350743282,0.745656149967736,0.944258405380543
"12164","GDPD3",15.5553522129457,-0.139289304423692,0.428795917113053,-0.32483822458358,0.745303512963453,0.944258405380543
"12165","ELAC2",87.7497510593124,-0.0653860790171297,0.200422155500482,-0.326241771294454,0.744241439784888,0.944258405380543
"12166","AC138150.1",24.8589159112945,-0.121314312834049,0.371589476514423,-0.326474027122616,0.744065737082128,0.944258405380543
"12167","AC091132.4",17.0839548188106,0.21827120414452,0.668212406706587,0.326649433554088,0.74393305006357,0.944258405380543
"12168","TM6SF2",6.03932562440529,0.228842920665701,0.704229576463325,0.324954998077419,0.74521513118245,0.944258405380543
"12169","ADNP-
AS1",10.1899914583943,-0.157176026653574,0.483109463194429,-0.325342471278229,0.744921890275523,0.944258405380543
"12170","TAFA5",52.452595540743,-0.11350324566811,0.348287765145895,-0.325889270386987,0.74450813392988,0.944258405380543
"12171","HDAC10",45.4364647943627,-0.0802135956885268,0.245410951814163,-0.326854181101373,0.743778177436602,0.944258405380543
"12172","FHL1",3147.95758567496,-0.0804225325179239,0.247943766917622,-0.324357952279736,0.745667049542481,0.944258405380543
"12173","SLC10A3",100.720326658087,-0.0824519491847536,0.253891648164391,-0.324752506751885,0.745368391931712,0.944258405380543
"12174","EIF4A3",396.679100458069,-0.0704706131291119,0.21737909630448,-0.324183025539883,0.745799472394652,0.944348518725444
"12175","PAX3",7.90187955994517,-0.258992724422798,0.799460456247105,-0.323959393362098,0.74596877712169,0.944407744688802
"12176","SVIL",323.597094544493,-0.076403015288157,0.235835676078016,-0.323967164589985,0.745962893569312,0.944407744688802
"12177","PSMC1",573.345581585219,-0.075148298760401,0.23220731743025,-0.323625885661307,0.746221287856629,0.944649844157834
"12178","OTULIN",102.281353804141,0.0914213225499729,0.282929890999653,0.323123591597061,0.746601644276704,0.94465853685704
"12179","PRDM1",27.6751827738699,0.155760124942087,0.481680765155222,0.323367956974353,0.746416593680073,0.94465853685704
"12180","ADAT2",36.940935288957,0.113392348195101,0.351105680706739,0.322957885406051,0.746727136937037,0.94465853685704
"12181","CLDN4",19.2080218453507,0.255585096253827,0.791360583355018,0.322969202193846,0.746718566290335,0.94465853685704
"12182","TCTN1",203.360082267974,-0.0816203389847388,0.252660872051656,-0.323043051035031,0.746662638439304,0.94465853685704

"12183","TGFB3",103.377471749784,0.0748490845778532,0.231473927783297,
0.32335859720636,0.746423681283388,0.94465853685704
"12184","IMP3",372.007077807079,-0.07312726104484,0.226478373925003,-0
.322888493843813,0.746779690570217,0.94465853685704
"12185","AP001020.2",7.96212225333049,0.222598082863848,0.688346632108
09,0.323380797523673,0.746406870320123,0.94465853685704
"12186","COL6A1",9286.91613832666,-0.0840028121536011,0.26003464148962
6,-0.32304469770791,0.746661391381787,0.94465853685704
"12187","CBFA2T3",2.70540978696533,0.297914348882274,0.92321911834777,
0.322690835752441,0.746929393189601,0.94469286150457
"12188","SIRT6",58.1187852995713,-0.0987139712951538,0.305839117450237
,-0.322764374021631,0.746873695535262,0.94469286150457
"12189","FGD3",2.66963954562088,0.312271183885961,0.967977036118642,0.
322601851318803,0.746996791487715,0.944700594042426
"12190","PRKAG1",190.204643914661,-0.0835899453344547,0.25934425772002
5,-0.322312689971698,0.747215820484254,0.944836152286
"12191","DNAJC14",51.3958813324123,0.0837984256488603,0.26000252833563
4,0.32229850296181,0.747226567143602,0.944836152286
"12192","ROGDI",39.6723689804801,-0.123806557245081,0.384482188529907,
-0.32200856356562,0.747446206984318,0.944985470513885
"12193","MT-
ND6",237.520795511186,-0.123355354024058,0.38311404001991,-0.321980771
097941,0.747467261886202,0.944985470513885
"12194","C2orf74",141.859331540651,-0.104729354696669,0.32572889304339
6,-0.321523073124239,0.747814029900736,0.945036339639272
"12195","TNFSF10",822.302693706135,-0.10219862684998,0.317791538508544
,-0.321590144689244,0.747763210941179,0.945036339639272
"12196","SLTM",420.060502138572,-0.0557847919460518,0.173475107606332,
-0.321572314989679,0.747776720087826,0.945036339639272
"12197","TBKBP1",19.6909079458108,0.124392030840847,0.386670389055659,
0.321700431069061,0.747679651250337,0.945036339639272
"12198","ZNF71",31.6185318509218,0.0983190727178286,0.305644273717281,
0.321678111361488,0.747696561783461,0.945036339639272
"12199","AP000866.2",4.27505558342313,-0.237109268315259,0.73774044397
7496,-0.321399308186081,0.747907807320989,0.945077371083945
"12200","NUP210",4.04806874600239,-0.252231308281349,0.78503835504664,
-0.321298069909417,0.747984518931323,0.945096832731667
"12201","S100A11",7222.94769094653,-0.0896228702891457,0.2791782622703
18,-0.321023813101777,0.748192344972851,0.945108372923541
"12202","AC091979.2",3.24654135367126,-0.261898066778219,0.81636170455
0756,-0.320811308661693,0.748353388951643,0.945108372923541
"12203","YBX3",3221.08087248587,-0.0511713199770516,0.159527178567732,
-0.320768664226863,0.748385707855585,0.945108372923541
"12204","SUCLA2",250.518790483539,-0.0812722803044185,0.25334927594429
2,-0.320791444938998,0.748368443000373,0.945108372923541
"12205","STK24",402.674776695138,0.0665035183498806,0.207307387803436,
0.320796663614023,0.74836448793244,0.945108372923541
"12206","NAPB",29.6913626635461,0.0989677826970123,0.308301240554029,0
.321010004757565,0.748202809129678,0.945108372923541
"12207","SNHG17",73.9595489737683,-0.104438917061016,0.325639248999229

,-0.320719684073657,0.74842282895087,0.945108372923541
"12208","BBOF1",65.4118243141569,-0.0756162685452691,0.235889187455188
,-0.320558434072499,0.748545041276511,0.94518527287659
"12209","C1GALT1C1L",36.2323724848149,-0.117520228028326,0.36702345676
8177,-0.320198139549854,0.748818133440326,0.945387234897497
"12210","FIZ1",19.6085205385514,0.140667866020813,0.439332279058009,0.
320185592377653,0.748827644378751,0.945387234897497
"12211","RFX5",26.7484033551799,0.101694069739287,0.319420900789616,0.
31837011757182,0.750204202659067,0.945541931771733
"12212","CYB5R1",189.290508451337,0.0733685450443958,0.230405112430981
,0.31843279982068,0.750156661435557,0.945541931771733
"12213","ANAPC13",262.699237923426,-0.0721163141719198,0.2265166151132
6,-0.318370968663208,0.750203557144189,0.945541931771733
"12214","PPP2R3A",70.3815119384106,-0.100574996511114,0.31595050567958
2,-0.318325163920171,0.750238298248464,0.945541931771733
"12215","AC005674.2",4.12931705899164,-0.269573024559133,0.84425728172
388,-0.319301983405692,0.749497528804463,0.945541931771733
"12216","PRDM8",122.667314608124,0.0592409861034833,0.185641140888653,
0.319115611011117,0.749638846188507,0.945541931771733
"12217","CLINT1",395.912556229639,0.0538493496114933,0.168811404421518
,0.318991183066238,0.749733198701293,0.945541931771733
"12218","LANCL2",36.9231411786085,0.106664896675426,0.334124972818654,
0.319236529301023,0.749547158602644,0.945541931771733
"12219","PURG",5.94939868402291,0.198641895742581,0.62128079817731,0.3
19729655777789,0.749173276841277,0.945541931771733
"12220","SLA",15.0520549901586,0.172016402192689,0.538866835756791,0.3
19218758287671,0.749560633441657,0.945541931771733
"12221","ANGPTL2",2960.91411980067,-0.0684624058508131,0.2145136000813
61,-0.31915181986059,0.749611390070189,0.945541931771733
"12222","CARD9",6.47578323899588,-0.196328632848287,0.614570446203232,
-0.31945667752361,0.749380237948018,0.945541931771733
"12223","AC016396.2",3.51263254861018,-0.256928318054349,0.80704337101
8851,-0.318357509993534,0.750213764965654,0.945541931771733
"12224","RAB30-
DT",76.700466029684,0.0966067846891204,0.302805072935103,0.31903951856
7857,0.749696545910812,0.945541931771733
"12225","CCDC89",31.9448113970711,-0.109283905293918,0.343046741833954
,-0.318568556312991,0.750053700454798,0.945541931771733
"12226","CMAS",177.11129687708,-0.0539481717712548,0.169316456172798,-
0.318623322213863,0.750012165942037,0.945541931771733
"12227","MIR17HG",15.6040522417798,-0.224742885798795,0.70442182183368
1,-0.319045888177863,0.749691715880381,0.945541931771733
"12228","CCPG1",621.235977162631,-0.0403458696610344,0.126564649731596
,-0.318776765444345,0.749895798306418,0.945541931771733
"12229","AC015871.4",3.0082480387514,-0.268294770772557,0.842739043754
916,-0.318360437623894,0.750211544480357,0.945541931771733
"12230","ERGIC3",600.564636333176,-0.0506441015100722,0.15839415350146
6,-0.319734664383326,0.749169479687193,0.945541931771733
"12231","RNF113A",59.4059127039958,-0.0814022194893951,0.2551405640424
19,-0.319048520547527,0.749689719775861,0.945541931771733

"12232","CRY2",129.903187516237,0.076508368699077,0.240495404514277,0.318128193981916,0.750387698006886,0.945652907519305
"12233","TMEM69",62.291114930392,-0.0855095390123723,0.268958538951915,-0.317928329569265,0.750539302764912,0.945766643678666
"12234","CGAS",37.9331796325283,0.0898795464667313,0.282818940273674,0.3177988941609,0.750637489584715,0.945813053943794
"12235","AL365436.2",4.7509248757966,0.228193895671256,0.718713992573038,0.317503065237827,0.750861913983398,0.945906851358791
"12236","USP34",321.877780981088,0.0603088561367301,0.190250646243755,0.316996852980256,0.751245990168715,0.945906851358791
"12237","MRPL14",306.505992084484,-0.08066944707034,0.254506124112551,-0.316964659894256,0.751270417971849,0.945906851358791
"12238","DOCK8",38.6734903425732,0.169520934482889,0.534724102628914,0.317025048336998,0.751224596008753,0.945906851358791
"12239","KAT5",146.658064212222,0.0707641713546439,0.223378871358339,0.316789904633038,0.751403024926931,0.945906851358791
"12240","IGHMBP2",21.1451695329998,0.119196448372561,0.376364530379688,0.316704786852022,0.751467616255102,0.945906851358791
"12241","AP002490.1",4.34169071833518,-0.248172992466253,0.783695598186499,-0.316670137028375,0.751493910650378,0.945906851358791
"12242","EEF1AKMT1",139.913208164261,-0.107760323972078,0.339955310896104,-0.316983793216901,0.751255899761128,0.945906851358791
"12243","AC015871.7",6.04590968747957,0.198052999853583,0.623906199294653,0.317440346124927,0.750909497230011,0.945906851358791
"12244","TELO2",69.5405312166642,-0.082423302133411,0.259613863039842,-0.317484209696313,0.750876219058731,0.945906851358791
"12245","ITGB3",69.5035008392755,0.108378976019541,0.342268060142404,0.316649400398181,0.751509647005586,0.945906851358791
"12246","ILF3-DT",203.769819058077,0.0812665495662163,0.256353952904539,0.317009153342287,0.751236656838469,0.945906851358791
"12247","BCAM",6.47005676000965,0.200870067064059,0.633090790086473,0.317284772120319,0.751027530989601,0.945906851358791
"12248","ANKRD31",2.45540285273422,0.299317465899878,0.945525846597874,0.31656190782818,0.751576043413175,0.945913186578551
"12249","CDCA8",2.64090564192999,-0.307740796686036,0.973750440793111,-0.316036618617994,0.751974713814038,0.946140861038227
"12250","ZSCAN12",9.34072551105183,0.170860767810978,0.540626964507068,0.316041890301875,0.75197071251897,0.946140861038227
"12251","DNAJC25",68.2465067629299,-0.0862401731440016,0.272823514649007,-0.316102419745422,0.751924770150095,0.946140861038227
"12252","LRFN3",49.9554536415381,-0.0793578268908736,0.25113230696942,-0.316000071231524,0.752002454066841,0.946140861038227
"12253","AC138969.1",9.74662948574336,-0.206796923090396,0.655017146170366,-0.315712228755321,0.75222094389565,0.946338517110213
"12254","NPIPB11",7.48678229019125,0.214940508671251,0.681573982464411,0.315359028075099,0.752489071679124,0.946598583314322
"12255","WWTR1",252.854302509914,0.0664697023671222,0.21093875949821,0.315113744506904,0.752675293140486,0.946755580886217
"12256","UROD",315.250939590839,-0.0852621250216899,0.270779901144933,

-0.31487612138559,0.752855712449442,0.946764101125324
"12257","CMC1",240.815368008311,-0.0811922237887895,0.257865939508489,
-0.31486214869458,0.752866321867936,0.946764101125324
"12258","AL606970.4",22.3941662591801,0.153078407148094,0.486117094554
869,0.314900275803438,0.752837372189219,0.946764101125324
"12259","DBI",1865.90853304995,0.084160422427621,0.267477644321056,0.3
14644697283944,0.753031438025777,0.946793341834238
"12260","TARS",212.804072645089,0.0619740316398185,0.197132203261832,0
.314378019493367,0.753233948396808,0.946793341834238
"12261","PACRG",5.25698037010837,-0.233567132460357,0.743831607744795,
-0.31400538781688,0.753516946698942,0.946793341834238
"12262","TTC26",34.4040259150234,-0.109956598937341,0.349687366716631,
-0.314442583298824,0.753184918233074,0.946793341834238
"12263","TMEM138",76.6131519205305,-0.066217531117336,0.21092251708687
2,-0.313942446884717,0.753564750996316,0.946793341834238
"12264","SNX6",696.251839127332,-0.0480155295469154,0.153087878761439,
-0.313646840856285,0.753789279505221,0.946793341834238
"12265","ARHGAP11A",5.33073928477095,0.237987854044013,0.7588045019199
09,0.313635268955128,0.753798069403482,0.946793341834238
"12266","RMC1",38.2238322122873,-0.103363939723025,0.32958505488008,-0
.313618406516139,0.753810877997509,0.946793341834238
"12267","TICAM1",46.4773554613761,-0.0915556191447497,0.29135808008196
4,-0.314237446646386,0.753340703751226,0.946793341834238
"12268","U2AF2",128.035037467539,-0.0617827966015977,0.196828501134942
,-0.313891515940775,0.753603434273265,0.946793341834238
"12269","SIRPA",230.149996414482,-0.0560701407430822,0.178692201004952
,-0.313780570320068,0.753687702281471,0.946793341834238
"12270","ZSWIM3",8.35944251156083,0.166493280304344,0.529577494628318,
0.314388889243107,0.753225693770176,0.946793341834238
"12271","TXNRD2",71.6870733299952,0.0898823615842886,0.286045558842381
,0.314223936732457,0.753350963844848,0.946793341834238
"12272","MAFF",1021.15336691402,-0.11689680752795,0.37145363876051,-0.
314700935271541,0.752988734067269,0.946793341834238
"12273","VBP1",246.333144029423,-0.0813537314225238,0.259104064764404,
-0.313980915337999,0.753535533690787,0.946793341834238
"12274","NEK11",78.0937773287133,0.0770990727290579,0.245916022133502,
0.313517891433697,0.753887229976037,0.946812094678231
"12275","ZSWIM7",192.633829368907,-0.104566219047623,0.334055362903151
,-0.313020626697553,0.754264991223767,0.947209355577546
"12276","PRDM16-
DT",3.0875628223013,-0.366108337297263,1.17174667563706,-0.31244666181
6401,0.754701093014303,0.947290713964704
"12277","NGF",4.07845803098906,-0.231268325024959,0.739622166022291,-0
.312684415975155,0.754520436551797,0.947290713964704
"12278","ECT2",16.2455762758727,0.132455940907184,0.423883493498448,0.
312481950674657,0.754674278080602,0.947290713964704
"12279","ADAMTS6",24.293530630729,0.207441679550657,0.664435334226746,
0.312207477334228,0.754882849843816,0.947290713964704
"12280","HIP1",80.6520112594211,0.0861905065560139,0.275529918430639,0
.312817232505773,0.754419522335283,0.947290713964704

"12281","NSMF",57.9136153319598,-0.0749753182544173,0.240092085933124,
-0.312277341266889,0.7548297586795,0.947290713964704
"12282","DKK1",90.0302618159632,-0.346182878035453,1.10693082786249,-0
.312741202360349,0.754477289650766,0.947290713964704
"12283","DNAJB12",227.848911759069,-0.0580020834297905,0.1856841189764
42,-0.312369650940096,0.754759612271425,0.947290713964704
"12284","ALG8",72.3396199119201,0.0695372360763129,0.222619348660835,0
.312359354632082,0.754767436367731,0.947290713964704
"12285","SPAG7",358.061822052529,-0.0732059387268704,0.234558036569858
,-0.312101600940319,0.754963309889739,0.947314564261321
"12286","FBXO11",207.048818408157,0.0631618615938222,0.202707341218309
,0.311591386943401,0.755351080812179,0.947346990937755
"12287","CRYGN",4.49214876478915,0.263958586104252,0.847700537377998,0
.311381878936513,0.755510328160317,0.947346990937755
"12288","CCL27",2.52298554871949,-0.294181524936466,0.943890093876035,
-0.311669257729388,0.755291893761387,0.947346990937755
"12289","GADD45G",135.383275996375,-0.0858695650123737,0.2753567133793
82,-0.311848452716182,0.755155698938904,0.947346990937755
"12290","OTUD1",170.496521439669,-0.0995383098574415,0.319279373651707
,-0.3117592869185,0.75522346729437,0.947346990937755
"12291","TMEM101",147.597092491093,-0.0836440743626901,0.2685265702850
9,-0.311492729653853,0.755426069070286,0.947346990937755
"12292","TRAPPC10",69.2412308814552,-0.0723625819386035,0.232391049934
562,-0.311382826313578,0.755509608034211,0.947346990937755
"12293","CCDC160",7.75563890688672,-0.212364149850581,0.68126660893191
1,-0.31171959269151,0.75525363655872,0.947346990937755
"12294","FAM122C",14.568341746819,0.146212987529631,0.469624998515939,
0.311339873285449,0.755542257968781,0.947346990937755
"12295","HNRNPK",1559.91862364141,0.0369262603247368,0.118657585937448
,0.31120016502108,0.755648457591547,0.947403088554185
"12296","APPL1",484.008950178844,0.0485215443784548,0.155993156075934,
0.311049187022253,0.755763229125098,0.947434198383391
"12297","HOXA4",16.5083444238655,0.164439679209699,0.5288725305336,0.3
10924976655131,0.755857656290557,0.947434198383391
"12298","MAGI2",109.968351327334,0.0789478707460291,0.253879045792394,
0.310966470271782,0.755826111620902,0.947434198383391
"12299","NTNG1",11.6706747314589,-0.175702125330357,0.565496571898363,
-0.310704138736913,0.756025550626969,0.947540251809323
"12300","UQCC1",55.2546190447028,0.0742842028902619,0.239123541514517,
0.310651985244842,0.756065202546524,0.947540251809323
"12301","AJAP1",3.24190535008834,-0.310923899004623,1.002457129279,-0.
310161791385783,0.756437924744176,0.947668651034638
"12302","BRAT1",80.3737943412227,-0.0668857180562022,0.215545311491517
,-0.310309315444492,0.756325747868849,0.947668651034638
"12303","C10orf143",15.8831433263672,-0.142983814441659,0.460996990661
822,-0.310162142786196,0.756437657534201,0.947668651034638
"12304","TMUB2",144.561011562222,0.0632708774693624,0.204001082746323,
0.31014971399951,0.756447108581743,0.947668651034638
"12305","MICALL1",14.493414149229,0.148762732098076,0.47970494906355,0
.310112981716118,0.756475040608578,0.947668651034638

"12306","C1orf198",48.8393400996795,-0.0991676579266423,0.31988051200681,-0.310014690499592,0.756549784951181,0.947673028381569
"12307","SCNN1A",3.29929631918874,0.274663139172954,0.886178523190782,0.309941091986747,0.756605753523256,0.947673028381569
"12308","SUPT6H",185.680855173531,0.068604481187597,0.22140058132873,0.309865858417665,0.75666296680638,0.947673028381569
"12309","AC091488.1",3.79455142437653,-0.22304962561206,0.720759055554619,-0.309464895228301,0.756967911962517,0.947723700117726
"12310","PYCR3",20.2342225957338,-0.13307053187589,0.429849490730102,-0.309574711022382,0.756884389821761,0.947723700117726
"12311","MORN4",48.0552739675009,-0.125539879696544,0.405674136384826,-0.309459904975199,0.756971707445948,0.947723700117726
"12312","MOB3A",112.796764442401,0.0568179631246134,0.183634155771885,0.309408469714066,0.757010828384662,0.947723700117726
"12313","USE1",251.949184718924,-0.0794347128271298,0.256636865416537,-0.309521832329906,0.7569246071941,0.947723700117726
"12314","S100A14",3.23379307757602,0.263144022822622,0.8509537988056,0.309234206594965,0.757143375098009,0.947735698508794
"12315","SMIM33",3.13783017175142,-0.256714858106488,0.830076643628958,-0.309266451570271,0.757118848626821,0.947735698508794
"12316","MTO1",56.1146427159914,0.0751179493152201,0.243254990089164,0.308803323161782,0.757471141097864,0.948068986685903
"12317","CDKN2B-AS1",4.79126436921871,-0.217463745065489,0.704405524069666,-0.308719533897326,0.75753488329173,0.948071789067306
"12318","KCNAB2",23.9606041536099,0.100948133393236,0.327187698519558,0.308532789741182,0.757676953750532,0.94817261260468
"12319","BORCS6",50.0080935585879,0.0829796973078546,0.269127803810445,0.308328222253468,0.757832593165865,0.948290398867749
"12320","AC010359.3",18.1163188547623,0.134964246355595,0.43826509654826,0.307951163390748,0.758119493508325,0.948457496081002
"12321","PCDHGA11",4.24725486567915,-0.224906519599268,0.730675124973729,-0.307806454485985,0.75822960992475,0.948457496081002
"12322","TNXB",7976.05309203377,0.0471455468196168,0.153210509609545,0.307717446667116,0.758297342968811,0.948457496081002
"12323","AC105206.1",3.06912228413279,-0.283498339878535,0.921685894369727,-0.307586718653646,0.758396827550725,0.948457496081002
"12324","MSC-AS1",66.7006214537703,-0.0775037949096271,0.251928477798589,-0.307642056137812,0.758354714998305,0.948457496081002
"12325","ZNF568",41.6880626256697,-0.078486137958424,0.254860632744741,-0.307957086636572,0.758114986311762,0.948457496081002
"12326","MED12",55.4082113373357,0.0765361954409242,0.248599763888063,0.307869139712402,0.75818190892346,0.948457496081002
"12327","GUCY1A1",12.7475510401939,0.383305205120404,1.24665943366536,0.307465852156134,0.758488811035612,0.948495580604685
"12328","PROX1",12.4020929460877,-0.198696932669138,0.646870211806766,-0.30716661401699,0.758716556066765,0.948634184132938
"12329","INSIG2",180.856322551202,-0.0499147953091895,0.162547795526698,-0.307077651514451,0.758784267944153,0.948634184132938

"12330","FBXO21",160.896238915613,-0.0679238736074114,0.221193678312499,-0.30707872903786,0.758783447799536,0.948634184132938
"12331","MKX",3.4196569518489,0.39867159062285,1.2989876279065,0.306909459380584,0.758912288789463,0.948717292327433
"12332","ZNF517",8.72135778656,-0.164416231207011,0.536036158041455,-0.306726008573279,0.759051931436278,0.948814914295347
"12333","ADGRB2",4.78026352608882,0.22875309870009,0.746169682460708,0.306569811233433,0.759170834953302,0.948886598622002
"12334","CEBPZ",281.064365713579,-0.0634258166322944,0.206989269130032,-0.306420796106343,0.759284276396612,0.948951444839774
"12335","SDC1",3.00143394355779,-0.354429047707847,1.15982761433138,-0.305587695385378,0.759918590250939,0.949282420479597
"12336","YTHDC1",359.941332589417,0.0667246981093965,0.218321518352839,0.305625843081394,0.75988954147745,0.949282420479597
"12337","NRF1",29.3030473411173,0.104860539215432,0.342723397820746,0.305962592230943,0.759633127847871,0.949282420479597
"12338","ERP44",438.810035625775,-0.0617414470952859,0.201979600933806,-0.305681597596186,0.759847086042399,0.949282420479597
"12339","MRVI1",3.46535067408414,-0.28708403093255,0.939131969982501,-0.305690829519838,0.759840056271919,0.949282420479597
"12340","ZNF579",45.8328920273113,-0.0859059042125701,0.280868423972246,-0.305858177283249,0.759712630541844,0.949282420479597
"12341","TBX19",19.1606188508531,0.131806128621899,0.431998481710207,0.305107851537119,0.760284011480631,0.949297490358069
"12342","RHOQ",591.391997392794,-0.0743749335630718,0.2446892321187,-0.303956708348295,0.76116087332451,0.949297490358069
"12343","MEIS1-AS2",9.05441023375684,-0.160200452157362,0.525413381050655,-0.304903639562841,0.760439543704458,0.949297490358069
"12344","AMT",22.5783577760285,0.124842904776819,0.409645326315993,0.304758523426964,0.760550073155572,0.949297490358069
"12345","TIMMDC1",311.690513007896,-0.0694304988616133,0.228059929412082,-0.304439710389278,0.760792918105782,0.949297490358069
"12346","FAM13A",382.080782303634,0.0812671627240628,0.266094490838793,0.30540715994491,0.760056069283024,0.949297490358069
"12347","AC099522.2",4.80717295037202,0.245131716154829,0.804176236444872,0.30482337707281,0.760500675991535,0.949297490358069
"12348","MOCS1",30.1603420738364,0.100715929236917,0.331214880215993,0.304080327463663,0.761066694043995,0.949297490358069
"12349","C7orf26",69.6524498623811,0.065475058648416,0.215158684264171,0.3043105551251,0.760891304430199,0.949297490358069
"12350","CDK13",229.114160514828,0.0823386934949056,0.270302300671167,0.304617065006317,0.760657821368933,0.949297490358069
"12351","AL583785.1",57.0317149878605,0.163686129877581,0.538313836182727,0.304071935134171,0.761073087632125,0.949297490358069
"12352","BET1L",104.05541015217,-0.0683067635491698,0.223848820991295,-0.305146854232599,0.760254307292358,0.949297490358069
"12353","TWF1",179.112264013941,-0.0527799909427123,0.173613468477669,-0.30400861986984,0.7611213240766,0.949297490358069
"12354","METTL25",39.7514658204622,0.0952091993364301,0.31208232751479

2,0.305077189389769,0.760307363812214,0.949297490358069
"12355","MED6",155.568565870915,-0.0591376123093853,0.193939358356638,
-0.30492836941657,0.760420708401437,0.949297490358069
"12356","SETD1A",51.353282102008,0.076626857077682,0.252093687545136,0
.303961823970552,0.761156975915113,0.949297490358069
"12357","GOSR1",253.549183301888,-0.06144352126054,0.201427855774001,-
0.305039841805588,0.760335808076325,0.949297490358069
"12358","RAPGEFL1",7.95180505320822,-0.179309624247849,0.5887352182179
09,-0.304567518129144,0.760695562090163,0.949297490358069
"12359","PRR22",2.3615812713662,-0.280695461071277,0.923477563112998,-
0.3039548249825,0.761162308194988,0.949297490358069
"12360","ZNF283",24.5576405754724,-0.116962895290704,0.384046102321705
,-0.304554308932232,0.760705623862191,0.949297490358069
"12361","WDR41",165.171406566136,-0.0547178334679437,0.180193898280067
,-0.303660856389811,0.761386282643476,0.949325327108538
"12362","AC079174.2",4.78412039875886,0.223795256219697,0.737019838011
018,0.303648890677962,0.761395399733894,0.949325327108538
"12363","CLCN7",92.3260574484796,-0.0581309197277237,0.191470672638345
,-0.303602211903872,0.761430966225752,0.949325327108538
"12364","PRSS53",10.4420515288986,-0.145721089559313,0.479733732038086
,-0.303754103219375,0.761315235788472,0.949325327108538
"12365","NFKB1",252.142207436479,0.0524199455127146,0.172742246376183,
0.303457588472938,0.761541164009205,0.949367733517844
"12366","AC073073.2",2.7782258296515,0.333692437580913,1.0998580224379
1,0.303395920903739,0.761588153920315,0.949367733517844
"12367","UBQLN4",36.5214062863117,0.0939019751095673,0.310165076116032
,0.302748382524017,0.762081623064482,0.949368693998626
"12368","CCDC149",66.212194438029,-0.0762565167067549,0.25165577800248
4,-0.303019137140583,0.761875277492879,0.949368693998626
"12369","DOLK",63.4238628252597,0.0948842123720635,0.312834443699737,0
.303304876694252,0.761657530068469,0.949368693998626
"12370","TIMM23",78.1237704483301,-0.07316461850100 2,0.241521301226034
,-0.302932363023869,0.76194140731932,0.949368693998626
"12371","PRKD1",33.4844711358082,0.08504147246768,0.280838329477288,0.
302812912418201,0.76203244246732,0.949368693998626
"12372","AP5M1",76.5701315130089,0.066196391739398,0.218559623511944,0
.302875666949437,0.761984615861027,0.949368693998626
"12373","AC087286.2",3.56453963846058,0.239249546977441,0.790131310063
473,0.302797198301408,0.762044418683088,0.949368693998626
"12374","EFHC2",4.1379125098464,0.241397897308078,0.796781763478243,0.
302966142515973,0.761915664045083,0.949368693998626
"12375","MCM4",48.7722456185686,-0.0835703717782322,0.276203858500503,
-0.302567720204676,0.762219317548627,0.949463497374714
"12376","DNAJB6",531.172812910341,-0.050491399352148,0.166938582436988
,-0.302454942500823,0.762305276575648,0.949480069654121
"12377","PLAGL2",35.110081241722,0.0823178069287945,0.272297959519513,
0.302307836144088,0.762417405266215,0.949480069654121
"12378","ACO2",140.273054331044,0.0610895654318798,0.202035243803167,0
.302370835315229,0.762369384877767,0.949480069654121
"12379","CENPE",16.1429044001412,0.153836603368965,0.509255386978003,0

.302081445386084,0.762589976546286,0.949618263871152
"12380","HOXA-
AS2",5.05472618679113,0.204770280316879,0.678614709480745,0.3017474827
116,0.762844568404027,0.94978184491948
"12381","AC011468.1",9.01092642936798,0.180357852463602,0.597646236967
13,0.301780286242346,0.762819559946203,0.94978184491948
"12382","GZMK",18.1332023136658,0.163089049842933,0.54065356023386,0.3
0165167093765,0.762917613931332,0.949796076461919
"12383","SHC1",198.167237216288,0.065956747903331,0.218843842699913,0.
30138726815254,0.76311920178628,0.94982763480519
"12384","ZNF416",10.7030520135849,-0.141781042646378,0.470219540954123
,-0.301520949892236,0.763017277193354,0.94982763480519
"12385","ARMCX1",415.748001276649,0.0641129502639855,0.212734113266139
,0.301375972474041,0.763127814275853,0.94982763480519
"12386","ETS1",139.572303910783,0.0969940242104548,0.322016892066381,0
.301207876357183,0.763255984104957,0.949910463020985
"12387","COL27A1",16.6610233978741,0.159052997602601,0.528860010410858
,0.300746879082494,0.763607518331597,0.950271243649113
"12388","STXBP5-
AS1",2.98340121006718,0.262212327459759,0.872294491599302,0.3006006916
07037,0.763719004012516,0.95032522067017
"12389","MAFB",824.087034527491,0.0827467471967198,0.275337594787469,0
.300528328725292,0.763774191299561,0.95032522067017
"12390","MMRN2",6.59879932915654,-0.17586767455394,0.585405694078115,-
0.300420163884624,0.763856685057288,0.950351154169337
"12391","IFI35",199.114879227875,-0.0814135381464743,0.271176727123336
,-0.300223175528798,0.764006928467366,0.95046136730889
"12392","ATAD3B",23.1872249557163,-0.108973200229458,0.363202854195459
,-0.30003398643672,0.764151231728547,0.950468222602272
"12393","KIF9-
AS1",5.1717700115403,0.19005824090299,0.633279213730889,0.300117604971
249,0.764087450998623,0.950468222602272
"12394","SFMBT1",30.8671949454605,-0.0906606328034019,0.30247963742638
1,-0.299724747010341,0.764387120561196,0.950468222602272
"12395","CC2D2A",114.028181556794,0.0747054980287082,0.249340952670566
,0.299611825608971,0.764473262821534,0.950468222602272
"12396","AC067838.1",8.92846481191575,-0.148482393038437,0.49501339863
8771,-0.299956311176113,0.764210480610677,0.950468222602272
"12397","CWF19L2",273.850119451272,0.0804297344986108,0.26847451790520
3,0.29958051559668,0.764497148227478,0.950468222602272
"12398","NAA30",73.1498741339615,0.0788831676296286,0.263366469667325,
0.29951864308798,0.764544349442234,0.950468222602272
"12399","SERPINF1",6812.31517055154,-0.0843331159064359,0.281590524764
327,-0.299488471698461,0.764567366867867,0.950468222602272
"12400","MYCBPAP",2.88642201340852,0.274197127033296,0.915315572260912
,0.299565674771603,0.764508469896048,0.950468222602272
"12401","WARS2",103.617994019175,0.0691237041819339,0.231013694751599,
0.299219075545544,0.764772895486446,0.950476549121723
"12402","RNF25",29.6564121387539,-0.104162346579567,0.348168711824286,
-0.299172048038987,0.764808775568769,0.950476549121723

"12403","SMUG1",95.1093106405264,0.0770686168968871,0.257679673614796,
0.299086908236684,0.764873735072948,0.950476549121723
"12404","CNEP1R1",54.0197665316779,0.0786802237922218,0.26292947403811
5,0.299244594315874,0.764753425909813,0.950476549121723
"12405","STARD3",105.231877909165,-0.066067010023217,0.220904043530227
,-0.299075602996724,0.764882360807978,0.950476549121723
"12406","RRAGC",390.744304959052,0.0665320295703519,0.222631820990737,
0.298843306739696,0.765059605956543,0.950620169742069
"12407","ARL6",35.2190589020915,0.085093052418787,0.284955013976584,0.
298619249513467,0.765230576272519,0.950702220091976
"12408","MBD4",204.144730196345,-0.0535302245073396,0.179273606823346,
-0.298595122036495,0.76524898779768,0.950702220091976
"12409","NFKBIA",3053.74868683362,-0.0833390311849013,0.27918016800390
3,-0.29851343589612,0.765311322959725,0.950703041616904
"12410","AC073611.1",17.4707447295537,0.16582972141456,0.5556929790498
86,0.298419680770653,0.765382869906952,0.950715305367903
"12411","CIAPIN1",95.6813281228377,0.0680889363725893,0.22836761753876
1,0.298154953431751,0.765584900951084,0.950824841117828
"12412","C18orf25",57.4165131690497,0.0810479784135236,0.2718431029351
47,0.298142485641282,0.76559441634476,0.950824841117828
"12413","WDR33",296.780864609856,-0.0478253073454645,0.160509468965147
,-0.297959414194121,0.765734140192711,0.950921757115173
"12414","RNF122",25.2727170443405,-0.177761208879912,0.596916840477481
,-0.297798950918722,0.765856615292875,0.950997238983379
"12415","ATF7IP2",15.7271353191831,0.161752928634651,0.544037873593067
,0.29731924280632,0.766222791900064,0.951375299004389
"12416","SATB2-
AS1",7.61463084467349,0.169596386080385,0.571709130413712,0.2966480279
18774,0.76673523943033,0.951398512100018
"12417","DNAJB2",152.495985420119,-0.0620401189076777,0.20909360761055
2,-0.296709782841524,0.766688087584622,0.951398512100018
"12418","MYOF",784.197257821536,0.0598031997953842,0.201492912103378,0
.296800513581845,0.766618813348718,0.951398512100018
"12419","PWWP2B",8.35471821439575,0.191630620586986,0.64529791954179,0
.296964572151508,0.766493556988312,0.951398512100018
"12420","BCL9L",203.550319363442,-0.0796556568513555,0.268500460062379
,-0.296668604712445,0.766719528298591,0.951398512100018
"12421","PIGBOS1",175.121632798348,0.0573274864555999,0.19311005552581
5,0.296864325886625,0.766570092846035,0.951398512100018
"12422","FAM104A",125.794961058564,0.063128778501479,0.21255337063323,
0.297002010899232,0.766464973897487,0.951398512100018
"12423","LINC01436",73.8414615996863,-0.277071012308268,0.933555770855
737,-0.29679106589882,0.766626026704705,0.951398512100018
"12424","MYOC",1955.78861437848,-0.219354840411181,0.740652885194619,-
0.29616416110165,0.76710471700578,0.951413646810541
"12425","RTN4IP1",12.858158526691,0.141261153958781,0.476549665546962,
0.296424830760605,0.766905664754045,0.951413646810541
"12426","CCN6",4.13045786236443,-0.213409721508204,0.72072925832236,-0
.296102480985658,0.767151819350668,0.951413646810541
"12427","AC004943.2",6.55833989488368,0.178751366512918,0.603176680673

593,0.296349929034556,0.766962859545893,0.951413646810541
"12428","PDRG1",74.413877950409,0.0878490661058684,0.296491971896327,0
.296294923413934,0.767004862524301,0.951413646810541
"12429","LINC01638",24.1402442620571,-0.108719109869342,0.366874769870
498,-0.296338475136129,0.766971605829245,0.951413646810541
"12430","OSM",4.65230949884504,-0.270191885175827,0.912606120150261,-0
.296066264744466,0.767179476474539,0.951413646810541
"12431","MOAP1",88.4841672977333,0.0687747566916775,0.232536310804298,
0.29575921478155,0.76741397210376,0.95162789638641
"12432","RIC3",2.37300382789172,-0.283840253469873,0.960637404687311,-
0.295470748989066,0.767634294308981,0.951747980919564
"12433","SPATA13",107.386139411588,0.0778910285383191,0.26360132607283
,0.295487999619542,0.767621118225758,0.951747980919564
"12434","GATA3",5.2118735453745,0.256204838854235,0.867484016211344,0.
295342431752444,0.767732305506128,0.951792945904533
"12435","PHLPP2",44.6722196181742,0.0854290977677136,0.28941213137929,
0.295181467896915,0.767855258076814,0.951868822135431
"12436","SENP5",114.943621782364,-0.0631044321889296,0.214075830680078
,-0.294776070649634,0.768164947473123,0.952040182387999
"12437","GPRIN3",8.03917107662372,-0.206641134792794,0.700784543554,-0
.294871136490571,0.76809232184366,0.952040182387999
"12438","ALG2",112.325138119631,-0.0534871407562801,0.181461217672341,
-0.294757973314497,0.768178773178199,0.952040182387999
"12439","AP001160.1",8.66070588697219,-0.189470767734894,0.64341931807
3356,-0.294474788699603,0.768395125516102,0.952231759774155
"12440","MAP4K4",385.847246483942,-0.0761546965306949,0.25913667866134
2,-0.293878492709321,0.768850753138171,0.952260514191299
"12441","BLOC1S4",174.13753581509,-0.0664491495596523,0.22603798336260
7,-0.293973378151474,0.768778246167014,0.952260514191299
"12442","KCNC1",4.53204702452449,-0.248740600935914,0.846383958904359,
-0.293886241957974,0.768844831452708,0.952260514191299
"12443","AL136295.2",5.19840583905405,-0.194372684990501,0.66135220498
8609,-0.293901923248065,0.76883284843964,0.952260514191299
"12444","OSER1-
DT",77.9828268310874,0.0842397808792534,0.286483917738987,0.2940471547
02776,0.768721871010346,0.952260514191299
"12445","PACSIN2",90.4093116562725,-0.0620518750037557,0.2108408685819
74,-0.294306675081971,0.768523572435582,0.952260514191299
"12446","SEPTIN6",108.848510175332,-0.0608364954786786,0.2067888319246
79,-0.29419623348343,0.768607958607552,0.952260514191299
"12447","CARD16",23.9848969475451,-0.108063982526606,0.367966242926717
,-0.293679065957491,0.769003152227913,0.952372747778041
"12448","NAA80",38.0430055228047,-0.0933942912873902,0.318294585129379
,-0.293420924045653,0.769200433862425,0.952387525139701
"12449","HAUS5",26.4567600434973,-0.0947787944044897,0.322961148919149
,-0.293468098939098,0.769164379940858,0.952387525139701
"12450","LINC01547",5.27588534988152,0.191868511238414,0.6536690525728
32,0.293525462897811,0.769120539588667,0.952387525139701
"12451","ALX1",5.7043204383019,0.306670704202583,1.04606108335541,0.29
3167109533303,0.769394422907477,0.952551203045438

"12452","THRAP3",705.361960259579,-0.0450710535650699,0.15384083950889
9,-0.292971968359955,0.769543578056243,0.952631460747329
"12453","HSPA1B",385.029872443757,0.0910179756568453,0.310725764203309
,0.292920594757285,0.769582846622542,0.952631460747329
"12454","AL020997.2",10.1383309022224,0.189284655576531,0.646686681567
165,0.292699170976929,0.769752103620068,0.952687970879434
"12455","FNIP1",341.615431566721,0.068720492422766,0.23472235441447,0.
292773530643017,0.769695261645939,0.952687970879434
"12456","TCEANC2",28.1570302313321,0.128727096522289,0.441092327114019
,0.291837079471605,0.770411193365977,0.952738752164985
"12457","BX664615.2",11.2135147386484,0.199688785327111,0.684218246143
79,0.291849547205944,0.770401660299452,0.952738752164985
"12458","ABL1",333.135509560814,0.0496804237002477,0.169863173013551,0
.292473187795004,0.769924857148838,0.952738752164985
"12459","DDX6",400.093777750366,0.0545926646670334,0.186741889719714,0
.29234289504606,0.770024465025958,0.952738752164985
"12460","DMAC2L",86.5486547213194,-0.0736884983123861,0.25222158862356
2,-0.29215777568654З,0.770165993980289,0.952738752164985
"12461","DET1",38.0963671625716,0.073617219090859,0.251958588528998,0
.292179836062176,0.770149127801137,0.952738752164985
"12462","DNAJA3",55.1624564666109,-0.070596572152758,0.241337471044325
,-0.292522217321951,0.769887375408066,0.952738752164985
"12463","ADCYAP1",2.60479987420045,-0.386676992030021,1.32389325176207
,-0.29207565754668,0.770228778057861,0.952738752164985
"12464","PGRMC1",914.758052399741,0.064382990390015,0.220504306457531,
0.291980648470533,0.770301419882388,0.952738752164985
"12465","DKC1",126.235923525796,-0.0628756996656437,0.21528539288209,-
0.292057435127892,0.770242710357384,0.952738752164985
"12466","AC009309.1",3.16843135051987,-0.345595504745223,1.18570880420
472,-0.291467435781604,0.77069384570661,0.953011842737637
"12467","CIDEB",26.0788002408102,0.0918905142243995,0.315531801187638,
0.291224256567897,0.770879812126752,0.953016689602335
"12468","PHB",569.591480822399,-0.0711014635777002,0.244150531631618,-
0.291219777825348,0.770883237278723,0.953016689602335
"12469","ZNF540",10.0859900267662,0.140457370613309,0.482288242805651,
0.291231172869188,0.770874522842219,0.953016689602335
"12470","NEURL2",18.5749081860834,0.130533375491285,0.448470003903352,
0.291063782092805,0.771002538972877,0.95303503040711
"12471","PTPN1",137.90592498154,0.0702895805282303,0.241512833314939,0
.291038697875615,0.771021723270002,0.95303503040711
"12472","MRPL19",233.328462214461,-0.0605039920482889,0.20811678958447
8,-0.290721340498717,0.771264448863772,0.953258617642322
"12473","NDUFB8",901.423474482301,-0.0823939920919151,0.28374337936751
1,-0.290382077902852,0.77152395307266,0.953426465978439
"12474","CCT8",437.951908393298,-0.0784539892265808,0.270131181461663,
-0.290429223320577,0.771487889699887,0.953426465978439
"12475","ZNF19",10.8209154964009,0.154851763095524,0.533456358895283,0
.290280096044222,0.77160196467143,0.953446435704216
"12476","PTAFR",3.9936802665191,-0.295746357500674,1.02034788708718,-0
.289848551894345,0.771932102360396,0.953539153551667

"12477","CD8A",5.53424760571128,-0.198637198535504,0.685282963548567,-0.289861574125395,0.771922139555517,0.953539153551667
"12478","FAM120A",442.909694510266,0.0488562019343163,0.168594738483217,0.289784855529047,0.771980834525554,0.953539153551667
"12479","TBC1D10C",8.49439522633491,0.191077984973901,0.658721920864024,0.290073821626082,0.771759762511603,0.953539153551667
"12480","ZNF41",16.1431638499144,0.111643618894171,0.385273292288385,0.28977772695078,0.77198628844144,0.953539153551667
"12481","POLD3",115.225869445174,-0.0609230151087184,0.210373686680657,-0.289594274217376,0.772126647901978,0.953582806156915
"12482","ALDH1A3",285.150207618578,-0.115688087555136,0.399517062034451,-0.289569829548758,0.772145351050964,0.953582806156915
"12483","DPH5",97.9991478200949,0.0739855290643664,0.256351807479772,0.288609352092063,0.772880337989177,0.953665103337418
"12484","MRPL55",270.970379649347,-0.0676133053169067,0.234327389557656,-0.288542049841214,0.772931847391516,0.953665103337418
"12485","SEPTIN10",506.386953542647,-0.0418967454880958,0.145240438699882,-0.288464740695732,0.77299101674929,0.953665103337418
"12486","NAIF1",20.2758115510993,0.101706656730683,0.352423842930986,0.288591872459094,0.772893715832612,0.953665103337418
"12487","TAF5",21.4404663888431,0.113896582287186,0.393962740443557,0.289104959923243,0.772501058124908,0.953665103337418
"12488","RRP8",75.9946841327071,-0.0674403019155793,0.233441317128892,-0.288896167760837,0.772660836436461,0.953665103337418
"12489","LTO1",38.0458547122422,0.0850545841482775,0.294968980590705,0.288350944488966,0.773078114259123,0.953665103337418
"12490","SQOR",242.867005319569,0.0571752943428576,0.197735203038,0.289150811107064,0.772465971773784,0.953665103337418
"12491","AC007950.2",5.16958630341232,-0.203833859923801,0.704784513458074,-0.28921444218982,0.772417280617151,0.953665103337418
"12492","L3MBTL4",24.0111870592941,0.103401893310909,0.3585775444394,0.288366895569458,0.77306590542518,0.953665103337418
"12493","ELL",53.5728878105279,0.111952780320897,0.388010425070145,0.288530341164566,0.772940808664044,0.953665103337418
"12494","GEMIN7-AS1",3.77689151666979,-0.222508867578836,0.770660529199604,-0.288724878397406,0.772791922908495,0.953665103337418
"12495","ZNF845",23.1338281673173,0.10272007479213,0.35622594460901,0.288356523006414,0.773073844498523,0.953665103337418
"12496","MRPL40",402.715257555606,-0.0857022838597735,0.296918821763614,-0.288638771199232,0.772857822553822,0.953665103337418
"12497","LINC00339",28.6622035084419,-0.0909027437981722,0.316105587776137,-0.287570822261291,0.773675282269656,0.953958207169067
"12498","WDR3",55.2570836548348,0.0822786230215351,0.28621175331482,0.287474647943729,0.77374891107589,0.953958207169067
"12499","RFESD",16.1334147216755,0.127267629583236,0.442361411947442,0.287700568236629,0.773575955014814,0.953958207169067
"12500","GIMAP1",5.38484144691201,-0.20727662956111,0.720610480999309,-0.287640320292966,0.773622077470828,0.953958207169067
"12501","AC092747.4",35.396343959344,0.0858940027576965,0.298294902035

041,0.28794995211687,0.773385049169166,0.953958207169067
"12502","SNRNP35",155.899449820099,0.0699576508728908,0.24332403873773
,0.28750817730876,0.773723241547721,0.953958207169067
"12503","ARMC6",46.1488204856273,0.0956295991462116,0.332283315087105,
0.287795368603275,0.773503382774987,0.953958207169067
"12504","GCNA",4.78138855750587,-0.193961958812319,0.675091022938096,-
0.287312306373395,0.77387320059994,0.954035139735131
"12505","SDHC",375.184126130208,0.0591146589741615,0.205916991120833,0
.28708004449945,0.774051031540314,0.954178060871167
"12506","KDM8",5.4714198528981,0.178926470571756,0.624630422542638,0.2
86451738683193,0.774532152305886,0.954618463883844
"12507","LINC00266-1",3.42508569298344,0.229789050810908,0.80215210238
4404,0.286465684161218,0.774521472713674,0.954618463883844
"12508","AL162274.2",3.44844844221748,0.271954830581761,0.950596436670
59,0.286088628245092,0.774810241070119,0.954884862975367
"12509","SLC9A5",2.5236340067312,-0.286970850616127,1.00343469077409,-
0.285988568319027,0.774886877218337,0.954902966849521
"12510","LZTFL1",65.4850502429933,-0.063710309852115,0.222846129319219
,-0.285893724278479,0.774959520531324,0.954916147800988
"12511","SETD6",29.2735339607672,-0.0992479794540928,0.347295398510248
,-0.285773954621412,0.775051257782784,0.954952852587452
"12512","AC119800.1",2.93942003990772,-0.265579186859082,0.93054282610
6345,-0.285402433298358,0.775335843526914,0.955117291096675
"12513","GATM",5.99284059819631,-0.208036515148206,0.729039304331144,-
0.285357063620964,0.77537059881828,0.955117291096675
"12514","CRISPLD2",449.436195508696,-0.0933106721873795,0.326880960841
098,-0.285457653903371,0.775293542572229,0.955117291096675
"12515","DNAAF1",3.00697188280701,-0.252619428278918,0.886056543214639
,-0.285105313214445,0.775563459558632,0.955278524098786
"12516","WDR63",2.60278708239291,-0.291280539986821,1.02225987475439,-
0.284937858934163,0.775691750460286,0.955280028330241
"12517","ARHGEF11",76.0349378435256,-0.0695599663002453,0.245303214276
549,-0.283567284290964,0.776742011678691,0.955280028330241
"12518","C1orf229",2.79535774505577,0.294584744085955,1.03878099838116
,0.283586958699703,0.77672693243214,0.955280028330241
"12519","LINC00954",7.08273937676799,-0.171553193183174,0.603176671389
669,-0.284416160837139,0.776091475615643,0.955280028330241
"12520","DNAJC27-
AS1",11.6167101184981,0.158589425198292,0.559594592937684,0.2834005674
8181,0.776869793424488,0.955280028330241
"12521","BABAM2",124.222741111096,0.0705324054293566,0.248765979839011
,0.28352914443929,0.776771243812103,0.955280028330241
"12522","CLSTN2",58.474946521206,-0.123908098029642,0.435771515617861,
-0.284341893833878,0.776148383829298,0.955280028330241
"12523","AC073475.1",2.98521262907753,-0.235334624244933,0.82748683289
6018,-0.284396820456121,0.776106295359147,0.955280028330241
"12524","KIAA0408",126.266657382877,0.103801568994046,0.36563350282003
8,0.283895125018497,0.776490752535719,0.955280028330241
"12525","ST7-
OT4",9.22710967855565,0.164206564085782,0.576711505141894,0.2847291282

06625,0.775851672848271,0.955280028330241
"12526","ZNF572",5.169308507489,0.213110782748313,0.751773567791492,0.28347735525522,0.776810937926576,0.955280028330241
"12527","CBWD6",3.84069704872172,0.224591898549083,0.790755784569976,0.284021821821031,0.776393657593077,0.955280028330241
"12528","POLL",71.8163701526216,-0.0662955416541673,0.232936417579353,-0.28460788717841,0.775944567966905,0.955280028330241
"12529","B3GAT3",173.393671351024,-0.0723365103684048,0.25472871169407 6,-0.283974703469154,0.776429766649426,0.955280028330241
"12530","SSPN",1961.43558093489,-0.0709148873927089,0.249475361562308,-0.284256076225778,0.776214144365108,0.955280028330241
"12531","ARMH4",71.2367577947782,0.0668107028851119,0.23520041267032,0.284058612510856,0.776365463456202,0.955280028330241
"12532","LPCAT4",48.569752944001,-0.092192097768438,0.324826946565747,-0.283819118897446,0.776549002011415,0.955280028330241
"12533","EIF2AK4",268.396191048411,-0.0545403567225143,0.191636522619952,-0.284603143371982,0.775948202746064,0.955280028330241
"12534","OAZ1",4000.53497822186,-0.0580727469744628,0.204968970439485,-0.283324577617509,0.7769280386102,0.955280028330241
"12535","ASXL1",284.913407671824,-0.0743737000863868,0.26126384984516,-0.284668928098798,0.775897797890706,0.955280028330241
"12536","UQCR10",1126.47874187001,0.0766221412379899,0.270362657108305,0.283405045865102,0.776866360844433,0.955280028330241
"12537","TBC1D8B",99.4523370962508,0.0598960866771232,0.210469143695809,0.284583695383353,0.775963104152385,0.955280028330241
"12538","BIRC2",340.614507855606,0.0402820849460041,0.142251392155465,0.283175330206823,0.777042438347383,0.955319380920977
"12539","ALKBH2",93.8934837023792,-0.0737962281831476,0.260652499795085,-0.283121121958022,0.777083990747203,0.955319380920977
"12540","SOGA3",3.68127248690676,-0.202639465097368,0.716032373546221,-0.283003216871013,0.777174371063568,0.955354300633565
"12541","SLC10A6",3.12729529836516,-0.253107877189624,0.895402835785277,-0.282674866634352,0.777426084306535,0.955380314134227
"12542","SPATA24",6.36167484025376,-0.168411397364729,0.596294731135774,-0.282429792803891,0.777613973097335,0.955380314134227
"12543","FKBP5",471.297803099915,0.193758061544907,0.686481526487484,0.28224803446104,0.777753328710374,0.955380314134227
"12544","TDRP",9.07128693098182,-0.151152207981485,0.535506138116154,-0.28226045832681,0.777743803002848,0.955380314134227
"12545","AC011029.1",2.9806866557547,-0.323750913260649,1.144957373579,-0.282762416078988,0.777358966629845,0.955380314134227
"12546","AC234917.3",2.358096565011,0.274862856924747,0.972604104260972,0.282605076125604,0.777479588711867,0.955380314134227
"12547","PXMP2",87.7958211620037,0.0755308696566848,0.267519774781786,0.282337519603157,0.777684718823855,0.955380314134227
"12548","MBOAT7",93.0306965839163,-0.0602940776619483,0.213490921412474,-0.282419867144877,0.777621582996937,0.955380314134227
"12549","TCN2",108.053588196255,0.0965305717382738,0.341329941485129,0.282807219660451,0.777324619684056,0.955380314134227
"12550","LINC02454",2.48700788337251,-0.282032647883734,1.000011932176

74,-0.282029282660488,0.777921056990612,0.955434076448911
"12551","L3MBTL2",53.7726394146015,0.0817365446625414,0.28976472637871
8,0.28207900141618,0.777882934149079,0.955434076448911
"12552","MAGI3",136.904082189204,0.0744120775418522,0.264251578004573,
0.281595584419043,0.778253626374725,0.955538004824084
"12553","CLUAP1",164.807952788001,0.0502738636229015,0.178524365293764
,0.281607855264884,0.778244216260661,0.955538004824084
"12554","PRR14",77.1292924529767,-0.0607880926291825,0.215819413862131
,-0.281661837280379,0.77820281958835,0.955538004824084
"12555","HCCS",49.2084312493024,-0.0835574707292906,0.296525394787663,
-0.281788582691627,0.778105626020158,0.955538004824084
"12556","MCPH1-
AS1",3.90394160767026,-0.217959332364762,0.774741108901061,-0.28133182
7962413,0.77845590051357,0.955710234662049
"12557","IFT88",68.7801373631773,-0.0618585413760832,0.219967877237144
,-0.281216249177122,0.778544542313212,0.955742941766199
"12558","MMGT1",115.512055430771,-0.0560266373900607,0.199507907351318
,-0.280824144435549,0.778845283926338,0.956035997111364
"12559","ABRAXAS2",74.0373656475763,0.059029190402817,0.21027974303236
8,0.280717436456685,0.778927133939329,0.956054379226567
"12560","SIX1",109.707733100932,0.0625973699398314,0.223049889607679,0
.280642909305777,0.778984301205688,0.956054379226567
"12561","CT70",2.42303012825439,0.312556442841718,1.11428312846126,0.2
80500022712661,0.779093908040423,0.956112777043478
"12562","ARHGEF28",3.72779939506781,0.274769819296062,0.98004970413888
2,0.280363147027821,0.779198908089095,0.956165512513405
"12563","C1orf56",127.796879208348,-0.094186423081598,0.33610994373841
8,-0.28022504194312,0.779304855318637,0.956219401793902
"12564","AKR7A3",2.9229114501048,0.284306772310224,1.02229766882006,0.
278105664310446,0.780931247707671,0.956311978345547
"12565","MACO1",178.189316280932,-0.0438447755142601,0.157571589124547
,-0.278253051567593,0.780818112877576,0.956311978345547
"12566","LDLRAP1",41.6298273076439,0.071570360641297,0.255603933667642
,0.280004926427927,0.779473725273511,0.956311978345547
"12567","SH3GLB1",725.990015488421,-0.0387347763738776,0.1390515335192
98,-0.278564179721914,0.780579305359424,0.956311978345547
"12568","PRCC",118.767019887989,0.0658005187802978,0.235835739517739,0
.279009953770592,0.780237185982408,0.956311978345547
"12569","MOGS",75.0938670704684,-0.0699474719512967,0.249939841539418,
-0.279857230925968,0.779587041307644,0.956311978345547
"12570","AC069148.1",4.3896834293638,0.21936577697873,0.78875848445664
1,0.278115267602917,0.780923876054904,0.956311978345547
"12571","CMTM6",351.01039118532,-0.0447042965544201,0.160475432234368,
-0.278574084094887,0.78057170356262,0.956311978345547
"12572","CEP19",28.638749310918,-0.0927063446070875,0.333176805206517,
-0.278249695532149,0.780820688927231,0.956311978345547
"12573","ERCC8",23.742362524762,-0.0992716217589247,0.355390406392665,
-0.279331180508123,0.779990679630424,0.956311978345547
"12574","AC010245.2",6.14723404418509,0.173722008064313,0.623995233411
981,0.278402780602038,0.780703185244172,0.956311978345547

"12575","C6orf226",115.348714070561,0.0832342285916373,0.299190996405597,0.278197638269841,0.780860647720457,0.956311978345547
"12576","MED23",74.6444612149375,-0.0671936157390915,0.240088679703654,-0.279869987298151,0.779577254084705,0.956311978345547
"12577","AL355304.1",5.97120072372039,0.204470672687633,0.734146604392591,0.278514770025811,0.780617228565038,0.956311978345547
"12578","YKT6",80.4873825468343,0.0636520014041387,0.227572471277089,0.279699917335946,0.779707741753622,0.956311978345547
"12579","CNOT7",210.686487120917,0.0472767925102545,0.169128971205651,0.279531012181045,0.779837341857569,0.956311978345547
"12580","CCDC88B",5.66293279748111,0.257443376765846,0.924306433489792,0.278526003323214,0.780608606676103,0.956311978345547
"12581","NECAP1",120.103639938636,-0.0572240449261987,0.205243692376754,-0.278810248751303,0.780390448859468,0.956311978345547
"12582","DNM1L",176.772223301416,-0.0626719754402446,0.224433554954504,-0.279245122027092,0.780056717924596,0.956311978345547
"12583","NPRL3",121.73318748204,-0.0685911976383785,0.246427631689826,-0.278342153304923,0.780749720409534,0.956311978345547
"12584","GOT2",50.4017721686388,-0.0780626764504637,0.280302681422778,-0.278494219371104,0.780633001871868,0.956311978345547
"12585","ILF3",290.299980268844,0.0518675571651367,0.18632133663049,0.278376905743221,0.780723045687681,0.956311978345547
"12586","LINC01270",3.11880652276449,0.233383876308672,0.838166552628606,0.278445704587886,0.780670238929842,0.956311978345547
"12587","SLC25A1",174.022249050557,-0.0496952316784438,0.177639265393366,-0.279753643252229,0.779666519402638,0.956311978345547
"12588","LINC00894",11.1561336123686,0.157874257185985,0.564905864269601,0.279470027081609,0.779884136931512,0.956311978345547
"12589","CDKL1",32.8490402295186,-0.0937026466234609,0.337161773435649,-0.277915985755559,0.781076852268492,0.956366856109079
"12590","LINC01273",3.51193226969717,0.241512471156479,0.869107403453025,0.277885644739571,0.781100143912637,0.956366856109079
"12591","FN3KRP",130.122126853136,0.0856660780206322,0.308534491922532,0.277654785002585,0.781277372583774,0.956507878514723
"12592","CPTP",48.3918323671582,0.0869750340412406,0.313533844161317,0.277402378278789,0.781471155657709,0.956580532713614
"12593","ATP1B1",78.6602558595509,0.121335178779122,0.437566743574522,0.277295248235559,0.781553407921998,0.956580532713614
"12594","ABI3BP",2581.15090477904,-0.0698586899798894,0.252215734045591,-0.276979904700401,0.781795536441559,0.956580532713614
"12595","ZNF92",69.718870012025,0.0694100307799945,0.250859856947598,0.276688473096329,0.782019323597597,0.956580532713614
"12596","BMT2",43.5354103086013,0.0776217648545629,0.28051813948415,0.276708540122585,0.782003913767885,0.956580532713614
"12597","SH2D4A",113.182130876939,-0.0712062988992299,0.257074079104182,-0.276987470488508,0.781789726995721,0.956580532713614
"12598","TMCO3",229.238189929785,-0.0468072202843881,0.168769429404429,-0.277344187567418,0.781515832996985,0.956580532713614
"12599","AL359220.1",2.71407990538355,-0.253477442463284,0.915701099795704,-0.276812425495433,0.781924139693311,0.956580532713614

"12600","SMAD3",402.913342138805,0.0594688071539267,0.21472510483224,0
.276953210479922,0.781816033891614,0.956580532713614
"12601","AP000915.1",6.67210974286608,-0.264794277025431,0.95637243324
4349,-0.276873598423531,0.781877165782386,0.956580532713614
"12602","AC120049.1",10.2684802464454,-0.137310012982578,0.49544979970
0715,-0.277142130374302,0.781670972937673,0.956580532713614
"12603","TMEM150A",66.2404122415713,0.0716323643454685,0.2592127409009
91,0.27634584664505,0.78228244579768,0.956598754717693
"12604","NUP35",49.9603155867879,-0.0806971564475154,0.291950579938377
,-0.276406905800798,0.782235553198774,0.956598754717693
"12605","AKT2",256.952094856753,0.0531297350455034,0.192161637008382,0
.276484608856584,0.782175879456045,0.956598754717693
"12606","KCNJ15",41.9115248827067,-0.133856740182705,0.484108282899012
,-0.276501652442555,0.782162790636902,0.956598754717693
"12607","ADCK5",23.7590064171565,-0.0900916837695005,0.326217246655516
,-0.27617081773925,0.782416869994724,0.956645563482825
"12608","AC087239.1",23.9726467679111,-0.11185020460232,0.405151759192
722,-0.276069897426054,0.782494380900659,0.956645563482825
"12609","COQ5",111.731618828337,-0.0793631956657398,0.287496597624116,
-0.276049164830473,0.782510304644879,0.956645563482825
"12610","ZNF527",20.4163688504099,0.101106803452399,0.366365111678574,
0.27597279388629,0.782568962407942,0.956645563482825
"12611","PSTPIP2",23.7028180120845,0.118588024201401,0.429907478393716
,0.275845455502396,0.782666769169127,0.956689259118396
"12612","GHITM",590.218288562037,-0.0517107742704917,0.187610959111934
,-0.275627684626032,0.782834043773864,0.95672940801061
"12613","AL049840.2",32.6335691312709,-0.0883832189224931,0.3210108012
24716,-0.275327866181744,0.783064357348249,0.95672940801061
"12614","AKAP13",1137.23428460183,-0.0724720596765922,0.26296327159605
4,-0.275597649955918,0.782857114853307,0.95672940801061
"12615","ZNF764",18.4725010768664,0.106757032080042,0.387612670753294,
0.275421935698254,0.782992093279929,0.95672940801061
"12616","BMP7",27.9820274092168,-0.155103967117261,0.563363152700681,-
0.275317912386914,0.783072003948743,0.95672940801061
"12617","GP1BB",59.0160486500125,0.121566322346855,0.441262299135732,0
.27549673422125,0.78293463449751,0.95672940801061
"12618","AL137796.1",7.4542605727099,0.176614168007237,0.6433360381033
75,0.274528640627556,0.783678396721573,0.956761511777811
"12619","DOK1",54.853218698278,-0.0761572665754295,0.277788608300078,-
0.274155470382578,0.783965146962626,0.956761511777811
"12620","AGAP1",223.187788450833,0.0903234984956499,0.330352704770917,
0.273415344240286,0.784533958997055,0.956761511777811
"12621","AC024230.1",2.98651550943024,0.250841245867858,0.914855676123
735,0.274186685850471,0.783941159351475,0.956761511777811
"12622","RAD1",109.719518514223,0.0506021629107179,0.185087900892274,0
.273395303889526,0.784549362288099,0.956761511777811
"12623","DTWD2",16.7809957345058,-0.0959569156544141,0.350842746720856
,-0.27350406001342,0.784465771839474,0.956761511777811
"12624","LEAP2",18.1742380842351,-0.141143548924347,0.513032025438105,
-0.275116448732059,0.78322677476064,0.956761511777811

"12625","HARS2",38.7621568364506,0.0850630452969383,0.310824210723649,
0.273669303619875,0.7843387696155,0.956761511777811
"12626","AC091729.3",22.2801829737182,-0.102023466562857,0.37335016660
3037,-0.27326482131005,0.784649655068506,0.956761511777811
"12627","MRM2",84.3898416964058,-0.0698168527996504,0.25433471887878,-
0.27450775539979,0.783694444503308,0.956761511777811
"12628","WIPI2",334.6741588577,-0.0424822905753952,0.155395351577206,-
0.273381990800983,0.784559594958923,0.956761511777811
"12629","ZNF250",22.5749892991896,-0.101685878537327,0.372077830513665
,-0.27329195721483,0.784628797312727,0.956761511777811
"12630","ISCA1",298.720712297843,-0.0627215222528342,0.228083745326191
,-0.274993389656654,0.783321316892072,0.956761511777811
"12631","SLC25A16",60.0438729311895,-0.0671292510662945,0.244506072458
767,-0.274550445276221,0.783661642573804,0.956761511777811
"12632","MCMBP",158.814607226728,0.0439016647689405,0.160209562872186,
0.274026493686678,0.784064261631331,0.956761511777811
"12633","ALDH3B1",14.676949892086,-0.122501149772148,0.445803329457861
,-0.274787426825907,0.783479558336622,0.956761511777811
"12634","BACE1",282.937892341224,0.0429429013204058,0.15678923514426,0
.273889347574752,0.784169658090719,0.956761511777811
"12635","ZNF385A",281.395067147389,-0.0983881233600424,0.3583546905103
49,-0.274555143173718,0.783658032839764,0.956761511777811
"12636","AL096870.8",54.6172830769886,0.0916998717569262,0.33508017365
1655,0.27366546566332,0.784341719310592,0.956761511777811
"12637","ZFYVE21",371.654779589983,0.0648414675247725,0.23675586684172
7,0.273874807791435,0.78418083211161,0.956761511777811
"12638","IQCK",102.642309860337,-0.0675333929091058,0.245686042214721,
-0.274876799269223,0.783410892300581,0.956761511777811
"12639","ECH1",618.67175515322,-0.0734149855163273,0.268440293073376,-
0.273487205202313,0.784478726360725,0.956761511777811
"12640","ZNF586",15.0356635341209,0.13097755058434,0.476843206780569,0
.274676347952279,0.783564904061963,0.956761511777811
"12641","ZNF334",5.14206934926331,0.252399575525072,0.919997297372986,
0.274348170636792,0.783817069207239,0.956761511777811
"12642","RUNX1",295.256269525392,0.0801472717208092,0.293295657957088,
0.273264433162988,0.784649953415186,0.956761511777811
"12643","AL359232.1",7.14246272329111,-0.149136812731023,0.54614373295
083,-0.273072460110882,0.784797516112851,0.956865752659938
"12644","AL353807.5",61.2708161606812,0.0824086996659422,0.30213750949
5644,0.272752296805208,0.785043631198923,0.95693876126296
"12645","TTC19",151.224646716105,-0.0452328042034072,0.165805365718693
,-0.272806636910349,0.78500185750258,0.95693876126296
"12646","CRLF3",29.4663455684349,0.0845760574470934,0.309900368546698,
0.272913703987251,0.784919552010691,0.95693876126296
"12647","NGDN",108.578327917051,-0.072640714463126,0.266832170145054,-
0.272233720632852,0.785442315286585,0.957121999220768
"12648","GPR65",7.70611722161425,-0.175851993101996,0.645517735631768,
-0.272420079875714,0.78529903483638,0.957121999220768
"12649","FOXK2",79.1032385641522,-0.0656623027598466,0.241127273131729
,-0.272313877675608,0.785380686450385,0.957121999220768

"12650","PHETA2",102.211334041043,0.0686613396619637,0.25213776297779,
0.272316763863777,0.785378467426319,0.957121999220768
"12651","ZNF14",21.891405358087,-0.105979518015841,0.389667115270193,-
0.271974497879698,0.78564162820907,0.95728920234312
"12652","CCDC84",68.1370370084453,0.083466877392933,0.307038429846064,
0.27184505025895 2,0.785741164022205,0.957334812156362
"12653","AGRN",13.0082523666877,-0.1391695229997,0.512234653203433,-0.
27169095673117,0.785859655313169,0.957403507994349
"12654","OARD1",147.058049142478,-0.0597473575143224,0.220073080124312
,-0.271488714024328,0.786015178778333,0.957517305268532
"12655","IFT46",52.9511894497127,-0.0606161667751012,0.223613832121079
,-0.271075211225215,0.786333186598085,0.957753324226413
"12656","PIGU",66.3033810844511,0.0630320177590351,0.232464832838349,0
.271146465422003,0.786278385412771,0.957753324226413
"12657","SPTLC1",304.31287048504,-0.04120309501352,0.152123287301745,-
0.27085330421365,0.78650386076092,0.957885518972078
"12658","AC114980.1",19.1004596156176,0.117325890078672,0.433552791068
501,0.270615003514381,0.786687155136957,0.957957381818169
"12659","CLASRP",76.8716987356428,-0.0570359734625406,0.21076350333661
6,-0.27061598692183,0.786686398702566,0.957957381818169
"12660","AC105114.2",4.06909318110546,0.216235124068125,0.799692614096
596,0.270397800675455,0.786854231913623,0.958085148890087
"12661","CDR2L",53.0033523465463,0.0636273271049674,0.235386203935157,
0.270310349719966,0.786921503712418,0.958091381385903
"12662","EHMT1",160.227733032408,-0.0486294526499461,0.180193406802725
,-0.269873651388286,0.787257458448149,0.958126694783852
"12663","FAR1",147.257089991304,-0.0482650756448285,0.178840714027523,
-0.269877448808444,0.787254536898517,0.958126694783852
"12664","FERMT3",26.4454981928573,-0.090356693723892,0.334817270128501
,-0.269868676992718,0.787261285509716,0.958126694783852
"12665","NLGN2",80.8489079715445,-0.0663843670201347,0.245919276766131
,-0.269943730695281,0.787203543333747,0.958126694783852
"12666","FLII",255.306116056316,0.0560410870990179,0.207495528798193,0
.270083347933355,0.787096132649769,0.958126694783852
"12667","MARCKSL1",175.716461069836,-0.0939251494754447,0.348200711343
182,-0.269744277985903,0.787356993814647,0.958167526616625
"12668","TVP23C",17.2240632930597,-0.103425362193219,0.383777372471808
,-0.269493121824989,0.787550234487949,0.958327033835786
"12669","KIF20B",58.0481050775037,-0.0719497922053918,0.26714992054583
8,-0.269323651896975,0.78768063279961,0.958334408414049
"12670","TARSL2",52.7353803678273,-0.0840002697864776,0.31182244121680
9,-0.269384940540801,0.787633473682759,0.958334408414049
"12671","MON1B",116.565087748488,-0.0626031829083625,0.232540056747511
,-0.269214619554067,0.787764530673765,0.958360842896069
"12672","EHD3",3.24802581255564,0.209213012770461,0.778827209693663,0.
268625710769338,0.788217724956322,0.958490948457015
"12673","SLCO4C1",5.28636593481198,0.233024916615305,0.867660567800085
,0.268566908838706,0.788262979874201,0.958490948457015
"12674","LINC00863",40.3085995063646,-0.0978456183714421,0.36452022122
3154,-0.268423019285787,0.788373722617704,0.958490948457015

"12675","LINC02823",6.30801746465079,-0.164816061196606,0.614167952266
195,-0.26835666137977,0.788424795567758,0.958490948457015
"12676","AL354696.2",2.98638543488092,-0.23354473224473,0.869888813266
603,-0.268476532498129,0.788332536362496,0.958490948457015
"12677","CLN5",258.833665571,0.0542559817676723,0.202162074102835,0.26
8378636341421,0.788407882243172,0.958490948457015
"12678","ADCK1",10.4703348752063,0.12222535499092,0.455472497982235,0.
268348485435201,0.788431088318842,0.958490948457015
"12679","CCP110",86.2977032317351,0.0691538586182908,0.257497347421719
,0.268561440771013,0.788267188223953,0.958490948457015
"12680","KIAA0895L",13.3005040827615,-0.128778139477586,0.479162092824
447,-0.268756943435354,0.788116728749859,0.958490948457015
"12681","AL139317.3",2.71641010191077,0.234332227273329,0.873521663581
471,0.268261494869582,0.788498042902575,0.958496753516536
"12682","METTL17",80.6292539644123,-0.063081787610444,0.23528304356453
4,-0.268110215911678,0.788614482464397,0.958562706764602
"12683","CDC37L1-
DT",6.56007141266493,0.176848591720077,0.659979595318835,0.26796069601
9158,0.788729572712646,0.958627009647989
"12684","MAEA",163.270908570085,-0.0541694328728591,0.202322599345895,
-0.267737924720164,0.78890105546618,0.95875983680315
"12685","AP3M1",71.0863005462493,-0.0629040187295672,0.235038432652049
,-0.267632905903054,0.788981899391726,0.958782497368818
"12686","ATP7B",7.45523332068417,0.15928570181448,0.595401893026331,0.
267526361068246,0.789063920374127,0.958801504458003
"12687","CAPN15",54.4679341256533,0.0788602785662895,0.294858794261699
,0.267450997226482,0.789121938829626,0.958801504458003
"12688","KIAA1217",122.273380255809,0.101585357200397,0.38020748862056
8,0.267183998844839,0.789327495531897,0.958975673362563
"12689","YTHDF1",98.0440286528002,0.0522974683709564,0.19581949762524,
0.267069770912412,0.789415441807192,0.959006937935052
"12690","ACSL5",79.3391469210548,-0.0823692012917033,0.308807443497303
,-0.266733211994039,0.789674580565709,0.959246151254564
"12691","SDF4",841.066554455777,0.0485691639335599,0.18392052146739,0.
264076915104721,0.791720651744512,0.959344006355789
"12692","FAAH",9.52931129667891,0.147002749361489,0.553612983433942,0.
265533420928213,0.790598567405938,0.959344006355789
"12693","QSOX1",169.431236439323,-0.0622789769982819,0.236040216923259
,-0.263849007639787,0.791896269556993,0.959344006355789
"12694","LMOD1",229.664791366064,0.0597916047157173,0.226403971032822,
0.264092561817519,0.791708595302513,0.959344006355789
"12695","LPGAT1",137.101952271848,-0.0532245385772323,0.20076987374527
6,-0.265102216703887,0.790930719734243,0.959344006355789
"12696","EPRS",399.210190448083,-0.0470123029691825,0.176768760776008,
-0.265953683008244,0.790274880295862,0.959344006355789
"12697","LINC01376",6.26574780033539,0.165505895100306,0.6228988515935
76,0.26570268138541,0.79046819814834,0.959344006355789
"12698","SLC35F6",334.880785712676,0.0545243206564648,0.20577902341774
2,0.26496539710842,0.791036118447081,0.959344006355789
"12699","MKRN2",84.7783227113906,0.0576536501053839,0.21739169999741,0

.265206307812445,0.790850535908941,0.959344006355789
"12700","LINC02158",4.22988649116087,0.249050483959813,0.947565707611716,0.262831888025507,0.792680156273349,0.959344006355789
"12701","USP19",68.5355034133713,-0.0594723729613436,0.224012984415189,-0.26548627579157,0.79063488098553,0.959344006355789
"12702","AC108865.2",2.54639705172723,0.257367603607795,0.972269981435853,0.264707960259879,0.791234444786583,0.959344006355789
"12703","AC008443.1",30.2388512443855,-0.0805652523638868,0.305013751468385,-0.264136459343333,0.791674770701821,0.959344006355789
"12704","RMND1",80.2187830825229,0.0685938591138984,0.259289039070957,0.264545926660352,0.791359280509332,0.959344006355789
"12705","TCTE3",9.54752170374877,-0.132361045674222,0.496675479634506,-0.266494020948294,0.789858763564516,0.959344006355789
"12706","AC092428.1",6.46744571925879,-0.17364894707689,0.653876747927466,-0.265568316394931,0.790571689437263,0.959344006355789
"12707","MDFIC",243.720392216616,-0.0712848638360011,0.270309615438093,-0.263715605234645,0.791999069855516,0.959344006355789
"12708","C5",17.3285067121623,0.0990005790957229,0.374346400678593,0.264462484255921,0.791423569219568,0.959344006355789
"12709","LINC01503",24.238208799496,0.116666962019302,0.443121459392916,0.263284387488563,0.792331392291374,0.959344006355789
"12710","ALDH18A1",79.283430840251,0.0688638063763631,0.261937000068996,0.262902172500349,0.792625981788288,0.959344006355789
"12711","DNHD1",11.8963728356518,0.159425712634452,0.603319177459372,0.264247712638287,0.791589047848559,0.959344006355789
"12712","KDM4D",6.44178664006795,0.162400107300213,0.617677311759426,0.262920628956928,0.792611755924457,0.959344006355789
"12713","PYROXD1",93.8875853917886,-0.0649766694263085,0.244034284962236,-0.26626041269719,0.790038659006862,0.959344006355789
"12714","TSPAN11",9.76740157371558,0.19523269193935,0.741802259784751,0.263186973838554,0.792406470359451,0.959344006355789
"12715","C12orf45",153.296767504008,0.0585260101775093,0.220906798726607,0.264935305363511,0.791059300077706,0.959344006355789
"12716","NUAK1",209.884312074893,0.123244211846482,0.465126811764993,0.264969055167587,0.791033300418498,0.959344006355789
"12717","C12orf49",100.889110174236,0.0537946584914043,0.202233976550504,0.266002080406949,0.790237606796659,0.959344006355789
"12718","HIP1R",4.58471618081087,0.216118678262357,0.820072204800383,0.263536158154468,0.792137358024622,0.959344006355789
"12719","RAB2B",95.206280665113,-0.0521061373109879,0.198037474846926,-0.263112511161151,0.792463861088707,0.959344006355789
"12720","RBM25",850.128073705329,0.0646920405119171,0.245131315276651,0.263907695509678,0.791851045652176,0.959344006355789
"12721","TDP1",20.0013881329019,-0.0939436474192741,0.354092490653027,-0.265308217200598,0.790772034858483,0.959344006355789
"12722","CMTM2",2.5470689668936,0.321325243159455,1.21703729442986,0.264022511577993,0.791762572308885,0.959344006355789
"12723","AC092376.3",6.40697365867975,-0.180258286042708,0.68540537897994,-0.262995143561579,0.79255432227472,0.959344006355789
"12724","DNAH9",4.47552358716506,0.210350913731643,0.795955880801364,0

.264274589591402,0.791568339005143,0.959344006355789
"12725","AC005899.8",3.44386857918642,-0.236145501320106,0.895157735025189,-0.263803229397846,0.79193154600155 4,0.959344006355789
"12726","FMNL1",171.002285418134,0.0626514384335402,0.238245352797052,0.262970243482186,0.792573514393043,0.959344006355789
"12727","TMED1",285.658566047302,0.0624857580439335,0.235423017584107,0.265419068556498,0.790686648198422,0.959344006355789
"12728","LIPE-AS1",26.8420285655712,0.122421722106924,0.464651402767978,0.263470036628847,0.792188315223987,0.959344006355789
"12729","SULT2B1",7.28507104872048,-0.165587643019887,0.623449425385218,-0.265599158933499,0.790547933413133,0.959344006355789
"12730","SLC6A16",2.73291797860072,-0.242580993157058,0.920700014341076,-0.263474518712446,0.792184861034444,0.959344006355789
"12731","LENG1",101.744655877089,-0.0707393303710365,0.268595497705483,-0.263367520957491,0.792267321682195,0.959344006355789
"12732","AL117335.1",9.52410002293541,-0.139949802788416,0.526335515163933,-0.265894659882161,0.79032033790159,0.959344006355789
"12733","LBP",3.32749686971106,-0.250613298697708,0.951909681564726,-0.26327424077225,0.792339212418304,0.959344006355789
"12734","SLPI",341.947970538865,0.112135427359525,0.423920083525479,0.264520204909767,0.791379097837919,0.959344006355789
"12735","APOBEC3D",31.9202936122626,0.0888623801065398,0.337162918045473,0.263559173771758,0.79211962102274,0.959344006355789
"12736","TENM1",67.1331083776967,-0.150422216571926,0.567829001973431,-0.264907597268102,0.791080645591336,0.959344006355789
"12737","ENOX2",86.5543517342145,-0.0785069501750299,0.297458825925664,-0.26392543549758,0.791837375647872,0.959344006355789
"12738","EFCAB2",70.7054815858525,-0.0686644650229407,0.261802305121285,-0.26227601392253,0.793108653035556,0.959711899406789
"12739","SLC41A2",124.892278023766,0.0580658606230775,0.221335395024722,0.262343312133117,0.793056772736946,0.959711899406789
"12740","ADPRHL2",126.186016346415,-0.0653696455257327,0.251226793462802,-0.260201726992195,0.794708172186127,0.959788475572578
"12741","RPF1",124.718685530906,-0.0519461180967134,0.200647167509604,-0.258892855261597,0.79571791097686,0.959788475572578
"12742","GLMN",31.9430469957456,-0.083843755985981,0.322631215782094,-0.259874903247457,0.794960270639501,0.959788475572578
"12743","NBPF20",20.3438498430831,0.128219213027831,0.490204385109594,0.261562762232666,0.793658556148279,0.959788475572578
"12744","MCL1",3030.43853198231,-0.0718458107878499,0.275575953657854,-0.260711465692871,0.794315023427558,0.959788475572578
"12745","CDC73",211.526261736127,0.0377132678882455,0.145416621230792,0.259346335852423,0.795368031190836,0.959788475572578
"12746","SH3YL1",147.820310156105,0.0516362680702697,0.197078603733072,0.262008493525797,0.793314893992036,0.959788475572578
"12747","RDH14",283.472674520105,-0.0535770118774245,0.206072462502737,-0.259991127522499,0.794870617538404,0.959788475572578
"12748","SF3B6",833.359808542205,-0.0620751876028972,0.240029126778481,-0.25861522906003 5,0.795932131812976,0.959788475572578

"12749","YPEL5",361.80290186664,0.0580152825298335,0.224000723412188,0
.258995960575888,0.795638357180351,0.959788475572578
"12750","PRKAR2A",235.033695057044,-0.0614561080771977,0.2358272691279
2,-0.260597972000693,0.794402553766627,0.959788475572578
"12751","ZBTB11-
AS1",11.7774618395098,-0.122780990926032,0.470971355318712,-0.26069736
4159111,0.794325898886962,0.959788475572578
"12752","C4orf33",39.2511614032677,0.077007374189659,0.297134577937282
,0.259166653454629,0.795506658973224,0.959788475572578
"12753","LRP2BP",3.90687359327153,0.188607330068519,0.723638727255139,
0.260637418873272,0.794372130653363,0.959788475572578
"12754","MCCC2",52.1531853658415,0.0640614501841537,0.247261598835994,
0.259083701172073,0.795570660136017,0.959788475572578
"12755","MSX2",19.6307311969623,-0.154363026756087,0.595925769687346,-
0.259030628658791,0.795611608526025,0.959788475572578
"12756","BPHL",108.948584982016,0.0612445853129224,0.236866834930539,0
.258561251645392,0.795973783439496,0.959788475572578
"12757","C6orf52",3.79797203401723,-0.225420737998957,0.86855399834781
7,-0.259535663214673,0.79522196915682,0.959788475572578
"12758","AL137003.2",3.20917454457971,0.239183102114729,0.915205624356
587,0.261343566679762,0.793827572257955,0.959788475572578
"12759","RARS2",232.485814441208,-0.0573521914941602,0.218984413731184
,-0.261900792467191,0.793397928638065,0.959788475572578
"12760","RFPL4B",16.1053743587412,0.31204037281975,1.19874849344503,0.
260305121988508,0.794628421982593,0.959788475572578
"12761","COX19",110.967297844167,0.0512039582011472,0.196608693078307,
0.260435881035806,0.794527568525476,0.959788475572578
"12762","VPS37D",28.7158896995286,-0.102024354190218,0.393235010917366
,-0.25944880633138,0.795288976504593,0.959788475572578
"12763","TMEM65",129.02409614105,-0.0607348298396119,0.234391064264729
,-0.259117513844368,0.795544572076051,0.959788475572578
"12764","LIN7C",150.77258611559,-0.0480639902225799,0.184484305211575,
-0.260531594638676,0.794453747529662,0.959788475572578
"12765","ARHGAP1",336.885828095989,-0.0438196847674793,0.1694574236569
66,-0.258588168177181,0.795953013247754,0.959788475572578
"12766","PCSK7",243.639062100869,-0.0725995363684246,0.278506123293734
,-0.260674830089303,0.794343277814381,0.959788475572578
"12767","AP001267.3",8.80738401378451,0.129166213677674,0.496479567358
758,0.260164208498712,0.794737111326874,0.959788475572578
"12768","HINFP",31.6773680514724,0.0774421468720367,0.298972704156824,
0.259027482426673,0.795614036036425,0.959788475572578
"12769","ATN1",305.600334974833,0.0413920316948937,0.159177958878344,0
.260036200907242,0.794835849552153,0.959788475572578
"12770","NANOG",2.53522459551008,-0.238161033022083,0.910164512433939,
-0.261668115784034,0.793577324154808,0.959788475572578
"12771","DGKA",35.0982318220201,-0.0786302488786739,0.301619654101593,
-0.260693385889864,0.79432896703603,0.959788475572578
"12772","VTI1B",470.513367219885,-0.0512744099369424,0.197377720107793
,-0.259778104179845,0.795034941587671,0.959788475572578
"12773","NOMO1",47.7365257903863,-0.0959628972828661,0.369729628050506

,-0.259548843269215,0.795211801292154,0.959788475572578
"12774","HEATR3",21.3075386197993,-0.089664279890968,0.34645368600825,-0.258805963140577,0.795784956678421,0.959788475572578
"12775","AC040169.1",10.252506036408,-0.15061097819881,0.581791700726736,-0.258874401286021,0.795732149883278,0.959788475572578
"12776","RAP1GAP2",3.99071374191922,0.221596899405346,0.850253924849385,0.260624376940806,0.794382189114209,0.959788475572578
"12777","LINC00910",25.1441023690378,-0.116920359287882,0.446631248954398,-0.261782756046745,0.793488934262732,0.959788475572578
"12778","DNM2",116.107932122409,-0.0541908709342312,0.209471350691604,-0.258703019555234,0.795864389593234,0.959788475572578
"12779","NOTCH3",37.8108445088183,0.141565434601655,0.544788037117108,0.2598541541969,0.794976276330558,0.959788475572578
"12780","RPS11",12143.840588238,-0.0476784855427246,0.183726870500799,-0.259507416703738,0.795243760283976,0.959788475572578
"12781","ZNF749",38.4247002811976,0.108538564589182,0.416436056716302,0.260636808073333,0.794372601725984,0.959788475572578
"12782","CPXM1",702.517011785438,0.121035996031059,0.462104944277445,0.261923178987648,0.793380669034523,0.959788475572578
"12783","PABPC1L",6.86039275835605,0.16921717333168,0.653107887372988,0.259095283648045,0.795561723687715,0.959788475572578
"12784","SLC25A17",58.7783308577795,-0.0642991353420864,0.248348699241218,-0.258906672507406,0.795707249770155,0.959788475572578
"12785","SERPINB6",534.813614600777,-0.0485256262070843,0.187910884764518,-0.258237442008186,0.796223663072939,0.959836252632944
"12786","KLHDC3",302.569202383209,-0.0594231606654273,0.230227318691009,-0.258106470610379,0.7963247379126,0.959836252632944
"12787","IL15RA",252.844319311531,-0.080634637081695,0.312268549538372,-0.25822208865061,0.796235511575888,0.959836252632944
"12788","ZNF641",53.3335914087272,-0.10001099202485,0.387431819959619,-0.258138301689506,0.796300172530805,0.959836252632944
"12789","AC116913.1",8.87450500226891,0.128629476888645,0.498129872470109,0.258224780318437,0.796233434356871,0.959836252632944
"12790","MITD1",188.578873843278,0.0542054144596294,0.210207782811863,0.257865877916345,0.796510419934211,0.959984997911326
"12791","DCPS",113.468840012773,0.0706526552109416,0.27425483960878,0.257616803815482,0.796702659780445,0.96014162305649
"12792","KIF18A",2.51878087209254,-0.245910096718084,0.955151543852745,-0.25745662905612,0.796826292042563,0.960215548142285
"12793","AC006064.2",3.8088339254139,-0.200399354695388,0.778712043737823,-0.257347187971397,0.79691076801475,0.960242280070927
"12794","TSPAN10",18.4517636724123,0.130835782887608,0.508933999722382,0.257078094524983,0.797118487424383,0.960417499112621
"12795","FAM200A",42.928160571034,0.0794798074580195,0.309335638926525,0.256937117668805,0.797227316433644,0.960473550826465
"12796","NECAP2",202.829110328668,0.0530719145941842,0.207462661491304,0.255814295510755,0.798094234895228,0.960534238903653
"12797","RGPD8",6.90518926823111,0.146973563978038,0.576172600519924,0.255086000003146,0.798656676953121,0.960534238903653
"12798","FARSB",64.8476140169291,-0.0601275080450035,0.234448984496071

,-0.256463077348118,0.797593287234594,0.960534238903653
"12799","C3orf52",4.23839297062278,-0.20046798949839,0.781242989110153,-0.256601329282616,0.797486548737153,0.960534238903653
"12800","PLSCR1",354.148849352953,0.0556595948147757,0.217728972013267,0.255637062445618,0.798231097351581,0.960534238903653
"12801","MEOX2",116.001302871829,-0.102022800035595,0.399418221078932,-0.255428507392589,0.798392155133301,0.960534238903653
"12802","HOXA10-AS",21.0993284984379,0.0941303345559964,0.36781454702991,0.255917921996549,0.798014215594421,0.960534238903653
"12803","PPP1R3B",118.571259464289,-0.0697597079731495,0.273175994460382,-0.255365439818199,0.798440861102873,0.960534238903653
"12804","AC019270.1",12.8608879496758,0.17963396187002,0.702856018500221,0.255577183863816,0.798277338035819,0.960534238903653
"12805","TMEM71",7.00218078864645,0.211545965667843,0.826582876161488,0.255928318585823,0.798006187572804,0.960534238903653
"12806","ZDHHC6",107.150699110713,-0.0584096398943353,0.229100324637116,-0.254952235387939,0.798759990956149,0.960534238903653
"12807","TNNT3",834.906340902442,0.078309233062863,0.30613196555881,0.255802209089528,0.798103568042873,0.960534238903653
"12808","HOXC-AS3",28.3841354643721,-0.0933165259383689,0.364192360452814,-0.256228674929768,0.797774268119232,0.960534238903653
"12809","MYL6B",356.700417241664,-0.0698969807188569,0.274015760508945,-0.255083797329881,0.798658378174137,0.960534238903653
"12810","AL049828.1",4.98404594755631,0.167030249622124,0.654655950764214,0.255142032127166,0.798613401211595,0.960534238903653
"12811","PIGH",118.108881647802,-0.0547344848359475,0.214637252701538,-0.255009250011498,0.798715954896038,0.960534238903653
"12812","EIF2B2",78.1719259391857,0.0649839278001447,0.253357541267205,0.256490994801725,0.797571733039127,0.960534238903653
"12813","FAM192A",146.335345558454,-0.0494755619744976,0.19393501425757,-0.255114127605591,0.798634952858111,0.960534238903653
"12814","TEKT3",35.7206216175546,0.101267728882223,0.396491415913531,0.255409637681307,0.798406727794532,0.960534238903653
"12815","KDSR",877.251851680594,-0.0475526545404745,0.186079477688966,-0.255550236549779,0.798298148082218,0.960534238903653
"12816","TECR",299.732633607519,-0.062294918390257,0.243145127550888,-0.256204675033923,0.797792798927256,0.960534238903653
"12817","BAX",271.751055626486,-0.0642114254334734,0.251478214046549,-0.255335937058897,0.798463645830143,0.960534238903653
"12818","RIN2",210.265202449918,-0.0790344448477498,0.308532112252891,-0.256162784063685,0.797825144070947,0.960534238903653
"12819","EDA",34.1489408780641,-0.0675682491945872,0.265040905999581,-0.254935172892497,0.798773169543038,0.960534238903653
"12820","KLRF1",3.20094599719436,0.240358299899259,0.943269098003617,0.25481413565648,0.798866656912016,0.960571725140306
"12821","AL592295.4",7.70660581983997,0.165927594453028,0.651771867880541,0.254579251775009,0.799048085970135,0.960677006455066
"12822","SOD2-

OT1",2.8773090595961,-0.205499477349159,0.807802023613843,-0.254393367857413,0.799191674156417,0.960677006455066
"12823","POLB",24.2740851561142,0.0801357283914713,0.315015469555681,0.254386644898741,0.799196867511012,0.960677006455066
"12824","PTPN2",135.123112403979,-0.0593751562252094,0.233413039017278,-0.254378060776692,0.799203498590968,0.960677006455066
"12825","ABRAXAS1",215.158527508494,-0.0487573711258784,0.191783786674357,-0.254230933549493,0.799317153979376,0.960738707882424
"12826","TMEM8B",76.1490515826912,0.0513825608014322,0.202202390853062,0.254114506681433,0.799407096435993,0.960771900168472
"12827","AC108488.1",30.9146615951729,-0.0878056275551487,0.34740292464623,-0.252748671141913,0.800462434491882,0.960884067017162
"12828","AL121658.1",6.03782634716648,-0.180557310470569,0.71334630306348,-0.25311312289018 9,0.800180798398756,0.960884067017162
"12829","LINC02245",3.78659993679744,-0.20369068533282,0.805289483891725,-0.252940947829646,0.800313846278159,0.960884067017162
"12830","SGO2",28.746249768001,0.0853193149276548,0.337842580489202,0.252541626943859,0.800622442865289,0.960884067017162
"12831","EOGT",22.5302586531695,0.116407234789039,0.459927494030177,0.253099099966833,0.800191634363479,0.960884067017162
"12832","AC093849.4",3.22705835457903,-0.240816678552577,0.951214004925912,-0.253167717575114,0.800138611692939,0.960884067017162
"12833","OSMR-
AS1",26.1180503036507,-0.10456456207635,0.412508019860556,-0.25348491918217,0.79989351345833,0.960884067017162
"12834","LARS",226.739566496346,0.0517815633240098,0.204244456622672,0.253527386643705,0.799860700807592,0.960884067017162
"12835","ECHDC1",305.687284838495,0.0567005859567017,0.223373801717573,0.253837224959766,0.799621313712179,0.960884067017162
"12836","DNAJC30",85.0773753759576,0.0681979469270973,0.269406121526165,0.253141786611088,0.800158649137676,0.960884067017162
"12837","TRIP6",399.157116018158,0.0632052727958654,0.250150458451737,0.252669026421392,0.800523984717404,0.960884067017162
"12838","AC026979.2",24.6558209046265,-0.11682633515395,0.461860906830095,-0.252947009427076,0.800309162094351,0.960884067017162
"12839","PITRM1",72.127543451565,0.0544006013079246,0.215186046197581,0.252807290571131,0.800417133610373,0.960884067017162
"12840","AL158211.1",3.27646260751472,0.202687415690034,0.80242622000436,0.252593211234962,0.800582576591947,0.960884067017162
"12841","RUBCNL",4.41566071738458,-0.196053155226754,0.773533492769535,-0.253451411036917,0.799919403907508,0.960884067017162
"12842","LINC00519",16.5320238415052,0.137085652417163,0.541079505428507,0.253355839653543,0.799993249430673,0.960884067017162
"12843","COPS2",371.459022754315,-0.0514641702701404,0.203009719641334,-0.253505942282292,0.799877269833403,0.960884067017162
"12844","LRRC37A2",15.6243975638766,-0.154449877357428,0.609886159527171,-0.253243781556166,0.800079836086438,0.960884067017162
"12845","MFN2",65.9441068862841,-0.0573623421597451,0.229331468658276,-0.250128525733292,0.802487956624733,0.960960048759806
"12846","ARHGEF19",18.006455897175,-0.0945533853933005,0.3759088382115

61,-0.25153275417293,0.801402241833271,0.960960048759806
"12847","TPR",833.331407560816,-0.0500869209029137,0.201223227789322,-0.24891222277457,0.803428680403268,0.960960048759806
"12848","FLVCR1",27.0896971451214,-0.082868701774777,0.331365753613262,-0.250082275766775,0.802523722456993,0.960960048759806
"12849","EPHX1",1628.4754951603,-0.065680211358386,0.262446034714367,-0.250261778311374,0.802384912607349,0.960960048759806
"12850","GFPT1",229.644775995817,0.0492390096911791,0.197718087945609,0.249036444782555,0.803332590438171,0.960960048759806
"12851","IL18RAP",2.43296210653289,0.269369494417336,1.07528736407911,0.250509308874869,0.802193506718077,0.960960048759806
"12852","GORASP2",243.606550733988,-0.0516296781515225,0.204614043758596,-0.2523271482403,0.800788205612917,0.960960048759806
"12853","CAMK1",182.428597172193,-0.0592416758135199,0.237966935825961,-0.24894918954979,0.803400085029703,0.960960048759806
"12854","ACAA1",267.222996528484,0.0595321779885437,0.238818061730463,0.249278373491421,0.803145458853808,0.960960048759806
"12855","ZBTB11",109.987338991385,0.0487181413283653,0.195427631667985,0.249289933631971,0.803136517380508,0.960960048759806
"12856","TPRA1",112.578555115182,0.0630057792105543,0.25243542471693,0.249591669953637,0.802903141163672,0.960960048759806
"12857","SEC62",1704.11467533803,-0.0423294310051495,0.16878762246617,-0.250785160586248,0.801980215155101,0.960960048759806
"12858","DCTD",268.305731699426,0.0509702558230535,0.204042592822726,0.249802039456227,0.802740442482763,0.960960048759806
"12859","FEM1C",245.944587698731,-0.101052766884954,0.402357782738453,-0.251151515442767,0.801696968382466,0.960960048759806
"12860","C7orf61",3.74394495472677,0.206073345751501,0.817503112741144,0.252076527342535,0.800981912706516,0.960960048759806
"12861","PCMTD1",669.690137357862,0.0469708482726697,0.18750027833683,0.250510818913507,0.802192339099536,0.960960048759806
"12862","GDF6",14.6549745212888,0.137230963616151,0.548955144159565,0.249985750340763,0.802598368431585,0.960960048759806
"12863","AP001330.4",7.28452174638369,-0.132865307363714,0.531512437626273,-0.249975913935502,0.802605975316632,0.960960048759806
"12864","AZIN1",389.09249997385,-0.045388740179912,0.181698708222246,-0.249802217219917,0.802740305004862,0.960960048759806
"12865","ABCA2",59.8753417594641,0.060809163759772,0.241730456182256,0.251557725576475,0.801382938023856,0.960960048759806
"12866","TWNK",7.79001767148349,0.150712666028635,0.605414424028581,0.248941320270757,0.803406172229827,0.960960048759806
"12867","RHOD",72.5372082580095,-0.0928367168853593,0.371989823945872,-0.249567893821923,0.802921530037931,0.960960048759806
"12868","TPCN2",21.0101746450557,-0.0906243334700985,0.360566938204505,-0.251338444731997,0.801552454288504,0.960960048759806
"12869","C11orf71",85.3951110708715,-0.0733689457082747,0.29280821293109,-0.250569971975279,0.802146600079931,0.960960048759806
"12870","ZNF384",73.3491969814527,-0.05551589286975,0.222975899012453,-0.248977100734324,0.803378494715204,0.960960048759806
"12871","RAPGEF3",3.22212984895058,0.21855985919643,0.870508863587605,

0.251071377143347,0.801758924977064,0.960960048759806
"12872","DDX23",151.971614267361,0.0454950156106343,0.181049066478839,
0.251285557531177,0.801593340429121,0.960960048759806
"12873","MMP19",194.091081453696,0.0920045512626791,0.366398547877464,
0.251105119809177,0.801732837664747,0.960960048759806
"12874","ANKRD52",36.3391840939809,0.0729263000013465,0.29229027059194
2,0.249499580857266,0.802974365081366,0.960960048759806
"12875","NUP37",94.8813360603263,0.0712537197996241,0.283522403478308,
0.251316012158015,0.801569796440293,0.960960048759806
"12876","CUL4A",147.436227231476,0.0487955680733636,0.194276749945018,
0.251165247962884,0.80168635160922,0.960960048759806
"12877","WDR25",28.176971016983,0.0804885798834659,0.322711740153337,0
.249413237476956,0.803041146610848,0.960960048759806
"12878","LINC02323",2.58222556442233,-0.277062258775717,1.105237684233
28,-0.250681154586147,0.802060632000935,0.960960048759806
"12879","AC090510.1",6.22448217816576,0.234897575720933,0.931635614671
002,0.252134602866041,0.800937024534428,0.960960048759806
"12880","ERCC4",76.2482152341649,0.0533342773345618,0.213386347778213,
0.249942313038675,0.802631960373215,0.960960048759806
"12881","ZNF785",49.4453910626768,0.0728065737183375,0.28939798964257,
0.251579403879963,0.80136617999971,0.960960048759806
"12882","SRCAP",83.5964908380953,0.0597075561943166,0.238921815473419,
0.249904162480965,0.802661464147925,0.960960048759806
"12883","AC079414.3",11.1815815666443,0.134954972947754,0.541144205161
178,0.249388188325065,0.803060520926624,0.960960048759806
"12884","RAD51D",54.942675341566,0.0577048772792882,0.230860519995633,
0.249955589116666,0.802621693370049,0.960960048759806
"12885","PLCD3",129.995223228236,0.0480863696979349,0.192498536924429,
0.249801221693507,0.802741074919656,0.960960048759806
"12886","MIER2",28.2829128021622,0.079254828361554,0.315170490397977,0
.251466526137888,0.801453439116875,0.960960048759806
"12887","NR2F6",157.708331104283,-0.0624676564222455,0.248917921590429
,-0.250956845626528,0.801847473850869,0.960960048759806
"12888","RYR1",4.46061029163917,0.269419806884665,1.07720004897637,0.2
50111209278802,0.802501347665038,0.960960048759806
"12889","MXRA8",3288.64372469674,-0.05103448271369,0.205401445959596,-
0.248462139471641,0.803776860082327,0.960961319400101
"12890","FAM229A",19.3631719781233,0.0970127140736719,0.39047002040643
7,0.248451120453991,0.803785384766336,0.960961319400101
"12891","RPL31",4390.09449260115,0.0480953757255957,0.193337207691792,
0.248764199606455,0.803543185258017,0.960961319400101
"12892","IFT80",87.6486244837074,0.0548156818805278,0.22065075827899,0
.248427344225208,0.803803778939014,0.960961319400101
"12893","HOXC6",224.41737658723,-0.040439516088371,0.162634456737621,-
0.24865281871733,0.803629347878502,0.960961319400101
"12894","GAS8",30.0379160113272,-0.0820968765381635,0.330410539917054,
-0.248469302942857,0.803771318191704,0.960961319400101
"12895","CCDC106",64.0033256236192,-0.092059951703548,0.37075008829049
2,-0.248307295429197,0.80389665480039,0.960997823477938
"12896","AC108471.2",17.2946424626873,0.107660646589392,0.433766040325

605,0.248199804919207,0.803979817281575,0.961022711181411
"12897","LINC01002",6.70294673321313,0.162761522421754,0.655989333117192,0.248116111352495,0.804044570251964,0.961025591256418
"12898","CD276",588.675581528325,0.0436144150428354,0.175853241710382,0.248015985481037,0.804122038517367,0.961043667525601
"12899","PAPOLA",524.213360057003,-0.0326840806937188,0.131902000250485,-0.247790637228026,0.804296399474411,0.961103023092872
"12900","SLC7A6OS",105.481886388992,0.0536804254018146,0.216571383958786,0.247864812149098,0.804239006295688,0.961103023092872
"12901","TP53I3",428.076483825191,0.0654733778953341,0.264575410672783,0.247465846235836,0.804547720383472,0.961328820224108
"12902","WNK3",4.96774516089117,-0.188470152727185,0.762567847632064,-0.247151979082812,0.804790607698399,0.961544506097568
"12903","GEMIN5",71.7686396525856,-0.0522964260370774,0.212034784397209,-0.24664078672634,0.805186236212795,0.961776251346395
"12904","SPTAN1",880.599104357834,0.0425061645737862,0.172332979460083,0.24665136474143,0.805178049034539,0.961776251346395
"12905","ZNF45",17.9092657222894,0.104009682529429,0.421810973934727,0.246578891865255,0.805234142061406,0.961776251346395
"12906","SUMO3",752.288020038574,-0.0466792712521203,0.189146680197356,-0.246788741961609,0.805071723674399,0.961776251346395
"12907","FASTKD1",15.3519523351834,0.0931625145912886,0.378200603639313,0.246330951602968,0.805426052053321,0.96178190350933
"12908","ANO7",3.5370427868345,-0.192417721921379,0.781048769071064,-0.246358139902365,0.805405007273471,0.96178190350933
"12909","ZNF699",35.53615057229,0.0717914101945549,0.291309785007779,0.246443524691894,0.80533891710898,0.96178190350933
"12910","GIGYF2",229.374908126415,0.0587355108445245,0.238917475373083,0.245840161975618,0.805805966149979,0.962161035492016
"12911","AEBP2",140.654495251997,0.0504247419719081,0.205218255678442,0.245712750092365,0.805904601589822,0.962204278019294
"12912","SPATA41",15.2080397538589,-0.112478167778599,0.458268464385448,-0.245441649425813,0.80611448344524,0.962380325457588
"12913","BCAT1",95.2442545978735,-0.0581517272461829,0.237019564569476,-0.245345684234169,0.806188781528925,0.962394491386075
"12914","ENO3",2.3707563878971,-0.242075932529808,0.987084048495263,-0.245243485495318,0.806267907667343,0.962414418204436
"12915","SH3D21",4.98348333406438,-0.184783792532296,0.754249806309622,-0.244990175650694,0.806464038301153,0.962573995386162
"12916","WASF2",1602.63238832581,0.0351156173398828,0.143510940180781,0.244689480088748,0.806696874152346,0.962641613317644
"12917","MFN1",122.822414948345,-0.0458239047812374,0.187284733859537,-0.244675066872269,0.806708035085133,0.962641613317644
"12918","ZCCHC10",133.193604284296,0.0503655642420801,0.205819829915867,0.244707054041722,0.806683265743657,0.962641613317644
"12919","AL592148.3",14.6581326703124,0.115673795114732,0.473381738307806,0.244356268427739,0.806954908016235,0.962678613449287
"12920","PKDCC",567.824404513694,0.0676230240741117,0.276651059408268,0.244434357919146,0.806894434857621,0.962678613449287
"12921","AC084198.2",9.47274582190065,0.145496960049011,0.595536421963

234,0.244312446196604,0.806988844825929,0.962678613449287
"12922","AC006333.2",49.9120969717273,-0.0761763528243053,0.311644083082129,-0.24443381716389,0.806894853619069,0.962678613449287
"12923","ARIH2",181.82265120015,0.0434411884043724,0.177908581272869,0.244177026726688,0.807093718659923,0.962729217143288
"12924","SLC66A1",34.3896016353381,0.0702070916575364,0.288833382719194,0.24307125096337,0.80795020181876,0.962736067139256
"12925","AL604028.1",8.78929653847634,0.125749895786695,0.517517734647335,0.242986640588053,0.808015746627497,0.962736067139256
"12926","SHISAL2A",3.90843953906413,0.178832850922475,0.733799374588345,0.243708099400874,0.807456900066663,0.962736067139256
"12927","FAM171B",91.6145165251392,0.0672523501017513,0.275565380571264,0.244052246194109,0.807190356378232,0.962736067139256
"12928","RFT1",84.5983492738797,-0.0561356165757647,0.230227761472282,-0.243826444807452,0.807365238338922,0.962736067139256
"12929","PAK2",383.365432691413,-0.044066330193468,0.181372676311523,-0.242960136496969,0.808036278731605,0.962736067139256
"12930","LINC02615",10.7896170093225,0.158366338603556,0.650956396892318,0.243282559875901,0.807786513807577,0.962736067139256
"12931","AL356776.2",3.4628676176165,-0.204060974029559,0.837790178157286,-0.243570501719643,0.807563476527114,0.962736067139256
"12932","NDUFAF6",41.2511930316187,-0.0723637527570413,0.296928051154892,-0.243708037942474,0.807456947668548,0.962736067139256
"12933","RAB14",618.467668786109,-0.0396781678992946,0.163250049738287,-0.243051490415496,0.807965509529184,0.962736067139256
"12934","AL135791.1",5.43394988055226,-0.16053586728148,0.6598764742777,-0.24328169519485,0.807787183605636,0.962736067139256
"12935","NUP98",113.443334840777,-0.0518074203939281,0.212905723706913,-0.24333502872495,0.80774587072283,0.962736067139256
"12936","YY1",764.216149035701,0.0289304085164004,0.118646818782873,0.243836360832765,0.807357558228402,0.962736067139256
"12937","PET100",541.899395125193,-0.0686628278804682,0.282049067696134,-0.243442846456923,0.807662355268717,0.962736067139256
"12938","NKILA",5.54525967946866,-0.164159229000243,0.675058659041959,-0.243177725078318,0.807867721822692,0.962736067139256
"12939","PIGF",149.36806179342,0.0678365734662591,0.279692217957464,0.242540081957431,0.808361702876131,0.962764428289336
"12940","MGAT4A",8.61868044129405,0.126653256187228,0.522128345254282,0.24257111750090,0.808337657922433,0.962764428289336
"12941","DHX16",76.2664207185668,-0.0669157445388921,0.276074915382791,-0.242382559263344,0.808483747218131,0.962764428289336
"12942","SLC22A3",2.97312672549066,-0.242966637291239,1.00248196006356,-0.242365096800182,0.808497276951376,0.962764428289336
"12943","ASH2L",123.511092565837,-0.0494406062813587,0.203908629441772,-0.242464511760533,0.808420252048218,0.962764428289336
"12944","RAB6A",309.787090779112,-0.0356054474260582,0.146727199048393,-0.242664261684127,0.808265495054056,0.962764428289336
"12945","ZNF222",16.9318384298605,0.109142074008463,0.449715994778465,0.24269111011323,0.808244694705778,0.962764428289336
"12946","HNRNPA2B1",2255.48489980941,0.0331915941462951,0.137143852110

603,0.2420202847994,0.808764445395456,0.96300818212351
"12947","CHCHD6",78.5936318385702,-0.062680062440398,0.259796701882562
,-0.24126581279208,0.809349105768685,0.963258130400944
"12948","AC113349.2",3.17957166244206,-0.265001107535355,1.09736076007
968,-0.241489505708327,0.809175749079368,0.963258130400944
"12949","FRAT1",22.192739105434,-0.109001561634858,0.451362762124906,-
0.241494360593029,0.809171986762999,0.963258130400944
"12950","OLMALINC",22.5872591352446,0.0873335339845352,0.3617838189260
27,0.241397015056641,0.809247425999411,0.963258130400944
"12951","AC025682.1",2.45922115916439,0.231902279381522,0.960004902003
15,0.241563640870619,0.809118298157596,0.963258130400944
"12952","TSPAN7",5.27087796543389,-0.173180811898289,0.717801583677092
,-0.241265575106611,0.809349289974248,0.963258130400944
"12953","AL161669.3",38.2707100343067,0.109576090138093,0.454489364512
,0.24109714922757,0.809479822206607,0.963339107489759
"12954","SLC35G1",21.5962691901778,-0.0912608249624262,0.3788371182013
97,-0.24089726317132,0.809634743360749,0.963374725504126
"12955","HNRNPA1",2415.68530216814,-0.0528587754825344,0.2193872729577
22,-0.240938203797814,0.809603011829822,0.963374725504126
"12956","NFX1",125.494548438381,-0.0547474832600819,0.227388122484418,
-0.240766679727669,0.809735955739072,0.963420790191247
"12957","IARS2",245.154705655434,0.037394616986,0.155407131844869,0.24
0623557890046,0.809846890008134,0.96347841394423
"12958","YPEL2",267.594972755678,-0.0753813758936341,0.31339841436466,
-0.240528900078999,0.809920261728911,0.963491343922763
"12959","AL133243.2",14.540293043656,-0.101950710404757,0.424273221645
784,-0.240294944868978,0.810101613636744,0.963558362207593
"12960","ZBTB20-
AS4",5.13322009223232,-0.162139777126136,0.67467589291808,-0.240322470
134297,0.810080276718321,0.963558362207593
"12961","GOLGA7",297.791888993519,0.0507555327769894,0.211296746225524
,0.240209722504748,0.810167676842419,0.963562590735737
"12962","SZRD1",267.779907965471,-0.0409045414866369,0.172462012245809
,-0.237180008246313,0.812517144980288,0.963591066349701
"12963","LAMTOR5",694.617733106928,-0.0597660412903136,0.2505809175799
12,-0.23850994667722,0.811485601598347,0.963591066349701
"12964","PIK3C2B",43.3879584361238,0.078360256904325,0.328318157018907
,0.238671712877008,0.811360152769656,0.963591066349701
"12965","NSL1",200.099332806392,0.0426412111547781,0.179912039555027,0
.237011437701677,0.812647917036676,0.963591066349701
"12966","GAREM2",28.8997023507776,0.070803556153629,0.297136949035428,
0.238285936446049,0.811659328334319,0.963591066349701
"12967","MEMO1",25.567709868647,-0.0908619113459025,0.379666319133648,
-0.239320442100943,0.810857115391645,0.963591066349701
"12968","OXER1",9.7571778696503,-0.12761255956054,0.53230032024646,-0
.23973789062717,0.810533458889139,0.963591066349701
"12969","RNF103",178.346240664992,-0.048985380758516,0.205601378707692
,-0.238254145309792,0.811683984077391,0.963591066349701
"12970","NHEJ1",48.5691038304849,0.0634082061044289,0.264278555626461,
0.239929440942049,0.810384956793932,0.963591066349701

"12971","BOK",35.9941135639776,-0.0864584386770669,0.360967384586304,-0.239518699940591,0.810703397940516,0.963591066349701
"12972","PRRT3-AS1",8.18394330397891,0.136020555082469,0.567933773276054,0.239500733153888,0.810717328027965,0.963591066349701
"12973","H1FX",1133.33086092693,0.0545896873612444,0.228367753521529,0.239042888146193,0.811072326631274,0.963591066349701
"12974","BOD1L1",741.248439988447,-0.0460738184730946,0.193733832079974,-0.237820198869938,0.812020551639654,0.963591066349701
"12975","FYB1",26.134444430408,0.108638924381942,0.454612060829414,0.238970616361863,0.811128367449836,0.963591066349701
"12976","GLRX",478.68656745164,0.0766951084491497,0.322898243510206,0.237520983748323,0.812252642230638,0.963591066349701
"12977","B3GALT4",110.961790461548,-0.0694933842514041,0.291672676803473,-0.238258122128554,0.811680899829451,0.963591066349701
"12978","LYRM2",207.093397392717,-0.0442710253242779,0.185505022815252,-0.238651356456091,0.811375938799622,0.963591066349701
"12979","PREP",51.7618279419166,0.0612914836844307,0.255986939205461,0.239432073662308,0.810770561956692,0.963591066349701
"12980","RP9",107.852211090713,0.0603378654073265,0.251888441844066,0.239542016956377,0.810685319779496,0.963591066349701
"12981","SPIDR",78.845153151868,-0.0507964704692923,0.21379616642128,-0.23759299018112,0.812196787878634,0.963591066349701
"12982","C8orf33",192.260910500598,0.052811007292065,0.222278380558547,0.237589490976855,0.812199502138857,0.963591066349701
"12983","PMPCA",111.277832767611,0.0418158317094704,0.176433363188619,0.237006374269285,0.812651845179021,0.963591066349701
"12984","FUT4",61.4733725388211,-0.059359360308905,0.248398223304503,-0.238968538177257,0.811129978925217,0.963591066349701
"12985","IFFO1",136.534692793805,0.0492152340867677,0.20758217535123,0.237087958074894,0.812588554140397,0.963591066349701
"12986","BLOC1S1",1121.19937522547,-0.0598378686596252,0.251445164997751,-0.237975817352305,0.81189985041175,0.963591066349701
"12987","AC025164.1",8.82736004041875,0.130690007529718,0.550595054671193,0.237361390046927,0.812376440168987,0.963591066349701
"12988","RHOF",11.7145838729052,-0.114225990486405,0.477111981920322,-0.239411280401423,0.810786683818318,0.963591066349701
"12989","DNAH10OS",4.0659488525889,-0.206568184478218,0.868783860620379,-0.237767060187688,0.812061768231271,0.963591066349701
"12990","LINC00539",4.82036738762367,0.17096629136447,0.720632700465446,0.237244703514072,0.812466957687957,0.963591066349701
"12991","TEX30",76.7570632566987,-0.0699656287483137,0.294573957127677,-0.237514644643173,0.812257559428885,0.963591066349701
"12992","EMC9",31.4512294731409,0.0920292307293168,0.388383403380586,0.236954591592409,0.812692017749755,0.963591066349701
"12993","LBHD2",33.0706906412643,0.234342382557024,0.987691142824696,0.237262816680555,0.812452906554328,0.963591066349701
"12994","NDN",884.721620889514,0.0575160004650912,0.242720878049324,0.236963548118853,0.812685069315653,0.963591066349701
"12995","SIN3A",117.157037514209,0.0550329233285713,0.231804242221137,

0.237411200076618,0.812337801680765,0.963591066349701
"12996","CMTM1",5.5883749434261,0.175516383557916,0.731667322194642,0.23988550292427,0.810419019761655,0.963591066349701
"12997","RND2",7.37789332583301,-0.153048091241564,0.641057766703488,-0.238743057476058,0.811304826950959,0.963591066349701
"12998","AC005944.1",3.18717004690878,0.232453522022677,0.972599111354686,0.239002400175859,0.811103721599619,0.963591066349701
"12999","ZNF211",10.7817573180546,0.107988002303502,0.452250025244247,0.238779427917512,0.811276623013946,0.963591066349701
"13000","NRSN2-AS1",15.2153224777032,-0.0911529159664837,0.383279954807663,-0.23782333206605,0.812018121417543,0.963591066349701
"13001","SLC16A2",42.8227387098435,-0.0698915655217758,0.293119905829236,-0.238440188236457,0.811539700392489,0.963591066349701
"13002","GPR153",58.5490107817753,0.0611216252306483,0.258084388173831,0.236828061019639,0.812790181191013,0.96363333664509
"13003","METTL18",34.0317758309291,0.0855076698127885,0.361383628896313,0.236611907611681,0.812957881556402,0.963683923730539
"13004","ZNF460",53.3308978014746,0.077868753497,0.329026400810781,0.236664150065518,0.812917349007613,0.963683923730539
"13005","C5orf15",421.314117233798,0.0518858877528618,0.21959701868245,0.236277742130424,0.813217157234155,0.963907522523684
"13006","ARMC1",168.881138042156,-0.0429795116383788,0.181968991812111,-0.236191403878066,0.813284149555689,0.963907522523684
"13007","PSMD5",121.293876362268,-0.0438884648817434,0.185868025328909,-0.236127030478099,0.813334099608534,0.963907522523684
"13008","ZDHHC18",49.2574231373504,0.0579086101442383,0.249601781361633,0.232003993835036,0.816534911798681,0.96396893526626
"13009","C1orf122",611.191807159191,0.0732355620057298,0.31469040544082,0.232722576664328,0.81597683685638,0.96396893526626
"13010","C1orf52",184.909686720717,-0.0489419174161929,0.211581488711203,-0.23131474173053,0.817070294971014,0.96396893526626
"13011","ST7L",63.2867957654497,-0.0592901238506071,0.25557350770249,-0.231988535836923,0.816546918009216,0.96396893526626
"13012","TUFT1",31.2946440154603,0.085005044293077,0.363577062199215,0.233801999991133,0.815138696836471,0.96396893526626
"13013","Z99127.4",21.2176349571109,0.117334351892082,0.505366974478821,0.232176532732649,0.816400903945309,0.96396893526626
"13014","SEC16B",19.1559376801076,0.106315350448274,0.455255165234679,0.233529147095935,0.815350539034549,0.96396893526626
"13015","AC104463.2",2.47811234034627,0.272252112098979,1.16739486845474,0.233213387736879,0.815595710535787,0.96396893526626
"13016","ZFP36L2",6639.2470022523,-0.0367629821531029,0.158132252252041,-0.232482505178689,0.816163273861875,0.96396893526626
"13017","ZNF502",25.842660010869,0.0782900986631752,0.333155253482899,0.234995840061679,0.814211961976605,0.96396893526626
"13018","POC1A",4.25674284479242,-0.16354359193699,0.701527742492597,-0.233124910150956,0.815664412237873,0.96396893526626
"13019","PCCB",44.8710269684012,0.0701053518066944,0.300302388679534,0.23344919803987,0.815412613808069,0.96396893526626

"13020","AADACL2",4.79947368309499,-0.228728114391461,0.989190356926444,-0.231227602240435,0.817137987487786,0.96396893526626
"13021","TMEM44",74.6474265995353,0.0555936365617943,0.237260619054775,0.234314640091872,0.814740721104147,0.96396893526626
"13022","TLR6",6.73574422196953,-0.163185200189689,0.701458860502927,-0.232636879193015,0.816043387421607,0.96396893526626
"13023","SCFD2",41.0598427690512,-0.0666103973594908,0.287405676215785,-0.231764376530544,0.816721027139504,0.96396893526626
"13024","AC110609.1",2.8327791445003,-0.209623538433171,0.892497369895102,-0.234873004116313,0.814307303100978,0.96396893526626
"13025","AC104118.1",2.72938156970985,-0.237421920980949,1.01500206549926,-0.233912746634821,0.815052717317661,0.96396893526626
"13026","LMBRD1",368.414033545639,0.0378742439228123,0.163493942738591,0.231655334065611,0.816805725929117,0.96396893526626
"13027","AC019205.1",30.6443247435931,-0.0880286466650826,0.376320238862566,-0.233919512092016,0.815047464943942,0.96396893526626
"13028","PEX7",40.2774186855113,-0.0762403946068828,0.32700741523792,-0.23314576689771,0.815648217115877,0.96396893526626
"13029","AC018647.1",3.47767169420368,0.216630275858658,0.925323940425706,0.234112905107583,0.814897327476897,0.96396893526626
"13030","BET1",153.234800718114,0.0547508719385558,0.233455282822725,0.234524022230548,0.814578186093063,0.96396893526626
"13031","FMC1",233.559511720472,-0.0596638055534268,0.257114712468013,-0.232051308852462,0.816498162543068,0.96396893526626
"13032","EPHB6",66.2241488710821,0.0873753966980786,0.37695475762911,0.231792794572043,0.816698953763016,0.96396893526626
"13033","PLIN2",1189.23191110504,-0.0683851313205469,0.291571771384353,-0.234539616081013,0.814566081527559,0.96396893526626
"13034","CTSL",1592.72744818544,-0.0768589597671337,0.331822187980373,-0.231626945247193,0.816827777309195,0.96396893526626
"13035","NOTCH1",9.14128450210595,-0.125600844849524,0.536133482351958,-0.234271592772991,0.81477413801099,0.96396893526626
"13036","NMT2",160.408590071471,0.0407669934774783,0.174286762524174,0.23390757213603,0.81505673455384,0.96396893526626
"13037","ARHGAP22",9.88260369580498,0.19361266237795,0.837229524046199,0.231253983307051,0.817117493755967,0.96396893526626
"13038","KIF11",4.13402265310713,0.178056823397216,0.759791710474133,0.234349520983986,0.814713643903731,0.96396893526626
"13039","MGMT",552.872538567814,0.0680687571303337,0.290808528256547,0.23406726597194,0.81493275805195,0.96396893526626
"13040","RPS3",10942.9445765858,-0.059039229004495,0.254510913814183,-0.231971305747616,0.816560300651821,0.96396893526626
"13041","PGR",33.1644738281114,0.0909306613356902,0.390077757213451,0.233109065190643,0.815676715794421,0.96396893526626
"13042","FDX1",267.182913340037,-0.0452552427019842,0.194434781985831,-0.232752814284443,0.815953355375139,0.96396893526626
"13043","PLEKHA5",546.987274478377,-0.0460342263391216,0.198393902052623,-0.232034482223709,0.816511231626153,0.96396893526626
"13044","PPFIBP1",372.751669807885,-0.0507886641889993,0.218470376659878,-0.232473916901186,0.816169943621229,0.96396893526626

"13045","PLBD2",304.747319007782,-0.0396770291279206,0.171378276379155
,-0.231517260916662,0.816912977386844,0.96396893526626
"13046","UBE2L5",5.16410740730607,-0.15833174677297,0.67131678929374,-
0.235852505550388,0.813547123670701,0.96396893526626
"13047","RFXAP",64.6657678157241,-0.0528787264620353,0.22808769972865,
-0.23183506399049,0.816666121762844,0.96396893526626
"13048","AC004846.2",2.36698502093341,0.240990977863621,1.031859424192
71,0.23355020287978,0.815334190904073,0.96396893526626
"13049","SRP14-
AS1",31.1626557457899,0.0816346081690747,0.346078476177748,0.235884672
952462,0.813522161906217,0.96396893526626
"13050","WDR24",25.7919620241495,0.0761062971880799,0.329009030765194,
0.231319781742998,0.817066379782949,0.96396893526626
"13051","TBL3",61.5011947848361,-0.052671631268338,0.227721818495495,-
0.231298132152322,0.817083197674254,0.96396893526626
"13052","ZNF646",29.1697436349602,-0.0725498803790326,0.31182426731948
9,-0.23266271417131,0.816023324468107,0.96396893526626
"13053","BEAN1",3.17015186912113,-0.205424491718469,0.886389483328786,
-0.23175420690576,0.816728926310192,0.96396893526626
"13054","AC009054.2",8.73261650044047,-0.115755460115711,0.50009006118
7566,-0.231469227444405,0.816950289282447,0.96396893526626
"13055","AC040977.1",8.748528435603,-0.14332406839123,0.61737883744743
3,-0.232149305576794,0.816422050430125,0.96396893526626
"13056","ALDOC",5.39016195883053,-0.163047403131304,0.69316807514373,-
0.235220589317383,0.814037526297819,0.96396893526626
"13057","MYO18A",34.3036841737885,0.0941471406291276,0.402597468930574
,0.233849310774884,0.815101966255226,0.96396893526626
"13058","AC099850.1",10.9275577896825,0.110834666688303,0.474635462621
572,0.233515351078333,0.815361250581328,0.96396893526626
"13059","RBBP8",43.0182526925431,0.0728057699190669,0.313251352619324,
0.232419650578632,0.816212087806317,0.96396893526626
"13060","ZNF823",25.5707130076399,0.088216617299968,0.378449618451037,
0.233100029697563,0.815683731843704,0.96396893526626
"13061","GTPBP3",58.411824016746,-0.0565967042242279,0.240695183742363
,-0.235138499010468,0.814101238358685,0.96396893526626
"13062","NIPSNAP1",66.72351996962,0.0550086052243604,0.236768363962101
,0.232330892116843,0.816281020341932,0.96396893526626
"13063","CCDC134",4.98524928041817,-0.160302946074766,0.68457698757673
1,-0.234163503862738,0.814858047092396,0.96396893526626
"13064","RABL2B",73.5242383862932,-0.0598749219718218,0.25468716053086
2,-0.235092031522203,0.814137303325443,0.96396893526626
"13065","CHST7",245.593778685998,0.0847380705347347,0.360497877353881,
0.235058445161251,0.814163371065169,0.96396893526626
"13066","DIAPH2",281.506926932527,-0.0503790832761819,0.21712838187518
8,-0.232024403447824,0.816519059740301,0.96396893526626
"13067","GPRASP2",32.5164720570108,0.0808472265508303,0.34444684041582
5,0.234716121806284,0.814429073866921,0.96396893526626
"13068","PDZD8",185.502751915364,0.044217516417686,0.191356795789171,0
.23107366652608,0.817257572607245,0.964036232150343
"13069","USP1",228.660321923089,-0.039744034344196,0.173546529163064,-

0.229010828023259,0.818860500211598,0.964187337278172
"13070","ASH1L-
AS1",12.210865033292,-0.108812296212179,0.475526508592649,-0.228824879887803,0.81900502842649,0.964187337278172
"13071","AL096803.2",13.4006148133402,-0.104213224513567,0.455789713909654,-0.228643212721172,0.819146235196784,0.964187337278172
"13072","TRIM11",57.9211454572989,-0.0598586830080254,0.261805802917802,-0.228637724377786,0.81915050128319,0.964187337278172
"13073","ZNF621",95.4543808667715,0.0713267822407908,0.310180766313077,0.229952305194829,0.818128831509614,0.964187337278172
"13074","SFXN1",104.248959225445,0.0530974454543031,0.230537746563403,0.230319963848957,0.817843148665389,0.964187337278172
"13075","ARMT1",95.4220777367688,-0.0528059646960985,0.229311941781364,-0.230280046847478,0.817874164316029,0.964187337278172
"13076","AC005076.1",8.9865316742623,-0.117429875462622,0.512279360257239,-0.229230151696244,0.818690038766352,0.964187337278172
"13077","AC083843.3",17.7053036439706,-0.113276207157754,0.495406512769817,-0.228653043990938,0.819138593369389,0.964187337278172
"13078","PHYH",387.101039197787,0.0640581368169811,0.277647682635097,0.230717347283501,0.817534395895098,0.964187337278172
"13079","TYSND1",69.5921720124949,-0.0518024015578283,0.2255420526645,-0.2296795694898,0.818340771712856,0.964187337278172
"13080","AS3MT",24.0152615670035,-0.0789639785971231,0.342695305646563,-0.230420368461546,0.8177651351888,0.964187337278172
"13081","LDLRAD3",17.7499772014467,0.0934868602029086,0.408999370228666,0.228574582280265,0.81919958198717,0.964187337278172
"13082","FADS1",31.4062242593071,0.104728339272767,0.457647662604246,0.228840542256482,0.818992854610946,0.964187337278172
"13083","KRAS",333.444078010201,-0.0408494244429086,0.178139737826288,-0.229311129236885,0.818627104047907,0.964187337278172
"13084","FAM216A",32.0036625044231,-0.0722670778958831,0.314654314758509,-0.22967133932788,0.818347167496253,0.964187337278172
"13085","NDUFAF1",61.3379995777217,-0.0659343900493593,0.2866663439349,-0.230003945159089,0.81808870410322,0.964187337278172
"13086","ALDH1A2",2.53195514380946,0.280826778343176,1.22418984370876,0.229398062552449,0.818559541877387,0.964187337278172
"13087","IQGAP1",970.891022781541,-0.0427290309050892,0.185763135167633,-0.2300188940423,0.818077087996838,0.964187337278172
"13088","AC009065.4",4.58824138479766,0.157361558392862,0.68422411951311,0.229985400843279,0.818103114114449,0.964187337278172
"13089","ZNF597",8.7222307920035,0.130560682344519,0.568831778248778,0.229524241325735,0.81846148161877,0.964187337278172
"13090","AC009053.2",5.42129442572065,0.157678415720662,0.689222908275993,0.228777096389723,0.819042169150604,0.964187337278172
"13091","CCDC144A",53.5738905841085,-0.21434654295974,0.936536565810206,-0.228871515309503,0.818968780460351,0.964187337278172
"13092","AC103702.1",10.3587653830411,0.115309408252678,0.500149024472864,0.230550101290729,0.817664336625704,0.964187337278172
"13093","AP000915.2",9.09984119061916,0.145978402349987,0.632602790239187,0.230758391525262,0.817502507593631,0.964187337278172

"13094","ATG4D",30.6426990209096,-0.0683184366449059,0.297717394705039
,-0.229474118274452,0.818500434576975,0.964187337278172
"13095","ZNF730",2.5150612206419,0.209001456657039,0.913807221600873,0
.228715041549896,0.819090403179835,0.964187337278172
"13096","PPP1R13L",16.0256277577618,-0.111444711918359,0.4851220716724
45,-0.229725090705842,0.81830539670525,0.964187337278172
"13097","ZNF274",32.7403216425263,0.0774611519679157,0.337722595704213
,0.229363249463351,0.818586597486486,0.964187337278172
"13098","CORO2B",104.023882215094,-0.0591264515079453,0.25883480827592
4,-0.228433153569188,0.819309517881734,0.964243107203156
"13099","IFI16",1329.41478516045,-0.049702262376466,0.218145487579266,
-0.227839974725152,0.819770648543039,0.96438306775782
"13100","B3GNT2",27.2394939695986,0.0860672168091352,0.37854789296868,
0.227361500110783,0.82014265482735,0.96438306775782
"13101","AC097382.3",35.2452130460942,0.0717737306263966,0.31552943331
4927,0.227470793682692,0.820057677271147,0.96438306775782
"13102","AL355353.1",4.37527871243211,-0.159693247945375,0.70044837445
3238,-0.227987177598962,0.819656208839611,0.96438306775782
"13103","GNB2",894.964431869772,-0.0384824386099708,0.168994257246463,
-0.227714475254904,0.819868218434047,0.96438306775782
"13104","RPS26",3854.62614832647,-0.152770227014076,0.670990318051721,
-0.227678735302258,0.819896005063777,0.96438306775782
"13105","HSP90B1",2025.70085743897,-0.0430400686263818,0.1886520047190
31,-0.22814530219535,0.819533282556861,0.96438306775782
"13106","NEURL4",21.8994324467077,-0.078114796774586,0.343156467031241
,-0.227636091053107,0.819929159851262,0.96438306775782
"13107","CHRNB1",55.3247508187606,0.0730653632917055,0.320567195679813
,0.22792526583002,0.819704340342942,0.96438306775782
"13108","AP001033.2",11.3605405920849,-0.102593801570264,0.44996141300
8294,-0.228005776949528,0.819641749448392,0.96438306775782
"13109","POLRMT",35.1602750668578,0.0741218593687733,0.326076208698549
,0.227314527682385,0.82017917731463,0.96438306775782
"13110","FDX2",145.593127170322,0.0675773157200136,0.297045061145792,0
.227498533250637,0.820036109635294,0.96438306775782
"13111","WARS2-
IT1",10.9895727666086,0.126590905158794,0.557626559554378,0.2270173523
65637,0.820410249168632,0.964507625910193
"13112","CCL4",214.197062126612,-0.148716018536509,0.655005977683067,-
0.227045284475964,0.820388529592661,0.964507625910193
"13113","AC016747.1",53.9636005437761,0.0776630994046656,0.34251682706
4441,0.22674243502219,0.820624027979088,0.964511298490912
"13114","VGLL4",510.814127378073,-0.044987489663567,0.198452493337825,
-0.226691481204949,0.820663651703328,0.964511298490912
"13115","KIF13A",158.415909105486,0.048588214419599,0.214270829433734,
0.226760752025863,0.820609784056998,0.964511298490912
"13116","AL158163.1",4.67599044513647,0.155269630358648,0.684929363588
095,0.226694369686893,0.820661405492116,0.964511298490912
"13117","AC017071.1",4.28566955483581,0.18142291548746,0.8006733657138
71,0.226587923685591,0.820744183578466,0.964532407552188
"13118","KYAT3",119.346514148176,-0.0600908213529851,0.265535260500986

,-0.226300722697285,0.820967536338841,0.964647806438237
"13119","MOB4",259.844710494206,-0.0564199610439379,0.249293549136521,-0.226319378256517,0.820953027693362,0.964647806438237
"13120","AATK",7.16072780544829,-0.127536728634906,0.564016412036121,-0.226122371465209,0.821106245258472,0.964737253861231
"13121","PPARGC1B",5.31403362354583,-0.159012753738601,0.703605789351216,-0.22599693769607,0.821203802085189,0.964771402691027
"13122","AL353751.1",2.91808281075273,0.198405492225281,0.878821505455505,0.225763128227552,0.821385656108793,0.964771402691027
"13123","AC130650.2",7.47294565873438,0.144056261221259,0.638067135600363,0.225769755537895,0.821380501339067,0.964771402691027
"13124","MMP28",101.228641010384,0.0822480982370786,0.36412158052262,0.22588086682209,0.821294079330628,0.964771402691027
"13125","LRRC14",28.8775351700325,0.0718048688990985,0.318464213518316,0.225472332058336,0.821611847054768,0.96496355217899
"13126","XPC",213.779276163481,-0.0495601876022711,0.220070183580096,-0.225201736991478,0.821822338232257,0.965098181846805
"13127","TMEM254-AS1",12.5521419756308,-0.127039544704902,0.564208946162744,-0.225164002749184,0.821851692059878,0.965098181846805
"13128","GSPT1",316.98177932527,-0.0359950709141596,0.159938274832923,-0.225056015839619,0.821935697486617,0.965123307187401
"13129","TRPC1",70.1339098773647,0.0622431095205396,0.276829326484399,0.224842903427168,0.822101488377425,0.965170940086672
"13130","PPP2CB",350.769396875845,0.0364147218566464,0.161946163266649,0.224856959387723,0.822090553292219,0.965170940086672
"13131","GCLC",44.2568454141822,0.0701910648889652,0.312389827175042,0.224690623006859,0.82221995986129,0.965216381573605
"13132","NDFIP2",161.649394417481,-0.0542075722198923,0.241403522335211,-0.224551703701407,0.822328040169066,0.965216381573605
"13133","AC010487.2",3.5332208216252,-0.20019652437373,0.891235556030221,-0.22462807169123,0.822268624857401,0.965216381573605
"13134","PEX10",70.7770056334667,0.0613345737078339,0.278835428926047,0.219966931548362,0.825896908755981,0.965307373531829
"13135","AGTRAP",399.706762817818,0.0552035538994946,0.24917502168002,0.221545295861897,0.824667871696937,0.965307373531829
"13136","MED18",29.663933264688,0.0657388520792965,0.296819139144994,0.221477807221803,0.824720414821478,0.965307373531829
"13137","SNHG12",80.1540992828717,-0.0586127507586146,0.266011017482166,-0.220339560794861,0.825606712086411,0.965307373531829
"13138","JUN",25224.4661579963,0.0331571868979331,0.147787249659155,0.22435756111846,0.822479090267215,0.965307373531829
"13139","COLGALT2",12.7442565665413,-0.175109320951194,0.795344596162972,-0.220167864088075,0.825740423304422,0.965307373531829
"13140","RAB29",133.737038695473,-0.067908103635766,0.306696946327118,-0.221417606040773,0.82476728497439,0.965307373531829
"13141","COQ8A",110.113812984095,0.054053171495988,0.245162494613029,0.220478958583396,0.825498157824283,0.965307373531829
"13142","C2orf81",30.5106415613997,-0.0867380644080742,0.388205757653512,-0.223433225030864,0.823198348016644,0.965307373531829

"13143","POLR1B",18.8191814631749,0.0812020740577989,0.364734605818131
,0.222633314093286,0.823820906202857,0.965307373531829
"13144","TANK",327.468211958015,0.0323297815697761,0.144854032001752,0
.223188689489741,0.823388654418384,0.965307373531829
"13145","TMBIM1",346.838291315131,0.031250556901676,0.142019389858521,
0.220044297703347,0.825836655487306,0.965307373531829
"13146","TXNRD3",32.3331700457986,0.0626692714816842,0.281682936879909
,0.222481603521488,0.823938992691192,0.965307373531829
"13147","LRRC34",33.8253843613292,0.0684970118140892,0.308453727023661
,0.222065761613686,0.82426269069827 5,0.965307373531829
"13148","APOD",14361.7590903856,0.0811470307011677,0.363689131812148,0
.22312195664698,0.823440590134487,0.965307373531829
"13149","LYAR",130.89409444397,-0.0457852963921614,0.205847247494168,-
0.222423651273056,0.8239841018557,0.965307373531829
"13150","PPAT",44.136813809078,0.0693089275741722,0.313078777749971,0.
221378555494181,0.824797688451048,0.965307373531829
"13151","AC114956.3",2.40586837192988,-0.239265831919052,1.08017545252
152,-0.221506451901419,0.824698113476518,0.965307373531829
"13152","SMN1",22.0028816675987,-0.0941863569627937,0.424027694922852,
-0.222123125660295,0.824218035826233,0.965307373531829
"13153","FAF2",195.052709347733,0.0336941907273614,0.151779143930245,0
.221994866059112,0.824317879918512,0.965307373531829
"13154","AL590062.1",13.7439881622904,-0.103532455387396,0.46261903442
8789,-0.223796358736581,0.822915763421862,0.965307373531829
"13155","HIST1H4J",6.6099986968959,0.164536152268116,0.743941595462549
,0.221168104151799,0.824961543497875,0.965307373531829
"13156","APOM",23.6831239466451,0.092066602701511,0.411534141156844,0.
223715588803172,0.822978615252656,0.965307373531829
"13157","PAPOLB",2.89186267687391,0.216706492686552,0.982227165901662,
0.220627671693055,0.825382353052032,0.965307373531829
"13158","EPHA1-
AS1",9.31096700890023,0.119933616257201,0.535336201710571,0.2240341973
39866,0.822730693830212,0.965307373531829
"13159","AC064807.4",3.3160001961518,-0.194287626570396,0.869649559457
604,-0.223409101352931,0.823217121473151,0.965307373531829
"13160","LINC01410",2.39905091006841,0.215289914773488,0.9791838458198
94,0.219866693770076,0.825974976119677,0.965307373531829
"13161","FBXW2",172.262244681174,-0.0398751496879302,0.177957560588866
,-0.224071118731806,0.82270196503432,0.965307373531829
"13162","RPL12",14958.7238453163,-0.0537277875602562,0.242487366839733
,-0.221569429618025,0.824649082607646,0.965307373531829
"13163","MED27",66.2950915487284,-0.0585429631584013,0.265602370137591
,-0.220415816048908,0.82554732885999,0.965307373531829
"13164","PCBD1",693.757807336437,-0.0817680438156272,0.369513870260684
,-0.221285452039842,0.824870176811696,0.965307373531829
"13165","ZMIZ1",359.523849632504,0.0641268956674234,0.286952572947545,
0.223475590438933,0.823165378780357,0.965307373531829
"13166","SMC3",477.537765653048,-0.0396431170339178,0.180075383708296,
-0.220147341727372,0.825756405718857,0.965307373531829
"13167","DDB2",78.2547404638107,0.0649336436860792,0.292742086863898,0

.221811781085882,0.824460407920158,0.965307373531829
"13168","AP000759.1",3.43851935906373,-0.204540425681086,0.927250330837022,-0.220588139878525,0.825413136603438,0.965307373531829
"13169","TIMM8B",481.60465997001,-0.0551301305884745,0.250722083120852,-0.219885420152244,0.825960391474897,0.965307373531829
"13170","USP28",57.3688540895572,0.0579844619724504,0.263626204165891,0.219949538612493,0.825910454629641,0.965307373531829
"13171","OGFOD2",34.3503688951449,0.064190378200854,0.287202676183308,0.223502019737046,0.823144811361885,0.965307373531829
"13172","MTMR6",100.70938438747,0.0435368224319622,0.197423958594061,0.220524513549451,0.825462683194696,0.965307373531829
"13173","NUBPL",61.9699189058584,-0.0506608647723194,0.229987204316982,-0.220276884197851,0.825655521779171,0.965307373531829
"13174","TRAPPC6B",131.369898160539,0.0395020814648601,0.179597936175115,0.219947301768234,0.825912196720531,0.965307373531829
"13175","VASH1-AS1",8.54615155716964,-0.114447909820194,0.516887097999603,-0.221417617625042,0.824767275955296,0.965307373531829
"13176","SNURF",2.5549997280242,-0.205568770569352,0.925863952708266,-0.22202913286346,0.824291204537899,0.965307373531829
"13177","TMEM62",25.1716802537271,0.085812786868591,0.382985680092298,0.224062651240408,0.822708553628192,0.965307373531829
"13178","ZNF689",56.6493461058516,0.060467300998561,0.271558472070102,0.222667702235972,0.823794140165032,0.965307373531829
"13179","SLC25A11",229.665625425214,-0.0528722803064868,0.240402885151979,-0.219931970754268,0.825924136788003,0.965307373531829
"13180","TMEM199",60.3133694329321,-0.0686920999329777,0.311086467123719,-0.220813526760259,0.825237630711191,0.965307373531829
"13181","WBP2",223.394437778729,-0.0342922324706712,0.155507600316221,-0.220518047998547,0.825467718036224,0.965307373531829
"13182","SERPINB2",3.05642676718178,0.240039116308939,1.07764820528347,0.222743484499004,0.823735155704713,0.965307373531829
"13183","MVB12A",385.67733916071,-0.0520806268048775,0.235198238076644,-0.221432895206919,0.824755381401962,0.965307373531829
"13184","KMT2B",118.468324925866,-0.0468393010092555,0.209068197755309,-0.224038383226872,0.822727436750019,0.965307373531829
"13185","TIMM50",114.291410022986,-0.0505839268806923,0.22694514107126,-0.222890548094216,0.823620692885316,0.965307373531829
"13186","EGLN2",229.88263839829,0.0517412806821978,0.233108683679843,0.221962047339517,0.824343428211446,0.965307373531829
"13187","FASTKD5",43.9461726131868,0.0542237144758323,0.246376692990226,0.220084594113711,0.825805272784997,0.965307373531829
"13188","BACH1",168.09975916596,-0.0693035728776761,0.309817360412506,-0.223691702703173,0.822997202648097,0.965307373531829
"13189","GRIPAP1",78.9004892835871,0.0509060466143514,0.228111644888321,0.2231628579912,0.823408758031487,0.965307373531829
"13190","SLC9A6",32.1033295090856,0.065355836959156,0.295844290168107,0.220912957022151,0.825160208409543,0.965307373531829
"13191","KIAA2013",182.281820189961,-0.0415156417446959,0.188892991817,-0.219783917578669,0.826039445316508,0.965309532981122

"13192","WDPCP",50.6515711405028,-0.0584014025075667,0.265965483655743
,-0.219582637960493,0.826196214311292,0.965327700771
"13193","GPBP1",470.623462439142,0.0299866041350817,0.136699202724258,
0.21936195338,0.82636810505184,0.965327700771
"13194","AL135925.1",66.6653424758586,-0.0680189221648296,0.3099590188
77923,-0.219444887943779,0.82630350652377,0.965327700771
"13195","MAP3K10",20.5806017318975,0.0848276416797794,0.38669298204324
6,0.219366902475341,0.82636425012098,0.965327700771
"13196","U2AF1",43.8743741839959,0.0717948524649377,0.327235665239224,
0.219398006059188,0.826340023129416,0.965327700771
"13197","SHC3",54.7685273291688,0.0726193868249702,0.331350119023117,0
.219162096694173,0.826523780171091,0.965436392463239
"13198","LINC01048",35.8127514566797,0.102178103276624,0.4664152410359
13,0.219071107217007,0.826594657205704,0.965446025217906
"13199","AC009630.1",6.19129459727448,-0.141943045397643,0.64845339891
831,-0.218894751163952,0.826732035290075,0.965533322524169
"13200","TMEM167B",174.901247635729,0.0368762047515808,0.1687040216827
17,0.218585214411391,0.826973171482283,0.965629774320172
"13201","ARHGEF2",120.984776993081,-0.0519297056098297,0.2375985827187
06,-0.21856067075665,0.826992292247715,0.965629774320172
"13202","HSD17B4",203.282826848186,-0.040114659418897,0.18361876736246
7,-0.218467098952417,0.827065190421617,0.965629774320172
"13203","PTBP1",346.892532378879,-0.0446455766816316,0.204340063331087
,-0.218486653834953,0.8270499558457,0.965629774320172
"13204","RNF19A",433.585085331853,0.04197109959934,0.192285460276827,0
.218274951933004,0.82721488940474,0.965658274909054
"13205","TNKS2-
AS1",3.83260919230552,0.211398779776965,0.96815277374643,0.21835270786
7501,0.827154310116342,0.965658274909054
"13206","C2orf42",24.6713753436909,0.0716858993019928,0.32870030740173
3,0.218088932951253,0.827359820103257,0.96575432582854
"13207","DDX20",34.075811697134,-0.0683466991739444,0.314588621420981,
-0.217257378430364,0.828007770849356,0.965802449100531
"13208","PTPN7",5.64425848015085,-0.139871251069643,0.64613619921666,-
0.216473324415526,0.828618816240951,0.965802449100531
"13209","GOLGA4",862.212990940937,-0.0440705866652499,0.20231964065528
2,-0.217826536872605,0.82756426755245,0.965802449100531
"13210","ADAMTS9",3.23503102857734,-0.191096985682847,0.87797746501455
6,-0.217655911794591,0.827697217357185,0.965802449100531
"13211","SEC24B-
AS1",9.16104525364908,0.124843683389805,0.5768369314876,0.216428034640
304,0.828654115588948,0.965802449100531
"13212","ADAM19",32.3468091245495,0.101414551942928,0.468012108565765,
0.216692154084892,0.828448262871384,0.965802449100531
"13213","HLA-
DRB1",275.427214075852,-0.091432547728815,0.421608541306045,-0.2168659
75830514,0.828312793931471,0.965802449100531
"13214","AC072061.1",4.2378422243508,0.174195722559606,0.8028305999974
95,0.216976934561474,0.828226320300168,0.965802449100531
"13215","TMEM168",96.3168778951397,0.0434405467531464,0.20046047212987

1,0.216703803456089,0.828439183709867,0.965802449100531
"13216","KLF14",8.94995180367999,0.182805828692947,0.842511135347348,0
.216977344302494,0.828226000979953,0.965802449100531
"13217","MTMR9",109.3739128537,-0.0482021567014505,0.221992270914506,-
0.217134391674448,0.828103612589849,0.965802449100531
"13218","ZNF79",5.91810985685776,-0.143971341627761,0.661737238355578,
-0.217565724403739,0.827767492703111,0.965802449100531
"13219","MSS51",7.32831987035747,0.119198292078392,0.548909896031355,0
.217154569338613,0.828087888262231,0.965802449100531
"13220","PIK3C3",109.891392708736,0.0440957646604355,0.203540045536265
,0.21664417212965,0.828485658772724,0.965802449100531
"13221","PDE4C",2.81561459958778,0.238847824420897,1.10140874882286,0.
216856661685472,0.828320052832533,0.965802449100531
"13222","ZNF226",88.4789289074407,0.0582110800699514,0.268421053537678
,0.216864807371678,0.828313704559291,0.965802449100531
"13223","IGLC2",199.976312982581,0.188003626674135,0.868402586031733,0
.216493628298874,0.828602991283722,0.965802449100531
"13224","LRRC75B",66.4240597841681,0.0510847189861951,0.23602913375297
4,0.2164339553085,0.828649500935498,0.965802449100531
"13225","MIATNB",20.1684693590522,-0.0900383972224091,0.41576490717022
5,-0.216560839237798,0.828550607208269,0.965802449100531
"13226","MECP2",228.301145919262,0.0412738644846944,0.18970260624287,0
.217571415080364,0.827763058403138,0.965802449100531
"13227","ZBTB8A",158.738375891835,0.0372105619864351,0.174133268446489
,0.213690136976152,0.830788703248452,0.965892817431176
"13228","ATP8B2",153.933921307345,-0.0555059596248456,0.25973930250228
,-0.213698732113744,0.830782000131474,0.965892817431176
"13229","RASAL2-
AS1",7.44259068867662,-0.167165770113773,0.784487053037022,-0.21308926
5739462,0.83125733707685,0.965892817431176
"13230","HSD11B1-
AS1",18.5659573536198,-0.112310693497342,0.522012019336798,-0.21514963
1305481,0.829650659651324,0.965892817431176
"13231","TSGA10",23.4186004051638,0.0690405349254054,0.324327301514273
,0.212873028582723,0.831426000291552,0.965892817431176
"13232","RBM45",32.5096278511368,0.0698849320858021,0.32800236495337,0
.213062281108056,0.83127838444453,0.965892817431176
"13233","UBA3",121.545269195868,-0.0449776565758319,0.211213355152776,
-0.212948923344825,0.831366802112818,0.965892817431176
"13234","MAP9",82.5109528748733,0.0485895486061547,0.226685945567672,0
.214347424514897,0.830276137912451,0.965892817431176
"13235","PCDHB10",21.1429813702553,-0.0954956128200258,0.4470408084472
87,-0.213617215734089,0.830845573062032,0.965892817431176
"13236","PWWP2A",115.013785013413,-0.0455066346101703,0.21261895691901
5,-0.21402905587343,0.830524398835827,0.965892817431176
"13237","LCP2",21.1235100499352,0.0980693424502717,0.454429410289082,0
.215807648514399,0.829137686148523,0.965892817431176
"13238","VEGFA",302.460655177233,0.0775291937858022,0.358681178202094,
0.216150716841126,0.828870267669221,0.965892817431176
"13239","SUPT3H",36.6069672687013,-0.0670460805151232,0.31478304738689

4,-0.212991395412466,0.831333674169648,0.965892817431176
"13240","RUNX2",15.7867279269205,-0.10215692242351,0.475542672694365,-0.2148217779168,0.829906272947142,0.965892817431176
"13241","RPA3",214.660758797198,-0.059291609089446,0.27821356921305,-0.213115446730932,0.831236916644789,0.965892817431176
"13242","SNHG26",53.5196773539706,0.0796039120639342,0.370308751867222,0.214966326511443,0.829793572356055,0.965892817431176
"13243","ZNF777",39.9947461051462,-0.0563775161835057,0.261947295165538,-0.215224654821794,0.829592169551327,0.965892817431176
"13244","RARRES2",917.690221428903,-0.0763067020212315,0.356979507656076,-0.213756533315485,0.830736922846363,0.965892817431176
"13245","GIMAP5",3.00678976290565,-0.202186270697875,0.942902452517205,-0.21442967950514,0.830211998984435,0.965892817431176
"13246","TM2D2",236.043186978827,-0.0433067942526311,0.201917585424526,-0.214477575896026,0.830174651945755,0.965892817431176
"13247","DLG5-AS1",4.17650554127841,0.158116947951773,0.739041773515701,0.213948593459872,0.830587145361054,0.965892817431176
"13248","API5",149.258418286355,0.0397574581851815,0.183979625891473,0.216097070491022,0.828912083178817,0.965892817431176
"13249","RCOR2",2.45070586516745,0.25086730951693,1.17171017315346,0.21410355159908,0.830466306240665,0.965892817431176
"13250","CCDC82",310.15410561721,-0.0349093825909749,0.163483018269227,-0.213535221948775,0.830909519428112,0.965892817431176
"13251","CD69",48.2972051180048,0.108219066778437,0.507884343639024,0.213078170520162,0.831265991068126,0.965892817431176
"13252","AC025159.1",37.1619151401206,-0.0817737603404659,0.382748426099219,-0.213648848079829,0.830820903521456,0.965892817431176
"13253","LINC00571",2.98959002071004,0.194815030177769,0.909692315050357,0.214154859785734,0.830426296085052,0.965892817431176
"13254","ACOT1",3.15799168524401,-0.193333981276272,0.902810014169231,-0.21414691711653,0.83043248975464,0.965892817431176
"13255","SPTLC2",96.421665594803,-0.0435918026084266,0.204037348663804,-0.213646192199123,0.830822974792256,0.965892817431176
"13256","CHST14",134.373785915116,-0.0486594709323766,0.227964616360186,-0.213451858052805,0.830974535481516,0.965892817431176
"13257","RPAP1",39.0229007201362,0.059537983116244,0.279267441809892,0.213193427527344,0.831176094506662,0.965892817431176
"13258","RNF111",65.465454951575,0.0500962981767264,0.234315855577733,0.213798157419643,0.830704461900632,0.965892817431176
"13259","AC116667.1",7.05635266042373,0.137816120764162,0.642818930466264,0.214393376163017,0.830240306640552,0.965892817431176
"13260","CMC2",129.097246176625,-0.0587263016974375,0.275701917982131,-0.213006504007142,0.831321889633963,0.965892817431176
"13261","TRPV1",28.0935516995148,0.0797229085699804,0.371194222766589,0.21477410929456,0.829943439637354,0.965892817431176
"13262","HEATR6",43.6106449125856,0.0591291676763341,0.27606034136518,0.214189286965042,0.830399449995834,0.965892817431176
"13263","CELF4",4.99135019079013,0.164185319270652,0.76222920387147,0.215401507101449,0.829454295087244,0.965892817431176

"13264","AC005253.1",9.30408995484017,0.127621551013923,0.592489917078
404,0.2153986883747,0.829456492532243,0.965892817431176
"13265","ZSCAN5A",17.6017049852709,0.0797906937177372,0.37271453037628
6,0.214079911607369,0.830484740868702,0.965892817431176
"13266","CRNKL1",133.51805044644,0.0533322159467194,0.247133682304607,
0.215803104819133,0.829141228048441,0.965892817431176
"13267","RBM38",35.6863793627244,-0.0689363706328291,0.323743382236595
,-0.212935227143731,0.831377485126628,0.965892817431176
"13268","MIS18A",75.9653663806261,-0.0560935404386202,0.26020902600771
2,-0.215571078756344,0.829322101547989,0.965892817431176
"13269","RAB9A",322.003529997924,-0.0530394648734366,0.248673088944232
,-0.213289926540187,0.83110083024426,0.965892817431176
"13270","KIF3A",97.3829121994168,0.0445128067610768,0.209254125133897,
0.212721286773172,0.831544362298828,0.965957524102218
"13271","GAR1",97.7827137815832,0.0468099733779468,0.220593096714851,0
.21220053607777,0.831950588533495,0.966106109962696
"13272","ZNF827",97.2083862510013,-0.0580182773724081,0.27328358416404
4,-0.21230063104552,0.831872503148711,0.966106109962696
"13273","REPS1",62.5302802323655,-0.0488628862123724,0.230417698994321
,-0.212062208873879,0.832058502112063,0.966106109962696
"13274","NCOR2",349.671796476566,-0.04859443038222,0.228943869849258,-
0.212254778493156,0.831908273114618,0.966106109962696
"13275","PEX11G",49.3718469618112,0.07061281062468,0.332915160068639,0
.212104521194293,0.832025492550072,0.966106109962696
"13276","AP1M1",174.450958966269,0.0454023706592218,0.214167240493578,
0.211994937015511,0.832110984234493,0.966106109962696
"13277","ZBTB46",7.43934953677517,-0.132202413212777,0.623293097022647
,-0.212103124267352,0.832026582344863,0.966106109962696
"13278","WBP4",288.735762777561,0.0362961655695812,0.171418125656234,0
.211740534617503,0.832309462963077,0.966118676454874
"13279","DDX24",859.612229138358,0.0367321535929658,0.173477578026777,
0.211740064686032,0.832309829602383,0.966118676454874
"13280","PIGA",22.5729275414192,0.0828534857591054,0.391179274849624,0
.211804385063487,0.832259647351163,0.966118676454874
"13281","TMEM79",18.3766289271394,0.0792104756474414,0.375062838277796
,0.211192545790882,0.832737027176507,0.966126132933502
"13282","AL133243.3",34.5916913216954,-0.0749470363088985,0.3543064305
66282,-0.211531685126663,0.832472410383901,0.966126132933502
"13283","TTC31",49.2780819256857,-0.0507437210251619,0.240422399726755
,-0.211060704338835,0.832839902880335,0.966126132933502
"13284","CDC23",57.7095074239494,-0.0482247390684749,0.228448297245501
,-0.211096951257423,0.832811619174176,0.966126132933502
"13285","AFDN",90.0867042005806,0.0528256367962516,0.250243714273174,0
.211096758013212,0.832811769963316,0.966126132933502
"13286","CDK20",15.0902633349487,0.0844787359459534,0.39972578448826,0
.211341722811565,0.83262062800653,0.966126132933502
"13287","EARS2",18.0654988852345,-0.0775848137636839,0.367138775644966
,-0.211322853674031,0.832635350929015,0.966126132933502
"13288","AC002553.2",3.7430169896537,0.161318350642772,0.7645096832013
97,0.211008904383329,0.832880323097847,0.966126132933502

"13289","ZNF337-
AS1",21.6783855331483,-0.0824456618914501,0.390256891771115,-0.2112599
76773977,0.832684411980127,0.966126132933502
"13290","FBXO5",33.8873932289887,-0.0749030062054157,0.355161975600394
,-0.210898157323271,0.832966742033426,0.966153674074135
"13291","CHCHD7",90.0141235280293,-0.0436116135022531,0.20700118039207
2,-0.210682921805809,0.833134701919641,0.966271405356971
"13292","AC104938.1",2.42622661611631,0.244925490212932,1.162947984720
77,0.210607433376945,0.833193611417766,0.966271405356971
"13293","TDP2",119.654260635231,-0.0467580924742389,0.22267707944709,
-0.210368356728952,0.833380187717918,0.966292018882272
"13294","AC025259.3",5.30416643787321,-0.262550300267067,1.24808123739
973,-0.210363149769055,0.833384251353126,0.966292018882272
"13295","XPNPEP3",66.4844731480163,-0.0671739988257142,0.3193535311685
47,-0.210343685820281,0.833399441520585,0.966292018882272
"13296","PPIE",197.418404403817,-0.0494876035493981,0.235451754740877,
-0.210181502379802,0.833526016076618,0.966300355637347
"13297","LOXL4",63.7699584388045,0.0827866657450886,0.393896163557011,
0.210173831086594,0.833532003172871,0.966300355637347
"13298","UBE2J1",281.28989795603,0.0358315231958118,0.170650484873223,
0.209970239594872,0.833690900620443,0.966364701931941
"13299","ZNF552",15.5311069964084,0.0973402905795728,0.463653099290266
,0.209942068172467,0.833712888160421,0.966364701931941
"13300","EMC2",271.355166170558,0.0507853412654149,0.242286744659361,0
.209608418061895,0.833973308935187,0.966409945892116
"13301","ERBB3",3.48498506751098,-0.178357952273814,0.851063102659643,
-0.209570772973744,0.834002692844815,0.966409945892116
"13302","FAM106A",30.5090150941108,-0.126497619568649,0.60301265190375
2,-0.209776062192539,0.833842456964284,0.966409945892116
"13303","SLC9A3R1",32.207471006418,0.0830042930809946,0.39599615783873
2,0.20960883442409,0.833972983944573,0.966409945892116
"13304","AL136454.1",17.1710206177335,0.100545348502981,0.481493684365
384,0.208819662163381,0.834589021511292,0.96644360302787
"13305","PREB",117.084983878115,0.0534555938383786,0.255629920584785,0
.209113212240931,0.834359860834093,0.96644360302787
"13306","AC012360.3",12.4468392646701,-0.0913247501301437,0.4373565937
95501,-0.208810731164705,0.834595993740318,0.96644360302787
"13307","AC109322.1",3.58442535206086,-0.167339374287736,0.80130231903
682,-0.208834256824417,0.834577627816919,0.96644360302787
"13308","SLC35F2",29.0187860972418,-0.0939285796830214,0.4497857263960
64,-0.208829614126775,0.834581252253478,0.96644360302787
"13309","FBRSL1",88.8906624362268,-0.0447526457672381,0.21378021833633
8,-0.209339508189805,0.834183211878531,0.96644360302787
"13310","ZNF774",4.09498907666089,0.174449084066508,0.833785203029175,
0.209225449711422,0.834272246059691,0.96644360302787
"13311","RAB3D",35.9706557206752,0.0653664573426101,0.312521973677229,
0.209157956394197,0.834324932422497,0.96644360302787
"13312","SHROOM4",4.8122498471254,-0.1546130934922,0.7387324542847,-0.
209295114348142,0.834217865510449,0.96644360302787
"13313","STK17B",113.281811117259,0.0816983150274013,0.391993233211546

,0.20841766698117,0.83490286316635,0.966726330331952
"13314","AL034417.2",10.2933910887269,-0.120128352804287,0.57928709098376,-0.207372742590003,0.835718768172142,0.96673841193759
"13315","RO60",316.143308149003,0.0265375265478046,0.128375517675171,0.206717971061683,0.836230121657605,0.96673841193759
"13316","ARID4B",565.395277946346,-0.0544300734501199,0.263115770291515,-0.206867392972359,0.836113422308759,0.96673841193759
"13317","IFT172",33.0581186677676,0.0615119607431792,0.297864392893498,0.206509949529862,0.836392593640516,0.96673841193759
"13318","LINC02614",27.7833917607978,0.0665358222782538,0.31951243954862,0.20824172721 4909,0.83504022905767,0.96673841193759
"13319","KIAA0232",150.096859721394,-0.0474744479762364,0.229661321788962,-0.20671503414868,0.836232415439234,0.96673841193759
"13320","TAPT1",89.6148027418919,-0.0521430047767249,0.252309407044064,-0.206662943675415,0.836273099262692,0.96673841193759
"13321","OSTC",1091.91369105162,-0.0582834025120112,0.280315666881615,-0.207920603084329,0.835290961776266,0.96673841193759
"13322","MGARP",2.52293875936804,0.226484432383213,1.09594466451742,0.206656813720556,0.836277886923201,0.96673841193759
"13323","AC010273.3",3.3021564062616,0.181620061771008,0.880499512500345,0.206269349605048,0.83658051917522,0.96673841193759
"13324","AP3B1",191.148534487541,-0.043244393661815,0.20832998711935,-0.207576423633343,0.835559714604065,0.96673841193759
"13325","ARHGEF37",5.74075537901338,-0.128279974087872,0.622248019127998,-0.206155696996256,0.836669293133921,0.96673841193759
"13326","STX7",267.888684913017,0.0338841423273102,0.163649661245044,0.20705293289042,0.835968519614647,0.96673841193759
"13327","NUP188",50.808348970815,0.0518342651047582,0.249341196382697,0.207884881667132,0.835318853982477,0.96673841193759
"13328","AC127035.1",3.30902698955998,-0.205196098193542,0.995249145943355,-0.206175608419182,0.836653740187632,0.96673841193759
"13329","C2CD3",31.6273432335804,0.0606187872795336,0.293234934383164,0.206724302501844,0.836225176694238,0.96673841193759
"13330","PCED1B-AS1",14.4518520253166,-0.117305715619308,0.565488476629938,-0.207441389996837,0.835665160984367,0.96673841193759
"13331","DHRS4L2",265.118590452489,-0.0568171808398386,0.275218648730702,-0.206443789699125,0.836444268210844,0.96673841193759
"13332","PSMA6",538.396428313899,-0.0521449047041277,0.252248597782342,-0.206720295623296,0.836228306134361,0.96673841193759
"13333","KTN1-AS1",12.0824833929533,0.102325436082683,0.494376543486833,0.206978744098543,0.836026458801291,0.96673841193759
"13334","DUOX1",6.27045036351684,-0.179173038571277,0.865835103686857,-0.206936676288974,0.836059312874462,0.96673841193759
"13335","PSMA4",750.254649375616,-0.0461933422673,0.223886769913239,-0.206324573288546,0.836537384752936,0.96673841193759
"13336","PRKCB",6.44521165934462,-0.135975546074716,0.653951901180796,-0.207928971273261,0.835284427707916,0.96673841193759
"13337","ATPAF2",56.0525982298642,-0.0612270817429854,0.29403109827401

7,-0.208233353894852,0.835046766707045,0.96673841193759
"13338","SEH1L",53.3560561661535,0.067057578466043,0.322691089515114,0
.207807344686232,0.835379397574761,0.96673841193759
"13339","ZIK1",4.69557937885192,0.173881179082074,0.838382776935996,0.
20740070510221,0.835696931979981,0.96673841193759
"13340","SERPIND1",4.40622479475483,-0.248735823850227,1.2041053255422
2,-0.206573144868551,0.836343235165432,0.96673841193759
"13341","MOSPD1",34.3200271848381,-0.0661737519441325,0.32054651638076
1,-0.206440402757421,0.836446913622901,0.96673841193759
"13342","DHX29",299.518081817834,-0.0366697222654768,0.178152665442448
,-0.205833138529846,0.836921254610253,0.966957063395071
"13343","PER3",220.308860938503,-0.173485949363808,0.869263031959783,-
0.199578197835789,0.841810480336272,0.967177857579687
"13344","SLC25A34",12.8401895705641,0.115350614138794,0.56445166384490
3,0.20435871045725,0.838073193551718,0.967177857579687
"13345","GNL2",106.331555351754,-0.0395174747076427,0.193334232634972,
-0.204399780468543,0.838041101723912,0.967177857579687
"13346","ARMH1",5.6320178201945,-0.125140347252644,0.622985732348783,-
0.20087193133756,0.840798715905488,0.967177857579687
"13347","ATG4C",78.2274760425956,-0.0528882535331234,0.257959786447264
,-0.205025187303509,0.837552446388635,0.967177857579687
"13348","RTCA",146.213970057173,0.037749675712898,0.18888493911655,0.1
99855403450694,0.841593669604973,0.967177857579687
"13349","CTTNBP2NL",139.152630021902,-0.0449783138595027,0.22421901406
3771,-0.200599909188389,0.841011429085023,0.967177857579687
"13350","CADM3-
AS1",3.57268380057008,-0.194943098952048,0.974418184569126,-0.20006102
3120426,0.841432856122244,0.967177857579687
"13351","CD48",23.6521044884469,0.0807958779383404,0.395357881218704,0
.204361369221436,0.838071116003291,0.967177857579687
"13352","ATP2B4",574.073705015306,-0.041037548370831,0.204426479396367
,-0.200744778719505,0.840898144001615,0.967177857579687
"13353","STAMBP",137.07432152774,-0.0381059546467542,0.186800578038555
,-0.203992701986657,0.838359201974116,0.967177857579687
"13354","LMAN2L",58.677977673384,0.0457574538832992,0.226968709573793,
0.201602476258616,0.840227509204261,0.967177857579687
"13355","ABCB11",2.36114753040158,0.225598698995829,1.12651645131156,0
.200262232063431,0.84127549863705,0.967177857579687
"13356","TLK1",253.284614785328,0.0321445668372725,0.160597061639589,0
.200156631192987,0.841358084073658,0.967177857579687
"13357","PJVK",20.6603804412829,0.0865897626466996,0.429190280661497,0
.201751452789755,0.840111036070856,0.967177857579687
"13358","DNAH7",2.94564013687381,-0.160494253816594,0.784550681768774,
-0.204568369572707,0.837909370166508,0.967177857579687
"13359","GOLGB1",654.623233641013,-0.0496799941621966,0.24631366474187
8,-0.201694023814141,0.840155934894424,0.967177857579687
"13360","SLC35G2",51.7435409916765,0.0613777586768617,0.30289014439106
6,0.20264032955003,0.839416165482016,0.967177857579687
"13361","GYG1",160.573618767005,0.0430565124573339,0.213087490111999,0
.202060254380506,0.839869619337018,0.967177857579687

"13362","EIF4G1",334.199393602882,0.051082638120297,0.250927431622851,
0.203575343635906,0.838685362569357,0.967177857579687
"13363","TACC3",6.36778563809268,-0.120691979278892,0.594897586910255,
-0.20287858269141,0.839229934698862,0.967177857579687
"13364","ENOPH1",115.430828976627,-0.04261802534858,0.20945681926044,-
0.203469266357896,0.83876826512162,0.967177857579687
"13365","ZSWIM6",73.4358944098405,0.0502398246096667,0.247248731615261
,0.203195479634832,0.838982245867343,0.967177857579687
"13366","GFM2",39.0172098926027,0.0518680215898365,0.257915612848879,0
.201104621069325,0.840616768721982,0.967177857579687
"13367","PCDHGA3",8.52664271074115,0.114258940471716,0.568271401430405
,0.201064034164156,0.840648504245706,0.967177857579687
"13368","UNC5CL",3.54699182839562,-0.175656601163498,0.857633752917537
,-0.204815401173218,0.837716353695613,0.967177857579687
"13369","RPL7L1",488.104037523206,0.0329096437237676,0.164792174518433
,0.199703922955919,0.841712145504316,0.967177857579687
"13370","ELOVL5",162.50777915016,-0.05189489839077,0.255888341632016,-
0.202802902468289,0.839289089238576,0.967177857579687
"13371","HIVEP2",197.308752379681,0.0615576970048091,0.306942485356684
,0.200551243120599,0.841049485718073,0.967177857579687
"13372","LINC00997",11.0100860781631,0.111233372157055,0.5441719598904
14,0.204408496497055,0.838034291113098,0.967177857579687
"13373","ARPC1A",499.771523552872,-0.0520418410100624,0.25527627224724
9,-0.203864779722485,0.838459168755966,0.967177857579687
"13374","CNPY4",231.988076825075,-0.0560682308030348,0.280325074561674
,-0.200011472005153,0.841471609044486,0.967177857579687
"13375","ATP6V0E2",49.2751915015888,0.0685808991011504,0.3387695707918
96,0.202441142930394,0.839571866784359,0.967177857579687
"13376","POLR1E",122.194354658845,0.0428547078704229,0.209198950432929
,0.204851447780865,0.837688189738627,0.967177857579687
"13377","EXOSC3",110.279131930319,-0.0535334603862214,0.26846184096709
9,-0.199408080468249,0.841943540114292,0.967177857579687
"13378","AL161457.2",11.5436962194878,-0.111486261020985,0.54541426463
5646,-0.204406573589455,0.838035793651345,0.967177857579687
"13379","PTGR1",262.505124983508,-0.0643608153142869,0.313850416820563
,-0.205068439820119,0.837518653784411,0.967177857579687
"13380","AL359091.4",2.83503116711814,-0.185321454587567,0.92182928662
0805,-0.201036631486193,0.8406699309664,0.967177857579687
"13381","CCSER2",393.669713394973,0.0333276775149969,0.16386723739611,
0.203382189414929,0.838836319683456,0.967177857579687
"13382","GBF1",107.392292596924,0.0516715693485116,0.254952628327105,0
.202671255784101,0.839391991455195,0.967177857579687
"13383","FANK1",8.6129018998982,-0.110463357569538,0.552127570099967,-
0.200068541314714,0.841426976328542,0.967177857579687
"13384","ELP4",66.4135226084777,-0.0438118904511205,0.217131312403158,
-0.20177601270964,0.840091834920595,0.967177857579687
"13385","VPS37C",40.2972566536159,0.05429532610051,0.27117086590583,0.
200225514341814,0.841304213629152,0.967177857579687
"13386","EIF1AD",58.8251420544831,-0.0475720077933131,0.23704051798979
6,-0.200691460669863,0.840939837269815,0.967177857579687

"13387","PHLDB1",352.300263847655,0.044056967122334,0.218993739080328,
0.201179117299667,0.840558519650853,0.967177857579687
"13388","ENO2",49.3724105898714,-0.0577655976772687,0.287082651833698,
-0.20121591224095,0.840529749776855,0.967177857579687
"13389","EPS8",547.307360435269,-0.0347812586564023,0.174180832362936,
-0.19968476545071,0.841727129224454,0.967177857579687
"13390","AC027288.3",20.5648222238564,0.122898798217301,0.615122102257
003,0.199795776751252,0.841640304401308,0.967177857579687
"13391","CHST11",28.9402124121171,0.0660923363767474,0.329285906293618
,0.200714136601444,0.8409221052520З,0.967177857579687
"13392","MLXIP",202.911148563899,0.0530594187267751,0.2608049874252,0.
203444800847578,0.838787385900605,0.967177857579687
"13393","SGCG",55.611569915808,0.114468649377462,0.570867749191414,0.2
00516931530284,0.841076317441417,0.967177857579687
"13394","AL121603.2",47.4674313522761,-0.0590550264724819,0.2905466053
7096,-0.203254918077885,0.83893579014678,0.967177857579687
"13395","GPHN",38.153731781811,0.0548598453298414,0.272552082732445,0.
201282062422159,0.840478027648753,0.967177857579687
"13396","AC078909.1",5.05355978269915,-0.1454120712157,0.7123736458992
21,-0.20412331653868,0.838257133954899,0.967177857579687
"13397","LINC01579",7.42372440241962,0.219354853446584,1.0866059011445
3,0.201871583078591,0.840017118114495,0.967177857579687
"13398","RGS11",20.8302634775949,0.0935112844376576,0.459683314030677,
0.203425448745823,0.838802510412829,0.967177857579687
"13399","ZNF469",36.1182920038187,0.073523192539775,0.365349413786039,
0.201240756835682,0.840510323969869,0.967177857579687
"13400","LLGL1",36.5641132858308,0.0891630683802509,0.439528826533699,
0.202860570223406,0.839244013846331,0.967177857579687
"13401","GGNBP2",430.956982461647,-0.0324099289483197,0.16191648150566
6,-0.200164483855743,0.841351942818147,0.967177857579687
"13402","NARF",143.190509782749,0.0389128251714684,0.194867693554993,0
.199688437121503,0.841724257484524,0.967177857579687
"13403","TGIF1",179.549544148211,0.047886608688896,0.24009624660388,0.
19944755224725,0.841912666284856,0.967177857579687
"13404","AC011503.2",2.71283480343608,-0.179098982582615,0.87384814444
0456,-0.204954354737798,0.837607787558109,0.967177857579687
"13405","ZNF599",38.7058144849526,0.0609674745708215,0.303908427594871
,0.200611332345462,0.841002496284474,0.967177857579687
"13406","AC074212.1",14.212219514613,0.102459050351221,0.5059427609821
42,0.202511149981326,0.839517142568377,0.967177857579687
"13407","NAPA",234.699985372759,-0.0402443480892463,0.197397017989201,
-0.203875157280481,0.838451058959632,0.967177857579687
"13408","ZSCAN18",176.154329638019,0.0451835257820774,0.22412572605163
8,0.201599015775937,0.840230214727423,0.967177857579687
"13409","CDH22",5.13766171356568,-0.205760254696641,1.027943419062,-0.
200166906933845,0.841350047826996,0.967177857579687
"13410","ARFRP1",164.80508992692,-0.0526609937200152,0.260740877755679
,-0.20196677319373,0.839942700023294,0.967177857579687
"13411","TTC3",1251.14848481498,-0.0382847723966266,0.191981104772098,
-0.199419481631146,0.84193462238743,0.967177857579687

"13412","GGA1",104.733833088224,-0.0464595867627364,0.227599952212229,-0.204128279954183,0.838253255374966,0.967177857579687
"13413","MAGED1",181.570580181067,0.0664852247906356,0.326936402897897,0.203358280697176,0.838855005636604,0.967177857579687
"13414","AMER1",14.035188969231,-0.0977064529770036,0.484609093359416,-0.201619107680545,0.84021450621876,0.967177857579687
"13415","PABPC5",59.2057938966389,-0.0507466968474957,0.248767391379349,-0.203992559338741,0.838359313447028,0.967177857579687
"13416","CAPN6",10.5335156204178,0.14616107212074,0.717465429012919,0.203718675913293,0.838573346969374,0.967177857579687
"13417","FMR1",162.735450803409,0.0457606423633515,0.228717823493025,0.200074666960737,0.84142185618916,0.967177857579687
"13418","MTM1",25.5488503497913,-0.0642403861953021,0.319743834215713,-0.200912040580469,0.840767352660782,0.967177857579687
"13419","GDI1",203.313138448406,-0.0414907897941243,0.203126720310156,-0.20426061982772,0.838149841993014,0.967177857579687
"13420","ZAP70",5.45248876383488,-0.162659530073865,0.81687612401381,-0.199123863817466,0.842165854377858,0.967293170692008
"13421","ZFP3",9.20598927650816,0.110962745262109,0.557267647068112,0.199119302629365,0.842169422241806,0.967293170692008
"13422","PECR",23.236172395778,0.0710696086667425,0.357070262770208,0.199035361038954,0.842235083820797,0.967296514461152
"13423","POLR3C",86.5391491257235,0.0415260963332716,0.209543841330108,0.198173785827725,0.842909096608883,0.967306046521953
"13424","ULK4",21.6514521862058,-0.0794981628638167,0.400814109837616,-0.198341727281069,0.842777706476939,0.967306046521953
"13425","HHIP-AS1",21.1287726527232,-0.216842637734002,1.09320915620132,-0.198354209259907,0.842767941291052,0.967306046521953
"13426","SCAMP1-AS1",39.3882245443033,-0.067172084648065,0.338266054069662,-0.198577669381604,0.842593122972919,0.967306046521953
"13427","RGMB",43.0412596051737,-0.0561314068224946,0.283148763209203,-0.198239985886932,0.842857304022465,0.967306046521953
"13428","TBC1D9B",246.998091165959,-0.0359943869917195,0.181524498280778,-0.198289417310738,0.842818631066917,0.967306046521953
"13429","MAP3K4",141.226054000251,0.0412883840399359,0.208145898055473,0.198362708204473,0.842761292217061,0.967306046521953
"13430","TRAM1",919.739597332202,-0.0256665625189383,0.129535935772282,-0.198142410180747,0.842933644043425,0.967306046521953
"13431","TRDMT1",44.6782013568771,0.0610429516674077,0.307552690571206,0.198479654182296,0.842669801705841,0.967306046521953
"13432","GDPD1",9.98246703586416,0.0972663065864797,0.489460402413466,0.198721502509441,0.842480602902363,0.967306046521953
"13433","TPGS2",245.591996795807,0.02838906777803,0.142787229194589,0.198820776466932,0.842402943165206,0.967306046521953
"13434","USP21",26.2352103270705,0.066544029805319,0.336229423728086,0.197912571325507,0.843113468230603,0.967383952193978
"13435","PRKAB1",35.9893079214215,0.0530519453067879,0.268080985437507,0.197895218939931,0.843127044938443,0.967383952193978

"13436","PIK3CD-
AS2",6.14007446221381,-0.153080913126491,0.774853862742928,-0.19756101
1807563,0.843388541568546,0.967405279193412
"13437","EIF2AK1",208.983400626246,-0.0339706763475009,0.1718657493630
67,-0.197658209814323,0.843312488294234,0.967405279193412
"13438","NAA16",68.1734441405812,0.0489167268865086,0.24761614778709,0
.197550633606372,0.843396662152466,0.967405279193412
"13439","COL18A1",456.934674634367,-0.0852508418307855,0.4314089642207
63,-0.197610269839364,0.843349999087333,0.967405279193412
"13440","AL050341.2",37.9023324304711,0.0578241931656288,0.29359821747
4174,0.19695008254168,0.84386660094421,0.967584324126376
"13441","ZBTB47",129.575611671498,-0.0505850126298525,0.25653906754895
3,-0.197182491981264,0.84368473102055,0.967584324126376
"13442","AGO2",265.472607187817,0.0457083417204134,0.231901198944246,0
.197102653753001,0.843747206771131,0.967584324126376
"13443","PARPBP",12.9750779680452,0.119973074623411,0.608850459600515,
0.197048507940898,0.84378957801352,0.967584324126376
"13444","HCFC1",100.066697546267,0.0421034077495039,0.213718551355155,
0.197003991850651,0.843824413955806,0.967584324126376
"13445","PRDX3",472.002861690792,-0.0454946387787051,0.231106599387463
,-0.196855645400376,0.843940504261797,0.967597089862075
"13446","GTF3C2",66.0402459335248,0.0420186609292949,0.21503814996961,
0.195400959947028,0.845079065166897,0.967624698366941
"13447","PARTICL",12.5008245143921,-0.0957374002461832,0.4880720338575
93,-0.196154242826617,0.844489441183546,0.967624698366941
"13448","REV1",183.424176686899,-0.0385524336406788,0.197481076118515,
-0.195220901153801,0.845220017094444,0.967624698366941
"13449","AC024060.2",6.14035052506814,-0.123314931757019,0.62809490786
9148,-0.196331685247016,0.844350562723871,0.967624698366941
"13450","TRMT10C",154.364171691479,-0.0424799492513696,0.2165001209874
9,-0.196212127076937,0.844444136516224,0.967624698366941
"13451","GPR171",2.73671502687691,-0.183718373053712,0.936163089344247
,-0.196246119020139,0.844417532046276,0.967624698366941
"13452","TMEM267",54.0038868606539,-0.0533026714099529,0.2722729113194
14,-0.195769278521511,0.844790756943587,0.967624698366941
"13453","AL035587.2",8.34486609894191,0.102516337970066,0.524434603508
65,0.195479736242033,0.845017399809236,0.967624698366941
"13454","C8orf58",35.1836746561395,-0.0628171823293639,0.3213349653823
94,-0.195488163744056,0.845010802894549,0.967624698366941
"13455","TERF1",282.42530657827,0.0322246172385571,0.164537086244842,0
.195850175629126,0.844727436009084,0.967624698366941
"13456","TIMM10B",93.5053587620961,-0.0461864820911123,0.2365661205315
78,-0.195237094759506,0.845207340365814,0.967624698366941
"13457","SYT7",3.43558719390258,0.156745335755653,0.798688719812776,0.
196253348604192,0.844411873691512,0.967624698366941
"13458","EHBP1L1",92.1379295102405,0.0477422197318966,0.24322297797115
7,0.196289923469147,0.844383247885349,0.967624698366941
"13459","MBD6",83.4894675391531,-0.0424470704377835,0.217283942401202,
-0.19535300201525,0.845116606658733,0.967624698366941
"13460","TMEM186",49.4231092865813,-0.0537746458778721,0.2741095085391

38,-0.196179425385362,0.84446973131256,0.967624698366941
"13461","ZC3H18",128.952719327691,0.0456250009116813,0.233087820304038
,0.195741677330752,0.844812361569599,0.967624698366941
"13462","TMX3",228.067221721028,-0.0322774659251464,0.165102026767972,
-0.195500119271752,0.845001444316063,0.967624698366941
"13463","BORCS8",82.5997912451851,-0.0552614650602477,0.28188839134189
9,-0.196040229954776,0.844578677932335,0.967624698366941
"13464","LINC00663",6.06803015170592,-0.12185840628195,0.6232350867917
26,-0.195525587157247,0.844981508572578,0.967624698366941
"13465","C5AR2",19.2061073957442,0.212103079004005,1.08562878223215,0.
195373485371217,0.845100572234892,0.967624698366941
"13466","DNAJC9",103.185857127285,0.0490688459740083,0.251454179375685
,0.195140307851861,0.845283107988947,0.967625063838528
"13467","GPSM2",141.734682419939,0.052680717589887,0.270196530703902,0
.194971850499508,0.845414984745661,0.967675702870667
"13468","HIST2H2AA4",17.9411120443274,0.137295003875714,0.705427273619
18,0.194626730508058,0.845685175286971,0.967675702870667
"13469","POLM",43.7316319090021,0.0501680671577294,0.257797393250125,0
.194602693709375,0.845703994101436,0.967675702870667
"13470","COMMD4",289.407469850936,0.0554727471722934,0.28465151299967З
,0.194879509290917,0.845487275854248,0.967675702870667
"13471","AC135050.3",5.00001677589689,0.158565171478762,0.814728420969
183,0.194623346133103,0.84568782496579,0.967675702870667
"13472","ZNF208",71.210205427938,0.102017493083939,0.524142305307666,0
.194637013747737,0.845677124392346,0.967675702870667
"13473","USF3",107.168091016326,0.0426170702665865,0.219465858539809,0
.194185421596481,0.846030697484376,0.967690376324231
"13474","ASAH2B",4.03062565405032,0.157272392118647,0.808675731438057,
0.194481404603266,0.845798954727646,0.967690376324231
"13475","AL158212.2",23.7094161135771,-0.100537070143002,0.51726765778
1713,-0.194361794383496,0.845892603100601,0.967690376324231
"13476","ABHD13",102.664097693537,0.036934687912947,0.190194820024162,
0.194193973885592,0.846024001199963,0.967690376324231
"13477","AC104532.2",16.5122306450296,-0.0967396730119715,0.4978879295
60402,-0.19430009700654,0.845940909681705,0.967690376324231
"13478","GPBAR1",2.82373293274821,-0.186207571145664,0.959890024661121
,-0.1939884428025,0.846184931318676,0.96774350751926
"13479","FBXO45",59.3116190365812,-0.0459369812673433,0.23749426721230
5,-0.193423537361761,0.846627283336216,0.96774350751926
"13480","AC246817.2",3.74720787702666,-0.165527948787325,0.85501210793
9354,-0.19359719850782,0.846491291959266,0.96774350751926
"13481","M6PR",223.4289720739,-0.0339517611909532,0.175132754431689,-0
.193863000105992,0.846283155664631,0.96774350751926
"13482","AL442125.1",7.53992759603698,0.108983244997134,0.563073306668
903,0.193550721915535,0.846527686617147,0.96774350751926
"13483","TOX4",303.899586919649,0.0318956166344009,0.164573769063224,0
.193807414243199,0.846326681361925,0.96774350751926
"13484","TMEM121",42.4425425837666,0.0703805979249996,0.36390355283003
,0.193404536387894,0.846642162984414,0.96774350751926
"13485","LINC00926",10.322499875541,-0.0950333490010723,0.491239474162

604,-0.193456255043575,0.846601662272602,0.96774350751926
"13486","DDX52",149.210663904061,0.0394440248903522,0.20371288657118,0
.193625575457005,0.846469070840556,0.96774350751926
"13487","ZFYVE19",82.1011853252258,-0.047806878552311,0.24730654056997
4,-0.193310207009201,0.846716033058652,0.96775618370276
"13488","AC017002.3",4.75946892795154,0.163717617114794,0.849163458157
181,0.192798707412689,0.847116615832606,0.967783490184512
"13489","RBPMS-
AS1",15.2449316308655,0.0981232854420415,0.508196748517126,0.193081293
275403,0.84689530279357,0.967783490184512
"13490","SLC2A3",1336.78030761922,-0.0425506454803379,0.22069577644772
4,-0.192802264570826,0.84711382989471,0.967783490184512
"13491","RIOX1",17.1314048429783,-0.0728204162063192,0.377334518777887
,-0.192986362451467,0.846969648511282,0.967783490184512
"13492","AL118558.3",13.9510528477939,-0.0781173527689841,0.4051217461
20893,-0.192824388019085,0.847096503029295,0.967783490184512
"13493","AL022238.2",3.89383601373566,0.152771035545177,0.790949856468
306,0.193148825169928,0.846842415562458,0.967783490184512
"13494","TMCO1",690.08203860532,0.033894599377217,0.176181290451738,0.
192384783255416,0.84744081097063,0.96795106497547
"13495","TACC1",1560.20121228468,0.0396720187894915,0.20622670151755,0
.192370912677937,0.8474516751806,0.96795106497547
"13496","AAAS",73.5323589276165,0.0410794454939272,0.213524990774116,0
.192387061322412,0.847439026664262,0.96795106497547
"13497","FBXO8",135.498222745762,-0.0434839025063295,0.226231920058437
,-0.192209403938655,0.847578179967457,0.968023830791905
"13498","DPH7",69.185437911249,-0.05519940863279,0.28764797375488,-0.1
91899174231028,0.84782118429061,0.968086189321464
"13499","ANG",399.182526685808,-0.0431751318061062,0.224922023425827,-
0.191955999454825,0.84777667175467,0.968086189321464
"13500","PAXBP1",126.856853176741,-0.0544620951158683,0.28368303358087
1,-0.19198220784798,0.847756142266748,0.968086189321464
"13501","SUDS3",178.043202526205,0.0394801439356275,0.206103495672596,
0.19155494576542,0.848090836924885,0.968274703677947
"13502","AC139099.2",7.07124790674399,0.126839037612389,0.662247843700
594,0.191528049232477,0.848111907172212,0.968274703677947
"13503","MISP3",13.0618861879538,0.0922744332850924,0.48226140330197,0
.191336965084296,0.848261602089906,0.968373887004066
"13504","PINLYP",31.8530609099541,0.0684024931299783,0.357689119020535
,0.191234481264864,0.848341889952591,0.968393826541705
"13505","INCENP",13.324827568147,-0.0911085412405899,0.477184633695368
,-0.190929327575022,0.848580962738072,0.968407400978474
"13506","FOXN3",853.599261713897,0.0414623112416577,0.217077262908704,
0.191002552206932,0.848523593598657,0.968407400978474
"13507","SNRPA1",171.156566943862,-0.052851219331766,0.276910685535978
,-0.190860165722638,0.848635149557209,0.968407400978474
"13508","CCR10",16.7712144542631,0.0820582075802725,0.430033053363923,
0.190818373002666,0.848667893598327,0.968407400978474
"13509","AC006115.2",34.2091636777405,-0.0578808359077521,0.3032058860
66563,-0.190896148681776,0.848606957595798,0.968407400978474

"13510","SEC24D",231.140018894699,0.0523744089294354,0.274818954966891
,0.190577862199298,0.84885633569269,0.968457029869028
"13511","L3MBTL3",31.0104561436767,0.0771983616856057,0.40513903502121
5,0.19054782435754,0.848879871183461,0.968457029869028
"13512","PPP1R3D",36.8701419551546,-0.0578471512479027,0.3036240292614
39,-0.190522309412121,0.848899862964016,0.968457029869028
"13513","SCP2",1028.5392974577,0.0427664484875442,0.22543142177815 7,0.
189709349966439,0.849536893653431,0.968487954851818
"13514","AC107204.1",5.79777978736841,0.119959081023587,0.632676358360
113,0.18960575883461,0.849618074178472,0.968487954851818
"13515","TMEM129",113.884588838733,0.0413668871181869,0.21764271453708
3,0.190067869747777,0.849255947328473,0.968487954851818
"13516","THEMIS",2.58611052907698,0.181449865152341,0.95468649706748,0
.190062251545091,0.849260349764762,0.968487954851818
"13517","MTFR2",2.83058398297087,-0.195736109853579,1.03108101752708,-
0.189835819422831,0.84943778639746,0.968487954851818
"13518","AGFG2",12.7867086314308,-0.0807345736238424,0.425309441104233
,-0.189825491327516,0.849445879876582,0.968487954851818
"13519","ENTPD2",2.93588260540564,-0.16147789565131,0.849150317754867,
-0.190164087882878,0.849180551310714,0.968487954851818
"13520","LINC00857",6.93865107959636,-0.103731355066713,0.545248528891
421,-0.190246006307648,0.849116361556945,0.968487954851818
"13521","MSANTD4",70.4253959635349,0.0435404929192872,0.22960570503654
1,0.189631581290012,0.849597837929392,0.968487954851818
"13522","SBNO1-
AS1",11.5406399184969,0.105609865637468,0.555387959285356,0.1901551228
68277,0.849187576190096,0.968487954851818
"13523","PTOV1-
AS1",4.12228564021862,-0.13405421940802,0.706395482895295,-0.189772192
283242,0.849487647239662,0.968487954851818
"13524","EVC",36.4326609940067,-0.0740471945952763,0.390795260581759,-
0.189478230838945,0.849718015324912,0.968530257781242
"13525","GOLPH3L",103.632204162859,0.0359429867007133,0.18997638083881
2,0.189197133570039,0.849938314173528,0.968709731089459
"13526","ACP2",99.147541936004,0.0546733788709553,0.28913904896156,0.1
89090263204898,0.850022072663368,0.96873356869036
"13527","RBFOX2",541.380729521934,-0.0249255412923414,0.13211171922635
6,-0.18867017580503,0.850351328002376,0.969037164275643
"13528","C12orf75",266.817954735765,0.0791148830372849,0.4199355511794
77,0.188397678679679,0.850564919262321,0.969065648959991
"13529","FBF1",15.0459809671691,0.0709173679766883,0.37633138141334,0.
188443939249374,0.850528658094255,0.969065648959991
"13530","PPP5C",76.8503314894646,-0.03742773697703 8,0.198636467995411,
-0.188423290822422,0.850544843244552,0.969065648959991
"13531","AC012063.1",29.852634195197,-0.0677642960696521,0.36012012010
9873,-0.188171369178087,0.850742315640366,0.969084215276663
"13532","C3orf35",5.79989294615927,0.149769270211498,0.796342319671847
,0.188071469406792,0.85082062610242,0.969084215276663
"13533","SLC18B1",57.0305952953195,0.0553439590951915,0.29429495488863
6,0.188056091943996,0.850832680477091,0.969084215276663

"13534","SLC24A3",3.24319064328895,-0.168672707296509,0.896704968302271,-0.188102791061653,0.850796073202508,0.969084215276663
"13535","HYPK",19.1443454043957,-0.0862742419025828,0.459591423918794,-0.187719433854855,0.851096595080826,0.969241578987215
"13536","CERS1",4.36718644571687,0.132196594794725,0.704078660410147,0.187758275073579,0.851066145636979,0.969241578987215
"13537","EMC3",367.324613632939,-0.0419456773384193,0.225081393296338,-0.186357818050266,0.852164169241585,0.969284387996282
"13538","MAGI1",21.8129251777738,0.0691208986140839,0.369979530654038,0.18682357505534,0.851798962121695,0.969284387996282
"13539","CENPC",256.594142359921,0.0468228370704699,0.251246649709925,0.186362035571534,0.852160862076957,0.969284387996282
"13540","UBA6-AS1",28.551892886774,-0.0622102853995742,0.333872344290809,-0.186329555182887,0.852186331604787,0.969284387996282
"13541","LIFR-AS1",20.8611964356441,0.0808996220622939,0.436923511558226,0.185157401518121,0.853105579974821,0.969284387996282
"13542","CLDN12",45.8652963079249,0.0512093303603358,0.276013211630124,0.185532170934483,0.852811649504471,0.969284387996282
"13543","PRKDC",423.020820687636,-0.0338647690108362,0.18299684728943,-0.185056570713894,0.853184664740082,0.969284387996282
"13544","TESK1",151.384040968915,0.0451957688918431,0.243932955582977,0.185279470680086,0.853009839274603,0.969284387996282
"13545","SECISBP2",204.189070014233,-0.0343859695530606,0.18338544392815,-0.187506537141154,0.851263498691681,0.969284387996282
"13546","BX255925.3",15.6125592236682,0.0925869224431824,0.497011699418153,0.186287209237877,0.852219537449556,0.969284387996282
"13547","TUBGCP2",200.719156278723,-0.031790451541815,0.170368379035046,-0.186598309626902,0.851975592191821,0.969284387996282
"13548","AKIP1",109.199736869123,0.042544064102713,0.228741632630848,0.185991783014736,0.852451205105148,0.969284387996282
"13549","CSNK2A3",2.54435754629306,-0.198760423669071,1.06634177685835,-0.186394670060341,0.852135271866607,0.969284387996282
"13550","SDHAF2",173.951663341126,-0.0502636352567081,0.270084981160433,-0.186103037054293,0.852363960296595,0.969284387996282
"13551","ATG16L2",24.7333280445134,-0.0673735570363368,0.359811947427468,-0.187246581215645,0.85146730412012,0.969284387996282
"13552","PPME1",78.0334769950307,-0.0374446589565251,0.201169730204999,-0.186134658123604,0.852339163556832,0.969284387996282
"13553","AP001528.1",143.990578071422,0.0571095307292282,0.306065302527882,0.186592633198027,0.851980043161725,0.969284387996282
"13554","CWC15",523.826331167302,-0.0416888560718223,0.22531437197379,-0.185025285811203,0.853209202771427,0.969284387996282
"13555","RHEBL1",5.27244953505459,0.132579300154837,0.71643723760663,0.185053614183622,0.853186983661865,0.969284387996282
"13556","CTSG",24.5805834042555,-0.141854522662602,0.763873828765143,-0.185704127201112,0.85267679158468,0.969284387996282
"13557","ATXN2L",157.163482127881,0.0385156993216216,0.207569365224781,0.185555798563589,0.852793119113965,0.969284387996282

"13558","AC012181.1",4.14010056416149,-0.152179519521706,0.813575080097221,-0.187050369713291,0.851621140495641,0.969284387996282
"13559","LINC01572",3.81643818456075,-0.190419395852885,1.02771209242962,-0.185284767256862,0.85300568513789,0.969284387996282
"13560","RNF167",201.865561950919,-0.0358169380022551,0.192221783003587,-0.186331317099409,0.852184949992307,0.969284387996282
"13561","MTMR4",45.706199804293,0.0634366173511335,0.341834597107877,0.185576936588177,0.852776541309161,0.969284387996282
"13562","ANKRD12",1160.3235570094,-0.0384934754768067,0.207876730871187,-0.18517452778618,0.853092147451782,0.969284387996282
"13563","AC005523.2",17.3832128394092,-0.0785583951928178,0.4228292710896,-0.185792234748504,0.852607694285011,0.969284387996282
"13564","BISPR",8.28499671862894,0.112475199543458,0.607181361109833,0.18524152213413,0.853039602664458,0.969284387996282
"13565","ZNF585B",27.8003795019058,-0.0539510701144134,0.288310271402956,-0.187128505175623,0.851559879004931,0.969284387996282
"13566","ZNF132",4.9394229539312,-0.12690154461541,0.684616286427326,-0.185361562573462,0.852945454571794,0.969284387996282
"13567","CEBPB-AS1",8.98876253515071,0.0961504338290098,0.513109155516862,0.187387874091149,0.85135652928352,0.969284387996282
"13568","NAP1L3",29.431355952893,0.0609120958267559,0.328694706176865,0.185315110593781,0.852981886756593,0.969284387996282
"13569","AC234775.3",4.08287483899941,0.143750512948366,0.771260991571756,0.186383746253543,0.852143837710854,0.969284387996282
"13570","MZF1-AS1",11.6509501259021,-0.0864408602065477,0.467398609825112,-0.184940345113332,0.853275825958218,0.969288640909796
"13571","GPLD1",4.85708691409944,0.127815914250317,0.6915896791401,0.184814664107248,0.853374405701775,0.969329191945535
"13572","MFAP2",517.104673033668,0.0554230853288454,0.300759171243693,0.184277291028769,0.853795927977976,0.969393877318812
"13573","ELP6",128.315724949658,0.0431009534097346,0.23401426507212,0.184180880582008,0.853871557978257,0.969393877318812
"13574","CYB5R4",60.9083677708881,0.0457323071227762,0.247674468402379,0.184646836703726,0.853506047161459,0.969393877318812
"13575","PRTFDC1",130.640420298322,-0.0450004941398956,0.244232731855626,-0.184252511110988,0.85381536666834,0.969393877318812
"13576","PHYHIPL",4.2786182759471,0.172850572973109,0.938351653863265,0.184206605552908,0.853851377673225,0.969393877318812
"13577","UCKL1",110.210078951394,-0.0363401500793945,0.197049505075083,-0.184421422756417,0.853682865116466,0.969393877318812
"13578","PDK3",38.0619125243387,0.0639531495728959,0.346709158899687,0.184457629489388,0.853654463536588,0.969393877318812
"13579","EAF1-AS1",4.9302392157103,-0.135595459255002,0.738119250582276,-0.183704000604287,0.854245670296194,0.969463346884861
"13580","AC009275.1",9.58838982638913,0.100860507795322,0.54840819612576,0.18391502626663,0.854080116627289,0.969463346884861
"13581","AC009318.1",11.7952283452134,0.115076349953705,0.625659978135

259,0.183927938457375,0.8540699869752,0.969463346884861
"13582","AC026124.1",11.7955752375651,0.106483350771653,0.579652501683077,0.183702046419999,0.854247203421153,0.969463346884861
"13583","SNAP23",205.914805216391,-0.0319911513346499,0.174096265699255,-0.183755528621811,0.854205244988239,0.969463346884861
"13584","ATXN7L2",5.87871744953408,-0.106836746412728,0.583000700181573,-0.183253204291957,0.854599350116783,0.969472367455568
"13585","ETV7",14.9400732732926,-0.0852497584041059,0.465620677493517,-0.183088429111469,0.854728634548544,0.969472367455568
"13586","PGM3",204.844044968798,-0.0347186269853854,0.189360203405027,-0.183347009356158,0.854525751367929,0.969472367455568
"13587","AC021054.1",20.6033516622917,0.0780827639982463,0.425667633120089,0.18343599071865,0.854455938429039,0.969472367455568
"13588","B3GLCT",39.7401282554409,0.0495765463351238,0.270767194884174,0.183096576216817,0.854722242147761,0.969472367455568
"13589","AL136418.1",2.73927174665124,0.191449665985517,1.04588361283027,0.183050641234768,0.854758283885088,0.969472367455568
"13590","FAM219B",114.574716797399,-0.0345949112390608,0.188602397673505,-0.18342773827801,0.85446241307556,0.969472367455568
"13591","CFDP1",685.451748188508,-0.0324972720333001,0.177443773695821,-0.183141235989536,0.854687201262233,0.969472367455568
"13592","GTPBP1",67.4725097792013,0.0403872889986425,0.220906704253497,0.182825094127958,0.854935258189654,0.969601751397405
"13593","ANKRD39",86.1169967349763,-0.0504643353327946,0.277344972105746,-0.181955111533637,0.855617953562711,0.969972767123997
"13594","AC079305.3",6.60633504538685,-0.11686499402741,0.642971204121297,-0.181757741681637,0.855772849288819,0.969972767123997
"13595","ABHD14B",287.922222435507,0.0427060656198399,0.235015383821793,0.181716043117513,0.855805575004889,0.969972767123997
"13596","PLK4",2.63327472663231,0.167081371536419,0.917768393481219,0.18205178204346,0.855542088641232,0.969972767123997
"13597","AC013643.2",4.43835573111245,-0.139177824013153,0.765196627872536,-0.181885046200618,0.855672940152046,0.969972767123997
"13598","DEPDC7",5.31835920678516,0.117781425652248,0.648267128593641,0.181686561692191,0.855828712659973,0.969972767123997
"13599","FAM174B",19.3470340899549,0.0716502228339136,0.393662958848308,0.182009054251718,0.855575620321771,0.969972767123997
"13600","AC135178.6",3.43986850982052,-0.136290573997873,0.749237288706106,-0.181905754094594,0.855656688726056,0.969972767123997
"13601","ZNF236-DT",7.09847118343698,0.111971334321277,0.615635693286614,0.181879211264555,0.855677519384516,0.969972767123997
"13602","TRIM62",9.21133674311259,0.0866285075179193,0.481562102036541,0.179890625012983,0.857238435295019,0.970293283809472
"13603","IRF2BP2",1093.36012200681,0.0381546108325741,0.211655280166165,0.180267701342579,0.856942410854024,0.970293283809472
"13604","GCFC2",81.7585733997951,-0.0358409373970327,0.198451574176715,-0.180602938251916,0.856679249419213,0.970293283809472
"13605","MRPL35",112.566656525018,-0.0436631101885277,0.242995425949885,-0.17968696331565,0.857398328625961,0.970293283809472

"13606","GORASP1",45.964735160988,-0.0550681302130705,0.306409473753786,-0.179720716655518,0.857371828717366,0.970293283809472
"13607","PROS1",556.388624771371,0.0360343038313607,0.19958790423916,0.180543525263846,0.856725887535741,0.970293283809472
"13608","DUBR",49.97078277218,-0.0556267425188528,0.308437139541609,-0.180350338488821,0.856877539102489,0.970293283809472
"13609","AC112236.2",3.09078888653589,-0.162629923219324,0.904613737876022,-0.179778303611848,0.857326617293582,0.970293283809472
"13610","THBS4",82.5320382876773,-0.273717810228608,1.51908960788547,-0.180185427382138,0.857006998457738,0.970293283809472
"13611","SPDL1",37.0625500294606,0.0576626196879812,0.318393753291655,0.181104745592044,0.856285359750057,0.970293283809472
"13612","TRIM26",72.7114856893765,-0.0426794567884462,0.237765308669884,-0.179502455708132,0.857543189271047,0.970293283809472
"13613","AL662797.1",3.70090016753615,-0.140448251234361,0.781091210531263,-0.179810308118606,0.857301490810817,0.970293283809472
"13614","AL355312.3",8.08861480105443,0.11747511457163,0.653622771283888,0.179729225683001,0.857365148264554,0.970293283809472
"13615","UBE2W",117.086325141197,-0.0404401544344873,0.224304967985923,-0.180290944055351,0.856924164779105,0.970293283809472
"13616","FBXO32",68.2913296637626,0.064768423226885,0.357352375940644,0.181245257027878,0.856175072829863,0.970293283809472
"13617","CSTF3",78.5889409243381,0.0507179519866817,0.280202832840101,0.181004422662722,0.856364104573538,0.970293283809472
"13618","AP5B1",51.1437229358622,0.0492394080882455,0.272514085860104,0.18068573568532,0.856614255772501,0.970293283809472
"13619","ORMDL2",94.4157281007642,-0.053282929829569,0.296528729316002,-0.179688929139729,0.857396785243664,0.970293283809472
"13620","PEBP1",2434.70356826805,-0.0391874500757801,0.218180453152787,-0.179610269891309,0.857458541597724,0.970293283809472
"13621","DTD2",44.4529769737771,-0.0471599138877938,0.262859650190364,-0.179411004517583,0.857614991224414,0.970293283809472
"13622","MAP1LC3B",857.185187034032,0.0375924687075833,0.209543382750004,0.179401841347732,0.857622185657091,0.970293283809472
"13623","B9D1",41.3696347874078,0.049446811055342,0.27504531626629,0.179776960853495,0.857327671485606,0.970293283809472
"13624","DCAF15",48.5042794815112,-0.0478160139672493,0.264427700158185,-0.180828309358834,0.856502341744527,0.970293283809472
"13625","AC007773.1",3.16811965209519,-0.164007595609576,0.913775943619394,-0.179483380750816,0.857558165678598,0.970293283809472
"13626","SELENON",253.0174654496,-0.0379014833832764,0.211811013698775,-0.178940097218823,0.857984737842893,0.970385853555271
"13627","MUC1",11.8533776001855,0.0997379046743667,0.557913720912679,0.178769406336176,0.858118768472352,0.970385853555271
"13628","ORMDL1",331.736438444102,0.031256745893481,0.174996936581092,0.178613103201363,0.858241505079958,0.970385853555271
"13629","TMF1",417.602220466635,-0.0291231530118745,0.163047026880247,-0.17861811754019,0.858237567530941,0.970385853555271
"13630","AL353135.2",7.3254157148768,-0.107947537511232,0.604758490118879,-0.17849693600831,0.858332727269341,0.970385853555271

"13631","FAM229B",220.078307495105,0.0582264400225464,0.325968474663639,0.178625985481048,0.858231389175585,0.970385853555271
"13632","NOL6",33.2610037489961,-0.0570725000117165,0.319746273196201,-0.178493089039684,0.858335748196773,0.970385853555271
"13633","NUDT13",6.6531536366492,0.115560128962368,0.647181058644276,0.178559195172437,0.858283837006561,0.970385853555271
"13634","TRIM6",13.3981269557025,-0.0801258834065153,0.449096416134057,-0.178415771152797,0.858396464422943,0.970385853555271
"13635","CACNA1C",34.4895034591196,0.0888058198070661,0.495760157885899,0.179130610627861,0.857835146918533,0.970385853555271
"13636","TRPV4",5.3560885920884,-0.120316872118783,0.672534683325752,-0.178900620446523,0.858015735600165,0.970385853555271
"13637","VRK2",64.8637204730225,-0.040557652879539,0.227676536033359,-0.178137165937892,0.858615254599805,0.970387685468351
"13638","ASNS",6.20739365173439,0.104291449479858,0.585592024479752,0.178095747756319,0.858647781448567,0.970387685468351
"13639","ANAPC2",41.3634653663769,-0.051992036461783,0.2915505872713,-0.178329383412979,0.858464304018136,0.970387685468351
"13640","LYRM1",211.963714088273,0.0428697872205446,0.240715646357457,0.178093064864109,0.858649888406637,0.970387685468351
"13641","INVS",42.4809971342658,0.0485556837234159,0.27279157572698,0.177995539613042,0.858726478680067,0.970403098662358
"13642","UGDH-AS1",6.57371298423482,-0.113453452582808,0.637872508854436,-0.177862270293732,0.858831142273742,0.970450231501959
"13643","DMAP1",143.866472413089,0.0480570432962945,0.270371268159736,0.177744638412919,0.858923527003874,0.970483483747322
"13644","GNL3L",119.891879719635,-0.052291436790223,0.294441131624511,-0.177595557053177,0.859040614034536,0.97054463979349
"13645","MORN1",14.947905235967,0.0858223994987352,0.485915933261758,0.176619850521558,0.859807000752393,0.970569785189001
"13646","SYTL1",7.25923902501122,-0.115456487452247,0.657974630506662,-0.17547255182672,0.860708336618884,0.970569785189001
"13647","TMEM59",2707.16848660421,-0.0416677299067799,0.238097924907485,-0.175002490773366,0.861077676434952,0.970569785189001
"13648","MCOLN2",2.79517431238581,0.157030116810393,0.890841677747186,0.176271632471776,0.860080547069202,0.970569785189001
"13649","RSBN1",184.555794402704,0.0326948439670566,0.185229726720317,0.176509702551272,0.859893526820417,0.970569785189001
"13650","PSMB4",596.441196287929,-0.0375346544298303,0.213755025910688,-0.175596593670332,0.860610878629134,0.970569785189001
"13651","CGREF1",68.0647876778951,0.0768875568647491,0.433920212043453,0.177192844976416,0.859356915621134,0.970569785189001
"13652","UMPS",82.0641226459421,-0.0463007928416297,0.262873716319778,-0.176133215179665,0.860189286880665,0.970569785189001
"13653","H1FX-AS1",23.3456555565699,-0.0615584691375172,0.351081255027311,-0.175339663556599,0.860812747479217,0.970569785189001
"13654","FOXD1",11.5658269722933,-0.122267186464484,0.698113512626184,-0.175139406777181,0.860970094649308,0.970569785189001

"13655","CKMT2-
AS1",32.9530580882822,0.0569882114042965,0.323400270952888,0.176215719
41292,0.860124471720457,0.970569785189001
"13656","FYN",1305.61268478058,-0.0305135704839992,0.174037198900185,-
0.175327864829056,0.860822017905588,0.970569785189001
"13657","HSF2",65.5941224287969,-0.0394937346257785,0.22310701332965,-
0.17701700200436,0.859495034804675,0.970569785189001
"13658","INHBA",14.4325521239792,-0.137521581953156,0.776415577639724,
-0.177123676950451,0.859411244440296,0.970569785189001
"13659","TP53I11",186.430417727201,0.045831101716087,0.26165974404217,
0.175155341085638,0.860957574429835,0.970569785189001
"13660","INTS5",48.4048908009501,-0.0444489793659588,0.251782119730829
,-0.176537473802658,0.859871711124181,0.970569785189001
"13661","SPINDOC",23.2189131596187,-0.0569356068910951,0.3250124924837
66,-0.175179749110533,0.860938396142325,0.970569785189001
"13662","DPP3",31.8132063176031,-0.0626226434385081,0.356510333833103,
-0.175654497206873,0.86056538533452,0.970569785189001
"13663","CCS",314.879619656333,0.0337248770556664,0.190978872972826,0.
176589569991153,0.85983078727113,0.970569785189001
"13664","BORCS5",76.2306229512593,0.0420309609630204,0.239732128263083
,0.175324689550561,0.860824512769888,0.970569785189001
"13665","PPP1R12A-
AS1",3.25478712304111,-0.145218597089365,0.8250853465104,-0.1760043342
22575,0.860290537386819,0.970569785189001
"13666","PARN",87.8053252923394,0.0422893834840138,0.241634162691001,0
.175014091604642,0.861068560978776,0.970569785189001
"13667","NOMO2",3.25243716509333,0.151885569235866,0.867037642570089,0
.175177595272154,0.860940088489467,0.970569785189001
"13668","ZFPM1",144.034739404877,-0.0544553537321755,0.307910166390705
,-0.176854679306293,0.859622538050049,0.970569785189001
"13669","LINC02693",40.2924337761356,0.0460086686515982,0.260056010731
014,0.17691830510769,0.859572560030385,0.970569785189001
"13670","SPOP",590.069551880429,0.0345711620781944,0.197490249620244,0
.17505250079268,0.861038380750332,0.970569785189001
"13671","C18orf32",630.4426681155,-0.0459852197669553,0.26222509146109
3,-0.175365444667137,0.860792490964377,0.970569785189001
"13672","RAC2",16.1800745786018,0.0882888363771878,0.504334177665272,0
.175060188833336,0.861032339854936,0.970569785189001
"13673","CHKB",160.31162817641,-0.0340985289013818,0.194675511990831,-
0.175155717083655,0.860957278993671,0.970569785189001
"13674","KRBOX4",77.5941960082053,0.036351190464327,0.207443423068329,
0.175234239421288,0.860895581386589,0.970569785189001
"13675","USP27X",18.6479803057969,0.0705914690323689,0.403247767940167
,0.175057308792948,0.861034602852256,0.970569785189001
"13676","MID2",4.5156071180246,-0.134661621216695,0.768714134183787,-0
.175177761444022,0.860939957922319,0.970569785189001
"13677","SLC41A1",62.7379965876704,-0.0624102155764304,0.3569447578568
84,-0.174845586614424,0.861200967088289,0.970592944825296
"13678","BCL2L11",81.2869406543108,-0.0600813494144014,0.3438407973815
45,-0.174735953010636,0.861287115943252,0.970592944825296

"13679","SLC16A12",10.9460074900584,0.120966702323076,0.692036638554286,0.174798118457693,0.861238266830255,0.970592944825296
"13680","MTPN",744.309708584548,-0.0234095418466696,0.13406761234123,-0.174609970580273,0.861386113560396,0.970633548284612
"13681","PIDD1",24.3002700461797,-0.0644055491855498,0.369384258995992,-0.174359214333085,0.861583165539815,0.970784628082469
"13682","TFDP1",133.16431527817,-0.0321968692643945,0.18489306824976,-0.174137784445774,0.861757179126252,0.970909729296242
"13683","AC099778.1",14.4929840778091,-0.076249998295156,0.438434953322672,-0.173914049774765,0.861933010772312,0.971036860414762
"13684","CYP39A1",8.58342075752352,-0.107472313211409,0.618576647165547,-0.173741303852758,0.862068775367955,0.971047875213521
"13685","PCCA-DT",30.1354130865197,-0.0517651888147508,0.297931214113309,-0.173748792884332,0.862062889498506,0.971047875213521
"13686","HCP5",4.80293230764593,0.130691837186162,0.752691033717216,0.173632780691875,0.862154068050605,0.971072991304989
"13687","SLC22A15",11.9934356854549,-0.0873017916054867,0.503470547064699,-0.173399997506245,0.862337027065072,0.971203840971874
"13688","ADAMTS10",55.6316483914296,-0.0564529135817858,0.325856902348136,-0.173244492214172,0.862459252615244,0.971203840971874
"13689","ZNF677",146.515028709385,0.0325333925063468,0.18774426204007,0.173285682091329,0.862426877477386,0.971203840971874
"13690","FYTTD1",214.491368670543,0.0259307450758226,0.149884296927074,0.173005081969588,0.86264743302594,0.971273842677296
"13691","RPL13A",18558.8848833653,0.0324743504500299,0.18767458721184,0.173035416954849,0.862623588794507,0.971273842677296
"13692","AC156455.1",5.55019265533554,0.105266264157483,0.609024783936548,0.172843974389802,0.862774070635478,0.9713454790276
"13693","TSTD1",5.33055920425428,0.115966415419911,0.67140437829408,0.17272216144101,0.862869823286446,0.971382335935191
"13694","UBE4B",106.059302167688,0.0384155707931388,0.230937317966719,0.166346310467999,0.867884423207373,0.971575092178001
"13695","AL031293.1",5.60846851743469,0.105811327515655,0.63387013060946,0.166929032314425,0.867425889162343,0.971575092178001
"13696","SAMD13",8.10977093891368,-0.100027032661693,0.597367574292331,-0.167446371323702,0.867018841145391,0.971575092178001
"13697","PSRC1",7.19904744403609,-0.0957299420643879,0.590125124509914,-0.16221973627015,0.871132818348995,0.971575092178001
"13698","S100A9",45.8180861988033,-0.0621477724204444,0.364951301898124,-0.170290589723099,0.864781611488628,0.971575092178001
"13699","MEF2D",164.073667379664,-0.0316353849453266,0.191262589603792,-0.165402889351549,0.868626879626213,0.971575092178001
"13700","SH2D2A",8.60814254289424,0.0836171074834027,0.503998104121508,0.165907583380996,0.868229679531058,0.971575092178001
"13701","KLHL12",46.7046410234202,-0.0455201182728396,0.271038701589859,-0.167946931585149,0.866625028393331,0.971575092178001
"13702","AKT3",242.824376781083,0.045225323374242,0.272668595858058,0.165861870641623,0.868265654622547,0.971575092178001
"13703","SILC1",2.58273592953666,-0.199503529770382,1.19852835056752,-

0.166457080198324,0.86779725693863,0.971575092178001
"13704","CYS1",205.636613728054,-0.0615022357197307,0.367631054413922,
-0.167293363771398,0.867139225501498,0.971575092178001
"13705","DDX1",403.977250132432,0.0357140705714059,0.218234961372911,0
.16364962949442,0.870006972654012,0.971575092178001
"13706","THUMPD2",67.5083407851184,-0.0377284474053597,0.2215866339165
6,-0.170264996306441,0.864801738173509,0.971575092178001
"13707","SRBD1",87.1819305831867,0.0363663217305547,0.221899801431946,
0.163886229261489,0.869820707979256,0.971575092178001
"13708","UGP2",1053.79887355559,0.0337816007729893,0.201879586360159,0
.16733539721407,0.867106153827291,0.971575092178001
"13709","AC073263.2",7.0308544261212,0.123327380185789,0.7549367874820
27,0.163361200872365,0.870234049679298,0.971575092178001
"13710","AC233263.6",6.36523883986356,0.112665746366195,0.670496016745
584,0.168033431299183,0.866556978619571,0.971575092178001
"13711","IL1R1",695.477307002683,0.0364171440789461,0.220612865841093,
0.165072621399956,0.868886822316448,0.971575092178001
"13712","POLR2D",68.2666192659993,0.0425663047721494,0.25693741472947,
0.16566798890293,0.868418239042855,0.971575092178001
"13713","AC012306.2",37.6873170564172,-0.0539116831776444,0.3226979555
64883,-0.167065462448536,0.867318541016526,0.971575092178001
"13714","MGAT5",29.5628825183974,-0.0516968484331778,0.316856118156014
,-0.163155594829712,0.870395927833372,0.971575092178001
"13715","UBXN4",982.036226231094,-0.0210579799068259,0.124149430951644
,-0.169618013916052,0.865310554779798,0.971575092178001
"13716","SP3",340.58833267628,0.0308412172288288,0.189479883321966,0.1
62767765570254,0.870701289106127,0.971575092178001
"13717","NFE2L2",910.595509375106,0.0267906089911624,0.163234023045994
,0.164123927666805,0.869633585667291,0.971575092178001
"13718","AC010136.1",2.66081222683753,-0.169614053670384,1.01537631618
107,-0.167045509105745,0.867334240872951,0.971575092178001
"13719","BRPF1",34.7716105217756,0.0452672603369104,0.277041310991204,
0.163395344091292,0.870207168499221,0.971575092178001
"13720","SEMA3B-
AS1",2.83406345947562,-0.151296289215242,0.91391433660291,-0.165547560
811467,0.868513018103225,0.971575092178001
"13721","C3orf38",114.823764965606,0.0431613378969192,0.25236465730086
6,0.171027664327271,0.864202013076727,0.971575092178001
"13722","LINC02066",2.74989346153812,-0.148824009512965,0.873605714231
459,-0.17035603944497,0.864730142170055,0.971575092178001
"13723","LINC00884",10.376156309631,-0.0981717189197241,0.604875598184
785,-0.162300676724825,0.871069081796747,0.971575092178001
"13724","PIGZ",41.3282919381068,0.0482068453729221,0.296398762643697,0
.162641857688427,0.870800428091852,0.971575092178001
"13725","NOP14-
AS1",36.1464368028038,-0.051122842045062,0.313464537985677,-0.16308971
4624746,0.870447797915071,0.971575092178001
"13726","AC110792.4",4.37356174475266,0.150128840999839,0.911768953662
01,0.164656671404378,0.869214222594636,0.971575092178001
"13727","CEP135",45.8083386622741,0.0492890475475238,0.287763579486898

,0.171283133311761,0.864001142145161,0.971575092178001
"13728","METAP1",70.1361062234283,-0.0347770301018219,0.211935541453009,-0.164092487099587,0.869658336073569,0.971575092178001
"13729","NUDT6",33.9675517672623,0.048657680633409,0.284504524082906,0.171026034788923,0.864203294383288,0.971575092178001
"13730","TMA16",187.102175389538,-0.0379726627324648,0.22753137108411,-0.166889789972864,0.867456766903618,0.971575092178001
"13731","NREP",108.474921439938,-0.0438698860773854,0.268894955905337,-0.163148787710356,0.870401287320884,0.971575092178001
"13732","APBB3",26.8560116515784,0.0604341008740994,0.354208532509104,0.17061729271738,0.864524699683885,0.971575092178001
"13733","PCDHB14",56.0098556341657,0.0496325379571057,0.29636886939074,0.167468796770517,0.867001197354005,0.971575092178001
"13734","AFAP1L1",37.936035399329,-0.0813620550925712,0.494250316622234,-0.16461710262431,0.869245368926082,0.971575092178001
"13735","SIMC1",26.9718287713071,-0.0535327898113594,0.3167448591346,-0.169009182840788,0.865789418445319,0.971575092178001
"13736","AL391422.4",53.6576897065613,0.0470262263537628,0.288665517229516,0.162909054067489,0.870590041950605,0.971575092178001
"13737","HLA-E",5205.3464245998,-0.0318774710173066,0.193231678478136,-0.16497021227766,0.868967427939056,0.971575092178001
"13738","AL512274.1",7.99742115841468,0.139116376451779,0.844494465719089,0.164733319280335,0.869153890252102,0.971575092178001
"13739","HECA",140.351671513649,-0.0445351177635812,0.270330594863855,-0.16474316488672,0.869146140470934,0.971575092178001
"13740","AHR",489.375114018672,0.0470451568586058,0.278698122865742,0.168803278525378,0.865951379444563,0.971575092178001
"13741","AC003986.2",2.43144684542489,-0.156398984547557,0.936066355245541,-0.167081087436939,0.867306246868718,0.971575092178001
"13742","TRGC2",2.44184711851787,0.176215649859665,1.03838681593419,0.169701355174788,0.865245008256341,0.971575092178001
"13743","VKORC1L1",189.560102551188,0.0292326729935509,0.17129553609159,0.1706563618676,0.864493977550791,0.971575092178001
"13744","ADAM22",37.2784786986788,-0.0677702734082627,0.413612849886748,-0.163849535687344,0.869849594756459,0.971575092178001
"13745","MEPCE",114.160451905611,0.0361824021680105,0.21269219206522,0.1701162690397,0.864918699159351,0.971575092178001
"13746","EPHB4",39.1192726029165,-0.0469906532594802,0.28330192848109,-0.1658677493352,0.868261028182721,0.971575092178001
"13747","ZNF783",45.9486732500031,0.0500833266746109,0.293274855470445,0.170772658277409,0.864402528763505,0.971575092178001
"13748","LINC01003",154.461519935994,-0.0483183734107632,0.288664642100082,-0.16738583935753,0.867066466542089,0.971575092178001
"13749","NOM1",75.5708964928495,0.0512497377192642,0.302449469534438,0.169448925792971,0.865443542642327,0.971575092178001
"13750","INTS9",33.9357878449434,0.0467202225497128,0.27931818898555,0.167265235104792,0.867161357106678,0.971575092178001
"13751","AC016405.1",2.72518282635967,0.176773262611272,1.06565548692607,0.165882186860579,0.868249666095382,0.971575092178001

"13752","OPLAH",16.2656255391669,-0.0666141710528129,0.39084119369698,-0.170437948013379,0.86466573050925,0.971575092178001
"13753","TDRD7",29.0624740684316,-0.050258001483699,0.307150084254103,-0.163626852344019,0.870024904491587,0.971575092178001
"13754","TRMO",38.3972133274249,0.048801534133087,0.296582256304038,0.164546371523516,0.869301044995475,0.971575092178001
"13755","UGCG",282.521954793533,-0.0624147624719591,0.36689717878766,-0.170115133286651,0.864919592341166,0.971575092178001
"13756","TTLL11",18.4383448204765,-0.0583774542009338,0.359629636885217,-0.162326594400133,0.871048673103096,0.971575092178001
"13757","WDR5",53.2968896183454,-0.0447914849005583,0.262219032655106,-0.170817062541269,0.86436761229123,0.971575092178001
"13758","SFTA1P",7.50155101765231,-0.0989865459896411,0.609870750724397,-0.162307416566651,0.87106377454679,0.971575092178001
"13759","ARL5B",180.254650451961,0.045631968815696,0.278641674508604,0.163765771563675,0.869915538167823,0.971575092178001
"13760","AL442003.1",4.46782441874944,-0.126102663467337,0.746753959788157,-0.168867753313435,0.865900664014945,0.971575092178001
"13761","SAR1A",551.255708023239,-0.0341754191706358,0.200013961280063,-0.170865168370836,0.864329785460047,0.971575092178001
"13762","FRA10AC1",241.10761001632,-0.0340784285166832,0.20442365716274,-0.166704915613331,0.867602237564698,0.971575092178001
"13763","TRIM8",421.905944512077,-0.0271330403558689,0.163941946550506,-0.165503953849358,0.868547338027841,0.971575092178001
"13764","DHX32",72.6606161489847,0.0403891425955896,0.240628929778718,0.167848240993846,0.866702670000853,0.971575092178001
"13765","MOB2",168.027759986449,-0.0419323545893065,0.245001725926656,-0.171151262019515,0.864104829209931,0.971575092178001
"13766","TRIM3",49.3253907262342,0.0479851693895668,0.291071113317989,0.16485720222310,0.869056379074106,0.971575092178001
"13767","LINC02709",14.6437141681819,0.102024469227155,0.608981556417399,0.167532937823863,0.866950733112641,0.971575092178001
"13768","COMMD9",178.558830966534,-0.0418481655993536,0.248659740249645,-0.168294897908843,0.866351287496891,0.971575092178001
"13769","YPEL4",52.0115442645306,0.0539810140800841,0.329383990152078,0.163884753643189,0.869821869646797,0.971575092178001
"13770","CLP1",30.3011618492485,0.0493338264291766,0.291053300715443,0.169501003108051,0.86540258341124,0.971575092178001
"13771","H2AFX",170.665728846493,0.0557807948454398,0.333512592628593,0.167252439872813,0.867171424416143,0.971575092178001
"13772","SC5D",86.7340726619497,-0.0407889263095445,0.239981454230672,-0.169966993659176,0.865036094164374,0.971575092178001
"13773","TMEM117",18.6138695211954,0.0614581729315849,0.376930690626166,0.163049002004823,0.870479852843853,0.971575092178001
"13774","ARHGAP9",6.28907090900325,0.131181042625952,0.783294622824649,0.167473436946239,0.866997546585858,0.971575092178001
"13775","TBK1",121.477764950147,0.0298135453382468,0.178357203601402,0.167156384694588,0.867247001475447,0.971575092178001
"13776","ANKRD13A",89.1626663424878,0.0359348797459239,0.210037397450444,0.171088007098366,0.864154565786617,0.971575092178001

"13777","WSB2",132.702865180311,0.0359563957683824,0.215652958194374,0.16673268045771,0.867580390169572,0.971575092178001
"13778","LINC00545",11.3339162778039,0.110559558970681,0.680247737196501,0.16252837448064,0.870889785715717,0.971575092178001
"13779","RFC3",31.3848584704146,-0.0560425492818722,0.333165069857891,-0.16821255993546,0.866416060337668,0.971575092178001
"13780","TPT1",35786.5403396089,-0.0415792117877846,0.253333461573697,-0.164128384499608,0.869630077203309,0.971575092178001
"13781","ALG11",33.6218048111354,-0.0548333167509662,0.326433628025856,-0.167976924076655,0.866601433032685,0.971575092178001
"13782","TDRD3",130.322828481095,-0.033000630273505,0.20318930155598,-0.162413227570513,0.870980455037237,0.971575092178001
"13783","PCCA",79.6512367806085,0.036174965165491,0.21017631754344,0.172117227993655,0.86334536889984,0.971575092178001
"13784","FGF14-AS2",46.2264350219096,-0.0514321343795489,0.303036280381842,-0.169722695628199,0.865228224491273,0.971575092178001
"13785","AL355075.4",43.5692404704424,-0.101823167624867,0.598513203374294,-0.170126852759152,0.864910375891949,0.971575092178001
"13786","CLEC14A",66.1624825344443,0.0740800432182389,0.453511051712737,0.163347823473027,0.870244581834371,0.971575092178001
"13787","FBXO33",101.733689731248,-0.0418535188624881,0.258210982891623,-0.162090389780418,0.871234673965391,0.971575092178001
"13788","STYX",47.0230555395249,-0.0527302327497014,0.319840798889801,-0.164863997753673,0.869051030209763,0.971575092178001
"13789","CDKN3",7.58683417188089,0.0965496046322918,0.571732369745673,0.168872027790276,0.865897301760059,0.971575092178001
"13790","BATF",4.31029088758253,-0.139575453882447,0.823005326529932,-0.169592406492609,0.865330694781491,0.971575092178001
"13791","FAH",30.7492306807058,-0.063931195601174,0.377807904284207,-0.169216140997097,0.865626634191349,0.971575092178001
"13792","AC012291.3",4.54705584278522,-0.117327930268335,0.7159520164337,-0.163876806790445,0.869828125741627,0.971575092178001
"13793","CHTF18",12.4566696701372,0.090876990876869,0.558731293068527,0.162648829597098,0.870794938406606,0.971575092178001
"13794","NTHL1",92.4573573841953,0.0534922015040321,0.321224799630324,0.166525752574498,0.867743218480745,0.971575092178001
"13795","BOLA2",110.236702536524,-0.0490622362856548,0.301354030412257,-0.162805973487519,0.870671204860993,0.971575092178001
"13796","CKLF",365.702481907143,-0.0414637529926699,0.247019578518319,-0.167856140154474,0.86669645554621,0.971575092178001
"13797","UTP4",55.0523343013859,-0.0396294957765924,0.232806699718504,-0.170224893976462,0.864833274856682,0.971575092178001
"13798","TMEM231",20.2463946584524,0.0626780142215219,0.377667446591282,0.165960859976775,0.868187752166936,0.971575092178001
"13799","ACAP1",26.2212504906487,-0.054614096411153,0.335092704246628,-0.162982051590586,0.870532566464348,0.971575092178001
"13800","AC087501.4",3.04107414813647,0.146344808835208,0.879662709093822,0.16636468423899,0.867869964515757,0.971575092178001
"13801","COX10",24.5323762949179,-0.0522963495200702,0.319445132405236

,-0.163709958972638,0.869959477202286,0.971575092178001
"13802","PGAP3",28.8232337940985,-0.0554530829715366,0.336707553944303
,-0.164692126214398,0.869186314724105,0.971575092178001
"13803","ORMDL3",62.8924010407071,-0.0352554099545324,0.20978373184973
5,-0.168055976713129,0.866539242187936,0.971575092178001
"13804","AC025048.2",3.72232205553385,-0.124121064522848,0.74411573962
9889,-0.166803439186199,0.867524712522669,0.971575092178001
"13805","TACO1",78.3689030698291,-0.0482457915531665,0.293375103824198
,-0.164450871680227,0.869376218839773,0.971575092178001
"13806","LIMD2",30.2214369561027,-0.0572795457934678,0.353341058979649
,-0.162108377551353,0.871220509127673,0.971575092178001
"13807","UNK",87.3321280382429,0.0338474301502473,0.203508517567014,0.
166319476722155,0.86790553930352,0.971575092178001
"13808","RIOK3",245.392718859423,0.0409992075747179,0.242128678186406,
0.169328176578712,0.865538514270051,0.971575092178001
"13809","DYM",161.07613482894,0.0314156994762744,0.184927984204088,0.1
69880722009081,0.865103942348871,0.971575092178001
"13810","RAB27B",7.18791841414501,-0.143287270355002,0.845837990435944
,-0.169402736664917,0.865479871068973,0.971575092178001
"13811","TIMM21",68.04769497818,-0.0355616280054247,0.213172464131087,
-0.166820926663196,0.867510952320572,0.971575092178001
"13812","ARHGAP45",12.1642296821045,0.112281406304535,0.65962157087268
2,0.170220943738979,0.86483638135586,0.971575092178001
"13813","GET3",437.025668983037,-0.0421532573426162,0.252566371483389,
-0.166899722615639,0.867448951408625,0.971575092178001
"13814","SYDE1",112.045813891165,0.0396878173075562,0.243615606713848,
0.162911637078217,0.870588008174074,0.971575092178001
"13815","SMIM7",478.446482653719,0.0388946260939865,0.23386948956566,0
.166309107554907,0.867913699066652,0.971575092178001
"13816","ZNF780B",31.189550954122,-0.0539721460344043,0.31679818473578
8,-0.170367598789802,0.864721052021426,0.971575092178001
"13817","CD79A",2.5578973272314,-0.149461267800887,0.920054302287012,-
0.162448311397888,0.87095282904615,0.971575092178001
"13818","ERF",90.7583713678255,0.0431833090185179,0.265769861308364,0.
162483845255929,0.870924848848769,0.971575092178001
"13819","PRR12",99.7425448261185,0.0397072219532776,0.241916455158695,
0.164136093707351,0.86962400844285,0.971575092178001
"13820","SH3BP1",11.313954485834,0.0742486043935435,0.44763407104819,0
.165868974673178,0.868260063861481,0.971575092178001
"13821","AL121672.2",7.39363824194411,0.0908444332906011,0.54833951717
4784,0.165671870155665,0.86841518445903,0.971575092178001
"13822","CXorf38",79.6947854251099,0.0377499104141016,0.23273167514972
2,0.162203577960826,0.871145542308141,0.971575092178001
"13823","ARMCX5",32.1201987175824,0.0512884915280086,0.300158195258015
,0.170871534871541,0.864324779342724,0.971575092178001
"13824","WDR35",49.842684858718,-0.0461116972983316,0.284872360183649,
-0.161867923123902,0.87140986329171,0.971700162228133
"13825","FAM189B",38.9876171459935,-0.0515896705267206,0.3195222924040
36,-0.161458751871639,0.87173209629653,0.971824071117753
"13826","CDC42SE2",93.2008876029444,0.0296711421585895,0.1844578583691

55,0.160855939784407,0.872206865266125,0.971824071117753
"13827","THOC3",58.9285727837121,-0.0457346665764839,0.284337720150044
,-0.16084628712768,0.872214467980157,0.971824071117753
"13828","LTV1",149.270031044038,0.0349470784824189,0.216465744726893,0
.161443920498878,0.871743776788518,0.971824071117753
"13829","LY6K",14.9687609849886,0.0883265778105555,0.548510106650723,0
.161029991498041,0.872069779085066,0.971824071117753
"13830","UQCC3",226.640528334484,-0.0414284705214219,0.25682630970098,
-0.161309293310551,0.871849804112288,0.971824071117753
"13831","AC009779.2",47.9182241595388,0.0544203076369434,0.33704207464
3832,0.161464433467103,0.871727621746435,0.971824071117753
"13832","ZFAND6",306.128862175543,0.0299844928140675,0.186227898937893
,0.161009671403034,0.872085783350119,0.971824071117753
"13833","DUS2",21.5064730790113,0.0576382169527555,0.357808546532662,0
.161086752989267,0.872025073570069,0.971824071117753
"13834","AJ009632.2",2.41056449397287,0.216226735089361,1.342546490529
58,0.161057167565249,0.872048375084776,0.971824071117753
"13835","AC007325.4",36.2162601837042,0.0771578403996002,0.47930551115
4519,0.160978412732513,0.872110403024648,0.971824071117753
"13836","ARL17A",30.0573412382416,-0.0706193214626004,0.43937137153426
,-0.160728090262235,0.872307564236497,0.971857552956462
"13837","AC022915.1",3.10362753025525,-0.170347945865306,1.06177720160
328,-0.160436620421009,0.872537144301848,0.972043078659607
"13838","ARMC2",21.5129390135176,0.0653150356921373,0.407324644641877,
0.16035129853133,0.872604351245976,0.972047700134176
"13839","ATP6V1FNB",3.037079461337,0.139739436455123,0.873931420447263
,0.159897485301086,0.872961829472275,0.972318388309625
"13840","CAMK1D",391.302295159649,-0.0450712190042502,0.28215502361613
6,-0.159739204450842,0.873086516732358,0.972318388309625
"13841","PPP1R13B",4.76612382142292,-0.111607603069016,0.6986340464520
35,-0.159751165343011,0.873077094314512,0.972318388309625
"13842","UBB",3502.98381629343,0.0441603404543746,0.276481597293452,0.
159722530854391,0.873099651701708,0.972318388309625
"13843","CCDC163",2.47312521454788,0.153170088891116,0.961786163684965
,0.159255866505985,0.873467290340338,0.972376563919716
"13844","NEDD9",87.7199069364781,0.0493324151910187,0.309610110138649,
0.159337223093027,0.873403195571288,0.972376563919716
"13845","ZMIZ2",75.3606585926862,-0.0432949448682928,0.271808763675579
,-0.159284580389645,0.873444668724497,0.972376563919716
"13846","CCDC171",50.246010353037,-0.0504234594116167,0.31639997031437
7,-0.159366195140649,0.873380370862823,0.972376563919716
"13847","MRPL10",168.131336314109,0.0430954853253644,0.270176996479419
,0.159508344111181,0.873268384833984,0.972376563919716
"13848","STX18",127.185517513224,0.0283859266536233,0.17846471797275З,
0.15905623798400，0.873624566237616,0.972481418872968
"13849","HENMT1",21.1895798443549,0.0626469120444751,0.3945574830323,0
.158777655319102,0.8738440539352,0.972515464938649
"13850","AL160408.2",2.61769966696381,-0.144100895646916,0.90700139699
7809,-0.158876156226322,0.873766446641601,0.972515464938649
"13851","PSMD1",282.34668628391,-0.0246927014498054,0.155561293660142,

-0.158732939723116,0.873879285040626,0.972515464938649
"13852","OSBPL7",8.51532903148723,0.0989748775883945,0.623742662968786,0.158679024964094,0.873921764434807,0.972515464938649
"13853","AC119403.1",5.73297069943583,-0.0966085584286239,0.609067935610618,-0.158617048739841,0.87397059589978,0.972515464938649
"13854","JOSD2",362.601393654442,-0.0410432719939938,0.258906275927104,-0.158525597137513,0.874042652081098,0.972525442603589
"13855","TMEM218",129.164162288491,0.0463521193459728,0.292819593732827,0.158295825614269,0.874223697350369,0.97265667951324
"13856","CCNE2",2.66528886656857,0.152921449628615,0.96742137924183,0.158071191013434,0.874400701419183,0.972713199998319
"13857","CSKMT",138.809453662045,-0.0372930953839044,0.235836522214598,-0.158131128434678,0.874353472249781,0.972713199998319
"13858","CHSY1",86.0303409210989,-0.042643009564383,0.269946510144255,-0.157968367665118,0.874481724654762,0.972733135052183
"13859","ZCCHC17",263.482368846417,-0.032930376141275,0.209035056155907,-0.15753518451333,0.874823080835421,0.972837434058871
"13860","FAM102B",170.29344990263,0.0525257301836765,0.334058681445936,0.157235040132245,0.875059613717861,0.972837434058871
"13861","LINC01118",5.50697156961166,-0.101768344149103,0.64731961189999,-0.157214986659212,0.875075417529474,0.972837434058871
"13862","MYMX",3.14247053350682,-0.131924512611194,0.841836216195561,-0.156710426652097,0.875473069337363,0.972837434058871
"13863","TMED4",449.482897807143,0.0234072389741127,0.148561684223815,0.157559057683061,0.874804267737548,0.972837434058871
"13864","TMEM273",6.91405437853248,-0.100858382146819,0.642262960462516,-0.157035962457164,0.875216505759895,0.972837434058871
"13865","BNIP3",336.950290475051,-0.0324212311097673,0.206440152225715,-0.157049056398287,0.875206186344755,0.972837434058871
"13866","ZFHX2",11.1766470929499,0.0856772226187899,0.543483443322081,0.1576445863651,0.874736867990694,0.972837434058871
"13867","MAP4K5",266.646028833122,0.0297780487127462,0.189286941504773,0.157316973247176,0.874995044098038,0.972837434058871
"13868","AC011330.2",3.37126485346035,0.13652339971325,0.869870220846518,0.156946859935487,0.875286728565972,0.972837434058871
"13869","CASC4",500.844642782227,0.0235183440969607,0.149918032330154,0.156874684995651,0.875343611254091,0.972837434058871
"13870","PALB2",31.0023616753235,0.0455388682071583,0.289427191827408,0.15734136077 4126,0.874975825004789,0.972837434058871
"13871","CENPN",9.79799796334609,-0.0918185552762001,0.586145081464062,-0.15664817155312,0.875522135757296,0.972837434058871
"13872","MRPS7",245.28555087592,0.0356988279039905,0.227646551178335,0.156816906380561,0.875389148340118,0.972837434058871
"13873","ZNF573",29.0748153592632,-0.0493724582534133,0.315001523878341,-0.156737204460261,0.875451964527691,0.972837434058871
"13874","WIPF3",2.62290762906425,-0.151766235407627,0.97039156799949,-0.15639690245918,0.875720178611918,0.972917229066863
"13875","ARMCX5-
GPRASP2",25.0456776459219,-0.0482613225848188,0.308551837538905,-0.156412364838805,0.875707991397602,0.972917229066863

"13876","SDR42E2",20.5938917841849,0.0629825292203329,0.402988513410111,0.156288646262821,0.875805505337803,0.972941904351559
"13877","BTD",194.542287934277,-0.0453413295412738,0.290823784967061,-0.155906538202882,0.876106691592664,0.973091369212504
"13878","FIP1L1",311.033766901642,-0.0249383594093729,0.160151265618328,-0.155717529381291,0.87625567927595,0.973091369212504
"13879","SQSTM1",1920.11845913884,-0.0234348444907265,0.150437499269377,-0.155777945023957,0.876208055695588,0.973091369212504
"13880","TNFRSF6B",11.3631227300046,-0.0782397912838036,0.502371938169088,-0.155740767625181,0.876237361311753,0.973091369212504
"13881","ST13",1252.63820495449,-0.0273011401042196,0.175103437252731,-0.155914358578897,0.876100527214595,0.973091369212504
"13882","LIMS1",938.667904565501,-0.0238398626739144,0.153423332741242,-0.155386160944123,0.876516893620608,0.973117145524408
"13883","AL590428.1",8.29912780670463,-0.088952650425421,0.573120461021626,-0.155207598533224,0.87665765811855,0.973117145524408
"13884","FDXACB1",9.18004790408627,0.0835839348875503,0.537854400425243,0.155402530538872,0.876503989320709,0.973117145524408
"13885","CLIP3",101.183858527675,0.0373996258720349,0.240447349308742,0.155541851384739,0.876394162764203,0.973117145524408
"13886","IQSEC2",46.6980711872615,0.0436628707704518,0.281180546094388,0.155284109718582,0.876597342266627,0.973117145524408
"13887","ABCB7",111.266659239071,-0.0338713685735759,0.218215974813041,-0.155219472830051,0.876648297239889,0.973117145524408
"13888","SYNJ2BP",163.958720526565,0.0253895210754121,0.163768710481521,0.15503279595205,0.876795462442684,0.973200032657977
"13889","PTPRC",55.3514131266891,0.0869956288864802,0.562805192893266,0.154575028775505,0.877156357501366,0.973374210410994
"13890","MARK4",66.7322480098139,0.0336958844456461,0.218037723074057,0.15454153515537,0.877182764246975,0.973374210410994
"13891","SNRPB2",510.505903469359,-0.0284786611228275,0.184289118826526,-0.15453251556124,0.877189875419466,0.973374210410994
"13892","KRTAP13-4",4.15120015238598,0.153629683638931,0.994280798306021,0.154513376805298,0.877204964711614,0.973374210410994
"13893","PTBP2",88.6826233282516,0.03815418948732,0.247177613961148,0.154359405270888,0.877326359881467,0.97337955757234
"13894","PLXNA2",30.6316014437577,-0.0502051297170871,0.325757829771475,-0.154117952444326,0.877516733390381,0.97337955757234
"13895","PEX3",81.4318793987651,-0.0372494358926439,0.241728934204652,-0.154095892637775,0.87753412680075,0.97337955757234
"13896","EXD2",50.0499949470608,0.0423278519136644,0.274311312383085,0.154305892622292,0.877368551323805,0.97337955757234
"13897","AMFR",182.004914149251,-0.0259373673654081,0.16839522614723,-0.154026737923857,0.877588653333791,0.97337955757234
"13898","SLC35C2",190.567646617191,-0.0311602200815053,0.20209725626116,-0.154184280667515,0.877464436188959,0.97337955757234
"13899","PINK1-AS",3.95768460432665,0.110665942971705,0.72186666962717З,0.153305239912049,0.878157568670927,0.973422741379567
"13900","IPO13",32.6078950581987,-0.0433738227033524,0.28379223590365,

-0.152836537494557,0.878527183460007,0.973422741379567
"13901","C2orf76",109.712594557533,-0.0443771033682945,0.290719884960577,-0.152645572814231,0.878677784198963,0.973422741379567
"13902","R3HDM1",68.32828434774,-0.0341047603097049,0.222281010769562,-0.153430831503017,0.878058532705587,0.973422741379567
"13903","PASK",5.31220755741141,0.109440929556179,0.718599510221911,0.152297528733887,0.878952273969632,0.973422741379567
"13904","ING5",92.5365551944028,0.0292367388752446,0.190475868564389,0.153493138504111,0.87800940087265,0.973422741379567
"13905","FANCD2",3.65826379549584,0.115565445695639,0.76073725464376,0.151912430987433,0.879256003580543,0.973422741379567
"13906","LINC02202",35.3957963869701,-0.0464553565020094,0.304839878357229,-0.152392648732035,0.878877254823189,0.973422741379567
"13907","AL139274.2",6.88657955319978,0.0922372991555504,0.603512026414868,0.152834235472458,0.878528998880704,0.973422741379567
"13908","PHF14",381.766805571659,0.0217889168893426,0.14275199971271,0.152634757714029,0.87868631345774,0.973422741379567
"13909","ZNF775",47.9579520281235,0.0461451928566507,0.302018399151567,0.152789343252869,0.878564401911155,0.973422741379567
"13910","TPRN",28.0193494190814,-0.0446283031279136,0.292243657500949,-0.152709227326067,0.878627583765543,0.973422741379567
"13911","CASC2",10.6087293857545,0.0795780348471198,0.52375459507177,0.151937635671178,0.879236123905015,0.973422741379567
"13912","EEF1G",5352.26875296881,-0.0341424633068277,0.224052914586131,-0.152385713749341,0.878882724261766,0.973422741379567
"13913","PRH1",78.0178586189491,-0.0310780932704208,0.204787710334702,-0.151757608987509,0.879378117915686,0.973422741379567
"13914","GAS6",1102.65640182829,0.0467447780712795,0.303815765715257,0.15385896107541,0.877720943039669,0.973422741379567
"13915","HAUS2",62.0288428117783,0.0422612050739133,0.277539997289273,0.152270683457079,0.87897344647277,0.973422741379567
"13916","FAM227B",6.82588910864938,-0.0928775901015844,0.610374641750226,-0.152164889804828,0.879056885323719,0.973422741379567
"13917","EFL1",34.0902121796278,-0.0421262263929904,0.27455095807364,-0.153436821668971,0.878053809173729,0.973422741379567
"13918","KCTD13",33.9257435632299,-0.0427075926567315,0.281461178954261,-0.151735286604735,0.879395724713062,0.973422741379567
"13919","AC027682.6",9.31076151077076,0.0731272659765895,0.481151081920615,0.151983999879386,0.879199555288914,0.973422741379567
"13920","TVP23B",47.7670204665736,-0.0453043414512835,0.294786316684851,-0.153685360842977,0.877857827997404,0.973422741379567
"13921","CCDC144NL",2.85204346955588,-0.176141269733457,1.16018922356651,-0.151821156545469,0.879327995054823,0.973422741379567
"13922","JPT1",204.554547056367,0.0358535170432732,0.234494084691017,0.152897319736299,0.87847924959886,0.973422741379567
"13923","PPP1R37",68.0527872262959,0.0357862897557422,0.234052039081625,0.152898859143294,0.878478035603333,0.973422741379567
"13924","ZNF324B",11.529795120311,-0.0737704665187008,0.48444952337385,-0.152276889457835,0.87896855187705,0.973422741379567
"13925","GSPT2",92.4192518967641,-0.032528460422976,0.212430639449954,

-0.153125088298006,0.87829963158259,0.973422741379567
"13926","MAMLD1",7.60474661154939,0.0841481952873248,0.549064243623099
,0.153257467162782,0.878195240645519,0.973422741379567
"13927","MTRR",54.616581904706,0.0355769174201069,0.234600798469958,0.
151648748223091,0.879463982494883,0.973428397369041
"13928","DCLRE1B",15.1213662744572,-0.0574891266664279,0.3795382251426
36,-0.151471242836799,0.879603993969908,0.97351346690451
"13929","P4HTM",195.553031304924,0.0359815521654402,0.238068040193126,
0.151139783972057,0.879865449883117,0.973593131142649
"13930","VIPR2",21.8264122190368,-0.171925096824673,1.13746616026677,-
0.151147438781257,0.879859411594951,0.973593131142649
"13931","RACGAP1",15.0993209792278,0.0599229417508743,0.39640742179548
,0.151165034901366,0.879845531400384,0.973593131142649
"13932","GYS1",23.4088825376224,-0.0490406810538438,0.324725179279031,
-0.151022107872035,0.879958276354946,0.973625956790948
"13933","CPNE8-
AS1",2.39641247278639,0.1469301366835,0.975548210553665,0.150612891391
714,0.880281091543947,0.973913229466012
"13934","WDFY4",6.24273090848972,0.0878387936325629,0.584339046798472,
0.150321622547427,0.880510874489968,0.973957744708873
"13935","CD63",12753.8370331232,-0.0417414443921923,0.277508614193396,
-0.150414950229627,0.880437246879248,0.973957744708873
"13936","CRYL1",292.530977663123,-0.0424371505493229,0.28226961379188,
-0.150342610312324,0.880494316836854,0.973957744708873
"13937","AL080317.2",6.74182237630483,0.116323882892562,0.775184940810
873,0.150059523564639,0.880717653837287,0.974082753524202
"13938","TMEM50B",245.355102736035,0.0226216988643214,0.15080385991442
2,0.150007426051022,0.880758756415501,0.974082753524202
"13939","ZBED4",29.8944338659575,0.0580288574264837,0.387018788618646,
0.149938088622522,0.880813461003818,0.974082753524202
"13940","PSMA3-
AS1",227.824887578278,-0.0314030533881154,0.209761313213228,-0.1497085
08719114,0.880994594854899,0.974166801865987
"13941","ZNF575",19.5349319950726,0.0514376248812854,0.34383070838172,
0.149601602263459,0.881078943988287,0.974166801865987
"13942","FBLN1",9225.2504476059,-0.0386117831365755,0.258097621569138,
-0.149601468242249,0.881079049731793,0.974166801865987
"13943","AC009506.1",13.3927188387601,-0.0712906368799808,0.4783909849
40999,-0.149021697992016,0.881536511614159,0.97432950641916
"13944","PRMT3",37.9382615977702,0.0434418310794827,0.291599483610036,
0.148977736660119,0.881571200474799,0.97432950641916
"13945","LINC00562",4.91364418427925,0.115643537297059,0.7759737157629
34,0.149030224797446,0.881529783337381,0.97432950641916
"13946","PRORP",57.0569530322188,-0.0331160770875756,0.221992877617973
,-0.149176304406329,0.881414517107623,0.97432950641916
"13947","TOMM34",32.0854089568542,-0.0416225231182338,0.27946895226160
3,-0.148934337003819,0.881605446353191,0.97432950641916
"13948","CHEK2",79.5544100484598,0.0403689546338292,0.270791519106062,
0.14907761796638,0.881492386796343,0.97432950641916
"13949","CHRD",81.4326862876343,-0.0428290631168157,0.288011666155157,

-0.148706001005331,0.881785625775606,0.974409948136765
"13950","C5orf30",32.6690369182585,0.0477375305812507,0.32137163832692
7,0.148543072530526,0.881914196009133,0.974409948136765
"13951","TSPYL1",303.972111503392,-0.0302556081556573,0.2038216602658
,-0.14844157461828,0.881994291686817,0.974409948136765
"13952","NSUN6",67.2375743136935,-0.0363850723285898,0.244942780259327
,-0.148545192024309,0.881912523452714,0.974409948136765
"13953","CUEDC1",174.293856762036,-0.0308455411590234,0.20776155987990
4,-0.148466064544633,0.881974965688688,0.974409948136765
"13954","AC073111.4",39.0511779147652,0.0421108499841392,0.28390864979
6101,0.148325350475875,0.882086009848,0.974441439143394
"13955","GPATCH11",126.100326752686,0.0313239739941696,0.2115963343996
33,0.14803646803734,0.882313987970703,0.974553605944998
"13956","ACVR1",226.228950453274,-0.0332646065365957,0.224651263216757
,-0.148072198928612,0.882285789599406,0.974553605944998
"13957","EEA1",1171.64100429001,0.0257590000529664,0.174120148372858,0
.147938077779526,0.882391637099017,0.97456954115364
"13958","HSD11B2",47.0454163166977,0.0803345002096861,0.54337394502531
7,0.147843857706396,0.882465996196619,0.97458184061978
"13959","DMRT2",57.8010887095032,0.0547388341733784,0.370491500258288,
0.147746531661906,0.882542807634715,0.974596846456704
"13960","DHRS3",867.489853062613,-0.0406640323690734,0.275672422270019
,-0.147508524916008,0.882730651425784,0.974664636611164
"13961","RNF14",87.9080443305782,0.0310746153198836,0.210625534628824,
0.147534891126306,0.8827098419053,0.974664636611164
"13962","NCF1",6.78119741777986,-0.107629350097754,0.730264027003908,-
0.147384159862469,0.882828807583397,0.974703199319443
"13963","SEMA4A",94.5503520618751,0.108693114791717,0.739458709930433,
0.146990106860655,0.883139829082125,0.974767325311539
"13964","CPVL",249.477225864412,-0.055189585829315,0.375413923702713,-
0.147009959793125,0.883124158959683,0.974767325311539
"13965","AP000880.1",3.58650792096892,-0.126089886642452,0.85741436637
92,-0.14705828545295,0.883086015213793,0.974767325311539
"13966","SIPA1L1",28.2938809636154,-0.0558840766662491,0.3795798593579
65,-0.147226137763931,0.882953530440212,0.974767325311539
"13967","THAP3",84.4013827823742,0.0445895529497665,0.303910889648068,
0.146719168245013,0.883353688267625,0.974786094780197
"13968","ATPAF1",112.538855035327,0.0310922660064608,0.212352274053491
,0.146418333144992,0.88359115551745,0.974786094780197
"13969","SH3PXD2B",302.960205860649,-0.0475695551160111,0.325089269107
611,-0.146327669463198,0.883662723870157,0.974786094780197
"13970","RNGTT",52.1959498577827,-0.0382959301390707,0.261221539839521
,-0.146603263125228,0.88344517791425,0.974786094780197
"13971","AL158152.1",67.3891728932422,-0.0427427448552662,0.2920768723
43213,-0.146340737328357,0.883652408263343,0.974786094780197
"13972","INPP5E",48.3601938310238,-0.0454722716689215,0.31027609115240
1,-0.146554223691655,0.883483887633987,0.974786094780197
"13973","FBXL19-
AS1",10.312105085768,-0.0897902089596728,0.612114205943567,-0.14668865
3992701,0.883377774526144,0.974786094780197

"13974","KCNE5",3.31582569526544,0.123699167596772,0.844158154109315,0.146535536018472,0.883498638990786,0.974786094780197
"13975","SUB1",1202.24545490665,0.0358354206136222,0.24506185792131,0.146230102544677,0.883739742579985,0.97480129745048
"13976","LRRC8B",10.7528307303778,-0.0664862805534284,0.455788077389984,-0.145871039308782,0.884023194290391,0.974806063886254
"13977","TBC1D14",71.1897704406074,-0.0354615541048993,0.24358174503327,-0.145583792004018,0.884249963757476,0.974806063886254
"13978","TMEM33",129.18664258878,-0.0291570783421328,0.200179520281276,-0.145654651890282,0.884194022024717,0.974806063886254
"13979","ZFAT",13.5388606943314,0.0691122024047493,0.473931847877925,0.145827301360324,0.884057722918081,0.974806063886254
"13980","ATAD1",178.906726878988,-0.0269400272006791,0.184453212454853,-0.146053445435508,0.883879197382771,0.974806063886254
"13981","MRPL21",315.929528953822,-0.0368231505612684,0.252542834724059,-0.145809524160538,0.884071757071208,0.974806063886254
"13982","CTDSPL2",143.258644889824,-0.0265805730769715,0.18246138230142,-0.145677801744708,0.884175746041329,0.974806063886254
"13983","ZNF285",3.37485101021433,0.111508155157362,0.764374694476734,0.145881536847183,0.884014907112455,0.974806063886254
"13984","ADA2",13.0330643083628,-0.0736392279590405,0.50681947516573,-0.145296760616708,0.884476572239645,0.974986152822807
"13985","STX2",219.026404760151,-0.0293887575595988,0.202833017755031,-0.144891388418293,0.884796625733119,0.975269215993996
"13986","MRPS24",773.450487204337,0.0366516730095273,0.253667034261631,0.144487332050111,0.885115659046708,0.975342079092831
"13987","ZKSCAN5",24.282554868077,0.0511947680140594,0.354320610017817,0.144487129923052,0.885115818646099,0.975342079092831
"13988","TMC8",7.96232395249243,0.10486261669957,0.725351179615256,0.144568065299338,0.885051912499577,0.975342079092831
"13989","EFCAB6",3.74455517862654,0.108307353541358,0.749513946313848,0.144503453303331,0.885102929730892,0.975342079092831
"13990","ARHGEF10L",126.802325778609,-0.0303697417065532,0.210459969705677,-0.144301748921776,0.885262197323175,0.97541603618589
"13991","LOH12CR2",18.8953593673628,0.053367760044854,0.369988023144309,0.144241858402099,0.885309488308582,0.97541603618589
"13992","LNCTAM34A",6.25412104862067,0.0968338116027966,0.675126599366675,0.143430597599968,0.885950119401771,0.975462259150484
"13993","CCDC28B",111.962145615792,0.045473802397096,0.315805795735081,0.143992931767606,0.885506051103152,0.975462259150484
"13994","DARS",279.581884768345,0.019887101113592,0.138202706518271,0.143898058255196,0.885580969018364,0.975462259150484
"13995","CRBN",377.217806182233,0.0252935265576361,0.176084124083563,0.143644560174163,0.885781151588968,0.975462259150484
"13996","ABHD6",31.0092238866595,-0.0425183887613554,0.296365210885169,-0.14346619373564,0.885922008512787,0.975462259150484
"13997","AC008549.2",8.12149095271852,0.0942436684309595,0.655444380311726,0.143785912675211,0.885669527331189,0.975462259150484
"13998","UBXN2B",35.0033408410264,-0.0490792959829239,0.34211804498677,-0.143457197602137,0.885929112902714,0.975462259150484

"13999","SEC23IP",102.343829515545,-0.0272399482873873,0.189935815441959,-0.143416596938303,0.885961176007729,0.975462259150484
"14000","MRPS35",145.982790692359,-0.0306860361017266,0.214007914266364,-0.143387389232407,0.885984241995843,0.975462259150484
"14001","HLF",110.665232334644,-0.0673952924587853,0.468027561134112,-0.143998554904491,0.885501610763228,0.975462259150484
"14002","RAPGEF2",70.4415376310095,-0.0355463314157244,0.24812781066724,-0.143258151192875,0.886086305367394,0.975504956237565
"14003","RPL7",8966.03718940051,-0.0288973342976002,0.202205463901716,-0.142910748997594,0.886360669242161,0.975597980461829
"14004","PGM5-AS1",2.4210604340435,-0.150636541232243,1.05354378879394,-0.142980807095532,0.886305339158565,0.975597980461829
"14005","CHCHD1",279.969479843958,0.0377949218820151,0.264281887641358,0.14300988319451,0.886282375766267,0.975597980461829
"14006","AC097662.1",11.5478075486537,0.066378428002304,0.465391016709474,0.142629371042934,0.886582899890512,0.975772911738701
"14007","AGK",40.1742361578104,0.0363962908106695,0.255697222757127,0.142341361467349,0.886810377390492,0.975953592309162
"14008","MRPS16",277.694459536985,-0.0269835715104817,0.190288177843028,-0.141803720106779,0.887235045546256,0.975965457244662
"14009","SAMD4A",169.757885023815,-0.0347627312459348,0.245085885703353,-0.141838976757768,0.887207196291916,0.975965457244662
"14010","MGRN1",92.5347945382625,-0.032163146202897,0.226784363312053,-0.141822591880556,0.88722013870106,0.975965457244662
"14011","TIMM22",123.413094312948,0.0421152223799074,0.297172516094613,0.141719775884318,0.887301353709166,0.975965457244662
"14012","AC022706.1",8.84886622614005,-0.0933828020589598,0.657059628540771,-0.142122264103105,0.886983432469175,0.975965457244662
"14013","P3H4",140.796321177597,-0.0348617862637628,0.245406198439347,-0.142057480558622,0.88703460308764,0.975965457244662
"14014","ZNF343",23.4631206991106,0.0513867236233847,0.362249718793015,0.141854419637925,0.88719499799347,0.975965457244662
"14015","BLCAP",179.437634321649,0.0334646272605467,0.236187877362877,0.141686472795265,0.887327660284394,0.975965457244662
"14016","SLC4A3",3.75874391985079,0.139033940133322,0.982144674059439,0.14156156807190З,0.887426325375668,0.976004138848205
"14017","TTF1",131.484798607563,0.0330323691076191,0.233474590122709,0.141481645134308,0.887489459243288,0.976004138848205
"14018","TMX4",432.013480585749,-0.0220883358806845,0.156210410559526,-0.1414011767946,0.887553024663952,0.976004413981654
"14019","PLPPR2",89.0104357291918,-0.0284772898860187,0.201666307144266,-0.141209953656993,0.887704082988929,0.976100894448559
"14020","ITPR1",27.0223481341496,-0.0608009785248624,0.431368548595698,-0.140949030064425,0.887910208353331,0.976126971952781
"14021","AC093627.5",6.40105855415268,-0.082549583568334,0.585709519479376,-0.140939460300578,0.887917768454226,0.976126971952781
"14022","CD300E",3.41718571363056,0.15315866460744,1.08594511128904,0.14103720622273 2,0.887840549783791,0.976126971952781
"14023","EEF1AKMT3",49.6669871231097,0.0347297861454711,0.246691338340

935,0.14078234922652,0.888041887454171,0.976193802688871
"14024","LMBR1",64.270039340952,0.0330836054042668,0.235417067302364,0
.14053188999154,0.888239757920236,0.97629780916971
"14025","TRPA1",2.59546872374905,0.152484564122867,1.08528196618756,0.
140502255518465,0.888263170522555,0.97629780916971
"14026","COPZ1",432.279053527849,0.0369410140996112,0.263093871608731,
0.140410013634028,0.888336046495074,0.976308295787934
"14027","CPLANE2",5.04291278503333,-0.0871095061997953,0.6216018430703
03,-0.140137142723921,0.88855163448509,0.976385030712046
"14028","RETSAT",81.6122232391642,0.0322808288071534,0.230443800601528
,0.140081133547055,0.888595886854316,0.976385030712046
"14029","GXYLT1",76.3997079335302,-0.0313535727381954,0.22370977870641
1,-0.140152893268661,0.888539190177637,0.976385030712046
"14030","TCEAL9",894.540707378433,-0.0320711280083684,0.22913678623086
4,-0.139964990065173,0.888687651927372,0.976416261900245
"14031","ABTB1",168.17409999386,0.0254936044555427,0.182498293460738,0
.139692289566682,0.888903118705941,0.976429690965406
"14032","PIGX",100.329288274778,-0.0274960296671406,0.19672814087773,-
0.139766631984947,0.888844378279817,0.976429690965406
"14033","STKLD1",4.72225146470806,-0.0998239827894145,0.71492376781971
7,-0.139628848952448,0.888953245735226,0.976429690965406
"14034","SRSF6",396.116409717564,0.0300122566440139,0.21477565494847,0
.139737702819319,0.888867236106673,0.976429690965406
"14035","BIN2",7.35849084026573,0.0939590523161779,0.673457841196624,0
.13951734847905,0.889041347904767,0.976456884784608
"14036","VMA21",127.832078103528,-0.02500373287245,0.179800441132091,-
0.139063801595909,0.889399732375846,0.976780911554122
"14037","KIAA1586",99.1791828727321,-0.0277162870509877,0.200234342421
622,-0.138419247746358,0.889909085937917,0.976950826218501
"14038","CLIP1",516.965908653997,0.0325049689980033,0.234321938350419,
0.138719273264945,0.889671987487687,0.976950826218501
"14039","DUOX2",2.78985908905347,-0.16970548209068,1.22631239197361,-0
.138386828023126,0.889934706568939,0.976950826218501
"14040","PGS1",48.0431640284834,0.0350533664271028,0.253162317599293,0
.13846202215049,0.889875282394495,0.976950826218501
"14041","SELENOO",72.8308492576404,-0.0279756684032006,0.2017331419009
94,-0.138676610791748,0.889705701372802,0.976950826218501
"14042","UPRT",37.1257393263352,-0.0431012453415267,0.311115905392383,
-0.138537582278755,0.889815569601249,0.976950826218501
"14043","ARCN1",265.769960775457,-0.0215903369300201,0.156166460445984
,-0.138252073257996,0.890041201680504,0.976998157367014
"14044","AL160006.1",14.7195976811838,0.0634702185486386,0.45984063390
8651,0.138026555002634,0.890219430552294,0.977054647345219
"14045","C1orf53",7.71779784246877,-0.0772353093160907,0.5593000015530
42,-0.138092810837881,0.890167067462528,0.977054647345219
"14046","ZBTB1",151.760512362896,0.0274300587851725,0.199020760377248,
0.137825112984084,0.890378636473895,0.977090245692681
"14047","SNW1",399.778669467747,-0.0253371881998683,0.183761096306672,
-0.137881133216489,0.890334361489824,0.977090245692681
"14048","AC104596.1",3.4172629530592,0.11757760625714,0.85422009596556

,0.137643221942979,0.890522394444828,0.977108883932452
"14049","AC009093.1",26.828900258986,-0.070806166161831,0.514257751880514,-0.13768614260633,0.890488471682707,0.977108883932452
"14050","TIMM9",259.474508957914,-0.0338740143823139,0.246535424685393,-0.137400190765854,0.890714480268629,0.977180536142688
"14051","CACNA1A",26.1165429902248,0.0512445822503075,0.372887530817548,0.137426376628778,0.890693783302532,0.977180536142688
"14052","RNF115",476.439534857234,0.0205348864047971,0.149745270499589,0.137132120008114,0.890926364196098,0.977188094050071
"14053","AC091978.1",2.60517730145046,0.13048556275093,0.952778134326534,0.13695272598081,0.891068162122364,0.977188094050071
"14054","POMK",5.32430033766613,-0.0977637381712802,0.713063863878248,-0.137103761841973,0.890948779031143,0.977188094050071
"14055","MSRB2",393.07309637232,0.0330534981971781,0.241761938571696,0.136719197374304,0.891252754713455,0.977188094050071
"14056","AC012470.1",5.51827386726499,0.0927499415084897,0.678491954785123,0.136700134547452,0.891267823177432,0.977188094050071
"14057","NAB2",64.2432468165031,0.0424476185566839,0.310514682448345,0.136700842040682,0.891267263929335,0.977188094050071
"14058","PLA2G4B",14.2553806028631,0.0642144605584749,0.467658218706881,0.137310664048702,0.890785241586009,0.977188094050071
"14059","UBL7-AS1",47.5916861613666,0.0388430940252924,0.284339862754293,0.136607979088947,0.891340669233884,0.977188094050071
"14060","CHD9",820.199713551321,0.0229909324344615,0.168005042319763,0.136846681010342,0.891151984594574,0.977188094050071
"14061","AP1B1",67.8099998178186,-0.0389495708948754,0.285157904900535,-0.136589483319641,0.891355289681352,0.977188094050071
"14062","TRABD2B",6.29271797969885,0.100644350282606,0.742340580397504,0.135577055788481,0.892155644777657,0.977851862621014
"14063","CYP21A2",6.04258245169096,0.0866609043850261,0.638809278705131,0.135660058915688,0.892090024107852,0.977851862621014
"14064","NFE2L3",3.1374353563843,-0.146375827855567,1.08024355079992,-0.135502616745247,0.892214495476131,0.977851862621014
"14065","ABCD1",31.4199396872708,0.0467551281694036,0.344929537903978,0.135549795049502,0.892177196752677,0.977851862621014
"14066","AL121821.2",10.296347506801,-0.0795810872995451,0.588054226354259,-0.135329504887536,0.892351358124092,0.977932332253866
"14067","AL353194.1",11.5636446996259,0.0600575902844458,0.444856512373973,0.13500440841913,0.892608388829511,0.9781444738613
"14068","AL355488.1",5.18098765193401,-0.0896749240787879,0.666309843842931,-0.134584420307509,0.892940460319915,0.978204091156979
"14069","ANKMY1",17.7224084547138,0.055366318119334,0.412854935105561,0.134105985932269,0.893318766313826,0.978204091156979
"14070","TIPARP-AS1",6.95482223077626,-0.0769620634983758,0.572119071228272,-0.134521059284298,0.892990559534141,0.978204091156979
"14071","RUBCN",45.790519316222,0.0436460468086733,0.325310359079418,0.134167405342349,0.893270199607995,0.978204091156979
"14072","CTNNA1",582.508334380813,-0.0161935700427144,0.12054475039394

8,-0.134336584461727,0.893136425197333,0.978204091156979
"14073","AC007938.3",9.82835369966136,0.0858209937471982,0.64020296189
9151,0.13405279084091,0.893360830055656,0.978204091156979
"14074","ETFRF1",227.616345558485,-0.032350373320721,0.240968473133652
,-0.134251476552196,0.893203721895832,0.978204091156979
"14075","COASY",97.6565762875565,-0.0269643350565079,0.200234559325426
,-0.134663742100008,0.892877741614998,0.978204091156979
"14076","FECH",75.9362049277204,-0.0272344702804409,0.202791929339194,
-0.134297604294143,0.89316724757888,0.978204091156979
"14077","CERS4",156.722676913561,-0.0296822144437602,0.22095949210636,
-0.134333285077758,0.893139034078572,0.978204091156979
"14078","RFXANK",146.078861623304,0.036534475893935,0.270943435181318,
0.134841709191019,0.89273702779974,0.978204091156979
"14079","SNTB2",873.599782954746,0.0271014388270833,0.202356591472877,
0.133929113105841,0.89345862874064,0.978241690605651
"14080","PYHIN1",31.2936360248665,0.0441774245039634,0.330104417665543
,0.133828637666774,0.893538081305927,0.978259199100204
"14081","USP48",220.136754973226,-0.0254907903525677,0.193406823697047
,-0.13179881591198,0.895143423147611,0.97843862550557
"14082","LAMTOR2",460.467842515836,0.0356156637794814,0.26678317407495
1,0.133500412471573,0.893797638085872,0.97843862550557
"14083","AL591895.1",34.7797483854748,-0.0439520194406026,0.3367717036
03656,-0.130509834912762,0.896163073558283,0.97843862550557
"14084","MBOAT2",73.2946802435176,0.0457648902693776,0.343858180496688
,0.133092341160161,0.894120352130549,0.97843862550557
"14085","LINC01943",14.576498904645,0.0722127649150223,0.5461177110741
45,0.132229304142121,0.894802923269619,0.97843862550557
"14086","EEF1B2",3483.00631077235,-0.032723898068228,0.246345029326532
,-0.132837663328097,0.894321767268589,0.97843862550557
"14087","MDH1B",2.95742729264013,0.115883897178238,0.870321631673702,0
.133150657137389,0.894074233173182,0.97843862550557
"14088","AC097468.3",5.76358728206291,-0.0839846305034851,0.6372733345
22387,-0.131787454383963,0.895152409947708,0.97843862550557
"14089","STK11IP",24.6784248341378,-0.0430990734866728,0.3255132710638
84,-0.132403429653762,0.894665202089682,0.97843862550557
"14090","PPP1R2",273.071464766024,0.0228034963835532,0.171600428471402
,0.132887176254071,0.894282608819899,0.97843862550557
"14091","ZNF330",179.993303878752,-0.0250518645769649,0.18995130526029
5,-0.131885719567105,0.895074684090956,0.97843862550557
"14092","NIPBL-
DT",79.1498599488283,0.0305202831259817,0.233037301072369,0.1309673729
72208,0.895801117644672,0.97843862550557
"14093","AL022068.1",3.05350818359652,0.122510064167546,0.942706270646
877,0.12995571153195,0.896601466536281,0.97843862550557
"14094","U91328.1",5.82768087303459,0.075022651767884,0.57776455300361
6,0.129849869428411,0.896685206780227,0.97843862550557
"14095","GTF2H4",55.2134439533598,0.0387178075810578,0.291079227087738
,0.133014670845568,0.894181777944054,0.97843862550557
"14096","AL646090.2",2.50275706494005,0.126049989870331,0.960383274636
429,0.131249672083314,0.895577803140999,0.97843862550557

"14097","COL12A1",1869.36139745144,0.0522041202103014,0.399164464870583,0.130783486018043,0.895946587028815,0.97843862550557
"14098","ZNRF2",60.7433966029014,-0.0399043178436401,0.300015059516171,-0.133007716039298,0.894187278205511,0.97843862550557
"14099","C7orf25",53.0542587642277,-0.0387899739721708,0.29268139054488,-0.132533106734105,0.894562638592807,0.97843862550557
"14100","MKRN1",260.997617265938,-0.0204730094804423,0.15651639401568,-0.130804249671068,0.895930161132438,0.97843862550557
"14101","TTI2",46.2490645498253,-0.0358690461831503,0.27590872934901,-0.130003303149491,0.896563813335876,0.97843862550557
"14102","VPS13B-DT",35.7241485224429,-0.0443726670972707,0.338038022080905,-0.131265313955276,0.895565429780667,0.97843862550557
"14103","LRRC24",10.3246192797677,-0.0643414079456773,0.48726999144912,-0.132044675590074,0.894948955088256,0.97843862550557
"14104","XPA",255.070545280257,-0.0254966781503299,0.19252419671973,-0.132433629563182,0.894641316382866,0.97843862550557
"14105","TRIM14",4.0293806821129,-0.105937189291972,0.813010831859818,-0.130302309810108,0.896327252687444,0.97843862550557
"14106","MRRF",55.81556993074,0.0340492263208749,0.262103301026228,0.12990765926091,0.896639484429543,0.97843862550557
"14107","C10orf25",8.96369278160039,-0.0734448499956481,0.562817280779193,-0.130495015885026,0.896174797173692,0.97843862550557
"14108","C10orf55",5.29993314467528,0.0892594060664489,0.683321710270713,0.13062574293313,0.896071377295669,0.97843862550557
"14109","ZBED5-AS1",39.5660284270867,-0.0421026819294763,0.323468005014578,-0.130160267095285,0.89643962935965,0.97843862550557
"14110","PLAAT5",26.8261686891714,0.0439505513355563,0.333785399985661,0.131673078982617,0.895242879942626,0.97843862550557
"14111","AVIL",21.7929760340816,-0.0673553467824148,0.517871976630526,-0.130061771676959,0.896517554937486,0.97843862550557
"14112","AC025165.5",2.91396268732085,-0.11703525086932,0.885069954492929,-0.132232769031654,0.894800182751835,0.97843862550557
"14113","NDUFB1",772.781634464111,-0.0345062466865912,0.261116445532338,-0.132148883293212,0.894866531643483,0.97843862550557
"14114","RASGRP1",8.39385158583348,0.087967455984285,0.664154673703396,0.132450255139769,0.894628166925653,0.97843862550557
"14115","TMED3",398.840517147513,-0.0345581550577398,0.264826106038947,-0.13049376277374,0.896175788534965,0.97843862550557
"14116","E4F1",57.1575515141856,-0.0294498871257906,0.223885185880554,-0.131540132992553,0.895348040831589,0.97843862550557
"14117","PDXDC1",148.7430938165,0.0330729423878726,0.252917800187762,0.130765578236565,0.895960753712606,0.97843862550557
"14118","AC133552.5",17.2641835235015,-0.0578947915636771,0.441396127267656,-0.131162889720078,0.895646452000269,0.97843862550557
"14119","INCA1",5.99776320075642,-0.0842404458498411,0.64813494179088,-0.129973621877373,0.896587296328138,0.97843862550557
"14120","INTS2",31.4170452832386,0.044438782232867,0.335411716179118,0.132490250308178,0.894596534171095,0.97843862550557

"14121","AC092329.4",16.0366587362415,0.0525520494679522,0.399506501978522,0.131542413471853,0.895346236946478,0.97843862550557
"14122","CEACAM19",2.80148635096588,0.116313079623665,0.889965228103577,0.130693959663476,0.896017410848941,0.97843862550557
"14123","ZNF583",32.8502571546459,0.0423164317318496,0.320076221447092,0.132207358423982,0.894820281032783,0.97843862550557
"14124","FP565260.6",10.1830442708935,-0.0754412733233824,0.565812155895571,-0.133332719237842,0.893930252394049,0.97843862550557
"14125","LIMK2",26.0143236935564,0.0436748659826412,0.335193899938222,0.130297317435343,0.896331202354667,0.97843862550557
"14126","TTLL1",42.7612935743228,-0.036164474233414,0.273588040199033,-0.132185874086838,0.894837273919692,0.97843862550557
"14127","AKAP17A",100.15126616389,-0.0247769488283369,0.190494097793729,-0.13006675332884,0.896513613632708,0.97843862550557
"14128","FANCF",29.2913480677956,0.04683189568718,0.36157965647281,0.129520272639292,0.896945984811429,0.978653904011055
"14129","AL445524.1",15.6246979614794,-0.0521923799459399,0.403414687526141,-0.129376499070966,0.897059742406337,0.978708750031402
"14130","KDM1A",154.843311123262,0.0217431893314945,0.16902749153867,0.128636999422784,0.897644888214368,0.97893487372005
"14131","AL390195.2",2.75896829302004,0.153397919855167,1.19661571196486,0.128193135290933,0.897996132492597,0.97893487372005
"14132","AC244033.2",13.1666720140576,-0.0539388996939165,0.4307205461554,-0.125229456025197,0.900341898261914,0.97893487372005
"14133","LINC01139",13.4804467241851,0.0810041924572885,0.64452630733461,0.125680195727425,0.89998507906641,0.97893487372005
"14134","FAM228A",2.43291153556521,-0.148376897903337,1.17076736906205,-0.126734739816167,0.899150349125538,0.97893487372005
"14135","KIF3C",24.4597877623503,0.0563436847684144,0.441267106989419,0.127686119984841,0.898397374821124,0.97893487372005
"14136","COMMD1",360.029046259766,0.0367417332122609,0.290226870702617,0.126596593634876,0.899259693134756,0.97893487372005
"14137","TMEM182",9.05454657463562,-0.0664025848042677,0.515995521079843,-0.128688296877664,0.897604296143561,0.97893487372005
"14138","ANKRD44",31.7010763251483,-0.0472150039601422,0.375931277607721,-0.125594774291195,0.900052699711507,0.97893487372005
"14139","GLB1L",49.6310650843565,0.0465386404261865,0.361076833638475,0.128888469407547,0.897445900644178,0.97893487372005
"14140","COL7A1",4.10346622054583,-0.104690677418003,0.82960427994121,-0.126193511713105,0.899578747211229,0.97893487372005
"14141","STIMATE",56.4647762091846,0.037784357481633,0.296464305738738,0.127449938323877,0.898584293397729,0.97893487372005
"14142","AC124016.1",9.3250498510779,-0.0667493780828874,0.525584250039485,-0.127000339294552,0.898940130006261,0.97893487372005
"14143","ARHGAP24",311.99046397871,-0.0314190181031457,0.251003157465485,-0.125173796299618,0.900385961584517,0.97893487372005
"14144","PDE4D",32.9820567447847,-0.0549086548347912,0.438596726480245,-0.125191665873649,0.900371815006183,0.97893487372005
"14145","PNRC1",4812.89626358045,0.0284305032980786,0.227677354549792,0.124871897577591,0.900624966684631,0.97893487372005

"14146","FAXC",4.83349246279647,-0.14075833174364,1.11468641889517,-0.126276170013046,0.899513318820613,0.97893487372005
"14147","SP4",40.2967832875811,0.0414491723186961,0.331466675693476,0.125047781144148,0.900485723297594,0.97893487372005
"14148","AC007036.2",5.03697086981969,-0.0917933509748912,0.733377893881444,-0.125165145746444,0.900392809869263,0.97893487372005
"14149","ALKBH4",82.3994371328373,-0.0319359208375532,0.252168245773043,-0.126645290883675,0.899221148583359,0.97893487372005
"14150","INSIG1",200.761408253993,-0.0343350159676371,0.272682049915955,-0.12591593754785,0.899798466749504,0.97893487372005
"14151","SLC39A14",149.361198679564,0.0432371649856933,0.345552545689758,0.125124718440107,0.900424814591965,0.97893487372005
"14152","LAPTM4B",197.403250367151,-0.0379924245454968,0.299721403877763,-0.126759130492367,0.899131043873391,0.97893487372005
"14153","KIF27",40.3965968997495,0.0446557859103222,0.349322908223148,0.127835263188053,0.898279343008214,0.97893487372005
"14154","ZMYND11",281.115737584004,0.0227470423741124,0.180367666620211,0.126114856395018,0.899641007664941,0.97893487372005
"14155","NUDT5",207.403623272571,0.0249372905389521,0.197607333234781,0.12619617972033,0.899576635332562,0.97893487372005
"14156","CUBN",12.7881123258038,-0.0584892193103982,0.455803683648623,-0.128321076394563,0.897894886438411,0.97893487372005
"14157","AL121749.1",3.95989780893134,-0.100736107151126,0.807264761476091,-0.124786949658163,0.900692219293455,0.97893487372005
"14158","IFITM3",5743.16618747783,-0.0279616516051065,0.222846835905507,-0.125474752609739,0.900147711556086,0.97893487372005
"14159","IPO7",171.770702286883,-0.0277249460222274,0.217075357445959,-0.127720374843236,0.89837026535438,0.97893487372005
"14160","CUL5",251.057493425848,-0.0232154068686911,0.180043221427621,-0.128943520809107,0.89740233946783,0.97893487372005
"14161","SORL1",6.98207639135427,-0.0886374436277794,0.695990448333767,-0.127354396658721,0.898659908454541,0.97893487372005
"14162","HOXC11",28.9535234086196,-0.0387346510013133,0.309635104828451,-0.125097737295562,0.900446174602547,0.97893487372005
"14163","DDIT3",319.993918382117,0.0421097206780564,0.329828037238742,0.127671743829272,0.898408752218072,0.97893487372005
"14164","UGGT2",152.421303249071,0.0268885224309875,0.212462248466003,0.126556706544928,0.899291264499617,0.97893487372005
"14165","SLC38A6",61.5433175092534,0.0284610884702836,0.227226255166663,0.125254400946793,0.900322150579561,0.97893487372005
"14166","SNRPN",326.198283173036,-0.0284996481345065,0.226679456291321,-0.12572664766709,0.899948307452195,0.97893487372005
"14167","LRRK1",113.004474259081,0.0290677789619135,0.226218529251843,0.128494244295759,0.897757852854546,0.97893487372005
"14168","FAM234A",172.857079662558,0.0272459434382811,0.217199906741366,0.125441782397838,0.900173811763025,0.97893487372005
"14169","TPSAB1",7.49532766632298,0.110174014491375,0.868381821876706,0.126872778443557,0.899041092158384,0.97893487372005
"14170","ARHGAP44",22.0562957764082,-0.0492804252671735,0.384989921116236,-0.128004455608319,0.898145446985393,0.97893487372005

"14171","TMEM98",649.279053292858,0.0274621889066487,0.217559727397259
,0.126228274116668,0.899551230857803,0.97893487372005
"14172","HSD17B1",10.887689770969,0.0570878581450003,0.443704219441864
,0.128661968139071,0.897625130243558,0.97893487372005
"14173","UBTF",360.064818445126,-0.0249874940695926,0.200022600139085,
-0.124923353922095,0.900584229442773,0.97893487372005
"14174","P2RY11",5.5511148834775,-0.0778339799996801,0.623046984406545
,-0.124924735931139,0.900583135329861,0.97893487372005
"14175","DOCK6",30.4356986680297,-0.0390245976918993,0.30725316073257,
-0.127011216414681,0.898931521034169,0.97893487372005
"14176","ZNF256",10.4057512684557,0.0669710902718034,0.529070120714906
,0.126582635551803,0.899270741195942,0.97893487372005
"14177","SNAI1",4.87315132530915,0.0920651983469307,0.728160174232865,
0.12643536629011,0.899387308521389,0.97893487372005
"14178","CSTF1",65.2351915628854,0.0303002176576681,0.237114923835725,
0.12778705434273,0.898317495204212,0.97893487372005
"14179","CBR3",454.530212812479,-0.0296515524284355,0.235111110935031,
-0.126117189062278,0.89963916120847,0.97893487372005
"14180","ADARB1",64.0402897392543,0.0472763315468411,0.369314585392205
,0.128011005838382,0.898140263302271,0.97893487372005
"14181","PRDX4",999.389405616874,-0.0381695666783705,0.298526730611591
,-0.127859795336158,0.898259928501082,0.97893487372005
"14182","TMSB15B-
AS1",10.1098379188837,0.0690199695957531,0.553125258537177,0.124781807
61133,0.900696290234931,0.97893487372005
"14183","FGF13",2.55357892642782,-0.14145677503232,1.12136402846451,-0
.126147059689455,0.899615516729985,0.97893487372005
"14184","IRAK2",5.91783510235685,-0.082954867257115,0.665934956539468,
-0.124569023509728,0.900864752993848,0.979048940172036
"14185","PANK4",35.2802443196383,0.0377183164855955,0.304526347926785,
0.123858959142228,0.901426948515806,0.979082824785134
"14186","TRAPPC12",161.137374677533,-0.0236301538021959,0.191273072940
782,-0.123541455359541,0.901678349071064,0.979082824785134
"14187","AC104695.3",3.4168622488827,-0.10157214855731,0.8227374401157
98,-0.123456334432786,0.901745749782393,0.979082824785134
"14188","RPN1",373.67880048364,-0.0182036089542842,0.147072040334413,-
0.123773416843152,0.901494680221896,0.979082824785134
"14189","SNX14",165.135356050102,0.0213756231818802,0.1723614807692,0.
124016242413832,0.901302414756376,0.979082824785134
"14190","THAP1",73.0864212473394,0.0273533995618515,0.221525267806032,
0.123477560066882,0.901728942769884,0.979082824785134
"14191","MFSD3",25.5168530487479,-0.048329857312041,0.391877982457388,
-0.123328840801349,0.901846703502512,0.979082824785134
"14192","CFAP300",31.342113790515,0.0447070409094289,0.360829312828485
,0.123900801071225,0.901393818673456,0.979082824785134
"14193","LINC02731",4.18933842423773,-0.09591783744283,0.7735875722688
3,-0.123990923434196,0.901322461655823,0.979082824785134
"14194","AC093525.4",10.473392681399,0.0748603511721649,0.602968392423
016,0.124153027111985,0.901194113333563,0.979082824785134
"14195","DHX38",142.039010113419,0.0280855520514973,0.227139456440457,

0.123648935731515,0.901593244648325,0.979082824785134
"14196","CEP76",22.76320843464,-0.0525254208785953,0.426122176325633,-0.123263758135077,0.90189823874579,0.979082824785134
"14197","KRI1",50.990482047168,0.0354370561368139,0.287682350610023,0.123181196419143,0.901963615244257,0.979082824785134
"14198","AKAP8L",119.764341809488,0.0216646038804509,0.174193938568886,0.124370595546776,0.901021853914606,0.979082824785134
"14199","ZNF146",128.437264394965,0.022404388774788,0.180588385416042,0.124063287476503,0.901265165885279,0.979082824785134
"14200","FITM2",39.4264046682975,-0.0376138327944033,0.302562208999799,-0.124317683027058,0.901063746878777,0.979082824785134
"14201","APOBEC3G",70.8865182376372,0.0319871824624716,0.259707992656029,0.123165953174329,0.90197568568107,0.979082824785134
"14202","AC139887.4",5.23579138142646,0.0915930234291147,0.74554374583519,0.122853989374572,0.902222720692066,0.979092823798465
"14203","AL359711.2",4.81348357405471,-0.082856133852133,0.675422361925052,-0.122673068768379,0.902365990769043,0.979092823798465
"14204","APOLD1",45.9048134256685,-0.0699279952916639,0.569239611777026,-0.122844569922613,0.902230179828015,0.979092823798465
"14205","ATF7",278.189781508842,0.0195186138192877,0.158623849489486,0.123049679364775,0.902067758402621,0.979092823798465
"14206","FES",90.927453745922,-0.0290011975452119,0.236306681114325,-0.122726947069182,0.902323324493185,0.979092823798465
"14207","SREBF2",71.3879692570276,0.0336634520154742,0.274286156983549,0.122731137384718,0.902320006190289,0.979092823798465
"14208","TM9SF2",429.135488679434,-0.016856453765931,0.137936318379573,-0.122204608358078,0.902736976870219,0.979288564282507
"14209","AC091057.2",3.25032110199735,-0.10585928960255,0.866185124616524,-0.122213238941752,0.902730141892073,0.979288564282507
"14210","AC002310.1",3.86343092416454,-0.0932405496123478,0.762368481295696,-0.122303783406522,0.902658435769986,0.979288564282507
"14211","AC080013.5",2.63465088392314,-0.118114516020761,0.967478151150819,-0.122084944120199,0.902831745531455,0.979322451436731
"14212","CASC3",168.27970843243,-0.0269102686920233,0.220574732607183,-0.122000685998551,0.902898474981586,0.97932592118218
"14213","RPE",103.63440553334,0.0263017666161584,0.215843759766075,0.121855580372875,0.903013395108885,0.979381656624461
"14214","CHML",40.7060906482968,0.0360418048141121,0.296180127896008,0.121688801575394,0.903145482371077,0.979456001881958
"14215","GMEB1",70.0362637268868,0.0272353498947851,0.225595296243019,0.120726585830257,0.903907600561845,0.979593339613389
"14216","PCAT6",12.0455236774431,0.0594072763777126,0.490916970744962,0.121012879810536,0.903680833556136,0.979593339613389
"14217","AHCTF1",152.516592480216,0.0295220357413666,0.244456619291742,0.120765949504252,0.903876421018184,0.979593339613389
"14218","SPDYA",2.6654592782452,-0.120314608816642,0.990945255226257,-0.121413981430459,0.903363143225804,0.979593339613389
"14219","OSGEPL1",35.2412675812832,-0.0343876347281541,0.284018097107047,-0.121075505675236,0.903631230067543,0.979593339613389
"14220","GIN1",34.4082893166938,-0.0394866086964459,0.326670054882973,

-0.120876119822466,0.903789157079729,0.979593339613389
"14221","KCTD9",170.032671972724,-0.0235495858369981,0.194175762052496
,-0.121279739489995,0.903469467095895,0.979593339613389
"14222","AP002381.2",2.92023718634393,0.113292640019043,0.937411951401
447,0.120856833380104,0.903804433441868,0.979593339613389
"14223","KMT5A",105.933000309143,-0.0229410228361625,0.189615952758993
,-0.120986776177641,0.903701509329876,0.979593339613389
"14224","TXNDC16",35.1272072616158,0.0400866334294044,0.33120014266135
3,0.121034469089563,0.903663733493395,0.979593339613389
"14225","THAP4",117.972883994524,-0.0239298193216553,0.198738066477674
,-0.120408836343105,0.904159291941434,0.979728348465993
"14226","SMN2",15.3540788527922,-0.0600094522439047,0.498169200426434,
-0.120459980650222,0.904118779561197,0.979728348465993
"14227","LINC01355",9.42466235977596,-0.09717334362117,0.8083650396588
69,-0.120209730571942,0.904317009792005,0.979777731049977
"14228","MCPH1",89.9832742305318,-0.0257984845001758,0.214741897275784
,-0.1201371731714,0.904374485695948,0.979777731049977
"14229","MLEC",451.430472944793,-0.0221603441635652,0.184622839857064,
-0.120030350419925,0.904459105601114,0.979777731049977
"14230","AC110597.1",4.82368872845754,0.0822224682199728,0.68484882363
0159,0.120059296859325,0.904436175500779,0.979777731049977
"14231","SDAD1",141.716278207988,-0.0260546939992473,0.217239990274899
,-0.119935072572399,0.904534581097712,0.979790637876553
"14232","NFYC",114.268773979714,0.0220842351177216,0.184713478507012,0
.119559413293618,0.904832172525949,0.979843151812898
"14233","C3orf67",11.9764735982369,-0.0645844481306553,0.5401696878320
81,-0.11956325870461,0.904829126183488,0.979843151812898
"14234","USP31",31.3151454385121,0.0378761728984629,0.316815128582608,
0.119552917399861,0.904837318589465,0.979843151812898
"14235","ACOX1",138.041751894556,0.0262439017180011,0.219400513510864,
0.119616409725046,0.904787019979759,0.979843151812898
"14236","KLHL23",23.2045747037654,0.0409741575401725,0.344060426557087
,0.119090003898993,0.905204050116549,0.980033742979815
"14237","KPNA2",150.656387607041,0.0554050059713853,0.464754211793111,
0.119213563998962,0.90510616076744,0.980033742979815
"14238","ZNF711",15.5574673823509,0.0526602460951418,0.441987400131074
,0.119144224653294,0.905161094046805,0.980033742979815
"14239","KYAT1",8.31371079524491,-0.0729807207199105,0.613902882882346
,-0.118879912042858,0.905370496757065,0.980076278617286
"14240","PPP2R1B",64.8327857550902,-0.0322646316306415,0.2712281130094
24,-0.118957549321299,0.905308987633222,0.980076278617286
"14241","COPG1",189.265437565573,-0.0235845858455925,0.199578625483605
,-0.118171902369024,0.905931452598627,0.980078845026611
"14242","SNX10",27.6582311913821,-0.0630568841377647,0.534335715041699
,-0.118009862269536,0.906059843528654,0.980078845026611
"14243","MKLN1-
AS",32.8953072275193,0.0390282264678557,0.329710218146988,0.1183712979
45205,0.905773466786607,0.980078845026611
"14244","AC067817.1",3.80450430936202,-0.0910479761564151,0.7716308562
87286,-0.117994213702772,0.906072242651588,0.980078845026611

"14245","ITPR2",77.5512491818592,-0.0355886189389337,0.300929542255644
,-0.118262297121698,0.90585983024507,0.980078845026611
"14246","MAP1LC3B2",4.92106590267316,-0.0921217050007301,0.77870801466
1576,-0.118300702273837,0.90582940097393,0.980078845026611
"14247","XPO4",41.7425057511083,-0.0299367987618443,0.252553245922227,
-0.118536582860089,0.905642510481354,0.980078845026611
"14248","FAM98B",86.8522518729482,-0.0350397154524755,0.29570913467267
1,-0.118493855427435,0.905676363456365,0.980078845026611
"14249","AC005332.5",24.1533523292682,-0.0389454677552337,0.3278426584
31966,-0.118793167251344,0.905439222095761,0.980078845026611
"14250","SLC9A8",26.6213233701201,0.0405027322841134,0.341600292258398
,0.118567616017951,0.905617922995989,0.980078845026611
"14251","NSDHL",45.5524433232276,-0.0312762013278209,0.264746425565066
,-0.118136444188306,0.905959547340507,0.980078845026611
"14252","AC012511.1",12.0802464717794,0.0605415088357957,0.51457691355
0686,0.117652982948743,0.906342622114055,0.980255621549238
"14253","FCHSD2",47.4853403624892,0.0368364871467655,0.313162332694031
,0.117627451647436,0.906362852672156,0.980255621549238
"14254","AC010210.1",2.96807591008635,0.10096309627757,0.8605617598471
4,0.117322313154495,0.906604643793451,0.980342378977101
"14255","CAVIN4",3.43948896309069,0.100729472237379,0.859536235627539,
0.117190489547933,0.906709103230079,0.980342378977101
"14256","PUS7L",168.047619880124,0.0215195055920148,0.183311494507849,
0.117393105379397,0.906548547406424,0.980342378977101
"14257","LRRC45",38.9761573530051,-0.0376557139432526,0.32156178631325
,-0.117102577314862,0.906778767388367,0.980342378977101
"14258","INSR",225.320455259405,0.0300013230441056,0.256323687445546,0
.1170446763742,0.906824650135224,0.980342378977101
"14259","GET1",170.994901693158,-0.0290009129044566,0.247282336177514,
-0.117278546267203,0.90663932529629,0.980342378977101
"14260","PCBP1-
AS1",85.0646996335786,-0.0315329524786011,0.272222831382184,-0.1158350
76427997,0.907783250651943,0.980959201968435
"14261","TBCCD1",33.778849564091,0.0361040938564081,0.310761148914715,
0.116179560998844,0.907510235008416,0.980959201968435
"14262","GSR",57.2985722199925,0.0303404558236058,0.262591434850144,0.
115542442733978,0.908015181285833,0.980959201968435
"14263","PRPF40B",31.6390664233503,-0.0343145570210515,0.2968606454447
96,-0.115591465381465,0.907976327202679,0.980959201968435
"14264","MAPK1IP1L",224.844332387719,0.0192777935883809,0.166408027533
459,0.115846536216559,0.907774168207555,0.980959201968435
"14265","ANKS3",73.1523281115142,-0.0286251665154653,0.2474200025667,-
0.115694633491682,0.907894559544778,0.980959201968435
"14266","LGALS3BP",2317.14633875325,0.0306915186315866,0.2650103094961
59,0.115812545896564,0.907801107238084,0.980959201968435
"14267","THOC1",95.5102857515781,0.024517493702958,0.212225590583456,0
.115525623632635,0.90802851172149,0.980959201968435
"14268","AC020911.2",10.3247788382639,-0.0722233338436871,0.6251526451
57621,-0.115529118213164,0.90802574199463,0.980959201968435
"14269","APOL4",52.6650857145267,-0.037087764727585,0.32104573560273,-

0.11552174850713,0.908031583061148,0.980959201968435
"14270","TNFRSF14",207.758879009082,0.0203200688119778,0.1763705820875
5,0.115212347611865,0.908276811872162,0.981155364751884
"14271","NIPAL3",30.575737203477,0.0354450037441758,0.308613611138705,
0.114852367053394,0.908562140742541,0.981394814627305
"14272","ODC1-
DT",4.24937970542737,0.0789213591397241,0.688929205072129,0.1145565590
17499,0.908796613827122,0.981510530522321
"14273","ZNF33A",114.16340769982,0.03425162988562,0.298882918807034,0.
114598820241493,0.908763114860242,0.981510530522321
"14274","SHOC2",204.857026762275,-0.0199432645161616,0.174346636656276
,-0.114388581842733,0.908929764952901,0.981585563034116
"14275","ATP1A1",627.055197042214,0.0265041615991558,0.232244951816723
,0.114121583232826,0.909141413051776,0.981745350766594
"14276","VPS26A",307.89198944611,0.019325599269296,0.169690319792654,0
.113887458594634,0.909327007469894,0.981809098628191
"14277","SPTY2D1",208.000305525071,0.0203496849616655,0.17868403280677
2,0.113886420862638,0.909327830107991,0.981809098628191
"14278","CROCC",166.531232809767,-0.0223264274635128,0.196521220055359
,-0.113608227433269,0.909548365034264,0.981836711406354
"14279","ALDH4A1",7.98519541869281,0.0628171405308695,0.56060565275757
3,0.112052278142179,0.910781955568786,0.981836711406354
"14280","CDC42-
AS1",3.89043105696526,0.0891083806963596,0.799408252038376,0.111467927
018699,0.911245297602014,0.981836711406354
"14281","DNAJB4",198.210200718975,-0.0258096871736326,0.23350483085301
5,-0.110531705401329,0.911987706636602,0.981836711406354
"14282","EFNA1",4.9675195725874,0.0696408351060725,0.626176111027321,0
.111216052288897,0.911445022714859,0.981836711406354
"14283","RUSC1-
AS1",5.10446433439453,0.0709020768484621,0.626167317648087,0.113231838
919306,0.909846754176573,0.981836711406354
"14284","EEF1AKNMT",81.24897524121,-0.0258663003272962,0.2312666126201
85,-0.111846236835652,0.910945325801793,0.981836711406354
"14285","AL391832.2",8.13730515711741,0.065857580533152,0.583016558712
168,0.112960051561187,0.910062226675634,0.981836711406354
"14286","HAT1",201.418302274311,-0.0210984578731691,0.188728904282611,
-0.111792403783447,0.91098801067068,0.981836711406354
"14287","MON1A",27.5097562956393,0.0368866277347529,0.327445272776306,
0.112649748832844,0.910308242173807,0.981836711406354
"14288","SRD5A3-
AS1",3.75588411833702,-0.0850438525774113,0.752152370169779,-0.1130673
19801458,0.90997718382827,0.981836711406354
"14289","AC146944.4",14.3469304734464,-0.0499835047030605,0.4437873935
46284,-0.112629392880326,0.910324381165478,0.981836711406354
"14290","SERINC5",69.3992561857514,-0.0300887445967351,0.2647026302970
46,-0.113669987196463,0.909499405026575,0.981836711406354
"14291","ZMAT2",320.049697139403,0.023378990159733,0.210969104910942,0
.110817127321094,0.911761363367549,0.981836711406354
"14292","DDR1",9.10459631291188,0.0578109056709592,0.514726970135906,0

.112313729462622,0.910574656144454,0.981836711406354
"14293","ZNF318",58.1146998560185,0.0332446076633768,0.29614479182644,
0.112257951451195,0.910618880882797,0.981836711406354
"14294","MRPS18A",180.111733763321,0.0325667850135276,0.28756424348308
8,0.113250467509681,0.909831985708306,0.981836711406354
"14295","AL122017.1",5.0026073049321,0.0763309545479986,0.685816148839
783,0.111299441806861,0.911378898030187,0.981836711406354
"14296","CCDC28A",113.922176083239,0.0247286163668873,0.22289463112870
8,0.110943077640161,0.91166148541319,0.981836711406354
"14297","TYW1B",26.352866805285,-0.047858476015636,0.431231048370432,-
0.110981053420173,0.911631371088986,0.981836711406354
"14298","NUDT18",55.5019902550441,-0.0389848694581196,0.34512998521975
8,-0.112957062926006,0.910064596096705,0.981836711406354
"14299","TNFRSF10B",68.5393213517866,0.037900328774419,0.3418745585631
92,0.110860337001104,0.911727098156667,0.981836711406354
"14300","IMPAD1",265.698824640488,-0.0166993843309185,0.15079454401708
5,-0.110742629580992,0.911820440348015,0.981836711406354
"14301","DECR1",501.445396907032,-0.0325224754429783,0.292480230058224
,-0.111195465883297,0.911461347038159,0.981836711406354
"14302","SLC31A2",136.867087731827,0.0291983135611692,0.26428543779679
6,0.110480220948152,0.912028535233707,0.981836711406354
"14303","NDOR1",5.2759192581997,-0.0689465808723243,0.618148603033679,
-0.111537226702376,0.911190347113198,0.981836711406354
"14304","CUTC",574.278318965045,-0.0326163461325033,0.290200152621039,
-0.112392587798173,0.910512132171353,0.981836711406354
"14305","AP000851.2",2.49682958224954,0.104872797782351,0.945436704362
797,0.110925244702693,0.911675626755984,0.981836711406354
"14306","AASDHPPT",148.251571347602,0.0216912035635869,0.1955524484874
7,0.110922689699673,0.911677652850365,0.981836711406354
"14307","CD3G",6.46642899052272,-0.0730119034741433,0.648605964385133,
-0.112567425344843,0.910373511667925,0.981836711406354
"14308","MARCH9",68.9038584368042,0.031713356958337,0.284763900894017,
0.11136719527571,0.911325172583735,0.981836711406354
"14309","LATS2-
AS1",5.47553631101202,0.104577867133944,0.936140839853523,0.1117116812
7897,0.911052016988209,0.981836711406354
"14310","NGRN",423.816143242837,-0.0212114187167641,0.187914506392328,
-0.112878026949548,0.910127256931505,0.981836711406354
"14311","TMEM159",635.977988755414,-0.0164479746182675,0.1472782873679
23,-0.111679561951844,0.911077485137689,0.981836711406354
"14312","AC007336.1",4.87627254315099,-0.0982342044743701,0.8691948970
23849,-0.113017465715373,0.910016708292059,0.981836711406354
"14313","TNFRSF11A",8.70762076844078,0.0683513833738369,0.611336302443
149,0.111806518115605,0.910976819223591,0.981836711406354
"14314","AC008946.1",4.11812779807693,-0.0804323125428415,0.7215734825
97795,-0.111467943989946,0.911245284144782,0.981836711406354
"14315","AC005498.3",4.54902909693939,0.0778156501016169,0.70345946370
7387,0.110618527600029,0.911918854755041,0.981836711406354
"14316","LINC00237",3.01511651414443,0.108872409830492,0.9597509725976
61,0.113438186507713,0.909683166629641,0.981836711406354

"14317","FAM207A",153.698702379343,-0.0288591648118083,0.259164040611083,-0.111354818916086,0.911334986448733,0.981836711406354
"14318","TCEAL8",648.515246302747,0.0250101861040924,0.226331331101378,0.110502536181744,0.912010838606981,0.981836711406354
"14319","TAZ",86.2080645580997,0.0243503855443383,0.216425909524118,0.112511416021679,0.91041791854065,0.981836711406354
"14320","CLCC1",121.194040449133,-0.0210037767076235,0.191460750416164,-0.109702780658538,0.912645095082596,0.981943415940297
"14321","NPRL2",65.6473123204583,-0.030987090397361,0.282514616029388,-0.109683140762309,0.912660671458382,0.981943415940297
"14322","HOXA3",33.1254662530751,0.0430847908126072,0.392962367110549,0.10964100997612,0.912694085442508,0.981943415940297
"14323","KIFC2",6.87220190181099,-0.0655991291956555,0.596548957039314,-0.109964368257763,0.9124376335054,0.981943415940297
"14324","ERC1",216.800356722608,-0.0284649341048818,0.25917946758705,-0.109827118520959,0.912546483666982,0.981943415940297
"14325","ATP6V0C",1076.00838221635,-0.0268540237550866,0.244946176956585,-0.109632344904269,0.912700957741977,0.981943415940297
"14326","N4BP1",197.018659497266,0.018561039244757,0.169179589673581,0.109712047892829,0.91263774526296,0.981943415940297
"14327","ABHD15",39.0072836553856,-0.0320799671971762,0.291888037722519,-0.109905042520697,0.912484683420141,0.981943415940297
"14328","ATAD5",12.6834415486174,0.0535384471827686,0.487504387638531,0.109821467335112,0.912550965550414,0.981943415940297
"14329","SMC5",234.771161425506,-0.0260836911214212,0.238303245406122,-0.109455878693422,0.912840915144406,0.982025452365903
"14330","LINC02227",3.98278815851781,0.0906972025531118,0.829313097806981,0.109364247101547,0.912913590291306,0.982035100791381
"14331","AC018816.1",3.17229573698035,-0.103207599619527,0.946113128061518,-0.109085897403187,0.913134360375488,0.982104565681348
"14332","TIMP4",51.5668671254496,-0.0491440349094794,0.45069824271559,-0.109039774846629,0.913170942635463,0.982104565681348
"14333","AL078587.2",2.3577787269546,-0.104293660792812,0.957158837281612,-0.10896170701303,0.913232862817962,0.982104565681348
"14334","CBY1",252.723479296112,0.0261920317111924,0.240378725176591,0.108961521831646,0.913233009696817,0.982104565681348
"14335","TMEM201",15.9906364956771,-0.0433597808955565,0.399827217499525,-0.108446296294494,0.913641678521123,0.982236685117443
"14336","AC079140.6",2.64376057468115,0.104339394658167,0.962983322389018,0.108350157507729,0.913717936831805,0.982236685117443
"14337","LRSAM1",29.8992619105626,-0.0309478656702887,0.285907564158667,-0.108244305327697,0.913801900828365,0.982236685117443
"14338","AC079866.2",21.8327313053694,0.0720484315209416,0.663506068151583,0.108587449277829,0.913529715910583,0.982236685117443
"14339","AL136419.3",8.73244956802553,-0.0671923128166913,0.619864942601956,-0.108398310984718,0.913679740881112,0.982236685117443
"14340","ZNF841",23.1429200773588,0.0403846780274741,0.372391257403411,0.108446901544,0.913641198433279,0.982236685117443
"14341","PDZD11",190.406299147118,0.0329486544882135,0.304273851648627,0.108286184664538,0.913768681213296,0.982236685117443

"14342","AEBP1",2840.00287551257,-0.0161467341289471,0.149635483764117
,-0.107907120174788,0.914069369022134,0.982437014788539
"14343","GINS4",5.00202525793805,-0.0723921316566928,0.671238081593435
,-0.107848665982781,0.914115738119495,0.982437014788539
"14344","TAPT1-
AS1",4.91684518430657,0.0751884060093136,0.701359068909125,0.107203869
376437,0.914627245804218,0.982849703316279
"14345","ZNHIT2",21.878867687908,0.0439366752611996,0.409801263389379,
0.107214592014209,0.914618739403345,0.982849703316279
"14346","AC027644.3",126.18822228498,-0.0299171695567765,0.27940606321
105,-0.107074160141537,0.914730146515298,0.982891761364374
"14347","PDSS1",14.7657323324704,-0.0431097957119799,0.402940328361133
,-0.106988039363841,0.91479846849471,0.98289666075458
"14348","GMPR",13.8722782797477,-0.0427528026159723,0.400000887652211,
-0.106881769355334,0.914882776280211,0.98291873406464
"14349","EPDR1",181.866811314128,0.0229692934599502,0.215180972336786,
0.106744073188778,0.914992016981113,0.982967589501976
"14350","FXR2",69.0657823131789,-0.0222811223869862,0.209594602261362,
-0.10630580247101,0.915339727974093,0.983272606740115
"14351","ZNF687",34.2833516713738,0.0345480119411482,0.328225473704163
,0.10525694898467,0.91617192288484,0.983329659183558
"14352","GJC2",23.9712629354897,0.0517263270656905,0.490451518065614,0
.105466748823011,0.91600545340117,0.983329659183558
"14353","TMEM232",5.35396784141801,-0.0689236407500486,0.6528361376088
34,-0.105575713076328,0.915918995192623,0.983329659183558
"14354","ETF1",225.404821469396,-0.0187656050935104,0.178107199079396,
-0.105361294717488,0.916089127412249,0.983329659183558
"14355","AC034213.1",7.09109734285764,0.0872856384430437,0.82544679952
7248,0.105743505811682,0.915785861188701,0.983329659183558
"14356","MCM7",80.5780356212356,0.03024474138841,0.285382068394818,0.1
05979824025128,0.915598360117612,0.983329659183558
"14357","IDO1",9.15415070260708,-0.0571244991886568,0.544503843774264,
-0.104911103643814,0.916446348159249,0.983329659183558
"14358","BNC2-
AS1",16.6549950499012,-0.0489438101262541,0.463039490165748,-0.1057011
57602636,0.91581946186425,0.983329659183558
"14359","AL353796.2",3.42368802328011,-0.0898645669990964,0.8556283526
67993,-0.105027570345096,0.916353931729665,0.983329659183558
"14360","RBM14",87.250038742797,-0.025282183902984,0.239613536968996,-
0.105512335499873,0.915969282313185,0.983329659183558
"14361","DDX47",13.0791104259875,-0.0526670833403488,0.502013825477016
,-0.104911619297146,0.916445938985388,0.983329659183558
"14362","RBM23",179.756374787176,-0.0181625271169001,0.172255720690082
,-0.10543932616077,0.916027212200364,0.983329659183558
"14363","PAPLN",96.8850422657917,0.0519259102160991,0.493539161068478,
0.105211327311257,0.916208122715217,0.983329659183558
"14364","ACYP1",109.969556141103,-0.0327640655238306,0.312419194685547
,-0.104872127196947,0.916477276256256,0.983329659183558
"14365","KIAA0556",26.6752895947797,0.0340061474000364,0.3226307555405
92,0.105402683457926,0.91605628683575,0.983329659183558

"14366","KIAA0100",127.175957687393,-0.0230096642366341,0.217661591780
25,-0.105713020144888,0.915810049658051,0.983329659183558
"14367","INE1",14.6759451953879,0.0487665581084763,0.464362866710915,0
.105018212274143,0.916361357325422,0.983329659183558
"14368","CEP85L",110.921147710441,0.0242685469075948,0.231983561395324
,0.104613218116083,0.91668272572136,0.983359700721625
"14369","SAAL1",30.7726722926162,0.0326507324850118,0.31240152881669,0
.104515277529805,0.916760444960783,0.983359700721625
"14370","METTL22",27.6851790630638,0.0350231663288793,0.33490895103623
5,0.104575187436809,0.916712904284936,0.983359700721625
"14371","CSNK1E",448.824101267699,-0.0160248312037561,0.15318053925264
,-0.104614014821598,0.916682093511268,0.983359700721625
"14372","AC025449.2",6.46401248701977,-0.0626455965333994,0.6024842460
60161,-0.10397881262964,0.917186162489618,0.983699577728836
"14373","PML",185.873890289821,0.0298172422367655,0.286827917209053,0.
103955160735046,0.917204932254074,0.983699577728836
"14374","DDAH1",17.0289323891894,0.0523707441570693,0.51769764280923,0
.101160870412477,0.91942275820993,0.983794312742258
"14375","AL121987.1",4.06562374947732,0.0777787508954492,0.77382641432
0042,0.100511884133334,0.919937948443195,0.983794312742258
"14376","SLAMF7",3.7624819902916,-0.0929169270980463,0.943684413385657
,-0.098461864771813,0.921565552276997,0.983794312742258
"14377","XCL1",4.84106738388454,0.102285191058031,1.03507857709742,0.0
988187692424861,0.921282165733759,0.983794312742258
"14378","AL356512.1",4.92710393656875,0.0701030191418726,0.70025126054
1305,0.100111235912209,0.920256015179844,0.983794312742258
"14379","CAB39",146.50639782763,-0.0219527747617908,0.211943805511354,
-0.103578279670999,0.917504025245479,0.983794312742258
"14380","PRRT3",23.378181069412,-0.0396530221680601,0.393616699895441,
-0.100740192625449,0.919756704550911,0.983794312742258
"14381","CMTM7",45.490787067404,-0.0325511939982246,0.314280788505776,
-0.103573604205929,0.917507735770188,0.983794312742258
"14382","ITGA9-
AS1",48.8436856236455,-0.0280568015621724,0.277227255238763,-0.1012050
62027571,0.919387678436168,0.983794312742258
"14383","KLHL18",40.5493893173686,0.0301121493372689,0.290829080094282
,0.103538990418383,0.917535205887775,0.983794312742258
"14384","LRTM1",5.2210114911935,-0.0914093982266206,0.885844530675735,
-0.103188985269111,0.917812981726219,0.983794312742258
"14385","COL8A1",105.288402485957,0.104906161703073,1.03291420459918,0
.101563286898336,0.919103321522759,0.983794312742258
"14386","P2RY12",3.54513973287046,0.0808310783364395,0.796810551958991
,0.10144328302093,0.919198578783781,0.983794312742258
"14387","CRIPAK",22.5537965117881,-0.0457055912130441,0.44831162682085
8,-0.101950492645393,0.918795971386477,0.983794312742258
"14388","PRKAA1",271.868983225059,-0.0178688796552009,0.17782250511621
3,-0.100487166365826,0.919957571022085,0.983794312742258
"14389","SNRNP48",90.4508729397167,-0.0228411938865553,0.2279198809325
44,-0.100215890746781,0.920172930533586,0.983794312742258
"14390","ZKSCAN4",26.8406462294654,0.0345061430815882,0.33591777767452

2,0.102721991436315,0.918183619329128,0.983794312742258
"14391","RGL2",131.456738748437,-0.0238149651132317,0.234008645723762,-0.101769595048827,0.918939559950837,0.983794312742258
"14392","MAPK13",20.005880992221,-0.0465223228224033,0.470794989734334,-0.0988165206444858,0.921283951117003,0.983794312742258
"14393","AGPAT4",96.6166864292014,-0.0231987026469545,0.23550254619759,-0.0985072264462502,0.92152953399591,0.983794312742258
"14394","AC004982.2",9.3411300468866,-0.0495804804767378,0.498295376013722,-0.0995001817463633,0.920741143604299,0.983794312742258
"14395","HOXA11-AS",21.4634990758811,0.0404156207452389,0.408127448194194,0.0990269606321808,0.921116863820643,0.983794312742258
"14396","AC016831.5",99.3046926046016,-0.046431653545674,0.454455350310746,-0.102169890868102,0.918621826270565,0.983794312742258
"14397","EZH2",17.6291944622956,-0.0517959361658549,0.521821694143494,-0.0992598367357486,0.920931966526123,0.983794312742258
"14398","CCDC107",554.147264528008,-0.0283468700990692,0.27424806264629,-0.103362152591136,0.917675549069066,0.983794312742258
"14399","GNE",69.8716794493317,-0.0290646306467282,0.280910012486644,-0.103465983250099,0.917593145998115,0.983794312742258
"14400","ABHD17B",42.8267920221018,-0.0279185541883904,0.27236324991389,-0.102504850405541,0.918355963064183,0.983794312742258
"14401","SLC31A1",66.4682037929539,-0.0241275611297535,0.244996321386677,-0.0984813200181607,0.921550104314941,0.983794312742258
"14402","AL356481.1",14.047546942257,0.0447307812979255,0.436605164823413,0.102451333382685,0.918398439843394,0.983794312742258
"14403","NDST2",63.4529776578815,-0.0242348850231777,0.236583315304772,-0.102436999802618,0.918409816530656,0.983794312742258
"14404","AL157394.3",12.3952030138137,-0.0471716424519215,0.461193036358989,-0.102281775163672,0.918533020747174,0.983794312742258
"14405","MARVELD1",338.936487524518,0.0181567762552686,0.180228321339789,0.100743191304752,0.919754324061354,0.983794312742258
"14406","PNPLA2",153.77431101726,0.0184514901916558,0.180485746909183,0.10232395120598,0.918572214867653,0.983794312742258
"14407","NRIP3",8.83327158297272,-0.0627078922865305,0.62564547296114,-0.10022911536423,0.92016243167628,0.983794312742258
"14408","TMEM134",150.600207980011,-0.0194677086514547,0.19501053904703,-0.0998290079427955,0.92048007807132,0.983794312742258
"14409","RDX",579.862787430982,-0.0124024390170532,0.125336295436545,-0.0989532918126837,0.921175355748593,0.983794312742258
"14410","ACAD8",51.0897712663631,-0.0278948065541816,0.272923207049949,-0.102207528834572,0.918591951908141,0.983794312742258
"14411","FKBP4",106.935179443114,-0.0274946789015785,0.278638448238207,-0.0986751077441883,0.921396233532069,0.983794312742258
"14412","REP15",4.08831809895479,0.0859337959161995,0.846451219458116,0.101522443279381,0.919135742438851,0.983794312742258
"14413","ASB8",219.846848819242,-0.0175518586887936,0.175597442588639,-0.0999550929105,0.920379977493135,0.983794312742258
"14414","MCF2L",76.9478726417738,-0.0308936531504526,0.305937547618597,-0.100980260157432,0.919566130179523,0.983794312742258

"14415","MIS18BP1",148.878090400538,0.0231818926532748,0.228251327888608,0.101563013313938,0.919103538688592,0.983794312742258
"14416","FRMD6-AS1",27.0878104213165,0.0435545905668275,0.441374287818949,0.0986794921427176,0.921392752278348,0.983794312742258
"14417","AL442663.3",3.71277266346338,-0.0930421232161911,0.935439045560604,-0.099463587347299,0.920770197585554,0.983794312742258
"14418","LINC02256",39.7950992924072,-0.0425796174542809,0.420213853607881,-0.101328447619468,0.919289734472657,0.983794312742258
"14419","ARPP19",273.736066451809,0.0144341527741359,0.139973495982721,0.103120613461835,0.917867245060541,0.983794312742258
"14420","SOX8",3.51790197558036,-0.0876993055919864,0.872230866616073,-0.100545978075996,0.919910882523835,0.983794312742258
"14421","UBE2I",812.223353999717,-0.0209159979636583,0.205486700740658,-0.101787599334986,0.918925268820975,0.983794312742258
"14422","TXNDC11",77.8148954663144,0.0288553050801991,0.281808077108785,0.102393463580748,0.918444371711315,0.983794312742258
"14423","ZNF821",30.3197751567694,-0.0297644372312557,0.295757052781333,-0.100638131707588,0.919837725630462,0.983794312742258
"14424","PIEZO1",94.617842985829,-0.0231407069649643,0.232395154202223,-0.0995748256645144,0.920681880682898,0.983794312742258
"14425","DPH1",3.65985460339046,-0.078950123249606,0.788465798702216,-0.100131322600872,0.920240068448893,0.983794312742258
"14426","SRR",34.8072504561439,-0.0406226990568386,0.396848070372024,-0.102363352853794,0.918468271018337,0.983794312742258
"14427","ZNF286A",63.5342970844652,0.0258501063439348,0.25503609745256,0.101358617866803,0.919265785399053,0.983794312742258
"14428","SLFN11",160.2902301436,-0.0212153391065565,0.214476235309888,-0.0989169689401874,0.921204195716697,0.983794312742258
"14429","VEZF1",257.957987334006,0.0186665362623207,0.187966212419075,0.0993079342403476,0.920893778947392,0.983794312742258
"14430","AP000897.1",3.21434821053304,-0.086556753225082,0.859379094136129,-0.100720105731791,0.919772650469544,0.983794312742258
"14431","AC010336.8",5.1376892334965,-0.0723040140297,0.728720463136973,-0.0992205072963752,0.920963192731972,0.983794312742258
"14432","MYO1F",15.9495163940972,0.0497898833819276,0.484402922573092,0.102786092035633,0.918132743743019,0.983794312742258
"14433","ZNF439",10.4897371252536,0.0512086404428144,0.520110581768682,0.0984572170569476,0.921569242685579,0.983794312742258
"14434","LINC01534",21.8222337336882,-0.0379914391555388,0.382154837125787,-0.0994137335569922,0.92080977897924,0.983794312742258
"14435","NELFCD",159.891929119366,0.0195916435301903,0.198052207367856,0.0989216115819473,0.921200509503551,0.983794312742258
"14436","TOM1",128.302704968665,-0.0221952923981632,0.223912470033673,-0.0991248606869698,0.921039133318905,0.983794312742258
"14437","SERHL2",12.4050732416961,0.0518126879358456,0.503053461706554,0.10299638483766,0.917965840176937,0.983794312742258
"14438","SLC25A43",115.168371683805,-0.0194930098028642,0.191370391111399,-0.101860113728446,0.918867709869198,0.983794312742258
"14439","PDZD4",2.61518908498256,0.105923923190219,1.06339376474567,0.

0996093137856165,0.920654499273171,0.983794312742258
"14440","IKBKG",83.2907243923921,-0.0223751237305059,0.218895175086061
,-0.102218441871589,0.918583289930761,0.983794312742258
"14441","NDUFV2-
AS1",16.8323841370477,0.0415589957682964,0.423230688370137,0.098194665
250623,0.9217777185068,0.983948724519238
"14442","ATR",83.8571855780122,0.0242789878272732,0.248033847366642,0.
0978857848839643,0.922022987794293,0.983950709756343
"14443","TLE4",146.374011114675,-0.0321617102367485,0.328387234975838,
-0.0979383691303191,0.921981232266876,0.983950709756343
"14444","MARCH5",170.252409963521,-0.0195351027325267,0.19960096721616
5,-0.0978707819154528,0.922034901228049,0.983950709756343
"14445","RTN4RL2",26.5880612000475,-0.0396165006588358,0.4045490009179
8,-0.0979275701310354,0.921989807401436,0.983950709756343
"14446","AP001267.1",2.57134003644302,0.0957297870807734,0.98109467864
6911,0.0975744636723546,0.922270202761002,0.984133682373034
"14447","LGALS8-
AS1",2.53965334349054,0.0854738402654904,0.877536153755783,0.097402072
7233509,0.922407098762142,0.984143509649669
"14448","AP001437.1",3.68612116877879,0.0721045021032831,0.73974193283
9897,0.0974725088605849,0.922351165005201,0.984143509649669
"14449","DSCC1",36.2805134735033,0.0313880186511735,0.323148453270911,
0.0971318857740575,0.922621659387879,0.984304303374916
"14450","TARDBP",198.733856824835,-0.0207991940318863,0.21451581260929
6,-0.0969587918899408,0.922759119513185,0.984314706753564
"14451","HLX-
AS1",5.23614518348607,0.101697648484269,1.04850644495178,0.09699286921
30706,0.922732057293608,0.984314706753564
"14452","CPT2",65.7100090903046,0.0233219325812123,0.241737901888962,0
.0964761106925005,0.923142446315102,0.984314920795967
"14453","AC011365.1",11.5364843389913,-0.056550285593487,0.58480634346
5291,-0.0966991658441942,0.922965302287445,0.984314920795967
"14454","KCNMB1",5.73392048413154,-0.0805805109320921,0.83456552251256
2,-0.0965538459934153,0.923080710727197,0.984314920795967
"14455","BTN2A2",22.0370657538393,0.0338521573621317,0.349869951252754
,0.0967564011739784,0.922919848239677,0.984314920795967
"14456","SERF2",8130.22644689851,0.0264208975711178,0.273733087800619,
0.0965206573432665,0.923107068333071,0.984314920795967
"14457","SCAF1",129.128728758699,-0.0181685106994385,0.187802316354705
,-0.0967427401966829,0.922930697227675,0.984314920795967
"14458","AC146507.3",4.40009209867468,0.080515683105098,0.835340075942
897,0.0963867117403834,0.923213445478888,0.98432253852933
"14459","ZMYM1",83.7845590584948,0.0197045213570639,0.204907265785165,
0.0961631169180848,0.923391023452543,0.984345050226315
"14460","AC124045.1",2.4688719179619,0.0957500733378017,1.008192743734
56,0.0949719921442039,0.924337073373163,0.984345050226315
"14461","TIPARP",461.464970056173,0.0366748344611694,0.387060665206633
,0.0947521609864191,0.92451168583695,0.984345050226315
"14462","GFM1",107.4655212871,-0.020317442276895,0.211884627968113,-0.
0958891754995676,0.923608591663808,0.984345050226315

"14463","GSTCD",26.5054605450315,0.0342563726377801,0.360364551046351,
0.0950603285986748,0.924266908522559,0.984345050226315
"14464","TBC1D22B",18.4442020962168,0.0386735072601596,0.4034684129056
58,0.095852626929686,0.923637619495698,0.984345050226315
"14465","AC102953.2",3.69651060837459,0.100337118080511,1.056555176392
28,0.0949662831837359,0.924341607969329,0.984345050226315
"14466","C8orf76",89.2621444228735,0.0233499566817777,0.24414095121239
1,0.0956412947759196,0.923805467022623,0.984345050226315
"14467","COMMD3",361.260043876537,0.0240691896215252,0.253514332598969
,0.0949421256572498,0.924360796188083,0.984345050226315
"14468","PSTK",21.7440492254898,0.0370097021808683,0.386750337236319,0
.0956940398432129,0.923763574692722,0.984345050226315
"14469","AMOTL1",103.080177161014,-0.0240306150314324,0.25203630012929
,-0.0953458490666032,0.924040126217685,0.984345050226315
"14470","LINC01852",17.0900030365825,-0.0411947538180288,0.43446082326
8763,-0.0948181092787399,0.92445930255354,0.984345050226315
"14471","LYSMD2",148.163500208474,-0.0242590275732883,0.25290371724885
4,-0.0959219889576305,0.923582530442262,0.984345050226315
"14472","FKBP10",556.304439287731,-0.0223934614554563,0.23481340267092
7,-0.0953670497541361,0.924023287247853,0.984345050226315
"14473","TXNL1",526.105405117527,-0.0246200587797064,0.259320840266628
,-0.0949405329490396,0.924362061270869,0.984345050226315
"14474","CDC34",121.963527090546,0.0210027669026928,0.22135862929555,0
.0948811752653683,0.924409209016182,0.984345050226315
"14475","TIMM13",583.304246720196,-0.0249054508997382,0.26023795722310
7,-0.0957026068199046,0.923756770462875,0.984345050226315
"14476","FEM1A",37.5449184980832,0.0317398745911041,0.334179774043087,
0.0949784429114247,0.924331949566898,0.984345050226315
"14477","ZNF784",10.8373742265303,-0.0426357979698572,0.44601469913640
5,-0.0955928090540754,0.92384397658934,0.984345050226315
"14478","PSMD10",108.305898309034,0.0166139249691243,0.173195652017573
,0.0959257624287166,0.923579533467873,0.984345050226315
"14479","CDC42-
IT1",6.78768877060668,0.0600325913647713,0.635792303559813,0.094421701
9121617,0.924774177184792,0.984368372270297
"14480","SKIV2L",37.889059653309,0.0306579793854203,0.324220671647834,
0.0945589904234138,0.924665124723048,0.984368372270297
"14481","AFMID",67.8193934235153,0.0282171701578522,0.298578435430139,
0.094505050631 6772,0.924707970579971,0.984368372270297
"14482","LIN7B",16.3594286381192,-0.0450081656209978,0.476766192523084
,-0.0944030141541937,0.924789021551634,0.984368372270297
"14483","TANGO6",25.7845979259973,-0.027738590389347,0.29424528652543,
-0.0942702964485709,0.924894444812464,0.984412612496315
"14484","DDX55",80.1845775544711,0.0198023089452174,0.210654088105311,
0.0940039147748116,0.925106046968937,0.984440325441303
"14485","EIF3C",27.8244548571304,0.041785090314244,0.444539579229219,0
.0939963329850057,0.925112069694629,0.984440325441303
"14486","DHX8",102.837143220454,0.0232814841479316,0.24740593516629,0.
0941023671573737,0.925027840008181,0.984440325441303
"14487","TM2D1",398.966543567288,-0.0188877351899538,0.201248849044313

,-0.093852637069218,0.925226217838562,0.984493832262127
"14488","TAMM41",55.658487526615,-0.027532753498762,0.293773936259501,-0.093720885689605,0.925330878919307,0.984537237613275
"14489","PTN",21.5875478111594,0.102145277543824,1.09267466670964,0.0934818758555213,0.925520747610382,0.984603335018222
"14490","RPP30",160.69252018457,0.0230679516474347,0.246669895645841,0.0935174987082928,0.925492448654333,0.984603335018222
"14491","HOXD9",9.68213373508125,-0.0525254967068005,0.564502316844402,-0.0930474422149776,0.925865871239554,0.98475591011175
"14492","YIPF5",361.171928006976,0.015529047734409,0.167381355018803,0.0927764489220705,0.926081161335183,0.98475591011175
"14493","EMC4",448.508986118256,-0.0193869024630859,0.208443314352743,-0.0930080320555539,0.92589718022727,0.98475591011175
"14494","AP001094.2",3.6599824859586,-0.0730126661522731,0.787042662069812,-0.0927683716156632,0.926087578418733,0.98475591011175
"14495","RASAL3",6.22439957992978,0.0615667575321559,0.661336960775269,0.0930943848352022,0.925828578317202,0.98475591011175
"14496","ITPKC",102.887546617031,-0.0332219451137793,0.358232687998794,-0.0927384524828484,0.926111347965621,0.98475591011175
"14497","MRPS26",315.839389570869,0.0255828701766804,0.275361374066076,0.0929065315113568,0.925977816798087,0.98475591011175
"14498","TMEM102",2.72603864276114,-0.0960234908099483,1.03651611338957,-0.0926406155867051,0.926189075902378,0.984770630779084
"14499","AL627309.5",4.28313036781956,-0.0716215464361012,0.786089593024486,-0.0911111749495841,0.927404253251567,0.984778763196905
"14500","PABPC4",371.285906626874,0.0187481890414824,0.20351338750721,0.0921226326735786,0.926600606638209,0.984778763196905
"14501","SF3B4",141.160136658813,-0.0229002529863791,0.248660514416405,-0.0920944486909189,0.926622999012814,0.984778763196905
"14502","OBSCN",11.0995028595495,-0.0452338887770073,0.49416627241944,-0.0915357670112572,0.927066887551695,0.984778763196905
"14503","RABL2A",45.5670886143521,0.0232044856795778,0.251077523019018,0.0924196057080754,0.92636466298811З,0.984778763196905
"14504","EXOSC7",205.731025368151,0.0228516342730462,0.247716864099626,0.0922490051539478,0.92650020351911,0.984778763196905
"14505","COMMD8",176.319982763444,-0.021106402467907,0.231192482995588,-0.0912936363433141,0.927259274336861,0.984778763196905
"14506","PKD2",349.02511503464,0.0246643823042864,0.269878711062249,0.0913906184270955,0.927182215964828,0.984778763196905
"14507","AC010226.1",121.448796502889,-0.029122354557947,0.319792012837878,-0.0910665475960805,0.927439713313075,0.984778763196905
"14508","VARS",70.0976948420568,0.0233301497733559,0.252538346558663,0.0923826028453719,0.926394061232131,0.984778763196905
"14509","GALNT12",157.427557909831,-0.0252712178784133,0.276750473832735,-0.0913140907346267,0.927243021978257,0.984778763196905
"14510","TAF1D",508.193020403672,0.0132975724458176,0.145424458406214,0.0914397247309901,0.92714319817483,0.984778763196905
"14511","RNF10",257.395680271484,-0.0132678319409647,0.145634741689316,-0.091103481127251,0.927410366608797,0.984778763196905
"14512","LYSMD4",32.2362757469748,0.0326203110317013,0.358135852094631

,0.0910836232700936,0.927426145284432,0.984778763196905
"14513","AC022167.2",5.50232560413867,0.059740217559716,0.656320912934
532,0.0910228767396829,0.927474413499362,0.984778763196905
"14514","ZKSCAN2",13.5895019662945,0.0420889224594945,0.46181550171019
5,0.0911379594310518,0.927382970872126,0.984778763196905
"14515","ADORA2A",4.89054326030516,-0.075445526801028,0.8176554506578
84,-0.0922705922395545,0.926483052665262,0.984778763196905
"14516","WBP2NL",2.60441431217222,0.0941921912621214,1.03133731063733,
0.0913301499815938,0.927230261872051,0.984778763196905
"14517","TIMM17B",110.083510496214,-0.0238127957794054,0.2590898747845
2,-0.0919094032493939,0.926770020374458,0.984778763196905
"14518","KDM5C-
IT1",2.3901093542937,-0.104745674511678,1.14252247396626,-0.0916793121
347136,0.926952834681925,0.984778763196905
"14519","HIST2H2AC",94.7805283707116,0.0252564634737439,0.279395960442
792,0.0903966665578021,0.927972005551231,0.984884907280469
"14520","SLC22A18",41.0106427802125,-0.03004080225107,0.33085429383051
,-0.0907976798586126,0.927653353642221,0.984884907280469
"14521","AP002807.1",7.15239275711884,-0.0653840050651846,0.7220395975
25906,-0.0905545974060497,0.927846509663087,0.984884907280469
"14522","AC026461.3",6.49022821725137,-0.0766528245789541,0.8479218670
17838,-0.090400811160283,0.927968712121273,0.984884907280469
"14523","METTL2A",75.80666730463,-0.0226883367381821,0.251064042900265
,-0.0903687221638306,0.927994211067478,0.984884907280469
"14524","AC020915.2",59.9108126071341,0.0211927707147068,0.23460399129
2563,0.0903342291746366,0.92802162038591,0.984884907280469
"14525","MMP24OS",365.562044685264,0.0230693002219531,0.25510499887970
4,0.0904306082721318,0.927945034446345,0.984884907280469
"14526","SLC35E2A",64.7017064080853,-0.0396904000737786,0.439969813253
837,-0.0902116437949338,0.928119031645897,0.984920478646668
"14527","DPP8",140.895104033133,-0.0163787667265639,0.181780641088174,
-0.0901018206807801,0.92820630241264,0.984945284758783
"14528","SLC25A19",29.0805992752997,0.0283800665324984,0.3156519432244
74,0.0899093673955813,0.928359237198196,0.985039760559622
"14529","AC009961.1",5.22701454137676,-0.0637810184489052,0.7124003765
99295,-0.0895297371309227,0.928660921657283,0.985076256632453
"14530","HIBCH",162.869818527225,-0.0169369140836104,0.188988538696835
,-0.0896187366725965,0.928590194607793,0.985076256632453
"14531","AP003071.4",7.30023667537183,-0.0543402151646649,0.6073974670
63242,-0.0894640134530015,0.92871315197142,0.985076256632453
"14532","AC092375.2",3.18296978472643,-0.0772081553785399,0.8599594392
2336,-0.0897811592698693,0.928461120441746,0.985076256632453
"14533","MAP1LC3A",371.596405841247,0.0179604639741759,0.2005625146672
61,0.0895504526554868,0.92864445918586,0.985076256632453
"14534","ZFX",231.285920163392,0.018495046150122,0.206993493318161,0.0
893508576218579,0.928803077125899,0.985103855366433
"14535","DTX1",119.65585295268,0.0290928445850261,0.326241164916824,0.
0891758849391168,0.928942130039556,0.985172253560315
"14536","NUSAP1",7.16126931064188,-0.0502643273002096,0.56407769346674
3,-0.0891088725584806,0.92899538616625,0.985172253560315

"14537","MGLL",514.955325724334,-0.0241778771027465,0.272491641333813,-0.0887288761754263,0.929297383169005,0.985424720475353
"14538","AK5",3.42542845013685,0.0812107970861368,0.918773281416668,0.0883904644690112,0.929566339883925,0.985642119226214
"14539","BNC1",3.20979802121818,0.0766129710580639,0.869973508901452,0.0880635677686393,0.929826152550913,0.985849793078776
"14540","PSEN2",26.6642727499943,-0.0270687313524028,0.30870475282624,-0.0876848545562854,0.930127157565973,0.985857281215045
"14541","HIST1H4E",3.4774492399685,-0.093369267745599,1.06364054072498,-0.0877827275011149,0.930049366219737,0.985857281215045
"14542","HCG25",11.5446315744348,0.0471233570896053,0.538131715118736,0.087568444240845,0.930219683649047,0.985857281215045
"14543","ADRA2A",20.8117220251501,0.101829329939113,1.16284153565363,0.0875693951556998,0.930218927832249,0.985857281215045
"14544","CHURC1",785.38203272225,-0.0149762669333381,0.171174098872,-0.0874914314258314,0.930280896046322,0.985857281215045
"14545","THAP10",12.1348320494015,-0.0463844468116546,0.528757641949566,-0.0877234542476435,0.930096477690473,0.985857281215045
"14546","C16orf72",141.274160120367,0.0214309453754881,0.244303337927734,0.0877226875296625,0.930097087093614,0.985857281215045
"14547","SPN",5.55609567137578,0.062052627674129,0.711421583249462,0.0872234257930435,0.930493919225532,0.985947468027329
"14548","ZNF30",9.87904514594733,0.0397918565704692,0.456154938544955,0.0872332034755503,0.930486147387034,0.985947468027329
"14549","AC009159.3",2.79959060847046,0.0771317841106291,0.886008227919252,0.0870553812934328,0.930627491248644,0.986021223286676
"14550","HYOU1",55.8004965882882,-0.0304662188319584,0.351274358692527,-0.0867305514281096,0.930885691384128,0.986226215443062
"14551","CATSPER2",8.41791125588208,-0.0674889200248869,0.778859075226254,-0.0866510029497721,0.930948923834706,0.986226215443062
"14552","TCHH",12.1863246038414,-0.0508234044129435,0.588955412440565,-0.0862941461091885,0.931232591847005,0.986261300987434
"14553","MRPS31",173.592091983202,0.0171275003613605,0.19844604896485,0.0863080945713067,0.931221503952085,0.986261300987434
"14554","TUBGCP3",73.4368160562606,0.0224963824620117,0.260577123551965,0.0863329142457334,0.931201774359015,0.986261300987434
"14555","ZNF587B",9.85756784447936,-0.0507221509557541,0.587828107094848,-0.0862873863014684,0.93123796535012,0.986261300987434
"14556","TMEM41A",106.99201157588,-0.0161000754727451,0.187056951916024,-0.0860704470367553,0.931410416269529,0.986274875242851
"14557","PFDN1",346.118551474034,0.0247723769738299,0.287796810372735,0.0860759260734902,0.931406060794601,0.986274875242851
"14558","PCYT2",34.4811884959463,-0.0240106681021286,0.279097099639692,-0.0860298015748849,0.931442726810602,0.986274875242851
"14559","ARL8A",208.425909841443,0.0176717838084498,0.205843206790387,0.085850702017314,0.931585100842397,0.986302070367082
"14560","IQCB1",66.4058498704509,0.019498387535116,0.228361281942107,0.0853839467412834,0.931956155197411,0.986302070367082
"14561","HIST1H1A",3.12490162591164,0.0782545049564314,0.91553393849369,0.0854741715912596,0.931884428396314,0.986302070367082

"14562","PDCD11",77.8657254526738,0.02482516631367,0.289768732690241,0.0856723431931068,0.931726888208588,0.986302070367082
"14563","ARL14EP",168.884726344167,0.0183052056278261,0.213336109462311,0.0858045347970497,0.931621801529807,0.986302070367082
"14564","NAA25",67.866478967453,0.0223218331270096,0.261479439413551,0.0853674505998379,0.931969269332254,0.986302070367082
"14565","HAGHL",26.4445667479358,0.037627114427681,0.440838021096844,0.0853536052404496,0.931980276157439,0.986302070367082
"14566","TBC1D3L",2.4075194453178,-0.100314532796777,1.17029508882919,-0.085717297931358,0.93169115097821,0.986302070367082
"14567","RAI14",27.5281560894795,-0.0351629915475908,0.413995884628669,-0.0849356064955334,0.932312584163553,0.9864031068289
"14568","CTSB",854.479983656469,0.0134866685364177,0.159133650701547,0.0847505758647602,0.93245968684468,0.9864031068289
"14569","UCK1",102.48653987034,-0.018326871443888,0.216115046677526,-0.0848014598041121,0.932419232967056,0.9864031068289
"14570","ZNF675",45.3471030494386,0.0242576096746642,0.286001375090985,0.0848164092460994,0.932407347857206,0.9864031068289
"14571","PHKA2",21.8357627268144,-0.0279642563243684,0.329266176639871,-0.0849290279667985,0.932317814172673,0.9864031068289
"14572","RTL3",2.85515418983344,-0.080114871713468,0.944037061797625,-0.0848641170516274,0.932369419284149,0.9864031068289
"14573","COMMD10",137.002509937371,0.0205703363544928,0.244207805233628,0.0842329193156363,0.932871245355178,0.986635351433967
"14574","C11orf74",168.902085514025,-0.0233278246263252,0.276764034110686,-0.0842877749678838,0.932827631975581,0.986635351433967
"14575","AC025034.1",2.73872885127154,-0.0765350071611941,0.908608367688894,-0.0842332184941968,0.932871007490572,0.986635351433967
"14576","PCDHB11",34.5442019217481,0.0371897351574536,0.442402956837638,0.0840630348026861,0.933006314533034,0.986642816665069
"14577","FAM98C",105.260164061219,-0.0182821106749278,0.217312625117025,-0.0841281571426541,0.932954537831706,0.986642816665069
"14578","CASP8AP2",160.754993315477,0.0154044914057227,0.183762584143238,0.0838282258466465,0.933193006017952,0.986704862320237
"14579","GCH1",22.6834881604342,0.049194346693433,0.586781473087852,0.0838375936352505,0.933185557822892,0.986704862320237
"14580","ISCA2",139.704161123645,0.0230683427794525,0.275527521936365,0.0837242777684483,0.933275654044198,0.986724568387607
"14581","HADH",131.609709197513,0.0177140185450514,0.212688602580226,0.0832861673364456,0.93362399894554,0.986839883463998
"14582","AL662884.3",8.28853927655918,0.0510879022459103,0.614746482724957,0.0831040171542835,0.933768831670832,0.986839883463998
"14583","AC090739.1",2.5561526260542,0.0869315287156208,1.04260857931508,0.0833788733761704,0.933550286613229,0.986839883463998
"14584","POLR3A",61.8165589034065,-0.0200869602999245,0.241443416944935,-0.0831953115727553,0.93369624064047,0.986839883463998
"14585","OSBPL8",759.690632656792,0.0154142445534869,0.184929666801973,0.08335192952028,0.933571710125899,0.986839883463998
"14586","AC011447.3",6.57341224122401,0.0499426669777421,0.600629130779433,0.083150590636077,0.933731799578428,0.986839883463998

"14587","OSCP1",46.3695870582369,-0.0248745977171471,0.301326720490835
,-0.082550255339548,0.934209156685713,0.987069267189191
"14588","RNPC3",112.475043373114,-0.0243039599037036,0.294162674299286
,-0.0826208150357523,0.934153049854813,0.987069267189191
"14589","LINC01142",3.30670696928168,-0.0943272208302036,1.14659770649
007,-0.0822670587044476,0.934434348946721,0.987069267189191
"14590","TGFB2",68.1317624620128,0.0341564301329422,0.41282281479114,0
.0827387172150915,0.934059298513984,0.987069267189191
"14591","HYAL2",127.237701807085,-0.0252505939314959,0.306256607864143
,-0.0824491399796903,0.934289560980197,0.987069267189191
"14592","AC005229.4",13.3225456603709,-0.0336319191515221,0.4085123514
34161,-0.0823277901719515,0.934386056064287,0.987069267189191
"14593","SERPING1",5982.15793111323,0.012814143294815,0.15589948996773
8,0.0821949019683567,0.934491727369521,0.987069267189191
"14594","FAM234B",15.2212036152158,0.0371850371783302,0.4524451885037,
0.0821868330643683,0.934498143714502,0.987069267189191
"14595","CYLD",121.281182064702,-0.020742635285685,0.252879808208996,-
0.0820256683702557,0.93462630182181,0.987136995038245
"14596","KRCC1",275.119664154881,0.0109509758541796,0.133901486112066,
0.0817838260959585,0.934818617889741,0.987171365142063
"14597","MAK16",52.5125704878296,-0.0213005825294027,0.260413112532726
,-0.0817953532456085,0.934809451266571,0.987171365142063
"14598","CLK3",120.293435713665,0.0157339299440784,0.192480097752494,0
.0817431522936479,0.934850962591232,0.987171365142063
"14599","AC096586.1",3.62347106790465,0.0884335330236382,1.08572193595
884,0.0814513643822984,0.935083001900755,0.9872160298597
"14600","CWF19L1",31.1438658821694,0.0234165070360023,0.28738850358543
2,0.0814803193024777,0.935059975752529,0.9872160298597
"14601","TLE3",152.596557156455,-0.0176042094578092,0.21619084193766,-
0.0814290249301377,0.935100767192739,0.9872160298597
"14602","GNAO1",8.03924128621645,-0.051105421369764,0.628078933278246,
-0.0813678323885507,0.935149430295903,0.9872160298597
"14603","IQCC",7.39881731975777,0.044274040714593,0.54629204603964,0.0
810446372696781,0.935406453826842,0.987352128577155
"14604","FAM98A",104.014747196473,-0.0173119968258771,0.21356859524497
1,-0.0810605922936356,0.93539376530687,0.987352128577155
"14605","VKORC1",1343.3142396369,-0.0209134676669995,0.258325553163087
,-0.080957796899776,0.935475515481614,0.987357416716815
"14606","NCAPD3",29.647990975215,-0.0243064954626325,0.300605233095416
,-0.0808585240261514,0.935554464944659,0.987373139608512
"14607","ABHD14A",387.956529669408,0.0228934868011723,0.28470841638943
,0.0804102916643606,0.935910941847055,0.987392038946908
"14608","ZDHHC11B",11.8314325948889,-0.0693964133728027,0.863763889555
979,-0.0803418783904895,0.935965351705447,0.987392038946908
"14609","ARSB",27.6612710960557,-0.0272669047531713,0.339680451748323,
-0.0802722223573054,0.936020750251779,0.987392038946908
"14610","FURIN",93.0092570071368,0.0147737828570645,0.183545975480737,
0.0804909114371455,0.935846824404812,0.987392038946908
"14611","FXYD1",1684.93809330921,0.022744812032017,0.283023437919758,0
.0803637048549512,0.935947992836376,0.987392038946908

"14612","ARFGAP1",60.6912833433626,-0.0197757094748857,0.245870641894725,-0.0804313574101014,0.935894188079033,0.987392038946908
"14613","TBL1X",105.882626054617,0.0190800577434417,0.237535391283131,0.0803251155138362,0.935978683458406,0.987392038946908
"14614","SHLD2",137.359353753753,-0.0143623100724834,0.179401045375373,-0.0800570032489622,0.93619191937298,0.987505025122108
"14615","HGS",159.99253857641,0.0154488401826152,0.193381526503434,0.0798878799953049,0.936326429381781,0.987579330066381
"14616","SHISA5",376.509044411887,0.0144792388856317,0.183166563457615,0.0790495743999815,0.936993191731522,0.987927710568382
"14617","POLR2J3",233.56450987122,-0.0208497978321038,0.263623800975234,-0.0790892087701234,0.93696166780203,0.987927710568382
"14618","ETFBKMT",50.0868499758998,-0.0202287637210159,0.256095509630064,-0.0789891386625124,0.937041262161551,0.987927710568382
"14619","ELF1",412.151717515151,0.0165528399836632,0.209053789670053,0.0791798130509295,0.936889601024704,0.987927710568382
"14620","SLC9A3R2",62.4272393540996,-0.0292290725794461,0.368787163529139,-0.0792572938269762,0.936827973986132,0.987927710568382
"14621","RAB12",109.733694766111,0.0236341672800411,0.297941105421546,0.0793249633903384,0.936774150947472,0.987927710568382
"14622","FANCL",48.1015782377351,-0.0213084907322279,0.270526508934589,-0.0787667382991293,0.937218160812843,0.988013072038063
"14623","IFITM2",1759.00766715853,-0.0192432125591305,0.244432165679375,-0.078726187716113,0.937250415336529,0.988013072038063
"14624","RAPGEF6",69.5952580402813,0.0217730536792679,0.277331003316184,0.0785092666125199,0.937422959285629,0.988127387676968
"14625","AC006059.5",2.4726744623735,-0.0752985398057159,0.967348135545119,-0.0778401663670791,0.937955195220845,0.988414362280836
"14626","UBE2K",377.931798359147,-0.0114562479359991,0.147211543789639,-0.0778216683358051,0.937969909878704,0.988414362280836
"14627","AC008966.1",6.50473516937452,-0.0549742615248085,0.711361548406663,-0.0772803388768231,0.93840053142913,0.988414362280836
"14628","ZNF484",22.9707197567726,0.0253770222014076,0.327842777028212,0.0774060738242947,0.938300509069158,0.988414362280836
"14629","PRKCQ-AS1",2.8733203414893,0.0671553709665548,0.861663186535648,0.0779369155093601,0.937878234374683,0.988414362280836
"14630","PAWR",52.2060909807814,-0.0437374818958823,0.564624253151952,-0.0774629882647135,0.938255233857044,0.988414362280836
"14631","AC108134.2",36.1669626452384,-0.03001189370383,0.386917665748718,-0.07756661522744,0.938172799530981,0.988414362280836
"14632","MYO15B",51.0746568882916,-0.027566834703194,0.355581905836618,-0.0775259771397378,0.938205126688947,0.988414362280836
"14633","CD7",7.56459703000407,-0.0539428805727268,0.692721191652959,-0.0778709836261963,0.937930681014605,0.988414362280836
"14634","MBD1",101.000011175894,0.0155447588936187,0.201070393558214,0.0773100336580291,0.938376909093539,0.988414362280836
"14635","RANBP3",117.994574753037,-0.0150715392740323,0.193975330523612,-0.077698227054691,0.938068104449678,0.988414362280836
"14636","DDX10",74.7026616589238,0.0172216704067466,0.22352779906082,0

.0770448708353304,0.938587849261154,0.988544116996494
"14637","NEMP2",19.4611958241887,-0.027182117179275,0.353722420422098,
-0.0768458983935439,0.938746137027312,0.988575741376965
"14638","AC048382.6",6.96476206042787,0.0441925635045076,0.57449934776
6089,0.0769236095329753,0.938684315500993,0.988575741376965
"14639","COPS4",225.21761830338,-0.0165852504279898,0.217610787287739,
-0.076215203458915 6,0.939247887259561,0.988622566108187
"14640","ITGA1",5.68900482647198,-0.0572719998104294,0.749534050700871
,-0.0764101374138716,0.939092804696097,0.988622566108187
"14641","VSTM5",2.36311195006746,-0.0759477057628387,0.997392574326239
,-0.0761462514538401,0.93930274358922,0.988622566108187
"14642","MSANTD2",53.8430770716406,0.0170648449058718,0.22493810742612
8,0.0758646238342522,0.939526801809756,0.988622566108187
"14643","AL079343.1",29.6562946576271,0.027833608697237,0.366839198273
39,0.0758741400271345,0.939519230807725,0.988622566108187
"14644","AC020765.2",9.812601279947,0.0411354223160992,0.5436776805909
03,0.0756614144457625,0.939688474772439,0.988622566108187
"14645","MAP3K14",33.2920110213947,-0.0257214905656246,0.3387159995270
02,-0.0759382214053758,0.939468248351207,0.988622566108187
"14646","CEP112",79.78617269879,0.016254098762117,0.211970115284419,0.
0766810865782065,0.938877250957228,0.988622566108187
"14647","AC132872.1",22.5901395972774,-0.0275043364039331,0.3610848691
31213,-0.0761713900394381,0.939282743984589,0.988622566108187
"14648","WIZ",88.2429027643788,0.0147266837378169,0.19408034670855,0.0
758793148691763,0.939515113749753,0.988622566108187
"14649","LINC00665",25.871338177889,-0.0363501720776999,0.478259117850
237,-0.0760051836357935,0.939414974184702,0.988622566108187
"14650","PIGP",291.130742592304,0.0256313913030096,0.337083081305709,0
.0760387949573886,0.939388233613352,0.988622566108187
"14651","BX890604.2",2.75654056655402,-0.0668583839872688,0.8823050914
46043,-0.075776944546123,0.93959655896193,0.988622566108187
"14652","MAGED2",694.281755206813,-0.021989445873579,0.290625186241639
,-0.0756625609705276,0.939687562592765,0.988622566108187
"14653","AC024909.1",3.1783578349897,-0.0724379331190653,0.95980544996
1806,-0.0754714751014884,0.939839592363516,0.988714073314925
"14654","ANKRD36",71.6666897661482,0.024735186672704,0.33329957322619,
0.0742130763423383,0.940840841127907,0.988822633530148
"14655","ZNF35",20.9260774246942,0.029885097094665,0.402374762327199,0
.074271798066 6078,0.940794116918849,0.988822633530148
"14656","PDHB",217.477668825426,-0.0164716825047871,0.221300715766055,
-0.0744312211000705,0.94066726684988,0.988822633530148
"14657","MED12L",2.65161838231768,0.0639092991282121,0.85791499504506,
0.0744937429667556,0.940617519724576,0.988822633530148
"14658","SORCS2",31.802914499995,-0.0412015756710751,0.55410198692143,
-0.0743573866247793,0.940726015440207,0.988822633530148
"14659","TEC",4.42216486713587,-0.0552701347895841,0.736558864729282,-
0.0750383132105786,0.940184228274801,0.988822633530148
"14660","ARMH3",46.2331476187559,0.0188253420275003,0.252410367857571,
0.07458228513863 2,0.940547069273144,0.988822633530148
"14661","AC016957.2",3.81914310134812,0.0613788860819565,0.82439599940

0093,0.0744531585871613,0.94064981169977,0.988822633530148
"14662","LACC1",26.9350531965544,0.0254794090325977,0.342092892860193,
0.0744809657387729,0.940627686234597,0.988822633530148
"14663","RCN2",401.637493221365,-0.0156263328038159,0.207735030100735,
-0.0752224253956511,0.940037742015183,0.988822633530148
"14664","CORO1A",28.0970759395332,0.0342045743451184,0.459799515526292
,0.0743901922253386,0.940699912651213,0.988822633530148
"14665","TAF1C",44.5003416720773,0.021460915137875,0.287106166468061,0
.074749056775353,0.940414375157532,0.988822633530148
"14666","DBP",125.173793826774,-0.051614471215948,0.692806961776636,-0
.074500508891521,0.940612136257055,0.988822633530148
"14667","CST3",18363.7084157985,-0.0217530210736484,0.289987730225743,
-0.0750135912878611,0.940203898077661,0.988822633530148
"14668","OXSM",18.1903064123414,0.0330477125197915,0.446020628449466,0
.0740945831018572,0.940935125473379,0.988854305915744
"14669","POLR3E",62.5498853018594,-0.0172435677886302,0.23308480543308
7,-0.0739798021436468,0.941026456767903,0.9888828707531
"14670","RAP1A",566.377671499285,0.0111714654370364,0.152084575826372,
0.0734556109739251,0.941443565915063,0.989118904619732
"14671","TBC1D23",197.510928781027,-0.0139034321802144,0.1890882270420
95,-0.0735288092638319,0.941385319639392,0.989118904619732
"14672","HSPH1",181.380999595096,-0.019020501101461,0.258840625279879,
-0.073483445965619,0.941421416663981,0.989118904619732
"14673","DRAM2",254.297901003851,0.018780289457562,0.256776946708576,0
.073138534040115,0.941695878024483,0.989181734906126
"14674","SLC36A1",36.3032315981548,-0.0204129038231484,0.2790492525452
95,-0.0731516162002082,0.941685467856455,0.989181734906126
"14675","NUP205",39.7475860441555,-0.0197739571180577,0.26978607416544
,-0.0732949511172019,0.941571409320418,0.989181734906126
"14676","CEP78",53.7575930886843,0.0243361395959517,0.33367752810019,0
.072933109204299,0.941859346757154,0.989218629846803
"14677","SNX12",75.9637262602986,-0.0161976619270052,0.221990584769695
,-0.0729655356501246,0.941833542946919,0.989218629846803
"14678","TDG",177.230831191137,-0.0137615718764905,0.189378444740614,-
0.0726670445273708,0.942071073849344,0.989373593363376
"14679","AL139089.1",3.06135730610473,-0.0681368861900435,0.9424847443
99154,-0.0722949486397062,0.942367184580994,0.98937801443727
"14680","CMA1",4.77181685732138,0.0630401757291448,0.869787094386666,0
.07247770878l823,0.942221744621781,0.98937801443727
"14681","CGNL1",8.2251375178789,-0.0627019310193069,0.864869340923775,
-0.0724987325280075,0.942205014112666,0.98937801443727
"14682","UBALD2",208.729908982731,0.0176344078215271,0.244046167445069
,0.072258491113147,0.942396197598601,0.98937801443727
"14683","MPST",317.870978723332,0.0163563890152508,0.226269249848378,0
.0722872817504415,0.942373285909931,0.98937801443727
"14684","AC079807.1",5.88181023212505,-0.0444538232564801,0.6169381231
35654,-0.0720555621211068,0.94255769054277,0.989451125464066
"14685","FBXW4",82.2630563710718,-0.0169520950446508,0.23541414576853,
-0.0720096704015353,0.942594211964957,0.989451125464066
"14686","ZNF143",79.0759338387268,-0.0206577547731995,0.28727840449978

1,-0.0719084847646988,0.942674737666782,0.989468274624366
"14687","CLMP",598.684516934878,0.0134913064843608,0.187868613949323,0
.0718124555280962,0.942751160336517,0.989481115039654
"14688","AC139768.1",8.96254011641057,-0.0356468553250516,0.4969485586
90072,-0.071731479449331,0.942815603704773,0.989481381475291
"14689","CIART",4.32771953602296,0.0878804453922917,1.22809953150133,0
.0715580807077252,0.942953601259551,0.989509642707611
"14690","PHYHD1",256.085559984236,0.0214141190649436,0.299625346655654
,0.0714696513628198,0.943023977478073,0.989509642707611
"14691","SPRYD4",31.2950040071696,-0.0238172153161524,0.33331457085944
6,-0.0714556680037724,0.943035106131529,0.989509642707611
"14692","PPP3R1",113.362336713686,-0.0152358437663536,0.21383409621700
3,-0.0712507688712655,0.943198176338586,0.989613387439376
"14693","LINC00710",15.647976435569,-0.0440277718593603,0.619360864238
061,-0.0710858150740979,0.943329457540339,0.989645422087288
"14694","C10orf88",33.623710886779,-0.0265258470637767,0.3733349324314
2,-0.0710510717307426,0.94335710879991,0.989645422087288
"14695","LRIG1",36.1146727667263,0.0244183449283347,0.344268355637002,
0.0709282294713183,0.943454876080827,0.989680633874511
"14696","GPR158",4.69135021838741,-0.0458550943085362,0.64755351840916
6,-0.0708128255116081,0.943546724169054,0.989709632081244
"14697","AL050343.2",4.54868646167089,0.0504846339134975,0.71878220453
4697,0.0702363436309315,0.944005547705706,0.989831031884043
"14698","CCDC150",10.2280331198772,0.0376168444575903,0.53294150003517
3,0.0705834401244934,0.943729290403269,0.989831031884043
"14699","BTBD9",27.2516082542242,-0.0230511860222965,0.327978822108906
,-0.0702825440803685,0.943968775952348,0.989831031884043
"14700","ARHGAP33",20.1396374760002,0.0259140863933508,0.3692341594656
03,0.070183339566568,0.944047734723269,0.989831031884043
"14701","ZNF773",16.335413757011,-0.0341018542441199,0.484899195104035
,-0.0703277188092742,0.943932820703522,0.989831031884043
"14702","SPTLC3",34.4525391408908,-0.0269234376483939,0.38355476654592
7,-0.0701945067476304,0.944038846522692,0.989831031884043
"14703","PRR26",2.91783066102419,0.0706117350019751,1.00833312564964,0
.0700281813676227,0.944171229204948,0.98989318494146
"14704","PEX14",39.5973658831959,0.0201760221946198,0.290073669783018,
0.0695548210553271,0.944547997376735,0.990153511020903
"14705","MYH9",380.56002773174,0.0141324978235206,0.202978402092213,0.
0696256236025553,0.944491641742299,0.990153511020903
"14706","ZNF831",5.37201673643029,-0.0623846760454069,0.89969489712205
5,-0.0693398131355008,0.944719135684062,0.990265570282185
"14707","LINC01409",11.3531825572954,0.0315496505523189,0.459453034681
659,0.0686678467020655,0.945254012384246,0.99027255235029
"14708","LEPROT",1536.75454896479,-0.00876335189164409,0.1308136553067
23,-0.0669911093845391,0.946588780984854,0.99027255235029
"14709","ZNF385D",86.2071569536483,0.0294589697563378,0.42742617165424
6,0.0689217734195456,0.945051887045612,0.99027255235029
"14710","NCKIPSD",42.1161319633224,-0.0204740114025501,0.3011007021929
72,-0.0679972223692409,0.94578784539686,0.99027255235029
"14711","ACAD9",91.8742373228433,-0.0169235779233427,0.248326499338782

,-0.0681505113969112,0.945665821508744,0.99027255235029
"14712","SENP2",110.287837745738,-0.0133879957377136,0.195307047314588
,-0.0685484518955891,0.945349051714715,0.99027255235029
"14713","LINC02362",3.15273926152254,0.0623402212950314,0.930513213918
276,0.0669955250098216,0.946585265722904,0.99027255235029
"14714","ZC3H12D",4.18757741481667,-0.0540616069358648,0.7942857818946
53,-0.0680631684063496,0.945735349690561,0.99027255235029
"14715","FSCN1",212.578828781943,-0.0237873303537294,0.35447614339689,
-0.0671055889002266,0.946497644628093,0.99027255235029
"14716","BRI3",2378.71189918526,-0.0169208051355274,0.249675210617617,
-0.0677712660927398,0.945967717493425,0.99027255235029
"14717","TPK1",42.8041175550051,0.0193852759328256,0.286150158648925,0
.0677451168447863,0.945988533734277,0.99027255235029
"14718","PPP3CC",73.1751146938267,0.0135995152681307,0.199354015801046
,0.0682179148159368,0.9456121662304 7,0.99027255235029
"14719","AC107959.1",31.8851501572961,0.0255403149194587,0.37967161324
0777,0.067269487706635,0.946367167132167,0.99027255235029
"14720","AP002784.1",7.21097303046561,-0.0482688412403528,0.7148032146
10037,-0.0675274540653626,0.9461618067183 7,0.99027255235029
"14721","TRHDE",11.6008989008798,-0.0615647115840951,0.909989960841826
,-0.0676542755780976,0.946060848669726,0.99027255235029
"14722","WSCD2",65.6811379486649,0.0232954574156304,0.344154487664903,
0.0676889543811871,0.946033242271604,0.99027255235029
"14723","IFI27L2",1193.40813942381,0.0183708061894733,0.26891464718518
4,0.0683146358213153,0.945535173646451,0.99027255235029
"14724","ARHGAP17",111.673698099777,-0.0137628776598828,0.205371992473
051,-0.0670143844550213,0.946570251804337,0.99027255235029
"14725","PSMB10",275.630071516503,-0.0182956797694184,0.26630374606679
8,-0.068702299684621,0.945226587647756,0.99027255235029
"14726","TNFSF9",201.389687375105,0.0241377805514468,0.353520863357933
,0.0682782349029504,0.945564149720304,0.99027255235029
"14727","TMEM147-
AS1",4.19440893489487,0.0462660998199788,0.678583472728613,0.068180410
633847,0.945642020729017,0.99027255235029
"14728","POU2F2",4.22389358633767,0.0625892440717456,0.930963716467502
,0.0672305944524214,0.946398129365597,0.99027255235029
"14729","ZNF615",28.972528967926,0.0216843308185063,0.322871357648918,
0.0671608995496134,0.94645361245049,0.99027255235029
"14730","ZNF816",13.0336428125814,-0.0305733286442653,0.44284964282057
3,-0.0690377177444232,0.944959596688559,0.99027255235029
"14731","ZNF761",35.8498860813291,-0.0221497865107144,0.32767866843922
4,-0.0675960587126918,0.946107192916655,0.99027255235029
"14732","TFPT",52.8171966612554,0.018993615493865,0.275299699603204,0.
0689925035197674,0.944995586606665,0.99027255235029
"14733","AL050309.1",45.9155795267529,-0.0191121638461182,0.2849932945
82872,-0.0670618018367467,0.9465325031262,0.99027255235029
"14734","NHSL2",62.8233743528506,0.0341952369152222,0.504141418490331,
0.0678286600962504,0.945922028836529,0.99027255235029
"14735","SASH3",3.75595286996824,-0.0810157693699014,1.20172431273272,
-0.0674162688659193,0.946250317971259,0.99027255235029

"14736","ELF4",44.9025891325052,-0.022701844392088,0.339374200878366,-0.0668932533272454,0.946666684074589,0.9902868441239
"14737","EIF4E2",361.174976740676,0.0143993996140396,0.216206846251867,0.0666001094029428,0.946900058649807,0.990335512643503
"14738","LRCH1",49.7244403039621,0.0169894058009543,0.255291613054176,0.0665490166233898,0.946940734548524,0.990335512643503
"14739","GNPTG",360.43012274391,0.0135841173185641,0.204235525759525,0.0665120197284316,0.946970188541371,0.990335512643503
"14740","NKRF",33.3673123563514,0.0176615149406316,0.265144399832667,0.066610929560563 2,0.946891444541667,0.990335512643503
"14741","DAB1",2.69531446564979,0.0684437388992629,1.03493962788894,0.0661330739058412,0.947271879309999,0.990516620510353
"14742","LINC02422",3.29317075443865,0.0540451912671206,0.816525831344658,0.0661891996461628,0.947227195352892,0.990516620510353
"14743","VIRMA",162.580773660385,0.0121659934686401,0.184280034446977,0.0660190535841291,0.947362655980234,0.990544349313932
"14744","GTF2IRD2",112.927914741653,-0.0208237820706036,0.31585880397229,-0.0659275024432452,0.947435544426805,0.990553372038741
"14745","PIAS3",63.7245615850418,-0.0166026620333564,0.254594429369012,-0.0652121968045589,0.948005050155264,0.990590992182684
"14746","EPC2",148.416641293478,0.0130784954540303,0.201304916536828,0.0649685843695607,0.94819901343215,0.990590992182684
"14747","UMAD1",61.5074242045863,0.0146389867096084,0.224793727058098,0.0651218648366685,0.94807697175927,0.990590992182684
"14748","NSD3",628.883823502164,0.00852395263641486,0.130144586246702,0.0654960216344065,0.947779073812497,0.990590992182684
"14749","CAMSAP1",42.2343999360648,0.0208614510157147,0.319940683863912,0.0652041208506894,0.948011480149127,0.990590992182684
"14750","NTF3",2.98090262654003,-0.0723756795277515,1.10886844718492,-0.0652698520834384,0.947959145696103,0.990590992182684
"14751","RILPL1",122.323832179438,0.0125297001848314,0.191154063897484,0.0655476526596425,0.947737966548407,0.990590992182684
"14752","AC022613.3",9.43463785031035,-0.0578165399199706,0.891006819544114,-0.0648889982116552,0.9482623802885 6,0.990590992182684
"14753","PIGB",25.3977749691178,0.0261376016979169,0.401312762842174,0.0651302528053317,0.948070293306612,0.990590992182684
"14754","G6PC3",109.209855867428,-0.0163306358440233,0.251820603423126,-0.0648502768321284,0.948293210543233,0.990590992182684
"14755","OGFOD3",164.486188112845,-0.0156691057369603,0.238944441037056,-0.0655763560305229,0.947715113739743,0.990590992182684
"14756","C22orf23",5.08573307857781,0.0422233610855437,0.647026743614085,0.0652575206547066,0.947968963821011,0.990590992182684
"14757","SUV39H1",7.13793289908518,0.0356888038229235,0.550472385107567,0.0648330502827124,0.9483069264768,0.990590992182684
"14758","TFDP2",380.56600002563,-0.0116572033275546,0.181198916767645,-0.0643337362910555,0.948704491407857,0.990939133693733
"14759","AL121944.1",21.5754091157987,0.0216923359806356,0.337728719952634,0.0642300601017228,0.948787042305439,0.990950898488634
"14760","AL133227.1",2.39477965888151,0.0682645730793232,1.06400520516087,0.0641581194793139,0.948844324469169,0.990950898488634

"14761","TRAF3IP2-
AS1",50.331012410841,-0.015097671922524,0.236200521837128,-0.063918876
2374309,0.949034821948614,0.991082703091788
"14762","HNRNPL",262.772562419462,-0.0149375352866749,0.23479836676339
8,-0.0636185655487425,0.949273948516967,0.991265270043967
"14763","ALG12",66.212641788355,0.013701656316303,0.215748350054076,0.
0635075833157879,0.949362320822935,0.991290400019342
"14764","NEXN",389.51703482948,0.0174435707763359,0.280006244510067,0.
0622970777200248,0.950326255600317,0.991475233101457
"14765","C1orf74",3.722564813071,-0.0456501329884189,0.728326083827466
,-0.0626781520009834,0.950022795356488,0.991475233101457
"14766","FHL2",101.238893241355,0.0322230884719346,0.519630092197146,0
.0620115904675306,0.950553601908506,0.991475233101457
"14767","OXNAD1",21.1752085140336,-0.0211044500515245,0.33737874589492
2,-0.0625541777848021,0.950121518743083,0.991475233101457
"14768","NADK2",110.498954487838,-0.014318450440854,0.23081111567460б,
-0.0620353590814057,0.950534673740992,0.991475233101457
"14769","VCAN",3371.96539770664,0.0177272739598446,0.283491089562881,0
.062532032266617б,0.950139153785414,0.991475233101457
"14770","PITX1",3.19446172497632,0.0721468391735193,1.16075656938992,0
.0621550125806643,0.950439387940609,0.991475233101457
"14771","MRAP2",5.98903379197052,-0.114345417190929,1.84851520861369,-
0.0618579802092533,0.950675930315858,0.991475233101457
"14772","SYNE1",703.456316020989,-0.0179092607681762,0.28711936943772,
-0.062375662092212,0.95026367604777,0.991475233101457
"14773","FNDC1",646.181363425044,-0.023945293149526,0.382333094576329,
-0.0626294021867509,0.950061615811013,0.991475233101457
"14774","POP1",17.4275844888246,-0.0260454601084494,0.422043201065204,
-0.06171278211025,0.950791560720762,0.991475233101457
"14775","PCAT1",6.03413941942956,0.052222767971392,0.842224606681505,0
.0620057494842828,0.950558253387614,0.991475233101457
"14776","SLC15A3",42.1157532751614,-0.0161876489310249,0.2568723074110
3,-0.06301827197403,0.949751954308245,0.991475233101457
"14777","FKBP3",398.218500347148,-0.0132892033792551,0.213488807169017
,-0.0622477756819083,0.950365516736994,0.991475233101457
"14778","NEMF",290.728380004473,-0.0125028886342567,0.19974725580437,-
0.0625935439458648,0.950090170522861,0.991475233101457
"14779","TRIM9",9.04369895251222,-0.0458258498965785,0.74060590537923,
-0.0618761605379223,0.950661452246487,0.991475233101457
"14780","VPS33B-
DT",2.43436581661527,-0.0563456105608403,0.908408607626195,-0.06202672
46345008,0.950541549791129,0.991475233101457
"14781","SLC16A5",23.2404525160119,0.0206136325021531,0.33425761395023
2,0.0616698966361384,0.950825713327203,0.991475233101457
"14782","ZSWIM4",13.2987278241795,0.0289197692036749,0.468701472307102
,0.061701895369183,0.950800230561164,0.991475233101457
"14783","NAGA",92.4037047735818,-0.0185486934232915,0.295988753525556,
-0.0626668858270995,0.950031766826107,0.991475233101457
"14784","CTNNB1",628.232954254949,0.013747869386766,0.226850660575704,
0.0606031710548099,0.951675248031866,0.991532025048369

"14785","RHOA",2514.09889622238,0.0127300535281131,0.209380355387227,0.0607987005493912,0.951519525220053,0.991532025048369
"14786","ATG3",251.397152221649,0.00971960422156941,0.158670066669661,0.0612566971551408,0.951154776666542,0.991532025048369
"14787","PCDHB6",7.01195266708445,0.0400597295411902,0.654787101345519,0.0611797780665985,0.951216034325268,0.991532025048369
"14788","NR3C1",1038.8319177858,-0.00868555089286193,0.143456626479557,-0.0605447869924617,0.951721746389457,0.991532025048369
"14789","HLA-F",127.338557758641,-0.0259638842990141,0.427785588232469,-0.0606936863074142,0.951603160009541,0.991532025048369
"14790","LINC00476",42.062973074437,0.0174705595035932,0.288611492616747,0.0605331386674637,0.951731023392596,0.991532025048369
"14791","BAG3",343.463946205135,-0.0187040307636154,0.306465757949098,-0.0610313885922684,0.951334211159846,0.991532025048369
"14792","DUSP8",25.5963284646239,0.0298505207658992,0.492615640702176,0.0605959663062061,0.9516809860 4399,0.991532025048369
"14793","CTSD",1232.56677953591,0.0134534635694393,0.222478773735539,0.0604707736542609,0.951780692483991,0.991532025048369
"14794","RBM19",63.8257177268445,0.0160847612335713,0.265887686312704,0.0604945699315102,0.951761740498129,0.991532025048369
"14795","PROCA1",10.7567804904762,-0.0352740142420462,0.576900714894013,-0.0611439946794426,0.951244531985846,0.991532025048369
"14796","BLMH",95.8378996145032,-0.0130657168727287,0.212382867633766,-0.0615196367687214,0.950945376144678,0.991532025048369
"14797","SARS2",24.266107958357,-0.0206282078995781,0.335813471216105,-0.0614275771155807,0.951018690462048,0.991532025048369
"14798","MIR4453HG",5.96537299724947,-0.0353449118420554,0.58732205375297,-0.0601797797583157,0.952012449937355,0.99170644112612
"14799","AL353796.1",3.66707774070318,-0.0500139981135735,0.834859776241159,-0.0599070640805749,0.952229653679515,0.991865674131341
"14800","SOCS4",61.9466715071806,0.0178568575516555,0.299803375814053,0.0595618962033665,0.952504566886705,0.991883935591336
"14801","AL162171.3",4.80295731945897,0.0425140223306843,0.713184365792312,0.0596115455832425,0.952465022677789,0.991883935591336
"14802","IFI27",3006.32609541438,-0.0269074132554542,0.451458675982432,-0.0596010547297607,0.952473378311209,0.991883935591336
"14803","ZNF592",58.8971736009891,0.0146609362360354,0.245820919344943,0.0596407184348,0.95244178745049,0.991883935591336
"14804","VAMP3",318.908642592789,0.0109887422035428,0.185324923832773,0.0592944649660745,0.952717569677192,0.991904723529239
"14805","SPR",93.2692485490074,-0.0138003321233041,0.232668736126901,-0.059313220817846 3,0.952702630965069,0.991904723529239
"14806","AC017033.1",3.12858696387442,-0.0539737247507183,0.908463918825358,-0.0594120730964266,0.95262389710657,0.991904723529239
"14807","NIFK-AS1",64.2070309968356,0.0149896042752715,0.253398767451721,0.0591542114668232,0.952829279694175,0.991954031639474
"14808","ZNF407",58.5581388783679,-0.015339137160569,0.259805001605391,-0.0590409617435581,0.952919482244901,0.991980944003589

"14809","WDTC1",107.77273438178,0.0134777088846267,0.231477196329595,0
.0582247802303434,0.953569582432518,0.992106475996741
"14810","ACTR2",713.574070915225,-0.00980835679403786,0.16719372172700
8,-0.0586646238430702,0.953219236871067,0.992106475996741
"14811","KIF5C",3.7797617651712,-0.0535490855608844,0.916436325867387,
-0.0584318670587412,0.953404631885311,0.992106475996741
"14812","UBP1",79.6070734949925,-0.0124943658915186,0.212874393023455,
-0.0586936066572459,0.9531961517092,0.992106475996741
"14813","C3orf33",31.8473511752382,-0.021014822388097,0.35963374462808
4,-0.0584339559398952,0.953402968042232,0.992106475996741
"14814","GOLGA1",69.6886493295815,0.0151882353658532,0.260721754805528
,0.0582545763286309,0.953545848870335,0.992106475996741
"14815","OS9",824.844317035146,-0.00864499213881676,0.148635534509704,
-0.0581623510645251,0.95361930942872,0.992106475996741
"14816","FGF14-
IT1",3.84900625306934,0.0453749515476282,0.778347177454348,0.058296545
3745601,0.953512419228996,0.992106475996741
"14817","AC011825.4",2.37439936200768,-0.0549871414221607,0.9403789651
03125,-0.0584733851592806,0.953371561777659,0.992106475996741
"14818","CASP3",110.491909390913,0.0118132283758666,0.204317709785208,
0.0578179365278,0.953893650795346,0.992314720379447
"14819","NDUFA2",674.989179378876,0.0152547937943341,0.264154892384472
,0.0577494274538404,0.953948221946353,0.992314720379447
"14820","ASF1A",123.115317116742,0.0130290554473005,0.226660425591264,
0.0574827097112919,0.95416067898438,0.992334831098652
"14821","PARP4",216.597228201153,0.00903470260165364,0.157110172228861
,0.057505522866418,0.954142506779735,0.992334831098652
"14822","CCDC113",14.0097412342628,-0.0276724942327715,0.4809437805346
85,-0.0575378980928018,0.954116717786392,0.992334831098652
"14823","KLHL17",8.07892046928699,-0.0277149874018764,0.50875462511414
3,-0.0544761384639173,0.956555819100922,0.992533124265062
"14824","SCNN1D",6.10129610543372,-0.0325226237921777,0.59477973695761
3,-0.0546801139503099,0.956393312425316,0.992533124265062
"14825","NBPF1",76.9521433611061,-0.0154980761508573,0.28122701878813,
-0.0551087737502676,0.956051806326765,0.992533124265062
"14826","CLSPN",2.76148972715861,-0.0589594930839731,1.05363810917539,
-0.0559580111715174,0.955375256949745,0.992533124265062
"14827","TMEM53",25.9443048709583,-0.0200293593928411,0.35639573244074
5,-0.0561997733689789,0.955182661704518,0.992533124265062
"14828","OSBPL9",254.959927444209,-0.00954351244448771,0.1671172098348
28,-0.0571067004644233,0.954460198937177,0.992533124265062
"14829","CYB561D1",28.8191912810477,0.0162071805912032,0.3010662407669
85,0.0538326069037646,0.957068530623307,0.992533124265062
"14830","CRTC2",74.6218130763337,-0.012876393905038,0.239327594359681,
-0.053802378866878,0.957092614205065,0.992533124265062
"14831","DPM3",294.651021853076,-0.0123354353232586,0.229443103577962,
-0.0537625020360097,0.957124385329705,0.992533124265062
"14832","AC231981.1",5.5509178519367,0.0357880395962482,0.657645513347
139,0.0544184349621761,0.956601791640573,0.992533124265062
"14833","CYTOR",536.611447514875,-0.014988768007004,0.278461943459544,

-0.0538269891418092,0.957073006459441,0.992533124265062
"14834","SPOPL",112.924961615433,-0.012917248142519,0.240611827459164,
-0.0536850090825701,0.957186126596372,0.992533124265062
"14835","CALCRL",20.2847753128015,0.0242286415948469,0.441799976946836
,0.0548407488888629,0.956265336313271,0.992533124265062
"14836","DOCK10",16.8235963080116,-0.0232373156242153,0.43015842428958
9,-0.0540203662466729,0.956918938013473,0.992533124265062
"14837","DIS3L2",60.1355808850552,0.0132503167786836,0.244211657108757
,0.0542575114372311,0.956730000620775,0.992533124265062
"14838","ABHD5",683.960492109659,-0.018082259940973,0.325555200920237,
-0.0555428384798043,0.955706002431913,0.992533124265062
"14839","MST1",5.15512612778148,0.050867366556606,0.914583350053585,0.
0556180763116076,0.955646063979631,0.992533124265062
"14840","CTBP1-
AS",4.46640211244461,0.0445789970975323,0.822853594562256,0.0541760981
444671,0.956794863628836,0.992533124265062
"14841","SDHA",331.761767570429,0.00927287005221886,0.168581214388177,
0.0550053580161489,0.956134195177789,0.992533124265062
"14842","AC008608.2",21.4071597659564,0.0197086957821979,0.34971553521
0785,0.0563563633806512,0.95505791865286,0.992533124265062
"14843","PTPRK",56.46346597321,-0.0161857810884233,0.29008485011615,-0
.0557967128650204,0.955503753620113,0.992533124265062
"14844","AL078581.2",3.1299236288856,0.0535957106547253,0.989238449546
143,0.0541787580934756,0.956792744409034,0.992533124265062
"14845","PDE10A",55.1864480197881,0.0217310926498707,0.382322122274273
,0.0568397468621527,0.954672851684126,0.992533124265062
"14846","LACTB2",82.7211921060455,-0.0147384120805157,0.27065832136955
3,-0.0544539403257144,0.956573504402012,0.992533124265062
"14847","TIGD5",55.9396846623881,-0.0137350482318263,0.252379010830024
,-0.0544223078878644,0.95659870606543,0.992533124265062
"14848","FXN",48.7197179738878,-0.0143437006216959,0.262698410754734,-
0.0546013985409595,0.956456024548166,0.992533124265062
"14849","RALGPS1",20.5538689241979,0.0190377143290802,0.35513902885992
9,0.0536063704127233,0.957248780956775,0.992533124265062
"14850","ZNF248",51.9734002534377,0.0154840008915189,0.278020058824376
,0.0556938263986917,0.955585717690973,0.992533124265062
"14851","PHRF1",74.4984806827052,-0.0155371844779348,0.275271367413466
,-0.0564431550724908,0.95498877895382,0.992533124265062
"14852","PATL1",67.8420422712253,0.0139727229487804,0.255748827380477,
0.054634553330691,0.956429610281229,0.992533124265062
"14853","ATL3",499.18428235715,0.00959712930450521,0.178835163540715,0
.0536646659107411,0.957202334734541,0.992533124265062
"14854","APOA1-
AS",2.45065996292439,0.0519305102561645,0.95001263779262,0.05466296782
83296,0.956406972621051,0.992533124265062
"14855","SLC38A2",996.478144655159,0.0174761470111055,0.31197938510272
8,0.0560169929348087,0.95532827001183,0.992533124265062
"14856","COQ10A",55.4074197086688,0.017914380471977,0.323630773928203,
0.0553543788637087,0.955856140468898,0.992533124265062
"14857","CPSF6",189.26632642082,-0.00994874083178281,0.178085332181052

,-0.0558650210544479,0.955449336448023,0.992533124265062
"14858","AL161891.1",3.38107339509251,-0.0493111966120254,0.884165032667263,-0.055771484723014,0.955523851469873,0.992533124265062
"14859","AEN",14.8026497802723,-0.0229899019274938,0.416665451641061,-0.0551759255223748,0.955998308361503,0.992533124265062
"14860","CDC27",232.249530850624,0.00931960462309406,0.164060473978369,0.0568059106322151,0.954699805539683,0.992533124265062
"14861","TBX2-AS1",4.53716623897651,-0.0408966647052491,0.718756365278338,-0.0568992035144091,0.954625488790268,0.992533124265062
"14862","SLC35E1",171.135826728658,-0.0111487314907139,0.199270563955423,-0.055947708830733,0.95538346417107,0.992533124265062
"14863","NDUFA3",475.672827870734,0.0179728216751948,0.32206135347868,0.0558055832563114,0.955496687082203,0.992533124265062
"14864","AL031668.2",5.54649834310008,0.0428471009215517,0.751504848931444,0.057015069140905,0.954533191229266,0.992533124265062
"14865","Z93930.2",18.5212913446119,-0.0246905165838009,0.44307686936518,-0.0557251309895196,0.955560778970546,0.992533124265062
"14866","LINC01521",2.42535117732579,0.0514698294766542,0.945148993126086,0.0544568420968396,0.95657119255394,0.992533124265062
"14867","LINC01315",74.2256510091661,-0.0247380427276237,0.451437398253208,-0.0547983902604105,0.956299082863495,0.992533124265062
"14868","VRK1",39.6742550717708,-0.0143631768522404,0.268747056480269,-0.0534449643480836,0.957377380013662,0.99259969820491
"14869","ITPKB",43.7718446475316,0.0149422472078893,0.281286745726156,0.0531210497292189,0.957635459871264,0.992652504448856
"14870","PRPF19",122.138344904357,0.0100247725697926,0.188940772471092,0.0530577515836419,0.957685893361232,0.992652504448856
"14871","FOXN3-AS1",29.2392657329096,-0.0177203767785377,0.333510118451545,-0.0531329509905477,0.957625977430034,0.992652504448856
"14872","GSS",84.900555955519,0.0141362718913227,0.265259157542382,0.0532923048625159,0.957499011320918,0.992652504448856
"14873","SLC25A44",39.8052684683422,0.0182448553008056,0.345857771780272,0.052752480324186,0.957929123951479,0.992837856902579
"14874","RORA",353.021508175667,-0.0147696086359088,0.282365996204097,-0.052306612107901,0.958284384831063,0.99313928950994
"14875","BRD1",164.235125863092,-0.00870770529453502,0.166813613457524,-0.0522002078490571,0.958369167321459,0.993160384151952
"14876","AC025171.1",35.2050771432972,0.0141025026180688,0.270912951433207,0.0520554759138039,0.958484489907746,0.99321312260876
"14877","WRAP73",76.532612050103,0.0120165839244003,0.231981273236144,0.05179801582122,0.958688213655321,0.993223927246238
"14878","AC242426.2",19.4501453811786,-0.0210022899474502,0.404835909611581,-0.0518785252217394,0.958625485619855,0.993223927246238
"14879","ERAP2",222.324725947256,0.0487985390542339,0.941562490169313,0.051827191040139,0.958666389344503,0.993223927246238
"14880","PLEKHM2",273.971691329085,-0.0125712036714175,0.243748538931783,-0.0515744780523005,0.958867755831435,0.993276423368159
"14881","AC091057.3",3.993919722985,-0.0475388294363988,0.920483883256

237,-0.0516454772333752,0.958811182075871,0.993276423368159
"14882","TNFRSF1B",117.408534289067,0.0107148465080329,0.211151262859491,0.05074488479457,0.95952880980571,0.993341104741869
"14883","MAP3K6",73.9293366829782,-0.01523210639655,0.298254874864845,-0.0510707709419752,0.959269126997259,0.993341104741869
"14884","TMEM177",20.4136047087559,-0.0206601438448325,0.408177463288779,-0.0506155917535695,0.959631838328187,0.993341104741869
"14885","ESPNL",5.29296656601488,-0.0358798045155639,0.706725472587881,-0.0507690834804349,0.959509526902868,0.993341104741869
"14886","CENPU",4.72766091443889,0.0429808742603652,0.844030119955844,0.0509233891589245,0.95938656783274,0.993341104741869
"14887","CSGALNACT2",160.965565869869,-0.00944804602769264,0.18568252449772,-0.0508827960695281,0.959418914497761,0.993341104741869
"14888","TCHP",65.2766150154989,-0.0140385229963271,0.27617267683854,-0.0508324109286689,0.959459064066806,0.993341104741869
"14889","METTL21C",3.17084314367169,-0.0471814508706172,0.932318872381872,-0.0506065599102149,0.959639035472975,0.993341104741869
"14890","PAQR5",2.76413133724806,0.0521201835976056,1.02627407078555,0.0507858330257832,0.959496179916932,0.993341104741869
"14891","ANTKMT",208.022749209473,0.0145734907236756,0.286955547981059,0.0507865794064994,0.959495585158789,0.993341104741869
"14892","METTL4",51.5140390177685,0.0124796707499029,0.246222622403195,0.0506845009938494,0.959576927189739,0.993341104741869
"14893","AC012358.3",14.0511333017516,-0.0205108310093344,0.406734102651081,-0.0504281073941069,0.959781238413387,0.993354893926572
"14894","SHC4",3.79511973183302,0.0383045370090369,0.759290393160922,0.0504478093678695,0.95976553849561,0.993354893926572
"14895","SPATS2",149.364596304145,0.00895410843746285,0.178317551542787,0.0502143976293571,0.959951538379084,0.993464448748814
"14896","LINC01140",327.955610333737,-0.014975545592971,0.306617268786635,-0.0488411681841444,0.961045873839394,0.993541686667471
"14897","PHGDH",312.168941675701,-0.0141498366175984,0.28979721131618,-0.0488266831600405,0.961057417443961,0.993541686667471
"14898","RTKN",36.9365692225175,-0.0147763974601939,0.300146362421662,-0.049230639815102,0.960735493822009,0.993541686667471
"14899","NOP58",199.6995043972,0.00855743336600602,0.171230306365107,0.0499761610410213,0.960141385310822,0.993541686667471
"14900","IL20RB",12.6254060652617,-0.0271473761719756,0.551219140877376,-0.0492496979128213,0.960720306083297,0.993541686667471
"14901","ARL10",48.335655984251,0.0145569815737693,0.29463155284049,0.049407408790498,0.960594624013594,0.993541686667471
"14902","RIPK1",96.0367309394415,-0.0116961164701337,0.235582390651651,-0.0496476686469679,0.960403159357271,0.993541686667471
"14903","SLC17A5",158.559144048527,-0.0103025367772845,0.209555458390574,-0.0491637719981616,0.960788782093178,0.993541686667471
"14904","SEPTIN7-AS1",3.05992158029639,0.0425072965698182,0.868957839839464,0.0489175591967399,0.960984995398478,0.993541686667471
"14905","HDHD3",37.0753792492177,0.0158326173712305,0.322933565906793,0.0490274751302879,0.960897400275181,0.993541686667471

"14906","AP002884.3",5.34457284727476,-0.0455518194291501,0.924299262833124,-0.049282544367207,0.960694130190169,0.993541686667471
"14907","MFSD5",90.1552664194708,0.0115056213653182,0.234175129060047,0.0491325505466804,0.960813663138523,0.993541686667471
"14908","SYNE3",459.376896516111,-0.0138040479339264,0.279266334440842,-0.0494296885500553,0.960576869031214,0.993541686667471
"14909","OPA3",28.86121315992,0.0188057161044724,0.379702886903983,0.0495274509441058,0.960498961348818,0.993541686667471
"14910","NTN5",4.57485432320432,0.0369122601907762,0.754449584121954,0.0489260793134846,0.960978205459067,0.993541686667471
"14911","BFSP1",4.65818019009506,0.0361803051356355,0.727655430267511,0.0497217551476725,0.960344119798858,0.993541686667471
"14912","CD300A",3.45415271205876,-0.0383591539496659,0.787856365269609,-0.048688004109149,0.961167935872835,0.993589306027344
"14913","GTF3C5",85.7611881862028,0.0100785278816543,0.208943894990381,0.0482355700419881,0.961528502374747,0.993811377275774
"14914","TRIM13",128.237280000293,-0.00938764380600005,0.195190167353845,-0.0480948601728587,0.961640642455105,0.993811377275774
"14915","AC105429.1",12.75467846914,0.034975464652936,0.726677898663982,0.0481306294263791,0.961612135733269,0.993811377275774
"14916","IGLC1",55.3357375375442,-0.0765681273775875,1.58827981670454,-0.0482082102739652,0.961550306944859,0.993811377275774
"14917","MTRES1",103.859608562337,-0.0123700818876727,0.260183585200053,-0.0475436675921021,0.962079927959405,0.994028679959115
"14918","MAMDC4",4.45272552901487,-0.0367975610714155,0.774205632370084,-0.0475294411883401,0.96209126616947,0.994028679959115
"14919","EPC1",486.681769720765,0.00814587183333919,0.17146539945948,0.0475073796755373,0.962108848847875,0.994028679959115
"14920","NDUFA6-DT",14.232519174143,-0.0219592659711955,0.461443096348929,-0.0475882425047499,0.96204440253604,0.994028679959115
"14921","IRF2",237.043885135546,0.00799816393331979,0.169578847853485,0.0471648677565622,0.962381827814039,0.994040384107754
"14922","AL359643.3",9.40291569069835,-0.0226755059804213,0.482378522322901,-0.0470077023148274,0.962507088757206,0.994040384107754
"14923","AC105219.1",2.88030980837094,0.0411672710814737,0.873535236785111,0.0471272014543826,0.96241184779303,0.994040384107754
"14924","SAV1",745.703049863567,-0.0094509013534097,0.200876677141802,-0.0470482760262813,0.962474751398209,0.994040384107754
"14925","ENTPD5",59.2247886740917,0.012861088339048,0.271800740957246,0.0473180768152176,0.962259721004079,0.994040384107754
"14926","NFS1",47.0969877909054,-0.0118438126987304,0.250133059853509,-0.0473500492324636,0.96223423926807,0.994040384107754
"14927","MFSD14A",153.545388934444,0.00845419781076592,0.183758471954344,0.0460071185880693,0.963304576106481,0.994139471462062
"14928","SATB2",17.8739010637267,0.019047508186612,0.410611123683484,0.0463881933244814,0.963000846745772,0.994139471462062
"14929","NUB1",323.385984464642,-0.00743497103579531,0.160756024969071,-0.0462500303626305,0.963110966647509,0.994139471462062
"14930","UBE2V2",326.125906832685,-0.0104133286503091,0.22344004479789

8,-0.04660457645239,0.962828384514166,0.994139471462062
"14931","DENND1A",53.9323377219175,0.0120221560557885,0.25951711295710
7,0.0463250994078974,0.963051134346164,0.994139471462062
"14932","CCAR1",258.910454500313,0.007782983873814,0.168704311342408,0
.0461338765552789,0.963203545157094,0.994139471462062
"14933","ARFGAP2",210.501609061655,-0.0088107786959728,0.1906387527730
42,-0.0462171440371421,0.963137178109816,0.994139471462062
"14934","LINC02334",3.43181755874366,0.0393583396041868,0.842009442824
32,0.046743347048661,0.962717782134989,0.994139471462062
"14935","PELP1",85.2520539811174,0.0125993417238425,0.272322235807988,
0.0462662980364415,0.963098000801432,0.994139471462062
"14936","SPAG5",7.15225918195509,-0.0258891988206421,0.560533560986113
,-0.0461867060646588,0.963161438191297,0.994139471462062
"14937","RPN2",742.227552413082,-0.00892392722070779,0.194010014649719
,-0.0459972503832843,0.963312441467974,0.994139471462062
"14938","TXLNA",257.381652516563,0.00923963339780212,0.212250253953818
,0.04353178960065,0.965277624100114,0.99457350274515
"14939","RNF220",112.94616406401,-0.0101508799734638,0.247849762228175
,-0.0409557785418357,0.967331149870768,0.99457350274515
"14940","GBP2",445.998310076809,-0.00814252021258287,0.200033309299809
,-0.0407058216508277,0.967530420437816,0.99457350274515
"14941","AC245297.2",8.4686627251181,-0.0205506867514259,0.50684099794
1449,-0.0405466148849307,0.9676573442717,0.99457350274515
"14942","TDRKH",4.39360689352926,0.0325848976878377,0.748110112743239,
0.0435562855424484,0.965258097686981,0.99457350274515
"14943","THEM4",72.3526544986274,-0.00929228066769787,0.22703981285458
8,-0.0409279788899812,0.967353312201328,0.99457350274515
"14944","RABGAP1L",277.950271697256,-0.00950798833364326,0.22143414864
9243,-0.0429382206477292,0.965750781161632,0.99457350274515
"14945","MIA3",328.402477102423,-0.00706647348980254,0.159436537815088
,-0.0443215437730975,0.964648099099133,0.99457350274515
"14946","TTC13",19.6852121375165,0.0152536748967189,0.346919332890612,
0.043968938743257,0.964929163204386,0.99457350274515
"14947","CRIPT",228.845574412237,-0.00775647190590194,0.19114804409559
9,-0.0405783482776454,0.967632045508372,0.99457350274515
"14948","IDH1-
AS1",4.09757279872539,-0.0340329439921088,0.809535387377716,-0.0420400
942599309,0.966466735757677,0.99457350274515
"14949","GALNT15",1065.2408435893,-0.0259113385531456,0.60204729286119
1,-0.0430387095173266,0.965670676695141,0.99457350274515
"14950","AC097359.2",4.22151371903665,0.0310513304396969,0.72262166351
6033,0.042970384099214,0.96572514210414,0.99457350274515
"14951","ZNF620",6.84305031299934,-0.0248086831048253,0.60839071568831
2,-0.0407775504541643,0.967473236612067,0.99457350274515
"14952","C3orf62",27.8455243214198,-0.0137392676467388,0.3311806625384
02,-0.0414857182222879,0.966908678260365,0.99457350274515
"14953","USP4",109.652389623191,-0.00890817843391114,0.215751013883369
,-0.0412891613975299,0.967065373655071,0.99457350274515
"14954","FOXP1",949.951622665589,-0.013510184965082,0.300820733624629,
-0.0449110830968861,0.96417818272572,0.99457350274515

"14955","GMPS",157.609728780165,-0.00799521147399563,0.184289943710674
,-0.0433838727876965,0.965395533248149,0.99457350274515
"14956","GOLIM4",715.313845920168,0.0110302644860206,0.245641118405553
,0.0449039825157026,0.964183842459986,0.99457350274515
"14957","C1QTNF7",250.8511834851,-0.0270551678358199,0.657419447243879
,-0.0411535861149897,0.967173455214552,0.99457350274515
"14958","TRIP13",3.52042068542053,-0.0339584675658704,0.81711149475294
7,-0.041559160266296,0.966850130480605,0.99457350274515
"14959","SLF1",38.5341590363947,0.0112546947348171,0.274236877296889,0
.0410400484637695,0.967263968684908,0.99457350274515
"14960","SMAD5-
AS1",12.4586744365097,0.0231166739845448,0.536581810592458,0.043081359
6886949,0.965636678315837,0.99457350274515
"14961","RNF145",249.575428782788,0.0103894525876034,0.237615623212877
,0.0437237772799798,0.965124585809752,0.99457350274515
"14962","SERPINB9P1",4.96877540054193,0.0499546393150028,1.17494298050
803,0.0425166498661943,0.966086839077636,0.99457350274515
"14963","SMIM8",121.957403791863,0.0107868367636089,0.252069811554723,
0.0427930528335685,0.965866501953995,0.99457350274515
"14964","STX11",19.7272474313136,-0.0194803636576824,0.452450607924757
,-0.0430552270601038,0.965657509807824,0.99457350274515
"14965","AL354892.3",3.13302428359104,0.0405880664434541,0.90866354073
675,0.0446678716860864,0.964372042806286,0.99457350274515
"14966","MRPL32",254.783458665064,-0.0110250423151731,0.25070483813137
1,-0.0439761848927541,0.964923387200478,0.99457350274515
"14967","ZNF138",75.079158092673,-0.0101892456687702,0.234798453243153
,-0.0433957103551208,0.965386097122619,0.99457350274515
"14968","VPS13A",83.4192360104238,0.0135572186850526,0.306818188280351
,0.0441864895984095,0.9647557512743,0.99457350274515
"14969","ZNF883",3.25407232942946,0.038347201891088,0.946089029230655,
0.0405323396702648,0.967668724889507,0.99457350274515
"14970","AL445222.1",4.52778331861805,0.030396712906338,0.732503172543
605,0.0414970392562068,0.96689965315405,0.99457350274515
"14971","UNC5B",8.92071831539149,0.0233694806369846,0.551710241697812,
0.042358250528517,0.966213109709532,0.99457350274515
"14972","EXOSC1",135.115960938993,0.0117910775368634,0.274844741240357
,0.0429008664442734,0.965780558065388,0.99457350274515
"14973","TPBGL",2.69634893073508,-0.0417887597091073,0.964114808134392
,-0.0433441736985355,0.965427178770744,0.99457350274515
"14974","AQP11",5.81269281048952,0.0252719376518173,0.614039838751339,
0.0411568371576806,0.967170863453611,0.99457350274515
"14975","FOXRED1",62.848153389777,-0.0115854480829407,0.25733012026896
7,-0.0450217334481923,0.964089985730086,0.99457350274515
"14976","LTA4H",137.05352786407,0.00901496888963405,0.214351015597064,
0.0420570383794231,0.966453228252806,0.99457350274515
"14977","ACADS",168.279037889525,-0.0100835321089908,0.245258832331057
,-0.0411138388499695,0.967205142125455,0.99457350274515
"14978","KCNRG",4.14963093888992,0.0327114911407167,0.752281226856766,
0.0434830618828469,0.965316466414194,0.99457350274515
"14979","CLYBL",25.9112048760133,-0.0145618928240337,0.324500332157692

,-0.0448748163898867,0.964207090226867,0.99457350274515
"14980","GTF2A2",539.290347153051,-0.0115950954872549,0.28237389657022
5,-0.0410629156168169,0.967245738703819,0.99457350274515
"14981","SPNS1",198.316548612519,0.00892434779698693,0.204066838644823
,0.0437324744003101,0.965117653143032,0.99457350274515
"14982","ORC6",5.82536259713694,0.0249817276682833,0.603492482303999,0
.0413952590973605,0.966980792251725,0.99457350274515
"14983","MLYCD",78.7283150023908,-0.00993979171350038,0.23548732810192
1,-0.0422094547236035,0.966331725499444,0.99457350274515
"14984","AC055811.3",2.92698988155773,-0.0366593102572562,0.9028881622
48423,-0.0406022714551547,0.967612973292302,0.99457350274515
"14985","SRSF2",882.422605210511,-0.00764675465188518,0.18861549747839
,-0.0405414971416189,0.967661424285291,0.99457350274515
"14986","TYMS",9.27587764492217,0.0247242111883659,0.567855551145617,0
.0435396134430422,0.965271387490167,0.99457350274515
"14987","TWSG1",186.714554357165,-0.00893695415831177,0.20422590886595
4,-0.0437601389947915,0.965095601101244,0.99457350274515
"14988","TIMM44",139.232809753643,0.0110103226499415,0.255043256698697
,0.0431704127074762,0.965565690331005,0.99457350274515
"14989","TMEM38A",10.9648319978602,-0.0198207782932999,0.4694317993272
12,-0.0422229135770242,0.966320996453041,0.99457350274515
"14990","XRCC1",78.2703919898286,0.0100395481529295,0.233170093871688,
0.0430567573492259,0.965656289944986,0.99457350274515
"14991","MOCS3",21.2843106135768,0.0153777371872249,0.340337734270777,
0.045183756130286,0.963960841756564,0.99457350274515
"14992","IL10RB",347.502997230589,0.00719795411337245,0.17081427457449
5,0.0421390667220454,0.966387837075006,0.99457350274515
"14993","TEF",68.0273156724561,0.0354115480810451,0.832957961378747,0.
0425130075261307,0.96608974261926,0.99457350274515
"14994","SLC9A7",6.84632434739999,0.0299972992308685,0.731331794537207
,0.0410173596374967,0.967282056518566,0.99457350274515
"14995","ZC4H2",26.9792895850538,-0.0139153438142732,0.332006926437799
,-0.0419128117704504,0.966568203057598,0.99457350274515
"14996","GLA",158.182939687375,0.00992056707913709,0.219582094523222,0
.0451793079972099,0.963964387232607,0.99457350274515
"14997","TMEM164",19.1667534203459,-0.0155180900448125,0.3641985053193
54,-0.0426088789991194,0.96601331750205,0.99457350274515
"14998","AC240274.1",76.4899415396209,0.0147709311991336,0.32850642215
5577,0.0449639039085156,0.96413608034742,0.99457350274515
"14999","NBPF26",72.2271885620353,-0.0179670741387476,0.45427691966992
1,-0.0395509288735218,0.968451149885469,0.994614925253725
"15000","AC092902.4",8.19600491229716,0.0224071554053155,0.56461314834
6912,0.0396858547678524,0.96834357905325,0.994614925253725
"15001","MCCC1",40.90691244759,-0.0100476385785333,0.25284604803594,-0
.0397381673812243,0.968301872526189,0.994614925253725
"15002","AC008443.5",14.1031759172604,0.0190833805613305,0.47381534462
2716,0.0402759867908583,0.967873098006253,0.994614925253725
"15003","CCZ1B",57.9158863436464,-0.0197919176412379,0.497414422349446
,-0.0397895934495715,0.968260872886675,0.994614925253725
"15004","COL14A1",2452.61799135167,-0.0115046353655726,0.2881734865144

8,-0.0399226018490585,0.96815483179539,0.994614925253725
"15005","MINPP1",75.7515490648364,-0.0107719715138629,0.26859392507693
5,-0.0401050452305554,0.968009379527151,0.994614925253725
"15006","CRACR2A",25.2844645669673,-0.0164989565955713,0.4170560952304
98,-0.0395605214364573,0.968443502113009,0.994614925253725
"15007","CERS3-
AS1",2.52135678131381,0.0399510726535332,1.00635353315849,0.0396988447
27205,0.968333222726557,0.994614925253725
"15008","AC005726.1",5.79340153064919,-0.0255453867943335,0.6342942271
25286,-0.0402737179401883,0.967874906819573,0.994614925253725
"15009","EPOP",3.72404842157289,0.0317173631428404,0.802755662516279,0
.0395106065566959,0.968483297311607,0.994614925253725
"15010","RAB36",12.294951434012,0.0202360040127701,0.506141562490495,0
.0399809174200156,0.968108339820408,0.994614925253725
"15011","ELAVL1",225.016138479656,-0.00690721315528553,0.1756433962998
52,-0.0393252083528024,0.968631108798632,0.994700455807802
"15012","MROH6",19.5161052131811,0.018300149387449,0.466880475858131,0
.0391966473942033,0.968733606576015,0.99473944480211
"15013","F3",697.030519300259,-0.0142578807398336,0.370618978909623,-0
.0384704549717903,0.969312587556089,0.994803830704201
"15014","TATDN3",64.9617767177068,0.0108898586682963,0.280354440907057
,0.0388431823411226,0.969015416266303,0.994803830704201
"15015","ANAPC10",116.16200084924,-0.0103027003970028,0.26532419388808
3,-0.0388306103790469,0.969025439678712,0.994803830704201
"15016","CNOT8",130.132686272552,-0.00776977733196212,0.20133454155978
9,-0.0385913776730398,0.969216176793567,0.994803830704201
"15017","HAUS6",60.6925867792099,-0.00904496633086482,0.23503642371492
8,-0.0384832537353244,0.969302383176694,0.994803830704201
"15018","AL354920.1",6.14123392801118,0.0240823134240791,0.61919104448
2869,0.0388931875527883,0.96897554800654,0.994803830704201
"15019","RAB18",310.465318762318,-0.00652162352468608,0.16869500384382
5,-0.0386592570976417,0.969162057234647,0.994803830704201
"15020","MVD",63.5278713163494,-0.00925931047408748,0.239459462737266,
-0.0386675488545915,0.969155446312839,0.994803830704201
"15021","TMEM204",133.396032434706,-0.0104683505381499,0.2734747725164
23,-0.0382790355462177,0.969465205744396,0.994894224522327
"15022","CLK2",46.0136899307604,-0.0113548508173787,0.29753271894763,-
0.0381633685785572,0.969557427246064,0.994922629543208
"15023","CBR3-
AS1",26.1031331313089,-0.0121488078049798,0.319119471849569,-0.0380697
791161633,0.969632046607206,0.994932969343678
"15024","LRRC29",13.6857457319423,-0.0161212099826503,0.42571941386193
1,-0.0378681578939661,0.969792801150187,0.99503168461995
"15025","SAMSN1",23.7439786368846,-0.0187935364786832,0.49832352711343
9,-0.0377135243594569,0.969916092789057,0.995091951437159
"15026","DNAJC8",797.95102778793,-0.00773992138975332,0.21168400420384
9,-0.0365635628391641,0.970832996733337,0.995092173552769
"15027","TRMT13",85.075495346381,0.0105391891309404,0.283440432777553,
0.0371830829767737,0.970339027113404,0.995092173552769
"15028","RNPEP",156.349999892633,0.00703440865831896,0.195329293710394

,0.0360130757895872,0.971271932745699,0.995092173552769
"15029","AC092620.1",4.99693186984136,-0.024172610607897,0.669992819653012,-0.0360789099507305,0.971219438799089,0.995092173552769
"15030","SCD5",53.0489597650508,-0.0131444459213512,0.355407772890048,-0.0369841261896591,0.970497662547113,0.995092173552769
"15031","AC012640.2",5.05789133106189,0.024971542192064,0.689270828716845,0.0362289264998366,0.971099821112842,0.995092173552769
"15032","TBCA",1019.5710306849,0.00785802035913439,0.21402818384331,0.0367148859464567,0.970712339376831,0.995092173552769
"15033","AC018645.3",15.9604135808955,-0.0159002806936985,0.43511219552065,-0.0365429442276891,0.970849437018164,0.995092173552769
"15034","BAZ1B",282.576598843242,0.00792753635364189,0.219086330896999,0.0361845320115792,0.971135219579591,0.995092173552769
"15035","CNOT4",281.985007633559,-0.00566550543917959,0.156428861913366,-0.0362177757344887,0.971108712301237,0.995092173552769
"15036","NSMCE2",137.643048441416,-0.0086532030774904,0.237094414866823,-0.0364968659525402,0.970886177653763,0.995092173552769
"15037","CD274",2.66210873752717,-0.0363315461042953,0.967019205378447,-0.0375706562002321,0.970030004356656,0.995092173552769
"15038","ATG13",103.621254114342,0.00722964531561714,0.197540950281346,0.0365982106764211,0.970805370249457,0.995092173552769
"15039","RAD51AP1",6.21582546650894,0.022854669567934,0.631698211532834,0.0361797281529047,0.971139049996151,0.995092173552769
"15040","PIBF1",135.296025481697,0.00706024721460712,0.193704978810687,0.036448455057561,0.970924778276563,0.995092173552769
"15041","PRMT5",80.0748904793038,-0.0079966184874507,0.21328977167935,-0.0374918048084952,0.970092874369969,0.995092173552769
"15042","WDHD1",10.64665866242,0.0173370866904306,0.473529448642135,0.0366124783583058,0.970793993910786,0.995092173552769
"15043","LINS1",50.3387598843175,-0.0101322689974924,0.28067945056706,-0.0360990766407089,0.971203358583484,0.995092173552769
"15044","ZFP90",122.40067766589,-0.00699929169462048,0.188449410898352,-0.0371414888550427,0.970372191512437,0.995092173552769
"15045","KLF8",60.2726951890117,-0.0109578973676924,0.303812082881369,-0.0360680104088264,0.971228129718731,0.995092173552769
"15046","RAP2C-AS1",42.8945975072098,0.0094578365493667,0.253837625529957,0.0372593957638109,0.970278180482542,0.995092173552769
"15047","BROX",166.896535477439,-0.00743971789684415,0.207642088149732,-0.0358295274485937,0.971418288678017,0.995109843166642
"15048","MAP7D2",11.0733417433049,-0.0200884203611165,0.560223488460024,-0.0358578688236309,0.971395690054132,0.995109843166642
"15049","ANKRD23",6.51337523961669,0.0208861773583236,0.585744724549891,0.0356574741230036,0.971555479704494,0.995184246105706
"15050","KDM7A",157.763478409385,-0.0131420738309053,0.369449751105944,-0.0355720197173354,0.971623619227269,0.995187912982615
"15051","SELENOF",1000.66928268815,0.00708050432336682,0.203597452305731,0.0347769790003779,0.972257577621467,0.995224448050132
"15052","THPO",5.48724824439474,-0.0286859134865981,0.824733120525479,-0.0347820558829029,0.972253529304476,0.995224448050132

"15053","ATOX1",534.522334398124,-0.00949471719875791,0.268001026826467,-0.0354279135090994,0.971738526916948,0.995224448050132
"15054","AL365203.2",4.21434309590324,0.0279555073884736,0.797549575988312,0.0350517487942133,0.972038476630042,0.995224448050132
"15055","IKZF5",59.934311532175,0.0101975643722795,0.292001173328299,0.0349230253291287,0.972141120252231,0.995224448050132
"15056","IFT81",62.1763025832088,-0.0088270460203344,0.251992045554585,-0.0350290660997168,0.972056563694447,0.995224448050132
"15057","TMEM8A",69.2447412128672,-0.00827008953508782,0.238210146177067,-0.0347176208394602,0.972304909999026,0.995224448050132
"15058","AC092718.4",4.98236230280437,-0.0264469504153346,0.751268924164903,-0.0352030405686386,0.971917837712355,0.995224448050132
"15059","UNC13D",6.57689201395889,0.0194985955404487,0.555786396830285,0.035082894528638,0.97201364120438,0.995224448050132
"15060","ZNF726",5.3686483082076,-0.022470065011905,0.64024981003317,-0.0350957777101736,0.972003368239039,0.995224448050132
"15061","GPR89B",22.6190389979064,-0.0119105952575592,0.346323157921836,-0.0343915645983091,0.972564909969732,0.995424479595208
"15062","URB2",10.0747001426633,-0.0160886501550465,0.471864270432964,-0.0340959279249608,0.972800655643902,0.995475639396884
"15063","TRIQK",175.402718547073,0.00836413993132789,0.244762934896472,0.0341724123175173,0.972739665469399,0.995475639396884
"15064","AP005482.3",3.77861713931071,-0.0258978744362291,0.759782003242897,-0.0340859277078055,0.972808630027548,0.995475639396884
"15065","SCOC-AS1",11.3708432858499,-0.0186529928262671,0.549654610640184,-0.0339358434645748,0.972928310688184,0.995532021855849
"15066","FGFR1OP",37.0542596374305,0.00895907550642371,0.266313804691635,0.0336410480740847,0.973163389149966,0.995645191105522
"15067","EDNRB",6.96682252603234,-0.0266839872522039,0.793336255606645,-0.0336351541526352,0.973168089159059,0.995645191105522
"15068","ADCY7",97.2342380350049,0.0074136283910322,0.225247082807993,0.032913315895646,0.973743714000363,0.996128993958032
"15069","CEBPA-DT",10.0285359437807,0.0161479938173506,0.491117715120958,0.0328800882561801,0.973770211479311,0.996128993958032
"15070","NSF",100.909537772883,-0.00671237648979732,0.204809821762781,-0.0327737040734885,0.973855048053674,0.996149672577796
"15071","AL138995.1",8.93925268713988,-0.017655730069413 8,0.540716274511098,-0.0326524850493498,0.973951715103655,0.996182448963098
"15072","C1orf159",17.5155351756909,-0.0123944000274869,0.420164290751869,-0.0294989372021777,0.976466666563743,0.996301760659144
"15073","LINC02606",8.04379091835172,0.0192136676656377,0.644792340313444,0.0297982256679687,0.976227973851638,0.996301760659144
"15074","PIGK",188.466117950809,0.00693842769287308,0.23815914400505,0.0291335767176169,0.976758056808332,0.996301760659144
"15075","GON4L",218.006541869089,0.00590790388904577,0.202010984114906,0.0292454586810254,0.976668825938838,0.996301760659144
"15076","C1orf131",137.196869490532,-0.00552053755525016,0.191293744595995,-0.0288589549381728,0.97697708119013,0.996301760659144

"15077","DNAJC27",49.4334494504189,0.00871424183165635,0.275904170311087,0.0315843063257467,0.974803558884251,0.996301760659144
"15078","AC093690.1",11.9643844896242,-0.0187681142433119,0.632012381774694,-0.0296958015135889,0.976309660353467,0.996301760659144
"15079","AC110769.2",6.44514567878977,0.0200028192036082,0.649607852157236,0.0307921450413234,0.975435304800181,0.996301760659144
"15080","GLI2",68.3561352955548,-0.00833716451992651,0.27516544337692,-0.030298733800328,0.975828806391493,0.996301760659144
"15081","PRPF40A",505.212511365145,0.00464581837905455,0.161732658964366,0.0287252952421819,0.977083682003409,0.996301760659144
"15082","CNPPD1",163.473127655987,-0.00612030276481918,0.213746544724383,-0.0286334582517395,0.977156927191646,0.996301760659144
"15083","CCDC174",173.19355586018,0.00577756365128694,0.206832149854704,0.0279335860278277,0.977715121111854,0.996301760659144
"15084","ATRIP",25.4075275787741,0.0100317129185836,0.331056752024377,0.0303020943002695,0.975826126331058,0.996301760659144
"15085","CNTN3",79.7528569612742,0.0243024292789298,0.74988337896707,0.0324082783544359,0.974146460784977,0.996301760659144
"15086","MBNL1",900.873111486956,-0.00704548589113771,0.224920067220222,-0.0313243988329391,0.97501083249825,0.996301760659144
"15087","TNK2",16.0589455473579,0.0137636753052082,0.482239239635005,0.0285411766069174,0.977230527210878,0.996301760659144
"15088","LINC02104",12.4967985042383,-0.0158921590331796,0.541370856364338,-0.0293554018402577,0.976581141636798,0.996301760659144
"15089","SREK1IP1",318.08988958416,0.0061777632486034,0.206186036327444,0.0299620835563884,0.976097292523162,0.996301760659144
"15090","CWC27",182.31114969788,-0.00546113365431586,0.180204259378423,-0.0303052418025683,0.975823616140402,0.996301760659144
"15091","OCLN",23.5087749226394,0.0202464047984533,0.665219567737572,0.0304356723409562,0.975719595611133,0.996301760659144
"15092","TRIM39",27.3067339336196,-0.00854404152977452,0.303786589394849,-0.0281251438610061,0.977562340102124,0.996301760659144
"15093","AKAP7",42.2258657722327,0.00976307483177141,0.309289380234087,0.031566149553477,0.974818038672581,0.996301760659144
"15094","SND1-IT1",22.2721082350971,0.0174578258143817,0.545496840129114,0.0320035324315528,0.974469233868874,0.996301760659144
"15095","TLN1",836.570385565186,-0.00537527843173983,0.185172842180853,-0.0290284383413523,0.97684190963059,0.996301760659144
"15096","C9orf64",80.4670498223183,-0.00665274792862768,0.230204243697933,-0.0288993279261924,0.976944881636409,0.996301760659144
"15097","TRIM32",34.7954399134415,0.00992938194115399,0.348401778615252,0.0284998026721306,0.977263525411262,0.996301760659144
"15098","EXOSC2",46.1700744663781,0.00736446513259481,0.263344704466804,0.027965115712145,0.977689973887531,0.996301760659144
"15099","DCLRE1C",89.2238055850365,0.00711555722420252,0.232652353917117,0.0305845056127713,0.975600899099632,0.996301760659144
"15100","PCGF6",51.7828990694285,-0.00841686731058636,0.282697808606641,-0.0297733730306272,0.976247794592898,0.996301760659144
"15101","ADD3-

AS1",18.9452617664978,0.0141913748309736,0.457028779116051,0.031051381
1808994,0.975228563149596,0.996301760659144
"15102","CASP7",146.837225256173,0.0080576745591429,0.277816250475191,
0.0290036113631245,0.976861710356044,0.996301760659144
"15103","THAP12",277.255199627364,0.00651685018572809,0.21250553307078
,0.0306667317860259,0.975535322865638,0.996301760659144
"15104","KANSL2",54.1239645119016,0.0065442098270815,0.22454036948720
2,0.0291449143066213,0.97674901456079,0.996301760659144
"15105","AC008147.2",33.0774319823161,-0.0148734857343317,0.5204379034
4822,-0.0285787903528658,0.977200528020969,0.996301760659144
"15106","TSPAN8",105.768275801652,-0.020524638380067,0.731659123043745
,-0.0280521867815756,0.977620528470333,0.996301760659144
"15107","TAOK3",279.766748078872,-0.00631337862952586,0.22048514232466
,-0.0286340320393541,0.977156469563009,0.996301760659144
"15108","CCDC92",130.709888302487,-0.0067804433261851 1,0.2150955544034
47,-0.0315229356784719,0.974852501205878,0.996301760659144
"15109","CDK8",30.7575675321265,-0.00925967585574388,0.290864933447632
,-0.0318349680244662,0.974603660309765,0.996301760659144
"15110","RPS29",12626.9588883097,-0.0082068262459763 2,0.28125838095068
8,-0.02917895715049,0.976721863848451,0.996301760659144
"15111","JKAMP",194.582257728674,0.00787086687646395,0.272932668175291
,0.0288381267405076,0.97699369277364,0.996301760659144
"15112","PNMA1",183.115683256023,0.00635129502613516,0.201889599581861
,0.0314592482192718,0.974903291256074,0.996301760659144
"15113","IGHM",27.6656424516601,-0.0319125455350866,0.997187243041133,
-0.0320025609611316,0.974470008593288,0.996301760659144
"15114","AC009754.1",2.47412665598653,-0.0291265134599227,0.9958691068
77206,-0.029247331058653,0.976667332636423,0.996301760659144
"15115","ZFHX3",444.164937871433,0.00760251675415711,0.262905903887026
,0.0289172538225844,0.976930584815538,0.996301760659144
"15116","PIK3R5",8.07167541045683,0.0246758886543572,0.783650749719316
,0.0314883749721359,0.974880062977484,0.996301760659144
"15117","RNF135",105.514987940588,-0.00527866064061477,0.1874209546707
44,-0.028164730298638,0.977530767202238,0.996301760659144
"15118","COA3",376.637571838923,0.00779631214744506,0.265808888931977,
0.0293305170446734,0.976600988285079,0.996301760659144
"15119","SRSF1",270.186794789121,0.00531101450712341,0.189232927022279
,0.0280660167905034,0.977609498062781,0.996301760659144
"15120","AC022211.2",2.62408893486904,-0.0279726220186225,0.9870168534
44152,-0.028340571815986,0.97739052195449,0.996301760659144
"15121","DNASE2",312.671418303204,0.00687399595781634,0.21711930270104
8,0.0316599946310677,0.974743198540539,0.996301760659144
"15122","NFIX",1289.02459059143,-0.00617708885638407,0.197935618451877
,-0.0312075658979279,0.975104006140614,0.996301760659144
"15123","FAM32A",346.594626377343,-0.00651574736443911,0.2023592983850
54,-0.0321989027261835,0.974313431224471,0.996301760659144
"15124","ZNF235",8.57259761376363,-0.0164552693370761,0.53270127159049
6,-0.0308902385157544,0.975357074745694,0.996301760659144
"15125","PNMA8A",7.14324399424421,0.0162318529497011,0.581700615685379
,0.0279041357564598,0.977738609872598,0.996301760659144

"15126","ZNF600",19.3801609545031,-0.0120708521757424,0.42099149584299
,-0.0286724370799269,0.977125839350484,0.996301760659144
"15127","GID8",198.33215794175,0.00435273373423409,0.151601958274784,0
.0287115930675817,0.977094610249513,0.996301760659144
"15128","HDAC6",58.652578852333,0.00641610040328976,0.230076091631326,
0.0278868628104606,0.977752386328351,0.996301760659144
"15129","NUCKS1",2601.38400307593,0.00432393620562091,0.15646654808180
3,0.0276348923052888,0.977953352249919,0.996434774876559
"15130","GFOD2",59.4808478193806,-0.00621786322175228,0.22560284472576
8,-0.0275611029165453,0.978012205246989,0.996434774876559
"15131","PISD",48.5568165295336,-0.00788365741456003,0.286915964422533
,-0.0274772351215355,0.978079096732344,0.996437068014611
"15132","STARD7-
AS1",10.148689812495,0.0146078135713775,0.557945566409544,0.0261814313
990893,0.979112626402976,0.996445652799236
"15133","LYG2",2.37160510582369,0.0257540772461014,0.993936656813966,0
.0259111856570974,0.979328178178816,0.996445652799236
"15134","KIAA1143",182.084672511125,0.00462182432246213,0.173581763820
813,0.0266262090021923,0.978757868906621,0.996445652799236
"15135","AC093849.2",32.5719244757779,0.011620881894015,0.446229603428
848,0.0260423822281615,0.979223533773038,0.996445652799236
"15136","FARS2",79.0521206080999,0.00764956260145074,0.284932262650869
,0.0268469513781381,0.978581804913752,0.996445652799236
"15137","QSOX2",20.9207828265785,0.00917244563849373,0.34348146968489,
0.0267043390926111,0.978695552272408,0.996445652799236
"15138","MICAL2",2.72415527490812,0.0236177536243305,0.863336954088745
,0.027356356649020З,0.97817550755735,0.996445652799236
"15139","FAR2",37.8102747245847,0.0134937417043427,0.503424158007919,0
.0268039216825397,0.97861612529276,0.996445652799236
"15140","GNPTAB",162.559031956335,0.0061300036274698,0.224761624071262
,0.0272733552838462,0.978241708364266,0.996445652799236
"15141","UCHL3",150.739701659724,-0.0070841951366556,0.2740948810917,-
0.0258457768654408,0.979380349371471,0.996445652799236
"15142","RSKR",8.91846074203526,0.015921697131298,0.615259402350111,0.
0258780232703177,0.979354629065675,0.996445652799236
"15143","MRM1",7.70236286481052,-0.0134512673203148,0.520296300386763,
-0.0258530904607928,0.979374515915919,0.996445652799236
"15144","TOP2A",3.98675777124747,0.0210746008267526,0.790057702321302,
0.0266747615583424,0.978719143326518,0.996445652799236
"15145","SEPTIN4",7.66089440566064,0.0156502030750887,0.60269594125374
5,0.0259669959657148,0.979283662973487,0.996445652799236
"15146","AC008982.2",11.5537928263224,0.0171700436519684,0.64415953338
0592,0.0266549554298434,0.97873494071357,0.996445652799236
"15147","ZNF335",70.0576263196954,-0.00739653405473078,0.2855291230238
07,-0.0259046572076435,0.979333385379958,0.996445652799236
"15148","SH3BGR",13.3568536493292,0.0127694319370224,0.478663418864015
,0.0266772672274129,0.978717144803063,0.996445652799236
"15149","AP000547.3",15.0449773062174,0.0142634012924916,0.53892978126
1202,0.0264661590218904,0.978885525327067,0.996445652799236
"15150","POLA1",21.9519184709087,-0.00936024463017312,0.35741523578091

8,-0.0261887118765989,0.979106819413521,0.996445652799236
"15151","GK",29.057788549106,0.00953969070766858,0.365161634157927,0.0
261245700952877,0.979157979646385,0.996445652799236
"15152","GNB1L",19.4071364429765,0.012361486929296,0.481902091325386,0
.0256514490221404,0.979535349166873,0.996537579686335
"15153","MYO1C",445.138373453316,-0.00471765277377316,0.18458009033709
2,-0.0255588387954382,0.979609217218124,0.996546959903477
"15154","AL049597.2",29.530092683444,-0.0112544357642256,0.45418131545
2442,-0.0247796097754798,0.980230755106605,0.996576308367802
"15155","MAP10",14.3800488477597,-0.0113467188939138,0.45574166087119,
-0.0248972605932571,0.980136912288117,0.996576308367802
"15156","SLC8A1",7.21178094376163,-0.0161145175568323,0.64847522313735
2,-0.0248498585325581,0.980174721962377,0.996576308367802
"15157","IL17RC",122.521335380966,-0.00733155583488659,0.2909381957978
87,-0.0251997020012449,0.97989567465036,0.996576308367802
"15158","SRF",52.4529110094273,0.00640236004113249,0.257213891878407,0
.0248911907299279,0.980141753837912,0.996576308367802
"15159","PLAAT4",515.740137820968,0.00747454299891992,0.30246430746673
8,0.024712148886334,0.980284564630619,0.996576308367802
"15160","AP002360.1",27.1313857219982,-0.00887116212753286,0.358908132
225582,-0.0247170830945595,0.980280628904234,0.996576308367802
"15161","LRMP",4.24023091447273,0.0204578332905237,0.821229328315669,0
.0249112307429185,0.980125769177524,0.996576308367802
"15162","AC092140.1",4.85371712805695,0.0211536148615403,0.85254250343
8975,0.0248123873897326,0.980204610392917,0.996576308367802
"15163","RCAN1",90.1098098882529,0.00617223809014177,0.244423507068815
,0.02525222784077,0.979853778426402,0.996576308367802
"15164","ADPRH",15.6746043919817,0.01037866532343,0.423688863013059,0.
0244959597229492,0.980457006423423,0.996616981221897
"15165","EXOC3",156.516373741247,0.00394187754639221,0.162506612360728
,0.0242567209366358,0.980647834652853,0.996616981221897
"15166","PSMB8",538.134033666503,-0.00630414075580852,0.25868411021706
5,-0.02437003475211,0.980557450027956,0.996616981221897
"15167","DAPK1",10.800327354349,-0.0139141435655303,0.573294231160483,
-0.0242705103405014,0.980636835538599,0.996616981221897
"15168","LINC02352",6.37894849163067,0.0204899431890643,0.842313887050
665,0.0243257810467891,0.980592748911684,0.996616981221897
"15169","DDA1",122.923935635496,0.0049867483390218,0.206868876230646,0
.0241058414870581,0.980768183844801,0.996673581249101
"15170","NIPSNAP2",292.749200279865,0.00493984312101169,0.206123984526
7,0.0239653970029471,0.980880209966197,0.996721716323594
"15171","C1D",318.157222133764,0.00578653456435238,0.244279006470498,0
.0236882188443452,0.981101303370704,0.996792544688915
"15172","OCIAD1",781.051835044609,-0.00522368697499202,0.2216559316697
49,-0.0235666464490331,0.981198277036061,0.996792544688915
"15173","LINC01176",7.14885274847917,0.0165207215682309,0.698342437414
017,0.0236570494403972,0.98112616598948,0.996792544688915
"15174","PARD3",140.225677320647,0.00589632509800403,0.25033492679284,
0.0235537452705647,0.981208567830659,0.996792544688915
"15175","ITGB7",5.78679262016772,0.0178931121006298,0.763927613359431,

0.0234225230083561,0.981313239170457,0.996833184962939
"15176","MRPS14",193.824463901623,0.00563700367991128,0.24358437818465
1,0.0231418932606512,0.98153708863444,0.996916124686127
"15177","WDR66",13.8052164627023,0.0118580892776958,0.513850243584185,
0.0230769361808293,0.981588903048072,0.996916124686127
"15178","GOLGA8A",91.9840618855354,-0.0105544656015632,0.4572162664053
17,-0.0230841865809884,0.981583119606386,0.996916124686127
"15179","AL359397.2",3.1117844609366,-0.018780538990843,0.820665153625
65,-0.0228845332446147,0.981742377848635,0.99694062941612
"15180","RANBP10",42.4338259430739,0.00688016248805882,0.3003128449562
55,0.022909984050336,0.981722076366264,0.99694062941612
"15181","ZFP69B",4.90117774122801,0.0139624479502347,0.689995456540526
,0.0202355650575427,0.983855456875106,0.997051545162714
"15182","ZNF684",42.5096072373681,0.00580819650291175,0.27946143365026
6,0.0207835350554326,0.9834183320256,0.997051545162714
"15183","ZRANB2",493.962149263343,-0.00413558098672614,0.2122130055352
82,-0.0194878771746087,0.984451907821344,0.997051545162714
"15184","CNN3",817.977212301836,-0.00484083659752154,0.24640242329140З
,-0.0196460592102076,0.984325720976129,0.997051545162714
"15185","HCN3",9.9316199316781,0.0114179695285966,0.565647922495561,0.
0201856474222022,0.983895277252134,0.997051545162714
"15186","CD55",1732.50165497068,0.00553032933153672,0.266541791378778,
0.0207484511262913,0.983446318915999,0.997051545162714
"15187","COA6",195.236690461342,0.0055173698946406,0.282818667022793,0
.0195085068207183,0.984435450873813,0.997051545162714
"15188","TBCE",19.1788347819138,-0.00826905336844938,0.376500333705501
,-0.0219629376873739,0.982477519842331,0.997051545162714
"15189","INPP1",136.088573833187,0.0043804911563491,0.212318193620547,
0.0206317276991244,0.983539430804574,0.997051545162714
"15190","CTLA4",4.05913176243287,-0.0164623885108223,0.734637252763939
,-0.0224088670277549,0.982121807263554,0.997051545162714
"15191","RAF1",176.32519074946,-0.00399094659774586,0.189029136241568,
-0.0211128648053793,0.983155622552232,0.997051545162714
"15192","COLQ",8.6105191250958,-0.0129260223271664,0.587228867226226,-
0.0220118986796791,0.982438464064324,0.997051545162714
"15193","COPB2",400.425950956882,-0.00338945059172212,0.17435855267682
2,-0.0194395430547336,0.984490465565126,0.997051545162714
"15194","PCOLCE2",4047.26785607525,0.00504521507793521,0.2507834994714
08,0.0201178111341827,0.983949391790135,0.997051545162714
"15195","HAUS3",65.1979822712463,-0.00569875659994821,0.25249890820879
1,-0.0225694306576404,0.98199372841517,0.997051545162714
"15196","FIG4",62.0548447021072,0.00473360106875853,0.223551046489837,
0.0211745869370096,0.983106386423263,0.997051545162714
"15197","SYTL3",6.41905734392539,0.0131038984722422,0.641922682925316,
0.0204135152422503,0.983713502493101,0.997051545162714
"15198","AC083880.1",4.7026332876178,0.0155897124851267,0.746493505520
304,0.0208839224585895,0.983338251847613,0.997051545162714
"15199","CASP2",68.4368411636449,0.00506252416487317,0.25971779280681,
0.0194924040827611,0.984448296557184,0.997051545162714
"15200","LEPROTL1",144.949768369624,0.00457694032289237,0.214842097257

278,0.021303740660339,0.98300335990188,0.997051545162714
"15201","LRRC69",5.2793047708504,-0.0139657514955366,0.669590308664834
,-0.0208571589445259,0.983359601392058,0.997051545162714
"15202","INTS8",62.9783499708989,-0.00524622362682237,0.23559309141936
,-0.0222681556373994,0.982234050700614,0.997051545162714
"15203","PKN3",4.46876911149384,0.0150576980825447,0.722933722203399,0
.0208286010460973,0.983382382349449,0.997051545162714
"15204","ZMYND19",42.536294405113,0.00557490187861768,0.27853398007366
4,0.0200151589301359,0.984031279910489,0.997051545162714
"15205","KIF1BP",149.477679771973,0.00462949549350658,0.21388106831506
8,0.021645185943652,0.982730988792848,0.997051545162714
"15206","AL121929.3",5.82358736115425,-0.0124493440855793,0.6242140690
67598,-0.0199440299450077,0.984088021303273,0.997051545162714
"15207","MS4A2",2.69513524584372,0.0273078578674038,1.22420064596909,0
.0223066847394013,0.982203316559206,0.997051545162714
"15208","AC026356.2",14.5101988886625,0.00925152899175279,0.4514516484
66897,0.0204928457414442,0.983650219150737,0.997051545162714
"15209","EIF4B",1023.70147046266,0.00503566208591506,0.230008538237213
,0.0218933702396807,0.98253301329132,0.997051545162714
"15210","CPB2-
AS1",7.36850940941403,0.0124731361253272,0.601231834414732,0.020745967
5475587,0.983448300098682,0.997051545162714
"15211","MNAT1",187.14432168232,-0.00351960209783498,0.181206142021047
,-0.0194231942614074,0.984503507552492,0.997051545162714
"15212","TMEM30B",20.6516272472365,-0.00817024545371292,0.389130607649
743,-0.0209961521738402,0.98324872512082,0.997051545162714
"15213","INAFM2",8.98989202438159,-0.0114459300727962,0.51545363646834
7,-0.0222055472364468,0.982283992627994,0.997051545162714
"15214","MRPS11",192.935586695093,-0.00524708499350119,0.2416933534887
35,-0.0217096784738259,0.982679543287571,0.997051545162714
"15215","MEF2A",321.213159423099,0.00523358726510959,0.233022556270122
,0.022459573651929,0.982081359410866,0.997051545162714
"15216","NRN1L",2.71333302650729,-0.0175040662730138,0.88122236839461,
-0.0198633930558325,0.98415234748924,0.997051545162714
"15217","PIEZO2",32.9336324939016,-0.0100409293746624,0.51248975962430
6,-0.0195924487974611,0.984368487665188,0.997051545162714
"15218","TRAPPC5",793.900672270491,0.00517801079886927,0.2482219055137
64,0.0208604103177435,0.983357007735863,0.997051545162714
"15219","AC024075.1",12.2870731521501,-0.0110313504324933,0.5514812145
45526,-0.0200031300097578,0.984040875679241,0.997051545162714
"15220","AC008555.4",5.0084802635946,0.021703184423219,1.0443390601307
,0.0207817415356492,0.983419762738336,0.997051545162714
"15221","PIGT",1128.19866753019,-0.003562986365424,0.178144401495598,-
0.0200005519988909,0.984042932222883,0.997051545162714
"15222","HAND2-
AS1",87.1853233998349,-0.00743297969009861,0.386581413368991,-0.019227
4626586971,0.984659649616327,0.997113314963146
"15223","AC139795.3",7.69756711595683,0.0130443230453349,0.68282437397
9011,0.0191034818650687,0.984758553811154,0.997113314963146
"15224","AC087284.1",3.27156808857534,-0.0150975380383892,0.7873912519

44836,-0.0191741246821052,0.984702199320216,0.997113314963146
"15225","RIDA",87.2862166537393,-0.00516255253394888,0.274639779244696
,-0.0187975410850778,0.985002615407212,0.997229431006317
"15226","TBC1D13",68.4019048057453,0.00410858984573788,0.2183434612238
64,0.0188170958851175,0.984987015693343,0.997229431006317
"15227","NEURL1",2.58786480089173,0.0167019961492662,0.898051846512268
,0.0185980310759687,0.985161773539096,0.997259570469212
"15228","IFT43",175.389051011,0.00510655753694494,0.274081894582459,0.
0186315026197712,0.985135071737686,0.997259570469212
"15229","RAB42",57.7893403733315,-0.00731963789208908,0.40673294554728
9,-0.0179961765384901,0.985641903598494,0.997365882187841
"15230","COL16A1",346.148313990256,0.00356794331118039,0.1993414284184
55,0.0178986542811894,0.985719702570986,0.997365882187841
"15231","AP003392.5",3.61100143638721,-0.0148783673873198,0.8276697469
13635,-0.0179762126654996,0.985657829888215,0.997365882187841
"15232","THSD4",4.99288481089105,-0.0182602953035751,1.00281945451648,
-0.0182089559803958,0.985472157982606,0.997365882187841
"15233","COIL",119.280108061728,-0.00397439486496591,0.21867900689914,
-0.0181745606097388,0.985499596977108,0.997365882187841
"15234","AC123912.4",3.59240114523317,-0.018891350773688,1.03386781620
012,-0.0182725010660659,0.985421464773973,0.997365882187841
"15235","ZNF582",19.0631113456752,0.00662515049250003,0.36785287023081
9,0.0180103270319541,0.985630614967798,0.997365882187841
"15236","PDE4DIP",627.564844033067,0.00454407708407198,0.2568043995399
43,0.0176947010729277,0.985882407918502,0.997371713833889
"15237","FAM161A",27.5900011231633,-0.00625181522280679,0.355883772924
487,-0.0175670140041292,0.98598427162599,0.997371713833889
"15238","LINC00607",6.85155963046977,-0.0103306970186895,0.58738936477
5059,-0.0175874771288133,0.985967946936768,0.997371713833889
"15239","LINC00839",2.57824353596779,-0.0228791583445784,1.30222200829
413,-0.0175693224341596,0.985982430049524,0.997371713833889
"15240","GRSF1",172.345355912663,0.00306352266575214,0.178264571429289
,0.0171852580756201,0.986288822805438,0.997614317817968
"15241","MTBP",9.2990791206567,0.00816231989665197,0.479986019908425,0
.0170053284014589,0.986432364937729,0.997628585849304
"15242","C14orf119",296.442588126854,0.00494561179146673,0.29023864903
5434,0.0170398112308707,0.98640485560648,0.997628585849304
"15243","GINS2",6.9534235286615,-0.0126504825979521,0.752413891226302,
-0.0168131964939324,0.986585642105835,0.997718144266971
"15244","LINC00115",4.77910125305689,-0.0104418052401053,0.72010076733
4062,-0.0145004778688998,0.988430698019908,0.997743194173247
"15245","BEND5",82.5701845610578,0.00414892625017947,0.27729941885429,
0.0149619002712717,0.988062576157161,0.997743194173247
"15246","CC2D1B",20.404448839513,0.00546578328376134,0.398292908140552
,0.0137230243673647,0.989050954387433,0.997743194173247
"15247","SELL",4.91849444778242,-0.0125884885759802,0.85320381974181,-
0.0147543743765582,0.988228139587971,0.997743194173247
"15248","AC012065.4",2.42150576181723,-0.0142155189694134,0.9750721690
63448,-0.0145789403291732,0.988368100651259,0.997743194173247
"15249","DNAJC10",266.697899739289,0.00281546525322681,0.1766069803571

6,0.0159419817242386,0.987280677678382,0.997743194173247
"15250","CRELD1",95.0054323629436,-0.00570037894473676,0.375627239994885,-0.0151756271584946,0.987892066133695,0.997743194173247
"15251","ZDHHC3",242.216269133927,-0.00289036474995529,0.178359187803501,-0.0162053033855459,0.987070604530117,0.997743194173247
"15252","CDV3",583.611060355157,0.00228293180213246,0.163884843766849,0.0139300971930039,0.98888574996932,0.997743194173247
"15253","AC079298.3",5.36322624492275,-0.0107369582954526,0.770900409324917,-0.0139278150142053,0.988887570707914,0.997743194173247
"15254","RPS10-NUDT3",7.18518530614607,0.0105258267470761,0.744106909296124,0.0141455839417387,0.988713833359641,0.997743194173247
"15255","IRAK1BP1",55.5699018036066,0.00412781052390192,0.281775690801397,0.0146492783396681,0.988311985031347,0.997743194173247
"15256","RNF216",208.150134554155,-0.00253291492417887,0.179810575537426,-0.0140865736990629,0.988760912030426,0.997743194173247
"15257","SKAP2",270.68607126867,0.0054067324603628,0.393145107257818,0.0137525111226099,0.989027429580654,0.997743194173247
"15258","PARP12",32.8427763296346,0.00413939832307418,0.302164028820032,0.0136991763686723,0.989069980548906,0.997743194173247
"15259","ZNF34",40.457718766484,-0.00355793937143806,0.252910954591461,-0.0140679527985862,0.988775767887363,0.997743194173247
"15260","ANK3",8.41572752605923,-0.0115708928128635,0.705553625930896,-0.0163997354525633,0.986915490799787,0.997743194173247
"15261","LRRC27",28.9200835898214,-0.00405520268224896,0.294617870983131,-0.0137642793653924,0.989018040770092,0.997743194173247
"15262","NRXN2",28.1462003289615,-0.00980111990885676,0.667759731057773,-0.0146776144966561,0.988289378479698,0.997743194173247
"15263","P3H3",199.906817952382,-0.00363284332985459,0.228883829573851,-0.0158719964473612,0.987336510786038,0.997743194173247
"15264","AL162377.1",24.1500612417334,0.00582332319357634,0.375076870543037,0.0155256792698129,0.987612797865706,0.997743194173247
"15265","TYRO3",26.9491492346826,-0.00613415339078181,0.391165877058109,-0.0156817190622958,0.987488311280174,0.997743194173247
"15266","OSGIN1",25.5526740098805,-0.00632713744488373,0.420990821399159,-0.0150291576995801,0.988008918526749,0.997743194173247
"15267","TRIM16L",10.9458397434908,-0.00733164473562839,0.477067013491682,-0.0153681653274823,0.987738460815323,0.997743194173247
"15268","ATP6V0A1",110.129646105954,-0.00355367312006526,0.214009943327368,-0.0166051776137768,0.986751593990121,0.997743194173247
"15269","TMEM200C",3.23058059330544,0.0119557987438571,0.810618960936412,0.0147489749438453,0.988232447243246,0.997743194173247
"15270","AC016588.2",7.46566104987118,-0.00790726157125746,0.548555785806399,-0.0144146899474107,0.988499139724671,0.997743194173247
"15271","ZNF700",25.1340961766512,0.00496138683314079,0.31976446443007,0.0155157542035938,0.987620715969043,0.997743194173247
"15272","SDHAF1",176.760070059304,0.00369416163160545,0.254040606530781,0.0145416186886558,0.988397875855645,0.997743194173247
"15273","CARD8-AS1",18.4659967403011,-0.00728329438415244,0.448236921381558,-0.016248

7605030479,0.987035935331796,0.997743194173247
"15274","ZNF432",31.9176264193503,0.00539527910060841,0.32951636745192
6,0.0163733265886877,0.986936559195725,0.997743194173247
"15275","WFDC2",6.19297808272245,0.0105037787843373,0.680383076239326,
0.0154380365284727,0.987682718275824,0.997743194173247
"15276","MTFP1",8.21209797864923,0.00915292836903248,0.557719603814955
,0.0164113441708413,0.98690622962907,0.997743194173247
"15277","AL033543.1",2.8446683080989,-0.013701011303912,0.934104816590
205,-0.0146675309457511,0.988297423123293,0.997743194173247
"15278","RPS6KA3",204.067288331316,0.00381531084499023,0.2310288289743
89,0.016514436150361,0.98682398527627,0.997743194173247
"15279","DLG3",19.3085894953437,0.00705550306381289,0.44255642521612,0
.0159426067769039,0.987280179021883,0.997743194173247
"15280","AL136169.1",21.1498639201643,0.00600846922065969,0.4372834772
6776,0.013740444203842,0.989037056679197,0.997743194173247
"15281","CLIC2",169.338181988862,0.00323637986575698,0.231020069543024
,0.0140090853238803,0.988822732708416,0.997743194173247
"15282","CCNG1",370.294491305081,0.00239987170828357,0.179252193815855
,0.0133882417681814,0.98931804771228,0.997797546812666
"15283","PPP1R26",11.6721556432261,-0.00588263970553165,0.434210164678
48,-0.0135479087871827,0.989190663416667,0.997797546812666
"15284","AL355574.1",2.87748965657868,0.0115035756111491,0.85690879688
3803,0.0134245040463845,0.989289117200333,0.997797546812666
"15285","SCAMP3",93.675311613148,0.00305187953563199,0.231640632059755
,0.0131750613374458,0.98948812608454,0.997838509982545
"15286","AP001269.4",5.62242547055252,-0.00909220256760761,0.689787066
265584,-0.0131811728753216,0.989483250206224,0.997838509982545
"15287","LRFN5",23.1895728242276,0.00592705616447097,0.454515544015245
,0.0130403816602412,0.989595575688573,0.997881585611261
"15288","COPB1",470.659302041993,0.00274115054373919,0.2117325074705,0
.0129462904703998,0.989670643259251,0.997892004568378
"15289","EDC4",54.188269933625,-0.00318699085856519,0.247763279655685,
-0.0128630475952455,0.989737055934017,0.997893695939752
"15290","SLC25A53",18.9693080151487,-0.00453033879820845,0.35711922701
5628,-0.0126857879819789,0.989878477103081,0.997971008799476
"15291","ATAD2",45.7890060711104,0.00370042540174698,0.298348667024635
,0.012403023075821,0.99010407310446,0.998133168982097
"15292","PPIB",3247.13096439655,0.00304264705258537,0.24948447985807,0
.0121957368022104,0.990269451112313,0.998169331648225
"15293","SYP",14.2622337215519,0.00558492739887811,0.455012524639415,0
.0122742278430775,0.990206829009936,0.998169331648225
"15294","SPACA6",32.4175099294095,0.00362085270450335,0.30341901897329
1,0.0119335060694468,0.990478665738072,0.998314936076395
"15295","GPN1",89.1803112725627,-0.00268584660537615,0.233182213101868
,-0.0115182310419312,0.99080998449035,0.998387757283223
"15296","CIAO1",277.924736034205,-0.00160895964356997,0.13761803090264
6,-0.0116914886299179,0.990671754244265,0.998387757283223
"15297","ILRUN",102.908086182916,0.00252033967198109,0.216125632025728
,0.0116614565720787,0.990695714726101,0.998387757283223
"15298","C21orf91",54.8038194792552,-0.00299917609016594,0.25911769042

8502,-0.0115745709418999,0.990765034750449,0.998387757283223
"15299","NAGK",257.975202583301,0.002115809436347,0.185583648632935,0.0114008397395605,0.990903643048163,0.998416867611441
"15300","LZTS2",243.322358146297,-0.00191720401190244,0.173353545723092,-0.0110595027284005,0.991175973404826,0.998418138609461
"15301","RAB11A",404.602406433994,0.00192299758703996,0.174613779189787,0.011012862764684,0.991213184445616,0.998418138609461
"15302","VAC14",51.0281515502267,-0.00260401596100783,0.236871878835872,-0.0109933520762595,0.991228750780357,0.998418138609461
"15303","LRRC37A",2.9412273184123,-0.00963160829109853,0.872002675353986,-0.0110453884641909,0.99118723427051,0.998418138609461
"15304","ENDOV",100.085422007754,-0.00275478414735753,0.244779626342557,-0.0112541398502764,0.991020685115596,0.998418138609461
"15305","NUDT16",133.157784286741,0.00271390231649093,0.253999637621658,0.0106846700330076,0.99147502894857,0.998600952057642
"15306","HBS1L",146.103837331215,-0.00194408389932549,0.183537325221351,-0.01059230811488,0.991548718926884,0.998609924360245
"15307","HTT",155.52009079919,0.0027608775253603,0.267578470057996,0.0103180107306911,0.991767564612734,0.998765075358025
"15308","EXO5",22.7171869047313,-0.00360079683725982,0.368482368811429,-0.00977196507087841,0.992203224028819,0.998877527325251
"15309","SGTB",68.7810332752028,0.00216524480081807,0.216905795375884,0.00998242023485742,0.992035313293845,0.998877527325251
"15310","LINC00265",6.19144456505371,0.00649412156693201,0.654414394677319,0.00992356161440207,0.992082273352333,0.998877527325251
"15311","SPAG9",575.898832313446,0.00185160521416117,0.186920296724223,0.00990585424167704,0.992096401097237,0.998877527325251
"15312","U2AF1L5",10.8613399979149,-0.00880439368878356,0.89743023567919,-0.00981067200406976,0.992172341844804,0.998877527325251
"15313","CCDC13",7.60754624065897,-0.00549470089699549,0.573206215668873,-0.00958590599123866,0.992351670742107,0.998961732155004
"15314","SCYL3",42.0262920154944,-0.00252595575297597,0.26877204832951,-0.00939813410165032,0.992501484284407,0.998975710720783
"15315","AC016065.1",20.1963426323053,-0.00370845137569063,0.391189756612206,-0.00947992965819625,0.992436223779982,0.998975710720783
"15316","HSBP1",899.441930821619,0.00215353608650702,0.230946516403112,0.00932482602486225,0.992559973104087,0.998975710720783
"15317","PDLIM5",299.398993432595,-0.0021065016860352,0.232631812661034,-0.00905508864819177,0.992775183303737,0.998996634938776
"15318","ALKBH3",89.4363202203022,-0.00219200654708675,0.240771201815999,-0.00910410601664027,0.992736074714316,0.998996634938776
"15319","IL2RG",11.4504370672489,0.00460567105123742,0.503562608698655,0.00914617362702873,0.992702511014903,0.998996634938776
"15320","THNSL2",84.7374469858338,-0.00374803884303999,0.434633420831635,-0.0086234483208135,0.99311956899995,0.999105157860196
"15321","TANC1",16.6241035622168,0.00458472272368443,0.560034450462489,0.00818650124094734,0.99346819001195,0.999105157860196
"15322","CHROMR",52.3630104836005,-0.00245248120824631,0.284631892609853,-0.00861632611074945,0.993125251490283,0.999105157860196
"15323","AGFG1",215.33747798334,0.00146237334533094,0.171042186204952,

0.00854978165198757,0.993178344330847,0.999105157860196
"15324","Z99289.3",3.58726859745971,0.00715195948383816,0.869548841915674,0.00822490829621706,0.993437546647208,0.999105157860196
"15325","DPYSL2",1973.44298648866,-0.00216840138300197,0.267454032891266,-0.00810756659587756,0.993531168656435,0.999105157860196
"15326","UBE2D1",174.79818316033,-0.00190959115650754,0.222503295663589,-0.00858230504322381,0.993152395371163,0.999105157860196
"15327","PLBD1",558.497148301712,-0.0019658434408359,0.23678469856415,-0.00830224019016718,0.993375846829656,0.999105157860196
"15328","SMYD4",152.57186959287,-0.00205181785201129,0.246411171611407,-0.00832680530916443,0.993356247377953,0.999105157860196
"15329","RAI1",42.9622677601872,0.0026497911417234,0.322878370345456,0.00820677810931814,0.993452011955206,0.999105157860196
"15330","UVSSA",50.4670500655663,-0.00257645968959547,0.322260518772526,-0.00799495916970863,0.993621013471115,0.999128598893879
"15331","RNF138",66.0675352363628,0.00226915714972736,0.286658956378147,0.00791587738404376,0.993684109610254,0.999128598893879
"15332","TTC5",117.027507945715,0.00176359170360828,0.226822577432094,0.00777520352503827,0.993796347656051,0.999176278314507
"15333","KLHL29",47.3567453779108,-0.00237041388700342,0.379179714206031,-0.00625142590227134,0.995012116277616,0.999177910390309
"15334","KIF15",2.62886590713355,0.00511556355027032,0.832019552413791,0.00614836939279785,0.995094341895039,0.999177910390309
"15335","C4orf36",11.4314570226556,0.00288548688580298,0.464343358989594,0.00621412329893501,0.995041878870788,0.999177910390309
"15336","KIFC1",6.61555623953988,0.00551201740678742,0.749737214262019,0.00735193251973361,0.994134059393727,0.999177910390309
"15337","AL135910.1",12.7453432716857,0.0033303194118135,0.533868570211274,0.00623808854395671,0.995022757742402,0.999177910390309
"15338","AC007384.1",2.5218035371264,-0.00740398255935271,1.12518978946762,-0.0065802077379816,0.994749791727261,0.999177910390309
"15339","ESYT2",355.078419845237,0.00130977051296129,0.171633254005401,0.00763121645948673,0.993911229303953,0.999177910390309
"15340","TEK",14.0020171469506,0.00382126648604029,0.564979178921976,0.00676355276194702,0.994603506819443,0.999177910390309
"15341","APBB1",86.0728392649616,-0.00138492617346202,0.191369835095536,-0.00723690947829177,0.994225832061001,0.999177910390309
"15342","RPL27A",11223.5291290484,0.00140713920974538,0.19661931066278,0.00715666841167369,0.994289853511203,0.999177910390309
"15343","ZP1",3.51830649783658,0.0064715657001234,0.927647018039032,0.00697632350913362,0.994433744331699,0.999177910390309
"15344","ZNF106",923.827077466056,-0.00140148405074096,0.20883687292792,-0.00671090325713067,0.994645514093094,0.999177910390309
"15345","AC005332.2",3.55756899766152,-0.00532984294232566,0.784493332134524,-0.00679399393723808,0.994579218833711,0.999177910390309
"15346","RPRD1A",76.7962796963438,-0.00162389570708593,0.240617330673269,-0.00674887258761506,0.994615219636565,0.999177910390309
"15347","ZNF627",14.3064263974818,-0.00270811670364135,0.414933016979407,-0.00652663584921648,0.99479253499233,0.999177910390309
"15348","AKAP8",86.7530326928847,-0.00173691299450418,0.24745951743541

9,-0.00701897834645812,0.994399711528811,0.999177910390309
"15349","DNTTIP1",134.164976863939,0.00151976825475182,0.222457567323377,0.00683172199101951,0.99454911690069,0.999177910390309
"15350","PRDM15",11.5460884790497,0.00358112556298304,0.489187930376436,0.00732055175651477,0.99415909694643,0.999177910390309
"15351","UBE2G2",227.991187401616,-0.00163538546385838,0.256437497340241,-0.00637732578433546,0.994911664708166,0.999177910390309
"15352","SFI1",30.0558435422076,-0.00231957404375613,0.33947918505453,-0.00683274305428636,0.994548302229088,0.999177910390309
"15353","BEND3",3.97226611601468,-0.00485890459007277,0.826385808547308,-0.00587970478173405,0.995308701363016,0.999203105103264
"15354","FAM210A",73.4814561499471,-0.001324960355545,0.221993670357803,-0.00596846006198949,0.995237886137866,0.999203105103264
"15355","SMIM11A",7.57571034030369,-0.0031616575854675,0.5383197415478,-0.00587319643224855,0.995313894184925,0.999203105103264
"15356","PENK",43.4051725834449,0.00326096114629976,0.56806559100009,0.0057404658862699,0.995419796052748,0.999244344630966
"15357","SUCO",137.367166603996,-0.000939109940546599,0.17016149358881,-0.00551893334232204,0.995596550647908,0.99927609702041
"15358","MIER3",40.6847231834314,0.00157546325883976,0.280550706679637,0.00561560966103284,0.995519415301239,0.99927609702041
"15359","ASB13",57.2760186718591,-0.00145783123354027,0.267144921937325,-0.00545708008585053,0.995645901663086,0.99927609702041
"15360","AC016831.1",59.947662482419,-0.00404333197541264,0.776981994775904,-0.00520389404464747,0.995847912025842,0.99928365863028
"15361","PGR-
AS1",4.83370776832404,-0.00331713987012588,0.634279906155628,-0.00522977290929973,0.99582726396026,0.99928365863028
"15362","RALBP1",570.796417002877,0.00084334454628144,0.159952944174153,0.00527245403725252,0.995793209816742,0.99928365863028
"15363","RAB1A",613.561730966647,0.000765773074955006,0.15493446880665,0.0049425610766488,0.996056422882338,0.999365164970666
"15364","C19orf38",2.74945519494525,0.00434814021025061,0.880264744680948,0.00493958236601489,0.996058799520552,0.999365164970666
"15365","SLC16A1",175.565350411801,0.00111317658768787,0.310955507945882,0.00357985808015211,0.997143692608762,0.99942788160094
"15366","ABL2",829.467808521219,0.00235431670065886,0.64487547719997,0.00365080823181746,0.997087082948137,0.99942788160094
"15367","AC017048.3",2.4711826729831,-0.00323771961429574,0.976005613777168,-0.00331731658977419,0.997353169164273,0.99942788160094
"15368","CHCHD4",35.914490362254,-0.00100535880928004,0.274996918835246,-0.00365589117702937,0.997083027371694,0.99942788160094
"15369","LINC01094",3.0781934335263,0.00356369857183822,0.854730444121115,0.00416938298658898,0.996673323325284,0.99942788160094
"15370","PLAC8",4.91079961357148,-0.00321070408831757,0.720173617210659,-0.00445823619692305,0.996442853953629,0.99942788160094
"15371","GOLPH3",319.406461563064,0.000476309376003649,0.139193511986631,0.00342192225201845,0.997269706395267,0.99942788160094
"15372","HOXA11",31.1052280373569,0.00136851253955511,0.321561355422031,0.00425583645696173,0.996604344048126,0.99942788160094

"15373","MPLKIP",261.566493438125,0.000946473736926772,0.208287512099121,0.00454407336948917,0.996374366492711,0.99942788160094
"15374","TYW1",55.9360767323186,-0.000854492602804153,0.254423227294448,-0.00335854792776147,0.997320271499544,0.99942788160094
"15375","IPMK",107.5363050677,0.00100325765447352,0.268200998666812,0.0037406932094233 9,0.997015365602061,0.99942788160094
"15376","AC037198.1",70.706519322283,0.00332524437370657,0.90572723083908,0.00367135298629148,0.997070690715594,0.99942788160094
"15377","HDGFL3",216.971365385062,-0.000997977044325438,0.235215532898779,-0.00424281947721072,0.996614730001529,0.99942788160094
"15378","LRRC36",6.31069908972728,-0.00274845411675754,0.73796727158053,-0.00372435773590756,0.997028399333381,0.99942788160094
"15379","FAM157C",5.80994679731344,0.0026272949627175,0.718890156127941,0.00365465424769277,0.997084014291921,0.99942788160094
"15380","COPZ2",1162.30131930094,0.000858279463545839,0.25465495264211,0.00337036234575833,0.997310845011181,0.99942788160094
"15381","ZNF223",19.7673324647604,-0.00145582194715095,0.38439956148647,-0.00378726224744195,0.996978209148826,0.99942788160094
"15382","ZNF460-AS1",2.47539890069809,-0.00450703060692895,0.951383869848036,-0.0047373418341104,0.996220162229433,0.99942788160094
"15383","ATXN10",393.971430380483,0.000787637846519238,0.222296352108858,0.00354318835665613,0.997172950629425,0.99942788160094
"15384","CNNM4",18.6825059426973,0.0012233256139125,0.380387704421334,0.00321599673094977,0.997434010283966,0.999443920211085
"15385","PRPF6",282.499441067194,0.00056965681812609,0.186268267743467,0.00305826013752721,0.997559865257094,0.999505058364517
"15386","RIOX2",84.3517675390638,-0.000641647808627544,0.232795130325294,-0.0027562767645996,0.997800812108788,0.999637063439276
"15387","ITSN1",190.567775807379,0.000579621588924101,0.21227001671908,0.00273058624992326,0.997821310096668,0.999637063439276
"15388","ITGA9",48.097866828079,0.000708406171343762,0.286591061665691,0.00247183623671459,0.99802776203828,0.999675423105244
"15389","ZNF425",15.6782239795828,0.00126159489241506,0.517309869226515,0.00243876053302715,0.998054152552041,0.999675423105244
"15390","EML3",61.4627886593694,0.000562814688728149,0.229727675709189,0.00244992113810707,0.998045247704161,0.999675423105244
"15391","NKAPD1",182.090796275204,0.000541768689110895,0.231781793530607,0.00233740830484753,0.998135019699483,0.999691464405661
"15392","WDR47",58.6597080867538,-0.000422073683062773,0.275934083332577,-0.00152961779119565,0.998779542056399,0.999797713807051
"15393","RBM15",28.9513022315626,0.00055366223391797,0.356175514353083,0.00155446461535566,0.998759717182588,0.999797713807051
"15394","OTUD7B",70.6694271109643,-0.000482767902975499,0.289857208174656,-0.00166553699325153,0.998671094362039,0.999797713807051
"15395","MARCO",5.30888944068439,0.00115670271674247,0.730564614850863,0.00158329967429178,0.998736710162569,0.999797713807051
"15396","RTP4",43.7527581895901,0.000333541971538184,0.359084293234439,0.000928868173357896,0.999258870532031,0.999797713807051
"15397","PAICS",77.6709838856903,0.000156384494950722,0.22209204495963

8,0.000704142712446738,0.999438175447564,0.999797713807051
"15398","GYPE",5.2283884009057,0.000492371168982393,0.639267640532088,
0.000770211313328128,0.9993854603453,0.999797713807051
"15399","TTC33",82.6332942284715,0.000229118930470534,0.21132440445263
1,0.00108420478488509,0.999134929910873,0.999797713807051
"15400","AC011374.2",9.76814309184407,-0.000428658492950823,0.47748579
8519046,-0.000897740821361255,0.999283706555248,0.999797713807051
"15401","AARS2",28.3205157954384,-0.000386023242756412,0.3465652117287
46,-0.00111385456385204,0.999111272824292,0.999797713807051
"15402","KMT2E-
AS1",117.994256324423,-0.000236659128031481,0.32637933366946,-0.000725
104513728668,0.999421450354225,0.999797713807051
"15403","CAPN5",82.2264865103137,0.000166286376224309,0.25196044334641
4,0.000659970168395387,0.999473420030273,0.999797713807051
"15404","TMEM126B",327.242332899794,-0.000375138343692037,0.2590418433
91773,-0.00144817662961378,0.998844522629797,0.999797713807051
"15405","IPO5",403.063963476282,0.00022676169373112,0.219699314867547,
0.00103214565720349,0.999176467061839,0.999797713807051
"15406","C15orf40",203.300030258014,0.000353668140925051,0.24738712688
6302,0.00142961416536276,0.998859333318101,0.999797713807051
"15407","C1QTNF1",134.437343244687,0.000681734211109054,0.357202297490
524,0.0019085381474265,0.998477207802931,0.999797713807051
"15408","GPATCH1",52.231454573484,-0.000317800071607032,0.316450007741
023,-0.00100426627850524,0.999198711576136,0.999797713807051
"15409","NRIP1",261.271726269095,0.000241864034857123,0.29738128397712
5,0.000813312901277699,0.999351070264511,0.999797713807051
"15410","NCAPH2",74.9985133732684,0.000168437888962756,0.2357372948884
62,0.000714515236303409,0.999429899373004,0.999797713807051
"15411","EHMT2",119.055857566775,6.93548783284078e-05,0.20212157726124
3,0.000343134460299438,0.99972621831722,0.999920818541393
"15412","WARS",111.623363313486,-0.000104451983086876,0.26777496891990
2,-0.00039007373806519,0.999688766194716,0.999920818541393
"15413","ZNF142",26.1228144668409,-8.33425110144964e-05,0.323009550373
781,-0.000258018720864612,0.999794130848508,0.999923864726514
"15414","AL109811.2",11.4922822286378,6.95619766910785e-05,0.537298599
849906,0.000129466141751552,0.999896700964638,0.999957593667592
"15415","FAM160A2",59.6498163500879,1.25248002816982e-05,0.23565689838
6346,5.31484559436255e-05,0.999957593667592,0.999957593667592
"15416","AL627309.1",1.00859034074865,-0.435797724977332,1.42291014608
741,-0.306272132625978,0.75939745530186,NA
"15417","AL627309.4",0,NA,NA,NA,NA,NA
"15418","LINC02593",1.14184429646053,0.332343452219126,1.4056282725974
5,0.236437654747077,0.813093080067267,NA
"15419","PLEKHN1",0.386471127445929,-0.0386990368869363,2.948184401817
32,-0.0131263963214382,0.989526951792023,NA
"15420","AL645608.7",1.52315122773972,-2.58211824294629,1.403324413960
56,-1.84000094152061,0.0657680990903939,NA
"15421","AL390719.2",0,NA,NA,NA,NA,NA
"15422","TTLL10",0.11921983002294,-0.0533240139618846,3.35247519753738
,-0.0159058638229612,0.987309491940711,NA

"15423","TNFRSF18",1.91369782089535,0.0759991728967967,1.0872288090647
1,0.0699017283787532,0.944271877449301,NA
"15424","AL162741.1",0.339262812684241,-0.0349910483728248,3.044545146
73679,-0.0114930298899752,0.990830090769536,NA
"15425","LINC01786",0.814733617497782,-0.193339265296354,1.55074217852
977,-0.124675312229952,0.900780602863336,NA
"15426","TAS1R3",0.106924501560529,0.385965786494404,3.34626872025989,
0.115342137395477,0.908173940404949,NA
"15427","AL391244.2",1.39165028575412,1.7292609753857,1.31489514934677
,1.31513221890337,0.188465470112326,NA
"15428","AL691432.4",1.11737048510541,1.16192750615122,1.4089065868925
9,0.824701592682522,0.409541027261111,NA
"15429","FO704657.1",1.23352163826391,1.42074007252515,1.4130372299748
1,1.005451266525,0.314679604482407,NA
"15430","AL109917.1",0.869022315416839,0.093937739887387,1.63454497395
29,0.0574702693314168,0.954170588589465,NA
"15431","CALML6",0,NA,NA,NA,NA,NA
"15432","TMEM52",2.09548613746448,0.147476341872636,1.01417897358913,0
.145414513328673,0.88438360647713,NA
"15433","CFAP74",0.0644961186387932,-0.0533240139618838,3.352475197537
38,-0.015905863822961,0.987309491940711,NA
"15434","PRKCZ",1.80199799011714,-1.78329358926266,1.17202411252997,-1
.52155025668643,0.128121809600567,NA
"15435","AL590822.2",0,NA,NA,NA,NA,NA
"15436","PRKCZ-
AS1",2.28399373463185,1.89996376012645,1.22083918964396,1.556276843210
24,0.119642275472047,NA
"15437","AL590822.1",0.33713047520475,-2.38103637458962,3.318158042575
58,-0.71757774766552,0.473017682228765,NA
"15438","PLCH2",0.661683056549067,-0.343917858269042,1.78926130366842,
-0.192212203753542,0.847575986925823,NA
"15439","AL139246.1",0.102230937222884,-1.35173207482145,3.35247519753
738,-0.403204198442509,0.686798011343181,NA
"15440","HES5",0.221906102043421,0.695830468846429,3.33747149841039,0.
208490310457437,0.834846147549618,NA
"15441","AL139246.3",0.204917209243366,-0.774663427007578,3.3524751975
3738,-0.231072082972194,0.817258802814834,NA
"15442","MMEL1",0.306195696679323,-0.0320258052725539,3.12823849898919
,-0.0102376482109348,0.991831681239967,NA
"15443","TTC34",1.64551406401988,-0.0989887317513684,1.1959363631575,-
0.0827709021991934,0.934033706394291,NA
"15444","AL513320.1",0.142120095111522,-0.0533240139618842,3.352475197
53738,-0.0159058638229611,0.987309491940711,NA
"15445","TP73",0.102230937222884,-1.35173207482145,3.35247519753738,-0
.403204198442509,0.686798011343181,NA
"15446","AL365330.1",0.453961805915417,0.696522223287419,2.14678704892
418,0.324448679544842,0.745598370244741,NA
"15447","LINC01134",0.427278646084383,1.70712042304806,2.8512221216445
2,0.598732876715838,0.549351029677302,NA
"15448","AL365255.1",0.102230937222884,-1.35173207482145,3.35247519753

738,-0.403204198442509,0.686798011343181,NA
"15449","CHD5",0.408923748891538,-2.68302595843898,3.30195635283568,-0.812556457972206,0.416472404531544,NA
"15450","AL031847.1",1.01805228843917,-1.66014650570637,1.53774197859947,-1.07960017272754,0.280320264501851,NA
"15451","LINC00337",0,NA,NA,NA,NA,NA
"15452","HES2",0,NA,NA,NA,NA,NA
"15453","ESPN",1.16355776238671,-1.91345529175563,1.45028686379071,-1.31936332013262,0.187047678228943,NA
"15454","PLEKHG5",0.448315791790845,0.611004695004865,2.47222544714815,0.247147644123514,0.804793962455449,NA
"15455","CAMTA1-DT",0.773029552193966,0.461207745456809,1.79633622052261,0.256749120898219,0.797372449241136,NA
"15456","Z98884.2",0.333884508879529,-1.5113465063233,2.58424426686441,-0.58483113446435,0.558661267333854,NA
"15457","Z98884.1",0.100823152638361,-1.35173207482145,3.35247519753738,-0.40320419844251,0.686798011343181,NA
"15458","UTS2",1.49278441597327,-0.414389095158588,1.3392266522287,-0.309424169888626,0.756998886985921,NA
"15459","TNFRSF9",0.320866135299661,-1.37464340165215,3.04127275254598,-0.451996092919114,0.651271797119151,NA
"15460","RERE-AS1",2.12285568356943,-0.352693634661574,1.07140161817639,-0.329189006884165,0.742012831553934,NA
"15461","ENO1-AS1",2.13436577996874,-0.887045901846178,1.01034669026297,-0.877961901983666,0.379964394138824,NA
"15462","CA6",0,NA,NA,NA,NA,NA
"15463","BX323043.1",1.03518062743572,-1.45969245692104,1.65231975987607,-0.88342008149229,0.3770093447387,NA
"15464","PIK3CD-AS1",0,NA,NA,NA,NA,NA
"15465","AL139424.3",1.01611858211772,1.06013142516061,1.55276973678511,0.682735759234677,0.49477382938523,NA
"15466","AL354956.1",0.168565237602375,-1.35173207482145,3.35247519753738,-0.40320419844251,0.686798011343181,NA
"15467","AL139423.2",0.102686272020481,-0.0533240139618846,3.35247519753738,-0.0159058638229612,0.987309491940711,NA
"15468","AL139423.1",0.273505233441565,-0.226106766776126,2.74490218220006,-0.082373342205914,0.934349833830783,NA
"15469","C1orf127",0,NA,NA,NA,NA,NA
"15470","MASP2",0,NA,NA,NA,NA,NA
"15471","AL109811.1",1.67650834192902,-1.15568133263117,1.29159545900255,-0.894770359074854,0.370909858358901,NA
"15472","ANGPTL7",0.850333730428894,2.76045730699051,2.38471934904185,1.15756066142527,0.247043370659998,NA
"15473","DISP3",0.341125932066361,1.37790492328387,3.03290540471936,0.454318463457443,0.649599628836418,NA
"15474","DRAXIN",0.535446817354266,2.03137562790896,1.82471980207838,1.11325345710349,0.265599589622981,NA

"15475","AL096840.2",0.738500907215511,0.657005433403435,1.54921663922
283,0.424088805122198,0.671501049531571,NA
"15476","TNFRSF8",0.713737522744808,-0.105587064070375,1.7267913944309
5,-0.0611463923267756,0.951242622512901,NA
"15477","AADACL4",0,NA,NA,NA,NA,NA
"15478","KAZN-
AS1",0.307603481263847,-0.0321837065735757,3.12361882183901,-0.0103033
399429408,0.99177926958558,NA
"15479","TMEM51-
AS1",1.67473541937872,0.0132358536717863,1.1666005477919,0.01134566042
92177,0.990947666920602,NA
"15480","TMEM51",0.833092690776395,-0.950572047199096,1.65013793020735
,-0.576056116157303,0.564577252127531,NA
"15481","AL031283.1",1.41532536934449,-0.455522434715539,1.27242117797
723,-0.357996583678121,0.720345870182927,NA
"15482","CTRC",0.306195696679323,-0.0320258052725539,3.12823849898919,
-0.0102376482109348,0.991831681239967,NA
"15483","CELA2A",0,NA,NA,NA,NA,NA
"15484","AL121992.3",0,NA,NA,NA,NA,NA
"15485","AGMAT",1.32088298460996,-0.199046777988837,1.21012139869447,-
0.164484966717866,0.869349380387406,NA
"15486","AL121992.1",0.622132058049334,1.13749906195951,2.000757867946
45,0.568534094096567,0.569672361897874,NA
"15487","AL121992.2",0.275368352823686,1.11865680170283,2.739778946225
96,0.408301846119294,0.683052084109357,NA
"15488","AL450998.3",0.221906102043421,0.695830468846429,3.33747149841
039,0.208490310457437,0.834846147549618,NA
"15489","CLCNKB",0.102230937222884,-1.35173207482145,3.35247519753738,
-0.403204198442509,0.686798011343181,NA
"15490","AL451042.2",0.798148503719929,-1.43435285640033,1.66949916261
992,-0.859151587802793,0.390256888870483,NA
"15491","SPATA21",1.694495451611,-0.25726189363024,1.29419227258181,-0
.198781818652821,0.842433418785842,NA
"15492","AL137802.2",0.963127969960388,1.09924799784837,1.513956723152
99,0.7260762352302,0.467792036799851,NA
"15493","AL137798.1",0.759926632001453,1.65892682353636,1.910665493964
78,0.868245555685395,0.385259920092006,NA
"15494","BX284668.2",0.362053593518754,0.143931775625763,2.98640704900
717,0.0481956321639453,0.96156033117261,NA
"15495","BX284668.6",1.22260477571273,0.833930219444167,1.394396853180
56,0.598058018807206,0.549801220814632,NA
"15496","PADI2",0.5415107767392,0.0702417634040783,1.89262582391733,0.
0371133916257641,0.970394594411954,NA
"15497","PADI4",0.257924125226034,-1.38354199007994,3.26926698797967,-
0.423196390862814,0.672151979809054,NA
"15498","PADI6",0,NA,NA,NA,NA,NA
"15499","IGSF21",0.730768952112098,-3.4752872980483,2.3415420726883,-1
.48418742442597,0.137759205065048,NA
"15500","PAX7",0,NA,NA,NA,NA,NA
"15501","AL137127.1",1.53931096496922,0.234186582201244,1.274671836122

81,0.183723038012335,0.854230734821922,NA
"15502","HTR6",0.388608220263676,-1.50962735921242,2.47349094131884,-0
.610322574461302,0.541648145797489,NA
"15503","PLA2G2D",0,NA,NA,NA,NA,NA
"15504","Z98257.1",0.311841710803895,0.171849028305229,2.6504567028403
6,0.064837515784003,0.948303371003042,NA
"15505","PLA2G2C",0.142120095111522,-0.0533240139618842,3.352475197537
38,-0.0159058638229611,0.987309491940711,NA
"15506","VWA5B1",0.102686272020481,-0.0533240139618846,3.3524751975373
8,-0.0159058638229612,0.987309491940711,NA
"15507","KIF17",0.386770038522978,-0.777140795601295,2.23651825660742,
-0.347477957448084,0.728232275507767,NA
"15508","AL031005.1",0,NA,NA,NA,NA,NA
"15509","AL031728.1",0,NA,NA,NA,NA,NA
"15510","LINC02596",0.205372544040962,0.695830627438559,3.337471494297
51,0.208490358233014,0.834846110249776,NA
"15511","RAP1GAP",0,NA,NA,NA,NA,NA
"15512","LINC01635",0.274106892219803,1.20013018856317,2.6989713142160
8,0.444662076340649,0.65656397946606,NA
"15513","AL445253.1",0.814451955508494,1.44466822453588,1.950734370161
39,0.740576598553679,0.458950201335191,NA
"15514","TEX46",0.38679665665695,-0.154274896056064,2.50238787680135,-
0.0616510723562424,0.950840704404243,NA
"15515","AL109936.6",0.102686272020481,-0.0533240139618846,3.352475197
53738,-0.0159058638229612,0.987309491940711,NA
"15516","E2F2",0.322273919884185,-1.38549380438952,2.60912169626995,-0
.531019233932344,0.595405450737739,NA
"15517","MDS2",0.376337829442688,0.498741423931096,2.21785614790155,0.
224875461108236,0.82207615960145,NA
"15518","CNR2",0,NA,NA,NA,NA,NA
"15519","MYOM3",0.370691815318116,0.409327796421713,2.565354855683,0.1
59559912545796,0.873227759460615,NA
"15520","IFNLR1",0,NA,NA,NA,NA,NA
"15521","RCAN3AS",0.49440128137563,0.471668930345083,2.14383053342663,
0.220012227175057,0.825861632100525,NA
"15522","NCMAP-
DT",1.05284964339392,-0.695596485407407,1.70287379667367,-0.4084838740
0532,0.682918467285881,NA
"15523","AL031432.1",0.102686272020481,-0.0533240139618846,3.352475197
53738,-0.0159058638229612,0.987309491940711,NA
"15524","AL031432.4",2.32457944006572,0.375476501357327,1.069487714422
16,0.351080705550876,0.725527802523938,NA
"15525","AL031432.5",0.271251509622856,-0.774665352300728,3.1828708973
8686,-0.243385728568736,0.8077065984266,NA
"15526","AL031432.3",0.970696002889987,-0.958051871205916,1.5370341577
6904,-0.623312023589963,0.533079498109511,NA
"15527","RHD",1.38684195603497,-0.1963154661365,1.56683905864099,-0.12
5293957317337,0.900290835849549,NA
"15528","RHCE",1.74267570825501,-0.510068090493125,1.0463804189144,-0.
487459514028665,0.625932727020553,NA

"15529","AL606491.1",1.36803476087338,-2.99672773675174,1.474696340480
64,-2.03209817132593,0.0421437184903384,NA
"15530","AL020996.1",0.839660360468155,1.85320889805573,1.656035440955
26,1.11906354913922,0.26311302894285,NA
"15531","AUNIP",0.221906102043421,0.695830468846429,3.33747149841039,0
.208490310457437,0.834846147549618,NA
"15532","SLC30A2",0,NA,NA,NA,NA,NA
"15533","TRIM63",0.388633157905099,0.432636944008462,2.25154176219256,
0.192151418762563,0.84762359891593,NA
"15534","FAM110D",0.25651634064151,-1.38285975574301,2.79187243968638,
-0.495316238695472,0.62037691003485,NA
"15535","CNKSR1",1.01480632211395,-1.19759671546903,1.47488335863673,-
0.811994188188553,0.416794966355299,NA
"15536","CRYBG2",0.155693188003149,-0.774663427007578,3.35247519753738
,-0.231072082972193,0.817258802814835,NA
"15537","ZNF683",0.49083915748656,-1.97471909140065,2.06099924310347,-
0.958136737802533,0.337993812366009,NA
"15538","AL513365.2",0.244806367132003,0.695807100884759,3.33664907448
574,0.208534696143316,0.834811494458112,NA
"15539","AL512408.1",0.166727055861678,-0.774663427007578,3.3524751975
3738,-0.231072082972193,0.817258802814835,NA
"15540","SFN",1.67438827562641,-1.48382520087609,1.17756855905666,-1.2
6007542360402,0.207642155049065,NA
"15541","TENT5B",0.907272818127666,1.85637767679242,1.68208111213471,1
.10361959563086,0.269758187189889,NA
"15542","FCN3",0.203054089861245,-1.95461545718866,3.33573296423696,-0
.585962808817275,0.557900507779092,NA
"15543","GPR3",0.481984566574001,1.85204954024787,1.89977319937515,0.9
74879286041629,0.329620142128351,NA
"15544","BX293535.1",0.100823152638361,-1.35173207482145,3.35247519753
738,-0.40320419844251,0.686798011343181,NA
"15545","LINC02574",1.47508906802049,-1.47739728876647,1.3934156445408
7,-1.06027034686644,0.289021625545623,NA
"15546","AL445490.1",0,NA,NA,NA,NA,NA
"15547","AL020997.3",0.605458929962323,1.3587872537337,2.2372671312890
7,0.607342428952948,0.543623686302899,NA
"15548","SMPDL3B",0,NA,NA,NA,NA,NA
"15549","MATN1",0.946742223007766,1.85421310415993,1.83918783512513,1.
0081695130578,0.313373091050922,NA
"15550","AL137857.1",0,NA,NA,NA,NA,NA
"15551","LINC01778",0.351019725660226,0.108771112860584,2.571025162754
53,0.0423065143182222,0.966254352262437,NA
"15552","NKAIN1",1.42589211255034,1.326619962987,1.43656688395965,0.92
3465505017357,0.355764668741914,NA
"15553","LINC01226",0.204461874445769,-1.96013641505178,3.335489479203
97,-0.587660799793491,0.556759992345141,NA
"15554","TINAGL1",1.48384839001968,-1.30126035393189,1.21671071456652,
-1.06949033846183,0.284848780051555,NA
"15555","AC114488.2",0,NA,NA,NA,NA,NA
"15556","AC114488.3",0.809498529142306,0.262301872428355,1.85437229081

376,0.14145049175279,0.887514068560467,NA
"15557","SPOCD1",1.3413121018751,-2.02981604173222,1.45970962760746,-1.39056152219754,0.164358431161503,NA
"15558","AL354919.1",0.762689251041947,1.26624442040671,1.94495607385266,0.651040112128842,0.515020593855944,NA
"15559","AL136115.2",2.12558713570254,-1.27837547043398,1.04442827066453,-1.2239954684687,0.220953937000978,NA
"15560","AL136115.1",0.102230937222884,-1.35173207482145,3.35247519753738,-0.403204198442509,0.686798011343181,NA
"15561","AL445248.1",1.31188114559971,1.15107175458679,1.59768470298478,0.720462399393551,0.471240342483413,NA
"15562","AL049795.2",1.33619011938986,1.59631742558297,1.33528149952764,1.19549130737427,0.231895130478488,NA
"15563","AL049795.1",1.15773584353188,1.08805168770263,1.60936468167442,0.676075286162359,0.498992846686479,NA
"15564","DCDC2B",0,NA,NA,NA,NA,NA
"15565","FAM167B",1.55102445354758,1.00508267661213,1.26597832858457,0.79391775824146,0.427243310802941,NA
"15566","AL031602.2",0.102686272020481,-0.0533240139618846,3.35247519753738,-0.0159058638229612,0.987309491940711,NA
"15567","AL020995.1",0,NA,NA,NA,NA,NA
"15568","AL662907.3",0.221906102043421,0.695830468846429,3.33747149841039,0.208490310457437,0.834846147549618,NA
"15569","AL513327.1",1.20316379544403,2.19479195209787,1.79807515044381,1.22063416067796,0.222224566249011,NA
"15570","GJA4",0.25651634064151,-1.38285975574301,2.79187243968638,-0.495316238695472,0.62037691003485,NA
"15571","DLGAP3",0.226144331583469,0.885845475851209,3.33280882803988,0.265795466094045,0.790396735192843,NA
"15572","AL590434.1",0.205372544040962,0.695830627438559,3.33747149429751,0.208490358233014,0.834846110249776,NA
"15573","AC004865.2",1.97816537723635,-0.696967730517225,0.995607211051616,-0.700042871104809,0.483900531505672,NA
"15574","AL139286.3",0.857972143732977,0.611371391372451,1.76500280791917,0.346385506373907,0.729053015967862,NA
"15575","AL139286.2",0.473094938280113,1.94288269051029,2.28130868096837,0.851652696857129,0.394406882262211,NA
"15576","TEKT2",0.244351032334407,-0.774664026145693,3.30062972833046,-0.234701887187309,0.814440122883676,NA
"15577","AL591845.1",0.621599531372288,2.26195232335986,1.93205642067774,1.17074858640329,0.2416998493121,NA
"15578","AL929472.2",0.221906102043421,0.695830468846429,3.33747149841039,0.208490310457437,0.834846147549618,NA
"15579","AL442071.2",0.37209959990264,0.409277752655724,2.56263907640071,0.159709479350703,0.873109933317219,NA
"15580","BMP8A",1.96038796150412,3.07726130724391,1.71351547543621,1.79587599374352,0.0725142411852532,NA
"15581","PABPC4-AS1",0,NA,NA,NA,NA,NA
"15582","HEYL",0.938108402948588,1.99335009475633,1.60009411765212,1.24577052859943,0.212848652213326,NA

"15583","HPCAL4",0,NA,NA,NA,NA,NA
"15584","OXCT2",0.302935778684673,1.24376146070571,3.12847474347089,0.397561611549346,0.690953362451962,NA
"15585","AL033527.4",0.275489739162904,-0.333877366261583,3.20869724067456,-0.104053870221608,0.917126598293383,NA
"15586","MYCL",0.567201673355407,-1.42775860435217,1.85112772890263,-0.771291241581995,0.440534322586199,NA
"15587","MFSD2A",1.05665798389887,-0.794657512640741,1.29938684838476,-0.611563456740049,0.540826622050684,NA
"15588","AL031985.3",1.07571392438411,1.40972506044708,1.60034182441303,0.880889969219005,0.378377377165009,NA
"15589","AL603839.2",0,NA,NA,NA,NA,NA
"15590","RIMS3",0.605160956143808,-0.0391432738249548,2.01725787708008,-0.0194041992695617,0.984518660507438,NA
"15591","KCNQ4",1.0636522115217,1.21277054648301,1.47947499838569,0.819730342051276,0.412369848806259,NA
"15592","SLFNL1-AS1",1.12837058808307,-0.0759367868709002,1.40833800980199,-0.0539194329361151,0.956999353942529,NA
"15593","SLFNL1",0.828263788430103,-0.491056259684204,1.87333965262632,-0.262128791752084,0.793222150035673,NA
"15594","AC093151.2",0.892100105243253,0.45410311552854,1.50989648598449,0.300751157277152,0.763604255763082,NA
"15595","EDN2",0,NA,NA,NA,NA,NA
"15596","RIMKLA",0,NA,NA,NA,NA,NA
"15597","AC098484.4",0.796190616132562,-1.08858648285374,1.69498447729603,-0.642239794779915,0.520717498248407,NA
"15598","AC098484.2",0.721942411571629,-0.513517873086733,1.75714301530339,-0.292245917728028,0.770098605956844,NA
"15599","TMEM269",0.767385890225299,0.191740252801486,1.89148073852936,0.101370449561419,0.919256393446812,NA
"15600","SLC2A1-AS1",0.700517427143603,2.49002744851278,2.12729313451281,1.17051449474218,0.24179398453585,NA
"15601","TIE1",1.87106540052227,-0.67522923700343,1.37762417365747,-0.490140380747499,0.624034565361461,NA
"15602","AL139289.2",1.26656862635967,-0.361220069740563,1.4665092420704,-0.24631284916459,0.805440064108653,NA
"15603","CDC20",0.769705660213809,-1.74478044762802,1.74023459483089,-1.00261220688902,0.31604800379142,NA
"15604","AL139289.1",0,NA,NA,NA,NA,NA
"15605","SZT2-AS1",0.284240190223044,0.69583062743856,3.33747149429751,0.208490358233014,0.834846110249776,NA
"15606","HYI-AS1",0.268005543297635,-0.150415693622294,2.77274999608506,-0.0542478383679276,0.956737707263394,NA
"15607","AL451062.1",1.41969340447111,0.33547428721514,1.53350461622719,0.218763141411658,0.826834560294508,NA
"15608","ARTN",0.312297045601491,1.32499568071923,3.05012201583773,0.4

34407434797427,0.663992599764673,NA
"15609","AL357079.1",1.36495959334706,-0.474668129054523,1.42510525391227,-0.333075839662678,0.739077036287248,NA
"15610","ERI3-IT1",0,NA,NA,NA,NA,NA
"15611","KIF2C",0.520455840138209,1.06350316989514,2.02880066843193,0.524202888161075,0.60013743231107,NA
"15612","AL592166.1",0,NA,NA,NA,NA,NA
"15613","BEST4",1.15401675151453,0.713639336182654,1.2940817600837,0.551463870518116,0.581315727224257,NA
"15614","TCTEX1D4",0.94931796096864,-0.726388017662717,1.50267383868846,-0.48339699471757,0.628813881652869,NA
"15615","LINC01144",0.660340002325551,1.18437385453593,2.01130762095729,0.588857637785025,0.555956777280083,NA
"15616","HPDL",0.362764736551061,1.46681914601225,2.23095446981539,0.657485020809783,0.510869099430787,NA
"15617","TSPAN1",1.20271431722475,-2.72427831310251,1.48779411242277,-1.8310855583816,0.0670877737151732,NA
"15618","RAD54L",0,NA,NA,NA,NA,NA
"15619","TMEM275",0,NA,NA,NA,NA,NA
"15620","MKNK1-AS1",0.380559755157402,1.49425147940121,2.95269759084475,0.506063162050306,0.612812310059885,NA
"15621","TEX38",0,NA,NA,NA,NA,NA
"15622","CYP4A22-AS1",0.792847424025729,1.67576347883257,1.89743998158755,0.883170743261397,0.37714402635372,NA
"15623","CYP4A22",0.203509424658842,-0.774663427007579,3.35247519753738,-0.231072082972194,0.817258802814834,NA
"15624","PDZK1IP1",0.0644961186387932,-0.0533240139618838,3.35247519753738,-0.015905863822961,0.987309491940711,NA
"15625","TAL1",0.204461874445769,-1.96013641505178,3.33548947920397,-0.587660799793491,0.556759992345141,NA
"15626","STIL",1.81340522701114,-0.44119568561017,1.21678348418765,-0.362591776880273,0.716909853928744,NA
"15627","SLC5A9",0,NA,NA,NA,NA,NA
"15628","AL109659.2",1.35757425316657,-1.3657235206921,1.32000263559694,-1.0346369650046,0.300838484150528,NA
"15629","AGBL4",0.167182390659274,0.576034712544328,3.34068880739852,0.172429922616139,0.863099549623161,NA
"15630","DMRTA2",0,NA,NA,NA,NA,NA
"15631","LINC01562",1.10393656944753,0.832095435118924,1.55016231919396,0.536779551932078,0.591419897791629,NA
"15632","TTC39A",1.43715706557088,0.461506051180293,1.39052471125418,0.331893455359049,0.739969712427517,NA
"15633","TTC39A-AS1",0.167182390659274,0.576034712544328,3.34068880739852,0.172429922616139,0.863099549623161,NA
"15634","AC104170.2",0.195582345891787,0.494635591052761,3.34300496985029,0.147961368742719,0.882373255878367,NA
"15635","AC104170.1",0.155693188003149,-0.774663427007578,3.3524751975

3738,-0.231072082972193,0.817258802814835,NA
"15636","AL050343.3",1.09488795070845,0.234942941196245,1.455823241551
77,0.16138150188193,0.871792935063535,NA
"15637","RAB3B",0.949923458971979,0.175218939741067,1.47801312512477,0
.118550327302591,0.905631620786316,NA
"15638","AL513218.1",0.593542675993841,2.12857656766778,2.290568643031
26,0.929278663681914,0.352744689640075,NA
"15639","ORC1",0.663976573087361,0.108456413585284,1.83558994298634,0.
0590853169574653,0.952884153579528,NA
"15640","AL445183.1",0.733809023370415,-1.52661665986802,1.81084514448
769,-0.843040977035022,0.399205526801777,NA
"15641","AL606760.2",0.748959734429773,0.170786021892052,1.69751024391
486,0.100609715024858,0.919860284367734,NA
"15642","AL606760.3",1.40061073626438,-0.477576986547089,1.20753257831
034,-0.395498221021372,0.692475234256767,NA
"15643","LINC01771",0,NA,NA,NA,NA,NA
"15644","LINC02812",0,NA,NA,NA,NA,NA
"15645","GLIS1",0.172682080803205,0.494635591052761,3.34300496985029,0
.147961368742719,0.882373255878367,NA
"15646","DIO1",0.156148522800746,0.494635591052761,3.34300496985029,0.
147961368742719,0.882373255878367,NA
"15647","SSBP3-
AS1",0.83989931506687,0.251764666598829,1.69208942051702,0.14878922091
5938,0.881719956422429,NA
"15648","FAM151A",0.155693188003149,-0.774663427007578,3.3524751975373
8,-0.231072082972193,0.817258802814835,NA
"15649","MROH7",0.221450767245824,-0.774663427007578,3.35247519753738,
-0.231072082972194,0.817258802814834,NA
"15650","TTC22",0.102686272020481,-0.0533240139618846,3.35247519753738
,-0.0159058638229612,0.987309491940711,NA
"15651","LEXM",0,NA,NA,NA,NA,NA
"15652","TMEM61",0.532091366774976,2.1203555181467,2.23641577107945,0.
948104348738011,0.343076334613843,NA
"15653","MIR4422HG",0,NA,NA,NA,NA,NA
"15654","LINC01767",0.102686272020481,-0.0533240139618846,3.3524751975
3738,-0.0159058638229612,0.987309491940711,NA
"15655","TACSTD2",2.21204951149255,-1.0263133699256,1.05949436673545,-
0.968682233854547,0.33270375944375,NA
"15656","AL136985.3",0.102686272020481,-0.0533240139618846,3.352475197
53738,-0.0159058638229612,0.987309491940711,NA
"15657","AL136985.2",0.203054089861245,-1.95461545718866,3.33573296423
696,-0.585962808817275,0.557900507779092,NA
"15658","AC093424.1",2.12894951802075,0.132059225165822,1.105774404254
32,0.119426914439095,0.904937139245032,NA
"15659","HOOK1",0.204917209243366,-0.774663427007578,3.35247519753738,
-0.231072082972194,0.817258802814834,NA
"15660","AC099792.1",0,NA,NA,NA,NA,NA
"15661","NFIA-
AS1",1.07511226560587,0.782036602129108,1.77122616592635,0.44152272429
8793,0.658834613953333,NA

"15662","AC099791.4",0.479024101463169,0.886663367987631,2.07888309986113,0.426509488699418,0.669736635334904,NA
"15663","L1TD1",0.618903882618642,1.29957250039615,1.9934577018339,0.651918773696876,0.51445357321842,NA
"15664","ANGPTL3",0.270796174825259,-2.10201442494854,3.17084321545501,-0.662919697417743,0.507381985665671,NA
"15665","AC103923.1",0.819683712759939,-0.41366848839902,1.84621070470723,-0.224063530421692,0.822707869532624,NA
"15666","DNAJC6",0.489482928677431,0.405462049251573,2.38477451752876,0.170021126220242,0.864993522216747,NA
"15667","AL139147.1",0.168565237602375,-1.35173207482145,3.35247519753738,-0.40320419844251,0.686798011343181,NA
"15668","TCTEX1D1",0,NA,NA,NA,NA,NA
"15669","IL23R",0.156148522800746,0.494635591052761,3.34300496985029,0.147961368742719,0.882373255878367,NA
"15670","IL12RB2",0.699994596269974,-1.36183499001207,1.72388286908074,-0.78998116080721,0.429538770640143,NA
"15671","DEPDC1",0.538662540889147,0.0325006829933678,2.09584401413129,0.015507205104116,0.987627536342964,NA
"15672","LRRC7",0.165319271277154,-0.774663427007578,3.35247519753738,-0.231072082972193,0.817258802814835,NA
"15673","AL353771.1",0,NA,NA,NA,NA,NA
"15674","AL354872.2",0.81248831292643,1.68104394447359,1.74997760047385,0.960608835232179,0.336748884601901,NA
"15675","AL031429.1",0.963686193694558,-3.28060944727923,1.91801176918261,-1.71042195881692,0.0871878733010214,NA
"15676","AL031429.2",0.142120095111522,-0.0533240139618842,3.35247519753738,-0.0159058638229611,0.987309491940711,NA
"15677","ZRANB2-AS1",0.362163077772823,-0.055843711240554,2.5915710022774,-0.0215482080913392,0.982808347881005,NA
"15678","NEGR1-IT1",0,NA,NA,NA,NA,NA
"15679","LRRIQ3",1.03237582966993,0.656000641556894,1.69749157713551,0.386452958231396,0.699161235511333,NA
"15680","AC105271.1",0.378036833949638,1.57816511636365,2.44326216676748,0.645925409818635,0.518327662756108,NA
"15681","ERICH3",0.128992237277586,0.513744972953418,3.34245151459318,0.153703044220807,0.877843884367224,NA
"15682","AC135803.1",1.75610595594819,1.45994885648468,1.22076340987692,1.19593104173386,0.231723470292331,NA
"15683","LHX8",0,NA,NA,NA,NA,NA
"15684","SLC44A5",1.67966412007125,0.490693903336841,1.58882503333498,0.308840742713414,0.757442674925092,NA
"15685","ST6GALNAC3",0,NA,NA,NA,NA,NA
"15686","AC093433.2",0.193488355916379,0.860398995964988,3.33340328940337,0.258114281791264,0.796318709677277,NA
"15687","AC093575.1",0,NA,NA,NA,NA,NA
"15688","AC096531.2",0.505523547776327,-3.00137143189983,2.57173918032013,-1.16705902949545,0.24318653187626,NA
"15689","LINC01781",0.309302485770796,1.17088912763199,2.6618389491026

3,0.439879778611974,0.660024182340411,NA
"15690","LINC01725",0.627876785024713,-1.41975607657452,1.989153746108
7,-0.713748788575005,0.475382531722742,NA
"15691","TTLL7-
IT1",1.11126913618324,0.358001360216399,1.34976531950164,0.26523230004
7967,0.79083051382488,NA
"15692","DNASE2B",1.05607144916154,-2.46071779388004,1.76920679578663,
-1.39085933862579,0.164268085031358,NA
"15693","AL359762.1",1.84417132918356,-0.919869767369912,1.46992542172
203,-0.625793495218469,0.531450398950271,NA
"15694","LPAR3",0.219614265997676,-1.35173207482145,3.35247519753738,-
0.40320419844251,0.686798011343181,NA
"15695","AL360219.1",0.849673402814065,-0.538900907285795,1.7409177724
1491,-0.309549891341657,0.756903266574559,NA
"15696","AC092807.2",0.297813283114671,-0.22601258613788,2.68166806192
223,-0.0842805973442787,0.932833338585223,NA
"15697","LINC02795",0.536829664297366,0.741943666966457,2.071899161747
1,0.358098347962467,0.720269715326313,NA
"15698","CLCA4-
AS1",1.28564139425395,0.997954405785614,1.40833099218638,0.70860785662
0643,0.478567859437644,NA
"15699","AL121989.1",0,NA,NA,NA,NA,NA
"15700","LINC02801",0,NA,NA,NA,NA,NA
"15701","PKN2-
AS1",0.673870366681226,-0.93221302514625,1.93010375512878,-0.482985965
220326,0.6291057014913,NA
"15702","GBP7",0.700571317406789,-1.62482876266137,1.92788135819891,-0
.842805370647567,0.399337303850669,NA
"15703","GBP6",0.258834794821227,1.09609913055011,3.27112624850828,0.3
35083102050849,0.737562399373167,NA
"15704","AC099568.1",0.626118881094856,1.41412238710867,2.192112242360
66,0.645095793811093,0.518865109467947,NA
"15705","AC099568.2",0.796870739290713,-1.11655482807898,1.74943056347
982,-0.638239008387978,0.523318108027411,NA
"15706","LINC01763",0.351730868692533,1.42565098196998,2.5401900302629
9,0.561237925110027,0.574635352232741,NA
"15707","BRDT",0,NA,NA,NA,NA,NA
"15708","EPHX4",0.531455976801571,-0.548448827074067,2.07372297825221,
-0.264475454448749,0.791413576162138,NA
"15709","AC104836.1",1.13537399132876,3.09650946109914,1.9999758072738
9,1.54827345902744,0.121556471703469,NA
"15710","GFI1",2.01194363627358,-0.249188165039233,1.05225728753799,-0
.23681296199171,0.81280189533543,NA
"15711","AC093577.1",0.594127088285279,-0.0669832411982057,1.851443120
84111,-0.0361789354715771,0.971139682050546,NA
"15712","AL049796.1",0.698474252585674,0.162818544639544,1.76123811159
036,0.0924455038578076,0.92634408733918,NA
"15713","ABCA4",0.0534622507802646,-0.0533240139618838,3.3524751975373
8,-0.015905863822961,0.987309491940711,NA
"15714","ARHGAP29-

AS1",1.91252881002651,-0.396355283272311,1.1541605160428,-0.3434143498
78532,0.731286765576927,NA
"15715","AC118469.2",0.333884508879529,-1.5113465063233,2.584244266864
41,-0.58483113446435,0.558661267333854,NA
"15716","AC092802.4",0,NA,NA,NA,NA,NA
"15717","DPYD-
AS1",0.512811910429638,0.896679482292354,2.11836189794499,0.4232890910
48237,0.672084352498009,NA
"15718","DPYD-
IT1",1.3837953017039,0.675047018815108,1.37123125469924,0.492292614029
695,0.6225125007409,NA
"15719","PLPPR5",0.954720642981483,2.90004056625135,1.99774167277724,1
.45165944414612,0.146596321248994,NA
"15720","LRRC39",0.995870313129785,0.30279217268156,1.48037161049892,0
.204537948805647,0.837933139901965,NA
"15721","AL445928.2",1.02112023315527,2.07124790967447,1.6739958173896
2,1.2373076970433,0.215972871412208,NA
"15722","GPR88",0,NA,NA,NA,NA,NA
"15723","AC093157.2",0.517856265775972,-0.238521814235107,1.9432285801
3899,-0.122745114328262,0.902308937887642,NA
"15724","AL109741.1",0.11921983002294,-0.0533240139618846,3.3524751975
3738,-0.0159058638229612,0.987309491940711,NA
"15725","COL11A1",2.07472517235168,0.160806301640616,1.55191494128542,
0.103617986632324,0.917472513231124,NA
"15726","VAV3-AS1",0,NA,NA,NA,NA,NA
"15727","LINC02785",0.155693188003149,-0.774663427007578,3.35247519753
738,-0.231072082972193,0.817258802814835,NA
"15728","AL160171.1",0.155693188003149,-0.774663427007578,3.3524751975
3738,-0.231072082972193,0.817258802814835,NA
"15729","FNDC7",0,NA,NA,NA,NA,NA
"15730","SPATA42",0.182454488057851,0.814003878402388,3.33451052748657
,0.244114952312343,0.80714179253596,NA
"15731","AL356488.2",1.43083291691937,1.71740447805871,1.3913969955749
4,1.23430227571324,0.217090288255334,NA
"15732","KIAA1324",1.49536421080977,-0.836921264012715,1.1907283133634
6,-0.702865006752595,0.482139880580005,NA
"15733","CELSR2",1.54255546537063,0.196750542026584,1.26859848117815,0
.155092840599858,0.876748126266658,NA
"15734","SYPL2",1.28022273829636,1.54800539243345,1.45336329438663,1.0
651193672032,0.286821916088735,NA
"15735","GNAT2",0.0644961186387932,-0.0533240139618838,3.3524751975373
8,-0.015905863822961,0.987309491940711,NA
"15736","AC000032.1",0.983727571763782,-0.209303721964489,1.3513273094
4746,-0.154887509858785,0.8769100005036,NA
"15737","ALX3",1.86176646646686,1.02780322731956,1.2670575677039,0.811
173267511509,0.417266175034448,NA
"15738","UBL4B",0.563153420449001,0.845131521674386,2.06534492446433,0
.409196309857822,0.682395602238133,NA
"15739","RBM15-
AS1",0.11921983002294,-0.0533240139618846,3.35247519753738,-0.01590586

38229612,0.987309491940711,NA
"15740","AL365361.1",2.22525256456104,0.687143734267363,1.08940090844229,0.630753773879162,0.528201532770674,NA
"15741","KCNA3",0.523957614698141,0.975101415462914,2.08106990456499,0.468557742017196,0.639385788682183,NA
"15742","AL360270.2",0.634406850610506,0.378594911230237,1.81193144713381,0.208945493952938,0.834490788856604,NA
"15743","AL360270.1",0.481382907795763,0.732552488271384,2.44638770409598,0.2994425156098,0.764602426668835,NA
"15744","AL360270.3",1.7979284369301,-1.79607550817325,1.15598116059172,-1.55372385762228,0.120250299718156,NA
"15745","AL355816.1",0.142120095111522,-0.0533240139618842,3.35247519753738,-0.0159058638229611,0.987309491940711,NA
"15746","AL355816.2",1.6248077163867,1.13430908054648,1.27945230872584,0.886558313123916,0.375316745962281,NA
"15747","AL390195.1",0.262617689563678,-0.0044714060693364,3.29137992303141,-0.00135852018724662,0.998916058050473,NA
"15748","TMIGD3",1.38050278319598,-1.97863217485623,1.36288801864639,-1.45179365273267,0.146558989323641,NA
"15749","ADORA3",0,NA,NA,NA,NA,NA
"15750","LINC01160",0.102686272020481,-0.0533240139618846,3.35247519753738,-0.0159058638229612,0.987309491940711,NA
"15751","INKA2-AS1",0.715293311802522,-1.02209367992566,1.78416657064663,-0.572868978009843,0.566733410563588,NA
"15752","AL049557.1",0.464970736132523,-0.953144535949503,2.13321814371253,-0.446810626826334,0.655011797800086,NA
"15753","AL354760.1",0.729894427980107,-0.042946622474472,1.75631959128327,-0.0244526239345157,0.98049157302585,NA
"15754","AL603832.1",0.410289753284328,0.547543542914136,2.92149338898395,0.18741905936832,0.85133208015149,NA
"15755","PPM1J",2.09342678633684,0.836545626105775,1.08427671520242,0.771524108538663,0.440396338131055,NA
"15756","TAFA3",0.269388390240736,-2.09481427187859,3.17548848369632,-0.659682528415343,0.509457580965155,NA
"15757","AL390242.1",0.67162967841879,1.3516220468258,2.03544777088786,0.664041625708837,0.506663669728473,NA
"15758","AL137856.1",0,NA,NA,NA,NA,NA
"15759","AP4B1-AS1",1.26967522202779,1.36717705162911,1.55723662347701,0.87795074365543,0.379970449785316,NA
"15760","BCL2L15",0.744010147288686,-0.43308392996085,1.75763416650217,-0.246401633636151,0.80537134175914,NA
"15761","AMPD1",0.100823152638361,-1.35173207482145,3.35247519753738,-0.40320419844251,0.686798011343181,NA
"15762","TSPAN2",2.24891536897063,-2.25167214438416,1.07979885524373,-2.0852699865809,0.0370448001946164,NA
"15763","LINC01765",0.219614265997676,-1.35173207482145,3.35247519753738,-0.40320419844251,0.686798011343181,NA
"15764","CASQ2",0.0534622507802646,-0.0533240139618838,3.3524751975373

8,-0.015905863822961,0.987309491940711,NA
"15765","MAB21L3",1.41946712947843,0.162766250216028,1.32086764036032,0.123226767953545,0.901927529340454,NA
"15766","LINC01779",1.43260250836565,-0.334846586232999,1.25288979696811,-0.267259408643361,0.789269437552959,NA
"15767","LINC01762",0.204461874445769,-1.96013641505178,3.33548947920397,-0.587660799793491,0.556759992345141,NA
"15768","AL390066.1",0.414527982824376,0.61066848644143,2.4538308962282,0.248863313026212,0.803466514579085,NA
"15769","AL157904.1",1.37375870065289,-1.03260162926247,1.39072994007889,-0.742488961734649,0.457791137209204,NA
"15770","CD101",1.50268193964397,-1.03851283613766,1.16473514207959,-0.891630035549055,0.372591265616885,NA
"15771","AL445231.1",0.417609834274426,0.175713628377686,2.43690601757665,0.0721052133772571,0.942518177352526,NA
"15772","AL358072.1",0.425397735807734,0.605016634891276,2.163955093459,0.279588350386782,0.779793345844935,NA
"15773","AL157902.1",0,NA,NA,NA,NA,NA
"15774","AL139420.2",1.7072719973378,1.63021586259385,1.47407683859753,1.10592325983826,0.268759736257601,NA
"15775","AL359915.1",0.49131942992558,0.845567594182551,2.13019309305047,0.396944106588801,0.691408678058255,NA
"15776","HAO2",0.154285403418625,-0.774663427007578,3.35247519753738,-0.231072082972193,0.817258802814835,NA
"15777","HSD3B2",0.409379083689134,-1.78470406805556,2.82546474996282,-0.631649737650787,0.527615778260201,NA
"15778","ADAM30",0,NA,NA,NA,NA,NA
"15779","AC245008.1",0.268005543297635,-0.150415693622294,2.77274999608506,-0.0542478383679276,0.956737707263394,NA
"15780","FCGR1B",0.851019157576901,-1.04181277285866,1.71702935517684,-0.6067530352452,0.544014820047089,NA
"15781","LINC02798",1.0835723618303,0.378699032796003,1.97282925253016,0.191957328446098,0.847775630730321,NA
"15782","AL592494.1",0,NA,NA,NA,NA,NA
"15783","AL592494.2",0.558448362018292,-1.79602275615255,2.64086766675917,-0.680088131169632,0.496448659172799,NA
"15784","AC239800.2",1.63642731234546,-1.46860109858336,1.08587588396281,-1.35245760613435,0.17622897185148,NA
"15785","FAM72C",0.442095408685363,0.693931517896884,1.96769736575265,0.352661710065081,0.724342068163414,NA
"15786","AC246785.3",0.744265955523396,-0.152784886352397,1.77806846567062,-0.0859274484094584,0.931524091527365,NA
"15787","FP700111.1",0.642711338520222,-2.39729897535534,1.90700735243388,-1.25710001710047,0.208717420978706,NA
"15788","PDZK1",2.05708015369457,0.0970746070870282,1.01388910239881,0.0957447977864189,0.923723260819204,NA
"15789","CD160",1.85062310867151,0.788615132560643,1.14847522350121,0.686662730221112,0.492295288174765,NA
"15790","ITGA10",0.860134174192147,0.705669694441825,1.73915495396171,0.405754353764938,0.684923100802983,NA

"15791","ANKRD34A",0.682387202141369,0.536148038604374,2.0003424200085
6,0.268028130204867,0.788677665889944,NA
"15792","AC239799.1",1.79605176981504,0.606216066052044,1.075043272203
99,0.563899223153328,0.572822718777857,NA
"15793","LINC00624",0.94521840054127,-1.38528639592171,1.6856999380240
2,-0.821787059887746,0.411198099698764,NA
"15794","AC239803.1",0.939596332405573,0.492527880011393,1.93426467215
918,0.254633136354393,0.799006463472466,NA
"15795","LINC02804",0.440258907437215,0.231940583716098,2.160672099604
37,0.107346498230142,0.914514097374778,NA
"15796","LINC02805",0.102686272020481,-0.0533240139618846,3.3524751975
3738,-0.0159058638229612,0.987309491940711,NA
"15797","LINC01731",0.559036577248171,-1.04308014632551,2.341988238812
35,-0.445382316204316,0.656043489529887,NA
"15798","AC245100.6",1.00125029742705,0.588576936718592,1.593348376872
01,0.36939626340478,0.711832382726508,NA
"15799","PPIAL4G",0.386926462243525,1.51070939514628,2.94063395657504,
0.513735955394397,0.607436610092053,NA
"15800","NUDT4B",1.0107199569805,-0.730461255579624,1.48464313486781,-
0.492011338229548,0.622711328226899,NA
"15801","AC239802.2",0.102686272020481,-0.0533240139618846,3.352475197
53738,-0.0159058638229612,0.987309491940711,NA
"15802","AC239804.1",0.839600403989821,0.860857480124555,1.73759420091
915,0.495430682071324,0.620296141184078,NA
"15803","AC243772.2",1.0461647378085,2.16026995294862,1.53948492110342
,1.40324203461523,0.160544676835167,NA
"15804","HIST2H3D",0.438294723048383,1.76325634544046,2.40772808339573
,0.732332009415971,0.463965946529121,NA
"15805","HIST2H4A",0.41353133857768,0.614206680598069,2.48673302608403
,0.246993414313272,0.804913320826333,NA
"15806","AC242988.2",0.102686272020481,-0.0533240139618846,3.352475197
53738,-0.0159058638229612,0.987309491940711,NA
"15807","AL356356.1",1.18004159658911,1.35112439304943,1.3784380474379
2,0.980185069296907,0.326994773221943,NA
"15808","HORMAD1",0.541914804015833,-1.80012731222464,2.66414887485842
,-0.675685705559643,0.499240213473887,NA
"15809","ANXA9",1.57121127898799,0.552435071893952,1.23274784015303,0.
448133068175056,0.654057163938574,NA
"15810","BNIPL",1.07549850198798,-0.12687668338143,1.42796335257186,-0
.0888514982915469,0.92919992978043,NA
"15811","AL592424.1",0.964640837058796,0.67832136723642,1.675136201634
36,0.404935053385277,0.685525251198272,NA
"15812","TNFAIP8L2",1.77628845266098,-2.56942218662681,1.3057998648822
5,-1.96769984109205,0.049102584133932,NA
"15813","TMOD4",0.100823152638361,-1.35173207482145,3.35247519753738,-
0.40320419844251,0.686798011343181,NA
"15814","CGN",0.0644961186387932,-0.0533240139618838,3.35247519753738,
-0.015905863822961,0.987309491940711,NA
"15815","AL391335.1",0.388179503600051,-2.37645961319578,2.86395305033
592,-0.829783020680815,0.406661472415033,NA

"15816","AL589765.5",0.142120095111522,-0.0533240139618842,3.35247519753738,-0.0159058638229611,0.987309491940711,NA
"15817","TDRKH-AS1",0.380559755157402,1.49425147940121,2.95269759084475,0.506063162050306,0.612812310059885,NA
"15818","LINGO4",0,NA,NA,NA,NA,NA
"15819","RORC",0.155693188003149,-0.774663427007578,3.35247519753738,-0.231072082972193,0.817258802814835,NA
"15820","C2CD4D",0.11921983002294,-0.0533240139618846,3.35247519753738,-0.0159058638229612,0.987309491940711,NA
"15821","C2CD4D-AS1",0.142120095111522,-0.0533240139618842,3.35247519753738,-0.0159058638229611,0.987309491940711,NA
"15822","AL450992.2",0.581797665103006,0.890341464216116,2.28794546315692,0.389144531001023,0.697169233531181,NA
"15823","AL450992.1",0,NA,NA,NA,NA,NA
"15824","AL589986.2",0.166727055861678,-0.774663427007578,3.35247519753738,-0.231072082972193,0.817258802814835,NA
"15825","HRNR",1.19941093854582,0.427694712104334,1.55873070627347,0.274386531543247,0.783787592251238,NA
"15826","FLG",0,NA,NA,NA,NA,NA
"15827","FLG2",0.572804441364428,2.13970439276979,2.21299260915756,0.966882755918616,0.333602647967332,NA
"15828","SPRR2D",0.11921983002294,-0.0533240139618846,3.35247519753738,-0.0159058638229612,0.987309491940711,NA
"15829","SPRR2A",0.287785067625315,-0.774666036310719,3.12039593109626,-0.248258891953677,0.803934102976972,NA
"15830","SPRR2B",0.937091202092338,0.555446476300087,1.8780443990384,0.295757904650437,0.767414972703242,NA
"15831","SPRR2E",0.341125932066361,1.37790492328387,3.03290540471936,0.454318463457443,0.649599628836418,NA
"15832","LOR",0.242943247749883,-0.774663951729422,3.30711343151841,-0.234241724020258,0.814797324817481,NA
"15833","S100A12",1.95740436109711,0.151960712875876,1.01849171108904,0.149201717816035,0.881394464545291,NA
"15834","S100A7",0.686746818020636,-0.480064233412545,1.94578543822942,-0.246720025744145,0.80512490727118,NA
"15835","BX470102.1",1.14716884787234,-0.900897553060941,1.32241064456538,-0.681254008929296,0.495710778893525,NA
"15836","S100A5",1.43724891866808,-0.167559689994054,1.36347087525835,-0.122892019943078,0.902192604889675,NA
"15837","AL358472.5",0.219614265997676,-1.35173207482145,3.35247519753738,-0.40320419844251,0.686798011343181,NA
"15838","AL358472.3",0.891525064598986,-0.0373622448920701,1.46730610000822,-0.0254631565232782,0.979685535787271,NA
"15839","NUP210L",0,NA,NA,NA,NA,NA
"15840","C1orf189",0.792072092439183,-1.17071181864754,1.64026021793154,-0.713735421885602,0.475390798630246,NA
"15841","AL162591.2",1.92969775800237,0.626207474334096,1.04764165788226,0.597730597693045,0.550019705734567,NA

"15842","UBE2Q1-AS1",0,NA,NA,NA,NA,NA
"15843","CHRNB2",0.102686272020481,-0.0533240139618846,3.3524751975373
8,-0.0159058638229612,0.987309491940711,NA
"15844","KCNN3",1.42831712479039,-0.685553317618192,1.16100573474658,-
0.59048228367953,0.55486735980671,NA
"15845","AL451085.1",1.83235006323475,-1.13223076018935,1.077110242682
6,-1.05117444373147,0.293178478395413,NA
"15846","DCST2",1.39246596148965,0.344501080867889,1.40495976031276,0.
245203521552247,0.806298849806707,NA
"15847","DCST1-
AS1",1.6791485351972,1.11577367736074,1.2659785558761,0.88135274660208
3,0.378126925149819,NA
"15848","TRIM46",0.55990745412378,1.08891901403085,1.87058799834173,0.
582126590674253,0.560481415483309,NA
"15849","AC234582.1",1.95058499135848,-0.951853648223689,1.07942950859
238,-0.881811772465761,0.377878604324253,NA
"15850","AC234582.2",0.168565237602375,-1.35173207482145,3.35247519753
738,-0.40320419844251,0.686798011343181,NA
"15851","ASH1L-
IT1",0.23843966004588,0.69583062743856,3.33747149429751,0.208490358233
014,0.834846110249776,NA
"15852","AL355388.2",1.34366186335932,0.433681321973178,1.201996628609
15,0.360800780676897,0.718248379161593,NA
"15853","PAQR6",1.22911527634152,0.158135708575816,1.30842386869153,0.
120859694140216,0.903802167495147,NA
"15854","TSACC",0.306195696679323,-0.0320258052725539,3.12823849898919
,-0.0102376482109348,0.991831681239967,NA
"15855","C1orf61",1.19066432410982,1.36712267795839,1.49127277969944,0
.916748898369841,0.359274236175292,NA
"15856","IQGAP3",0.221450767245824,-0.774663427007578,3.35247519753738
,-0.231072082972194,0.817258802814834,NA
"15857","TTC24",0,NA,NA,NA,NA,NA
"15858","HAPLN2",0.307439366388676,-0.151735553622885,2.6698589116342,
-0.0568327985279224,0.954678386705447,NA
"15859","BCAN",2.08474040359699,-1.11450251038858,1.01205275218813,-1.
10122966216824,0.270796715051885,NA
"15860","NTRK1",2.18618846253983,1.79827288608761,1.28019949546449,1.4
0468176441137,0.160115930711692,NA
"15861","INSRR",0.362309401753464,0.230443291991928,2.25634854133181,0
.102131070519765,0.918652639244649,NA
"15862","LRRC71",0,NA,NA,NA,NA,NA
"15863","FCRL5",0.213849003121058,0.96816891762709,3.33094636315095,0.
290658813464423,0.771312274283839,NA
"15864","FCRL3",0.833689031701294,1.72077075607155,1.75011234222833,0.
983234455612477,0.325492060483836,NA
"15865","AL356276.1",0,NA,NA,NA,NA,NA
"15866","FCRL2",0,NA,NA,NA,NA,NA
"15867","FCRL1",0.0534622507802646,-0.0533240139618838,3.3524751975373
8,-0.015905863822961,0.987309491940711,NA
"15868","KIRREL1-

IT1",0.322729254681782,-0.0522366723701696,2.65678688704254,-0.0196615967298446,0.984313326223208,NA
"15869","CD1D",1.45687679315789,-1.13880864915152,1.17922548071411,-0.965725951292953,0.334181330162888,NA
"15870","AL138899.1",0.273651557422207,0.0572713106177806,2.75828745228977,0.0207633582824144,0.98343442728806,NA
"15871","CD1A",0,NA,NA,NA,NA,NA
"15872","CD1B",0.462841334469399,-1.25399534357129,2.39492373119974,-0.52360554419121,0.600552924549524,NA
"15873","CD1E",1.2321306962297,-1.08661917969217,1.38414477296945,-0.785047345416773,0.432425809942905,NA
"15874","SPTA1",0.0534622507802646,-0.0533240139618838,3.35247519753738,-0.015905863822961,0.987309491940711,NA
"15875","AIM2",0.897024754742917,-0.0451760603322506,1.52069705859127,-0.0297074687407499,0.976300355358326,NA
"15876","FCRL6",0.915682173982535,-0.227793768545685,1.85529581642371,-0.12278029548128,0.902281078114654,NA
"15877","SLAMF8",2.04229337983529,-2.45728687017369,1.32356740702527,-1.85656344900216,0.0633732881660021,NA
"15878","AL590560.2",1.51674607637787,0.885285797561202,1.30692679516098,0.677379789624836,0.498165017954561,NA
"15879","VSIG8",1.51783689792051,-1.43021624026846,1.34725369159378,-1.06157901009463,0.288426846771441,NA
"15880","CFAP45",1.10230209035548,-1.6797658682374,1.39410650857694,-1.20490497526768,0.228239987726985,NA
"15881","IGSF9",0.448315791790845,0.611004695004865,2.47222544714815,0.247147644123514,0.804793962455449,NA
"15882","SLAMF9",0.8896243411109,-2.02959952923652,1.82570360675144,-1.11168073598095,0.266275445428978,NA
"15883","KCNJ10",1.27348397052482,-0.379734296554388,1.26848678764674,-0.29936007237321,0.764665323645378,NA
"15884","KCNJ9",0.102686272020481,-0.0533240139618846,3.35247519753738,-0.0159058638229612,0.987309491940711,NA
"15885","ATP1A2",0,NA,NA,NA,NA,NA
"15886","ATP1A4",0.221450767245824,-0.774663427007578,3.35247519753738,-0.231072082972194,0.817258802814834,NA
"15887","AL121987.2",0.909455421213975,-1.32577976934175,1.50630050294288,-0.880156228290141,0.378774682140878,NA
"15888","NHLH1",0.90115552890486,-0.529830151762229,1.94356569813406,-0.272607276548922,0.785155117859169,NA
"15889","SLAMF6",1.59532436401311,-0.283414432789058,1.25982450204647,-0.224963423340851,0.822007728883487,NA
"15890","SLAMF1",2.15088081492834,0.916850026460695,1.0326746911602,0.88784012459008,0.374626756055105,NA
"15891","AL121985.1",0.389088492702695,1.57791456375365,2.49326979048727,0.632869563403832,0.526818825201591,NA
"15892","LY9",0.709474355563337,-1.60502703880771,2.16107021564283,-0.742700087757344,0.457663276616414,NA
"15893","CD244",1.00430352609433,2.97257147930481,1.52056438253652,1.95491326342008,0.0505933087859853,NA

"15894","ITLN1",0,NA,NA,NA,NA,NA
"15895","F11R",1.24044745531629,-2.00701451078626,1.68034330285288,-1.19440742101852,0.232318635349334,NA
"15896","NECTIN4",0.271251509622856,-0.774665352300728,3.18287089738686,-0.243385728568736,0.8077065984266,NA
"15897","AL590714.1",0.106924501560529,0.385965786494404,3.34626872025989,0.115342137395477,0.908173940404949,NA
"15898","NR1I3",0.394588182846626,1.58555090478059,2.48513843699939,0.63801311072836,0.523465145618432,NA
"15899","MPZ",1.51422407928716,1.27656954238535,1.30637339780248,0.977185806548673,0.328477173444652,NA
"15900","CFAP126",0,NA,NA,NA,NA,NA
"15901","FCGR3B",1.78650767869423,0.519152717576585,1.32678226235319,0.391287050111608,0.695585069893263,NA
"15902","FCRLA",0.100823152638361,-1.35173207482145,3.35247519753738,-0.40320419844251,0.686798011343181,NA
"15903","NOS1AP",0.578690876011532,-0.31293706568785,1.89703372327304,-0.164961256011794,0.868974477428428,NA
"15904","C1orf226",0.270796174825259,-2.10201442494854,3.17084321545501,-0.662919697417743,0.507381985665671,NA
"15905","SH2D1B",0.973425382838799,0.997596014160322,1.43895141806662,0.693279843666087,0.488133949820036,NA
"15906","AL596325.1",2.21200133741057,-1.84951992377798,1.08241031027938,-1.70870501344412,0.0875056049870058,NA
"15907","RGS4",1.36970597487488,-1.86329294144729,1.35361766004393,-1.37652824460551,0.168658149020915,NA
"15908","AL499616.1",0.102686272020481,-0.0533240139618846,3.35247519753738,-0.0159058638229612,0.987309491940711,NA
"15909","NUF2",1.94597844358649,0.946074664509912,1.13865802805078,0.830868128273291,0.406048130363105,NA
"15910","AL357568.1",1.09890980004962,-1.40394849584417,1.56301836776381,-0.898229045032131,0.36906345165628,NA
"15911","AL356441.1",1.94513639416679,0.175110178468113,1.04772089352728,0.167134376674099,0.867264317750483,NA
"15912","AL451074.5",0.160386752340794,0.715986771710925,3.33695183420161,0.214563112470645,0.830107956063492,NA
"15913","TMCO1-AS1",0.723350196156153,-0.523717511742404,1.64105843411093,-0.319133981372295,0.749624916437823,NA
"15914","AL596087.2",0.842703898263923,-1.02542863398743,1.78279131971754,-0.575181527218733,0.565168534744015,NA
"15915","LINC01675",0.0534622507802646,-0.0533240139618838,3.35247519753738,-0.015905863822961,0.987309491940711,NA
"15916","ILDR2",0,NA,NA,NA,NA,NA
"15917","GPA33",0.102686272020481,-0.0533240139618846,3.35247519753738,-0.0159058638229612,0.987309491940711,NA
"15918","DUSP27",0.100823152638361,-1.35173207482145,3.35247519753738,-0.40320419844251,0.686798011343181,NA
"15919","AL451050.2",0.586617232088785,0.835130349831921,2.00890957573881,0.41571326052582,0.677619836708347,NA

"15920","AL136984.1",0.512976025304809,0.936381618809862,2.77607406361
731,0.33730426398989,0.735887545663924,NA
"15921","AL359962.3",0.256971675439106,-0.226175799809587,2.7950284843
5046,-0.0809207494935953,0.935504978368658,NA
"15922","AL359962.2",0.11921983002294,-0.0533240139618846,3.3524751975
3738,-0.0159058638229612,0.987309491940711,NA
"15923","AL031733.2",0,NA,NA,NA,NA,NA
"15924","ADCY10",0.273505233441565,-0.226106766776126,2.74490218220006
,-0.082373342205914,0.934349833830783,NA
"15925","Z99943.1",0.544617565830674,1.03740947538299,1.87235359000574
,0.554067074146938,0.579532942722168,NA
"15926","AL023755.1",0.413120198239852,0.610630740268671,2.45617295473
343,0.248610644088353,0.803661974175172,NA
"15927","CCDC181",0.554784958553778,0.0622611564471181,1.8838388238726
3,0.0330501503940379,0.973634595232349,NA
"15928","F5",1.4701459922496,-3.67991055942792,1.69993128602649,-2.164
74076903987,0.0304075430579819,NA
"15929","SELP",0,NA,NA,NA,NA,NA
"15930","SELE",0.306692811668653,-2.34839005368482,3.32010114242453,-0
.707324853353681,0.479364624090735,NA
"15931","LINC01681",0.997136529071923,-1.91142367307772,1.675756522233
13,-1.14063328873728,0.254022558404452,NA
"15932","BX284613.2",1.37231015024065,1.46209960355072,1.4921386711370
5,0.979868447774072,0.327151059516962,NA
"15933","MROH9",0,NA,NA,NA,NA,NA
"15934","AL021026.1",0.345629519770364,-0.054409187889382,2.6169885026
1154,-0.0207907630603215,0.98341256615796,NA
"15935","MYOCOS",0.269413327882159,-0.15046778595343,2.76844275514859,
-0.0543510555432646,0.956655473192921,NA
"15936","FASLG",0.978578672628541,-1.49156434101235,1.4413357303776,-1
.03484865432538,0.300739596729849,NA
"15937","ANKRD45",1.82801918793534,0.768048239374715,1.19444032635176,
0.643019347580642,0.520211545808557,NA
"15938","GAS5-
AS1",1.89788789167081,0.50695601214035,1.19245532608797,0.425136272235
451,0.670737337373502,NA
"15939","SERPINC1",0,NA,NA,NA,NA,NA
"15940","RC3H1-
IT1",1.78679669233492,-0.607147407763249,1.17885752197807,-0.515030354
766266,0.606531808538388,NA
"15941","RABGAP1L-
DT",1.15045022588528,-0.905834398286242,1.60402192127174,-0.5647269443
59998,0.572259504367878,NA
"15942","GPR52",0.45325066288311,-0.61028385474067,2.15474811642802,-0
.283227468717946,0.77700247307112,NA
"15943","RABGAP1L-
IT1",2.1488959083523,-0.401203200030266,1.16139755109359,-0.3454486361
30227,0.729757118576975,NA
"15944","Z99127.1",0.11921983002294,-0.0533240139618846,3.352475197537
38,-0.0159058638229612,0.987309491940711,NA

"15945","TNN",0.63073600684676,0.90092384666817,2.279079558907,0.395301622160236,0.692620302389344,NA
"15946","TNR",0,NA,NA,NA,NA,NA
"15947","LINC02803",0.916692228091891,0.113090001206458,1.45500508536784,0.0777248150839746,0.938046954232061,NA
"15948","AL590723.1",0.5415107767392,0.0702417634040783,1.89262582391733,0.0371133916257641,0.970394594411954,NA
"15949","AL591043.2",0.432869479777192,-1.22608949904548,2.41171321006996,-0.508389427866474,0.611180265515232,NA
"15950","ASTN1",0.479479436260766,1.92433648503242,2.30972246837453,0.833146194567123,0.40476228343553,NA
"15951","CLEC20A",0.0534622507802646,-0.0533240139618838,3.35247519753738,-0.015905863822961,0.987309491940711,NA
"15952","TEX35",2.12038722010363,1.21105857007619,1.09875700643185,1.1022078248302,0.270371330946469,NA
"15953","AL449106.1",0.106924501560529,0.385965786494404,3.34626872025989,0.115342137395477,0.908173940404949,NA
"15954","AXDND1",0.267550208500038,-1.3833228396413,2.75673373075219,-0.501797770386714,0.615809781659427,NA
"15955","AL160286.2",1.61683336291124,-0.825603120910292,1.12678413608834,-0.73270744099788,0.463736884618282,NA
"15956","AL353708.3",1.81482223168591,-0.0975289802016085,1.19267513312427,-0.0817732989419565,0.934826989303642,NA
"15957","AL353708.1",2.32383394985985,0.299742293021733,1.09387464094008,0.274018869990563,0.78407012031963,NA
"15958","AL162431.1",1.37237819072441,1.55265438186892,1.46289832765008,1.06135495032182,0.288528621866844,NA
"15959","KIAA1614-AS1",0.442095408685363,0.693931517896884,1.96769736575265,0.352661710065081,0.724342068163414,NA
"15960","LINC01732",0,NA,NA,NA,NA,NA
"15961","TEDDM1",0,NA,NA,NA,NA,NA
"15962","RGSL1",0,NA,NA,NA,NA,NA
"15963","AL355999.1",1.52141659813538,-0.133248064185411,1.27746207353994,-0.104306865108074,0.916925830065597,NA
"15964","SHCBP1L",1.9010507773072,0.621529484904203,1.27320732446027,0.488160469205341,0.62543618243418,NA
"15965","AL445228.2",1.60747134270071,0.924108506537641,1.19797356272639,0.77139307184252,0.440473980391792,NA
"15966","AL713852.1",2.03378874544442,1.71000929727492,1.28134980066446,1.33453745135651,0.182027781467976,NA
"15967","AL713852.2",1.29244087487932,3.30941696805297,1.79355059495774,1.84517625393832,0.0650119035931815,NA
"15968","AL078644.2",0.297557474879961,-0.207493288647577,3.12446022598217,-0.0664093230959124,0.947051947833083,NA
"15969","OCLM",0.102686272020481,-0.0533240139618846,3.35247519753738,-0.0159058638229612,0.987309491940711,NA
"15970","AL096803.4",0.322729254681782,-0.0522366723701696,2.65678688704254,-0.0196615967298446,0.984313326223208,NA
"15971","PACERR",0.782186349000945,0.048083683185238,1.97357006400699,

0.0243638085427849,0.980562416349661,NA
"15972","ERVMER61-1",0.632984190239505,0.281945149292095,1.86776027198647,0.150953606584762,0.880012313004837,NA
"15973","RGS18",1.42767565258425,-2.13763397367293,1.45421639018044,-1.46995590759893,0.14157369618535,NA
"15974","AL390957.1",1.80548599697247,-0.798905186515799,1.14432633684787,-0.698144542156077,0.485086804629276,NA
"15975","AL136987.1",0.168565237602375,-1.35173207482145,3.35247519753738,-0.40320419844251,0.686798011343181,NA
"15976","RGS13",1.88237299736663,0.158547644187828,1.29808665170905,0.122139491981591,0.902788545921892,NA
"15977","B3GALT2",1.10676554847923,1.1047296092113,1.45747227854294,0.757976412639368,0.448465103500574,NA
"15978","LINC01031",0,NA,NA,NA,NA,NA
"15979","AL138778.1",0.0534622507802646,-0.0533240139618838,3.35247519753738,-0.015905863822961,0.987309491940711,NA
"15980","AL136456.1",0.0534622507802646,-0.0533240139618838,3.35247519753738,-0.015905863822961,0.987309491940711,NA
"15981","CFHR3",1.34665736585148,3.32837573717673,1.67488332587414,1.9872284151134,0.0468970903310933,NA
"15982","CFHR1",0.220189306641942,-0.267602342861375,2.93506496557825,-0.0911742486111047,0.927354136344172,NA
"15983","ASPM",1.28823824620949,-2.74983184695231,1.6137524618777,-1.703998544952,0.0883813590757661,NA
"15984","CRB1",1.09630672126497,1.40071524868976,1.47282735601268,0.951038316182456,0.341584925573029,NA
"15985","AL450352.1",0,NA,NA,NA,NA,NA
"15986","AL157402.2",0.142120095111522,-0.0533240139618842,3.35247519753738,-0.0159058638229611,0.987309491940711,NA
"15987","MIR181A1HG",1.80975656335452,0.177374815555825,1.10558467717593,0.160435305605813,0.87253817995772,NA
"15988","NR5A2",0,NA,NA,NA,NA,NA
"15989","LINC00862",0.156148522800746,0.494635591052761,3.34300496985029,0.147961368742719,0.882373255878367,NA
"15990","AL359834.1",0,NA,NA,NA,NA,NA
"15991","KIF14",1.23937017525271,-1.2806093999731,1.32843709441285,-0.963997019775417,0.335047420027023,NA
"15992","DDX59-
AS1",0.730668272497458,2.57215377853561,2.00978913957091,1.2798127564192,0.200610996405684,NA
"15993","INAVA",1.03881398732745,2.11596473666169,1.66124881501914,1.27371933543701,0.202762901447626,NA
"15994","AL358473.1",0,NA,NA,NA,NA,NA
"15995","ASCL5",0.100823152638361,-1.35173207482145,3.35247519753738,-0.40320419844251,0.686798011343181,NA
"15996","IGFN1",0.59577915332575,-0.644815732345979,2.02351755945697,-0.318660803970993,0.749983740158533,NA
"15997","PKP1",0.343766400388244,-1.37533059850399,2.97715619562404,-0.461961183133594,0.644109157014252,NA
"15998","LAD1",0,NA,NA,NA,NA,NA

"15999","TNNI1",0,NA,NA,NA,NA,NA
"16000","AC096677.1",1.83410603537742,0.181893640301199,1.087675892752
01,0.167231471721761,0.867187922238295,NA
"16001","AL645504.1",1.96880373601315,0.102669394546298,1.168649089747
4,0.0878530565308443,0.92999346773292,NA
"16002","ELF3",1.15021353543957,0.519688382262577,1.45527057076104,0.3
57107738384899,0.721011151538132,NA
"16003","GPR37L1",0.324137039266305,-0.0524131773797337,2.653492295942
42,-0.0197525266833754,0.984240788704199,NA
"16004","LGR6",0.377143955248973,-0.842802200139846,2.52682562173735,-
0.333541892598178,0.738725272299152,NA
"16005","SYT2",0.760381966799049,2.57820255871369,2.09567840506692,1.2
3024723281975,0.218604537446964,NA
"16006","AC098934.4",0.183715948661733,0.576034712544328,3.34068880739
852,0.172429922616139,0.863099549623161,NA
"16007","PPFIA4",0.493265823419883,-0.648609686894505,2.11554841579398
,-0.306591748055588,0.759154135443565,NA
"16008","ADORA1",0.0534622507802646,-0.0533240139618838,3.352475197537
38,-0.015905863822961,0.987309491940711,NA
"16009","CHIT1",1.93973361858401,-4.21010556225138,1.40777332949463,-2
.99061324294497,0.00278417874491021,NA
"16010","LINC01353",0.506146292266465,1.88603467024434,2.3772538781346
5,0.793366954868217,0.427564057735052,NA
"16011","LINC01136",0.773241165968907,-0.0845784313169009,1.7760747606
2526,-0.0476209860034977,0.96201830658999,NA
"16012","LAX1",1.15341509273629,0.0934942471635033,1.30882426498822,0.
0714337666748138,0.943052536322074,NA
"16013","LINC00303",0.171420620199322,0.765902268429694,3.335691013369
87,0.22960827767316,0.818396174063793,NA
"16014","AL592146.2",0.527482349049977,-0.142990433014554,2.1253022112
4961,-0.0672800471658475,0.946358760946984,NA
"16015","LINC00628",0,NA,NA,NA,NA,NA
"16016","AL512306.3",0.851432712182764,1.80931134214735,1.774480988049
21,1.01962847409057,0.307904695460201,NA
"16017","LRRN2",2.24562392200695,3.21927918978889,1.44440812198284,2.2
2878779258701,0.0258280275608975,NA
"16018","AL512306.2",0.350443004523411,0.278677423508893,2.58156446456
357,0.107949046918727,0.914036110590592,NA
"16019","NFASC",0.721949772887255,0.207588645048869,1.9839119438927,0.
104636017585313,0.916664633668953,NA
"16020","CNTN2",1.41564409363298,0.157408748031358,1.4359577716106,0.1
09619343370247,0.912711269317349,NA
"16021","NUAK2",1.4975346605163,0.430069940592014,1.32436685284963,0.3
2473626145704,0.745380688036259,NA
"16022","CDK18",0.944962163169096,-0.303505699229135,1.63732288083884,
-0.185367042005571,0.852941157086008,NA
"16023","MFSD4A",0.536724581783482,1.14856381622338,2.05196674310039,0
.559738026985748,0.57565814039046,NA
"16024","SLC45A3",0.891087306127186,-0.221970617700672,1.6016069496804
4,-0.138592441638044,0.889772216366387,NA

"16025","PM20D1",1.97386625964219,-0.176598727727501,1.13641326455468,-0.15540009364173,0.876505910346733,NA
"16026","RHEX",0.779641425968125,0.116907547868288,1.66393294406158,0.0702597711557548,0.943986901310178,NA
"16027","C1orf147",0,NA,NA,NA,NA,NA
"16028","AC244034.2",0.735670462259986,0.609585602697333,1.80069033414392,0.338528835935103,0.734964700539956,NA
"16029","IL19",0.819007607108961,0.880823849669997,1.95896784458704,0.449636706444091,0.652972416228046,NA
"16030","IL20",0.886256245048343,-0.910192809934758,1.46596164723595,-0.620884462871806,0.534675644659674,NA
"16031","IL24",0,NA,NA,NA,NA,NA
"16032","PIGR",0,NA,NA,NA,NA,NA
"16033","C4BPB",0.203054089861245,-1.95461545718866,3.33573296423696,-0.585962808817275,0.557900507779092,NA
"16034","AL445493.3",1.00316345182967,2.05021249447615,1.62128394059007,1.26456103286262,0.206028729127021,NA
"16035","CR2",0.219614265997676,-1.35173207482145,3.35247519753738,-0.40320419844251,0.686798011343181,NA
"16036","CR1",2.15601300938355,0.229490625069378,1.06542361859219,0.215398477248532,0.829456657123683,NA
"16037","CR1L",0.688273128667403,-0.778369704036361,2.23065293250479,-0.348942541752711,0.727132446378234,NA
"16038","AL356275.1",1.71293100516495,2.06923238588434,1.29744343751586,1.59485363758607,0.110744969274736,NA
"16039","IRF6",0.271251509622856,-0.774665352300728,3.18287089738686,-0.243385728568736,0.8077065984266,NA
"16040","KCNH1",0.156148522800746,0.494635591052761,3.34300496985029,0.147961368742719,0.882373255878367,NA
"16041","AL590101.1",0.907898888758143,-0.547047299148904,1.52854405070641,-0.357887820698454,0.720427265549768,NA
"16042","RD3",0,NA,NA,NA,NA,NA
"16043","NEK2",0.102230937222884,-1.35173207482145,3.35247519753738,-0.403204198442509,0.686798011343181,NA
"16044","DTL",0.675342902335073,1.54188956692058,1.9061555504141,0.808900179518727,0.418572565654836,NA
"16045","AL360091.2",0,NA,NA,NA,NA,NA
"16046","AL360091.3",0.244351032334407,-0.774664026145693,3.30062972833046,-0.234701887187309,0.814440122883676,NA
"16047","LINC01740",0.306692811668653,-2.34839005368482,3.32010114242453,-0.707324853353681,0.479364624090735,NA
"16048","AC092803.4",1.49973466982706,-0.665275763265011,1.25894197733609,-0.528440369168344,0.597193724730533,NA
"16049","AL590648.2",0,NA,NA,NA,NA,NA
"16050","LINC02773",0.522550044682349,0.493660772099988,2.35823272327543,0.209335052994399,0.834186689569368,NA
"16051","PROX1-AS1",0.593980764304638,-0.173140762338544,2.07201272934259,-0.083561630624479,0.933404974524611,NA
"16052","AC011700.1",0.205372544040962,0.695830627438559,3.33747149429

751,0.208490358233014,0.834846110249776,NA
"16053","KCNK2",0.806782323779106,-2.10793032846405,1.8330235478209,-1
.14997449485576,0.250154376296835,NA
"16054","TGFB2-
AS1",1.57635210725929,0.534267174429745,1.23896973356717,0.43121890709
2683,0.666309207559588,NA
"16055","LYPLAL1-
DT",0.857611252984382,0.834016047886343,1.5621276893102,0.53389748712
1948,0.593412460733196,NA
"16056","AL360093.1",0.314802175914727,1.16511964632437,2.651977911478
1,0.439339875826864,0.660415284707152,NA
"16057","AC096642.2",0.249044596672051,0.88628321168114,3.274626073559
89,0.270651729929472,0.786658905412859,NA
"16058","SLC30A10",0,NA,NA,NA,NA,NA
"16059","C1orf115",0.324713760403121,-0.225893185321799,2.608892863089
65,-0.0865858420319639,0.931000720053001,NA
"16060","LINC01352",0.508429709064854,0.69367862620079,1.9134119917202
8,0.362534900587264,0.716952347855726,NA
"16061","LINC02817",1.33084229365254,2.41976142120617,1.40430596699122
,1.72310128852518,0.0848702098608272,NA
"16062","LINC01655",0,NA,NA,NA,NA,NA
"16063","LINC02257",0.79325123673363,1.38226839926984,1.74229022473479
,0.793362885038422,0.427566428223141,NA
"16064","AL356108.1",0.635437440621101,1.33082457431499,1.987972500071
06,0.669438120631657,0.503216041245057,NA
"16065","AL513314.2",1.74868372121005,0.385705656550384,1.156838633349
81,0.333413533600199,0.738822148701562,NA
"16066","HHIPL2",0.0644961186387932,-0.0533240139618838,3.352475197537
38,-0.015905863822961,0.987309491940711,NA
"16067","FAM177B",0.204461874445769,-1.96013641505178,3.33548947920397
,-0.587660799793491,0.556759992345141,NA
"16068","AL392172.2",0,NA,NA,NA,NA,NA
"16069","AL929091.1",1.01018849481762,1.27675073970446,1.6738050754404
8,0.762783407959537,0.445592577872426,NA
"16070","SUSD4",0.237178199441998,0.945890913069552,2.83590635755811,0
.333540954393156,0.738725980375699,NA
"16071","CAPN8",0.106924501560529,0.385965786494404,3.34626872025989,0
.115342137395477,0.908173940404949,NA
"16072","AC138393.3",0.205372544040962,0.695830627438559,3.33747149429
751,0.208490358233014,0.834846110249776,NA
"16073","AC092809.2",0.168565237602375,-1.35173207482145,3.35247519753
738,-0.40320419844251,0.686798011343181,NA
"16074","AC092809.4",0.594795717350366,-1.08733999000724,2.06601432875
907,-0.526298377930583,0.598680909866317,NA
"16075","AC096537.1",1.37023600479965,-4.34823958238606,1.629545711496
96,-2.66837533412403,0.00762190584143897,NA
"16076","AC099066.2",0.644601076036314,-1.07007166783785,2.59791101942
449,-0.411896966384516,0.680414944411643,NA
"16077","LEFTY1",1.91317557629588,-2.087273760941,1.2486630474696,-1.6
7160689600836,0.0946018640526724,NA

"16078","AL117348.1",0,NA,NA,NA,NA,NA
"16079","LEFTY2",2.18998657036649,-0.464343597498494,1.285401725685,-0.361243950603101,0.717917089420544,NA
"16080","ACBD3-AS1",0,NA,NA,NA,NA,NA
"16081","MIXL1",0,NA,NA,NA,NA,NA
"16082","ITPKB-IT1",0,NA,NA,NA,NA,NA
"16083","ITPKB-AS1",0.322850641021,-1.51171106944378,2.60963996021438,-0.579279552923307,0.562400559885429,NA
"16084","AL359732.1",0.551499178800657,-1.10038970849099,2.33611426399405,-0.471034197877658,0.637616313896921,NA
"16085","AL353689.3",0.11921983002294,-0.0533240139618846,3.35247519753738,-0.0159058638229612,0.987309491940711,NA
"16086","AL627308.3",0.23843966004588,0.69583062743856,3.33747149429751,0.208490358233014,0.834846110249776,NA
"16087","AL731702.1",0.909346701339299,0.21518242632736,1.9061626014089,0.112887760030709,0.910119540377548,NA
"16088","PRSS38",0,NA,NA,NA,NA,NA
"16089","LINC02809",0.204917209243366,-0.774663427007578,3.35247519753738,-0.231072082972194,0.817258802814834,NA
"16090","IBA57-DT",0,NA,NA,NA,NA,NA
"16091","OBSCN-AS1",0.347492639152484,1.36515954890128,3.01727533496929,0.452447787273339,0.650946427429436,NA
"16092","AL353593.3",0,NA,NA,NA,NA,NA
"16093","AL670729.3",1.40044163645666,0.840355124553822,1.71927035647916,0.48878590931723,0.62499327482658,NA
"16094","TRIM17",2.19973439549071,1.54440933303664,1.10240548587578,1.40094489080823,0.161230553366332,NA
"16095","HIST3H2BB",1.54341298253372,1.05628338858784,1.30051348776915,0.812204870246864,0.416674085428085,NA
"16096","AL117350.1",0.5834093779906,2.18793633594127,1.91232893192176,1.14412133781951,0.252573319657131,NA
"16097","ACTA1",1.01044905839058,-0.472224713405119,1.46739991432481,-0.321810509047495,0.747596252413276,NA
"16098","LINC01736",1.2392133223947,-1.05384953111067,1.50598115531535,-0.699776041281205,0.484067179390576,NA
"16099","AL136988.2",0.221906102043421,0.695830468846429,3.33747149841039,0.208490310457437,0.834846147549618,NA
"16100","AL136988.1",0.766086123971027,0.718273629940659,1.98382457304103,0.362065093709172,0.717303387691052,NA
"16101","PGBD5",0.873010625531556,0.989975263229481,1.70853692986584,0.57942865964695,0.562299970637541,NA
"16102","AL158214.2",0,NA,NA,NA,NA,NA
"16103","AL118511.1",0.911356652823131,0.144770127370962,1.70928039499607,0.0846965353342713,0.932502650474362,NA
"16104","TRIM67",0.102686272020481,-0.0533240139618846,3.35247519753738,-0.0159058638229612,0.987309491940711,NA
"16105","DISC1-IT1",0.571773851353833,1.09689045870771,1.86497812632338,0.58815191622

1196,0.556430328685596,NA
"16106","SLC35F3",0.166727055861678,-0.774663427007578,3.3524751975373
8,-0.231072082972193,0.817258802814835,NA
"16107","LINC01132",0.591662310124921,-2.31771523900407,1.934570314882
47,-1.19805169198251,0.230896893683207,NA
"16108","AL391832.4",0.102230937222884,-1.35173207482145,3.35247519753
738,-0.403204198442509,0.686798011343181,NA
"16109","AL391832.3",0.273505233441565,-0.226106766776126,2.7449021822
0006,-0.082373342205914,0.934349833830783,NA
"16110","LINC01348",0.410289753284328,0.547543542914136,2.921493388983
95,0.18741905936832,0.85133208015149,NA
"16111","AL732292.2",0.865656643244949,0.69210724554715,1.566282041366
92,0.441879066009808,0.65857671987911,NA
"16112","LINC02768",0.339409136664882,0.229924359511437,2.292888735885
26,0.100277155150604,0.920124293611764,NA
"16113","EDARADD",0.355931708695347,-1.7667699881487,2.95338520765146,
-0.598218608115003,0.549694076769176,NA
"16114","AL359921.1",0.347037304354888,-0.0545756175730095,2.614005304
4848,-0.0208781586936244,0.983342849664216,NA
"16115","ACTN2",0.788986389059609,0.727627646135318,1.79707776708003,0
.404894912988434,0.685554757793496,NA
"16116","RYR2",0,NA,NA,NA,NA,NA
"16117","AC093426.1",0,NA,NA,NA,NA,NA
"16118","CHRM3",0,NA,NA,NA,NA,NA
"16119","CHRM3-
AS2",0.450178911172965,1.76774888517765,2.81742879482692,0.62743338480
2275,0.530375189704012,NA
"16120","FMN2",1.85796098055614,0.617655149736452,1.15860699159804,0.5
33101521236752,0.593963306081474,NA
"16121","AL358176.4",0,NA,NA,NA,NA,NA
"16122","RGS7",0,NA,NA,NA,NA,NA
"16123","AL359764.2",0.483367413517102,0.406160622257373,2.14632006088
113,0.189235813269448,0.849907999795882,NA
"16124","KMO",0.788652440601231,-1.20948029634058,1.60780505566527,-0.
752255562376075,0.451897385773939,NA
"16125","AL133390.1",0.249044596672051,0.88628321168114,3.274626073559
89,0.270651729929472,0.786658905412859,NA
"16126","EXO1",0.591233593461296,-3.10691786786195,1.92131913827195,-1
.61707537596088,0.105862011419001,NA
"16127","AL591686.2",0.500251223803273,2.01684082651871,2.023551627873
85,0.996683652019204,0.318918087339069,NA
"16128","AL591686.1",0.669173727497874,-0.264031774240659,2.0359672181
1723,-0.129683706049464,0.896816674382602,NA
"16129","AL591721.1",0.203054089861245,-1.95461545718866,3.33573296423
696,-0.585962808817275,0.557900507779092,NA
"16130","C1orf100",0.659431013222907,-2.38972712282497,2.1832352162675
,-1.09458069612421,0.273700376459465,NA
"16131","AL451007.3",0.275489739162904,-0.333877366261583,3.2086972406
7456,-0.104053870221608,0.917126598293383,NA
"16132","AL451007.2",0.439677569991484,0.279268974458065,2.42445800272

399,0.11518820872306,0.908295944540891,NA
"16133","BX323046.2",1.49404298569627,1.35347873025308,1.4426408382417
3,0.938195214203613,0.348144097385103,NA
"16134","BX323046.1",0.358747277864394,-1.79344174898354,2.50947896308
126,-0.714666978830322,0.474814846659672,NA
"16135","KIF26B",0.816376290182766,0.474101816753772,1.99992636764037,
0.237059636007073,0.812610525679466,NA
"16136","SMYD3-
IT1",0.769996880136235,-0.79786074961557,1.95273053537325,-0.408587224
485157,0.682842607711289,NA
"16137","AL591848.3",0.711389030720852,1.18313728735065,1.984975132820
55,0.596046402691941,0.55114422804883,NA
"16138","ZNF695",0.931252808151773,1.04490676705204,1.57883322780852,0
.661822128295596,0.508085222784455,NA
"16139","AC104335.1",1.64671265872013,-0.37701793959803,1.146689731404
19,-0.328788101325673,0.742315857305814,NA
"16140","AC104335.2",0.32696748422183,0.17244460144307,2.6132994629272
4,0.0659873098699178,0.947387928690436,NA
"16141","OR2B11",0.220042982661301,-0.774663427007579,3.35247519753738
,-0.231072082972194,0.817258802814834,NA
"16142","GCSAML",0.710882140147861,-1.64349215327227,1.91355343949796,
-0.858869221704856,0.390412671815044,NA
"16143","OR2C3",1.0400455979918,1.36555348805387,1.84848084003838,0.73
8743652882829,0.460062673108148,NA
"16144","AL390860.1",0.717248847233984,-1.67130669419584,1.75775715616
471,-0.950817744268214,0.341696903343228,NA
"16145","TRIM58",2.2953987809993,-0.44913291190075,1.16069953573176,-0
.386950195183453,0.69879307969553,NA
"16146","OR2L2",1.59295978229549,0.382276872702557,1.30363527387099,0.
293239129352054,0.769339377086112,NA
"16147","FAM110C",0,NA,NA,NA,NA,NA
"16148","AC079779.2",1.13407311231168,0.93814876998218,1.7167057478835
6,0.546481988039464,0.584734665693255,NA
"16149","AC093326.1",2.27949400803013,-0.333946998842868,1.44633738727
979,-0.230891493077519,0.817399099735423,NA
"16150","AC116609.3",0.786687932056155,2.64144186709427,2.064733744885
1,1.27931355490161,0.200786661195631,NA
"16151","LINC01115",0,NA,NA,NA,NA,NA
"16152","TPO",0,NA,NA,NA,NA,NA
"16153","AC144450.1",0.676887511997439,0.545158577848217,2.26665313391
533,0.240512573225764,0.809932917264621,NA
"16154","MYT1L",0.0644961186387932,-0.0533240139618838,3.3524751975373
8,-0.015905863822961,0.987309491940711,NA
"16155","EIPR1-IT1",0,NA,NA,NA,NA,NA
"16156","TRAPPC12-
AS1",0.853049174725862,0.297456876961083,1.90988164272999,0.1557462359
47845,0.87623305081758,NA
"16157","AC114810.1",0,NA,NA,NA,NA,NA
"16158","SOX11",0,NA,NA,NA,NA,NA
"16159","LINC00487",0.0644961186387932,-0.0533240139618838,3.352475197

53738,-0.015905863822961,0.987309491940711,NA
"16160","NRIR",0.320866135299661,-1.37464340165215,3.04127275254598,-0
.451996092919114,0.651271797119151,NA
"16161","AC017076.1",0.102686272020481,-0.0533240139618846,3.352475197
53738,-0.0159058638229612,0.987309491940711,NA
"16162","RNF144A-
AS1",1.19521837901198,-2.13242382799994,1.62365089215593,-1.3133511879
3207,0.189064637370983,NA
"16163","AC010904.2",0.420894148103731,1.71663245073883,2.394105599680
25,0.717024533490962,0.473358958865676,NA
"16164","AC013460.1",0.417033113137611,0.413097229319217,2.22161725051
422,0.185944374182187,0.852488383400628,NA
"16165","AC092580.4",0.156148522800746,0.494635591052761,3.34300496985
029,0.147961368742719,0.882373255878367,NA
"16166","LINC00298",0,NA,NA,NA,NA,NA
"16167","LINC00299",0.965529504828934,1.99706896345856,1.6726601319273
9,1.19394784710828,0.232498369410133,NA
"16168","U91324.1",1.17337364141132,-0.156571218621887,1.6433645521694
9,-0.0952747936635174,0.924096563304857,NA
"16169","LINC01814",0.20961077358101,0.88584532733971,3.33280883148308
,0.265795421258984,0.790396769724465,NA
"16170","AC092617.1",0,NA,NA,NA,NA,NA
"16171","AC082651.4",0.102686272020481,-0.0533240139618846,3.352475197
53738,-0.0159058638229612,0.987309491940711,NA
"16172","AC082651.3",1.28745873853718,-1.73139353727922,1.420433867927
67,-1.21891879401976,0.222875015650851,NA
"16173","AC010969.1",0.142120095111522,-0.0533240139618842,3.352475197
53738,-0.0159058638229611,0.987309491940711,NA
"16174","RRM2",0.912188640387888,0.857900703074758,1.579471548061,0.54
3156794516457,0.587021851402053,NA
"16175","AC007240.3",0.546025350415198,1.03737835727126,1.871390844990
42,0.554335487986511,0.579349267503447,NA
"16176","ATP6V1C2",0.913962848143078,-2.14023770325648,1.5484338145106
1,-1.38219514660555,0.166911789940007,NA
"16177","AC092687.3",1.15680738304215,-0.0960227690127695,1.3878629033
7692,-0.0691875031598068,0.944840370304166,NA
"16178","C2orf50",0.392871387445147,0.542201704530288,2.19605406031056
,0.246898158988677,0.804987041144592,NA
"16179","LINC00570",1.25575772049498,2.47796943396374,1.52415006924032
,1.62580410155992,0.103991335563797,NA
"16180","AC099344.3",0.645029792699939,-0.175674016925042,2.0411932112
4105,-0.086064374483311,0.931415243553722,NA
"16181","MIR3681HG",0.0534622507802646,-0.0533240139618838,3.352475197
53738,-0.015905863822961,0.987309491940711,NA
"16182","AC009486.1",0,NA,NA,NA,NA,NA
"16183","LINC00276",0.414363867949205,0.555913947657834,2.169634880190
48,0.256224654541426,0.797777372334948,NA
"16184","MYCN",0,NA,NA,NA,NA,NA
"16185","AC079148.1",2.07201924529623,0.74191890620609,1.2074789210407
6,0.614436321229204,0.538927040524367,NA

"16186","NT5C1B",0.102686272020481,-0.0533240139618846,3.3524751975373
8,-0.0159058638229612,0.987309491940711,NA
"16187","AC130814.1",0.388179503600051,-2.37645961319578,2.86395305033
592,-0.82978302068815,0.406661472415033,NA
"16188","AC010096.1",0.142120095111522,-0.0533240139618842,3.352475197
53738,-0.0159058638229611,0.987309491940711,NA
"16189","AC013400.1",1.32485350383014,0.424305043108221,1.247214759777
05,0.340202070078188,0.733704359368856,NA
"16190","AC079145.1",0.944276507588873,1.97853957241215,1.829123712256
44,1.08168712654836,0.279391575321103,NA
"16191","MATN3",2.0568164397365,0.879415413863609,1.16504384281362,0.7
54834609262252,0.450348225018614,NA
"16192","LAPTM4A-
DT",1.38340067976014,-0.0120863125702601,1.25698715324527,-0.009615303
17876026,0.992328216260997,NA
"16193","AC012065.3",1.26201903384676,-0.76515929673513,1.293825336982
82,-0.591393038042885,0.554257104019742,NA
"16194","GDF7",1.01912707290953,-0.274993679882011,2.4072271607536,-0.
11423669704521,0.909050162273575,NA
"16195","APOB",1.17781813626906,3.19078666396819,1.47234443035835,2.16
714689727294,0.0302236549643285,NA
"16196","TDRD15",0,NA,NA,NA,NA,NA
"16197","AC018742.1",0,NA,NA,NA,NA,NA
"16198","AC011752.1",0.142120095111522,-0.0533240139618842,3.352475197
53738,-0.0159058638229611,0.987309491940711,NA
"16199","AC018467.1",0.657240359980972,1.4267240323098,1.9516414724466
3,0.731037976212514,0.464755957067319,NA
"16200","AC009242.1",1.23307971332897,1.0050007493412,1.44393520112039
,0.696015131815745,0.486419353438946,NA
"16201","MFSD2B",0.828436515975984,0.262917691454743,1.55958466638407,
0.168581864852726,0.866125546134242,NA
"16202","AC012073.1",0.6939448031232,-0.871242795360431,1.964811995437
62,-0.443422982648464,0.657459815331197,NA
"16203","EFR3B",1.40769550045362,-0.59194624145956,1.23292230001122,-0
.480116420519096,0.631144612782562,NA
"16204","AC012074.1",0.574187288307529,0.882860482742878,2.06239570861
431,0.428075213236386,0.668596362152897,NA
"16205","ADGRF3",0.117958369419058,0.451348601316665,3.34428110054629,
0.134961322851401,0.892642454377335,NA
"16206","OTOF",0.474785871923121,0.808293718624075,2.42713866470911,0.
333023296269292,0.739116698027793,NA
"16207","KCNK3",0.219614265997676,-1.35173207482145,3.35247519753738,-
0.40320419844251,0.686798011343181,NA
"16208","DPYSL5",0.102686272020481,-0.0533240139618846,3.3524751975373
8,-0.0159058638229612,0.987309491940711,NA
"16209","AC013472.1",0,NA,NA,NA,NA,NA
"16210","AGBL5-
AS1",0.67617564627533,1.22266143822716,1.90148485699089,0.643003510510
213,0.52022182200525,NA
"16211","AC013472.3",0.0534622507802646,-0.0533240139618838,3.35247519

753738,-0.015905863822961,0.987309491940711,NA
"16212","AC013403.2",0.416777304902901,0.279134849681388,2.45910155107754,0.113510907900113,0.909625515721006,NA
"16213","TCF23",1.99893257269711,0.555628150067095,1.23163801739938,0.451129424569333,0.651896271644896,NA
"16214","SLC30A3",0.11921983002294,-0.0533240139618846,3.35247519753738,-0.0159058638229612,0.987309491940711,NA
"16215","TRIM54",0,NA,NA,NA,NA,NA
"16216","GTF3C2-AS1",0.7422941162666,1.18686327460034,2.21287357323326,0.536344818319737,0.591720261052158,NA
"16217","KRTCAP3",2.26597721751864,0.280262699677929,1.08771168210982,0.257662673195076,0.7966672560458,NA
"16218","GCKR",0.375762788798422,-0.225710046048203,2.51055325657251,-0.0899045042989249,0.928363101737299,NA
"16219","LINC01460",0.258834794821227,1.09609913055011,3.27112624850828,0.335083102050849,0.737562399373167,NA
"16220","AC104695.2",0.273076516777941,-0.774665331385065,3.18476194606587,-0.243241204367569,0.807818548712931,NA
"16221","PLB1",1.27852597371525,-0.841264311208228,1.26460258661901,-0.665240068389707,0.505896956293773,NA
"16222","AC097724.1",0.182454488057851,0.814003878402388,3.33451052748657,0.244114952312343,0.80714179253596,NA
"16223","TOGARAM2",2.2873527734694,-0.207267053424543,1.06631432912972,-0.194377068526975,0.845880644145967,NA
"16224","PCARE",0.449858372577248,-0.329289749654812,2.43155560776834,-0.13542349128385,0.892277051856705,NA
"16225","ALK",0.424986810038638,-0.0437719833216291,2.82908759553679,-0.0154721201954596,0.987655526691838,NA
"16226","AC016907.2",0.805212616737662,-1.03576761455337,1.66096800812814,-0.623592754035433,0.532895070478084,NA
"16227","LINC01936",0.345629519770364,-0.054409187889382,2.61698850261154,-0.0207907630603215,0.98341256615796,NA
"16228","GALNT14",0,NA,NA,NA,NA,NA
"16229","CAPN14",0,NA,NA,NA,NA,NA
"16230","AL121652.1",0.370691815318116,0.409327796421713,2.565354855683,0.159559912545796,0.873227759460615,NA
"16231","AL121655.1",1.04787783985905,-0.441137205070226,1.54398597488299,-0.285713220357236,0.775097778231398,NA
"16232","NLRC4",0.769679042079837,-1.8196180135069,1.86625310590983,-0.975011378544931,0.329554615807526,NA
"16233","AL133243.1",0.477867723373171,0.426489552005014,2.14898565583742,0.198460864941799,0.842684500975616,NA
"16234","LINC00486",0.117958369419058,0.451348601316665,3.34428110054629,0.134961322851401,0.892642454377335,NA
"16235","AC019127.1",0,NA,NA,NA,NA,NA
"16236","RASGRP3",1.13075986447916,-1.30025391216228,1.47118363095445,-0.883814831000214,0.376796178963991,NA
"16237","AC020594.1",0.82934740013991,1.52909169060165,1.71500249816337,0.891597354662275,0.372608788568835,NA

"16238","AC017050.1",0.172682080803205,0.494635591052761,3.34300496985
029,0.147961368742719,0.882373255878367,NA
"16239","AC007378.1",0.339262812684241,-0.0349910483728248,3.044545146
73679,-0.0114930298899752,0.990830090769536,NA
"16240","AC006369.1",0.168565237602375,-1.35173207482145,3.35247519753
738,-0.40320419844251,0.686798011343181,NA
"16241","LINC00211",0,NA,NA,NA,NA,NA
"16242","RMDN2-
AS1",0.541375438730553,2.04168176237738,2.69418093198324,0.75781167409
3638,0.448563732625456,NA
"16243","AC009229.3",0.167182390659274,0.576034712544328,3.34068880739
852,0.172429922616139,0.863099549623161,NA
"16244","AC009229.1",0.589337999366887,2.17289977712762,2.235459776972
33,0.972014705659591,0.331043232718552,NA
"16245","AC074366.1",0,NA,NA,NA,NA,NA
"16246","CDKL4",0.324592374063902,1.32629053141379,3.07347552214963,0.
431527930466861,0.666084548480396,NA
"16247","AC007684.2",1.43868312796322,0.753846616518977,1.403483734181
6,0.537125296260421,0.59118106844706,NA
"16248","SLC8A1-
AS1",0.824291874856757,-1.63168403955387,2.15013856802825,-0.758873899
48555,0.447927993595444,NA
"16249","AC007877.1",0.334364781318548,1.40513136139298,2.996844298340
15,0.468870325419052,0.639162328433279,NA
"16250","AC009413.2",0,NA,NA,NA,NA,NA
"16251","LINC01913",0.271251509622856,-0.774665352300728,3.18287089738
686,-0.243385728568736,0.8077065984266,NA
"16252","C2orf91",0.814196689080552,0.14511898792536,1.78235361193124,
0.0814198635747252,0.935108052705447,NA
"16253","AC083949.1",0.205372544040962,0.695830627438559,3.33747149429
751,0.208490358233014,0.834846110249776,NA
"16254","LINC01819",0.916127958850886,1.76515372751106,1.6649443564909
4,1.06018781986884,0.289059161200435,NA
"16255","AC010883.2",0,NA,NA,NA,NA,NA
"16256","LINC01126",1.8867999820663,-0.566216883616986,1.2384986911735
4,-0.457180041975231,0.647541642314599,NA
"16257","AC010883.1",2.0289229711405,-0.725330179190661,0.939615006868
622,-0.771944013120766,0.440147587872846,NA
"16258","ABCG5",0.308058816061443,1.2759273050851,3.31959886931736,0.3
84361892901681,0.700710240394097,NA
"16259","ABCG8",0.233061356241168,-0.774663445836492,3.35085807648355,
-0.231183603767975,0.817172167312465,NA
"16260","AC019129.2",0.52781864966426,2.06805563436852,2.2049002157441
3,0.937936156748289,0.348277220570322,NA
"16261","LINC01833",0,NA,NA,NA,NA,NA
"16262","SIX3",0.824377277097584,0.311162165546047,2.09199381019275,0.
148739524959387,0.88175917171552,NA
"16263","AC093833.1",0.472228463144689,0.896335102595912,2.40092843029
805,0.373328538778909,0.70890393847627,NA
"16264","U51244.1",0,NA,NA,NA,NA,NA

"16265","AC018682.1",0.768070629692366,0.447947284716244,1.77116557503251,0.252911015791408,0.800336976782738,NA
"16266","AC016722.2",0.672206399555605,1.19626085154926,2.00630469852492,0.596250834895006,0.551007670182554,NA
"16267","STPG4",0,NA,NA,NA,NA,NA
"16268","AC073283.1",0.172682080803205,0.494635591052761,3.34300496985029,0.147961368742719,0.882373255878367,NA
"16269","EPCAM-DT",0,NA,NA,NA,NA,NA
"16270","KCNK12",0.90847392940241,0.0827954130361913,1.45331757434631,0.0569699386408589,0.954569141724813,NA
"16271","AC092650.1",0.171420620199322,0.765902268429694,3.33569101336987,0.22960827767316,0.818396174063793,NA
"16272","STON1-GTF2A1L",0.220042982661301,-0.774663427007579,3.35247519753738,-0.231072082972194,0.817258802814834,NA
"16273","GTF2A1L",0,NA,NA,NA,NA,NA
"16274","NRXN1",0.30714814646625,-1.38503406788992,3.08534410716656,-0.448907486420329,0.653498395299422,NA
"16275","GPR75",0.102686272020481,-0.0533240139618846,3.35247519753738,-0.0159058638229612,0.987309491940711,NA
"16276","TSPYL6",0.142120095111522,-0.0533240139618842,3.35247519753738,-0.0159058638229611,0.987309491940711,NA
"16277","EML6",1.00474984168377,-0.858497039252114,1.49059088800687,-0.575944107910149,0.564652960827923,NA
"16278","AC012358.1",0.361521066841708,1.49138941530967,2.96515696371608,0.502971489725318,0.614984317080534,NA
"16279","AC011306.1",0.142120095111522,-0.0533240139618842,3.35247519753738,-0.0159058638229611,0.987309491940711,NA
"16280","AC007743.1",1.61089153326364,-0.614396494778223,1.23293151506883,-0.498321672590161,0.618257334657278,NA
[truncated: 421,268 more chars]
