# Supplementary material for: PPARγ agonist treatment reduces fibroadipose tissue in secondary lymphedema by exhausting fibroadipogenic PDGFRα+ mesenchymal cells
Source: JCI Insight. 2023 Dec 22;8(24):e165324. doi: 10.1172/jci.insight.165324 (PMC10807713; doi:10.1172/jci.insight.165324)
Supplement: Supplemental data set 6 [file jciinsight-8-165324-s188.pdf]

```
"","gene","baseMean","log2FoldChange","lfcSE","stat","pvalue","padj"
"1","PDGFD",232.540594679186,2.19144220621941,0.262755016485639,8.3402
4878204062,7.41343989175337e-17,1.05760133495754e-12
"2","PLP2",182.919792792422,-1.14913395351755,0.179482189105421,-6.402
49575317245,1.52857381697366e-10,1.09033170364731e-06
"3","MARCKS",2000.70308713887,1.52176658845643,0.249987765983322,6.087
36424548855,1.14784629881525e-09,5.20620173620647e-06
"4","CAV1",589.213511640006,-1.53962099986205,0.25453545209081,-6.0487
4875863171,1.45975094243838e-09,5.20620173620647e-06
"5","VGLL3",239.244638456318,1.8559414866169,0.312016909310445,5.94820
803372009,2.71093914066089e-09,7.73485155613366e-06
"6","FXYD5",148.062545382342,-1.33150653784775,0.228648575928908,-5.82
33755991629,5.76707456954251e-09,1.37121809681822e-05
"7","CRIP2",95.8471518879719,-1.79609406031615,0.310264177430218,-5.78
891857639643,7.08410252809524e-09,1.44374009522581e-05
"8","CXCL11",17.3518470188453,-4.07411236917316,0.725245863088964,-5.6
175603013035,1.93672592759556e-08,3.45366651038478e-05
"9","CAPG",65.7478249172921,-1.77736484894977,0.320486640758617,-5.545
83131684555,2.92560775445437e-08,4.1898104775043e-05
"10","GPC1",37.3646772469712,-1.62729810783388,0.293462993110674,-5.54
515610498179,2.93692028424527e-08,4.1898104775043e-05
"11","ADAMTSL1",69.1207252358132,1.98764021355066,0.365979020760498,5.
43102227395543,5.60321394955435e-08,7.26685910948567e-05
"12","C1QC",9.49146084289217,-3.11255079283117,0.595249098698013,-5.22
898866985141,1.70439809069415e-07,0.000202624526348689
"13","HAS2",177.2637888453,-1.3846097589004,0.267575922347848,-5.17464
257154052,2.28347689884633e-07,0.000244556042601738
"14","ABCA1",236.66819011081,1.77556410749377,0.343745515925324,5.1653
4478337603,2.39996116390322e-07,0.000244556042601738
"15","VEGFD",162.533004414448,1.68503435613211,0.327471833891311,5.145
58560994098,2.6668754359596e-07,0.000253637633129331
"16","RNASE1",20.2390416684896,-2.57812083468068,0.506331466015673,-5.
09176499530621,3.54745627388215e-07,0.000316300070020017
"17","SPP1",11.7230111759224,-2.95521766706879,0.601963890297849,-4.90
929392061468,9.14048958562964e-07,0.000695902850046228
"18","A2M",88.848404097229,-1.85052842605233,0.375978993109695,-4.9218
9313755735,8.57110147733752e-07,0.000695902850046228
"19","CDH13",12.3122278020897,-2.99384050860492,0.610169749507366,-4.9
0656987014198,9.26829815707158e-07,0.000695902850046228
"20","C1QA",23.5072394236866,-2.36194628665096,0.489967468033898,-4.82
061859357476,1.43113747157631e-06,0.000887678572587287
"21","VEGFC",58.4543921024007,1.89379827536055,0.392150991026102,4.829
25791008519,1.37042814127055e-06,0.000887678572587287
"22","KCP",25.9259466271012,3.10339439075846,0.642343075870968,4.83136
583444991,1.35599581315887e-06,0.000887678572587287
"23","CD9",298.672638925406,-2.03493624566776,0.420634705883793,-4.837
77543127871,1.31300367469419e-06,0.000887678572587287
"24","C1QB",20.8298667854752,-2.5961319680902,0.548055167455502,-4.736
99021969533,2.16915629668107e-06,0.00119019937417123
"25","FAM43A",93.7211595154989,1.17215632478406,0.24714878071465,4.742
```

71538542362,2.10872435962714e-06,0.00119019937417123
"26","KLK1",3.68266449287741,-5.61562318791213,1.18429349046634,-4.7417496027111,2.11880401548731e-06,0.00119019937417123
"27","KANK4",5.64127356192144,5.71928691146333,1.22275787261248,4.67736666396906,2.90582398301693e-06,0.00128100512219588
"28","FCGR3B",5.68969789587281,5.71702346885688,1.22649753907591,4.66125963299061,3.14279961284562e-06,0.00128100512219588
"29","IGSF10",100.561793335473,2.24163624124471,0.480350461414706,4.66666823769201,3.06123007729431e-06,0.00128100512219588
"30","SMOC2",485.256574323404,1.57053697319232,0.336524090505819,4.66693772452278,3.0572194066083e-06,0.00128100512219588
"31","STEAP4",369.681354984972,1.19550219664506,0.254435033995555,4.69865402523909,2.61881650247064e-06,0.00128100512219588
"32","RDH10",121.943104032762,1.36711078799352,0.291486708501935,4.69013079539592,2.73030463935206e-06,0.00128100512219588
"33","VIM",21169.3275218876,-0.883671824619671,0.18865288944703,-4.68411497544431,2.81172155100195e-06,0.00128100512219588
"34","PLXNC1",44.8921870748536,1.9323943724817,0.414537549035318,4.66156655043344,3.13811559246197e-06,0.00128100512219588
"35","SYNE2",215.708838230308,1.14487526725608,0.244742096537788,4.67788453009068,2.89849680608332e-06,0.00128100512219588
"36","DBNDD2",157.50494505081,-1.69309974515657,0.363762953392312,-4.65440399954801,3.24919176683329e-06,0.00128758249293455
"37","PTGDR",12.8580127878855,-2.06604337421448,0.447297392559988,-4.6189479495734,3.85690583873904e-06,0.00148709780257976
"38","CRIP1",1684.1673554874,-0.97987647843123,0.213458871417094,-4.59046968592171,4.42249687562561e-06,0.00166029843230724
"39","EBF2",93.0114826962804,1.08138498666787,0.236049808470967,4.58117290445026,4.62375355847941e-06,0.00169134534013506
"40","GPX3",1952.90852185392,1.1462590407219,0.250515443464657,4.57560230566632,4.7485184419471e-06,0.00169355910232043
"41","ARHGAP20",100.538732655185,1.22188695494217,0.268017880879596,4.55897550914182,5.14037647602915e-06,0.00178860026358614
"42","LMNA",1676.87414244046,-1.00996574231766,0.224011950459993,-4.50853510379142,6.52767697215795e-06,0.00221723427820965
"43","ITGA7",14.2862032588143,-2.44811387396108,0.547069992853437,-4.47495550101747,7.64272283551601e-06,0.00253560660398771
"44","PCSK5",214.747053693892,1.83266552028547,0.411290694076234,4.45588861280137,8.35464048094612e-06,0.00270880229775403
"45","AFF3",31.4820934874813,-1.83591171947154,0.417107930235901,-4.40152676654508,1.07491804332513e-05,0.00340772906801695
"46","PLEKHA2",75.2707905750473,1.30805240904522,0.297583939712729,4.39557460764833,1.10480108393922e-05,0.00342632440510367
"47","SERTAD4-AS1",10.563085490638,-2.51414177409241,0.572652352617195,-4.3903456653972,1.13170614974618e-05,0.00343508934729341
"48","ANOS1",37.5758536386033,2.05942998479853,0.471042732943537,4.37206614340314,1.2307622385656e-05,0.003604278659214
"49","TNMD",43.5100131180916,-2.40382768054411,0.549975512974249,-4.37079037854666,1.23797598697242e-05,0.003604278659214

"50","AC007262.2",15.5446342258454,3.64618517506655,0.845766718786505,4.31110032361884,1.6244417829313e-05,0.00463485729505959
"51","TRIL",36.613623033299,1.39463984814501,0.326927600119573,4.26589816104521,1.99099590086819e-05,0.00556932304348738
"52","AQP7",6.33021666117912,-3.66110744001882,0.859847663440035,-4.25785589202121,2.06396920897516e-05,0.00566242014139224
"53","MAN1C1",145.577364577896,0.953139268791468,0.224480838077237,4.2459716248187,2.17648175686311e-05,0.00583788172588067
"54","VEPH1",8.40361379625439,4.32121851067826,1.01903597216915,4.2404965366237,2.23025876427337e-05,0.00583788172588067
"55","EYA2",9.16105291995663,3.65951755861815,0.863409548980723,4.2384492538197,2.25069041723985e-05,0.00583788172588067
"56","SEMA3E",18.1102257361582,-2.68933320917511,0.636657354624635,-4.22414535800142,2.39849214565366e-05,0.0061101587410527
"57","SYCE1L",19.473437027087,-1.74175331277579,0.41393437181059,-4.2078006355384,2.57868212380055e-05,0.006342668823817
"58","ABCA6",337.122038608566,1.08766335732039,0.258348659508882,4.21005999949072,2.55302878334377e-05,0.006342668823817
"59","TUBA1C",137.393703270693,-1.12935885879064,0.268860109267858,-4.20054452059114,2.66273799533216e-05,0.00635346694885698
"60","RASL12",24.8125401012491,-1.76504916173143,0.420275199826001,-4.19974617217999,2.67214367679391e-05,0.00635346694885698
"61","FBN1",3161.42107021302,1.22398925110008,0.292369358309108,4.186448464295,2.83353232234738e-05,0.00662674952632914
"62","PARP16",35.4492393652813,-1.86787710842478,0.447809361525541,-4.17114350191681,3.03074918697813e-05,0.00697365611313388
"63","NBL1",272.88718792591,-0.896475671448073,0.216214137726958,-4.14623983830406,3.37979802976674e-05,0.00765336487184957
"64","CX3CL1",13.3003992070515,-2.5625720125861,0.620115997094614,-4.13240752470883,3.58983212609325e-05,0.00800196017356974
"65","RBMS3",362.920538247437,0.862000366205262,0.209173821507005,4.12097632483324,3.77270197899397e-05,0.00828021022035814
"66","FABP4",221.934994979726,-2.93789950522033,0.713818237005827,-4.11575293669101,3.85917521033513e-05,0.00834166568949105
"67","GALNT16",94.5402855024311,1.17560654995031,0.286128637161498,4.10866441616178,3.97953842638774e-05,0.00847344704341008
"68","FOLR2",5.91166636537792,-3.0731723899858,0.749280485613269,-4.10149796904223,4.10484110582747e-05,0.00861171517878451
"69","RAB13",279.264355189497,-1.10821570231058,0.271277331873618,-4.08517620936669,4.40433795367555e-05,0.00897604074959078
"70","MS4A7",10.8445567564058,-2.5397758608598,0.621484253507082,-4.08662946249669,4.37685298992397e-05,0.00897604074959078
"71","COL6A6",41.4843952304346,1.61251701942134,0.396985326240924,4.06190585100551,4.86737081792716e-05,0.00977998761810547
"72","MID1",55.4327139483599,1.21082002093621,0.298363046203637,4.05821041292695,4.94502038798344e-05,0.00979800845207941
"73","TWIST1",272.335733056896,-1.40603584848551,0.347426157409647,-4.04700630191083,5.18768451661575e-05,0.0101380147005535
"74","MIR4435-2HG",83.0452785647121,0.941121261484531,0.233524613748426,4.03007308899092,5.57595123880437e-05,0.0107495297801058

"75","SLC4A4",19.0050192523061,2.2529388416439,0.562023833260649,4.008
6179772363,6.10751249100343e-05,0.0116173030928873
"76","PIM3",101.796513409975,-1.05373277687342,0.263940314388774,-3.99
231462353004,6.54314795229822e-05,0.0122821774588798
"77","UBD",6.06685230544464,-4.18639885655764,1.05035653873533,-3.9856
9314529923,6.72834196667392e-05,0.0124657826618922
"78","RGCC",161.477995251839,-1.31987331033918,0.333991409138534,-3.95
181814329759,7.7559673023808e-05,0.0141048666274077
"79","EMILIN2",142.523907925339,0.921952897746181,0.233397924183314,3.
95013323692659,7.81077010770508e-05,0.0141048666274077
"80","FLRT2",148.246057737176,1.04391515515312,0.26488936484442,3.9409
4778311033,8.11602938072887e-05,0.0144729093931848
"81","PRELP",410.035870368389,0.871731511683122,0.221584550348992,3.93
408073942952,8.35157085794014e-05,0.0147090752912808
"82","LAMC1",694.355235285706,1.05900494077008,0.270388551790722,3.916
60421181492,8.98050049891396e-05,0.0156238805021349
"83","APBB2",57.375407853498,1.30643861111312,0.333855469078974,3.9131
8619017167,9.10862478599642e-05,0.0156558603851837
"84","GSTM1",3.44631976168186,5.03633448424772,1.28982802118841,3.9046
5581574772,9.43595809949998e-05,0.0160254021723175
"85","CXXC5",141.589872186265,-1.00658670283155,0.259272506457646,-3.8
8235033704192,0.000103451687871372,0.0173628444608587
"86","EHBP1",100.782357863493,0.932557761011996,0.240562731983369,3.87
656788449038,0.000105940262017677,0.0175737648598161
"87","AKAP12",357.997594072012,0.884191392138025,0.22875820109476,3.86
517898771096,0.000111007763205271,0.018202721263062
"88","PERP",38.4986076914245,-1.50496957781734,0.390995223811022,-3.84
907407090152,0.000118565153859654,0.0192210282382025
"89","ADIRF",398.485741162177,-1.5229365806617,0.395978883844961,-3.84
600452901418,0.0001200595442248,0.0192446006506854
"90","TXNIP",2822.17316767796,0.85727811685442,0.224182979790618,3.824
01071506454,0.000131298204874578,0.0208122243415636
"91","LARP6",165.479996334543,-0.688999558609596,0.180646267464102,-3.
81408134406383,0.000136690443230218,0.0214288556387064
"92","IER3",374.496779835478,-1.47192573750129,0.386379886470922,-3.80
952991871657,0.00013923125840836,0.0215899253527572
"93","TBC1D2B",121.450137880286,0.885307105121327,0.232626490073279,3.
80570202835648,0.000141402537197817,0.0216908451146673
"94","TMEM37",108.243892495331,1.28986646679024,0.339732730735679,3.79
670944273484,0.000146629483036976,0.0222533638830372
"95","APOC1",154.277922487898,-1.57855410256464,0.416693153711007,-3.7
8828902876439,0.000151688323143799,0.0226904712798742
"96","DYNLT3",37.3383463367349,-1.23992042008564,0.327445037285931,-3.
78665204506647,0.000152690680139347,0.0226904712798742
"97","MYADM",637.201462340738,-0.827179385056587,0.218709388461021,-3.
78209363062624,0.00015551484140859,0.0228719044075768
"98","SLCO2B1",6.43260253645564,-2.91619132645708,0.772810003537825,-3
.77349065502145,0.000160979251976196,0.0234339796805348
"99","NTM",106.055814199504,1.02986890031037,0.273369256806872,3.76731
792133432,0.000165010808198279,0.0237782241389561

"100","ID1",170.10942366158,-1.31006039303008,0.348023674774357,-3.764
2852713381,0.000167026122107543,0.0238279465798621
"101","NT5DC1",27.3946561771077,-1.13382556769613,0.303388543047193,-3
.73720627782493,0.000186076262973693,0.0262828115602248
"102","ABRACL",49.9462989092115,-1.18795791767657,0.319341428408167,-3
.7200244377882,0.000199203502177276,0.0275906520588448
"103","MID1IP1",128.171076493685,-1.0020257138959,0.269193686034203,-3
.72232249819035,0.000197398672496604,0.0275906520588448
"104","TNFSF14",181.792109590263,1.7415759669942З,0.468792408506617,3.
71502595902137,0.000203182834676388,0.0278712146105129
"105","RGS16",366.767423122539,-1.24924815494289,0.337559722349104,-3.
7008211354402,0.000214902921422729,0.0291981435906348
"106","TRIO",167.244829935881,0.988653067973312,0.268465336334196,3.68
260976062324,0.000230858417433167,0.0310700583311469
"107","HMGA1",37.3311293547247,-1.36320110183419,0.370522897685763,-3.
67912782272988,0.000234032954256971,0.0312029357516818
"108","ABCA8",740.333106739536,1.08709159091443,0.295929116906152,3.67
348641552962,0.000239263402827397,0.0316049231919967
"109","FBLN2",1774.10813615749,0.639033698008505,0.174164230044555,3.6
6914433489027,0.000243363657471810,0.0318516141054389
"110","GNG4",8.06239754208558,2.50870680357834,0.689859801859254,3.636
54585586386,0.000276318471541334,0.0350737817293738
"111","ADD3",764.646211711755,0.73174442342332,0.201288632779151,3.635
29928799394,0.000277658100010774,0.0350737817293738
"112","DKK3",16.3375593138117,-1.87705590870305,0.516016259095693,-3.6
3759062939711,0.000275200370297014,0.0350737817293738
"113","MEG8",101.018006257583,1.49223201648965,0.410500605957211,3.635
15179961804,0.000277817000940645,0.0350737817293738
"114","MAZ",159.630623847577,-0.685040410481306,0.188729339404778,-3.6
2975048098941,0.000283695322199607,0.0355017321622771
"115","PTGS1",22.2312543928274,1.88625789027071,0.520886842531601,3.62
124311127378,0.000293190839768193,0.036057418277009
"116","ADGRD1",157.00805840159,0.999427388656846,0.275959397340003,3.6
2164651137239,0.000292733941107304,0.036057418277009
"117","CYP26B1",157.513806149254,-1.38575823884287,0.383670216626923,-
3.61184730737221,0.000304023567841916,0.0361433351569397
"118","GPD1",6.21211048014053,-3.05496134839627,0.845785385253172,-3.6
1198171742093,0.000303865993729040,0.0361433351569397
"119","CEP152",28.1814446533169,1.38407527691983,0.383001417281045,3.6
1376019636032,0.000301788201898683,0.0361433351569397
"120","ALPK2",11.3235659138336,2.0035376430139,0.553775414484704,3.617
96062195758,0.000296933559676362,0.0361433351569397
"121","S100A6",8870.80252518344,-0.742531435474157,0.206157266971006,-
3.60177182392793,0.000316055717330912,0.0372632302763867
"122","SEMA3B",32.9166582261549,1.52353958482255,0.424320913522032,3.5
9053616324628,0.000329998438972275,0.038274563130003
"123","AKR1C1",486.180836198435,-1.01326044676871,0.282203161910709,-3
.59053541394875,0.000329999387704357,0.038274563130003
"124","RECK",158.116360028745,0.755337091890884,0.210723479357191,3.58
44942110629,0.000337732054974597,0.0385446839701408

"125","MAF",330.644407326412,0.897038659819323,0.250155283847586,3.58592729292846,0.000335882538668807,0.0385446839701408
"126","ECHDC3",4.42817537783155,-3.34886486969834,0.936278905177907,-3.57678128939795,0.000347850974970163,0.0392791358877906
"127","SPHK1",28.2780109530924,-1.85255404175112,0.518136927732363,-3.57541403169017,0.000349674068256653,0.0392791358877906
"128","F13A1",6.38129691814861,-2.50534090726032,0.701135214825392,-3.57326355071787,0.000352559598924715,0.0392938690489062
"129","HS6ST1",53.6221330056975,1.21822162763445,0.34129953192551,3.56936213994087,0.000357851470016025,0.0395744889244078
"130","FZD10-AS1",12.6141367937133,-2.16380195933217,0.607861981372567,-3.55969286719701,0.000371288770587327,0.0407446584707601
"131","S100A8",36.4138139022786,1.73743248169932,0.490342381807755,3.54330473187713,0.000395145817288866,0.0429996813896805
"132","OLFML2A",69.0350881060214,0.87700864527172,0.247637960237931,3.54149519091939,0.000397866111274207,0.0429996813896805
"133","SEPTIN1",54.2423830667006,-0.925644430670564,0.262698287175988,-3.52360283967307,0.000425721852141467,0.04566427024549
"134","INHBB",48.3337446367711,1.9212706584179,0.545852617701856,3.51976082208201,0.000431936115075304,0.0459850792363007
"135","BHLHE41",59.0823762414569,-1.52114680250738,0.434071394042726,-3.5043700722597,0.000457688844232203,0.0483658448282712
"136","SLC6A6",18.1512010511647,-1.52693302220565,0.436290687674012,-3.49980658616886,0.000465595850162947,0.0488396352825338
"137","KCND2",38.5621022454441,1.02531427295359,0.293343233321638,3.49527160161005,0.000473579577393612,0.0493144981831917
"138","SEC14L2",8.31498165701079,-2.11985280935461,0.609652397901093,-3.47714995734097,0.000506774295694765,0.0523887108868226
"139","MUC20",11.4804738068105,2.35738008117883,0.680819799866433,3.4625609913832,0.000535060630191581,0.0549149277000942
"140","C6",19.188071671588,-1.32084169941549,0.382221032598313,-3.45570124814037,0.000548863291018429,0.0559291693547779
"141","NEGR1",494.884286036005,0.810421905320945,0.235109283345879,3.44700087460477,0.000566846747079351,0.056948138688972
"142","WDR86-AS1",21.914813563896,2.02055920204645,0.585934151907708,3.44844074281697,0.000563833172116024,0.056948138688972
"143","MMP23B",138.441668168725,1.57967050020743,0.458662573037002,3.44407979431972,0.00057300656489578,0.0571644171664559
"144","SCX",6.29171928866722,-3.43831472557041,1.00037555115422,-3.43702394725993,0.000588143600992801,0.057956755570713
"145","RASSF4",65.5770724137441,0.902796281726144,0.26270074652382,3.4365957983461,0.000589073991150525,0.057956755570713
"146","IL7",18.1685853787555,-1.21020558893942,0.352769925759984,-3.43058038842806,0.000602291483002429,0.0580560155169774
"147","AGAP2-AS1",11.2399559497886,-1.69645037846667,0.494045266309986,-3.43379543161586,0.000595193203471635,0.0580560155169774
"148","HS3ST3A1",6.52086877297971,-2.62329991328577,0.76431722693958,-

3.43221351138425,0.000598676031605372,0.0580560155169774
"149","CKB",146.15208384525,-0.800694778992398,0.233559107705313,-3.42
823188039686,0.000607526308071543,0.058167585979521
"150","MT-
CYB",16622.743946947,-0.527597024612377,0.154131863135269,-3.423023727
08845,0.000619286714188333,0.0588982950974051
"151","NHSL1",68.2027769312809,0.793221896052039,0.23194332772369,3.41
989529872138,0.000626452419195478,0.0591852331936602
"152","PFKP",65.6070375268198,-1.06587355805922,0.311974147623042,-3.4
165444995369,0.000634212976624242,0.0595242258192199
"153","PHLDA2",33.329005733625,-1.47075810212572,0.431266397171073,-3.
41032390135952,0.000648857708205002,0.0605006801650494
"154","PHLDA3",161.791772520394,-0.778791233579093,0.228541190067388,-
3.40766245834922,0.000655218916047606,0.0606970977684101
"155","CPB1",183.61319118095,1.30913578644313,0.3850029287269,3.40033
244133438,0.000673039713042186,0.06121103304286
"156","KCTD16",5.46972655210974,4.18035129394335,1.23355943045231,3.38
8852770888,0.000701856840418951,0.06121103304286
"157","HRH2",8.8713335409733,2.28460263666983,0.672632858828097,3.3965
0762921427,0.000682516547434969,0.06121103304286
"158","NT5E",301.620374092584,0.689983591619438,0.203269881695924,3.39
442117967874,0.000687738338243477,0.06121103304286
"159","PLAT",68.5297700514561,0.897422176941773,0.264891701122827,3.38
788332415764,0.00070434219558364,0.06121103304286
"160","PXDNL",16.8552834233654,2.27487390779003,0.670334744071426,3.39
363866770964,0.000689706300406922,0.06121103304286
"161","KCNQ1OT1",593.230391928944,1.43574940542654,0.423648305868302,3
.38901250291526,0.000701448120833712,0.06121103304286
"162","MS4A4A",6.02917312098939,-2.56354126084399,0.756248779004578,-3
.3898120988946,0.000699405451134691,0.06121103304286
"163","KRT222",164.849842668793,1.28630939472114,0.379792724695409,3.3
8687213072011,0.000706943288458033,0.06121103304286
"164","SSC5D",148.080880235367,-0.828045241035814,0.243601876175983,-3
.39917431685797,0.000675896235219659,0.06121103304286
"165","JAM2",28.0308568246126,-1.28703367915817,0.380051014763529,-3.3
8647610231741,0.00070796442254815,0.06121103304286
"166","SPECC1L",86.5451151909235,0.846401589647993,0.250655898460692,3
.37674714557226,0.000733484440541399,0.0630354760768892
"167","MME",132.143794546892,1.09087285899579,0.324439441510305,3.3623
3120707408,0.000772873609655148,0.0657275297226238
"168","CD36",79.5517814992789,-2.52861189965796,0.752500041449048,-3.3
6028140913953,0.000778631187657607,0.0657275297226238
"169","CD163",11.2414730887365,-1.84137856848355,0.547946804056709,-3.
36050608353029,0.000777998173312067,0.0657275297226238
"170","SERTAD4",8.85551538356965,-2.00239523718494,0.596695755529867,-
3.35580606804686,0.000791340388383899,0.0664074234157924
"171","ABCA9",340.912687230172,0.993650351341702,0.296626552718745,3.3
4983615672418,0.000808593741399945,0.0674584696772609
"172","SVEP1",835.268581321452,1.29690882373358,0.387756199032103,3.34
465013575762,0.000823864254967384,0.0679378465974838

"173","PTGER2",20.7503601929942,-1.37793288136151,0.411859278963267,-3.34564000799022,0.000820929021006337,0.0679378465974838
"174","HRCT1",41.9701318739696,-1.26741855813033,0.37936869328217,-3.34086228139979,0.000835186396089058,0.0682639347055767
"175","CCDC34",47.5425088509065,-1.05358677290681,0.315432769799462,-3.34013100026553,0.000837388796682737,0.0682639347055767
"176","ARHGEF10",121.429788251313,0.807779102507461,0.242584941151081,3.32988147852252,0.000868829582760477,0.069633274312702
"177","TJP2",30.4742576641833,-1.10863169304365,0.332899861553156,-3.33022575579122,0.000867755972013944,0.069633274312702
"178","TNFRSF12A",79.8722134870044,-1.41684205768275,0.425261614269335,-3.33169514985994,0.000863187557628619,0.069633274312702
"179","MLIP",3.29179483404767,-4.07984671271575,1.22588186917713,-3.32809124214746,0.000874432222357488,0.0696907825930275
"180","UBE2E3",316.280229817075,-0.766813642455315,0.230993086369932,-3.31963893164869,0.000901339427888044,0.0702650725587477
"181","FSTL1",2942.44404903821,0.91352985523948,0.275115434882132,3.32053290878014,0.000898457660111544,0.0702650725587477
"182","MUC12",9.49345599993702,-2.07472403167007,0.624874312794186,-3.32022614658737,0.000899445555384177,0.0702650725587477
"183","TET1",40.4404977236299,0.988432031752117,0.297557497498946,3.32181860669002,0.000894328136294744,0.0702650725587477
"184","PLXNA4",61.5129850945806,1.51245924694148,0.455849611140001,3.31789083500385,0.000906999250723274,0.0703220179935773
"185","EMCN",21.0015864745397,-1.45896724491457,0.440786069361465,-3.30992140252543,0.000933221722452153,0.0715771026478625
"186","DENND2A",49.4375770334148,0.834653061438473,0.252115206821902,3.31060181557427,0.000930955775124273,0.0715771026478625
"187","MIR497HG",25.7305164915609,1.43181830402132,0.43345399202542,3.30327631158914,0.000955621758746539,0.0729032086111129
"188","PODN",688.577510919805,0.686754472311473,0.208598988233366,3.29222340974723,0.000993986165589591,0.0742419195722571
"189","RAP2B",103.820910979084,-0.661097815225162,0.200535193714299,-3.29666729804557,0.000978393250109307,0.0742419195722571
"190","KANK1",50.8171846895558,0.970131692346282,0.294636295469007,3.29264149483691,0.000992509424094156,0.0742419195722571
"191","SDC4",52.4643612650945,-1.06038234475547,0.3220539932924,-3.29256077192225,0.000992794391640414,0.0742419195722571
"192","DNASE1L3",48.4757221828263,1.79354784488426,0.545735536099983,3.28647802139033,0.00101448690338557,0.07537849043593
"193","SCARA5",953.420552407449,0.737685806098377,0.224850305228264,3.28078632292489,0.00103518135679438,0.0762136739977037
"194","SQLE",30.2137962113307,-1.43260796684606,0.436710647834185,-3.28045119566218,0.0010364119413679,0.0762136739977037
"195","BDKRB2",128.805585349719,1.26225188115781,0.385183650691484,3.2770131309878,0.00104911490276765,0.0767521702711962
"196","PTX3",1874.66691743885,2.17139658528324,0.664473126601326,3.26784710826394,0.00108368890207712,0.0784766795788437
"197","MRC1",6.93178595433076,-2.58523396906549,0.790906032743901,-3.26869926645585,0.0010804306974769,0.0784766795788437

"198","SERPINA5",35.6776848723528,-1.16430474269248,0.356814803688577,-3.26305055355459,0.00110219856743242,0.0794139634494492
"199","PLEKHO1",60.3441170374922,-1.08116727889059,0.331833504143917,-3.25816189561645,0.00112136412625596,0.0803888473626512
"200","MAP4K3-DT",50.1580561871691,1.03329752715946,0.317369068109488,3.25582304953231,0.0011306419203610 1,0.0804436104400074
"201","AGTR1",205.466456877104,0.802611914834644,0.246568332124558,3.25512975619753,0.00113340569875519,0.0804436104400074
"202","LPIN1",64.5327263778534,0.799649288721773,0.245953472519185,3.25122178813474,0.00114910178321944,0.080754118420732
"203","LINC00882",31.0597781251972,1.20369319081983,0.37019110419352,3.25154542392943,0.00114779433174186,0.080754118420732
"204","SEPTIN11",295.915010155863,1.06567246392237,0.328130378686062,3.24771046249867,0.00116337589351473,0.0809596121799081
"205","CAV2",66.4404520263553,-0.827460388598895,0.254709967776952,-3.24863763998233,0.00115959092842555,0.0809596121799081
"206","NID1",353.893908048488,1.06830341622793,0.329172909905119,3.24541717766465,0.0011727867400713,0.081218328319695
"207","CNTNAP4",4.09853979245174,-2.65587186857194,0.822274216757607,-3.22991018622057,0.001238291109999,0.0853403911847618
"208","PXN",157.756498563531,0.747751117619103,0.232074185510736,3.22203486774496,0.00127283644392165,0.0872994457162802
"209","TBC1D12",65.1082262639909,0.835793712471487,0.260362209866839,3.21011913710116,0.00132679967922069,0.0901339248750586
"210","DUSP6",61.4610977485836,-1.21912799278662,0.379650631820603,-3.21118389014745,0.00132189315552586,0.0901339248750586
"211","IL17RD",31.5159573357862,1.10360311130318,0.344100267140222,3.20721375916124,0.00134027366018409,0.0906177442473282
"212","MEOX1",5.7105511892442,-3.5101459839772,1.09525781852863,-3.20485818461695,0.00135129041644909,0.0909316466087862
"213","SERPINA3",177.621755618678,-1.08179201409447,0.338046733252104,-3.2001256266769,0.0013736769860634,0.0920041121276081
"214","SHTN1",20.1581420968906,-1.36432523570461,0.426937534601003,-3.19560854957305,0.00139536283531962,0.0926777743325895
"215","GRK5",53.9287333603129,0.916775819439802,0.286933023538131,3.19508646350695,0.0013978895460747,0.0926777743325895
"216","C11orf96",1824.85701095262,-1.20154106765927,0.376188466158682,-3.19398699255294,0.00140322439757741,0.0926777743325895
"217","RALGPS2",78.0513682823779,0.834176848723529,0.261483180873631,3.19017401400922,0.00142187150762985,0.0934765849209557
"218","PAM",393.006584042235,0.710289614509423,0.222787251207993,3.18819685892304,0.00143163033778743,0.0936864146737409
"219","NABP1",506.96073029908,1.03922089849854,0.32758052733028,3.17241353436968,0.00151177569333793,0.0979576761662787
"220","TUSC1",131.518804734917,-0.810346866995496,0.255494144668008,-3.17168468987213,0.00151557463099257,0.0979576761662787
"221","ACP5",6.49279037764799,-2.5652553491948,0.808892997104047,-3.17131605586744,0.00151749939946359,0.0979576761662787
"222","FBLN5",797.027998601678,0.776777734634331,0.245113549882975,3.1

6905260849589,0.00152936710089807,0.0980197950784523
"223","EMP2",320.548582007195,-0.582825194902671,0.183942752371789,-3.
16851404791776,0.0015322034419245,0.0980197950784523
"224","FCGR2B",8.07166475311183,-1.86401303844389,0.588868931795233,-3
.1654124335635,0.00154863267408567,0.0982983881422092
"225","CMKLR1",37.5498397154787,-1.58004066414147,0.499208443515035,-3
.16509202652115,0.00155033908117181,0.0982983881422092
"226","CDO1",107.794746091562,-0.934597248838117,0.297009987101093,-3.
14668627125999,0.00165132040954477,0.104237774170645
"227","MAST4",63.6794456670411,0.864497702895075,0.275413529576287,3.1
3890789688172,0.00169578739068539,0.106573140596995
"228","ADAMTS16",24.8476746944107,2.18255113401205,0.698416141390842,3
.1250009910505,0.00177804460780544,0.111252563048037
"229","IL6ST",851.046378485217,0.726947984823661,0.233010004895831,3.1
1981446954885,0.00180964981707848,0.112245496914963
"230","DHRS4",36.8239068571591,-1.28395915154126,0.411540994414738,-3.
11988154027574,0.00180923783253657,0.112245496914963
"231","SPAG17",11.8569114614898,-2.40817426253522,0.772992934440639,-3
.11538974709755,0.00183702008029498,0.113347768484717
"232","ENPP5",2.06898082241324,4.25194870490489,1.36615586227042,3.112
34524722426,0.00185607296527013,0.113347768484717
"233","H19",796.319167026571,1.27725941329929,0.410333276091261,3.1127
3661611402,0.00185361359889804,0.113347768484717
"234","ABCG1",10.1543812066565,2.454209311439,0.788666197796906,3.1118
4797610788,0.00185920214674216,0.113347768484717
"235","LMO3",138.25909650314,0.737941388910756,0.23724078294171,3.1105
1658049901,0.00186760410416328,0.113375489999972
"236","HFE",15.9887316547908,1.36838881759012,0.440981130329104,3.1030
5526354129,0.00191533870636057,0.115780601631101
"237","AC025171.2",20.5155539578941,1.16350426250649,0.375852927004384
,3.09563709342329,0.0019639056294124,0.11771881390419
"238","FRMD6",211.769672421402,0.816109688145713,0.263578483894903,3.0
9626823891711,0.00195972992215551,0.11771881390419
"239","STAB1",6.28558320509186,-2.19862941720201,0.712576991503519,-3.
08546226361163,0.0020323601205306,0.120487730129142
"240","PRSS12",7.60319994620476,2.17768389261635,0.706701118468004,3.0
8147792002531,0.00205975752352982,0.120487730129142
"241","SNHG18",32.8360771982708,-1.04817294734908,0.339702485293502,-3
.08556160972286,0.00203168128301438,0.120487730129142
"242","KCNB2",4.03728321136146,3.82810525396944,1.24214147755425,3.081
85929150913,0.00205712052348544,0.120487730129142
"243","TEAD1",135.889635999668,0.877711011565663,0.284848013515116,3.0
8133099028647,0.00206077429913856,0.120487730129142
"244","GALNT8",10.5792852876256,1.78187235168002,0.577687537663372,3.0
8449159018963,0.00203900374548954,0.120487730129142
"245","CTHRC1",456.276197123955,-1.01327539199207,0.329476011026567,-3
.07541477400721,0.00210209995812488,0.122402277561671
"246","MGAT4C",4.39688413994659,3.33850192373929,1.08882566071013,3.06
614919560394,0.00216835087918934,0.125746722124045
"247","DDIT4",257.644015665467,1.39019445915178,0.453873930350216,3.06

295287345341,0.00219164571734667,0.126583068031043
"248","TUBB6",236.680751502129,-0.661974249836755,0.216414292498548,-3
.0588287039369,0.00222204163517674,0.127821153094481
"249","DYRK3",49.424177802968,-1.25391520956551,0.410381717344203,-3.0
554850680977,0.00224696791074775,0.128735920541074
"250","FBXL7",56.2843331279952,1.05594735636772,0.347263351738967,3.04
076819819865,0.00235975424806799,0.134657016411752
"251","EGFR",360.487313304374,0.60042211453868,0.197715369011956,3.036
80041434904,0.00239103716026464,0.135898550312093
"252","EPB41L3",145.752740911298,0.637288130020097,0.209951474056081,3
.03540679047516,0.00240211458807328,0.135986375847037
"253","CSF1R",4.89894570498815,-2.21924076639859,0.731734293307462,-3.
03285056706521,0.00242255530173347,0.136601478002094
"254","EN1",28.4311601970922,1.13443245743584,0.37504854379556,3.02476
166406401,0.00248829126024009,0.138714896062961
"255","LINC02762",41.7380561758536,-0.872174981204172,0.28835562509308
6,-3.02465048470138,0.00248920604178593,0.138714896062961
"256","PTPRT",3.61825334619496,-3.25621660715516,1.07649384301596,-3.0
2483532839573,0.00248768532102679,0.138714896062961
"257","PTPRD",80.371179839967,0.983163895821993,0.325381333981092,3.02
157435951481,0.00251463868527716,0.139586908498693
"258","LHFPL6",1151.52848885475,0.642652147745429,0.212892438915653,3.
01867060670973,0.00253886406528152,0.140385406028319
"259","GJA1",226.759801750789,0.718773451427377,0.238802578762314,3.00
99065728381,0.00261328060604729,0.143942320949307
"260","KCNAB1",26.6252713681579,1.90460820139631,0.633393632645004,3.0
0698981365318,0.00263848604105513,0.144771699468048
"261","SCRN1",68.9592066132144,0.730475624763989,0.243482625488932,3.0
0011396417768,0.00269878609200326,0.146949932780605
"262","SLC35F2",10.6408905481641,-1.38598817940023,0.461829974105822,-
3.00107887558344,0.0026902486979951,0.146949932780605
"263","FAM107A",20.5737551114408,-1.26775908706497,0.42309181089014,-2
.99641603650457,0.00273173455281077,0.148178422549044
"264","LINGO2",2.13337038205128,4.36417149110713,1.45704588768765,2.99
52189755898,0.0027424788213423,0.14819773812602
"265","S100A12",2.70842785983272,3.87623736955499,1.29628362410984,2.9
902694884515,0.00278731417585294,0.149606906225162
"266","LOX",243.644195408462,0.999495740952601,0.334276521145668,2.990
02675248331,0.00278953014551333,0.149606906225162
"267","SAMD9L",98.9255415105475,0.888905620454992,0.297521659372324,2.
98770053356888,0.0028108482554383,0.150185622517164
"268","IFIT5",60.0163622281361,0.70705609501508,0.236887809851866,2.98
477197056795,0.00283789793444316,0.151065119152113
"269","ALKAL2",7.28264718181262,2.50349134362195,0.839115814539611,2.9
8348726152363,0.00284983898338697,0.151136813892188
"270","AC097518.2",2.49856045249543,3.70300193379948,1.24517581877413,
2.97387877114822,0.00294061227307624,0.154427580989098
"271","ACTN1",96.5049303620984,-1.17064838389451,0.393695416323034,-2.
97348746101217,0.00294436436485593,0.154427580989098
"272","ADAMTS5",596.886991649488,0.943792353394734,0.317355900408072,2

.97392407760863,0.00294017813235573,0.154427580989098
"273","FXYD6",7.5335048467097,-1.86590502487809,0.629329450917215,-2.96490974982758,0.00302771738351222,0.157640205084618
"274","NAV3",65.5262576611024,0.841940031646273,0.283886333385126,2.96576457769835,0.00301931515671877,0.157640205084618
"275","MYLIP",118.549851115382,0.819917179350064,0.277140537757708,2.95848880854407,0.00309151467211194,0.160376539317632
"276","TM4SF1",3.67951890511555,-2.49991333426523,0.847241373116935,-2.95065068065343,0.00317105328027118,0.163906688754886
"277","MCC",48.4466247752763,0.81412708695967,0.276318672702808,2.94633395201379,0.00321565017742774,0.164497983807783
"278","ITPR3",34.2529697167754,1.0076154650232,0.342134110751387,2.94508917222634,0.00322861597267484,0.164497983807783
"279","CABLES1",19.1570150968584,1.1774378602228,0.399571197103302,2.94675359174698,0.00321128985864941,0.164497983807783
"280","AL592183.1",6.90953183669981,-2.01390261370645,0.683731208481026,-2.94545954422722,0.00322475315812048,0.164497983807783
"281","LUZP2",9.77853880547664,2.06441555554558,0.701317780112905,2.9436235813289,0.0032439428178895,0.164690705480469
"282","TAGLN2",638.667227942359,-0.791514395457661,0.269764221689274,-2.93409700701289,0.00334519639961168,0.165180529218507
"283","PXDN",122.529115361549,0.731726261315314,0.249018504211823,2.93844131636452,0.00329867099875071,0.165180529218507
"284","PDLIM4",131.288830397374,-0.758538596712126,0.25853376430672,-2.93400205867196,0.00334621988953796,0.165180529218507
"285","GEM",169.114972131591,-1.17808489787811,0.401348907462633,-2.93531357871653,0.0033321076422916,0.165180529218507
"286","C16orf89",50.0449668015045,1.47307804351667,0.502053450837749,2.9341060021769,0.00334509945156749,0.165180529218507
"287","CYGB",330.738492829636,-0.742349696512352,0.252673575564782,-2.93797914899899,0.00330359241388514,0.165180529218507
"288","PKN1",95.1128958303775,-0.700226615227229,0.238142359061932,-2.94036986105915,0.00327820677503169,0.165180529218507
"289","SERTAD3",59.2872581615817,-0.816494046873744,0.278139155734449,-2.93555952134004,0.00332946728810988,0.165180529218507
"290","PPP1R12B",75.9184471714409,0.696386607263349,0.237821840224826,2.92818610185262,0.00340945904687953,0.167721871595805
"291","STAC",17.642915900835,1.29918149644099,0.444208056607287,2.92471394229926,0.00344772993899275,0.168675822331778
"292","AIF1",26.5407364574088,-1.25502281704909,0.429172715692481,-2.92428379335364,0.0034524982560549,0.168675822331778
"293","FABP5",97.1834154094392,-1.44878282033908,0.496563904130441,-2.91761605764744,0.0035271836074001,0.171736523355528
"294","PRR16",11.6261822125361,-1.48890831237563,0.511550576236743,-2.91057889784601,0.00360759870838453,0.175054432563992
"295","ROR1",43.36033419117,1.24419418305967,0.427674656255297,2.90920718555966,0.00362346640777672,0.175228378892687
"296","GALC",44.1214767213222,0.83670467529833,0.288156883349721,2.90364285444767,0.00368848655098655,0.177770098433696
"297","FCER1G",24.8724310534049,-1.40136379802283,0.483426936829329,-2

.89881198431764,0.0037457943929141,0.179924251883207
"298","S100A4",5034.63583399469,-0.610477859606307,0.210862249939813,-2.8951500791657,0.00378977294457933,0.180911730052048
"299","NOX4",63.3987712948836,0.970854533189815,0.335356868925745,2.89498925815884,0.00379171507679535,0.180911730052048
"300","TRIM73",9.67574930672991,1.62279819606769,0.561837817012215,2.88837480662575,0.00387238144518651,0.184144645656769
"301","SH3BGRL3",1194.2511388436,-0.780763563004397,0.27076501841369,-2.88354665450727,0.00393224394040629,0.184148323658305
"302","AC079781.5",7.56975179516692,2.09906617062921,0.728608199529953,2.88092581442726,0.00396508954946682,0.184148323658305
"303","EGR3",65.5872639704045,-1.32145669238018,0.457772083768621,-2.88671314664112,0.0038928896851637,0.184148323658305
"304","GASAL1",14.6968719723141,-1.73774483370229,0.603580759689229,-2.87905935669159,0.00398863255365317,0.184148323658305
"305","AIF1L",29.1046456450301,1.62468125158796,0.563919646081636,2.88105098461627,0.00396351520984527,0.184148323658305
"306","ZEB1",251.490112431369,0.731863347345088,0.254135518006768,2.87981527763308,0.00397908231651713,0.184148323658305
"307","SPI1",5.9709653076606,-1.77673133578152,0.616890534816059,-2.88014037419345,0.00397498146439154,0.184148323658305
"308","TBC1D4",40.6015495973965,0.920701456700824,0.31941825956869,2.8824321375492,0.00394618127895966,0.184148323658305
"309","ICAM2",37.52604114643,-0.82393316233873,0.285545770879917,-2.8854679227073,0.00390832287682823,0.184148323658305
"310","SLC46A3",16.4997507008468,1.16426573110251,0.405258379539422,2.87289736593653,0.0040672622593051,0.187172785133053
"311","MLF1",68.0587666921109,-0.755618180683766,0.263407001525535,-2.86863362138274,0.00412249025146458,0.189104327740816
"312","HSD11B1",100.356988563564,-1.22874603824704,0.429531343643541,-2.86066676257916,0.00422751152243152,0.193300254419898
"313","APOE",1272.22772910188,-1.03886937513796,0.363368043906633,-2.85900037870391,0.00424978277369069,0.193697766931219
"314","DTNA",5.33278498176698,-2.28185664265171,0.799909713683544,-2.85264274657182,0.00433573344828556,0.196985902462553
"315","VPS35L",39.7336621874797,0.782403570631119,0.274428492164764,2.85102893092228,0.00435780042486394,0.197359939241616
"316","HPSE2",5.31351321032558,-2.66589024448326,0.935983060139102,-2.84822488570152,0.00439638461447657,0.198477287690262
"317","TMEM64",86.6031154268526,1.41602386038793,0.497479646881335,2.84639556465252,0.00442172304014535,0.198991485459664
"318","LAMA4",563.53246375219,0.641189485777541,0.225432837206528,2.8442594864302,0.0044514778993407З,0.199700577710676
"319","DOCK4",35.9031825503576,1.22337475813461,0.430353860165152,2.84271821720184,0.00447305983070463,0.200039722711073
"320","HES4",16.0449928630511,-1.32854070606409,0.467806653851394,-2.83993546292337,0.00451226616360691,0.200994579407165
"321","MRPS6",288.358165856833,-0.730743047099636,0.257375810535608,-2.83920639464499,0.0045225893726132,0.200994579407165
"322","UCP2",46.2712500786902,-1.04471761774851,0.368184018172112,-2.8

3748768600853,0.00454701007058482,0.20145231573591
"323","GSTT2B",4.73115769039843,-2.47499251295819,0.872908492725579,-2
.83534016862438,0.00457769144477349,0.20155971034302
"324","MT-
CO2",12932.4108795062,-0.557963195230319,0.196780844014578,-2.83545483
313907,0.00457604851719628,0.20155971034302
"325","AC093627.6",3.3400170323297,-3.67297763538371,1.29760351971275,
-2.83058544430952,0.00464628990777746,0.203950682536472
"326","MAN2A1",58.6415898242997,0.758627721765424,0.268285677136696,2.
82768625541978,0.00468857322242539,0.205175415923683
"327","CRIM1",83.4367590230579,0.83599215825885,0.296007907402525,2.82
422238512037,0.00473954871891683,0.206141469585572
"328","NECAB2",2.13219830875951,4.25780492555219,1.50711722732734,2.82
513187982252,0.00472611594400658,0.206141469585572
"329","SNAI2",207.965830439337,0.761720450474894,0.269890313709442,2.8
2233341391772,0.0047675583454944,0.206729444853565
"330","LYZ",64.1877379105108,-1.26821494903754,0.449932758478774,-2.81
867662475919,0.00482220724093085,0.208465480300362
"331","MDGA2",2.21676800214295,3.61119750631996,1.28167272641109,2.817
56600722241,0.00483891672735269,0.208555849040524
"332","MMP9",4.03446323369083,-2.37357164083865,0.843730993538663,-2.8
1318531500631,0.00490533725500894,0.210781750843246
"333","ATP11A",50.7115779726307,0.806967773407761,0.287173792925233,2.
81003278602744,0.00495364534496038,0.211582947578457
"334","SHISA6",5.9943932691927,1.94645876966132,0.692561982298591,2.81
05192306414,0.00494616330001479,0.211582947578457
"335","OSCAR",2.94548753980943,-2.91128308066133,1.03674096588373,-2.8
0811039253158,0.0049833140982045,0.212214802761151
"336","KLF10",173.601366680011,-0.812387911433079,0.289837603764748,-2
.80290721728596,0.00506442366467752,0.2150269880961
"337","IER5L",136.762146932464,-0.714761507912178,0.255111895503939,-2
.80175687809564,0.00508251600565724,0.215154817022867
"338","INKA1",5.76536031169863,-1.80998285361002,0.647024352921576,-2.
79739525326553,0.0051516468447547,0.217436076589558
"339","PDE5A",47.9627038718145,-1.06310355488626,0.380197198865055,-2.
79618986688956,0.00517090125593678,0.217604947838331
"340","ANTXR2",288.466599320138,0.498561834507179,0.17859643083782,2.7
9155542005155,0.00524553738473993,0.219102116797653
"341","HELLPAR",160.54933568607,-0.74193872026809,0.265820875773511,-2
.7911228495848,0.00525255319955119,0.219102116797653
"342","NRK",10.3688620748539,4.12275992192746,1.47672320723823,2.79182
984442823,0.00524109091475113,0.219102116797653
"343","CDA",20.737096208929,-1.1754773589062,0.421868705758124,-2.7863
5827417868,0.00533039213816004,0.221700799542248
"344","TNFSF12",138.859807719924,-0.622028567090777,0.22351711352219,-
2.78291249063138,0.00538733347073563,0.223417730504403
"345","ALPL",271.446386957777,1.69198665571474,0.609754434409201,2.774
86568400953,0.00552245101562655,0.227697358927539
"346","NID2",174.726892588337,0.962850061580733,0.346908139991493,2.77
551879181718,0.00551137142205512,0.227697358927539

"347","PLAGL1",363.25605376582,0.830677538084357,0.299543068112895,2.7
7314892752345,0.00555167071824433,0.228214541129279
"348","EGLN3",10.1502000903039,-1.8350455020632,0.661933103046547,-2.7
7225220134392,0.0055669886662687,0.228214541129279
"349","NIPAL2",46.8838543794313,0.867198517871566,0.312919319525983,2.
77131664221057,0.00558301060261361,0.228215556609988
"350","PI16",518.1068962287,0.964213227261091,0.348538693320969,2.7664
452921247,0.00566710927128628,0.230977149112486
"351","MT-
CO3",18333.3773640357,-0.408699257026102,0.14778307287663,-2.765534976
84601,0.00568295102610981,0.230977149112486
"352","ENTPD1",17.5449279642671,1.75831146102817,0.636463668236466,2.7
6262660192396,0.00573383201937945,0.231724780703873
"353","BGN",309.53623019336,-1.11310804530029,0.402874621378394,-2.762
91428209576,0.00572878091069287,0.231724780703873
"354","TPPP",6.41622005053536,-1.85161224323969,0.670739639288994,-2.7
6055288040297,0.0057703615500788,0.232542310376905
"355","NLRP3",12.3320232473023,-1.26284781769367,0.45789256708584,-2.7
5795657861581,0.00581639233893713,0.233103443437999
"356","MEG3",1043.06281660554,1.04395402520809,0.378528879079249,2.757
92438280389,0.00581696522248197,0.233103443437999
"357","SMARCE1",282.948015074039,0.442730083185195,0.160824671374675,2
.7528741666368,0.00590745975167879,0.23606672497885
"358","PCBP4",24.516894371544,-0.953876366924648,0.346648096983228,-2.
7517138424412,0.00592842995617077,0.236242965795341
"359","TPP1",126.145090460005,0.63519607388766,0.23098403551491,2.7499
5660402105,0.00596031576529863,0.236851990829388
"360","MIR155HG",11.7307802954142,-1.36001067390724,0.495052800199377,
-2.74720327480122,0.00601058689549319,0.238186201808627
"361","ATP8B4",66.0277173281962,0.978406412398499,0.357061180704556,2.
74016461399668,0.00614084195618424,0.242072977003877
"362","RNF24",103.324747002332,-0.805690351632597,0.294040038650354,-2
.7400702140114,0.00614260603360462,0.242072977003877
"363","PHACTR2",196.406235190346,0.720003418426739,0.263028473332863,2
.73735922694413,0.00619346210720905,0.243404767001224
"364","CCL18",2.90217982333832,-2.78435495550557,1.0178867889659,-2.73
542695090313,0.0062299412246169,0.244165773380178
"365","LILRB5",4.97930482729308,-2.8596836241717,1.04609104177044,-2.7
3368522431076,0.00626298863347928,0.244788481767714
"366","CDKN1C",517.607065881309,0.884984448343784,0.324092446291991,2.
73065435022962,0.00632087266665862,0.246375872848503
"367","GIMAP7",18.5078997162235,-1.03160701314364,0.378192287652755,-2
.72773149221602,0.00637714939678771,0.247892134317639
"368","RBP5",36.339522173854,-1.28222851756805,0.470303577250765,-2.72
638478546882,0.00640323030425754,0.248229574784071
"369","TGFBR3",548.752428173421,0.692820412657637,0.254668718625477,2.
720477082529,0.00651877903062655,0.249129549587628
"370","ZNF595",23.3734952260003,1.0582198377791,0.389073382256143,2.71
984639926468,0.00653122469828773,0.249129549587628
"371","EGFLAM",30.2959231877149,-1.02905195009566,0.377867517937973,-2

.72331412795472,0.00646305723355143,0.249129549587628
"372","AC011389.1",2.07769135601824,4.27215102858948,1.57016134044155,2.72083569920789,0.00651171174021934,0.249129549587628
"373","SYNJ2",23.1538238964598,-1.19601845710092,0.439627004917813,-2.72053000321151,0.00651773568435075,0.249129549587628
"374","SEMA3C",488.073044357333,0.989139166187669,0.363668071084995,2.71989554440838,0.00653025411918032,0.249129549587628
"375","GNS",163.54588109822,0.738295914330595,0.271755852621544,2.71676178160832,0.00659240379710704,0.250792620185411
"376","COL8A2",36.3799488498268,-0.856599535744139,0.315460796458375,-2.71539140635235,0.006619748231294,0.251163107094788
"377","CLSTN1",112.992309034825,0.596931855740054,0.220109047730077,2.71198236463264,0.00668821510159094,0.253087736443757
"378","MAOA",24.3731307803066,-1.2582669119831,0.464270798212664,-2.71020041929653,0.00672425628080097,0.253778412968007
"379","LIFR",127.975726900605,0.72558909820946,0.267859708997357,2.70884001526567,0.0067518888811722,0.254148936091827
"380","PLD1",25.6596901396399,0.968410913679126,0.358127318941937,2.70409673448044,0.00684903472254102,0.255674294456414
"381","FNDC3B",267.840662168921,0.635407809914457,0.235118539449201,2.70249981733891,0.00688202222565982,0.255674294456414
"382","ECM2",397.222961795499,0.554051040836204,0.205002456760848,2.70265561491562,0.00687879764447266,0.255674294456414
"383","RERG",43.6135524709098,-1.26402591917797,0.467324902238423,-2.70481181962154,0.00683430932204371,0.255674294456414
"384","KLHL13",15.553848202115,1.37669556346413,0.508844532640148,2.70553278094808,0.00681949172158664,0.255674294456414
"385","ENPEP",14.2166570610668,1.46248252106718,0.541693713430193,2.69983292182999,0.00693743061305467,0.257063337989189
"386","TUBB2A",155.686080002827,-0.724251040463305,0.268706737802533,-2.6953214734628,0.00703207495796685,0.258555622036998
"387","HGF",44.4930954793908,-1.05940576541233,0.393042622353941,-2.69539664443393,0.00703048851631296,0.258555622036998
"388","SUSD1",14.0684793150913,1.32722733576142,0.492404062201725,2.6954028970169,0.00703035657349685,0.258555622036998
"389","EDN1",4.07661575461451,2.32598783802814,0.863656662102187,2.69318577635534,0.00707728218325687,0.258883352888058
"390","MT1F",112.217786408111,-1.33040598212928,0.493968913811066,-2.69329900107467,0.00707487897197015,0.258883352888058
"391","MSANTD3",38.8542767758485,-0.70767503298527,0.263005324971825,-2.69072511387775,0.00712969148325952,0.259666562675734
"392","APBB1IP",148.974533494805,0.8106758358369,0.30131358396143,2.69047224880731,0.00713509691356286,0.259666562675734
"393","NRN1",73.5295769836071,-0.722678389127894,0.269045202230864,-2.6860853980506,0.00722946085324232,0.262431268530165
"394","CMTM8",32.8418864455547,0.90463164318151,0.337118197197044,2.68342572635662,0.00728721598653007,0.26326433162288
"395","OPHN1",34.6506706483714,0.830474848770733,0.309494199646946,2.68332928280431,0.00728931802825161,0.26326433162288
"396","STK38",66.8323162745249,0.715523479034046,0.266748865251954,2.6

823862150574,0.0073099014122536,0.263341044311136
"397","FNDC10",8.60671526106388,1.9931902667027,0.743746545499046,2.67993213382293,0.00736370879419569,0.264080952728347
"398","ACVR2A",91.7472819966942,0.861719200654962,0.321565627789253,2.6797615360175,0.00736746244118059,0.264080952728347
"399","ZNF43",34.9722220329156,0.782961839741438,0.29242261901603,2.67750094837403,0.00741736425614187,0.265203304456441
"400","NMUR1",4.66174931260723,2.57996375311267,0.964758347093959,2.67420723633439,0.00749061470366201,0.265823655130453
"401","AC108863.2",10.9944664487197,1.39209061643926,0.520295733768762,2.67557568914827,0.00746010261341523,0.265823655130453
"402","SREBF1",51.2121879024567,0.922211970698999,0.344753842399295,2.67498677978733,0.00747321970629969,0.265823655130453
"403","NLGN1",14.3733819123356,1.25078005490868,0.468040442280458,2.67237602121397,0.00753161996835624,0.266615609103152
"404","AC016885.2",2.67266546909947,3.76642271458397,1.41003334819677,2.6711586072774,0.00755899203148734,0.266922228517818
"405","AQP3",41.0681996876978,-1.09067340242902,0.409019889268884,-2.6665534636435,0.00766334164478935,0.268676053663404
"406","CYP11A1",11.4112532802295,1.64818110623641,0.618112626039163,2.66647377323107,0.00766515868785962,0.268676053663404
"407","PLEKHG2",41.9231884877852,1.45769748616699,0.546387114521473,2.66788408332735,0.00763305880689264,0.268676053663404
"408","ANKRD36B",26.6621191988952,-1.02810332628804,0.387150745038725,-2.65556334183265,0.00791760624838225,0.276844536126032
"409","PROSER2",8.92642801805325,1.52725221870212,0.575342495625909,2.65450967087116,0.00794237629193453,0.277031638583712
"410","ODF2L",226.0660588878,0.457271239669189,0.1723270258080З,2.65350856910038,0.007965974791647,0.277177064335698
"411","PYCARD-AS1",4.17496781535619,2.9838572278568,1.12655162862538,2.64866443049548,0.00808105270940776,0.280124430197099
"412","LGALS2",6.05786404617461,-2.05634937948909,0.776481254104999,-2.64829236844785,0.00808995270161255,0.280124430197099
"413","VAMP8",24.6635382020748,-0.828111379449361,0.312970554442453,-2.64597217755714,0.00814565158507558,0.280800772423428
"414","TGFBR2",788.254587938652,0.477653511624665,0.180585764610133,2.64502305957434,0.00816853501722434,0.280800772423428
"415","GNAL",24.3387829932175,1.11417504349775,0.421164390471154,2.64546354987735,0.00815790755893157,0.280800772423428
"416","ISLR",735.299595157412,0.646453758509868,0.244607604873614,2.64281954293238,0.00822188425937564,0.281279138715235
"417","NATD1",17.9725984793198,1.06879113739468,0.404360718308096,2.6431626243683,0.0082135574897236,0.281279138715235
"418","GPR34",2.44295253902972,-2.76772394955126,1.04761574122223,-2.64192665368145,0.00824359058246719,0.281347041266691
"419","DACT1",36.5184528299043,-0.970049488774578,0.367427871972631,-2.64010861115848,0.00828794614246677,0.28218577486499
"420","SETX",145.683779512486,0.565906783991595,0.214631470313818,2.63664402598636,0.0083730646944954,0.28440509745636

"421","SULT1A1",60.5162659046315,1.24094931116895,0.470832544509569,2.
63564897040318,0.00839765551715988,0.284562835172928
"422","TXN",763.247732139383,-0.830467620575786,0.315552734259193,-2.6
3178711642424,0.00849370654812857,0.28713558676683
"423","TFPI",359.442074856377,0.55493313436951,0.211287047288766,2.626
44180743878,0.00862827444607069,0.290308875584067
"424","ISM1",139.706112468454,0.932183484982531,0.354884294850336,2.62
672510029123,0.00862109501943636,0.290308875584067
"425","OSR2",332.29544223688,0.887457814616191,0.338197035795654,2.624
08513583902,0.00868820649643563,0.29095294337594
"426","SLC3A2",179.800318156666,-0.66250962913984,0.252462498367336,-2
.62419025963959,0.00868552520641302,0.29095294337594
"427","ANXA5",1261.79835690617,-0.51747036522838,0.197461474150439,-2.
62061431200568,0.00877714986158637,0.293243137998574
"428","ELMO1",17.9473144781173,0.956889806833127,0.365395408405232,2.6
1877895786778,0.00882451076061348,0.294136613343252
"429","EFHD1",63.4122100157683,0.891125929787824,0.34040712873962,2.61
782393655291,0.00884924506929078,0.294273496872966
"430","SLC43A2",66.8960792231744,0.902817591174215,0.345028736489816,2
.61664463186203,0.00887987359035876,0.294605294511763
"431","LMO4",348.17371384807,-0.599718649696135,0.229269793685741,-2.6
1577698507535,0.00890246826035438,0.294669633879851
"432","STMN2",2.91757404571231,4.02176092234068,1.53832575451135,2.614
37534315884,0.00893907736898554,0.295196476263768
"433","KDM7A-
DT",41.4624692490966,-0.912101571412218,0.349428591083882,-2.610266001
94048,0.00904718432865699,0.298076516472565
"434","IL10",4.29567422105767,-1.94425836181919,0.745339118323345,-2.6
0855537301308,0.0090925299519942,0.298880258744583
"435","CACHD1",47.3675879639495,0.815015335428751,0.312903905551191,2.
60468252703038,0.00919594246860301,0.300892924901584
"436","FAM168B",58.8170002147704,0.640693146759307,0.245970436258003,2
.60475671997944,0.00919395155105951,0.300892924901584
"437","SCIN",55.7644912182032,2.12291698967958,0.815431274887216,2.603
42846179551,0.0092296527291557,0.300938578897664
"438","PRXL2C",100.040741398158,-0.517167615944526,0.198676650247581,-
2.60306188623604,0.00923952737678232,0.300938578897664
"439","AL354822.1",4.81949697419815,2.08821477868896,0.802916723048428
,2.60078625683701,0.00930103846208328,0.302251969704055
"440","LINC02019",2.56163756842665,-2.98652291616573,1.14873809476989,
-2.59982926461925,0.00932701526388826,0.302407272169614
"441","WASF1",38.3614536695205,0.785276126613314,0.302491020352439,2.5
9603120019355,0.00943075012287854,0.304157521734655
"442","SRGAP1",265.623662739931,0.589433202041673,0.22709691767726,2.5
9551388046292,0.00944495879212477,0.304157521734655
"443","FPR2",2.70109665911379,3.14436519888,1.21144260955931,2.5955544
0271648,0.00944384512175325,0.304157521734655
"444","ABCA10",142.814913867609,1.01824714125451,0.393002233019875,2.5
9094492524936,0.00957128077893345,0.307531287369965
"445","HLA-

DPA1",143.034855833855,-0.670632793096138,0.259314795150649,-2.58617250398895,0.00970483466062514,0.311121733187591
"446","DANCR",57.9852005520914,-0.777302215727326,0.300913515901651,-2.58314158271766,0.00979051357094228,0.313164723325252
"447","IL33",169.699354776143,0.52221535248165,0.202423589267795,2.57981470623361,0.00988533407792409,0.314786107043895
"448","TIMP1",2811.74160422532,-0.802968213925086,0.311243841110097,-2.57986860418243,0.00988379141578321,0.314786107043895
"449","FHIT",18.1967140123625,1.04374223466909,0.404750438804614,2.57873033504712,0.00991641645803728,0.315062507371358
"450","TRIM47",120.823765845914,-0.728025101267648,0.282402171687389,-2.57797274333128,0.0099381836756702,0.315062507371358
"451","NCEH1",12.9063004747324,-1.126508362128,0.437134741896783,-2.57702775405116,0.00996539487626144,0.315224663646886
"452","SMS",98.4019535420298,-0.591147479286354,0.229873638032787,-2.57161927894506,0.0101224139345436,0.31948309112876
"453","RAB3GAP2",62.3314710269613,0.664077019789409,0.25847720787562,2.56918985332341,0.0101936593411319,0.319711902925887
"454","P2RY14",2.18845244169715,-2.98989454919706,1.16469739861957,-2.5670998773937,0.010255306889938,0.319711902925887
"455","FAM171A1",37.2518779163384,0.710826402326979,0.276918086010958,2.56691938243012,0.0102606464449035,0.319711902925887
"456","TMCC3",13.0380394888908,1.49197460868862,0.580517596348413,2.5700764594794,0.0101676071359298,0.319711902925887
"457","MRC2",461.485117799479,0.614523150546948,0.239271163896918,2.56831262296068,0.0102194945176521,0.319711902925887
"458","INAFM1",90.6228039349371,-0.571458911611623,0.222634613216025,-2.56680173561839,0.0102641281045883,0.319711902925887
"459","CISD1",87.9205371481802,-0.734870218194099,0.286468824321411,-2.56527117718608,0.0103095196371814,0.320426159355185
"460","S100A10",6569.95138422086,-0.67439411482749,0.263535187114979,-2.55902872861245,0.0104965066708683,0.322128100119343
"461","ADH1B",1279.45587946044,0.690872689127572,0.269970198728606,2.55907019508509,0.0104952546902575,0.322128100119343
"462","ADGRA2",73.067709799912,0.568672230337097,0.222356453809342,2.55748021069224,0.0105433556776062,0.322128100119343
"463","NECAB1",15.2011100630479,2.97730132713749,1.16218393681611,2.56181593362409,0.0104126487646713,0.322128100119343
"464","SWAP70",105.458283272487,0.721381714454857,0.282073058727686,2.55742862401929,0.0105449195819244,0.322128100119343
"465","ACADVL",284.038686608347,-0.494453534956045,0.193033251862045,-2.56149409589502,0.0104223012741929,0.322128100119343
"466","TLE2",38.9187118418142,-0.730071365771641,0.285281413108234,-2.55912699610281,0.0104935399354853,0.322128100119343
"467","PLAUR",76.5586615937456,-0.678814176066274,0.265295105400645,-2.55871353163918,0.0105060276309762,0.322128100119343
"468","ATP1A1-AS1",13.8736595654735,1.57973623272416,0.618487558196381,2.55419241953863,0.0106434419702844,0.323854751111854
"469","CFH",2075.09180400006,-0.699747403625312,0.273972267954298,-2.5

5408114423478,0.0106468441238932,0.323854751111854
"470","SLITRK2",13.9720289593144,-1.36190470730401,0.533514266461173,-2.55270532189812,0.0106889887623867,0.324444922732359
"471","GAB1",56.9398725426266,0.676228752724096,0.265342288922204,2.54851480881874,0.0108182693683129,0.327060553872506
"472","TMEM192",34.7337869748712,0.793616584374319,0.311414370794264,2.54842633739154,0.010821013698852,0.327060553872506
"473","ZMAT3",93.0390774189281,0.774447829432339,0.304047651139646,2.54712649984145,0.010861405275664,0.32758733121062
"474","DUXAP8",91.567396661988,0.861133554164844,0.338211495870385,2.5461392196286,0.0108921738332738,0.327822261403975
"475","CCNG2",119.428106802851,0.569948562282329,0.224078965607821,2.54351657120661,0.0109742851331431,0.329598214125093
"476","VHL",30.6082038451379,0.738677461044486,0.290970867794095,2.53866466648204,0.0111276423371605,0.331615159625726
"477","COL5A1",339.961212589044,0.863509697376865,0.340075840893541,2.53916801354667,0.0111116447079721,0.331615159625726
"478","FAM111A",58.3826446752139,0.604833213862824,0.238268567046432,2.53845155221402,0.0111344218043406,0.331615159625726
"479","GPRC5B",83.3785206386396,0.83703498227357,0.329620974036,2.53938628972728,0.0111047136977604,0.331615159625726
"480","AMOT",22.2555124804621,1.06526316422918,0.41982555303334,2.53739477393026,0.011168093652217,0.331925050088601
"481","B9D2",12.9619733831343,-1.23347723182726,0.486268864435042,-2.53661569152765,0.0111929752336847,0.331972941130449
"482","MTX3",34.0250958683531,0.841319184737537,0.331957992674311,2.53441460457007,0.0112635376601099,0.332682460163827
"483","GATM",2.39005820935923,-2.92608957347351,1.15441745804876,-2.53468929551645,0.0112547101013521,0.332682460163827
"484","CLHC1",6.36238108845204,1.66718026306594,0.65853743299231,2.53164084460694,0.0113530212023743,0.33463264560552
"485","DMD",38.3985186832802,0.864488859122149,0.34185340746106,2.52882914212467,0.0114443723924539,0.336629724846903
"486","FGF10",32.3004672461446,-1.01172984678689,0.400214451083449,-2.52796930257756,0.0114724382358201,0.336760913317304
"487","FZD5",3.53058296819701,-2.11339286561757,0.837323472872675,-2.52398617032314,0.0116032493268243,0.338158140761041
"488","PTPRG-
AS1",2.73759494740539,3.0245895676383,1.19941537473325,2.52171985731879,0.011678267147447,0.338158140761041
"489","CD86",2.2031323528641,-2.65667235721227,1.05251184977507,-2.52412584027442,0.0115986400939369,0.338158140761041
"490","ARL9",4.16897920685048,-2.14888692062265,0.850871091232695,-2.52551407935303,0.011552915215428,0.338158140761041
"491","UPP1",47.8766012814419,-0.829019756206984,0.328781947737912,-2.52148806195356,0.0116859640680775,0.338158140761041
"492","METTL7A",395.194197412957,0.710132666591708,0.28156147962072,2.52212293936053,0.0116648932476307,0.338158140761041
"493","FZD10",3.23559058764577,-2.23143581210126,0.884365600530422,-2.52320512100753,0.0116290546187536,0.338158140761041

"494","ROR1-
AS1",2.4525099219714,-3.24893272847571,1.29045176448207,-2.51767080172
863,0.0118133676506649,0.338412656434508
"495","REEP5",345.660361351518,-0.469928368480581,0.186470098699906,-2
.52012720407713,0.0117312431007555,0.338412656434508
"496","FREM1",33.1558021456153,0.890596939502411,0.353731503155239,2.5
1772016786292,0.0118117121921494,0.338412656434508
"497","ITGA8",4.57006053309029,-2.3621972540772,0.937510448552147,-2.5
1964898921956,0.0117471913779869,0.338412656434508
"498","BCL7A",21.7030670423372,1.17589491047551,0.467025713820429,2.51
783761724016,0.0118077744370732,0.338412656434508
"499","ACE",91.8059579101659,0.708637183729577,0.28155776763026,2.5168
4473027984,0.0118410999212552,0.338527317588429
"500","VIT",283.43854495265,0.53271946556949,0.211917941089104,2.51380
068545258,0.0119437913841617,0.338787439910768
"501","CPNE2",19.0640957456796,-0.929706857731237,0.369776520163024,-2
.51423983686513,0.0119289279515366,0.338787439910768
"502","JUNB",6503.09929152382,-0.45877180434595,0.182504263268725,-2.5
1375938363939,0.011945190121626,0.338787439910768
"503","SSBP4",194.319298158245,-0.579047139608825,0.230304658805647,-2
.51426585381184,0.0119280479018539,0.338787439910768
"504","OSBPL10",29.0683014357947,0.954951808022611,0.380098857779487,2
.51237747359036,0.0119920740304683,0.339442317695755
"505","SNX33",49.4979612479119,0.676317363351488,0.269611797629234,2.5
0848579067579,0.0121249844644224,0.342524808652375
"506","QPCT",9.24031801782799,-1.51734383347619,0.605163096866527,-2.5
0733040618777,0.0121646941800584,0.342967445005362
"507","NFKBIZ",728.585823462481,0.790629479886595,0.315681549616338,2.
50451596188464,0.0122619072340898,0.344175325177468
"508","SHMT1",15.195161061271,-1.00746226383335,0.402669981311876,-2.5
0195522534632,0.0123509543902582,0.344175325177468
"509","MARCH2",84.2369479850831,-0.714632292380466,0.285582820094985,-
2.50236443544741,0.0123366861587314,0.344175325177468
"510","MIR99AHG",215.028141917011,0.592034715402791,0.236307295727389,
2.50535944554915,0.0122327006872947,0.344175325177468
"511","CLDN5",1.99419823417121,-2.97750477616161,1.18932487149514,-2.5
0352519107624,0.0122962926519022,0.344175325177468
"512","FOXRED2",22.1072915120364,0.898950977407916,0.359304883584446,2
.50191694707829,0.0123522898143042,0.344175325177468
"513","GGA2",40.4795085481038,0.782743385510452,0.312974577213794,2.50
098072654559,0.0123849918313168,0.344413827418257
"514","CD52",99.5101315882278,-0.752940726728986,0.301256787795901,-2.
4993319892898,0.0124427684001188,0.344500586302156
"515","AMOTL2",160.348550247479,0.690230638819784,0.276111379146973,2.
49982684868767,0.0124254020619847,0.344500586302156
"516","POLR3G",9.91802226903487,1.43973267192656,0.576163695531328,2.4
9882573840212,0.0124605567455427,0.344500586302156
"517","MT1H",13.5471688175194,-1.96879747253172,0.788253031245136,-2.4
9767193336609,0.0125011826458423,0.344955264266125
"518","OGN",804.30250284778,0.677157168659416,0.271191730734018,2.4969

6835086599,0.0125260135732689,0.344973184625972
"519","AMPH",24.2015240012596,1.49237237922521,0.599976708947341,2.4873838550226,0.0128686465323834,0.353046368136503
"520","LARP4B",43.8414214217933,0.660166339650509,0.265339249415402,2.48800861955024,0.0128460619546941,0.353046368136503
"521","PDLIM3",93.3291216098288,-2.12481308340759,0.854702070464649,-2.48602777135249,0.0129177884462763,0.353714337763105
"522","GRAMD1A",53.3465955725295,-0.603439158421953,0.24301487925684,-2.4831366715788,0.0130231110750972,0.355915139075357
"523","LYVE1",4.53265088455828,-2.56556107918014,1.03353131885529,-2.48232543356469,0.0130528005779065,0.356044460887982
"524","BEND6",23.0652148802886,-1.0881686017424,0.438693655252501,-2.48047490250588,0.013120750019453,0.357214923239536
"525","RIC3",2.65776680096906,-2.76700036288054,1.11601332022183,-2.47936141329438,0.0131617866738211,0.357649616549965
"526","KAZN",55.2799893596143,0.685364030046471,0.27659591926445,2.47785300617976,0.0132175586635373,0.357802071905167
"527","ANXA1",2914.19689095321,-0.613582399319463,0.247571396781765,-2.47840585502024,0.0131970933670973,0.357802071905167
"528","PLOD1",38.3568261990375,0.687128357078528,0.277685288158296,2.47448599684844,0.0133428051342591,0.360508443267689
"529","ID4",193.02499746333,-0.797220852204362,0.322535423170481,-2.47173114930381,0.0134460594097858,0.360647661516692
"530","MSRA",25.7397622090411,-0.79808342658233,0.322836220248323,-2.47210002015403,0.0134321929642617,0.360647661516692
"531","NR4A1",258.1782146281,-1.12051547034234,0.452995927968197,-2.47356631960941,0.0133771973032679,0.360647661516692
"532","RFX2",31.6625898037206,-0.911752350324686,0.368883954748105,-2.47165087716348,0.0134490786434095,0.360647661516692
"533","S100A3",7.72922259784017,-1.35722966343504,0.549385471255008,-2.47045059334132,0.0134942957921609,0.360992969867534
"534","UBAC1",68.2651672530683,-0.616191943205067,0.24947378983851,-2.46996665903838,0.0135125645527312,0.360992969867534
"535","AL133243.2",6.66365414896879,1.90050294414036,0.770774512679857,2.4657054856843,0.0136743716543466,0.364632871067118
"536","ANGPTL1",160.474727812971,-0.592450951331114,0.240631018201363,-2.46207224554627,0.0138136838455319,0.364937062482145
"537","CHL1",366.1396731195,0.609916878793741,0.247567848406607,2.46363525279749,0.013753599349557,0.364937062482145
"538","MSMO1",28.7024477732189,-0.782056593894483,0.317533049134056,-2.46291400541527,0.0137812965127543,0.364937062482145
"539","ANGPT1",126.222568530368,1.1977816981008,0.486397612948413,2.4625566947999,0.0137950361027552,0.364937062482145
"540","STX16",104.740694784552,0.638010142917299,0.259070024102862,2.46269380306222,0.013789762477962,0.364937062482145
"541","ARHGEF35",13.0223927116846,-1.01593427571411,0.413523647454437,-2.4567743150071,0.0140190726263943,0.368996476177381
"542","VPS51",116.621993967839,-0.547793591715127,0.222914299232443,-2.4574179117326,0.0139939788363622,0.368996476177381
"543","GPR183",49.7323095833759,-0.900149160962483,0.366735620570468,-

2.45449067522341,0.0141084323543372,0.370664633456675
"544","RBP4",3.29078228683911,-2.16663366505764,0.882982285087667,-2.45376798793028,0.014136815910929,0.370727602546532
"545","PDE7A",37.8292711310271,0.831952991658223,0.339177669848574,2.45285307853447,0.0141728212622624,0.37087368183958
"546","LGALS3",2455.75747222473,-0.638556094355369,0.260390030114094,-2.45230623490298,0.0141943803648122,0.37087368183958
"547","NDUFA4L2",5.4894156268203,-1.79400760990441,0.731906481899623,-2.45114321880052,0.0142403280678697,0.371394004051607
"548","SLC25A45",21.1625023749031,1.20763313919925,0.493312206349823,2.44800984783028,0.0143647729355511,0.373955931931702
"549","ZBTB6",16.0640794516831,1.02116085733871,0.417337337560559,2.4468475869129,0.0144111765669349,0.374480591810371
"550","FGFBP2",53.6036142918818,-1.26674542927448,0.518992977791054,-2.44077566264195,0.0146557558454803,0.379453743904939
"551","TMEM140",105.577493700576,0.655513342167911,0.268498199172976,2.44140684811672,0.0146301621710519,0.379453743904939
"552","POLR3K",37.9501255477415,-0.819161713565943,0.335873335031789,-2.4389007048994,0.0147320156195794,0.380737200777028
"553","CXCR2",2.76145094005965,3.21360532100921,1.31844549409322,2.43741992779111,0.0147924899229499,0.381608790670531
"554","PILRA",10.9516061702406,1.21853577488737,0.50012273071044,2.43647348953006,0.0148312565574495,0.381918241964935
"555","GLUL",614.32780409849,0.593669388738702,0.243932315262707,2.43374637796284,0.0149434616381229,0.382455374740827
"556","TMOD2",45.2626959639313,0.736324021192479,0.30259202497908,2.43338872279726,0.0149582324410933,0.382455374740827
"557","ETS2",30.8059014177925,-0.79163968095434,0.325327589246215,-2.43336165490474,0.014959350841538,0.382455374740827
"558","VSIG4",7.89723249713236,-1.83909076381674,0.75537377709324,-2.43467647353838,0.0149051097583625,0.382455374740827
"559","SLC2A10",53.3086527075716,0.579518917946697,0.238241915404409,2.43248093838979,0.0149957808042043,0.382700910470086
"560","DLGAP2",2.89704112731113,2.76184620832363,1.13731426884,2.42839317503734,0.0151658921439525,0.385817700754806
"561","SBF2",97.4916462502726,0.704984847895481,0.290326639730181,2.428247192716,0.015171998466525,0.385817700754806
"562","ACSL1",71.2123130441503,0.709263825865215,0.292274462278757,2.42670474982776,0.0152366500156373,0.386772329400501
"563","SMAD5",141.555292110784,0.469263260855596,0.193482581377695,2.42535145807029,0.0152935729362061,0.386840623240986
"564","GFRA1",135.21732689139,0.783671402453656,0.323045865312254,2.42588278198875,0.0152712018048371,0.386840623240986
"565","LINC00968",8.80995471485568,1.56078449123568,0.643721726353648,2.42462608816503,0.0153241608579987,0.386928281062317
"566","POLG2",40.7533949301732,1.31305092761095,0.541703025078179,2.42393131812666,0.0153535089061324,0.386984378188842
"567","ACKR4",121.829111321669,0.703201585682368,0.290531148772308,2.42039997657351,0.0155034437890102,0.389160020240802
"568","EHD4",44.8964225029834,0.6983911892799,0.288526167338636,2.4205

4714039235,0.0154971698216444,0.389160020240802
"569","JMJD8",73.3432296207504,-0.554845868918568,0.229338237912423,-2.41933431585206,0.0155489423480483,0.389160020240802
"570","NOL4L",8.27794441151641,1.38388376567902,0.57189351225884,2.41982770570873,0.0155278624010662,0.389160020240802
"571","RPL39L",14.652519535007,-0.987822051319228,0.408574338290462,-2.41772906113592,0.0156177007630109,0.389514194204743
"572","PKM",324.700432935813,-0.568736996780413,0.235208197462464,-2.41801520064442,0.0156054248957098,0.389514194204743
"573","S100A9",48.6472959197636,1.18174757586928,0.489285201954431,2.41525304903732,0.0157242811911615,0.390126252996713
"574","RHOBTB3",352.642210711009,0.716300921179536,0.296495210061527,2.41589373747688,0.0156966414507806,0.390126252996713
"575","LTBP2",194.004169548251,0.912270865246169,0.377691326573356,2.41538738398587,0.0157184823443397,0.390126252996713
"576","SURF2",46.4431880445176,-0.676659128331596,0.280253403364966,-2.41445463358175,0.015758785296809,0.390303526118537
"577","MFSD4B",17.0549106488997,1.12549516304721,0.466764324861165,2.41127074864167,0.0158970423850611,0.392365409455506
"578","IQCN",17.1691589745466,1.62191860355657,0.672577615306297,2.41149655689617,0.015887201857684,0.392365409455506
"579","GATA6",23.7855618447725,0.905964910553717,0.375819634767216,2.4106375152934,0.0159246667399578,0.392368386376923
"580","PIP4K2A",76.4213386954101,0.552479912939646,0.229423191100451,2.4081258319598,0.016034653318378,0.393718354974148
"581","MT-ATP6",23586.3481002518,-0.43952023565105,0.182467864285611,-2.40875420651103,0.0160070743498871,0.393718354974148
"582","ZNF727",18.2026916665869,0.852803805487961,0.354310769140533,2.40693729845312,0.0160869315618506,0.394323308696495
"583","TGFB1",89.4970627308247,-0.587476065915109,0.244219857863577,-2.40552128338097,0.0161494112344652,0.395175815902025
"584","HIST2H3PS2",2.48267829025305,3.05499286129309,1.27244016703286,2.40089313465865,0.0163551120348613,0.396507236781162
"585","CLIP4",44.2059573685086,0.63224669619278,0.263267909310641,2.40153347154349,0.0163265153589066,0.396507236781162
"586","ARHGEF4",13.0373613227506,1.1062039165741,0.460380585387852,2.40280314088867,0.0162699433415753,0.396507236781162
"587","CD44-AS1",2.26036422281889,3.56866322454529,1.48660329817814,2.40054843744714,0.0163705240114619,0.396507236781162
"588","AL109628.2",3.49221224242298,2.3457336427204,0.977109418779397,2.40068675793821,0.016364337943766,0.396507236781162
"589","CSF3",11.5303755139527,5.98450083202335,2.49297374111952,2.4005470789018,0.016370584779483,0.396507236781162
"590","FZD1",58.6908283731266,0.659833575797369,0.275178930103328,2.3978346581608,0.0164923078402937,0.398778412965474
"591","PLPP3",1277.29979885313,0.549806329368294,0.229516672009215,2.39549625983693,0.0165978835754393,0.399300855121783
"592","RALGAPA2",17.7566995944732,1.10977860097402,0.463232715264004,2

.39572587255962,0.016587490637379,0.399300855121783
"593","DIP2A",48.7495036268894,0.755351079357901,0.315203977839987,2.39638815643803,0.0165575457558624,0.399300855121783
"594","C6orf120",79.9494501478441,0.542433920160046,0.22684054824997,2.39125643252415,0.0167908207934856,0.401909143355478
"595","ARC",27.2550785548556,-1.48788296435351,0.622071411679176,-2.39182019366109,0.0167650533554265,0.401909143355478
"596","SMCHD1",141.881091652723,0.486778423542682,0.203543411467302,2.39152139601865,0.0167787059643403,0.401909143355478
"597","PCSK6",15.3798833777857,1.23647221554421,0.51727580731008,2.39035384618908,0.0168321470706255,0.402223467520172
"598","KIRREL1",99.2279679980463,0.626964818191628,0.26236946424954,2.3896257134379,0.0168655507668654,0.402347737859702
"599","PPL",185.245960492408,-0.916969043998491,0.384212730578706,-2.38661806603165,0.0170041464305124,0.404976883101318
"600","MX2",213.021318293679,0.791201787746606,0.331766324930709,2.3848164454661,0.017087644851614,0.406287235755208
"601","FMO3",19.5877107353812,1.24391730629518,0.521904222748201,2.38342065857422,0.0171525814904907,0.407152624864126
"602","NPL",8.77038412637193,-1.16439859381847,0.488851587850605,-2.38190613011636,0.0172232871832465,0.407474983343606
"603","MAP3K5",28.6943681379821,0.888714037085838,0.37304107440509,2.38234901747246,0.0172025846047164,0.407474983343606
"604","PIK3CA",63.7230667435978,0.629440995500354,0.264510431995779,2.37964525917223,0.0173293115114363,0.409304566261838
"605","DLG2",10.1967190327506,1.31743167675813,0.554369859955804,2.37644895929796,0.0174801795793619,0.412185523767235
"606","TMEM238",10.7672043058244,-1.25454227298615,0.528608232673454,-2.37329310336553,0.0176302672204292,0.415038600935055
"607","THBS2",359.31230387162,0.588166359536375,0.248222310007805,2.36951448690443,0.0178114572964864,0.417924752946833
"608","INTS10",92.1777893627629,-0.507680349165827,0.214239313969959,-2.36968808272517,0.0178030975069097,0.417924752946833
"609","SLC9A9",53.1498389349229,0.56481036848172,0.23857919787515,2.36739151406356,0.0179139710914013,0.419639920508918
"610","SMURF2",151.329178626643,0.680190206722185,0.287597291454475,2.3650786253314,0.0180262434873928,0.421577687854336
"611","NIBAN2",30.4459248974916,-0.704245678217717,0.298005815356748,-2.36319441409106,0.018118162064752,0.422342647084562
"612","ERLIN1",54.1470441593923,0.534734131916162,0.226271594589451,2.36324021531023,0.0181159228587945,0.422342647084562
"613","F10",403.567256580195,0.6332274484785,0.268029552275577,2.36252847158973,0.018150747159434,0.422412004855605
"614","ID2",601.682081202376,0.508924672465566,0.215756808217954,2.35878847425041,0.0183347032845118,0.424615060157216
"615","FAIM",56.8392157289343,-0.659095102922042,0.279379602929675,-2.35913823346634,0.0183174310619049,0.424615060157216
"616","NRG2",2.05766266560465,-2.60507640784254,1.10429507714708,-2.35904013497252,0.0183222740396714,0.424615060157216
"617","ZNF462",47.1425902759995,0.822169310398033,0.348670057017338,2.

35801524636556,0.0183729383448487,0.424810921276518
"618","RXRG",3.7834998577193,-2.0385069310608,0.864880903495727,-2.356
97992963128,0.0184242426204905,0.425286717417826
"619","GCH1",50.8520759669934,1.0094835626912,0.428401143691292,2.3563
9791713217,0.0184531387972546,0.425286717417826
"620","APP",683.070779519769,0.411198408777478,0.174550312210303,2.355
75865531569,0.0184849230311807,0.425332116069071
"621","CLPTM1L",103.984855581899,-0.470837118661175,0.200164877148771,
-2.35224643487892,0.0186604075294218,0.425333811966161
"622","TMEM200A",4.32009023840716,-1.82451992131994,0.774911564581677,
-2.35448792444449,0.0185482463470851,0.425333811966161
"623","IGF2R",110.763030355831,0.552613069426658,0.234903893669991,2.3
5250706488078,0.0186473355330882,0.425333811966161
"624","CTNNAL1",56.1593777443265,-0.634095053081487,0.269577932347565,
-2.35217715174086,0.0186638838000012,0.425333811966161
"625","NET1",37.0442626770372,0.945548755171386,0.401834703971297,2.35
307888001362,0.0186186839966851,0.425333811966161
"626","PGF",20.2504793745497,-1.26227961590433,0.536272884358286,-2.35
38009336698,0.0185825595980119,0.425333811966161
"627","LSAMP",70.7188081858292,2.13454899025817,0.908430309856969,2.34
971132853796,0.0187879755698601,0.425443269015276
"628","HEBP2",168.487929892936,-0.560099414153605,0.238358999634293,-2
.34981441864141,0.018782773173984,0.425443269015276
"629","CCN3",31.7699134237827,-0.897851291810461,0.382009453680371,-2.
35033788604011,0.0187563760651359,0.425443269015276
"630","ANPEP",145.17453303502,0.721438464151787,0.306995831142739,2.34
999433531836,0.0187736967779537,0.425443269015276
"631","PTMS",734.487492190903,-0.513447397083527,0.218600573904393,-2.
34879253934661,0.0188343975561103,0.425818566617225
"632","KL",4.26834843229383,1.93259484146808,0.823528354334304,2.34672
532074537,0.0189392110755413,0.42751073608176
"633","STXBP6",57.1332010973342,-0.84513927669112,0.36037704010838,-2.
34515294436336,0.0190192759532635,0.427909723369526
"634","ULK2",30.9968649082604,0.746584647322474,0.318368855100399,2.34
503041161811,0.0190255276910037,0.427909723369526
"635","ADAMTS1",315.038508410306,0.91470877282194,0.390132201892319,2.
34461233496027,0.0190468718869795,0.427909723369526
"636","CALN1",2.35063762923587,-3.55038483001802,1.51479610366679,-2.3
4380377756701,0.0190882108193405,0.428164175391055
"637","TKT",192.212160975037,-0.480239013298937,0.205193580474474,-2.3
40419286941,0.0192621014022744,0.431386402833354
"638","MS4A6A",16.9299115902804,-1.2433438246335,0.531415914895048,-2.
33968119844331,0.0193002067606781,0.431562303523251
"639","CHPF",58.6525185470189,-0.657037038303211,0.281267960334023,-2.
3359825183179,0.0194921526691847,0.434492265591546
"640","GSN",18670.0948897801,-0.667255840470103,0.285578554932422,-2.3
3650541661995,0.0194649156307442,0.434492265591546
"641","PTGFR",263.812508134031,-0.863565401224681,0.369812831835001,-2
.33514179845976,0.0195360143901328,0.434790610436248
"642","FOXO4",4.81375796943349,1.93906808611486,0.831320688319659,2.33

251513328061,0.0196736077406354,0.437170853626019
"643","PTGER3",21.224172014002,-1.26664030656244,0.543356820506716,-2.33113905772124,0.0197460283649557,0.437610185544086
"644","F2R",30.5005040960013,-1.05747174335065,0.453660884265732,-2.33097403815675,0.0197547286899195,0.437610185544086
"645","FHDC1",12.873920447449,1.050326143604,0.450728186005495,2.33028724676027,0.0197909743532052,0.437733395539264
"646","UBXN11",21.4374180808732,-0.794499388720711,0.341056098463521,-2.32952699658496,0.0198311645342569,0.437943333197691
"647","CSF1",361.692869079458,0.465307725037383,0.199839702724796,2.32840480991993,0.0198906184942024,0.438577377802615
"648","FMO4",16.0163119405667,0.913381528672128,0.392776811902855,2.32544666842001,0.0200480885058054,0.440687258280155
"649","ZNF706",300.300379483135,-0.527211972922328,0.226698242862886,-2.3256112013237,0.0200393014754467,0.440687258280155
"650","CYP27C1",12.4653856688434,1.04129749589989,0.448364800528135,2.32243364036011,0.0202095977278407,0.443180379474239
"651","TEX26-AS1",12.4023594378332,-1.32510399748622,0.5706311159587,-2.32217269690937,0.0202236385137901,0.443180379474239
"652","IGSF3",12.7305795306099,1.24741017637505,0.53800806588234,2.31857151496284,0.0204182811126205,0.445393269652361
"653","YIPF4",153.299031485678,0.404616907904807,0.17447706342649,2.31902635199542,0.0203936075181316,0.445393269652361
"654","RNF213",219.231314495297,0.658000292084047,0.28378937289948,2.31862203070273,0.0204155394949988,0.445393269652361
"655","SLC26A7",2.29525695521336,-2.89591062040029,1.24933697370973,-2.31795798998992,0.0204516043160381,0.445439064385648
"656","SASH1",294.390646380268,0.627155419346269,0.270940423889763,2.31473550658295,0.020627411349497,0.447219833300797
"657","PCNX4",75.3002280678034,0.604265855383127,0.26099149696226,2.31527027668072,0.0205981453494021,0.447219833300797
"658","PLP1",47.9855899975144,0.709478100734028,0.306453171439145,2.31512729139733,0.0206059668596161,0.447219833300797
"659","MAN2B1",73.9832475914682,-0.497920364839445,0.215223280874388,-2.31350606131708,0.0206948318924839,0.448000715900114
"660","DIRC3",11.3493864977692,1.52469854674865,0.659962017139573,2.31028226951158,0.0208725318929658,0.448300327550278
"661","TTC26",14.1462735924653,-1.01514297042204,0.43938992006969,-2.31034651468797,0.0208689776609935,0.448300327550278
"662","LINC01235",5.82161828614801,4.38367491272628,1.89685555790058,2.31102199346064,0.0208316401080649,0.448300327550278
"663","CDYL2",4.08210364226707,1.91803155526938,0.829580827569652,2.31204903913759,0.0207749810255346,0.448300327550278
"664","LASP1",131.13478694646,0.559996426602486,0.242439875486398,2.30983630675023,0.0208972184088697,0.448300327550278
"665","HUNK",17.2787938231039,1.36675729099822,0.591288869950451,2.31148827664007,0.0208058999673793,0.448300327550278
"666","RRAS2",56.9989108066596,0.63112240411424,0.273466440010631,2.30786053341575,0.0210068947857073,0.449301890574063

"667","RIPOR3",81.3136483676261,0.632011529484304,0.273803104127181,2.30827013995698,0.0209841161532909,0.449301890574063
"668","PCYOX1",237.055977354014,0.659670963680566,0.286665867636058,2.30118419440874,0.0213812219006686,0.456623520411583
"669","AL591846.2",6.08667442777458,-1.68135918024326,0.731726477842027,-2.29779737532779,0.0215733242304027,0.45792182395486
"670","HMCES",31.4711043949123,-0.708153171526457,0.308163962581107,-2.29797529080018,0.0215631954955788,0.45792182395486
"671","ARHGEF26",28.3064662581588,-1.02739141774517,0.44721980615254,-2.29728514616534,0.0216025085883654,0.45792182395486
"672","TSNARE1",3.04668091840598,-1.88829911980223,0.821653268408681,-2.29817027742049,0.0215520996547723,0.45792182395486
"673","MAP2K3",60.6905048044545,-0.538402793579387,0.234304962311952,-2.29787191985529,0.0215690799034952,0.45792182395486
"674","IL1RN",4.09732239840972,-2.023618149249,0.881711046665916,-2.29510354543143,0.0217271909536707,0.459881463123244
"675","CD320",64.4114517351488,-0.802472054274212,0.349965558397934,-2.29300293991144,0.0218478356963284,0.461749961546401
"676","WDR81",28.6010153148243,0.794157812567765,0.34642893670228,2.29241188720404,0.0218818867257256,0.461785497084618
"677","ALCAM",29.0904084475948,-0.976352123654778,0.426308726926329,-2.29024662641613,0.0220070239194912,0.462542917672633
"678","MAPK11",5.37080102138935,-1.4566433538728,0.635974041668629,-2.29041322197829,0.0219973737819991,0.462542917672633
"679","IL3RA",1.89206583692218,3.34390067500525,1.46014965033904,2.29010819146435,0.0220150456399634,0.462542917672633
"680","TCEA3",60.3823930451973,0.723620699793571,0.316086486977485,2.28931235470726,0.0220612103787875,0.462831216564385
"681","IL17RE",7.64308784165113,-1.23531065852736,0.539897833104456,-2.28804522408291,0.0221348876166338,0.463695017237736
"682","EDEM3",83.7358999051589,0.562457367559947,0.245968505010955,2.2867048264366,0.0222130578017993,0.464650267742624
"683","EPB41L5",13.5436038204009,1.01221733813719,0.442905132804989,2.28540439738564,0.0222891264066888,0.464878183213191
"684","PLCD3",31.4327088987526,0.722624859949968,0.316178478682691,2.28549666935167,0.0222837215012957,0.464878183213191
"685","TMEM131",68.9590473086933,0.551987221547034,0.241995870173108,2.28097785781293,0.0225497571547749,0.467319179308203
"686","CXCL3",63.1605608024192,-1.10583662756338,0.485254546543576,-2.27887947766828,0.0226742306768189,0.467319179308203
"687","DAAM2",8.03418801243975,1.35711787580765,0.595637336399934,2.27842983116228,0.0227009807416644,0.467319179308203
"688","ZNF696",9.42822469121381,1.24062407579057,0.544426570156771,2.27877209489119,0.0226806165291758,0.467319179308203
"689","KLF4",1476.46255387137,0.783889360451701,0.344044483313322,2.27845350956496,0.0226995713984676,0.467319179308203
"690","ZSWIM8",46.8895109299453,0.697132133305297,0.30569258290271,2.28050064769536,0.0225780124197112,0.467319179308203
"691","CD82",24.5487789162744,-0.838112270463413,0.36782642030085,-2.2785537530934,0.0226936057261474,0.467319179308203

"692","SMG6",69.6948051293537,0.486571625424639,0.213490963699102,2.27
912046952218,0.0226599050309368,0.467319179308203
"693","KCNB1",2.22420976203796,2.87993571252876,1.26349300440084,2.279
34440673413,0.0226466002410617,0.467319179308203
"694","ARHGAP29",132.322485362294,0.699752476409363,0.307715059804546,
2.27402739681909,0.0229643393410134,0.47138023746604
"695","STARD10",29.8901923281856,-0.913996498331902,0.401863707251461,
-2.27439423326671,0.0229422938661987,0.47138023746604
"696","LRRN4CL",289.914107571358,0.624240881824561,0.274775002976829,2
.27182558479383,0.0230970469156741,0.473423090946849
"697","DOCK7",60.2672189990307,0.574946684059325,0.253531372158481,2.2
6775360841707,0.0233442284111331,0.474399946600036
"698","CCL26",41.5859576087264,-2.55531088215019,1.126315718957,-2.268
73410282906,0.0232845004264585,0.474399946600036
"699","CXCL12",2998.06592254127,0.511228481775914,0.225280417685642,2.
26929835725574,0.0232501883753341,0.474399946600036
"700","TAFA2",5.07984728709863,1.9614394204194,0.864869887451353,2.267
9011593287,0.0233352316790509,0.474399946600036
"701","WASHC4",99.3818944529003,0.487440898793197,0.214714973812904,2.
27017655144041,0.0231968731224234,0.474399946600036
"702","MBP",173.255760924415,0.403241638513156,0.177741227047315,2.268
70065663388,0.0232865356535522,0.474399946600036
"703","PTGER4",101.811198143062,0.676310335404431,0.298581432855405,2.
26507833704432,0.0235078727327756,0.476368341485479
"704","BCORL1",23.6625612090113,0.848799320847475,0.374695769374503,2.
265302654109,0.0234941132920915,0.476368341485479
"705","KCTD10",54.6242583456954,0.557323161380999,0.246175587951865,2.
26392537951397,0.0235787046869108,0.477125958955275
"706","NMB",40.0908984318385,-0.772330053625655,0.341230011719617,-2.2
6337082642152,0.0236128395719727,0.477139899906179
"707","OGT",103.868907071314,0.535323035378632,0.236754091270494,2.261
09307132108,0.0237534943455945,0.479303182933878
"708","MN1",26.1925876555546,0.8573574756766,0.379394735642357,2.25980
330018286,0.023833461501673,0.480237516642467
"709","GLIPR1L2",4.46660811772701,1.71508406489009,0.759450903205323,2
.25832118659869,0.0239256423470857,0.481414969990867
"710","MERTK",2.75786530026828,-2.2314661814359,0.989709112172443,-2.2
5466872436666,0.0241541301849594,0.484373913328797
"711","CP",217.590668091722,-0.767051989366202,0.340255127576121,-2.25
434365921378,0.0241745567285927,0.484373913328797
"712","TUG1",114.192826633249,0.625837943386429,0.277588882224514,2.25
454974410773,0.0241616049660866,0.484373913328797
"713","PINK1",122.904998682303,-0.568514752370139,0.25264152266164,-2.
25028232248087,0.0244310293667661,0.488141547543817
"714","ZDHHC7",67.2857028924233,0.641521664540336,0.285036155661626,2.
25066768477576,0.0244065929693303,0.488141547543817
"715","ZNF234",17.816409152822,1.00939960029791,0.448690715654672,2.24
965564269616,0.0244708133421439,0.48825261977486
"716","CTSO",152.902186373302,0.438770889387514,0.195251962045054,2.24
720348411285,0.0246270256270186,0.489316361552992

"717","DDX11",9.94795512981399,1.25151745156628,0.556861865864028,2.24744685941894,0.0246114831276751,0.489316361552992
"718","TRAFD1",37.7642065629611,0.607719959672754,0.270320814087134,2.24814342071664,0.0245670461021147,0.489316361552992
"719","ASAP1-IT2",7.10577070517615,1.54016702753005,0.686513365921808,2.24346255147124,0.0248670021391262,0.493397291400242
"720","RGPD2",3.39678007636858,2.59383171407647,1.15688542394618,2.24208176573683,0.0249560882534566,0.49379133845189
"721","JUND",3554.54200944715,-0.391576131825574,0.174616525956941,-2.24249182418241,0.0249296031261993,0.49379133845189
"722","CFAP36",128.716820991799,-0.54781613342586,0.244549406015782,-2.24010412599615,0.0250841637452019,0.495638060926663
"723","CAB39L",131.262262396425,-0.698572520299521,0.312141529350199,-2.23799928754682,0.0252211015868704,0.497654543898054
"724","SNX18",93.5072982781711,0.52940754261032,0.236636309764341,2.23722024374679,0.0252719489002711,0.497969092557
"725","LPP",435.16085941119,0.530617714926269,0.237327351676494,2.23580514920828,0.0253645375054661,0.498416655720357
"726","SCARB2",297.278878457868,0.392707255017997,0.175643935680123,2.23581448170908,0.0253639259264411,0.498416655720357
"727","LRRFIP2",85.9266267285647,-0.465048111070936,0.208118573456067,-2.2345343971383,0.0254479319040658,0.499367533072081
"728","CNTN1",28.985532587078,1.21822202569587,0.545477359537632,2.23331363693716,0.0255282688567163,0.500255883942191
"729","FLCN",52.8604775129981,0.728148302863796,0.326916937975975,2.22731898619859,0.0259259625047491,0.506657234373632
"730","ETV2",4.12058290004445,-1.83645575940108,0.824463160280259,-2.22745641997736,0.0259167852982752,0.506657234373632
"731","GRIA1",10.5470155114075,1.25191578441971,0.56293795084475,2.22389658139245,0.0261554031376162,0.510441834693888
"732","PER2",81.9787300205234,-0.758278023236914,0.341058582434995,-2.22330726241566,0.0261950881480112,0.510517933769847
"733","SAA1",3.66354847674786,-2.06850079430694,0.93084328890993,-2.22217941403356,0.0262711830972336,0.510669707782035
"734","RNF215",19.2804219765128,-0.792366727550839,0.356579699505323,-2.22213078492712,0.026274468352167,0.510669707782035
"735","OLA1",95.4343637643143,-0.499730887158058,0.224997463729802,-2.22105120152901,0.0263474936605382,0.511392305525494
"736","VLDLR",13.615371908486,1.54448715638225,0.695982571510603,2.21914631142271,0.0264767722386282,0.513203305375366
"737","SLC25A21",4.0965054645563,-1.70272039448307,0.767495912565553,-2.21854001644294,0.0265180342744334,0.513305667515695
"738","OGA",133.264927774335,0.632229083441877,0.285201460759856,2.21678066359633,0.0266380834890417,0.513602761003074
"739","ANGPTL4",482.638145539936,-0.865029066498275,0.390227117079599,-2.21673233006466,0.0266413881355863,0.513602761003074
"740","NFATC2",26.2938149236337,-1.05301889634118,0.474986529955771,-2.21694475512649,0.0266268669106376,0.513602761003074
"741","ASIP",9.45769838848154,-1.25496235650634,0.56645187237024,-2.21

547922730862,0.0267271886027806,0.51456150149429
"742","POLR2J",194.102778442969,-0.479048224747567,0.216311645056374,-2.21462059808533,0.0267861169882285,0.515001003981224
"743","FCGR1A",2.3395279520936,-2.94370226621578,1.33120293025988,-2.21130993577448,0.0270143817824199,0.515913213531462
"744","PBX1",194.763505613731,0.535012501869739,0.241831946566749,2.21233178438676,0.0269437484835234,0.515913213531462
"745","PDE7B",14.429491040522,1.1671371528103,0.527351447102072,2.21320555622632,0.0268834772179547,0.515913213531462
"746","TMEM233",13.1790076426011,-1.21220627065694,0.547879154171523,-2.21254315194741,0.0269291580293538,0.515913213531462
"747","AC000403.1",3.88812772005901,-1.74170623688475,0.78761702522731,-2.21136184350775,0.0270107899114733,0.515913213531462
"748","SOD3",1368.79541690434,-0.612646587725888,0.277461981290668,-2.2080379620878,0.0272416269696986,0.519557553943477
"749","AOPEP",17.3936728494894,-0.934302860756507,0.423446735062623,-2.20642357915059,0.0273543555867117,0.52101099706279
"750","UBAC2",71.8493528393333,-0.491882325836642,0.223154892405982,-2.20421932288075,0.0275089234920824,0.523256403384062
"751","IGFBP7",2318.39270048968,-0.768989930516091,0.34921970549562,-2.20202330628715,0.0276636622120928,0.524266330180156
"752","CREBBP",129.103702987199,0.513403200917559,0.233059897358025,2.20288091918651,0.0276031428140563,0.524266330180156
"753","GPC3",1982.9599657636,0.584900772086879,0.265634392913485,2.2019015146031,0.0276722659908634,0.524266330180156
"754","XCL1",7.58462344645444,-1.66727889958329,0.758070950296819,-2.19937051925083,0.0278515871095529,0.526527145080613
"755","TSPAN32",4.92579563615393,1.75302357739845,0.797315629186779,2.19865698504674,0.0279023217216419,0.526527145080613
"756","ADAM33",92.6531198866794,0.696622305360095,0.316804901742346,2.19890002183947,0.0278850320718343,0.526527145080613
"757","DHCR7",15.7929068324726,-0.943877748176583,0.429486401233513,-2.19768948554763,0.0279712413430069,0.527130421399389
"758","FCGR2A",13.0663056045401,-1.29855496431988,0.591296904553033,-2.19611324585147,0.0280838387389129,0.527618241818024
"759","LATS1",53.4964923279902,0.578495238916529,0.26343171847229,2.19599690679382,0.028092164783791,0.527618241818024
"760","DIXDC1",84.2227845579313,-0.564614590152641,0.257136860409184,-2.19577461299857,0.028108079614587,0.527618241818024
"761","PLCXD3",4.10326553088135,-1.94777296483387,0.887612379741117,-2.19439589767998,0.0282069605769882,0.528778580277679
"762","FGFR2",5.91862086064882,-1.69113521904972,0.771537437447882,-2.19190299390231,0.0283865118613518,0.53144616563523
"763","SYDE2",28.0822124405753,0.79507929102163,0.362989996041503,2.19036144161594,0.0284980340967657,0.532834802653289
"764","STK3",93.6313995073908,0.450161435849236,0.205796475517173,2.18741081312479,0.0287125475076127,0.534857158300395
"765","SLC43A1",37.9981614765783,0.673574052049833,0.307943942893317,2.18732684176608,0.02871867259625,0.534857158300395
"766","AC087645.2",4.99752058176311,-1.5251590948239,0.697087717367325

,-2.18790125952001,0.0286767955435762,0.534857158300395
"767","EBP",45.7548697815676,-0.59326364247875,0.271381695002059,-2.18608569923719,0.0288093360519564,0.535846138353599
"768","RGS2",76.1295403954453,-0.81948435490606,0.37563038566419,-2.18162424069354,0.0291372763349431,0.541240083586325
"769","DUSP23",52.1657394783789,-0.645688621090818,0.296047801441929,-2.181028259443,0.0291813263549295,0.541353448347755
"770","GALNT17",9.75592914455295,1.21391364237687,0.55683536686197,2.18002252482245,0.0292557919220262,0.54165754568876
"771","CCDC85B",271.764380000696,-0.639767969729293,0.293500951329212,-2.17978158786846,0.029273655385254,0.54165754568876
"772","FPR1",4.96689539728218,1.9529624096905,0.897772262350197,2.17534278078275,0.0296044389011326,0.547068556170411
"773","GLB1",59.9721755326445,0.472423766050826,0.217680072765786,2.17026648350643,0.0299866641341688,0.551275451724294
"774","DINOL",5.527990114147,-1.63270265021895,0.751817565225237,-2.1716739881301,0.0298802618848942,0.551275451724294
"775","AC018926.2",17.277893609084,-1.00855436674949,0.464592694304466,-2.17083561389053,0.0299436007825953,0.551275451724294
"776","LINC01426",13.5552627429664,1.43821263793165,0.662540626065722,2.17075388489307,0.02994978155094,0.551275451724294
"777","TUBB3",54.1416536469559,-1.15843211200343,0.533911196662851,-2.16970934350894,0.0300288717837066,0.55134090716391
"778","SKIL",112.792748690231,-0.585198599528802,0.269850544569041,-2.16860262581045,0.0301128656586483,0.551463596259663
"779","C9orf16",240.219586739338,-0.630102929334094,0.290518562166855,-2.16889043038911,0.0300910034478501,0.551463596259663
"780","AC254633.1",9.39985996246543,1.41636028392936,0.653671659511283,2.16677633689718,0.030251912600627,0.552284278393883
"781","AQP1",20.5789588304707,-1.33213307155952,0.614880993335832,-2.1664892653983,0.0302738192698736,0.552284278393883
"782","CALCOCO1",111.385462951895,0.572544922367208,0.264228364292598,2.16685640052326,0.0302458053071733,0.552284278393883
"783","RUNX1T1",294.832584638845,0.649495023522272,0.299946611000617,2.1653687679803,0.0303594558540172,0.553139204614826
"784","IDH1",42.115008610235,0.689458027921342,0.318816289162968,2.16255583970151,0.0305753578855582,0.554946635617523
"785","COCH",16.9040140455867,-0.991673919948744,0.458464894801017,-2.16303130554664,0.0305387718833794,0.554946635617523
"786","PRRG1",38.0361254029844,0.574663299204929,0.265716832273311,2.16269061424698,0.0305649834735766,0.554946635617523
"787","PMS1",30.403616523141,0.642120311590828,0.29761189713854,2.15757608403644,0.0309608057956447,0.556952122980007
"788","AC009570.1",2.06789342910631,-2.81985081269579,1.30629183496381,-2.15866832909807,0.0308759075315649,0.556952122980007
"789","SLC12A7",35.4984390799992,0.750163931250264,0.347442385136115,2.15910310124189,0.0308421691200058,0.556952122980007
"790","CCM2",75.6228485902784,-0.560879789026437,0.259623786376376,-2.16035593985726,0.030745125532427,0.556952122980007
"791","CCL19",210.569568660043,-2.30577295786138,1.06917280177466,-2.1

565952239284,0.031037217003302,0.556952122980007
"792","EIF4EBP2",176.639486143608,0.478018097724682,0.221631107087184,
2.15681861633548,0.0310198000130101,0.556952122980007
"793","CADM1",3.73563638305591,-2.05440869552375,0.951755402813876,-2.
15854692229733,0.0308853343825501,0.556952122980007
"794","NIPA2",63.8542146725735,-0.484585860670044,0.224394727073314,-2
.15952427666324,0.0308095160037496,0.556952122980007
"795","FTX",209.510108649637,0.757090443088962,0.350899582079056,2.157
57009057478,0.0309612722089975,0.556952122980007
"796","MSL1",83.0960064699286,0.503165441530661,0.233554305618938,2.15
438306820006,0.0312101432913706,0.559351638435544
"797","MAOB",19.7614789211052,-0.797903808112767,0.370480397420482,-2.
15370047556706,0.0312636688346458,0.559607904134326
"798","ESR1",13.2850869999902,1.15663359914876,0.537308170268098,2.152
64472634324,0.0313466105926005,0.560389406909823
"799","LAMA2",322.7989750509,0.594824062268209,0.276482200972299,2.151
40092265037,0.0314445684363729,0.561437062970333
"800","CMC4",51.7498405946579,-0.55190204643341,0.25662030717933,-2.15
065616786022,0.0315033484476822,0.561783461193292
"801","RFTN2",37.0160450893354,0.608635822879235,0.283521742245758,2.1
4669893764855,0.031817257409215,0.566672901622798
"802","SLC35E2B",45.440697332528,0.657939249221138,0.30679450500947,2.
14456008330667,0.0319880366864886,0.568435566499308
"803","AC147067.1",9.86402134169304,-1.18334060678931,0.55231443967843
2,-2.1425125286934,0.0321522615842558,0.568435566499308
"804","MAP3K7",52.4502946114046,0.508403689212829,0.2371987030087,2.14
336622740378,0.0320837027342337,0.568435566499308
"805","TPST1",168.744240292175,0.486738785220351,0.227290365408129,2.1
41484459081,0.0322349904176322,0.568435566499308
"806","ADM",809.249185661614,0.920679914138072,0.429915103151833,2.141
53889311703,0.0322306055381607,0.568435566499308
"807","ARID3A",14.4872304663848,0.998517222469754,0.465735845204775,2.
14395613468558,0.0320364016865513,0.568435566499308
"808","POLR2I",162.200174780549,-0.512064431362823,0.239045338983389,-
2.14212263472916,0.0321836149360049,0.568435566499308
"809","PABPC1L",5.70030232618756,1.54827294891625,0.721989364759446,2.
14445395526304,0.0319965310242435,0.568435566499308
"810","FZD7",65.4318613446128,0.530829361328862,0.248059510336887,2.13
9927473887,0.0323606284048408,0.569946573856123
"811","PALMD",37.2956115091463,-0.812008952055603,0.380350418838253,-2
.13489695774705,0.0327694278943211,0.576434843822916
"812","FXYD7",2.87229922001581,-1.82986248508739,0.857757007097843,-2.
13331103091608,0.0328992199187564,0.578005260296772
"813","RADIL",13.6071686554308,0.901868484064707,0.423217284966665,2.1
3098215999317,0.0330906119306729,0.580502072687946
"814","ZNF823",5.67817796236556,-1.47569732052818,0.692622935680554,-2
.13059262768739,0.0331227174518427,0.580502072687946
"815","AC007563.2",9.84934258102977,1.09296138405679,0.514106916691989
,2.12594180037379,0.0335081058148184,0.586535751600245
"816","KLHL24",77.9698849861379,0.733247047790926,0.345155556024188,2.

12439589916246,0.0336370526262769,0.58750155776054
"817","SPATA22",3.08405470053931,-1.85032790119179,0.871032311725671,-2.12429306729846,0.0336456450785337,0.58750155776054
"818","S100A2",2.60609297935726,-1.96935879106736,0.927767752133073,-2.12268510792654,0.0337802477488605,0.588280470231468
"819","TMC3-AS1",2.82592492486467,2.24501114404263,1.05766288078035,2.12261504571877,0.0337861231274786,0.588280470231468
"820","TYROBP",43.8450634507596,-0.867851821354498,0.40892366098365,-2.12228321361233,0.033813962259204,0.588280470231468
"821","HDAC9",3.72279749487191,-1.64959874971082,0.777826447484068,-2.12077996967905,0.0339403230840914,0.58904215221125
"822","ADAMTS8",2.59465582257007,-2.03533257638962,0.959562304076502,-2.12110518279317,0.0339129519034048,0.58904215221125
"823","HDGF",175.081007975926,-0.478805702296307,0.225832722076174,-2.12017859012833,0.0339909872451904,0.589204646464018
"824","MAVS",72.9046138296761,0.65449102613387,0.308914278349369,2.11868169263989,0.0341173765438158,0.590677783706402
"825","CENPF",9.14462842593349,-1.08184224455467,0.511103813536192,-2.11667809142273,0.0342871774510161,0.592723676898942
"826","CARF",15.2427159226374,0.923283593928788,0.436270952288253,2.11630774198039,0.0343186427252577,0.592723676898942
"827","EPHA3",3.4230068658959,2.58315532998411,1.22131023330402,2.11506893133605,0.0344240727374493,0.593825661030775
"828","NPLOC4",59.5056249797844,0.534346456386809,0.252706356657715,2.11449550954735,0.0344729678931831,0.593950917831099
"829","ETHE1",72.5326372829447,-0.619480856365465,0.293116611895675,-2.11342800518569,0.0345641510765544,0.594803593797496
"830","C3",5201.71489198329,0.405312369464176,0.191886320345372,2.11225255002369,0.0346647933669434,0.595816797798572
"831","PRKACB",44.4472716671266,0.54081044660174,0.256334583998254,2.10978338609754,0.0348770181426036,0.597863915653786
"832","WDR66",5.75917891114397,1.49714536985004,0.709747640213878,2.10940520971494,0.034909620197645,0.597863915653786
"833","ADAMTSL3",40.560031308235,0.864180215031408,0.40950913975325,2.11028309539592,0.034833978781528,0.597863915653786
"834","RCC1",17.4839030067855,-0.826112227726334,0.392308105017841,-2.10577405146591,0.0352239838568344,0.598933675448868
"835","CD1D",2.18450993855669,-2.19294846871215,1.04075843987577,-2.10706767746597,0.0351117136120333,0.598933675448868
"836","PTMA",3702.57667098619,-0.541004101746697,0.256884466816931,-2.10602107807571,0.0352025214595103,0.598933675448868
"837","PCDHB2",14.1097961650231,1.00864525908605,0.4785939610349,2.10751773153379,0.0350727263440276,0.598933675448868
"838","TP53INP1",27.7680089044459,0.697341113998655,0.331019081034862,2.10664929592144,0.0351479903291268,0.598933675448868
"839","SEC14L5",2.47974459540972,2.32881303457566,1.10524402349944,2.10705779453314,0.0351125701653393,0.598933675448868
"840","RTN4RL2",13.1804191495683,-0.947608711037877,0.450211020693317,-2.10481011677274,0.0353078401843002,0.599201260247206

"841","PAN3",40.5066832906961,-0.543221871326248,0.258108291957297,-2.10462774057689,0.035323724931158,0.599201260247206
"842","CLU",1214.46989174521,-0.824993837308484,0.39210553884121,-2.10400964940865,0.0353776052939517,0.599402514398473
"843","ARID1A",112.611126634644,0.484166009734642,0.230168624143334,2.10352740968349,0.0354196918776586,0.599403706200092
"844","SLC25A3",836.297900406358,-0.444936443390787,0.211594098924942,-2.10278285477431,0.0354847554334654,0.599793271343386
"845","COX18",14.712349503777,0.852359352775882,0.405810706801232,2.10038655582681,0.0356948506938854,0.602630461537241
"846","KAT6A",97.2240970302715,0.468045229012855,0.223063358403704,2.09826137453637,0.0358820625926874,0.605075064949502
"847","FAM107B",126.439792588807,0.467331715683542,0.223123415008602,2.09449875830166,0.0362155735632294,0.60997800761869
"848","AC097448.1",19.1852587639598,-0.885107296178137,0.422803577616219,-2.09342432996523,0.0363112924234031,0.610868983151259
"849","ULBP2",2.47248309545306,2.53534602628545,1.21219918147325,2.09152593487493,0.0364809440677326,0.613000174405504
"850","FAM53C",101.553878684368,0.662476701379263,0.316917458214467,2.09037616643684,0.0365840221238779,0.613287496614856
"851","POLH",11.8928569059059,1.03982864194436,0.497385086457821,2.09059071181568,0.0365647690751908,0.613287496614856
"852","PCDH18",291.648299656262,0.494619926390402,0.236675298146678,2.08986713131281,0.0366297368943284,0.613333129735316
"853","TSHR",2.74986640186221,3.06109622557689,1.46614639959223,2.08785168140661,0.0368112159288594,0.615649245534711
"854","TSC22D1",174.150647650277,-0.737293228359221,0.353403431523759,-2.08626505175758,0.0369546205301038,0.617323906888128
"855","SRGAP2",42.8290967628273,0.595854011231885,0.286933013377041,2.07663107224581,0.0378356227781586,0.617602509919638
"856","DESI2",142.47257323517,-0.408462963106773,0.1970737519296,-2.0726401111635,0.0382057851582974,0.617602509919638
"857","ST3GAL5",138.136922860873,0.449425892478438,0.21766248392835,2.06478344070713,0.038943495359314,0.617602509919638
"858","POLR1A",29.199046258351,0.726516537490174,0.350968308000575,2.0700345898154,0.038449105398375,0.617602509919638
"859","NPAS2",36.2940257184191,1.06100141507487,0.511522647491798,2.07420222795097,0.038060533413923,0.617602509919638
"860","ERCC3",28.8719948997867,0.647264330546507,0.311573805407755,2.0774029116454,0.0377643876952914,0.617602509919638
"861","BHLHE40",95.7617754248671,-0.910330011246101,0.441220064272732,-2.06321082144487,0.0390926024433922,0.617602509919638
"862","HPGDS",1.91162657250309,-2.62265961331162,1.26772590795193,-2.06879073533225,0.0385657281950317,0.617602509919638
"863","CAMK2D",246.715659285349,0.45651951516518,0.219354488913815,2.08119522616448,0.0374160403388456,0.617602509919638
"864","HPGD",32.1368603854873,0.959946278241818,0.464226122869029,2.06784200834955,0.0386548820452119,0.617602509919638
"865","ELL2",69.0139295590359,1.08670327683032,0.524141806238189,2.073300133469,0.0381443562972797,0.617602509919638

"866","SPOCK1",171.297537262388,-0.519470731950385,0.250160256753541,-2.07655180200015,0.0378429453052228,0.617602509919638
"867","HIST1H2AC",29.4299809679843,0.968152368192942,0.465000325298,2.08204664711254,0.0373382091260517,0.617602509919638
"868","ENPP4",3.67555635583153,1.84178719694493,0.890822824565996,2.06751235616604,0.0386859011192378,0.617602509919638
"869","ARMC2",7.1806531078478,1.19774631810493,0.575089103588296,2.08271433179925,0.0372772703009297,0.617602509919638
"870","STX7",74.6103853526444,0.435238118502043,0.21017171738583,2.07086911557677,0.0383710290698827,0.617602509919638
"871","LINC00242",2.71264690049976,-2.1749690414727,1.05251164901577,-2.06645602783263,0.0387854402969221,0.617602509919638
"872","GTF2I",264.941886262676,0.382650382262548,0.184402600501935,2.07508126903304,0.037979003402131,0.617602509919638
"873","TRMT9B",7.9533792673404,1.37210665705362,0.660036208209894,2.07883543355138,0.0376324788624133,0.617602509919638
"874","R3HCC1",63.7260387289727,-0.615369999601049,0.29679307066946,-2.07339746245757,0.038135304911197,0.617602509919638
"875","ASPH",365.179744125951,0.526416800946309,0.253612537434541,2.07567341217182,0.0379241666001869,0.617602509919638
"876","ZBTB26",4.36057571679333,1.56058813702872,0.755587587182699,2.06539673692571,0.0388854770749813,0.617602509919638
"877","HTRA1",950.274995955737,-0.47509376909455,0.227907480937878,-2.08459049759779,0.0371064877783627,0.617602509919638
"878","OLR1",4.66609145446258,-1.67284208826721,0.804624350187262,-2.07903487867088,0.037614144774228З,0.617602509919638
"879","HDAC7",167.082812028277,0.403214319783127,0.194352782477823,2.07465164451215,0.0380188319877466,0.617602509919638
"880","CACNB3",8.44854102754384,-1.07744584677265,0.521899670490709,-2.06446929878227,0.0389732418900487,0.617602509919638
"881","TNFRSF19",8.38109784620544,1.44753736973243,0.699665382056124,2.06889951519185,0.038555517101034,0.617602509919638
"882","GTF3A",253.812032185373,-0.603555537851607,0.292196577553092,-2.06558044897682,0.0388681120750829,0.617602509919638
"883","NYNRIN",27.4540322090272,0.694906412728557,0.334223834383137,2.07916474302653,0.0376022110170044,0.617602509919638
"884","RASGRP1",17.0205208603483,0.863425038612535,0.415360222242625,2.07873790598123,0.0376414468988456,0.617602509919638
"885","MAP1A",190.247407106563,-0.502562685548227,0.24217093520704,-2.07523947957904,0.0379643453462053,0.617602509919638
"886","TMOD3",191.554583481456,-0.442683519692082,0.214467210726968,-2.06410815989792,0.0390074624725847,0.617602509919638
"887","ADCY9",25.0012908916708,0.66429744390837,0.319215883170689,2.08102879252146,0.0374312707131447,0.617602509919638
"888","KIF22",31.937488036653,-0.595963139733519,0.286306436205596,-2.08155690675971,0.0373829610626586,0.617602509919638
"889","CRNDE",164.601951786749,-0.384056539833572,0.186117613633358,-2.06351528120355,0.0390636974592432,0.617602509919638
"890","AC137932.3",4.3771339277791,1.71704921003311,0.829309572005001,2.0704562783254,0.0384096363212943,0.617602509919638

"891","KIF1C",29.2803350337987,-0.788431843160335,0.379155437141619,-2
.07944227070611,0.0375767186825939,0.617602509919638
"892","SNAPC2",57.9956053436238,-0.676390773162331,0.327733427589814,-
2.06384432047894,0.0390324793414874,0.617602509919638
"893","SLC44A2",44.6590990077623,0.674867736938604,0.325258904900287,2
.07486321441528,0.0379992138425543,0.617602509919638
"894","RAD23A",242.604592624778,-0.431543048533184,0.208121183075977,-
2.07351814051357,0.0381240846483382,0.617602509919638
"895","NUMBL",87.3743923497077,-0.5311879251197,0.256423631777995,-2.0
7152484908095,0.0383097747301757,0.617602509919638
"896","NR1H2",96.4977557509085,-0.430455976797677,0.206479257074339,-2
.08474198763074,0.0370927271456603,0.617602509919638
"897","ZNF524",47.7036529439092,-0.695662166765624,0.334528465448708,-
2.07953055902888,0.0375686120332828,0.617602509919638
"898","OXT",7.88796220804749,-1.17839661595106,0.570751718952274,-2.06
463962669133,0.0389571109117289,0.617602509919638
"899","GMEB2",12.1640180432308,0.9522839865875,0.460481487522306,2.068
0179603124,0.0386383342568202,0.617602509919638
"900","AP000525.1",6.46477501043956,1.40719427837394,0.680488610823412
,2.06791745812056,0.0386477854685835,0.617602509919638
"901","TTC28",80.5828735289473,0.552606239026783,0.265313184571736,2.0
8284499663592,0.0372653545798089,0.617602509919638
"902","MXRA5",203.061865297071,0.673540258933148,0.324292796636044,2.0
7695103289348,0.0378060789123431,0.617602509919638
"903","PNMA6A",7.04942588483307,1.35859058454016,0.656419155319516,2.0
69699784856,0.0384804669203791,0.617602509919638
"904","RHOB",1222.40502319214,0.601567394732796,0.291978802149796,2.06
031187984726,0.0393687349467623,0.620226247603336
"905","AC009950.1",12.0562056161389,1.09986994116345,0.534116755144801
,2.05923130208726,0.0394720858929926,0.620226247603336
"906","NMNAT3",17.9253434569756,0.909704565455081,0.441653275206348,2.
05977090293296,0.0394204474722902,0.620226247603336
"907","FAM110A",4.68829627997644,-1.45850306491691,0.708289758071339,-
2.05918982774563,0.0394760572566822,0.620226247603336
"908","GAS2L1",33.2051399143826,-0.654067885381691,0.317510547975091,-
2.05998789505728,0.0393996980554141,0.620226247603336
"909","MFAP5",3931.89995833386,0.758281093535266,0.368653162985245,2.0
5689566690526,0.0396962628864843,0.622315259712731
"910","NCOA6",40.4138863775339,0.578536854674921,0.281247913675371,2.0
5703518690878,0.0396828413146404,0.622315259712731
"911","CDKN2C",77.7493216014642,-0.592369590765615,0.28813062836849,-2
.05590635788999,0.0397915431749115,0.622440959356675
"912","LINC02381",79.0460077805162,0.50010709412407,0.243237397027091,
2.05604524730369,0.0397781550457464,0.622440959356675
"913","FCRL6",3.4624619682588,1.85740991319055,0.903936182589204,2.054
80204130146,0.0398981289775118,0.622874427690646
"914","ZNF777",13.968262961229,0.833770821036339,0.40596259932929,2.05
381190881587,0.039993899885366,0.622874427690646
"915","LURAP1L",50.4958934425432,0.713714862344537,0.347504492309491,2
.05382916808136,0.0399922288082892,0.622874427690646

"916","BDNF",9.96921535055504,-1.36017752898158,0.66208452896107,-2.05
438651635003,0.0399382970518459,0.622874427690646
"917","FRZB",15.0071759341452,1.28760114314037,0.62713195763489,2.0531
5823482558,0.0400572335475523,0.62318047305276
"918","SLC12A6",27.9895841767091,0.649712054332615,0.317138934497859,2
.04866695210834,0.0404946928734905,0.629299878576488
"919","OLFM2",22.5364356289916,-1.03077918804217,0.503498530114071,-2.
04723375817729,0.0406351383495962,0.630795303259346
"920","SLC25A33",29.3099807878364,-0.816871177917626,0.399517618220103
,-2.04464369195251,0.0408899986690628,0.634061653274836
"921","ELP5",67.3546473754174,-0.609334648090881,0.29808307074082,-2.0
4417730459001,0.0409360344100275,0.634086283271936
"922","EIF4G2",437.211584260583,0.300619807302624,0.14722706028253,2.0
4187876009839,0.0411635591426841,0.636228965037412
"923","LINC01232",5.49617572601735,1.39695231124838,0.684138644915302,
2.04191405006996,0.0411600578332762,0.636228965037412
"924","ESR2",3.24074316672668,2.55847668194118,1.25376190507364,2.0406
3999040624,0.0412866241725323,0.63744045502743
"925","CLDN23",13.5867914864244,-0.892455284082074,0.437957371485625,-
2.03776746822348,0.0415731932286112,0.637504998028265
"926","STRBP",10.6953913208533,1.01712950572285,0.498789003714634,2.03
919793369136,0.0414302770596052,0.637504998028265
"927","STARD5",8.97463865462698,1.17945198267376,0.57880938631712,2.03
772089837457,0.0415778529762037,0.637504998028265
"928","LINC00921",5.55709583404896,1.32295661992913,0.648557471445782,
2.03984485288552,0.0413657808575491,0.637504998028265
"929","NXN",18.9317376698777,0.796219101859152,0.390760536397159,2.037
61390339043,0.0415885604971507,0.637504998028265
"930","PAFAH1B3",13.2418101363207,-0.986553313929337,0.483874343694914
,-2.03886262370498,0.0414637401101017,0.637504998028265
"931","ZNF776",5.25009817682402,1.36687564734969,0.670871223746719,2.0
3746352349992,0.0416036137084196,0.637504998028265
"932","ZNF586",6.19830352160652,1.26750457531861,0.622517354759394,2.0
3609516365775,0.0417408002959366,0.638920876632866
"933","IL18RAP",3.89879686134909,1.60272308538464,0.787334541545574,2.
03563161631195,0.041787360553366,0.638948001773119
"934","ARPC1A",170.293066233031,-0.564091087943122,0.277348624107777,-
2.03387015081754,0.0419646892197732,0.640972437269041
"935","LRP6",103.008210200358,0.641382215416273,0.315513461983643,2.03
282044253796,0.0420706670389413,0.641499080380164
"936","SNHG9",117.943085126278,-0.736015926411811,0.362243554497726,-2
.03182614921153,0.0421712589700686,0.641499080380164
"937","XYLT1",46.4100995228288,-0.561106294114914,0.276071571330746,-2
.03246676725972,0.0421064248183199,0.641499080380164
"938","FAM174C",113.807888213859,-0.52634946281162,0.259062202546045,-
2.03174935455151,0.0421790366883915,0.641499080380164
"939","CCDC18-
AS1",68.9324083542694,0.621710016128206,0.306115399072808,2.0309661585
51,0.0422584276512854,0.642022075477356
"940","MGA",51.7616345627512,0.510965698536014,0.251851198906388,2.028

83965117013,0.0424746251896715,0.644620215910482
"941","FOSL1",25.0164140102254,-0.930159469136375,0.45867620170403,-2.02792180121998,0.0425682296930867,0.645354266526648
"942","UGGT1",62.1682479814087,0.523841144543355,0.258447767449085,2.02687432634354,0.0426752667394511,0.646290186098736
"943","XIAP",120.442430606387,0.473594096424741,0.233719327748625,2.02633689300233,0.0427302730695947,0.646436983680634
"944","PDZRN3",43.3217996864263,0.933563878492094,0.461204746035139,2.02418532445233,0.0429510869948342,0.648402335522016
"945","STK17A",82.2839260007057,0.564071045854246,0.278637908486468,2.02438730938737,0.04293031648422,0.648402335522016
"946","AC026461.3",3.22325281690462,2.04277611874617,1.0100607566276,2.0224289532509,0.0431320564567154,0.650446001492073
"947","CMPK1",232.050901518954,-0.387681416580503,0.191769611591595,-2.02159984245123,0.0432177083581065,0.650627780741268
"948","LDLR",79.1356202473489,-0.791989150859738,0.391796574560851,-2.02142949245395,0.0432353242774935,0.650627780741268
"949","CYP4B1",68.2073762601765,0.765971515125026,0.37902383274381,2.02090594034693,0.0432895028654861,0.650756636331955
"950","SIPA1L2",54.1787165582968,0.713959942460365,0.353536110768307,2.01948236888385,0.0434371080156382,0.65159958812716
"951","TMEM17",6.35668990232573,1.28977571048951,0.638805018801441,2.01904442283414,0.0434826025443051,0.65159958812716
"952","MKLN1",122.489009780869,0.487849251172988,0.241580657546489,2.01940526252235,0.0434451150285586,0.65159958812716
"953","AOX1",162.365293260249,0.507898131344592,0.251674615114457,2.01807453291867,0.0435834995409094,0.652384228218433
"954","IRF4",33.7293998600561,1.2382960472205,0.613976678945412,2.01684541072054,0.043711648330203,0.652384228218433
"955","LINC02550",2.64552518038039,2.2930586540946,1.13698677489128,2.01678568716321,0.0437178832312367,0.652384228218433
"956","PPAN",46.1848899002472,-0.622259048115711,0.308503887045837,-2.01702174346756,0.0436932442766483,0.652384228218433
"957","CREB3L1",68.498961288938,0.702045508784635,0.34837757618537,2.01518569728805,0.0438851955768865,0.654196656321696
"958","AC074387.1",1.85766929263237,-2.65289665102066,1.31753252686652,-2.01353408505976,0.044058472895215,0.656094127685947
"959","CCL2",1493.91935468432,-0.709113741779375,0.352318920866803,-2.01270411488193,0.04414576624204,0.656708551834143
"960","ZNRF1",74.0188997893646,-0.550367397040197,0.273808687827395,-2.0100435870287,0.0444265758771443,0.659510438567472
"961","KCTD11",28.2895675683077,-0.697490202846905,0.346941815463518,-2.01039532209472,0.0443893651987335,0.659510438567472
"962","FAM43B",14.9019933721155,-0.846975089543978,0.42164641963121,-2.00873302869447,0.0445654539970937,0.660397405407694
"963","CTXN1",8.30640770864802,-1.27099020021279,0.632772202273853,-2.00860624984081,0.044578907991561,0.660397405407694
"964","AADAC",254.829767950265,-1.25182404714233,0.623903728237261,-2.00643783725921,0.0448095547862307,0.661752700393756
"965","RBM3",668.195055125477,-0.496945048411599,0.247595271108983,-2.

00708618620127,0.0447404868324909,0.661752700393756
"966","SRPX2",172.547075391526,0.447740874132247,0.223136257082186,2.0
0658055300863,0.0447943437084988,0.661752700393756
"967","IL1B",41.347379571358,-1.74064743919227,0.868603424601968,-2.00
3961059663,0.0450742318996934,0.664059185991784
"968","SH3BP5",430.394226940217,0.359664843166837,0.179541893599808,2.
00323632526966,0.0451519283900204,0.664059185991784
"969","NBR1",82.6449496925478,0.490915949636437,0.24503680840255,2.003
43757673318,0.0451303415391953,0.664059185991784
"970","MED16",37.2989950513219,0.579958142630346,0.289503384099531,2.0
0328622905132,0.0451465747454342,0.664059185991784
"971","IL17D",42.2039960971324,-0.613229888883787,0.306353887272095,-2
.00170428501576,0.0453165452816998,0.665793856837003
"972","DISP1",12.7206279294179,0.906841434980136,0.453138439983931,2.0
012458775563,0.0453658991358569,0.665833248016599
"973","SEL1L3",12.0583777263802,0.943182274890808,0.471838583389154,1.
99895114154518,0.0456136405134525,0.666725610209952
"974","ARSI",5.75123787869341,-1.7139174312366,0.857366220327533,-1.99
904940339479,0.0456030087778385,0.666725610209952
"975","EPB41L2",446.518342858774,0.359526338663581,0.179749102013557,2
.00015652170805,0.0454833650250096,0.666725610209952
"976","CCND1",109.339645981194,-0.635963419683143,0.318079185344852,-1
.99938709913901,0.0455664866916534,0.666725610209952
"977","HTR2B",5.46652318499142,1.51064211095039,0.756158794667133,1.99
778422416602,0.0457400583964861,0.667860212908971
"978","TNKS1BP1",116.468579177248,0.471011905460381,0.235815930181366,
1.99737102195821,0.0457848933285415,0.667860212908971
"979","SNED1",216.139950737495,0.608825547389422,0.304916741236449,1.9
96694392445,0.04585839166427,0.668249045436645
"980","SLC37A3",67.1966707799154,0.534448821398473,0.267897259338871,1
.99497681580398,0.046045408525804,0.669606318072498
"981","MT-
ND4",14107.4359050529,-0.38081738869786,0.190878672769901,-1.995075631
92733,0.0460346316294643,0.669606318072498
"982","PEX16",46.5593196755483,-0.575251590110276,0.288464548311831,-1
.99418470476458,0.0461318730412591,0.670180550719554
"983","RPH3AL",4.7299234461848,1.5425931026053,0.773723013681876,1.993
7278267899,0.0461818066460967,0.670223452302356
"984","MTRNR2L12",17.9870138510118,-0.851028378521933,0.42724593158486
9,-1.99189346371315,0.0463827482892082,0.670411638392953
"985","EXOC4",52.5213997115834,0.514118142772695,0.258070427362352,1.9
9216217071951,0.0463532673821828,0.670411638392953
"986","UBN2",49.7684542770474,0.593997589131571,0.298110870923223,1.99
253917608577,0.0463119312295484,0.670411638392953
"987","SMCR5",5.73599387868224,1.3663950862578,0.685851138598961,1.992
26189089522,0.0463423307021395,0.670411638392953
"988","SERINC1",341.616540638191,0.334391037287542,0.168020787775448,1
.99017658299781,0.0465714869224475,0.671099830743066
"989","AP001372.2",6.52058956948062,-1.16529034642831,0.58536596275170
8,-1.9907039708125,0.0465134419206434,0.671099830743066

"990","STK24",125.302897943742,0.482457355731561,0.242400460426121,1.9
9033184542405,0.0465543922050861,0.671099830743066
"991","AC108734.4",19.1280378047768,0.939525259980244,0.47237517339885
7,1.98893869298872,0.0467079702742599,0.672110286825985
"992","HLA-
DMB",10.1506881378378,-1.00094966608838,0.503429976777262,-1.988259961
1887,0.046782946503449,0.672110286825985
"993","ERVK13-1",14.2654980069807,0.935719424062479,0.470576122243031,
1.98845495942784,0.0467613956270139,0.672110286825985
"994","PIM1",223.376779806466,-0.858766530376542,0.432316806928093,-1.
98642874071602,0.0469857379762181,0.674344605602342
"995","AL356218.2",3.15414411016804,-1.90454644514166,0.95946219118068
1,-1.98501458697188,0.0471428486051291,0.674737555678646
"996","GALT",34.1409731890531,0.649432991820279,0.327185881248885,1.98
490530624782,0.0471550079217447,0.674737555678646
"997","PUDP",12.3049615028037,0.879870535577582,0.443199872652812,1.98
526802435894,0.0471146595876171,0.674737555678646
"998","RAB30",62.1018156569714,0.593558972388986,0.299187614786607,1.9
8390221738402,0.0472667416939305,0.675658654314242
"999","C17orf49",148.956197919707,-0.611536973430067,0.308641826863954
,-1.98138074687986,0.0475485912231334,0.679007209598819
"1000","PDGFC",7.84717972181979,-1.47486353477998,0.744876313077361,-1
.98001132387574,0.0477022562754468,0.67946209653238
"1001","RIN1",10.5778101374251,-0.965480335406516,0.487659724668609,-1
.97982381272641,0.0477233296456922,0.67946209653238
"1002","ADGRG4",3.43477826321377,1.93008528176719,0.974493250538226,1.
98060405313344,0.0476356940553646,0.67946209653238
"1003","DPT",1221.67523186256,-0.564683498284172,0.286619720266535,-1.
97014880120271,0.048821319314395,0.679528217165804
"1004","STAT1",303.105499610013,0.4294060726305,0.217552684398343,1.97
38026851659,0.04840418341407,0.679528217165804
"1005","EPHA4",9.91735206676346,1.06894273619255,0.540627755241501,1.9
7722504223826,0.0480161981552449,0.679528217165804
"1006","ROBO2",17.8009342043728,0.943635422304556,0.47807357397504,1.9
7382887001787,0.0484012049273488,0.679528217165804
"1007","FILIP1L",577.50348268063,-0.579803773832355,0.294303133185501,
-1.9700903879501,0.0488280123386739,0.679528217165804
"1008","ADGRA3",21.7537397992324,0.69412071728445,0.351237423627304,1.
9762151484774,0.0481304149122438,0.679528217165804
"1009","CXCL9",104.496085152003,-1.77226185163073,0.896218856564404,-1
.97748779625613,0.0479865186201985,0.679528217165804
"1010","C5orf22",14.6991056955365,-0.826423721821716,0.419303316955196
,-1.97094487070328,0.0487301819559937,0.679528217165804
"1011","HSPA1B",126.190659073526,0.740477608203838,0.376010799654029,1
.96929877781478,0.0489187914642703,0.679528217165804
"1012","PRKAR2B",16.5974351607,-0.770793877527651,0.390848962948329,-1
.97210163156951,0.0485980057581123,0.679528217165804
"1013","DEFB1",18.3498337397801,2.9986335358937,1.51931150052428,1.973
67921907979,0.0484182295661945,0.679528217165804
"1014","FBXO10",6.41550850270163,1.16520666515548,0.591406082709432,1.

97023111398732,0.0488118891752617,0.679528217165804
"1015","NXF1",46.8358341498072,0.517278368867325,0.261924277790631,1.9749157017083,0.0482777152133762,0.679528217165804
"1016","NUDT8",26.6672977201987,-0.692229167618562,0.351252128572519,-1.97074725335265,0.0487527926762237,0.679528217165804
"1017","TNFAIP2",1174.57482267429,0.695830155530139,0.35324463191596,1.96982513720317,0.0488584146141285,0.679528217165804
"1018","CLN6",19.5879755183384,-0.656717972096484,0.332835991850096,-1.97309782648824,0.0484844178723257,0.679528217165804
"1019","SLC7A6",55.4636711997102,0.578209647109018,0.292461485742159,1.977045441186,0.048036493971805,0.679528217165804
"1020","VMO1",3.36878801902765,-1.61595617839123,0.8205723503236,-1.96930371557605,0.0489182247800209,0.679528217165804
"1021","RAB34",261.122114685609,-0.450662577991283,0.228780741292137,-1.96984490672586,0.0488561481405487,0.679528217165804
"1022","CCL3L1",14.9643025958966,-1.31150753155886,0.66570209088236,-1.97011178051208,0.0488255610771577,0.679528217165804
"1023","EPOP",2.85247581758679,-1.7996815093892,0.911216036284053,-1.9750327449551,0.048264432139885,0.679528217165804
"1024","SPINT2",3.46876054934999,-1.47623134274352,0.748350948046613,-1.97264578416966,0.0485359328653757,0.679528217165804
"1025","AL035681.1",4.10759300390354,1.59024208525583,0.805966243486373,1.9730877044886,0.0484855708782209,0.679528217165804
"1026","CLCN5",14.1920582541923,0.89464953310154,0.453913623593867,1.97096867465255,0.0487274589814911,0.679528217165804
"1027","BEX2",2.10247094776054,-1.97674284031451,1.00111308975053,-1.97454499451914,0.0483198065186388,0.679528217165804
"1028","LINC01550",8.24490289555228,1.06418073908429,0.540964099405361,1.96719290661628,0.0491609754871312,0.682228089785422
"1029","FOXF2",17.7249613472686,0.769315996879472,0.392058476088822,1.96224809256561,0.0497336072602523,0.682834104393406
"1030","FAM120B",21.720307626782,0.684067696232081,0.348670461374171,1.96193188702034,0.0497704147944204,0.682834104393406
"1031","PUS7",25.8085985422331,-0.687967253491604,0.350041958723099,-1.96538510983428,0.0493696815660139,0.682834104393406
"1032","RNF170",51.2023930706015,0.487582119101646,0.248076228966061,1.96545280107571,0.0493618533894003,0.682834104393406
"1033","ZFAND5",477.84939645853,0.609687545393364,0.310300973957957,1.96482639940399,0.0494343336084092,0.682834104393406
"1034","HMCN2",60.6327289687974,-0.970054011778236,0.493955592848116,-1.96384862490364,0.0495476492146981,0.682834104393406
"1035","AKR1C2",270.278969131844,-0.633061572862246,0.322264903127849,-1.96441364454477,0.0494821417813932,0.682834104393406
"1036","GDF10",124.20617728113,-0.715365097904056,0.364411787026431,-1.96306794503376,0.049638279612155,0.682834104393406
"1037","UBE4A",63.7919738315426,0.516788576052874,0.263388077324487,1.96208036940185,0.0497531280336595,0.682834104393406
"1038","CCNB1IP1",60.922135248187,-0.515597558636246,0.262810843822297,-1.96185800835857,0.0497790178444653,0.682834104393406
"1039","MMP14",246.660052575385,0.615423151324013,0.3133507952236,1.96

400698739207,0.0495292815994635,0.682834104393406
"1040","PIK3IP1",82.654663427406,0.503745026213848,0.256229709126615,1
.96598992338131,0.0492997746064868,0.682834104393406
"1041","FAP",88.88202262935,-0.828205237882096,0.422274958844332,-1.96
129375075591,0.0498447658679172,0.683079183354185
"1042","CDC14B",51.7158159080193,0.555323629720069,0.283332858337181,1
.95996903775701,0.0499994093316891,0.683113177423913
"1043","RARG",70.3641863135454,-0.483005542975912,0.246477638316681,-1
.95963230690864,0.050038782448338,0.683113177423913
"1044","DNASE1",18.4223415623203,0.833113353606868,0.425127552197188,1
.95967857011639,0.0500333714645372,0.683113177423913
"1045","CYBB",4.20268724568142,-1.50417806005368,0.767572862079219,-1.
95965508209752,0.0500361185817309,0.683113177423913
"1046","TPGS1",127.66879307766,-0.569084533112683,0.290580497312976,-1
.95844022009412,0.050178379204093,0.684364013121979
"1047","PTPN23",38.6731287990922,0.506532404990515,0.258710299869847,1
.95791356295186,0.0502401561384135,0.684552117927991
"1048","MKNK1",46.6150173119796,0.501290479235256,0.25674920678471,1.9
5245191022381,0.0508845751214845,0.685476249936825
"1049","GULP1",66.5800609783297,0.533594319437979,0.272914328118195,1.
95517151157739,0.0505628298976136,0.685476249936825
"1050","ESRG",6.88890420082306,1.18753525112164,0.60714408451308,1.955
93645958689,0.0504726396870328,0.685476249936825
"1051","CPLANE1",44.2474305057761,0.640268809594739,0.32784175653135,1
.95298126867348,0.0508218147754252,0.685476249936825
"1052","CCDC71L",862.801939412258,-0.662782491723051,0.339301987091997
,-1.95337049866244,0.0507757093307391,0.685476249936825
"1053","SCAI",18.0958482958953,0.823870470052471,0.421472556872843,1.9
5474285719872,0.0506134288474902,0.685476249936825
"1054","FBXL15",56.7675496706935,-0.49031631911564,0.250842046989322,-
1.9546815416337,0.0506206700876375,0.685476249936825
"1055","LDLRAD3",13.9109661388045,-0.780078569509252,0.399465794528128
,-1.95280442079083,0.0508427745134909,0.685476249936825
"1056","CLCF1",12.1812778372143,-1.01397270779811,0.519164977813981,-1
.95308380019698,0.0508096662122464,0.685476249936825
"1057","AC068888.1",55.6427423930144,-0.508253026493065,0.259832528658
579,-1.9560792835177,0.0504558151581934,0.685476249936825
"1058","CMTM2",2.23013323889328,2.30270164116689,1.17688823478749,1.95
660180219465,0.0503943029754696,0.685476249936825
"1059","PAF1",63.9055431109699,0.445017986322964,0.227715234611518,1.9
542741050337,0.0506688095480538,0.685476249936825
"1060","HLA-F-
AS1",33.7450694276368,-0.694121741334644,0.355851775278273,-1.95059232
398615,0.0511055610559033,0.685862590802931
"1061","ST6GALNAC4",50.1903497460987,-0.689932963671475,0.353698803179
534,-1.95062283917674,0.0511019282755578,0.685862590802931
"1062","LINC00944",4.06836168866913,1.48182385703464,0.759489558588625
,1.95107864259299,0.0510476914033119,0.685862590802931
"1063","THOC6",39.490315691409,-0.605469711143846,0.310287529513905,-1
.95131822439778,0.0510192024641017,0.685862590802931

"1064","Z82215.1",2.19473764128491,2.29137836403715,1.17507726259116,1
.9499810242132,0.0511783807979965,0.686194342541559
"1065","SH2D2A",12.1550770102636,0.970519086927897,0.497855334025848,1
.94939979668373,0.0512476987995862,0.686478564389574
"1066","ATXN7L1",15.9546397860846,0.882683858685401,0.452901941140866,
1.94895137005134,0.0513012324816515,0.686551015556511
"1067","MARCKSL1",122.773980782887,-0.560671972051606,0.28782682195370
4,-1.94794900713523,0.0514210650019199,0.687104285256526
"1068","CRABP2",213.830508329262,-0.768359831553294,0.394990447049236,
-1.94526180897109,0.0517434761325624,0.687104285256526
"1069","NCSTN",89.0689897505618,0.473491904327824,0.243541817586423,1.
94419138782933,0.0518723759442926,0.687104285256526
"1070","FIGN",14.5436201585968,1.07520261834861,0.552688381955707,1.94
540477681829,0.0517262802907451,0.687104285256526
"1071","KCNMB1",2.68304842858848,-2.06120879248694,1.06018767514468,-1
.94419237349241,0.051872257127564,0.687104285256526
"1072","AL360178.1",3.15685025071237,1.80399100796462,0.92699257667290
1,1.94606845120527,0.0516465176797253,0.687104285256526
"1073","PHF21A",60.2432517582724,0.596682059701268,0.306351091854003,1
.94770665281528,0.0514500736157337,0.687104285256526
"1074","PICALM",101.427720556778,0.422533307364028,0.217163943051887,1
.9456881350836,0.0516922127446843,0.687104285256526
"1075","AL139384.1",4.32987257015795,1.5540682704854,0.799276670871652
,1.94434333832189,0.0518540617643241,0.687104285256526
"1076","MED1",72.7472769745519,0.468957878995363,0.241119374843096,1.9
44919935615,0.05178461526413,0.687104285256526
"1077","MXRA7",341.937152420762,-0.435626178987128,0.223815311413803,-
1.94636451025336,0.0516109695237428,0.687104285256526
"1078","SUCNR1",7.23209926796927,-1.64077005080123,0.845356914281828,-
1.94091989203773,0.0522679948011159,0.688545120762491
"1079","C6orf226",35.6813726245524,-0.754855625112357,0.38865154374738
1,-1.94224270366723,0.0521077256407916,0.688545120762491
"1080","C8orf82",57.0468088052928,-0.464467297389492,0.239265480680545
,-1.94122150871243,0.0522314153057322,0.688545120762491
"1081","HMG20B",98.8056945253464,-0.473918830339818,0.24401673607807,-
1.94215707478438,0.0521180878046953,0.688545120762491
"1082","ZNF44",22.3586162105303,0.738418511067884,0.380452135458575,1.
94089726997547,0.0522707392251351,0.688545120762491
"1083","CALR",853.571336791243,-0.41699426516449,0.214831214195154,-1.
94103201774808,0.0522543939077975,0.688545120762491
"1084","TTN",5.57162778415596,1.60186863370206,0.825623405902484,1.940
19285578644,0.052356256436128,0.689035382211994
"1085","STXBP5",42.0125549958978,0.694894288247425,0.358234848080705,1
.93977300636837,0.052407282568441,0.689071237899889
"1086","AKIRIN1",130.375966212385,-0.347397156015871,0.179237130437634
,-1.93819860409308,0.0525989969996542,0.689819949049546
"1087","SATB1",31.9236980351516,0.662646323610665,0.342251968940909,1.
93613589911903,0.0528510587628735,0.689819949049546
"1088","FAXDC2",64.4209053192643,0.547240644116711,0.28231615962805,1.
93839645891222,0.0525748721234288,0.689819949049546

"1089","NAV2",24.3339571350611,-1.01782258670434,0.525608218049242,-1.93646627231575,0.052810619521802,0.689819949049546
"1090","IQSEC3",3.7644965441466,1.75684267427223,0.90715968467394,1.93664103900703,0.052789237712841,0.689819949049546
"1091","ZNF98",2.29445794943122,2.56303039120037,1.32251081785342,1.93800334681607,0.0526228142236071,0.689819949049546
"1092","HIC2",25.8460200575769,0.732955900001762,0.37844564946551,1.93675340445038,0.0527754941984633,0.689819949049546
"1093","TYMP",813.261623194635,-0.549052198196619,0.283350890691861,-1.93771121331573,0.0526584651142284,0.689819949049546
"1094","SPG11",55.5190111447526,0.586395535518218,0.303067182676511,1.93486978807642,0.0530062764418567,0.6912134732354
"1095","AGO3",108.731979182772,0.521830057129456,0.269829819242889,1.93392286513644,0.0531226123647016,0.691466412404044
"1096","SMARCA1",148.363981575329,0.398991896252953,0.206280395670773,1.93422111178102,0.0530859477453459,0.691466412404044
"1097","SPTBN1",893.360561701771,0.388596749722567,0.201107183817569,1.93228676542493,0.0533241210135605,0.691505570532573
"1098","MXD4",185.730336309959,0.40592089171543,0.209988129464033,1.93306589639848,0.0532280806094956,0.691505570532573
"1099","GLI3",109.52803275401,0.515777457889886,0.266975047075811,1.93193133043413,0.0533679821362935,0.691505570532573
"1100","TMEM25",10.7040865274242,-0.924589183518398,0.478283997843811,-1.93313844428542,0.0532191452757468,0.691505570532573
"1101","KCTD12",383.719734965607,0.66022530932825,0.341688941007717,1.93224079006215,0.053329792734881,0.691505570532573
"1102","GPM6B",4.1926180827809,-1.40668470112578,0.728515390818052,-1.93089222115982,0.0534963823691134,0.692540282103241
"1103","GREM2",5.52189789137119,1.69640201803453,0.87920810268303,1.92946586008218,0.0536730544021392,0.693568654076918
"1104","HIST1H2BD",3.78255061016628,2.17329398095355,1.12634297309401,1.92951350775831,0.0536671448072236,0.693568654076918
"1105","GAL",259.117764359654,1.48956427215045,0.772241423715457,1.92888418881204,0.0537452411158583,0.693872950007996
"1106","ADCY3",201.380867457113,0.501131717172134,0.259864218702325,1.9284367800793,0.053800820681298,0.693962484484084
"1107","NDRG1",296.232436111509,0.36835346663581,0.19111612879656,1.92738032606299,0.0539322496298969,0.69502933443551
"1108","AC023157.3",8.30267530877938,-0.973132235190818,0.505078525811237,-1.92669493051167,0.0540176600243959,0.695501749014469
"1109","LDHA",1416.50690831246,-0.418518734382033,0.217356425817017,-1.92549510698324,0.0541674475156462,0.695547080340422
"1110","STARD9",40.2913610973045,0.751800340682319,0.390424933736291,1.92559510348823,0.0541549505994747,0.695547080340422
"1111","APCDD1L",3.06684691705969,1.91117621741296,0.992169445185861,1.92625990115524,0.0540719296433492,0.695547080340422
"1112","ZSCAN31",4.20064490582083,1.58602838452786,0.824205678600098,1.9243114015202,0.0543155623019594,0.696195697933291
"1113","ZC3H7B",48.4595774984846,0.577546283355147,0.300129947370244,1.92432074311691,0.054314392084416,0.696195697933291

"1114","EVA1A",3.53338860790946,-1.91161072122011,0.993655084277587,-1
.92381717908675,0.0543775033233392,0.696363969848076
"1115","YDJC",14.908570491635,-0.745695317276015,0.387705728008599,-1.
92335388261139,0.0544356218918763,0.696483033102697
"1116","VEGFB",336.485757222378,-0.427794882431449,0.222473230333784,-
1.92290497957716,0.0544919842863051,0.696579433538018
"1117","PELI2",21.1761466575233,-0.787784653601534,0.409941291412377,-
1.92170115600546,0.054643371721668,0.697889293626961
"1118","MAATS1",4.52555111554055,-1.30747938853296,0.68058195757951,-1
.92111967408453,0.0547166218188655,0.698199755695827
"1119","CCL28",6.41856033702632,-1.21716368132577,0.634140575277046,-1
.91939095017538,0.0549348753863941,0.70035829514057
"1120","DENND4C",53.34587945895,0.525495490962584,0.273870543893047,1.
91877331345208,0.0550130285888166,0.700691568938399
"1121","DPEP2",2.47430469653899,-1.81382665623481,0.945485160587203,-1
.91840838105626,0.0550592491784625,0.700691568938399
"1122","C1orf162",12.9765408939851,-0.902290060774289,0.47213458236984
3,-1.91108657248811,0.0559934564218825,0.700842374942646
"1123","C2CD6",4.40139921313302,1.40566926400444,0.733967225971069,1.9
1516625574756,0.055471304394447,0.700842374942646
"1124","C4orf36",6.57803280493484,1.25016154015501,0.652740479150145,1
.91525051699367,0.055460562847531,0.700842374942646
"1125","CYP2U1",42.1682058994306,0.589173406866987,0.307506984417674,1
.9159675608107,0.0553692248704905,0.700842374942646
"1126","CYSTM1",246.484309660108,-0.484868395813287,0.253337680503187,
-1.91392135133718,0.0556302057904265,0.700842374942646
"1127","TMEM70",85.7022734038396,-0.53553036289023,0.280074731532619,-
1.91209810310167,0.0558636121207313,0.700842374942646
"1128","ZNF704",98.3904807055245,0.473325670901034,0.247479423610451,1
.9125859596557,0.055801078517297,0.700842374942646
"1129","COX15",27.6371297352167,0.604706974453433,0.315638198359008,1.
91582317221833,0.055387607190711,0.700842374942646
"1130","SPON1",52.8733390263142,1.46591600840745,0.766056972983132,1.9
1358614320678,0.0556730569874949,0.700842374942646
"1131","TMEM135",42.6716836006044,0.608161753351322,0.318275786544204,
1.91080119526107,0.0560301340617485,0.700842374942646
"1132","TBX5",6.05257790654185,-1.23308969234071,0.645259738059149,-1.
91099741640424,0.0560049129130205,0.700842374942646
"1133","MBNL2",156.383231110916,0.50792568031217,0.265177280143487,1.9
1541930001444,0.0554390517549974,0.700842374942646
"1134","COL4A2",360.238387370481,-0.5901953096582,0.308819830701705,-1
.91113151094329,0.0559876826033411,0.700842374942646
"1135","ZFYVE1",23.1159565167923,0.618215755025372,0.323568403895097,1
.9106184274587,0.0560536345022823,0.700842374942646
"1136","SPIRE1",22.6809219468948,-0.631228266800174,0.329158786265235,
-1.91770140473033,0.0551488835266788,0.700842374942646
"1137","EMP3",820.812307566353,-0.494538576968429,0.257923280913094,-1
.91738634534143,0.0551888676540382,0.700842374942646
"1138","TOB2",97.4625977347388,0.488091347562105,0.255357990968761,1.9
1140032747914,0.0559531546557078,0.700842374942646

"1139","UXT",273.256932421915,-0.463238141274982,0.241970867097067,-1.
91443766281646,0.0555642570810217,0.700842374942646
"1140","SPIN4",5.10106993379532,1.36934495529044,0.715596944220366,1.9
1357015475007,0.0556751015523614,0.700842374942646
"1141","MAP7D3",281.223744815286,0.461377486627754,0.241032382555195,1
.9141722026587,0.0555981562917551,0.700842374942646
"1142","BICC1",307.393032634208,0.488981765896495,0.256015583149856,1.
90996876002769,0.0561372357524519,0.701273034364692
"1143","FGFR1",628.044128140028,0.360289285786181,0.18882554112407,1.9
0805377091147,0.0563842668584022,0.703127579547172
"1144","CREBL2",83.9476316054159,0.400382047397074,0.209834672790136,1
.90808335950042,0.0563804430969897,0.703127579547172
"1145","TSPAN15",38.691495190453,-0.930898649813266,0.488139536431626,
-1.90703391210283,0.0565161961991466,0.704157253255044
"1146","USP49",9.82975608823448,1.08493711097631,0.569158098440179,1.9
0621395698255,0.0566224521606705,0.704865534488766
"1147","STMN1",19.4057219003877,-0.823031170774609,0.431852714169144,-
1.90581451446488,0.0566742751261803,0.704895561421176
"1148","ASS1",68.8347664295016,-0.715066858987157,0.375384032081187,-1
.90489418269263,0.0567938275980822,0.705767199054217
"1149","ARHGAP5",102.484230054632,0.480500237072045,0.252305687662936,
1.90443680252647,0.056853319980003,0.705891612562857
"1150","KREMEN1",73.72628043546,0.540854181374328,0.284250064623516,1.
90274075079166,0.0570743817503749,0.708020113087694
"1151","C7",234.89271765937,-0.878419262400721,0.461770764606584,-1.90
228427117752,0.0571340008380052,0.708143923505632
"1152","CTNS",14.5284617413415,0.774164150098443,0.407137833969313,1.9
0147926698651,0.0572392656369121,0.708832780882107
"1153","SVBP",137.169485734661,0.499467961684049,0.262758002117389,1.9
0086679628851,0.0573194621744726,0.709210275265418
"1154","CD247",23.3198603646466,0.900789744126196,0.474274844636274,1.
89929901261582,0.0575251726291693,0.709294825175219
"1155","ATP6V1A",47.3778559915094,0.46871539860683,0.24672607950407,1.
89973998512426,0.0574672502375166,0.709294825175219
"1156","ZFAND1",66.6732930654897,-0.527647048248695,0.277758503972493,
-1.8996611830144,0.057477597451105,0.709294825175219
"1157","RPP25",24.0557794248539,-0.715257450542959,0.376494673615563,-
1.8997810611082,0.0574618573161098,0.709294825175219
"1158","CPXM2",74.1239539524184,0.778105531909974,0.40985179188547,1.8
9850464805924,0.0576296360667301,0.709969247088059
"1159","TRANK1",41.464036684986,0.551582222805208,0.290604849689573,1.
89804892586759,0.0576896372741053,0.710095224635363
"1160","GIPC2",2.74135646603172,-1.71568144073838,0.904394071209846,-1
.8970507385605,0.0578212418794913,0.711101583321399
"1161","ITGB3BP",30.31886321767,-0.684249618534957,0.361051386974837,-
1.89515853759245,0.0580714013506477,0.712213816616468
"1162","BBS7",29.8653086051426,-0.572003039562614,0.301871380954257,-1
.89485680210702,0.0581113754760668,0.712213816616468
"1163","CTNND2",3.3340156671688,2.07147387930604,1.09259139692854,1.89
592732024919,0.0579696557575755,0.712213816616468

"1164","CD2AP",40.1524431653769,0.584685009145165,0.308449953250572,1.
89555875429227,0.0580184156214824,0.712213816616468
"1165","NCOA1",134.506251327201,0.426522073625674,0.225155780920344,1.
89434209453662,0.0581796170560383,0.712438126112826
"1166","SLA2",3.5892780296224,1.63785112202469,0.864941773264166,1.893
59697109284,0.0582785258658465,0.713037264152802
"1167","CARD19",52.3001122190927,-0.527801042542568,0.279086554617911,
-1.89117330738188,0.0586012132894729,0.716370958686907
"1168","SLC39A1",216.457058205064,-0.391373933312299,0.207166131246689
,-1.88917913829389,0.0588678291085281,0.717095165147109
"1169","MOCS2",78.2781625184658,-0.416982256183443,0.220790717330508,-
1.88858599322022,0.0589473252928129,0.717095165147109
"1170","WDR86",37.8510562078396,0.835732744416236,0.442543437695239,1.
88847618839118,0.0589620516414944,0.717095165147109
"1171","ANGPT2",12.9983488622236,-0.959861209553867,0.507776444745878,
-1.89032244304723,0.0587148489827508,0.717095165147109
"1172","ALDH6A1",45.3398072081898,0.51019402429764,0.270010339707273,1
.88953513724977,0.0588201591565506,0.717095165147109
"1173","Z83843.1",29.2280544743463,0.802018266766225,0.424488666126352
,1.88937498398956,0.0588416004715905,0.717095165147109
"1174","SELENOM",1380.48762430637,-0.400497043621201,0.212184326285561
,-1.88749588922137,0.0590936587336333,0.718083590710402
"1175","DDX17",510.917714342444,0.371339269244193,0.1970283176336,1.88
469999492533,0.0594703530603045,0.722046005751748
"1176","PCDHB13",7.13272810914206,1.14865589822278,0.609584987038799,1
.88432445458121,0.059521101527084,0.722047648286888
"1177","WBP1L",109.517443475635,0.508807971498042,0.270132630653555,1.
88354872296264,0.0596260434187247,0.722706147333498
"1178","SH3D19",226.644377422366,0.380924734950318,0.202304295231487,1
.88292954687118,0.0597099164042936,0.722942253479526
"1179","HMOX1",33.8522626528584,0.778831194732071,0.413687262594199,1.
8826569371464,0.0597468748669817,0.722942253479526
"1180","LYPLA2",29.5905850021811,-0.637384674621943,0.338733362016358,
-1.88167079506967,0.0598807274113919,0.723318301907877
"1181","LYPLAL1-
AS1",14.3049404428757,1.22784638078886,0.653171423442614,1.87982256528
824,0.0601322640240344,0.723318301907877
"1182","ITGB1BP1",307.667435977589,-0.49824030773882,0.264933859449532
,-1.88062148331678,0.0600234272109891,0.723318301907877
"1183","RGS22",9.4795425518875,-0.873496431094109,0.464670713317831,-1
.87981812939574,0.0601328687833129,0.723318301907877
"1184","KLRF1",6.79861372498446,1.17836909177846,0.626170372040648,1.8
818665724126,0.0598541340936936,0.723318301907877
"1185","AL161421.1",7.4896623006883,-1.210970915952,0.644035560836102,
-1.88028579412585,0.0600691383230404,0.723318301907877
"1186","VAT1",303.017209151175,0.344781687642137,0.18328233149876,1.88
115070788735,0.0599514207907184,0.723318301907877
"1187","CMAS",59.6437550351718,-0.45414302450978,0.241704681853808,-1.
87891695364206,0.0602558335492686,0.723577206577328
"1188","GABPB1-

AS1",143.842018336712,0.738183036381401,0.392845994375317,1.8790646893
453,0.0602356608476692,0.723577206577328
"1189","LAMB3",15.6275669392265,-1.07556575273543,0.572610282301173,-1
.87835563904478,0.060332529816603,0.723813652488529
"1190","LINC01088",15.2657307500169,-1.25745214336048,0.67008759400737
9,-1.87654890883808,0.0605799453996428,0.723813652488529
"1191","NUMA1",158.3553821186,0.45163170734965,0.240660517609203,1.876
63398980565,0.0605682754833086,0.723813652488529
"1192","RHOJ",20.0122768771937,0.860702544297998,0.45856731199998,1.87
693828533953,0.0605265528004613,0.723813652488529
"1193","PYCARD",102.915705145862,-0.494508845462566,0.263476041165204,
-1.8768645652775,0.0605366585461485,0.723813652488529
"1194","APCDD1",10.844946578469,1.11171027119318,0.592331873594656,1.8
7683682197717,0.0605404620350047,0.723813652488529
"1195","LEPR",232.231279104782,0.752971781201209,0.401649769093572,1.8
7469740839261,0.0608343636407591,0.724226759921256
"1196","GALNT2",120.536567477911,0.395916872217487,0.211160984387199,1
.87495276822307,0.0607992216143216,0.724226759921256
"1197","DDIT4L",12.7683856623068,-1.06355118483717,0.567211441265393,-
1.87505241866154,0.0607855125158765,0.724226759921256
"1198","VENTX",1.93922548888359,-2.50452915376699,1.33519276957797,-1.
87578094401951,0.0606853656995866,0.724226759921256
"1199","WDR76",4.15417648650866,-1.29389239950238,0.69027822170392,-1.
87445056039645,0.0608683502835824,0.724226759921256
"1200","SRM",304.528605138883,-0.60021182071878,0.320675656563632,-1.8
7170996124453,0.0612467406751912,0.726910151807219
"1201","EYA4",2.10092102795537,2.50874459276908,1.34000759424563,1.872
18684695694,0.0611807581377476,0.726910151807219
"1202","AC036176.1",21.9357205855389,0.69704079893367,0.37235542271566
7,1.87197703164897,0.0612097812044139,0.726910151807219
"1203","EFHD2",80.2622656426385,-0.542112089178774,0.289803229524982,-
1.87062128350796,0.0613975924198133,0.72748818739126
"1204","SPATS2L",150.468246127273,-0.356278925328942,0.190547390809618
,-1.86976543638381,0.0615163980607819,0.72748818739126
"1205","ZDHHC14",63.5248704486027,0.665541920809563,0.356135716083396,
1.86878734918492,0.0616524056186761,0.72748818739126
"1206","ZNF438",28.4505658114484,0.611209835754477,0.326763811415252,1
.87049426650783,0.0614152124510093,0.72748818739126
"1207","PARVA",214.223391661992,0.360652191210599,0.192931631663628,1.
86932639350393,0.0615774182504712,0.72748818739126
"1208","ALDH2",187.121695759227,-0.479207366347987,0.256365268498194,-
1.8692366916752,0.0615898915841705,0.72748818739126
"1209","AAR2",27.8745224105213,0.634019139668618,0.339205453537262,1.8
6913014828333,0.0616047095100704,0.72748818739126
"1210","MEIS2",85.2810592247584,0.434382962307415,0.232599762933631,1.
86751248938874,0.0618300543610622,0.727584749041862
"1211","LINS1",23.1748712417847,-0.611028528798588,0.327060165969977,-
1.86824502759739,0.0617279251612768,0.727584749041862
"1212","TRADD",82.9201505610564,-0.526390940422096,0.281862156533827,-
1.86754741003666,0.0618251826127153,0.727584749041862

"1213","ARL4D",136.346045901977,0.766375827358856,0.410426928267682,1.86726497355705,0.0618645941811144,0.727584749041862
"1214","SLC39A8",110.607139440758,0.646942271690062,0.346946855848882,1.86467253063059,0.0622273194002174,0.731247890085257
"1215","TMEM154",11.0454752953759,0.874894211175754,0.469460941209068,1.86361448712329,0.0623758617328341,0.731819118662268
"1216","PTPRS",30.5809910154363,0.614445301569669,0.329709580974201,1.86359553081276,0.0623785257460618,0.731819118662268
"1217","MICAL2",3.56233286526531,-1.46929907099418,0.78887330949655,-1.86252856232627,0.06252862329156,0.732977271879535
"1218","GFOD1",4.19274414580949,-1.25150091919008,0.672303933609259,-1.86151063027611,0.0626721008609166,0.733695662649858
"1219","RPS6KA2",13.8216710552634,0.839900740852034,0.451228596023944,1.86136416940974,0.0626927669122513,0.733695662649858
"1220","NOD1",21.0591078426053,0.715139028405217,0.384380043534492,1.86049988919637,0.0628148341170944,0.734521658618417
"1221","ATF5",46.6888589214033,-0.637459336244808,0.342699604783353,-1.86011109247644,0.0628698101343029,0.734562417179333
"1222","DBT",34.9198947944192,0.589897096117665,0.317325313633869,1.85896640063922,0.0630319010439574,0.734930561738419
"1223","HDAC4",49.7495284958499,0.534576618302372,0.287599534521915,1.85875341971959,0.0630620976988054,0.734930561738419
"1224","AL117339.4",1.93378042666436,2.41151866500393,1.29760779577431,1.85843416851925,0.0631073838587945,0.734930561738419
"1225","GAS8",11.8765092533714,-0.791884426679824,0.426040800373479,-1.85870561219873,0.0630688775415541,0.734930561738419
"1226","IBA57",18.4973850005905,0.699280668105062,0.3765766925196,1.85694091534533,0.0633195606250773,0.735001506816398
"1227","GLI1",8.39760934113444,-1.1184822812586,0.602209954411529,-1.85729623541605,0.0632690197230467,0.735001506816398
"1228","KCNJ12",20.2826815738816,0.764258047893713,0.411463027667319,1.85741608967025,0.0632519791159609,0.735001506816398
"1229","ZNF266",15.9379225571102,0.764220072000948,0.411421109078198,1.85751303260352,0.063238198763555,0.735001506816398
"1230","C12orf10",107.04346920736,-0.431097363732312,0.232287052963992,-1.85588201422117,0.0634703771411221,0.736153170971746
"1231","SPART",232.261714705135,0.316178370187429,0.170401123798394,1.85549463019685,0.0635256253322425,0.736195427286573
"1232","LINC01140",154.580555165025,-0.70237088735336,0.378861615571714,-1.85389825330671,0.0637537178571754,0.737488590554975
"1233","TRA2A",194.113786363235,0.369874381997557,0.199540706725458,1.85362870597855,0.0637922978231347,0.737488590554975
"1234","MBD2",180.631084458413,-0.356514149431517,0.192267959063469,-1.85425669034032,0.063702445075959,0.737488590554975
"1235","LRRC28",21.8592954618363,-0.684863073084024,0.369900736912674,-1.85147799055536,0.0641008182278088,0.740455281650138
"1236","DUSP12",41.7349805389999,-0.572315387701804,0.309490264145917,-1.84921935842211,0.064426144538725,0.742683920519678
"1237","FGFRL1",16.0255082028038,-0.816563091889994,0.441644641050021,-1.8489142989454,0.0644701886363805,0.742683920519678

"1238","SGCD",73.9136074660239,0.714103391742745,0.386079921335642,1.8
4962582170113,0.0643674984845062,0.742683920519678
"1239","CAPN1",108.687123391899,-0.44856586328075,0.242639305016074,-1
.84869414809375,0.0645019891717286,0.742683920519678
"1240","CRIM1-
DT",15.3360852644279,0.756465965156359,0.409654769451089,1.84659381891
239,0.0648060307037594,0.744767162639816
"1241","RMDN2",19.7113381470169,0.711706040153289,0.385463797165154,1.
84636286309491,0.0648395356791428,0.744767162639816
"1242","RETREG3",47.5651403854931,0.494045368541102,0.267525947156168,
1.84671944457299,0.064787812071362,0.744767162639816
"1243","ZFHX4",357.557886289168,0.502020938486086,0.271955683690145,1.
84596597384619,0.0648971461538905,0.744829193106518
"1244","SVIP",8.72197016320563,-1.12801943253442,0.611332027938659,-1.
84518294638998,0.0650109303827582,0.744878716961749
"1245","AC110048.2",2.57006382671078,1.87691398263481,1.01716279192237
,1.8452444363282,0.065001989132587,0.744878716961749
"1246","PIAS1",91.8544045022651,0.411953986402124,0.223298399865555,1.
84485865841473,0.0650581018739899,0.744878716961749
"1247","C21orf62-
AS1",8.33457214543138,0.982531988090166,0.532719286779742,1.8443709707
4055,0.0651290949217311,0.745093559064487
"1248","LRRFIP1",163.983723576235,-0.561866174148332,0.30492914546871,
-1.84261223467072,0.065385646312843,0.747010606533751
"1249","MBTPS1",148.608654103152,0.337326352394954,0.183080342729825,1
.84250448390712,0.0654013912491697,0.747010606533751
"1250","UBR3",45.281991794095,0.492651766284879,0.267569488466725,1.84
121055471594,0.0655907092236792,0.748573646228006
"1251","DHRS3",378.444633534651,0.544974789428856,0.296154901488752,1.
84016805627495,0.0657435681818227,0.749718420209338
"1252","PRG4",411.53227350775,-1.63204688641433,0.887563736732182,-1.8
3879401430164,0.0659454889968749,0.750049061191046
"1253","CCNI",1326.70493090578,-0.257910522067736,0.14025142843389,-1.
83891547449947,0.0659276193801218,0.750049061191046
"1254","HHIP-
AS1",7.1092881709062,-1.37641770808145,0.748309051071242,-1.8393706532
2281,0.0658606875123131,0.750049061191046
"1255","MLLT3",31.5587464707267,0.580443207377349,0.315770085995424,1.
83818301074207,0.0660354423703879,0.750049061191046
"1256","PRIMA1",4.35286772103129,1.51420838292438,0.82367483631268,1.8
3835697798295,0.0660098202162055,0.750049061191046
"1257","LINC01750",5.58186743016911,1.41212006017603,0.768364583598204
,1.83782554573658,0.0660881160697266,0.75005017012786
"1258","POU2F1",52.8842631695986,0.562952107229015,0.306725541346054,1
.83536103566244,0.0664522129926797,0.7512140874081
"1259","CRISPLD1",24.7758658421602,0.949923365828547,0.517307669232115
,1.83628316827122,0.0663157878066298,0.7512140874081
"1260","WDR24",8.59584673021862,0.940014189291875,0.512171834402417,1.
835349244436,0.0664539589449756,0.7512140874081
"1261","ATP6V0D1",138.334760018624,-0.352946799639449,0.19219813698461

2,-1.83636951521391,0.0663030250048456,0.7512140874081
"1262","HSD17B14",9.24262068756361,-1.02831419807743,0.560136874395686
,-1.83582664359823,0.0663832996207039,0.7512140874081
"1263","HAAO",13.8599405820139,0.77386369118001,0.421990289596904,1.83
384241357597,0.0666773897791504,0.751288691215312
"1264","PTPRG",114.924614540827,0.484977177631105,0.26456635425852,1.8
3310224382202,0.0667873675066844,0.751288691215312
"1265","LVRN",38.5079475447765,0.644939682468982,0.351867853993483,1.8
3290310595104,0.066816981785932,0.751288691215312
"1266","PHKG1",20.9951785231308,-0.739311581444395,0.403091690242256,-
1.83410275959813,0.0666387418756439,0.751288691215312
"1267","ANKFY1",67.4060196860873,0.415601389926999,0.226519302548239,1
.83472836642031,0.0665459470805174,0.751288691215312
"1268","PRPS2",34.4107404079381,-0.688499235924062,0.375650019875118,-
1.83282097563298,0.0668291987349103,0.751288691215312
"1269","CA5B",30.1006966144452,-0.608591369216901,0.332037278267436,-1
.83290072847398,0.0668173354116554,0.751288691215312
"1270","PRUNE2",5.50076445470984,-1.43503500096836,0.783686211351147,-
1.83113468143611,0.0670804431326891,0.751989971001125
"1271","PDS5B",89.6371530495734,0.430130815854902,0.23491217294779,1.8
3102821134135,0.0670963323887631,0.751989971001125
"1272","LINC01578",309.056048514522,-0.368376581411433,0.2011637609099
67,-1.83122735300373,0.067066615645992,0.751989971001125
"1273","CARHSP1",307.376492261789,0.443126279418103,0.242014929073241,
1.83098737385742,0.0671024276660895,0.751989971001125
"1274","MYZAP",2.99226830454894,-1.75608583690638,0.95953593697471,-1.
83014076830003,0.0672288921110097,0.752815835836471
"1275","HPRT1",49.5048838520521,-0.687510621758873,0.37575441675117,-1
.82968074654503,0.0672976915616371,0.752995190445737
"1276","MIIP",25.4927380448622,-0.621592296364063,0.340002149681843,-1
.82820107739236,0.0675193793931242,0.75302867450918
"1277","LINC01137",3.23874042340971,-1.64020335084625,0.89723804664088
5,-1.8280581803089,0.067540820379778,0.75302867450918
"1278","SPRED2",38.7733222516622,0.545506941509111,0.298281492371264,1
.82883268141267,0.0674246774664682,0.75302867450918
"1279","HIBCH",54.992637809427,-0.507597407234627,0.278077091529076,-1
.82538376118463,0.0679431380806879,0.75302867450918
"1280","USP40",29.6684140862448,0.629497006534547,0.344739180497933,1.
82600946496803,0.0678488361471472,0.75302867450918
"1281","AC091607.2",11.0588220951308,-0.906947651523402,0.496841424248
618,-1.82542680070406,0.0679366479986077,0.75302867450918
"1282","SETD7",164.825034699446,0.38281034802579,0.209597917024536,1.8
26403398756,0.0677895202980002,0.75302867450918
"1283","THBS4",66.6647340026711,-2.22737189045833,1.22041507339235,-1.
82509372345507,0.0679868871980811,0.75302867450918
"1284","FAM184A",23.8519661700929,-0.642479827881262,0.351931447681067
,-1.82558231756402,0.067913201308177,0.75302867450918
"1285","TSC1",41.0233249437465,0.560637557718524,0.306754682684864,1.8
2764139999936,0.0676033881619575,0.75302867450918
"1286","SPRY2",158.081335310889,0.516461538733296,0.282883007166481,1.

82570718512389,0.0678943803146383,0.75302867450918
"1287","MCRIP2",18.0988528268632,-0.724837279835091,0.396373670233187,-1.82867161536908,0.067448817121597,0.75302867450918
"1288","ALKBH5",84.8523538277708,-0.387127780567517,0.211984233513915,-1.82621025229268,0.0678185976363199,0.75302867450918
"1289","FAM89B",138.144383939792,-0.463952257528425,0.25428702418158,-1.82452195121497,0.0680732008336821,0.753103286733002
"1290","NINJ2",65.3859995106838,0.519784641922654,0.284915007746067,1.82434981587883,0.0680992036930866,0.753103286733002
"1291","PPA2",35.8656864843554,-0.493350008710292,0.270557138182442,-1.82345959165792,0.068233811834932,0.754007404831247
"1292","MAN2B2",73.7528173689092,0.427564719853499,0.234908261858841,1.82013487507914,0.0687384677524667,0.757049269379325
"1293","DNAJC18",39.1660298635853,0.920859627010643,0.505994964860324,1.81989879536615,0.0687744184155057,0.757049269379325
"1294","ZNF487",11.5258671377334,-0.796044434644544,0.437380126899879,-1.82002881632244,0.0687546166686008,0.757049269379325
"1295","PDPR",39.1060676225996,0.551025668832017,0.302689823355481,1.82043011133839,0.068693530353245,0.757049269379325
"1296","C2CD2",36.6299435449554,0.620354239521139,0.340612153629941,1.82129214389433,0.068562460012169,0.757049269379325
"1297","PGM5-AS1",3.71619240425468,-1.50783695903835,0.829038255315861,-1.81877850553938,0.0689452289420642,0.758344360900145
"1298","AAMP",118.541404611431,-0.394579859341005,0.21702024107806,-1.81817077237085,0.0690380357125247,0.758484428939028
"1299","SLC39A7",154.691322587041,-0.378052657577722,0.207949880182647,-1.81799891995932,0.069064297854465,0.758484428939028
"1300","YWHAQ",508.344811287898,-0.332357299982693,0.182976916501227,-1.81638922732892,0.069310686530319,0.759116506527716
"1301","KLRD1",29.6628514552136,0.826599512456905,0.454989949679875,1.81674235450363,0.0692565731606964,0.759116506527716
"1302","CPB2-AS1",2.47073765290435,1.87750627561037,1.0337182570142,1.81626498600632,0.0693297335833666,0.759116506527716
"1303","ARHGEF6",36.7406919863746,0.512234506747612,0.282031335191,1.81623260550361,0.069334698444246,0.759116506527716
"1304","SEC61A1",153.78828054747,0.315050622784399,0.173605569571624,1.81474951271318,0.0695624123119286,0.759544010813418
"1305","AK1",105.137318048382,-0.648583400267641,0.35737767433785,-1.81484028477531,0.0695484575621203,0.759544010813418
"1306","RBM7",116.254044544663,-0.402204739774705,0.221650295355984,-1.81459149029664,0.0695867112107905,0.759544010813418
"1307","SINHCAF",17.9599934544888,-0.713964278349269,0.393344164333841,-1.81511343776619,0.0695064785326443,0.759544010813418
"1308","C1GALT1C1L",9.82261059340373,-0.863761815902232,0.476453037124111,-1.81290022016846,0.0698472122856532,0.76122255956236
"1309","UBE2O",23.5946696670304,0.678986223638225,0.374522066446375,1.8129405032946,0.0698409983080701,0.76122255956236
"1310","UHRF2",33.2595909739394,0.689060421995428,0.380240828426643,1.

81216842190939,0.0699601767437921,0.761290527404224
"1311","ARCN1",103.700525402657,0.355647647857058,0.196222029924742,1.81247563279954,0.0699127357102245,0.761290527404224
"1312","LRRK2",42.9121496312489,0.61278997200672,0.338304621015544,1.81135560657495,0.07008582317424,0.762076488874777
"1313","RSU1",98.1208088628533,0.40626375664633,0.224403073849704,1.81041974905579,0.0702307189768585,0.76307040131292
"1314","ZBTB20",558.00825603591,0.449775571768073,0.248609915589603,1.80916183773839,0.0704258644661502,0.763179126680155
"1315","TSPAN33",2.32945036065379,-1.7595656669006,0.972565656765451,-1.80919987731476,0.0704199566983525,0.763179126680155
"1316","FAM204A",179.588605686903,-0.357239186995628,0.1974079524108,-1.80964942208723,0.0703501705641918,0.763179126680155
"1317","INSYN1",8.05483362350452,-1.03247181176773,0.570749272150708,-1.80897613391104,0.0704547111900858,0.763179126680155
"1318","ASXL2",49.4152683438702,0.522810541362097,0.289286764159448,1.80723975699744,0.0707249045225729,0.763727001331603
"1319","LINC02615",3.02892847603945,1.92878154168982,1.06742347130786,1.80695065598154,0.0707699732353439,0.763727001331603
"1320","PHYHIP",7.72665531650263,-1.05586823268191,0.584291024186747,-1.80709302209722,0.0707477764670731,0.763727001331603
"1321","NEDD4",30.8086035610195,0.527701974886241,0.291969436478975,1.80738772266748,0.0707018468758381,0.763727001331603
"1322","ZHX3",59.3389392463519,0.555974361118612,0.307689786323277,1.80693148044398,0.0707729633927084,0.763727001331603
"1323","FBRSL1",36.0942279048534,0.491753436563104,0.272365088020381,1.80549364875393,0.0709974684906581,0.765570586158525
"1324","ANKRD10",110.976785976831,-0.444050801711003,0.245993613142315,-1.80513142613221,0.0710541184336248,0.765602759497048
"1325","LINC01871",12.2037229360679,0.951438518319928,0.527388608959755,1.80405587484452,0.0712225481028438,0.766281657247773
"1326","PPP1CC",247.532560461245,-0.365271544868807,0.202473847982302,-1.80404308264411,0.0712245533093052,0.766281657247773
"1327","SIGIRR",91.7487046154118,-0.426429612749796,0.236577476324292,-1.80249455432207,0.0714676307177088,0.768317422621578
"1328","EGR2",35.3731930780982,-1.54343001940525,0.856646693806258,-1.80171128957199,0.0715908409369091,0.768483774872796
"1329","TBCEL",15.1380177717945,0.737639213557918,0.40936273933642,1.80192074821865,0.0715578753400675,0.768483774872796
"1330","CFLAR",289.566154635029,0.451062641332897,0.250407185785681,1.80131668313605,0.0716529797773829,0.768572488348981
"1331","GGCT",96.7002642134112,-0.536926323674672,0.29825191816239,-1.80024432695293,0.0718220674051374,0.769558560916347
"1332","DNAJC4",74.8979681586516,-0.516528295965484,0.287058826779332,-1.79938133852456,0.0719583794742298,0.769558560916347
"1333","FLI1",14.1945665188117,-0.70929410761969,0.394095272048244,-1.79980364629408,0.0718916480176583,0.769558560916347
"1334","NEK9",42.9141031890494,0.459486719366339,0.25536023550445,1.79936675911442,0.0719606841625127,0.769558560916347
"1335","NOTCH2",188.86069380439,0.346888391041998,0.19311090800752,1.7

9631691767763,0.0724441287759078,0.77011023928249
"1336","NISCH",58.1777876589047,0.492469179613501,0.274059818830977,1.79694046983672,0.0723450709510828,0.77011023928249
"1337","ZNF107",18.5022226124346,0.70731286789982,0.393460485171112,1.79767192528169,0.0722290129329193,0.77011023928249
"1338","TRMT112",382.646581393437,-0.39605523470204,0.220415039860704,-1.79686120762147,0.0723576564323891,0.77011023928249
"1339","APPL2",77.2228360175202,0.444664925669895,0.247513298899997,1.79652942951382,0.072410356567468,0.77011023928249
"1340","PRPSAP2",23.6825701489403,-0.631306964867535,0.351238344921708,-1.79737484245422,0.072276131846285,0.77011023928249
"1341","AC005332.7",2.83783901974232,1.75657409685015,0.977702753526534,1.796634089977,0.0723937287496478,0.77011023928249
"1342","LINC01638",6.65540139004195,-1.05156842535268,0.584671901050781,-1.79856159234399,0.0720880574541257,0.77011023928249
"1343","COX20",133.292639324194,-0.440068889656318,0.245203199859902,-1.79471103928396,0.0726997507870796,0.770395412177923
"1344","BOP1",40.1544524943561,-0.692917531389914,0.3861040318605,-1.79463946038322,0.072711161804964,0.770395412177923
"1345","ZDHHC17",57.4470910230952,0.49242199182955,0.274209161591458,1.79578971385065,0.0725279672616263,0.770395412177923
"1346","AL121839.2",8.51666781912823,1.05551947235179,0.588212529296401,1.7944525486636,0.0727409659472636,0.770395412177923
"1347","ICAM4",11.816946865698,1.17552426344266,0.654955598013477,1.79481520122601,0.0726831480404278,0.770395412177923
"1348","SDSL",8.88035706252414,1.01154646789413,0.563842796551493,1.79402215312642,0.0728096329976736,0.770550611531759
"1349","MELTF",27.1754063163533,1.09428518160035,0.61067551709383,1.79192574611144,0.0731448611910024,0.772974022192518
"1350","STBD1",14.6062261901053,-0.768740593727661,0.429390366846596,-1.79030703313915,0.0734045662448369,0.772974022192518
"1351","TMEM243",134.014544881625,-0.443630636564996,0.24760578519379,-1.79168122512883,0.0731840436789516,0.772974022192518
"1352","GPR137",35.4161112411181,-0.494773792424799,0.276427396613784,-1.78988695941771,0.0734720856647311,0.772974022192518
"1353","MON2",69.7465246417044,0.524287602469109,0.292915462020262,1.78989391291622,0.0734709675995476,0.772974022192518
"1354","LATS2",106.443941962005,0.442022641838236,0.246856819259465,1.79060332691736,0.0733569727906043,0.772974022192518
"1355","PLEKHM1",37.9272207590123,0.45923704162467,0.256486758942523,1.79049025188697,0.0733751329714233,0.772974022192518
"1356","MSN",212.612686789856,-0.325626453039143,0.181800474483536,-1.79111992949519,0.0732740516654321,0.772974022192518
"1357","DOK2",13.6254900073386,-0.736676399145233,0.41209288637047,-1.78764648337793,0.0738330609722141,0.775627722996765
"1358","SHB",6.236810915493,-1.08457576861965,0.606687534638418,-1.78770076307246,0.0738242985676293,0.775627722996765
"1359","SLC43A3",229.512621717742,0.346720114971849,0.19400717591900,1.78715098206778,0.0739130893707046,0.775897080914255
"1360","IFRD1",189.577829872304,0.54040784092109,0.302537170869059,1.7

8625270861339,0.0740583502823107,0.776850312593709
"1361","FKBP1B",10.8994608551201,-0.99897914160974,0.559597865135202,-1.78517325359771,0.0742332188947404,0.777314259793244
"1362","NKD2",11.4268504736358,1.21005030841866,0.678292978285004,1.78396407917734,0.0744295022345137,0.777314259793244
"1363","EPB41L4A",20.0994740667405,0.703530770920816,0.394188289916833,1.78475816993257,0.0743005510201839,0.777314259793244
"1364","HMBOX1",40.3527198462313,0.632587792888054,0.354503164457097,1.78443482685642,0.0743530361685137,0.777314259793244
"1365","WSB1",588.558437328639,0.577645673459349,0.323790046158542,1.78401306745701,0.0744215418117301,0.777314259793244
"1366","LANCL3",2.60583807490854,-2.00400147931104,1.12301896509495,-1.78447696931068,0.0743461938757925,0.777314259793244
"1367","DDI2",69.5079766297865,0.578686216882742,0.324628775320242,1.78260912425855,0.0746499535159669,0.777908135031982
"1368","ACSL3",99.9971549799975,0.472781358111097,0.265176693047759,1.78289182460673,0.0746039141632838,0.777908135031982
"1369","GALNT10",46.2023129548737,0.508266232885274,0.285070198752183,1.78295112961674,0.0745942589506452,0.777908135031982
"1370","PLA2G5",42.1679332837537,-0.884845405543718,0.496527117478758,-1.7820686411585,0.0747380388541607,0.778257563717851
"1371","EMX2",78.2135563311661,-0.533214712709618,0.299585063769262,-1.77984411506008,0.0751014756725422,0.78090207867674
"1372","ARMC7",7.87596578268234,0.949922256086045,0.5336216151671,1.78014201277919,0.0750527224545813,0.78090207867674
"1373","MAP4K3",44.7278651027938,0.547068690620094,0.307481154839674,1.77919421079755,0.0752079271769201,0.78143939483317
"1374","MYO10",9.70466952875959,1.05912925445597,0.596393390509978,1.77589032894933,0.0757509952858028,0.781447593100882
"1375","ADAMTS2",158.500722560314,0.344733464845112,0.194163312518495,1.77548199180149,0.0758183364140546,0.781447593100882
"1376","C6orf62",145.60581593755,0.352088595754698,0.198137299736593,1.7769930054703,0.0755693906556458,0.781447593100882
"1377","HSP90AB1",1365.98723672508,-0.309991406201934,0.174619505664242,-1.77523928396628,0.0758583858404457,0.781447593100882
"1378","DDX58",50.7403631997401,0.528478945576542,0.29758058716918,1.77591875398812,0.0757463093737044,0.781447593100882
"1379","MARCH8",21.2247543995841,0.627624913980554,0.352975727319989,1.77809652450006,0.0753880031729102,0.781447593100882
"1380","POSTN",338.957997783189,-1.88716961887441,1.06173323244997,-1.77744235670173,0.0754954870101324,0.781447593100882
"1381","TVP23A",2.01015730963602,2.71569975813737,1.52916079145526,1.77594127008247,0.0757425977282333,0.781447593100882
"1382","ALYREF",72.4615890707778,-0.523217146151526,0.294455414379921,-1.77689769180622,0.0755850742377936,0.781447593100882
"1383","ECSIT",55.1164718579242,-0.428291513565873,0.241264771896784,-1.77519291440153,0.0758660392853442,0.781447593100882
"1384","ZNF429",24.9671579443091,0.618603519127776,0.348047294405726,1.7773547706613,0.0755098874312484,0.781447593100882
"1385","PLCXD1",3.86327575608257,1.53090886007149,0.862181195941248,1.

77562311411836,0.0757950576338483,0.781447593100882
"1386","ACTR8",20.1346275652325,-0.611778217799011,0.344937233031324,-1.77359287202102,0.0761305175843259,0.783606034529576
"1387","PIK3R1",579.068751884389,0.599020203382578,0.337920308601685,1.77266707011876,0.0762838907679241,0.784618590984286
"1388","IGF1R",64.3927941889325,0.562666858844691,0.317594419021776,1.77165222417246,0.076452304885965,0.785784280621885
"1389","COBLL1",62.7343179276947,-0.499149629137023,0.282199657010982,-1.76878184199068,0.0769302881273432,0.786404251670844
"1390","SCRN3",15.6564429056238,0.756314824737184,0.427462438969824,1.76931294024309,0.0768416651312704,0.786404251670844
"1391","TTN-AS1",20.5897536382102,0.669208826536985,0.377943329303959,1.77065918260665,0.076617393930854,0.786404251670844
"1392","FLNB",71.0774817524555,0.530286213749167,0.300292395990106,1.76589957265065,0.0774126989222444,0.786404251670844
"1393","DLG1",65.6455814291172,0.470227420363298,0.266364893678221,1.76535058306652,0.0775048631606061,0.786404251670844
"1394","ABHD18",13.3641336844717,0.795791148565798,0.450398297001941,1.76686091813169,0.077251523558293,0.786404251670844
"1395","ESCO2",2.9481288033851,-1.90035988076124,1.07604666675906,-1.76605712323325,0.0773862658634741,0.786404251670844
"1396","TTLL11",6.16101653985044,1.18738683582467,0.672130543418243,1.76660151432191,0.0772949872109096,0.786404251670844
"1397","ADAM8",6.68010074951333,1.163968146765,0.657903196402539,1.76920883365465,0.0768590305685667,0.786404251670844
"1398","LAYN",9.06559731553743,-1.10521969660051,0.624422706207707,-1.76998639801685,0.0767294066580462,0.786404251670844
"1399","UBASH3B",5.51680269736266,1.37021898265506,0.776172173529652,1.76535442700036,0.0775042175313237,0.786404251670844
"1400","RXYLT1",66.8161982353558,-0.448119100621316,0.253629295261253,-1.7668270542633,0.0772571963900912,0.786404251670844
"1401","MMAB",39.4462837455016,-0.563692227409006,0.319121369617751,-1.76638821801312,0.0773307403801513,0.786404251670844
"1402","SLC7A8",20.9499739637461,0.785520017664218,0.444858665825672,1.76577434139957,0.0774337148498174,0.786404251670844
"1403","BCL2A1",15.620452468239,-1.14277716038886,0.645963107362616,-1.76910592472482,0.0768761993749133,0.786404251670844
"1404","AP3S2",40.0021001874121,0.506025835865301,0.28593408597284,1.76972897142934,0.0767723012228588,0.786404251670844
"1405","SOX8",2.60679332251622,-1.63889698936778,0.927454788144487,-1.76709097879223,0.0772129930763647,0.786404251670844
"1406","PLS3",228.981120441103,0.356064188122212,0.20160904470109,1.76611217344004,0.0773770315453517,0.786404251670844
"1407","AFF1",120.573357549234,0.474451380829209,0.268899513935543,1.76441888601903,0.0776614807920464,0.787433322657665
"1408","PTP4A2",419.483872720391,-0.263719556856925,0.150125464425456,-1.75666105591216,0.0789755992769775,0.791147093972486
"1409","LRIG2-DT",4.56600080646928,1.36426136586214,0.77531581202593,1.7596202021177

3,0.0784722238656856,0.791147093972486
"1410","SOCS5",72.5026173104937,-0.453987397472036,0.25817975767507,-1
.75841592524615,0.0786767654004689,0.791147093972486
"1411","ZNF385B",16.0994705630042,0.95680089455486,0.54423081309239,1.
75807924053067,0.0787340274622246,0.791147093972486
"1412","MFSD6",7.89620507190907,-1.03753490175818,0.589133722283108,-1
.76111952603453,0.0782181748107282,0.791147093972486
"1413","ARL13B",58.9144457213137,-0.403049147381039,0.229371045954607,
-1.75719278648973,0.0788849543931704,0.791147093972486
"1414","AC079298.3",2.40302346843037,1.92187902273315,1.09436110965234
,1.75616531488743,0.0790601852705752,0.791147093972486
"1415","EBF1",697.987949288362,0.335576866252217,0.19086944610439,1.75
814868802356,0.0787222133205477,0.791147093972486
"1416","NQO2",108.768590399632,-0.440320053747602,0.250434333204425,-1
.75822559196856,0.0787091323987966,0.791147093972486
"1417","SEMA3D",65.5108606473988,0.658636150579781,0.37393910589214,1.
76134600581133,0.0781798578024005,0.791147093972486
"1418","TBXAS1",3.83489561504673,-1.3485659713333,0.766451963184661,-1
.75949183524812,0.0784940058297416,0.791147093972486
"1419","STIM1",55.3077373050273,0.423167304249492,0.24102850347936,1.7
5567328403435,0.0791442110625724,0.791147093972486
"1420","PELI3",11.6016498852656,-0.740720226184915,0.421968652925405,-
1.75539159378234,0.0791923489550476,0.791147093972486
"1421","AMIGO2",10.9347594761647,0.840142530206602,0.477138472874753,1
.76079393712428,0.0782732863944807,0.791147093972486
"1422","AC026124.2",5.20346711520611,-1.10577509668092,0.6298253786445
47,-1.75568520128654,0.0791421750547109,0.791147093972486
"1423","SSH1",32.5860823380549,0.616036612696356,0.35010339769769,1.75
958478765835,0.0784782326855738,0.791147093972486
"1424","CFL2",83.6267097086298,0.45687055267967,0.260200030075308,1.75
584358136869,0.0791151205891227,0.791147093972486
"1425","CDH5",4.10314129559917,1.52558799745348,0.868365201700762,1.75
685068271448,0.0789432636055129,0.791147093972486
"1426","ZNF625",4.15257056065031,1.33222743115464,0.757469280420994,1.
75878740642023,0.0786136246141072,0.791147093972486
"1427","DPY19L3",30.8698252835131,0.504588943934619,0.287048544202529,
1.75785230103311,0.0787726436004419,0.791147093972486
"1428","RBM12",51.8494912664118,0.474673931394592,0.270060509013257,1.
75765769356263,0.0788057703762867,0.791147093972486
"1429","CCL20",17.2288550779839,1.82855060820136,1.04274174953992,1.75
359873047009,0.0794992887207258,0.793657699712998
"1430","RICTOR",78.5886029703192,0.498414218082987,0.284620400543779,1
.75115422903891,0.0799193472122201,0.794967607238483
"1431","TMEM176B",883.491243816499,0.490216791853941,0.279864208820037
,1.75162373895823,0.0798385278631494,0.794967607238483
"1432","ZBTB5",9.43725712122604,0.898769573973777,0.512950934356613,1.
75215505767835,0.0797471491542497,0.794967607238483
"1433","RECQL",119.693972875889,-0.381939109893252,0.21805250713837,-1
.75159237976972,0.0798439238233856,0.794967607238483
"1434","SERP2",13.4309281133855,-0.800954385577404,0.457515957459803,-

1.75065890602903,0.0800046819709307,0.794967607238483
"1435","ZNF649",10.0498184007832,0.848242025885689,0.484465262450203,1
.75088306970797,0.0799660536534783,0.794967607238483
"1436","SLC7A4",3.61995544963218,1.73194741053758,0.989363839743543,1.
75056671869727,0.080020572269344,0.794967607238483
"1437","FAAP20",115.078376168169,-0.402166577715494,0.230106616605112,
-1.74774017213793,0.0805090292451741,0.795389065936048
"1438","EFEMP1",2332.21081128812,0.548383548194534,0.313411801792482,1
.74972207510435,0.08016628262l016,0.795389065936048
"1439","PCSK1",4.44516326095781,-1.5343481433932,0.877509646316451,-1.
74852567129487,0.0803730443616987,0.795389065936048
"1440","RILPL2",54.5538081195436,-0.409113793021244,0.233884615295597,
-1.74921207409979,0.0802543677606467,0.795389065936048
"1441","FTO",52.6529877971208,0.42499202277349,0.24315564651893,1.7478
1885124927,0.080495399974491,0.795389065936048
"1442","CDK12",103.187222394806,0.399047177824222,0.22818386064435,1.7
4879667956088,0.0803261709770568,0.795389065936048
"1443","EXOC7",94.63856390037,0.386831833864142,0.221106329001745,1.74
952854407478,0.0801996991998048,0.795389065936048
"1444","MT-
ND1",13731.6963958411,-0.314504173052425,0.179908525214282,-1.74813379
564882,0.0804408621681203,0.795389065936048
"1445","ZNF471",10.3596952330926,0.970203624890416,0.555305636097699,1
.74715249012838,0.0806108903673602,0.795844264346547
"1446","PRKG2",8.94417928454489,1.13422239026357,0.649367054855208,1.7
4665835259609,0.0806966186916836,0.796139669609653
"1447","PROCR",631.059249436415,0.644555760798956,0.36945762952084,1.7
4459994677847,0.0810545301139336,0.798566247655647
"1448","MT-
ND3",17122.5380080551,-0.667533557005132,0.382587318958439,-1.74478746
138904,0.0810218721953649,0.798566247655647
"1449","SOS1",97.3297018794868,0.461312755888484,0.264514489060044,1.7
4399806047587,0.081159428038729,0.798616816355872
"1450","RTL10",10.5805583059817,0.939759788198966,0.538875298134406,1.
74392812484155,0.0811716237008281,0.798616816355872
"1451","ZFP36",5914.396208874,-0.385721489318435,0.221243295859853,-1.
74342679094227,0.0812590919162338,0.798926399226045
"1452","SLC27A4",13.4543725538811,-0.742823701912374,0.426228844278911
,-1.7427813999052,0.0813718065355067,0.799483603330261
"1453","LGALSL",28.9913815868702,-0.59263077146353,0.340845957454112,-
1.7387055897335,0.0820865623143382,0.799597264563625
"1454","SEMA4C",25.1120994883861,-0.51561478960308,0.296065154404561,
-1.7415585114611,0.0815857264285746,0.799597264563625
"1455","PID1",113.205145114862,0.538511605907214,0.309591100410084,1.7
394285727009,0.0819594059939674,0.799597264563625
"1456","POPDC2",8.84511154992723,-1.42540433284337,0.819325066358345,-
1.73972992084676,0.0819064528787113,0.799597264563625
"1457","UBXN7",72.5916694514404,0.540371208687224,0.310383282491318,1.
74098039156584,0.0816870156803467,0.799597264563625
"1458","SLC38A9",15.6331378865822,0.661797338641909,0.380243549498078,

1.7404564509128,0.0817789005870611,0.799597264563625
"1459","FAM135A",15.4282909959753,0.72081490638621,0.414206390795956,1
.74023125283283,0.0818184199463567,0.799597264563625
"1460","NCS1",32.0809909883459,-0.571898477931683,0.328316707879146,-1
.74191097865845,0.0815240225829009,0.799597264563625
"1461","BMI1",93.6881748607771,0.385915323449425,0.221951865390313,1.7
3873430967013,0.0820815080785904,0.799597264563625
"1462","SMPD1",46.201135658021,0.543895904953624,0.312842580385666,1.7
3856098579394,0.082120140604031,0.799597264563625
"1463","ANGPTL5",84.7774938498406,-0.637530472057763,0.366281867851613
,-1.74054608762681,0.0817631748086241,0.799597264563625
"1464","ACAT1",104.185571007317,0.347088622927316,0.199627718894478,1.
73867950227285,0.0820911534964257,0.799597264563625
"1465","VAMP7",45.5097988151824,0.448167743266949,0.257420993048731,1.
74099143181422,0.0816850804231915,0.799597264563625
"1466","SMIM1",5.27680515697614,1.2056957955246,0.694655539870864,1.73
5674339758,0.0826214331449738,0.799698321335865
"1467","ENO1",561.466321095843,-0.430493899876585,0.248007571108489,-1
.73580950755841,0.0825975224557047,0.799698321335865
"1468","MLLT11",6.46220801143186,-1.05189847952871,0.605641683324919,-
1.7368330293151,0.0824166471954702,0.799698321335865
"1469","CYP1B1-
AS1",12.8543238908742,0.820454539194232,0.472902554808673,1.7349336155
0176,0.0827525641914758,0.799698321335865
"1470","AC104162.2",5.46085661062127,1.22495075816083,0.70590362173405
,1.73529462159684,0.0826886339189186,0.799698321335865
"1471","THEM6",8.04623990001885,-0.920398770259671,0.529604939148532,-
1.73789687788682,0.0822289859237617,0.799698321335865
"1472","PRF1",23.5834009453397,0.876513201429914,0.50495577286831,1.73
582172642772,0.0825953612589079,0.799698321335865
"1473","PHC1",35.9292488334365,0.491793285683834,0.283013475043024,1.7
377027210774,0.0822632090187265,0.799698321335865
"1474","POLR3B",11.2833523820622,0.834859399570693,0.480912987727704,1
.73598846542983,0.0825658740926399,0.799698321335865
"1475","QPRT",16.957597351884,1.32547722259272,0.764098697125358,1.734
69373469598,0.0827950666348712,0.799698321335865
"1476","ZNF347",17.1582289997742,0.65575312647551,0.377923011420287,1.
73515003495315,0.0827142338413901,0.799698321335865
"1477","NINL",10.0366457928703,1.17133543218912,0.674558986935111,1.73
644626322619,0.0824849581483702,0.799698321335865
"1478","WDR26",76.8910819030646,0.430671980911132,0.248610439710397,1.
7323165568301,0.0832172156494022,0.802688842768338
"1479","ARHGAP26",47.3565856412873,0.586486478998799,0.338541128548516
,1.73239358394456,0.0832035095753144,0.802688842768338
"1480","BARD1",17.5076588229398,-0.704039252019232,0.406727521406898,-
1.73098503288863,0.0834544339800061,0.804045610234491
"1481","METRN",69.1070900446109,-0.546339979941956,0.31564026957707,-1
.73089441557632,0.0834705978380262,0.804045610234491
"1482","SIL1",118.860675410564,0.390069687240706,0.225429808109443,1.7
3033766258334,0.0835699642767138,0.804459588644803

"1483","SLC22A23",6.49592997489481,1.04965079055214,0.607284193489035,
1.72843423524919,0.0839104020874708,0.805627368346121
"1484","PPP1R9A",5.05798098265009,1.35185105299049,0.781766954211057,1
.72922511716392,0.0837688126838768,0.805627368346121
"1485","IL4R",80.9946219267898,0.393453895049242,0.227621674296314,1.7
2854319021066,0.0838908846824513,0.805627368346121
"1486","PQBP1",82.8401552377312,-0.373718425154903,0.216222624578318,-
1.7283964889601,0.083917164542432,0.805627368346121
"1487","SNRK",55.4443243905754,0.463207724707787,0.268206928277109,1.7
2705353915841,0.0841580486057097,0.806407226768241
"1488","SPSB4",9.14371821069903,1.02639341617865,0.594430513744733,1.7
2668359454274,0.0842245035668497,0.806407226768241
"1489","DAB2",836.984339930464,0.31943420815829,0.184948709196625,1.72
715024368561,0.0841406840969834,0.806407226768241
"1490","AL359220.1",3.15874588732231,1.56901599380688,0.908547524036052,1.72694983178956,0.084176673785352,0.806407226768241
"1491","PLPP1",354.720897429353,0.543603186948778,0.314955958826997,1.
72596571588403,0.0843535807878975,0.807101397397817
"1492","PRSS23",382.424302230047,-0.567086321875603,0.328760206028155,
-1.72492385476556,0.0845411959421657,0.808354357447008
"1493","ASAP3",75.159331561707,0.424140832213878,0.246034952318885,1.7
239047875773,0.0847250329106057,0.808549096736255
"1494","NR2C1",33.0148036634416,0.517330955471096,0.300098857872397,1.
72386845834337,0.0847315925711974,0.808549096736255
"1495","YIPF2",71.0546263564433,-0.405399562698392,0.235112825879398,-
1.72427667942855,0.0846579071878812,0.808549096736255
"1496","KLF7",70.6449567247572,0.524596722998861,0.304603523615665,1.7
2222801880904,0.0850282211398497,0.810181789588253
"1497","ADO",28.0937252435761,-0.522672266238235,0.303529736182265,-1.
72198043200742,0.0850730632835556,0.810181789588253
"1498","CCDC91",63.6499584822764,0.398076090746199,0.231104993355794,1
.72249021955725,0.0849807530064465,0.810181789588253
"1499","AC108463.3",11.9277543837115,0.792279807743233,0.460568596778034,1.72022107735032,0.0853922631174628,0.810516317786909
"1500","GSEC",6.96627970248863,1.05394028531208,0.612591403932428,1.72
046208703956,0.0853484795660034,0.810516317786909
"1501","SIPA1L1",12.7581538700818,-0.811525702072424,0.471734837162684
,-1.7203005547639,0.0853778226733937,0.810516317786909
"1502","ZNF844",18.0780225451325,0.852016519261178,0.495069937311031,1
.72100233734429,0.0852503997537845,0.810516317786909
"1503","SERTAD1",426.838641638716,-0.564549728953287,0.328178171152626
,-1.72025374804935,0.0853863268599191,0.810516317786909
"1504","CPE",1291.45101190809,0.448877094020008,0.261243833388405,1.71
823039111756,0.0857546002582367,0.811890618427036
"1505","PM20D2",22.2391580079584,0.655838134516847,0.38151567726328,1.
719033249751,0.0856083177583095,0.811890618427036
"1506","PTDSS1",31.6178703316946,-0.508004238012802,0.295641362369108,
-1.71831246460892,0.0857396370353935,0.811890618427036
"1507","KLF6",1508.0891948823,-0.511506796692849,0.297703546521631,-1.
71817501897205,0.0857646966192026,0.811890618427036

"1508","LETM1",38.0358623499716,-0.524991075644839,0.305898341718065,-1.71622726915173,0.0861204540476267,0.812327224582347
"1509","TRIM2",44.1468883152272,0.500249697951548,0.29151117210376,1.71605669292665,0.0861516665666461,0.812327224582347
"1510","CD164",442.399577723315,0.264925532963779,0.154287266177834,1.71709266439669,0.0859622422984405,0.812327224582347
"1511","FKBP9",275.255813002322,0.420167068546697,0.244845138763684,1.71605232053321,0.0861524667596446,0.812327224582347
"1512","MOB3A",38.2957537966994,0.476386871841336,0.277472756162685,1.71687800427522,0.0860014645912342,0.812327224582347
"1513","JAG1",114.689395195928,0.383776343055175,0.223629896639724,1.71612270461965,0.0861395864769988,0.812327224582347
"1514","SET",510.993907934634,-0.372573319707039,0.217287314848302,-1.71465747996908,0.086408043568112,0.813124768827629
"1515","HERC4",63.2281361360128,0.454183436641646,0.264881972668811,1.71466344827296,0.0864069486920489,0.813124768827629
"1516","AC027279.1",14.8478819170233,0.712236619651266,0.415276786185683,1.71508893187399,0.0863289232545605,0.813124768827629
"1517","CCDC126",32.2721477639636,0.510011254731293,0.29757085243604,1.71391536017767,0.086544271629182,0.813869860950502
"1518","CLDN11",27.385043799748,1.48947100256892,0.869413844939291,1.71318988217047,0.0866776124155884,0.814420355339469
"1519","FZD6",8.94235534318493,-1.00879510549996,0.588913799252588,-1.71297583242277,0.0867169858236825,0.814420355339469
"1520","HDX",21.0260197496352,0.570963827123117,0.333419623126657,1.71244818097051,0.0868141064132952,0.81479608032373
"1521","CLOCK",61.4083462814351,0.418292165375727,0.244352521902276,1.71183895348956,0.0869263512592418,0.815242164685679
"1522","KLHL8",38.4165579110103,0.509817431031866,0.297872488537913,1.71152909600437,0.0869834847242034,0.815242164685679
"1523","AL161772.1",23.4426264593783,-0.629467939586925,0.367848227617609,-1.71121645376331,0.0870411623773633,0.815242164685679
"1524","DUS3L",27.5302449309602,-0.546510479221371,0.319419190195122,-1.7109506754667,0.0870902186303781,0.815242164685679
"1525","TMEM158",7.25498639246302,-1.14724153940361,0.671505785168382,-1.70846114023565,0.0875508109516688,0.815525600544972
"1526","OSMR",228.05070050029,0.372159810573238,0.217727032563452,1.70929537867458,0.0873962489208538,0.815525600544972
"1527","SAYSD1",53.1955528930479,0.551951461093278,0.322924653300931,1.70922676683628,0.0874089525413339,0.815525600544972
"1528","LRP11",66.6775627324964,0.488822250490636,0.285916308966506,1.7096690015955,0.0873270980234742,0.815525600544972
"1529","ZNF32",90.5998333611566,-0.36279441899083,0.212244826522409,-1.70932043402493,0.0873916102434673,0.815525600544972
"1530","LCOR",51.7436202256914,0.515572412535261,0.301509120660176,1.70997285722693,0.0872708923590975,0.815525600544972
"1531","ZNF740",17.0683527857951,0.65100056773925,0.381078106987999,1.70831269443603,0.0875783370786417,0.815525600544972
"1532","AP4E1",17.0891600154385,0.77492645825257,0.453702076988332,1.70800729720376,0.0876349884785814,0.815525600544972

"1533","RAI2",40.1148366841667,-0.583737846250787,0.341696289994524,-1
.7083528950816,0.0875708820317873,0.815525600544972
"1534","RAVER2",8.02075289605939,-0.955574082951005,0.560306534937434,
-1.70544875593448,0.088110761763773,0.817379238907894
"1535","USP53",390.754972021665,0.458233004641784,0.268572610263229,1.
70617921236521,0.0879747178672945,0.817379238907894
"1536","KIAA1109",94.3845714812076,0.483292749425005,0.283449105924902
,1.70504241968944,0.0881865132761592,0.817379238907894
"1537","IL6",206.587649058928,1.35200870935215,0.793068777030453,1.704
78115960457,0.0882352465945715,0.817379238907894
"1538","ZBED5-
AS1",11.3551413676287,-0.815396182708341,0.478214170905027,-1.70508578
021683,0.0881784272583392,0.817379238907894
"1539","RNF157",3.40665143051895,1.62672044329976,0.953715406563256,1.
70566652494552,0.0880701856266846,0.817379238907894
"1540","TIMP2",1806.10908102579,0.251525718183383,0.147428679205184,1.
70608405053485,0.0879924316849926,0.817379238907894
"1541","DENND2D",13.4829907736295,0.897580076702756,0.5269450462142,1.
70336562256607,0.0884996669297787,0.818594535611776
"1542","LTC4S",20.6121757119518,-0.675584446714941,0.396789003188707,-
1.70262895716805,0.0886375274690748,0.818594535611776
"1543","BZW2",99.9611881236491,-0.460651037713612,0.270789309535146,-1
.70114188962775,0.0889163465708629,0.818594535611776
"1544","SRRM3",18.8963370876489,-0.68303899706517,0.401309926843673,-1
.70202367640719,0.0887509299932219,0.818594535611776
"1545","DEPTOR",66.3380817481194,0.634636247089907,0.372932667034698,1
.70174485420142,0.0888032081144119,0.818594535611776
"1546","S1PR3",97.017828907802,-0.738563867525752,0.434208152112085,-1
.70094426816543,0.0889534529265306,0.818594535611776
"1547","NRAV",5.35580387239185,-1.14896274912006,0.675578644814409,-1.
70070910017545,0.0889976254544977,0.818594535611776
"1548","GLRX5",200.08263533623,-0.463449890192565,0.27221285385934,-1.
70252757583609,0.0886565136471117,0.818594535611776
"1549","TMEM100",59.2697046767975,-0.697475512495915,0.410072909125256
,-1.70085732799011,0.0889697811829161,0.818594535611776
"1550","TMEM205",109.078220784488,-0.352617417847277,0.207060004860437
,-1.70297213160479,0.0885732838317118,0.818594535611776
"1551","ZGPAT",42.6940009914436,-0.460813115383304,0.270913948148199,-
1.70095751264613,0.0889509656891805,0.818594535611776
"1552","SLC25A37",119.99294636033,0.350456711877505,0.206159795778972,
1.69992752734988,0.0891445583286198,0.818671232080206
"1553","RIPK3",4.59751104082835,-1.2257021970496,0.721107703672471,-1.
69974913706694,0.0891781224346446,0.818671232080206
"1554","TMEM221",11.0583396982503,1.04836846751634,0.616661111543134,1
.70007228912636,0.0891173289012929,0.818671232080206
"1555","GNG11",307.114128020874,-0.420345245252382,0.247421168270493,-
1.6989057492156,0.0893369435900873,0.818931366376349
"1556","MATN2",47.1215753955115,-0.929285770456292,0.54706206362808,-1
.69868435821217,0.0893786721889791,0.818931366376349
"1557","PTTG1IP",238.994901635942,0.400767076244287,0.235909993659001,

1.69881347554772,0.0893543337664373,0.818931366376349
"1558","KDM5B",110.922598505323,0.38565420025772,0.227083966824646,1.6
9828898821167,0.0894532320425663,0.819088452066271
"1559","PCDHGA8",4.23929621522512,1.40271832398171,0.826299994354309,1
.69758965698388,0.0895852363037311,0.819245500710916
"1560","COL15A1",151.401047847682,-0.597098505038098,0.35169632059976,
-1.69776727837199,0.0895516940210719,0.819245500710916
"1561","LINC01011",2.95720016130582,1.49181707396779,0.879639056320476
,1.69594228820177,0.0898968100617254,0.819575042545003
"1562","GTF2H4",23.9022088225711,-0.615255464146359,0.3626554314703,-1
.69652902109298,0.0897857389489325,0.819575042545003
"1563","TMEM123",185.572842975421,0.293440684501845,0.173031466747013,
1.69588046624422,0.0899085196679468,0.819575042545003
"1564","TGFB3",41.2368617611516,-0.536403536681055,0.316120385021238,-
1.69683311199629,0.0897282166882845,0.819575042545003
"1565","OCRL",23.7899791708486,0.583099019240199,0.343737786110375,1.6
9634832945879,0.0898199328897827,0.819575042545003
"1566","NDUFV1",149.080448354824,-0.364314792479765,0.214862752110958,
-1.69556979467352,0.0899673821027091,0.819587913842431
"1567","IGFBP5",3697.65619742544,1.08483160461789,0.640003535271099,1.
69504001904983,0.0900678293684734,0.819979357862567
"1568","AL133245.1",2.53105417798749,2.03323717594093,1.2003934611347,
1.69380893996121,0.0903015947521961,0.821059624432651
"1569","GDPD5",26.1878629552957,0.665285549218499,0.392709657444555,1.
69409011621373,0.090248160185255,0.821059624432651
"1570","LAGE3",45.1467834799518,-0.48056023322498,0.283810928750966,-1
.69324076186881,0.0904096486735877,0.821518501896434
"1571","CAMK2N1",249.991083361141,-0.529919208791726,0.314131725300415
,-1.6869331115313,0.0916162143426405,0.82291321192232
"1572","EVA1B",216.317388363379,-0.414048594710016,0.245320793609355,-
1.68778434399385,0.0914526340068115,0.82291321192232
"1573","ZNF687-
AS1",1.9639034392264,-1.75807620320932,1.03906370638495,-1.69198114841
862,0.0906495680691779,0.82291321192232
"1574","MPC2",196.576529073248,-0.454239155252441,0.269146113293294,-1
.68770468090486,0.0914679328043403,0.82291321192232
"1575","BATF3",36.6707395123742,-0.925528399676894,0.549045698727591,-
1.68570376167557,0.0918528719885304,0.82291321192232
"1576","AL445524.1",8.56007287652381,0.996213703374225,0.5910652986764
01,1.68545456078219,0.0919009045656008,0.82291321192232
"1577","STRADB",19.2711822818803,0.64970257884613,0.384816958741416,1.
68834185731068,0.091345624621356,0.82291321192232
"1578","SP110",107.734151512563,0.344521060441958,0.204395938539558,1.
68555727136075,0.0918811050263398,0.82291321192232
"1579","RBPJ",606.85293379951,0.365103922046623,0.2163929185349,1.6872
2675639563,0.0915597584091898,0.82291321192232
"1580","LRP2BP",1.90877519439544,2.05258076126912,1.21637850337618,1.6
8745234774536,0.091516405385789,0.82291321192232
"1581","PPDPFL",12.4642648369562,0.876494618205287,0.520048221281995,1
.68541027992481,0.0919094416530024,0.82291321192232

"1582","AL512625.1",4.47007239116006,1.30623074513003,0.773662035599494,1.6883738441655,0.091339488102849,0.82291321192232
"1583","ZNF143",28.1787925198819,0.546525616262346,0.32434014237639,1.68503846689478,0.0919811501453998,0.82291321192232
"1584","SCN2B",2.2286816805236,1.88811627643805,1.11767627178309,1.68932303933217,0.0911575405235375,0.82291321192232
"1585","FGFR1OP2",157.640049257194,-0.345005203036973,0.204264040633451,-1.68901585402435,0.0912163917761825,0.82291321192232
"1586","AC099518.5",23.4578442634545,-0.805199063573804,0.476204165443765,-1.69086942535132,0.0908617439158371,0.82291321192232
"1587","AC012645.1",2.43876711925552,-1.69242434719579,1.0034120642362,-1.68666932312008,0.0916669539486099,0.82291321192232
"1588","AC009061.2",4.92547222333096,1.13417844107685,0.671552712973189,1.6888896719,0.0912405748806165,0.82291321192232
"1589","SSH2",50.5056027562202,0.569023925272146,0.337086707962361,1.68806396642517,0.0913989505487718,0.82291321192232
"1590","ENOSF1",67.3816654054343,0.408173054538107,0.241731696774827,1.6885375810617,0.0913080811859899,0.82291321192232
"1591","MIB1",66.7608378871847,0.452296623734881,0.268305913295195,1.68574974058531,0.0918440119234038,0.82291321192232
"1592","SDCBP2-AS1",5.57155296910356,1.0410817903235,0.616507360421937,1.6886769845067,0.0912813485916637,0.82291321192232
"1593","Z95331.1",14.9245098051088,1.06324202765683,0.628723964991068,1.69111102305752,0.0908156002982369,0.82291321192232
"1594","KLHL15",22.6455795683885,0.711053201664816,0.422011650322789,1.68491367743271,0.0920052273248353,0.82291321192232
"1595","PIM2",9.52359163814124,0.9067759406405,0.535986179518615,1.69178977983145,0.090686062942088,0.82291321192232
"1596","DPYD",100.193162955644,0.362195989274057,0.21504163616819,1.68430633122031,0.0921224824639907,0.823445698515846
"1597","AL020997.5",4.89666317830205,1.31633189216455,0.781716862115033,1.6838985519681,0.0922012762489498,0.823500804773526
"1598","SQOR",110.291590369467,0.424184556362858,0.251939380892285,1.68367706096815,0.092244096875655,0.823500804773526
"1599","DACT2",9.45873550883997,1.15321481966132,0.685374788083035,1.68260467077701,0.0924516469032967,0.823622131539699
"1600","RPS6KB2",38.8741067486931,-0.45429450827633,0.269976163032721,-1.68272081198987,0.0924291508811355,0.823622131539699
"1601","TMEM106C",60.5053327463228,0.734549312622331,0.436508368082208,1.68278403424328,0.0924169068674466,0.823622131539699
"1602","KIAA0753",12.4798543216806,0.754799518399782,0.448640817083981,1.68241383676531,0.0924886201266366,0.823622131539699
"1603","PTHLH",13.8758178573774,-0.840247283664519,0.499672301280835,-1.68159668148639,0.0926470745479168,0.824518506238666
"1604","AL645933.2",4.0334801180056,-1.24444296819772,0.740238898650623,-1.68113695519948,0.0927363157427493,0.824798179791809
"1605","PTGES2",52.0929610359032,-0.490667956702584,0.29194906229451,-1.68066289662411,0.0928284113525859,0.825102876234262
"1606","ACADL",14.4810872510357,0.730297072448293,0.434992599132559,1.

67887240818491,0.0931769129940675,0.82643844607256
"1607","NRBP2",35.0374834865614,0.595462786881224,0.354632790995335,1.67909680661498,0.093133178498073,0.82643844607256
"1608","ANO8",9.05590787219543,0.986188236074398,0.587471158662367,1.67870068433637,0.0932103925228339,0.82643844607256
"1609","BCAM",3.50800531157532,-1.2689505560649,0.755617754516594,-1.67935513489451,0.0930828515893176,0.82643844607256
"1610","UTRN",185.988921509981,0.459547616825728,0.273859164820418,1.67804359268777,0.0933385892053465,0.826719514391159
"1611","ARHGEF17",18.4656948441905,0.774644110329921,0.461662618171891,1.6779441952597,0.0933579936691544,0.826719514391159
"1612","OTUD4",57.7381121178347,0.432224770670252,0.257699923034144,1.6772405889038,0.0934954449611091,0.827048605839161
"1613","DSE",222.342832252017,0.414540627513554,0.247168133751871,1.67716048675476,0.0935111034080027,0.827048605839161
"1614","LRRN2",4.03977181518919,1.98045179688163,1.18172879196697,1.67589366557212,0.0937590227069611,0.828214376431893
"1615","KBTBD11",2.77063474580579,1.84591753331173,1.1013649755577,1.67602708845631,0.0937328867841626,0.828214376431893
"1616","JAGN1",119.18837418113,-0.439379390234123,0.262305045117495,-1.67507029854234,0.0939204396667069,0.829126851661658
"1617","SCARA3",145.393549719841,-0.400529445040357,0.239193603489986,-1.67449897989068,0.094032574721473,0.82948242953301
"1618","GEMIN8",26.8958499250325,-0.603771492404079,0.360617163587887,-1.67427275617438,0.0940770062375165,0.82948242953301
"1619","SLX4",9.5514927454281,0.817644712617333,0.48861572086362,1.67339010536165,0.0942505244462485,0.830499062229883
"1620","TRAF3IP3",15.3037997174305,-0.71645903989221,0.428396313386524,-1.67242111452481,0.094441311381544,0.830641028464307
"1621","FAM200B",127.397942322151,-0.377889910080372,0.225899551654705,-1.67282275379629,0.0943621941304443,0.830641028464307
"1622","ZNF208",20.4434576593792,0.814656445235739,0.487060292771104,1.67259876718916,0.0944063097660054,0.830641028464307
"1623","FOSL2",163.77535845503,0.430947706767694,0.258055503026244,1.66998068909178,0.0949231843488738,0.83271964593497
"1624","ADH1C",70.2628264774685,-0.564049815501697,0.337627818243099,-1.67062601191105,0.0947955712879964,0.83271964593497
"1625","MRPL2",69.0348087412413,-0.431588986402345,0.258612652658237,-1.66886260964463,0.0951446111645872,0.83271964593497
"1626","AC026979.2",14.6693209803623,0.736250703383947,0.441150824562426,1.66893194433951,0.0951308679527768,0.83271964593497
"1627","ANP32B",278.64218804375,-0.438168071415115,0.262240927665383,-1.67086074365254,0.0947491870236709,0.83271964593497
"1628","FKTN",27.3907703312342,0.656572987311157,0.393316261343667,1.66932581192585,0.0950528274599696,0.83271964593497
"1629","N4BP2L1",64.8217800613002,0.443489863169751,0.265608744210513,1.66971108006239,0.0949765404794716,0.83271964593497
"1630","MC1R",4.42012159489914,-1.1479150758133,0.687261550801515,-1.67027396552965,0.0948651716665605,0.83271964593497
"1631","UBR4",35.6884503511095,0.621896189629608,0.372826641040063,1.6

6805727159069,0.0953043582008737,0.833386050489445
"1632","KDM4A",34.0513316841563,0.477943764125675,0.286707564182272,1.66700786387982,0.0955128411993721,0.833386050489445
"1633","VGLL4",163.895661193111,-0.402716055206959,0.241554232929693,-1.66718691004754,0.095477244764324,0.833386050489445
"1634","TASOR2",101.143583034571,0.360024572581079,0.215886913807029,1.66765352393192,0.0953845264805456,0.833386050489445
"1635","RAB21",136.824360487062,0.3373154737602,0.202293517356117,1.66745567613218,0.0954238309219438,0.833386050489445
"1636","HLA-DMA",32.8985589844243,-0.655626381345652,0.393616751418108,-1.66564654320119,0.0957838350704489,0.834545588790854
"1637","SYBU",5.77953334590551,-1.19408979776636,0.716779395864887,-1.66590976896809,0.0957313875891307,0.834545588790854
"1638","C20orf194",31.167822881009,0.574303951875369,0.344860789979552,1.66532110510279,0.0958487100963403,0.834545588790854
"1639","SPIN3",11.2572259962272,0.751880943098417,0.451535246722997,1.6651655625007‍6,0.0958797294285861,0.834545588790854
"1640","AC008280.3",2.22040073200162,1.9132654242175,1.14955697451589,1.66435023807605,0.096042458286001,0.834943150461969
"1641","PHC3",80.3063329687038,0.391042558873248,0.23492176133169,1.66456507331021,0.0959995583476653,0.834943150461969
"1642","MEGF6",54.0196957245281,0.704088015362755,0.423981790682602,1.66065626127289,0.0967825025523495,0.835656135403946
"1643","CSF3R",9.30425810647931,1.35736110128511,0.817861695621964,1.65964625626936,0.0969856370246395,0.835656135403946
"1644","WDR78",3.76775226690905,-1.21698314782096,0.732717123070325,-1.66091812174582,0.0967298921733079,0.835656135403946
"1645","AL357568.2",5.52696262686001,1.1372480328536,0.685707356710115,1.65850347342033,0.0972158870307179,0.835656135403946
"1646","CREG1",228.979077896806,0.293308582074285,0.176383350896023,1.66290401324323,0.0963316509349709,0.835656135403946
"1647","SPRTN",29.0328512280116,-0.490960797423011,0.297494788044402,-1.65031730690264,0.0988780543215683,0.835656135403946
"1648","DYSF",2.22056944015315,-1.69903078720333,1.02451504513473,-1.65837563369302,0.0972416715715848,0.835656135403946
"1649","BOLA3-AS1",10.7239622399604,-0.767794403528866,0.464944769073245,-1.65136690334054,0.0986636786559291,0.835656135403946
"1650","PARD3B",52.0297650789347,0.59449246217265,0.360818070134114,1.64762386194151,0.0994298807087386,0.835656135403946
"1651","PLEKHM3",20.4208141816344,0.711786802583981,0.431697422109394,1.64880948120096,0.0991866719537536,0.835656135403946
"1652","C3orf14",43.3941562802649,-0.55946913577211,0.336842433864329,-1.660922376536,0.0967290375324437,0.835656135403946
"1653","AC096992.2",2.18202853333312,1.98142889974422,1.19816271486761,1.65372271658708,0.0981838648388344,0.835656135403946
"1654","POLN",3.75913209257051,1.40206773406798,0.851634186575044,1.64632627032808,0.0996966036785375,0.835656135403946
"1655","ADD1",199.617613599603,0.28519780093716,0.173037638040049,1.64

818362159539,0.0993149967228512,0.835656135403946
"1656","C4orf46",15.1461391161573,0.731968103300244,0.442017801053635,
1.65596974048433,0.0977279468700274,0.835656135403946
"1657","DDX60",33.3897371771094,0.465379047083878,0.281170772317433,1.
65514730869139,0.0978946204410345,0.835656135403946
"1658","STOX2",3.85992404911867,1.53791653696946,0.925096004023583,1.6
6243993086176,0.0964245981454384,0.835656135403946
"1659","AC022424.1",2.34634633617993,1.95449879690504,1.18446383175651
,1.65011268770157,0.0989198902113228,0.835656135403946
"1660","HMGCR",19.4885462301975,-0.566467225255541,0.343170620769744,-
1.65068683322872,0.0988025377516568,0.835656135403946
"1661","DHFR",20.623653506854,0.590875362756725,0.356987262568373,1.65
517211596186,0.0978895896935167,0.835656135403946
"1662","NR2F1",10.879186832497,1.2325608757392,0.747906874840406,1.648
01383327599,0.0993498325517406,0.835656135403946
"1663","AC011379.2",7.99577792000222,0.995731415727669,0.6025073370166
69,1.6526461248722,0.0984029049517489,0.835656135403946
"1664","CCDC69",67.1003431644438,0.540358129275728,0.325631954283633,1
.65941370976469,0.097032455551336,0.835656135403946
"1665","GFPT2",289.354887985527,0.526863612968347,0.319577404260298,1.
64862598526901,0.0992242818068251,0.835656135403946
"1666","CARD11",3.57746831084673,1.49492082200332,0.900244137429244,1.
66057268228621,0.0967992992209455,0.835656135403946
"1667","INMT",81.3668457041653,0.752981045675415,0.456520059844869,1.6
4939311961732,0.0990671233800521,0.835656135403946
"1668","BCL7B",67.3478257171311,-0.437926425053184,0.264656639802612,-
1.65469653578237,0.0979860701381447,0.835656135403946
"1669","SERPINE1",123.208773045822,-1.18408165631634,0.713379562233227
,-1.65981998784712,0.0969506714819194,0.835656135403946
"1670","WNT2",30.3017742102116,-0.743316208327291,0.451122386014979,-1
.64770410728988,0.0994134048015933,0.835656135403946
"1671","CA13",4.44242299801421,-1.16013822629698,0.703695127146019,-1.
64863757264052,0.0992219064893111,0.835656135403946
"1672","ZNF618",47.8493771922318,0.471925404684909,0.286701427761468,1
.64605181205287,0.0997530922712913,0.835656135403946
"1673","SNHG7",266.380264250435,-0.439383236001224,0.266272878266933,-
1.65012388366776,0.0989176007489444,0.835656135403946
"1674","ARHGAP12",95.9415207659433,0.32908916469308,0.198432810171429,
1.65844128503131,0.0972284293897974,0.835656135403946
"1675","ACTA2",105.82452064585,0.568655684717854,0.345257874283999,1.6
4704624303541,0.099548541223958,0.835656135403946
"1676","PSD",4.05354711893987,-1.51892763167404,0.918948243266055,-1.6
5289791106796,0.0983516423601228,0.835656135403946
"1677","EBF3",92.1722799770307,0.535571748484446,0.323404668735006,1.6
5604210532683,0.0977132923119302,0.835656135403946
"1678","SF3B2",240.942354084508,-0.280369402845408,0.170140086620249,-
1.64787386920279,0.099378556607457,0.835656135403946
"1679","ACER3",47.2545255961193,0.509837333165949,0.309514274150225,1.
64721751384718,0.0995133452065177,0.835656135403946
"1680","SLC37A4",8.71959114927444,-0.988449371485376,0.5959791604459,-

1.65853009146467,0.0972105190112142,0.835656135403946
"1681","APLP2",562.497223791367,0.31494076109727,0.191145075657356,1.64765301964581,0.0994238938215553,0.835656135403946
"1682","IGFBP6",2943.06543545882,-0.382490375909799,0.230140358036641,-1.66198740270015,0.0965153003372369,0.835656135403946
"1683","PPP1R1A",10.2622283773793,-1.24473848426441,0.755832871356753,-1.64684354363956,0.0995902086212054,0.835656135403946
"1684","RASSF9",15.899464666519,-0.663134884942948,0.398774272325043,-1.66293296976397,0.0963258538505065,0.835656135403946
"1685","DCN",24596.2966589918,0.314249796702624,0.189299718298682,1.66006478787672,0.0969014196798207,0.835656135403946
"1686","HCFC2",47.2668047592614,0.410092477737977,0.247447059893145,1.65729379817674,0.0974600905613494,0.835656135403946
"1687","AC130895.1",3.03059652906228,1.64347447317193,0.988251012141037,1.66301319500939,0.096309794244789,0.835656135403946
"1688","AL355001.2",9.94980673394616,-0.816761748418888,0.495707750836916,-1.6476678991602,0.0994208387544868,0.835656135403946
"1689","INTS6",249.3850280966,0.467601674672771,0.282381920994615,1.65591930611481,0.0977381613405439,0.835656135403946
"1690","LMO7",15.7946462621742,1.00788939828225,0.607328277126333,1.65954630509094,0.0970057579551395,0.835656135403946
"1691","TUFM",255.757277974922,-0.411680658210736,0.25010420023253,-1.64603656327236,0.0997562315009757,0.835656135403946
"1692","ZNF594",3.41132763709921,1.40501373541542,0.848599636128903,1.65568505523374,0.0977856153444421,0.835656135403946
"1693","COPS3",79.2652179104672,-0.465840911154349,0.282306449754372,-1.65012493182379,0.0989173864137535,0.835656135403946
"1694","KSR1",26.1781452525294,0.64982642364675,0.393543898379371,1.65121712297601,0.0986942479479749,0.835656135403946
"1695","KRT24",3.68650775150236,1.53998749040341,0.929633941925949,1.65655256434911,0.0976099694569552,0.835656135403946
"1696","TBX21",5.2095783872614,1.16513172233405,0.707743542388991,1.64626259732607,0.0997097064871979,0.835656135403946
"1697","AC015813.1",8.26261056474284,1.01522480308814,0.615862926986031,1.64845902976584,0.0992585113730004,0.835656135403946
"1698","RNF126",56.0793403792298,-0.477163442795718,0.287105305480146,-1.66198058234321,0.0965166678930369,0.835656135403946
"1699","SIN3B",41.8542020322224,0.505587531404949,0.305020010788795,1.65755528661047,0.0974072610137657,0.835656135403946
"1700","MED29",91.0927878013083,0.359469481150567,0.217650238994481,1.65159240261474,0.0986176698381374,0.835656135403946
"1701","SNTA1",46.8118547327716,-0.44232319993443,0.268240005902185,-1.64898296377061,0.099151124931004,0.835656135403946
"1702","ZNFX1",106.606728057488,0.380833444414197,0.229769623451773,1.65745775569907,0.0974269628941454,0.835656135403946
"1703","PMM1",81.5349256236752,-0.433430398067061,0.262093229254604,-1.65372603977501,0.0981831893164394,0.835656135403946
"1704","NRXN2",20.8957847791658,-0.847478971354477,0.51506120523651,-1.64539468851148,0.0998884441923563,0.836272620216053
"1705","EPB41L4A-

AS1",235.884190221906,-0.338462431932593,0.205764013874759,-1.64490585
870182,0.0999892265527648,0.836625399414512
"1706","ACTR1B",34.5633319571943,-0.48881198900072,0.297285543794172,-
1.64425078583422,0.100124410473422,0.836890292486645
"1707","HOXB7",8.68340254592684,-0.971748652545732,0.591021858688282,-
1.64418394727809,0.100138211781488,0.836890292486645
"1708","CHRAC1",69.0378315113362,-0.354396815332376,0.215615408079276,
-1.64365255010938,0.100247992484675,0.837317248703964
"1709","AC020656.1",2.59635194262631,-1.85164249293155,1.1276818492184
,-1.64199015370774,0.100592044654251,0.838717772669518
"1710","IFI30",82.3529834998349,-0.531348482881915,0.323571001865367,-
1.64213875723944,0.100561251182559,0.838717772669518
"1711","LBP",35.5438626672059,-0.943334099567362,0.574325983754589,-1.
64250639227643,0.100485102510866,0.838717772669518
"1712","IQSEC1",66.7861392759136,0.541429672227892,0.329886327126959,1
.64126133066291,0.100743179447909,0.839487265189177
"1713","LINC00893",5.81366510193434,1.21831015114033,0.742600944635913
,1.6405987090922,0.100880743144425,0.840142838119303
"1714","SLC7A6OS",44.826433580524,0.511229934006708,0.311780431442996,
1.639711420119,0.101065183309294,0.84069732075241
"1715","AC002398.2",2.17269607868091,2.02950058447346,1.23769886856429
,1.63973696350522,0.101059869869814,0.84069732075241
"1716","ANTXR1",182.8623089667,0.387687066567757,0.23679842813305,1.63
720287176031,0.10158808721013,0.841142198767436
"1717","OCIAD2",9.94825560942853,-0.806932845519387,0.492841087522662,
-1.63730838590498,0.101566049597298,0.841142198767436
"1718","SAP30L-
AS1",1.86292174417731,-1.87253580418606,1.14266216900052,-1.6387484026
2188,0.101265669013282,0.841142198767436
"1719","AC127502.2",22.192215405305,-0.580970551896281,0.3545747813069
24,-1.63849935902065,0.101317567650231,0.841142198767436
"1720","SNAI3-
AS1",3.48314058699516,1.41217912733178,0.861825743210359,1.63859009603
417,0.101298656350795,0.841142198767436
"1721","AC005921.2",26.4554235056844,-0.798446923519258,0.487692901599
426,-1.63719201345907,0.101590355283632,0.841142198767436
"1722","CEP131",24.9525724272907,0.772303686763651,0.471652510626323,1
.63744211970394,0.10153812351676,0.841142198767436
"1723","C19orf54",6.15476163984411,1.01473836495039,0.619616967586042,
1.63768653544737,0.101487100806647,0.841142198767436
"1724","LMO2",16.2352351399195,-0.849770108231621,0.51930274631277,-1.
63636744512769,0.101762708295569,0.841592345822947
"1725","ZNHIT3",82.6890707091719,-0.40115567742471,0.245146303928644,-
1.63639292535072,0.101757378882169,0.841592345822947
"1726","TCEAL7",32.4607187156723,-0.604606869050932,0.369569761629564,
-1.63597494119921,0.101844832034195,0.841783530590861
"1727","KLHL20",58.2852086212448,0.362718094701673,0.221980130945667,1
.63401153588135,0.102256428378735,0.842090806029377
"1728","SMARCAD1",39.7318290810529,0.431567180487533,0.263910997237438
,1.63527547167448,0.101991313074077,0.842090806029377

"1729","KIF3A",31.7790562029687,-0.506203027676608,0.309705644958743,-1.63446496993635,0.102161255865504,0.842090806029377
"1730","ZNF138",27.7776325970541,0.491356691325658,0.300666844740606,1.63422306090838,0.102212022036579,0.842090806029377
"1731","HILPDA",151.385772436266,0.731612340746122,0.447790608864321,1.63382689646311,0.102295203059646,0.842090806029377
"1732","TPI1",458.500426067106,-0.346747555578968,0.212105799344012,-1.63478583165273,0.102093951924458,0.842090806029377
"1733","IGHG2",15.2254849922109,1.20064977080415,0.734426778402713,1.634812082173,0.102088447178392,0.842090806029377
"1734","KDM5A",146.75972976512,0.395748122289557,0.242500009050724,1.63195095884214,0.102689817680244,0.844851752610362
"1735","NES",27.5975027068885,-0.562664860471845,0.345288095978197,-1.62955186415513,0.103196244936868,0.845137935706933
"1736","PARP9",85.0855843365936,0.380020481002614,0.233247145464697,1.62926101515841,0.103257775265469,0.845137935706933
"1737","AREG",60.7025883316467,-1.19337236118392,0.731715951077136,-1.63092298237751,0.10290657166019,0.845137935706933
"1738","VPS13B",50.028277332509,0.462616644879679,0.283853427664852,1.62977297362741,0.103149487805837,0.845137935706933
"1739","ERMP1",6.31604278569596,1.06218689880395,0.651377177448423,1.63067871515664,0.102958130090871,0.845137935706933
"1740","IFNG",6.83866757276478,1.12431704680254,0.689889308663346,1.62970643650196,0.103163556372074,0.845137935706933
"1741","INO80C",18.8693685728486,-0.705040523263622,0.432734054298538,-1.62926979344506,0.103255917755221,0.845137935706933
"1742","TXNL4A",146.673658569139,-0.390813202358556,0.239700924818939,-1.63042008558691,0.103012742426455,0.845137935706933
"1743","UBE2S",131.774028729485,0.445909561085858,0.273392107595293,1.63102572714332,0.102884891059436,0.845137935706933
"1744","ZNF91",73.4975512138576,0.479878230379436,0.294649947122303,1.62863844051616,0.103389581588392,0.845731520034402
"1745","AC019270.1",4.53095910006273,1.47061432148651,0.903328825857159,1.62799445715801,0.103526061076286,0.846362628833407
"1746","FRMD8",21.2892227445099,-0.56205937839926,0.345376673647482,-1.62738083166827,0.103656240011638,0.846456737267331
"1747","TOX2",3.10020753558433,-1.37680971048144,0.845945385186632,-1.62753971425435,0.103622521039306,0.846456737267331
"1748","ATG2B",36.0622377099875,0.538380938388486,0.330893491694815,1.62705206328155,0.103726040812433,0.846542161458909
"1749","AKR1B1",107.124288548516,-0.326080921183538,0.200540270657814,-1.6260121725872,0.103947065906126,0.846892542670931
"1750","ZNF280D",107.593030010297,0.338370789444273,0.208034517710302,1.62651272091044,0.103840629462272,0.846892542670931
"1751","KLF16",16.1071297936706,-0.617092644007002,0.379497902352293,-1.62607656111402,0.103933369493199,0.846892542670931
"1752","NMD3",64.0480048735508,-0.388662201304246,0.23928298842321,-1.62427844898374,0.104316394578527,0.847185704823342
"1753","POU5F2",12.9139689790673,0.829612752058191,0.51056602222232,1.62488829250166,0.104186363237688,0.847185704823342

"1754","USP54",10.7991137164868,0.779287652272703,0.479806462440555,1.62417081318335,0.10433935815047,0.847185704823342
"1755","AC004585.1",2.67311204661449,-1.69221101057237,1.0418614950957,-1.62421878391516,0.104329123333393,0.847185704823342
"1756","CNN2",108.633817828815,0.435141929468636,0.267873600166307,1.62443006402453,0.104284055060344,0.847185704823342
"1757","MAGT1",124.458424077475,0.332590601585769,0.204676566520645,1.6249569124574,0.104171740096189,0.847185704823342
"1758","DLL1",7.35775151330296,0.948946331155748,0.584544968581941,1.62339320695517,0.104505375939107,0.847568898889884
"1759","DOCK9",26.7700281994756,0.538982117487829,0.331986727617316,1.62350501586654,0.104481491995797,0.847568898889884
"1760","NDUFAF6",18.7274213816313,-0.635967666589652,0.391832897707618,-1.62305837593097,0.104576926451636,0.84760545749925
"1761","ASCL2",3.39893122753295,1.46271937553131,0.901346326775065,1.62281614966446,0.104628712369002,0.84760545749925
"1762","FBLL1",3.37805511240979,1.60829891421145,0.991244053688662,1.62250548512909,0.104695159609403,0.847662398971474
"1763","Z97200.1",46.2117520206566,0.530941831708241,0.327455805143695,1.62141523640191,0.104928615268306,0.847961610116502
"1764","MTDH",572.685838881171,-0.242000460148212,0.149270332068995,-1.62122276271453,0.104969872666882,0.847961610116502
"1765","FJX1",21.3700652554516,-0.7391547429408,0.455909938443074,-1.6212735907118,0.104958976259331,0.847961610116502
"1766","ACVR1B",42.2418076053712,0.487074962078951,0.300289188830768,1.62201964038555,0.104799142863437,0.847961610116502
"1767","TLR5",30.0703743864677,0.642260136630527,0.396231497375519,1.62092145850243,0.105034484119839,0.848003367545911
"1768","ITIH4",8.63259226560035,1.24575768933144,0.770493353574782,1.61683119465161,0.105914723818266,0.849872093452207
"1769","SUCLG2-AS1",18.001063929144,0.709640481852546,0.43885795364196,1.61701633971411,0.105874753902926,0.849872093452207
"1770","WDFY3-AS2",46.0089982506097,0.446952174196037,0.276270612324583,1.61780571026108,0.105704475403365,0.849872093452207
"1771","TARS",103.097925472696,0.342464648230035,0.211646759222351,1.61809540334256,0.105642038988689,0.849872093452207
"1772","SKP2",13.5249114381107,0.6829065457517,0.421991654684697,1.61829395953802,0.105599261850855,0.849872093452207
"1773","THOC3",29.3756832329497,-0.49367575547863,0.305272246753984,-1.61716553249755,0.105842554225996,0.849872093452207
"1774","CYP51A1",67.9291953543105,-0.369675415175269,0.228461728476666,-1.61810653206638,0.105639641042122,0.849872093452207
"1775","DCAF12",27.3145649837764,0.503679688760017,0.311137130045649,1.61883504127623,0.105482760426305,0.849872093452207
"1776","WDR34",31.0033066194131,-0.462320814690458,0.285996677106655,-1.61652512668197,0.105980825336568,0.849872093452207
"1777","POGLUT3",141.659370236642,0.340941013978658,0.210783916707429,1.61749064778927,0.105772412809339,0.849872093452207

"1778","AC008014.1",3.49667294160893,1.38097339358326,0.85420816434372
,1.61667079668367,0.105949360898273,0.849872093452207
"1779","MEG9",3.97485234918534,1.81517653838347,1.12268953549525,1.616
81077536966,0.105919132753632,0.849872093452207
"1780","CCDC66",92.2552881914789,-0.370668297191567,0.22947383358042,-
1.61529657394103,0.106246485240161,0.850941955018655
"1781","NPY1R",26.20944208273,1.00925547481849,0.624894701167512,1.615
08086551681,0.106293184063034,0.850941955018655
"1782","RNASE6",3.02969438166007,-1.3639925535392,0.844507874095527,-1
.61513302051807,0.106281891509749,0.850941955018655
"1783","KIAA1324L",12.4658532050213,-0.751626363944232,0.4657004027811
45,-1.61396975277571,0.106533987263592,0.850959609351849
"1784","BMP1",94.9798911034071,0.391633632607828,0.242529352383094,1.6
1478859676009,0.106356483430652,0.850959609351849
"1785","AL583785.1",19.3859224142664,0.852899259099084,0.5283609821028
64,1.61423588794268,0.106476270421535,0.850959609351849
"1786","ARRB2",19.4949386239005,-0.667533936683184,0.413581721255819,-
1.61403152599746,0.106520588270239,0.850959609351849
"1787","CHMP4B",166.388209218482,-0.322841659471294,0.200094864938842,
-1.61344300149816,0.106648297143803,0.851395974848069
"1788","WEE2-
AS1",2.59482620889573,1.68297010841683,1.0434892873709,1.6128293110292
7,0.106781596209999,0.85198336215428
"1789","DHX57",21.6590214833448,0.609102827178626,0.377892940787535,1.
61183965466316,0.106996836546563,0.852746854845397
"1790","USP51",17.9489117179188,0.6060756969402,0.375982206920846,1.61
197973144457,0.1069663503829,0.852746854845397
"1791","CEBPG",61.5345014555294,-0.511281250660231,0.317268216950962,-
1.6115110916996,0.107068371657392,0.852840530465856
"1792","NBPF9",16.2110882734002,0.946447889733457,0.587885348049152,1.
60991916684803,0.107415504092109,0.852921479054867
"1793","C1orf21",278.685792140417,0.34145124607303,0.21202246448491,1.
6104484348041,0.107299994038947,0.852921479054867
"1794","AP2M1",378.106039488245,-0.296800406339206,0.184339487552994,-
1.61007503210011,0.107381477062949,0.852921479054867
"1795","PDZRN4",5.67287094122813,1.39073013414862,0.863309318328016,1.
61092913585373,0.107195168751045,0.852921479054867
"1796","SOX9",59.9457088848566,-0.635682676411937,0.394878219075035,-1
.60981954867241,0.107437256263956,0.852921479054867
"1797","RRP7A",79.8188035791014,-0.525656513161682,0.326390231296854,-
1.610515458974,0.107285373374571,0.852921479054867
"1798","COL24A1",6.19724603341834,1.31829750439653,0.824526926246294,1
.59885318772809,0.109853227644037,0.85312297714767
"1799","RPAP2",59.2111243880496,0.388750927444278,0.242100493391428,1.
60574198754625,0.108330611740484,0.85312297714767
"1800","GSTM3",238.293075626063,0.493984283892437,0.308133329019204,1.
60315109522492,0.108901299572725,0.85312297714767
"1801","ADAM15",53.6580603072066,-0.405261977350023,0.25306389575483,-
1.6014215545889,0.109283581831522,0.85312297714767
"1802","LINC01341",4.45798912352782,1.12563912879089,0.701846602114309

,1.60382500307034,0.108752631653453,0.85312297714767
"1803","FNDC4",82.3415099558521,-0.517564115099894,0.322065799809272,-1.60701358357949,0.108051388371162,0.85312297714767
"1804","ACTR3",225.737797619984,-0.28397835009911,0.177494552945932,-1.59992712669676,0.109614750676857,0.85312297714767
"1805","TEX41",4.44325054857345,-1.16468607467573,0.725202397739951,-1.60601520114303,0.108270570028679,0.85312297714767
"1806","LINC01117",5.17451039081764,1.06095197210309,0.663100879048616,1.59998577233873,0.109601739723169,0.85312297714767
"1807","PTH2R",2.2924216065237,1.8387926067315,1.14893533517252,1.60043176533987,0.109502832935517,0.85312297714767
"1808","SUMF1",61.7438350199356,0.404881848866807,0.253166951757838,1.59926817483701,0.109761027786608,0.85312297714767
"1809","RFTN1",170.981023222394,0.429216054986293,0.266959822249524,1.60779270591928,0.107880586483479,0.85312297714767
"1810","GPD1L",18.6215640131832,0.660932039735934,0.413186773896985,1.5995963121044,0.109688167121319,0.85312297714767
"1811","ARHGAP31",7.78555133928242,-0.948328659782341,0.591129091293164,-1.60426660394561,0.108655299083826,0.85312297714767
"1812","ATP11B",44.3963258038406,0.476221461615274,0.296760746095155,1.60473198656326,0.108552799414937,0.85312297714767
"1813","CTBP1-AS",1.8988153882775,2.00604662117361,1.24964821395828,1.60528907156953,0.108430203082118,0.85312297714767
"1814","CLIC1",791.998246264911,-0.412976981252848,0.258100611826988,-1.60006200035542,0.109584829818971,0.85312297714767
"1815","HLA-DPB1",163.2968534079,-0.369279304925686,0.229479919156709,-1.6092009544134,0.107572407798548,0.85312297714767
"1816","BMPER",59.2579632917775,0.543687200656935,0.33950476704875,1.60141256743787,0.109285571038866,0.85312297714767
"1817","AC087071.1",2.21554304552318,1.84634314045861,1.15259866845547,1.60189595128788,0.109178619958415,0.85312297714767
"1818","GIMAP1",6.0598667443797,-0.945473048071563,0.588116369457284,-1.60762919920772,0.107916413255453,0.85312297714767
"1819","TMUB1",34.2434397944348,-0.525413392135275,0.327595636307825,-1.60384734686017,0.10874770523703,0.85312297714767
"1820","SLA",30.8677569921432,0.726083227717135,0.454217042835337,1.59853805393285,0.109923283446687,0.85312297714767
"1821","AL158071.1",12.5747282981199,0.898459185541387,0.560128408223873,1.60402359949987,0.108708850788836,0.85312297714767
"1822","ECPAS",73.2024421850768,0.352203121626069,0.219881870413098,1.60178336196784,0.109203523508523,0.85312297714767
"1823","ZEB1-AS1",22.181227220783,0.534135729254759,0.332379438847032,1.60700593005267,0.108053067264808,0.85312297714767
"1824","CWF19L1",11.099436300215,0.723382761762995,0.451846476385356,1.60094810863604,0.109388412879954,0.85312297714767
"1825","WDR11",67.0295524459851,0.466517314978966,0.29125659624715,1.60173991246913,0.109213135272772,0.85312297714767

"1826","TMEM223",66.7576952827731,-0.485280624372376,0.303407542060298
,-1.59943494178511,0.109723993532799,0.85312297714767
"1827","ALOX5AP",32.8955525172166,0.590964218645302,0.367401515984047,
1.60849695206745,0.107726383205024,0.85312297714767
"1828","NDRG2",64.9676027333093,-0.36219987944359,0.226637635477807,-1
.59814533309962,0.110010636639179,0.85312297714767
"1829","HEATR5A",40.0199994544644,0.460590507259398,0.287675979864062,
1.60107391474619,0.109360548966362,0.85312297714767
"1830","DYNC1H1",282.782217249859,0.290241196243636,0.180668991871035,
1.60648041059982,0.108168395705679,0.85312297714767
"1831","GPR132",4.12408642293793,1.41630619538645,0.885813678280214,1.
59887596016374,0.109848166586167,0.85312297714767
"1832","AC022467.1",5.86356696124141,1.24014101422203,0.77154133170318
9,1.60735525533596,0.107976459575174,0.85312297714767
"1833","SLC38A7",17.5905042115411,0.617687071616922,0.384780594271006,
1.60529683880544,0.108428494540293,0.85312297714767
"1834","MYH10",77.4627620788729,0.539000141316131,0.335746023580043,1.
60538056584796,0.108410078639091,0.85312297714767
"1835","IKZF3",19.1562601598494,0.850000583286215,0.529793097316372,1.
60440101539984,0.108625687346575,0.85312297714767
"1836","PSMC5",309.11454564614,-0.443128606912085,0.277202418242948,-1
.59857410235041,0.109915267917191,0.85312297714767
"1837","WDR7",17.0958872364365,-0.614195533960657,0.384407668397361,-1
.59777128411956,0.11009388762995,0.85312297714767
"1838","ERCC1",127.567528001639,-0.397263349083396,0.248535784189802,-
1.5984150949467,0.109950627411038,0.85312297714767
"1839","ZNF587",22.0490375192155,0.618507336300393,0.386556016364058,1
.60004581513972,0.109588420064554,0.85312297714767
"1840","KLHL22",22.8908332917063,0.509481983989564,0.318855148756011,1
.59784775619045,0.110076863417302,0.85312297714767
"1841","CLCN4",14.9746488555795,0.825336466527521,0.515321657520432,1.
60159476024894,0.109245250250163,0.85312297714767
"1842","IMPDH1",32.8429044164002,-0.48165433939813,0.301528641400312,-
1.597375085701,0.110182122634359,0.853343192997702
"1843","CCSER2",132.263094261107,0.31868752103408,0.199562276112583,1.
59693268308031,0.110280713514989,0.853643330984716
"1844","LUM",4376.77396784165,-0.429459921419321,0.269136935940156,-1.
59569298773177,0.110557354990058,0.855320621631324
"1845","PAGR1",55.5816042265134,-0.404661349472102,0.253652701543196,-
1.59533624917135,0.110637063662738,0.855473360548844
"1846","EVA1C",30.0075149357772,-0.594838496049773,0.372978402998302,-
1.59483361842933,0.11074944708972,0.855878446469095
"1847","TPT1-
AS1",65.1046664740822,0.419267325447731,0.263099160044298,1.5935715088
4533,0.111032040094153,0.857597771512282
"1848","NFE2L1",169.050107920214,0.425962948026154,0.267426360153321,1
.59282333941179,0.111199827734598,0.858428973193605
"1849","MAL",2.81603932265313,-1.58440711343604,0.994915381351983,-1.5
9250439095936,0.111271417280671,0.858457424799715
"1850","PDE1B",8.0598435903361,-1.05920695910098,0.665217836529623,-1.

59227083360657,0.111323863443115,0.858457424799715
"1851","FXYD2",2.89538046406031,-1.73367349287681,1.08946408118187,-1.59130853675881,0.111540156553911,0.859660655536518
"1852","BMS1",59.933672162252,-0.445494316295693,0.280064654617507,-1.59068382586199,0.111680748715123,0.860219401969764
"1853","VSIR",230.058049662756,0.453901793215869,0.285480664802951,1.58995634092826,0.111844646502598,0.860219401969764
"1854","RTN1",2.55489463760684,-1.54958117375277,0.974664231517811,-1.58986153758784,0.111866019067286,0.860219401969764
"1855","GPR108",80.1702102004399,-0.40364652204784,0.253921933753911,-1.58964810987551,0.111914146225703,0.860219401969764
"1856","POFUT1",36.5349679458073,0.442224943698301,0.278172409307693,1.58975127978687,0.111890879753989,0.860219401969764
"1857","TIMP4",24.1575512742973,-0.654798306967504,0.412761200410315,-1.58638531508433,0.112651929553579,0.860721967754195
"1858","PARP14",237.107952894269,0.361482049520751,0.22786552241375,1.58638325663145,0.112652396218295,0.860721967754195
"1859","HES1",108.491375761334,-0.436283605857963,0.27462591290998,-1.58864690238089,0.112140132860199,0.860721967754195
"1860","CHIC2",95.5795240366622,-0.365129751955938,0.23019763208079,-1.58615772306377,0.112703535382366,0.860721967754195
"1861","TMEM30A",126.477299427846,0.333796683735794,0.21042976215756,1.58626175457948,0.112679944227,0.860721967754195
"1862","AC108134.2",9.97487964402805,1.12807279858259,0.710893193473854,1.58683865443997,0.112549191678766,0.860721967754195
"1863","KIFC3",16.0512825817099,-0.786887362635566,0.496095130201295,-1.58616223931946,0.112702511153332,0.860721967754195
"1864","BCL2",39.0855570562272,0.478499983425192,0.301322125929952,1.58800148495045,0.112286003337782,0.860721967754195
"1865","CCDC61",19.0638265303935,0.58476693200796,0.368657758198877,1.58620541410796,0.112692720033781,0.860721967754195
"1866","TM9SF4",57.7760390495031,0.371861997149393,0.234013057588529,1.5890651614973,0.112045682156015,0.860721967754195
"1867","TAFA5",10.3986913086956,1.05189009719969,0.662438917942186,1.58790504106749,0.112307813416308,0.860721967754195
"1868","XIST",954.512481020908,0.466902230323007,0.294035202884115,1.58791269121277,0.112306083269956,0.860721967754195
"1869","TSPAN5",13.8678245519533,-1.14722618371542,0.72359202855939,-1.58545995317203,0.112861868827738,0.861469995022213
"1870","GSAP",8.98194592029253,-0.827504754753319,0.522302335969367,-1.58434052035687,0.113116249477681,0.862949954571442
"1871","PTK2B",16.2275138827802,0.729381896558953,0.46054985182299,1.58371975079755,0.113257508164742,0.863565799828014
"1872","PAWR",40.4068321429887,0.856040808081515,0.540700175124651,1.58320793568111,0.113374078316349,0.863696352669807
"1873","AC106739.1",15.8354249324068,-0.80482132625945,0.508378956342717,-1.58311298337237,0.11339571488508,0.863696352669807
"1874","ZBTB40",20.653631562157,0.57586515689355,0.365321054967864,1.57632621789129,0.114950642465042,0.86425059212316
"1875","LSM10",113.284956444226,-0.453798009843065,0.287454499504268,-

1.5786777059523,0.114410000966605,0.86425059212316
"1876","PPM1G",150.753402655618,-0.317395088285367,0.201414808119015,-1.57582797039342,0.115065454475212,0.86425059212316
"1877","BOK",19.6900518689213,-0.569937422760904,0.360787270288215,-1.57970491116721,0.114174460406527,0.86425059212316
"1878","RBM5",138.278001004384,0.384178943589447,0.243861614607257,1.57539735890035,0.115164753653871,0.86425059212316
"1879","RASSF1",60.011571209856,-0.383047207352447,0.242926509715997,-1.57680282732529,0.114840900902152,0.86425059212316
"1880","AC108673.3",5.10572713042501,-1.05435013410557,0.668701185020968,-1.57671342256184,0.1148614804862,0.86425059212316
"1881","HIVEP1",81.3950163503429,0.403380076368856,0.255956605514572,1.57597056562734,0.115032586805802,0.86425059212316
"1882","HLA-DQB1",43.0585706936044,-0.62646457966612,0.396279925758257,-1.58086377569446,0.113909188496107,0.86425059212316
"1883","VNN2",3.09922410560149,1.65219260560682,1.0484712782737,1.57581103063418,0.115069359517352,0.86425059212316
"1884","MBLAC1",7.6466227132628,-0.880850901983373,0.557890308826856,-1.57889622394704,0.114359862281049,0.86425059212316
"1885","FLNC",51.7024272666439,0.530542998892677,0.336678917019953,1.57581295433844,0.115068916049711,0.86425059212316
"1886","ZNF775",13.6825784448662,-0.659124323392692,0.418187076145813,-1.576147042772,0.114991919708391,0.86425059212316
"1887","SH2D4A",32.3379367525977,0.496387260090036,0.314231003903978,1.57968899924885,0.114178106133623,0.86425059212316
"1888","PIWIL2",4.42730979648531,1.30915582494246,0.829616066376289,1.57802612316896,0.114559608548427,0.86425059212316
"1889","AKR1C3",249.278191418132,-0.481933248368847,0.305143285465813,-1.57936704270965,0.114251892268103,0.86425059212316
"1890","HSD17B12",126.161770106049,-0.277899686258898,0.176289920824092,-1.57637875699199,0.114938541045869,0.86425059212316
"1891","NOC4L",41.7573876090072,-0.496202813062879,0.313773878740171,-1.58140255350502,0.113786023993179,0.86425059212316
"1892","ITGBL1",93.9107101529553,-0.643695287992427,0.407735857312449,-1.57870659753911,0.114403370835151,0.86425059212316
"1893","ZNF668",14.5070384814765,-0.666762432039098,0.421829904318068,-1.58064287338042,0.113959717055037,0.86425059212316
"1894","CAMTA2",32.1320584291984,-0.500259009324801,0.316644211153281,-1.5798773250986,0.11413496292843,0.86425059212316
"1895","KRT27",13.2946755471899,1.15954452775319,0.733794521635116,1.58020330428384,0.114060315320002,0.86425059212316
"1896","SAMD1",57.6535871717372,-0.392455380288462,0.249101962750606,-1.57548088322924,0.115145487644147,0.86425059212316
"1897","ZNF525",7.59059593412592,-0.839198181223723,0.531613940926145,-1.57858573039248,0.114431109818735,0.86425059212316
"1898","C20orf27",51.8004310814526,-0.562987832642292,0.356171787693294,-1.5806637473687,0.113954941644207,0.86425059212316
"1899","MORC2-AS1",5.87746532585448,-0.921191487630793,0.583431624931973,-1.57891935

96391,0.114354554837514,0.86425059212316
"1900","PATZ1",21.8429774302812,0.51000232239056,0.323724568733242,1.5
7542050140413,0.115159415270241,0.86425059212316
"1901","PRRG3",49.2218010478923,0.606452356493103,0.383890178493522,1.
57975481131861,0.114163027914637,0.86425059212316
"1902","PTGDS",1009.54812947743,1.55877547846133,0.990040602946375,1.5
7445611202449,0.115382040217251,0.865425965162623
"1903","NADK2",38.0227259225753,-0.436725362905708,0.277623745301266,-
1.57308360793055,0.115699459999919,0.865734918540948
"1904","PPP1R11",46.8195711851941,-0.387203332270678,0.246038893632044
,-1.5737484694178,0.115545611494335,0.865734918540948
"1905","GLT8D2",166.112308866074,0.458506474932056,0.291318249384797,1
.57390234185577,0.115510028445098,0.865734918540948
"1906","CMTR2",27.0018296975909,0.534567118833914,0.339769530571682,1.
57332271064587,0.115644113258148,0.865734918540948
"1907","APPBP2",79.3594897804686,0.344124753891883,0.218774418319086,1
.57296614721184,0.115726657062778,0.865734918540948
"1908","PLA2R1",66.7963017366652,0.533324117558142,0.339296796497455,1
.57185132032964,0.115985036326472,0.866758789016996
"1909","PHKB",67.8982967990585,0.383357758959041,0.243853595174295,1.5
7208163646321,0.115931619691972,0.866758789016996
"1910","MAPK13",20.4877126790191,0.620069621245966,0.394734647929193,1
.57085177219405,0.116217083150729,0.868038171847277
"1911","RSRP1",359.766552834899,0.362965272492617,0.231704935683589,1.
56649780213691,0.117232122716623,0.868417555495217
"1912","CTPS1",25.2648690012816,-0.528070274460295,0.336760442917709,-
1.56808878704716,0.116860413003281,0.868417555495217
"1913","PLK3",119.611143113933,-0.482823379612642,0.307965631186875,-1
.56778331969018,0.116931708975917,0.868417555495217
"1914","VASH2",26.3836520507095,0.622708896900164,0.396546038051883,1.
57033190889853,0.116337914429577,0.868417555495217
"1915","CALCRL",9.13812789648039,-0.869080809349703,0.555067221702477,
-1.5657217276929,0.117413777344369,0.868417555495217
"1916","SRRT",52.0940461092718,-0.461648741529188,0.294530462016381,-1
.56740575616084,0.117019879365049,0.868417555495217
"1917","UBE2H",142.522870228646,0.385859892418217,0.246092866851837,1.
5679442372888,0.116894146609384,0.868417555495217
"1918","NOL8",76.4772760207759,0.386842888887112,0.247059194475367,1.5
6579029454288,0.117397719108023,0.868417555495217
"1919","AC022387.2",2.10521321783907,-1.56766814877113,0.9987280106888
07,-1.56966474554963,0.116493127095946,0.868417555495217
"1920","GRID1",6.37908789129684,1.05828270608012,0.67592697991233,1.56
567608267004,0.117424468284752,0.868417555495217
"1921","SH3PXD2A",154.213470707915,0.479281888224234,0.305721813143112
,1.56770589346164,0.116949785655112,0.868417555495217
"1922","PTPMT1",98.7766250942797,-0.487578991171907,0.310889337904945,
-1.56833616249968,0.116802700681415,0.868417555495217
"1923","PRG2",13.6109326357816,-0.704035668207597,0.448913722696616,-1
.56830952722601,0.116808913575289,0.868417555495217
"1924","CTSC",216.241748221618,0.417259762629483,0.265770556320703,1.5

6999995938594,0.116415120725766,0.868417555495217
"1925","GUCY1A2",27.8609743338255,0.8399630467865,0.535811816172313,1.56764562003682,0.116963859193146,0.868417555495217
"1926","KLRB1",49.0459047054942,-0.582607205414436,0.371951999763936,-1.56635051238922,0.117266581642068,0.868417555495217
"1927","AL627171.2",64.7096086803325,0.617594003821364,0.394288895804828,1.56634896491498,0.117266943721007,0.868417555495217
"1928","SLFN5",226.308993021634,0.438735275704864,0.280104047564223,1.56632965328453,0.117271462341561,0.868417555495217
"1929","AL118522.1",3.5671132400568,-1.26490534595121,0.807355572975974,-1.56672646884529,0.117178641132082,0.868417555495217
"1930","DDR2",446.81030182883,0.391249555411393,0.249933687665485,1.56541344652605,0.117485997557295,0.868422404742158
"1931","SAMD11",19.7074784938218,-0.744747847965505,0.47625232462424,-1.56376737594532,0.117872208418874,0.868597845489652
"1932","TOR1AIP1",117.582222887744,0.334361131155928,0.213951160260466,1.56279185749156,0.118101560019892,0.868597845489652
"1933","FAM71A",9.99112574280969,1.15506727842578,0.739884919767235,1.56114450716087,0.11848965881151,0.868597845489652
"1934","MYO1B",115.232425443937,0.312074997667702,0.199931342724334,1.56091082776347,0.118544792174986,0.868597845489652
"1935","SATB2-
AS1",2.64511835663951,-1.59895315269702,1.02270562025067,-1.56345396078405,0.117945856508029,0.868597845489652
"1936","MSX1",248.156654981084,-0.404490283025892,0.258640700196081,-1.56390808839923,0.117839154739208,0.868597845489652
"1937","HSD17B11",150.530122365904,-0.282730038871781,0.1809480245471,-1.56249309479578,0.118171871317901,0.868597845489652
"1938","MGST2",45.5870174950549,-0.63443276280231,0.40605034192686,-1.56244853727173,0.118182360371003,0.868597845489652
"1939","AGFG2",5.30929400581429,-0.945282628821826,0.60425806634602,-1.56436906922567,0.117730920218143,0.868597845489652
"1940","ERLIN2",50.4771170206093,0.493445239409058,0.316017622281994,1.56144849089694,0.118417968223197,0.868597845489652
"1941","PHB2",287.06301788941,-0.360005861507629,0.230259834617448,-1.56347659202371,0.117940537280723,0.868597845489652
"1942","GLIPR1",103.102160882596,0.355153489611289,0.226980866159177,1.56468470501926,0.117656856523598,0.868597845489652
"1943","MIPEP",14.6230438919677,-0.670234954731485,0.428377334016496,-1.564590144038,0.117679041347421,0.868597845489652
"1944","MLKL",14.6019250752037,-0.651332097430783,0.417003194327002,-1.56193551102639,0.118303181805466,0.868597845489652
"1945","C17orf113",2.07065487596537,1.91279015674114,1.22393629542031,1.56281839495925,0.118095316236411,0.868597845489652
"1946","USP9X",101.552764986507,0.364793202676133,0.233703270352196,1.56092468079878,0.118541523185557,0.868597845489652
"1947","DKC1",78.5251441514559,-0.367842194334229,0.23546565534982,-1.56219043404756,0.118243133465257,0.868597845489652
"1948","AC079848.1",1.85482747800113,-1.73687087682949,1.11368336983995,-1.55957332565637,0.118860744123219,0.870019176840352

"1949","RCE1",25.2608937405644,-0.573895609035442,0.367947881343115,-1
.55971983570216,0.118826102570111,0.870019176840352
"1950","ARL3",98.3361312663603,-0.39558576062513,0.25378942642945,-1.5
5871647684699,0.119063500220239,0.870509581627853
"1951","CEP76",9.6375577580075,0.748628588128736,0.480346837102232,1.5
5851674312036,0.119110802140584,0.870509581627853
"1952","BEX5",4.95021810783764,-1.43810805095194,0.92268489288621,-1.5
5861233021109,0.11908816289983,0.870509581627853
"1953","IRX1",31.7066404854262,-0.64614630753623,0.414792787958481,-1.
55775685184022,0.119290897937152,0.871379390666368
"1954","TIPIN",13.3443493822689,-0.712497699853472,0.457725038466778,-
1.55660634655272,0.119563976072635,0.872799474181612
"1955","SF3A2",45.0321022987611,-0.563600341749456,0.362112700385848,-
1.55642246501963,0.119607666621692,0.872799474181612
"1956","PGGHG",9.06794491831207,0.830042306330473,0.533452610865482,1.
55598133634363,0.119712530498341,0.873118077755283
"1957","AL022069.3",3.9050768450953,-1.24116339002842,0.79808796928941
7,-1.55517115629935,0.119905311812071,0.873630836726761
"1958","CFAP298",109.006251419495,-0.355069006794132,0.228315024843716
,-1.55517144365414,0.119905243393286,0.873630836726761
"1959","TNFRSF25",4.62576717306783,-1.09442394980467,0.70432977219502,
-1.55385160901822,0.120219816794572,0.873812759425956
"1960","GMPPB",39.9147169824667,-0.537200537281667,0.345733263327294,-
1.5538005574347,0.12023199755219,0.873812759425956
"1961","AKT1",86.8901731576524,-0.336092249935072,0.216288152769335,-1
.55390966001501,0.12020596717155,0.873812759425956
"1962","MPI",26.0196900770617,0.499504839563727,0.321307902998887,1.55
459867280469,0.120041680072384,0.873812759425956
"1963","SGCA",9.02599615159612,1.30688376127599,0.841098787739577,1.55
378152997722,0.120236537694739,0.873812759425956
"1964","PAPOLG",22.4131232690057,0.505499940088994,0.325458243462746,1
.55319445809907,0.120376684916793,0.873940858535863
"1965","JCAD",10.3400261153796,-0.9328891231732,0.600601413482765,-1.5
5325828782781,0.120361441135828,0.873940858535863
"1966","TLCD3A",26.1740366595162,0.543559950394056,0.350065550044023,1
.552737623927,0.120485829926286,0.87428832641322
"1967","CISD2",64.7684111049217,-0.351537874526794,0.226489770297444,-
1.55211369619532,0.120635021394454,0.874925884704262
"1968","ARHGEF7",52.7878028804742,0.420136473093541,0.270804199179622,
1.55144002333164,0.12079627004555,0.8756501973932
"1969","AFF4",204.616552688936,0.421340572842363,0.271625783303065,1.5
5118033243646,0.120858474031244,0.875656165835312
"1970","GBP1",401.326289104297,0.555567531763249,0.358567595382194,1.5
4940808628029,0.121283651135947,0.87651473994309
"1971","SLC4A5",3.11295741114073,1.32402281683668,0.857355007098244,1.
54431105653409,0.122512995334819,0.87651473994309
"1972","INO80D",70.8961851733148,0.453811777989224,0.29346955017108,1.
54636751146642,0.122015835778574,0.87651473994309
"1973","CSRNP1",182.164500624854,-0.474949279279148,0.30741963746763,-
1.54495426249131,0.122357326853769,0.87651473994309

"1974","LRCH3",73.8579749881642,0.357920096692883,0.231044332819096,1.54914034170718,0.121347986971693,0.87651473994309
"1975","PRDM6",14.9212676810068,0.804043765599118,0.519353405437509,1.54816307581883,0.121583038887539,0.87651473994309
"1976","LMNB1",33.6209187680359,0.790724722192691,0.511205952448138,1.54678308890175,0.121915559411848,0.87651473994309
"1977","LTB",57.7875900194665,-0.405839987185132,0.262178277317179,-1.54795428262789,0.121633303940211,0.87651473994309
"1978","DTX2",21.6695782482029,0.571694342090062,0.369769449572027,1.54608322226659,0.122084470204107,0.87651473994309
"1979","SLCO5A1",2.3306813730592,1.69237377598739,1.09351934675268,1.54763953743761,0.121709106677766,0.87651473994309
"1980","ZDHHC21",36.9551661789442,0.474284744374923,0.306766050911996,1.54607963614261,0.122085336175592,0.87651473994309
"1981","HEBP1",214.174055569765,-0.365017028455478,0.235879983815164,-1.54746927887491,0.121750126875151,0.87651473994309
"1982","ATG101",92.5320058485483,-0.42673340828844,0.276190065370713,-1.54507153512441,0.122328961239655,0.87651473994309
"1983","PYGO1",25.3791089163124,0.500654166456525,0.323959789975488,1.54542070327434,0.122244535740297,0.87651473994309
"1984","IGDCC4",28.6064604145522,0.627177007003198,0.405792173338006,1.54556210841649,0.122210358295264,0.87651473994309
"1985","PTPN9",36.6183233498305,0.400859789353903,0.259499682054554,1.54474096530736,0.122408931824405,0.87651473994309
"1986","DCUN1D3",93.5143073063788,0.550430094379886,0.355573113384417,1.54800819764121,0.12162032283744,0.87651473994309
"1987","TGFB1I1",115.296460374988,-0.409140293989719,0.264285949338912,-1.54809703282807,0.121598936377302,0.87651473994309
"1988","AC025280.2",2.18795738434304,1.79771404042861,1.16296229644945,1.54580595253782,0.122151439021608,0.87651473994309
"1989","TRIM16",12.0302386580439,-0.732923870310453,0.473858316099578,-1.54671522142588,0.121931931027665,0.87651473994309
"1990","SS18",85.806952947007,0.335922029470279,0.217496494015816,1.54449399743359,0.122468704303035,0.87651473994309
"1991","RTTN",10.197090385832,0.891076120050418,0.575781396719156,1.5475944952856,0.121719957579608,0.87651473994309
"1992","CCDC124",119.830439222801,-0.376323695226635,0.242926651902257,-1.54912477605813,0.121351728032865,0.87651473994309
"1993","ZNF284",5.90567209152236,0.956309496183588,0.618372694453297,1.54649373227752,0.121985372631622,0.87651473994309
"1994","AC011043.1",6.02107903234551,1.04653063376273,0.675373999855018,1.54955718459311,0.121247836152573,0.87651473994309
"1995","CCDC17",2.80347788054956,1.40344713686189,0.909156319052992,1.54368078123657,0.122665684411569,0.876570271956541
"1996","PPP1CA",137.915765537328,-0.33564602965452,0.217463741945149,-1.54345743640878,0.122719827089362,0.876570271956541
"1997","CSNK1G1",46.1987964704703,0.46642847455752,0.302234891259534,1.54326481834617,0.122766536055598,0.876570271956541
"1998","CBARP",2.73500479310887,-1.50615163825644,0.97585266613462,-1.5434211439134,0.122728626785643,0.876570271956541

"1999","DNAAF2",27.8063302587075,-0.534410838087647,0.346386809141503,
-1.542815211157,0.122875617707742,0.876910236227437
"2000","GNB1",237.597114612229,0.278226733891147,0.180927552018228,1.5
3777979521393,0.12410246540428,0.877534555593311
"2001","GSTM5",102.320875279894,0.542306908172901,0.35230036287434,1.5
3933110868334,0.123723483068794,0.877534555593311
"2002","ANKRD35",117.804137356805,-0.38641674028637,0.251916218682404,
-1.5339097351788,0.125051860673131,0.877534555593311
"2003","ARNT",74.2821419696728,0.326709453839345,0.21219671415811,1.53
965368943418,0.123644790893578,0.877534555593311
"2004","MGST3",668.31827336997,-0.372352158485172,0.242015677700036,-1
.53854561003557,0.123915265542784,0.877534555593311
"2005","CENPA",6.11081749530615,2.52796467838585,1.64040049470192,1.54
106554256143,0.123300835051465,0.877534555593311
"2006","TRMT44",15.6574777772009,0.634106353692075,0.411757417546603,1
.53999983162491,0.123560394465912,0.877534555593311
"2007","HSD17B13",2.17263553496603,-1.72057570584195,1.12050483577583,
-1.53553617164948,0.12465217908073,0.877534555593311
"2008","SYNPO2",70.5610976859632,0.621465454700794,0.405220138590725,1
.53364898610944,0.125116030146979,0.877534555593311
"2009","ZDHHC11B",2.88332149123265,-1.54116034092118,1.00000673716347,
-1.54114995794198,0.123280293459281,0.877534555593311
"2010","ADAMTS12",16.7747323441929,-0.608510810528904,0.39545408553346
9,-1.53876475876591,0.1238617362306,0.877534555593311
"2011","DMXL1",47.7350152528381,0.419202692534666,0.273164367567423,1.
53461703760172,0.124877925104433,0.877534555593311
"2012","MXD3",3.69086591374903,1.24548562611582,0.811909098699682,1.53
402102293291,0.125024480942484,0.877534555593311
"2013","BICRAL",38.4158167236997,0.468351158343699,0.304941701602738,1
.53587113826052,0.124569988011106,0.877534555593311
"2014","ADAM9",119.483737723927,0.378565838586421,0.246248460349061,1.
5373328143851,0.124211829721486,0.877534555593311
"2015","NCOA2",75.9810181435986,0.415696459586401,0.270592817948321,1.
53624350689822,0.124478669188253,0.877534555593311
"2016","NIPSNAP3A",29.4006664401238,-0.438557583860055,0.2855041540367
45,-1.53608127118042,0.124518449079195,0.877534555593311
"2017","AL138756.1",2.85529227808063,1.411641135014,0.9167000190333,1.
53991611836403,0.123580801318665,0.877534555593311
"2018","PSMC3",212.999308626398,-0.370826405431198,0.241791059771479,-
1.53366466808852,0.125112170139042,0.877534555593311
"2019","DCP1B",16.830434144211,0.593789656132918,0.385614815964402,1.5
3985176800814,0.123596489848051,0.877534555593311
"2020","PRPF40B",12.2600374958707,0.697485541507142,0.454070665989669,
1.5360726727126,0.124520557683882,0.877534555593311
"2021","TRPV4",2.35295951039261,-1.64236967488872,1.07027007076305,-1.
53453760854752,0.124897448413543,0.877534555593311
"2022","RASA3",73.4645733993168,0.361013599216183,0.235293290235923,1.
53431319207702,0.12495262184728,0.877534555593311
"2023","CEP128",9.865399833474,1.1563779924272,0.751646900050745,1.538
45907213763,0.123936408278159,0.877534555593311

"2024","TMEM121",17.3475986935498,-0.620646633950472,0.403172585569854
,-1.53940683509826,0.123705006428016,0.877534555593311
"2025","SNRNP25",47.3163296245019,-0.578765714939554,0.376138969027434
,-1.53870181660795,0.123877108646982,0.877534555593311
"2026","ADGRG1",4.52797497079604,1.61240948516138,1.04993096184106,1.5
3572905625529,0.124604845641809,0.877534555593311
"2027","CTC1",7.8133511613792,0.869060930004034,0.564181184805356,1.54
039332294263,0.123464507993632,0.877534555593311
"2028","SNHG30",31.0087220635988,-0.506693569867963,0.329631016947029,
-1.53715379869543,0.124255651179711,0.877534555593311
"2029","MYO5B",4.43903169339642,1.18778973581977,0.77254197507301,1.53
750834795419,0.124168872364361,0.877534555593311
"2030","POLR3F",20.1931672734275,-0.545136460463531,0.354943348871928,
-1.53584075373174,0.124577441747688,0.877534555593311
"2031","MRPL39",36.602706794647,-0.552893245958549,0.359902272431362,-
1.53623160593962,0.124481586943834,0.877534555593311
"2032","NCF4",5.04185542700596,-1.03363989901642,0.670725296206227,-1.
54107785238297,0.123297839419843,0.877534555593311
"2033","ZNF81",3.78092294414466,1.2342265885493,0.804094791347412,1.53
49267298214,0.124801826855859,0.877534555593311
"2034","UBQLN2",68.8185842773188,0.352823929469361,0.229731450017149,1
.53581031000772,0.124584910354523,0.877534555593311
"2035","DENND5A",78.6486602529678,0.324917695958339,0.211943995563811,
1.53303562619926,0.125267077177305,0.878162222610043
"2036","AASS",89.472658123151,0.455664418326402,0.297601393680316,1.53
112326757407,0.12573893031035,0.878878775015904
"2037","KIAA1549",2.69230753156407,1.50249756740949,0.980894859947003,
1.53176209679666,0.125581152546152,0.878878775015904
"2038","OMD",161.991452681725,0.280825689687102,0.18338624521508,1.531
33453033919,0.125686735640978,0.878878775015904
"2039","CHP1",94.5124769207245,-0.35214739534582,0.229827844292517,-1.
53222250519662,0.125467536769492,0.878878775015904
"2040","TRMU",20.9891270396099,-0.571051680447381,0.372905325125543,-1
.53135834210769,0.125680853753989,0.878878775015904
"2041","ELK1",28.0961631307474,-0.543552326608925,0.354942289904162,-1
.53138226148169,0.125674945502656,0.878878775015904
"2042","AMPD2",20.7977676959628,-0.6747746108974,0.441878321489162,-1.
52705977659949,0.126746147808471,0.879072488178762
"2043","AAK1",131.307493430348,0.308208629103813,0.201639732840442,1.5
2851139387146,0.12638561680977,0.879072488178762
"2044","MAP4",222.717182212714,-0.315158606104475,0.206340902275342,-1
.52736855673882,0.126669390654181,0.879072488178762
"2045","POC1A",3.79455586695604,1.31036371834786,0.85852814329018,1.52
629093010986,0.12693742644387,0.879072488178762
"2046","HAND2",15.7406400303949,-0.722335301992124,0.472899327764622,-
1.52746104632159,0.126646406462088,0.879072488178762
"2047","NPR3",45.1349903334639,1.38484700989665,0.906697481085997,1.52
735288096086,0.126673286496765,0.879072488178762
"2048","SLC30A5",52.3625692348305,-0.365416193276307,0.239051439752782
,-1.52860904604551,0.12636139211893,0.879072488178762

"2049","MAT2B",58.883788930487,0.451217404482311,0.295197145624541,1.5
2852902262209,0.126381243357188,0.879072488178762
"2050","SDC2",482.102848295853,-0.380072373596613,0.248826618828924,-1
.52745865930817,0.126646999607802,0.879072488178762
"2051","AC091563.1",8.19530740976805,0.809367529731997,0.5302506055668
9,1.52638680886881,0.126913560847457,0.879072488178762
"2052","AL121929.3",2.54993439104498,-1.43037401611997,0.9370989673655
15,-1.52638522283426,0.126913955605764,0.879072488178762
"2053","GLG1",315.678535538848,0.323440918031836,0.211492515749503,1.5
2932559757777,0.126183746918114,0.879072488178762
"2054","COL1A1",7236.21836087967,0.5771142084529,0.377815169836879,1.5
27504066875,0.12663571671405,0.879072488178762
"2055","SCPEP1",400.816725770415,0.329785109340157,0.215996035422076,1
.52681093750507,0.126808031045699,0.879072488178762
"2056","FZR1",23.9852732582139,0.522083445042538,0.34130041746398,1.52
968885570624,0.126093763432906,0.879072488178762
"2057","LINC00662",33.5392249869818,0.44390444633105,0.290767151539666
,1.52666642012515,0.126843981543401,0.879072488178762
"2058","ZNF444",20.0164011623942,0.52996878091814,0.346761244146764,1.
52833913784735,0.12642835738184,0.879072488178762
"2059","MATN4",8.29637769550898,1.17351705448128,0.768364364350568,1.5
2729240049173,0.126688318356211,0.879072488178762
"2060","ADRM1",152.11403012866,-0.480635603198779,0.314397940463566,-1
.52874921028459,0.126326627729129,0.879072488178762
"2061","TMEM53",11.5890672003827,-0.6824020068563,0.447264865732611,-1
.52572236081754,0.12707902326286,0.879199488781745
"2062","MEX3D",34.371460567064,-0.442333198620938,0.289898043116192,-1
.52582333383896,0.127053867912206,0.879199488781745
"2063","NTRK1",3.00332327842212,1.47004316043585,0.964601695575574,1.5
2398981587802,0.127511255244156,0.880155584770738
"2064","ZNF638",156.053459322041,0.389833310519798,0.255982226200734,1
.52289210194657,0.127785702086116,0.880155584770738
"2065","AUP1",152.254078059894,-0.312031042688752,0.204702558379902,-1
.52431432786327,0.127430209693204,0.880155584770738
"2066","STAT4",9.19341661099859,0.818558538612128,0.537488597317551,1.
52293191464399,0.127775740223668,0.880155584770738
"2067","PRRC1",83.4631334690496,0.315235169416707,0.206884936057716,1.
52372219758312,0.12757812199055,0.880155584770738
"2068","CSAD",80.7373169849754,0.432547365898637,0.283936629929285,1.5
233940263585,0.127660155637476,0.880155584770738
"2069","CSPG4",2.9438012739084,-1.27619858801221,0.837829592044516,-1.
52321975749025,0.127703734665758,0.880155584770738
"2070","ACTG1",3864.81258764425,-0.408777119230582,0.268365169643565,-
1.52321226995854,0.127705607315373,0.880155584770738
"2071","LGI4",34.3360440255707,0.69094430835716,0.453191529634359,1.52
46187608904,0.127354215217526,0.880155584770738
"2072","CIC",38.4408621551652,-0.380077761114592,0.249608037833323,-1.
52269840512263,0.127834177179656,0.880155584770738
"2073","TCTA",26.3506595961603,0.530326609368073,0.348525284537395,1.5
2163023142492,0.128101758159431,0.881147387609666

"2074","RASA2",47.2164993552732,0.53831604814957,0.353739213369138,1.5
217878815935,0.128062238910115,0.881147387609666
"2075","AC007620.2",2.87267504568295,1.42147234080844,0.93454978761447
3,1.52102366256686,0.128253899366446,0.881343992467108
"2076","FGF18",65.200362491086,-0.50774117188925,0.333776306984945,-1.
52120195850855,0.128209164137026,0.881343992467108
"2077","RUFY3",137.147848938165,0.375640272711113,0.247010697788657,1.
52074495588248,0.128323852397291,0.881400133991212
"2078","BCHE",11.88173511057,0.940223438679275,0.618382374490264,1.520
45639957689,0.128396308846076,0.88147340808379
"2079","MRPS35",70.4127520515203,-0.403351183351626,0.265422620432124,
-1.51965639814326,0.128597355396251,0.882162094788999
"2080","RAB8B",144.850555532387,0.316781440335909,0.208468479258836,1.
51956517101366,0.128620297011154,0.882162094788999
"2081","NRXN1",2.19423913255126,-4.87374694274808,3.2119758540244,-1.5
1736724192419,0.129173989785037,0.882223742583199
"2082","RIF1",144.143329066959,0.418884727730679,0.275728514630657,1.5
1919263153389,0.128714015505706,0.882223742583199
"2083","ZNF518B",18.9572559337152,0.540483544039362,0.356554408940755,
1.51585152360062,0.12955690037234,0.882223742583199
"2084","TIFA",37.2396132615435,-0.510120410497202,0.335818818250247,-1
.51903461859326,0.128753782309081,0.882223742583199
"2085","AC096733.2",2.99917394516683,1.39520389724326,0.91998544682253
8,1.5165499650696,0.129380346208765,0.882223742583199
"2086","ERGIC1",170.215377223959,0.287013256671312,0.1892378251788,1.5
166801689891,0.129347453538307,0.882223742583199
"2087","AUTS2",95.6310108987872,0.416952828923038,0.274983881054819,1.
51628098099291,0.129448318686989,0.882223742583199
"2088","IMMP2L",40.5721597308798,-0.438955173746006,0.289274402204575,
-1.51743524625997,0.129156830715934,0.882223742583199
"2089","AC087854.1",3.98287290105711,1.38949484050527,0.91545823870744
6,1.51781346407142,0.129061429906422,0.882223742583199
"2090","ARAP1",69.2472309077982,0.419195596026329,0.27654007597227,1.5
1585839612036,0.129555162204955,0.882223742583199
"2091","VWA5A",37.8056438860253,-0.421141190630392,0.277821248708996,-
1.51587105949379,0.129551959487816,0.882223742583199
"2092","AL110292.1",3.47708738903966,-1.27397961156316,0.8390392025293
18,-1.51837912665189,0.128918850620616,0.882223742583199
"2093","PRKCH",14.9138702695994,0.841550262249821,0.554748455733535,1.
51699433058728,0.129268115433411,0.882223742583199
"2094","POLR3E",35.1060904622292,0.417434982701389,0.275123403196374,1
.51726453602872,0.129199908218039,0.882223742583199
"2095","IRAK1",61.1091263960696,-0.402867924383886,0.265464629844189,-
1.51759548765628,0.129116405101308,0.882223742583199
"2096","TNXB",1935.9741652117,0.26783915880916,0.176859007278078,1.514
42192812963,0.129918861167199,0.883755082723659
"2097","TAGLN",1036.15373562658,0.592460512360982,0.391262017189722,1.
51422956058037,0.129967626773744,0.883755082723659
"2098","B3GNT9",21.435628184027,0.539576181919867,0.356274931408009,1.
51449382022879,0.129900640004696,0.883755082723659

"2099","RNF130",172.872712690922,-0.27462886234233,0.181418100996262,-1.51378975325064,0.130079172296252,0.884092173405586
"2100","SLC30A7",83.6388127057772,0.351122041949222,0.232193062537928,1.5121986768742,0.130483327539017,0.884469287762152
"2101","LGI2",9.48097141042676,-1.15176720501533,0.761481880315396,-1.51253396146246,0.130398079731283,0.884469287762152
"2102","KLHL42",51.1120708462097,0.381777752379903,0.252480648987924,1.51210698289263,0.130506648726996,0.884469287762152
"2103","PPTC7",58.885794528393,-0.379652025636336,0.250958676176312,-1.51280693467481,0.130328706826086,0.884469287762152
"2104","OAZ2",96.8915188892737,0.361843437604198,0.239262937118232,1.51232548577046,0.130451080650894,0.884469287762152
"2105","PTRH2",51.8775527168482,-0.546639927302882,0.361408227935985,-1.51252762125744,0.130399691359617,0.884469287762152
"2106","OXSM",8.09557269112867,-0.741598438986146,0.490649206153784,-1.51146364792794,0.130670363672542,0.885158313462715
"2107","SND1-IT1",15.4066770830301,0.668013520605277,0.442059934034579,1.51113790048438,0.130753320142019,0.885299888536327
"2108","TTLL3",66.3053320339418,0.503106466836015,0.333176032961477,1.51003198628692,0.13103526256521,0.885753971988189
"2109","SRPK2",115.671389596234,0.344094156178927,0.227891896518225,1.5099007969834,0.13106873930093,0.885753971988189
"2110","WNK1",180.396320099949,0.356037320169145,0.235784022392005,1.51001461658505,0.131039694564894,0.885753971988189
"2111","LENG9",17.4845912933667,-0.557381598588145,0.369047984868757,-1.5103228345397,0.13096106788818,0.885753971988189
"2112","SLC25A44",25.147221174142,0.525785027756708,0.348958651945209,1.50672586802423,0.13188093812253,0.885794632032736
"2113","KIDINS220",197.906718695538,0.292955585666656,0.194361657102763,1.50727046699218,0.13174134434583,0.885794632032736
"2114","PNISR",596.863418151143,0.266853626269612,0.176974225297922,1.50786718133889,0.131588523651128,0.885794632032736
"2115","ST7-AS1",10.4606568537055,-0.717363422843451,0.476151332105015,-1.50658703331158,0.131916543117143,0.885794632032736
"2116","CDK5",9.53751129587091,-0.742738419208841,0.492604435018305,-1.50777858746083,0.131611204172828,0.885794632032736
"2117","PPP2R2A",112.466422337339,0.274523074556019,0.181894743296055,1.50924138642754,0.131237107386606,0.885794632032736
"2118","GNG10",203.728774835124,-0.325462697294368,0.215701600934707,-1.50885619709835,0.131335535798863,0.885794632032736
"2119","TCF7L2",284.683874730445,0.337648773213237,0.224130957808315,1.50647985675413,0.131944034282179,0.885794632032736
"2120","AP001160.3",6.67217172071723,-0.888759625120023,0.589471690577403,-1.50772232038736,0.131625610427367,0.885794632032736
"2121","ALG10B",12.8965716692853,0.632406378822773,0.419210229140911,1.50856619152344,0.131409679421937,0.885794632032736
"2122","DLEU2",41.3206940064376,0.554574667037398,0.368053386673643,1.50677778582471,0.131867625404496,0.885794632032736

"2123","RASIP1",1.84905745956683,-2.08589114130528,1.38373707176103,-1
.50743315610577,0.131699665445734,0.885794632032736
"2124","ZNF552",5.71976447288715,0.938821945792472,0.622899296174885,1
.50718093848815,0.131764284780134,0.885794632032736
"2125","AL359555.4",3.89434677032942,-1.29669523857807,0.8605248671598
33,-1.50686550506997,0.131845134876746,0.885794632032736
"2126","PIK3CD",7.96045427959285,0.949313088492473,0.630341843804063,1
.50602898700718,0.132059732583779,0.886154348560768
"2127","LNCTAM34A",1.99463813354802,2.0481195669207,1.36027644381986,
1.50566421717093,0.132153394178373,0.886193562761216
"2128","PDE1A",23.6227327337026,-0.748789406186157,0.497601475823763,-
1.50479739825241,0.132376172875659,0.886193562761216
"2129","ZNF451",84.0490088634423,0.390408321185026,0.259439856687532,1
.50481243001623,0.13237230712732,0.886193562761216
"2130","MCAM",2.39856281511387,1.50693390756196,1.00137238883726,1.504
86864263526,0.132357851592159,0.886193562761216
"2131","A4GALT",145.994646212859,-0.426610094333709,0.28344660846698,-
1.50508096265829,0.132303262791232,0.886193562761216
"2132","RPS6KC1",32.5661989063287,0.471438493778416,0.313628238929101,
1.50317616611363,0.132793621876726,0.886618469876884
"2133","GPATCH2",68.2438842498867,-0.34565842566312,0.22996326454194,-
1.50310279492523,0.132812538211615,0.886618469876884
"2134","BTN3A2",84.1206636269695,0.51108850020052,0.339883376299713,1.
50371726256431,0.132654182463032,0.886618469876884
"2135","RPL30",6757.1142422323,-0.356554188625692,0.237204746389797,-1
.50314946919219,0.132800504553732,0.886618469876884
"2136","NFATC4",58.1074570820234,0.490703200365745,0.326201751961565,1
.50429357725695,0.132505792103464,0.886618469876884
"2137","CCNQ",29.704149752615,-0.53783512872629,0.357807813594544,-1.5
0313969760243,0.132803023815727,0.886618469876884
"2138","LMNB2",29.0727184264913,-0.520868535124162,0.346597228697265,-
1.50280640466146,0.132888973878559,0.886713798574148
"2139","SYNGR1",29.371119893527,0.486287442719617,0.323642529706869,1.
50254493178032,0.132956433022665,0.886749169472342
"2140","ETNK2",15.7301529955257,-0.564887255029594,0.376231248892288,-
1.50143630198914,0.133242750402284,0.888243494036907
"2141","SCML1",63.2859563085687,0.469275478384796,0.312604262970133,1.
50118067465264,0.133308836984188,0.888268971703139
"2142","CNIH3",10.5547538734722,-0.753190992851106,0.501815964424733,-
1.50093071214771,0.133373483582031,0.888284835098624
"2143","C2orf81",10.0688142756081,0.734232152764647,0.489788245508427,
1.49908079562521,0.13385267301197,0.888934776756976
"2144","VARS2",12.7424083222363,0.716985004797941,0.478182899957001,1.
49939490697474,0.133771214069128,0.888934776756976
"2145","CDK9",45.492312646708,-0.356751111912704,0.238013310713433,-1.
49887042385722,0.133907250473205,0.888934776756976
"2146","ENTR1",45.3263210235969,-0.385855175514497,0.257275138260241,-
1.49977637996328,0.133672337712287,0.888934776756976
"2147","ST8SIA1",34.5054948535039,0.599942652893385,0.399951687012358,
1.50003781050397,0.133604608564328,0.888934776756976

"2148","PCNX1",83.0020233532906,0.407692251136413,0.271962131968416,1.49907727294828,0.133853586770314,0.888934776756976
"2149","ACBD4",12.0786396545205,0.674183916185687,0.449748533843973,1.49902415561754,0.133867365628196,0.888934776756976
"2150","KCNJ8",26.0504897938689,-0.541519544304846,0.361363747293503,-1.49854419088979,0.133991920241108,0.889083132167274
"2151","AC092868.2",2.52542028549114,1.72067402892303,1.14908991679447,1.49742331193982,0.134283146208475,0.890601284895444
"2152","CCDC51",28.8314697544907,0.610718133692145,0.407950323938094,1.4970404430536,0.134382734998109,0.890648737410714
"2153","ST3GAL6",3.34927374299523,-1.20139007555585,0.802576913281473,-1.4969158166334,0.134415164141684,0.890648737410714
"2154","NDUFS5",614.714009885316,-0.352335134878942,0.235414102329236,-1.49666112349627,0.13448145687084,0.890674309990435
"2155","AC068491.3",9.64597906731408,0.726520030922252,0.485758984825077,1.49563889422215,0.134747781869122,0.891898862861763
"2156","CENPH",10.5735185238004,-0.719466055981387,0.481096413286567,-1.49547166869613,0.134791388499226,0.891898862861763
"2157","VPS18",16.339427169721,0.626090174873946,0.418782653750768,1.49502413547089,0.134908143332554,0.892257567353832
"2158","ABL1",128.033667848582,0.334533626728949,0.223825028728354,1.49462117185722,0.13501333746694,0.89253951450573
"2159","WDR92",29.1991400054337,0.449692022914263,0.301263081422488,1.49268878480208,0.135518670445536,0.892997029812116
"2160","AGA",27.3293954679894,-0.515373737478016,0.345101637511191,-1.49339696326797,0.135333307403112,0.892997029812116
"2161","PRPF4",38.5040481216881,-0.440244859321213,0.294852931888473,-1.49309981929478,0.135411059852382,0.892997029812116
"2162","ARHGAP21",231.510938545746,0.364691408246385,0.24431972866668,1.4926809645566,0.1355207184595,0.892997029812116
"2163","DIO3OS",113.030961952989,0.492594653088243,0.329810703673003,1.49356781815255,0.135288616123698,0.892997029812116
"2164","ANXA2",2900.20978051602,-0.330540370361288,0.221274173848832,-1.4938045620593,0.135226708803716,0.892997029812116
"2165","ABAT",6.34185083777418,0.922339629197371,0.61756324799612,1.49351444113651,0.135302576964664,0.892997029812116
"2166","ATOH8",34.2800311415049,0.640447351409735,0.429725162986733,1.49036502065276,0.136128284646798,0.895166923432868
"2167","ARHGEF3",39.8444492486541,0.583647954818172,0.391659445211208,1.49019246683412,0.136173636564474,0.895166923432868
"2168","SESN1",54.5808726638186,0.402431465769702,0.26999666810024,1.49050530364432,0.136091422989517,0.895166923432868
"2169","FAM20C",175.102760716124,-0.416456632275594,0.279523834671618,-1.48987878892275,0.136256109671575,0.895166923432868
"2170","ZKSCAN5",9.54205009001138,-0.768131547631282,0.515198074070187,-1.49094413642283,0.135976162235523,0.895166923432868
"2171","FAM86C1",5.71996054709662,-0.911337829743069,0.611313039018899,-1.49078748787312,0.13601729779629,0.895166923432868
"2172","ZNF423",47.1485807397123,0.44733415012897,0.300274088554732,1.48975275316649,0.13628925821507,0.895166923432868

"2173","RGL1",72.154330662657,0.363188465899345,0.243933578673142,1.48888262073176,0.136518280777213,0.895846271190302
"2174","POLI",24.7170758594388,0.503892400556596,0.338414378758626,1.48898046946166,0.136492511762539,0.895846271190302
"2175","NCK1-DT",6.29152359184005,0.940593769752606,0.631870879479949,1.48858540613036,0.136596576941311,0.895947938687236
"2176","SEC24A",40.4370094170337,0.407607429879634,0.274022722836759,1.48749499917368,0.136884123027004,0.897421369073178
"2177","ZNF669",11.2879185907941,-0.726016980634478,0.488301002552187,-1.48682262956625,0.137061663074112,0.898172570241285
"2178","PGD",97.3558073544885,0.416646314436014,0.280555424049661,1.48507666835293,0.137523515835843,0.898902579641174
"2179","IGSF8",42.1309033862251,-0.407463456612136,0.274214192293634,-1.48593131961534,0.137297288335525,0.898902579641174
"2180","NOCT",11.8148436965002,-0.840965133589135,0.566108014979847,-1.48552062740018,0.137405963347535,0.898902579641174
"2181","ARHGAP22",3.27289108869616,-1.43203989217585,0.964029398844024,-1.48547325827721,0.137418502157935,0.898902579641174
"2182","HYLS1",6.51394463063606,-0.808241002918558,0.544280135300925,-1.48497244433088,0.137551123745737,0.898902579641174
"2183","MESD",200.006637024904,0.265168844711906,0.178531901605236,1.48527429735352,0.137471177605769,0.898902579641174
"2184","ELOVL4",13.6039925529047,-0.764198673056925,0.514924930798428,-1.48409724864551,0.137783123040615,0.899594523248243
"2185","FKRP",19.328785613555,0.538406834668762,0.36276553591543,1.4841730577027,0.137763015440782,0.899594523248243
"2186","STX18-AS1",7.19527904527184,1.08454094137614,0.731247346179104,1.48313829382501,0.138037671648277,0.90002076039046
"2187","SND1",95.2790836716286,-0.317627757679161,0.214108172306751,-1.48349198564966,0.137943744192819,0.90002076039046
"2188","CAPN12",2.71138126710463,-1.27566656459199,0.860046321272998,-1.4832533237324,0.138007118555923,0.90002076039046
"2189","AC020916.1",339.452375127735,-0.519211883097733,0.350286181667457,-1.48225054333044,0.138273642443,0.901147456871559
"2190","GSK3B",48.2318913938854,0.375533633747584,0.253470075105863,1.48156989968437,0.138454773095332,0.901504241432225
"2191","CLTB",299.32082384453,-0.393153084946441,0.265357954706089,-1.48159524888522,0.138448023973551,0.901504241432225
"2192","EEF1D",1587.71990247229,-0.291933885317887,0.197158979471488,-1.48070296417874,0.138685743759102,0.902596177220509
"2193","MED13",110.809586179706,0.327986352771909,0.221561674578637,1.48033884197557,0.138782842374533,0.902816246837708
"2194","C1orf56",55.7693105894657,-0.497219347772638,0.33600441493997,-1.47980004328654,0.138926617132712,0.903339617144607
"2195","TMEM245",66.9735202782848,0.46998948909676,0.317684293633329,1.47942312073892,0.139027264516637,0.903582212115875
"2196","MT-ND5",3303.85469651254,-0.313750956162416,0.212147854449576,-1.47892589

805564,0.139160120807687,0.904033826704217
"2197","TMEM97",14.7201149791741,-0.651567250127029,0.440765975524107,
-1.47826122320867,0.139337872427155,0.904776553502862
"2198","FHL2",41.7218727105157,0.730673576064479,0.495029168775282,1.4
760212572366,0.139938185751626,0.905796326625605
"2199","AC018816.1",2.48297649929898,-1.38792557819998,0.9410102207672
82,-1.47493145937171,0.140230971461732,0.905796326625605
"2200","PLSCR4",228.58916551356,0.385565635219595,0.261544832956221,1.
47418563334468,0.140431616945113,0.905796326625605
"2201","TREM2",2.31765321922341,-1.59907812074767,1.08341971431006,-1.
47595442433498,0.139956127567897,0.905796326625605
"2202","TMEM168",46.6179168470546,0.342927877868916,0.232570630782888,
1.47451067537866,0.140344145541713,0.905796326625605
"2203","AC022034.1",13.8963225700783,0.635308142491034,0.4301686835950
17,1.47688143446803,0.139707422373946,0.905796326625605
"2204","PLXDC2",225.573188941916,0.444619014400504,0.301588267956988,1
.47425832381489,0.140412051720838,0.905796326625605
"2205","AC012470.1",2.10000327942797,1.69299196933211,1.1482422799678,
1.4744205111308,0.140368405258714,0.905796326625605
"2206","NHLRC3",75.7675830677106,0.395729668051726,0.26793103856291,1.
47698329456074,0.13968011534131,0.905796326625605
"2207","ADAL",17.5706235331618,0.563783423328256,0.382164022223898,1.4
7523940125885,0.140148191897205,0.905796326625605
"2208","REXO5",5.88638012227228,0.884193634477985,0.59901090063563,1.4
7608938925775,0.139919896998283,0.905796326625605
"2209","FUS",588.276764397911,0.32029263324937,0.217046491961648,1.475
68675427367,0.140028003434241,0.905796326625605
"2210","CPD",73.1171749405029,0.352383100035344,0.238616793485528,1.47
67740982854,0.139736201902634,0.905796326625605
"2211","ZNF850",4.54031949324309,1.07500296034115,0.729247035518834,1.
47412729566507,0.140447320517022,0.905796326625605
"2212","TAB3",15.4671576805203,0.687474799243826,0.465875274532243,1.4
7566277247505,0.140034444511455,0.905796326625605
"2213","TRIM52",26.4227162007593,0.487650249008343,0.330940898353535,1
.4735266974691,0.140609070496887,0.906429733261903
"2214","AC242426.2",8.02593935496309,-0.737449977903042,0.500684574373
316,-1.47288335940063,0.140782489819722,0.907002913374664
"2215","ARPC5L",96.4003768274458,-0.370857102433771,0.251829359955724,
-1.47265236467652,0.140844797265473,0.907002913374664
"2216","AQP5",2.05445429685725,-1.58068098741518,1.07347515879216,-1.4
7248958158819,0.140888718354006,0.907002913374664
"2217","CFD",45438.8483717427,0.370758323680785,0.251880853333952,1.47
195913771668,0.141031912378881,0.907515228686115
"2218","PPM1B",61.1948385741343,0.40718447685275,0.276850087129628,1.4
7077604733459,0.141351692193915,0.907535841528498
"2219","CUTA",240.910822006913,-0.419680266434399,0.285313147007572,-1
.47094611950448,0.141305688791415,0.907535841528498
"2220","AGPAT5",21.3968711970499,-0.57333264754701,0.389817885608871,-
1.47077050261945,0.141353192196574,0.907535841528498
"2221","PRELID3B",45.6689417151903,-0.387764274349209,0.26361883362625

7,-1.47092781276378,0.141310640091594,0.907535841528498
"2222","PAK3",23.9547905720844,0.564006633911314,0.383345598342871,1.47127457925539,0.141216875161423,0.907535841528498
"2223","TP53BP2",48.0142194066499,0.435411139311447,0.296498118797833,1.46851231662732,0.141965113122994,0.907934889916959
"2224","RANBP2",97.0396708220921,0.359263496379183,0.24497633803062,1.46652325390821,0.142505790861449,0.907934889916959
"2225","NKTR",234.8494120624,0.405243119923763,0.276032336136737,1.46810017114451,0.142077015117957,0.907934889916959
"2226","ATP13A3",103.300525185469,0.440856562951878,0.300573586567934,1.46671757816696,0.142452899018605,0.907934889916959
"2227","USP46-AS1",9.58311600281801,-0.738025967298499,0.502353461424379,-1.46913682092663,0.14179568240952,0.907934889916959
"2228","FAM13A-AS1",7.89714257490189,0.936651797185259,0.638420328340987,1.4671396814999,0.14233806141334,0.907934889916959
"2229","AC097534.2",12.0221934503254,-0.676438522193577,0.460528630607709,-1.46883055088444,0.141878755374749,0.907934889916959
"2230","ANKHD1",151.266201151708,0.338193963774713,0.23014867378323,1.46945866867453,0.141708424403009,0.907934889916959
"2231","DPYSL3",217.627196870469,-0.344067045729243,0.234559567988458,-1.46686425405666,0.142412986254578,0.907934889916959
"2232","HELLS",8.9314859644159,-0.797772230535026,0.54406393004144,-1.46632075108207,0.142560924815224,0.907934889916959
"2233","ITGAL",15.754784424906,0.819632187501325,0.5585340550232,1.46747038990717,0.142248138421215,0.907934889916959
"2234","USP22",126.739342161608,0.270536947736357,0.184293213064381,1.46797021571189,0.142112313479557,0.907934889916959
"2235","NFIC",659.179258328248,-0.282803986436833,0.19253907295598,-1.46881348338833,0.141883385877419,0.907934889916959
"2236","MFSD12",40.3796513427094,-0.425543152471035,0.28982042341357,-1.46829939539419,0.142022915105259,0.907934889916959
"2237","C19orf53",367.720107090841,-0.357989639491413,0.243544988849456,-1.46991174477705,0.141585658236348,0.907934889916959
"2238","RPRD1B",33.5129292602875,0.410900382331617,0.280206807116344,1.4664182735611,0.142534371043997,0.907934889916959
"2239","IFNAR1",159.741113282506,0.308538737782351,0.210307313850009,1.46708515331236,0.142352892374938,0.907934889916959
"2240","MOSPD2",40.4925663614922,0.394596736775783,0.268656348482573,1.46877875399017,0.141892808506693,0.907934889916959
"2241","CAPN2",256.611488999057,-0.270155646868795,0.184300471060636,-1.46584349629748,0.142690928234139,0.907952177604025
"2242","WAPL",80.6541824217755,0.321555324155617,0.219340766610818,1.46600802543086,0.142646100494434,0.907952177604025
"2243","GALNT13",8.09782204998296,-0.996862931923685,0.680301111794422,-1.46532603672258,0.142831986171916,0.908230415358701
"2244","ENPP2",97.1697985099274,-0.456045547224064,0.311302569449132,-1.46495914900755,0.142932063492466,0.908230415358701
"2245","LRIG3",77.5859278049527,0.468034512774123,0.319439686345858,1.

46517334188521,0.142873630772616,0.908230415358701
"2246","SGCG",11.7181031378053,0.800803594859849,0.546717147547132,1.4
6474936528456,0.142989311152085,0.908230415358701
"2247","PRDM16",3.3754732972814,-1.34305259034486,0.92006413455640З,-1
.45973801162503,0.144362090775058,0.908523232341914
"2248","NFIA-
AS2",3.29736346758158,-1.52236075033991,1.04132380234186,-1.4619475199
8968,0.143755591583655,0.908523232341914
"2249","CCN1",1087.08802145997,0.518426168946782,0.355051199492442,1.4
6014481767106,0.144250277452868,0.908523232341914
"2250","RNASEH1-
AS1",14.1300245809528,-0.68312280324581,0.468539950411997,-1.457981977
08675,0.144845510848763,0.908523232341914
"2251","BOLA3",82.7686464416429,-0.521857665464256,0.357664046267576,-
1.45907219612967,0.144545237871887,0.908523232341914
"2252","FMNL2",60.5780213754425,0.48168049973838,0.329363480268628,1.4
624587381258,0.143615543468337,0.908523232341914
"2253","MARCH7",111.295377044939,0.256991249473133,0.176335455375235,1
.45739975506495,0.145006064664748,0.908523232341914
"2254","AC064836.4",13.0321907248572,-0.62759876041401,0.4309151935549
42,-1.45643219315726,0.145273181466761,0.908523232341914
"2255","RETREG2",87.3558296963483,-0.307535541435371,0.21026541104456,
-1.46260642636176,0.143575103791235,0.908523232341914
"2256","BOC",203.838594985726,0.503791575704474,0.345556538150252,1.45
791359758738,0.144864360149841,0.908523232341914
"2257","AASDH",40.1810672350553,0.369031474017983,0.252830130363856,1.
45960243538576,0.144399369552713,0.908523232341914
"2258","ANXA3",4.28244034967486,1.37598838218092,0.945415991385308,1.4
5543167739811,0.14554979205519,0.908523232341914
"2259","TMEM173",196.925270608018,-0.290757569637448,0.199766127635497
,-1.45548984244205,0.145533700249318,0.908523232341914
"2260","UBE2D2",265.009695531927,-0.300357868720005,0.20511963691936,-
1.46430577408874,0.14311042022441,0.908523232341914
"2261","NDST1",44.5647557469287,0.431536959848761,0.294809634518355,1.
46378174021953,0.143253593113863,0.908523232341914
"2262","TRIM38",142.262580446559,0.361888912995315,0.247896159987732,1
.45984073740079,0.144333849651285,0.908523232341914
"2263","ZSCAN26",17.3391290078985,0.573301368257021,0.393738036853496,
1.45604771344542,0.145379430135264,0.908523232341914
"2264","RPS10",2940.41939447478,-0.461583369836897,0.31546642435027,-1
.46317748643954,0.143418819513207,0.908523232341914
"2265","AC004951.1",7.7973817694389,0.954468036113652,0.65301409945040
5,1.46163465217207,0.143841353312456,0.908523232341914
"2266","AC005089.1",2.20160888522177,1.90846779056976,1.30594486162919
,1.46136934769889,0.143914107955405,0.908523232341914
"2267","ABCB1",2.59998970285524,-1.43678128909146,0.986351602491799,-1
.45666239651434,0.145209594614834,0.908523232341914
"2268","PDAP1",287.648524970422,-0.342491737741613,0.234525815725838,-
1.46035836899929,0.144191608040404,0.908523232341914
"2269","AC104051.2",2.57910739351892,1.6939777224511,1.1633062988431,1

.45617514848475,0.145344207633333,0.908523232341914
"2270","MTAP",58.4338023656357,0.331119634859264,0.22717827992398,1.45
753209756701,0.144969557867847,0.908523232341914
"2271","TMEM38B",32.6704231646591,-0.501073911608217,0.344165588886645
,-1.4559093871911,0.145417670320623,0.908523232341914
"2272","FPGS",34.2034903446228,-0.418565694612433,0.286618888060373,-1
.46035628511777,0.144192180461232,0.908523232341914
"2273","AKR1C8P",2.87775682524968,1.3973490853374,0.955779151895575,1.
46199996365904,0.143741219830903,0.908523232341914
"2274","PYROXD2",17.2827480821091,0.635353686516513,0.435507152165643,
1.45888232456596,0.144597498808865,0.908523232341914
"2275","ASIC1",10.5868002501702,0.701563572735203,0.479643538338939,1.
46267700210202,0.143555781977201,0.908523232341914
"2276","PPP2R3C",66.092979089553,-0.365327310885562,0.250938609334069,
-1.45584337083502,0.145435923205861,0.908523232341914
"2277","DLST",71.114727309684,0.319367314259972,0.218546463406866,1.46
132455900423,0.143926393175784,0.908523232341914
"2278","ULK3",20.935012324061,-0.508336518836198,0.348808797300438,-1.
45735005186339,0.145019777156055,0.908523232341914
"2279","NPEPPS",140.686651015611,0.361515445609895,0.247785695331801,1
.45898432565207,0.144569421860813,0.908523232341914
"2280","ZSCAN30",21.0877824118824,0.585134065868439,0.400741575519701,
1.460128176393,0.144254850115148,0.908523232341914
"2281","LIPG",2.52035041935885,-1.57787015133838,1.07981134408889,-1.4
6124613338799,0.143947906701229,0.908523232341914
"2282","ZNF559",26.8896911940583,0.443821704319302,0.304748017055169,1
.45635633205435,0.145294140518114,0.908523232341914
"2283","MAU2",41.0284370228038,0.472350882387918,0.324194234046932,1.4
5699964028212,0.145116479515052,0.908523232341914
"2284","IFT52",70.9959518591995,-0.388295299241329,0.266743908034994,-
1.45568572531519,0.145479517767342,0.908523232341914
"2285","DDX3X",643.77345723025,0.33615944926485,0.230743294188209,1.45
685468540922,0.145156496850325,0.908523232341914
"2286","INTS6L",6.08909689791539,1.1033084733408,0.758124828688224,1.4
5531241240291,0.145582791892164,0.908523232341914
"2287","SPN",9.74633168088174,0.997614905862826,0.686145958036129,1.45
393978377163,0.145963001645068,0.909543282627207
"2288","MYOM1",6.22604690077966,0.952981887838019,0.655717508007884,1.
45334214230949,0.146128781983847,0.909543282627207
"2289","ZNF728",3.0234319518506,1.31716812731856,0.905768964877291,1.4
5419878401,0.145891202046048,0.909543282627207
"2290","PPP1R37",24.4745330019754,-0.459137267718432,0.315777841703445
,-1.4539882381919,0.145949567138116,0.909543282627207
"2291","ARHGAP35",58.5649041171289,0.385423183918887,0.265197498559188
,1.45334396445246,0.146128276318957,0.909543282627207
"2292","LAMP2",302.58007334927,0.26595547523637,0.182953090101552,1.45
36812419443,0.146034701135134,0.909543282627207
"2293","ZWILCH",11.3797184508246,0.663586309527541,0.456717389937128,1
.45294732398714,0.146238380031669,0.909828490855554
"2294","FOXN2",30.854370307698,-0.485848083008967,0.334495381646307,-1

.45248069081773,0.146367994330498,0.909841310291456
"2295","HES6",4.51240808822274,-0.988535274288776,0.680553278653871,-1
.45254648724063,0.146349713074764,0.909841310291456
"2296","CITED4",44.17603407253,0.698756822781508,0.481944323397484,1.4
4987042871591,0.147094655008728,0.910186013745234
"2297","DIRAS3",3.9565100193135,-1.27063650211934,0.877875634200836,-1
.44739921307424,0.14778514603439,0.910186013745234
"2298","SLC25A24",57.1562341894338,-0.372077513379864,0.25652425933256
2,-1.45045741228511,0.146931007006492,0.910186013745234
"2299","AL391001.1",2.32379431929351,1.52771675325227,1.05562834583978
,1.4472108098205,0.147837889895225,0.910186013745234
"2300","CHI3L1",605.102069270109,-1.14802684046243,0.79300594782918,-1
.44769007547182,0.14770374677314,0.910186013745234
"2301","PPP1CB",238.349181165403,-0.299520272361952,0.206496507634252,
-1.45048589825289,0.146923068808921,0.910186013745234
"2302","RGS12",26.5355402811799,0.610515628293559,0.420906320394805,1.
45047864266068,0.146925090696963,0.910186013745234
"2303","UGDH",287.188660640154,-0.372001454008412,0.256933395007124,-1
.44785170490624,0.147658528799872,0.910186013745234
"2304","PAIP1",141.119940263201,-0.31140459888315,0.215206851076142,-1
.44700132605431,0.147896552174086,0.910186013745234
"2305","KCTD20",87.4851902282764,0.31212536678414,0.21501051175314,1.4
5167491691058,0.146592016920287,0.910186013745234
"2306","RSPH9",1.85309832758112,-1.67767236538658,1.1595167560923,-1.4
4687203231157,0.147932767509617,0.910186013745234
"2307","NRF1",13.9736150881142,0.648087404107811,0.447376654260697,1.4
4863930188488,0.147438339491868,0.910186013745234
"2308","NUDT2",58.0616014688655,-0.4178462594619,0.288813154342248,-1.
44677018058099,0.147961301074935,0.910186013745234
"2309","CALHM2",171.529073393738,-0.324393574672726,0.223771483916873,
-1.44966449251966,0.147152101975099,0.910186013745234
"2310","DDB1",122.997995654678,0.295491649477065,0.203961491033948,1.4
4876195981467,0.147404070486519,0.910186013745234
"2311","FAM124A",3.78452036983235,1.14947550568103,0.793454630950748,1
.44869720440561,0.147422161533631,0.910186013745234
"2312","SNHG14",73.5700511081834,0.510569850244695,0.352812526819914,1
.44714206960487,0.147857137434678,0.910186013745234
"2313","MT1G",344.918539369679,-1.00103972297559,0.689888563480179,-1.
45101654957982,0.146775251919481,0.910186013745234
"2314","PAFAH1B1",164.418476543913,0.240706964391423,0.166302469736294
,1.44740462828431,0.147783630248323,0.910186013745234
"2315","IMPA2",58.1476116081476,-0.480442910624422,0.332104747644633,-
1.44666077203605,0.147991956351637,0.910186013745234
"2316","FHOD3",4.03202329871894,1.41492447445593,0.978126358165785,1.4
4656614418329,0.148018474126521,0.910186013745234
"2317","ZNF442",2.7542738822968,1.60171625251269,1.10465528863042,1.44
996929720812,0.147067081225206,0.910186013745234
"2318","ZNF146",57.0030328408029,0.347506939776406,0.239997194075417,1
.44796251104171,0.147627535430253,0.910186013745234
"2319","PHF20",113.178217586521,-0.264371473400605,0.182730650725762,-

1.44678231238485,0.147957902152958,0.910186013745234
"2320","BCL2L13",47.0003421500149,0.378130191400495,0.260668641676035,
1.45061634176329,0.146886722246349,0.910186013745234
"2321","BCL10",59.5151063371615,-0.346229738653322,0.239504276107644,-
1.44560984162851,0.148286664691238,0.910682195541345
"2322","CHROMR",20.2636654615334,0.582253823391811,0.40284119258365,1.
44536813541208,0.1483545087928,0.910682195541345
"2323","SPATA5",13.9724006230233,0.574170314343743,0.397236729027848,1
.44541094110029,0.148342492010658,0.910682195541345
"2324","UROS",89.7718147846732,-0.396887199642821,0.2745752258963,-1.4
4545888416282,0.148329033903143,0.910682195541345
"2325","ANKRD16",21.5598624601491,0.485220454799757,0.335782679835865,
1.44504313038701,0.148445771262324,0.91085048293691
"2326","SHPK",13.6280339076865,0.613551377002024,0.424704292932998,1.4
4465546313378,0.148554685557037,0.911126889147328
"2327","SEC11C",22.7772958054241,-0.481983645561823,0.333889155681073,
-1.44354387484872,0.148867322295093,0.912652006816413
"2328","MRPL35",45.5381956352611,-0.373190742804845,0.258580728705103,
-1.44322720673607,0.148956477792256,0.912806319666803
"2329","RPL41",11242.8041071412,-0.327850424772106,0.227202717626785,-
1.44298637004268,0.149024310814604,0.912829891833892
"2330","PPCS",152.847955373505,-0.320956826946593,0.222565745835341,-1
.44207647830967,0.149280799832248,0.912888613620616
"2331","TMEM127",48.6805249264748,0.362947494708808,0.251718430908913,
1.44187890174854,0.149336539105308,0.912888613620616
"2332","FAM114A1",251.269505344608,-0.289020668146799,0.20048108730618
9,-1.44163557785072,0.149405206202465,0.912888613620616
"2333","TRIM61",4.50648314966692,1.04246880086873,0.723116470443921,1.
44163332392188,0.149405842383919,0.912888613620616
"2334","ZMYND19",19.9450219261171,-0.510345411885055,0.35407111358778,
-1.441364156239,0.149481831026059,0.912888613620616
"2335","ABCD2",3.07723561433286,1.36030389479802,0.943631411734675,1.4
4156275202558,0.1494257626616,0.912888613620616
"2336","STK35",21.4088145735224,-0.614415095971914,0.425883058546163,-
1.44268498979354,0.149109229487198,0.912888613620616
"2337","RBM8A",319.179742742076,-0.373488195097231,0.260370186954132,-
1.43445069293984,0.151443690247203,0.913142724034913
"2338","PIGC",71.3575487451362,-0.362488282178006,0.251595124618363,-1
.44076035943802,0.149652395976869,0.913142724034913
"2339","OSR1",69.6741342670937,-0.466235346797854,0.324345818567224,-1
.43746371961081,0.150586274525066,0.913142724034913
"2340","STON1",8.00677497095886,1.01889797333246,0.710053918934322,1.4
3495859421727,0.151298896970901,0.913142724034913
"2341","CNPPD1",51.990784099237,0.353757942672705,0.245509123993033,1.
44091566504365,0.149608509932015,0.913142724034913
"2342","ZNF718",7.44533472637466,0.857416558266877,0.597554138964096,1
.43487677912042,0.151322213815617,0.913142724034913
"2343","METAP1",28.9423896332917,0.424721667252312,0.296038620229421,1
.43468330896545,0.151377362617694,0.913142724034913
"2344","C5orf38",42.4056166816387,-0.605671972862492,0.42194522604454,

-1.4354279548089,0.151165184719343,0.913142724034913
"2345","TRIM23",50.3616852447129,0.342519841613962,0.237856209327757,1
.440029009888,0.149859191764588,0.913142724034913
"2346","TNPO1",119.745913667672,0.290892844081416,0.202586431578005,1.
43589499955928,0.151032221599311,0.913142724034913
"2347","AC073349.1",4.10210256864468,-1.11366777587735,0.7737353013568
,-1.43933949236186,0.150054358727575,0.913142724034913
"2348","GLIS3",61.4634173343765,-0.437801215004838,0.304433378920819,-
1.43808545750401,0.15040980831897,0.913142724034913
"2349","RABGAP1",58.213128668954,0.368870277210742,0.256424854363162,1
.43851218372276,0.150288783027949,0.913142724034913
"2350","CELF2",415.510124265632,0.388975982735113,0.27061503890165,1.4
3737755415906,0.150610743082164,0.913142724034913
"2351","TNKS2",75.5142971151491,0.309919631172727,0.215341040600574,1.
43920374076571,0.15009280585454,0.913142724034913
"2352","FAM66C",8.81004655744753,0.714004685107411,0.496043448050494,1
.43939948791488,0.150037369375047,0.913142724034913
"2353","ADCY6",21.3216149697688,0.515234179152227,0.359003642128833,1.
43517813941099,0.151236341346833,0.913142724034913
"2354","MNAT1",73.8503426954487,-0.359187123123441,0.250015084538503,-
1.43666180697239,0.150814112252858,0.913142724034913
"2355","TEDC1",12.2212687704141,-0.654830820390658,0.45553712015702,-1
.4374916805132,0.150578335068982,0.913142724034913
"2356","DBF4B",3.97574662611691,1.1575937801753,0.805527455728317,1.43
706309691041,0.150700065781926,0.913142724034913
"2357","SGSH",29.7633175262662,0.506276083138239,0.352524547042489,1.4
3614419871084,0.150961313484039,0.913142724034913
"2358","CAPS",18.1204922571765,-0.709986367851919,0.493951856972616,-1
.43735944673507,0.150615885465025,0.913142724034913
"2359","CCDC159",11.2217147483651,-0.625159084328055,0.435292270234751
,-1.4361823700451,0.150950454302147,0.913142724034913
"2360","ZNF737",19.4558134845118,0.531132422410414,0.369274768112034,1
.43831224950976,0.150345477809713,0.913142724034913
"2361","BCL3",105.068704916599,-0.479283795173644,0.333727959543067,-1
.43615115685803,0.150959333949371,0.913142724034913
"2362","FCGRT",919.641391870231,0.36898837011226,0.256606259885337,1.4
3795545080288,0.150446694730468,0.913142724034913
"2363","ZNF528-
AS1",12.2690115869201,0.652961133644404,0.455146882275758,1.4346162943
6978,0.151396468744569,0.913142724034913
"2364","ZNF667-
AS1",44.7898203175166,-0.39055002857033,0.271483441802805,-1.438577712
05808,0.150270204889943,0.913142724034913
"2365","APOBEC3D",9.88322881095871,0.688220157751016,0.47917569081254,
1.43625849755441,0.150928798925471,0.913142724034913
"2366","TSC22D3",1240.49898221778,0.448746911138904,0.312492773599543,
1.43602332293921,0.150995704786654,0.913142724034913
"2367","DDX60L",49.9661647006433,0.403600412572447,0.281508443992533,1
.43370623931676,0.151656110939447,0.913820634575478
"2368","PTN",16.4445648507705,-1.45020540496488,1.01169036295577,-1.43

34478789814,0.151729883903018,0.913820634575478
"2369","TOB1",103.439012416635,0.509033947267103,0.355127548797241,1.43338343924913,0.15174828846974,0.913820634575478
"2370","PYGO2",13.6319648876426,0.598891373824005,0.418314933564577,1.43167581592339,0.152236620723953,0.914916581521085
"2371","ZNF862",6.80517004606248,0.814247340508201,0.568935062370684,1.43117799264354,0.15237920914721,0.914916581521085
"2372","MRE11",47.9880012918922,0.361603186750709,0.252529087604586,1.4319268729823,0.15216475055051,0.914916581521085
"2373","MIPOL1",20.9475681056105,-0.530192141453385,0.370215180572487,-1.43211885756147,0.152109808502476,0.914916581521085
"2374","TNK1",2.30064987848006,-1.94033860994612,1.35538856419824,-1.43157369126388,0.152265863370719,0.914916581521085
"2375","ZNF135",8.0691886354285,0.757664411451505,0.52934940404348,1.43131248597622,0.152340677041539,0.914916581521085
"2376","SLC16A2",22.5492202778618,-0.549985014254378,0.384001457717779,-1.43224720427647,0.15207308672966,0.914916581521085
"2377","STPG1",5.57412205271522,-0.961311380471275,0.672306951731323,-1.42986976111984,0.152754402298341,0.914939715516253
"2378","SNHG3",82.9751020173354,-0.413552744869479,0.291278250001495,-1.41978587439109,0.155670028546735,0.914939715516253
"2379","BMP8B",6.91527781501267,1.11390494860565,0.779673672022494,1.42868098356605,0.153095945937021,0.914939715516253
"2380","SRSF11",410.991054027456,0.297246049281315,0.208201993363956,1.42768109218677,0.153383670833929,0.914939715516253
"2381","AC092164.1",5.94997356171771,0.928635452475896,0.651176824234679,1.42608799624789,0.153842943252737,0.914939715516253
"2382","IFRD2",70.9607448546287,-0.516443770693346,0.361557670860499,-1.42838560018439,0.15318090156357,0.914939715516253
"2383","CD47",335.053680559229,-0.309507624312787,0.218169318151026,-1.41865788890871,0.155998779961908,0.914939715516253
"2384","ZNF148",147.083872370584,0.293157781840955,0.206379575953391,1.42047865195324,0.155468379294159,0.914939715516253
"2385","IGF2BP2",25.3908779672243,-0.603481318638311,0.42398625481019,-1.42335113884406,0.154634389254183,0.914939715516253
"2386","FAM184B",2.74476476561568,1.93495143816003,1.36363797213922,1.41896271421993,0.155909886714915,0.914939715516253
"2387","INTU",29.9148003051689,0.531949908109476,0.374534504923079,1.42029613057608,0.155521487198746,0.914939715516253
"2388","ANKRD37",17.5074392285606,-0.603796635251288,0.421990872399673,-1.43082866180912,0.152479326478812,0.914939715516253
"2389","ANKRD33B",22.4880387678725,0.555622261987227,0.388746160108045,1.42926752468192,0.152927356275817,0.914939715516253
"2390","PARP8",39.8690614631282,0.457251226318614,0.32023122060152З,1.42787834821262,0.153326876663176,0.914939715516253
"2391","NR2F1-AS1",7.6451433619558,1.00479548433823,0.70841661301948,1.41836804201343,0.156083340860699,0.914939715516253
"2392","AC116366.1",13.5505470258159,0.837738858459674,0.589680648908475,1.42066533811202,0.155414073813004,0.914939715516253

"2393","PRELID1",355.969132245712,-0.418076738215786,0.293223838092138
,-1.42579382677754,0.153927863363448,0.914939715516253
"2394","TCF21",5.18807593090749,-1.30574654806398,0.919046667676718,-1
.42076196344285,0.155385971948131,0.914939715516253
"2395","PLEKHG1",20.1983611188244,0.633583741122561,0.445354516908614,
1.42265030906282,0.154837552164875,0.914939715516253
"2396","ZKSCAN1",138.823777917419,0.374560981117585,0.263921934590545,
1.41921125918802,0.155837434408754,0.914939715516253
"2397","CLN8",34.2914503759987,0.399544014956462,0.280481563176252,1.4
244929699903,0.154303818666153,0.914939715516253
"2398","AC107375.1",6.20256854881297,-0.812946585229781,0.570670729638
585,-1.42454578973173,0.154288539871033,0.914939715516253
"2399","IDNK",21.2656449153742,-0.552922280843248,0.387984016309762,-1
.42511613262388,0.154123634009901,0.914939715516253
"2400","NEK6",58.0060533786064,0.442755165927112,0.31125482192425,1.42
248451988598,0.154885642276975,0.914939715516253
"2401","COQ4",62.8041512656047,-0.338393311944686,0.238581428518607,-1
.41835562828938,0.156086963258341,0.914939715516253
"2402","DPH7",32.8075422059815,0.478507108425309,0.335623101787659,1.4
2572756725206,0.153946995913969,0.914939715516253
"2403","UNC5B",6.56652816760895,0.86068611242225,0.604669759490517,1.4
2339863853527,0.15462062694247,0.914939715516253
"2404","SAMD8",61.6264013643933,0.364181083125298,0.256515690459401,1.
4197224445533,0.155688501212054,0.914939715516253
"2405","PGAM1",268.49610864841,-0.336613487736149,0.236293705578553,-1
.42455545699776,0.154285743613436,0.914939715516253
"2406","HPS6",14.9683561399206,-0.601964482587611,0.421759207286231,-1
.42727051878937,0.153501934877254,0.914939715516253
"2407","RRM1",40.1491498865324,-0.427782944995904,0.300385565161893,-1
.42411285564055,0.154413805579338,0.914939715516253
"2408","DCHS1",36.4259825876025,-0.430389228807157,0.302406534612259,-
1.42321404978565,0.154674113937977,0.914939715516253
"2409","FTH1",14684.1763728879,-0.38030553495035,0.266808996487854,-1.
42538497560618,0.154045948668771,0.914939715516253
"2410","ANKRD13D",46.2656508272745,-0.356334499796259,0.25115494332019
,-1.41878354089164,0.155962132636104,0.914939715516253
"2411","AP003307.1",9.33508785350261,-0.723113619476559,0.509402374161
527,-1.41953327301782,0.155743603498894,0.914939715516253
"2412","NPAT",31.6255281727454,0.429827060497873,0.301028569957553,1.4
2786135069665,0.153331769976804,0.914939715516253
"2413","MAP3K12",56.1209835123128,0.351346361119696,0.246551101946625,
1.42504478116572,0.154144256851431,0.914939715516253
"2414","CAPS2",9.26454175519269,0.822112234305942,0.578370004795223,1.
42142958225681,0.155191911549965,0.914939715516253
"2415","RPL6",4373.78724861458,-0.333100392816553,0.23481719157317,-1.
41855198328934,0.156029673189238,0.914939715516253
"2416","GRTP1",6.82373812969138,0.804450867256675,0.567193255802954,1.
41830118575342,0.156102850663575,0.914939715516253
"2417","CCDC88C",11.3998503856536,0.778978120110667,0.54777942071958,1
.42206532528618,0.155007287758814,0.914939715516253

"2418","KLF13",134.482175378972,-0.390929739703462,0.273537613191971,-1.42916264838907,0.15295749052159,0.914939715516253
"2419","CAPN3",7.80597283658312,0.756034643810843,0.529880383598389,1.42680247696028,0.15363683703308,0.914939715516253
"2420","PIGB",10.5224123768581,0.668059639448017,0.467579134348106,1.42876272778814,0.15307244169993,0.914939715516253
"2421","MAN2A2",25.1506372834237,0.487718752179975,0.34132325832719,1.42890570824345,0.153031336567855,0.914939715516253
"2422","RPUSD1",17.7352520424031,-0.543563885695252,0.38295904570388,-1.41937863015138,0.155788659195196,0.914939715516253
"2423","PSMB6",317.479537246704,-0.404601782034699,0.284304340794063,-1.42312910490443,0.154698732545695,0.914939715516253
"2424","ZFP3",3.71235326432675,-1.12529920241664,0.790291064975961,-1.42390475141063,0.154474046217224,0.914939715516253
"2425","MTMR4",21.5255658835328,-0.477662418403683,0.335142635696564,-1.42525112452763,0.154084622791152,0.914939715516253
"2426","RAD51C",32.6466058887173,0.443306287512676,0.310456404461339,1.42791799796123,0.153315462594844,0.914939715516253
"2427","SLC25A19",12.834337908937,-0.586009249843567,0.41008708703305,-1.4289873257978,0.153007876284085,0.914939715516253
"2428","ANKRD29",6.4812284151832,-0.857342477529798,0.603025488878403,-1.42173505654696,0.155103179058966,0.914939715516253
"2429","ZNF787",47.492421275543,-0.504411994570965,0.353764925123929,-1.42583947347031,0.15391468385485,0.914939715516253
"2430","PSMF1",133.417230427651,-0.297454966484896,0.209549638596135,-1.41949644235932,0.155754333332417,0.914939715516253
"2431","OSER1-DT",20.8844482355346,0.56879154385044,0.400678448891484,1.41957109353911,0.155732585873559,0.914939715516253
"2432","H1F0",102.645390453751,-0.285035195961649,0.199632026587035,-1.42780294742628,0.153348584251542,0.914939715516253
"2433","AL021707.2",2.64956254183615,1.44762119308658,1.02066733868248,1.41830853033392,0.156100707298613,0.914939715516253
"2434","PLXNB2",52.6306774770396,0.392624658093234,0.275532196773899,1.4249683437737,0.154166352016222,0.914939715516253
"2435","IPO13",13.1031985098189,-0.579877043998406,0.410362701299167,-1.41308418665384,0.157630972229922,0.915368559105224
"2436","WNT2B",10.7868484870456,0.731411971178076,0.517013726424936,1.41468578839419,0.157160643032132,0.915368559105224
"2437","AL133243.3",12.2781638503634,0.615046677595535,0.435541980298741,1.41214097702745,0.157908455345434,0.915368559105224
"2438","LINC00852",2.72226427397676,-1.29466419859874,0.915036384789143,-1.41487728807316,0.157104478167287,0.915368559105224
"2439","INTS12",22.6521628672306,-0.556481151104209,0.393452809785536,-1.41435297261579,0.157258290647021,0.915368559105224
"2440","LEF1",4.67429421971856,-1.05375030091533,0.74345787091121,-1.41736383747449,0.156376579803704,0.915368559105224
"2441","KMT2C",154.913598921687,0.365228122235373,0.258463366249968,1.41307500375952,0.15763367196626,0.915368559105224
"2442","PABPC1",993.945361781638,-0.246170448111608,0.173821785517335,

-1.41622321608851,0.156710160639276,0.915368559105224
"2443","APBA1",5.12833852151587,0.957942844723284,0.676265508665061,1.41651885605441,0.156623647389598,0.915368559105224
"2444","STAMBPL1",20.5984457698177,0.553249033436948,0.390760049890312,1.4158280346014,0.156825859349724,0.915368559105224
"2445","MARVELD1",88.6703216843603,0.302523316884158,0.213908140831333,1.41426743137699,0.157283395735622,0.915368559105224
"2446","COX6A1",447.739930124079,-0.338201947746161,0.239480969598474,-1.41222890617659,0.157882571815416,0.915368559105224
"2447","PTGR2",13.2740350507276,-0.553745113497423,0.391851028004216,-1.41315212650524,0.157610999257896,0.915368559105224
"2448","GPR176",17.9924735113134,-0.617255174389866,0.436815548487465,-1.41307967751422,0.157632297895606,0.915368559105224
"2449","NDUFAB1",254.870583104705,-0.407099114224381,0.288252924668683,-1.41229829564539,0.157862148043386,0.915368559105224
"2450","MMP2",2182.35776921477,-0.351026791395006,0.247923592881888,-1.41586682943175,0.156814498381152,0.915368559105224
"2451","NOB1",84.8203519814914,-0.362200927324039,0.255593772223911,-1.41709605900231,0.1564548446335,0.915368559105224
"2452","RTN4RL1",1.99800078480233,1.62511680228915,1.14710152997965,1.41671574818489,0.156566050856511,0.915368559105224
"2453","MMD",35.5401100256722,0.562631098582436,0.396853173559193,1.41773113097839,0.156269277568092,0.915368559105224
"2454","FSTL3",121.288999406315,-0.353499964504647,0.249844423946403,-1.41488034401953,0.15710358201334,0.915368559105224
"2455","EIF3G",521.538526698794,-0.305200160952593,0.216125825840036,-1.41214109774408,0.157908419808116,0.915368559105224
"2456","ICAM3",42.7003418796182,-0.41838974966523,0.295750954660719,-1.41466914331756,0.157165525578868,0.915368559105224
"2457","SLC5A3",195.687031807041,-0.631097812610464,0.446446948492729,-1.4136009098979,0.157479113983109,0.915368559105224
"2458","ARSA",54.270648175295,0.482184033397894,0.34022503677678,1.41725029399953,0.156409762043652,0.915368559105224
"2459","KLHL4",6.9602635696014,-1.11701660163789,0.790869978668695,-1.41238968701051,0.157835251389898,0.915368559105224
"2460","CHRDL1",524.59230048443,0.304879188611154,0.215641127873036,1.41382672043275,0.157412786019631,0.915368559105224
"2461","BCAP31",181.770301646075,-0.267486383917262,0.189113702061501,-1.41442095946212,0.157238339679233,0.915368559105224
"2462","TMEM265",22.0035206434112,-0.554809721853684,0.392965447242614,-1.41185370303346,0.157993042029027,0.915486895851382
"2463","AC005162.3",2.12946445768133,1.52128389399059,1.07824804219172,1.41088491187828,0.158278551752914,0.915783023968502
"2464","AL513534.2",9.26235955272903,0.885069657880731,0.627272766399096,1.41098052600233,0.158250356217993,0.915783023968502
"2465","AFAP1L2",5.41320250528865,-0.878323375848705,0.622333044475405,-1.41133976999259,0.158144453177064,0.915783023968502
"2466","ZNF268",27.6575707563776,0.435992277395422,0.309084730052589,1.41059144953955,0.158365114266809,0.915783023968502
"2467","ASCC2",57.0195741194754,-0.359485389167892,0.254830749831561,-

1.41068293133974,0.158338126058166,0.915783023968502
"2468","SLC49A3",31.1968673041785,-0.516897777986059,0.366578401469068,-1.41006064709373,0.158521775980051,0.915946397785097
"2469","ATP8B1",6.31006714248486,-0.846430509289576,0.600214845527882,-1.41021255238222,0.15847693047965,0.915946397785097
"2470","VPS13D",62.9100284872663,0.351176834837167,0.249521125572621,1.40740321698717,0.159307857827243,0.916175273529893
"2471","DNPEP",129.894229829328,-0.328995508456946,0.233665699786315,-1.40797519172822,0.159138416105343,0.916175273529893
"2472","GNB4",103.294439324632,0.435130736930222,0.309216134299413,1.40720579770552,0.15936637296355,0.916175273529893
"2473","MALINC1",6.14584755259524,0.860619211402824,0.611718173955462,1.40688841372479,0.159460479754292,0.916175273529893
"2474","LYRM4",74.2433861726444,-0.305286930552203,0.216792334013822,-1.40819984221739,0.159071902984462,0.916175273529893
"2475","HMGN3-AS1",11.1354417783239,-0.67633122091541,0.480642325778258,-1.40714037162726,0.159385768860214,0.916175273529893
"2476","EEPD1",9.96340129139352,-0.645881921455364,0.458736538625993,-1.40795830955587,0.159143415324025,0.916175273529893
"2477","DEPP1",1604.64512418681,0.86783987652495,0.616552162973667,1.40756926768257,0.159258652936544,0.916175273529893
"2478","PRPF31",106.369578246673,-0.32864004445165,0.233168501495697,-1.40945300220028,0.158701261074418,0.916175273529893
"2479","RRBP1",600.470828076576,0.274695469403618,0.194909481503366,1.40934893102609,0.158732016860374,0.916175273529893
"2480","ZNF217",84.7711771172382,0.368462041942208,0.261727541955939,1.40780767353952,0.159188027544737,0.916175273529893
"2481","RBM38",24.4703985347798,0.589290149263358,0.41879372283527,1.40711313740286,0.159393843112174,0.916175273529893
"2482","PI4KA",35.6953164545243,0.390380003861316,0.277453906479301,1.40700849670841,0.159424869283995,0.916175273529893
"2483","GTPBP1",34.0301539249318,0.40208804611063
7,0.285779673587035,1.40698616197481,0.159431492168044,0.916175273529893
"2484","LPAR4",9.5027345917221,0.839570571539157,0.596864661490903,1.40663474604444,0.159535724392192,0.916238584613131
"2485","AL160006.1",5.7626562933431,0.88769287411627,0.631362710696173,1.40599509454313,0.159725581426013,0.916590967266092
"2486","VWF",1.97014079947954,-1.48673425866408,1.05731756774526,-1.40613785679789,0.159683192893175,0.916590967266092
"2487","NRBP1",153.027952995473,-0.238803928391794,0.170110309068921,-1.4038180854462,0.160373027052959,0.916728662079708
"2488","BIRC6",102.010237563334,0.295075994095823,0.209936163920685,1.40555104268412,0.159857482348077,0.916728662079708
"2489","POMGNT2",28.7073793378703,0.475440652753804,0.338698513759653,1.40372819318359,0.160399803723482,0.916728662079708
"2490","LIMD1",14.6068912784675,0.553564441313151,0.394452068001556,1.40337568546089,0.160504839619604,0.916728662079708
"2491","SLC12A8",2.16935396807863,-1.46891971877944,1.04703827227036,-1.40292839114113,0.160638193672829,0.916728662079708

"2492","RNF14",28.4367402392124,0.451763618507908,0.321509878028696,1.4051313797195,0.159982214495619,0.916728662079708
"2493","B3GALT4",28.905651747166,-0.415350471372013,0.296067434357078,-1.40289144692311,0.1606492117762,0.916728662079708
"2494","COL1A2",8711.89187485189,0.430748826626703,0.306621646882076,1.40482197198675,0.160074223721371,0.916728662079708
"2495","FIS1",387.925701215193,-0.370682160656248,0.263939181075741,-1.40442263685692,0.160193033977413,0.916728662079708
"2496","NDUFB2",407.742656199246,-0.339740296871505,0.241827129984834,-1.40488909119837,0.160054260943532,0.916728662079708
"2497","LY6K",3.8479933643974,1.26148721285562,0.898959617669159,1.40327461663565,0.160534964446053,0.916728662079708
"2498","CASP1",27.5188084105616,0.564769245450721,0.402376921208895,1.4035826004979,0.16044317933387,0.916728662079708
"2499","LINC00324",7.8475220389968,0.77558008706546,0.552629325045325,1.40343635763782,0.160486757571301,0.916728662079708
"2500","SLC38A5",4.7170714739385,-1.07481083043862,0.765724241453358,-1.40365261049933,0.160422320541294,0.916728662079708
"2501","CASP9",7.17103894686402,-0.761821846244841,0.543789434271944,-1.40095007043454,0.161229004358466,0.916824735965291
"2502","ALG14",44.1696508294159,-0.41094195393369,0.293240047955053,-1.40138414517201,0.161099230842224,0.916824735965291
"2503","WNT5A",9.9393055824584,0.881420568391516,0.6285213443314,1.40237173540883,0.160804269094705,0.916824735965291
"2504","RBM47",20.5470840304626,0.564786572605643,0.403062194585666,1.40123926330085,0.161142536796196,0.916824735965291
"2505","SLC1A3",84.7483350180825,0.371116609332522,0.264749478227242,1.4017652152424,0.160985368962657,0.916824735965291
"2506","STN1",27.9076443555502,-0.458720313951438,0.327493349318059,-1.40070115899035,0.161303455987202,0.916824735965291
"2507","SPA17",14.1754558456214,-0.613384864499744,0.437963845904735,-1.40053767048426,0.161352370980687,0.916824735965291
"2508","IPO8",28.8923692832077,0.472716831447805,0.337415589663678,1.40099285844791,0.161216208697489,0.916824735965291
"2509","TPCN1",54.6677338713242,0.446175902830345,0.318289295312353,1.40179361794901,0.160976884807497,0.916824735965291
"2510","BRI3BP",14.0644448476193,0.57782014452138,0.412590514291908,1.40046880504037,0.16137297855102,0.916824735965291
"2511","CES1",53.972780384406,0.751772721116113,0.536447644170197,1.40139066558675,0.161097282062681,0.916824735965291
"2512","ABCB10",13.1243200431382,0.6069574370859,0.433481948573557,1.40019080167742,0.161456189595228,0.916932325145513
"2513","NCDN",20.2827759843027,0.537535121309183,0.384080653452883,1.39953709325566,0.161651983097203,0.917520288987546
"2514","NGF",4.95839934308364,1.01812201095077,0.731687677853592,1.3914707624125,0.164082719656409,0.917520288987546
"2515","SRGAP2C",59.0579395959574,0.341037424114957,0.243883466806979,1.39836221200213,0.162004324640191,0.917520288987546
"2516","MPZL1",291.883235881258,0.283549943455418,0.202807490249676,1.39812362505123,0.162075946536239,0.917520288987546

"2517","KCTD3",77.482282419576,0.318909849927737,0.228752350552901,1.39412709489945,0.163279228497451,0.917520288987546
"2518","SLC16A14",2.70866414817012,1.32887853509604,0.950083020957842,1.39869727779822,0.161903780901176,0.917520288987546
"2519","CIP2A",5.72945608625646,0.82883567714823,0.594355525109256,1.39451160481073,0.163163167604322,0.917520288987546
"2520","SHOX2",76.6872230713793,-0.317427836758346,0.227337335440683,-1.39628555135049,0.162628523017368,0.917520288987546
"2521","MRPS18C",149.36896050699,-0.368777228123982,0.264398698900888,-1.39477701538245,0.163083092081186,0.917520288987546
"2522","AC098487.1",21.5502862990881,0.493630622506458,0.354529559091972,1.39235392324058,0.16381525002966,0.917520288987546
"2523","NFKB1",176.889931706333,0.330850009380539,0.236985929181201,1.39607448646273,0.162692065896354,0.917520288987546
"2524","NSA2",462.364156754509,-0.338475181507455,0.243271447147138,-1.39134775361753,0.164119999568921,0.917520288987546
"2525","SLC12A2",22.5215210070809,-0.511621914442322,0.367384934011433,-1.39260450573185,0.163739419752991,0.917520288987546
"2526","TSPAN17",21.9869224416849,-0.471727850201073,0.33875148670516,-1.39254842772588,0.163756387557991,0.917520288987546
"2527","TENT5A",292.847722288916,-0.368821687636476,0.264594820869968,-1.39391121271315,0.163344417890584,0.917520288987546
"2528","AC016831.5",51.2570738697801,0.686202995439887,0.492392941665418,1.39360851339369,0.163435856274062,0.917520288987546
"2529","MICU3",43.0554232449364,0.446178135931484,0.319746300843671,1.39541297195375,0.162891341797537,0.917520288987546
"2530","SFRP1",596.518162487953,1.07545410458965,0.771244643161924,1.39443964262823,0.16318488401556,0.917520288987546
"2531","EFR3A",94.0550867792585,-0.272901135730645,0.19611101578836,-1.39156454130631,0.164054302650148,0.917520288987546
"2532","RAD23B",203.473093785806,0.235002231835783,0.168207259763959,1.39709922250417,0.162383735597964,0.917520288987546
"2533","RPS13",4790.99309320394,-0.332058326122958,0.238230808744482,-1.39385131533979,0.163362508424478,0.917520288987546
"2534","PPP6R3",72.0580871725381,0.312254563068857,0.223645284088682,1.39620454927652,0.162652907166276,0.917520288987546
"2535","CLEC7A",8.25405173373614,-0.859820155895441,0.617498841410953,-1.39242391763974,0.1637940659398,0.917520288987546
"2536","LARP4",77.1324143646272,0.405298635875202,0.289878390493237,1.39816781508126,0.162062679239453,0.917520288987546
"2537","MSRB3",51.9163917646946,-0.364572725690436,0.261517768816741,-1.39406483674121,0.163298026421661,0.917520288987546
"2538","DHRS12",41.153468206857,-0.416509355973283,0.299032220597776,-1.3928577834879,0.163662800731688,0.917520288987546
"2539","MLH3",52.9046035870805,0.329599446277517,0.236791674217074,1.39193849347661,0.163941024034182,0.917520288987546
"2540","BTBD6",94.2325708343544,-0.376455150366471,0.269732444034583,-1.39566136255453,0.162816494562695,0.917520288987546
"2541","CHD2",141.549499182354,0.35137291795293,0.252636048478626,1.39082652720741,0.164278036928411,0.917520288987546

"2542","HAGHL",14.352580011282,-0.649878155128393,0.466621981086593,-1
.39272940724966,0.16370163243319,0.917520288987546
"2543","RBL2",103.353009766565,0.340926998762209,0.243748736046734,1.3
9868211951197,0.161908328455332,0.917520288987546
"2544","FAAP100",25.0981501696951,0.451117169340156,0.322730893946809,
1.39781216425629,0.162169480536124,0.917520288987546
"2545","ZNF532",54.0787255492101,0.403447421654949,0.28879896218372,1.
39698362696433,0.162418494775864,0.917520288987546
"2546","PLCB1",34.0037092607699,0.552810884532135,0.397371587415786,1.
39116862412638,0.164174299227165,0.917520288987546
"2547","AHCY",60.6463825371874,-0.387210745169382,0.278427447814408,-1
.3907060823525,0.164314572458099,0.917520288987546
"2548","MYL9",421.856440778422,0.48312909863794,0.345390079006375,1.39
879263477346,0.161875175590035,0.917520288987546
"2549","SYCP2",2.18214383259036,1.57192990842122,1.12422475597225,1.39
82345612571,0.162042641401755,0.917520288987546
"2550","TOM1",56.3615547797322,0.351561380223977,0.252519637035702,1.3
9221402482165,0.163857597043032,0.917520288987546
"2551","TMSB4X",8086.02258320845,-0.377638740419466,0.271275053854471,
-1.39208797511494,0.163895759139024,0.917520288987546
"2552","ARHGEF9",23.3142354690044,0.517572886070227,0.371123756193113,
1.39460995808878,0.163133490536038,0.917520288987546
"2553","ENOX2",29.8202615535751,-0.473468496173584,0.33893049233642,-1
.39694865725926,0.162429011150837,0.917520288987546
"2554","AL683813.1",6.97002826145301,0.944712869939347,0.6793217071991
35,1.39067081167541,0.164325272561558,0.917520288987546
"2555","MT-
ND2",9533.35769061564,-0.307854241528514,0.220804567031573,-1.39423855
976899,0.163245577414457,0.917520288987546
"2556","HSDL1",20.7284184517367,0.465618179904757,0.335011761692267,1.
38985621744368,0.164572542959726,0.91854143108899
"2557","PNO1",69.6108045012855,-0.522269570181713,0.376106809516802,-1
.38862035189602,0.164948225339827,0.919091572409147
"2558","NCKIPSD",11.8132244951437,0.656491970336498,0.473104378474478,
1.38762607197455,0.165250938120669,0.919091572409147
"2559","CHSY3",9.93565854700356,0.74012322145294,0.533153188273551,1.3
8819993527488,0.165076172000746,0.919091572409147
"2560","IRF7",51.6813602015543,-0.450092783278063,0.324089017426862,-1
.38879369270708,0.164895493749425,0.919091572409147
"2561","NR1H3",43.5333634751837,0.424398873123179,0.305492294856543,1.
38922938571159,0.164763008673585,0.919091572409147
"2562","DYNC2H1",61.1979392047878,0.447436508560306,0.322302221638024,
1.38825139425449,0.165060507322616,0.919091572409147
"2563","C12orf66",8.11381725843628,-0.711861534738745,0.51297082676195
7,-1.38772323415009,0.165221338259486,0.919091572409147
"2564","ABCC3",4.6500802730583,-0.968711947037386,0.698082423286952,-1
.38767560208172,0.165235848578458,0.919091572409147
"2565","MICOS13",236.547005403357,-0.394564310549626,0.284302824516262
,-1.38783113119249,0.165188472763447,0.919091572409147
"2566","PEAR1",33.3854249957744,0.696653412850132,0.502268924502247,1.

38701277117735,0.165437868563759,0.919238631260364
"2567","SBF2-
AS1",8.28481763980568,0.770772626991923,0.555750063014726,1.3869051544
6959,0.165470685901908,0.919238631260364
"2568","NBDY",123.936056003232,-0.348020318717526,0.250899938150321,-1
.38708810087078,0.165414899951864,0.919238631260364
"2569","TUBB4B",494.769746286569,-0.281589396624345,0.203101085063419,
-1.38644949403602,0.165609692239103,0.91965273237954
"2570","NIT1",44.7170405388921,0.367804791993078,0.26546436119723,1.38
551476489838,0.165895120934433,0.920163217438035
"2571","SLC25A25-
AS1",2.0117134154749,-1.39100309216203,1.00381029669376,-1.38572307610
667,0.165831479044021,0.920163217438035
"2572","ANAPC5",170.807824972305,-0.249240349577193,0.179847727573075,
-1.38584097191844,0.165795468419439,0.920163217438035
"2573","CHST2",6.23950729079318,-0.863144798932565,0.623255732099156,-
1.38489668763326,0.166084060023688,0.92052976116787
"2574","MSH5",1.90759084697768,-1.48254115938983,1.07066862136782,-1.3
8468722236001,0.166148127845316,0.92052976116787
"2575","SLC30A4",22.1293808222176,0.503362442070953,0.363526392494523,
1.38466546711196,0.166154783051119,0.92052976116787
"2576","MICALL2",32.3751287952835,-0.440488296042067,0.318391750748983
,-1.38347898463407,0.166518046761618,0.920826217052783
"2577","TMCO3",68.0999797980245,0.356796897494721,0.257894508333507,1.
38349939981395,0.166511791229501,0.920826217052783
"2578","ADSSL1",4.51222388946064,1.0170840570804,0.735116680000346,1.3
8356819366406,0.166490713012932,0.920826217052783
"2579","ZNF213",13.0359367718917,-0.560817837200061,0.405527730888086,
-1.38293338404231,0.166685292827377,0.920826217052783
"2580","CNP",95.6973744171055,0.327549968120641,0.236838529894799,1.38
30096322002,0.166661912456762,0.920826217052783
"2581","HOXB3",14.7183034760091,0.579336979599113,0.418778927092427,1.
38339573010857,0.166543559086763,0.920826217052783
"2582","MAP3K3",53.9687828392745,0.402845005235569,0.291193096294579,1
.38342910722046,0.166533330728552,0.920826217052783
"2583","SNRPB",300.590729415095,-0.36883211163647,0.266727495302387,-1
.38280499060784,0.166724668347633,0.920826217052783
"2584","NDUFA8",103.457458340745,-0.379346189792933,0.274463054055031,
-1.38213935970002,0.166928915201667,0.920884727094731
"2585","PAPSS2",84.573619861135,0.413804119555822,0.299350219245028,1.
38234112739069,0.166866983537096,0.920884727094731
"2586","FUT8",67.8315360287308,0.380411186769166,0.27516377397666,1.38
249007589725,0.166821275559231,0.920884727094731
"2587","FN1",1379.69830743497,-0.441640716715733,0.319780200969308,-1.
381075862036,0.167255636442597,0.921482778241216
"2588","CCDC136",8.71361432495596,1.01319287276768,0.733588033427762,1
.38114694705887,0.167233783164066,0.921482778241216
"2589","SLC35E3",45.8492871951336,0.377093552822477,0.273002371872128,
1.38128306445303,0.167191943327747,0.921482778241216
"2590","PCOTH",2.04939091165319,-1.43548629668277,1.03949520077549,-1.

38094557397846,0.167295695755275,0.921482778241216
"2591","ARMH3",20.4122562613873,0.473990625613251,0.343299058175671,1.38069305558859,0.1673733574182,0.921554734437682
"2592","CMSS1",86.7388093045812,-0.349369733567419,0.253226713456539,-1.37967171314009,0.167687746127039,0.922877124110328
"2593","FKBP1A",727.717774054536,-0.403869482066224,0.292766679964621,-1.37949264620902,0.167742912015848,0.922877124110328
"2594","RSAD2",47.4095690629794,0.71299541733068,0.51711268786161,1.37880085727367,0.167956162335706,0.922982516132968
"2595","GOT1",22.5962444765825,-0.582845942076792,0.422604222702227,-1.37917680601946,0.167840247427328,0.922982516132968
"2596","RPS17",1826.11263570689,0.268953542314892,0.195055060810083,1.37885959583874,0.167938047726564,0.922982516132968
"2597","TAF1B",58.4414655454535,-0.38995572378606,0.28324706216775,-1.37673351596887,0.168594652495525,0.92364489727387
"2598","CCDC74A",14.6615295056758,-0.629415359041767,0.457105189325388,-1.37695955710037,0.168524752021047,0.92364489727387
"2599","NOL7",288.101418440874,-0.30601012694284,0.222255185262738,-1.37684133929695,0.16856130672732,0.92364489727387
"2600","SNX13",67.0689841913713,0.307986080919959,0.223611417774894,1.37732716864219,0.168411119070802,0.92364489727387
"2601","RNF6",41.0886135568364,-0.351537018928371,0.255199607221739,-1.37749827578271,0.168358247506481,0.92364489727387
"2602","ABCA3",5.59685047276746,0.919234771004887,0.667093309345934,1.37797030509296,0.168212456541393,0.92364489727387
"2603","SLFN11",75.2513106457593,0.297785467646395,0.216107359531833,1.37795153432769,0.168218252269541,0.92364489727387
"2604","NFATC1",35.9956984328395,-0.515288305541687,0.374031404288022,-1.37766053768279,0.168308120604262,0.92364489727387
"2605","SCAMP1",72.4775229952018,0.311373815885996,0.226280414884072,1.376052876894,0.168805263262121,0.923734516953366
"2606","GGH",18.8617387879159,0.614663874768062,0.446597637083293,1.3763258551532,0.168720771653774,0.923734516953366
"2607","AL121603.2",19.2697677879428,-0.48305364920976,0.350997663641112,-1.37623038341267,0.168750318239942,0.923734516953366
"2608","GABPB2",47.7660084740724,0.525371294690163,0.381854610467149,1.37584117171569,0.168870811621452,0.923738879828079
"2609","ELOA",63.1853391871958,0.337049820768154,0.245302792406556,1.37401542583967,0.169436893505582,0.924129848437644
"2610","HACL1",24.5776910473516,-0.495023334595024,0.360394821998011,-1.37355839867687,0.169578819568918,0.924129848437644
"2611","PDS5A",87.9772158171982,0.28615892922125,0.208132146260908,1.3748905892823,0.169165367260622,0.924129848437644
"2612","AMD1",246.734178092286,0.3243314536253,0.235922125323341,1.37473945345647,0.169212234992453,0.924129848437644
"2613","FDFT1",50.6407916420872,-0.365256193957207,0.265880262122001,-1.37376197481559,0.169515589650898,0.924129848437644
"2614","FAM92A",91.8122560203091,-0.34464743269995,0.25071606771665,-1.37465235411022,0.169239249218337,0.924129848437644
"2615","CD274",4.1186205311884,1.03074203698118,0.750111263063076,1.37

411886440973,0.169404783865518,0.924129848437644
"2616","UBXN1",339.783637629129,-0.294252252790511,0.214018098857644,-
1.37489424661339,0.169164233230533,0.924129848437644
"2617","ATF7IP",87.4968260306094,0.422212644418555,0.30720607438675,1.
37436294272951,0.169329034597929,0.924129848437644
"2618","C1QTNF6",30.2829478161719,0.682496585110731,0.496895204381603,
1.37352218152339,0.169590070321727,0.924129848437644
"2619","DCUN1D4",38.0386325685297,0.382848382503199,0.278907987862729,
1.37266912087017,0.169855232992935,0.925221364596107
"2620","LINC00545",2.2481216829573,1.56320555039014,1.13924140571955,1
.37214601097018,0.170017988475854,0.925754436487228
"2621","IL18",9.32916805113797,-0.827159949991789,0.603015147852435,-1
.37170675220617,0.17015474557254,0.926145593413907
"2622","PCMT1",181.463798018115,-0.396939294346917,0.28956081661578,-1
.37083221060817,0.170427267353537,0.927116078116785
"2623","SLC7A5",8.7641060920993,1.00241863558986,0.731309405313988,1.3
7071754896885,0.170463022073484,0.927116078116785
"2624","AC139887.2",11.9606159059428,0.659169379786016,0.4810036460499
61,1.37040412312706,0.170560785733261,0.927294271825726
"2625","HNRNPA0",429.794945313444,-0.267856409016535,0.195573712722291
,-1.36959310782673,0.170813952261891,0.927610141974926
"2626","AL121821.2",3.26387218092666,1.33715825708211,0.97630314462948
3,1.36961379714655,0.170807490387331,0.927610141974926
"2627","OIP5-
AS1",125.995473994309,0.299058749538688,0.218326759350567,1.3697759744
5345,0.170756844054192,0.927610141974926
"2628","COL12A1",720.084252893581,0.432691969674963,0.316121862378064,
1.36875053949128,0.17107726638427,0.928005817033961
"2629","ROM1",18.2322069743768,-0.510429159815268,0.372876834270784,-1
.36889480091593,0.171032161163508,0.928005817033961
"2630","SPINDOC",12.8531708358688,-0.626312557255311,0.457584792458652
,-1.36873551651502,0.171081964026309,0.928005817033961
"2631","TMEM120A",57.2782962773654,-0.335811005860912,0.24549979099883
4,-1.3678667688255,0.171353783298122,0.928068744317013
"2632","PIGS",31.104932189887,0.430890955773102,0.314989896247764,1.36
795167370757,0.171327203474323,0.928068744317013
"2633","PKIG",115.231271330975,-0.284395521016565,0.207853238469949,-1
.36825157553502,0.17123334267727,0.928068744317013
"2634","PRMT2",169.116002710835,0.241850109952498,0.176731117400428,1.
36846364980835,0.171166992666036,0.928068744317013
"2635","TPBG",135.231581701473,-0.336527553694485,0.246070758291311,-1
.36760481428715,0.171435808703674,0.928160625034766
"2636","ZNF500",13.8472833410336,0.621185661859054,0.454395188802072,1
.367060385249,0.171606378921717,0.928731639490596
"2637","PTPN12",171.6496032089,0.296013883859148,0.21657592229078,1.36
679036491283,0.171691023701191,0.928837369784296
"2638","CA12",84.6007648531266,-0.622564161366419,0.45562585536499,-1.
36639339939938,0.171815519425934,0.929158529238203
"2639","PLK2",81.7368317134688,-0.51850053256584,0.379766886498787,-1.
36531264572878,0.172154806091012,0.929789117288539

"2640","PAXIP1-
AS2",28.3087977274332,0.472704756102686,0.346446208002545,1.3644391111
3385,0.172429405496076,0.929789117288539
"2641","POMT1",18.2494839543455,0.500662623066257,0.366810092344095,1.
36490961812632,0.172281458893498,0.929789117288539
"2642","KCTD21",8.72141341922718,0.734770771268875,0.538207655140417,1
.36521798649849,0.172184546816917,0.929789117288539
"2643","CCND2-
AS1",6.24521716567265,-0.866876315571786,0.635315158704571,-1.36448234
186538,0.172415808026285,0.929789117288539
"2644","TOGARAM1",41.2542165599246,0.405274599415606,0.296865429508589
,1.36517950266715,0.17219663904609,0.929789117288539
"2645","EXOSC6",89.8220565619329,-0.345107266300436,0.252919742442155,
-1.36449319048063,0.172412395910167,0.929789117288539
"2646","SPECC1",15.8735471171848,-0.721248371023231,0.528634001605573,
-1.36436243002275,0.172453526170298,0.929789117288539
"2647","MICOS10",384.950699241638,-0.299699737058524,0.219766072790556
,-1.36372158474228,0.172655208012838,0.930022967072988
"2648","DARS",134.812495348141,-0.24851574823614,0.182564214668308,-1.
36125115586128,0.173434332908396,0.930022967072988
"2649","D2HGDH",34.4012544316595,0.452503589514304,0.332109390329765,1
.36251368582199,0.173035828033148,0.930022967072988
"2650","RASGEF1B",14.2240842925127,-0.619825983711994,0.45483597241481
3,-1.36274617950998,0.17296251843389,0.930022967072988
"2651","TYW1B",13.3295286175319,-0.690837183485207,0.507639954734226,-
1.36088024010422,0.17355153898243,0.930022967072988
"2652","ADAMTS13",2.13963539294342,1.53677793574787,1.12862798656278,1
.36163373055111,0.17331350469761,0.930022967072988
"2653","ENTPD7",10.4287640950186,-0.608109781676724,0.446443649592438,
-1.36211990523747,0.173160047387473,0.930022967072988
"2654","CDKN1B",76.7749090112727,0.325159379384238,0.238962916912943,1
.36071062232094,0.173605156407919,0.930022967072988
"2655","SLC8B1",33.0963645408199,0.415703115646849,0.305174230662583,1
.36218289055498,0.173140173989028,0.930022967072988
"2656","TMED8",20.7871135965585,0.525851309988646,0.386362341007993,1.
36103148308071,0.173503740404959,0.930022967072988
"2657","CES4A",6.18576981438266,0.862302866373385,0.633378164900479,1.
36143447020924,0.173376429054194,0.930022967072988
"2658","ATMIN",85.0595640405217,0.290741714596037,0.213346461114366,1.
36276792723636,0.172955662157652,0.930022967072988
"2659","TMEM199",26.7986960016637,-0.447801162652346,0.328331767572737
,-1.36386791312584,0.172609141149682,0.930022967072988
"2660","DLGAP1-
AS1",23.9463138339142,0.558376113349758,0.410238642170909,1.3611007251
6556,0.173481860535357,0.930022967072988
"2661","SMIM26",214.874123024425,-0.345028064446377,0.253208588791971,
-1.36262385921609,0.173001085410464,0.930022967072988
"2662","AP001347.1",8.41940878587588,0.741724648475301,0.5441193227457
47,1.36316542616497,0.172830380832354,0.930022967072988
"2663","AP000547.3",4.70038022088597,-1.02130900691959,0.7494851405306

44,-1.36268079470727,0.172983133135271,0.930022967072988
"2664","CRAT",66.356967429696,-0.278326815226061,0.204662148144235,-1.35993302987278,0.173851117527972,0.930991007002272
"2665","PGK1",274.918902643629,-0.316052818700611,0.232465291467597,-1.35956992420378,0.173966061005001,0.931256970468045
"2666","BCAR1",31.9877736785656,-0.40670888179097,0.299227925396857,-1.35919427056002,0.174085036359623,0.931530873470192
"2667","SUZ12",71.1221917848216,0.308831988193958,0.22725009065093,1.35899610560923,0.174147822763564,0.931530873470192
"2668","FNDC5",20.593503878049,0.611047959486181,0.450120011104928,1.35752231496266,0.174615307974793,0.931977604708921
"2669","AZIN2",10.8421110920647,-0.669919682505687,0.494234546859842,-1.35546915277792,0.175268130213494,0.931977604708921
"2670","GBP5",15.0364752972564,0.521394516429204,0.38466736215207,1.35544256604511,0.175276595642369,0.931977604708921
"2671","FAM117B",14.9802382920135,0.551663327656389,0.406980555447932,1.35550291106467,0.175257381743948,0.931977604708921
"2672","WWC2",54.9098362601422,0.361622277906855,0.266582480219713,1.35651179180572,0.174936386106503,0.931977604708921
"2673","ANKH",80.4788021509635,-0.326528541390133,0.240765727819864,-1.35620856152099,0.175032818745063,0.931977604708921
"2674","FBXL4",32.2318017125154,0.430114749682234,0.316719376753695,1.35803105604342,0.174453829944588,0.931977604708921
"2675","GRB10",20.6121313931079,0.548898288394613,0.404863534243884,1.355761242908,0.175175146459718,0.931977604708921
"2676","WWP1",61.8797338070262,-0.344553867870834,0.253966648381556,-1.35668943172877,0.174879911864761,0.931977604708921
"2677","AC120053.1",4.68851629049265,0.994194025030233,0.733057569841866,1.35622912296602,0.175026278585294,0.931977604708921
"2678","ZFPM2",51.6719843805937,0.365957060227437,0.26942557087972,1.3582862941796,0.174372857562635,0.931977604708921
"2679","FRMD4A",28.251970138502,0.477287857849775,0.351836449041421,1.35656171823626,0.17492051241373,0.931977604708921
"2680","ZNF37A",101.069785558523,0.420455502710889,0.309591729231996,1.35809668996621,0.174433005394489,0.931977604708921
"2681","SESN3",98.3830939600447,0.420392131325374,0.309838946009801,1.35680855082716,0.174842049842649,0.931977604708921
"2682","AL162377.1",8.95364950514373,-0.694958208818128,0.512700830058866,-1.35548485212777,0.175263131557894,0.931977604708921
"2683","NAGLU",104.260403754159,0.412783043966521,0.304353325611885,1.35626263697511,0.175015618880373,0.931977604708921
"2684","GJC2",5.50860549936941,0.872482149369312,0.643923588563345,1.35494671241335,0.175434535322173,0.932469851306303
"2685","RPL5",4438.63280164519,-0.316324111116289,0.233536680609157,-1.35449433592697,0.175578719222843,0.932541328530558
"2686","PCDH7",41.0794944817221,0.686754134456966,0.506979716952725,1.35459883599447,0.175545404542923,0.932541328530558
"2687","KLHL21",38.5311131467716,0.636810906286193,0.470388745694967,1.35379707128271,0.175801128305343,0.933140931366686
"2688","ARSK",24.4739565747297,0.473069140957628,0.349455970275901,1.3

5373031567935,0.175822432602948,0.933140931366686
"2689","C2orf69",45.5592795974143,-0.366793811150779,0.271180246442894,-1.35258307329557,0.176188862998843,0.934390453361152
"2690","ENDOD1",23.3944644125056,-0.464311431534561,0.343235299638285,-1.35274965023665,0.176135622848549,0.934390453361152
"2691","ADAMTSL4-AS1",13.8151245513676,0.718223802608774,0.531381275454721,1.35161669367097,0.176497967179796,0.935154663219023
"2692","PTH1R",42.1601984335351,0.450380331817669,0.333196469290737,1.35169599118615,0.17647258802887,0.935154663219023
"2693","TBC1D1",23.9210815198545,-0.478701371084081,0.354484331067131,-1.35041616548469,0.176882528360782,0.935154663219023
"2694","NUP43",15.6564439188027,0.561741570378053,0.415764934731766,1.35110376910565,0.176662194276692,0.935154663219023
"2695","ZRANB1",45.2995686259387,0.392369787817655,0.290363401068833,1.35130593722671,0.176597450925896,0.935154663219023
"2696","CARD16",15.4131507060876,-0.507464298015361,0.375814903080106,-1.35030381673606,0.176918548581069,0.935154663219023
"2697","RTRAF",385.340257378379,-0.394463114280012,0.292105511650365,-1.35041311631314,0.17688350588592,0.935154663219023
"2698","AC009996.1",4.73408336349743,1.15581238989872,0.855973342135226,1.35029017027042,0.176922924157307,0.935154663219023
"2699","KDM6B",109.46874470695,-0.488286863165957,0.361430632737666,-1.35098361604664,0.176700681084952,0.935154663219023
"2700","TMEM115",85.9045030671261,-0.332755136304191,0.246483728664353,-1.3500085304102,0.177013246619415,0.935285546767621
"2701","MYCBP",23.2128599962919,-0.564472237430425,0.42042701152635,-1.34261648741626,0.179396188865737,0.935407866371236
"2702","LMX1A",3.946752226393,1.21783858407524,0.904352550543908,1.34664139924501,0.178095754026995,0.935407866371236
"2703","SMYD2",20.2123728200351,-0.510669906710988,0.379047437476153,-1.34724537411789,0.177901218848667,0.935407866371236
"2704","ZFAND2B",38.7651283739934,-0.358291185257426,0.266919534170284,-1.34231908642869,0.179492557437131,0.935407866371236
"2705","THUMPD3-AS1",75.5766224305638,-0.407520290662355,0.303373187042547,-1.34329699547641,0.179175824601284,0.935407866371236
"2706","TUSC2",54.3226782176098,-0.364103170589562,0.269976243117746,-1.34864892697527,0.177449757388221,0.935407866371236
"2707","FAM162A",140.77547456895,-0.420441136040615,0.312846532742136,-1.34392135452293,0.178973819803034,0.935407866371236
"2708","MYLK",25.7585446301195,-0.817553869794929,0.60786268374501,-1.3449647291359,0.178636625039466,0.935407866371236
"2709","SH3BP2",24.7387948629144,0.473284492176304,0.351919075100904,1.34486740180424,0.178668059001026,0.935407866371236
"2710","PAPSS1",56.4156879050308,0.333802641596788,0.248514477570342,1.34319193336455,0.179209833000128,0.935407866371236
"2711","AC026748.3",2.1634100948461,1.44963325169188,1.07500448726939,1.34849041921125,0.177500699612999,0.935407866371236
"2712","ISOC1",28.3098145848837,-0.395634770388874,0.294243898801913,-

1.34458104993782,0.178760566377323,0.935407866371236
"2713","RPS14",6748.4696108859,-0.302939478829643,0.225162931532293,-1.3454234085871,0.178488539997636,0.935407866371236
"2714","ECI2",140.187056480351,-0.33167265497794,0.246540089023311,-1.34530922046751,0.178525397185166,0.935407866371236
"2715","THSD7A",4.43495804505939,1.17494444141948,0.874804806032857,1.34309326299626,0.179241776774596,0.935407866371236
"2716","KCTD7",18.4302566440267,0.468014386259938,0.348692778943051,1.34219695537881,0.179532143416454,0.935407866371236
"2717","DMTF1",48.7196567078812,0.437798611846877,0.3261514929185,1.34231674958568,0.179493314810313,0.935407866371236
"2718","MTMR9",42.1759537487884,0.344228195979813,0.256102550215003,1.34410296067269,0.17891509485542,0.935407866371236
"2719","ATAD2",19.6480744883438,0.501473629752776,0.373633445220357,1.34215401797616,0.179546062132765,0.935407866371236
"2720","PAXX",84.0514535595936,-0.317827363400679,0.235528774198217,-1.34942053039006,0.177201930114961,0.935407866371236
"2721","MPP7",5.11893013548451,0.925117412363613,0.687988467405727,1.34466994171,0.178731845641268,0.935407866371236
"2722","CCDC186",133.914947305732,-0.364979882107982,0.270856914949412,-1.34750069857419,0.177819028596024,0.935407866371236
"2723","ZNRD2",85.9960271353867,-0.37990220544109,0.282033281317486,-1.34701196846848,0.177976378064795,0.935407866371236
"2724","UNC93B1",26.6672516155202,-0.463627723929596,0.344769859011624,-1.34474552171907,0.178707428606778,0.935407866371236
"2725","PANX1",45.3958180765509,-0.402585299433966,0.300020127859833,-1.3418609688149,0.179641079242215,0.935407866371236
"2726","TAS2R14",5.73424897635915,0.840201312440087,0.623483054124827,1.34759286059421,0.17778936811361,0.935407866371236
"2727","EMP1",1146.8529166666,-0.448727091241092,0.332900905700959,-1.34792992015521,0.177680923678592,0.935407866371236
"2728","CKAP4",188.907120477865,0.263039553610309,0.195517225829655,1.34535232122966,0.178511484627157,0.935407866371236
"2729","ERO1A",56.310953293718,0.326849521281984,0.243589394182118,1.34180522259363,0.179659158408607,0.935407866371236
"2730","IQCH-AS1",25.5741225187893,0.636426178388096,0.471742929995612,1.34909531849055,0.177306351777134,0.935407866371236
"2731","HAPLN3",57.7730293641267,-0.565073936435407,0.420482128360643,-1.34387147115739,0.178989952812568,0.935407866371236
"2732","DUSP14",52.1540674012748,-0.439313153829548,0.327291050994966,-1.34227059522109,0.179508273973382,0.935407866371236
"2733","AC007114.1",3.29277196187831,1.22461619915218,0.911774192535004,1.34311346951747,0.179235234723723,0.935407866371236
"2734","HELZ",87.6359643277065,0.438825382579874,0.326256198148403,1.34503309077447,0.178614548633359,0.935407866371236
"2735","ZNF358",110.858512284049,-0.270639978019287,0.201385645028169,-1.34388912368322,0.178984243604052,0.935407866371236
"2736","ZNF614",8.71330986234138,0.685580638972316,0.509561566383651,1.34543239561388,0.178485639441193,0.935407866371236

"2737","ABHD12",54.2253830205336,-0.358456211331439,0.265864154108552,
-1.34826830090484,0.177572103717337,0.935407866371236
"2738","RBL1",11.9784402604757,0.735984794353242,0.545607144837302,1.3
4892807273026,0.177360070237989,0.935407866371236
"2739","ZDHHC8",39.7464744791785,-0.336528997230157,0.249620393145061,
-1.34816307670259,0.177605937480334,0.935407866371236
"2740","ZNF674-
AS1",5.52726997386025,-0.898704789536709,0.668304793803094,-1.34475287
005273,0.178705054771229,0.935407866371236
"2741","AKR1A1",179.293182118752,-0.329739514001459,0.246227893510746,
-1.33916393183564,0.180517311428338,0.935481939575963
"2742","NGF-
AS1",3.54424940569215,1.05720266327187,0.790360041841752,1.33762159940
21,0.181019819772503,0.935481939575963
"2743","SFT2D2",205.153070270956,0.474136568905376,0.353750094568642,1
.34031503082291,0.180142946839719,0.935481939575963
"2744","BTG2",549.006562625504,0.367707531717648,0.274995669147737,1.3
37139355166,0.18117715300942,0.935481939575963
"2745","EPAS1",109.446821909996,0.384330543008601,0.286998981476474,1.
33913556428459,0.180526544516274,0.935481939575963
"2746","STK36",7.30998023976324,0.800173091598759,0.597248452751983,1.
33976586780886,0.180321475612618,0.935481939575963
"2747","ACAD9",39.362022873115,-0.442958268668171,0.330466732904462,-1
.34040199682136,0.180114686895748,0.935481939575963
"2748","TRA2B",349.141682271416,0.253722431962927,0.18977990835923,1.3
3692988976821,0.181245523165718,0.935481939575963
"2749","SAP30",52.5214143397755,-0.374850721738148,0.280029542025163,-
1.3386113444576,0.180697231044605,0.935481939575963
"2750","PCDHB16",15.7964386878677,0.571826113396285,0.427125290580977,
1.33877840063857,0.180642824370273,0.935481939575963
"2751","SMPDL3A",43.8884805154365,0.511428116377309,0.382142079125283,
1.33831929095053,0.180792375942984,0.935481939575963
"2752","PDE1C",10.4994170842932,0.860164167318228,0.641899617056104,1.
34002910184483,0.180235883809397,0.935481939575963
"2753","EPHX2",21.3792749902201,-0.473996767064247,0.354532066446179,-
1.33696444390935,0.181234243267914,0.935481939575963
"2754","ASAP1",115.236537889739,0.328381350162055,0.24495090018787,1.3
406007077753,0.180050127350053,0.935481939575963
"2755","TPM2",224.923054489039,-0.485247333091423,0.36295782165309,-1.
3369248550186,0.181247166759285,0.935481939575963
"2756","LMX1B",7.48181359439662,0.722102852184843,0.539012419702042,1.
33967757660206,0.180350190681139,0.935481939575963
"2757","LINC01465",1.90867891952675,-1.56429249573055,1.16946347134414
,-1.33761552546195,0.181021800777817,0.935481939575963
"2758","AC048341.2",4.61509970765454,-0.920759458744798,0.687262049943
47,-1.33975018527581,0.180326575815965,0.935481939575963
"2759","FOS",12214.7809910999,-0.162312106941735,0.121033138593738,-1.
34105509307294,0.17990256593885,0.935481939575963
"2760","NUFIP2",213.281061348344,0.32805015587424,0.245249410126656,1.
33761853170135,0.181020820295907,0.935481939575963

"2761","ZNF576",33.3074555773902,-0.44369617068964,0.331240837910334,-
1.33949718726936,0.180408869507124,0.935481939575963
"2762","CD93",2.89602962845665,-1.24397387253447,0.928391062312534,-1.
33992443813047,0.180269912100359,0.935481939575963
"2763","TRIOBP",353.962844833737,0.290189881065301,0.216666271023746,1
.33934035830384,0.180459895907012,0.935481939575963
"2764","NUDT11",8.76646655538951,-0.914606646189692,0.683941223252569,
-1.33725913147941,0.181138066253436,0.935481939575963
"2765","ALKBH8",14.0995964280293,-0.514692702676987,0.385091776029591,
-1.33654555800599,0.181371020049102,0.935782630025494
"2766","AKNA",51.0985687334488,0.531839346710368,0.398090308014612,1.3
3597662641625,0.181556913190989,0.936403081555548
"2767","IL15",8.58064136592599,-0.706389058477898,0.528831592691825,-1
.33575427081102,0.181629604245203,0.936439441330709
"2768","CDKN2AIPNL",34.7027426036967,-0.435935908574724,0.326835533977
608,-1.33380817951267,0.182266729640264,0.936567841207851
"2769","VOPP1",41.7099434272215,-0.333813478740964,0.250291127948714,-
1.33370080464603,0.182301930984254,0.936567841207851
"2770","SYPL1",236.955859900681,-0.280195863295868,0.209972655385763,-
1.3344397763656,0.18205977149713,0.936567841207851
"2771","SEC24C",30.9292393308522,0.415349867300175,0.311382709827818,1
.33388866559048,0.182240346714135,0.936567841207851
"2772","EMX2OS",119.066137836294,-0.317360749906572,0.237734382716434,
-1.33493837231409,0.181896517502353,0.936567841207851
"2773","TMEM260",33.5684087717426,0.503188922877263,0.376868123960617,
1.33518568137072,0.181815582035025,0.936567841207851
"2774","BRF1",18.3178841930638,0.498002084182664,0.373267293390083,1.3
3417015902925,0.18214809687375,0.936567841207851
"2775","TCF4",786.140446775116,0.364219254413266,0.27290838973496,1.33
458430782207,0.182012436750425,0.936567841207851
"2776","CPT1C",4.00289421999478,-0.969445095707365,0.726898585636133,-
1.33367310772654,0.182311011848745,0.936567841207851
"2777","MAPK8IP2",3.04236188762136,-1.13638961598585,0.85204833509291,
-1.33371496566792,0.18229728820326,0.936567841207851
"2778","TMEM181",49.8918062234954,0.378830077034746,0.284131939716871,
1.3332893071164,0.182436881361862,0.936672590571398
"2779","CNOT7",83.7286082511289,0.265559910464193,0.199188275342637,1.
33321055171237,0.18246271759413 4,0.936672590571398
"2780","OSGIN2",35.7120093622989,-0.50557531561405,0.379401141479455,-
1.33256139831996,0.182675780017564,0.937091937335693
"2781","FAM102A",47.1082118262733,-0.365106500380328,0.273965261301519
,-1.33267443706486,0.182638665655005,0.937091937335693
"2782","WLS",54.4266085639438,0.399136279508737,0.299631408896404,1.33
209092123829,0.182830313286138,0.937361395783931
"2783","DISC1",5.32197770576469,0.851701691939697,0.6394150000978,1.33
200142600569,0.182859719926166,0.937361395783931
"2784","APOOL",50.4715503913371,0.353623846007202,0.265559201275993,1.
33161963248897,0.182985210285739,0.937667747821966
"2785","RER1",310.570301403597,-0.297602188005762,0.22436501298742,-1.
32641976591265,0.184700695213036,0.937940240068499

"2786","LRRC42",33.712528672696,-0.396911159577434,0.299804662451655,-1.32389922268616,0.185536516424366,0.937940240068499
"2787","TTC4",42.3759712744779,-0.391483236256092,0.296511337006063,-1.32029769994288,0.186735643219459,0.937940240068499
"2788","OLFML3",546.085210760408,0.339110932467479,0.257132638100899,1.31881714811486,0.187230249954148,0.937940240068499
"2789","ADAMTSL4",172.202824458158,0.388232403115874,0.291743574037782,1.33073163443729,0.183277330069962,0.937940240068499
"2790","UBAP2L",76.8545874669529,0.330273979551977,0.24947908193051,1.32385439691481,0.185551406083751,0.937940240068499
"2791","UHMK1",64.528746039848,0.312705018087954,0.236957461992496,1.31966731690372,0.186946116640994,0.937940240068499
"2792","NME7",40.487096082513,-0.340166784772864,0.256703758983308,-1.32513363310345,0.185126832661693,0.937940240068499
"2793","TMSB10",6908.68358720954,-0.294772028205718,0.222448246894177,-1.32512632633129,0.185129255707936,0.937940240068499
"2794","AC012511.1",3.23625430265982,1.20435016029817,0.907245699555102,1.32747960215051,0.184350083060649,0.937940240068499
"2795","AC009309.1",2.07854969275341,-1.69637491160542,1.28744767156667,-1.31762629974789,0.187628777125409,0.937940240068499
"2796","RBM43",73.7906448729568,0.310837000248596,0.235360228695573,1.32068617527836,0.186606025583492,0.937940240068499
"2797","STAM2",57.1930332794091,0.328793839730132,0.24787162748066,1.32646823306062,0.184684650682539,0.937940240068499
"2798","CD302",303.884165025502,0.332789342835366,0.251588665160178,1.32275173296655,0.185917953352624,0.937940240068499
"2799","TMEM43",181.244027706629,-0.270834891209708,0.203956996753605,-1.32790193776434,0.184210504506619,0.937940240068499
"2800","RBSN",40.4807817649349,0.377331878755859,0.285630841431133,1.32104739413036,0.186485561923238,0.937940240068499
"2801","EIF1B-AS1",8.04045490209946,0.841770292728855,0.638633139537554,1.31808113393301,0.187476489292395,0.937940240068499
"2802","CEP70",15.7600036025078,-0.544315628226745,0.41316502307583,-1.3174291090145,0.187694829004019,0.937940240068499
"2803","LMLN",13.3812138522964,0.658240296868949,0.497287898236613,1.32366039713227,0.185615856672453,0.937940240068499
"2804","RAB28",49.4669926032651,-0.333567520659452,0.251992284651834,-1.3237211651949,0.185595666532367,0.937940240068499
"2805","CXCL1",94.3451059534184,1.67791435458645,1.2717661235906,1.3193576424643,0.187049575455757,0.937940240068499
"2806","SEC24B",34.7901240325996,0.358302010560027,0.270431328165488,1.32492789570877,0.185195067547615,0.937940240068499
"2807","AC095055.1",1.9847862997952,-1.38205149613944,1.04881101915783,-1.3177316703339,0.187593488763419,0.937940240068499
"2808","NOP16",58.0057960737707,-0.468439235339901,0.352412433023072,-1.3292358368901,0.183770175290478,0.937940240068499
"2809","AL590004.3",2.89393397780472,-1.4313752770727,1.08018479324093,-1.32512074418126,0.185131106856826,0.937940240068499
"2810","FAM8A1",72.7832395667501,0.336348231589267,0.25304292555349,1.

32921412781472,0.183777335358737,0.937940240068499
"2811","ZNF391",3.47116359901334,1.15786971516113,0.871523292315081,1.328558542693,0.183993657213617,0.937940240068499
"2812","SKIV2L",17.3532657540211,0.484842482990181,0.367338924001558,1.31987777856104,0.186875827849641,0.937940240068499
"2813","AC004540.1",9.19336313527201,0.767424044568312,0.579544487253988,1.32418487527082,0.185441652706807,0.937940240068499
"2814","ZNF736",13.3470942780341,0.567118006262583,0.430307806448997,1.3179356678248,0.187525184406654,0.937940240068499
"2815","LRRC17",101.19951779478,0.599578852786628,0.454848983770851,1.31819323375402,0.187438969988787,0.937940240068499
"2816","PAG1",14.4587732402885,0.624772479131519,0.47251408879017,1.3222303714397,0.186091450485643,0.937940240068499
"2817","ASPN",482.118625420231,0.488047892995989,0.369967129702986,1.31916555232298,0.187113771899465,0.937940240068499
"2818","INIP",38.2291271277992,0.361444228158177,0.273923100482425,1.31950984609042,0.186998720620065,0.937940240068499
"2819","RHOBTB1",8.65615520147203,0.825306643151084,0.622635869031193,1.32550449500322,0.185003879547606,0.937940240068499
"2820","CH25H",54.9468399762038,1.12776271196857,0.848209246327236,1.32958078074697,0.18365643393087,0.937940240068499
"2821","FGFBP3",7.99600249639194,-0.733415636370292,0.553301880412713,-1.32552529158808,0.18499698657407,0.937940240068499
"2822","MYOF",216.95615479159,0.353277087294274,0.266677443534628,1.32473554047851,0.185258880930284,0.937940240068499
"2823","SLF2",45.1478253248312,0.371933297423032,0.280649990270964,1.32525676221807,0.185086004543761,0.937940240068499
"2824","PPFIBP2",16.1280525781716,0.631381466539089,0.47866078860519,1.31905825914616,0.18714963631509,0.937940240068499
"2825","APLNR",3.22307836576969,-1.43392007237177,1.08418815739841,-1.32257492630484,0.185976777140889,0.937940240068499
"2826","KCNE3",8.52103847568842,0.844486114277767,0.634544190991336,1.33085469265496,0.183236827680007,0.937940240068499
"2827","JAML",9.2910575530175,0.705087429183103,0.531132614440018,1.32751672560437,0.1843378109175,0.937940240068499
"2828","C2CD5",25.702095250889,0.486789000888387,0.368212604584363,1.32203242047586,0.18615735535159,0.937940240068499
"2829","LRP1",1552.15868822417,0.266601136186414,0.202389892905389,1.31726506872081,0.187749789741306,0.937940240068499
"2830","CSRP2",49.512227744074,-0.593999026658935,0.447771812907403,-1.32656636602932,0.184652167972234,0.937940240068499
"2831","IKBIP",149.322043680621,-0.260405047364688,0.197725596847793,-1.31700220667506,0.187837884892451,0.937940240068499
"2832","POP5",56.3967059144306,-0.409177869336547,0.307935358583209,-1.32877845278681,0.183921072999719,0.937940240068499
"2833","PXMP2",32.4616534844373,-0.455632184703826,0.345301016531587,-1.31952170103776,0.186994760030137,0.937940240068499
"2834","N4BP2L2",323.738025229773,0.221987711123408,0.16853871245265,1.31713187963135,0.187794422700498,0.937940240068499
"2835","CUL4A",52.9641497161804,0.360575864974975,0.27177199555865,1.

32673331524581,0.184596916310218,0.937940240068499
"2836","HOMEZ",7.01296343384858,0.76742919909322,0.577110760529065,1.3
2977801070574,0.183591423004598,0.937940240068499
"2837","EXOC5",66.0757355132514,0.301093251165711,0.227250214643435,1.
3249415479679,0.185190539061689,0.937940240068499
"2838","GPR135",4.22219586314373,0.997991346215944,0.756849410332903,1
.31861283445669,0.187298580738501,0.937940240068499
"2839","PATL2",6.81944252525067,0.861986396954524,0.647875008389316,1.
33048255572863,0.183359330129567,0.937940240068499
"2840","NME3",194.418855481592,-0.258739893081749,0.195526540762633,-1
.32329806517601,0.185736274887803,0.937940240068499
"2841","ABCC1",31.7810999501163,0.420686752946679,0.319378543746372,1.
31720418038088,0.187770193044585,0.937940240068499
"2842","NLRP1",19.7274595076529,0.526695433549206,0.398688044425493,1.
32107155183991,0.186477507566561,0.937940240068499
"2843","FBXL20",51.1497418434945,0.409594356451769,0.310726273392973,1
.31818385352229,0.187442109297424,0.937940240068499
"2844","AC015802.6",7.40451360975503,0.977496328699577,0.7409167804081
65,1.31930650586842,0.187066663695446,0.937940240068499
"2845","CXXC1",35.6565327236327,-0.350642070659628,0.265847000289916,-
1.3189619227497,0.187181842581213,0.937940240068499
"2846","ZNF426",31.2095287136123,0.415291664400677,0.314291793622115,1
.32135700908563,0.186382353500306,0.937940240068499
"2847","ZNF846",15.6162108018104,-0.623050448750483,0.468157748075735,
-1.33085578805734,0.18323646717788,0.937940240068499
"2848","ZNF253",14.5686747486751,0.626252184562989,0.474206646253316,1
.32063139458499,0.186624299535447,0.937940240068499
"2849","ZNF528",11.5062957856964,0.662456346083068,0.501144624553063,1
.32188656452989,0.186205926983812,0.937940240068499
"2850","LENG8",108.496680407514,0.368460804477097,0.278895710446779,1.
32114188449453,0.186454059609742,0.937940240068499
"2851","SLX4IP",12.812960081691,0.566862766191272,0.427243053463827,1.
32679223593103,0.184577419494857,0.937940240068499
"2852","CST7",40.9317717032853,0.604899817060534,0.455675932654549,1.3
2747809070512,0.18435058272186,0.937940240068499
"2853","PFKL",88.613864802165,-0.261205149962974,0.197577152586361,-1.
32204127118798,0.186154408268621,0.937940240068499
"2854","MCM3AP",38.4084495337872,0.387232773316446,0.293995357244074,1
.31713907643435,0.1877920107817,0.937940240068499
"2855","RGL4",3.52440279543531,1.05152836481466,0.793285045460914,1.32
553660355932,0.184993237330639,0.937940240068499
"2856","SLC25A6",1055.56057263658,-0.320666156255307,0.241488103018323
,-1.32787558578394,0.184219211336795,0.937940240068499
"2857","GPR82",6.54481628891289,0.851925167666863,0.644506409758796,1.
32182574877059,0.186226182066367,0.937940240068499
"2858","AK2",88.4103192605711,-0.349466626553469,0.265875008337333,-1.
31440193923784,0.188710978224871,0.938091561071115
"2859","PGM1",64.1408381247663,-0.311302425968963,0.236955272104494,-1
.31376028566135,0.188926886276977,0.938091561071115
"2860","GBA",37.4583891015732,0.398137067515114,0.30264685200302,1.315

51696269136,0.188336220322242,0.938091561071115
"2861","SLC30A1",58.6201949074778,0.367781562874419,0.280040159972663,1.31331721461065,0.18907608017243,0.938091561071115
"2862","PELI1",28.5172032305177,-0.574045865971849,0.437657026536215,-1.31163406769695,0.189643632561972,0.938091561071115
"2863","CYBRD1",987.448584237812,0.307134257798103,0.234104955709952,1.31195111554422,0.18953662907217,0.938091561071115
"2864","TASOR",86.5345604167818,0.307297746110494,0.234078349326235,1.31279867187637,0.189250797695807,0.938091561071115
"2865","CYFIP2",10.643608931047,0.694963551620781,0.5289335096402,1.31389586584053,0.188881250146619,0.938091561071115
"2866","MYO6",47.2062750178729,-0.377902270776511,0.288039645414394,-1.31198005827578,0.189526863131404,0.938091561071115
"2867","LPL",24.891422246189,0.565166520044151,0.429343395012776,1.31635079660963,0.188056328851226,0.938091561071115
"2868","PARG",19.6718655206594,0.463044522416341,0.352495538404695,1.31361810850702,0.188974751673874,0.938091561071115
"2869","RPLP2",4387.30734667819,-0.181671199202155,0.138050580909082,-1.31597562289,0.188182224707209,0.938091561071115
"2870","NCAPD2",9.79076609909979,0.657886821785242,0.499793061568323,1.31631843731649,0.188067185107009,0.938091561071115
"2871","ARID2",46.8860349848351,0.366664630801134,0.279487925072001,1.31191582143191,0.189548538616158,0.938091561071115
"2872","APAF1",20.5312953349413,0.498228452748746,0.378404272844697,1.31665651923869,0.18795378444262,0.938091561071115
"2873","RBM26",105.681343775478,0.293056551349777,0.222701883572459,1.3159141119452,0.188202871671967,0.938091561071115
"2874","SMAD3",176.343577979846,0.299780742225859,0.228497876648766,1.31196292334333,0.189532644805425,0.938091561071115
"2875","PEAK1",69.1626648432496,0.525120390313067,0.399164331941072,1.31554938227945,0.188325332361122,0.938091561071115
"2876","LINC02126",1.87086122032778,-1.48106067748233,1.12888822718212,-1.31196396757479,0.189532292456815,0.938091561071115
"2877","HSD11B1L",13.8316918288515,-0.599358093659027,0.455724854125464,-1.31517534809286,0.188450978153548,0.938091561071115
"2878","ZNF121",27.0419390902859,0.43923156115296,0.334868208421681,1.31165500368987,0.189636565302478,0.938091561071115
"2879","LYL1",3.30101238336187,-1.17691875290872,0.894911987843895,-1.31512234599099,0.188468787652666,0.938091561071115
"2880","ZNF100",18.5253847486026,0.550544477490005,0.419036738371893,1.31383343529512,0.188902263185684,0.938091561071115
"2881","CD79A",2.08401341309902,1.64424722079085,1.25073059512124,1.31462940716779,0.18863448193949,0.938091561071115
"2882","ZNF415",7.71354368512382,0.783074305594622,0.596227786883303,1.31338109833497,0.189054563453286,0.938091561071115
"2883","AC008735.2",3.17896549343862,1.40918064922787,1.07407206648942,1.31199822916328,0.189520732047116,0.938091561071115
"2884","ATRX",321.576698928976,0.274105169005712,0.208548271564594,1.31434879296428,0.188728854337588,0.938091561071115
"2885","COPA",207.166089006112,0.236598531182924,0.180524374032093,1.3

1061820572142,0.189986785274169,0.938487354127873
"2886","C8orf48",7.7369367171814,-0.744716586282589,0.568186114333601,-1.31069128142287,0.18996208543755,0.938487354127873
"2887","CERS5",47.7542607917228,-0.309919979427302,0.236465773865263,-1.3106335617259,0.18998159469589,0.938487354127873
"2888","AC026401.3",4.91014266266634,0.946024725543512,0.721753206848953,1.31073158604129,0.189948463351096,0.938487354127873
"2889","TTC27",28.9870281128484,0.413467820349311,0.315543005268342,1.3103374609673,0.190081699963593,0.938535673493407
"2890","NFIB",920.024176585183,0.297036185843009,0.226710546160532,1.31020012466769,0.190128143585865,0.938535673493407
"2891","STK17B",91.6849442309216,0.486821285721066,0.371739447639276,1.30957661021077,0.190339105337031,0.938602722688589
"2892","NPM1",1413.33522418197,-0.345250186036147,0.26360908673952,-1.309705178628,0.1902955910116,0.938602722688589
"2893","CHST7",50.553198491698,-0.411761832880202,0.31435515753927,-1.3098618648519,0.190242570044401,0.938602722688589
"2894","FNDC3A",139.336558736861,0.239103495838078,0.182639899009705,1.30915258459145,0.190482669879095,0.938986098305171
"2895","S100A16",340.020950023392,-0.371400617194116,0.283750001765418,-1.30890084540391,0.190567940225949,0.939081946550392
"2896","KCNMA1",5.8728621027383,-1.12443767615626,0.859359203042174,-1.30846062062953,0.19071712288726,0.939168268936708
"2897","DCLRE1A",2.90385401882408,1.16388429245713,0.889474227511693,1.30850816859877,0.190701005767885,0.939168268936708
"2898","SAMD4B",191.534570329636,0.245699042158244,0.187860728567357,1.3078786824259,0.190914461085975,0.939510649931158
"2899","AC244090.1",13.048850565599,0.605610969873427,0.463052349414355,1.30786717881763,0.190918363532204,0.939510649931158
"2900","FERMT2",303.594816437897,0.259704483890554,0.198665772550183,1.30724321838052,0.191130121788695,0.939904280399006
"2901","ZNF785",17.0957933254611,0.571730485730443,0.437326755650305,1.30733022469727,0.19110058341906,0.939904280399006
"2902","CIB1",217.883552786014,-0.327390309027583,0.250481132119312,-1.30704578926782,0.191197160810742,0.939909957314284
"2903","FHL3",47.4527601115134,-0.420425068376526,0.32176294471884,-1.30662985056871,0.191338453563717,0.939933460483731
"2904","TRIT1",18.2960240179137,0.467597167704,0.357914346498583,1.30644991540134,0.191399600638248,0.939933460483731
"2905","ANKRD52",15.6175071202541,-0.541479082425613,0.414377760534148,-1.30672814517755,0.19130505633822,0.939933460483731
"2906","FANCE",3.97457619733161,-1.00263290658746,0.768110795754827,-1.30532328425636,0.191782788722425,0.94050119492272
"2907","RELN",4.90526216049967,-1.45477165138798,1.11477043234579,-1.30499662457564,0.191893997164223,0.94050119492272
"2908","CACNA1C",20.2562377235081,0.619917651213182,0.474976643242459,1.30515396921683,0.191840424633559,0.94050119492272
"2909","LINC01579",2.2903041562852,2.02662829207778,1.55242023952677,1.30546371431975,0.191734995102828,0.94050119492272
"2910","NF1",114.991288802563,0.333944655370596,0.255906605794229,1.30

494738240214,0.191910765345579,0.94050119492272
"2911","SUPT5H",87.398786840246,0.336724295079641,0.257899410397762,1.30564197320306,0.191674339535311,0.94050119492272
"2912","MUSK",2.05716429537585,-1.39024916344957,1.06605328204184,-1.30410851583965,0.192196585771785,0.941255232619391
"2913","MAN1B1-DT",5.71030391914013,-0.969128670525475,0.743040341221294,-1.304274635927,0.192139960138962,0.941255232619391
"2914","GPR157",7.0020367389127,-0.744874293368724,0.572776366158504,-1.30046268906737,0.193442436112079,0.941761406616285
"2915","FAM76A",55.2648837258392,-0.359363670978279,0.276738709373907,-1.29856669416181,0.194092674163975,0.941761406616285
"2916","PPIH",48.2521079367044,-0.377529607971009,0.291855720272814,-1.29354876998166,0.195821327827492,0.941761406616285
"2917","CYB5RL",7.62272080297853,0.758182880049823,0.585993109072359,1.29384265499307,0.195719775630263,0.941761406616285
"2918","SLC16A1-AS1",41.3599540346853,0.474508696400797,0.364656147680726,1.30124968252627,0.193173004339829,0.941761406616285
"2919","BX470102.2",4.00579349132129,-0.904602468182445,0.698353677761706,-1.29533572599172,0.195204439960221,0.941761406616285
"2920","MNDA",12.0842901048048,0.703832561945206,0.54459273934964,1.29240166291187,0.196218081132456,0.941761406616285
"2921","ACKR1",2.67691076643387,-1.33931454980488,1.03824235439112,-1.28998257886554,0.197056706863639,0.941761406616285
"2922","NIBAN1",180.809217441501,0.367826147631799,0.283387769247024,1.29796055986866,0.194300888081865,0.941761406616285
"2923","MALL",11.6299224553506,0.612207656505834,0.474484148283454,1.29025945064893,0.196960590742442,0.941761406616285
"2924","PDCD1",3.4357848541055,1.07298498661772,0.824858046950848,1.30081168582169,0.1933229210117,0.941761406616285
"2925","VILL",12.8972630497098,-0.596980993207519,0.461057743551094,-1.29480743259952,0.195386667341305,0.941761406616285
"2926","IFT57",209.863570946112,-0.296653016122138,0.229006209205392,-1.29539289415543,0.195184728080912,0.941761406616285
"2927","PHLDB2",111.684182292162,0.403455171521403,0.310067400843891,1.30118538880045,0.193195005323393,0.941761406616285
"2928","HPS3",23.69288960111,0.454374808673543,0.350010367783073,1.29817528421087,0.194227109124543,0.941761406616285
"2929","TNIK",8.27553482866795,0.695222330808446,0.539037443731527,1.28974775109447,0.19713825430336,0.941761406616285
"2930","TBL1XR1",200.146913070344,0.286622619718897,0.220978853950311,1.29705903798084,0.194610874087404,0.941761406616285
"2931","MED28",116.726232601399,0.248910116984075,0.191423802674264,1.30030912303854,0.193495042498407,0.941761406616285
"2932","GUF1",22.6369659188056,-0.40470586271826,0.313304348957108,-1.29173394517312,0.196449297653521,0.941761406616285
"2933","EXOC1",76.4875276929062,0.316078152867215,0.242844666369726,1.30156514282259,0.193065082112141,0.941761406616285
"2934","NUDT9",61.8075480855431,-0.323124120964602,0.250086609977062,-

1.29204886656762,0.196340222185015,0.941761406616285
"2935","TBCK",25.9060492743212,0.5940074892428,0.45661096764833,1.3009
0499643953,0.193290975670964,0.941761406616285
"2936","HHIP",5.01171124378825,-1.2079241536673,0.933461437414144,-1.2
9402683951623,0.195656150203547,0.941761406616285
"2937","CCDC112",26.8912579303199,-0.455775904026379,0.349944364666465
,-1.3024238994698,0.192771517022187,0.941761406616285
"2938","FCHSD1",11.2321258104799,-0.649980278239188,0.503878714012004,
-1.28995383246871,0.197066688151173,0.941761406616285
"2939","MPC1",160.829346526309,-0.28364551102782,0.218296975141251,-1.
29935612183487,0.193821742530539,0.941761406616285
"2940","C7orf31",3.67689463048625,-0.992348913624823,0.76666058154613,
-1.29437842183505,0.195534740318226,0.941761406616285
"2941","CREB5",160.007986051223,0.52621347593949,0.404832093068861,1.2
9983142381447,0.193658752829467,0.941761406616285
"2942","C8orf37",8.41210612721338,0.682002694072922,0.527803246954156,
1.29215327493459,0.196304069331722,0.941761406616285
"2943","AC027031.2",3.90313231905904,-1.05108438543583,0.8124637947215
41,-1.29369996825036,0.195769076324433,0.941761406616285
"2944","PLEC",140.200080684676,-0.36140007772883,0.278584928168853,-1.
29727074649847,0.194538046073936,0.941761406616285
"2945","CBWD1",43.9844650323887,-0.477540128567369,0.367744208007783,-
1.29856600911377,0.194092909393169,0.941761406616285
"2946","AL158152.1",28.8680021058712,-0.410947596542867,0.317314693661
308,-1.29507900123119,0.195292977986228,0.941761406616285
"2947","ANKS6",15.9567175708867,0.490649492994026,0.380518604865156,1.
28942313653204,0.197251022218524,0.941761406616285
"2948","LINC00963",85.2905114255383,-0.291618748746505,0.2256218100910
97,-1.29251134289173,0.196180120349729,0.941761406616285
"2949","ITGB1",840.751003225339,0.242517197535845,0.186454316732058,1.
3006789104505,0.193368383986066,0.941761406616285
"2950","ARNTL",28.159777650832,0.741731862376227,0.570811578565421,1.2
9943380658179,0.193795096133611,0.941761406616285
"2951","BBOX1-
AS1",4.46349635121701,-0.921870959226807,0.707420762676572,-1.30314376
94009,0.19252568306255,0.941761406616285
"2952","PPP1R14B",433.889811734494,-0.422708719028057,0.32596236161509
2,-1.29680223487645,0.19469924154471,0.941761406616285
"2953","ARL2",277.275417078877,-0.333137176997718,0.256070910000257,-1
.300956742792,0.193273261728671,0.941761406616285
"2954","SAC3D1",24.5550429851308,-0.460665982722874,0.354821721925165,
-1.29830265245157,0.194183355307211,0.941761406616285
"2955","WNT11",300.726765662607,-0.497883327655142,0.383892402132942,-
1.29693457043915,0.194653700424466,0.941761406616285
"2956","C1R",3498.12943454594,0.279478428751676,0.216026660538191,1.29
372193254021,0.195761486696409,0.941761406616285
"2957","ITGA5",78.6830964485888,-0.401627509244825,0.31135242206237,-1
.28994502944439,0.197069744800164,0.941761406616285
"2958","NABP2",50.1346377278255,-0.40076147307777,0.309945702872978,-1
.29300541792641,0.196009185252371,0.941761406616285

"2959","PCDH9",45.086903491384,-0.577497825699199,0.446646962371621,-1
.29296261779725,0.196023988490251,0.941761406616285
"2960","AL161431.1",7.26893269554579,-0.729478562650453,0.559913644267
102,-1.30284119724445,0.192628982685593,0.941761406616285
"2961","PLEKHG3",11.885613117734,0.675991524426607,0.520886048749391,1
.29777237468658,0.194365565133773,0.941761406616285
"2962","HHIPL1",8.24464358375726,0.761717789947004,0.589829548519593,1
.29142019395066,0.196558011957139,0.941761406616285
"2963","EXOC3L4",14.3638006585647,0.611805012318739,0.472314504884284,
1.29533394801972,0.195205053037591,0.941761406616285
"2964","NR2F2-
AS1",10.9370897431836,0.742924293509476,0.574916971202693,1.2922288447
24,0.196277905280644,0.941761406616285
"2965","HSF4",11.5538490382104,0.599058232582632,0.464169155990623,1.2
9060327436908,0.196841280022203,0.941761406616285
"2966","PHLPP2",21.6556512521928,0.483416609483414,0.371075111765129,1
.30274597825734,0.19266149934151,0.941761406616285
"2967","IFT20",113.294900634345,-0.295023920566892,0.228466749148969,-
1.29132104197152,0.196592377122864,0.941761406616285
"2968","ASPSCR1",33.8710090635222,-0.37304343406424,0.289015831661576,
-1.29073702267306,0.196794882148313,0.941761406616285
"2969","ZADH2",50.7762590556993,0.340002849591439,0.263076506407782,1.
29241053955771,0.196215008681358,0.941761406616285
"2970","ZNF700",8.07427151638168,0.65454017828823,0.506396317412479,1.
29254529660231,0.196168369893565,0.941761406616285
"2971","DDX39A",53.9515004838283,-0.421229493069152,0.32663296913226,-
1.289611070763,0.197185729956242,0.941761406616285
"2972","ZNF333",19.6001804717557,0.546497712149541,0.421885981151282,1
.29536826670137,0.195193219575815,0.941761406616285
"2973","HCST",73.74123708307,-0.360618229713545,0.276923703396178,-1.3
022295502008,0.192837926387829,0.941761406616285
"2974","PSMC4",150.029246412512,-0.397689426089771,0.307255732625308,-
1.29432711536987,0.195552454240044,0.941761406616285
"2975","AC011462.5",3.65543225565776,1.36714814299487,1.05383885353813
,1.29730284512176,0.194527005850567,0.941761406616285
"2976","ZNF28",7.56446551271486,0.70488896930185,0.54449636596635,1.29
457056715308,0.195468411233116,0.941761406616285
"2977","ZNF160",33.6954394211592,0.378480172057149,0.292756848931418,1
.29281406545612,0.196075374496205,0.941761406616285
"2978","AL117332.1",6.94114656864571,-0.735434020246333,0.566361938776
469,-1.29852302899294,0.194107668162188,0.941761406616285
"2979","BCL2L1",47.524108901821,0.327965299945545,0.254040966210098,1.
29099375127675,0.1967058443094,0.941761406616285
"2980","CBFA2T2",33.4170881048501,0.469564757065527,0.363708144082093,
1.29104823388156,0.196686952612298,0.941761406616285
"2981","SLC9A8",12.7958693899207,-0.526442324574054,0.405505888203876,
-1.29823595634048,0.19420626599202,0.941761406616285
"2982","AJ011932.1",2.27678797814232,-1.26344983440162,0.9780819070420
7,-1.29176281179003,0.19643929762931,0.941761406616285
"2983","SHOX",2.95469864702233,4.00375153468669,3.10255362617096,1.290

46972819869,0.196887615766624,0.941761406616285
"2984","TBC1D8B",29.2376145260655,0.420358358810756,0.324151777229686,
1.29679486073865,0.194701779463568,0.941761406616285
"2985","DANT2",3.96119287504428,1.09323206631213,0.845537002643799,1.2
9294408511259,0.196030398625524,0.941761406616285
"2986","IDS",176.048412963435,-0.33429390501656,0.257982306777826,-1.2
9580167412199,0.195043821161982,0.941761406616285
"2987","ZFP92",2.02102030608256,-1.42257682842779,1.09143008572937,-1.
30340628046471,0.192436093485733,0.941761406616285
"2988","AC009509.1",2.02201747323851,4.22265248029569,3.24337682060457
,1.3019308929724,0.192940010665182,0.941761406616285
"2989","EIF5A2",25.70860350159,-0.376839822518579,0.292370802857252,-1
.28891058490053,0.197429173753383,0.942296618523173
"2990","BRWD1",90.7161176734233,0.331349228386342,0.257136572819821,1.
28861182504179,0.197533070338522,0.942477184431224
"2991","SIRT7",31.1424895110374,-0.368002443064519,0.285650598760521,-
1.28829571743008,0.197643043328369,0.942686611876467
"2992","PIGX",40.6030824420881,-0.375565473716467,0.291701860526266,-1
.28749769726837,0.19792087170224,0.943696241879732
"2993","FBLIM1",17.4213056493224,-0.508827688241898,0.398615028957344,
-1.2764889712584,0.201782722980323,0.944011862325955
"2994","RUNX3",32.4824912399569,0.637440053241688,0.498017962380869,1.
27995394020385,0.200561335379991,0.944011862325955
"2995","PTPRU",10.7208052536388,-0.681830764696254,0.533921976513043,-
1.27702322565776,0.201594047648629,0.944011862325955
"2996","PRDX1",806.185535162506,-0.349859788265191,0.274986693042373,-
1.27227897610042,0.203274015712965,0.944011862325955
"2997","AC092807.3",5.39057589714271,-0.907317727274799,0.714196975040
487,-1.27040264658551,0.203941241013118,0.944011862325955
"2998","DIPK1A",24.9064936057408,0.460862730718512,0.358867001946038,1
.28421595805515,0.199066403039927,0.944011862325955
"2999","POLR3GL",116.809304664111,-0.247233015205583,0.193024500936383
,-1.28083747921237,0.200250756620031,0.944011862325955
"3000","S100A13",1615.77699875311,-0.281013930255021,0.219988130139464
,-1.27740496760834,0.201459311880797,0.944011862325955
"3001","RPS7",3539.29217379194,-0.338371468786846,0.264055585697295,-1
.28144029937221,0.200039056839786,0.944011862325955
"3002","XPO1",106.502566897232,0.262966051735928,0.204576937722387,1.2
8541396045715,0.198647665331297,0.944011862325955
"3003","WBP1",67.9824848419503,0.274653698308995,0.215528553247592,1.2
7432627450286,0.20254780855288,0.944011862325955
"3004","CHCHD5",105.829556096805,-0.336242816888806,0.264610181650753,
-1.27071004899047,0.203831819273401,0.944011862325955
"3005","AC009404.1",7.59137958529347,0.827590185687728,0.6488355737576
14,1.27550063399713,0.202132099887185,0.944011862325955
"3006","MBD5",49.2665243821913,0.353203706521449,0.27731941310581,1.27
363498489262,0.202792807568061,0.944011862325955
"3007","METTL5",126.897454978568,-0.323997579669775,0.254319123893516,
-1.27398040190416,0.202670361821486,0.944011862325955
"3008","FAM126B",36.1145198544913,0.410830959951284,0.320350460616444,

1.28244223267459,0.199687557200712,0.944011862325955
"3009","AC112715.1",3.86703107998908,-0.898872344152197,0.70720525696619,-1.27102045028374,0.203721373422558,0.944011862325955
"3010","RPL14",4476.67439323711,-0.28274627886264,0.221836479376719,-1.27457070927674,0.202461230385976,0.944011862325955
"3011","COX17",159.029024836009,-0.347628402544706,0.271680027436263,-1.27955082243306,0.200703154767264,0.944011862325955
"3012","KPNA4",149.576192117335,-0.223603290644721,0.173890158044232,-1.28588813282834,0.198482105896251,0.944011862325955
"3013","DHX15",95.7599524338498,0.271412975627008,0.213474292601762,1.27140824461393,0.203583451076799,0.944011862325955
"3014","SMIM14",356.963357476095,0.271146042545757,0.211233377588973,1.28363256621955,0.199270549354269,0.944011862325955
"3015","PYURF",269.169039618099,-0.273296327072098,0.21276780660462,-1.28448157375592,0.19897350678768,0.944011862325955
"3016","ARSJ",17.2765833818771,0.630324556279112,0.491839286790264,1.28156609935046,0.199994898730138,0.944011862325955
"3017","LMBRD2",20.1876344941744,0.472688015727579,0.372078407865754,1.27039894209106,0.203942559910879,0.944011862325955
"3018","NIPBL",164.737486294557,0.33660660880657,0.261645344143208,1.28649951677463,0.198268787358307,0.944011862325955
"3019","AC010245.2",2.41881583137142,1.42881085930014,1.11837760935958,1.27757462894695,0.201399451020144,0.944011862325955
"3020","ZFYVE16",102.410336695262,0.274831737791951,0.216318073397596,1.2704982689394,0.203907199080236,0.944011862325955
"3021","MGAT4B",58.9723236932401,-0.308141843367561,0.242103456462479,-1.27276928578389,0.203099923168913,0.944011862325955
"3022","ZKSCAN8",25.5251383684832,0.498773486134561,0.392303313957241,1.27139758546349,0.203587241185429,0.944011862325955
"3023","ANKS1A",25.4457742756018,0.437061061495166,0.340377869106233,1.28404664687162,0.19912563435611,0.944011862325955
"3024","NFKBIE",18.657993564195,-0.510056649996523,0.398281575633426,-1.28064334682148,0.200318967289687,0.944011862325955
"3025","IL20RA",3.85733599758436,1.12230959586995,0.875601117494802,1.28175898071146,0.199927207643934,0.944011862325955
"3026","RMND1",24.101623182463,0.420000318006301,0.329751754214081,1.27368637964433,0.202774585398853,0.944011862325955
"3027","SDHAF3",49.7511210435164,-0.439299035764359,0.345021867007609,-1.27324983652317,0.202929401086899,0.944011862325955
"3028","MOSPD3",36.8905418737474,-0.500771716677827,0.393090532388769,-1.27393482014102,0.20268651686601,0.944011862325955
"3029","IFT22",72.1653936634954,-0.342216338279681,0.267910541609568,-1.277353015763,0.201477644415639,0.944011862325955
"3030","AC018638.7",2.0159167281735,1.60296618679133,1.25579764290975,1.2764526162648,0.201795566654058,0.944011862325955
"3031","KLHDC10",51.4021233496511,0.383728739799496,0.299793098715617,1.2799785633608,0.200552675168154,0.944011862325955
"3032","ZC3HAV1",303.137123484037,0.538217113893399,0.423659643303752,1.27039977113778,0.203942264747834,0.944011862325955
"3033","NCALD",82.3590952298394,-0.428941010161915,0.336230743040089,-

1.27573405775918,0.202049544917955,0.944011862325955
"3034","LRRC6",10.741451492996,0.638732579421333,0.498777325230377,1.2
8059666530814,0.200335371910858,0.944011862325955
"3035","SLC52A2",62.53433637208,-0.356026965890355,0.27885195404155,-1
.27675980293581,0.201687060970535,0.944011862325955
"3036","PSIP1",206.124496808174,-0.220789315076681,0.173072767751465,-
1.27570222597779,0.202060801414854,0.944011862325955
"3037","AL158206.1",8.53519880002393,0.73527471666291,0.57821957537698
4,1.27161851305973,0.203508695744868,0.944011862325955
"3038","UBAP1",59.3926236628727,0.329104719809418,0.257696895635579,1.
2771000558533,0.201566925161579,0.944011862325955
"3039","ABITRAM",19.8612625832101,0.429146198483581,0.337499818088484,
1.27154497716224,0.203534837197254,0.944011862325955
"3040","VDAC2",309.201888541244,-0.308058919668958,0.242051457191226,-
1.27270012436068,0.203124473493406,0.944011862325955
"3041","HRAS",55.9944238834825,-0.336635723102529,0.263757362397977,-1
.27630834658783,0.201846540842017,0.944011862325955
"3042","IMMP1L",21.2418096421029,-0.449475781837963,0.350464242809899,
-1.28251538083949,0.199661912941272,0.944011862325955
"3043","TPCN2",7.89393204692227,-0.722344299573347,0.567010574044038,-
1.27395207892057,0.202680399915656,0.944011862325955
"3044","SLC36A4",32.282620823641,-0.39836146649227,0.311574227839518,-
1.27854434320368,0.201057559978504,0.944011862325955
"3045","ZBTB44",109.230513917029,0.315168168967241,0.246945806881983,1
.27626450898946,0.20186203165262,0.944011862325955
"3046","PRR4",3.97911240134761,-0.972097106703681,0.762897727437898,-1
.27421680749837,0.202586590227929,0.944011862325955
"3047","DCLK1",564.180603757295,0.313497940125118,0.244928456468377,1.
27995719503338,0.200560190608072,0.944011862325955
"3048","ITM2B",3747.78419556335,-0.255550622753627,0.198846041008722,-
1.28516827117729,0.1987334885574,0.944011862325955
"3049","ZFYVE26",11.636352032722,0.583518019908006,0.458429027881311,1
.2728644662944,0.203066140346964,0.944011862325955
"3050","DCAF5",93.8408182201452,0.333009366902227,0.260926041866144,1.
27625960414125,0.201863764923623,0.944011862325955
"3051","SMAD6",23.4344698205252,0.517574910086494,0.403397030393307,1.
28304095243801,0.199477728957141,0.944011862325955
"3052","RPLP1",11716.4553616263,-0.26801512048491,0.20890307055235,-1.
28296400706924,0.19950468632472,0.944011862325955
"3053","MYO9A",111.180453988226,0.328004477224578,0.257172716086728,1.
27542486705301,0.202158901662745,0.944011862325955
"3054","UBN1",73.7685618868791,0.309918677087889,0.242503033128348,1.2
7799917836022,0.201249715974635,0.944011862325955
"3055","ORC6",2.95022534184213,-1.16435214167904,0.912418882142724,-1.
2761157889945,0.201914591018493,0.944011862325955
"3056","IRX3",58.2206651648713,0.34751407911175,0.272569658799248,1.27
495510924677,0.202325131460719,0.944011862325955
"3057","CCDC102A",32.3547678720445,-0.423146889647643,0.33064744474129
2,-1.27975248675738,0.20063219883413,0.944011862325955
"3058","HSD11B2",13.7177687681689,-0.604848174747191,0.470990473945251

,-1.28420468821945,0.199070345245356,0.944011862325955
"3059","IST1",91.9322536281131,0.320928516530376,0.252199159241403,1.27252016817069,0.203188362914165,0.944011862325955
"3060","CCL3",48.2845522660513,-0.66845942376022,0.525021434119113,-1.27320406429076,0.202945638740504,0.944011862325955
"3061","RAMP2",406.760034513425,-0.440370276002119,0.344338045374392,-1.27888939929166,0.200936006148523,0.944011862325955
"3062","ASB16-AS1",9.16858595820803,0.693579268272392,0.540103607747163,1.28415966552305,0.19908609482133,0.944011862325955
"3063","DCAKD",21.8066027965302,-0.409138110034794,0.319183201777046,-1.28182845386889,0.199902830364331,0.944011862325955
"3064","DLX3",2.37105627705539,-1.27254827172581,0.997033202085966,-1.27633489944308,0.20183715833041,0.944011862325955
"3065","SNHG25",49.4318790924003,-0.607244528916727,0.474154434940915,-1.28068933699291,0.200302806575986,0.944011862325955
"3066","PRCD",10.9309581725529,-0.614934693863801,0.482192759053559,-1.27528811314128,0.202207283506066,0.944011862325955
"3067","AP001269.4",2.02714877703686,-1.37641153611386,1.07689865027204,-1.27812541669186,0.20120520843206,0.944011862325955
"3068","LINC00683",2.76725055305235,1.24001966263261,0.972874952505986,1.27459305991844,0.202453315192089,0.944011862325955
"3069","RPS15",5032.2771373265,-0.256259785155751,0.199935334084628,-1.28171334161116,0.199943223026422,0.944011862325955
"3070","MCOLN1",41.2545463923246,-0.364137168437021,0.28466364677952,-1.2791839511529,0.200832286006676,0.944011862325955
"3071","ZNF177",4.37511825640855,0.919849083517992,0.723446711700957,1.27148146316853,0.203557417906486,0.944011862325955
"3072","HOOK2",22.8907052014632,0.424840349541362,0.33403356057327,1.27184929805331,0.20342666930778,0.944011862325955
"3073","WIZ",41.622032499408,0.33673152531143,0.261768621258904,1.28637085565112,0.198313664694344,0.944011862325955
"3074","HSPB6",219.718981052292,0.443276245108926,0.345422778671356,1.28328608441503,0.19939186612149,0.944011862325955
"3075","PTPRH",3.2257547640497,1.16798168945005,0.911826585943041,1.2809252411105,0.200219926024372,0.944011862325955
"3076","FIZ1",9.16165881905776,0.621169773713444,0.486278936077802,1.27739395566593,0.201463197624179,0.944011862325955
"3077","AC012313.1",5.20151198768247,0.959745296175772,0.749384423350873,1.28071156307769,0.200294996783326,0.944011862325955
"3078","COMMD7",94.5057657240027,-0.286757195643466,0.224720137687858,-1.2760636345007,0.201933025382275,0.944011862325955
"3079","CTSA",202.950356968945,0.287695929366375,0.225406277397192,1.27634390971029,0.201833974604318,0.944011862325955
"3080","PCMTD2",74.9143057594963,0.284619672291607,0.222619181898096,1.27850470864587,0.201071525584262,0.944011862325955
"3081","HSD17B10",127.954965488079,-0.375534890609342,0.293854041815111,-1.2779640133234,0.201262115305199,0.944011862325955
"3082","DCAF12L2",10.9906720057236,-0.572703080649855,0.450707402692719,-1.27067600227616,0.203843936302136,0.944011862325955

"3083","KTI12",20.7335481467357,-0.533708765454729,0.420529938129374,-1.26913381679507,0.20439334098728,0.944272873906758
"3084","SLAMF7",4.0597035366421,0.999880662276388,0.787416576430086,1.26982424831536,0.204147241236115,0.944272873906758
"3085","MAD2L1BP",23.9508059732608,-0.487933887355659,0.384464463727629,-1.26912610498465,0.204396091029305,0.944272873906758
"3086","PGP",60.0383977706704,-0.39353178566256,0.31002793504936,-1.26934298872102,0.204318760261004,0.944272873906758
"3087","BCKDK",48.8898346908185,-0.345088830252388,0.271785330826099,-1.26971102231118,0.204187585110942,0.944272873906758
"3088","ARHGAP27",6.46592017980481,0.830301187878354,0.654083080632201,1.26941242246448,0.204294007878822,0.944272873906758
"3089","MBOAT1",41.840868807486,-0.357095979171196,0.281430102887124,-1.26886205671616,0.204490267237444,0.944402121207308
"3090","CETN3",50.1467318174953,0.342905809987988,0.270395589295636,1.26816347441626,0.204739577794655,0.94518018164403
"3091","LINC00648",5.55756421878132,0.874408316962815,0.689586256935475,1.2680187694701,0.204791247824316,0.94518018164403
"3092","PPP2R5D",36.3263415208233,0.360436999156318,0.284366085325569,1.26751050057062,0.204972811369969,0.945456832240345
"3093","TMEM159",182.127968251296,0.278164402399952,0.219462570024373,1.26747992775743,0.204983736304457,0.945456832240345
"3094","LRRC41",88.2879317376262,0.25269739675004,0.19966525688393,1.26560524697063,0.205654446637866,0.945493501687332
"3095","KIF21B",2.7440266560533,1.36423056647834,1.07735080756723,1.26628258585419,0.205411929258971,0.945493501687332
"3096","OSBPL6",3.61558301017735,-1.11106434946875,0.877044563381768,-1.26682770278472,0.205216904209116,0.945493501687332
"3097","EIF2A",152.553684706941,-0.236796973465718,0.187086412561751,-1.26570909251659,0.205617251826498,0.945493501687332
"3098","AC108519.1",4.64408628462587,-0.912767611706914,0.721095706045931,-1.26580647208676,0.205582377403237,0.945493501687332
"3099","UBE2D1",89.3379707121505,0.315461766874356,0.249164540264808,1.26607809658264,0.205485123579711,0.945493501687332
"3100","UEVLD",27.4217324224458,0.42160355361573,0.333075104273219,1.26579125310396,0.205587827475045,0.945493501687332
"3101","GPCPD1",83.1003486002487,0.375105873450894,0.296276140835296,1.26606844679883,0.205488578064738,0.945493501687332
"3102","TNFRSF6B",15.4009227418713,-0.768013501524659,0.606666289023404,-1.26595710923874,0.205528438376617,0.945493501687332
"3103","RNF185",29.6078579814146,0.408234705571531,0.322157435056527,1.26719007897459,0.205087332332798,0.945493501687332
"3104","KHDRBS3",204.102428813885,-0.473687373771975,0.374342145432293,-1.26538617025063,0.205732930336491,0.945549608305534
"3105","CPS1",2.42096453181119,-1.16461923635208,0.920601863292907,-1.26506287113775,0.205848791203267,0.945777409116203
"3106","TBC1D5",96.2133820643812,0.323770754578859,0.256150196438355,1.26398792224537,0.206234362003456,0.94632985874917
"3107","ACTRT3",4.65452054530747,-0.819003204511399,0.647889912334466,-1.2641085914741,0.206191053335211,0.94632985874917

"3108","MAP9",37.8452919863324,-0.344343605472497,0.27241050480677,-1.264061405109,0.206207987923619,0.94632985874917
"3109","SUGT1",133.606418445139,-0.233233222877568,0.184521721440175,-1.26398789832007,0.206234370590997,0.94632985874917
"3110","GPX7",59.5157779521853,0.381050637123402,0.30186049912071,1.26234018108817,0.206826404038407,0.94661067693677
"3111","SHISA5",174.090307245497,0.233738409310129,0.185108687804261,1.26270901751133,0.206693772125817,0.94661067693677
"3112","KIF7",20.6301365365525,-0.431935086524628,0.341986599508019,-1.26301757772383,0.206582862709596,0.94661067693677
"3113","MT1X",4193.96511706294,-0.475019087203265,0.375984637426452,-1.2634002560708,0.206445372174605,0.94661067693677
"3114","ACAP1",32.7282978463708,0.394324563036778,0.312314903007967,1.26258644476763,0.206737841879123,0.94661067693677
"3115","HEXIM1",89.0360404848389,0.416810317996554,0.329973808751549,1.26316182358094,0.206531029552821,0.94661067693677
"3116","NUP85",36.3064908119402,0.378066647799659,0.299320263262127,1.26308404141877,0.206558978541144,0.94661067693677
"3117","RPL28",6298.94119665859,-0.24517029526686,0.194191561312469,-1.26251776137873,0.206762539257987,0.94661067693677
"3118","PUSL1",20.6060123061351,-0.453497974092224,0.359785049925556,-1.26046920011284,0.207500151931363,0.947186043556631
"3119","TRAK1",30.7926884487655,0.515481055098246,0.408687156595241,1.26130965159929,0.207197305205009,0.947186043556631
"3120","TMEM42",47.1470871617984,0.349967274151466,0.277547850931456,1.26092590152282,0.207335545180252,0.947186043556631
"3121","GLIDR",6.89576346035564,0.708766718678863,0.561851252267625,1.2614846292827З,0.207134294431631,0.947186043556631
"3122","WAC-AS1",65.3307299260295,0.318769625495736,0.25289499034483,1.26048216716782,0.207495476969144,0.947186043556631
"3123","MAP6",3.327945759544,-1.02555350211634,0.813316234602829,-1.26095294607903,0.207325800609655,0.947186043556631
"3124","DYRK2",63.3012820071013,-0.383267415204502,0.303760757892353,-1.26174104207471,0.207041983387421,0.947186043556631
"3125","MVK",9.24908365767888,-0.6323471017883,0.501730629893014,-1.26033186756634,0.207549668593721,0.947186043556631
"3126","HIF1A-AS3",2.49060307043044,1.45688683521456,1.15588074651854,1.26041275417264,0.207520503058308,0.947186043556631
"3127","PES1",39.1743182246042,-0.367889671396019,0.291961710668552,-1.26006136405217,0.20764722648744,0.947328216523769
"3128","ZMYM5",42.0711983106233,0.306873369029064,0.243638173633821,1.2595455155984,0.207833360949803,0.947874273436666
"3129","JTB",366.191614385654,-0.256430622052191,0.203787750242899,-1.25832206178509,0.208275305611387,0.948219896533401
"3130","ARIH2OS",9.53678851601574,0.645561248228866,0.512979545641366,1.25845416978904,0.20822755183633,0.948219896533401
"3131","ABCC5",29.9186678726253,-0.374854258399264,0.298166972553782,-1.25719577587236,0.208682752514297,0.948219896533401

"3132","TTC1",119.184859744032,-0.305934463816814,0.243313774537445,-1
.25736598512935,0.208621140330162,0.948219896533401
"3133","CCZ1",82.2296196072737,-0.429201634056382,0.340972349488836,-1
.25875788667267,0.208117795800561,0.948219896533401
"3134","AC104232.3",2.31601282629032,1.30072695374972,1.03459235814507
,1.25723618921929,0.208668122535962,0.948219896533401
"3135","BTAF1",37.9082476085459,0.401465234602488,0.318951122202929,1.
25870456836663,0.208137060728182,0.948219896533401
"3136","LHPP",11.2984856361586,0.676119855929097,0.537491233260337,1.2
5791792328939,0.208421440972612,0.948219896533401
"3137","ZCCHC8",40.6328487270174,0.349346492587434,0.277459926634184,1
.25908810265069,0.207998511226618,0.948219896533401
"3138","TM6SF2",1.91244839454219,1.37308449791253,1.09223791501683,1.2
5712949443928,0.20870674857107,0.948219896533401
"3139","TMEM184B",29.7030829172496,0.365763598169849,0.290845420692532
,1.2575876123438,0.208540935683222,0.948219896533401
"3140","LINC01278",22.1060134530752,0.496216245438539,0.39466340192516
7,1.25731507663998,0.208639566754448,0.948219896533401
"3141","UQCRH",495.203957967745,-0.293880544050899,0.233923244205015,-
1.25631185156331,0.209002926651901,0.948961092175693
"3142","TMEM185A",36.5890169306184,0.339791670439539,0.270456276544068
,1.25636452139862,0.208983838673485,0.948961092175693
"3143","PRDM2",170.06036417811,0.353735806577718,0.283211665774764,1.2
49015663285,0.211659344982924,0.949780120053927
"3144","AL360012.1",21.0271569415097,0.678496787860381,0.5431008163820
9,1.24930172703522,0.211554736503326,0.949780120053927
"3145","EIF3I",360.980227424236,-0.287958925626781,0.230829943350872,-
1.24749381058016,0.212216488302711,0.949780120053927
"3146","MTF1",31.610485043766,0.405958451664764,0.325234631409446,1.24
820179790046,0.211957165722006,0.949780120053927
"3147","SHISAL2A",3.29300297165308,-1.0276491799872,0.820732616126569,
-1.25211202746781,0.210529045659245,0.949780120053927
"3148","GBP2",318.632930548198,0.28765007163518,0.229349404245878,1.25
420021290659,0.20976924251656,0.949780120053927
"3149","ZBTB37",25.6369797224132,0.56394670137279,0.451047700895232,1.
25030390411807,0.21118855280064,0.949780120053927
"3150","ADI1",207.337052835172,0.372364057543667,0.297056563837768,1.2
5351230328991,0.210019324390212,0.949780120053927
"3151","BAZ2B",105.885478790964,0.362415503130123,0.29101855128677,1.2
453347098584,0.213008741934181,0.949780120053927
"3152","C2orf88",17.1964821176343,0.621483847229238,0.50027677611464,1
.24228002757982,0.214133261690856,0.949780120053927
"3153","HDLBP",422.543417162997,0.219974692994211,0.176980676937514,1.
24293056621021,0.21389342088918,0.949780120053927
"3154","AC093495.1",3.2760740353529,1.20877064071758,0.968565223437739
,1.24800128217207,0.212030587648029,0.949780120053927
"3155","PLXNB1",9.36537611414312,0.663331347798145,0.532307996781338,1
.2461419926228,0.212712270568618,0.949780120053927
"3156","EIF4E3",35.1675909336898,-0.357509452641683,0.287329663426772,
-1.24424832569644,0.213408182862904,0.949780120053927

"3157","EPHB3",6.3759543403277,1.10518409807711,0.884156841930625,1.24998647939415,0.211304486428033,0.949780120053927
"3158","WDR19",27.6209759290333,0.40600575226225,0.325685536575451,1.24661892121879,0.212537260533354,0.949780120053927
"3159","GPRIN3",13.9937456357256,0.683407770239624,0.550418977257319,1.24161374966571,0.214379106257239,0.949780120053927
"3160","EDNRA",2.69507753372399,1.14124456344474,0.919220949069938,1.24153454574707,0.21440834460832,0.949780120053927
"3161","MIR4453HG",2.31534415712675,-1.32528686823384,1.05749042718475,-1.25323769763289,0.21011921446181,0.949780120053927
"3162","RAI14",22.9293231797268,-0.469364592457509,0.375546296667083,-1.24981818919012,0.211365970037065,0.949780120053927
"3163","RANBP3L",2.72765772138872,-1.16315620294678,0.928296559947308,-1.25300066070783,0.210205466245923,0.949780120053927
"3164","MAP1B",458.680444806625,-0.374876330358671,0.299051568741774,-1.25355079037345,0.210005327143169,0.949780120053927
"3165","TCF7",41.2045699018184,0.578630831542578,0.464516342818307,1.24566302238564,0.212888134500835,0.949780120053927
"3166","MAP3K4",41.9246342577546,0.365208995152798,0.293273740494732,1.2452836538884,0.213027502059997,0.949780120053927
"3167","PHF10",60.332591058871,0.327834808679474,0.261713538830921,1.25264749444724,0.210334022104225,0.949780120053927
"3168","HOTAIRM1",15.1535746213715,-0.661677911543261,0.527158891095379,-1.25517737198431,0.209414377973192,0.949780120053927
"3169","ANKIB1",107.000566118284,0.327672062414986,0.263807540545424,1.24208755268148,0.214204260571471,0.949780120053927
"3170","LMTK2",13.564887810837,0.524740642801619,0.420395218596275,1.24820792337686,0.211954923073595,0.949780120053927
"3171","FAM3C",327.251837002057,-0.220112540648038,0.17706916331364,-1.24308793540836,0.213835431080981,0.949780120053927
"3172","FAM180A",22.926229174447,0.586330705344668,0.471256214478406,1.24418668089848,0.213430864573421,0.949780120053927
"3173","KRBA1",5.57206990574902,-0.736577052061797,0.59228127853854,-1.24362710548493,0.213636835449615,0.949780120053927
"3174","YTHDF3",74.4893334497064,0.307297298855358,0.247244146123515,1.24289008930404,0.213908338272509,0.949780120053927
"3175","EIF3H",428.583300202107,-0.269068255236333,0.215212667336509,-1.25024357797497,0.211210582290283,0.949780120053927
"3176","GCNT1",4.40711580483742,-0.9562898435545,0.770306272252608,-1.24144107091069,0.214442854808194,0.949780120053927
"3177","AL158151.1",5.90006974839298,0.912760364933461,0.734021703504071,1.24350596252962,0.213681445138834,0.949780120053927
"3178","MRPL23",36.3698499886014,-0.510529247366846,0.409295119603283,-1.24733773483956,0.212273686764086,0.949780120053927
"3179","OSBPL5",23.9591530823679,0.395478872297499,0.318498826721903,1.24169648085646,0.21434856887375,0.949780120053927
"3180","OLFML1",161.70552894786,0.258175348695914,0.207760508594305,1.24265843611335,0.213993726317285,0.949780120053927
"3181","CYB5R2",7.50988689417096,-0.647374689556776,0.519219161800589,-1.24682357120981,0.212462195648228,0.949780120053927

"3182","SYT7",2.59159173578975,1.27053461513497,1.0134705186163,1.25364733536565,0.209970217968515,0.949780120053927
"3183","DRAP1",310.485136863345,-0.354680853838916,0.284907983860484,-1.24489615570969,0.213169924185689,0.949780120053927
"3184","PAAF1",29.8739241426267,0.369181865060479,0.294030545110446,1.25559017999917,0.20926459355193,0.949780120053927
"3185","INTS4",17.0982043987569,0.451513334148092,0.36310353603843,1.24348371562073,0.213689638072319,0.949780120053927
"3186","ARHGAP42",40.8065127175108,0.338281972217484,0.269864439902413,1.25352555653428,0.210014504282986,0.949780120053927
"3187","VPS11",38.1675653765307,0.350444931867797,0.280591264854858,1.24895168083395,0.211682747354917,0.949780120053927
"3188","CRTAM",6.29623993548885,-0.94430692944845,0.756258991634139,-1.24865547371275,0.211791113140226,0.949780120053927
"3189","BBS10",18.1078632389678,0.45915365305882,0.367385704964984,1.24978638758572,0.211377589976144,0.949780120053927
"3190","DYNLL1",1043.6352553737,-0.360031007775692,0.287180810689518,-1.25367362433187,0.209960658561823,0.949780120053927
"3191","AKAP11",69.4380813531837,0.278716150868694,0.223836862689699,1.24517538138959,0.213067289908767,0.949780120053927
"3192","LMO7-AS1",7.15935612654754,1.09096058820236,0.872676025322946,1.25013241632098,0.211251179897027,0.949780120053927
"3193","DZIP1",48.4786204721399,0.345040134904476,0.277388746758572,1.24388656330311,0.213541315296141,0.949780120053927
"3194","MCF2L",12.4945123763498,-0.710171896689326,0.568661518790572,-1.2488481692936,0.211720611939957,0.949780120053927
"3195","ATG14",42.7654899584099,0.416921013392951,0.335569127140575,1.24242959102223,0.214078103426956,0.949780120053927
"3196","EML1",69.8451865992353,-0.311882013602566,0.249489387973155,-1.25008128055581,0.211269857203681,0.949780120053927
"3197","AL049840.5",12.6281595911286,0.55410861757619,0.445190108438942,1.24465617513195,0.213258161751775,0.949780120053927
"3198","DUOX1",2.51224529659313,1.50121955597162,1.20596121515153,1.24483236866203,0.213193375236084,0.949780120053927
"3199","SHF",12.2245783873661,-0.543387127487185,0.437092186806248,-1.24318654940418,0.213799098069224,0.949780120053927
"3200","PLIN1",7.06818877453207,-0.90855318399047,0.730719669994062,-1.24336762961075,0.213732393080017,0.949780120053927
"3201","LYSMD4",9.97252841066287,0.635504389810034,0.510018849059163,1.24604098648973,0.212749348349268,0.949780120053927
"3202","TSR3",107.549408642049,-0.270235500943465,0.217528482254607,-1.24229939060195,0.214126120125727,0.949780120053927
"3203","UQCRC2",168.44076979225,-0.275469432907445,0.221551146338316,-1.2433672199862 8,0.213732543957509,0.949780120053927
"3204","ZNF843",2.01199505260709,1.35242203927719,1.08304943199176,1.24871681691393,0.211768667768191,0.949780120053927
"3205","TPPP3",329.771913177448,-0.383374244280763,0.307131780606256,-1.24824022940254,0.211943095533231,0.949780120053927
"3206","AC040169.1",2.71920115016994,-1.22163314392371,0.9772419452930

28,-1.2500825919393,0.211269378206754,0.949780120053927
"3207","ASPA",45.7222838486209,0.42557417575243,0.340488828805395,1.24989174313165,0.211339096045442,0.949780120053927
"3208","TVP23B",19.8361345086837,-0.468820733916895,0.373710486458114,-1.25450248495888,0.209659423089639,0.949780120053927
"3209","TAOK1",147.64695479569,0.337682342993873,0.269291522797032,1.25396573752672,0.209854459196345,0.949780120053927
"3210","BECN1",69.6503749550167,-0.277746654233143,0.223302589585516,-1.24381295688816,0.213568410580835,0.949780120053927
"3211","PRAC2",3.25570886663173,-1.28054087998279,1.02626691266744,-1.24776592149351,0.21211679203305,0.949780120053927
"3212","BAHCC1",30.7180338511054,-0.526577836887599,0.422583466663502,-1.24609190474294,0.212730656467069,0.949780120053927
"3213","AC130371.2",2.15555446577799,1.40267398675568,1.12556708417505,1.24619314697154,0.212693494382904,0.949780120053927
"3214","PGPEP1",22.4906395617783,0.421672568421614,0.339206576151894,1.24311436766719,0.213825692030752,0.949780120053927
"3215","LPAR2",7.07806772083143,-0.755144601672579,0.607750418235241,-1.24252419910353,0.214043217722579,0.949780120053927
"3216","POP4",56.292588278391,-0.362610406454161,0.289710453911941,-1.25163038322524,0.210704578060962,0.949780120053927
"3217","PLEKHA4",109.099061944809,-0.381283449536501,0.30422878371197,-1.25327868351038,0.210104303328186,0.949780120053927
"3218","NUCB1",443.053766418835,0.225374819206996,0.180010043788025,1.25201246810645,0.210565320803456,0.949780120053927
"3219","ELMO2",21.8582230475721,0.425266244551478,0.341455565700952,1.24545120147178,0.212965942446908,0.949780120053927
"3220","ASPHD2",5.4564377525339,-0.777469146077316,0.619662777521481,-1.25466491498332,0.209600427316001,0.949780120053927
"3221","FBLN1",3090.58611018252,0.322702229731013,0.257209753805231,1.25462671985362,0.209614298985526,0.949780120053927
"3222","PCLAF",2.80514502963485,-1.10732050269481,0.892218063229865,-1.24108729505685,0.214573502430855,0.950063806852444
"3223","C1orf112",4.63815955218301,0.966824330567519,0.779383631667997,1.24049863415578,0.214791019072901,0.950436934892681
"3224","LINC01985",7.85861889305343,-0.712896884589829,0.574681188408865,-1.24050847490527,0.21478738150249,0.950436934892681
"3225","CENPS",15.3688290777239,-0.541472614396043,0.440731522690567,-1.22857700554404,0.21923044120417,0.951387542637888
"3226","RCAN3",21.8089128527955,-0.423775470801999,0.346613660109189,-1.22261618503005,0.221474697119054,0.951387542637888
"3227","USP33",157.817265227318,0.318964212513641,0.257641329283905,1.23801648361378,0.215709948892779,0.951387542637888
"3228","PKN2",125.358837323223,0.267207516588355,0.217191060427647,1.23028782152555,0.218589343161529,0.951387542637888
"3229","NUDT17",3.14877917431733,1.0646693060691,0.862715099061159,1.23409142511556,0.217168838425444,0.951387542637888
"3230","KIAA0040",25.5301410398468,0.442896332142036,0.359257761000346,1.23280936481038,0.217646896596064,0.951387542637888
"3231","TFB2M",24.9194956225202,-0.420457249762239,0.340949495399234,-

1.23319510788513,0.217502980144812,0.951387542637888
"3232","TTC32",35.3057984120376,-0.571999770636094,0.467640127590995,-1.2231622927286,0.221268404464332,0.951387542637888
"3233","DNAJC27",24.6451316921254,-0.414649103096748,0.338946446196351,-1.22334695569147,0.221198679037757,0.951387542637888
"3234","C2orf49",61.6281671452374,0.315685978003518,0.25486656257123,1.23863238401582,0.215481669515097,0.951387542637888
"3235","GTDC1",32.5457998687765,0.383138173759188,0.311437336851174,1.2302255652227,0.218612648970988,0.951387542637888
"3236","CLK1",190.761175924655,0.284618785139801,0.231197624846864,1.23106275563307,0.218299393933918,0.951387542637888
"3237","ALS2",15.8107849642239,0.543612217601097,0.440456001075296,1.23420322636986,0.217127185499512,0.951387542637888
"3238","ARPC2",686.319090912505,-0.243415130243712,0.197166437996681,-1.23456675850587,0.216991786875815,0.951387542637888
"3239","MRPL44",39.2000025914316,0.381097969020319,0.311252790401093,1.2244001685229,0.22080130578066,0.951387542637888
"3240","SP100",284.380137037121,0.179716081599377,0.145466288931635,1.23544831534018,0.21666370069163,0.951387542637888
"3241","TWF2",103.644061822164,-0.318615739316621,0.259563710427118,-1.22750494971863,0.219632862591391,0.951387542637888
"3242","LINC02018",2.48125732406156,-1.260537157843,1.02462549222652,-1.2302418468077,0.218606553744779,0.951387542637888
"3243","NPHP3",66.8302724705512,0.400396037568742,0.326934416910364,1.22469833966278,0.22068889994802,0.951387542637888
"3244","TIPARP-AS1",3.16854183925023,-1.05303869130117,0.854560927713971,-1.23225700725418,0.217853094253307,0.951387542637888
"3245","TTC14",107.229315240234,0.313940793935228,0.255094799489114,1.23068284639266,0.21844150616532 9,0.951387542637888
"3246","AC072022.2",9.00563945489734,0.617319183777806,0.498347026506831,1.2387335550187,0.215444187805079,0.951387542637888
"3247","PCYT1A",49.2667328199048,0.32517779654936,0.264283234239707,1.23041401958333,0.21854210606564,0.951387542637888
"3248","USP46",42.0469198679021,0.307769561454813,0.250881064199892,1.22675484670933,0.219914746498286,0.951387542637888
"3249","USO1",145.103658488031,0.236740776438518,0.192515338417722,1.22972423072511,0.218800389917041,0.951387542637888
"3250","NDUFC1",259.553478824385,-0.285683083356905,0.233314040027245,-1.22445731651445,0.220779758706858,0.951387542637888
"3251","GPX8",190.770050806397,0.274200872928719,0.223026447941053,1.22945451295172,0.218901442374804,0.951387542637888
"3252","RGMB",20.6397109306483,-0.42826118527236,0.348695747405916,-1.22818012108941,0.219379359364474,0.951387542637888
"3253","PCDHB10",6.43198147978843,-0.82850558196834,0.676429515905349,-1.22482174784974,0.220642389009617,0.951387542637888
"3254","AC008443.5",4.39223637923574,0.878428370358545,0.7159497915802,1.22694130327175,0.219844653083372,0.951387542637888
"3255","GNL1",91.5360567726404,-0.239334663173392,0.194977377587991,-1.22749965218597,0.219634852460525,0.951387542637888

"3256","CFB",722.079733629844,-0.458893910640591,0.37235452284032,-1.23241127068942,0.217795492863717,0.951387542637888
"3257","RCAN2",18.8227437685168,-0.43672552902478,0.353368610383311,-1.23589225582614,0.216498615997961,0.951387542637888
"3258","ZBTB2",21.521551358162,-0.419174875828222,0.341846291021485,-1.22620864066031,0.220120170512194,0.951387542637888
"3259","PLEKHA8",20.1445011983711,0.452061060904656,0.365726889627811,1.23606186399011,0.216435569037021,0.951387542637888
"3260","LIMK1",16.995140080014,-0.462239848727506,0.377878504932769,-1.22324991417479,0.221235318212313,0.951387542637888
"3261","OR2A1-AS1",8.91699570228968,-0.848428351578814,0.689061473434646,-1.23128107474911,0.218217757608,0.951387542637888
"3262","PRKAG2",60.4449655896897,0.397885726006323,0.321745892805887,1.2366458590549,0.216218586743937,0.951387542637888
"3263","AC026979.4",4.85660321870304,0.946604809326191,0.773884302839177,1.22318647096646,0.221259274298756,0.951387542637888
"3264","MTBP",2.80174621773962,1.0865864317371,0.880090770859061,1.23462995831268,0.216968254119451,0.951387542637888
"3265","MAF1",185.717238759708,-0.246454434808996,0.20101288994045,-1.22606284045768,0.220175028150218,0.951387542637888
"3266","SMC5",103.755868152031,-0.289353337204511,0.236330016589441,-1.22436134596979,0.220815944292768,0.951387542637888
"3267","RFK",56.4928230571635,-0.294376911845183,0.239920999594256,-1.22697434715186,0.219832232782841,0.951387542637888
"3268","NINJ1",67.7873661540598,-0.460669471924432,0.374069966594325,-1.23150617013855,0.218133610396134,0.951387542637888
"3269","CCDC180",9.24470462182118,0.693793902886242,0.56396097692684,1.23021615195238,0.218616173008656,0.951387542637888
"3270","AL157392.3",9.05392331561897,0.686466787614837,0.559221990846466,1.22753897173423,0.219620083486612,0.951387542637888
"3271","AL132656.3",2.1762472999972,-1.39195160351654,1.13689482744269,-1.22434509324628,0.220822072784908,0.951387542637888
"3272","CACUL1",67.0168555145935,0.272790384977131,0.220626909885649,1.23643296784838,0.216297667934675,0.951387542637888
"3273","SSRP1",98.1139261895532,-0.295350997894771,0.24089599759805,-1.22605190970247,0.220179141265424,0.951387542637888
"3274","RASGRP2",9.57544916954774,-0.561720509303406,0.453581656418414,-1.23841099249666,0.215563706762137,0.951387542637888
"3275","CFL1",1088.14768449782,-0.311774088976181,0.251698089831825,-1.23868277738817,0.215462999252703,0.951387542637888
"3276","PCF11-AS1",3.13509658326592,1.18561359799316,0.964245529018548,1.22957645362372,0.218855751933361,0.951387542637888
"3277","PHLDB1",138.399267571734,-0.252596297040555,0.204945251119161,-1.23250622135025,0.217760044086322,0.951387542637888
"3278","CD27",8.71193383710133,-0.649832474194805,0.527179797043168,-1.23265815162032,0.217703331232112,0.951387542637888
"3279","GAPDH",1503.46769236345,-0.402584110462143,0.328476692764148,-1.22560936386195,0.220345712379518,0.951387542637888

"3280","IFT81",21.3440122445718,-0.503733536752906,0.411161085547535,-1.22514886369194,0.220519137320754,0.951387542637888
"3281","PGAM5",17.7351745255469,-0.518614676626934,0.422845205697206,-1.22648825063966,0.220014994095929,0.951387542637888
"3282","RPL36AL",971.067352044872,-0.311463329785639,0.253511070325567,-1.22859853569964,0.219222364779924,0.951387542637888
"3283","EME2",4.98416539044423,-0.853673719873242,0.692657699233906,-1.23246117211636,0.217776862201571,0.951387542637888
"3284","GLYR1",69.9154518264364,0.330964140084514,0.267289397952661,1.23822397229212,0.215633025150213,0.951387542637888
"3285","LINC02185",2.1629295028342,-1.37896464213559,1.11440813085131,-1.23739642951294,0.215939943705026,0.951387542637888
"3286","ORAI3",68.3381497404563,0.460625809544249,0.376418644159911,1.22370614923252,0.221063099102766,0.951387542637888
"3287","CNOT1",77.400782382743,0.373298506025584,0.302806747545996,1.23279454322226,0.217652427731367,0.951387542637888
"3288","ACD",22.7699678505441,-0.374554338222521,0.306321753334125,-1.22274808806664,0.221424858013621,0.951387542637888
"3289","LYRM9",37.4742950158513,-0.374431247139361,0.302703437900906,-1.23695736571692,0.216102911081649,0.951387542637888
"3290","ERN1",38.993757253477,-0.338410718715675,0.274868451819577,-1.23117337211841,0.218258028238025,0.951387542637888
"3291","C17orf58",368.118768799542,0.50371734448376,0.409252343279333,1.23082336058843,0.218388936430634,0.951387542637888
"3292","NAPG",49.7824967283953,-0.293737807296655,0.239482508483335,-1.22655224031568,0.219990929193474,0.951387542637888
"3293","HDHD2",38.5780447087258,0.372456158464369,0.304442109530667,1.22340552375804,0.221176568074853,0.951387542637888
"3294","ZBTB7C",9.74343834067807,0.674539377567928,0.548010633506165,1.23088738853886,0.218364985052022,0.951387542637888
"3295","AC068473.5",2.67943444967438,1.19172971006087,0.972088263845462,1.22594804853062,0.220218225758605,0.951387542637888
"3296","TBXA2R",10.3890218881782,-0.653462481426044,0.531250711942307,-1.23004537544414,0.218680113545034,0.951387542637888
"3297","RPL36",3261.95298571553,-0.263364159626869,0.214180578215633,-1.22963604739977,0.218833424993347,0.951387542637888
"3298","ZNF562",21.3196843865859,0.469238057824317,0.383655436784207,1.22307157108853,0.221302665100142,0.951387542637888
"3299","HOMER3",97.3310712263999,-0.323297176551333,0.263135380389451,-1.22863438612033,0.219208916990397,0.951387542637888
"3300","CILP2",5.94884134367327,-0.851076372296589,0.695123212613905,-1.22435326119559,0.220818992841978,0.951387542637888
"3301","LINC01534",6.26908284339075,0.711471108947512,0.577374123244965,1.23225319650436,0.217854517311626,0.951387542637888
"3302","NOVA2",6.56401320314,0.863905824732678,0.697143765051696,1.23920756096645,0.215268641211143,0.951387542637888
"3303","ISOC2",127.367437892919,-0.423906919506597,0.344536561817151,-1.23036846153811,0.218559158015816,0.951387542637888
"3304","CDC25B",28.8208489270261,0.425410477674049,0.344243653007802,1.23578306805967,0.216539210405538,0.951387542637888

"3305","NXT1",92.2476878065578,-0.406544778335038,0.332463316506134,-1
.22282597252361,0.221395433415529,0.951387542637888
"3306","RGS19",19.5775575615412,-0.413508088456532,0.336430818698493,-
1.22910288081282,0.219033235197148,0.951387542637888
"3307","USP25",47.1714111020255,0.302968865913177,0.246501180599749,1.
22907673373425,0.219043037479348,0.951387542637888
"3308","PSMG1",56.5706889583161,-0.41341505097210 1,0.335836809418244,-
1.23099981710832,0.218322932685952,0.951387542637888
"3309","NDUFV3",107.597546035398,-0.302116660793054,0.243976028440533,
-1.23830469216238,0.215603104642021,0.951387542637888
"3310","PKNOX1",38.4353631865196,-0.384783460404474,0.313253062023171,
-1.22834700455701,0.21931673285089,0.951387542637888
"3311","CFAP410",57.144042086419,-0.361720753676672,0.291949823419269,
-1.23898260817649,0.215351938907328,0.951387542637888
"3312","LGALS1",3378.54690903669,-0.349929104651143,0.285567358893926,
-1.22538201146835,0.220431321345176,0.951387542637888
"3313","AL031846.2",4.73522759947639,0.906758797612465,0.7411080127608
87,1.22351773560573,0.221134209619731,0.951387542637888
"3314","GTPBP6",94.9415996313886,-0.291054596948371,0.23557323313292,-
1.2355164170292,0.216638370365888,0.951387542637888
"3315","XG",154.783900033041,-0.337545641157796,0.272555749956552,-1.2
384462305844,0.215550647684841,0.951387542637888
"3316","BX890604.2",3.07916887154276,1.07651154814414,0.87416840245480
4,1.23146929712985,0.218147393003206,0.951387542637888
"3317","ACOT9",84.3814773521023,-0.311038593105932,0.253844765023278,-
1.22531025241907,0.220458346985174,0.951387542637888
"3318","TSPYL2",79.4510091878753,0.397420604879356,0.323184808176741,1
.22970076199255,0.218809181380957,0.951387542637888
"3319","IGBP1",203.766081473463,-0.297858381155294,0.243177650446231,-
1.22485919494955,0.220628277075613,0.951387542637888
"3320","RTL5",17.2568222657856,0.527630869249809,0.426632172251744,1.2
3673483522117,0.216185541565152,0.951387542637888
"3321","G6PD",57.9089861791915,-0.284511471331913,0.232250771618631,-1
.22501841156031,0.22056828354586,0.951387542637888
"3322","SMARCC1",71.0057129589689,0.310696645734546,0.254206856992859,
1.22221976782976,0.221624530366017,0.951458185435328
"3323","GOLGA7",106.840148131631,0.268432701287626,0.219597282322366,1
.22238626293002,0.2215615916055,0.951458185435328
"3324","ALX1",2.00061084924625,1.49415701800371,1.22292224765071,1.221
79232643289,0.221786171144625,0.951579403774201
"3325","ZFAS1",961.463425864907,-0.219551857174576,0.179685364553841,-
1.22186833479579,0.221757421731862,0.951579403774201
"3326","DUSP19",4.19404135491555,0.878902010695975,0.719854603101195,1
.22094379463518,0.222107301217224,0.951582098025616
"3327","MFF",131.104460957205,-0.239816385833059,0.196330825514526,-1.
22149125184275,0.221900075870449,0.951582098025616
"3328","AMT",7.60647086529988,0.760143272568129,0.622400726378815,1.22
130845989,0.221969251489864,0.951582098025616
"3329","ANAPC10",42.1362643487761,-0.355744688159173,0.291376804024889
,-1.22090943151668,0.222120313081824,0.951582098025616

"3330","NUPR1",1162.79148678593,0.207948947921616,0.170317069657804,1.22095188896463,0.222104236315271,0.951582098025616
"3331","ERRFI1",431.752822661789,0.414625866562799,0.339726349736269,1.22047014276248,0.222286701317093,0.951631341273565
"3332","SLC9A1",23.1302609165289,-0.465168421584863,0.381408382631521,-1.21960723142853,0.222613803761554,0.951631341273565
"3333","SGIP1",4.96065261221616,-1.10779504759159,0.908769929690371,-1.21900495537856,0.222842311682564,0.951631341273565
"3334","HS2ST1",34.2629826804014,0.396965090053615,0.325644668469561,1.21901301783702,0.222839251618895,0.951631341273565
"3335","RPL32",7480.58006302571,-0.282656388748943,0.232199541003369,-1.21729951544065,0.223490278609396,0.951631341273565
"3336","UBA3",50.5636913288902,-0.27870842150616,0.2287559464506,-1.21836579914371,0.223084995772154,0.951631341273565
"3337","AC092903.2",4.2446306551627,0.856450787756046,0.702757105140775,1.21870100137157,0.222957697766263,0.951631341273565
"3338","ZBED3",27.5889524247919,-0.419926194135648,0.344839970635528,-1.21774222797241,0.223321944498182,0.951631341273565
"3339","KIAA1586",36.5758148266551,-0.321267740572084,0.263824052240454,-1.21773484200476,0.223324752145879,0.951631341273565
"3340","PRDM1",47.0900026283067,0.545860530561656,0.448258931535708,1.21773486741596,0.223324742486208,0.951631341273565
"3341","AL359711.2",2.68280782477304,-1.09487506793708,0.897510488713365,-1.2199022537404,0.22250193137454,0.951631341273565
"3342","AC083862.3",15.6876746947106,0.490547498650946,0.402402954934912,1.21904546831743,0.222826935513997,0.951631341273565
"3343","EXTL3",11.7511160906252,0.629279311100447,0.515680214750432,1.2202898096546,0.222355031442548,0.951631341273565
"3344","TBC1D2",3.33619306505687,-0.965200195180845,0.791613679412947,-1.21928185462463,0.22273723325134,0.951631341273565
"3345","GZMH",28.9819247816854,0.733529510280612,0.600997448459887,1.2205201738549,0.222267746670726,0.951631341273565
"3346","THRA",87.4098585964688,0.313332314333237,0.2572057697711,1.2182165066207,0.223141708553404,0.951631341273565
"3347","RFNG",92.4620875340444,-0.297208532236433,0.243926416060467,-1.21843520286363,0.223058634414193,0.951631341273565
"3348","TSHZ1",53.7760803215241,0.380866738812192,0.31251966714349,1.2186968656834,0.222959268038245,0.951631341273565
"3349","ZNF101",7.75915193693473,-0.6556279689589,0.53864143385166,-1.21718814735566,0.223532638764034,0.951631341273565
"3350","ZNF615",11.0242838352557,0.552695131971222,0.453899816748715,1.21765885681598,0.223353638065781,0.951631341273565
"3351","ACSS1",13.5100433358557,0.5245200494738,0.430847827653812,1.2174137034184,0.223446851837784,0.951631341273565
"3352","AL359921.2",2.29005933262417,-1.20617214673966,0.991246204062788,-1.21682397551281,0.223671195879301,0.951884193954668
"3353","STARD7",70.8586262374416,0.287900500210183,0.236693247491428,1.21634437509929,0.223853764007675,0.951884193954668
"3354","AC005726.1",2.99062697014236,1.05645112529836,0.868520876156904,1.21637965684028,0.223840329777397,0.951884193954668

"3355","RRAS",307.641911969824,0.315017843418114,0.258990268188676,1.21633081281889,0.223858928271268,0.951884193954668
"3356","AC124852.1",21.8667621753899,0.503643313149935,0.414226288731104,1.21586516078625,0.224036291565537,0.95235450997436
"3357","FBXO42",36.0733885454997,0.377907724422522,0.311198045468564,1.21436406791538,0.224608730251805,0.95251728471232
"3358","SYT11",15.11650764818,0.514888923847429,0.423921087550358,1.21458672137008,0.224523755858644,0.95251728471232
"3359","RRP36",76.9252692799374,-0.331285851480496,0.272729888741923,-1.21470313726407,0.22447933555884,0.95251728471232
"3360","DIS3",88.3514763050274,0.306132577847074,0.251933162286793,1.21513410568229,0.224314947501764,0.95251728471232
"3361","FGF14-AS2",16.1145146877856,-0.471121208030961,0.387904711058045,-1.21452819365337,0.224546090394434,0.95251728471232
"3362","AC103702.1",2.69486834667927,1.29624297404375,1.0671683513329,1.21465649953425,0.22449713015782,0.95251728471232
"3363","MATK",9.83118703885933,-0.603243398847733,0.496702841625954,-1.2144955661478,0.224558541939988,0.95251728471232
"3364","COPE",341.461745388273,-0.340124353234876,0.279818415060597,-1.21551811792379,0.224168542860702,0.95251728471232
"3365","RPL22L1",311.787420915533,-0.349650002680999,0.288042110325023,-1.21388501940379,0.224791634232086,0.952726516326481
"3366","ADPRHL1",4.15045251729829,0.921026354296576,0.758733309734963,1.21389998630521,0.224785918157355,0.952726516326481
"3367","RABGGTB",67.5626314675239,-0.279563259078611,0.231111644796407,-1.20964592383429,0.226414788209868,0.952856525839923
"3368","SIKE1",58.4918265844338,0.278881494036105,0.23108417841846,1.20683941213444,0.227493994603307,0.952856525839923
"3369","CHTOP",83.8194251737569,0.287600395345594,0.237498930380678,1.21095448676173,0.225912848406375,0.952856525839923
"3370","EMILIN1",276.659425063519,0.289507761960582,0.239836083606366,1.2071067772927,0.227391025190511,0.952856525839923
"3371","GGCX",74.1347440992763,0.323233869994906,0.266340624093315,1.21361084549257,0.224896363565464,0.952856525839923
"3372","DPP4",95.9480286785454,0.544231960100359,0.449258065845045,1.21140164523629,0.225741509147945,0.952856525839923
"3373","NCKAP1",146.463130224333,0.268940795742747,0.221907949026472,1.21194755267944,0.225532457690479,0.952856525839923
"3374","PLCL1",13.1364926810546,0.582410149467352,0.482181439519035,1.20786513485109,0.227099142353506,0.952856525839923
"3375","CCR2",2.16701719306664,1.52026881781299,1.25633949122278,1.21007803100524,0.226248952219994,0.952856525839923
"3376","ITGB5",217.351185533024,-0.261904001296028,0.216629328040294,-1.20899604714332,0.226664364008508,0.952856525839923
"3377","RNF5",70.042421206045,-0.304195156539681,0.251458615575674,-1.20972254556987,0.22638537565956,0.952856525839923
"3378","UBR2",68.0491751651185,0.317855181453453,0.263343171723925,1.20699989816586,0.227432183190047,0.952856525839923
"3379","FBXL18",15.3298694408763,0.477586322773732,0.394208594273033,1

.21150662291992,0.225701297935276,0.952856525839923
"3380","GARS-
DT",81.0918924111554,0.289684238413077,0.239639914917207,1.20883133560
267,0.226727650265518,0.952856525839923
"3381","PDK4",948.004222211524,0.447757097967119,0.370631811525614,1.2
0809138353246,0.227012113678219,0.952856525839923
"3382","ADCK2",14.8415993894304,-0.519916636987422,0.42983788881526,-1
.20956446724704,0.226446059689537,0.952856525839923
"3383","AC004918.1",4.19035547546959,0.866666260198142,0.7171285248049
68,1.20852292193208,0.226846184333947,0.952856525839923
"3384","DAPK1",5.03979451422251,-0.884870410096783,0.732030641764276,-
1.20878875775493,0.226744011779929,0.952856525839923
"3385","HABP4",43.4367757350636,-0.381695251262287,0.314997682106747,-
1.21173987284432,0.22561197094217,0.952856525839923
"3386","ZNF189",16.6380496919901,0.525229793722771,0.434878444428281,1
.20776230795544,0.227138703544187,0.952856525839923
"3387","SURF1",93.3377436189859,-0.289032225829181,0.239053687381377,-
1.20906825991799,0.226636622043802,0.952856525839923
"3388","MRPL41",201.843112234916,-0.29643464784685,0.244606895893763,-
1.21188181046047,0.225557625892447,0.952856525839923
"3389","LINC00702",10.5740951324606,-0.808037305873736,0.6692159951165
04,-1.20743872198253,0.227263230721621,0.952856525839923
"3390","ERCC6",25.9114750476028,0.397122898310312,0.328341795544893,1.
20948019319707,0.226478416046319,0.952856525839923
"3391","CPNE8",63.9244411604549,0.310768578271207,0.256198328159104,1.
21300002425548,0.225129811239417,0.952856525839923
"3392","DAZAP2",194.776401674648,0.208752431708774,0.172180151906172,1
.21240705968555,0.225356599852549,0.952856525839923
"3393","BAZ2A",83.5645454946407,0.33523396316453,0.276368271241877,1.2
1299728676572,0.225130857860684,0.952856525839923
"3394","ATP5F1B",375.137249797982,-0.267249885546157,0.22115880410685,
-1.20840717431732,0.226890681559817,0.952856525839923
"3395","HERC1",73.2494782447913,0.283511822613468,0.23477751281638,1.2
0757656562792,0.227210177726007,0.952856525839923
"3396","PLEKHO2",27.1284010715062,0.399042565918912,0.330101973391204,
1.20884635077903,0.226721880540514,0.952856525839923
"3397","TSPOAP1-
AS1",2.13619228537,-1.23619226361135,1.02061991046338,-1.2112170759534
8,0.225812219951812,0.952856525839923
"3398","AFMID",21.6487866441974,-0.448004305992351,0.371218148514742,-
1.20684914728666,0.227490244755086,0.952856525839923
"3399","MLLT1",51.7734386080111,0.308699351949185,0.255250691116234,1.
20939673306746,0.226510463155353,0.952856525839923
"3400","LRP3",25.3462394635906,-0.401059529108179,0.3305742731893,-1.2
1322063341123,0.225045477423437,0.952856525839923
"3401","ZNF264",52.8369898369622,0.332177381403949,0.275006210435631,1
.20789047228335,0.227089394890061,0.952856525839923
"3402","SLC2A4RG",189.016760185208,-0.328249154584784,0.27083722749097
2,-1.21197945210736,0.225520246302493,0.952856525839923
"3403","MTFP1",8.90595243956698,-0.630120706188686,0.52121617923454,-1

.20894310517007,0.226684704252753,0.952856525839923
"3404","SLC35E4",9.48172649020028,-0.662529691139129,0.547946626936797
,-1.20911354969532,0.226619224316542,0.952856525839923
"3405","PIR",26.8461660605097,0.452707983470815,0.373523329042041,1.21
19938656358,0.225514728828988,0.952856525839923
"3406","RTL8B",31.3467142751779,-0.363009942067976,0.300348031874599,-
1.20863099985133,0.226804641212701,0.952856525839923
"3407","RALY-
AS1",5.29658818197145,0.741779648774227,0.614820952917941,1.2064970220
2526,0.227625906640249,0.953129199920691
"3408","ZNF346",26.6736115795145,0.397211899108158,0.329291569938399,1
.20626197379564,0.227716494809964,0.953228730915182
"3409","NOC2L",58.304769581899,-0.323224821761121,0.2684304331138,-1.2
0412882403722,0.228539790718416,0.953675192565129
"3410","DTNB",5.59428947054992,0.78284269385659,0.651798421163079,1.20
10503070254,0.229731687513227,0.953675192565129
"3411","SLC25A12",34.7708808993598,-0.312578919431112,0.26018030663256
2,-1.20139346239049,0.229598610907487,0.953675192565129
"3412","AC112220.2",4.55645312475629,-0.97147353528655,0.8076908809389
26,-1.20277888263048,0.229061898769521,0.953675192565129
"3413","TNIP2",89.9628975875793,-0.315361701648134,0.26280104728481,-1
.20000169293984,0.230138682953467,0.953675192565129
"3414","RNF150",6.31714063377563,1.00465483796407,0.835899126710854,1.
20188525847281,0.229407986652456,0.953675192565129
"3415","TLL1",2.09300390616872,-1.39895229228789,1.16246897977263,-1.2
0343193378073,0.22880921651181,0.953675192565129
"3416","PDE4D",29.75226026462,0.486625596892716,0.405541816078246,1.19
993938380654,0.230162882938577,0.953675192565129
"3417","AC008906.1",3.55246836147731,1.05268574401762,0.87506305266182
1,1.2029827345761,0.228983001903841,0.953675192565129
"3418","HIGD2A",323.57433985615,-0.32694111160237,0.271567897623141,-1
.20390191353203,0.228627492115862,0.953675192565129
"3419","HLA-
DRA",361.222435093089,-0.436553877992851,0.362999989540502,-1.20262779
771828,0.229120385681175,0.953675192565129
"3420","LZTS1",2.01586140841013,-1.2538953245965,1.04312152218407,-1.2
0206064004041,0.229340034545394,0.953675192565129
"3421","FAM160B2",32.4729010050789,0.337913895723587,0.281353139657192
,1.20103118854586,0.229739103326071,0.953675192565129
"3422","AC104211.1",2.19975255732845,1.19832921748124,0.99847364214445
9,1.20016109279314,0.230076782542487,0.953675192565129
"3423","ZHX1",99.5015609930215,0.254563713851883,0.21138634173049,1.20
425809807732,0.228489836737752,0.953675192565129
"3424","MROH6",5.63169354641133,-0.764015741098693,0.634285826883004,-
1.20452910772609,0.228385138624423,0.953675192565129
"3425","MED22",18.8564181349515,0.540263444052193,0.449325218453591,1.
20238843016997,0.229213069693977,0.953675192565129
"3426","AC132217.1",17.286043740444,1.22392190780746,1.01961479412488,
1.20037676469566,0.229993048620113,0.953675192565129
"3427","TMEM134",55.6173122157642,-0.309833192586258,0.257249225575095

,-1.20440865038022,0.228431670227668,0.953675192565129
"3428","AP000766.1",5.98055368745424,0.792345574505209,0.6584992432432
1,1.20325965843603,0.228875854988148,0.953675192565129
"3429","TRIM29",1.86560506820632,-1.27353690781453,1.06120793852246,-1
.20008234162637,0.230107362832159,0.953675192565129
"3430","CRACR2A",9.97920738382921,0.555814323693092,0.462132819996737,
1.20271553900244,0.229086418608379,0.953675192565129
"3431","ANO6",133.632664433122,0.270555897566503,0.225166089299676,1.2
0158367722245,0.229524868695883,0.953675192565129
"3432","AC074032.1",17.8418652455908,-0.439818881254683,0.364948350415
73,-1.20515377245483,0.228143944458078,0.953675192565129
"3433","HOXC10",63.4633053937819,-0.319957872072892,0.26658967999844,-
1.20018851470456,0.23006613487079,0.953675192565129
"3434","GOLGA6L4",15.1080370969128,0.62727464323133,0.521559920962824,
1.2026895051164,0.229096496670231,0.953675192565129
"3435","DECR2",14.3436311371177,0.574810472777176,0.477364704138756,1.
20413274754829,0.228538274483691,0.953675192565129
"3436","ARHGAP17",40.3170988832668,0.347527436813444,0.288781582817134
,1.20342659467141,0.228811281545406,0.953675192565129
"3437","RAB31",97.3075155440717,-0.273138532310171,0.227419044814199,-
1.20103631836694,0.229737113517597,0.953675192565129
"3438","KIAA1328",32.1786542210702,0.373653561273603,0.310594692716342
,1.20302622689967,0.228966171563842,0.953675192565129
"3439","ZNF431",46.3181832107435,0.388202945806644,0.323446698548958,1
.20020685803316,0.230059012523485,0.953675192565129
"3440","RPS5",3509.33405105372,-0.26132488442196,0.216730917740745,-1.
2057572917887,0.2279110872999,0.953675192565129
"3441","SDCBP2",9.02243625071293,-0.573066572976798,0.476119460914399,
-1.20361930150095,0.228736755779188,0.953675192565129
"3442","NCOA5",22.7063190494453,-0.379884715935123,0.315254937495853,-
1.20500798164381,0.228200220645148,0.953675192565129
"3443","ODF3B",59.8418119700653,-0.416009908295402,0.346225489837196,-
1.20155771457214,0.229534932865536,0.953675192565129
"3444","LINC02482",11.7184991779657,0.593606374991998,0.49508452202549
5,1.1990000668238,0.23052791962145,0.954135832458668
"3445","UBTD2",83.9706373577987,0.309422150960235,0.258099136012118,1.
19885000678851,0.230586273946304,0.954135832458668
"3446","DPY19L4",50.3558407924033,0.305710334796961,0.255064903610628,
1.19855899604144,0.230699470147405,0.954135832458668
"3447","ZBED5",50.0050064509142,0.33031762676002,0.275660439530683,1.1
9827722585944,0.230809109618314,0.954135832458668
"3448","MYLPF",2.15083778568566,-1.16637689899626,0.972649526740565,-1
.19917490003299,0.230459944914419,0.954135832458668
"3449","IRX6",5.55687913231037,-0.784216246501353,0.654384517149793,-1
.19840281355838,0.230760237671011,0.954135832458668
"3450","LINC01572",2.42270335912184,-1.27501919016582,1.06333680944547
,-1.19907368844941,0.230499293983752,0.954135832458668
"3451","IFNAR2",52.0193449555055,-0.412696791008439,0.344269830507222,
-1.19875967754828,0.230621405628244,0.954135832458668
"3452","ZNF703",114.166646778814,0.394020460673744,0.328920018306899,1

.19792180087411,0.230947461684823,0.954237504891143
"3453","CTNND1",90.5693542085478,0.274426392302293,0.22912808817165,1.
19769860819817,0.231034371365064,0.954237504891143
"3454","GNPTG",130.00960492279,0.258827977991954,0.216098837478753,1.1
9772961766813,0.23102229510267 8,0.954237504891143
"3455","LINC00824",4.19818677892701,-0.99043855215103,0.82742186848728
,-1.19701761564724,0.231299688752908,0.955056833501879
"3456","ATP8B2",47.2493436912138,0.306571189186985,0.256211509871617,1
.19655510144959,0.231480009485194,0.955248427918939
"3457","AC092821.3",13.7507697738918,-0.55908542436207,0.4672085306884
88,-1.19665071940829,0.231442722660463,0.955248427918939
"3458","PTCD3",65.049986459328,0.275495031811059,0.230281095827322,1.1
9634236940422,0.231562980989788,0.955314484326292
"3459","TSIX",15.8881233835397,0.920477018714594,0.769606401275569,1.1
9603607401,0.23168248193713,0.955531161409394
"3460","IGF1",766.896852061934,-0.343256341297882,0.287080479215834,-1
.19567983944953,0.23182152169428,0.95582827413023
"3461","P3H1",67.4970318286045,0.29954874344076,0.251039295516305,1.19
323448077994,0.23277755417324,0.956112410483644
"3462","NCKAP5",5.09573889074923,0.84149654971706,0.705809025103735,1.
19224396371722,0.233165599568492,0.956112410483644
"3463","AGPS",82.2363598773182,-0.314955302186381,0.264042402654311,-1
.19282092201958,0.232939514387072,0.956112410483644
"3464","BCS1L",26.9346087863105,-0.365757783937417,0.306657950408394,-
1.19272232613019,0.232978138862149,0.956112410483644
"3465","RPSA",2513.82597833856,-0.263276757555092,0.220541041747373,-1
.19377670237303,0.232565327351791,0.956112410483644
"3466","PLOD2",49.8246226902813,0.384946150389592,0.32274090971058,1.1
927404887555,0.232971023397961,0.956112410483644
"3467","MAPK14",71.6426408603366,0.303862746647422,0.254378772214109,1
.19452871009088,0.232271217041661,0.956112410483644
"3468","SLC25A27",9.92263559548357,0.615304949753574,0.515007425434074
,1.19474966644406,0.232184851183441,0.956112410483644
"3469","PHIP",211.674515865569,0.248257357204556,0.208005133926005,1.1
9351552780842,0.232667534553103,0.956112410483644
"3470","PEG10",42.1211969876302,0.56793494165453,0.4756568368572,1.194
00142633718,0.232477410111362,0.956112410483644
"3471","TRIM35",12.4433186440514,0.523741385245878,0.43918535766394,1.
19252925013643,0.233053788630206,0.956112410483644
"3472","SGMS1",42.5566293265911,0.33811681478236,0.28356369011166,1.19
238402719763,0.233110700407432,0.956112410483644
"3473","CD27-
AS1",47.1115397742642,0.349415357230952,0.292390230113312,1.1950308910
648,0.232074961061876,0.956112410483644
"3474","SH2B3",22.6411619629068,-0.465604950341177,0.389990746641436,-
1.19388717386482,0.232522105387695,0.956112410483644
"3475","EBPL",162.020396053187,-0.369148888158307,0.309383979031418,-1
.19317389773703,0.232801275050627,0.956112410483644
"3476","DLEU1",9.21395033649005,-0.562989484788918,0.472275892010849,-
1.19207754262414,0.233230841755438,0.956112410483644

"3477","LONP2",103.698825788489,0.294673053204731,0.246683642607706,1.1945382761894,0.232267477440523,0.956112410483644
"3478","FDXR",12.5694790241136,0.654758766342686,0.549118520802717,1.19238150151181,0.233111690291898,0.956112410483644
"3479","QTRT1",57.2413301866665,-0.415090603374254,0.347755606861825,-1.19362734973583,0.232623770624508,0.956112410483644
"3480","TMEM91",49.5347778992345,0.297847585834768,0.249855014502247,1.19208168156293,0.233229219007178,0.956112410483644
"3481","IFFO2",11.2302137074508,0.6338073218779,0.533605431482083,1.18778274073693,0.23491901584415,0.956170243054452
"3482","LDLRAD2",5.41772223201443,1.01009048551927,0.852424871495621,1.18496130192332,0.236032747766613,0.956170243054452
"3483","SFPQ",540.897217022643,0.215691517401474,0.181957413439959,1.1853956006725,0.235861070333143,0.956170243054452
"3484","CCDC30",10.9686552038351,-0.669573525228944,0.565981846388562,-1.18303003797981,0.236797243005144,0.956170243054452
"3485","MFSD14A",68.6919207452882,0.272478478306268,0.228883729895436,1.19046678604349,0.233862976946623,0.956170243054452
"3486","PRKAB2",29.5935373619992,0.418451414468361,0.352002207080115,1.18877497371237,0.234528228600899,0.956170243054452
"3487","MTIF2",30.8054147539236,-0.362110040818014,0.304827345501678,-1.18791849275222,0.234865523213041,0.956170243054452
"3488","PTPN4",27.3281943178103,-0.387737975602621,0.326673505907465,-1.18692813647537,0.235255967916215,0.956170243054452
"3489","ANKRD28",165.943488944467,-0.428849857126386,0.361809487644271,-1.18529190574468,0.235902052701858,0.956170243054452
"3490","DZIP3",36.9065667719049,0.345341873024719,0.290912342024741,1.18709942184354,0.235188406383584,0.956170243054452
"3491","SIAH2",32.6225270399799,-0.436290229338078,0.368520122332916,-1.18389798249318,0.236453448910496,0.956170243054452
"3492","CEP19",11.5776491581605,-0.597055160380405,0.504440368627042,-1.18359908824394,0.236571801495849,0.956170243054452
"3493","CLK4",48.0494072766009,0.282059364345857,0.238419553179057,1.18303788672075,0.236794132525009,0.956170243054452
"3494","HIST1H3D",1.98781569036184,1.46376617417546,1.23643722348465,1.1838580611882,0.236469254050483,0.956170243054452
"3495","ZNRF2",45.1494850032323,-0.308323649173081,0.260197359600884,-1.18496071461301,0.236032979989047,0.956170243054452
"3496","IKZF1",33.0243475428407,0.532127956111665,0.448596265815472,1.18620683376476,0.235540628163766,0.956170243054452
"3497","DCAF10",70.7948860404074,0.308885723780625,0.259964133551288,1.18818592226948,0.234760168732495,0.956170243054452
"3498","FAM78A",3.66542693532716,0.97406602495504,0.818857688506853,1.18954250369341,0.234226255801066,0.956170243054452
"3499","NRARP",1.87230860870683,-1.61478839462519,1.36081182640585,-1.18663606774357,0.2353712027325,0.956170243054452
"3500","NMT2",52.7403542263684,0.323321839172755,0.271591320348147,1.19047191478099,0.233860962268311,0.956170243054452
"3501","SLC29A3",12.441325378596,0.496701469349883,0.41701903697218,1.19107624667748,0.233623653825233,0.956170243054452

"3502","HSPA12A",48.4984674611698,0.337159631859957,0.283891273646138,
1.18763647621031,0.234976660549382,0.956170243054452
"3503","MTG1",63.2288434307229,-0.345657001080965,0.291413230699652,-1
.18614038302612,0.23556686503272,0.956170243054452
"3504","FANCF",12.3175456403158,-0.506203826306131,0.425379957274958,-
1.19000394270793,0.234044842403993,0.956170243054452
"3505","FOLH1",5.91052736219355,0.85822592725276,0.72390285262392,1.18
555400651063,0.235798474811998,0.956170243054452
"3506","FAU",3602.11793210883,-0.272658739429984,0.230255104841868,-1.
18415936800723,0.236349982908104,0.956170243054452
"3507","VSIG10L2",3.2916044689518,-1.1140310098414,0.936489302205455,-
1.1895822058168,0.234210643111432,0.956170243054452
"3508","GIT2",47.4748029834189,0.284395133726564,0.240187292762826,1.1
8405570276106,0.236391013594716,0.956170243054452
"3509","NEK5",3.82762714150119,0.918949344372499,0.771860474487461,1.1
9056406532892,0.233824765648635,0.956170243054452
"3510","ZBTB25",28.49506077616,-0.363367454935985,0.306102436702498,-1
.18707795615865,0.235196872521881,0.956170243054452
"3511","AC004825.2",7.29512923786421,-0.632862470002051,0.532607122943
283,-1.18823508499988,0.234740804602776,0.956170243054452
"3512","GTF2A1",57.6974042123965,0.2940255444395,0.247752541647137,1.1
8677105181132,0.235317940221089,0.956170243054452
"3513","AC005632.3",4.68061433393509,0.786357662354839,0.6644442296494
48,1.18348181422196,0.236618249705104,0.956170243054452
"3514","ARL2BP",136.812411783563,-0.213343158941755,0.179277023676708,
-1.19001952713404,0.234038717171143,0.956170243054452
"3515","SGSM2",27.3399946165516,0.450702986498225,0.378365874140221,1.
19118297209646,0.233581762736168,0.956170243054452
"3516","TNFSF13",21.3994362675425,-0.490903248261355,0.414696716369683
,-1.18376449314283,0.236506301259069,0.956170243054452
"3517","RSKR",3.51812455218028,1.13384443496253,0.958418074650887,1.18
303740815358,0.23679432218182,0.956170243054452
"3518","SUPT6H",76.3664169044254,0.264434577396837,0.223107952371939,1
.18523151947539,0.235925920918664,0.956170243054452
"3519","WIPF2",80.6353866317528,0.292958396859183,0.246652030384261,1.
18773965250876,0.234935996420935,0.956170243054452
"3520","MIF4GD",30.0330692334603,-0.365001459631778,0.306986966585446,
-1.18898031304591,0.234447414010875,0.956170243054452
"3521","RANBP3",43.5804122787139,-0.290232839910086,0.244123415890478,
-1.18887751447937,0.234487869570373,0.956170243054452
"3522","NACC1",18.2028775849696,-0.532497501418308,0.449498826214862,-
1.18464714558292,0.236156988227202,0.956170243054452
"3523","GATAD2A",56.5736670044616,0.283618253333463,0.239231835585964,
1.18553725359663,0.235805094323925,0.956170243054452
"3524","LYPD3",2.17268466440765,-1.15091934442143,0.969347815132886,-1
.18731308458528,0.235104148840026,0.956170243054452
"3525","ZNF611",12.2437726934604,0.538301266303868,0.454910567713092,1
.1833122914906,0.236685403227795,0.956170243054452
"3526","RBBP9",23.618984440433,0.39115842724601,0.32872592534897,1.189
92265922064,0.234076791542169,0.956170243054452

"3527","DTD1",101.542184347803,-0.363161768966394,0.304930965725111,-1
.19096388949155,0.2336677612091,0.956170243054452
"3528","ZNF335",26.7025599204148,0.447693013695412,0.375836240988049,1
.19119170763963,0.233578334159766,0.956170243054452
"3529","C21orf91",26.4973425878425,0.39869334433704,0.335400056323275,
1.18870983120158,0.234553870603703,0.956170243054452
"3530","DENND6B",8.17998261013393,-0.624930732366319,0.524707803327808
,-1.19100712511397,0.23365078776253,0.956170243054452
"3531","AC234772.2",4.4853268835333,-0.924855497342789,0.7802087939950
12,-1.1853948641198,0.235861361416161,0.956170243054452
"3532","DLG3",8.35037894276526,-0.636194408296458,0.534807429572798,-1
.18957660854609,0.23421284416947,0.956170243054452
"3533","RENBP",8.46352760758024,0.647371671483035,0.545518535822707,1.
18670884483645,0.235342485018768,0.956170243054452
"3534","RHOC",254.77893832259,-0.336876043606297,0.287783525712955,-1.
17058835376945,0.241764280837298,0.956412311591315
"3535","NOTCH2NLC",10.7152249345454,0.610298369046718,0.52191526572368
8,1.16934377882294,0.24226515140269,0.956412311591315
"3536","ECM1",366.92864599366,-0.416023724729506,0.353392070903562,-1.
17722993519862,0.239103743511346,0.956412311591315
"3537","MTX1",86.8631825502694,-0.299331958769132,0.255006724905561,-1
.17381986251534,0.240467186410483,0.956412311591315
"3538","TSTD1",13.5664200901259,-0.560667524977007,0.478198457130609,-
1.17245782920599,0.24101329358304,0.956412311591315
"3539","LCLAT1",10.4586273330044,-0.537138789600358,0.459737161170181,
-1.1683606089905,0.242661337011773,0.956412311591315
"3540","CDC42EP3",11.5579940879491,0.70185413976598,0.597692474199677,
1.17427300838241,0.240285691028399,0.956412311591315
"3541","LINC01118",2.74438483114249,1.05575737859512,0.8981952037184,1
.17542085976906,0.239826382057261,0.956412311591315
"3542","CALM2",859.030764882604,-0.236277515135721,0.202675775578763,-
1.16579060551763,0.243699116435248,0.956412311591315
"3543","APLF",7.21474035259808,0.637042075746121,0.539307846227574,1.1
8122159765001,0.237514703605322,0.956412311591315
"3544","TSGA10",10.4152387757505,-0.51147127122601,0.434112728716922,-
1.17819920355188,0.238717200026522,0.956412311591315
"3545","PAX8",3.55248779783278,-1.16799790839418,1.00025639877441,-1.1
6769851192684,0.242928397263275,0.956412311591315
"3546","WIPF1",115.620868999719,0.281906825620451,0.24051778990442,1.1
7208305353412,0.241163712968263,0.956412311591315
"3547","GPR1",7.47986136326566,0.713175369503877,0.606286492673832,1.1
7630093713394,0.239474641429268,0.956412311591315
"3548","TRAF3IP1",34.4948444368892,-0.367559324426562,0.31410789613528
5,-1.17016900545619,0.241932963193016,0.956412311591315
"3549","KLF15",7.98568292735574,0.869706843435804,0.745566442104011,1.
16650481341604,0.243410403610218,0.956412311591315
"3550","ATP2C1",59.4403033239148,0.299179979560604,0.254896016034964,1
.17373344713071,0.240501808730195,0.956412311591315
"3551","LDB2",63.8476025408453,0.340549114850961,0.292089356031087,1.1
6590730822357,0.243651923864604,0.956412311591315

"3552","C4orf33",15.8853214960571,0.501716535045403,0.426089338406223,
1.17749140807433,0.238999424864673,0.956412311591315
"3553","SCOC-
AS1",3.8805654549137,0.86319753886527,0.73774940521178,1.1700416601725
1,0.24198420406882,0.956412311591315
"3554","TMEM144",5.2567392104432,0.80882185224441,0.693022221247732,1.
16709367671962,0.243172541178652,0.956412311591315
"3555","HPF1",114.215479396183,-0.31030282125623,0.262841979372794,-1.
18056796709829,0.237774395185569,0.956412311591315
"3556","LPCAT1",38.838001632725,0.35455375178245,0.301975674617569,1.1
741136176994,0.240349519668488,0.956412311591315
"3557","MED10",164.737290959728,-0.279796832886732,0.238891697771899,-
1.17122878482738,0.241506827962587,0.956412311591315
"3558","PGGT1B",57.413776453325,0.343109991597084,0.290129287469845,1.
18261067191552,0.236963481064665,0.956412311591315
"3559","MIR3936HG",3.96319125133988,-0.862906440067434,0.7315133346607
89,-1.17961819584269,0.238152102650485,0.956412311591315
"3560","PCDHB4",17.6192566281715,0.440632295562777,0.375942647342113,1
.17207318371038,0.241167675198606,0.956412311591315
"3561","PXDC1",308.245430445702,-0.272629565560438,0.232528023692731,-
1.17245896314286,0.241012838568164,0.956412311591315
"3562","BRD2",327.648349702873,0.327884214266927,0.279825030215496,1.1
7174726654874,0.241298540047757,0.956412311591315
"3563","HMGCLL1",12.4839145100152,-0.707193148102205,0.606120508616565
,-1.16675337337839,0.243309981636895,0.956412311591315
"3564","PPIL6",7.06543916201344,-0.625223650317178,0.535221996914115,-
1.16815761295683,0.242743194551267,0.956412311591315
"3565","Z97989.1",9.14378784952806,-0.606794829742163,0.51915763277145
3,-1.16880652703278,0.242481590058601,0.956412311591315
"3566","CCDC170",10.2416132692649,0.617774962893309,0.529917851150127,
1.16579383304884,0.243697811190708,0.956412311591315
"3567","SYTL3",14.0202623661852,0.868755094981656,0.742020135930187,1.
17079719661865,0.241680304932293,0.956412311591315
"3568","RFC2",35.5747256484655,-0.402404927110708,0.344941948883356,-1
.16658738785866,0.243377039059981,0.956412311591315
"3569","ARMC10",91.0982257258209,-0.278357748227919,0.238372516633357,
-1.16774262469219,0.242910597730848,0.956412311591315
"3570","RINT1",20.8866821092805,0.389544847905753,0.330723928502868,1.
17785504565442,0.23885439941422,0.956412311591315
"3571","CYREN",27.2110591177555,-0.412074171690296,0.351236590447363,-
1.17320969083957,0.240711726813045,0.956412311591315
"3572","HIPK2",39.0941581620296,0.365501455955443,0.310125822744686,1.
17855860154008,0.23857398450959,0.956412311591315
"3573","ZNF398",16.5759282984438,0.535532007213705,0.459254969657282,1
.16608864921668,0.24357860542041,0.956412311591315
"3574","DPYSL2",791.90489252217,0.251391973255795,0.215148950450644,1.
16845549434118,0.242623081437291,0.956412311591315
"3575","SULF1",63.9423524317666,0.393711298750547,0.335476264837011,1.
173589132876,0.240559636059081,0.956412311591315
"3576","RBIS",318.288038701759,-0.279363047328653,0.23966617867519,-1.

16563400339963,0.243762453732372,0.956412311591315
"3577","ZNF34",13.040348181248,-0.504564450873666,0.426781298180966,-1.18225529802789,0.237104416968927,0.956412311591315
"3578","AL441992.1",2.39280945109254,-1.13518571841973,0.967420638816101,-1.17341482378227,0.240629495506205,0.956412311591315
"3579","KYAT1",2.96544768741548,-1.00741161563801,0.859487020095711,-1.17210800405785,0.241153696811192,0.956412311591315
"3580","GPSM1",18.6772016471237,0.416762467860514,0.352582168803884,1.18202933879033,0.237194059782503,0.956412311591315
"3581","KIF20B",24.8161459300106,-0.378585822461858,0.321799346079689,-1.1764654809712,0.239408918560531,0.956412311591315
"3582","PPP1R3C",1.87981403826356,-1.35696037583512,1.14954978337146,-1.18042767304549,0.237830161036587,0.956412311591315
"3583","AL158212.2",5.77161956303306,0.804049480047501,0.683107969640523,1.17704596605808,0.239177160091634,0.956412311591315
"3584","CHST15",8.98866138421465,0.708945761561235,0.60667119911955,1.16858318408738,0.242571606584745,0.956412311591315
"3585","TNNT3",350.702167651923,0.366846033698832,0.310427282001489,1.18174546816112,0.23730671120956,0.956412311591315
"3586","TRIM5",28.4327765975781,0.392631802560866,0.336361488622119,1.16729119070454,0.243092794989875,0.956412311591315
"3587","BDNF-AS",12.2399244767742,0.555789644112648,0.471401432259872,1.1790156034279,0.238391962673928,0.956412311591315
"3588","LRRC4C",22.1157223945854,-0.523767531268148,0.446347557404768,-1.17345221807313,0.240614507450835,0.956412311591315
"3589","NECTIN1",14.3122536254881,0.490696304582912,0.417528442224126,1.17524042666178,0.239898540745689,0.956412311591315
"3590","NTF3",2.98872990404885,1.29725153624617,1.10702421161686,1.17183664334809,0.241262647790821,0.956412311591315
"3591","AC048341.1",11.0990636866823,0.631871851241953,0.535369643269148,1.18025341777604,0.237899439080975,0.956412311591315
"3592","FICD",20.5086071278212,-0.495877632718786,0.420014245432554,-1.18062098633846,0.237753322833391,0.956412311591315
"3593","RBM23",74.7234675786514,0.260750472483244,0.222885235139203,1.16988670120024,0.24204656636984,0.956412311591315
"3594","RALGAPA1",67.5923091338528,0.307736007235375,0.260408377899276,1.18174388135241,0.23730734102611,0.956412311591315
"3595","SGPP1",25.3229305504411,0.385936207084811,0.331035678271514,1.16584474851762,0.243677221137181,0.956412311591315
"3596","BTBD7",109.631927308354,0.311880394732557,0.264160313383409,1.18064818570943,0.237742513029593,0.956412311591315
"3597","CCDC85C",4.18194788624741,0.945898316172102,0.803612256509008,1.17705810048393,0.239172317115536,0.956412311591315
"3598","APBA2",4.21605638274721,0.878111980653165,0.742837511684999,1.18210505910144,0.237164017264317,0.956412311591315
"3599","GOLGA8N",6.16739246179074,0.746580448208655,0.636822213670725,1.17235302441049,0.241055351217833,0.956412311591315
"3600","SRP14-AS1",9.14895862721426,-0.61824789693521,0.525306326617207,-1.176928328

49837,0.239224114056222,0.956412311591315
"3601","CGNL1",4.82123270227647,-0.874777422895691,0.743471781683773,-1.17661146589121,0.239350619231363,0.956412311591315
"3602","TXNL4B",23.4895899911785,-0.381245671627431,0.325821901447571,-1.17010449553459,0.241958919579203,0.956412311591315
"3603","C16orf74",10.0575142948628,0.706761157412996,0.604406274752607,1.1693478160899,0.242263525453409,0.956412311591315
"3604","SLC25A11",92.0030748294837,-0.275860496907116,0.236306495010554,-1.16738431965146,0.243055200594169,0.956412311591315
"3605","AC004148.1",26.6872578634217,-0.571436740333949,0.485947688291268,-1.17592233506303,0.239625912752654,0.956412311591315
"3606","OMG",8.67019621176657,0.834899873883011,0.710786880204938,1.17461351234042,0.240149374865775,0.956412311591315
"3607","PCGF2",44.9031086577715,-0.352424175161353,0.30033409664738,-1.17344044214577,0.240619227304969,0.956412311591315
"3608","LSM12",102.432951239163,-0.267452732069148,0.22872051205564,-1.16934301023288,0.242265460941787,0.956412311591315
"3609","TMUB2",50.8032896195184,0.329130087798142,0.280523523724813,1.17327090230412,0.240687187001694,0.956412311591315
"3610","USP32",24.0125145601524,0.406039293154202,0.347302853874308,1.16912167183386,0.242354613519849,0.956412311591315
"3611","CENPX",109.330150892071,-0.320048097810113,0.272877531507148,-1.1728635041604,0.240850546987199,0.956412311591315
"3612","AC124283.1",4.5585120309474,0.849605086176593,0.726543286921128,1.1693798586688,0.242250621053078,0.956412311591315
"3613","LINC01915",11.7520198622417,-0.655672851591931,0.561932629430839,-1.1668175458258,0.243284059729837,0.956412311591315
"3614","DSEL",80.8075567320794,0.283033842436333,0.240640834234164,1.1761671427756,0.239528091575387,0.956412311591315
"3615","AP3D1",83.083919469198,0.300203421348186,0.255559353781882,1.17469158105794,0.240118128790489,0.956412311591315
"3616","CDKN2D",24.5820282425038,-0.383038835476447,0.324755261859698,-1.17946922024601,0.238211386059125,0.956412311591315
"3617","AP1M1",66.6154508944224,0.285857751626087,0.244834807917579,1.16755356012254,0.242986891876637,0.956412311591315
"3618","CCDC8",35.3810109765961,0.432526911368613,0.366280938795787,1.18086109746967,0.237657907832594,0.956412311591315
"3619","STRN4",35.9164948217307,-0.359035848024419,0.307460243648085,-1.16774723055046,0.2429087393166,0.956412311591315
"3620","VRK3",35.7177770131087,0.322842243336639,0.27542910727073,1.17214279396877,0.241139731212062,0.956412311591315
"3621","ZNF134",11.6907235008664,0.596432901172995,0.507674370868432,1.17483358506503,0.240061300722379,0.956412311591315
"3622","SMOX",9.45388698569154,-0.588517463863214,0.500705831648619,-1.17537569300015,0.239844443685634,0.956412311591315
"3623","BMP2",3.32401508629466,-0.947897971045372,0.812510202941551,-1.16662900676653,0.243360223983652,0.956412311591315
"3624","TASP1",58.7227390756524,0.375727171205941,0.320984871312036,1.17054479754807,0.24178179742223,0.956412311591315
"3625","TRPC4AP",59.1107613127744,0.268802715730242,0.228424076472766,

1.1767705045851,0.239287118251239,0.956412311591315
"3626","EIF6",170.041484995238,-0.350725449512846,0.297254353998494,-1
.17988330463487,0.238046630914632,0.956412311591315
"3627","NORAD",385.453690771151,0.191635996793998,0.162728620271267,1.
17764162490005,0.238939508063518,0.956412311591315
"3628","NEURL2",6.3874735634719,-0.69422407699102 9,0.587745436378105,-
1.1811645553032,0.23753735890993 9,0.956412311591315
"3629","HMGN1",245.857233685628,-0.286653537613195,0.245695063124817,-
1.16670450747954,0.243329721897294,0.956412311591315
"3630","DGCR2",36.6331500185831,0.35713187666152,0.303272527472117,1.1
775938943047,0.238958545158776,0.956412311591315
"3631","CRKL",36.8017681703304,0.314952031901974,0.266658229313626,1.1
8110748996067,0.237560024875091,0.956412311591315
"3632","MAPK1",106.033019658311,0.260719333842369,0.222814265177789,1.
17011957755189,0.24195285096252,0.956412311591315
"3633","ARFGAP3",114.293286914489,-0.240298583118952,0.20367584836318,
-1.17980892211859,0.238076220165228,0.956412311591315
"3634","SLC9A7",2.54617238836857,1.24780890056265,1.05791700635524,1.1
7949602196266,0.238200719804758,0.956412311591315
"3635","ZXDA",5.32180321525126,-0.795888374487114,0.675524142303445,-1
.17817902373295,0.23872524322557,0.956412311591315
"3636","RPS4X",8148.28167375143,-0.278590037169585,0.237522362307411,-
1.17290024595251,0.2408358109271,0.956412311591315
"3637","BCKDHB",23.6694391940106,-0.393511633760958,0.337659340734112,
-1.1654101820652,0.24385299772991,0.956504499756638
"3638","SLC25A22",12.9026332950885,-0.492097911412026,0.42237590664757
2,-1.16507097982421,0.243990262591258,0.956516923915054
"3639","ZBED4",15.3140444547663,0.482815306111311,0.414359695200873,1.
16520817951961,0.243934735500536,0.956516923915054
"3640","ZBTB7A",270.402890346647,-0.235975125979087,0.20264029511402,-
1.16450247886932,0.244220439362095,0.957156260422979
"3641","SNX7",98.893792001429,-0.296390137347509,0.254950620890285,-1.
16253938238125,0.245016437281956,0.957382770272359
"3642","OLFML2B",135.01605923647,0.304530969477068,0.261634822730295,1
.1639542714503,0.244442543978123,0.957382770272359
"3643","RAB4A",129.711601729826,-0.271824105659549,0.23359247477253,-1
.16366807588407,0.244558551588971,0.957382770272359
"3644","FAM228B",32.2466810578289,-0.366146960272413,0.31490486506885,
-1.16272246283765,0.244942124793031,0.957382770272359
"3645","TSTD2",35.8478544670265,0.387817622981869,0.333367402038827,1.
16333396909846,0.244694028603794,0.957382770272359
"3646","CKAP5",48.1976408759054,0.294328316631634,0.253013380519325,1.
16329150666857,0.244711250473431,0.957382770272359
"3647","TAX1BP3",240.090358505592,-0.284521588957166,0.244606503888836
,-1.16318080032112,0.244756154642845,0.957382770272359
"3648","ATP5F1D",449.083694249088,-0.284685430019158,0.244823310132073
,-1.16281995315553,0.244902559856865,0.957382770272359
"3649","APMAP",90.2944114183445,0.27310339024819,0.234817490473356,1.1
630453493803,0.244811103459711,0.957382770272359
"3650","ZSWIM1",2.80594714136634,1.02829698169524,0.884111082487055,1.

16308572764701,0.244794722173292,0.957382770272359
"3651","ZMYM3",15.3093028870882,0.5005074249871,0.430504989066363,1.16260539993404,0.244989638886274,0.957382770272359
"3652","COMMD6",676.937417194807,-0.261612735389418,0.225095831249887,-1.16222825601329,0.245142759980783,0.957614078281996
"3653","TRPC1",17.6856470716547,0.444430652786879,0.38255912578369,1.16173062628278,0.245344901254459,0.957617061914122
"3654","KAT8",51.6757093270501,-0.311286659522663,0.26789734232675,-1.1619624771902,0.245250706973512,0.957617061914122
"3655","TMEM35A",14.7544175235173,0.614081289948675,0.528584910771295,1.16174578092406,0.245338743589375,0.957617061914122
"3656","PRKCE",20.6795777638801,0.472449884560088,0.407025269035523,1.16073846147094,0.245748275614216,0.957833105101888
"3657","SERTAD2",76.595097899825,-0.24038715201419,0.207240623165467,-1.1599422369149,0.246072324713449,0.957833105101888
"3658","SEMA4F",7.31943398031967,-0.618787121291215,0.5337116265013,-1.15940348788656,0.246291755731709,0.957833105101888
"3659","GBE1",60.5605617043371,-0.281246156406263,0.242294098164458,-1.16076354536447,0.245738071784118,0.957833105101888
"3660","PFN2",55.3168241163334,-0.314853362667635,0.271468328654305,-1.15981619008152,0.246123650961745,0.957833105101888
"3661","OARD1",51.1251579145764,-0.286607097060535,0.247430253629652,-1.15833489581885,0.246727396073242,0.957833105101888
"3662","PEBP4",5.44290653860017,0.752095836700869,0.649121186490398,1.15863701933259,0.246604172581659,0.957833105101888
"3663","MEGF9",33.695485999041,0.398829460571866,0.343867320401988,1.15983531120557,0.246115864360586,0.957833105101888
"3664","RAPGEF1",50.7830077461336,0.3671389779262,0.317144784495411,1.15763839064966,0.247011635614037,0.957833105101888
"3665","HSPA8",814.947698005712,-0.311613234431649,0.268896967711259,-1.15885737605736,0.24651432552778,0.957833105101888
"3666","SPRYD3",81.5260684342548,-0.316143445791945,0.273035806542084,-1.15788273265623,0.246911895015676,0.957833105101888
"3667","LTA4H",48.8103383958054,-0.316068934785146,0.272991227889475,-1.15779886858896,0.246946125213403,0.957833105101888
"3668","CRY1",33.0699953730155,-0.365236099953904,0.314986708436961,-1.15952860921114,0.246240781932895,0.957833105101888
"3669","KLHL33",3.24753213959035,1.08500205762594,0.936443207341895,1.15864160166822,0.246602303971447,0.957833105101888
"3670","GANC",16.6308715058823,0.429972875064644,0.370492599903914,1.16054376032384,0.245827487832465,0.957833105101888
"3671","NLRC5",26.550841370602,0.383485556806576,0.331228488731158,1.15776743200925,0.246958957314245,0.957833105101888
"3672","GLOD4",82.4155804727877,-0.296709909848096,0.255887424118639,-1.15953298943886,0.246238997584107,0.957833105101888
"3673","PROCA1",3.7636139523742,0.845298990141513,0.728760547432223,1.15991321582914,0.246084141429498,0.957833105101888
"3674","SLC38A10",104.948988926769,0.228326381843839,0.19659364180954,1.16141285009126,0.245474045702748,0.957833105101888
"3675","DOT1L",14.0155222283076,-0.485192485490371,0.419024862748197,-

1.15790858401148,0.246901344122719,0.957833105101888
"3676","CEBPB-
AS1",3.0479926803844,-1.03718806086551,0.89332432588441,-1.16104311817
399,0.245624364992305,0.957833105101888
"3677","SYNJ1",18.2529152284439,0.418708457408189,0.361687299084918,1.
1576531950874,0.247005591627964,0.957833105101888
"3678","WDR45",125.960987936303,-0.295491260078455,0.255055855974249,-
1.15853548607913,0.246645578919627,0.957833105101888
"3679","GPC4",65.0287290159109,0.318044032712009,0.274313646114838,1.1
594174668906 7,0.246286060388743,0.957833105101888
"3680","KIAA0319L",45.1600083691061,0.324811265285181,0.280661758903236,1.15730502992097,0.24714775923382,0.957902077949519
"3681","PSMD6-
AS2",3.65039458470133,0.906898937499568,0.783883285477525,1.1569310818
3459,0.247300518694678,0.957902077949519
"3682","FOPNL",51.4148376520902,0.284050616921906,0.245498805291166,1.
15703462012785,0.247258216223008,0.957902077949519
"3683","AKAP1",23.7087725883232,-0.436327307887832,0.377193573133314,-
1.15677291175271,0.247365151771066,0.957902077949519
"3684","KCNK6",9.74417646263082,-0.595594347619007,0.514667831879061,-
1.15724028339693,0.247174203746135,0.957902077949519
"3685","RNF216",88.5330050815502,0.234926642550086,0.203146272623696,1
.15644082225057,0.247500892063255,0.958059568258105
"3686","AC007611.1",2.91013888232821,-0.994941281129946,0.860419183710
962,-1.15634483745329,0.247540135188516,0.958059568258105
"3687","B3GALNT2",25.7074997857419,-0.382828101447875,0.33112493675506
7,-1.15614397755568,0.247622270310587,0.958117523257617
"3688","SNRNP27",91.3049747391204,0.236923836210975,0.205309017506501,
1.1539865081838,0.248505700330883,0.958308700211886
"3689","HECW2",24.1701965659226,0.434196670520793,0.375899981690203,1.
1550856389204,0.248055358554091,0.958308700211886
"3690","PPARG",72.9154881650864,-0.330036002898984,0.285959104175531,-
1.15413707093024,0.248443977168869,0.958308700211886
"3691","DIPK2A",41.9057535841808,0.445935235784986,0.386746185816261,1
.15304365534673,0.248892466520742,0.958308700211886
"3692","MOXD1",12.5795707655124,-0.818512406099523,0.709634072344382,-
1.15342884170633,0.248734409004283,0.958308700211886
"3693","TMEM60",52.9703889502026,0.295264624026502,0.25610415241972,1.
1529083821437,0.248947991236874,0.958308700211886
"3694","ZNF277",53.5922311715869,-0.303272007072814,0.263008619162875,
-1.15308771263122,0.248874384481854,0.958308700211886
"3695","ZNF517",3.51612519458856,0.87538382735526,0.758279706572713,1.
15443393746067,0.248322308225023,0.958308700211886
"3696","CDC26",191.999822213542,-0.316010622765641,0.273844936889667,-
1.1539765034727,0.248509802139825,0.958308700211886
"3697","ENTPD1-
AS1",22.9186078608908,0.391761097380935,0.339061781579733,1.1554268828
4026,0.247915658498539,0.958308700211886
"3698","SFR1",24.1344207877808,-0.395466814126344,0.342253424829882,-1
.15547949395368,0.247894125211091,0.958308700211886

"3699","MED13L",210.069873996249,0.274263873695631,0.237732674908797,1.15366503069403,0.248637525849645,0.958308700211886
"3700","WASF3-AS1",2.98201842406644,1.04404857328676,0.905343614760489,1.15320697717956,0.248825440404444,0.958308700211886
"3701","TYRO3",9.02867584897621,-0.666883671708994,0.57829763076163,-1.15318416717477,0.248834800709485,0.958308700211886
"3702","TMEM170A",46.3248183394393,-0.301637135032082,0.261429504678627,-1.15379912991413,0.24858253098749,0.958308700211886
"3703","BSG",390.495718879556,-0.260327279225384,0.225506174921229,-1.15441308565638,0.248330852848965,0.958308700211886
"3704","GSK3A",58.0945565790754,-0.286317687988986,0.248287598338707,-1.15316950949117,0.248840815760996,0.958308700211886
"3705","UBE2M",175.26653609007,-0.346068441604725,0.299930954653748,-1.15382702663765,0.248571091449693,0.958308700211886
"3706","GAB3",10.2044436241775,0.581094944096509,0.503729631961316,1.15358499327102,0.248670353712308,0.958308700211886
"3707","FILIP1",206.612635971034,0.388740635905427,0.337261140996861,1.15263986463547,0.24905823353768,0.958474442850969
"3708","RPL19",6758.8706730501,-0.271425459921596,0.235538086904384,-1.15236335443185,0.249171792976683,0.958652858307812
"3709","UPF1",68.1886051845282,0.302490621117484,0.262537543997366,1.15218043298417,0.249246936520534,0.958683417741154
"3710","ELOC",213.028966129282,-0.303195458225853,0.263233177547722,-1.1518132366536,0.249397827405753,0.959005230665896
"3711","LIPT1",22.4845764480427,0.409159453438287,0.355288928661277,1.15162455238894,0.249475387690574,0.959044969224933
"3712","YBX1",1671.63110831807,-0.264788328729594,0.230234764175764,-1.15007970094148,0.250111046149666,0.959203943048339
"3713","DNTTIP2",101.955758533743,-0.235776987939314,0.204991839620205,-1.15017743328781,0.250070798831614,0.959203943048339
"3714","G0S2",460.274974658257,0.921615907219536,0.800648949719244,1.15108613774203,0.249696800321401,0.959203943048339
"3715","RPS27A",6968.40671219759,-0.260945166331061,0.226885601609674,-1.15011778834684,0.250095360774725,0.959203943048339
"3716","LAMB2",460.439772165894,0.201226641135143,0.17497156206927,1.15005340728157,0.250121874957229,0.959203943048339
"3717","ADAMTS9",2.57555298017236,1.46359602671452,1.27161793119761,1.15097152281905,0.249743951215758,0.959203943048339
"3718","ANXA6",231.414650282957,0.221939920131855,0.192834855722014,1.15093259100314,0.249759968607803,0.959203943048339
"3719","TCEA1",293.271847429831,-0.201779291700806,0.175408553315339,-1.15033895375706,0.250004292738382,0.959203943048339
"3720","EIF3F",585.703194314473,-0.220449644447132,0.191591746344578,-1.15062182298111,0.249887851032225,0.959203943048339
"3721","SEC61G",468.433434804121,-0.342256962589863,0.297667586740711,-1.1497958724273,0.250227955683931,0.959352866376501
"3722","NENF",493.730359110241,-0.259201860744593,0.225474687409387,-1.14958296970147,0.250315675790696,0.959370330187339
"3723","PBX3",50.1522192998038,0.316714518183062,0.275584108625361,1.1

4924811798062,0.250453684723311,0.959370330187339
"3724","PPP2R5C",129.524874219517,0.245578765397107,0.213708033355937,
1.14913211983982,0.250501505674179,0.959370330187339
"3725","SNHG16",253.42733837528,-0.232223715381873,0.202033933129829,-
1.14942926559096,0.250379018251071,0.959370330187339
"3726","ECT2",8.70957683996568,0.572160496473479,0.498126397128834,1.1
4862512762096,0.250710591174511,0.959913390685874
"3727","PPP1R7",129.138147688353,-0.325235575255904,0.283283580248519,
-1.14809186953434,0.250930640241339,0.960191571996818
"3728","MAMDC2",21.5870873089585,-0.741927419850956,0.646300880237456,
-1.1479597855079,0.250985165566811,0.960191571996818
"3729","KXD1",114.201802309893,-0.268417883493928,0.233759390557817,-1
.14826567118183,0.250858906150799,0.960191571996818
"3730","ARHGAP18",55.7448561633612,-0.380987509806703,0.33199056404636
4,-1.14758535653289,0.251139777735513,0.960525487714431
"3731","LRRC8D",10.3405897968988,0.643819717725999,0.561217572737108,1
.14718381783028,0.251305658107489,0.960902309986985
"3732","LRRC4",2.64809942841749,1.09570267747786,0.95558679527073,1.14
662810631182,0.25153535516889,0.961265303198334
"3733","TNFRSF13C",2.01747249388659,1.38702641105819,1.209538642165,1.
1467400566678,0.251489069981689,0.961265303198334
"3734","TLR2",4.47793123439514,-0.779289879931646,0.679910568084073,-1
.146165270129,0.251726775010907,0.961739199867597
"3735","ZMYM2",76.331404452559,0.289565833215814,0.25271691624582,1.14
581104232117,0.251873345204815,0.962041537534644
"3736","SCYL2",34.5803014606712,0.318412542495117,0.278013119535833,1.
14531480754122,0.25207877423132,0.962053449219907
"3737","NPC2",482.12452397849,-0.209300286659283,0.18273336000483,-1.1
4538629757451,0.252049171910959,0.962053449219907
"3738","ALDH16A1",52.6561466405364,-0.304463926461644,0.26578745042923
1,-1.14551656208731,0.251995238710323,0.962053449219907
"3739","POU3F1",4.34341232678582,1.15024399005818,1.01184732694411,1.1
3677623039441,0.255631842424616,0.962090187801935
"3740","VCAM1",199.438530867891,0.389493002014823,0.341447600609014,1.
14071090650546,0.253990246406895,0.962090187801935
"3741","LRIG2",23.8932888942387,0.419761779338644,0.36832505585321,1.1
3965035141658,0.254431999205691,0.962090187801935
"3742","ZBTB41",38.0788019979995,0.301794484709486,0.265246579284743,1
.13778841379707,0.255208844115721,0.962090187801935
"3743","ENAH",169.454580991938,-0.253113479136311,0.223272847500039,-1
.13365096549086,0.25694098559801,0.962090187801935
"3744","AGT",5.21067839082604,1.11217010574158,0.972436256264787,1.143
69461090799,0.252750309116626,0.962090187801935
"3745","SPR",32.5120609656825,-0.38499291080898,0.338599394252057,-1.1
3701594670422,0.255531619347596,0.962090187801935
"3746","CNNM3",27.0102678709154,0.412051265672997,0.360439163244759,1.
14319227123827,0.252958770481337,0.962090187801935
"3747","PLEKHB2",50.1817711679928,0.303825175531355,0.266984062918138,
1.13798993172306,0.255124686526511,0.962090187801935
"3748","NBEAL1",111.404039511745,0.262971949431007,0.231496577206164,1

.13596474127051,0.255971320952674,0.962090187801935
"3749","RAB5A",102.678576023998,-0.216486952389142,0.189530671857319,-1.14222648116879,0.253359891346477,0.962090187801935
"3750","TRAIP",3.15935643997176,1.04413991225777,0.912161808962388,1.14468716185948,0.252338771423374,0.962090187801935
"3751","SLMAP",51.751378540626,0.332303524942525,0.290858709299304,1.14249123137163,0.253249888799416,0.962090187801935
"3752","NSUN3",24.8312235046418,0.369110821196233,0.325664534296752,1.1334081004347,0.257042913937427,0.962090187801935
"3753","ZXDC",19.7593961451639,0.4786539118162,0.420738659997371,1.13765136728626,0.255266088281877,0.962090187801935
"3754","XRN1",116.725028245867,0.263874848742659,0.232606709947282,1.1344249218024,0.25661635008562,0.962090187801935
"3755","USP13",21.0663947429833,-0.373034229015917,0.327686523373019,-1.13838745999108,0.254958727995876,0.962090187801935
"3756","TICAM2",24.7262242592703,0.350821522698855,0.309213049233258,1.13456247583591,0.256558682947452,0.962090187801935
"3757","JADE2",36.8801749855166,0.367531475509044,0.324075539195826,1.13409199725796,0.256755960165504,0.962090187801935
"3758","AL357054.4",4.33428351664197,-0.90142422413203,0.796096636791033,-1.13230502739662,0.257506217230026,0.962090187801935
"3759","HLA-C",1496.23760690218,-0.302620948434381,0.265911114164009,-1.13805302717708,0.255098340672315,0.962090187801935
"3760","CSNK2B",237.67193987156,-0.242984923665215,0.212796161919267,-1.14186704061609,0.253509290566119,0.962090187801935
"3761","FBXO30",23.7296466688588,0.403812525064997,0.356474358269303,1.13279543310078,0.257300169744527,0.962090187801935
"3762","TMED4",141.866246844474,0.244640016616736,0.214952450570838,1.13811224746244,0.255073614637144,0.962090187801935
"3763","GTF2IRD2B",31.3257123977383,0.33586161925981,0.296234671980733,1.13376876857161,0.256891554781808,0.962090187801935
"3764","GATAD1",132.169868054521,0.263269793713963,0.230290852546417,1.1432056063143,0.252953235132436,0.962090187801935
"3765","LAMB1",188.558098167026,0.51453052803987,0.4541725441875,1.13289659321073,0.257257680834069,0.962090187801935
"3766","GALNT11",115.57522192706,0.282459065185697,0.248175207493899,1.13814376560012,0.255060455660674,0.962090187801935
"3767","PNMA2",4.61394148077502,1.11125264453545,0.98131514981629,1.13241158535409,0.257461436405709,0.962090187801935
"3768","TNFRSF11B",20.3674852653768,0.668180229053697,0.588801068943751,1.13481490489198,0.256452879862294,0.962090187801935
"3769","C8orf31",5.62636253052018,0.760725765208796,0.669621671180592,1.13605308482263,0.255934348107195,0.962090187801935
"3770","ZNF251",16.1176717864942,0.494389358282028,0.436327164775478,1.13307031556568,0.257184725947544,0.962090187801935
"3771","ZNF169",5.76451933756388,0.784490657733469,0.691408997376373,1.13462604726046,0.256532034772981,0.962090187801935
"3772","TRUB2",45.2109239099712,-0.329180488251282,0.287818154286226,-1.14370995487631,0.25274394354749,0.962090187801935

"3773","TRIM34",16.1731449177243,0.509288634428391,0.446401565686047,1
.14087555594859,0.25392171290694,0.962090187801935
"3774","HIPK3",140.80670532957,-0.243768559381995,0.214645610820444,-1
.13567921771255,0.256090841392847,0.962090187801935
"3775","MPEG1",4.28559732933605,-1.02713693947757,0.907205705309111,-1
.13219850081035,0.257550990271666,0.962090187801935
"3776","HNRNPUL2",104.688108850599,0.268055975239358,0.234107832294508
,1.14501070986016,0.252204720924276,0.962090187801935
"3777","ZFPL1",46.7080049099995,-0.372863240894281,0.327353210997021,-
1.13902423549978,0.254693045938097,0.962090187801935
"3778","CD248",919.12256880976,0.329133622873435,0.290314372333056,1.1
3371453238231,0.256914311763073,0.962090187801935
"3779","AP001267.3",3.55630714421411,-0.840631033646111,0.736139677937
494,-1.14194501239409,0.253476876874919,0.962090187801935
"3780","ZFC3H1",78.2894722965521,0.283785040416773,0.248348360719474,1
.14268940449068,0.253167570509486,0.962090187801935
"3781","SLC25A30",6.73773726473603,0.639154253342833,0.559158837805277
,1.1430638489978,0.253012082476473,0.962090187801935
"3782","KLF5",20.5205846486613,-0.458886769160472,0.404393848926326,-1
.13475209966429,0.256479201206118,0.962090187801935
"3783","DDHD1",68.4640565398832,0.29574857876992,0.26018634455022,1.13
667986412268,0.255672139930683,0.962090187801935
"3784","CGRRF1",43.5894444947527,-0.298965758930622,0.262319754739786,
-1.13969975012819,0.254411411308674,0.962090187801935
"3785","MPP5",23.1074831175131,0.436206098707888,0.384257806382552,1.1
3519124780934,0.2562951958041,0.962090187801935
"3786","CCDC32",79.9090025667731,-0.28949261861903,0.254602689744137,
-1.1370367646266,0.25552291686406,0.962090187801935
"3787","TRIM69",60.5819750655775,0.359933840519271,0.317676328293811,1
.13302065171937,0.257205580866534,0.962090187801935
"3788","SPPL2A",253.439704936546,0.215822179434905,0.19007559717096,1.
13545443311583,0.25618496371504,0.962090187801935
"3789","CYP19A1",70.6779321313989,0.592951511306574,0.521286212437006,
1.13747783302101,0.255338586146803,0.962090187801935
"3790","C16orf91",40.8141534876479,-0.410453627594297,0.35899694065563
7,-1.14333461127743,0.252899689927231,0.962090187801935
"3791","ALDOA",28.1753120992898,-0.493071664039708,0.43317909350798,-1
.13826283730986,0.255010746900215,0.962090187801935
"3792","ZNF764",7.16087529753998,-0.610946610454336,0.53515263220523,-
1.14163058104896,0.253607606951852,0.962090187801935
"3793","PSMD7",231.848427749931,-0.261967295288251,0.231169085093693,-
1.13322806629647,0.25711849080096,0.962090187801935
"3794","AC129507.1",2.08078345415702,1.29094168342862,1.13569496280692
,1.13669755146048,0.255664743281806,0.962090187801935
"3795","RABEP1",96.6250966097854,0.217229512532843,0.190271945507874,1
.14167914745925,0.25358741159178,0.962090187801935
"3796","PIP4K2B",49.3568752543587,0.279104767472516,0.245071537488923,
1.13887059400005,0.254757132199203,0.962090187801935
"3797","AC100793.2",2.90949781666585,1.04418915806338,0.92029771521633
9,1.13462104794851,0.256534130338895,0.962090187801935

"3798","HOXB5",2.23292218970742,-1.14171220026378,1.00339600714304,-1.1378480601239,0.255183932702888,0.962090187801935
"3799","SNF8",209.891764216171,-0.319761359478887,0.281613898430288,-1.1354601504444,0.256182569444328,0.962090187801935
"3800","NME1",206.241777313083,-0.444617990120601,0.390584608407056,-1.13833976186085,0.2549786368585,0.962090187801935
"3801","AC091271.1",17.3627769702166,-0.494425600610585,0.435764570496188,-1.13461633663242,0.256536105196213,0.962090187801935
"3802","GNA13",58.8032896006023,0.312605370405395,0.273367925890069,1.14353346094854,0.25281717022596,0.962090187801935
"3803","AFG3L2",65.4921608616836,-0.288566202747766,0.252357564577842,-1.14348148521125,0.252838737584564,0.962090187801935
"3804","DSG3",1.9992102246139,-1.53096625018457,1.34430269818827,-1.13885529817642,0.254763512939138,0.962090187801935
"3805","SLC14A2-AS1",2.67216060490439,-1.18228458883618,1.03625676772839,-1.14091856927304,0.253903811209592,0.962090187801935
"3806","PIGN",21.0789399746415,0.476717375715018,0.421007195390192,1.13232595769104,0.257497420878262,0.962090187801935
"3807","ZNF799",6.17654768553423,0.737897757408282,0.64606334651481,1.14214459215009,0.253393922611018,0.962090187801935
"3808","NFIX",411.556311642144,0.282538997127372,0.248344544571893,1.13768956597949,0.255250131835513,0.962090187801935
"3809","ZNF529-AS1",4.2237994077002,0.825298507065208,0.724669229441529,1.13886235752169,0.254760568079245,0.962090187801935
"3810","ZNF473",6.17881469441778,-0.710980021656885,0.626558280939898,-1.1347388475823,0.256484755324337,0.962090187801935
"3811","ZNF480",29.8901454314814,-0.368161366607142,0.323941025981936,-1.13650737967247,0.255744278814705,0.962090187801935
"3812","DSTN",1137.96416290971,-0.167678636540305,0.14752175527314,-1.13663666914649,0.25569020421662,0.962090187801935
"3813","NAPB",14.6073940262734,0.449527172416817,0.393120348176347,1.14348487556581,0.252837330716138,0.962090187801935
"3814","AGPAT3",40.2773123262077,0.382119358371165,0.336875130169071,1.13430563478933,0.256666366378952,0.962090187801935
"3815","BID",46.2321713577807,-0.368860676582083,0.325463326705345,-1.13334021475184,0.257071409982634,0.962090187801935
"3816","LIMK2",10.692667867056,0.558505174632155,0.490961279229121,1.13757479104887,0.255298077973058,0.962090187801935
"3817","AMER1",4.7503816918177,0.806846992168088,0.706374674649599,1.1422365794305,0.253355694952777,0.962090187801935
"3818","MBNL3",23.0258357426563,0.434220388242922,0.38312318061911,1.13337018016305,0.257058831276405,0.962090187801935
"3819","SLC9A6",13.6850446694831,0.506548835282817,0.447139711771142,1.13286478912006,0.257271038550616,0.962090187801935
"3820","MED19",65.2050412625718,-0.252936948710033,0.223461146723667,-1.13190571344743,0.257674076392958,0.962298003618307
"3821","AGO2",105.148020681778,0.296587946811365,0.262133874952266,1.13143693032949,0.257871235072797,0.962530360949377

"3822","PPIF",72.7644046173302,0.358356636774975,0.316718349660246,1.1
3146787093137,0.257858218990691,0.962530360949377
"3823","AL592295.5",27.2837918539057,-0.390614182350677,0.345351419380
768,-1.1310629128181,0.258028612682458,0.962614066037642
"3824","PRKAB1",12.9994878786457,0.475656876175687,0.420487317295163,1
.13120385945386,0.257969297895826,0.962614066037642
"3825","LARS2",9.90950608636092,0.510254274694622,0.451273415955533,1.
1306987220025,0.258181919443198,0.96268250464628
"3826","EIF5A",432.115657139832,-0.349381015275266,0.308978694701923,-
1.13076086237053,0.258155756878976,0.96268250464628
"3827","FAM120AOS",115.258715262656,-0.20624956043928,0.18246966776031
,-1.13032244192063,0.258340381523818,0.962770084330925
"3828","TLNRD1",70.3238210734875,0.307210764997696,0.271783441679788,1
.1303512940264,0.258328228707844,0.962770084330925
"3829","SLC35E2A",21.040601889822,0.572682062571765,0.507131097840324,
1.12925842057527,0.258788835555264,0.962866812130124
"3830","ACADM",67.3094988880819,-0.257371211812784,0.227935613940133,-
1.12913996792262,0.258838793251018,0.962866812130124
"3831","CHL1-
AS2",8.57914153819309,0.656156253764341,0.580726721385904,1.1298881721
8265,0.258523348468093,0.962866812130124
"3832","FXR1",205.110943997217,0.229215014720054,0.202966646582713,1.1
2932355428479,0.25876136810143,0.962866812130124
"3833","CFAP70",4.15728273343741,-0.773830040082722,0.685251536951713,
-1.12926421664816,0.25878639121884,0.962866812130124
"3834","NUP93",25.2554831149253,0.358018350870404,0.316974681580172,1.
12948563931233,0.258693024199906,0.962866812130124
"3835","ZNF507",36.4548465785361,0.341500921686843,0.302388782316881,1
.12934388329583,0.258752795593725,0.962866812130124
"3836","ZNF697",9.47683284047673,0.701702658077282,0.621740485961102,1
.12861020622225,0.259062303317438,0.963223955529919
"3837","AC096642.1",3.27883372729586,1.06300979064468,0.94234709160767
6,1.12804485747512,0.259300975248054,0.963223955529919
"3838","KALRN",2.28757805926686,-1.16661231067041,1.03409379508192,-1.
12814941567074,0.259256822689138,0.963223955529919
"3839","SMIM20",120.817224184358,-0.326347219804655,0.289281302488904,
-1.12813105097649,0.259264577306737,0.963223955529919
"3840","PAXIP1",16.2769841525534,0.46132952133709,0.409113185589694,1.
1276329817435,0.259474951710464,0.963223955529919
"3841","AC064807.1",6.17497784382615,-0.647566541348554,0.574189677182
07,-1.1277920295025,0.259407760299377,0.963223955529919
"3842","PCAT1",3.61622544225719,-0.875235711329004,0.775880509393204,-
1.12805477226577,0.259296788232917,0.963223955529919
"3843","RDH11",105.977622597519,-0.248589923030243,0.220421460196934,-
1.12779364953005,0.259407075963575,0.963223955529919
"3844","ZNF681",5.39166625275696,0.747780802913315,0.663310943773067,1
.12734579450742,0.259596307529278,0.963423757339407
"3845","RAB11FIP4",3.94676455792844,0.984900058333006,0.87397509074562
3,1.12692005614571,0.259776282774868,0.963840949302019
"3846","DEGS1",168.860104263643,-0.24155993955759,0.214731041565335,-1

.12494187052174,0.260613667636802,0.964274752691162
"3847","ATL2",34.3440613648323,0.385147730308417,0.342196996051305,1.1
2551464435027,0.260371015165721,0.964274752691162
"3848","IGFBP3",395.940200945862,-0.338969309020312,0.301437875201462,
-1.12450802273525,0.260797568954494,0.964274752691162
"3849","NDUFA5",198.314787009938,-0.261731949835098,0.232596494891593,
-1.12526179707516,0.260478113237694,0.964274752691162
"3850","ZMYND11",104.910759718295,0.242593895977665,0.215731533490462,
1.12451755222141,0.260793528589875,0.964274752691162
"3851","PLCE1",30.6242036161666,0.350409109165439,0.311155231979037,1.
1261552856969,0.260099796265048,0.964274752691162
"3852","ABCC2",52.6145580383864,0.982055796993652,0.872382404587136,1.
12571710734861,0.260285280242904,0.964274752691162
"3853","FADS2",8.20758074277594,0.879426325455749,0.781872165323178,1.
12476996171394,0.260686526368253,0.964274752691162
"3854","TBC1D10C",13.7279011927946,-0.501066940896111,0.445339024161252,-1.12513593849049,0.260531434295518,0.964274752691162
"3855","SCG5",7.09274400083541,-0.645287754357632,0.573253689632244,-1
.12565826618857,0.260310195089483,0.964274752691162
"3856","ADAM10",65.7847219181327,0.328414946004569,0.29207779897093,1.
12440913743415,0.260839497450946,0.964274752691162
"3857","CLTC",227.50658830749,0.196383969834745,0.174354814378863,1.12
634670017206,0.260018797889285,0.964274752691162
"3858","CYP4F12",10.6766220126571,0.63681539459079,0.565959115956971,1
.12519681481587,0.260505642580601,0.964274752691162
"3859","APOBEC3C",91.0876115692388,0.243594101830921,0.216291217108964
,1.12623205457391,0.26006730887983,0.964274752691162
"3860","SELPLG",10.8720445166992,0.552420887305321,0.491605851371357,1
.12370690007923,0.261137388227385,0.96512590167147
"3861","ATP6V0B",197.464919965007,-0.303601440636679,0.272077994245036
,-1.11586180087484,0.264481295061991,0.965909878643543
"3862","TCEANC2",9.60554800143458,0.546020412064904,0.487703568699791,
1.11957436260018,0.262895185715359,0.965909878643543
"3863","CD58",41.2969518682291,-0.290922416753414,0.260227820208458,-1
.11795278660202,0.263587158413094,0.965909878643543
"3864","PRUNE1",13.590123122827,0.486835899670467,0.43565545139317,1.1
1747918708151,0.263789493710043,0.965909878643543
"3865","RCOR3",45.0520771428704,0.30432828452966,0.271321281411233,1.1
2165283514344,0.262010077581118,0.965909878643543
"3866","PRADC1",42.2559565079584,-0.424094835441616,0.378903576325962,
-1.11926849451739,0.263025612285567,0.965909878643543
"3867","IMP4",115.288562339286,-0.330223844625452,0.295859254204846,-1
.11615181858335,0.264357154432289,0.965909878643543
"3868","RAB6B",8.33552127545743,0.642687487005535,0.573145631988723,1.
12133365611723,0.262145864550291,0.965909878643543
"3869","EIF4A2",610.440175337224,0.213439740767275,0.190827152263941,1
.11849775168294,0.263354466225917,0.965909878643543
"3870","BCL6",204.096745833392,0.322853137516081,0.287908365056278,1.1
2137463408878,0.262128428747376,0.965909878643543
"3871","LINC00887",1.99857372050298,-1.43150830336614,1.28230208001583

,-1.1163580919626,0.264268884594803,0.965909878643543
"3872","USP38",28.4043723677082,0.386128287012125,0.344794047153131,1.11988095560315,0.26276449484349,0.965909878643543
"3873","SCAMP1-AS1",15.1251626842544,-0.427732888933468,0.383336358274396,-1.11581612257946,0.264500851090473,0.965909878643543
"3874","DMGDH",7.98746144802443,0.593159323463198,0.529478538380206,1.12027075786264,0.262598399286018,0.965909878643543
"3875","MRPL22",111.027140726249,-0.323897850166639,0.290420074017885,-1.11527362997191,0.264733182054355,0.965909878643543
"3876","SLIT3",981.083268250728,0.405482325485793,0.362435671643909,1.11877046662277,0.263238074134916,0.965909878643543
"3877","RANBP17",3.16402083212477,-1.07250121250301,0.958511462187441,-1.11892372163754,0.263172681964383,0.965909878643543
"3878","TJAP1",24.344656501815,0.377823986794094,0.33833732438757,1.11670797030153,0.264119208867679,0.965909878643543
"3879","FOXO3",235.888750895303,0.347969948048829,0.311330941943002,1.11768507774128,0.263701518155507,0.965909878643543
"3880","LINC01625",3.54314711905687,0.976189373453523,0.875380766168167,1.11515972383838,0.26478198200241,0.965909878643543
"3881","ARPC1B",591.968783107093,-0.252930013194631,0.226678645923476,-1.11580873515548,0.264504013926341,0.965909878643543
"3882","BUD31",224.880490989814,-0.301099842243669,0.270055926376714,-1.11495365527972,0.264870282157125,0.965909878643543
"3883","VIPR2",11.8696435016336,0.840248038167862,0.751611745630396,1.1179282961619,0.263597618805389,0.965909878643543
"3884","AC009812.4",2.00086635569457,1.21752851868578,1.0861580587417,1.12094967107852,0.262309286078306,0.965909878643543
"3885","GPAA1",138.924843378768,-0.283038372290001,0.252678783284303,-1.12015092288749,0.262649453499281,0.965909878643543
"3886","FBXL6",14.0087771160657,-0.469458235003124,0.418345145309524,-1.12217923469814,0.261786239846636,0.965909878643543
"3887","YME1L1",205.721658767145,0.207941730904193,0.185581484901573,1.12048748297536,0.26250608362983,0.965909878643543
"3888","AL450306.1",2.41506326975471,-1.09611299595912,0.978060504176478,-1.12070060213917,0.262415325812798,0.965909878643543
"3889","UBTD1",15.4164215428503,-0.503742392298669,0.448652230235552,-1.12279034483835,0.261526546939925,0.965909878643543
"3890","SORL1",13.4837348911608,0.571739132714455,0.509799405257981,1.12149823404586,0.262075842795604,0.965909878643543
"3891","PCBP2",674.326585041576,-0.174473656518187,0.156117035686181,-1.11758243263667,0.263745375118365,0.965909878643543
"3892","RNF31",20.7100186271602,-0.377730866939333,0.337231078654933,-1.12009506492087,0.262673253438936,0.965909878643543
"3893","IRF9",125.599244073286,-0.308993202164954,0.2770047127942,-1.11547994634489,0.264644807265077,0.965909878643543
"3894","PRKD1",10.631802769804,0.505598092938523,0.452408633079328,1.11756950679115,0.26375089827449,0.965909878643543
"3895","SAMD4A",76.8507901268572,0.29691749767826,0.265086510364972,1.12007773337641,0.26268063835904,0.965909878643543

"3896","AL136038.5",9.97954707485807,0.568353266321012,0.506281761457556,1.12260268804619,0.26160627324915,0.965909878643543
"3897","ITPK1",21.6873340460737,-0.391604536337185,0.351156229454996,-1.11518607243553,0.264770693119984,0.965909878643543
"3898","POLG",25.3685725945759,0.380357013172456,0.340172293465068,1.11813048998805,0.263511266063385,0.965909878643543
"3899","E2F4",63.8614332427047,-0.279068162821428,0.249318644863385,-1.11932327794556,0.263002248557214,0.965909878643543
"3900","NQO1",46.4181105288574,0.402472326421871,0.359689249435248,1.11894455298232,0.263163794332283,0.965909878643543
"3901","TXNDC17",141.056459168612,-0.349039644782972,0.312755260791563,-1.11601526349893,0.264415601147867,0.965909878643543
"3902","CORO6",4.39000777670764,0.825117577464845,0.737690178151111,1.11851506486484,0.263347076072272,0.965909878643543
"3903","PIGW",8.66723175019566,-0.590687781502378,0.526367116178366,-1.12219734734002,0.261778540268745,0.965909878643543
"3904","TRIM25",100.205790533328,0.336613202379157,0.300362722216936,1.12068901192086,0.262420261005478,0.965909878643543
"3905","TMC6",11.9159482413539,0.526777840133701,0.470909188254368,1.11863996981336,0.263293764477119,0.965909878643543
"3906","R3HDM4",51.2462827278537,-0.32874984865045,0.294660531686212,-1.11569013593086,0.26455479431321,0.965909878643543
"3907","ZNF493",36.3037572212276,0.423442273794944,0.378542978601623,1.11861082553739,0.26330620309261,0.965909878643543
"3908","AC020910.5",15.6992911049884,-0.534678003880337,0.47760652485157,-1.11949476411886,0.2629291233594,0.965909878643543
"3909","EGLN2",88.5551819037211,-0.271651158536146,0.243616516471439,-1.11507693513873,0.264817454432497,0.965909878643543
"3910","ZNF337-AS1",7.16229850737063,-0.6100894941979,0.546115768857006,-1.11714315716389,0.263933120287228,0.965909878643543
"3911","TXNRD2",26.0464691547394,0.406781152173322,0.364445129765841,1.11616569669811,0.264351214982015,0.965909878643543
"3912","FAM199X",26.6312917732209,0.367656997082184,0.329530369370623,1.11569989067891,0.264550617389122,0.965909878643543
"3913","PIK3CD-AS2",2.56354329127398,-1.17230018434076,1.0595082645166,-1.10645685701717,0.268528827732925,0.966153621353896
"3914","MAD2L2",86.4773166653867,-0.382775332556208,0.35290564214933,-1.08463931102082,0.278081436860876,0.966153621353896
"3915","EPHA2",8.80435622320571,0.656449180184727,0.595354324961346,1.10261931871805,0.270192517158454,0.966153621353896
"3916","AL031280.1",3.02189253973155,0.991523572323634,0.898875992686604,1.10307047956651,0.269996559396106,0.966153621353896
"3917","SESN2",38.0344996817841,0.293255844868169,0.263734443041513,1.11193608800656,0.266165630908925,0.966153621353896
"3918","C1orf216",32.7909163451394,-0.334996415420633,0.308744252738765,-1.08502883033123,0.277908886419145,0.966153621353896
"3919","DHCR24",13.5463132309577,-0.630101666162514,0.581782388434122,-1.08305386805957,0.278784513616565,0.966153621353896

"3920","DDAH1",5.80293340666563,0.715913139074689,0.655501320450947,1.09216124626901,0.274762233007443,0.966153621353896
"3921","KCNC4",4.33841648502627,0.81003776149088,0.737564482137876,1.09826026213049,0.272090858645008,0.966153621353896
"3922","CHD1L",6.64445390517937,-0.681830166226265,0.617159359242859,-1.10478785748748,0.269251523193171,0.966153621353896
"3923","RPRD2",75.8679662885604,0.259687705422652,0.239065220912282,1.08626300568386,0.277362649158636,0.966153621353896
"3924","POGZ",90.5819948827146,0.283851471200422,0.258080761335535,1.09985521482317,0.271395210448219,0.966153621353896
"3925","AL162258.2",2.56880654386374,-1.17179619714755,1.05853953916095,-1.10699331843227,0.268296817128355,0.966153621353896
"3926","SLAMF8",2.31386794542782,-1.00043284088517,0.915703620424354,-1.09252908754641,0.274600612474173,0.966153621353896
"3927","PEA15",145.874878208234,0.190525610890452,0.174363489830127,1.09269211734676,0.27452900190397,0.966153621353896
"3928","SLC19A2",83.4175931220653,0.378401139264402,0.345249603862064,1.09602193610505,0.273069174863528,0.966153621353896
"3929","GLRX2",55.2672320673011,-0.316278839112831,0.290728160253038,-1.08788511865364,0.27664582645149,0.966153621353896
"3930","PLXNA2",11.2371789085266,0.519789166782789,0.478258532807964,1.08683720441116,0.277108762323788,0.966153621353896
"3931","AL392172.1",11.4468600790728,-0.527277530844895,0.480592716686412,-1.09714007836066,0.272580162588256,0.966153621353896
"3932","SRP9",329.885133705479,-0.288039460424776,0.264305493389897,-1.08979747916123,0.275802364650734,0.966153621353896
"3933","COA6-AS1",8.27007078966195,-0.663093114289741,0.607116562304153,-1.09220066699077,0.274744909391787,0.966153621353896
"3934","IAH1",165.007592757267,-0.276007897139612,0.253849056184822,-1.0872914057189,0.276908045564569,0.966153621353896
"3935","LRATD1",2.15663698581834,-1.12819076063545,1.01708355765274,-1.10924097842967,0.267326239059881,0.966153621353896
"3936","DDX1",159.508704441602,0.232607480602078,0.210452387084393,1.10527366225026,0.269041026702851,0.966153621353896
"3937","KCNS3",10.6905983130565,-0.517290661033937,0.47197366168559,-1.09601594967504,0.273071794603668,0.966153621353896
"3938","KLHL29",20.4770128635937,0.495743055906259,0.452318765875903,1.0960037330007,0.273077140833611,0.966153621353896
"3939","PAIP2B",12.5238670533656,0.496961450253334,0.456211081530736,1.08932349601388,0.27601125498295,0.966153621353896
"3940","TRABD2A",16.8558057388597,-0.481088032510773,0.440819070948406,-1.09135031630036,0.275118765367816,0.966153621353896
"3941","TCF7L1",126.664452834362,0.287800830692582,0.262349461910172,1.09701323035732,0.27263560858971,0.966153621353896
"3942","USP39",32.883030602016,0.310978295879609,0.281655916719754,1.10410709457608,0.269546684094463,0.966153621353896
"3943","SLC35F5",64.382483850884,0.259078893216653,0.234494810268806,1.10483849480364,0.269229577051448,0.966153621353896
"3944","ZRANB3",6.89509203196443,0.657616470816722,0.594147030539357,1

.10682446770751,0.268369827392649,0.966153621353896
"3945","PSMD14",121.437867350252,-0.323230977306316,0.292630207460987,
-1.1045714661888,0.269345320712275,0.966153621353896
"3946","KCNE4",84.203849414343,0.521079811068984,0.471850736391418,1.1
043318805732,0.269449198254613,0.966153621353896
"3947","COPS9",335.494434918253,-0.244436948244379,0.225837795980064,-
1.08235624238007,0.279094263831894,0.966153621353896
"3948","CIDEC",2.95387274962722,-0.963595791763945,0.883341207209312,-
1.09085343681427,0.275337378688619,0.966153621353896
"3949","NUP210",7.45884751053595,0.638410414392029,0.590079809567393,1
.08190520001026,0.279294654035011,0.966153621353896
"3950","RPL29",5110.50909621669,-0.281296974674758,0.258393928979679,-
1.08863616024384,0.276314364153372,0.966153621353896
"3951","KCTD6",24.5068128038449,-0.376905311304504,0.346656196752242,-
1.08725969659755,0.276922054970619,0.966153621353896
"3952","NDUFB4",381.507190574685,-0.263495450071981,0.240511466310432,
-1.09556294389675,0.273270085727702,0.966153621353896
"3953","DTX3L",83.6703335092607,0.239361859438882,0.216815649375303,1.
10398792766361,0.269598374559209,0.966153621353896
"3954","MYLK-
AS1",3.18699694148346,-0.895799306053697,0.824857801422825,-1.08600452
648748,0.277476989629216,0.966153621353896
"3955","ZBTB38",162.706843689488,0.253065009296993,0.230533969483789,1
.09773414245048,0.272320596394088,0.966153621353896
"3956","SENP2",39.0521435428868,0.313652200031851,0.289326631150017,1.
08407649439371,0.278330883865783,0.966153621353896
"3957","RFC4",18.5249714992449,-0.401495212286603,0.364437005202423,-1
.10168618047884,0.270598126981143,0.966153621353896
"3958","MFSD10",142.208119846185,-0.210753722736553,0.190158381155602,
-1.10830625216618,0.267729576249459,0.966153621353896
"3959","KLF3",142.81804012502,-0.249273478048436,0.228436039750915,-1.
09121782324821,0.275177047082036,0.966153621353896
"3960","ARHGAP24",99.7396388227021,-0.274322680629494,0.25202570199978
6,-1.08847105058248,0.276387209892241,0.966153621353896
"3961","HERC6",17.9835084268117,-0.439659285759089,0.403120185183943,-
1.09064071192186,0.275431008029335,0.966153621353896
"3962","NAP1L5",19.6082320734923,0.377631487271047,0.344520723947264,1
.09610673907922,0.273032065818313,0.966153621353896
"3963","ANK2",460.127421082621,-0.330640595177661,0.298611195612639,-1
.10726121470198,0.268181008032725,0.966153621353896
"3964","ARFIP1",69.1817191861702,0.234463607160448,0.214947294878071,1
.09079580319188,0.27536274356991,0.966153621353896
"3965","TMEM131L",13.2978315607093,0.44539505242361,0.406188350792236,
1.09652345163249,0.27284976613982,0.966153621353896
"3966","ANXA2R",18.723529979242,0.459420401697462,0.416873418367219,1.
1020621163539,0.270434667700226,0.966153621353896
"3967","MTREX",86.4790008389439,0.236570292282379,0.213148668211905,1.
10988398035492,0.267049024463646,0.966153621353896
"3968","SMN2",6.51470440234419,-0.794566814120348,0.72955350579624,-1.
08911383168963,0.276103691101412,0.966153621353896

"3969","ENC1",18.1525019339075,-0.514137590770208,0.465886355248964,-1
.1035686814555,0.269780283044466,0.966153621353896
"3970","FER",56.5403386337796,0.274015892211278,0.249896204277691,1.09
651882469885,0.272851789832113,0.966153621353896
"3971","SLC25A46",55.3556295909958,0.24648047057355,0.224819784334975,
1.09634688647464,0.2729269981169,0.966153621353896
"3972","H2AFY",171.921191487446,-0.212151607418792,0.19441183642056,-1
.09124841020408,0.275163591588969,0.966153621353896
"3973","AC114296.1",2.83629844391259,-1.00210545066081,0.9043950321799
09,-1.10803953472123,0.267844742288707,0.966153621353896
"3974","ZNF300",6.70802181008939,-0.629518156278106,0.567878330519283,
-1.10854407087951,0.267626917112196,0.966153621353896
"3975","PANK3",55.7456331747905,0.324240774185402,0.292794501436872,1.
10740048940198,0.268120814429887,0.966153621353896
"3976","NHP2",225.125212161078,-0.302637868412131,0.273319651376561,-1
.10726713899974,0.268178447401431,0.966153621353896
"3977","ZNF879",2.85667961022582,-0.960024301661036,0.862617226352904,
-1.11292039195642,0.26574262121161,0.966153621353896
"3978","CAP2",2.95170001364938,-1.05625462537316,0.96035674496281,-1.0
9985651781314,0.271394642639803,0.966153621353896
"3979","PPP1R18",93.6655287573751,-0.238554424558245,0.217930671509763
,-1.09463446749191,0.27367680900069,0.966153621353896
"3980","COL21A1",6.19956393977218,0.909485617663613,0.820590978312018,
1.10833002275318,0.267719313989581,0.966153621353896
"3981","UBE2J1",120.734469162086,-0.203535854304734,0.185150826403648,
-1.0992975740816,0.271638290166708,0.966153621353896
"3982","CRYBG1",32.3768500044954,0.538016385910876,0.496576470905671,1
.08345122540669,0.278608189018605,0.966153621353896
"3983","CDC40",109.433899121606,0.205494132057511,0.185916697560904,1.
10530218508315,0.269028671430652,0.966153621353896
"3984","IFNGR1",275.100581624056,-0.229835908128653,0.212347467950304,
-1.08235765816827,0.27909363497605,0.966153621353896
"3985","FBXO5",17.6771522582227,-0.384269824102969,0.349123121564075,-
1.10067136883239,0.271039711577913,0.966153621353896
"3986","SNX8",16.802330075256,0.443308512026847,0.398840645592078,1.11
149281530411,0.266356280911554,0.966153621353896
"3987","ANKMY2",32.159619672648,0.309510690782318,0.280332378306953,1.
10408470349228,0.269556396049504,0.966153621353896
"3988","AC007036.3",5.37564444552814,0.742720744522018,0.6816023268871
82,1.08966873384068,0.275859093660697,0.966153621353896
"3989","MINDY4",25.233461423342,-0.449357385023634,0.405557848697612,-
1.10799824603735,0.267862573389727,0.966153621353896
"3990","SMIM30",65.5134015811955,0.309485627368101,0.282092612953244,1
.09710645779795,0.272594857580617,0.966153621353896
"3991","AC083862.2",8.24029652558371,0.620075754629995,0.5568208690061
88,1.1136000626857,0.265450799527658,0.966153621353896
"3992","TNFRSF10B",55.1203892704975,0.343648914206576,0.30933687014331
1,1.11092128800349,0.266602231424037,0.966153621353896
"3993","AC044849.1",22.4864557590392,-0.341318811178414,0.314824479858
491,-1.08415588054599,0.2782956897592,0.966153621353896

"3994","UBXN8",22.2884538011841,0.452780239934337,0.414035994337456,1.0935769984416,0.274140541846345,0.966153621353896
"3995","IDO1",65.7850308183506,-0.81439685297704,0.751582204283152,-1.08357655135515,0.278552592231957,0.966153621353896
"3996","LYPLA1",63.5954995032325,-0.241904706850498,0.221430736103987,-1.09246218978785,0.27463000089506,0.966153621353896
"3997","CHCHD7",36.0958457595217,-0.307390576806652,0.283263956090176,-1.08517363468861,0.277844759137953,0.966153621353896
"3998","YTHDF3-AS1",5.32922631899951,-0.709431228416525,0.643188884166476,-1.10299049918422,0.270031291064353,0.966153621353896
"3999","CYP7B1",245.396953217409,0.260307918693685,0.23769642658863,1.09512760637411,0.273460735798452,0.966153621353896
"4000","LACTB2",33.774122183337,-0.363779340627743,0.33568892008898,-1.08367991571279,0.278506743672843,0.966153621353896
"4001","PIP4P2",115.925083940476,-0.265687125004879,0.238590243464415,-1.11357078624426,0.265463365034962,0.966153621353896
"4002","DPYS",6.08704980958021,2.39685610513758,2.17560761939864,1.10169503166205,0.270594277652717,0.966153621353896
"4003","LY6E-DT",6.57613491065113,0.628849847574005,0.579253434189696,1.08562126775077,0.277646586477259,0.966153621353896
"4004","SLC39A4",22.9366588216202,-0.412837563757707,0.37841991257017,-1.0909509516922,0.2752944654741,0.966153621353896
"4005","AL391834.2",3.95104859968351,-0.746185384027439,0.681098949272335,-1.09556090906415,0.273270976643137,0.966153621353896
"4006","C9orf40",13.2252542279291,0.494351881303045,0.449957904402444,1.09866251146217,0.271915300190067,0.966153621353896
"4007","IPPK",10.619676100544,-0.485822749232177,0.445705188230291,-1.09000918558112,0.275709097821761,0.966153621353896
"4008","CAVIN4",1.86568427266601,-1.17766574508596,1.08841711122665,-1.08199855821702,0.279253168596228,0.966153621353896
"4009","TMEM250",41.5851222797565,-0.281457801621132,0.259698231909501,-1.08378790087109,0.278458850984431,0.966153621353896
"4010","NPDC1",147.106644669498,0.3096872830404,0.278990374752637,1.11002855677362,0.266986721100626,0.966153621353896
"4011","ATP5F1C",342.897330550887,-0.299818142510718,0.272461456264649,-1.10040571103568,0.271155391271668,0.966153621353896
"4012","CAMK1D",93.2032697153283,0.332370559097132,0.30260568987901,1.09836189540925,0.272046494301932,0.966153621353896
"4013","HSPA14.1",37.8637478970322,-0.384574895269498,0.351011665052974,-1.09561856074344,0.27324573564484,0.966153621353896
"4014","VIM-AS1",8.68477776565496,-0.591216954595434,0.532296909723192,-1.11069018774293,0.26670172724063,0.966153621353896
"4015","KIF5B",256.55055228048,-0.200012515304498,0.184100453666505,-1.08643140916327,0.277288171716964,0.966153621353896
"4016","AGAP5",5.52516419424579,0.710798895908128,0.639045383903822,1.11228234146059,0.266016773891774,0.966153621353896
"4017","SNCG",40.708899260926,-0.548163058337094,0.492704369623097,-1.

11255976632889,0.265897548172249,0.966153621353896
"4018","LIPA",79.3708249361279,-0.232883917041938,0.21392108978l045,-1
.08864402888141,0.276310892867998,0.966153621353896
"4019","LOXL4",16.7050893755273,0.43383513758554,0.399304452397425,1.0
8647708529367,0.277267973525226,0.966153621353896
"4020","PAMR1",79.1839882480384,0.496222233826619,0.458588880439266,1.
08206337962513,0.279224366474386,0.966153621353896
"4021","PRDM11",10.092847897518,-0.517024758122194,0.466378534250946,-
1.1085946718208,0.267605077709518,0.966153621353896
"4022","PTPRJ",9.86102115573413,0.54944835751621,0.507156954117115,1.0
8338918170356,0.278635715486329,0.966153621353896
"4023","TTC9C",37.2254826741554,0.324092549045196,0.294672755245975,1.
09983886625237,0.271402334790223,0.966153621353896
"4024","EFEMP2",375.539939057091,0.286565264066934,0.260894258915427,1
.09839620564372,0.272031518524337,0.966153621353896
"4025","FIBP",135.850557422596,-0.299222441419904,0.276015754209404,-1
.08407740086058,0.278330481986512,0.966153621353896
"4026","SRSF8",68.1155559266199,-0.261618332396285,0.236274631147207,-
1.10726374273033,0.268179915352893,0.966153621353896
"4027","REXO2",343.104921684037,-0.261087086755226,0.239555566824268,-
1.08988110865632,0.275765519276764,0.966153621353896
"4028","ST3GAL4",65.2272172983173,-0.334329508143298,0.302964305757967
,-1.1035277152761,0.269798062545528,0.966153621353896
"4029","TSPAN9",26.750811033629,0.347963051424632,0.320920528835666,1.
08426548057576,0.278247105971263,0.966153621353896
"4030","LRRC23",14.7459324128757,-0.414764600739238,0.382139256855784,
-1.08537553600719,0.277755363021507,0.966153621353896
"4031","C1RL-
AS1",29.8474113218764,0.423151090664725,0.384788811719426,1.0996969708
4974,0.271464175018468,0.966153621353896
"4032","ABCC9",99.8272564603607,0.333480806124849,0.299239768519658,1.
1144267614381,0.265096147864852,0.966153621353896
"4033","LRMP",3.15495168166692,1.11372169217806,1.02843309290068,1.082
93062510934,0.278839217251354,0.966153621353896
"4034","ZCRB1",195.293199913084,-0.264916853101822,0.242123406013016,-
1.09413979203473,0.27389367268212,0.966153621353896
"4035","PCBP2-
OT1",5.96698168847848,0.756642582571815,0.697049167080983,1.0854938479
3097,0.277702986995337,0.966153621353896
"4036","ATP5MC2",632.62433011798,-0.274733963579325,0.25332520199958,-
1.08451098197399,0.27813830041819,0.966153621353896
"4037","AVIL",14.6528417880365,0.504708987466158,0.462396516439528,1.0
9150689834871,0.275049898128155,0.966153621353896
"4038","CNOT2",142.469180860744,0.186316392859139,0.170929633471051,1.
09001809151305,0.27570517480341,0.966153621353896
"4039","NEDD1",33.8048821478631,0.310683230879206,0.279018987923525,1.
11348418683376,0.265500536073502,0.966153621353896
"4040","AACS",28.1920548929683,0.340261069192242,0.312553806012137,1.0
8864797883482,0.276309150339237,0.966153621353896
"4041","SPATA13",44.4370854180563,0.388224030154015,0.357620371047582,

1.08557582728519,0.277666699139211,0.966153621353896
"4042","MTIF3",121.985891568861,-0.246211463167851,0.22363761822829,-1.10093939078048,0.270923036696097,0.966153621353896
"4043","MTRF1",8.59037915972422,0.571627428336197,0.517917749857531,1.10370310438953,0.269721948548898,0.966153621353896
"4044","MYCBP2-AS1",1.87654748316985,1.41899214254021,1.30143816175634,1.09032621313734,0.275569472223796,0.966153621353896
"4045","ERCC5",56.4416400718999,0.297331821397092,0.272241178677267,1.09216329007144,0.274761334830826,0.966153621353896
"4046","IPO4",28.587950297974,-0.350525610397579,0.318091182761221,-1.10196581796077,0.27047653239597,0.966153621353896
"4047","GZMB",32.4068849977245,0.97486001743437,0.88895806474051,1.09663217659085,0.272802215817299,0.966153621353896
"4048","SPTSSA",94.0568548717157,-0.273415323909432,0.248237031949864,-1.10142842815109,0.270710238441083,0.966153621353896
"4049","DHRS7",193.367910435929,-0.195966409796698,0.180961030683522,-1.08292049982528,0.278843711868217,0.966153621353896
"4050","FNTB",8.35853375977507,0.673061529033628,0.604179246076328,1.11400968074398,0.265275033598261,0.966153621353896
"4051","SRSF5",733.728512040249,0.197790030620735,0.181897499883198,1.08737080359951,0.2768729689079,0.966153621353896
"4052","ENTPD5",20.9002120947081,0.413489680031756,0.374375966834035,1.10447709431909,0.269386234402976,0.966153621353896
"4053","STON2",17.3620240322122,0.45742080752215,0.418008892281143,1.0942848728072,0.273830057702439,0.966153621353896
"4054","AVEN",47.604634638571,-0.2796787461065,0.254579358302111,-1.09859160605866,0.271946240646092,0.966153621353896
"4055","RPUSD2",14.9377675082038,-0.490650679085903,0.440335601368099,-1.11426529574597,0.265165390570048,0.966153621353896
"4056","JMJD7",9.4347883043984,0.521791533196914,0.477692796650764,1.09231610117494,0.274694185603775,0.966153621353896
"4057","CELF6",4.47474334578629,0.845405939585268,0.774543005386078,1.09148999307517,0.275057332765754,0.966153621353896
"4058","IDH3A",48.3236743496528,-0.35034606548751,0.316504692583744,-1.10692218376766,0.268327573761449,0.966153621353896
"4059","TTC23",24.0195301559881,0.367934788502156,0.338886706033053,1.08571620530394,0.277604568830749,0.966153621353896
"4060","ADAMTS17",4.22583206904499,0.86158348749722,0.781611662974459,1.10231657012182,0.27032406802697,0.966153621353896
"4061","MPG",187.595538117451,-0.311196937701829,0.287042089257615,-1.08415089406117,0.278297900318544,0.966153621353896
"4062","MRPL28",75.7405994350264,-0.321267649660278,0.293999427788454,-1.09274923450342,0.274503916304679,0.966153621353896
"4063","TSC2",45.7824789733955,0.34437496313362,0.315621167316005,1.09110224153257,0.27522789663412,0.966153621353896
"4064","KCTD13",15.0654368304795,-0.413909288867259,0.376674611210249,-1.09885104158567,0.271833044446225,0.966153621353896
"4065","C16orf87",31.4004590435421,-0.310076539032312,0.278756965594249,-1.11235440653939,0.265985799767728,0.966153621353896

"4066","KATNB1",17.5119985719743,-0.38126187119484,0.35100840156766,-1
.08619015810466,0.2773948706562,0.966153621353896
"4067","KIAA0895L",6.08267173717268,0.714634675707847,0.65459299304236
9,1.09172368678501,0.274954570602149,0.966153621353896
"4068","SF3B3",81.2975319313449,0.234185643740729,0.213547330855375,1.
09664514561098,0.27279654425897,0.966153621353896
"4069","NPIPB15",15.4856643256092,0.533979701529489,0.486035574898,1.0
9864324569565,0.271923706808794,0.966153621353896
"4070","TERF2IP",242.466300866149,-0.180119611748363,0.166449145139373
,-1.08212998989898,0.279194771609658,0.966153621353896
"4071","GEMIN4",21.8184337921851,0.36010361658506,0.332243619632355,1.
08385412181439,0.278429483989215,0.966153621353896
"4072","ABR",37.8679007533066,0.329722918045589,0.303945922350161,1.08
480783521001,0.278006774557236,0.966153621353896
"4073","CRK",136.553775623718,0.246608363213273,0.223923488569145,1.10
130636490652,0.270763341943499,0.966153621353896
"4074","PFN1",687.328796473951,-0.283056236108065,0.257154368054673,-1
.10072497795521,0.271016371787005,0.966153621353896
"4075","SENP3",10.8700083493387,-0.504915795204107,0.465627725544205,-
1.08437656845709,0.278197868535822,0.966153621353896
"4076","AC015726.1",2.56847951180227,1.09682729826901,0.99348751722294
5,1.10401719121235,0.269585680418914,0.966153621353896
"4077","ALDOC",4.55770915929246,-0.738423993646928,0.681478986439968,-
1.08356091433492,0.278559528675895,0.966153621353896
"4078","TTC25",2.28566827994749,-1.07065903900551,0.977228541913362,-1
.09560762205043,0.273250524689493,0.966153621353896
"4079","TUBG1",21.8814027229046,-0.406551051454364,0.37467948060689,-1
.08506356098244,0.277893504870653,0.966153621353896
"4080","AARSD1",58.851833109736,-0.279725649319909,0.255018273492486,-
1.09688472707879,0.27269178598935З,0.966153621353896
"4081","C17orf67",8.9300094737098,-0.534021529992912,0.48377284703131,
-1.10386834083383,0.269650254006265,0.966153621353896
"4082","COIL",53.5570197501514,0.351234887192582,0.320426240510195,1.0
9614895032733,0.273013595828369,0.966153621353896
"4083","TSPAN10",7.82732213052689,-0.602524122265627,0.554693169560046
,-1.08622956858026,0.277377438538092,0.966153621353896
"4084","MRPL4",105.250724473197,-0.317514444024348,0.286884794982995,-
1.10676637304242,0.268394950409522,0.966153621353896
"4085","EPOR",8.15826067881434,-0.545917195528584,0.496337185862851,-1
.09989178944862,0.271379272507215,0.966153621353896
"4086","ZSWIM4",5.11456788104948,-0.781311946662108,0.708329944264339,
-1.10303390812253,0.270012440251062,0.966153621353896
"4087","SYDE1",42.1363638315568,0.323793651665484,0.294602652375192,1.
09908600297704,0.271730554577707,0.966153621353896
"4088","TPM4",373.46231421713,-0.328819207296208,0.29789922066624,-1.1
0379344585332,0.269682748615284,0.966153621353896
"4089","RAB8A",95.3872523402884,-0.232356795314752,0.211436461029715,-
1.09894383486724,0.271792564969512,0.966153621353896
"4090","EPS15L1",36.5452727089039,0.34884444200353,0.31533179133478,1.
10627742457204,0.268606460012095,0.966153621353896

"4091","ZNF626",38.081347418108,0.300488337823218,0.272242540849063,1.10375232645883,0.26970059018748,0.966153621353896
"4092","AC092279.1",10.3721942389324,0.659826890450921,0.602875678202934,1.09446593104859,0.273750681497093,0.966153621353896
"4093","C19orf12",76.995566897928,0.249907024259539,0.227343138653956,1.0992503479066,0.271658883251314,0.966153621353896
"4094","CEBPA",58.0262149269677,-0.312439563616527,0.282950704509019,-1.10421906939118,0.269498119508934,0.966153621353896
"4095","LSR",8.77006428287238,-0.64029733552113,0.580594643826878,-1.10283024883029,0.270100889375746,0.966153621353896
"4096","MEIS3",39.6412180946095,-0.305129770772414,0.275558908204494,-1.10731230850275,0.268158924552336,0.966153621353896
"4097","CARD8",29.2445954335555,0.364131605141221,0.334947139680393,1.08713155600814,0.276978673680763,0.966153621353896
"4098","ZNF175",7.21554483613634,0.612819385461817,0.554196823216582,1.10577931844681,0.268822048712482,0.966153621353896
"4099","SPACA6",11.9691627477197,-0.461620203458512,0.418053406727059,-1.1042134713661,0.269500547285223,0.966153621353896
"4100","AC005261.1",112.594459218247,0.385708136115938,0.354834734252979,1.08700783458518,0.277033347273933,0.966153621353896
"4101","ZNF304",5.08330008370831,0.679275127285612,0.622515184513568,1.09117840686311,0.275194387387366,0.966153621353896
"4102","IDH3B",100.286500466702,-0.289703654753458,0.26455365352385,-1.09506578682475,0.27348781619003,0.966153621353896
"4103","CDS2",43.8117236213503,0.271482719642566,0.249501027613438,1.0881026111972,0.276549811119196,0.966153621353896
"4104","CPNE1",146.472753970137,0.261361943534467,0.235278049415023,1.11086412091692,0.266626841246988,0.966153621353896
"4105","NNAT",4.07959817086469,-0.996407465279851,0.908725054856242,-1.09648948266039,0.272864623463991,0.966153621353896
"4106","KCNK15-AS1",8.55828076316764,-0.674284480982383,0.612309484980277,-1.10121514940129,0.27080302982606,0.966153621353896
"4107","YWHAB",809.776832653608,-0.215303934602065,0.195263582212618,-1.10263230942688,0.270186873394556,0.966153621353896
"4108","TP53RK",53.2162042784829,-0.350908101380556,0.322765167505386,-1.08719321881194,0.276951427087034,0.966153621353896
"4109","SULF2",76.4970946594581,0.293029261609577,0.268851664552945,1.08992913284296,0.275744362361103,0.966153621353896
"4110","PSMA7",756.089541208183,-0.268581132360176,0.244494164082362,-1.09851755917454,0.271978554499089,0.966153621353896
"4111","LINC02246",3.08167045957418,0.942536123725321,0.849991967728998,1.10887650649639,0.267483460074115,0.966153621353896
"4112","LINC00323",2.64068092093607,-1.00316946741603,0.925591699691068,-1.08381424309536,0.278447168748366,0.966153621353896
"4113","TANGO2",16.2875340035866,-0.425090381033854,0.389149731650323,-1.09235686539244,0.274676274625693,0.966153621353896
"4114","LRP5L",5.27975538747915,-0.713991766843685,0.648716679259812,-1.10062187341684,0.27106126160784,0.966153621353896
"4115","APOL1",287.148148340051,0.281219024837597,0.2526521568037,1.11

306797612692,0.265679236082253,0.966153621353896
"4116","GCAT",8.65502279101736,-0.553854568442861,0.50423000815369,-1.
09841651525437,0.272022654023894,0.966153621353896
"4117","XPNPEP3",24.3885916117101,0.361279437070976,0.324488665938668,
1.11338075869578,0.265544935198603,0.966153621353896
"4118","MCAT",28.5414600009174,-0.326784563946089,0.299812950264543,-1
.0899614698356,0.275730117016976,0.966153621353896
"4119","PIN4",99.4431251540696,-0.266843585554787,0.24245120623343,-1
.10060737457362,0.271067574546233,0.966153621353896
"4120","ZMAT1",16.4647688845435,0.532249166028829,0.491768915415496,1.
08231559446805,0.279112318975295,0.966153621353896
"4121","TCEAL3",69.0014026427629,-0.310706590005352,0.280706385333151,
-1.10687396596481,0.268348423163722,0.966153621353896
"4122","DOCK11",62.9304148124845,-0.300965593409175,0.271618285665973,
-1.10804614155945,0.267841889108057,0.966153621353896
"4123","SLC25A5-
AS1",2.95775584157362,0.984156558798223,0.898729025709389,1.0950537154
638,0.273493104329605,0.966153621353896
"4124","HAUS7",19.182519547047,-0.485663911611882,0.44216250771803,-1.
09838329377667,0.272037154247318,0.966153621353896
"4125","UFC1",221.291157591679,-0.252352981776163,0.233380270545692,-1
.08129526624555,0.279565792460782,0.966154456212577
"4126","WDFY1",62.4198670144467,0.278865105335344,0.257843109122927,1.
08153018431993,0.279461341389929,0.966154456212577
"4127","TXLNB",2.3971259660563,-1.06484017138714,0.984464177133752,-1.
08164440730327,0.279410564281053,0.966154456212577
"4128","SLC25A5",422.543916341295,-0.309180907162574,0.28593520076421,
-1.08129711324886,0.279564971128551,0.966154456212577
"4129","SPESP1",7.79381967006791,-0.590471050511195,0.546223865268826,
-1.08100558773753,0.279694628069421,0.966365600396793
"4130","CD84",13.4943348508544,-0.521454829507785,0.482461589448376,-1
.08082143928596,0.279776549765904,0.96641459054731
"4131","LINC01359",5.60205277394321,0.716978390840915,0.66363657922128
2,1.08037804619243,0.279973867929113,0.966628073542285
"4132","HOXA5",10.9950140356346,0.566282587921593,0.524078933504591,1.
08052919459055,0.27990659346319,0.966628073542285
"4133","ANGEL1",6.73469858934755,0.614198427018796,0.56873804366042,1.
07993202470823,0.28017245116173,0.967079648747456
"4134","ST3GAL3",21.8013134591589,0.399368164832803,0.370475695994232,
1.07798748784595,0.281039339958098,0.967095875811858
"4135","SEPTIN2",399.124061490687,0.210090452342551,0.194857996665178,
1.07817208396916,0.280956967514716,0.967095875811858
"4136","FARP2",36.7009691231667,0.322569223571402,0.299014119262377,1.
0787758931489,0.280687643869557,0.967095875811858
"4137","KLHDC8B",34.5991704351744,-0.338084665937321,0.313675777287464
,-1.07781566323334,0.2811160280934,0.967095875811858
"4138","HSPBAP1",13.0016473542378,0.495667102260369,0.459232790413046,
1.07933734830771,0.280437369206877,0.967095875811858
"4139","EREG",9.92513397193075,-0.947431884470729,0.878963244209536,-1
.07789704599396,0.28107970386559,0.967095875811858

"4140","SF1",313.892925225035,-0.164484573053567,0.152583330268437,-1.
07799831583301,0.281034507726082,0.967095875811858
"4141","ATP23",9.63559941494981,-0.503622848094349,0.467268828919121,-
1.07780107921883,0.281122537831578,0.967095875811858
"4142","DENND4A",47.5193182664387,0.314409290890367,0.291459856117296,
1.07873960784443,0.280703823649788,0.967095875811858
"4143","STUB1",299.485212266748,-0.249291642492501,0.231298291712277,-
1.07779283905221,0.281126215967459,0.967095875811858
"4144","CHMP1A",72.3926731233366,-0.243792564253765,0.225879001746656,
-1.0793060106012,0.280451334330573,0.967095875811858
"4145","LRRC59",154.369275583505,-0.277493989269999,0.257369293038431,
-1.07819385130985,0.280947255341161,0.967095875811858
"4146","ZNF554",5.21143146504956,0.706004992823783,0.653878869216333,1
.07971831796602,0.280267634238317,0.967095875811858
"4147","TMEM255A",9.81449457779327,0.546684808820392,0.506590705249599
,1.07914496487068,0.280523109112377,0.967095875811858
"4148","ZNF839",8.50200923640309,-0.647746645564972,0.601171541804518,
-1.0774738997469,0.28126860495023,0.967352439300862
"4149","EAF1",27.9809451093183,-0.359970135256911,0.334465812678291,-1
.07625389983625,0.281813719956136,0.967370921370624
"4150","CLEC3B",582.648065421445,0.472679427514884,0.438971785551648,1
.07678771864774,0.281575113253701,0.967370921370624
"4151","CHMP2B",158.072088264818,-0.202473545688065,0.188212359805934,
-1.07577178192142,0.282029335202544,0.967370921370624
"4152","SERPINI1",12.8724936837401,-0.557588582700512,0.51870537781688
,-1.0749620238126,0.282391731093515,0.967370921370624
"4153","JARID2",25.9863046752923,0.404811735890233,0.376336825868443,1
.07566336341409,0.282077838088752,0.967370921370624
"4154","MDGA1",18.7458364549134,0.415409676150103,0.386426928743078,1.
07500188328307,0.282373885161619,0.967370921370624
"4155","DPM2",84.0265353766956,-0.377249368079719,0.35086580438313,-1.
07519559719698,0.282287166214448,0.967370921370624
"4156","IGF2",80.835813216922,0.7779501830052,0.723628561513902,1.0750
6837676176,0.282344116320097,0.967370921370624
"4157","IGF2-
AS",1.92526988013219,1.21998098254449,1.13435905315112,1.0754804478842
2,0.282159681323517,0.967370921370624
"4158","MARCH9",26.4907186589462,-0.324870770366283,0.302035525545901,
-1.07560449976575,0.282104174124828,0.967370921370624
"4159","WSCD2",7.31747306195166,0.64354795294065,0.59876541982054,1.07
479144860024,0.282468109878653,0.967370921370624
"4160","NUFIP1",34.4157101028731,-0.347952448047518,0.323310054546088,
-1.07621907563632,0.281829290465564,0.967370921370624
"4161","CORO2B",26.52747334258,0.34297660054936,0.319024867160275,1.07
507795113994,0.282339830087845,0.967370921370624
"4162","DCTN5",40.4678611104611,0.324115119259382,0.300964832395464,1.
07692023908461,0.281515900421676,0.967370921370624
"4163","CENPV",17.9102895912029,-0.519910886477446,0.483758453147792,-
1.0747324064198,0.282494550569888,0.967370921370624
"4164","NXPH3",25.6848912082056,0.361160816113632,0.335836361210392,1.

07540712629201,0.282192492661192,0.967370921370624
"4165","HSH2D",4.29047059426776,0.789779963812286,0.734546294496624,1.07519426580664,0.282287762169723,0.967370921370624
"4166","CRELD2",131.338182999014,-0.317078670025739,0.294797349289074,-1.07558182185287,0.28211432083745,0.967370921370624
"4167","AL035530.2",6.15071509840085,-0.685771097259808,0.638236035582347,-1.07447881195566,0.28260813613532,0.967527638614466
"4168","ECE1",83.5070848476993,-0.262471895427144,0.244480464760524,-1.07359046328811,0.283006273769712,0.967972984337168
"4169","INPP4B",8.3854455978611,0.673974981989785,0.627778238357255,1.07358767923115,0.283007522117743,0.967972984337168
"4170","GIMAP6",2.79565905507604,0.976654025926239,0.909428976416982,1.07392006550541,0.282858509240283,0.967972984337168
"4171","PHLDA1",164.910153202413,-0.490786471802081,0.457148145202057,-1.07358298825681,0.283009625520141,0.967972984337168
"4172","SCAMP3",38.5445394169122,0.294782975916415,0.274865075839313,1.07246428094323,0.283511549115909,0.968116325878008
"4173","CLASP1",35.4278902652217,0.351335087536154,0.327368323323381,1.07321039485271,0.283176727816039,0.968116325878008
"4174","AP001816.1",20.1645921240269,-0.401510319948731,0.374445516433292,-1.07227968376612,0.283594429121281,0.968116325878008
"4175","AC008914.1",1.96052321588435,1.15770847817859,1.0796554162212,1.07229441985349,0.283587812344931,0.968116325878008
"4176","SLC45A4",5.07171603299191,0.702071301026465,0.654434777383478,1.07279033035721,0.283365200299442,0.968116325878008
"4177","RBM18",35.9739813752674,0.309551052314384,0.288485892090015,1.07301972402102,0.283262266543759,0.968116325878008
"4178","MLST8",27.8620163095142,-0.361587062508398,0.336932779122601,-1.07317270658556,0.283193634134486,0.968116325878008
"4179","JADE3",5.85525563645705,0.794385748006967,0.740803743875627,1.07232955364267,0.283572037044217,0.968116325878008
"4180","STT3B",122.065550077721,0.219360040617319,0.204758216902551,1.07131251646774,0.284028933794988,0.969367648210358
"4181","IL1RL1",2.95355952016226,-1.01215130848593,0.945856117353012,-1.07009014364515,0.284578735222808,0.969388786219813
"4182","LINC01184",47.1263371613669,-0.265959303117687,0.248334654444384,-1.07097136206276,0.284182306554706,0.969388786219813
"4183","CASC15",3.17209433578266,-0.839467991926507,0.784037284618306,-1.07069907056166,0.284304760843204,0.969388786219813
"4184","ZNF655",95.9082419877511,0.333678904397634,0.311813243230684,1.07012422224406,0.284563397527483,0.969388786219813
"4185","ENDOG",54.0219913830979,-0.302219289245308,0.282277304704144,-1.07064678671941,0.284328277895015,0.969388786219813
"4186","CTDNEP1",206.540626544058,-0.181899439022957,0.1699671882043,-1.07020326066885,0.284527826995044,0.969388786219813
"4187","IER2",1028.12529586727,-0.262034192633629,0.244818879080627,-1.07031857027387,0.284475938340591,0.969388786219813
"4188","MED14",19.9477829538897,0.361972444054065,0.338080327633392,1.07066993985696,0.284317863548955,0.969388786219813
"4189","ADAM17",105.841081394083,-0.319283822602677,0.298608390154291,

-1.06923928841284,0.284961859472638,0.969669417615839
"4190","MRPL53",9.95002919194355,-0.507173000486509,0.474482361775366,
-1.06889747932637,0.285115867911368,0.969669417615839
"4191","FAM198B-
AS1",5.05357397869868,0.8522717848834,0.797047525989671,1.069286030121
18,0.284940803500996,0.969669417615839
"4192","HBEGF",12.2538598547818,-0.663014865164149,0.619840872570966,-
1.06965334895407,0.284775372174155,0.969669417615839
"4193","NDOR1",3.26792075480695,0.888637594129957,0.83145816248638,1.0
6877006471689,0.285173291331822,0.969669417615839
"4194","FADS3",35.6910392755498,-0.343019414379868,0.320733795997036,-
1.06948322459613,0.284851983902335,0.969669417615839
"4195","NSMCE1-
DT",2.26867708568941,1.09844890617915,1.02783656290032,1.0686999721819
4,0.285204884082158,0.969669417615839
"4196","ZNF77",8.04354462709425,0.586838878519866,0.549049315298053,1.
06882726591017,0.285147510839008,0.969669417615839
"4197","AC025181.2",17.3813300563064,-0.429031236785069,0.401703374395
819,-1.06802995476539,0.285506999918934,0.969772109724644
"4198","CA3",2.87877035181836,-1.02073044204971,0.955585938609443,-1.0
6817231272267,0.285442791563889,0.969772109724644
"4199","PEX11A",13.3564140915798,-0.487184192870795,0.456036545360247,
-1.06830077068921,0.28538486096431,0.969772109724644
"4200","MIEN1",88.8531008106164,-0.290830008414356,0.272190930942834,-
1.06847795188091,0.285304970729731,0.969772109724644
"4201","H6PD",159.023136587625,0.281117065601796,0.264563233914133,1.0
6257041631505,0.287976808933148,0.971073540040176
"4202","NEXN-
AS1",2.97318793373253,0.946753037821679,0.890493693875081,1.0631777005
6493,0.287701373018947,0.971073540040176
"4203","HMCN1",106.635869649537,0.379014266752478,0.358640138402917,1.
05680939239063,0.290598586882489,0.971073540040176
"4204","RABIF",24.2435225278894,-0.366051714760338,0.344715926758126,-
1.06189382719523,0.288283887700268,0.971073540040176
"4205","MAP1LC3C",2.54905139060633,1.00720442401265,0.95136257813367,1
.05869670214328,0.289737930276526,0.971073540040176
"4206","ZNF692",8.75168509313827,-0.520161666449302,0.490094690279249,
-1.06134932037913,0.28853117947813,0.971073540040176
"4207","AC093702.1",1.87039701088484,1.14761574084143,1.08440966404744
,1.05828616148448,0.289925000030575,0.971073540040176
"4208","SMYD5",14.5218904801471,0.426133582410651,0.403188520338884,1.
05690901628965,0.290553113141221,0.971073540040176
"4209","KCNIP3",7.4733460336893,0.565196956836841,0.53504232544481,1.0
563593382392,0.290804075634944,0.971073540040176
"4210","FBLN7",34.2837613728706,0.347166171164019,0.328522905079838,1.
05674875570594,0.290626267092272,0.971073540040176
"4211","SLC39A10",57.019618242466,0.245015701384566,0.230985148376436,
1.06074222999508,0.28880706258871,0.971073540040176
"4212","MREG",3.22580877335049,-0.908152652999561,0.85296835872607,-1.
06469676595731,0.287013174051375,0.971073540040176

"4213","GNL3",182.410248831871,-0.284046790010092,0.267297888587661,-1.06266005882399,0.287936140072154,0.971073540040176
"4214","MUSTN1",36.4978806365239,-0.388615125132249,0.36748735796572,-1.05749250064951,0.290286875426382,0.971073540040176
"4215","ZIC1",15.5345186545428,-0.601733223319686,0.566770129484463,-1.06168831421484,0.288377206137954,0.971073540040176
"4216","ADH5",389.827385744576,0.22694342453624,0.213643556180632,1.0622526070684,0.288121023305192,0.971073540040176
"4217","MRPL36",138.56580605773,-0.307911727752051,0.291216817374177,-1.05732811218942,0.290361867517442,0.971073540040176
"4218","ST8SIA4",9.70016694757312,-0.53505777009203,0.50644557955349,-1.05649608110661,0.290741630322259,0.971073540040176
"4219","SPARC",1874.57406995324,0.332587229976448,0.313435223741785,1.06110355436772,0.288642842743369,0.971073540040176
"4220","ITK",17.946664307345,0.475415844929022,0.446690329759458,1.06430744803684,0.287189445048356,0.971073540040176
"4221","PSORS1C1",12.5957946442793,-0.50511475662228,0.476145610003953,-1.06084094027053,0.288762193087537,0.971073540040176
"4222","SNHG32",311.179683336274,0.233354051994998,0.219288953582052,1.06413956646331,0.287265479125416,0.971073540040176
"4223","DEF6",5.15474723581484,-0.733444596890679,0.693221156106076,-1.05802396598878,0.290044516355291,0.971073540040176
"4224","GNA12",66.6236587613068,0.276990370503589,0.259930030078731,1.06563435713715,0.286588961840595,0.971073540040176
"4225","ARL4A",95.9229236116102,-0.367028631434386,0.344828059884747,-1.0643815690552,0.287155879735722,0.971073540040176
"4226","CCT6A",207.381469268088,-0.326695297890835,0.308819074708985,-1.05788574814783,0.290107533410402,0.971073540040176
"4227","MLXIPL",41.0944073169268,0.532977155565336,0.500299677501672,1.06531580877054,0.286733041219876,0.971073540040176
"4228","CFAP69",26.3214077366743,-0.472866439441433,0.443494195211035,-1.06622915146933,0.286320067347812,0.971073540040176
"4229","CDK6",50.3249985443096,0.379001209658207,0.357395408027786,1.0604534953307,0.288938336082363,0.971073540040176
"4230","FOXP2",27.7938666142446,0.435839846106793,0.409009375716098,1.06559866835258,0.286605101440172,0.971073540040176
"4231","POT1",15.555088202966,0.403847017758709,0.380322751183792,1.0618534297559,0.288302229585337,0.971073540040176
"4232","SLC25A32",36.1211629719118,-0.291282220031912,0.273866215620876,-1.06359311013063,0.287513064942337,0.971073540040176
"4233","ZNF707",3.64721172375477,0.836863115644754,0.792244195721631,1.05631965518218,0.290822199079918,0.971073540040176
"4234","AK3",244.021600421166,0.217200497980241,0.204635312759742,1.06140281973352,0.288506876019937,0.971073540040176
"4235","CCNY",143.438976708516,-0.182679565767779,0.172685659101319,-1.0578734025655,0.290113162526,0.971073540040176
"4236","ADD3-AS1",3.83428024792453,0.783001091142552,0.736649234600508,1.06292256119316,0.287817070725211,0.971073540040176
"4237","NHLRC2",46.5303987974411,0.288813602890127,0.270798919228216,1

.06652420812185,0.286186741459847,0.971073540040176
"4238","TRUB1",18.0286760623403,0.403017619634006,0.381183484388906,1.05727985639279,0.290383883721885,0.971073540040176
"4239","ABTB2",3.78117956904676,-0.810074688889126,0.760171953175053,-1.06564664153372,0.286583406589098,0.971073540040176
"4240","ZPR1",69.2237102491301,-0.317188688118707,0.297366388052588,-1.0666595178962,0.286125613686352,0.971073540040176
"4241","KLRC3",4.73805678506176,0.862958476121224,0.812816844702728,1.06168872082964,0.288377021483974,0.971073540040176
"4242","PCED1B-AS1",31.7494135799919,-0.354196716734306,0.334483936356682,-1.05893490907917,0.28962942454823,0.971073540040176
"4243","POC1B",17.309094049572,-0.376250370339729,0.35606514436619,-1.05668969932305,0.290653227610667,0.971073540040176
"4244","UBE3B",27.8463079229207,0.307084786661045,0.290520759869233,1.05701495066744,0.290504764208141,0.971073540040176
"4245","ZDHHC20",39.9927035956484,0.380689966495038,0.360387247824997,1.05633584093935,0.290814806874223,0.971073540040176
"4246","HTR2A",6.36354756939435,-0.601692056760873,0.56551060961629,-1.06398013853203,0.287337697097898,0.971073540040176
"4247","MDP1",66.5928576109186,-0.32173412972383,0.304373048403286,-1.0570388259132,0.290493868184645,0.971073540040176
"4248","GPATCH2L",69.7202240240196,0.34130236930094,0.320781583023021,1.06397121082991,0.28734174154789,0.971073540040176
"4249","TRMT61A",34.4263265514162,-0.379405763807187,0.35847256741174,-1.05839553231811,0.289875155427626,0.971073540040176
"4250","IGHG4",53.2656709466079,0.937781921362463,0.886293792552162,1.05809374864517,0.290012704154855,0.971073540040176
"4251","GALK2",40.5726943101352,0.309167208208614,0.290517627208873,1.06419431818618,0.287240680410275,0.971073540040176
"4252","METTL26",175.958435035958,-0.248648768554334,0.235122569177143,-1.0575282901362,0.29027055040946,0.971073540040176
"4253","RPS2",7756.31926764378,-0.221928383904577,0.209780362861753,-1.05790828501345,0.290097257647161,0.971073540040176
"4254","AC005736.1",4.7937861230093,0.907406062239858,0.853567188868099,1.0630751440237,0.28774787542344,0.971073540040176
"4255","ITPRIPL2",151.293076600903,-0.307100696503095,0.288148745962957,-1.06577141426315,0.286526985968831,0.971073540040176
"4256","ADORA2B",1.99435223808741,-1.06394017264176,1.00671693346612,-1.05684143901167,0.290583958547247,0.971073540040176
"4257","RASD1",1116.39598958418,0.396333797238434,0.373618776937758,1.0607973198961,0.288782020478796,0.971073540040176
"4258","UNC119",18.9863956874011,-0.3722081001042,0.350017629788566,-1.06339815034185,0.28760143122647,0.971073540040176
"4259","AC073508.3",7.71380922599985,0.629683939389041,0.596156798879358,1.05623879585489,0.290859130278762,0.971073540040176
"4260","DHX58",20.6355135528744,0.428191515335602,0.403161034087348,1.06208556663944,0.2881968418514,0.971073540040176
"4261","TUBD1",15.1332259403128,0.451501510036906,0.423774853897319,1.06542779941895,0.286682382292145,0.971073540040176

"4262","FTSJ3",48.9585938440497,-0.305352260098308,0.286664503240324,-1.06519034148541,0.286789803478199,0.971073540040176
"4263","FOXK2",40.3536582114392,-0.279342966557259,0.262969290448466,-1.06226459401731,0.288115583025641,0.971073540040176
"4264","PEX11G",14.5381532472536,0.555645426489004,0.526153699032014,1.05605154446552,0.290944666453221,0.971073540040176
"4265","RNASEH2A",7.55350899233955,-0.619978034359814,0.583510228902273,-1.06249728565367,0.288009989596541,0.971073540040176
"4266","RFX1",30.6816651854081,-0.307522044713364,0.291197615137488,-1.05605962661531,0.290940974189119,0.971073540040176
"4267","AC024075.2",13.8660104671284,0.423870385123075,0.397637750771996,1.06597118684066,0.286436667005687,0.971073540040176
"4268","KIAA0355",43.9586945652366,0.343555728895164,0.322527477985793,1.06519832369228,0.286786192047221,0.971073540040176
"4269","LTBP4",730.518403777013,0.370556880276712,0.349473867617326,1.06032786600934,0.288995466125436,0.971073540040176
"4270","PPP1R15A",734.212513386525,-0.192729358042582,0.182525358109175,-1.05590456054497,0.291011820354588,0.971073540040176
"4271","NAT14",18.4146018703558,-0.430550882937024,0.406947203868075,-1.05800182147609,0.290054611986105,0.971073540040176
"4272","OSBPL2",27.6019679335936,0.339162833914905,0.320281891269186,1.05895101521631,0.289622089023571,0.971073540040176
"4273","MICALL1",9.25524522543849,0.504390974485883,0.474018933904585,1.0640734755701,0.287295415619314,0.971073540040176
"4274","APOBEC3F",11.9731141677407,0.521578712383299,0.493620849025609,1.05663833570417,0.290676677583642,0.971073540040176
"4275","TAF9B",17.0507520326554,0.428256730216535,0.403905059259181,1.06029058165802,0.289012422681124,0.971073540040176
"4276","UBE2A",121.810821646596,-0.224889588258616,0.213005645135268,-1.05579168155755,0.29106339949613,0.971073540040176
"4277","EXOSC10",41.9366707467942,0.29161940335838,0.276376571244488,1.05515240327809,0.291355628538019,0.971104504284898
"4278","MRPL21",137.754516385676,-0.288875349000745,0.273808523508524,-1.05502686804325,0.291413036789825,0.971104504284898
"4279","NUBPL",23.4301213427152,-0.402684189034817,0.381586639713522,-1.05528901467078,0.291293163711627,0.971104504284898
"4280","ZFP82",12.1642130408728,0.529117978734984,0.501464477556175,1.05514548371118,0.291358792712469,0.971104504284898
"4281","UBOX5",12.6519500672597,0.435852218050051,0.413092388835915,1.05509622019005,0.291381320566757,0.971104504284898
"4282","RPS6KA1",4.82909337365764,-0.695169114905263,0.659863088050288,-1.05350507930258,0.292109567588548,0.971155824036293
"4283","DOK1",15.0672510454244,-0.396271634630285,0.376231686303755,-1.05326491376473,0.292219594732523,0.971155824036293
"4284","BRPF1",16.0639577237618,0.406230305978264,0.385836450381949,1.05285621816219,0.292406894618598,0.971155824036293
"4285","SLC25A38",27.8796342777344,-0.358638169577582,0.340484271806281,-1.05331787478756,0.292195329283409,0.971155824036293
"4286","DAG1",57.940166901522,0.258093398945831,0.244857856202424,1.05405398441643,0.291858201954148,0.971155824036293

"4287","SLC25A36",175.508027419587,0.185469409717599,0.176178380857137
,1.05273648682238,0.292461781203606,0.971155824036293
"4288","FRAS1",5.35011867467741,1.11709808753554,1.05949080629847,1.05
437261078114,0.291712356875204,0.971155824036293
"4289","C6orf89",133.585891957134,0.207969139796725,0.197560251986852,
1.05268715596985,0.292484397195349,0.971155824036293
"4290","TBC1D22B",6.59627542890463,-0.628523810464246,0.59705656789835
7,-1.05270395513218,0.292476695398143,0.971155824036293
"4291","ZNF292",178.113951524489,0.289957517433578,0.274996807124717,1
.05440321458742,0.291698351151226,0.971155824036293
"4292","RHBDD2",41.9182872390185,-0.304649297736872,0.289141725075025,
-1.05363311939093,0.292050919902016,0.971155824036293
"4293","PKN3",2.40034109523622,-0.991236104958837,0.941671879714086,-1
.05263428409883,0.292508637889812,0.971155824036293
"4294","SSSCA1-
AS1",4.11948016587459,1.02723526484155,0.975889185364011,1.05261466183
621,0.29251763464769,0.971155824036293
"4295","C2CD3",13.1848887013162,0.479633600742317,0.455649943629188,1.
05263614634099,0.292507784066139,0.971155824036293
"4296","BTBD1",54.6494603172026,-0.294620595490584,0.279564532696614,-
1.05385541094481,0.291949119914099,0.971155824036293
"4297","YIF1B",34.3995345197868,-0.361347774535328,0.343262465800585,-
1.05268653155119,0.29248468347093,0.971155824036293
"4298","E2F6",25.9455363361774,-0.332791246834174,0.316251860554453,-1
.05229814696022,0.292662781593441,0.971185680905333
"4299","ZNF425",4.21688287735488,0.760985188108812,0.723111858422154,1
.0523754786283,0.292627314482566,0.971185680905333
"4300","DGAT1",36.2657438748995,-0.293412349619444,0.278939660128322,-
1.05188466023248,0.292852470596282,0.97136325169183
"4301","ECI1",113.365934070187,-0.250819600327575,0.238426295542452,-1
.05197960550839,0.292808906700722,0.97136325169183
"4302","ZNF786",4.71088462246765,-0.723117306474504,0.687570274737016,
-1.05169948882837,0.29293744561096,0.971419246649457
"4303","DDOST",268.413268324338,-0.264969138522636,0.252118247720352,-
1.050971680624,0.293271596599318,0.971552953929161
"4304","MRPS9",47.7320837092527,-0.301428292876854,0.286798122417407,-
1.05101208590953,0.293253039038741,0.971552953929161
"4305","SNHG8",311.678519293325,-0.256682362355652,0.24424035645098,-1
.05094164652175,0.293285391336705,0.971552953929161
"4306","PON3",10.2510832835586,0.495239722265132,0.471068445299575,1.0
5131160281853,0.293115499846683,0.971552953929161
"4307","ZNF202",5.55822877541252,-0.658267156071554,0.626444437592539,
-1.05079894810993,0.293350939018984,0.971552953929161
"4308","SAMM50",66.591630260435,-0.247387011711688,0.235444826977345,-
1.05072179706667,0.29338638199403,0.971552953929161
"4309","NFKBID",7.48596750695265,-0.610058995423436,0.58077631642998,-
1.0504198917295,0.293525104088765,0.971786756771947
"4310","FNBP1",103.775287653184,0.281266359781485,0.26782312427708,1.0
5019445404758,0.293628718853298,0.971904246673122
"4311","LINC01914",12.8056656506145,-0.496557506667967,0.4729014846611

36,-1.05002315022077,0.293707469236535,0.971939400632896
"4312","PLA2G2A",12589.6867447997,-0.404415954195133,0.387936568168359
,-1.04247958913639,0.297189383681409,0.972101975282525
"4313","GPN2",25.7740106252194,0.413518496871001,0.395643609492944,1.0
4517926474527,0.29594012495165,0.972101975282525
"4314","IL6R",34.6585940134866,0.38616349861264,0.369250773474389,1.04
580281573716,0.295652080896898,0.972101975282525
"4315","USP21",9.55104004865636,0.540908844211171,0.519330986767863,1.
04154933557422,0.297620668531573,0.972101975282525
"4316","DENND1B",27.1500643858092,0.386483743880495,0.36914014283434,1
.04698378483843,0.295107056893791,0.972101975282525
"4317","ATF3",1225.52359709984,-0.304410250988172,0.291691680921216,-1
.04360278643117,0.296669203207255,0.972101975282525
"4318","SNRPG",266.580556108788,-0.293972128413811,0.280778330617988,-
1.04699008561944,0.295104150853522,0.972101975282525
"4319","AC092683.1",34.8315627844121,-0.449168210024998,0.429524761840
372,-1.04573298196002,0.295684330676339,0.972101975282525
"4320","BIN1",219.452897115915,0.281084355839459,0.268628368290601,1.0
4636884640338,0.295390770457638,0.972101975282525
"4321","ARF4-
AS1",4.70955034352099,-0.74588595056338,0.711421556276176,-1.048444405
40655,0.294433903711728,0.972101975282525
"4322","AFAP1",73.3045431982202,0.247908574494967,0.237778472716221,1.
0426031072663,0.297132149553501,0.972101975282525
"4323","SPATA18",2.6469794286818,1.14871521556878,1.10230074012488,1.0
4210690762908,0.297362116366594,0.972101975282525
"4324","UNC5C",2.08014079421974,1.46062153467176,1.40049373481203,1.04
293328728657,0.296979191471067,0.972101975282525
"4325","EXOSC9",38.1553164858147,-0.316172949049521,0.302091783164096,
-1.04661221082527,0.295278467810574,0.972101975282525
"4326","ANKRD50",28.1054604163507,0.347929317920646,0.33216461992119,1
.04746049715709,0.29488724223189,0.972101975282525
"4327","MRPS30-
DT",9.05861376463818,0.514635736612813,0.491375982044072,1.04733596150
138,0.294944655715863,0.972101975282525
"4328","KLHL3",3.59631265022583,0.818046566979555,0.782500295003104,1.
04542652853097,0.295825881090135,0.972101975282525
"4329","NDFIP1",488.705797886133,-0.218796901341082,0.210102866338177,
-1.04137989716386,0.29769926870249,0.972101975282525
"4330","AFAP1L1",8.89410849947174,0.527026975382176,0.504737011611998,
1.04416154008399,0.296410657538527,0.972101975282525
"4331","ZNF354B",15.3876311353979,0.441412860274231,0.423212244511941,
1.04300588179645,0.296945568645535,0.972101975282525
"4332","RACK1",3749.61451668943,-0.23561227932319,0.225250105592053,-1
.04600296947209,0.29555966139988,0.972101975282525
"4333","ORC3",33.9830232733952,0.306829507022717,0.292461423719571,1.0
491281315683,0.294119150203182,0.972101975282525
"4334","GOPC",153.25002111353,0.185174762341413,0.177207136359888,1.04
496221848168,0.296040431677209,0.972101975282525
"4335","RPS12",9544.29481299367,-0.221067397449067,0.212267225720677,-

1.04145798626478,0.297663042508955,0.972101975282525
"4336","FKBP14",102.64010082279,0.256289245202458,0.245695201396575,1.04311864352932,0.296893347043603,0.972101975282525
"4337","PHTF2",43.775150679765,0.269403519390901,0.257461171487891,1.04638504452533,0.295383294803706,0.972101975282525
"4338","SEM1",468.518821985728,-0.247169977143928,0.235534644823943,-1.0493996640226,0.293994212785509,0.972101975282525
"4339","ZSCAN25",12.3638725099467,0.428224108032043,0.410159629111728,1.04404255718544,0.296465700493313,0.972101975282525
"4340","MTUS1",89.4308336965499,0.283022478959678,0.270971032082451,1.04447503773599,0.296265662405888,0.972101975282525
"4341","SPAG1",9.79744827684062,-0.516538748804299,0.495652820887028,-1.04213822061961,0.297347600649895,0.972101975282525
"4342","OPLAH",5.95055563755614,-0.620901237028035,0.595542596618455,-1.04258073318948,0.297142516398109,0.972101975282525
"4343","UBE2R2",219.593871783153,-0.164696713741774,0.157390480597273,-1.04642106127877,0.295366673035463,0.972101975282525
"4344","FAM149B1",20.2232658872184,0.359553620420998,0.344485505785306,1.04374092489419,0.296605269948094,0.972101975282525
"4345","NUTM2A-AS1",90.6676423068411,-0.273411177179875,0.260478072314593,-1.04965141499382,0.293878409016977,0.972101975282525
"4346","FAM53B",19.7229127243882,0.444494489232497,0.426840623785482,1.04135938442421,0.297708785224264,0.972101975282525
"4347","TSSC4",99.0030358061009,-0.261734732803126,0.249690518430505,-1.04823657080905,0.294529625152711,0.972101975282525
"4348","EMSY",28.7329845638201,0.353154862498034,0.337616272893503,1.04602440952079,0.295549762764222,0.972101975282525
"4349","KCNA1",7.84957244828302,-0.821218325242327,0.783454003855663,-1.04820234653319,0.29454538967268,0.972101975282525
"4350","KLRC2",3.27094878248028,0.983161838328492,0.942644850301882,1.04298224088705,0.296956517874087,0.972101975282525
"4351","GNPTAB",66.4664058950862,0.256278028566303,0.244501504674048,1.04816544547631,0.294562387815627,0.972101975282525
"4352","NAXD",25.020820331463,0.327268907477995,0.311810448947229,1.04957646090103,0.293912884201223,0.972101975282525
"4353","C14orf93",8.7773322923433,-0.501474831371942,0.481427893164686,-1.04164058313173,0.297578345778345,0.972101975282525
"4354","KIAA0586",44.6980200546869,-0.278296110843364,0.265650346480395,-1.04760304110464,0.294821535748667,0.972101975282525
"4355","JDP2",256.531835557485,-0.210711551112986,0.202088142562472,-1.04267152164976,0.29710045181145,0.972101975282525
"4356","AC103691.1",3.20982338243481,0.915704625105059,0.875861879999383,1.04548975816336,0.295796671696985,0.972101975282525
"4357","TLE3",68.2700250274823,-0.296402879666021,0.282818666793046,-1.04803152856567,0.29462408096947,0.972101975282525
"4358","AC024337.2",2.13129231736875,1.26620131989105,1.21262424161825,1.04418275376162,0.296400844548155,0.972101975282525
"4359","AC009065.4",2.44882877223841,-0.900013088564996,0.858963553303415,-1.04778961238077,0.294735549430316,0.972101975282525

"4360","LCAT",16.8300000289245,0.38386141479892,0.368454360784948,1.04181536617222,0.297497288544404,0.972101975282525
"4361","RPL13",12325.4934943259,-0.223665470912908,0.213995451958832,-1.04518796481683,0.295936104736169,0.972101975282525
"4362","NDEL1",49.2871608669843,-0.254925079725032,0.244081252161539,-1.04442712198279,0.2962878207458,0.972101975282525
"4363","MILR1",9.78432234122644,0.626443199728835,0.600968980673581,1.04238857557457,0.297231561006891,0.972101975282525
"4364","ARHGDIA",264.373657128026,-0.217850758687823,0.208942614399052,-1.04263440617125,0.297117647868891,0.972101975282525
"4365","ELANE",40.7204682486575,-0.486242160880824,0.465082191881791,-1.04549726772684,0.295793202727247,0.972101975282525
"4366","TNPO2",45.1167774846526,0.320687346862889,0.306794749455396,1.04528303509807,0.295892176098642,0.972101975282525
"4367","DGCR6L",117.772045425468,-0.318835787389936,0.303893945416444,-1.04916794888104,0.294100827248033,0.972101975282525
"4368","LINC00899",11.2035414586861,-0.521639026525199,0.497654009737389,-1.04819616906225,0.294548235223787,0.972101975282525
"4369","ZBED1",61.3681296288097,0.320790370494682,0.306375611515418,1.04704930300413,0.295076839604039,0.972101975282525
"4370","LINC01128",25.3395045992096,-0.403459179360726,0.390467744302457,-1.03327146799661,0.301476875656953,0.972274281519332
"4371","CDK11A",49.5793778237939,-0.357037518463696,0.345385047930435,-1.03373762298942,0.301258839478196,0.972274281519332
"4372","HMGN2",507.788064763773,0.178532490937163,0.172601130250068,1.03436455299279,0.300965769117455,0.972274281519332
"4373","DCLRE1B",5.00573354286624,0.742366327851248,0.714454018118629,1.03906802820722,0.298773099016187,0.972274281519332
"4374","ASH1L",231.177546377555,0.229675306886282,0.220923454227281,1.03961486429592,0.298518868238037,0.972274281519332
"4375","AL590560.3",1.85940157719872,1.15104508834539,1.10605362633509,1.04067746892109,0.298025263538272,0.972274281519332
"4376","PRDX6",804.205249075472,-0.316342910858541,0.306761272686747,-1.03123483641815,0.302430707993988,0.972274281519332
"4377","CENPL",18.5473635768502,-0.373856525002953,0.361981218676072,-1.03280641567624,0.301694500752355,0.972274281519332
"4378","ARPC5",297.59822558597,-0.227045115662348,0.218431834502193,-1.03943235279686,0.298603703998775,0.972274281519332
"4379","ROCK2",196.622083872591,0.194370862133799,0.187573326782588,1.03623934952697,0.30009049334069,0.972274281519332
"4380","LINC02580",4.37047277490549,0.848381198337574,0.817729007889096,1.03748453357134,0.299510100065713,0.972274281519332
"4381","TIA1",74.3348954439415,0.225131790277804,0.217499638843575,1.03509040968899,0.300626690911852,0.972274281519332
"4382","PCGF1",20.1322971124372,0.406771050625988,0.392804540400799,1.03555587776796,0.300409385202113,0.972274281519332
"4383","DUSP2",89.0296629589888,-0.455398043578939,0.43875271503257,-1.03793783599751,0.299298997120464,0.972274281519332
"4384","KLHL41",2.35188080090098,-3.07569852509133,2.96487380840324,-1.03737923562682,0.299559151532454,0.972274281519332

"4385","ITM2C",115.182962688766,0.302751262212551,0.293554239214342,1.03132989331997,0.302386144604069,0.972274281519332
"4386","ZNF662",16.7596272380714,0.389799628373425,0.377188403713435,1.03343481542866,0.301400460680253,0.972274281519332
"4387","DVL3",61.0411764712755,0.254611451590875,0.246675524291873,1.03217152298261,0.30199177286121,0.972274281519332
"4388","PDGFRA",924.850012745916,0.21841411338652,0.210676665276097,1.03672664981802,0.299863268346395,0.972274281519332
"4389","JADE1",94.9117764509182,0.423912887969516,0.408507495197114,1.03771140787752,0.299404432285871,0.972274281519332
"4390","ZSWIM6",30.3584898067615,0.410974170385561,0.39643849945844,1.03666563905115,0.299891710989992,0.972274281519332
"4391","CDKAL1",21.8523171771621,0.349161715015116,0.338479452515782,1.03155955973084,0.302278493292849,0.972274281519332
"4392","TREM1",4.93388738410012,-0.770919759742458,0.74404949166848,-1.03611354940076,0.300149171769523,0.972274281519332
"4393","MDFI",7.01528003969065,0.914754189704538,0.883061776487011,1.03588923681377,0.300253819499907,0.972274281519332
"4394","GUCA1B",2.97752212058565,0.983095826089772,0.94633677726538,1.03884351713627,0.298877518795915,0.972274281519332
"4395","NUS1",55.6737943225,0.273482555030064,0.263905151898217,1.03629108057558,0.3000663660401,0.972274281519332
"4396","SUN1",130.032557254539,0.209182763858652,0.202860897414525,1.03116355357144,0.302464128794532,0.972274281519332
"4397","SEPTIN7",640.60600395971,-0.15405259711472,0.14818568353648,-1.03959163556306,0.298529664621208,0.972274281519332
"4398","CPED1",101.38991642891,0.26742159485658,0.258115709504148,1.03605315372051,0.300177345552811,0.972274281519332
"4399","TNPO3",50.175562415141,0.262217429735599,0.252590820532704,1.03811147682482,0.29921815888953,0.972274281519332
"4400","MRPS28",71.6942591496339,-0.307806503721731,0.296144431910711,-1.03937967611201,0.298628192389586,0.972274281519332
"4401","HSDL2",73.8851786444701,-0.242817896404691,0.234380981649712,-1.03599658426036,0.30020373605419,0.972274281519332
"4402","PMPCA",50.3333703329358,-0.284493680199027,0.274978752081741,-1.03460241216913,0.300854627034195,0.972274281519332
"4403","MICU1",101.35406814736,0.22203026586588,0.214578202373853,1.03472889328732,0.300795538601874,0.972274281519332
"4404","MCU",25.3250350434988,0.370460364967899,0.357154671009492,1.03725471074142,0.299617166496299,0.972274281519332
"4405","SLC35G1",7.39106861808609,0.635121230849432,0.614140227149131,1.03416321350206,0.301059868383731,0.972274281519332
"4406","ZNF518A",53.0216008753826,0.282571389642913,0.271471653982782,1.04088727311778,0.29792786907394,0.972274281519332
"4407","C11orf58",485.243794295436,-0.185394388300113,0.179538122068436,-1.03261851112291,0.301782461905042,0.972274281519332
"4408","RHNO1",12.2514697976766,0.435084686559133,0.421232243345007,1.03288552439415,0.301657473791059,0.972274281519332
"4409","KLRK1",10.3393798251286,0.544830656035713,0.525684609191421,1.03642116681662,0.300005699684979,0.972274281519332

"4410","LDHB",386.656081687288,-0.280415585023329,0.270856649035519,-1
.03529149467753,0.300532800699969,0.972274281519332
"4411","INTS13",26.013780600083,0.371731481517905,0.360126782176331,1.
03222392756086,0.301967228392198,0.972274281519332
"4412","MPHOSPH9",12.9545869741137,0.444955026858445,0.430149976802166
,1.03441834442569,0.300940632138493,0.972274281519332
"4413","PIBF1",53.4333777419666,0.255716725040796,0.247631936726903,1.
03264840723194,0.301768465912303,0.972274281519332
"4414","DET1",13.1267459532768,-0.438679298744992,0.4252988713283,-1.0
3146123424899,0.3023245781776,0.972274281519332
"4415","RAB40C",12.1406417677659,0.524387568324214,0.504124803401777,1
.04019394559781,0.298249803786348,0.972274281519332
"4416","IFT140",13.6696036764576,0.46225033190979,0.447836450864989,1.
03218559145188,0.301985183552573,0.972274281519332
"4417","TRAP1",42.7934035625228,-0.286705675225061,0.277977622599333,-
1.03139840014499,0.302354030798378,0.972274281519332
"4418","PMP22",1045.28444514697,0.193651274552346,0.187394794477851,1.
03338662683736,0.301423002271128,0.972274281519332
"4419","AC104564.5",2.1729790395668,1.08182874252951,1.04810787692896,
1.03217308670492,0.301991040449227,0.972274281519332
"4420","MMP28",34.9859049968716,-0.480049606381455,0.462034660975543,-
1.03899046311347,0.298809171677131,0.972274281519332
"4421","TSPOAP1",2.56438298199049,0.933213637370756,0.897530884795096,
1.03975657348416,0.298453009478767,0.972274281519332
"4422","PYCR1",30.1635550309727,0.395225642230602,0.382236664963398,1.
03398150532851,0.301144809458952,0.972274281519332
"4423","ADNP2",32.6850490025601,0.325960460005346,0.315449127956886,1.
03332179776986,0.301453329691271,0.972274281519332
"4424","WDR83",18.7483237053524,-0.371566370544223,0.359811785174539,-
1.0326687058457,0.301758963274757,0.972274281519332
"4425","DHPS",84.037261528407,-0.251387478666833,0.242038434525204,-1.
03862627916913,0.298978579051475,0.972274281519332
"4426","GTPBP3",20.0976578229137,-0.363100800499028,0.351410936072921,
-1.03326551118967,0.301479662533219,0.972274281519332
"4427","PLD3",298.662733402565,0.230323018625466,0.222954097327108,1.0
3305129345771,0.301579895128924,0.972274281519332
"4428","RPL18",4535.83216606303,-0.24697624747962,0.237900306480674,-1
.03815018624065,0.299200139764254,0.972274281519332
"4429","PTPRA",357.190222466341,0.204061283774407,0.196266984622876,1.
03971273704799,0.298473381244677,0.972274281519332
"4430","CEBPB",973.171158354407,-0.325552517307941,0.313594758823358,-
1.03813124469762,0.29920895690932,0.972274281519332
"4431","URB1",34.894335033344,0.362769863076526,0.351666277673273,1.03
157421142771,0.302271626482689,0.972274281519332
"4432","SCARF2",11.2192748512646,-0.642971789027366,0.620055146891438,
-1.0369590386449,0.299754947345161,0.972274281519332
"4433","RPS19BP1",247.811630798181,-0.243903922201044,0.23634191373181
,-1.03199605330189,0.302073966379259,0.972274281519332
"4434","TSPO",605.375506227044,-0.272131489651777,0.262197091122644,-1
.03788904936587,0.29932171228684,0.972274281519332

"4435","MAP7D2",3.52233392763483,-0.799966078757191,0.7751008459137,-1
.03207999704113,0.302034643590863,0.972274281519332
"4436","ALG13",81.1437707664672,-0.233979724324039,0.225494240722644,-
1.03763060011733,0.299442066029841,0.972274281519332
"4437","LINC00894",4.30399793844469,0.885128520943463,0.85600302731475
9,1.03402498904714,0.301124481161601,0.972274281519332
"4438","F8A1",36.7083228578075,-0.313137195701502,0.302522840084071,-1
.03508612974307,0.300628689508302,0.972274281519332
"4439","BIN2",17.7405524284513,-0.431144467179696,0.418196272454379,-1
.03096200415495,0.302558638073037,0.972358984174352
"4440","P4HA2",141.257395654728,-0.213353567702147,0.207047321354744,-
1.03045799533238,0.302795060670109,0.972899625117067
"4441","ILF2",202.127045573214,-0.30893181087245,0.30056857153078,-1.0
2782472997452,0.304032281192271,0.973503727582122
"4442","HHAT",4.44736204656027,0.732216595689929,0.713198166615491,1.0
2666640208105,0.304577574973203,0.973503727582122
"4443","NBAS",51.0897714205812,0.272967744290607,0.265930258872706,1.0
2646365046134,0.304673088936808,0.973503727582122
"4444","CCDC36",9.33489192395935,-0.548096687164024,0.533503301675975,
-1.02735388036439,0.304253859819294,0.973503727582122
"4445","ZNF639",40.5126441469376,-0.275313931769691,0.268246479932016,
-1.02634685770887,0.304728117687705,0.973503727582122
"4446","WDFY3",53.32554821193,0.311346769527735,0.302327326857196,1.02
983336889953,0.303088233514812,0.973503727582122
"4447","DNAJB14",115.591215336707,0.189796494864022,0.184823591502327,
1.02690621538773,0.304464627336721,0.973503727582122
"4448","ALPK1",44.1960439646514,0.301257902014737,0.293181617754795,1.
02754703491232,0.304162949632491,0.973503727582122
"4449","CD14",42.1338576169948,-0.49887864992944,0.485553692537375,-1.
02744280930587,0.304212002251648,0.973503727582122
"4450","PGM3",88.4246183059937,-0.223115396499526,0.21675510812366,-1.
02934319947947,0.303318430112658,0.973503727582122
"4451","PYCR3",13.6134889068274,-0.465065183586201,0.452414291366555,-
1.02796306938367,0.303967199982896,0.973503727582122
"4452","CIZ1",96.0297544933868,-0.20122930065169,0.195708935190675,-1.
02820701801702,0.303852457894168,0.973503727582122
"4453","YIF1A",176.549641582942,-0.29366698939805,0.286065882392814,-1
.02657117633762,0.304622432253359,0.973503727582122
"4454","NDUFS8",171.85496404366,-0.253366289122726,0.246946821813762,-
1.02599534289129,0.304893779253955,0.973503727582122
"4455","DGAT2",2.92845312467035,-0.856845369709493,0.832216560913683,-
1.02959423057956,0.3032005247176,0.973503727582122
"4456","GVQW3",9.16815233663926,0.603889736689484,0.588068688972709,1.
02690340093504,0.30446595273065,0.973503727582122
"4457","FAIM2",8.59045148216553,-0.610221871238458,0.593720248307153,-
1.02779359972707,0.30404692756546,0.973503727582122
"4458","GGACT",9.43206846667489,0.548258468956507,0.532511385462983,1.
02957135551164,0.303211267518686,0.973503727582122
"4459","BATF",9.9080709537508,0.614605497066042,0.59767743760332,1.028
32306926392,0.3037978828875,0.973503727582122

"4460","MYEF2",2.75744434846312,0.93414374490918,0.910327613605965,1.0
2616215409404,0.304815157180028,0.973503727582122
"4461","CBX1",113.26466535258,0.211251598286273,0.205359568117122,1.02
869128632853,0.303624765872344,0.973503727582122
"4462","EIF3K",664.338835056085,-0.255014234091043,0.248268134564671,-
1.02717263549831,0.304339180998553,0.973503727582122
"4463","ROMO1",250.545837247264,-0.262768374823675,0.255892897071818,-
1.02686857599618,0.304482352954673,0.973503727582122
"4464","GATD3B",71.8655814949127,-0.35580540940552,0.346544857815193,-
1.0267225191241,0.304551142390202,0.973503727582122
"4465","MIF",1086.62225046664,-0.293549213292994,0.285803404502777,-1.
02710187726312,0.304372494811154,0.973503727582122
"4466","TOMM22",155.349186577304,-0.251558787119517,0.245167991781384,
-1.02606700528767,0.304860001419076,0.973503727582122
"4467","PDHA1",78.8804392504423,-0.263494973003739,0.256557926342183,-
1.02703890992751,0.304402142612238,0.973503727582122
"4468","CHIC1",15.4461917005406,0.392333209074574,0.381981309388491,1.
02710053982132,0.30437312451786,0.973503727582122
"4469","MEAK7",18.917047599005,-0.431181060055229,0.42051296222229,-1.
02536924849251,0.305188992427884,0.974228276119084
"4470","RNF44",27.1766704115538,0.478933520669765,0.467301035352573,1.
02489291578054,0.305413717637413,0.974291613554413
"4471","VCPIP1",44.3918503521567,0.289400862875102,0.282361923036045,1
.02492878559323,0.305396791084098,0.974291613554413
"4472","LUC7L3",266.56814552245,0.2584704730339,0.252126569635859,1.02
516158216567,0.305286952175456,0.974291613554413
"4473","PPP6R2",49.6566351225245,0.289115219495887,0.282145835609652,1
.02470135301191,0.305504124447063,0.974362137125375
"4474","SFRP2",805.677336837542,0.626405396335502,0.611669458276196,1.
02409134191665,0.305792133483374,0.974755088447121
"4475","TNFAIP8",47.5016896868391,-0.273563556712596,0.267084098134209
,-1.02425999385082,0.305712488601387,0.974755088447121
"4476","LIME1",30.8268007249694,-0.346434024273185,0.338312404969506,-
1.02400627108075,0.305832312904059,0.974755088447121
"4477","CCNL1",1171.82440605594,0.30944225755919,0.302273923048924,1.0
2371469704684,0.305970051471739,0.974976268549437
"4478","GMDS-
DT",23.6567608418473,0.338308199677001,0.330523436609744,1.02355283228
054,0.306046533578584,0.975002199203233
"4479","CYP4X1",12.9747760643066,0.510097451277795,0.498767731001279,1
.02271542357757,0.306442416760762,0.975421076913475
"4480","CSRP1",113.973297491946,-0.267626670366126,0.261874983050557,-
1.02196348520415,0.306798183055124,0.975421076913475
"4481","DHRS9",5.94267420859837,-0.879123515506301,0.86038076626585,-1
.02178424945713,0.306883025632531,0.975421076913475
"4482","SERP1",364.020657484508,-0.193569261181237,0.189468938584022,-
1.02164113351697,0.306950781787748,0.975421076913475
"4483","ATP10D",40.068321149339,0.300000351612403,0.293414808127237,1.
02244448235997,0.306570576270191,0.975421076913475
"4484","TMEM176A",707.480352296765,0.316185727659913,0.309419967666051

,1.02186594499668,0.30684435251243,0.975421076913475
"4485","BNC2-
AS1",6.57131307253653,-0.669994169217753,0.655959287221546,-1.02139596
50692,0.307066876238498,0.975421076913475
"4486","SIDT2",64.4050421574769,0.236217406432661,0.23107995333356,1.0
2223236167823,0.306670937511407,0.975421076913475
"4487","MEDAG",514.782843105018,0.2446014055935,0.239093880085313,1.02
30349915532,0.306291301658981,0.975421076913475
"4488","USP10",49.3529536843344,0.294324171852018,0.288005434198785,1.
02193964732232,0.306809465998328,0.975421076913475
"4489","ZNF345",13.0850759880033,0.45096918682386,0.441492253445019,1.
02146568440305,0.307033859130594,0.975421076913475
"4490","SELENOW",479.590135655165,-0.275872667308411,0.269733256733956
,-1.02276104418415,0.306420840980251,0.975421076913475
"4491","AC008969.1",8.34987580465588,0.536737891738211,0.5253688779322
55,1.0216400595534,0.306951290277593,0.975421076913475
"4492","UBE4B",35.66341694428,0.334493257186533,0.32946317313673,1.015
26751533992,0.309978324837565,0.975572404911451
"4493","FBXO6",29.3928483992133,0.351494159540356,0.355944745174269,0.
987496414277071,0.323399339887498,0.975572404911451
"4494","EIF4G3",98.8756292321358,0.237112728602032,0.234996015907673,1
.00900744076951,0.312971065709954,0.975572404911451
"4495","CDC42-
IT1",4.21154574372264,0.775652039242246,0.776904110860102,0.9983883833
27681,0.318091064442909,0.975572404911451
"4496","ID3",608.846154853186,-0.367381646419137,0.366106026691717,-1.
00348429043615,0.315627252886742,0.975572404911451
"4497","LAPTM5",50.544635777061,-0.346377884638343,0.339483549393617,-
1.02030830435537,0.307582266108728,0.975572404911451
"4498","ZBTB8A",44.4620364599248,0.257360191852376,0.254081626527097,1
.01290359074795,0.311106213111297,0.975572404911451
"4499","TRAPPC3",100.193205613942,-0.253936523398503,0.250790565803342
,-1.01254416243723,0.311277942268072,0.975572404911451
"4500","DPH2",17.8526907286472,-0.438847175112698,0.430254838724381,-1
.01997034225993,0.307742526553808,0.975572404911451
"4501","NASP",213.82720392685,0.238064630925308,0.239162105800758,0.99
5411167367943,0.319536329429054,0.975572404911451
"4502","OMA1",25.1955061525921,0.332846929845131,0.331731707391934,1.0
0336182049634,0.315686318289059,0.975572404911451
"4503","NEXN",134.228382188411,0.369471099409277,0.370005361616488,0.9
98556069012412,0.318009790410827,0.975572404911451
"4504","AC253572.2",18.1999530086553,-0.495110705429058,0.496094835171
17,-0.998016246748926,0.318271480506966,0.975572404911451
"4505","NBPF11",15.4535308100981,0.483804734033773,0.476161964305266,1
.01605077746951,0.309605206990632,0.975572404911451
"4506","MINDY1",13.440190687505,0.420254361891327,0.411901497072463,1.
02027879208556,0.307596258522196,0.975572404911451
"4507","ADAR",210.148145773259,0.188592906175888,0.185269000410427,1.0
1794097101025,0.308706009351453,0.975572404911451
"4508","MUC1",10.2266287343677,-0.579648336382993,0.576369144785905,-1

.00568939476853,0.314565006274154,0.975572404911451
"4509","DCAF8",89.3271391653561,0.278263444142294,0.276758896135497,1.00543631307902,0.314686801680622,0.975572404911451
"4510","FAM78B",3.14611408497694,-0.801435556171212,0.794589316814822,-1.00861607274539,0.313158796022016,0.975572404911451
"4511","FMO1",48.033507078914,-0.797760399662234,0.783138911915711,-1.01867036297655,0.308359487217013,0.975572404911451
"4512","MARK1",13.2838357022667,0.4215847089079,0.425811175444618,0.990074317489895,0.322137795403086,0.975572404911451
"4513","RNF187",127.630888287651,-0.241886550646352,0.240144359771609,-1.00725476491057,0.31381236161786,0.975572404911451
"4514","RHOU",85.8847554369053,0.266248207817786,0.263757710282821,1.00944236865074,0.312762527680426,0.975572404911451
"4515","ERO1B",26.4637652453545,0.37566833235349,0.373528363222585,1.00572906729878,0.314545916703755,0.975572404911451
"4516","KIF3C",5.87121378807114,0.619135752871795,0.623512091083721,0.992981149404305,0.320719141002342,0.975572404911451
"4517","MORN2",60.8339283614002,-0.283841361224444,0.286405402870636,-0.991047509507529,0.3216623820733,0.975572404911451
"4518","AC010883.3",4.7182110840523,-0.741323617685473,0.748902723212834,-0.989879719631881,0.322232913244731,0.975572404911451
"4519","PREPL",58.2069893304067,0.231757916186835,0.229866599744366,1.00822788715095,0.313345073012871,0.975572404911451
"4520","MSH6",45.0825675701567,-0.275297279751204,0.275852068093675,-0.997988819346887,0.318284780276508,0.975572404911451
"4521","ARHGAP15",16.1691008052159,-0.430757691954431,0.434577347043313,-0.991210643824695,0.321582734297029,0.975572404911451
"4522","AC009951.6",17.0974789534613,-0.389154345969094,0.387370782040299,-1.00460428099249,0.315087435064534,0.975572404911451
"4523","LY75",6.99792965549743,-0.600685909656622,0.591306562159899,-1.01586207239518,0.309695072144978,0.975572404911451
"4524","SCN7A",2.16373694319567,-1.47122891237245,1.48856501653529,-0.988353814599788,0.322979398326421,0.975572404911451
"4525","ITGA6",3.90105011807618,-0.825601981328552,0.817043686079609,-1.01047470948586,0.312267910207904,0.975572404911451
"4526","ITGA4",33.0800574556281,-0.362122910024013,0.363369246780721,-0.996570054379258,0.318973247382266,0.975572404911451
"4527","CDK15",3.07461148440181,-1.02382974575254,1.01219837145791,-1.01149120036409,0.311781390680166,0.975572404911451
"4528","SLC11A1",5.69788617005548,0.843596297379026,0.827095280929623,1.01995056292772,0.307751907555524,0.975572404911451
"4529","AGAP1",39.5499828015665,0.331542410414949,0.329368766995808,1.0065994217939,0.314127312438809,0.975572404911451
"4530","TRNT1",36.4904993406479,-0.330074238521046,0.324175620319658,-1.01819574894488,0.308584939292719,0.975572404911451
"4531","RPL15",5239.74222850612,-0.215261844295679,0.217532102446633,-0.989563571880106,0.322387482747678,0.975572404911451
"4532","SLC26A6",3.30809573828008,-0.79237950171827,0.785195020621477,-1.00914993206542,0.312902734291586,0.975572404911451
"4533","PRKAR2A",119.339640891238,0.232252901702172,0.230682322577305,

1.00680840693522,0.314026853867468,0.975572404911451
"4534","MAPKAPK3",17.8458825581359,-0.349411385225966,0.349650447604445,-0.999316281789095,0.317641500558822,0.975572404911451
"4535","TEX264",107.374315958409,-0.26490059134914,0.262409461460409,-1.00949329294328,0.312738116626329,0.975572404911451
"4536","ADAMTS9-AS2",6.48912499048382,0.820003869263395,0.803650079096569,1.02034939159741,0.307562786449024,0.975572404911451
"4537","PPP4R2",116.224529764673,0.184182129065679,0.186253071596181,0.988881028845569,0.322721353465786,0.975572404911451
"4538","BBX",223.479374127835,0.200537740577873,0.202554762408909,0.990042091298926,0.322153546035684,0.975572404911451
"4539","ATG3",106.348130625356,0.219227128067576,0.217956279883493,1.00583074818841,0.314496993518897,0.975572404911451
"4540","GTPBP8",37.8493047170477,-0.289880700492954,0.288496174922246,-1.00479911240099,0.314993591375972,0.975572404911451
"4541","LRRC58",114.207870575738,0.275639075156073,0.270787453124285,1.01791671650887,0.308717536688001,0.975572404911451
"4542","PLXND1",72.3796106688538,0.374282113683915,0.372067177775985,1.00595305375005,0.314438153518281,0.975572404911451
"4543","NCK1",106.283852470729,0.226887224819133,0.224603151160544,1.01016937494771,0.312414149074132,0.975572404911451
"4544","CLSTN2",26.7234776249737,0.463385814926053,0.456732328847827,1.01456758293202,0.310311998548605,0.975572404911451
"4545","MFN1",46.0064072633303,-0.243862073465338,0.243463951553571,-1.00163523966988,0.316519794639099,0.975572404911451
"4546","DCUN1D1",62.1722692145869,0.250460444029873,0.252975957391723,0.990056314490178,0.322146594352921,0.975572404911451
"4547","LINC00884",3.70066320762958,-0.940167243013059,0.925167359108323,-1.01621315728129,0.309527892247147,0.975572404911451
"4548","TFRC",44.69585384459,-0.396146345868276,0.389681060475641,-1.0165912230498,0.309347931255788,0.975572404911451
"4549","RPL35A",4880.08051714203,-0.228040144862158,0.22499368117815,-1.01354021885439,0.310802195692522,0.975572404911451
"4550","SLBP",93.8830272594082,-0.251710331185159,0.25420091351221,-0.990202307723293,0.322075244813186,0.975572404911451
"4551","AC006160.1",2.14290715630145,1.01355004524485,1.00225696332668,1.01126765124254,0.311888344339367,0.975572404911451
"4552","STIM2",29.993455329481,-0.310593676161628,0.306212810241495,-1.01430660564683,0.310436473028223,0.975572404911451
"4553","ANKRD17",138.224914492679,0.202146535065986,0.198474304698312,1.01850229617006,0.308439310174431,0.975572404911451
"4554","SLC9B2",44.1880667445479,-0.352643128996384,0.352424981277164,-1.00061899051091,0.317011045409017,0.975572404911451
"4555","C4orf3",458.516035164601,-0.242751206778687,0.24137064600418,-1.00571967137414,0.314550437752275,0.975572404911451
"4556","LRBA",25.1123893107679,0.365938253778461,0.360557591180461,1.01492317102625,0.31014245198854,0.975572404911451
"4557","TLR3",52.9289013303616,0.260052017546475,0.255506379430576,1.01779070301896,0.308777431178596,0.975572404911451

"4558","WDR70",56.2727695486644,0.298677978556854,0.301831649678127,0.989551555893375,0.322393358502617,0.975572404911451
"4559","PELO",26.6274100713752,-0.317942726011102,0.321032580028457,-0.990375263416937,0.321990731576987,0.975572404911451
"4560","FST",342.29635462982,0.313767621111396,0.309165747576715,1.01488481039944,0.31016073962416,0.975572404911451
"4561","TRAPPC13",17.4151005433447,0.394698653996936,0.388344185347037,1.01636298131829,0.309456567065324,0.975572404911451
"4562","POLK",91.7957112807314,0.223057762092363,0.220660959255222,1.01086192521428,0.312082519524764,0.975572404911451
"4563","GPR150",2.68000785699633,-0.949733265398605,0.937414831530689,-1.01314085659153,0.310992885498771,0.975572404911451
"4564","PITX1",2.8735996941876,1.18624469616563,1.16781404148591,1.0157821828005,0.309733122368734,0.975572404911451
"4565","WDR55",46.0257447047886,-0.271838583003253,0.27159431922907,-1.00089936996796,0.316875461179346,0.975572404911451
"4566","HARS2",11.847303801115,-0.455464263242889,0.455109020366848,-1.00078056654591,0.31693290678652З,0.975572404911451
"4567","HDAC3",56.7819004035728,-0.275195980521296,0.276492910194773,-0.995309356494661,0.319585828531177,0.975572404911451
"4568","RPL26L1",79.0165751611977,-0.340736378915903,0.339211989937095,-1.00449391243243,0.3151406040111,0.975572404911451
"4569","ZFP62",18.9041377986067,-0.404020276501042,0.400390782897406,-1.00906487801086,0.31294352061278,0.975572404911451
"4570","TRIM7",7.72089502928844,-0.594920087944984,0.598566302428948,-0.99390842005444,0.320267454724556,0.975572404911451
"4571","RREB1",53.2482335348326,0.27506282425628,0.277752983699076,0.990314561496446,0.322020391373058,0.975572404911451
"4572","ALDH5A1",7.85854952936215,0.542421144471236,0.548856951546158,0.988274163137058,0.323018395406662,0.975572404911451
"4573","ACOT13",83.1033499296609,-0.299891106514123,0.300585575107519,-0.997689614369726,0.318429890842633,0.975572404911451
"4574","NCR3",4.41265184001204,-0.761008627057106,0.77064868013757,-0.987490988658096,0.323401998405252,0.975572404911451
"4575","PPT2",6.61277936085755,0.620166988827581,0.609724171896614,1.01712711650988,0.30909296206052,0.975572404911451
"4576","ZBTB22",13.7489998946105,-0.443813072247921,0.434686766032704,-1.02099513242262,0.307256744880132,0.975572404911451
"4577","GLO1",169.517131624991,0.215548849645979,0.215427252867687,1.00056444473331,0.317037426752125,0.975572404911451
"4578","CCND3",48.9871823682858,-0.260363179357818,0.261932817520274,-0.994007478034572,0.320219226820218,0.975572404911451
"4579","PAQR8",9.71032050332483,0.460614384552805,0.461764118383949,0.997510127388919,0.318516960509878,0.975572404911451
"4580","DST",431.976317263161,0.292115571939316,0.295436067279435,0.988760697464272,0.322780237780131,0.975572404911451
"4581","UBE3D",13.905457866543,0.411173521119488,0.409462342939552,1.00417908559711,0.315292301070871,0.975572404911451
"4582","ANKRD6",15.2773110281179,0.422097206095677,0.41977290577972,1.00553704225298,0.314638322114738,0.975572404911451

"4583","COQ3",14.1831749001378,-0.410140210105437,0.414244987525852,-0
.990090942451877,0.322129670107235,0.975572404911451
"4584","AK9",10.8429801177555,-0.46869394665204,0.470987181290853,-0.9
95131004133642,0.319672553184336,0.975572404911451
"4585","TBC1D32",13.3586662524617,0.407242657261185,0.410834036824879,
0.991258320290476,0.321559459440839,0.975572404911451
"4586","HBS1L",61.3406109646545,-0.233601316370708,0.234533994678884,-
0.996023270274945,0.319238838815216,0.975572404911451
"4587","HIVEP2",76.8756563191252,0.30535059607067,0.307098792563998,0.
994307380765871,0.320073243503931,0.975572404911451
"4588","MTRF1L",46.1452990348289,-0.252448990846344,0.251911387018174,
-1.00213409895651,0.316278830946902,0.975572404911451
"4589","MRPL18",192.233330533229,-0.264625605000114,0.265800803619918,
-0.995578649109412,0.319454912931075,0.975572404911451
"4590","QKI",338.65727169158,0.172275413650174,0.172403285226145,0.999
258299655934,0.317669580514951,0.975572404911451
"4591","PDE10A",10.1109427763602,0.527219229039561,0.532418514016042,0
.990234590196231,0.322059469175863,0.975572404911451
"4592","AVL9",27.7437059778801,0.327195363182558,0.330068059914189,0.9
91296653386037,0.321540746660026,0.975572404911451
"4593","POLM",18.0294592860525,-0.354823248856467,0.35093425173633,-1.
0110818396919,0.311977261437679,0.975572404911451
"4594","PURB",75.1382950767849,0.253649924109816,0.250297483523797,1.0
1339382457555,0.310872087921326,0.975572404911451
"4595","SBDS",342.524339477365,-0.200036006509911,0.202470043222054,-0
.9879782871905,0.323163282126682,0.975572404911451
"4596","SEMA3A",26.9761806747471,0.482019334177663,0.474136257920967,1
.01662618313829,0.309331293590779,0.975572404911451
"4597","GTPBP10",24.6739831352651,0.353038052305999,0.35325916621165,0
.9993740745413,0.31761351393632,0.975572404911451
"4598","TFPI2",4.10585362141257,1.12999449838416,1.13578662353631,0.99
4900340405387,0.319784737307847,0.975572404911451
"4599","TRIM4",43.4969651700077,0.259042768922194,0.257560946709995,1.
00575328764367,0.314534262767869,0.975572404911451
"4600","TRIM56",184.282791312398,0.298008905979546,0.298818803629942,0
.997289669724402,0.318623926512056,0.975572404911451
"4601","LINC00513",24.3977795971771,0.474478351002384,0.47817870469120
7,0.992261567375294,0.3210699460308,0.975572404911451
"4602","AC246817.1",1.88368986853779,1.37189657473349,1.38853906800112
,0.988014386018262,0.323145602717141,0.975572404911451
"4603","TNKS",51.8574639638269,0.255379742549792,0.252767064686672,1.0
1033630653724,0.312334192206776,0.975572404911451
"4604","LONRF1",40.0154644884991,-0.271605961038223,0.273140020899079,
-0.994383613738453,0.320036142603356,0.975572404911451
"4605","ASAH1",215.5938449786,0.20792755788438,0.206870938697015,1.005
107625044,0.314845028862552,0.975572404911451
"4606","AC021546.1",4.02863691587232,-0.751453555384871,0.737469036878
225,-1.01896285512656,0.308220601302835,0.975572404911451
"4607","RPL8",5163.63222991769,-0.21698503558507,0.216660889408857,-1.
00149609916722,0.316587025069974,0.975572404911451

"4608","ENHO",2.11250940408713,-0.909602881699518,0.917808735264741,-0
.991059298904084,0.321656625653305,0.975572404911451
"4609","DCTN3",247.439304252953,-0.251594853512244,0.25255639797267,-0
.99619275350716,0.319156499635131,0.975572404911451
"4610","LERFS",10.6875581379065,1.98892549730411,1.98068839359025,1.00
415870751831,0.315302121755278,0.975572404911451
"4611","NMRK1",28.8219936174179,-0.412888541653872,0.405017708377245,-
1.01943330652915,0.307997300679894,0.975572404911451
"4612","OSTF1",73.2945204376773,-0.284070410407885,0.284403062719617,-
0.998830349052675,0.317876881473273,0.975572404911451
"4613","ROR2",7.47408154996784,0.643845944179156,0.651508808022258,0.9
88238280513254,0.323035964417971,0.975572404911451
"4614","BICD2",53.1226355449216,0.246677097428365,0.245248453709514,1.
0058252914432,0.31449961887394,0.975572404911451
"4615","FBP1",4.37449271434633,0.769247106627702,0.777780476307629,0.9
89028562763059,0.322649167117097,0.975572404911451
"4616","SEC61B",685.293811981387,-0.278479431414219,0.273969617269969,
-1.01646100100146,0.309409909723932,0.975572404911451
"4617","ZBTB34",12.3342775119651,0.498832416773829,0.490086978174927,1
.01784466633141,0.308751781356278,0.975572404911451
"4618","RXRA",92.7587803167628,0.279462406913865,0.274121359691599,1.0
1948424314061,0.307973129947456,0.975572404911451
"4619","STAM",53.5241941080556,0.250007229849012,0.248659084667465,1.0
0542166067791,0.314693854088799,0.975572404911451
"4620","MLLT10",59.5028414756134,0.238383115366617,0.238045093121847,1
.00141999249108,0.316623802543605,0.975572404911451
"4621","FZD8",47.5015858446313,0.409303017342667,0.41286828067114,0.99
136464704269,0.321507556453952,0.975572404911451
"4622","VSTM4",56.458677849484,0.293460254261393,0.290256145156818,1.0
1103890187353,0.311997811009305,0.975572404911451
"4623","SIRT1",58.5836851088547,0.337018137210852,0.335495975650798,1.
00453704864001,0.315119822874116,0.975572404911451
"4624","H2AFY2",13.783610981031,0.519845445729769,0.518797926486216,1.
00201912766045,0.316334354781215,0.975572404911451
"4625","R3HCC1L",22.4460380494311,0.357119476067027,0.352933930165294,
1.01185929020701,0.311605336411312,0.975572404911451
"4626","AL138762.1",3.41716464475444,0.842741744469556,0.8407683012743
76,1.00234719029272,0.316175938280124,0.975572404911451
"4627","SHOC2",91.4073919253279,0.213694449539093,0.21113986901046,1.0
1209899646431,0.311490722177605,0.975572404911451
"4628","SFXN4",29.5471682060936,-0.307697922865725,0.311473397167464,-
0.98787866207492,0.323212076822227,0.975572404911451
"4629","TSPAN4",506.032759189906,0.22449382359927,0.226859967259773,0.
98957002555769,0.322384326961786,0.975572404911451
"4630","SCUBE2",5.20993232202699,0.696542501567483,0.68499261547107,1.
01686132935677,0.309219401801012,0.975572404911451
"4631","AC132192.2",4.0352724502484,-0.720557666126567,0.7289540673790
49,-0.988481577059209,0.32291685268014,0.975572404911451
"4632","PSMA1",279.352028360389,-0.268634996169821,0.264803799644078,-
1.01446805722158,0.310359463968842,0.975572404911451

"4633","TCP11L1",13.1464502084683,0.440694665373637,0.444232523156843,
0.992036022581001,0.321179953450482,0.975572404911451
"4634","FBXO3",47.9546435073669,-0.240165018312408,0.239812979987506,-
1.00146797026966,0.316600617634878,0.975572404911451
"4635","TTC17",101.863961370441,0.207362372944332,0.207911214396364,0.
997360212369376,0.318589696679921,0.975572404911451
"4636","CELF1",123.073808590843,0.258335897148226,0.256920218394166,1.
00551018819347,0.31465124612418,0.975572404911451
"4637","SELENOH",320.286081585394,-0.253436192170265,0.252522300998174
,-1.00361905134113,0.315562268125287,0.975572404911451
"4638","VWCE",8.61114962155144,0.539174420987237,0.536821276735454,1.0
0438347799121,0.31519381059541,0.975572404911451
"4639","MRPL49",25.1346299847739,-0.289555848801868,0.292225427994612,
-0.990864658113212,0.321751671710588,0.975572404911451
"4640","BANF1",232.419050504007,-0.282453658391648,0.280197424259612,-
1.00805230147278,0.31342935452588,0.975572404911451
"4641","CPT1A",60.92013474134,0.287686684292454,0.289810058604344,0.99
2673220791176,0.320869229291918,0.975572404911451
"4642","CLPB",21.8860558535982,-0.35332515382761,0.350213808065707,-1.
00888413217939,0.31303020602985,0.975572404911451
"4643","NARS2",18.7694625377201,-0.366446506942309,0.360712300379498,-
1.01589689776805,0.309678486305132,0.975572404911451
"4644","NKAPD1",75.7793192444837,0.23370955369922,0.229894783942035,1.
01659354636835,0.309346825559587,0.975572404911451
"4645","ATP5MG",690.279948906384,-0.238423616235629,0.237879887158796,
-1.00228572950545,0.316205612803253,0.975572404911451
"4646","ARHGAP32",33.5796672619872,-0.335735742833514,0.33084929674896
5,-1.01476940145427,0.310215762745529,0.975572404911451
"4647","RIMKLB",36.7008886085324,0.313482667064899,0.307373844515641,1
.01987424323265,0.307788106466109,0.975572404911451
"4648","RASSF8-
AS1",88.204891033369,-0.240110172955255,0.240213418318689,-0.999570193
18839,0.317518553894324,0.975572404911451
"4649","PFDN5",1615.24702062293,-0.22471821190299,0.226469727953478,-0
.992266003645096,0.321067782527457,0.975572404911451
"4650","GDF11",27.6514455276991,0.461801497739471,0.459685608218393,1.
0046029057322,0.315088097546115,0.975572404911451
"4651","UHRF1BP1L",49.5481481279103,-0.26906785319428,0.26781993096571
,-1.00465955697946,0.315060808627832,0.975572404911451
"4652","ALDH1L2",56.109511458099,0.33246524547817,0.329163504250671,1.
01003070262913,0.31248058057356,0.975572404911451
"4653","ISCU",397.864392016462,-0.205641822627626,0.204691391166154,-1
.00464324100812,0.315068667875581,0.975572404911451
"4654","TESC",2.94567052048697,0.838023669940999,0.841932515071526,0.9
9535729401044,0.319562521319157,0.975572404911451
"4655","MLXIP",88.0219868507721,0.303638886588863,0.299802002935808,1.
0127980587704,0.311156628137142,0.975572404911451
"4656","CCDC92",49.5538204876257,-0.24450103904678,0.241335283301573,-
1.0131176643443,0.311003960839963,0.975572404911451
"4657","SCARB1",11.1895312331481,0.434679639104464,0.438893524584166,0

.9903988433557,0.321979210574329,0.975572404911451
"4658","SUCLA2",104.14704939195,-0.257782483824641,0.258012257553124,-0.999109446463272,0.317741675535397,0.975572404911451
"4659","NDFIP2",57.3350198938494,-0.294813293299764,0.292766824369915,-1.00699009846574,0.313939532433219,0.975572404911451
"4660","AL356966.1",2.71928279484818,0.939216101805209,0.950596510009088,0.988028140137217,0.323138866799981,0.975572404911451
"4661","TPP2",66.8973092018157,0.247819818767181,0.248375951398749,0.997760924000749,0.318395302656355,0.975572404911451
"4662","LINC00641",11.6957315154671,0.524702029069257,0.524095233657658,1.00115779608863,0.316750526705787,0.975572404911451
"4663","NOP9",14.2182130748473,0.427619280813538,0.41937594861757,1.01965618730197,0.307891547289336,0.975572404911451
"4664","WDHD1",4.38664321454376,-0.711152111526662,0.715745131478184,-0.993582883418242,0.320425980697342,0.975572404911451
"4665","SLC39A9",37.761656099806,0.310970165889791,0.311937658961509,0.996898440941888,0.318813808522219,0.975572404911451
"4666","AREL1",13.6416068040592,0.45738264836431,0.45038754317032,1.0155313025417,0.309852632973076,0.975572404911451
"4667","LINC02328",6.96380130159482,0.596825768770864,0.58584745361848,1.01873920435188,0.308326795103265,0.975572404911451
"4668","TMEM251",35.8437186158271,-0.313583615112888,0.314817360358525,-0.996081076201669,0.319210753692642,0.975572404911451
"4669","SERPINA1",6.68384763072922,0.745120877546839,0.747472797959037,0.996853503674488,0.318835623466549,0.975572404911451
"4670","SETD3",47.992739151756,-0.24562027066298,0.247020411065668,-0.994331883763582,0.320061318130545,0.975572404911451
"4671","SLC25A29",22.4577471342966,0.412517910791503,0.406961006713918,1.01365463517612,0.310747577746169,0.975572404911451
"4672","IGHA1",53.5155296961023,0.550579504777267,0.553927600931321,0.993955715244329,0.320244427740077,0.975572404911451
"4673","USP8",151.405188802715,0.216774616165006,0.213816048042794,1.01383697879225,0.310660547016177,0.975572404911451
"4674","RFX7",18.1655524923085,0.423808416209689,0.420636914937119,1.00753975973089,0.313675461029347,0.975572404911451
"4675","AQP9",3.4882594342872,0.871887205973732,0.866767759987844,1.00590636410607,0.31446061459172,0.975572404911451
"4676","TRIP4",28.7104230293908,-0.322315175753357,0.317724498116864,-1.01444861086791,0.310368738808976,0.975572404911451
"4677","COX5A",188.996369970324,-0.309023239666005,0.303968024997738,-1.01663074485649,0.309329122691966,0.975572404911451
"4678","RCN2",135.12883024654,-0.25297869583082,0.250188161725287,-1.01115374159308,0.311942851969627,0.975572404911451
"4679","HCFC1R1",200.94389702354,-0.315275416601989,0.308937396506186,-1.02051554835213,0.307484019058701,0.975572404911451
"4680","VASN",221.695911447162,-0.282176654317658,0.27971771568768,-1.00879078618218,0.313074980814017,0.975572404911451
"4681","ZNF48",7.02394869211435,-0.527977145221011,0.525409866851283,-1.00488623935655,0.314951631217511,0.975572404911451
"4682","BBS2",37.236969128466,0.34518844847063,0.34583857162273,0.9981

20154299013,0.318221098195866,0.975572404911451
"4683","C16orf86",15.5664577501793,0.473429234633119,0.477580935328645,0.991306812336072,0.321535787560494,0.975572404911451
"4684","NFATC3",39.1782179773923,0.269100310159618,0.269088116644897,1.00004531420738,0.31728857893659,0.975572404911451
"4685","NFAT5",116.386409089123,0.27589003858499,0.272305533228744,1.0131635421203,0.310982051413803,0.975572404911451
"4686","GPS2",114.153181141144,-0.251328393645351,0.247907732359789,-1.01379812260392,0.310679091325856,0.975572404911451
"4687","PLSCR3",70.8834473892651,-0.247172540044282,0.248411661840778,-0.995011821154797,0.319730515034523,0.975572404911451
"4688","LLGL1",12.8235930877301,0.569137141954311,0.560135079028337,1.01607123578404,0.309595465364662,0.975572404911451
"4689","RPL23A",4707.52745995627,-0.214710010097718,0.213958404192713,-1.00351285993107,0.315613475294945,0.975572404911451
"4690","EVI2B",23.6842105245782,-0.391128986852656,0.39432856396835,-0.991886012305335,0.321253133227566,0.975572404911451
"4691","CDK5R1",2.47787794599004,1.07112831660563,1.073836252804,0.997478259658959,0.318532421270048,0.975572404911451
"4692","AC006449.2",2.16620835793978,-0.948615863899004,0.957383962258019,-0.990841607229001,0.321762929021894,0.975572404911451
"4693","IGFBP4",3635.44037350761,-0.263717968409927,0.260393296157965,-1.01276788727289,0.311171042738263,0.975572404911451
"4694","GRN",347.838410556961,0.287079757251551,0.286792793477498,1.00100059618156,0.316826520156193,0.975572404911451
"4695","FZD2",12.0191482793752,0.804442961079222,0.813135495830254,0.98930985697266,0.322511562852838,0.975572404911451
"4696","ARL17B",8.33458560627785,0.697414101968196,0.70290852113311,0.992183308354183,0.321108113366857,0.975572404911451
"4697","TLK2",54.6940287645272,0.291714560813256,0.293807680631783,0.992875884612594,0.320770443227617,0.975572404911451
"4698","FAM20A",68.1087305154543,0.292662339376344,0.295206266345831,0.991382544141163,0.321498820593478,0.975572404911451
"4699","PTPRM",33.9434089068381,0.30432390114153,0.306443463738518,0.993083348650577,0.320669337942425,0.975572404911451
"4700","NDUFS7",170.492575237595,-0.232794750696495,0.234300351238898,-0.993574057680911,0.320430279264329,0.975572404911451
"4701","MKNK2",107.576498202632,0.25285636534282,0.254315787292914,0.994261378872192,0.32009563296804,0.975572404911451
"4702","LRRC8E",2.94412981106102,-0.983625506460998,0.974097910894927,-1.00978094240785,0.312600252625961,0.975572404911451
"4703","PIN1",127.520218106975,-0.264898658095093,0.261132893226611,-1.01442087521779,0.310381967503754,0.975572404911451
"4704","ZNF653",3.92569905508666,-0.749252115308956,0.740802639964451,-1.01140583859813,0.311822227858034,0.975572404911451
"4705","ZNF441",12.7636693917152,0.464332739134384,0.462467175226681,1.00403393799093,0.315362255561877,0.975572404911451
"4706","TRIR",499.247930764749,-0.220677300870167,0.218275553094225,-1.01100328342728,0.312014858286218,0.975572404911451
"4707","AC008764.7",2.08476796658391,1.20847859194865,1.21309895060913

,0.996191276352056,0.319157217213862,0.975572404911451
"4708","OCEL1",35.977833900758,-0.303427220405458,0.303595153553359,-0
.999446851684106,0.31757827329696,0.975572404911451
"4709","URI1",158.61651484827,-0.161241887957814,0.163303447476688,-0.
987375897136719,0.323458395852458,0.975572404911451
"4710","DEDD2",18.2358807023611,-0.344999130686853,0.347935526831305,-
0.991560516480758,0.32141195787774,0.975572404911451
"4711","XRCC1",25.3307084406419,-0.311930581693866,0.311287573630682,-
1.00206563999868,0.316311891505314,0.975572404911451
"4712","ZNF223",5.96330188441724,-0.591554463483555,0.59763459100407,-
0.98982634604483,0.322259005019797,0.975572404911451
"4713","FOSB",3832.69561759663,0.326128217667692,0.323063480573232,1.0
0948648571798,0.312741379663212,0.975572404911451
"4714","SAE1",51.8030027318051,-0.31415588487752,0.309343467710951,-1.
01555687340735,0.309840450510878,0.975572404911451
"4715","CYTH2",64.6195018335521,-0.272036272089197,0.275216872462539,-
0.988443294392226,0.322935592991445,0.975572404911451
"4716","LMTK3",2.1020918462611,-1.0885596763153,1.08233834889257,-1.00
574804304874,0.31453678624927,0.975572404911451
"4717","CENPB",57.5085770237512,-0.253952721528289,0.256117746659948,-
0.991546758629992,0.321418672106468,0.975572404911451
"4718","SLC23A2",26.1086505785777,0.355678212599606,0.357443327491946,
0.995061832865297,0.319706192173479,0.975572404911451
"4719","RPS21",2863.60819339353,-0.259995291810289,0.256701817311214,-
1.01282996175707,0.311141386786528,0.975572404911451
"4720","LSS",33.9694467891213,0.324175741608223,0.323014323370308,1.00
35955626543,0.315573594269805,0.975572404911451
"4721","YPEL1",11.2304565955593,0.463562689768625,0.469438254679055,0.
987483838711766,0.323405501853865,0.975572404911451
"4722","SMTN",29.01655931873,0.37746098825234,0.371742247905257,1.0153
8361695317,0.309922999475587,0.975572404911451
"4723","PRR5",31.205936120233,-0.302407145262787,0.304116711517065,-0.
994378584965785,0.320038589909078,0.975572404911451
"4724","P2RY8",6.6611936965423,0.884046243452698,0.884197568943202,0.9
99828855568236,0.31739333883464,0.975572404911451
"4725","TRAPPC2",25.1174112089599,-0.329395052876584,0.325788648503488
,-1.01106976682479,0.311983039290066,0.975572404911451
"4726","FAM104B",46.2189930624676,-0.328327603672792,0.330082997977882
,-0.994681960852743,0.319890970706403,0.975572404911451
"4727","ARMCX4",14.1135829707271,0.427511229980439,0.420535212632415,1
.01658842622085,0.309349262304941,0.975572404911451
"4728","RAP2C-
AS1",12.0323304787158,0.452163816807106,0.454340061027484,0.9952100983
22705,0.31963409137271,0.975572404911451
"4729","CXorf40A",10.7903411292922,-0.537636302645972,0.53115595878370
8,-1.01220045403821,0.311442219168755,0.975572404911451
"4730","F8",9.17036255352004,0.495139675698894,0.493495870315255,1.003
33094050532,0.315701212388179,0.975572404911451
"4731","EPHB2",24.6133447249551,-0.455994428244395,0.462550116471824,-
0.985827074745039,0.324217977313569,0.975704053334232

"4732","MED18",11.0816549614195,0.440026793674882,0.447428634731193,0.
983456934845582,0.32538260061012,0.975704053334232
"4733","COL16A1",129.505093654055,0.261711384501594,0.265538570726425,
0.985587079819095,0.324335780722301,0.975704053334232
"4734","NBPF14",34.8504995119968,0.500428199401488,0.509279757353156,0
.982619458512013,0.325794763899049,0.975704053334232
"4735","SHC1",104.891837514601,-0.222956221307248,0.22642365753129,-0.
984686069195032,0.324778297624197,0.975704053334232
"4736","C1orf220",2.56135948782558,0.936937562493519,0.952113835403998
,0.984060442831356,0.325085795021465,0.975704053334232
"4737","SDE2",32.4807131884471,0.306571788992285,0.311682186410459,0.9
83603819400047,0.325310346473442,0.975704053334232
"4738","CDC42BPA",88.5989447480774,0.216653699183741,0.22055524799908,
0.982310333348516,0.325946985333464,0.975704053334232
"4739","CXCR6",4.90946632806435,0.792554839951628,0.807426623845769,0.
981581256482098,0.326306185227648,0.975704053334232
"4740","SETD2",96.8632898149694,0.2532864076428,0.257080916734557,0.98
5240020379754,0.32450618688419,0.975704053334232
"4741","TMEM167A",199.351594131444,-0.230709168442932,0.23498597052055
5,-0.981799755669887,0.326198508570646,0.975704053334232
"4742","LRRC1",2.36843242040339,-0.85900258869212,0.873476464026558,-0
.983429576032631,0.325396059865942,0.975704053334232
"4743","LUC7L2",134.772392973147,-0.206394910203483,0.210221606040601,
-0.981796848053866,0.326199941295614,0.975704053334232
"4744","ZNF395",46.045829670929,0.357197254056328,0.362989771104251,0.
984042203089356,0.325094762755104,0.975704053334232
"4745","GLI4",23.3650581999947,-0.342867514606466,0.348987237222974,-0
.982464336904681,0.325871144116571,0.975704053334232
"4746","PLIN2",1171.84358959335,0.419970489862486,0.427411090533029,0.
982591465604545,0.32580854644594,0.975704053334232
"4747","DNAJA1",950.207201397121,0.155394880618003,0.157762987891193,0
.984989462326719,0.324629247096034,0.975704053334232
"4748","STOML2",177.559227854489,-0.253031478389391,0.257307980061639,
-0.983379832715554,0.325420532178174,0.975704053334232
"4749","RNF38",37.877250882386,0.319112084035883,0.323324804835889,0.9
86970622924696,0.323657040398991,0.975704053334232
"4750","STX17",89.7999787829203,0.213186630679049,0.216615753073299,0.
984169561328766,0.325032149290036,0.975704053334232
"4751","GTF3C5",33.304455990895,-0.279949137618554,0.284764432029196,-
0.983090253314542,0.325563020864338,0.975704053334232
"4752","PARD3",55.4434063092542,0.240194469539605,0.24402888834198,0.9
84287029177457,0.324974405220811,0.975704053334232
"4753","BBIP1",68.2042721848777,-0.229472072615638,0.23366200296239,-0
.982068413804422,0.326066145183929,0.975704053334232
"4754","AMPD3",4.03561817239245,0.744863099310477,0.754823044820124,0.
986804926561271,0.323738279111192,0.975704053334232
"4755","CEP295",24.1541883426069,0.378228247015461,0.383714341019985,0
.985702661021371,0.32427904329565,0.975704053334232
"4756","BCO2",5.59465851432113,0.594659330268532,0.603749144573659,0.9
84944385616404,0.324651389498609,0.975704053334232

"4757","EI24",178.61173009028,-0.196947108281386,0.19964699236605,-0.986476710454448,0.323899238300221,0.975704053334232
"4758","KMT2D",27.0581649518886,0.328530205064166,0.334664897556131,0.981669148641631,0.326262869091221,0.975704053334232
"4759","PCID2",91.5668916522992,-0.219879572606673,0.223630879058885,-0.983225454069679,0.325496489750625,0.975704053334232
"4760","TMEM8A",26.7282213603787,0.293180178641888,0.298652264181969,0.981677401458616,0.326258802024498,0.975704053334232
"4761","LINC02175",3.47700598196282,0.78905632693338,0.80253527291622,0.983204543852808,0.325506778908067,0.975704053334232
"4762","LAT",15.8255173783858,-0.407053896074502,0.413934634328868,-0.98337723475224,0.325421810335913,0.975704053334232
"4763","NIP7",61.8189938076967,-0.301761120837731,0.306120884299032,-0.985758033231596,0.324251864031473,0.975704053334232
"4764","MRPS23",73.7133417805076,-0.292806859459684,0.298230047745655,-0.981815419583086,0.326190790263008,0.975704053334232
"4765","KATNAL2",5.29866320633521,0.707021709884837,0.719854313714694,0.982173332040428,0.326014463198064,0.975704053334232
"4766","SCAMP4",46.6080928131318,0.290564530810982,0.295260443242488,0.984095694025463,0.325068463910138,0.975704053334232
"4767","CHMP2A",317.332750951791,-0.228517170748285,0.232737645503771,-0.981865955778874,0.326165889637918,0.975704053334232
"4768","RLIM",82.663912984479,0.250682880649984,0.254551115540289,0.9848037008909,0.324720502412775,0.975704053334232
"4769","RPL36A",2362.69730759066,-0.243966707845414,0.247180062222255,-0.986999945109035,0.323642665507386,0.975704053334232
"4770","TCEAL4",272.291971449936,-0.21514540005198,0.219036571623773,-0.982235059912842,0.325984058970119,0.975704053334232
"4771","ZNF75D",11.8490050561073,0.431684846899815,0.438710691630235,0.983985243887466,0.325122768296527,0.975704053334232
"4772","AL034417.3",17.5566335804342,1.00813801987518,1.04182573067311,0.967664735275669,0.333211835532405,0.975978052195351
"4773","AL021155.5",22.7738276073966,0.531613184824552,0.547752317118864,0.970535711506977,0.331779533264526,0.975978052195351
"4774","SLC6A9",2.54616010913788,-0.958529080037435,0.978143837831002,-0.979946959705781,0.327112301031831,0.975978052195351
"4775","MAGOH",157.034029630549,-0.276999690945775,0.283877496256992,-0.975771924855252,0.32917750041805,0.975978052195351
"4776","AC103591.3",85.0425232407503,-0.477573109921641,0.490005247750348,-0.974628561865849,0.329744540547785,0.975978052195351
"4777","MCOLN3",19.9025358958092,0.538109295143632,0.548934551246259,0.980279514054909,0.326948164109562,0.975978052195351
"4778","PRMT6",18.3667832154109,0.356870068708461,0.366798404767454,0.972932444825417,0.330586878439623,0.975978052195351
"4779","ENSA",218.589392187581,-0.218790514162857,0.224180041755016,-0.975958932160213,0.329084816065649,0.975978052195351
"4780","PYHIN1",25.4823844738919,0.328802309470826,0.335415013581513,0.980285008592558,0.32694545265173,0.975978052195351
"4781","SUCO",51.5713950809695,0.243329535725678,0.249306511755419,0.976025592000564,0.329051782278938,0.975978052195351

"4782","TGFB2",21.4007586811064,-0.47956917931217,0.495783841570855,-0
.967294895680122,0.333396634659633,0.975978052195351
"4783","NUP133",31.9199952350569,0.268166276002705,0.276759611228029,0
.968950183203414,0.332570045310111,0.975978052195351
"4784","CCDC121",11.5462794430869,0.44038265639336,0.450497186955916,0
.977548071651898,0.328297891109703,0.975978052195351
"4785","FAHD2B",19.813203582186,-0.4326576848512,0.441248157852774,-0.
980531424667295,0.326823865661016,0.975978052195351
"4786","AC017002.3",3.36468861802989,-0.765330568964842,0.784543865443
978,-0.975510232983259,0.329307228248044,0.975978052195351
"4787","MCM6",36.8392179251341,0.27065501469945,0.279674624861672,0.96
7749629889794,0.33316942525582,0.975978052195351
"4788","ORC4",52.3056630070644,0.27520592485407,0.281071154645477,0.97
9132580151081,0.327514475544904,0.975978052195351
"4789","MMADHC",100.844248344294,-0.238447100841131,0.245988665507613,
-0.969341820482179,0.332374669924453,0.975978052195351
"4790","AC007364.1",3.73040776751338,0.746474996996284,0.7626628720518
91,0.978774533743783,0.327691395291577,0.975978052195351
"4791","GPD2",28.4366646486364,0.323238572086535,0.332881029527687,0.9
71033322461081,0.331531685200687,0.975978052195351
"4792","TTC21B",28.5173224165911,0.297007017939113,0.305189726510689,0
.973188125743517,0.330459811282721,0.975978052195351
"4793","GORASP2",107.690253588882,-0.239981636551571,0.246221712018292
,-0.974656680698177,0.329730587693868,0.975978052195351
"4794","IGFBP2",2.76254389603806,0.950862957698845,0.977603561510445,0
.972646781513066,0.330728883503664,0.975978052195351
"4795","TMBIM1",153.029377788079,0.206105319437648,0.213087075337476,0
.967235197682588,0.333426470378331,0.975978052195351
"4796","CNTN4",37.4809291066144,0.501201777039152,0.514066372706003,0.
974974835254964,0.329572742769694,0.975978052195351
"4797","TADA3",95.5392154412619,-0.234796958319674,0.242295532427143,-
0.969051950597875,0.332519269661777,0.975978052195351
"4798","NEK11",28.5974921735716,0.28606323493938,0.295358635457243,0.9
68528428148129,0.332780528424562,0.975978052195351
"4799","ZNF721",68.8911836047424,0.266030075965254,0.274593104563709,0
.968815573092921,0.332637215138273,0.975978052195351
"4800","GRK4",17.0390385634941,0.384134063306973,0.392481913033763,0.9
78730612928724,0.327713101942382,0.975978052195351
"4801","PAQR3",24.8947409350765,-0.34869306541798,0.360504602827867,-0
.96723609818783,0.333426020313233,0.975978052195351
"4802","CDS1",6.81467802689988,0.753106606872936,0.771138555176189,0.9
7661646122838,0.328759066531353,0.975978052195351
"4803","DCLK2",3.75869547159461,0.908916949452373,0.931425978806394,0.
975833796924082,0.329146833579814,0.975978052195351
"4804","SELENOP",1901.49508928682,0.227195291107296,0.234860373425348,
0.967363237117186,0.333362481258991,0.975978052195351
"4805","GM2A",31.8131398749614,0.291241072082371,0.299824322365519,0.9
71372401626963,0.331362866602444,0.975978052195351
"4806","TMED9",322.018944351533,-0.232903911226084,0.240083568151621,-
0.970095175688984,0.331999053489864,0.975978052195351

"4807","ZFP2",2.42282811991483,-0.981667291091864,1.0030116411947,-0.978719738409604,0.327718476517482,0.975978052195351
"4808","CDYL",64.1710507256888,-0.252714591767785,0.259809585496652,-0.972691562879392,0.330706619791168,0.975978052195351
"4809","BTN3A1",43.024076210818,0.323748454647643,0.330333178981161,0.980066415508645,0.327053335766584,0.975978052195351
"4810","TRIM26",28.9467021912602,0.289275342253478,0.298733641885756,0.968338686019517,0.332875250094619,0.975978052195351
"4811","HSD17B8",12.5146969277747,-0.475575644558268,0.488091312183352,-0.974357938130269,0.329878846379628,0.975978052195351
"4812","UHRF1BP1",14.7996567939361,0.458560535358733,0.468462395267571,0.978863063484141,0.327647644746489,0.975978052195351
"4813","VEGFA",145.92142063491,-0.272383837832835,0.278864662027652,-0.976759966115124,0.328687999907404,0.975978052195351
"4814","CENPQ",22.1268281076907,0.322883431073725,0.332148334373414,0.97210612747113,0.330997754112947,0.975978052195351
"4815","TSTD3",30.4299317372502,-0.283827970708487,0.289327283019513,-0.980992762750772,0.326596310505963,0.975978052195351
"4816","CD24",6.45588839230473,0.787028117137249,0.813186691860109,0.967832018176509,0.333128270381068,0.975978052195351
"4817","HOXA13",3.86374796704148,0.762881645318419,0.788125659481779,0.967969556809051,0.333059573931225,0.975978052195351
"4818","SFRP4",571.527979084114,-0.701272256871975,0.724591797723121,-0.967816995825205,0.333135774152023,0.975978052195351
"4819","ZMIZ2",28.0232471713604,0.309427625585536,0.317604450602286,0.974254690067334,0.32993009592367,0.975978052195351
"4820","PCOLCE",1214.1944267334,0.330844327893248,0.339913481043656,0.973319230756711,0.330394667562088,0.975978052195351
"4821","TES",42.5649366648333,-0.416402700375242,0.427232202233842,-0.974651953195529,0.329732933502584,0.975978052195351
"4822","ELP3",30.3297810074994,0.289625805707059,0.298527129949615,0.970182528321433,0.331955517971277,0.975978052195351
"4823","TOPORS",114.968694030211,-0.255520504779538,0.26416665881641,-0.967270078383055,0.333409037578222,0.975978052195351
"4824","GOLGA2",174.150808138826,-0.249243190483635,0.257708725238936,-0.967150763919803,0.333468671418567,0.975978052195351
"4825","PLPP7",11.6809018531638,0.482011693949426,0.493008442828009,0.97769460333072,0.328225391690537,0.975978052195351
"4826","LGI1",2.726008514472,1.08754268715929,1.11294375367158,0.977176684420486,0.328481688738127,0.975978052195351
"4827","TAF5",7.80179522514201,0.515450887760744,0.532483231246132,0.968013371152499,0.333037691892518,0.975978052195351
"4828","TENM4",3.26569715452222,-0.917914113043207,0.949177587972886,-0.967062565187146,0.33351275791759,0.975978052195351
"4829","RPS25",4004.99412623247,-0.186582546345124,0.191712542010674,-0.97324120992948,0.330433433705007,0.975978052195351
"4830","MARS",40.5929692634101,-0.295092079218279,0.305126163354132,-0.967114966394384,0.333486564505378,0.975978052195351
"4831","AC079866.2",10.0389169161038,-0.698281196408176,0.718001311970151,-0.97253470817781,0.330784606655055,0.975978052195351

"4832","TDG",77.8301211893519,0.227475037267481,0.232314100508159,0.97
9170169911801,0.327495905094457,0.975978052195351
"4833","HMGB1",1608.64045457623,-0.180337652199834,0.185199276254486,-
0.973749227572725,0.330181068225052,0.975978052195351
"4834","SPRYD7",33.1655200286826,-0.299068858874638,0.308362669759029,
-0.969860778246428,0.332115892634463,0.975978052195351
"4835","CHD8",75.7597875414724,0.263874299562424,0.268921545354104,0.9
8123153061227,0.326478578616721,0.975978052195351
"4836","ACIN1",105.790532974433,0.208677402230455,0.212950938080649,0.
979931828952193,0.327119770302601,0.975978052195351
"4837","EFS",48.5009435264577,0.267698110407663,0.273822462703589,0.97
7633857224654,0.328255445756435,0.975978052195351
"4838","AL135999.1",3.17651483686223,0.792722103818276,0.8139622619625
49,0.97390522984068,0.330103596776947,0.975978052195351
"4839","SDR39U1",34.7458333299816,-0.297634134964176,0.304458876404895
,-0.977584028682931,0.328280099702501,0.975978052195351
"4840","G2E3",28.4038913990049,0.27754136851372,0.284526095581139,0.97
5451365706359,0.329336414933221,0.975978052195351
"4841","COA8",95.8745385515628,-0.218058030851146,0.224098039470758,-0
.973047472285451,0.330529708696025,0.975978052195351
"4842","GLDN",7.80617414423943,-0.645387884274981,0.662046466414984,-0
.974837744803306,0.329640750980783,0.975978052195351
"4843","C15orf65",4.62118012647221,-0.626781530140247,0.64183847226900
2,-0.97654091678935,0.328796481720877,0.975978052195351
"4844","IREB2",70.4362868639218,0.215378979466714,0.220232378502221,0.
977962372887607,0.328092934285887,0.975978052195351
"4845","SNHG21",9.94979166377017,-0.478593453055039,0.493035948646708,
-0.970707013086346,0.331694198548595,0.975978052195351
"4846","NUBP1",49.0276587989305,-0.270476770017589,0.279186931743211,-
0.968801685411144,0.332644145526352,0.975978052195351
"4847","MIS12",22.4998782563735,-0.398881075297536,0.410259042932739,-
0.972266381859941,0.33091804387185,0.975978052195351
"4848","ZBTB4",80.4418295705068,0.252343725558975,0.259624860192522,0.
971955171672899,0.331072850610642,0.975978052195351
"4849","NSRP1",166.826082327754,-0.228063936301961,0.234491737750033,-
0.972588367036948,0.330757926574004,0.975978052195351
"4850","CCT6B",9.22320876084691,0.478917623560248,0.491580467722401,0.
97424054657659,0.329937116771165,0.975978052195351
"4851","ERBB2",34.1596899351763,0.274753703313882,0.283951642812767,0.
967607373538074,0.3332404933528,0.975978052195351
"4852","PSMD3",134.640830793648,-0.265282774922413,0.271070626207645,-
0.978648179752245,0.327753844784192,0.975978052195351
"4853","KANSL1",109.142194587192,0.254500554255088,0.260934071742147,0
.975344279709796,0.329389512994081,0.975978052195351
"4854","SLC35B1",87.8286332114342,-0.351652694971139,0.361272505235973
,-0.973372426283725,0.330368238033015,0.975978052195351
"4855","SPATA20",46.2496881585007,-0.303872038071799,0.313865243220974
,-0.968160841746533,0.332964048007057,0.975978052195351
"4856","AC004687.1",6.68797830754194,0.749118545779798,0.7678480864734
02,0.975607752335979,0.329258881361727,0.975978052195351

"4857","SLC39A11",15.372922032851,-0.38834726893069,0.398057903269162,
-0.975604970385664,0.329260260497422,0.975978052195351
"4858","MOCOS",3.09776180352065,-0.779209120636201,0.801343654727074,-
0.972378225047015,0.330862420632311,0.975978052195351
"4859","VPS4B",148.413175596637,0.215224304897132,0.219778405675803,0.
979278670419562,0.327442306477345,0.975978052195351
"4860","SH3GL1",116.593851107916,-0.203280338677568,0.207260002904522,
-0.980798686812777,0.326692025977389,0.975978052195351
"4861","VAV1",3.0903784247253,0.814526451740556,0.838893343601025,0.97
0953528185273,0.331571420752614,0.975978052195351
"4862","PRKCSH",157.087597184715,0.208554246956755,0.213698349301923,0
.975928207391533,0.329100042680114,0.975978052195351
"4863","RPL18A",5824.50866860158,-0.186872055523557,0.193149071897452,
-0.967501700566146,0.333293291542858,0.975978052195351
"4864","IRGQ",21.5896329304183,0.350115133732849,0.361259537214878,0.9
69151254613382,0.332469727914295,0.975978052195351
"4865","ZNF880",17.8464767979411,0.462733427953479,0.476852660140578,0
.970390786573494,0.331851739405454,0.975978052195351
"4866","LPIN3",26.1586991962066,-0.371625584373819,0.383575059766029,-
0.968847100227272,0.33262148245712,0.975978052195351
"4867","TCEA2",77.2981550995104,-0.268230513922572,0.273810936754276,-
0.97961943048056,0.327274010024915,0.975978052195351
"4868","POFUT2",38.215732077877,0.273518472595687,0.281732899943133,0.
970843208765805,0.331626362132554,0.975978052195351
"4869","UFD1",122.678068920944,-0.293718596543682,0.300229591028847,-0
.978313281969135,0.3279194028091,0.975978052195351
"4870","POLR2F",195.201008220152,-0.221229626633711,0.226718841766434,
-0.975788447532835,0.329169310785774,0.975978052195351
"4871","AL022322.1",3.17696101213506,0.793883781995747,0.8181651347205
07,0.970322185956929,0.331885921922458,0.975978052195351
"4872","TRO",5.45630485889211,-0.665657295119536,0.682835393216931,-0.
974842988122706,0.329638149693196,0.975978052195351
"4873","SLC25A53",7.98272424207072,-0.49962714002059,0.516360363158719
,-0.967593904699098,0.333247222590998,0.975978052195351
"4874","THOC2",158.880979381245,0.216247139912913,0.222020114006785,0.
973997968068356,0.330057548121855,0.975978052195351
"4875","CD40LG",10.3808458201848,-0.514735240221392,0.524859134739409,
-0.980711215928358,0.326735171320935,0.975978052195351
"4876","RBPMS-
AS1",4.9608119292745,0.696922092481109,0.721245211705653,0.96627621392
8654,0.333905985045156,0.976861412362861
"4877","MTA2",18.3830741728211,-0.349483201543235,0.361714545200625,-0
.966185093135789,0.33395157073417,0.976861412362861
"4878","COL6A2",4694.93574016741,-0.182894260808914,0.189333486850968,
-0.965990030875399,0.334049169500197,0.976946587144284
"4879","AL009176.1",4.59112579230507,0.892820080967405,0.9248560351687
74,0.965361144888325,0.334363955787443,0.977230916460463
"4880","HOTAIR",4.44430205177939,0.841595206073751,0.871643513550244,0
.96552683865666,0.334280999906443,0.977230916460463
"4881","PNN",264.703871492652,0.18279892870625,0.18938071479366,0.9652

45742711549,0.334421740629596,0.977230916460463
"4882","CWC25",81.3819145995916,-0.235771354817339,0.244294383675656,-0.965111646325719,0.334488894229387,0.977230916460463
"4883","COX11",83.5064313200337,-0.23008397633562,0.238362486667223,-0.965269239940591,0.334409974439929,0.977230916460463
"4884","PRPF38A",80.7728794612283,-0.239917046592877,0.248873341086328,-0.964012640106986,0.335039588720457,0.977438603821277
"4885","NDUFS6",309.031767393251,-0.281579882930345,0.291948747983278,-0.964483954377064,0.334803349161873,0.977438603821277
"4886","TTC37",118.525822821812,0.20479173101829,0.212410124484633,0.964133567150683,0.334978965524474,0.977438603821277
"4887","SARAF",356.387123167114,-0.202009754581422,0.209532575865676,-0.964097127841941,0.334997232549954,0.977438603821277
"4888","AC093249.6",1.94006229961625,-1.14104996108367,1.18291080313977,-0.964612004603404,0.334739184358572,0.977438603821277
"4889","FBXO27",5.33412211356501,0.712853477538418,0.738938400606577,0.964699462029925,0.334695364792731,0.977438603821277
"4890","NCOA3",69.5263473076001,0.283756127399319,0.294301671366052,0.96416757024252,0.334961920353357,0.977438603821277
"4891","PARL",94.9755825854988,-0.275408833753587,0.28573109255333,-0.963874219261539,0.33510899059159,0.97744118989565
"4892","TUB",17.3528238111346,0.395266558784329,0.410219562359415,0.96354877985564,0.335272196885462,0.977717326403925
"4893","SNX25",6.70567691799162,0.648934306129039,0.674887180650318,0.961544869623586,0.336278276117426,0.977857838124674
"4894","GRK6",25.8687551162738,-0.2962677310944,0.307807225240325,-0.962510645625956,0.335793158259738,0.977857838124674
"4895","STEAP1",164.429576516637,-0.287466482147188,0.298766330053405,-0.962178308699652,0.335960043183101,0.977857838124674
"4896","AC067930.8",2.39770477098875,1.02514371437656,1.06576593777555,0.961884479547378,0.336107635658201,0.977857838124674
"4897","AL161729.1",3.33629504980788,0.766059762473496,0.795678178918092,0.962775884485272,0.335660005319365,0.977857838124674
"4898","PRRC2B",148.570670046635,0.210954072342537,0.21945102034787,0.961280890870937,0.33641095342238,0.977857838124674
"4899","NELFB",61.5160487996407,-0.215384540924241,0.224062789858755,-0.961268674107,0.336417094456463,0.977857838124674
"4900","SUV39H2",7.84946323466503,0.506655425878556,0.526475374927223,0.962353511688164,0.335872057329909,0.977857838124674
"4901","FZD4",90.0754405688761,0.315001576863143,0.327400290056667,0.962129803882038,0.335984404623203,0.977857838124674
"4902","STARD13",14.9925289959341,0.403958638478043,0.419473880040317,0.96301261580153,0.335541192205125,0.977857838124674
"4903","MZT1",38.3406338789896,-0.284580840336211,0.295418612483777,-0.963313847910779,0.33539004593623,0.977857838124674
"4904","AL137779.1",2.00492109852643,1.1151664843604,1.15963289999205,0.961654748125929,0.33622306044784,0.977857838124674
"4905","AC009120.2",11.7365357096884,0.428223437472098,0.44528394553376,0.961686226883362,0.336207242953958,0.977857838124674
"4906","GAA",104.828592328095,0.220331510522115,0.228980172152845,0.96

2229648316637,0.335934259209665,0.977857838124674
"4907","NRSN2",22.0749511153111,0.398073439517733,0.413998320267338,0.961533948400269,0.336283764522064,0.977857838124674
"4908","EMID1",5.52283717349723,0.693340881977408,0.721131745500956,0.961462154874013,0.336319845424398,0.977857838124674
"4909","MIGA1",65.7245787379637,0.258507658688452,0.269040653732961,0.960849801327931,0.33662769400616,0.978071422136838
"4910","SH2D1A",7.75418175702805,0.48615826575916,0.505911988228223,0.96095423131157,0.336575181091196,0.978071422136838
"4911","ATAD3A",27.1919387783531,-0.390123782367099,0.408813027744542,-0.954284124748778,0.339939841652579,0.978771448053539
"4912","AL645728.1",6.2272305447215,-0.620311695018991,0.650874480491782,-0.953043503180984,0.340568029413334,0.978771448053539
"4913","C1orf167",15.8017971576238,0.477730874885681,0.501502282134806,0.95259960304082,0.340792978437699,0.978771448053539
"4914","HDAC1",23.366921096661,-0.434378773171856,0.455387038672643,-0.953867230033553,0.340150853051652,0.978771448053539
"4915","RNF19B",31.9757236498267,-0.405278515189632,0.422652180607135,-0.95889370452899,0.337612296579946,0.978771448053539
"4916","HECTD3",24.7501859591669,0.303848314519456,0.318289063273596,0.954630081833106,0.339764799082922,0.978771448053539
"4917","CTH",2.00008832797031,-0.974713034952173,1.02254484154363,-0.953222778456103,0.340477207578399,0.978771448053539
"4918","AC105942.1",5.68072464857785,-0.625218583250782,0.656844306273491,-0.951852025326774,0.341172032905671,0.978771448053539
"4919","TSPAN2",4.50604135326286,-0.727638027353142,0.758382948350226,-0.959459899429483,0.337327111907577,0.978771448053539
"4920","AC239809.3",4.99501718818476,0.668750294035925,0.700113785584706,0.955202293977703,0.339475405978149,0.978771448053539
"4921","DSTYK",50.7015954846944,0.281779242000248,0.295971592169084,0.952048269008439,0.341072502690646,0.978771448053539
"4922","RASSF5",42.7036758571069,0.275854356400576,0.287735268154943,0.958708879065986,0.337705424182686,0.978771448053539
"4923","FH",68.6774154092489,-0.315831909809389,0.332292702209192,-0.950462973485825,0.341877059137164,0.978771448053539
"4924","RBKS",7.34918648036233,-0.611633552904503,0.639660781565148,-0.9561842315984,0.338979165015162,0.978771448053539
"4925","ERLEC1",188.332528253636,0.163492688497386,0.171216005041151,0.954891386807513,0.33963262593011,0.978771448053539
"4926","AC012358.3",3.88780937213192,0.748310474569841,0.785983526281979,0.952068904178759,0.341062038094248,0.978771448053539
"4927","USP37",19.0189196496566,0.416963627767571,0.434883908908544,0.958792954225534,0.337663059369392,0.978771448053539
"4928","TIGD1",4.44879011511494,-0.614704997441374,0.646398337963252,-0.950969334757666,0.341619942857154,0.978771448053539
"4929","WDR82",137.28611318953,0.19245055088253,0.202099936300598,0.952254386643072,0.340967984657829,0.978771448053539
"4930","TRMT10C",79.8830915823385,-0.237943837010817,0.249736298098804,-0.952780348000028,0.340701373376359,0.978771448053539
"4931","PSMD2",181.25918541354,-0.188262090609724,0.197308560465202,-0

.954150646914916,0.340007392368528,0.978771448053539
"4932","SEPSECS",10.9958682520341,0.452859299345865,0.473290172198136,
0.956832247841991,0.33865193268682,0.978771448053539
"4933","BBS12",5.85005860628062,-0.564211863914155,0.587942125419218,-
0.959638439773061,0.337237215684066,0.978771448053539
"4934","AC106897.1",3.91393903967983,-0.655738156206623,0.689325499109
818,-0.951275060988504,0.341464763442661,0.978771448053539
"4935","NNT-
AS1",52.6388385721545,0.277480801003966,0.29028510953634,0.95589057753
315,0.339127519905834,0.978771448053539
"4936","LARP1",136.102898014253,0.215237965626718,0.226432608958764,0.
950560816379215,0.341827367568222,0.978771448053539
"4937","SIRT5",12.1831226096304,-0.400126996111434,0.418592656474716,-
0.955886325099931,0.339129668553438,0.978771448053539
"4938","AC004160.1",7.50588154015068,0.619287187937337,0.6494340461904
34,0.953579800089111,0.340296384693758,0.978771448053539
"4939","AC005076.1",2.91943546790006,-0.772196049832898,0.809459106937
549,-0.953965485365123,0.340101113523442,0.978771448053539
"4940","PVT1",19.5072494172811,-0.380881942188156,0.400534251722418,-0
.95093475913795,0.341637495465292,0.978771448053539
"4941","WASHC1",34.3448958133395,0.31326760741139,0.329052305265352,0.
952029821395013,0.341081858097825,0.978771448053539
"4942","RMRP",3.37683768408496,0.992580157685731,1.04304557923935,0.95
1617242277731,0.341291133817548,0.978771448053539
"4943","FAM225B",3.51032738347138,0.742022280713177,0.777754002772854,
0.954057810140113,0.340054380453079,0.978771448053539
"4944","NTNG2",6.15060270535606,0.652492634534574,0.681977553544365,0.
956765557962205,0.338685600079447,0.978771448053539
"4945","AL117336.2",3.66206023022078,0.781724042840409,0.8196315156399
83,0.953750591483815,0.34020990471807,0.978771448053539
"4946","CD81",2900.9053250768,-0.196187260970395,0.206218948681906,-0.
951354190409606,0.341424606568304,0.978771448053539
"4947","GAS2",3.26538886521669,0.845129789891935,0.885012268131671,0.9
54935677531419,0.339610226086372,0.978771448053539
"4948","MDK",158.931257070185,0.302883624989663,0.317161601988825,0.95
4982012609254,0.339586793333377,0.978771448053539
"4949","C11orf53",3.25888087557958,-0.994737264797379,1.04395726820766
,-0.95285247307604,0.340664823387918,0.978771448053539
"4950","EPS8",223.512804156682,-0.211100000786146,0.22071571007076,-0.
956433960765496,0.338853033882341,0.978771448053539
"4951","DCTN2",194.856772161581,-0.243217365434406,0.254298463908579,-
0.956424831263798,0.338857644404731,0.978771448053539
"4952","XPOT",55.4261520752631,-0.250356248717878,0.262236464138378,-0
.954696554273891,0.339731172898579,0.978771448053539
"4953","WASHC3",150.356548240311,-0.265678689840242,0.279393385109186,
-0.950912598508426,0.341648745801299,0.978771448053539
"4954","HECTD4",36.2345134724519,0.345539913103055,0.361016978020405,0
.957129260229763,0.338502016495585,0.978771448053539
"4955","CDK8",12.4370774317613,0.404132063018631,0.421896822004662,0.9
57893119693055,0.338116656238459,0.978771448053539

"4956","PLD4",2.5830813401261,1.29312912439061,1.3507358797403,0.957351576859891,0.338389830668667,0.978771448053539
"4957","HAUS2",31.5731471505726,-0.299991854210369,0.315399244122205,-0.951149566148401,0.341528456200063,0.978771448053539
"4958","MINDY2",94.6065173705058,0.236113971733937,0.247160181247822,0.955307487403039,0.33942222196387,0.978771448053539
"4959","CD276",167.607967816719,0.205505187838065,0.214742966371341,0.956982160163972,0.338576259525934,0.978771448053539
"4960","ZNF205",25.5449507207318,-0.34908898325679,0.364012601188745,-0.959002468916682,0.337557501418645,0.978771448053539
"4961","TFAP4",12.7831767458706,-0.379656392459999,0.39943872053217,-0.950474685964808,0.341871110465158,0.978771448053539
"4962","MIR193BHG",26.2518693260276,-0.305608417096533,0.320156335229726,-0.954559955458155,0.339800275983378,0.978771448053539
"4963","STX4",147.214480688612,-0.212391619016884,0.222631610386674,-0.95400477339222,0.340081226157512,0.978771448053539
"4964","CCDC113",8.03715512701289,-0.499144085297974,0.522827616540069,-0.954701070691663,0.339728888271316,0.978771448053539
"4965","PRMT7",30.1835750663857,0.281165525650466,0.295434249944912,0.951702538561097,0.341247861580354,0.978771448053539
"4966","COG4",38.5134359853143,0.267675443093321,0.280400421492965,0.954618547533233,0.339770634116674,0.978771448053539
"4967","RANGRF",51.1503357670511,-0.287125542697342,0.300919345176554,-0.954161130880041,0.340002086312176,0.978771448053539
"4968","AC015908.2",3.07610488560611,-0.779696066940276,0.818315673248218,-0.952805979928693,0.340688383893379,0.978771448053539
"4969","PSMB3",308.341884303627,-0.308526278007981,0.323171199324733,-0.954683705270293,0.339737672613337,0.978771448053539
"4970","RBBP8",35.926402538439,0.329538395424886,0.346282301657738,0.951646658946488,0.341276209850597,0.978771448053539
"4971","TNFRSF11A",4.68200035267316,-0.643050883456007,0.670591448383398,-0.958930933292121,0.337593540201195,0.978771448053539
"4972","SMARCA4",66.2619601907431,0.243782521405585,0.255931685056577,0.952529661779446,0.340828430262409,0.978771448053539
"4973","RAB3A",2.73858500584271,-1.01194601532892,1.05461154969305,-0.959543839268074,0.33728484573499,0.978771448053539
"4974","ZNF675",15.9674957671487,-0.406553830780336,0.423956587630576,-0.958951559291717,0.337583148818384,0.978771448053539
"4975","AC073548.2",2.35110185314424,1.38924778548425,1.44737195958878,0.959841577889184,0.337134953052067,0.978771448053539
"4976","EMC10",89.1032010494368,-0.207873075975874,0.218621949239404,-0.950833512824646,0.341688897338771,0.978771448053539
"4977","SLC27A5",15.3082624150426,-0.383949981599345,0.403495542110282,-0.951559413993242,0.341320473075505,0.978771448053539
"4978","AL050403.2",10.523675451113,0.524876718924998,0.54864874817572,0.95667167868374,0.338732997275655,0.978771448053539
"4979","KCNK15",17.6939467513588,-0.47646526749304,0.50109224994854,-0.950853395840729,0.341678802513792,0.978771448053539
"4980","SUN2",86.4024451135133,-0.218316418685956,0.228962901638021,-0.953501275202668,0.34033615038328,0.978771448053539

"4981","PHETA2",29.2265540867766,0.310096039196964,0.325249904140302,0
.953408549086608,0.340383111533654,0.978771448053539
"4982","TMEM164",8.66959836401472,0.528220755899498,0.550949171239129,
0.958746801835615,0.337686314773816,0.978771448053539
"4983","WDR44",18.4282074767544,0.346402669691226,0.360971658800628,0.
959639520848234,0.337236671402598,0.978771448053539
"4984","CHAMP1",35.6442464003706,0.31952151266809,0.336245983747006,0.
950261201955354,0.341979547639333,0.978786294255869
"4985","MFGE8",280.191784052153,0.158784482958683,0.167109435949691,0.
950182627667328,0.342019464241239,0.978786294255869
"4986","SSU72",231.131931051385,-0.225233811006521,0.23791524249428,-0
.946697692191521,0.343792846772056,0.979210039987908
"4987","RPL22",2877.98427551985,-0.211215360830718,0.223837811343208,-
0.943608944187108,0.345369519943915,0.979210039987908
"4988","LINC01138",11.266800461168,0.473497022679468,0.502599185761506
,0.94209667682222,0.346143145744861,0.979210039987908
"4989","PMVK",105.461696695878,-0.242852419835794,0.257722552135141,-0
.942301780825337,0.346038156686722,0.979210039987908
"4990","SLC50A1",35.617864399294,-0.325949974344039,0.343286286772268,
-0.949498966034347,0.342366897637906,0.979210039987908
"4991","ARV1",28.7983500825969,-0.291888387858547,0.307856617310259,-0
.948130952677821,0.343062792499298,0.979210039987908
"4992","MBOAT2",23.2395589841935,0.356655390432895,0.376542481845929,0
.94718499937765,0.343544517795193,0.979210039987908
"4993","PDCL3",36.0545226821084,-0.33063781730316,0.35048258418172,-0.
94337873613637,0.345487215564157,0.979210039987908
"4994","PPP1R1C",3.06021395476841,0.873251655113875,0.920504450881739,
0.948666412506099,0.342790301799201,0.979210039987908
"4995","TBC1D23",77.4243804421414,0.207637784462905,0.219899867404067,
0.944237879331547,0.345048102385031,0.979210039987908
"4996","KLHL5",31.1294670953108,-0.330298393884119,0.350193651776004,-
0.943187839668175,0.345584832226183,0.979210039987908
"4997","CFI",22.8808406582266,0.576670370297154,0.609762228567458,0.94
5729898114469,0.344286368903883,0.979210039987908
"4998","CMBL",176.370683631626,-0.215438738893334,0.227722475062123,-0
.9460583055518,0.344118848384854,0.979210039987908
"4999","AC012640.2",2.05423374500519,-0.882056630141117,0.933499166230
201,-0.944892788392273,0.344713613651368,0.979210039987908
"5000","ACSL6",2.42736600388828,0.900298358584278,0.949152073338303,0.
948529096520645,0.342860167478432,0.979210039987908
"5001","C6orf47",35.5082769103529,-0.248895570723251,0.264160782996171
,-0.942212420406319,0.346083896187278,0.979210039987908
"5002","LSM2",156.072617869563,-0.247052891403295,0.261057954083581,-0
.946352668205609,0.343968738324788,0.979210039987908
"5003","EFHC1",60.2536504836328,-0.26045921858616,0.27527775546909,-0.
946168781935617,0.344062506117516,0.979210039987908
"5004","C7orf26",31.4538796820471,0.267902340505594,0.282690342495828,
0.947688336786914,0.343288140218906,0.979210039987908
"5005","RBM48",22.5066170195839,0.306448849270331,0.324891840008369,0.
94323344428237,0.345561510289885,0.979210039987908

"5006","TNFRSF10D",14.7219218124936,0.622503851862475,0.659118105963622,0.944449630847847,0.344939929625153,0.979210039987908
"5007","AC087623.2",3.29052642798849,0.775011889278586,0.819565096055172,0.945637988988264,0.344333261036674,0.979210039987908
"5008","MFSD3",10.8049937503867,-0.440799275963569,0.464719962232278,-0.948526665061243,0.342861404674683,0.979210039987908
"5009","NANS",124.350638678757,-0.219079018583189,0.230831388439215,-0.949086777428792,0.342576478825289,0.979210039987908
"5010","ZER1",33.3173497299029,0.286415351667924,0.302990194091252,0.945295779379789,0.344507892577256,0.979210039987908
"5011","LINC00710",2.69102156176758,-0.882605123900275,0.932107212356134,-0.946892280416188,0.343693671973856,0.979210039987908
"5012","FAM168A",34.310407579624,0.30415739084642,0.321739655953792,0.945352508520441,0.344478939462128,0.979210039987908
"5013","HSPB2",146.065609017447,-0.197162008522677,0.207604286818239,-0.949701046854082,0.342264177695818,0.979210039987908
"5014","KMT2A",234.806488923128,0.230876720903097,0.24394308491696,0.946436833746238,0.343925825843777,0.979210039987908
"5015","AC021054.1",7.50723890901829,0.513568606992327,0.541520599290187,0.948382402563266,0.342934814676335,0.979210039987908
"5016","NCKAP5L",55.4150005797711,0.351525285513676,0.372438852695668,0.943846977750516,0.345247850352776,0.979210039987908
"5017","AC009779.2",19.5273925039018,-0.432965081677033,0.457721481671918,-0.945913834097414,0.34419253681239,0.979210039987908
"5018","TBX5-AS1",3.01441742661754,-0.826762814948057,0.873746989590294,-0.946226796541791,0.344032921388145,0.979210039987908
"5019","GAS6",670.435303425442,0.325748173536065,0.345419479329268,0.943050965650807,0.345654834831833,0.979210039987908
"5020","PNP",15.4974557945508,-0.461757554708275,0.490034493582887,-0.942296023555679,0.346041103452968,0.979210039987908
"5021","AP1G2",8.23770995980736,0.512573169885743,0.540892858366499,0.947642702167891,0.343311379413992,0.979210039987908
"5022","IFI27L1",56.6167840464927,0.320571530595475,0.33976524284417,0.943508900180534,0.345420665063928,0.979210039987908
"5023","FAM214A",43.3673116359945,0.337064705803707,0.356699362969628,0.944954605462548,0.344682051842953,0.979210039987908
"5024","SNX1",103.407107430822,0.192448623220966,0.203109533906455,0.947511520112104,0.343378188798261,0.979210039987908
"5025","NDUFB10",363.333013000035,-0.220684518410272,0.233597074164345,-0.944722955969094,0.344800334115907,0.979210039987908
"5026","AC008915.2",9.1598357081376,0.443910565077829,0.469544074105311,0.945407661514365,0.344450792260906,0.979210039987908
"5027","RANBP10",15.2116936808044,0.376339712440119,0.398527656456605,0.944325208910811,0.345003487655694,0.979210039987908
"5028","POLR2A",139.693524051324,0.275958716172467,0.290981719181088,0.94837131675866,0.342940456259556,0.979210039987908
"5029","AATF",73.5618285592251,-0.229354637774999,0.24239850482224,-0.946188335374402,0.344052534598981,0.979210039987908
"5030","AC104532.2",4.08326249537509,-0.67635395033005,0.7160210282801

29,-0.944600680170862,0.344862779655264,0.979210039987908
"5031","ZNF571",11.0895921480463,0.502799774408621,0.533709008054759,0
.942085980975298,0.346148621313544,0.979210039987908
"5032","EID2",41.9373414010737,-0.289153201457987,0.30468448611607 3,-0
.949025023045742,0.342607885487729,0.979210039987908
"5033","AKT2",97.5535899506726,0.229022046114215,0.242635985756011,0.9
43891506449972,0.345225092779366,0.979210039987908
"5034","KDELR1",436.132120658957,-0.203675065843666,0.216133840440824,
-0.942356205896554,0.346010300875749,0.979210039987908
"5035","ZNF816",2.96606597876582,0.845219995451902,0.895612237350738,0
.943734308445921,0.3453054372624,0.979210039987908
"5036","UBE2V1",261.675946821633,-0.168875242939441,0.178256352972046,
-0.947372927381293,0.343448781368513,0.979210039987908
"5037","FAM217B",31.1805097673402,0.296076966423955,0.313887318201258,
0.943258772353831,0.345548558093724,0.979210039987908
"5038","FP565260.1",10.5742456526769,-0.460325854236984,0.488411846883
321,-0.942495267415072,0.345939132971968,0.979210039987908
"5039","PCBP3",10.4996146224618,0.586810235051453,0.621843324967675,0.
943662513514891,0.34534213588331,0.979210039987908
"5040","MED15",44.7392710127827,0.278271948481341,0.295247378355562,0.
942504384056622,0.345934467648497,0.979210039987908
"5041","SRRD",33.7131401809389,-0.310837595310351,0.328576918787363,-0
.946011656745458,0.344142640753735,0.979210039987908
"5042","ZC4H2",8.39561717250828,-0.468999303931476,0.495423008788677,-
0.94666435674474,0.343809838514119,0.979210039987908
"5043","AL136169.1",7.26333405220439,0.660158784291268,0.6956991797708
63,0.948914133417117,0.342664285669817,0.979210039987908
"5044","PKD2",104.837098116397,-0.223613086620706,0.23748038804215,-0.
941606540498902,0.346394119904233,0.979516058385289
"5045","PUF60",164.628489325896,-0.262346337056036,0.278577713520298,-
0.941734834925769,0.346328415586804,0.979516058385289
"5046","EIF2AK2",224.499046894328,0.174569092662009,0.18559679533409,0
.940582472600185,0.346918867469016,0.979834599745195
"5047","IFT80",31.4281080566946,-0.28260335743837,0.300281317555349,-0
.941128671404208,0.346638924174898,0.979834599745195
"5048","NDUFB6",170.035524104052,-0.254270825827604,0.270320424385375,
-0.940627503103905,0.346895782541956,0.979834599745195
"5049","CBWD5",17.7564723723353,-0.431225370277042,0.458400935553906,-
0.940716601627293,0.346850108987464,0.979834599745195
"5050","SGPL1",22.2843668409584,0.331542264996067,0.352422476526192,0.
940752327331842,0.346831796404337,0.979834599745195
"5051","RADX",2.01676155024181,-0.908767814935698,0.96586762482882,-0.
940882364803104,0.346765145897081,0.979834599745195
"5052","MFAP2",140.114454880772,-0.290036681592499,0.309710154258968,-
0.936477792555619,0.349027241303582,0.979869443782536
"5053","PITHD1",129.610753441948,-0.181761339800292,0.193616232282816,
-0.938771184922102,0.347848236278064,0.979869443782536
"5054","PAQR7",16.4062797857854,-0.356037321925838,0.3810181340323,-0.
934436684569076,0.350078681977515,0.979869443782536
"5055","RPA2",84.8318199980041,-0.249055171182364,0.265596115502679,-0

.937721437344787,0.34838758405193,0.979869443782536
"5056","SCMH1",27.1302757348495,0.314385872372123,0.335932334599994,0.935860707622779,0.349344909961058,0.979869443782536
"5057","VAV3",4.42299357817915,0.683320079774008,0.7304794237533,0.935440558014651,0.349561303448338,0.979869443782536
"5058","MEX3A",3.21549349112243,0.782431509058016,0.834142388830147,0.93800713107908,0.348240745408955,0.979869443782536
"5059","MR1",56.1205824043112,0.244050288104576,0.261034967653789,0.934933316781756,0.349822665871087,0.979869443782536
"5060","LINC00342",103.733627565288,-0.322659304364487,0.344923917993775,-0.935450653121451,0.349556103075939,0.979869443782536
"5061","AC012306.2",12.8080213424758,-0.396706133827852,0.424410354443106,-0.934723033203076,0.349931053481045,0.979869443782536
"5062","ATP5MC3",505.207653688255,-0.291386964508321,0.311719628179343,-0.934772591030668,0.349905507701136,0.979869443782536
"5063","ABI3BP",817.212907770127,-0.193218256282198,0.205533129936602,-0.940083267071333,0.347174851121646,0.979869443782536
"5064","GAP43",5.2750991459517,-0.779437773773523,0.831776407125438,-0.937076078494708,0.348719425387036,0.979869443782536
"5065","ST6GAL1",4.38181048721882,0.763196888074488,0.815213832592861,0.936192269514211,0.349174202669163,0.979869443782536
"5066","UCHL1",22.4407725202547,0.319061897644714,0.340767632051084,0.936303415099249,0.349116990332272,0.979869443782536
"5067","TXNDC15",162.32506817238,-0.203206960557244,0.216423271542157,-0.938933041300327,0.34776512367183,0.979869443782536
"5068","IK",171.602532999776,-0.189759498923515,0.202624836298022,-0.93650661187662,0.349012409913959,0.979869443782536
"5069","PCDHB7",6.87554944008519,-0.607377419512982,0.647826981593259,-0.937561164894992,0.348469976865632,0.979869443782536
"5070","CLINT1",181.48315750024,0.1592711658287,0.169449645793881,0.939932126045504,0.34725237722982,0.979869443782536
"5071","WDR46",100.174723797149,-0.26382977155983,0.282781210818848,-0.932981971453688,0.350829276860958,0.979869443782536
"5072","LEMD2",30.5205287550079,0.305353040542521,0.326249405846648,0.935949721502483,0.349299075250739,0.979869443782536
"5073","HOXA11",11.4138073420438,0.451587097579393,0.482263379565877,0.936391019334502,0.349071900131905,0.979869443782536
"5074","TRBC2",52.3307131022101,0.358069643867635,0.38244637771728,0.93626104136443,0.349138801566102,0.979869443782536
"5075","ATP6V1H",47.6744031148311,0.249258179530043,0.265503444584129,0.938813354834128,0.347826580979412,0.979869443782536
"5076","RAB2A",422.177810011601,-0.165489210804224,0.176660074533656,-0.936766336372717,0.348878765032535,0.979869443782536
"5077","VPS28",369.043889763017,-0.237256949711629,0.253915794367793,-0.934392247250149,0.350101595404801,0.979869443782536
"5078","C9orf116",5.8308052281583,-0.571545665061605,0.609231804577622,-0.938141542787406,0.348171675134527,0.979869443782536
"5079","EDF1",810.030088927369,-0.231065610835468,0.246749186438576,-0.936439200349654,0.349047102774647,0.979869443782536
"5080","DLG5",33.4293353493822,0.276034773248569,0.293926066859364,0.9

39129952637527,0.347664027512805,0.979869443782536
"5081","INTS5",23.0682130749905,0.298099468512566,0.318066383456219,0.937224063962102,0.348643314022172,0.979869443782536
"5082","AP000866.1",3.03908415223905,0.740298863248268,0.791693906950826,0.935082178539805,0.34974595012513,0.979869443782536
"5083","C12orf73",12.198000591428,0.456858370787755,0.487050487611563,0.93801029340538,0.348239120281246,0.979869443782536
"5084","MRPL57",167.661706925416,-0.217352432605007,0.231843682682718,-0.937495600871976,0.348503685567809,0.979869443782536
"5085","DHRS1",34.0056778300284,0.273769930556603,0.292174577124852,0.937008049265066,0.348754417482535,0.979869443782536
"5086","NPAS3",4.44391162615678,0.703153319180589,0.753051805077881,0.933738309156391,0.350438898649695,0.979869443782536
"5087","FAM177A1",134.123204717474,-0.169738450202621,0.181883254367471,-0.933227474914685,0.350702531824742,0.979869443782536
"5088","ACTR10",142.965606882844,-0.261639116452866,0.278838241475847,-0.938318628994543,0.348080688969695,0.979869443782536
"5089","TRPM7",67.2431042904827,0.230605910992497,0.245694039486442,0.938589765850716,0.347941409260938,0.979869443782536
"5090","ICE2",52.7698521854223,0.21686314668089,0.232435671432259,0.933002862015919,0.350818490648314,0.979869443782536
"5091","NPIPB5",27.5606607318183,0.430673480199172,0.458557192574599,0.939192508967371,0.347631914408939,0.979869443782536
"5092","TERF2",36.4924674114202,0.274365467018477,0.292160646815344,0.9390911130885,0.34768396662786,0.979869443782536
"5093","BORCS6",16.4687651205061,-0.356952298932942,0.382295139348562,-0.933708703545631,0.350454174188076,0.979869443782536
"5094","FLOT2",38.7376552084796,0.309930882480083,0.332065770582496,0.933341855549925,0.350643490931693,0.979869443782536
"5095","SEPTIN4",9.44702467116371,-0.504464940425174,0.537657904309646,-0.938263785171927,0.348108865884249,0.979869443782536
"5096","CANT1",39.6298950203256,0.263331019431247,0.281581012771721,0.935187414943815,0.349691723100726,0.979869443782536
"5097","TAF4B",17.5046384259589,0.425486586051148,0.454658922652497,0.935836876506996,0.349357181642414,0.979869443782536
"5098","CCDC102B",27.9901496598723,-0.498876130926613,0.530984441369729,-0.93953060025584,0.34745838891549,0.979869443782536
"5099","AC022150.4",3.1319258965838,0.773194123828306,0.825865167125399,0.936223193090433,0.349158284119626,0.979869443782536
"5100","ZNF677",47.8011735180806,-0.239736935134655,0.256966809239759,-0.932949028880114,0.350846286193831,0.979869443782536
"5101","DHX35",8.07116200344033,0.472254105912601,0.502717672403403,0.939402236756149,0.347524265044939,0.979869443782536
"5102","HPS4",37.5549649859643,0.312763686104034,0.335116118549343,0.933299440975655,0.350665383713116,0.979869443782536
"5103","LINC01422",1.94005712138211,1.11073452360946,1.18645085265106,0.936182498522867,0.349179232584576,0.979869443782536
"5104","RTL6",16.1995199713243,-0.393412525969551,0.421566044668318,-0.933216825560707,0.350708029119382,0.979869443782536
"5105","HDAC10",16.9923205619947,-0.357927577248579,0.382955667351591,

-0.93464494134243,0.349971310219449,0.979869443782536
"5106","PNPLA4",22.0434112682661,-0.326266261744071,0.349289736714758,-0.934084879827175,0.350260110788957,0.979869443782536
"5107","WDR13",112.256205202545,-0.213261397281988,0.228071879132183,-0.935062218513965,0.349756235882972,0.979869443782536
"5108","TSR2",73.1192691224412,-0.221879232332009,0.237624179816253,-0.933740129070962,0.350437959646368,0.979869443782536
"5109","LINC02802",201.191785629301,0.256270896944697,0.275207028403932,0.931193140055125,0.351753665750971,0.979980787102788
"5110","DUSP10",8.73957143799087,-0.513047419720895,0.550590771723091,-0.931812602153314,0.35143338070636,0.979980787102788
"5111","TAF1A-AS1",1.96535191880345,1.1175184169995,1.1990174590303,0.932028477636423,0.351321808443862,0.979980787102788
"5112","THADA",34.6074443730136,-0.28643337281985,0.307551111532327,-0.931335840058388,0.351679868135575,0.979980787102788
"5113","RAB24",20.0232332376053,-0.452320263141007,0.485815099668398,-0.931054352673984,0.351825449352693,0.979980787102788
"5114","CCHCR1",18.998793819767,0.321494173504746,0.345364302535837,0.93088420298269,0.351913466824294,0.979980787102788
"5115","SOBP",83.9131032250329,-0.19770328372414,0.212108684369304,-0.932084814499711,0.351292695207231,0.979980787102788
"5116","INSIG1",76.226241605356,0.312500278512145,0.335207446873071,0.93225935589216,0.351202507045976,0.979980787102788
"5117","FZD3",7.51850393041706,0.512248885308801,0.549935046384525,0.931471614105181,0.351609661398283,0.979980787102788
"5118","ANXA11",279.884728297856,-0.179683974103433,0.193026292356733,-0.930878233786714,0.351916554908705,0.979980787102788
"5119","TMEM62",8.92385589378927,-0.457768102319065,0.491343261381014,-0.93166592989228,0.351508856251311,0.979980787102788
"5120","TMEM219",239.082048179021,-0.22601325619092,0.242396433818452,-0.932411639191846,0.351123831962257,0.979980787102788
"5121","AC092376.2",7.23907078116489,0.530849445880051,0.569940971733127,0.931411272760051,0.351640861913002,0.979980787102788
"5122","GEMIN7",22.1448555618231,0.353466541461374,0.379670355136207,0.930982724038509,0.351862500740872,0.979980787102788
"5123","RPS6KA6",2.12795661214707,-0.965844678504171,1.0367727407636,-0.931587647446062,0.351549669321975,0.979980787102788
"5124","FAM174B",7.28231256334547,0.497859483022028,0.53503976585,0.930509309398891,0.352107446352686,0.980321005009254
"5125","AURKAIP1",283.893545798275,-0.189608269411707,0.255155968421426,-0.743107326020069,0.457416704598765,0.980372848261233
"5126","CCNL2",93.5697570780541,0.229979433882789,0.271522516476886,0.846999493326979,0.396995403861561,0.980372848261233
"5127","MRPL20",341.499979240928,-0.20010379843542,0.265821969542333,-0.752773740936235,0.451585888874162,0.980372848261233
"5128","NADK",64.8780060361659,0.194783808636732,0.243122974533259,0.80117401085057,0.423030914596273,0.980372848261233
"5129","TNFRSF14",97.3170624300021,0.204402152131142,0.221228897468006,0.92393965919711,0.355517733046956,0.980372848261233

"5130","WRAP73",30.8550617190838,-0.270095493558819,0.299038217698297,
-0.903213962542146,0.366412348883996,0.980372848261233
"5131","RNF207",12.0174046315688,-0.490269671380023,0.550050876735403,
-0.891316952878637,0.37275915636004,0.980372848261233
"5132","CAMTA1",133.040126517478,-0.23488322240584,0.257419464520286,-
0.91245323209555,0.361530178208206,0.980372848261233
"5133","RERE",121.679875308722,0.205300905258661,0.254546194944552,0.8
06536924676412,0.419933326211252,0.980372848261233
"5134","RBP7",2.76382388188244,-0.876597870192169,1.0616008403527,-0.8
25732080148818,0.408956090080905,0.980372848261233
"5135","KIF1B",109.101260696714,0.207815282971606,0.267965539083856,0.
775529882245692,0.43802661501501,0.980372848261233
"5136","MTOR",22.4831468451961,0.281693228524001,0.369478001946043,0.7
6240866043532,0.44581613898694,0.980372848261233
"5137","UBIAD1",21.6125962175627,-0.265701450161361,0.311818507269431,
-0.852102886669836,0.394156989953767,0.980372848261233
"5138","TNFRSF1B",87.2554175816042,0.2386059112258,0.27204479584349,0.
877083167446702,0.380441466694108,0.980372848261233
"5139","PDPN",297.840815217784,0.543823019535892,0.624724316144464,0.8
70500804086095,0.384026779977171,0.980372848261233
"5140","ARHGEF19",10.0770294051373,-0.448828665999534,0.59528761768933
8,-0.753969430343104,0.450867577993974,0.980372848261233
"5141","RCC2",50.8440012431111,0.233082442576235,0.320061082816504,0.7
28243623139477,0.466464466422267,0.980372848261233
"5142","MRTO4",104.743627002735,-0.221412594995689,0.293602655660498,-
0.754123270777616,0.450775205280783,0.980372848261233
"5143","AKR7A2",164.197112665151,-0.224919105914807,0.293103419508294,
-0.767371142554828,0.44286088090693,0.980372848261233
"5144","USP48",82.0448100562636,0.194086362665786,0.213612707886703,0.
908589964454392,0.363566605846541,0.980372848261233
"5145","HSPG2",687.073500639089,0.160273866995325,0.186384689200401,0.
85990897472806,0.389839220919346,0.980372848261233
"5146","CDC42",612.883654325618,-0.167729565666243,0.221721120936561,-
0.756488894507409,0.449356128325634,0.980372848261233
"5147","LUZP1",89.9578860107621,-0.184976884389979,0.220204890964397,-
0.840021688800211,0.400896225989867,0.980372848261233
"5148","HNRNPR",353.777068833513,-0.165164167128165,0.207373714012453,
-0.796456618982318,0.425766680500306,0.980372848261233
"5149","RPL11",7581.82539004883,-0.177265998126854,0.235359105414016,-
0.753172467302796,0.451346281946264,0.980372848261233
"5150","HMGCL",52.1290534024751,0.286843791637373,0.316175449995404,0.
907229804342946,0.364285285817404,0.980372848261233
"5151","PNRC2",301.85408009974,0.126637645747865,0.147086051651412,0.8
60976580213002,0.389250941932071,0.980372848261233
"5152","SYF2",235.763021898636,-0.156185054385631,0.186456157483011,-0
.837650289987671,0.402227139647038,0.980372848261233
"5153","NUDC",281.576221283121,-0.22232098724294,0.244585282702679,-0.
908971238114913,0.363365308139456,0.980372848261233
"5154","PHACTR4",67.4963350662976,-0.198812532317929,0.269619688439003
,-0.737381359161783,0.460890468157188,0.980372848261233

"5155","RAB42",16.6602685988323,-0.331922758980196,0.401259553051701,-0.827202135016657,0.408122501741224,0.980372848261233
"5156","MATN1-AS1",2.34976459287039,-0.673924003972408,0.90294624904058,-0.746361153488906,0.455449278985033,0.980372848261233
"5157","PUM1",185.289888684171,0.178513066750722,0.194543841430388,0.917598138487454,0.358829292820484,0.980372848261233
"5158","SERINC2",1.9056196959785,-0.799656234447796,1.01867403746109,-0.78499716792708,0.432455229074264,0.980372848261233
"5159","KPNA6",88.3328685055823,0.197755800139995,0.229879190949465,0.860259683893998,0.389645911249084,0.980372848261233
"5160","TMEM234",21.2669600835726,-0.315667506000097,0.390877333060245,-0.807587136170529,0.419328294472928,0.980372848261233
"5161","BSDC1",67.5070590402696,0.203112954754415,0.218471471972501,0.929700124783251,0.352526369860607,0.980372848261233
"5162","ZBTB8OS",76.2205349348648,-0.248590626431213,0.32221258557178,-0.77151122446095,0.440403971895941,0.980372848261233
"5163","YARS",57.9354296463992,-0.287369281213919,0.335799244631823,-0.855777032878666,0.392121121372404,0.980372848261233
"5164","S100PBP",34.3521894808159,0.234409920850945,0.306226768371936,0.76547821765286,0.443986832333356,0.980372848261233
"5165","TRIM62",3.76986356678393,0.578695002341119,0.763595823871847,0.757855116869053,0.44853772219877,0.980372848261233
"5166","TMEM35B",293.47053160292,0.21827485150257,0.237644746130165,0.918492224452606,0.358361227858965,0.980372848261233
"5167","MAP7D1",79.9081609160326,-0.187295232097874,0.217056140295007,-0.86288842989337,0.388198812670426,0.980372848261233
"5168","MRPS15",191.423411761045,-0.231697160449714,0.257843732062726,-0.898595279381655,0.368868274065234,0.980372848261233
"5169","YRDC",26.0468343826805,-0.284308870734856,0.381802154848482,-0.744649727940072,0.456483497506274,0.980372848261233
"5170","C1orf122",222.3764267706,0.237884107948933,0.305219378852521,0.779387301170925,0.435751602032919,0.980372848261233
"5171","INPP5B",16.9191238721047,0.329163824141419,0.450127369434357,0.731268184280941,0.464615359766969,0.980372848261233
"5172","MFSD2A",11.3221905559298,0.625438051741183,0.78408267953506,0.797668496021429,0.425062890530462,0.980372848261233
"5173","AL050341.2",11.5714001515718,-0.357043518379102,0.446308606599631,-0.799992456115448,0.423715167979242,0.980372848261233
"5174","ZMPSTE24",79.0007857791992,-0.198088896789743,0.247602988362233,-0.800026276338583,0.423695573260157,0.980372848261233
"5175","SMAP2",56.9223810379809,0.220137707872305,0.296912540874096,0.741422734197181,0.458437164286116,0.980372848261233
"5176","ZFP69B",1.87728233789699,-1.0611899488052,1.1557947483335,-0.918147404922274,0.358541699596225,0.980372848261233
"5177","EBNA1BP2",126.92352837297,-0.22709779835075,0.30966900863248,-0.733356558196218,0.463340987661846,0.980372848261233
"5178","KDM4A-AS1",4.28030705808941,0.656569927414156,0.748545976064654,0.877127054861686,0.380417631110738,0.980372848261233

"5179","MUTYH",17.7792167109092,-0.30666719490519,0.374681790449612,-0.818473709483438,0.413086748130649,0.980372848261233
"5180","MMACHC",4.97180339072164,0.532077487790908,0.623752622079982,0.853026454649005,0.393644633907557,0.980372848261233
"5181","TMEM69",23.8569683578548,-0.280604733315744,0.32684406302087,-0.858527857970687,0.390601054534035,0.980372848261233
"5182","SPATA6",78.1031434116917,0.228682512817161,0.283037508930863,0.807958329201591,0.419114571110853,0.980372848261233
"5183","FAF1",44.8139777045218,-0.217411913184685,0.272327456291867,-0.798347387167873,0.424668925912496,0.980372848261233
"5184","NRDC",118.174186367574,0.159280406626922,0.190445579444335,0.836356543909585,0.402954351972598,0.980372848261233
"5185","ZFYVE9",38.0619738701261,0.260696312085729,0.307151746576552,0.848754125579278,0.396018121479152,0.980372848261233
"5186","ECHDC2",102.487090339157,0.159775263446779,0.211199559697868,0.75651324119873,0.449341536555581,0.980372848261233
"5187","LRP8",2.41102660200653,0.679543355874001,0.891251664531627,0.762459564360103,0.445785767756666,0.980372848261233
"5188","YIPF1",30.0027271857814,0.249754883659752,0.323689018086555,0.7715889934609,0.440357895225604,0.980372848261233
"5189","HSPB11",80.1828504008907,-0.208052309146579,0.284867146731418,-0.730348555576814,0.465177155433036,0.980372848261233
"5190","PARS2",2.32352493804788,0.761936997404102,0.921670134883164,0.826691642233466,0.408411859709558,0.980372848261233
"5191","AL603840.1",2.457611205118,0.930482344863131,1.1042429049186,0.842642810488983,0.399428240658018,0.980372848261233
"5192","MYSM1",57.5232610470916,0.221305926082123,0.253045719470797,0.874568937759339,0.381808495210515,0.980372848261233
"5193","HHLA3",19.5289383518005,0.277097896134059,0.343019082318972,0.807820644439797,0.419193838964729,0.980372848261233
"5194","TYW3",92.7927807690002,-0.212306388718815,0.240124961862274,-0.884149598910026,0.37661546167817,0.980372848261233
"5195","IFI44L",93.007363154694,0.32205169516838,0.389917907635496,0.825947433708128,0.408833911453364,0.980372848261233
"5196","IFI44",56.9484758591628,0.269695641392236,0.296834016691423,0.908573904023276,0.363575086662222,0.980372848261233
"5197","ADGRL4",4.50006899046594,-0.598921752243848,0.822439974936034,-0.728225488176727,0.466475565783743,0.980372848261233
"5198","ADGRL2",34.1733073404297,0.259261846785519,0.353884054073525,0.732618053289436,0.463791416988808,0.980372848261233
"5199","GNG5",416.781361222422,-0.190992491621609,0.24686309507075,-0.773677781066753,0.439121362587368,0.980372848261233
"5200","SSX2IP",5.56874733238941,-0.531148776476727,0.70845701461045,-0.749726187366191,0.453419632152672,0.980372848261233
"5201","AL049597.2",10.9518788688563,-0.463245422438408,0.50273506942659,-0.921450383333666,0.356815334349428,0.980372848261233
"5202","KYAT3",47.447443269994,-0.265324962024291,0.302606695179751,-0.876798055861539,0.380596335269866,0.980372848261233
"5203","RBMXL1",28.1395553262835,0.233955438832464,0.31385509548887,0.745425013629454,0.456014827041769,0.980372848261233

"5204","LRRC8B",6.57369814324944,0.528498036867513,0.663848722881872,0
.796112154246858,0.42596685028986,0.980372848261233
"5205","BTBD8",17.3369853665914,0.364466160544126,0.409084983415166,0.
890930186440607,0.372966624758068,0.980372848261233
"5206","PTBP2",31.5454436134999,-0.218149384271792,0.29309204409035,-0
.744303329518369,0.45669298742438,0.980372848261233
"5207","AGL",69.5201708546893,-0.232164938450052,0.280266046378853,-0.
82837340252133,0.407459064598726,0.980372848261233
"5208","CDC14A",20.7803190205439,0.284690461336526,0.370844048267258,0
.767682433267356,0.442675875101898,0.980372848261233
"5209","EXTL2",51.2816173944496,0.206127659978848,0.258767758681582,0.
796573966668281,0.425698501844967,0.980372848261233
"5210","HENMT1",11.0253456966285,0.403756659363238,0.472556091406065,0
.854410019690746,0.392877846167901,0.980372848261233
"5211","TMEM167B",64.9779828468086,0.19607147870621,0.226793517810809,
0.864537402121751,0.387292743093457,0.980372848261233
"5212","SARS",189.855859363791,-0.178278532896689,0.199439142305562,-0
.893899416311906,0.371375710111419,0.980372848261233
"5213","PSRC1",2.20078352742495,0.748569013993056,1.00861942849842,0.7
42171916227599,0.457983181633939,0.980372848261233
"5214","ATXN7L2",2.39565468189176,-0.772280538696814,0.947350942187332
,-0.815200053439226,0.414957807297642,0.980372848261233
"5215","CYB561D1",12.3213545879924,0.376522751058224,0.457954665702053
,0.82218345888235,0.410972491462314,0.980372848261233
"5216","RBM15",14.4663370982021,0.326649172705137,0.393131762845026,0.
830889802292325,0.406035885053386,0.980372848261233
"5217","CD53",38.525912085286,0.234690356322396,0.312792474706377,0.75
0306913689991,0.45306987962847,0.980372848261233
"5218","CHI3L2",709.0742677458,-0.760331523655564,0.899327689996288,-0
.845444360396267,0.397862785576565,0.980372848261233
"5219","ATP5PB",234.809577404005,-0.197470985967571,0.221742450448429,
-0.890542093172625,0.37317487676181,0.980372848261233
"5220","AL445426.1",24.6748841592522,0.303685587144746,0.3330743212273
61,0.91176523613613,0.361892314262404,0.980372848261233
"5221","PHTF1",18.4616179069705,-0.247852120312066,0.340872720998776,-
0.727110458079032,0.467158292707216,0.980372848261233
"5222","PTPN22",8.46354547006967,0.446507057271574,0.536055263539072,0
.832949674486367,0.404873112475602,0.980372848261233
"5223","AP4B1",13.7080368302882,0.406192494526541,0.447824077640686,0.
90703585360243,0.364387837600746,0.980372848261233
"5224","TRIM33",64.1569308918221,0.177421542874266,0.239553615781901,0
.740633959104159,0.458915411774871,0.980372848261233
"5225","CSDE1",440.214639074677,-0.133588438033649,0.162632217377422,-
0.821414355580169,0.411410289284668,0.980372848261233
"5226","TTF2",29.1443653690348,0.264869383963913,0.305080151198035,0.8
68196055770211,0.385287013106305,0.980372848261233
"5227","WDR3",35.8436051228321,-0.231946983008225,0.309305200865524,-0
.74989680858638,0.453316856753314,0.980372848261233
"5228","SEC22B",149.519239872326,0.15292375693604,0.208596820774018,0.
733106843951897,0.463493266229249,0.980372848261233

"5229","LINC00623",69.830270948992,-0.185712013943431,0.25430814846761
,-0.730263717708142,0.46522900139893,0.980372848261233
"5230","NBPF15",12.7085338762763,0.349740389166613,0.468967153194457,0
.745767345930926,0.455807968803248,0.980372848261233
"5231","NBPF10",9.55537527008231,0.529383698569056,0.602885635191725,0
.878083118369052,0.379898613592867,0.980372848261233
"5232","AC245407.2",13.9308798656001,-0.360320334790996,0.450010373386
086,-0.80069339753166,0.423309165790108,0.980372848261233
"5233","AC245297.3",150.030834802139,-0.193630043608416,0.263968814926
492,-0.733533783762816,0.463232930426233,0.980372848261233
"5234","NBPF19",41.9305093298671,0.387046672644429,0.474794332343533,0
.815188064132967,0.414964668982915,0.980372848261233
"5235","HIST2H2BE",5.16966852222331,0.488555396201365,0.67355747387393
,0.72533586984265,0.468246005308078,0.980372848261233
"5236","HIST2H2AC",31.9917579325132,0.272662598527041,0.34843228639689
,0.782541139762401,0.433896617718337,0.980372848261233
"5237","CIART",2.74797466016549,0.831375870024725,0.958373601542514,0.
867486196079082,0.385675671916693,0.980372848261233
"5238","TARS2",20.6899960488733,-0.26671496541244,0.335208344347278,-0
.795669230525245,0.426224315657508,0.980372848261233
"5239","CTSS",173.939800654529,-0.182750660434938,0.249249840969557,-0
.733202716294847,0.463434798893971,0.980372848261233
"5240","PSMD4",203.236428084962,-0.230447107558727,0.25529933221998,-0
.902654564564863,0.366709257579113,0.980372848261233
"5241","SELENBP1",178.972979292637,0.209688152433335,0.264854263439868
,0.791711448061859,0.428528945588265,0.980372848261233
"5242","SNX27",119.550959241128,0.147680627417126,0.198863487803754,0.
742623138355407,0.457709875826947,0.980372848261233
"5243","THEM4",38.8905274074734,-0.237520145709958,0.29567181382654,-0
.803323599351619,0.42178772114242,0.980372848261233
"5244","GATAD2B",63.2991294498444,0.223684726933057,0.276797826498912,
0.808115908142568,0.419023860603325,0.980372848261233
"5245","HAX1",151.209325827396,-0.227288449242794,0.255926541047104,-0
.888100344391251,0.374486777428888,0.980372848261233
"5246","TDRD10",5.88555765797848,0.450389053623312,0.592654690870803,0
.759951891988813,0.44728334171497,0.980372848261233
"5247","AL451085.2",6.86763868656102,-0.438622286820101,0.561375737380
075,-0.781334599295543,0.434605725570089,0.980372848261233
"5248","ZBTB7B",13.5014845221755,0.332735533087726,0.43623366007872,0.
762746123322264,0.445614817705773,0.980372848261233
"5249","EFNA1",2.42125397455914,-0.944858847249841,1.06751786024207,-0
.885098865732874,0.376103311169445,0.980372848261233
"5250","CLK2",18.83559818402,0.332665993754763,0.369277112401066,0.900
857330668953,0.367664180632721,0.980372848261233
"5251","FDPS",132.343241733201,-0.218975613651659,0.280003377581189,-0
.782046329381028,0.434187346706498,0.980372848261233
"5252","ASH1L-
AS1",5.69934077715128,-0.532282316550718,0.630863174336641,-0.84373654
7327269,0.398816639957358,0.980372848261233
"5253","RIT1",28.3675267456093,-0.256278524342941,0.299716422285067,-0

.855070010475399,0.392512390760252,0.980372848261233
"5254","ARHGEF2",41.0442874694202,-0.217561070542733,0.278014696508627
,-0.78255240918885,0.433889997605894,0.980372848261233
"5255","SSR2",744.042295219058,-0.210279813853443,0.247769659117359,-0
.848690733976596,0.396053403558532,0.980372848261233
"5256","TMEM79",5.61061032040898,0.478515208252256,0.656345878058711,0
.729059516100826,0.46596525721999,0.980372848261233
"5257","CCT3",272.622081077118,-0.262345684665347,0.329622289815911,-0
.795897889101684,0.426091388290285,0.980372848261233
"5258","GPATCH4",105.512473368409,-0.301636206866922,0.342416501109564
,-0.880904412870007,0.378369558814048,0.980372848261233
"5259","PEX19",51.2951449023347,-0.22527812989513,0.304051108783126,-0
.740921915386983,0.458740786683206,0.980372848261233
"5260","PFDN2",192.108034212906,-0.252194412745402,0.298841133128827,-
0.843907965764149,0.398720836553669,0.980372848261233
"5261","PPOX",14.650808579756,0.304092276106246,0.388637421381661,0.78
2457528215256,0.433945736313223,0.980372848261233
"5262","TOMM40L",11.4117684949042,-0.427569112882489,0.530140952019742
,-0.80651968359269,0.419943263158135,0.980372848261233
"5263","FCGR3A",10.1257307402213,0.595269113044209,0.657284806577294,0
.905648673280581,0.365121837509423,0.980372848261233
"5264","AL596325.2",11.8169594631858,0.514672655639533,0.6572024967222
31,0.783126446120397,0.433552862555792,0.980372848261233
"5265","GPR161",22.9734339512881,0.319291519929005,0.3939382474417,0.8
10511601761285,0.417646199803991,0.980372848261233
"5266","TBX19",7.47868783904949,0.480310460790618,0.585021113741327,0.
821013890796072,0.411638355991483,0.980372848261233
"5267","SELL",8.2710938477286,0.583838207783258,0.690443188420219,0.84
5599200014007,0.397776371985828,0.980372848261233
"5268","SCYL3",17.6169869027191,0.373014538421695,0.418400548224422,0.
891524975300983,0.372647599005069,0.980372848261233
"5269","KIFAP3",121.822338340056,0.162447134585755,0.195606486103979,0
.830479284308611,0.406267855499744,0.980372848261233
"5270","PRRX1",1433.81642628745,0.140870147850067,0.190397207780028,0.
739875072184979,0.459375801354021,0.980372848261233
"5271","DNM3",2.51853669897017,-0.802356650590014,0.930160909376021,-0
.86259983891202,0.388357518970053,0.980372848261233
"5272","TNFSF4",5.85739329342536,0.558939532668647,0.718087858631269,0
.778372069587737,0.436349699586989,0.980372848261233
"5273","GAS5",688.53191468265,-0.156830335133526,0.180575995974715,-0.
868500457588425,0.385120421907832,0.980372848261233
"5274","Z99127.4",8.43960773801237,-0.429929900832543,0.59287291012002
7,-0.725163679253795,0.468351622057127,0.980372848261233
"5275","SEC16B",5.19452230758419,0.529871722584989,0.717664665403398,0
.738327728991854,0.460315320156729,0.980372848261233
"5276","TOR3A",54.8022903563891,-0.240682234493359,0.283784120581686,-
0.848117343563905,0.396372623638326,0.980372848261233
"5277","TOR1AIP2",119.622947641629,0.163231844322426,0.196222108328373
,0.831872849155514,0.40548071860644,0.980372848261233
"5278","CEP350",114.316410413225,0.194387383817732,0.24467804695883,0.

794461890773717,0.426926586164865,0.980372848261233
"5279","LINC01686",3.79648042712243,-0.701684467044964,0.757915569371768,-0.925808223766385,0.354545652636765,0.980372848261233
"5280","SMG7",35.2274687920714,0.231685594850559,0.316654971133641,0.731665743383446,0.464372610207288,0.980372848261233
"5281","COLGALT2",4.16748793807567,-0.608542374122515,0.805980360643268,-0.755033749999598,0.45022873203723,0.980372848261233
"5282","RNF2",26.4138370828923,-0.24746581474384,0.319838629174367,-0.773720845986143,0.439095889765775,0.980372848261233
"5283","AL096803.2",6.66214624295656,0.52779930285238,0.634180477875471,0.832254100000883,0.405265532906086,0.980372848261233
"5284","PTPRC",122.830769142092,0.313223604301408,0.364884130168774,0.85841936769497,0.390660936819097,0.980372848261233
"5285","IPO9",57.3620657372106,0.235412981333708,0.261140948272642,0.901478618695703,0.367333897068622,0.980372848261233
"5286","ARL8A",75.8219070731084,-0.188774222259507,0.25012904478966,-0.754707324846068,0.450424610536425,0.980372848261233
"5287","PTPN7",18.1107992042876,0.42722997917269,0.459797922617281,0.929169006986359,0.352801506591125,0.980372848261233
"5288","PIK3C2B",11.8187466762467,-0.36166721936323,0.424966760385583,-0.851048253833029,0.394742548470981,0.980372848261233
"5289","TMCC2",2.12203558010834,-0.772469359402445,1.01175373921144,-0.763495433191584,0.445167984856742,0.980372848261233
"5290","ELK4",72.1331353740225,0.21087347392002,0.248046839685978,0.850135700930441,0.395249644981223,0.980372848261233
"5291","FAM72A",4.06453668520496,0.765555282981275,0.910957440390758,0.840385345173632,0.400692363221488,0.980372848261233
"5292","PFKFB2",6.02617007525649,0.494105728073864,0.58253174641208,0.848203949599574,0.396324398042437,0.980372848261233
"5293","CR1",2.22928548579882,0.845891729477134,0.935327003400705,0.904380742137885,0.365793546135471,0.980372848261233
"5294","NSL1",68.6290385475253,-0.180377202091743,0.230194974320202,-0.783584448897818,0.433283983676932,0.980372848261233
"5295","SPATA17",2.24949264721919,-0.706016002816939,0.952330876012035,-0.741355783583789,0.458477746701928,0.980372848261233
"5296","AIDA",196.064004819783,0.138977763738487,0.185876029547767,0.747690619799754,0.454646790386236,0.980372848261233
"5297","NVL",20.2143684454764,0.323818151287629,0.354251635684768,0.914090772401626,0.360669150801325,0.980372848261233
"5298","DNAH14",9.54174922161436,-0.367415206270549,0.491716371955716,-0.747209625762957,0.454937034596392,0.980372848261233
"5299","TMEM63A",27.3282791691071,0.303227401883895,0.355819128089824,0.852195337310104,0.394105684119398,0.980372848261233
"5300","ITPKB",17.4649926672551,0.377056954816423,0.443037232717653,0.851072837611192,0.394728892956623,0.980372848261233
"5301","SNAP47",34.8990018022812,-0.217619922768577,0.281263491296487,-0.773722610657557,0.439094845983941,0.980372848261233
"5302","C1orf35",54.7862197969822,-0.212324145302111,0.273250884351228,-0.777030038919093,0.437141047984694,0.980372848261233
"5303","GUK1",782.614094638485,-0.208832910800736,0.281106348382962,-0

.742896458945262,0.457544369733138,0.980372848261233
"5304","AL353593.1",2.32302234737513,0.842826706582769,0.972873791161804,0.866326870185564,0.386310934959494,0.980372848261233
"5305","AL162595.1",2.93122200755291,0.787381847733358,0.863609249797035,0.911733921236264,0.361908802693983,0.980372848261233
"5306","TTC13",9.69685827810899,0.435270152622962,0.47863863472684,0.909392015275516,0.363143235082143,0.980372848261233
"5307","EXOC8",16.1861445806585,0.299557603994441,0.405414551544753,0.738892086761649,0.459972527852301,0.980372848261233
"5308","HEATR1",45.4055025741358,0.246023902919204,0.288941458023103,0.851466261029016,0.394510397514666,0.980372848261233
"5309","LINC01139",3.66691326805986,-0.800360294191413,0.926979711200566,-0.863406485083515,0.38791401509704,0.980372848261233
"5310","ACP1",129.185136329468,-0.210269808832232,0.23872831532743,-0.880791239798406,0.378430822082561,0.980372848261233
"5311","TMEM18",109.015267013618,-0.174473367316205,0.21779697527168,-0.801082600428988,0.423083828432542,0.980372848261233
"5312","GREB1",4.34155899980612,0.558811044535382,0.705579814002884,0.791988423485564,0.428367426348872,0.980372848261233
"5313","SMC6",51.7689543162251,-0.212367911373535,0.251418046078224,-0.844680462226887,0.398289270874553,0.980372848261233
"5314","GEN1",5.19554448711764,0.612475808959546,0.683575319186632,0.895988769296578,0.37025876141654,0.980372848261233
"5315","SDC1",2.00962182022529,0.929093103070463,1.23099789834047,0.754747919816101,0.450400248015505,0.980372848261233
"5316","ATAD2B",45.8335146015725,0.26190461731973,0.359021177667015,0.729496290501952,0.465698136061994,0.980372848261233
"5317","FAM228A",1.91638143273621,0.868010092386776,1.16221825676157,0.74685635622815,0.455150273003187,0.980372848261233
"5318","POMC",5.08492416550612,-0.615509194760307,0.748614774593281,-0.822197498165476,0.41096450243197,0.980372848261233
"5319","DNMT3A",29.7001651711956,0.296556974700935,0.336534485045977,0.881208279919425,0.378205098585038,0.980372848261233
"5320","TMEM214",70.353486835965,0.206087713638913,0.259613864184408,0.793823990434217,0.427297904298975,0.980372848261233
"5321","PREB",88.5969781375361,0.252497456519684,0.33633895284645,0.750723204620778,0.452819254932275,0.980372848261233
"5322","ZNF513",12.9851375909087,0.337771771492214,0.410567250892582,0.822695358087842,0.410681255707,0.980372848261233
"5323","ZNF512",21.9588133188813,0.330114670248407,0.369471066219429,0.893479085185934,0.371600667545681,0.980372848261233
"5324","GPN1",37.8676331573628,-0.239403552347918,0.284310849240811,-0.842048599225784,0.399760750818649,0.980372848261233
"5325","AC104695.4",21.5322342965819,0.31926028439092,0.388174738979022,0.822465380489824,0.410812082115557,0.980372848261233
"5326","FEZ2",169.256917030647,0.17682943392546,0.200738813538754,0.88089309091847,0.378375687380084,0.980372848261233
"5327","PRKD3",65.0426323174537,0.206544916152739,0.260703333504669,0.792260357303948,0.428208881605951,0.980372848261233
"5328","MTA3",33.8141610742263,0.229770032846656,0.294483268392741,0.7

80248175391139,0.435244810778997,0.980372848261233
"5329","LRPPRC",89.1100441378367,0.190620284442789,0.257001603098081,0.741708542456218,0.458263942978649,0.980372848261233
"5330","LINC01119",3.62022300676839,-0.714866472623716,0.823967355595132,-0.867590770155434,0.385618401006329,0.980372848261233
"5331","MCFD2",157.705597016832,0.140520312522762,0.19085393171265,0.736271510163749,0.461565480700734,0.980372848261233
"5332","TTC7A",31.0481794428929,0.242292729123903,0.32796386854713,0.738778726440972,0.46004137172266,0.980372848261233
"5333","PSME4",41.2406286422959,0.260119103386846,0.324508248483154,0.801579326882191,0.422796340045758,0.980372848261233
"5334","RTN4",1382.37426081889,-0.13646077799758,0.17425685867414,-0.783101331195014,0.433567609520399,0.980372848261233
"5335","VRK2",31.2184861893406,0.290043172174271,0.336284108495281,0.862494435053988,0.388415494098844,0.980372848261233
"5336","KIAA1841",14.2000508611474,-0.311995253486714,0.398085285886756,-0.78373972751022,0.433192846435734,0.980372848261233
"5337","CCT4",238.625586683597,-0.186024149145305,0.227368462899441,-0.818161616492865,0.413264909184361,0.980372848261233
"5338","MDH1",200.641255893578,-0.23475930500473,0.321808821444461,-0.729499284547258,0.465696305266525,0.980372848261233
"5339","UGP2",522.400271937828,0.248428066874837,0.27555806563822,0.901545256167526,0.367298482834967,0.980372848261233
"5340","RAB1A",266.507173480682,0.165820617508478,0.197959845447582,0.837647741811288,0.402228571198322,0.980372848261233
"5341","MXD1",28.624145853104,0.419217229351689,0.455848251952406,0.919642068508927,0.357759835215643,0.980372848261233
"5342","PCBP1",505.841565669382,-0.13122381543561,0.166125266491301,-0.789908833298886,0.429581012287122,0.980372848261233
"5343","FAM136A",58.1455829562902,-0.206374905537052,0.247314782522474,-0.834462475037448,0.404020424579929,0.980372848261233
"5344","TEX261",61.3840582537681,0.205475417984486,0.244913579098839,0.838971112751418,0.401485520125825,0.980372848261233
"5345","AC007878.1",3.32456680028101,-0.773543958739032,0.896354124830183,-0.862989233061857,0.388143386785455,0.980372848261233
"5346","EXOC6B",24.8484594072719,0.254540223616051,0.328715425300518,0.77434827825115,0.438724860994551,0.980372848261233
"5347","CCT7",263.605545500228,-0.203618292548625,0.281190319584451,-0.724129809481124,0.468986045280251,0.980372848261233
"5348","DGUOK",147.804435498871,-0.239400518251621,0.264529920435649,-0.905003554446153,0.365463504139993,0.980372848261233
"5349","AC073263.2",2.58238821533186,0.83523525385804,0.915477989641753,0.91234880937431,0.361585127902865,0.980372848261233
"5350","AC005041.4",2.66795440141073,0.65941648643909,0.865586617692777,0.761814557850681,0.446170690481339,0.980372848261233
"5351","RTKN",15.3166242261556,-0.405398048618775,0.46707846536647,-0.867944207833901,0.385424876217898,0.980372848261233
"5352","TTC31",16.577522921452,-0.28522039930993,0.39091576016393,-0.729621131648219,0.465621801730598,0.980372848261233
"5353","LOXL3",8.97493000668675,-0.393198858311702,0.52766116082265,-0

.745173015384883,0.456167133620854,0.980372848261233
"5354","POLE4",204.082548006821,-0.194533371474084,0.256239361273219,-0.759186139504385,0.447741216193554,0.980372848261233
"5355","RETSAT",26.6139609763009,0.250071520907565,0.30908994576402,0.809057442128794,0.418482106142564,0.980372848261233
"5356","ELMOD3",29.360287347861,0.251591616006757,0.346103662972943,0.726925609066511,0.467271528281791,0.980372848261233
"5357","PARTICL",4.49434230570437,-0.648078731789043,0.787866113189324,-0.822574700116985,0.410749890823593,0.980372848261233
"5358","C2orf68",40.8299274470607,0.292808255217167,0.323217488630192,0.905917116236808,0.364979724048599,0.980372848261233
"5359","GNLY",76.4746154958034,0.950585691292874,1.04658899721588,0.908270289312809,0.363735435678556,0.980372848261233
"5360","RMND5A",42.7172015725137,0.24590342519203,0.308924838816533,0.795997583535422,0.426033439940489,0.980372848261233
"5361","LINC01943",8.81117348583274,-0.425167797147126,0.487094831314182,-0.872864522089103,0.382736925939033,0.980372848261233
"5362","PLGLB2",2.93822784094697,0.72432482343528,0.95909990144391,0.755213114238486,0.450121121180635,0.980372848261233
"5363","MRPS5",103.52947341319,0.18437214876398,0.238086255426289,0.774392240467075,0.438698870924757,0.980372848261233
"5364","FAHD2A",48.9364306770999,0.229950117509987,0.286638584237723,0.802230160749325,0.422419832742354,0.980372848261233
"5365","ANKRD36C",68.8640710608503,-0.34970107081529,0.399488396154965,-0.875372286607387,0.38137137383421,0.980372848261233
"5366","SNRNP200",117.439259441565,0.174392848521628,0.230329987513814,0.757143480985816,0.448963906730299,0.980372848261233
"5367","ARID5A",41.5156325943043,-0.284359537366016,0.359942384912774,-0.790014039149421,0.429519569403357,0.980372848261233
"5368","KANSL3",25.7468432585185,0.287958280627864,0.335145166907197,0.85920463447292,0.390227626944149,0.980372848261233
"5369","AC159540.2",6.02156143486214,-0.599220065935104,0.671286269817708,-0.892644603170308,0.372047525203273,0.980372848261233
"5370","COX5B",536.08712740353,-0.194597399618879,0.249527416251108,-0.779863802312808,0.435471046758397,0.980372848261233
"5371","UNC50",125.017349030016,-0.239176661662582,0.283032959973963,-0.84504879461595,0.398083596073203,0.980372848261233
"5372","CHST10",8.31718769505688,-0.36910090080133,0.502456275798528,-0.734593075217812,0.462587356588966,0.980372848261233
"5373","RPL31",1836.28256218876,0.139082602287896,0.173657267358629,0.800902861155062,0.423187883608043,0.980372848261233
"5374","IL18R1",9.32413814796856,0.383863499712688,0.514404930846176,0.746228266282843,0.455529535781027,0.980372848261233
"5375","MFSD9",9.61488725141306,0.442092223860725,0.51940700250599,0.851147985553421,0.39468715241346,0.980372848261233
"5376","ST6GAL2",2.46806398859039,-0.725323894035113,0.962917157739569,-0.753256797020627,0.451295614843783,0.980372848261233
"5377","PAX8-AS1",39.6787730610796,-0.816261937820469,0.945278693575488,-0.863514583972039,0.387854604474409,0.980372848261233

"5378","CBWD2",18.0257539639892,-0.431694783843912,0.471577556509059,-0.915426906741731,0.359967558357599,0.980372848261233
"5379","INSIG2",62.9239671212783,0.178425070208452,0.243617066018448,0.732399717000701,0.463924631473495,0.980372848261233
"5380","STEAP3",2.15693464284984,0.858727085091094,1.06454503935822,0.806661111876297,0.419861754653091,0.980372848261233
"5381","C2orf76",40.7212909831154,-0.222084751921013,0.279357397619933,-0.794984324070633,0.426622620865302,0.980372848261233
"5382","POLR2D",36.4025501681243,-0.240427567811378,0.321861218125537,-0.746991418262772,0.455068741035168,0.980372848261233
"5383","MZT2B",614.443145216263,-0.17676877648292,0.243210751012856,-0.726813168195746,0.467340415219954,0.980372848261233
"5384","PTPN18",47.1151500578812,0.222007464457756,0.261580092118458,0.848716974827042,0.396038798369117,0.980372848261233
"5385","MZT2A",215.825404855233,-0.180320299314408,0.236196733396346,-0.763432655149487,0.445205411219329,0.980372848261233
"5386","CXCR4",225.560332901557,0.288436531235783,0.373055249202721,0.773173764079766,0.439419550808633,0.980372848261233
"5387","ZEB2-AS1",2.82933675970556,-0.727183730365316,0.820155536834912,-0.886641249989745,0.375272077901306,0.980372848261233
"5388","EPC2",64.7807769113926,0.220453862489626,0.267536821436006,0.824013163146436,0.409932078683578,0.980372848261233
"5389","NMI",84.673158557498,-0.221291302651784,0.262661850636337,-0.842495025888508,0.399510922769094,0.980372848261233
"5390","TNFAIP6",1217.98060348982,0.558012965843827,0.758704131091364,0.735481649534646,0.462046211755531,0.980372848261233
"5391","NEB",2.53949123957528,0.851888748895969,1.17603856699226,0.724371438833582,0.468837729431414,0.980372848261233
"5392","ARL5A",196.73759456982,-0.149073885245254,0.181012741173029,-0.823554652999564,0.410192651175646,0.980372848261233
"5393","CACNB4",56.7641988940433,0.319872183079157,0.402855556008149,0.794012092693308,0.427188391495981,0.980372848261233
"5394","PRPF40A",236.615009407575,0.144604706346871,0.185271982365529,0.780499590389095,0.435096868543055,0.980372848261233
"5395","NR4A2",93.8159630815394,-0.401419254098888,0.451597088824521,-0.888888046518983,0.37406325025285,0.980372848261233
"5396","RBMS1",586.086787037994,0.173904413440853,0.198246670251743,0.877212279126913,0.380371347795628,0.980372848261233
"5397","CERS6",8.91693940784223,0.458962513254687,0.542840196378529,0.845483654888827,0.397840854858095,0.980372848261233
"5398","PHOSPHO2",9.07838695277584,-0.387537088646993,0.478615706207563,-0.809704077030284,0.418110274240812,0.980372848261233
"5399","DYNC1I2",279.266583754944,0.179257194464492,0.218832516261645,0.819152462014215,0.412699433327901,0.980372848261233
"5400","RAPGEF4",1.98864088273391,0.959992241265273,1.09818523793997,0.874162398199849,0.382029820214308,0.980372848261233
"5401","ATF2",56.6285401527489,0.219210363380837,0.260110369809524,0.842759031642464,0.399363224807286,0.980372848261233
"5402","TTC30A",4.50639223764344,-0.537237875080247,0.645735794314284,

-0.831977845754622,0.405421449587959,0.980372848261233
"5403","PJVK",5.49334294535593,0.492889376590616,0.584305081538342,0.843547989165097,0.39892203852228,0.980372848261233
"5404","LINC01934",2.40554831821434,-0.859578797491313,1.01458319397917,-0.847223571800026,0.396870517278605,0.980372848261233
"5405","DNAJC10",111.959117192538,-0.142118925574744,0.190336447376543,-0.746672156245459,0.455261480995148,0.980372848261233
"5406","COL3A1",5975.57872626279,0.305323968967734,0.402465157045817,0.758634539220436,0.448071205368615,0.980372848261233
"5407","COL5A2",579.00289157549,0.247847653992581,0.304467949041358,0.814035286055393,0.415624736788814,0.980372848261233
"5408","WDR75",38.6433521228221,0.201064279735462,0.260306650997505,0.772413147973652,0.439869769094842,0.980372848261233
"5409","ANKRD44",27.429416030311,0.327905731826849,0.388218419869648,0.844642384400385,0.39831053697589,0.980372848261233
"5410","COQ10B",160.897786204832,-0.177900185081488,0.201005519392815,-0.885051244457751,0.376128993656922,0.980372848261233
"5411","CASP10",5.98647560884223,0.478395317959848,0.610348956052509,0.783806236114362,0.433153814119334,0.980372848261233
"5412","AC064836.3",4.93353622631,-0.493675780248106,0.635656518585953,-0.776639216012935,0.437371657796873,0.980372848261233
"5413","BMPR2",143.325435796788,0.219493630620105,0.24274236369583,0.904224657279612,0.365876288090014,0.980372848261233
"5414","ICOS",4.77516879053309,0.566569166903274,0.686588281355932,0.825194927277766,0.409260932924105,0.980372848261233
"5415","NDUFS1",100.88837012882,0.172432315784945,0.203730884578907,0.846372979439746,0.397344707228807,0.980372848261233
"5416","FASTKD2",53.2251904057876,-0.240567601394853,0.272121783834636,-0.884043893895101,0.376672518489153,0.980372848261233
"5417","PIKFYVE",34.4169046866206,0.270795913357059,0.325986087871838,0.83069776113735,0.406144391457019,0.980372848261233
"5418","PECR",6.98544748297004,-0.493572890591726,0.583643138490892,-0.845675821475333,0.397733614920226,0.980372848261233
"5419","CNOT9",41.1414493501348,-0.23856954780476,0.261019828062501,-0.913990134679099,0.360722029513141,0.980372848261233
"5420","TTLL4",6.89454789017793,-0.424863448234004,0.55792149353896,-0.761511168066042,0.44635181085971,0.980372848261233
"5421","GLB1L",17.8048205186061,-0.433721669999177,0.501283933351251,-0.865221566347843,0.386917190559007,0.980372848261233
"5422","STK16",45.3382682331626,-0.218082230177407,0.296689369124599,-0.73505239106097,0.462307587521557,0.980372848261233
"5423","TMEM198",3.72763983731195,-0.642752662007609,0.753849294886338,-0.852627529623835,0.393865890968818,0.980372848261233
"5424","RHBDD1",18.658968794402,0.272768951126839,0.345196982889571,0.790183473921317,0.429420625935968,0.980372848261233
"5425","COL4A4",20.1854029339452,-0.928618295231515,1.26948094445293,-0.731494473618658,0.464477178858438,0.980372848261233
"5426","AC097662.1",3.41473264544421,0.710879265173467,0.813715137187911,0.873621778292302,0.382324262029364,0.980372848261233
"5427","SLC19A3",6.27280634493069,0.638080648418957,0.809471020606723,

0.788268674449514,0.430539566815179,0.980372848261233
"5428","TRIP12",119.591261661394,0.175267141848667,0.241791644316883,0
.72486848064513,0.468532719069395,0.980372848261233
"5429","DGKD",19.3201472982945,0.328941127627272,0.409107122456762,0.8
04046445468663,0.42137015197876,0.980372848261233
"5430","ACKR3",671.011955087718,0.281541830529931,0.341957085273788,0.
823325038884673,0.410323178448885,0.980372848261233
"5431","AC012076.1",3.33965901961448,-0.760972598106956,0.906631518220
147,-0.83934055105525,0.401278233781152,0.980372848261233
"5432","ASB1",33.0274025156042,0.219773753661286,0.279146858282711,0.7
87305130400951,0.431103267119479,0.980372848261233
"5433","TWIST2",227.644019967036,0.20342849203022,0.240919786986869,0.
8443826660087,0.398455605451867,0.980372848261233
"5434","CAPN10-
DT",2.54410671040963,-0.692982992662288,0.876711311049032,-0.790434643
569383,0.429273976779745,0.980372848261233
"5435","PASK",2.9167901580129,-0.716275500546295,0.896791020932186,-0.
798709491762918,0.424458881726574,0.980372848261233
"5436","ITPR1",10.7954876282851,0.392932916773331,0.494634704344597,0.
794390109149289,0.426968360444428,0.980372848261233
"5437","RPUSD3",30.1505038701876,-0.238900556910697,0.293063934892431,
-0.815182383319822,0.41496792023297,0.980372848261233
"5438","BRK1",486.281391641092,-0.158579610649677,0.205464090025984,-0
.771811807258498,0.440225897548217,0.980372848261233
"5439","HRH1",38.9947076562765,-0.314759087037611,0.400290701117197,-0
.786326252793608,0.431676376037556,0.980372848261233
"5440","ATG7",27.1797069541313,-0.248277120677311,0.290417837635016,-0
.854896251205252,0.392608586147449,0.980372848261233
"5441","NR2C2",35.9657483148695,0.266580996276189,0.346820289039362,0.
768643025512079,0.442105256146688,0.980372848261233
"5442","SH3BP5-
AS1",6.12298879917565,0.61311252953312,0.667094183805293,0.91907941099
9739,0.358054037529083,0.980372848261233
"5443","DPH3",73.7228318160391,-0.20321299286527,0.280547841383776,-0.
724343455515255,0.468854904697837,0.980372848261233
"5444","NGLY1",46.4284328144715,-0.217276090800544,0.275741759290118,-
0.787969480429475,0.430714557906384,0.980372848261233
"5445","AC096921.2",11.7999258358878,0.342014141571716,0.4634116426102
56,0.738035280350867,0.460493010385015,0.980372848261233
"5446","CNOT10",24.2763439662983,-0.266598673127361,0.329475318740587,
-0.809161287547817,0.418422379206861,0.980372848261233
"5447","EPM2AIP1",77.2542511733142,0.192879126036389,0.241223355447768
,0.799587277435716,0.42394996112219,0.980372848261233
"5448","ACVR2B",10.5004914924698,0.360050385104624,0.472558153047167,0
.761917623858425,0.446109170728241,0.980372848261233
"5449","SEC22C",70.7817221356598,-0.178690524755899,0.235808873370608,
-0.757776932656225,0.448584533929528,0.980372848261233
"5450","ZBTB47",42.9919647315061,0.257165980897904,0.282073842748866,0
.911697371127256,0.361928048248622,0.980372848261233
"5451","ZNF501",6.70038593083772,0.611889086198493,0.688647686559409,0

.888537198542823,0.374251855576471,0.980372848261233
"5452","ZDHHC3",90.7439318091246,0.15167181979379,0.207138894378543,0.732222793062767,0.464032594483733,0.980372848261233
"5453","CCR5",2.53932057004063,0.935556335344019,1.00642935010926,0.92957974172996,0.352588720393201,0.980372848261233
"5454","LRRC2",26.7028822726413,-0.413188581582302,0.494879945372443,-0.834926905901066,0.403758864570636,0.980372848261233
"5455","NBEAL2",7.56023851980887,0.4747941711496,0.654553096107244,0.725371515272472,0.468224143075668,0.980372848261233
"5456","ZNF589",10.1247901916507,0.389188734299008,0.474098555431082,0.820902594704452,0.411701752993337,0.980372848261233
"5457","TMA7",869.477104450535,-0.180267104401212,0.231922487537541,-0.777273072202763,0.436997678546881,0.980372848261233
"5458","UQCRC1",114.106598884089,-0.138030213630752,0.190595012150946,-0.724206851338985,0.468938752973014,0.980372848261233
"5459","WDR6",40.468153069746,0.267337427132964,0.311599208795547,0.857952843225526,0.390918503185714,0.980372848261233
"5460","DALRD3",69.8647988933481,-0.189137167872147,0.252332212539022,-0.749556174255387,0.453522054328839,0.980372848261233
"5461","IMPDH2",237.566167529672,-0.170195506652515,0.19511048246561,-0.872303243279171,0.383042968780678,0.980372848261233
"5462","QRICH1",51.236195508714,0.199367087829609,0.250836030937173,0.79481040696081,0.426723796356069,0.980372848261233
"5463","AC121247.1",3.4540162797677,0.744157143188814,0.98564843898118,0.754992463598903,0.450253504171824,0.980372848261233
"5464","NICN1",10.6668180193523,0.404834515201687,0.454656924220532,0.890417573417008,0.37324170968782,0.980372848261233
"5465","HYAL1",3.87734022720548,-0.674964247175513,0.838209020328198,-0.80524574516179,0.420677883376933,0.980372848261233
"5466","HEMK1",22.5336395396501,0.338400520729973,0.377918739184449,0.895431968947197,0.370556217575304,0.980372848261233
"5467","CISH",14.857813364107,-0.362962195514513,0.456752350004718,-0.794658627395708,0.426812104859914,0.980372848261233
"5468","RAD54L2",31.1097244473665,-0.233157219102347,0.313909202363722,-0.742753692299185,0.457630816218161,0.980372848261233
"5469","NT5DC2",26.209784542541,-0.316041231831544,0.376137975143196,-0.840226865450708,0.400781197991344,0.980372848261233
"5470","PRKCD",13.7430458770424,-0.378766071278493,0.420573521975173,-0.900594192187048,0.367804123694587,0.980372848261233
"5471","DCP1A",86.4569463605493,0.19102524116658,0.24948880609859,0.76566658101323,0.443874716679436,0.980372848261233
"5472","SELENOK",290.169006029156,-0.188787480038482,0.216441071493053,-0.872235009447094,0.383080184172943,0.980372848261233
"5473","C3orf67",3.14552909058164,-0.598962409899706,0.755965662119814,-0.792314307266479,0.428177431384136,0.980372848261233
"5474","THOC7",218.444862231473,-0.182713789117672,0.226571660497035,-0.806428256370852,0.419995959802601,0.980372848261233
"5475","ATXN7",80.1855347398432,0.32424916472851,0.382812455181416,0.84701832539604,0.396984907194135,0.980372848261233
"5476","PSMD6",90.4353519503277,-0.203009585367643,0.277623985300263,-

0.731239360129778,0.464632962536079,0.980372848261233
"5477","PSMD6-
AS1",1.99655584882096,0.969610650517247,1.06989500029301,0.90626711055
917,0.364794489265185,0.980372848261233
"5478","AC092040.1",34.6751560287674,-0.362426740629434,0.401644811043
532,-0.902356337401189,0.366867607313002,0.980372848261233
"5479","PRICKLE2",65.129964330163,0.23120826323839,0.311586451851344,0
.74203567537878,0.458065720880144,0.980372848261233
"5480","SLC25A26",48.7640128692504,-0.225957461612743,0.28388127234309
8,-0.795957618999435,0.426056669159747,0.980372848261233
"5481","LRIG1",12.740419124699,0.353963529279405,0.482535654463508,0.7
33548963698753,0.463223675631881,0.980372848261233
"5482","ARL6IP5",756.540325499641,-0.13587666766969,0.187213294073863,
-0.72578535804237,0.467970364349901,0.980372848261233
"5483","ROBO1",80.6814803267186,-0.208682460933709,0.25122532077068,-0
.830658551031147,0.40616654793853,0.980372848261233
"5484","CPOX",17.2940096253008,0.303929917288499,0.383624089432854,0.7
92259729408093,0.428209247646814,0.980372848261233
"5485","PCNP",212.426969460058,-0.107883050714403,0.145070286302357,-0
.743660562505213,0.457081854037048,0.980372848261233
"5486","RPL24",4323.74143684693,-0.178866988563594,0.230370620409589,-
0.776431422746428,0.437494297249332,0.980372848261233
"5487","NECTIN3",63.3087697688547,-0.200371865875095,0.247434807936307
,-0.809796598733487,0.418057087768264,0.980372848261233
"5488","C3orf52",2.72959256533294,-0.775607673180162,0.939575707457629
,-0.825487150235989,0.409095074957779,0.980372848261233
"5489","AC078785.1",2.72253754935992,-0.673247929660364,0.855490491591
383,-0.786973012882923,0.431297664333898,0.980372848261233
"5490","TIGIT",5.69433127445855,0.56645643213013,0.759489962318787,0.7
45837944191772,0.455765315557847,0.980372848261233
"5491","ZBTB20-
AS2",5.82991959066143,0.49569380705794,0.609734969067855,0.81296601343
981,0.416237544299958,0.980372848261233
"5492","SEC22A",21.1304164530197,0.29150417527583,0.382023660334173,0.
763052673284264,0.445431983154957,0.980372848261233
"5493","HEG1",122.521912591123,0.265708553904374,0.304078006218554,0.8
73817074798227,0.382217880201057,0.980372848261233
"5494","TXNRD3",15.7647529278292,0.323852074719046,0.384566382841428,0
.842122684583645,0.39971928487538,0.980372848261233
"5495","TPRA1",48.3528425722675,0.21740249101105,0.254925145242283,0.8
52809128752007,0.393765160730299,0.980372848261233
"5496","ABTB1",45.0389431232848,0.22371873205501,0.260831512556837,0.8
57713586299354,0.391050636016967,0.980372848261233
"5497","RPN1",173.395411592637,0.156188036726073,0.190311787813071,0.8
20695546612618,0.411819708109848,0.980372848261233
"5498","CNBP",589.026658577482,-0.150276170824366,0.177346125948485,-0
.847360888323089,0.39679399780741,0.980372848261233
"5499","H1FX-
AS1",6.96307815909026,0.452339996502777,0.573349144461865,0.7889433530
54682,0.430145115645566,0.980372848261233

"5500","PIK3R4",22.0282079704873,0.282776656262126,0.340026215649082,0
.831631924974753,0.405616736291061,0.980372848261233
"5501","DNAJC13",53.8320709712077,0.191243564232156,0.263319250414629,
0.726280224218393,0.467667000105527,0.980372848261233
"5502","ACAD11",20.665409511901,-0.256429957068015,0.350882718149958,-
0.730813869717071,0.464892850623124,0.980372848261233
"5503","MSL2",28.0821918187051,0.290845507111817,0.339429602154216,0.8
56865474507656,0.391519235501884,0.980372848261233
"5504","DBR1",24.0045195341506,0.289792042045463,0.337061544565263,0.8
59760025188374,0.389921339027329,0.980372848261233
"5505","AC046134.2",8.03273078836908,0.535094537293856,0.5874882063969
21,0.91081749636406,0.362391542123229,0.980372848261233
"5506","RBP1",8.40535092170018,0.472500760169869,0.522365326564954,0.9
04540818735057,0.365708700255958,0.980372848261233
"5507","PLSCR1",331.923175087752,0.178551089605254,0.241749804998521,0
.738578008806862,0.460163281945584,0.980372848261233
"5508","ZIC4",6.00710887739471,-0.53621348979276,0.686626715119459,-0.
780938868216728,0.434838450275473,0.980372848261233
"5509","COMMD2",85.6985114915307,-0.199247759440008,0.222831263317503,
-0.894164294873237,0.371233992896305,0.980372848261233
"5510","TSC22D2",140.396793558404,-0.350824477756475,0.451479658969721
,-0.777054892255961,0.437126385338645,0.980372848261233
"5511","MBNL1-
AS1",5.54727936032945,-0.549605250530765,0.664503922248352,-0.82709105
5642191,0.408185453451766,0.980372848261233
"5512","P2RY1",5.18890653078269,-0.595555874323518,0.730540139588574,-
0.815226764485416,0.414942520349663,0.980372848261233
"5513","DHX36",246.627815436615,0.167286638158738,0.192749513737218,0.
867896550892501,0.385450967312395,0.980372848261233
"5514","SLC66A1L",55.4849860732908,0.347097186745379,0.397557376547451
,0.873074447164611,0.382622500723503,0.980372848261233
"5515","RSRC1",142.495084130163,-0.185223582577375,0.222065977925974,-
0.834092571528989,0.404228820834284,0.980372848261233
"5516","B3GALNT1",18.438187874781,0.284868629967083,0.366425742276631,
0.777425265477181,0.436907910953276,0.980372848261233
"5517","ACTL6A",55.0838634973331,-0.236559611891279,0.272388253535341,
-0.868464806470029,0.385139930561095,0.980372848261233
"5518","DNAJC19",165.211416871838,-0.196294931710256,0.250464177472662
,-0.783724577666127,0.43320173780308,0.980372848261233
"5519","B3GNT5",5.39711189453458,-0.698997453543412,0.823575454430991,
-0.848735170266033,0.396028671303214,0.980372848261233
"5520","EIF2B5",42.6604259678092,-0.232320433651363,0.284126789910592,
-0.81766465501007,0.413548697961455,0.980372848261233
"5521","EEF1AKMT4",11.2977686062945,-0.498843005888983,0.6305833754906
53,-0.791081759015351,0.428896282877306,0.980372848261233
"5522","FAM131A",16.0285318294678,-0.305596298785415,0.377673705584179
,-0.809154289183898,0.418426404174537,0.980372848261233
"5523","POLR2H",123.972975757303,-0.233488977421079,0.296431086328109,
-0.787666976204507,0.430891527000831,0.980372848261233
"5524","CHRD",30.8766411851593,0.247200023642955,0.317145300189649,0.7

79453529644399,0.435712601666608,0.980372848261233
"5525","AC107294.2",2.32049935929944,0.975850438358017,1.0938280889395
8,0.892142420025126,0.372316600280899,0.980372848261233
"5526","MAP3K13",48.6241168748434,-0.230259799409861,0.264230942049622
,-0.871433896513976,0.383517284570555,0.980372848261233
"5527","TMEM41A",38.8707187021027,-0.227652268398456,0.27709602251257З
,-0.821564547676342,0.411324773471907,0.980372848261233
"5528","DNAJB11",149.839032608108,-0.30158907370043,0.332434633364006,
-0.907213158414213,0.364294086672663,0.980372848261233
"5529","FGF12",7.69029715793533,0.821745301872613,0.945846788303864,0.
868793246468812,0.38496022771664,0.980372848261233
"5530","ACAP2",137.410642359591,0.15657313055968,0.20051708139827,0.78
0846845903825,0.434892577803873,0.980372848261233
"5531","PPP1R2",138.861265797505,-0.186178087447378,0.211736337907441,
-0.879292091699274,0.379242922704681,0.980372848261233
"5532","TCTEX1D2",29.7856102348683,0.296176199940373,0.387746565828505
,0.763839646928988,0.444962806961702,0.980372848261233
"5533","WDR53",8.14135314768688,-0.4165870845181,0.540185309322605,-0.
771192917187071,0.440592591785457,0.980372848261233
"5534","NCBP2",121.839023097965,-0.189178792796482,0.223274716090039,-
0.847291606095662,0.396832604134509,0.980372848261233
"5535","AC024560.5",8.878664366425,-0.374879910545809,0.50156673240796
1,-0.747417813669691,0.454811395834733,0.980372848261233
"5536","FYTTD1",112.850890471037,0.159440537598166,0.18359367901559,0.
868442412903699,0.385152184856385,0.980372848261233
"5537","PIGG",26.8419297434172,0.23675021766211,0.313377748190042,0.75
5478712287311,0.449961800505957,0.980372848261233
"5538","MYL5",27.3998032871675,0.302371204407099,0.35271288163695,0.85
7272926930784,0.391294067001862,0.980372848261233
"5539","GAK",42.6053220106948,0.245448842177149,0.282799296696985,0.86
7925928543392,0.385434883587271,0.980372848261233
"5540","DGKQ",9.15772900231886,-0.352975690583949,0.475364681471774,-0
.74253663417127,0.457762264425761,0.980372848261233
"5541","UVSSA",19.7802997483084,-0.340841655852966,0.406257174213185,-
0.838980029123394,0.401480516525203,0.980372848261233
"5542","TMEM129",37.631197721297,0.271924276553277,0.294723530844081,0
.922641893487409,0.356193856098328,0.980372848261233
"5543","C4orf48",36.2138346880203,0.304225670279447,0.351853196181332,
0.864638075143873,0.38723746755165,0.980372848261233
"5544","HAUS3",23.8046509886093,-0.267001634672878,0.334032226647008,-
0.79932896700723,0.424099686690612,0.980372848261233
"5545","TBC1D14",22.665663995702,-0.241972711692423,0.322821589482781,
-0.749555542676398,0.453522434839681,0.980372848261233
"5546","AC097382.3",11.5257388141861,-0.378168634196894,0.485786559832
713,-0.778466646601175,0.436293961998458,0.980372848261233
"5547","WDR1",109.292902700519,-0.202716008036185,0.218976749941772,-0
.925742153402539,0.354579995666887,0.980372848261233
"5548","TAPT1-
AS1",2.01565056374445,0.976080371861597,1.16916474660003,0.83485272259
5398,0.403800636613309,0.980372848261233

"5549","PI4K2B",22.9643388883866,-0.228905553061764,0.315865325386273,
-0.724693515446289,0.46864007416491,0.980372848261233
"5550","DTHD1",2.93039022140014,0.758238213332126,0.989189365491717,0.
766524833144777,0.443364081584897,0.980372848261233
"5551","UGDH-
AS1",2.56773735315837,-0.664565004718795,0.870892794847744,-0.76308474
3208812,0.445412858269671,0.980372848261233
"5552","FRYL",45.592600456465,0.217054003923256,0.277651828837904,0.78
1748871713626,0.434362174007887,0.980372848261233
"5553","SGCB",108.21975538362,-0.167684928386571,0.216198282007715,-0.
775607127075076,0.437980990947455,0.980372848261233
"5554","SCFD2",23.3614986953171,-0.358575712100627,0.423881271844724,-
0.845934312077795,0.397589389876944,0.980372848261233
"5555","CEP135",19.9366738639022,-0.261449388173896,0.351926015397574,
-0.742910091140986,0.457536115795822,0.980372848261233
"5556","NOA1",55.2794833482655,-0.237223854022595,0.267005397926656,-0
.888460892044431,0.374292883441642,0.980372848261233
"5557","YTHDC1",144.018761305005,0.188793034149033,0.231325257329715,0
.816136708668783,0.414421950774602,0.980372848261233
"5558","JCHAIN",22.794376178896,-0.484494878680524,0.554456352494017,-
0.873819691128442,0.382216455158014,0.980372848261233
"5559","CXCL8",193.662601384681,-0.91640260265683,1.02986922971973,-0.
889824238079453,0.373560269663317,0.980372848261233
"5560","CXCL10",574.058315512118,-0.805928008457752,1.02279172912381,-
0.787968836185414,0.430714934753125,0.980372848261233
"5561","MRPL1",81.7186788366734,0.209128487467302,0.232009893881685,0.
901377454075075,0.367387664692108,0.980372848261233
"5562","AC124016.1",3.81274885786207,-0.620733044249944,0.777443039217
782,-0.798428968988506,0.424621597857119,0.980372848261233
"5563","ENOPH1",43.9714119704376,-0.217733379190545,0.256756624759828,
-0.848014649648142,0.396429812179678,0.980372848261233
"5564","SEC31A",337.395454225524,0.164611824067823,0.189451162422289,0
.86888790738006,0.384908444410388,0.980372848261233
"5565","HPSE",6.62442623003687,0.527178367150142,0.597592519931954,0.8
82170290903523,0.377684725248241,0.980372848261233
"5566","HELQ",23.1425172701465,-0.295162075638306,0.354843984086436,-0
.831808030783489,0.405517310197831,0.980372848261233
"5567","MAPK10",27.1920654845382,0.310605512357158,0.372834616702602,0
.833091935250524,0.404792881589256,0.980372848261233
"5568","SPARCL1",196.986403159942,-0.537954924802329,0.691900114447823
,-0.777503737272322,0.4368616303756,0.980372848261233
"5569","HERC5",27.9732033856715,0.408081409325187,0.45566589787359,0.8
95571538773341,0.370481642079894,0.980372848261233
"5570","AP002026.1",3.03842887452167,0.709338778766347,0.8733371817300
97,0.812216396605414,0.4166674726619,0.980372848261233
"5571","DAPP1",3.42214794074759,-0.633013224381411,0.7557149632711,-0.
837634895624434,0.402235788160998,0.980372848261233
"5572","H2AFZ",320.322411903209,-0.208213731640215,0.246450885694228,-
0.844848786214328,0.39819527173479,0.980372848261233
"5573","MANBA",36.1189920295117,0.245822804479455,0.315105741227494,0.

780127977109693,0.435315550317118,0.980372848261233
"5574","CASP6",24.2923562043144,0.274579009529984,0.310570711030723,0.884111088964928,0.376636247725017,0.980372848261233
"5575","GAR1",58.7044888333589,-0.21134873281402,0.28810186882995,-0.733590287603331,0.463198482092564,0.980372848261233
"5576","FAM241A",49.2425841521064,-0.283714122912724,0.305005169110527,-0.93019447421205,0.352270402415475,0.980372848261233
"5577","TRAM1L1",6.31268034793003,0.613462953960612,0.662454502808069,0.926045413474002,0.354422379972408,0.980372848261233
"5578","METTL14",33.6755112540046,0.213345474483062,0.282466215599155,0.755295545807214,0.450071670660574,0.980372848261233
"5579","SPRY1",510.606292880372,0.181929410792646,0.250050026485114,0.727572051680932,0.466875594238783,0.980372848261233
"5580","SCLT1",32.8485407075633,-0.238985661126418,0.319783853521214,-0.747334984224161,0.454861380013309,0.980372848261233
"5581","AC107223.1",2.68158768263521,0.795608855625252,0.883792593124654,0.900221230427348,0.368002530107295,0.980372848261233
"5582","ZNF827",29.4332533841411,0.314918940089243,0.401942678589406,0.783492166580648,0.433338151932735,0.980372848261233
"5583","ARHGAP10",118.781029202201,0.18961410497705,0.216962815211863,0.873947477091377,0.382146857627359,0.980372848261233
"5584","ETFDH",44.8537721836059,0.188519061148885,0.246704163915229,0.764150301142316,0.444777679434307,0.980372848261233
"5585","SH3RF1",34.4332159110696,0.251088342918002,0.298830614162116,0.840236344666435,0.400775884146465,0.980372848261233
"5586","SCRG1",2.87933886637121,0.818507059644684,0.893244286260246,0.916330585299945,0.35949353081672,0.980372848261233
"5587","HAND2-AS1",22.6509945218414,0.39245134082319,0.434551524153854,0.903118086140327,0.366463226043719,0.980372848261233
"5588","FBXO8",45.9671470784507,0.232412678529558,0.260961886015598,0.890600087537944,0.373143752237545,0.980372848261233
"5589","SPCS3",155.215731841563,-0.198785271705767,0.250949038172743,-0.792134025112028,0.428282532430155,0.980372848261233
"5590","CDKN2AIP",60.9682085840871,0.230191676043276,0.256457181646678,0.89758327127065,0.369407760872364,0.980372848261233
"5591","TRAPPC11",36.4800371420406,0.237460678992214,0.266216664802505,0.891982773386394,0.372402165910048,0.980372848261233
"5592","PRIMPOL",17.6911944467221,0.344186813429295,0.395197188038795,0.870924247050837,0.383795516576503,0.980372848261233
"5593","CENPU",2.77370767397123,0.62933214945321,0.842039329447192,0.747390445368358,0.454827911141586,0.980372848261233
"5594","FRG1",160.336892715878,-0.210004187208841,0.227653361252423,-0.92247347481941,0.356281659940277,0.980372848261233
"5595","AC116351.2",1.85869857539529,1.003788537026,1.27081614961307,0.789877070205333,0.429599563742545,0.980372848261233
"5596","SRD5A1",6.43531546629776,-0.534148239076596,0.575129302500722,-0.928744608828073,0.353021456610967,0.980372848261233
"5597","ADCY2",2.13327607209925,-0.884633431807004,1.05826002025831,-0.835932015641178,0.403193150117381,0.980372848261233

"5598","MTRR",26.2857234057909,0.278205603555205,0.340434422589284,0.817207617958322,0.413809789792753,0.980372848261233
"5599","SEMA5A",20.10003489304,0.599191423746075,0.667173455663979,0.898104411467859,0.36912988737588,0.980372848261233
"5600","AC010457.1",1.87411276505037,-0.935399315661201,1.06303873979798,-0.879929658856046,0.378897416496059,0.980372848261233
"5601","LINC02104",4.6968284781357,-0.654289188417794,0.773942621695298,-0.845397539916581,0.397888917588225,0.980372848261233
"5602","GHR",95.7798715573845,0.229154629701226,0.295574129424052,0.775286491235721,0.438170389926647,0.980372848261233
"5603","CCDC152",30.2045499395691,0.370825583201563,0.511049664775156,0.725615549253346,0.468074486122666,0.980372848261233
"5604","AC025171.3",2.06029786121556,-0.936908055766747,1.06111898896061,-0.882943445093252,0.377266828734468,0.980372848261233
"5605","FGF10-AS1",3.70098488564373,-0.617825412114176,0.763884161217289,-0.808794635995122,0.418633282124887,0.980372848261233
"5606","SETD9",17.7274476413161,-0.327631824632558,0.393242356956417,-0.833154971321844,0.404757334139165,0.980372848261233
"5607","MIER3",17.1271514095297,0.266583354431025,0.347198882828579,0.767811671106799,0.4425990796589,0.980372848261233
"5608","IPO11",14.6142560811587,0.315283518644209,0.383247386511717,0.822663192863208,0.410699551917114,0.980372848261233
"5609","PPWD1",51.8410602426993,0.238887920107233,0.290896895574667,0.821211651761733,0.411525720753792,0.980372848261233
"5610","SHLD3",4.99297358349645,0.652433004424712,0.744652926446685,0.876157175045258,0.380944593987065,0.980372848261233
"5611","ERBIN",91.0559034733819,0.245351711754273,0.280365423506259,0.875114016150409,0.381511871472374,0.980372848261233
"5612","PTCD2",13.9643949982622,0.328361677961838,0.398183171119794,0.824649813899468,0.409570431654887,0.980372848261233
"5613","BTF3",1733.8764225732,-0.222788766748047,0.240166487942352,-0.927643022375062,0.35359277314734,0.980372848261233
"5614","IQGAP2",21.1249710363652,-0.415864091859139,0.478984902579914,-0.868219623664979,0.385274113437777,0.980372848261233
"5615","ARSB",13.0564888312862,-0.365446015218595,0.410522216695055,-0.890197900032428,0.373359632059584,0.980372848261233
"5616","BHMT2",77.7514332836671,0.202627685127902,0.231847925865827,0.873968073560272,0.382135640664213,0.980372848261233
"5617","CMYA5",42.4240869311863,0.399165520129664,0.471210670583937,0.847106283979111,0.396935882828616,0.980372848261233
"5618","RPS23",7843.76638457456,-0.182458673530069,0.219166567704109,-0.832511433844241,0.405120327061364,0.980372848261233
"5619","VCAN",1683.22207064888,0.208577031057878,0.258457100482131,0.807008322343607,0.419661688386355,0.980372848261233
"5620","RASA1",37.057674772003,0.192939656519273,0.265393476369329,0.726994721794792,0.467229189138947,0.980372848261233
"5621","TMEM161B",24.3507673352976,0.251905004730194,0.334026097421564,0.754147674911377,0.450760552926477,0.980372848261233
"5622","CAST",685.95079474353,-0.157299080098299,0.197106481425419,-0.

798041134724521,0.424846619624123,0.980372848261233
"5623","RIOK2",45.8494695707794,-0.252567993256096,0.283655105933392,-0.890405242045612,0.373248328652369,0.980372848261233
"5624","FAM174A",37.8645610610353,-0.276593878930164,0.310568959875981,-0.89060374559201,0.373141789080155,0.980372848261233
"5625","PPIP5K2",65.5747157711181,0.175542502719595,0.236217655092478,0.743138791428907,0.457397656229072,0.980372848261233
"5626","C5orf30",9.16750484221015,-0.383726821279862,0.513015912241763,-0.747982298644623,0.454470834676952,0.980372848261233
"5627","AC008467.1",4.53730477896265,0.670474767863645,0.730355646397645,0.918011343063681,0.358612927411327,0.980372848261233
"5628","EPB41L4A-DT",3.43367891429774,0.627144586821407,0.800841961516614,0.78310655155199,0.433564544210903,0.980372848261233
"5629","ATG12",147.528829009026,0.142196509574955,0.177834945224238,0.799598242042104,0.423943606345017,0.980372848261233
"5630","PPIC",512.753805882561,0.307274261494646,0.349829411882642,0.878354566704436,0.379751332006644,0.980372848261233
"5631","CEP120",22.6972983552297,0.309822231081741,0.402345719012867,0.770039835000291,0.441276263343395,0.980372848261233
"5632","PHAX",129.801674063575,-0.175970856920516,0.202741818117399,-0.867955405325497,0.385418746001796,0.980372848261233
"5633","SLC22A4",7.64760102027894,0.510910506332195,0.612298463064903,0.834414157721052,0.404047641940047,0.980372848261233
"5634","IRF1",968.944913327248,-0.270800622620324,0.335599700399188,-0.806915567261274,0.41971512933887,0.980372848261233
"5635","SEPTIN8",75.757469763185,0.200381000656672,0.233668372570945,0.857544384171347,0.391144096521238,0.980372848261233
"5636","AC109454.2",2.55186758472911,0.769337756314157,0.964161173049721,0.797934803660137,0.424908325164575,0.980372848261233
"5637","SLC25A48",4.33779297738264,0.593787896800307,0.744373448525412,0.797701607945028,0.425043670531042,0.980372848261233
"5638","AC106791.1",4.72023484480348,0.592176946865158,0.757438765234171,0.781814945373279,0.434323336466279,0.980372848261233
"5639","EGR1",3388.90427542408,0.210422422057416,0.257160554927661,0.818253103072542,0.413212678544315,0.980372848261233
"5640","LRRTM2",2.20604758772161,-0.832194079963348,1.12700203998499,-0.738413996104593,0.460262912047969,0.980372848261233
"5641","MZB1",2.4027051181144,-1.02970692273327,1.1623953992086,-0.885849103871483,0.375698845351815,0.980372848261233
"5642","PURA",217.141956804956,-0.152524478128324,0.193467189238791,-0.788373877392033,0.430478046053829,0.980372848261233
"5643","AC011379.1",6.95678089971613,-0.399988836411054,0.532684150145341,-0.750893069189158,0.452717011794487,0.980372848261233
"5644","PFDN1",135.496976016428,0.20840800251819,0.266100047346757,0.783194157972518,0.43351310500219,0.980372848261233
"5645","EIF4EBP3",72.4818839300933,0.269823884087286,0.297683962603749,0.90641054938674,0.364718591118561,0.980372848261233
"5646","HARS",65.8486963342142,-0.193823625804597,0.228113478857638,-0.849680723713655,0.395502618295241,0.980372848261233

"5647","TAF7",230.833103076327,0.161480328681427,0.187970350523739,0.859073402967526,0.390300020095526,0.980372848261233
"5648","PCDHGA9",2.86802011795402,-0.744306555831007,1.00119704260654,-0.743416654421255,0.457229464273784,0.980372848261233
"5649","PCDHGB7",11.6113462217169,0.375831210101649,0.491637917507709,0.764447160639833,0.444600813621496,0.980372848261233
"5650","PCDHGA12",10.4066326283205,0.425357331794406,0.500428882716988,0.849985575342904,0.39533310596787,0.980372848261233
"5651","SPRY4",22.4098658676897,0.314189475064391,0.407554579865723,0.770913861814304,0.440757990202512,0.980372848261233
"5652","STK32A",3.01778204536994,-0.722895653905044,0.951839693147676,-0.759472061429244,0.447570220719599,0.980372848261233
"5653","AC011346.1",4.28560855763359,0.60376256441634,0.792515487988387,0.761830618539516,0.446161103592507,0.980372848261233
"5654","GRPEL2",10.8621161153667,0.370830490820387,0.444317722324401,0.834606571352649,0.403939260998382,0.980372848261233
"5655","PDGFRB",441.72192932633,-0.161980285194182,0.207113786968781,-0.782083547236755,0.434165475204687,0.980372848261233
"5656","DCTN4",50.4856531302054,0.250381329951442,0.276787951035535,0.904596204476031,0.365679346845805,0.980372848261233
"5657","SMIM3",58.2722870097736,-0.268722635859645,0.32262787535929,-0.832918220598222,0.404890852826767,0.980372848261233
"5658","ADAM19",17.686995929677,0.367320760207347,0.43075915274346,0.852728857571382,0.393809683993878,0.980372848261233
"5659","PWWP2A",45.131459675408,-0.191765367369886,0.256006964890681,-0.749063086825677,0.453819182227769,0.980372848261233
"5660","NUDCD2",120.03962766328,-0.227524698143481,0.25114833799512,-0.905937502751471,0.364968932857756,0.980372848261233
"5661","SPDL1",20.0797818546771,-0.246021856384164,0.329550999376665,-0.746536520446021,0.455343378864584,0.980372848261233
"5662","CREBRF",211.709322019423,0.208295464508839,0.265521120182976,0.784477951755017,0.432759714260676,0.980372848261233
"5663","CPEB4",27.3975788227672,0.335613914752407,0.38544752260805,0.870712341025169,0.38391123840691,0.980372848261233
"5664","MSX2",9.23395448155664,0.483479294745923,0.660420577510983,0.732077877658018,0.464121035579584,0.980372848261233
"5665","FAM193B",20.8225918879969,0.294166358469137,0.346041789554812,0.850089114518759,0.395275543143154,0.980372848261233
"5666","HNRNPAB",307.435176421284,-0.207418682398913,0.230037888622781,-0.901671823023209,0.367231225213672,0.980372848261233
"5667","ZNF454",3.98874148420525,-0.550826483873442,0.760820714637771,-0.723989861574277,0.46907195934793,0.980372848261233
"5668","MRNIP",45.5995625532319,0.217127432581004,0.268113181543202,0.809834978389592,0.418035026238172,0.980372848261233
"5669","CNOT6",22.4504205947152,0.287767359606829,0.327679612131395,0.878197327368167,0.379836642153893,0.980372848261233
"5670","AL365272.1",2.22747071488018,-0.738011471900723,0.997275006490378,-0.740028043516243,0.45928297830153,0.980372848261233
"5671","DUSP22",80.9672581102713,0.184496283425142,0.229372030514843,0.804353883126142,0.421192626876408,0.980372848261233

"5672","PSMG4",73.2111471819786,-0.232516915909623,0.297756160483101,-0.780897078778724,0.434863030342597,0.980372848261233
"5673","AL391422.4",20.3084631651683,0.309178996524169,0.379545370639919,0.814603524218697,0.415299292520445,0.980372848261233
"5674","SSR1",204.695170027467,0.145318444038036,0.173316976902396,0.838454758646482,0.401775346411852,0.980372848261233
"5675","RIOK1",70.9804240449572,-0.209786944926098,0.289194913328003,-0.725417133074394,0.468196165362977,0.980372848261233
"5676","EEF1E1",54.6551807227127,-0.277429391887979,0.321411255286842,-0.863160164196453,0.388049412570743,0.980372848261233
"5677","SLC35B3",45.3093257624068,0.276091810615196,0.304447030705883,0.906863206959374,0.364479139985013,0.980372848261233
"5678","PAK1IP1",79.2339027951595,-0.284165214565123,0.321414624999006,-0.884107916887733,0.376637959910271,0.980372848261233
"5679","TMEM14B",235.062135805409,-0.210607697911711,0.239628850342846,-0.878891242061994,0.379460247710134,0.980372848261233
"5680","RANBP9",56.7747943079038,0.224474594621253,0.278153899712142,0.807015809785732,0.419657374662028,0.980372848261233
"5681","MCUR1",72.5174868799564,-0.191401090543748,0.257985335369689,-0.741906861758145,0.458143768138821,0.980372848261233
"5682","ATXN1",69.3463389524362,0.242634998914284,0.290163202104315,0.836201824196355,0.403041372356457,0.980372848261233
"5683","TPMT",20.2121038359361,-0.348057310713554,0.391114188349876,-0.889912258570878,0.373513001095705,0.980372848261233
"5684","MRS2",24.6474661731315,-0.298028537870606,0.335315898880234,-0.888799304971379,0.374110949484647,0.980372848261233
"5685","U91328.1",2.56261544430981,0.778080844895014,0.902134525098745,0.862488712323528,0.388418641914705,0.980372848261233
"5686","HIST1H4H",7.40458777704816,0.607884063101708,0.679088072126696,0.895147607582035,0.370708187492196,0.980372848261233
"5687","BTN2A1",27.1978401908902,-0.236472107745859,0.30648738123447,-0.771555771051313,0.440377578540407,0.980372848261233
"5688","ZKSCAN4",8.7657990842634,0.395438772361949,0.503716810933533,0.78504184053156,0.432429037408098,0.980372848261233
"5689","ZKSCAN3",4.74439291979619,0.502374003161706,0.688097855045995,0.730090930349035,0.465334604832424,0.980372848261233
"5690","RPP21",113.809213013551,-0.239640252325636,0.277247789904749,-0.864354058180107,0.387393422299894,0.980372848261233
"5691","MDC1",18.7076832678232,-0.277397250859204,0.377739626918399,-0.734361001841961,0.462728748279087,0.980372848261233
"5692","TUBB",887.376417323342,0.189893437137205,0.23626912714648,0.803716674415429,0.421560621940342,0.980372848261233
"5693","FLOT1",201.788379677438,-0.143478673617649,0.183900214050486,-0.78019851340825,0.435274037229031,0.980372848261233
"5694","DDX39B",284.273179898235,-0.131689847932639,0.174631897233723,-0.754099623371719,0.450789403549442,0.980372848261233
"5695","ATP6V1G2",2.14138719272618,0.812859153606081,1.07898268816729,0.753356993138382,0.451235419051693,0.980372848261233
"5696","TNF",11.3592029763896,-0.47869329545338,0.558311502455075,-0.857394650384975,0.391226814801478,0.980372848261233

"5697","PRRC2A",106.679627548182,0.164662912311895,0.195184371104035,0.843627547536215,0.398877565646693,0.980372848261233
"5698","APOM",9.4896915446022,-0.412455407792371,0.492050230924273,-0.838238419312618,0.401896813617044,0.980372848261233
"5699","DDAH2",563.646027825038,-0.163745659665939,0.210986589667298,-0.776095105969282,0.43769283312599,0.980372848261233
"5700","EHMT2",39.0728097664249,0.247019999924104,0.281668147100287,0.876989473133975,0.380492355846486,0.980372848261233
"5701","NELFE",106.387095671246,-0.156762519689508,0.211128744824777,-0.742497284392095,0.457786096522938,0.980372848261233
"5702","DXO",11.2220905300113,-0.37834616074524,0.492171660446953,-0.768728049887421,0.442054769549269,0.980372848261233
"5703","AGPAT1",58.8956368551709,0.181693026494742,0.243397564020219,0.746486626627244,0.455373507197005,0.980372848261233
"5704","AGER",2.45046772868994,-0.698812911013826,0.918099977337203,-0.761151212573403,0.446566754627049,0.980372848261233
"5705","HLA-DRB5",27.1995550782239,1.12970453867707,1.481770962232,0.762401590712363,0.445820357147526,0.980372848261233
"5706","HLA-DQA1",38.7458677907548,-0.348991795062778,0.41949040351198,-0.831942261708525,0.405441535672741,0.980372848261233
"5707","TAP2",85.8370130327103,-0.225251752957611,0.259024273556952,-0.869616387161045,0.384510079332835,0.980372848261233
"5708","PSMB9",325.865834633659,0.266783617685075,0.305064634696358,0.874515061211914,0.381837821725744,0.980372848261233
"5709","HLA-DOA",3.46828479380802,-0.713586610844835,0.873381535812414,-0.817038810170243,0.413906249385485,0.980372848261233
"5710","HCG25",4.58601069080794,0.676232042769882,0.749714037514695,0.901986636146756,0.367063966822177,0.980372848261233
"5711","VPS52",23.6629311340283,0.283223820797186,0.348367846221033,0.813002186824919,0.416216804372518,0.980372848261233
"5712","DAXX",72.8630618986821,-0.229545555941052,0.252977960411563,-0.9073737315599,0.364209195719862,0.980372848261233
"5713","PHF1",50.0253517353294,-0.219359413101148,0.244169400003175,-0.898390269617305,0.36897752218496,0.980372848261233
"5714","NUDT3",69.5912292917582,0.223818229350738,0.252000924450993,0.888164318596633,0.374452369085015,0.980372848261233
"5715","RPS10-NUDT3",2.40287230372369,0.877137188276885,1.09979381741576,0.797546935059102,0.425133455617032,0.980372848261233
"5716","ZNF76",17.2966276312684,0.308902600661015,0.357232215580263,0.864710928042295,0.387197469931005,0.980372848261233
"5717","TEAD3",27.1850065749959,0.268167308355112,0.30358521160758,0.883334556828645,0.377055538081909,0.980372848261233
"5718","Z84485.1",2.59146967973092,-0.695586714243197,0.878626531679905,-0.791675062342197,0.42855016668727,0.980372848261233
"5719","PPIL1",23.720723793384,-0.33577931227545,0.45717457870482,-0.734466280314,0.462664603936088,0.980372848261233

"5720","FOXP4",16.0831494626009,-0.386855687829101,0.466751547190738,-0.828825721430362,0.407203031299881,0.980372848261233
"5721","TFEB",20.5037829890615,-0.303061924325876,0.3562798266514 3,-0.850628920459141,0.394975518948579,0.980372848261233
"5722","TOMM6",366.933803283438,-0.220835766153887,0.257317521061824,-0.858222810645015,0.39076944264002,0.980372848261233
"5723","C6orf132",55.0431420492212,-0.227254194838152,0.298408669088106,-0.761553595385175,0.446326479697719,0.980372848261233
"5724","MRPS10",113.272019287331,-0.234232064874236,0.302762989261079,-0.773648276646697,0.439138814888164,0.980372848261233
"5725","TBCC",40.0208433749392,-0.275362372735824,0.322954935859776,-0.852634043207143,0.393862277714807,0.980372848261233
"5726","KLC4",7.79584898442385,0.481885651426896,0.521509264104775,0.924021267875468,0.355475242810991,0.980372848261233
"5727","ZNF318",22.9812249133215,0.271302765785462,0.365668548581254,0.741936288581779,0.458125937977807,0.980372848261233
"5728","CDC5L",124.301156392614,-0.206702189264383,0.227477185344037,-0.90867217717577,0.363523194819538,0.980372848261233
"5729","PLA2G7",7.44942329315507,0.598216397509129,0.690740403966894,0.866050970919895,0.386462210484517,0.980372848261233
"5730","TRAM2",132.408348587625,0.236968702401891,0.325547716690549,0.727907739027834,0.466670065436935,0.980372848261233
"5731","AL445250.1",4.40322101701457,0.543130142540496,0.696250422535073,0.780078725931597,0.435344537718945,0.980372848261233
"5732","COX7A2",456.748879118756,-0.184882653767749,0.23009339044098,-0.803511362987943,0.421679231713325,0.980372848261233
"5733","IBTK",70.4613444954065,0.178600142630468,0.23569013080734,0.757775228087347,0.448585554547781,0.980372848261233
"5734","TBX18",31.3097216755213,0.274309019633917,0.314003036290743,0.873587156590192,0.3823431230422,0.980372848261233
"5735","SNX14",61.3003566894251,0.204703789496298,0.258334187890387,0.792399144565236,0.428127978056605,0.980372848261233
"5736","SNHG5",592.907577137956,0.274652989112869,0.369195328418346,0.743923251384297,0.456922907832578,0.980372848261233
"5737","AL139274.2",2.33476048985912,-0.762757719534955,0.938517850900844,-0.812725851514508,0.416375256055969,0.980372848261233
"5738","SRSF12",3.49093153724606,0.618928277950198,0.814881672722181,0.759531473916472,0.44753469376806,0.980372848261233
"5739","MANEA",25.4137354074681,-0.311248881943875,0.344589701907993,-0.90324487418077,0.366395946450238,0.980372848261233
"5740","CCNC",74.4727682096329,-0.196828215754159,0.253916958085036,-0.7751676659904,0.438240591731613,0.980372848261233
"5741","BVES",4.17711193226792,0.642702535507296,0.726530733066133,0.884618511311887,0.376362419283772,0.980372848261233
"5742","PDSS2",28.1853124860015,-0.268058170126819,0.306134218242496,-0.875623024651508,0.381235004169735,0.980372848261233
"5743","SEC63",276.362057704356,0.125976650657988,0.139727355117924,0.901589030663818,0.367275220201448,0.980372848261233
"5744","CEP57L1",37.0958287221191,0.202339056931762,0.255068790733487,0.793272498567569,0.427619076144879,0.980372848261233

"5745","RFPL4B",3.16843757085207,0.778873608730599,0.924818816861147,0
.84219048588794,0.399681338415245,0.980372848261233
"5746","FRK",3.77405697477597,-0.645608469674908,0.86278396706491,-0.7
48285195738154,0.454288152169099,0.980372848261233
"5747","RWDD1",453.456390654236,-0.173444396437862,0.235986654679008,-
0.734975444580895,0.462354449002388,0.980372848261233
"5748","ZUP1",13.3363414165416,0.406552562089823,0.439631283716482,0.9
24758035081071,0.355091783887737,0.980372848261233
"5749","CEP85L",42.596657452002,0.225262871943308,0.277992517075276,0.
810319911892846,0.417756334111118,0.980372848261233
"5750","RNF217",43.3058652847052,0.25805589273379,0.327901603855663,0.
786991858836353,0.431286631936205,0.980372848261233
"5751","THEMIS",5.25933107989609,0.558955363362001,0.647167213732583,0
.863695427551384,0.387755226122815,0.980372848261233
"5752","MED23",34.147112970983,0.233479030037926,0.289069757538603,0.8
07690960223491,0.419268508808968,0.980372848261233
"5753","TBPL1",44.2574099707593,-0.195281420889674,0.254441940048366,-
0.767489120907323,0.442790758996995,0.980372848261233
"5754","SGK1",159.31400266144,-0.374410830168561,0.460493690538077,-0.
813063974299126,0.416181380076482,0.980372848261233
"5755","BCLAF1",436.383723077519,0.175440772560357,0.22000085486797,0.
797454958371161,0.425186851835071,0.980372848261233
"5756","AL121933.2",7.27857958821935,-0.538470000461277,0.590560163205
261,-0.911795332652875,0.361876467797418,0.980372848261233
"5757","TNFAIP3",247.428892477562,0.306060348781299,0.366866984468764,
0.834254271270785,0.404137714515001,0.980372848261233
"5758","CITED2",301.761468748862,0.297756009758687,0.345474427385126,0
.861875687912368,0.388755928957831,0.980372848261233
"5759","SF3B5",383.241739170927,-0.202916340380884,0.276788020130734,-
0.733110993333605,0.46349073566161,0.980372848261233
"5760","EPM2A",4.30571042088679,0.616884409864639,0.722318072849779,0.
854034300195244,0.393085984671101,0.980372848261233
"5761","AL356599.1",23.455505600096,0.354907589049288,0.42068872689198
3,0.843634655179184,0.398873592642193,0.980372848261233
"5762","SHPRH",88.532131700658,0.204151568005426,0.223004840647999,0.9
15458011638719,0.359951235662685,0.980372848261233
"5763","GINM1",291.900234708562,-0.125311181499975,0.155504543026294,-
0.805836145113695,0.420337333796066,0.980372848261233
"5764","SYNE1",221.588385812243,0.213614335925939,0.259260267358596,0.
823937806214165,0.409974897462635,0.980372848261233
"5765","IPCEF1",6.51797833108747,0.527862044445338,0.723070624680984,0
.73002833530712,0.465372864687872,0.980372848261233
"5766","TIAM2",11.414595596805,0.35602368469837,0.477715008208935,0.74
5263763081645,0.456112282834879,0.980372848261233
"5767","TFB1M",15.2342681325798,-0.348179355392106,0.436534256699667,-
0.797599157565432,0.425103140065797,0.980372848261233
"5768","AL139393.3",12.6438284071347,0.400093642535424,0.5046262231202
38,0.792851469472868,0.427864365101219,0.980372848261233
"5769","CCR6",4.48418354647718,-0.503247864991968,0.671983286096355,-0
.748899378011922,0.453917855245002,0.980372848261233

"5770","PRKAR1B",3.76388576777808,0.571461965617512,0.746543609693893,
0.765477003884381,0.443987554832262,0.980372848261233
"5771","ADAP1",31.3480430997211,-0.468563608558928,0.528927098743224,-
0.885875595469158,0.375684568203917,0.980372848261233
"5772","COX19",51.4144703536994,-0.225816382895877,0.260459852512256,-
0.866991134018443,0.385946867514452,0.980372848261233
"5773","GPER1",5.41776939049935,-0.519412376632398,0.638299291709842,-
0.813744247218298,0.41579148035311,0.980372848261233
"5774","BRAT1",26.1163971687513,0.246398744811787,0.328531450702825,0.
750000477228795,0.45325441733088,0.980372848261233
"5775","IQCE",19.6197976536735,-0.331066756259784,0.360570984128351,-0
.918173593640958,0.358527990932391,0.980372848261233
"5776","AC073316.1",2.42826857985796,0.944921643396007,1.1835590728777
8,0.798373030167784,0.424654049308722,0.980372848261233
"5777","SDK1",78.1513516471858,0.276103489939097,0.316365503598273,0.8
72735765431928,0.382807118535788,0.980372848261233
"5778","TNRC18",83.6907850112099,0.225669855833682,0.245488004898849,0
.919270397454519,0.357954157485377,0.980372848261233
"5779","RAC1",885.808138780708,-0.159908995748931,0.219785634912917,-0
.727568004215969,0.466878072656464,0.980372848261233
"5780","MIOS",22.4441007530342,-0.309791232844109,0.414140301771319,-0
.748034498258443,0.45443934922923,0.980372848261233
"5781","NDUFA4",762.42084578612,-0.224529902918904,0.274021434521317,-
0.819388101194093,0.412565021492439,0.980372848261233
"5782","MEOX2",42.9271633248961,-0.396730171142437,0.438875144976092,-
0.903970470152847,0.366011059914549,0.980372848261233
"5783","AC004130.2",10.0031262208649,-0.574607819059836,0.619604661402
348,-0.927378141021936,0.353730235861287,0.980372848261233
"5784","ITGB8",15.6140515952922,0.4865778295672,0.531569669351697,0.91
5360408280314,0.360002455839824,0.980372848261233
"5785","ABCB5",9.83886629233245,0.700552342730372,0.787352300923814,0.
889757154336632,0.373596297311096,0.980372848261233
"5786","FAM126A",109.985425733856,-0.214147146569429,0.290276033056896
,-0.737736230973828,0.460674750849823,0.980372848261233
"5787","NUP42",46.0863453944867,-0.213509296601732,0.280232479888115,-
0.761900607263574,0.446119327543485,0.980372848261233
"5788","MPP6",27.3981364549189,-0.284825088356977,0.310616818184961,-0
.916966087095045,0.359160411253626,0.980372848261233
"5789","CBX3",245.972676248666,-0.135874279094925,0.180891458906696,-0
.751137062612831,0.452570172595378,0.980372848261233
"5790","AC004540.2",3.09794549547367,0.855059859076371,1.1049722081652
9,0.773829289784694,0.439031749215572,0.980372848261233
"5791","HOXA10-
AS",8.08600498408074,-0.49723400072873,0.554900527102421,-0.8960777228
40138,0.370211254034552,0.980372848261233
"5792","RP9",42.9665781965435,-0.228239157724862,0.296350746841057,-0.
770165623531478,0.441201652808673,0.980372848261233
"5793","TRGC1",3.41807371241194,0.613919876233691,0.806501249198771,0.
761213794577005,0.446529380188863,0.980372848261233
"5794","COA1",56.4023604762896,-0.211676463982379,0.289542036250883,-0

.731073341623409,0.46473435645401,0.980372848261233
"5795","URGCP",34.0038667303762,0.315009937541786,0.361175201875766,0.872180415227235,0.383109961983809,0.980372848261233
"5796","H2AFV",208.738750022241,0.179490097168658,0.227639845800382,0.788482774347216,0.430414370475429,0.980372848261233
"5797","MYO1G",13.9204814109591,0.372221467705142,0.501104245669303,0.742802462605325,0.457601284329293,0.980372848261233
"5798","SNHG15",83.3628614671082,-0.254093819875931,0.335750467214306,-0.75679364494747,0.449173500772067,0.980372848261233
"5799","ADCY1",15.303840235123,0.394908886567493,0.438392254831459,0.900811732450237,0.367688428432275,0.980372848261233
"5800","CHCHD2",1186.61758521562,-0.246685973581611,0.271181336966203,-0.909671647545404,0.362995700885759,0.980372848261233
"5801","ZNF117",23.3106009173674,0.361299711206856,0.402921527735033,0.896699943628855,0.369879050721714,0.980372848261233
"5802","GUSB",144.307575937265,-0.23322660795601,0.261231062803909,-0.892798143730249,0.371965280607047,0.980372848261233
"5803","AC008267.5",7.34686412395337,-0.554107077147574,0.608901900321291,-0.910010425086859,0.362817011868188,0.980372848261233
"5804","AC027644.3",35.584272292881,-0.232525701787192,0.299314539594428,-0.776860696784944,0.437240961775821,0.980372848261233
"5805","AC006480.2",3.02236414116287,-0.645884069667686,0.881368287955469,-0.73281972870383,0.463668386807035,0.980372848261233
"5806","CLDN4",3.08910649042981,1.09937089158116,1.24459732139332,0.883314524854051,0.377066358200695,0.980372848261233
"5807","METTL27",13.53997360371,0.516070556590167,0.637167549230293,0.809944820971482,0.417971889925039,0.980372848261233
"5808","ELN",426.237897355699,0.212997446287896,0.288553343398058,0.738156223662489,0.460419521224869,0.980372848261233
"5809","CASTOR2",21.1916604666182,0.308331562942741,0.352168735985201,0.875522246687162,0.381289810990414,0.980372848261233
"5810","MDH2",212.640060929992,-0.223000327338509,0.28271061921724,-0.788793600876913,0.430232650205812,0.980372848261233
"5811","YWHAG",174.112645146588,-0.164332169754752,0.203390515856446,-0.807963778757206,0.419111433874776,0.980372848261233
"5812","FGL2",517.273547379971,-0.186161827387902,0.250951243241974,-0.741824686671902,0.458193561337541,0.980372848261233
"5813","GNAI1",41.3031494851131,-0.310618916068066,0.341639585311714,-0.909200600348043,0.363244247375551,0.980372848261233
"5814","TP53TG1",62.2631608890589,-0.22721909672942,0.281200468873276,-0.808032424838582,0.419071916429364,0.980372848261233
"5815","CROT",22.124819507657,-0.251353511274157,0.320354949235886,-0.784609421123938,0.432682604633595,0.980372848261233
"5816","ADAM22",16.6432037295916,-0.428700752851407,0.543659993418296,-0.788545705112353,0.430377575310448,0.980372848261233
"5817","SRI",470.668094578392,-0.196267094312563,0.234936516188633,-0.835404804227957,0.403489825760079,0.980372848261233
"5818","AKAP9",330.717494214602,0.209931287658531,0.258336428174819,0.812627507245971,0.416431655608327,0.980372848261233
"5819","KRIT1",80.8141052942147,0.170893231535363,0.195748938733299,0.

873022518748868,0.382650803728152,0.980372848261233
"5820","SGCE",312.231594089191,0.202346559718082,0.245777272829083,0.823292395545446,0.410341737003631,0.980372848261233
"5821","MCM7",23.5801006499225,-0.28129094857283,0.348373744090864,-0.807440151113863,0.419412942383996,0.980372848261233
"5822","TAF6",36.4931596620793,-0.202466286742457,0.265782293186698,-0.761774926067911,0.446194347844955,0.980372848261233
"5823","CNPY4",75.090498508529,0.227971885483425,0.281445255593702,0.810004364800977,0.417937667131553,0.980372848261233
"5824","LAMTOR4",390.419567380733,-0.222793177839711,0.253408894431871,-0.879184522465956,0.379301235020382,0.980372848261233
"5825","AC005072.1",2.74505301627622,0.910470271406358,1.05037220419921,0.866807278188101,0.386047613610379,0.980372848261233
"5826","ORAI2",42.1343422532167,-0.30288605699379,0.343487646187098,-0.881796071433694,0.377887096506596,0.980372848261233
"5827","DNAJC2",97.6511732873026,-0.216271740629516,0.281365904449654,-0.768649424856714,0.44210145616698,0.980372848261233
"5828","ORC5",16.0633685903325,0.291009932827264,0.379447140818315,0.766931415531745,0.443122294090365,0.980372848261233
"5829","AC005070.3",3.28445912009637,0.727769146032359,0.856976492240009,0.849228832555346,0.395753972541944,0.980372848261233
"5830","HBP1",156.471469348826,0.159245743085393,0.200333497346347,0.794903224846519,0.426669798231083,0.980372848261233
"5831","COG5",37.768376796393,0.191279620099689,0.253719603269624,0.753901620665942,0.450908297375136,0.980372848261233
"5832","TFEC",2.25503174795002,-1.01000877936629,1.16630925095753,-0.865987111511876,0.386497229740274,0.980372848261233
"5833","CAPZA2",197.611307727604,-0.18930455708708,0.217799657160412,-0.869168296934716,0.384755085095979,0.980372848261233
"5834","ST7-OT4",3.91114486491418,0.602989164108281,0.731248664383636,0.824602072424345,0.409597544434626,0.980372848261233
"5835","LSM8",186.245707769855,-0.182172986973289,0.221801355370093,-0.821333966464358,0.411456065273054,0.980372848261233
"5836","ING3",43.9758936226236,0.1964630818882,0.263398363827465,0.745878140749919,0.455741031066965,0.980372848261233
"5837","ZNF800",79.5485896760245,-0.194242971933016,0.227653169713315,-0.853240796856142,0.393525783693874,0.980372848261233
"5838","GCC1",11.9408820263978,-0.384138179558306,0.452741638167918,-0.848471064231633,0.396175680579563,0.980372848261233
"5839","ARF5",173.975374011654,-0.369531383409394,0.444088938052676,-0.832111209591897,0.405346175149766,0.980372848261233
"5840","FSCN3",2.44339091284461,-0.797840883062551,1.01555250703612,-0.785622483854671,0.432088688256854,0.980372848261233
"5841","FAM71F2",4.55177662558623,0.880548543353997,1.00690551664932,0.874509602732343,0.381840793006296,0.980372848261233
"5842","KLF14",4.05314274648645,-0.602128458138328,0.71324747060455,-0.844206930909916,0.398553782212536,0.980372848261233
"5843","AC016831.7",14.553056154839,0.629204299111651,0.788644474123796,0.797830099311496,0.42496909181315,0.980372848261233

"5844","AC058791.1",56.4333698940429,-0.399327038152791,0.434514252026
711,-0.919019425232208,0.358085411859537,0.980372848261233
"5845","CREB3L2",92.8244775783651,-0.211298696623421,0.25934077069603,
-0.814753099006873,0.415213652320842,0.980372848261233
"5846","TRIM24",20.6169677198372,0.297498423586547,0.346990791509617,0
.857366912511571,0.391242139357268,0.980372848261233
"5847","AC004889.1",32.0331133305296,0.247823080174543,0.3285770934904
39,0.754231153310007,0.450710434150374,0.980372848261233
"5848","ARHGEF5",12.4754182053683,-0.349500430604582,0.446536994767448
,-0.782690873768697,0.433808662731661,0.980372848261233
"5849","CUL1",62.5680485245335,-0.178262105808813,0.224868125430207,-0
.792740658409325,0.427928936558052,0.980372848261233
"5850","AC073111.4",11.7339381762227,0.380698713002267,0.4659752921115
3,0.816993345885704,0.413932230712129,0.980372848261233
"5851","GIMAP2",10.2459229830016,0.431727658345497,0.493255264738166,0
.875262139522567,0.381431289328614,0.980372848261233
"5852","AC144652.1",5.78001982593051,-0.527240558646691,0.689907667615
113,-0.764219015668091,0.444736736462544,0.980372848261233
"5853","AC009403.1",4.69519756076377,0.542011566152022,0.6558610850238
59,0.82641214508451,0.40857033627367,0.980372848261233
"5854","RBM33",78.4698735582379,0.234348941454291,0.266665157301185,0.
878813504643973,0.379502402765387,0.980372848261233
"5855","NCAPG2",8.07780893681503,-0.47486076776164,0.559315896830956,-
0.849002809418018,0.395879729001968,0.980372848261233
"5856","ZNF596",4.20322521869609,0.605400375585233,0.786382801524322,0
.769854547189646,0.441386178598158,0.980372848261233
"5857","AC004908.2",1.95015164895553,0.952352696052489,1.0845236238706
7,0.878129968855395,0.379873191095989,0.980372848261233
"5858","AC246817.2",3.23842643148976,0.974466488524267,1.3068120755656
,0.7456821885446,0.455859421201848,0.980372848261233
"5859","NEIL2",24.1893654414068,-0.26706266852781,0.36127671690929,-0.
739219152600042,0.459773932662233,0.980372848261233
"5860","MSR1",11.8724993678368,-0.371692046101028,0.431895819678166,-0
.860605796967428,0.389455192100596,0.980372848261233
"5861","SLC7A2",40.1569166268716,0.363403572515585,0.431289461492484,0
.842597848920355,0.399453394539847,0.980372848261233
"5862","XPO7",32.7010968762333,0.252524307167021,0.316960720770572,0.7
96705366371902,0.42562216657247,0.980372848261233
"5863","NUDT18",16.1376489051013,0.331987484736986,0.394629264958533,0
.841264230041,0.400199924817904,0.980372848261233
"5864","SLC39A14",407.659384400529,0.380413628707942,0.424085037927543
,0.897022046726717,0.369707152855706,0.980372848261233
"5865","C8orf58",10.7378235654654,-0.37689775051412,0.478189634095678,
-0.788176329306858,0.430593572774547,0.980372848261233
"5866","RHOBTB2",10.6953098948173,0.432441274853004,0.550632208725691,
0.785354122044164,0.432245971585249,0.980372848261233
"5867","DCTN6",122.529104406238,-0.209076074710935,0.267454315333958,-
0.781726308845948,0.4343754367313,0.980372848261233
"5868","WRN",47.2706961949559,0.263868877474271,0.311876897957967,0.84
6067404164813,0.397515143335746,0.980372848261233

"5869","TTI2",22.3688368557358,0.263070391097578,0.350370679302643,0.750834492261675,0.452752268344529,0.980372848261233
"5870","BRF2",24.5946386814446,0.337649324211763,0.366016266170489,0.922498138523951,0.356268800845487,0.980372848261233
"5871","TACC1",493.190269344939,0.221633160422094,0.292255587730619,0.758353885183471,0.448239156766058,0.980372848261233
"5872","AC009630.1",1.95822892888244,-0.726007963309083,0.973770486045651,-0.745563737772853,0.45593099501661,0.980372848261233
"5873","SMIM19",210.468629084669,-0.23848803898547,0.297422214637947,-0.801850121638634,0.4226396614456,0.980372848261233
"5874","PLAG1",2.19901242339346,-0.837567296446492,0.928747492079624,-0.901824557901132,0.367150072126018,0.980372848261233
"5875","PENK",10.0005107508269,-0.942147837848839,1.23863627641584,-0.76063317035657,0.446876201653113,0.980372848261233
"5876","TOX",4.1728715731898,0.591943482189493,0.765830617180805,0.772943088079411,0.43955606288899,0.980372848261233
"5877","AC100814.1",1.99660648601833,0.918026740641701,1.04650639774402,0.877229936310674,0.380361759022733,0.980372848261233
"5878","MTFR1",25.6789428375209,-0.271801666488654,0.359461047349647,-0.756136634254761,0.449567279512618,0.980372848261233
"5879","SNHG6",543.491758847985,-0.209778232795272,0.227502015564325,-0.922093952771854,0.35647957099547,0.980372848261233
"5880","TRPA1",2.91512948586903,-0.730367528780383,0.947816746496087,-0.770578839718146,0.440956607563605,0.980372848261233
"5881","C8orf89",3.02725680515218,0.856093982880295,0.977079558058239,0.876176331619935,0.380934181346163,0.980372848261233
"5882","PEX2",79.0656426326573,0.233520614967302,0.2761345191452,0.845677011661515,0.397732950782613,0.980372848261233
"5883","AC062004.1",35.9213362913007,0.368988330053386,0.501315636449538,0.736039938164841,0.46170639288036,0.980372848261233
"5884","LRRCC1",36.4402678490625,0.257459039707342,0.317213305889148,0.811627491430997,0.417005411591444,0.980372848261233
"5885","C8orf88",10.8802904239881,-0.386757943406824,0.433374040700477,-0.892434495572678,0.372160088420256,0.980372848261233
"5886","OTUD6B-AS1",117.113554523307,0.167928782953794,0.211964045752284,0.79225126298093,0.428214183289324,0.980372848261233
"5887","PLEKHF2",15.1234774809499,-0.350466297710586,0.396622532224197,-0.883626796857018,0.376897708623492,0.980372848261233
"5888","AP003117.1",3.20853258930383,-0.759095113010397,0.891312207865045,-0.851660177334105,0.394402729200347,0.980372848261233
"5889","YWHAZ",444.844710845158,-0.165009480304445,0.19914122996587 4,-0.828605308567804,0.407327783126899,0.980372848261233
"5890","RRM2B",26.2404824319499,0.293913448660853,0.324649090364221,0.905326573781906,0.365292402300064,0.980372848261233
"5891","UBR5",93.6408167385045,0.239881576188863,0.290994777311119,0.824350108292122,0.409740654637031,0.980372848261233
"5892","EIF3E",885.309730232806,-0.203915402045536,0.220676606966021,-0.924046299465416,0.355462210547943,0.980372848261233
"5893","TRPS1",42.1895103691597,0.29022742849253,0.387465037738434,0.7

49041591433738,0.453832137560894,0.980372848261233
"5894","UTP23",76.1035434945314,-0.171337594973729,0.23572862750291,-0
.72684254258263,0.467322418448433,0.980372848261233
"5895","SAMD12",2.71760052110448,-0.810218300028166,0.89633788108485,-
0.903920627618178,0.366037490413297,0.980372848261233
"5896","DSCC1",13.0370908236219,0.340848640146727,0.42214154659564 2,0.
807427373343133,0.419420301508751,0.980372848261233
"5897","HAS2-
AS1",13.5488137625658,-0.424309844024283,0.480314670512865,-0.88339971
7046364,0.377020343609971,0.980372848261233
"5898","ZHX2",41.2335588016413,0.248629815988719,0.3096858840855,0.802
845169139435,0.422064231072294,0.980372848261233
"5899","WDYHV1",26.5268466869798,-0.325809850707391,0.353285306044671,
-0.922228706183988,0.35640929260088 9,0.980372848261233
"5900","NDUFB9",270.779107107586,-0.192778744030457,0.253632865689316,
-0.760070046547512,0.447212716029352,0.980372848261233
"5901","PTK2",69.9873274311607,0.179334010397185,0.22545638504116,0.79
5426620383561,0.426365380011196,0.980372848261233
"5902","JRK",8.48819155473091,0.580482485121517,0.633476223655333,0.91
6344550032157,0.359486208649138,0.980372848261233
"5903","TOP1MT",14.8017576065501,-0.338078084190203,0.39403826434313,-
0.857982878271445,0.390901917846596,0.980372848261233
"5904","SCRIB",23.3921118009429,0.302554757355394,0.332416481489584,0.
910167738974982,0.36273405501136,0.980372848261233
"5905","EXOSC4",39.906713588026,-0.24407606952662,0.326403806310276,-0
.747773355604207,0.454596875986317,0.980372848261233
"5906","SHARPIN",91.946153304055,-0.216952613221271,0.275279288382773,
-0.788118185337651,0.430627578971124,0.980372848261233
"5907","MROH1",13.7766387432901,0.338035613964711,0.414291119009921,0.
81593738908175,0.414535946381967,0.980372848261233
"5908","HSF1",60.3541218126509,0.203076243767409,0.222698285655425,0.9
11889569197775,0.361826853037121,0.980372848261233
"5909","TONSL",2.06423069885058,0.790826732173472,1.07897805158944,0.7
32940518121295,0.463594709069317,0.980372848261233
"5910","KIFC2",5.38142643958909,-0.551230062178369,0.610409551040345,-
0.903049536559325,0.36649960482802,0.980372848261233
"5911","COMMD5",69.9976588924816,-0.241664343063103,0.280921082141828,
-0.860257055898333,0.389647359574302,0.980372848261233
"5912","PLPP6",6.13840702807429,-0.436343982903654,0.594570653372596,-
0.733880793524824,0.463021393756148,0.980372848261233
"5913","RCL1",32.447302436351,-0.277344030362286,0.338286220474122,-0.
819850214334998,0.412301501136436,0.980372848261233
"5914","PLGRKT",65.3368976860532,-0.276217157590946,0.333986476962972,
-0.82703096275832,0.408219512138619,0.980372848261233
"5915","RIC1",30.1133430516947,0.226780393155886,0.298677912234347,0.7
59280763212082,0.447684622419227,0.980372848261233
"5916","MPDZ",83.7448934339764,0.273232037460609,0.311241629657731,0.8
77877544083929,0.380010176806575,0.980372848261233
"5917","SNAPC3",37.0045137534971,0.248371842233844,0.277433255526763,0
.895248991553877,0.370654000891429,0.980372848261233

"5918","BNC2",105.861412240421,0.287380604358084,0.341898560845721,0.84054347478743,0.400603736494418,0.980372848261233
"5919","RPS6",5895.75862877174,-0.179056679197228,0.222771400673026,-0.803768700364009,0.421530569312721,0.980372848261233
"5920","FOCAD",22.5093946565835,0.258156629294344,0.33715164892006,0.765698848340955,0.443855512482793,0.980372848261233
"5921","LINC01239",21.690552353197,0.392939126122961,0.449981497127563,0.873233963243534,0.382535566286272,0.980372848261233
"5922","DNAJB5",5.38886671188594,-0.539831340282591,0.701020251691399,-0.770065257002352,0.441261183888604,0.980372848261233
"5923","FANCG",15.4219773194204,0.348586403437425,0.437053474382095,0.797582959225426,0.42511254318691,0.980372848261233
"5924","UNC13B",9.36364239467157,0.438003247709247,0.562666418770945,0.778442133913012,0.436308407801078,0.980372848261233
"5925","CD72",3.30688045829023,0.690454890053169,0.833408000708896,0.82847163630043,0.407403451606294,0.980372848261233
"5926","SPAG8",3.29542081881234,0.701526128540355,0.758442153469099,0.924956669841713,0.354988446850684,0.980372848261233
"5927","TOMM5",357.518356095755,-0.238765435722849,0.307077213491783,-0.777542016250054,0.436839055477248,0.980372848261233
"5928","ZNF658",9.39545536203868,0.42752697029818,0.495459807856662,0.862889307101708,0.388198330323041,0.980372848261233
"5929","PTAR1",54.3393280690192,0.219929681926027,0.245651707537677,0.895290670398842,0.370631726262886,0.980372848261233
"5930","AL162390.1",13.8872889833902,-0.363413261651049,0.442764559338604,-0.820782183185373,0.411770348891207,0.980372848261233
"5931","C9orf64",28.3013538029862,-0.290010365708818,0.317035382689558,-0.914757095086756,0.360319163404616,0.980372848261233
"5932","NTRK2",963.706729132265,0.275415209027837,0.327485548186123,0.840999581671031,0.400348168770345,0.980372848261233
"5933","AGTPBP1",10.0115980176923,-0.449826088920256,0.500664743863095,-0.898457689369994,0.368941592498832,0.980372848261233
"5934","GAS1",682.593484541128,0.271806365475882,0.315045085583357,0.86275386576048,0.388272809288499,0.980372848261233
"5935","GAS1RR",23.2766199183627,-0.286640321320488,0.370031055123778,-0.774638553579254,0.438553269177431,0.980372848261233
"5936","SEMA4D",14.1284966211529,0.372314904427849,0.509222770638782,0.731143471767392,0.464691523763341,0.980372848261233
"5937","AUH",33.8008099424103,0.22020259601099,0.303235959612238,0.726175735531411,0.467731044974458,0.980372848261233
"5938","FANCC",4.42691134674973,0.478401217504924,0.659815370704978,0.725053156906268,0.46841942023223,0.980372848261233
"5939","NCBP1",20.4780478110755,0.315037264110105,0.357961350220547,0.880087372326663,0.378811979342212,0.980372848261233
"5940","TMEFF1",4.26346748147398,-0.713958676082703,0.775230468383942,-0.920963126708672,0.357069678592749,0.980372848261233
"5941","NIPSNAP3B",5.08465259557313,-0.5593389620799,0.676985496081603,-0.826220008135119,0.408679300313009,0.980372848261233
"5942","DNAJC25",36.2825104173423,0.230152857440147,0.292089034592327,0.787954459712514,0.430723344235405,0.980372848261233

"5943","FKBP15",46.4939094040751,0.217343185003417,0.274095997840536,0
.792945488864318,0.427809582880543,0.980372848261233
"5944","RGS3",11.7905718725804,0.559465378058763,0.616095073231625,0.9
08082863127243,0.363834443751452,0.980372848261233
"5945","CDK5RAP2",46.9789283323233,0.262451795297695,0.282274836508102
,0.929773969739461,0.352488126465528,0.980372848261233
"5946","C5",6.04932363605995,0.470739092180166,0.597556994443329,0.787
772708808632,0.430829667192029,0.980372848261233
"5947","AL359644.1",2.68281053653461,-0.740718379597462,0.943904014374
233,-0.784739092447366,0.432606557390253,0.980372848261233
"5948","PDCL",63.3248336463837,0.188415244879464,0.23884250938467,0.78
8868134759087,0.430189081663012,0.980372848261233
"5949","RC3H2",54.8281693819054,0.208328450719114,0.255868867950762,0.
814200072043167,0.415530344211725,0.980372848261233
"5950","DENND1A",22.9175503011344,0.287940244211402,0.340376471934383,
0.84594637982766,0.397582657436451,0.980372848261233
"5951","PSMB7",258.588552382375,-0.21415215788979,0.257349966848033,-0
.832143716638787,0.405327828490839,0.980372848261233
"5952","GAPVD1",53.1545511304065,0.240359669265601,0.278595856515244,0
.862753927040007,0.388272775589022,0.980372848261233
"5953","ZNF79",1.99898269053321,-0.900031133020372,0.997648552483482,-
0.902152497269598,0.36697586486709,0.980372848261233
"5954","NAIF1",8.347496838211,-0.405664691110262,0.51903656949018,-0.7
81572465132318,0.434465874140957,0.980372848261233
"5955","DNM1",131.065097213939,-0.20906550679542,0.260785138441399,-0.
801677227640022,0.422739691915027,0.980372848261233
"5956","SWI5",86.9667182674978,-0.20587784351897,0.237313030507446,-0.
867537037805054,0.385647827350449,0.980372848261233
"5957","CERCAM",169.261592455473,0.266076495427287,0.33701904694169,0.
789499874982801,0.429819902812802,0.980372848261233
"5958","ODF2",24.8383367713499,-0.246984106143318,0.326426929117995,-0
.756629077174081,0.449272115940372,0.980372848261233
"5959","LRRC8A",42.0230856773981,0.240638205739627,0.291387540624061,0
.825835604447106,0.408897353922636,0.980372848261233
"5960","PHYHD1",75.3072543143508,0.253669063186436,0.31694992969993,0.
800344279699307,0.423511354796225,0.980372848261233
"5961","PTPA",44.3576840264965,-0.216194045410593,0.263480064446161,-0
.820532839420075,0.411912416320482,0.980372848261233
"5962","NTMT1",85.5805110088553,-0.194961603636186,0.267327182442132,-
0.729299586578287,0.465818425117585,0.980372848261233
"5963","TTF1",47.4632723798255,-0.215891372317317,0.276081493610493,-0
.781984223187031,0.434223845544605,0.980372848261233
"5964","RALGDS",106.178308368532,-0.249545374699446,0.288940175834943,
-0.863657585790352,0.38777601989183,0.980372848261233
"5965","SURF6",47.3857647049954,0.246167559247152,0.339502846766684,0.
725082459813143,0.468401444301061,0.980372848261233
"5966","RPL7A",6389.51433296198,-0.180847367049548,0.231225877951694,-
0.782124252923492,0.434141554767689,0.980372848261233
"5967","SLC2A6",7.03929613692573,-0.514574642202034,0.568623571599989,
-0.904947786026751,0.365493049476492,0.980372848261233

"5968","BRD3",85.8197135632569,0.189924146042091,0.239072471807572,0.794420807239404,0.426950494987412,0.980372848261233
"5969","WDR5",27.6461935384709,-0.268014677419651,0.31308321800351,-0.856049324932665,0.39197049684302,0.980372848261233
"5970","QSOX2",15.8124229528511,0.374041976331075,0.458448563691131,0.815886461328467,0.414565076147984,0.980372848261233
"5971","AGPAT2",86.8469472659809,-0.220960636790981,0.28097796319808,-0.786398457288309,0.431634086992601,0.980372848261233
"5972","TMEM141",72.3775296378544,-0.226069530750822,0.277303591660113,-0.815241985858993,0.41493380918323,0.980372848261233
"5973","AJM1",2.18685786605786,-0.990660172061183,1.11579365054468,-0.887852490984856,0.374620103135417,0.980372848261233
"5974","FBXW5",118.391949301405,-0.197509298619944,0.21475994436647,-0.91967475221036,0.3577427502414,0.980372848261233
"5975","SSNA1",122.36731949153,-0.198151050717541,0.253553138150647,-0.781497133747996,0.434510161848849,0.980372848261233
"5976","TPRN",10.2319405030361,-0.422124204025298,0.465546935646742,-0.906727489117461,0.364550922992561,0.980372848261233
"5977","ARRDC1",20.4955045378805,-0.252790624902156,0.325979004823472,-0.775481307573934,0.43805530667309,0.980372848261233
"5978","ASB13",16.8803939132184,0.323425803948239,0.438618757980986,0.737373397884318,0.460895308257377,0.980372848261233
"5979","IL2RA",1.85664893130033,0.894364413505022,1.1626142217269,0.769270147217507,0.441732955337582,0.980372848261233
"5980","RBM17",171.901285601443,-0.160089324857263,0.208241715135257,-0.76876683787051,0.442031738740862,0.980372848261233
"5981","USP6NL",14.8149151338728,-0.300395043900986,0.412899304696745,-0.72752615585442,0.466903698437491,0.980372848261233
"5982","DHTKD1",23.3194137982167,0.303477689530983,0.372714458233091,0.814236429060639,0.415509519919394,0.980372848261233
"5983","SEPHS1",52.7882633099458,-0.214072452306155,0.27545679725518,-0.777154364819834,0.437067702653491,0.980372848261233
"5984","MINDY3",32.219159375523,0.232239921749803,0.296251036389817,0.78392948284648,0.433081488925214,0.980372848261233
"5985","PTER",35.4768975972658,0.276387328205243,0.317137015282399,0.871507628837113,0.383477042246362,0.980372848261233
"5986","ARL5B",66.3037743797682,0.271269970703632,0.32911605307761,0.824238040554231,0.409804316251204,0.980372848261233
"5987","THNSL1",7.0066652453879,0.495077889543812,0.653082732238471,0.758063052512368,0.448413237067124,0.980372848261233
"5988","ABI1",88.1503197396759,0.21431844846421,0.236370781766837,0.906704487171433,0.36456308990933,0.980372848261233
"5989","ACBD5",41.4769159027815,0.195487341792545,0.265698936136482,0.735747551853685,0.461884345041238,0.980372848261233
"5990","BAMBI",7.12675859871169,0.590568830460096,0.748665720633733,0.788828464004187,0.430212270757232,0.980372848261233
"5991","AL365203.2",1.99149489779541,-0.922787745748568,1.11488595400004,-0.827696987694347,0.407842125518661,0.980372848261233
"5992","ZNF248",19.2145308436584,0.360798975374228,0.443740533938292,0.813085458234027,0.416169063219109,0.980372848261233

"5993","ZNF25",27.8785566846289,0.302706785970922,0.347059209365862,0.
872205023817175,0.383096539326221,0.980372848261233
"5994","AL022345.4",2.20573684192133,-0.865170703534927,0.995711365342
269,-0.868897085690621,0.38490342373374,0.980372848261233
"5995","HNRNPF",446.78313807451,-0.184096128260313,0.252252131363261,-
0.729810001070722,0.465506330416904,0.980372848261233
"5996","ZNF22",179.680350665025,0.172169847618408,0.204334970870819,0.
842586302700257,0.39945985426043,0.980372848261233
"5997","TIMM23",40.9935550008268,0.271100842729384,0.320039866224844,0
.847084602075549,0.396947967058388,0.980372848261233
"5998","NCOA4",115.929351027502,0.176756187578885,0.221191962985593,0.
799107640228311,0.424227999864316,0.980372848261233
"5999","AGAP9",7.80979963825859,0.515452529015763,0.579634782825093,0.
889271217478512,0.373857336252175,0.980372848261233
"6000","TMEM273",3.23292089975037,0.64306831560671,0.78941974073421,0.
814608860691293,0.415296236902348,0.980372848261233
"6001","CCDC6",85.0173402807968,0.23023561083496,0.248392175564326,0.9
26903636605639,0.353976568905221,0.980372848261233
"6002","ANK3",4.18419600278431,0.645288255192256,0.781907765926205,0.8
25274135022668,0.409215972766331,0.980372848261233
"6003","NRBF2",112.454790657739,-0.241490724440521,0.270964668793021,-
0.891225876481285,0.372808004923243,0.980372848261233
"6004","REEP3",98.2532770211428,-0.177602091954586,0.19335308758015,-0
.918537656560385,0.358337453851174,0.980372848261233
"6005","VPS26A",125.949093967194,-0.18361972990613,0.200055541850762,-
0.917843755826103,0.358700670859179,0.980372848261233
"6006","SPOCK2",26.0447599617166,0.315959144269565,0.386695996773744,0
.8170737398516,0.413886288889779,0.980372848261233
"6007","ANAPC16",215.196612276488,-0.158639879547986,0.216431135422662
,-0.732980858960904,0.463570103881503,0.980372848261233
"6008","AC016394.2",2.15440210358124,-0.808623403088902,0.943257005285
454,-0.857267317982115,0.391297166114779,0.980372848261233
"6009","DNAJC9",35.945018456631,-0.251085549907403,0.304577433869617,-
0.824373449856062,0.409727395881786,0.980372848261233
"6010","AC016394.1",4.03520103841435,-0.633632747427484,0.685917438014
946,-0.923774075873076,0.355603955224783,0.980372848261233
"6011","ANXA7",270.289210420255,-0.191584395248901,0.221607641451213,-
0.864520708736833,0.38730190923045,0.980372848261233
"6012","PPP3CB-
AS1",2.13026337563233,-0.818256097193946,1.01134324018342,-0.809078525
155855,0.418469979784227,0.980372848261233
"6013","AC073389.1",2.63390336938727,0.723789353997263,0.9077856024411
32,0.797313101299377,0.425269213328547,0.980372848261233
"6014","LRMDA",35.9344156106398,0.264498290232858,0.310910005122134,0.
850722993391467,0.394923247295779,0.980372848261233
"6015","RPS24",6399.0666093135,-0.169984137393313,0.224145251247895,-0
.758365999042819,0.448231906748112,0.980372848261233
"6016","ZMIZ1-
AS1",3.66113562421538,-0.532038744793738,0.721303902079528,-0.73760691
3341053,0.460753353123041,0.980372848261233

"6017","ZCCHC24",235.775026690826,0.188006676526427,0.224420431190005,
0.837743139203096,0.402174979559688,0.980372848261233
"6018","EIF5AL1",12.0191324477062,-0.481760180961832,0.523177096247585
,-0.920835763677712,0.357136179944421,0.980372848261233
"6019","BMPR1A",47.8725746874753,0.258723705448988,0.320307960170005,0
.807734235863725,0.419243590602436,0.980372848261233
"6020","MMRN2",2.20207164871217,-0.802531100526754,0.977916791880878,-
0.820653768490062,0.411843511500128,0.980372848261233
"6021","RNLS",11.0959994589235,-0.330096851416867,0.454488403216377,-0
.72630423368517,0.467652284527712,0.980372848261233
"6022","PDLIM1",343.529036055882,0.183236511581381,0.244307273566908,0
.750024790118248,0.453239774427431,0.980372848261233
"6023","TM9SF3",283.68955095725,0.152350154517059,0.167666894835747,0.
90864779637213,0.363536068349318,0.980372848261233
"6024","EXOSC1",53.9660819718152,-0.240640573027196,0.301113689874312,
-0.799168490571259,0.424192719897911,0.980372848261233
"6025","PI4K2A",35.0015355405246,0.21234691428048,0.291997026886441,0.
727222864372049,0.467089441834955,0.980372848261233
"6026","ZFYVE27",20.3349839311792,-0.2548142338737,0.339503406657754,-
0.750549858636832,0.452923607010585,0.980372848261233
"6027","CRTAC1",2.68300504027179,-1.84758282483169,2.42627931568689,-0
.761488099447705,0.446365584284306,0.980372848261233
"6028","BLOC1S2",95.5942045842,-0.194409603800641,0.260247255961437,-0
.747018842071667,0.455052187297969,0.980372848261233
"6029","NPM3",48.0557629028188,-0.252677271142048,0.291585437911444,-0
.866563409174046,0.386181269453041,0.980372848261233
"6030","CUEDC2",121.392927263638,-0.18327534455357,0.227157684437449,-
0.806819918980281,0.41977024140034,0.980372848261233
"6031","BORCS7",201.546030045217,-0.160395677285411,0.184687807434363,
-0.86846922660238,0.385137511787078,0.980372848261233
"6032","AS3MT",7.10603440772325,-0.430748263943703,0.560440327967399,-
0.768588986995167,0.442137345375834,0.980372848261233
"6033","PDCD4",226.000489993715,0.189399633339905,0.20979863659652,0.9
02768656710361,0.366648689322534,0.980372848261233
"6034","GPAM",15.8153323268081,0.302454775810494,0.39105791531953,0.77
3427065306582,0.439269677357196,0.980372848261233
"6035","RGS10",135.242643055537,-0.252399763166654,0.33015006702299,-0
.764500111850889,0.444569270056045,0.980372848261233
"6036","NSMCE4A",67.8317955086905,-0.248400646907113,0.275412726067811
,-0.901921456040315,0.367098592737626,0.980372848261233
"6037","ABRAXAS2",28.2499603170506,-0.260355895684311,0.33423519870246
1,-0.778960135542403,0.436003198033689,0.980372848261233
"6038","PTPRE",31.58278175084,0.360212491173892,0.432624328731046,0.83
2621901386016,0.405058003050579,0.980372848261233
"6039","C10orf143",7.40486376732481,-0.4336965198369,0.571799714580424
,-0.7584762789802,0.448165908423738,0.980372848261233
"6040","STK32C",14.6252372862032,-0.28218277048927,0.389624310676357,-
0.724243233178707,0.468916420821035,0.980372848261233
"6041","ANO9",2.0428523392187,0.937197395292048,1.17348815414192,0.798
642399571004,0.424497794971784,0.980372848261233

"6042","PTDSS2",35.015999007396,-0.265022297570725,0.319697363759565,-0.828978676752681,0.407116473267205,0.980372848261233
"6043","DEAF1",50.784802231975,-0.255572412813349,0.308609986695915,-0.82814044856291,0.407590964684495,0.980372848261233
"6044","EPS8L2",39.315824355168,0.204588492903518,0.282316703070395,0.724677253164521,0.468650053062463,0.980372848261233
"6045","GATD1",27.719178515167,0.284847675246147,0.311118762562006,0.915559296072275,0.359898088573761,0.980372848261233
"6046","AP006623.1",7.63125023833216,0.495014325580562,0.581279752360293,0.85159395896821,0.394439493680676,0.980372848261233
"6047","CHID1",153.450968268137,0.19667465204604,0.269483530845547,0.729820673749308,0.465499805811305,0.980372848261233
"6048","AP2A2",87.7785564319305,0.248503874532537,0.286809885581201,0.866441106201622,0.386248309952927,0.980372848261233
"6049","TOLLIP",49.6760017204373,-0.217940612508455,0.27303106733062,-0.798226423971546,0.424739105956143,0.980372848261233
"6050","DUSP8",9.98401323584172,0.382719150458201,0.513727131485445,0.744985279153091,0.456280619127515,0.980372848261233
"6051","LSP1",483.953861003469,0.164932325285641,0.224135581011612,0.735859628093125,0.461816128673337,0.980372848261233
"6052","DNHD1",4.97079834824956,0.534122813787721,0.681760979892843,0.783445854985237,0.433365337580341,0.980372848261233
"6053","ZNF215",3.89741453913862,-0.714606369074122,0.78352624347324,-0.912038843659393,0.361748269995077,0.980372848261233
"6054","TMEM9B",109.518250616813,-0.2202718444403,0.253895978410976,-0.867567284125117,0.385631262884465,0.980372848261233
"6055","AC132192.1",2.89524107815366,-0.72009793085722,0.99343408465303,-0.724857282412174,0.468539589670111,0.980372848261233
"6056","RNF141",33.4352172635014,0.230039883884959,0.289129906107005,0.795628120875961,0.42624821672468,0.980372848261233
"6057","MRVI1",3.08319093703677,-0.832536281407838,1.08471744302142,-0.767514422086598,0.442775721751312,0.980372848261233
"6058","CTR9",91.7052236437361,0.17858528929617,0.222557161445331,0.802424366560038,0.422307522776102,0.980372848261233
"6059","SERGEF",26.123231431776,-0.276131989008002,0.346442729144181,-0.797049456601766,0.425422308843117,0.980372848261233
"6060","SAAL1",12.5088721861947,-0.40093885403499,0.490868438672387,-0.816794934136277,0.414045627724251,0.980372848261233
"6061","HPS5",21.6518408104263,0.260246510329675,0.337609462156907,0.770850759534467,0.440795396376783,0.980372848261233
"6062","LGR4",62.5436751714893,-0.360065898390066,0.422319805365988,-0.852590605070079,0.393886374358118,0.980372848261233
"6063","METTL15",31.617580641932,0.235874300991422,0.302042079777321,0.780931919040285,0.434842537645582,0.980372848261233
"6064","EIF3M",262.438711956894,-0.187629112991518,0.25745174299556,-0.728793329609557,0.466128092467655,0.980372848261233
"6065","LINC00294",17.0478480991618,0.383498559383996,0.419223341682628,0.914783413167684,0.360305344154872,0.980372848261233
"6066","AL356215.1",1.92845580066483,-0.8824307347451,1.08047228604469,-0.816708347027976,0.414095120066999,0.980372848261233

"6067","AL133330.1",50.7546831006729,0.766670698286171,0.835719027621078,0.917378536263011,0.358944316045143,0.980372848261233
"6068","COMMD9",61.1100360802153,-0.215295817209117,0.273727812737251,-0.78653248661939,0.431555594471908,0.980372848261233
"6069","API5",64.7063894001726,-0.231720869652892,0.259426133730169,-0.893205577715261,0.371747091662848,0.980372848261233
"6070","EXT2",97.7113814414376,0.151976514370204,0.186807974230267,0.813544041663189,0.415906206145163,0.980372848261233
"6071","LINC02716",2.79353019066166,0.742433050597467,0.843749503327887,0.879921170524177,0.378902015164438,0.980372848261233
"6072","SLC35C1",26.9108266347756,0.26897172738687,0.293470294671931,0.916521134404941,0.359393627748393,0.980372848261233
"6073","LRP4",8.44692912968145,0.462462658934804,0.534183740560941,0.865737056034646,0.386634373933113,0.980372848261233
"6074","C11orf49",32.8882578677751,-0.263495127640754,0.327507480192429,-0.804546899160703,0.421081195193142,0.980372848261233
"6075","PACSIN3",8.71994701155861,-0.494326259511857,0.533094925523539,-0.927276242643638,0.353783125994957,0.980372848261233
"6076","KBTBD4",15.6130950875043,-0.344917640240136,0.373209258116297,-0.924193686890411,0.355385481954841,0.980372848261233
"6077","TIMM10",105.564750817674,-0.254615774438657,0.308157061450247,-0.826253252936621,0.408660445398971,0.980372848261233
"6078","YPEL4",12.0415511647348,-0.398441175129906,0.476504514720176,-0.836175026303556,0.403056445677512,0.980372848261233
"6079","ZDHHC5",48.8065465152136,0.203275953889473,0.247344351656469,0.821833822070855,0.411171481443463,0.980372848261233
"6080","TMX2",102.894775452064,-0.21544273011756,0.255893040581336,-0.841924929369389,0.399829975215636,0.980372848261233
"6081","DTX4",18.6623444844307,0.404467051437098,0.44368161828854,0.911615525108504,0.361971146807914,0.980372848261233
"6082","STX3",13.7167814272042,-0.408623754979494,0.503715877760634,-0.811218730678313,0.417240070953763,0.980372848261233
"6083","CD6",12.4343106445312,0.396762783275516,0.433523357277655,0.915205090140979,0.360083972948198,0.980372848261233
"6084","VPS37C",17.0854045353253,0.342204980395889,0.396563258238709,0.862926590616977,0.38817782970852,0.980372848261233
"6085","PPP1R32",2.64170157621756,0.866111457675295,0.960220676164845,0.901992093249413,0.367061067913812,0.980372848261233
"6086","DAGLA",5.65584332738062,-0.536738378282827,0.603222346280484,-0.889785303201046,0.373581179565772,0.980372848261233
"6087","TMEM258",514.931545686405,-0.202358556351595,0.222844403419505,-0.908071072220986,0.363840672873104,0.980372848261233
"6088","FEN1",14.0484977048928,-0.341051496193647,0.41601599964067,-0.819803797181424,0.412327966042812,0.980372848261233
"6089","FADS1",13.2964112724099,-0.448590606106349,0.483684165681571,-0.927445299918448,0.353695379913214,0.980372848261233
"6090","BEST1",10.5543993160094,-0.412393638946809,0.464306165737742,-0.888193328838421,0.374436766653497,0.980372848261233
"6091","INCENP",4.26801629766129,0.584737860541507,0.709489026199789,0.824167589558809,0.40984433991503,0.980372848261233

"6092","GANAB",151.872429929981,0.150619560879306,0.176179197298329,0.854922506113238,0.392594050173093,0.980372848261233
"6093","POLR2G",123.687518070141,-0.18623176502334,0.229999398712143,-0.809705442997352,0.418109488980179,0.980372848261233
"6094","STX5",93.5215869879622,-0.179211094042822,0.230178820234235,-0.778573345108179,0.436231085709978,0.980372848261233
"6095","ATL3",183.093095548739,0.168250264031055,0.19338731561605,0.870017061331461,0.384291080448981,0.980372848261233
"6096","C11orf95",33.1053926709585,0.232776045127277,0.318984953752989,0.729739890200373,0.465549193064567,0.980372848261233
"6097","COX8A",391.108277388198,-0.212832287952115,0.245571361553123,-0.866682037376227,0.386116250070026,0.980372848261233
"6098","MACROD1",35.3278790363262,0.216381055128369,0.26885327191707,0.804829539865573,0.420918052770507,0.980372848261233
"6099","BAD",182.39085596492,-0.198664361679339,0.259955569552172,-0.764224294257594,0.444733591348345,0.980372848261233
"6100","RPS6KA4",27.095089859185,-0.256644879854163,0.306756173826492,-0.83664128631141,0.402794231201934,0.980372848261233
"6101","ZNHIT2",10.2628067002667,-0.334290762310972,0.452724651608401,-0.738397525125555,0.46027291806985,0.980372848261233
"6102","SYVN1",53.5131497466391,-0.212022948508122,0.271767231213633,-0.780163773098357,0.435294482835571,0.980372848261233
"6103","POLA2",7.69540681555198,-0.424282633817453,0.5811483935795,-0.730076239571351,0.465343584093157,0.980372848261233
"6104","SCYL1",71.3108765810891,-0.188782801456873,0.227914493464594,-0.828305381492553,0.407497575928476,0.980372848261233
"6105","SIPA1",26.342208627036,0.402364504176618,0.43698409064496,0.920776094119937,0.35716733849917,0.980372848261233
"6106","RELA",81.2103878228006,-0.20395820263445,0.227424311013668,-0.896817942309573,0.369816072197021,0.980372848261233
"6107","MUS81",27.7502586075635,0.257764164643234,0.334199308201019,0.771288743925795,0.440535802697506,0.980372848261233
"6108","C11orf68",54.2726725366538,-0.216494789558688,0.261135693620675,-0.829050929641076,0.407075588883268,0.980372848261233
"6109","SART1",57.569673776389,-0.190159654481988,0.233261794501652,-0.815219890116389,0.414946454574899,0.980372848261233
"6110","PACS1",70.8920767133991,0.193920459602344,0.220038653309929,0.881301792595515,0.378154496113078,0.980372848261233
"6111","BRMS1",59.8377741942025,-0.210125164563943,0.252294414016462,-0.832856983310904,0.404925392687562,0.980372848261233
"6112","AP001107.9",2.12874181318411,-0.830320403078781,0.975092068693904,-0.851530260307584,0.394474861175397,0.980372848261233
"6113","BBS1",22.6880485075462,0.268710135409653,0.346433753140246,0.775646521085005,0.437957724229285,0.980372848261233
"6114","POLD4",166.356389519221,-0.229696895632462,0.273450746486106,-0.839993668271566,0.400911936652097,0.980372848261233
"6115","AIP",160.412361184153,-0.193997256876002,0.22821301500306,-0.850070960560252,0.395285635508672,0.980372848261233
"6116","TCIRG1",52.6100273958726,-0.194828048346131,0.245649696500865,-0.793113328130835,0.427711798231687,0.980372848261233

"6117","AP002807.1",2.61496482444639,0.716633183968246,0.916921153080646,0.781564676047141,0.43447045326329,0.980372848261233
"6118","MRGPRF",104.135104056828,-0.195573406177935,0.241737250728817,-0.809032971080371,0.418496181453933,0.980372848261233
"6119","AP003555.1",7.47954199480222,-0.40277409782687,0.552355875089319,-0.729193108992892,0.465883545853757,0.980372848261233
"6120","ANO1",2.916767732733,-1.04472784645189,1.13176337521017,-0.923097415356707,0.355956441452303,0.980372848261233
"6121","AP000487.1",3.35414340681004,-0.689213980242192,0.776911270685505,-0.88712058409716,0.375013982928373,0.980372848261233
"6122","IL18BP",16.9017657440181,-0.466199161525696,0.527683566517464,-0.883482433615387,0.376975669483254,0.980372848261233
"6123","LAMTOR1",324.446772157286,-0.215551982478059,0.251665354368113,-0.856502409794435,0.391719940477987,0.980372848261233
"6124","SPCS2",554.672164067097,-0.187838612900093,0.254029932811467,-0.739434958790866,0.459642920895831,0.980372848261233
"6125","LRRC32",94.7912160103611,-0.24014198846761,0.271924008110365,-0.883121685857706,0.37717052851124,0.980372848261233
"6126","GAB2",18.8802854328373,0.433255673869386,0.466221872145614,0.929290751365838,0.352738426944532,0.980372848261233
"6127","PCF11",137.649100639069,0.178220534867843,0.198238266642508,0.899021858323832,0.368641017944411,0.980372848261233
"6128","ANKRD42",20.6435924075065,-0.280361736638725,0.385794890070132,-0.726711897577906,0.467402463517582,0.980372848261233
"6129","CCDC89",10.3463087430566,-0.467336768500661,0.517245021245026,-0.903511390744305,0.366254545564785,0.980372848261233
"6130","TAF1D",254.076738558467,0.157680523105001,0.184645819543069,0.853962052838256,0.393126015422727,0.980372848261233
"6131","FAM76B",35.4155219267525,-0.251668718725712,0.318796260016508,-0.789434351308514,0.429858185241216,0.980372848261233
"6132","CASP4",304.850630885694,0.198916184562686,0.273734249024594,0.726676275517185,0.467424290166021,0.980372848261233
"6133","MSANTD4",28.420604611941,0.262201626374781,0.330947377112869,0.792275885858908,0.428199829070818,0.980372848261233
"6134","KBTBD3",15.0829837864145,-0.34799380783871,0.393536042882243,-0.884274297444312,0.376548159516149,0.980372848261233
"6135","ATM",97.7380914545722,0.206781510023527,0.248393381979851,0.832475923373434,0.405140362715439,0.980372848261233
"6136","EXPH5",5.21808640683563,-0.557374846316477,0.698721204440834,-0.797707072254274,0.425040498791281,0.980372848261233
"6137","C11orf1",21.4493794729443,-0.296064879313439,0.388738605694308,-0.761604005819414,0.446296383293762,0.980372848261233
"6138","PTS",42.7205429332604,-0.238265931443322,0.310124883121517,-0.76829027405054,0.442314751567896,0.980372848261233
"6139","ZW10",14.5564918448504,-0.328336672665403,0.432985637223415,-0.758308462079505,0.448266342611138,0.980372848261233
"6140","USP28",27.4135595482226,0.26496413213628,0.319424654630665,0.829504323774394,0.406819091169269,0.980372848261233
"6141","BACE1-AS",8.07807498809866,0.471540712037505,0.558312901691478,0.84458143562

3133,0.39834457770771,0.980372848261233
"6142","SIAE",11.9760301397506,0.33605242005539,0.446769262784201,0.752183393193079,0.451940779058797,0.980372848261233
"6143","ROBO3",91.2613492868604,-0.227454200332948,0.2613700464886,-0.870238205902716,0.384170240823927,0.980372848261233
"6144","PUS3",33.3155495889726,-0.310037973024267,0.394672417993207,-0.785557740773269,0.43212663029104,0.980372848261233
"6145","FAM118B",14.1550929001994,-0.367602293140647,0.456672683658608,-0.804957919084674,0.420843963537189,0.980372848261233
"6146","ETS1",110.207008745002,0.211403344746808,0.264090876116527,0.800494692794797,0.423424237238146,0.980372848261233
"6147","PRDM10",5.65147654625764,0.509551918700143,0.638813131430596,0.797654108266406,0.425071242140758,0.980372848261233
"6148","ADAMTS15",33.2560239889657,0.378642497424438,0.468628946088658,0.807979320493796,0.419102486781346,0.980372848261233
"6149","SNX19",33.7974469906741,0.276519691143486,0.310216039243844,0.891377801797438,0.372726522427426,0.980372848261233
"6150","NCAPD3",14.4460519944372,-0.348385735841774,0.407961393929013,-0.853967412177228,0.39312304584012,0.980372848261233
"6151","VPS26B",55.1483462381053,0.191083902169864,0.227300812800492,0.840665283223529,0.400535474688407,0.980372848261233
"6152","THYN1",90.9518920770535,-0.230484661513439,0.306450569424646,-0.752110403795851,0.451984667911187,0.980372848261233
"6153","RAD52",5.67899158863024,0.532504053533948,0.675057309485771,0.788827920313293,0.430212588570679,0.980372848261233
"6154","ADIPOR2",41.9866071502517,0.229212622651033,0.264207892508017,0.867546463034134,0.385642665555684,0.980372848261233
"6155","FKBP4",67.9560808149376,-0.252955660512596,0.294552549957836,-0.858779394538617,0.390462237883707,0.980372848261233
"6156","TULP3",88.945249149179,0.168849305396166,0.197258196421761,0.85598118840723,0.392008184815336,0.980372848261233
"6157","TEAD4",7.40302038394751,-0.675335690984592,0.777348232899014,-0.86876854208058,0.384973742706308,0.980372848261233
"6158","ING4",54.7211656327826,0.23200514651861,0.293160222640059,0.79139367690911,0.428714298640053,0.980372848261233
"6159","MLF2",184.122255817611,-0.203485369232135,0.262095396251687,-0.77637902894231,0.437525223162684,0.980372848261233
"6160","SPSB2",16.803682179442,0.293346023412716,0.380185417769609,0.771586730321368,0.440359236054169,0.980372848261233
"6161","C12orf57",749.271399442727,-0.19953439990699,0.238833301152581,-0.835454683011379,0.403461752066972,0.980372848261233
"6162","C1RL",117.37103663565,0.201501424922638,0.262420983541728,0.767855612013576,0.442572970919526,0.980372848261233
"6163","CLSTN3",72.7325338954924,-0.294937220779881,0.351547367292658,-0.83896865179579,0.40148690114693,0.980372848261233
"6164","C3AR1",3.29083726222915,-0.598525343089212,0.823630219503746,-0.726691819843419,0.467414765646323,0.980372848261233
"6165","CLEC4E",2.53003332216724,0.771234569302669,0.965387564129959,0.798885958301869,0.424356541683782,0.980372848261233
"6166","CD69",98.9205601512664,0.314467165953612,0.355663024588539,0.8

84171657476663,0.376603555711501,0.980372848261233
"6167","CLEC12A",4.3286653963187,0.715624653886065,0.895253630993758,0
.799354092640429,0.424085121655885,0.980372848261233
"6168","TAS2R30",2.25594826401932,0.853140850303288,1.11598925608962,0
.76447048719148,0.444586917604278,0.980372848261233
"6169","LINC01252",5.11484603092707,0.759806616550619,0.86996779667131
1,0.873373266755168,0.382459657371264,0.980372848261233
"6170","ETV6",53.3919628323379,0.200906779557066,0.239741357175344,0.8
38014691850291,0.402022452176657,0.980372848261233
"6171","DUSP16",25.6652270719934,0.234503954631269,0.296883788345517,0
.789884674869316,0.429595122143344,0.980372848261233
"6172","ERP27",2.24530076795992,0.876269783821173,1.00886892006784,0.8
68566536634162,0.38508426438174,0.980372848261233
"6173","STRAP",196.362311434486,-0.232954533107837,0.308929070926718,-
0.754071257874908,0.450806434966947,0.980372848261233
"6174","DERA",92.5165429556103,-0.224041013390708,0.258568418549965,-0
.866467044378877,0.386234091320834,0.980372848261233
"6175","PYROXD1",34.7256831759707,-0.207066417521826,0.268902849120418
,-0.770041738862717,0.441275134027645,0.980372848261233
"6176","ETFRF1",82.8306460775389,-0.190874717199728,0.25429583906749,-
0.750601023987144,0.452892804707829,0.980372848261233
"6177","AC009318.1",6.47624082232021,0.506618788380425,0.5985182917816
19,0.846454979466652,0.397298978812287,0.980372848261233
"6178","TMTC1",36.6019030393554,0.228053406873052,0.309068403050266,0.
737873573041893,0.460591279348561,0.980372848261233
"6179","BICD1",22.8083822346492,0.318198291126698,0.399273576726831,0.
796943022714468,0.425484122922486,0.980372848261233
"6180","FGD4",38.8151956625713,0.314045487150348,0.341622021420504,0.9
19277644469496,0.357950367864653,0.980372848261233
"6181","YAF2",57.449926248633,-0.203784724111392,0.232299582983906,-0.
877249633829564,0.380351062416569,0.980372848261233
"6182","TMEM117",5.73490599089629,0.436417950013897,0.593772310622663,
0.734992087381516,0.462344313079786,0.980372848261233
"6183","DBX2",10.9667009230859,0.474165578989876,0.586393405542919,0.8
08613423186204,0.418737541274577,0.980372848261233
"6184","DDX23",72.3261960379136,0.207621659593746,0.240144943980244,0.
864568106879762,0.387275883825354,0.980372848261233
"6185","LMBR1L",34.6245967724599,0.273211534214515,0.366343892347038,0
.745778870405464,0.455801005925367,0.980372848261233
"6186","TUBA1B",971.199794133357,-0.233920639655591,0.282957000986541,
-0.826700307255228,0.408406947175231,0.980372848261233
"6187","RACGAP1",5.15682119766949,-0.48736371556305,0.643938496132656,
-0.756848237044442,0.449140789857301,0.980372848261233
"6188","COX14",178.50916609088,-0.190581559917171,0.262808989300973,-0
.725171389396098,0.468346892595783,0.980372848261233
"6189","LIMA1",798.871227938293,-0.140203435106523,0.155064862443547,-
0.904159929575056,0.365910604240592,0.980372848261233
"6190","DIP2B",26.1195745132484,0.365311759436509,0.405673320627248,0.
900507232942181,0.367850377901192,0.980372848261233
"6191","CSRNP2",24.5892302791587,0.257122494503783,0.318508631040352,0

.807270100229116,0.419510886317883,0.980372848261233
"6192","ITGB7",6.44267086650746,-0.54173363111042,0.652675889727705,-0
.83001937046645,0.406527831896216,0.980372848261233
"6193","SP1",87.3660203187066,0.222490432326142,0.275426337319274,0.80
7803765215929,0.419203557276107,0.980372848261233
"6194","TARBP2",15.7754616563026,-0.354645840126418,0.389711580131824,
-0.910021303463591,0.362811274955287,0.980372848261233
"6195","IKZF4",23.3109241894505,0.323603757710001,0.421846823165178,0.
76711199406921,0.443014931339821,0.980372848261233
"6196","STAT2",123.187640474045,0.311732197875136,0.341572109372986,0.
912639496378594,0.361432174555011,0.980372848261233
"6197","TIMELESS",2.70189877453693,0.79818592234306,0.886591828588636,
0.900285674427758,0.367968242734579,0.980372848261233
"6198","PTGES3",470.698347896258,-0.172036262391294,0.214559297016634,
-0.801812201957188,0.422661599270646,0.980372848261233
"6199","NACA",3840.6404931304,-0.214165046852168,0.261936590675612,-0.
817621724020203,0.413573219022783,0.980372848261233
"6200","NAB2",25.6987483357029,-0.339364103794906,0.372012307703818,-0
.912238914592834,0.361642962778474,0.980372848261233
"6201","SHMT2",64.290606812296,-0.227969356051935,0.256942136348969,-0
.887240058369859,0.374949669706177,0.980372848261233
"6202","CTDSP2",264.351065816752,-0.151627702431305,0.206777132581775,
-0.733290478198019,0.463381281278393,0.980372848261233
"6203","CAND1",103.22590734883,0.163614149164299,0.192815079311577,0.8
48554738293624,0.396129101541527,0.980372848261233
"6204","NUP107",47.7076428152642,-0.195559687707461,0.25714971096293,-
0.760489626743746,0.446961967488451,0.980372848261233
"6205","ATXN7L3B",134.187885921585,-0.159277452702656,0.21595350263387
6,-0.737554384439375,0.460785283556016,0.980372848261233
"6206","OSBPL8",383.967436756971,0.189481472977437,0.227267298784715,0
.833738395231812,0.404428416955887,0.980372848261233
"6207","ATP2B1-
AS1",47.0514200862071,-0.263555532259223,0.325971819386193,-0.80852244
45337,0.418789890779831,0.980372848261233
"6208","NUDT4",117.158860821433,0.311001881929055,0.407681014844642,0.
762855935412079,0.445549317939613,0.980372848261233
"6209","ELK3",80.1513199858836,-0.207985892498094,0.247743757197838,-0
.839520215768765,0.401177449805241,0.980372848261233
"6210","TMPO",54.5216183359406,0.224049671154823,0.251697227022979,0.8
90155500737275,0.373382394984375,0.980372848261233
"6211","NUP37",36.8923117637047,0.230271736065545,0.274280540172066,0.
839548208272771,0.401161748614372,0.980372848261233
"6212","SLC41A2",47.8566737135926,-0.259077205067382,0.302705845712573
,-0.855871165809538,0.392069045635167,0.980372848261233
"6213","PRDM4",29.5856601543822,0.246180018025396,0.299167772535036,0.
822882812340908,0.410574637687116,0.980372848261233
"6214","ALKBH2",53.4835315545542,-0.244964010049021,0.309742716978997,
-0.790862856883997,0.429024025156672,0.980372848261233
"6215","ATP2A2",140.170562240661,0.183330134887136,0.210393695977952,0
.871367053252147,0.383553769152417,0.980372848261233

"6216","FAM216A",16.7887128548655,0.300271268692588,0.396974138122335,
0.756400077125561,0.44940936177198,0.980372848261233
"6217","VPS29",217.42152612938,-0.219010698398262,0.2906603396492,-0.7
53493574880521,0.451153370833875,0.980372848261233
"6218","MAPKAPK5",35.8916793132693,-0.233474701890662,0.26394594085763
,-0.884555000664305,0.37639668582327,0.980372848261233
"6219","OAS2",24.1959478901456,0.326217525089708,0.428204693458264,0.7
6182613145856,0.446163781992986,0.980372848261233
"6220","DDX54",73.6294619995438,-0.21227801702067,0.237227949922773,-0
.894827178204652,0.370879479428464,0.980372848261233
"6221","RITA1",15.2855797258685,-0.342165682574151,0.41914292816155,-0
.816346070957137,0.414302231475089,0.980372848261233
"6222","IQCD",3.50191416964017,0.761785994318488,0.829332142853966,0.9
18553562505087,0.358329130719818,0.980372848261233
"6223","BICDL1",4.14572243524066,-0.701380032975469,0.763106293893166,
-0.919111844035795,0.358037074812459,0.980372848261233
"6224","PXN-
AS1",12.9465640688051,0.395371437473473,0.42611969448636,0.92784126758
1047,0.3534899139968,0.980372848261233
"6225","SIRT4",1.84875304991332,0.856153347493492,1.09495001963575,0.7
81910892862762,0.434266942910523,0.980372848261233
"6226","MLEC",217.931936056713,0.157101504752577,0.183397245897237,0.8
56618669402516,0.391655664474887,0.980372848261233
"6227","P2RX7",9.10734437925114,0.465359055067165,0.533044681327384,0.
873020726720939,0.38265178047592,0.980372848261233
"6228","ORAI1",35.0383601084359,0.289204071825292,0.36281009673876,0.7
97122446218835,0.425379921371434,0.980372848261233
"6229","TMEM120B",14.1548837013016,0.331594208194815,0.420199726411229
,0.789134755100481,0.43003325056583,0.980372848261233
"6230","SETD1B",27.9970803158574,0.246528997251581,0.329332525415203,0
.748571666101828,0.454115415001662,0.980372848261233
"6231","KNTC1",4.46718294258973,0.571667894118419,0.683673914834022,0.
836170404800664,0.403059045221904,0.980372848261233
"6232","VPS37B",29.1569093068801,0.297051767518304,0.362360465024485,0
.819768700479596,0.412347977221694,0.980372848261233
"6233","ARL6IP4",513.040658999779,-0.156914591450713,0.209562730111411
,-0.748771460303517,0.453994964133454,0.980372848261233
"6234","RAN",548.960606284913,-0.250652167537379,0.317429366480154,-0.
789631313311555,0.429743115495125,0.980372848261233
"6235","ULK1",32.5222311886489,0.326584630564923,0.376249363802034,0.8
68000485807487,0.38539406669351,0.980372848261233
"6236","PUS1",19.582325224736,0.345851400681321,0.414157801770547,0.83
5071557755974,0.403677419676721,0.980372848261233
"6237","EP400",49.248727777468,0.2584914634355,0.292989883007912,0.882
253888024247,0.377639526534007,0.980372848261233
"6238","PSPC1",61.9613604153477,0.168662642868909,0.231677418354849,0.
728006398148725,0.466609669696153,0.980372848261233
"6239","IFT88",26.1986825886555,-0.269072761312774,0.301257564458099,-
0.893165161833464,0.371768731618756,0.980372848261233
"6240","XPO4",18.831783021285,0.337535613452653,0.379613632495591,0.88

9155669235808,0.373919423865261,0.980372848261233
"6241","LATS2-
AS1",2.26879974540833,1.08848625870066,1.19884895342751,0.907942785943
695,0.363908450670109,0.980372848261233
"6242","LINC00539",3.28814506284788,-0.603741619435007,0.7890581591950
69,-0.765142103151044,0.444186931025534,0.980372848261233
"6243","POLR1D",228.757958027079,-0.174890175498433,0.195222555033148,
-0.895850253925511,0.370332745801986,0.980372848261233
"6244","PAN3-
AS1",1.88080339813453,-0.893634144816949,0.983734510264583,-0.90840987
6336045,0.363661709641332,0.980372848261233
"6245","SLC7A1",23.0334844299279,-0.272328605649871,0.357223805648766,
-0.762347305368649,0.445852747335127,0.980372848261233
"6246","RFC3",13.6165592198272,0.44998509944283,0.503677488765766,0.89
3399267347631,0.371643394880861,0.980372848261233
"6247","SUPT20H",44.4474559907628,0.223401729738279,0.255097511476194,
0.875750329532817,0.38116577793408,0.980372848261233
"6248","LINC01048",6.66220820490751,-0.706004877841994,0.8496470040486
21,-0.830939054075206,0.406008059777242,0.980372848261233
"6249","COG6",29.2250374287932,0.290286190888819,0.382874058821315,0.7
58176701191172,0.448345207141003,0.980372848261233
"6250","FOXO1",121.117988208206,0.276286602555782,0.325211205529105,0.
849560525155571,0.395569466608434,0.980372848261233
"6251","DNAJC15",266.673828858918,-0.196698001717544,0.262091019155933
,-0.750495008760741,0.452956628766461,0.980372848261233
"6252","CCDC122",20.8386830627217,-0.340257049647211,0.429523642933364
,-0.792173039238257,0.428259786668444,0.980372848261233
"6253","GPALPP1",44.6001414876907,0.208110536375636,0.258294038600948,
0.80571172878348,0.420409085117517,0.980372848261233
"6254","LCP1",46.9977570561203,0.256912866138827,0.337453424345493,0.7
61328371869752,0.446460958390927,0.980372848261233
"6255","ESD",363.41483792538,-0.177213112101492,0.231396479576442,-0.7
65841867714976,0.443770398900212,0.980372848261233
"6256","LINC00562",2.05094632416065,0.884522826627341,1.14154109138637
,0.774849747680231,0.438428449262546,0.980372848261233
"6257","RB1",42.2307133470007,0.215969604914596,0.262659868402459,0.82
2240589048336,0.410939982213775,0.980372848261233
"6258","VPS36",115.868699851342,0.15098609458011,0.206481297027686,0.7
31233757020935,0.464636384370506,0.980372848261233
"6259","AL136441.1",2.29452267309432,0.733769909853558,0.9848934303806
81,0.745024677004843,0.456256802029436,0.980372848261233
"6260","EDNRB",20.0920034418712,-0.506864144911765,0.564879223425571,-
0.897296490810925,0.369560728520813,0.980372848261233
"6261","AL590807.1",3.69282856524515,0.620777034424778,0.8539729797922
52,0.726928192243033,0.467269945763965,0.980372848261233
"6262","TGDS",20.3118829886026,0.261341604904219,0.337698821384327,0.7
73889597342693,0.438996081824218,0.980372848261233
"6263","DNAJC3",227.257730736416,-0.190956694259549,0.226514222989688,
-0.843022975507556,0.399215594315777,0.980372848261233
"6264","RAP2A",45.001979311486,0.1965022197704,0.263538922711953,0.745

628834436635,0.455891659610322,0.980372848261233
"6265","UBAC2-
AS1",3.04341263286101,-0.670310195118841,0.845607547069044,-0.79269656
1711399,0.427954634018009,0.980372848261233
"6266","POGLUT2",19.8087587865248,-0.329902121222207,0.398043237401211
,-0.828809762919497,0.407212062917647,0.980372848261233
"6267","IRS2",63.6449354217434,0.282948839099198,0.379107290932515,0.7
4635557233207,0.455452649546431,0.980372848261233
"6268","PARP2",23.5920334585392,-0.234854299662502,0.31031328092273,-0
.756829675365979,0.449151911635681,0.980372848261233
"6269","OSGEP",51.5763742252248,-0.179854734947214,0.241713064620228,-
0.744083631680383,0.456825881158431,0.980372848261233
"6270","APEX1",211.706225968441,-0.197903935708207,0.263883222093268,-
0.749967861307451,0.453274061313712,0.980372848261233
"6271","HNRNPC",472.390247001846,0.156321298894668,0.175950307306269,0
.888440044736982,0.374304092938253,0.980372848261233
"6272","METTL3",28.8542246233648,0.269029565240132,0.290442362448177,0
.926275227113725,0.354302966635274,0.980372848261233
"6273","OXA1L",98.480164036002,-0.1898106735851,0.239776869136592,-0.7
91613779379911,0.428585909888197,0.980372848261233
"6274","HAUS4",47.5239939984603,-0.242279298162609,0.274223705029171,-
0.883509681035183,0.376960954226412,0.980372848261233
"6275","AL136295.7",2.21338249271791,0.915878923488842,1.0826036502614
7,0.845996522612537,0.397554684165863,0.980372848261233
"6276","GMPR2",61.4090041519081,-0.188023640562515,0.234336459369037,-
0.802366140842014,0.422341193095637,0.980372848261233
"6277","AL096870.8",19.2201239936282,0.289524893290375,0.3823894388199
26,0.757146677962301,0.448961991611175,0.980372848261233
"6278","LINC00645",5.99622388830053,0.593747728606938,0.81667090245706
5,0.727034264133285,0.467204966066901,0.980372848261233
"6279","PRORP",28.3650349948158,-0.233624129060162,0.292836056105924,-
0.797798372805758,0.424987505741327,0.980372848261233
"6280","C14orf28",16.8177817443666,0.27425343936389,0.372570874666578,
0.736110785925833,0.46166327927825,0.980372848261233
"6281","MIS18BP1",71.6966018342029,0.186933773047951,0.250402445640824
,0.746533335844842,0.455345301849284,0.980372848261233
"6282","MAP4K5",96.4937122826957,0.181052904135747,0.239662614322922,0
.755449090995042,0.44997956744246,0.980372848261233
"6283","NIN",63.7705463931513,0.236159594515471,0.265460252057727,0.88
9623183451646,0.373668253457016,0.980372848261233
"6284","TRIM9",3.00837927342851,0.752559009397691,0.952904634597586,0.
789752701450023,0.429672206657032,0.980372848261233
"6285","FRMD6-
AS1",9.61523827781806,0.429233380785749,0.496414959777926,0.8646664898
61162,0.387221867034133,0.980372848261233
"6286","AL133453.1",20.8707831801342,-0.39268072603448,0.4905405035727
52,-0.800506223593097,0.423417559163468,0.980372848261233
"6287","AL139317.5",15.0931458275558,-0.408814375472174,0.508994663996
151,-0.803180081029818,0.421870656758158,0.980372848261233
"6288","MAPK1IP1L",113.032150986611,-0.156609536125725,0.1849171411882

49,-0.84691735508875,0.397041188243214,0.980372848261233
"6289","FBXO34",23.7011012000644,0.334762646952943,0.376249952733689,0
.889734721614407,0.37360834539261,0.980372848261233
"6290","KTN1",362.062165463493,-0.161310823272049,0.219297041976656,-0
.735581391422599,0.461985490666571,0.980372848261233
"6291","ARMH4",21.571791742061,0.325289165582275,0.360764711438015,0.9
01665698636838,0.367234479535601,0.980372848261233
"6292","TIMM9",103.699329933469,-0.257360077977289,0.296929768896028,-
0.866737205010269,0.386086015308538,0.980372848261233
"6293","DAAM1",127.471210922352,-0.203073752628939,0.262970168818341,-
0.772231137628475,0.439977542554438,0.980372848261233
"6294","SIX4",2.74487300329321,-0.712512933496339,0.840240542060332,-0
.847986853561249,0.396445292216727,0.980372848261233
"6295","TRMT5",30.3759651375622,0.270160965977,0.302354164689433,0.893
52487092248,0.371576159330657,0.980372848261233
"6296","WDR89",12.3366770803457,-0.319554444238137,0.420706380429251,-
0.759566431847535,0.447513790684009,0.980372848261233
"6297","ZBTB1",62.7683245665821,0.181315625099245,0.232699102107798,0.
779184893525075,0.435870807539363,0.980372848261233
"6298","PIGH",46.2877390387125,-0.201002024763418,0.273431681105117,-0
.735108762638759,0.462273258125616,0.980372848261233
"6299","ACOT2",33.934067634895,-0.364581389474303,0.437278108474957,-0
.833751752965203,0.404420888132896,0.980372848261233
"6300","ELMSAN1",125.916362454921,0.248659117826838,0.302553600569297,
0.821867984247918,0.41115203608714,0.980372848261233
"6301","ZNF410",39.2058111862111,0.225749543251257,0.263497399827581,0
.856742963683798,0.391586953452646,0.980372848261233
"6302","ERG28",38.5129836503481,-0.257433449136658,0.288086997567246,-
0.893596209862152,0.37153797498071,0.980372848261233
"6303","IFT43",66.1883324916359,0.227705761776905,0.261187025015272,0.
871811154338889,0.383311408462238,0.980372848261233
"6304","IRF2BPL",94.5208558804255,0.198571068812315,0.239425639577489,
0.829364261750455,0.40689831787034,0.980372848261233
"6305","AC007686.3",5.29067202585057,-0.491872581673706,0.673451148067
058,-0.730376038537435,0.465160360784373,0.980372848261233
"6306","VIPAS39",21.1894793883212,0.300913995708435,0.35244445216742,0
.853791267979709,0.393220653565813,0.980372848261233
"6307","AHSA1",147.797516876881,-0.218506945207064,0.260133970988097,-
0.839978509446819,0.40092043611681,0.980372848261233
"6308","SLIRP",226.363355360205,-0.226658974211436,0.271662554812758,-
0.834340140722225,0.404089338174107,0.980372848261233
"6309","TTC8",23.2219397909893,-0.350621881771574,0.420013633604355,-0
.834786906231365,0.403837699462076,0.980372848261233
"6310","NRDE2",37.7150745599797,0.291611280120544,0.323516313563892,0.
901380449437376,0.36738607262724,0.980372848261233
"6311","CALM1",1237.49221266968,-0.147868017538901,0.185236729420466,-
0.798265106501946,0.424716662511269,0.980372848261233
"6312","TRIP11",183.064483092107,0.162738887478004,0.199752622783392,0
.814702131117824,0.415242833207016,0.980372848261233
"6313","ATXN3",101.247938971809,0.209040272504049,0.23464910503449,0.8

90863284875189,0.37300251920194,0.980372848261233
"6314","MOAP1",33.7040438403618,0.289467356386663,0.324824366086871,0.891150377275724,0.372848501710436,0.980372848261233
"6315","GON7",26.7775162710937,-0.246868331802171,0.325298695947066,-0.758897391468002,0.447913939496028,0.980372848261233
"6316","GSC",61.3077392102707,-0.225484661620121,0.307947460021254,-0.732217962130807,0.464035542624455,0.980372848261233
"6317","DICER1",114.062618315721,0.185622396822047,0.249234832771878,0.744769078854824,0.456411330669455,0.980372848261233
"6318","CLMN",4.90226908392956,-0.603137278631689,0.679482205715059,-0.887642492413723,0.374733088870103,0.980372848261233
"6319","SYNE3",149.759802178697,-0.240676993860562,0.275682710932506,-0.873021717780066,0.382651240297325,0.980372848261233
"6320","SNHG10",11.8321204120836,-0.380185770084763,0.422117500634191,-0.900663368643967,0.367767330859258,0.980372848261233
"6321","WARS",228.739245574402,-0.292982816323856,0.350091333367208,-0.83687537622232,0.402662622693518,0.980372848261233
"6322","DIO3",27.4790483358109,0.435013265600232,0.469432746062068,0.926678569506344,0.354093447570705,0.980372848261233
"6323","HSP90AA1",2212.41931223484,0.130920392699669,0.153976132729178,0.850264196009773,0.395178217743205,0.980372848261233
"6324","TECPR2",25.8748334730411,-0.232912022388941,0.311684700669271,-0.747268062528627,0.454901766776052,0.980372848261233
"6325","MARK3",106.526174361093,0.193581313347453,0.21910504146165,0.883509170104312,0.376961230156766,0.980372848261233
"6326","AC123768.4",2.85480174741447,-0.601607752839979,0.808862849418938,-0.743769791469784,0.457015758661275,0.980372848261233
"6327","KATNBL1",98.5038093356992,-0.157865882316463,0.201420926330149,-0.7837610778222,0.433180316224179,0.980372848261233
"6328","GOLGA8B",18.7395331770663,-0.41288864607661,0.523188758813288,-0.789177212089068,0.430008438834115,0.980372848261233
"6329","LINC01852",4.80133635766066,-0.530192034818983,0.673674330306164,-0.78701534401352,0.431272883971712,0.980372848261233
"6330","LINC02694",1.97921988592355,0.874719492144956,1.05255236094073,0.831046059659361,0.405947609851182,0.980372848261233
"6331","CCDC9B",3.93705380000471,0.785112535811453,1.0378369758918,0.756489269556827,0.449355903544188,0.980372848261233
"6332","GCHFR",30.9380490698047,-0.322172903324984,0.410089121101785,-0.785616800707619,0.432092018730979,0.980372848261233
"6333","INO80",57.0696193285841,-0.252505103338088,0.278589678502179,-0.906369197508202,0.364740470729517,0.980372848261233
"6334","NDUFAF1",25.8274837444956,-0.291166867243983,0.347605820583032,-0.837635189064484,0.402235623306084,0.980372848261233
"6335","LTK",2.46282729671112,0.853132470692409,1.06010846046978,0.804759609516133,0.42095841365992,0.980372848261233
"6336","TTBK2",41.2573172001502,0.36467908295828,0.405625572007452,0.899053482139879,0.368624174108136,0.980372848261233
"6337","CCNDBP1",121.84311706957,-0.195953787224794,0.220451534278099,-0.888874681078876,0.374070434034142,0.980372848261233
"6338","ZSCAN29",15.0893279970801,0.359643639944673,0.403773799921007,

0.89070573676408,0.373087056345266,0.980372848261233
"6339","PDIA3",680.202142588431,-0.193267824328983,0.245381819475683,-0.787620797424789,0.430918545926745,0.980372848261233
"6340","SECISBP2L",82.2042662174537,0.221357229560925,0.275464104955978,0.803579216233271,0.421640030278566,0.980372848261233
"6341","AC022306.2",3.07226620533642,0.688981327113594,0.890448852022275,0.77374610068716,0.439080952050807,0.980372848261233
"6342","FGF7",1072.67775500771,0.16163844123615,0.197848213626616,0.81698206050623,0.413938680080699,0.980372848261233
"6343","TNFAIP8L3",6.99269288014459,0.498359508652306,0.568835633009464,0.876104589326974,0.380973178085183,0.980372848261233
"6344","GNB5",26.3618053996311,-0.304235290423011,0.338431379156624,-0.898957097835222,0.368675512906266,0.980372848261233
"6345","RSL24D1",348.199540120621,-0.206590233192484,0.222686510112666,-0.927717772791726,0.353553986813124,0.980372848261233
"6346","PIGBOS1",71.7552075184454,-0.201555696292119,0.23214623653841,-0.868227283360549,0.38526992103131,0.980372848261233
"6347","DNAAF4",12.2561272814049,-0.40229574784913,0.455836173671247,-0.882544587475563,0.377482379111867,0.980372848261233
"6348","TCF12",92.3226572191552,0.193513928964462,0.250596541046642,0.772213088641333,0.439988230694492,0.980372848261233
"6349","RNF111",27.294903815323,0.225187901088975,0.309212489212027,0.728262631508922,0.466452832656429,0.980372848261233
"6350","TPM1",318.87807419777,0.231569579318272,0.295781193431214,0.782908394654664,0.433680907537598,0.980372848261233
"6351","AC011939.2",21.3637103883642,0.390155742819204,0.472080752341381,0.826459754785907,0.4085433386997,0.980372848261233
"6352","AAGAB",27.9901435745038,-0.277119628789821,0.351088013686645,-0.789316689794933,0.429926934349379,0.980372848261233
"6353","ANP32A",118.261279430389,-0.138852136722136,0.178787706524371,-0.776631343515826,0.437376303778943,0.980372848261233
"6354","PAQR5",2.73668211081316,-1.00986991043198,1.10293567435283,-0.915619953107905,0.359866262302167,0.980372848261233
"6355","KIF23",3.21915560544156,0.726411602912893,0.841977380396816,0.862744795555603,0.388277797289836,0.980372848261233
"6356","PARP6",22.7683173390212,-0.282799125638263,0.344258915423517,-0.821472191331213,0.411377357732317,0.980372848261233
"6357","ADPGK",71.2200114843646,0.230246400257667,0.251236273722949,0.91645365076371,0.359429006779157,0.980372848261233
"6358","FBXO22",40.7121122048752,0.2700164349881,0.331090984246654,0.815535450481928,0.414765881007571,0.980372848261233
"6359","CIB2",18.196090153155,-0.340677984663498,0.378046776420045,-0.901152994583329,0.36750697937512,0.980372848261233
"6360","DNAJA4",21.5380513683826,0.364377044201806,0.402491337082311,0.90530406652526,0.365304322667755,0.980372848261233
"6361","WDR61",115.517611873543,-0.174368523339165,0.237072780661331,-0.735506298330629,0.462031205592142,0.980372848261233
"6362","MTHFS",56.370814888421,-0.224996122820195,0.277772143794347,-0.810002470898503,0.41793875562587,0.980372848261233
"6363","HOMER2",2.37366522578312,0.905401488189246,0.989128603506904,0

.915352649775967,0.360006527535076,0.980372848261233
"6364","AC021739.2",2.07007640310948,1.00091469617283,1.19462496269517
,0.83784846912514,0.402115813072831,0.980372848261233
"6365","ISG20",54.5379603425857,-0.291998630952666,0.338400021626047,-
0.862880059964484,0.388203415031392,0.980372848261233
"6366","ABHD2",73.9624791899606,-0.231068083499084,0.317926012691518,-
0.726798293549158,0.467349528598962,0.980372848261233
"6367","IQGAP1",321.124482374989,0.159848908595198,0.190893207768372,0
.837373474226267,0.402382671061531,0.980372848261233
"6368","CRTC3",56.9713181688788,0.234702706109433,0.26387583177674,0.8
89443737719831,0.373764647710706,0.980372848261233
"6369","PRC1",4.54213131136782,-0.510246456522164,0.635049945378267,-0
.803474530209173,0.4217005123241,0.980372848261233
"6370","LRRK1",39.9878241115099,0.254655489925398,0.30541117654218,0.8
33811954128757,0.404386957974811,0.980372848261233
"6371","AXIN1",22.3191489490185,0.305404468889261,0.333908355335797,0.
91463559988527,0.360382963118851,0.980372848261233
"6372","CHTF18",4.30843641515332,-0.578173819803575,0.674966387525906,
-0.85659646241478,0.391667941472375,0.980372848261233
"6373","CLCN7",36.4091638743116,-0.207485768003238,0.27968370319649,-0
.741858626841299,0.458172995252201,0.980372848261233
"6374","TELO2",25.4662820221815,-0.311947528948172,0.370209870130596,-
0.842623479590453,0.399439055269282,0.980372848261233
"6375","CRAMP1",20.0621694109887,0.299375904751846,0.380990266768408,0
.785783603584359,0.431994274030779,0.980372848261233
"6376","MRPS34",144.146962591928,-0.214827172767457,0.246713748739013,
-0.870754766872409,0.383888067952099,0.980372848261233
"6377","SPSB3",86.1176855213159,-0.179041840571579,0.218139201772988,-
0.82076875277972,0.411778000329773,0.980372848261233
"6378","FAHD1",44.6492577457768,-0.248489487641678,0.311238061961537,-
0.798390422031311,0.424643959716288,0.980372848261233
"6379","MSRB1",22.1546389377191,-0.265430198147485,0.355844036224515,-
0.745917230941074,0.455717415678849,0.980372848261233
"6380","PKD1",72.9558156518935,0.234402166588588,0.257439496524452,0.9
1051361486144,0.36255170489169,0.980372848261233
"6381","PDPK1",56.7496017399792,0.210256794426207,0.244559166459415,0.
859737941824803,0.389933514811016,0.980372848261233
"6382","AC093525.7",2.66082295515204,0.73854176710299,0.99591786861713
2,0.741568948982191,0.458348542539355,0.980372848261233
"6383","SRRM2",727.42359325908,0.148405063837134,0.194603307897477,0.7
62602986765872,0.445700202798125,0.980372848261233
"6384","ZSCAN32",9.88404373619118,-0.375668969858721,0.481114407707496
,-0.780830845720832,0.434901989509133,0.980372848261233
"6385","ZNF597",5.19549323519127,0.600269108642184,0.665749400791401,0
.901644234194761,0.367245885261684,0.980372848261233
"6386","LINC01569",2.0773857597687,0.699163234267307,0.955968413257255
,0.731366459991141,0.4645553460572,0.980372848261233
"6387","PAM16",70.2633738294728,-0.278603998074978,0.337321571848817,-
0.825929976988974,0.408843814534169,0.980372848261233
"6388","CDIP1",26.9113611481687,0.243778738285849,0.327657821457494,0.

744004025911751,0.456874039511249,0.980372848261233
"6389","MGRN1",47.295357816896,0.200104796807503,0.239824694648256,0.834379449960277,0.404067193653937,0.980372848261233
"6390","ALG1",24.6735934910376,-0.262744547586329,0.354585134055726,-0.740991435769095,0.458698633077429,0.980372848261233
"6391","DEXI",104.858850034297,-0.188991773903447,0.25349378625577,-0.745547954823466,0.455940532332494,0.980372848261233
"6392","CLEC16A",13.4925540774756,0.304526784215685,0.41098794583451,0.740962812418608,0.458715988550951,0.980372848261233
"6393","MRTFB",27.2864148231556,-0.242525712665987,0.335049674290442,-0.723850017701408,0.469157818244739,0.980372848261233
"6394","NOMO1",22.3047628976135,-0.390578462582844,0.429351710945219,-0.90969350447675,0.362984170733403,0.980372848261233
"6395","NPIPA1",31.3551797364791,0.366317337202572,0.4766051671952,0.768597074509984,0.442132542751447,0.980372848261233
"6396","NDE1",27.8212589242192,0.221183737328785,0.305202876907039,0.72471052557003,0.468629636500738,0.980372848261233
"6397","NOMO3",3.3681643995577,-0.584642743597979,0.80301582477875,-0.728058807258328,0.4665775883952,0.980372848261233
"6398","NOMO2",2.34232602705708,-0.860014559658794,0.957071172448112,-0.898589973678703,0.368871101179688,0.980372848261233
"6399","RPS15A",4927.60335311524,-0.178536877124142,0.205864799134337,-0.867253060624699,0.385803369296519,0.980372848261233
"6400","GDE1",74.8159596292719,-0.169109316243295,0.220232330048235,-0.767867806721461,0.442565725243196,0.980372848261233
"6401","METTL9",221.495083361254,-0.175087188298879,0.200142829140367,-0.874811198836829,0.381676642790594,0.980372848261233
"6402","NPIPB4",7.59608137438963,0.532640012621521,0.662429537400705,0.804070444551034,0.421356292501866,0.980372848261233
"6403","CDR2",40.8126848597008,-0.209659112045979,0.256472170116072,-0.817473147090749,0.413658088932477,0.980372848261233
"6404","LCMT1",57.7889798910204,-0.217930642898014,0.275747038233766,-0.790328136591851,0.429336158916995,0.980372848261233
"6405","NSMCE1",96.400873893396,0.210777117984745,0.261616108476188,0.805673317336764,0.420431238582861,0.980372848261233
"6406","AC009093.2",2.94159043936826,-0.620024395266249,0.774855777585784,-0.800180386081727,0.423606292059824,0.980372848261233
"6407","HIRIP3",26.4588884266403,-0.261254516908838,0.294576064185193,-0.886883045407901,0.375141870694164,0.980372848261233
"6408","PPP4C",139.209836724312,-0.192910534852917,0.257091543983075,-0.750357370235473,0.453039498490146,0.980372848261233
"6409","ZNF688",46.822793059833,-0.205268174638788,0.260234378296754,-0.788781927976917,0.430239473794228,0.980372848261233
"6410","ZNF689",23.7728531877207,-0.319396738898907,0.368856971239568,-0.865909454891291,0.386539817728226,0.980372848261233
"6411","BCL7C",235.127968206271,-0.188207478433669,0.24821197847124,-0.758253004519991,0.448299535373362,0.980372848261233
"6412","ARMC5",14.2082399924879,0.354506790658511,0.461437607129559,0.768265926272834,0.442329213554717,0.980372848261233
"6413","VPS35",140.477353821146,0.173175271452691,0.189546382258129,0.

913630053971998,0.360911268821313,0.980372848261233
"6414","ITFG1",92.6395159845854,-0.178593983501061,0.199424493422905,-0.895546883111943,0.37049481551148,0.980372848261233
"6415","N4BP1",78.2320183208079,0.200873584472947,0.242014635266985,0.830005938489413,0.406535426108034,0.980372848261233
"6416","ADCY7",25.7939625338849,0.33600856607381,0.367726164619645,0.913746690887111,0.360849963831085,0.980372848261233
"6417","NKD1",2.13694112818518,1.02393418806293,1.15135039801695,0.889333247138771,0.373824008445659,0.980372848261233
"6418","IRX5",19.9343468985511,-0.296313278179505,0.385015178599592,-0.769614536386018,0.441528579588188,0.980372848261233
"6419","MT1E",2547.0949600502,-0.387667406335211,0.418141669329883,-0.927119765309423,0.353864354947994,0.980372848261233
"6420","MT1M",3036.67786937407,-0.316215884615466,0.424841706721022,-0.744314599091642,0.456686171121716,0.980372848261233
"6421","POLR2C",100.412895940811,-0.251203032475112,0.293006122939219,-0.857330317725889,0.391262357716934,0.980372848261233
"6422","DOK4",12.4301838048034,0.352217495559592,0.423048456775558,0.832570099047675,0.405087228383165,0.980372848261233
"6423","ZNF319",8.10579697607072,0.4670476172173,0.545458428382812,0.856247869525114,0.391860689526849,0.980372848261233
"6424","GOT2",22.8657766590514,-0.25233254305694,0.332697763697379,-0.758443760645356,0.448185368829465,0.980372848261233
"6425","CDH11",243.098996625353,0.30305933309847,0.35638480113878,0.85037109363274,0.395118801943434,0.980372848261233
"6426","TK2",88.9304201798831,0.187086717310985,0.253648737335558,0.737581898795275,0.460768558412837,0.980372848261233
"6427","NAE1",94.1113335440836,-0.200381690946805,0.236452537585052,-0.847449949124473,0.396744373540141,0.980372848261233
"6428","CIAO2B",252.709862865074,-0.211285649184964,0.275613054579257,-0.766602472830999,0.443317904787686,0.980372848261233
"6429","CBFB",54.4865472625547,0.200763882637656,0.251722631569638,0.797559922942866,0.425125915936465,0.980372848261233
"6430","TMEM208",174.408767249773,-0.221401946415613,0.294622434484903,-0.751476875149362,0.452365712736538,0.980372848261233
"6431","PARD6A",7.12826135593487,-0.397926857703334,0.531620980450937,-0.748516090102012,0.454148923568366,0.980372848261233
"6432","PSKH1",19.3308870678305,0.342891930679113,0.375176358230388,0.913948662161037,0.360743822094543,0.980372848261233
"6433","PSMB10",187.114651792376,-0.242742463042161,0.316757109051067,-0.766336275038508,0.443476239402486,0.980372848261233
"6434","SLC12A4",48.2208624110107,0.212925845336859,0.247154149631416,0.861510298954712,0.388957051354636,0.980372848261233
"6435","DUS2",8.63018308714603,0.360792636226194,0.476884073588298,0.756562561444801,0.449311978139342,0.980372848261233
"6436","TANGO6",10.7892879789824,0.385941151676211,0.469889606854227,0.821344303101261,0.411450179110743,0.980372848261233
"6437","AARS",50.2758454480477,0.225548199675469,0.259938783256711,0.867697374164909,0.385560023733358,0.980372848261233
"6438","IL34",46.4026609278252,-0.32614315987449,0.367874902954612,-0.

88655996170179,0.37531585804073,0.980372848261233
"6439","ATXN1L",28.7059072580185,0.238799150478951,0.307976537950077,0.775380982163196,0.438114569421931,0.980372848261233
"6440","ZNF821",7.7308567082315,0.482498239333465,0.534006899327067,0.903543081449865,0.366237734263563,0.980372848261233
"6441","DHODH",15.0303370766914,-0.33768866969661,0.402641110126377,-0.838684032017048,0.401646641229484,0.980372848261233
"6442","LDHD",2.80185675178087,0.657651271858689,0.847694160085381,0.775811964768578,0.437860018365637,0.980372848261233
"6443","GABARAPL2",409.942768735235,-0.227239272533757,0.254181977528771,-0.894002300017653,0.371320660552058,0.980372848261233
"6444","WWOX",28.1784173159496,0.267158225690663,0.33006318826806,0.809415394344707,0.418276250253238,0.980372848261233
"6445","GCSH",154.458658597333,-0.274953154134581,0.298098772472042,-0.922355875049328,0.356342977809897,0.980372848261233
"6446","PLCG2",3.1726581572432,0.764735987395982,0.857065922337773,0.892272073203019,0.372247119180092,0.980372848261233
"6447","KLHL36",57.568489094792,0.200314234163441,0.243837673357891,0.821506502276334,0.411357821893302,0.980372848261233
"6448","CRISPLD2",354.18331618173,-0.262417184909079,0.312928367875532,-0.838585477854332,0.401701962683683,0.980372848261233
"6449","IRF8",30.5510031498764,-0.358711599941014,0.393848379655807,-0.9107860244455,0.362408127549892,0.980372848261233
"6450","C16orf95",11.308846217683,-0.334086302491506,0.44963417414063,-0.743018039342836,0.457470758939887,0.980372848261233
"6451","MAP1LC3B",301.55959401815,0.168231900803151,0.212777686218914,0.790646349213831,0.429150391882287,0.980372848261233
"6452","ZCCHC14",48.4922671791007,0.2383737744648,0.267601400486319,0.890779248657133,0.37304760986763,0.980372848261233
"6453","CTU2",22.4842966204989,-0.284246553026317,0.381460735479153,-0.745152846909067,0.456179324591084,0.980372848261233
"6454","APRT",487.719274469718,-0.265808162118474,0.291026267689872,-0.913347665241439,0.361059720913902,0.980372848261233
"6455","ACSF3",29.3487395821459,0.261328637659782,0.295526246689628,0.884282328852633,0.376543825067246,0.980372848261233
"6456","FANCA",2.34559737026694,0.805374421482076,1.01685233341341,0.792026919757918,0.428344979909792,0.980372848261233
"6457","RFLNB",85.9540602134687,0.379168052079274,0.47529332661209,0.79775589272839,0.425012161726291,0.980372848261233
"6458","VPS53",46.2407412456691,0.278577757307515,0.324666112047364,0.858043839410241,0.390868256446687,0.980372848261233
"6459","PRPF8",124.615786761098,0.173635458656392,0.218516968251623,0.794608583697949,0.426841223657847,0.980372848261233
"6460","TLCD2",45.9260873871206,0.26248044547042,0.282837869042497,0.928024406204893,0.353394909678562,0.980372848261233
"6461","DPH1",1.97604511767092,-0.781358013868242,1.02935037335508,-0.759078768603803,0.447805438898439,0.980372848261233
"6462","AC027796.1",2.86470067120016,0.969807990774904,1.0701054142656,0.906273323960769,0.364791201347961,0.980372848261233
"6463","EMC6",90.8699013763315,-0.247531788577769,0.32458096754288,-0.

762619541286157,0.445690327071693,0.980372848261233
"6464","UBE2G1",49.1501483651832,-0.195738826806182,0.234282516542067,
-0.835482005636711,0.403446374341017,0.980372848261233
"6465","ENO3",1.9708389745814,0.996362696284259,1.09033279060404,0.913
8152175835,0.360813948877527,0.980372848261233
"6466","AC012146.1",13.8961566694108,0.313441084400973,0.4243863455474
67,0.738574856824452,0.460165196514839,0.980372848261233
"6467","DERL2",123.161842990021,-0.212596257802831,0.242548574034608,-
0.876510029584826,0.380752826380265,0.980372848261233
"6468","XAF1",127.744265488698,0.203132497919352,0.272153067075129,0.7
46390625328785,0.455431480641548,0.980372848261233
"6469","AC040977.1",2.46342168964661,-0.784851581171039,1.028698876898
35,-0.762955611983811,0.445489868470143,0.980372848261233
"6470","CLEC10A",9.10564290313862,-0.592825887733602,0.641619577444586
,-0.92395230534374,0.355511148515939,0.980372848261233
"6471","NLGN2",47.3799878766998,-0.274511515888501,0.295770416948532,-
0.928123639681888,0.353343438429644,0.980372848261233
"6472","TMEM88",5.67697072037618,-0.68420199568904,0.799224225559734,-
0.856082653412891,0.39195206284917,0.980372848261233
"6473","CYB5D1",13.9810269258605,-0.473089408079798,0.521278350985371,
-0.907556216722827,0.364112735453294,0.980372848261233
"6474","PER1",132.606633994678,0.245294353126266,0.304618395214026,0.8
05251281538403,0.420674689178071,0.980372848261233
"6475","ZNF18",8.73564748092107,0.388646272031846,0.506542091930444,0.
767253656158575,0.442930716725511,0.980372848261233
"6476","AC005838.2",9.36071509336422,0.399188553316655,0.5333709340102
81,0.748425772501808,0.454203381942325,0.980372848261233
"6477","ZNF286A",25.3507543160998,-0.267374606116538,0.323108430218723
,-0.827507366290453,0.407949548639677,0.980372848261233
"6478","NCOR1",201.546227300517,-0.165360219572521,0.184536135205375,-
0.896085850007032,0.370206913750696,0.980372848261233
"6479","AC002553.1",2.43957490612954,0.793154165706058,1.0643287874118
4,0.745215364920077,0.456141535757059,0.980372848261233
"6480","PIGL",23.6127287061508,0.296019389565301,0.374633902823692,0.7
9015643628124,0.429436414002005,0.980372848261233
"6481","LRRC75A",58.0394525677389,-0.360285728997391,0.455081097769903
,-0.791695657681563,0.428538154874074,0.980372848261233
"6482","MED9",12.9913739150476,0.314005762877885,0.426497753713711,0.7
36242477583277,0.461583145786409,0.980372848261233
"6483","FLII",88.6799258745759,-0.192754233548697,0.241231306002555,-0
.799043195275226,0.424265365793489,0.980372848261233
"6484","TRIM16L",4.8831955296238,-0.569511035900985,0.633964619213031,
-0.898332523048281,0.369008298517942,0.980372848261233
"6485","AC007952.4",67.1334653549528,-0.306202991286743,0.414355704677
847,-0.738985822639536,0.459915606286853,0.980372848261233
"6486","SARM1",7.68570654427503,0.465271986505181,0.577770388086577,0.
805288737704331,0.420653079309388,0.980372848261233
"6487","KIAA0100",52.3881701872285,0.171821176366835,0.233157957050476
,0.736930356314787,0.461164702475238,0.980372848261233
"6488","COPRS",253.419958700385,-0.208268340573048,0.239389219035748,-

0.869998830406591,0.384301043377489,0.980372848261233
"6489","AC116407.2",4.94796321067429,-0.510555144527855,0.684741229729
668,-0.745617647018888,0.455898419600047,0.980372848261233
"6490","ZNF207",193.530450769851,0.14668835989361,0.174710826309523,0.
839606583015828,0.401129007000639,0.980372848261233
"6491","RFFL",17.752174168552,0.326365496218908,0.415213902318858,0.78
6017747470025,0.431857089832882,0.980372848261233
"6492","RAD51D",18.6936037228248,0.311699983434979,0.339036308569666,0
.919370508574689,0.357901809451969,0.980372848261233
"6493","CCL5",174.469362846762,0.27692752659411,0.308819082475658,0.89
6730617726441,0.369862678631454,0.980372848261233
"6494","DHRS11",7.28234178583236,-0.483648596399514,0.599633786571082,
-0.806573290616574,0.419912367052909,0.980372848261233
"6495","SYNRG",71.6910495428557,0.176953976529041,0.233455769779324,0.
757976453939467,0.448465078775709,0.980372848261233
"6496","CISD3",83.3835947299263,-0.214147322701746,0.246974327377871,-
0.867083332001954,0.385896352524394,0.980372848261233
"6497","PLXDC1",83.4233799040233,0.279646724639446,0.362004560146242,0
.77249503300863,0.439821287597,0.980372848261233
"6498","GSDMB",2.1088229738546,1.02395081190996,1.23941079644554,0.826
159345106976,0.408713706912009,0.980372848261233
"6499","RARA",73.5443858701126,-0.243502374431404,0.272517243859647,-0
.893530152377491,0.371573332334922,0.980372848261233
"6500","CCR7",5.20351623250909,-0.560685509713088,0.673252411758514,-0
.8328013385776,0.404956779690099,0.980372848261233
"6501","KRT10",505.68867487884,-0.195613772704329,0.258919603115153,-0
.755500048473852,0.449949003246088,0.980372848261233
"6502","GHDC",30.3855567807521,0.275578803441811,0.313329006299339,0.8
79518965373211,0.37911995460554,0.980372848261233
"6503","STAT5B",53.491102416613,0.260617378327928,0.290965664221237,0.
895698050921108,0.370414051667961,0.980372848261233
"6504","TUBG2",18.1928874793104,0.276594133477717,0.363189329357952,0.
761570098897678,0.44631662652348,0.980372848261233
"6505","CCR10",7.8127083824764,-0.38546831509414,0.517406965195556,-0.
745000243567365,0.456271572640195,0.980372848261233
"6506","EZH1",81.2102935448191,0.234377710389579,0.285697660122729,0.8
20369723325333,0.412005369942371,0.980372848261233
"6507","RPL27",2645.26532108281,-0.191021582601901,0.212571678284477,-
0.898621980799642,0.368854046567042,0.980372848261233
"6508","RND2",4.14626368480729,-0.487677312714058,0.654869953191244,-0
.744693370550229,0.456457107780492,0.980372848261233
"6509","G6PC3",57.0895499600107,-0.229902592673188,0.280355554067907,-
0.820039372637153,0.412193662267721,0.980372848261233
"6510","EFTUD2",30.8974848823847,-0.221686479468228,0.279962051236844,
-0.791844746417738,0.428451207738746,0.980372848261233
"6511","NMT1",175.155255750955,0.178823426946553,0.218404775223636,0.8
18770682845404,0.412917260501371,0.980372848261233
"6512","HEXIM2",13.0568817595204,-0.435736250743411,0.526372297031085,
-0.8278099991225,0.407778111015348,0.980372848261233
"6513","MAP3K14",11.6786584207046,-0.336821318855073,0.441624252142215

,-0.762687549927868,0.445649757301487,0.980372848261233
"6514","KANSL1-
AS1",13.6966440865107,-0.327397529815094,0.394247717185872,-0.83043608
2552481,0.40629227201629,0.980372848261233
"6515","AC005670.3",34.0984129042956,-0.233178427250626,0.317075961441
707,-0.735402413322007,0.462094452528759,0.980372848261233
"6516","SCRN2",46.7664685854519,0.244575295219089,0.308918802196753,0.
791713853219323,0.428527542859354,0.980372848261233
"6517","AC018521.5",22.7500115060976,-0.304102825991051,0.392074143759
87,-0.775625811676329,0.437969955441201,0.980372848261233
"6518","CDK5RAP3",78.1249828306942,-0.167949874150554,0.22539252792418
1,-0.745143930445868,0.456184714265362,0.980372848261233
"6519","HOXB2",56.1655918338749,-0.232186188175625,0.265176256658402,-
0.875591921771207,0.381251918561662,0.980372848261233
"6520","ABI3",2.66092878126831,-0.67966756002639,0.915597552385197,-0.
742321294171011,0.457892693089418,0.980372848261233
"6521","KAT7",36.0556987042109,0.208980436347274,0.279076245879183,0.7
48829179957313,0.453960169769321,0.980372848261233
"6522","MRPL27",161.261269236787,-0.262941555932133,0.285405477389729,
-0.921291204138589,0.356898412119516,0.980372848261233
"6523","ACSF2",12.7934776190204,0.320701862149647,0.410850662373553,0.
780580126844382,0.435049483939594,0.980372848261233
"6524","NME2",1730.84666387376,-0.211387441370112,0.261696715544431,-0
.807757334402701,0.419230290770639,0.980372848261233
"6525","AC015912.3",14.9241851919675,-0.493985356715533,0.576211158842
223,-0.857299184743479,0.391279558968401,0.980372848261233
"6526","MSI2",26.9541558522972,0.299779157130514,0.366128349077417,0.8
18781604554545,0.412911028086152,0.980372848261233
"6527","TRIM37",27.7213814399612,0.22449391435201,0.290595132840134,0.
772531570497816,0.439799655913231,0.980372848261233
"6528","YPEL2",90.7322944501947,0.356508698196724,0.407420579733067,0.
875038512856422,0.381552950827922,0.980372848261233
"6529","RNFT1",42.9953182108972,-0.240849269227002,0.291022052410253,-
0.82759800239288,0.407898199943776,0.980372848261233
"6530","CYB561",82.7988045810853,-0.146635991762574,0.198145509613989,
-0.740041962334843,0.459274532879481,0.980372848261233
"6531","LIMD2",35.6603476134274,-0.28114523482803,0.313609123937922,-0
.896482957184885,0.36999487861536,0.980372848261233
"6532","CD79B",3.96755594176436,-0.622771474797509,0.741195655013558,-
0.840225479716441,0.400781974807776,0.980372848261233
"6533","CEP95",55.1760729235485,-0.272580075567583,0.298096592865924,-
0.914401848565179,0.360505730851053,0.980372848261233
"6534","NAT9",33.9605313958126,0.246829122088252,0.319404505726505,0.7
72779086277523,0.439653132555461,0.980372848261233
"6535","KCTD2",21.2281420036762,0.306362567062499,0.346958639851135,0.
88299448946983,0.37723924884314,0.980372848261233
"6536","TRIM65",21.0866143304143,0.295357117483957,0.37627629585753,0.
784947446160118,0.432484382157837,0.980372848261233
"6537","ACOX1",56.759044680359,0.207375019617485,0.278750747199572,0.7
43944264547621,0.45691019465582,0.980372848261233

"6538","TEN1",46.9550534514049,-0.262519460858714,0.337339225008669,-0.778206153915153,0.436447489490827,0.980372848261233
"6539","METTL23",68.6060249122277,-0.305957178050207,0.328909784480366,-0.930216103280658,0.352259205863332,0.980372848261233
"6540","SEC14L1",61.6361912866801,-0.211112937560274,0.238410521088853,-0.885501766432508,0.375886067320659,0.980372848261233
"6541","TNRC6C-AS1",4.52550542911146,0.694391813790386,0.894933745816582,0.775914213802261,0.437799639555076,0.980372848261233
"6542","SYNGR2",5.33884081664282,-0.507753072072404,0.591388989963766,-0.858577147510835,0.390573850525407,0.980372848261233
"6543","SOCS3",2433.44609622923,-0.219713641916835,0.284316671761325,-0.772777904847163,0.439653831867114,0.980372848261233
"6544","ENGASE",12.8519744140518,0.383403465379473,0.484610683019627,0.791157683504771,0.428851981634689,0.980372848261233
"6545","TBC1D16",83.4492779994019,0.205280761403539,0.245785949107418,0.835201369927875,0.403604338505008,0.980372848261233
"6546","EIF4A3",222.46021766109,0.190233713107026,0.213475537464031,0.891126521412686,0.372861298250107,0.980372848261233
"6547","RNF213-AS1",5.78918935376408,0.596948180249374,0.655646439566089,0.910472694161868,0.362573275804805,0.980372848261233
"6548","SLC25A10",3.33328513530347,-0.670198321326248,0.729568355760345,-0.91862306805752,0.358292761855187,0.980372848261233
"6549","LRRC45",12.581801543335,0.409166543540585,0.475796059081683,0.859962027281821,0.389809974793446,0.980372848261233
"6550","CD7",17.3207616206479,-0.392048518501748,0.456729230644441,-0.858382805822545,0.390681118764137,0.980372848261233
"6551","SECTM1",42.4272045504533,0.30071736052343,0.385601376150322,0.779865890328667,0.435469817601699,0.980372848261233
"6552","OGFOD3",67.7977071853063,0.205324982376938,0.252546453316917,0.813018673120225,0.416207352195229,0.980372848261233
"6553","B3GNTL1",7.23169350759972,-0.487143327232399,0.553595266546265,-0.87996295609888,0.37887937759689,0.980372848261233
"6554","METRNL",456.597017752608,0.220453730465303,0.279929116680114,0.787534119636522,0.430969263232103,0.980372848261233
"6555","AP000915.1",2.65812463416201,-0.680586305331663,0.869791651304674,-0.782470496596276,0.433938117673441,0.980372848261233
"6556","AP001020.2",3.52683001181662,0.697707688259114,0.808655250345348,0.862799923652443,0.388247481154632,0.980372848261233
"6557","DLGAP1-AS2",3.5427929867306,0.780869985193538,0.844051250960483,0.925145225843754,0.354890370714693,0.980372848261233
"6558","TMEM200C",2.24766430005074,-0.85275725132354,0.96818462356152,-0.880779585392119,0.378437131236004,0.980372848261233
"6559","NDUFV2-AS1",4.49513684539935,-0.553082877295384,0.705022110610741,-0.784490115942979,0.432752579367566,0.980372848261233
"6560","CHMP1B",105.803133985223,-0.191049769881725,0.230306616271897,-0.829545294765541,0.406795917487552,0.980372848261233

"6561","MPPE1",43.4792988392938,0.213225858430839,0.261602692821423,0.
815075166586272,0.415029285471082,0.980372848261233
"6562","CEP192",25.7804134871432,0.297082060113839,0.320510374738876,0
.926903100580989,0.353976847237005,0.980372848261233
"6563","ZNF519",15.8695679720191,-0.363070333134135,0.439954490438901,
-0.825245203820817,0.409232394448546,0.980372848261233
"6564","ROCK1",217.682131972315,-0.164325088198513,0.217461410542826,-
0.75565171672678,0.449858039886927,0.980372848261233
"6565","TMEM241",5.94321710187561,0.565118642583198,0.680452980120577,
0.830503589657374,0.406254119129552,0.980372848261233
"6566","RIOK3",101.886711422759,0.188630906729835,0.211544534556217,0.
891684141712991,0.372562255907486,0.980372848261233
"6567","RMC1",18.7605709462443,0.348032689870368,0.391349226269307,0.8
8931487916337,0.373833877151098,0.980372848261233
"6568","TRAPPC8",35.0979260570394,0.251463711397566,0.299355408251158,
0.840017265318919,0.400898706140494,0.980372848261233
"6569","SLC39A6",110.238146202601,0.216919740549653,0.267026674454487,
0.81235232769461,0.416589492554188,0.980372848261233
"6570","ELP2",70.4481851381497,0.191354191327858,0.224486922100238,0.8
52406855319681,0.393988316598711,0.980372848261233
"6571","ATP5F1A",294.709587742042,-0.163659882019708,0.184528072753392
,-0.886910482387292,0.375127097602003,0.980372848261233
"6572","HAUS1",51.4123988944706,-0.268157511824388,0.304794064390622,-
0.879798995956562,0.378968208690747,0.980372848261233
"6573","C18orf25",28.430390936401,-0.250109976367021,0.325183289063759
,-0.769135391572909,0.441812940031885,0.980372848261233
"6574","PIAS2",36.6681318601849,0.237238634946283,0.320558756792809,0.
740078472102449,0.459252380522181,0.980372848261233
"6575","RPL17",5659.72685925984,-0.164519533570947,0.22564433499925,-0
.729109966671636,0.465934398467282,0.980372848261233
"6576","ACAA2",208.28452186382,-0.201529471077544,0.241267564557079,-0
.835294505697498,0.403551910158686,0.980372848261233
"6577","FECH",25.2740615630771,-0.235918693687921,0.310484783452849,-0
.759839793320334,0.447350353416466,0.980372848261233
"6578","NEDD4L",4.78271608335122,0.644103252018037,0.776382121951142,0
.82962143744285,0.406752852372237,0.980372848261233
"6579","MALT1",44.9131428646661,0.246981361034445,0.318712543325223,0.
774934549037874,0.438378335720141,0.980372848261233
"6580","RNF152",6.42278879570805,0.499098771580081,0.65826401924513,0.
758204545574931,0.448328540429243,0.980372848261233
"6581","CTDP1",17.1131107067554,-0.318622220066423,0.355962465698102,-
0.895100609671167,0.370733308091865,0.980372848261233
"6582","SLC66A2",75.0177916963218,-0.226198806224735,0.251189049522103
,-0.900512210445032,0.36784773023625,0.980372848261233
"6583","TMEM259",131.237473206923,-0.159820070771189,0.199683124063841
,-0.800368441351571,0.423497359934333,0.980372848261233
"6584","GPX4",2185.77084791856,-0.233625868563816,0.28435638586943,-0.
821595294403171,0.411307268316924,0.980372848261233
"6585","CIRBP",930.605573269473,-0.147663821936776,0.198617507743788,-
0.743458235953998,0.457204297737819,0.980372848261233

"6586","C19orf25",45.5266000633658,-0.240097898482473,0.300928468847166,-0.797857043576733,0.424953453842903,0.980372848261233
"6587","MBD3",62.6629260541003,-0.19179423649066,0.247725601875534,-0.774220488470241,0.438800414196606,0.980372848261233
"6588","REXO1",34.0281817243023,-0.227861341376761,0.274828186764821,-0.829104700136706,0.407045164372947,0.980372848261233
"6589","LSM7",251.442468501293,-0.227218735040047,0.286700429546797,-0.79253015211461,0.428051617713813,0.980372848261233
"6590","TIMM13",249.961769356799,-0.228328565456859,0.266960014614297,-0.855291253211637,0.392389928593058,0.980372848261233
"6591","PIAS4",24.4742437157988,0.229566269231762,0.306650968072079,0.748623983400575,0.454083872484968,0.980372848261233
"6592","STAP2",8.76527605133872,-0.442100657645384,0.558640402308565,-0.79138683098898,0.428718292316627,0.980372848261233
"6593","HDGFL2",70.7504023428069,-0.160542303880332,0.217806248094404,-0.737087688185825,0.461069025765629,0.980372848261233
"6594","MYDGF",412.584998452164,-0.188447324170012,0.248340308106828,-0.758826972578886,0.447956068409261,0.980372848261233
"6595","DPP9",33.6385844223471,0.24570638263308,0.289242132189365,0.849483374960802,0.395612377212603,0.980372848261233
"6596","KDM4B",42.5190079173845,0.265923254522649,0.298126934770221,0.891979970637699,0.372403668204953,0.980372848261233
"6597","NDUFA11",468.615930098208,-0.196533104143602,0.253654391804872,-0.774806628598759,0.438453931838213,0.980372848261233
"6598","VMAC",13.0316635706369,-0.328549673757102,0.425164428330433,-0.772759082991197,0.43966497297656,0.980372848261233
"6599","AC008760.2",119.445692041935,-0.361335455422494,0.389113886878846,-0.928611050920931,0.353090692720221,0.980372848261233
"6600","SH2D3A",4.40169087703158,-0.492494052631448,0.664550369262007,-0.741093640770029,0.458636665132456,0.980372848261233
"6601","INSR",71.5484879919906,0.244268923761343,0.324616891716105,0.752483712323169,0.451760221170951,0.980372848261233
"6602","CDC37",216.23013858271,-0.196541148015495,0.228250256919838,-0.86107744485265,0.389195390777204,0.980372848261233
"6603","TIMM29",29.1827389663711,0.286285911046014,0.330702156950405,0.865691090998684,0.386659586920607,0.980372848261233
"6604","KANK2",170.463867526018,0.223477658063589,0.245344183545731,0.910874082417094,0.362361722962531,0.980372848261233
"6605","SWSAP1",5.14478736585186,-0.56396236791728,0.733603825233706,-0.768756034958811,0.442038153024192,0.980372848261233
"6606","FBXW9",11.5076685031607,-0.431503579644287,0.495070419705376,-0.87160040767752,0.38342640820801,0.980372848261233
"6607","STX10",85.9190379681831,-0.218766153772221,0.239067234356143,-0.91508212893081,0.360148516077738,0.980372848261233
"6608","AC011446.2",1.9520581727886,-1.01419909384077,1.11476517719365,-0.909787204148189,0.362934744092228,0.980372848261233
"6609","MISP3",2.61981152445797,-0.689458305479717,0.94274248843969,-0.731332589688222,0.464576029038319,0.980372848261233
"6610","GIPC1",73.8915019123752,-0.193508371269237,0.241616943706532,-0.800889077979033,0.423195863622239,0.980372848261233

"6611","RASAL3",7.07186360110992,-0.571913214049562,0.716806477176803,-0.797862787599361,0.424950120158245,0.980372848261233
"6612","C19orf44",7.31947618211587,0.430002143605663,0.553328515669472,0.777119073802664,0.43708852166049,0.980372848261233
"6613","CHERP",36.923831247557,0.226783695846312,0.298435326991489,0.759909016578256,0.447308971623738,0.980372848261233
"6614","MED26",15.9398608205213,0.285469432332702,0.381825806571636,0.747643106933748,0.454675456230413,0.980372848261233
"6615","HAUS8",11.3626077496213,-0.349563899189903,0.473420612706971,-0.738379128004445,0.460284094355467,0.980372848261233
"6616","MYO9B",47.0150208883191,-0.22927734437775,0.290005949589766,-0.790595312620584,0.429180183017949,0.980372848261233
"6617","SLC27A1",52.3166985154557,0.237956496572946,0.302247391278535,0.787290489311976,0.431111835867351,0.980372848261233
"6618","PDE4C",2.53411779888808,1.96899709046798,2.49851328968386,0.788067487412532,0.430657231531441,0.980372848261233
"6619","ISYNA1",18.1747198522701,-0.312661621149768,0.351153729858271,-0.89038388194242,0.373259794034597,0.980372848261233
"6620","UBA52",3090.20791591867,-0.183712696883682,0.205396925560378,-0.894427686210322,0.371093104665405,0.980372848261233
"6621","CRTC1",26.0762269867612,0.282053810717456,0.337603110964022,0.835459750095473,0.403458900183082,0.980372848261233
"6622","TMEM161A",38.7455430312941,-0.214458952138753,0.29451831218325,-0.728168481440013,0.466510457263688,0.980372848261233
"6623","NR2C2AP",25.5729325233755,-0.361829203668138,0.393002329161751,-0.920679540093049,0.357217761200985,0.980372848261233
"6624","PBX4",9.03984561112199,-0.399575224855366,0.477121217265445,-0.837471087841108,0.402327821862218,0.980372848261233
"6625","GMIP",4.44016255171233,-0.511101301131108,0.656239581327872,-0.778833395109934,0.436077862904982,0.980372848261233
"6626","ZNF90",5.16361430211261,0.637566635965466,0.688997759938775,0.925353713809056,0.354781947017318,0.980372848261233
"6627","ZNF486",9.77722927258082,0.431397767152008,0.533410721801902,0.808753460550463,0.41865697070291,0.980372848261233
"6628","ZNF714",13.4657309912644,0.459910262407547,0.539142124760825,0.853040861185858,0.393636644972326,0.980372848261233
"6629","ZNF257",3.09697200391942,-0.685546823413696,0.937291057036804,-0.731412956804491,0.464526953501866,0.980372848261233
"6630","ZNF726",2.47361444849765,0.858854926931776,1.10390091934768,0.778018128148032,0.436558326241862,0.980372848261233
"6631","PLEKHF1",72.8015934218102,0.276117838107059,0.340238416426751,0.811542215035272,0.417054360220216,0.980372848261233
"6632","ANKRD27",21.0327954553962,0.321445904210813,0.36296986015221,0.885599438135212,0.375833414458705,0.980372848261233
"6633","UBA2",146.646063897093,-0.174657469721953,0.204939885205966,-0.852237569794778,0.394082248386386,0.980372848261233
"6634","WTIP",61.1570273373662,-0.181306212113009,0.229984796040592,-0.788339991314071,0.430497861456933,0.980372848261233
"6635","COX6B1",557.008613543329,-0.220175402910754,0.250714062875025,-0.878193270795924,0.379838843201644,0.980372848261233

"6636","ALKBH6",12.2309897727317,-0.393370588307824,0.441845056536363,
-0.890290798750739,0.373309760494829,0.980372848261233
"6637","ZNF790",11.9089118973251,-0.363061824275384,0.467648262218914,
-0.776356620150187,0.437538450537544,0.980372848261233
"6638","ZNF585A",34.1058251952703,-0.239168536580142,0.287276946873625
,-0.832536474586501,0.405106198974828,0.980372848261233
"6639","ZNF793",3.42673683446848,-0.735753670707834,0.894776325131649,
-0.822276640589013,0.410919468294751,0.980372848261233
"6640","PSMD8",383.403361323491,-0.249125407377056,0.272626033656016,-
0.913799038324373,0.360822451860458,0.980372848261233
"6641","RINL",11.5579780260589,-0.419658400535498,0.464804257653978,-0
.902871248756744,0.366594231466277,0.980372848261233
"6642","MRPS12",114.007402744535,-0.282356291560563,0.332037162817832,
-0.850375569904125,0.395116314061635,0.980372848261233
"6643","PAK4",13.5215569584357,0.38512781860638,0.426345771186873,0.90
3322712769616,0.366354645514766,0.980372848261233
"6644","GMFG",41.9853206268548,-0.213144231651714,0.290863205415617,-0
.732798881684433,0.463681103491795,0.980372848261233
"6645","RPS16",4719.68649350643,-0.16673506429838,0.191679524771552,-0
.869863719127533,0.384374884601306,0.980372848261233
"6646","ZNF780A",17.712539777843,0.328619350414525,0.362648652041637,0
.90616454401379,0.364848766608821,0.980372848261233
"6647","C19orf47",5.86554957916651,-0.521039679794356,0.57607943595224
8,-0.904458043938138,0.365752572096409,0.980372848261233
"6648","BLVRB",298.544971117957,-0.263235321246304,0.294447843986301,-
0.893996429664981,0.371323801441205,0.980372848261233
"6649","HNRNPUL1",190.949630658037,-0.155829728540012,0.20841775991035
6,-0.747679701610057,0.454653377546077,0.980372848261233
"6650","ARHGEF1",59.3842597533417,-0.199842253384418,0.242226323459204
,-0.825022856849311,0.409358614226778,0.980372848261233
"6651","RABAC1",575.995969748609,-0.207554021695328,0.246515919579062,
-0.841949769612189,0.399816070272076,0.980372848261233
"6652","MEGF8",25.019753789128,0.356349108370716,0.410368563151097,0.8
68363564778007,0.385195334334136,0.980372848261233
"6653","CNFN",3.01453769558384,-0.667725931712489,0.903925354275468,-0
.738695876328967,0.460091690350472,0.980372848261233
"6654","ZNF230",8.48122104584866,0.379790607975104,0.51953365230138,0.
73102215090927,0.464765623084014,0.980372848261233
"6655","PVR",14.3781232360017,-0.325327819966796,0.391038301770701,-0.
831958962827032,0.405432108337907,0.980372848261233
"6656","MARK4",23.7889278950538,0.238698055354302,0.303799966390993,0.
785707971564079,0.432038591997675,0.980372848261233
"6657","IRF2BP1",36.2997270582932,-0.270819538119506,0.30383394829935,
-0.891340614290683,0.372746466278788,0.980372848261233
"6658","TMEM160",128.250023209421,-0.176297116181156,0.221622994986985
,-0.795482058129886,0.426333143633797,0.980372848261233
"6659","BBC3",19.7867942544168,0.333219149321045,0.380434576293608,0.8
75890810365976,0.381089395796123,0.980372848261233
"6660","C5AR1",19.2634460604719,-0.404179250557129,0.480163220239666,-
0.841753873516987,0.399925735878512,0.980372848261233

"6661","EHD2",156.914040097617,0.189411430737076,0.261655735101095,0.723895582353253,0.469129842301549,0.980372848261233
"6662","NOP53",871.300671349535,-0.186367101118767,0.215148102833242,-0.866227025312035,0.38636567571411,0.980372848261233
"6663","CA11",10.9062530287361,-0.327408222342205,0.451030015574985,-0.725912269773924,0.467892554174302,0.980372848261233
"6664","BAX",117.798757689124,0.268691520495327,0.311431096645971,0.86276394165214,0.38826726827298,0.980372848261233
"6665","RUVBL2",63.4380058439545,-0.247736767648994,0.315235521361506,-0.785878338135931,0.431938766301552,0.980372848261233
"6666","SNRNP70",189.642847131128,0.18114953958886,0.226095516069678,0.801208014815445,0.423011232050147,0.980372848261233
"6667","TEAD2",40.189907701049,-0.252181091196743,0.334575900005804,-0.753733580907554,0.451009213403399,0.980372848261233
"6668","FLT3LG",54.3702175542365,-0.254122343903736,0.289005208952409,-0.87930022031396,0.379238516479759,0.980372848261233
"6669","RPL13A",7593.34910293479,0.147712212097448,0.163745762516647,0.902082654398042,0.36701296233118,0.980372848261233
"6670","NOSIP",88.6707636150958,-0.191438160146052,0.260006255688741,-0.73628290072846,0.461558550131562,0.980372848261233
"6671","AP2A1",112.334998469033,-0.153791377248306,0.185395808451596,-0.829529958270113,0.406804591900229,0.980372848261233
"6672","AKT1S1",51.4172555542841,0.229039044263156,0.255166076578026,0.897607735850884,0.369394713377249,0.980372848261233
"6673","CLDND2",4.17725680721651,0.675514176440713,0.79355788995357,0.851247508206662,0.394631877169842,0.980372848261233
"6674","HAS1",139.29162402593,-0.766017464138426,0.845006471505669,-0.906522600677251,0.364659308002846,0.980372848261233
"6675","AC011468.1",3.03539292336405,0.649148756697996,0.885803344197471,0.732836199987615,0.463658339458999,0.980372848261233
"6676","ZNF616",10.6230014697603,0.432157236594781,0.466212672195863,0.926953003141933,0.353950935833447,0.980372848261233
"6677","ZNF836",12.3313631563673,0.310345646809516,0.42332510091692,0.733114209710952,0.463488774107087,0.980372848261233
"6678","ZNF701",15.1287397237956,0.366445730339737,0.403361975942349,0.908478617707166,0.363625405769775,0.980372848261233
"6679","ZNF845",7.26791561092518,-0.440133527216236,0.53929512043612,-0.816127405084448,0.414427271302985,0.980372848261233
"6680","LAIR1",3.32246015761707,-0.582945628753514,0.794694022592804,-0.733547267477324,0.463224709766869,0.980372848261233
"6681","LAIR2",2.47823235165004,-0.962242438109223,1.10967063127479,-0.867142385307432,0.385863999522491,0.980372848261233
"6682","LILRB4",1.99040890888299,-1.1307134150307,1.2370300617891,-0.914054920698831,0.360687988029364,0.980372848261233
"6683","PPP6R1",32.7028384816788,0.229846241019093,0.279730488277027,0.821670324299681,0.411264553106842,0.980372848261233
"6684","ZNF580",64.420271673847,0.217913643997946,0.235030806023438,0.927170559829568,0.353837985711439,0.980372848261233
"6685","U2AF2",62.3610928161159,0.184583638249182,0.245548283076288,0.751720337591751,0.452219257313338,0.980372848261233

"6686","AC006115.2",11.0509461858077,0.449380046047311,0.50253710447107,0.894222619681571,0.371202792050314,0.980372848261233
"6687","ZNF543",4.4572130303219,0.511846864272151,0.70239594253704,0.728715576606793,0.46617566256191,0.980372848261233
"6688","ZNF550",5.55122621757608,0.600715811811547,0.662144043531214,0.907228295233058,0.364286083692171,0.980372848261233
"6689","ZNF329",10.1268999679746,0.340239269371335,0.450970882666914,0.754459506031202,0.450573351824443,0.980372848261233
"6690","ZNF274",14.0927682348648,0.345524017698449,0.434138220561782,0.795884815788242,0.426098987620665,0.980372848261233
"6691","ZNF544",9.60743386912897,0.435285440848343,0.584412372736694,0.744825847560316,0.456377007101488,0.980372848261233
"6692","A1BG",13.0411181012294,-0.500548805154333,0.573514825229267,-0.872773960035358,0.382786295656326,0.980372848261233
"6693","C20orf96",11.9414340582024,-0.344590784529173,0.434349603019362,-0.79334891095506,0.427574567540244,0.980372848261233
"6694","TRIB3",4.01518989183684,0.852129514669679,0.917358271354978,0.92889500348762,0.352943502648035,0.980372848261233
"6695","SRXN1",14.3427876237111,-0.326330114287071,0.433823590065439,-0.752218463357067,0.451919692068658,0.980372848261233
"6696","SIRPA",116.154016362257,0.213820003061477,0.254433151119862,0.84037792292541,0.400696523450019,0.980372848261233
"6697","PCED1A",48.0767032762041,0.226036735424505,0.27103722185816,0.833969348840196,0.404298256317495,0.980372848261233
"6698","RASSF2",32.2593468495313,-0.472610964813282,0.515505995591247,-0.916790432808124,0.35925246692888,0.980372848261233
"6699","TRMT6",41.1290327578993,-0.258917063928758,0.307430913290846,-0.842195929996825,0.399678291596435,0.980372848261233
"6700","KAT14",12.0643103217983,0.349182457880788,0.427116489072053,0.817534482546945,0.413623051727932,0.980372848261233
"6701","ZNF133",11.995807593443,0.383007425985815,0.443518450178073,0.86356593695716,0.387826383066825,0.980372848261233
"6702","NAA20",146.659445086956,-0.204507645945616,0.255002833502531,-0.801981856972528,0.422563453259682,0.980372848261233
"6703","GZF1",15.3172979896316,0.330128874119998,0.414522287237614,0.796408020229706,0.425794918096591,0.980372848261233
"6704","ZNF337",15.0430230226465,0.379900747475187,0.410873198381752,0.924617981828575,0.355164656101876,0.980372848261233
"6705","AL390198.1",3.75810446601167,0.742237814953886,0.983790043152801,0.754467703876327,0.450568431018573,0.980372848261233
"6706","REM1",10.391305257206,-0.422387260647822,0.57050642936462,-0.740372481197521,0.45907401187324,0.980372848261233
"6707","HM13",250.089997634201,-0.158074134579148,0.18764495565472,-0.842410785984652,0.399558057679518,0.980372848261233
"6708","PDRG1",33.4471449280731,0.293800118328375,0.366901522095289,0.800760151254077,0.423270512301752,0.980372848261233
"6709","MAPRE1",106.09964307967,-0.175407782830892,0.217402521634124,-0.806834168768722,0.419762030473831,0.980372848261233
"6710","ZNF341",4.19366983254731,0.584639946400305,0.727214893794451,0.803943856746084,0.42142940001027,0.980372848261233

"6711","DLGAP4",141.238622941171,0.199100508597217,0.215578882581693,0.923562207081059,0.355714298303194,0.980372848261233
"6712","SOGA1",34.8451070182353,0.272294229989316,0.300252595518193,0.906883850643738,0.364468222034405,0.980372848261233
"6713","SLPI",172.96621391137,0.274375118287749,0.317157897633284,0.865105741762096,0.386980753551872,0.980372848261233
"6714","SYS1",82.8805788294684,-0.162532116640263,0.19519604087563 5,-0.832660928526809,0.405035985935562,0.980372848261233
"6715","TNNC2",15.5517667023679,0.471130540502931,0.562557156220569,0.837480308077725,0.402322641232709,0.980372848261233
"6716","CD40",172.399178303283,-0.245074749618335,0.277123449928442,-0.884352261353622,0.376506084687073,0.980372848261233
"6717","SPATA2",13.846005719205,-0.323225566428753,0.414470657295476,-0.779851506347592,0.435478285090767,0.980372848261233
"6718","CASS4",2.99640995600551,0.801372978825002,0.914481134397846,0.876314391496636,0.380859143458071,0.980372848261233
"6719","RTF2",189.123981321412,-0.164601571072696,0.212154302338113,-0.775857803771374,0.437832949503148,0.980372848261233
"6720","APCDD1L-DT",3.01905533604361,-0.924286890719036,1.20252856860506,-0.768619486347187,0.442119234043301,0.980372848261233
"6721","PPDPF",481.234572677326,-0.190919742007263,0.255908518673941,-0.746046841256264,0.455639119686279,0.980372848261233
"6722","DNAJC5",24.5538768363776,0.304884125070526,0.34195053933067,0.891602995179662,0.372605764180075,0.980372848261233
"6723","AF165147.1",1.93084422630625,0.817851275695482,1.08506626034213,0.75373394748963,0.451008993239163,0.980372848261233
"6724","IL10RB",127.379569200356,0.126829245393954,0.169257064699841,0.74932910847102,0.453658867500488,0.980372848261233
"6725","LINC00310",8.74444564036111,-0.589792226487375,0.717202375441461,-0.822351189403603,0.410877050723185,0.980372848261233
"6726","CBR1",162.978606872897,-0.217334568362939,0.249429220256318,-0.871327618069775,0.383575294774042,0.980372848261233
"6727","CBR3",150.120098500014,-0.243741796603856,0.264479486475265,-0.921590554534942,0.356742187196794,0.980372848261233
"6728","GET1",46.9416597160248,-0.268425911342937,0.320388137027408,-0.837814763784384,0.402134745648954,0.980372848261233
"6729","LCA5L",2.1931654862251,-0.843376421146073,1.11927999108108,-0.753499059990772,0.451150075961312,0.980372848261233
"6730","ZBTB21",54.9796275577882,-0.308548463637825,0.353021549615964,-0.874021611353417,0.382106484598614,0.980372848261233
"6731","ICOSLG",6.50405448820276,-0.454291176443034,0.56348638116986,-0.806215006474291,0.420118887463717,0.980372848261233
"6732","LINC01547",1.99139577929889,-0.826734908245866,1.01702377939864,-0.812896340275061,0.416277492967999,0.980372848261233
"6733","LINC00205",13.9950418235262,0.457978428102708,0.562163434391755,0.814671321691757,0.415260473260818,0.980372848261233
"6734","MCM3AP-AS1",2.48999946297664,0.930335835642128,1.09378691252694,0.850564058672819,0.395011561855762,0.980372848261233

"6735","ADA2",8.78075198099441,-0.399527743456018,0.545593513833321,-0
.73228096252272,0.463997096613013,0.980372848261233
"6736","SLC25A18",4.1842659288514,-0.572820761155512,0.698423998146032
,-0.820161911211622,0.412123812134723,0.980372848261233
"6737","ATP6V1E1",114.042909067154,0.181856489420373,0.210934476593582
,0.862146825674049,0.388606726526928,0.980372848261233
"6738","TUBA8",2.67992053547111,-0.882229645515951,1.05197570533506,-0
.838640703432363,0.401670962320949,0.980372848261233
"6739","USP18",32.9282152240431,0.410556842731682,0.456680082294016,0.
899003172350661,0.368650970904871,0.980372848261233
"6740","SEPTIN5",4.69703230565459,-0.545694464707838,0.637323440742456
,-0.856228454538131,0.391871426380017,0.980372848261233
"6741","SNAP29",72.305568660797,0.213799070455989,0.261240823159346,0.
818398395282887,0.413129737745212,0.980372848261233
"6742","THAP7",63.744871332084,-0.25338842023591,0.303919031963023,-0.
833736599512263,0.404429429084191,0.980372848261233
"6743","SDF2L1",156.065748438792,-0.35911671996682,0.421502226788094,-
0.851992462064411,0.394218275805448,0.980372848261233
"6744","PPM1F",24.9993560243793,0.288149284545646,0.37473931850685,0.7
68932616128401,0.441933313744971,0.980372848261233
"6745","SLC2A11",13.6179321722747,0.299341397617049,0.396551204336694,
0.754861905205289,0.450331845219962,0.980372848261233
"6746","SUSD2",7.68768095290932,0.530664054954037,0.695594693112745,0.
762892615783694,0.445527440371041,0.980372848261233
"6747","GGT5",353.723734675031,-0.23603797878752,0.276833383976288,-0.
852635528985685,0.393861453517631,0.980372848261233
"6748","ZNRF3",20.8904935605457,0.259741508818054,0.33005360528301,0.7
86967646044448,0.431300806104398,0.980372848261233
"6749","NIPSNAP1",21.0280505596585,-0.296929913452612,0.40753884268714
,-0.728592915204797,0.466250713561134,0.980372848261233
"6750","MTMR3",49.5403176279201,0.266147734353781,0.327824308632408,0.
811860888120456,0.4168714592779,0.980372848261233
"6751","AC003681.1",16.3418636789244,0.521563323124426,0.5841770014491
28,0.892817282828011,0.371955029465324,0.980372848261233
"6752","OSM",3.76150164340983,0.913280644616034,0.983735042861357,0.92
8380717189463,0.353210117367683,0.980372848261233
"6753","TBC1D10A",23.6685747599808,0.251382981584098,0.335423141187579
,0.749450323236692,0.453585829484748,0.980372848261233
"6754","SF3A1",67.5287448126636,0.198853136099282,0.235494785156636,0.
844405688079326,0.398442744943711,0.980372848261233
"6755","TCN2",33.2577072796369,0.301347581104244,0.409223050276207,0.7
36389557970519,0.461493657539498,0.980372848261233
"6756","RTCB",113.567456813576,-0.224252684546891,0.261132788380873,-0
.858768774068346,0.390468098445569,0.980372848261233
"6757","FBXO7",102.079523779187,-0.147591277265562,0.192623131174579,-
0.766217828386338,0.443546701930689,0.980372848261233
"6758","TIMP3",2253.96618020127,-0.164447721647939,0.224836992769447,-
0.731408651318192,0.464529582539494,0.980372848261233
"6759","Z95114.1",3.7706741210448,-0.733515285050134,0.892943865918239
,-0.821457331246504,0.411385818883264,0.980372848261233

"6760","MYH9",164.129632841605,0.217845351701859,0.238021017327644,0.915235781057044,0.360067864221732,0.980372848261233
"6761","EIF3D",278.904764209184,-0.188394982345742,0.249837411030721,-0.754070343462599,0.450806984011085,0.980372848261233
"6762","KCTD17",28.6354172349714,-0.271086745366779,0.355019268477627,-0.763583189524437,0.445115670202052,0.980372848261233
"6763","CDC42EP1",80.9611723924097,-0.186555652145381,0.254507513840461,-0.733006461500087,0.463554488439013,0.980372848261233
"6764","NOL12",33.8438992301271,-0.281664511810102,0.339130206421348,-0.830549760761067,0.406228025909579,0.980372848261233
"6765","DNAL4",24.602940233079,-0.320245741182034,0.359932552931025,-0.88973819837686,0.373606478090449,0.980372848261233
"6766","TAB1",20.4726000070536,0.312058856472724,0.362898599510448,0.859906477714966,0.389840597473317,0.980372848261233
"6767","TNRC6B",319.778348032588,0.203471134009275,0.243386216284062,0.836001056739379,0.403154308566374,0.980372848261233
"6768","RBX1",373.486410691741,-0.219348521714799,0.279076374739483,-0.785980260491629,0.431879051643414,0.980372848261233
"6769","POLR3H",39.5811914852995,-0.23453427666228,0.323093932029617,-0.725901211418545,0.467899333818582,0.980372848261233
"6770","CCDC134",2.93991444373694,-0.710886394859811,0.898810934071782,-0.790918721515061,0.428991422753944,0.980372848261233
"6771","TCF20",19.8551044350526,0.323940093801879,0.421148078590868,0.769183359177987,0.441784467738177,0.980372848261233
"6772","CYB5R3",416.727679106173,-0.168252403202547,0.184365455596043,-0.912602649225134,0.36145156050168,0.980372848261233
"6773","NUP50",48.8144209671607,0.24800770128134,0.267669910071744,0.926543073948304,0.354163822965587,0.980372848261233
"6774","FAM118A",27.2096526622908,0.323926355865169,0.363183084477713,0.891909259295492,0.372441571272113,0.980372848261233
"6775","BX537318.1",3.2020431387906,-0.853462391363685,0.923943196474795,-0.923717382865071,0.355633479312076,0.980372848261233
"6776","CERK",34.9671003016748,0.268633726387286,0.31914705493095,0.841723970930601,0.399942477391099,0.980372848261233
"6777","Z98885.3",2.88224544863272,0.953427824646395,1.08974291198844,0.874910783229312,0.381622451336591,0.980372848261233
"6778","ALG12",25.6019285106813,0.218152534839033,0.29321070224739,0.744012865720609,0.456868691634474,0.980372848261233
"6779","LINC00685",11.5492271194074,0.417875019675015,0.499609462665083,0.83640333280705,0.402928038294394,0.980372848261233
"6780","ARSD",29.1425211341343,0.332040347237185,0.36259297672964,0.915738496183735,0.359804068781209,0.980372848261233
"6781","PIGA",8.42324673010573,0.497822757818987,0.612519412728731,0.812746090121816,0.416363649956208,0.980372848261233
"6782","REPS2",9.29372925438956,0.459726722307581,0.527477136827868,0.871557628207882,0.383449754588506,0.980372848261233
"6783","RPS6KA3",107.200213854667,0.201449878797678,0.251122993943177,0.802196069879852,0.422439549428648,0.980372848261233
"6784","APOO",11.5755516934045,-0.433741784771517,0.562173105465484,-0.771544886360894,0.440384027513745,0.980372848261233

"6785","EIF2S3",257.405395800497,-0.132536192151995,0.165105409914743,-0.802736822617951,0.422126864966882,0.980372848261233
"6786","RPGR",38.2608270734486,-0.340485042078389,0.428263126019164,-0.795037026052886,0.426591964487387,0.980372848261233
"6787","ATP6AP2",433.310872987771,-0.159889928053142,0.172692248333082,-0.92586627133809,0.354515481545621,0.980372848261233
"6788","KRBOX4",27.8439177966178,0.282461622356365,0.314365124371245,0.898514499409916,0.368911318685702,0.980372848261233
"6789","NDUFB11",319.689524507074,-0.211856198030165,0.246383308331027,-0.859864247563094,0.389863878569162,0.980372848261233
"6790","INE1",4.54714987512044,0.654302333238219,0.756216760768552,0.865231197167918,0.386911905579815,0.980372848261233
"6791","USP11",71.0029203262046,0.197766907307043,0.219386424195739,0.901454627523321,0.367346647607764,0.980372848261233
"6792","SUV39H1",2.88893050762189,-0.743758520311015,0.895959688033306,-0.830124982457209,0.406468123619346,0.980372848261233
"6793","GPKOW",30.1256753328256,-0.25246191440878,0.291901227812103,-0.8648881551512,0.387100179412863,0.980372848261233
"6794","USP27X",4.80680291645295,-0.630144706857771,0.67858432784236,-0.928616652349446,0.353087788780145,0.980372848261233
"6795","MAGEH1",114.732112561365,-0.239985740645814,0.289231873939676,-0.829734763934998,0.406688761706337,0.980372848261233
"6796","LAS1L",36.6317340648134,-0.26822159967754,0.288387694455221,-0.930072970638446,0.35233330441494,0.980372848261233
"6797","AR",137.211894960183,0.225750882694084,0.273282653973406,0.8260710272378,0.40876380174921,0.980372848261233
"6798","CITED1",4.65988865962879,0.801777983184185,1.03326707009717,0.775963936515256,0.437770279656367,0.980372848261233
"6799","UPRT",14.0356148987058,-0.291294184169148,0.400918622447584,-0.726566858857328,0.467491336394479,0.980372848261233
"6800","MAGEE1",4.99968597259442,0.499195049494946,0.659823616807071,0.756558323738976,0.449314517821249,0.980372848261233
"6801","COX7B",330.262621956122,-0.213081425158922,0.253607892474744,-0.840200291401191,0.400796095058765,0.980372848261233
"6802","ATP7A",24.9683739722739,0.300479053037876,0.346887115630167,0.866215663536582,0.38637190519889,0.980372848261233
"6803","CHM",67.0542962802683,0.190506595734555,0.246332574459299,0.773371512690589,0.439302544255633,0.980372848261233
"6804","SYTL4",28.7592849142156,0.341786809321335,0.39429488419897,0.866830443452727,0.386034919030504,0.980372848261233
"6805","ARMCX5-GPRASP2",11.8317097583071,-0.342384413268524,0.448460679793984,-0.763465848167134,0.445185622326554,0.980372848261233
"6806","BEX3",281.028978168299,0.26120101327453,0.301714897990879,0.865721298530066,0.38664301721083,0.980372848261233
"6807","TCEAL1",53.3691533043928,0.177027843318946,0.22715885653701,0.779312970745226,0.435795375843032,0.980372848261233
"6808","MORF4L2",377.769966244918,-0.185393253947709,0.204270942216367,-0.90758505314641,0.36409749420106,0.980372848261233
"6809","LONRF3",12.8521274129463,-0.376476092317375,0.471622036882105,

-0.798258060217583,0.424720750684824,0.980372848261233
"6810","RPL39",6082.05236096261,-0.165149781270391,0.21506996853579,-0
.767888619665224,0.442553359064005,0.980372848261233
"6811","UPF3B",40.3652798595393,-0.225814636885223,0.277088947639128,-
0.814953605364718,0.415098867229372,0.980372848261233
"6812","NDUFA1",365.999222787982,-0.189834378441846,0.252226234382303,
-0.7526353430552,0.451669073231373,0.980372848261233
"6813","SASH3",5.11335812558982,0.604880090372518,0.806244204824416,0.
750244264396604,0.453107603923601,0.980372848261233
"6814","AIFM1",25.37180814578,-0.263794199819792,0.317003696769553,-0.
83214865475704,0.405325041508676,0.980372848261233
"6815","MIR503HG",8.11714126474,-0.679947838761961,0.831739596045897,-
0.81750086444657,0.413642255480336,0.980372848261233
"6816","MOSPD1",13.6965262296947,-0.343215728247597,0.394619163468546,
-0.869739130839128,0.384442982475462,0.980372848261233
"6817","RTL8A",94.9247861978788,-0.188250044717961,0.227276970968164,-
0.828284730811245,0.407509268112768,0.980372848261233
"6818","ATP11C",63.7502869030012,0.169522319864339,0.22652481362593,0.
748360928548336,0.454242482862924,0.980372848261233
"6819","CD99L2",46.7159095752037,0.189988798880969,0.254805706851565,0
.74562222812241,0.455895651464476,0.980372848261233
"6820","HMGB3",4.01436770114668,0.699379958838445,0.758738723917663,0.
921766527517239,0.35665037056511,0.980372848261233
"6821","NSDHL",22.6240684374828,0.289814113457859,0.317038472776696,0.
914129162052796,0.360648980767,0.980372848261233
"6822","ZNF275",24.085008103199,0.364989282267776,0.395640902618069,0.
922526664590382,0.356253928367905,0.980372848261233
"6823","NAA10",115.927258665515,-0.244886479867976,0.291058761896054,-
0.84136439759691,0.400143824128414,0.980372848261233
"6824","EMD",91.1222537612846,-0.151115108826103,0.201626833415753,-0.
749479155457969,0.45356845759261,0.980372848261233
"6825","DNASE1L1",4.3154441389404,-0.668747286285776,0.738252376830883
,-0.905851856727542,0.365014269195257,0.980372848261233
"6826","SLC10A3",36.2944936080165,0.249830978503704,0.28848555766583,0
.866008615908248,0.386485436931804,0.980372848261233
"6827","FAM3A",57.3457796307097,-0.21757502035658,0.290548058159748,-0
.74884348473929,0.453951546842147,0.980372848261233
"6828","LINC00115",3.03139650460508,-0.536797057847504,0.7802828076605
74,-0.687951922786709,0.491483057555646,0.98045720611026
"6829","ACAP3",34.4900244416499,-0.203325609084561,0.31315269714175,-0
.649285830651888,0.51615364258148,0.98045720611026
"6830","AL691432.2",4.8725281256556,-0.408604997700345,0.6362040147859
89,-0.642254667062726,0.520707843332934,0.98045720611026
"6831","KIAA2013",69.5753291400797,-0.14814461586581,0.217708406070316
,-0.680472649356323,0.496205234138742,0.98045720611026
"6832","SPEN",113.74407804804,0.180300281075741,0.277012269351144,0.65
0874712149268,0.515127366583034,0.98045720611026
"6833","ZBTB17",21.8041573446367,-0.205063536560787,0.325272052583272,
-0.630436998605312,0.528408710284697,0.98045720611026
"6834","NBPF1",26.6339582073232,0.239580318021466,0.346150150194382,0.

69212830873229,0.488856755347852,0.98045720611026
"6835","ZNF436-
AS1",4.14199356113753,0.542488616279873,0.7595299413711,0.714242568634
72,0.475077198436237,0.98045720611026
"6836","PAFAH2",8.60044237978483,-0.367351207054847,0.530811664061892,
-0.69205564219104,0.488902386655136,0.98045720611026
"6837","ZNF593",122.267357731291,-0.231953195001418,0.36238045192147,-
0.640081973990373,0.522119307481917,0.98045720611026
"6838","GPATCH3",11.8067393331681,0.308068479115527,0.443869923513505,
0.694051258704295,0.487650063875907,0.98045720611026
"6839","TMEM222",72.2979056481612,-0.185050857844551,0.270985431011001
,-0.68288120565802,0.494681910847128,0.98045720611026
"6840","WASF2",518.473933441271,0.125660596763504,0.176396967591056,0.
712373905739835,0.476233269683472,0.98045720611026
"6841","XKR8",11.5409102518901,-0.309576222021383,0.467096244425453,-0
.662767525356951,0.50747945518236,0.98045720611026
"6842","TRNAU1AP",49.7211404196656,-0.173890854760052,0.25026844811643
4,-0.694817329426808,0.487169786581959,0.98045720611026
"6843","YTHDF2",166.219443346793,-0.148818226452219,0.209751229802747,
-0.709498707550701,0.478015053253335,0.98045720611026
"6844","PEF1",74.0514256451057,-0.179902577962732,0.282934860403762,-0
.635844511015723,0.524877772547634,0.98045720611026
"6845","KHDRBS1",281.918231111808,0.112456318663823,0.16342296837742,0
.688130437112787,0.491370644827572,0.98045720611026
"6846","RBBP4",125.64069139746,0.146537987910833,0.212269359631526,0.6
90339802999383,0.489980522761726,0.98045720611026
"6847","CSMD2",4.47592864425913,0.495771456977205,0.702444590566304,0.
705780162073023,0.480324860703297,0.98045720611026
"6848","SMIM12",66.5898544412155,-0.191235423743103,0.290309371694905,
-0.658729763447245,0.51006931677411,0.98045720611026
"6849","MED8",78.7174237860276,-0.180244692980292,0.276977898672646,-0
.65075478528819,0.515204791665764,0.98045720611026
"6850","B4GALT2",64.3327171028107,-0.215837836549192,0.301879393975422
,-0.714980355919109,0.47462118174348,0.98045720611026
"6851","ERI3",87.9270640669159,-0.183683420321278,0.281574196575605,-0
.65234464860475,0.514178863102344,0.98045720611026
"6852","UROD",102.685975194338,-0.173052449080043,0.270557911828503,-0
.639613337900591,0.522424009000497,0.98045720611026
"6853","POMGNT1",30.6462274726696,-0.197812901497857,0.297273935876923
,-0.665422957160144,0.505780005440171,0.98045720611026
"6854","TRABD2B",9.52197948587808,0.375806230612933,0.60252913473367,0
.623714620503865,0.532815019583843,0.98045720611026
"6855","EPS15",153.186512295952,0.160188651636699,0.230082043323268,0.
696224048269738,0.486288529579378,0.98045720611026
"6856","ZYG11B",83.6023828485425,0.177328438038114,0.276968469837902,0
.64024774423564,0.522011547551299,0.98045720611026
"6857","CZIB",187.477086315488,-0.172713922094739,0.26474400599145,-0.
652380859192395,0.514155509024276,0.98045720611026
"6858","PRKAA2",5.70306597212106,0.426795145686565,0.617058974700141,0
.69166021917753\5,0.489150734672422,0.98045720611026

"6859","JUN",7875.07919820304,0.129462459907883,0.192553646411667,0.672344888401132,0.501364159132507,0.98045720611026
"6860","LINC01358",7.43575217753424,-0.407740496609897,0.588546745960428,-0.69279203293278,0.48844007274148,0.98045720611026
"6861","PDE4B",29.5389909134829,0.316892302676226,0.458448963534931,0.691227002091545,0.489422897485748,0.98045720611026
"6862","SLC35D1",18.3652888187609,-0.252461466332738,0.363626935765107,-0.694287032949125,0.487502221304319,0.98045720611026
"6863","TTLL7",12.1219984251776,0.32424399722678,0.509682630419401,0.636168426928677,0.52466664963575,0.98045720611026
"6864","GBP4",142.359274244853,0.196577638091536,0.302636942796058,0.649549378457761,0.515983340840418,0.98045720611026
"6865","SASS6",17.1354388878658,0.252568334801034,0.399716838812899,0.631868138332939,0.527473044605992,0.98045720611026
"6866","AMY2B",2.79093197975731,-0.636585456489195,0.982475761011187,-0.647940113895539,0.517023684388191,0.98045720611026
"6867","AL356488.3",5.46642953155584,0.439826032758006,0.644080957426062,0.682873833928706,0.494686569377906,0.98045720611026
"6868","DDX20",20.1708984212919,-0.241836107308518,0.334738671391352,-0.722462410164081,0.470010228133472,0.98045720611026
"6869","SLC16A1",80.7856773327474,0.189612072235645,0.264614363929823,0.716560013673072,0.473645624724806,0.98045720611026
"6870","HIPK1",76.8575087411386,0.171380013625658,0.271192728856412,0.631949146823909,0.527420107297389,0.98045720611026
"6871","BCAS2",117.083744946854,-0.178525531321315,0.259857347291434,-0.687013598738448,0.492074159035515,0.98045720611026
"6872","LINC02591",15.4229510621289,0.293511042663804,0.430400863207635,0.681948080857375,0.495271781163485,0.98045720611026
"6873","AC245297.2",3.25705468233846,-0.519524606607935,0.757493852203827,-0.685846631093371,0.492809827361451,0.98045720611026
"6874","BOLA1",38.2757983391565,0.229210074444434,0.339654168190576,0.674833686468488,0.499781435577006,0.98045720611026
"6875","SF3B4",63.9836310013936,-0.209694211362517,0.319454863598333,-0.656412643089939,0.511558657812642,0.98045720611026
"6876","AC244033.2",3.43456916420017,0.554069655807084,0.815960489935938,0.679039809697875,0.497112638219933,0.98045720611026
"6877","GOLPH3L",38.6082577032901,0.184588534860139,0.272321645051871,0.677832769499388,0.497877731101352,0.98045720611026
"6878","CERS2",166.233760647967,-0.169172365825136,0.271473334155692,-0.623163841676303,0.533176860164131,0.98045720611026
"6879","SCNM1",41.2500409460103,-0.237499418458833,0.328367369306623,-0.723273506013507,0.46951186657945,0.98045720611026
"6880","VPS72",48.7243652936273,0.160017994504578,0.254550233511584,0.628630319041902,0.529591106318249,0.98045720611026
"6881","ZNF687",12.6062473511418,0.276304695553894,0.421599321207733,0.655372723946469,0.512227808486972,0.98045720611026
"6882","TUFT1",4.90699613629318,0.489024413391791,0.680269584501817,0.718868555250664,0.472221912624102,0.98045720611026
"6883","SPRR2E",2.94894724466797,2.11301421733709,3.16539125786096,0.667536505033813,0.504429500003304,0.98045720611026

"6884","SNAPIN",60.1461512941295,-0.182386125921581,0.271441389417449,-0.671917154244629,0.501636438999803,0.98045720611026
"6885","SLC27A3",131.534318135182,-0.176615244936461,0.256351645255216,-0.688956939443974,0.490850365849054,0.98045720611026
"6886","UBE2Q1",99.9070005396896,0.247310404044818,0.343792972013682,0.719358521485353,0.47192004669304,0.98045720611026
"6887","PMF1",113.003894913095,0.16147859609637,0.253107130411958,0.637985171865948,0.523483332596238,0.98045720611026
"6888","BGLAP",13.9425179078234,-0.280728449034867,0.404179672788789,-0.694563502161987,0.487328891720914,0.98045720611026
"6889","GLMP",64.8174893094613,0.191738992355603,0.307528821641448,0.623483000169508,0.532967170127451,0.98045720611026
"6890","PRCC",56.3439990196525,0.169254829644776,0.270484878014815,0.625745996918562,0.531481558123225,0.98045720611026
"6891","B4GALT3",33.7562599311657,0.264220872324838,0.410478542206809,0.643689852590922,0.519776571381582,0.98045720611026
"6892","ADAMTS4",93.7218118918764,0.60667282777739,0.941627305182862,0.644281261214675,0.51939306466701,0.98045720611026
"6893","ATF6",87.1665340976584,0.170570019321847,0.236628255177862,0.72083538457247,0.471010801884403,0.98045720611026
"6894","DCAF6",78.5685182616336,0.160616008196119,0.237796242295579,0.675435434326477,0.499399159210772,0.98045720611026
"6895","FMO2",80.8536887991381,0.320035338915425,0.442531616718905,0.723192031539547,0.469561913731225,0.98045720611026
"6896","RABGAP1L",76.2925779731876,0.181015498725464,0.264338547800359,0.68478661259112,0.493478584403517,0.98045720611026
"6897","AL137796.1",2.8057919455627,-0.677812991926922,0.977519617126623,-0.693400909865445,0.488057991428837,0.98045720611026
"6898","KIAA1614",12.9573633556501,0.311195426839091,0.468801395812222,0.663810794120885,0.506811416237457,0.98045720611026
"6899","UCHL5",47.4528827736159,-0.158003931070301,0.244799008207777,-0.64544350986991,0.518639814974444,0.98045720611026
"6900","CDC73",82.5121013003708,0.136966240725843,0.217605649282809,0.629424103543546,0.529071442015683,0.98045720611026
"6901","NEK7",70.8858353600393,0.178897937428768,0.273900934881853,0.65314832717141,0.513660659075093,0.98045720611026
"6902","ZC3H11A",2.63621500199237,0.697620939552516,0.975281283445274,0.71530229421414,0.474422271241518,0.98045720611026
"6903","ARF1",433.28633508662,-0.132704055289114,0.188535529571291,-0.703867624266196,0.481515215326317,0.98045720611026
"6904","OBSCN",4.75116749921191,0.44992447575587,0.664189273316469,0.677404007910697,0.498149656111415,0.98045720611026
"6905","FAM89A",40.060561912107,0.18556332698209,0.281224539915846,0.659840450046139,0.509356222343461,0.98045720611026
"6906","LINC01354",9.47220962194497,0.323032107824603,0.475138607166818,0.679869206484389,0.496587281188685,0.98045720611026
"6907","AL391832.2",2.31514834328879,0.748440299845875,1.12851113267948,0.663210382398992,0.507195822939347,0.98045720611026
"6908","TBCE.1",6.9156136786115,0.386154945212549,0.578299287728055,0.667742384275836,0.504298049914667,0.98045720611026

"6909","LGALS8",71.3593559191539,0.196086772020986,0.282636859213086,0
.693776362244216,0.487822468637192,0.98045720611026
"6910","CEP170",94.2661124962379,0.135778326329209,0.211126991969842,0
.64311211495214,0.520151353964792,0.98045720611026
"6911","SCCPDH",120.650200321721,0.184116887506672,0.270128054240973,0
.681591136559356,0.495497521071373,0.98045720611026
"6912","AHCTF1",77.3248576346013,-0.167710549761206,0.241140608848651,
-0.695488622019975,0.486749139202118,0.98045720611026
"6913","AL390728.5",2.45126029288575,0.710223245909889,1.0350973160697
9,0.686141520109985,0.492623870674593,0.98045720611026
"6914","COLEC11",3.70988740738839,0.602951944018512,0.965945089350816,
0.624209337224064,0.532490115607288,0.98045720611026
"6915","ODC1",79.6109683907784,-0.211888789129816,0.310752512859288,-0
.681857041734565,0.495329351211386,0.98045720611026
"6916","PDIA6",504.850166257326,-0.157143879695198,0.2512222456368,-0.
625517375250225,0.531631548280939,0.98045720611026
"6917","WDR35",20.7542081044022,-0.25706531829581,0.390875787578803,-0
.657664983262706,0.510753427912044,0.98045720611026
"6918","LAPTM4A",2382.81663181343,-0.151158582212364,0.231507559315243
,-0.652931518346368,0.513800428493095,0.98045720611026
"6919","PUM2",73.1283572921344,0.158653403122377,0.251800940326775,0.6
30074704711128,0.528645708632453,0.98045720611026
"6920","WDCP",6.65732628612125,0.380656116486315,0.581925350428446,0.6
54132211642017,0.513026630321805,0.98045720611026
"6921","PTRHD1",75.4289994465538,-0.188821736486732,0.302917825889188,
-0.623343099510446,0.533059080999381,0.98045720611026
"6922","HADHA",331.205513920057,-0.147015824311087,0.209610727448077,-
0.701375478731186,0.483068720832347,0.98045720611026
"6923","LBH",32.628789776508,0.248555658864128,0.38551743100911,0.6447
32608363575,0.519100480954367,0.98045720611026
"6924","GPATCH11",44.1190089738013,-0.17389943776946,0.275522673432955
,-0.631161986063465,0.527934614684276,0.98045720611026
"6925","COX7A2L",271.352311690903,-0.146162642575787,0.231411967021151
,-0.631612290657498,0.527640253358361,0.98045720611026
"6926","OXER1",3.4102408848729,0.544603112957264,0.78502071727037,0.69
3743618449876,0.48784300651016,0.98045720611026
"6927","DYNC2LI1",60.6123341182374,-0.138994436106111,0.22034686296047
2,-0.630798343296793,0.528172386780362,0.98045720611026
"6928","SRBD1",34.3831512749031,0.19035218859001,0.28968273794419,0.65
7105735539833,0.51111293123241,0.98045720611026
"6929","MSH2",22.9320138333339,0.227767754075181,0.362747161925973,0.6
27896722515673,0.530071598181192,0.98045720611026
"6930","AC016747.1",16.0110932029532,0.246077040980718,0.3917795017597
53,0.628100857434899,0.529937871429681,0.98045720611026
"6931","AC016727.1",3.10384988243878,-0.609894155055956,0.849708368968
078,-0.717768798483929,0.472899854852448,0.98045720611026
"6932","FAM161A",9.97702620857728,0.348338490388497,0.516542728990831,
0.674365296108312,0.500079100506653,0.98045720611026
"6933","MEIS1",22.5370938960558,0.268376364815786,0.381936221052787,0.
702673247580501,0.482259403325991,0.98045720611026

"6934","PPP3R1",44.5744298936024,0.19056821160671,0.274047543313929,0.695383761891303,0.486814834033526,0.98045720611026
"6935","PLEK",12.9926121218022,-0.382411240012545,0.590708529563009,-0.647377210373859,0.517387841724603,0.98045720611026
"6936","MPHOSPH10",126.02485113982,-0.176062679819577,0.280257010444053,-0.628218646665126,0.529860716679103,0.98045720611026
"6937","TPRKB",91.6732159555119,-0.200670790291204,0.316585434350274,-0.633859832190446,0.526172301660562,0.98045720611026
"6938","HK2",13.5499058764911,0.414196266188592,0.632997426410859,0.654341153544832,0.512892037772107,0.98045720611026
"6939","CHMP3",263.902233823337,-0.117747901409188,0.169146778474611,-0.696128548654931,0.486348329249342,0.98045720611026
"6940","EIF2AK3-DT",2.66727472642167,0.556760252726031,0.857584876435638,0.649218833056014,0.516196940349566,0.98045720611026
"6941","GPAT2",11.7906817131719,-0.445147944826798,0.700046004365095,-0.635883844848916,0.524852133100608,0.98045720611026
"6942","ANKRD23",3.03950957094211,0.503184033875776,0.779274625689141,0.645708223119407,0.518468334105557,0.98045720611026
"6943","INPP4A",21.3640363460376,0.267707624170093,0.383372419501195,0.698296514179103,0.484991778762276,0.98045720611026
"6944","EIF5B",352.864776292181,-0.207233097834546,0.297226770045532,-0.697222184269608,0.485663757684413,0.98045720611026
"6945","IL1R1",402.05020345743,0.12550776485662,0.199499329739499,0.629113716925787,0.529274610912821,0.98045720611026
"6946","LIMS1",382.945218321957,-0.124625205847083,0.186684834063345,-0.66757005984105,0.504408074647019,0.98045720611026
"6947","AC079922.2",8.48054109587645,-0.376931891518691,0.57061084690398,-0.660576106402196,0.508884196257239,0.98045720611026
"6948","LIMS2",39.6769771966445,0.196043482250384,0.291516396185217,0.672495560509835,0.5012682654429,0.98045720611026
"6949","SFT2D3",20.1394182653427,0.256552923955909,0.411501763696878,0.623455223256083,0.532985418155171,0.98045720611026
"6950","SMPD4",30.1436299698137,0.20087964062247,0.302788369770847,0.663432485119879,0.507053606379456,0.98045720611026
"6951","C2orf27A",25.864770111423,0.198867629891954,0.317234945568234,0.626878068353224,0.530739164497506,0.98045720611026
"6952","RND3",849.93236952416,-0.211555471716596,0.324247221776716,-0.65245114686681,0.514110178447818,0.98045720611026
"6953","TANK",148.598703780328,0.10657778975524,0.16494050694951,0.646158980145883,0.518176401788665,0.98045720611026
"6954","SLC4A10",2.01376222426432,0.913113993331869,1.31255500494745,0.695676744890722,0.48663129230158,0.98045720611026
"6955","ERICH2",2.93344888005926,-0.581949111256124,0.867755104164766,-0.670637497219061,0.502451488915879,0.98045720611026
"6956","GPR155",13.7578860094384,0.3193261338818,0.466940808456021,0.683868550572135,0.494058174692936,0.98045720611026
"6957","LNPK",91.1866299194976,0.152636284755561,0.233158080815787,0.654647199966256,0.512694927342934,0.98045720611026
"6958","HOXD9",1.85531878427795,0.69396008129519,1.05686933441913,0.65

6618617548023,0.511426174799946,0.98045720611026
"6959","LINC01116",58.624146342715,0.295563844910038,0.421321140337158
,0.701516768594891,0.482980573568743,0.98045720611026
"6960","HNRNPA3",444.969430466125,-0.124904927150997,0.199870293895866
,-0.624929921882607,0.532017052910371,0.98045720611026
"6961","GLS",161.760195899388,0.188017483273765,0.296118292128633,0.63
4940455458559,0.525467250688635,0.98045720611026
"6962","HSPD1",434.213985956825,-0.180313804474603,0.285196798832418,-
0.632243437558904,0.527227817430459,0.98045720611026
"6963","MAIP1",37.9242978580791,-0.218785899428421,0.334805989742659,-
0.653470684907954,0.513452882384211,0.98045720611026
"6964","PPIL3",57.366990351681,-0.18935499163347,0.294007181953659,-0.
644048864300588,0.519543748086823,0.98045720611026
"6965","ABI2",82.4034560019115,-0.147087806569407,0.214645878894776,-0
.685258004145109,0.493181127326858,0.98045720611026
"6966","CTLA4",7.63858970391975,0.444718066610538,0.678071838096421,0.
655856860032726,0.51191622749798,0.98045720611026
"6967","METTL21A",23.2297380696934,0.215102229976469,0.343841919636898
,0.625584658797916,0.531587403824627,0.98045720611026
"6968","AC007038.2",3.8126998064319,0.52351653793175,0.737533862145355
,0.709820341548703,0.477815553186424,0.98045720611026
"6969","LANCL1",28.6211664282627,0.225889134818832,0.321020518134307,0
.703659492332902,0.481644852457218,0.98045720611026
"6970","SPAG16",56.0915258832415,-0.184240042562696,0.265947022905977,
-0.692769712364225,0.488454082366414,0.98045720611026
"6971","TNS1",131.888635932895,-0.194898498694616,0.280277168709017,-0
.695377720534058,0.486818619087535,0.98045720611026
"6972","GMPPA",40.4659196009138,-0.208516911844607,0.31851058172884,-0
.654662431347808,0.512685118541786,0.98045720611026
"6973","STK11IP",10.4422250619052,0.297865943860844,0.459456490729066,
0.648300654950353,0.516790510503588,0.98045720611026
"6974","FBXO36",10.1202315653562,0.314204619898054,0.494004280139351,0
.636036229907606,0.52475280816429,0.98045720611026
"6975","SP140L",38.491623847592,0.213049220064497,0.304700762378144,0.
699208030861752,0.484422032472076,0.98045720611026
"6976","NCL",776.774770525494,-0.154883957354259,0.220645129692481,-0.
701959556370563,0.482704385784396,0.98045720611026
"6977","ANKMY1",7.65193742849528,0.368356444241882,0.540888708332864,0
.681020769276614,0.495858348659257,0.98045720611026
"6978","DUSP28",8.94646174228847,-0.377187392804607,0.535098721654417,
-0.704893092695905,0.480876769023906,0.98045720611026
"6979","AC024060.2",3.33493565030307,0.533962149268178,0.7771036888120
94,0.687118279009086,0.492008196330421,0.98045720611026
"6980","AC083855.2",2.76300299742756,-0.585695551952374,0.907646690265
382,-0.645290241493775,0.518739115406476,0.98045720611026
"6981","EAF1-
AS1",1.88554565808752,0.715377889394582,1.02949114058564,0.69488493993
9967,0.487127411390299,0.98045720611026
"6982","AZI2",215.183757131257,0.10657676899397,0.168792048922313,0.63
1408704820107,0.527773325794588,0.98045720611026

"6983","FBXL2",8.75580488464792,0.389139329138519,0.55564369078694,0.700339688168497,0.483715191736024,0.98045720611026
"6984","PDCD6IP",133.719236995292,0.125060839799627,0.176899494399295,0.706959848722582,0.479591429752155,0.98045720611026
"6985","C3orf35",2.00944579588048,0.706745861254116,1.1139690573669,0.634439400789691,0.525794102928627,0.98045720611026
"6986","WDR48",45.4286491142779,0.200342460135627,0.28822951427448,0.695079616117457,0.487005408344122,0.98045720611026
"6987","ACKR2",20.9049180359439,0.335379792916725,0.533196072338934,0.628998993645129,0.529349715051901,0.98045720611026
"6988","ZNF35",11.217755891012,0.315296428942782,0.480445114341063,0.656258997191002,0.511657494700077,0.98045720611026
"6989","DHX30",65.0990946227756,0.153505672661524,0.231755997719472,0.66235900762894,0.507741168294918,0.98045720611026
"6990","IP6K2",108.227964189978,-0.134450957320709,0.213896197972138,-0.628580398321166,0.52962379642416,0.98045720611026
"6991","NDUFAF3",220.920404142513,-0.168310571542976,0.257493462242034,-0.653649883292068,0.513337398428653,0.98045720611026
"6992","RNF123",12.3895870146111,0.316144140806623,0.441726433138741,0.71570120574452,0.474175866141162,0.98045720611026
"6993","MON1A",12.0740956555194,0.327784204809264,0.454151043223514,0.721751517915025,0.470447261817987,0.98045720611026
"6994","GNAI2",456.225326324107,-0.132272800916,0.188099129883015,-0.703207936146567,0.481926174186014,0.98045720611026
"6995","RRP9",45.159231433636,-0.241844470679126,0.349551637698865,-0.691870512383272,0.489018650186798,0.98045720611026
"6996","DUSP7",16.8582533214119,-0.241962981692627,0.355237265966653,-0.681130626974649,0.495788839226404,0.98045720611026
"6997","GLT8D1",161.820218121891,-0.151145919044646,0.236299634726282,-0.639636702019141,0.522408815769273,0.98045720611026
"6998","RPP14",27.5215803597347,0.219422998820812,0.322387551731962,0.680618707645522,0.496112786425751,0.98045720611026
"6999","ACOX2",26.4009454892566,0.222241841400969,0.356826843908075,0.62282825744529,0.533397387044004,0.98045720611026
"7000","TMF1",168.373999621754,-0.138277045114657,0.214337894765183,-0.645135780894674,0.5188391981995,0.98045720611026
"7001","MITF",104.678440078082,0.261415446284711,0.363114470465021,0.71992571915388,0.471570731685698,0.98045720611026
"7002","CGGBP1",133.268097179675,0.116281060026868,0.171226193364097,0.67910789664994,0.49706949938675,0.98045720611026
"7003","LNP1",5.19306314483084,0.49100486154274,0.704111166301895,0.697339972779549,0.485590057970045,0.98045720611026
"7004","TMEM45A",111.150516257857,0.479412620477481,0.673266545042122,0.712069571862489,0.476421695450852,0.98045720611026
"7005","AC026329.1",2.7878735046101,0.587652320275202,0.874377867606886,0.672080506661917,0.501532445606746,0.98045720611026
"7006","HCLS1",22.4407697381395,0.2315557117448,0.360270079047893,0.642728123181215,0.520400529255236,0.98045720611026
"7007","EAF2",4.34683006433965,-0.4563011774146,0.697002591294019,-0.654662096115676,0.512685334425637,0.98045720611026

"7008","CSTA",12.1038379555541,-0.364287087503702,0.527774962399812,-0.690231847769503,0.490048398454836,0.98045720611026
"7009","ALG1L",2.8785902424316,0.635044387652496,0.932491632685323,0.681018858929319,0.495859557424901,0.98045720611026
"7010","TMCC1-AS1",3.80405383405809,-0.484491180292728,0.766121275650018,-0.63239489058917,0.527128871808134,0.98045720611026
"7011","UBA5",59.393216783038,0.169972393697939,0.252288458176265,0.67372243235632,0.500487797437107,0.98045720611026
"7012","CDV3",285.503879337213,0.133673719581555,0.203271429323091,0.657611942941013,0.510787518348331,0.98045720611026
"7013","CEP63",72.7281299095483,-0.15856667922082,0.220774818416055,-0.718228103904481,0.47261665208094,0.98045720611026
"7014","PPP2R3A",27.6174345485628,-0.212837907337796,0.31168316369815,-0.682866231247315,0.494691373882363,0.98045720611026
"7015","MRPS22",87.6559111655594,-0.19674624533547,0.307979190449965,-0.638829672381497,0.522933742919136,0.98045720611026
"7016","COPB2",181.837664734727,-0.148260037770264,0.206297035062152,-0.718672654338424,0.472342636031178,0.98045720611026
"7017","RNF13",187.519975104976,-0.134867895765653,0.212223808871479,-0.635498422551298,0.525103395207544,0.98045720611026
"7018","C3orf33",14.2278004627858,-0.285036127962107,0.450726599277787,-0.632392515593331,0.527130423341073,0.98045720611026
"7019","LINC00886",2.30116001525394,0.743797195523228,1.08003233213473,0.688680489826705,0.491024356509678,0.98045720611026
"7020","MFSD1",63.5844528184479,-0.168831474085505,0.259945844516687,-0.649487105283068,0.516023578462244,0.98045720611026
"7021","AC080013.1",4.120213070927,0.543043129402188,0.836639943509422,0.64907626466447,0.516289082657011,0.98045720611026
"7022","PRKCI",42.1644353704588,0.182851289599118,0.286532089863027,0.638152919229843,0.52337414126734,0.98045720611026
"7023","NDUFB5",203.807429138961,-0.176714053283278,0.267449016114984,-0.660739216207487,0.508779569638542,0.98045720611026
"7024","ALG3",67.3826055829335,-0.228567320623007,0.316160535760355,-0.722947030923105,0.469712427504879,0.98045720611026
"7025","MASP1",39.7604954962252,-0.36526938260896,0.544145047150991,-0.671272089163394,0.502047211766587,0.98045720611026
"7026","TPRG1",20.7640312147505,-0.284690006584549,0.420706651033169,-0.676694808331194,0.498599611151463,0.98045720611026
"7027","CCDC50",196.418328737108,0.135265069079461,0.190120426366817,0.711470469871983,0.47679274373877,0.98045720611026
"7028","RNF168",56.3318816467418,-0.180877442702303,0.257309642112682,-0.702956333921961,0.482082962373214,0.98045720611026
"7029","SPON2",603.023261970526,-0.15411202564025,0.217695810004443,-0.707923710782975,0.478992634402959,0.98045720611026
"7030","CTBP1-DT",15.6150650673064,0.313037151879,0.476293264740583,0.657236150608803,0.511029084205941,0.98045720611026
"7031","LYAR",77.5152713367602,-0.16912315218984,0.264782321768341,-0.638725240644301,0.523001689644867,0.98045720611026

"7032","STX18",46.575787120486,-0.179258107574249,0.263785656518256,-0
.679559722618364,0.49678328002659,0.98045720611026
"7033","STK32B",10.4549763239539,0.461398332411058,0.658642344018795,0
.700529409627346,0.483596745238799,0.98045720611026
"7034","MRFAP1",583.857750082682,0.142320447067473,0.226679759135139,0
.627848060234728,0.530103478886859,0.98045720611026
"7035","AC093323.1",24.5973576858905,0.223096189496947,0.3238810816289
55,0.688821305569586,0.490935726368372,0.98045720611026
"7036","CPEB2",14.4550966746746,-0.276176587071809,0.428186670416979,-
0.644991089523784,0.518932960065406,0.98045720611026
"7037","QDPR",71.1348514963597,-0.168907695621511,0.253183481862123,-0
.667135527085824,0.504685568864595,0.98045720611026
"7038","TMEM156",2.67556821523154,0.612547741910266,0.959513119641759,
0.638394337056033,0.523217016343109,0.98045720611026
"7039","PPAT",19.5926980200912,-0.256712832062911,0.396033581512463,-0
.648209758077882,0.516849291395529,0.98045720611026
"7040","SRP72",233.699921648661,-0.130109474446721,0.208284482444636,-
0.624671953088515,0.532186384551309,0.98045720611026
"7041","MOB1B",19.6525952339459,0.236873620423602,0.3663225254203 7,0.6
46625866514159,0.517874112954476,0.98045720611026
"7042","CXCL2",384.027842228019,-0.284962490548954,0.448129384626082,-
0.635893338676567,0.524845944720465,0.98045720611026
"7043","AC098818.2",17.3604908945805,-0.379450105693868,0.550601974002
229,-0.689155004177904,0.490725729100304,0.98045720611026
"7044","PRDM8",44.5232041129216,-0.165903589105616,0.234874189578265,-
0.706350874072239,0.47996996317845,0.98045720611026
"7045","THAP9",4.30184303034967,0.552826741290803,0.785935894791118,0.
703399278433174,0.481806956088816,0.98045720611026
"7046","ABRAXAS1",61.4937281048431,-0.171660758333737,0.24750323808270
2,-0.693569747464786,0.487952071332844,0.98045720611026
"7047","GPAT3",5.00518173136479,-0.505012679553701,0.788291311671155,-
0.640642199243689,0.521755175722133,0.98045720611026
"7048","SLC9B1",2.63984836413377,0.568846594582744,0.90789237065503,0.
626557302351082,0.530949464061238,0.98045720611026
"7049","TET2",30.0959850951438,0.219008720538166,0.322293833736497,0.6
79531215348119,0.496801336003779,0.98045720611026
"7050","DKK2",26.0379706130254,0.333542153116769,0.524610956635079,0.6
35789529170627,0.524913613101697,0.98045720611026
"7051","MCUB",165.400377416465,-0.134583240804374,0.198235812896195,-0
.678904779303667,0.497198197276191,0.98045720611026
"7052","AP1AR",29.4883626248453,-0.192634174132558,0.307814861319841,-
0.625811805533318,0.531438387519955,0.98045720611026
"7053","FAT4",55.3896416495532,0.223242525394487,0.317798302560662,0.7
02466072334902,0.482388552950883,0.98045720611026
"7054","LINC02511",93.8808664165254,-0.167596969741492,0.2371169975349
95,-0.706811285077769,0.479683760618195,0.98045720611026
"7055","TBC1D9",29.8684508801693,0.240765751936251,0.343446858587195,0
.701027672597346,0.483285745724601,0.98045720611026
"7056","RNF175",2.50059386532367,-0.771390470761482,1.08469299144685,-
0.711160187116672,0.476984976683948,0.98045720611026

"7057","FNIP2",55.8071283752149,-0.196703677094173,0.290193538091915,-0.677836172326722,0.497875573309264,0.98045720611026
"7058","KLHL2",13.405669243488,0.271020515431617,0.40918372763465,0.662344314125816,0.507750582871777,0.98045720611026
"7059","WDR17",2.78060194137116,-0.605467446793007,0.971995329672631,-0.622911888884208,0.533342424903367,0.98045720611026
"7060","SORBS2",10.1455185874357,0.370808742078831,0.586053881717829,0.632721245684653,0.526915693311227,0.98045720611026
"7061","FAM149A",23.51836350021,0.230050931902114,0.339792559016895,0.677033459966602,0.498384725061938,0.98045720611026
"7062","AC110772.2",2.10472122350198,-0.65217451118436,0.932972580156163,-0.69902859425429,0.484534161183767,0.98045720611026
"7063","BRD9",75.9592828475906,0.190732895987654,0.294729824131275,0.647144877685335,0.517538182651823,0.98045720611026
"7064","TENT4A",26.9997212044941,0.214959803915688,0.325277772051723,0.660849963893341,0.508708537074851,0.98045720611026
"7065","C5orf49",2.25817107591606,-0.600081139482899,0.887434279700493,-0.676197835951779,0.498915046353269,0.98045720611026
"7066","MARCH6",125.512213076148,0.149235567221631,0.209652853728789,0.711822255540033,0.476574849355584,0.98045720611026
"7067","DROSHA",23.7826529393356,0.222469402589857,0.331639627425365,0.670816706426084,0.502337303326492,0.98045720611026
"7068","IL7R",108.11394821966,0.204279558559706,0.307888352916681,0.66348582732841,0.507019453478019,0.98045720611026
"7069","LIFR-AS1",7.38076968179385,0.375847050341444,0.558758844908908,0.672646265497089,0.501172360547325,0.98045720611026
"7070","CARD6",83.8808061369298,0.157261408552725,0.23046417896613,0.682368120105264,0.495006208905799,0.98045720611026
"7071","EMB",24.0664613986232,-0.231685363196694,0.354002687864143,-0.654473457799306,0.512806821839868,0.98045720611026
"7072","SREK1IP1",113.881364916811,0.150445209671948,0.240478810924858,0.625606926004627,0.531572794811954,0.98045720611026
"7073","RAD17",31.9802115325858,0.195709847245017,0.288147746665072,0.67919964500885,0.497011372209273,0.98045720611026
"7074","OCLN",9.17329833118905,0.46377647506841,0.657990233828774,0.704837931058254,0.480911100365772,0.98045720611026
"7075","NAIP",21.6641793185536,-0.249661969932719,0.355061670043897,-0.703151004449038,0.481961649256995,0.98045720611026
"7076","HEXB",226.548620578255,-0.123450159972119,0.175902604958142,-0.701809731592634,0.482797829014224,0.98045720611026
"7077","LHFPL2",48.8215952467582,0.208152378693262,0.327881411718285,0.63484043698124,0.5255324872879,0.98045720611026
"7078","ATP6AP1L",7.94189509110503,0.380216082254099,0.535933172620519,0.709446814786591,0.478047245067717,0.98045720611026
"7079","XRCC4",21.4784980319348,-0.27573364772418,0.390730763672992,-0.705687069869281,0.480382763698792,0.98045720611026
"7080","EDIL3",31.1701632103124,-0.373901696933863,0.535790620620255,-0.697850396300364,0.485270758341906,0.98045720611026
"7081","COX7C",973.287797570375,-0.151412465259968,0.215332962396792,-

0.703155074702227,0.481959112968589,0.98045720611026
"7082","MEF2C",23.4413741106226,-0.268409777902735,0.385265107624813,-0.696688520685147,0.485997744296421,0.98045720611026
"7083","LNPEP",89.736842825821,0.186879474662603,0.262165682174224,0.712829662192059,0.475951168288578,0.98045720611026
"7084","EFNA5",101.15952959281,0.155279089223441,0.233313442329001,0.665538546229489,0.505706097687685,0.98045720611026
"7085","WDR36",49.8438303250949,0.229892993676477,0.321954411356118,0.71405449208829,0.475193484547034,0.98045720611026
"7086","MARCH3",224.339275298684,0.278260222559732,0.416129171810752,0.668687132288529,0.503695077539182,0.98045720611026
"7087","HINT1",671.336635010089,-0.164408007157749,0.259605825075962,-0.633298606106555,0.526538663380782,0.98045720611026
"7088","C5orf56",92.8686594122246,0.159862582057334,0.22939094617739,0.696900138045164,0.485865291382456,0.98045720611026
"7089","HSPA4",102.368508458033,0.181921407647443,0.261931259228868,0.694538743420789,0.487344412607253,0.98045720611026
"7090","AC008608.2",6.86210549918122,0.457603677694728,0.645130603707472,0.709319438676364,0.478126268201673,0.98045720611026
"7091","PPP2CA",94.5352834163752,-0.152568198441067,0.218439406361775,-0.698446315077354,0.4848981203363,0.98045720611026
"7092","C5orf24",124.610617032519,0.151893268826126,0.231085233659394,0.657304088282885,0.510985408266327,0.98045720611026
"7093","TGFBI",29.4448825361819,0.277245144738177,0.402529752462877,0.688756900680891,0.490976262064614,0.98045720611026
"7094","SMIM33",1.87056063111045,0.784471869794247,1.16882730931873,0.671161482573069,0.502117663111932,0.98045720611026
"7095","PCDHGA7",2.23914854415531,0.676647653207681,1.00390294333504,0.674017003037972,0.500300503839357,0.98045720611026
"7096","ADRB2",7.178551399364,-0.369554212058441,0.57500437076895,-0.642698092127958,0.520420019236122,0.98045720611026
"7097","HMGXB3",45.9683885655764,0.166340733409988,0.250674318878749,0.663573094180611,0.506963582578397,0.98045720611026
"7098","TCOF1",36.9225357196202,-0.189611893349733,0.304481812792969,-0.622736352002276,0.533457790118312,0.98045720611026
"7099","SYNPO",7.85575862771146,-0.359337208492288,0.510778791229724,-0.70350847502334,0.481738927039757,0.98045720611026
"7100","TNIP1",96.0460808228333,-0.186596324085281,0.263935990257796,-0.706975672029516,0.479581596291566,0.98045720611026
"7101","BOD1",157.245349248335,-0.18319909976554,0.260398728060497,-0.703533005441477,0.481723645403919,0.98045720611026
"7102","UIMC1",35.0631413753679,-0.185325031917038,0.285983328810676,-0.648027396169394,0.516967231041917,0.98045720611026
"7103","ZNF354C",15.6177066890114,-0.257690224098616,0.381534382712226,-0.675404985172674,0.499418499116615,0.98045720611026
"7104","GMDS",16.8691130523551,0.303443486944553,0.432345736991351,0.701853773454977,0.482770359815203,0.98045720611026
"7105","TMEM14C",371.110224766275,-0.174203214970472,0.24714542034611,-0.704861189523608,0.480896624666039,0.98045720611026
"7106","RIPOR2",27.0650207699866,0.272719376237986,0.39074735332777,0.

69794299031175,0.485212847581173,0.98045720611026
"7107","HIST1H1C",28.2337008978438,0.291785921426179,0.428128674009665
,0.681537909370659,0.49553118789463,0.98045720611026
"7108","ZNF184",6.47510027952338,-0.365564356222368,0.575810981538723,
-0.634868677296639,0.525514067250264,0.98045720611026
"7109","DHX16",32.237400081078,-0.199969925792734,0.281704362169935,-0
.709857398914199,0.477792570523006,0.98045720611026
"7110","HLA-
B",2768.92376324959,0.22992487130641,0.362126357637504,0.6349299531976
36,0.525474100546145,0.98045720611026
"7111","GPANK1",35.005191361354,-0.199710506370301,0.306257371451069,-
0.652100243086586,0.514336507270341,0.98045720611026
"7112","ABHD16A",67.8323553699722,-0.172425039195259,0.264741794748417
,-0.651295120814279,0.514855997422739,0.98045720611026
"7113","AL662884.5",6.71064043226355,-0.405034931642823,0.599681047267
619,-0.675417263040612,0.499410700730211,0.98045720611026
"7114","EGFL8",13.4267292892612,-0.329784150740232,0.479862636994733,-
0.68724698552401,0.49192710035003,0.98045720611026
"7115","GPSM3",26.4415309286446,-0.203275257013481,0.317220550212698,-
0.640801035359104,0.521651960174062,0.98045720611026
"7116","ZBTB9",4.03630012578678,-0.43495867493458,0.697066371327726,-0
.623984591461641,0.532637704396314,0.98045720611026
"7117","RPL10A",4324.52194124607,-0.152566965707339,0.232327529990225,
-0.656689139310173,0.511380819237073,0.98045720611026
"7118","MED20",10.3626270527729,0.297779904229395,0.432950388409417,0.
687792209457037,0.4915836427603,0.98045720611026
"7119","ABCC10",9.46038517634395,0.319924834395086,0.478896434903225,0
.668045972110308,0.50410424766602,0.98045720611026
"7120","YIPF3",193.792919947299,-0.143205104544353,0.218677677643832,-
0.654868416782785,0.512552476348304,0.98045720611026
"7121","TDRD6",2.8111889975225,0.750752183103129,1.09429566959214,0.68
6059722216524,0.492675448565351,0.98045720611026
"7122","MMUT",51.4626348426019,0.187554554826839,0.264646464647189,0.7
08698508694896,0.478511590491795,0.98045720611026
"7123","TRAM2-
AS1",18.4329166782013,0.25821756860738,0.363343371980779,0.71067092045
6588,0.47728818371352,0.98045720611026
"7124","FBXO9",89.0034953631936,0.129043247028097,0.19618755627649,0.6
57754495123198,0.510695898937119,0.98045720611026
"7125","AL391840.3",6.26480863264107,-0.359517150068576,0.557156947527
613,-0.64527087325023,0.518751664518935,0.98045720611026
"7126","DOP1A",19.8455217394804,0.277035751100102,0.395523448984087,0.
700428133430964,0.483659971819578,0.98045720611026
"7127","RWDD2A",11.5457391055334,0.285456799678699,0.439547795729183,0
.649432900022042,0.516058604351126,0.98045720611026
"7128","PNRC1",2413.67182685119,0.154857891243587,0.231587257464145,0.
668680535100521,0.503699286783402,0.98045720611026
"7129","RTN4IP1",3.98051898359656,0.543180228960266,0.753816238301946,
0.720573796849799,0.471171780378534,0.98045720611026
"7130","SCML4",7.80575383855209,0.380166563522631,0.529199274238074,0.

718380734875316,0.472522562145954,0.98045720611026
"7131","CDK19",25.805317442861,0.226075548426711,0.341556889350807,0.6
6189719919399,0.508037106892389,0.98045720611026
"7132","GTF3C6",196.870338060042,-0.19931780022277,0.302583425153397,-
0.658720153365058,0.510075489016074,0.98045720611026
"7133","FAM229B",68.5581104953498,0.209798779061661,0.310102006767781,
0.676547634271739,0.498693013498383,0.98045720611026
"7134","CALHM6",15.2851980233974,0.335119328085188,0.508147243950247,0
.659492562588807,0.509579520459907,0.98045720611026
"7135","KPNA5",39.3448635026347,-0.179908879929385,0.263062726037439,-
0.683901070438159,0.494037638053197,0.98045720611026
"7136","MCM9",11.2073345648559,0.323445653341414,0.489718544160113,0.6
6047254529872,0.508950631273103,0.98045720611026
"7137","SOGA3",3.24345218571961,-0.517189317989516,0.783018466270737,-
0.660507178652785,0.508928413280761,0.98045720611026
"7138","L3MBTL3",12.3687378696688,-0.297216684383189,0.454958039740747
,-0.653283728214924,0.513573380577752,0.98045720611026
"7139","TAB2",64.1162061509035,0.166030633200544,0.254392702179913,0.6
52654859112754,0.513978810207508,0.98045720611026
"7140","ARMT1",37.1889329338186,0.219564703593154,0.311401624379271,0.
705085286664192,0.480757162321353,0.98045720611026
"7141","AL080276.2",5.60459679370051,-0.42973636062501,0.6057936409920
34,-0.70937747038956,0.47809026489909,0.98045720611026
"7142","CNKSR3",6.47209594238218,-0.355205162885412,0.556558980258982,
-0.638216569104906,0.523332712888487,0.98045720611026
"7143","SERAC1",4.42149758096282,-0.457029648574703,0.687478756138757,
-0.664790940074457,0.5061842182271,0.98045720611026
"7144","CAHM",5.0005146245806,-0.402651837281265,0.61688296280691,-0.6
52719983461918,0.513936817048734,0.98045720611026
"7145","AFDN",43.4229954918889,0.203937904190667,0.288297995618402,0.7
07385785854039,0.479326767708941,0.98045720611026
"7146","C7orf50",83.1009412962991,0.164169352793053,0.250170562576495,
0.656229698259781,0.511676343162917,0.98045720611026
"7147","GPR146",8.86200274149242,0.430913428410803,0.614877255400979,0
.700812112703358,0.483420277860576,0.98045720611026
"7148","PSMG3-
AS1",13.2018740882132,0.284432882451522,0.405238227501423,0.7018905501
71562,0.482747422585883,0.98045720611026
"7149","FOXK1",48.8129724952775,0.185715581735922,0.260362306759392,0.
71329672888306,0.475662161222697,0.98045720611026
"7150","ZNF12",52.1960500062655,0.18037597688375,0.278876229057973,0.6
46795811507668,0.517764103533309,0.98045720611026
"7151","AC004982.2",2.81841281409449,0.566147896427809,0.8440644932539
06,0.670740092673824,0.502386117143618,0.98045720611026
"7152","PHF14",152.216158824737,-0.114806015809603,0.173332611264181,-
0.662345158088134,0.507750042116826,0.98045720611026
"7153","HIBADH",98.0480613256411,-0.158886770021525,0.245158246379891,
-0.648098819304318,0.51692103761161,0.98045720611026
"7154","GARS",104.423627515597,-0.151992849942604,0.226381515127886,-0
.67140132822568,0.501964899014334,0.98045720611026

"7155","C7orf25",18.1749811809536,0.281370798968349,0.411696934116521,
0.683441569882359,0.494327859881871,0.98045720611026
"7156","PSMA2",402.200948687437,-0.178442584713365,0.2735198651597,-0.
652393509367877,0.514147350402821,0.98045720611026
"7157","MRPL32",126.790669659276,0.155071323147594,0.248636698443593,0
.623686383057303,0.532833567482055,0.98045720611026
"7158","BLVRA",70.2241764039965,-0.196945075374737,0.308147964876369,-
0.639125023764972,0.522741602126657,0.98045720611026
"7159","POLD2",101.389603373772,-0.1965618344533,0.309895601502301,-0.
634284041142932,0.525895469562357,0.98045720611026
"7160","PPIA",1318.72815026274,-0.16817083257041,0.260337453286806,-0.
645972488580585,0.518297172547277,0.98045720611026
"7161","SUMF2",147.565942766688,-0.147211222151109,0.206391006337998,-
0.713263745175151,0.47568256740765,0.98045720611026
"7162","VKORC1L1",72.6918518503208,0.152879232979932,0.213439314872627
,0.71626557211901,0.47382738079708,0.98045720611026
"7163","ASL",28.2438060807604,-0.237476021051353,0.358030696620394,-0.
663283967807765,0.50714870249378,0.98045720611026
"7164","TBL2",52.8823541848577,-0.18055567527442,0.268209928011824,-0.
673187889101781,0.500827765053279,0.98045720611026
"7165","DNAJC30",38.5001483628824,-0.206097094847045,0.330474176111243
,-0.623640543634094,0.532863678016112,0.98045720611026
"7166","LAT2",2.37816124478545,-0.616752373747975,0.989997204383147,-0
.622983954921634,0.533295065780225,0.98045720611026
"7167","GTF2IRD2",41.219255775956,0.227161716836996,0.337467408754564,
0.67313675615475,0.500860291839341,0.98045720611026
"7168","MAGI2-
AS3",185.184288169787,0.140753402345394,0.195727663294236,0.7191288138
65215,0.472061555252055,0.98045720611026
"7169","CACNA2D1",50.2519568457913,0.185436622487045,0.279971139055793
,0.662341922501128,0.507752115267533,0.98045720611026
"7170","MTERF1",10.8090852075492,-0.320349733661599,0.472718402230461,
-0.677675614382833,0.497977391320876,0.98045720611026
"7171","CASD1",45.9467804740996,0.176589269344389,0.253821256691536,0.
695722933713918,0.486602360327278,0.98045720611026
"7172","TMEM225B",9.22431590450954,-0.378394527499779,0.54141744285286
6,-0.698896078238488,0.484616978567294,0.98045720611026
"7173","CASTOR3",11.4880852379003,0.310963420264181,0.433137003583376,
0.717933165930309,0.472798496925972,0.98045720611026
"7174","POP7",70.2857830238838,-0.176022332458677,0.273502012331035,-0
.643586973852414,0.519843299367389,0.98045720611026
"7175","SH2B2",3.75906247379386,-0.568457014117622,0.833598101851899,-
0.681931752069437,0.495282106671125,0.98045720611026
"7176","UPK3BL1",60.8563692696495,-0.264637749373811,0.398626197555482
,-0.663874454304969,0.506770667516733,0.98045720611026
"7177","KMT2E-
AS1",41.6358644048222,0.307499449822702,0.430199918670945,0.7147826777
20368,0.474743340478367,0.98045720611026
"7178","CDHR3",5.78603246727852,-0.388749408108472,0.583747884590036,-
0.665954290149573,0.505440317663294,0.98045720611026

"7179","LRRN3",27.8147147605061,0.456745447256644,0.651160230501977,0.
701433266132578,0.483032667641072,0.98045720611026
"7180","ZC3HC1",14.5387175720595,0.295759450372052,0.419991876122726,0
.704202788640675,0.481306494637587,0.98045720611026
"7181","MEST",276.329345420531,0.444107853138438,0.640272450678327,0.6
93623242211866,0.487918514045036,0.98045720611026
"7182","SLC35B4",40.4224415529749,0.181006757914796,0.25551667996374,
0.70829808834339,0.478760163997303,0.98045720611026
"7183","ZC3HAV1L",2.55781844411134,-0.579783897680782,0.89731135356541
8,-0.646134583472105,0.518192200093068,0.98045720611026
"7184","PARP12",23.0067799932855,0.217402188442705,0.3401805921837,0.6
39078752397802,0.522771701561204,0.98045720611026
"7185","EPHB6",22.8631189417639,0.253922967838692,0.373122253137423,0.
68053557702218,0.496165402894761,0.98045720611026
"7186","ZYX",193.62280010236,0.148491119709505,0.232497720157589,0.638
677745351033,0.523032593145475,0.98045720611026
"7187","FASTK",69.7949478027132,-0.198871978417954,0.277081046921463,-
0.71773937852315,0.472917998065669,0.98045720611026
"7188","SMARCD3",32.3330983427794,0.261105353755441,0.386601823575348,
0.67538572720817,0.499430731097665,0.98045720611026
"7189","DNAJB6",213.88512687757,0.104156595705972,0.153904114240699,0.
676762906695763,0.498556396444699,0.98045720611026
"7190","PTPRN2",31.9656134089098,0.210028254569662,0.308333409945944,0
.681172548270015,0.495762316052307,0.98045720611026
"7191","MCPH1",39.1579312626297,-0.194550004031733,0.272231641527033,-
0.714648756259342,0.474826109419614,0.98045720611026
"7192","PINX1",32.9543486554196,-0.237126518498219,0.376676614896115,-
0.62952280317061,0.529006844794067,0.98045720611026
"7193","NAT1",4.9260168039862,0.432322053483109,0.63545607473043,0.680
33349695572,0.496293319311239,0.98045720611026
"7194","CCAR2",23.6175807532082,0.242561966862505,0.338777286459643,0.
715992413179093,0.473996033586666,0.98045720611026
"7195","BIN3",34.2864530444186,-0.202926781899838,0.304756981118027,-0
.665864260616391,0.505497866206921,0.98045720611026
"7196","GNRH1",2.82207627050413,0.664932965586838,0.955838688205796,0.
695653956877371,0.486645566708025,0.98045720611026
"7197","ASH2L",40.9017408417936,-0.208980224801842,0.33199409388593,-0
.629469706390758,0.529041595231613,0.98045720611026
"7198","DDHD2",34.1140518808089,-0.231962919167769,0.32073150880568,-0
.723230841994721,0.469538073347694,0.98045720611026
"7199","HTRA4",2.51565322117603,0.583001000537815,0.918855475476959,0.
634486071093161,0.525763654169929,0.98045720611026
"7200","ADAM32",8.84754913557591,0.360296310079979,0.555021006242528,0
.649157970649025,0.516236274832008,0.98045720611026
"7201","ANK1",4.56963611962477,-0.475959311453906,0.73933534841222,-0.
643766475491893,0.519726876016394,0.98045720611026
"7202","IKBKB",26.6186372007206,0.243732877240045,0.380586248461705,0.
640414303525655,0.521903286215638,0.98045720611026
"7203","THAP1",27.4102835206077,-0.227646362146574,0.340980702777922,-
0.667622420541604,0.504374642343698,0.98045720611026

"7204","PCMTD1",206.086645166486,0.134782732007893,0.192694881277497,0
.699461921947967,0.484263401698661,0.98045720611026
"7205","ADHFE1",4.12617473970564,0.618012444048311,0.875252984149636,0
.706095786292862,0.480128571958713,0.98045720611026
"7206","TRAM1",350.269873895166,0.0991583468076856,0.145281210295412,0
.682526987530314,0.494905783887282,0.98045720611026
"7207","MSC",47.3449901474135,-0.227327474634885,0.324051572395524,-0.
7015163449274,0.48298083787139,0.98045720611026
"7208","UBE2W",43.0015501996549,0.180416001912765,0.268848990085903,0.
671068177920692,0.502177097980125,0.98045720611026
"7209","GDAP1",7.42877506000766,-0.392342923915839,0.605050803674225,-
0.648446248700608,0.516696365631235,0.98045720611026
"7210","ZBTB10",61.638657160676,0.169457409734199,0.254642245443311,0.
665472492355648,0.505748331896519,0.98045720611026
"7211","IMPA1",29.1767405827521,-0.204452151722761,0.298716094792089,-
0.684436343696386,0.493699672961657,0.98045720611026
"7212","DECR1",189.536852109681,-0.191130407426363,0.284866541177275,-
0.670947197366437,0.502254167880924,0.98045720611026
"7213","TMEM67",20.4757897562492,-0.243357880096504,0.33939673980712,-
0.717030694622477,0.473355157331196,0.98045720611026
"7214","MTERF3",23.6483954825683,-0.252234746008237,0.359978671318348,
-0.700693585774065,0.483494259840971,0.98045720611026
"7215","EXT1",92.7449680318528,0.160547768137635,0.252251152074646,0.6
36459999556812,0.524476644731411,0.98045720611026
"7216","RNF139-
AS1",2.95242355921453,0.512117918039579,0.79385175831179,0.64510522610
5,0.51885899736658,0.98045720611026
"7217","TMEM71",4.66627389651243,0.483004686727092,0.738650658957393,0
.653901382026592,0.513175343504667,0.98045720611026
"7218","PHF20L1",127.62893675167,0.150486980826186,0.233991325322484,0
.643130597336402,0.520139362151583,0.98045720611026
"7219","LY6E",749.060346874926,-0.182488618450866,0.25689241768405,-0.
710369811986071,0.477474838270022,0.98045720611026
"7220","PUM3",116.203450496212,0.199335031974067,0.28288692195589,0.70
4645625170148,0.481030797590991,0.98045720611026
"7221","KIAA2026",87.4245696990099,0.157334681549817,0.245134150022614
,0.641830938428216,0.520982959215503,0.98045720611026
"7222","RRAGA",223.810072950961,-0.168156752191691,0.237121987242911,-
0.709157147959582,0.478226962426061,0.98045720611026
"7223","C9orf72",15.2900028540528,-0.266853298660328,0.382394976446151
,-0.697847291667825,0.485272700126621,0.98045720611026
"7224","B4GALT1",307.845950228042,-0.21725039589912,0.313247718238016,
-0.693541830475668,0.487969584171748,0.98045720611026
"7225","VCP",263.999035360616,0.12902308010796,0.180521330515168,0.714
724845755109,0.474779082026749,0.98045720611026
"7226","CCDC107",176.540202744786,0.186544342576985,0.269176770111004,
0.693017984055821,0.488298265500575,0.98045720611026
"7227","CLTA",546.833343887418,-0.17300055650256,0.260998334930577,-0.
662841609884511,0.507432001202069,0.98045720611026
"7228","AL590399.1",3.74236859298157,0.605568805703642,0.9567349367520

68,0.632953582482764,0.526763955351402,0.98045720611026
"7229","FAM122A",30.4958646530437,-0.215742645305249,0.313129353838646
,-0.688988888012143,0.490830260333673,0.98045720611026
"7230","TRPM3",2.20412090254411,0.910683252573778,1.27029682003174,0.7
16905874448325,0.473432177011784,0.98045720611026
"7231","ABHD17B",16.7428172830682,0.265353437341925,0.397530582290717,
0.667504461701692,0.504449960703141,0.98045720611026
"7232","ISCA1",131.58453584888,-0.15902834784983,0.223033410379899,-0.
713024777673232,0.475830424888834,0.98045720611026
"7233","PHF2",38.4912734307706,0.208984061119498,0.297276207337793,0.7
02996257221591,0.482058081959022,0.98045720611026
"7234","LINC00476",14.3412217505708,0.320310677180169,0.46986754134698
7,0.681704201703147,0.495426010090183,0.98045720611026
"7235","ZNF510",13.3737175463201,0.275968367179708,0.404438266881848,0
.682349791742948,0.495017795531642,0.98045720611026
"7236","LPAR1",181.652680359379,-0.153501666972329,0.237106235022742,-
0.647396163823386,0.517375578071761,0.98045720611026
"7237","ZNF883",2.10226684694076,-0.593452110811759,0.942359561983923,
-0.629751248623595,0.528857346539459,0.98045720611026
"7238","WDR31",5.05603768530013,-0.464204087736635,0.692387384691566,-
0.670439840470839,0.502577444523536,0.98045720611026
"7239","ALAD",24.0108531866906,0.265701539306275,0.370128601491499,0.7
17862759688345,0.472841911788118,0.98045720611026
"7240","COL27A1",24.7911819473987,0.352216140950708,0.49242272825479,0
.715271901033097,0.474441047798201,0.98045720611026
"7241","TMEM268",9.09398750191063,0.355704865866021,0.529705934341718,
0.671513839670432,0.501893245947873,0.98045720611026
"7242","ASTN2",7.36543851589713,-0.38473693409679,0.565363104696562,-0
.680512985196095,0.496179702617696,0.98045720611026
"7243","FBXW2",66.1330446970662,-0.154518727148683,0.218441490950716,-
0.70736894568965,0.479337230068884,0.98045720611026
"7244","STOM",565.727976238176,0.152216155344295,0.230745603457737,0.6
59670880239219,0.5094650575161,0.98045720611026
"7245","RABEPK",38.1688381788769,-0.207687685876708,0.305659723499453,
-0.679473512240743,0.496837885154219,0.98045720611026
"7246","SLC2A8",15.2418428649991,-0.314881393938886,0.461124069099819,
-0.682856122764487,0.494697761963355,0.98045720611026
"7247","PTRH1",42.3295313379077,-0.217878397111228,0.330184954550623,-
0.659867732034482,0.509338713052478,0.98045720611026
"7248","ZDHHC12",26.1986468454489,-0.203710792522886,0.318217824143809
,-0.640161477663883,0.522067624220597,0.98045720611026
"7249","NUP188",33.077954673043,0.204883419572214,0.328140868147337,0.
624376417143567,0.532380408944023,0.98045720611026
"7250","GPR107",43.7852840592651,0.186885180064671,0.259818834019478,0
.719290350023895,0.47196204044519,0.98045720611026
"7251","FUBP3",54.6324628423698,-0.165128891787815,0.243449553986554,-
0.678287920777769,0.497589155941765,0.98045720611026
"7252","DDX31",9.86275416757879,-0.304225923742323,0.480374893097921,-
0.63330937589261,0.526531631766543,0.98045720611026
"7253","ADAMTSL2",14.2486700515539,-0.416442839298975,0.63429300273040

6,-0.656546481683285,0.511472570634195,0.98045720611026
"7254","NACC2",26.7149423030429,0.230259215315242,0.346936985142775,0.663691751458793,0.506887619761187,0.98045720611026
"7255","DNLZ",8.09201969160773,-0.388579201414172,0.569814962252264,-0.68193927354639,0.495277350451541,0.98045720611026
"7256","CARD9",2.70860988837825,-0.57774978117306,0.808006078561339,-0.71503147872569,0.474589592311717,0.98045720611026
"7257","PHPT1",418.748537778641,-0.191394935898311,0.290468154038201,-0.658918828923116,0.509947894139458,0.98045720611026
"7258","CLIC3",12.4380598432923,-0.364932932198658,0.549909985039995,-0.66362303308989,0.506931611621419,0.98045720611026
"7259","GTPBP4",106.009036888933,-0.174301174736705,0.263647369450795,-0.661114787907014,0.508538702427493,0.98045720611026
"7260","UPF2",84.3679265377346,-0.171369583920912,0.241610185984918,-0.709281288048052,0.478149937940077,0.98045720611026
"7261","CDNF",3.29219357705354,-0.509221275188862,0.75355711475117,-0.675756708045959,0.499195125120764,0.98045720611026
"7262","DCLRE1C",30.5518054246427,-0.189833745538096,0.292964344098534,-0.647975596218796,0.517000734373138,0.98045720611026
"7263","ANKRD26",51.5252866167589,0.15650458921321,0.24341538944501,0.642952730187035,0.520254772529002,0.98045720611026
"7264","WAC",218.859967327308,0.134810895634007,0.208861235610024,0.645456756205921,0.518631233320202,0.98045720611026
"7265","EPC1",177.503962961064,0.131305157030395,0.193699107221406,0.677882097207128,0.49784645202948,0.98045720611026
"7266","SLC25A16",24.5369171222073,0.208741334189549,0.326454671996668,0.63941904373076,0.522550363509169,0.98045720611026
"7267","PSAP",1222.87977864936,0.113881664901775,0.163488618579872,0.69657243354918,0.48607041241232,0.98045720611026
"7268","KAT6B",131.794445496813,0.162472280298452,0.234088220553848,0.694064314359971,0.487641876675844,0.98045720611026
"7269","COMTD1",26.3989078920125,-0.235920004787252,0.368611899381202,-0.640022758850966,0.522157803406235,0.98045720611026
"7270","ZNF503-AS2",2.93621816662102,-0.619996158607456,0.930812152409669,-0.666080859604617,0.50535941796043,0.98045720611026
"7271","POLR3A",22.7459941307657,0.251596889765784,0.35000009563999,0.718848060043322,0.472234541943974,0.98045720611026
"7272","PRXL2A",54.8457431934873,0.227038107381644,0.344512364409136,0.659012943616789,0.509887456939333,0.98045720611026
"7273","GHITM",251.083304297442,-0.12507824337664,0.1792414753692,-0.697819760292672,0.485289919740938,0.98045720611026
"7274","GLUD1",134.600980168529,-0.134708326782031,0.214197681138045,-0.628897222725839,0.529416344402748,0.98045720611026
"7275","IFIT2",179.279267488025,0.274057699094494,0.407064230397372,0.673254190934345,0.500785590674438,0.98045720611026
"7276","IFIT3",421.217580528556,0.266135651254703,0.380506918191926,0.699423948766327,0.484287125494818,0.98045720611026
"7277","SLC16A12",2.84146869366607,0.649296665721294,1.00461858718573,0.64631619159057,0.518077564558416,0.98045720611026

"7278","NOC3L",100.150070630639,-0.209883730841559,0.321332305071633,-0.653167227598764,0.513648475537786,0.98045720611026
"7279","SORBS1",6.03324225646763,-0.418119438300274,0.624085532146714,-0.669971368927648,0.502876041937823,0.98045720611026
"7280","TCTN3",56.5277322533337,-0.17585264541569,0.268970777334362,-0.653798331396741,0.513241741658272,0.98045720611026
"7281","ZDHHC16",25.3686963939439,-0.226488771092069,0.360917141782282,-0.627536752545534,0.530307452684733,0.98045720611026
"7282","SFXN3",59.4502532871231,-0.201874754883098,0.280171527899628,-0.720539864976646,0.471192663940856,0.98045720611026
"7283","NOLC1",115.941433360969,0.147609133552255,0.210449227864711,0.701400214436269,0.483053288204779,0.98045720611026
"7284","TRIM8",219.605371458834,-0.171460565468005,0.263622867602316,-0.650400957350403,0.515433259082665,0.98045720611026
"7285","ATP5MD",399.253096711107,-0.177754766378082,0.278855544780182,-0.637443901351158,0.523835740001002,0.98045720611026
"7286","PDCD11",57.0664902863452,0.207744914860325,0.316065921754163,0.657283498668071,0.510998644762756,0.98045720611026
"7287","ZDHHC6",39.6857326324648,-0.196672870040622,0.298853442137014,-0.658091366237147,0.510479423354369,0.98045720611026
"7288","ABLIM1",292.841289216344,0.2304452420623,0.353139453190841,0.652561587157933,0.514038956474085,0.98045720611026
"7289","PRDX3",188.333545726956,-0.158887423837725,0.240320602256624,-0.66114774324699,0.508517569873748,0.98045720611026
"7290","EEF1AKMT2",55.8315037365805,0.190716346596547,0.289363411104106,0.659089364024439,0.509838385155028,0.98045720611026
"7291","EDRF1-DT",10.9763629418366,-0.30521653802578,0.480886297656319,-0.634695851209122,0.525626800025762,0.98045720611026
"7292","FANK1",3.24571492147543,-0.53130190907717,0.852192866425038,-0.623452659614471,0.532987102354022,0.98045720611026
"7293","PPP2R2D",55.2067962767837,-0.151709105663601,0.242571617175193,-0.625419855093892,0.531695534148594,0.98045720611026
"7294","RIC8A",72.5750525514528,-0.164445709402362,0.252282335605974,-0.651832039716014,0.514509530222825,0.98045720611026
"7295","SIRT3",21.5450379547966,-0.203178943557739,0.32598195266975,-0.623282798000718,0.533098699899162,0.98045720611026
"7296","PSMD13",192.486465698161,-0.158174240779083,0.22241620722758,-0.711163285943617,0.476983056623296,0.98045720611026
"7297","AC136475.1",5.7105669244738,-0.372180987415679,0.581050207547449,-0.640531545434971,0.52182708744991,0.98045720611026
"7298","TMEM80",39.4301331822676,0.21497999350285,0.337220751706482,0.637505231854679,0.523795803166625,0.98045720611026
"7299","CD151",390.390596330955,-0.176704305118945,0.283316953075019,-0.623698311029613,0.532825732498073,0.98045720611026
"7300","TOLLIP-AS1",3.97758338687827,-0.543216989877594,0.803349387488277,-0.676190208566657,0.49891988838565,0.98045720611026
"7301","ILK",178.494655544693,-0.141920987188374,0.208685656138271,-0.680070637410458,0.496459735375007,0.98045720611026

"7302","ZNF214",7.37155311967053,0.342668367283362,0.539520940339011,0.635134508529075,0.525340692267886,0.98045720611026
"7303","USP47",109.568123507562,0.151248138261394,0.222178357712953,0.680750995813916,0.496029062452917,0.98045720611026
"7304","CD44",1360.93875778916,-0.14641069360227,0.205050672457171,-0.714022011475483,0.475213568612086,0.98045720611026
"7305","TRIM44",154.452249846521,0.177642043514805,0.252658089301777,0.70309264194042,0.481998017363881,0.98045720611026
"7306","PRR5L",6.0582809489734,0.432136236370995,0.616964243899202,0.700423469664793,0.483662883509985,0.98045720611026
"7307","ACCS",26.2586539379897,0.273699554676624,0.389431787284615,0.702817704186513,0.482169362588833,0.98045720611026
"7308","TP53I11",70.3665662252743,0.186742920614934,0.286804773169734,0.651115107154852,0.514972185162617,0.98045720611026
"7309","HARBI1",1.89116625275222,0.654361818218203,1.03403479845538,0.632823788131382,0.526848720619824,0.98045720611026
"7310","ZNF408",7.72452574123234,0.444280587061562,0.648678624089549,0.684900920983994,0.493406444802038,0.98045720611026
"7311","CLP1",15.9171927428635,0.254891112971172,0.390785557688402,0.65225315510358,0.514237874444347,0.98045720611026
"7312","LPXN",10.0055061779923,-0.33641872252781,0.479043417514593,-0.702271882313385,0.482509624868371,0.98045720611026
"7313","CCDC86",25.186443166466,-0.255545128787351,0.395369552258196,-0.646344988701779,0.518055958319785,0.98045720611026
"7314","PRPF19",59.9767370105025,0.143156392174288,0.223022116563801,0.641893254265367,0.520942494483772,0.98045720611026
"7315","AHNAK",1864.53772110601,0.125302338451375,0.198920773941272,0.629910772860597,0.528752964151607,0.98045720611026
"7316","PLAAT3",213.247090081138,-0.196127515103212,0.298798203957677,-0.656387864804543,0.511574596441989,0.98045720611026
"7317","PYGM",5.3504080364062,0.503584596967212,0.708142055235474,0.711134995081966,0.477000586049041,0.98045720611026
"7318","NAALADL1",24.9640336391218,-0.237684736869704,0.363835322932654,-0.65327559444716,0.513578623325525,0.98045720611026
"7319","DPP3",17.3148062808639,0.252104172777743,0.359470423355509,0.701321044508903,0.483102683333116,0.98045720611026
"7320","ZDHHC24",51.042065544684,0.192672006954506,0.26932263611168,0.715394776080428,0.474365139522093,0.98045720611026
"7321","RBM4B",18.798610835357,-0.253758899277605,0.356812284245129,-0.711183192065419,0.476970722714623,0.98045720611026
"7322","PC",6.22700396298913,-0.41378690034402,0.57587526798918,-0.718535633226384,0.472427085023056,0.98045720611026
"7323","LRFN4",17.7699750177145,-0.263742970612235,0.394646212127691,-0.668302298380856,0.503940646518918,0.98045720611026
"7324","CHKA",11.3507285252976,-0.313248509204641,0.437000532740411,-0.716814936678164,0.473488294059929,0.98045720611026
"7325","AP003071.4",2.35854193032396,0.729110207425592,1.09013734803627,0.668824170402915,0.503607646471877,0.98045720611026
"7326","CTTN",227.103444719751,-0.131850131993047,0.186732480155716,-0.706091044702543,0.480131520460821,0.98045720611026

"7327","FCHSD2",18.2179443190406,0.256003554941951,0.39991402551028,0.
640146478021862,0.522077374894738,0.98045720611026
"7328","MRPL48",30.4045594172826,-0.208551101243758,0.306364269102653,
-0.680729191607782,0.49604286161681,0.98045720611026
"7329","RNF169",53.4373866103026,0.183077935478616,0.267859973722825,0
.683483735677732,0.494301224049134,0.98045720611026
"7330","UVRAG",59.6637292263526,0.166042074814239,0.233701975025823,0.
710486399594578,0.477402561874582,0.98045720611026
"7331","AP002360.1",10.3898647174607,0.358659012520268,0.5385599604717
66,0.665959296725457,0.505437117468609,0.98045720611026
"7332","CLNS1A",119.918614102136,-0.171695993920887,0.252687195107614,
-0.679480390162887,0.496833528602841,0.98045720611026
"7333","RSF1",212.430613069572,0.114105581072081,0.182908561538259,0.6
23839475377505,0.532733011994486,0.98045720611026
"7334","RAB30-
DT",33.6344262138622,-0.222756093692603,0.323706810814799,-0.688141510
312701,0.491363672348389,0.98045720611026
"7335","CREBZF",85.5612745138036,-0.171193189672874,0.238999941624136,
-0.716289671493314,0.473812503033676,0.98045720611026
"7336","PIWIL4",7.37676686245247,0.427943811423105,0.653533152565913,0
.654815765264402,0.512586379047806,0.98045720611026
"7337","DDX10",54.5511349701765,-0.163873059569245,0.262848456024148,-
0.623450721560219,0.532988375571506,0.98045720611026
"7338","COLCA1",18.839288730572,0.280539590281875,0.432141700313286,0.
649184260807265,0.516219283697028,0.98045720611026
"7339","SDHD",244.533420809894,-0.17447318398551,0.272550880934552,-0.
640149037079817,0.522075711345731,0.98045720611026
"7340","TTC12",8.61682014502946,0.384667928288931,0.558005703714406,0.
689362000654044,0.490595490028621,0.98045720611026
"7341","PAFAH1B2",107.647110708829,0.140410448043107,0.219180030464567
,0.640616974755856,0.5217715681707,0.98045720611026
"7342","CD3E",37.3190644881075,0.200711450520031,0.314680978654692,0.6
37825175764047,0.523587489306364,0.98045720611026
"7343","AP004609.3",4.65475726357025,0.508671477429047,0.7596884641138
84,0.669579046487657,0.503126175064307,0.98045720611026
"7344","DPAGT1",29.3512673460268,-0.252628670085798,0.372809923857794,
-0.677633973558497,0.498003799708631,0.98045720611026
"7345","TMEM218",43.6873078663934,-0.250955529167979,0.36343738452475,
-0.69050554470653,0.489876324285258,0.98045720611026
"7346","SRPRA",122.769243163128,-0.118581408665214,0.183630251322663,-
0.645761838319605,0.518433605823634,0.98045720611026
"7347","CCDC77",9.69611240757657,0.363010592126659,0.5079806947199,0.7
14614936945237,0.474847012392072,0.98045720611026
"7348","VAMP1",9.35431933591037,0.377476345319956,0.561334687197544,0.
672462176183164,0.501289511711011,0.98045720611026
"7349","LPCAT3",74.6849062983315,0.246493455163911,0.391540101936496,0
.62954842669957,0.528990075287841,0.98045720611026
"7350","SMIM10L1",73.8067583870461,-0.191863681140028,0.28928622974380
2,-0.663231296249207,0.507182430513181,0.98045720611026
"7351","BORCS5",24.8666139021305,-0.227842016495275,0.351659937079097,

-0.647904388505955,0.517046792151946,0.98045720611026
"7352","WBP11",106.485668677572,0.117413317694192,0.184407011723557,0.636707447275406,0.524315421723467,0.98045720611026
"7353","PDE3A",6.44341244058436,0.409348815610773,0.581114286244826,0.70442049920334,0.481170943673692,0.98045720611026
"7354","GOLT1B",65.7259638315505,-0.168743305849818,0.242096113114838,-0.697009562354165,0.485796809536766,0.98045720611026
"7355","ITPR2",35.0077157156172,0.258782381274542,0.387042222776543,0.668615375909384,0.503740861698097,0.98045720611026
"7356","AC092747.4",14.6900821587512,0.324051075372099,0.451847112349548,0.717169738425625,0.473269369145536,0.98045720611026
"7357","ARNTL2",7.29020181708587,-0.505033691354217,0.731857435332232,-0.690071135404881,0.490149454000039,0.98045720611026
"7358","FAR2",10.0115793900344,0.34833760156688,0.485362328175868,0.71768569859147,0.472951103327029,0.98045720611026
"7359","AC010198.2",2.98035140415032,-0.604646174464203,0.916359387241852,-0.659835194447155,0.509359595367399,0.98045720611026
"7360","DENND5B",32.5744468843979,0.209492701351832,0.321097856331633,0.652426346737941,0.514126172538756,0.98045720611026
"7361","RESF1",91.5277518872336,0.175062809519982,0.256106420245108,0.683554943107001,0.494256244565785,0.98045720611026
"7362","AC026356.2",5.20976095856453,0.454006449204461,0.628596872133487,0.722253751698672,0.470138481383677,0.98045720611026
"7363","GXYLT1",34.4453918382914,0.180695200448636,0.266386754624913,0.678319012906874,0.497569446150033,0.98045720611026
"7364","PUS7L",66.7040788798708,-0.167990207265039,0.248491472376302,-0.676040129902905,0.499015166712778,0.98045720611026
"7365","SCAF11",347.606902019571,0.12032840858832,0.169437040049242,0.710165903236681,0.477601262229752,0.98045720611026
"7366","ZNF641",21.7890622885063,-0.283331326960602,0.4197148655367,-0.675056687826148,0.499639749908342,0.98045720611026
"7367","RND1",5.59303727453118,0.455046058165429,0.68994581184591,0.659538836750062,0.509549815563295,0.98045720611026
"7368","FKBP11",126.870202322207,0.17661678761233,0.249107499914531,0.70899827453179,0.478325547599561,0.98045720611026
"7369","TUBA1A",840.405570780163,-0.212972080242041,0.294526264092236,-0.723100470847467,0.469618160041886,0.98045720611026
"7370","SLC11A2",44.5382970638183,0.181094619585215,0.280929134749379,0.644627406647487,0.519168669875426,0.98045720611026
"7371","LETMD1",91.3635721855825,-0.165097278070798,0.23549866970146,-0.701053973171444,0.48326933278051,0.98045720611026
"7372","POU6F1",10.905569067686,0.336084783991939,0.516261095963804,0.650997695971075,0.515047974195179,0.98045720611026
"7373","AC023509.3",4.4671080617547,-0.441662516132361,0.676415297111539,-0.652945783482971,0.513791231627157,0.98045720611026
"7374","ATF7",90.841023912934,0.135006699865212,0.201232548575795,0.670898921773388,0.502284923290022,0.98045720611026
"7375","HOXC6",73.1794571571468,0.136999485807416,0.210573967807082,0.650600296105586,0.515304539175877,0.98045720611026
"7376","PYM1",39.416503102222,-0.196070058933866,0.293975182971916,-0.

666961261667441,0.504796878006945,0.98045720611026
"7377","CDK2",16.6702096716914,-0.26731244910132,0.402756201812846,-0.663707840867801,0.506877319995279,0.98045720611026
"7378","R3HDM2",100.696602475883,-0.162748836242778,0.239393393537195,-0.679838461028754,0.496606750709912,0.98045720611026
"7379","CDK4",183.011166797843,-0.181582523706528,0.256458002844786,-0.708039997552448,0.478920419330411,0.98045720611026
"7380","HMGA2-AS1",4.65767700938801,-0.455214489273056,0.729000872630209,-0.624436137683428,0.53234119850315,0.98045720611026
"7381","IRAK3",66.856077970315,0.194323697147918,0.296728845489742,0.65488643959502,0.512540871595644,0.98045720611026
"7382","MDM2",69.9189998307277,0.165731269625769,0.246252985145329,0.673012225731846,0.500939513048751,0.98045720611026
"7383","CPSF6",85.3737592223905,-0.143298662767687,0.229881851295017,-0.62335787692864,0.533049372264829,0.98045720611026
"7384","TSPAN8",47.4521984559001,0.397909014397618,0.564348547284817,0.705076705365914,0.480762502313906,0.98045720611026
"7385","AC011611.4",2.93255278687828,0.540132946165901,0.867206137623326,0.622842623838196,0.533387945322679,0.98045720611026
"7386","CEP290",66.8334483183038,0.138576002420742,0.212387393843246,0.652468114576606,0.51409923578711,0.98045720611026
"7387","BTG1",1205.67181252801,-0.150318727488091,0.223194650727252,-0.673487142269297,0.500637426019654,0.98045720611026
"7388","MRPL42",92.3927430077407,-0.16557086047353,0.24564944294562,-0.674012765867265,0.500303197648891,0.98045720611026
"7389","METAP2",238.604649521546,-0.139088896466259,0.199264136290299,-0.698012693381147,0.485169255894156,0.98045720611026
"7390","SNRPF",264.491947129152,-0.173015505644702,0.251990428630183,-0.68659554485943,0.492337636727757,0.98045720611026
"7391","PARPBP",5.67834855782182,-0.386435181405057,0.57773680485793,-0.668877554892983,0.50357358904367,0.98045720611026
"7392","RIC8B",17.0150998702963,0.237978602434067,0.374657165209941,0.635190314058758,0.525304299649385,0.98045720611026
"7393","C12orf76",61.5827604963209,-0.198817900402634,0.295342028517811,-0.673178488684496,0.500833744780339,0.98045720611026
"7394","ARPC3",510.41747082176,-0.165274645632935,0.239301852386772,-0.690653431991867,0.489783360591236,0.98045720611026
"7395","PHETA1",12.0135981999475,0.300496677847537,0.424007330704108,0.708706326724426,0.478506737906402,0.98045720611026
"7396","ATXN2",95.0326297861608,0.159363702418046,0.22820752609565,0.698327987444421,0.484972100253544,0.98045720611026
"7397","ACAD10",15.8051756245201,0.288839208155352,0.450447343207848,0.641227465342324,0.521374907625052,0.98045720611026
"7398","MAP1LC3B2",2.26500548348899,0.65221346492247,0.980514598075008,0.665174660533282,0.505938785742583,0.98045720611026
"7399","RFC5",9.48170709694163,-0.357349698379742,0.507621364217382,-0.703968988639161,0.481452086352697,0.98045720611026
"7400","RPLP0",4523.76965743062,-0.110667904772651,0.165992135957724,-0.666705709485156,0.504960131117488,0.98045720611026

"7401","SRSF9",364.079462503315,-0.173920566479327,0.245940237788134,-0.707165968625074,0.479463344289456,0.98045720611026
"7402","MORN3",1.9154612316935,-0.693260956242787,1.06183101893627,-0.652891979871984,0.513825919762166,0.98045720611026
"7403","AC156455.1",3.4263713975985,-0.553393623669785,0.820971428004696,-0.67407172136887,0.500265716986761,0.98045720611026
"7404","VPS33A",21.8855732115735,0.253138965895717,0.363092452389002,0.697174959793751,0.485693307515787,0.98045720611026
"7405","CLIP1",238.289368256374,0.187955310585058,0.273736664073454,0.68662819144542,0.492317058553645,0.98045720611026
"7406","SBNO1",78.7024277409656,0.17203697916764,0.249865307422385,0.688518870196014,0.491126091317356,0.98045720611026
"7407","SNRNP35",50.0742809034664,0.200898369865827,0.310983645710547,0.646009436949027,0.51827324386162,0.98045720611026
"7408","RILPL1",45.1457986188381,0.175578028896176,0.254274632347208,0.690505487218352,0.489876360424851,0.98045720611026
"7409","GTF2H3",47.0162169701206,-0.17212124774599 7,0.260940377764386,-0.659619064020106,0.50949831720477,0.98045720611026
"7410","TCTN2",9.42436248171308,-0.355619229094177,0.496458107755657,-0.716312662717562,0.47379830962676,0.98045720611026
"7411","TMEM132C",21.0366149388528,-0.245305003408741,0.379488983184077,-0.64640876093563,0.518014668127178,0.98045720611026
"7412","SACS",44.8736115967142,0.240238401127015,0.350013863819374,0.686368244119014,0.492480924347738,0.98045720611026
"7413","KBTBD7",19.896023886745,0.306807064595529,0.426335653676255,0.719637360727301,0.4717483026195,0.98045720611026
"7414","EPSTI1",175.600337832657,0.249468910837577,0.358004761041528,0.69683126590777,0.485908396832844,0.98045720611026
"7415","NUDT15",33.7612676729952,-0.199756495610151,0.299178426919346,-0.667683487967542,0.504335652273221,0.98045720611026
"7416","CDADC1",45.2743123665369,0.166612876472016,0.251210918197905,0.663238993222253,0.507177501714124,0.98045720611026
"7417","SETDB2",20.8957529877779,0.276717935041324,0.406588005261595,0.68058558408108,0.496133751207129,0.98045720611026
"7418","RCBTB1",12.9873002126508,0.273380750616581,0.421952564829237,0.647894510908394,0.517053181237288,0.98045720611026
"7419","OLFM4",8.95440026701689,0.445406158393044,0.671680774301024,0.663121791533411,0.507252555185979,0.98045720611026
"7420","BORA",3.88715563948737,0.458137709093587,0.708292948703832,0.64681952563833,0.517748753775926,0.98045720611026
"7421","ABCC4",8.76080085868128,0.372661783805567,0.541840858589849,0.687769808972003,0.491597751153757,0.98045720611026
"7422","BIVM",32.0839219783917,0.204587632767018,0.319013652148573,0.641313095502686,0.52131928264273,0.98045720611026
"7423","EFNB2",2.66470236736691,0.762959498758423,1.06942350768969,0.713430641156064,0.475579318021417,0.98045720611026
"7424","COL4A1",452.314358511554,-0.233331745163139,0.323785028227581,-0.720637845549597,0.471132362637271,0.98045720611026
"7425","ING1",47.6395558918991,-0.196284105945803,0.288455255899503,-0.680466387529399,0.496209197772611,0.98045720611026

"7426","TTC5",41.0966017192182,-0.179202883345364,0.283053420700357,-0
.633106227446267,0.526664275861131,0.98045720611026
"7427","AL356019.2",2.14722072451135,0.683329165002181,1.0454973673766
2,0.653592430095546,0.513374422526058,0.98045720611026
"7428","DAD1",641.508046114081,-0.192333400617104,0.289410687404701,-0
.664569101928679,0.506326137291195,0.98045720611026
"7429","NGDN",45.8847223001855,-0.21328704596779,0.324346832548448,-0.
657589421459607,0.510801993867677,0.98045720611026
"7430","MIA2",39.8874147628655,-0.179526016063693,0.276357908867222,-0
.649614178944839,0.515941471931387,0.98045720611026
"7431","L2HGDH",3.835186236692,0.485271570044162,0.745965818024286,0.6
50527890580052,0.515351291989737,0.98045720611026
"7432","SAV1",276.013618821791,0.150253936513645,0.237776589801266,0.6
31912235932175,0.52744422743614,0.98045720611026
"7433","TMX1",95.1571405069963,-0.146112635364383,0.217759401488811,-0
.670981984545409,0.502232006286613,0.98045720611026
"7434","AL365295.1",15.459486533886,0.340791240087913,0.47294030078456
7,0.720579826930736,0.471168069184714,0.98045720611026
"7435","BMP4",65.7787266152536,0.180754061109252,0.256579408666483,0.7
04476099811293,0.481136328951808,0.98045720611026
"7436","PPM1A",74.0602783760066,0.17273069930769,0.242450256202711,0.7
12437685210244,0.476193786336131,0.98045720611026
"7437","LINC01303",3.82598046439542,-0.566871872940065,0.8067270032136
95,-0.702681168080232,0.482254466190419,0.98045720611026
"7438","HSPA2",9.5013279299032,0.411957796313789,0.593528056681586,0.6
94083104709563,0.487630093380158,0.98045720611026
"7439","ZFP36L1",2286.17853173467,0.186769578402661,0.277469801952821,
0.673116775548852,0.500873002244017,0.98045720611026
"7440","AC004816.2",11.2168136095173,-0.311556008325295,0.476110318028
356,-0.654377770293857,0.512868452528302,0.98045720611026
"7441","FAM161B",10.4685998354534,0.306570635940391,0.454317387820574,
0.674793974782816,0.499806668946078,0.98045720611026
"7442","EIF2B2",36.845844957837,-0.229842179906778,0.337546868038788,-
0.680919308308816,0.495922549911836,0.98045720611026
"7443","CIPC",8.79644926668385,-0.319424965554513,0.505580460640488,-0
.63179847803029,0.527518568284262,0.98045720611026
"7444","SEL1L",132.885629012233,0.131292740038295,0.193472550296045,0.
678611719530212,0.497383915176615,0.98045720611026
"7445","PSMC1",246.578166858699,-0.162669496656788,0.248596423493299,-
0.654351717417901,0.512885233409288,0.98045720611026
"7446","AL133467.1",20.6510673379999,-0.279209605843468,0.419418051756
985,-0.665707173722806,0.505598287317588,0.98045720611026
"7447","TRAF3",36.9761160573796,-0.185000296680664,0.29167918889835,-0
.634259500581431,0.525911482317927,0.98045720611026
"7448","AL161669.3",9.86423744030758,-0.363144266091389,0.575789687312
899,-0.630689076398908,0.528243842884071,0.98045720611026
"7449","AL049840.4",4.55844797046113,0.550909460816295,0.7849182294701
49,0.701868602527145,0.482761111016498,0.98045720611026
"7450","TUBGCP5",27.2730725094464,0.209228679706789,0.318729244803622,
0.656446445118961,0.511536915079244,0.98045720611026

"7451","FAM98B",37.2441615925085,0.1776390283202,0.266449767782212,0.666688621269121,0.504971048488565,0.98045720611026
"7452","CHST14",42.8832788569631,0.192098265924331,0.298191855850047,0.644210303385792,0.519439070494563,0.98045720611026
"7453","RAD51-AS1",18.5622558582631,0.335262972102496,0.491890493959012,0.681580506677635,0.495504244499058,0.98045720611026
"7454","PLA2G4B",5.30426742808167,0.411193728787819,0.632044289854191,0.650577396850906,0.515319325165525,0.98045720611026
"7455","VPS39",34.4872818538063,0.187012289629266,0.267241211566247,0.699788361732176,0.484059483999064,0.98045720611026
"7456","UBR1",51.7984706939792,0.201628221514942,0.281143821228242,0.717171092838116,0.473268533533028,0.98045720611026
"7457","PPIP5K1",1.88702236402499,-0.76459160656758,1.21099216162361,-0.631376181281362,0.527794586159458,0.98045720611026
"7458","LEO1",54.6871053463884,-0.196206188370726,0.299585676553048,-0.65492513069457,0.512515959151551,0.98045720611026
"7459","SLTM",191.437588298143,-0.116735526388238,0.185173127669099,-0.630412889049657,0.528424480122323,0.98045720611026
"7460","DPP8",50.4230355635022,0.184565252392462,0.282620379263967,0.65305004852491,0.5137240135884,0.98045720611026
"7461","HACD3",123.070015851934,0.143065405631122,0.220168984882147,0.649798179828566,0.515822594836033,0.98045720611026
"7462","RPL4",1861.44004031886,-0.110737120217459,0.161349335254825,-0.686319035914018,0.492511947545173,0.98045720611026
"7463","CALML4",7.2862922048265,-0.394615784665075,0.546624475741073,-0.721913858924966,0.470347440022188,0.98045720611026
"7464","GLCE",49.3627600548779,0.208215406324648,0.294577615645992,0.706826979599394,0.479674006166989,0.98045720611026
"7465","HEXA",216.336501813538,0.210935010958147,0.305885999721807,0.689587006760639,0.490453940647007,0.98045720611026
"7466","LOXL1-AS1",8.20857629349515,0.347939026994655,0.502719616069906,0.692113488060654,0.488866061874245,0.98045720611026
"7467","SCAMP2",97.0056818741461,-0.16321393312874,0.231250409713452,-0.705788730627471,0.480319531286317,0.98045720611026
"7468","SIN3A",54.0096085850777,-0.173004023100764,0.267141929420646,-0.647610891618398,0.517236650963678,0.98045720611026
"7469","SNUPN",70.9289983436865,0.142064531011001,0.217863185759494,0.652081399231121,0.514348662801657,0.98045720611026
"7470","ODF3L1",1.8625164584069,-0.681843208260082,1.08646170477679,-0.627581446508659,0.530278166031975,0.98045720611026
"7471","SCAPER",40.5117695048996,0.23719891862103,0.333003123182975,0.712302384295347,0.476277547914549,0.98045720611026
"7472","TSPAN3",236.755032946209,0.104780418875792,0.16331707942653,0.641576614299725,0.521148121135726,0.98045720611026
"7473","HDGFL3",108.803397125135,0.16688116720338,0.241361915293966,0.691414662500204,0.489304992346967,0.98045720611026
"7474","SEC11A",684.386941332467,-0.159249371790192,0.25328166147856,-0.628744184875273,0.529516546206311,0.98045720611026

"7475","AC012291.3",1.97946321905429,0.852188435170713,1.2526231765192
4,0.680323062150865,0.496299925006549,0.98045720611026
"7476","ZNF592",21.6811373768191,0.224360418831613,0.355127260165591,0
.631774701629487,0.527534106849687,0.98045720611026
"7477","GDPGP1",3.50579680706585,0.585896008593576,0.819854846225166,0
.714633829745838,0.474835335106256,0.98045720611026
"7478","FES",28.5299709260978,0.216105356280244,0.33583310356275,0.643
490334894471,0.519905984205285,0.98045720611026
"7479","VPS33B",9.93220175660311,0.323580810657555,0.471083685397508,0
.686886047400501,0.492154540013134,0.98045720611026
"7480","LINC02254",6.5395650110362,0.356753476173748,0.565197829202056
,0.63120107286578,0.527909060566756,0.98045720611026
"7481","AC015712.2",7.15005164621639,0.411468484668932,0.5728218646492
41,0.718318398898562,0.472560988146663,0.98045720611026
"7482","TARSL2",27.53478673559,-0.251474271141544,0.369744693169704,-0
.680129494180794,0.496422470638834,0.98045720611026
"7483","HBA2",4.47939575803919,0.675269701653982,1.06964839215432,0.63
130062795117,0.527843976423653,0.98045720611026
"7484","WDR90",15.0231451036858,-0.299646276459235,0.463942453524146,-
0.645869491319661,0.518363879159339,0.98045720611026
"7485","UNKL",36.474935624995,0.216813686238078,0.301701743630568,0.71
863584091037,0.472365324103883,0.98045720611026
"7486","JPT2",14.4314863589144,0.295282927463377,0.444458385992386,0.6
64365746647055,0.506456250482311,0.98045720611026
"7487","AC093525.6",3.79755372790334,0.513472747460499,0.7514838765388
8,0.683278462108072,0.494430901128965,0.98045720611026
"7488","ZNF200",13.5975842549148,-0.262083205788091,0.40634166519635,-
0.644982359024023,0.518938617821279,0.98045720611026
"7489","ZNF263",25.6443982880875,0.220053341668236,0.322711683554346,0
.681888363149946,0.495309544209274,0.98045720611026
"7490","ZNF75A",19.3281110378777,0.248151111522961,0.381614376166718,0
.650266675002174,0.515519979232909,0.98045720611026
"7491","ZNF174",8.75847598833348,0.340581292376504,0.532299644584592,0
.639830020255404,0.522283113397581,0.98045720611026
"7492","ANKS3",27.7135585231927,-0.201919764162332,0.319728688621916,-
0.631534708482492,0.527690962378247,0.98045720611026
"7493","TXNDC11",35.1550440720361,0.188354862132663,0.275886457584515,
0.682726016281399,0.494779987016674,0.98045720611026
"7494","ZC3H7A",64.5978294176297,0.158656384417769,0.24475823786345,0.
648216729302828,0.516844783143969,0.98045720611026
"7495","ERCC4",29.047189278988,-0.200708586925832,0.291032566879723,-0
.689643049496865,0.490418688065869,0.98045720611026
"7496","MARF1",53.8243930994833,0.206215074293268,0.289414028574902,0.
712526187167525,0.476139001247019,0.98045720611026
"7497","AC138969.1",3.13570834591021,-0.58098985981213,0.9207410138706
5,-0.631002476331254,0.528038905280053,0.98045720611026
"7498","IGSF6",4.65151288262151,-0.522587010078101,0.723727639447589,-
0.722076899642778,0.470247199757101,0.98045720611026
"7499","MOSMO",81.9644169646341,-0.142191603568104,0.210028380571901,-
0.677011379038017,0.498398734668049,0.98045720611026

"7500","EEF2K",66.6328536696886,0.201937272108655,0.313464052125885,0.644211898427059,0.519438036319276,0.98045720611026
"7501","UBFD1",41.2683655674718,0.190410345795998,0.268452380675552,0.70928909371873,0.478145095027142,0.98045720611026
"7502","PALB2",11.7502958769777,0.283343828578761,0.43805654699455,0.646820211962924,0.517748309533081,0.98045720611026
"7503","IL21R",3.15777672600714,0.759408613460728,1.05333837089089,0.720954096468013,0.470937757771469,0.98045720611026
"7504","SH2B1",25.6548513392318,0.211613239684699,0.305880128934361,0.691817544414952,0.489051917386811,0.98045720611026
"7505","NFATC2IP",71.3418715823349,0.161105841569486,0.250141596965488,0.644058579316231,0.519537448533422,0.98045720611026
"7506","PRRT2",16.2501434605557,-0.306300564462562,0.44151930221563,-0.693742182789033,0.487843907009635,0.98045720611026
"7507","INO80E",40.7025804576795,-0.183828955201947,0.27110764797365,-0.67806628317551,0.49772966756053,0.98045720611026
"7508","MAPK3",130.261959595542,-0.138210894576906,0.212318836389225,-0.650959175018915,0.515072840769149,0.98045720611026
"7509","SEPHS2",118.965930936961,-0.205038150984338,0.31442998917684,-0.652094768444689,0.514340038760543,0.98045720611026
"7510","MIR762HG",7.69916616504973,-0.317593342442093,0.50610932604679,-0.627519245540895,0.530318924739049,0.98045720611026
"7511","PRSS53",3.44549953674526,0.513845981775045,0.791493489847008,0.649210623165541,0.51620224619088,0.98045720611026
"7512","CNEP1R1",22.1196304620123,-0.216266307906058,0.346995683676453,-0.623253596744189,0.53311788605108,0.98045720611026
"7513","HERPUD1",360.36146764578,-0.176749002460307,0.277196020728215,-0.637631817354281,0.523713378907436,0.98045720611026
"7514","USB1",33.0117488683368,-0.220980666666136,0.351880606954303,-0.627998992552703,0.530004599956085,0.98045720611026
"7515","GINS3",4.04237560164669,0.529383220192514,0.832870677314078,0.635612748307721,0.525028858249621,0.98045720611026
"7516","CMTM1",2.35069719464187,0.692066225790575,0.980432982260767,0.705878156194571,0.480263912843643,0.98045720611026
"7517","NOL3",15.8115855146158,0.318723523480694,0.497128790273547,0.641128676746554,0.521439084064617,0.98045720611026
"7518","ZDHHC1",34.7658103639203,-0.191344796190777,0.287972023711187,-0.664456198643381,0.506398374241046,0.98045720611026
"7519","CTCF",92.1672694209109,0.141523885382871,0.2255140947755,0.62756115321199,0.530291463536624,0.98045720611026
"7520","VPS4A",81.6072628269682,0.173357722931213,0.2468178486681,0.702371096201921,0.482447765786821,0.98045720611026
"7521","DDX19A",50.3758872956732,-0.19322470355799,0.28497278693593,-0.678046158847555,0.497742426829137,0.98045720611026
"7522","ST3GAL2",32.9164582249048,0.21250821638612,0.315790917484976,0.67293960851876,0.500985712242347,0.98045720611026
"7523","MTSS2",9.12177787102924,0.360472557796113,0.574434866416079,0.627525554019927,0.530314790881208,0.98045720611026
"7524","TMEM231",10.4080652872653,-0.342703432219045,0.476112581843162,-0.7197949503715,0.471651254450735,0.98045720611026

"7525","MON1B",47.2579633019113,-0.179998206949216,0.265005108154646,-0.67922542400268,0.496995040578241,0.98045720611026
"7526","AC079414.3",4.13400576239871,0.521369363957421,0.738352943555812,0.706124853307381,0.480110497193741,0.98045720611026
"7527","TAF1C",19.0737348051252,-0.242691629919856,0.34253495019872,-0.708516400382094,0.478624631201838,0.98045720611026
"7528","COTL1",201.910607242651,-0.15446109337816,0.21873914969524,-0.706142881113709,0.480099287134855,0.98045720611026
"7529","FBXO31",21.6505128020821,0.23167366199307,0.336689921824339,0.688092072800972,0.491394802154955,0.98045720611026
"7530","GALNS",29.2845418491586,0.242738376151269,0.340926401519037,0.711996416439794,0.476466995105588,0.98045720611026
"7531","SPG7",100.649142382618,0.168716766880244,0.269564570527663,0.625886282273619,0.531389532750899,0.98045720611026
"7532","CDK10",33.1230818318074,-0.179108355920061,0.280631187370597,-0.638233966788351,0.523321389391309,0.98045720611026
"7533","TCF25",357.285029217088,-0.110173871469586,0.160177837196236,-0.687822194368944,0.491564757842706,0.98045720611026
"7534","C17orf97",15.397750945424,-0.491593334299236,0.740959867453166,-0.663454737419107,0.507039358967487,0.98045720611026
"7535","MRM3",25.1655932499028,0.231082841825923,0.328487711684823,0.70347484428167,0.481759878303555,0.98045720611026
"7536","ITGAE",88.1514215602674,-0.181904568219993,0.284365559654497,-0.639685651247664,0.522376985775515,0.98045720611026
"7537","ZZEF1",37.5296141692372,0.239441564159098,0.338562155714104,0.707230740701251,0.479423097973198,0.98045720611026
"7538","MYBBP1A",28.845846875234,0.223229075273401,0.344861855643607,0.647299988735474,0.517437808821348,0.98045720611026
"7539","DLG4",15.684058845852,-0.242936292248226,0.388642824736375,-0.625088839381031,0.531912752481542,0.98045720611026
"7540","GABARAP",1100.09703912276,-0.142534377849715,0.223473538622805,-0.637813222666574,0.523595271143371,0.98045720611026
"7541","RPL26",6484.05583021418,-0.162940107441543,0.242488484595021,-0.671949877181459,0.501615606013996,0.98045720611026
"7542","STX8",116.315470807546,-0.196961350471602,0.289231415306127,-0.680981871430306,0.495882961446942,0.98045720611026
"7543","CDRT4",4.47302157090592,0.466958871134051,0.694698654273222,0.672174716708748,0.501472474810523,0.98045720611026
"7544","ZSWIM7",73.0019340740801,-0.207885909027984,0.300592776618867,-0.691586509051648,0.489197036312875,0.98045720611026
"7545","SNHG29",1179.53505156379,-0.155776675445096,0.244146666582835,-0.638045473343554,0.52344407937624,0.98045720611026
"7546","PLD6",5.30309004909342,0.428053909767631,0.639980733180955,0.668854369474585,0.50358838037626,0.98045720611026
"7547","PEMT",49.6880631791512,-0.197366848709943,0.287840271858574,-0.685681845127345,0.492913757591372,0.98045720611026
"7548","AKAP10",26.1562270202421,0.228744557944191,0.32569159662648,0.702334847793225,0.48247036588569,0.98045720611026
"7549","LINC02693",17.3055601665697,-0.294043964972642,0.454144425601952,-0.64746796040246,0.51732912412919,0.98045720611026

"7550","POLDIP2",60.1770315363736,-0.175513041437906,0.279421522046196,-0.628130002845268,0.529918779960373,0.98045720611026
"7551","VTN",1.92374037243193,-0.70982186099468,1.05704862729608,-0.671512967960988,0.501893801076409,0.98045720611026
"7552","FAM222B",23.7537024555971,0.244804406312757,0.344024204058585,0.711590648055299,0.476718299804545,0.98045720611026
"7553","MYO18A",10.5936111790286,0.338327941687302,0.538785051190301,0.627946044419491,0.530039286322847,0.98045720611026
"7554","AC079915.1",2.19367881531023,0.754398781153112,1.18026564897269,0.639177105433631,0.522707724167069,0.98045720611026
"7555","LIG3",22.0653346990324,0.220065024669197,0.332863924360712,0.661126089563016,0.508531455208602,0.98045720611026
"7556","SLFN12",18.2716712270146,0.234095659604782,0.373278055925455,0.627134801761643,0.530570876238568,0.98045720611026
"7557","PEX12",5.06124170981873,0.455599791014595,0.632832419888361,0.719937501139666,0.471563477130154,0.98045720611026
"7558","MLLT6",102.252670238033,0.202013439621458,0.28701055227326,0.703853701619732,0.481523886598268,0.98045720611026
"7559","GJD3",5.44500013182414,-0.433185157650726,0.656266768311162,-0.660074802759685,0.509205827531637,0.98045720611026
"7560","NKIRAS2",36.4827337093194,0.18900332076832,0.275529140368675,0.685964905619136,0.492735239058544,0.98045720611026
"7561","KAT2A",13.813648414588,0.266312554295966,0.417767572308255,0.637465834948684,0.523821457231401,0.98045720611026
"7562","AC003102.1",2.57606196856749,-0.560886568332398,0.882238213730884,-0.635754107680819,0.524936703674661,0.98045720611026
"7563","FAM171A2",5.06655754650331,-0.462068531442025,0.701143730358491,-0.659021127102958,0.509882201964775,0.98045720611026
"7564","AC142472.1",5.33875820306283,-0.395984531606591,0.607910510828505,-0.651386223059237,0.514797201720654,0.98045720611026
"7565","HOXB4",16.4269950024566,-0.260945247076657,0.392057626441012,-0.6655788064766,0.505680356585285,0.98045720611026
"7566","MBTD1",24.8611729853292,0.27349627166286,0.41079680958211,0.665770194128525,0.50555799893219,0.98045720611026
"7567","AC006141.1",9.09805434406854,-0.368189984285508,0.5509672394277,-0.668261119604777,0.503966927128945,0.98045720611026
"7568","STXBP4",21.3851483498537,0.204343378886305,0.326197936349445,0.626439827220116,0.531026493300542,0.98045720611026
"7569","VEZF1",91.1244499645401,0.179927255896129,0.253897629324908,0.708660637653609,0.478535097110217,0.98045720611026
"7570","DHX40",68.1075731617955,0.174807361573777,0.254312280629991,0.687372867486927,0.491847791014565,0.98045720611026
"7571","AC080038.1",12.6905125208105,-0.356363478353626,0.556932112051384,-0.639868793058115,0.522257903818224,0.98045720611026
"7572","AC002546.1",3.21525600855839,-0.600772894780703,0.932059872271411,-0.644564703034186,0.519209314869634,0.98045720611026
"7573","CDC42EP4",267.733151728569,-0.177558420675003,0.276835847995241,-0.641385217849589,0.521272434647511,0.98045720611026
"7574","MRPL38",77.0413117408879,-0.174349689655747,0.25583648012533,-0.681488775839693,0.495562266515242,0.98045720611026

"7575","FBF1",6.91712148531613,0.374446228250756,0.569269222031373,0.657766507935537,0.510688178600367,0.98045720611026
"7576","SRSF2",450.270329793353,-0.134754326054201,0.18658038140989,-0.722232021587338,0.470151839045925,0.98045720611026
"7577","MFSD11",40.3254732190706,0.197859204130205,0.31494076339694,0.628242600278551,0.529845027190453,0.98045720611026
"7578","BAIAP2",20.0655734345134,-0.289176097362881,0.407119159930131,-0.710298423224564,0.477519097406447,0.98045720611026
"7579","AC139530.1",10.9124440173477,0.295382123457886,0.474023080820128,0.623138693894045,0.533193384258956,0.98045720611026
"7580","THOC1",46.7851773627716,-0.163404182476767,0.25194921460518,-0.648559999414293,0.516622817515487,0.98045720611026
"7581","YES1",83.4740453504191,0.138666722532077,0.209676601996489,0.661336177769605,0.508396745411414,0.98045720611026
"7582","ZBTB14",17.9478944832321,-0.274394393841422,0.39917868244192,-0.687397413516304,0.491832327094286,0.98045720611026
"7583","L3MBTL4",9.15818304100138,-0.326594048472287,0.485329905523411,-0.672932050457652,0.500990520823309,0.98045720611026
"7584","NDUFV2",324.684021829051,-0.208313477580682,0.296666663543276,-0.70218026890067,0.4825667489404,0.98045720611026
"7585","AP001033.2",4.88101430366158,-0.425805711647792,0.640364030457589,-0.664943206356423,0.506086819282245,0.98045720611026
"7586","GATA6-AS1",2.29692223312857,0.824589879488879,1.16125120371692,0.710087427121314,0.477649922506396,0.98045720611026
"7587","TTC39C",62.3276307416414,-0.144601888677342,0.228247592646244,-0.633530838160724,0.526387049255105,0.98045720611026
"7588","ASXL3",2.46843291777917,-0.632559222152521,1.00537721619499,-0.629176006739581,0.529233834861625,0.98045720611026
"7589","MAPRE2",55.6671350969671,-0.150417046150189,0.239239864927209,-0.628729021377585,0.529526475058301,0.98045720611026
"7590","EPG5",28.0466387000261,0.20878128060451,0.323075054903741,0.646231510095126,0.518129435782385,0.98045720611026
"7591","PSTPIP2",11.9170563050399,-0.308973589394718,0.428277348911114,-0.721433412671198,0.470642895332656,0.98045720611026
"7592","LMAN1",334.045280147207,-0.107291067637332,0.148478846784803,-0.722601703613946,0.469924621290755,0.98045720611026
"7593","RELCH",22.4675274248751,0.242035068926832,0.337447249521309,0.717253049980924,0.47321797111713,0.98045720611026
"7594","SERPINB2",11.282984521008,1.22315181131835,1.76825932647688,0.691726486609509,0.489109110238263,0.98045720611026
"7595","SERPINB8",23.8108967158024,-0.235201052152026,0.374076115437949,-0.628751856762266,0.529511522795145,0.98045720611026
"7596","CNDP2",76.5821087015943,0.167720129080679,0.241434076236698,0.694682920054122,0.487254034170145,0.98045720611026
"7597","PARD6G-AS1",3.41159749976558,-0.471550572200155,0.737250757349709,-0.639606765404079,0.522428283007883,0.98045720611026
"7598","PARD6G",3.25709831628553,0.63879190794543,0.889855100165574,0.717860590815933,0.472843249222799,0.98045720611026

"7599","ARHGAP45",12.4925117031656,0.381181103560487,0.536122241850138
,0.710996623167592,0.477086328362776,0.98045720611026
"7600","ABHD17A",154.543463552067,-0.146488267669986,0.21199691281864,
-0.690992457023678,0.489570281286456,0.98045720611026
"7601","SLC39A3",74.7960490172464,-0.208502335383327,0.317851567104462
,-0.655973910346658,0.511840910938298,0.98045720611026
"7602","SGTA",47.5304693845398,-0.18391032918848,0.282702320848149,-0.
650544108151364,0.515340819985771,0.98045720611026
"7603","MRPL54",169.904631737804,-0.171022578229803,0.274318565995561,
-0.623445145278905,0.532992038954763,0.98045720611026
"7604","MAP2K2",351.982236035509,-0.163221136245868,0.232583592201898,
-0.70177407916282,0.482820066290897,0.98045720611026
"7605","UBXN6",117.861593754835,-0.148277978573924,0.20879352551202,-0
.710165596420221,0.477601452470558,0.98045720611026
"7606","LONP1",39.5064961125755,0.160780647059212,0.256485206607113,0.
626861288360766,0.53075016469363,0.98045720611026
"7607","CLPP",87.7488722126504,0.180170743646387,0.265674105442738,0.6
78164487826687,0.497667406139574,0.98045720611026
"7608","TIMM44",60.7998921674383,-0.189523350775021,0.290389180567738,
-0.652652934260446,0.513980051408354,0.98045720611026
"7609","AC010336.8",2.06619877273019,0.69556018577454,1.0702310472238,
0.649915910754818,0.515746540100001,0.98045720611026
"7610","ZNF561-
AS1",9.39442408141904,0.323310100800403,0.461988226907984,0.6998232465
01903,0.48403769522465,0.98045720611026
"7611","ILF3",148.411909283617,-0.145382317571883,0.218036000486115,-0
.666781252856183,0.504911869230765,0.98045720611026
"7612","ZNF136",33.0050224109489,0.217280522904677,0.312278800354699,0
.695790180626675,0.486560239549018,0.98045720611026
"7613","ZNF791",84.9632439161845,0.157702423544686,0.241341741487751,0
.653440314852002,0.513472455631969,0.98045720611026
"7614","DNASE2",88.217195867714,-0.155701853496612,0.231075182036746,-
0.673814695824201,0.500429130584621,0.98045720611026
"7615","GCDH",31.3987949328403,0.212917820623994,0.331823854522855,0.6
41659174655055,0.521094502242498,0.98045720611026
"7616","CC2D1A",19.8538345527497,0.266153130177188,0.408648104586316,0
.65130151636607,0.514851869738167,0.98045720611026
"7617","AC020911.2",3.5233688849737,-0.721435306569992,1.0000475176812
8,-0.721401027265904,0.470662814752194,0.98045720611026
"7618","SLC35E1",70.1728114554333,0.170883932389182,0.263738271798937,
0.647929977032143,0.517030241022157,0.98045720611026
"7619","USE1",88.8138010114038,-0.191272718024313,0.274564486812193,-0
.696640414953398,0.486027856763965,0.98045720611026
"7620","MRPL34",157.584633824438,-0.185582962709361,0.282425999004243,
-0.657102969852904,0.511114709437757,0.98045720611026
"7621","ARRDC2",15.9189864747853,-0.268826204298529,0.407761992262875,
-0.659272343669595,0.509720898604255,0.98045720611026
"7622","COMP",56.7581954840158,-0.814393437776274,1.30181503819989,-0.
625583062016544,0.531588451444732,0.98045720611026
"7623","SLC25A42",9.65140629594244,-0.302471626883308,0.47574446809808

3,-0.635785904337512,0.524915976035271,0.98045720611026
"7624","MEF2B",9.36904037946726,-0.335927198466691,0.470952579656104,-0.713293042607367,0.475664441804305,0.98045720611026
"7625","NDUFA13",780.992215698536,-0.175345888771304,0.25597895202229,-0.685001197895504,0.49334316506649,0.98045720611026
"7626","YJEFN3",6.87102414916974,0.390810994424662,0.579456974965696,0.674443507126302,0.500029390386787,0.98045720611026
"7627","LINC00663",3.49542907262949,0.50820984107463,0.80346045781622,0.632526263278628,0.527043052527445,0.98045720611026
"7628","AC011477.2",2.35924507366461,0.580886949801849,0.905384388179124,0.641591524424347,0.521138437532411,0.98045720611026
"7629","UQCRFS1",209.880304563624,-0.166249526489354,0.260647063005247,-0.637833876094766,0.523581825158659,0.98045720611026
"7630","AC008736.1",3.62159837262589,-0.508343922750423,0.744456951834953,-0.682838573133673,0.49470885260115,0.98045720611026
"7631","PROSER3",9.03136231929065,-0.356021668225494,0.570260803356395,-0.624313763334338,0.5324215468397,0.98045720611026
"7632","TBCB",173.761293361764,-0.184213947214751,0.272828259848535,-0.675201122189542,0.499547993778086,0.98045720611026
"7633","ZNF565",13.4629256594919,0.282438869498641,0.435937719967866,0.647888119246622,0.517057315551074,0.98045720611026
"7634","ZNF568",14.6562101837183,-0.262517185634913,0.400124858922488,-0.656088167933019,0.511767396949355,0.98045720611026
"7635","ZNF781",4.97755047718845,0.552890625796799,0.829115905742352,0.666843588414537,0.504872047161285,0.98045720611026
"7636","ACTN4",167.341420341668,-0.136511897322013,0.202919508521549,-0.672739148229882,0.501113257134579,0.98045720611026
"7637","SHKBP1",25.890176939544,-0.230924767058764,0.334918483799519,-0.689495439126001,0.490511542398419,0.98045720611026
"7638","EXOSC5",31.8971445935669,-0.234877620117895,0.330223642714405,-0.711268333748682,0.476917970574899,0.98045720611026
"7639","LIPE-AS1",7.18148907377041,0.346026014259332,0.537353138922194,0.643945273965236,0.519610922107246,0.98045720611026
"7640","LIPE",2.87837566839422,-0.533141483443911,0.803127953365516,-0.663831312569531,0.506798282242979,0.98045720611026
"7641","ZNF428",173.876648196319,-0.178461445868398,0.2578833761544,-0.692023846320167,0.488922353748328,0.98045720611026
"7642","PPP1R13L",5.50324555004543,-0.479234855177834,0.68795921423069,-0.696603585306635,0.486050911487665,0.98045720611026
"7643","OPA3",16.56224335904,0.31471917659034,0.475294845351805,0.662155669618909,0.507871461316154,0.98045720611026
"7644","SNRPD2",410.904844792999,-0.146034074885908,0.217224112263523,-0.672273779205265,0.501409419211821,0.98045720611026
"7645","AC074212.1",3.35222080780109,-0.551492478761812,0.848637107627459,-0.649856662883413,0.515784813778255,0.98045720611026
"7646","AC011484.1",4.73527468780087,-0.474482748535102,0.701432911627326,-0.676447798028611,0.498756378731796,0.98045720611026
"7647","PRKD2",19.2465917985028,-0.26847720820978,0.391884839605009,-0.685092101241746,0.493285804274475,0.98045720611026

"7648","CCDC9",17.8502972992803,-0.270197722829204,0.388339520391814,-0.695777042101171,0.486568468841336,0.98045720611026
"7649","AC010331.1",1.88187886697253,-0.620206845999639,0.961776043693097,-0.644855785363632,0.519020646815793,0.98045720611026
"7650","BICRA",11.89434944453,0.313373340893605,0.464934119405867,0.674016657013816,0.500300723826233,0.98045720611026
"7651","LIG1",7.47534672098324,-0.377692709262954,0.596792221034684,-0.632871367874287,0.526817646735929,0.98045720611026
"7652","TMEM143",5.9272160960827,-0.413154721629475,0.591777088264032,-0.698159374235757,0.485077529904355,0.98045720611026
"7653","RPS11",4721.42134586904,0.101532023990932,0.154572174649373,0.65685835255436,0.511271999525621,0.98045720611026
"7654","VSIG10L",2.63130793699425,-0.535124041893526,0.839929098051852,-0.637106207100937,0.524055665708937,0.98045720611026
"7655","ZNF350",15.1410338136595,0.246490905518005,0.395027577835771,0.623984044021557,0.532638063921219,0.98045720611026
"7656","PPP2R1A",149.707945807777,0.128644113036606,0.202515260182221,0.63523170017338,0.525277311238038,0.98045720611026
"7657","ZNF578",2.18700435593912,-0.683209408064769,0.996812839339555,-0.685393868439169,0.493095412195048,0.98045720611026
"7658","ZNF761",13.9260752903599,0.296062850277056,0.430490535125289,0.68773370404274,0.491620491411184,0.98045720611026
"7659","ZNF581",50.5970591282375,-0.172162657181464,0.250701557661442,-0.686723524127281,0.492256969993957,0.98045720611026
"7660","ZNF71",12.0481296488286,-0.314245539793942,0.494204754570823,-0.635861020938254,0.524867010607557,0.98045720611026
"7661","ZNF419",4.12934874829554,-0.427709976428174,0.669856045154572,-0.638510288176138,0.523141559061231,0.98045720611026
"7662","ZNF773",5.66736080147562,0.432339236728338,0.670353805579282,0.644941869696309,0.518964857140226,0.98045720611026
"7663","ZNF417",6.63024969926663,0.412817404107417,0.620664509011832,0.665121652863104,0.505972686394186,0.98045720611026
"7664","ZNF324",21.1976898403039,-0.250913117715943,0.355818069067198,-0.705172501142871,0.48070289210223,0.98045720611026
"7665","ZNF343",10.5556322596191,0.330773216787031,0.524686288006786,0.630420928367681,0.528419221652035,0.98045720611026
"7666","PRNP",279.236880138992,0.166721027359322,0.255795389993813,0.651774949358371,0.514546364173186,0.98045720611026
"7667","SLC24A3",2.7448069713839,0.540533154024562,0.837164904212128,0.645671063496466,0.518492404288605,0.98045720611026
"7668","XRN2",213.705735571219,-0.14322828149505,0.2187078172694,-0.654884143069403,0.512542350303527,0.98045720611026
"7669","NANP",12.569897221121,-0.330397999818217,0.477552355463416,-0.691857125272892,0.489027058015057,0.98045720611026
"7670","AL035071.1",12.0452593291363,0.332757946604661,0.529802465791772,0.628079271219256,0.529952011533875,0.98045720611026
"7671","DSN1",16.0966472944024,-0.271312964736654,0.385055497997408,-0.7046074297022,0.481054573580903,0.98045720611026
"7672","CTNNBL1",88.6984660674495,-0.168139230463504,0.236958471996178,-0.709572563694689,0.4779692384416,0.98045720611026

"7673","TOP1",269.662900798552,0.129826030416061,0.20317789938982,0.63897712697076,0.522837811828415,0.98045720611026
"7674","PLCG1",14.1295978250818,0.291847933196373,0.42215387341365,0.691330700903943,0.489357742678727,0.98045720611026
"7675","L3MBTL1",4.51808717797225,0.5576567955909,0.832305147733236,0.670014834234373,0.502848333794783,0.98045720611026
"7676","PLTP",851.292796118013,-0.181961353089862,0.264542286522697,-0.687834657671072,0.491556908395011,0.98045720611026
"7677","PCIF1",30.4534305836245,-0.210002025023189,0.335787486529553,-0.62540158120129,0.531707524625837,0.98045720611026
"7678","TMEM189",43.4008267532253,0.217239708535846,0.30807010540179,0.705163223327101,0.480708665171875,0.98045720611026
"7679","DPM1",87.9847287806795,0.171549191314131,0.256004445491515,0.67010239210794,0.502792520094638,0.98045720611026
"7680","ZFP64",11.2939499574477,0.333623639108309,0.473297371042668,0.704892229537088,0.480877306223935,0.98045720611026
"7681","FAM210B",123.793611150717,0.157026769202954,0.218089467900862,0.720010786006112,0.471518354613135,0.98045720611026
"7682","RAB22A",110.521259713868,-0.127549511706666,0.183018218382258,-0.696922485827405,0.485851304875912,0.98045720611026
"7683","GNAS",1890.33090005324,-0.132129072185149,0.185638230091063,-0.711755720361772,0.47661605674808,0.98045720611026
"7684","NELFCD",64.7200413781504,0.177152562862842,0.254648522519574,0.695674811344035,0.486632503466005,0.98045720611026
"7685","ATP5F1E",1370.82842854591,-0.175288448422907,0.259779499559933,-0.674758588417663,0.499829154514793,0.98045720611026
"7686","TCFL5",17.3843326117185,-0.282946384486491,0.436558962346703,-0.648128681096193,0.516901724915093,0.98045720611026
"7687","PRPF6",112.770737653794,-0.145436070841033,0.211108091762964,-0.688917557003599,0.490875150174689,0.98045720611026
"7688","HSPA13",46.1852011501976,-0.18062289414496,0.254548843642607,-0.709580493708936,0.477964319398329,0.98045720611026
"7689","NCAM2",22.4454525257431,0.31585455186538,0.494947529674312,0.638157648899107,0.523371062766184,0.98045720611026
"7690","ATP5PF",538.387618072399,-0.175169770824091,0.247524179601645,-0.707687512007924,0.479139334151657,0.98045720611026
"7691","PAXBP1",58.3248702912835,0.221321168264817,0.340612303763223,0.649774438032834,0.515837932853554,0.98045720611026
"7692","MORC3",84.9991164912649,0.14565272493263,0.219069101525699,0.664871147588759,0.506132911304126,0.98045720611026
"7693","KCNJ15",50.8840969562008,-0.245020431384491,0.385327981442426,-0.635875003074752,0.524857896485058,0.98045720611026
"7694","PDXK",78.8188567070487,-0.169474753560294,0.244325493829866,-0.693643348075276,0.48790590194646,0.98045720611026
"7695","RRP1",47.3969257755425,-0.230157317615038,0.335837034154255,-0.685324410974053,0.493139231068467,0.98045720611026
"7696","MICAL3",17.5103671106177,-0.26718032044273,0.411707482385919,-0.648956678888544,0.516366377818581,0.98045720611026
"7697","SLC25A1",62.3749782934778,-0.172158430432424,0.24012207088116,-0.716962125974782,0.473397466428137,0.98045720611026

"7698","COMT",335.495628507242,0.182593814228042,0.264523881977089,0.6
90273456080073,0.490022237076701,0.98045720611026
"7699","ZNF74",7.68310604516099,0.377051140090624,0.561021496089437,0.
672079666677363,0.501532980328395,0.98045720611026
"7700","AC245452.1",14.2935531067626,0.31599639600231,0.43764005123088
4,0.722046337197782,0.470265989180464,0.98045720611026
"7701","AC245060.5",19.1722749644555,0.317937568600892,0.4990984302159
74,0.637023779985265,0.524109354121681,0.98045720611026
"7702","CHEK2",33.7776824011077,-0.242660117912888,0.371432098326285,-
0.653309498576838,0.513556770070147,0.98045720611026
"7703","Z93930.2",11.6909716763698,0.285520262628897,0.458022278596073
,0.623376363927257,0.533037226469196,0.98045720611026
"7704","CCDC157",2.28209455756319,0.595326375825178,0.952165683566115,
0.625234017671716,0.531817478398824,0.98045720611026
"7705","APOL6",452.24732215142,0.183409193302434,0.270364620492017,0.6
78377196574983,0.497532563720606,0.98045720611026
"7706","APOL3",263.714959004868,0.166547673142532,0.260503426878273,0.
639330066165907,0.522608233154917,0.98045720611026
"7707","IL2RB",9.72129943701173,0.35975662200868,0.522749640811375,0.6
88200610621734,0.49132645946249,0.98045720611026
"7708","RAC2",35.886093289284,-0.267838087204406,0.372076044901881,-0.
719847705527609,0.471618768789835,0.98045720611026
"7709","MFNG",6.43875542921877,-0.397077137876969,0.586265721967179,-0
.677298915830523,0.498216318644055,0.98045720611026
"7710","CBX6",128.636164669741,-0.139648529159779,0.210739737665251,-0
.662658740619691,0.507549140200077,0.98045720611026
"7711","RPL3",6462.7090830135,-0.144550150233281,0.227104710824362,-0.
636491201387154,0.524456313975475,0.98045720611026
"7712","MRTFA",37.5636990577159,0.178188436524527,0.273147993770797,0.
652351254953928,0.514174602285431,0.98045720611026
"7713","EP300",98.1545429748936,0.159874295398133,0.237719091285901,0.
67253452187336,0.501243470471084,0.98045720611026
"7714","CHADL",8.62339738731632,0.324356524746511,0.510350453329962,0.
635556454648236,0.525065559347262,0.98045720611026
"7715","PHF5A",79.9868097561355,-0.19482614996285,0.292690273277235,-0
.66563930458431,0.505641677345095,0.98045720611026
"7716","NAGA",30.5057857651059,0.188264153144715,0.283588280018126,0.6
63864363974004,0.506777126196886,0.98045720611026
"7717","SMDT1",263.258379526109,-0.195739675827908,0.270642697194258,-
0.723240190321531,0.469532330982283,0.98045720611026
"7718","PARVB",41.3635913296658,-0.266457247740104,0.418266849326676,-
0.637050840077442,0.524091728380348,0.98045720611026
"7719","PARVG",6.98417523299723,-0.420206466517147,0.582087582183398,-
0.721895603649474,0.470358664417171,0.98045720611026
"7720","PPP2R3B",13.8497529952454,-0.271511316226543,0.409794295865993
,-0.662555137944941,0.507615510377251,0.98045720611026
"7721","FANCB",3.24058850762034,-0.570782894137969,0.839496299133886,-
0.679911149967962,0.496560721186603,0.98045720611026
"7722","SYAP1",159.929961270154,-0.116433311174595,0.171444247319222,-
0.679132213504959,0.497054093089244,0.98045720611026

"7723","RBBP7",172.178925902631,-0.13024613679633,0.190573919918419,-0
.683441558278729,0.494327867211907,0.98045720611026
"7724","AL732509.1",3.4605346362103,0.480383924199153,0.75881070894930
1,0.633074782068276,0.526684809385025,0.98045720611026
"7725","BCLAF3",33.642719370369,0.201727459829086,0.311828103944579,0.
646918790440193,0.517684503897029,0.98045720611026
"7726","CNKSR2",16.3476721846071,-0.414507519429578,0.598137019152804,
-0.69299760114611,0.488311056932136,0.98045720611026
"7727","PDK3",16.6223903926347,-0.281360034761632,0.400328524523459,-0
.702822850548947,0.482166154996454,0.98045720611026
"7728","SRPX",858.351620898819,-0.197118654888415,0.277581735059506,-0
.710128333357949,0.477624557650674,0.98045720611026
"7729","KDM6A",37.5214347337329,0.243399942915329,0.34546200250087,0.7
04563573282465,0.481081874200372,0.98045720611026
"7730","MIR222HG",65.5885608543445,-0.515623151522523,0.73232982464642
8,-0.704085965325075,0.481379239746006,0.98045720611026
"7731","UBA1",128.297763761999,0.123479818275922,0.192824847443109,0.6
4037295977819,0.521930158027354,0.98045720611026
"7732","LINC01560",4.31206672475024,-0.567585602443176,0.8557106554692
69,-0.663291497909318,0.507143880719987,0.98045720611026
"7733","FTSJ1",32.5039976408717,-0.228780875097553,0.348247653948837,-
0.656948790618887,0.511213844474285,0.98045720611026
"7734","TBC1D25",16.4525781376309,-0.233637118309711,0.368815227551821
,-0.633480130038512,0.526420152461488,0.98045720611026
"7735","TFE3",49.3123083035514,0.16927357633656,0.263859418453419,0.64
1529407321283,0.521178781024462,0.98045720611026
"7736","SHROOM4",2.10933850575685,-0.735948358549402,1.0343211959775,-
0.711527871043849,0.476757185999576,0.98045720611026
"7737","PHF8",20.94867245741,0.246972791952421,0.387054302013888,0.638
083056220777,0.52341961558299,0.98045720611026
"7738","FAM120C",13.7159036955161,0.279073742069512,0.422406866346493,
0.660675202757222,0.508820629656978,0.98045720611026
"7739","FGD1",14.4409184926408,-0.281719465219371,0.443869260584141,-0
.634690189738804,0.525630493156971,0.98045720611026
"7740","APEX2",14.2639264072709,-0.257949890081005,0.405032553189183,-
0.636862118982623,0.52421465924292,0.98045720611026
"7741","AL050309.1",19.7581638861077,-0.267327156537462,0.412622621938
422,-0.647873243792621,0.517066937496108,0.98045720611026
"7742","PJA1",34.0203951108517,-0.205684762052615,0.289577655373439,-0
.710292241945824,0.477522929740702,0.98045720611026
"7743","RPA4",4.35014486643964,-0.506373549025973,0.701674196555788,-0
.721664771929109,0.47050060581603,0.98045720611026
"7744","CSTF2",6.05818871861784,-0.378420590897347,0.592216333803524,-
0.638990465641046,0.522829134394456,0.98045720611026
"7745","AMMECR1",27.661076110496,0.207966413805907,0.298669977544376,0
.696308398707426,0.486235714575319,0.98045720611026
"7746","LRCH2",13.9471505535859,0.30758764229775,0.444604837835478,0.6
91822526707571,0.489048788143203,0.98045720611026
"7747","NKAP",93.5593593302851,-0.146290942667474,0.205197926299279,-0
.712926028570631,0.47589153174063,0.98045720611026

"7748","AC002377.1",1.87036504666109,-0.733018890766326,1.16844512336649,-0.627345586119073,0.530432727748181,0.98045720611026
"7749","STAG2",170.136090667513,0.150606623503243,0.236850614501767,0.635871787033594,0.524859992823679,0.98045720611026
"7750","RAB33A",2.98571871843796,-0.549359462070056,0.84189961165471,-0.652523714781527,0.514063379458748,0.98045720611026
"7751","FAM122C",7.17776947167132,-0.375034704698107,0.522831488641719,-0.717314685219938,0.473179947999144,0.98045720611026
"7752","RTL8C",187.066168202708,0.114223795023047,0.1769138863677,0.645646293619048,0.518508449322345,0.98045720611026
"7753","SLITRK4",18.5571310905821,-0.280992800636076,0.407072962430184,-0.690276256518188,0.490020476317935,0.98045720611026
"7754","MTM1",9.42678158675849,0.325773765955236,0.508711686572301,0.640389781784451,0.521919224298267,0.98045720611026
"7755","TMEM187",21.5998982798514,0.314096467280408,0.44568009912237,0.704757667885383,0.480961056713297,0.98045720611026
"7756","RPL10",14898.0577700826,-0.161817146423465,0.228262013029651,-0.708909661645915,0.478380539068806,0.98045720611026
"7757","FAM50A",121.775237256391,-0.164906605869569,0.240293322111559,-0.686272112851347,0.492541531055885,0.98045720611026
"7758","FUNDC2",233.967487794569,-0.137060512375899,0.211243966638698,-0.648825689825837,0.516451050464022,0.98045720611026
"7759","CLIC2",67.1805610290538,-0.166828519364405,0.25401412046529,-0.656768683011861,0.511329663799497,0.98045720611026
"7760","MT-ND4L",860.15281934106,-0.229692882618773,0.343120826759674,-0.669422735972983,0.503225852310947,0.98045720611026
"7761","MAFIP",2.49232837610892,0.694248035492805,1.02121790385162,0.679823603634821,0.496616159281835,0.98045720611026
"7762","PWP2",2.2274491370739,2.07312131182163,3.16266714886281,0.655497785331932,0.512147311829544,0.98045720611026
"7763","ADSS",77.81987851279,-0.166533432194787,0.267519330369187,-0.622509902237586,0.533606634597605,0.980478136162988
"7764","TRIB2",10.1977723290816,-0.376920734391236,0.605470524751234,-0.622525323666415,0.533596497491304,0.980478136162988
"7765","KATNA1",61.209082007073,-0.152752279165654,0.245551989856797,-0.622077138347512,0.533891146850451,0.980722127459479
"7766","BAALC-AS1",2.15962528678403,-0.578941592687256,0.930782891014824,-0.621994235472078,0.533945658487156,0.980722127459479
"7767","PRDX5",598.978767574349,-0.146950492167605,0.236178791602929,-0.622200203372463,0.533810232332952,0.980722127459479
"7768","WDR47",17.2257509885936,-0.21598218136432,0.347660289192589,-0.62124489934102,0.534438501576242,0.980843541417782
"7769","ECHDC1",125.721840983089,0.170513915711517,0.274357104344913,0.621503555078902,0.534268356126036,0.980843541417782
"7770","TMOD1",5.64127151765279,-0.464944867238843,0.748330519149,-0.621309508755005,0.534395998515423,0.980843541417782
"7771","DCTPP1",81.658060485353,-0.218553667683756,0.351803593169939,-0.621237735847069,0.534443214158953,0.980843541417782

"7772","MRPL10",61.2441584379798,0.158664273596733,0.255282009705168,0
.621525479919163,0.534253935078813,0.980843541417782
"7773","LIN7B",4.83338849216756,-0.39628196085906,0.637968775513591,-0
.621162000507058,0.53449303876222,0.980843541417782
"7774","RRP1B",69.3604686473938,-0.150436225988432,0.242140119300977,-
0.621277574417325,0.534417006186536,0.980843541417782
"7775","PIGM",33.2052640911928,0.224442752764921,0.361860233046655,0.6
20247079584408,0.535095130126142,0.980945642280752
"7776","FGD5-
AS1",174.410452603973,0.113065393789361,0.182316505491786,0.6201599437
4385,0.535152490329175,0.980945642280752
"7777","PDCD10",104.744879586004,-0.151944241511823,0.244935357130288,
-0.620344254467925,0.535031165029611,0.980945642280752
"7778","AC007920.2",6.68805589723648,-0.41988868946501,0.6769604222744
01,-0.620255890372881,0.535089330290217,0.980945642280752
"7779","BDP1",134.663404459497,0.143523862147207,0.231438921253011,0.6
20137102999652,0.535167526557626,0.980945642280752
"7780","RARS2",85.2916145520474,-0.159488407051319,0.257070889246274,-
0.620406330405344,0.534990305734897,0.980945642280752
"7781","YLPM1",66.2144706120463,0.161190325835957,0.25990920659901,0.6
20179361651635,0.535139707546394,0.980945642280752
"7782","PLA2G15",18.4472427320931,0.223392718749056,0.359762154519425,
0.620945577356428,0.534635431680102,0.980945642280752
"7783","UQCR11",467.468192707825,-0.143220744731042,0.23087888838389,-
0.620328457632316,0.535041562990225,0.980945642280752
"7784","OTUD5",53.6424898947363,-0.14680243202813,0.236788646839555,-0
.619972426835151,0.535275940380365,0.981018315193511
"7785","CXCL16",37.50337538986,-0.210416373254743,0.339504577670791,-0
.619774775051129,0.535406078171594,0.981130778573662
"7786","C1orf174",47.9777072864777,-0.18800233203321,0.305013716170916
,-0.616373369674503,0.537648125114224,0.98115673833913
"7787","TRMT13",40.4333472828913,0.168550565195718,0.27288712490286,0
.617656715234576,0.536801650265165,0.98115673833913
"7788","YY1AP1",51.7632038070753,0.139712600329357,0.225645974120149,0
.619167263560239,0.535806175386994,0.98115673833913
"7789","TET3",7.09369476726334,0.409180598588247,0.661339592044657,0.6
1871480780878,0.536104253212192,0.98115673833913
"7790","CNOT11",27.2771554727279,0.192372973994218,0.311272435041953,0
.618021232648788,0.53656134249656,0.98115673833913
"7791","AC108471.2",4.67901432886463,0.407126665124163,0.6602386731088
97,0.616635592106574,0.537475112781556,0.98115673833913
"7792","AC099343.3",22.3842737077217,-0.202787299453473,0.328550354816
259,-0.617218324317077,0.537090730741745,0.98115673833913
"7793","SLC17A5",42.9231990325902,0.179753836540458,0.291250801174571,
0.617178856901125,0.537116759890501,0.98115673833913
"7794","HDAC2",107.537199244879,-0.134663354152898,0.218379468874024,-
0.616648418677953,0.537466650626515,0.98115673833913
"7795","BCAP29",153.234009234771,-0.133387063142912,0.215396120954275,
-0.619263998589965,0.535742457186956,0.98115673833913
"7796","CDKN2A",11.4599207877574,-0.441597237308748,0.713682447472863,

-0.618758719473115,0.536075320552704,0.98115673833913
"7797","PGM5",22.4036694011935,0.288734172257924,0.466331764484309,0.619160422359861,0.535810681747863,0.98115673833913
"7798","LRSAM1",10.0409444667168,-0.323556319490641,0.523583870870584,-0.617964642326837,0.536598646065056,0.98115673833913
"7799","GLE1",19.4847853187596,-0.214943492266044,0.347636311453391,-0.61829988751006,0.536377676041934,0.98115673833913
"7800","SLC39A13",65.8690074699399,-0.152155956889811,0.246882125310303,-0.616310138688933,0.53768984861456,0.98115673833913
"7801","ME3",23.777952785911,-0.222943963915644,0.361233042244019,-0.617174892226613,0.537119374667357,0.98115673833913
"7802","VDR",16.7118961436451,0.369207692780423,0.596750984350787,0.61869641184101,0.5361163742406,0.98115673833913
"7803","DIABLO",2.04070913252327,0.640338223657258,1.03488283739508,0.618754317415356,0.536078220959312,0.98115673833913
"7804","MED4",162.279740742832,-0.149197862960313,0.241606064054436,-0.61752532389542,0.536888283152514,0.98115673833913
"7805","UPF3A",140.647332023202,-0.140887208331406,0.227879138408313,-0.618254085544963,0.536407862772045,0.98115673833913
"7806","IVD",36.2850266358672,0.179482851983395,0.291070994322746,0.61662912308047,0.537479380658443,0.98115673833913
"7807","CPPED1",21.8979677583521,-0.236752987115411,0.383543888961582,-0.617277432724591,0.537051749351895,0.98115673833913
"7808","XPO6",38.8883432032381,0.189898291227423,0.307837770698391,0.616877814559927,0.537315321134146,0.98115673833913
"7809","KIAA0513",8.40448323094068,0.367588612224467,0.595246967575705,0.617539663782858,0.536878827805127,0.98115673833913
"7810","ANKRD11",295.52253395781,0.133991651040157,0.217015117607206,0.617430032144947,0.536951118171727,0.98115673833913
"7811","AC005899.7",2.26377550049863,-0.571316810488797,0.924937543930411,-0.617681501024442,0.536785308541301,0.98115673833913
"7812","CACNB1",5.59944885156582,0.379720259780768,0.615377177792682,0.617052879898472,0.537199847203698,0.98115673833913
"7813","COG1",21.2327611490519,0.194693298345499,0.315832564302798,0.616444661985014,0.537601084230763,0.98115673833913
"7814","HSBP1L1",17.3117976525831,-0.284461076819061,0.460328271976911,-0.617952652782814,0.536606549576449,0.98115673833913
"7815","ZNF607",3.17132856033429,-0.522013041406414,0.844224595397355,-0.61833432033654,0.5363549829401,0.98115673833913
"7816","SEC23B",28.8856569662607,-0.222126285009056,0.358559169620121,-0.619496874795837,0.535589080087643,0.98115673833913
"7817","TOMM34",15.2849731527605,-0.260874941395097,0.423104641243323,-0.616573102645477,0.537516340306181,0.98115673833913
"7818","SLC35C2",64.0627914336333,-0.130068631553656,0.21059529682953,-0.617623629358361,0.536823464777396,0.98115673833913
"7819","FLAD1",24.4725801051625,0.203611265535473,0.331100661110317,0.614952760446566,0.538585918058233,0.98125635021561
"7820","AL592295.4",3.72080940251749,-0.490340637492643,0.795909271609436,-0.616076046583938,0.537844330420923,0.98125635021561
"7821","AC018809.2",2.10136496830957,-0.620882548538049,1.010152476452

39,-0.614642405984649,0.538790902827578,0.98125635021561
"7822","PBRM1",103.096727929933,0.122477283883275,0.198920086569772,0.615710992264804,0.538085281131544,0.98125635021561
"7823","C5orf51",26.4374990807421,0.194531277476178,0.315965523605243,0.615672479884924,0.538110704028815,0.98125635021561
"7824","AL606970.4",4.63266709352521,0.477698858205982,0.777376668386354,0.614501151928794,0.538884212100834,0.98125635021561
"7825","RNF208",5.88211898795412,0.399871865280232,0.651245889725041,0.614010578168961,0.539208337070984,0.98125635021561
"7826","DNAJB12",98.9412429691497,-0.15278496317281,0.248726064249002,-0.614270014821829,0.53903691357933,0.98125635021561
"7827","KCTD21-
AS1",4.40300423116594,-0.492845063617619,0.800632931330009,-0.615569313141925,0.538178809710851,0.98125635021561
"7828","PIH1D2",2.90070500662677,-0.520627563204374,0.847645511866873,-0.614204353017493,0.539080297217957,0.98125635021561
"7829","CDON",107.076809503855,0.20214137942800７,0.328479343548428,0.615385361053017,0.538300256693114,0.98125635021561
"7830","TMBIM4",618.929691167399,-0.149571712334362,0.243532850117338,-0.614174688393357,0.539099897608525,0.98125635021561
"7831","PSMD9",161.619333730221,-0.151411726311983,0.246185175811185,-0.615031858896777,0.53853368089617,0.98125635021561
"7832","UBE3A",131.357652861797,0.116109772468405,0.188915794514777,0.614611249242705,0.538811483578863,0.98125635021561
"7833","NEO1",29.2853883503733,0.20429198776482,0.332499503170978,0.614412911347325,0.538942505967791,0.98125635021561
"7834","TRPV3",3.25937453338491,0.497904671885802,0.809449692988447,0.615115029629035,0.538478757111747,0.98125635021561
"7835","CACTIN",19.2147970951719,0.219761463176703,0.357690164530598,0.614390567504418,0.538957267353488,0.98125635021561
"7836","ZNF606",6.56741942117924,0.390984798059827,0.635656891020193,0.6150877990677,0.538496739155548,0.98125635021561
"7837","TRIM28",126.813190224427,-0.131432141920099,0.214071275715103,-0.613964397984041,0.539238853620324,0.98125635021561
"7838","STAU1",199.881320169272,-0.126203517808734,0.20556463617205,-0.613935938393149,0.539257660569913,0.98125635021561
"7839","NR0B1",2.21095298610091,-0.780885419749512,1.27159593994856,-0.614098704798557,0.539150104091699,0.98125635021561
"7840","HUWE1",105.226789896936,0.158268165118613,0.257242711275196,0.615248394537793,0.538390692266269,0.98125635021561
"7841","AC118553.1",2.06969480512756,-0.644972714786698,1.0516284422885,-0.613308549722315,0.539672341236613,0.981476135809195
"7842","IER5",155.986487621741,0.140704117444978,0.229471367630154,0.613166334859498,0.539766362183713,0.981476135809195
"7843","SIVA1",254.427436377099,-0.171934396524905,0.280428277025518,-0.613113621595512,0.539801214006627,0.981476135809195
"7844","FBRS",39.6731408859844,0.162356766615868,0.264732393147503,0.613286363204543,0.5396870086251,0.981476135809195
"7845","NUDT7",15.5253029787631,0.279009742286952,0.455012783787634,0.613190996447197,0.539750057351991,0.981476135809195

"7846","PSMC3IP",6.98481846858633,-0.382729960611382,0.624330446777715,-0.613024661197804,0.539860033484842,0.981476135809195
"7847","OAZ1",1467.60166692251,-0.128766367482518,0.209895783907048,-0.613477627256878,0.53956057153707,0.981476135809195
"7848","VWA1",35.0676282810193,0.19211129543306,0.314553368693055,0.610743087035651,0.541369676487347,0.981542913157654
"7849","AL357873.1",1.86154255784315,0.6540633006566,1.07392063534783,0.609042492646355,0.54249627372421,0.981542913157654
"7850","LRRC40",33.0140324143007,0.190710379867468,0.311997963275925,0.611255207774568,0.541030639544675,0.981542913157654
"7851","RPS27",9514.97220001328,-0.160719643961327,0.26282588654373,-0.61150614224062,0.54086455328426,0.981542913157654
"7852","ODR4",73.8036204722305,0.137742361744339,0.225250850580855,0.611506511026008,0.540864309214638,0.981542913157654
"7853","NPHP1",18.6961731933197,-0.254559824206216,0.416992593696391,-0.610466056362524,0.541553122039918,0.981542913157654
"7854","SLC4A3",3.36610180105754,-0.477917829249637,0.782145125174723,-0.611034722159619,0.541176593619527,0.981542913157654
"7855","NPRL2",18.2473772073957,0.243420235126738,0.399032597948789,0.610025938677766,0.541844625160227,0.981542913157654
"7856","TBC1D19",14.456414826974,-0.246129340425623,0.403652530988722,-0.609755474151851,0.542023800746797,0.981542913157654
"7857","PPM1K",34.8925783853408,0.190552251511984,0.312767240160597,0.609246196673732,0.542361263795179,0.981542913157654
"7858","AC010359.3",7.20248284039292,-0.354598042624223,0.58039361761514,-0.610961306020698,0.541225197003053,0.981542913157654
"7859","FIG4",17.8497967237847,-0.214746396829694,0.351193525306272,-0.61147595657527,0.540884530954264,0.981542913157654
"7860","GSDME",16.7981907701189,0.269386020138955,0.440492497393936,0.611556432249607,0.540831270847989,0.981542913157654
"7861","AEBP1",1024.25585444338,0.0992207286614093,0.162894448035636,0.609110561212639,0.542451157719089,0.981542913157654
"7862","KMT2E",229.652276844949,0.132279236678291,0.215997250686951,0.612411668470752,0.540265423633802,0.981542913157654
"7863","PDGFRL",575.033789057498,-0.0991438426438663,0.162482828898216,-0.610180431471763,0.541742291039533,0.981542913157654
"7864","CCDC25",145.246055960363,-0.130357897289935,0.212792755338189,-0.612604959613212,0.540137578071442,0.981542913157654
"7865","ALDH1A1",201.527706451272,0.386347410412821,0.632977926272757,0.610364744767323,0.541620216805729,0.981542913157654
"7866","CDK20",6.97857342821519,-0.327358075787513,0.538066409514807,-0.608397160645473,0.542924094190877,0.981542913157654
"7867","TNFSF15",6.28671965784025,-0.499778656200978,0.820954541025028,-0.608777503778668,0.542671926778828,0.981542913157654
"7868","AL356481.1",2.99631025444253,-0.533644166057732,0.875972229100837,-0.609202150855289,0.542390454841246,0.981542913157654
"7869","MMS19",24.196935499093,0.202374949167091,0.331226119204707,0.610987290655113,0.541207994253479,0.981542913157654
"7870","TAF10",209.978645235707,-0.173034743180301,0.284239844148313,-0.608763151059193,0.542681441568792,0.981542913157654

"7871","FAM180B",73.9642755658999,0.198820827839355,0.326272581982083,
0.609370320458838,0.542279005872891,0.981542913157654
"7872","NELL2",4.6021661587697,0.477422208147636,0.783564380559151,0.6
09295445266345,0.542328625575924,0.981542913157654
"7873","VASH1",11.7210200136409,0.274294517845786,0.450795967969518,0.
608467105598277,0.54287771632295,0.981542913157654
"7874","KLC1",109.098550804659,0.129160995314806,0.211734574398114,0.6
10013719686379,0.541852719280969,0.981542913157654
"7875","AC020659.1",1.99143010848757,-0.695262181833371,1.137341460680
97,-0.61130470124345,0.540997879232929,0.981542913157654
"7876","NPTN",159.595934941119,-0.128405444056852,0.211048260039595,-0
.60841744932065,0.542910641330103,0.981542913157654
"7877","HAGH",58.9059996171433,0.177160406006098,0.290812172461517,0.6
09191852275516,0.54239728026313,0.981542913157654
"7878","NTAN1",204.705229007534,-0.132589602427741,0.216728186434695,-
0.61177830446938,0.540684445790807,0.981542913157654
"7879","DHX33",34.0754844533753,-0.196559177720317,0.322554903025363,-
0.609382080001622,0.542271213043255,0.981542913157654
"7880","AC016876.1",25.5619810865343,0.210241666779601,0.3443680481332
51,0.610514442089731,0.541521079501265,0.981542913157654
"7881","MYO1D",50.4981161506017,-0.232765049097501,0.3825417870851,-0.
608469602422075,0.542876060809468,0.981542913157654
"7882","PRPSAP1",47.1036157040958,-0.165178521688217,0.269914963802452
,-0.611965040252862,0.54056088789108,0.981542913157654
"7883","CBX8",4.96212077416217,0.427513700952065,0.701105929657969,0.6
09770482415724,0.5420138573949,0.981542913157654
"7884","SEH1L",37.7258827226939,0.182899360580985,0.298874417655188,0.
61196057533434,0.540563842039345,0.981542913157654
"7885","ZNF790-
AS1",6.06885218837078,0.379957649323355,0.623774336196036,0.6091267743
40302,0.542440411895935,0.981542913157654
"7886","ZNF225",5.56262865629864,-0.391008252971671,0.641664895834632,
-0.609365192813104,0.542282403885549,0.981542913157654
"7887","C19orf48",40.2911926171156,-0.219873710452267,0.36029547747742
3,-0.610259423714373,0.541689971279532,0.981542913157654
"7888","ZNF835",4.35137987647369,0.468401287045378,0.767926499160128,0
.609955884524968,0.541891031345121,0.981542913157654
"7889","NSFL1C",108.732671827302,-0.136141762048837,0.223764749714012,
-0.608414695446158,0.542912467338563,0.981542913157654
"7890","TSHZ2",646.62143345592,-0.156866850462182,0.256749421938183,-0
.610972555568014,0.54121774937022,0.981542913157654
"7891","AP1S2",53.5653379306912,-0.184168944552006,0.300935030581646,-
0.611989053570948,0.5405449999676,0.981542913157654
"7892","LTO1",15.9121489348097,-0.247753334237756,0.407295586681734,-0
.608288776846861,0.542995963310495,0.981548455725739
"7893","PNKD",117.529026946536,-0.155848045546413,0.2562649847508,-0.6
08151931868355,0.54308671176008,0.981588119849145
"7894","ENTPD4",47.3826131860372,0.191241355385913,0.314745362526951,0
.607606586640454,0.543448431271963,0.982061469870044
"7895","CEP126",137.801592708477,0.149002326969394,0.24547573770117,0.

606994110150235,0.543854820885735,0.982061469870044
"7896","CRYAB",315.453903045222,-0.142459991380159,0.234780585806262,-0.606779265376379,0.543997410219308,0.982061469870044
"7897","AL049775.3",1.99841484452894,-0.611811763538989,1.008308556512
8,-0.606770377566682,0.544003309329038,0.982061469870044
"7898","PTPN21",32.0763415445211,0.208377155180173,0.343251674971047,0
.607068137971212,0.543805694010798,0.982061469870044
"7899","NMRAL1",40.1035999617574,0.171705728058389,0.28300671569178,0.
606719623732859,0.544036996802394,0.982061469870044
"7900","CORO1A",57.0869533975602,-0.171518501964273,0.282501294426433,
-0.607142357745686,0.543756441966604,0.982061469870044
"7901","PLEKHG4",21.4099297014972,-0.280254726192034,0.461424193750702
,-0.607368945945322,0.543606092373343,0.982061469870044
"7902","ZNF181",22.2593652064901,0.217697094758893,0.358480483179918,0
.607277397162044,0.543666835863014,0.982061469870044
"7903","ITPA",125.698474650434,-0.185161196135657,0.304779402310859,-0
.607525294464625,0.543502361451303,0.982061469870044
"7904","RUSC1-
AS1",2.0911706968118,0.651063482361574,1.07346223984164,0.606508043038
028,0.544177443064053,0.982178221854565
"7905","AC008622.2",2.0556056790075,-0.589229542972061,0.9718165958495
05,-0.606317638007603,0.544303848428636,0.982178221854565
"7906","C5AR2",11.0916481984935,-0.512932485497872,0.84598899949909,-0
.606311058183474,0.544308216878045,0.982178221854565
"7907","WNT4",16.0029856791704,0.767337996670744,1.26585928014921,0.60
6179540414868,0.544395537262143,0.982184239055819
"7908","TRBC1",38.724282684802,-0.245498027108305,0.405046318896084,-0
.606098650093617,0.544449247333059,0.982184239055819
"7909","DERL1",117.42532177013,-0.155131256036305,0.256108119708235,-0
.605725645141725,0.54469695160277,0.982506854414605
"7910","C15orf61",200.447702775927,-0.141757220888846,0.23412195574541
3,-0.60548452381371,0.544857104686192,0.982671486150849
"7911","TMEM240",15.8540344385368,-0.25179595362835,0.421402171784204,
-0.597519354402597,0.550160689020617,0.982717452900852
"7912","DFFB",8.74068607499393,-0.284528312329652,0.501610145760994,-0
.567229978767661,0.570557942764947,0.982717452900852
"7913","ICMT",60.8732927455954,0.143224919209762,0.251336178964573,0.5
69853969292461,0.568776747341347,0.982717452900852
"7914","UTS2",2.70913991959208,-0.49455588208344,0.873255824397307,-0.
566335623841692,0.571165648002715,0.982717452900852
"7915","CTNNBIP1",29.735644334185,-0.18695086412195,0.316452055999031,
-0.590771526295667,0.554673515670846,0.982717452900852
"7916","TARDBP",91.5204697306264,-0.133138355364408,0.238982890053072,
-0.557104131324389,0.577456285048722,0.982717452900852
"7917","SLC25A34",2.73624989777039,0.487740025313343,0.874860606018141
,0.55750598662026,0.577181769956108,0.982717452900852
"7918","CROCC",46.3444490125898,0.149766827670195,0.260027090869991,0.
575966247090289,0.564637996108982,0.982717452900852
"7919","SDHB",105.028806771721,-0.123303481073643,0.219816497511713,-0
.560938248354508,0.574839634589047,0.982717452900852

"7920","UBXN10-
AS1",2.11563625381961,-0.674314889283411,1.17564524388882,-0.573570039
76208,0.566258790648143,0.982717452900852
"7921","FUCA1",40.9229381453365,-0.157181511819067,0.269164054158672,-
0.583961748942929,0.55924604732382,0.982717452900852
"7922","AL020997.2",2.74096945907442,0.476435961573188,0.8498085559890
19,0.560639167746087,0.575043544806757,0.982717452900852
"7923","MECR",40.8329156125517,-0.165301953901014,0.29573852253275,-0.
558946303259186,0.57619836732554,0.982717452900852
"7924","SNRNP40",86.9729474949437,-0.192129119544596,0.337713650524503
,-0.568911322495258,0.569416321120105,0.982717452900852
"7925","TMEM39B",31.4259565406972,-0.15216395606639,0.272277416829683,
-0.558856323224092,0.576259779685771,0.982717452900852
"7926","SNIP1",35.4829328096258,-0.167454368552691,0.300908722921708,-
0.556495560935469,0.577872128214669,0.982717452900852
"7927","RRAGC",125.931801779972,0.171677314826265,0.29952877783557,0.5
73157998596414,0.566537720062418,0.982717452900852
"7928","C1orf50",42.2862617053631,-0.198143887073823,0.331081467157508
,-0.598474716132506,0.549523224616134,0.982717452900852
"7929","BTBD19",10.897368338291,-0.305441809707442,0.527321394534701,-
0.57923272765551,0.56243215058553,0.982717452900852
"7930","RNF11",97.8821275370578,0.120776186769467,0.211648528150294,0.
57064505869704,0.568240268370531,0.982717452900852
"7931","FAM151A",1.98251413554109,-0.707802100916234,1.19970547052143,
-0.589979889487876,0.555204132205141,0.982717452900852
"7932","AC093425.1",17.9313395263543,0.286431163574534,0.508330443837б
73,0.563474344389259,0.573111925320807,0.982717452900852
"7933","JAK1",404.182018400734,-0.0989536615567383,0.16954195013605,-0
.58365296304149,0.55945381931692,0.982717452900852
"7934","RPF1",57.9421769949827,0.168646391917557,0.287135333172812,0.5
87341133026138,0.556974620936656,0.982717452900852
"7935","ZNHIT6",60.4948285514913,0.14122091510172,0.252428799980638,0.
559448506321593,0.575855664965296,0.982717452900852
"7936","AC099063.4",2.26966929621564,0.602444539121928,1.0103744891902
8,0.596258660098131,0.551002443386506,0.982717452900852
"7937","ZNF644",128.332593367909,0.107787602318933,0.188522653447637,0
.571748807624709,0.567492163308544,0.982717452900852
"7938","MTF2",45.0872571614145,0.160068145434299,0.269124134177804,0.5
94774400011802,0.551994281882956,0.982717452900852
"7939","TMED5",173.084701922496,0.142124604598451,0.23899778622069,0.5
94669125793547,0.552064663366287,0.982717452900852
"7940","CNN3",378.958312486818,0.145441103210279,0.255605802192258,0.5
6900548408085,0.569352418260162,0.982717452900852
"7941","RTCA-
AS1",15.4987186108178,0.316046181515648,0.567780309564729,0.5566346282
03179,0.577777089526653,0.982717452900852
"7942","GSTM2",17.6256584901719,0.226684065824179,0.395890531365614,0.
572592794887612,0.566920439331313,0.982717452900852
"7943","DRAM2",94.3571189383691,0.158744887034193,0.262691888642954,0.
604300680368385,0.545643754087612,0.982717452900852

"7944","NBPF26",27.5315537345065,0.225622195646467,0.373625798308308,0.603872100556312,0.545928678931213,0.982717452900852
"7945","NOTCH2NLA",11.3165315302705,0.258375828933486,0.462630277292849,0.5584931242404,0.576507698360678,0.982717452900852
"7946","NBPF12",28.8208799048622,0.261601949611726,0.442336654070511,0.59140916133535,0.554246303481389,0.982717452900852
"7947","BCL9",14.6686599143384,0.249562961604743,0.432651522246634,0.576822104563124,0.564059635617127,0.982717452900852
"7948","PI4KB",67.1935058131047,-0.147603376432452,0.255572964755365,-0.577539085848686,0.563575342831617,0.982717452900852
"7949","FLG-AS1",3.29052545207955,0.531032578002279,0.935953634560934,0.567370602980127,0.570462418049979,0.982717452900852
"7950","FCER1A",5.72787371363409,-0.513689326557067,0.891179582829694,-0.576415053098488,0.564334672106145,0.982717452900852
"7951","SLAMF6",4.16968694425467,0.445704723636989,0.774846065244661,0.575217121992168,0.565144464405169,0.982717452900852
"7952","SLAMF1",7.01025048542593,-0.300362030554338,0.528829306741989,-0.567975387757551,0.57005168028184 2,0.982717452900852
"7953","ALDH9A1",187.165233613734,0.125566852703712,0.211210215669222,0.594511265971924,0.552170209414166,0.982717452900852
"7954","GORAB",19.1569893249362,-0.199405130603083,0.35843133164653,-0.556327287815698,0.577987136041131,0.982717452900852
"7955","RC3H1",64.9560070830372,0.178296150696686,0.311674791657382,0.572058297524054,0.567282480225151,0.982717452900852
"7956","XPR1",21.8564203654903,0.22868411063186,0.394752668809564,0.579309853234156,0.562380118215883,0.982717452900852
"7957","SWT1",12.9549711512961,0.273540875525466,0.46112136386991,0.593207985918945,0.553041968804473,0.982717452900852
"7958","RGS13",1.92703712970348,0.715865620702151,1.23931593814205,0.577629641215911,0.56351419048386,0.982717452900852
"7959","ZNF281",36.423739832578,0.173336124349935,0.309032753820901,0.560898876273779,0.574866476131657,0.982717452900852
"7960","DDX59",35.3907940637537,-0.192464280617359,0.327634385365717,-0.587436145942141,0.556910823769942,0.982717452900852
"7961","TMEM183A",78.7183411743393,-0.129729013772744,0.223254105073058,-0.581082322003851,0.56118497243491,0.982717452900852
"7962","IKBKE",10.1238794664735,-0.273320168477763,0.467858919576,-0.584193561438267,0.559090092850437,0.982717452900852
"7963","ACBD3",169.403204217232,-0.100898363126936,0.178217082040612,-0.566154276411858,0.571288909324358,0.982717452900852
"7964","STUM",4.41675153531952,0.41126333312283,0.736866251831595,0.558124805011182,0.576759163464147,0.982717452900852
"7965","ZNF678",17.9329351712772,0.212155629283733,0.357617153590845,0.593247910939035,0.553015253084766,0.982717452900852
"7966","HIST3H2A",24.2983196035367,0.290173926264272,0.499580757481423,0.58083487387935,0.561351748696213,0.982717452900852
"7967","AC104794.3",15.6909019748751,-0.24421549522275,0.426422520406572,-0.572707780512864,0.566842568488434,0.982717452900852
"7968","HPCAL1",24.9408439017322,-0.20392144366425,0.360183479109845,-

0.566159903192174,0.571285084627846,0.982717452900852
"7969","UBXN2A",67.3502778545213,0.126025325219282,0.226135836582822,0.557299219458856,0.577323008908135,0.982717452900852
"7970","HADHB",201.169861733334,-0.148650413514715,0.254037775843431,-0.585150822633288,0.558446308299801,0.982717452900852
"7971","ATRAID",410.544412832277,-0.150771066081828,0.257242347543185,-0.586105155398322,0.557804852055959,0.982717452900852
"7972","CAD",23.4907161416945,0.192979930640363,0.340090341012712,0.56743725818767,0.570417142451916,0.982717452900852
"7973","LTBP1",60.1978191013421,-0.185499993748757,0.329727763874479,-0.562585302399263,0.573717302560919,0.982717452900852
"7974","STRN",67.8001166430214,0.147829524836231,0.259176916518253,0.570380753124749,0.568419480836393,0.982717452900852
"7975","CYP1B1",270.439401587289,0.348476144275057,0.616414292737311,0.565327813421683,0.571850814045583,0.982717452900852
"7976","AC019171.1",2.47465722929896,-0.551150099309313,0.936063510984339,-0.588795624273121,0.55599838153908,0.982717452900852
"7977","AC009236.1",2.89710130276596,0.532423109655765,0.943499142511238,0.564306935392284,0.572545262287469,0.982717452900852
"7978","B3GNT2",11.1753075443036,0.265436958497133,0.472027929570159,0.562333162655961,0.573889047750447,0.982717452900852
"7979","ETAA1",26.844308813554,0.165857339550558,0.295825138313998,0.560660059168169,0.575029300129647,0.982717452900852
"7980","LBX2-AS1",9.58328736505261,0.279070714512775,0.478132409064361,0.583668266827756,0.559443521014615,0.982717452900852
"7981","HTRA2",59.452919354197,-0.145163557428041,0.260952029840057,-0.55628445395506,0.578016412960259,0.982717452900852
"7982","KCMF1",93.5625212749278,-0.115884263598131,0.204909897901728,-0.565537657208278,0.571708118267572,0.982717452900852
"7983","RNF103",51.1314621082236,0.16080850148037,0.266329741600549,0.603794756507352,0.545980106011113,0.982717452900852
"7984","STARD7-AS1",2.69434854527101,-0.513322373333751,0.917724857510254,-0.559342344421586,0.57592810160999,0.982717452900852
"7985","ANKRD39",29.5903442077597,0.177213225881665,0.309170660204878,0.573189014003565,0.566516722028738,0.982717452900852
"7986","ANKRD36",26.0576957499631,-0.22305534109573,0.387728350486349,-0.575287674517326,0.565096755958726,0.982717452900852
"7987","TMEM182",2.88503253537912,-0.551663665781781,0.913812934604839,-0.603694306450518,0.546046900165965,0.982717452900852
"7988","UXS1",64.7794796787291,0.150536185670676,0.259375099038154,0.580380253266072,0.561658218528593,0.982717452900852
"7989","MGAT5",11.2122405216018,-0.279091014171392,0.475968892730877,-0.586363979734273,0.557630944694255,0.982717452900852
"7990","IFIH1",45.0143501193037,0.217271460648527,0.368331761461079,0.589879786056641,0.555271246966092,0.982717452900852
"7991","SESTD1",267.900733038137,0.120717877951287,0.211448204338473,0.570909922498321,0.568060704510665,0.982717452900852
"7992","CWC22",88.7879168918492,0.142270034008638,0.236247555246883,0.

602207433892652,0.547036067934155,0.982717452900852
"7993","LINC01473",12.075209708607,-0.248783273639953,0.436918508344254,-0.569404291392327,0.569081805217837,0.982717452900852
"7994","ITGAV",171.621341737138,-0.121278696219266,0.214717095287024,-0.564830182977031,0.572189275165476,0.982717452900852
"7995","HSPE1",462.847229855894,-0.212076810776578,0.351926094707403,-0.602617464194868,0.546763199615841,0.982717452900852
"7996","BZW1",506.172104620353,0.122357276639417,0.218171836767092,0.560829841525508,0.574913541347386,0.982717452900852
"7997","NOP58",139.559768541284,0.133250945606981,0.2256488005598,0.590523615797675,0.554839657888583,0.982717452900852
"7998","ADAM23",4.30912591189094,0.470620338547556,0.818866518207339,0.574721676956383,0.565479544688949,0.982717452900852
"7999","ZNF142",9.47903203130122,0.293277472340212,0.513868588807905,0.570724653593966,0.568186304323641,0.982717452900852
"8000","OBSL1",47.6843045592606,0.159164589526887,0.28034807619099,0.5677391894013,0.570212076904632,0.982717452900852
"8001","SERPINE2",83.4552696499769,-0.191441289239486,0.335259174678728,-0.571024758451251,0.567982860153771,0.982717452900852
"8002","COPS7B",23.9420818680286,-0.210526103258538,0.353305580500024,-0.595875397610719,0.551258469844871,0.982717452900852
"8003","DTYMK",61.2324433415668,-0.175775580637116,0.298774249142577,-0.588322391041251,0.556315919110371,0.982717452900852
"8004","SETD5",124.427780066024,0.141035549640355,0.24016950009945,0.587233389676685,0.557046970360416,0.982717452900852
"8005","SEC13",118.186286898331,-0.156306108908644,0.274256506059757,-0.569926712603081,0.568727406207139,0.982717452900852
"8006","NR1D2",72.0494684226613,-0.44559622681368,0.752640886595335,-0.592043609043603,0.553821386654366,0.982717452900852
"8007","MLH1",32.1521865632491,0.194177399906875,0.343036124228589,0.566055252470962,0.571356220951868,0.982717452900852
"8008","TTC21A",3.92826884421609,0.423240564092909,0.731236283775006,0.578801371709744,0.562723205476983,0.982717452900852
"8009","SS18L2",106.41061792827,-0.186851462999848,0.319089592570557,-0.585576801470049,0.558159941670004,0.982717452900852
"8010","FYCO1",37.6526389360022,0.207320857995559,0.360655457657523,0.574844643533525,0.565396370805793,0.982717452900852
"8011","KIF9",16.7155104817146,-0.24688228237327,0.427035261217319,-0.578130905793354,0.563175742675467,0.982717452900852
"8012","SCAP",39.9536553200926,0.164451886763909,0.290060322548084,0.56695753944991,0.570743029930932,0.982717452900852
"8013","SUCLG2",134.297760662292,-0.129692874517283,0.227381129967303,-0.570376594293169,0.568422300949227,0.982717452900852
"8014","RYBP",83.3508324789856,0.166397894501874,0.292416144835934,0.569044826834834,0.569325719272519,0.982717452900852
"8015","ZNF654",28.485995969288,0.182486017996902,0.32268036113399,0.56553183886244,0.571712074570056,0.982717452900852
"8016","COL8A1",53.8081870266129,0.592761929087058,1.01754714742759,0.582540013586194,0.560202997457525,0.982717452900852
"8017","NIT2",85.1785680539063,-0.182906580602128,0.314315340571038,-0

.581920628722192,0.560620144756361,0.982717452900852
"8018","TFG",221.411630216857,-0.118750968125915,0.199116647502936,-0.596388948966031,0.550915421326216,0.982717452900852
"8019","CBLB",168.99204674727,0.14641752999601 7,0.247684450296479,0.5911454264519,0.554422984967065,0.982717452900852
"8020","CCDC80",5697.31042980925,-0.128098690442641,0.228020000080968,-0.561787081822448,0.57426109462748,0.982717452900852
"8021","POGLUT1",43.0657505953293,-0.170917458570688,0.283833149515205,-0.602175816540878,0.547057111552492,0.982717452900852
"8022","GTF2E1",13.4757623014636,0.242175369947546,0.409211690128982,0.59180951030801,0.553978154041049,0.982717452900852
"8023","AC083798.2",6.16337480548149,0.452168748324619,0.788792369522668,0.573241788074403,0.566480993814668,0.982717452900852
"8024","SLC41A3",46.8341202055128,-0.14639348742155,0.262068824269672,-0.558607029392057,0.576429941588293,0.982717452900852
"8025","CHCHD6",26.3878039666361,-0.216171578166017,0.371290296016187,-0.58221715053008 8,0.560420422573414,0.982717452900852
"8026","RUVBL1",52.6410315495296,-0.190022993843131,0.316199473555021,-0.600959235341883,0.547867137897557,0.982717452900852
"8027","ISY1",67.5467419939297,-0.131941685538827,0.22460767111832 7,-0.587431786643288,0.556913750777371,0.982717452900852
"8028","TF",2.40698747599314,-0.535994535793756,0.955563620549773,-0.560919779977996,0.574852225155756,0.982717452900852
"8029","PIK3CB",11.6141006520823,-0.304880365266755,0.529571861538729,-0.57571103642269,0.564810514341459,0.982717452900852
"8030","TIPARP",193.262143891567,0.228171947657128,0.405489826322736,0.562706960434371,0.573634443804326,0.982717452900852
"8031","GFM1",59.3405942430556,-0.148433350783014,0.251318157537521,-0.590619286077065,0.554775539639672,0.982717452900852
"8032","SMC4",44.7463060116993,-0.166070496811299,0.286336567837719,-0.579983541974349,0.561925716482429,0.982717452900852
"8033","TRIM59",3.33520424891062,-0.445878402900043,0.792244958021575,-0.562803711636749,0.57356855262241,0.982717452900852
"8034","TNFSF10",542.758394347017,-0.176704323373123,0.296453613236513,-0.596060616175209,0.551134733101526,0.982717452900852
"8035","YEATS2",22.6736605830757,0.209878608365937,0.372625542745043,0.563242677406952,0.573269645612635,0.982717452900852
"8036","EHHADH",1.85194804887793,0.712802817881472,1.19102015408781,0.598480903480092,0.549519097300212,0.982717452900852
"8037","ZFYVE28",14.7393889963536,0.2584819073934,0.440994886863927,0.586133570009299,0.557785758628236,0.982717452900852
"8038","WFS1",31.6297169307483,0.195679098194921,0.325194717579916,0.601729018389832,0.547354530640401,0.982717452900852
"8039","LINC02447",2.42876915468042,0.569356695457207,0.956365036321995,0.595334076250684,0.551620182183696,0.982717452900852
"8040","SORCS2",13.4051697992997,-0.357864166590654,0.609516280779467,-0.587128150429398,0.557117642704423,0.982717452900852
"8041","PGM2",21.9950357000087,-0.210733571624596,0.357043650851616,-0.590217949883598,0.555044539546842,0.982717452900852
"8042","COMMD8",62.980103188864,-0.164868435773154,0.277406684693709,-

0.594320342190702,0.552297875464076,0.982717452900852
"8043","TXK",3.22807612068828,0.476778199935305,0.851680916725737,0.559808480584801,0.575610078482789,0.982717452900852
"8044","TMEM165",298.190847466608,-0.111065980518337,0.199138987332875,-0.557730969740657,0.577028106503055,0.982717452900852
"8045","CENPC",99.3372386841686,-0.135668835742979,0.229031983118574,-0.592357599561722,0.553611152768077,0.982717452900852
"8046","DCK",19.4398872870757,-0.217608985193365,0.361033199056584,-0.602739542407733,0.546681971626331,0.982717452900852
"8047","ART3",2.22464946500792,0.551554837852318,0.962382583917692,0.573113901965094,0.566567574981833,0.982717452900852
"8048","LINC01091",13.9134759938895,0.254459781104202,0.429357030217609,0.592653114297989,0.55341332514707,0.982717452900852
"8049","FBXW7",57.8922079020375,0.181769091933926,0.314745967695785,0.577510470633299,0.563594667449005,0.982717452900852
"8050","GUCY1B1",14.9339418412812,-0.263319357547141,0.449182813466117,-0.586218683469295,0.557728567853115,0.982717452900852
"8051","ING2",40.5857928636318,-0.190673982014153,0.315415721249366,-0.604516418074821,0.545500357067646,0.982717452900852
"8052","MIR3945HG",9.11003074164334,0.485396858779311,0.822993492311239,0.589794285512703,0.555328574298796,0.982717452900852
"8053","CCDC110",2.7848007752332,-0.521802657010244,0.90781310650784,-0.574790838851738,0.565432763258681,0.982717452900852
"8054","EXOC3-AS1",8.6795579367806,0.321011120170136,0.552795784275644,0.580704718272722,0.561439481210718,0.982717452900852
"8055","TRIP13",2.51287173279169,-0.503257652679133,0.904471126927778,-0.556410965144406,0.577929944647744,0.982717452900852
"8056","IRX2",37.011335163767,-0.229319342777476,0.396458022495946,-0.57842023559965,0.562980435307633,0.982717452900852
"8057","ATPSCKMT",13.9788118730861,0.22921633343988,0.407369882316804,0.562673735565023,0.573657072008618,0.982717452900852
"8058","FYB1",58.1988500751243,0.218294922780985,0.376777550995733,0.57937348497564,0.562337191143351,0.982717452900852
"8059","RPL37",6754.8017386267,-0.145975259675029,0.246401113194676,-0.592429383870914,0.553563094723616,0.982717452900852
"8060","AC008966.1",3.21546430424291,0.456721820112497,0.767372502557751,0.595176160978123,0.551725723548355,0.982717452900852
"8061","KIF2A",66.247580796745,-0.147116487124523,0.263069124576352,-0.559231294669946,0.576003877948541,0.982717452900852
"8062","ADAMTS6",11.6077977262462,-0.340328560542603,0.567191313232806,-0.600024282111869,0.548490052823864,0.982717452900852
"8063","MRPS27",41.2219398115603,-0.179429450740396,0.297718094199627,-0.602682383893282,0.546720002821868,0.982717452900852
"8064","FOXD1",4.22265811293351,-0.502867232109561,0.855253090708018,-0.587974761591641,0.556549233389614,0.982717452900852
"8065","AC008549.2",4.18453515987371,0.460189665021713,0.760706137589304,0.604950640309101,0.545211793965982,0.982717452900852
"8066","ZNF608",3.73904334586839,0.492386120483046,0.862346417756085,0.57098413160257,0.568010399472481,0.982717452900852

"8067","LYRM7",48.5002085989599,0.144227581854531,0.248055977515435,0.581431591768663,0.560949610751875,0.982717452900852
"8068","SLC22A5",8.97337025348731,0.29192396848991,0.500046168229887,0.583794031505714,0.55935889428707,0.982717452900852
"8069","CDKL3",9.84757212638885,-0.256505332552272,0.452736998389231,-0.566565872603473,0.57100916682725,0.982717452900852
"8070","SAR1B",105.47364064165,0.116358096055024,0.197927182491285,0.58788335482999,0.556610589687001,0.982717452900852
"8071","SMAD5-AS1",2.70355193357249,0.509243003112602,0.909118802976492,0.560150116184287,0.575377049017242,0.982717452900852
"8072","FAM13B",39.1219811136012,0.155795974176792,0.277864098277488,0.560691270094226,0.575008019478254,0.982717452900852
"8073","CTNNA1",216.104934527028,0.121598097848488,0.205772847354612,0.590933640719543,0.554564884578825,0.982717452900852
"8074","TMCO6",8.97090737032226,0.304308880099429,0.524938080943961,0.579704333037165,0.56211402083107,0.982717452900852
"8075","MFAP3",6.59200396336459,-0.356749847823672,0.603831390366345,-0.590810371099176,0.554647485247443,0.982717452900852
"8076","C5orf58",3.42340180841402,-0.506708464349949,0.886386849212032,-0.571656116965629,0.567554969552643,0.982717452900852
"8077","ARL10",13.7851159807083,0.268065210095016,0.467044104699204,0.573961232778349,0.565994035289061,0.982717452900852
"8078","FAF2",86.6936576352884,-0.11719914336417,0.197452764522673,-0.593555342957542,0.552809556975033,0.982717452900852
"8079","B4GALT7",52.0005363493699,-0.193323256650763,0.331016833848949,-0.584028474935451,0.559201154494806,0.982717452900852
"8080","E2F3",24.0707932152694,0.174884713631411,0.302855687801486,0.577452300470064,0.563633952294591,0.982717452900852
"8081","HIST1H1D",4.64949971313482,-0.379544776178944,0.659781377315104,-0.575258395020866,0.5651165548632,0.982717452900852
"8082","BTN2A2",11.5963516763917,-0.2673718942442,0.461483437019265,-0.579374843810567,0.562336274467083,0.982717452900852
"8083","HLA-A",2249.94955445303,0.13625436503801,0.238601582839661,0.571053902561794,0.567963104918337,0.982717452900852
"8084","C6orf136",9.63322381421264,-0.290691374183592,0.491020417088829,-0.592014841067197,0.553840650348354,0.982717452900852
"8085","HCG27",2.01654456714052,0.598913232475044,1.06364711460891,0.563075125433174,0.573383728842299,0.982717452900852
"8086","MICB",3.11446408053551,-0.465881822164492,0.787861830977289,-0.591324270128173,0.554303170860353,0.982717452900852
"8087","TSBP1-AS1",2.83406829296483,-0.497884906160538,0.847388201304479,-0.587552323001534,0.556832820612127,0.982717452900852
"8088","TAP1",119.457669932138,0.159559960132271,0.264851934804769,0.602449667773385,0.546874857184512,0.982717452900852
"8089","RPS18",11011.1079650617,-0.14146488287029,0.242348724395684,-0.583724478942667,0.559405695259575,0.982717452900852
"8090","PFDN6",78.1090089420827,-0.188142843725236,0.314655447854818,-

0.597932897739132,0.54988470783508,0.982717452900852
"8091","SNRPC",123.546019494912,-0.160912809694099,0.279420005734818,-0.575881491630964,0.56469528659985,0.982717452900852
"8092","GCLC",24.0256761930325,-0.211680309547342,0.366059952225457,-0.578266779144875,0.56308401951555,0.982717452900852
"8093","AL359715.1",2.38016317695931,0.621623082797278,1.0346787656429,0.600788479901812,0.547980878030191,0.982717452900852
"8094","CEP162",39.9943406561062,0.150428181632671,0.260293217968833,0.577918175534958,0.563319363610331,0.982717452900852
"8095","SIM1",11.3985052482279,0.390183890975318,0.654574260708497,0.596088044392384,0.551116410622811,0.982717452900852
"8096","TRAF3IP2-AS1",19.2688380979435,0.226850861616414,0.38373469763237,0.591165883658881,0.554409279272843,0.982717452900852
"8097","FYN",459.480064818903,0.109518940362191,0.187365096781267,0.584521569084158,0.558869458086807,0.982717452900852
"8098","HSF2",25.2517776563734,-0.172097885796118,0.306086509205867,-0.562252437203525,0.573944039104401,0.982717452900852
"8099","NCOA7",292.836481813676,0.130690202573823,0.232808248504236,0.561364141577847,0.574549323817337,0.982717452900852
"8100","CCN2",840.843773111515,-0.257649369901885,0.435462680935065,-0.591668083585572,0.554072872884913,0.982717452900852
"8101","FUCA2",144.61459890174,-0.13992145565493,0.233951113522793,-0.598079887494523,0.549786629576483,0.982717452900852
"8102","STX11",18.505223915842,0.220613603603252,0.395723323227562,0.557494569195226,0.577189568570436,0.982717452900852
"8103","AL023806.1",2.72691555730295,-0.554697189641013,0.92886803585503,-0.597175452517763,0.550390246414878,0.982717452900852
"8104","ZC3H12D",4.93316243897292,-0.413295782110665,0.728721303464029,-0.567152051334349,0.570610881424423,0.982717452900852
"8105","TULP4",41.5963615237331,-0.191893147868016,0.332919592394805,-0.576394878077505,0.564348305641049,0.982717452900852
"8106","SLC22A3",3.40090771984417,-0.533532508788732,0.949431969407664,-0.561949171694308,0.574150650286044,0.982717452900852
"8107","AL135910.1",5.2863016916368,0.424512997788782,0.732049120051982,0.579896875989193,0.561984162573129,0.982717452900852
"8108","TCTE3",4.1816642963372,-0.456452466173616,0.771890138291022,-0.591343824114932,0.554290071677356,0.982717452900852
"8109","TBP",15.915568374028,0.267405110644804,0.446020148673332,0.599535943477417,0.548815548649597,0.982717452900852
"8110","PDCD2",118.576879522471,-0.127584270974243,0.227602323047955,-0.560557859277041,0.575098986033494,0.982717452900852
"8111","PSMG3",75.8452544913152,-0.194073554850308,0.336319263284726,-0.577051557959696,0.563904627026917,0.982717452900852
"8112","RBAK",15.6732035885268,0.259158504386925,0.44971059908912,0.576278399734953,0.564427020507762,0.982717452900852
"8113","USP42",42.9602843671501,0.16387688760914,0.286039966464642,0.572916049580776,0.566701537063052,0.982717452900852
"8114","C1GALT1",175.289554128316,0.115319907078808,0.206413947949155,0.558682725777886,0.57637827057187,0.982717452900852

"8115","TWISTNB",96.5551192605819,-0.176940928122206,0.314034379411971
,-0.563444449787719,0.573132276590747,0.982717452900852
"8116","FAM221A",7.83259407654949,0.300445904706593,0.535076373385196,
0.561500973787727,0.574456066659221,0.982717452900852
"8117","HOXA3",10.2685120590849,0.299215516428049,0.531216673413422,0.
563264542329571,0.573254758960545,0.982717452900852
"8118","RALA",144.304613583331,0.131997351978315,0.220169665001764,0.5
99525606660013,0.548822439552345,0.982717452900852
"8119","LINC02604",3.42589476581717,-0.456520770839993,0.7597707692362
65,-0.600866457785545,0.547928935544184,0.982717452900852
"8120","CDK14",73.2925018978077,0.142787908321477,0.244329825782024,0.
584406377176662,0.558946937253039,0.982717452900852
"8121","AC002074.1",5.57264589732385,-0.391796234497246,0.654127689393
225,-0.598959868004793,0.549199646866551,0.982717452900852
"8122","TSC22D4",69.5772035160028,0.144677311824946,0.252145123698933,
0.573785880537966,0.566112704510403,0.982717452900852
"8123","PSMC2",126.93184897618,-0.154293783844022,0.267759552135747,-0
.576240072906156,0.564452922540128,0.982717452900852
"8124","DUS4L",7.85586455953736,0.30813963270431,0.512225587105125,0.6
01570168420871,0.547460291202968,0.982717452900852
"8125","CHCHD3",91.6144573453748,-0.135507763627068,0.235853120222087,
-0.574543018550995,0.565600398677621,0.982717452900852
"8126","STMP1",169.118282853089,-0.105050255952522,0.180754240033905,-
0.581177271043915,0.561120984595621,0.982717452900852
"8127","KDM7A",71.7215112601119,0.190394920219915,0.33156739427093,0.5
74226909852118,0.56581426170246,0.982717452900852
"8128","EPHA1-
AS1",3.76586228244604,0.444075564839749,0.788731732855291,0.5630248490
60085,0.573417963251323,0.982717452900852
"8129","ATP6V0E2",13.9339213941402,0.237820061143137,0.402031160692918
,0.591546338679928,0.554154416417445,0.982717452900852
"8130","REPIN1",47.6627200545248,-0.145329252579409,0.258838994920735,
-0.56146583564008,0.574480014165783,0.982717452900852
"8131","AGAP3",47.8387256888491,-0.160098381322261,0.265581266460017,-
0.602822568987047,0.546626731095621,0.982717452900852
"8132","KIF13B",35.4407557222705,-0.262192714722905,0.45491883833501,-
0.576350532509321,0.564378273297301,0.982717452900852
"8133","DUSP4",12.0371445530092,0.425886837744687,0.762206105645257,0.
558755479115647,0.576328610565202,0.982717452900852
"8134","GSR",24.8602388596582,0.207177386502186,0.348198995771413,0.59
4997082180544,0.551845421382912,0.982717452900852
"8135","RAB11FIP1",13.9077178167069,0.363776842139014,0.63710734780099
5,0.570982022722869,0.568011829015276,0.982717452900852
"8136","AP3M2",21.859643257008,-0.219244151011382,0.365081046719676,-0
.600535560477141,0.548149369002717,0.982717452900852
"8137","COPS5",130.92919015663,-0.177294962947232,0.314791282186784,-0
.563214335910462,0.573288942089044,0.982717452900852
"8138","UBR5-
AS1",2.68581802125858,0.517248454714736,0.904223332466534,0.5720361730
81035,0.567297468566326,0.982717452900852

"8139","EBAG9",73.0212844580209,-0.121900869917995,0.213464784181251,-0.571058455311721,0.567960018882095,0.982717452900852
"8140","SNTB1",9.97877542233217,0.287512362873047,0.4855751895905,0.59210678188792,0.553779085826423,0.982717452900852
"8141","AC040970.1",2.51888917185552,0.677065014202192,1.19787262265324,0.565222880461633,0.57192217580965,0.982717452900852
"8142","CYC1",183.243349088664,-0.150870618955338,0.265819414773429,-0.567568095370054,0.570328276180586,0.982717452900852
"8143","LRRC24",2.45142957878603,0.586856758283915,0.997445708745926,0.588359600064611,0.556290948803868,0.982717452900852
"8144","ARHGAP39",4.02025084927139,-0.437747188264029,0.751464257991832,-0.582525627278453,0.560212684733864,0.982717452900852
"8145","KDM4C",18.5582643383002,0.200991170457107,0.360939077383283,0.556856220485303,0.577625668374976,0.982717452900852
"8146","C9orf92",1.95473043299821,-0.627754491722216,1.07903748538558,-0.58177264481029,0.560719832173013,0.982717452900852
"8147","B4GALT1-AS1",9.96395001741042,0.327734441905346,0.583093229156675,0.562061820507411,0.574073899879085,0.982717452900852
"8148","FAM27C",3.48320076067953,-0.477995617394852,0.853501558217842,-0.560040708528913,0.575451671069863,0.982717452900852
"8149","SUSD3",4.23720476794198,-0.400979169308006,0.701669164240163,-0.571464715486288,0.567684671771616,0.982717452900852
"8150","AL691447.2",2.68654218923925,-0.588387042205407,1.02930757720953,-0.571633839323845,0.567570065151302,0.982717452900852
"8151","SLC44A1",186.047287570715,-0.12495841922268,0.218213667745655,-0.572642495374438,0.566886780418409,0.982717452900852
"8152","PTGR1",105.39103645628,-0.18762420159622,0.31918673728107,-0.587819541608966,0.556653425922988,0.982717452900852
"8153","UGCG",222.664125915932,-0.144771288483675,0.259722388812487,-0.557407812031932,0.577248829232934,0.982717452900852
"8154","STXBP1",60.6392964316377,0.137938473274878,0.229058879453628,0.602196577595689,0.547043293534306,0.982717452900852
"8155","MED27",36.694676288826,-0.188138749200859,0.327092997346158,-0.575184277032243,0.565166675213478,0.982717452900852
"8156","MRPS2",63.4341916171614,-0.153517278161359,0.265030574301044,-0.579243653552896,0.562424779344475,0.982717452900852
"8157","EGFL7",13.9044830287756,-0.250072257986047,0.416572647364223,-0.600308876658914,0.548300403972957,0.982717452900852
"8158","DPP7",276.47655934425,0.125284235755936,0.214197216591425,0.584901324814656,0.558614067742467,0.982717452900852
"8159","ARRDC1-AS1",14.8226347575004,-0.213679223867133,0.378761723778722,-0.564152105274415,0.572650619823711,0.982717452900852
"8160","PRKCQ",4.56273547134959,0.367939789688306,0.661088352634449,0.556566740621067,0.577823483038527,0.982717452900852
"8161","GATA3",9.56384232813134,-0.318277220263686,0.551993180864733,-0.576596290130042,0.564212205916026,0.982717452900852
"8162","HACD1",2.843765984937,0.510547811996464,0.892918123488386,0.571774498205831,0.567474756222193,0.982717452900852

"8163","SVIL",117.60953238209,-0.143918082946855,0.242196631975749,-0.594220001214821,0.552364976821481,0.982717452900852
"8164","CCDC7",10.2465296129639,0.283117364683911,0.501433009627943,0.564616527527736,0.572334621161508,0.982717452900852
"8165","ZNF33A",47.5706834772079,0.196158611501858,0.329776084112539,0.594823642320033,0.55196136226117,0.982717452900852
"8166","WDFY4",1.99705132853961,0.570739729606116,1.01150349581724,0.564248894804848,0.57258475619474,0.982717452900852
"8167","CDH23",5.57574924999893,0.439036642861036,0.739222950625192,0.593916412483845,0.552568021095629,0.982717452900852
"8168","FUT11",42.0000523249728,0.155670093307561,0.259180131797778,0.600625102810814,0.548089714370917,0.982717452900852
"8169","MINPP1",31.0905026507446,-0.187273943989285,0.335795996606446,-0.557701538677875,0.577048206820555,0.982717452900852
"8170","ATAD1",78.0370692677297,-0.122360696567179,0.212886661833623,-0.574769201194988,0.565447398871262,0.982717452900852
"8171","PCGF5",69.2950125244432,-0.131403065633923,0.234243480764519,-0.560967866448419,0.574819443125138,0.982717452900852
"8172","FAM160B1",64.4684052357232,0.139480006426329,0.234949121442471,0.593660472403521,0.552739225749,0.982717452900852
"8173","PLEKHA1",67.1493635946769,-0.139252482241476,0.244585385434714,-0.569340976747138,0.569124763639637,0.982717452900852
"8174","IFITM1",258.902954040307,-0.36931482098912,0.630760856965718,-0.585506879367425,0.558206942291575,0.982717452900852
"8175","TRIM21",27.0490500170022,0.176544767933653,0.314682442855848,0.561025160258228,0.574780385321637,0.982717452900852
"8176","TRIM22",161.668666281703,0.112738241941278,0.198943405346409,0.566684991367136,0.570928219596814,0.982717452900852
"8177","TMEM9B-AS1",6.32249115054956,0.378336847022422,0.674618380495096,0.560816096864666,0.574922912141959,0.982717452900852
"8178","NRIP3",3.77887345882125,-0.561947430269211,1.00096330417581,-0.561406624923098,0.574520368785631,0.982717452900852
"8179","BTBD10",30.2278023148347,-0.172465788786026,0.294702410857927,-0.585220148976554,0.558399698417347,0.982717452900852
"8180","ZDHHC13",8.0911510057722,-0.296625520381154,0.506768995517121,-0.585326890565729,0.558327937000654,0.982717452900852
"8181","DNAJC24",30.140878747339,-0.181795584788875,0.323566614730935,-0.561849018138195,0.574218891447281,0.982717452900852
"8182","RCN1",396.176559425307,-0.122681719049216,0.211124508509285,-0.581087055763687,0.561181782186715,0.982717452900852
"8183","CAPRIN1",161.161571104081,0.111310665381059,0.189591840059353,0.587106836170864,0.557131956609322,0.982717452900852
"8184","UBE2L6",237.987768969483,0.166372556933381,0.292478512686087,0.568836853707426,0.569466861866258,0.982717452900852
"8185","TMEM109",198.045711947693,-0.122332722847888,0.202313857541933,-0.604668035764835,0.545399590672207,0.982717452900852
"8186","TMEM179B",204.049295792679,-0.13534830092454,0.241484179865719,-0.560485167184873,0.575148554212173,0.982717452900852
"8187","AP001462.1",2.59835960250892,0.555458628258384,0.9312256501931

74,0.596481237542329,0.550853784371576,0.982717452900852
"8188","AP002748.4",3.89643273629176,-0.56911950300566,0.9735868363697
52,-0.584559570595424,0.558843899055308,0.982717452900852
"8189","NDUFC2",472.366650342382,-0.172986692844028,0.287443277937056,
-0.601811578567889,0.547299566973808,0.982717452900852
"8190","ALG8",37.0480830427801,0.167319439460487,0.286107386792274,0.5
84813420360824,0.558673179511768,0.982717452900852
"8191","AP000873.2",18.4092363551761,-0.219657829096078,0.387748715002
26,-0.566495311518434,0.5710571192334,0.982717452900852
"8192","C11orf54",43.9664255610132,0.161008075315755,0.289115766494032
,0.556898287728207,0.577596924581387,0.982717452900852
"8193","SIK2",58.9580037886064,-0.170791007230948,0.290572785137086,-0
.587773583649179,0.556684277359347,0.982717452900852
"8194","AC005906.2",2.33585454725029,-0.633057720377879,1.057795520967
78,-0.598468898600263,0.54952710525794,0.982717452900852
"8195","PLEKHG6",2.38651455959305,-0.551585947566081,0.97725597643228,
-0.564423202178599,0.572466152063986,0.982717452900852
"8196","ENO2",12.3540109220886,0.262757041477898,0.437303402096367,0.6
00857528704968,0.547934883243711,0.982717452900852
"8197","MGP",15951.4023369033,-0.1732025468171,0.300220407357392,-0.57
6917966175812,0.563994873245805,0.982717452900852
"8198","AEBP2",51.0826643689006,0.155079103900663,0.271154260808768,0.
571922061774398,0.567374776991099,0.982717452900852
"8199","KRAS",110.813885952182,0.134420783599443,0.240712984443288,0.5
58427639083641,0.57655240370932,0.982717452900852
"8200","MED21",87.8762770012462,-0.148194228537619,0.252084390153344,-
0.587875466812809,0.556615884630678,0.982717452900852
"8201","ALG10",6.26785991513106,-0.316081314161803,0.546245704725891,-
0.578643111382294,0.562830008929129,0.982717452900852
"8202","CCNT1",51.8851578312714,0.176242975985299,0.300910960107044,0.
585698094621091,0.558078414740579,0.982717452900852
"8203","SMARCD1",47.4552593553818,0.168554951012553,0.296641873667497,
0.568210242635822,0.569892217315938,0.982717452900852
"8204","TFCP2",36.9629889328343,0.180327404262492,0.298743273957074,0.
603619963970815,0.546096336724377,0.982717452900852
"8205","RNF41",45.4780963282361,-0.145473508883446,0.244684739919454,-
0.594534456588235,0.552154703404664,0.982717452900852
"8206","ARHGAP9",8.23784251774168,0.32279108065006,0.557127346774445,0
.579384735857785,0.562329601268451,0.982717452900852
"8207","PIP4K2C",6.24922593698419,0.40089058020346,0.674804802742145,0
.59408376848297,0.552456086596877,0.982717452900852
"8208","LINC02384",20.5555760505677,-0.279980677350971,0.5017137618555
06,-0.558048629791434,0.576811177544854,0.982717452900852
"8209","LIN7A",2.84918413063275,-0.495202372063906,0.835668490877727,-
0.592582318789811,0.55346071491425,0.982717452900852
"8210","PPFIA2",2.09963622132828,-0.632297118376586,1.133156468175,-0.
557996301600722,0.576846909641707,0.982717452900852
"8211","SOCS2",19.2048440394946,0.22755171275219,0.389657462333983,0.5
8397883974605,0.559234548572085,0.982717452900852
"8212","NDUFA12",228.052142706393,-0.151165799600248,0.270838799882689

,-0.558139379090899,0.576749212220626,0.982717452900852
"8213","NTN4",49.562070161166,0.192487195478465,0.339777062550503,0.56651027009763,0.571046953415198,0.982717452900852
"8214","MTERF2",42.0229904965003,-0.186220644563686,0.313751108641414,-0.593529837615706,0.552826620619711,0.982717452900852
"8215","FBXO21",63.3922355992153,0.148912975750168,0.252495065892728,0.589765884032891,0.555347617870914,0.982717452900852
"8216","PEBP1",885.964067494673,-0.124026981889867,0.212837127267223,-0.582731892139043,0.560073800382211,0.982717452900852
"8217","RAB35",43.2835684999436,0.160610712238244,0.284995475627969,0.563555305165277,0.573056811585816,0.982717452900852
"8218","CDK2AP1",335.804482335132,-0.120283716811144,0.213097077417165,-0.564455028051242,0.572444498015065,0.982717452900852
"8219","KPNA3",76.0204091701394,-0.121362781417811,0.217975260738041,-0.556773190713889,0.577682403106617,0.982717452900852
"8220","ALG11",12.6298696131014,-0.280636909944467,0.464669344408841,-0.60394969739503,0.545877086181745,0.982717452900852
"8221","OBI1",31.1696245576532,0.176388780140271,0.308663047318087,0.571460632145241,0.567687438981058,0.982717452900852
"8222","MIR17HG",10.3285773036484,0.37978841771576,0.660757639603496,0.574777187508057,0.565441996944244,0.982717452900852
"8223","GPC6",59.5627956803353,0.229335101676067,0.407726431771111,0.562472981405363,0.57379380708813,0.982717452900852
"8224","FARP1",75.6539307342546,0.148882050612746,0.265674007933595,0.560393738818284,0.575210901379777,0.982717452900852
"8225","PCCA-DT",10.1080297125612,-0.270716237611723,0.471615361955269,-0.57401912543595,0.565954859189756,0.982717452900852
"8226","TMTC4",12.5347285711456,0.247399766353983,0.426563819057974,0.579983006764009,0.561926077410315,0.982717452900852
"8227","ABHD4",23.1841731987766,0.196775377320671,0.34339390195562,0.573031076556804,0.56662365264016,0.982717452900852
"8228","DHRS4-AS1",40.082367326755,0.158969162825999,0.266841662253581,0.595743413841163,0.551346650991227,0.982717452900852
"8229","PSME2",452.223246522926,-0.210762920929521,0.357508854357293,-0.589532030775621,0.555504431733341,0.982717452900852
"8230","BRMS1L",38.6892731206491,0.156628879114564,0.26607634377656,0.588661422851236,0.556088421162116,0.982717452900852
"8231","KLHDC2",101.037762631904,-0.122459387077995,0.215359880287232,-0.568626741966362,0.569609472803912,0.982717452900852
"8232","AL627171.1",5.2724865763655,-0.392114530886383,0.683477520739324,-0.573705087567809,0.566167384983596,0.982717452900852
"8233","AP5M1",30.3576925091119,-0.167930038692403,0.283945337289355,-0.591416785693767,0.554241196174837,0.982717452900852
"8234","ARID4A",89.9481523170003,0.161132775866054,0.266553466679141,0.60450452163886,0.545508263930115,0.982717452900852
"8235","AC004846.1",5.7760727321446,-0.366390383023682,0.621039066449628,-0.589963502808724,0.555215118450892,0.982717452900852
"8236","ISCA2",55.1815300304637,0.173057020417621,0.299328250042067,0.

578151311789982,0.563161966869173,0.982717452900852
"8237","ZC2HC1C",6.07255180932058,0.401707396005279,0.696503067041169,
0.576748926191785,0.564109076011287,0.982717452900852
"8238","FLVCR2",8.32811472942519,0.297779434560434,0.508138014652008,0
.586020777769135,0.557861552160597,0.982717452900852
"8239","SNW1",158.766626760926,-0.128756279572189,0.229815181855797,-0
.560260112201727,0.575302030279261,0.982717452900852
"8240","SPATA7",33.9663560287609,-0.184843999871185,0.318221099346036,
-0.58086657437565,0.561330381706327,0.982717452900852
"8241","FOXN3",265.953680472834,0.14017160882558,0.237771891409884,0.5
89521360133965,0.555511587610397,0.982717452900852
"8242","TC2N",36.8551374512903,0.242278548515573,0.427653403259963,0.5
66530154252732,0.571033440319587,0.982717452900852
"8243","LBHD2",21.3475094964176,0.575296641119056,1.00278388733629,0.5
7369952627303,0.566171148946755,0.982717452900852
"8244","CDCA4",13.97744902284,-0.25075603034841,0.427900051268636,-0.5
86015424875436,0.557865149294293,0.982717452900852
"8245","FAN1",19.953940609493,0.194723225944826,0.333221956665019,0.58
4364931691992,0.558974815153925,0.982717452900852
"8246","PLCB2",4.93488305060875,0.393052145345387,0.693960224779697,0.
566390019644375,0.571128677801386,0.982717452900852
"8247","INAFM2",5.67955436984668,0.395312932104651,0.661544993946928,0
.597560159508009,0.550133454400212,0.982717452900852
"8248","DNAJC17",38.5832565627205,-0.198107766713749,0.341562744482667
,-0.58000402536232,0.561911903257415,0.982717452900852
"8249","FAM227B",2.48403971396013,0.576493842218436,0.97806118968517,0
.589425128303071,0.555576124015172,0.982717452900852
"8250","MYO5A",29.9663791840643,-0.195806861771408,0.348142970913023,-
0.562432328470956,0.573821497969687,0.982717452900852
"8251","RAB27A",41.8288294153746,-0.146944718842375,0.25907299979446,-
0.567194261690552,0.570582206255581,0.982717452900852
"8252","DAPK2",11.6693220377127,-0.280899571737835,0.487421441129319,-
0.576297117925326,0.564414370590747,0.982717452900852
"8253","ZNF609",41.1214824303298,0.221736448508879,0.37698111806675,0.
588189800184148,0.556404902894673,0.982717452900852
"8254","MAP2K5",22.8784125764809,0.207789959970437,0.344498026393285,0
.603167345095963,0.546397368891409,0.982717452900852
"8255","THAP10",3.3855146430911,-0.444590147544241,0.764488450256906,-
0.581552471322277,0.560868165032312,0.982717452900852
"8256","AC068338.2",1.87759419753277,-0.624329549148137,1.042836740909
34,-0.598683882775099,0.549383706937113,0.982717452900852
"8257","EFL1",13.1393637182904,0.234197944894204,0.395327486723699,0.5
92415029967013,0.553572704188765,0.982717452900852
"8258","CPEB1",12.617690503119,0.251603743495375,0.426995499767759,0.5
89242143376736,0.555698850153946,0.982717452900852
"8259","ZSCAN2",4.58562068944181,-0.404459035577378,0.669612545598148,
-0.604019500883283,0.545830677157298,0.982717452900852
"8260","TM2D3",113.578061239578,0.128484157701523,0.222089962724342,0.
57852302790017 5,0.562911054909131,0.982717452900852
"8261","FAM234A",65.3531651605727,0.127153999086426,0.223553350652908,

0.56878592387482,0.569501428342008,0.982717452900852
"8262","CIAO3",17.3563960445639,-0.209789167592466,0.364035704118935,-0.576287340002028,0.564420978579938,0.982717452900852
"8263","TBC1D24",17.7526671352117,0.241895192401883,0.426653062796061,0.566959934183123,0.570741402895877,0.982717452900852
"8264","EEF2KMT",26.486598258522,-0.222612424075573,0.392979979984064,-0.566472684141824,0.571072496913516,0.982717452900852
"8265","USP7",109.086567380664,0.139167887232003,0.237012377445145,0.587175609696638,0.557085771376529,0.982717452900852
"8266","CIITA",9.37574854611035,-0.32113806581624,0.557826183820207,-0.575695575308001,0.564820966616283,0.982717452900852
"8267","RSL1D1",322.135209346332,-0.133261920038423,0.234128525697911,-0.569182758235822,0.569232120257542,0.982717452900852
"8268","AC130650.2",2.57648172934411,-0.675612710851238,1.1411932365544,-0.592023059031712,0.553835147378163,0.982717452900852
"8269","KNOP1",60.7717781739601,0.172604444858412,0.288481284071856,0.598321119561501,0.549625687242264,0.982717452900852
"8270","CLN3",67.4538124903693,0.129107555449442,0.228896841420527,0.564042538325145,0.572725182608854,0.982717452900852
"8271","SGF29",36.8113811976528,-0.179249202474557,0.303633322349079,-0.590347597845268,0.554957634634408,0.982717452900852
"8272","CCDC189",3.03276899624886,-0.503175611361157,0.896044567076139,-0.561551991775428,0.574421297494512,0.982717452900852
"8273","AKTIP",32.2222498237145,-0.163620884268792,0.277084753858619,-0.590508434658509,0.554849832625471,0.982717452900852
"8274","MT2A",16254.1550974717,-0.225713022811311,0.387696907156199,-0.582189382079267,0.560439124542918,0.982717452900852
"8275","MT1A",1273.77718052136,-0.386844104100173,0.689097546646316,-0.561377857144982,0.574539975733148,0.982717452900852
"8276","COQ9",46.4790968619953,-0.15537421119853,0.278793974721273,-0.557308354148853,0.577316768821006,0.982717452900852
"8277","CYB5B",66.5688330350014,0.151163384311745,0.262230760461878,0.576451763498281,0.564309864978963,0.982717452900852
"8278","SPATA33",19.9111507254559,-0.243983004529875,0.403116177666261,-0.605242404168329,0.545017944381945,0.982717452900852
"8279","YWHAE",535.071509763389,-0.115304572767818,0.199644615532901,-0.577549123776976,0.563568564026851,0.982717452900852
"8280","AC087500.1",5.0921056363724,0.386085235098239,0.650151884527034,0.593838523407657,0.5526201202719,0.982717452900852
"8281","C17orf100",2.32838032221409,-0.604989732941109,1.00887648518687,-0.599666799478483,0.548728318917723,0.982717452900852
"8282","FXR2",21.6054496604757,0.223719578330822,0.384887822798586,0.581259174956793,0.561065790935059,0.982717452900852
"8283","VAMP2",246.1820957778,0.158264599247659,0.263652883039744,0.600276383944573,0.548322054913929,0.982717452900852
"8284","KRBA2",8.92396036257917,-0.307473245785213,0.538338637863939,-0.571152104194543,0.567896541698596,0.982717452900852
"8285","SCO1",64.1555421336399,0.161387795781155,0.270778712830788,0.596013601268604,0.55116614049239,0.982717452900852
"8286","HS3ST3B1",2.86937548809182,0.491674407996743,0.855792252854682

,0.57452542524971,0.56561230038756,0.982717452900852
"8287","FAM106A",13.5789065654249,-0.683440446877418,1.1442082434447,-0.597304250159819,0.550304267384528,0.982717452900852
"8288","AC006449.6",3.87116737028117,-0.432916139545267,0.735603041148047,-0.588518691915166,0.556184191289239,0.982717452900852
"8289","EIF1",5588.6600552644,-0.120709695906414,0.203583412081247,-0.592925006376456,0.55323134193753,0.982717452900852
"8290","ACLY",71.2027661238257,-0.143961199368097,0.249613068960347,-0.576737427922759,0.564116844602259,0.982717452900852
"8291","RAB5C",244.334918188344,-0.130448113229797,0.225958890337366,-0.577309054027627,0.563730698483905,0.982717452900852
"8292","AC067852.3",4.53474521906068,-0.38818274139652,0.661086284550015,-0.587189222448847,0.557076629880345,0.982717452900852
"8293","COASY",39.4115277478504,-0.163466697465371,0.281855626500375,-0.579966060976093,0.561937505138737,0.982717452900852
"8294","VPS25",94.4290409852321,-0.170984761547638,0.295281615951246,-0.579056576200358,0.56255099896376,0.982717452900852
"8295","GPATCH8",99.049129694564,0.133158507227825,0.233247320242072,0.570889762375957,0.568074371068915,0.982717452900852
"8296","CCDC43",55.226958558397,0.130746079075426,0.222805361543333,0.586817472298563,0.557326300909581,0.982717452900852
"8297","COPZ2",346.440612695261,0.150155562982033,0.259552128087069,0.578517941997616,0.562914487578429,0.982717452900852
"8298","HOXB6",2.63044067907181,-0.520219128306352,0.873237524973096,-0.595736112373755,0.551351529458356,0.982717452900852
"8299","UTP18",57.2316956813474,-0.165046344856958,0.29121846747621,-0.566744088337877,0.570888062241117,0.982717452900852
"8300","VMP1",755.104534687384,-0.169400727834126,0.303750422619704,-0.557697093466158,0.577051242762941,0.982717452900852
"8301","PECAM1",5.45785221005848,-0.370473649360645,0.650181014196351,-0.569800780508125,0.568812826110566,0.982717452900852
"8302","ATP5PD",405.54598915535,-0.142061543814971,0.254842108318735,-0.557449256530603,0.577220519645875,0.982717452900852
"8303","TSEN54",16.2771725762372,-0.23785318532504,0.417480272106637,-0.569735149698967,0.568857346003331,0.982717452900852
"8304","TMC8",13.2993378821606,-0.276010309913504,0.468987208301923,-0.588524175132331,0.556180511991467,0.982717452900852
"8305","CYTH1",48.2232069015135,0.183973780269474,0.322486270412922,0.570485621089877,0.568348371849946,0.982717452900852
"8306","LGALS3BP",790.827569735057,0.160518255384346,0.275805858255929,0.581997265755666,0.560568522600129,0.982717452900852
"8307","SLC26A11",22.0234948949428,0.190109293925562,0.338609245525042,0.561441533088624,0.574496577225055,0.982717452900852
"8308","BAIAP2-DT",7.95698928067418,0.335201670184159,0.566262227160056,0.591954847253854,0.553880824631386,0.982717452900852
"8309","NDUFAF8",196.379437645803,-0.150003987213743,0.2570375460377,-0.583587843589753,0.559497640860632,0.982717452900852
"8310","OXLD1",44.6419015959526,0.182197723774372,0.311143564251812,0.585574457284667,0.558161517366187,0.982717452900852

"8311","CYBC1",79.1319378439744,-0.157240200590201,0.277729123969537,-0.566163887829957,0.571282376153744,0.982717452900852
"8312","TBCD",39.8556324575561,0.160089836040704,0.278876226718444,0.574053363832736,0.565931690597728,0.982717452900852
"8313","LAMA1",5.52422309825567,0.374308119594538,0.667503155579886,0.560758576892962,0.574962128723276,0.982717452900852
"8314","AP001094.2",1.94537784716357,0.615844938936945,1.02768349674017,0.599255452569216,0.549002549348718,0.982717452900852
"8315","RNF125",24.9223845250336,0.332409855940617,0.563652412457451,0.589742629666666,0.555363210472035,0.982717452900852
"8316","RNF138",32.3051854396556,0.190263272212877,0.325434848740721,0.58464320262291,0.558787651880894,0.982717452900852
"8317","ZNF24",94.5038042895068,-0.117263081411639,0.200900927931499,-0.583686111453017,0.559431513034074,0.982717452900852
"8318","RPRD1A",28.0786340067048,-0.192209500063814,0.326709396077957,-0.58831947403787,0.556317876681995,0.982717452900852
"8319","PIK3C3",39.32458187228,0.163520242536487,0.28504044400619,0.573673827609307,0.566188542322702,0.982717452900852
"8320","AC120049.1",3.72834459063498,-0.447224668539132,0.772929191905202,-0.578610140777272,0.562852260679386,0.982717452900852
"8321","IER3IP1",173.791507782554,-0.128757968950724,0.225838200766225,-0.570133699763253,0.568587019867427,0.982717452900852
"8322","CFAP53",2.09483225672761,-0.576185419479749,0.963353850071708,-0.598103614198314,0.549770798847363,0.982717452900852
"8323","AC027575.2",2.07143588701448,0.615154135741507,1.0699801399306,0.574921078237404,0.565344673776074,0.982717452900852
"8324","GZMM",27.5525255958343,0.260236852633089,0.44646660219316,0.58288089490846,0.559973482707987,0.982717452900852
"8325","POLR2E",224.694570140107,-0.131976466335292,0.233515542526485,-0.565172086223441,0.57195672097044,0.982717452900852
"8326","AC027307.2",5.96995973912207,0.367018694767001,0.632066513251785,0.580664672264955,0.561466475903335,0.982717452900852
"8327","SIRT6",25.481553523432,0.182146939989951,0.32437777375704,0.561527190597156,0.574438199570504,0.982717452900852
"8328","PSPN",2.61257133728535,-0.509774929621201,0.858054055574267,-0.594105844858487,0.552441321849827,0.982717452900852
"8329","EVI5L",6.28485168173651,0.337929012979328,0.576410823690699,0.586264169738526,0.557698005173401,0.982717452900852
"8330","WDR83OS",443.023842776707,-0.137374396974405,0.234546160802781,-0.585703029647612,0.558075097795774,0.982717452900852
"8331","PRDX2",377.89469867264,-0.152442601789889,0.268166683966681,-0.56846212040579,0.569721219707958,0.982717452900852
"8332","DNAJB1",529.283236730024,0.1683968720195,0.294454532073705,0.571894311945414,0.567393577780111,0.982717452900852
"8333","PIK3R2",31.6164842766299,0.21399324445451,0.361862728785388,0.591365806511189,0.554275345887752,0.982717452900852
"8334","AC011447.3",2.35579177388063,0.549491890567994,0.967241542947657,0.568102036739886,0.569965684865305,0.982717452900852
"8335","FXYD1",515.490486705909,0.165640670415554,0.280532031276586,0.590451898351114,0.554887725317623,0.982717452900852

"8336","LIN37",22.5907142193918,0.205910797995807,0.355237143828969,0.579643208974072,0.56215524824521,0.982717452900852
"8337","THAP8",9.34885599995639,0.287797503305146,0.517315896354214,0.556328358230241,0.57798640442234,0.982717452900852
"8338","ZNF569",9.25109879103275,-0.277306728699694,0.489361144787514,-0.566670917079253,0.570937783503318,0.982717452900852
"8339","PRX",4.67320544324947,0.37565617609228,0.666026872738293,0.564025554326077,0.572736741016584,0.982717452900852
"8340","ZNF526",8.38224231008212,0.320664081559452,0.569946970144446,0.562620907482294,0.573693052092586,0.982717452900852
"8341","SMG9",30.7622708696583,-0.161857040068989,0.284319136090154,-0.569279445255722,0.569166513691285,0.982717452900852
"8342","ZNF235",2.33620750947713,-0.51633322828078,0.878511610127961,-0.587736374031041,0.556709256671533,0.982717452900852
"8343","RELB",78.5801818220072,-0.191858435407353,0.343961848591399,-0.557789871734486,0.576987879630454,0.982717452900852
"8344","TRAPPC6A",53.9474483802144,-0.197982506216186,0.351506670736782,-0.56323968418921,0.573271683548185,0.982717452900852
"8345","DMWD",48.4404007898094,0.182923072921644,0.308056176003028,0.593797778363144,0.552647375159716,0.982717452900852
"8346","AP2S1",319.793370263891,-0.142527323840476,0.245201006543257,-0.58126728698943,0.561060324516575,0.982717452900852
"8347","PNKP",43.545840498579,-0.151354216241656,0.271013468349382,-0.558474887478784,0.576520148052643,0.982717452900852
"8348","IL4I1",4.2507231013039,-0.411806939337697,0.724317303346148,-0.568544942161759,0.569664998092775,0.982717452900852
"8349","ZNF813",7.84435991640083,0.336086653815576,0.57054142127469,0.589066176938915,0.555816881545235,0.982717452900852
"8350","ZNF579",18.3194684348012,-0.223491277851552,0.395370279393045,-0.565270809416039,0.571889580237767,0.982717452900852
"8351","ZNF416",3.24536533107126,0.539148284532415,0.932560756877262,0.578137435610937,0.563171334468183,0.982717452900852
"8352","AC010642.2",66.1489771084174,-0.158199705431474,0.272902881579848,-0.579692323201751,0.56212212119158,0.982717452900852
"8353","MRPS26",133.975629296846,-0.149739241987084,0.267389128518835,-0.56000497408606,0.575476044920574,0.982717452900852
"8354","AL353194.1",5.7305615713715,-0.353910608294475,0.609313138294648,-0.580835347297784,0.56135142959652,0.982717452900852
"8355","SHLD1",9.89827894956454,-0.29543600055827,0.502610037076412,-0.58780362261917,0.556664112197293,0.982717452900852
"8356","PYGB",36.6001367959349,-0.176513703228894,0.301305624516529,-0.585829433194701,0.557990142333008,0.982717452900852
"8357","KIF3B",50.8231278417061,-0.168952766723144,0.279187315403901,-0.605159179523323,0.545073235825342,0.982717452900852
"8358","ASXL1",112.899446204782,-0.133350922624367,0.23872943905556,-0.558586000754319,0.57644429630853,0.982717452900852
"8359","PIGU",23.1489290159335,0.184071199322091,0.328729502755899,0.559947305547366,0.575515380670664,0.982717452900852
"8360","RAB5IF",163.29912933193,-0.133312120263459,0.234661328310923,-0.568104345198379,0.569964117465592,0.982717452900852

"8361","AL391097.1",3.00127772172856,0.602590427116964,1.0209168641248
9,0.590244365914647,0.555026831957926,0.982717452900852
"8362","SS18L1",14.5526409250529,0.24311810426665,0.434119400295064,0.
560025891727959,0.575461777296527,0.982717452900852
"8363","YTHDF1",50.3964340707787,0.141182480068654,0.246993891368356,0
.571603124621817,0.567590878118932,0.982717452900852
"8364","RWDD2B",32.068880354975,0.164267628057859,0.29424177042766l,0.
558274332767597,0.576657069122431,0.982717452900852
"8365","HDHD5",29.0690275653771,-0.244206444009273,0.421273489089592,-
0.579686237880821,0.562126225634921,0.982717452900852
"8366","CASTOR1",11.1932876256312,0.266977956148948,0.458044540505743,
0.582864618043845,0.559984440853619,0.982717452900852
"8367","PRR14L",47.7268157120482,0.149761136948567,0.263297343648571,0
.56879091476311,0.569498040942832,0.982717452900852
"8368","HMGXB4",84.1611668190091,0.129353002644531,0.217779727038186,0
.593962552914074,0.552537159388316,0.982717452900852
"8369","MIEF1",32.2981613130271,0.176418878939156,0.307857200235689,0.
573054256337332,0.566607958301252,0.982717452900852
"8370","L3MBTL2",28.1538699352664,-0.198233227756182,0.336829554015966
,-0.588526824302317,0.55617873437413,0.982717452900852
"8371","XRCC6",296.200365268275,-0.111943394693461,0.195496263751133,-
0.572611427684188,0.566907820435691,0.982717452900852
"8372","MEI1",3.26129073928978,-0.431720874619307,0.750099533133434,-0
.575551450906594,0.564918404419395,0.982717452900852
"8373","KIAA0930",110.991785031037,-0.156749349191697,0.27215135050604
2,-0.575963885169906,0.564639592612247,0.982717452900852
"8374","TTC38",18.1190349860403,-0.229363399097474,0.385529743214803,-
0.594930490148151,0.551889935333235,0.982717452900852
"8375","TRABD",51.4339155601972,-0.139284928714969,0.240814986727971,-
0.578389786314703,0.56300098806232,0.982717452900852
"8376","OFD1",50.6420304441721,0.16989847218668,0.287089539794326,0.59
1796107612965,0.553987129994605,0.982717452900852
"8377","TXLNG",89.5175465013589,0.112181766341978,0.194612383197749,0.
576436938383251,0.564319883017349,0.982717452900852
"8378","TMEM47",71.6163090086586,0.128779467577915,0.213443502803764,0
.603342176671046,0.546281080460805,0.982717452900852
"8379","CXorf38",40.319972344852,0.162453853633051,0.273889955819678,0
.59313549175935,0.553090479693688,0.982717452900852
"8380","RP2",21.6626428029928,-0.192885639648118,0.329075754575708,-0.
586143576261987,0.557779034921498,0.982717452900852
"8381","GPR173",4.48321305629102,0.418188802517684,0.712663398394896,0
.586797081847552,0.557339996911516,0.982717452900852
"8382","EDA2R",7.06114955878842,0.384401851672262,0.667832916877484,0.
575595844346172,0.564888390602565,0.982717452900852
"8383","SNX12",25.3114975482527,-0.195662655495107,0.337680589485389,-
0.57943115946726,0.562298284301773,0.982717452900852
"8384","GCNA",2.96639837445558,0.558992367921086,0.936635438673117,0.5
96808902205303,0.550634973733118,0.982717452900852
"8385","HDAC8",47.692586644529,0.165068158961654,0.282763717450527,0.5
83767112874143,0.559377007242274,0.982717452900852

"8386","ARMCX2",51.1857066444277,0.181083347125267,0.323382855204931,0
.559965824442093,0.575502748781313,0.982717452900852
"8387","TMSB15B.1",7.36491169430016,0.312870626839116,0.55792551595925
3,0.560774902544457,0.574950997917479,0.982717452900852
"8388","RBM41",26.4774819665863,0.212371680202852,0.373368772000142,0.
568798721610203,0.569492742324705,0.982717452900852
"8389","CUL4B",41.9287819448821,0.158985013236843,0.277183622057276,0.
573572897478014,0.566256856360309,0.982717452900852
"8390","C1GALT1C1",92.3764817950831,-0.170964193801419,0.3039944543028
48,-0.562392475854509,0.573848644326574,0.982717452900852
"8391","HCFC1",49.0593077188988,0.162941583010101,0.286934417597021,0.
567870471498965,0.570122923733953,0.982717452900852
"8392","POR",103.839439493812,-0.125609463126524,0.225923320926503,-0.
555982722861033,0.578222665752703,0.982757668312506
"8393","C12orf43",43.9666065651923,0.150969906627789,0.271554240485894
,0.555947520309968,0.578246731236168,0.982757668312506
"8394","PNPO",23.7456133947896,0.183791794015764,0.330527677084447,0.5
56055685372474,0.578172787966525,0.982757668312506
"8395","AL451165.2",15.925112862854,-0.222162355748054,0.3996914369481
69,-0.555834664471092,0.578323885931425,0.982771716104552
"8396","PCOLCE2",865.147824462538,0.180863004763637,0.325503112524873,
0.555641398820161,0.578456024606326,0.982795062827712
"8397","LINC00891",13.8321195369205,0.242948625078081,0.43726227024260
2,0.555613053335902,0.578475406039836,0.982795062827712
"8398","FERMT3",15.2620570519402,-0.225352088731629,0.40569386936529,-
0.555473241644479,0.578571007778655,0.982840437838805
"8399","NAA80",10.5467650066152,-0.245426241673753,0.442097609132902,-
0.555140395703822,0.578798634148265,0.982876004613635
"8400","BTN3A3",47.5752470918311,0.187831984186729,0.338264702240941,0
.555281065220158,0.578702428078337,0.982876004613635
"8401","STK4",99.052902539135,0.138498161745895,0.249473458465037,0.55
516191020098,0.578783919562359,0.982876004613635
"8402","LDLRAP1",17.0957593704204,0.199174974633211,0.359332354966649,
0.55429179109601,0.579379167363079,0.982898210280146
"8403","RFX5",15.5472293361646,-0.211010771600787,0.380295447895134,-0
.554860103555521,0.578990352706203,0.982898210280146
"8404","EMC3",160.52244567512,-0.122123936211083,0.220354846856516,-0.
554214885459742,0.579431792265248,0.982898210280146
"8405","TFAM",108.336819446223,-0.122915616718556,0.221582360845597,-0
.554717515642889,0.579087893635285,0.982898210280146
"8406","TMEM263",133.799658956578,0.111103927914351,0.200269604837792,
0.554771793774394,0.579050762373801,0.982898210280146
"8407","SFSWAP",97.5334899738424,0.127154722244439,0.229385878517849,0
.554326722577848,0.579355265228874,0.982898210280146
"8408","PAPOLA",233.32329768544,0.105439020388817,0.190124693486219,0.
554578253121337,0.579183167192675,0.982898210280146
"8409","DYNLRB1",371.498162805287,-0.156764827663959,0.282645705225872
,-0.554633680135643,0.579145247072146,0.982898210280146
"8410","ARHGAP6",95.6013983751287,-0.129027012225822,0.232747179871082
,-0.554365523557751,0.579328715903525,0.982898210280146

"8411","CYBA",590.349576977143,0.1562154927511,0.28191982600671,0.5541
13185169823,0.579501387069418,0.982899392216421
"8412","INPP5A",17.5728888368191,0.215687178914746,0.389434923198672,0
.553846524967954,0.579683884665559,0.983092046913797
"8413","CEP97",25.1563547609969,0.199038375086896,0.35944560226702,0.5
5373712692981l,0.579758762574829,0.983102164137942
"8414","DPCD",56.388162402851,-0.15945171832604 2,0.288045138248176,-0.
55356503947885,0.579876557674185,0.983185045374366
"8415","ZNF582",6.48580571693738,0.318667503286524,0.575912171217555,0
.553326564730901,0.580039813906491,0.98334497744385
"8416","NAB1",39.0790003325696,-0.165152902071235,0.298641544237058,-0
.553013822953375,0.580253944883728,0.98347425183691
"8417","ALOX5",3.95077583689596,-0.47403518527616,0.857100964126662,-0
.553068080793931,0.580216792460086,0.98347425183691
"8418","SRRM1",344.112716931467,-0.100545353685381,0.18258296336566,-0
.550683107733433,0.581850926419611,0.983709533992603
"8419","PPT1",102.178913540596,-0.110135704384955,0.199751930968352,-0
.551362401610048,0.581385269360855,0.983709533992603
"8420","MIA3",133.694000660633,0.109147628402594,0.197813643983687,0.5
51769969980408,0.581105964266499,0.983709533992603
"8421","PPM1M",34.3977001054889,0.184247162811208,0.334795997892816,0.
550326658534892,0.582095342631764,0.983709533992603
"8422","MRFAP1L1",67.2358040472813,0.131334090297838,0.237623571989228
,0.55269807283173,0.58047017321561,0.983709533992603
"8423","MTHFD2L",13.2500057909729,-0.226605727902274,0.410303416326118
,-0.552288182075877,0.580750926134298,0.983709533992603
"8424","HNRNPD",330.63249364788,-0.113637609574533,0.205736401195468,-
0.552345666173906,0.580711548814597,0.983709533992603
"8425","FAT1",69.5516190088454,-0.175047293512478,0.317880336493301,-0
.550670404604176,0.581859636095368,0.983709533992603
"8426","ETF1",142.219080739,0.115758100615634,0.210276988605536,0.5505
02940827197,0.58197446036569,0.983709533992603
"8427","KDM1B",21.5576090611213,0.216708057668245,0.393689539456126,0.
550454192833325,0.582007887212248,0.983709533992603
"8428","PITRM1",30.8678948304503,0.153080702353781,0.277257342340777,0
.55212497191735,0.580862733929321,0.983709533992603
"8429","EIF3A",354.179263189677,-0.128636468258075,0.233803750711999,-
0.550189925808892,0.582189112602934,0.983709533992603
"8430","ARHGDIB",102.968616345882,-0.143436253596848,0.260415211677009
,-0.550798291210232,0.5817719556882,0.983709533992603
"8431","TBX3",35.1994952014965,-0.183514801202689,0.332503065865604,-0
.551919125091212,0.581003764517584,0.983709533992603
"8432","JKAMP",71.7104993879479,-0.16767001382373,0.304146604330592,-0
.551280242607874,0.581441580224005,0.983709533992603
"8433","NR2F2",171.930206546852,0.165694370563375,0.300480749628415,0.
551430901208408,0.581338322450539,0.983709533992603
"8434","ZNF23",14.3001608135084,-0.249388464491335,0.453102167825704,-
0.55040227613139,0.582043487850814,0.983709533992603
"8435","MPHOSPH6",38.9893514484865,-0.156637741838652,0.28438857838000
9,-0.550787738139568,0.581779190751076,0.983709533992603

"8436","ORMDL3",23.3138067285262,0.177108733825344,0.321875910803094,0
.550239169447164,0.582155340982752,0.983709533992603
"8437","TEX2",16.3626004870085,0.209070536479801,0.380062930049524,0.5
50094523695215,0.582254542621165,0.983709533992603
"8438","LAMA3",2.84462687973762,-0.459478522979005,0.833892539924255,-
0.551004477172491,0.581630605421696,0.983709533992603
"8439","DDA1",49.9843011404008,-0.149288756306863,0.271172355072131,-0
.550530883825353,0.581955300057318,0.983709533992603
"8440","ETFB",286.132675369215,-0.138054492423041,0.250898842870021,-0
.550239653733919,0.582155008860164,0.983709533992603
"8441","RBM39",670.110855844057,0.0850700829491587,0.154379926870019,0
.551043679537329,0.581603732155994,0.983709533992603
"8442","ZNF512B",19.0673214095355,-0.191421591633498,0.347049070175414
,-0.551569239291564,0.581243516417196,0.983709533992603
"8443","FAM227A",3.51094610529623,-0.438191475827508,0.796065962634677
,-0.550446189631396,0.582013375150525,0.983709533992603
"8444","GRAMD4",12.5453848090028,0.273616425433049,0.496514222355812,0
.551074698595382,0.581582468967798,0.983709533992603
"8445","MTMR11",12.9000852770212,-0.229246751044194,0.417181345756586,
-0.549513427136679,0.582653152570667,0.983734577067387
"8446","KLF11",40.9909001542177,0.173033008163119,0.314743127177677,0.
549759448966265,0.582484375487666,0.983734577067387
"8447","SMARCA2",154.948688567652,0.119124268754668,0.216760765336213,
0.549565640118946,0.582617331260794,0.983734577067387
"8448","HDHD3",16.6420820514904,0.216005374501262,0.392784708100279,0.
549933258720742,0.582365151440049,0.983734577067387
"8449","DIP2C",48.3828632763896,0.188839878111085,0.343690222518899,0.
549447920650991,0.582698095493249,0.983734577067387
"8450","SLC9A3R2",44.4432763644018,0.22027557796966,0.400960868731013,
0.549369265551529,0.582752061600763,0.983734577067387
"8451","FAM98C",35.0326388554936,-0.153493351843932,0.279175104840213,
-0.549810313250476,0.582449484173121,0.983734577067387
"8452","RPS6KB1",59.3105635354189,0.123731465887274,0.225267048234732,
0.54926571310307,0.582823113602498,0.983738113896501
"8453","SH3BP4",21.8296056200872,-0.197358181925625,0.359843249822937,
-0.548455979159638,0.583378847869451,0.984002499748895
"8454","EIF1B",421.131860849669,-0.136866656521097,0.249574464688583,-
0.5484000804805,0.583417221201721,0.984002499748895
"8455","CD96",13.0406420253778,0.282649734432971,0.515220065611366,0.5
48600012496749,0.583279977267308,0.984002499748895
"8456","CPQ",392.447492347098,0.113056451326064,0.206231064224257,0.54
8202821681246,0.583552644846452,0.984002499748895
"8457","TNS2",163.798690261576,0.131936429466423,0.240681207236284,0.5
4817919097812,0.583568868962887,0.984002499748895
"8458","ZNF385A",154.748447645045,-0.240196575353003,0.437619125957532
,-0.548871292650751,0.58309378025809,0.984002499748895
"8459","E4F1",20.6008972176827,-0.194125181071558,0.354124293829345,-0
.548183743544881,0.58356574329375,0.984002499748895
"8460","ZNF708",38.9238311901355,0.146866979607872,0.267887586225527,0
.548241080063446,0.583526378259527,0.984002499748895

"8461","ZNF814",5.76735272722465,0.34182873046006,0.623623612800121,0.
548133078100141,0.583600529256652,0.984002499748895
"8462","RDH14",112.837472971855,-0.139590318027321,0.254920357361044,-
0.547584035548872,0.583977553723745,0.984086310542322
"8463","AC137630.3",1.91576703455627,-0.654820653690183,1.195783796524
78,-0.547607900017746,0.583961163768368,0.984086310542322
"8464","UST",56.8399505644996,0.158340793724147,0.288989010333009,0.54
7912855031015,0.583751741591972,0.984086310542322
"8465","ZCWPW1",18.3469153018486,0.225531778006195,0.412138473233953,0
.547223306372008,0.584225326159775,0.984086310542322
"8466","DMRT2",20.1310554052502,0.203108992266628,0.371271727630394,0.
547062911477131,0.584335511595223,0.984086310542322
"8467","RORB",4.81606383777303,0.375182850823285,0.685821269246172,0.5
47056307010822,0.584340048829666,0.984086310542322
"8468","ZNF782",5.16774187938694,0.381322642991443,0.696579150527407,0
.547421843881961,0.584088951534708,0.984086310542322
"8469","DLAT",25.2426578636331,0.172544228919728,0.315172828500316,0.5
47459086942056,0.584063371075776,0.984086310542322
"8470","SLC25A39",65.2884048911738,-0.149521605811505,0.27302200831942
6,-0.54765403980389,0.583929475883548,0.984086310542322
"8471","MT-
ATP8",170.475145819181,-0.210345773062822,0.384357337687347,-0.5472661
82892355,0.584195873194106,0.984086310542322
"8472","ZNF627",4.02878773182785,0.398592823068862,0.728856307204337,0
.546874355245327,0.584465055204773,0.984180651269039
"8473","DDX46",190.67965973436,-0.10239865468355,0.18737488997581,-0.5
46490806194843,0.584728605766801,0.984484239040381
"8474","RNF19A",233.481242983145,0.137806608456385,0.252249506208498,0
.546310716432007,0.5848523710828,0.984484239040381
"8475","C11orf98",3.29118573781045,-0.42125067005747,0.771053292357241
,-0.546331458840718,0.584838115400054,0.984484239040381
"8476","CTSG",8.86698203692282,-0.422108780899995,0.773309909667714,-0
.545846853406253,0.585171213479645,0.984788549192004
"8477","SUMO2",1160.9902610621,-0.126728634366425,0.232135011141598,-0
.545926414732514,0.585116520209908,0.984788549192004
"8478","NPHP4",12.0595856248806,0.240858078583496,0.443638062226459,0.
542915721375926,0.587187830992562,0.984837651926531
"8479","S100A14",2.1571294059509,0.559881870876912,1.02718391951128,0.
545064871287409,0.585708902011225,0.984837651926531
"8480","SEMA4A",49.3693411333552,0.527067288806587,0.970629513341177,0
.543015930962448,0.587118833748628,0.984837651926531
"8481","LAMC2",12.7426736163208,0.232407380562505,0.427717759280109,0.
543366216435971,0.586877681412685,0.984837651926531
"8482","RRP15",87.2740099819158,-0.163962725528534,0.303458800072074,-
0.540312969963604,0.588981215317667,0.984837651926531
"8483","GNPAT",41.5014612302123,0.145090057016645,0.267547133270433,0.
54229718421236,0.587613795075956,0.984837651926531
"8484","TARBP1",32.494550757757,0.184601821721627,0.341664908196759,0.
540300795583366,0.588989609816972,0.984837651926531
"8485","FMN2",2.00533582166488,-0.597397747196228,1.10188704026333,-0.

542158792477911,0.587709120005853,0.984837651926531
"8486","SLC35F6",118.949552028679,0.107220748247124,0.196851065373308,0.54467954259628,0.585973937170926,0.984837651926531
"8487","FBXO11",75.0142018209412,0.133361578666538,0.246855391164721,0.540241710085033,0.589030351334254,0.984837651926531
"8488","DENND6A",43.4657072704417,0.153103872635935,0.282708468645802,0.541560970455945,0.588120985019459,0.984837651926531
"8489","SH3TC1",2.0111181688768,0.568642051254373,1.04643970959089,0.543406415145205,0.586850009741479,0.984837651926531
"8490","FAM114A2",38.3120203271053,0.150566145919498,0.277884055501902,0.541830820942755,0.587935057024186,0.984837651926531
"8491","GTPBP2",23.5164362583995,-0.188636841629341,0.349152665791347,-0.540270374856796,0.589010585809309,0.984837651926531
"8492","DYNLT1",286.50222080819,-0.146028638581739,0.268160275135825,-0.544557311882884,0.586058021009853,0.984837651926531
"8493","PDGFA",9.97081872326394,-0.285122377846502,0.52467256514326,-0.543429172380398,0.586834344562914,0.984837651926531
"8494","FSCN1",41.5679571144223,0.253645722498275,0.468177305558082,0.541772784556316,0.587975042017509,0.984837651926531
"8495","ZNF853",16.1058988455832,-0.219278504116789,0.406027183971878,-0.540058677775566,0.589156566765921,0.984837651926531
"8496","YAE1",29.4110673943428,0.199368433215967,0.365383965832183,0.545640892483867,0.585312809335225,0.984837651926531
"8497","NSUN5",33.058533640075,0.182475546402817,0.336768610298143,0.541842502011309,0.587927009337979,0.984837651926531
"8498","PTPRZ1",2.41315084586875,0.560574917533361,1.03620882992352,0.540986431832217,0.588516934786795,0.984837651926531
"8499","LYN",14.6775357870443,0.286583763894272,0.530597281297616,0.540115402011502,0.589117449529349,0.984837651926531
"8500","ZFHX4-AS1",9.77063396641883,0.317032433546699,0.586103823105174,0.54091514344176,0.588566072570152,0.984837651926531
"8501","FAM49B",36.5751611151433,-0.157344010766567,0.289086438434692,-0.544280152395017,0.586248702772848,0.984837651926531
"8502","DMRT3",5.18471854350147,-0.406138699393585,0.748060471406773,-0.542922283581989,0.587183312606056,0.984837651926531
"8503","TRMT10B",33.8410794501808,0.156196235887642,0.288105081501398,0.542150228915292,0.587715018866301,0.984837651926531
"8504","TRIM14",5.1999035694223,0.40504358474926,0.74964522084741,0.540313702382298,0.588980710300847,0.984837651926531
"8505","DOLPP1",7.72889344547782,-0.277272608610704,0.509210658353599,-0.54451454238447,0.586087443935489,0.984837651926531
"8506","SEC61A2",6.43422133157431,-0.329897788688506,0.609890692972008,-0.540912974226428,0.588567567800303,0.984837651926531
"8507","CDC123",105.202637026544,-0.165623458999265,0.305236747819511,-0.542606551086699,0.587400727473367,0.984837651926531
"8508","ARID5B",1099.0483984261,-0.112538242245993,0.206508910112836,-0.544955867446606,0.5857838709302,0.984837651926531
"8509","KIF1BP",55.7950827081108,0.146832665906325,0.271968107693954,0.539889280222356,0.589273391058802,0.984837651926531

"8510","DNMBP",40.9433642818328,0.18469717734632,0.338861507677139,0.545052102885337,0.585717683428373,0.984837651926531
"8511","GLRX3",111.63888641994,-0.152701846079581,0.282799138595053,-0.53996573977631,0.589220659733673,0.984837651926531
"8512","TRIM3",14.7812377121678,0.21039988906071,0.388878695602941,0.54104246758618,0.588478311696936,0.984837651926531
"8513","AP002387.2",5.36684577903458,-0.334576626947409,0.615425098026446,-0.5436512550761,0.586681481809912,0.984837651926531
"8514","CUL5",97.5397875093626,0.103683927127545,0.191351545412979,0.541850482073567,0.587921511493789,0.984837651926531
"8515","BCL9L",86.28223531322,0.189872258527846,0.349061159528826,0.543951262822084,0.586475011415353,0.984837651926531
"8516","PA2G4",378.955997868988,-0.132134563982707,0.243957972780486,-0.541628389827628,0.588074530273257,0.984837651926531
"8517","SMARCC2",118.496467450108,0.12107389085221,0.222520423732664,0.544102374160803,0.586371026799399,0.984837651926531
"8518","HELB",23.9590945982168,0.259081792434985,0.478196805521123,0.541789048867958,0.587963836362033,0.984837651926531
"8519","CRYL1",75.7598562315739,-0.183987088183235,0.340019303433556,-0.541107773368488,0.588433300643727,0.984837651926531
"8520","N4BP2L2-IT2",4.3010203501878,0.414346346252096,0.762133413283553,0.543666422479679,0.586671042538367,0.984837651926531
"8521","DIO2",6.42514654161482,0.360662811596853,0.667362446513002,0.540430186746845,0.5889003946279,0.984837651926531
"8522","PPP4R3A",77.7365674159729,0.133044166113026,0.245146486090731,0.542712923340802,0.587327474881947,0.984837651926531
"8523","CPSF2",45.6846127875758,0.161151262187128,0.297692667695484,0.541334334616441,0.588277158955959,0.984837651926531
"8524","UBL7-AS1",14.1050849385851,-0.206078085675168,0.381399239295923,-0.540321176454352,0.588975556795406,0.984837651926531
"8525","ELOB",663.322578903512,-0.135964662657332,0.249576067158285,-0.54478245532694,0.585903146723349,0.984837651926531
"8526","CENPT",35.8339823555239,0.162940972756666,0.300827040671152,0.541643372195336,0.588064207030234,0.984837651926531
"8527","TP53",61.1862963328597,-0.198374641483965,0.364111310496646,-0.544818674304246,0.585878233766188,0.984837651926531
"8528","MLX",59.1012087015845,-0.135155708171307,0.248097478134789,-0.54476856914232,0.585912698363675,0.984837651926531
"8529","AC016588.2",3.95254648459578,-0.399394362039418,0.734665875277223,-0.543640824325356,0.586688661034527,0.984837651926531
"8530","THOP1",21.1572775798087,-0.234003983197098,0.43105606015347,-0.542862065583268,0.587224776125054,0.984837651926531
"8531","SUGP2",76.254743911244,0.160183715395855,0.296573853898947,0.540114083861335,0.589118358516832,0.984837651926531
"8532","ZNF226",31.5494769373263,0.178386249798574,0.328061650125213,0.543758314117143,0.586607798105453,0.984837651926531
"8533","BTG3",108.664970647655,-0.124368884247199,0.227990421230026,-0.545500480135172,0.585409350394377,0.984837651926531

"8534","NDUFA6-
DT",8.59557311864159,-0.283476072972428,0.520784071209532,-0.544325544
201167,0.586217471889396,0.984837651926531
"8535","ASB9",1.87960365257389,-0.598393953512308,1.09973856387315,-0.
544123824670508,0.586356266700459,0.984837651926531
"8536","LDOC1",21.0709384284451,-0.183626310049393,0.33904093638499,-0
.541605128888863,0.588090557834151,0.984837651926531
"8537","TIMM17A",74.9652053785929,-0.152348232960663,0.282288008597116
,-0.539690770847072,0.589410305791092,0.984854878293854
"8538","STAT6",124.863577109207,0.144333635469373,0.267469605117659,0.
539626307841151,0.589454769996175,0.984854878293854
"8539","SMIM25",2.7776246130874,-0.475774335896643,0.881759082632235,-
0.539574068776652,0.589490803711708,0.984854878293854
"8540","B3GALT6",36.5483809305984,-0.154767343428341,0.310193128676708
,-0.498938658275839,0.61782259887988,0.985086336678093
"8541","DNAJC11",25.7873753917694,-0.160755112437339,0.310023053521534
,-0.518526317999036,0.604091102663557,0.985086336678093
"8542","MIR34AHG",3.40894033395914,0.403076902454485,0.840431909684467
,0.479606851917148,0.631506973268305,0.985086336678093
"8543","MTHFR",53.2882300142463,0.135386370249803,0.284290036728048,0.
476226222374841,0.63391321989933,0.985086336678093
"8544","SZRD1",119.652638585907,0.107625104345593,0.211361628063509,0.
509198880287078,0.610612827864582,0.985086336678093
"8545","OTUD3",14.0109619713122,0.198597325242585,0.403408851728747,0.
492297886849845,0.622508773766719,0.985086336678093
"8546","MUL1",32.6058465144776,0.17020753906125,0.320667615590996,0.53
0791170625554,0.595563499203397,0.985086336678093
"8547","HP1BP3",341.061464247392,0.0866809529333664,0.185034663618416,
0.468457916145499,0.639457159297842,0.985086336678093
"8548","DHDDS",26.7649789050768,-0.172385129252075,0.320695331019553,-
0.537535512924459,0.590897761159732,0.985086336678093
"8549","GMEB1",32.9331353061824,-0.140790038610186,0.290245816299359,-
0.485071724393006,0.627625468580095,0.985086336678093
"8550","ZMYM4",76.1700451138799,0.118268708530412,0.22146062945228,0.5
34039430949492,0.593314253596504,0.985086336678093
"8551","HIVEP3",40.1743299791531,-0.224975739134818,0.458071518741665,
-0.491136711037684,0.623329758123667,0.985086336678093
"8552","FOXJ3",49.0941598735398,0.143270761403152,0.281641232674465,0.
508699525430467,0.610962855101989,0.985086336678093
"8553","HYI",117.643315412364,-0.109868931903225,0.230774769139261,-0.
476087279008064,0.634012199367654,0.985086336678093
"8554","RNF220",42.4554577883898,0.146015032247773,0.277873156958468,0
.525473686792989,0.599253940687776,0.985086336678093
"8555","PIK3R3",3.29280247426174,0.412362357409944,0.805268394198863,0
.512080643398641,0.608594570987528,0.985086336678093
"8556","MOB3C",31.1287873512653,-0.146265364055138,0.29156555397217,-0
.501655158033855,0.615910112592651,0.985086336678093
"8557","EFCAB14",138.653563996729,0.115792486595811,0.228365104949535,
0.507049825416188,0.612119858843598,0.985086336678093
"8558","CC2D1B",6.3324046897793,0.307204267721194,0.574115670277856,0.

535091243150558,0.592586764925873,0.985086336678093
"8559","TUT4",120.269665306666,0.111375064033501,0.228649852187508,0.487098779937834,0.626188330990972,0.985086336678093
"8560","CPT2",20.3364403589421,0.192535732736768,0.380084529475121,0.506560298580559,0.612463370909536,0.985086336678093
"8561","SSBP3",83.5371390855686,0.127928643576233,0.251002163157478,0.509671478392681,0.610281637974882,0.985086336678093
"8562","GNG12",237.853997064553,0.118654714543302,0.22503392322894 3,0.527274789688425,0.598002778552967,0.985086336678093
"8563","SAMD13",2.3561930901789,-0.472643302037439,0.981511923006768,-0.481546164604441,0.630128377720311,0.985086336678093
"8564","MCOLN2",3.38864889058209,0.439224315727924,0.874673236071436,0.502158174749561,0.615556261249565,0.985086336678093
"8565","BCAR3",6.27151885033511,0.326843928737876,0.625232201426598,0.522756070452087,0.601144013535314,0.985086336678093
"8566","TLCD4",16.2399028862269,-0.281136051007448,0.525510744962007,-0.534976789157324,0.592665907493867,0.985086336678093
"8567","NTNG1",10.4931006604799,-0.304961392682602,0.630867361688953,-0.48340017442995,0.628811624368921,0.985086336678093
"8568","AL359258.2",3.56088492935225,-0.383365996618072,0.759279827236068,-0.504907391012351,0.613623882694114,0.985086336678093
"8569","TAF13",42.7402922392799,0.113911462707117,0.241024668995452,0.472613293825399,0.636489096357873,0.985086336678093
"8570","CEPT1",47.4610004660595,0.130579190465249,0.269584984282723,0.48437115595543,0.628122485094446,0.985086336678093
"8571","ST7L",27.5925180679349,-0.154146460358403,0.299712397686521,-0.514314594752368,0.607032059396177,0.985086336678093
"8572","CAPZA1",176.248529553751,0.0921237266688773,0.191519413800869,0.48101508270416,0.630505779129225,0.985086336678093
"8573","TRIM45",3.18688494921314,-0.394558999603045,0.797072225614111,-0.495010347775013,0.620592816195153,0.985086336678093
"8574","TENT5C",22.7878891831523,0.260554095965181,0.515360625231695,0.505576257107422,0.613154152785813,0.985086336678093
"8575","TBX15",76.4039750279136,-0.128147352764034,0.246231115536928,-0.52043525240341,0.602760245023845,0.985086336678093
"8576","WARS2-
AS1",10.126190341825,0.222081905331473,0.475561252312973,0.4669890666057,0.640507700448856,0.985086336678093
"8577","NBPF20",9.31379125761572,-0.27091751546004,0.56432168970908,-0.480076382674045,0.63117308097767,0.985086336678093
"8578","RNF115",189.767550109027,0.0892828725332207,0.175442640811974,0.508900641942041,0.610821869991812,0.985086336678093
"8579","APH1A",171.48743165353,-0.0968736655684045,0.199940002467905,-0.484513675966143,0.628021360958045,0.985086336678093
"8580","MRPL9",83.9560888817533,-0.1432987533756,0.287008182137508,-0.499284558051187,0.617578932130294,0.985086336678093
"8581","INTS3",32.6737920279752,0.156054594591102,0.321556780921387,0.485309605799461,0.627456742611366,0.985086336678093
"8582","AL358472.4",2.65309832799266,-0.461578854141571,0.861488403789213,-0.535792300989009,0.592102103731157,0.985086336678093

"8583","TPM3",317.910247287974,-0.0855302026892917,0.183235339467497,-0.466777876679534,0.640658805668121,0.985086336678093
"8584","EFNA4",15.6182696552883,-0.203748042935591,0.423528550068263,-0.481072746814239,0.630464796758405,0.985086336678093
"8585","KRTCAP2",608.518663596662,-0.136630606516708,0.262063721649857,-0.521364062360604,0.602113181452356,0.985086336678093
"8586","AL353807.5",18.9157243285901,0.188014113854213,0.390849170570256,0.481040073795979,0.630488017609288,0.985086336678093
"8587","ISG20L2",52.0549312521277,-0.124878357292023,0.245825041701813,-0.507996892535876,0.611455522470994,0.985086336678093
"8588","CADM3",78.3079332114141,-0.234468364450587,0.488562904382769,-0.479914382257092,0.631288274068604,0.985086336678093
"8589","LINC01133",147.599950924814,0.188440555019273,0.359164056993562,0.524664290176037,0.599816585732983,0.985086336678093
"8590","LY9",5.12039034464442,-0.312787769529726,0.666549446077243,-0.469264165427689,0.63888082592461,0.985086336678093
"8591","KLHDC9",3.26514358641634,-0.399851464389472,0.744586284850119,-0.537011589556689,0.591259608580615,0.985086336678093
"8592","RGS5",3.09783635295223,-0.472814676260059,0.891608537712555,-0.530294020594596,0.595908091776204,0.985086336678093
"8593","CACYBP",150.2250801249,-0.107343011480499,0.230159969424534,-0.466384366268761,0.640940399915354,0.985086336678093
"8594","RASAL2",26.8764651411817,-0.213366802025231,0.421660924884583,-0.506015116491132,0.612846037796785,0.985086336678093
"8595","SOAT1",67.9988551684213,0.107564911624451,0.211286283517746,0.509095573236378,0.61068523456383,0.985086336678093
"8596","QSOX1",61.2779583802813,-0.13494028217635,0.280541663310783,-0.480999080792018,0.630517152025047,0.985086336678093
"8597","RNASEL",28.9973575947296,0.179215182917542,0.340266093131493,0.526691276430784,0.598407994549983,0.985086336678093
"8598","TSEN15",29.5796197143734,-0.163561745473562,0.319002689245349,-0.512728422009524,0.60814130531824,0.985086336678093
"8599","PTGS2",65.3753578222754,-0.227705248069601,0.481838437218048,-0.472575931020124,0.636515757616173,0.985086336678093
"8600","LMOD1",70.7950071222257,0.189257366661439,0.361578393450613,0.523420010956183,0.600682001644683,0.985086336678093
"8601","SOX13",21.9119190034868,0.171845577335032,0.344996906713692,0.498107588766422,0.618408212025345,0.985086336678093
"8602","RAB29",52.4976016480767,0.177688723140732,0.378308374967275,0.469692808561541,0.638574506529204,0.985086336678093
"8603","MAPKAPK2",69.8429699085291,-0.123776185789112,0.244657285072483,-0.505916616185951,0.612915187071159,0.985086336678093
"8604","CD55",342.354604982212,0.21170791315768,0.422123263080046,0.5015310258263,0.615997448179601,0.985086336678093
"8605","CD46",155.97001264832,0.104272897626338,0.219414059901485,0.475233436148785,0.634620597449317,0.985086336678093
"8606","TRAF5",29.0403633694637,0.149633019290039,0.308670282415526,0.484766522125397,0.627841972812319,0.985086336678093
"8607","LIN9",5.32107959570378,-0.33938707275765,0.701185060786654,-0.484019257878789,0.628372201956155,0.985086336678093

"8608","PSEN2",12.3995571649554,0.236109740709355,0.444259471931331,0.531468107326815,0.595094436218018,0.985086336678093
"8609","WNT9A",5.50557380366295,0.345738021508958,0.702771880351114,0.491963368449265,0.622745239724006,0.985086336678093
"8610","TBCE",10.7554551744703,0.224878192068181,0.438042255703073,0.513371002775165,0.607691825414387,0.985086336678093
"8611","OPN3",10.6243064513494,-0.214238474052413,0.457034060731423,-0.468758222766925,0.639242464836542,0.985086336678093
"8612","ZBTB18",6.72801526048137,0.326097230315299,0.615637848422501,0.529690029862335,0.596326861723316,0.985086336678093
"8613","HNRNPU",658.291923380273,0.10568903889373,0.219733464132658,0.480987451369371,0.630525417355463,0.985086336678093
"8614","AC010969.2",11.2774234511109,-0.241145220031238,0.508857928995545,-0.473894983826319,0.635574796441628,0.985086336678093
"8615","NOL10",51.1508840378545,-0.150067709944525,0.3198271324818,-0.469215068715487,0.638915915630553,0.985086336678093
"8616","EIF2B4",38.3335680061114,-0.13847116542914,0.27017826511940 1,-0.512517782908794,0.608288677858645,0.985086336678093
"8617","SUPT7L",38.8835633753124,0.137740342838708,0.26429786809234,0.521155709022159,0.602258305386097,0.985086336678093
"8618","TRMT61B",25.8166862690851,-0.175949835413641,0.343822890694053,-0.511745553236618,0.608829100344517,0.985086336678093
"8619","MEMO1",12.2032145418821,-0.230418342437774,0.444438218301667,-0.518448533337823,0.604145360004326,0.985086336678093
"8620","SPAST",26.3444905921409,0.16961833087821,0.347887267564205,0.487566941054853,0.625856616548511,0.985086336678093
"8621","HEATR5B",20.1536783072494,0.204775045353585,0.386991753238779,0.529145759928471,0.59670433973149,0.985086336678093
"8622","GALM",37.8249520673261,-0.144777320284922,0.288877402417052,-0.501172189564026,0.616249944863756,0.985086336678093
"8623","GEMIN6",43.3221810506939,0.184383185185896,0.342387236565021,0.538522367351392,0.590216467394523,0.985086336678093
"8624","CRIPT",80.8858617970487,-0.129184790441816,0.24629802594846,-0.524505992057155,0.59992665324741,0.985086336678093
"8625","PPP1R21",24.2413238996553,0.150953109391981,0.309951483069057,0.487021736102967,0.626242927467246,0.985086336678093
"8626","AC007098.1",5.26062090683347,0.325914236189806,0.661920409768622,0.492376774276731,0.622453015120708,0.985086336678093
"8627","WDPCP",17.6790960616789,-0.196914771190408,0.385162185588645,-0.511251567672621,0.6091749138213,0.985086336678093
"8628","VPS54",28.6240364325283,0.168932704075826,0.325684466745771,0.518700525584768,0.603969595123933,0.985086336678093
"8629","C1D",115.449293909634,-0.13334338506494,0.250654457629824,-0.531980904412508,0.594739221165825,0.985086336678093
"8630","NFU1",76.0478615277473,-0.143628933098658,0.287963729008746,-0.498774389375599,0.617938331722206,0.985086336678093
"8631","MCEE",51.0814610035911,-0.136469891751595,0.292381329364675,-0.466753099618689,0.640676534494008,0.985086336678093
"8632","MOGS",31.6682923052666,0.140833810388113,0.293140138194926,0.480431684501917,0.630920469076745,0.985086336678093

"8633","AC019069.1",3.11302168208282,-0.538916502217982,1.01380829876793,-0.531576337334111,0.595019457120847,0.985086336678093
"8634","CD8A",11.0244472104447,0.283133152257027,0.547312405172152,0.517315430056753,0.604935984511647,0.985086336678093
"8635","RPIA",49.9190730067513,0.136601223375374,0.261929775091368,0.521518499864033,0.602005621579669,0.985086336678093
"8636","CIAO1",123.057030062729,-0.0994417001530868,0.20589630614028,-0.48296981144157,0.629117171421297,0.985086336678093
"8637","LMAN2L",20.8993839476756,-0.184174166387618,0.342803251202606,-0.537259100494254,0.591088652577904,0.985086336678093
"8638","MAP4K4",173.39027761536,0.174760639922285,0.35176937763697,0.496804585709674,0.619326861140448,0.985086336678093
"8639","SH3RF3",11.9703425599888,0.23054267115855,0.489543498324117,0.470933986352144,0.637687876944583,0.985086336678093
"8640","SOWAHC",36.9921481480544,0.15894782696879,0.333077983971531,0.477209045982383,0.63321327023389,0.985086336678093
"8641","RGPD5",17.4015207074831,0.226179465986151,0.468035344444538,0.483252960851877,0.628916135209702,0.985086336678093
"8642","SLC20A1",122.523978985577,0.163430400468747,0.330564318294381,0.494398189471876,0.621024992402815,0.985086336678093
"8643","TSN",82.5852560409657,-0.116329584979671,0.242233028131286,-0.480238330326376,0.631057934360036,0.985086336678093
"8644","SAP130",15.5761551511621,-0.208938660393469,0.428740591583783,-0.487331184625281,0.626023651744584,0.985086336678093
"8645","CCNT2",46.6317285244458,0.14190800718452,0.269255737049107,0.527038007582503,0.598167194921886,0.985086336678093
"8646","SPOPL",33.7121069060308,0.159754431587322,0.30590553893888,0.522234517692865,0.601507055491907,0.985086336678093
"8647","ACVR1",67.0778445668053,0.14458924930894,0.284410372557478,0.50838247567683,0.611185140110973,0.985086336678093
"8648","STK39",20.4465709504794,-0.167968050049934,0.353605613290187,-0.475015225259139,0.634776121293689,0.985086336678093
"8649","NOSTRIN",8.4937738356257,0.292029924785944,0.553631247635208,0.52748092892756,0.597859656640552,0.985086336678093
"8650","PDK1",9.79720360243948,0.246211749137218,0.509028782653465,0.483689248088812,0.628606424919249,0.985086336678093
"8651","MTX2",71.8294323916313,0.1639445995833,0.315194541294998,0.520137813649065,0.602967524486209,0.985086336678093
"8652","TTC30B",4.83989061865341,-0.311354032480983,0.629521711826724,-0.494588235213535,0.62089080844655,0.985086336678093
"8653","FAM171B",35.9968696056169,-0.179054475867777,0.336699153679409,-0.531793661822701,0.594868913067848,0.985086336678093
"8654","ASNSD1",67.6040015974478,-0.137847377557944,0.289054513948481,-0.476890589511822,0.633440033405347,0.985086336678093
"8655","ANKAR",18.9014555908497,0.172320509645734,0.367894145304741,0.468396988223324,0.639500721422403,0.985086336678093
"8656","SF3B1",349.116840063989,0.0918631288515361,0.174867302466124,0.525330508082447,0.599353452717521,0.985086336678093
"8657","SGO2",11.31870270428,-0.240851656055532,0.448884216350138,-0.536556304015072,0.591574134008469,0.985086336678093

"8658","CASP8",31.7971486332208,0.190291703835415,0.369299081528304,0.515278031691586,0.606358747924837,0.985086336678093
"8659","ICA1L",20.6054345413307,0.173904301145785,0.372556940853486,0.466785830770969,0.640653114289882,0.985086336678093
"8660","NRP2",18.5935168484262,-0.322557051787025,0.637708033247081,-0.505806787699741,0.612992293028779,0.985086336678093
"8661","CYP27A1",75.9050040977435,0.14411742560964,0.271934643434191,0.529970818685031,0.596132163386141,0.985086336678093
"8662","PDE6D",20.669103053185,-0.156089999149733,0.333802679771238,-0.467611581958253,0.640062380892347,0.985086336678093
"8663","UBE2F",63.3856307723143,0.133629349312269,0.248613019577094,0.537499401839776,0.590922697994342,0.985086336678093
"8664","THAP4",39.8992231007926,-0.141443385644257,0.290137233354818,-0.487505116143715,0.625900418104155,0.985086336678093
"8665","LMCD1",169.794580157742,0.341908709834678,0.713918294500765,0.478918543576153,0.631996578380643,0.985086336678093
"8666","PRRT3",8.57736271228013,-0.288262323324683,0.574309654229577,-0.501928395599373,0.615717890243298,0.985086336678093
"8667","PRRT3-AS1",2.20481580285573,-0.551389169732901,1.04891366361078,-0.525676410615913,0.599113056434466,0.985086336678093
"8668","AC022007.1",1.97489454928786,0.461819601132734,0.987689345471739,0.46757576483946,0.640087999339485,0.985086336678093
"8669","TATDN2",24.2347659186827,-0.157248634767289,0.319212223205913,-0.4926147037479,0.622284856633789,0.985086336678093
"8670","CCDC174",70.1282761241352,0.103357761141517,0.211770600593315,0.488064730665829,0.62550399195574,0.985086336678093
"8671","COLQ",4.43525229530236,0.408022150900808,0.785698843212298,0.519311125917694,0.603543795981702,0.985086336678093
"8672","UBP1",35.1759190696958,0.141661324383274,0.284566532938578,0.497814422941472,0.618614849198237,0.985086336678093
"8673","PLCD1",17.0723829952874,0.195468659405557,0.394324651152337,0.495704894011414,0.62010263369014,0.985086336678093
"8674","CTNNB1",258.291343087229,-0.102274092087352,0.212243210340534,-0.481872149988958,0.62989677133044,0.985086336678093
"8675","SACM1L",42.4031419521099,0.130937791174171,0.259725035481034,0.504140045381694,0.614162968021108,0.985086336678093
"8676","CCR1",10.7417488383155,-0.354620943405759,0.70033552685176,-0.506358637837348,0.612604905599536,0.985086336678093
"8677","CCDC12",122.858872801116,-0.113844154348859,0.233587464116889,-0.48737270546287,0.625994232527594,0.985086336678093
"8678","CCDC71",15.7426046968165,0.174940865969342,0.372096984006107,0.470148572788406,0.638248873346213,0.985086336678093
"8679","UBA7",44.927506863677,0.133361342596657,0.258460331037735,0.515983795506267,0.605865726981773,0.985086336678093
"8680","BAP1",34.8527728196976,-0.148739263808312,0.300293254009799,-0.495313370587602,0.620378934271777,0.985086336678093
"8681","HESX1",1.85806216821222,0.520254168287512,1.02118664097281,0.509460413418553,0.610429539390289,0.985086336678093
"8682","AC137936.1",2.17720204187676,-0.550502497943017,1.085604066804

22,-0.507093253218528,0.612089388687463,0.985086336678093
"8683","ABHD6",9.34099452414215,-0.287387426395468,0.549018006581342,-0.523457196212905,0.600656130531646,0.985086336678093
"8684","C3orf38",48.3675926102227,0.128612596778691,0.277250769350926,0.463885445944069,0.642729820678515,0.985086336678093
"8685","CRYBG3",117.334229820058,0.138361302737987,0.264114450131542,0.523868734440983,0.600369842299868,0.985086336678093
"8686","AC012020.1",2.0723996862516,0.454937896336512,0.975442896965135,0.466391110901464,0.640935573051652,0.985086336678093
"8687","ABHD10",23.5669124974027,0.193098303701174,0.361138596780048,0.534693066381881,0.592862116816819,0.985086336678093
"8688","IQCB1",28.5548949150912,0.153957912736042,0.29732868601003,0.517803763915494,0.604595191945909,0.985086336678093
"8689","WDR5B",8.18158147568039,0.255676349308213,0.540573773956173,0.472972167031066,0.636233036484177,0.985086336678093
"8690","TMCC1",28.925467331356,0.148075523293112,0.301444057245056,0.491220575540276,0.623270447831651,0.985086336678093
"8691","MRPL3",172.242875823175,-0.14925434001999,0.298769085111637,-0.499564203452375,0.617381968584455,0.985086336678093
"8692","SRPRB",123.309647164792,-0.175226351990816,0.331738065225643,-0.528206951082414,0.597355705384255,0.985086336678093
"8693","RYK",90.9943530809474,-0.116634686645409,0.234220752801335,-0.497969053768426,0.618505854300708,0.985086336678093
"8694","GMPS",71.5922704415871,-0.12762106630817,0.262866827808571,-0.485497038070958,0.627323813230638,0.985086336678093
"8695","RARRES1",396.749561506295,-0.309976059834602,0.609696465947282,-0.508410458559891,0.611165519743815,0.985086336678093
"8696","NAALADL2",38.183007296251,0.138207908235726,0.27330139200058,0.505697783769185,0.613068824335971,0.985086336678093
"8697","TNK2",8.16337338601028,-0.276662152911241,0.586851976572204,-0.471434303633468,0.63733062407478,0.985086336678093
"8698","IQCG",11.6674089036502,-0.241461682857067,0.478764480934297,-0.504343351423776,0.614020118576449,0.985086336678093
"8699","RNF212",29.7721981475,-0.163876589761351,0.323567047283433,-0.506468724603469,0.612527639903776,0.985086336678093
"8700","MAEA",60.7622717929127,0.137749505337861,0.270521528826816,0.509199788775578,0.610612191132549,0.985086336678093
"8701","NOP14-AS1",12.691618067374,-0.224380323382901,0.422021522373688,-0.531679811306445,0.594947776921597,0.985086336678093
"8702","GRPEL1",64.378139293105,-0.145829980606655,0.304629863734937,-0.478712030457866,0.632143506068093,0.985086336678093
"8703","ACOX3",17.1043744038556,0.203990701768387,0.394150842728023,0.517544756105334,0.604775934477624,0.985086336678093
"8704","C1QTNF7",65.3506864434798,-0.225042847023369,0.470221676500258,-0.478588840689579,0.632231158690077,0.985086336678093
"8705","DCAF16",40.3350980678128,-0.147254673618735,0.305732374763612,-0.481645667170807,0.630057679191033,0.985086336678093
"8706","RELL1",38.8789686275095,0.144093044881959,0.305818121803081,0.471172355753142,0.637517658143281,0.985086336678093

"8707","LIAS",29.1943388489777,0.152408313987602,0.303271190963883,0.502547945629799,0.615282135093506,0.985086336678093
"8708","LIMCH1",74.5350184104227,0.123458376750062,0.231033340555679,0.534374720346082,0.593082305395896,0.985086336678093
"8709","SLAIN2",80.7160596530068,0.0991721956695392,0.2085928203313,0.475434367836955,0.634477403145302,0.985086336678093
"8710","KIT",2.37985766051192,0.537971376452145,1.04736976029276,0.513640355915729,0.607503459219644,0.985086336678093
"8711","HOPX",17.6026713081957,-0.206505706871453,0.420229763660599,-0.491411424722971,0.623135485402524,0.985086336678093
"8712","GRSF1",73.154872284355,-0.110296981294367,0.219228272958848,-0.503114766201122,0.614883585575278,0.985086336678093
"8713","THAP6",27.6708735409561,0.154262072687994,0.304040262735033,0.507373830361511,0.611892544120198,0.985086336678093
"8714","TMEM150C",47.3154135874692,-0.228412158082993,0.445262468360816,-0.512983182534712,0.607963084720628,0.985086336678093
"8715","LIN54",12.4786983186094,0.197678499259282,0.419624132541043,0.471084677762061,0.637580266484683,0.985086336678093
"8716","PTPN13",65.6491910631289,0.135072696701938,0.272277657889733,0.496084393221275,0.619834869847241,0.985086336678093
"8717","UBE2D3-AS1",13.5442224789952,-0.19251985457836,0.395037429947488,-0.48734585632544,0.626013256175043,0.985086336678093
"8718","RPL34",6933.94452916371,-0.119872404833179,0.226130185740469,-0.530103508475232,0.596040166663876,0.985086336678093
"8719","ELOVL6",18.6999225512289,0.223545727611971,0.427109502066986,0.523392072829395,0.600701439529988,0.985086336678093
"8720","SEC24D",116.283730559258,0.102074051957458,0.202631591748794,0.503742042770908,0.614442659999231,0.985086336678093
"8721","NUDT6",14.0674275668318,-0.189891662430992,0.385904026000266,-0.492069658871249,0.622670100425836,0.985086336678093
"8722","AC093772.1",9.1798545873932,-0.364034707499197,0.757824684637616,-0.480367972802673,0.630965763542527,0.985086336678093
"8723","MFSD8",23.2038172281009,0.175786623457937,0.351601718149347,0.499959512095642,0.617103586515089,0.985086336678093
"8724","PGRMC2",192.330996932334,0.100564227035384,0.200173965198719,0.502384148385885,0.61539732728084,0.985086336678093
"8725","ELF2",150.348857442457,-0.104120353163139,0.22091794798894,-0.471307805051454,0.637420942764272,0.985086336678093
"8726","AC104596.1",1.88844842354378,-0.507306882044267,0.965338914997236,-0.52552204636412,0.599220331522668,0.985086336678093
"8727","SMARCA5",152.196313251605,0.0896089324534165,0.171538041587575,0.522385190037679,0.601402165465659,0.985086336678093
"8728","TMEM184C",28.901703710472,0.159300449015111,0.3076553179222,0.517788706176029,0.604605698973062,0.985086336678093
"8729","NR3C2",11.5518829917566,0.219562130872649,0.458639222426755,0.478725150698844,0.632134170990833,0.985086336678093
"8730","RPS3A",5979.32039518798,-0.119008329671513,0.235361745793565,-0.505640070225749,0.613109346536411,0.985086336678093
"8731","GUCY1A1",4.54334665106582,-1.0107637712514,1.94203253159406,-0

.520466961705189,0.602738149301311,0.985086336678093
"8732","NAF1",26.7119837670518,-0.16198977341713,0.332565805737977,-0.48709088734384,0.626193923918228,0.985086336678093
"8733","PDCD6",254.652117054778,-0.11664978222625,0.235370368631035,-0.495600966700737,0.620175970631167,0.985086336678093
"8734","SLC9A3-AS1",2.97263421602193,-0.449229854351459,0.94782773383925,-0.473957279696616,0.635530371511809,0.985086336678093
"8735","CCT5",246.846295736941,-0.161960705534134,0.312018766541553,-0.519073603582638,0.603709414978648,0.985086336678093
"8736","C1QTNF3",64.5954191757418,0.269327788488778,0.509433722550862,0.528680722470014,0.597026952256672,0.985086336678093
"8737","RAD1",46.744229943858,0.119273417181467,0.25594134249232,0.466018565113394,0.641202211900996,0.985086336678093
"8738","SPEF2",17.920891741649,-0.203550882989477,0.419927186327816,-0.484728994970513,0.627868596018647,0.985086336678093
"8739","OSMR-AS1",16.0280025621901,0.203202247864291,0.418719837103168,0.485294055495689,0.627467771630338,0.985086336678093
"8740","NDUFAF2",149.087683937583,-0.135427954975718,0.283855894437146,-0.477101084140795,0.633290142719262,0.985086336678093
"8741","DIMT1",81.5616961550369,-0.135898262091314,0.280004113072019,-0.485343806561725,0.627432486088298,0.985086336678093
"8742","CCDC125",10.5660941783298,0.21891155803631,0.455825520984382,0.480252965133582,0.631047529287273,0.985086336678093
"8743","AC146944.4",6.22461791542782,0.329104821287414,0.69605718337622,0.472812908403722,0.636346663857345,0.985086336678093
"8744","GFM2",14.8551428471171,0.186170726702267,0.39319819691371,0.473478078392925,0.63587213713423,0.985086336678093
"8745","ATG10",25.4762009953283,0.167781126878732,0.329334560771418,0.509454964233722,0.610433358056125,0.985086336678093
"8746","LUCAT1",8.46860891167141,0.371860952048689,0.774682149699492,0.480017452568047,0.631214983173133,0.985086336678093
"8747","AC114980.1",7.57348583258413,0.343475084334544,0.686598246573565,0.500256279488973,0.616894634771079,0.985086336678093
"8748","NUDT12",17.7145202692782,0.191277315620322,0.358866007579237,0.533004830718296,0.594030236339307,0.985086336678093
"8749","CSNK1G3",105.857157004054,0.131434577287481,0.263063590341139,0.499630439609819,0.617335320288227,0.985086336678093
"8750","VDAC1",263.13350040046,-0.120241244560678,0.224289994282401,-0.536097229595017,0.59189135471693,0.985086336678093
"8751","HSPA9",222.442960261767,-0.094614588000348,0.17895120577218\5,-0.528717242178282,0.597001614404579,0.985086336678093
"8752","APBB3",7.73752164870377,0.251953621872627,0.522374936046428,0.48232333614535,0.629576272206844,0.985086336678093
"8753","NDUFA2",267.976133897273,0.133256802891351,0.260832776894341,0.510889791068441,0.609428230127371,0.985086336678093
"8754","PCDHB5",6.29913410762388,0.341857109697835,0.682972918531395,0.500542701507015,0.61669299654524,0.985086336678093
"8755","PCDHGA5",13.3176802955577,0.195061291790831,0.407303477340199,

0.478908977317437,0.632003384156501,0.985086336678093
"8756","PCYOX1L",7.15016429590426,0.31525294121549,0.605195156029283,0.520911210334005,0.602428625664636,0.985086336678093
"8757","CD74",371.755988752029,-0.171684896795069,0.360705410885869,-0.475969840245595,0.634095864647884,0.985086336678093
"8758","LSM11",7.41541727905024,0.265314820559093,0.571858795436966,0.463951630500607,0.642682400606801,0.985086336678093
"8759","C1QTNF2",38.3172042174753,-0.162052593887445,0.307404045748077,-0.527164805177124,0.598079146864461,0.985086336678093
"8760","CCNG1",126.551119797909,0.0910433000499078,0.193390989881609,0.47077322529681,0.637802686672893,0.985086336678093
"8761","DOCK2",8.22145416749284,-0.295515298716044,0.630545224804829,-0.468666301941331,0.639308177435351,0.985086336678093
"8762","DUSP1",3606.28250227429,-0.158127420986786,0.313471049438464,-0.50444027054507,0.613952025201302,0.985086336678093
"8763","LMAN2",286.711588934442,-0.129275035505708,0.26169497848007,-0.493991272803705,0.621312342937003,0.985086336678093
"8764","PDLIM7",83.3086852193632,-0.143802290669126,0.269416063894084,-0.533755443497456,0.593510744367336,0.985086336678093
"8765","TRIM41",15.2332187767662,-0.177382312224919,0.381258773874422,-0.465254374141549,0.641749304289457,0.985086336678093
"8766","SERPINB6",161.149699297667,-0.102706809947276,0.20933220661173,-0.490640267972605,0.623680900471052,0.985086336678093
"8767","TUBB2B",6.94737173693446,-0.283738392147026,0.596324796636377,-0.475811828968839,0.634208441825048,0.985086336678093
"8768","FAM50B",30.0529022867917,-0.165971509402281,0.357021294358946,-0.464878459701664,0.642018496811964,0.985086336678093
"8769","PRPF4B",236.520565845641,0.106935446999413,0.22623200994463,0.472680444405659,0.636441180396107,0.985086336678093
"8770","AL359643.3",3.67174557085883,-0.370359635313721,0.787239782589016,-0.470453403784688,0.638031117460068,0.985086336678093
"8771","TDP2",40.9260215555062,0.144938293680994,0.27495498276748,0.527134632084712,0.598100098480512,0.985086336678093
"8772","HIST1H4J",2.44974947106824,-0.583999366102472,1.14168720000102,-0.511523091528004,0.60898482334587,0.985086336678093
"8773","AL121944.1",7.50445617189357,-0.323505430943836,0.648063998588606,-0.49918747476852,0.617647317543273,0.985086336678093
"8774","TRIM39",10.5429840250294,0.263289289309152,0.498057530263318,0.528632283041587,0.597060560921561,0.985086336678093
"8775","ATAT1",15.4261751411238,-0.207818389848204,0.393724692668573,-0.527826660907803,0.597619650806542,0.985086336678093
"8776","IER3-AS1",4.17843787413599,-0.40648714514487,0.813603469479159,-0.499613337938552,0.617347364375119,0.985086336678093
"8777","MICA",38.1655338510095,-0.16294849915911,0.339813143263953,-0.479523827695323,0.631566021204033,0.985086336678093
"8778","BAG6",81.7049276240431,0.106903287439628,0.223878170212049,0.477506526600486,0.633001474457796,0.985086336678093
"8779","FKBPL",10.8024852678332,-0.221574167543241,0.456296355081561,-0.485592674751118,0.627255991125695,0.985086336678093

"8780","TAPBP",298.313952177845,0.0819869118824366,0.171593270179253,0.47779794508718,0.632794023876656,0.985086336678093
"8781","SRSF3",837.482528237848,0.127688065265298,0.250732601200398,0.509259923336587,0.610570045297847,0.985086336678093
"8782","ZFAND3",97.2067757973968,0.0986152752953458,0.201960397168125,0.488290163210819,0.625344328050318,0.985086336678093
"8783","FRS3",8.31414870240034,-0.281525001229929,0.53788513343615,-0.523392419179676,0.600701198555863,0.985086336678093
"8784","TRERF1",32.3532776085932,0.187302362478911,0.386597515944977,0.484489306717556,0.628038651503272,0.985086336678093
"8785","POLR1C",37.9979996114594,0.153221919490247,0.318630751264622,0.480876120343441,0.630604545524805,0.985086336678093
"8786","RAB23",36.1476850648368,-0.144001552536381,0.292162240700555,-0.492882147231243,0.622095862418722,0.985086336678093
"8787","AC019205.1",9.17510438132325,-0.248897645127345,0.473513356200961,-0.525640178609263,0.599138235003958,0.985086336678093
"8788","HMGN3",244.057626920392,-0.128897997442134,0.245862965091927,-0.524267643945235,0.600092398193651,0.985086336678093
"8789","RNGTT",25.1421398572645,0.18643004869524,0.358240695221206,0.520404440874936,0.602781715507958,0.985086336678093
"8790","MTRES1",39.412513989633,-0.163613484832006,0.312817656827842,-0.52303148898673,0.600952340385349,0.985086336678093
"8791","SNX3",564.357775364419,-0.108071585402399,0.21743707789726,-0.497024640174128,0.619171675565118,0.985086336678093
"8792","RAB32",188.453452549435,0.172354180328,0.34791718611837,0.495388521190674,0.62032589585433,0.985086336678093
"8793","PPIL4",126.016737103394,-0.0948041839583046,0.201098367097092,-0.471431893390423,0.637332344913251,0.985086336678093
"8794","RAET1G",3.70208666111667,0.506731818619973,1.06689717094564,0.474958442499976,0.634816594296107,0.985086336678093
"8795","SCAF8",48.4876234021728,0.128949277402129,0.247094175192178,0.521862877997177,0.601765806449818,0.985086336678093
"8796","ARID1B",219.278765106577,0.112617870197833,0.230413092553532,0.488765065169498,0.625008033502877,0.985086336678093
"8797","TAGAP",22.9870108813087,0.209114390425406,0.413749093536637,0.505413531273124,0.613268416937605,0.985086336678093
"8798","FGFR1OP",14.3199894857006,0.226625000293855,0.423089091141749,0.535643686019614,0.592204830283449,0.985086336678093
"8799","AFDN-DT",2.79155221780513,-0.453555587461372,0.843080003387031,-0.537974552402186,0.590594617220529,0.985086336678093
"8800","MRM2",31.9929650764284,-0.150616186069348,0.299621718484357,-0.502687811922458,0.615183780111982,0.985086336678093
"8801","EIF3B",179.781595479114,-0.112408217688228,0.234890445881786,-0.478555938136369,0.632254570559669,0.985086336678093
"8802","AC092428.1",3.18082597551192,-0.375812567395309,0.741627608669143,-0.506740260209174,0.612337077853572,0.985086336678093
"8803","PMS2",19.4399503827685,0.196321245734277,0.412385597514779,0.476062323508379,0.634029977679261,0.985086336678093
"8804","AIMP2",40.4057958436046,-0.181745614621799,0.360264718637789,-

0.504477971945197,0.613925537874699,0.985086336678093
"8805","HNRNPA2B1",981.750714636524,0.0673053530495521,0.1333889701675
9,0.504579598785339,0.613854141886913,0.985086336678093
"8806","HOXA7",21.9121866263208,0.273758323872811,0.551789631740266,0.
496128067882349,0.61980405748296,0.985086336678093
"8807","LSM5",157.800246391151,-0.13928393488916,0.274648559396439,-0.
507135137337866,0.612060002257984,0.985086336678093
"8808","AC018647.2",11.3347091986157,-0.219250232464142,0.467137964063
24,-0.469347921451445,0.638820966872961,0.985086336678093
"8809","EPDR1",53.1392047961194,0.165546372824498,0.337714456422651,0.
490196287651117,0.623995007516847,0.985086336678093
"8810","TRGC2",7.50800061004773,0.313712039127723,0.597232370819913,0.
525276348797107,0.599391096441045,0.985086336678093
"8811","DBNL",101.716661521155,-0.116651108770451,0.228687193778335,-0
.510090254041598,0.609988232734775,0.985086336678093
"8812","PSPH",7.87814063323141,-0.256503134106742,0.528161585013417,-0
.485652764958713,0.627213378915892,0.985086336678093
"8813","ZNF92",27.7514076881367,-0.175073152817448,0.334785274945047,-
0.522941616372422,0.60101488276811,0.985086336678093
"8814","BUD23",101.079643680397,-0.123925286494996,0.255512353933719,-
0.485007024463257,0.627671362707497,0.985086336678093
"8815","GTF2IRD1",3.43791077852354,0.372983281494604,0.733020721451217
,0.508830474473055,0.61087105659866,0.985086336678093
"8816","STYXL1",15.1667796985878,0.182923807062121,0.386820040139953,0
.472891236441468,0.636290777531845,0.985086336678093
"8817","STEAP2",93.1873685265558,-0.184469238894406,0.352629538169138,
-0.523124749708078,0.600887443325896,0.985086336678093
"8818","CLDN12",19.0632635036791,0.195104036517012,0.380676066464145,0
.512519839582266,0.608287238840929,0.985086336678093
"8819","VPS50",28.1031918532884,0.176292806199654,0.34362438821198,0.5
13039272669144,0.607923849362721,0.985086336678093
"8820","ASNS",2.65347824327954,-0.419379560071,0.860639597729575,-0.48
728824606415,0.626054076102992,0.985086336678093
"8821","TRRAP",39.3315897216051,0.144620294363364,0.303751952517262,0.
476113134960491,0.633993779795815,0.985086336678093
"8822","ZNF3",19.139591337796,0.211536598417376,0.410264288643871,0.51
5610557079219,0.606126435425634,0.985086336678093
"8823","PILRB",30.0374783374006,0.154456008506974,0.32295400077501,0.4
78260086997893,0.632465100514991,0.985086336678093
"8824","PPP1R35",39.1143795918303,-0.141832194359828,0.29816828358029,
-0.475678340622823,0.634303553918985,0.985086336678093
"8825","GNB2",305.296014585411,0.101143044918978,0.207759235597621,0.4
86828152924416,0.62638011762535,0.985086336678093
"8826","GIGYF1",53.3660336713542,0.114654851483168,0.24642695013282,0.
46526912507488,0.641738742098255,0.985086336678093
"8827","AP1S1",71.1613982618865,-0.152177087989615,0.304814952631889,-
0.499244169866536,0.617607381143586,0.985086336678093
"8828","PLOD3",58.7670827170614,0.128485099652475,0.252405179864375,0.
509043038346177,0.610722057112471,0.985086336678093
"8829","LRWD1",13.3935541632795,-0.24638818419878,0.465451035312557,-0

.529353606514866,0.596560175048987,0.985086336678093
"8830","NAPEPLD",16.6628060657947,0.192424591187662,0.382441119607057,
0.50314827910077,0.61486002514995,0.985086336678093
"8831","CTTNBP2",4.03137251422862,-0.36227849080452,0.747141430666269,
-0.484886095101774,0.627757146376438,0.985086336678093
"8832","CADPS2",28.2094124167446,-0.160600643132926,0.331051774103862,
-0.48512243611339,0.627589497829894,0.985086336678093
"8833","WASL",148.196384946562,0.101435966162381,0.194119898287823,0.5
22542856539013,0.601292415321004,0.985086336678093
"8834","MKLN1-
AS",9.29802254863192,-0.277141528072076,0.550753484632514,-0.503204311
556914,0.614820633788016,0.985086336678093
"8835","SSBP1",327.14367513341,-0.129345061351337,0.273836862082334,-0
.472343498124248,0.636681627080269,0.985086336678093
"8836","TPK1",15.5430552974321,-0.213975198067025,0.413389953968224,-0
.517611025650305,0.604729687521399,0.985086336678093
"8837","LINC01006",6.16469594703402,-0.291940275596042,0.5839754514148
37,-0.499918746393772,0.617132291768975,0.985086336678093
"8838","UBE3C",46.6164967188587,0.140732262718336,0.273515685491195,0.
514530866723789,0.606880885619266,0.985086336678093
"8839","AC100810.1",108.445145295746,0.141532598338286,0.2693690408141
29,0.525422661455541,0.59928940344585,0.985086336678093
"8840","FAM66D",3.27194420854556,0.459598778245032,0.885953197951026,0
.518761915762549,0.6039267788844,0.985086336678093
"8841","VPS37A",61.5293866138107,-0.121717349670337,0.229505978713923,
-0.530345006053445,0.595872747738069,0.985086336678093
"8842","DOCK5",4.82327319098452,-0.525763165611039,1.01101412189178,-0
.520035431975219,0.603038879765287,0.985086336678093
"8843","INTS9",10.6796136100233,0.233659385801137,0.486376389183713,0.
480408570393987,0.630936901395673,0.985086336678093
"8844","LEPROTL1",73.7776130143615,-0.167829851242838,0.31553849096580
1,-0.53188392556846,0.594806391080711,0.985086336678093
"8845","LSM1",106.043655439069,-0.125848891266616,0.261643056179906,-0
.480994577513579,0.630520352615617,0.985086336678093
"8846","SPIDR",33.8930103624837,-0.144413674684008,0.29099394224132,-0
.496277254336264,0.619698811848025,0.985086336678093
"8847","IMPAD1",123.469699007994,0.0966144986527391,0.189206260501452,
0.510630559457613,0.609609773134473,0.985086336678093
"8848","UBXN2B",17.1738926213257,-0.184173282402927,0.376603081647226,
-0.489038171428048,0.624814672834368,0.985086336678093
"8849","SDCBP",895.723992061595,0.13330542815337,0.262454065187807,0.5
07919083127857,0.611510091164431,0.985086336678093
"8850","NSMAF",39.0299837195865,0.130703095464067,0.276323323226751,0.
473007829877653,0.636207593034926,0.985086336678093
"8851","TPD52",4.49217221227453,-0.305612429015283,0.657318505469578,-
0.464938118236848,0.641975772162918,0.985086336678093
"8852","NBN",118.801985391108,-0.106614570622317,0.207379126975117,-0.
514104636167696,0.607178836212552,0.985086336678093
"8853","LINC00535",5.05635328148024,-0.429946380088733,0.8073089433978
53,-0.53256734439129,0.59433311237142,0.985086336678093

"8854","COX6C",677.51424863798,-0.136603282870531,0.266499837898651,-0
.512582986720168,0.608243056653999,0.985086336678093
"8855","POLR2K",169.405948674611,-0.152991636779736,0.307251309349973,
-0.4979364843177,0.618528810887969,0.985086336678093
"8856","AP001330.4",2.27600634073875,-0.506780671121393,0.992174420148
926,-0.510777803609697,0.609506653339157,0.985086336678093
"8857","OXR1",105.901837374921,-0.0980658639685757,0.20469130481627,-0
.479091498569513,0.631873537440343,0.985086336678093
"8858","MED30",70.098234668796,-0.119345094783107,0.251321691133113,-0
.474869854030607,0.634879739609337,0.985086336678093
"8859","COL14A1",649.965174762611,0.160551483846221,0.3180511533969,0.
504797678396929,0.613700946595517,0.985086336678093
"8860","TBC1D31",7.62559629040796,0.271025584398737,0.545625058605711,
0.496724958144912,0.619383019825871,0.985086336678093
"8861","FBXO32",32.6096585013116,-0.294013955853248,0.564015683977102,
-0.521286843975751,0.602166964381118,0.985086336678093
"8862","RNF139",86.1990467645845,0.113445416537782,0.225722807003084,0
.502587301850417,0.61525445881746,0.985086336678093
"8863","PTP4A3",6.7395314908433,-0.252693261450518,0.537417691100671,-
0.470199000953957,0.638212847853757,0.985086336678093
"8864","NAPRT",45.8004669406464,0.166461800309951,0.324932267495013,0.
512296921426883,0.608443219521876,0.985086336678093
"8865","TIGD5",17.4469214537566,0.207844588417299,0.410538003842124,0.
506273685924648,0.612664533046208,0.985086336678093
"8866","LRRC14",17.0227252933111,-0.185319858949466,0.387337668153533,
-0.478445227991638,0.632333349264741,0.985086336678093
"8867","ZNF7",30.6096645348292,-0.143565218721462,0.302262827009934,-0
.474968159802012,0.634809668024988,0.985086336678093
"8868","LINC01230",2.39951195576901,0.59848986266591,1.11193572433655,
0.538241419505615,0.590410388259494,0.985086336678093
"8869","RANBP6",20.8392068943598,0.162212817324444,0.338714728108967,0
.478906890851993,0.632004868546187,0.985086336678093
"8870","CCDC171",15.6841648025925,0.179935466311471,0.386379383578543,
0.465696343953336,0.641432869695311,0.985086336678093
"8871","HACD4",38.3696933033283,-0.15250833966737,0.327499614305711,-0
.465674868016816,0.64144824419361,0.985086336678093
"8872","KLHL9",45.0189097590595,0.126300815076612,0.265901889861071,0.
474990287367275,0.634793896120753,0.985086336678093
"8873","PLAA",31.4916734703814,0.15092878295641,0.313470512639509,0.48
1476811600388,0.630177656396397,0.985086336678093
"8874","IFT74",47.0290621950604,-0.11773784759608,0.253319358849885,-0
.464780299976405,0.642088796789013,0.985086336678093
"8875","SMIM27",25.6651653059719,-0.166825800235564,0.330697043047664,
-0.50446716637717,0.613933129334615,0.985086336678093
"8876","PIGO",15.7335899438588,-0.216434328391103,0.419115192453188,-0
.516407737749275,0.605569662630468,0.985086336678093
"8877","SPAAR",2.01694836710347,-0.494153229118818,0.94318820995173,-0
.523917945437536,0.600335612601617,0.985086336678093
"8878","BX664615.2",4.84463015994943,0.41989379214474,0.79275979033351
,0.529660809320428,0.596347124845123,0.985086336678093

"8879","KLF9",705.369111397125,-0.137934913365908,0.28164058691981 5,-0.489755098419742,0.624307207648823,0.985086336678093
"8880","GNA14",5.99632817526056,0.374095530223116,0.727424341516005,0.514274143539758,0.607060336598808,0.985086336678093
"8881","CENPP",6.87789178276837,-0.286800114768831,0.557494519873721,-0.514444724647328,0.606941096783419,0.985086336678093
"8882","TDRD7",8.41196137250559,-0.262754647021734,0.489479743457293,-0.53680392403135,0.591403060856918,0.985086336678093
"8883","RAB14",230.67715966102,-0.0850632125721218,0.182895143280513,-0.465092790581418,0.641865008592477,0.985086336678093
"8884","MRRF",24.3740447683923,-0.158982041675996,0.307767328081632,-0.516565688330074,0.605459372814846,0.985086336678093
"8885","RPL35",3471.11356673061,-0.104509754594541,0.219754548235729,-0.475574933186068,0.634377237145346,0.985086336678093
"8886","ZBTB43",58.062222455166,0.144824903688626,0.274751234561689,0.527112840528871,0.598115230325741,0.985086336678093
"8887","ANGPTL2",708.706475718899,0.130802650538243,0.25703174898508,0.508896862176492,0.610824519520209,0.985086336678093
"8888","ENG",158.21253496357,-0.11970960461886,0.237194535085897,-0.504689556087406,0.613776897616314,0.985086336678093
"8889","URM1",79.1861252623626,-0.1455468732939,0.29534846512867,-0.492797120955047,0.622155945214168,0.985086336678093
"8890","SPTAN1",282.859246810435,0.0850559271024143,0.168431128688298,0.504989355381098,0.613566312392215,0.985086336678093
"8891","LINC01503",20.3916595253856,0.19802939347697,0.389739674236675,0.508106837890755,0.611378420120482,0.985086336678093
"8892","TOR1B",24.5854172164986,0.170183898577136,0.325438154042472,0.522937757798755,0.601017568016645,0.985086336678093
"8893","C9orf78",140.22951536422,-0.0920530588214436,0.187016569566853,-0.49221873246123,0.622564723281203,0.985086336678093
"8894","SURF4",222.333540844942,-0.0750582669481011,0.15843050841465,-0.473761447205963,0.635670029564239,0.985086336678093
"8895","EXD3",11.5761574523569,0.210344987720817,0.430710314479958,0.48836765837565,0.625289445709838,0.985086336678093
"8896","IDI1",79.9223995758548,-0.134222283090103,0.257879780815476,-0.520483935055554,0.60272632205606,0.985086336678093
"8897","ITIH5",487.660920572934,-0.166552709040178,0.322639515967299,-0.516219188281509,0.605701330057501,0.985086336678093
"8898","BEND7",18.8718673404477,-0.210917431721645,0.414703550838256,-0.50859808481338,0.611033971675958,0.985086336678093
"8899","RPP38",30.8981415585207,-0.163545314790073,0.319181471728947,-0.512389750896814,0.608378262558199,0.985086336678093
"8900","COMMD3",133.459928591683,0.120711589905402,0.253004154691732,0.477113073706164,0.633281605547446,0.985086336678093
"8901","KIAA1217",55.3558251059692,-0.213692965486768,0.429830011981026,-0.49715692141153,0.619078397100072,0.985086336678093
"8902","PRTFDC1",40.2906163870021,-0.140684743792747,0.281221383190135,-0.50026332349567,0.61688967551446,0.985086336678093
"8903","MAP3K8",109.547002602585,0.163738355319858,0.342363349638746,0.478259006089964,0.632465869752052,0.985086336678093

"8904","C10orf25",2.43730798752478,0.446599382604377,0.891470574698814
,0.500969291953649,0.616392734742731,0.985086336678093
"8905","ZFAND4",9.89542009902575,0.232318248022807,0.470176164089516,0
.494108944192621,0.621229241516804,0.985086336678093
"8906","SGMS1-
AS1",7.84347672338854,0.281739034354646,0.523595677722464,0.5380851033
38046,0.590518296308899,0.985086336678093
"8907","CSTF2T",63.0149703138919,-0.146186783711696,0.298852963620392,
-0.489159558402055,0.624728738506784,0.985086336678093
"8908","PHYHIPL",3.92758125840829,0.607411788469539,1.22552525195755,0
.495633841489035,0.620152771927689,0.985086336678093
"8909","JMJD1C-
AS1",3.13933450968634,-0.399458477471324,0.805086134375655,-0.49616862
1486822,0.619775447614094,0.985086336678093
"8910","PBLD",9.73337525333885,0.297158189052099,0.61615166325023,0.48
2280916819366,0.629606401715231,0.985086336678093
"8911","LRRC20",3.53579697335514,0.370104674598999,0.759228116762212,0
.487474932010341,0.625921803361742,0.985086336678093
"8912","NDST2",32.1604566636023,0.161583474919245,0.31912832160826,0.5
06327592941103,0.612626695604893,0.985086336678093
"8913","ADK",95.6104014876296,0.127063435125851,0.24806669572933,0.512
214808812917,0.608500679993283,0.985086336678093
"8914","ZNF503",240.202383877348,0.0917900119460425,0.171482593444298,
0.535273056596605,0.592461054655303,0.985086336678093
"8915","PLAC9",2599.12699163061,-0.130280602475587,0.248946870999447,-
0.523326932981924,0.600746761527951,0.985086336678093
"8916","IDE",22.4339498313208,0.167290371227378,0.328416565019722,0.50
938469323961,0.610482603333807,0.985086336678093
"8917","ARHGAP19",13.7174443442308,0.206074651994072,0.436542125745122
,0.472061319723333,0.636883020556075,0.985086336678093
"8918","RRP12",20.8738593563918,-0.189734005527152,0.358460579710294,-
0.529302289474881,0.596595767638965,0.985086336678093
"8919","ANKRD2",2.72998140514786,-0.497276985797524,0.926991936737915,
-0.536441543976576,0.591653425943844,0.985086336678093
"8920","SCD",2.405272953026,0.516806457839221,1.01376024998748,0.50979
1598009099,0.610197472603014,0.985086336678093
"8921","LDB1",22.4991144939868,0.194380051885194,0.374852584767536,0.5
18550651066574,0.604074130003854,0.985086336678093
"8922","GBF1",49.1589839297809,0.137341513286716,0.260325051172298,0.5
2757701446034,0.597792950033762,0.985086336678093
"8923","ITPRIP",15.7607743218933,-0.256695081395995,0.483660243740864,
-0.530734301026253,0.595602912964497,0.985086336678093
"8924","ACSL5",55.5818286342514,-0.139495931030228,0.270111586623287,-
0.516438160887842,0.60554841881594,0.985086336678093
"8925","TIAL1",134.831146313448,0.0822419814869456,0.173649077788092,0
.473610240460404,0.635777871606356,0.985086336678093
"8926","ATE1",42.9828248166483,-0.140228736320716,0.286081436542042,-0
.490170694106215,0.624013116513862,0.985086336678093
"8927","ADAM12",47.5694569711685,0.386745370214434,0.792249376183354,0
.48816154607117,0.62543569699046,0.985086336678093

"8928","DOCK1",94.0220277624432,-0.125088031096546,0.249408506627786,-
0.501538751776523,0.61599201228098,0.985086336678093
"8929","IFITM2",1098.49948594038,-0.112694698736087,0.224164275409508,
-0.502732643416146,0.615152255747031,0.985086336678093
"8930","AP006621.3",12.485804361353,-0.218011752252199,0.4589120624055
47,-0.475062152669108,0.63474267370462,0.985086336678093
"8931","PIDD1",6.05934919767623,0.321218801807936,0.646499375717531,0.
496858641899576,0.619288738365152,0.985086336678093
"8932","PNPLA2",63.5113794809906,-0.109680477403766,0.222612405882349,
-0.492697057780921,0.622226656877439,0.985086336678093
"8933","IFITM10",2.19533386381219,-0.438653524155793,0.945195822687073
,-0.464087455347354,0.642585089051391,0.985086336678093
"8934","ARFIP2",44.5630802104813,-0.14390327564242,0.310066984935345,-
0.464103831217072,0.642573356992663,0.985086336678093
"8935","NUCB2",303.580929460823,-0.115184887142097,0.218160598982683,-
0.527982081453854,0.59751177271827,0.985086336678093
"8936","NCR3LG1",2.43300395120384,0.510949812620981,0.954171758030253,
0.535490396064291,0.592310796875982,0.985086336678093
"8937","APIP",41.5472285236658,-0.15144812528485,0.315947840286088,-0.
479345341141485,0.631692971184681,0.985086336678093
"8938","ATG13",34.7700850606195,0.133994232921333,0.278973945054642,0.
480310922566938,0.631006323360164,0.985086336678093
"8939","ARFGAP2",77.8658017329404,-0.128349794223627,0.2568213879222,-
0.499762871239171,0.617242056978459,0.985086336678093
"8940","NDUFS3",136.786983883328,-0.157834472941933,0.301165339833386,
-0.524079142139172,0.600223495174531,0.985086336678093
"8941","MTCH2",58.3404454721364,-0.151149451553815,0.302140520501881,-
0.500262100901737,0.616890536267223,0.985086336678093
"8942","NUP160",41.1942529213561,-0.120465378714902,0.255830562318907,
-0.470879544738425,0.637726756201836,0.985086336678093
"8943","FAM111A-
DT",10.0516794711873,-0.256534885509331,0.497027087163868,-0.516138641
403163,0.6057575812739,0.985086336678093
"8944","OSBP",62.8042829677422,-0.118539583972025,0.223685421251841,-0
.529938801145938,0.596154362812444,0.985086336678093
"8945","UQCC3",84.5761921226291,-0.148419334302856,0.296413440333299,-
0.500717289121463,0.616570102796035,0.985086336678093
"8946","NEAT1",4394.68436106251,0.196372689847799,0.378906662451402,0.
518261380196728,0.604275914395917,0.985086336678093
"8947","MRPL11",163.803881336815,-0.138644727866548,0.268411021240934,
-0.516538878416978,0.60547809234229,0.985086336678093
"8948","KDM2A",96.0834098386583,0.13563026031689,0.265966098759864,0.5
09953189332404,0.610084256979873,0.985086336678093
"8949","GSTP1",1134.73162726083,-0.13748917737016,0.267885711174944,-0
.51323818940224,0.607784715114997,0.985086336678093
"8950","KMT5B",78.7487938995634,0.120688380042631,0.250235289852203,0.
482299599364714,0.62959313184081,0.985086336678093
"8951","PGM2L1",17.0218145017426,-0.270607592021979,0.503707538503188,
-0.537231570577867,0.591107666389744,0.985086336678093
"8952","TSKU",70.4540522681903,0.171935132180437,0.347217500324257,0.4

95179914664068,0.62047312721733,0.985086336678093
"8953","PAK1",25.3122187071097,-0.173859471940969,0.355952832890944,-0
.488434016745796,0.625242452136147,0.985086336678093
"8954","TMEM126A",87.7332502378092,-0.15240978056259,0.322519237029754
,-0.472560278779679,0.636526926843164,0.985086336678093
"8955","NAALAD2",19.2260331782401,0.202178494545978,0.425886830949609,
0.474723517736335,0.634984053033256,0.985086336678093
"8956","SMCO4",30.3216861308265,-0.171736215836404,0.356026950545855,-
0.482368583538692,0.629544134664263,0.985086336678093
"8957","JRKL",21.1569748317307,0.168080510795498,0.356723512564664,0.4
71178671647076,0.637513148244239,0.985086336678093
"8958","DCUN1D5",82.9261425812821,-0.148343898831141,0.318237637178978
,-0.466141906237546,0.641113928938605,0.985086336678093
"8959","CWF19L2",103.823545460261,0.145120098468378,0.308201635965942,
0.470860896028517,0.637740074331438,0.985086336678093
"8960","CD3G",35.7473400135876,-0.164042576091046,0.32171554857108,-0.
509899433893239,0.610121918582643,0.985086336678093
"8961","USP2",2.60892767357259,0.409709109274744,0.878970791414571,0.4
66123690658001,0.641126966649566,0.985086336678093
"8962","GRAMD1B",2.5256293915666,0.46944292852991,0.918529784122592,0.
511080790894914,0.609294486039501,0.985086336678093
"8963","TBRG1",85.5341700930676,-0.105980566898777,0.223839359972327,-
0.473467074387092,0.635879986085135,0.985086336678093
"8964","MSANTD2",22.8869685707569,0.16466306967588,0.349685868242769,0
.470888544919873,0.637720328690052,0.985086336678093
"8965","ACAD8",23.720878822394,-0.206190989732049,0.406172991310816,-0
.507643280432364,0.61170353235071,0.985086336678093
"8966","WNT5B",13.9075759271586,0.193473704534966,0.405993680869501,0.
47654363516351,0.63368712825043,0.985086336678093
"8967","C1S",3827.07328670317,0.0995284919134616,0.194067275534012,0.5
1285561483558,0.608052323232098,0.985086336678093
"8968","PEX5",21.0993530400343,0.170519537266542,0.332205415636649,0.5
13295476955886,0.607744647411429,0.985086336678093
"8969","M6PR",123.047722765806,-0.111114436699615,0.230975894338074,-0
.481065078319298,0.63047024675709,0.985086336678093
"8970","KLRG1",11.81690260835,0.212240102812414,0.433225793024609,0.48
9906432695612,0.62420011089205,0.985086336678093
"8971","PRH1",32.8020151345371,0.141461753898174,0.281366878990291,0.5
02766190554558,0.615128666718157,0.985086336678093
"8972","MANSC1",23.2272477292686,0.165314352829423,0.346048586481975,0
.47772006385015,0.6328494619653,0.985086336678093
"8973","APOLD1",15.8842485142274,-0.327804854697372,0.646173846465824,
-0.507301334602544,0.611943402301428,0.985086336678093
"8974","GPRC5A",191.52920953349,-0.330196575291674,0.711316943721262,-
0.464204569012861,0.642501188024753,0.985086336678093
"8975","ETNK1",81.139323709304,0.120612311808032,0.228621808332906,0.5
27562583322774,0.597802968518937,0.985086336678093
"8976","SOX5",29.9702533512982,0.168640124029981,0.324338524157043,0.5
19950950841491,0.60309776205889,0.985086336678093
"8977","PPFIBP1",128.934263170365,-0.133642642563209,0.262437754508073

,-0.5092355816476,0.610587105228671,0.985086336678093
"8978","PRICKLE1",145.312139251746,0.163620989938357,0.303447825699657
,0.53920633493121,0.589744489685592,0.985086336678093
"8979","TWF1",90.806886539642,-0.103262704806439,0.19780001752500б,-0.
522056095335707,0.601631274266034,0.985086336678093
"8980","SLC38A2",396.842608388123,0.16759853039215,0.315786312207284,0
.530733992935507,0.595603126491574,0.985086336678093
"8981","FMNL3",19.0872657921496,-0.275415708126407,0.526326115023041,-
0.523279579456836,0.6007797094028,0.985086336678093
"8982","FIGNL2",2.16721849949511,0.514604544658115,1.00598116665677,0.
51154490930315,0.608969550138488,0.985086336678093
"8983","HOXC11",6.55971025431446,-0.278369053588047,0.552065369009792,
-0.504232051518357,0.614098319699799,0.985086336678093
"8984","HOXC-
AS2",11.2766402186784,0.237171637979099,0.455979593732847,0.5201365175
96125,0.602968427751263,0.985086336678093
"8985","SMUG1",32.5221104799312,0.160916677175684,0.331106675164345,0.
485996475594496,0.626969664773457,0.985086336678093
"8986","HNRNPA1",1221.98162282068,-0.110059475505778,0.205197950432697
,-0.536357577030853,0.591711444890344,0.985086336678093
"8987","SUOX",14.329134478877,0.208351865673138,0.427437991278519,0.48
7443488703313,0.625944081070113,0.985086336678093
"8988","ZC3H10",9.2135181293553,0.248951287165782,0.496983106680837,0.
500925049200231,0.616423872657682,0.985086336678093
"8989","COQ10A",16.4568809633066,-0.207647574404465,0.402187150668436,
-0.51629589373841,0.605647763734161,0.985086336678093
"8990","RBMS2",37.0321590280189,0.14821905727372,0.291614963920036,0.5
0826972416396,0.611264199341989,0.985086336678093
"8991","ZBTB39",6.40184091030848,0.31347551282458,0.642800553412326,0.
487671504264404,0.625782538876946,0.985086336678093
"8992","USP15",101.936733121801,0.093818110323752,0.195715336442708,0.
479360033960423,0.631682520390072,0.985086336678093
"8993","AC025159.1",12.587365887263,0.255608560162206,0.50307921467512
9,0.508088095683438,0.611391563329303,0.985086336678093
"8994","PPP1R12A",211.009721938728,0.0925872126074462,0.18805778598788
5,0.49233384366979,0.622483358756213,0.985086336678093
"8995","TMTC2",11.309510179665,0.230149791596617,0.464516522134641,0.4
95460937619583,0.620274788980355,0.985086336678093
"8996","POC1B-
AS1",8.45467622390989,0.270163961981313,0.558244106473707,0.4839530930
07776,0.628419159177869,0.985086336678093
"8997","UBE2N",143.869489435725,-0.121237135066923,0.242504729415614,-
0.49993719858198,0.617119298549741,0.985086336678093
"8998","VEZT",53.0157226291547,0.120953681105087,0.236084649036124,0.5
1233183351358,0.608418789598969,0.985086336678093
"8999","ARL1",225.533055050377,-0.12250506103273,0.235526256985822,-0.
520133349888475,0.602970635441131,0.985086336678093
"9000","DRAM1",127.322275781926,0.137010703291265,0.26511403767172,0.5
16799127253005,0.605296389348647,0.985086336678093
"9001","USP30",14.5959714836409,0.213730222665823,0.427703555028753,0.

499715796497072,0.61727520806665,0.985086336678093
"9002","C12orf49",42.9932781609217,-0.11767126097983 8,0.252034168600472,-0.46688614338785,0.64058133957841,0.985086336678093
"9003","FBXW8",18.7793849398694,-0.180650244612736,0.385593845377832,-0.468498776052097,0.639427946059617,0.985086336678093
"9004","HSPB8",74.3529318214864,-0.135799250971233,0.284044939796328,-0.478090724195287,0.632585633802791,0.985086336678093
"9005","AC004812.2",6.04759509802973,-0.277990402372691,0.582438890680068,-0.477286813811665,0.633157899361068,0.985086336678093
"9006","COQ5",41.4019326974531,-0.150118010789726,0.313390209136465,-0.479013084688801,0.63192932015564,0.985086336678093
"9007","P2RX4",28.9897131779301,0.140926852662599,0.29648849192182 3,0.475319806678225,0.634559043671002,0.985086336678093
"9008","TMED2",331.176564594562,-0.0948114584523012,0.183920023019407,-0.515503733067153,0.606201061636337,0.985086336678093
"9009","NCOR2",122.304835019244,0.143213779113661,0.274409942764673,0.521897193923757,0.601741912176036,0.985086336678093
"9010","SLC15A4",60.1173133685465,0.128075031540305,0.247927654845432,0.516582273244797,0.605447792833756,0.985086336678093
"9011","CHFR",20.2980020082247,0.172078536939708,0.364640553510413,0.471912778990432,0.636989046304631,0.985086336678093
"9012","ZNF891",17.3496340532694,0.183914177918547,0.379625485207022,0.48446214778824,0.628057921632002,0.985086336678093
"9013","RPL21",6021.24337387116,-0.102634708224483,0.199037599634771,-0.515654873314464,0.606095477743086,0.985086336678093
"9014","B3GLCT",16.4731176815983,-0.181761139607814,0.389290126831857,-0.466904057102687,0.640568522484503,0.985086336678093
"9015","BRCA2",9.81086518957584,0.301855729300868,0.59640776573287,0.506123069893205,0.612770256209208,0.985086336678093
"9016","ALG5",120.868818139634,-0.143873099474583,0.303128938509875,-0.474626738647375,0.635053044398556,0.985086336678093
"9017","NAA16",31.711332476795,0.169541500074622,0.338732309819181,0.500517651136158,0.616710630601564,0.985086336678093
"9018","LACC1",14.1958675771033,-0.243874227540888,0.456020637098541,-0.534787699724627,0.592796669540112,0.985086336678093
"9019","RUBCNL",1.8990021523264,-0.584930930063643,1.1100657474067,-0.526933590582488,0.59823970634636,0.985086336678093
"9020","WDFY2",63.2223574692831,0.181244098846057,0.351225527829736,0.516033387339426,0.605831090677785,0.985086336678093
"9021","ATP7B",2.83399899623027,-0.539317378839703,1.06648920189788,-0.505694176631094,0.613071356968078,0.985086336678093
"9022","MYCBP2",487.006642476006,0.110488085212819,0.224556218997672,0.492028614063743,0.622699115538103,0.985086336678093
"9023","TM9SF2",164.647679938404,-0.0773986359726322,0.162313642208481,-0.476846153653671,0.633471677567609,0.985086336678093
"9024","LAMP1",496.027776991781,0.0937860716396529,0.184090287339769,0.509456924615231,0.610431984263754,0.985086336678093
"9025","CDC16",60.1297098010077,-0.11217689368211,0.217390682625178,-0.516015186702016,0.60584380240196,0.985086336678093
"9026","TOX4",117.756620294107,-0.090912945600262,0.177815401427242,-0

.511277115877171,0.609157026716054,0.985086336678093
"9027","SALL2",4.85398394508009,-0.349968013582118,0.6808304615999,-0.514031074284954,0.607230265240615,0.985086336678093
"9028","MRPL52",131.847927433445,-0.144428367561073,0.300444718331337,-0.480715282209735,0.630718868192867,0.985086336678093
"9029","SLC22A17",39.3239012683544,0.151306008249769,0.290000424595899,0.52174409213575,0.601848520675001,0.985086336678093
"9030","DCAF11",28.3203507740077,-0.159679666822675,0.305958029292238,-0.521900559995293,0.601739568394241,0.985086336678093
"9031","PSME1",527.006430839748,-0.122514841706848,0.233244180370025,-0.525264302468286,0.599399469456937,0.985086336678093
"9032","EMC9",11.5334833851913,0.245182031496437,0.459304942329241,0.533811002017663,0.593472301112019,0.985086336678093
"9033","RABGGTA",16.2932664740787,-0.209368140589785,0.395608419676934,-0.529230749842892,0.596645387873877,0.985086336678093
"9034","CBLN3",4.53407266256633,0.375399547058621,0.771757770671065,0.486421467103857,0.626668373268698,0.985086336678093
"9035","NFKBIA",1564.93044704568,-0.134440751946063,0.270388992827084,-0.497212370002201,0.619039299203926,0.985086336678093
"9036","KLHL28",36.0328504642021,-0.148494696591889,0.287923595870693,-0.515743408048357,0.606033632771608,0.985086336678093
"9037","MGAT2",68.4902366183364,-0.124493726043561,0.264661561018874,-0.470388391741869,0.638077556164723,0.985086336678093
"9038","KLHDC1",17.5684738744877,0.176060930491605,0.375157173857236,0.469299117171098,0.638855846202796,0.985086336678093
"9039","GNPNAT1",62.0684295913471,-0.141158217791437,0.274839295504943,-0.513602749316094,0.60752975700306,0.985086336678093
"9040","PSMA3-AS1",97.2568088457076,0.140216052832963,0.295588613886088,0.474362158235899,0.635241673257004,0.985086336678093
"9041","AL122035.2",2.34145959145006,-0.485321007738915,1.01978662136843,-0.475904466257535,0.634142440164267,0.985086336678093
"9042","GPHN",17.1080330079956,-0.226192253032546,0.420937372263777,-0.537353696622605,0.591023320960054,0.985086336678093
"9043","ERH",428.890116101476,-0.143125508440641,0.282676888780327,-0.506321931935038,0.6126306690277,0.985086336678093
"9044","NUMB",85.1738880809636,0.125028717206239,0.244463832425264,0.51144055120899,0.609042606004654,0.985086336678093
"9045","DNAL1",27.2891975653423,0.193384869517069,0.36906777425603,0.523981997363209,0.600291061318555,0.985086336678093
"9046","BBOF1",21.5257761824685,-0.177487769035287,0.339368964046371,-0.522993519852438,0.600978762760151,0.985086336678093
"9047","AL136040.1",1.85222814799915,0.55531661330883,1.14932606256242,0.483167163259792,0.628977048635502,0.985086336678093
"9048","GPR65",11.9328810223812,-0.249173344523429,0.518235581135142,-0.480810954696782,0.63065086376461,0.985086336678093
"9049","LGMN",202.164642051639,-0.0859665485399291,0.179910474049459,-0.477829592713409,0.632771496781957,0.985086336678093
"9050","UBR7",30.7700108694326,-0.158042722791152,0.333964041877063,-0.473232752552833,0.636047133158853,0.985086336678093

"9051","GSKIP",15.9087898597605,-0.189464909326195,0.365804210937154,-0.517940755358734,0.604499605476764,0.985086336678093
"9052","CCNK",76.435067815363,0.111310400231744,0.210027282406764,0.529980671826085,0.596125331766409,0.985086336678093
"9053","BAG5",87.2093258157848,-0.10784447055137,0.217032421913842,-0.496904884534633,0.619256126861032,0.985086336678093
"9054","ZFYVE21",115.344523422854,-0.121877910739555,0.233305037989213,-0.522397252069585,0.601393768881179,0.985086336678093
"9055","ATP5MPL",410.964747872324,-0.125024072012836,0.248356614394721,-0.503405445099728,0.614679244099671,0.985086336678093
"9056","CLBA1",6.87830991450631,-0.305072272005776,0.582193272384048,-0.524005148250035,0.600274959099326,0.985086336678093
"9057","IGHA2",6.72654302886715,-0.764189806737654,1.60264907680021,-0.476829156051683,0.633483782268099,0.985086336678093
"9058","NIPA1",7.7772321472736,0.23505775166909,0.499530711455849,0.47055715750495,0.637957008158337,0.985086336678093
"9059","NSMCE3",84.5952305653625,-0.136691082455648,0.291019082082773,-0.469698005633768,0.63857079294615,0.985086336678093
"9060","LINC02352",3.19897555073548,-0.433067849554241,0.827866040016976,-0.523113436982342,0.600895315309823,0.985086336678093
"9061","ARHGAP11A",2.1916482032823,-0.509994180296773,1.01122299561944,-0.504334041557637,0.614026659671562,0.985086336678093
"9062","ZNF770",56.5492939342718,0.13319300051417,0.266553742052466,0.499685352336766,0.617296647941764,0.985086336678093
"9063","SPRED1",87.2995473805006,0.122759992805784,0.238594421421777,0.514513256740293,0.606893194341757,0.985086336678093
"9064","BMF",6.57771042071458,0.318969966375102,0.659437229502289,0.483700270632043,0.628598601115121,0.985086336678093
"9065","RMDN3",42.4136501569461,-0.135529722717005,0.286552390942663,-0.472966644148937,0.636236976790499,0.985086336678093
"9066","CATSPER2",3.45868643988104,-0.410926022215663,0.827844930437814,-0.496380429603332,0.619626029995075,0.985086336678093
"9067","SERF2",2607.71038487204,0.130287271352964,0.274759881705151,0.474185934803894,0.635367322394633,0.985086336678093
"9068","HYPK",7.32204697360102,-0.306355842416909,0.646047329445224,-0.47420030770808,0.635357073971331,0.985086336678093
"9069","EIF3J",182.70585674894,-0.112005437184861,0.224650432266647,-0.498576548706202,0.618077729567493,0.985086336678093
"9070","SORD",17.0178578199887,-0.231451173982882,0.452709966116193,-0.511257076950407,0.609171056582088,0.985086336678093
"9071","ARPP19",114.565728948368,0.0934812837799406,0.197509585340444,0.473299985004821,0.635999172830968,0.985086336678093
"9072","TEX9",4.99162268882502,0.320247192560037,0.619784894475531,0.516706998532183,0.605360709856569,0.985086336678093
"9073","LIPC",6.0446716322474,0.284712395268899,0.598066252100003,0.476054942523812,0.634035235937171,0.985086336678093
"9074","RORA",131.0239061973,0.157939610541969,0.326687745922634,0.483457406998584,0.628770995451393,0.985086336678093
"9075","PDCD7",135.023706527315,-0.0869488949702571,0.183247641160459,-0.474488481377619,0.635151610007519,0.985086336678093

"9076","CLPX",57.2327528093214,0.124445077802012,0.23505148062009,0.529437540549471,0.59650196197325,0.985086336678093
"9077","SENP8",3.40343753754568,0.417807601047014,0.796723111004057,0.52440753290121,0.599995118270355,0.985086336678093
"9078","C15orf39",11.4428493690247,0.225341995249771,0.468603980531322,0.480879387738595,0.630602223173843,0.985086336678093
"9079","COMMD4",106.864106320129,-0.15778232983682,0.303399816645525,-0.520047545121506,0.603030437265861,0.985086336678093
"9080","UBE2Q2",73.870943182303,-0.113617142201361,0.220381889223896,-0.515546638616624,0.606171087744984,0.985086336678093
"9081","MORF4L1",794.692456891421,-0.0812935303610886,0.165706323015991,-0.49058798047944,0.623717889255507,0.985086336678093
"9082","ABHD17C",3.74776508485026,-0.36837042640865,0.708456551086966,-0.519961916991901,0.60309011864312,0.985086336678093
"9083","AC048382.6",2.92756560291613,0.437544843377284,0.905388541299497,0.483267485083563,0.628905823740081,0.985086336678093
"9084","AEN",16.7950434837019,0.216841210331229,0.425476627377894,0.509643059990315,0.610301550925149,0.985086336678093
"9085","MESP2",4.18015819725317,0.477788014561234,0.913002993455159,0.523314839038039,0.600755176235676,0.985086336678093
"9086","ARPIN",44.9943509677989,-0.124641602662169,0.265402865086881,-0.469631714870023,0.638618161883523,0.985086336678093
"9087","ZNF710",8.15739323031813,0.253261372568159,0.531319033544796,0.476665349024818,0.633600441104225,0.985086336678093
"9088","SEMA4B",6.90525907050756,0.277697785361906,0.57579935819335,0.482282207179287,0.629605485192048,0.985086336678093
"9089","HDDC3",37.0537939079163,0.165334003058245,0.324223093412978,0.509939009334079,0.610094191529452,0.985086336678093
"9090","SPATA41",4.7434502976876,0.328957413606087,0.675079559861391,0.487286881673072,0.626055042860382,0.985086336678093
"9091","ASB7",11.6641143473057,0.232957231328103,0.450125493404439,0.517538408158522,0.604780364547807,0.985086336678093
"9092","AC015712.1",2.09300293540044,0.461655200722878,0.980312241766607,0.470926691572204,0.637693086424209,0.985086336678093
"9093","ALDH1A3",156.630309424772,-0.208533627883849,0.440924555395161,-0.472946279204067,0.636251506271735,0.985086336678093
"9094","NHLRC4",4.87941959009574,0.314013024577414,0.624524538000892,0.502803341534941,0.615102544073554,0.985086336678093
"9095","UBE2I",336.837625106645,-0.118870010657759,0.220603933468564,-0.538839035137596,0.589997926441118,0.985086336678093
"9096","MAPK8IP3",15.6675265455959,0.227598414931418,0.472540742477919,0.48164823574351,0.630055854214496,0.985086336678093
"9097","RNPS1",174.911728136477,0.0912582673391227,0.174185808750089,0.523913331367049,0.600338821973637,0.985086336678093
"9098","GLIS2",66.6428375295645,-0.157668895392694,0.321927968491337,-0.489764515123006,0.624300543372503,0.985086336678093
"9099","HMOX2",74.5969992088017,-0.143579649999993,0.275504917667867,-0.521150951552466,0.602261619280983,0.985086336678093
"9100","SNN",14.7274928369652,0.193144705936154,0.416148247775815,0.464124760751615,0.642558362711015,0.985086336678093

"9101","NPIPB2",5.81955033066314,-0.336930068557441,0.664932011515904,
-0.506713562773602,0.612355812790782,0.985086336678093
"9102","PARN",38.3224276594049,-0.125651639816055,0.270279140893244,-0
.464895808832269,0.642006072054902,0.985086336678093
"9103","PDXDC1",63.0506988441245,0.137357337917713,0.280344543186491,0
.489959020983475,0.62416289689186З,0.985086336678093
"9104","MYH11",1.85885759780361,0.584133008434681,1.09492967495483,0.5
33489064910747,0.593695078646765,0.985086336678093
"9105","AC009152.6",1.85844808640811,0.625020382802,1.2701169548684,0.
492096716295518,0.622650973498548,0.985086336678093
"9106","SMG1",109.048140447324,0.129314980564125,0.275766950392274,0.4
68928493353454,0.639120748837253,0.985086336678093
"9107","TNRC6A",194.902052774612,0.131105415601124,0.248861801012885,0
.526820167126959,0.598318476741732,0.985086336678093
"9108","ATXN2L",67.6481347711787,-0.105430059002866,0.209506914018333,
-0.503229497207143,0.614802928392618,0.985086336678093
"9109","SPNS1",64.6274389674433,0.107311523669278,0.230233241838645,0.
46609917322228,0.641144515057686,0.985086336678093
"9110","C16orf54",7.97309235464941,-0.279277133700344,0.57304351955369
8,-0.487357633706167,0.62600491141677,0.985086336678093
"9111","MVP",195.143355179978,0.0965672952694542,0.186826732323737,0.5
16881572933153,0.605238831740479,0.985086336678093
"9112","TBC1D10B",36.9125633692527,-0.141699866647203,0.28395172809514
7,-0.499028012957618,0.617759649550897,0.985086336678093
"9113","CTF1",12.3781401780231,0.199416893948954,0.429620540158219,0.4
64169831999916,0.642526073381367,0.985086336678093
"9114","HSD3B7",101.61996831677,-0.2091636236374,0.388957873329792,-0.
537753926528831,0.590746943997557,0.985086336678093
"9115","ZNF720",50.7787976578637,-0.141701779667535,0.264517964782847,
-0.535698132200068,0.592167194710772,0.985086336678093
"9116","FBXL8",17.4189306719276,-0.193896877746311,0.376438168293927,-
0.515082938122563,0.606495064844274,0.985086336678093
"9117","SLC9A5",2.32514542210504,-0.47873545581941,0.906186246163489,-
0.528296978514325,0.597293228414754,0.985086336678093
"9118","LRRC36",2.8522658586597,-0.429988983470648,0.873945587176185,-
0.492008873069536,0.622713070914282,0.985086336678093
"9119","DDX28",9.73420391434795,0.25694971114017,0.53935861645383,0.47
639863960932,0.633790403665824,0.985086336678093
"9120","ZFP90",38.6724946141315,0.123254608376592,0.26420290939141,0.4
66514954965895,0.640846945510263,0.985086336678093
"9121","FCSK",6.64915178607033,-0.294854754178885,0.607054971234336,-0
.485713433133331,0.627170358109626,0.985086336678093
"9122","AC116667.1",3.33715297344254,0.407841244117906,0.8129752963377
42,0.501664990258785,0.615903195175321,0.985086336678093
"9123","GAN",4.92212201628619,0.345235692351408,0.687541737368194,0.50
2130523265275,0.615575710598222,0.985086336678093
"9124","GSE1",51.292648771522,-0.159712251833471,0.321809323900385,-0.
496294668835977,0.61968652705761,0.985086336678093
"9125","GINS2",2.07076054142021,-0.532662624456157,1.09927215181568,-0
.484559372832609,0.627988938525244,0.985086336678093

"9126","ZNF778",8.76643698235356,0.29724710804398,0.577659945765031,0.514571090177141,0.606852771342008,0.985086336678093
"9127","TIMM22",49.0561366166585,-0.161118153432632,0.300359928270165,-0.536416939371856,0.591670426806561,0.985086336678093
"9128","SMYD4",49.8788332473096,0.170044476859961,0.33936891668065,0.501060847066245,0.616328300725497,0.985086336678093
"9129","TSR1",52.4431587074915,-0.135710174511387,0.264978082846334,-0.512156224596461,0.608541677324079,0.985086336678093
"9130","CAMKK1",4.99881597603796,-0.316209078910844,0.670374609700531,-0.471690118234192,0.637147991535506,0.985086336678093
"9131","NUP88",25.5980551421032,0.176223001114947,0.354926854149555,0.496505122265817,0.61953807432472,0.985086336678093
"9132","C1QBP",290.036272692324,-0.142207658161409,0.285836177889054,-0.497514552607145,0.618826243228854,0.985086336678093
"9133","TMEM102",1.86932265251214,-0.525813212628896,1.08968709718894,-0.482535962833124,0.629425257466378,0.985086336678093
"9134","MPDU1",57.8642819269299,-0.171126639804914,0.338209511230387,-0.5059782002651 7,0.612871953355749,0.985086336678093
"9135","WRAP53",7.35963759292961,0.294634800251387,0.566869124357677,0.519758066882263,0.603232209459847,0.985086336678093
"9136","ARHGAP44",9.94146651938272,-0.242362759307497,0.519200329331574,-0.466800087780217,0.64064291304789,0.985086336678093
"9137","CCDC144A",24.2105104191959,-0.341905622304952,0.655030317580522,-0.521969156432095,0.601691805873796,0.985086336678093
"9138","MPRIP",105.039126915159,-0.123580494474445,0.261266563415276,-0.473005396706724,0.636209328952848,0.985086336678093
"9139","TOM1L2",26.9648577263285,0.180945369020787,0.35391537493035,0.511267330661735,0.609163877626956,0.985086336678093
"9140","MFAP4",1367.26342823841,-0.11021186388852,0.214836640874923,-0.513003105241648,0.607949148548132,0.985086336678093
"9141","TRAF4",13.2617309358258,-0.205349903656122,0.415935613675767,-0.49370598935103,0.621513834733097,0.985086336678093
"9142","GIT1",30.5605801673535,0.162513116917181,0.32105282358426,0.5061880942297,0.612724612147385,0.985086336678093
"9143","CCL8",51.3299392416966,-0.490608095310707,0.960822387915851,-0.510612680846145,0.609622294626577,0.985086336678093
"9144","SOCS7",16.0645973461615,0.208324585426204,0.435855087392479,0.477967543461554,0.632673306048198,0.985086336678093
"9145","CNTNAP1",23.4851708097191,0.197848084259428,0.397025970843669,0.498325295544284,0.618254781488491,0.985086336678093
"9146","MPP3",4.03963952464774,0.360844797221573,0.735748564305591,0.490445805439177,0.623818470339888,0.985086336678093
"9147","ATXN7L3",23.5895455456862,-0.166928062358105,0.318594079460664,-0.523952179653467,0.600311800824809,0.985086336678093
"9148","CDC27",96.0777288754462,-0.0922155162081201,0.193102484358145,-0.4775470212858,0.632972645986771,0.985086336678093
"9149","SP2-AS1",7.46328721757673,-0.298367229018344,0.572037148045601,-0.521587155725347,0.601957808153638,0.985086336678093
"9150","ANKRD40",50.5757475927878,-0.128064968263363,0.267610949934516

,-0.478549058977969,0.63225946548352,0.985086336678093
"9151","SRSF1",127.086295735768,0.108644130699511,0.218197666705581,0.497916097545199,0.618543180700232,0.985086336678093
"9152","INTS2",13.1414705843449,-0.232531355039506,0.453380337338797,-0.512883634090515,0.608032722185326,0.985086336678093
"9153","DDX5",2410.24138747562,0.104247318868424,0.197117403070207,0.528859031443784,0.596903243791736,0.985086336678093
"9154","PRKCA",23.0282847355048,0.195628956187911,0.366485700135618,0.533796969746755,0.593482010517388,0.985086336678093
"9155","AC005332.4",6.86543808197521,0.296390344638104,0.584861405262213,0.506770222776492,0.612316051910567,0.985086336678093
"9156","FAM104A",49.8321310819128,-0.124355747847282,0.266726738134529,-0.46622902794455,0.64105157353872,0.985086336678093
"9157","NT5C",166.075233997819,-0.137376084199376,0.275257898559449,-0.499081352136771,0.617722074060419,0.985086336678093
"9158","GALK1",55.5874237489733,-0.166915776371606,0.321334768632101,-0.519445116636941,0.603450376270042,0.985086336678093
"9159","WBP2",76.2319431362148,-0.0976971445964159,0.20304706832593,-0.481155159746474,0.630406227163813,0.985086336678093
"9160","CDK3",7.0454287397202,-0.273594909580763,0.578817861069979,-0.472678761285988,0.63644238138424,0.985086336678093
"9161","SRP68",75.1297246247281,0.131462919980818,0.260589301434805,0.504483181991675,0.613921877567428,0.985086336678093
"9162","JMJD6",37.2154453769026,-0.137161398137503,0.289651860165127,-0.473538813316473,0.635828816788521,0.985086336678093
"9163","USP36",52.4975213882842,0.150630600282694,0.301781982976605,0.499137154567546,0.617682764370371,0.985086336678093
"9164","TEPSIN",13.2577020885219,-0.201875006906502,0.435040960166743,-0.464036781339227,0.642621393669048,0.985086336678093
"9165","CCDC137",36.3416986094209,-0.154909008786505,0.315263310751121,-0.491363896475714,0.623169094673276,0.985086336678093
"9166","MRPL12",83.131131303029,-0.149008140948321,0.285351650931849,-0.522191269830461,0.601537163864794,0.985086336678093
"9167","MCRIP1",204.462021494832,-0.139167053123067,0.277244945344449,-0.501964257455322,0.615692663420828,0.985086336678093
"9168","AC145207.5",5.367186477208,0.381880115514825,0.74364650383427,0.513523715294614,0.607585026074307,0.985086336678093
"9169","MAFG-DT",2.59482266891072,-0.435498295234795,0.837918656749647,-0.519738153252402,0.603246090779317,0.985086336678093
"9170","CCDC57",17.962080037072,0.209239330473448,0.429670772514569,0.486975944975064,0.626275377941903,0.985086336678093
"9171","AP000915.2",3.10893891667657,-0.521626659647129,0.970092137350848,-0.53770836765217,0.590778401475775,0.985086336678093
"9172","MYL12A",720.110826262728,-0.132056957107561,0.283970617001769,-0.465037398945885,0.641904674595558,0.985086336678093
"9173","SNRPD1",251.857115211153,-0.17843179633239,0.333424245370095,-0.535149434422006,0.592546528724296,0.985086336678093
"9174","LINC01894",6.06686004498596,0.298951118794866,0.571620915291638,0.522988419068506,0.60098231238922,0.985086336678093

"9175","GALNT1",160.009798856611,-0.0806119994042056,0.172387642752156
,-0.467620521501664,0.640055986887704,0.985086336678093
"9176","C18orf21",36.7611726620366,-0.154513592653084,0.29054909221147
9,-0.531798573098347,0.594865511151836,0.985086336678093
"9177","TPGS2",75.5634365322318,-0.100145136345212,0.21139666959325,-0
.473730908523306,0.635691809411195,0.985086336678093
"9178","MBD1",37.8437452420664,0.143887045106954,0.280947795428268,0.5
12148689003298,0.60854695083383,0.985086336678093
"9179","TXNL1",229.22077880391,-0.126702115631085,0.266427855573818,-0
.475558816318964,0.634388721585521,0.985086336678093
"9180","ZCCHC2",37.8484313148221,0.153631441872376,0.329845348003173,0
.465768102543917,0.641381499252107,0.985086336678093
"9181","PALM",166.216020993495,0.0860243630675644,0.181007276162147,0.
475253619034097,0.634606213464451,0.985086336678093
"9182","AC027307.3",2.97518054043068,0.399395425647044,0.8088866111256
69,0.493759471542289,0.621476058831798,0.985086336678093
"9183","ADAMTSL5",4.10222498958731,0.357735219346746,0.708792818530643
,0.504710558563991,0.613762144004276,0.985086336678093
"9184","PLEKHJ1",117.548670445662,0.122522972729751,0.235067754222352,
0.521224074884621,0.602210684911436,0.985086336678093
"9185","TNFAIP8L1",2.43649638785125,0.460376170991898,0.91736877644146
,0.501844168686154,0.615777140981062,0.985086336678093
"9186","PLIN3",161.047336542536,0.123500184325569,0.265080384510156,0.
465897107225743,0.641289151872113,0.985086336678093
"9187","SAFB",83.6583642085097,0.12815522186745,0.252160766798492,0.50
8228236670384,0.611293290729148,0.985086336678093
"9188","ALKBH7",164.401522577999,-0.163292831015383,0.31920178186418,-
0.511566163765539,0.608954671433698,0.985086336678093
"9189","GTF2F1",82.604518906356,-0.109928197318955,0.226334174966783,-
0.48568978739109,0.627187125545874,0.985086336678093
"9190","KHSRP",136.079143820948,0.123695215504267,0.236672840985012,0.
522642205119348,0.601223264367304,0.985086336678093
"9191","TNFSF9",47.341310110817,0.191428772893333,0.407133377640193,0.
470186880778195,0.638221506335847,0.985086336678093
"9192","NDUFA7",76.5382158675527,-0.158354859445145,0.295602451696929,
-0.535702117949619,0.592164439630103,0.985086336678093
"9193","AC136469.1",2.18759452136882,-0.602700756775865,1.137853102421
65,-0.529682395287371,0.596332155924859,0.985086336678093
"9194","ZNF561",20.4909058920644,0.191022544403062,0.362597088304591,0
.526817645713133,0.598320227868334,0.985086336678093
"9195","DNMT1",83.7127371293243,-0.110556823792331,0.213748131044546,-
0.51722942910453,0.604996010741593,0.985086336678093
"9196","S1PR5",3.97973899773947,0.508700101203762,1.01345237763082,0.5
01947711043873,0.615704302845214,0.985086336678093
"9197","KRI1",24.782351871095,0.200060923029118,0.404609422387619,0.49
4454434225849,0.620985278841175,0.985086336678093
"9198","ILF3-
DT",71.4526084995714,-0.140447821242419,0.282710813383964,-0.496789703
801211,0.619337356689043,0.985086336678093
"9199","DOCK6",11.2078374177508,-0.211884704375838,0.439821403527143,-

0.481751689837354,0.629982351730868,0.985086336678093
"9200","RAB3D",11.6457084286388,-0.199667796434577,0.424974171413807,-0.469835133204262,0.638472811317737,0.985086336678093
"9201","ZNF440",6.49958571616025,0.290065774941174,0.593646633705845,0.48861689508864,0.625112949501678,0.985086336678093
"9202","ZNF763",3.98664404547104,-0.355008836818816,0.690492473791493,-0.514138604392692,0.607155088798185,0.985086336678093
"9203","ZNF564",6.85596106983346,-0.299330124256634,0.565494157512674,-0.529324875031844,0.596580102577584,0.985086336678093
"9204","GET3",154.820780515907,-0.130208992849171,0.243591435526967,-0.534538468347571,0.592969042073927,0.985086336678093
"9205","DCAF15",15.3847962291436,-0.204181477295831,0.403971511801944,-0.505435337222333,0.613253104513222,0.985086336678093
"9206","NDUFB7",403.364027703404,-0.121456399259348,0.258684108361746,-0.469516276158343,0.638700653653673,0.985086336678093
"9207","SMIM7",185.187708120815,-0.127653381662116,0.250278375187858,-0.51004559050018,0.61001952220246,0.985086336678093
"9208","AC024075.1",5.27172311227538,0.352557575097818,0.74063588543282,0.476020109249482,0.634060051621184,0.985086336678093
"9209","IL12RB1",2.57761467292403,0.460272196422484,0.952407810500985,0.483272177472349,0.62890249239693,0.985086336678093
"9210","LSM4",113.31861357218,-0.143453927184132,0.30212457718506,-0.474817138415925,0.634917316234269,0.985086336678093
"9211","KLHL26",8.66866284459276,-0.252465475487913,0.543389553238377,-0.464612309867429,0.642209115292345,0.985086336678093
"9212","ZNF93",4.29347395843758,0.358589946169735,0.727214249275676,0.493100824862687,0.621941348066602,0.985086336678093
"9213","GPATCH1",18.7799318672494,-0.196703923755539,0.367509865562406,-0.535234403720074,0.592487779177196,0.985086336678093
"9214","AC008555.4",3.57370056461601,-0.419672799492183,0.795575587015718,-0.527508393094887,0.597840589518613,0.985086336678093
"9215","ZNF573",9.45571887426874,-0.263099524807214,0.52557501660665,-0.500593666924854,0.616657120431635,0.985086336678093
"9216","FBXO17",111.616511765532,0.117837500280768,0.23253201821376,0.506758171136859,0.612324508994214,0.985086336678093
"9217","AC243960.1",3.13065462274015,0.446688941095703,0.898533745649777,0.497130957249334,0.619096705314029,0.985086336678093
"9218","PINLYP",12.2861699157162,-0.206805787416007,0.438976851854416,-0.471108639424552,0.637563155891204,0.985086336678093
"9219","ZNF224",36.113652160557,0.157192692845915,0.300557029416613,0.523004546428439,0.600971089412258,0.985086336678093
"9220","ERCC2",11.2765656896951,0.229179536876366,0.458605718493394,0.499731092820352,0.617264435967517,0.985086336678093
"9221","SYMPK",45.2153135817112,0.120709608513882,0.258369717861672,0.467197199087042,0.640358797394089,0.985086336678093
"9222","PTGIR",42.6857196292438,-0.292870352515943,0.58877998113182,-0.497419005233423,0.618893606116199,0.985086336678093
"9223","GYS1",11.1250667199624,-0.236585189153493,0.477256777402782,-0.495718867400862,0.620092773570719,0.985086336678093
"9224","IRF3",81.8744968567355,-0.100049209918015,0.206188911944802,-0

.485230796236022,0.627512638979993,0.985086336678093
"9225","MED25",29.0202295313526,-0.149399408561665,0.308517190645841,-0.484249867078447,0.628208550318191,0.985086336678093
"9226","ZNF613",3.41226162426946,0.377109725427965,0.809214128283263,0.466019700160202,0.641201399453818,0.985086336678093
"9227","ZNF841",9.90874061553141,0.247012350313456,0.516214315438064,0.478507362012693,0.632289135659757,0.985086336678093
"9228","ZNF468",11.4016711806598,0.243444901000494,0.454439519924804,0.535703631235191,0.592163393599079,0.985086336678093
"9229","LENG1",34.6736812297819,-0.191288366319744,0.367786901022668,-0.520106523064979,0.602989332169161,0.985086336678093
"9230","LENG8-AS1",4.1590035193303,-0.370155155524609,0.716362048135941,-0.516715195183493,0.605354987165494,0.985086336678093
"9231","ZNF582-AS1",7.08179126031754,0.371831172747636,0.694898125311153,0.535087315973319,0.592589480407376,0.985086336678093
"9232","PEG3",18.5875723442127,0.239092661832949,0.482687780764649,0.49533605647566,0.620362923232788,0.985086336678093
"9233","ZNF548",13.8285218500782,0.220660270433371,0.411404969209376,0.536357815165499,0.591711280341884,0.985086336678093
"9234","ZNF211",3.87646209961365,0.364303053244864,0.751320264737526,0.484883837616351,0.627758747816849,0.985086336678093
"9235","ZNF584",16.1132822881502,-0.203496684221505,0.420732659214722,-0.483672184140215,0.628618536996789,0.985086336678093
"9236","ZBTB45",10.3173095464466,0.21436324898551,0.454910137269956,0.471221086151134,0.637482862280419,0.985086336678093
"9237","TMEM230",340.756756345106,-0.136752652519028,0.276984700771766,-0.493719155382923,0.62150453512028,0.985086336678093
"9238","PCNA",59.9025628656153,-0.123963520318207,0.263137003239665,-0.471098776652485,0.637570198696478,0.985086336678093
"9239","LINC00654",4.76203468378593,0.359817269495861,0.693697618719681,0.518694687405668,0.603973667000614,0.985086336678093
"9240","TMX4",170.831852069966,0.10259886647739,0.208448368993539,0.492202778907665,0.622576000160298,0.985086336678093
"9241","RIN2",72.3781991982022,-0.161785667035351,0.339828475617891,-0.476080371844017,0.634017120013954,0.985086336678093
"9242","KIZ",50.1457383681088,-0.134298931357486,0.276810060905374,-0.485166366129284,0.627558338181965,0.985086336678093
"9243","ENTPD6",23.3713194056521,-0.196591142031613,0.385364408857295,-0.510143483708203,0.609950943117854,0.985086336678093
"9244","RALY",172.040397021894,-0.114917321678819,0.236425810265827,-0.486060813536436,0.626924049333279,0.985086336678093
"9245","ACSS2",16.4757706199649,-0.180602845761269,0.375645925726667,-0.480779461169192,0.630673249158389,0.985086336678093
"9246","OSER1",83.6741125727986,-0.15443577860157,0.320521172684044,-0.481827073413981,0.629928795209107,0.985086336678093
"9247","ACOT8",23.6664008805984,0.182295001317045,0.338478000309048,0.538572672819504,0.590181747747867,0.985086336678093
"9248","AL162458.1",5.09773899378963,0.366899233193802,0.6819849168600

18,0.537987313389675,0.590585807201767,0.985086336678093
"9249","PTGIS",428.141614964365,0.155787308900111,0.318068874638154,0.489791115453659,0.624281718271493,0.985086336678093
"9250","PFDN4",95.1358611769524,-0.110893559071556,0.22904432788496,-0.484157630514443,0.628274003731118,0.985086336678093
"9251","CSTF1",22.7154457262704,-0.157798166034256,0.315199359974985,-0.500629715894026,0.616631745014311,0.985086336678093
"9252","BMP7",11.1509876278391,0.321750274800111,0.611939879273335,0.525787394641092,0.599035933715659,0.985086336678093
"9253","AL121832.2",2.47873459178095,0.44622064930975,0.890739272808437,0.500955400678415,0.616402511305559,0.985086336678093
"9254","CABLES2",4.27799720992373,0.363933119644984,0.680010162525093,0.535187765861594,0.592520025235727,0.985086336678093
"9255","GID8",69.4472583725922,0.111449276345574,0.231881878864857,0.480629520905889,0.630779830366591,0.985086336678093
"9256","NRIP1",136.991348287369,-0.157148560001934,0.300746207797937,-0.522528816414928,0.60130218815049,0.985086336678093
"9257","GABPA",67.4669160785435,-0.123360510961974,0.244699706171591,-0.504130196525325,0.614169888520137,0.985086336678093
"9258","ATP5PO",409.598825178014,-0.12922904059284,0.275559964663128,-0.468968853116318,0.639091899542764,0.985086336678093
"9259","CLIC6",34.4639158193655,0.168442451400354,0.358347678908079,0.470053139212775,0.638317052694094,0.985086336678093
"9260","MX1",124.970591652422,0.227412257437224,0.436625503567966,0.520840527131106,0.602477868346934,0.985086336678093
"9261","COL18A1",176.808824976075,-0.199844365694956,0.373874842968217,-0.534522098647714,0.592980364432342,0.985086336678093
"9262","DGCR6",7.19264598240042,0.262388668942089,0.55963631365386,0.468855688132452,0.639172791635355,0.985086336678093
"9263","GNB1L",9.3130113031183,0.29011971313207,0.558021020974308,0.519908215331242,0.603127549160205,0.985086336678093
"9264","LINC01637",3.20934711059639,-0.432508567896799,0.927394409139794,-0.466369608911028,0.640950961218639,0.985086336678093
"9265","AC245060.6",3.71342192408471,0.499936662540238,0.936220129281321,0.533994780612129,0.59334514516788,0.985086336678093
"9266","MMP11",10.7909869267663,0.224276309245356,0.470178702940102,0.477002271355382,0.633360504237573,0.985086336678093
"9267","LRRC75B",15.0493881779511,0.19207450068045,0.413038955612568,0.465027567183314,0.641911715236899,0.985086336678093
"9268","KIAA1671",15.9175058161603,0.243615345929288,0.524680274866739,0.464311996465205,0.642424230277962,0.985086336678093
"9269","AL022323.4",7.5019810557382,0.298539684845582,0.56112604891538,0.53203675969533,0.594700536006014,0.985086336678093
"9270","GRK3",9.69893064907015,-0.224309778602519,0.483169501155643,-0.464246559573847,0.642471106812037,0.985086336678093
"9271","NF2",43.761356753182,-0.124339174839464,0.252247194354726,-0.492925898175146,0.622064947321525,0.985086336678093
"9272","LARGE1",20.5702297744361,-0.188687567468929,0.356486339220991,-0.52929817137245,0.596598623923774,0.985086336678093
"9273","Z82217.1",4.37121531928078,-0.363006807546221,0.71267588882092

2,-0.509357497903841,0.610501662052487,0.985086336678093
"9274","APOL2",171.952930752844,-0.178091791033933,0.352663835034809,-0.504990229622935,0.613565698353279,0.985086336678093
"9275","PDXP",9.11068827027317,-0.296512331591363,0.550093071633478,-0.539022116222773,0.589871594271265,0.985086336678093
"9276","EIF3L",302.164502596096,-0.097168755388347,0.206896645105232,-0.469648772404816,0.638605973056704,0.985086336678093
"9277","PICK1",7.48657508551838,-0.28215772526764,0.5640291928327,-0.500253761424247,0.616896407591515,0.985086336678093
"9278","GRAP2",4.18699916403348,0.394278747683127,0.791221468669204,0.498316543844904,0.618260948995305,0.985086336678093
"9279","TEF",17.3681524849839,0.324747043968827,0.686630726815648,0.472957342696984,0.636243612944876,0.985086336678093
"9280","PACSIN2",29.3828122467853,0.161143888936584,0.324090165379089,0.497219311632285,0.619034404599403,0.985086336678093
"9281","AL031595.3",3.67512737617903,0.413687936163898,0.810293289726097,0.510540987330324,0.609672507156926,0.985086336678093
"9282","ATXN10",170.281232427635,-0.12337593205446,0.256855419559395,-0.480332212830459,0.630991186935027,0.985086336678093
"9283","SELENOO",27.3445142450679,-0.150978393045834,0.32495443603984,-0.464614039081232,0.642207876740534,0.985086336678093
"9284","NCAPH2",28.4395585189754,-0.155921997894662,0.294442284599182,-0.529550292366858,0.596423766180874,0.985086336678093
"9285","MSL3",41.7975048792926,-0.138769759046951,0.274943878115496,-0.504720308733906,0.613755294854495,0.985086336678093
"9286","RAB9A",104.483501510809,0.135905419576502,0.258014551760822,0.526735483130755,0.598377291207763,0.985086336678093
"9287","NHS",6.17096770406942,-0.323645463079088,0.663672259351448,-0.487658567190017,0.625791703925937,0.985086336678093
"9288","ADGRG2",38.0030776013824,0.453426676771018,0.968933157332969,0.467964867689217,0.639809713770792,0.985086336678093
"9289","FUNDC1",36.7460770662999,-0.144824638067992,0.302430511451526,-0.478869137154519,0.632031728197237,0.985086336678093
"9290","KCND1",2.79916095586656,-0.480159911230068,0.952781376754769,-0.503956020703849,0.61429228237654,0.985086336678093
"9291","PRICKLE3",8.10969630434101,-0.247675793355843,0.506796472016022,-0.488708598089872,0.625048015713022,0.985086336678093
"9292","SPIN2A",9.43312421791775,-0.231675260308488,0.475130696196839,-0.48760322615003,0.625830909991494,0.985086336678093
"9293","AL034397.3",2.11371039799914,-0.516466257471131,0.975129755042512,-0.529638496621011,0.596362597902333,0.985086336678093
"9294","MED12",20.9456808599575,0.181554092677351,0.341391813238987,0.531805642774031,0.594860614182489,0.985086336678093
"9295","ZNF711",5.07074018770544,0.31694727011757,0.630622590520179,0.502594221777134,0.615249592608969,0.985086336678093
"9296","ZCCHC18",2.62719141666417,-0.41170093979402,0.854674038041481,-0.481705213296813,0.630015372118494,0.985086336678093
"9297","MORC4",75.4630217548918,-0.132455577731782,0.25321603782492,-0.523093161355622,0.600909424263762,0.985086336678093
"9298","IL13RA1",255.458628791169,0.124398608539939,0.256932267750247,

0.484168880885221,0.6282660200252,0.985086336678093
"9299","SEPTIN6",65.3045433517716,0.118140942470436,0.245254380668361,
0.481707776833514,0.630013550772286,0.985086336678093
"9300","TENM1",21.2789354381735,-0.286685969807371,0.617102475329172,-
0.464567849374528,0.642240960524409,0.985086336678093
"9301","FAM122B",19.298434768911,-0.171437604380473,0.367110406683315,
-0.466991949177738,0.640505638087647,0.985086336678093
"9302","SMIM10",64.6231857236247,-0.148009396205897,0.287616836153889,
-0.51460616209096,0.606828258220294,0.985086336678093
"9303","RBMX",347.654882539331,0.0819513047586051,0.17071572193093,0.4
80045445326717,0.631195078801072,0.985086336678093
"9304","CXorf40B",18.4907476522471,-0.228728621019417,0.44156385931127
4,-0.517996697864211,0.604460573262114,0.985086336678093
"9305","GABRE",46.8730060176389,0.239752711957265,0.447890521101963,0.
53529311441419,0.592447186938055,0.985086336678093
"9306","ZNF185",5.61217336354826,0.306839027012484,0.61564886036701,0.
498399407138618,0.618202554565953,0.985086336678093
"9307","PLXNA3",22.3302594881757,0.16272136225791,0.344041107324332,0.
472970696796737,0.636234085423468,0.985086336678093
"9308","VBP1",114.668110411943,-0.134534861275902,0.250069456758418,-0
.537989976944167,0.590583968326233,0.985086336678093
"9309","RTN3",234.125639736252,0.0828729620435267,0.178721498452837,0.
463698898906648,0.642863486187184,0.985086632567791
"9310","LYRM1",78.69471580446,0.142147992685258,0.306556633914236,0.46
3692437088236,0.642868116445124,0.985086632567791
"9311","HGH1",20.3350106955456,0.174131982022883,0.375672398851288,0.4
63520829731795,0.642991087886805,0.985169247104839
"9312","AC025449.2",2.08885765517365,0.482708131593378,1.0426076092824
,0.462981592783131,0.643377561222961,0.98523231949407
"9313","TMEM30B",13.7918517420127,-0.18166597391388,0.392377365473799,
-0.462987903735264,0.6433730375794,0.98523231949407
"9314","B4GALT6",6.64645569008772,-0.268825123775668,0.580430689642649
,-0.463147673912925,0.643258519907478,0.98523231949407
"9315","PPP5C",27.9549416897092,-0.173216975340581,0.373953350083941,-
0.463204769529942,0.64321759782239,0.98523231949407
"9316","LILRB2",3.93698233161656,-0.401275206431545,0.866685414783584,
-0.462999837757447,0.64336448339717,0.98523231949407
"9317","EIF1AD",25.3858069168186,0.163640784618719,0.3536025924924,0.4
62781631393827,0.643520898903237,0.985346049560329
"9318","NUTF2",82.0788774189895,-0.126359804176677,0.273179498627898,-
0.462552295510264,0.643685309338509,0.985492017924787
"9319","CD28",7.27866736682313,-0.253031384923547,0.54775851029381,-0.
461939668975338,0.644124585581255,0.985529959019969
"9320","AGGF1",65.9436463985,0.0985427888403513,0.213294415739167,0.46
2003604261524,0.644078735766009,0.985529959019969
"9321","DDX41",36.2611168827978,-0.123716984049404,0.267705215898332,-
0.462138862831829,0.6439817424551,0.985529959019969
"9322","CNPY3",71.1577272512301,-0.12015453762292,0.260047230642539,-0
.462048902909044,0.64404625163499,0.985529959019969
"9323","LINC01588",3.3888188581539,0.400325882500407,0.865863027528542

,0.462343199527838,0.643835225029935,0.985529959019969
"9324","CCDC144NL-AS1",4.3064228995026,-0.328195727309934,0.710342781411575,-0.462024442140106,0.644063792622227,0.985529959019969
"9325","BAG2",44.6977425020123,0.131925692332064,0.285863055415262,0.461499623098972,0.644440191742525,0.985907107281379
"9326","GOLGA8O",2.38469843359163,0.532122811099098,1.15448600451541,0.460917507027254,0.644857790820973,0.986440193421831
"9327","BBS4",36.5011300082169,0.124523394353118,0.270305375926015,0.460676721380493,0.645030558666583,0.986598686602066
"9328","LINC01355",5.95289403532782,0.302354396180467,0.656654693717755,0.460446562056291,0.645195719858169,0.98662325214069
"9329","AC007406.5",6.36812574070371,0.30320372728766,0.658615629247692,0.460365217318023,0.645254096626429,0.98662325214069
"9330","RUNDC1",19.5546077809539,0.160340251589723,0.348189172071729,0.460497523905458,0.645159148378475,0.98662325214069
"9331","CPTP",16.9358739543367,-0.177269244060271,0.388337856462254,-0.456482006866877,0.648043406740708,0.986627023387189
"9332","MORN1",5.5445552675564,0.291409599499858,0.638657284521449,0.4562847814665,0.648185206084143,0.986627023387189
"9333","TNFRSF14-AS1",3.23507970466696,0.34728438147914,0.755296774907831,0.459798575892925,0.645660805510702,0.986627023387189
"9334","USP24",32.621027575154,0.164161747840805,0.359712143922251,0.456369768478785,0.648124101321936,0.986627023387189
"9335","ATP1A1",277.925201150539,0.104611341514412,0.228919155179355,0.456979414555544,0.647685841743097,0.986627023387189
"9336","TOMM20",450.251297418735,-0.092481986309908,0.201428849066411,-0.459129795650159,0.646140961238244,0.986627023387189
"9337","PEX13",30.1456180975025,-0.13308996823712,0.289734328418834,-0.45935174117417,0.645981597449473,0.986627023387189
"9338","CCDC138",4.50199724307301,-0.306125096914665,0.668828946754236,-0.457703121852399,0.647165744844941,0.986627023387189
"9339","ARAP2",11.3677807143452,-0.222124932124911,0.487154802235564,-0.455963753422064,0.648416043227653,0.986627023387189
"9340","AIMP1",147.000604011048,-0.0977470653580703,0.213931502787823,-0.456908235039212,0.647737004655516,0.986627023387189
"9341","PDZD2",3.1969892151223,-0.343683423788691,0.752126062871726,-0.456949228001032,0.647707539243439,0.986627023387189
"9342","TMEM161B-AS1",39.8601707986216,0.127038117694258,0.277584275938536,0.457656029920035,0.647199582527468,0.986627023387189
"9343","RMND5B",42.6069709235788,-0.12655216650239,0.276527558751867,-0.457647574345194,0.647205658317516,0.986627023387189
"9344","EXOC2",19.8074582160706,0.190795184533262,0.416229585154668,0.458389291242629,0.646672783881146,0.986627023387189
"9345","FKBP5",223.123657971569,0.287830852208696,0.629576909388831,0.457181398993985,0.64754066701274,0.986627023387189
"9346","AL356417.2",2.18189548538579,-0.521601801284495,1.1436765486236,-0.456074579750922,0.648336349070073,0.986627023387189

"9347","INTS1",55.776542591937,0.117894679050236,0.256237057161823,0.4
60100035319175,0.645444418743227,0.986627023387189
"9348","AGMO",2.6357991274548,0.409913917497188,0.893455973073027,0.45
8795877862113,0.64638075518995,0.986627023387189
"9349","POM121",52.4890806216026,0.112198103161079,0.243829371551486,0
.460150073172737,0.645408504609873,0.986627023387189
"9350","LMBR1",24.7131776341372,0.168084283016241,0.366312407144216,0.
458855009380194,0.646338288825558,0.986627023387189
"9351","SORBS3",124.163635382831,-0.0894332981211607,0.194623404827668
,-0.459519748924085,0.645860973463486,0.986627023387189
"9352","BNIP3L",267.76609814027,0.0862929456932083,0.187890577963774,0
.459272341531919,0.646038606995848,0.986627023387189
"9353","TUT7",54.3391039699859,0.12193414362016,0.266711613007303,0.45
7175982122762,0.647544560171518,0.986627023387189
"9354","AKR1E2",2.2352729696343,-0.42549445905169,0.927035844085542,-0
.458983826533063,0.646245780457125,0.986627023387189
"9355","HSPA14",18.8749327918265,0.162079274007149,0.353135241471722,0
.458972243414929,0.646254098499485,0.986627023387189
"9356","MRPL43",177.828723132843,-0.110921675201607,0.242378334154043,
-0.45763857396227,0.647212125607611,0.986627023387189
"9357","AKIP1",37.106238871634,0.126771707464264,0.277594272538604,0.4
56679838185904,0.647901184583826,0.986627023387189
"9358","GTF2H1",47.1139946316739,0.113716351598585,0.247592174955882,0
.459288956199234,0.646026677366478,0.986627023387189
"9359","PKNOX2",5.51173318026987,-0.276973109773879,0.60371995397518,-
0.458777464534932,0.646393979287645,0.986627023387189
"9360","FBXL14",9.14520931581955,0.254338329739612,0.556577323419753,0
.456968545137435,0.647693654432092,0.986627023387189
"9361","TMTC3",46.0399053683857,-0.114955028631968,0.25165798714638,-0
.456790702077351,0.647821489465032,0.986627023387189
"9362","STRN3",83.2569369194378,-0.10976176185934,0.239367106333271,-0
.458549896603248,0.646557423423356,0.986627023387189
"9363","EID1",1320.07823206214,-0.0845961074963236,0.185083178537456,-
0.457070751457858,0.647620192401139,0.986627023387189
"9364","CTSH",220.093310744993,0.173920259317444,0.379308692915236,0.4
58519044160978,0.646579583617664,0.986627023387189
"9365","ATF7IP2",8.16017895114583,-0.258023177113569,0.564987142338234
,-0.456688582408662,0.647894898604563,0.986627023387189
"9366","PITPNA",71.4925666645085,-0.107613684390862,0.236022945870591,
-0.455945857272137,0.648428912556311,0.986627023387189
"9367","RAPGEFL1",2.85424452546428,0.402261743829494,0.879723006215771
,0.457259547593132,0.647484501916642,0.986627023387189
"9368","SMARCD2",97.6394823867021,0.122861533768256,0.267216244281585,
0.459783177099022,0.64567185952902,0.986627023387189
"9369","PSMG2",288.693109449239,-0.124013167558716,0.271506573531586,-
0.456759355567829,0.647844022666801,0.986627023387189
"9370","SMAD4",89.2907774682704,0.103991726592433,0.228014687947535,0.
45607468329567,0.648336274613915,0.986627023387189
"9371","STK11",45.4053900874094,-0.11675468769948,0.25574390822935,-0.
456529692174623,0.648009124303486,0.986627023387189

"9372","CD70",20.8630459916161,0.308388933651862,0.672788595442141,0.4
58374199177969,0.646683624724808,0.986627023387189
"9373","RPS9",5502.84847077216,-0.107584787742535,0.236115436277766,-0
.455644872010708,0.648645370275372,0.986627023387189
"9374","ZNF446",10.3591375273267,-0.221626933807839,0.482859681024452,
-0.458988278618806,0.646242583347237,0.986627023387189
"9375","MACROD2",4.20396191920383,-0.358730332475817,0.787298637834196
,-0.455647088965706,0.648643775812935,0.986627023387189
"9376","ARVCF",4.84542086573587,0.343425547164004,0.75366351122184,0.4
5567490272581,0.648623771937195,0.986627023387189
"9377","DDT",201.490870553308,0.109039245497037,0.23895998853182,0.456
30754406618,0.648168839783574,0.986627023387189
"9378","AL121672.2",2.28499894190995,-0.450422772794381,0.988382193807
097,-0.455717206983891,0.648593346867033,0.986627023387189
"9379","MT-
CO1",14219.9953883545,-0.10024586684244,0.21880844320703,-0.4581444178
89718,0.646848689138636,0.986627023387189
"9380","PEX7",12.0286597754439,-0.199904705723796,0.438832752890576,-0
.45553734174815,0.648722709351364,0.98663946392394
"9381","B4GAT1",47.6028458833974,0.124841003882137,0.274125589090622,0
.455415360150365,0.648810446859015,0.986667715050709
"9382","ATP13A2",11.8513761868261,0.191394264517959,0.42925267250699,0
.445877863497385,0.655685473291132,0.986674667778914
"9383","ADPRHL2",59.1741268603809,-0.155141897652523,0.343987469706521
,-0.451010316698118,0.651982113455948,0.986674667778914
"9384","RPS8",7574.344156894,-0.090040383692126,0.200938447691799,-0.4
48099329553051,0.654081511892497,0.986674667778914
"9385","SERBP1",695.58525835816,-0.0811773456642135,0.180033894399554,
-0.450900348153635,0.652061372589556,0.986674667778914
"9386","FPGT",14.1405143735669,0.173413136449709,0.387736537829293,0.4
47244764242613,0.654698343720689,0.986674667778914
"9387","GDAP2",18.2735671492832,0.163863858333068,0.367380850000853,0.
446032661562755,0.655573653109274,0.986674667778914
"9388","VPS45",30.0967527475932,0.133181402388061,0.296493251760512,0.
449188646275282,0.653295577112216,0.986674667778914
"9389","SETDB1",20.4799453195933,0.15860867025072,0.349198155332321,0.
454208213384681,0.649678971995471,0.986674667778914
"9390","C1orf43",176.757574368058,-0.110017073644566,0.245606558418021
,-0.44794029260942,0.654196288109783,0.986674667778914
"9391","GORAB-
AS1",3.85581881502473,-0.338238184715145,0.744486331787176,-0.45432423
7092691,0.649595473860591,0.986674667778914
"9392","SNRPE",355.16063424699,-0.120619508712396,0.270220798160168,-0
.446373889551246,0.655327190374729,0.986674667778914
"9393","H3F3A",2056.37658024704,-0.0907921525633308,0.199466675023917,
-0.455174542576821,0.648983673636687,0.986674667778914
"9394","AL356512.1",3.43026839391209,-0.347697588716264,0.776782936454
518,-0.447612289609843,0.654433032158371,0.986674667778914
"9395","AL390728.6",26.0159644306595,0.197904573100856,0.4364695640784
32,0.453421244889582,0.650245441324961,0.986674667778914

"9396","COMMD1",150.609349893302,-0.137285652395804,0.304238261595678,-0.451243876019288,0.651813790092136,0.986674667778914
"9397","ZDBF2",14.9641019557975,0.193122067560761,0.42851253431082,0.450680090073354,0.652220134027612,0.986674667778914
"9398","CAPN10",15.9301177020546,-0.171741090092516,0.377463285809864,-0.454987535341451,0.649118206240623,0.986674667778914
"9399","OXNAD1",17.607247770949,0.170546911486668,0.379834222073374,0.449003543060747,0.653429100757581,0.986674667778914
"9400","XYLB",4.38122152242476,0.394280578160371,0.874971004603431,0.450621307547298,0.652262506980533,0.986674667778914
"9401","LIMD1-AS1",2.53608015607997,0.423621648271237,0.946968248754197,0.447345144706321,0.654625876099153,0.986674667778914
"9402","DNAH1",2.79948417355215,0.370312868266492,0.817636595549416,0.452906426011494,0.650616123510776,0.986674667778914
"9403","CNTN3",39.8486731983062,0.259777932792549,0.581180524672299,0.446983203607908,0.654887187350492,0.986674667778914
"9404","EEFSEC",23.6489581779789,-0.162179568677354,0.363703726513431,-0.445911209742211,0.65566138459126,0.986674667778914
"9405","OPA1",49.070859570992,0.117450680947524,0.263422551096457,0.44586418459108,0.655695354779281,0.986674667778914
"9406","CBR4",36.1183248908689,0.137977282754349,0.309460857002605,0.445863441634516,0.655695891484477,0.986674667778914
"9407","ZNF622",69.5458038473386,-0.114348846740539,0.256604468306257,-0.445622975684366,0.65586961128189,0.986674667778914
"9408","DHX29",143.56630987793,-0.0973578113218123,0.217310636632642,-0.448012176626187,0.654144408738774,0.986674667778914
"9409","SGTB",22.7286781005042,-0.168676627201675,0.377559430549499,-0.446755168997325,0.655051843610637,0.986674667778914
"9410","HOMER1",9.70087074732372,0.231238951307777,0.508284205214336,0.454940265574978,0.64915221382183,0.986674667778914
"9411","GRAMD2B",28.0779649711697,0.157457438485142,0.347434332600166,0.453200572628344,0.650404320161238,0.986674667778914
"9412","TBC1D9B",92.8585704111142,0.103148849567943,0.230311451716741,0.447866785603024,0.654249340535233,0.986674667778914
"9413","PPP1R3G",5.96381386741218,-0.284915577262238,0.627204231681647,-0.45426284273996,0.649639656643793,0.986674667778914
"9414","CGAS",17.7446203917695,-0.170433514906897,0.376735713174639,-0.45239542986436,0.650984138716693,0.986674667778914
"9415","LINC01176",3.42530303513682,0.371762669618357,0.825696552593083,0.450241276230165,0.652536476848943,0.986674667778914
"9416","NCF1",8.45256606022725,0.270691097254014,0.607408389092216,0.445649256933326,0.655850624015,0.986674667778914
"9417","RSBN1L",136.630025565631,-0.11312349409518,0.249344121616432,-0.453684223080255,0.650056123974984,0.986674667778914
"9418","LINC01004",4.25840690415035,-0.399540037028786,0.880076395746179,-0.453983357535721,0.649840805277185,0.986674667778914
"9419","AGBL3",3.27773093346635,-0.357020693511524,0.786109107025891,-0.454161757344665,0.649712405988449,0.986674667778914
"9420","FAM66B",3.74389572917626,0.332518828802557,0.731979108071543,0

.454273660458157,0.649631871524032,0.986674667778914
"9421","CDKN2B-AS1",3.31061092489782,0.393015272255028,0.867142831014758,0.453230146405187,0.650383026815495,0.986674667778914
"9422","FAM219A",15.1374120932077,-0.170338049923254,0.380692984786276,-0.44744205102409,0.654555919653254,0.986674667778914
"9423","PTBP3",79.879297042737,0.130676857705032,0.288159961225981,0.453487212966943,0.650197948947341,0.986674667778914
"9424","OPTN",358.745529944776,0.0867350733744287,0.193328462705777,0.44864099243591,0.653690657709259,0.986674667778914
"9425","TMEM254-AS1",4.89810623782738,0.318842767296911,0.713684026499425,0.44675620506853,0.655051095459901,0.986674667778914
"9426","IFIT1",179.426951669235,0.158696150417592,0.355543200531649,0.446348432990117,0.655345575887749,0.986674667778914
"9427","PAOX",11.4910887897433,-0.199796976689187,0.442743110816426,-0.451270661943804,0.651794486935654,0.986674667778914
"9428","TRIM68",6.50919366537912,0.248287250679075,0.554211869273921,0.448000601294156,0.654152762651924,0.986674667778914
"9429","C11orf74",52.6561342500471,-0.161456331717942,0.359151794577954,-0.449549004502879,0.653035665638905,0.986674667778914
"9430","TSPAN18",12.6363854189428,0.209585685272733,0.469702559050274,0.446209374921248,0.655446011598039,0.986674667778914
"9431","MAP3K11",15.4654232387478,-0.178796844580407,0.394316413856617,-0.453434952990371,0.650235572339249,0.986674667778914
"9432","EED",30.1788008068253,0.147335498836771,0.326064833770996,0.451859518650969,0.651370188954995,0.986674667778914
"9433","FUT4",22.912541213521,-0.139388082539572,0.31135511080518,-0.447682012282078,0.654382705234017,0.986674667778914
"9434","MTMR2",25.8086452124679,-0.133053981751927,0.297848280584355,-0.44671730684792,0.655079184242185,0.986674667778914
"9435","C11orf65",1.92309749195861,-0.49284456960343,1.09672012329162,-0.449380438214482,0.653157240286049,0.986674667778914
"9436","GABARAPL1",209.217139948832,0.134665008633126,0.30108232539542,0.447269724173501,0.65468032410544,0.986674667778914
"9437","AC087239.1",7.79718573403901,0.251735309728044,0.553356654181295,0.454924157549877,0.649163802685286,0.986674667778914
"9438","CCT2",201.057971583668,-0.116819583588394,0.260300030189115,-0.44878820606944,0.653584447411275,0.986674667778914
"9439","C12orf29",51.7312206177627,-0.130075121213569,0.2917029393039,-0.445916388514876,0.655657643575456,0.986674667778914
"9440","RNF10",94.4653149470549,0.0849171131629078,0.19023897331081,0.446370749826174,0.655329457969648,0.986674667778914
"9441","EIF2B1",83.6829649694059,-0.106024357476907,0.235444828421992,-0.450315083102518,0.652483264777564,0.986674667778914
"9442","DDX51",27.6438965199754,-0.204472371684345,0.455779415741892,-0.448621338792839,0.653704837764334,0.986674667778914
"9443","RBM26-AS1",6.00231936910385,0.300117105180757,0.667329515859269,0.449728504506982,0.652906215423228,0.986674667778914

"9444","IPO5",155.103900768682,0.124169802571865,0.274983502922253,0.451553643226999,0.651590571997453,0.986674667778914
"9445","RAB20",8.24586469985384,-0.22685785751806,0.50653917216485,-0.447858467783475,0.654255343889323,0.986674667778914
"9446","LRP10",267.840736728417,0.0733634508914534,0.162356029483387,0.451867732445134,0.651364271341537,0.986674667778914
"9447","EIF2S1",127.955745772429,-0.120128352038143,0.266316221137086,-0.451074108536207,0.651936137693194,0.986674667778914
"9448","AL118558.3",7.07879453904759,0.257311997251645,0.57424626500092,0.448086496916498,0.65409077284286,0.986674667778914
"9449","INF2",33.7967823730074,0.144648095286365,0.320855713042388,0.450819759183329,0.652119459076172,0.986674667778914
"9450","IGHG1",42.2612992346912,0.499579009549874,1.10737193774429,0.451139307871131,0.651889148892771,0.986674667778914
"9451","AC015871.1",9.6811605795737,-0.212430193465247,0.47012578498259,-0.451858205295235,0.651371135161844,0.986674667778914
"9452","RHBDF1",49.5307110952772,0.132658234502066,0.293531463589824,0.451938721933538,0.651313128005598,0.986674667778914
"9453","UBALD1",20.242620517949,-0.165730190281454,0.368852980597383,-0.44931232496222,0.653206368051174,0.986674667778914
"9454","AC009133.1",5.25546729718548,-0.285356033375744,0.630575985737422,-0.452532351104422,0.650885520822178,0.986674667778914
"9455","RSPRY1",64.2786046731647,-0.100229409265929,0.224240058084762,-0.446973703637033,0.654894046630706,0.986674667778914
"9456","CYB5D2",66.3840707409177,-0.15793391042626,0.35384533474037,-0.446335997455337,0.655354557290184,0.986674667778914
"9457","GOSR1",103.371043544753,0.096080858609395,0.213786278570724,0.449424814594028,0.653125233926382,0.986674667778914
"9458","NR1D1",13.5533811275716,-0.31071453496124,0.694737074310132,-0.447240469021718,0.654701444640105,0.986674667778914
"9459","TMEM106A",11.9454072781551,-0.232046860017218,0.510943615324103,-0.454153556395896,0.649718308210565,0.986674667778914
"9460","NSF",44.0927407687269,-0.118773619086975,0.262738947893584,-0.45205943024131,0.651226169242393,0.986674667778914
"9461","DDX42",134.194366714793,0.0929684955385049,0.204932150996769,0.453655002820766,0.650077158452444,0.986674667778914
"9462","RGS9",37.2631436913724,0.148757956049665,0.330184009800937,0.450530466752004,0.652327991109283,0.986674667778914
"9463","H3F3B",4096.60965553019,-0.0805357756475975,0.17889711858286,-0.450179277819368,0.652581176815474,0.986674667778914
"9464","RHBDF2",4.46332884713206,-0.308564688769949,0.69175629627711,-0.446059819665655,0.655554035933958,0.986674667778914
"9465","RAC3",3.9568021079999,-0.308848235890179,0.686134991349192,-0.450127511035212,0.652618500881452,0.986674667778914
"9466","MYL12B",1085.77763935413,-0.124109196541008,0.274708358941831,-0.451785293389226,0.651423665409316,0.986674667778914
"9467","ME2",45.8687394578482,-0.126207963686746,0.277389162677032,-0.454985200102037,0.649119886279221,0.986674667778914
"9468","PHLPP1",17.29784387148,0.174983805080386,0.390143791739275,0.448511058705565,0.653784406894621,0.986674667778914

"9469","TLE5",433.807454225243,-0.0836686787798726,0.186233351048496,-0.449267965747366,0.653238363647959,0.986674667778914
"9470","PNPLA6",16.7138793667817,-0.166917875418003,0.368290520659862,-0.453223382233509,0.650387897045716,0.986674667778914
"9471","FBXL12",51.4272054232635,-0.128357999298691,0.287414860333083,-0.446594859952397,0.655167607519099,0.986674667778914
"9472","ZNF443",2.66157423557665,-0.397044702164577,0.89084981997843,-0.445692072064617,0.655819692082898,0.986674667778914
"9473","GADD45GIP1",286.941785223588,-0.131992265771006,0.293441248963874,-0.449808151502436,0.652848779658598,0.986674667778914
"9474","RBCK1",170.973866817359,-0.117694254022061,0.261729244316124,-0.449679417099859,0.652941614804753,0.986674667778914
"9475","AL117335.1",3.75950684721142,-0.325708236575921,0.729302524052905,-0.446602371216109,0.655162183228055,0.986674667778914
"9476","CTSZ",323.220210191034,-0.0890818265659788,0.195886603487418,-0.454762219467963,0.649280313201305,0.986674667778914
"9477","FP565260.6",4.47958103958696,0.301002959216869,0.665414404582527,0.452354137728224,0.651013880688737,0.986674667778914
"9478","ITGB2",44.476394508275,0.132188819490184,0.290810655277064,0.454552875183491,0.649430944044128,0.986674667778914
"9479","RAB36",5.11371650186882,-0.277519328270935,0.618503729769649,-0.448694672179732,0.653651928543409,0.986674667778914
"9480","BRD1",64.8555239843492,-0.118070617513433,0.263801567245636,-0.447573601424029,0.654460958578595,0.986674667778914
"9481","PBDC1",126.619553895468,-0.131704547875281,0.293250864417495,-0.449119043986093,0.653345783211366,0.986674667778914
"9482","ARMCX1",119.769141957647,0.104783128303889,0.233059169674406,0.449598822695007,0.652999737150975,0.986674667778914
"9483","TMSB15B-AS1",4.11953412202192,0.30280700871373,0.67578665924201,0.448080773085059,0.654094903587077,0.986674667778914
"9484","AL450998.2",7.94967471977058,-0.225916672005555,0.507684036358791,-0.444994634115095,0.656323631888957,0.986863929736557
"9485","GNAI3",208.73721523935,-0.0879152036012715,0.197986626476191,-0.444046172036997,0.657009202315057,0.986863929736557
"9486","ACP6",2.7095983634575,-0.389054407462025,0.877500971430959,-0.443366355284582,0.657500767368506,0.986863929736557
"9487","COG2",32.2153494891679,-0.136877016609561,0.308571314062467,-0.443583088808611,0.657344034586425,0.986863929736557
"9488","PLEKHA3",110.266237259664,-0.086359600031351,0.194756935394532,-0.443422463268929,0.657460190933805,0.986863929736557
"9489","FAM13A",146.840186573755,0.137345925761608,0.308709682261882,0.444903200817317,0.656389709408935,0.986863929736557
"9490","CERT1",117.581078405397,0.109848170314384,0.247683059316336,0.443502961476618,0.657401977626412,0.986863929736557
"9491","LINC00174",7.30726227802961,0.241617012734483,0.543215515624128,0.444790337876998,0.656471277527995,0.986863929736557
"9492","ZNF212",9.69028186342634,0.223471697353561,0.503316258809329,0.443998566392863,0.657043620396292,0.986863929736557
"9493","ZNF467",3.51617040412522,-0.357548133916153,0.805645767776206,

-0.44380315545265,0.657184906845716,0.986863929736557
"9494","ZFP41",6.18040205255517,0.265184607239132,0.597386481191342,0.
44390794835244,0.657109137728048,0.986863929736557
"9495","EHD1",66.783451562649,0.102326792213133,0.229879269926152,0.44
5132752709736,0.656223820679255,0.986863929736557
"9496","MED17",40.5329683145037,-0.121075533879908,0.272934899327673,-
0.443605908142036,0.657327533458434,0.986863929736557
"9497","ITFG2",32.5764581207523,0.149926717279496,0.337067244380637,0.
444797647291379,0.656465994756005,0.986863929736557
"9498","B2M",10943.0498583071,0.120637805702355,0.272026845978474,0.44
3477573944673,0.657420336720159,0.986863929736557
"9499","TAOK2",21.4645219874533,0.170183850961466,0.383498149170038,0.
44376707248725,0.657210996971362,0.986863929736557
"9500","RNF167",68.8030353362997,-0.119394791879986,0.268890510451171,
-0.444027539981435,0.657022672891678,0.986863929736557
"9501","FOXO3B",3.19455009238866,-0.338961266238966,0.763482540203099,
-0.44396727939423,0.657066240767055,0.986863929736557
"9502","CDC6",11.9092665049385,-0.234562090374037,0.529091811976267,-0
.443329654824744,0.657527309124882,0.986863929736557
"9503","SLC16A5",7.82559918587536,0.254100102396634,0.573268651587164,
0.443247858910699,0.657586465447618,0.986863929736557
"9504","LSM14A",139.647196557974,0.0769282812786634,0.173272719083191,
0.443972263410543,0.65706263732281,0.986863929736557
"9505","ZNF600",8.90523274486576,-0.27237520345076,0.614271465668614,-
0.443411779113471,0.657467917473539,0.986863929736557
"9506","TGM2",10.3680784400505,0.341864128552733,0.770731432442822,0.4
43558046502916,0.657362143383484,0.986863929736557
"9507","TRIM27",43.9917442063099,-0.122681437809903,0.276969754814306,
-0.442941641379416,0.65780794668999,0.986962702325326
"9508","PHF3",193.329715425212,0.103495555740656,0.233793878492222,0.4
42678638158179,0.657998195837388,0.986962702325326
"9509","CRCP",69.3215691762468,-0.130776360114354,0.295270649101471,-0
.442903351593922,0.657835643067856,0.986962702325326
"9510","AC023794.2",4.99809868797694,0.301174649484108,0.6801345525178
46,0.442816275645996,0.657898629978822,0.986962702325326
"9511","AC007336.1",2.96067079790118,0.414479631856313,0.9362986035425
55,0.442678895694278,0.657998009532167,0.986962702325326
"9512","MAGOHB",50.9917045816058,-0.166459203559363,0.376180048819799,
-0.442498755799519,0.658128330443077,0.987054117125835
"9513","RILP",47.0870296356425,-0.156335722351174,0.353393827422667,-0
.442383851159329,0.658211462805208,0.987075026635036
"9514","GTF2H2C",22.0161825658321,-0.154916344042842,0.350496131714812
,-0.441991594272123,0.658495288585657,0.987189342892285
"9515","BMP5",11.0516550981001,0.389082219017284,0.880113595019709,0.4
42081819004934,0.658430000209658,0.987189342892285
"9516","TBC1D22A",36.3528166243594,0.129751886921551,0.293444041114996
,0.442169097823672,0.658366846032457,0.987189342892285
"9517","PON2",52.7341723710635,0.105192968044233,0.238096029191411,0.4
41808997829468,0.658627426898261,0.987283689411641
"9518","NAMPT",1323.56943580013,0.143869627972143,0.325906336119052,0.

441444709806403,0.658891080553747,0.987485573670442
"9519","MSRB2",144.948232443562,-0.11415197830131,0.258594920576288,-0
.441431633873234,0.658900545056003,0.987485573670442
"9520","NTPCR",99.1945935426211,0.128941524129209,0.29339440062391,0.4
39481884640646,0.660312405370734,0.987528224522586
"9521","RNF181",152.226839509662,-0.123569831296073,0.280276598622609,
-0.440885296536867,0.659296038756355,0.987528224522586
"9522","CCDC14",62.1263543937203,-0.116902457107702,0.265913289949835,
-0.43962622977496,0.660207840049466,0.987528224522586
"9523","MB21D2",11.6468015554124,0.213236147149839,0.484664616135206,0
.439966401612354,0.659961441797373,0.987528224522586
"9524","CAMLG",314.894638509298,-0.0886242704258743,0.200996430760277,
-0.440924597967483,0.659267585261879,0.987528224522586
"9525","HMGN4",101.733028744281,-0.115841398338585,0.263389964716435,-
0.439809460710848,0.660075114951508,0.987528224522586
"9526","CUL7",15.9049966364932,-0.195148293733023,0.443667286963953,-0
.43985278939188,0.660043730966437,0.987528224522586
"9527","AL021368.2",20.7854143747626,0.173890809272061,0.3952100774816
9,0.439995888718493,0.659940085000771,0.987528224522586
"9528","AL355312.2",4.23930618818438,0.328309313162485,0.7457647955586
02,0.44023171262705,0.659769293430692,0.987528224522586
"9529","JAK2",40.0529017935725,0.124361880177964,0.282528457857646,0.4
4017470353597,0.659810579696043,0.987528224522586
"9530","SLC35D2",41.2393653446091,-0.1397162428759,0.317709401385251,-
0.439761122166105,0.660110128404758,0.987528224522586
"9531","CORO2A",2.87272201075913,-0.414614614207208,0.942831486685621,
-0.439754738850229,0.660114752139762,0.987528224522586
"9532","KIAA1958",3.11060634310446,0.430047922073355,0.977654647334223
,0.439877131711049,0.660026099513235,0.987528224522586
"9533","MASTL",10.1946470449263,-0.215237843674482,0.489260818885308,-
0.439924546103779,0.659991757203982,0.987528224522586
"9534","KAZALD1",52.8044267278736,0.198355292032588,0.450593253621876,
0.44020919185586,0.659785602958718,0.987528224522586
"9535","ACP2",41.1239567707947,0.135848786814455,0.308639840777728,0.4
4015311332502,0.659826215705135,0.987528224522586
"9536","FAM219B",38.0373401663492,0.131158180344476,0.2981744091739,0.
439870680746388,0.660030772011085,0.987528224522586
"9537","HOXB-
AS1",32.7800887637791,0.159973432004117,0.36262476547107,0.44115418260
6097,0.659101380168022,0.987528224522586
"9538","MRPL58",47.7728373059246,-0.128602007222809,0.291662706817333,
-0.440927153924249,0.659265734814465,0.987528224522586
"9539","AC008982.2",4.52204187878514,0.338610984912547,0.7704802221642
2,0.439480437228375,0.660313453926886,0.987528224522586
"9540","SMYD3",24.8954709899708,-0.143095451552227,0.326076791834879,-
0.438839730810063,0.660777669568953,0.987610635529928
"9541","PDIA4",145.013150739819,0.0884423926471615,0.201584736561834,0
.438735561806947,0.660853156229405,0.987610635529928
"9542","INPP5F",9.59350776011552,0.214399013408136,0.488213105503623,0
.439150467267711,0.660552513129856,0.987610635529928

"9543","WASF3",51.7996903562481,0.112097416552954,0.255303601556857,0.439074951819625,0.660607228021093,0.987610635529928
"9544","ERI2",9.72937976633627,0.217350012050584,0.49520736101572,0.438907070373061,0.660728873408011,0.987610635529928
"9545","DYNC1LI2",184.437433584539,-0.0959169876917805,0.21860788398178,-0.438762710405151,0.660833482510908,0.987610635529928
"9546","STARD3",40.8771077762457,0.118224156461412,0.26926259246018,0.439066397531233,0.660613426164781,0.987610635529928
"9547","AL121658.1",2.92027393623343,-0.355945153047367,0.811966081698102,-0.438374411284474,0.661114892719866,0.987624161881353
"9548","ZNF717",9.05591474020887,-0.219261479803734,0.50020757886125,-0.438340978964961,0.661139124209093,0.987624161881353
"9549","PHACTR1",23.1985868776935,0.229578286175557,0.523600173857174,0.438461057956372,0.661052093528381,0.987624161881353
"9550","TIGAR",17.1345221421289,-0.187397045654621,0.427338462134789,-0.438521364818113,0.661008386135265,0.987624161881353
"9551","CC2D2A",31.0346696564871,0.162253641156601,0.37033437331486,0.438127413624262,0.661293923111649,0.987648566489822
"9552","KLF3-AS1",15.3401638032386,0.169170252750381,0.386114863932134,0.438134525637208,0.661288767866908,0.987648566489822
"9553","WNK3",2.88631605445099,0.359478823108671,0.820991152619613,0.437859557879094,0.661488093889388,0.987835145757983
"9554","ZNF784",4.30069039109986,0.350864015010281,0.801923985091405,0.437527772623348,0.661728639257113,0.988090932346867
"9555","AK6",116.3726368827,-0.12374518755574,0.282901397731225,-0.437414549903731,0.661810734082591,0.988110092351883
"9556","ME1",15.3597417081642,-0.20896603599734,0.478304944549831,-0.436888722097602,0.662192051313589,0.988575952704025
"9557","ZNF746",13.642706730895,0.187618557577932,0.429746661248013,0.436579442020737,0.662416374448496,0.988807366106753
"9558","ATR",54.2801578481254,0.117457708589863,0.269221553817216,0.436286422555933,0.662628931585417,0.988814261296816
"9559","ENY2",340.69755545611,-0.113488620679945,0.260095565823531,-0.436334315506727,0.662594188047109,0.988814261296816
"9560","AC008147.2",13.9586835281447,0.247104330732278,0.566278098159161,0.43636568593339,0.662571431028483,0.988814261296816
"9561","SNHG26",23.5674054238708,0.159228631625695,0.365299820839631,0.435884778863873,0.662920329190122,0.989145634999087
"9562","WDTC1",37.7364597768067,0.133930625887308,0.315632594638635,0.424324446087844,0.671329213222972,0.989248586300928
"9563","MEAF6",140.93626587498,0.0948176597423582,0.217728411264747,0.435485930345877,0.663209749348788,0.989248586300928
"9564","SF3A3",96.744123913597,-0.0897985968862378,0.211184208313625,-0.425214544228041,0.670680282618052,0.989248586300928
"9565","ZNF684",13.219785361968,-0.170165344367242,0.391313129755059,-0.434857231787127,0.663666059796602,0.989248586300928
"9566","EIF2B3",60.2860271427064,0.113196864133988,0.264113367538287,0.428591953482168,0.668220202323501,0.989248586300928
"9567","ZRANB2",220.326059775229,0.102776793953432,0.239087397561535,0

.429871231196865,0.667289313713992,0.989248586300928
"9568","CRYZ",24.8863118235448,-0.161367603368632,0.379357585015465,-0
.425370704956521,0.670566458147947,0.989248586300928
"9569","STRIP1",21.9179873453299,0.14216771057624,0.328117231860682,0.
433283280399623,0.664808983161009,0.989248586300928
"9570","PIFO",10.2103727449398,0.220085003093871,0.515996747114258,0.4
26524012650679,0.669726054445624,0.989248586300928
"9571","SLC22A15",3.07881896888176,0.395964966160717,0.923399447448915
,0.428812219083142,0.668059885821044,0.989248586300928
"9572","WARS2-
IT1",2.7241788679983,0.385632866694586,0.911762544672999,0.42295317892
5432,0.672329422154549,0.989248586300928
"9573","PDE4DIP",193.743110958777,0.108657078311764,0.253749837976198,
0.42820550814285,0.668501506437187,0.989248586300928
"9574","SEMA6C",14.4542793060767,0.170465428430294,0.39674471985653,0.
429660232130972,0.667442815631707,0.989248586300928
"9575","PIP5K1A",39.1381760231346,0.126068199445316,0.294284973237023,
0.428388164229434,0.668368540280782,0.989248586300928
"9576","MSTO1",12.5575733844274,-0.19963554536012,0.461691548218218,-0
.432400259720073,0.665450529387831,0.989248586300928
"9577","AC092803.1",5.34320482842833,0.285929174336907,0.6702432336624
69,0.426605088983112,0.669666990476692,0.989248586300928
"9578","ZNF496",18.5722231818744,0.148534316602935,0.344250930203625,0
.431471068255578,0.666125884891391,0.989248586300928
"9579","SH3YL1",53.6771710130722,-0.101612410601838,0.238133116888281,
-0.42670423975305,0.669594762087839,0.989248586300928
"9580","AC007390.2",4.01471600578422,-0.292932183584696,0.690285250422
889,-0.424363961717619,0.671300398947808,0.989248586300928
"9581","ACTR2",288.428400932871,-0.0678437961424953,0.159993036039172,
-0.424042182222762,0.67153505035356,0.989248586300928
"9582","MAP3K2",136.62449819356,-0.11059265022949,0.255322399879825,-0
.433149031505043,0.664906503994027,0.989248586300928
"9583","MAP3K2-
DT",5.79270825651133,-0.28770896036871,0.676901699842686,-0.4250380231
51034,0.670808956705483,0.989248586300928
"9584","INPP1",50.5298205915427,-0.112037851166744,0.260036413593146,-
0.430854470028337,0.666574190700026,0.989248586300928
"9585","BHLHE40-
AS1",2.5111049584249,0.387222610807904,0.914502699016231,0.42342424054
566,0.671985762011864,0.989248586300928
"9586","PLCL2",29.4991176583951,0.140772487152497,0.33320096126192,0.4
22485237195459,0.672670873998242,0.989248586300928
"9587","CLASP2",32.9642237771519,0.142973613315794,0.332809738821339,0
.429595641708506,0.667489807976226,0.989248586300928
"9588","ITGA9-
AS1",15.3394733994089,-0.180983059821156,0.419005900609257,-0.43193439
4140982,0.665789096208924,0.989248586300928
"9589","DCAF1",23.8154121153752,0.149458528273573,0.348924395357235,0.
428340724415541,0.668403073523957,0.989248586300928
"9590","PPM1L",13.8945478273223,-0.218671532740615,0.512989699271825,-

0.426268856959532,0.669911948255808,0.989248586300928
"9591","TMEM175",32.7795719957373,-0.132387662844164,0.309002030949338
,-0.428436222368613,0.668333557650717,0.989248586300928
"9592","NOP14",47.6920427636122,-0.155132046653041,0.364785114148734,-
0.425269674216446,0.670640097889215,0.989248586300928
"9593","BOD1L1",286.360724415846,0.0840504810603802,0.19340461657552,0
.434583633775671,0.663864676655185,0.989248586300928
"9594","RPL9",5925.23590060581,-0.0807567952331816,0.1903721606228,-0.
42420485731205,0.67141641872179,0.989248586300928
"9595","POLR2B",70.9073346069083,-0.0904750796581217,0.209714311178842
,-0.431420627183451,0.666162554255971,0.989248586300928
"9596","COQ2",21.9735963437057,0.163761818243305,0.383008809772295,0.4
27566714041549,0.668966604166328,0.989248586300928
"9597","HADH",50.8317905888443,-0.101567141393035,0.240023485057019,-0
.423155014889095,0.672182165565642,0.989248586300928
"9598","PABPC4L",6.07704437110393,0.32312205739317,0.763184427053451,0
.423386596920877,0.672013222170697,0.989248586300928
"9599","RETREG1",46.0663362280714,-0.185569438380889,0.43847618473621,
-0.423214406712941,0.672138836547793,0.989248586300928
"9600","MRPS30",54.7709217096949,-0.108061617688791,0.253476607094506,
-0.426317911256014,0.669876208152556,0.989248586300928
"9601","MRPS36",130.15289676868,-0.104348663961561,0.244334413400901,-
0.427073135172107,0.669326059578697,0.989248586300928
"9602","PDE8B",2.39465432555451,0.406498527496494,0.951559030633011,0.
427192128297156,0.669239394309023,0.989248586300928
"9603","NR3C1",358.067940544099,0.082699645336975,0.19390255616806,0.4
26501057909197,0.669742777306393,0.989248586300928
"9604","LINC02202",11.9917407409496,0.199210287351807,0.45740945488466
9,0.435518516778443,0.663186101466272,0.989248586300928
"9605","ATP6V0E1",728.850902632826,-0.102717097218903,0.24309754940110
5,-0.422534482441132,0.67263493711398,0.989248586300928
"9606","ZNF354A",22.2770555340356,0.146290227001002,0.343495230337407,
0.425887214961631,0.670190031466221,0.989248586300928
"9607","NUP153",53.8892085628909,0.122600551034609,0.28288403831164,0.
433395082191047,0.66472777263999,0.989248586300928
"9608","AL138724.1",11.5864010470447,0.216740634001355,0.4995324524283
22,0.433886993623212,0.664370505051481,0.989248586300928
"9609","UQCC2",105.106568997753,-0.144298495696863,0.334924091268726,-
0.430839403490643,0.666585146514789,0.989248586300928
"9610","CUL9",13.528521656243,0.178715304893656,0.421338083535917,0.42
4161289655699,0.671448189853055,0.989248586300928
"9611","EEF1A1",16831.3595807834,-0.0750313556573379,0.176974965298113
,-0.423965929480184,0.67159066086903,0.989248586300928
"9612","TMEM242",79.5733122827849,-0.11981221204504,0.28046504006496,-
0.427191253559763,0.669240031383135,0.989248586300928
"9613","HUS1",21.1436826565034,0.156137222888503,0.363356228575305,0.4
29708398011247,0.667407773705569,0.989248586300928
"9614","ZNHIT1",278.547881675607,-0.100613801701811,0.232444734038783,
-0.432850424071226,0.665123438185528,0.989248586300928
"9615","CHPF2",39.223542269112,-0.114151687803545,0.26856570743254,-0.

42504193441085,0.670806105509124,0.989248586300928
"9616","MFHAS1",45.9733152326344,-0.153861324186831,0.35448219397398,-0.4340452829575,0.664255558192016,0.989248586300928
"9617","TCIM",28.2940731586156,-0.232419594127268,0.539523126100711,-0.430787083784585,0.666623191972898,0.989248586300928
"9618","FNTA",155.747808915794,-0.0896598176548035,0.209818087176484,-0.42732168070614 5,0.669145043493304,0.989248586300928
"9619","VPS13B-DT",7.79630718388987,-0.234583714312425,0.545699083897355,-0.429877420055464,0.667284811526649,0.989248586300928
"9620","CDC37L1-DT",2.8782148944663,-0.359994858341524,0.851395370656591,-0.422829240971675,0.67241985171649,0.989248586300928
"9621","HAUS6",29.037126938094,-0.133651705638979,0.307448007389796,-0.434713195163204,0.663770619387401,0.989248586300928
"9622","RUSC2",22.8278080673034,0.144096367432945,0.331388194644964,0.434826495818066,0.663688371213668,0.989248586300928
"9623","INPP5E",15.1598615891448,0.193947380405772,0.44816967951972,0.432754354586448,0.665193237306932,0.989248586300928
"9624","GDI2",331.119439402268,-0.0737405404119567,0.173108395502039,-0.425978995403999,0.67012315157309,0.989248586300928
"9625","WASHC2C",50.9933722962431,0.120963273966851,0.278176758327505,0.434843207944921,0.663676239747486,0.989248586300928
"9626","RTKN2",3.51891951578849,-0.345618889519991,0.795084103809688,-0.434694754761087,0.663784006184085,0.989248586300928
"9627","ADIRF-AS1",3.9983315350502,-0.304884155492171,0.707874727975061,-0.430703546041712,0.666683940110448,0.989248586300928
"9628","LZTS2",91.6627110121598,-0.0886063265089969,0.209288399232313,-0.423369507502624,0.672025688652862,0.989248586300928
"9629","RASSF7",2.98911320668828,-0.341107436002025,0.80357339151399,-0.424488216763069,0.671209796958884,0.989248586300928
"9630","PGAP2",17.2073093498418,-0.159745761816311,0.372149924691814,-0.42925109268422,0.667740504418808,0.989248586300928
"9631","TRIM6",3.02495030684144,0.377566622222275,0.889000588263421,0.424709080294103,0.671048763583557,0.989248586300928
"9632","CSTF3",37.4844961519154,0.110881124936945,0.259553869520703,0.427198889932561,0.669234469795806,0.989248586300928
"9633","CD59",810.172070215914,0.0934196438724711,0.221199654884065,0.422331779502251,0.672782865062797,0.989248586300928
"9634","NUDT22",53.5820222746613,-0.129213498689876,0.300642641274574,-0.429790990865684,0.667347686951687,0.989248586300928
"9635","MAP4K2",5.72190445165523,-0.289459361502184,0.675567441003485,-0.428468490240179,0.668310069525504,0.989248586300928
"9636","MEN1",18.1102859966457,0.158063805718884,0.369371529409702,0.427926337396625,0.668704751410353,0.989248586300928
"9637","FEZ1",116.469305443886,0.126877526794622,0.299418110716469,0.423747002113602,0.671750332836804,0.989248586300928
"9638","PTPN6",6.91014189767864,-0.266257985529952,0.627284584082309,-0.424461229059975,0.671229474945365,0.989248586300928

"9639","FAM234B",9.50489365272215,-0.200545627171117,0.471654385331851,-0.425196146602168,0.670693693017473,0.989248586300928
"9640","AC008035.1",2.12525062760539,0.433733473568408,1.01930081765763,0.425520578473716,0.670457223492641,0.989248586300928
"9641","KCNMB4",12.8635309214039,0.192303629066928,0.446952077944109,0.430255587917806,0.667009729805623,0.989248586300928
"9642","KRR1",168.792926574958,0.0753625419163574,0.17786894529413,0.423697019126838,0.671786789387475,0.989248586300928
"9643","UBC",3063.30692011266,-0.0756309338143103,0.178444849596527,-0.423833660569727,0.671687127791706,0.989248586300928
"9644","FGF9",1.99245466153751,0.418804459314289,0.966643031892223,0.433256585416512,0.66482837442935,0.989248586300928
"9645","RASL11A",24.6057559334797,0.186799204615334,0.43648737413398,0.427960155745527,0.668680129309254,0.989248586300928
"9646","PCCA",28.1168932085113,0.120253667677197,0.283262730245654,0.424530497086254,0.671178968866795,0.989248586300928
"9647","METTL17",32.1526482483948,-0.136415638328753,0.314863501339191,-0.433253259741266,0.664830790219622,0.989248586300928
"9648","SOS2",52.5084524026931,0.117366222877443,0.276285027357347,0.424801242398276,0.670981571918903,0.989248586300928
"9649","WDR25",16.8670367199425,-0.163566187932776,0.380513077807159,-0.429856941778148,0.667299708833256,0.989248586300928
"9650","PPP1R13B",4.0761138729867,-0.336850740357509,0.795952539593714,-0.423204555047279,0.672146023707491,0.989248586300928
"9651","C15orf41",11.5733468798503,0.192025942416077,0.451841025608573,0.424985628866795,0.670847151094755,0.989248586300928
"9652","MAPK6",61.3664306972023,0.123497230997828,0.287824312891267,0.429071574104589,0.667871138172121,0.989248586300928
"9653","AC103740.1",2.19092435094971,0.449845983092037,1.04629779044823,0.429940679602626,0.66723879301185,0.989248586300928
"9654","AC007950.2",3.68492855439767,0.333664003783984,0.785022860211702,0.42503730871481,0.670809477509536,0.989248586300928
"9655","CORO7",22.3232598742854,0.157407959673204,0.365791800848438,0.430321180814069,0.666962021667125,0.989248586300928
"9656","CCP110",34.228781157409,0.148649890169868,0.346499369884831,0.429004792185555,0.667919737229571,0.989248586300928
"9657","IQCK",30.1908640700741,0.14244047634236,0.33575719371423,0.42423655846848,0.671393301446098,0.989248586300928
"9658","AC105429.1",4.0562807778073,-0.408716647802723,0.953362943238447,-0.428710440972635,0.66813396137446,0.989248586300928
"9659","AC090617.10",4.81743940648688,0.340677578788449,0.782445667878075,0.435400939355108,0.663271428650901,0.989248586300928
"9660","MIEF2",9.65516335925758,0.200399373786893,0.470696774092508,0.425750472102252,0.670289680069101,0.989248586300928
"9661","TP53I13",108.468462120447,-0.122282102479352,0.285708087711207,-0.427996643213524,0.668653564304172,0.989248586300928
"9662","BLMH",43.473974439759,-0.110795880871982,0.258170239524086,-0.429158221630132,0.667808084436567,0.989248586300928
"9663","MKS1",7.73537500363589,-0.225066333319544,0.532927884752798,-0.422320429759352,0.672791148218916,0.989248586300928

"9664","UNK",31.8637030526615,0.134131795845326,0.309470833859089,0.43342306017245,0.664707450625457,0.989248586300928
"9665","MAFG",87.997589323389,-0.0974519680156709,0.229580428252363,-0.424478553148043,0.671216843123058,0.989248586300928
"9666","DUS1L",78.1298529253594,-0.108984123647982,0.254163583940837,-0.428795195433467,0.668072275649774,0.989248586300928
"9667","PPP4R1",40.9753552587375,-0.118116897025542,0.272968513745217,-0.432712533049837,0.665223623580145,0.989248586300928
"9668","OSBPL1A",221.251525330831,0.0866867097063719,0.205063829568584,0.422730375653009,0.672491990785514,0.989248586300928
"9669","ZNF397",47.8967825131431,0.122265412590367,0.287990799935616,0.424546244594275,0.671167486938289,0.989248586300928
"9670","CDC34",42.8988183100711,0.114884173346979,0.26963867008932,0.426067126458245,0.670058933429429,0.989248586300928
"9671","GNG7",2.72689321525613,-0.397856891757646,0.927069601600907,-0.429155363384376,0.667810164355536,0.989248586300928
"9672","ZNF709",6.4091597296908,-0.259421614437946,0.598082949176973,-0.433755242136459,0.664466186625182,0.989248586300928
"9673","ILVBL",53.8897438996122,-0.119498635745302,0.281673372723752,-0.424245410880562,0.671386846101456,0.989248586300928
"9674","NUDT19",15.6361908314291,0.153141745041423,0.362017042348208,0.423023579354373,0.672278057595664,0.989248586300928
"9675","PDCD2L",12.5950965451615,-0.195366311539276,0.461308019343227,-0.423505127479515,0.671926758333978,0.989248586300928
"9676","ZNF302",72.1888477286365,-0.107367226118791,0.253040440664593,-0.424308564420762,0.671340794060898,0.989248586300928
"9677","U2AF1L4",26.878823870569,-0.149817246876551,0.353168603697228,-0.424208848997771,0.671413507867675,0.989248586300928
"9678","ZNF420",15.3842557027334,0.16735588497526,0.393456821619663,0.425347524250159,0.670583353925968,0.989248586300928
"9679","HNRNPL",118.30483549549,-0.0984609519030384,0.226246744974863,-0.435192788802234,0.663422496428704,0.989248586300928
"9680","AXL",260.332547402792,-0.0697764372187376,0.164678447198965,-0.42371323270029,0.671774963459709,0.989248586300928
"9681","CLASRP",29.7431149762799,-0.138810937944519,0.318613805498495,-0.435671447843697,0.663075124305844,0.989248586300928
"9682","KIF16B",27.1279575269289,-0.151744650417399,0.353275166683324,-0.429536703193809,0.667532689449563,0.989248586300928
"9683","MAP1LC3A",165.029467885707,-0.0867748055370828,0.199871507949344,-0.434152953702011,0.664177374152125,0.989248586300928
"9684","UQCC1",22.5684336359986,0.135002379955501,0.314512082992297,0.429243858204351,0.667745768677921,0.989248586300928
"9685","SAMHD1",387.15423945544,0.100789312259918,0.238426266353514,0.422727385708746,0.672494172505479,0.989248586300928
"9686","STMN3",92.8284705114363,-0.108295084816813,0.255592439886858,-0.423702222431742,0.671782994169126,0.989248586300928
"9687","LINC01697",34.2087830078428,0.182135016554524,0.43133492718701,0.422258910824436,0.672836046044996,0.989248586300928
"9688","BACH1",79.6823017171454,-0.147827495380427,0.339889880997878,-0.434927615221797,0.663614969179107,0.989248586300928

"9689","AP000240.1",2.11623323600963,0.439187848365909,1.0155286167873
1,0.432472154015026,0.665398286434081,0.989248586300928
"9690","KRTAP13-4",9.4604871026277,0.271106742217894,0.630334616619373
,0.430099720164348,0.667123103586231,0.989248586300928
"9691","CHAF1B",2.37575898543866,-0.460219138585673,1.07465388043378,-
0.428248710552191,0.668470055911935,0.989248586300928
"9692","VPS26C",32.0575375357109,0.119571308480495,0.28024184122843,0.
426671862975059,0.669618347272301,0.989248586300928
"9693","LRRC3",5.21908109267415,-0.306669806631508,0.724390152056967,-
0.423348944985921,0.672040688829411,0.989248586300928
"9694","CYTH4",6.87749389479697,-0.268348719631522,0.625120060035409,-
0.429275489281727,0.667722752052202,0.989248586300928
"9695","DESI1",56.0076745777727,-0.139619228859059,0.326504871783306,-
0.427617597546051,0.668929551885307,0.989248586300928
"9696","POLDIP3",58.9801654024411,-0.0964186056573835,0.22687332196024
8,-0.424988733026432,0.670844888200526,0.989248586300928
"9697","PPARA",38.0329334265733,0.130886771142878,0.307550849022484,0.
425577661576572,0.670415620555406,0.989248586300928
"9698","SCML2",4.29920466260535,-0.335155365881552,0.793516624880671,-
0.422367163299134,0.672757041868043,0.989248586300928
"9699","ZNF41",5.40418864475677,-0.283846238548502,0.666229394778208,-
0.426048806572091,0.670072282317581,0.989248586300928
"9700","CCDC22",21.4626265395112,-0.176174575269297,0.409014885722303,
-0.430729006251644,0.666665425369354,0.989248586300928
"9701","GPR174",3.64335631093215,-0.332761124211259,0.780400156111299,
-0.42639807489198,0.669817803936897,0.989248586300928
"9702","SH3BGRL",346.273306856441,-0.0938279101636823,0.21760711871926
6,-0.431180334154091,0.666337252074015,0.989248586300928
"9703","TSPAN6",44.1328851030332,0.145493361196994,0.335563478062833,0
.43357925015234,0.664594005402094,0.989248586300928
"9704","GALNT4",20.5167536585138,0.144102823649569,0.341386679713284,0
.422110270296997,0.672944531873961,0.989270291363541
"9705","SNU13",364.155324655889,-0.114781676756196,0.271963133002589,-
0.422048663320492,0.672989497944986,0.989270291363541
"9706","C11orf52",3.14538384905175,-0.337691401021822,0.80042395477448
7,-0.421890673070827,0.673104818163337,0.98933786687803
"9707","COL6A3",2999.67532242731,-0.102400385164178,0.24298773482328,-
0.42142203283903,0.673446933176913,0.989366345985078
"9708","ADRA2A",36.265887957896,-0.185490947866837,0.439808637322664,-
0.421753763173032,0.673204757631466,0.989366345985078
"9709","SARS2",9.63612787652257,0.200352103693099,0.475543450809586,0.
421311876658191,0.67352735879644,0.989366345985078
"9710","AL118506.1",3.19683106889339,0.355183021014658,0.8424111933446
8,0.421626663820137,0.673297540908167,0.989366345985078
"9711","LTN1",79.009068860963,0.0935510849683429,0.222056454406202,0.4
21294148906891,0.67354030227163,0.989366345985078
"9712","PAXBP1-
AS1",2.4037275685139,0.412028843906583,0.977624925450316,0.42145902091
9289,0.673419928818004,0.989366345985078
"9713","NAP1L3",8.30422967485395,-0.236604652243837,0.561805349740077,

-0.421150585969507,0.67364512472853,0.98941844428881
"9714","PCBD2",14.3403071135888,-0.18197781596234,0.4325109353739,-0.4
207473177644,0.67393960484768,0.989647185049615
"9715","TINF2",75.5570390719839,-0.103315122203971,0.245542490714445,-
0.420762703446393,0.673928368783805,0.989647185049615
"9716","GAS2L3",3.14643187319087,-0.330564421380172,0.786589085325384,
-0.420250455475655,0.674302499248367,0.990078165322891
"9717","SKP1",863.220397325533,-0.0861992121382567,0.205168184028563,-
0.420139275231174,0.674383712586223,0.990095507230118
"9718","GSTA4",61.2504982989244,0.122454594987797,0.291839131359853,0.
419596215275204,0.674780453677724,0.990376553365949
"9719","LFNG",10.9778523556753,-0.210305201769804,0.501224428961134,-0
.419582904619582,0.674790179124574,0.990376553365949
"9720","AC016831.1",39.2359265211642,0.330929847589379,0.7888726778338
86,0.419497159539176,0.674852830174568,0.990376553365949
"9721","ZNF276",29.4085262961842,0.166293253001864,0.396367919062261,0
.419542664793066,0.674819580733454,0.990376553365949
"9722","CCT8",221.772045804952,-0.117846572705147,0.281057387473716,-0
.419297189675072,0.674998950192794,0.99048909930574
"9723","ATP6V1D",102.301071301504,-0.105653345572124,0.252034622316894
,-0.41920171364108,0.675068719825858,0.990489597555867
"9724","DFFA",71.0635192637986,0.108496114746089,0.263566634794921,0.4
11645862650669,0.680599010879785,0.990608592989741
"9725","NECAP2",81.3619229299495,0.104020695720093,0.257831123381474,0
.403445070385041,0.686620836209635,0.990608592989741
"9726","ZNF436",56.8313422387778,0.111973193725322,0.27919638445109,0.
401055314328173,0.688379399444362,0.990608592989741
"9727","PDIK1L",10.3412840353235,-0.209756928149784,0.512029578130705,
-0.409657834446899,0.682056965247496,0.990608592989741
"9728","PPP1R8",45.3627455214996,0.118966671594838,0.289641755987871,0
.41073729576415,0.681265176200822,0.990608592989741
"9729","TAF12",95.4479789045934,-0.12175277765164,0.2998267587776,-0.4
06077089810225,0.684685958677018,0.990608592989741
"9730","PIGK",57.7561798262193,0.112716238285785,0.271776326689277,0.4
14738986499932,0.678332988901751,0.990608592989741
"9731","C1orf52",79.3927338716785,0.105496626564427,0.258921119919112,
0.407446973029409,0.683679732975666,0.990608592989741
"9732","GBP3",142.682041151852,0.10188858779856,0.249056601823057,0.40
9098120879954,0.682467655233317,0.990608592989741
"9733","WDR77",22.8788492440351,0.143435905211664,0.349262663596366,0.
410682045812458,0.681305693737972,0.990608592989741
"9734","CTSK",1036.93712593075,0.0860478380649115,0.206426683470179,0.
416844550415607,0.676792113924853,0.990608592989741
"9735","SHE",22.1928633623937,-0.172449900735597,0.412905392745237,-0.
417649911494373,0.676203098883123,0.990608592989741
"9736","IFI16",1071.98021801993,0.109580916279606,0.265504765510212,0.
4127267398347,0.679806831360912,0.990608592989741
"9737","FMOD",105.71759130644,-0.14926835020398,0.369172880224359,-0.4
04331840717186,0.685968714184261,0.990608592989741
"9738","HLX",12.8267463755874,0.174848514375697,0.421429221736379,0.41

489413965003,0.678219400097162,0.990608592989741
"9739","TRIM11",19.5272848551491,0.135518032677055,0.333269990520219,0.406631369555708,0.684278753869388,0.990608592989741
"9740","ITSN2",64.9209195247296,0.110927644336275,0.275247209190502,0.403010968440013,0.686940155536214,0.990608592989741
"9741","SNX17",158.320932799423,-0.098125069328754,0.239659168375009,-0.40943590847821,0.682219792229599,0.990608592989741
"9742","RAB11FIP5",22.4475593731917,0.135744298495087,0.331764599758186,0.409158477408461,0.682423364087705,0.990608592989741
"9743","THNSL2",31.4816039120246,-0.167518386652482,0.407811362456333,-0.410774201198034,0.681238112123058,0.990608592989741
"9744","MITD1",76.5026632394776,0.101292383742924,0.248102378415861,0.40826849137714,0.68307656909781,0.990608592989741
"9745","RABL2A",16.0105530187193,0.155182864869107,0.382208813057772,0.4060159252415,0.684730899197281,0.990608592989741
"9746","MARCO",2.50303771111163,0.376846198507327,0.910565248256323,0.41385963194726,0.678976909122328,0.990608592989741
"9747","METAP1D",14.216747132002,0.188634775822853,0.462605588605208,0.407765881928927,0.683445564081602,0.990608592989741
"9748","NFE2L2",438.960267665773,0.0705960266879026,0.172848829642533,0.408426408404971,0.682960648601284,0.990608592989741
"9749","AC019080.1",4.06781026285291,-0.287835505726801,0.711169746902256,-0.404735306838581,0.685672086590824,0.990608592989741
"9750","PRKRA",99.0329092467012,-0.0996986210088759,0.248200542495289,-0.401685749783436,0.687915313267531,0.990608592989741
"9751","ZC3H15",273.624328664333,-0.0937243130488927,0.231468070227859,-0.404912491630615,0.685541835941958,0.990608592989741
"9752","IKZF2",18.373581735347,-0.173490545097559,0.418097375644674,-0.414952485243539,0.678176686734797,0.990608592989741
"9753","SGPP2",2.037255567585,-0.408658945428767,0.99045942434226,-0.412595342510015,0.679903114173499,0.990608592989741
"9754","DOCK10",19.9896667181611,-0.148150966298103,0.364204699331768,-0.406779392385451,0.684170023566572,0.990608592989741
"9755","IL17RC",43.6778282522264,-0.135191946043299,0.328690449519864,-0.411304758750311,0.680849080610461,0.990608592989741
"9756","LSM3",240.851330318248,-0.0979323233815492,0.236142826033503,-0.414716487587282,0.67834946113419,0.990608592989741
"9757","UBE2E2",76.7703120611163,-0.081063227430115,0.200532506165889,-0.404239836124403,0.68603636257896,0.990608592989741
"9758","C3orf62",10.0871639374669,0.235454943978947,0.585681711722078,0.402018603733825,0.687670334911514,0.990608592989741
"9759","CYB561D2",44.2985750123394,-0.148328168011187,0.363836322072198,-0.407678285571922,0.683509881430631,0.990608592989741
"9760","CD200",26.0170780592628,-0.194822362120946,0.482215330673294,-0.404015280577921,0.686201482532934,0.990608592989741
"9761","KPNA1",54.3012343206028,0.109674452662105,0.263304997896648,0.416530083128746,0.677022158832007,0.990608592989741
"9762","TOPBP1",45.6337400392196,0.0992885803076285,0.246022572936962,0.403575083059834,0.686525211581833,0.990608592989741
"9763","ATP1B3",199.307708676575,-0.10240319436021,0.246789481282955,-

0.414941487083885,0.678184738135698,0.990608592989741
"9764","HLTF",55.0118402955255,0.0954448073900896,0.229398885784049,0.416064825528138,0.677362567917391,0.990608592989741
"9765","GOLIM4",213.904083730035,-0.0889678502285212,0.216482914702503,-0.410969384585308,0.681094983971296,0.990608592989741
"9766","EIF4G1",170.334478681113,0.0940014816109445,0.233868815320805,0.401941068893686,0.687727397125056,0.990608592989741
"9767","MAGEF1",58.4916650273402,-0.0982161507039348,0.237971661573786,-0.4127220445258,0.679810271809407,0.990608592989741
"9768","LRPAP1",310.567239534678,-0.0885690045112879,0.218973235735924,-0.404474109420841,0.685864112954089,0.990608592989741
"9769","SEPSECS-AS1",6.69559357544165,0.265352393736947,0.648828193899844,0.408971737405585,0.682560402162518,0.990608592989741
"9770","ANAPC4",26.9987797893973,-0.123526404317592,0.307022284197324,-0.402336933426635,0.687436076979798,0.990608592989741
"9771","NAAA",53.3028493482443,-0.125591949584426,0.307113839887502,-0.408942656672299,0.682581743832981,0.990608592989741
"9772","CNOT6L",69.3459072556315,0.111185960730119,0.271242130656037,0.409914051556816,0.681868997281211,0.990608592989741
"9773","AC124016.2",3.80259699877206,-0.312942324646212,0.748060985870611,-0.418337983877079,0.675700021902818,0.990608592989741
"9774","SGMS2",53.0931350690309,0.107079715020858,0.259623134722737,0.412442886244378,0.68001483469499,0.990608592989741
"9775","RWDD4",82.5612346423096,-0.0971223937763205,0.240281114173787,-0.404203193872637,0.686063305298802,0.990608592989741
"9776","ZFR",142.09666868823,0.0804351359494226,0.198402072465202,0.405414797083485,0.685172636033119,0.990608592989741
"9777","MAP3K1",22.4089440847241,-0.147892678336041,0.363113616802166,-0.407290367236812,0.683794736848249,0.990608592989741
"9778","SMIM15",108.311500627253,-0.0899107359360451,0.216123646341018,-0.416015264679444,0.677398833355477,0.990608592989741
"9779","TSLP",4.06192670971878,0.320227978768973,0.793476440179687,0.403575912974121,0.686524601194039,0.990608592989741
"9780","DCP2",60.7382575545063,0.0983294030877791,0.244899577709351,0.401509075709706,0.688045357552893,0.990608592989741
"9781","HSD17B4",87.0094492824378,-0.0917476317754773,0.222945233763212,-0.41152542365145,0.680687302982818,0.990608592989741
"9782","CXCL14",10820.9553761566,-0.172043434025695,0.418797824866619,-0.410803074444066,0.681216938622665,0.990608592989741
"9783","CSNK1A1",324.194232252796,0.0680200950257669,0.165297195476288,0.411501809391101,0.680704614771578,0.990608592989741
"9784","MAPK9",40.888045231225,0.1011958289754,0.251908276223671,0.401716968145769,0.687892335362247,0.990608592989741
"9785","TRIM52-AS1",37.4466442797928,-0.122276544557046,0.295439766732176,-0.413879776272273,0.678962155521263,0.990608592989741
"9786","ABT1",50.4488637672956,-0.112117388887177,0.275978016524887,-0.406254781808198,0.684555406239568,0.990608592989741
"9787","HIST1H2BK",2.14443923610675,0.416269867769705,1.01911598730481

,0.408461718739775,0.682934729736788,0.990608592989741
"9788","NOTCH4",6.50979916231957,-0.255260462795372,0.619300966352698,
-0.412175140463125,0.680211056805897,0.990608592989741
"9789","BTBD9",9.35010667779432,-0.199115103524572,0.491895103003444,-
0.404791798716438,0.68563055772475,0.990608592989741
"9790","PEX6",14.0289487276098,0.184925797275826,0.455194866344723,0.4
06256333162993,0.684554266482126,0.990608592989741
"9791","SDHAF4",13.9286221226453,0.159417423799492,0.390850685990922,0
.407872953824609,0.683366949999952,0.990608592989741
"9792","RSPO3",39.7304591307429,-0.138177973958527,0.336711998023597,-
0.410374369697523,0.68153134474616,0.990608592989741
"9793","PEX3",33.9079234733913,0.118006142677924,0.290454794262717,0.4
06280581380892,0.684536451768151,0.990608592989741
"9794","MTHFD1L",31.2688478506437,-0.154491170508146,0.381501219943079
,-0.404955901664474,0.685509926134551,0.990608592989741
"9795","WTAP",364.391591252185,-0.139601257534317,0.336924581967855,-0
.414339781083816,0.678625283824881,0.990608592989741
"9796","RNASET2",103.493871658535,-0.102183687220124,0.244734253947991
,-0.417529158962109,0.67629140076329,0.990608592989741
"9797","AC007566.1",5.31501034230154,-0.25842402162701 5,0.638108192174
033,-0.404984648052495,0.685488795575038,0.990608592989741
"9798","CPSF4",32.8321636063304,0.114740071918805,0.278000789056525,0.
41273290017704,0.679802317431477,0.990608592989741
"9799","SLC12A9",17.9268571390822,-0.16466447332097,0.397195573372558,
-0.414567745362356,0.678458364330226,0.990608592989741
"9800","DLD",60.0574542695654,0.116082978669444,0.279566460551007,0.41
5224982426906,0.677977211998322,0.990608592989741
"9801","NUP205",20.3336432883891,0.135959618833869,0.337125179384348,0
.403291202045947,0.686734013215379,0.990608592989741
"9802","RNF122",10.4696097477689,-0.252745815760688,0.623420708164508,
-0.405417741904706,0.68517047177944,0.990608592989741
"9803","ZC2HC1A",83.6034103607508,-0.109203841320877,0.261983133512833
,-0.416835388815317,0.676798815561249,0.990608592989741
"9804","AC009812.1",5.402268723603,-0.277575942784655,0.66969993546394
6,-0.414478079040518,0.678524017722398,0.990608592989741
"9805","RIPK2",60.1845605571157,-0.139629428467266,0.338603475014737,-
0.412368563143628,0.680069301488442,0.990608592989741
"9806","FSBP",5.11334816494758,0.310561237495078,0.774544934779858,0.4
0095961325129,0.688449858706805,0.990608592989741
"9807","ZFAT",6.32870842978341,0.245916879327131,0.60283588851597,0.40
7933376250236,0.683322588300489,0.990608592989741
"9808","RFX3",11.1960395066178,0.187333088186829,0.459146540994995,0.4
08002830165873,0.683271597098448,0.990608592989741
"9809","AL355377.3",2.98020951117277,0.403743666836479,1.0008523287340
6,0.403399837563608,0.686654106232268,0.990608592989741
"9810","RMI1",5.79444744566737,0.260370721648412,0.631217014924432,0.4
12490024020634,0.679980291205946,0.990608592989741
"9811","DOLK",25.0404014329651,0.145699136255917,0.34790947201875,0.41
878462063851,0.675373545553646,0.990608592989741
"9812","SKIDA1",2.37982324966063,0.425196483823569,1.06012811776627,0.

401080281428131,0.688361018032767,0.990608592989741
"9813","CSGALNACT2",67.1165851422709,0.0986863825509128,0.238920894573
746,0.413050448044644,0.679569652944054,0.990608592989741
"9814","CAMK2G",43.5286508511537,0.10233575688218,0.249118858694658,0.
410790886801595,0.681225876106451,0.990608592989741
"9815","AP3M1",31.6187465133213,0.122715354255066,0.301719262157137,0.
406720318012559,0.684213415915451,0.990608592989741
"9816","CHUK",17.8702667922892,-0.156752982684596,0.383490364848985,-0
.408753379622259,0.682720656037316,0.990608592989741
"9817","NDUFB8",373.807561202281,-0.118784462621956,0.287173418909667,
-0.413633208369193,0.679142749057928,0.990608592989741
"9818","TWNK",8.6162042054076,-0.208013481085317,0.517885372470386,-0.
401659309458893,0.687934774580146,0.990608592989741
"9819","TMEM86A",4.13802758279058,0.287683903576019,0.691767452176929,
0.415867937513834,0.677506642304384,0.990608592989741
"9820","MAPK8IP1",15.9739295121819,-0.182763187770871,0.44642474497933
8,-0.409393049615406,0.682251239462536,0.990608592989741
"9821","AP000662.1",3.48240316482175,0.325316541763057,0.7953882466600
4,0.409003456021776,0.682537124900844,0.990608592989741
"9822","TAF6L",9.94910447708537,-0.184459291857373,0.458503722311739,-
0.402307075997866,0.687458047703547,0.990608592989741
"9823","RHOD",33.7061251927449,-0.132747299917516,0.319683059744525,-0
.415246588366619,0.677961396876935,0.990608592989741
"9824","YAP1",113.438804659109,0.092407057454365,0.221078552896055,0.4
17982912606688,0.67595961050895,0.990608592989741
"9825","IL10RA",17.3184514978378,-0.213706465007952,0.511055168797391,
-0.418167113955317,0.675824938166789,0.990608592989741
"9826","MPZL3",2.83437806622637,0.334539894371482,0.800682774218923,0.
417818273532648,0.676079989357566,0.990608592989741
"9827","AP003396.5",3.81196572832304,-0.351170975648792,0.847189114901
452,-0.414513087422803,0.678498384407217,0.990608592989741
"9828","CACNA2D4",5.88150348285744,0.303233027129168,0.747874122558727
,0.405459980473328,0.685139429439901,0.990608592989741
"9829","C12orf4",25.6704927346986,-0.13101530193892,0.32213516539066,-
0.406709096102672,0.684221658948199,0.990608592989741
"9830","IFFO1",32.5992863836825,0.11696532997411,0.286599083646894,0.4
08114807925267,0.683189389073717,0.990608592989741
"9831","ZNF384",27.4568060936306,0.157598181715992,0.389887874751572,0
.404214113651035,0.686055276041717,0.990608592989741
"9832","P3H3",63.7943356369828,0.124736376859052,0.311117703863232,0.4
00929858089614,0.688471766291839,0.990608592989741
"9833","U47924.2",2.0276811763658,0.442315220759182,1.08173689326698,0
.408893533642302,0.682617794653013,0.990608592989741
"9834","AC073611.1",8.67463549053441,-0.258246384913781,0.621377604868
581,-0.415602981006049,0.677700544977816,0.990608592989741
"9835","USP44",6.90517635838432,0.262176474953838,0.634620158994572,0.
413123458557011,0.679516163107362,0.990608592989741
"9836","TXNRD1",138.243692156253,0.0808029536254032,0.200409270145815,
0.403189700589259,0.686808675895177,0.990608592989741
"9837","SPPL3",86.2301652485173,0.087773872299402,0.212400772903847,0.

413246482578182,0.679426035424152,0.990608592989741
"9838","POLE",14.7772139014029,0.174809534497305,0.432997266004516,0.403719718857259,0.686418837508527,0.990608592989741
"9839","EEF1AKMT1",56.0645941152077,-0.144325029252182,0.346416015540301,-0.416623431878805,0.676953867487589,0.990608592989741
"9840","USP12",40.2030782224046,-0.121688187139533,0.302999449749485,-0.401611908008885,0.687969664776581,0.990608592989741
"9841","TRIM13",46.4979612208298,0.106626362418456,0.255094672961802,0.417987412988521,0.675956320089351,0.990608592989741
"9842","NEMF",130.526137609451,0.111544837518766,0.271975524852959,0.410128218629494,0.681711893532955,0.990608592989741
"9843","RAB15",2.73001463457007,-0.383724948807547,0.939882548140989,-0.408269043367731,0.683076163890848,0.990608592989741
"9844","PAPLN",40.7038398593725,-0.22346987321004,0.545067396296529,-0.409985764564915,0.681816390168211,0.990608592989741
"9845","FCF1",42.2360244928312,0.11245248299783,0.268642313913663,0.418595571783118,0.675511726415965,0.990608592989741
"9846","ACYP1",40.910247662162,-0.173365004136888,0.429728794834348,-0.403428874724852,0.686632748507121,0.990608592989741
"9847","VASH1-AS1",4.05551229272772,0.296650175418695,0.716679272451558,0.413923196639883,0.678930355083436,0.990608592989741
"9848","AL049775.1",3.34498724831558,0.339173349642178,0.842637413876198,0.402513992444217,0.687305792726229,0.990608592989741
"9849","DICER1-AS1",3.31873911962541,0.319203831206504,0.795865797592215,0.401077458250137,0.688363096518708,0.990608592989741
"9850","YY1",307.974159004363,-0.0631798379284533,0.151998943974926,-0.415659716286421,0.677659022696421,0.990608592989741
"9851","GABPB1-IT1",96.6555922305473,0.10073152149577,0.246051796632927,0.409391530052701,0.682252354435574,0.990608592989741
"9852","DMXL2",25.2854777747889,0.185235612830486,0.451308747905509,0.410440997853801,0.681482476947332,0.990608592989741
"9853","LACTB",62.0945765170644,-0.151290631480219,0.375168444441675,-0.403260545287517,0.686756563463605,0.990608592989741
"9854","CIAO2A",53.458918837361,0.0979318908940166,0.242529737539636,0.403793332263067,0.686364700061152,0.990608592989741
"9855","ST20-AS1",3.56323599740876,-0.29419263660354,0.726636145656148,-0.404869257278543,0.685573617166853,0.990608592989741
"9856","SLCO3A1",215.09834800464,0.114373526780177,0.278883656035575,0.410111974312283,0.681723809181477,0.990608592989741
"9857","AC090825.1",3.48743416390368,-0.381235925899981,0.911498444425654,-0.418251866727214,0.675762977640429,0.990608592989741
"9858","SNRPA1",97.6857153262564,-0.130528702131657,0.314882082659904,-0.414532008391973,0.67848453053241,0.990608592989741
"9859","KCTD5",18.9598207357123,0.148260046963633,0.365028521700058,0.406160171465881,0.684624916417499,0.990608592989741
"9860","TIGD7",4.05264434757209,0.296567917453208,0.737236413237513,0.

402269763305443,0.687485504787693,0.990608592989741
"9861","NPIPB13",2.25579493129604,-0.375163478853393,0.90820916602663,
-0.413080480672436,0.679547649884989,0.990608592989741
"9862","ZNF771",22.2222723829813,-0.164301912535777,0.407859449561381,
-0.402839538749122,0.687066272212815,0.990608592989741
"9863","AC135050.6",8.37666658167032,-0.216771560450849,0.520063555381
561,-0.416817441267939,0.676811944120943,0.990608592989741
"9864","ITGAM",5.40243495375739,-0.272863939980496,0.674377654697769,-
0.40461592711399,0.685759849313984,0.990608592989741
"9865","CMTM3",189.946948568358,0.1241440584412,0.304148407421245,0.40
8169352237509,0.683149346945374,0.990608592989741
"9866","CES2",49.0678424245593,0.126275460489323,0.308497600090001,0.4
09323963792532,0.682301931608932,0.990608592989741
"9867","METTL16",40.0573991184167,0.112323677509676,0.277429900444189,
0.404872284241303,0.685571392052733,0.990608592989741
"9868","ATAD5",7.03430151934004,-0.242920453073853,0.604603416206886,-
0.401784784144735,0.687842421193563,0.990608592989741
"9869","AP2B1",178.165012368834,0.0936528430664023,0.229487567005595,0
.408095498542276,0.683203564712071,0.990608592989741
"9870","AC067852.2",7.75411940548912,0.215740717982562,0.5241492297504
45,0.411601707561947,0.680631379833906,0.990608592989741
"9871","AC060780.1",17.5280729667306,0.174874931444666,0.4340653446588
22,0.402876971397288,0.687038733175695,0.990608592989741
"9872","ARL17A",14.8188444990759,-0.168483937583206,0.420072250931192,
-0.401083235585593,0.68835884311937,0.990608592989741
"9873","PRR11",2.45395256688196,-0.35170200986561,0.872819004720797,-0
.402949532449875,0.68698535150642,0.990608592989741
"9874","GDPD1",1.97762685872416,0.410902751407114,1.01950040095422,0.4
03043246498503,0.68691641032402,0.990608592989741
"9875","GGA3",27.0508761706774,0.129143493627845,0.315309835075066,0.4
09576484022771,0.682116650298424,0.990608592989741
"9876","CCDC40",3.67464700482081,-0.318848840629023,0.795665539685551,
-0.400732248320109,0.688617265421414,0.990608592989741
"9877","GAREM1",32.8809245488775,-0.144603326552466,0.360806718612189,
-0.400777810093641,0.688583717482918,0.990608592989741
"9878","ZNF407",21.8300170977732,0.14758652765292,0.364074969984537,0.
40537400211607,0.685202617969024,0.990608592989741
"9879","MIDN",277.617276650098,-0.137420716892769,0.333080126560368,-0
.412575551450267,0.679917616739214,0.990608592989741
"9880","PWWP3A",82.0941243542888,0.108198604082186,0.264255871805947,0
.409446357209576,0.682212125668194,0.990608592989741
"9881","MPV17L2",11.6236832388664,-0.244265101969582,0.587950469757908
,-0.415451835713577,0.677811166947363,0.990608592989741
"9882","KMT2B",50.3594961417686,0.112020134656742,0.272316329105118,0.
411360328720869,0.680808338819363,0.990608592989741
"9883","TOMM40",102.397834708679,-0.129436821773129,0.309269598453508,
-0.4185242339382,0.675563871995941,0.990608592989741
"9884","PNMA8B",8.66527895897886,0.207836930110903,0.509265863360174,0
.40811086126916,0.683192286431666,0.990608592989741
"9885","ZNF665",6.60341006918428,0.256916657432377,0.630798642960986,0

.40728790446727,0.683796545448084,0.990608592989741
"9886","AC022137.4",7.96830047944167,0.238247198090151,0.5827410594364
49,0.408838873170449,0.682657910188346,0.990608592989741
"9887","NDUFA3",193.734102525859,-0.124367206467421,0.304738000270897,
-0.408111907136177,0.683191518629044,0.990608592989741
"9888","CNOT3",38.3350783025208,0.112703879898103,0.272938965986294,0.
412927042098351,0.679660067755057,0.990608592989741
"9889","ZNF837",3.70058008558389,-0.282392480225731,0.704699801152768,
-0.400727344840719,0.688620875976396,0.990608592989741
"9890","CSNK2A1",125.726938710716,0.0869411862412574,0.209094035015053
,0.415799457095934,0.677556756166041,0.990608592989741
"9891","MKKS",28.3875767834243,-0.141124184511282,0.336848094623854,-0
.418954973365279,0.675249039561581,0.990608592989741
"9892","HCK",2.27854925945812,-0.452103089116155,1.1047729240682 9,-0.4
09227162674572,0.682372962434595,0.990608592989741
"9893","CDK5RAP1",28.5339641982363,0.127385204557477,0.316306166066471
,0.402727541298411,0.687148670732056,0.990608592989741
"9894","NFS1",21.6522777745875,-0.138991600685552,0.338347412065803,-0
.410795518833526,0.681222479323634,0.990608592989741
"9895","SLC13A3",3.35130110128708,-0.318499277210695,0.791761128533253
,-0.402266877891213,0.687487628078636,0.990608592989741
"9896","ATP9A",32.788536258296,-0.140393267415646,0.340781054449211,-0
.411974978017946,0.680357763451024,0.990608592989741
"9897","LSM14B",20.4517859427184,0.146413365280162,0.361510058724438,0
.405004955593133,0.685473868293272,0.990608592989741
"9898","MAP3K7CL",5.38274263188916,-0.256219116858706,0.63776068433210
3,-0.401748058720541,0.687869451799876,0.990608592989741
"9899","SON",680.854134283617,0.0878998906365699,0.211772852658782,0.4
15066848904369,0.678092966934398,0.990608592989741
"9900","DOP1B",11.090698526428,0.174279296472179,0.428694027891211,0.4
06535396187992,0.684349254690503,0.990608592989741
"9901","AP000692.2",10.4160541180871,0.237985627499751,0.5728756973874
18,0.415422802162978,0.677832417154497,0.990608592989741
"9902","WDR4",11.0843922154336,0.198344115726916,0.49294251580647,0.40
236763794338,0.687413483197804,0.990608592989741
"9903","SUMO3",300.863296208606,-0.0915213811320767,0.223128683098759,
-0.410173088735385,0.681678980502301,0.990608592989741
"9904","S100B",2.14757229868981,-0.501879655228624,1.22044995475883,-0
.411225100440762,0.680907484680665,0.990608592989741
"9905","TBX1",7.79577537103807,-0.27484274076105,0.663871196167791,-0.
414000098735394,0.678874034523704,0.990608592989741
"9906","GUCD1",53.8008946996097,0.110358003171487,0.270011216230799,0.
408716366349595,0.682747821682169,0.990608592989741
"9907","TTC28-
AS1",14.949930425015,0.180751835955008,0.443500643063667,0.40755710004
4745,0.683598865308774,0.990608592989741
"9908","IFT27",44.1014726835055,-0.106927934444772,0.265117415128486,-
0.403322936718248,0.686710670377741,0.990608592989741
"9909","RABL2B",30.7608510693369,-0.130603265465344,0.317305724581222,
-0.41160072241909,0.680632102023378,0.990608592989741

"9910","PHKA2",9.23504964172483,0.192508596722867,0.476987060634295,0.403592911864044,0.686512098845602,0.990608592989741
"9911","MED14OS",7.83033335450474,-0.233321220828401,0.570935414603711,-0.408664824182171,0.682785651397115,0.990608592989741
"9912","WAS",13.1974298483231,-0.189204857228432,0.469203012125361,-0.403247320112857,0.686766291615747,0.990608592989741
"9913","CCDC120",5.75163305837621,-0.250558541702615,0.615649617521274,-0.406982372069705,0.684020935301107,0.990608592989741
"9914","KLF8",15.4862514668846,0.188323576294786,0.458286738872109,0.410929578189999,0.681124173104653,0.990608592989741
"9915","SPIN2B",25.9176546850262,-0.147974509935293,0.355235614793148,-0.416553137616727,0.677005292564065,0.990608592989741
"9916","YIPF6",99.1879833980719,-0.0785644256004597,0.194146978058992,-0.404664684384568,0.685724004600821,0.990608592989741
"9917","MCTS1",136.915437545659,-0.12260450670254,0.29983713672707,-0.408903673643809,0.682610352964602,0.990608592989741
"9918","ZNF829",9.2839987513886,0.195444156628182,0.488193218213043,0.400341810039015,0.688904777132023,0.990917075072136
"9919","PARK7",853.908081645376,-0.102891598545415,0.257200735492078,-0.400043951462902,0.689124145041101,0.990960407788673
"9920","GPR158",2.49884178697047,-0.399091472494287,0.99752260840893,-0.400082633847113,0.689095654628341,0.990960407788673
"9921","QSER1",62.8171898182506,0.122551501705553,0.306437248322806,0.399923646280937,0.689212755227759,0.990960407788673
"9922","CLUH",19.6901707351272,0.1497214093226,0.374286814218601,0.400017856988025,0.689143364435354,0.990960407788673
"9923","DNM3OS",35.2942788644846,-0.160807592905398,0.402554757719153,-0.39946762476867,0.68954867431661,0.990975815560983
"9924","PACC1",6.9658150033483,-0.223725031143678,0.560384215765261,-0.39923506917153,0.689720005293001,0.990975815560983
"9925","LINC01121",3.03718727326811,0.437211584761576,1.09401721716034,0.399638669212551,0.689422670686596,0.990975815560983
"9926","IFT122",23.5692255935343,0.138309812792317,0.346419036695927,0.399255809124949,0.689704724874934,0.990975815560983
"9927","TCAF1",102.80762539574,0.101690314993049,0.254886330368119,0.398963392215591,0.68992017834357,0.990975815560983
"9928","CEMIP2",87.3411871394456,-0.188337954795377,0.471652582260746,-0.399315008289845,0.689661109851563,0.990975815560983
"9929","FKBP3",142.891894755877,-0.0911589210913277,0.228463096988311,-0.399009390544993,0.689886284999483,0.990975815560983
"9930","NPTN-IT1",4.04240505645857,-0.272205569175032,0.682438528491083,-0.398871924445557,0.689987577174207,0.990975815560983
"9931","ZNF227",14.2643110796272,0.153515051366307,0.384754716674965,0.398994592432753,0.689897188751743,0.990975815560983
"9932","PTOV1",107.135965397627,-0.0767977813951511,0.192430055107098,-0.399094524773736,0.689823556455734,0.990975815560983
"9933","ZNF587B",7.52177813037689,0.219615446454441,0.550159985461505,0.399184695830278,0.689757119004142,0.990975815560983
"9934","AC090114.2",15.1838356573855,0.167022398301349,0.4190677623435

77,0.39855701943595,0.690219636502728,0.991046364408319
"9935","BLOC1S6",197.822985689831,0.0700374432865207,0.175742775581099,0.398522460197522,0.690245105619028,0.991046364408319
"9936","CDKL5",19.8701825650333,0.151585015986812,0.380239168117568,0.398657026148191,0.690145936548817,0.991046364408319
"9937","TBC1D7",16.9014655884192,0.15062619499147,0.37823376801646,0.398235714863288,0.690456441808486,0.991150291692479
"9938","CD37",41.0149703266937,-0.128154477587331,0.321743235849954,-0.39831288837754,0.69039956124136,0.991150291692479
"9939","HECTD2",20.2245390837876,-0.149843207530548,0.376591985795306,-0.397892714615532,0.6907092705259,0.991381707531538
"9940","COA4",151.679794932416,-0.102820821078218,0.258455166333793,-0.397828461070209,0.69075663625848,0.991381707531538
"9941","ALKBH3",30.0318383217793,-0.118023142554205,0.29691070131654,-0.397503835432254,0.690995958777957,0.991532173717031
"9942","RNFT1-DT",4.3422337546128,0.28702475605525,0.722079034502476,0.397497700861811,0.691000481641296,0.991532173717031
"9943","RHEB",314.040961575639,-0.109156234147675,0.274690623570852,-0.397378813767626,0.69108813626107,0.991558217027097
"9944","C8orf76",34.3714130752714,-0.122755289806683,0.309066334722166,-0.397181045023987,0.691233958940847,0.991567989768741
"9945","SRP14",1550.72340423507,-0.0838724245441565,0.211129440593927,-0.397255940754711,0.691178734022496,0.991567989768741
"9946","SMG5",22.1419816319452,0.136641452592694,0.344536834328261,0.396594613342582,0.691666425427937,0.99159011407446
"9947","C14orf119",111.628155986432,0.131355170401696,0.331157468407401,0.396654712434564,0.69162210047374,0.99159011407446
"9948","SIX1",63.2566361139384,0.127899322535262,0.322277243730375,0.396861165420247,0.691469843009888,0.99159011407446
"9949","CD300E",3.64704263360967,0.436163051716513,1.09856642967844,0.397029291932926,0.69134586023587,0.99159011407446
"9950","KCTD15",7.20670822078298,0.250123013478977,0.630420105839033,0.396756085604354,0.691547336994627,0.99159011407446
"9951","AC020915.2",18.8173454569594,0.164241413847341,0.413981366465236,0.396736247454106,0.69156196754379,0.99159011407446
"9952","COA6",92.6076073192699,-0.117863359888332,0.297298073603244,-0.396448448050239,0.691774231297444,0.991619321111337
"9953","TMEM108",6.84155170289056,0.312844267070297,0.78968568391411,0.396163022127578,0.691984768433695,0.991619321111337
"9954","SPACA9",11.4978465597602,-0.179793567152773,0.453723404986368,-0.396262492031186,0.691911394298301,0.991619321111337
"9955","CSK",38.7374392859353,-0.11203164976247,0.282839770563195,-0.396095816155526,0.69203434466455,0.991619321111337
"9956","LINC01290",2.72876383511508,-0.341944904020402,0.863018375779644,-0.396219725578255,0.691942940687149,0.991619321111337
"9957","GLYCTK",6.17353825873108,-0.248104201812043,0.627338331158132,-0.395487075297977,0.692483458261648,0.991725112677559
"9958","RCHY1",36.5176973840281,-0.121978137344991,0.308461764258738,-0.395440056041031,0.692518152379959,0.991725112677559

"9959","NR4A3",26.3338658473649,-0.245503878778885,0.620524914070406,-0.395639035938901,0.692371335413676,0.991725112677559
"9960","POLE3",85.6360799506812,-0.111631081601399,0.28232508392766,-0.39539909117676,0.692548379666914,0.991725112677559
"9961","P4HA3",5.29540314988074,-0.308475276057976,0.780081289054077,-0.395439911694371,0.692518258890081,0.991725112677559
"9962","NCBP3",86.4627133970419,0.0837027605968692,0.211775983403476,0.395241987555305,0.692664308335847,0.991725112677559
"9963","PSME3",51.6492474246697,-0.128442405250454,0.324700095884202,-0.39557242784511,0.692420480791415,0.991725112677559
"9964","FITM2",13.540788069873,0.157401393618822,0.398227569708685,0.395254888389486,0.692654788382407,0.991725112677559
"9965","CDK11B",44.5941655889932,-0.115371357008606,0.294509845360806,-0.391740238317886,0.695250155593028,0.991853795908803
"9966","TXLNA",124.476747079785,0.0878417923767099,0.22547281681034,0.389589280070951,0.696840280301341,0.991853795908803
"9967","LURAP1",9.63378179333543,-0.211794909292584,0.545508884516513,-0.388251988746799,0.697829563264871,0.991853795908803
"9968","TATDN3",26.4907479740335,0.124122986688135,0.314982900056513,0.394062619481455,0.693534808041843,0.991853795908803
"9969","MARC2",30.5531668485781,0.16696237043514,0.427331127550103,0.39070959186226,0.696011908058116,0.991853795908803
"9970","SFXN5",16.990310625182,0.13908103190804,0.356062312173405,0.390608686044554,0.696086504212003,0.991853795908803
"9971","ARL6IP6",32.5420776281398,0.113894723804424,0.291613349590206,0.390567592205489,0.696116884297435,0.991853795908803
"9972","NUP35",25.5060025706463,-0.155324468536481,0.396086812483311,-0.392147538471823,0.694949204121293,0.991853795908803
"9973","SUMO1",302.54061532497,-0.0998615268953791,0.256623575307975,-0.389136215468648,0.697175384555776,0.991853795908803
"9974","ZNF502",11.7174668358702,-0.178893955816199,0.459307126971007,-0.389486566420058,0.696916246142313,0.991853795908803
"9975","CLDND1",89.9779763488749,-0.0923701425394223,0.235416372008057,-0.392369238177968,0.694785411828925,0.991853795908803
"9976","NCBP2AS2",88.8925009133182,-0.11112300566555,0.282340714132106,-0.393577688599157,0.69389285632708,0.991853795908803
"9977","LNX1",16.6551208991819,-0.152107935006602,0.387930550883272,-0.392100943481431,0.694983630426904,0.991853795908803
"9978","CLCN3",69.1558710065265,-0.0903654418303895,0.231200290447322,-0.390853496142033,0.695905529727791,0.991853795908803
"9979","DAP",75.1215011035028,-0.104139717484926,0.266823585882993,-0.390294273050482,0.696318957630493,0.991853795908803
"9980","BRIX1",91.4999455881573,-0.117809438899127,0.298359077142316,-0.394857900847214,0.692947760887365,0.991853795908803
"9981","C5orf34",3.29601571760617,0.305072679161796,0.776056427275366,0.393106310881114,0.694240963150637,0.991853795908803
"9982","AC008771.1",13.0743619135964,-0.163582826508511,0.418171475932002,-0.391185998863087,0.695659756698305,0.991853795908803
"9983","THG1L",40.2949953636033,-0.118459525458095,0.30497147292541,-0.388428216979717,0.697699166145127,0.991853795908803

"9984","RARS",81.6003856785231,0.0890499749893654,0.226735261620624,0.
392748681227911,0.694505111426627,0.991853795908803
"9985","CANX",406.495595318822,0.0536451406884762,0.138168411799413,0.
388259081723801,0.697824314763374,0.991853795908803
"9986","TCF19",7.48348004226597,-0.224470279014707,0.571242367885227,-
0.392951033806735,0.694355647546932,0.991853795908803
"9987","SYNGAP1",22.6146621610706,-0.145489766416901,0.368532236196246
,-0.394781655788252,0.693004034173512,0.991853795908803
"9988","SLC35B2",63.6021202316194,0.0918172765247302,0.235350736331464
,0.390129548587502,0.696440753949769,0.991853795908803
"9989","ATP6V1F",359.302275478053,-0.0942353817117995,0.24158688261697
4,-0.390068288025413,0.696486051653504,0.991853795908803
"9990","SLC4A2",38.0550925032674,0.100860865443559,0.25709649011031,0.
39230743834847,0.694831068264196,0.991853795908803
"9991","PPP2CB",137.559793692401,-0.069595658243946,0.178933283665787,
-0.388947527358505,0.697314963074105,0.991853795908803
"9992","ST3GAL1",58.7909205398604,0.105623667842016,0.269548894788591,
0.391853462893465,0.695166489859129,0.991853795908803
"9993","GNAQ",112.878040405626,-0.0924614298830903,0.236782010720381,-
0.390491784412961,0.696172929189835,0.991853795908803
"9994","MFSD14C",41.8127266823953,0.109011487626727,0.277792234302386,
0.392420932501894,0.694747221982396,0.991853795908803
"9995","AL137026.3",10.1034526364758,0.224349508031994,0.5763411965327
66,0.389265090508308,0.697080057533248,0.991853795908803
"9996","PTPN20",2.25807342415634,-0.372805593310992,0.946685514311569,
-0.393800884956074,0.693728051002441,0.991853795908803
"9997","SLC16A9",2.50078340505632,0.439657326794311,1.12774607350666,0
.389854894752346,0.696643848818128,0.991853795908803
"9998","NFKB2",78.1904969780376,-0.1169370267865,0.298242295705205,-0.
392087334594839,0.694993685353728,0.991853795908803
"9999","VTI1A",32.3009032774079,0.112153181802952,0.288464718467506,0.
388793410850294,0.697428975493993,0.991853795908803
"10000","SPTY2D1",89.4316064338882,0.100797362189802,0.259345138057833
,0.388661082851395,0.697526874646339,0.991853795908803
"10001","EEF1G",2094.27313108034,0.0781830630781619,0.199563829565124,
0.391769707208631,0.695228379606573,0.991853795908803
"10002","PGR",13.36537029967,0.201689559023268,0.519326572294604,0.388
367493178942,0.697744096693432,0.991853795908803
"10003","MIR100HG",105.524518200625,0.0937197572690532,0.2406470756852
6,0.389448976274402,0.696944048143612,0.991853795908803
"10004","FOXRED1",22.6685042127941,-0.135679996650218,0.34410667840182
6,-0.394296319037955,0.693362280607715,0.991853795908803
"10005","EMG1",77.8910300494306,-0.117950954533542,0.301208734145516,-
0.391592079386913,0.695359641190657,0.991853795908803
"10006","CLEC2B",373.296509378249,-0.100788443156274,0.256969162298621
,-0.392219993460339,0.694895672629358,0.991853795908803
"10007","AC139768.1",3.99984510435428,-0.318406772203122,0.80835848069
1638,-0.393893031134765,0.693660015671101,0.991853795908803
"10008","ANKS1B",5.28333187443778,-0.282035017485414,0.717871254557639
,-0.392876878263091,0.694410419748089,0.991853795908803

"10009","GPN3",52.5465598759189,0.109708438998486,0.282503273628142,0.388343956477102,0.697761512174829,0.991853795908803
"10010","NAA25",39.898236915932,0.125562017690504,0.323350781599013,0.388315182259907,0.697782803260457,0.991853795908803
"10011","RHOF",9.85238824633341,0.190055624601734,0.483642923268233,0.392966826263944,0.69434398324896,0.991853795908803
"10012","ZNF84",36.5571508909019,0.106421314908312,0.272645492265788,0.390328532571399,0.696293627321546,0.991853795908803
"10013","UGGT2",59.9735664939085,0.094826215010711,0.242176960799863,0.391557539980346,0.695385165827158,0.991853795908803
"10014","SOCS4",28.895168072172,-0.131020530566397,0.335917138542239,-0.390038243166097,0.696508268024462,0.991853795908803
"10015","KTN1-AS1",6.05948070720331,0.231814697606093,0.590662043464658,0.392465878197171,0.694714018401664,0.991853795908803
"10016","EIF3J-DT",42.5734808859157,-0.107562140931363,0.276165962803853,-0.389483699726452,0.6969183663597,0.991853795908803
"10017","HYKK",13.7474674246043,-0.171933562445807,0.436549779409238,-0.393846407799076,0.69369443929779,0.991853795908803
"10018","ENKD1",22.6593196391652,0.122054395458861,0.310360919472122,0.393265993883695,0.694123031928961,0.991853795908803
"10019","TMEM220",46.5024454016059,-0.121324654844363,0.311677084062882,-0.389263956344913,0.697080896436826,0.991853795908803
"10020","ZNF624",14.3816780790553,0.15781630997575,0.403990256484607,0.390643852030039,0.696060506890847,0.991853795908803
"10021","ANKRD13B",17.1362638061561,-0.150993882587388,0.382361272606488,-0.394898472740428,0.69291781716204,0.991853795908803
"10022","UTP6",59.3590069087665,-0.108692749616592,0.279129622472814,-0.389398834325364,0.696981134202291,0.991853795908803
"10023","CASC3",65.4889336214192,0.10006109864352,0.253869636566864,0.394143624250101,0.69347500490968,0.991853795908803
"10024","METTL2A",33.9349907670629,-0.136540813608283,0.35140637796124,-0.38855530853041,0.697605132427109,0.991853795908803
"10025","ANKRD12",441.883580024673,-0.077475763392917,0.197966825085277,-0.391357306253425,0.695533145439188,0.991853795908803
"10026","WDR18",45.2569457773541,-0.115388051286374,0.292469065404856,-0.394530789526907,0.693189200012762,0.991853795908803
"10027","COLGALT1",70.6480507819199,0.0849606037048266,0.218110451182075,0.389530181815555,0.696883988323544,0.991853795908803
"10028","ZNF676",7.52387611205407,0.211370952269168,0.541005093718693,0.390700484567109,0.696018640643252,0.991853795908803
"10029","BCAT2",17.9194251006758,0.136175719739588,0.349161221844312,0.390008143001367,0.696530525551523,0.991853795908803
"10030","TSKS",6.81011649486636,0.226762849769328,0.57755898451217,0.392622841736003,0.694598066362416,0.991853795908803
"10031","GSS",37.3996529774431,0.117340071115801,0.297671445054862,0.394193239106877,0.693438376850473,0.991853795908803
"10032","PPP1R16B",2.72077607327551,0.387417579752845,0.995142471122656,0.38930865780031,0.697047832512346,0.991853795908803

"10033","CSTB",539.860087039021,-0.0892319197976379,0.228654619254017,-0.390247614890773,0.696353455610361,0.991853795908803
"10034","ZMAT5",32.7726124057928,-0.137367740528928,0.348233730482712,-0.394469945052459,0.69323411243345,0.991853795908803
"10035","EIF4ENIF1",25.9862805234896,0.120300481028092,0.306055854543321,0.393067079888401,0.694269937679735,0.991853795908803
"10036","PISD",23.0464283016142,-0.124235222741686,0.315233010081582,-0.394106006568075,0.693502776558362,0.991853795908803
"10037","APOL4",53.6119871549348,0.145000770178098,0.370695170537398,0.391159048465321,0.695679676189957,0.991853795908803
"10038","VCL",183.679810362731,-0.0786571409544682,0.202691436539452,-0.388063463841297,0.69796906897855,0.991953251449293
"10039","C1orf198",27.9148516311655,0.144609455412167,0.37331952994271,0.387361077611875,0.698488914485049,0.992031990728129
"10040","ZNF619",6.18373700745309,-0.220875209716971,0.57011443177287,-0.38742258993536,0.698443382731289,0.992031990728129
"10041","INTS8",22.3153844618834,-0.122211671471272,0.31547059055864,-0.387394816280204,0.698463940806352,0.992031990728129
"10042","PML",114.637650328294,0.103910619313131,0.268291403759693,0.387305064034787,0.698530376980839,0.992031990728129
"10043","AC087190.1",7.08073639201224,0.230625022963492,0.595395403015527,0.387347671472495,0.698498837925754,0.992031990728129
"10044","PRSS36",5.81462888413963,-0.246236245218449,0.635879990987859,-0.387236976643837,0.698580777993466,0.992031990728129
"10045","AC005332.5",8.47307653639536,0.197919418440453,0.510249191782837,0.387887764699659,0.698099093000476,0.992031990728129
"10046","ADAMTS10",21.3201845244795,0.158575661146418,0.409426109295408,0.38731203884216,0.698525214024234,0.992031990728129
"10047","GSTM4",28.6840386560636,0.134105822174801,0.346865826388387,0.386621604010774,0.699036361708904,0.992283854342211
"10048","RFC1",171.621248245168,-0.0773706999489459,0.200076886643561,-0.386704837559686,0.698974734410849,0.992283854342211
"10049","TRMT11",55.1123611285873,-0.10405987642557,0.268970136051366,-0.386882640404723,0.698843093311889,0.992283854342211
"10050","MTSS1",43.6330666040843,0.165248228524825,0.427274470800498,0.386749594974005,0.698941596207667,0.992283854342211
"10051","POLR1B",13.9257266762851,0.185031518963958,0.479016323772752,0.386273932183861,0.699293804290995,0.992286471819633
"10052","PRKCQ-AS1",8.12277267792334,-0.239815361627548,0.620891838263156,-0.38624337903747,0.699316429810359,0.992286471819633
"10053","STT3A",71.8428792740598,0.0915377873655338,0.236877418211697,0.386435262831709,0.699174338545553,0.992286471819633
"10054","NOTCH3",39.3961983731754,0.179783573518155,0.465287344285297,0.386392571657652,0.699205950741439,0.992286471819633
"10055","ARHGEF11",33.0259240991342,-0.105895414429091,0.275125653172944,-0.384898366284024,0.70031271635175,0.992332949256648
"10056","GCC2",254.590169179698,0.0730555612476802,0.189759983206658,0.384989290224162,0.700245350306859,0.992332949256648
"10057","HLA-

DQA2",4.97534045208867,-0.84829562165238,2.20595501820483,-0.384548014194192,0.700572316172988,0.992332949256648
"10058","TBRG4",36.1369688844214,-0.131401740976138,0.34092076263287,-0.385431910809264,0.699917443981868,0.992332949256648
"10059","GSTK1",233.437849211625,-0.0850832775995406,0.221218709078477,-0.384611581696544,0.7005252120654,0.992332949256648
"10060","REEP4",7.93233939794771,-0.200300998424724,0.520238984351693,-0.385017279461156,0.700224613402632,0.992332949256648
"10061","AC090739.1",2.28928472177452,0.361328864486416,0.937695101532325,0.385337263568887,0.69998755673661,0.992332949256648
"10062","UBQLN1",87.3155741001059,0.0817638934257324,0.212465860188579,0.384833089669846,0.700361081606366,0.992332949256648
"10063","SFMBT2",23.6007215651458,0.187791938981506,0.48763479686948,0.385107749051326,0.700157587050924,0.992332949256648
"10064","GPR180",21.4028566425525,0.132831961996039,0.345248118445416,0.384743478383473,0.700427479073576,0.992332949256648
"10065","AP4S1",18.2699427150805,0.134274874701729,0.349211297496948,0.384508965386214,0.700601252272043,0.992332949256648
"10066","NDN",306.893731146548,0.0907707818201911,0.235764135448801,0.38500674263878,0.700232419987086,0.992332949256648
"10067","RAP1GAP2",3.81257282714876,0.307245991367112,0.797189261629911,0.385411603185579,0.69993248724184,0.992332949256648
"10068","HDAC5",36.5876236902134,0.114943496809936,0.298074233676849,0.385620371784801,0.69977784353346,0.992332949256648
"10069","P2RY11",3.28546852082132,0.359030628274431,0.932129788010291,0.385172357854598,0.700109721651561,0.992332949256648
"10070","DIDO1",60.5009600280191,0.093289684836724,0.241865951721886,0.385708216359427,0.699712777072184,0.992332949256648
"10071","Z93241.1",8.53403455995003,0.230713748917819,0.597715587014866,0.385992525425107,0.699502204512354,0.992332949256648
"10072","APLN",5.02798711027064,0.268221752663076,0.696935413880366,0.384858836731632,0.700342004748796,0.992332949256648
"10073","PER3",55.3096126586385,-0.340611915955564,0.887976613136672,-0.3835820796590 5,0.701288224194398,0.992556425867662
"10074","ZSWIM5",2.1934152534617,0.401350429810263,1.05802982835208,0.379337537614967,0.704437227984802,0.992556425867662
"10075","HIST2H2AA4",10.6919505735302,-0.230647033941601,0.608062698702445,-0.379314558241744,0.704454290117044,0.992556425867662
"10076","AC234582.1",2.6993291847589,-0.316649519946875,0.830410625963408,-0.3813167968311,0.702968191443094,0.992556425867662
"10077","ATP1B1",22.3708459087821,-0.193587172748803,0.510343123869215,-0.379327483206011,0.704444693342235,0.992556425867662
"10078","MRPL19",94.2106021600424,-0.0893480110577278,0.234842587826014,-0.380459148763607,0.703604615205679,0.992556425867662
"10079","TLK1",92.8044166169032,0.0759416357780531,0.199221994797437,0.381191021881236,0.703061510614948,0.992556425867662
"10080","GIGYF2",97.2896588174136,0.0852542349427144,0.22472065546742,0.37937872139695,0.704406649492687,0.992556425867662
"10081","CMTM7",35.4374025989188,-0.13089984985514,0.343556201482637,-0.38101437054617,0.703192585256684,0.992556425867662

"10082","MRPL47",105.308080887837,-0.121234546900384,0.315638502310683
,-0.384093024180723,0.700909501590562,0.992556425867662
"10083","OSTC",446.366100800012,-0.111370965883336,0.292018660716114,-
0.38138304452949,0.702919040526802,0.992556425867662
"10084","FBXL17",31.3934609442495,-0.1216225640856,0.319198665954052,
-0.381024662875839,0.703184948143802,0.992556425867662
"10085","AP3S1",352.822335231826,0.0847421926930247,0.222056810994002,
0.381623929091343,0.702740332379652,0.992556425867662
"10086","SNX2",197.146680114877,0.0877997601729688,0.231075344282926,0
.379961611419121,0.703973911500947,0.992556425867662
"10087","AIG1",80.9521852844772,-0.0878108473340936,0.2308954867632,-0
.380305603046066,0.70371857681455,0.992556425867662
"10088","CCZ1B",19.0092684339128,-0.207325455563127,0.54329381887155,-
0.381608345910787,0.702751892778583,0.992556425867662
"10089","CDCA7L",9.05251999526451,0.20280764919928,0.53470092720278,0.
379291747744375,0.704471227006436,0.992556425867662
"10090","AC145124.1",16.590652031299,0.151460273525705,0.3977553169191
84,0.380787552254087,0.703360896532261,0.992556425867662
"10091","GBA2",17.1132269001913,0.151358582070064,0.395654710735173,0.
382552205150855,0.702051813914194,0.992556425867662
"10092","SLC25A51",10.4030370789641,-0.175016723749656,0.4605568789658
66,-0.380011094704824,0.703937179483564,0.992556425867662
"10093","SHC3",12.6395931311333,0.203799110301545,0.532682102292862,0.
382590497079436,0.702023417372685,0.992556425867662
"10094","CNTRL",34.920401617533,0.106964193730186,0.279184627284741,0.
383130671521869,0.701622878968541,0.992556425867662
"10095","MAPKAP1",73.9128187222742,0.0872821160566345,0.22899892710737
7,0.381146397317871,0.703094621044012,0.992556425867662
"10096","EXOC6",9.58523768140262,0.200940638969742,0.525739646894386,0
.382205603394619,0.702308865882093,0.992556425867662
"10097","CASP7",59.9272104750213,0.104494372947234,0.27532231276819,0.
379534705693156,0.704290837249727,0.992556425867662
"10098","IFITM3",3601.27212005704,-0.0856815639944093,0.2249445582246,
-0.380900808050929,0.703276852870102,0.992556425867662
"10099","KCNC1",1.92292125868297,0.485630032852116,1.26855130100837,0.
382822541324177,0.701851346822348,0.992556425867662
"10100","AP001160.2",1.92613811398382,0.435437376628202,1.147782187272
89,0.379372830016462,0.704411023746754,0.992556425867662
"10101","HIKESHI",136.201963873818,-0.114865465677658,0.30295830047152
9,-0.379146125057078,0.704579355912355,0.992556425867662
"10102","JAM3",77.228747996588,-0.104307150752572,0.273107021259697,-0
.381927752246936,0.702514954590979,0.992556425867662
"10103","CD4",11.8209458714912,0.168495970921302,0.443540732602211,0.3
79888381237845,0.704028272381856,0.992556425867662
"10104","SPATS2",55.3127197274964,-0.100081884449785,0.263989292806765
,-0.379113423069939,0.704603638870646,0.992556425867662
"10105","SARNP",114.88506293082,-0.0928032444549997,0.241687586188356,
-0.383980186647546,0.700993132703434,0.992556425867662
"10106","ESYT1",38.5998092233612,0.134325945185081,0.351307785655259,0
.382359716094923,0.702194566314756,0.992556425867662

"10107","SPRYD4",15.6854891585819,-0.154792082716863,0.404698501548214
,-0.382487412542154,0.702099863786541,0.992556425867662
"10108","SEC23A",111.4109378187,0.0767614239130259,0.201786468703524,0
.380409174144417,0.70364170563001,0.992556425867662
"10109","EFCAB11",18.1907665393879,-0.139756406185581,0.36601640074625
7,-0.381830994186702,0.702586727447202,0.992556425867662
"10110","RIN3",21.06323847269,-0.135178700480581,0.353910143378508,-0.
381957689005841,0.702492748743669,0.992556425867662
"10111","MRPL46",47.299452458404,-0.137459480390048,0.362338518469817,
-0.37936756205371,0.704414935131584,0.992556425867662
"10112","NTN1",8.62996534418202,-0.209345835449575,0.546225439254396,-
0.383259036297055,0.701527708897191,0.992556425867662
"10113","AC104984.3",2.83626961206473,-0.340369088093222,0.88859471582
4507,-0.383041989820299,0.701688630610994,0.992556425867662
"10114","TGIF1",71.0596565599957,0.0926954588778462,0.242298570121664,
0.382567089980357,0.702040775567813,0.992556425867662
"10115","ZNF516",21.9328421372458,0.14553478862889,0.382387486896364,0
.3805950602885,0.70350374724618,0.992556425867662
"10116","RBFA",50.9715757160511,-0.0914950329250801,0.241383705286852,
-0.379043949202581,0.704655227897637,0.992556425867662
"10117","GAMT",54.3251132051006,-0.122629507853078,0.322366127133837,-
0.380404445539544,0.703645215167231,0.992556425867662
"10118","DAPK3",41.3391215088014,-0.0985084064039556,0.259553401361255
,-0.379530400631692,0.704294033497603,0.992556425867662
"10119","TRIP10",210.020871697209,-0.094623336687162,0.248992653212225
,-0.380024613041539,0.703927144785417,0.992556425867662
"10120","ZNF283",6.36611912290168,0.235121538337383,0.615121767930988,
0.38223576305588,0.702286497070844,0.992556425867662
"10121","ZNF667",14.8112200210383,0.172682733316579,0.455330456833306,
0.379247051729284,0.704504414408842,0.992556425867662
"10122","CEP250",11.7993205117384,0.185490462175757,0.486731462783213,
0.381094045400498,0.703133465714994,0.992556425867662
"10123","CNBD2",9.05312775083254,-0.214216018361542,0.560866123084945,
-0.381937880618079,0.702507441756521,0.992556425867662
"10124","TIAM1",22.8473601396209,0.138444624519606,0.361312890863558,0
.383171007789718,0.701592973020995,0.992556425867662
"10125","PCNT",29.0230792593476,-0.157278265821545,0.414166362919007,-
0.379746594371068,0.704133529171114,0.992556425867662
"10126","PLA2G6",7.21160859456215,0.198383580562586,0.520938677666567,
0.380819449711822,0.70333722603797,0.992556425867662
"10127","CSNK1E",137.072881654596,0.0697518638109755,0.18190793907889,
0.383445957137284,0.701389133537535,0.992556425867662
"10128","Z68871.1",2.85376848203315,0.316796935685836,0.83095998281095
4,0.381242108211014,0.703023606391029,0.992556425867662
"10129","PWWP2B",4.57937024136676,0.271749820566956,0.717232840086916,
0.37888647225638,0.704772170168923,0.992623139463901
"10130","HINFP",10.906321637877,0.177809627382146,0.469752262055034,0.
37851787366447,0.705045918504144,0.992910668645619
"10131","C1orf53",5.30946692187758,-0.28315702410876,0.748874983285863
,-0.378109872046123,0.705348974953344,0.992945379582041

"10132","ITGA1",7.21801256896199,-0.248968191222708,0.658286139839307,-0.378206643213668,0.705277090795313,0.992945379582041
"10133","PDSS1",7.16946687050204,0.211264222293782,0.558606994296673,0.378198311963098,0.70528327936297,0.992945379582041
"10134","ATG4D",13.0242167712614,-0.155725762259731,0.411588047681474,-0.378353460789139,0.705168035878356,0.992945379582041
"10135","ZNF605",26.5933837708434,-0.14630456861764,0.387327740568277,-0.377728092501162,0.70563259645203,0.993246632558921
"10136","GALE",4.22921590519629,0.282642105075386,0.748714551110871,0.377503154781791,0.705799720347606,0.993285864701485
"10137","CXorf56",20.7744511541376,0.144628559032187,0.383031445371016,0.377589257435757,0.705735746247641,0.993285864701485
"10138","ABLIM3",15.1035296753359,-0.165116555222671,0.437599802778151,-0.377323193873512,0.705933437657675,0.993290053655807
"10139","UCHL3",83.9801733173601,0.122393238462902,0.324462846390722,0.377218038442264,0.706011576059855,0.993290053655807
"10140","NLRC3",5.87788386218672,0.243278486039157,0.644775504775927,0.377307270882911,0.705945269439308,0.993290053655807
"10141","VAMP3",109.973733781462,-0.0846548773023236,0.232748270320481,-0.363718609748458,0.716068145256611,0.993514238127841
"10142","EMC1-AS1",10.6521784919875,0.189896727943475,0.526085286302316,0.360961868517932,0.71812795251882,0.993514238127841
"10143","CAPZB",309.199203105252,-0.0832404908066344,0.228397919016198,-0.364453805731615,0.71551916287352,0.993514238127841
"10144","TMEM54",92.6741946985203,0.0967615605031695,0.265076373677033,0.365032760788644,0.71508695154425,0.993514238127841
"10145","PSMB2",234.23719637881,-0.108644259988986,0.296716593395392,-0.366154985623642,0.714249429474295,0.993514238127841
"10146","MACF1",276.226564678283,0.0900186602697115,0.245051916654389,0.3673452609500З,0.713361497001585,0.993514238127841
"10147","CAP1",275.917326211259,-0.0619000406776836,0.171169386204491,-0.361630324500513,0.717628300573928,0.993514238127841
"10148","ZFP69",3.05059888289702,-0.314638359745478,0.87055804847838,-0.36142145867863,0.717784408906509,0.993514238127841
"10149","SZT2",24.9423210809662,0.125834482619339,0.338193722798822,0.372078114217964,0.709834690724124,0.993514238127841
"10150","FAAH",4.27081214049322,-0.283243136200009,0.763686791811587,-0.370889138370602,0.710720105436992,0.993514238127841
"10151","BEND5",27.202739439018,-0.134178043539357,0.363354396935955,-0.369275959423734,0.711922042819897,0.993514238127841
"10152","NFIA",1000.52785766667,0.0747379104501368,0.207058112563252,0.360951375074985,0.71813579703245,0.993514238127841
"10153","FUBP1",85.6127117505146,0.0913615227736314,0.248426946067265,0.367760117088482,0.713052109990168,0.993514238127841
"10154","SPATA1",2.55349169503193,-0.413453273887124,1.10360046070175,-0.374640359994251,0.707927952621823,0.993514238127841
"10155","EVI5",79.1404344189568,0.0774400453460738,0.209457500570627,0.369717222515801,0.711593197971803,0.993514238127841
"10156","GPSM2",37.4470458579015,-0.0997936027278926,0.27647600235775,

-0.360948515881545,0.7181379344659,0.993514238127841
"10157","SLC16A4",28.0068886453564,0.125382419772622,0.340427935603232
,0.368308257518427,0.712643396021547,0.993514238127841
"10158","HIPK1-
AS1",4.12380108511879,-0.257422890846631,0.688377946152585,-0.37395574
9578258,0.708437238270185,0.993514238127841
"10159","OTUD7B",23.1374394018439,-0.141564128100008,0.378675554846828
,-0.373840154950774,0.708523242655555,0.993514238127841
"10160","NPR1",86.1481681683417,-0.102805664907382,0.283140980756163,-
0.363090021913558,0.716537638100322,0.993514238127841
"10161","DHX9",112.707123113427,0.0943526355478273,0.254317363688409,0
.371003513796362,0.710634914563918,0.993514238127841
"10162","KLHL12",17.8832837259522,0.156611143648233,0.418249138327158,
0.374444629520625,0.708073544329897,0.993514238127841
"10163","NUCKS1",1082.22826564714,-0.0563845668917425,0.15555061154628
3,-0.362483736523052,0.716990574759653,0.993514238127841
"10164","PROX1",3.29698795027964,0.402388303197637,1.11040039150381,0.
362381269203882,0.717067134694626,0.993514238127841
"10165","ARID4B",252.329298204297,0.0987928919041163,0.267545172647783
,0.369256865771131,0.71193627329446,0.993514238127841
"10166","LYST",70.4382195483908,0.100116779085239,0.272251114551231,0.
367736893383404,0.713069428271941,0.993514238127841
"10167","HS1BP3",30.4904292726061,-0.110045742998743,0.296600282952733
,-0.371023728983698,0.710619857951411,0.993514238127841
"10168","CEBPZ",144.773027216426,0.0788705759317501,0.209975501693398,
0.375617990173516,0.707200914347672,0.993514238127841
"10169","INO80B",23.7375885189897,-0.114345886480944,0.308017097627251
,-0.371232270421951,0.710464539363115,0.993514238127841
"10170","CCDC142",9.19181175586524,0.205596511586413,0.549345151545132
,0.374257442717272,0.7082127909162,0.993514238127841
"10171","IGKC",347.050241500515,0.253319848832428,0.672879839230627,0.
376471152891243,0.706566657401583,0.993514238127841
"10172","ANAPC1",25.884256585408,-0.119520478865722,0.326427989668308,
-0.366146539661535,0.714255731454654,0.993514238127841
"10173","GALNT5",7.76780322384552,-0.587335829058837,1.61192213350093,
-0.364369851900478,0.715581845083502,0.993514238127841
"10174","SATB2",6.80010225168173,0.22603168655184,0.611328024731214,0.
369738793917096,0.711577123576204,0.993514238127841
"10175","TYW5",20.5944455233643,0.124558947233673,0.343032494291092,0.
363111219218707,0.71652180406630７,0.993514238127841
"10176","ANKZF1",22.4334943774109,0.139458619112888,0.377420387161963,
0.369504732273621,0.711751546515559,0.993514238127841
"10177","TUBA4A",30.2951102061797,0.162311333070786,0.431752599294247,
0.37593597198049,0.706964497005011,0.993514238127841
"10178","DNAJB2",47.0050334462329,0.0951896613628442,0.254082309869963
,0.374641042155046,0.707927445223562,0.993514238127841
"10179","PSMD1",148.360229931952,0.0774518746248437,0.206468795480935,
0.375126296661112,0.707566540765497,0.993514238127841
"10180","HDAC11",9.61856153712703,-0.178829660368386,0.494219935716745
,-0.361842263827414,0.717469907126335,0.993514238127841

"10181","ZNF385D",19.9185226454727,-0.174193097765231,0.467921809606303,-0.372269670250661,0.709692078060712,0.993514238127841
"10182","THRB-IT1",3.2261794888452,-0.292860323415282,0.810507784260027,-0.361329439522479,0.717853188652632,0.993514238127841
"10183","MYL3",9.6007582795375,-0.214022144067799,0.580718825495959,-0.36854693643695,0.712465453887154,0.993514238127841
"10184","SMIM4",48.9957803665403,-0.0960848801688332,0.262776286655433,-0.365652781656151,0.714624184354378,0.993514238127841
"10185","STAG1",120.383732479207,0.0907369551931226,0.249196046699308,0.364118758684042,0.715769329539103,0.993514238127841
"10186","WWTR1",101.29263075829,-0.0791500288519251,0.21900874556132,-0.36140122463632,0.717799532651388,0.993514238127841
"10187","APOD",8511.81113726708,0.142011647056488,0.379244065218424,0.374459774274113,0.708062278710133,0.993514238127841
"10188","RHOH",15.9077582206103,0.153353676332442,0.420098278106464,0.365042382519783,0.715079769337853,0.993514238127841
"10189","ATP8A1",9.12844108419709,-0.208085469328159,0.563928121941812,-0.368992893299318,0.712133021915932,0.993514238127841
"10190","UTP3",52.6801008969064,-0.0989697654181592,0.273468780920248,-0.361905169157213,0.717422896989844,0.993514238127841
"10191","SDAD1",80.8674338182207,0.111945113928566,0.304101909239156,0.36811710327188,0.712785918400508,0.993514238127841
"10192","PLA2G12A",62.4406019873625,-0.0887619317418437,0.23754472175399,-0.373664087698689,0.708654246852222,0.993514238127841
"10193","GTF2H2",21.7228319917964,0.130019142548503,0.357287807533016,0.36390590388811,0.71592827583461,0.993514238127841
"10194","APC",79.0096656673046,0.0958011195492664,0.26299436338009,0.36427061902772,0.715655937513515,0.993514238127841
"10195","YTHDC2",42.2313625987753,0.0976221833545934,0.270565039496012,0.360808563946165,0.718242560355478,0.993514238127841
"10196","FNIP1",118.455606535458,-0.113949586393915,0.314951247121726,-0.361800714984546,0.717500957813031,0.993514238127841
"10197","PPARGC1B",5.33331876524659,-0.247591796184219,0.668622920824529,-0.370301089706728,0.71115816196955,0.993514238127841
"10198","LCP2",21.3142893203616,0.152881648730882,0.419052293045307,0.364827137968561,0.715240446120403,0.993514238127841
"10199","C6orf52",2.00566058197975,-0.35381832776982,0.969634192041967,-0.364898773861005,0.715186969615415,0.993514238127841
"10200","RNF144B",5.107039809047,-0.244716870647931,0.674978237921591,-0.362555200892801,0.71693718080852,0.993514238127841
"10201","LINC00472",13.2416507117155,0.175915381423733,0.471001293004411,0.373492353495695,0.708782035313812,0.993514238127841
"10202","BEND3",3.48795940638275,-0.296105918915963,0.788037533279364,-0.375751035212421,0.707101992815706,0.993514238127841
"10203","DCBLD1",5.94367656690886,-0.210307380992519,0.576671181980185,-0.364692024786745,0.715341312501509,0.993514238127841
"10204","RSPH3",6.59151921969887,-0.208793682175958,0.566927043463079,-0.368290214029198,0.712656848608095,0.993514238127841
"10205","AL078604.2",1.91508741404045,-0.521300433597838,1.40517385130

237,-0.370986432116337,0.710647637375351,0.993514238127841
"10206","SOD2",4359.67868841687,0.148089230588794,0.396776011886332,0.
373231309737593,0.708976295254974,0.993514238127841
"10207","ZDHHC4",58.5421378235074,-0.0898761869569896,0.24270316436213
,-0.370313206229516,0.711149135015499,0.993514238127841
"10208","CRPPA",6.78096123563457,-0.212114240883353,0.570156516277321,
-0.372028091984801,0.70987193374102,0.993514238127841
"10209","HOXA10",81.3514213184103,-0.0729731508164553,0.19761239751328
5,-0.369274153518376,0.711923388754526,0.993514238127841
"10210","TMEM248",174.596960322747,0.0667468710166871,0.18498197267810
1,0.360829058369042,0.718227238713543,0.993514238127841
"10211","HIP1",22.8317960482854,-0.137186399942456,0.364568868087266,-
0.376297627008617,0.706695643264613,0.993514238127841
"10212","AC002456.1",2.746494541291,-0.320575558826404,0.8643580350192
17,-0.370882835397344,0.710724800220064,0.993514238127841
"10213","ZNF394",43.7290355261208,-0.117043177059792,0.322372915675715
,-0.363067650439746,0.716554349350959,0.993514238127841
"10214","COPS6",216.373782653555,-0.0861878188338588,0.229049987785302
,-0.376283883126186,0.706705859775155,0.993514238127841
"10215","STAG3",2.06437028765543,-0.349813315459346,0.961029168278217,
-0.363998645416843,0.71585902090135 8,0.993514238127841
"10216","PRKRIP1",42.8766114908985,-0.099717799860385,0.27541812894929
7,-0.362059680823489,0.717307432548911,0.993514238127841
"10217","TSPAN12",6.76213278236579,0.220065230137939,0.602044902288698
,0.365529596382849,0.714716118241193,0.993514238127841
"10218","AC007938.3",3.73557459678924,-0.388843815879205,1.03302368523
931,-0.376413262769601,0.706609687551837,0.993514238127841
"10219","BPGM",24.7081678340818,0.127123745622468,0.347743427603625,0.
365567644221215,0.714687722475954,0.993514238127841
"10220","ATP6V1B2",95.3629803107366,-0.0866713787801048,0.238007283876
838,-0.364154312289681,0.715742781589563,0.993514238127841
"10221","NKX3-1",2.39945954193747,-0.375681837257922,1.03988405438739,
-0.361272812745687,0.7178955154908,0.993514238127841
"10222","PLPBP",76.6080959044271,-0.0972887377498661,0.261269971059494
,-0.372368616857666,0.709618416709398,0.993514238127841
"10223","BHLHE22",20.4631747069211,0.210434597023787,0.577786353873963
,0.364208319585358,0.715702454889813,0.993514238127841
"10224","LRP12",30.4775989558776,0.111873120789308,0.307423062269789,0
.363906077713618,0.715928146027857,0.993514238127841
"10225","MRPL13",100.757815138501,-0.118391189416744,0.327646988456851
,-0.361337639556346,0.717847059442013,0.993514238127841
"10226","CPSF1",25.3089063789419,0.111317113419861,0.308312090195818,0
.361053351327093,0.718059564579972,0.993514238127841
"10227","CNTLN",73.833769306741,-0.0944513423980828,0.259678199508304,
-0.363724573633539,0.716063691338972,0.993514238127841
"10228","HINT2",87.0008421699268,-0.107055522697795,0.296837389228079,
-0.360653767290541,0.718358290080519,0.993514238127841
"10229","CKS2",24.1754050814899,-0.137816936860072,0.376260697058451,-
0.366280448469649,0.714155817284708,0.993514238127841
"10230","ELP1",22.3957776837791,0.115912877687889,0.31764510397357,0.3

64913156972612,0.7151762327286,0.993514238127841
"10231","GARNL3",5.91879961135068,-0.249916273018457,0.668531134643115,-0.37382892144862,0.708531600771978,0.993514238127841
"10232","MAN1B1",70.275349817931,-0.104547596777994,0.284255663433202,-0.367794243798988,0.713026661434312,0.993514238127841
"10233","AL135791.1",2.06105276151734,-0.341897775927841,0.936839820029734,-0.364947954408033,0.715150256932601,0.993514238127841
"10234","AL137026.1",21.216211560245,0.136262913591859,0.370551999522816,0.367729532609009,0.71307491734793,0.993514238127841
"10235","AGAP4",8.61873752859461,0.194639991173839,0.527603000590316,0.368913730505822,0.712192028662341,0.993514238127841
"10236","TSPAN14",78.7602900715308,0.111732056638879,0.30287921169694,0.368899720825599,0.712202471445118,0.993514238127841
"10237","CCNJ",10.3485786822689,0.173800889264987,0.471518744935592,0.368598048607225,0.712427350299538,0.993514238127841
"10238","POLR2L",641.291120510793,-0.111708937307583,0.300199763132656,-0.372115341271004,0.709806974543266,0.993514238127841
"10239","COPB1",177.19133807144,0.0685476432564347,0.186198368886741,0.368143092048945,0.712766540881959,0.993514238127841
"10240","ZBTB3",4.06194969651762,0.263053341400383,0.710104206825843,0.370443293916294,0.711052220688692,0.993514238127841
"10241","ESRRA",52.1609819178102,-0.115873038625432,0.313538055484279,-0.369566107203348,0.711705808405198,0.993514238127841
"10242","AP5B1",24.0464561431575,0.124269189180538,0.331129127134959,0.375289211963191,0.707445388437419,0.993514238127841
"10243","AP001528.1",49.0739929562741,-0.136019398760429,0.371274588789394,-0.366357954106001,0.714097989785385,0.993514238127841
"10244","GPR162",1.98484432555,0.379829970306468,1.0459930324955,0.363128585474687,0.716508831853059,0.993514238127841
"10245","BCAT1",41.007118371724,0.116184186868831,0.318696244033521,0.364560891582426,0.715439212452915,0.993514238127841
"10246","TCP11L2",34.0818965673126,-0.128795262776736,0.348004735883422,-0.370096293229415,0.711310743915568,0.993514238127841
"10247","PLBD2",92.2642944081875,0.082329128887281,0.224505503777153,0.366713187437053,0.71383296871741,0.993514238127841
"10248","ACADS",53.7701978285545,-0.101690933257504,0.277437845861112,-0.366535909842711,0.713965221974675,0.993514238127841
"10249","LINC01089",7.37946564219221,-0.198427805788748,0.548094958000086,-0.362031802870036,0.717328264882211,0.993514238127841
"10250","MICU2",66.6407122293389,-0.0970345643503091,0.266413535389944,-0.364225354422333,0.715689735314421,0.993514238127841
"10251","WBP4",110.158975543769,-0.0774820285407987,0.210123796506725,-0.368744663045905,0.712318054754295,0.993514238127841
"10252","LRCH1",25.3262670094895,-0.128509086094603,0.349725547564421,-0.367456958719698,0.713278191582852,0.993514238127841
"10253","RNASEH2B",92.0866061928779,-0.0817376256788304,0.219050332508761,-0.373145407919257,0.709040224624215,0.993514238127841
"10254","RBM25",426.07321257485,0.0733868118624414,0.200310853276228,0.366364631082876,0.714093008122886,0.993514238127841
"10255","ATP10A",11.6779851147454,0.164641276846519,0.450800259385945,

0.365220013561624,0.714947179975526,0.993514238127841
"10256","TMEM87A",176.234677333348,-0.0667041679782957,0.1774567281877
6,-0.375889765688223,0.706998849319289,0.993514238127841
"10257","CASC4",177.298047840696,0.0699124491066045,0.191401260967855,
0.365266397687663,0.714912558830023,0.993514238127841
"10258","LOXL1",240.017308785383,0.136090101491203,0.375558208718027,0
.362367532734136,0.717077398312175,0.993514238127841
"10259","AC027020.2",2.57673900048772,0.390598451166009,1.062459285078
27,0.367636159476205,0.713144548841297,0.993514238127841
"10260","SELENOS",328.033040443982,-0.0882482534447413,0.2365306376598
09,-0.373094387762421,0.709078195529076,0.993514238127841
"10261","RAB11FIP3",17.7441517080201,-0.1385356875517,0.37914657291482
3,-0.36538820985947,0.714821640929213,0.993514238127841
"10262","ARL6IP1",234.821612796177,0.0609228252840572,0.16504527046781
2,0.36912796780771,0.71203234340438,0.993514238127841
"10263","SRCAP",37.0320196011823,-0.101223775099096,0.278685529014683,
-0.363218626589554,0.716441574422035,0.993514238127841
"10264","AC010542.2",2.201477942026,-0.348895848483942,0.9574923891933
53,-0.364384983548404,0.715570547242612,0.993514238127841
"10265","EXOC3L1",2.73608206971016,-0.347279215257763,0.93328724450495
2,-0.372103248279121,0.70981597794148,0.993514238127841
"10266","CHTF8",2.97992187710169,-0.31142735084393,0.831251446593225,-
0.374648792637022,0.707921680343943,0.993514238127841
"10267","AC087392.1",2.96533628050506,-0.328163713961251,0.90281014828
0997,-0.363491388068791,0.716237844964556,0.993514238127841
"10268","PITPNA-
AS1",38.2710434402261,-0.113305858021262,0.304286765615539,-0.37236538
300329,0.709620824127358,0.993514238127841
"10269","SRR",13.4879759455408,-0.149520607034853,0.4067704139112,-0.3
67579848291262,0.713186543146019,0.993514238127841
"10270","AC233723.1",4.32739019566209,-0.341770013387431,0.94332946934
6749,-0.362301851572712,0.717126474678468,0.993514238127841
"10271","ALOX12-
AS1",21.127738373735,-0.133632727873866,0.370497993915731,-0.360684079
450807,0.718335627467297,0.993514238127841
"10272","NEURL4",7.83733939971948,-0.182031447361184,0.500383443241777
,-0.363783913755975,0.716019375783689,0.993514238127841
"10273","CHD3",115.237826072816,0.0993251260290388,0.269878342496767,0
.368036668337804,0.712845892748387,0.993514238127841
"10274","GAS7",256.770068297782,-0.0978806020845398,0.263300713477285,
-0.371744537991858,0.710083061086639,0.993514238127841
"10275","SMCR8",19.151646922266,0.132399070679433,0.358177475225793,0.
369646557466992,0.711645856457046,0.993514238127841
"10276","DHRS13",3.70201834517505,-0.266017286216218,0.724368577941807
,-0.367240234207935,0.71343983021308,0.993514238127841
"10277","PHF12",32.0440930479014,-0.107217931260438,0.292343068567355,
-0.366753799862148,0.713802672102831,0.993514238127841
"10278","HSD17B1",4.50747565849098,-0.261161756679452,0.70282960946598
3,-0.371586161371154,0.710200994120333,0.993514238127841
"10279","IFI35",163.163049296883,0.101030710104605,0.274702557889414,0

.367782196426713,0.713035645217891,0.993514238127841
"10280","CEP112",33.3910256402974,-0.120554070863739,0.323966450120616
,-0.372118998182853,0.709804251930248,0.993514238127841
"10281","JPT1",96.6907573874266,0.105347473442543,0.284464659820063,0.
370335891668159,0.711132234201933,0.993514238127841
"10282","MYO15B",19.6106598381171,-0.150404499943165,0.409412554533773
,-0.367366604364248,0.713345578585532,0.993514238127841
"10283","AC027601.6",2.18088403057563,-0.381952370102985,1.04425751418
845,-0.365764540751061,0.714540781473567,0.993514238127841
"10284","METTL4",19.1471602842907,-0.127281484693127,0.342517319800768
,-0.371605981172463,0.710186235190215,0.993514238127841
"10285","TWSG1",74.7203027086102,-0.0797373445689135,0.213365759241454
,-0.373711999771618,0.708618596651137,0.993514238127841
"10286","SMAD7",81.347308486951,-0.132436898072466,0.359582308827837,-
0.368307602518552,0.712643884364693,0.993514238127841
"10287","MEX3C",35.0231417500142,0.12754785170798,0.340939100267969,0.
374107433285683,0.708324388630379,0.993514238127841
"10288","CSNK1G2",69.5301335687751,-0.092898732258851,0.25414249986384
2,-0.36553796515192,0.714709872449022,0.993514238127841
"10289","FDX2",50.103619132916,-0.111097172144035,0.303409152370968,-0
.366162890195877,0.714243531471293,0.993514238127841
"10290","RAVER1",19.7490146560374,0.139252278790046,0.376346177948962,
0.370011141202369,0.71137418914358,0.993514238127841
"10291","ZNF69",4.03481048689425,0.297690170331247,0.81381610956867,0.
365795376659508,0.714517770044056,0.993514238127841
"10292","CACNA1A",11.3638355716331,-0.206665553114188,0.55969178484438
5,-0.369248859301462,0.711942240535795,0.993514238127841
"10293","NR2F6",62.2843740169107,-0.0931690765070429,0.247579280274738
,-0.376320168649225,0.706678887057786,0.993514238127841
"10294","PEPD",145.512149985324,-0.106384430073517,0.28875172857919,-0
.368428721091935,0.712553584938442,0.993514238127841
"10295","SCN1B",59.973609393121,-0.113364914442905,0.309864126118514,-
0.365853627081523,0.714474301123991,0.993514238127841
"10296","PSENEN",139.978273738285,-0.113683487288129,0.312584524976989
,-0.363688788805199,0.716090416123292,0.993514238127841
"10297","ZNF546",11.1661593293515,0.168168145699557,0.45034371534379,0
.373421766463819,0.708834561929284,0.993514238127841
"10298","RPS19",4765.02807182448,-0.0759793485490378,0.204832428485384
,-0.370934178298137,0.710686557670354,0.993514238127841
"10299","DHX34",6.88877850504969,-0.199735782522006,0.534547795419892,
-0.373653739166789,0.708661947025404,0.993514238127841
"10300","THBD",37.8164042762947,0.193509272759563,0.5207023845614,0.37
1631239834941,0.710167426338088,0.993514238127841
"10301","MROH8",2.450927962272,-0.319932096215564,0.869979016437201,-0
.367746911328704,0.713061957714587,0.993514238127841
"10302","SERINC3",252.43327820124,-0.0654888273452634,0.18019069342569
9,-0.363441785478602,0.716274892347793,0.993514238127841
"10303","AL133342.1",1.92222312061824,-0.360701899303101,0.98602009054
3722,-0.365815973490154,0.714502399715047,0.993514238127841
"10304","LINC01436",26.3129081795317,-0.398600226803225,1.061550307583

32,-0.375488777079874,0.707296991487144,0.993514238127841
"10305","AC008079.1",2.05094974395954,0.379515737048639,1.045605051445
04,0.362962799887148,0.716632673396259,0.993514238127841
"10306","BCR",23.0602598435299,0.123087405448731,0.341142835708989,0.3
60809000115513,0.718242234273851,0.993514238127841
"10307","SMARCB1",98.3516418403213,-0.10865105969627,0.28839491163591
4,-0.376744024642995,0.706363842465621,0.993514238127841
"10308","PRR34-
AS1",8.39403232927645,-0.177294731750179,0.484404398927792,-0.36600561
8740486,0.714360882904266,0.993514238127841
"10309","CD99",1475.33333845661,-0.0863358141345097,0.236661805861824,
-0.364806707276278,0.715255697977122,0.993514238127841
"10310","EIF1AX",655.846398007504,-0.0709294822360638,0.18963744288332
1,-0.374026780564136,0.708384391842494,0.993514238127841
"10311","ZXDB",12.4082970168056,-0.165821941675325,0.441600815041761,-
0.375501892268118,0.707287239400853,0.993514238127841
"10312","TAF1",48.1488507543095,0.104157338783397,0.282175828586295,0.
369122115473983,0.712036705368789,0.993514238127841
"10313","DIAPH2",105.154360767341,0.0943025378382882,0.258410046666079
,0.364933713123573,0.715160887811225,0.993514238127841
"10314","HNRNPH2",127.022662633329,-0.0922716345395392,0.2513867788311
57,-0.367050466888368,0.713581374051275,0.993514238127841
"10315","ACSL4",227.647109162555,0.124447712843929,0.333939753853448,0
.372665163125633,0.709397667450803,0.993514238127841
"10316","SLC35A3",60.0176595559291,0.105413743769432,0.292888296658869
,0.359911082047121,0.718913629359384,0.993542712711909
"10317","LXN",107.023860377936,0.135162310644031,0.375192669551595,0.3
60247738330197,0.718661877849089,0.993542712711909
"10318","HIST1H2AG",2.04259125565372,0.36131686861524,1.00371562159459
,0.359979321674023,0.718862597312342,0.993542712711909
"10319","NSD3",222.805639587133,0.0586715230291349,0.162827418307039,0
.360329504939394,0.718600737400016,0.993542712711909
"10320","PRRX2",301.258097942545,-0.109624859663836,0.304692926793686,
-0.359788003014804,0.71900567545477,0.993542712711909
"10321","BX255925.3",5.68076217519817,0.218465250799304,0.606395102148
759,0.360268824773111,0.718646110426489,0.993542712711909
"10322","CLEC2D",28.3832065061477,0.134316451351179,0.373170554582433,
0.359933145050726,0.718897129714736,0.993542712711909
"10323","PSMC6",181.040134486161,-0.0792794093708243,0.220072800002317
,-0.360241744413619,0.718666359831996,0.993542712711909
"10324","TTI1",18.6697534419647,0.148544869886469,0.412833313372431,0.
359818030848836,0.718983218411544,0.993542712711909
"10325","PPP6C",62.6743077042893,-0.087099739501953,0.2422208437034,-0
.359588126976417,0.719155163774394,0.993653033065909
"10326","ELOA-
AS1",7.79431413070526,-0.192834667962451,0.536637563453141,-0.35933874
3865792,0.719341693754112,0.993733613098899
"10327","MTHFD2",54.0606492776168,-0.145941473047289,0.40615576597131,
-0.359323898057372,0.719352798434903,0.993733613098899
"10328","MAPK7",21.3799675753174,0.124489224396159,0.346605209239741,0

.359167205447429,0.719470008288767,0.993799296886866
"10329","ABHD5",283.175100136534,-0.0911828046749611,0.253989294565555
,-0.359002550996994,0.719593180899341,0.993813672979073
"10330","RARA-
AS1",21.743278874233,-0.125249985630696,0.348917782612609,-0.358967045
740274,0.71961974217537,0.993813672979073
"10331","CHAF1A",9.88161265812755,0.190755305193928,0.531804704383906,
0.358694279350006,0.719823808413532,0.993903063378576
"10332","TST",17.0043506486368,0.145025435937043,0.404245019525171,0.3
58756271400428,0.719777428201964,0.993903063378576
"10333","RNASEH1",91.2918592763012,-0.082815281572889,0.23106898406444
6,-0.358400682411757,0.720043481037364,0.993917784274701
"10334","KIAA1191",34.1760048829352,0.0969750739916237,0.2705711296663
92,0.358408800344633,0.720037406795303,0.993917784274701
"10335","ZFP1",13.2770618065071,-0.155599595937122,0.434085216283332,-
0.358454031835907,0.720003562662294,0.993917784274701
"10336","ANKRD65",53.1167935554679,-0.0959339118240781,0.2679106079515
37,-0.358081796602216,0.720282101275328,0.993958643528132
"10337","MIER1",177.602503286285,0.0638117200710193,0.178119371521007,
0.358252555722125,0.720154319913698,0.993958643528132
"10338","AL590062.1",6.65393248086768,-0.222429201995785,0.62116261276
0109,-0.358085302345275,0.720279477801755,0.993958643528132
"10339","ABCA2",23.983413694124,-0.116314859929595,0.325331551507177,-
0.357527142359043,0.720697210447589,0.994435284287195
"10340","PANK4",12.2394605124071,0.154709751192879,0.433202919352728,0
.357129982928182,0.720994499403076,0.994471784856034
"10341","SLC25A40",11.8862885355485,-0.15356648895112,0.43012669428329
1,-0.357026176222343,0.721072209627844,0.994471784856034
"10342","LRRC61",4.66734222683818,-0.263470910428863,0.737671501475793
,-0.357165635247886,0.720967810560897,0.994471784856034
"10343","AC144831.1",8.1357516282855,0.184499321605168,0.5163267309678
69,0.357330563264308,0.720844352111324,0.994471784856034
"10344","ZNF551",5.64130164814503,0.227494579937955,0.637036313754259,
0.357113990248462,0.721006471416254,0.994471784856034
"10345","ATP5MC1",252.554323953397,-0.0997352134298474,0.2795134554898
71,-0.356817217457574,0.721228645948697,0.994591383577005
"10346","TNFRSF18",5.44285247912683,-0.257940634394251,0.7280136287802
32,-0.354307425297001,0.723108503645989,0.994624859391346
"10347","FBXO2",4.22007757598129,-0.246184465081287,0.696420459050606,
-0.353499759925056,0.723713808714404,0.994624859391346
"10348","TMEM200B",19.3373871077679,-0.133376212305509,0.3827647047992
31,-0.348454835655415,0.727498626817613,0.994624859391346
"10349","AL355816.2",2.21714109143492,-0.390724612738019,1.10852764427
658,-0.352471690494472,0.724484545661208,0.994624859391346
"10350","DENND2C",13.67685518561,0.141835339693014,0.408562708830612,0
.347156841844364,0.728473492075199,0.994624859391346
"10351","POLR3C",35.0438773837108,-0.104701634007577,0.298793358606588
,-0.350414863622972,0.726027373121139,0.994624859391346
"10352","CD48",51.2156404423188,-0.122904118769237,0.344881917067166,-
0.356365795616074,0.721566641298707,0.994624859391346

"10353","UCK2",19.6981644766015,-0.153752655537942,0.439257871002719,-0.350028230995526,0.726317510936327,0.994624859391346
"10354","PLA2G4A",10.9960397899739,0.151702897751704,0.435393841883266,0.348426833727191,0.727519653169706,0.994624859391346
"10355","CPSF3",25.0234622355504,0.119144922264784,0.338837159860877,0.351628854148417,0.725116622982618,0.994624859391346
"10356","STAMBP",53.1446754340797,-0.0819863549080123,0.231097807928224,-0.35476907220806,0.722762599987786,0.994624859391346
"10357","TBC1D8",6.03982572707329,0.234903137990183,0.671675475879903,0.349727132261985,0.726543489394601,0.994624859391346
"10358","MTMR14",34.0435863267056,0.100033213448214,0.287755845117065,0.347632255419583,0.728116379073614,0.994624859391346
"10359","CRELD1",36.406691607842,-0.127529514398741,0.363111283770606,-0.351213305944267,0.725428328496295,0.994624859391346
"10360","HIGD1A",132.597271931375,0.119628604661232,0.344903346847742,0.346846749254776,0.728706453865443,0.994624859391346
"10361","P4HTM",63.7352439690685,-0.104731099717245,0.29609871379652,-0.353703325402545,0.723561230197241,0.994624859391346
"10362","CDHR4",2.14858638379171,-0.386143537394925,1.11318065209156,-0.346883083773868,0.728679155707079,0.994624859391346
"10363","AC084198.2",4.38550995698362,0.236399434381401,0.681672687654466,0.34679317312069,0.728746706292993,0.994624859391346
"10364","RAB7A",301.256024396938,-0.0703993752392307,0.198042002192947,-0.355476992050618,0.722232278546503,0.994624859391346
"10365","U2SURP",224.849356671328,0.0527331020165283,0.148920666296053,0.354101974750067,0.723262462212643,0.994624859391346
"10366","AC106707.1",10.4636037949003,-0.184624471160134,0.531743405301117,-0.347205944294852,0.72843660540926,0.994624859391346
"10367","MYNN",28.4878097050652,-0.116697124393683,0.334894495643649,-0.348459368283727,0.727495223334187,0.994624859391346
"10368","VPS8",22.0827319841032,0.115364608666527,0.331855972376451,0.347634571227966,0.728114639668837,0.994624859391346
"10369","BANK1",4.82628340468828,-0.296630131402091,0.852119333029507,-0.348108674342001,0.727758570094835,0.994624859391346
"10370","LARP7",177.330290741695,-0.0726218438542005,0.208822398559376,-0.347768459490956,0.728014078511954,0.994624859391346
"10371","FGF2",62.2642051533833,0.085896798116559,0.241602999296403,0.35552869114501,0.722193554615665,0.994624859391346
"10372","CFAP97",174.39293524279,-0.0836320754484092,0.235715873769824,-0.354800353963755,0.722739163184111,0.994624859391346
"10373","UFSP2",70.3170002973155,-0.0800576367128061,0.227427055967402,-0.352014567362122,0.724827337651054,0.994624859391346
"10374","KIAA0825",10.3691774734556,0.165486955099792,0.468542898433914,0.353194884935671,0.72394234232699,0.994624859391346
"10375","NREP",34.8501810333612,-0.102596807835297,0.29213661526139,-0.35119462085743,0.725442345375287,0.994624859391346
"10376","SRP19",160.401768176181,-0.0909598849510783,0.258340231276579,-0.352093378958451,0.724768233698888,0.994624859391346
"10377","DTWD2",4.80192955781152,0.239148379079599,0.673471403082517,0.355098045715086,0.72251614066776,0.994624859391346

"10378","PAIP2",248.008401184699,0.0578823771202821,0.164355303763992,
0.352178334344471,0.724704524111184,0.994624859391346
"10379","SLC36A1",10.6568940136422,-0.156352771804234,0.44435759034143
1,-0.351862498138262,0.724941385047285,0.994624859391346
"10380","SAP30L",66.0743717035087,-0.0890649908180547,0.25041011798466
4,-0.355676485977733,0.722082856469545,0.994624859391346
"10381","STK10",26.6670195306423,-0.149781714068444,0.427751469668628,
-0.350160606542106,0.726218168927756,0.994624859391346
"10382","FAM153CP",13.0491440689734,-0.199427785979897,0.5751420048492
06,-0.346745298201936,0.728782675958064,0.994624859391346
"10383","RIPK1",48.8230282835474,-0.088469533659195,0.248620006438287,
-0.355842375384843,0.721958612440953,0.994624859391346
"10384","TAF8",28.4674264881366,-0.112565458578705,0.32333513595407,-0
.348138652629125,0.727736057228973,0.994624859391346
"10385","XPO5",18.1592410229117,0.128820055966821,0.367410551660733,0.
350616103387725,0.725876373828783,0.994624859391346
"10386","TSPYL1",122.8656002315,-0.0721982683244467,0.20714306305491,-
0.348543017852876,0.727432413077506,0.994624859391346
"10387","RPA3",85.1217501156224,-0.0890380283145097,0.252100056446597,
-0.353185277185255,0.723949544675383,0.994624859391346
"10388","MRPS24",288.060794821808,0.0858502844047711,0.246872206859732
,0.347751921922704,0.728026499339803,0.994624859391346
"10389","UBE2D4",47.3289263594848,0.109612107101221,0.30872313192439,0
.355049867555978,0.72255223282814,0.994624859391346
"10390","MEPCE",44.0328443560992,-0.100680521028157,0.285448259771636,
-0.352710228847441,0.724305690100023,0.994624859391346
"10391","CLDN15",8.32583463102846,0.19573109305308,0.557802776498279,0
.350896591590708,0.72566592862518,0.994624859391346
"10392","MTPN",309.764003982123,-0.0506601932984523,0.145893634630655,
-0.347240600501207,0.728410571406761,0.994624859391346
"10393","ZNF282",17.8565476697092,-0.137959232333047,0.388388572294261
,-0.355209298559183,0.722432799130863,0.994624859391346
"10394","FAM86B1",4.12800459918738,0.240337052790255,0.678651253701412
,0.354139260009379,0.723234520909394,0.994624859391346
"10395","ZDHHC2",24.2307153840729,0.130888057409546,0.373352980011122,
0.350574561921661,0.725907543396162,0.994624859391346
"10396","AC037459.2",3.16671852599296,0.2958261012428,0.84560783050922
5,0.349838412760031,0.726459969181457,0.994624859391346
"10397","DENND3",6.46328668885749,0.271508261852087,0.778212696839364,
0.348886959766644,0.727174175408363,0.994624859391346
"10398","CYHR1",81.0561375417593,-0.0850372922431982,0.240306453932296
,-0.353870197207257,0.723436162897383,0.994624859391346
"10399","GLIPR2",158.198700191227,-0.0880132537934624,0.25378110170252
1,-0.346807753623162,0.728735751702892,0.994624859391346
"10400","PTCH1",20.3732541352914,0.142622605965426,0.400430086891274,0
.356173550975333,0.721710598110638,0.994624859391346
"10401","ERCC6L2",50.9175009339242,0.0859830474507176,0.24366341065775
9,0.352876319093663,0.724181164864311,0.994624859391346
"10402","WHRN",4.57734509986755,0.272557360251972,0.76828775917563,0.3
54759472602329,0.722769792221772,0.994624859391346

"10403","CCDC183",3.88386644837715,-0.252652806551429,0.72337697525415
7,-0.349268521385631,0.726887728455593,0.994624859391346
"10404","TRAF2",17.688563926797,-0.124887134382446,0.358885355302966,-
0.347986153620054,0.727850582215989,0.994624859391346
"10405","AL450384.2",2.29163628406958,-0.354778972069683,1.01274394356
513,-0.350314582796481,0.726102622333938,0.994624859391346
"10406","CHST3",19.552434214357,0.157192547120633,0.451778115810575,0.
347941924629526,0.727883798811951,0.994624859391346
"10407","NUTM2B-
AS1",67.5405149398441,-0.109541954322491,0.307760587405076,-0.35593236
6928816,0.721891215714578,0.994624859391346
"10408","DUSP5",43.5695271430395,-0.200783301110203,0.571116927362868,
-0.351562511090889,0.725166384304159,0.994624859391346
"10409","AL158163.1",2.4194073137973,-0.402479079786557,1.137110025432
36,-0.353949108516148,0.723377022917429,0.994624859391346
"10410","TUBGCP2",84.1641523585244,-0.088468698964222,0.25485014374811
3,-0.347140078726665,0.728486084982194,0.994624859391346
"10411","ARL14EP",78.6518648158162,0.0895033017264379,0.25749589413534
8,0.347591180150593,0.728147230960347,0.994624859391346
"10412","ELP4",24.2493144607095,0.107525731977011,0.303173296580361,0.
354667555453747,0.722838659794243,0.994624859391346
"10413","TRAF6",18.8824272179241,0.125217050831132,0.358097709031367,0
.349672862107486,0.726584222372745,0.994624859391346
"10414","AC090559.2",2.70373743015188,0.296014683870514,0.853354103664
551,0.346883764429491,0.728678644333152,0.994624859391346
"10415","FNBP4",137.569362647599,0.102220979155416,0.29316930531515,0.
34867558541141,0.727332875342821,0.994624859391346
"10416","PATL1",36.760869469404,0.12270885448604,0.352303413063526,0.3
48304472610699,0.7276115352461,0.994624859391346
"10417","PPP2R5B",9.87707768817178,0.158239079567064,0.453293578074595
,0.349087406530661,0.72702369072396,0.994624859391346
"10418","RELA-
DT",2.42668189247677,-0.377082769212194,1.06043482625215,-0.3555925926
58337,0.722145691747639,0.994624859391346
"10419","BIRC2",107.511682645075,0.0727242767410588,0.206701082495748,
0.351833071520343,0.724963454840519,0.994624859391346
"10420","PPP2R1B",30.5565407244395,0.126586390862856,0.364368884284372
,0.347412735616747,0.728281266858267,0.994624859391346
"10421","CHD4",158.769898816501,0.0841450574079204,0.242028134294503,0
.347666429992522,0.728090710677787,0.994624859391346
"10422","H2AFJ",448.269812502022,0.0919953927001453,0.261780398116428,
0.3514220062391,0.725271775306235,0.994624859391346
"10423","TMBIM6",884.488007060627,0.0532656027482577,0.150063800441039
,0.354953043916719,0.722624769104617,0.994624859391346
"10424","ORMDL2",43.752726806262,0.11265244852982,0.319981630128863,0.
352059111907309,0.724793931722596,0.994624859391346
"10425","CHPT1",152.33150027211,0.0919738158794908,0.259669369745922,0
.354195860564857,0.723192105568255,0.994624859391346
"10426","ATP6V0A2",29.9845221794174,-0.108253462254321,0.3117373047428
65,-0.347258607190479,0.728397044770217,0.994624859391346

"10427","KATNAL1",36.1042695014641,0.107341587758657,0.30941998768271,
0.346912261753202,0.728657234512846,0.994624859391346
"10428","CKAP2",12.0078347403697,0.159063918031595,0.457272481380306,0
.34785368573121,0.7279500689968,0.994624859391346
"10429","TUBGCP3",26.6631950994084,-0.10895836348589,0.312994269764954
,-0.348116160617615,0.727752948086552,0.994624859391346
"10430","VCPKMT",15.8718733906009,-0.146915957555401,0.41674602247878,
-0.352531157181907,0.724439956255036,0.994624859391346
"10431","ACOT4",5.62119567024962,-0.225635274159553,0.648073028269504,
-0.348163346285291,0.727717513151519,0.994624859391346
"10432","IFI27",1237.24553375487,0.217940612914809,0.612142422125698,0
.356029258939445,0.721818653484233,0.994624859391346
"10433","NUDT14",14.1273645726082,0.204896216648106,0.586268288101532,
0.34949223897408,0.726719796342154,0.994624859391346
"10434","BAHD1",12.6375646824626,0.148309977588682,0.427294098656775,0
.347091097337651,0.728522881545099,0.994624859391346
"10435","LRRC57",24.9112970343881,-0.122721513916068,0.350418344169153
,-0.350214296591813,0.726177878226193,0.994624859391346
"10436","SDR42E2",9.32660188350824,0.179278174289955,0.507671253201444
,0.353138321619359,0.723984744769875,0.994624859391346
"10437","COG7",21.0601148269267,0.115519316192815,0.330699030238433,0.
349318581640611,0.726850149919674,0.994624859391346
"10438","AC020765.2",3.43323279891977,-0.278012773537113,0.78709327807
8876,-0.35321451888864,0.723927624017997,0.994624859391346
"10439","FHOD1",22.0190527551056,-0.113337114211478,0.320966372001203,
-0.353112114221902,0.724004391317985,0.994624859391346
"10440","LINC00672",3.16706124945341,0.319435753352976,0.9201228906339
,0.34716640201496,0.728466310249523,0.994624859391346
"10441","CCDC47",164.807970330438,-0.0634319377415376,0.17837560668569
2,-0.355608812887225,0.722133542808193,0.994624859391346
"10442","UBALD2",83.3516423422496,0.0999486167680183,0.28108636024119,
0.355579746673784,0.722155313430295,0.994624859391346
"10443","AC009831.1",2.25765768752507,0.324946619130932,0.926638631042
051,0.350672428544787,0.725834112425337,0.994624859391346
"10444","ZNF414",30.4901551184258,-0.12031920155199,0.341621739206381,
-0.352200073190608,0.724688222061711,0.994624859391346
"10445","UBL5",531.542585732139,-0.0829754625001149,0.235975808077315,
-0.351626987428003,0.725118023120406,0.994624859391346
"10446","BABAM1",92.7134468187569,-0.0970574686180489,0.27275537630543
4,-0.355840716809054,0.721959854606569,0.994624859391346
"10447","CARD8-
AS1",6.39567428023812,-0.194793379265673,0.552423759791399,-0.35261586
022156,0.724376445701095,0.994624859391346
"10448","ZNF628",9.67430373611222,0.175942742446432,0.499689636436104,
0.352104045425664,0.724760234616681,0.994624859391346
"10449","UCKL1",40.4761185748872,-0.0890722560636704,0.251895237031827
,-0.353608337788522,0.723632424936424,0.994624859391346
"10450","RANBP1",243.088427541876,-0.109436413295488,0.311796007014887
,-0.350987218672954,0.725597937198018,0.994624859391346
"10451","MAFF",335.298810395534,0.142535187406507,0.401922187281896,0.

354633787127899,0.722863960779958,0.994624859391346
"10452","HDAC6",22.2244403025285,0.13511553638737,0.380289821182569,0.355296221095814,0.722367686163301,0.994624859391346
"10453","ATP6AP1",102.827974547793,-0.0781037380009483,0.221633400013703,-0.352400576790859,0.724537869478689,0.994624859391346
"10454","KCND3",37.7779970509967,0.120996984679277,0.349143976908439,0.346553263644035,0.728926962490184,0.994716349137926
"10455","SHISA4",36.6086186533523,-0.111090776116683,0.320893437721292,-0.346192109460243,0.729198344264996,0.994716349137926
"10456","RFX8",4.4168263633418,0.227390735819583,0.656333125327639,0.346456284232295,0.728999832311071,0.994716349137926
"10457","LPAR6",40.237083598421,0.107118096204003,0.309296541825654,0.346328140533765,0.729096122568367,0.994716349137926
"10458","TP53BP1",59.9687488024411,0.0892492545105651,0.257785560583744,0.346215103392386,0.729181064942761,0.994716349137926
"10459","TRMT2A",24.6825706933029,-0.13389970010737,0.386918084611011,-0.346067308386441,0.729292131318032,0.994749167739081
"10460","TSEN2",14.911647737286,-0.140210969112606,0.405872804664832,-0.345455441954017,0.729752002853778,0.995085557232182
"10461","AC008393.1",3.44139653640105,-0.281245746821369,0.814503875583235,-0.345297002571019,0.729871099862158,0.995085557232182
"10462","NAP1L1",592.167047360974,-0.0630047010570066,0.182407685374875,-0.345405956594002,0.729789199713134,0.995085557232182
"10463","AC090517.5",8.75464873224674,-0.179858197223218,0.52091263515761,-0.34527516724335,0.729887513730377,0.995085557232182
"10464","C16orf58",42.0274297612155,0.0981179094637631,0.283959931725131,0.34553434658077,0.729692693611331,0.995085557232182
"10465","KLHL17",2.1655692380077 6,0.319899581127078,0.930665352108422,0.343732127130708,0.731047747775762,0.99511030171561
"10466","PRXL2B",5.90866676956712,0.205538090617096,0.603643085772474,0.340496057126327,0.733482991559455,0.99511030171561
"10467","SLC45A1",4.22980125561062,-0.369174403282806,1.08367219730674,-0.340669811590919,0.733352167487523,0.99511030171561
"10468","SNHG12",44.1543046315803,-0.102498355763687,0.301630645181927,-0.339814131624012,0.733996505051574,0.99511030171561
"10469","EFCAB7",15.2302738206783,0.142950757591742,0.415840697106481,0.343763269411645,0.731024325352588,0.99511030171561
"10470","DLEU2L",4.99811504291061,0.243082013210739,0.706913159146167,0.343864037704916,0.730948538222683,0.99511030171561
"10471","SLC44A3",3.89157746454086,-0.241785955503901,0.703066019858244,-0.343902206442364,0.730919832467387,0.99511030171561
"10472","LIX1L",56.4753861275841,0.104755541417622,0.303910479078363,0.344692100566268,0.730325857440864,0.99511030171561
"10473","TMCO1",292.104384880567,-0.0689333124211325,0.200648340410008,-0.34355286607541,0.731182576695869,0.99511030171561
"10474","RO60",124.628541643567,-0.0614390371534457,0.178518484761082,-0.344160646644922,0.730725475952891,0.99511030171561
"10475","IARS2",104.077679283364,0.0707419350666657,0.206767583925996,0.342132619260014,0.73225109045234,0.99511030171561
"10476","TGOLN2",292.072203074744,0.0570517165443068,0.167786021493053

,0.340026636525671,0.733836468735224,0.99511030171561
"10477","KIF9-
AS1",4.12357464048175,0.252243867928987,0.738124718711278,0.3417361071
03132,0.732549496694923,0.99511030171561
"10478","RIOX2",40.0263360627322,0.0919131669259255,0.268689282831495,
0.342079765732851,0.732290864505657,0.99511030171561
"10479","PPARGC1A",2.46360307247906,-0.365715234731363,1.0708160497592
2,-0.341529467001915,0.73270502547631,0.99511030171561
"10480","ZCCHC4",13.2913945040929,0.151909645382037,0.443177668912734,
0.342773691090354,0.731768719677399,0.99511030171561
"10481","G3BP2",124.651824609129,0.0665409167897835,0.194023131238387,
0.342953525000211,0.731633423724698,0.99511030171561
"10482","CENPE",7.24823444398658,-0.185642831499207,0.539005497019776,
-0.344417325102708,0.730532461444983,0.99511030171561
"10483","NAA15",79.3638888411364,-0.0969680555099534,0.283018802489266
,-0.342620542017279,0.731883946179009,0.99511030171561
"10484","UQCRQ",358.517879451301,-0.0905921239171559,0.265579546230182
,-0.341111072757984,0.733019965756111,0.99511030171561
"10485","TIGD6",10.0190223635349,-0.168481729658913,0.490638629355076,
-0.343392712229722,0.731303041395004,0.99511030171561
"10486","GEMIN5",29.2910483663123,0.107030665025392,0.315415650164041,
0.33933213196532,0.734359539267588,0.99511030171561
"10487","AL354740.1",13.5106502303891,0.142729594739346,0.415170590064
225,0.343785417741817,0.731007667522956,0.99511030171561
"10488","AL355353.1",1.94082755703973,0.379942881318733,1.119687128388
11,0.33932950704336,0.734361516478282,0.99511030171561
"10489","HDDC2",164.313815759974,-0.0965602383239395,0.27995801324528,
-0.344909714155386,0.730162247403432,0.99511030171561
"10490","CENPW",21.3970715241423,0.15416914632455,0.450962163922299,0.
341867142430853,0.732450877941996,0.99511030171561
"10491","SLC18B1",22.6028246822325,-0.117868061217643,0.34643486756330
5,-0.340231518976954,0.733682183794482,0.99511030171561
"10492","THAP5",41.821473633988,-0.0973066881067082,0.282594317255457,
-0.344333492094768,0.730595499478794,0.99511030171561
"10493","DNAJB9",109.391253314224,-0.0967446460144192,0.28355747598315
3,-0.341181785734921,0.732966734416706,0.99511030171561
"10494","GTF2E2",53.7909760223639,0.0938771011233579,0.274972740418592
,0.341405118850867,0.732798622070122,0.99511030171561
"10495","EIF4EBP1",60.2165417104709,-0.103171053988935,0.3039839517524
9,-0.33939638390167,0.734311142333885,0.99511030171561
"10496","PKIA",6.00320050058658,-0.227470378557335,0.666741363245735,-
0.341167341786021,0.732977607433227,0.99511030171561
"10497","ERP44",186.459311691899,-0.0887004293503146,0.260887758183716
,-0.339994601386593,0.733860593488815,0.99511030171561
"10498","C10orf88",13.6953983843281,0.139428586315477,0.40699065395543
5,0.342584246002722,0.731911255508695,0.99511030171561
"10499","NAA40",7.50938163241075,0.203837390104869,0.59515537889689,0.
342494409582045,0.73197885040759,0.99511030171561
"10500","ALDH3B1",5.61197634046955,0.267860778822383,0.788072121900698
,0.339893737360418,0.733936553038844,0.99511030171561

"10501","CCDC82",100.644971275666,0.0791038596687547,0.231288241500068
,0.34201418608966,0.73234021638815,0.99511030171561
"10502","ALG9",21.632577130226,0.111945915472015,0.328910407191934,0.3
40353825917977,0.733590086746494,0.99511030171561
"10503","MBD6",31.6938861304914,0.0972041960114036,0.284639450298942,0
.341499380740495,0.732727670978033,0.99511030171561
"10504","AC135279.4",2.0690193286544,-0.326147183375138,0.953734325464
404,-0.341968590903265,0.732374529643807,0.99511030171561
"10505","TMEM19",24.2335361214189,-0.122850577180335,0.358797456730738
,-0.342395339977363,0.732053394968646,0.99511030171561
"10506","C12orf75",74.0505890681887,0.134495138184044,0.39636859340136
3,0.339318352722901,0.734369918439783,0.99511030171561
"10507","NBEA",7.73757167105451,-0.206563051913602,0.598680443819699,-
0.345030565213872,0.730071392359379,0.99511030171561
"10508","SRP54",88.4278374603302,0.0727192934925713,0.212580805115142,
0.342078361464402,0.732291921274609,0.99511030171561
"10509","GSTZ1",37.5455377169129,-0.115509848921035,0.334967604152173,
-0.34483886647307,0.730215511993672,0.99511030171561
"10510","LINC02308",3.13930579929607,0.30980640210895,0.91039261402437
2,0.340299775433653,0.733630786253778,0.99511030171561
"10511","DGLUCY",36.9690660377205,0.11471085344145,0.335341266242631,0
.342071987521074,0.732296717931814,0.99511030171561
"10512","GOLGA8A",43.6347721229002,-0.156568840237294,0.45999332334916
1,-0.340371984309105,0.733576413816453,0.99511030171561
"10513","ITGA11",136.15005332935,0.136637104825795,0.401139199172852,0
.340622669406382,0.733387661249235,0.99511030171561
"10514","RAMAC",37.9847213261333,0.110525450572655,0.322845205305899,0
.342348124600243,0.732088922895946,0.99511030171561
"10515","AKAP13",400.323679650594,-0.0933719890483346,0.27357902022280
5,-0.341298060693001,0.7328792077054,0.99511030171561
"10516","EIF4A1",1804.74522941487,-0.0825994796996736,0.24130422299748
6,-0.342304327183425,0.732121879440557,0.99511030171561
"10517","CCL4",360.391597707894,0.132531301466734,0.389759861307565,0.
34003322205144,0.733831509395328,0.99511030171561
"10518","HGS",61.0235477843135,0.0820990268507453,0.239824023521452,0.
34233028720495,0.732102345064291,0.99511030171561
"10519","PRKACA",88.4167910496134,0.0838785070092394,0.245502135826945
,0.341661007252358,0.732606019730641,0.99511030171561
"10520","MAP1S",31.9175056451178,0.101378613790545,0.298559574233949,0
.339559078119215,0.734188599754739,0.99511030171561
"10521","LINC00665",9.87227564252641,0.169478644746237,0.4962825617946
64,0.341496272070019,0.732730010843418,0.99511030171561
"10522","NECTIN2",85.6872095790202,0.093027913687451,0.272698680542892
,0.341138114428166,0.732999609166577,0.99511030171561
"10523","WFDC2",4.57071833225559,0.304384191568343,0.89242917602022,0.
34107377901487,0.733048040270216,0.99511030171561
"10524","FAM3B",2.78199886174639,-0.307611243123049,0.904717825302072,
-0.340007938961899,0.73385054930765,0.99511030171561
"10525","ESS2",16.1430987559176,-0.120964593564638,0.356343546859251,-
0.339460598152539,0.734262774840725,0.99511030171561

"10526","CBX7",43.2257613499486,0.11786703734322,0.346071460096653,0.340585835394521,0.733415394294516,0.99511030171561
"10527","LINC01315",20.324187759682,-0.157590784653092,0.460092525011571,-0.342519767407934,0.73195977041094,0.99511030171561
"10528","RNF113A",22.9938615706864,-0.126986709903815,0.369450051865526,-0.343718208354823,0.731058216308376,0.99511030171561
"10529","FBXO28",30.9021897854717,0.108025715245136,0.318529956849289,0.339138322541662,0.734505530081341,0.995116811676066
"10530","EDEM1",34.6027350212174,-0.116963044078178,0.345000198002734,-0.339023121596155,0.734592312038618,0.995116811676066
"10531","FARS2",28.0969540689677,-0.116005141273362,0.342229918662421,-0.338968438898501,0.734633506216122,0.995116811676066
"10532","ASB6",14.1394928548617,0.135119980062792,0.398652712368644,0.338941579652024,0.734653740401818,0.995116811676066
"10533","TIMM23B",22.1187256588668,0.143928039978355,0.424836900662394,0.338784224614072,0.734772286186397,0.99518289516141
"10534","CMPK2",17.1476657988487,-0.177157004596753,0.523228503249642,-0.33858439189852,0.734922842277011,0.995196154097539
"10535","IQCA1",2.27099053316622,0.36570770517488,1.08055912344389,0.338443031242306,0.735029351049261,0.995196154097539
"10536","GAL3ST4",11.7415251288883,0.168018782855082,0.49650812335599,0.338400873926114,0.735061115640388,0.995196154097539
"10537","SYNJ2BP",61.9678853469567,0.0862504601815373,0.254722434610788,0.338605668218139,0.73490681198714,0.995196154097539
"10538","TESK2",6.06054307663444,0.201087046640774,0.600768051613915,0.334716611678284,0.737838869268591,0.99532754619453
"10539","RNPEP",47.1790335823268,-0.108037619273014,0.32132451602163,-0.336225883448437,0.73670053577262,0.99532754619453
"10540","ANGEL2",40.9054661442371,0.092969759397382,0.277439770735796,0.335098890655863,0.737550489661931,0.99532754619453
"10541","AC010896.1",2.84529654486817,0.321293754931289,0.951624782733741,0.337626510743348,0.735644661468709,0.99532754619453
"10542","CCDC93",48.5027430366563,0.0823486858126952,0.245185682389087,0.335862538996121,0.736974527270239,0.99532754619453
"10543","GCA",20.5295853508417,-0.115649347280552,0.344886254359388,-0.335326055529138,0.737379140901833,0.99532754619453
"10544","ING5",35.3009605445969,-0.0904275210337898,0.268987413926747,-0.336177517429927,0.736737005784327,0.99532754619453
"10545","RAD18",15.9710405280384,-0.139837102706135,0.41760903147922,-0.334851720545448,0.737736943039783,0.99532754619453
"10546","SLC35A5",35.6756582714678,-0.0923158598813496,0.275847057910875,-0.334663202792529,0.73787916224263,0.99532754619453
"10547","SLC33A1",47.0739756745875,-0.0968584956819352,0.286787321518447,-0.337736323799464,0.735561899080683,0.99532754619453
"10548","FIP1L1",117.936128102017,0.0596501257015517,0.1769641062258,0.337074715170998,0.736060577568123,0.99532754619453
"10549","PRDM5",26.4687885339777,0.113044679655054,0.336172653748807,0.336269706635695,0.736667491758823,0.99532754619453
"10550","RFESD",5.43536612935285,-0.205200802426524,0.610828096689099,-0.335938709333746,0.736917085821317,0.99532754619453

"10551","UBE2B",372.990222440687,0.0690075320858781,0.204853050972145,0.336863579811957,0.7362197413875,0.99532754619453
"10552","HEIH",37.6904893215633,-0.099586789844651,0.296909487823267,-0.33541127491328,0.7373148639257,0.99532754619453
"10553","BLOC1S5",42.9531667250233,0.095194913338558,0.28194841529794,0.337632375900974,0.735640241020108,0.99532754619453
"10554","LMBRD1",127.738275809632,0.0652061546458479,0.193826368964317,0.336415292688335,0.736557718950959,0.99532754619453
"10555","HOXA-AS2",2.26549761914308,0.31462662529216,0.933915826501443,0.336889702866251,0.736200047979858,0.99532754619453
"10556","ZNF783",16.8260113655338,0.143574687256282,0.428456462127786,0.335097495188347,0.737551542292931,0.99532754619453
"10557","AL162231.1",8.76616944753379,-0.177221045581652,0.529492570492529,-0.334699777594239,0.73785156923696,0.99532754619453
"10558","PHF19",32.9932649452075,0.0947196616593967,0.282217154261397,0.335626875365857,0.737152254842315,0.99532754619453
"10559","AK8",1.89839611343168,0.332364951205063,0.984722536085385,0.337521422558612,0.735723865754254,0.99532754619453
"10560","ANAPC2",16.4273520289002,-0.146302785154803,0.43449630129554,-0.336718137113184,0.736329389558194,0.99532754619453
"10561","SEC31B",6.803318931072,-0.19755287865869,0.589131421009973,-0.335329048177429,0.73737688365676,0.99532754619453
"10562","PITPNM1",6.79304473823884,0.180507291440267,0.538240476356263,0.335365509227866,0.737349382602844,0.99532754619453
"10563","LTBR",139.815950261196,-0.0719413401376696,0.214864288351171,-0.334822229835094,0.737759190460028,0.99532754619453
"10564","SMAGP",3.85647150066,-0.248349709990965,0.739390372895105,-0.335884424649113,0.736958022747363,0.99532754619453
"10565","TESPA1",3.04501048154033,-0.363902536474399,1.07959055197343,-0.337074584257158,0.736060676253658,0.99532754619453
"10566","RAB5B",72.1013927485713,0.0728433391296876,0.215715666306903,0.337682192382132,0.735602695716399,0.99532754619453
"10567","HSPH1",128.074160003859,0.0892063348694338,0.265321804937988,0.336219387962793,0.736705433607451,0.99532754619453
"10568","MEX3B",17.8981378074047,0.135182915780504,0.403815781690877,0.334763825263242,0.73780325078132,0.99532754619453
"10569","CIAPIN1",41.8544617219829,0.107486848064926,0.320738977379018,0.335122500368604,0.73753268042516,0.99532754619453
"10570","MYO19",24.5156766784537,-0.115328060590066,0.341626659075738,-0.337585072845553,0.735675892608889,0.99532754619453
"10571","CSNK1D",90.6835226551855,0.080372778975925,0.23845161964556,0.337061157711543,0.736070797482018,0.99532754619453
"10572","PIEZO2",5.30777315011168,0.242544082315935,0.721189200581748,0.336311306547972,0.736636124612385,0.99532754619453
"10573","RPS28",5355.21446073711,-0.0873377502968739,0.259081758338289,-0.337104977428921,0.736037765374795,0.99532754619453
"10574","ZNF682",7.0941647110737,0.190431275357345,0.566231976256975,0.336313177888988,0.736634713595019,0.99532754619453
"10575","ZNF155",6.14229848474779,0.198906309866896,0.591319898563913,

0.336376824710216,0.736586723528135,0.99532754619453
"10576","YWHAH",218.042324491152,-0.0857938345369277,0.254196375442508
,-0.337510062397927,0.735732428002208,0.99532754619453
"10577","EGLN1",63.2346552130733,0.0774910994840948,0.233058379103333,
0.332496517749044,0.739514368719188,0.995425607500164
"10578","AP1S3",2.30041668108898,0.355987132131979,1.06773455922769,0.
333404149051306,0.738829231665969,0.995425607500164
"10579","OXSR1",36.1261001904594,0.113769066904352,0.342231204708094,0
.332433353064317,0.739562057088565,0.995425607500164
"10580","AC006059.1",2.36811101658188,0.291772143009238,0.878155675312
516,0.332255602522187,0.739696261398376,0.995425607500164
"10581","SMN1",11.6562908793764,0.160077386552786,0.480540989822145,0.
333119109385514,0.739044375185127,0.995425607500164
"10582","TMEM63B",24.3536354756729,0.132107027864554,0.397102512417418
,0.332677391186306,0.739377817585529,0.995425607500164
"10583","MFSD14B",67.8229673065546,-0.0825202011393778,0.2478413550327
7,-0.332955737465472,0.739167694987857,0.995425607500164
"10584","SNX30",15.0171769848585,0.145010706842738,0.434059950828492,0
.334079904321869,0.738319263785144,0.995425607500164
"10585","CUTC",175.28481716811,-0.0920063301710539,0.276800285914727,-
0.332392467973816,0.739592925239458,0.995425607500164
"10586","BCCIP",75.8804678784592,-0.084150466437137,0.252043334338571,
-0.333873009012399,0.738475387919355,0.995425607500164
"10587","MRPL17",92.4429286975163,-0.104453364699352,0.312318768337653
,-0.33444472535325,0.738043994481841,0.995425607500164
"10588","RPL27A",4334.37921652519,0.0596787753499963,0.17936452336394,
0.332723407230898,0.739343078897294,0.995425607500164
"10589","AP002495.1",7.44792987541447,0.193057450993628,0.578095443894
653,0.333954285633167,0.738414054928015,0.995425607500164
"10590","ETFBKMT",14.5459505764614,-0.129839563045558,0.3900427758348,
-0.332885445109617,0.739220756595347,0.995425607500164
"10591","DTX3",17.0063158956921,0.139847115337708,0.420729953993996,0.
332391630332324,0.739593557661276,0.995425607500164
"10592","NEDD8",471.909899702827,-0.0879531471996287,0.264463548555009
,-0.332571908984025,0.73945745080278,0.995425607500164
"10593","IGHG3",31.810057460612,0.359831821129707,1.08289826070549,0.3
32285898118704,0.739673387210816,0.995425607500164
"10594","DTWD1",180.526761033292,0.0674919417721734,0.202944451619183,
0.332563621393401,0.739463707592809,0.995425607500164
"10595","ETFA",109.79343307876,0.0756607794885373,0.227493749850714,0.
33258399203577,0.739448328626623,0.995425607500164
"10596","NME4",122.803450921525,-0.0977866970846091,0.29416408275532,-
0.332422286802248,0.739570412049999,0.995425607500164
"10597","BRD7",137.589035940155,-0.0706098257924717,0.211521476628362,
-0.333818706818747,0.738516366386768,0.995425607500164
"10598","FAM215B",11.7877832113234,-0.154152282134605,0.46199097286359
8,-0.333669467996549,0.738628991412557,0.995425607500164
"10599","ADA",68.9167843304959,0.0757839961671923,0.227802299961255,0.
332674411891722,0.739380066749492,0.995425607500164
"10600","MPP1",17.1252287020736,-0.123813717264248,0.372123402194324,-

0.332722200576873,0.739343989824469,0.995425607500164
"10601","AC007325.4",11.5608389878493,0.200580681369949,0.601839133420
845,0.333279559655502,0.738923267295468,0.995425607500164
"10602","CTIF",27.4338993246548,-0.119161942432593,0.358841765604329,-
0.332073782526155,0.739833546406961,0.995516447183711
"10603","PSMB1",689.621304639456,-0.0980636047297562,0.295907223212278
,-0.331399834262941,0.740342490066882,0.996013387711631
"10604","POM121C",54.3235523678786,0.0835967327889171,0.25219336211720
5,0.331478719689958,0.74028291249701,0.996013387711631
"10605","ZFHX2-
AS1",7.93334318854617,0.187570310421525,0.56631860890798,0.33120986573
8322,0.740485968623491,0.996112477923878
"10606","LRATD2",2.68705572130669,0.30972362835599,0.9358953902009,0.3
3093829887281,0.740691092077282,0.996294467242552
"10607","TCF23",2.82173373477068,0.390811291282232,1.18341754787551,0.
330239560824345,0.741218956792198,0.996320807907136
"10608","GK5",47.0014844185222,0.113079985205062,0.342874178354576,0.3
29800236774094,0.741550908456753,0.996320807907136
"10609","NCAPG",2.89745588212544,0.271083495290701,0.823570935442243,0
.329156219124141,0.742037612786578,0.996320807907136
"10610","ADAMTS3",14.94464467493,0.195368732595243,0.591037428880341,0
.330552217251874,0.740982744053531,0.996320807907136
"10611","HAVCR2",2.29107796416388,-0.344528568544126,1.04621476477551,
-0.329309602716276,0.74192168662926,0.996320807907136
"10612","AHI1",151.475541732259,-0.0817104356888386,0.247290366767605,
-0.330423043796231,0.741080331974805,0.996320807907136
"10613","TIMM8B",211.268623410996,-0.0857226500498676,0.25999091462893
4,-0.329714021631153,0.741616057954825,0.996320807907136
"10614","C12orf45",70.4869017340232,-0.103091474976147,0.3128931705585
89,-0.329478188328958,0.741794277654652,0.996320807907136
"10615","PIP4P1",39.2588195151572,-0.0887292296760239,0.26852846862773
3,-0.330427645640177,0.741076855303641,0.996320807907136
"10616","PSMA6",287.465804050481,-0.0961372920218341,0.291612532601901
,-0.329674761108699,0.741645726249249,0.996320807907136
"10617","LINC00640",6.15632657663001,-0.263422230315208,0.797425319652
894,-0.330340940804176,0.741142361282401,0.996320807907136
"10618","UBE2Z",104.592792607014,-0.0722026129286011,0.218288201354105
,-0.330767363882735,0.740820214395388,0.996320807907136
"10619","GNA11",122.243320165284,0.0992559128469937,0.300958367419963,
0.329799479236576,0.741551480890921,0.996320807907136
"10620","ZFP14",20.4433433216116,0.115635624272889,0.3509187610945,0.3
29522490938434,0.741760797027073,0.996320807907136
"10621","ZNF805",23.9724456922561,0.113968782962166,0.344902023024154,
0.330438139976304,0.741068926902496,0.996320807907136
"10622","TAF4",11.0401503886667,-0.1440892323361,0.437734224028807,-0.
329170588970484,0.742026751917019,0.996320807907136
"10623","SPATC1L",42.914054295902,0.119092558656737,0.361242348247738,
0.329674965392109,0.741645571875873,0.996320807907136
"10624","SLC25A14",8.42443505835492,0.164733735579381,0.49964955147897
6,0.32969855590136,0.741627745013389,0.996320807907136

"10625","MMGT1",41.593909555306,-0.0816157272656816,0.247806111885419,-0.329353165039849,0.741888763622545,0.996320807907136
"10626","KIN",69.3017099670658,0.0878557940410707,0.26706185910284,0.328971700924314,0.742177077999979,0.996411165416408
"10627","CREM",76.8930750581377,-0.112510177865052,0.3420985363026,-0.328882371380662,0.742244599388768,0.996411165416408
"10628","XPC",79.0878688347411,-0.0799283930165893,0.24332180889569,-0.328488405455074,0.742542409484757,0.996623390131672
"10629","ZSWIM8-AS1",3.1043642092411,0.377643779581835,1.14943022987668,0.328548675479285,0.742496847153751,0.996623390131672
"10630","MMS22L",12.0785041036512,0.138888622199202,0.423673774931562,0.327819729275519,0.74304796911337,0.996910239000032
"10631","LOH12CR2",6.33939088404974,0.201921524263879,0.616314767843265,0.327627269050333,0.743193501232167,0.996910239000032
"10632","RFXAP",24.0301784616253,0.103730616640736,0.31631090669944,0.3279387920041,0.742957942374279,0.996910239000032
"10633","SNAPC5",61.0878375439082,-0.101567106212741,0.310008426117871,-0.327626921257048,0.743193764230365,0.996910239000032
"10634","ZNF652",95.6519747511512,0.0743834852458631,0.227084385740124,0.327558783944695,0.743245289640007,0.996910239000032
"10635","ST13",503.082316003887,-0.0538280430507964,0.164297579551068,-0.327625295502697,0.743194993611793,0.996910239000032
"10636","PDZD4",2.55816784173759,-0.284551982516502,0.868542345314778,-0.327620160434866,0.743198876710125,0.996910239000032
"10637","TPRG1L",52.7112217685233,-0.0766385005937961,0.238898979859953,-0.320798777117939,0.74836288617688,0.996931670688095
"10638","KCNAB2",13.9372813336329,0.131807023519305,0.40345549715896,0.326695321906529,0.743898338858362,0.996931670688095
"10639","GLMN",17.5795964155982,0.116383546438834,0.356249440871723,0.32669116940661,0.743901479901492,0.996931670688095
"10640","AKNAD1",3.707588296852,-0.250001162666956,0.773413655575127,-0.323243791811576,0.746510618420135,0.996931670688095
"10641","SRGAP2B",45.3408020763813,0.0987874449609115,0.305350986716053,0.323520961970148,0.746300735013064,0.996931670688095
"10642","CAMSAP2",56.9520638625379,0.0922086994344925,0.284017410655924,0.324658615898022,0.745439459051156,0.996931670688095
"10643","MIR29B2CHG",8.94078896416623,0.164409048411238,0.511384853988103,0.32149768834386,0.747833263793705,0.996931670688095
"10644","LYPLAL1",63.017946276931,0.0943791745401006,0.293923650613257,0.321100987767344,0.74813386178349,0.996931670688095
"10645","ZAP70",7.70772080609571,-0.226447547617322,0.69494460471267,-0.325849781524599,0.744538012211242,0.996931670688095
"10646","ECRG4",7.34043376982477,0.265395496700812,0.827251223232605,0.32081608252417,0.748349771015799,0.996931670688095
"10647","PKP4",12.864079475625,-0.158521118165253,0.484451894550344,-0.327217459459805,0.743503416390516,0.996931670688095
"10648","AC009506.1",3.3343373353615,-0.257711776184515,0.802953888395679,-0.320954639997359,0.748244765786945,0.996931670688095
"10649","DCAF17",12.6548452847544,0.144041776129713,0.446773715862016,

0.322404320164169,0.747146411801345,0.996931670688095
"10650","ZNF660",11.9523425415081,-0.155831409386141,0.481369892316466
,-0.323724877424807,0.746146334768938,0.996931670688095
"10651","IP6K1",42.3832867820984,0.0827368480252366,0.253367395372192,
0.326548914881876,0.744009086955305,0.996931670688095
"10652","SFMBT1",16.0775951406648,0.13707946660858,0.426448448939613,0
.321444401895318,0.747873639120719,0.996931670688095
"10653","CFAP44",10.4190731885328,0.169627603140967,0.520510649700601,
0.325886902099962,0.744509925825464,0.996931670688095
"10654","PARP15",6.47078780725278,-0.210296511764109,0.652167102345651
,-0.322458018823298,0.747105736716085,0.996931670688095
"10655","NELFA",31.9747382157484,0.0992461035591051,0.30643390674693,0
.323874419161675,0.746033111571328,0.996931670688095
"10656","PDLIM5",109.157624550906,0.0762159570760434,0.234949117361791
,0.324393459877019,0.74564017053665,0.996931670688095
"10657","MAD2L1",5.07652834790018,-0.201993465516531,0.62192543818442,
-0.324787270490509,0.745342079388413,0.996931670688095
"10658","HMGB2",107.437854181594,0.102640508704752,0.315749884328679,0
.325069030264186,0.745128827437578,0.996931670688095
"10659","SRA1",103.545351219456,-0.11170634908608,0.343429332098302,-0
.32526735093817,0.744978738692052,0.996931670688095
"10660","DIAPH1",94.9364398500425,-0.0905542610832001,0.27804119469252
5,-0.325686491109134,0.744661565989466,0.996931670688095
"10661","ZSCAN12",3.66996451321253,-0.233374735794176,0.72299120321878
6,-0.32279056059767,0.746853862223918,0.996931670688095
"10662","RING1",63.078516424805,0.0719714837087487,0.223489997205973,0
.322034474063814,0.747426577986757,0.996931670688095
"10663","BAK1",16.5100948114532,-0.118213730045748,0.368186003256809,-
0.321070678950537,0.748156829721528,0.996931670688095
"10664","CCDC167",52.3695088194898,-0.10333150172603,0.319743555991989
,-0.323169927242002,0.746566554554698,0.996931670688095
"10665","SENP6",143.570155662114,0.0738266152744711,0.227033856355059,
0.325178880629211,0.745045691674148,0.996931670688095
"10666","SUMO4",5.59750235908292,-0.196763291204618,0.602404509040734,
-0.326629844650305,0.743947867804774,0.996931670688095
"10667","KLHL7",35.1691888525588,0.0923118482736825,0.28435054723464,0
.324641007979172,0.745452786938498,0.996931670688095
"10668","DPY19L1",26.2023818261216,-0.0992372991276631,0.3054408186965
08,-0.324898615552321,0.745257804728771,0.996931670688095
"10669","TMEM68",18.6075552350271,0.112636616444193,0.346567450357504,
0.325006333768511,0.745176278051135,0.996931670688095
"10670","DMAC1",142.776264055215,0.0936829938246413,0.289811568575012,
0.323254845502805,0.746502247799711,0.996931670688095
"10671","TLE1",22.8870904914124,0.138445469633215,0.431688452542695,0.
320706909850738,0.748432510379188,0.996931670688095
"10672","POLL",22.1813086332253,-0.118615945357425,0.369644388838087,-
0.320892049059025,0.748292199501968,0.996931670688095
"10673","SMNDC1",74.6910547702768,-0.0670095218836918,0.20845253153185
2,-0.321461780249249,0.747860471405627,0.996931670688095
"10674","TSG101",99.0259167499664,-0.0738366861476406,0.22929480762750

4,-0.322016389780576,0.747440278066994,0.996931670688095
"10675","NEU3",5.64683453526376,-0.19745906114724,0.608485651196216,-0.324508985148717,0.745552720846563,0.996931670688095
"10676","AP000866.6",3.14230603517986,-0.264436214968472,0.809870511772618,-0.326516660533401,0.744033486139258,0.996931670688095
"10677","RPAP3",40.9786178255686,-0.0938937592700565,0.290220872302702,-0.323525177652022,0.746297542889433,0.996931670688095
"10678","CS",46.371010673146,0.0943201834468255,0.289057939182351,0.326301999224191,0.744195875774023,0.996931670688095
"10679","EEF1AKMT3",16.5479031787441,-0.117838181051608,0.361932158535075,-0.325580853407886,0.744741500312038,0.996931670688095
"10680","EXOSC8",75.0539368351069,-0.094659251865211,0.295009832065674,-0.320868125656701,0.74831032978777,0.996931670688095
"10681","DTD2",18.9608922933328,0.113499094108124,0.349400434048871,0.324839591047128,0.745302478715507,0.996931670688095
"10682","IFI27L2",401.212388969361,0.0877909370138822,0.269940694881654,0.32522305335389,0.745012262188221,0.996931670688095
"10683","EMC7",264.724749539491,-0.0763566702686604,0.23505792158482,-0.324841935782655,0.745300704034841,0.996931670688095
"10684","VPS13C",164.718143790251,0.0718017428810031,0.222923961703844,0.322090735927222,0.747383956291143,0.996931670688095
"10685","DIS3L",38.9225652708395,0.09741362192651,0.301310566584057,0.323299720387782,0.746468265736593,0.996931670688095
"10686","AC133552.5",5.84698377803994,-0.214912564318798,0.664165555636904,-0.323582821323378,0.746253895410875,0.996931670688095
"10687","AC009093.1",6.23177548243825,0.222305255868979,0.692442716355385,0.321044976888579,0.748176306848541,0.996931670688095
"10688","FBXL19-
AS1",3.41545139744233,0.309186199796848,0.959391598138721,0.322273199386662,0.747245734722608,0.996931670688095
"10689","SNTB2",306.714662401451,0.0620110271144468,0.192727356961917,0.321755188738982,0.747638164599696,0.996931670688095
"10690","AC009054.2",2.21643795209394,0.322081993805284,0.985613972450415,0.326783104550081,0.743831939115109,0.996931670688095
"10691","COX4I1",999.685033507664,-0.0747590740971275,0.232996812049966,-0.320858785317181,0.748317408376611,0.996931670688095
"10692","INPP5K",19.247703104663,0.117036666032822,0.363879002782241,0.321636217363334,0.747728303111523,0.996931670688095
"10693","TRPV1",8.79577413397479,-0.1571775766419,0.485557537298731,-0.323705358413989,0.746161113688521,0.996931670688095
"10694","C17orf75",15.8321891267477,0.131668304627122,0.408349973698004,0.322439850882659,0.747119498294372,0.996931670688095
"10695","CCL14",2.14308555759369,0.360295053900692,1.11781623165436,0.322320470662212,0.747209926632408,0.996931670688095
"10696","AC103703.1",3.42862353065016,0.240279168527776,0.742606706661785,0.323561807848322,0.746269806607111,0.996931670688095
"10697","SKA2",51.9584504059394,-0.111232089148379,0.346772646655092,-0.320763734456291,0.7483894440054,0.996931670688095
"10698","RNF165",2.30274441279219,0.317852061839236,0.98186286410505,0.323723478562307,0.746147393921779,0.996931670688095

"10699","ELAC1",24.0809056199496,-0.110228275915701,0.34179471758855,-0.322498477136774,0.747075091251279,0.996931670688095
"10700","POLRMT",15.5355328354864,-0.120438150455586,0.370311544613923,-0.325234663102797,0.745003476123513,0.996931670688095
"10701","MRI1",26.642934355154,0.12322673427326,0.378950841469366,0.325178679628876,0.745045843790333,0.996931670688095
"10702","PODNL1",1.84978853957519,-0.351362342936248,1.08110340476554,-0.325003456087023,0.745178455991161,0.996931670688095
"10703","AC010618.3",7.14220204644333,-0.170232839631266,0.530580226102339,-0.320842789189116,0.748329531113008,0.996931670688095
"10704","ZNF724",4.15912487277343,0.240992356003512,0.738380332614277,0.326379706174277,0.744137089748109,0.996931670688095
"10705","GPI",115.33451377583,-0.0662003726037223,0.204646280405284,-0.323486810865159,0.746326594465721,0.996931670688095
"10706","IGFLR1",17.5400981791262,-0.140020749104273,0.435351919058861,-0.321626580645306,0.747735604509738,0.996931670688095
"10707","ZNF260",20.733786317675,0.108468750005893,0.331965674055917,0.326746885244595,0.743859335559652,0.996931670688095
"10708","KMT5C",11.1563829517256,-0.153558471912143,0.469119271431725,-0.32733354023912,0.743415627027547,0.996931670688095
"10709","GGT7",30.7416127954702,0.0982772784289152,0.301034878052244,0.326464757388874,0.744072749427161,0.996931670688095
"10710","IL2RG",32.1582328102012,0.139928463396956,0.434090440342191,0.322348640727151,0.747188588008672,0.996931670688095
"10711","LINC02609",42.7320952900708,0.110037142095094,0.343705901352002,0.320149120693744,0.748855290660834,0.997007738873304
"10712","AL031963.3",4.9467178437682,0.21612555343226,0.675616227558325,0.319893964378768,0.749048713391566,0.997007738873304
"10713","LINC01003",38.1230082898212,-0.10496734883478,0.328050210786144,-0.319973422919727,0.748988477688469,0.997007738873304
"10714","AC068587.4",13.3538701386111,0.143453293663154,0.448184080208612,0.320076727393758,0.748910167218602,0.997007738873304
"10715","PREX1",16.1946044472588,0.196914588004927,0.615422697590683,0.319966404839841,0.74899379787271,0.997007738873304
"10716","CBR3-
AS1",7.56391138431789,-0.179154061100864,0.559163997809553,-0.320396273370022,0.748667950198982,0.997007738873304
"10717","BEX4",77.2523931291779,0.0931708565279383,0.291086290530148,0.320079851092433,0.748907799323628,0.997007738873304
"10718","BRCC3",29.3077901251313,0.0949881599345336,0.296762548385139,0.320081359495733,0.748906655891332,0.997007738873304
"10719","SH3KBP1",151.057557573866,0.0699525737412375,0.218742370287348,0.319794348252445,0.749124232262198,0.997015234392436
"10720","ETV3",26.6670362411812,0.134884345069589,0.422181783190627,0.319493522553731,0.749352302477691,0.997225741338315
"10721","P4HA1",119.739939173829,0.0747768740943374,0.23419925998424,0.319287405516863,0.74950858222591,0.997340680350232
"10722","LAMTOR5",315.850633010843,-0.0894777605077967,0.284718411585299,-0.314267560041476,0.753317834313508,0.997438912742878
"10723","RRNAD1",23.8784842155262,-0.131332745084331,0.419837938559279

,-0.312817716128786,0.754419154885389,0.997438912742878
"10724","BLZF1",44.1001170350338,-0.0806816764875611,0.256434162830402
,-0.314629203835534,0.753043203010144,0.997438912742878
"10725","C1orf131",64.9521009899424,-0.0843384127212106,0.270020538767
585,-0.312340731953739,0.754781587681651,0.997438912742878
"10726","RNF144A",16.4849206750336,0.16693625988774,0.526973090952032,
0.316783271772287,0.751408058187372,0.997438912742878
"10727","USP34",134.356734242979,0.0755444082473499,0.238272765337265,
0.317050117500505,0.751205574111965,0.997438912742878
"10728","RGPD8",2.70162341551712,0.280526723427553,0.885175289381399,0
.316916577758963,0.751306902696484,0.997438912742878
"10729","CRBN",124.025854028503,0.0636585685562704,0.201908860881408,0
.315283679370864,0.752546275551638,0.997438912742878
"10730","NKIRAS1",23.76611764982,-0.101441997049001,0.324071592937821,
-0.313023415997045,0.75426287208928,0.997438912742878
"10731","APEH",33.1432924854565,-0.0895400050738003,0.285144623777942,
-0.314016108343429,0.753508804440876,0.997438912742878
"10732","PLAC8",8.53558113195582,-0.173040717267556,0.553486714240944,
-0.312637526457823,0.754556064284576,0.997438912742878
"10733","AADAT",8.20226260701597,0.155190578413993,0.48770164147478,0.
318208029697636,0.750327142274154,0.997438912742878
"10734","NDUFS4",278.105825428801,-0.0817029038975671,0.25951842171147
2,-0.314825064666904,0.752894479874946,0.997438912742878
"10735","JMY",44.8532621523472,0.107703982413231,0.341407335401549,0.3
15470616021046,0.752404357886882,0.997438912742878
"10736","AC011374.2",2.54074850771642,-0.265939092081233,0.83666278407
4248,-0.317856963574028,0.750593438920801,0.997438912742878
"10737","SMIM29",27.2484085893954,0.110961861884698,0.349960252522479,
0.317069898895363,0.751190564557461,0.997438912742878
"10738","LCA5",48.9269201673345,0.0888900993977853,0.280806055375641,0
.316553356653491,0.751582532831784,0.997438912742878
"10739","ASCC3",75.4609103671445,0.0852640080488583,0.267376942714901,
0.318890653708214,0.749809431880971,0.997438912742878
"10740","HECA",54.9847009597924,0.102050246116566,0.321960194111762,0.
316965413684468,0.751269846000257,0.997438912742878
"10741","SNX10",24.0655856310281,0.188210757408288,0.598459260161007,0
.314492180065278,0.753147254809771,0.997438912742878
"10742","DDX56",56.4213019601998,-0.084733539253561,0.267044087093884,
-0.317301686682812,0.751014697654537,0.997438912742878
"10743","AGK",16.9591644622487,0.120517907744608,0.380823670909772,0.3
16466430399917,0.751648501472592,0.997438912742878
"10744","AC005229.4",4.53140288786161,-0.226727750475681,0.72107840413
9144,-0.314428707300365,0.753195455670424,0.997438912742878
"10745","AC087672.2",3.59212227605244,-0.26199625995497,0.826802943413
119,-0.316878721879515,0.751335628129276,0.997438912742878
"10746","AZIN1",123.462160603409,-0.0707767796674893,0.226694031572664
,-0.312212805853261,0.754878800542281,0.997438912742878
"10747","TAF2",40.6453134023628,0.0825173394006614,0.260316804506237,0
.316988138960827,0.751252602258,0.997438912742878
"10748","PPP1R16A",11.9745617853846,-0.15308486280906,0.48798047368512

1,-0.313711041864026,0.753740513653136,0.997438912742878
"10749","UBAP2",40.6163607448134,0.0880776272222802,0.282128255834549,
0.312190024929415,0.754896112497186,0.997438912742878
"10750","TMEM203",79.6435066684428,0.0728148476798219,0.232799833883,0
.312778778512433,0.754448739310977,0.997438912742878
"10751","CORO1B",105.788981587005,-0.0749327314186777,0.23842643118906
,-0.314280304599535,0.753308155600326,0.997438912742878
"10752","INPPL1",28.9870485381621,0.0989700989468985,0.31650604200607,
0.312695765046408,0.754511813345173,0.997438912742878
"10753","THAP12",114.624268407754,0.0681934832218704,0.215576493817841
,0.316330792908678,0.751751440868652,0.997438912742878
"10754","COLCA2",12.1315721784616,0.140085399494056,0.44349509080469,0
.315866854895464,0.752103570844545,0.997438912742878
"10755","TLCD5",19.790894980976,0.117474166438488,0.369883169928159,0.
317598030916911,0.750789867967451,0.997438912742878
"10756","NDUFA9",107.976508166741,-0.0957114267919896,0.30221425699894
2,-0.316700567810488,0.751470817905198,0.997438912742878
"10757","AC010168.2",3.88870088786363,0.252032030889424,0.794308359170
305,0.317297467639248,0.751017898700422,0.997438912742878
"10758","AMN1",7.48930913640191,0.185860841029357,0.589762162769976,0.
315145414138493,0.752651248465707,0.997438912742878
"10759","ATF1",64.0471883547629,-0.0732525380161535,0.234477046485853,
-0.312408140216715,0.75473036474775,0.997438912742878
"10760","CNPY2",234.597569270241,-0.0853673398959313,0.271475812291472
,-0.314456522573274,0.75317433279021,0.997438912742878
"10761","TCTN1",62.8078868022884,0.0984017553785775,0.311735043028128,
0.315658305279778,0.752261877280077,0.997438912742878
"10762","GATC",49.8515679811805,-0.0864604331570776,0.274229418508664,
-0.315285039902989,0.752545242639197,0.997438912742878
"10763","THSD1",17.9374789901217,0.14327392790973,0.455258335775116,0.
314709070984486,0.752982556327917,0.997438912742878
"10764","ARHGEF40",50.9929106318996,-0.0975831843617207,0.309876506026
682,-0.314909915607859,0.752830052800194,0.997438912742878
"10765","TTC9",2.50825421554452,-0.301277347270374,0.959104864600362,-
0.314123469070203,0.753427265298986,0.997438912742878
"10766","GOLGA5",57.3015700305252,-0.0739060804188414,0.23247103635307
5,-0.317915218937612,0.750549248014389,0.997438912742878
"10767","BCL11B",35.3097596022797,0.118472500658626,0.372620513549139,
0.317944118347641,0.750527326025523,0.997438912742878
"10768","LINC00926",3.40062505626874,-0.241402076726,0.756927107852749
,-0.318923809468008,0.749784289015251,0.997438912742878
"10769","UBAP1L",4.32578105544785,0.221153747513674,0.704490142730777,
0.31392028660107,0.753581582188753,0.997438912742878
"10770","RBBP6",251.301314037911,0.0731448867317762,0.233810912754212,
0.312837779341241,0.754403911190625,0.997438912742878
"10771","FAM192A",60.2632477164454,-0.0743058951227749,0.2371661366396
11,-0.313307355660506,0.754047162258649,0.997438912742878
"10772","AC004943.2",2.64917747474167,-0.26659770086034,0.847739812388
775,-0.314480571708808,0.753156070058492,0.997438912742878
"10773","ZMYND15",3.36568248373271,-0.323973883731723,1.02668065605021

,-0.315554677905491,0.752340542918366,0.997438912742878
"10774","FGF11",5.27007279861786,-0.217879382684222,0.696064657815856,
-0.313016011138813,0.754268497839327,0.997438912742878
"10775","PFAS",14.5636729915937,-0.14294513321779,0.455706226829285,-0
.313678253229882,0.753765419147012,0.997438912742878
"10776","TOP3A",26.8274116771092,-0.104591520835897,0.334782726791464,
-0.312416120862314,0.754724300398213,0.997438912742878
"10777","CAVIN1",1018.05142603818,-0.0666159112659847,0.20979790843525
3,-0.31752419155572,0.750845886246909,0.997438912742878
"10778","TYMS",7.52809381645041,0.185645789090566,0.582956591552613,0.
31845559648983,0.750139371583742,0.997438912742878
"10779","SMAD2",76.848380602561,0.072252888348663,0.22924191354584,0.3
15181841012748,0.75262359222525,0.997438912742878
"10780","EEF2",1590.69539986074,-0.0408579714025866,0.128698428409615,
-0.3174706319843,0.750886520072522,0.997438912742878
"10781","RAB11B",135.842601004959,-0.0653623134105411,0.20516097604787
2,-0.318590380440038,0.750037148928656,0.997438912742878
"10782","ZNF317",15.5043213207984,0.122162793591089,0.388868991030813,
0.314148971527044,0.75340789690311,0.997438912742878
"10783","CARM1",56.5149413035467,0.0899513468279592,0.287001116913243,
0.313418107202525,0.753963029174651,0.997438912742878
"10784","ABHD8",20.739489298559,-0.12548927555661,0.400220760321383,-0
.313550140317161,0.753862733230362,0.997438912742878
"10785","TSHZ3",33.3839977599378,-0.0855105808430049,0.2726013844928,-
0.313683589693079,0.753761365674606,0.997438912742878
"10786","RBM42",93.1465382337674,-0.0833527314661319,0.261217536360883
,-0.319093168963115,0.749655863500589,0.997438912742878
"10787","BCKDHA",40.311001549588,-0.0890335208837398,0.283644068967918
,-0.313891706629727,0.753603289439267,0.997438912742878
"10788","AP5S1",17.5478820224181,-0.126689172632094,0.401523157626891,
-0.315521459287332,0.752365760388601,0.997438912742878
"10789","MMP24OS",118.017438086784,0.0734102707046553,0.23214857897445
9,0.316221064238053,0.751834720508805,0.997438912742878
"10790","SNHG17",44.8803189310684,0.106146659621769,0.333895486017972,
0.317903847361552,0.750557874112962,0.997438912742878
"10791","SNAI1",4.17161316952953,-0.236754269558582,0.748269854445999,
-0.316402255351939,0.751697205307615,0.997438912742878
"10792","DOK5",39.8196927880904,-0.130654804841256,0.417220334807841,-
0.313155409602535,0.754162593757797,0.997438912742878
"10793","USP16",197.118985189471,-0.0631818225533033,0.200774843901978
,-0.314689934881234,0.752997087084291,0.997438912742878
"10794","AP001505.1",5.55157081117874,-0.204205877677081,0.65140417972
4118,-0.313485673001923,0.753911703837469,0.997438912742878
"10795","TMEM191B",3.26225014931959,0.323397677299894,1.0245744534406,
0.315640972907239,0.752275034455876,0.997438912742878
"10796","NUP50-
DT",8.44110971374091,-0.165563717238465,0.52366713286562,-0.3161621321
01865,0.751879448818678,0.997438912742878
"10797","SYP",4.68176150728711,0.24161766002299,0.768825976359479,0.31
4268335686432,0.753317245257446,0.997438912742878

"10798","MAN1A2",134.276663015369,0.0612387072686118,0.196575906441702
,0.311527024736235,0.755400001515781,0.99747119446006
"10799","SIMC1",11.717534080431,-0.137565202619781,0.440847744553094,-
0.312046969320977,0.755004827857493,0.99747119446006
"10800","PAXIP1-
AS1",19.2017664273554,-0.108360530134727,0.347652843847494,-0.31169176
9684651,0.755274783424295,0.99747119446006
"10801","TRHDE-
AS1",2.7530385556057,0.31858447352588,1.02173319732356,0.3118078911015
28,0.755186526601108,0.99747119446006
"10802","KITLG",58.3898184282755,-0.0932641540372047,0.299390028145583
,-0.311513895819715,0.755409980719648,0.99747119446006
"10803","EID3",30.9181287038555,-0.123489178182163,0.396214217777185,-
0.311672758425869,0.755289233029629,0.99747119446006
"10804","ARHGAP23",32.3378897293318,0.102361122202921,0.32819099811792
9,0.311894972104445,0.755120343726162,0.99747119446006
"10805","KLHL18",19.7139489893599,-0.120472218489915,0.387489349524884
,-0.310904592958826,0.755873152726002,0.99747821945422
"10806","RNF4",49.9324523366865,-0.0725196339866016,0.233386161186305,
-0.310728080953829,0.756007347749291,0.99747821945422
"10807","TMA16",93.95095978521,-0.0858329767861046,0.275965736709255,-
0.311027658033266,0.755779595624874,0.99747821945422
"10808","KIF13A",51.6447165682555,0.0865210002210879,0.27796698192591,
0.311263588292474,0.755600245644352,0.99747821945422
"10809","AL662884.3",2.47640840270701,-0.277592909336882,0.89350362304
4474,-0.31067910882222,0.756044580608333,0.99747821945422
"10810","DMAC2L",36.722123448078,-0.0842030562490618,0.270540396562805
,-0.311240233690995,0.755617998809203,0.99747821945422
"10811","SIAH1",65.3464038638532,0.0788904919583162,0.25363690685309,0
.311037115761747,0.755772405774865,0.99747821945422
"10812","CST3",5593.39920190142,0.091209068851116,0.29350258170724,0.3
10760703774982,0.755982545367527,0.99747821945422
"10813","MOCS3",8.44485285870083,-0.151838585714871,0.48791695164666,-
0.311197602793742,0.755650405318854,0.99747821945422
"10814","EOGT",9.52952917697719,0.161315431550783,0.519748888131295,0.
310371864634048,0.756278187192799,0.997579437114132
"10815","METTL22",11.2129345957685,0.147021179877721,0.473799835378936
,0.31030230257496,0.756331080318691,0.997579437114132
"10816","PLAGL2",14.79120775086,0.125889484053519,0.405464615025606,0.
310482047972469,0.756194409071538,0.997579437114132
"10817","GPR89A",17.1212338518252,0.119646876888435,0.388872241740867,
0.307676568409231,0.758328451069349,0.997610151244476
"10818","EEF1B2",1797.30115728916,-0.0730073965670338,0.23731388225731
4,-0.307640648210684,0.758355786440458,0.997610151244476
"10819","SEC24B-
AS1",2.94220518712293,-0.248812256983374,0.802262401378565,-0.31013824
9724564,0.756455826231482,0.997610151244476
"10820","PLRG1",80.0515433623198,-0.0838976378382651,0.272376324966531
,-0.308021036147596,0.758066325505914,0.997610151244476
"10821","FAM151B",1.92452173122456,0.374936382612983,1.21085139107272,

0.30964690248307,0.756829485010186,0.997610151244476
"10822","ZNF316",19.8643394566468,0.130187671350376,0.421336722581274,
0.308987240781661,0.757331232625809,0.997610151244476
"10823","CYP3A5",14.6760383575846,-0.132770695783955,0.431531347174291
,-0.307673351318161,0.758330899272294,0.997610151244476
"10824","POLR2J2",4.32962145608777,-0.235065602085745,0.76273232733320
9,-0.308188854283416,0.757938632986005,0.997610151244476
"10825","RPS20",3535.33368811824,0.0494719495652897,0.159978959862217,
0.309240350155407,0.757138702107415,0.997610151244476
"10826","DOCK8",22.1197711270909,-0.131305227973471,0.424235736812081,
-0.309510059101018,0.756933561520415,0.997610151244476
"10827","APTX",43.6062071551188,-0.0784807082633586,0.254716119543375,
-0.308110489450176,0.757998259825995,0.997610151244476
"10828","PTEN",201.451323292104,0.0632286113467336,0.204648404910548,0
.308962150838023,0.75735031839674,0.997610151244476
"10829","LIN7C",59.6333729792594,-0.0790752121782383,0.255819698717008
,-0.309105250982695,0.757241464952421,0.997610151244476
"10830","CCS",109.308807706398,0.0650689219969745,0.210972127241441,0.
308424258918849,0.757759525149109,0.997610151244476
"10831","ANAPC15",22.5748045446714,-0.128033092630783,0.41534620753792
5,-0.308256317999708,0.757887301825613,0.997610151244476
"10832","NFRKB",28.6375822172924,0.0887617646634034,0.287869816038416,
0.308339949929166,0.757823670228567,0.997610151244476
"10833","AC022075.1",2.50735219380563,-0.28810064641794,0.935775954343
404,-0.307873530069586,0.75817856806815,0.997610151244476
"10834","YBX3",1463.22377050199,-0.0518996472799097,0.168721295125032,
-0.307605790018676,0.758382313910441,0.997610151244476
"10835","MYL6B",137.773353578785,-0.074589123500759,0.240915487441275,
-0.309607009051008,0.75685982558507,0.997610151244476
"10836","USPL1",23.4697741459814,0.111219521515459,0.358800142114422,0
.309976247110825,0.756579019371394,0.997610151244476
"10837","COQ7",28.4715858476703,-0.111554934363876,0.360609664429208,-
0.309350928074684,0.757054594486936,0.997610151244476
"10838","ATP2A3",8.83245837663751,-0.176608936337024,0.569618008800557
,-0.31004802096919,0.756524438951651,0.997610151244476
"10839","KLHL11",4.24251335274758,0.21193183636955,0.68762010640115,0.
308210644797392,0.757922053107184,0.997610151244476
"10840","TIMM21",27.2547501412776,-0.0979603034672128,0.31820282032214
5,-0.30785491897287,0.758192730268348,0.997610151244476
"10841","S1PR2",84.0149111991162,-0.0841706019067596,0.272795270008153
,-0.308548611947135,0.757664916279643,0.997610151244476
"10842","REX1BD",261.686461016174,-0.0675407626399907,0.21814007373172
6,-0.309621068172252,0.756849133009754,0.997610151244476
"10843","ZNF222",5.0365215432696,-0.19090684319247,0.617469178962198,-
0.309176311461138,0.757187412443617,0.997610151244476
"10844","SETD4",23.0355110475062,0.107511529270638,0.347467592624409,0
.309414551321487,0.757006202766839,0.997610151244476
"10845","TBL1X",42.5461168005192,0.0863710908670595,0.280369078732542,
0.308062113188499,0.758035069448603,0.997610151244476
"10846","LINC01409",4.10449802330383,-0.0783796352400775,0.70627833692

7438,-0.110975561817536,0.911635725854157,0.997645679347435
"10847","FAM87B",2.2876327485256,-0.157514158956955,0.90999499201042,-0.173093434952828,0.862577985212243,0.997645679347435
"10848","AGRN",4.09729450160647,-0.224154736024051,0.8219311970856З,-0.272717152991454,0.785070648167633,0.997645679347435
"10849","C1orf159",10.2593485472023,-0.0668362968162961,0.502137526256397,-0.133103568885964,0.894111472700672,0.997645679347435
"10850","TNFRSF4",5.30333304965577,-0.135873061799388,0.781513677974673,-0.173858840387168,0.861976400529482,0.997645679347435
"10851","SDF4",314.878418132072,0.0435917387816687,0.209090803582352,0.208482334157273,0.834852374894376,0.997645679347435
"10852","UBE2J2",78.7456502518327,0.0604819269942075,0.26278309776255,0.230159121759265,0.817968125275053,0.997645679347435
"10853","SCNN1D",3.01605100548354,0.138953852001436,0.847109646605306,0.164032900059959,0.869705244083955,0.997645679347435
"10854","INTS11",74.4093925513459,-0.075600256878626,0.246496718222362,-0.306698837306336,0.759072614856931,0.997645679347435
"10855","DVL1",36.4306346413501,-0.0665570948009457,0.266892027751597,-0.249378354841277,0.803068126687075,0.997645679347435
"10856","MXRA8",937.80489878224,0.0492925660040998,0.207719694279611,0.237303285926019,0.812421513102255,0.997645679347435
"10857","SKI",125.795769514685,-0.0413055064961508,0.229637182309827,-0.179872902465861,0.857252348906448,0.997645679347435
"10858","PEX10",27.6908744737821,-0.0357733164976017,0.328749699273812,-0.108816271396211,0.913348217749104,0.997645679347435
"10859","PRDM16-DT",2.53880348567115,0.212478949705714,1.08293909080988,0.196205817583711,0.844449074786026,0.997645679347435
"10860","CEP104",25.0687102678251,0.0559608559901968,0.350383158038563,0.159713315855318,0.873106911022215,0.997645679347435
"10861","GPR153",33.8659706122625,0.0512862516324561,0.310676024935265,0.165079528242138,0.868881386030308,0.997645679347435
"10862","ACOT7",10.6359443751323,-0.123757937777512,0.486948710732842,-0.254149841759022,0.799379798987493,0.997645679347435
"10863","PHF13",25.5932487051946,0.0446226939582329,0.345509971580771,0.129150234808205,0.89723877318818,0.997645679347435
"10864","THAP3",28.3060065573068,-0.0373275675578281,0.314513025059883,-0.118683693785721,0.905525955703069,0.997645679347435
"10865","SLC2A5",27.6173375173975,-0.112548678054679,0.981541315724267,-0.114665247658608,0.908710460540837,0.997645679347435
"10866","LINC02606",1.98223140275209,0.29323409052256,1.06609464572967,0.27505446321969,0.783274395736306,0.997645679347435
"10867","TMEM201",7.40371502135406,0.128443791216496,0.511566505481011,0.251079360826651,0.801752752568685,0.997645679347435
"10868","PEX14",18.2045235532119,0.0600412156427724,0.346624479003355,0.173216894015703,0.862480944857752,0.997645679347435
"10869","CASZ1",2.69038927421923,0.169934444229833,0.882543481038716,0.192550789712737,0.847310787703943,0.997645679347435
"10870","AL109811.2",6.001031550443,-0.105114327746113,0.711407490136453,-0.147755441436175,0.882535775837118,0.997645679347435

"10871","FBXO44",16.4778995646552,0.124984421827023,0.444302724184653,
0.281304648888624,0.778476744927658,0.997645679347435
"10872","AGTRAP",188.150733471238,0.0352119957591114,0.304278429055751
,0.115722944503107,0.907872121336989,0.997645679347435
"10873","MFN2",30.0300884606996,0.090861930616436,0.318873523915677,0.
284946613004043,0.77568504359729,0.997645679347435
"10874","AL031293.1",2.75758596197689,0.128313346733879,0.884977396657
996,0.144990535598353,0.884718344325898,0.997645679347435
"10875","DNAJC16",33.3194002619879,0.0694830385472528,0.29211563933720
5,0.237861412367055,0.811988585080642,0.997645679347435
"10876","PLEKHM2",128.076816329916,0.0600782931154231,0.26408021477918
8,0.227500167574681,0.820034838945421,0.997645679347435
"10877","BX284668.5",34.8041493290583,0.0583116492519956,0.32011428298
5637,0.182158848734069,0.855458066580597,0.997645679347435
"10878","ALDH4A1",3.16355111600204,-0.185087954839922,0.81277180841088
5,-0.227724378385862,0.819860519117659,0.997645679347435
"10879","EMC1",55.936014008701,0.0366000345819782,0.237108329725574,0.
154359969657492,0.877325914899092,0.997645679347435
"10880","SLC66A1",14.7877106628894,0.049621173532692,0.391977210841338
,0.12659198586108,0.899263340264268,0.997645679347435
"10881","TMCO4",23.0800876195061,-0.0866276385955898,0.321857225791029
,-0.269149273820667,0.787814813889413,0.997645679347435
"10882","NBPF3",2.34376924542524,0.132217284738733,1.00194150634894,0.
131961081461263,0.895015074974678,0.997645679347435
"10883","LINC00339",9.24828754317406,-0.117867194987345,0.542116513198
833,-0.217420410774529,0.827880726128102,0.997645679347435
"10884","KDM1A",62.688710456207,0.0281962289623821,0.238508052627581,0
.11821919072229,0.905893984585426,0.997645679347435
"10885","NIPAL3",11.3811877429232,0.081416244739131,0.463141137017281,
0.175791434255803,0.860457799317104,0.997645679347435
"10886","TMEM50A",489.044209865348,-0.0425165822958871,0.2224100517345
38,-0.191163043056316,0.848397856996941,0.997645679347435
"10887","MACO1",55.8909504903653,-0.0589242291355605,0.220667270885749
,-0.267027497548871,0.789447989560548,0.997645679347435
"10888","SELENON",91.2666791262784,-0.0383119581424325,0.2186429337136
46,-0.175226143793923,0.860901942350484,0.997645679347435
"10889","MTFR1L",61.3016315162007,-0.0375056412697443,0.26251161845482
3,-0.142872309768639,0.886391027791767,0.997645679347435
"10890","CEP85",3.66673623902012,0.18730289132333,0.732654014598586,0.
255649853261162,0.798221219853625,0.997645679347435
"10891","PIGV",26.5080843017785,-0.0428865744899526,0.319365917274495,
-0.134286635392879,0.893175920932919,0.997645679347435
"10892","ZDHHC18",22.6921305705316,0.092725353488431,0.328464541990176
,0.282299431550832,0.777713921414679,0.997645679347435
"10893","SYTL1",9.98919011244366,-0.113295441390552,0.473529859269052,
-0.23925722776054,0.810906129601567,0.997645679347435
"10894","MAP3K6",31.2952574482243,0.0417606363079478,0.353742815260457
,0.118053666410722,0.906025135492168,0.997645679347435
"10895","AHDC1",55.2020504573734,0.0733442022057487,0.332216391854722,
0.220772376089805,0.825269673562279,0.997645679347435

"10896","IFI6",683.492211069199,0.0807504241517919,0.330314739483781,0
.244465094951529,0.806870632157919,0.997645679347435
"10897","STX12",131.156850812113,-0.0369731592634626,0.227065572689826
,-0.162830317363735,0.870652037016657,0.997645679347435
"10898","THEMIS2",5.90243972333065,0.202426427045098,0.807246792544921
,0.250761512977871,0.801998499166762,0.997645679347435
"10899","EYA3",28.4500633504965,0.0396115414123511,0.339740273354319,0
.116593599637921,0.907182109880613,0.997645679347435
"10900","EPB41",12.9808493911624,0.162639924324591,0.536711313029235,0
.30303054989961,0.761866580054337,0.997645679347435
"10901","SDC3",42.864719347479,0.0530006138390987,0.307889831301064,0.
17214148845102,0.863326296502099,0.997645679347435
"10902","ZCCHC17",101.004394349083,-0.0294095629778406,0.2276432044382
33,-0.12919148212843,0.89720613600462,0.997645679347435
"10903","FABP3",18.5655410945413,0.0998701935955781,0.526384138435949,
0.189728729084283,0.849521707135182,0.997645679347435
"10904","LCK",20.2667920841229,0.0432473802251995,0.357498305184848,0.
120972266435887,0.903713002016577,0.997645679347435
"10905","SYNC",58.786798312077,0.0587662377244171,0.380329937664501,0.
154513836289838,0.877204602446351,0.997645679347435
"10906","KIAA1522",7.71840828133125,0.101394969377811,0.60354394135061
6,0.167999316091069,0.866583817112679,0.997645679347435
"10907","ZNF362",29.1157700928487,0.0903999629389107,0.305563847060709
,0.295846396124703,0.767347389099707,0.997645679347435
"10908","PHC2",138.536536291303,0.0407472830288076,0.253889927978258,0
.160491924013217,0.872493582872063,0.997645679347435
"10909","ZSCAN20",3.45918908878818,-0.0852740871546737,0.7559608418776
7,-0.112802254337498,0.910187331049946,0.997645679347435
"10910","ZMYM6",23.2144126789813,0.0396190508628369,0.328663653860427,
0.120545884515913,0.904050734033992,0.997645679347435
"10911","ZMYM1",27.6602350966969,0.0724796977560264,0.316858319750718,
0.228744815073969,0.81906726070881,0.997645679347435
"10912","AGO1",32.3764688317319,0.0713558409642011,0.327491192840401,0
.21788628984284,0.827517709645927,0.997645679347435
"10913","OSCP1",12.5289440987734,-0.0474159676860521,0.430667661230755
,-0.110098741917486,0.912331065897497,0.997645679347435
"10914","ZC3H12A",33.6038988234382,0.099186705062725,0.381714322926295
,0.259845384638283,0.794983041140537,0.997645679347435
"10915","DNALI1",17.0780756423627,0.0762325805535977,0.370937046008439
,0.205513526820568,0.837170930774184,0.997645679347435
"10916","C1orf109",21.5490558008051,0.0812300257521464,0.3310565619771
04,0.245366004126401,0.806173049334369,0.997645679347435
"10917","MANEAL",2.41674144272134,0.299204676170812,1.01255041638544,0
.295496077359685,0.767614948446731,0.997645679347435
"10918","UTP11",87.5971824181031,-0.0700785905777626,0.285480519009198
,-0.245475911354584,0.806087957629696,0.997645679347435
"10919","AL139260.1",3.49215727223433,-0.132634409260846,0.73121604301
885,-0.181388811866409,0.856062400054417,0.997645679347435
"10920","PPIE",74.59761511827,-0.0343600600627502,0.287564940688641,-0
.119486262756743,0.904890122797721,0.997645679347435

"10921","RLF",65.7771853127901,-0.0426292527031288,0.290296433676323,-0.146847317975184,0.883252535155184,0.997645679347435
"10922","EXO5",6.41109298089009,-0.104294705322435,0.570429955131568,-0.18283525327554,0.854927286713014,0.997645679347435
"10923","NFYC",42.271695739796,0.0500446789563922,0.267594173739317,0.18701707237148,0.851647247285,0.997645679347435
"10924","ERMAP",10.3513799634109,0.0601583936744369,0.469806750407653,0.128049232205023,0.898110012028391,0.997645679347435
"10925","ZNF691",6.85332886451408,0.113383436765381,0.552246959495272,0.2053129217207 6,0.837327649030486,0.997645679347435
"10926","SLC2A1",4.7779301963407,0.113748319215465,0.701059306917313,0.162252063546003,0.871107362138737,0.997645679347435
"10927","PTPRF",17.8185678577576,-0.0929049576336158,0.576093206581796,-0.16126723344797,0.871882929354569,0.997645679347435
"10928","DMAP1",55.5303937404569,-0.0309041824276219,0.262179079257794,-0.117874326643868,0.906167235776965,0.997645679347435
"10929","ARMH1",4.13692078378202,-0.0865486496792819,0.746339063748481,-0.115964249874035,0.907680875059934,0.997645679347435
"10930","PTCH2",22.9974123008556,-0.101579360777637,0.384967899232231,-0.263864496183251,0.791884334272769,0.997645679347435
"10931","TOE1",21.1010332232039,-0.0988579682052851,0.386165980303114,-0.25599864630149,0.797951882591222,0.997645679347435
"10932","IPP",25.8824433205476,-0.0839796034255396,0.307179173736181,-0.273389639030883,0.78455371639218,0.997645679347435
"10933","AL604028.1",2.18890704362857,-0.182206633823849,1.01378481214679,-0.179729101916618,0.857365245433691,0.997645679347435
"10934","MAST2",26.7347185221957,-0.0358918488804046,0.308609774019661,-0.116301724384523,0.907413419026908,0.997645679347435
"10935","NSUN4",27.9009943144651,0.0995369477621985,0.323720996052122,0.307477577840432,0.758479887244271,0.997645679347435
"10936","OSBPL9",84.5851338626669,0.0359589410269197,0.214346205751995,0.167761033608056,0.866771278640945,0.997645679347435
"10937","TXNDC12",129.009698744236,0.0504390243887336,0.262358042524189,0.19225263271311,0.847544319823983,0.997645679347435
"10938","COA7",24.1428852931918,0.101359191635975,0.393285001363097,0.257724528737866,0.796619514243973,0.997645679347435
"10939","SLC1A7",4.36821336933235,0.238204154683555,0.837275995163134,0.284498965764739,0.776028026505715,0.997645679347435
"10940","TMEM59",982.046729079069,-0.0241370104859963,0.205435840359821,-0.117491721228975,0.906470404109469,0.997645679347435
"10941","MRPL37",73.3781595735082,-0.0622417013113279,0.273510639878211,-0.227565923355094,0.819983714100245,0.997645679347435
"10942","AC119674.1",6.74571708709371,0.183176289884567,0.605116201960309,0.302712585270658,0.76210890587747,0.997645679347435
"10943","AC099791.2",2.0881267148623,-0.198086985703195,0.993212065282616,-0.199440776675251,0.841917965949008,0.997645679347435
"10944","PATJ",10.0369098344385,-0.16650873380612,0.589110257494414,-0.282644431475883,0.777449417078103,0.997645679347435
"10945","AK4",44.9707190710477,-0.0826436775143848,0.308236194768598,-0.26811801766638,0.788608477330651,0.997645679347435

"10946","GADD45A",167.027393584488,-0.0788885784219092,0.378651017450182,-0.2083411235843,0.834962624079581,0.997645679347435
"10947","GNG12-AS1",2.18383675391717,0.115418383127325,0.945392966078605,0.122085087649921,0.90283163186158,0.997645679347435
"10948","ANKRD13C",43.2766350817746,0.0287993476830419,0.265887104398917,0.108314195034572,0.913746462853127,0.997645679347435
"10949","ZRANB2-AS2",2.86101191933119,0.360499209639806,1.17836115422632,0.30593270012919,0.75965588776473,0.997645679347435
"10950","AL513166.1",2.71683170610782,0.16204944549892,0.911628363614294,0.177758231277984,0.858912851455917,0.997645679347435
"10951","TNNI3K",2.19371038008406,0.208120550025126,0.981574026268585,0.212027360601918,0.832085688888723,0.997645679347435
"10952","AC118549.1",46.1617719762797,0.0596167150091599,0.243320457665674,0.24501316322146,0.806446239174233,0.997645679347435
"10953","DNAJB4",59.1168491270172,0.025139138969228 7,0.232098459880335,0.10831239027691,0.913747894419321,0.997645679347435
"10954","AL359504.2",3.08628643544329,0.110096282041486,0.804504221070011,0.136849850079165,0.891149479609636,0.997645679347435
"10955","CTBS",79.5852110512095,0.0679315993073671,0.23974636790976,0.283347772479859,0.776910259934282,0.997645679347435
"10956","SH3GLB1",283.943216756717,-0.0341020102421776,0.153016649372757,-0.22286470382123 7,0.823640807777743,0.997645679347435
"10957","SELENOF",409.223592648459,-0.0263058636519009,0.217482403034257,-0.120956285588574,0.903725659940696,0.997645679347435
"10958","GTF2B",120.222491888098,0.0476854805491501,0.284529695792685,0.167594037649746,0.866902662121401,0.997645679347435
"10959","LRRC8C-DT",3.87767404483741,0.0900875322667931,0.708868635692245,0.127086356668635,0.898872049702847,0.997645679347435
"10960","LRRC8C",10.6657081290046,0.125344746336266,0.491221901284806,0.255169295197186,0.798592345128935,0.997645679347435
"10961","ZNF326",75.5254098513038,0.0500004348005468,0.217498508544136,0.229888632962283,0.818178309379366,0.997645679347435
"10962","CDC7",6.16743944946806,0.119068811864663,0.618484246013461,0.192517129792941,0.847337151238551,0.997645679347435
"10963","GFI1",2.19819748897409,0.142260830944315,0.967387856683786,0.147056663944477,0.883087295076189,0.997645679347435
"10964","FNBP1L",21.8286479451965,-0.0952176662177946,0.387010073517851,-0.246034076974492,0.805655853497999,0.997645679347435
"10965","GCLM",62.1527846815408,0.0951567376485494,0.327359317055534,0.290679790343058,0.771296229478543,0.997645679347435
"10966","F3",325.605986379549,-0.0595932909997535,0.353863379100776,-0.168407624296105,0.866262610448169,0.997645679347435
"10967","AC092802.1",4.78462753812173,0.171718396516533,0.723214214208032,0.237437806313827,0.812317162956707,0.997645679347435
"10968","FRRS1",9.20268471090079,0.0680864757787468,0.513481375536362,0.132597751393858,0.894511510845496,0.997645679347435
"10969","RTCA",55.0402606674353,0.0588844219215621,0.232679239758174,0

.253071232236968,0.800213168770454,0.997645679347435
"10970","AC104506.1",7.635262687537,-0.0976136051769088,0.531416331483814,-0.183685745796245,0.854259991844016,0.997645679347435
"10971","RNPC3",38.3801411806148,0.0512870259870851,0.320384874738256,0.160079423315426,0.872818509952491,0.997645679347435
"10972","PRPF38B",159.853461874012,-0.0275263196854566,0.186439750560122,-0.147641903632455,0.882625383160586,0.997645679347435
"10973","CLCC1",54.6236698116012,0.0783139725512183,0.257445026368952,0.3041968751767,0.760977905356788,0.997645679347435
"10974","SORT1",10.3788699799941,-0.112416663241628,0.52431980654302,-0.214404761824317,0.830231428608204,0.997645679347435
"10975","PSMA5",197.595680778609,-0.0583901856884466,0.313930040268514,-0.185997445922995,0.852446764239887,0.997645679347435
"10976","AMIGO1",3.13544466157702,-0.214007836710046,0.852406366923915,-0.251063160734401,0.801765277325051,0.997645679347435
"10977","AHCYL1",276.718580008636,0.048366045130476,0.17897644212347,0.27023693485375,0.786977979453152,0.997645679347435
"10978","AL355488.1",2.00347301621958,0.226718782000268,1.12026731036976,0.202379182094884,0.839620301954729,0.997645679347435
"10979","INKA2",2.25068492884031,-0.180256040987316,1.03075384256713,-0.174877874370453,0.861175596046845,0.997645679347435
"10980","MOV10",26.0464861210634,0.088282415408796,0.336963971553552,0.261993633924052,0.793326350209666,0.997645679347435
"10981","MAGI3",42.6514931165829,-0.0868734807019676,0.349582422852175,-0.248506432311968,0.803742593881381,0.997645679347435
"10982","RSBN1",79.4045959859956,0.064478752061945,0.236772597310574,0.272323540791202,0.785373257043495,0.997645679347435
"10983","NRAS",34.9244781676166,0.0304242502713816,0.277836029515619,0.109504337232372,0.912802481844627,0.997645679347435
"10984","CD2",51.2072697513002,-0.0483246018699578,0.268155691501021,-0.180210987130116,0.856986933162458,0.997645679347435
"10985","PTGFRN",76.5285496942878,-0.0608218763603291,0.266355761916597,-0.228348265953316,0.819375504746556,0.997645679347435
"10986","WARS2",33.350216663124,-0.0604760379703906,0.270283027776408,-0.223750778833288,0.822951231683007,0.997645679347435
"10987","AC245014.3",3.03999425475346,-0.188568166633888,0.791354342194923,-0.238285375563708,0.811659763327247,0.997645679347435
"10988","CD160",2.45069461491832,0.247752365506082,1.0394422096869,0.238351264935364,0.811608663149025,0.997645679347435
"10989","PIAS3",20.0573424665375,-0.104330907733443,0.35647463229198,-0.2926741436344,0.769771235285457,0.997645679347435
"10990","FMO5",5.77116599920279,0.110221101488879,0.580398150604967,0.189906017746597,0.849382776803972,0.997645679347435
"10991","MCL1",1348.34727526695,-0.0542021458237227,0.265824876747022,-0.203901705841118,0.838430312016145,0.997645679347435
"10992","CDC42SE1",72.333799931732,-0.0683403053813376,0.231472993920352,-0.295240944629822,0.767809825971912,0.997645679347435
"10993","LYSMD1",10.5681093928537,0.059893242218106,0.521099577684849,0.114936270883582,0.908495635581183,0.997645679347435
"10994","PSMB4",252.039378784711,0.0619373046184214,0.24066539031614,0

.257358586280561,0.796901969715651,0.997645679347435
"10995","S100A11",3170.33542761895,0.0360541137315641,0.30924804952702
8,0.116586390073296,0.907187823316637,0.997645679347435
"10996","SPRR2F",6.20279495889995,0.361579159786226,1.71052912536993,0
.211384392363403,0.832587334676737,0.997645679347435
"10997","DENND4B",19.4512319006582,-0.0759003563732818,0.3508153156918
7,-0.216354169781877,0.828711687412795,0.997645679347435
"10998","CRTC2",29.2389950996133,-0.0849561867949146,0.30920560472658,
-0.274756296445656,0.783503476617544,0.997645679347435
"10999","CREB3L4",10.1542344639176,-0.121853358061192,0.46884926775452
1,-0.259898791449093,0.794941843533326,0.997645679347435
"11000","PBXIP1",71.8248770870212,0.038069217175900 5,0.215983526743649
,0.176259818282738,0.860089828123169,0.997645679347435
"11001","CKS1B",205.645190499094,-0.0335628042369423,0.271589679318975
,-0.123579085630583,0.901648552781202,0.997645679347435
"11002","THBS3",95.0300740231138,0.0319996337833103,0.266642821574396,
0.120009357815703,0.904475735071743,0.997645679347435
"11003","HCN3",2.78116833507464,-0.115613545322975,0.981427080448799,-
0.11780146240728,0.906224970784118,0.997645679347435
"11004","RUSC1",38.1380292925389,0.031222308327355,0.286386001292258,0
.109021768474963,0.913185224499304,0.997645679347435
"11005","KHDC4",26.4127014162843,0.0516688054312952,0.369000257055949,
0.140023765412881,0.888641213283965,0.997645679347435
"11006","LAMTOR2",172.372401376369,0.0559478525181025,0.27409382320115
9,0.204119348129352,0.838260235006343,0.997645679347435
"11007","MEF2D",54.6062023332979,0.0833427888945154,0.305032475436764,
0.273225953319168,0.784679530850393,0.997645679347435
"11008","ARHGAP30",13.3222255117057,0.0999554966131934,0.5081447244752
07,0.196706748685473,0.844057028593116,0.997645679347435
"11009","DEDD",29.4196253284093,0.0529742092072867,0.297765552074092,0
.177905767938211,0.858796980951623,0.997645679347435
"11010","NDUFS2",125.873900744572,-0.0482753802755995,0.26511083820745
2,-0.18209508370919,0.855508106865141,0.997645679347435
"11011","HSPA6",3.46455048735596,-0.172418106708029,0.873188271292821,
-0.197458111127341,0.843469058531877,0.997645679347435
"11012","AL359541.1",3.75626156060392,-0.0898968052713289,0.7548055472
2918,-0.119099290673381,0.905196692705147,0.997645679347435
"11013","HSD17B7",16.5079316477281,0.0858996429007345,0.37386785780214
6,0.229759368472358,0.818278759342664,0.997645679347435
"11014","AL592435.1",2.29679968322021,0.281109122353484,0.996716605160
097,0.282035155126498,0.777916554133042,0.997645679347435
"11015","POGK",33.1431249309704,0.0654450521358204,0.323210048046401,0
.202484584038752,0.839537908962134,0.997645679347435
"11016","TADA1",8.78554733754356,-0.140414966797683,0.505609154765212,
-0.277714446968207,0.781231569610858,0.997645679347435
"11017","RCSD1",11.3913567752053,-0.0773026677916282,0.454151592847373
,-0.170213358290714,0.864842346620488,0.997645679347435
"11018","XCL2",14.3568204169351,0.114928249738362,0.598672621078124,0.
191971781725031,0.847764309216917,0.997645679347435
"11019","Z99572.1",5.2230201574184,-0.136575731651083,0.88158015747850

5,-0.154921512800058,0.876883193639647,0.997645679347435
"11020","VAMP4",40.0177862940742,0.0560184017894987,0.291600448923966,0.192106706269527,0.847658621909599,0.997645679347435
"11021","AL645568.1",6.41112978247374,0.0786631105115471,0.563860819844474,0.139508027057536,0.889048713293962,0.997645679347435
"11022","MRPS14",79.3209502469457,0.0781169682577575,0.272769382635168,0.286384664961609,0.774583518655981,0.997645679347435
"11023","RASAL2-AS1",2.99006142039003,-0.224635295105677,1.02698408893759,-0.218732984790505,0.826858052948963,0.997645679347435
"11024","FAM20B",45.1917691453244,-0.0799657442848287,0.317140393534833,-0.252146197441247,0.80092806284653,0.997645679347435
"11025","ABL2",267.937701431319,0.111067465786186,0.648765850672979,0.171198076580285,0.86406801983576,0.997645679347435
"11026","ACBD6",109.408321312301,-0.0369227160289038,0.228832160447467,-0.16135282714066,0.87181551824862,0.997645679347435
"11027","NMNAT2",2.68511682364826,-0.213883133381536,0.88411100006078,-0.241918869199492,0.808843028830994,0.997645679347435
"11028","IVNS1ABP",59.3671044018694,-0.102709015191082,0.343194700565975,-0.299273313433166,0.764731514802416,0.997645679347435
"11029","TPR",332.814985700179,-0.0240261599551507,0.21751146695746,-0.11045927964731,0.91204514233164,0.997645679347435
"11030","RGS18",2.20367452443518,0.141375600617005,0.99673214722736,0.14183910994571,0.887207091086927,0.997645679347435
"11031","AL136454.1",7.0539844270153,0.149540062916418,0.713837290260905,0.209487603066746,0.834067612017206,0.997645679347435
"11032","KCNT2",17.5719418083811,0.077307206656513,0.495688527158687,0.155959241380151,0.876065148683134,0.997645679347435
"11033","NAV1",256.52626423133,-0.0508234332376532,0.259646713812366,-0.195740714339951,0.844813115345567,0.997645679347435
"11034","IPO9-AS1",2.73716619518434,-0.209214591929577,1.02348422141108,-0.204414086268114,0.838029923332577,0.997645679347435
"11035","UBE2T",4.10158962413631,-0.138126001832567,0.786232937386194,-0.175680762360023,0.860544749643001,0.997645679347435
"11036","ADIPOR1",53.3265055458089,-0.0411330228592862,0.234491499340054,-0.175413705720889,0.860754571945992,0.997645679347435
"11037","CYB5R1",61.2398507602065,-0.0604501858945243,0.280286396145191,-0.215672921432871,0.829242710036125,0.997645679347435
"11038","ATP2B4",168.015078897792,-0.0307168103112744,0.22275834242018,-0.137892973962495,0.890325003328211,0.997645679347435
"11039","LAX1",3.24199810560079,-0.265985119517884,0.900004780190574,-0.295537451991713,0.767583346728034,0.997645679347435
"11040","PLEKHA6",19.7953032512078,-0.145720850695854,0.543871575928564,-0.267932462635248,0.788751305369795,0.997645679347435
"11041","PPP1R15B",181.144068083012,-0.0647487365992772,0.451523606665846,-0.143400556789038,0.885973843265619,0.997645679347435
"11042","MDM4",143.116002418866,0.0617109313011905,0.267891068160509,0.230358300950205,0.817813360871229,0.997645679347435
"11043","RBBP5",37.8695244113639,-0.0718730079363385,0.315906061817869

,-0.227513861312911,0.82002419205797,0.997645679347435
"11044","NUAK2",1.89953650950408,0.189762573607018,1.06526894860745,0.178135834950489,0.858616299857462,0.997645679347435
"11045","RAB7B",28.4623436907041,-0.0504120434512089,0.30296909794012,-0.166393350985164,0.867847406162283,0.997645679347435
"11046","EIF2D",56.7687871269571,-0.0560324754343049,0.27537459746893,-0.20347728493957,0.83876199829868,0.997645679347435
"11047","FCMR",8.53487445273981,0.0789679260538324,0.548264888396624,0.144032433455242,0.885474858461676,0.997645679347435
"11048","YOD1",6.14266926749823,0.140677762944258,0.602773142057,0.233384258734863,0.81546303542685,0.997645679347435
"11049","CD34",530.245005928543,-0.0241031087657331,0.200496264911983,-0.120217245824077,0.904311056659339,0.997645679347435
"11050","LINC02767",5.74946103634371,-0.0816890026293937,0.624560684022902,-0.130794340276466,0.8959380003394,0.997645679347435
"11051","LINC00467",31.9279747081047,0.0624477081880498,0.430642437249215,0.14501057672565 4,0.884702521040827,0.997645679347435
"11052","LPGAT1",62.164993760853,0.0699419069053279,0.244051731521725,0.286586398995091,0.774429029820775,0.997645679347435
"11053","INTS7",13.2573977121886,-0.0985242418794496,0.463986858309706,-0.212342742288804,0.831839652116566,0.997645679347435
"11054","PPP2R5A",41.6207436699562,-0.0690326599955102,0.27402880670787,-0.251917529492083,0.801104809887418,0.997645679347435
"11055","FLVCR1-
DT",8.33290011796222,-0.109043856080952,0.51419772856213,-0.212066001119599,0.832055543614863,0.997645679347435
"11056","EPRS",159.375650699221,0.0370734792963717,0.189740798536492,0.195390130021201,0.845087542807244,0.997645679347435
"11057","BPNT1",15.309862833926,-0.0583531474498788,0.430102594620577,-0.135672623647751,0.892080090737655,0.997645679347435
"11058","TAF1A",12.3983120172056,0.0699111678821552,0.465581794480121,0.150158723367222,0.880639390575351,0.997645679347435
"11059","AL592148.3",6.62198871527529,-0.12274486608574,0.627550202305925,-0.195593700129034,0.844928191609535,0.997645679347435
"11060","CNIH4",168.680374795027,-0.0428986709872433,0.238234233290321,-0.180069297324559,0.857098165774366,0.997645679347435
"11061","AL591895.1",13.0820815698999,-0.0505159663385944,0.442781011196716,-0.114087923965085,0.909168094997161,0.997645679347435
"11062","PARP1",108.248157024226,-0.0534795876315071,0.190846582885953,-0.28022292473251,0.779306479567351,0.997645679347435
"11063","COQ8A",44.2666998398498,-0.0320310882030321,0.26630862732156,-0.120278071819113,0.904262874146791,0.997645679347435
"11064","JMJD4",27.100635254477,-0.0524724839229442,0.336018366450616,-0.156159570910407,0.875907243453829,0.997645679347435
"11065","MRPL55",103.769751858672,0.0588655111101209,0.243650298986467,0.241598353685543,0.809091397872548,0.997645679347435
"11066","TAF5L",23.4340991435405,-0.0675515720893335,0.348274773837933,-0.193960565518214,0.846206759629025,0.997645679347435
"11067","PCNX2",43.0873656938433,0.0978785698575633,0.328569690071336,0.29789287574369,0.765784925578699,0.997645679347435

"11068","MAP3K21",3.28183733954043,-0.164189398814175,0.825128290400646,-0.198986510006161,0.84227329703936,0.997645679347435
"11069","KCNK1",24.326503957229,0.0902600760913878,0.440150995601906,0.205066163642223,0.837520432123839,0.997645679347435
"11070","IRF2BP2",301.987118335293,0.0526185041296732,0.254627892006971,0.20664862641298,0.836284281441276,0.997645679347435
"11071","RBM34",9.21004167748055,-0.0933806078063775,0.488003890880728,-0.1913521788481,0.848249683458903,0.997645679347435
"11072","GGPS1",66.3205765200293,-0.059022979016421,0.268417932974239,-0.219892085310432,0.825955200472581,0.997645679347435
"11073","GPR137B",40.3359483963886,0.0529166559285307,0.313290370654023,0.168906104002055,0.865870497886092,0.997645679347435
"11074","MTR",89.5435133735324,0.0560131945826529,0.259586317410332,0.215778686417096,0.829160262733073,0.997645679347435
"11075","CHML",11.606952611936,-0.062396757094282,0.459854265478154,-0.135688112035673,0.892067846019866,0.997645679347435
"11076","SDCCAG8",76.7383608440648,0.0500450135321677,0.225551146886414,0.22187878103484,0.824408249123853,0.997645679347435
"11077","AKT3",89.759713470539,-0.0836822043142464,0.296264658020014,-0.282457600152204,0.777592653606009,0.997645679347435
"11078","CNST",37.684914103878,-0.0312637081377701,0.284656170243495,-0.109829722331426,0.912544418619039,0.997645679347435
"11079","ZNF124",8.82137435279937,0.0961021516148586,0.601163803145748,0.159860176397812,0.87299121963505,0.997645679347435
"11080","SH3BP5L",9.4099094982494,-0.0986545842724891,0.517808982816072,-0.190523122515106,0.848899225870169,0.997645679347435
"11081","PGBD2",8.7446423122037,-0.0822836123765909,0.555688009238211,-0.148075198688186,0.882283422233558,0.997645679347435
"11082","EIPR1",18.2172662971179,-0.0837361309428124,0.36577554321928,-0.228927637440793,0.818925159343215,0.997645679347435
"11083","TRAPPC12",61.3384061045583,-0.0511214510007905,0.240545629915055,-0.212522883990215,0.831699126672734,0.997645679347435
"11084","AC108488.1",10.0651591376298,0.104892229764928,0.476784145501505,0.219999408022675,0.825871615739992,0.997645679347435
"11085","ASAP2",68.6807461863542,-0.0881561374259875,0.302091134337622,-0.291819677592599,0.770424499231242,0.997645679347435
"11086","SLC66A3",150.243168885238,-0.0665502282305691,0.228463941992504,-0.291294230722643,0.770826299447048,0.997645679347435
"11087","FAM49A",35.7567845975342,0.0941994176789167,0.328162814641026,0.287050858525701,0.774073378574271,0.997645679347435
"11088","LINC00954",2.99207485829921,0.107849269983021,0.826746492356709,0.13045022988315,0.896210228371538,0.997645679347435
"11089","PFN4",2.88708000727942,0.123784331014501,0.830288435865383,0.149085938894818,0.881485821026282,0.997645679347435
"11090","CENPO",2.77965761545869,0.230725709897664,0.878989815780378,0.262489628156639,0.792943980640262,0.997645679347435
"11091","DNAJC27-AS1",3.41175354427068,-0.180615689751907,0.801773253163961,-0.225270285631455,0.821769014228446,0.997645679347435
"11092","RAB10",156.237708070269,-0.0420456370715162,0.168223355459028

,-0.24993935566668,0.802634247449961,0.997645679347435
"11093","OST4",813.455805003164,-0.0719767014717652,0.242347326266357,
-0.296998124884026,0.76646794071072,0.997645679347435
"11094","KHK",4.10003728592084,0.130845737718776,0.731466810021277,0.1
78881305243323,0.858030902268927,0.997645679347435
"11095","CGREF1",32.6735403717173,0.0625997946687511,0.415591201115283
,0.150628296510508,0.880268938675731,0.997645679347435
"11096","SLC5A6",16.1177127637686,0.145298574359762,0.477495956658562,
0.304292784752644,0.760904841630802,0.997645679347435
"11097","UCN",1.94140112677333,-0.268677186175269,1.08764196946711,-0.
247027232966108,0.804887148198992,0.997645679347435
"11098","GTF3C2",27.8427978825979,0.0626160875341589,0.314963203444346
,0.198804453502528,0.842415712140133,0.997645679347435
"11099","IFT172",11.5005976929361,0.0761539146733382,0.466626590203476
,0.163200975409761,0.870360198238541,0.997645679347435
"11100","SLC4A1AP",53.8421723686538,-0.0494006789798491,0.230797920606
08,-0.214042998524951,0.83051352613068,0.997645679347435
"11101","MRPL33",198.371900712346,-0.0272153325731646,0.24678618681879
1,-0.110278994638983,0.912188115496033,0.997645679347435
"11102","BABAM2",48.0050611728563,0.0814271951736927,0.293735804936343
,0.277212358198345,0.781617050880339,0.997645679347435
"11103","AC093690.1",5.08868298689839,-0.107158960740955,0.61277155738
2234,-0.174875872500903,0.861177169069782,0.997645679347435
"11104","WDR43",117.149840929762,-0.06736875228521,0.269788655424067,-
0.249709359273526,0.80281211980159,0.997645679347435
"11105","DPY30",107.163168047625,-0.0859126461229374,0.280321875884435
,-0.306478564514014,0.759240298155557,0.997645679347435
"11106","SLC30A6",31.2212499228105,-0.0316672024654648,0.2836783711250
48,-0.111630655308245,0.911116264509999,0.997645679347435
"11107","NDUFAF7",25.662577704104,0.095217191252047,0.312085295787565,
0.305099895885068,0.760290070494666,0.997645679347435
"11108","HNRNPLL",37.9190588052423,0.03427589658336,0.28724922555343,0
.11932459179767,0.905018201248275,0.997645679347435
"11109","SRSF7",597.597373506833,0.0197009071909078,0.16890942395896,0
.11663592669462,0.907148566630119,0.997645679347435
"11110","THUMPD2",38.4929545529627,0.0691967292878701,0.31465783613815
2,0.219911031414736,0.825940444784627,0.997645679347435
"11111","SLC8A1",2.38099588277733,0.148758177596283,0.915585471260173,
0.162473283233229,0.870933165617527,0.997645679347435
"11112","EML4",84.5582380402123,0.055567619731095,0.2470376026573,0.22
4935876698013,0.822029158804978,0.997645679347435
"11113","ZFP36L2",2877.85814881278,0.0261050731558583,0.18689538480579
3,0.13967746278478,0.888914833914195,0.997645679347435
"11114","PLEKHH2",124.498888924242,0.143510715711323,0.660436978621476
,0.217296608695158,0.827977199829052,0.997645679347435
"11115","CAMKMT",10.2697834244036,-0.0849661740206708,0.44248256040394
3,-0.19202152044841,0.847725348220166,0.997645679347435
"11116","SIX2",12.2994371695094,0.050431352418245,0.439162939791976,0.
114835173573921,0.908575768985302,0.997645679347435
"11117","CHAC2",3.52255063917286,0.162599319990102,0.902626900484845,0

.180140122018036,0.857042564978698,0.997645679347435
"11118","C2orf73",2.18270046619034,0.229461464084155,1.04642335022737,
0.219281674127682,0.826430636451714,0.997645679347435
"11119","CCDC85A",13.5784042922304,0.0868646541548336,0.60679927799942
5,0.143152204203704,0.886169976468665,0.997645679347435
"11120","REL",69.2411624701441,0.0626053265084511,0.321223579675891,0.
194896422521718,0.845474034905694,0.997645679347435
"11121","PUS10",5.1441852910279,0.182142839826785,0.617163878445572,0.
295128808065601,0.767895483665204,0.997645679347435
"11122","C2orf74",46.943877317433,0.0610502928648904,0.362154676296218
,0.168575188616246,0.866130797844122,0.997645679347435
"11123","AFTPH",45.1594973861075,0.0527681007851592,0.285929656298088,
0.184549240076543,0.853582602462136,0.997645679347435
"11124","SLC1A4",6.3069215114574,-0.150068677322466,0.660714383670927,
-0.227130937408513,0.820321928059019,0.997645679347435
"11125","CEP68",52.2012520490557,0.0599836024141103,0.281358329268504,
0.213192915134448,0.831176494150878,0.997645679347435
"11126","FBXO48",9.34853603797011,0.140187395375112,0.478727781904461,
0.292833214770663,0.769649638833867,0.997645679347435
"11127","ARHGAP25",6.11001945104263,0.122296040014174,0.57270281255103
7,0.213541888277833,0.830904320363992,0.997645679347435
"11128","ANXA4",289.347361356473,-0.0718580103574615,0.249503819350395
,-0.288003648780007,0.773343945631638,0.997645679347435
"11129","GMCL1",38.6896615750944,-0.0711268335352378,0.25816396115055,
-0.275510312199462,0.782924204283547,0.997645679347435
"11130","ASPRV1",2.72215825946038,0.238329624850527,0.851587143734795,
0.279865221784922,0.779580910381932,0.997645679347435
"11131","PCBP1-
AS1",34.6431454068469,0.0474685636132313,0.316579375478747,0.149942059
685496,0.880810327971131,0.997645679347435
"11132","C2orf42",11.1944072326512,-0.107948255793578,0.45019684989895
8,-0.239780122446005,0.810500717487101,0.997645679347435
"11133","ANKRD53",2.35378226259581,-0.288703112175106,0.97769929881348
9,-0.295288247138429,0.767773693882037,0.997645679347435
"11134","NAGK",86.6847546357393,-0.0636940233950464,0.228066113987877,
-0.279278768254156,0.780030898787698,0.997645679347435
"11135","ALMS1",21.7305861518722,0.0897139382363982,0.407196593869916,
0.220320944691051,0.825621209393868,0.997645679347435
"11136","ALMS1-
IT1",2.43819851422719,0.187661830529706,0.989302966010714,0.1896909611
89005,0.849551304139334,0.997645679347435
"11137","DCTN1",76.9244520909162,0.070718256251951,0.245317367597249,0
.288272522017491,0.773138139030398,0.997645679347435
"11138","WDR54",19.7740379710062,0.0654845693919336,0.340574579300578,
0.192276738699688,0.847525438259761,0.997645679347435
"11139","LBX2",3.86380418827115,0.201512058932075,0.744766169066074,0.
270570908429915,0.786721073165426,0.997645679347435
"11140","GCFC2",36.7503943360739,0.0308161014293245,0.279873736674038,
0.110107156875585,0.912324392306029,0.997645679347435
"11141","MAT2A",250.388420462129,0.0718195501692505,0.241583152642809,

0.297287080591413,0.766247344220677,0.997645679347435
"11142","VAMP5",391.159377837631,-0.0300705006418197,0.268752839871818
,-0.111889052618614,0.910911376840521,0.997645679347435
"11143","TMEM150A",25.847616746508,0.0798792175323534,0.32563806814493
6,0.245300612386634,0.806223677617655,0.997645679347435
"11144","CD8B",8.3198377149159,0.113162063449986,0.586624512972129,0.1
92903741571677,0.847034354721459,0.997645679347435
"11145","EIF2AK3",24.8654386352976,0.0642638877082318,0.32166562770317
4,0.199784752157396,0.841648926936564,0.997645679347435
"11146","AC233263.6",2.93510552833358,0.282143588647856,0.955055887795
714,0.295421024312042,0.767672274542004,0.997645679347435
"11147","ZNF2",4.80955280082636,-0.183174127201866,0.767301884977829,-
0.238724980073728,0.811318845401388,0.997645679347435
"11148","CNNM4",5.12542113382839,0.0943303256586224,0.689756237644074,
0.136758930924375,0.891221346928767,0.997645679347435
"11149","COA5",76.8264025107723,-0.0516901369223932,0.27382184451938,-
0.188772875345725,0.850270831930678,0.997645679347435
"11150","MRPL30",39.6747739295711,-0.0289282457039958,0.26628303034060
5,-0.108637210816602,0.913490245444428,0.997645679347435
"11151","REV1",69.2176813147784,-0.0507128417651827,0.267341423119785,
-0.189693169032246,0.849549573945859,0.997645679347435
"11152","LONRF2",9.59753339174248,0.146846288896178,0.599145133196247,
0.245093017968452,0.806384408940719,0.997645679347435
"11153","IL1R2",4.52732421968562,0.225668420489366,0.886452883536521,0
.25457463637442,0.799051651107564,0.997645679347435
"11154","IL1RL2",2.23260558402171,-0.245144649274693,0.927174722116134
,-0.264399625472088,0.79147200009051,0.997645679347435
"11155","TGFBRAP1",28.446544094523,-0.0743811871340657,0.3729270073122
19,-0.19945240134296,0.841908873461325,0.997645679347435
"11156","AC012360.3",6.00838184308333,-0.186463626567188,0.62804129806
8091,-0.29689707848953,0.766545086690684,0.997645679347435
"11157","SEPTIN10",165.026996047621,0.0316211582552252,0.1739931226667
37,0.181737977746349,0.855788360318013,0.997645679347435
"11158","MTLN",150.382895354862,-0.106287709027393,0.379010621123478,-
0.280434645109233,0.779144059992032,0.997645679347435
"11159","BCL2L11",37.3224791815099,0.0823969856233843,0.38574585593371
5,0.21360433133867,0.830855621435604,0.997645679347435
"11160","TMEM87B",39.0745026588076,-0.0821329463075065,0.2826648908922
02,-0.290566493943632,0.771382888847105,0.997645679347435
"11161","TTL",42.5374525804912,-0.11002293982356,0.408728828425058,-0.
269183214326986,0.787788696728837,0.997645679347435
"11162","PSD4",4.67523046463551,-0.188133547829976,0.698467979198839,-
0.269351714656655,0.787659039551624,0.997645679347435
"11163","DDX18",262.751441283925,-0.0406994993214523,0.276929948098541
,-0.146966767591962,0.883158251063848,0.997645679347435
"11164","TMEM177",9.17311583549855,-0.177496765362934,0.61288450222907
4,-0.289608832850846,0.772115508836961,0.997645679347435
"11165","TMEM185B",49.3920274873768,-0.0563748518958356,0.249543165278
322,-0.225912225778491,0.821269688825087,0.997645679347435
"11166","NIFK-

AS1",21.7347563124885,0.100319245934016,0.352965961364745,0.284217904599443,0.776243395131871,0.997645679347435
"11167","NIFK",179.051937233794,0.074782319642766,0.303561857656682,0.246349525661891,0.805411675008622,0.997645679347435
"11168","GYPC",545.225158060524,0.0491984141376552,0.22362940059017,0.219999758564018,0.825871342735734,0.997645679347435
"11169","WDR33",115.182162744544,-0.05020439111316,0.19925204676869,-0.251964242914111,0.801068702298046,0.997645679347435
"11170","AMMECR1L",21.5525180456081,-0.0995243847665428,0.328963200367702,-0.302539568727743,0.762240774295022,0.997645679347435
"11171","CCDC115",80.8496899060908,-0.0502821818317395,0.257063291982975,-0.195602341524007,0.844921427413724,0.997645679347435
"11172","R3HDM1",24.0791272623985,0.0409341334339281,0.348444807552928,0.117476663582396,0.906482335744994,0.997645679347435
"11173","UBXN4",438.879811232762,0.0161292563399012,0.140134278853683,0.11509857881912,0.908366986356796,0.997645679347435
"11174","HNMT",200.437371616523,0.0453482551662739,0.215188612067052,0.210737244553367,0.833092310230144,0.997645679347435
"11175","LRP1B",18.1861882259205,0.133889666728994,0.498153392628027,0.268771966045748,0.788105167627979,0.997645679347435
"11176","ZEB2",317.20500374415,-0.0464236084233744,0.216724457035958,-0.214205674146282,0.830386671448638,0.997645679347435
"11177","CYTIP",33.3626516857747,-0.0843158271839561,0.355436905142278,-0.237217424426441,0.812488119320288,0.997645679347435
"11178","DAPL1",1.9154824757443,-0.208449525971389,1.03058763916947,-0.202262784889769,0.839711292015714,0.997645679347435
"11179","TANC1",9.2208039127851,0.138522402432104,0.595663041806935,0.232551615107592,0.81610960274667,0.997645679347435
"11180","WDSUB1",16.4092934991397,-0.101432597969606,0.377871094034699,-0.268431747151031,0.788367005206612,0.997645679347435
"11181","LINC02478",3.41213198546876,0.118255922111136,0.800245499214088,0.14777455446769,0.882520691426808,0.997645679347435
"11182","AC008063.1",2.98661885685895,-0.136132949375462,1.06012930859185,-0.128411645892787,0.897823215368524,0.997645679347435
"11183","BBS5",21.9809259031042,-0.0385977958538575,0.355249651859033,-0.108649778126098,0.913480277185866,0.997645679347435
"11184","FASTKD1",7.09636284105384,-0.118537251116495,0.654838113931823,-0.181017641756931,0.856353728645975,0.997645679347435
"11185","SSB",348.595310504866,-0.0594938952887384,0.225730308969342,-0.263561838728616,0.792117567278786,0.997645679347435
"11186","SP5",10.1933092435843,-0.132379699002621,0.619760494549208,-0.213598156331196,0.83086043725398,0.997645679347435
"11187","METTL8",42.3867191242254,0.0371250283442569,0.310278399174516,0.119650702217836,0.904759853631403,0.997645679347435
"11188","HAT1",93.7657259332324,-0.0733078082834548,0.261016913852165,-0.280854628160131,0.778821901933536,0.997645679347435
"11189","MAP3K20",268.1904347458,0.0396738684178164,0.223586283240672,0.177443212717619,0.859160266976808,0.997645679347435
"11190","HOXD-
AS2",52.6111424527285,0.0554868969327497,0.403377852096067,0.137555635

849673,0.890591619583949,0.997645679347435
"11191","HOXD8",28.3640867332268,-0.125672704086296,0.420761695533432,-0.298679051397421,0.76518494240478,0.997645679347435
"11192","HOXD4",2.18567583536809,-0.230440070281584,1.13228514628999,-0.203517701381704,0.838730411485846,0.997645679347435
"11193","AC079305.3",2.10721369519456,-0.26058851943673,0.931788935260395,-0.279664749790044,0.779734725136008,0.997645679347435
"11194","RBM45",14.768818219764,0.105958467073863,0.408691077986613,0.259262980723387,0.795432340174584,0.997645679347435
"11195","ITPRID2",39.8754961517826,0.0373192724719327,0.288171656149484,0.129503619372524,0.896959161224923,0.997645679347435
"11196","FSIP2",5.19948747795935,0.161231927578657,0.733732585697669,0.219742084134576,0.826072027215111,0.997645679347435
"11197","SLC40A1",116.947440689375,0.10667110091855,0.386662764130273,0.27587632121362,0.782643061349611,0.997645679347435
"11198","ORMDL1",140.730872483185,0.0548257360393757,0.202392884416893,0.270887665825467,0.786477431725213,0.997645679347435
"11199","NEMP2",10.1931578092416,-0.0843231036953089,0.497953707062949,-0.169339242783565,0.865529810401821,0.997645679347435
"11200","AC108047.1",2.39291220193552,-0.145830185640521,1.07863238581618,-0.135199153630246,0.892454416255652,0.997645679347435
"11201","AC006460.2",2.62444753297234,-0.163192904754031,1.12039191940382,-0.145656981211421,0.884192183106069,0.997645679347435
"11202","CAVIN2",11.5143693184138,-0.151187502165271,0.559849169071048,-0.270050418072666,0.787121465967925,0.997645679347435
"11203","AC114760.2",4.67183254724733,-0.109197961582298,0.774997510221995,-0.140901048251134,0.887948114025535,0.997645679347435
"11204","GTF3C3",29.02057456002,0.0570135158352265,0.289246859425712,0.197110232928456,0.843741275802243,0.997645679347435
"11205","PGAP1",19.2850876162479,0.0406604239124339,0.340429648400577,0.119438550970712,0.904927920619191,0.997645679347435
"11206","MOB4",123.445260624444,-0.0557102543860846,0.251856369684243,-0.221198512691696,0.824937867279364,0.997645679347435
"11207","MARS2",3.1509972082317,-0.184526479145445,0.842727310052928,-0.21896344991342,0.826678519611529,0.997645679347435
"11208","ORC2",18.522985852934,0.0492691331024968,0.373538285823152,0.131898482625213,0.895064588833074,0.997645679347435
"11209","NDUFB3",167.094842736403,-0.064031269825087,0.289856873062262,-0.220906508611003,0.825165229473522,0.997645679347435
"11210","TRAK2",47.5574679263487,-0.0368319814350752,0.287554856727365,-0.128086800043152,0.898080282039767,0.997645679347435
"11211","TMEM237",60.2322282142512,-0.0819809748944293,0.272927672141188,-0.300376192165739,0.763890221603947,0.997645679347435
"11212","CREB1",91.9785335056331,0.0308286634608382,0.217474997883526,0.141757276748425,0.887271731401408,0.997645679347435
"11213","CCNYL1",19.6265451084417,0.0390824746647482,0.355269894656551,0.110007842636175,0.912403155143426,0.997645679347435
"11214","LINC01963",2.83663399473336,0.207899187439083,0.909265227280893,0.228645263451671,0.819144641166428,0.997645679347435
"11215","SMARCAL1",26.8189650614487,0.0844468626102601,0.3364624751259

97,0.250984489663036,0.801826100913001,0.997645679347435
"11216","RPL37A",5795.9757440772,-0.0668748501240316,0.251801601375213
,-0.26558548380469,0.790558466461463,0.997645679347435
"11217","GPBAR1",2.67805115328494,-0.187633686891714,0.87756016042646,
-0.213812904633833,0.830692961217444,0.997645679347435
"11218","AC012510.1",2.66226649798034,0.104214423523989,0.887896803399
919,0.117372225156046,0.90656509303218,0.997645679347435
"11219","NHEJ1",17.9876971205962,-0.0579168826360587,0.396471962331355
,-0.146080651694745,0.883857720268368,0.997645679347435
"11220","ATG9A",38.8382001862912,-0.050600240027948,0.257941226632127,
-0.196169649530719,0.844477382661439,0.997645679347435
"11221","IRS1",38.736215880182,-0.0457101423886658,0.309628911239761,-
0.147628792820545,0.882635730689881,0.997645679347435
"11222","ARMC9",12.6336630590799,-0.0924656738016172,0.421013448551091
,-0.219626413645065,0.826162118515373,0.997645679347435
"11223","ATG16L1",15.4395733702854,0.135953743558554,0.455246901568884
,0.298637383560495,0.765216738383179,0.997645679347435
"11224","ARL4C",155.192172687573,0.0598180642310535,0.369581420159156,
0.161853548279818,0.871421183493955,0.997645679347435
"11225","AC012063.1",6.85418168781428,0.0932737516341149,0.65198265846
9045,0.143061706354485,0.886241447694411,0.997645679347435
"11226","AC012485.3",4.66614123883419,0.198786909655255,0.832573645498
102,0.238761953047802,0.811290174100849,0.997645679347435
"11227","HDAC4-
AS1",5.33999480137359,-0.108905368643162,0.734904276391294,-0.14818986
926833,0.882192926697851,0.997645679347435
"11228","NDUFA10",186.395502425186,-0.0523095477686076,0.2362925967380
8,-0.221376160280597,0.824799553294456,0.997645679347435
"11229","ATG4B",41.4652644536327,-0.0570240962000465,0.263873036643144
,-0.216104293661366,0.828906452934384,0.997645679347435
"11230","ARL8B",99.1141551849535,0.0601878847996226,0.207051875569122,
0.290689879694084,0.771288512365589,0.997645679347435
"11231","LMCD1-
AS1",3.99910720058762,0.115767098157174,0.761710744606033,0.1519830184
58077,0.879200329357894,0.997645679347435
"11232","SRGAP3",5.13210208143007,0.201725954701333,0.718670475612228,
0.28069325448424,0.77894568298455,0.997645679347435
"11233","THUMPD3",62.2355538511194,0.0676241090290689,0.24248323771205
2,0.278881582360642,0.780335703165291,0.997645679347435
"11234","IRAK2",3.5807366985251,-0.207450379668628,0.733835159687139,-
0.282693431801594,0.777411851628665,0.997645679347435
"11235","MKRN2",29.7569601401974,-0.0665453069319186,0.336996065137656
,-0.197466124433044,0.843462788280935,0.997645679347435
"11236","CAPN7",52.6520158454877,0.0723204023675087,0.255250152093366,
0.283331476100572,0.776922750966066,0.997645679347435
"11237","METTL6",20.2518812208575,0.058613785377253,0.379474533129119,
0.15446039262208,0.877246738496428,0.997645679347435
"11238","BTD",58.0015335587207,0.0399818374014018,0.320316478616725,0.
124819795641054,0.900666215299044,0.997645679347435
"11239","GALNT15",191.574077620627,0.203923980097709,0.717190430920517

,0.2843372851977,0.776151915306024,0.997645679347435
"11240","UBE2E1",106.481086080771,-0.0362748869074259,0.190967755003016,-0.18995294209147,0.849346005834353,0.997645679347435
"11241","THRB",54.4174945360004,0.0860602693118886,0.305726242856786,0.281494543967567,0.778331112259024,0.997645679347435
"11242","TOP2B",81.0343609210192,-0.063478965253041,0.228720392610604,-0.277539595523141,0.781365806624061,0.997645679347435
"11243","ZCWPW2",13.6472717407762,0.118432195125614,0.43620037938848,0.271508693531279,0.785999814273471,0.997645679347435
"11244","CMTM6",159.190907860757,0.0311243770345151,0.196932516790583,0.158045900909358,0.874420629514706,0.997645679347435
"11245","DYNC1LI1",97.8575571026088,0.0481451288257534,0.207190405200267,0.232371420767371,0.816249544376206,0.997645679347435
"11246","CRTAP",350.851415530967,0.0389040405677108,0.153096636775603,0.254114273096235,0.799407276888652,0.997645679347435
"11247","GOLGA4",364.095743047001,-0.0251962170955428,0.200860731156682,-0.125441229604449,0.900174249371744,0.997645679347435
"11248","CTDSPL",101.540504780786,-0.0670097961081463,0.261573508375563,-0.256179597560525,0.797812161876573,0.997645679347435
"11249","ACAA1",104.735546003892,-0.0677372718808961,0.266597131790497,-0.254081022650037,0.799432964119814,0.997645679347435
"11250","MYD88",42.766254172305,0.0390485671035876,0.273935725224636,0.142546457098892,0.886648386536356,0.997645679347435
"11251","EXOG",36.2303090009505,-0.0751637554648916,0.286615365359789,-0.262246078016572,0.793131730967811,0.997645679347435
"11252","AC092053.3",3.39743968044819,0.192610835096065,0.903996488147009,0.213065910787855,0.831275553373976,0.997645679347435
"11253","ENTPD3-AS1",17.3931774681496,0.0426220496384144,0.373687247665159,0.114058079061359,0.909191753354261,0.997645679347435
"11254","ZNF620",2.30577319080348,-0.197880769990526,0.922659552593964,-0.21446780606585,0.830182269904583,0.997645679347435
"11255","KRBOX1",9.46836678312845,-0.134555694834973,0.557570111530916,-0.241325157235426,0.809303114324502,0.997645679347435
"11256","GASK1A",18.6859995504593,0.0840309631265074,0.445848345712057,0.188474318531569,0.850504845583726,0.997645679347435
"11257","ANO10",30.4413396059787,0.0832675694336557,0.304932383558033,0.273068961918926,0.784800205117461,0.997645679347435
"11258","TCAIM",11.8067309589701,-0.0801950856238194,0.450757059608248,-0.177911990315841,0.858792094165015,0.997645679347435
"11259","ZNF445",27.7350677301657,0.112374486015557,0.366193487236587,0.306871885853488,0.758940889096138,0.997645679347435
"11260","ZNF852",2.07924126713731,0.117933005252546,0.961327648071027,0.12267722195360,0.902362701847818,0.997645679347435
"11261","ZKSCAN7",4.11636579754691,0.172753979644435,0.68744929929763,0.251297048118223,0.801584457217414,0.997645679347435
"11262","ZNF197",25.6564940390347,0.0775838976057683,0.305007149826269,0.254367471877167,0.799211678345428,0.997645679347435
"11263","LZTFL1",21.3997435302806,-0.039707963115347,0.340342720553696,-0.11667052273293,0.907121150162642,0.997645679347435

"11264","AC099778.1",4.72942227320487,-0.123625182382462,0.67820329163
058,-0.182283371237014,0.855360347891991,0.997645679347435
"11265","ELP6",49.8651515989706,0.0478069493152616,0.287986912078061,0
.166003895698924,0.86815388438792,0.997645679347435
"11266","SLC25A20",21.4690304738923,-0.0862470611055546,0.331315533845
318,-0.260316985758419,0.794619271405609,0.997645679347435
"11267","USP19",23.5310409155277,0.0357807755324109,0.323932107017999,
0.110457638366865,0.912046443919316,0.997645679347435
"11268","RHOA",790.605680528198,0.0372783954412223,0.222356008900851,0
.167651846358894,0.866857180907313,0.997645679347435
"11269","RBM6",110.378405465589,-0.0263171759432327,0.241749354600684,
-0.108861411384956,0.913312413942316,0.997645679347435
"11270","HYAL2",52.0833355320308,0.0723051578542889,0.365710002925821,
0.197711731360421,0.843270610602132,0.997645679347435
"11271","C3orf18",7.99136103790042,0.126186304357024,0.50766381177101,
0.248562732720336,0.803699038828716,0.997645679347435
"11272","RBM15B",55.3247679629512,-0.0372160524768079,0.23883775166487
4,-0.155821482229609,0.876173737086476,0.997645679347435
"11273","ALAS1",33.2109356282372,0.0757155953379035,0.278429452223452,
0.27193816865731,0.785669562387309,0.997645679347435
"11274","PHF7",7.61884098983624,-0.0672594513207625,0.530531221312808,
-0.12677755543647,0.899116460545583,0.997645679347435
"11275","SPCS1",416.400853421603,-0.0522059837593781,0.250629421789111
,-0.208299502056491,0.834995120420624,0.997645679347435
"11276","STIMATE",26.5464278180684,-0.0639929851347333,0.3128765188170
75,-0.204531121020773,0.83793847491729,0.997645679347435
"11277","RFT1",41.9077121356505,0.0320090572922364,0.29505968078807,0.
108483331937268,0.913612301666502,0.997645679347435
"11278","CHDH",4.92913934050271,-0.0963220091125062,0.722204166316976,
-0.133372270065568,0.893898974742767,0.997645679347435
"11279","AC012467.2",3.05129520665743,0.166354891015296,0.869725346417
646,0.19127290207248,0.848311790143241,0.997645679347435
"11280","APPL1",244.395186604569,-0.0253933891715203,0.199535948241755
,-0.127262227158958,0.89873285553219,0.997645679347435
"11281","PDE12",46.992602969864,0.0425461950770717,0.258078839505723,0
.164857355831873,0.869056258166265,0.997645679347435
"11282","ARF4",407.559137831794,-0.0446760715010755,0.214112721948631,
-0.208656781785223,0.834716180588452,0.997645679347435
"11283","PXK",115.931227639984,0.0251992772215927,0.223091944016449,0.
112954671369643,0.910066492148052,0.997645679347435
"11284","PDHB",105.890420323477,-0.0753187547707306,0.259855883166556,
-0.289848179894602,0.771932386963551,0.997645679347435
"11285","AC135507.1",4.25364050799796,0.170585948097929,0.752470234699
615,0.226701257048429,0.820656049581342,0.997645679347435
"11286","AC012557.2",2.53946337566206,0.109127766201225,0.998982348378
266,0.109238933378935,0.913012980698495,0.997645679347435
"11287","MAGI1",7.26419266941834,-0.18285458456385,0.677519644478878,-
0.269888240221425,0.787246234528684,0.997645679347435
"11288","FOXP1",447.592671793129,0.0368834170575841,0.295481061622936,
0.124824978139043,0.900662112351985,0.997645679347435

"11289","SHQ1",16.9616995676392,0.0712451801401127,0.391720635865309,0.181877526014764,0.855678841962134,0.997645679347435
"11290","EBLN2",6.77131663878712,0.173668253965588,0.719446781931415,0.241391383389555,0.809251790390031,0.997645679347435
"11291","LINC00960",2.82733755246919,0.173592128565203,1.08512591708863,0.159974179799287,0.872901413779446,0.997645679347435
"11292","PROS1",214.638938066758,-0.0459703883833903,0.192190660662521,-0.23919158311294,0.810957028972124,0.997645679347435
"11293","DHFR2",14.2913700160117,-0.0663190487589036,0.417921898204373,-0.158687661603394,0.873914959609376,0.997645679347435
"11294","DCBLD2",63.3321697669938,0.0654360254807905,0.281207889045767,0.232696265040206,0.815997269680503,0.997645679347435
"11295","TOMM70",80.4351032956131,0.0487940442730372,0.218521205259306,0.223292033444243,0.823308227147685,0.997645679347435
"11296","SENP7",49.9691621594177,0.0289147467128266,0.244372252602943,0.118322544416724,0.905812095011508,0.997645679347435
"11297","ZBTB11",45.056656103923,0.0626132638223526,0.279161646179384,0.224290351770309,0.822531383155142,0.997645679347435
"11298","DUBR",17.56607634036,-0.117281036302699,0.411362609046773,-0.285103783677538,0.775564631347303,0.997645679347435
"11299","TRAT1",7.74565695044789,0.123698125288774,0.6897750221879,0.179331117117601,0.857677714951681,0.997645679347435
"11300","NEPRO",46.2315263853163,-0.0477719191538196,0.289219422982951,-0.16517534908655,0.868805967322727,0.997645679347435
"11301","SPICE1",17.4800741412866,-0.0810925894700466,0.373937211280274,-0.216861513173306,0.828316271864223,0.997645679347435
"11302","NAA50",137.873847443224,0.0338696195776459,0.229520361731953,0.1475669492766,0.882684540132595,0.997645679347435
"11303","TMEM39A",42.2840636373055,-0.054540948691609,0.27158841203628,-0.200822075885635,0.840837700507379,0.997645679347435
"11304","TIMMDC1",127.042552156915,0.0624213369346229,0.281284660655172,0.221915182965293,0.824379910906797,0.997645679347435
"11305","ADPRH",6.43837175210652,0.109203276642955,0.638202488039311,0.171110703404572,0.864136719894617,0.997645679347435
"11306","GOLGB1",270.14532139322,-0.0716622990643545,0.252719231084645,-0.283564882485544,0.77674385252331,0.997645679347435
"11307","PDIA5",30.9569572783054,0.0855510675657318,0.339420288232811,0.252050541855211,0.801001997818373,0.997645679347435
"11308","HACD2",31.2057118729549,0.0721867821227148,0.305550871602443,0.236251272150315,0.813237695904212,0.997645679347435
"11309","UMPS",41.3297337336294,-0.05929857882737,0.336465385922453,-0.176239759893272,0.860105585744571,0.997645679347435
"11310","SNX4",65.6707685864248,0.0737294438415088,0.240808617807667,0.306174440569217,0.759471831989143,0.997645679347435
"11311","OSBPL11",45.7875173845722,0.0589001478956901,0.270497008925942,0.217747871333454,0.827625562547783,0.997645679347435
"11312","AC092902.4",1.90262060152884,-0.177654330633093,1.07823434994917,-0.164764117041409,0.869129648424071,0.997645679347435
"11313","LINC02614",8.49337612776317,0.0921422678430881,0.512088060221364,0.179934419488822,0.857204053332672,0.997645679347435

"11314","MCM2",3.83586553878247,0.138137921971423,0.697211171012177,0.19812924364204,0.842943945222986,0.997645679347435
"11315","PODXL2",16.2586781666504,0.0963034580987325,0.402783880768765,0.239094617974595,0.811032215002778,0.997645679347435
"11316","RAB43",12.7162172035715,-0.0448055718622016,0.414742115892963,-0.108032365523652,0.913970018470791,0.997645679347435
"11317","COPG1",78.9850520189558,0.0388946112988884,0.250311397220524,0.155384899492301,0.876517888035875,0.997645679347435
"11318","NUDT16",55.347128176485,0.0850451317038281,0.281251572151512,0.302381000231401,0.762361636873861,0.997645679347435
"11319","ANAPC13",90.716500873084,-0.0514330083280044,0.290262246798128,-0.177194963848589,0.859355251339199,0.997645679347435
"11320","SLC35G2",21.9918852215097,-0.101946483242945,0.350850858201082,-0.290569285666438,0.771380753449852,0.997645679347435
"11321","DZIP1L",12.5925022859672,-0.100776740778424,0.526417806880455,-0.191438700327456,0.848181902233897,0.997645679347435
"11322","ARMC8",51.2025407165211,0.040756067184133,0.24097892643313,0.169127100810794,0.865696668617807,0.997645679347435
"11323","PXYLP1",21.9524761012738,-0.0512360332806096,0.44387070645587,-0.115430084786872,0.908104234120333,0.997645679347435
"11324","RNF7",292.835076699821,-0.0816199628316869,0.272751759304622,-0.299246329482077,0.764752102068062,0.997645679347435
"11325","TFDP2",132.4868828263,0.0566845607020386,0.247666225825431,0.228874811303472,0.818966218622357,0.997645679347435
"11326","AC026304.1",3.53290721744863,0.217379777851634,0.732124825392993,0.296916277541808,0.766530428595181,0.997645679347435
"11327","SELENOT",129.318686949537,-0.0724241528880262,0.264099335996618,-0.274230726914639,0.783907316225175,0.997645679347435
"11328","MBNL1",357.522330643662,0.0518453899645751,0.235404029957927,0.220240027215512,0.825684224641037,0.997645679347435
"11329","AC084036.1",4.25280766671719,0.246641779709249,0.898330401387349,0.27455575290377,0.783657564340458,0.997645679347435
"11330","SSR3",351.132156228867,0.0453253194069726,0.239154392928329,0.189523256721259,0.849682729163176,0.997645679347435
"11331","AC092944.1",22.8447540749214,-0.042230953976793,0.350951396110441,-0.120332770990042,0.904219545218464,0.997645679347435
"11332","SEC62",669.188798111636,-0.0256612599616795,0.175611064766674,-0.146125530277801,0.883822292487609,0.997645679347435
"11333","KCNMB2",2.15395691148524,0.176402688267615,1.02177829322077,0.172642822261934,0.862932189962413,0.997645679347435
"11334","KCNMB3",1.95413490784991,0.10918074458852,0.998511234777127,0.109343531435467,0.912930020468886,0.997645679347435
"11335","CCDC39",6.73858427457465,-0.0716616775532724,0.600568952316292,-0.119322980778286,0.905019477537342,0.997645679347435
"11336","KLHL6",2.44981481315941,0.148119784491256,0.890723247192088,0.166291589400173,0.867927484582999,0.997645679347435
"11337","ABCF3",33.4729114833916,-0.0797120536386203,0.294019681037486,-0.271111285330785,0.786305442082518,0.997645679347435
"11338","ETV5",13.7057669273548,0.0851957765096195,0.55909284626644,0.152382161708109,0.878885525665565,0.997645679347435

"11339","TBCCD1",15.9422701708363,-0.0792639610714533,0.460024119847574,-0.172303924189273,0.863198599436661,0.997645679347435
"11340","RTP4",19.7302096414827,0.102351177754566,0.426299811067383,0.240092008247191,0.810258929541354,0.997645679347435
"11341","LPP-AS2",9.81516178936664,0.100601467652204,0.456301674294528,0.220471397146943,0.825504046111584,0.997645679347435
"11342","TMEM44-AS1",20.2514386782339,0.0388145480466829,0.350911696862494,0.11061058492414,0.911925153441351,0.997645679347435
"11343","AC046143.1",2.13059975125979,0.236321337577151,1.05373014440029,0.22427121292022,0.822546274466667,0.997645679347435
"11344","LSG1",55.6614219142075,-0.0578210685648594,0.293050096757512,-0.19730779550878,0.843586679325762,0.997645679347435
"11345","AC016949.1",10.7752540129922,-0.060180910427788,0.494669119528672,-0.12165891916829,0.903169149243901,0.997645679347435
"11346","PIGZ",14.5119042381434,-0.107573098058621,0.393563404040765,-0.273331048959722,0.784598750085791,0.997645679347435
"11347","MELTF-AS1",6.88468074679973,0.116496366137624,0.549477686885377,0.212012915024747,0.832096958622072,0.997645679347435
"11348","PCGF3",41.1825665454258,0.0586754143351145,0.256330404177045,0.228905402476516,0.818942441477682,0.997645679347435
"11349","CTBP1",74.9805837432671,0.0587169667067832,0.267329881420882,0.219642362442602,0.826149696473661,0.997645679347435
"11350","CRIPAK",7.37107158577372,0.0787589850786793,0.50875813367227,0.154806340903464,0.876973992160401,0.997645679347435
"11351","TACC3",5.23028848621192,-0.0941778617814623,0.63305023432732,-0.148768386258534,0.881736397104916,0.997645679347435
"11352","FAM193A",39.9629089566008,0.0425068342735409,0.33994246320586,0.125041261020104,0.900490885090503,0.997645679347435
"11353","HTT",48.1766327926195,-0.0723398252400008,0.292887974518846,-0.246988034789889,0.804917484118793,0.997645679347435
"11354","EVC2",25.4709779682572,0.0780839101206051,0.404702734285822,0.192941395017752,0.847004865536992,0.997645679347435
"11355","EVC",11.1048168871964,0.110826057185559,0.562780492975615,0.19692590373839,0.843885522328247,0.997645679347435
"11356","BLOC1S4",75.4139955395816,-0.0502002040627791,0.268155528939914,-0.187205552916373,0.851499471210819,0.997645679347435
"11357","KIAA0232",58.3373940610821,-0.0419216833131171,0.299717723080171,-0.139870551805519,0.888762268783837,0.997645679347435
"11358","CCDC96",4.886552547457,-0.122349388587924,0.703464662160461,-0.173924000975639,0.861925190041782,0.997645679347435
"11359","HTRA3",476.756100510156,-0.0683648244187471,0.387225891335968,-0.176550240953366,0.859861681929559,0.997645679347435
"11360","AC104825.1",8.27434267736892,0.0792299510932655,0.609248635607924,0.130045348422008,0.896530548447438,0.997645679347435
"11361","FBXL5",83.6968011555419,0.0367870137467222,0.227658812424495,0.161588358275931,0.87163002557557,0.997645679347435
"11362","BST1",77.8350188674913,-0.055462385354484,0.31891940362198,-0

.173907215191661,0.86193838213517,0.997645679347435
"11363","CD38",27.2802229459895,-0.124179938641708,0.418161609006484,-0.296966378469676,0.766492177924305,0.997645679347435
"11364","TAPT1",32.1043110311995,-0.0493806838911815,0.283951563766288,-0.173905307074925,0.86193988174312,0.997645679347435
"11365","LAP3",345.520131401321,0.0667956646663011,0.285030778867468,0.23434544483829,0.814716808106505,0.997645679347435
"11366","SLIT2",89.5433389221358,0.0924778492618693,0.316350829297831,0.292326874777386,0.770036712669046,0.997645679347435
"11367","CCDC149",27.9179871884235,-0.103992799248284,0.360666646949186,-0.288334948983889,0.77309035715169,0.997645679347435
"11368","NSUN7",2.752354688838,-0.236404665230338,1.00613378073072,-0.234963450942522,0.814237101053107,0.997645679347435
"11369","TMEM33",60.6046514865968,0.0518430703565566,0.247774323718196,0.209235039283247,0.834264760346923,0.997645679347435
"11370","NFXL1",18.1491052775963,0.0862886666580215,0.390482940543946,0.220979350692813,0.825108511304931,0.997645679347435
"11371","OCIAD1",309.354682504765,-0.064942848696134,0.248607171271195,-0.261226771392248,0.793917634084852,0.997645679347435
"11372","SRD5A3",35.5504433831582,-0.0427056784728132,0.313503901724932,-0.136220564521979,0.891646918561583,0.997645679347435
"11373","SRD5A3-AS1",2.56392086257294,0.190797196153384,0.890320756543718,0.214301637641327,0.830311841050724,0.997645679347435
"11374","REST",107.847081289932,-0.0504981841804616,0.201672948913972,-0.250396418817691,0.802280798797295,0.997645679347435
"11375","UBA6-AS1",10.0671458305786,0.12347511546306,0.513494284831924,0.240460544762394,0.80997324674385,0.997645679347435
"11376","NUP54",82.1629880205006,0.071505889843678,0.237972475746082,0.300479665219667,0.763811305019064,0.997645679347435
"11377","BMP2K",36.489129612918,0.031602811218923,0.264492542922323,0.119484696505051,0.904891363596768,0.997645679347435
"11378","SCD5",25.267273870558,-0.0625809255829353,0.422493033599879,-0.148122976252911,0.882245717028524,0.997645679347435
"11379","AC093827.4",15.8127464520056,0.0607011899211859,0.475705385613022,0.127602486238332,0.898463563485681,0.997645679347435
"11380","HERC3",25.605845553384,0.0986762360966868,0.332111123960407,0.297118129980044,0.766376323321714,0.997645679347435
"11381","GSTCD",9.52076779299078,0.0790330521554038,0.467296166821859,0.169128398148262,0.865695648191373,0.997645679347435
"11382","LARP1B",58.2835040997721,-0.0541802610325134,0.274176962655272,-0.197610552352042,0.843349778033307,0.997645679347435
"11383","AC097376.3",19.024457417445,0.0814926371631193,0.448533408631276,0.181686883507292,0.855828460091908,0.997645679347435
"11384","MAML3",11.7556242010475,-0.0994482567715698,0.545024918054658,-0.18246552309301З,0.855217408724251,0.997645679347435
"11385","SCOC",105.730012837334,0.0469679574470328,0.300418086135727,0.156341976780363,0.875763470441485,0.997645679347435
"11386","ELMOD2",26.4217363723462,-0.0659338742191089,0.29450726572197

3,-0.223878599590657,0.822851768148599,0.997645679347435
"11387","SMAD1",25.7554250129369,0.0790402042413316,0.329155303389061,0.24013042909385,0.810229145219504,0.997645679347435
"11388","MMAA",13.3732473993049,-0.0502289364710279,0.413832588972345,-0.121375014461185,0.903394006121654,0.997645679347435
"11389","PRMT9",14.8295144346166,0.0906559927093203,0.443939583224398,0.204207951115493,0.838190998185806,0.997645679347435
"11390","GATB",13.5371840375463,0.0557553976715638,0.45369509548343,0.122891779581955,0.902192795227383,0.997645679347435
"11391","LINC02273",3.36087813553462,-0.127233881789041,0.80233241452877,-0.158580009339107,0.873999779710594,0.997645679347435
"11392","GASK1B",81.098297892931,0.128196464970426,0.467861378478794,0.274005230752845,0.784080601885791,0.997645679347435
"11393","PPID",59.5662437982076,-0.0308347864838571,0.270611820098376,-0.113944714139418,0.909281619608473,0.997645679347435
"11394","RAPGEF2",26.2018908216896,-0.108522730110747,0.356381602967655,-0.304512716725718,0.760737305939087,0.997645679347435
"11395","PALLD",247.716711914025,0.0719140003103288,0.236445950223421,0.304145620774541,0.761016951733391,0.997645679347435
"11396","NEK1",50.8495339799564,-0.0402874658401829,0.248594060754735,-0.162061256483238,0.87125761566307,0.997645679347435
"11397","GALNT7",18.3670887160184,0.098226441736458,0.398955225500665,0.246209187041452,0.80552030419315,0.997645679347435
"11398","AC093849.2",14.5702761645068,-0.0958355728507326,0.458143074540017,-0.209182629131638,0.834305672402813,0.997645679347435
"11399","DCTD",107.230015201411,-0.0458324267341744,0.201589772811459,-0.227354920316519,0.820147770793399,0.997645679347435
"11400","WWC2-AS2",3.2672897876016,0.215473776837834,0.777301384598089,0.277207504202822,0.781620777822716,0.997645679347435
"11401","IRF2",100.36114600371,0.0281958482454513,0.258539618834417,0.109058133420973,0.913156381448783,0.997645679347435
"11402","CASP3",50.0393532361841,0.0620810533423593,0.250093762673491,0.248231114117824,0.803955593977937,0.997645679347435
"11403","SLC25A4",65.0450857735879,0.0394449197431668,0.31483317789686,0.125288319378109,0.900295299104576,0.997645679347435
"11404","CYP4V2",48.5765225641441,0.0362429630870436,0.277402206818969,0.13065131493599,0.896051147156117,0.997645679347435
"11405","TRIML2",1.88239467958446,0.796946165418026,3.15858674197661,0.252310995555976,0.800800689795357,0.997645679347435
"11406","CCDC127",60.6608846476902,0.0654819255762521,0.259402019190942,0.252434139797701,0.800705514628487,0.997645679347435
"11407","SDHA",124.581332109477,0.0495802887145853,0.20982599186804,0.2362924072141,0.813205778311692,0.997645679347435
"11408","EXOC3",54.9005808283096,0.0323117891897038,0.23038013322666,0.140254234326333,0.888459122538702,0.997645679347435
"11409","CEP72",2.24268639743901,0.180783105030623,0.953871069837214,0.18952572391306,0.849680795667757,0.997645679347435
"11410","ICE1",64.7421149319221,0.0382929941943554,0.253668073209997,0.150957090144549,0.880009565015246,0.997645679347435

"11411","BASP1",789.445634377809,-0.0344690275422182,0.205729645544896
,-0.167545262866339,0.866941036203994,0.997645679347435
"11412","GOLPH3",106.803296162621,0.0207548882958341,0.186731137882717
,0.111148512943085,0.911498579271348,0.997645679347435
"11413","SUB1",629.578283999348,0.0322176053060451,0.26225218063081 9,0
.122849713693702,0.902226106544549,0.997645679347435
"11414","AMACR",17.7498238480814,0.0876122407974455,0.368159910914642,
0.237973332239747,0.811901777886356,0.997645679347435
"11415","NIPBL-
DT",25.8613533135226,-0.0460844964057136,0.301606708583355,-0.15279665
5691686,0.878558635138209,0.997645679347435
"11416","NUP155",21.9270836723691,-0.0386066785265774,0.34793878392249
5,-0.110958249871843,0.911649454005759,0.997645679347435
"11417","LINC00603",5.25275710902321,0.150620864645629,0.8845031776320
35,0.170288664252022,0.864783125677689,0.997645679347435
"11418","PRKAA1",104.403744369325,-0.0600483862057809,0.20584105795110
5,-0.291722102497377,0.7704991085794,0.997645679347435
"11419","OXCT1",28.766291664363,-0.0558694675143396,0.382561153302088,
-0.146040618688282,0.883889323079236,0.997645679347435
"11420","FBXO4",25.5305130795511,-0.0925416941099516,0.364058556334972
,-0.254194531345676,0.799345275240331,0.997645679347435
"11421","AC008875.3",46.2846574680891,0.0413097170886328,0.27121003267
6613,0.152316330929726,0.878937444986139,0.997645679347435
"11422","AC025171.1",8.50009560335352,0.0974306652068277,0.49702838654
6788,0.19602635954809,0.844589534298771,0.997645679347435
"11423","ZNF131",83.1882051360231,0.051448598753618,0.241860718511917,
0.212719945058309,0.831545408883492,0.997645679347435
"11424","NNT",60.7972279618112,0.0624619347351683,0.257096248959705,0.
242951559923218,0.808042922832512,0.997645679347435
"11425","AC093297.2",10.6838016638581,-0.083795044313357,0.49341075232
344,-0.169828168354199,0.865145273563905,0.997645679347435
"11426","ITGA2",3.15914530840429,-0.10644954008134,0.954866399179376,-
0.111481082770138,0.911234865848266,0.997645679347435
"11427","GZMA",72.7914823052453,0.100870655321566,0.337679710185347,0.
298716956568695,0.765156017992721,0.997645679347435
"11428","AC016596.1",4.0092614714114,-0.197623520413322,0.802774628257
254,-0.246175593319813,0.805546307988292,0.997645679347435
"11429","GPBP1",203.943833664204,0.0497393148681377,0.168589774506415,
0.29503162344075,0.767969722322219,0.997645679347435
"11430","RNF180",4.17611742649738,0.263819988451555,0.877804446384414,
0.300545286069354,0.763761258735466,0.997645679347435
"11431","CWC27",80.2024226858028,-0.0356019873789502,0.221118521216365
,-0.161008617383586,0.872086613505471,0.997645679347435
"11432","NLN",20.1832237284835,0.0697219108106849,0.421274090946299,0.
165502489493408,0.868548490522095,0.997645679347435
"11433","CCNB1",6.88442357569583,-0.157373113354323,0.564696119179506,
-0.278686373093874,0.780485521062627,0.997645679347435
"11434","CDK7",15.1859186821148,0.101298965863623,0.40906201759864,0.2
47637183374514,0.804415138431897,0.997645679347435
"11435","MCCC2",26.7197426781511,0.0550002741470781,0.310215840915892,

0.177296794337431,0.85927526865587,0.997645679347435
"11436","UTP15",18.2850074125146,-0.0933738144984373,0.401108330912792
,-0.232789516702256,0.815924853779504,0.997645679347435
"11437","WDR41",55.413848421931,0.0599576128001892,0.266954803781197,0
.224598366281253,0.822291735940298,0.997645679347435
"11438","TBCA",395.636453490602,0.0513881827177081,0.227267819650477,0
.226112886535102,0.821113622120073,0.997645679347435
"11439","AP3B1",71.3516652672138,-0.0622517549402575,0.227083270166791
,-0.274136244799249,0.783979921016547,0.997645679347435
"11440","TENT2",42.5363415346431,-0.0577979845548231,0.269153129431436
,-0.214740154338598,0.829969914167814,0.997645679347435
"11441","SERINC5",30.6425651819893,-0.0681417743115529,0.3310517188515
08,-0.205834225987262,0.836920405131865,0.997645679347435
"11442","MSH3",42.6191490194032,-0.0322386319081445,0.265206548091384,
-0.12156046726657,0.90324712377973,0.997645679347435
"11443","MBLAC2",12.5074823499711,-0.0892344856758694,0.41395792867606
,-0.215564141895442,0.829327509244955,0.997645679347435
"11444","LYSMD3",42.4349721487998,0.0675968124443629,0.268429172948257
,0.251823643838417,0.801177380992591,0.997645679347435
"11445","ARRDC3",133.253974873601,-0.0378543016978456,0.32711951528406
1,-0.115720095956287,0.907874378981208,0.997645679347435
"11446","SLF1",18.760832979831,0.0923835748242385,0.349207412666924,0.
264552158611691,0.791354479121299,0.997645679347435
"11447","GLRX",227.911842156034,0.0732741926179415,0.354563977971026,0
.206660002624207,0.83627539630246,0.997645679347435
"11448","ERAP1",54.4517540087205,0.0604264731147902,0.308915913201082,
0.195608159154485,0.844916873573034,0.997645679347435
"11449","ERAP2",99.4303478048949,-0.262058368771438,0.85453880928231,-
0.306666433314514,0.759097281778725,0.997645679347435
"11450","GIN1",11.3029820390153,-0.0703061736772002,0.485359685946115,
-0.144853756323317,0.88482633835374,0.997645679347435
"11451","LINC01023",4.61499868722232,-0.127513107954355,0.660091034469
351,-0.193175033890384,0.846821890533237,0.997645679347435
"11452","CAMK4",6.48624478578439,-0.165430754285846,0.573783353182423,
-0.288315709001147,0.773105083428673,0.997645679347435
"11453","STARD4",3.2293942605094,-0.119459972840942,0.84019880772224,-
0.14218060266569,0.886937352955714,0.997645679347435
"11454","STARD4-
AS1",5.15798408771453,0.118817878008051,0.758831450784723,0.1565800651
58316,0.875575814430094,0.997645679347435
"11455","FEM1C",86.0136324773422,-0.0504584407613343,0.358043923238264
,-0.140928074703718,0.887926763074104,0.997645679347435
"11456","AC010226.1",41.2431810455197,0.0491917746290158,0.29573277532
3848,0.166338595967753,0.867890493916652,0.997645679347435
"11457","TMED7",161.429874277476,-0.0421842998505991,0.17916817654306,
-0.23544527083168,0.813863152412727,0.997645679347435
"11458","AC034236.2",5.08015956147513,-0.0872559415080569,0.6919492284
04097,-0.126101653020559,0.899651458996991,0.997645679347435
"11459","COMMD10",62.2878448921009,0.0547854207260934,0.32154869843856
9,0.170379855344244,0.86471141361396,0.997645679347435

"11460","SRFBP1",85.0833261199199,-0.0760553258392862,0.29002228576935
,-0.262239591821477,0.793136731273668,0.997645679347435
"11461","SNCAIP",12.437043370705,0.115186648667857,0.439349319558925,0
.262175548111685,0.793186103994316,0.997645679347435
"11462","SNX24",28.1530368513951,-0.0658837229552581,0.381598785578429
,-0.172651815061181,0.862925120889988,0.997645679347435
"11463","ALDH7A1",62.8058020485848,0.0517506712090607,0.25029942312080
3,0.206755055859973,0.836201157886106,0.997645679347435
"11464","C5orf63",7.50935921393198,0.150758065320256,0.586860137228166
,0.256889258200958,0.797264263099805,0.997645679347435
"11465","RAPGEF6",34.8404015969328,0.0794496861350903,0.32082302327950
4,0.247643343432597,0.804410371837896,0.997645679347435
"11466","RAD50",136.254203451374,0.0483797101781422,0.188380541679245,
0.256819041642413,0.797318469868262,0.997645679347435
"11467","SHROOM1",8.4487328905329,-0.0708074231149888,0.54029883724674
3,-0.13105233295672,0.895733908640348,0.997645679347435
"11468","LEAP2",5.85739792831614,0.131527589552239,0.725292996981695,0
.181344077634267,0.856097510573758,0.997645679347435
"11469","NME5",3.20603746051701,0.12893102103464,0.77199422046246,0.16
701034491865,0.867361909180758,0.997645679347435
"11470","BRD8",45.8389521506923,0.0927063465779738,0.32097957177347,0.
2888231985162,0.772716678462176,0.997645679347435
"11471","KDM3B",42.3378092153273,-0.0699176633073449,0.268122781111717
,-0.260767335835639,0.794271935353772,0.997645679347435
"11472","SNHG4",5.5246541981158,-0.136574355343475,0.657969397467905,-
0.20756946427761,0.83556514902128,0.997645679347435
"11473","MATR3.1",15.2738210044846,0.11183108368867,0.418874706903403,
0.266979795737487,0.789484717308534,0.997645679347435
"11474","SLC35A4",40.0007777625367,0.0688115571532256,0.27539660776707
1,0.249863488556206,0.802692919681059,0.997645679347435
"11475","ZMAT2",141.394185873304,0.0417826882414078,0.185429809416157,
0.225328863643685,0.821723447030868,0.997645679347435
"11476","PCDHB11",14.9173255733435,-0.10494801257874,0.520183908746556
,-0.201751747437987,0.840110805711846,0.997645679347435
"11477","PCDHB14",21.5791175403654,-0.0794090933513822,0.3870820269375
93,-0.205147973362723,0.837456516089318,0.997645679347435
"11478","PCDHGA2",2.22115654267019,-0.130475029354188,0.96016897750751
1,-0.135887570220074,0.891910161852906,0.997645679347435
"11479","PCDHGA6",9.0916175197366,0.0996314115726069,0.57182917995978,
0.174232821731159,0.861682492004372,0.997645679347435
"11480","PCDHGB6",10.1636296402301,0.0840404477002308,0.54351609506317
7,0.154623659655308,0.877118016613897,0.997645679347435
"11481","DELE1",31.8291848051504,-0.0761547736724346,0.287724596349341
,-0.264679400505514,0.791256447720666,0.997645679347435
"11482","GNPDA1",53.0403100431176,0.04851918221799,0.27121680577168,0.
178894453387358,0.858020578087848,0.997645679347435
"11483","YIPF5",134.212658256948,0.0580991976489316,0.191050203073111,
0.304104349089323,0.761048393568076,0.997645679347435
"11484","PRELID2",17.4429099670679,-0.0640965027566338,0.4629030830353
1,-0.138466355281856,0.88987185804015,0.997645679347435

"11485","LARS",140.872781256956,0.0453539763019736,0.26722293661623,0.169723366101273,0.865227697180943,0.997645679347435
"11486","TCERG1",95.1777151346534,0.0459763453443811,0.233013580715208,0.197311869991706,0.843583491028783,0.997645679347435
"11487","RBM22",73.0441406911179,0.0396298035238595,0.226153984729564,0.175233717731081,0.8608959912931,0.997645679347435
"11488","ATOX1",236.78710120136,-0.0500290424975721,0.261424553662203,-0.191370863205973,0.848235045973555,0.997645679347435
"11489","MED7",28.4268427983665,-0.0631435338818368,0.353666652425374,-0.178539688287859,0.858299155122163,0.997645679347435
"11490","ADRA1B",6.59523398788924,0.0734455570340003,0.604329517677518,0.121532301311802,0.903269431567763,0.997645679347435
"11491","ZBED8",6.37188461620046,0.148884087265697,0.591148060439338,0.251855833130953,0.801152499335034,0.997645679347435
"11492","SLU7",112.43063118343,0.038436608734519,0.194822501735833,0.197290397115609,0.843600293657417,0.997645679347435
"11493","PTTG1",9.05865104878943,-0.0757017331507051,0.56586374141655,-0.133780851484136,0.893575869371379,0.997645679347435
"11494","AC011365.1",5.30138874328269,0.078168719315477,0.710020904260065,0.11009354632585,0.912335186331798,0.997645679347435
"11495","FBXW11",72.3952467516052,0.0710983089636098,0.267956520826893,0.265335244479985,0.790751216035252,0.997645679347435
"11496","SH3PXD2B",98.1433620968451,-0.0722772561334886,0.363174499832915,-0.19901522867586,0.842250832110007,0.997645679347435
"11497","BNIP1",23.5154817265805,0.0399199656907362,0.33498596479543,0.119169069411952,0.905141411027395,0.997645679347435
"11498","SFXN1",41.0201072198195,-0.0303213520470216,0.275196221181312,-0.1101808444784,0.912265953662792,0.997645679347435
"11499","NSD1",82.2648405016104,0.0477884656552874,0.257457681295648,0.185616779483111,0.85274529411315,0.997645679347435
"11500","RGS14",10.6659114117794,-0.10078369507944,0.503459848609296,-0.200182190015419,0.841338095569653,0.997645679347435
"11501","PRR7",3.54072569876904,-0.159413734555888,0.734895757152756,-0.216920199911771,0.828270535138286,0.997645679347435
"11502","DBN1",242.959171460498,-0.0643032691360795,0.216727252543949,-0.296701353342907,0.766694523723741,0.997645679347435
"11503","AC139795.3",2.08264967940122,-0.238593870965744,1.14828104269873,-0.20778351474391,0.835398005024939,0.997645679347435
"11504","PHYKPL",121.334439621613,0.0494506279485754,0.203640585413773,0.242832870707466,0.808134870425856,0.997645679347435
"11505","RUFY1",69.6379014219362,0.0414757722899339,0.230080230730392,0.180266562486784,0.856943304884511,0.997645679347435
"11506","MAML1",35.9604220922787,0.0352511291816131,0.294452923818391,0.119717368482831,0.904707041254948,0.997645679347435
"11507","MGAT1",223.485343814978,0.0381692508461132,0.208264277312654,0.183273153411769,0.854583698081263,0.997645679347435
"11508","LINC00847",11.9350686364461,0.0836444564490684,0.452614534791743,0.18480285103428,0.853383671576858,0.997645679347435
"11509","AC008443.1",10.6492999899368,-0.0976491701661325,0.529975316089807,-0.184252298553439,0.853815533410213,0.997645679347435

"11510","FOXC1",33.515419226694,0.0775677759263485,0.360303358005942,0
.215284632248888,0.829545410416091,0.997645679347435
"11511","WRNIP1",30.5750825193778,-0.0607843343365062,0.28593653427056
6,-0.212579810731669,0.831654720219005,0.997645679347435
"11512","SERPINB9",29.9951753745371,0.107646457714147,0.47556519562550
3,0.226354785220481,0.820925491453977,0.997645679347435
"11513","RPP40",17.0252809491731,0.117355238996631,0.386637930037732,0
.303527486258727,0.761487903839913,0.997645679347435
"11514","SNRNP48",32.3195462606382,0.0828195708774595,0.37548890824291
8,0.220564626702316,0.825431446516869,0.997645679347435
"11515","HULC",3.65836779007206,-0.165376937066306,0.70522482644538,-0
.234502432224165,0.814594945185795,0.997645679347435
"11516","ELOVL2",13.7608630071623,-0.127452203425194,0.531420529594263
,-0.239833044317094,0.81045968879029,0.997645679347435
"11517","SMIM13",30.5857929025763,0.0345101167475949,0.308019535995924
,0.112038727141163,0.910792700047024,0.997645679347435
"11518","NEDD9",29.1099738906951,0.0445012257396228,0.357667640351283,
0.124420609300623,0.900982256273089,0.997645679347435
"11519","TMEM170B",6.42277046996994,-0.108364495869988,0.6183346267978
94,-0.17525218736522,0.860881479210477,0.997645679347435
"11520","CD83",42.9071866494012,0.0632725516423638,0.44323463521504,0.
142751821756137,0.88648618794259,0.997645679347435
"11521","GMPR",13.2084300489652,0.0719031993855576,0.58947136882716,0.
121979120934439,0.90291555385936,0.997645679347435
"11522","NHLRC1",2.19306227204855,-0.136442119230474,0.928284723583927
,-0.146983049234827,0.883145399749319,0.997645679347435
"11523","DEK",524.46484601633,-0.065101396839509,0.216463696165273,-0.
30074972382345,0.763605348920193,0.997645679347435
"11524","SOX4",377.556644308798,0.0677671276642957,0.300701320197925,0
.225363585433182,0.821696437612973,0.997645679347435
"11525","HIST1H4C",278.287428422285,-0.0999143769244352,0.356702661609
114,-0.280105498719055,0.779396566139125,0.997645679347435
"11526","HIST1H1E",10.6525374029257,0.0994524620408106,0.4767195109337
13,0.208618400883196,0.834746144802898,0.997645679347435
"11527","HCG11",62.9049826210745,-0.0649514439339271,0.268089857984387
,-0.242274901491088,0.808567160242164,0.997645679347435
"11528","ZSCAN16",2.38718963240727,0.231698280999877,0.973574263463919
,0.237987269893011,0.811890967737722,0.997645679347435
"11529","ZSCAN9",8.7287201165353,0.0749597690805736,0.573042326425344,
0.130810178627075,0.895925470809071,0.997645679347435
"11530","NKAPL",2.4512734632969,0.12487004495708,0.926386784606842,0.1
34792558607228,0.892775889513417,0.997645679347435
"11531","GABBR1",32.8929312270543,-0.0666258019522944,0.37926373907617
8,-0.175671426207482,0.860552084748806,0.997645679347435
"11532","HLA-
F",100.73291873163,0.0939623908937325,0.331219941949937,0.283685789993
691,0.776651185484687,0.997645679347435
"11533","HLA-
E",2016.15256193994,-0.0505126559682827,0.1915095122048,-0.26376055887
1167,0.791964428042593,0.997645679347435

"11534","PRR3",15.4030247700532,-0.0577570546035473,0.408985847732713,
-0.141220178946861,0.887696005331969,0.997645679347435
"11535","ABCF1",162.487996350645,-0.07080172280658,0.258754389548653,-
0.273625204697318,0.784372662415002,0.997645679347435
"11536","PPP1R10",123.320435911974,0.0349899569493371,0.2299726609836,
0.152148332761312,0.879069943891216,0.997645679347435
"11537","MRPS18B",113.405291974301,-0.0516915622042635,0.2415644986108
73,-0.213986585369614,0.830557518221498,0.997645679347435
"11538","NRM",16.5439370309673,0.0938364567930609,0.375798978669741,0.
24969854129254,0.8028204863571,0.997645679347435
"11539","AL662844.4",14.9303495669067,0.058800465235012,0.510965443868
459,0.115077185630873,0.908383942981614,0.997645679347435
"11540","LST1",17.8832588214824,-0.0842820308067476,0.370290128711207,
-0.227610795621505,0.819948826539295,0.997645679347435
"11541","VARS",48.5049359103549,0.031173838655947,0.265672943670816,0.
117339154771339,0.906591298304612,0.997645679347435
"11542","HSPA1A",320.645141671488,0.0626165146901242,0.296157180196084
,0.211430007027573,0.832551743726713,0.997645679347435
"11543","STK19",59.8847425003503,0.0564185227620023,0.282419166385648,
0.199768746165627,0.84166144554748,0.997645679347435
"11544","ATF6B",51.2360416794822,-0.0690625533956779,0.266638977042095
,-0.259011469972654,0.795626390654155,0.997645679347435
"11545","PBX2",30.7077690749293,0.0410518177802607,0.31553430243233,0.
130102551335333,0.896485291605564,0.997645679347435
"11546","HLA-
DRB1",199.660310639326,0.0536794589418978,0.413857087406149,0.12970530
3051191,0.896799586786755,0.997645679347435
"11547","HLA-
DOB",3.13048465007131,-0.179289111714885,0.930520827965172,-0.19267608
6688944,0.847212652573827,0.997645679347435
"11548","PSMB8",343.636090962969,0.0656238255540882,0.294360802624711,
0.222936698666887,0.823584773656443,0.997645679347435
"11549","RGL2",36.5799242433863,0.0460923424077713,0.29997989591053,0.
1536514381001,0.877884576736339,0.997645679347435
"11550","ILRUN",43.8131517661644,-0.0300516249356268,0.275103151610804
,-0.109237661435961,0.913013989524998,0.997645679347435
"11551","TAF11",80.6486160066806,0.0451582161754195,0.226345376502706,
0.19951022138453,0.841863648566487,0.997645679347435
"11552","PPARD",18.2990525203326,0.07160112662298,0.348833323456855,0
.205258849451216,0.837369892888742,0.997645679347435
"11553","SRPK1",43.7914417749332,-0.0320928764728098,0.252207724290209
,-0.127247793711034,0.898744278887992,0.997645679347435
"11554","BRPF3",25.0315269316924,0.0912495707763114,0.344986250201965,
0.264502051090126,0.791393084533554,0.997645679347435
"11555","CDKN1A",718.656262828918,0.0400762906362709,0.329753619641719
,0.121534043143527,0.903268052013063,0.997645679347435
"11556","MTCH1",465.613739261856,0.0220799869130771,0.186553929439983,
0.118357125895761,0.905784695512199,0.997645679347435
"11557","CMTR1",27.8631977214844,0.0415575337920628,0.318731761043468,
0.13038403721051,0.896262595239704,0.997645679347435

"11558","MOCS1",13.669845649258,0.135912150701125,0.448953453036859,0.302731051029397,0.762094832195365,0.997645679347435
"11559","NFYA",16.0810796684102,0.107424062225902,0.425167234037,0.252663078492435,0.800528581385273,0.997645679347435
"11560","AL512274.1",2.95957498899041,0.179397746825467,1.03432797741059,0.173443772907105,0.862302620647388,0.997645679347435
"11561","RPL7L1",228.901041333292,-0.0330095151518025,0.19610802738230 1,-0.168323120641321,0.866329085717122,0.997645679347435
"11562","AL035587.2",2.41025819468875,0.13494446140634,0.952215332041327,0.141716329138548,0.887304076336035,0.997645679347435
"11563","MEA1",87.510921368497,-0.0654696239883079,0.227422613942685,-0.28787649061499,0.773441283257145,0.997645679347435
"11564","PTK7",37.7763609406131,0.0483268359906787,0.322364173499599,0.149913793043565,0.880832629424165,0.997645679347435
"11565","DNPH1",129.514229948739,-0.0687692446328339,0.312888129055024,-0.219788602528734,0.826035796470686,0.997645679347435
"11566","MRPS18A",66.4213035486364,0.0427190166385314,0.307079061932708,0.139114065184596,0.889360013890166,0.997645679347435
"11567","MRPL14",155.602458675104,0.0391555202098792,0.305992619254025,0.127962302833761,0.898178805707903,0.997645679347435
"11568","AARS2",14.2392711048231,-0.106822685184693,0.417154733110101,-0.256074489166826,0.797893320069924,0.997645679347435
"11569","SUPT3H",13.1037410472206,-0.0708737634543586,0.417456869192173,-0.169775056262717,0.865187044340367,0.997645679347435
"11570","ELOVL5",117.726019822728,0.0885489162084171,0.296962900426735,0.298181746208609,0.765564452875065,0.997645679347435
"11571","KHDC1",4.67252997040724,-0.1215915787615,0.633237746700512,-0.192015683517057,0.847729920346059,0.997645679347435
"11572","IRAK1BP1",19.5568745863165,0.0861954082288215,0.423836984566283,0.203369246591414,0.838846435187208,0.997645679347435
"11573","SH3BGRL2",10.8324224366907,0.122890143814706,0.44476024192744,0.276306495567458,0.782312667307912,0.997645679347435
"11574","CYB5R4",22.9116924160153,-0.0902881613759704,0.34788053700193,-0.259537834896091,0.795220293792411,0.997645679347435
"11575","SYNCRIP",219.768032080767,0.0538948465561405,0.191684432655577,0.281164442044076,0.77858427612412,0.997645679347435
"11576","SMIM8",37.9506851729626,-0.0415319078843348,0.261438406246804,-0.158859245206413,0.873779770478102,0.997645679347435
"11577","SLC35A1",42.5192109990091,-0.0493798534041199,0.295576244557442,-0.16706299749513,0.867320480508993,0.997645679347435
"11578","AKIRIN2",104.374388012934,0.0582275203916018,0.256675586337884,0.2268525854849,0.820538372142634,0.997645679347435
"11579","RRAGD",12.2861332916519,-0.119972107996401,0.452024837076184,-0.26541043357797,0.790693299465517,0.997645679347435
"11580","MDN1",52.1459355549463,0.075738028362138,0.331614638636628,0.228391691855103,0.819341747729715,0.997645679347435
"11581","UFL1",113.516641558694,-0.0332159324147502,0.186685673304575,-0.177924378592024,0.858782364964134,0.997645679347435
"11582","NDUFAF4",42.2411433797154,-0.0542152665957833,0.436413393939508,-0.124229153707638,0.901133839708037,0.997645679347435

"11583","AL513550.1",4.62350756389751,0.109032445442309,0.708549179722784,0.153881266907919,0.877703355004813,0.997645679347435
"11584","AL133338.1",2.67152861125581,0.290127623272395,1.01406908723443,0.286102423320713,0.774799675559176,0.997645679347435
"11585","HACE1",12.715700469296,0.108427164397533,0.472600738286374,0.229426565837971,0.818537390204822,0.997645679347435
"11586","QRSL1",28.076137602064,-0.0697251022047051,0.312012039364451,-0.223469268515186,0.823170298552594,0.997645679347435
"11587","OSTM1",53.1593477111817,-0.0650745754683547,0.239440440458947,-0.271777713671188,0.785792942349731,0.997645679347435
"11588","AFG1L",4.05233657967503,-0.176037491863251,0.678198603363569,-0.259566284846624,0.795198345893062,0.997645679347435
"11589","MICAL1",15.6207459297621,-0.10398347394882,0.425869941451926,-0.244167206528611,0.807101323918523,0.997645679347435
"11590","REV3L",351.126255311921,-0.0487544669560191,0.280745629667324,-0.173660644383999,0.8621321687097,0.997645679347435
"11591","AL080317.2",3.58118676160745,-0.221534360854476,0.883200743084892,-0.250831266378568,0.801944567090508,0.997645679347435
"11592","TRAF3IP2",51.093618125665,0.036720922650637,0.293316966710772,0.125191948704577,0.900371591101275,0.997645679347435
"11593","TUBE1",37.8921247335686,0.0471920133125668,0.289418911146458,0.163057808232461,0.870472919275474,0.997645679347435
"11594","TSPYL4",28.6650424556685,-0.0958477938708402,0.323490105992792,-0.296292814201173,0.767006473159118,0.997645679347435
"11595","CALHM5",17.1731615585986,-0.042627695825718,0.384169788475534,-0.110960562502516,0.911647620117676,0.997645679347435
"11596","FAM162B",4.62803459865755,0.124676274449396,0.776346640521944,0.160593564706579,0.872413523729728,0.997645679347435
"11597","ASF1A",37.2496839761606,-0.0616242206931457,0.30684294698723,-0.200833101422763,0.840829079025976,0.997645679347435
"11598","MAN1A1",331.292053632608,-0.044066897266463,0.196051994779658,-0.22477148123889,0.822157053076536,0.997645679347435
"11599","HINT3",19.3161425950897,-0.102130258517757,0.377386255500524,-0.270625273255652,0.786679255689234,0.997645679347435
"11600","KIAA0408",33.3246189764574,0.0423484414450746,0.377607624426899,0.112149328312286,0.910705005757003,0.997645679347435
"11601","PTPRK",24.9638302848968,0.109826366402288,0.362308447707513,0.303129466335131,0.761791199050153,0.997645679347435
"11602","WAKMAR2",9.31338894815781,-0.117802018246593,0.491920496837362,-0.239473693419894,0.810738292705476,0.997645679347435
"11603","AL355304.1",3.41562475762366,0.210905217787679,0.836609378689999,0.252095210930965,0.800967471574583,0.997645679347435
"11604","ADAT2",14.764548156833,0.123006518708959,0.426737498798744,0.288248675251693,0.773156391678357,0.997645679347435
"11605","LTV1",102.375185174961,0.0422317916239646,0.257161984732515,0.164222529499808,0.869555965904167,0.997645679347435
"11606","HYMAI",120.867666755958,0.0697730214963461,0.338047580768371,0.206400002442716,0.836478468923207,0.997645679347435
"11607","SAMD5",1.95457000862476,0.125035095023757,1.1148921251872,0.112149949039027,0.910704513593575,0.997645679347435

"11608","AL355312.3",2.68735999478385,-0.15068444435803,0.962002761374295,-0.156636186930239,0.875531581505008,0.997645679347435
"11609","SNX9",405.513136623045,-0.0393664007644365,0.204892156410443,-0.192132297566223,0.847638576382998,0.997645679347435
"11610","GTF2H5",207.539394260119,0.081784676320665,0.279123846651923,0.293004977187253,0.769518347330072,0.997645679347435
"11611","EZR",34.7236269984433,-0.10174257752643,0.38001487467889,-0.267733144952293,0.788904734902152,0.997645679347435
"11612","ACAT2",41.0835580227008,-0.107506902957841,0.351962270428695,-0.305450078006645,0.760023386184374,0.997645679347435
"11613","TCP1",215.679198896056,-0.0687026486229562,0.235297464380695,-0.291982103605673,0.770300307303258,0.997645679347435
"11614","AGPAT4",28.8836659820971,-0.0891877948499417,0.35983407869126,-0.247858110533398,0.804244191657615,0.997645679347435
"11615","PRKN",6.21441576144727,0.0884780199453616,0.618344376228256,0.143088581940464,0.886220222440627,0.997645679347435
"11616","SFT2D1",158.576926255877,0.0442222793140677,0.281096466180575,0.157320651927582,0.874992145033402,0.997645679347435
"11617","Z94721.3",2.24529443356334,0.166774899467368,0.916229548403862,0.182023052801454,0.855564634588459,0.997645679347435
"11618","BX322234.1",19.7849328538727,0.0877207260643987,0.529651641494184,0.165619662419874,0.868456272621673,0.997645679347435
"11619","ERMARD",15.4829401085058,-0.092826859567078,0.408675008765075,-0.227141023004025,0.820314085842689,0.997645679347435
"11620","DNAAF5",26.3888025885393,0.0927658219543818,0.319346118121966,0.290486768713288,0.771443871648952,0.997645679347435
"11621","MAD1L1",29.9653333831324,-0.0683733346929058,0.294488341084548,-0.232176711787975,0.816400764879051,0.997645679347435
"11622","NUDT1",109.573826513589,0.0756687553333288,0.292628540911424,0.258582963567567,0.795957029383154,0.997645679347435
"11623","CHST12",101.636988215626,-0.0583233274833785,0.211424300299284,-0.275859148644779,0.782656251500676,0.997645679347435
"11624","TTYH3",19.4009024656755,-0.087385499356372,0.457475024717851,-0.191016983736472,0.848512287054963,0.997645679347435
"11625","AP5Z1",28.2890328620371,-0.09863505429042,0.338996569219217,-0.290961806833615,0.771080530067649,0.997645679347435
"11626","WIPI2",112.704917569059,-0.0204300409928334,0.178700872333561,-0.114325356815824,0.908979882445816,0.997645679347435
"11627","EIF2AK1",74.7160853680606,0.0352569964165708,0.220340227692127,0.160011618331602,0.872871922039177,0.997645679347435
"11628","CYTH3",43.6433126658612,0.0788719924896398,0.276549968130317,0.285199788750195,0.775491082196093,0.997645679347435
"11629","DAGLB",9.98219075573052,0.0837436806609085,0.448450388163138,0.186740123035514,0.851864395779495,0.997645679347435
"11630","KDELR2",524.621352039009,-0.0340061201302003,0.202556099123952,-0.16788494781088,0.866673791958141,0.997645679347435
"11631","GLCCI1",12.8624025510533,-0.0970677699126853,0.446957871505558,-0.217174315748623,0.828072500074988,0.997645679347435
"11632","TMEM106B",133.922871929545,-0.0336235638048922,0.19983243086846,-0.16825879392432,0.866379689319876,0.997645679347435

"11633","AHR",162.533307293458,0.0803670166959809,0.266692092488828,0.301347580072505,0.763149462441973,0.997645679347435
"11634","TOMM7",1226.29457696388,-0.0258528711666496,0.212959884390144,-0.121397845611557,0.903375923214019,0.997645679347435
"11635","MALSU1",103.714036800396,0.0401634839177063,0.282026002941602,0.142410570297742,0.886755713593122,0.997645679347435
"11636","NFE2L3",2.12859043593593,-0.320696867976198,1.05934255659117,-0.30273197841514,0.762094125390191,0.997645679347435
"11637","SKAP2",96.6657395145315,-0.0658832201956363,0.377021932142337,-0.174746386294481,0.861278917514874,0.997645679347435
"11638","HOXA6",5.22630563095623,0.138473994482302,0.706166764928936,0.196092483191611,0.844537779592267,0.997645679347435
"11639","HOXA9",13.0962273261452,0.0757470279595832,0.479063231325807,0.158114885481721,0.874366271240979,0.997645679347435
"11640","HOXA11-AS",7.49922608457246,-0.114363941629334,0.514039510826981,-0.222480838963812,0.82393958780768,0.997645679347435
"11641","TAX1BP1",286.949905817809,-0.0573936553237947,0.209272386151529,-0.274253361273558,0.783889923129001,0.997645679347435
"11642","JAZF1",66.828001869042,0.0623210188098719,0.23674310308676,0.263243228619137,0.792363113775176,0.997645679347435
"11643","CPVL",83.3895467283431,-0.079769834659789,0.328450617943087,-0.242867056117432,0.808108386988324,0.997645679347435
"11644","WIPF3",1.93181944443493,-0.196234281942921,1.05938087914712,-0.185234872372725,0.853044818154374,0.997645679347435
"11645","MTURN",272.85273471878,0.0611213067263249,0.225023185473044,0.27162226238081,0.785912479910981,0.997645679347435
"11646","AC018645.3",4.27684735810597,0.190897053232803,0.715402930795826,0.266838511578987,0.78959350101531,0.997645679347435
"11647","KBTBD2",45.7154621251957,-0.0548260679491484,0.276015789461294,-0.198633810247427,0.842549203819536,0.997645679347435
"11648","NT5C3A",76.1647770964568,-0.0280140775970107,0.248509596396018,-0.112728353364545,0.910245921788206,0.997645679347435
"11649","BBS9",19.1793038214566,0.0923318151944157,0.371749799007782,0.248370854378009,0.80384748201627,0.997645679347435
"11650","HERPUD2",63.3725092744291,-0.0386167169061102,0.235699998353858,-0.163838426711122,0.86985834025987,0.997645679347435
"11651","AOAH",3.96437126054472,0.120961928000107,0.765355360881338,0.158046750807121,0.874419959810997,0.997645679347435
"11652","STARD3NL",49.4871181032871,-0.0448744870124644,0.23967411821764,-0.187231259454204,0.851479316689801,0.997645679347435
"11653","VPS41",40.9544055590047,-0.069836238657803,0.304193502932637,-0.229578337421848,0.81841944152516,0.997645679347435
"11654","LINC00265",2.52699652329554,0.194690939526934,0.886609337179997,0.219590445715562,0.826190133020334,0.997645679347435
"11655","AC072061.1",1.90740345998501,0.283210086642239,0.974234468556325,0.29070012998197,0.771280672178903,0.997645679347435
"11656","CDK13",97.3866862031449,0.0791784542341707,0.273946289435156,0.289029117340582,0.772559095585955,0.997645679347435
"11657","MPLKIP",102.910123547785,-0.0520224752969082,0.26785444148770

2,-0.194219199830953,0.84600424981545,0.997645679347435
"11658","SUGCT",3.55798397167953,0.193886118269002,0.770041035944112,0.251786735016385,0.801205910997079,0.997645679347435
"11659","INHBA",2.2247581153934,0.144797476510453,1.18217913602075,0.122483532400889,0.902516087032551,0.997645679347435
"11660","YKT6",43.5867999710673,0.045719708783321,0.27568569449525,0.165839975364078,0.868282885902935,0.997645679347435
"11661","OGDH",68.1692392032145,0.0436807313640119,0.253051522057097,0.172615959820846,0.862953306093668,0.997645679347435
"11662","AC004854.2",2.32074728120912,-0.206373262984375,0.948051998139926,-0.217681375482862,0.827677375925196,0.997645679347435
"11663","NACAD",6.60630934831211,-0.173114217812882,0.586042238253214,-0.295395462157941,0.767691799402181,0.997645679347435
"11664","TNS3",22.5487176838428,-0.112923150502878,0.503559202691916,-0.224249998608339,0.822562780704588,0.997645679347435
"11665","FIGNL1",12.2591224269628,0.092275049329942,0.415767059750596,0.221939297897468,0.824361138012471,0.997645679347435
"11666","LANCL2",21.207340198101,0.0709069906460193,0.351235224035097,0.201878928404214,0.840011375606894,0.997645679347435
"11667","ZNF680",36.0474157765456,-0.0427185343098045,0.32608944598666,-0.131002505096568,0.895773325704522,0.997645679347435
"11668","ZNF273",16.8991971274994,-0.0524544169177016,0.367389789705234,-0.142775924610717,0.886467151633384,0.997645679347435
"11669","ERV3-1",16.5625040407241,0.0468396540956592,0.38533197371938,0.12155662465158,0.903250167174311,0.997645679347435
"11670","RABGEF1",52.0145004196116,0.066264498095601,0.267559440711999,0.247662717186377,0.804395380660001,0.997645679347435
"11671","TYW1",30.3600725854921,-0.0655788286851461,0.351786978932463,-0.186416304788064,0.852118307162902,0.997645679347435
"11672","BAZ1B",114.390576122635,-0.0453102328760728,0.250373618336521,-0.180970475951554,0.856390750129882,0.997645679347435
"11673","VPS37D",6.60270440849278,-0.153385880289548,0.602742599225033,-0.254479906492027,0.799124825540231,0.997645679347435
"11674","CLIP2",9.88395230668915,0.0735268182882603,0.44443095608609,0.165440362066088,0.868597387021448,0.997645679347435
"11675","RCC1L",59.9436733580451,-0.0582672167694593,0.289675868682536,-0.201146257140652,0.840584213108307,0.997645679347435
"11676","HSPB1",558.078939515806,-0.0533199723653225,0.244149448129528,-0.218390714268724,0.827124699879406,0.997645679347435
"11677","POMZP3",7.25943696860426,-0.137628844835591,0.56324363802545,-0.244350464957,0.806959402318833,0.997645679347435
"11678","CCDC146",73.0236610088179,0.0602699548913743,0.406841394268337,0.148141157071206,0.882231369119898,0.997645679347435
"11679","APTR",23.8346526273106,-0.055965397924055,0.333237690239208,-0.167944381933152,0.866627034232786,0.997645679347435
"11680","MAGI2",31.7927750453494,-0.0829132199283221,0.315748310359211,-0.262592758878095,0.792864481831955,0.997645679347435
"11681","ABCB4",3.99614445677107,0.16922859764634,0.800744436725354,0.21133908633621,0.83262268515173,0.997645679347435
"11682","DBF4",21.2116493400741,-0.107215817606964,0.352516611057417,-

0.304144015470241,0.761018174688251,0.997645679347435
"11683","PEX1",27.4271349749216,-0.0920675969735195,0.319025116700172,
-0.288590434276205,0.772894816532958,0.997645679347435
"11684","AC002451.1",11.9218382078571,0.0656750416565348,0.43305206789
117,0.151656224565217,0.87945808543996,0.997645679347435
"11685","SLC25A13",30.5012197364714,0.0379870858162795,0.3136928382575
88,0.121096439521155,0.903614649273967,0.997645679347435
"11686","BRI3",745.06626623279,0.0525989251518157,0.219577418809251,0.
239546149312871,0.810682115889205,0.997645679347435
"11687","SMURF1",17.392109571557,0.103748208758937,0.398640436108153,0
.260255105507635,0.794667000221054,0.997645679347435
"11688","PTCD1",11.531962244099,0.125440807821965,0.486152817125783,0.
258027524274346,0.796385664989626,0.997645679347435
"11689","ZNF789",5.70080529760465,0.180702688338589,0.593182477180418,
0.304632546122275,0.760646029249459,0.997645679347435
"11690","FAM200A",12.287562691901,0.0478438071363829,0.429042488931555
,0.11151298151269,0.911209572044791,0.997645679347435
"11691","MAP11",5.89693118258133,-0.0847876048019501,0.585900119082536
,-0.144713411109592,0.884937150091293,0.997645679347435
"11692","GPC2",2.25723478641333,0.187841617365329,0.932297995619417,0.
201482378218058,0.840321407044534,0.997645679347435
"11693","SAP25",5.99180712682293,-0.186529883566917,0.666254082639079,
-0.279968090894181,0.779501986065304,0.997645679347435
"11694","EPHB4",12.0010860330013,-0.0640679407807996,0.482458310452354
,-0.132794770849173,0.894355689991006,0.997645679347435
"11695","POLR2J3",120.778213021881,0.0400793217598656,0.32095946448633
2,0.124873469065662,0.900623722556435,0.997645679347435
"11696","RASA4",4.76333362788572,0.130395702192152,0.690117046812939,0
.188947226842662,0.850134178509847,0.997645679347435
"11697","PMPCB",85.2712548776321,0.0314224912114778,0.223308162651628,
0.140713580902541,0.888096215841864,0.997645679347435
"11698","AC007384.1",2.41896872829375,0.198529661969243,0.901052722263
872,0.220330794263006,0.825613539023509,0.997645679347435
"11699","AC002467.1",6.80004636799556,-0.183329883494974,0.62807258361
8185,-0.291892829390596,0.770368566188079,0.997645679347435
"11700","CBLL1",26.4234298100316,0.0708964074264058,0.31858537660872,0
.222535033406381,0.8238974041822,0.997645679347435
"11701","LSMEM1",8.18913317848112,-0.101576835012756,0.723553572474761
,-0.140386059687791,0.888354971535266,0.997645679347435
"11702","AC006333.2",14.6623281517952,0.142430896112936,0.478913132808
594,0.297404448438587,0.766157747887863,0.997645679347435
"11703","RBM28",69.8344282347815,-0.0703701357317485,0.30011704138382,
-0.234475641260745,0.81461574160333,0.997645679347435
"11704","CALU",473.712824488894,-0.0267895142563416,0.178509864429413,
-0.15007301888874,0.880707006690308,0.997645679347435
"11705","IRF5",2.23014425716782,-0.12094614829765,1.06339208279247,-0.
113736175259125,0.909446934869243,0.997645679347435
"11706","SMO",21.1873253735099,-0.0533887974543407,0.348775787942804,-
0.153074838621241,0.878339257879539,0.997645679347435
"11707","AC078846.1",6.36464401253083,0.167002118591601,0.615636465423

456,0.271267424805207,0.78618535868414,0.997645679347435
"11708","TMEM209",17.5533944199469,0.0854815175055697,0.417004617826502,0.204989378657516,0.83758042342452,0.997645679347435
"11709","CEP41",17.2046069264901,0.0652091465291054,0.38798385659775,0.168071803556281,0.866526791282527,0.997645679347435
"11710","AUXG01000058.1",41.6034857633061,0.191221166735132,0.815337990176626,0.234529936098903,0.814573595508569,0.997645679347435
"11711","LINC-PINT",189.493355902182,0.0812156797604797,0.390417820609412,0.208022470986873,0.835211421821769,0.997645679347435
"11712","AC009275.1",3.23544408755581,0.178575710276321,0.971060146796855,0.183897682203695,0.854093723134226,0.997645679347435
"11713","CALD1",1584.39952140173,0.0632915736346791,0.208198530859358,0.303996254793141,0.761130744462091,0.997645679347435
"11714","WDR91",9.26473085160404,-0.0563845900523344,0.490103088326584,-0.115046387985138,0.908408353821464,0.997645679347435
"11715","CNOT4",112.525519639929,-0.0208832919816695,0.189595480321209,-0.110146570721461,0.912293134727586,0.997645679347435
"11716","MKRN1",84.682843437895,-0.0285071023674865,0.193284044038427,-0.14748813079376,0.882746747533777,0.997645679347435
"11717","BRAF",57.9799676010325,0.0594834622685385,0.301126584904689,0.197536402464651,0.843407797556319,0.997645679347435
"11718","MRPS33",98.2343657086307,-0.0790671717479124,0.295691381973257,-0.267397619843596,0.789163031833363,0.997645679347435
"11719","DENND11",16.8710719557685,0.0982485968260619,0.417812711086018,0.235149851163419,0.814092427634216,0.997645679347435
"11720","TCAF2",5.28400360659812,-0.143530727802541,0.628059218253049,-0.228530564684286,0.819233797528662,0.997645679347435
"11721","EZH2",9.96575408223502,0.147838764603685,0.579734679359415,0.255011076389361,0.798714544277552,0.997645679347435
"11722","RARRES2",678.775379817554,0.0868790358682685,0.318669005024827,0.272630957194911,0.785136912679597,0.997645679347435
"11723","GIMAP4",11.1008937740346,0.0631917649076403,0.457390539059581,0.13815713162228,0.890116234133972,0.997645679347435
"11724","GIMAP5",3.73790490470716,0.144899896549536,0.730756412431827,0.19828754710114,0.842820094229133,0.997645679347435
"11725","ABCB8",26.1104803354008,-0.0841051537820403,0.383350709443862,-0.219394804053067,0.82634251720657,0.997645679347435
"11726","AC021097.2",5.05518847905339,0.0750824167118987,0.653120795959861,0.114959464124173,0.908477251906532,0.997645679347435
"11727","NUB1",175.971028245152,0.056520775554748,0.216563337862132,0.260989584445406,0.79410053938627,0.997645679347435
"11728","PRKAG2-AS1",31.713581446751,0.0991316644892561,0.475572286835141,0.208447100963266,0.834879882664862,0.997645679347435
"11729","ACTR3B",9.6389329733193,-0.0655927197399264,0.561023618467489,-0.11691614680876,0.906926502605603,0.997645679347435
"11730","WDR60",87.9753757520954,-0.0409474402530392,0.263527677658809,-0.155381934136171,0.876520225656957,0.997645679347435
"11731","FBXO25",40.4127270616558,0.0412635434318996,0.263383846085475

,0.15666694842974,0.875507336692378,0.997645679347435
"11732","ERICH1",92.4582761161928,0.0316860784439441,0.214486189066801
,0.147730157274022,0.882555730700213,0.997645679347435
"11733","MYOM2",5.68009090102997,-0.191246338886486,0.634187360112338,
-0.301561259203604,0.762986544481439,0.997645679347435
"11734","AC016065.1",6.48747838080091,-0.11732304147081,0.631723955172
951,-0.185718842082995,0.852665251534329,0.997645679347435
"11735","PRAG1",2.33662917466631,0.253567066901031,1.02613689657983,0.
247108419691549,0.804824317791897,0.997645679347435
"11736","ERI1",21.2904873858914,-0.0421174758081535,0.322309661993286,
-0.130673947370001,0.896033242580585,0.997645679347435
"11737","PPP1R3B",33.211453163741,0.0928985775092514,0.375055744366078
,0.2476927200949,0.804372164911829,0.997645679347435
"11738","CTSB",309.276568761084,0.0601625959290274,0.215075504842096,0
.279727791285194,0.77968635479639,0.997645679347435
"11739","DLC1",336.193766356903,-0.0523806420877753,0.249252365397663,
-0.210151033087313,0.833549796033786,0.997645679347435
"11740","TUSC3",70.6195250564302,0.0735673942101297,0.290339104656092,
0.253384380644387,0.7999711963618,0.997645679347435
"11741","PCM1",213.647595874131,0.0543456047211091,0.224852832966293,0
.241694107226373,0.809017195969011,0.997645679347435
"11742","PSD3",42.6899428168881,-0.0638610632951142,0.33782282718805,-
0.189037146561933,0.850063702886079,0.997645679347435
"11743","PPP3CC",31.0740422061954,-0.0638724250842969,0.28993250614180
5,-0.220301013964461,0.825636730530896,0.997645679347435
"11744","TNFRSF10A",3.08026201904789,-0.245437454813706,0.828646893862
838,-0.296190641190446,0.76708449561072,0.997645679347435
"11745","KCTD9",53.9843217479378,0.0605463085462372,0.244609695898362,
0.247522112007346,0.804504180891112,0.997645679347435
"11746","AC013643.2",2.37833177542642,0.267160379382817,1.030568165950
51,0.259236010008528,0.795453148541042,0.997645679347435
"11747","RBPMS",120.060279470099,-0.0581618810460876,0.256103555873911
,-0.227102981243739,0.820343665916619,0.997645679347435
"11748","FUT10",19.6296443686938,-0.103565920160065,0.339555351865374,
-0.30500452898509,0.760362702943725,0.997645679347435
"11749","STAR",1.85363881284648,0.217511693021828,1.20887873741164,0.1
79928462872585,0.857208729709182,0.997645679347435
"11750","BAG4",40.9237300773916,-0.0590067334238907,0.268344883975943,
-0.219891404485209,0.825955730717008,0.997645679347435
"11751","GINS4",3.63728340076829,-0.194374353331139,0.712049675196398,
-0.272978641943101,0.784869633537963,0.997645679347435
"11752","GPAT4",42.0917332208061,0.0575316271081619,0.275506719469647,
0.208821139531227,0.834587868164823,0.997645679347435
"11753","SLC20A2",27.1292695448896,0.0364041960124075,0.30767823406848
6,0.118319048868125,0.905814864600891,0.997645679347435
"11754","HOOK3",174.222823751822,0.0621746847235472,0.217129447336398,
0.286348468557653,0.774611238970421,0.997645679347435
"11755","HGSNAT",109.873381490244,0.031258766652555,0.259384593830254,
0.120511269350913,0.904078153054706,0.997645679347435
"11756","CEBPD",3310.77064261112,-0.0586925602714524,0.231376918514087

,-0.253666444554533,0.799753259639969,0.997645679347435
"11757","PRKDC",206.415961434525,-0.0554425966321492,0.21641309996509,
-0.256188727212413,0.797805112627826,0.997645679347435
"11758","MCM4",18.9093718019452,-0.0731439819481672,0.380020813497644,
-0.192473620786617,0.84737122914846,0.997645679347435
"11759","UBE2V2",143.215344683289,0.0406381223926998,0.248617861512828
,0.163456165801679,0.870159283539468,0.997645679347435
"11760","RB1CC1",230.702868144028,0.0879518778010342,0.319298789964089
,0.275453213621405,0.782968066038731,0.997645679347435
"11761","FAM110B",42.1837687119071,0.0587155931494764,0.28244270188422
2,0.207884971917401,0.835318783512473,0.997645679347435
"11762","AC090152.1",2.1191218614738,-0.163539106197308,1.020252410886
73,-0.160292790737118,0.872650437606911,0.997645679347435
"11763","CHD7",9.51607070720562,0.176831706113339,0.673763267366811,0.
262453764813314,0.792971626573803,0.997645679347435
"11764","ARMC1",60.7237965223597,-0.0482333952375705,0.239676370277799
,-0.201243848868644,0.840507906338697,0.997645679347435
"11765","RRS1",41.5974692391008,-0.0858881482839828,0.406564713095859,
-0.211253327004137,0.832689600643911,0.997645679347435
"11766","MYBL1",17.0025972672172,0.0602141421327187,0.40355487630786,0
.149209303784458,0.881388478818388,0.997645679347435
"11767","CSPP1",39.7345208367423,0.0437454211022359,0.28782752407173,0
.151984843156744,0.879198890177257,0.997645679347435
"11768","ARFGEF1",65.3105214807098,0.0677066641958456,0.26554517081857
6,0.254972304663389,0.79874449009115,0.997645679347435
"11769","AC021321.1",1.86383998883135,-0.13982307469545,1.020214326777
03,-0.1370526476894,0.890989180851705,0.997645679347435
"11770","PREX2",17.7875981499918,-0.113955092514894,0.477321991317367,
-0.238738408428214,0.811308432133887,0.997645679347435
"11771","MSC-
AS1",33.3147635515426,0.0370029346612529,0.280127029558329,0.132093410
334571,0.894910408031581,0.997645679347435
"11772","TERF1",112.006157403568,0.0311042101123581,0.189182395425402,
0.164413871821509,0.869405344038616,0.997645679347435
"11773","STAU2",31.9357815753077,0.0858080136140504,0.310444473034203,
0.276403740660561,0.782237983963864,0.997645679347435
"11774","LY96",91.6193257058735,-0.0441744172104363,0.360928463407289,
-0.12239105997187,0.902589318391977,0.997645679347435
"11775","SNX16",21.0801502379774,0.0657282943800218,0.347599772279514,
0.189091879862246,0.850020805613091,0.997645679347435
"11776","E2F5",4.4880715974613,-0.209125804182336,0.698688161610779,-0
.299312076077274,0.764701941334923,0.997645679347435
"11777","RMDN1",44.9489019395116,-0.0463878640392794,0.282444695997013
,-0.164236980537138,0.869544590094513,0.997645679347435
"11778","CPNE3",210.703423538784,0.0493671727012931,0.166163763004099,
0.29709951080053,0.766390537842431,0.997645679347435
"11779","MMP16",9.49471219869949,-0.0693509870044528,0.619795637511545
,-0.111893312581054,0.910907999092666,0.997645679347435
"11780","AF117829.1",55.8590321849518,0.0619875595675768,0.40258845786
1732,0.153972520466213,0.877631402669559,0.997645679347435

"11781","OTUD6B",18.7122072500535,0.0449186793611736,0.382667371132345
,0.117383092340106,0.906556481799286,0.997645679347435
"11782","VIRMA",64.4996504966431,-0.0582595450262317,0.225436513869928
,-0.258429941211059,0.796075111452443,0.997645679347435
"11783","AC023632.2",2.34062161057747,0.230717641720331,1.038915460790
17,0.222075472382376,0.824255131339172,0.997645679347435
"11784","GDF6",3.21008349105798,0.227062926632688,0.796454255581206,0.
285092238557996,0.77557347615872,0.997645679347435
"11785","UQCRB",883.979604604027,-0.0462510612107754,0.207058905091728
,-0.223371514450373,0.823246372441702,0.997645679347435
"11786","LAPTM4B",87.95963248568,-0.036393548360521,0.259478774209386,
-0.140256359971676,0.888457443118996,0.997645679347435
"11787","RIDA",29.4573411010029,0.0588483486961415,0.390839816617167,0
.150568970187048,0.880315740481348,0.997645679347435
"11788","POP1",12.5476710815349,0.145978198854687,0.572472200158299,0.
254996135732566,0.798726083840281,0.997645679347435
"11789","ANKRD46",24.6707289174435,0.0553186340242229,0.39001627793468
1,0.141836731320962,0.887208969959935,0.997645679347435
"11790","AP003469.4",2.72804737499109,-0.135493189170072,0.86572971551
5914,-0.156507495054998,0.875633011845181,0.997645679347435
"11791","ATP6V1C1",54.8375437368338,0.0296772446169228,0.2459997597370
37,0.120639323585708,0.903976720577375,0.997645679347435
"11792","DCAF13",94.6725546710555,-0.0479096359753748,0.28434782539374
9,-0.168489545889905,0.866198167274902,0.997645679347435
"11793","EMC2",125.709029938809,0.0754108515897648,0.281820553352915,0
.267584640980142,0.789019054693834,0.997645679347435
"11794","AC104248.1",3.67085692934084,0.206958914937809,0.810910340096
588,0.25521799970285,0.798554729550187,0.997645679347435
"11795","NUDCD1",35.4986098597239,0.0561318650095451,0.343350406606438
,0.163482739293464,0.87013836236694,0.997645679347435
"11796","RAD21",159.234905220712,0.0586381686434762,0.206831116094692,
0.283507480647303,0.776787848077691,0.997645679347435
"11797","FAM91A1",48.2424526209876,0.0663076718834378,0.27927875846472
4,0.237424687247789,0.812327339531028,0.997645679347435
"11798","TRMT12",12.4633144063692,-0.099998822412207,0.480861740426159
,-0.207957535410457,0.835262124278864,0.997645679347435
"11799","TATDN1",103.141686967257,-0.064667954054959,0.267068972586207
,-0.24213952459073,0.80867205307849,0.997645679347435
"11800","WASHC5",48.9168287037289,-0.0764450599550722,0.27586053412848
1,-0.277114884144566,0.78169189331542,0.997645679347435
"11801","TRIB1",62.7072218768516,-0.169002865505496,0.762632158092994,
-0.221604693313875,0.824621628598143,0.997645679347435
"11802","AC103706.1",7.0619060632469,-0.113540799107692,0.617720954473
186,-0.183805969808041,0.854165672703463,0.997645679347435
"11803","AC083843.3",5.89246555980231,0.158094692332855,0.700545235009
532,0.225673781552027,0.821455151349429,0.997645679347435
"11804","TRAPPC9",15.1606389078572,0.0458667430084553,0.42438464881356
9,0.108078233123377,0.913933634451315,0.997645679347435
"11805","LYNX1",4.62734621478829,-0.123841651296887,0.777661048920589,
-0.15924888030432,0.873472794284751,0.997645679347435

"11806","MINCR",10.4671309781792,0.0998452327092903,0.45758917099145,0
.218198416918297,0.827274518484266,0.997645679347435
"11807","GSDMD",139.010136708012,0.044718145045342,0.241137997801118,0
.185446281602719,0.852879010432577,0.997645679347435
"11808","TSTA3",59.344979648882,-0.0442786416785271,0.282441112969844,
-0.15677123352525,0.875425144800209,0.997645679347435
"11809","PARP10",42.5721324289518,-0.0373435617789364,0.26359038086243
,-0.141672703141721,0.88733853716234,0.997645679347435
"11810","GRINA",294.121007699428,0.0503511225235984,0.17152861499458,0
.293543573037009,0.769106699084901,0.997645679347435
"11811","ZNF250",8.37225053681008,0.053329727555276,0.48267413845124,0
.110488056655357,0.912022321285917,0.997645679347435
"11812","ZNF16",9.36789884418015,0.110033983360732,0.482470566513207,0
.228063618794288,0.819596782793755,0.997645679347435
"11813","SLC1A1",2.05198181382251,-0.181499554080813,1.18757802518733,
-0.152831687881883,0.878531007962361,0.997645679347435
"11814","CDC37L1",64.6698014150275,-0.0647762730416765,0.2559325663241
33,-0.253098986080727,0.8001917223671,0.997645679347435
"11815","PDCD1LG2",18.0255911622902,-0.0412797792977814,0.372964635033
285,-0.110680143424584,0.911869992474529,0.997645679347435
"11816","TTC39B",26.2315379535069,0.0490341349948596,0.331256328477314
,0.148024749354238,0.882323236240928,0.997645679347435
"11817","CDKN2B",20.7837686225513,-0.109607768100753,0.446749656244038
,-0.24534494110697,0.806189356879017,0.997645679347435
"11818","CAAP1",35.5014636952073,-0.0353329837243811,0.294878961191121
,-0.119821989272,0.904624162517904,0.997645679347435
"11819","TEK",2.62479576144603,-0.191022995466623,0.836869723594344,-0
.228258939331897,0.81944494362614,0.997645679347435
"11820","MOB3B",13.0050524332719,-0.0741010432342946,0.597041514964193
,-0.124113719694582,0.901225235407464,0.997645679347435
"11821","BAG1",206.91330762453,-0.0836833444625156,0.276363349014603,-
0.302801890195989,0.762040842838746,0.997645679347435
"11822","NOL6",21.6148182621753,0.0822251817223589,0.400981887418204,0
.205059590725609,0.83752556743747,0.997645679347435
"11823","CNTFR",60.9411075678721,0.0801464225253084,0.536508077412694,
0.14938530452666,0.881249606905863,0.997645679347435
"11824","RPP25L",37.1818167974896,0.0572299054865537,0.388529208624235
,0.147298849652005,0.882896140420269,0.997645679347435
"11825","SIGMAR1",59.4907951366863,-0.0300992611385037,0.2599636199821
01,-0.115782589658415,0.907824849144675,0.997645679347435
"11826","IL11RA",163.772630332668,-0.0499779321608187,0.30240979908644
4,-0.165265584355395,0.868734946003448,0.997645679347435
"11827","TESK1",51.8191733335958,0.0729484640158755,0.267521099851693,
0.27268302969865,0.785096880853932,0.997645679347435
"11828","SIT1",6.69722807516024,-0.0679106271887032,0.552291567199446,
-0.122961550061435,0.90213754545863,0.997645679347435
"11829","TLN1",325.721017371329,0.04565640524094,0.211528604416575,0.2
15840336898486,0.829112205011917,0.997645679347435
"11830","CREB3",76.7110087855413,-0.0622996500774598,0.267720080847366
,-0.232704434722543,0.81599092533498,0.997645679347435

"11831","RGP1",16.2800316957398,-0.0744724398547976,0.383009513854918,-0.194440182713078,0.845831229006015,0.997645679347435
"11832","GNE",23.3008925458641,-0.072224070186438,0.305297297313947,-0.236569634981628,0.812990679338158,0.997645679347435
"11833","EBLN3P",91.3628567600003,0.0424067460089042,0.227749693185976,0.18619891608054,0.852288773938402,0.997645679347435
"11834","ZCCHC7",76.7667285497589,-0.0237727576165396,0.199564547909358,-0.119123150206705,0.905177790118668,0.997645679347435
"11835","FRMPD1",3.21194477662915,-0.102408156691175,0.936940562151442,-0.109300590483585,0.91296407826444,0.997645679347435
"11836","ALDH1B1",21.910241228607,0.0391584688904738,0.347633835397494,0.112642858384883,0.910313705185072,0.997645679347435
"11837","CBWD6",2.71126347837208,-0.182770491583435,0.931139212213404,-0.196286966745791,0.844385561996708,0.997645679347435
"11838","CBWD3",7.47437489410794,-0.0809199554474082,0.541989127899999,-0.149301805667103,0.881315490527176,0.997645679347435
"11839","AL161457.2",5.2249079775454,-0.176240944318619,0.786848183330624,-0.223983416435703,0.822770207198649,0.997645679347435
"11840","FXN",31.0040292038885,-0.0610539203224792,0.308323904972632,-0.198018769669833,0.843030378429652,0.997645679347435
"11841","VPS13A",32.4843935980816,0.0823063231813309,0.342518271007795,0.240297613727759,0.810099544795935,0.997645679347435
"11842","CEP78",24.659323021841,-0.111653327273809,0.392483105658869,-0.284479320673878,0.776043079382015,0.997645679347435
"11843","TLE4",59.6722110022097,0.0806955463229888,0.319590225199805,0.25249691623872,0.800656997393962,0.997645679347435
"11844","GKAP1",11.7432081717241,-0.105646309035875,0.512597824006469,-0.206099800054052,0.836712954952364,0.997645679347435
"11845","KIF27",14.6208793701471,-0.132272768660035,0.558045778481142,-0.23702852662025,0.812634659719654,0.997645679347435
"11846","HNRNPK",736.08330747293,-0.047422595999748,0.160977500362458,-0.294591454662738,0.768305990825571,0.997645679347435
"11847","AL354733.3",6.46821944168954,-0.090688442487854,0.7953268471183,-0.114026632970388,0.909216681075821,0.997645679347435
"11848","NAA35",33.1164192984423,-0.0461306221036796,0.348489269161352,-0.132373149436406,0.894689151409526,0.997645679347435
"11849","CTSL",1214.54178360784,-0.0618694058884497,0.408726643682733,-0.15137111036117,0.87968297743486,0.997645679347435
"11850","SPIN1",83.3560184021794,0.0564726700410824,0.218509985599135,0.258444344711476,0.796063996569339,0.997645679347435
"11851","SECISBP2",87.9058826936737,-0.0422964359050964,0.191225550398594,-0.221186111463311,0.824947522909306,0.997645679347435
"11852","GADD45G",36.5460345461854,-0.105878292365919,0.472303170806519,-0.224174426322649,0.822621581931092,0.997645679347435
"11853","NFIL3",368.131557964613,-0.065495884558528,0.293991980637251,-0.222781194291628,0.82370580498906,0.997645679347435
"11854","SPTLC1",119.305586980704,-0.0453012426169551,0.205488535504032,-0.220456301884862,0.825515801214276,0.997645679347435
"11855","FGD3",5.29320093213623,0.208524223987661,0.771485017038052,0.270289402104326,0.786937617938087,0.997645679347435

"11856","FAM120A",187.582111497133,0.0516415313104143,0.18143903837612
2,0.284621941190858,0.775933799590145,0.997645679347435
"11857","PTPDC1",11.5607022956342,0.0751081979305343,0.462598093770366
,0.162361667594372,0.87102105509628‍5,0.997645679347435
"11858","TRMO",17.8237109068372,-0.0459173641972109,0.360732379164188,
-0.127289278283255,0.89871144596993,0.997645679347435
"11859","AL499604.1",17.0335304207447,0.0695974181087684,0.40265909346
2205,0.172844520932944,0.862773641022683,0.997645679347435
"11860","GALNT12",40.17347826765,-0.0713535107164108,0.372870511119199
,-0.191362707933749,0.848241434876949,0.997645679347435
"11861","ALG2",59.665225616762,-0.0440544826760626,0.246475782306781,-
0.178737571146967,0.858143766689277,0.997645679347435
"11862","INVS",10.8035118628246,0.0967962305919329,0.474100939308894,0
.204167978939326,0.838222233393209,0.997645679347435
"11863","TEX10",21.6108269054065,0.0835982162511395,0.339862758659968,
0.245976395238936,0.805700505089073,0.997645679347435
"11864","RNF20",40.2057998361055,0.0586464191433532,0.303826456460265,
0.19302604462631‍1,0.846938570981578,0.997645679347435
"11865","SMC2",40.3738061937749,-0.0740996752504953,0.279508938033031,
-0.265106639422524,0.790927312765202,0.997645679347435
"11866","PALM2-
AKAP2",152.91209825994,-0.0596005456437786,0.37227673300859,-0.1600974
2312422,0.872804331042751,0.997645679347435
"11867","ZFP37",15.1492508778516,0.0625062922917978,0.474042353410341,
0.131858033026199,0.895096583506713,0.997645679347435
"11868","FAM225A",4.09360764623132,0.16420937598295,0.705581177357432,
0.23272924682876‍7,0.815971657025093,0.997645679347435
"11869","SLC31A1",36.6065940891043,-0.0625398190202554,0.2830055934059
55,-0.22098439210191,0.825104585867551,0.997645679347435
"11870","TNFSF8",5.59325808344977,-0.105014392354276,0.668662052981752
,-0.157051520848219,0.875204244099053,0.997645679347435
"11871","TNC",135.961528678283,0.179886170592408,0.754843784564077,0.2
38309136633207,0.81164133545412,0.997645679347435
"11872","TRIM32",14.4966498003253,0.082719587121369,0.455571620947402,
0.181573178218051,0.855917699597713,0.997645679347435
"11873","TLR4",37.6727776180424,0.130153073463094,0.433631246533069,0.
300146897862371,0.764065107981799,0.997645679347435
"11874","PSMD5",47.3363476371357,-0.076358882358976,0.280751198230014,
-0.271980610734265,0.785636927954776,0.997645679347435
"11875","TRAF1",7.19870318184717,0.101800257954945,0.580659025727449,0
.175318480286101,0.860829391486826,0.997645679347435
"11876","DAB2IP",13.5639376220673,0.0868091439309091,0.505627958832231
,0.171685806559033,0.863684544847681,0.997645679347435
"11877","AL596244.1",2.35112607872918,-0.241273360879932,0.91303463952
7031,-0.264254334320674,0.791583945791413,0.997645679347435
"11878","HSPA5",891.843777801629,-0.0437413824601263,0.357948345650451
,-0.122200264344401,0.902740417108238,0.997645679347435
"11879","MVB12B",6.99926585520795,0.102509366228719,0.719977828912621,
0.142378504048573,0.886781040683643,0.997645679347435
"11880","RALGPS1",6.03390582293728,0.153721483208486,0.6096510798555,0

.252146659438249,0.800927705759998,0.997645679347435
"11881","RPL12",6615.13717908175,0.0497280869020494,0.229711341531909,
0.21648076481736,0.828613017143177,0.997645679347435
"11882","SH2D3C",2.93564299978492,0.154351327914674,0.878266695415542,
0.175745395698563,0.860493969699774,0.997645679347435
"11883","ST6GALNAC6",158.604458664708,-0.0319270986471524,0.2161693543
18109,-0.147694842073541,0.882583602422474,0.997645679347435
"11884","PIP5KL1",2.48132249768998,0.259578430016723,0.905393832486618
,0.286702229132491,0.774340330613634,0.997645679347435
"11885","SLC25A25",53.4112567127553,0.122930003615651,0.42966351801889
2,0.286107613191042,0.774795700700005,0.997645679347435
"11886","AL359091.5",3.20540112090728,0.256234171884965,0.872920362116
305,0.293536710798855,0.769111943477293,0.997645679347435
"11887","TBC1D13",22.1557702861917,-0.0413125161363031,0.3740471168303
85,-0.110447358841792,0.912054595915518,0.997645679347435
"11888","SPOUT1",22.2523004671242,-0.0374849079839783,0.34230619597400
2,-0.109506951451224,0.912800408468539,0.997645679347435
"11889","MIGA2",10.2118053166031,-0.0957700290677228,0.490783709066709
,-0.195136935677515,0.845285747851491,0.997645679347435
"11890","PTGES",291.354188587097,-0.0535817614699349,0.281701732939749
,-0.190207425814434,0.849146592389138,0.997645679347435
"11891","USP20",12.8658957015082,-0.0859049774536797,0.437181773652904
,-0.196497161205726,0.844221054387434,0.997645679347435
"11892","EXOSC2",25.775526264989,0.0623631846163857,0.371621847586631,
0.167813558383022,0.866729955720466,0.997645679347435
"11893","AL157938.3",8.84676398235718,0.104057793884827,0.680138823051
864,0.152994933325386,0.87840227087237,0.997645679347435
"11894","UCK1",37.5830124168232,0.0402054339035638,0.271321859897216,0
.148183540827837,0.882197920925801,0.997645679347435
"11895","GTF3C4",32.7078662149886,0.0532414454778513,0.306629749867715
,0.173634311415708,0.862152864982486,0.997645679347435
"11896","GBGT1",6.54893075677593,0.0994924181470095,0.604195501964325,
0.164669246665269,0.869204324095285,0.997645679347435
"11897","REXO4",52.5295880020847,-0.0712179912369914,0.247733685146989
,-0.287478027845649,0.773746323467581,0.997645679347435
"11898","CACFD1",4.29933759412397,-0.183754366435609,0.72628625843797,
-0.2530054290588,0.800264017878751,0.997645679347435
"11899","SARDH",3.90914315758465,0.0997489555968394,0.8077583371164,0.
123488611647057,0.901720191856943,0.997645679347435
"11900","FCN1",5.87672443769845,-0.168442071244969,0.67885864505165,-0
.248125397640261,0.804037385467139,0.997645679347435
"11901","OLFM1",42.7975989977984,0.0940444430554114,0.385709820010509,
0.243821749347344,0.807368875049769,0.997645679347435
"11902","PPP1R26",3.43226531717304,-0.0856004579685674,0.7601707810754
59,-0.112606877427548,0.91034223233613,0.997645679347435
"11903","CAMSAP1",14.331743377054,0.0613753184070175,0.432173704855368
,0.142015392693911,0.887067847307813,0.997645679347435
"11904","SEC16A",45.5336511384415,-0.0523448689043061,0.26549133056263
3,-0.197162253070095,0.843700568467913,0.997645679347435
"11905","NOTCH1",8.85231623586308,0.0587103397800441,0.494122283263062

,0.118817429953443,0.905419999401457,0.997645679347435
"11906","RABL6",80.4335596060873,-0.0489733239869604,0.234305543754361
,-0.209014789843397,0.834436692983458,0.997645679347435
"11907","UAP1L1",9.1168982732847,0.175216535704403,0.577701636041846,0
.303299358653212,0.761661734905234,0.997645679347435
"11908","NSMF",27.7541348154048,0.0530483716167626,0.314064970674006,0
.168908909207279,0.865868291357573,0.997645679347435
"11909","EHMT1",70.7143819943728,-0.0273394453464664,0.246469274117961
,-0.110924355355474,0.911676332000253,0.997645679347435
"11910","AL954642.1",1.95279880585033,0.349433747146819,1.604546533120
35,0.217777259764026,0.827602663405412,0.997645679347435
"11911","WDR37",15.1298420855289,0.107180500072185,0.439464280403453,0
.243888991327776,0.807316795430486,0.997645679347435
"11912","AL137186.2",13.0137772393204,0.0896718470508787,0.46480075634
7942,0.192925346669944,0.847017434152934,0.997645679347435
"11913","IL15RA",133.393676831325,-0.084435556350841,0.338416358436002
,-0.249501994351165,0.802972498406366,0.997645679347435
"11914","PFKFB3",56.58757566055,0.101488378241684,0.520509486586939,0.
194978921339473,0.845409449254905,0.997645679347435
"11915","NUDT5",99.5047411304097,0.0787475505879621,0.271802676455845,
0.28972323457144,0.772027979715008,0.997645679347435
"11916","CCDC3",3.04506231082511,0.221266155926473,0.943391745972848,0
.234543239190945,0.814563269132201,0.997645679347435
"11917","PHYH",114.680113281839,0.0398116168134777,0.285793789062299,0
.139301896462135,0.889211591333998,0.997645679347435
"11918","CUBN",4.5044092472142,-0.152418619606963,0.748983995314678,-0
.203500502761645,0.838743852755803,0.997645679347435
"11919","TRDMT1",19.1484488098958,0.0468091605198935,0.356978413052255
,0.131126025575786,0.895675613478281,0.997645679347435
"11920","DNAJC1",189.640105369896,-0.0464315216385372,0.21988491661054
8,-0.211162831695159,0.832760212794582,0.997645679347435
"11921","OTUD1",53.4446720954433,0.102596289341266,0.383754755197936,0
.267348581226957,0.789200785120267,0.997645679347435
"11922","MTPAP",33.3325611119869,0.0316108986718516,0.286756975663427,
0.110235849010188,0.912222332113182,0.997645679347435
"11923","NRP1",272.823316863243,-0.0611863342605143,0.311967898857874,
-0.196130225207528,0.844508239370923,0.997645679347435
"11924","CUL2",55.6540933535097,0.0278183838699533,0.23800802753657,0.
116880023576847,0.906955128516061,0.997645679347435
"11925","AL450326.1",11.2274036536223,0.0736072911532101,0.45410088913
4567,0.162094576149129,0.871231377320134,0.997645679347435
"11926","ZNF485",5.43146284098988,-0.0824765920787392,0.60384761023826
5,-0.136585109687187,0.891358746934865,0.997645679347435
"11927","MAPK8",33.4924720906143,0.0441215786768738,0.290990921002099,
0.151625275884657,0.879482496628053,0.997645679347435
"11928","PRKG1",33.0110313503988,0.040775824844372,0.358753005116863,0
.113659883716066,0.909507414529855,0.997645679347435
"11929","IPMK",33.213831355619,0.0684122431631519,0.365656778792324,0.
187094147110033,0.851586817063957,0.997645679347435
"11930","FAM13C",36.0449519895331,0.135118744595848,0.529092467994602,

0.255378318100016,0.798430915370405,0.997645679347435
"11931","RUFY2",63.2112795956726,0.0687263726839327,0.245129534674681,
0.28036757290443,0.779195512848444,0.997645679347435
"11932","DDX50",106.475228518925,-0.0437147380639854,0.238915651497464
,-0.182971428577376,0.85482043683177,0.997645679347435
"11933","DDX21",565.491117693545,-0.0628428545911218,0.283879966831138
,-0.2213712200054,0.824803399655482,0.997645679347435
"11934","HK1",55.772545107361,0.0281553239153065,0.240480045590104,0.1
1707966807066,0.906796921444024,0.997645679347435
"11935","AIFM2",56.4295840867001,-0.05026664724231,0.27800840827474,-0
.180809809150212,0.856516863427212,0.997645679347435
"11936","TYSND1",26.9328487990319,0.054326121136069,0.384659887897421,
0.141231573255583,0.887687004200671,0.997645679347435
"11937","SAR1A",279.892924038078,0.0219048403298949,0.202387260436355,
0.108232308113995,0.913811417311035,0.997645679347435
"11938","PPA1",786.926969580516,-0.0599511651385393,0.325543687491883,
-0.184157050012017,0.853890252279916,0.997645679347435
"11939","PCBD1",175.325872776092,-0.0669346003555872,0.304265524559152
,-0.219987461453506,0.825880919831309,0.997645679347435
"11940","NUDT13",2.31133888808959,0.257364682410284,1.00494548709692,0
.256098152302527,0.797875048671181,0.997645679347435
"11941","ECD",29.4552997471154,-0.0687617178154598,0.276791530135399,-
0.24842421219256,0.803806202003767,0.997645679347435
"11942","MRPS16",125.726685598531,0.0391716954603316,0.239668121439377
,0.163441408999569,0.870170901532627,0.997645679347435
"11943","CHCHD1",118.572446967747,-0.0790964989240495,0.29172392107314
1,-0.271134772332292,0.786287378426954,0.997645679347435
"11944","PLAU",268.139473201964,-0.0440740019682546,0.37708257183968,-
0.116881567220754,0.906953905250611,0.997645679347435
"11945","TMEM254",17.7661396703418,-0.0719659096330463,0.4112510968593
55,-0.174992626603639,0.861085427308454,0.997645679347435
"11946","AC067750.1",2.27208117777986,0.179328355417633,0.938875644827
178,0.191003309549736,0.848523000249167,0.997645679347435
"11947","SHLD2",48.059765994002,-0.0299584672210685,0.249094132771031,
-0.120269662267021,0.904269535641962,0.997645679347435
"11948","AL157893.2",3.83412253630003,0.0857168955898744,0.69338429041
5082,0.123621052242994,0.901615323065784,0.997645679347435
"11949","LINC00863",15.2955037339583,0.118669574086898,0.4284111370417
86,0.276999274356686,0.781780663333358,0.997645679347435
"11950","RPP30",63.0672528013935,0.0562623953541199,0.310064593687354,
0.181453788983243,0.856011402026573,0.997645679347435
"11951","CPEB3",9.16817790187116,0.0881778607889389,0.525015502021184,
0.167952870819005,0.866620355935632,0.997645679347435
"11952","MARCH5",69.457658877282,-0.039860115747195,0.233767158909568,
-0.170512042551771,0.864607464458211,0.997645679347435
"11953","FRA10AC1",87.0693388744974,0.049944386947212,0.21757667911525
5,0.229548438510523,0.818442677019593,0.997645679347435
"11954","ALDH18A1",35.7961704340737,0.0827572831117003,0.3226236878709
1,0.256513350454334,0.797554473048074,0.997645679347435
"11955","FRAT1",6.15593601370444,-0.0903612558592658,0.574589813867253

,-0.157262195880385,0.875038212799713,0.997645679347435
"11956","MORN4",13.5822541357001,-0.146335423191766,0.704841143064111,
-0.207614757781606,0.835529780399823,0.997645679347435
"11957","AVPI1",19.9277890324308,0.0731390946868577,0.428722282457513,
0.170597838459926,0.864539997668802,0.997645679347435
"11958","HPS1",57.8144530544589,-0.0289582639843278,0.216474542340637,
-0.133772145542916,0.893582753828386,0.997645679347435
"11959","OLMALINC",4.7568517513023,-0.133003528871621,0.63218384029353
,-0.210387422762762,0.833365308170158,0.997645679347435
"11960","BTRC",20.8430308967771,-0.0938377707337202,0.36068740426207,-
0.260163703042813,0.794737501202117,0.997645679347435
"11961","RPARP-
AS1",12.2353088864541,0.126036305263547,0.526014420004582,0.2396061789
75947,0.810635574169763,0.997645679347435
"11962","SUFU",16.9673264498083,0.0490742301500174,0.361900792377547,0
.135601333800954,0.89213645097331,0.997645679347435
"11963","AL391121.1",12.2442380857524,0.0661168220910763,0.52060536687
837,0.126999885705218,0.89894048901128,0.997645679347435
"11964","NT5C2",38.0989655315793,0.0802098066358907,0.287841648511662,
0.278659488821823,0.780506154661896,0.997645679347435
"11965","PCGF6",25.6137413270141,0.0956096088913327,0.325428689868879,
0.293795881764007,0.768913882165347,0.997645679347435
"11966","AL139339.1",4.43347573904408,-0.204510587190783,0.69910071904
8829,-0.292533796087397,0.769878523835046,0.997645679347435
"11967","SLK",151.222435380823,-0.0478869515634308,0.244007938088021,-
0.196251613528068,0.844413231677102,0.997645679347435
"11968","GSTO1",336.643899401302,-0.0732697235498993,0.325019497699475
,-0.225431778919452,0.821643391815661,0.997645679347435
"11969","XPNPEP1",48.6206689280627,0.0565917639446119,0.25979866783047
1,0.217829307660424,0.82756210861574,0.997645679347435
"11970","MXI1",85.8461525946136,-0.0874391803014175,0.306535156339803,
-0.285250087936043,0.775452548969189,0.997645679347435
"11971","AL158212.3",3.76096058470712,0.176999841164967,0.771268084363
06,0.229491981781069,0.818486551962397,0.997645679347435
"11972","AL133482.1",17.6726948430373,-0.0866500094662114,0.5175277893
61162,-0.167430640919152,0.867031217481312,0.997645679347435
"11973","NANOS1",7.47611384819322,-0.228817330684281,0.824469861634761
,-0.277532680491899,0.781371115568226,0.997645679347435
"11974","DENND10",84.2042110553933,-0.0507482756814191,0.2234200510523
76,-0.227142888216073,0.820312635519155,0.997645679347435
"11975","BAG3",137.108946673633,0.0447840067323339,0.279630574214034,0
.160154185064382,0.872759618478381,0.997645679347435
"11976","SEC23IP",54.4864711056597,-0.0754510017040266,0.2670416580240
12,-0.282543938134335,0.777526460592355,0.997645679347435
"11977","TACC2",25.9268869740381,-0.105275666870986,0.381447685647226,
-0.275989790558981,0.782555907770745,0.997645679347435
"11978","FAM24B",8.06827792214945,0.0938052564347574,0.53517664971889,
0.175279053157551,0.860860370126981,0.997645679347435
"11979","PSTK",7.88120799383484,0.0723253331275036,0.537357505872314,0
.134594441013893,0.89293253704135,0.997645679347435

"11980","IKZF5",21.4896638939123,0.0930279375673609,0.382124549671089,
0.243449256655805,0.807657390007268,0.997645679347435
"11981","ACADSB",21.5000083078407,-0.0717603757427011,0.34359467380927
9,-0.208851828077328,0.834563910416254,0.997645679347435
"11982","BUB3",112.558144391775,-0.0366898656878682,0.196187447429023,
-0.187014338423165,0.851649390845098,0.997645679347435
"11983","OAT",161.891928759566,0.0416579813673645,0.188405653235348,0.
221107916094891,0.825008406644478,0.997645679347435
"11984","CTBP2",72.1981759725952,0.0523474331841929,0.214167620233075,
0.2444227242532,0.806903443993166,0.997645679347435
"11985","MGMT",191.676703099057,0.0316703041252073,0.274140807953413,0
.115525683175885,0.908028464528824,0.997645679347435
"11986","DPYSL4",2.08986480048894,-0.295680264122664,1.10509924642081,
-0.267559918333408,0.789038086867164,0.997645679347435
"11987","LRRC27",9.79827325216447,0.0888933534208074,0.475274726094554
,0.187035725950053,0.851632621921923,0.997645679347435
"11988","ZNF511",85.8559707241245,-0.0571315372619926,0.25088953709277
8,-0.227715902081902,0.819867109127997,0.997645679347435
"11989","FUOM",63.1859470827219,-0.0965024143389222,0.357074429221433,
-0.270258541193601,0.786961358261102,0.997645679347435
"11990","ECHS1",211.261877845845,-0.0604304056264859,0.263696193697038
,-0.229166772486351,0.81873929710016,0.997645679347435
"11991","BET1L",46.8716107801876,0.075099617039073,0.250774080756048,0
.29947120855815,0.764580536823812,0.997645679347435
"11992","PHRF1",36.8647130551194,0.0835259961661879,0.296540339389426,
0.281668242297717,0.778197907875687,0.997645679347435
"11993","TALDO1",236.301520276891,-0.0435501762991517,0.26659254687413
2,-0.163358566508287,0.870236123737636,0.997645679347435
"11994","CTSD",390.769076484504,0.0670222900755821,0.225905135824729,0
.296683339362334,0.766708277915434,0.997645679347435
"11995","SLC22A18",15.0551666108392,0.0965224254551434,0.4244506299721
05,0.227405541750491,0.820108411471439,0.997645679347435
"11996","CARS",41.2837528405275,0.0445681701560201,0.276877338024251,0
.160967201122529,0.872119233442825,0.997645679347435
"11997","ZNF195",27.1124163270488,-0.0811340619103565,0.31572473249810
8,-0.256977213246488,0.79719636370368,0.997645679347435
"11998","NUP98",63.4430529249059,0.0810530306955517,0.285719048580552,
0.283680878465128,0.776654949761419,0.997645679347435
"11999","HBB",11.0063773254209,0.552704684975824,1.91722006342622,0.28
8284425726329,0.773129027812169,0.997645679347435
"12000","FAM160A2",22.0707966721865,-0.087560443607503,0.3143475861724
42,-0.278546575380635,0.780592817081807,0.997645679347435
"12001","CAVIN3",382.527084910059,-0.0639526009083741,0.25551848621953
,-0.250285612812487,0.802366481785871,0.997645679347435
"12002","RRP8",30.5401248619337,-0.0850805584961596,0.317848734589672,
-0.267676253630503,0.788948529854031,0.997645679347435
"12003","ST5",76.9937908819176,0.0307656957610992,0.243095806078452,0.
126557904298729,0.899290316453145,0.997645679347435
"12004","IPO7",105.026154095554,-0.0311675674310794,0.222710504486764,
-0.139946553050584,0.888702219166975,0.997645679347435

"12005","WEE1",42.4965530422194,-0.100275173445928,0.338973182183709,-0.295820373753294,0.767367262984708,0.997645679347435
"12006","LINC02709",4.31010225252096,0.102513939661631,0.698298016499683,0.146805428684298,0.883285599644036,0.997645679347435
"12007","PDE3B",5.90674403824228,-0.0821907036547407,0.720382543205558,-0.114093136250927,0.909163963173708,0.997645679347435
"12008","PLEKHA7",2.01019129631183,-0.199602852573524,0.997815949209926,-0.200039749546567,0.841449493705826,0.997645679347435
"12009","PIK3C2A",61.9038804020273,0.0637509655516415,0.281945029089234,0.2261113301326,0.821114832606608,0.997645679347435
"12010","AC124798.1",1.94795566518239,-0.197093051766023,1.0686754931358,-0.184427408536987,0.853678169688714,0.997645679347435
"12011","TPH1",2.11410702711312,-0.277858256518254,0.984715516074505,-0.28217109610084б,0.777812320136419,0.997645679347435
"12012","SPTY2D1OS",4.83151861915352,0.104684427101055,0.752580472655492,0.139100642263111,0.889370620708397,0.997645679347435
"12013","PRMT3",24.7795319184954,0.051760484455814,0.325246799391154,0.159142179270348,0.873556857399877,0.997645679347435
"12014","FIBIN",130.269686813649,-0.0731866376896444,0.352990375211108,-0.207333238607071,0.835749617424536,0.997645679347435
"12015","PRRG4",2.22016158052747,-0.170651692446261,1.08492473712167,-0.157293576786721,0.875013482230211,0.997645679347435
"12016","KIAA1549L",4.07431621554507,0.102914915026113,0.922909601923411,0.111511371007118,0.911210849077658,0.997645679347435
"12017","NAT10",31.8409829285809,0.0426256000092848,0.340902128647708,0.125037646958592,0.900493746239889,0.997645679347435
"12018","CAT",156.336662182894,0.0290875574802727,0.235983311234771,0.123261078624897,0.901900360505602,0.997645679347435
"12019","PDHX",22.87216548485,-0.0543455939484259,0.336231599589507,-0.161631429094632,0.871596105892212,0.997645679347435
"12020","SLC1A2",7.40071246890342,0.219913231800144,0.810934558830066,0.271184930282675,0.786248802753798,0.997645679347435
"12021","DGKZ",40.0840622894542,-0.0526804246955172,0.313659469797697,-0.167954198001721,0.866619311827677,0.997645679347435
"12022","AMBRA1",16.2802454343819,-0.100640015193605,0.393236629567191,-0.255927366950462,0.798006922404182,0.997645679347435
"12023","ARHGAP1",99.5904884852343,0.053688856903056,0.211572766245669,0.25376071720268,0.799680423340315,0.997645679347435
"12024","C1QTNF4",57.5254661329377,0.126351625309125,0.414186664396084,0.305059617246139,0.760320746878287,0.997645679347435
"12025","SERPING1",2304.80531766866,-0.0204375332221536,0.149714258259863,-0.136510266020753,0.891417909411607,0.997645679347435
"12026","ZFP91",164.894781411862,-0.0298796085903592,0.200006059288921,-0.149393516859389,0.881243127122585,0.997645679347435
"12027","MS4A1",1.93345846253152,0.127888968871806,1.08752620200038,0.117596218497144,0.906387601358623,0.997645679347435
"12028","TMEM132A",6.15349667932264,0.122520334106917,0.633441402380269,0.193420154802836,0.84662993221154,0.997645679347435
"12029","SLC15A3",28.6666074048403,-0.0591328905533955,0.300722046405645,-0.19663636657231,0.844112109765176,0.997645679347435

"12030","CD5",8.28928452501633,0.162053193889621,0.59199755442658,0.27
373963402026,0.78428471711951,0.997645679347435
"12031","TKFC",13.304420528281,0.078658764706743,0.428819016548922,0.1
83431148505908,0.854459737499816,0.997645679347435
"12032","TMEM216",42.7424954118054,-0.0765824838273594,0.3042340918914
92,-0.251722229258493,0.801255773684551,0.997645679347435
"12033","RAB3IL1",91.6960310304625,-0.0360862464848476,0.2047831414567
99,-0.17621688107788,0.86012355912552,0.997645679347435
"12034","EML3",37.7948940868478,0.0518863232454763,0.28266106534256,0.
183563743321333,0.854355708368581,0.997645679347435
"12035","B3GAT3",76.6859714559197,-0.0464610127295412,0.31005994127196
9,-0.149845260690377,0.880886699594516,0.997645679347435
"12036","CSKMT",55.4238043992461,0.0348930296638893,0.303659722116218,
0.114908323766874,0.908517787389117,0.997645679347435
"12037","AP001160.1",3.40081598685709,0.136824185522351,0.780147012556
533,0.17538256677287,0.860779037979734,0.997645679347435
"12038","SNHG1",35.9820072155948,-0.0821730021488434,0.282350149683748
,-0.291032259911613,0.771026647018664,0.997645679347435
"12039","PLAAT5",8.06604783464513,-0.106217831453053,0.50407993510912,
-0.210716245688414,0.833108696997706,0.997645679347435
"12040","PLAAT4",384.568915839006,-0.040154359062753,0.292876341067178
,-0.137103457781667,0.890949019366676,0.997645679347435
"12041","MARK2",41.9733663388141,-0.037363934221699,0.259792735386608,
-0.143822090198543,0.885640958769389,0.997645679347435
"12042","OTUB1",75.423563141205,0.0567861488481002,0.210577578222677,0
.269668543666369,0.787415262760673,0.997645679347435
"12043","STIP1",87.0872862269867,-0.0713449626442798,0.320851298561783
,-0.222361458295739,0.824032512587062,0.997645679347435
"12044","TRPT1",32.7449395549752,-0.0652139384170129,0.315870397477168
,-0.206457898359173,0.836433248490035,0.997645679347435
"12045","PLCB3",18.3847694671107,-0.0615220790288785,0.364276982024936
,-0.168888186914503,0.865884591198588,0.997645679347435
"12046","CCDC88B",5.80582625641395,0.170352791547537,0.669887137873201
,0.254300735028864,0.799263231993612,0.997645679347435
"12047","ATG2A",24.9481058658662,-0.113436272027678,0.418172465306771,
-0.271266717535937,0.786185902617746,0.997645679347435
"12048","AP003068.2",2.32199626220198,-0.10858935837755,0.910427529594
532,-0.11927292930818,0.905059129661696,0.997645679347435
"12049","DPF2",53.6942166387408,0.0324345071474968,0.236611635314046,0
.137079087866696,0.890968281882408,0.997645679347435
"12050","MALAT1",75824.7259097323,-0.0260713491859026,0.16295787567553
1,-0.159988273520538,0.872890311604306,0.997645679347435
"12051","LTBP3",295.740316850821,0.0569798243248796,0.190652774119409,
0.298867008823025,0.765041520386355,0.997645679347435
"12052","EHBP1L1",43.7573250747768,-0.0733364610366671,0.2845383633294
21,-0.25773839484612,0.796608812106324,0.997645679347435
"12053","KLC2",13.5950403982503,0.0577019183799253,0.393956270150343,0
.146467825877996,0.88355208720519,0.997645679347435
"12054","RAB1B",114.262933755787,0.0273209951506754,0.229895973417158,
0.118840685830978,0.905401574439523,0.997645679347435

"12055","CTSF",342.066659420947,-0.0429547257576144,0.216144537033956,
-0.198731489340701,0.842472790364289,0.997645679347435
"12056","RBM14",37.1610670464803,0.0486366514401952,0.27657465927188 9,
0.175853607008813,0.860408953501484,0.997645679347435
"12057","RBM4",265.228698542636,-0.0412402937514167,0.203207783726652,
-0.202946427519193,0.839176905374096,0.997645679347435
"12058","RAD9A",21.121535115099,-0.0691442663995249,0.357390024608892,
-0.193470051312127,0.846590858527828,0.997645679347435
"12059","C11orf24",50.5948123705974,-0.0498786163740153,0.268898650820
555,-0.185492252273519,0.852842956612485,0.997645679347435
"12060","LRP5",38.4928757062612,-0.116152617950304,0.379236731811826,-
0.30627997819562,0.759391482267108,0.997645679347435
"12061","IGHMBP2",9.48883752973383,-0.058204286173344,0.45695945823395
1,-0.127372976146048,0.89864520391831,0.997645679347435
"12062","RNF121",36.0524078788267,0.0862962238040429,0.292708069030843
,0.294820105539864,0.768131306728485,0.997645679347435
"12063","ATG16L2",9.50825491370974,0.143561790528989,0.535612246488404
,0.268033062108294,0.78867386964103,0.997645679347435
"12064","PPME1",35.6643451609704,-0.0562001046074297,0.279491482441894
,-0.201079847286987,0.840636139691606,0.997645679347435
"12065","POLD3",51.4894091078031,-0.0426714584199648,0.242648330310856
,-0.175857210166246,0.860406122709259,0.997645679347435
"12066","XRRA1",65.308093078819,-0.08581170172196,0.428471048041859,-0
.200274212491428,0.84126612942233,0.997645679347435
"12067","RPS3",4957.35368867563,-0.0562924856569897,0.23681601248407,-
0.237705571791841,0.812109461861024,0.997645679347435
"12068","KLHL35",5.39633254303638,0.0956006486968468,0.643589033762472
,0.148543004435545,0.881914249744944,0.997645679347435
"12069","AAMDC",136.469161972545,-0.0472546493418497,0.242010366427632
,-0.195258781842224,0.845190363279254,0.997645679347435
"12070","USP35",4.19566905912812,-0.125920596133485,0.802433599472354,
-0.156923384335208,0.875305230142142,0.997645679347435
"12071","CCDC90B",124.443121841876,0.0679807007060174,0.24805843736951
7,0.27405115273201,0.784045311633959,0.997645679347435
"12072","SYTL2",21.1170536672518,-0.0866865387538536,0.39127534047865,
-0.221548689083777,0.824665229932636,0.997645679347435
"12073","AP003120.1",1.90859165601569,0.302317049481204,1.088369851731
57,0.2777705106405,0.781188529768419,0.997645679347435
"12074","AMOTL1",40.3957649665055,0.0305226907913986,0.280099382879201
,0.108970931951543,0.913225545950145,0.997645679347435
"12075","AP000787.1",5.61032121257561,0.161680488503648,0.726919174361
316,0.222418797310861,0.823987880133759,0.997645679347435
"12076","MAML2",116.252505406453,-0.046213004785749,0.290274166076118,
-0.159204676773167,0.873507619327249,0.997645679347435
"12077","AP001527.2",2.24419820815557,-0.256610965329673,0.92995434535
7932,-0.2759393153122,0.782594676480955,0.997645679347435
"12078","BIRC3",53.6538622807113,-0.0889533332224079,0.378165890762453
,-0.235223047332644,0.814035618598034,0.997645679347435
"12079","MMP27",8.28788046294542,0.173682252338502,0.655695439121893,0
.264882507908088,0.791099973842285,0.997645679347435

"12080","AASDHPPT",67.470251440884,0.0381201532296911,0.293573991933467,0.129848536577213,0.896686261313892,0.997645679347435
"12081","AP002433.1",2.10060526985173,0.190221454105515,1.04495916086615,0.18203721372981,0.855553521452697,0.997645679347435
"12082","RDX",241.904098030034,0.0244173254656096,0.157449235728989,0.155080622351437,0.876757758482591,0.997645679347435
"12083","FDXACB1",4.46613381435381,0.147939727157888,0.673893925159378,0.219529693969121,0.82623745151207,0.997645679347435
"12084","NNMT",4099.03080785018,-0.0766580959382968,0.31964898653033,-0.239819612038792,0.810470102372673,0.997645679347435
"12085","C11orf71",24.6978329163597,0.0654238648792332,0.403335155281802,0.162207196725817,0.871142692686698,0.997645679347435
"12086","LINC00900",2.89416343454154,-0.265752747212829,0.931081831077833,-0.285423620505182,0.775319613291324,0.997645679347435
"12087","BUD13",18.2230159169495,0.0580684772314867,0.412194292810835,0.140876470742732,0.887967530376769,0.997645679347435
"12088","SIK3",56.4009047103029,0.0262558575364281,0.239970797313791,0.109412719507263,0.912875145608078,0.997645679347435
"12089","CD3D",52.8641602403609,-0.0759620716475335,0.380640705477906,-0.199563710749644,0.841821811445888,0.997645679347435
"12090","IFT46",15.4242689173387,-0.0540375534579169,0.377414024837676,-0.143178445690136,0.886149252268609,0.997645679347435
"12091","CCDC84",29.1255463498643,0.0659525812251291,0.336325703604661,0.196097355980422,0.844533965706855,0.997645679347435
"12092","AP003392.5",1.86251080566161,-0.248856995352487,1.06909441881402,-0.232773636241176,0.815937185865789,0.997645679347435
"12093","HMBS",19.7691174040967,-0.0811669790247394,0.415752570003309,-0.195229049393714,0.845213638459,0.997645679347435
"12094","H2AFX",66.4222190992358,0.0786316988790623,0.303335772360667,0.259223296570406,0.795462957225816,0.997645679347435
"12095","NLRX1",11.0511261591845,-0.137121792260205,0.49152899772126,-0.278969893731406,0.780267929226535,0.997645679347435
"12096","CBL",19.9181315932699,-0.12365947300705,0.406939411632346,-0.303876865873026,0.761221703232439,0.997645679347435
"12097","RNF26",26.2884186182772,0.0944496275165877,0.325966295282965,0.289752740953165,0.77200540475865,0.997645679347435
"12098","THY1",1184.18116726346,-0.0823403594006956,0.345026223632698,-0.238649568527731,0.811377325308841,0.997645679347435
"12099","OAF",460.616137752141,0.0707758774639872,0.30451017566571 8,0.23242532801821,0.81620767858151,0.997645679347435
"12100","ARHGEF12",99.5229471447685,-0.041073998468649,0.264709760579999,-0.15516616530744,0.876690321358247,0.997645679347435
"12101","SC5D",45.6604297631175,-0.0314087494230149,0.284314315675082,-0.110471923822891,0.91203511510554,0.997645679347435
"12102","ROBO4",11.962504521144,0.0555244105901218,0.50021356976273,0.111001408091466,0.911615230138458,0.997645679347435
"12103","CHEK1",4.91160806008067,-0.078581241952456,0.63305857637326,-0.124129495887476,0.901212744417451,0.997645679347435
"12104","AP000821.2",4.50918886840326,0.11438751522758,0.906304615125037,0.12621310022988 1,0.899563241808506,0.997645679347435

"12105","RPUSD4",29.2799469080867,-0.0960713548853276,0.395175175500183,-0.243110804629182,0.807919561388067,0.997645679347435
"12106","TIRAP",5.52775883646013,-0.163622638618228,0.623226837104325,-0.262541066714107,0.792904328713944,0.997645679347435
"12107","DCPS",42.1570602505113,0.0870809822538358,0.330424916397948,0.263542420478242,0.792132531949685,0.997645679347435
"12108","PARP11",17.815438060921,0.0912021726806791,0.412322495450296,0.221191357946836,0.82494343797982,0.997645679347435
"12109","CCND2",91.564058643237,-0.0535451223659782,0.298303657437818,-0.179498712238015,0.857546128394636,0.997645679347435
"12110","RAD51AP1",2.28656555979427,0.123849304918164,0.904833281702469,0.13687527572498,0.891129381991464,0.997645679347435
"12111","DYRK4",9.25569985913788,-0.196421795965014,0.647406951016944,-0.303397724810454,0.761586779353632,0.997645679347435
"12112","TAPBPL",49.6458511448363,0.0961109149906557,0.341256782892582,0.281638108921952,0.778221015850282,0.997645679347435
"12113","MRPL51",322.597335443321,-0.0716196241796923,0.271319930349408,-0.263967428000811,0.791805017498698,0.997645679347435
"12114","NOP2",21.2375294915145,-0.105694314884584,0.36600727862992,-0.288776538215935,0.772752387351805,0.997645679347435
"12115","LAG3",6.55011608972256,-0.142985765084355,0.59113698283914,-0.241882624899589,0.808871113751246,0.997645679347435
"12116","USP5",37.2742236704097,-0.0410981142969233,0.299968420656728,-0.137008136412981,0.89102436383522,0.997645679347435
"12117","ATN1",140.255799530917,0.0481748035500449,0.236215769216389,0.203944062286179,0.838397211894048,0.997645679347435
"12118","SLC2A3",501.367868984335,-0.083566851473598,0.296065953862745,-0.2822575523572,0.777746031083859,0.997645679347435
"12119","LINC00987",2.10321219214057,-0.178045723849449,1.13000942606331,-0.157561273156561,0.874802521851493,0.997645679347435
"12120","KLRC1",6.03604317598557,-0.445831595013404,1.53532071363877,-0.290383364891083,0.771522968598502,0.997645679347435
"12121","PLBD1-
AS1",3.92801820172671,-0.136417795088555,0.722106563275659,-0.188916431488628,0.850158315001333,0.997645679347435
"12122","HIST4H4",2.78171673791626,-0.20641054792309,0.910620184495084,-0.226670297273873,0.820680125309279,0.997645679347435
"12123","AC092828.1",3.24135177242899,-0.173359746252388,0.817501655840978,-0.21206042215786,0.832059896008413,0.997645679347435
"12124","RASSF8",110.144634800137,-0.0645704647458859,0.228301282225109,-0.282830057354729,0.777307112209891,0.997645679347435
"12125","SSPN",603.648629216576,-0.0685924987730107,0.239821662238934,-0.286014608241153,0.774866932965572,0.997645679347435
"12126","TM7SF3",68.430632211618,0.0649730530023117,0.227589062605414,0.285484074930964,0.775273303357758,0.997645679347435
"12127","STK38L",26.5091635245194,-0.0502522538737803,0.322165149260996,-0.155982898799116,0.876046500999731,0.997645679347435
"12128","REP15",2.15847258431102,-0.144249069824829,1.04171015843142,-0.138473325480512,0.889866349688486,0.997645679347435
"12129","ERGIC2",172.695524782433,-0.0294347019946717,0.22887142859279

2,-0.128608023184239,0.897667817453307,0.997645679347435
"12130","TSPAN11",7.76528256612644,0.108307502069771,0.583335590822499,0.185669285011494,0.852704116470863,0.997645679347435
"12131","AC026356.1",2.16312106570529,-0.218634680667525,1.06780711412589,-0.204751099496559,0.837766594436241,0.997645679347435
"12132","DNM1L",81.9514835855228,0.0656258172106518,0.246100327778844,0.266662859830182,0.789728752245972,0.997645679347435
"12133","SLC2A13",27.2512373536166,0.055150377347564,0.395987421815634,0.139273053408351,0.889234382598824,0.997645679347435
"12134","AC079630.1",5.03492961360925,0.178714085360599,0.680676883067138,0.262553481404145,0.792894758806215,0.997645679347435
"12135","IRAK4",27.8664624108331,-0.0359764503102309,0.319689185435621,-0.1125357126523,0.910398654974372,0.997645679347435
"12136","SLC38A1",26.4597739873476,0.0711863647491028,0.500116411913187,0.142339589450345,0.88681177700478,0.997645679347435
"12137","PCED1B",5.5467472038438,-0.137206892076726,0.639401822542053,-0.214586332474368,0.830089850819345,0.997645679347435
"12138","SENP1",20.0800315686224,0.0552020701659364,0.359899612503453,0.153381854962144,0.878097153209309,0.997645679347435
"12139","ASB8",74.0224365703551,-0.0314895068353046,0.23461318596848 7,-0.134218827920159,0.893229538092333,0.997645679347435
"12140","KANSL2",23.3488533946394,-0.0464431643340314,0.341136827599299,-0.136142335205696,0.891708760417739,0.997645679347435
"12141","ARF3",135.639071513414,-0.0579842152345869,0.188825588085821,-0.307078165742205,0.758783876545547,0.997645679347435
"12142","PRKAG1",69.6200436913167,-0.0686303384121946,0.270298879942772,-0.253905374771531,0.799568662370312,0.997645679347435
"12143","MCRS1",50.2411435396542,-0.0734988413447229,0.292157937920718,-0.251572289521937,0.801371679616654,0.997645679347435
"12144","BCDIN3D",14.3557425739132,-0.0722053722992907,0.401140505848026,-0.180000202539123,0.857152409192237,0.997645679347435
"12145","C12orf81",16.8394238765017,0.0623549533948678,0.439626765899308,0.141836116978259,0.887209455228647,0.997645679347435
"12146","ACVRL1",183.313847865362,-0.0373772839904729,0.245408836985209,-0.152306186075629,0.87894544606091,0.997645679347435
"12147","GRASP",40.234473837786,-0.0389374572572309,0.32290380637694,-0.120585315156606,0.904019500765892,0.997645679347435
"12148","AC025259.3",2.89743888611722,0.217778880934854,1.06826356112056,0.203862500660805,0.838460949786716,0.997645679347435
"12149","EIF4B",398.308391572848,-0.0246191599124129,0.192097219452325,-0.12815989727807,0.898022435629984,0.997645679347435
"12150","MFSD5",35.6769246778044,0.0619073673011692,0.333977929209407,0.185363647974333,0.852943819003866,0.997645679347435
"12151","AAAS",28.3841795390411,-0.0949428740819425,0.325781083992592,-0.291431512592368,0.770721316414683,0.997645679347435
"12152","HOXC-AS3",7.08324072990993,-0.118426380448745,0.534636341351877,-0.221508287575987,0.824696684315008,0.997645679347435
"12153","HOXC9",98.2728213990371,0.0384858576263461,0.206734487353762,0.186160800353012,0.85231866334641,0.997645679347435

"12154","HOXC-
AS1",11.3093092000638,0.134999060841944,0.455042219656599,0.296673704131941,0.766715634720825,0.997645679347435
"12155","HOXC8",59.052493020817,0.0325909052252062,0.261450421484892,0.124654246262477,0.900797280968917,0.997645679347435
"12156","HOXC4",22.0519457115165,0.0612764345397109,0.422542709189877,0.145018321715203,0.88469640606891,0.997645679347435
"12157","HOXC5",13.5326891975718,0.116576703473352,0.453093318583802,0.257290714058036,0.796954360332963,0.997645679347435
"12158","CBX5",154.950783418873,0.0466410265993683,0.214468322873096,0.217472799593656,0.827839902528847,0.997645679347435
"12159","COPZ1",177.822217164888,-0.0328932561184651,0.277429032910461,-0.118564577662934,0.905620330272292,0.997645679347435
"12160","BLOC1S1",358.76217799882,0.0535863801691581,0.262524603638962,0.204119459381617,0.838260148069965,0.997645679347435
"12161","RDH5",21.7412221033039,-0.0584242841683399,0.336164658475276,-0.173796628215863,0.862025294362156,0.997645679347435
"12162","CD63",4996.86445731925,-0.043760115014578,0.274826744163457,-0.159228007986556,0.873489238174731,0.997645679347435
"12163","MMP19",87.879143861307,-0.0692767641955563,0.389591726588394,-0.177818879271909,0.858865220121983,0.997645679347435
"12164","DGKA",15.9489433104181,0.0458791327108403,0.394119525054582,0.116409185016871,0.90732825629487,0.997645679347435
"12165","RPS26",1811.41575056583,-0.17805773834826,0.612172476910472,-0.290862044708195,0.771156830787992,0.997645679347435
"12166","MYL6",2065.61687926701,0.0471491766479414,0.261484274077555,0.180313622355573,0.856906361854977,0.997645679347435
"12167","AC073896.2",10.9879132862605,0.162271774368931,0.536150943446606,0.302660615172622,0.762148515321431,0.997645679347435
"12168","TSPAN31",88.5307577724903,0.0454888065527201,0.257118940054136,0.176917369615565,0.859573294854678,0.997645679347435
"12169","METTL1",32.3472557745377,-0.135437033156427,0.460345769520101,-0.294207185389402,0.768599590357181,0.997645679347435
"12170","SLC16A7",95.1941105529612,0.0662387895379137,0.325172594934314,0.203703481073779,0.838585221732591,0.997645679347435
"12171","TBK1",57.409988987765,0.0635424451928461,0.255180296302528,0.249009998473838,0.803353047312037,0.997645679347435
"12172","RASSF3",8.41614705276308,-0.0807338362322526,0.512611844875845,-0.157495065787655,0.874854696298527,0.997645679347435
"12173","LEMD3",43.0116095066644,0.0715063271050023,0.259569451198176,0.275480518893607,0.782947090698886,0.997645679347435
"12174","LLPH",102.432026768355,-0.0528129353659176,0.285687955000432,-0.184862310228787,0.853337033496782,0.997645679347435
"12175","MDM1",20.8517460469189,-0.0953963223229315,0.341049663644199,-0.279713873057661,0.779697033870006,0.997645679347435
"12176","RAP1B",545.564950046237,0.0344026082800712,0.218219990304422,0.157651039357479,0.874731782832246,0.997645679347435
"12177","CPM",16.9029203660994,-0.0567524459744636,0.476783271225002,-0.119031957284595,0.905250037508594,0.997645679347435
"12178","YEATS4",37.5192489255821,-0.080925664565589,0.276099552977129

,-0.293103207495206,0.76944326516117,0.997645679347435
"12179","RAB3IP",3.77621943444324,-0.139307092311749,0.723719473899023
,-0.192487693555122,0.847360206789264,0.997645679347435
"12180","THAP2",26.3939473271799,0.0997059071027448,0.4463039225951,0.
223403609188533,0.823221395583047,0.997645679347435
"12181","CCDC59",160.191459952705,-0.0548746834332575,0.20592964632338
3,-0.266472964980888,0.789874977740633,0.997645679347435
"12182","AC024941.2",2.37071474478903,0.244228928787006,0.940495747229
999,0.259681055981723,0.79510980660047,0.997645679347435
"12183","LINC02823",2.80491396042571,-0.14370792073679,0.8991273595516
01,-0.159830439158761,0.87301464532981,0.997645679347435
"12184","AC025164.1",8.96677983829916,0.081755642793679,0.517449151478
708,0.157997442956563,0.87445881363355,0.997645679347435
"12185","CRADD",14.2142753485957,-0.0891382576859713,0.417730531581488
,-0.213386982628496,0.831025133054206,0.997645679347435
"12186","CEP83",35.0993630310531,0.0732819799245924,0.262295825775235,
0.279386756186463,0.77994803362559,0.997645679347435
"12187","FGD6",8.33180951353257,0.123436570229019,0.589752338136544,0.
209302383809184,0.83421219098007,0.997645679347435
"12188","CDK17",28.9450316294348,0.0967721023345014,0.327410686333025,
0.295567940736271,0.767560059838208,0.997645679347435
"12189","NT5DC3",7.3893188724114,0.0706230760914116,0.552283884176844,
0.127874591518585,0.898248218973413,0.997645679347435
"12190","HSP90B1",1239.14050931625,-0.0459553861797101,0.2083112805826
06,-0.220609205853768,0.825396732427197,0.997645679347435
"12191","NFYB",154.063904313146,0.0291737190039883,0.244401758309569,0
.119367876916154,0.90498390980769,0.997645679347435
"12192","CHST11",28.5448565722463,-0.106393195996443,0.387288723868143
,-0.274712867789731,0.783536844325344,0.997645679347435
"12193","AC007541.1",10.9673355872871,0.0552327522906632,0.48088622433
9134,0.114856174902011,0.908559122492346,0.997645679347435
"12194","USP30-
AS1",7.03168180685815,-0.153082712365142,0.642447736697242,-0.23828041
3519899,0.811663611648414,0.997645679347435
"12195","UNG",18.6717533903087,-0.0954138018115263,0.374093940199238,-
0.25505305368141,0.79868212293986,0.997645679347435
"12196","GLTP",112.606107188384,-0.0230019783590188,0.212034647221437,
-0.10848216864764,0.91361322439178,0.997645679347435
"12197","TCHP",26.0985500020089,-0.0998788104792723,0.355191211653061,
-0.281197302192348,0.778559073756816,0.997645679347435
"12198","HVCN1",10.2175033374531,-0.0664502194692155,0.479178006759388
,-0.138675436960492,0.889706628992076,0.997645679347435
"12199","BRAP",28.5954927882468,0.0483452068398065,0.349439877675247,0
.138350571667543,0.889963359346632,0.997645679347435
"12200","MAPKAPK5-
AS1",44.9045259236819,0.0843938771173365,0.306534297621642,0.275316262
395879,0.783073271489678,0.997645679347435
"12201","TMEM116",12.0876028443665,0.120483484314248,0.478985265458718
,0.251539020096709,0.801397398033331,0.997645679347435
"12202","ERP29",309.531107429991,-0.0445549615946106,0.246426473143056

,-0.180804282211779,0.856521201790208,0.997645679347435
"12203","OAS1",64.022136394151,0.107914733394529,0.433724431038587,0.24880944136838,0.803508187576991,0.997645679347435
"12204","OAS3",30.4223178818105,-0.112697936781004,0.385717587576223,-0.292177334949067,0.770151040012512,0.997645679347435
"12205","DTX1",39.7311921006307,-0.120192088183609,0.391673324686371,-0.306868200125326,0.758943694625224,0.997645679347435
"12206","RBM19",31.4126751221491,0.0450762023902411,0.387335750735632,0.116375011355476,0.907355338863577,0.997645679347435
"12207","WSB2",59.3529914420384,-0.0310609379715512,0.272367681728888,-0.114040468290467,0.909205713628285,0.997645679347435
"12208","VSIG10",34.9559953291148,0.0463800367964262,0.361489228931833,0.128302679815592,0.897909444449031,0.997645679347435
"12209","TAOK3",99.5690966586411,0.0479102383705278,0.2792332092935,0.171577866729203,0.863769409176,0.997645679347435
"12210","SUDS3",63.0446812326294,-0.0702246762377302,0.2501987716564,-0.280675543580088,0.778959268406265,0.997645679347435
"12211","CIT",2.28046014341191,0.162846333190227,1.00183611244936,0.162547877009632,0.870874429144492,0.997645679347435
"12212","GCN1",37.2197904022253,0.0637315426983534,0.286329951668812,0.222580775524558,0.823861800034715,0.997645679347435
"12213","TRIAP1",60.8449803596791,-0.0558746701192936,0.340403094820769,-0.164142661948221,0.869618837868245,0.997645679347435
"12214","UNC119B",18.6187156409172,-0.0962244285348249,0.361755399851519,-0.265993067620607,0.79024454800288,0.997645679347435
"12215","OASL",34.3456835821667,-0.121122768209475,0.565263850188065,-0.214276515593872,0.830331430567963,0.997645679347435
"12216","CAMKK2",92.7866550098171,-0.0360509430364169,0.238253814327536,-0.151313183120151,0.879728670392324,0.997645679347435
"12217","RNF34",30.6801236793127,0.0738948280701664,0.301427325828647,0.245149731753828,0.806340497112798,0.997645679347435
"12218","KDM2B",18.5229419735877,0.0568705778812305,0.3783714072087,0.150303582135799,0.880525106960908,0.997645679347435
"12219","RSRC2",315.503895187317,0.0398796426306616,0.170159519713886,0.234366215288555,0.81470068459359,0.997645679347435
"12220","DENR",122.833581294499,0.0574571721873871,0.204063469461264,0.281565202919839,0.778276925101928,0.997645679347435
"12221","PITPNM2",11.9113554451934,-0.12750890611295,0.428578972306644,-0.297515544047082,0.766072942541244,0.997645679347435
"12222","C12orf65",37.0511459787577,0.0514928201159517,0.279128822166535,0.184476901082002,0.853639346434558,0.997645679347435
"12223","SBNO1-AS1",3.77072537783911,-0.0988888892707235,0.73384112580284,-0.134755174919553,0.892805447710445,0.997645679347435
"12224","KMT5A",43.6963900025258,-0.0285425934915826,0.260099157343151,-0.109737354719401,0.912617674522087,0.997645679347435
"12225","TMED2-DT",2.23370104514847,0.282842577418407,0.986631271942723,0.286675058313808,0.774361136938342,0.997645679347435
"12226","ZNF664",41.779223211629,0.045736765385936,0.284460343794559,0

.160784328584541,0.872263268617248,0.997645679347435
"12227","STX2",70.2988452396963,0.0564028162401178,0.237035745603917,0
.237950677423843,0.81191934920836,0.997645679347435
"12228","MMP17",18.6483958471911,0.0407372320542566,0.355388413297963,
0.114627350048416,0.908740500390276,0.997645679347435
"12229","ANKLE2",79.3192549474925,-0.0760567091279379,0.25120506048556
5,-0.302767424274513,0.762067110548675,0.997645679347435
"12230","GOLGA3",95.9984818033933,-0.0552219581298838,0.26186841073467
2,-0.21087674521321,0.83298345071671,0.997645679347435
"12231","ZNF26",19.6818446135736,0.0819255532104292,0.392546804818583,
0.208702636742366,0.834680381646228,0.997645679347435
"12232","ZNF140",19.823080179985,-0.0593048053814953,0.367470207085427
,-0.161386703569436,0.871788838414519,0.997645679347435
"12233","ZNF10",7.90483353447865,0.0848023612492926,0.506407948654676,
0.167458590400444,0.867009227467368,0.997645679347435
"12234","MPHOSPH8",299.108928567254,0.028924321095551,0.18728240536776
5,0.154442276831892,0.877261021424616,0.997645679347435
"12235","SAP18",583.650888963055,-0.0730181052465649,0.258864522605464
,-0.282070731483943,0.777889275246495,0.997645679347435
"12236","PARP4",70.7899002506996,0.0555412379435279,0.244508475748508,
0.227154652915408,0.82030348771533,0.997645679347435
"12237","CENPJ",12.4336135106811,-0.089328847174207,0.447403938642414,
-0.199660395134793,0.841746190139553,0.997645679347435
"12238","LNX2",6.40249861109235,0.147580982478291,0.688987589285,0.214
1997690139,0.830391276202013,0.997645679347435
"12239","POMP",466.466334359628,-0.0502899771315547,0.291276969146072,
-0.172653462026154,0.862923826242608,0.997645679347435
"12240","UBL3",212.577676848166,-0.0299325313677008,0.220359389555942,
-0.135835062113847,0.891951672380026,0.997645679347435
"12241","FRY",3.00391238835507,0.0886408550562768,0.789853077498461,0.
112224485263779,0.910645415367591,0.997645679347435
"12242","MAB21L1",6.49013044942658,0.0997635690429378,0.72729693678225
1,0.137170341297349,0.890896153498479,0.997645679347435
"12243","SMAD9",29.3759762686249,0.123201954989043,0.450141433445908,0
.273696100458719,0.784318174756869,0.997645679347435
"12244","UFM1",168.145362334431,-0.0482280054261493,0.166982600543388,
-0.28882054339319,0.77271871040135,0.997645679347435
"12245","PROSER1",18.3048579825585,0.0524414588274203,0.34375824215202
1,0.15255331333766,0.878750544490225,0.997645679347435
"12246","SLC25A15",4.83561628521829,0.104915383741251,0.66238603033534
2,0.158390091180118,0.874149421314449,0.997645679347435
"12247","KBTBD6",20.5596436913077,0.0589007307438279,0.371802574635109
,0.158419372974041,0.874126349111145,0.997645679347435
"12248","VWA8",14.9429130212834,-0.0533129803379716,0.402458721482496,
-0.132468194853843,0.894613978126763,0.997645679347435
"12249","DGKH",38.4789607055197,0.12369443367292,0.437352119599709,0.2
82825732698249,0.777310427501377,0.997645679347435
"12250","GTF2F2",82.5753357666571,-0.0593768782415176,0.26545251930649
7,-0.223681728079438,0.823004964609151,0.997645679347435
"12251","COG3",31.5716877262591,0.0805823410505061,0.30950624719091,0.

260357720665978,0.794587852610538,0.997645679347435
"12252","ZC3H13",198.37803171402,-0.0266486130481976,0.20961750033937,-0.127129714861848,0.898837733209035,0.997645679347435
"12253","INTS6-AS1",6.8481834838261,-0.167254536866168,0.547590249590683,-0.305437390441464,0.760033048014692,0.997645679347435
"12254","NEK3",15.8473353507277,-0.0883716188746794,0.39027669258187,-0.226433247371392,0.820864471686034,0.997645679347435
"12255","TDRD3",47.077432268228,0.0654288037349907,0.268226712367432,0.243930975992289,0.80728427830046,0.997645679347435
"12256","FBXL3",104.669605788384,0.0362673395415315,0.196100227064237,0.184942873776742,0.853273842587432,0.997645679347435
"12257","LINC00377",4.6280259678293,0.151659778103365,0.911323563127653,0.166417049047729,0.867828757832271,0.997645679347435
"12258","AL354811.1",2.5146811929789,0.139849043751394,0.997138935531346,0.140250308926982,0.888462223900761,0.997645679347435
"12259","TEX30",38.3369123466619,0.0882164617707989,0.364793746557292,0.241825586659129,0.80891531194271,0.997645679347435
"12260","TNFSF13B",193.609170541994,0.0301434031839036,0.238331956463102,0.126476548219711,0.899354711769329,0.997645679347435
"12261","CARS2",66.1912658866351,-0.0604537099215267,0.244377454789562,-0.247378425205323,0.804615369592869,0.997645679347435
"12262","AL442128.2",2.45422584049466,-0.23287201883919,0.987203827322835,-0.235890514596877,0.81351762883581,0.997645679347435
"12263","AL442125.1",2.43806002881972,-0.202531989439911,0.91564196923309,-0.221191247502061,0.824943523972439,0.997645679347435
"12264","TFDP1",53.1169506404671,0.0758960348872345,0.274002891900791,0.276989904598213,0.781787857949336,0.997645679347435
"12265","GAS6-DT",3.77006905838967,0.175121178770598,0.752753496630812,0.232640804133635,0.816040339379493,0.997645679347435
"12266","AL355075.4",18.392961733303,-0.271858462806042,0.976763223812762,-0.278325858486821,0.780762227814764,0.997645679347435
"12267","TEP1",11.0169654881133,0.113530943813852,0.463347514878619,0.245023314398467,0.806438379215637,0.997645679347435
"12268","ANG",117.954927527804,-0.0416396791117037,0.222675522405483,-0.186997109794042,0.851662899026895,0.997645679347435
"12269","RNASE4",527.753037080915,-0.0360679109673387,0.17828176893125,-0.202308464760899,0.839675582876569,0.997645679347435
"12270","ZNF219",18.2150483102623,0.0953206402856479,0.365417147821209,0.260854316372384,0.794204855625805,0.997645679347435
"12271","TRDC",11.4758184821658,-0.115564246428651,0.624444450046861,-0.185067296890826,0.85317625179262,0.997645679347435
"12272","ZFHX2",5.30320356830579,0.119896390468512,0.719181740361178,0.166712228272507,0.867596483423792,0.997645679347435
"12273","AL136295.2",2.9813681492453,-0.194170777871925,0.884862315264645,-0.219436147886864,0.826310314182611,0.997645679347435
"12274","REC8",8.83365784821157,0.111751275665828,0.599990308626223,0.186255134556591,0.852244689191252,0.997645679347435
"12275","CIDEB",9.14895939839941,0.0814175544451559,0.541534888548518,

0.150345907838699,0.880491715359353,0.997645679347435
"12276","LTB4R",8.66969452145512,0.156346071085899,0.578702897065789,0.270166387413342,0.787032250429334,0.997645679347435
"12277","CMA1",2.40882319062438,-0.286895207370533,1.13773476669609,-0.252163523317178,0.800914671366138,0.997645679347435
"12278","NOVA1",1082.0495194289,-0.038579709486851,0.207483702415519,-0.185940915058422,0.852491096079401,0.997645679347435
"12279","AL079343.1",7.27499398459729,0.139904860468535,0.598451595336581,0.233778072543777,0.815157273496985,0.997645679347435
"12280","SCFD1",122.705903182628,-0.0418682238506575,0.175479542241265,-0.238593190499057,0.811421045851961,0.997645679347435
"12281","HECTD1",135.857862758025,-0.0324411880470009,0.220385846889446,-0.147201775907482,0.882972758897298,0.997645679347435
"12282","ARHGAP5-AS1",3.30593971092795,-0.223358067270778,0.744160379842305,-0.300147754867183,0.764064454308311,0.997645679347435
"12283","EAPP",163.453737519751,-0.0577756007682175,0.202065034944714,-0.28592577030472,0.77493497549506,0.997645679347435
"12284","SNX6",278.165906134674,0.0434631322455949,0.175819005603346,0.247203833831534,0.804750478399845,0.997645679347435
"12285","BAZ1A",110.679914704361,0.0834895850206517,0.27373582462406,0.305000579063093,0.760365711292052,0.997645679347435
"12286","CLEC14A",16.4684004129785,-0.105803816399858,0.548494698592683,-0.19289852148312,0.847038442973851,0.997645679347435
"12287","GEMIN2",28.6104051401932,-0.0726270183650995,0.300498004605964,-0.241688854008644,0.809021266779233,0.997645679347435
"12288","TRAPPC6B",43.7242346017162,0.0487666827551646,0.272142738118068,0.17919523810335,0.857784402653464,0.997645679347435
"12289","LRFN5",8.61559571330206,0.119425148402891,0.570309445644904,0.209404121420163,0.834132775658777,0.997645679347435
"12290","FANCM",14.2567273611349,0.113893277079068,0.441066850270953,0.258222255898628,0.796235382506888,0.997645679347435
"12291","LRR1",16.1400682316173,-0.117029043949738,0.402925767709055,-0.2904481503259,0.77147341183263,0.997645679347435
"12292","POLE2",2.66398339846365,0.2303015787387,0.949795402481636,0.242474935272339,0.808412176208376,0.997645679347435
"12293","ARF6",186.934331349285,0.0410406093350346,0.187969194325084,0.218336890161144,0.827166633534774,0.997645679347435
"12294","ATL1",44.8794581262551,-0.0363221534617352,0.250252401349218,-0.145142077622061,0.884598696876079,0.997645679347435
"12295","AL606834.1",8.64101209330609,0.161255280598828,0.57042389475279,0.282693768760709,0.777411593305231,0.997645679347435
"12296","PYGL",50.22709279476,-0.0741890075614886,0.256552984489677,-0.289176162612421,0.772446572434704,0.997645679347435
"12297","GNG2",49.9128496831824,0.0352613417171849,0.303325849319237,0.116249049648498,0.907455164232672,0.997645679347435
"12298","TXNDC16",10.4944115661202,-0.0560632009885131,0.504435542600124,-0.111140465438922,0.911504960713408,0.997645679347435
"12299","STYX",19.9552032024202,0.0431820850420249,0.33879618840383,0.127457411033662,0.898578379269421,0.997645679347435

"12300","CDKN3",31.9362210252817,-0.121247250520453,0.620236663958745,-0.195485461543947,0.845012918132678,0.997645679347435
"12301","GMFB",59.5726537426721,0.0635636149560904,0.22943440388635,0.277044827974345,0.781745685025946,0.997645679347435
"12302","AL138995.1",2.95655184799064,0.142815719189985,0.841990168200716,0.16961684896532,0.865311471000875,0.997645679347435
"12303","NAA30",28.5503706194997,0.0598893004890068,0.30661054254076,0.19532694470558,0.845137004480624,0.997645679347435
"12304","PSMA3",308.780081879048,-0.0729196229596387,0.29605653571984,-0.24630303391999,0.805447661550524,0.997645679347435
"12305","L3HYPDH",32.136472180512,-0.0660780970949261,0.291816853594493,-0.226436877380454,0.82086164866564,0.997645679347435
"12306","SLC38A6",29.1185584153469,-0.075952000876271,0.294710786730177,-0.257717071434542,0.796625269954552,0.997645679347435
"12307","MTHFD1",26.018011715796,-0.0952292061636488,0.315129244629963,-0.302190951130133,0.762506501936541,0.997645679347435
"12308","CHURC1",319.170048417539,-0.047200677559123,0.179143310763732,-0.263479989053986,0.792180645233613,0.997645679347435
"12309","AL359232.1",2.17960355693982,0.215183089660611,0.933822751535194,0.230432476941531,0.817755727131218,0.997645679347435
"12310","VTI1B",176.51932887174,0.0620354496995465,0.205636009285031,0.301676004680481,0.7628990620335,0.997645679347435
"12311","RAD51B",20.0465464928815,0.0453450695906848,0.377493312983855,0.120121517470759,0.904386887329802,0.997645679347435
"12312","EXD2",15.4583015494019,-0.0951849413986246,0.390519428585818,-0.24373932365751,0.807432715776088,0.997645679347435
"12313","SUSD6",35.1599880079062,0.0675361739267913,0.374550552586677,0.180312573190404,0.856907185469239,0.997645679347435
"12314","SMOC1",65.8523038641681,0.110759702855958,0.498528529865809,0.222173248310927,0.824179018463196,0.997645679347435
"12315","COX16",214.33694560249,-0.030821802661863,0.282935085837199,-0.108935951052737,0.913253291497877,0.997645679347435
"12316","MED6",63.2617532799739,-0.0720568687484316,0.241749501532934,-0.298064187481334,0.765654174158144,0.997645679347435
"12317","DCAF4",12.548717678222,-0.0832496559159628,0.422251895798133,-0.197156381639461,0.843705163019081,0.997645679347435
"12318","PSEN1",53.2027634541335,0.0376733676734324,0.263032324414954,0.143227140455937,0.886110795809342,0.997645679347435
"12319","RIOX1",5.55132434799871,-0.20704691172323,0.696102994829296,-0.297437179930541,0.766132761860876,0.997645679347435
"12320","COQ6",24.8243829852561,-0.0553675310837358,0.316833796752235,-0.174752604208551,0.86127403151256,0.997645679347435
"12321","ABCD4",54.2546580416287,0.067689727540827,0.245703791728066,0.275493215081284,0.782937337806403,0.997645679347435
"12322","POMT2",12.8856572844008,0.111758455582051,0.439251123327482,0.254429527090202,0.799163742005913,0.997645679347435
"12323","SAMD15",1.85708854625942,-0.162768850060101,1.11575711282189,-0.145881973943628,0.884014562051261,0.997645679347435
"12324","SPTLC2",39.0850100166078,-0.0659741360841462,0.305378500423312,-0.216040539830714,0.828956147434903,0.997645679347435

"12325","ALKBH1",8.15897455475673,0.083637175448447,0.521542429997145,
0.160365045369185,0.872593522978224,0.997645679347435
"12326","ZC3H14",82.2615138298544,0.0257468043651277,0.208704554653307
,0.123364841787461,0.901818196557114,0.997645679347435
"12327","FOXN3-
AS1",8.55883440845573,0.142045548574519,0.563676114091753,0.2519985236
61759,0.801042204934424,0.997645679347435
"12328","TDP1",7.63357892679849,-0.134661628907356,0.524528506956505,-
0.25672890438064,0.797388056734757,0.997645679347435
"12329","TTC7B",14.7113181503878,-0.0912376556333442,0.405825240034796
,-0.22482006201862,0.822119258332297,0.997645679347435
"12330","RPS6KA5",54.3742906903702,-0.0529119057173648,0.3040043299131
37,-0.174049842423242,0.861826291382281,0.997645679347435
"12331","NDUFB1",316.184021604249,0.0768016786265296,0.279851013289669
,0.274437736435971,0.783748246313867,0.997645679347435
"12332","DDX24",438.684394077247,-0.0232574270773096,0.18704058611378,
-0.124344280354007,0.901042688673766,0.997645679347435
"12333","C14orf132",51.0245439169645,0.0520038576180962,0.270327086638
932,0.192373832251432,0.847449388405378,0.997645679347435
"12334","VRK1",17.8233616664628,0.123936771015213,0.410502495802078,0.
301914780744642,0.76271702783772,0.997645679347435
"12335","CYP46A1",11.5456647951488,0.0869241401746886,0.57732903535416
5,0.150562564589125,0.880320793803636,0.997645679347435
"12336","EVL",126.129819422794,-0.0323799553867151,0.190784323674253,-
0.169720209517848,0.865230179755507,0.997645679347435
"12337","DEGS2",2.16584816341888,0.241795742251308,1.05330452348249,0.
229559198560992,0.81843431498797,0.997645679347435
"12338","ANKRD9",40.2358352112142,0.0457364396792079,0.329168873809998
,0.138945214199104,0.889493441703719,0.997645679347435
"12339","RCOR1",46.1323927743996,-0.052298427038746,0.285475410722486,
-0.183197659323401,0.854642930878359,0.997645679347435
"12340","CDC42BPB",93.5261285127583,0.0780858306503028,0.2592828275707
98,0.301160826507036,0.763291859809817,0.997645679347435
"12341","AL049840.2",11.6757796805481,-0.106592882740617,0.44314622866
3448,-0.240536590059914,0.809914300963197,0.997645679347435
"12342","ZBTB42",3.09803139460349,0.248793925426699,0.853561047012166,
0.291477599988408,0.770686073120475,0.997645679347435
"12343","AL928654.1",2.69153543241796,0.221682890942813,0.826884309865
864,0.268094204107918,0.788626807041844,0.997645679347435
"12344","IGHM",16.1688575149857,0.139512798869413,0.677613026411151,0.
205888602242958,0.836877928810822,0.997645679347435
"12345","CYFIP1",97.7767980456031,-0.0481201050596913,0.18333277583407
,-0.262474098484407,0.792955951948594,0.997645679347435
"12346","HERC2",85.6122720573841,0.0648910919706391,0.260838163144041,
0.248779132579632,0.80353163351069,0.997645679347435
"12347","TJP1",120.480908831884,-0.0649779546912336,0.279994397263329,
-0.232068767540813,0.816484602599224,0.997645679347435
"12348","AC022613.3",3.49619251618673,0.171880516556061,1.068678693284
11,0.160834605982329,0.87222366840679,0.997645679347435
"12349","OTUD7A",6.0160988345502,-0.139739135832707,0.661240315260887,

-0.211328820411644,0.832630695287761,0.997645679347435
"12350","LINC02256",16.0715639515684,-0.11265088746265,0.487843015078331,-0.230916266054484,0.817379853725412,0.997645679347435
"12351","GREM1",14.2228827175639,0.193595178231754,1.05842424079327,0.182908866568152,0.854869525749976,0.997645679347435
"12352","FMN1",5.54837895668682,0.203142656426373,0.677010769108053,0.300058234958373,0.764132735803024,0.997645679347435
"12353","NOP10",264.224495343698,0.04726925591094,0.286370441544736,0.165063320278311,0.868894143118777,0.997645679347435
"12354","LPCAT4",18.9382416322543,-0.0958370923967571,0.445525597397257,-0.215110182123392,0.829681415541401,0.997645679347435
"12355","THBS1",255.955968876547,-0.219753260018359,0.755060821765698,-0.29104047473218,0.771020364333438,0.997645679347435
"12356","EIF2AK4",107.138783375899,0.0599476494380845,0.1962537897363,0.305459830959872,0.760015959144207,0.997645679347435
"12357","NUSAP1",2.114822572953,0.131304195290632,0.964066603428499,0.13619826143098,0.891664549538797,0.997645679347435
"12358","RTF1",249.400518868895,0.0567340527341385,0.200044037372931,0.283607816954686,0.77671094593079,0.997645679347435
"12359","ZNF106",309.45976994614,0.0297677694138907,0.244542171970465,0.121728572106924,0.903113984235083,0.997645679347435
"12360","SNAP23",76.4185543557101,0.0656170951606231,0.225521131050456,0.290957636009473,0.771083719980254,0.997645679347435
"12361","AC018362.1",2.64372308168585,0.200551456565531,0.888336383561635,0.225760714383277,0.821387533616174,0.997645679347435
"12362","CDAN1",6.77172065090862,0.13038464667738,0.611462560292967,0.21234063938288,0.831144400009251,0.997645679347435
"12363","TUBGCP4",38.1906727083819,-0.0663772583077712,0.307905653103861,-0.215576614585186,0.829317786044909,0.997645679347435
"12364","MFAP1",57.377004137427,0.0473833679606114,0.248388198777598,0.190763362324784,0.848710994132004,0.997645679347435
"12365","CTDSPL2",61.726199791826,0.039657757643888,0.253673789482703,0.156333682422449,0.875770007997536,0.997645679347435
"12366","C15orf48",44.587947679658,0.431367476026575,1.40417763737208,0.307202924007449,0.758688920037521,0.997645679347435
"12367","DUT",269.590196885193,-0.0512832298718027,0.225806805551527,-0.227111090591555,0.820337360321886,0.997645679347435
"12368","COPS2",156.814851343459,-0.0338554452110954,0.214874759302595,-0.157558967470064,0.874804338829187,0.997645679347435
"12369","GABPB1",28.9780712406283,0.0858740435832517,0.336312438043721,0.255340076277786,0.798460449136643,0.997645679347435
"12370","CCPG1",247.553546693822,-0.0444607377511965,0.168833430844925,-0.263340841495036,0.792287883277042,0.997645679347435
"12371","PRTG",9.73830356489796,-0.139743490764729,0.474895289111634,-0.294261690879565,0.768557943615755,0.997645679347435
"12372","MNS1",36.5654844276151,-0.0381701331431634,0.292112152005291,-0.13066944624225,0.896036803429864,0.997645679347435
"12373","MYO1E",40.5527842519385,0.0813420376112066,0.397856471625065,0.204450708766809,0.838001307071805,0.997645679347435
"12374","TLN2",94.7983784902524,0.0441219732502618,0.244278846398719,0

.180621342783995,0.856664802297322,0.997645679347435
"12375","RPS27L",527.322569424236,-0.0542124854622835,0.219684054848106,-0.246774785269541,0.805082525521412,0.997645679347435
"12376","APH1B",52.3265062018814,0.0435095409398214,0.255937913015742,0.170000374024872,0.865009842502549,0.997645679347435
"12377","PPIB",1387.96974185856,-0.0715517067225368,0.242127649974972,-0.295512332977803,0.767602532448027,0.997645679347435
"12378","RBPMS2",6.60545600382073,-0.15022975514217,0.610216168280497,-0.24619104335681,0.80553434859604,0.997645679347435
"12379","SPG21",95.4887755073848,0.0611273374965489,0.211660766213256,0.288798621445794,0.772735487108734,0.997645679347435
"12380","MTFMT",14.3239646298867,-0.119067260903148,0.401147143686287,-0.29681692310955,0.766606284635551,0.997645679347435
"12381","CILP",965.656538695861,0.0744732601372736,0.342881734051361,0.217198097015335,0.828053967650351,0.997645679347435
"12382","INTS14",21.1168755833438,0.0883775976220495,0.321734193204894,0.274691343004898,0.78355338269439,0.997645679347435
"12383","AC116913.1",4.21968714001075,-0.171135356544154,0.666022860469171,-0.256951174954565,0.797216464542964,0.997645679347435
"12384","FEM1B",70.2215302960189,0.0342201433809395,0.248276856718843,0.137830580881292,0.890374314965487,0.997645679347435
"12385","UACA",171.728444548026,0.0770905856420679,0.258581437595836,0.298128846211153,0.765604825972081,0.997645679347435
"12386","TMEM202-
AS1",3.31772214048503,0.0887069002737368,0.774852172035821,0.114482353505793,0.908855434285346,0.997645679347435
"12387","UBL7",29.8864181679146,0.0652326051286899,0.31161084786288,0.209339968669494,0.834182852431857,0.997645679347435
"12388","ARID3B",4.25389310849421,-0.0892988936638732,0.686967004022949,-0.129990076875497,0.896574277582706,0.997645679347435
"12389","CLK3",43.6378871175464,-0.0538429996135778,0.240372064715533,-0.223998573533484,0.822758413190871,0.997645679347435
"12390","EDC3",18.3514380012153,-0.0485376721435893,0.375629124556009,-0.129217009466347,0.897185937436196,0.997645679347435
"12391","MAN2C1",43.5088580671924,0.0849636993169347,0.292199989318339,0.29077242444513,0.771225376630819,0.997645679347435
"12392","HMG20A",43.1416103110859,0.0509965114751326,0.278040809810747,0.18341376400768,0.854473376940572,0.997645679347435
"12393","LINGO1",2.1026257940097,-0.170875492911618,1.15968723493243,-0.147346187630991,0.882858777842962,0.997645679347435
"12394","ADAMTS7",15.5542501556667,0.128531103697774,0.433893112075655,0.29622757338301,0.767056292780718,0.997645679347435
"12395","TMED3",152.301318360729,0.0554138857421988,0.239852230648998,0.231033439181527,0.817288823962778,0.997645679347435
"12396","ZFAND6",116.430558698421,-0.0604972176741958,0.222168881303343,-0.272302841510889,0.785389171538707,0.997645679347435
"12397","GOLGA6L9",4.0592169600985,0.144663447292512,0.927234391483544,0.156016050117658,0.876020369976967,0.997645679347435
"12398","WHAMM",40.0916153283703,-0.0551806854798338,0.270223741070781,-0.204203691582303,0.838194326674027,0.997645679347435

"12399","C15orf40",76.9487595205208,0.0715716297613469,0.258078120505398,0.277325445571237,0.781530222775627,0.997645679347435
"12400","AC048382.5",3.34626822570862,0.166008640190972,0.807432934958802,0.205600531020501,0.837102962693479,0.997645679347435
"12401","WDR73",48.7528091637887,0.0850458412945539,0.28554520512595,0.297836698945939,0.765827803230438,0.997645679347435
"12402","ALPK3",2.90267998160303,-0.284676919865424,0.943661411961496,-0.3016727358531,0.762901554159736,0.997645679347435
"12403","KLHL25",5.48812526832627,-0.125136720593731,0.613782757751152,-0.203877868860672,0.838448939931845,0.997645679347435
"12404","MRPS11",88.294207938148,-0.0742997085364135,0.309740028737468,-0.239877644614636,0.810425111957527,0.997645679347435
"12405","FANCI",4.33339161376211,-0.0736950362348572,0.665208472122685,-0.110784873198767,0.911786940944713,0.997645679347435
"12406","NGRN",178.487751978362,-0.0265307662836784,0.19568508054163,-0.135578891401658,0.892154193569333,0.997645679347435
"12407","FURIN",36.1123531439481,0.0378360655592909,0.285750581289835,0.132409408892555,0.89466047298365,0.997645679347435
"12408","UNC45A",48.3433168133577,-0.0504387010835469,0.236182661582264,-0.213558017957972,0.830891740871376,0.997645679347435
"12409","RCCD1",14.9965359257734,0.0462040527279133,0.396005837897639,0.116675180783209,0.907117458786466,0.997645679347435
"12410","RGMA",70.3744915757811,0.0501045648480812,0.254911105142901,0.196557010805759,0.844174214649274,0.997645679347435
"12411","MCTP2",6.1855248123823,0.0967756386471546,0.679728954258422,0.142373865407478,0.886784704460759,0.997645679347435
"12412","AC087477.2",10.3263280868648,0.0868026001081551,0.542612397873457,0.159971649096743,0.872903407315414,0.997645679347435
"12413","ARRDC4",25.1330432522038,0.0786575718008948,0.35395593339591,0.22224193408037,0.824139361322025,0.997645679347435
"12414","MEF2A",111.319725417579,0.0747796852835371,0.262305599790784,0.285086118417531,0.775578164860675,0.997645679347435
"12415","LUC7L",71.0514522368882,-0.0451750832940639,0.215920345562254,-0.209221058703054,0.834275673728422,0.997645679347435
"12416","CAPN15",29.8173360565127,-0.0833073776456792,0.29820752249268,-0.279360416361476,0.779968245411576,0.997645679347435
"12417","RHBDL1",2.09897774161046,0.169558469953427,1.23293165371569,0.137524630373815,0.890616125482179,0.997645679347435
"12418","ANTKMT",83.537862845427,-0.0587964660532379,0.288093323177357,-0.204088263500093,0.838284525690929,0.997645679347435
"12419","LMF1",33.3356661473423,0.0467456395994049,0.391847541408626,0.119295477601728,0.905041266266183,0.997645679347435
"12420","TPSAB1",5.38416375439365,-0.28061472483227,0.970781211211197,-0.289060729226682,0.772534904883681,0.997645679347435
"12421","TMEM204",55.1831653418945,0.0324400534278665,0.300411786981462,0.107985288306508,0.914007362194797,0.997645679347435
"12422","NUBP2",81.8737024816059,-0.0684113664440873,0.263965604725538,-0.259167729504831,0.795505828764431,0.997645679347435
"12423","GFER",61.5293488846441,-0.0696930884551268,0.304872776988091,-0.228597282917947,0.819181936573535,0.997645679347435

"12424","ZNF598",32.0205909577196,0.0751951765768077,0.311864688260228
,0.241114750747488,0.809466180553167,0.997645679347435
"12425","NTHL1",33.1260038964149,-0.0934205764281692,0.352479830376151
,-0.265038077011314,0.790980129147461,0.997645679347435
"12426","SNHG19",53.9102009107971,-0.0363695519872847,0.32766232528116
8,-0.110997051479983,0.911618684863271,0.997645679347435
"12427","TRAF7",64.8884800590737,0.0562517119877303,0.239038066882483,
0.235325330067135,0.813956236525151,0.997645679347435
"12428","CCNF",2.29660207043102,0.197297024444551,0.963992191114963,0.
204666621019363,0.83783260089403,0.997645679347435
"12429","ATP6V0C",398.201283928444,0.055895286612147,0.256532001571228
,0.217888163152336,0.827516250023375,0.997645679347435
"12430","AC093525.4",3.84923100378782,-0.235605210481847,0.81681409073
6467,-0.288444106380948,0.773006809659624,0.997645679347435
"12431","FLYWCH1",30.358136926494,0.0941980134605754,0.311089941287988
,0.3027999332623,0.762042334280474,0.997645679347435
"12432","NAA60",47.2291176437187,-0.0504705484186996,0.242676620398068
,-0.20797449847419,0.835248879254344,0.997645679347435
"12433","CLUAP1",55.5823564328602,0.0727994904601637,0.261147455506969
,0.278767757161704,0.780423059973705,0.997645679347435
"12434","DNAJA3",29.0445853115066,-0.0662360825246927,0.33922816981578
3,-0.195255254186768,0.845193124794329,0.997645679347435
"12435","NAGPA",21.3588285863034,0.0508428169186857,0.335035521018753,
0.15175351187864,0.879381349509134,0.997645679347435
"12436","PMM2",46.57191790438,-0.0393747309237202,0.31739961291942,-0.
124054123952938,0.901272421271641,0.997645679347435
"12437","C16orf72",50.419359186219,-0.0473156830910228,0.2832758734826
51,-0.167030402234098,0.867346127423135,0.997645679347435
"12438","GSPT1",196.841812651608,-0.038684969372079,0.196314814928656,
-0.197055781990462,0.843783885755362,0.997645679347435
"12439","AC007216.3",4.14524159004281,0.089454588474057,0.773741400171
323,0.115613030987162,0.907959234930805,0.997645679347435
"12440","SNX29",82.8798003205408,0.10135412282045,0.348557564267029,0.
290781590219062,0.771218366128644,0.997645679347435
"12441","BFAR",108.372268854938,-0.034149417088757,0.2400733250662,-0.
142245778781713,0.886885873158967,0.997645679347435
"12442","RRN3",44.3730312370401,0.0396721049673371,0.270918323914802,0
.146435665163103,0.883577474028288,0.997645679347435
"12443","NPIPA5",1.85788537958639,0.22611974883824,1.24363382099354,0.
181821807208165,0.855722570097616,0.997645679347435
"12444","BMERB1",95.3596537459192,-0.038274377006203,0.273575609135943
,-0.139904200988853,0.888735682027901,0.997645679347435
"12445","ACSM5",2.99980819776972,0.219581862787305,0.883489437708321,0
.248539318542254,0.803717152402336,0.997645679347435
"12446","THUMPD1",131.539234899929,0.0517116498289444,0.17454674941735
1,0.296262462644314,0.767029650294314,0.997645679347435
"12447","AC092338.3",17.9271340601508,-0.0963477580240524,0.3687090116
98075,-0.261311101620019,0.79385260606231,0.997645679347435
"12448","USP31",12.301585966,0.0912913256062121,0.463298244324994,0.19
7046560664653,0.843791101842725,0.997645679347435

"12449","PRKCB",10.9183821016496,-0.10705890201037,0.618341028830143,-0.17313892660967,0.862542227964442,0.997645679347435
"12450","ZKSCAN2",3.64517426348343,0.0909031512306587,0.761056901641985,0.119443304481616,0.904924154823935,0.997645679347435
"12451","ZKSCAN2-DT",3.76729922808294,0.114565721806074,0.829530464775052,0.138109119159525,0.890154178797863,0.997645679347435
"12452","KDM8",2.27998200115749,0.256149066330607,0.897663384701345,0.2853509129325,0.77537531056748,0.997645679347435
"12453","GTF3C1",49.0099956556774,0.0729574727527224,0.243641076884169,0.299446520618555,0.764599371237098,0.997645679347435
"12454","KIAA0556",12.4687284351073,-0.0714281247700006,0.395371446093147,-0.180660807642574,0.856633823479713,0.997645679347435
"12455","EIF3C",14.6451048334373,-0.0847596294888458,0.439405819862357,-0.19289601015161,0.847040409791961,0.997645679347435
"12456","RABEP2",23.3038166905934,0.0819678615188352,0.320242034263861,0.255955973135302,0.797984833431161,0.997645679347435
"12457","NPIPB11",3.72682093247356,-0.0894740981360616,0.776262136438387,-0.115262736562913,0.908236873373897,0.997645679347435
"12458","BOLA2",48.9660932463702,0.0914214991588826,0.306132864426267,0.298633403278078,0.765219775685946,0.997645679347435
"12459","CDIPT",178.683705117515,0.0268158164990081,0.177651374975639,0.150946293000464,0.880018082295776,0.997645679347435
"12460","YPEL3",235.365002363595,0.0385169510032861,0.241027770687275,0.159802959192036,0.873036292943261,0.997645679347435
"12461","GDPD3",5.07406662032491,0.150820061288,0.640020548613538,0.235648779737961,0.81370521841425,0.997645679347435
"12462","SULT1A3",4.26649871904825,0.23274878556089,0.825176673989257,0.282059336984992,0.777898012177605,0.997645679347435
"12463","CD2BP2",114.621085219853,-0.0575682737979229,0.2770363900243,-0.20780040410169,0.835384817074007,0.997645679347435
"12464","ZNF747",15.6709999225694,0.0501951981049718,0.373786226425947,0.134288517222601,0.893174432927422,0.997645679347435
"12465","PRR14",25.8380809138903,-0.0846533808126832,0.315544401072292,-0.26827723935209,0.788485924630436,0.997645679347435
"12466","FBXL19",8.79153899386701,0.0945503351497537,0.514098311259589,0.183914891527452,0.854080222330728,0.997645679347435
"12467","SETD1A",21.0133860100326,0.0781590359560448,0.429709142443872,0.181888231447749,0.855670440385987,0.997645679347435
"12468","AC026471.1",6.55197690726285,-0.171152006025738,0.598410503138421,-0.286011032774517,0.774869671444623,0.997645679347435
"12469","ZNF267",37.989614959268,-0.039458694489242,0.295683596510866,-0.133449048086751,0.893838257491576,0.997645679347435
"12470","DNAJA2",156.303336672606,0.0632434319737359,0.232102608710768,0.272480487509496,0.785252592588819,0.997645679347435
"12471","SNX20",2.54515786696514,-0.224828872057275,0.918616527329638,-0.244747253471303,0.80665213749377,0.997645679347435
"12472","HNRNPA1P48",45.3212859607688,0.0585166016758937,0.350519947889472,0.166942286817712,0.867415459934275,0.997645679347435
"12473","AC007906.2",3.58831215039979,0.229282131336802,0.890023975813

643,0.257613432410286,0.796705261975921,0.997645679347435
"12474","CHD9",291.689520751196,0.0361807170905051,0.189245919300324,0
.191183605037677,0.848381747985487,0.997645679347435
"12475","RPGRIP1L",18.1809952887413,0.106538126679498,0.38528241948275
3,0.276519564070759,0.782149035261152,0.997645679347435
"12476","GNAO1",3.13142902786991,0.139411988349325,0.78720419016853,0.
177097619766835,0.85943171152659,0.997645679347435
"12477","AMFR",63.7213162903629,-0.0515050738507364,0.246184888223461,
-0.209212979002941,0.834281980838141,0.997645679347435
"12478","AC009107.2",34.0643951685456,-0.0613539696182126,0.4467208024
16255,-0.137342987580513,0.890759693182678,0.997645679347435
"12479","CKLF",134.692955081441,-0.0442471583196712,0.262068874839417,
-0.168837899375818,0.865924146850526,0.997645679347435
"12480","C16orf70",10.7391148618935,-0.0756091851207136,0.447233178696
817,-0.169059874629672,0.865749545969617,0.997645679347435
"12481","LRRC29",6.97748228903236,-0.119128600738195,0.56359866928216,
-0.211371330755493,0.832597526090528,0.997645679347435
"12482","GFOD2",24.3330314595878,-0.0499222900164092,0.324525533636912
,-0.153831624454745,0.877742497907572,0.997645679347435
"12483","THAP11",42.7912169905978,-0.0392069826472729,0.28079903763896
2,-0.139626485108127,0.888955113514084,0.997645679347435
"12484","UTP4",36.3003237874659,0.0585340424977428,0.334352418340052,0
.175066903324178,0.861027063935167,0.997645679347435
"12485","COG8",21.7702142724746,0.0888754569726319,0.341153564436452,0
.2605145196693,0.794466916800221,0.997645679347435
"12486","WWP2",20.2247095397396,0.0583184474579228,0.373756436002032,0
.15603329291594,0.876006778656831,0.997645679347435
"12487","AC009060.1",2.02437062217051,0.348229348847107,1.137332126793
22,0.306180877725631,0.759466931068078,0.997645679347435
"12488","VAC14",24.654602116707,-0.0889933353159228,0.327302623403153,
-0.271899242329951,0.78569949383152,0.997645679347435
"12489","ZNF19",4.1568332923317,0.137572616551559,0.790178026953912,0.
174103318314094,0.861784265392409,0.997645679347435
"12490","DHX38",58.2470266198628,-0.0811522140227331,0.268167013302698
,-0.302618181942949,0.762180856623606,0.997645679347435
"12491","RFWD3",12.3347040094241,-0.0499063642075243,0.452605490608734
,-0.110264601828852,0.912199529688086,0.997645679347435
"12492","CFDP1",231.018554104948,0.05808480421489,0.197307211083676,0.
294387639944172,0.768461710578774,0.997645679347435
"12493","ADAT1",17.8968610991649,0.0518395091208551,0.367971300803096,
0.140879218046939,0.887965359989739,0.997645679347435
"12494","CENPN",7.87518708264575,0.0612165165550084,0.555249355779667,
0.110250495417594,0.912210716769608,0.997645679347435
"12495","C16orf46",3.99340732341128,-0.166636895426039,0.7207629181930
48,-0.231195156160083,0.817163192905041,0.997645679347435
"12496","CMIP",27.4653571918747,0.0971913838834806,0.341201276965016,0
.28485058657458,0.775758614418885,0.997645679347435
"12497","HSBP1",355.918394907914,-0.0532817939579454,0.246420097296597
,-0.216223410925019,0.828813605871161,0.997645679347435
"12498","MLYCD",31.0168265300046,-0.0701184787084906,0.293047936401036

,-0.23927306764083,0.810893847837442,0.997645679347435
"12499","OSGIN1",9.03483057642254,-0.119300888782599,0.502344538081901
,-0.237488177413305,0.812278089948612,0.997645679347435
"12500","EMC8",60.6016809637773,-0.069992593701306,0.253815237312719,-
0.27576198514461,0.782730883401505,0.997645679347435
"12501","MTHFSD",11.1394477974718,-0.113134695190592,0.436946231220801
,-0.258921320535253,0.795695947584509,0.997645679347435
"12502","KLHDC4",25.383574143786,-0.0678585538839315,0.328023860597878
,-0.206870786046625,0.836110772340637,0.997645679347435
"12503","BANP",25.5641568877915,-0.0536218885394966,0.311812998084601,
-0.17196809904938,0.863462608609214,0.997645679347435
"12504","ZNF469",12.7574211555324,0.10263860185151,0.411129738756372,0
.24965015219279,0.802857910445956,0.997645679347435
"12505","ZFPM1",52.5328685056368,0.0602416892332083,0.329626752540681,
0.182757281588586,0.854988468355335,0.997645679347435
"12506","MVD",28.1536078643459,0.0854491880695013,0.33646846603196,0.2
53958978911815,0.799527249397644,0.997645679347435
"12507","SNAI3",1.89617754255009,-0.236574110045486,1.06428723212769,-
0.22228408168736,0.824092743122254,0.997645679347435
"12508","RNF166",36.1512630359526,0.0588044760759739,0.30223916493884,
0.19456272679068,0.845735285063414,0.997645679347435
"12509","CDT1",2.44712261377814,0.111528720651385,0.944047018835521,0.
118138946923381,0.905957564333161,0.997645679347435
"12510","AC009113.1",7.76090484936563,-0.161692042858993,0.54497768481
3197,-0.296694795704188,0.766699530660788,0.997645679347435
"12511","VPS9D1",8.66219659037794,-0.0787725505276553,0.47409397820136
7,-0.166153872754312,0.868035858986584,0.997645679347435
"12512","SPIRE2",2.82641406776343,-0.151360876329309,0.975077849387687
,-0.155229530056864,0.876640368827707,0.997645679347435
"12513","DEF8",60.0389820576112,-0.0535123892161636,0.285939701938734,
-0.187145712376903,0.85154638795532,0.997645679347435
"12514","CENPBD1",6.14273147718067,-0.14302273094999,0.560340665109828
,-0.255242462051112,0.798535836908042,0.997645679347435
"12515","FAM157C",3.45621458001031,0.223093653017471,0.854758342783947
,0.261002018758724,0.794090950458294,0.997645679347435
"12516","LINC02193",3.37163281183202,-0.191722627831804,0.749222674335
025,-0.255895389180484,0.798031615021472,0.997645679347435
"12517","MIR22HG",160.15333401577,0.0866599760565466,0.330822624358157
,0.261952991349003,0.793357684398697,0.997645679347435
"12518","SERPINF2",7.11188007504241,-0.0796010561644463,0.586820956536
626,-0.135647943853686,0.892099601995919,0.997645679347435
"12519","SERPINF1",2566.22008870444,0.0293952655499601,0.2709553879565
07,0.108487473792839,0.913609016333559,0.997645679347435
"12520","RPA1",48.4947466985931,-0.0335680444159142,0.248188722291801,
-0.135252094075602,0.89241256033451,0.997645679347435
"12521","SPNS3",1.95693290478169,0.17001193365681,1.0306367254262,0.16
4958155926865,0.868976917512032,0.997645679347435
"12522","PELP1",35.2014907590063,-0.0487194405130555,0.277006420539625
,-0.175878380068401,0.860389490778791,0.997645679347435
"12523","PLD2",17.125770209026,0.0889969032461642,0.386251583770065,0.

230411749713741,0.817771831807149,0.997645679347435
"12524","INCA1",1.84914259776557,0.118738249349902,1.06634470379646,0.11135071888777,0.911338237579317,0.997645679347435
"12525","ZNF232",6.86177023978921,0.113300217824466,0.549695521914792,0.206114500314283,0.836701472341812,0.997645679347435
"12526","MED31",55.3862848770193,-0.0920834708783065,0.318554590099941,-0.289066532833248,0.772530463751668,0.997645679347435
"12527","DVL2",32.6393566510751,0.0455246624735875,0.308652646338058,0.147494806909012,0.882741478389095,0.997645679347435
"12528","PHF23",33.9323145437083,0.0693723173612523,0.301966848019667,0.229734879229968,0.818297789990277,0.997645679347435
"12529","CD68",139.060930584427,-0.0573018865734343,0.232141834129213,-0.246839983789993,0.80503206514106,0.997645679347435
"12530","SOX15",5.44275016892831,0.169076702612898,0.710448057112528,0.237986015895484,0.811891940346162,0.997645679347435
"12531","SAT2",268.556950860406,-0.0463217673774958,0.219516724648078,-0.211017030487118,0.832873982158109,0.997645679347435
"12532","RNF227",4.70748915826789,-0.138543564962257,0.674987851791383,-0.205253419886846,0.837374134751537,0.997645679347435
"12533","CNTROB",8.72270810427934,-0.139246566758994,0.487573380710609,-0.285590994643822,0.775191401554311,0.997645679347435
"12534","SLC25A35",4.93329229355935,-0.0894363450200673,0.661260155371816,-0.135251374657193,0.892413129121071,0.997645679347435
"12535","PIK3R5",5.59137035546398,-0.102871518330935,0.695156228989655,-0.147983307983084,0.882355941494874,0.997645679347435
"12536","ADPRM",12.739416191565,0.0699620914888515,0.46613835553871,0.150088682163898,0.880694649163608,0.997645679347435
"12537","ELAC2",33.1400017273416,0.0521837850038749,0.265803624862998,0.196324579963015,0.844356123707545,0.997645679347435
"12538","COX10-AS1",11.1372821829266,-0.133812957768911,0.498643952587852,-0.268353716262778,0.788427062324214,0.997645679347435
"12539","COX10",13.1899606337995,0.110794458692833,0.410868593524594,0.269659108627393,0.787422522033539,0.997645679347435
"12540","TEKT3",12.6946050976669,-0.0864633708808411,0.490737723374585,-0.176190593798803,0.860144210252757,0.997645679347435
"12541","AC015922.3",30.8909738555962,-0.0879829672054348,0.290040928168097,-0.303346730274022,0.761625637191028,0.997645679347435
"12542","AC002553.2",2.07067778991366,-0.144853082994911,0.9727558022597,-0.14891001694199,0.881624636967844,0.997645679347435
"12543","UBB",1312.73244984835,-0.029506148085434,0.264495838120321,-0.111556190430533,0.911175310089244,0.997645679347435
"12544","TRPV2",35.1091930810986,-0.0876036504983861,0.328740115626149,-0.266482994725266,0.789867254311092,0.997645679347435
"12545","DRC3",7.8646050076966,0.120960441901606,0.534054741918466,0.226494462846794,0.820816865356206,0.997645679347435
"12546","ATPAF2",21.5219181415757,0.0518679452928854,0.369354129811698,0.140428767696001,0.888321229716705,0.997645679347435
"12547","DRG2",53.8265977943739,-0.0829986814956927,0.292742694556684,-0.283520931654271,0.776777538508975,0.997645679347435

"12548","EPN2",101.280164776302,0.055274446814956,0.286952106676603,0.
192626035944216,0.847251853046977,0.997645679347435
"12549","RNF112",3.74279581014946,-0.136811805072986,0.712976215650444
,-0.191888315584516,0.84782969017937,0.997645679347435
"12550","ALDH3A2",136.858483790065,0.0260471326666073,0.17295162094791
5,0.150603576444371,0.880288439980512,0.997645679347435
"12551","TMEM11",70.161833418545,-0.0912876312723781,0.320932409617825
,-0.284445037449118,0.776069348799837,0.997645679347435
"12552","LGALS9",20.5251392133284,-0.0565268695038948,0.39345906334872
,-0.143666456740877,0.885763860002563,0.997645679347435
"12553","NLK",9.444334725157,0.114465017361929,0.571100294963495,0.200
428923555793,0.841145140674582,0.997645679347435
"12554","TNFAIP1",22.0545797874865,0.104584430688427,0.357855395131451
,0.292253329448925,0.770092939486678,0.997645679347435
"12555","SDF2",139.362231200376,-0.0604376387363051,0.272104190819543,
-0.22112120192911,0.82422660295559,0.997645679347435
"12556","NEK8",4.87409261669761,0.19152425396156,0.673447615147305,0.2
84393692476982,0.776108692209374,0.997645679347435
"12557","ERAL1",33.03407116752,-0.069181377971895,0.324905572098148,-0
.212927643946336,0.831383400021238,0.997645679347435
"12558","ABHD15",14.2366537094267,-0.0760051478537507,0.47388412929988
5,-0.160387620421136,0.872575740852787,0.997645679347435
"12559","AC005562.1",22.411731451846,0.0585619130438112,0.319471756285
477,0.183308577023256,0.85455590493465,0.997645679347435
"12560","TEFM",25.9217708543138,0.0661783416498167,0.334802719529045,0
.197663692047984,0.84330819872489,0.997645679347435
"12561","RNF135",37.9710852497402,0.0289838598722357,0.266053402111587
,0.108940008442664,0.91325007332169,0.997645679347435
"12562","EVI2A",10.9256980982306,0.0651012132800155,0.549411544044722,
0.118492619941594,0.905677342335218,0.997645679347435
"12563","LRRC37B",6.04321457826553,0.165780422805478,0.596484193168964
,0.277929280782329,0.781066646243917,0.997645679347435
"12564","RHBDL3",2.88250046883097,-0.189474884894153,0.879529269899386
,-0.215427605855377,0.829433948880316,0.997645679347435
"12565","AC005899.6",4.50105779429517,0.162327596768656,0.712634085144
763,0.227785339141729,0.81981312477729,0.997645679347435
"12566","PSMD11",175.012569430643,-0.0483469174027352,0.23281120902987
7,-0.207665763191543,0.835489951884839,0.997645679347435
"12567","CCL13",36.0890535215145,-0.221895939743394,0.819405331469543,
-0.270801191085052,0.786543943742859,0.997645679347435
"12568","ZNF830",48.4464331658485,0.0346346683421294,0.309724240686098
,0.11182420938512,0.910962791598871,0.997645679347435
"12569","SLFN13",7.19140331901776,0.160546138711744,0.61937658740102,0
.259206017756363,0.795476288245908,0.997645679347435
"12570","SLFN12L",5.73243184921658,0.150481517143792,0.846234599841378
,0.177824822067071,0.858860552835123,0.997645679347435
"12571","TAF15",90.8313434340638,-0.0572277074584597,0.268147089754472
,-0.213419088422179,0.831000093049805,0.997645679347435
"12572","AC243829.1",2.40202934336366,0.255616353344225,1.040636910054
12,0.245634525236021,0.805965160604093,0.997645679347435

"12573","CCL4L2",50.1936326295163,0.194533486367357,0.766092992373234,
0.253929337957685,0.799550149052074,0.997645679347435
"12574","GGNBP2",166.431042819489,0.0618272588905002,0.202730723455757
,0.304972319126525,0.76038723479316,0.997645679347435
"12575","MRM1",4.08554222079087,-0.16874698306444,0.697632925580713,-0
.241885061436821,0.808869225723781,0.997645679347435
"12576","TADA2A",17.0502225171217,-0.0834761721934808,0.41564691541556
5,-0.200834335820876,0.840828113782546,0.997645679347435
"12577","DDX52",72.6184281122287,0.0421797313728335,0.258859940155875,
0.16294421293702,0.87056235252265,0.997645679347435
"12578","MRPL45",50.6111788226208,-0.0613628234953453,0.26964531593917
,-0.22756866100796,0.81998158560314,0.997645679347435
"12579","RPL23",3548.88928415702,-0.0242065420921745,0.145012761783238
,-0.166926977974242,0.867427505609916,0.997645679347435
"12580","PGAP3",11.8677868447686,0.131776175835675,0.479119635078624,0
.275038145356014,0.783286932229561,0.997645679347435
"12581","MED24",34.3687521198462,0.0565914207791774,0.269268463674942,
0.210167280664155,0.83353711548799,0.997645679347435
"12582","TMEM99",39.8278198235238,0.0937758783300087,0.336846805437718
,0.278393254192068,0.780710497295528,0.997645679347435
"12583","JUP",23.1318452464737,0.101183194871107,0.42923381910961,0.23
5729782618243,0.813642357873012,0.997645679347435
"12584","P3H4",46.1245081810242,0.0527409711011345,0.28996289716916,0.
181888688573719,0.855670081635926,0.997645679347435
"12585","FKBP10",187.036273338845,0.0550037718393308,0.210153467755212
,0.261731450005858,0.793528491899145,0.997645679347435
"12586","NT5C3B",87.6917347635986,0.0369816215549957,0.250838429749343
,0.147432040584653,0.882791017110276,0.997645679347435
"12587","DNAJC7",145.722042680453,0.0219919478448614,0.188683389942024
,0.116554763255096,0.907212886995367,0.997645679347435
"12588","STAT5A",52.3778536978218,0.0472745336964492,0.307385962310413
,0.153795356629556,0.877771095151361,0.997645679347435
"12589","STAT3",568.223225601128,0.0360074045526605,0.175318070069181,
0.205383304404685,0.837272663394385,0.997645679347435
"12590","ATP6V0A1",39.4394022566121,-0.0562049562409508,0.323903136941
607,-0.173523963897526,0.862239593125241,0.997645679347435
"12591","PLEKHH3",51.183958469598,0.0524435245879755,0.269459948221072
,0.194624562701057,0.845686872495966,0.997645679347435
"12592","RAMP2-
AS1",33.3073754604646,-0.086370812592906,0.472117017800417,-0.18294365
4510286,0.854842229523311,0.997645679347435
"12593","COA3",143.698745962421,0.0903352342055896,0.294907354847356,0
.306317332276596,0.759363043837579,0.997645679347435
"12594","BRCA1",13.1401090843438,0.0922086246309258,0.43368748565927,0
.212615368623688,0.831626983090108,0.997645679347435
"12595","NBR2",6.29774623343469,-0.142798046361196,0.695426805274995,-
0.20533871469727,0.837307498481598,0.997645679347435
"12596","LINC00910",7.89472344591571,-0.119658158668642,0.746185362606
766,-0.16035983103531,0.872597630262046,0.997645679347435
"12597","MPP2",3.62836459637311,-0.108113670002721,0.73027023673563,-0

.148046113019748,0.882306376256845,0.997645679347435
"12598","TMEM101",56.1814272190357,0.04838115181255,0.298577063436604,
0.162039077133675,0.871275081384336,0.997645679347435
"12599","AC138150.1",5.94352145504495,0.0892517569532594,0.64597138660
4988,0.13816673432292,0.890108645066941,0.997645679347435
"12600","AC138150.2",2.63407716947178,-0.121985135218753,0.86217325242
0473,-0.141485640938513,0.88748630280364,0.997645679347435
"12601","AC091132.4",7.06383516288236,0.208133875590691,0.680370985545
371,0.305912333142565,0.759671395355661,0.997645679347435
"12602","MAPT",3.26556607101083,0.178413183584487,0.836598145350012,0.
213260314496446,0.831123925949283,0.997645679347435
"12603","LRRC37A2",4.25647809276395,0.203028786024463,0.77982923926929
2,0.260350312351333,0.794593566611719,0.997645679347435
"12604","AC002558.3",18.997264913063,-0.0981581722981241,0.37973143787
5412,-0.2584936681759,0.796025934986338,0.997645679347435
"12605","ITGB3",20.7776497246746,0.0708481494928874,0.340697067985231,
0.207950570023569,0.835267562975028,0.997645679347435
"12606","KPNB1",255.912911340624,-0.0320934755068489,0.206983602997194
,-0.155053226642711,0.876779355865745,0.997645679347435
"12607","TBKBP1",7.37466641065135,0.133098777140485,0.535840103326945,
0.248392713262961,0.803830570930579,0.997645679347435
"12608","SP2",23.0177014868343,0.106286016626843,0.369655821315152,0.2
8752696562089,0.773708857634399,0.997645679347435
"12609","SNX11",20.0815609638555,-0.0848960405367907,0.367327733892361
,-0.231117970966135,0.817223154220928,0.997645679347435
"12610","PHB",255.776522089895,-0.0448123696102738,0.290570412293031,-
0.154222067059884,0.877434643340341,0.997645679347435
"12611","NGFR",85.2088373273257,0.0484786572016663,0.399141024461479,0
.121457465483719,0.903328702849393,0.997645679347435
"12612","SPOP",183.429949784698,0.0343548042282616,0.193742083234133,0
.1773223641182,0.859255185116968,0.997645679347435
"12613","FAM117A",19.2750803979073,-0.0671074131360261,0.4582658873011
87,-0.146437723155075,0.883575849499932,0.997645679347435
"12614","PPP1R9B",29.1729984627418,-0.0419799532540351,0.3234379655216
93,-0.129792905376223,0.896730276123099,0.997645679347435
"12615","XYLT2",17.1773371980413,0.0531121665092718,0.430787943324181,
0.123290745092425,0.901876869266661,0.997645679347435
"12616","HLF",24.5704932510031,-0.119473441449845,0.63722943211485,-0.
187488893997464,0.851277330601091,0.997645679347435
"12617","PCTP",16.5967566246929,0.0848384501455931,0.373877995609257,0
.22691479878976,0.820489994420383,0.997645679347435
"12618","CUEDC1",54.7654043829387,0.062009937871214,0.264984831567422,
0.234013160317202,0.814974761845666,0.997645679347435
"12619","DYNLL2",73.8457242681279,0.0262789864944197,0.231013319246939
,0.113755287271246,0.909431784019093,0.997645679347435
"12620","AC099850.1",3.11472696071251,-0.214342200336906,0.81163677217
1782,-0.264086359423277,0.791713374498894,0.997645679347435
"12621","BCAS3",20.2232375408315,-0.0424720380463068,0.339884846126384
,-0.124960081422735,0.900555152973193,0.997645679347435
"12622","TBX2-

AS1",1.94249750817457,-0.269307026237779,0.984072795927148,-0.273665756590751,0.784341495715661,0.997645679347435
"12623","TBX2",7.68552974216008,0.103419462288749,0.583122935913004,0.177354475221976,0.859229963887676,0.997645679347435
"12624","NACA2",2.12284593378995,0.156158875367465,1.1605676954395,0.134553870473129,0.892964615853413,0.997645679347435
"12625","TANC2",24.0903296911191,-0.108420089141753,0.516779843680918,-0.209799376789734,0.83382425948912,0.997645679347435
"12626","DCAF7",86.3114195607714,0.0309406632323347,0.20561307562307,0.150480037023789,0.88038589954113,0.997645679347435
"12627","STRADA",26.9111591105359,0.0771554761500423,0.305375703683515,0.25265754681651,0.800532856371416,0.997645679347435
"12628","LRRC37A3",5.98152736932215,0.0869781574646581,0.568804443107038,0.152913990948363,0.878466102488618,0.997645679347435
"12629","AXIN2",6.65641444617471,0.140490051130703,0.623121572433886,0.225461703375082,0.821620114679669,0.997645679347435
"12630","PSMD12",151.606874074073,-0.0418429046794003,0.22179944164963,-0.188651983829149,0.850365587066124,0.997645679347435
"12631","PITPNC1",28.401651213761,0.0460398215171523,0.320584771803777,0.143612003957981,0.885806861136684,0.997645679347435
"12632","NOL11",60.852586202614,-0.0457398524579308,0.27732129983488,-0.164934509124127,0.868995530003061,0.997645679347435
"12633","BPTF",276.969052374125,0.0474477574790423,0.248976427080399,0.190571284339784,0.848861489618431,0.997645679347435
"12634","KPNA2",90.4921883085531,0.102093302828555,0.387136293542507,0.263714109298164,0.792000222649219,0.997645679347435
"12635","AC005332.6",34.6578424513213,0.0765187683177552,0.296958572689054,0.257674892577956,0.796657824731152,0.997645679347435
"12636","AC005332.3",4.38136710062925,-0.175583951190421,0.677522644685172,-0.259155841605871,0.795515000689729,0.997645679347435
"12637","ARSG",22.7543029909493,-0.0700113081456929,0.392317749738322,-0.178455622240877,0.858365170037345,0.997645679347435
"12638","PRKAR1A",249.319327620085,0.0495478666990509,0.177280501261504,0.279488530021491,0.779869939175202,0.997645679347435
"12639","SSTR2",3.54061056352007,-0.166557270845987,1.05490499772339,-0.157888408155652,0.87454473244253,0.997645679347435
"12640","C17orf80",16.9099128900635,0.0814172768639303,0.375003246463603,0.217110858723812,0.828121951758279,0.997645679347435
"12641","TTYH2",5.05096213271938,-0.0809145227463307,0.681003076331316,-0.118816677278804,0.905420595724745,0.997645679347435
"12642","CD300A",5.81987507557115,0.101566393725273,0.654380849470072,0.15520991148736,0.876655834743289,0.997645679347435
"12643","RAB37",5.76749044931823,0.0728723878973632,0.649658449154442,0.112170307324117,0.910688371882707,0.997645679347435
"12644","SLC9A3R1",19.0674387692666,-0.0739486932621965,0.430502438408888,-0.171772995143782,0.863615996687471,0.997645679347435
"12645","TMEM104",9.08250793003884,-0.0589690284169805,0.486678256794748,-0.121166350856414,0.90355927583489,0.997645679347435
"12646","CDR2L",15.2864092460088,-0.109417151045134,0.419052053128225,-0.261106347596522,0.794010496762097,0.997645679347435

"12647","MRPS7",117.255720747733,-0.0599894860594504,0.272744592570434,-0.219947480879785,0.825912057225494,0.997645679347435
"12648","GRB2",96.0770495123389,-0.0447882342058065,0.184599851744391,-0.242623348732822,0.808297191904152,0.997645679347435
"12649","TMEM94",18.0763666053551,0.08110141655238,0.376906914399585,0.215176250299295,0.829629906748834,0.997645679347435
"12650","SNHG20",10.8949295723595,0.0646210747418043,0.4876207449234,0.1325232271485,0.894570452439352,0.997645679347435
"12651","SEPTIN9",79.7936391311355,-0.0518031011784878,0.200703828448787,-0.258107189976728,0.79632418274512,0.997645679347435
"12652","TNRC6C",111.723116148551,0.0696480267529609,0.298963516669118,0.23296497020419,0.81578860713545,0.997645679347435
"12653","CBX4",38.8760584221721,0.0924798313606729,0.311610094473883,0.29678060178639,0.766634016133529,0.997645679347435
"12654","AC087741.1",5.59102099666558,0.15570641276234,0.663184282103872,0.234786042076239,0.814374801771185,0.997645679347435
"12655","ENDOV",32.0630962330397,0.0450377782209146,0.327967160016912,0.137324048598622,0.890774662472116,0.997645679347435
"12656","RPTOR",14.6620601740638,0.0799824258245233,0.45900666924949,0.174251119172844,0.861668112703016,0.997645679347435
"12657","ARL16",88.8444014703558,-0.0327572266662591,0.259835179813608,-0.126069251630042,0.899677106884649,0.997645679347435
"12658","P4HB",573.206727774763,-0.0587848306937758,0.2181875551204,-0.269423389713209,0.787603889006981,0.997645679347435
"12659","ANAPC11",307.147995653936,-0.0292782509077613,0.24513822124833,-0.119435683096117,0.904930192589043,0.997645679347435
"12660","NPB",9.50819006564783,-0.151002148234041,0.512773849986258,-0.294480984625264,0.768390391651265,0.997645679347435
"12661","PCYT2",12.391628558867,-0.116169159852692,0.464616120344741,-0.250032564015418,0.8025621658036,0.997645679347435
"12662","GPS1",60.6553219331931,-0.0402648135585238,0.231081499574268,-0.17424507644578,0.861672861459821,0.997645679347435
"12663","FASN",28.0452934547512,-0.0586322990118735,0.322388199048919,-0.181868626658312,0.85568582614991,0.997645679347435
"12664","AC132872.1",7.35488898781014,-0.10860900644017,0.586963820605199,-0.185035265594712,0.853201375202786,0.997645679347435
"12665","HEXD",32.4782612812956,-0.0508737764304772,0.290839764815899,-0.174920291462483,0.861142265803359,0.997645679347435
"12666","HEXD-IT1",2.12080442336738,0.194308904521277,0.941042383420739,0.206482628141523,0.836413933104758,0.997645679347435
"12667","WDR45B",101.676748014783,-0.0301750427756272,0.212738787981017,-0.141840813619375,0.887205745357409,0.997645679347435
"12668","RAB40B",42.6717427463734,0.0776512013141547,0.272720175019452,0.284728481523658,0.77585216832955,0.997645679347435
"12669","FN3KRP",43.8021311120182,-0.0825924278569626,0.346951401104382,-0.2380518643074,0.811840868292606,0.997645679347435
"12670","FN3K",4.77398666715539,0.188433913524477,0.68931972476015,0.27336213770764,0.784574854460076,0.997645679347435
"12671","USP14",109.69738130789,-0.0491422271131421,0.197503355803727,

-0.248817175349558,0.803502204838675,0.997645679347435
"12672","NDC80",3.5589218028444,-0.177549252573842,0.737499965780452,-0.240744760423077,0.809752945225813,0.997645679347435
"12673","LPIN2",130.797159417805,0.05457394822897,0.185877643343753,0.293601464098851,0.76906245687822,0.997645679347435
"12674","AP005329.3",6.37696180396051,-0.113946362059032,0.622605937594207,-0.183015219063488,0.854786077214757,0.997645679347435
"12675","LINC00526",6.18895357389981,0.165203598736136,0.594351071426341,0.277956256290875,0.781045938413299,0.997645679347435
"12676","LINC00667",78.7434600262524,-0.0598777845532555,0.223559766556008,-0.267837927529122,0.788824074997555,0.997645679347435
"12677","ARHGAP28",35.433235837397,0.102800004232786,0.425664198788848,0.241504933995588,0.809163792868834,0.997645679347435
"12678","RAB12",44.6107914910954,-0.0797061972970545,0.30162241301926,-0.264258204485503,0.791580963803816,0.997645679347435
"12679","PRELID3A",5.38417161321975,-0.0759948879191094,0.692961044073336,-0.109666897683597,0.912673553848029,0.997645679347435
"12680","PTPN2",115.614344168483,0.0627730808666194,0.278234030870134,0.2256125200440З,0.82150280231763,0.997645679347435
"12681","FAM210A",37.3596610581339,0.0392838474889625,0.298596013704196,0.131561861799933,0.895330853119345,0.997645679347435
"12682","RNMT",139.019840024839,0.0503222681009081,0.200212642773108,0.251344107963932,0.801548076178299,0.997645679347435
"12683","ABHD3",15.8567883869568,0.0714264894579967,0.434429984597117,0.164414271552265,0.869405029381392,0.997645679347435
"12684","IMPACT",42.4553988686005,-0.049717890707571,0.281228856592132,-0.176788012830693,0.859674905120372,0.997645679347435
"12685","ZNF521",76.8902647992697,0.0525305881918224,0.267490327676515,0.19638313148784,0.844310298314915,0.997645679347435
"12686","KCTD1",10.7943717666076,0.0983061177942151,0.488852039935957,0.201095852657368,0.840623624855858,0.997645679347435
"12687","ZNF396",5.0993217107923,0.128171793624849,0.649210404923812,0.197427201801996,0.843493244551761,0.997645679347435
"12688","C18orf32",224.976128379858,-0.0559830173864374,0.253081124757932,-0.221205818648009,0.824932178854429,0.997645679347435
"12689","RAB27B",4.1625878362519,-0.115759358784338,0.699728838899537,-0.165434597445481,0.868601924008783,0.997645679347435
"12690","NARS",167.495160235733,-0.038385110624618,0.259128963385659,-0.148131301584724,0.882239146851902,0.997645679347435
"12691","AC027097.2",7.449224893563,0.150123594297226,0.613023058719599,0.244890615714821,0.806541128070745,0.997645679347435
"12692","CCBE1",36.585393052579,0.13905293670412,0.602637704557159,0.230740518976158,0.817516393188437,0.997645679347435
"12693","PMAIP1",12.2430279831857,0.0698714458881334,0.538309590399525,0.129797884218031,0.89672633690394,0.997645679347435
"12694","KDSR",268.306769329063,-0.0395967742490521,0.185787206016375,-0.213129714893081,0.831225787930265,0.997645679347435
"12695","DOK6",13.6124912543994,0.0861148707700471,0.568880199916679,0.151376108331174,0.87967903505923,0.997645679347435
"12696","CD226",7.08506777592033,-0.107643379737522,0.723903286127417,

-0.148698564850244,0.881791493731763,0.997645679347435
"12697","SOCS6",26.4866989398767,-0.0342948518709507,0.307466320908018
,-0.111540190059419,0.911187997358432,0.997645679347435
"12698","CYB5A",600.102962355869,-0.0640075334075942,0.25949897714509,
-0.246658133730548,0.805172809979622,0.997645679347435
"12699","LINC00909",20.8313121507508,-0.071375590403994,0.319630255641
035,-0.223306740035753,0.823296781916709,0.997645679347435
"12700","ZNF236",27.8330195241094,0.100320925377431,0.349908132855644,
0.286706469377262,0.774337083618638,0.997645679347435
"12701","ATP9B",36.0321708060449,0.0530754947851817,0.303532099991711,
0.174859577575588,0.861189973266883,0.997645679347435
"12702","MIER2",10.658195021308,0.117427760069253,0.462555631301269,0.
253867323458809,0.799598060030679,0.997645679347435
"12703","SHC2",62.5835009692256,-0.0716608169453643,0.302449966313844,
-0.236934451733428,0.812707642218262,0.997645679347435
"12704","PTBP1",158.565810895346,-0.0316447078157377,0.215994884298277
,-0.146506746761827,0.883521364242335,0.997645679347435
"12705","ABCA7",2.68485902949623,0.23387109246973,0.902283893194276,0.
25919901068141,0.795481694389567,0.997645679347435
"12706","DAZAP1",87.1678885169986,-0.0677931429428451,0.22819327529073
4,-0.29708650641204,0.766400465885601,0.997645679347435
"12707","TCF3",41.5958530000619,0.0340720822206166,0.288917352624126,0
.117930203607201,0.906122961211665,0.997645679347435
"12708","BTBD2",58.0762393692951,-0.0292079200802091,0.254031188468949
,-0.114977693314927,0.908462802924606,0.997645679347435
"12709","IZUMO4",3.02463767520567,0.124360659344979,0.791515670620104,
0.1571171158842,0.8751525485892,0.997645679347435
"12710","GADD45B",1795.86660194723,-0.0524764740769767,0.3190698939744
54,-0.164467018255186,0.869363508787151,0.997645679347435
"12711","AC092068.2",3.67699120542323,-0.177961308483838,0.97344375451
1872,-0.18281622092596,0.854942220648358,0.997645679347435
"12712","ZNF57",4.79673451686632,-0.0952988779340898,0.764870252318647
,-0.124594828528366,0.900844322730213,0.997645679347435
"12713","AC119403.1",2.34059793059796,0.231526397818398,0.969797145488
317,0.238736934724446,0.811309574943282,0.997645679347435
"12714","S1PR4",8.86573157636424,-0.129903606699211,0.52333198116293,-
0.248224093644237,0.803961025562208,0.997645679347435
"12715","NCLN",42.0003090136449,0.0561905210384354,0.308819198560822,0
.181952810253695,0.855619759573611,0.997645679347435
"12716","DOHH",34.460499182357,-0.0909487340481596,0.389817182599883,-
0.23331249754507,0.815519723594872,0.997645679347435
"12717","PIP5K1C",35.0032160426195,-0.0749718229681141,0.2802920629865
76,-0.267477509599352,0.789101528286062,0.997645679347435
"12718","APBA3",16.327751169271,-0.0414215711448071,0.379711244436393,
-0.109087027976455,0.913133463659831,0.997645679347435
"12719","YJU2",28.9747959608098,-0.05325335755351,0.349910508135123,-0
.152191364121438,0.879036005054957,0.997645679347435
"12720","AC005523.2",7.75711023610111,-0.167766215646289,0.60280881613
9382,-0.278307501739486,0.780776317961911,0.997645679347435
"12721","TICAM1",17.528894425036,0.0436500247411035,0.39020844507826,0

.111863352245872,0.91093175484282,0.997645679347435
"12722","SAFB2",91.9959725044227,0.0836942956012785,0.279016376070037,
0.29996194768249,0.764206181168257,0.997645679347435
"12723","ZNF557",14.4333637857656,-0.107995770282126,0.458830242067173
,-0.235371953242602,0.813920052710824,0.997645679347435
"12724","STXBP2",5.64319665934739,0.0757969671990277,0.653443327481009
,0.115996237181304,0.907655523960097,0.997645679347435
"12725","TRAPPC5",321.998280892516,-0.0536368888670199,0.2729770800689
63,-0.196488616749324,0.844227741529702,0.997645679347435
"12726","ELAVL1",100.227954130546,-0.0422525694266699,0.19774649819019
2,-0.213670380074349,0.830804111172252,0.997645679347435
"12727","CERS4",45.2449250409343,0.0637401689815018,0.294264917978323,
0.216608114278159,0.828513761590242,0.997645679347435
"12728","MYO1F",18.8996675826206,-0.0703849131728396,0.380590803689175
,-0.184935927223093,0.853279291156485,0.997645679347435
"12729","ZNF558",13.1847004415353,0.0907484632485952,0.415181042668541
,0.2185756427252,0.826980628304379,0.997645679347435
"12730","ZNF699",12.3243489559197,0.143562509622549,0.510815690396693,
0.281045614536742,0.778675413824309,0.997645679347435
"12731","COL5A3",76.6995165488486,0.0503850521342643,0.375373478180897
,0.13422645728312,0.893223505329187,0.997645679347435
"12732","SHFL",92.370637787319,0.0640036342197882,0.232908783949067,0.
274801289734889,0.783468907168887,0.997645679347435
"12733","TYK2",29.039631973347,-0.0572520622763431,0.298119674598995,-
0.192043890941964,0.847707825239406,0.997645679347435
"12734","PDE4A",54.2486957327633,-0.103671619135878,0.339561531553267,
-0.30531025897324,0.760129863194117,0.997645679347435
"12735","TMED1",106.441056119132,-0.0717115717482365,0.269449806975872
,-0.266140742697426,0.790130817995751,0.997645679347435
"12736","PLPPR2",40.1820283609623,0.0723640224415978,0.294326150977537
,0.245863380475222,0.805787991966841,0.997645679347435
"12737","ELOF1",83.5558412814354,-0.0470060463893102,0.250553174232332
,-0.187609063558391,0.851183120718181,0.997645679347435
"12738","ZNF439",2.53051340836129,0.193852436252203,0.842305271549001,
0.23014510629348,0.817979015712093,0.997645679347435
"12739","ZNF433-
AS1",10.701575558979,-0.0790902058156941,0.473850215837577,-0.16690971
7822739,0.867441086698004,0.997645679347435
"12740","ZNF20",4.7476950548735,0.105017908836052,0.651065034725305,0.
16130171831507,0.871855769963039,0.997645679347435
"12741","ZNF563",7.57889024431383,-0.107628411812404,0.528106866247629
,-0.20380044019715,0.838509448833466,0.997645679347435
"12742","FARSA",42.4706068996439,-0.0683099065022887,0.322790108663489
,-0.211623295351662,0.832400933862631,0.997645679347435
"12743","CCDC130",36.0492593968462,-0.0562761289694583,0.3245442777471
53,-0.173400465909006,0.862336658910327,0.997645679347435
"12744","IL27RA",7.70937906026435,-0.15171907699804,0.523093145491435,
-0.290042181408251,0.771783967793093,0.997645679347435
"12745","AC022098.1",5.9848791067399,0.134387951270396,0.6193906296757
03,0.216968008283816,0.828233276752299,0.997645679347435

"12746","ADGRE5",80.9905349782375,0.0355377668955828,0.262497019171917
,0.135383506478251,0.892308663993273,0.997645679347435
"12747","TECR",121.492293245129,-0.0615820039227022,0.250510111098217,
-0.24582642054938,0.80581660387585,0.997645679347435
"12748","BRD4",225.444938154424,0.0565782050741839,0.219565620998194,0
.257682440524918,0.796651998996567,0.997645679347435
"12749","KLF2",1243.7419202159,-0.0535506253075162,0.249083324705752,-
0.214990808279827,0.829774484936652,0.997645679347435
"12750","TMEM38A",2.73068391612344,-0.105409396437934,0.86677302171777
4,-0.121611302840314,0.903206861563628,0.997645679347435
"12751","BST2",229.270198791807,-0.128039586964098,0.677902268132543,-
0.188876174314649,0.850189867600376,0.997645679347435
"12752","JAK3",11.4608892773721,-0.0910640260380601,0.482827995923556,
-0.188605521649324,0.85040200483781,0.997645679347435
"12753","MAST3",6.75702778267268,0.205249398829226,0.685201342790336,0
.299546112962055,0.764523393154316,0.997645679347435
"12754","GDF15",23.3197096474191,-0.168988580467826,0.621983658809165,
-0.271692958608218,0.785858115924066,0.997645679347435
"12755","FKBP8",374.01769148784,-0.0336735961221184,0.220264863330673,
-0.152877747330793,0.878494684666501,0.997645679347435
"12756","CRLF1",51.3579854683465,-0.119488905049821,0.492498357109932,
-0.242617875419937,0.808301432315778,0.997645679347435
"12757","BORCS8",43.4999018851611,0.0487563514714411,0.321554169265368
,0.151627178658051,0.879480995786639,0.997645679347435
"12758","ZNF14",8.39154172734314,-0.1760969362591,0.5781472063435 8,-0.
304588406424728,0.760679651041347,0.997645679347435
"12759","ZNF66",7.90253340760887,-0.129551917170389,0.499760934155145,
-0.259227779356942,0.795459498658076,0.997645679347435
"12760","ZNF85",10.2219283792826,0.0989869612995351,0.457630976261781,
0.216303018008359,0.828751556719871,0.997645679347435
"12761","ZNF430",50.9196550288117,0.0407563127269208,0.272689518446886
,0.149460503502482,0.881190272896431,0.997645679347435
"12762","ZNF738",14.2735516507942,0.0495954918628115,0.423465832935025
,0.117118048271019,0.906766507709894,0.997645679347435
"12763","ZNF254",29.2827962022642,-0.0687959854998519,0.31321890441570
5,-0.219641868769663,0.826150080980105,0.997645679347435
"12764","AC006504.5",5.8206547387191,0.0753987360848818,0.613019875944
421,0.12299558145439,0.90211059688945,0.997645679347435
"12765","PDCD5",377.512892650671,-0.0353764903645418,0.311337763842881
,-0.11362736703664,0.909533192090765,0.997645679347435
"12766","CEP89",39.6303114759842,0.0385775807047596,0.248085941662658,
0.155500873794843,0.876426465131859,0.997645679347435
"12767","FAAP24",8.52523026514006,-0.108253840529822,0.506337380792164
,-0.213797844355199,0.830704706045779,0.997645679347435
"12768","ZNF599",13.0987681840822,-0.0845874479463713,0.41204092082056
7,-0.205288949888564,0.837346376921163,0.997645679347435
"12769","USF2",100.735308220158,-0.0429107718350205,0.188428015484806,
-0.227730317726987,0.819855901508928,0.997645679347435
"12770","TMEM147-
AS1",2.21976321461398,-0.248043786249699,0.91985140333844,-0.269656365

527592,0.787424632564582,0.997645679347435
"12771","TMEM147",189.659596544417,-0.0766877062012941,0.255574279553553,-0.300060343847022,0.764131127222769,0.997645679347435
"12772","LRFN3",15.3848991136587,0.0835050089576714,0.450071767777336,0.185537096383668,0.852807786626196,0.997645679347435
"12773","SDHAF1",58.7039968634893,0.033366311050313,0.296429316414063,0.112560766438181,0.910378791153245,0.997645679347435
"12774","CLIP3",37.4433860501882,-0.072015527465112,0.35415692422621 2,-0.20334355348962,0.838866515789275,0.997645679347435
"12775","CAPNS1",296.008239208974,0.0425880245223389,0.229836596834071,0.185296967971924,0.852996116059908,0.997645679347435
"12776","ZNF529",11.991227705344,0.0998589181811058,0.45946613099026,0.217336842578333,0.827945846988418,0.997645679347435
"12777","ZNF461",2.7664928003451,0.129185678809314,0.961956661429976,0.134294697452665,0.893169546081804,0.997645679347435
"12778","ZNF567",23.8627652760975,-0.0336628666767267,0.299561378505775,-0.112373854215248,0.91052698531364,0.997645679347435
"12779","ZNF585B",8.25828832519086,-0.152048162672139,0.502070852084977,-0.302842043191156,0.762010241152529,0.997645679347435
"12780","ZNF383",18.2895463325092,0.097955296272993,0.425579169529237,0.230169386300904,0.817960149440797,0.997645679347435
"12781","ZNF875",15.3011106138709,0.104381516235507,0.471286597832375,0.221482038138995,0.824717120827614,0.997645679347435
"12782","ZNF540",3.71196268581725,0.200246139885889,0.781703659143443,0.256166307453787,0.797822423553409,0.997645679347435
"12783","AC093227.1",4.7113937539635,0.203639557379892,0.731249426836891,0.278481664267089,0.780642638373657,0.997645679347435
"12784","SIPA1L3",9.28369753259016,-0.131713560161121,0.529277033541055,-0.248855612116607,0.803472471665969,0.997645679347435
"12785","MAP4K1",4.73143477246225,-0.123393497895455,0.689504296796316,-0.17895972290352,0.857969327540624,0.997645679347435
"12786","ECH1",232.526312521008,-0.0511074816735412,0.25985424746042,-0.196677491990296,0.844079924844328,0.997645679347435
"12787","SIRT2",82.4944307774537,0.0396796814487331,0.20936969069855,0.189519702285198,0.84968551471465,0.997645679347435
"12788","NFKBIB",36.2567349067234,-0.0963408284195235,0.374266355826786,-0.257412473548948,0.796860374746183,0.997645679347435
"12789","TIMM50",61.194944856136,0.0308229929330842,0.283407176187594,0.108758688991988,0.913393890795435,0.997645679347435
"12790","FBL",191.809881628735,-0.0547953586701499,0.18267926898587,-0.299953897201046,0.764212321956831,0.997645679347435
"12791","ZNF780B",12.2514795824487,-0.0601989135102012,0.420630619554644,-0.143115861546025,0.886198678191946,0.997645679347435
"12792","COQ8B",20.1343290813714,0.0769871297264562,0.361216281645965,0.213133055286563,0.831223182534047,0.997645679347435
"12793","ITPKC",73.3691841874572,0.049560805182568,0.394984112222543,0.125475439768181,0.900147167582037,0.997645679347435
"12794","RAB4B",25.5597477723619,0.0406345442223651,0.340950795919187,0.119180083192991,0.905132685484684,0.997645679347435
"12795","B3GNT8",10.1158419301647,-0.152048918542317,0.534568632150728

,-0.284432922916892,0.776078631584962,0.997645679347435
"12796","DMAC2",40.0419829675167,-0.0612079990269406,0.319126405712131
,-0.191798603723671,0.84789996472694,0.997645679347435
"12797","ZNF574",9.26234505363747,0.0686338817845152,0.462045414643644
,0.148543583832446,0.881913792525331,0.997645679347435
"12798","POU2F2",7.17429460747278,-0.143184811894744,0.645389185692576
,-0.221858089768099,0.824424356987221,0.997645679347435
"12799","ERF",38.1730971145512,-0.0350789973521214,0.282652075470665,-
0.124106632840777,0.901230846516956,0.997645679347435
"12800","ZNF575",5.90055305506737,-0.138241047382916,0.567436597412855
,-0.243623777551899,0.807522211239053,0.997645679347435
"12801","ZNF221",2.29876150170207,-0.168583795062378,0.883311944268571
,-0.190854200666306,0.848639823083656,0.997645679347435
"12802","CLPTM1",102.709926125561,-0.0247430003303952,0.18301499049449
8,-0.135196577414455,0.892456453078142,0.997645679347435
"12803","BLOC1S3",9.83201352906894,-0.132642077969754,0.45328383893309
3,-0.292624767478933,0.769808980338435,0.997645679347435
"12804","PPM1N",3.46452527109544,-0.222378894620495,0.843554737867379,
-0.263621179086373,0.792071837118203,0.997645679347435
"12805","VASP",40.5141425360016,-0.0694609465698841,0.292360237969227,
-0.237586845093468,0.812201554497259,0.997645679347435
"12806","EML2",10.7430616393583,-0.106259210704759,0.47979471393658,-0
.221468072945056,0.824727993484924,0.997645679347435
"12807","DMPK",9.95415966616296,-0.156153336838678,0.519061818200232,-
0.300837648548521,0.763538298070798,0.997645679347435
"12808","MYPOP",13.1193115397978,-0.103673338087872,0.393599914923147,
-0.263397765490155,0.792244012698124,0.997645679347435
"12809","HIF3A",16.7101644763336,-0.0670847210548588,0.530330234894119
,-0.126496127584075,0.89933921416545,0.997645679347435
"12810","PPP5D1",6.95844191960353,0.160925261222076,0.559104953556966,
0.287826570303637,0.77347949746411,0.997645679347435
"12811","CALM3",296.477260619532,0.0414363971186311,0.186261268492161,
0.22463840464906,0.82395281912729,0.997645679347435
"12812","SLC1A5",68.8053235884401,-0.0830448727569543,0.27553287615603
7,-0.301397328389681,0.763111531294539,0.997645679347435
"12813","NAPA-
AS1",7.54018129150488,-0.143884570939622,0.609340533188174,-0.23613162
6082371,0.813330533658987,0.997645679347435
"12814","PLA2G4C",19.3848300171854,0.0715501158125,0.600356308052684,0
.119179418709833,0.905133211913581,0.997645679347435
"12815","GRIN2D",2.89193295728737,-0.229738559251563,0.838090082522621
,-0.274121558102749,0.783991207178892,0.997645679347435
"12816","KCNJ14",3.94273526405204,-0.114541104896063,0.724539191911923
,-0.15808821134135,0.874387289786503,0.997645679347435
"12817","SPHK2",15.678926284877,-0.0887965270306669,0.398326044786543,
-0.222924230521385,0.823594477641999,0.997645679347435
"12818","DBP",30.3556434128333,-0.0927661649035961,0.563588742776052,-
0.164599038026666,0.869259588432688,0.997645679347435
"12819","FTL",13232.7666051728,0.0637283288426255,0.267572068990753,0.
238172575646631,0.811747246610658,0.997645679347435

"12820","SLC6A16",2.60255595790767,-0.206590569446353,0.87915684720446
2,-0.234987158552275,0.814218700181497,0.997645679347435
"12821","PIH1D1",189.096157300778,-0.0350119738595659,0.26087709189464
2,-0.134208694237154,0.893237551105025,0.997645679347435
"12822","RCN3",200.748469160479,-0.0327640363752684,0.270206172019154,
-0.121255692016339,0.903488513639812,0.997645679347435
"12823","SCAF1",48.9707346524515,-0.0665585949347185,0.25499140133528,
-0.261022899541631,0.794074847964512,0.997645679347435
"12824","PRMT1",239.416687242474,-0.100447805289315,0.335621823081137,
-0.299288658786149,0.764719807219036,0.997645679347435
"12825","AC006942.1",2.32837545486471,0.158539515497639,1.053318364004
24,0.150514337274956,0.880358840082334,0.997645679347435
"12826","TBC1D17",45.5143321795571,0.0581210892764094,0.27856826770472
7,0.208642175059278,0.834727584124064,0.997645679347435
"12827","POLD1",7.16658427255873,-0.126175124596278,0.587401559360778,
-0.214802161461036,0.829921567631645,0.997645679347435
"12828","CTU1",35.0603735459398,0.0620078396813644,0.320460756501891,0
.193495891223107,0.846570623583014,0.997645679347435
"12829","NKG7",117.414782116274,0.107646293914441,0.39740339107603,0.2
70874120180436,0.786487850250654,0.997645679347435
"12830","ZNF577",9.66440566042963,0.0558826169348262,0.466743941725605
,0.119728639065398,0.904698112849059,0.997645679347435
"12831","ZNF766",30.9014851221617,-0.0640201496994753,0.33480483366423
9,-0.191216324444343,0.848356114531059,0.997645679347435
"12832","AC010332.3",2.33384739701268,-0.229165360975271,1.11826231072
612,-0.20492987984766,0.837626909868015,0.997645679347435
"12833","ZNF83",73.667159816923,0.0717046638170654,0.239973585010734,0
.298802319488031,0.765090881054668,0.997645679347435
"12834","ZNF888",2.95731928281018,-0.15485034358835,0.805777828898451,
-0.192174986745465,0.847605138395271,0.997645679347435
"12835","ZNF765",18.7543867648501,0.0831678213909781,0.349271663629794
,0.238117860826897,0.811789682170021,0.997645679347435
"12836","ZNF331",81.3702057780222,-0.0822957733135144,0.33684969878430
8,-0.244310069477634,0.806990685414884,0.997645679347435
"12837","TSEN34",78.2702524775781,-0.0640337722471851,0.25654778496194
3,-0.249597837130747,0.802898371379336,0.997645679347435
"12838","CDC42EP5",94.7080022520442,0.0694097828787094,0.2468112219663
45,0.281226203272775,0.778536908022398,0.997645679347435
"12839","RDH13",17.7291649869286,0.116927283974997,0.440726214342574,0
.265305943167952,0.790773786528233,0.997645679347435
"12840","HSPBP1",30.5409521016007,0.0433236402883951,0.351035256454207
,0.123416777921413,0.901777071780723,0.997645679347435
"12841","ZNF865",13.5033793349544,-0.0939425179202738,0.42774405208947
1,-0.219623200980534,0.826164620769019,0.997645679347435
"12842","ZSCAN5A",7.16220413111475,-0.0698728615785383,0.5190454140186
83,-0.134618011625517,0.892913900021911,0.997645679347435
"12843","ZNF583",14.4525121699905,0.0754252598585143,0.444629607916459
,0.169636161235322,0.865296282134001,0.997645679347435
"12844","ZFP28",3.23091444462071,-0.157860746047369,0.811954765427789,
-0.194420616478799,0.845846548289556,0.997645679347435

"12845","AURKC",2.42524662737544,0.167634963215865,0.882946940982781,0
.189858478958289,0.849420029599396,0.997645679347435
"12846","ZNF460",21.4226180063578,0.0914036336153685,0.387945454676103
,0.235609497452887,0.813735703017893,0.997645679347435
"12847","TRAPPC2B",27.4285636628053,-0.117372597567852,0.3822797000903
03,-0.307033299283549,0.758818026372822,0.997645679347435
"12848","ZNF549",4.20402643718888,0.125763888137875,0.674591114349289,
0.186429802383607,0.852107723162852,0.997645679347435
"12849","ZNF256",3.66727859196388,0.218594628531815,0.731430505875538,
0.298859053287849,0.765047590740985,0.997645679347435
"12850","ZNF8",11.3941465334182,0.0829260426869767,0.467618049864084,0
.177337129546388,0.859243587794581,0.997645679347435
"12851","ERVK3-1",79.7350568037736,0.0808500581545048,0.26752103093810
9,0.302219447461719,0.762484780057165,0.997645679347435
"12852","ZSCAN22",5.14185310142465,0.0998803274386109,0.61118786231632
,0.163420011418548,0.870187747844082,0.997645679347435
"12853","A1BG-
AS1",3.70508310176123,0.118200218794298,0.858714357984471,0.1376478891
90687,0.89051870563067,0.997645679347435
"12854","MZF1-
AS1",4.35490598905002,0.196023028095089,0.771678891032971,0.2540214982
84982,0.799478949492058,0.997645679347435
"12855","MZF1",20.9076666500564,0.0833784357201085,0.377715541839356,0
.220743989813291,0.825291777314253,0.997645679347435
"12856","NRSN2-
AS1",5.0721210991934,-0.11809123196819,0.672641738928575,-0.1755633424
65623,0.860637003440323,0.997645679347435
"12857","SOX12",8.33475117646541,0.143800466013495,0.538955737699053,0
.266813127600085,0.789613046198295,0.997645679347435
"12858","NOP56",179.247365730936,0.0285913087558912,0.18806015043763,0
.152032786793784,0.879161076101798,0.997645679347435
"12859","CPXM1",299.041122921395,0.111382213678439,0.393021069079021,0
.283400108649251,0.776870145109569,0.997645679347435
"12860","HSPA12B",30.7425188144181,-0.0459321173951552,0.3270445309097
66,-0.140446064844387,0.88830756401774,0.997645679347435
"12861","PANK2",45.6365355667818,-0.0363688789861493,0.253730005615118
,-0.14333692579236,0.886024094347164,0.997645679347435
"12862","MCM8",8.25342424170365,-0.0682617830454821,0.543027804815306,
-0.125705870749472,0.899964754549386,0.997645679347435
"12863","CRLS1",62.3411081247291,0.0323608140477141,0.236135376431113,
0.137043481314858,0.890996426198263,0.997645679347435
"12864","SPTLC3",21.5196940715209,0.118146977474575,0.397973933980067,
0.296871145034571,0.766564886501678,0.997645679347435
"12865","ESF1",140.074709858909,0.0376757616363824,0.252227888274194,0
.149371910830992,0.881260174961024,0.997645679347435
"12866","NDUFAF5",21.4150146264243,0.0993731155448167,0.32934778536802
6,0.301726988793239,0.762860192497004,0.997645679347435
"12867","SNX5",156.439911102795,-0.0460082712771311,0.233076059198801,
-0.197395954931126,0.843517694845279,0.997645679347435
"12868","CRNKL1",49.710291098497,-0.0429897783039357,0.325572401118455

,-0.132043681086759,0.894949741699573,0.997645679347435
"12869","FAM182A",8.9507828290634,-0.128835982142273,0.494597735547249
,-0.260486397091411,0.794488606796217,0.997645679347435
"12870","ACTL10",2.05378324973862,-0.10880444209548,0.957261314755926,
-0.113662215758945,0.909505565809156,0.997645679347435
"12871","ITCH",59.523064378866,0.084967244782641,0.278975951849137,0.3
04568347986456,0.760694929968672,0.997645679347435
"12872","TP53INP2",20.5873135964404,-0.100780270098572,0.4711788110955
65,-0.213889648102473,0.830633113053937,0.997645679347435
"12873","EDEM2",46.9644622584928,0.0950241047973144,0.391855173846578,
0.242498022584535,0.808394288889624,0.997645679347435
"12874","SCAND1",244.257131730825,-0.0647747844807782,0.27395608858357
9,-0.236442215304212,0.813089541572178,0.997645679347435
"12875","EPB41L1",27.1262786140694,-0.0866681868731326,0.3345067077283
75,-0.259092523022015,0.795563853643664,0.997645679347435
"12876","TGIF2",7.43824381120394,0.139828829439511,0.631558955089002,0
.221402654990151,0.824778925341698,0.997645679347435
"12877","NDRG3",30.7593736334091,0.0839661037638341,0.308372557916314,
0.27228785963056,0.785400690307352,0.997645679347435
"12878","BLCAP",80.28818729339,0.0313843057718043,0.238752966204417,0.
131450956487525,0.895418580887416,0.997645679347435
"12879","KIAA1755",25.5767591802033,-0.0871853590197563,0.422461385065
824,-0.206374741223206,0.83649819972915,0.997645679347435
"12880","AL031651.2",3.13911123038551,0.369329972537029,1.207029147073
39,0.305982646262124,0.759617858777844,0.997645679347435
"12881","RALGAPB",35.1549734236367,-0.0681829867528567,0.2941213375934
42,-0.231819246134072,0.816678407956775,0.997645679347435
"12882","MAFB",258.762549458679,-0.0443077004672224,0.258496655159058,
-0.171405314470932,0.863905076277295,0.997645679347435
"12883","PLCG1-
AS1",4.23971619040774,0.168390151821488,0.830070852074509,0.2028623838
56328,0.839242596247848,0.997645679347435
"12884","CHD6",86.7499465702821,0.060576688394965,0.217573932086166,0.
278418870377241,0.78069083548771,0.997645679347435
"12885","SRSF6",188.234344858184,-0.0241273198810985,0.175914556037473
,-0.137153629719868,0.890909362568068,0.997645679347435
"12886","TTPAL",28.1040304737288,0.0367587918953454,0.324027039273133,
0.113443594021671,0.909678879730375,0.997645679347435
"12887","CCN5",3296.7875008819,0.04170264482851,0.233980710315004,0.17
8231123293738,0.858541468167513,0.997645679347435
"12888","PIGT",348.108636980044,0.0455201505907667,0.180988397317624,0
.251508667215178,0.801420862048583,0.997645679347435
"12889","WFDC3",4.16090617670377,-0.115985967838283,0.853237818052129,
-0.135936271675193,0.89187166095624,0.997645679347435
"12890","SNX21",127.45578156773,-0.0487350793228194,0.236472264028354,
-0.206092158516214,0.836718923894659,0.997645679347435
"12891","ZMYND8",49.8987746283356,0.0834375613974276,0.318463399584299
,0.262000473229707,0.793321077345851,0.997645679347435
"12892","ARFGEF2",30.1473899882754,0.0850796478634026,0.31102153750982
7,0.273549055620351,0.784431188739606,0.997645679347435

"12893","CSE1L",55.6690362976103,0.052625223918909,0.239886135302113,0.219375846180659,0.82635728373405,0.997645679347435
"12894","B4GALT5",190.37186640944,0.092439596650089,0.382176867465665,0.24187648316625,0.808875872871322,0.997645679347435
"12895","ADNP-AS1",3.56276274182792,-0.163208559171293,0.735223544871725,-0.221984946360454,0.824325602057771,0.997645679347435
"12896","RAE1",33.0606523210436,0.0860021463963775,0.305980434329837,0.281070737691907,0.778656144744369,0.997645679347435
"12897","ZBP1",3.78685881761143,-0.087170159048892,0.766497047757415,-0.113725368289325,0.909455501997475,0.997645679347435
"12898","PMEPA1",47.1652999615219,-0.0498224014985022,0.302668453406589,-0.164610486946168,0.869250576436114,0.997645679347435
"12899","NPEPL1",52.5344022902109,-0.0770384059267478,0.270248875249608,-0.285064668097484,0.775594598233957,0.997645679347435
"12900","ZNF831",11.4290909620274,-0.0966966359932108,0.459965240701414,-0.210225963696203,0.833491316226786,0.997645679347435
"12901","PPP1R3D",11.1145107978042,-0.0582399154235665,0.464215931803199,-0.125458674365913,0.900160439561828,0.997645679347435
"12902","MTG2",34.113386169359,0.0952555450059726,0.352940047720893,0.269891573997012,0.787243669694482,0.997645679347435
"12903","LAMA5",2.45716769606188,-0.279153489175798,1.07225210827533,-0.260343147867347,0.794599092557846,0.997645679347435
"12904","MRGBP",25.3888445099186,0.0570970443287048,0.356740360833406,0.160052101184504,0.872840032371866,0.997645679347435
"12905","OGFR",85.0207888053853,0.0687437440268811,0.233112357412996,0.294895323395879,0.768073844561772,0.997645679347435
"12906","ARFRP1",73.2612363608602,0.0410141897434915,0.280517749663972,0.14620889335032,0.883756485129601,0.997645679347435
"12907","TPD52L2",74.5293165061656,-0.0457161741357508,0.247508805762251,-0.184705243092096,0.853460233547218,0.997645679347435
"12908","U2AF1L5",4.01602020352791,-0.19258244455438,1.09114935877778,-0.17649503526272,0.85990504874666,0.997645679347435
"12909","CU638689.4",3.87460373568176,0.213792931072775,0.754092396621481,0.283510259526047,0.776785718193677,0.997645679347435
"12910","SAMSN1",28.8001069556891,0.13774050109332,0.460105708475794,0.299367077078043,0.764659979608085,0.997645679347435
"12911","LINC01684",10.787625265543,0.0675518348503307,0.503726986702857,0.134104061591957,0.893320287970689,0.997645679347435
"12912","SOD1",655.415053787473,-0.0484870787168003,0.224573383889248,-0.21590750371697,0.829059847929103,0.997645679347435
"12913","SCAF4",39.876500450932,0.0573692353427433,0.29506650036916,0.194428155249641,0.845840645840394,0.997645679347435
"12914","MIS18A",30.0578528615056,-0.0902024495655837,0.308541755412505,-0.292350866562575,0.770018370749467,0.997645679347435
"12915","IL10RB-DT",2.06443283120513,0.156930784256051,0.963144995367074,0.162935783304612,0.870568996281837,0.997645679347435
"12916","IFNGR2",271.244668132938,-0.0494053908977501,0.220346954405858,-0.224216354752742,0.822588958167085,0.997645679347435

"12917","TMEM50B",96.3856398684921,0.0265497551281391,0.204465742352299,0.129849405688671,0.896685573684755,0.997645679347435
"12918","GART",49.7649077027069,-0.0421218088239527,0.288102323860483,-0.146204335527508,0.883760083084197,0.997645679347435
"12919","DONSON",9.6273661980419,-0.133284396428072,0.493162398333044,-0.270264717826402,0.7869566067569,0.997645679347435
"12920","CRYZL1",37.3019043415143,0.0401742299159603,0.275689431018215,0.145722778590326,0.884140238604044,0.997645679347435
"12921","ITSN1",86.3507115378928,0.045145161909545 7,0.277163073587228,0.162883032451788,0.870610530550988,0.997645679347435
"12922","LINC00649",5.1394964667935,0.172888363487876,0.640127524855762,0.270084251613509,0.787095437441476,0.997645679347435
"12923","RCAN1",42.1865375811071,0.091177085883115,0.386438201209898,0.235942216886553,0.813477508535446,0.997645679347435
"12924","RUNX1",218.025845525671,0.0313029673939679,0.258280470585436,0.12119757766824,0.903534542715976,0.997645679347435
"12925","HLCS",12.0115453079474,-0.0932098981218421,0.453342319844198,-0.205605993620618,0.837098695325731,0.997645679347435
"12926","PIGP",92.0978557823628,0.0572016091410073,0.3029554211951,0.188811967501219,0.850240191843864,0.997645679347435
"12927","TTC3",394.395456847373,-0.0423349648264967,0.212529843053795,-0.19919538930719,0.842109905954519,0.997645679347435
"12928","SH3BGR",4.19603255966882,0.0950557458237716,0.704793615146324,0.134870327683143,0.89271440023909,0.997645679347435
"12929","BACE2",102.316299174197,0.0514473470298121,0.192079084279169,0.267844607979483,0.788818932573473,0.997645679347435
"12930","PRDM15",5.78086926133101,0.0917478541306056,0.61662926604056,0.148789328018321,0.88171987190779,0.997645679347435
"12931","PDE9A",6.89434515930083,0.194094542891228,0.734436571581283,0.264276794486598,0.791566640127532,0.997645679347435
"12932","U2AF1",18.3461989992755,-0.0893547402234266,0.366394856571377,-0.243875531058443,0.807327220481932,0.997645679347435
"12933","TRAPPC10",38.2727837012592,-0.0501044810702655,0.308219485910688,-0.162561042895205,0.870864062164762,0.997645679347435
"12934","UBE2G2",144.315319997105,0.054934403975541,0.22686390345977,0.2421469574391,0.808666293863847,0.997645679347435
"12935","FAM207A",87.4325869887633,0.0590065631918701,0.272503564082733,0.216535014470326,0.828570734891768,0.997645679347435
"12936","SLC19A1",4.62819495070734,-0.181155609024738,0.720930686867778,-0.251280202555676,0.801597480294348,0.997645679347435
"12937","YBEY",57.5118498608428,0.0701356558877996,0.323360152216417,0.216896409180497,0.828289076056845,0.997645679347435
"12938","IL17RA",49.6149395427977,-0.0415170197587232,0.314713030842053,-0.131920243809541,0.895047376322909,0.997645679347435
"12939","DGCR11",2.55433991756077,0.192586936698177,0.943357032227929,0.204150634509337,0.838235786821868,0.997645679347435
"12940","HIRA",20.1946068626055,0.0936332669818185,0.370946097149989,0.252417447443742,0.800718415566521,0.997645679347435
"12941","C22orf39",62.3919495532457,0.0633084179644333,0.2481210270204,0.255151361916732,0.798606195520174,0.997645679347435

"12942","MRPL40",149.770400514141,-0.0696200139012763,0.279891074997999,-0.248739670965836,0.803562160049314,0.997645679347435
"12943","GP1BB",12.926752210774,-0.121951725174084,0.502780744339501,-0.242554486318466,0.808350542997682,0.997645679347435
"12944","DGCR8",26.7639328175122,-0.0936326746743755,0.331029445863676,-0.282853008529444,0.777289517855067,0.997645679347435
"12945","LZTR1",37.6860265098901,0.0509601120227821,0.289749494637565,0.175876448331777,0.860391008426564,0.997645679347435
"12946","THAP7-AS1",12.0711784384235,0.13003048417668,0.450939220029079,0.288354790182799,0.773075170789597,0.997645679347435
"12947","UBE2L3",264.32771278351,-0.0320314671090694,0.255267703461472,-0.125481863450477,0.900142082415797,0.997645679347435
"12948","PPIL2",27.4909464431693,0.060492580674659,0.309795327964801,0.195266278132936,0.845184495048134,0.997645679347435
"12949","TOP3B",11.4557488579321,-0.0947389421336225,0.41838514402911,-0.226439546159007,0.820859573185723,0.997645679347435
"12950","ZNF70",6.07973950589769,0.144955942743506,0.619168897170776,0.234113734404079,0.81489668368092,0.997645679347435
"12951","CABIN1",52.9143385554323,-0.0533556987728212,0.29828438220095,-0.178875267887396,0.85803564292758,0.997645679347435
"12952","ADORA2A",5.62838487045018,-0.121579553886303,0.644739339691616,-0.18857163880283,0.850428562935641,0.997645679347435
"12953","SNRPD3",232.614862806153,-0.0782389579829781,0.267876494469295,-0.292071008835552,0.770232332312003,0.997645679347435
"12954","MIAT",11.299720226377,-0.101038208060796,0.597254558703303,-0.169171095621538,0.865662064432828,0.997645679347435
"12955","MIATNB",7.98387892956766,0.157736248471253,0.563518096245959,0.279913368394129,0.779543970552666,0.997645679347435
"12956","XBP1",355.257067204406,-0.0350772869350808,0.252939558608981,-0.138678533037637,0.889704182319749,0.997645679347435
"12957","RHBDD3",22.8469772779431,0.0704789424683662,0.358306143251426,0.196700346326216,0.844062039059798,0.997645679347435
"12958","EWSR1",148.065536215272,0.0231646217619312,0.200855308255823,0.115329895749766,0.908183643075582,0.997645679347435
"12959","AP1B1",28.1776418839712,-0.0468980656630778,0.338883281688736,-0.138390024522229,0.889932180437265,0.997645679347435
"12960","THOC5",31.1470975535823,-0.0642149850041013,0.280653042175869,-0.228805590369697,0.819020021559343,0.997645679347435
"12961","LIF",5.78927759141362,-0.0978430979217209,0.666016101154703,-0.146908006806571,0.883204631979157,0.997645679347435
"12962","DUSP18",17.5638560012058,-0.101476378455726,0.399732405190427,-0.253860775704148,0.799603118719688,0.997645679347435
"12963","MORC2",18.8261925729919,0.120831768320109,0.42155575948826,0.286632943805085,0.774393386862633,0.997645679347435
"12964","SFI1",12.3846731568864,-0.0531583883449624,0.432772919743421,-0.12283205792192,0.902240087924611,0.997645679347435
"12965","DEPDC5",12.6039420195068,0.0800176706102652,0.436835753054661,0.183175644508779,0.85466020389567,0.997645679347435
"12966","MCM5",19.6326155798035,-0.0953851694125506,0.331409194866587,

-0.287816906983975,0.77348689483913,0.997645679347435
"12967","RBFOX2",188.405403487957,0.0362539407935821,0.198222244140124,0.182895421000047,0.854880075810461,0.997645679347435
"12968","TXN2",175.63962192083,-0.031413439854379,0.240215457345545,-0.130771933669495,0.895955725983359,0.997645679347435
"12969","CSF2RB",2.22612673401456,0.309293638689574,1.08667190895422,0.284624674790048,0.775931705074022,0.997645679347435
"12970","MPST",89.1167473280745,0.0403406271285453,0.237257525366888,0.170028862377132,0.864987438234621,0.997645679347435
"12971","GGA1",44.1377507765729,-0.0755854814288258,0.266906263132183,-0.28319111189756,0.777030341283934,0.997645679347435
"12972","SH3BP1",14.1933163077638,-0.0647209344435063,0.405006079722297,-0.159802377504762,0.873036751174147,0.997645679347435
"12973","ANKRD54",21.1491664430964,0.105696270248605,0.353314290473229,0.299156510502407,0.764820630190671,0.997645679347435
"12974","AL022311.1",13.6635215358329,0.117943800149589,0.406527972459404,0.290124685482416,0.771720851299481,0.997645679347435
"12975","KDELR3",94.685654300201,-0.0722087922052871,0.335939950084569,-0.214945534721635,0.829809782931092,0.997645679347435
"12976","CBY1",82.8025305230346,0.0693943726798646,0.247295567590577,0.280613087229909,0.779007177050241,0.997645679347435
"12977","AL021707.6",5.19060719372816,-0.195115802422235,0.660849894610243,-0.295249804855171,0.767803058037506,0.997645679347435
"12978","APOBEC3A",2.00873639966798,-0.197473581702037,1.17383641415188,-0.168229217735348,0.866402956068313,0.997645679347435
"12979","APOBEC3G",46.8850557086945,0.0412690345280306,0.280674010796142,0.147035467982837,0.88310402515205,0.997645679347435
"12980","ADSL",75.0706965937371,0.0332736691117836,0.284011673049668,0.117155991352386,0.906736440497625,0.997645679347435
"12981","RANGAP1",27.5660897031403,-0.0914088105730866,0.323073003716178,-0.282935465116702,0.777226307634099,0.997645679347435
"12982","SREBF2",28.8660542113788,-0.0957813669378909,0.314666823003274,-0.3043897860719,0.760830948385764,0.997645679347435
"12983","NDUFA6",173.680495186072,-0.0713140496821652,0.300827674209244,-0.23705947223646,0.812610652728966,0.997645679347435
"12984","NFAM1",2.0977158125332,0.185980203592994,1.04682656032803,0.177660952292533,0.858989252876229,0.997645679347435
"12985","SERHL2",3.82989025557723,0.180365436020721,0.758340708116596,0.237842218003401,0.812003472828459,0.997645679347435
"12986","TTLL1",9.95001203956856,0.0655527639935803,0.530311757087987,0.123611749348231,0.901622689205288,0.997645679347435
"12987","TTLL12",13.9434850301404,-0.107698616204759,0.378419061122322,-0.28460145713946,0.775949494765148,0.997645679347435
"12988","CDPF1",9.02561541896143,0.132519177456713,0.528061771311372,0.250953931256221,0.801849727099751,0.997645679347435
"12989","TUBGCP6",16.1457852654341,-0.0835532350672909,0.39268861361798,-0.212772237772532,0.831504618952909,0.997645679347435
"12990","LMF2",122.630452173712,-0.0343254862340039,0.200387762332949,-0.171295321801993,0.863991558761497,0.997645679347435
"12991","CHKB-

DT",5.64583175180986,-0.0812990910654397,0.716165742649483,-0.113519938505674,0.909618356614397,0.997645679347435
"12992","SHANK3",2.96171262557028,-0.110848858821266,0.823064011351744,-0.134678296332281,0.892866233846431,0.997645679347435
"12993","ASMTL",58.8085559223974,-0.0654246915143207,0.235357122067385,-0.277980504433551,0.781027324385841,0.997645679347435
"12994","AKAP17A",47.3462136638506,-0.0650239161796047,0.307282044817029,-0.211609878534631,0.832411401902359,0.997645679347435
"12995","DHRSX",48.3025074496131,0.0999113981892807,0.345486942315872,0.289190084926375,0.772435918938917,0.997645679347435
"12996","PRKX",18.4792726081924,-0.0642820347685655,0.424619788542928,-0.151387279874891,0.879670223007985,0.997645679347435
"12997","STS",22.1543161771016,0.070564878147313,0.370213996212983,0.190605646650693,0.848834565916364,0.997645679347435
"12998","WWC3",49.7700891111095,-0.0627770657938352,0.29147286490989,-0.215378765406663,0.829472024273211,0.997645679347435
"12999","HCCS",25.7554415036446,-0.0987598359437198,0.381762796157461,-0.258694238772774,0.795871165083168,0.997645679347435
"13000","CTPS2",12.239341575815,-0.0463371465164576,0.402519289440404,-0.115117828466996,0.908351728776221,0.997645679347435
"13001","YY2",5.22837725560072,0.197871925644603,0.672185442989875,0.294371036606312,0.76847439637298,0.997645679347435
"13002","PRDX4",377.442981713206,-0.0372365378326025,0.283031718146473,-0.131563126834188,0.895329852465629,0.997645679347435
"13003","POLA1",11.3302453186904,-0.0904889058208284,0.450511240733289,-0.200858264210103,0.840809402912946,0.997645679347435
"13004","GK",7.53343323031459,-0.102072227764022,0.578602030545612,-0.176411803580726,0.859970432173296,0.997645679347435
"13005","TSPAN7",2.62833476437863,-0.339629632732889,1.17418696056978,-0.289246639707258,0.772392643088243,0.997645679347435
"13006","BCOR",15.4155861442734,-0.107154245885454,0.458701488152663,-0.233603440697344,0.815292856359459,0.997645679347435
"13007","CASK",44.1089540913809,0.0585626211998854,0.264875347012828,0.221095023981409,0.82501844468054,0.997645679347435
"13008","CDK16",50.8591567167053,0.0484621368215293,0.275667753092669,0.175799077976444,0.860451794024741,0.997645679347435
"13009","ARAF",33.3027243071978,-0.0603985266405959,0.272617675262647,-0.221550295968178,0.82466397890702,0.997645679347435
"13010","ZNF182",7.07746071191555,0.13886010219166,0.630950748476139,0.220080731383603,0.825808281053525,0.997645679347435
"13011","GRIPAP1",33.4143157663897,-0.0556989632052134,0.329612555470099,-0.168983135747892,0.865809906355427,0.997645679347435
"13012","PRAF2",55.5715672635392,-0.0834009210573798,0.310365336533706,-0.26871854308486,0.788146281158375,0.997645679347435
"13013","PPP1R3F",11.399388755016,-0.0619324574161226,0.475865957432006,-0.13014685427455,0.896450240980353,0.997645679347435
"13014","GSPT2",34.6707515708287,-0.0488645280448193,0.274260170681223,-0.178168517592062,0.858590633434284,0.997645679347435
"13015","MAGED1",61.7242256071805,0.0858483652018727,0.34847761462641,0.246352596547436,0.805409298029998,0.997645679347435

"13016","KDM5C",53.1735503926107,-0.0304955913516286,0.256785196015024
,-0.118759148988653,0.905466173954848,0.997645679347435
"13017","SMC1A",114.392558531717,-0.0371204131409336,0.283296919982256
,-0.131030062533891,0.895751525955206,0.997645679347435
"13018","GNL3L",60.4082681766671,0.0925777480305427,0.340956768108524,
0.271523420825821,0.785988488842716,0.997645679347435
"13019","RRAGB",32.6978928981461,-0.067832410866714,0.290897613916208,
-0.233183111932478,0.815619219147134,0.997645679347435
"13020","ZC3H12B",2.54821514657375,-0.158915560562497,0.96013378405076
,-0.165513976491943,0.86853944989995,0.997645679347435
"13021","HEPH",15.9841922970993,0.0544703425151212,0.41752039437448,0.
130461513375239,0.89620130173157,0.997645679347435
"13022","STARD8",4.35324714982452,0.20913359008343,0.701573358791708,0
.298092262858459,0.765632746634088,0.997645679347435
"13023","EFNB1",29.720529059889,0.0651477564192651,0.328356316639584,0
.198405674317433,0.842727678230808,0.997645679347435
"13024","EDA",6.48623608377934,-0.140399752234758,0.623315941352208,-0
.225246528959579,0.821787494456088,0.997645679347435
"13025","PDZD11",69.6094410968145,0.0802613639808003,0.308868303747143
,0.259856265622214,0.794974647586003,0.997645679347435
"13026","NONO",180.33237451631,0.0361631763236085,0.175362687300853,0.
206219332517223,0.836619587224802,0.997645679347435
"13027","NHSL2",31.5422751414959,0.106921111436601,0.510907805824017,0
.209276723153903,0.834232221687779,0.997645679347435
"13028","JPX",203.792223117464,0.024370676941922,0.192863996660791,0.1
26361982349589,0.899445394566701,0.997645679347435
"13029","ZDHHC15",7.36826604031296,0.0780516331871159,0.63849009636055
9,0.122244078071087,0.902705718947509,0.997645679347435
"13030","P2RY10",5.97759860472552,-0.101652965672996,0.663117461327009
,-0.153295564664473,0.87816519822171,0.997645679347435
"13031","BRWD3",25.9857452285756,0.097657415162062,0.372742120805553,0
.261997262211766,0.793323552926432,0.997645679347435
"13032","TRMT2B",7.78411808722405,-0.0693057760567167,0.50258625153390
4,-0.137898272873948,0.890320815415463,0.997645679347435
"13033","TIMM8A",24.7856360814366,-0.0962565231851743,0.38586790977725
7,-0.249454595073061,0.803009158761741,0.997645679347435
"13034","GLA",60.100820645431,0.0326278342132672,0.296482607222095,0.1
100497412613,0.912369926625101,0.997645679347435
"13035","ARMCX6",63.7572868156327,0.062351820640419,0.296216335205228,
0.210494200453934,0.833281977639222,0.997645679347435
"13036","ARMCX3",173.735935902593,0.0257639131809265,0.177687761349344
,0.144995428977651,0.884714480799687,0.997645679347435
"13037","GPRASP1",14.3572615733517,0.0637098503914451,0.45256703036029
8,0.140774396094926,0.888048170564508,0.997645679347435
"13038","LINC00630",8.7968544832002,0.0860178016328879,0.5203352810769
32,0.165312260692486,0.868698208953942,0.997645679347435
"13039","TCEAL8",199.523281751085,0.0599494159518044,0.246293141165292
,0.243406761829276,0.807690306123379,0.997645679347435
"13040","TCEAL9",307.491091028448,0.0561744075761225,0.240138918116574
,0.233924630029577,0.815043491630299,0.997645679347435

"13041","RAB9B",12.064131904771,-0.108892226725043,0.452306478655811,-0.240748766298142,0.809749840295737,0.997645679347435
"13042","TMSB15B",2.98155888784312,-0.112319857517661,0.80768298419258,-0.139064286008135,0.889399349590073,0.997645679347435
"13043","PRPS1",43.2803421743867,-0.0461267457783602,0.344760716881109,-0.133793508134127,0.893565860799531,0.997645679347435
"13044","MID2",4.46586890006683,0.084086074588096,0.68920815907581,0.122003887331878,0.902895939626359,0.997645679347435
"13045","PSMD10",39.931275046485,0.0774713518788477,0.260529890273386,0.297360705128894,0.766191140314558,0.997645679347435
"13046","NXT2",14.1105208076101,-0.0725751798557239,0.430261032368661,-0.168677092266955,0.866050638293983,0.997645679347435
"13047","ZBTB33",18.8154416016341,-0.0469567837859459,0.352238297906795,-0.133309705574296,0.893948452173597,0.997645679347435
"13048","XPNPEP2",37.9549210287813,0.0783627952268384,0.341582078668261,0.229411319037446,0.818549239421961,0.997645679347435
"13049","UTP14A",45.8545687737506,0.0486064794410602,0.361959220998425,0.134287169993859,0.893175498211745,0.997645679347435
"13050","ZNF280C",9.05487235310759,0.135963674910862,0.479133387834146,0.283769986319397,0.776586656843643,0.997645679347435
"13051","RBMX2",81.0333947874797,-0.0504895115650393,0.220025192766408,-0.229471502468547,0.818502467453906,0.997645679347435
"13052","STK26",6.09789768901465,-0.087115832412622,0.619090790710651,-0.140715762081717,0.888094492657464,0.997645679347435
"13053","RAP2C",42.0116266037079,0.0354533812928239,0.262854334654016,0.134878435006558,0.892707990100596,0.997645679347435
"13054","PHF6",38.5670164425406,0.0531396346799223,0.263115261985781,0.201963330742836,0.839945391250751,0.997645679347435
"13055","ZNF449",10.3801774814005,-0.0587797146091974,0.475984841516506,-0.123490728028067,0.90171851605578,0.997645679347435
"13056","HTATSF1",109.963406534329,0.0231740997401208,0.189760436007076,0.12212292629458 6,0.9028016652181,0.997645679347435
"13057","LINC00632",122.878674360169,0.051498353425918,0.288272137168043,0.178644921884691,0.858216519268338,0.997645679347435
"13058","CDR1",3.18310418978152,0.169925761973189,0.819732245335557,0.20729422679186,0.83578008258375,0.997645679347435
"13059","FMR1",63.7919719513197,-0.0409141564352557,0.252430869818225,-0.162080638016729,0.871242353208205,0.997645679347435
"13060","VMA21",60.6089371466399,0.0528414467316663,0.248945369574975,0.212261215470216,0.831903251553084,0.997645679347435
"13061","CETN2",93.4041164943113,-0.0236909593707349,0.212897905959728,-0.111278498790008,0.911395504980415,0.997645679347435
"13062","IDH3G",148.933574876933,-0.074858051350648,0.26320611815214,-0.284408477569576,0.776097362979791,0.997645679347435
"13063","ARHGAP4",9.73985281643252,0.139862219288799,0.53803980181542,0.259947719140638,0.794904101586515,0.997645679347435
"13064","MECP2",110.425654911733,-0.0395002602075604,0.258078068353726,-0.153055470616049,0.878354531339274,0.997645679347435
"13065","FLNA",361.85222711287,-0.0234790963594123,0.209460751036974,-0.112093059168244,0.910749620724101,0.997645679347435

"13066","TAZ",29.7529181691527,0.0496182683085751,0.310232555977792,0.15993894693672,0.872929168192013,0.997645679347435
"13067","GDI1",76.3581615791794,-0.0408101558476691,0.245234745080255,-0.166412617568989,0.867832245017996,0.997645679347435
"13068","UBL4A",32.2633108663139,-0.037479677800571,0.329012831666805,-0.113915550377461,0.909304738394143,0.997645679347435
"13069","TMLHE",17.0682868453356,0.120164000230741,0.408514170794705,0.294148915316645,0.768644114292386,0.997645679347435
"13070","MT-ND6",122.116275152338,0.0396682419039022,0.349359432107152,0.11354564456624,0.909597977907346,0.997645679347435
"13071","TMX3",91.5576950569816,-0.0241801131288574,0.224324949151184,-0.107790565518244,0.91416182691034,0.997661614343858
"13072","ZFP30",6.31797069692801,-0.0640219029782357,0.593676241126824,-0.10783975935557,0.914122803374364,0.997661614343858
"13073","AC092329.4",5.41771233576249,-0.0709544495333299,0.658855161832932,-0.107693547297914,0.914238788254424,0.997669284268157
"13074","MEIS1-AS2",2.05754729532289,-0.104563448292188,0.988595028043211,-0.105769749317026,0.915765038671515,0.997719123318987
"13075","KANSL1L",32.7159784056953,0.0336156120662962,0.316642531435789,0.106162655767907,0.915453299837123,0.997719123318987
"13076","FRMD4B",20.6592387939813,0.0453124298533203,0.426565606054149,0.106226168285045,0.915402909117312,0.997719123318987
"13077","CCDC58",31.3390510410028,0.0346923348361224,0.326846426866283,0.106142616178317,0.915469199282914,0.997719123318987
"13078","CCDC28A",33.7926089436782,0.0304974255066592,0.286218329647464,0.106553013373472,0.915143596177701,0.997719123318987
"13079","CASP2",25.5379182269997,0.0330840736490095,0.309293689321262,0.106966533076093,0.91481553012199,0.997719123318987
"13080","C8orf33",95.9547218321359,-0.0274867286616799,0.26005307737798,-0.105696609856797,0.915823070227263,0.997719123318987
"13081","MOB2",56.1516261223971,0.0261328144263566,0.247051749933097,0.105778706013755,0.915757932127555,0.997719123318987
"13082","DDIT3",90.7682686154955,0.0377627874250287,0.356751212556151,0.105851882477022,0.915699871716519,0.997719123318987
"13083","ATP2B1",238.319758931628,-0.0185281510146519,0.173869519801866,-0.106563537046434,0.915135247037183,0.997719123318987
"13084","IDH2",37.2659708573005,-0.0347119474791721,0.324600644916239,-0.106937395297318,0.914838646118279,0.997719123318987
"13085","CMTM4",5.80622087839299,-0.0740489346390684,0.70057721140102,-0.105697035864162,0.915822732215948,0.997719123318987
"13086","SKAP1",14.6801661777336,0.0478859537511069,0.452689958005011,0.10578090568242,0.915756186837599,0.997719123318987
"13087","AC027097.1",9.6791059995889,0.0527534782728159,0.494670761602464,0.106643615041898,0.915071716061759,0.997719123318987
"13088","COX7A1",230.497588324568,-0.0303202353782525,0.284361558321802,-0.106625647844918,0.915085970536586,0.997719123318987
"13089","CCDC97",38.2787841078639,0.0279843499329423,0.263527128992766,0.106191533448196,0.915430388294047,0.997719123318987

"13090","PHLDB3",6.2375228480429,0.0707334008407782,0.66607827979638,0.106193825840412,0.915428569513744,0.997719123318987
"13091","FBXO46",6.96310560149179,-0.0587365278310764,0.547067515522171,-0.107366140676463,0.914498515025597,0.997719123318987
"13092","SIX5",33.7949507502754,0.0388479270700384,0.365490201821535,0.106289927545055,0.915352322974093,0.997719123318987
"13093","DNTTIP1",48.9920605354019,0.0296151560847869,0.279329294249875,0.106022378226805,0.91556459699609 2,0.997719123318987
"13094","ATF4",407.576476522529,-0.0170800499650727,0.159021259581518,-0.107407336666938,0.914465834360404,0.997719123318987
"13095","CHKB",52.9359560336768,0.0278539729423843,0.260130497607559,0.107076921770263,0.914727955649766,0.997719123318987
"13096","AGO4",21.5839409306856,0.0373435408725981,0.362774460735548,0.102938726163032,0.918011601908866,0.997745356405695
"13097","STK40",46.1558871872932,0.0282420897975517,0.273611727655661,0.103219587988912,0.917788693979514,0.997745356405695
"13098","PNPT1",67.8378551618116,-0.026448282651541,0.255469266999373,-0.103528236340092,0.917543740535671,0.997745356405695
"13099","CAB39",64.475621485075,-0.0239643941992071,0.229994425540203,-0.10419554362207,0.917014170398585,0.997745356405695
"13100","SCLY",11.0968508766607,-0.0557372646860769,0.528113969488333,-0.105540220305247,0.915947156992614,0.997745356405695
"13101","EOMES",4.64643139293462,-0.0715021318012409,0.689203661672263,-0.103746012648497,0.917370910830528,0.997745356405695
"13102","MRAS",38.4299089325827,0.0271785402074632,0.26028869273204,0.104416906943564,0.916838506220842,0.997745356405695
"13103","SENP5",57.4005204999531,0.0268965338499573,0.258317127465135,0.104122146734492,0.917072415829245,0.997745356405695
"13104","UBE2K",168.564290873902,-0.0202991481948806,0.196196058620694,-0.103463588094423,0.917595046854277,0.997745356405695
"13105","ABCE1",133.591225511313,0.0260635479741633,0.250053420365318,0.10423191946779,0.916985303851866,0.997745356405695
"13106","FCHO2",59.9923321109281,0.0274996951790879,0.26700333608668,0.102993826152645,0.917967870912578,0.997745356405695
"13107","ANKRA2",23.0647642021782,0.0335317091313501,0.321170207939358,0.104404793167122,0.916848119075103,0.997745356405695
"13108","PCDHB12",8.94107483455275,0.059922816270201,0.575592314615739,0.104106352271581,0.917084949869817,0.997745356405695
"13109","ZSCAN16-AS1",73.2150121273951,0.0317109351566576,0.302679246786118,0.104767457608567,0.916560332969269,0.997745356405695
"13110","ABHD11",20.344388270257,-0.0377989487834552,0.366711921445285,-0.103075320361776,0.917903192172866,0.997745356405695
"13111","TMEM8B",22.9532987565612,-0.0347697948449062,0.337786842010334,-0.102934130405951,0.918015249416876,0.997745356405695
"13112","GOLM1",115.080190583977,-0.0202454353026508,0.19602868771160 2,-0.103277920895109,0.917742398475194,0.997745356405695
"13113","NOXA1",2.24280001616067,-0.10674419554836,1.0183776498877,-0.104817889080865,0.916520314810289,0.997745356405695
"13114","PPP3CB",97.344074097764,-0.019225430990665,0.185610906709917,

-0.103579209495009,0.917503287322319,0.997745356405695
"13115","TRAPPC4",93.3080455037729,0.0317186400230739,0.30626257116615
1,0.103566818179248,0.917513121273927,0.997745356405695
"13116","NUAK1",68.5081784512332,0.0456592253669888,0.438200385571491,
0.104197136448068,0.917012906387576,0.997745356405695
"13117","PIGQ",21.0627941857255,0.0344533810748794,0.32728185675082,0.
105271283342513,0.916160548911759,0.997745356405695
"13118","RHOT1",44.3457941597741,-0.025363100851109,0.244214513612349,
-0.103855829352423,0.917283760551014,0.997745356405695
"13119","MAP2K6",14.3187922997396,-0.0517733528849965,0.49126226400413
9,-0.105388418119085,0.916067605887187,0.997745356405695
"13120","ARHGEF18",19.5180826634633,-0.0369736436505493,0.352575042948
356,-0.104867444222265,0.916480992239587,0.997745356405695
"13121","PET100",202.730554217214,-0.0286539794070353,0.27219331322459
6,-0.10527069554935,0.91616101531136,0.997745356405695
"13122","FUZ",21.8591422265402,0.0346339745061168,0.33613282655898,0.1
03036572954411,0.917933944394198,0.997745356405695
"13123","ATRN",40.4290941222373,0.0303909286288147,0.292753896280949,0
.103810500952818,0.917319732942572,0.997745356405695
"13124","TFIP11",23.2654044675799,-0.0365332390142871,0.34753286332129
8,-0.105121681630758,0.916279255167716,0.997745356405695
"13125","ZFX",85.8537774797459,0.0232624757226204,0.221116707779865,0.
105204513743844,0.916213529151382,0.997745356405695
"13126","ITM2A",1621.05528956523,-0.0310624139994703,0.298367570474408
,-0.104107875899786,0.917083740760522,0.997745356405695
"13127","DPH5",39.6240258763149,-0.0302770864433739,0.294825131764463,
-0.102695066265797,0.918204989491821,0.997842203315303
"13128","TCHH",3.26342111817692,0.0827603663548971,0.811718669345963,0
.101956958094337,0.918790839446153,0.997842203315303
"13129","METTL18",13.0932244048045,0.0528747901061762,0.51858455801746
9,0.101959823694548,0.918788564881401,0.997842203315303
"13130","ATP6V1E2",19.5503923599859,-0.0344554045303728,0.338480850110
979,-0.101794250750303,0.918919989182563,0.997842203315303
"13131","ACYP2",31.3499524651242,0.0290738139438617,0.287653224554456,
0.101072442309291,0.919492953848917,0.997842203315303
"13132","RAF1",71.9017307750216,-0.0288046914209885,0.285656149952265,
-0.100836937786223,0.919679904155221,0.997842203315303
"13133","CHCHD4",19.8378004241266,-0.0372476034010231,0.36299998004877
8,-0.102610483328451,0.918272122269254,0.997842203315303
"13134","B4GALT4",42.8734466805548,-0.0282687212380756,0.2758920614022
72,-0.102462974448756,0.918389200240221,0.997842203315303
"13135","IL20RB",2.72230684505483,-0.0939766191715151,0.92434579492457
4,-0.101668249790852,0.919020004519608,0.997842203315303
"13136","HNRNPDL",513.354796887715,-0.0249307360159133,0.2457370280890
58,-0.101452907645966,0.919190938859354,0.997842203315303
"13137","COPS4",90.3638495412537,-0.0272498391885931,0.267499164575838
,-0.101868875859115,0.918860754877984,0.997842203315303
"13138","MIR4458HG",37.5884774289793,0.0283627751352077,0.277503567740
244,0.102206884639972,0.9185924632234,0.997842203315303
"13139","SERPINB1",249.994608327688,0.0221754377824371,0.2200050803342

1,0.100795116861621,0.919713103280031,0.997842203315303
"13140","PIK3CG",3.18589458466749,0.0812781052239935,0.805580450653464
,0.10089384015968,0.919634732997172,0.997842203315303
"13141","NDUFB2-
AS1",2.29247761436759,0.0952180165884926,0.938593919041503,0.101447510
639883,0.919195222944148,0.997842203315303
"13142","AC107959.1",11.9643797580379,-0.0438657601811193,0.4350619222
40227,-0.100826475356071,0.9196882096381 5,0.997842203315303
"13143","ATP6V1G1",423.10324574087,-0.0217158757461044,0.2150318037317
02,-0.100989134487285,0.919559085500975,0.997842203315303
"13144","ANKRD13A",37.965057177075,-0.0286570108057975,0.2812057870100
22,-0.101907614030632,0.918830006307707,0.997842203315303
"13145","PPCDC",14.2803861716797,-0.0439923575665796,0.433259587010756
,-0.101538105296415,0.919123310200908,0.997842203315303
"13146","OGFOD1",36.6885767835543,-0.0289806928888206,0.28620662611518
3,-0.10125793830209,0.919345704893623,0.997842203315303
"13147","B9D1",16.5872191298675,-0.0398884422275996,0.39518908167165,-
0.100935081654765,0.919601994171767,0.997842203315303
"13148","DDRGK1",114.542161872696,-0.0216899502599328,0.21440526241906
,-0.101163329739264,0.919420805966065,0.997842203315303
"13149","CAPN6",4.83154257486019,0.092359115746257,0.913733425804927,0
.101078841090766,0.91948787437304,0.997842203315303
"13150","ISG15",335.320642987304,0.0686486565466219,0.698150251669603,
0.0983293444103915,0.921670777612581,0.99786267871228
"13151","AL365361.1",3.30660175181543,0.0780557927035284,0.78874032178
5671,0.0989625996637445,0.921167965432154,0.99786267871228
"13152","BROX",51.2171905201293,-0.0263078434453291,0.263168371667945,
-0.0999658252190093,0.920371457025045,0.99786267871228
"13153","CNRIP1",94.4419215475235,0.024233608043488,0.243951197472064,
0.0993379343680535,0.920869960085978,0.99786267871228
"13154","GRIP2",2.3581847173372,-0.0934671881404855,0.945230498751044,
-0.0988829584572079,0.921231199766458,0.99786267871228
"13155","CMC1",97.2803349560395,0.0298170507930532,0.297236838598716,0
.10031411629063 8,0.920094950843149,0.99786267871228
"13156","PCDHGA10",11.8466293105157,0.0445312813082136,0.4512645425795
96,0.098681099679705,0.921391475879219,0.99786267871228
"13157","HIST1H2BC",3.90995898351276,0.0762239506340423,0.774686089279
933,0.0983933385261792,0.921619964100313,0.99786267871228
"13158","ENPP1",36.1664504754893,0.0481042090526334,0.479755314967073,
0.100268214966904,0.920131391075349,0.99786267871228
"13159","NIPSNAP2",110.271205095226,0.0204024699788575,0.2032642255919
49,0.100374130860662,0.920047306612642,0.99786267871228
"13160","SMU1",97.1482686532703,-0.0222706127035558,0.224152010062332,
-0.0993549542445005,0.920856447039113,0.99786267871228
"13161","SUPV3L1",25.6322568205004,-0.0319569005700505,0.3217636104939
34,-0.099317945000039,0.920885830810078,0.99786267871228
"13162","SMC3",174.720833499711,-0.0217955977483021,0.216562410473274,
-0.100643494411935,0.919833468425847,0.99786267871228
"13163","FAR1",58.9944502579806,-0.0228515028951568,0.232803296613477,
-0.098157986710545,0.921806843049102,0.99786267871228

"13164","CPSF7",59.0890320747292,-0.023701620792637,0.236290550713081,-0.100307103780113,0.920100517937793,0.99786267871228
"13165","PPP1R14B-AS1",2.86870990356817,0.0802051960310329,0.817725541032579,0.0980832712278427,0.921866171095617,0.99786267871228
"13166","FADD",25.0771851140542,-0.0363508759368378,0.371777107053686,-0.0977759933227643,0.922110170577876,0.99786267871228
"13167","KIF21A",27.2598789031884,-0.0362757462507836,0.370343931212787,-0.0979515072165199,0.921970799751603,0.99786267871228
"13168","PFKM",27.2973809794353,0.029392537892143,0.297693053322945,0.0987343761100707,0.921349174018876,0.99786267871228
"13169","DHX37",18.8518257796166,0.0386925492757081,0.388642740930061,0.0995581422236601,0.920695126324556,0.99786267871228
"13170","PCK2",16.8178978722504,0.0411925430360597,0.412258930563855,0.0999191041894951,0.920408549301764,0.99786267871228
"13171","MMP25-AS1",9.57415426519051,0.0447180047185634,0.457015746033596,0.0978478424576558,0.922053116837717,0.99786267871228
"13172","NUDT21",95.4591278234226,0.0190516782726234,0.194190609867417,0.0981081334758202,0.92184642910696,0.99786267871228
"13173","CFAP20",62.2699521504982,-0.0313947157589475,0.31400410242143,-0.0999818649401342,0.920358722975421,0.99786267871228
"13174","HIC1",210.817012724156,-0.0183527326313417,0.185041679178027,-0.0991816152602285,0.920994071774714,0.99786267871228
"13175","ACACA",21.3375298983147,-0.0352281496614758,0.359174799985537,-0.0980807942619982,0.921868137945003,0.99786267871228
"13176","LDLRAD4",22.513231094164,0.0389394129114856,0.398132676989602,0.0978051166408093,0.922087044374691,0.99786267871228
"13177","ESCO1",39.2218709303373,0.0255976747153131,0.259896077004845,0.0984919626733571,0.921541653787662,0.99786267871228
"13178","ICAM1",223.552531917915,0.0309038917534878,0.310002321227479,0.0996892269423063,0.920591053589651,0.99786267871228
"13179","DACT3",13.7078650656722,-0.0563946631283104,0.570991406457805,-0.0987662204553298,0.921323889491058,0.99786267871228
"13180","ZNF17",4.42045275878364,0.0659809579176046,0.665011532757155,0.0992177649070924,0.920965370098148,0.99786267871228
"13181","IGLC3",19.8438972901718,-0.111991523481896,1.11763324545835,-0.100204180518957,0.92018222714765,0.99786267871228
"13182","MAGED2",226.515831805114,-0.0270362244905727,0.270714195730574,-0.0998699917365258,0.920447540339348,0.99786267871228
"13183","AC240274.1",21.0205386696272,0.042633577428139,0.435279383330898,0.097945317561087,0.921975714748496,0.99786267871228
"13184","CCSAP",6.3739153842421,0.0631904056130523,0.651927589459254,0.096928564820314,0.922783124161157,0.997915823141226
"13185","MAP10",4.01008965766406,-0.0718706803058778,0.742096019860587,-0.0968482223087245,0.922846928031815,0.997915823141226
"13186","ZNF141",40.7989200992328,0.0333276877787353,0.342620399516496,0.0972729231118961,0.922509658236041,0.997915823141226
"13187","CNOT8",50.0243140125979,0.0238720714604521,0.246314184446966,0.09691716095867,0.922792180488477,0.997915823141226

"13188","AC091729.3",6.99972853538471,0.0577136312282085,0.59311005907
5173,0.0973067820131064,0.922482770294539,0.997915823141226
"13189","TSPYL5",25.7444818376031,0.033238707960569,0.343004827674851,
0.0969044901959144,0.922802242931577,0.997915823141226
"13190","VAV2",16.0006896788685,0.0420283000066665,0.431745259646469,0
.0973451336584008,0.922452314693932,0.997915823141226
"13191","MGST1",1620.63229300162,-0.0265174001825122,0.273873681333093
,-0.0968234700517316,0.922866585095195,0.997915823141226
"13192","PSMA4",402.899883567468,-0.0258557126412967,0.267499288544364
,-0.0966571267609505,0.922998688234157,0.997915823141226
"13193","IL32",711.064144114667,0.0335523336376136,0.346872588534951,0
.0967281207757726,0.922942307388173,0.997915823141226
"13194","EDC4",21.5702857237388,-0.0317922490030911,0.328618621963866,
-0.0967451230033668,0.922928804899281,0.997915823141226
"13195","AMZ2",37.627399741681,0.0271510431612778,0.279737000285917,0.
0970591774900242,0.922679399389496,0.997915823141226
"13196","TMEM98",218.456191272065,-0.019593925006188,0.203024425763135
,-0.0965101855726854,0.92311538477204,0.997966359439066
"13197","FOXP1-
IT1",2.66753943087144,-0.0859412907013417,0.895026380071854,-0.0960209
582810757,0.923503927170581,0.998031356427687
"13198","MAFK",29.6506864293471,-0.0327815159282187,0.340410687171483,
-0.0962999023344555,0.92328238865355,0.998031356427687
"13199","RNF214",38.6234652102577,-0.0285142495924933,0.29642299992468
2,-0.0961944572443384,0.923366132880106,0.998031356427687
"13200","PDE8A",40.7892205382537,0.0253751029175449,0.264265054005852,
0.0960214093119669,0.923503568955194,0.998031356427687
"13201","TRAPPC1",169.686884840108,0.0300690663328942,0.31323886468555
6,0.0959940471086782,0.923525300448752,0.998031356427687
"13202","FAM172A",78.8190950429985,0.0258468773544237,0.26954355927555
,0.0958912816314073,0.923606918921814,0.998043955865671
"13203","B3GAT2",7.83658632250311,-0.063370543730115,0.662282409100025
,-0.0956850776336173,0.923770692838056,0.998086351828091
"13204","RPAIN",60.7412517238128,0.0246960731914417,0.258149697021513,
0.095665706667026,0.923786078055384,0.998086351828091
"13205","WASHC2A",57.6109209654015,0.026286813707823,0.275310783461825
,0.0954805088899397,0.923933171175418,0.998169679665923
"13206","TTC33",29.1831079746595,0.0293385077077552,0.307577832006169,
0.0953856378933278,0.924008523362449,0.998175495554195
"13207","EFCAB2",31.5589986161351,-0.0269929710938262,0.28367592668885
2,-0.095154253689751,0.924192305314504,0.998298434740419
"13208","TOP2A",2.202097002879,0.0970475420746262,1.02235294015746,0.0
949256741607055,0.924373863565791,0.998343367221559
"13209","EID2B",13.6061227656827,0.036698551547495,0.38638962575187,0.
0949780974995999,0.924332223925389,0.998343367221559
"13210","CXCR3",5.7569092025662,0.0646278852586849,0.683778808268292,0
.0945157768523985,0.924699450431076,0.998619406498844
"13211","MRPL24",96.4103718173731,-0.0229905660401184,0.24434024932604
3,-0.0940924227732955,0.925035739429176,0.998661110972001
"13212","DIS3L2",22.3463951722198,-0.0312867034749567,0.33268692593175

9,-0.0940424796896717,0.925075412329142,0.998661110972001
"13213","ABHD14A",134.662573613244,0.0234016706534302,0.24885627470140
3,0.0940368921037228,0.925079850908044,0.998661110972001
"13214","LSM6",137.98463967795,0.026790505633516,0.28492496377879,0.09
40265299263688,0.925088082258166,0.998661110972001
"13215","PGLS",296.752212027445,0.0239174832446289,0.253868465377752,0
.0942121078686952,0.924940666867159,0.998661110972001
"13216","ZC3H6",81.2452310721779,0.0239771535772217,0.255457442198345,
0.0938596792126539,0.925220623714639,0.998728618183493
"13217","SP3",128.159412218699,0.0198247495320547,0.21176024301342,0.0
936188457754948,0.925411938599559,0.998859553307203
"13218","ZFAND2A",36.0240277423933,0.0289320422678437,0.31058541858677
5,0.0931532536185702,0.925781811048068,0.998908197314309
"13219","AC007032.1",11.2754116653053,-0.0424098177437572,0.4562211299
06162,-0.0929589073449542,0.925936206998933,0.998908197314309
"13220","RBM12B",30.1614579396754,0.0299861280930267,0.321293369145182
,0.0933294333860869,0.925641849664611,0.998908197314309
"13221","AL359095.1",1.95110633282093,0.0910176461922644,0.97692339801
4367,0.093167638708686,0.92577038310532,0.998908197314309
"13222","RCBTB2",24.1786424965118,-0.0300489132396027,0.32193115070527
6,-0.0933395639837043,0.925633801747658,0.998908197314309
"13223","LCMT2",6.40745784364822,0.0566495091829663,0.609494066026772,
0.0929451365330897,0.925947147152981,0.998908197314309
"13224","SBNO2",55.3015712703084,-0.0305728615875293,0.3286470410919,-
0.0930264319007825,0.92588256264897,0.998908197314309
"13225","PRRC2C",545.915225501362,-0.017276203488802,0.19036487506982,
-0.0907531049646931,0.927688773029917,0.998911328420716
"13226","AC007405.3",8.31323497874494,0.0497573142894706,0.54625530438
5485,0.0910880203633822,0.92742265143527,0.998911328420716
"13227","CIR1",212.723797444911,-0.0176594290913852,0.195989465753306,
-0.0901039707593947,0.928204593847724,0.998911328420716
"13228","ATIC",59.129311588329,0.024435415790363,0.264238905356802,0.0
924747086632374,0.926320884666426,0.998911328420716
"13229","SP140",4.5614135480523,-0.0638657929896552,0.706715077347976,
-0.0903699313014779,0.927993250246566,0.998911328420716
"13230","MLPH",1.92196643691126,0.098811872942148,1.0907253721713,0.09
05928068267483,0.927816147750427,0.998911328420716
"13231","CPA3",3.76324593461432,-0.0984560016290899,1.09696676465345,-
0.0897529485865452,0.928483538803244,0.998911328420716
"13232","PAK2",157.832906717137,0.0172509375257627,0.18791995276419,0.
0917993926244218,0.92685742664133,0.998911328420716
"13233","LCORL",23.3620415127268,0.0327611409086055,0.357263875584826,
0.0917001218076606,0.92693630061244,0.998911328420716
"13234","PAICS",71.9431291321227,0.0294653636279301,0.31982223828623,0
.0921304402902705,0.92659440344152,0.998911328420716
"13235","NEU1",168.255499239402,0.0239869814176215,0.266675174577384,0
.0899483105453482,0.928328290449803,0.998911328420716
"13236","SMAP1",131.877945326821,0.021081341574845,0.228797723656124,0
.0921396473617442,0.926587088375634,0.998911328420716
"13237","LYRM2",81.7446308392019,0.022788251594659,0.2472062581139,0.0

921831500890215,0.926552525317844,0.998911328420716
"13238","PREP",27.6388328908302,-0.0276767240394805,0.307994372371029,
-0.0898611355344488,0.92839756550829,0.998911328420716
"13239","EIF4H",260.88776425966,-0.0141674506301437,0.156069520024208,
-0.0907765374555277,0.927670153461792,0.998911328420716
"13240","FAM133B",311.004950921161,0.020926885023134,0.228455328858225
,0.0916016497742581,0.927014540632924,0.998911328420716
"13241","CHMP7",25.5439085218464,0.0293830300384405,0.318104845829866,
0.092369011109487,0.926404859696706,0.998911328420716
"13242","SLC31A2",52.0502332925834,-0.0248313496599674,0.2736688111915
99,-0.090735036820044,0.927703130090211,0.998911328420716
"13243","SFTA1P",3.60352229992069,0.0638568887558484,0.711499013268363
,0.0897497924312129,0.928486046928623,0.998911328420716
"13244","MSS51",3.39650910084803,-0.0663018786988947,0.738043294460244
,-0.0898346739230028,0.928418593771129,0.998911328420716
"13245","AL391988.1",4.07795168331088,-0.0651955673678523,0.7155986549
62594,-0.0911063302253694,0.927408102771959,0.998911328420716
"13246","GRK2",48.9562423851472,0.0228462102660534,0.249349607170033,0
.0916232053675324,0.926997413780305,0.998911328420716
"13247","RAB6A",111.058119401736,-0.0182146194288907,0.198767387504541
,-0.0916378670443338,0.926985764462287,0.998911328420716
"13248","FOXJ2",31.8279447428254,0.0287915525213693,0.315075920655385,
0.0913797298805965,0.927190867566595,0.998911328420716
"13249","DNAJC14",17.8303186422096,-0.0330886768400602,0.3696726687651
16,-0.0895080422109437,0.9286781624796,0.998911328420716
"13250","FGF14",12.697701902029,-0.0410007079205549,0.449629280039948,
-0.0911878068014435,0.927343363349868,0.998911328420716
"13251","ARGLU1",427.984800895199,0.018319633645584,0.204282079651242,
0.0896781238807631,0.928543000498621,0.998911328420716
"13252","TM9SF1",138.966585395873,0.0206825495856586,0.227300406155939
,0.0909921365097314,0.927498839292818,0.998911328420716
"13253","SNRPN",116.460695616472,0.0233626131026321,0.256626879404687,
0.0910372801042032,0.927462968793691,0.998911328420716
"13254","TENT4B",55.95057002442,-0.0281614482213999,0.305365439367806,
-0.0922221200922479,0.926521563641694,0.998911328420716
"13255","SPATA2L",14.8260076034221,-0.0352843297681553,0.3931584979280
3,-0.0897458148662839,0.928489207810673,0.998911328420716
"13256","RECQL5",6.83023168205316,-0.0482267715826187,0.53835876909792
7,-0.0895811015829228,0.928620102769973,0.998911328420716
"13257","NPC1",21.3233308253657,0.0349604506479992,0.38946910379012,0.
0897643749087706,0.928474458568914,0.998911328420716
"13258","BISPR",4.77919063368009,0.058017764809986,0.636603355917997,0
.0911364419785739,0.92738417660621,0.998911328420716
"13259","EPN1",108.267884046664,-0.0190539979461136,0.207250066688017,
-0.0919372343305268,0.92674790800576,0.998911328420716
"13260","ZNF749",18.4837923381939,0.0425300967689304,0.475171842299798
,0.0895046654344831,0.928680845986538,0.998911328420716
"13261","SNRPB2",224.887093776975,0.0234983574305039,0.254447111903086
,0.0923506549347431,0.926419443470026,0.998911328420716
"13262","URB1-

AS1",7.99531654298928,0.0502181333859846,0.545385901294202,0.092078165
6929834,0.926635935996933,0.998911328420716
"13263","SLC35A2",24.5815577945059,0.0313530681136917,0.34111941037703
6,0.0919122956944533,0.926767722264493,0.998911328420716
"13264","RPL7",3885.43338811188,0.0155358901439383,0.173963114076521,0
.0893056566986064,0.928838998639874,0.999006118410467
"13265","PTPN14",26.5012248971937,-0.0364605750554379,0.40902276683987
6,-0.0891406982969032,0.928970093553159,0.999071794544242
"13266","GOSR2",64.6393285155802,0.0282177153135883,0.317533048482781,
0.0888654439228189,0.929188846618403,0.999156409577005
"13267","AL022238.2",2.8216345474233,-0.0739389087343515,0.83128600699
4738,-0.0889452103273759,0.929125453266981,0.999156409577005
"13268","GPBP1L1",54.4749872082457,-0.0238587859616188,0.2691055340482
44,-0.0886595886851644,0.929352449566699,0.999234693861394
"13269","AC239799.2",9.89248416134132,-0.0549798445383593,0.6293134951
42678,-0.0873647950706892,0.930381551812828,0.999234693861394
"13270","GPR89B",7.77049181566804,0.0506384868780963,0.604599295520034
,0.083755451343258,0.933250868186421,0.999234693861394
"13271","HIST2H2BF",2.16753670649733,0.0835932016187198,0.971507838170
926,0.0860448041017375,0.931430800748686,0.999234693861394
"13272","SDHC",145.709253880676,-0.0208318240690204,0.241125184069253,
-0.0863942277511642,0.931153035362977,0.999234693861394
"13273","STX6",24.8443485312271,0.0270144961891673,0.306430705454991,0
.0881585810699223,0.929750636610779,0.999234693861394
"13274","SLC41A1",30.3661750323317,-0.0328606521066759,0.3730849400768
31,-0.0880782057295284,0.929814518355732,0.999234693861394
"13275","ASB3",30.6904532690689,0.0275640997745248,0.319076766065708,0
.0863870475885683,0.931158742965173,0.999234693861394
"13276","LINC01266",2.47716427552729,0.095546191592464,1.0813860303909
3,0.0883553041256911,0.92959428444543,0.999234693861394
"13277","AC097359.2",1.90906337947333,0.0871458138311701,1.01267761658
353,0.0860548435198697,0.931422820053538,0.999234693861394
"13278","AC092053.4",2.43802610544946,-0.0782506501019322,0.8881964593
37081,-0.0881006102640127,0.929796711346691,0.999234693861394
"13279","MANF",220.253249633195,-0.0327268291212832,0.382235314511305,
-0.0856195853152005,0.931768828800431,0.999234693861394
"13280","NXPE3",13.8120563055715,0.0398687630277492,0.473663769632631,
0.0841710208460128,0.932920458419209,0.999234693861394
"13281","H1FX",331.17106478006,0.0184013605953148,0.222038175847299,0.
0828747602753225,0.933951123369305,0.999234693861394
"13282","SLC30A9",47.5491778432629,-0.0232838656420105,0.2682235090450
98,-0.0868076990152852,0.930824367799475,0.999234693861394
"13283","PPP2R2B",2.13741153230744,-0.0811911744759896,0.9803700199555
45,-0.0828168679410161,0.933997156525015,0.999234693861394
"13284","DTNBP1",51.8698930273262,-0.0208251738264243,0.24930226200490
8,-0.0835338342257575,0.933427075572108,0.999234693861394
"13285","LY6G5B",8.39778365324064,-0.0483530040194418,0.54983391361465
6,-0.087941108800592,0.929923483056415,0.999234693861394
"13286","RUNX2",8.26865652722859,0.0538087588967799,0.645924701084571,
0.0833050025899765,0.933609022631759,0.999234693861394

"13287","AL353135.2",2.97730841232927,-0.0805175866195461,0.917235028002032,-0.0877829391175057,0.930049198023581,0.999234693861394
"13288","VTA1",54.4064483061027,-0.0185509429085223,0.220263628058705,-0.08422154430135,0.932880289167069,0.999234693861394
"13289","NUDCD3",56.2065958573934,0.0219578144976965,0.257877199893714,0.0851483361334255,0.932143463130445,0.999234693861394
"13290","LRCH4",20.4433498686835,0.0284487486985017,0.335664561919278,0.0847535067027515,0.932457356757742,0.999234693861394
"13291","AC105446.1",2.36583942822892,0.0761959804221657,0.890438821766884,0.0855712695353637,0.931807238252259,0.999234693861394
"13292","FAM185A",6.393769718942,-0.0499777328139648,0.581963665554935,-0.0858777545266649,0.93156359548506,0.999234693861394
"13293","PNPLA8",123.265800024085,0.0182243900717824,0.214769402077512,0.0848556167475154,0.932376177169273,0.999234693861394
"13294","COPG2",13.8416884569359,0.0326333859143757,0.39039066305832,0.0835916147653886,0.933381134050554,0.999234693861394
"13295","ESYT2",114.891958378774,0.0162198888835147,0.194620295457612,0.08334119956696,0.933580241705516,0.999234693861394
"13296","POLB",10.7503004984473,-0.042253836774429,0.508567447109385,-0.0830840373574695,0.933784718301903,0.999234693861394
"13297","PDP1",46.2806378820871,0.0245813770407105,0.286854719034988,0.0856927755046353,0.93171064531585,0.999234693861394
"13298","RAD54B",1.98833027759776,0.0839445295748648,1.00325722075434,0.0836719914278292,0.933317226636935,0.999234693861394
"13299","LINC00861",5.41591680508042,-0.0596213172617904,0.689203053783571,-0.0865076220055652,0.931062897303517,0.999234693861394
"13300","ZNF623",17.5202347104871,-0.0292566894511751,0.350580744918129,-0.0834520716704149,0.933492085662239,0.999234693861394
"13301","ZNF483",14.4138861556019,-0.0353949716220431,0.414299503009168,-0.0854332949109521,0.931916924403106,0.999234693861394
"13302","SH3GLB2",64.1677316883455,0.0214264760834368,0.245746374564757,0.087189388333339,0.930520974117627,0.999234693861394
"13303","ASCC1",24.8825142399836,0.0261711076982791,0.314789434492629,0.0831384564747579,0.93374144783172,0.999234693861394
"13304","ZMIZ1",136.467787787173,0.024361707087602,0.2788988945793,0.0873496007373924,0.930393628966677,0.999234693861394
"13305","FRAT2",15.6864365930084,0.0383810153733607,0.452302155537974,0.0848570251178879,0.932375057490738,0.999234693861394
"13306","HIF1AN",26.5093535439721,-0.0268016714093127,0.30854688758361,-0.0868641768491344,0.930779474585493,0.999234693861394
"13307","RAB11FIP2",75.9086446652403,-0.0197962891972594,0.227486107985621,-0.0870219697042364,0.930654049051663,0.999234693861394
"13308","APBB1",21.4000037610268,-0.0299833415184528,0.355940151549007,-0.0842370308265842,0.932867976461935,0.999234693861394
"13309","BSCL2",96.9774603214768,-0.0237645686023897,0.282145745149971,-0.0842279885870969,0.932875165576126,0.999234693861394
"13310","ANKRD49",37.8622843386025,0.0242860263641624,0.287508956333684,0.0844705037152858,0.932682353630293,0.999234693861394
"13311","BACE1",105.51069071264,0.0188362396101811,0.22112057510705,0.0851853772588191,0.932114015579612,0.999234693861394

"13312","CEP164",57.4261308104452,0.0209701603385926,0.242930091906603
,0.0863217898367847,0.931210617340925,0.999234693861394
"13313","AC011603.2",4.46899923165254,-0.0722281281504074,0.8302811253
85524,-0.0869923763675463,0.930677571912442,0.999234693861394
"13314","METTL25",12.8100682769484,-0.0349294697192657,0.4049004756124
56,-0.0862668033828093,0.931254327133263,0.999234693861394
"13315","PPP1R3E",8.29367746885342,-0.045114135071O825,0.5286991322351
32,-0.085330450383676,0.931998683903744,0.999234693861394
"13316","AL049838.1",3.05700679008758,-0.0729490695172905,0.8252383526
73684,-0.088397575416779,0.929560688291042,0.999234693861394
"13317","AC005520.2",6.35341459724508,0.0507701860018688,0.59620566492
0227,0.0851554907796152,0.932137775207272,0.999234693861394
"13318","NEIL1",10.3075600719543,-0.0458448835014109,0.554411521286007
,-0.0826910728605885,0.934097183387433,0.999234693861394
"13319","NPRL3",48.7593517663542,-0.0229722833351945,0.266434431303344
,-0.0862211510082186,0.931290617243485,0.999234693861394
"13320","LITAF",224.79051952997,-0.018960958441933,0.229534304490315,-
0.0826062077476226,0.934164665079869,0.999234693861394
"13321","PIEZO1",45.9372030544565,-0.0217857707584108,0.25693994878482
2,-0.0847893481003829,0.932428862028434,0.999234693861394
"13322","MED11",51.3386095658973,-0.0270504857405789,0.316380609598326
,-0.0854998217966707,0.931864037139655,0.999234693861394
"13323","DHRS7B",78.3932235035406,-0.0269937642888473,0.31739094226687
2,-0.0850489434136095,0.932222480415555,0.999234693861394
"13324","WIPI1",71.4592147642871,0.0197461809227825,0.233127442708084,
0.0847012290505331,0.932498918839846,0.999234693861394
"13325","AC012615.1",6.06368478923205,0.0559934195910704,0.66588833661
4702,0.0840883020653799,0.932986225302412,0.999234693861394
"13326","MAP2K7",51.4910238648537,0.0283190590241881,0.320998237176344
,0.0882218521612338,0.929700349518898,0.999234693861394
"13327","ZNF433",5.99505255092747,0.0531362945421146,0.640878617782941
,0.0829116357882786,0.933921801878216,0.999234693861394
"13328","AC005253.1",3.56876563496362,-0.0791912912895294,0.9154127149
41494,-0.0865088391246463,0.931061929809978,0.999234693861394
"13329","ARMC6",25.8875647233985,-0.0289879510642997,0.342700112152199
,-0.0845869319454021,0.932589788627798,0.999234693861394
"13330","ZNF30",2.7839601917801,0.0668967342222047,0.808540030778203,0
.082737689756459,0.934060115506167,0.999234693861394
"13331","ZC3H4",33.1066156716871,0.0274444418719042,0.316721956469514,
0.0866515292397981,0.930948505489532,0.999234693861394
"13332","TRPM4",6.04530016650209,-0.0510161376740883,0.589274049424522
,-0.0865745534253714,0.931009693362257,0.999234693861394
"13333","ZNF324B",5.26758777782114,-0.0592629856207565,0.688331750992,
-0.0860965450676199,0.931389670064726,0.999234693861394
"13334","VPS16",46.9893852270927,-0.0251263682442272,0.286693942202958
,-0.0876417829102213,0.930161391971065,0.999234693861394
"13335","RPN2",327.107622699744,-0.0170821338201123,0.203842749465031,
-0.083800546572998,0.933215013447675,0.999234693861394
"13336","ZNF674",3.85146802529602,0.0624692562424345,0.73493286589673,
0.0849999491670745,0.932261431123294,0.999234693861394

"13337","FGF16",6.19599166000513,0.0618292616428609,0.737525606555715,
0.0838333762153791,0.933188911032534,0.999234693861394
"13338","LZIC",117.537900347647,0.0179882212079995,0.218026590773253,0
.0825047125866734,0.934245371010861,0.999246098578568
"13339","PHKG2",19.4013216116415,0.0289496197683333,0.351500161317762,
0.0823601891384691,0.93436029292126l,0.999294095420549
"13340","CCDC28B",47.124413661018,-0.0325494489827327,0.39614092416253
8,-0.0821663377787687,0.934514441464164,0.999305100451774
"13341","ADCK1",4.04169651122464,-0.0626590989762003,0.76336286129176,
-0.0820829806550568,0.934580726919078,0.999305100451774
"13342","SRC",20.385043985368,-0.0277982231706337,0.337951150654164,-0
.082255151718896,0.934443817256898,0.999305100451774
"13343","AL161891.1",1.93852776386469,-0.082910720711915,1.01369740482
108,-0.0817904044319307,0.93481338666251,0.999341584569109
"13344","RAB2B",33.1752359197805,-0.0252598712092546,0.308397757056574
,-0.0819067928714566,0.934720832661968,0.999341584569109
"13345","ZNF530",2.01836788214799,0.0823623555004038,1.00717271654646,
0.0817758008604719,0.934824999724853,0.999341584569109
"13346","ZCCHC9",69.7179460499027,-0.0173826818579729,0.21428244360215
5,-0.081120420160255,0.935346186265282,0.99937452765582
"13347","ABCF2",69.1651823405331,-0.0308580753097282,0.3802931679647,-
0.0811428600594595,0.935328340645912,0.99937452765582
"13348","SERPINH1",395.37550436396,-0.0211484372464315,0.2590381508197
51,-0.0816421719329962,0.934931264858085,0.99937452765582
"13349","ZBTB16",399.127499659914,-0.0365277728539691,0.44986199018555
7,-0.0811977309727862,0.935284703981013,0.99937452765582
"13350","ERC1",76.5237625576202,-0.0203567510801009,0.250212324364977,
-0.0813579072564273,0.935157323236998,0.99937452765582
"13351","HNRNPA1L2",9.38752412945833,-0.0402860828761519,0.49639778675
5128,-0.0811568543435608,0.935317211527371,0.99937452765582
"13352","SOCS1",190.161957523073,0.0339083440080013,0.416609119508083,
0.0813912668259376,0.935130794132622,0.99937452765582
"13353","MRPL20-
AS1",14.2881213094548,-0.0205261109661409,0.411810490574752,-0.0498435
84454328,0.960247034454647,0.999451080313105
"13354","MIB2",35.691874944059,-0.0140056176429917,0.269938258178927,-
0.051884522547775,0.958620706881781,0.999451080313105
"13355","AL590822.3",2.19776281602589,0.0457797800102383,0.98118382844
8797,0.0466576992841534,0.962786044685111,0.999451080313105
"13356","NOL9",43.9371545740422,-0.0110446668085383,0.254544088959696,
-0.0433899952408129,0.965390652833005,0.999451080313105
"13357","ZBTB48",12.9745650546973,0.0309075302371317,0.397879564442493
,0.0776806174512611,0.938082112542577,0.999451080313105
"13358","SPSB1",156.012526925155,-0.0305093955829582,0.380209703868092
,-0.0802436004987998,0.936043513758937,0.999451080313105
"13359","CLCN6",19.2724178769977,0.0180146565871248,0.374431431205366,
0.0481120308974387,0.961626958040695,0.999451080313105
"13360","ARHGEF10L",38.0991806442209,-0.0197688271220179,0.29431307981
6097,-0.0671693800845362,0.94644686120775,0.999451080313105
"13361","DNAJC8",343.654561609259,0.0144616150317891,0.210705927060501

,0.0686341159621798,0.945280862275024,0.999451080313105
"13362","TSSK3",1.88978637253076,-0.0719691125766866,1.08229755200217,
-0.066496604786317,0.946982460716827,0.999451080313105
"13363","THRAP3",283.986453232832,-0.0105707345564589,0.16614490132031
8,-0.0636235868356807,0.949269950209694,0.999451080313105
"13364","SH3D21",2.56696999639078,-0.047839001470806,1.00425501080505,
-0.0476363084635808,0.962006094894557,0.999451080313105
"13365","GNL2",60.153277849061,0.0233116697428499,0.340489783577807,0.
0684651078158498,0.945415394806354,0.999451080313105
"13366","AL050343.2",2.71575615560721,-0.0718763014638232,0.9065410185
61922,-0.0792863201908317,0.93680488695232,0.999451080313105
"13367","SCP2",386.738198424843,0.00885659129577864,0.218211646828954,
0.0405871612468097,0.967625019564448,0.999451080313105
"13368","FGGY",11.8788988054733,-0.0181445493887982,0.453534550512504,
-0.0400069837420203,0.968087558531175,0.999451080313105
"13369","USP1",84.683075440389,-0.0152158920656504,0.229351178814452,-
0.0663432040955859,0.947104587075584,0.999451080313105
"13370","ALG6",21.2884228420188,-0.0279793752215113,0.352746073772766,
-0.07931874314648,0.936779098394941,0.999451080313105
"13371","ABCD3",40.1833050727617,-0.020746232291966,0.259439903506517,
-0.0799654641077401,0.93626472362803,0.999451080313105
"13372","S1PR1",6.7555850944001,-0.0482773027300128,0.622072324175219,
-0.0776072184114954,0.938140500240256,0.999451080313105
"13373","STXBP3",76.0199293080377,0.0122375906054517,0.220941398334052
,0.0553884002623588,0.955829036901687,0.999451080313105
"13374","VANGL1",31.5292860564402,0.0201506000513597,0.299202025788728
,0.0673478062130116,0.946304819396974,0.999451080313105
"13375","PHGDH",110.408279007484,0.0149981681063081,0.309472981606602,
0.0484635783984968,0.961346790544099,0.999451080313105
"13376","PEX11B",18.9590968956968,-0.0214592996213553,0.34335191447096
,-0.0624994319732277,0.950165114276974,0.999451080313105
"13377","ANP32E",91.7514791332506,-0.0148740129852867,0.21721466957565
3,-0.0684760979281205,0.945406646496522,0.999451080313105
"13378","MRPS21",285.924690043258,0.00935267169840336,0.22198000885108
2,0.0421329458756699,0.96639271647036,0.999451080313105
"13379","UBQLN4",15.0474393368498,0.0172369689265399,0.381219968586698
,0.0452152834240103,0.963935712298472,0.999451080313105
"13380","CD1C",3.63080780810788,-0.0609819444421119,0.894567239519734,
-0.068169212718824,0.945650934633394,0.999451080313105
"13381","VANGL2",9.56109514176574,-0.0206605943707668,0.49932569810737
6,-0.041376989906744,0.966995356478627,0.999451080313105
"13382","UAP1",561.5884467821,0.0256072073323809,0.375336830438867,0.0
682246058891672,0.94560683993554,0.999451080313105
"13383","DARS2",8.43954801148143,-0.0402986064088293,0.517072067607016
,-0.0779361503616493,0.937878843022922,0.999451080313105
"13384","RGS1",77.6963548226152,0.0291287324206888,0.389804558724766,0
.0747265052927615,0.940432318454011,0.999451080313105
"13385","LBR",56.4151135204288,0.014502730645983,0.289463203182163,0.0
501021562898144,0.960040981427376,0.999451080313105
"13386","EPHX1",597.560885799889,-0.0187391224769281,0.249698306604486

,-0.0750470547107484,0.940177273177855,0.999451080313105
"13387","URB2",13.311636324619,-0.0373953152540949,0.470593229412285,-0.0794642016010244,0.936663404524634,0.999451080313105
"13388","ZNF670",6.96836298535776,-0.0341838475102698,0.56896140034025 5,-0.0600811364177376,0.952091013776798,0.999451080313105
"13389","ZNF672",21.2450243408876,0.0231789638799661,0.40096918120996, 0.057807345217958,0.953902087327444,0.999451080313105
"13390","GRHL1",19.9840729260141,-0.0353348431481335,0.560558588021308 ,-0.0630350580710224,0.949738587515861,0.999451080313105
"13391","RN7SL832P",7.74485969967704,-0.027496470711329,0.591532454705 884,-0.0464834524168257,0.962924922887649,0.999451080313105
"13392","LDAH",9.75555084948113,0.018502461932951,0.462121531931736,0. 0400380866383956,0.968062761877997,0.999451080313105
"13393","TP53I3",117.170438267855,0.0173615189038798,0.278043152522211 ,0.0624418143241018,0.950210996891969,0.999451080313105
"13394","GAREM2",16.3540743042608,-0.0218780126597307,0.42657883344395 9,-0.051287150098611,0.959096707365328,0.999451080313105
"13395","MAPRE3",18.7473720205607,-0.0165128251691346,0.36132229102586 5,-0.045701097273161З,0.963548489227366,0.999451080313105
"13396","MPV17",75.2194568191797,-0.0132753218643609,0.268235316532003 ,-0.049491327376263,0.960527748483032,0.999451080313105
"13397","YPEL5",147.152592448534,-0.0176867251638998,0.250148609274311 ,-0.070704871057287,0.943632643999597,0.999451080313105
"13398","CEBPZOS",76.2640237716024,-0.0165825238483578,0.2432768322456 47,-0.0681631855170399,0.945655732484761,0.999451080313105
"13399","PKDCC",269.39435916174,0.0136362577905494,0.30799873560391,0. 0442737460068205,0.964686198799373,0.999451080313105
"13400","RHOQ",205.368112150997,0.0180646579163822,0.264146958011762,0 .068388665356418,0.945476244441364,0.999451080313105
"13401","CCDC88A",118.312827115985,-0.0149427105075257,0.2613215473194 14,-0.0571813180382757,0.954400759853632,0.999451080313105
"13402","FANCL",17.8915800461019,-0.0273554393143151,0.409754754427236 ,-0.0667605171599609,0.946772355992868,0.999451080313105
"13403","DUSP11",55.6902763953423,-0.0204236387739025,0.29522727890153 2,-0.0691793754625043,0.944846839767265,0.999451080313105
"13404","IMMT",76.2290425734579,-0.0131652980932459,0.219898401002478, -0.0598699128016742,0.952259243000765,0.999451080313105
"13405","CYTOR",219.092424760638,0.0186673434387559,0.262487738159869, 0.07111701129211,0.94330462939725,0.999451080313105
"13406","MGAT4A",9.85318509842065,-0.0224003231343681,0.53262553947791 3,-0.0420564195181576,0.966453721596157,0.999451080313105
"13407","TXNDC9",68.3534702714327,0.0185582911157928,0.318780489144876 ,0.0582165212355848,0.953576160997972,0.999451080313105
"13408","NCK2",53.9296913496594,0.0119473661331558,0.239020898358604,0 .0499846089408931,0.960134653275639,0.999451080313105
"13409","LINC01886",7.1986427940029,0.042085634704311,0.55030646825897 3,0.0764767218482077,0.939039833002927,0.999451080313105
"13410","ZC3H8",31.3787336642007,0.0166358869749333,0.305447232436652, 0.0544640291621679,0.95656546660З187,0.999451080313105
"13411","RALB",75.9092091918773,-0.0170513770622053,0.211433585943145,

-0.0806464923070002,0.935723091079391,0.999451080313105
"13412","IWS1",93.8760514795667,-0.0132893632474167,0.251768455251622,
-0.0527840679410573,0.95790395574439,0.999451080313105
"13413","CCDC74B",7.77343466158608,0.0322970798313844,0.51454726808633
1,0.0627679551219882,0.949951283641228,0.999451080313105
"13414","RAB3GAP1",81.2258077720997,0.0108362031397999,0.2280566655689
16,0.0475153975998362,0.962102458686298,0.999451080313105
"13415","PPIG",333.623245977415,-0.00679209021584414,0.161978709371412
,-0.0419319936688104,0.966552911560266,0.999451080313105
"13416","AC009948.2",2.01201350325396,0.0744313770313896,0.98433622596
6959,0.0756158059287844,0.939724761154553,0.999451080313105
"13417","AC010680.3",1.93238996039307,0.0945072518364606,1.22461261357
446,0.0771731817791817,0.938485775845815,0.999451080313105
"13418","CCDC150",2.71820350041311,0.0589801546073278,0.94008807173768
7,0.0627389671036961,0.94997436723713,0.999451080313105
"13419","CYP20A1",61.1167886284752,0.0107120081683971,0.23517184727136
2,0.0455497045785272,0.963669157459378,0.999451080313105
"13420","RAPH1",29.649754060864,-0.0160846729690325,0.355897148899392,
-0.045194722741596,0.963952100596293,0.999451080313105
"13421","XRCC5",387.507746508951,-0.0113611805370506,0.191161183723136
,-0.0594324659210382,0.952607654688029,0.999451080313105
"13422","CTDSP1",139.450459760004,0.00826342275462942,0.18151927868446
9,0.0455236645634405,0.963689912855133,0.999451080313105
"13423","INPP5D",12.5586615771787,0.031651154375903,0.571154007198161,
0.0554161469183594,0.955806932223477,0.999451080313105
"13424","ILKAP",55.922377398364,-0.0134055915765613,0.24171934834606,-
0.0554593236672517,0.955772535059918,0.999451080313105
"13425","RNPEPL1",58.0417999955922,0.0171502528846448,0.23649833899424
2,0.0725174348267299,0.942190131012102,0.999451080313105
"13426","MTERF4",48.7269217133886,0.0189981453650447,0.294091867755584
,0.0645993563508998,0.948492997198771,0.999451080313105
"13427","SETMAR",23.2627171682807,0.0274374554957583,0.366672817556527
,0.0748281688252728,0.940351429159811,0.999451080313105
"13428","OGG1",25.670031430488,-0.019376879831019,0.322586317009052,-0
.0600672713296617,0.952102056572292,0.999451080313105
"13429","ARPC4",169.274997221278,-0.0130210875355471,0.227328276512969
,-0.0572787852671913,0.954323119507585,0.999451080313105
"13430","FANCD2",2.88718804886418,0.0491773962276908,0.932640623361542
,0.0527292024343089,0.957947671206984,0.999451080313105
"13431","TAMM41",19.4230014492745,-0.0203337689775078,0.35845480808322
7,-0.0567261716650949,0.954763325604931,0.999451080313105
"13432","FGD5",37.1515050598521,0.0254580264815813,0.327256390780374,0
.077792297412051,0.937993273656861,0.999451080313105
"13433","MRPS25",89.0031761960804,-0.0153322482601368,0.24449700291517
2,-0.062709350533251,0.949997951401943,0.999451080313105
"13434","NEK10",1.89302455110146,-0.0843602994186211,1.0536983683669,-
0.0800611464829057,0.936188624127888,0.999451080313105
"13435","GORASP1",22.0376031304204,0.0219468964164507,0.34305019585472
2,0.0639757583049014,0.948989529326576,0.999451080313105
"13436","ZNF621",28.6689434118909,-0.0193075020841364,0.37712403176344

9,-0.0511966898366399,0.959168789515259,0.999451080313105
"13437","ULK4",8.50827520629676,0.0334187885138462,0.519228599880564,0
.0643623801183783,0.948681684207761,0.999451080313105
"13438","EXOSC7",88.325895774935,0.0145523799907234,0.317267199836404,
0.0458678993549512,0.963415539840402,0.999451080313105
"13439","NME6",33.72918723361,0.0173165874584333,0.290864351608087,0.0
595349253447388,0.95252604839774 9,0.999451080313105
"13440","ATRIP",8.36225052678929,-0.0214409726902686,0.50369075220328,
-0.042567731483018,0.966046118709548,0.999451080313105
"13441","ARIH2",78.628695788446,-0.0153610625902748,0.246883731714576,
-0.0622198250309741,0.950387774984469,0.999451080313105
"13442","QARS",84.8177206225072,-0.0117225998494574,0.218075891221095,
-0.0537546804638416,0.957130617030106,0.999451080313105
"13443","PARP3",15.5531338507352,-0.0290405612405857,0.423705189540819
,-0.0685395457913974,0.945356141084249,0.999451080313105
"13444","NEK4",16.9672573816616,0.0213588936855375,0.42320825811007,0.
0504689908011728,0.959748659657868,0.999451080313105
"13445","ARL6",12.8017636777026,0.0263816973667002,0.455821918744723,0
.0578772022182524,0.953846442669323,0.999451080313105
"13446","USF3",45.6802043826204,-0.0128919997363684,0.261896853766576,
-0.0492254853426337,0.960739601515696,0.999451080313105
"13447","CCDC191",11.7573580166874,0.0186103235707331,0.44367098233284
6,0.0419462266224377,0.966541565289116,0.999451080313105
"13448","QTRT2",26.5918562755094,0.0297980436167597,0.375019798160677,
0.0794572546913717,0.936668929885443,0.999451080313105
"13449","RABL3",35.566015985158,-0.0202014040449631,0.288315732752235,
-0.070066950048554,0.944140372068691,0.999451080313105
"13450","SLC49A4",15.9722202887007,0.0178507264078486,0.41308903374960
9,0.0432127821109593,0.965531915956101,0.999451080313105
"13451","LINC02035",5.58325697258968,-0.0291881517266034,0.65911049033
4385,-0.0442841559262628,0.964677901023775,0.999451080313105
"13452","MGLL",116.112847861024,-0.0144812288465448,0.267527696108496,
-0.0541298305079857,0.956831725772081,0.999451080313105
"13453","ASTE1",18.5332082806743,0.0242176094901686,0.347685884688713,
0.0696537034048733,0.944469291562961,0.999451080313105
"13454","PCCB",16.7050594275871,0.0242409225676483,0.383416837368378,0
.0632234169318918,0.949588598063609,0.999451080313105
"13455","GYG1",62.7169233257075,0.0120770188958894,0.223658918586854,0
.0539974840806517,0.956937168731885,0.999451080313105
"13456","MCCC1",16.3816484924215,0.0203242489642232,0.383881917343221,
0.052944012327759,0.957776516783146,0.999451080313105
"13457","CLCN2",3.99781208389584,-0.0534084248606417,0.761235197520795
,-0.070160214654529,0.944066140362321,0.999451080313105
"13458","XXYLT1",25.6889536184407,0.0168569393023756,0.294860911338593
,0.0571691216236478,0.954410475291665,0.999451080313105
"13459","RUBCN",15.6479551783521,-0.0216630906362788,0.429147792528098
,-0.0504793244039823,0.959740425131719,0.999451080313105
"13460","IDUA",32.6030392576538,0.0176854313083625,0.340009343821173,0
.0520145449816346,0.95851710388319,0.999451080313105
"13461","TLR1",4.05338648446849,0.0482087395051652,0.704113223715078,0

.0684673116218494,0.945413640540062,0.999451080313105
"13462","N4BP2",36.775033774475,0.0264760717724954,0.331602994485082,0.0798426799902933,0.936362378933464,0.999451080313105
"13463","GNPDA2",52.1840262577695,-0.0136821436879512,0.248064776180383,-0.0551555279174423,0.95601455854998,0.999451080313105
"13464","TEC",3.53652991566078,0.045621373090958,0.843900454126664,0.0540601357279404,0.956887252858027,0.999451080313105
"13465","UBA6",47.6510372296424,0.0158055601465047,0.258656907140688,0.0611062751860238,0.951274571616565,0.999451080313105
"13466","THAP9-AS1",23.7923750828349,-0.0256175768846853,0.392429807867976,-0.0652793859464003,0.947951554962486,0.999451080313105
"13467","RAP1GDS1",23.2385977652643,0.0215665422881659,0.334511621847986,0.0644717279747203,0.948594618071939,0.999451080313105
"13468","EIF4E",97.4476241690425,-0.0139360272318979,0.225898021745306,-0.0616916745185596,0.950808370113747,0.999451080313105
"13469","TRMT10A",23.2361992099089,-0.0230446457287269,0.349302201626128,-0.0659733766962982,0.947399021582323,0.999451080313105
"13470","UBE2D3",514.357867038161,-0.0108470352863584,0.152453462465201,-0.0711498126114018,0.943278523861147,0.999451080313105
"13471","BDH2",242.685132809626,0.0112883771213861,0.249680277205035,0.0452113288552471,0.963938864364392,0.999451080313105
"13472","ZGRF1",2.75889269358504,0.038078990097301,0.86453886378719,0.0440454347309415,0.964868187309409,0.999451080313105
"13473","NDNF",18.732248660224,0.0384751273112826,0.526080144053545,0.0731354865721116,0.941698303057693,0.999451080313105
"13474","HSPA4L",4.86641588102713,0.0405511905655629,0.642621485228368,0.063102761886575,0.949684675016964,0.999451080313105
"13475","RAB33B",26.0232267182172,-0.0164933144724983,0.311877106291786,-0.0528840179024473,0.957824318442201,0.999451080313105
"13476","ZNF330",71.1090069385087,-0.0158070167067777,0.210136374053833,-0.075222658513791,0.940037556539313,0.999451080313105
"13477","SLC10A7",14.6472451835366,-0.023962683166457,0.390302698636045,-0.0613951255018147,0.951044534324489,0.999451080313105
"13478","TENM3",8.39161108705867,0.0348110157107326,0.572557999873925,0.0607991080700957,0.951519200665987,0.999451080313105
"13479","NSUN2",27.5217564031596,0.023047334106863,0.334039352856174,0.0689958650374538,0.944992910879681,0.999451080313105
"13480","FASTKD3",12.2850456156462,-0.0359014302695503,0.480101751828276,-0.0747787945635149,0.940390714056553,0.999451080313105
"13481","OTULIN",53.1759895924671,-0.0135729670890142,0.269332348665607,-0.0503948640267713,0.959807729100908,0.999451080313105
"13482","MTMR12",21.2720400566638,-0.0149019212240769,0.325588059403754,-0.0457692498040824,0.963494168216689,0.999451080313105
"13483","HMGCS1",46.7370404228064,-0.0121787145812728,0.30031331412786,-0.0405533621332807,0.967651965170665,0.999451080313105
"13484","GZMK",54.8113428643626,-0.0224870172289142,0.377101048927569,-0.0596312773270311,0.952449306981294,0.999451080313105
"13485","ERCC8",11.6016083035046,0.0220141117491083,0.439183771818612,0.0501250573488868,0.960022731955931,0.999451080313105

"13486","CENPK",4.2040181341271,0.0332288227227015,0.70282887519362,0.
0472786817609726,0.962291118569659,0.999451080313105
"13487","CKMT2-
AS1",13.2503826877304,0.0362543435783825,0.485095035784533,0.074736579
234931,0.940424303024666,0.999451080313105
"13488","SSBP2",121.718482081894,0.0121313667431017,0.200211465018727,
0.0605927674619779,0.951683533671084,0.999451080313105
"13489","CHD1",150.33615937555,0.0243269281893051,0.313326458359682,0.
0776408360681084,0.938113757920348,0.999451080313105
"13490","PJA2",210.833078560586,0.0153378062150333,0.201713677886179,0
.0760375120604758,0.939389254262129,0.999451080313105
"13491","SEMA6A",25.6054771520965,0.0236303993641618,0.467374322880744
,0.0505599007205009,0.959676216522732,0.999451080313105
"13492","ZCCHC10",61.4309361781553,0.0165082499895534,0.23811807944683
1,0.0693279990662761,0.944728539317781,0.999451080313105
"13493","C5orf15",167.825191085908,0.0124267893036487,0.22600217255316
,0.0549852648019374,0.956150203017142,0.999451080313105
"13494","SPATA24",2.26424933209827,-0.0658742103236942,0.8973448263497
42,-0.0734101411066916,0.941479747934746,0.999451080313105
"13495","IGIP",16.435197824149,0.0242927594200936,0.449477938603852,0.
0540466112653954,0.956898028065058,0.999451080313105
"13496","PCDHB3",4.6448399358627,-0.0395245598960296,0.820337047839528
,-0.048180878823068,0.961572088976143,0.999451080313105
"13497","RBM27",130.137092658769,0.0111279894349891,0.211580269284293,
0.0525946463374466,0.958054882672418,0.999451080313105
"13498","FBXO38",23.4940704605689,0.0180830500353517,0.348516208593627
,0.0518858222070146,0.95861967129861,0.999451080313105
"13499","RNF145",95.0223492939594,-0.0218921991397748,0.28728219084966
,-0.0762045119296356,0.939256393129005,0.999451080313105
"13500","UBLCP1",74.4628461084848,-0.0227777305193036,0.28453931510626
2,-0.0800512593867642,0.936196487650193,0.999451080313105
"13501","HNRNPH1",338.05612614416,0.0125233287038542,0.223080556625927
,0.0561381453106823,0.955231756274115,0.999451080313105
"13502","SERPINB9P1",1.9544922641646,0.105277867458822,1.3824016205323
9,0.0761557754961812,0.93929516650443,0.999451080313105
"13503","BPHL",26.6910318231266,-0.016514887992832,0.310973495198466,-
0.053107059758556,0.957646606518848,0.999451080313105
"13504","GMNN",23.6376910730223,0.0269537738794433,0.3795267361316,0.0
710194337141431,0.943382288675762,0.999451080313105
"13505","CARMIL1",50.1345066442149,0.0141887222578139,0.32134387461228
9,0.044154326186964,0.964781388941648,0.999451080313105
"13506","ZNF322",72.650847270659,-0.0136251750327574,0.240870571276568
,-0.0565663748815247,0.95489062059525,0.999451080313105
"13507","HIST1H2BN",2.77091170714975,0.0636178651123799,0.896151522435
273,0.0709900764766875,0.943405653389326,0.999451080313105
"13508","ZNRD1",88.9024328988767,-0.0190860534932014,0.265078601986069
,-0.0720014869182253,0.942600724534844,0.999451080313105
"13509","DDR1",5.58090638267524,0.0271206204020752,0.643546583445144,0
.0421424355279589,0.96638515154238,0.999451080313105
"13510","HCP5",6.75458832496677,0.0285420189470083,0.588486889884717,0

.0485006878447192,0.961317216267756,0.999451080313105
"13511","PSMB8-
AS1",30.5839372461442,0.0142227261806972,0.312360145153145,0.045533101
4580109,0.963682391102365,0.999451080313105
"13512","TMEM217",5.88430088683373,0.0459716351762761,1.05595811868166
,0.043535472063675,0.965274688702588,0.999451080313105
"13513","SRF",28.9336161066876,0.0233005559188327,0.317111439245003,0.
0734775004468713,0.941426147711976,0.999451080313105
"13514","SLC29A1",76.4352384787619,-0.0199136273659176,0.3290298648085
18,-0.0605222488770327,0.951739696286677,0.999451080313105
"13515","MCM3",42.8051020890998,-0.0192802064036097,0.287027048674961,
-0.0671720888070144,0.946444704830084,0.999451080313105
"13516","ICK",59.2928779799349,-0.0154049945983346,0.270577028076056,-
0.0569338598619114,0.954597881778239,0.999451080313105
"13517","PRIM2",15.3475417676967,-0.0360757764634636,0.50497053378687,
-0.0714413496425712,0.943046501407902,0.999451080313105
"13518","OGFRL1",116.517260022457,0.018446337239713,0.238265009316287,
0.077419413335788,0.938289897522654,0.999451080313105
"13519","MTO1",23.9880462678339,0.0131089082295344,0.32565937878619,0.
0402534337515302,0.967891078147099,0.999451080313105
"13520","AL080250.1",2.96739512789129,0.0536569698957308,0.90368275276
5227,0.0593758924042126,0.952652714249105,0.999451080313105
"13521","ATG5",61.7481923615836,-0.00973200338042858,0.236601468503387
,-0.0411324724313335,0.967190287244532,0.999451080313105
"13522","SMPD2",11.7429505492366,0.0341076840821258,0.465563534119179,
0.0732610730491503,0.941598367632037,0.999451080313105
"13523","ZBTB24",22.94000352497,-0.0214258482235511,0.315991442771306,
-0.0678051533156791,0.945940741453401,0.999451080313105
"13524","RNF146",126.933159111368,0.0112621664991294,0.207836292302967
,0.0541876799972561,0.956785636201629,0.999451080313105
"13525","SAMD3",11.6938477747176,0.0287283572303427,0.477464485596247,
0.0601685739924036,0.952021374672405,0.999451080313105
"13526","AKAP7",26.340497074062,-0.0248618288924678,0.323699550675264,
-0.0768052622890699,0.938778464405935,0.999451080313105
"13527","LINC00271",4.34406501823938,-0.0282858164006009,0.66193388706
8769,-0.0427320869246603,0.965915101255871,0.999451080313105
"13528","FNDC1",132.474370734918,0.0194681061185771,0.455766663859606,
0.0427150725630386,0.965928664368267,0.999451080313105
"13529","WDR27",23.874642607702,-0.0242606045419825,0.328311706684276,
-0.0738950334333126,0.941093907792229,0.999451080313105
"13530","ACTB",4283.51131567014,-0.0219264666208306,0.299821537055457,
-0.073131726413553,0.941701295217624,0.999451080313105
"13531","UMAD1",23.7967050538843,0.016882428692819,0.326339755002396,0
.0517326756364544,0.95874170069718,0.999451080313105
"13532","ETV1",13.5021224698386,-0.0364855008329573,0.543493393076486,
-0.0671314523741095,0.946477054990948,0.999451080313105
"13533","GPNMB",1010.43185257036,-0.00930571472895456,0.19598640665889
9,-0.0474814293888785,0.962129530841411,0.999451080313105
"13534","CYCS",250.401224785802,-0.0164434942295793,0.244068654478078,
-0.0673724131627736,0.946285230383563,0.999451080313105

"13535","ADCYAP1R1",4.22814164500873,-0.0551154010332989,0.855001268470198,-0.064462361713116,0.94860207575429,0.999451080313105
"13536","LINC00997",3.84652437451437,-0.046557307137516,0.769454851889214,-0.0605068731754768,0.951751941879265,0.999451080313105
"13537","MRPS17",64.6471559375321,-0.0224013202123461,0.330204565184379,-0.067840734424243,0.945912417056626,0.999451080313105
"13538","SAMD9",68.288041884125,0.011102252112807,0.27741429563086 7,0.0400204758286135,0.968076802018156,0.999451080313105
"13539","ATP5MF",331.695848391544,-0.011242276194495,0.250277732427016,-0.0449192027012365,0.964171710750248,0.999451080313105
"13540","ZSCAN21",16.5084559759014,-0.0288132513188264,0.370327858402333,-0.0778047091653662,0.937983400435142,0.999451080313105
"13541","AP4M1",23.289336430107,-0.0134084019605904,0.31071537779881,-0.0431533259009551,0.965579310936978,0.999451080313105
"13542","CUX1",89.6160893972244,-0.0109742513293791,0.21262758014442,-0.0516125486727792,0.958837420272986,0.999451080313105
"13543","METTL2B",49.0417020909106,-0.0209032353636936,0.271689980533919,-0.076937822008066,0.938673009093652,0.999451080313105
"13544","AHCYL2",13.3670014471707,-0.0192104175032586,0.470454362724873,-0.0408337535483607,0.967428430349958,0.999451080313105
"13545","AC106845.1",4.91148325588205,0.071230177932258,1.00644251914317,0.0707742137056168,0.943577454829582,0.999451080313105
"13546","CSGALNACT1",17.8264755541626,0.0362094933953369,0.600994147162589,0.0602493278283807,0.95195705911492,0.999451080313105
"13547","POLR3D",51.7533734307619,0.0162696555350412,0.255775143933596,0.0636092127046759,0.949281395922783,0.999451080313105
"13548","PLPP5",56.2133294091971,-0.0171228723022097,0.289666611050855,-0.0591123438082531,0.95286262689821 8,0.999451080313105
"13549","AC087623.3",2.96115213941844,0.0661949240772953,0.969666749818925,0.0682656429022203,0.945574173295802,0.999451080313105
"13550","MRPL15",103.197561654585,-0.0228256525037786,0.330959312949979,-0.0689681529137949,0.945014969409859,0.999451080313105
"13551","TRIQK",53.792413248684,-0.0135274499739044,0.275956512964793,-0.0490202236162849,0.960903179197721,0.999451080313105
"13552","AZIN1-AS1",16.7356061565563,0.0206494631503189,0.431941084727095,0.0478062029301184,0.961870692987331,0.999451080313105
"13553","TMEM65",41.3972917497156,-0.0233058651847069,0.305650588263602,-0.076250025616202 2,0.939220183813019,0.999451080313105
"13554","NSMCE2",49.8236376602065,0.017333402026123,0.272520684239719,0.0636039868844446,0.949285557097903,0.999451080313105
"13555","ZC3H3",19.7916920257296,-0.0134302780456269,0.334066025961038,-0.0402024659855511,0.967931711649009,0.999451080313105
"13556","AL162253.2",10.7431633910512,0.0241978296438994,0.531014259809657,0.0455690769068483,0.963653716610984,0.999451080313105
"13557","PTPRD-AS1",15.3855280810592,0.0248334339432205,0.48767942000365,0.0509216360678796,0.959387964784625,0.999451080313105
"13558","NFX1",51.1570731471541,0.0197843556872918,0.287694429552709,0.0687686435849713,0.945173777773282,0.999451080313105

"13559","MYORG",3.4951789586157,-0.0532087056342455,0.789633820255175,
-0.0673840256956709,0.946275985930783,0.999451080313105
"13560","AL162231.2",23.5407469543968,0.0186863555826289,0.32385487025
2417,0.0576997825231546,0.953987766930636,0.999451080313105
"13561","FAM214B",12.223452233405,-0.0271850132047379,0.48600713185003
8,-0.055935420332733,0.955393253644034,0.999451080313105
"13562","NPR2",27.1286533471033,0.0256243991657561,0.413539932071385,0
.0619635425227396,0.950591865038923,0.999451080313105
"13563","EXOSC3",50.2485645223493,-0.0162449629947889,0.36759992957467
9,-0.0441919643825414,0.964751387291396,0.999451080313105
"13564","C9orf85",41.3155910826056,0.0134854762867737,0.26399872860706
1,0.0510815955740668,0.959260501448842,0.999451080313105
"13565","LINC01504",10.83949845262,0.0222271643262624,0.46945782836372
,0.0473464558120896,0.962237103190655,0.999451080313105
"13566","CARNMT1",18.7973229629479,0.0189238365773583,0.35227925944431
7,0.0537182819312402,0.957159616956832,0.999451080313105
"13567","SYK",1.94355515274041,0.0512171246421461,1.03868041683625,0.0
493098009858991,0.96067240896349,0.999451080313105
"13568","IARS",87.7123683110034,-0.0195035263283518,0.270649594042204,
-0.072061908673362,0.942552639856494,0.999451080313105
"13569","TGFBR1",63.5885627202114,0.0187149150848113,0.27446563452332,
0.0681867335315567,0.945636987499903,0.999451080313105
"13570","MRPL50",69.4672966333416,0.0145251905967106,0.289681754768473
,0.0501418897034777,0.960009318547074,0.999451080313105
"13571","GOLGA1",23.9583676837366,0.0158747993482303,0.317942164229876
,0.049929833580527,0.960178303186918,0.999451080313105
"13572","TOR2A",18.9069616697761,0.0246287571565501,0.368040536558423,
0.0669185992033801,0.946646506204061,0.999451080313105
"13573","NUP214",46.5809404783572,-0.0178427655028601,0.25054607247101
8,-0.0712155067005655,0.943226240189536,0.999451080313105
"13574","BRD3OS",18.1048899136576,-0.024779409967343,0.340247104723548
,-0.072827687946019,0.941943237659901,0.999451080313105
"13575","SNAPC4",17.6146562910798,-0.0194789044843399,0.39908360330281
4,-0.0488090824156458,0.961071444082168,0.999451080313105
"13576","MAMDC4",1.86976952151279,-0.0825586804172641,1.02789807175749
,-0.0803179640916206,0.935984371091133,0.999451080313105
"13577","TAF3",67.1004720072531,0.0187334935281808,0.257536678639374,0
.072741069843543,0.942012166088072,0.999451080313105
"13578","ZNF33B",9.1500530965044,0.0269852365493517,0.534323002314146,
0.0505036025633914,0.959721078639742,0.999451080313105
"13579","ZNF239",5.76385855494483,0.0413738359727656,0.715079382426512
,0.0578590810888294,0.953860877050106,0.999451080313105
"13580","ZWINT",5.18243544667561,0.0436180370313553,0.706770307378505,
0.0617145861618603,0.950790124034309,0.999451080313105
"13581","JMJD1C",231.894880151186,-0.0128641447261078,0.31554958734354
2,-0.0407674268707011,0.967481307351842,0.999451080313105
"13582","HNRNPH3",235.987707989708,-0.00935224653473477,0.176353422644
002,-0.0530312731928871,0.957706990358996,0.999451080313105
"13583","CCAR1",122.658730166928,-0.0155190642531567,0.200005655665832
,-0.0775931270617959,0.938151709709245,0.999451080313105

"13584","SRGN",270.313475700915,0.0162861676844611,0.37721421478397,0.0431748514402835,0.96556215203364,0.999451080313105
"13585","AL135925.1",24.4842694458352,-0.0285101255514026,0.35643857886311,-0.0799860824334392,0.936248325111708,0.999451080313105
"13586","MCMBP",74.6812917025168,-0.0095451304078717,0.218654734813737,-0.0436538930474238,0.965180292170799,0.999451080313105
"13587","EDRF1",27.1472816293387,0.0241003126365786,0.331899881952471,0.0726132003868258,0.942113922062343,0.999451080313105
"13588","BNIP3",113.661362409187,0.00955722097368253,0.224694005064714,0.0425343834648814,0.966072702501247,0.999451080313105
"13589","NAP1L4",128.640912574492,-0.0137471132596551,0.195338263027239,-0.0703759368318851,0.943894443377322,0.999451080313105
"13590","RHOG",90.5978600938948,0.0153547105559584,0.233299584314088,0.0658154218366998,0.947524778007668,0.999451080313105
"13591","TRIM66",6.6678251260735,-0.0321183974014607,0.591038391758746,-0.054342319973304,0.956662432883789,0.999451080313105
"13592","CRY2",50.9602281434156,0.020152640774923,0.292954903393352,0.0687909317833264,0.945156036377844,0.999451080313105
"13593","DDB2",24.7842317268916,0.0259102071367787,0.389878196595243,0.0664571842258666,0.947013844351791,0.999451080313105
"13594","MADD",23.3664424529702,0.0268371683695004,0.384764981253721,0.0697495086014688,0.9443930355388,0.999451080313105
"13595","MRPL16",87.0799396738859,0.0117573607653626,0.271154136119615,0.0433604330496955,0.965414217870711,0.999451080313105
"13596","TMEM138",42.5321220295616,0.020850924131737,0.337895115936373,0.0617082732135829,0.950795151456186,0.999451080313105
"13597","SDHAF2",76.9755698860665,0.0187066979800349,0.312982544029796,0.0597691415603487,0.952339503089436,0.999451080313105
"13598","TUT1",8.84201050975943,-0.0259918501370625,0.491237265063089,-0.0529109902395626,0.957802827718981,0.999451080313105
"13599","WDR74",111.564203155535,-0.0155247481763148,0.284210717929683,-0.0546240771263096,0.956437956619416,0.999451080313105
"13600","FKBP2",40.8809849846787,-0.0220967543671519,0.379860215654039,-0.0581707519149008,0.953612617849457,0.999451080313105
"13601","CATSPERZ",2.15017698733891,0.0443776913895538,1.06780063109027,0.0415599036912371,0.966849537825359,0.999451080313105
"13602","SNX15",38.6660111782029,0.0126557917316837,0.309148336889822,0.0409376025082553,0.967345640094863,0.999451080313105
"13603","CDC42EP2",55.7198862515448,0.0171359359051184,0.314983389255985,0.0544026653138594,0.956614355388185,0.999451080313105
"13604","RNASEH2C",261.543468811032,-0.0149835442031931,0.268970540403279,-0.0557070085843888,0.955575216131906,0.999451080313105
"13605","SSH3",19.1274546533716,0.020776328967029,0.361282366225301,0.0575071769599531,0.954141189184474,0.999451080313105
"13606","PPFIA1",96.75657010209,0.0128068554632935,0.231999267472229,0.0552021375016907,0.955977426054098,0.999451080313105
"13607","NADSYN1",25.0555473005299,-0.0210134967253536,0.336459863923993,-0.0624546906733002,0.950200743065106,0.999451080313105
"13608","LRTOMT",11.8906313015128,0.0315442386608896,0.434619512332214,0.0725789748638295,0.942141158214209,0.999451080313105

"13609","ARRB1",38.0891953443372,0.0161327153567275,0.292251530928192,
0.0552014742420337,0.955977954453045,0.999451080313105
"13610","CAPN5",21.3527808172763,0.0218429583650394,0.352860292039505,
0.0619025684040241,0.950640422132187,0.999451080313105
"13611","PRCP",110.944346104812,-0.0104572981475534,0.235694273376813,
-0.0443680620565393,0.964611019354858,0.999451080313105
"13612","CEP57",91.9926301728627,0.0141198701907269,0.263075479953222,
0.0536723156154181,0.957196239937051,0.999451080313105
"13613","C2CD2L",4.95230855421489,0.0323289002857312,0.642347957199963
,0.050329264572826,0.959860003557513,0.999451080313105
"13614","CLEC4A",2.98038644312653,-0.0640025647348417,0.79670712233492
9,-0.0803338679178214,0.93597172254545,0.999451080313105
"13615","DDX47",7.44642365119402,-0.0360583064936488,0.558716427389498
,-0.0645377596326007,0.948542041926503,0.999451080313105
"13616","AC007529.1",3.09196447986425,0.0392458196983295,0.80379039663
6568,0.048825937536144,0.961058011657029,0.999451080313105
"13617","CAPRIN2",23.4878501777227,-0.0207157510517537,0.3442638399781
14,-0.060174054449258,0.952017009809335,0.999451080313105
"13618","AC016957.2",2.71715310990908,-0.0637746849590942,0.8454668113
54075,-0.0754313287081661,0.939871533502317,0.999451080313105
"13619","YARS2",35.1176479940223,-0.0162593101201862,0.322160394023853
,-0.0504696120994388,0.959748164564514,0.999451080313105
"13620","PPHLN1",114.375033750313,-0.0139865168724333,0.20947438906963
5,-0.0667695794915711,0.946765141396115,0.999451080313105
"13621","SLC48A1",27.5515290558605,0.0154403574057448,0.3128733237327,
0.0493501881896972,0.960640223821401,0.999451080313105
"13622","PRR13",151.177136215343,-0.0158590208321018,0.206440086981961
,-0.0768214209941096,0.938765609604182,0.999451080313105
"13623","NPFF",4.25701084529853,0.0550118471489167,0.721776443581314,0
.0762172936483749,0.939246224366486,0.999451080313105
"13624","PAN2",12.7253483634721,0.023323980751755,0.532940264462591,0.
0437647186880777,0.965091950532046,0.999451080313105
"13625","PRIM1",12.0471589167159,0.0266787147980703,0.45052644528793,0
.0592167564792341,0.952779463322298,0.999451080313105
"13626","ARHGEF25",23.0192319081438,0.0306290485065623,0.4058650730175
22,0.0754660860044985,0.939843880013165,0.999451080313105
"13627","TSFM",52.0369959422271,-0.0153060205662375,0.284932303869452,
-0.0537180949944176,0.957159765895789,0.999451080313105
"13628","GIHCG",36.26327462824,-0.0223616918325544,0.343750979437668,-
0.0650520090710294,0.948132590661849,0.999451080313105
"13629","AC026124.1",5.56949244835227,-0.0464498388070839,0.7135530336
75851,-0.0650965472990826,0.948097129459963,0.999451080313105
"13630","FRS2",56.6486459039137,-0.0097282539060681,0.239396135486152,
-0.04063663720516,0.967585576002223,0.999451080313105
"13631","AC121761.2",2.62063404501571,0.0393365006276067,0.87693474318
0287,0.0448568162380579,0.964221437822399,0.999451080313105
"13632","AC027288.3",2.60261048924257,-0.049748065648393,0.98575074270
8058,-0.050467185560241,0.959750098198567,0.999451080313105
"13633","EEA1",413.155900794862,-0.0106102274934464,0.177563057108669,
-0.0597547016041346,0.952351003951618,0.999451080313105

"13634","TMEM119",107.521679731529,-0.0160216425202707,0.266046363860043,-0.0602212422218973,0.951979427570266,0.999451080313105
"13635","ACACB",47.5473212048302,0.0245548986389231,0.317159054084465,0.0774214020463806,0.938288315509518,0.999451080313105
"13636","ANAPC7",34.7853742248717,0.0181142595355408,0.268265349056476,0.0675236649058515,0.946164823145394,0.999451080313105
"13637","HIP1R",3.35046526031509,-0.0455163679819516,0.831737292544214,-0.0547244525284191,0.956357988248924,0.999451080313105
"13638","OGFOD2",12.3324331837394,-0.0192549087791734,0.423370224174941,-0.0454800731834582,0.963724657757307,0.999451080313105
"13639","MTMR6",42.3062327293884,0.0213455400628481,0.284580050912048,0.0750071552606654,0.940209018857931,0.999451080313105
"13640","MRPS31",64.5943752467074,0.015903639148871,0.220423121394902,0.07215050330577,0.942482135093479,0.999451080313105
"13641","ELF1",178.69553984794,-0.0118790649651653,0.212784210306684,-0.0558268160407399,0.955479772140669,0.999451080313105
"13642","ENOX1",4.66108187704862,-0.0468689693917914,0.687973100474197,-0.068126165629857,0.945685201578921,0.999451080313105
"13643","TPT1",14665.5625257096,-0.0120437522693589,0.2355349019318,-0.0511336204128519,0.959219045809379,0.999451080313105
"13644","KLF12",36.4869278960604,0.021650372585852,0.341018414980797,0.0634874001952977,0.949378392191155,0.999451080313105
"13645","CLN5",90.4259132559934,-0.0150332624895521,0.237756059636342,-0.0632297764042108,0.949583534070869,0.999451080313105
"13646","SUPT16H",175.955226528894,-0.0126629768344173,0.243601061118539,-0.0519824370890379,0.958542687664317,0.999451080313105
"13647","PRMT5",42.6661404475765,0.011448973827963,0.277877317951633,0.0412015414297177,0.967135224834627,0.999451080313105
"13648","PSMB5",291.599150868692,0.0167110177759264,0.289481698257153,0.0577273723227974,0.953965790086823,0.999451080313105
"13649","PABPN1",154.789321450913,0.0076807113590212,0.189465902987653,0.0405387525560298,0.967663612348977,0.999451080313105
"13650","DHRS4L2",102.55941446974,-0.018102091907436,0.273034567394012,-0.0662996340727551,0.947139274553197,0.999451080313105
"13651","AL136419.3",2.15658480020132,-0.0726951262852147,1.04339842473212,-0.0696714932302856,0.944455131735597,0.999451080313105
"13652","CHMP4A",131.272955498767,-0.0123402259445064,0.246017851478023,-0.0501598801484078,0.959994982288806,0.999451080313105
"13653","KHNYN",33.6861718224075,-0.0233900206997395,0.340972132715888,-0.0685980420553282,0.945309577410901,0.999451080313105
"13654","MBIP",70.7769861467673,-0.0147080550715324,0.267056262962465,-0.0550747430836309,0.956078917696149,0.999451080313105
"13655","PRPF39",22.4356287006466,-0.0190009397484633,0.377463864184357,-0.0503384338247093,0.95985269681467,0.999451080313105
"13656","RPS29",5427.46742037826,0.0173677298716744,0.268849869111585,0.0646001053638824,0.94849240081856,0.999451080313105
"13657","CDKL1",11.6643690552045,-0.0257121868775153,0.516393295387691,-0.049791868150053,0.960288246922949,0.999451080313105
"13658","LINC00519",6.03167314156015,0.0476419217598626,0.708082388901585,0.0672830203188182,0.946356394077378,0.999451080313105

"13659","SNAPC1",43.1499595057973,0.0145799127558339,0.270351929725049
,0.0539293829737478,0.956991426495387,0.999451080313105
"13660","PNMA1",68.9476902369393,0.0193444402023704,0.244348177334059,
0.0791675240365051,0.936899375555844,0.999451080313105
"13661","TTLL5",19.0864818964496,-0.0189302899852802,0.394925670678001
,-0.0479338047404745,0.961768998058623,0.999451080313105
"13662","BDKRB1",30.2264271648981,0.0519321256500518,0.896594247623211
,0.0579215467729344,0.953811120090042,0.999451080313105
"13663","MTA1",57.5559900911528,-0.0140619240160272,0.261748764804529,
-0.0537229813731059,0.957155872751336,0.999451080313105
"13664","MTMR10",18.3934019687352,0.0240687073718438,0.372372304278765
,0.0646361372617698,0.948463711482293,0.999451080313105
"13665","KNSTRN",5.81789813174065,-0.0518979868434994,0.76382984048184
6,-0.0679444348636086,0.945829866550829,0.999451080313105
"13666","RPAP1",15.1565847388004,-0.017926615564501,0.386226308617184,
-0.0464147966219186,0.96297964322441,0.999451080313105
"13667","POLR2M",60.0689662247151,-0.0167714455223064,0.22671732771557
8,-0.0739751376363545,0.941030168336261,0.999451080313105
"13668","GTF2A2",231.968303586438,-0.0156173594309628,0.28471738670276
8,-0.0548521451809564,0.956256257053843,0.999451080313105
"13669","PIF1",2.34137622986524,-0.0513444573097012,1.0444164172889,-0
.0491609059947383,0.960791066071278,0.999451080313105
"13670","SLC24A1",4.66511845716326,0.0321701594332084,0.66942986535433
9,0.0480560565014235,0.961671567546982,0.999451080313105
"13671","STOML1",30.2942955236709,0.0254746475060045,0.33076321014847,
0.077017778049045,0.93860940213659,0.999451080313105
"13672","IMP3",163.029467750884,-0.0133588132737295,0.272373832433462,
-0.0490458762296592,0.960882735963563,0.999451080313105
"13673","PSTPIP1",9.53689476623037,-0.0392554550258076,0.5083868655214
67,-0.0772157144255531,0.938451940665909,0.999451080313105
"13674","AC015871.7",4.4029452857054,-0.0455975903488937,0.68853437211
2362,-0.0662241308433221,0.94719938530703,0.999451080313105
"13675","FAH",18.6526676686136,-0.020267627246478,0.368165285579585,-0
.0550503484177539,0.956098352339285,0.999451080313105
"13676","IL16",25.999105941745,-0.0312231155283062,0.477600860065138,-
0.0653749147856388,0.947875496444908,0.999451080313105
"13677","GOLGA6L10",3.79655176952269,-0.0360827285740808,0.89528287284
4825,-0.0403031596699994,0.967851434775068,0.999451080313105
"13678","AP3B2",1.89234128200266,-0.0493548592947256,1.21370177107175,
-0.0406647336858898,0.967563176768807,0.999451080313105
"13679","MESP1",4.52394363758195,0.031483020920437,0.653078164473348,0
.0482071253229319,0.961551171605228,0.999451080313105
"13680","RGS11",6.40701760747221,-0.0442414581434627,0.628296433840538
,-0.0704149439032009,0.943863397257541,0.999451080313105
"13681","TPSB2",13.2709512069288,-0.0438081002940662,0.746043789538623
,-0.0587205481881412,0.953174692489361,0.999451080313105
"13682","ROGDI",13.1254804374418,-0.0177199432756452,0.441560969246977
,-0.0401302300469723,0.967989301114817,0.999451080313105
"13683","AF001548.2",2.68831111932587,0.0790738554039301,1.07800516864
748,0.0733520188063105,0.941525998129439,0.999451080313105

"13684","APOBR",4.34713796817258,0.0511042463185139,0.72127514840596,0
.0708526370712424,0.943515038727368,0.999451080313105
"13685","BOLA2B",9.85038377632159,0.0389671836224599,0.64336715083731,
0.0605675679458394,0.951703603115146,0.999451080313105
"13686","ZNF629",16.05919469213,0.0283175901898315,0.383633475558785,0
.0738141794028407,0.94115824427356,0.999451080313105
"13687","ZNF646",12.5368125819868,-0.0294184110553911,0.41806523653114
7,-0.0703679916069733,0.943900767072067,0.999451080313105
"13688","VKORC1",416.021753134673,-0.0126176857890781,0.27015346954297
8,-0.0467056218468101,0.962747849651815,0.999451080313105
"13689","LPCAT2",17.3229114589264,-0.0234097678144641,0.39118828543342
2,-0.0598427117737612,0.952280907436758,0.999451080313105
"13690","CSNK2A2",97.5072053659784,0.0131996752081938,0.21490078300382
1,0.0614221829427171,0.951022986277555,0.999451080313105
"13691","SETD6",13.8722252769652,0.0242953952191604,0.393963412454695,
0.0616691663517214,0.950826294902908,0.999451080313105
"13692","AP1G1",49.379178556107,-0.0187953938035023,0.243488596196032,
-0.0771920907062529,0.938470733576235,0.999451080313105
"13693","HP",147.79819464446,-0.048052287803286,0.702018268912054,-0.0
684487711092684,0.945428399105217,0.999451080313105
"13694","ZFHX3",152.850876678795,-0.015230505455982,0.263927871565261,
-0.0577070749127607,0.953981958124362,0.999451080313105
"13695","WDR59",27.0888016003658,0.0152896112563191,0.299779285575122,
0.0510028944361056,0.959323214125484,0.999451080313105
"13696","KARS",94.7109178300116,-0.0172227987491692,0.245793501883341,
-0.0700701955796353,0.94413778885861,0.999451080313105
"13697","CMC2",75.670104374841,-0.0224784056226362,0.28635898848626,-0
.0784972936992851,0.937432482897098,0.999451080313105
"13698","ZC3H18",60.1087371294836,-0.0220258711202281,0.27734700666521
8,-0.0794162929142958,0.936701509700032,0.999451080313105
"13699","TRAPPC2L",175.514615335554,0.0203503494884136,0.3198675860695
4,0.0636211681792272,0.94927187611654,0.999451080313105
"13700","SCARF1",2.84268431601831,-0.0422580978911856,0.86053437315691
4,-0.0491068099187714,0.960834176426538,0.999451080313105
"13701","MINK1",31.4525257070933,0.0115321714096166,0.286257312715039,
0.0402860325217142,0.967865089172743,0.999451080313105
"13702","SPAG7",124.60688899536,0.0201251253923964,0.267239731853918,0
.0753073850687647,0.939970145735043,0.999451080313105
"13703","RNASEK",654.159295530419,-0.0132839362354059,0.23298482315095
5,-0.0570163157228442,0.954532198216133,0.999451080313105
"13704","TMEM256",139.122445391114,-0.0137995499793091,0.2640469722226
89,-0.0522617239771681,0.958320151457674,0.999451080313105
"13705","MAP2K4",27.1797285194339,0.0203040054796486,0.327077491611537
,0.0620770490185954,0.950501473971053,0.999451080313105
"13706","TVP23C",6.6880003854016,0.0456924661215018,0.599398259126268,
0.0762305619440852,0.939235668508005,0.999451080313105
"13707","NT5M",2.68380306546595,0.0379600805703228,0.944715469639451,0
.0401814956886545,0.967948430016297,0.999451080313105
"13708","SLC46A1",6.09689173921772,-0.0450475110043727,0.5800156942447
49,-0.07766602085316,0.93809372386357,0.999451080313105

"13709","TLCD1",6.92726286242009,0.0393774388273519,0.582274005998657,
0.067626990766685,0.946082569054466,0.999451080313105
"13710","CRLF3",18.0261302937006,-0.017809833351023,0.386658954529226,
-0.0460608325305883,0.963261763967269,0.999451080313105
"13711","AC009283.1",4.19233511824385,0.0579363438460882,0.73558877815
5562,0.0787618647355653,0.93722203731076,0.999451080313105
"13712","AC100793.4",12.2512832685631,-0.0330963479627273,0.5570447321
84274,-0.0594141655966308,0.952622230477094,0.999451080313105
"13713","AOC3",8.11302935524653,0.035187598811502,0.651745797894994,0.
0539897593895516,0.956943323166064,0.999451080313105
"13714","UBTF",148.635434430448,0.013748516800781,0.212726235610349,0.
0646300949261574,0.9484685225092,0.999451080313105
"13715","CALCOCO2",207.527656610185,0.0104344828664916,0.1944554988193
31,0.0536600041132617,0.957206048959097,0.999451080313105
"13716","SUPT4H1",141.525742318071,-0.0149424289384335,0.2362831792535
96,-0.0632394950230299,0.949575795222791,0.999451080313105
"13717","HEATR6",14.8245955868094,0.0333542968590679,0.425380301553654
,0.0784105346139563,0.937501493922625,0.999451080313105
"13718","PPM1D",15.6586270865945,0.0268516325954248,0.405886820472016,
0.0661554680790037,0.947254050389011,0.999451080313105
"13719","ABCA5",29.3512932258231,0.0230149180316547,0.3739756927907,0.
0615412137080665,0.950928192796733,0.999451080313105
"13720","LLGL2",2.58551466444731,0.0470973535975822,0.86521221502601,0
.0544344529349558,0.956589030062815,0.999451080313105
"13721","UNC13D",3.91439558734159,0.0384024185352055,0.707216711362573
,0.0543007792635679,0.956695528708601,0.999451080313105
"13722","PGS1",21.5694565197114,0.0240660443978052,0.362251188182804,0
.066434687263636,0.947031754749186,0.999451080313105
"13723","DCXR",46.6315761121707,-0.012296966120306,0.2573419405111,-0.
0477845394959072,0.961887958175478,0.999451080313105
"13724","COLEC12",340.456090118326,0.0201877648118668,0.25329488815545
6,0.0797006404624985,0.93647535006029,0.999451080313105
"13725","VAPA",341.334844603097,-0.0143605367447694,0.194925151202879,
-0.0736720564593686,0.941271334063555,0.999451080313105
"13726","SETBP1",113.00968044925,-0.0238269156007026,0.307274411133533
,-0.0775427915159133,0.938191751022729,0.999451080313105
"13727","DYM",50.9847594108037,0.013137065219444,0.233676316576584,0.0
562190700876551,0.955167289454109,0.999451080313105
"13728","ADAT3",2.31511643797253,-0.0687002507109058,1.02039237518237,
-0.0673272874061089,0.946321153960919,0.999451080313105
"13729","SPPL2B",44.3265934412679,0.0204798552418534,0.283652207655145
,0.0722005846919129,0.942442279873089,0.999451080313105
"13730","ZNF555",5.01998506381566,0.0335176062788101,0.623049146983322
,0.0537960872606849,0.957097626920962,0.999451080313105
"13731","GNA15",2.51306837483284,0.0622155398616407,0.85686091811734,0
.0726086795956783,0.942117519635461,0.999451080313105
"13732","MPND",20.9711940428545,-0.0141740867853268,0.327490005916438,
-0.0432809750809416,0.965477556695842,0.999451080313105
"13733","FEM1A",19.3695260811422,-0.0206399894318337,0.343142625414303
,-0.0601498849258772,0.952036259430698,0.999451080313105

"13734","SLC25A23",25.7729638079846,0.0170642633332541,0.305668916490741,0.0558259685975334,0.955480447249682,0.999451080313105
"13735","DENND1C",3.24113567508654,0.0491695906885151,0.867865635618702,0.0566557640612906,0.954819412544555,0.999451080313105
"13736","XAB2",47.1234512918447,0.0113470374565409,0.274039020407189,0.0414066487308289,0.966971712423952,0.999451080313105
"13737","KEAP1",64.743237262665,-0.0122485039704982,0.253024600050369,-0.0484083522632184,0.961390802966981,0.999451080313105
"13738","DNM2",44.8735695854626,0.0177905517141499,0.241845069864182,0.073561771278368,0.941359090787654,0.999451080313105
"13739","ZNF490",10.1593956387877,-0.0199096261233631,0.485101913488513,-0.0410421512877305,0.96726229228657,0.999451080313105
"13740","AKAP8L",53.0316307637153,-0.0170677818638774,0.226478756186642,-0.0753615135973802,0.939927079796931,0.999451080313105
"13741","FAM32A",138.786001704308,-0.0170277524567871,0.242918890065868,-0.0700964525738201,0.944116890195104,0.999451080313105
"13742","ELL",46.6453461948716,0.0184268677772208,0.330865415056312,0.0556929402067744,0.95558642367408,0.999451080313105
"13743","DDX49",63.9965503029048,-0.0181471873862864,0.304981531969179,-0.0595025779728865,0.952551812192008,0.999451080313105
"13744","RFXANK",53.3983362939514,-0.0222379644972135,0.323993965731528,-0.0686369712071756,0.945278589478821,0.999451080313105
"13745","ATP13A1",29.8529080049888,-0.0245451456276345,0.311870200492488,-0.0787030809255714,0.937268794859956,0.999451080313105
"13746","ZNF506",54.7206975478247,0.0174250760397297,0.236780596224086,0.073591655387331,0.941335311171556,0.999451080313105
"13747","HAUS5",10.1326077587275,-0.0232055613724921,0.466276169073668,-0.0497678477083519,0.960307388730799,0.999451080313105
"13748","ARHGAP33",7.7716393250945,-0.0285196919024995,0.519641976358308,-0.0548833489210548,0.956231397518994,0.999451080313105
"13749","ZNF566",29.3301924746215,-0.0128744793406322,0.310514796189087,-0.0414617258135176,0.966927804976182,0.999451080313105
"13750","AC010632.2",2.30609355703049,0.0531310146387107,0.921785445498418,0.0576392422967605,0.954035990785076,0.999451080313105
"13751","ZNF527",7.3924768791212,0.0366557876120019,0.516364294348557,0.070988230621652,0.943407122462226,0.999451080313105
"13752","MAP3K10",11.1263908677913,0.0271083792445955,0.435726936987098,0.0622141459328649,0.950392297487494,0.999451080313105
"13753","ZNF45",6.19828428257167,0.0250500490021509,0.595394540029009,0.0420730243863681,0.966440484544449,0.999451080313105
"13754","RTN2",44.1400089486827,0.0244583658222683,0.324344035378176,0.0754087115977038,0.939889528094483,0.999451080313105
"13755","GRWD1",59.9542236532968,-0.0184939790208146,0.325115127872471,-0.0568844001256964,0.954637281086192,0.999451080313105
"13756","PRR12",33.421026481915,-0.0135185971509768,0.318633980955042,-0.0424267277157869,0.966158521891612,0.999451080313105
"13757","NUP62",56.1706598253314,-0.0109029085678342,0.246549554058005,-0.0442219764277856,0.964727464530885,0.999451080313105
"13758","CLEC11A",137.949852257697,0.0144477435469109,0.284314123961752,0.0508161302210034,0.959472037425483,0.999451080313105

"13759","ZNF432",12.6541717996569,0.0300725664721309,0.523068360462494,0.0574926123337701,0.954152790879995,0.999451080313105
"13760","ZNF808",11.293396061618,0.0195483849395375,0.435929306268395,0.0448430161919462,0.964232437597458,0.999451080313105
"13761","TFPT",22.4865501071214,0.0145067307659988,0.360514349787333,0.0402389829269107,0.96790259890276,0.999451080313105
"13762","MBOAT7",36.1262752619819,0.0117321190828551,0.293688978873622,0.0399474271314199,0.968135039874302,0.999451080313105
"13763","CCDC106",18.8403085226357,-0.0251042439624601,0.381247427576597,-0.0658476415750147,0.947499126021349,0.999451080313105
"13764","ZNF470",3.59205729834449,0.0402854826534004,0.719382425026276,0.056000092929611,0.955341733131872,0.999451080313105
"13765","ZSCAN18",59.9149969615943,0.0140648841942898,0.244646602881094,0.0574906171949821,0.954154380141777,0.999451080313105
"13766","ZCCHC3",34.8752893522002,0.0230130060458517,0.332216215897832,0.0692711702336921,0.944773773418536,0.999451080313105
"13767","EBF4",3.07327032223523,-0.0359171521498654,0.817510362167036,-0.0439347974191509,0.964956377741008,0.999451080313105
"13768","LZTS3",8.79949441759313,-0.0218245283320172,0.536121439786576,-0.0407081804837077,0.96752853992019,0.999451080313105
"13769","DZANK1",4.1151361311912,-0.0351823009532197,0.813515042015013,-0.0432472654298756,0.965504427945616,0.999451080313105
"13770","E2F1",2.58366444092508,-0.0606405163970542,0.913007943218926,-0.0664183886322592,0.947044730516232,0.999451080313105
"13771","PXMP4",25.2413294734899,0.0261434577642829,0.328141882533341,0.0796712006478678,0.936498765174533,0.999451080313105
"13772","EIF2S2",381.970124625982,-0.00899374919395899,0.221557486219281,-0.0405932985945581,0.967620126701755,0.999451080313105
"13773","ERGIC3",227.245281025246,-0.0114949243698169,0.164574006216558,-0.0698465367288384,0.944315806643818,0.999451080313105
"13774","MANBAL",84.5796533770182,0.0137372977794322,0.299405863216538,0.0458818595997135,0.963404412891161,0.999451080313105
"13775","SNHG11",18.3644706725307,0.0271592666912986,0.378470140221946,0.0717606590453124,0.942792381600502,0.999451080313105
"13776","ACTR5",10.6515039138852,-0.0332575098982832,0.468019914413237,-0.0710600315800207,0.943349977898817,0.999451080313105
"13777","PTPN1",65.5190906811611,-0.0136008933317463,0.269645441726045,-0.0504399156339701,0.959771828775851,0.999451080313105
"13778","BCAS4",4.07133628426739,-0.0380591422858146,0.75421154045796,-0.050462158485012,0.959754104120123,0.999451080313105
"13779","HAR1A",2.88967132957666,0.0404044966311789,0.849271860860058,0.0475754566862268,0.962054592601386,0.999451080313105
"13780","ARFGAP1",27.5821630915624,0.0142698452224163,0.335360215118334,0.0425507993468488,0.966059616369945,0.999451080313105
"13781","HELZ2",78.5094645434156,-0.0153309897079024,0.312694412395214,-0.0490286653684286,0.960896451743247,0.999451080313105
"13782","MHENCR",7.04306489636325,0.0326150452843357,0.536817396820581,0.0607563120672047,0.951553283927195,0.999451080313105
"13783","RTEL1",5.79897768964237,0.0382635467521168,0.672031049165615,0.056937170982835,0.954595244164555,0.999451080313105

"13784","N6AMT1",12.5849916855007,-0.0226116421420265,0.453479738845391,-0.0498625191052598,0.960231945551087,0.999451080313105
"13785","DYRK1A",51.2290436919582,-0.0189749474211527,0.283349951351073,-0.0669664749567669,0.94660839237516,0.999451080313105
"13786","ADARB1",32.4562816303355,-0.0248508665313092,0.361380050251462,-0.0687665700251495,0.945175428327311,0.999451080313105
"13787","COL6A1",3140.00319372853,0.0166174905217878,0.258165554912775,0.0643675742389501,0.94867754847489,0.999451080313105
"13788","C21orf58",6.19393066303451,-0.0457003903862488,0.609214216110258,-0.0750153052534442,0.940202534373283,0.999451080313105
"13789","PEX26",46.7508144285475,0.0180267067925795,0.259811899085473,0.0693836843348313,0.944684215633662,0.999451080313105
"13790","IGLC2",84.8756713267198,-0.0487047871573659,0.816050530658022,-0.0596835432704061,0.952407678935218,0.999451080313105
"13791","PITPNB",151.678037988708,0.0106710853862171,0.218628006682405,0.0488093247893838,0.961071250926123,0.999451080313105
"13792","UQCR10",407.91367049357,-0.0229420130352098,0.284700586832015,-0.0805829495839659,0.935773626362307,0.999451080313105
"13793","DRG1",64.2089910474944,0.00982297816389231,0.240788087205191,0.0407951168926456,0.967459232275143,0.999451080313105
"13794","JOSD1",69.7123952750109,-0.0171494878915981,0.243896326444528,-0.0703146625519126,0.943943212364159,0.999451080313105
"13795","SGSM3",44.6651929776794,0.0117757633627801,0.255231226550265,0.0461376279146668,0.963200555189135,0.999451080313105
"13796","SREBF2-AS1",3.32724915356198,-0.0570871300356622,0.767713523734091,-0.074359938011767,0.940723985348089,0.999451080313105
"13797","MIRLET7BHG",16.4902048201047,0.0330457051560169,0.453843463631027,0.0728130022885664,0.94195492409271,0.999451080313105
"13798","AL118516.1",39.7429308981096,-0.0277521623355625,0.394674537575902,-0.0703165765544866,0.943941688981734,0.999451080313105
"13799","SBF1",32.6394975649054,-0.0187511598609322,0.275115596096303,-0.0681573859388487,0.945660349142104,0.999451080313105
"13800","TCEANC",3.87402646698983,0.0569392662722179,0.758424028028344,0.0750757678659542,0.940154427843131,0.999451080313105
"13801","ZRSR2",30.3694427447286,0.0181746130845944,0.326447334326505,0.0556739515796338,0.955601550936174,0.999451080313105
"13802","SAT1",1550.77238588018,-0.0174266773366193,0.309754339683239,-0.0562596713073985,0.955134945557913,0.999451080313105
"13803","RGN",11.1177762014846,-0.0302054419859977,0.518605664663205,-0.0582435635476791,0.953554620904043,0.999451080313105
"13804","PORCN",12.4459486650314,-0.0172177754051179,0.408526875997838,-0.0421460041351331,0.966382306733317,0.999451080313105
"13805","KANTR",15.0269100178024,-0.0382099889438145,0.531731031442613,-0.0718596182738271,0.942713626879034,0.999451080313105
"13806","IQSEC2",12.0198224280545,-0.0241270492021773,0.537367205838632,-0.0448986260047707,0.964188112031189,0.999451080313105
"13807","ABCB7",47.0291572969195,-0.0117439567842695,0.242328935723362,-0.0484628744364073,0.961347351565364,0.999451080313105
"13808","ARMCX5",13.171495201552,-0.0271506200769001,0.441011140989232

,-0.0615644766161656,0.950909666809976,0.999451080313105
"13809","GPRASP2",9.96508782604532,0.0216181582463094,0.500956432893165,0.0431537691241101,0.965578957625192,0.999451080313105
"13810","ATG4A",14.6767016403687,-0.0237918583683089,0.415240805236377,-0.0572965326824404,0.954308982336228,0.999451080313105
"13811","PGRMC1",292.091165474441,0.0146880765863197,0.218377498183515,0.067260027743227,0.946374698034667,0.999451080313105
"13812","GRIA3",19.1618015449891,0.0230001650591155,0.411334924384179,0.05591590622544З,0.955408799318992,0.999451080313105
"13813","ZDHHC9",101.919291551509,0.0125690014825883,0.287777398393988,0.043676124507111,0.965162570934353,0.999451080313105
"13814","ELF4",15.8616556937485,-0.0319721003123906,0.420036285387278,-0.0761174722867381,0.939325639592773,0.999451080313105
"13815","FHL1",916.825104416425,0.0219885481361542,0.279970175733201,0.0785388946467937,0.937399392304006,0.999451080313105
"13816","LINC00892",6.04154092244407,-0.0334379821608333,0.652447778082178,-0.0512500513974035,0.959126268969818,0.999451080313105
"13817","MAMLD1",3.06457636453077,0.0409477768602694,0.801432554464882,0.051093228784062,0.959251231594896,0.999451080313105
"13818","ABCD1",8.26838880279978,-0.0365061067575639,0.525874950825367,-0.0694197483646391,0.944655509916345,0.999451080313105
"13819","SSR4",678.732998593938,0.018594429971458,0.260254105762935,0.0714472108593577,0.943041836753588,0.999451080313105
"13820","DDX27",116.877693269096,0.0097122634042172,0.243689775345323,0.0398550303986056,0.968208703137573,0.999454801661405
"13821","SRSF10",250.434626429193,-0.00636807950466042,0.160937195432772,-0.0395687242314381,0.968436962350082,0.999521899868837
"13822","PCAT6",3.98519791218062,-0.033500707934044,0.845383490395425,-0.0396278237210123,0.96838984473505,0.999521899868837
"13823","HIF1A",183.146444831139,0.0103597532559984,0.262206788797837,0.039509859006686,0.968483893304846,0.999521899868837
"13824","SMG8",11.3600745389158,-0.0181304481626785,0.460582620648217,-0.0393641604130916,0.968600053597214,0.999569470820157
"13825","ATAD3B",13.2738949504993,0.00623472236106001,0.414298200063366,0.0150488762927438,0.987993187144803,0.999765644820604
"13826","LRRC47",56.3686416808079,-0.00693805640129047,0.265375833910331,-0.0261442660360506,0.979142269925187,0.999765644820604
"13827","AL034417.4",3.45663712607013,0.033130113317954,0.903156173025168,0.0366825963299161,0.970738085420148,0.999765644820604
"13828","AL034417.2",3.54137276707969,0.0355527163448998,1.02437555969702,0.0347067206049071,0.972313601889726,0.999765644820604
"13829","NMNAT1",18.4304043835713,-0.00090633455855488,0.393710922855405,-0.0023020305151З443,0.998163247015742,0.999765644820604
"13830","CLIC4",174.65456223806,-0.00355790750141713,0.200251866207619,-0.0177671627675536,0.985824600938688,0.999765644820604
"13831","FGR",5.76925859061849,-0.00700699557070861,0.644564898062126,-0.0108708922744242,0.991326453727022,0.999765644820604
"13832","ATP5IF1",292.790415467892,-0.00402181365928145,0.229025805564435,-0.0175605262008343,0.985989447345692,0.999765644820604
"13833","SRSF4",196.100402094878,0.0080418667335517,0.217832873230122,

0.0369175993242129,0.970550707080949,0.999765644820604
"13834","FAM229A",8.04523797427727,0.00354645749169601,0.500733467559327,0.00708252537818599,0.99434900959361,0.999765644820604
"13835","AC004865.2",2.29383826837203,-0.013068434882192,1.00259174164135,-0.0130346524307069,0.989600146563875,0.999765644820604
"13836","PABPC4",194.644285587689,0.00270082990144934,0.209133090613036,0.0129144072491462,0.989696080262692,0.999765644820604
"13837","ELOVL1",85.9105557968683,0.00806743635423762,0.231508089279567,0.0348473195012009,0.972201488019266,0.999765644820604
"13838","ATPAF1",44.2558517359316,0.00444687583660164,0.243159141429987,0.0182879237459474,0.985409161311681,0.999765644820604
"13839","BTF3L4",171.221924469841,0.000315347180113946,0.266190627009653,0.00118466673171971,0.999054772926158,0.999765644820604
"13840","NDC1",12.0328097382569,0.00803320890849531,0.418607736903233,0.0191903020424878,0.984689294028649,0.999765644820604
"13841","TM2D1",157.062688945154,0.00309680262350322,0.199076682731733,0.0155558279403135,0.987588745611651,0.999765644820604
"13842","ATG4C",27.3561640533337,0.00669237117716715,0.299897375648519,0.0223155376491545,0.982196254717056,0.999765644820604
"13843","LEPROT",551.93096076364,-0.0025475756063900 8,0.147812751308577,-0.0172351545034955,0.986249017111486,0.999765644820604
"13844","AL078459.1",3.80039707806847,-0.0208419064204881,0.788720278191106,-0.0264249658551801,0.978918381227605,0.999765644820604
"13845","CCDC18",18.4062201104072,-0.00270623213175595,0.448653228928671,-0.00603190160520653,0.995187268021217,0.999765644820604
"13846","DR1",67.9946095691416,0.00867091239416301,0.241983477234135,0.0358326630118358,0.971415788466002,0.999765644820604
"13847","RWDD3",48.6726706457534,-0.00523371289155628,0.272488140358726,-0.0192071217656159,0.984675876304299,0.999765644820604
"13848","PLPPR4",2.84125705880146,-0.0182487517432608,0.947368080417115,-0.0192625782106001,0.984631636647694,0.999765644820604
"13849","AC093157.1",14.8340359244804,0.00799399836982505,0.433199310986709,0.0184533958551709,0.985277155947179,0.999765644820604
"13850","FAM102B",54.031402973832,-0.00474841859279391,0.330685045042421,-0.0143593387846879,0.988543298992408,0.999765644820604
"13851","LRIF1",23.3547530215791,-0.00534586599276048,0.375958067627021,-0.0142193144743578,0.988655010822152,0.999765644820604
"13852","RAP1A",178.756053509992,0.00671152918811299,0.190419382179621,0.0352460401419749,0.97188355029464,0.999765644820604
"13853","CTTNBP2NL",42.8332644614869,-0.00851405572470335,0.263683280073732,-0.0322889480225011,0.974241622811048,0.999765644820604
"13854","LINC01719",4.4159489418569,0.0299828494096474,0.770135130463279,0.038931933142189,0.968944656895281,0.999765644820604
"13855","C1orf54",56.0214674463055,0.0106784806419067,0.410470743966045,0.0260152052220074,0.979245210542427,0.999765644820604
"13856","PRPF3",32.8127069925381,-0.000319622280920219,0.341363631776965,-0.000936310289577212,0.999252932584982,0.999765644820604
"13857","DPM3",104.333531114057,0.00319388289361689,0.256993708963639,0.0124278641158052,0.990084254349705,0.999765644820604
"13858","FAM189B",16.2664809988366,0.00783592938393053,0.3919420097563

84,0.0199925733625774,0.984049296980973,0.999765644820604
"13859","DAP3",89.2598960982918,-0.0069749348780959,0.211227610602099,-0.0330209429449778,0.973657886692009,0.999765644820604
"13860","GON4L",89.6437862054518,-0.00310175587813625,0.220209197419813,-0.0140854964936954,0.988761771430693,0.999765644820604
"13861","NAXE",92.0625344009761,-0.000287225798926185,0.292086653974349,-0.000983358174767578,0.999215393821065,0.999765644820604
"13862","USF1",10.490919652298,-0.0115116154890229,0.564790821990216,-0.0203820866784947,0.983738573642666,0.999765644820604
"13863","TIPRL",122.218748198815,-0.0020166674563139,0.238428935748332,-0.0084581489657889,0.993251453992805,0.999765644820604
"13864","MYOC",955.10205865862,-0.00677536260837377,0.859126955465846,-0.00788633456937683,0.993707680630207,0.999765644820604
"13865","EEF1AKNMT",32.7566326356751,-0.00466677354272946,0.306301684147629,-0.0152358729457074,0.987844002507065,0.999765644820604
"13866","COP1",41.9011012928399,0.00513877640322256,0.28156635665571,0.018250676196752,0.985438875596868,0.999765644820604
"13867","NCF2",4.81125193902975,-0.00367672366989489,0.821455211714555,-0.0044758662644805 3,0.996428787335266,0.999765644820604
"13868","TRMT1L",25.9028200976315,0.00609804623524544,0.302649824726 81,0.0201488510384895,0.983924630548727,0.999765644820604
"13869","TMEM9",103.95805083309,-0.00611366561249497,0.228079013237751,-0.0268050335965022,0.978615238432429,0.999765644820604
"13870","HSD11B1-AS1",4.30409006867565,0.0220673819570448,0.798996933145333,0.0276188568962015,0.977966141773559,0.999765644820604
"13871","UTP25",33.2426751354957,-0.00782744828410499,0.318794734697051,-0.0245532545935659,0.98041130547638,0.999765644820604
"13872","FLVCR1",13.5763878536996,-0.00747054204990348,0.514442861892056,-0.0145216166911672,0.98841383345567,0.999765644820604
"13873","PYCR2",50.8641966084829,-0.0104657727565034,0.282214607738725,-0.0370844473302127,0.970417672731341,0.999765644820604
"13874","TSNAX",101.732694933938,-0.00326882740360698,0.231479898479986,-0.0141214309539263,0.98873310273103,0.999765644820604
"13875","CYS1",44.8883027275236,-0.0121244549909861,0.429901498966786,-0.0282028674478355,0.977500350242601,0.999765644820604
"13876","SF3B6",331.931888509257,0.00100621017910491,0.246600896912815,0.0040803184080091,0.996744385972878,0.999765644820604
"13877","SELENOI",14.6615953773547,0.00318141736443246,0.395946787101276,0.00803496193951614,0.993589096903868,0.999765644820604
"13878","AGBL5",6.46216218776956,-0.0198003363877097,0.544489013313083,-0.036364987912666,0.970991331301838,0.999765644820604
"13879","AC074117.1",3.90182441037371,-0.00929548008888833,0.744507254702622,-0.0124854123719738,0.990038341046885,0.999765644820604
"13880","FAM98A",49.2024630970064,-0.000405900431209881,0.257451228555879,-0.0015766109701115,0.998742046969705,0.999765644820604
"13881","PIGF",59.3089795656002,-0.000418888324528785,0.277157228337553,-0.00151137434531787,0.998794098203375,0.999765644820604
"13882","PPP4R3B",111.294714239139,-0.00394122116259942,0.182682371146707,-0.0215741734566952,0.982787635331908,0.999765644820604

"13883","AC015982.2",3.26567285155844,-0.0129946522798346,0.860389687717496,-0.0151032171414185,0.987949834347677,0.999765644820604
"13884","GFPT1",77.15172505386,0.00212543662266723,0.23811671613871,0.00892602861795351,0.992878154147551,0.999765644820604
"13885","MOB1A",183.826764335304,-0.000901215333900512,0.176797578876778,-0.00509744160313773,0.995932847658691,0.999765644820604
"13886","SUCLG1",157.206976559388,0.00503460990534991,0.230543625945938,0.0218379922007929,0.982577188012144,0.999765644820604
"13887","KDM3A",32.6865442373391,0.00880426465488357,0.354567850270432,0.0248309728255635,0.980189785928109,0.999765644820604
"13888","KRCC1",107.668068600417,-0.00201275371643412,0.191335667146943,-0.0105194904141336,0.991606815809179,0.999765644820604
"13889","ZNF514",16.5525369469734,-0.0145554328606931,0.477401338331807,-0.0304888815593907,0.975677160490667,0.999765644820604
"13890","RNF149",119.291389540105,-0.000562173011279578,0.232992196730635,-0.0024128405121203,0.998074833675688,0.999765644820604
"13891","THORLNC",2.98449035267762,-0.035603994958026,0.940424787765486,-0.0378594816100323,0.96979971886255,0.999765644820604
"13892","DBI",626.144384365679,0.00487611451958772,0.261482219774569,0.0186479773798446,0.985121929064323,0.999765644820604
"13893","GLI2",21.372559687659,0.00754117108979682,0.391030028963839,0.0192854014556877,0.984613429714942,0.999765644820604
"13894","KIF5C",3.4363477042754,0.0115701930314671,0.806833558889606,0.0143402476309865,0.988558529960959,0.999765644820604
"13895","GALNT3",2.85114499497342,0.00940439076589883,0.985145124908598,0.00954619834998562,0.992383351406435,0.999765644820604
"13896","ABCB11",11.1786112092692,0.0142396198541358,1.25656203621894,0.0113322060063056,0.990958401306828,0.999765644820604
"13897","KLHL23",5.63224293813782,0.0119902441823944,0.627455146218946,0.01910932479341,0.984753892679702,0.999765644820604
"13898","FKBP7",92.3190492495511,0.00301603717375251,0.254096517065238,0.011869651770859,0.99052961048882,0.999765644820604
"13899","OSGEPL1",11.1368614179539,0.00269727463420983,0.454254554232214,0.00593780427533366,0.995262345483316,0.999765644820604
"13900","KCTD18",27.6017596289617,-0.0057519733414151,0.358214426275294,-0.0160573469952732,0.987188641290739,0.999765644820604
"13901","NIF3L1",27.9239565095345,0.012219052898121,0.318704547773619,0.0383397506671302,0.969416797621969,0.999765644820604
"13902","WDR12",39.2747228815131,0.00888154813237264,0.310269585873622,0.0286252618263081,0.977163464313358,0.999765644820604
"13903","AC008269.1",2.25341418212146,0.0296960699875002,0.916007347944475,0.0324190303212505,0.974137886462125,0.999765644820604
"13904","RPE",44.3772172123629,0.00842887699831212,0.266759895085229,0.0315972421402367,0.974793242746507,0.999765644820604
"13905","RNF25",13.6180282134631,0.000704137065986698,0.40652519976841,0.00173208712864007,0.998617995113124,0.999765644820604
"13906","ABCB6",48.3242539774221,0.00499887363883397,0.320437605132856,0.0156001466705551,0.9875533886714,0.999765644820604
"13907","FARSB",42.7348967350887,-0.0021891334302618,0.256201288285215,-0.00854458400624727,0.993182491300656,0.999765644820604

"13908","CUL3",109.376405342073,0.00324607784375383,0.189800363947342,
0.0171025901965838,0.986354772537285,0.999765644820604
"13909","AGFG1",86.5610457724947,0.00407274631294913,0.201664112408997
,0.0201956920559527,0.983887264427379,0.999765644820604
"13910","DNER",4.59420089967888,-0.0150043769582901,0.756804018151206,
-0.0198259742263846,0.984182197517317,0.999765644820604
"13911","EIF4E2",139.028846310845,-0.000748571094131516,0.239465816082
652,-0.00312600397992983,0.997505813749563,0.999765644820604
"13912","COPS8",147.550424834382,-0.00468373904703914,0.25747821670981
9,-0.0181908167102065,0.985486628629437,0.999765644820604
"13913","STK25",92.5353612470998,-0.0045899506982059,0.214135927846353
,-0.0214347528897592,0.982898851131237,0.999765644820604
"13914","CAMK1",58.7838264392534,-0.00257316016633775,0.26483612255821
6,-0.00971604681975407,0.992247838220041,0.999765644820604
"13915","KAT2B",31.1227255554586,-0.0109760726512975,0.353597768569196
,-0.0310411253320722,0.975236742190318,0.999765644820604
"13916","SLC4A7",97.996015744551,-0.00959117535620693,0.30680238605406
5,-0.0312617365189486,0.975060805317034,0.999765644820604
"13917","LINC02084",1.89561294449151,0.00768801435196687,0.97461087855
8293,0.00788829113352344,0.99370611956644,0.999765644820604
"13918","RBMS3-
AS3",4.11173417500167,0.00899733873525413,0.752305704504232,0.01195968
43163423,0.990457780009517,0.999765644820604
"13919","ITGA9",14.7353206132119,-0.00635164828932915,0.45961813092396
8,-0.0138194032436459,0.988974062461571,0.999765644820604
"13920","KIAA1143",75.0085408920048,-0.0065348786615389,0.22599954205
8737,-0.0289154509191682,0.976932022723056,0.999765644820604
"13921","USP4",42.674184947396,0.000791219677702698,0.290008204818521,
0.00272826652679644,0.997823160961042,0.999765644820604
"13922","ABHD14B",108.988741390635,0.00728465816980449,0.2230504953134
77,0.0326592333254699,0.973946333628509,0.999765644820604
"13923","ACY1",16.9519656509715,0.00314841866999789,0.520895295821216,
0.00604424477482421,0.995177419776275,0.999765644820604
"13924","CACNA2D3",2.18728605276619,-0.0233126573411046,1.043572864813
44,-0.022339271293023,0.982177322728552,0.999765644820604
"13925","GXYLT2",100.755043100816,-0.00263296324788858,0.2371498311739
92,-0.0111025305599177,0.991141644270059,0.999765644820604
"13926","ZBTB11-
AS1",5.8470579634113,-0.0211913217227948,0.603689295015357,-0.03510302
71660783,0.971997587571994,0.999765644820604
"13927","PLXNA1",66.4047096121091,0.000269474051880129,0.2423143453954
87,0.00111208460002776,0.999112685050226,0.999765644820604
"13928","MBD4",100.274068719305,0.000863076269643248,0.219069146258555
,0.00393974361238715,0.996856547530048,0.999765644820604
"13929","AC083799.1",6.85848539104972,-0.00728514951905784,0.596452397
889764,-0.0122141340110838,0.990254773356634,0.999765644820604
"13930","ERICH6-
AS1",5.36838307938072,-0.0113164936667066,0.588332899423939,-0.0192348
476139734,0.984653758364033,0.999765644820604
"13931","GPR171",8.37153881298405,0.020634223204421,0.579181340858907,

0.0356265330886198,0.971580151402269,0.999765644820604
"13932","LRRC34",10.8726610919685,0.0156860525182841,0.52095711087426,0.0301100650914622,0.975979273589479,0.999765644820604
"13933","THPO",2.56526395168936,0.0044154576162438,0.938382675094921,0.00470539123689295,0.996245654833527,0.999765644820604
"13934","C3orf70",3.84320004071818,0.0258880234082057,0.81380468171845,0.0318111015944759,0.974622693325915,0.999765644820604
"13935","IL1RAP",11.9853707705501,-0.0106848231462881,0.453048694827369,-0.0235842708924687,0.981184218672129,0.999765644820604
"13936","TMEM44",21.3230581830177,-0.00734042690019885,0.364875827501125,-0.0201176026114698,0.983949558133524,0.999765644820604
"13937","FBXO45",23.6097763687419,-0.00669669861385795,0.312633779503428,-0.0214202656683313,0.982910407608229,0.999765644820604
"13938","ATP5ME",459.377737254218,0.00824545888559739,0.274230467572505,0.030067625084063,0.976013120491219,0.999765644820604
"13939","NSD2",13.551788238292,0.0135705177617565,0.414278379157197,0.032757002162083,0.973868367099548,0.999765644820604
"13940","TMEM128",60.0656215263137,-0.000387606483436007,0.253860628605381,-0.00152684756815335,0.998781752372014,0.999765644820604
"13941","ZBTB49",5.00787154659482,-0.00216892000334332,0.631471299860476,-0.00343470875687074,0.997259504300413,0.999765644820604
"13942","TADA2B",26.2725016410847,-0.00151340671782473,0.309278698807517,-0.00489334287702309,0.996095692849027,0.999765644820604
"13943","PACRGL",24.167592580654,-0.00230261925592122,0.313927733266353,-0.00733487045557571,0.99414767258424,0.999765644820604
"13944","TLR6",2.20782229506391,0.00106402208006717,0.963508241033307,0.0011043206843006,0.999118879754913,0.999765644820604
"13945","AC019131.2",1.98955752950235,0.0323467970372039,0.973575893946514,0.0332247308487497,0.97349537664135,0.999765644820604
"13946","LAMTOR3",72.6560361409115,0.000638571632759035,0.207550028677066,0.00307671185029133,0.997545142989624,0.999765644820604
"13947","PPP3CA",168.158223471683,-0.000635277783655585,0.242029701369291,-0.00262479265999784,0.997905720866048,0.999765644820604
"13948","CEP44",34.7672194814765,-0.00543587876419821,0.292376083925299,-0.018592077338265,0.985166523113273,0.999765644820604
"13949","LINC01060",4.44305378755572,0.000978724313758552,0.983146971372483,0.000995501529534535,0.999205704830523,0.999765644820604
"13950","OTULINL",8.1593909141318,0.00883403035517829,0.516139103070108,0.017115599850179,0.986344393854681,0.999765644820604
"13951","DNAJC21",132.741118377568,-0.00756156991666027,0.220387731432923,-0.0343103033344744,0.97262970883534,0.999765644820604
"13952","TMEM267",21.5418694209612,-0.00492607049095985,0.361295771227504,-0.0136344537723857,0.989121616886619,0.999765644820604
"13953","ARL15",11.3702352574781,0.00359756126368574,0.455606826347422,0.00789619701822119,0.993699811779551,0.999765644820604
"13954","SREK1",180.055367701335,0.00431650799158861,0.165650370780503,0.0260579434338137,0.979211121939418,0.999765644820604
"13955","TAF9",119.285148625214,-0.00418057425011118,0.279068902796194,-0.0149804374769921,0.98804778726437,0.999765644820604
"13956","POC5",13.9536977212754,0.0013644717598134,0.444260444959636,0

.0030713329878769,0.997549434680621,0.999765644820604
"13957","CCNH",91.1488568523249,-0.00512381627068225,0.24577690391103,-0.0208474278467477,0.983367363996891,0.999765644820604
"13958","CDC42SE2",54.24304658613,-0.00721930322178918,0.268836396745328,-0.0268538907275569,0.978576270109493,0.999765644820604
"13959","CDC23",24.3413166570254,0.00986154613818878,0.3244579444302,0.0303939117764776,0.975752900312799,0.999765644820604
"13960","PCDHB9",4.23548392377421,-0.00428872495167225,0.809838342443248,-0.00529577907948067,0.99577459938549,0.999765644820604
"13961","PCDHB15",4.8097903316598,0.00551907773135503,0.739868984363551,0.00745953384720222,0.994048208310138,0.999765644820604
"13962","PCDHGC3",23.6603339109306,0.0018671429538338,0.343006934284946,0.00544345541505209,0.995656772416101,0.999765644820604
"13963","G3BP1",231.441627818789,-0.00181814765870386,0.215229024140054,-0.00844750221754828,0.993259948565374,0.999765644820604
"13964","STC2",4.05468824295689,-0.0029212919416203,0.863436288035737,-0.00338333236869862,0.997300496489127,0.999765644820604
"13965","SQSTM1",810.236671629322,-0.000245634815339094,0.189203141423021,-0.0012982597090706,0.998964138913207,0.999765644820604
"13966","AL512308.1",3.25413010613132,0.0277722402703856,0.853678546726263,0.0325324331704108,0.974047451781769,0.999765644820604
"13967","TXNDC5",196.834571225653,-0.00498459481317047,0.20155057535583,-0.0247312358417749,0.980269340096599,0.999765644820604
"13968","LINC00240",2.00763116286815,0.0342370614776801,1.12570205404718,0.0304139637611829,0.975736908536892,0.999765644820604
"13969","PGBD1",6.33772105439273,0.0117608803397519,0.601778252265395,0.0195435449777025,0.984407499798219,0.999765644820604
"13970","HCG18",62.479557403588,-0.00349559235647626,0.289465807657172,-0.012076011273208,0.990364971229047,0.999765644820604
"13971","NFKBIL1",53.8091260818623,-0.00872092273140617,0.308340239878065,-0.0282834401856044,0.977436088134405,0.999765644820604
"13972","C2",172.570966766456,0.00908811922343393,0.297300291335789,0.0305688204427935,0.975613408205639,0.999765644820604
"13973","RXRB",37.5062284022514,0.00241930731738245,0.263219069272292,0.0091912311827216,0.99266656179794,0.999765644820604
"13974","ETV7",34.380596457428,-0.0109395425587788,0.435879100429685,-0.0250976533355115,0.979977071960986,0.999765644820604
"13975","RNF8",54.7238133495503,0.00833343821652553,0.301333617256525,0.0276551892629732,0.977937163807928,0.999765644820604
"13976","BYSL",27.8620040344924,-0.00022448488111208,0.368667040044926,-0.000608909549073668,0.999514160501891,0.999765644820604
"13977","KLHDC3",104.875549447534,-0.00754776442763022,0.259801624176083,-0.0290520294150072,0.976823094612504,0.999765644820604
"13978","CYP39A1",2.88568250923257,0.0178345139188841,0.859999265198972,0.0207378246012313,0.983454795832368,0.999765644820604
"13979","TNFRSF21",12.3918249252337,0.016792944168401,0.62268044930619,0.0269687994654596,0.978484619395342,0.999765644820604
"13980","TMEM14A",58.981688587032,-0.00606712258345615,0.301708843400202,-0.0201091970493169,0.98395626344534,0.999765644820604
"13981","CD109",95.8321608214355,0.00812307346613541,0.337746197524629

,0.0240508213731794,0.98081207082026,0.999765644820604
"13982","MRAP2",2.32345290526075,0.0695196841175749,2.4764436404276,0.0280723869433876,0.977604417418009,0.999765644820604
"13983","CASP8AP2",58.1418406309976,0.0046892359455169,0.293801217119443,0.0159605735860873,0.987265845405962,0.999765644820604
"13984","BACH2",11.5071409356803,0.00539413099088772,0.497946535378273,0.010832751324979,0.991356884009898,0.999765644820604
"13985","USP45",16.591639385433,-0.00109292491572322,0.37984095476765,-0.00287732247406488,0.997704231989264,0.999765644820604
"13986","Z95118.2",2.09564621519755,0.00547263579738897,1.0922899518128,0.00501024090563719,0.996002422860369,0.999765644820604
"13987","RPF2",116.726518026438,-0.00494170255591882,0.305121729303831,-0.0161958395004966,0.987078154627025,0.999765644820604
"13988","TPD52L1",2.82528295986282,-0.0023408603697023б,0.919336605354734,-0.00254624949780948,0.997968389033033,0.999765644820604
"13989","SLC2A12",4.01293300780894,0.0245973256369385,0.77778718052349,0.0316247506424358,0.974771305100703,0.999765644820604
"13990","REPS1",21.6220877180773,0.000184628550190266,0.331339530328434,0.000557218602945615,0.999555403902725,0.999765644820604
"13991","RGS17",4.59549118017064,0.00924522697369582,0.813919007374117,0.011358902900576,0.990937101637977,0.999765644820604
"13992","AC073332.1",16.3028713642666,0.0131212818347176,0.482094385091794,0.0272172467476865,0.978286459886238,0.999765644820604
"13993","SP4",15.9017265837867,0.00573001158139342,0.412739929214224,0.0138828622476682,0.988923434358858,0.999765644820604
"13994","OSBPL3",5.65845055674494,-0.0224476250649279,0.617407497236248,-0.0363578757391383,0.970997002245121,0.999765644820604
"13995","HOXA4",4.85832469640476,-0.0246896770944797,0.654664190117981,-0.03771349871761,0.969916113233746,0.999765644820604
"13996","TRG-AS1",6.08225508665568,-0.00035876083239298,0.613097482136906,-0.000585161157639314,0.999533108973383,0.999765644820604
"13997","ZNF713",5.74740553993183,0.0118932310622105,0.633471962991101,0.018774676318828,0.985020855632254,0.999765644820604
"13998","BET1",57.4752346070987,0.00807402541206261,0.279051406584588,0.0289338280386527,0.976917366035577,0.999765644820604
"13999","TECPR1",12.0616674149606,0.00371807019810131,0.45151219547393,0.00823470602870125,0.99342972945246,0.999765644820604
"14000","TRIP6",137.846802358135,0.000749628826801663,0.249620686397426,0.003003071731035,0.99760389903234,0.999765644820604
"14001","ALKBH4",27.1387448307124,0.0105314834766334,0.319495007262152,0.0329629047003919,0.973704169315883,0.999765644820604
"14002","BMT2",14.278704071586,-0.000389809634853243,0.393190816792745,-0.000991400658929215,0.99920897685025,0.999765644820604
"14003","MDFIC",117.684475277063,0.00877780501591694,0.264811088349166,0.033147422453636,0.973557025859243,0.999765644820604
"14004","ST7",23.0627703279771,0.012272327351638,0.346883193835193,0.0353788467407525,0.971777652006491,0.999765644820604
"14005","FMC1",70.8606685297081,-0.0025106247452504,0.313844325268507,-0.00799958623786634,0.993617321722927,0.999765644820604

"14006","AC093673.1",18.3257902594989,0.0151460111182768,0.486715708209242,0.0311188048029168,0.975174792867497,0.999765644820604
"14007","NOM1",40.6681645900636,-0.00262517342630863,0.333405860497172,-0.00787380708423658,0.993717675806854,0.999765644820604
"14008","TDRP",3.11435491971389,-0.0131228632783203,0.780441782634681,-0.0168146600685819,0.986584474507275,0.999765644820604
"14009","PDLIM2",376.937856107651,0.000233917559420251,0.304861880569098,0.000767290285632259,0.999387790987511,0.999765644820604
"14010","LOXL2",45.6367559136764,0.0133379281343548,0.441056531321892,0.0302408584549914,0.975874963085358,0.999765644820604
"14011","MAK16",34.4819591715134,0.0130107417805266,0.353458825617414,0.0368097804823511,0.970636675637648,0.999765644820604
"14012","TM2D2",107.663010570311,0.00203692045916218,0.257464385768983,0.00791146493165804,0.993687630127648,0.999765644820604
"14013","VDAC3",173.576005298187,0.00259538704314214,0.269701280989466,0.00962319138277849,0.992321922675978,0.999765644820604
"14014","TGS1",100.886760775842,-0.00424203002085618,0.268102247693936,-0.0158224336324806,0.987376051225526,0.999765644820604
"14015","SGK3",30.0059840488849,-0.00568799017344482,0.348778333182567,-0.0163083243203283,0.986988416578049,0.999765644820604
"14016","MYC",940.378758179256,0.00486121221706215,0.334508751280863,0.0145323917489397,0.98840523711054,0.999765644820604
"14017","ADCK5",8.52008355833893,-0.0126384791876032,0.497521867659347,-0.0254028616813617,0.979733628554096,0.999765644820604
"14018","ACO1",117.012114502864,0.000615888873559171,0.229471903364179,0.00268394023202804,0.997858528097776,0.999765644820604
"14019","CHMP5",212.980190639143,-0.00316700782481707,0.22430250779503,-0.0141193598589237,0.988734755061023,0.999765644820604
"14020","GRHPR",173.483638435502,-0.00835575332221732,0.24680825373408,-0.0338552426663175,0.972992583888086,0.999765644820604
"14021","POLR1E",61.4818645889336,0.00516382665236314,0.289697149935903,0.0178249135468045,0.985778529779478,0.999765644820604
"14022","ZNF484",12.6809113050248,-0.0120755704792796,0.437830245625544,-0.0275804849023777,0.977996746536312,0.999765644820604
"14023","XPA",100.096917088768,0.000167317933640134,0.226476598233924,0.000738786854557549,0.999410533428647,0.999765644820604
"14024","FSD1L",9.30286642981677,0.00328723520480006,0.499404083789798,0.00658231542652678,0.994748110071531,0.999765644820604
"14025","PAPPA",21.1868363575899,-0.0179719124863854,0.516134633830547,-0.034820202537088,0.97222311110366,0.999765644820604
"14026","TOR1A",59.7309690907389,0.00352527176369038,0.282387084465342,0.0124838279001498,0.99003960517397,0.999765644820604
"14027","PNPLA7",13.1586794780221,-0.0177017402969924,0.495588442032318,-0.0357186302093744,0.971506715271372,0.999765644820604
"14028","FBH1",83.0003967895602,-0.000243893310844854,0.229952298976644,-0.00106062566858541,0.999153743312907,0.999765644820604
"14029","NSUN6",28.4347221478867,-0.00240898494144283,0.327688239797752,-0.0073514537565634,0.994134441381146,0.999765644820604
"14030","AL512598.1",2.29409585605705,-0.00659579422755462,0.932712216119474,-0.00707162843325485,0.994357703880001,0.999765644820604

"14031","RAB18",123.989320188135,-0.000868550348008666,0.217314882302592,-0.00399673661925872,0.996811074047833,0.999765644820604
"14032","ASAH2B",2.41112420084914,-0.0320921564173902,1.04198820918614,-0.0307989631115463,0.975429867346176,0.999765644820604
"14033","AL157394.3",6.36284660690835,0.00945395474230521,0.561275744463413,0.0168436901746811,0.986561315113668,0.999765644820604
"14034","FAS",61.0204652574383,-0.00131322561491992,0.21488096699803,-0.0061114096481703,0.995123830950608,0.999765644820604
"14035","PIK3AP1",3.64343453926267,-0.0155347319392011,0.875026918452637,-0.0177534331934292,0.985835553826348,0.999765644820604
"14036","SLC25A28",97.2615022168799,-0.00516254559667991,0.219673382347901,-0.023501006546637,0.981250635599579,0.999765644820604
"14037","FBXW4",34.4030021814154,0.00489763908306159,0.30204156657039,0.0162151161466719,0.987062776108124,0.999765644820604
"14038","PPRC1",40.1328409544976,-0.00234770674672382,0.319435165513962,-0.00734955634250984,0.994135955257627,0.999765644820604
"14039","ACTR1A",71.8022041125944,-0.00147301973050621,0.258657306244688,-0.00569486998798613,0.995456175721359,0.999765644820604
"14040","CNNM2",8.35922058359588,0.0161277360399885,0.629459475480549,0.0256215636879182,0.979559186379084,0.999765644820604
"14041","PDZD8",75.0152255883705,0.00375375903639302,0.241231807628753,0.0155607963696476,0.987584781858358,0.999765644820604
"14042","CASC2",3.5851792202535,0.0240618473542092,0.733542571046979,0.0328022507539896,0.973832283337951,0.999765644820604
"14043","DHX32",33.9158943884368,0.00265972805123857,0.286994873530094,0.00926751066499339,0.99260570216885,0.999765644820604
"14044","LINC01001",3.09550409752585,0.0283230533447366,0.862673642705005,0.032831712878032,0.973808788618826,0.999765644820604
"14045","RNH1",623.034279945717,0.000500516896258557,0.234634481940469,0.0021331770680898 8,0.998297972242743,0.999765644820604
"14046","TIMM10B",46.3228263795396,-0.00738126786141991,0.303772426284086,-0.0242986763206644,0.9806143689636,0.999765644820604
"14047","TMEM41B",40.7022448045444,0.00540823288888551,0.29988287996428,0.0180344836275072,0.985611343923106,0.999765644820604
"14048","CYP2R1",6.89561662905887,0.00860892322810052,0.554364974993406,0.0155293418892543,0.987609875870474,0.999765644820604
"14049","HTATIP2",57.5913871178442,-0.0126571186538825,0.363544762584218,-0.034815846510649,0.97222658460381,0.999765644820604
"14050","CYB561A3",50.1068811780689,-0.00542968194419985,0.257297458240036,-0.0211027422553644,0.983163697379577,0.999765644820604
"14051","PCNX3",17.7616949617586,-0.00965192337423242,0.349776200756964,-0.0275945686222914,0.977985513628972,0.999765644820604
"14052","KAT5",51.939673876175,-0.00536300773479474,0.298517472605174,-0.0179654734712564,0.985666397141983,0.999765644820604
"14053","CTSW",21.3297836615974,-0.0113135385134388,0.416485948090209,-0.0271642742457864,0.978328710206137,0.999765644820604
"14054","C11orf80",3.78689588798028,0.0275731289337895,0.896876663322254,0.030743501376936,0.975474098462443,0.999765644820604
"14055","CDK2AP2",45.0403527398284,-0.0063067644353989,0.358614398045774,-0.0175864785958591,0.985968743527593,0.999765644820604

"14056","RELT",6.41064049258671,-0.0140400122015709,0.776529939181601,-0.0180804518836297,0.985574672540507,0.999765644820604
"14057","TMEM126B",120.554730567885,-0.000392198059068837,0.256952762515638,-0.00152634303375107,0.998782154931755,0.999765644820604
"14058","CHORDC1",70.659891824559,0.00152922076131237,0.25049151833117,0.00610488040273929,0.995129040437549,0.999765644820604
"14059","AP002784.1",3.68818270526959,0.00215239195048984,0.860019597984963,0.00250272430480994,0.998003117001869,0.999765644820604
"14060","CWC15",194.714585606412,0.00861025491443213,0.238908179792703,0.0360400172229479,0.971250450537509,0.999765644820604
"14061","CFAP300",9.96950523029976,0.000581862997841197,0.47668387381273,0.00122064753981962,0.999026064415653,0.999765644820604
"14062","ZC3H12C",17.5583057533408,-0.00725533252002502,0.390001298068142,-0.0186033547989816,0.985157526557459,0.999765644820604
"14063","FDX1",119.3277183275,-0.00805880625058717,0.275885294024471,-0.0292107133839195,0.976696536835983,0.999765644820604
"14064","PCSK7",129.352502555837,0.00301507531045818,0.255252516959396,0.0118121276388346,0.990575504888166,0.999765644820604
"14065","DDX6",166.772765040373,-0.00488838766579709,0.194213092104005,-0.02517022726346,0.979919184631405,0.999765644820604
"14066","HYOU1",50.2727851981564,0.00607492905963501,0.357994098750242,0.0169693553073712,0.986461063173084,0.999765644820604
"14067","JHY",12.1521216358089,0.0180202822000415,0.46392097570684,0.0388434305488891,0.969015218374505,0.999765644820604
"14068","CLMP",239.387945720055,-0.00426740185509918,0.233440753464512,-0.0182804492864521,0.98541512407072,0.999765644820604
"14069","CCDC15",4.77757706866886,0.0170807194504808,0.794336559753042,0.0215031264024801,0.982844309532059,0.999765644820604
"14070","AP003486.1",7.81062426150727,-0.011847085653602,0.575060006316705,-0.0206014772779684,0.983563562019608,0.999765644820604
"14071","AC004803.1",2.03954386976506,-0.0115825860027088,0.967284195990953,-0.0119743360335199,0.990446090467647,0.999765644820604
"14072","TNFRSF1A",482.87988301311,-0.00857363223836747,0.22486540881613,-0.038127839597499,0.969585754654587,0.999765644820604
"14073","COPS7A",70.6148096831609,-0.00152275497299798,0.250882483028323,-0.00606959463497525,0.995157193885252,0.999765644820604
"14074","NECAP1",57.0299363633803,-0.00428558223861065,0.253566558345398,-0.0169012123151232,0.986515425618277,0.999765644820604
"14075","A2M-AS1",10.0365705193429,-0.0110492963377783,0.568420244922298,-0.0194386045122104,0.984491214272242,0.999765644820604
"14076","PLBD1",194.377516516097,0.00334690154136701,0.202200345353285,0.016552402695056,0.986793696496495,0.999765644820604
"14077","C12orf60",1.94645729182196,0.0305905472183948,1.04416700621896,0.0292966039304063,0.976628035412271,0.999765644820604
"14078","PLEKHA5",170.007748543683,0.00720070695045945,0.2262159406982,0.0318311208672341,0.974606728342246,0.999765644820604
"14079","AC008124.1",34.9782876929565,-0.000279614242218009,0.286244347406789,-0.000976837603086855,0.999220596482038,0.999765644820604
"14080","AC004241.1",2.93417541020654,-0.0182250233902713,0.8281964926

92524,-0.0220056756471167,0.98244342812352,0.999765644820604
"14081","WNT10B",16.7550740288897,0.00686135156600684,0.513283560663059,0.013367565400192,0.989334543590886,0.999765644820604
"14082","NCKAP1L",9.40349282094239,0.0103866699359158,0.557506900258093,0.0186305674981017,0.985135817727345,0.999765644820604
"14083","NEMP1",8.40709957934811,0.0063874350568365,0.55027224304469,0.01160777258452,0.990738545451959,0.999765644820604
"14084","OS9",277.960249891212,0.0024101308077001 3,0.156577289189043,0.0153925950575774,0.987718971016208,0.999765644820604
"14085","AVPR1A",3.24764660226316,-0.00480486139315101,0.954991753450042,-0.00503131192054044,0.995985610834799,0.999765644820604
"14086","TBC1D15",82.9400112100294,-0.00176968420017322,0.26076838164713,-0.00678642168576994,0.994585260476965,0.999765644820604
"14087","TRHDE",6.94741533049757,-0.0172360433672344,0.750608611196709,-0.0229627573013779,0.981679980478337,0.999765644820604
"14088","ACSS3",23.2349461488319,-0.00454968797676348,0.339153333959421,-0.0134148407849879,0.989296826673187,0.999765644820604
"14089","AC008149.2",5.31589598029741,-0.00737410619594409,0.725925513588015,-0.0101582132848538,0.99189505784546,0.999765644820604
"14090","ACTR6",47.1427978874482,0.00176020381896101,0.299666320804593,0.00587387936767442,0.995313349290692,0.999765644820604
"14091","UTP20",43.5981631501662,-0.00533346865800421,0.293246342677686,-0.0181876732350805,0.985489136344835,0.999765644820604
"14092","PWP1",104.941811532367,0.00615200615164027,0.231301871489016,0.0265973038265383,0.978780923735008,0.999765644820604
"14093","SART3",42.3959739814444,0.00796678329191153,0.293221284457144,0.0271698669714951,0.978324249503055,0.999765644820604
"14094","CORO1C",47.0664219702918,-0.00784669304939317,0.292921177131739,-0.0267877287884317,0.978629040715389,0.999765644820604
"14095","PTPN11",74.2496794090823,-0.00125029107831857,0.245907875915785,-0.00508438810128532,0.995943262711316,0.999765644820604
"14096","DDX55",37.1723998330256,0.0104958594632494,0.274472614157171,0.0382400972697376,0.969496251263576,0.999765644820604
"14097","NUP58",47.9276677309255,0.00242916751289533,0.274192262111745,0.00885935837206574,0.992931347204105,0.999765644820604
"14098","PHF11",97.9976804376308,-0.000407629595574208,0.225063149700152,-0.00181117875635032,0.998554889223537,0.999765644820604
"14099","DNAJC3-DT",4.38666720774724,0.00712410478747029,0.696835047686919,0.0102235167578297,0.991842955918175,0.999765644820604
"14100","CLYBL",8.81242943244459,0.00566587340503813,0.593718602698801,0.00954302826167715,0.992385880655743,0.999765644820604
"14101","LIG4",11.9749673782304,-0.0023075684092451,0.433936442023916,-0.00531775667072903,0.995757064051664,0.999765644820604
"14102","ABHD13",40.8778943374512,0.000155953848683874,0.263048166847998,0.000592871832381907,0.99952695674612,0.999765644820604
"14103","LINC00346",3.73122555851627,-0.0364397472411907,0.98467728779338,-0.0370067916594793,0.970479590490199,0.999765644820604
"14104","ANKRD10-IT1",4.19820917752792,-0.0127121864414918,0.787504079723026,-0.0161423

753461225,0.987120807274432,0.999765644820604
"14105","DCUN1D2",9.39791342965774,-0.00994676222636874,0.496486447785
367,-0.0200343076245835,0.984016004525775,0.999765644820604
"14106","TRAC",114.259344940796,-0.00316794221487345,0.482879802721004
,-0.00656051919550632,0.99476550057225,0.999765644820604
"14107","SLC7A7",9.87809585031762,-0.0144997166184934,0.51300888030430
4,-0.0282640655457904,0.977451540682725,0.999765644820604
"14108","BCL2L2",21.4594592843729,0.0036474593883048,0.328924576629994
,0.0110890448675953,0.991152403633407,0.999765644820604
"14109","SLC25A21-
AS1",9.79185297986579,-0.0141540874283153,0.476991654605384,-0.0296736
584207642,0.976327320202864,0.999765644820604
"14110","FBXO33",40.988135023011,0.00794118382782487,0.329272858124763
,0.0241173350061423,0.980759016008709,0.999765644820604
"14111","CNIH1",200.195763493142,-0.00407559167939544,0.23951560858495
6,-0.0170159752989535,0.986423871171575,0.999765644820604
"14112","PPP2R5E",126.970755890355,0.00474338264517976,0.1933904252688
48,0.0245274947742919,0.980431852650474,0.999765644820604
"14113","MAX",101.108586567086,-0.00063502539987397,0.195699118626205,
-0.00324490679534894,0.997410943510291,0.999765644820604
"14114","TMEM229B",3.14287471821964,-0.0164421056285347,0.974835598607
657,-0.0168665420631117,0.986543084534495,0.999765644820604
"14115","LIN52",12.2162135827802,0.0125473711526513,0.438969044384763,
0.0285837266047702,0.977196591070139,0.999765644820604
"14116","TMED10",360.732633504307,-0.000403739510357528,0.191343505854
366,-0.0021100246311198,0.998316445173171,0.999765644820604
"14117","WDR20",39.7577026088053,0.00374656098217223,0.308927385739027
,0.0121276427896141,0.990323778255813,0.999765644820604
"14118","MOK",29.3018616130502,-0.0126683114961683,0.353727146579119,-
0.0358137949509474,0.971430833343806,0.999765644820604
"14119","CINP",71.3023087955737,-0.0096614774018781,0.25999675875465,-
0.0371599917174172,0.970357438548586,0.999765644820604
"14120","EIF5",524.781227271405,0.00766863067540824,0.207309304168689,
0.0369912518213279,0.970491981003377,0.999765644820604
"14121","XRCC3",3.77008297012673,0.024470970891654,0.849964386040677,0
.0287905838098055,0.977031610799707,0.999765644820604
"14122","CEP170B",12.6216191363503,0.0076666258054077,0.42501311881965
3,0.0180385627311916,0.985608089798607,0.999765644820604
"14123","AHNAK2",170.323224755898,-0.0125710069673052,0.33302378383639
1,-0.0377480755953489,0.969888544507413,0.999765644820604
"14124","PACS2",50.2465367952308,-0.00686634158862695,0.25247695300420
1,-0.0271959143475274,0.978303474380756,0.999765644820604
"14125","PGBD4",2.87621747302549,-0.0362557430161001,0.968921249539299
,-0.037418668476245,0.970151187802139,0.999765644820604
"14126","EMC4",174.218524638106,-0.00612160002369057,0.233710337057115
,-0.0261931076766817,0.979103313275251,0.999765644820604
"14127","AQR",51.6131193765033,0.000963653047007773,0.276262594819719,
0.0034881777883706,0.997216842441283,0.999765644820604
"14128","DPH6",32.1610792550356,-0.00579175539519988,0.299660848247666
,-0.0193277013966572,0.984579685534661,0.999765644820604

"14129","AC037198.1",29.1851265330491,0.0353530843099793,1.05474103941972,0.0335182599222925,0.973261304700451,0.999765644820604
"14130","ZFYVE19",22.5399148534972,0.00525483824199883,0.342420979043033,0.0153461340385294,0.987756037167889,0.999765644820604
"14131","MAPKBP1",16.1920460472153,-0.00577461441074991,0.386585718949972,-0.014937474737646,0.988082062735649,0.999765644820604
"14132","SPATA5L1",15.7854376871282,-0.0153931163478903,0.455605860653957,-0.0337860367419236,0.973047770655341,0.999765644820604
"14133","LYSMD2",44.6771097713314,-0.00924556214509214,0.318803438145591,-0.0290008231996227,0.976863934053251,0.999765644820604
"14134","BNIP2",124.538330470203,0.00499073879194577,0.191803095811,0.0260201159467393,0.979241293676905,0.999765644820604
"14135","USP3",63.158193020033,-0.0028361387239139,0.237619981024596,-0.0119356070633653,0.990476989506842,0.999765644820604
"14136","ANKDD1A",12.5700003950198,-0.00164570660216473,0.440188598458184,-0.00373863977379021,0.997017003995193,0.999765644820604
"14137","RAB11A",144.529973708175,0.00602548160952694,0.211602083438404,0.028475530635694,0.977282883837662,0.999765644820604
"14138","MAP2K1",49.468853796844,-0.00453563948170321,0.257941425356395,-0.0175839901459657,0.985970728716369,0.999765644820604
"14139","LRRC49",14.9173049587837,-0.012689968572401,0.382458059226242,-0.0331800265840242,0.973531025828762,0.999765644820604
"14140","ARIH1",94.6391381596291,0.0100134503843481,0.311787352564677,0.0321162815039809,0.974379319345427,0.999765644820604
"14141","SYNM",7.66856029905552,0.0209333718875172,0.625983911412678,0.0334407506420991,0.973323113508668,0.999765644820604
"14142","CHSY1",37.0926983309689,0.00124574434662952,0.314920259767396,0.00395574532914981,0.996843780106785,0.999765644820604
"14143","RHOT2",42.8465806047196,0.00426599994263389,0.307730667863711,0.0138627715341106,0.988939462886562,0.999765644820604
"14144","AC009041.2",10.2977515420061,-0.00393825128617881,0.529468300602678,-0.0074381247785675,0.994065289801572,0.999765644820604
"14145","TBL3",28.9790388542751,0.0033135639854387,0.32358232486101,0.0102402502573711,0.991829605216113,0.999765644820604
"14146","AMDHD2",31.7488255248526,-0.00475646084605062,0.289894835666344,-0.0164075390826385,0.986909265241473,0.999765644820604
"14147","FLYWCH2",92.1472419315038,0.00822063469315343,0.332375228713551,0.0247329944682435,0.980267937344773,0.999765644820604
"14148","ZNF213-AS1",8.4832446367443,0.0152631080459962,0.524789124193891,0.0290842689803094,0.976797382026417,0.999765644820604
"14149","NUDT16L1",38.1227158284882,-0.00494959803266754,0.32410098101205,-0.0152717773862077,0.987815358240983,0.999765644820604
"14150","TMEM186",18.5658229951728,0.00806318341779522,0.398017365665136,0.0202583708987688,0.983837264175833,0.999765644820604
"14151","RMI2",2.27294921131296,-0.0257296357184781,0.964996787569836,-0.026662923700786,0.978728585212035,0.999765644820604
"14152","MPV17L",8.53763088643864,0.0218269987843114,0.565667110296727,0.0385862964047207,0.969220228041632,0.999765644820604
"14153","CRYM",2.66361878747791,0.0125097236752785,0.900659030688177,0

.0138895222820561,0.988918120932555,0.999765644820604
"14154","EARS2",10.56306599096,-0.018014313202224,0.51835941003172,-0.0347525536405748,0.972277054465639,0.999765644820604
"14155","ZNF768",16.7473975299323,0.0135565211683624,0.417431292241137,0.0324760539526856,0.974092412131976,0.999765644820604
"14156","RNF40",42.1135630903828,0.00996647030311771,0.276031482280536,0.03610628114147,0.971197613968554,0.999765644820604
"14157","ITGAX",5.08639846312657,0.0284730837822165,0.749673900344332,0.0379806256682253,0.96970312935841,0.999765644820604
"14158","HEATR3",11.0833848040374,-0.00894968131073392,0.432411695703814,-0.0206971305347488,0.983487258032368,0.999765644820604
"14159","CYLD",81.3878514289188,-0.00751165302281499,0.248258839863322,-0.0302573436134259,0.975861815848252,0.999765644820604
"14160","PDP2",6.65758561929188,-0.0176563488567491,0.579708300057274,-0.0304572987052362,0.975702348264867,0.999765644820604
"14161","RRAD",14.4049633673312,-0.0373541394937434,1.18599683851551,-0.0314959857232831,0.974873993487125,0.999765644820604
"14162","RIPOR1",37.9337469149043,-0.00998695314126954,0.280461148242688,-0.0356090431913502,0.971594097472285,0.999765644820604
"14163","DDX19B",48.3424694424358,-0.00503696320814093,0.29701475631235,-0.0169586294993502,0.986469619898403,0.999765644820604
"14164","DYNLRB2",4.50727203420095,0.00334292124717544,0.67921870281504,0.00492171554363949,0.99607305500899,0.999765644820604
"14165","MYO1C",158.164277729663,-0.00252659415640036,0.193508965804138,-0.0130567291592974,0.989582533381838,0.999765644820604
"14166","MNT",30.2322401903034,-0.00068946567464556,0.351382777414203,-0.00196214988030797,0.998434431909102,0.999765644820604
"14167","CHRNB1",20.4859849521555,0.00888185282692421,0.387815021795587,0.0229022918859645,0.981728212215531,0.999765644820604
"14168","NAA38",240.387256083842,-0.00256687353977937,0.273411413552159,-0.00938831889433792,0.992509315341297,0.999765644820604
"14169","TMEM107",43.4278350281029,-0.000953336714450441,0.239667120114799,-0.0039777534523459,0.996826220303229,0.999765644820604
"14170","RCVRN",2.78739676705839,0.0068534642043037,0.85822807027821,0.00798559781676918,0.993628482511661,0.999765644820604
"14171","TMEM220-
AS1",5.06090819615592,0.0203221270279734,0.644158243463433,0.0315483458205981,0.974832236924674,0.999765644820604
"14172","TTC19",70.3599795146089,0.00197061762489291,0.203848706015363,0.00966705976904456,0.99228692239592,0.999765644820604
"14173","ZNF287",13.1777574432313,0.00100302813605548,0.409622468020181,0.00244866484229588,0.998046250080186,0.999765644820604
"14174","RAI1",17.8077567444106,-0.00601905926382867,0.420973407597255,-0.0142979560114806,0.988592270332028,0.999765644820604
"14175","GID4",25.770017268864,0.00856783249591892,0.316332852671255,0.0270848646404202,0.978392046600304,0.999765644820604
"14176","SPAG5",2.25068284520366,0.011557225747851,0.967693821483474,0.0119430603888054,0.99047104303739,0.999765644820604
"14177","AC022706.1",2.38314048605676,-0.040585129555898,1.07366786323764,-0.0378004510943577,0.969846784709448,0.999765644820604

"14178","DHX8",45.5694260277752,0.0117424585285714,0.301398604240223,0
.0389598968388466,0.968922362008136,0.999765644820604
"14179","DUSP3",43.1412225891964,0.00564237922200495,0.260195712164551
,0.0216851352970668,0.982699121300393,0.999765644820604
"14180","AC008105.3",17.7797421450123,0.0102017262409891,0.38424794728
9809,0.0265498522840375,0.978818771222695,0.999765644820604
"14181","FMNL1",69.7607710966047,0.00780592437541851,0.234989740042203
,0.0332181497541834,0.973500624698253,0.999765644820604
"14182","EFCAB13",10.3790387678435,0.000557443070698445,0.509403053909
79,0.00109430649545568,0.999126869916753,0.999765644820604
"14183","OSBPL7",4.3106498294365,0.0100292429855316,0.717382748222874,
0.0139803236283231,0.9888456789741,0.999765644820604
"14184","PDK2",34.6682480637997,-0.00426641082439622,0.32349345931292,
-0.0131885535907212,0.989477361759207,0.999765644820604
"14185","RSAD1",36.176604679865,0.00429409440498746,0.283153903911506,
0.0151652311540423,0.987900359990704,0.999765644820604
"14186","SPAG9",226.037069967736,0.00618257958069535,0.222106254530472
,0.0278361345283373,0.977792845934751,0.999765644820604
"14187","DGKE",10.1728260811227,0.0079079631026549,0.486937981668166,0
.0162401854042348,0.98704277636807,0.999765644820604
"14188","TACO1",31.1810221287249,-0.00730324291282995,0.33597442164047
5,-0.0217374967926729,0.982657352717097,0.999765644820604
"14189","RPL38",2571.60380375809,-0.00234178191398084,0.22291943120628
7,-0.0105050596141786,0.99161832928548,0.999765644820604
"14190","CASKIN2",8.99365263250142,0.00448909722168209,0.5083256087633
67,0.00883114512487964,0.992953857237852,0.999765644820604
"14191","SAP30BP",81.7652824698405,0.0094141136138187,0.2621087300524
59,0.0359168172670086,0.971348686279146,0.999765644820604
"14192","C1QTNF1",95.0073818729487,0.0147605746085962,0.44538248202196
4,0.0331413452580928,0.973561872097091,0.999765644820604
"14193","CHMP6",42.6288462714518,-0.00747905454819158,0.32835788729768
1,-0.022777142981832,0.981828040552618,0.999765644820604
"14194","AATK",2.74335724000431,-0.013048328514107,0.919293862749645,-
0.0141938601385621,0.988675318394276,0.999765644820604
"14195","SLC16A3",6.78064561160634,-0.0103251403025262,0.6035602687620
14,-0.0171070576327109,0.986351208560377,0.999765644820604
"14196","AC132938.3",2.59649330169683,0.0171906636144206,0.89040234673
5521,0.0193066243338719,0.98459649946027,0.999765644820604
"14197","NARF",53.6756040807056,0.00432373281403707,0.27337204359971,0
.0158162947355662,0.987380948743749,0.999765644820604
"14198","RALBP1",195.913393450929,-0.00694155955184069,0.1898570844185
24,-0.0365620254471969,0.970834222575002,0.999765644820604
"14199","C18orf54",4.22969055736113,-0.0255861603996125,0.690670629032
914,-0.0370453865041845,0.970448817360638,0.999765644820604
"14200","ZNF236-
DT",2.70544156388299,0.0174470314158778,0.875931103755395,0.0199182690
751325,0.984108571421438,0.999765644820604
"14201","RAB11B-
AS1",28.2151683048434,-0.0068480946204449,0.291893978094637,-0.0234608
972242128,0.981282629387498,0.999765644820604

"14202","HNRNPM",309.123657557111,-0.00294702157800381,0.2066238594325
66,-0.0142627360949359,0.988620368894478,0.999765644820604
"14203","AC011511.5",2.52146572944644,-0.00889976643740084,0.999777285
190797,-0.00890174898872843,0.992897525719229,0.999765644820604
"14204","TRMT1",27.4176725934701,-0.014267674199641З,0.373992731412157
,-0.0381496029234795,0.969568402657165,0.999765644820604
"14205","ADGRL1",11.4468428533469,0.0114700005152434,0.473049376485796
,0.0242469414090599,0.980655635292602,0.999765644820604
"14206","AKAP8",39.1906418569629,-0.00768783396055653,0.29925713538201
,-0.0256897264980593,0.979504818221098,0.999765644820604
"14207","MVB12A",125.825978686485,-0.00447352131026915,0.2701952321498
71,-0.01655662564685,0.986790327530125,0.999765644820604
"14208","SUGP1",20.7615890885533,0.00119759259924717,0.348114430119922
,0.00344022682091807,0.997255101548316,0.999765644820604
"14209","CEBPA-
DT",5.134429666699,0.00766748942472383,0.735056253642445,0.01043116004
62263,0.991677289379235,0.999765644820604
"14210","ZNF570",17.0947404807371,0.0124314541691261,0.412877859696379
,0.0301092777856092,0.975979901483977,0.999765644820604
"14211","DYRK1B",17.3172310871248,-0.00684973167558461,0.4033968509756
36,-0.0169801317462399,0.986452466057575,0.999765644820604
"14212","SNRPA",71.4468978054358,-0.00520556833257745,0.27922647682658
8,-0.0186428178005854,0.985126045097421,0.999765644820604
"14213","ZNF112",2.74315124663203,0.0263200950230071,0.989288981797824
,0.0266050623298926,0.97877473553481,0.999765644820604
"14214","ZNF180",10.448448660173,-0.0169368429372З,0.502737028739459,-
0.0336892688801872,0.973124936309573,0.999765644820604
"14215","CD3EAP",17.3953139902429,-0.0105275642449004,0.38836222539133
6,-0.027107590688801З,0.978373920492539,0.999765644820604
"14216","KPTN",11.8777164942261,-0.0141471410769123,0.417975525057162,
-0.0338468169278036,0.97299930280408,0.999765644820604
"14217","NAPA",100.23985730281,-0.00587802560996424,0.231713169622162,
-0.0253676803072917,0.97976169018621,0.999765644820604
"14218","ZSWIM9",9.91440527556105,-0.000795675682276426,0.469963624803
199,-0.00169305801615949,0.998649135793726,0.999765644820604
"14219","MAMSTR",4.19206532776911,-0.0136628121823377,0.71717787360603
5,-0.0190507999272759,0.984800580267754,0.999765644820604
"14220","SLC17A7",25.2415878804423,-0.00541898886108374,0.308671247053
45,-0.0175558589042969,0.985993170735554,0.999765644820604
"14221","BCL2L12",21.0268350475865,0.00381934385928293,0.3166459313848
27,0.012061875681078,0.99037624897838,0.999765644820604
"14222","JOSD2",114.923864297961,-0.00598640642423177,0.25231410596937
4,-0.0237260077126183,0.981071160687835,0.999765644820604
"14223","LILRB3",3.73588527936632,0.0283720811591772,0.85834992693038,
0.0330542128204531,0.973631355655049,0.999765644820604
"14224","PPP1R12C",56.8170476026545,-0.00501785868382564,0.24361975272
2268,-0.0205970929194158,0.983567059489486,0.999765644820604
"14225","TBC1D20",58.1658903446723,-0.00247923034663241,0.229710892457
785,-0.0107928288471911,0.991388735676506,0.999765644820604
"14226","FASTKD5",15.3042293315712,-0.0117763748324492,0.3905846128275

11,-0.0301506368804391,0.975946916676205,0.999765644820604
"14227","BTBD3",11.5363845406031,0.0163852094230375,0.463289433148757,
0.0353671123290575,0.971787008856713,0.999765644820604
"14228","MGME1",20.1041657294281,0.00663219328597462,0.321860842581264
,0.020605778673745,0.983560130730713,0.999765644820604
"14229","PET117",30.5287503039835,-0.00106584185393814,0.3399245704881
94,-0.00313552460302413,0.997498217428615,0.999765644820604
"14230","NECAB3",25.5406304847684,-0.00871504154663414,0.3643920889360
04,-0.0239166595852324,0.980919085656479,0.999765644820604
"14231","ZSWIM3",3.38664006471735,0.022592585123109,0.818607588161874,
0.0275987975799724,0.977982140693549,0.999765644820604
"14232","RNF114",107.068261408309,-0.00302287757648931,0.2532400221139
86,-0.0119368082156014,0.990476031194288,0.999765644820604
"14233","PARD6B",1.90466669264867,-0.00205703897186945,1.0698067492042
3,-0.00192281360479317,0.9984658176568,0.999765644820604
"14234","ADNP",76.3047507608766,-0.00750178906274566,0.210466157975394
,-0.0356436832168652,0.971566476265245,0.999765644820604
"14235","VAPB",54.6878770022422,0.00100497496957612,0.225064865618728,
0.00446526812087412,0.996437243345921,0.999765644820604
"14236","ZBTB46",4.66256633867198,-0.0112220570403057,0.66398895861633
7,-0.0169009693530009,0.986515619446319,0.999765644820604
"14237","AP000254.2",4.70017277080024,0.000759546043122811,0.686478090
638909,0.00110643887034457,0.999117189688004,0.999765644820604
"14238","SMIM11A",4.02578374588136,0.0243829498687213,0.71086020304194
6,0.0343006258676187,0.972637425794487,0.999765644820604
"14239","SLC37A1",9.61314434391243,0.00398576667428282,0.4775523271505
57,0.008346240710552,0.993340740710101,0.999765644820604
"14240","ITGB2-
AS1",4.82908891551712,0.00738508308484542,0.704385336195252,0.01048443
61535634,0.991634783520137,0.999765644820604
"14241","IGLC1",19.8759169250771,0.016024767625792,1.4195478013703,0.0
11288642489054,0.990993157741463,0.999765644820604
"14242","CHCHD10",77.8796705816033,0.0113205967920905,0.33826184523410
4,0.0334669633941592,0.973302210458712,0.999765644820604
"14243","TPST2",24.8581022761358,0.00424416987888283,0.351446684872856
,0.0120762837197291,0.990364753864024,0.999765644820604
"14244","HSCB",49.5358759236742,-0.011428779294561,0.312267914268387,-
0.036599275085106,0.970804521542799,0.999765644820604
"14245","CCDC117",36.1686489952812,0.00235276184109749,0.2814350924207
96,0.00835987374872103,0.993329863498907,0.999765644820604
"14246","AC004832.6",2.15913343037349,0.0373165121589719,1.11012379917
399,0.0336147303451542,0.973184375789986,0.999765644820604
"14247","SLC16A8",3.50159666340737,-0.00323390724033088,0.763199661232
464,-0.004237301724045,0.996619132492016,0.999765644820604
"14248","SLC25A17",21.304772693864,-0.00981241695669743,0.385973395034
261,-0.0254225215596179,0.9797179473051,0.999765644820604
"14249","ACO2",60.1990253866583,0.00290355587241694,0.236971010194075,
0.0122527893603483,0.990223933158087,0.999765644820604
"14250","SCO2",71.327022079831,-0.00203829465522171,0.245825129009805,
-0.0082916448104067,0.993384300428608,0.999765644820604

"14251","MBTPS2",24.4994514146879,-0.00957483273582505,0.338740174970902,-0.0282660087090276,0.97744999088198,0.999765644820604
"14252","RBM10",43.0796707362748,0.00124180157180666,0.244575769774597,0.00507736957324561,0.995948862614197,0.999765644820604
"14253","CFP",3.52987250398403,0.0228346988903347,0.762650273722874,0.0299412452563181,0.976113911624872,0.999765644820604
"14254","TIMM17B",42.0592626659821,0.00783304743208043,0.310470623533821,0.0252295928771732,0.979871832762233,0.999765644820604
"14255","CYSLTR1",5.46502112686453,0.0143068341504858,0.666062994232004,0.0214797012810809,0.982862995758854,0.999765644820604
"14256","HMGN5",12.2077478724878,0.0134813573300225,0.421089150298526,0.0320154468963759,0.974459732370241,0.999765644820604
"14257","PABPC5",20.2389242948657,0.0107536399031225,0.357184852366188,0.0301066515891827,0.975981995936045,0.999765644820604
"14258","SLC25A43",36.9323650973406,0.00587118672933702,0.275914703899342,0.0212789918274124,0.983023102138265,0.999765644820604
"14259","NKRF",15.9201282135208,-0.00432774905686599,0.389040858185413,-0.011124150499389,0.991124395119373,0.999765644820604
"14260","MTMR1",12.7970678567998,0.0105085600485751,0.429975800941967,0.0244398871414473,0.980501732480196,0.999765644820604
"14261","SLC6A8",13.4635157108693,0.00495033837693491,0.490000924715456,0.0101027123159198,0.991939338939416,0.999765644820604
"14262","IKBKG",32.9228446093611,0.00732119762016622,0.309958249791591,0.0236199476061335,0.981155760700613,0.999765644820604
"14263","F8A3",4.44448406093999,0.00396412646490899,0.698262217720069,0.00567713154787675,0.995470328720066,0.999765644820604
"14264","PRPF18",73.7528034100203,-9.98156699936155e-05,0.227613766191831,-0.000438530901112069,0.999650102975782,0.999790267039576
"14265","SLC26A2",52.8196941069131,-9.57357618904657e-05,0.279352124955208,-0.000342706402916449,0.999726559857577,0.999796642336361
"14266","NLE1",24.4554098113756,-3.56089399664728e-05,0.376474221270005,-9.45853340139704e-05,0.999924531822424,0.999924531822424
"14267","AL627309.1",0.762283148000877,-0.0918621627677792,1.83690915589464,-0.0500090940659714,0.960115141374421,NA
"14268","AL627309.5",1.59361626682723,0.317518589406224,1.16630843198213,0.272242385203889,0.78543565345616,NA
"14269","AL627309.4",0,NA,NA,NA,NA,NA
"14270","FAM41C",0.767927417484193,-3.26474361228513,2.13016689189438,-1.5326233943021,0.125368673927802,NA
"14271","LINC02593",1.38683969536061,-1.43831911158076,1.41551387553334,-1.0161109237017,0.309576567765102,NA
"14272","PLEKHN1",0.32157802385943,0.28926759090872,2.49200598520945,0.116078208730469,0.907590558967189,NA
"14273","AL645608.7",0.443006446529585,0.0156912000299491,2.07856718469127,0.00754904635535259,0.993976789672886,NA
"14274","AL390719.2",0,NA,NA,NA,NA,NA
"14275","TTLL10",0.208975155481072,-1.67232023626263,3.33344910552292,-0.501678646748169,0.615893587270659,NA
"14276","C1QTNF12",1.50817024394429,-0.111345779042653,1.26533800346183,-0.087996866242872,0.929879166857639,NA

"14277","AL162741.1",0.166184609419855,-1.47576652541587,3.34432018311
399,-0.441275489370681,0.659013568361442,NA
"14278","LINC01786",0.145281756542051,0.889415776427968,3.347292588293
49,0.265711990501974,0.790461028169891,NA
"14279","TAS1R3",0.395027128116867,-0.367769408968258,2.14971981283592
,-0.171077833851797,0.864162564937158,NA
"14280","AL391244.2",0.581123682123901,-1.02256937886395,1.94288731764
869,-0.526314300152762,0.598669848868385,NA
"14281","ATAD3C",1.47820994529062,-0.61342365344327,1.35771371228143,-
0.451806332877427,0.651408507082868,NA
"14282","AL691432.4",0.543085775466451,-1.16497294805769,1.83479813925
7,-0.634932488284211,0.525472447090202,NA
"14283","FO704657.1",0.585410575844208,-0.487284902844901,1.7830506650
2614,-0.273287188301941,0.784632462881175,NA
"14284","AL109917.1",0.530231637612684,0.266831785494427,2.20486737269
773,0.121019426745813,0.903675647968152,NA
"14285","CALML6",0.188817094997298,0.903560157643627,3.31675525104427,
0.272422922179483,0.785296849615702,NA
"14286","TMEM52",0.536492256167186,0.627077206024893,2.1708584013332,0
.288861404152285,0.77268744024222,NA
"14287","CFAP74",0,NA,NA,NA,NA,NA
"14288","PRKCZ",1.13853258335671,0.606710715265102,1.27459263926034,0.
476003623885027,0.634071796155683,NA
"14289","AL590822.2",0.122207625915474,0.359191432502738,3.35247519753
738,0.10714215955023,0.914676201160951,NA
"14290","PRKCZ-
AS1",0.655472230854279,2.45625708339888,2.06389658188364,1.19010666763
019,0.234004469955238,NA
"14291","AL590822.1",0,NA,NA,NA,NA,NA
"14292","AL513477.2",1.81932062385794,-0.142561673490753,1.24350580650
026,-0.114644960035997,0.908726541673932,NA
"14293","PLCH2",1.45545821677149,0.225543919509374,1.20137899933992,0.
187737524655664,0.851082412830257,NA
"14294","AL139246.1",0.0786722874602274,0.359191432502738,3.3524751975
3738,0.10714215955023,0.914676201160951,NA
"14295","HES5",0,NA,NA,NA,NA,NA
"14296","AL139246.5",1.65527025976458,-0.419317169147028,1.15111978044
711,-0.364268928628921,0.715657199676482,NA
"14297","AL139246.3",0.0830923047099275,-0.939216628356831,3.352475197
53738,-0.280156175069319,0.779357688030112,NA
"14298","MMEL1",0.757059964798087,-0.796146116360636,1.81854733342432,
-0.437792353120387,0.661536814732215,NA
"14299","TTC34",0.657797515970437,0.31297100092801,1.68045044159814,0.
186242327164595,0.852254732301545,NA
"14300","AL354743.1",0.589339709201867,0.542045719070027,1.95605444402
836,0.277111775045341,0.781694280574298,NA
"14301","AL513320.1",0.218618355933563,-1.71512321784709,3.15240431335
981,-0.544068287997971,0.586394481847521,NA
"14302","TP73",0,NA,NA,NA,NA,NA
"14303","AL365330.1",0.200580936021446,-0.939216628356831,3.3524751975

3738,-0.280156175069319,0.779357688030112,NA
"14304","LINC01134",0,NA,NA,NA,NA,NA
"14305","AJAP1",0.845405531119312,0.420123978381466,2.01073605650418,0
.208940391267407,0.8344947723017,NA
"14306","AL365255.1",0.192803065653254,-0.362148402639638,3.3015497885
8522,-0.109690425960478,0.912654893573412,NA
"14307","CHD5",0.207447245666404,1.02558430340912,3.22299417080721,0.3
18208550514461,0.750326747237409,NA
"14308","AL031847.1",0.0786722874602274,0.359191432502738,3.3524751975
3738,0.10714215955023,0.914676201160951,NA
"14309","LINC00337",0,NA,NA,NA,NA,NA
"14310","AL031848.2",1.05467152354498,0.231149529649618,1.573320255749
41,0.14691829511819,0.883196511205992,NA
"14311","HES2",0,NA,NA,NA,NA,NA
"14312","ESPN",0,NA,NA,NA,NA,NA
"14313","PLEKHG5",0.406198238384026,-2.49026032580906,2.22267811297646
,-1.12038729821938,0.262548755277703,NA
"14314","CAMTA1-
DT",0.398102996278655,-0.431923741096099,2.14061825090882,-0.201775230
549735,0.840092446418267,NA
"14315","Z98884.2",0.279253223481674,-0.362151360256245,2.919906114567
08,-0.124028426273541,0.901292767920445,NA
"14316","Z98884.1",0,NA,NA,NA,NA,NA
"14317","TNFRSF9",1.24205530767902,0.540905711686745,1.61999540092932,
0.333893362522172,0.738460028590631,NA
"14318","RERE-
AS1",1.22588733159815,-0.468248730054181,1.35953489568151,-0.344418323
899995,0.730531710412676,NA
"14319","ENO1-
AS1",0.598223698697794,0.61774554178125,1.78989540714181,0.34512940773
8799,0.729997086004739,NA
"14320","CA6",0.0786722874602274,0.359191432502738,3.35247519753738,0.
10714215955023,0.914676201160951,NA
"14321","BX323043.1",0,NA,NA,NA,NA,NA
"14322","PIK3CD-AS1",0,NA,NA,NA,NA,NA
"14323","AL139424.3",0.464390923632661,1.21738489597677,2.274583905332
56,0.535212129621913,0.592503179695434,NA
"14324","AL139424.2",1.02301370107925,0.448943068026852,1.535609989215
15,0.292354876029627,0.770015305491061,NA
"14325","CORT",0.686353827977922,-1.20920410696587,1.66244652515814,-0
.727364212121555,0.467002871789165,NA
"14326","AL354956.1",0,NA,NA,NA,NA,NA
"14327","AL139423.2",0.200879913375701,0.981067669524494,3.25622950176
531,0.301289472683859,0.763193767774683,NA
"14328","AL139423.1",0.188817094997298,0.903560157643627,3.31675525104
427,0.272422922179483,0.785296849615702,NA
"14329","C1orf127",0.104487577740536,-0.939216628356831,3.352475197537
38,-0.280156175069319,0.779357688030112,NA
"14330","MASP2",0,NA,NA,NA,NA,NA
"14331","AL109811.1",0.302947873190324,0.185474739119599,2.52471436804

289,0.0734636525490903,0.941437166955118,NA
"14332","ANGPTL7",0.249151526962087,1.39724561189142,3.03826011163213,
0.459883472959405,0.645599863589248,NA
"14333","DISP3",0,NA,NA,NA,NA,NA
"14334","DRAXIN",1.12138749793055,0.178595398214435,1.42557473880679,0
.125279575565375,0.900302221119822,NA
"14335","AL096840.2",0.249769334282587,0.171289752160848,2.67665743248
737,0.0639939015287703,0.94897508273126,NA
"14336","TNFRSF8",0.39596341759935,0.249663980796492,2.37164573576477,
0.105270351735726,0.916161288119135,NA
"14337","AADACL4",0.0786722874602274,0.359191432502738,3.3524751975373
8,0.10714215955023,0.914676201160951,NA
"14338","KAZN-AS1",0,NA,NA,NA,NA,NA
"14339","TMEM51-
AS1",0.730025286222851,1.15906315847665,2.04431502581427,0.56696895725
011,0.570735272443141,NA
"14340","TMEM51",1.06946727771414,0.244280803653162,1.35502373355783,0
.180277878241855,0.856934421739881,NA
"14341","FHAD1",1.4796945254961,-0.463673844332293,1.31772267593981,-0
.351875134881166,0.724931907633332,NA
"14342","AL031283.1",0.642649147358452,-0.800741172425247,1.7054580692
1582,-0.469516774923369,0.638700297229736,NA
"14343","CTRC",0.0786722874602274,0.359191432502738,3.35247519753738,0
.10714215955023,0.914676201160951,NA
"14344","CELA2A",0.0786722874602274,0.359191432502738,3.35247519753738
,0.10714215955023,0.914676201160951,NA
"14345","AL121992.3",0,NA,NA,NA,NA,NA
"14346","AGMAT",1.2744529154924,1.63083286122374,1.60710953559795,1.01
476148644527,0.31021953659298,NA
"14347","AL121992.1",0.203160351946096,0.359191432502738,3.35247519753
738,0.10714215955023,0.91467620116095,NA
"14348","AL121992.2",0.188817094997298,0.903560157643627,3.31675525104
427,0.272422922179483,0.785296849615702,NA
"14349","AL450998.3",0.2876474429413,-1.09428600047155,2.9164898464785
5,-0.3752065181344,0.707506882962305,NA
"14350","UQCRHL",0.465462769835662,1.23753369484536,2.00327320546017,0
.617755826550419,0.536736305864805,NA
"14351","HSPB7",1.13830757362486,-0.838017975740292,1.69070738593098,-
0.49566155037093,0.620133497903912,NA
"14352","CLCNKB",0.425484639289007,-2.53349637189639,2.51768132661992,
-1.00628159136316,0.314280133148881,NA
"14353","FAM131C",1.14413095213341,-2.34831495151824,1.80225688982838,
-1.3029856979722,0.192579644345619,NA
"14354","AL451042.2",0.370467444766316,-0.574627864891087,2.1789845793
8634,-0.263713598676737,0.792000616142746,NA
"14355","AL109627.1",1.28699714460163,-0.354923388666163,1.24523469867
149,-0.28502529607055,0.775624761924895,NA
"14356","CPLANE2",1.10629944856748,2.51237922640747,1.46562408868231,1
.71420437601177,0.0864911975045575,NA
"14357","SPATA21",0.589339709201867,0.542045719070027,1.95605444402836

,0.277111775045341,0.781694280574298,NA
"14358","LINC01772",1.57724370244556,1.29549285613945,1.28804383284845
,1.00578320636381,0.314519867376086,NA
"14359","AL137802.2",0.562014903489812,0.428902987522946,1.82175391581
83,0.23543409668479,0.813871824391365,NA
"14360","AL137798.1",0.122207625915474,0.359191432502738,3.35247519753
738,0.10714215955023,0.914676201160951,NA
"14361","BX284668.2",0,NA,NA,NA,NA,NA
"14362","BX284668.6",0.564295342060207,0.0919589059942438,1.8539237575
0639,0.0496023127282928,0.96043930361197,NA
"14363","PADI2",0.122207625915474,0.359191432502738,3.35247519753738,0
.10714215955023,0.914676201160951,NA
"14364","PADI4",0.104487577740536,-0.939216628356831,3.35247519753738,
-0.280156175069319,0.779357688030112,NA
"14365","PADI6",0,NA,NA,NA,NA,NA
"14366","IGSF21",0.205299930625402,-0.362148984940857,3.22997826280445
,-0.11212118332537,0.910727321463183,NA
"14367","PAX7",0,NA,NA,NA,NA,NA
"14368","AL137127.1",0.133218938163648,0.832405827491147,3.34883738676
897,0.248565615870131,0.803696808386323,NA
"14369","AKR7A3",0.826561541314942,0.36518013665375,1.48082323132713,0
.246606164009509,0.805213033672621,NA
"14370","HTR6",0.0830923047099275,-0.939216628356831,3.35247519753738,
-0.280156175069319,0.779357688030112,NA
"14371","PLA2G2D",0,NA,NA,NA,NA,NA
"14372","Z98257.1",0.0666094690818238,0.359191432502738,3.352475197537
38,0.10714215955023,0.91467620116095,NA
"14373","PLA2G2C",0.161764592170155,-0.362147980542956,3.3524751975373
8,-0.108024059599003,0.913976607082588,NA
"14374","UBXN10",1.63532047009122,-1.38454682919287,1.08859058021587,-
1.27187103614135,0.203418944318492,NA
"14375","VWA5B1",0.0666094690818238,0.359191432502738,3.35247519753738
,0.10714215955023,0.91467620116095,NA
"14376","PINK1-
AS",1.69307556414281,1.33826515914359,1.15895241384181,1.1547196788756
6,0.248205238218298,NA
"14377","KIF17",0.0786722874602274,0.359191432502738,3.35247519753738,
0.10714215955023,0.914676201160951,NA
"14378","AL031005.1",0,NA,NA,NA,NA,NA
"14379","AL031728.1",0.261832152660991,0.224500406754452,3.01038077252
586,0.074575418765409,0.940552532631568,NA
"14380","LINC02596",0.427114395948375,1.8494659039979,2.27071224058689
,0.814487133569986,0.415365940104337,NA
"14381","RAP1GAP",0,NA,NA,NA,NA,NA
"14382","LINC01635",0.0666094690818238,0.359191432502738,3.35247519753
738,0.10714215955023,0.91467620116095,NA
"14383","CDC42-
AS1",0.913672059495267,1.35965175751732,1.81075443447901,0.75087584027
2909,0.452727381452723,NA
"14384","AL445253.1",0.846934206985457,0.0447417471125121,1.6757875141

2996,0.0266989381023889,0.978699860103152,NA
"14385","TEX46",0,NA,NA,NA,NA,NA
"14386","AL109936.6",0,NA,NA,NA,NA,NA
"14387","AL109936.2",0.74718290437606,0.682189367866817,1.71320216828854,0.398195484744402,0.690486094021378,NA
"14388","E2F2",0.236338404108501,-0.362150168278439,3.07941143273211,-0.117603696741858,0.90638167569981,NA
"14389","MDS2",0,NA,NA,NA,NA,NA
"14390","CNR2",0.366765545441301,-2.20455412572815,2.64323073774586,-0.834037715378638,0.404259731170285,NA
"14391","MYOM3",0,NA,NA,NA,NA,NA
"14392","IL22RA1",0.980251231472745,-0.587950850536677,1.41824710231485,-0.414561644142989,0.678462831546029,NA
"14393","IFNLR1",0.546567557523572,-2.15623886945886,1.88144157350458,-1.14605677892107,0.251771659511826,NA
"14394","RCAN3AS",0.114130778193026,-0.939216628356831,3.35247519753738,-0.280156175069319,0.779357688030112,NA
"14395","NCMAP-DT",0.607398579631806,-0.0334224041116684,1.9694437973739,-0.0169704787495,0.986460166925014,NA
"14396","AL031432.1",0,NA,NA,NA,NA,NA
"14397","AL031432.4",1.27893888543236,-1.04579851168296,1.1775709319103,-0.888098103768943,0.374487982576639,NA
"14398","AL031432.5",0,NA,NA,NA,NA,NA
"14399","AL031432.3",0.0852396197509296,0.359191432502738,3.35247519753738,0.10714215955023,0.91467620116095,NA
"14400","RHD",0.813293753204145,1.19574702281935,1.68270416626438,0.710610365619952,0.477325717929034,NA
"14401","RHCE",0.834993437638466,-0.423291004109638,1.49589252125516,-0.282968861796612,0.777200706563392,NA
"14402","AL606491.1",0.769163171801961,-0.203125383628923,1.58720029335753,-0.127977158572241,0.898167049202466,NA
"14403","AL020996.1",0.86498117106306,-1.55385065069277,1.47669601021347,-1.05224815395022,0.292685711739155,NA
"14404","AUNIP",0.223954044002279,1.30188442273399,3.13819752384994,0.414851013309333,0.678250972486497,NA
"14405","SLC30A2",0,NA,NA,NA,NA,NA
"14406","TRIM63",0.22669520365601,-0.362149828162381,3.12343089148524,-0.115946163287824,0.90769520937636,NA
"14407","FAM110D",0.305068513761982,-1.76759864781942,2.83680500104418,-0.623094871578693,0.533222179627375,NA
"14408","CNKSR1",0.857487509932115,-0.782205384159798,1.67447922648552,-0.467133525329859,0.640404349616448,NA
"14409","CRYBG2",0.114130778193026,-0.939216628356831,3.35247519753738,-0.280156175069319,0.779357688030112,NA
"14410","ZNF683",1.61396331564045,-3.76321719674859,1.67080078533575,-2.25234344499806,0.0243005766885605,NA
"14411","AL513365.2",0,NA,NA,NA,NA,NA
"14412","AL512408.1",0.122207625915474,0.359191432502738,3.35247519753738,0.10714215955023,0.914676201160951,NA

"14413","SFN",0.525552199779843,-2.02442560976877,2.14561092051569,-0.
943519437942738,0.345415277656031,NA
"14414","TRNP1",1.25787017872969,-1.92015978867558,1.35845631318105,-1
.41348659507438,0.157512700070981,NA
"14415","TENT5B",0.249151526962087,1.39724561189142,3.03826011163213,0
.459883472959405,0.645599863589248,NA
"14416","FCN3",0,NA,NA,NA,NA,NA
"14417","GPR3",0.320798820614111,-0.518518230315316,2.51109187135226,-
0.20649114284938,0.836407282650016,NA
"14418","BX293535.1",0,NA,NA,NA,NA,NA
"14419","LINC02574",1.17353592135127,0.925069049942807,1.4148559420012
6,0.653825610425281,0.513224164647847,NA
"14420","AL445490.1",0.192803065653254,-0.362148402639638,3.3015497885
8522,-0.109690425960478,0.912654893573412,NA
"14421","AL020997.3",0.297130845374805,1.62930537364398,2.484682928871
4,0.655739754441847,0.511991585412106,NA
"14422","SMPDL3B",0.0830923047099275,-0.939216628356831,3.352475197537
38,-0.280156175069319,0.779357688030112,NA
"14423","PTAFR",1.57009095928281,-0.196864246028182,1.14153378007069,-
0.172455909290737,0.863079121260559,NA
"14424","AL353622.1",1.7682134866587,0.71241124096669,1.14267064170527
,0.623461577610428,0.532981243637764,NA
"14425","LINC01715",1.36957988076774,-1.21476883586697,1.3077357377872
4,-0.92891002422433,0.352935717557762,NA
"14426","MATN1",0,NA,NA,NA,NA,NA
"14427","AL137857.1",0.0852396197509296,0.359191432502738,3.3524751975
3738,0.10714215955023,0.91467620116095,NA
"14428","LINC01778",0.18074024727485,-0.362147980542956,3.352475197537
38,-0.108024059599002,0.913976607082588,NA
"14429","NKAIN1",1.28507105727357,-0.0164976486520804,1.59966015166859
,-0.0103132209893907,0.991771386070038,NA
"14430","LINC01226",0,NA,NA,NA,NA,NA
"14431","TINAGL1",1.15182544805127,-2.67162821559632,1.4455896992231,-
1.84812344542309,0.0645844867960932,NA
"14432","AC114488.2",0.114130778193026,-0.939216628356831,3.3524751975
3738,-0.280156175069319,0.779357688030112,NA
"14433","AC114488.3",0.0786722874602274,0.359191432502738,3.3524751975
3738,0.10714215955023,0.914676201160951,NA
"14434","ADGRB2",1.32795668974698,1.01132827809448,1.30818417188446,0.
773077904342508,0.439476276823543,NA
"14435","SPOCD1",0.232794078501679,-0.90181358082427,3.12933488781614,
-0.288180592091764,0.773208504171299,NA
"14436","AL354919.1",0.435522329564127,-0.493802246910433,2.1089806936
729,-0.234142611353379,0.814874266126724,NA
"14437","AL136115.2",1.55510509524999,0.577875893172798,1.483855144690
75,0.389442254683987,0.696949019533712,NA
"14438","AL136115.1",0,NA,NA,NA,NA,NA
"14439","AL445248.1",0.279253223481674,-0.362151360256245,2.9199061145
6708,-0.124028426273541,0.901292767920445,NA
"14440","AL049795.2",0.838810066130199,-0.645296653960657,1.5132887223

4953,-0.426420050866941,0.669801793415198,NA
"14441","AL049795.1",0.205299930625402,-0.362148984940857,3.2299782628
0445,-0.11212118332537,0.910727321463183,NA
"14442","IQCC",1.75034701024758,-0.0158636357216872,1.12680014289888,-
0.0140784821706495,0.988767367495872,NA
"14443","DCDC2B",0,NA,NA,NA,NA,NA
"14444","FAM167B",0.963978064969018,2.20688379325892,1.79556795369822,
1.2290728338705,0.219044499526852,NA
"14445","HPCA",0.617473114916467,-1.46771072693399,2.12270844231073,-0
.691433028520993,0.48929345398345,NA
"14446","AL031602.2",0.0786722874602274,0.359191432502738,3.3524751975
3738,0.10714215955023,0.914676201160951,NA
"14447","AL020995.1",0.0786722874602274,0.359191432502738,3.3524751975
3738,0.10714215955023,0.914676201160951,NA
"14448","AL662907.3",0.203160351946096,0.359191432502738,3.35247519753
738,0.10714215955023,0.91467620116095,NA
"14449","AL513327.1",0.183159865200764,-0.362147980542956,3.3524751975
3738,-0.108024059599003,0.913976607082588,NA
"14450","GJA4",0.0666094690818238,0.359191432502738,3.35247519753738,0
.10714215955023,0.91467620116095,NA
"14451","DLGAP3",0.451559350152457,-0.0685741236573337,2.2920944376269
5,-0.0299176694169416,0.976132713999908,NA
"14452","AL590434.1",0,NA,NA,NA,NA,NA
"14453","TFAP2E",1.25601926895814,0.614171460907794,1.2826669638861,0.
478823793081127,0.632063988619461,NA
"14454","CLSPN",0.916725366914407,0.132268038035474,1.45381342523887,0
.0909800636995371,0.927508432212036,NA
"14455","AL139286.3",0.488388176961483,-0.569776754727876,2.0546301214
2295,-0.277313541151276,0.781539362821031,NA
"14456","AL139286.2",0.431102312314427,0.62586471926526,2.092321156929
61,0.29912459528139,0.764844980612087,NA
"14457","TEKT2",0.0830923047099275,-0.939216628356831,3.35247519753738
,-0.280156175069319,0.779357688030112,NA
"14458","AL591845.1",0.0786722874602274,0.359191432502738,3.3524751975
3738,0.10714215955023,0.914676201160951,NA
"14459","CDCA8",1.41856758216851,-1.07213207247234,1.23493125278676,-0
.86817146302918,0.385300473996343,NA
"14460","AL929472.2",0.247349716356674,0.113410451520479,3.07142756361
078,0.0369243451690434,0.970545328342794,NA
"14461","GJA9",1.73782728759109,2.2111093072224,1.45988111248041,1.514
58176170635,0.129878353800233,NA
"14462","RHBDL2",1.10933422371763,-1.01943721110791,1.46663760496958,-
0.695084598713158,0.487002285981207,NA
"14463","AL356055.1",1.31311092475516,-0.461452616132444,1.19686622396
465,-0.385550704742817,0.699829447466647,NA
"14464","AL442071.2",0.236016862380682,1.34721548542771,3.328855936460
28,0.404708257474268,0.68569197172232,NA
"14465","BMP8A",0.893754059081962,0.169876571774655,1.78003375781188,0
.0954344663572434,0.923969740807608,NA
"14466","PABPC4-AS1",0,NA,NA,NA,NA,NA

"14467","HEYL",1.10629596661686,-1.78130439956924,1.50642604830933,-1.18247052456934,0.237019054308327,NA
"14468","HPCAL4",0.531109553536106,-0.766311851250473,2.20042046387639,-0.348257009890052,0.727647176489307,NA
"14469","OXCT2",0.358224488296156,1.80762754813311,2.7207094319684,0.664395663459482,0.50643710764916,NA
"14470","AL033527.4",0.336663848921714,-0.262928595387691,2.80598959981227,-0.0937026264834628,0.925345383827667,NA
"14471","MYCL",0.0852396197509296,0.359191432502738,3.35247519753738,0.10714215955023,0.91467620116095,NA
"14472","COL9A2",0.444227294824038,-0.875458133402646,2.04713937525031,-0.427649501537041,0.6689063204918,NA
"14473","AL031985.3",0.984844351427976,1.2286562866755,1.45594732499847,0.843887869828525,0.398732067178273,NA
"14474","AL603839.3",0.474936628123929,2.15173459801757,1.98692767986213,1.08294560482789,0.278832567839841,NA
"14475","AL603839.2",0.0786722874602274,0.359191432502738,3.35247519753738,0.10714215955023,0.914676201160951,NA
"14476","RIMS3",0.170479239501859,1.00912327877525,3.3428978888652,0.301870805607469,0.762750551898993,NA
"14477","NFYC-AS1",1.46635702415374,-0.287760448135784,1.08876920514782,-0.264298849356889,0.791549646845807,NA
"14478","KCNQ4",0.293304672737834,0.185328792052959,2.54859534931574,0.0727180139062551,0.942030513475199,NA
"14479","SLFNL1-AS1",1.13558808718447,2.44044320663023,1.73493500587688,1.40664820201537,0.159531732315892,NA
"14480","SLFNL1",0.104487577740536,-0.939216628356831,3.35247519753738,-0.280156175069319,0.779357688030112,NA
"14481","FOXO6",1.23619340822341,-1.21420112183444,1.29766199078906,-0.935683660655062,0.349436085706134,NA
"14482","AC093151.2",0.189727197491466,-0.362148248616811,3.32022295329944,-0.109073472989797,0.913144214822408,NA
"14483","EDN2",0,NA,NA,NA,NA,NA
"14484","RIMKLA",0,NA,NA,NA,NA,NA
"14485","ZMYND12",1.61099986723079,1.10462692137821,1.23660825284346,0.893271510066526,0.371711791049655,NA
"14486","AC098484.4",0.218618355933563,-1.71512321784709,3.15240431335981,-0.544068287997971,0.586394481847521,NA
"14487","AC098484.2",0.825352461466519,-0.0646007924410741,1.66523750227314,-0.0387937410446802,0.969054835002692,NA
"14488","TMEM269",0,NA,NA,NA,NA,NA
"14489","AL512353.1",1.49475256221305,0.868003538490762,1.1556203714727,0.751114777757507,0.452583582866133,NA
"14490","SLC2A1-AS1",0.387865951213436,1.95337401117089,2.31193104826563,0.844910151034252,0.398161006382902,NA
"14491","CFAP57",1.68965181824916,-0.767344338711233,1.26918825233235,-0.604594580276889,0.545448408655471,NA

"14492","TIE1",1.80608205115193,1.24484013714288,1.26521853602537,0.98
3893376280667,0.325167940812512,NA
"14493","AL139289.2",0.776160380695767,-0.207055045494139,1.6126402730
9548,-0.128395060540498,0.897836339922832,NA
"14494","CDC20",0.567521185505047,-0.950634849551681,2.1519928464073,-
0.441746287000323,0.658672810872432,NA
"14495","AL139289.1",0,NA,NA,NA,NA,NA
"14496","SZT2-
AS1",0.183159865200764,-0.362147980542956,3.35247519753738,-0.10802405
9599003,0.913976607082588,NA
"14497","HYI-
AS1",0.230521376292981,1.33222568121774,2.70690437450901,0.49215838348
9031,0.622607381833459,NA
"14498","AL451062.1",0.0786722874602274,0.359191432502738,3.3524751975
3738,0.10714215955023,0.914676201160951,NA
"14499","ARTN",0.0786722874602274,0.359191432502738,3.35247519753738,0
.10714215955023,0.914676201160951,NA
"14500","AL357079.1",1.76331518332001,-0.725791724386504,1.22563842814
557,-0.592174419240956,0.553733797296766,NA
"14501","CCDC24",1.46159344817854,0.0245990307835402,1.15461836076369,
0.021304901792199,0.983002433662918,NA
"14502","AL139220.2",1.32668172230455,-1.62409527320753,1.376087517024
39,-1.18022673203186,0.237910049695042,NA
"14503","ERI3-IT1",0,NA,NA,NA,NA,NA
"14504","KIF2C",0.909381543139959,0.579055918476396,1.49360874782253,0
.387689158436289,0.698246079830225,NA
"14505","AL592166.1",0.0666094690818238,0.359191432502738,3.3524751975
3738,0.10714215955023,0.91467620116095,NA
"14506","BEST4",1.04982211814034,-1.0659534848298,1.51639714536178,-0.
702951392443755,0.482086041977465,NA
"14507","TCTEX1D4",0.655807774647628,0.0455051337724639,1.703268896497
79,0.0267163533990611,0.978685969661714,NA
"14508","LINC01144",0.0786722874602274,0.359191432502738,3.35247519753
738,0.10714215955023,0.914676201160951,NA
"14509","HPDL",1.26411791500267,-1.24762694654774,1.44762508607934,-0.
861843966746071,0.388773386846704,NA
"14510","CCDC163",1.12036845694152,0.748056852828522,1.56376395588248,
0.478369417593059,0.63238729654788,NA
"14511","TSPAN1",0.612274660472925,0.395279535236294,1.79116277824809,
0.22068320089976,0.825339112733224,NA
"14512","RAD54L",0.286119533126631,1.51303924540838,2.55102271431352,0
.593110848021414,0.553106971009519,NA
"14513","TMEM275",0,NA,NA,NA,NA,NA
"14514","MKNK1-AS1",0,NA,NA,NA,NA,NA
"14515","TEX38",0,NA,NA,NA,NA,NA
"14516","CYP4Z1",0.423758488539979,1.03843179813776,2.08152161655449,0
.498881102112531,0.617863148010363,NA
"14517","CYP4A22-
AS1",0.279552200835929,1.4935861496343,2.9278366319717,0.5101330222200
51,0.609958271749706,NA

"14518","CYP4A22",0.0666094690818238,0.359191432502738,3.3524751975373
8,0.10714215955023,0.91467620116095,NA
"14519","LINC00853",1.12324300527892,1.12867572643934,1.42631726229895
,0.79132164790611,0.428756318990261,NA
"14520","PDZK1IP1",0.0666094690818238,0.359191432502738,3.352475197537
38,0.10714215955023,0.91467620116095,NA
"14521","TAL1",0,NA,NA,NA,NA,NA
"14522","STIL",1.22146842820419,-1.24229137055372,1.42429207479968,-0.
872216726143365,0.38309015643998,NA
"14523","FOXD2-
AS1",0.599916974856296,0.47947300759283,1.95763411730399,0.24492472998
6393,0.806514713003374,NA
"14524","FOXD2",1.15664271735873,-1.6049982976561,1.45925681248701,-1.
09987377404852,0.271387122907593,NA
"14525","SLC5A9",0.200879913375701,0.981067669524494,3.25622950176531,
0.301289472683859,0.763193767774683,NA
"14526","AL109659.2",0.674579328952796,0.80330678079002,1.748768624747
34,0.459355668567122,0.64597877760546,NA
"14527","AGBL4",0.0666094690818238,0.359191432502738,3.35247519753738,
0.10714215955023,0.91467620116095,NA
"14528","DMRTA2",0,NA,NA,NA,NA,NA
"14529","LINC01562",0.615907528237902,1.41330086095942,1.9691703684116
7,0.717713857384206,0.472933737195936,NA
"14530","TTC39A",0.908448876292476,0.761922682702784,1.75499351952979,
0.434145581863414,0.664182727022842,NA
"14531","TTC39A-
AS1",0.247004211921084,0.251392038816408,2.68232836300611,0.0937215749
881831,0.925330331348742,NA
"14532","AC104170.2",0.122207625915474,0.359191432502738,3.35247519753
738,0.10714215955023,0.914676201160951,NA
"14533","AC104170.1",0,NA,NA,NA,NA,NA
"14534","AL050343.3",0.491861108801167,-0.348510412713336,2.2262332757
7053,-0.156547122220474,0.875601778910774,NA
"14535","RAB3B",0.247004211921084,0.251392038816408,2.68232836300611,0
.0937215749881831,0.925330331348742,NA
"14536","AL513218.1",0.465968072290237,-1.14678452840732,2.24831902565
936,-0.51006308060352,0.610007269259714,NA
"14537","AL513218.2",0.415953943702442,-0.914127789450739,2.0824529527
7954,-0.438966838713265,0.66068556477833,NA
"14538","ORC1",0.114130778193026,-0.939216628356831,3.35247519753738,-
0.280156175069319,0.779357688030112,NA
"14539","AL445183.1",0.478127169188492,0.820639743463464,2.26544923431
717,0.362241506466956,0.717171565021905,NA
"14540","AL445183.2",0.692283360659368,-1.06417100741925,1.81814409599
462,-0.585306197547058,0.558341848381904,NA
"14541","AL606760.2",0.432929199483351,-0.132978516444521,2.3126597728
2594,-0.0575002505803213,0.954146706506299,NA
"14542","AL606760.3",0.514626717908003,0.135691109987533,2.03577038884
92,0.0666534451678697,0.946857597216965,NA
"14543","AL606760.1",1.65108337999085,0.519518053846579,1.156919757012

91,0.449052797912226,0.653393569835272,NA
"14544","LINC01771",0,NA,NA,NA,NA,NA
"14545","LINC02812",0.0830923047099275,-0.939216628356831,3.3524751975
3738,-0.280156175069319,0.779357688030112,NA
"14546","GLIS1",0.0786722874602274,0.359191432502738,3.35247519753738,
0.10714215955023,0.914676201160951,NA
"14547","DIO1",0,NA,NA,NA,NA,NA
"14548","SSBP3-
AS1",0.157344574920455,0.946214072007857,3.34552927999138,0.2828294098
83477,0.777307608562567,NA
"14549","ACOT11",1.25493154421332,-1.35671967394732,1.38689870470119,-
0.978239917124751,0.327955678250116,NA
"14550","MROH7",0.205299930625402,-0.362148984940857,3.22997826280445,
-0.11212118332537,0.910727321463183,NA
"14551","TTC22",0.452437266075879,-1.35655134596369,2.52419268884531,-
0.537419885557249,0.590977610385391,NA
"14552","LEXM",0,NA,NA,NA,NA,NA
"14553","TMEM61",1.71932880585322,-0.0200102106154612,1.18514802722009
,-0.0168841445590536,0.986529041774423,NA
"14554","MIR4422HG",0,NA,NA,NA,NA,NA
"14555","LINC01767",0,NA,NA,NA,NA,NA
"14556","DAB1",0.651970806083125,0.141706873052888,1.87949568222727,0.
075396216529067,0.93989946533915,NA
"14557","TACSTD2",1.137016380343,0.741435344646988,1.54378723517892,0.
480270420529198,0.631035118924815,NA
"14558","AL136985.3",0.281832639406324,0.978331721828331,2.92526312973
549,0.334442297475233,0.738045826283596,NA
"14559","LINC01135",1.51831360093153,-0.299888820705708,1.140891758566
13,-0.262854752393519,0.79266253256504,NA
"14560","AL136985.2",0.0786722874602274,0.359191432502738,3.3524751975
3738,0.10714215955023,0.914676201160951,NA
"14561","LINC02777",1.27929615831397,-2.10428241048718,1.4333329329824
5,-1.46810441737959,0.142075861873541,NA
"14562","AC093424.1",1.21432307182841,-0.950869780918212,1.39494805011
196,-0.681652467876418,0.495458729806038,NA
"14563","HOOK1",0.364924944116251,0.250598050714205,2.42552639564272,0
.10331697530251,0.917711403372903,NA
"14564","AC099792.1",0.0666094690818238,0.359191432502738,3.3524751975
3738,0.10714215955023,0.91467620116095,NA
"14565","NFIA-
AS1",0.602639740127104,2.47153947902377,2.33661959126709,1.05774148614
56,0.290173315981708,NA
"14566","AC099791.4",0.350282709813197,-1.04293746194225,2.42920893677
904,-0.429332136133629,0.66768153326385,NA
"14567","L1TD1",1.34595058246896,1.53326200163798,1.63966050468134,0.9
3510943104406,0.349731906732897,NA
"14568","ANGPTL3",0.0786722874602274,0.359191432502738,3.3524751975373
8,0.10714215955023,0.914676201160951,NA
"14569","AC103923.1",0.338084822195305,0.593167528157078,2.45253308361
61,0.241859134182399,0.808889316328949,NA

"14570","AL357078.2",0.547951685787622,1.37220967592162,1.97317189755499,0.695433417444253,0.486783724251009,NA
"14571","DNAJC6",0.163911907211157,0.976662149432262,3.34440272959326,0.29202886984578,0.770264550640504,NA
"14572","AC119800.1",0.618211929516068,2.37068580563268,2.07947231156693,1.14004201568152,0.254268797157276,NA
"14573","AL139147.1",0.351219486776709,0.638256129566455,2.43123438004485,0.262523487988303,0.79291787939026,NA
"14574","TCTEX1D1",0,NA,NA,NA,NA,NA
"14575","IL23R",0.701367127350418,0.726431713710624,1.83259755204787,0.396394567317227,0.691813973219326,NA
"14576","IL12RB2",0.650895337245123,-0.500290806403631,1.69377611772322,-0.295370091223227,0.767711178351129,NA
"14577","DEPDC1",0,NA,NA,NA,NA,NA
"14578","LRRC7",0,NA,NA,NA,NA,NA
"14579","AL353771.1",0.22669520365601,-0.362149828162381,3.12343089148524,-0.115946163287824,0.90769520937636,NA
"14580","AL354872.2",0.0852396197509296,0.359191432502738,3.35247519753738,0.10714215955023,0.91467620116095,NA
"14581","AL031429.1",0.420866381104947,-0.176908445248513,2.32613701973685,-0.0760524611179295,0.939377361077942,NA
"14582","AL031429.2",0.218618355933563,-1.71512321784709,3.15240431335981,-0.544068287997971,0.586394481847521,NA
"14583","ZRANB2-
AS1",0.323087539291176,1.63986284743208,2.81636913523065,0.582261333189118,0.560390666306499,NA
"14584","NEGR1-IT1",0,NA,NA,NA,NA,NA
"14585","LRRIQ3",0.397174443157869,0.810641013013347,2.36543366266254,0.342702915667855,0.731821969031526,NA
"14586","AC105271.1",0.104487577740536,-0.939216628356831,3.35247519753738,-0.280156175069319,0.779357688030112,NA
"14587","ERICH3",0.247004211921084,0.251392038816408,2.68232836300611,0.0937215749881831,0.925330331348742,NA
"14588","AC135803.1",0.540196379792993,-1.00066942262807,1.9719251571295,-0.507458114731258,0.611833418188896,NA
"14589","LHX8",0,NA,NA,NA,NA,NA
"14590","SLC44A5",0.122207625915474,0.359191432502738,3.35247519753738,0.10714215955023,0.914676201160951,NA
"14591","ST6GALNAC3",0.0852396197509296,0.359191432502738,3.35247519753738,0.10714215955023,0.91467620116095,NA
"14592","AC093433.2",0,NA,NA,NA,NA,NA
"14593","AK5",1.33841607638088,-0.20134693649824,1.6198308071582,-0.124301214428363,0.901076785785153,NA
"14594","AC093575.1",0,NA,NA,NA,NA,NA
"14595","AC096531.2",0.614882696597264,0.416223563650788,1.79735972490946,0.23157499185187,0.816868133153151,NA
"14596","LINC01781",0.249276914129782,-1.90751516048253,3.31840217494316,-0.57482940882993,0.565406675152336,NA
"14597","LINC01725",0.444996187852394,0.316958161582557,2.58192383274946,0.122760461622538,0.902296784429941,NA

"14598","TTLL7-
IT1",0.122207625915474,0.359191432502738,3.35247519753738,0.1071421595
5023,0.914676201160951,NA
"14599","DNASE2B",0.104487577740536,-0.939216628356831,3.3524751975373
8,-0.280156175069319,0.779357688030112,NA
"14600","AL359762.1",0.384039778576465,0.765086127903674,2.37979135013
116,0.321492944270726,0.747836858366337,NA
"14601","LPAR3",0.122207625915474,0.359191432502738,3.35247519753738,0
.10714215955023,0.914676201160951,NA
"14602","WDR63",0.586598549548135,1.55901388382422,1.94641219670537,0.
800967999719235,0.423150171673033,NA
"14603","AL360219.1",0.236338404108501,-0.362150168278439,3.0794114327
3211,-0.117603696741858,0.90638167569981,NA
"14604","AC092807.2",0.0786722874602274,0.359191432502738,3.3524751975
3738,0.10714215955023,0.914676201160951,NA
"14605","LINC02795",0.281832639406324,0.978331721828331,2.925263129735
49,0.334442297475233,0.738045826283596,NA
"14606","CLCA2",1.46590430458637,2.32046557591626,1.39314701779329,1.6
6562864240403,0.0957874026222756,NA
"14607","CLCA4-
AS1",0.232794078501679,-0.90181358082427,3.12933488781614,-0.288180592
091764,0.773208504171299,NA
"14608","AL121989.1",0,NA,NA,NA,NA,NA
"14609","LINC02801",0.357925510941901,0.224037573956491,2.729715667382
88,0.082073593463779,0.93458819162738,NA
"14610","PKN2-
AS1",0.199828407245471,1.19974298654701,3.33462853032117,0.35978309896
8286,0.719009343087826,NA
"14611","GBP7",0.328441621742815,0.592619608108833,2.47302666356015,0.
239633327388271,0.810614525899142,NA
"14612","GBP6",0.0666094690818238,0.359191432502738,3.35247519753738,0
.10714215955023,0.91467620116095,NA
"14613","AC099568.1",0.266437876327295,1.49131777161396,3.323586424892
4,0.448707384422009,0.6536427569794,NA
"14614","AC099568.2",0.168331924460857,-0.362147980542956,3.3524751975
3738,-0.108024059599003,0.913976607082588,NA
"14615","LINC01763",0.0852396197509296,0.359191432502738,3.35247519753
738,0.10714215955023,0.91467620116095,NA
"14616","HFM1",1.7181284935925,0.899621123171215,1.26156491920295,0.71
309934944893,0.475784282040292,NA
"14617","BRDT",0.197223082902954,-1.61249994371822,3.25614921967676,-0
.495216845092344,0.620447061165921,NA
"14618","EPHX4",0.395051090824639,0.895241747444736,2.32538612313806,0
.384986277563498,0.700247582365865,NA
"14619","AC104836.1",0,NA,NA,NA,NA,NA
"14620","AC093577.1",0,NA,NA,NA,NA,NA
"14621","AL049796.1",0.487132570031725,0.90912826423112,2.071428964552
37,0.438889423576049,0.660741660639156,NA
"14622","ABCA4",0.133218938163648,0.832405827491147,3.34883738676897,0
.248565615870131,0.803696808386323,NA

"14623","ARHGAP29-
AS1",0.200580936021446,-0.939216628356831,3.35247519753738,-0.280156175069319,0.779357688030112,NA
"14624","AC118469.2",0,NA,NA,NA,NA,NA
"14625","AC092802.4",0,NA,NA,NA,NA,NA
"14626","DPYD-AS1",0,NA,NA,NA,NA,NA
"14627","DPYD-
IT1",0.426065601599966,-0.484326382888175,2.1116803201741,-0.22935592014621,0.818592293622712,NA
"14628","PLPPR5",0,NA,NA,NA,NA,NA
"14629","LRRC39",0.534651654862384,-0.493590974306223,1.96186874846923,-0.251592250853352,0.801356248920214,NA
"14630","AL445928.2",0.247004211921084,0.251392038816408,2.68232836300611,0.0937215749881831,0.925330331348742,NA
"14631","GPR88",1.54045326228796,2.07543014740831,1.51077903718207,1.37374830887212,0.169519833683542,NA
"14632","AC093157.2",0.161764592170155,-0.362147980542956,3.35247519753738,-0.108024059599003,0.913976607082588,NA
"14633","AL109741.1",0.294871025467877,-1.1768104696752,2.89552204169079,-0.406424282989752,0.684430880475328,NA
"14634","COL11A1",1.00086283972708,1.09451485938706,1.95677994303803,0.559344888668347,0.575926365563067,NA
"14635","VAV3-AS1",0,NA,NA,NA,NA,NA
"14636","LINC02785",0.187579882450464,-1.57167522621382,3.3176487217629,-0.473731656972615,0.635691275621751,NA
"14637","AL160171.1",0,NA,NA,NA,NA,NA
"14638","FNDC7",0,NA,NA,NA,NA,NA
"14639","SPATA42",0,NA,NA,NA,NA,NA
"14640","AL356488.2",0.294214775232002,-1.09422072206501,2.89496022075552,-0.377974354956573,0.705449645017841,NA
"14641","KIAA1324",1.1140514105178,1.64940185815792,1.49227804498141,1.10529124495594,0.269033410333576,NA
"14642","CELSR2",0.620829509805893,0.226270873698512,1.79030189467765,0.126386993373122,0.899425597375665,NA
"14643","SYPL2",0.472930172974016,1.69172405687485,2.55922718335019,0.661029262224498,0.508593547768755,NA
"14644","GNAT2",0,NA,NA,NA,NA,NA
"14645","AC000032.1",0.327507556540876,0.329023874713084,2.82936419574862,0.116288979413634,0.907423519508415,NA
"14646","ALX3",0.443916549023753,-0.968812640529986,2.0532768015345,-0.471837328413759,0.63704290443868,NA
"14647","UBL4B",0.5767036648742,-0.0794718716872259,1.97672894188345,-0.0402037274829923,0.96793070593277,NA
"14648","RBM15-
AS1",0.244415251830948,1.06920175933036,3.34018855838734,0.320102215979858,0.748890845867476,NA
"14649","LAMTOR5-
AS1",0.806131177966505,-0.137000303201376,1.58094267656885,-0.0866573502201299,0.930943878424148,NA
"14650","KCNA2",1.26686318475218,0.999265322912733,1.32571868616203,0.

753753668363551,0.450997149238715,NA
"14651","KCNA3",0.930944128224497,2.06051564264749,1.61690115804431,1.27436091711366,0.202535536589044,NA
"14652","AL360270.2",0.183159865200764,-0.362147980542956,3.35247519753738,-0.108024059599003,0.913976607082588,NA
"14653","AL360270.1",0.149701773791751,-0.362147980542956,3.35247519753738,-0.108024059599003,0.913976607082588,NA
"14654","AL360270.3",0.667758088160106,-1.0131521351585,1.6996349226843,-0.596099857467269,0.551108519396038,NA
"14655","AL355816.1",0.0786722874602274,0.359191432502738,3.35247519753738,0.10714215955023,0.914676201160951,NA
"14656","OVGP1",0.358676929272824,-1.50882299437205,2.3731366179591,-0.635792723838055,0.524911530586359,NA
"14657","AL390195.2",1.30109225556379,0.00373985978054401,1.50630893786873,0.00248279731104531,0.998019016393138,NA
"14658","AL390195.1",0.0786722874602274,0.359191432502738,3.35247519753738,0.10714215955023,0.914676201160951,NA
"14659","TMIGD3",0.648598184847625,-1.00499396260284,1.90927636119589,-0.526374276154215,0.598628185012032,NA
"14660","ADORA3",0.285820555772376,-0.362151522613874,2.89750072012302,-0.12498755223726,0.900533404934662,NA
"14661","LINC01160",0.114130778193026,-0.939216628356831,3.35247519753738,-0.280156175069319,0.779357688030112,NA
"14662","INKA2-AS1",0.200580936021446,-0.939216628356831,3.35247519753738,-0.280156175069319,0.779357688030112,NA
"14663","AL049557.1",0.114130778193026,-0.939216628356831,3.35247519753738,-0.280156175069319,0.779357688030112,NA
"14664","AL354760.1",0.483342616029585,0.093245494467271,1.89783124080355,0.0491326586171015,0.960813577014817,NA
"14665","AL603832.1",0,NA,NA,NA,NA,NA
"14666","PPM1J",0.789151890101264,1.83670669987652,1.69971867236015,1.08059452999134,0.279877516806024,NA
"14667","TAFA3",0.390026148556208,-1.56756401867122,2.32155075682576,-0.675222807023417,0.499534218629175,NA
"14668","AL390242.1",0.449412035111455,-0.926992265007577,2.25633404573974,-0.410839993642724,0.681189865182341,NA
"14669","AL137856.1",0,NA,NA,NA,NA,NA
"14670","AP4B1-AS1",0.659466829400919,2.52476358919949,1.84246034894677,1.37032180401752,0.170586469639931,NA
"14671","BCL2L15",0.552717207472912,0.275082191568991,1.81943356243398,0.151191116426914,0.879824957805078,NA
"14672","AMPD1",0,NA,NA,NA,NA,NA
"14673","LINC01765",0.104487577740536,-0.939216628356831,3.35247519753738,-0.280156175069319,0.779357688030112,NA
"14674","CASQ2",0,NA,NA,NA,NA,NA
"14675","MAB21L3",0.401460849397148,0.249241608674987,2.348406986036,0.106132203726619,0.915477460553842,NA
"14676","LINC01779",0.529466914574968,-0.436562107430769,1.96587250463

222,-0.222070407110374,0.824259074403978,NA
"14677","LINC01762",0.216311242873575,0.112131870992111,3.212840643262
62,0.0349011617576657,0.972158554230368,NA
"14678","AL390066.1",0.104487577740536,-0.939216628356831,3.3524751975
3738,-0.280156175069319,0.779357688030112,NA
"14679","AL157904.1",0.350282709813197,-1.04293746194225,2.42920893677
904,-0.429332136133629,0.66768153326385,NA
"14680","CD101",0.525850411062373,1.34055528244467,2.20289248116653,0.
608543219383447,0.542827250376821,NA
"14681","AL445231.1",0,NA,NA,NA,NA,NA
"14682","AL358072.1",0.479647342641463,-2.72955610610946,2.37942110460
007,-1.14715133896749,0.251319078849523,NA
"14683","AL157902.1",0.0852396197509296,0.359191432502738,3.3524751975
3738,0.10714215955023,0.91467620116095,NA
"14684","AL139420.1",1.67636153084081,-1.02099675175106,1.170207869921
93,-0.872491783719732,0.382940148516198,NA
"14685","AL139420.2",0.839872230261406,-1.40130281422202,1.47314012287
417,-0.951235250783888,0.341484967606996,NA
"14686","AL359915.1",0,NA,NA,NA,NA,NA
"14687","HAO2",0.600831247341104,-0.044343087747977,1.83507935231055,-
0.0241641254870775,0.980721693483281,NA
"14688","HSD3B2",0.0786722874602274,0.359191432502738,3.35247519753738
,0.10714215955023,0.914676201160951,NA
"14689","LINC00622",1.34809635584783,1.59420175013108,1.42492022042109
,1.11880070707395,0.263225169990607,NA
"14690","ADAM30",0.0786722874602274,0.359191432502738,3.35247519753738
,0.10714215955023,0.914676201160951,NA
"14691","AC245008.1",0,NA,NA,NA,NA,NA
"14692","AC253572.1",1.49233948856142,0.0721939197941339,1.34735287389
614,0.0535820431253254,0.957268163467226,NA
"14693","FCGR1B",0.967705544134445,3.02307647370599,1.91543145154489,1
.57827442546572,0.114502578605214,NA
"14694","AC244453.2",0.557273344512294,2.41416305425686,2.421393227657
54,0.997014044097382,0.318757693080667,NA
"14695","FAM72B",1.71259873094915,3.16558262790807,1.50069091615745,2.
10941679850612,0.0349086207578787,NA
"14696","LINC02798",0.493723865030199,2.12796054367112,2.1607247173661
7,0.98483648868747,0.324704394192333,NA
"14697","AL592494.1",0,NA,NA,NA,NA,NA
"14698","AL592494.2",0.637464407071036,-0.745281012313631,1.7349017317
8616,-0.429581110364292,0.6675003803415,NA
"14699","AC239800.2",0.163911907211157,0.976662149432262,3.34440272959
326,0.29202886984578,0.770264550640504,NA
"14700","FAM72C",0.0852396197509296,0.359191432502738,3.35247519753738
,0.10714215955023,0.91467620116095,NA
"14701","AC246785.3",0.26597986702578,0.184881689379099,2.626378402746
53,0.070394155383611,0.943879943038437,NA
"14702","FP700111.1",0.270672187160391,-1.99840686264331,2.90174147617
329,-0.688692248793554,0.491016955027494,NA
"14703","FAM72D",0.565530330326498,1.48282830783749,1.88708709790281,0

.785776294843738,0.43199855660619,NA
"14704","PDZK1",0.275895370363181,-1.03718220655362,2.58196919069566,-0.401702007247487,0.687903347118004,NA
"14705","ITGA10",0.40681764361036,0.810191890652543,2.34976289835272,0.344797294748556,0.730246766986374,NA
"14706","LIX1L-AS1",1.56291932736071,0.222796114832379,1.18373868096833,0.188213934725971,0.850708949367308,NA
"14707","ANKRD34A",0.200879913375701,0.981067669524494,3.25622950176531,0.301289472683859,0.763193767774683,NA
"14708","AC239799.1",0.70271127643833,0.112099866791551,1.59142802129108,0.0704397970199164,0.943843616456836,NA
"14709","LINC00624",1.00928198574712,2.1934801242144,1.97861107892094,1.10859589718392,0.267604548857109,NA
"14710","AC239803.1",0.573627796712412,-0.0239759898493979,1.84302843035367,-0.0130090179047304,0.989620598222326,NA
"14711","LINC02804",0.149701773791751,-0.362147980542956,3.35247519753738,-0.108024059599003,0.913976607082588,NA
"14712","LINC02805",0.183159865200764,-0.362147980542956,3.35247519753738,-0.108024059599003,0.913976607082588,NA
"14713","LINC01731",0.0786722874602274,0.359191432502738,3.35247519753738,0.10714215955023,0.914676201160951,NA
"14714","AC245100.6",0.496661041337746,-1.02938001091997,2.00415939433332,-0.513621827600388,0.607516415754968,NA
"14715","PPIAL4G",0.0852396197509296,0.359191432502738,3.35247519753738,0.10714215955023,0.91467620116095,NA
"14716","NUDT4B",0.449857850151228,-2.56583329666803,2.47715854800066,-1.03579696129621,0.300296875519202,NA
"14717","AC239802.2",0,NA,NA,NA,NA,NA
"14718","AC239804.1",0,NA,NA,NA,NA,NA
"14719","AC243772.2",0.441316793006084,1.08877394635344,2.30755650597506,0.471829809382447,0.637048271779016,NA
"14720","HIST2H3D",0.351491789661621,-0.398343995216617,2.203447149187,-0.180782187293938,0.85653854520998,NA
"14721","HIST2H4A",0.0830923047099275,-0.939216628356831,3.35247519753738,-0.280156175069319,0.779357688030112,NA
"14722","HIST2H4B",0.828083312673747,0.665427604957525,1.77255168140703,0.375406602773532,0.707358095090641,NA
"14723","AC239868.1",1.26136756073792,0.944103402899804,1.41327631225694,0.668024642252802,0.504117862785644,NA
"14724","HIST2H2AB",0.956769728721768,0.667754953013017,1.46552416730357,0.455642402841863,0.648647146135135,NA
"14725","SV2A",1.55233383786355,-0.713938874145979,1.32625783618274,-0.538310767837459,0.590362518682682,NA
"14726","AC242988.2",0.114130778193026,-0.939216628356831,3.35247519753738,-0.280156175069319,0.779357688030112,NA
"14727","FALEC",1.73749438686657,1.29151267356764,1.21934644349845,1.05918435277683,0.289515829691978,NA
"14728","AL356356.1",0.0830923047099275,-0.939216628356831,3.35247519753738,-0.280156175069319,0.779357688030112,NA

"14729","HORMAD1",0.145281756542051,0.889415776427968,3.34729258829349
,0.265711990501974,0.790461028169891,NA
"14730","ANXA9",0.151849088832753,0.903480691260343,3.34685071118547,0
.269949504541936,0.787199101303289,NA
"14731","BNIPL",0.371492276406953,0.286980760168805,2.41816197757207,0
.118677227923725,0.905531078509693,NA
"14732","AL592424.1",0.151849088832753,0.903480691260343,3.34685071118
547,0.269949504541936,0.787199101303289,NA
"14733","TNFAIP8L2",0.89047908960619,-1.3249683105613,1.42746251242013
,-0.928198323271507,0.353304704047708,NA
"14734","TMOD4",0.122207625915474,0.359191432502738,3.35247519753738,0
.10714215955023,0.914676201160951,NA
"14735","AL391069.2",1.00274259200482,-2.06340986520399,1.470844801916
27,-1.4028739555089,0.16065442854838,NA
"14736","CGN",0.283673240731374,-1.61851549881525,2.89644275100986,-0.
558794230699344,0.576302160315398,NA
"14737","AL365436.2",1.31132707632821,0.520409928687472,1.204403087708
19,0.432089500598788,0.665676365350835,NA
"14738","AL391335.1",0.22669520365601,-0.362149828162381,3.12343089148
524,-0.115946163287824,0.90769520937636,NA
"14739","OAZ3",1.30553066717616,-0.135850554290261,1.16547340146882,-0
.116562552280517,0.90720671432568,NA
"14740","AL589765.5",0.0786722874602274,0.359191432502738,3.3524751975
3738,0.10714215955023,0.914676201160951,NA
"14741","TDRKH",1.289320621914,0.269272806245112,1.21329050751166,0.22
193597046874,0.824363728329679,NA
"14742","TDRKH-
AS1",0.254189351532287,-0.999830626761722,2.64981024805783,-0.37732159
4063025,0.705934626414087,NA
"14743","LINGO4",0,NA,NA,NA,NA,NA
"14744","RORC",0.486401491793822,-1.40804846406995,2.54357594248975,-0
.55357044409364,0.579872858005747,NA
"14745","C2CD4D",0.114130778193026,-0.939216628356831,3.35247519753738
,-0.280156175069319,0.779357688030112,NA
"14746","C2CD4D-AS1",0,NA,NA,NA,NA,NA
"14747","AL450992.2",0.0830923047099275,-0.939216628356831,3.352475197
53738,-0.280156175069319,0.779357688030112,NA
"14748","AL450992.1",0.163911907211157,0.976662149432262,3.34440272959
326,0.29202886984578,0.770264550640504,NA
"14749","AL589986.2",0.0830923047099275,-0.939216628356831,3.352475197
53738,-0.280156175069319,0.779357688030112,NA
"14750","HRNR",0.716283610248479,0.970720521450426,1.71547385222289,0.
565861449996791,0.571487969304634,NA
"14751","FLG",0.157344574920455,0.946214072007857,3.34552927999138,0.2
82829409883477,0.777307608562567,NA
"14752","FLG2",0.412747176291805,0.921514569013548,2.35763429573849,0.
3908640838315,0.695897703228618,NA
"14753","SPRR2D",0,NA,NA,NA,NA,NA
"14754","SPRR2A",0.358546030023975,0.325387423573685,2.74973617638295,
0.11833405196047,0.905802977374664,NA

"14755","SPRR2B",3.10869601890478,-3.6521478507988,3.18181295474853,-1
.14781978159601,NA,NA
"14756","LOR",0.392879813075865,-1.44104481813441,2.3048714659393,-0.6
25216997750086,0.531828647372181,NA
"14757","S100A7",0.32352918434031,-0.320695617766887,2.8435136113956,-
0.112781460402255,0.910203817008388,NA
"14758","BX470102.1",0.285820555772376,-0.362151522613874,2.8975007201
2302,-0.12498755223726,0.900533404934662,NA
"14759","S100A5",0.882357173031475,-1.8938673644956,1.61289635254294,-
1.17420276976241,0.240313816894074,NA
"14760","S100A1",0.696331874865024,-0.728728435978554,1.73702074904989
,-0.419527766940694,0.674830466115747,NA
"14761","AL358472.5",0.161764592170155,-0.362147980542956,3.3524751975
3738,-0.108024059599003,0.913976607082588,NA
"14762","AL358472.3",0.259412534735078,0.171500712501937,2.64651507850
536,0.0648024694417359,0.948331275255137,NA
"14763","AL358472.2",0.651698503198213,0.806580849807377,1.72700068382
712,0.467041418895071,0.640470244981237,NA
"14764","NUP210L",0,NA,NA,NA,NA,NA
"14765","C1orf189",0.804903160010435,-2.26048469844923,2.2265078549382
,-1.01526014985111,0.309981834899699,NA
"14766","IL6R-
AS1",1.36271343898661,-1.70282307970722,1.37394545516014,-1.2393673077
1364,0.215209502737395,NA
"14767","AL162591.2",0.370925454067831,0.79044230939703,2.734957675161
95,0.289014457728391,0.772570313793063,NA
"14768","UBE2Q1-
AS1",0.0786722874602274,0.359191432502738,3.35247519753738,0.107142159
55023,0.914676201160951,NA
"14769","CHRNB2",0,NA,NA,NA,NA,NA
"14770","KCNN3",0.266252169910691,-1.00025133134918,2.61314057122404,-
0.382777467987741,0.701884769397932,NA
"14771","AL451085.1",1.44510901462436,0.217689751189133,1.271468597046
86,0.171211268366945,0.864057647445009,NA
"14772","DCST2",0.266252169910691,-1.00025133134918,2.61314057122404,-
0.382777467987741,0.701884769397932,NA
"14773","DCST1-
AS1",0.527812019686771,0.229276883089463,1.83272096641129,0.1251019043
76866,0.900442875668077,NA
"14774","EFNA3",1.60156570364019,0.0304301692371851,1.05186094623748,0
.0289298403425228,0.976920546425399,NA
"14775","TRIM46",0.371677982843806,-1.17349104784383,2.39674677994784,
-0.4896182849443,0.624404035108053,NA
"14776","AC234582.2",0.0786722874602274,0.359191432502738,3.3524751975
3738,0.10714215955023,0.914676201160951,NA
"14777","ASH1L-IT1",0,NA,NA,NA,NA,NA
"14778","AL355388.2",0.452005165192231,-1.61777514371583,2.23501924951
201,-0.723830519164007,0.46916979030637,NA
"14779","PAQR6",0.725926810700969,0.945502363486489,1.70717264855853,0
.553841091751813,0.579687603344252,NA

"14780","TSACC",0.18074024727485,-0.362147980542956,3.35247519753738,-0.108024059599002,0.913976607082588,NA
"14781","C1orf61",0.407113909203042,0.933638270160703,2.33752397647948,0.399413344870518,0.689588662590381,NA
"14782","IQGAP3",0.32536797786157,1.05056297657179,2.83388144670233,0.370715217390017,0.710849654876456,NA
"14783","TTC24",0.104487577740536,-0.939216628356831,3.35247519753738,-0.280156175069319,0.779357688030112,NA
"14784","HAPLN2",0.244415251830948,1.06920175933036,3.34018855838734,0.320102215979858,0.748890845867476,NA
"14785","BCAN",1.28029028340093,-1.58913308800082,1.42640596877104,-1.11408191131588,0.26524404798466,NA
"14786","INSRR",0.359914141819658,0.66011251553761,2.40654147005796,0.274299248008268,0.783854662360472,NA
"14787","LRRC71",0,NA,NA,NA,NA,NA
"14788","FCRL5",0.290539550376331,0.291375383648554,2.56622020713621,0.113542626949274,0.909600370146826,NA
"14789","FCRL3",1.77874679729636,1.3753510257925,1.17128815235215,1.17422089776163,0.240306557621748,NA
"14790","AL356276.1",0,NA,NA,NA,NA,NA
"14791","FCRL2",0.170479239501859,1.00912327877525,3.3428978888652,0.301870805607469,0.762750551898993,NA
"14792","FCRL1",0,NA,NA,NA,NA,NA
"14793","KIRREL1-IT1",0.340666183830051,1.75463701655981,2.40175875280775,0.73056339006096,0.465045880536311,NA
"14794","AL138899.1",0,NA,NA,NA,NA,NA
"14795","CD1A",0,NA,NA,NA,NA,NA
"14796","CD1B",0.104487577740536,-0.939216628356831,3.35247519753738,-0.280156175069319,0.779357688030112,NA
"14797","CD1E",1.47081180003874,-0.723741691518549,1.26744145315492,-0.571025738283225,0.567982195972685,NA
"14798","SPTA1",0.647278485948513,1.68642759805619,1.86077381381153,0.906304455457584,0.364774727915364,NA
"14799","AIM2",1.60698617332576,-0.505688393288432,1.28023414437585,-0.394996802350532,0.69284524785732,NA
"14800","CADM3-AS1",0.661624802463148,-0.914424598231422,1.69279553399452,-0.540186088554734,0.589068705512823,NA
"14801","AL590560.2",0.458782932679034,-0.167890714862898,2.0691423521862,-0.0811402437756443,0.935330421277699,NA
"14802","VSIG8",0.896688661534244,-0.868864223876859,1.54818416576767,-0.561215030542591,0.574650957690667,NA
"14803","CFAP45",0.580035331004377,-1.14826377325826,2.13175578274559,-0.538646960666083,0.590130477749913,NA
"14804","IGSF9",0.456324871837746,-1.11966888142586,2.26024084063085,-0.495375918043033,0.62033479053545,NA
"14805","SLAMF9",0.275584624562896,-1.09440651193773,2.95796138001227,-0.369986748080257,0.71139236438397,NA
"14806","KCNJ10",0.0830923047099275,-0.939216628356831,3.3524751975373

8,-0.280156175069319,0.779357688030112,NA
"14807","AL121987.1",0.751104301372021,0.340324651255951,1.81001186301
631,0.188023436867875,0.850858278867766,NA
"14808","KCNJ9",0,NA,NA,NA,NA,NA
"14809","ATP1A2",0.0666094690818238,0.359191432502738,3.35247519753738
,0.10714215955023,0.91467620116095,NA
"14810","ATP1A4",0.114130778193026,-0.939216628356831,3.35247519753738
,-0.280156175069319,0.779357688030112,NA
"14811","AL121987.2",0.32794920159001,-1.34539026578307,2.784258536305
42,-0.483213124154892,0.628944417620445,NA
"14812","AL139011.1",1.03842880423105,0.334544655654782,1.44847190516,
0.230963855400306,0.817342882093985,NA
"14813","NHLH1",0.249151526962087,1.39724561189142,3.03826011163213,0.
459883472959405,0.645599863589248,NA
"14814","AL121985.1",0.228374061251979,0.170807695949971,2.75008424109
352,0.0621099868133684,0.95047524402806,NA
"14815","CD244",1.17582575388408,0.501685165459197,1.51883139499761,0.
330309978521339,0.741165753911424,NA
"14816","ITLN1",0,NA,NA,NA,NA,NA
"14817","F11R",1.61395340779072,1.00815973681353,1.14621753816578,0.87
9553577959404,0.379101196349091,NA
"14818","NECTIN4",0,NA,NA,NA,NA,NA
"14819","AL590714.1",0.192803065653254,-0.362148402639638,3.3015497885
8522,-0.109690425960478,0.912654893573412,NA
"14820","NR1I3",0.157344574920455,0.946214072007857,3.34552927999138,0
.282829409883477,0.777307608562567,NA
"14821","MPZ",0.782001371219058,-0.324783469147969,1.4899928561989,-0.
217976527737535,0.827447399855537,NA
"14822","CFAP126",0,NA,NA,NA,NA,NA
"14823","FCRLA",0.0786722874602274,0.359191432502738,3.35247519753738,
0.10714215955023,0.914676201160951,NA
"14824","FCRLB",1.77913651599498,-0.200912566566784,1.11297062921649,-
0.180519199062982,0.856744983305946,NA
"14825","NOS1AP",0.741863442653418,-1.34814560365334,1.64623334433841,
-0.818927406791857,0.412827832153447,NA
"14826","C1orf226",0.427433713395649,-0.154291225047592,2.318247322255
7,-0.0665551184148305,0.946935876793192,NA
"14827","SH2D1B",0.934606611442539,2.1991832888482,1.82013411046076,1.
20825343374917,0.226949794162524,NA
"14828","AL596325.1",0.390607110867167,0.781661342677902,2.14746417132
541,0.36399272831428,0.715863439440428,NA
"14829","RGS4",0.406198238384026,-2.49026032580906,2.22267811297646,-1
.12038729821938,0.262548755277703,NA
"14830","AL499616.1",0,NA,NA,NA,NA,NA
"14831","NUF2",1.35840103413888,-0.913623079077334,1.24876099451143,-0
.731623651838022,0.464398307960509,NA
"14832","AL357568.1",0.192803065653254,-0.362148402639638,3.3015497885
8522,-0.109690425960478,0.912654893573412,NA
"14833","AL356441.1",0.450156827505484,-0.759200852052902,2.2783000655
5285,-0.333231282187878,0.73895970645481,NA

"14834","AL451074.5",0.0666094690818238,0.359191432502738,3.3524751975
3738,0.10714215955023,0.91467620116095,NA
"14835","TMCO1-
AS1",0.743602360134408,-0.233248669711809,1.88446289419268,-0.12377461
5266031,0.901493731315322,NA
"14836","AL358115.1",1.47354410947504,-0.802638461116734,1.16305370802
78,-0.690112980661723,0.490123140724729,NA
"14837","AL596087.2",0.6081739606705,-0.345620196654101,1.901778350599
24,-0.181735267175166,0.855790487618776,NA
"14838","LINC01675",0.0830923047099275,-0.939216628356831,3.3524751975
3738,-0.280156175069319,0.779357688030112,NA
"14839","ILDR2",0,NA,NA,NA,NA,NA
"14840","GPA33",0.458252069610855,-2.67346529121449,2.40827692786756,-
1.11011539423821,0.266949304409175,NA
"14841","DUSP27",0,NA,NA,NA,NA,NA
"14842","AL451050.2",0.471559836897789,0.782889488900411,2.06967487415
934,0.378266895286345,0.705232335306444,NA
"14843","AL136984.1",0.371060175523305,0.28120847625078,2.697031688148
12,0.10426591481536,0.916958326480743,NA
"14844","AL359962.3",0.0852396197509296,0.359191432502738,3.3524751975
3738,0.10714215955023,0.91467620116095,NA
"14845","AL359962.2",0.122207625915474,0.359191432502738,3.35247519753
738,0.10714215955023,0.914676201160951,NA
"14846","AL031733.2",0,NA,NA,NA,NA,NA
"14847","ADCY10",0,NA,NA,NA,NA,NA
"14848","Z99943.1",0.26719040510327,-0.362151043418806,2.9631418910391
,-0.122218596589652,0.902725898914264,NA
"14849","AL023755.1",0.157344574920455,0.946214072007857,3.34552927999
138,0.282829409883477,0.777307608562567,NA
"14850","CCDC181",0.412771138999577,2.00835164696736,2.25023360443715,
0.892508068054432,0.372120670236776,NA
"14851","F5",1.0479122066848,-0.472241379044323,1.41491552644168,-0.33
3759415469802,0.7385611107058,NA
"14852","SELP",0,NA,NA,NA,NA,NA
"14853","SELE",0.114130778193026,-0.939216628356831,3.35247519753738,-
0.280156175069319,0.779357688030112,NA
"14854","LINC01681",0.104487577740536,-0.939216628356831,3.35247519753
738,-0.280156175069319,0.779357688030112,NA
"14855","LINC01142",1.47493889574006,0.246666034317767,1.2492944193150
4,0.197444277749202,0.843479882896077,NA
"14856","BX284613.2",0.466859780401481,1.07598643108137,1.888495627506
76,0.569758497403523,0.568841508169891,NA
"14857","MROH9",0.122207625915474,0.359191432502738,3.35247519753738,0
.10714215955023,0.914676201160951,NA
"14858","AL021026.1",0.0786722874602274,0.359191432502738,3.3524751975
3738,0.10714215955023,0.914676201160951,NA
"14859","MYOCOS",0,NA,NA,NA,NA,NA
"14860","FASLG",1.31723621267295,0.722629529401438,1.26134230059138,0.
572905173371761,0.566708901577403,NA
"14861","ANKRD45",0.692573360548216,1.54397195077024,2.41211513475046,

0.640090486779342,0.522113773414446,NA
"14862","GAS5-
AS1",0.747323541960645,0.157444437301274,1.89523957104073,0.0830736333
849432,0.933792990872608,NA
"14863","SERPINC1",0,NA,NA,NA,NA,NA
"14864","RC3H1-
IT1",0.622967142775102,-0.175755420859613,1.75781448888537,-0.09998519
2960299,0.920356080838905,NA
"14865","RABGAP1L-
DT",0.321256482131612,1.70014465421147,2.80178187083304,0.606808357178
063,0.543978101327833,NA
"14866","GPR52",0,NA,NA,NA,NA,NA
"14867","RABGAP1L-
IT1",1.04257254277545,2.33612313369984,1.67993583191849,1.390602598809
96,0.164345967861416,NA
"14868","Z99127.1",0,NA,NA,NA,NA,NA
"14869","TNN",0.249276914129782,-1.90751516048253,3.31840217494316,-0.
57482940882993,0.565406675152336,NA
"14870","TNR",0,NA,NA,NA,NA,NA
"14871","LINC02803",0.356714972864411,0.659068085576625,2.423310720244
76,0.27197011100172,0.785645001342075,NA
"14872","AL590723.1",0.364492843232603,0.248526632325804,2.40422023837
841,0.103370992539947,0.917668533390713,NA
"14873","AL591043.2",0.151849088832753,0.903480691260343,3.34685071118
547,0.269949504541936,0.787199101303289,NA
"14874","PAPPA2",4.74958288931055,0.530055507665663,1.05025802273955,0
.504690748548663,NA,NA
"14875","ASTN1",0.211891225623875,1.25257216600867,3.19422177898822,0.
392136881117075,0.694957078160183,NA
"14876","CLEC20A",0.0852396197509296,0.359191432502738,3.3524751975373
8,0.10714215955023,0.91467620116095,NA
"14877","TEX35",0.487786595987056,0.238184214187548,2.00633502571415,0
.11871607240808,0.905500302659623,NA
"14878","AL449106.1",0,NA,NA,NA,NA,NA
"14879","AXDND1",0.122207625915474,0.359191432502738,3.35247519753738,
0.10714215955023,0.914676201160951,NA
"14880","AL160286.2",0.719793525004011,1.06829593706466,1.703099484904
23,0.627265727301147,0.530485065228402,NA
"14881","AL353708.3",1.08113484021844,-1.78311579354831,1.337214480891
77,-1.33345534245125,0.182382421257897,NA
"14882","AL353708.1",0.965005608391476,1.30705566144465,1.599543520423
47,0.817142919061444,0.413846758251735,NA
"14883","LHX4",0.954284993411137,-1.51419278851152,1.45449422264132,-1
.04104420969221,0.297855030509283,NA
"14884","AL162431.1",0.819376401703739,1.19494204416206,1.683053999755
08,0.709984376220813,0.47771382486056,NA
"14885","KIAA1614-AS1",0,NA,NA,NA,NA,NA
"14886","LINC01732",0,NA,NA,NA,NA,NA
"14887","TEDDM1",0,NA,NA,NA,NA,NA
"14888","RGSL1",0,NA,NA,NA,NA,NA

"14889","AL355999.1",1.06383595633548,0.0928589706809822,1.31764679312603,0.0704733401738722,0.943816919238685,NA
"14890","SHCBP1L",0.681933810728222,-0.57198520467025,1.55763147517499,-0.367214719133735,0.713458860848289,NA
"14891","AL354953.1",1.78594544461691,-0.279139699614643,1.06928615504883,-0.261052383682921,0.794052111025809,NA
"14892","SMG7-AS1",1.42134852680788,0.794456215404806,1.33115709080072,0.596816274273776,0.550630051243893,NA
"14893","AL445228.2",1.14280116534408,-1.76080144771915,1.32961887437358,-1.32429035241299,0.1854066333736,NA
"14894","AL078645.1",1.07269200730324,1.36194472673386,1.50665990992185,0.903949668910024,0.366022090252584,NA
"14895","AL713852.1",0.325676499381312,0.658136172855001,2.4872279853395,0.264606291314773,0.791312773077208,NA
"14896","AL713852.2",0.348442108488148,1.43515432875047,2.41256076779889,0.59486763935892,0.551931950038862,NA
"14897","AL078644.2",0.183159865200764,-0.362147980542956,3.35247519753738,-0.108024059599003,0.913976607082588,NA
"14898","OCLM",0,NA,NA,NA,NA,NA
"14899","AL096803.4",0,NA,NA,NA,NA,NA
"14900","PACERR",0.412771138999577,2.00835164696736,2.25023360443715,0.892508068054432,0.372120670236776,NA
"14901","ERVMER61-1",0.475069751653322,0.858501406476679,2.07745888219353,0.413245919731616,0.679426447756419,NA
"14902","BRINP3",0.62477926180677,2.38563028136541,2.07650035317563,1.14887063597992,0.250609327394505,NA
"14903","AL390957.1",0.840648721724904,0.476847768046987,1.6079483561034,0.296556643897786,0.766805015618809,NA
"14904","AL136987.1",0.346161669917753,1.77269564705361,2.39335804484567,0.740672984918108,0.458891743200608,NA
"14905","B3GALT2",0.249769334282587,0.171289752160848,2.67665743248737,0.0639939015287703,0.94897508273126,NA
"14906","LINC01031",0,NA,NA,NA,NA,NA
"14907","AL138778.1",0.151849088832753,0.903480691260343,3.34685071118547,0.269949504541936,0.787199101303289,NA
"14908","AL136456.1",0,NA,NA,NA,NA,NA
"14909","CFHR3",0.401759826751403,1.92565540342568,2.61480155185955,0.736444187153026,0.461460421927211,NA
"14910","CFHR1",0.870599122658928,0.907918717220901,1.99409747232718,0.455303078119512,0.648891212082982,NA
"14911","ASPM",1.12994346111926,-1.45936136126767,1.62642862246891,-0.897279684522746,0.369569694154634,NA
"14912","CRB1",0,NA,NA,NA,NA,NA
"14913","LHX9",1.49109169965597,1.44890099399864,1.42697976934509,1.01536197297571,0.30993331288496,NA
"14914","AL450352.1",0,NA,NA,NA,NA,NA
"14915","AL157402.2",0.0786722874602274,0.359191432502738,3.35247519753738,0.10714215955023,0.914676201160951,NA
"14916","MIR181A1HG",0.877739680898892,1.17441242241464,1.580086426169

86,0.743258345216863,0.457325285424259,NA
"14917","NR5A2",0.122207625915474,0.359191432502738,3.35247519753738,0
.10714215955023,0.914676201160951,NA
"14918","LINC00862",0.0786722874602274,0.359191432502738,3.35247519753
738,0.10714215955023,0.914676201160951,NA
"14919","AL359834.1",0,NA,NA,NA,NA,NA
"14920","KIF14",0.311024720912772,1.59651094038275,2.84467532261099,0.
56122782367915,0.574642237573076,NA
"14921","DDX59-
AS1",0.45554726649826,2.14587417004055,2.48624002273772,0.863100163465
966,0.38808239808636,NA
"14922","INAVA",0.290539550376331,0.291375383648554,2.56622020713621,0
.113542626949274,0.909600370146826,NA
"14923","AL358473.1",0,NA,NA,NA,NA,NA
"14924","ASCL5",0,NA,NA,NA,NA,NA
"14925","IGFN1",1.55441241562242,-3.67230573670533,1.47974802102739,-2
.48171018614077,0.0130753571625837,NA
"14926","PKP1",0,NA,NA,NA,NA,NA
"14927","LAD1",0,NA,NA,NA,NA,NA
"14928","TNNI1",0,NA,NA,NA,NA,NA
"14929","AC096677.1",0.765659939227017,0.901057826203211,1.69299991652
687,0.532225558552715,0.594569783134739,NA
"14930","AL645504.1",0.475069751653322,0.858501406476679,2.07745888219
353,0.413245919731616,0.679426447756419,NA
"14931","AL513217.1",0.619005134826917,1.62366785380049,2.074219725509
53,0.782784887170831,0.433753443753556,NA
"14932","ELF3-
AS1",1.74756620351451,-0.310474994915874,1.3600913678131,-0.2282751014
11084,0.819432379778707,NA
"14933","ELF3",0.508228865708078,-1.16345072849723,2.21105170969501,-0
.526197882842694,0.598750724870706,NA
"14934","GPR37L1",0.294871025467877,-1.1768104696752,2.89552204169079,
-0.406424282989752,0.684430880475328,NA
"14935","LGR6",0.435821306918382,1.07561819445238,1.91537867230443,0.5
61569474488451,0.57440938311558,NA
"14936","SYT2",0.0786722874602274,0.359191432502738,3.35247519753738,0
.10714215955023,0.914676201160951,NA
"14937","AC104463.2",1.05620117013892,1.23505694477784,1.7633523689318
9,0.700402804645306,0.48367578524445,NA
"14938","AC098934.4",0.0852396197509296,0.359191432502738,3.3524751975
3738,0.10714215955023,0.91467620116095,NA
"14939","PPFIA4",0.205299930625402,-0.362148984940857,3.22997826280445
,-0.11212118332537,0.910727321463183,NA
"14940","ADORA1",0.59771902261793,-0.324908649704985,1.94143825300075,
-0.167354614138665,0.867091034137532,NA
"14941","CHIT1",1.05631965281952,-2.98874519731107,1.79391737915312,-1
.66604395054247,0.0957046608878007,NA
"14942","LINC01353",0.332861638992515,-0.46854418161012,2.236949960888
52,-0.209456711058486,0.834091725397205,NA
"14943","LINC01136",0.313613681002908,0.620599635142673,2.254562456021

01,0.275263891441688,0.783113503721555,NA
"14944","ZBED6",1.5594011887422,0.629559755259212,1.08170639622903,0.582006131658219,0.560562550740324,NA
"14945","LINC00303",0,NA,NA,NA,NA,NA
"14946","AL592146.2",0,NA,NA,NA,NA,NA
"14947","LINC00628",0.0786722874602274,0.359191432502738,3.35247519753738,0.10714215955023,0.914676201160951,NA
"14948","AL512306.3",0.933670321960056,1.48525917433713,1.56928941522352,0.94645331825269,0.343917421485252,NA
"14949","AL512306.2",0.586164502954391,1.46636231842891,2.45130558844167,0.598196457162689,0.549708855115315,NA
"14950","NFASC",0.488388176961483,-0.569776754727876,2.05463012142295,-0.277313541151276,0.781539362821031,NA
"14951","CNTN2",0.665610773119104,-1.73638064922068,1.67843473953746,-1.0345237788031,0.300891366236283,NA
"14952","TMEM81",1.46484290652689,2.1142143862508,1.3910549277868,1.51986405714011,0.128545145588191,NA
"14953","CDK18",0.888452015792811,0.114231701899071,1.45544524692785,0.0784857432048313,0.937441670512581,NA
"14954","MFSD4A",0.421177126905233,-0.0731535521177196,2.10367812760873,-0.0347741183205027,0.972259858734036,NA
"14955","SLC45A3",1.34777774643788,1.14007615120613,1.52956765774601,0.745358432124647,0.456055065816262,NA
"14956","PM20D1",0.507627284733652,-0.0751594349487679,2.02901340178876,-0.0370423551084029,0.970451234405568,NA
"14957","RHEX",0.497375127178098,-0.446481214919928,2.25888255406181,-0.197655789636821,0.843314381961664,NA
"14958","C1orf147",0.197223082902954,-1.61249994371822,3.25614921967676,-0.495216845092344,0.620447061165921,NA
"14959","AC244034.2",0.323087539291176,1.63986284743208,2.81636913523065,0.582261333189118,0.560390666306499,NA
"14960","IL19",0.17109704682236,-0.362147980542956,3.35247519753738,-0.108024059599003,0.913976607082588,NA
"14961","IL20",0.482291109899355,0.217842837747772,2.03694567985525,0.106945825753808,0.914831957941454,NA
"14962","IL24",0.145281756542051,0.889415776427968,3.34729258829349,0.265711990501974,0.790461028169891,NA
"14963","PIGR",0.114130778193026,-0.939216628356831,3.35247519753738,-0.280156175069319,0.779357688030112,NA
"14964","C4BPB",0,NA,NA,NA,NA,NA
"14965","AL445493.3",0.363740314456628,1.88607282613257,2.34476894513503,0.804374703974917,0.421180605787472,NA
"14966","CR2",0.104487577740536,-0.939216628356831,3.35247519753738,-0.280156175069319,0.779357688030112,NA
"14967","CR1L",0.645892271310227,0.679736437362413,2.07169005745265,0.328107206441014,0.742830605397839,NA
"14968","AL356275.1",0.145281756542051,0.889415776427968,3.34729258829349,0.265711990501974,0.790461028169891,NA
"14969","C1orf74",1.10078759822739,0.531351968301436,1.38862033969737,0.382647404125766,0.701981216994315,NA

"14970","IRF6",0.0830923047099275,-0.939216628356831,3.35247519753738,
-0.280156175069319,0.779357688030112,NA
"14971","KCNH1",0.203160351946096,0.359191432502738,3.35247519753738,0
.10714215955023,0.91467620116095,NA
"14972","AL590101.1",0.314711714214473,-1.82631342394583,2.81227742011
208,-0.649407277847091,0.51607516109671,NA
"14973","RD3",0,NA,NA,NA,NA,NA
"14974","NEK2",0.0830923047099275,-0.939216628356831,3.35247519753738,
-0.280156175069319,0.779357688030112,NA
"14975","DTL",0.74258580860044,0.773357384389378,1.8719011063516,0.413
140086175107,0.679503981408036,NA
"14976","AL360091.2",0.122207625915474,0.359191432502738,3.35247519753
738,0.10714215955023,0.914676201160951,NA
"14977","AL360091.3",0.475069751653322,0.858501406476679,2.07745888219
353,0.413245919731616,0.679426447756419,NA
"14978","LINC01740",0.200879913375701,0.981067669524494,3.256229501765
31,0.301289472683859,0.763193767774683,NA
"14979","AC092803.4",0.444535954250087,2.08667833413236,2.245305408571
53,0.929351671343417,0.35270686503969,NA
"14980","AL590648.2",0,NA,NA,NA,NA,NA
"14981","LINC02773",0.47983415950312,-1.41026983303372,2.5537563468799
2,-0.552233510748482,0.580788377831152,NA
"14982","PROX1-
AS1",0.464413488006224,-1.72499908679082,2.02555745614535,-0.851616961
818253,0.39442672224818,NA
"14983","AC011700.1",0,NA,NA,NA,NA,NA
"14984","KCNK2",0.80596720995688,1.25335235081225,1.63470959433837,0.7
66712543410216,0.443252444178907,NA
"14985","ESRRG",0.720590356560528,0.747804539346605,1.85827188930202,0
.402419335755805,0.687375442223274,NA
"14986","TGFB2-
AS1",0.166184609419855,-1.47576652541587,3.34432018311399,-0.441275489
370681,0.659013568361442,NA
"14987","LYPLAL1-
DT",0.0786722874602274,0.359191432502738,3.35247519753738,0.1071421595
5023,0.914676201160951,NA
"14988","AL360093.1",0,NA,NA,NA,NA,NA
"14989","AC096642.2",0.114130778193026,-0.939216628356831,3.3524751975
3738,-0.280156175069319,0.779357688030112,NA
"14990","ZC3H11B",1.26485700817272,1.56451342065239,1.73622102164452,0
.901102682866097,0.367533726606106,NA
"14991","SLC30A10",0,NA,NA,NA,NA,NA
"14992","C1orf115",0.596581686111367,-2.28989626475573,1.8570293855571
8,-1.23309640793254,0.217539797483107,NA
"14993","MARC1",1.78695063011258,0.903093060117908,1.27011051954382,0.
711035021143097,0.477062534167983,NA
"14994","AL445423.1",1.72943412572326,1.14435048336264,1.1517117560658
6,0.993608407082367,0.320413549633096,NA
"14995","LINC01352",0.0666094690818238,0.359191432502738,3.35247519753
738,0.10714215955023,0.91467620116095,NA

"14996","HLX-
AS1",1.14873229593136,0.801459608138716,1.44831063408434,0.55337549091
837,0.580006318016265,NA
"14997","LINC02817",0.323105933674099,-2.17962193225865,2.750473705762
67,-0.792453288207046,0.428096418402325,NA
"14998","LINC01655",0,NA,NA,NA,NA,NA
"14999","LINC02257",0,NA,NA,NA,NA,NA
"15000","AL356108.1",0.589040731847611,-1.07542667595708,1.80436111598
618,-0.596015213600578,0.551165063390905,NA
"15001","AL513314.2",0.635155486227508,0.394277340536777,1.97938594774
974,0.199191744785806,0.84211275674027,NA
"15002","HHIPL2",0,NA,NA,NA,NA,NA
"15003","FAM177B",0,NA,NA,NA,NA,NA
"15004","AL392172.2",0,NA,NA,NA,NA,NA
"15005","AL929091.1",0.271475353113481,0.224450700853462,2.97462372112
04,0.0754551573228636,0.939852575048144,NA
"15006","SUSD4",0.163911907211157,0.976662149432262,3.34440272959326,0
.29202886984578,0.770264550640504,NA
"15007","CAPN8",0.122207625915474,0.359191432502738,3.35247519753738,0
.10714215955023,0.914676201160951,NA
"15008","AC138393.3",0,NA,NA,NA,NA,NA
"15009","AC092809.2",0.189727197491466,-0.362148248616811,3.3202229532
9944,-0.109073472989797,0.913144214822408,NA
"15010","AC092809.4",0.114130778193026,-0.939216628356831,3.3524751975
3738,-0.280156175069319,0.779357688030112,NA
"15011","AC096537.1",0.166184609419855,-1.47576652541587,3.34432018311
399,-0.441275489370681,0.659013568361442,NA
"15012","AC099066.2",0.157344574920455,0.946214072007857,3.34552927999
138,0.282829409883477,0.777307608562567,NA
"15013","LEFTY1",0.783709127585163,-0.522583225854821,1.80200426920409
,-0.290001103097073,0.771815393700973,NA
"15014","AL117348.1",0.0786722874602274,0.359191432502738,3.3524751975
3738,0.10714215955023,0.914676201160951,NA
"15015","LEFTY2",1.48411294140903,-0.199377352696371,1.24866487576203,
-0.159672428180296,0.873139121326623,NA
"15016","AL512343.2",0.888743256785369,0.158019890073934,1.54667317203
22,0.102167602652802,0.918623642498142,NA
"15017","LINC01703",1.27386261792653,1.23146812281698,1.29018236201945
,0.954491519237199,0.339834900125444,NA
"15018","ACBD3-AS1",0,NA,NA,NA,NA,NA
"15019","MIXL1",0,NA,NA,NA,NA,NA
"15020","ITPKB-
IT1",0.0830923047099275,-0.939216628356831,3.35247519753738,-0.2801561
75069319,0.779357688030112,NA
"15021","ITPKB-
AS1",0.282462702653884,-1.0372525382335,2.56395923547331,-0.4045511035
75567,0.685807506504499,NA
"15022","AL359732.1",0.268399484951693,0.250807602063781,2.61654731113
631,0.0958544112679779,0.923636202325112,NA
"15023","AL353689.3",0,NA,NA,NA,NA,NA

"15024","AL627308.3",0,NA,NA,NA,NA,NA
"15025","AL731702.1",0.0786722874602274,0.359191432502738,3.3524751975
3738,0.10714215955023,0.914676201160951,NA
"15026","PRSS38",0,NA,NA,NA,NA,NA
"15027","LINC02809",0,NA,NA,NA,NA,NA
"15028","IBA57-DT",0,NA,NA,NA,NA,NA
"15029","OBSCN-AS1",0,NA,NA,NA,NA,NA
"15030","AL353593.3",0.122207625915474,0.359191432502738,3.35247519753
738,0.10714215955023,0.914676201160951,NA
"15031","AL670729.3",0.157344574920455,0.946214072007857,3.34552927999
138,0.282829409883477,0.777307608562567,NA
"15032","TRIM17",0.972641727339591,0.867773097867524,1.81199587060699,
0.478904567026875,0.632006521804184,NA
"15033","HIST3H2BB",0.557728009769505,0.0772003433572516,1.99426358628
262,0.0387112034177768,0.969120641070043,NA
"15034","AL117350.1",0.268399484951693,0.250807602063781,2.61654731113
631,0.0958544112679779,0.923636202325112,NA
"15035","ACTA1",0.692539975152788,-1.0464991740723,2.32318204657459,-0
.450459392803634,0.652379227839796,NA
"15036","LINC01736",0.437349216733051,-0.980388118241331,2.06143510558
725,-0.475585244271875,0.634369889792838,NA
"15037","AL136988.2",0,NA,NA,NA,NA,NA
"15038","AL136988.1",0,NA,NA,NA,NA,NA
"15039","PGBD5",0.0786722874602274,0.359191432502738,3.35247519753738,
0.10714215955023,0.914676201160951,NA
"15040","AL158214.2",0,NA,NA,NA,NA,NA
"15041","AL118511.1",0.278042685404183,0.250550992867118,2.58936724201
233,0.0967614746961895,0.922915819060029,NA
"15042","TRIM67",0,NA,NA,NA,NA,NA
"15043","AL136171.2",0.696331874865024,-0.728728435978554,1.7370207490
4989,-0.419527766940694,0.674830466115747,NA
"15044","DISC1-
IT1",0.199370397943956,-0.362148716949221,3.26311252083995,-0.11098260
1622331,0.911630143386501,NA
"15045","SLC35F3",0.575740700922373,-2.92445222774639,1.99408593359668
,-1.46656278873179,0.142495028916197,NA
"15046","AL160408.2",0.803673117125218,-0.907617885542496,1.6542631022
4277,-0.548653889645482,0.583242995710902,NA
"15047","LINC01132",0.305068513761982,-1.76759864781942,2.836805001044
18,-0.623094871578693,0.533222179627375,NA
"15048","AL391832.4",0,NA,NA,NA,NA,NA
"15049","AL391832.3",0.114130778193026,-0.939216628356831,3.3524751975
3738,-0.280156175069319,0.779357688030112,NA
"15050","LINC01348",0,NA,NA,NA,NA,NA
"15051","AL732292.2",0.845379622701444,-0.347857622310169,1.7129964699
9412,-0.203069666752648,0.839080580174086,NA
"15052","LINC02768",0.151849088832753,0.903480691260343,3.346850711185
47,0.269949504541936,0.787199101303289,NA
"15053","EDARADD",0.876548995433392,-1.83715809847443,1.53043428096005
,-1.20041619645501,0.229977741710139,NA

"15054","LGALS8-
AS1",0.877467240107675,-0.527006662091602,1.7118356060396,-0.307860556
37133,0.758188440455328,NA
"15055","AL359921.1",0.497375127178098,-0.446481214919928,2.2588825540
6181,-0.197655789636821,0.843314381961664,NA
"15056","ACTN2",0.37807583504373,-0.00692142756391464,2.40002309063613
,-0.00288390040534157,0.997698983581232,NA
"15057","RYR2",0,NA,NA,NA,NA,NA
"15058","AC093426.1",0,NA,NA,NA,NA,NA
"15059","CHRM3",0,NA,NA,NA,NA,NA
"15060","CHRM3-
AS2",1.38443827460545,-0.431761463792007,1.40392576104663,-0.307538671
752933,0.758433392454737,NA
"15061","AL358176.4",0,NA,NA,NA,NA,NA
"15062","RGS7",0.0852396197509296,0.359191432502738,3.35247519753738,0
.10714215955023,0.91467620116095,NA
"15063","AL359764.2",0.0666094690818238,0.359191432502738,3.3524751975
3738,0.10714215955023,0.91467620116095,NA
"15064","KMO",0.205299930625402,-0.362148984940857,3.22997826280445,-0
.11212118332537,0.910727321463183,NA
"15065","AL133390.1",0,NA,NA,NA,NA,NA
"15066","EXO1",0.166184609419855,-1.47576652541587,3.34432018311399,-0
.441275489370681,0.659013568361442,NA
"15067","PLD5",1.75715989992932,1.69438586491887,1.35950528757473,1.24
632532172166,0.212644985229032,NA
"15068","AL591686.2",0.334098851539349,1.73481287665147,2.412525677212
15,0.719085766853338,0.472088076436264,NA
"15069","AL591686.1",0.351491789661621,-0.398343995216617,2.2034471491
87,-0.180782187293938,0.85653854520998,NA
"15070","AL591721.1",0,NA,NA,NA,NA,NA
"15071","AKT3-
IT1",0.877739680898892,1.17441242241464,1.58008642616986,0.74325834521
6863,0.457325285424259,NA
"15072","C1orf100",0,NA,NA,NA,NA,NA
"15073","CATSPERE",0.97369574565977,1.42983940809896,1.75140396486327,
0.816396123786661,0.414273612876663,NA
"15074","AL451007.3",0.458782932679034,-0.167890714862898,2.0691423521
862,-0.0811402437756443,0.935330421277699,NA
"15075","AL451007.2",0.477217066694324,1.98730650884634,2.027410691945
53,0.980219013711173,0.326978020932393,NA
"15076","BX323046.2",0.805079671836275,-0.0163621674716061,1.559888195
41377,-0.0104893206575397,0.991630886464122,NA
"15077","BX323046.1",0.330442021066602,-0.554189166955134,2.4863221641
0539,-0.222895156128948,0.823617106411498,NA
"15078","KIF26B",0.168331924460857,-0.362147980542956,3.35247519753738
,-0.108024059599003,0.913976607082588,NA
"15079","SMYD3-IT1",0,NA,NA,NA,NA,NA
"15080","AL591623.1",0.863097446595839,0.138809283051583,1.46333117355
999,0.0948584199938064,0.924427283574716,NA
"15081","AL591848.3",0.453832052361155,-1.82912497444579,2.18784610131

088,-0.836039140664346,0.403132884045081,NA
"15082","AL591848.4",0.998783295818211,0.515279799586181,1.47970945362447,0.348230389637662,0.727667166728303,NA
"15083","ZNF695",0.0852396197509296,0.359191432502738,3.35247519753738,0.10714215955023,0.91467620116095,NA
"15084","C1orf229",1.00730178858979,0.139379437821794,1.30562228226979,0.10675326219118,0.914984726872926,NA
"15085","AC104335.1",0.274966817242395,0.284464995938123,2.96042306445425,0.0960893053947896,0.923449645061639,NA
"15086","AC104335.2",0.428954997273425,-0.505718626621086,2.11715173722603,-0.238867445223221,0.811208369850691,NA
"15087","OR2B11",0,NA,NA,NA,NA,NA
"15088","GCSAML",0.416422401147476,-0.444123842470041,2.34323182696576,-0.189534743152211,0.849673727454178,NA
"15089","OR2C3",0.71568976563575,2.7907969576574,1.95754562889753,1.42566125481793,0.153966145551619,NA
"15090","AL390860.1",0.161764592170155,-0.362147980542956,3.35247519753738,-0.108024059599003,0.913976607082588,NA
"15091","TRIM58",0.842008749374875,0.0612245331592683,1.78326889631457,0.0343327544633337,0.97261180597385,NA
"15092","OR2L2",0.197223082902954,-1.61249994371822,3.25614921967676,-0.495216845092344,0.620447061165921,NA
"15093","FAM110C",0.404040265321798,1.66181225674076,2.34790920529425,0.707783867022446,0.479079486440851,NA
"15094","AC079779.2",0.452311878908183,1.9387780844847,2.24938708539565,0.861913939611546,0.388734877595126,NA
"15095","AC093326.1",0.244856896880082,-0.902701635500065,3.0767047150267,-0.293398853354776,0.769217301797547,NA
"15096","AC116609.3",0.510983679623078,1.28620824799311,2.18742008787865,0.588002393833946,0.55653068604458,NA
"15097","LINC01115",0,NA,NA,NA,NA,NA
"15098","TPO",0,NA,NA,NA,NA,NA
"15099","AC144450.1",0.0786722874602274,0.359191432502738,3.35247519753738,0.10714215955023,0.914676201160951,NA
"15100","MYT1L",0,NA,NA,NA,NA,NA
"15101","EIPR1-IT1",0.0786722874602274,0.359191432502738,3.35247519753738,0.10714215955023,0.914676201160951,NA
"15102","TRAPPC12-AS1",0.200879913375701,0.981067669524494,3.25622950176531,0.301289472683859,0.763193767774683,NA
"15103","AC114810.1",0,NA,NA,NA,NA,NA
"15104","AC231981.1",1.75188054828195,-1.11622914980205,1.10937877265689,-1.00617496684992,0.314331411795135,NA
"15105","AC108488.3",1.59892082250631,1.48620989461831,1.23044223999501,1.20786644533948,0.227098638193381,NA
"15106","SOX11",0,NA,NA,NA,NA,NA
"15107","SILC1",1.60157121572135,0.422947505391425,1.16366770600191,0.363460722687383,0.716260748369862,NA
"15108","LINC00487",0.0830923047099275,-0.939216628356831,3.3524751975

3738,-0.280156175069319,0.779357688030112,NA
"15109","NRIR",0.0666094690818238,0.359191432502738,3.35247519753738,0
.10714215955023,0.91467620116095,NA
"15110","AC017076.1",0,NA,NA,NA,NA,NA
"15111","RNF144A-
AS1",0.157344574920455,0.946214072007857,3.34552927999138,0.2828294098
83477,0.777307608562567,NA
"15112","AC010904.2",0.0830923047099275,-0.939216628356831,3.352475197
53738,-0.280156175069319,0.779357688030112,NA
"15113","AC013460.1",0.0786722874602274,0.359191432502738,3.3524751975
3738,0.10714215955023,0.914676201160951,NA
"15114","AC092580.4",0.157344574920455,0.946214072007857,3.34552927999
138,0.282829409883477,0.777307608562567,NA
"15115","LINC00298",0.200879913375701,0.981067669524494,3.256229501765
31,0.301289472683859,0.763193767774683,NA
"15116","LINC00299",1.83808835595648,-0.115797095147829,1.054844645572
9,-0.109776444933214,0.912586672342591,NA
"15117","U91324.1",0.502131798645951,-0.0949818643491546,2.03215692487
082,-0.0467394339416935,0.962720900933852,NA
"15118","LINC01814",0.911516602253902,2.03926327258307,1.6558946368372
5,1.231517529689,0.218129364483578,NA
"15119","AC092617.1",0.0830923047099275,-0.939216628356831,3.352475197
53738,-0.280156175069319,0.779357688030112,NA
"15120","ID2-
AS1",1.42250250615752,2.72917898074443,1.42607981341055,1.913763139397
82,0.0556504273249601,NA
"15121","AC073195.1",1.61425872317246,0.757877495384806,1.239539457031
31,0.611418612845063,0.540922483563984,NA
"15122","AC082651.1",0.973249852588299,-0.685478368335218,1.3547218981
3561,-0.505991945120679,0.612862304273474,NA
"15123","AC082651.4",0,NA,NA,NA,NA,NA
"15124","AC082651.3",0.0666094690818238,0.359191432502738,3.3524751975
3738,0.10714215955023,0.91467620116095,NA
"15125","AC010969.1",0.0830923047099275,-0.939216628356831,3.352475197
53738,-0.280156175069319,0.779357688030112,NA
"15126","AC104794.5",0.695340778194538,-1.12837436648824,1.78889790176
203,-0.630765101449788,0.528194125074038,NA
"15127","RRM2",0.897345399251148,-0.168124511596778,1.47199974907701,-
0.114215040934754,0.909067328991854,NA
"15128","AC007240.3",0.161764592170155,-0.362147980542956,3.3524751975
3738,-0.108024059599003,0.913976607082588,NA
"15129","ODC1-
DT",0.857918218389575,0.974967590829583,1.65820406993085,0.58796598591
767,0.556555123870503,NA
"15130","ATP6V1C2",0.966966729534845,-0.259756044581426,1.643966221981
96,-0.1580057066308,0.874452301965124,NA
"15131","AC092687.3",0.713827773687735,0.707220950883431,1.78239644396
732,0.396780948075319,0.691529001193477,NA
"15132","C2orf50",0.0666094690818238,0.359191432502738,3.3524751975373
8,0.10714215955023,0.91467620116095,NA

"15133","LINC00570",0.892304862919374,0.164284468726726,1.50613755325766,0.109076669904015,0.913141679182589,NA
"15134","AC099344.3",0,NA,NA,NA,NA,NA
"15135","MIR3681HG",0.17109704682236,-0.362147980542956,3.35247519753738,-0.108024059599003,0.913976607082588,NA
"15136","AC009486.1",0,NA,NA,NA,NA,NA
"15137","LINC00276",0.166184609419855,-1.47576652541587,3.34432018311399,-0.441275489370681,0.659013568361442,NA
"15138","MYCN",0,NA,NA,NA,NA,NA
"15139","AC079148.1",0.931844548646872,0.10632020698868,1.44934803683094,0.0733572642918491,0.941521824082706,NA
"15140","NT5C1B",0,NA,NA,NA,NA,NA
"15141","AC130814.1",0,NA,NA,NA,NA,NA
"15142","LINC01376",1.3225196371132,-0.786481763826799,1.45495402163522,-0.540554376380136,0.588814771760343,NA
"15143","AC010096.1",0.0830923047099275,-0.939216628356831,3.35247519753738,-0.280156175069319,0.779357688030112,NA
"15144","AC013400.1",0.95122500379116,0.87398308055839,1.44903305200617,0.60314916857719,0.546409459624389,NA
"15145","AC079145.1",0.421618909860674,1.82758340282461,2.27552689244673,0.803147354087971,0.421889570170481,NA
"15146","MATN3",1.35634728585616,-1.20266991938314,1.16761105905326,-1.03002614617089,0.302997732009595,NA
"15147","LAPTM4A-DT",0.0786722874602274,0.359191432502738,3.35247519753738,0.10714215955023,0.914676201160951,NA
"15148","AC012065.3",0.314711714214473,-1.82631342394583,2.81227742011208,-0.649407277847091,0.51607516109671,NA
"15149","GDF7",0.31910916709061,0.630344098013162,2.86281997001412,0.220182933127316,0.82572868787641,NA
"15150","AC012065.4",0.285227825015386,-1.13200789171034,2.56421258353021,-0.441464135610736,0.658877020046736,NA
"15151","APOB",0.742559134110847,1.79018173429313,1.71098836844967,1.04628515734167,0.295429396118101,NA
"15152","TDRD15",0.0852396197509296,0.359191432502738,3.35247519753738,0.10714215955023,0.91467620116095,NA
"15153","AC018742.1",0.0852396197509296,0.359191432502738,3.35247519753738,0.10714215955023,0.91467620116095,NA
"15154","AC011752.1",0,NA,NA,NA,NA,NA
"15155","AC018467.1",0.483342616029585,0.093245494467271,1.89783124080355,0.0491326586171015,0.960813577014817,NA
"15156","AC009242.1",0.85850794925536,1.41356368720976,1.75075663556501,0.807401587687588,0.419435152519494,NA
"15157","MFSD2B",0.360824244313826,-0.544159242155938,2.43169962691388,-0.223777326826563,0.822930573166964,NA
"15158","AC009228.1",0.66315382270262,-0.102865502420274,1.80512019134992,-0.0569854034724126,0.954556822587482,NA
"15159","AC012073.1",0.122207625915474,0.359191432502738,3.35247519753738,0.10714215955023,0.914676201160951,NA
"15160","EFR3B",1.11008383426502,-2.1936146468527,1.55410713440836,-1.

4114951268709,0.158098671499744,NA
"15161","AC012074.1",0.0852396197509296,0.359191432502738,3.3524751975
3738,0.10714215955023,0.91467620116095,NA
"15162","ADGRF3",0.114130778193026,-0.939216628356831,3.35247519753738
,-0.280156175069319,0.779357688030112,NA
"15163","OTOF",0.0852396197509296,0.359191432502738,3.35247519753738,0
.10714215955023,0.91467620116095,NA
"15164","KCNK3",0.340504440121218,0.631065267568559,2.80473121548499,0
.225000265296165,0.82197906787763,NA
"15165","DPYSL5",0,NA,NA,NA,NA,NA
"15166","AC013472.1",0,NA,NA,NA,NA,NA
"15167","AGBL5-
AS1",0.244856896880082,-0.902701635500065,3.0767047150267,-0.293398853
354776,0.769217301797547,NA
"15168","AC013472.3",0,NA,NA,NA,NA,NA
"15169","AC013403.2",0.122207625915474,0.359191432502738,3.35247519753
738,0.10714215955023,0.914676201160951,NA
"15170","ABHD1",1.35883864712393,-1.85108351448748,1.34207360634506,-1
.37927123053157,0.167811143302726,NA
"15171","SLC30A3",0,NA,NA,NA,NA,NA
"15172","TRIM54",0,NA,NA,NA,NA,NA
"15173","GTF3C2-
AS1",0.207447245666404,1.02558430340912,3.22299417080721,0.31820855051
4461,0.750326747237409,NA
"15174","KRTCAP3",1.54796572184979,-0.551201784333171,1.09443980560558
,-0.503638282809145,0.614515585393954,NA
"15175","GCKR",0.314711714214473,-1.82631342394583,2.81227742011208,-0
.649407277847091,0.51607516109671,NA
"15176","LINC01460",0,NA,NA,NA,NA,NA
"15177","AC104695.2",0,NA,NA,NA,NA,NA
"15178","AC104695.3",1.73856568571393,1.47032952348708,1.3536609656358
3,1.08618742861987,0.277396077994842,NA
"15179","PLB1",1.32292763738277,-2.75824689910328,1.42897236831992,-1.
93023109491348,0.0535782103585183,NA
"15180","SPDYA",0.963310386543126,-0.858625220704587,1.46359125017554,
-0.586656431979631,0.557434474070276,NA
"15181","AC097724.1",0.151849088832753,0.903480691260343,3.34685071118
547,0.269949504541936,0.787199101303289,NA
"15182","TOGARAM2",1.33681002895317,1.29136780916268,1.40824124549286,
0.917007517920498,0.359138700636067,NA
"15183","PCARE",0.371492276406953,0.286980760168805,2.41816197757207,0
.118677227923725,0.905531078509693,NA
"15184","ALK",0.236016862380682,1.34721548542771,3.32885593646028,0.40
4708257474268,0.68569197172232,NA
"15185","AC016907.2",0.508228865708078,-1.16345072849723,2.21105170969
501,-0.526197882842694,0.598750724870706,NA
"15186","LINC01936",0.309787508365938,-1.00169890791137,2.498784887506
29,-0.400874406164283,0.688512594116363,NA
"15187","GALNT14",0,NA,NA,NA,NA,NA
"15188","CAPN14",0,NA,NA,NA,NA,NA

"15189","EHD3",1.55420502440233,0.405408601381907,1.27083478405265,0.319009682823656,0.749719170276289,NA
"15190","AL121652.1",0.22669520365601,-0.362149828162381,3.12343089148524,-0.115946163287824,0.90769520937636,NA
"15191","AL121655.1",0.719840052085346,-0.342800071699753,1.71571516180752,-0.199800106294223,0.841636918192243,NA
"15192","NLRC4",0.0830923047099275,-0.939216628356831,3.35247519753738,-0.280156175069319,0.779357688030112,NA
"15193","AL133243.1",0.31193482340694,0.289908158656661,2.51389477307914,0.115322312517309,0.90818965351672,NA
"15194","LINC00486",0,NA,NA,NA,NA,NA
"15195","AC019127.1",0,NA,NA,NA,NA,NA
"15196","RASGRP3",0.44195459261534,1.01338285886586,2.26779613028657,0.446858006913438,0.654977585671061,NA
"15197","AC020594.1",0.104487577740536,-0.939216628356831,3.35247519753738,-0.280156175069319,0.779357688030112,NA
"15198","AC017050.1",0,NA,NA,NA,NA,NA
"15199","AC007378.1",0,NA,NA,NA,NA,NA
"15200","AC006369.1",0.605550241945059,0.519763556274597,1.80619015792033,0.287767904168551,0.773524407337488,NA
"15201","LINC00211",0,NA,NA,NA,NA,NA
"15202","RMDN2-AS1",0.0830923047099275,-0.939216628356831,3.35247519753738,-0.280156175069319,0.779357688030112,NA
"15203","AC009229.3",0.0666094690818238,0.359191432502738,3.35247519753738,0.10714215955023,0.91467620116095,NA
"15204","AC009229.1",0.0852396197509296,0.359191432502738,3.35247519753738,0.10714215955023,0.91467620116095,NA
"15205","LINC02613",1.66600529330745,0.892681121535791,1.18610236210824,0.752617269852741,0.451679936809989,NA
"15206","AC074366.1",0.0786722874602274,0.359191432502738,3.35247519753738,0.10714215955023,0.914676201160951,NA
"15207","ARHGEF33",1.52528670919101,-0.52348526439541,1.18972112484642,-0.440006698597528,0.659932255736681,NA
"15208","SOS1-IT1",0.713827773687735,0.707220950883431,1.78239644396732,0.396780948075319,0.691529001193477,NA
"15209","CDKL4",0.0786722874602274,0.359191432502738,3.35247519753738,0.10714215955023,0.914676201160951,NA
"15210","AC007684.2",0.249769334282587,0.171289752160848,2.67665743248737,0.0639939015287703,0.94897508273126,NA
"15211","TMEM178A",0.835883200039614,1.28881756245533,1.68728083490839,0.763842945282615,0.444960841149828,NA
"15212","SLC8A1-AS1",0.0786722874602274,0.359191432502738,3.35247519753738,0.10714215955023,0.914676201160951,NA
"15213","AC007877.1",0.528836851327408,1.18173249322263,2.00084496813575,0.590616720456702,0.554777259071908,NA
"15214","AC009413.2",0.0786722874602274,0.359191432502738,3.35247519753738,0.10714215955023,0.914676201160951,NA

"15215","LINC01913",0,NA,NA,NA,NA,NA
"15216","C2orf91",0.242584194671384,1.37348808658286,3.06276733356427,
0.448446759742755,0.653830801606371,NA
"15217","AC083949.1",0.104487577740536,-0.939216628356831,3.3524751975
3738,-0.280156175069319,0.779357688030112,NA
"15218","LINC01819",0,NA,NA,NA,NA,NA
"15219","AC010883.2",0.200580936021446,-0.939216628356831,3.3524751975
3738,-0.280156175069319,0.779357688030112,NA
"15220","LINC01126",0.827695793161337,-0.73196586328071,1.516331468391
39,-0.48272154112664,0.629293466082054,NA
"15221","AC010883.1",1.03987645199423,0.0991408080298446,1.66215021826
848,0.0596461179863293,0.952437486887772,NA
"15222","ABCG5",0.122207625915474,0.359191432502738,3.35247519753738,0
.10714215955023,0.914676201160951,NA
"15223","ABCG8",0.200580936021446,-0.939216628356831,3.35247519753738,
-0.280156175069319,0.779357688030112,NA
"15224","AC019129.2",0.377926485168263,1.86589722618286,2.683960516108
33,0.695202934240017,0.48692813374274,NA
"15225","SLC3A1",0.874436634968404,1.03764523622023,1.98525552667339,0
.522675908606572,0.601199806082878,NA
"15226","LINC01833",0,NA,NA,NA,NA,NA
"15227","SIX3",0.322036033160946,1.68990356201465,2.79747458123059,0.6
04081829144367,0.545789239709996,NA
"15228","AC093833.1",0.133218938163648,0.832405827491147,3.34883738676
897,0.248565615870131,0.803696808386323,NA
"15229","U51244.1",0,NA,NA,NA,NA,NA
"15230","AC018682.1",0.104487577740536,-0.939216628356831,3.3524751975
3738,-0.280156175069319,0.779357688030112,NA
"15231","AC016722.2",0.104487577740536,-0.939216628356831,3.3524751975
3738,-0.280156175069319,0.779357688030112,NA
"15232","STPG4",0,NA,NA,NA,NA,NA
"15233","AC073283.1",0,NA,NA,NA,NA,NA
"15234","EPCAM-DT",0,NA,NA,NA,NA,NA
"15235","KCNK12",0.904752742504481,-0.985076927785743,1.76172416360454
,-0.559155030132666,0.576055920839302,NA
"15236","AC079807.1",1.08687644218249,-0.949101128426368,1.27760930504
19,-0.742872742614569,0.457558729549164,NA
"15237","AC092650.1",0.381321183296296,-1.22487377445586,2.38326253840
343,-0.513948318625616,0.607288124201822,NA
"15238","AC093635.1",1.05575359653188,-1.11277234729422,1.481566445529
91,-0.751078259535106,0.452605558780169,NA
"15239","STON1-GTF2A1L",0,NA,NA,NA,NA,NA
"15240","GTF2A1L",0,NA,NA,NA,NA,NA
"15241","LHCGR",1.54621109061833,0.458653546074776,1.49911485656804,0.
305949570218244,0.759643042813501,NA
"15242","GPR75",0.315010691568728,0.251864597445819,2.50466424555899,0
.100558227671593,0.919901158045867,NA
"15243","TSPYL6",0.0786722874602274,0.359191432502738,3.35247519753738
,0.10714215955023,0.914676201160951,NA
"15244","AC093110.1",1.34384387297881,1.91671567826133,1.4907500382812

3,1.28573914408295,0.198534115118838,NA
"15245","EML6",0.2876474429413,-1.09428600047155,2.91648984647855,-0.3752065181344,0.707506882962305,NA
"15246","AC012358.1",0.163911907211157,0.976662149432262,3.34440272959326,0.29202886984578,0.770264550640504,NA
"15247","AC011306.1",0,NA,NA,NA,NA,NA
"15248","AC007743.1",0.592875754702019,-1.05859626041584,1.93677656051293,-0.546576348556946,0.584669821697499,NA
"15249","AC132153.1",0.682526541485211,-0.237581084600011,1.7041416495846,-0.139413929973441,0.889123065327509,NA
"15250","AC068193.1",0.971544320502738,-0.256480523908974,1.38542980240934,-0.185127043941845,0.853129390219637,NA
"15251","AC007381.1",0.249276914129782,-1.90751516048253,3.31840217494316,-0.57482940882993,0.565406675152336,NA
"15252","BCL11A",1.02409314573764,-0.521227299223693,1.37708497074068,-0.378500463151046,0.705058849772676,NA
"15253","LINC01185",0,NA,NA,NA,NA,NA
"15254","AC016747.3",0.0786722874602274,0.359191432502738,3.35247519753738,0.10714215955023,0.914676201160951,NA
"15255","AC016727.3",0.104487577740536,-0.939216628356831,3.35247519753738,-0.280156175069319,0.779357688030112,NA
"15256","AC107081.2",0.0786722874602274,0.359191432502738,3.35247519753738,0.10714215955023,0.914676201160951,NA
"15257","AC107081.4",0.122207625915474,0.359191432502738,3.35247519753738,0.10714215955023,0.914676201160951,NA
"15258","AC092155.1",0.684680538706802,1.80025402564596,1.81851789054911,0.989956730699178,0.322195268673864,NA
"15259","AC092567.2",0,NA,NA,NA,NA,NA
"15260","OTX1",0.161764592170155,-0.362147980542956,3.35247519753738,-0.108024059599003,0.913976607082588,NA
"15261","AC009501.3",0,NA,NA,NA,NA,NA
"15262","AC012368.2",0,NA,NA,NA,NA,NA
"15263","AC012368.1",0.530976430006713,0.533797293450798,2.00063014592334,0.266814580665253,0.789611927362065,NA
"15264","LINC00309",0.35183729409721,-0.646357439767805,2.44929799598929,-0.263894977592033,0.791860845810361,NA
"15265","LGALSL-DT",0.484870525844254,-1.92743735114259,1.95396127304051,-0.986425564178842,0.323924325441137,NA
"15266","AC008074.2",0.939162185412756,0.855249945202882,1.42786669260071,0.59897044285355,0.54919259489351,NA
"15267","AC007365.1",0.792671349022282,0.101886039892796,1.63252080212438,0.0624102552079045,0.950236128406916,NA
"15268","LINC01800",0,NA,NA,NA,NA,NA
"15269","LINC02245",0.982388864441955,-0.715512950693432,1.33636509543477,-0.535417269680069,0.592361350896539,NA
"15270","LINC02576",0.0786722874602274,0.359191432502738,3.35247519753738,0.10714215955023,0.914676201160951,NA
"15271","AC012370.1",0.230521376292981,1.33222568121774,2.70690437450901,0.492158383489031,0.622607381833459,NA

"15272","AC007389.1",0.690834443067227,-0.58825335056864,1.89373297081
278,-0.310631625279336,0.756080682254126,NA
"15273","AC007389.5",1.1758168474027,1.1937960441526,1.39085422505733,
0.858318594893288,0.390716564363853,NA
"15274","LINC01873",1.51370982295507,0.604114760283766,1.2219648132310
6,0.49437983298913,0.621037953873916,NA
"15275","LINC01829",0.228374061251979,0.170807695949971,2.750084241093
52,0.0621099868133684,0.95047524402806,NA
"15276","LINC02831",0,NA,NA,NA,NA,NA
"15277","AC017083.2",1.43382539949127,0.484630835481806,1.152217618757
29,0.420607034289666,0.674042056315157,NA
"15278","AC017083.1",1.51293870562592,1.30182239001611,1.3480857365924
8,0.965682192667279,0.33420323276005,NA
"15279","PROKR1",0,NA,NA,NA,NA,NA
"15280","LINC01888",0.114130778193026,-0.939216628356831,3.35247519753
738,-0.280156175069319,0.779357688030112,NA
"15281","AC114802.1",0.166184609419855,-1.47576652541587,3.34432018311
399,-0.441275489370681,0.659013568361442,NA
"15282","LINC01816",1.21443522889007,2.58175323678099,1.50347158429722
,1.71719456739038,0.0859436278352792,NA
"15283","TGFA",0.594996938998401,1.5014158107164,1.95704783884303,0.76
7184010996895,0.442972117910759,NA
"15284","ADD2",0.418101258743444,-0.0192148541779159,1.95022085726453,
-0.00985265546019617,0.992138845512297,NA
"15285","CLEC4F",0,NA,NA,NA,NA,NA
"15286","VAX2",0.905899761103086,-1.15835257930285,1.54223523605139,-0
.751086833075219,0.452600399346182,NA
"15287","ATP6V1B1",0.203160351946096,0.359191432502738,3.3524751975373
8,0.10714215955023,0.91467620116095,NA
"15288","AC007881.3",0.0852396197509296,0.359191432502738,3.3524751975
3738,0.10714215955023,0.91467620116095,NA
"15289","AC007881.4",0,NA,NA,NA,NA,NA
"15290","AC010913.1",1.7035449329168,-1.23650702190079,1.1121960184792
7,-1.11177076824235,0.266236723362158,NA
"15291","FBXO41",1.40033268737351,0.607329757822541,1.30698714368853,0
.46467921337662,0.642161196285642,NA
"15292","EGR4",0.673556721592703,0.501600363638976,2.11554809871546,0.
23710184795304,0.812577778780519,NA
"15293","AC074008.1",0.504438911705938,-1.7988204971545,1.992330124170
83,-0.902872709362428,0.366593456184751,NA
"15294","AC092653.1",0.285820555772376,-0.362151522613874,2.8975007201
2302,-0.12498755223726,0.900533404934662,NA
"15295","AC073046.4",0.161764592170155,-0.362147980542956,3.3524751975
3738,-0.108024059599003,0.913976607082588,NA
"15296","ACTG2",1.79845566520924,4.05865332088616,3.24545969763181,1.2
5056346373604,NA,NA
"15297","DGUOK-
AS1",0.0830923047099275,-0.939216628356831,3.35247519753738,-0.2801561
75069319,0.779357688030112,NA
"15298","AC073046.1",0.0786722874602274,0.359191432502738,3.3524751975

3738,0.10714215955023,0.914676201160951,NA
"15299","DCTN1-
AS1",0.0786722874602274,0.359191432502738,3.35247519753738,0.107142159
55023,0.914676201160951,NA
"15300","AC005041.5",0.187579882450464,-1.57167522621382,3.31764872176
29,-0.473731656972615,0.635691275621751,NA
"15301","AC005041.3",0,NA,NA,NA,NA,NA
"15302","TLX2",0,NA,NA,NA,NA,NA
"15303","M1AP",0.197223082902954,-1.61249994371822,3.25614921967676,-0
.495216845092344,0.620447061165921,NA
"15304","TACR1",0,NA,NA,NA,NA,NA
"15305","AC007681.1",0.469412521856787,-0.403068222503402,2.0825922224
751,-0.193541595975216,0.846534832951393,NA
"15306","AC005034.3",0.0786722874602274,0.359191432502738,3.3524751975
3738,0.10714215955023,0.914676201160951,NA
"15307","AC005034.5",0.242584194671384,1.37348808658286,3.062767333564
27,0.448446759742755,0.653830801606371,NA
"15308","AC005034.6",0.326022003816901,0.54804389107956,2.473248030269
42,0.221588730435523,0.824634056225657,NA
"15309","AC005034.4",0.0830923047099275,-0.939216628356831,3.352475197
53738,-0.280156175069319,0.779357688030112,NA
"15310","LRRTM4",0.713429945728822,0.724717115109267,2.03618162718781,
0.355919680952127,0.72190071640209,NA
"15311","AC079117.2",0,NA,NA,NA,NA,NA
"15312","AC013716.1",0,NA,NA,NA,NA,NA
"15313","CTNNA2",0,NA,NA,NA,NA,NA
"15314","DNAH6",0.17109704682236,-0.362147980542956,3.35247519753738,-
0.108024059599003,0.913976607082588,NA
"15315","AC022210.2",0.371492276406953,0.286980760168805,2.41816197757
207,0.118677227923725,0.905531078509693,NA
"15316","TCF7L1-
IT1",0.114130778193026,-0.939216628356831,3.35247519753738,-0.28015617
5069319,0.779357688030112,NA
"15317","AC062037.3",0.0830923047099275,-0.939216628356831,3.352475197
53738,-0.280156175069319,0.779357688030112,NA
"15318","SH2D6",0.18074024727485,-0.362147980542956,3.35247519753738,-
0.108024059599002,0.913976607082588,NA
"15319","AC105053.1",0,NA,NA,NA,NA,NA
"15320","ST3GAL5-
AS1",0.275895370363181,-1.03718220655362,2.58196919069566,-0.401702007
247487,0.687903347118004,NA
"15321","REEP1",0,NA,NA,NA,NA,NA
"15322","AC015971.1",0.261832152660991,0.224500406754452,3.01038077252
586,0.074575418765409,0.940552532631568,NA
"15323","RGPD1",0,NA,NA,NA,NA,NA
"15324","PLGLB1",1.07528764957397,1.6953444591418,1.45390981033807,1.1
6605888968284,0.243590636510922,NA
"15325","AC068279.2",0.423918286558964,-1.54639415640956,2.05070676925
79,-0.754078632592198,0.450802006950613,NA
"15326","AC133644.2",0.952331887220161,-1.08159753759812,1.60211040516

738,-0.675107991377857,0.49960715681033,NA
"15327","AC108479.3",0.122207625915474,0.359191432502738,3.35247519753
738,0.10714215955023,0.914676201160951,NA
"15328","AC062029.1",0.431534413198075,0.710545556532873,2.10149402096
596,0.338114479243804,0.735276919596293,NA
"15329","AC233266.2",1.63450483833336,-0.315486450584038,1.31251653708
585,-0.240367600460491,0.81004529308891,NA
"15330","TEKT4",0.52333362887801,-0.295339489193173,1.99202387940142,-
0.148261018478312,0.882136778056901,NA
"15331","AC092835.1",1.07469046091694,0.77407585729528,1.3765700487262
8,0.562322170245909,0.573896535793468,NA
"15332","PROM2",0.793438296340542,1.59256277722304,1.72565237595378,0.
922875776961058,0.356071945286293,NA
"15333","AC009237.14",1.60454718272828,-0.666903822075912,1.2412353987
8212,-0.53729036629979,0.591067058877952,NA
"15334","AC009237.15",0.240436879630382,0.224611920977265,3.0970535943
4052,0.0725243894350796,0.942184596610118,NA
"15335","ADRA2B",0.555889354154551,2.25597272966728,1.80110236668507,1
.25255108837562,0.210369124709022,NA
"15336","ASTL",0,NA,NA,NA,NA,NA
"15337","AC021188.1",0,NA,NA,NA,NA,NA
"15338","ITPRIPL1",1.43722978146156,0.579639351288058,1.31604364105861
,0.440440828255363,0.659617859717029,NA
"15339","NCAPH",0.796177093787174,0.00467006285323055,1.58915989035682
,0.00293869917153646,0.99765526067703,NA
"15340","NEURL3",0.122207625915474,0.359191432502738,3.35247519753738,
0.10714215955023,0.914676201160951,NA
"15341","AC013270.1",0.0786722874602274,0.359191432502738,3.3524751975
3738,0.10714215955023,0.914676201160951,NA
"15342","FER1L5",0.0852396197509296,0.359191432502738,3.35247519753738
,0.10714215955023,0.91467620116095,NA
"15343","CNNM3-
DT",0.0666094690818238,0.359191432502738,3.35247519753738,0.1071421595
5023,0.91467620116095,NA
"15344","FAM178B",1.15201226491293,-1.92962167874119,1.32345281623822,
-1.45802075832664,0.144834821355989,NA
"15345","C2orf92",1.5997003735559,-0.179629156152541,1.10026130466657,
-0.163260450395442,0.870313372064086,NA
"15346","LINC02611",0.871229185906487,-0.457529224873494,1.62586783277
046,-0.28140616085249,0.778398893294434,NA
"15347","KIAA1211L",0.0830923047099275,-0.939216628356831,3.3524751975
3738,-0.280156175069319,0.779357688030112,NA
"15348","C2orf15",0.292067460191,-2.07593642622498,2.83478040256232,-0
.732309431922336,0.463979723733664,NA
"15349","AC079447.1",0.392887549437562,0.285760355102374,2.38126940153
705,0.120003370856705,0.904480477700097,NA
"15350","LYG2",0.622359643901105,-1.78763603363055,1.90555598711395,-0
.938117822682294,0.34818386357502,NA
"15351","LYG1",0.0852396197509296,0.359191432502738,3.35247519753738,0
.10714215955023,0.91467620116095,NA

"15352","AC018690.1",0.122207625915474,0.359191432502738,3.35247519753
738,0.10714215955023,0.914676201160951,NA
"15353","AC092667.1",0.0852396197509296,0.359191432502738,3.3524751975
3738,0.10714215955023,0.91467620116095,NA
"15354","AC092168.2",0.584780512596648,1.22196114944416,1.831453349686
17,0.66720844931898,0.504638994839217,NA
"15355","AC016738.2",0,NA,NA,NA,NA,NA
"15356","AC016738.1",0.223954044002279,1.30188442273399,3.138197523849
94,0.414851013309333,0.678250972486497,NA
"15357","TBC1D8-
AS1",0.817114357516268,-0.823070817204654,1.55729273780633,-0.52852671
6411758,0.597133809058932,NA
"15358","LINC01127",0.510983679623078,1.28620824799311,2.1874200878786
5,0.588002393833946,0.55653068604458,NA
"15359","AC007271.1",0,NA,NA,NA,NA,NA
"15360","AC007278.2",0.57009175121226,1.45830783985701,1.9427457685761
3,0.750642654044137,0.452867743666427,NA
"15361","AC007278.1",0.537727523024172,-0.538582983551186,1.9854646440
037,-0.271262943501795,0.786188805084083,NA
"15362","PANTR1",0.668476206084791,-3.15224642275581,1.74364937298661,
-1.8078442097315,0.0706307508607436,NA
"15363","POU3F3",0.955025615814526,-1.27860221354627,1.5038073685951,-
0.850243349147023,0.395189805468548,NA
"15364","AC010884.1",0,NA,NA,NA,NA,NA
"15365","LINC01918",0,NA,NA,NA,NA,NA
"15366","AC012360.2",0.0830923047099275,-0.939216628356831,3.352475197
53738,-0.280156175069319,0.779357688030112,NA
"15367","AC108058.1",0.367072259157253,1.5178050344468,2.3903218115619
1,0.63497936851232,0.525441870944802,NA
"15368","AC010978.1",0.319430708818428,-1.03763655696582,2.47298779156
487,-0.419588224618456,0.674786292054358,NA
"15369","RGPD3",0.616841593439841,1.4437097198664,1.77986294222159,0.8
11135332737692,0.417287957192668,NA
"15370","LINC01885",0,NA,NA,NA,NA,NA
"15371","SULT1C2",0.183159865200764,-0.362147980542956,3.3524751975373
8,-0.108024059599003,0.913976607082588,NA
"15372","SULT1C4",0.876034010907025,-0.576197286179666,1.7526054166561
1,-0.328766121971153,0.74233247162682,NA
"15373","GCC2-
AS1",0.580804086106178,0.268322717311844,1.80740167916098,0.1484577116
45152,0.881981557293908,NA
"15374","SH3RF3-
AS1",0.922194665993545,1.46928002806213,1.6284930251076,0.902232926643
98,0.36693314721362,NA
"15375","AC011753.2",0.114130778193026,-0.939216628356831,3.3524751975
3738,-0.280156175069319,0.779357688030112,NA
"15376","LINC01123",0.253571544211787,0.285278815139288,3.040904660736
53,0.0938137978552049,0.925257070922706,NA
"15377","LINC01106",1.23402255287966,0.86527849362543,1.40466512749895
,0.616003399448014,0.537892276169962,NA

"15378","RGPD6",0.0830923047099275,-0.939216628356831,3.35247519753738
,-0.280156175069319,0.779357688030112,NA
"15379","BUB1",0,NA,NA,NA,NA,NA
"15380","ACOXL",0,NA,NA,NA,NA,NA
"15381","ACOXL-
AS1",1.16676700408191,-0.300575770578237,1.29462630177959,-0.232171839
985844,0.816404548646209,NA
"15382","AC017002.1",0.286119533126631,1.51303924540838,2.551022714313
52,0.593110848021414,0.553106971009519,NA
"15383","AC093675.1",0.104487577740536,-0.939216628356831,3.3524751975
3738,-0.280156175069319,0.779357688030112,NA
"15384","AC092645.2",0.999814183284974,2.3255085426025,1.7142776420940
9,1.35655303755916,0.174923272285504,NA
"15385","AC012442.1",0,NA,NA,NA,NA,NA
"15386","CKAP2L",0.207447245666404,1.02558430340912,3.22299417080721,0
.318208550514461,0.750326747237409,NA
"15387","IL1A",0.662807207842227,-2.44605433618553,1.97287397422936,-1
.23984317707927,0.215033404598227,NA
"15388","AC079753.2",0,NA,NA,NA,NA,NA
"15389","AC016745.2",0.507010591268892,-1.8731473907886,1.928987806828
57,-0.971051960078601,0.33152240457723,NA
"15390","PGM5P4-
AS1",1.21092837370038,0.490824972128876,1.38970219504814,0.35318716044
1861,0.723948132910078,NA
"15391","AC104653.1",0.187579882450464,-1.57167522621382,3.31764872176
29,-0.473731656972615,0.635691275621751,NA
"15392","AC110769.2",1.84500308547575,-0.896139423221436,1.17249729615
288,-0.764299778056453,0.444688617621747,NA
"15393","LINC01191",0.764101184952364,0.331990145489534,2.050297345383
33,0.161922926075615,0.871366548662531,NA
"15394","AC009303.2",0,NA,NA,NA,NA,NA
"15395","LINC01956",0.285227825015386,-1.13200789171034,2.564212583530
21,-0.441464135610736,0.658877020046736,NA
"15396","SCTR",0,NA,NA,NA,NA,NA
"15397","AC013275.1",0.665158332142437,0.554519356926691,2.10767472034
183,0.263095320912117,0.792477110314479,NA
"15398","CFAP221",0,NA,NA,NA,NA,NA
"15399","AC073257.2",0.0786722874602274,0.359191432502738,3.3524751975
3738,0.10714215955023,0.914676201160951,NA
"15400","AC073257.1",0.0852396197509296,0.359191432502738,3.3524751975
3738,0.10714215955023,0.91467620116095,NA
"15401","AC017033.1",1.59264884495511,0.119394051526124,1.124722591732
94,0.106154221853199,0.915459991315833,NA
"15402","TFCP2L1",0.375159764900927,-2.38708443157501,2.57959192441785
,-0.92537288901372,0.354771976045552,NA
"15403","AC012447.1",0,NA,NA,NA,NA,NA
"15404","AC011246.1",1.02823682803834,-0.830437259913532,1.50901486893
505,-0.55031747997261,0.582101636979435,NA
"15405","AC012508.2",0,NA,NA,NA,NA,NA
"15406","AC012508.1",0.200879913375701,0.981067669524494,3.25622950176

531,0.301289472683859,0.763193767774683,NA
"15407","AC110926.2",0,NA,NA,NA,NA,NA
"15408","AC068282.1",0.200879913375701,0.981067669524494,3.25622950176
531,0.301289472683859,0.763193767774683,NA
"15409","AC068282.2",0.149701773791751,-0.362147980542956,3.3524751975
3738,-0.108024059599003,0.913976607082588,NA
"15410","PROC",0.31910916709061,0.630344098013162,2.86281997001412,0.2
20182933127316,0.82572868787641,NA
"15411","AC010976.1",0.200580936021446,-0.939216628356831,3.3524751975
3738,-0.280156175069319,0.779357688030112,NA
"15412","MYO7B",0.39659348084691,-1.56773382283473,2.31042849421982,-0
.678546783316107,0.497425071567152,NA
"15413","AC010976.2",0,NA,NA,NA,NA,NA
"15414","AC012306.3",1.79481055224889,0.590588080857233,1.039367838364
41,0.568218545021178,0.569886580509254,NA
"15415","LINC02572",0.151849088832753,0.903480691260343,3.346850711185
47,0.269949504541936,0.787199101303289,NA
"15416","RAB6C",0.0830923047099275,-0.939216628356831,3.35247519753738
,-0.280156175069319,0.779357688030112,NA
"15417","AC073869.3",0,NA,NA,NA,NA,NA
"15418","AC093787.2",0.912577652549618,0.1653498935057,1.5439063072515
6,0.107098398865961,0.91471091738276,NA
"15419","AC097532.2",0.237088708583683,1.35169743040986,3.083754038640
28,0.438328548085457,0.661148134105955,NA
"15420","AC097532.3",0.247004211921084,0.251392038816408,2.68232836300
611,0.0937215749881831,0.925330331348742,NA
"15421","GPR39",0.413681241493744,0.966624584743449,2.08484017904813,0
.463644453161286,0.642902500073556,NA
"15422","LYPD1",0.0786722874602274,0.359191432502738,3.35247519753738,
0.10714215955023,0.914676201160951,NA
"15423","AC092623.1",0.0786722874602274,0.359191432502738,3.3524751975
3738,0.10714215955023,0.914676201160951,NA
"15424","TMEM163",0.0786722874602274,0.359191432502738,3.3524751975373
8,0.10714215955023,0.914676201160951,NA
"15425","AC110620.2",0,NA,NA,NA,NA,NA
"15426","CCNT2-
AS1",1.45095780317466,-0.594760896674825,1.258909415976,-0.47244137594
5801,0.636611776993928,NA
"15427","MAP3K19",0.29729064339379,-1.13154245073425,2.53302761211231,
-0.446715403070812,0.655080558990623,NA
"15428","AC011893.1",0.411041506299937,-1.68775176004836,2.60288165529
253,-0.648416633394218,0.516715514972491,NA
"15429","DARS-
AS1",0.374257398768456,0.18632766577431,2.39786170675018,0.07770575978
16346,0.938062112319522,NA
"15430","AC068492.1",0,NA,NA,NA,NA,NA
"15431","THSD7B",0,NA,NA,NA,NA,NA
"15432","AC114763.1",0.686070867092033,0.314534374425757,1.76967668765
563,0.177735501981684,0.858930702577702,NA
"15433","AC092620.1",1.4771820036106,0.324413651046863,1.3810401715507

8,0.234905296550916,0.814282238534095,NA
"15434","LINC02631",0.526257435402758,-0.0164562389832786,2.0178193835
3051,-0.00815545688459273,0.993492958997721,NA
"15435","KYNU",1.36789391907255,0.452536906820582,1.41220105658557,0.3
20447931057869,0.748628795810312,NA
"15436","AC079793.1",0.424534980023725,0.597456655482955,2.30916619628
67,0.258732635374495,0.795841537337236,NA
"15437","AC092652.3",0.240436879630382,0.224611920977265,3.09705359434
052,0.0725243894350796,0.942184596610118,NA
"15438","AC092652.1",0.183159865200764,-0.362147980542956,3.3524751975
3738,-0.108024059599003,0.913976607082588,NA
"15439","AC092652.2",0,NA,NA,NA,NA,NA
"15440","AC016910.1",0.234941393542681,0.184365908906169,2.72598185286
798,0.0676328452855251,0.946077908494717,NA
"15441","AC009951.5",0.0786722874602274,0.359191432502738,3.3524751975
3738,0.10714215955023,0.914676201160951,NA
"15442","LINC01412",1.21093240578471,-1.0516389434019,1.19573840877526
,-0.879489138831829,0.379136119527865,NA
"15443","AC018678.1",0,NA,NA,NA,NA,NA
"15444","AC105402.3",0,NA,NA,NA,NA,NA
"15445","LYPD6B",0.114130778193026,-0.939216628356831,3.35247519753738
,-0.280156175069319,0.779357688030112,NA
"15446","LYPD6",0.145281756542051,0.889415776427968,3.34729258829349,0
.265711990501974,0.790461028169891,NA
"15447","LINC02612",0,NA,NA,NA,NA,NA
"15448","AC079790.2",0.151849088832753,0.903480691260343,3.34685071118
547,0.269949504541936,0.787199101303289,NA
"15449","AC012066.2",0.364791820586858,1.8285189661938,2.3658880172308
9,0.772867926493814,0.439600548129325,NA
"15450","AC012443.2",0.367072259157253,1.5178050344468,2.3903218115619
1,0.63497936851232,0.525441870944802,NA
"15451","RPRM",0.917149731436358,-0.588183708380956,1.50729205620779,-
0.390225441684323,0.696369850194037,NA
"15452","AC073225.1",1.01893056037445,0.0928053230493908,1.46620718587
457,0.0632961862030666,0.949530652644784,NA
"15453","LINC01876",0.203160351946096,0.359191432502738,3.352475197537
38,0.10714215955023,0.91467620116095,NA
"15454","AC074099.1",0,NA,NA,NA,NA,NA
"15455","ERMN",0.804282840279758,0.399031841389285,1.79643822258763,0.
222123887352224,0.824217442893235,NA
"15456","ACVR1C",0.749727283094958,1.19822906445624,2.03754948822068,0
.588073600854038,0.556482891776656,NA
"15457","AC019186.1",0.200879913375701,0.981067669524494,3.25622950176
531,0.301289472683859,0.763193767774683,NA
"15458","UPP2",0,NA,NA,NA,NA,NA
"15459","AC013731.1",0.290539550376331,0.291375383648554,2.56622020713
621,0.113542626949274,0.909600370146826,NA
"15460","AC091488.1",0.694598210081052,-1.32149467578765,1.71800095417
171,-0.769204855549557,0.441771708405013,NA
"15461","CCDC148",0.240436879630382,0.224611920977265,3.09705359434052

,0.0725243894350796,0.942184596610118,NA
"15462","PKP4-
AS1",0.170479239501859,1.00912327877525,3.3428978888652,0.301870805607
469,0.762750551898993,NA
"15463","AC008277.1",0.122207625915474,0.359191432502738,3.35247519753
738,0.10714215955023,0.914676201160951,NA
"15464","AC009961.1",1.67288526084459,0.933580722253013,1.158561622222
18,0.80581015661675,0.420352320856439,NA
"15465","AC009961.4",1.72277092436204,-1.01541284158399,1.150780560645
24,-0.882368781946273,0.377577411920289,NA
"15466","LINC01806",0,NA,NA,NA,NA,NA
"15467","TBR1",0.0852396197509296,0.359191432502738,3.35247519753738,0
.10714215955023,0.91467620116095,NA
"15468","AC007750.1",0.0786722874602274,0.359191432502738,3.3524751975
3738,0.10714215955023,0.914676201160951,NA
"15469","GRB14",0.32536797786157,1.05056297657179,2.83388144670233,0.3
70715217390017,0.710849654876456,NA
"15470","AC019197.1",0.0852396197509296,0.359191432502738,3.3524751975
3738,0.10714215955023,0.91467620116095,NA
"15471","SLC38A11",0,NA,NA,NA,NA,NA
"15472","SCN3A",0,NA,NA,NA,NA,NA
"15473","SCN2A",0,NA,NA,NA,NA,NA
"15474","CSRNP3",0.208975155481072,-1.67232023626263,3.33344910552292,
-0.501678646748169,0.615893587270659,NA
"15475","AC009495.3",0.114130778193026,-0.939216628356831,3.3524751975
3738,-0.280156175069319,0.779357688030112,NA
"15476","TTC21B-
AS1",0.247004211921084,0.251392038816408,2.68232836300611,0.0937215749
881831,0.925330331348742,NA
"15477","SCN9A",1.67528043203289,-3.85086457478818,1.4632988103114,-2.
63163241003981,0.0084975747491275,NA
"15478","B3GALT1",0,NA,NA,NA,NA,NA
"15479","CERS6-AS1",0,NA,NA,NA,NA,NA
"15480","SPC25",0.358546030023975,0.325387423573685,2.74973617638295,0
.11833405196047,0.905802977374664,NA
"15481","CCDC173",1.62422883952762,-0.678216479599831,1.09376218255786
,-0.620076731866667,0.535207270340084,NA
"15482","MYO3B",0.385606131306508,-0.587268273578269,2.69175660882552,
-0.21817287330243,0.827294419953835,NA
"15483","AC007405.1",1.11636033138158,-1.29880270904794,1.314271453872
79,-0.988230175144356,0.323039933091328,NA
"15484","LINC01124",0.942371177103937,0.150364127974467,1.446780552624
53,0.103930155614616,0.917224775967051,NA
"15485","AC007405.2",0,NA,NA,NA,NA,NA
"15486","AC007405.4",0,NA,NA,NA,NA,NA
"15487","AC010092.1",0,NA,NA,NA,NA,NA
"15488","DLX1",0,NA,NA,NA,NA,NA
"15489","DLX2",0,NA,NA,NA,NA,NA
"15490","ITGA6-
AS1",0.888649146302366,-2.93213297795161,1.60976512370864,-1.821466333

67013,0.0685359997820649,NA
"15491","AC078883.1",0.200879913375701,0.981067669524494,3.25622950176531,0.301289472683859,0.763193767774683,NA
"15492","MAP3K20-AS1",0.526556412757013,1.37157529929017,1.99149875613711,0.688715117226885,0.491002561051744,NA
"15493","CDCA7",1.25721252650844,0.635650417669595,1.30675325660757,0.486434921401913,0.626658836057968,NA
"15494","AC016737.1",0.122207625915474,0.359191432502738,3.35247519753738,0.10714215955023,0.914676201160951,NA
"15495","AC016737.2",0.775556504436522,2.74917978627385,1.93088150685903,1.42379518189386,0.154505770851586,NA
"15496","LINC01960",1.59059869417256,0.446691272589716,1.10230288321898,0.405234604199959,0.68530507109709,NA
"15497","LINC01305",1.11332444239593,1.45742596171301,1.58074498502973,0.921986769222991,0.356535477023393,NA
"15498","SP9",0,NA,NA,NA,NA,NA
"15499","AC010894.2",0.56908753823509,-2.02534562340146,2.10010400536034,-0.964402533508787,0.334844152539751,NA
"15500","AC010894.3",0,NA,NA,NA,NA,NA
"15501","CHRNA1",0.737210425834054,0.395945811088848,1.67484509935176,0.236407421344276,0.813116537987912,NA
"15502","CHN1",1.7519052770412,-0.0891905135155669,1.00034652193067,-0.089159617752685,0.928955057880259,NA
"15503","EVX2",0,NA,NA,NA,NA,NA
"15504","HOXD13",0.236016862380682,1.34721548542771,3.32885593646028,0.404708257474268,0.68569197172232,NA
"15505","HOXD11",0.436597798402128,0.63390542127264,2.60659656112817,0.24319276359295,0.807856072434437,NA
"15506","HOXD10",0.436597798402128,0.63390542127264,2.60659656112817,0.24319276359295,0.807856072434437,NA
"15507","HOXD3",1.71778933749048,-1.45261432252666,1.14718527139572,-1.26624213084547,0.205426408108453,NA
"15508","HAGLR",0.398702352972538,-1.57377945263065,2.63628592463865,-0.596968423615264,0.550528462681198,NA
"15509","HAGLROS",0.0852396197509296,0.359191432502738,3.35247519753738,0.10714215955023,0.91467620116095,NA
"15510","HOXD1",0.133218938163648,0.832405827491147,3.34883738676897,0.248565615870131,0.803696808386323,NA
"15511","AC017048.3",0.771926069862671,2.00791096211713,1.78850765568796,1.12267395430565,0.261575993678674,NA
"15512","AC092162.2",0.0852396197509296,0.359191432502738,3.35247519753738,0.10714215955023,0.91467620116095,NA
"15513","AC074286.1",0,NA,NA,NA,NA,NA
"15514","AC019080.4",0,NA,NA,NA,NA,NA
"15515","AC019080.5",0.32536797786157,1.05056297657179,2.83388144670233,0.370715217390017,0.710849654876456,NA
"15516","AC019080.6",0.70650123044047,0.793103664363703,1.64194263702145,0.483027632318767,0.629076116337501,NA
"15517","PDE11A",1.47139422234923,0.306677993536363,1.11905416194252,0

.274051072741655,0.784045373104786,NA
"15518","AC009948.3",1.71861032769548,0.393640783192436,1.04856122541471,0.375410394406631,0.707355275646647,NA
"15519","AC010680.2",0.680254953132254,-0.865399758196823,1.59311355150333,-0.543212853459312,0.586983257763485,NA
"15520","AC010680.4",0.622967142775102,-0.175755420859613,1.75781448888537,-0.099985192960299,0.920356080838905,NA
"15521","AC010680.5",0.122207625915474,0.359191432502738,3.35247519753738,0.10714215955023,0.914676201160951,NA
"15522","AC010680.1",0.283673240731374,-1.61851549881525,2.89644275100986,-0.558794230699344,0.576302160315398,NA
"15523","CCDC141",0.537543762297416,0.550854802750083,1.99728275421372,0.275802112438978,0.782700061129726,NA
"15524","SCHLAP1",1.34949991431885,-1.43668261524598,1.21970972774522,-1.17788895387583,0.238840879348549,NA
"15525","AC104076.1",0.203160351946096,0.359191432502738,3.35247519753738,0.10714215955023,0.91467620116095,NA
"15526","AC068196.1",0.369344961365951,-0.910724457509032,2.70222203740393,-0.337027988412078,0.736095801433169,NA
"15527","CERKL",0,NA,NA,NA,NA,NA
"15528","AC009962.1",0.0666094690818238,0.359191432502738,3.35247519753738,0.10714215955023,0.91467620116095,NA
"15529","AC012500.1",0,NA,NA,NA,NA,NA
"15530","AC021851.1",1.69550966052611,-0.771010588973207,1.28041460746505,-0.602156976715256,0.547069651000003,NA
"15531","AC021851.2",0.207447245666404,1.02558430340912,3.22299417080721,0.318208550514461,0.750326747237409,NA
"15532","AC096667.1",0.0830923047099275,-0.939216628356831,3.35247519753738,-0.280156175069319,0.779357688030112,NA
"15533","ZNF804A",0.303857975684492,-1.13129237031979,2.51681897225932,-0.44949294438298,0.653076096718193,NA
"15534","AC009315.1",1.26058263555399,0.456495720966964,1.35487084997666,0.33692932501635,0.736170178328959,NA
"15535","FSIP2-
AS1",0.166184609419855,-1.47576652541587,3.34432018311399,-0.441275489370681,0.659013568361442,NA
"15536","FSIP2-
AS2",0.0830923047099275,-0.939216628356831,3.35247519753738,-0.280156175069319,0.779357688030112,NA
"15537","AC097500.1",1.479506451671,0.0655951680007585,1.36679817652975,0.0479918462924073,0.961722740836879,NA
"15538","AC017071.1",1.79001778939418,0.385914605399843,1.18157678472215,0.326609840672005,0.743962999620899,NA
"15539","AC007319.1",0.740916495149709,1.8547384697507,1.65937858406369,1.11773075027195,0.263682005364598,NA
"15540","DIRC1",0.421177126905233,-0.0731535521177196,2.10367812760873,-0.0347741183205027,0.972259858734036,NA
"15541","AC133106.1",0,NA,NA,NA,NA,NA
"15542","OSGEPL1-
AS1",0.163911907211157,0.976662149432262,3.34440272959326,0.2920288698

4578,0.770264550640504,NA
"15543","AC013468.1",0,NA,NA,NA,NA,NA
"15544","MSTN",1.74392498870641,4.00969349344802,3.2461280184615,1.235
22346335201,0.216747349202392,NA
"15545","AC093388.1",1.09557883358714,-0.157091691383777,1.36891240714
46,-0.114756569203324,0.908638074310854,NA
"15546","AC006460.1",0.0786722874602274,0.359191432502738,3.3524751975
3738,0.10714215955023,0.914676201160951,NA
"15547","AC005540.1",0.469412521856787,-0.403068222503402,2.0825922224
751,-0.193541595975216,0.846534832951393,NA
"15548","AC067945.3",0,NA,NA,NA,NA,NA
"15549","AC067945.4",0.161764592170155,-0.362147980542956,3.3524751975
3738,-0.108024059599003,0.913976607082588,NA
"15550","AC092614.1",0.163911907211157,0.976662149432262,3.34440272959
326,0.29202886984578,0.770264550640504,NA
"15551","AC098617.1",0,NA,NA,NA,NA,NA
"15552","TMEFF2",0.748590572963105,-3.35336565959189,1.85030733760797,
-1.81232900688112,0.0699353751189276,NA
"15553","AC096647.1",0.479055722309278,-0.434223832109352,2.0692019359
7973,-0.209850872724877,0.833784066272884,NA
"15554","LINC01821",0,NA,NA,NA,NA,NA
"15555","LINC01825",0.856056088535255,-3.52885527895244,1.608152695391
56,-2.19435336524012,0.0282100157484368,NA
"15556","LINC01827",0,NA,NA,NA,NA,NA
"15557","DNAH7",1.0810354516592,-0.830525240613795,1.48942724812911,-0
.55761383555795,0.577108106734762,NA
"15558","HECW2-
AS1",0.275895370363181,-1.03718220655362,2.58196919069566,-0.401702007
247487,0.687903347118004,NA
"15559","C2orf66",0.535307905098259,-0.603028671269492,1.9591146837619
1,-0.307806723244783,0.75822940540787,NA
"15560","AC013264.1",1.56996285442527,-0.509525676674413,1.30875116395
731,-0.389322042804336,0.697037932258437,NA
"15561","ANKRD44-
IT1",1.8273688513807,0.802351071683368,1.15280801681499,0.695997130467
677,0.486430626805795,NA
"15562","AC011997.1",1.67079115508576,-0.71149252119411,1.102390354404
22,-0.645408877492068,0.518662251947519,NA
"15563","AC011997.2",0,NA,NA,NA,NA,NA
"15564","FTCDNL1",1.04061296428159,-0.966597941249374,1.40242594212309
,-0.689232787426956,0.490676786869745,NA
"15565","AC097717.1",0.286252656656024,-0.362151520384174,2.8978095924
9269,-0.124974229266959,0.900543952427349,NA
"15566","AC012459.1",1.78988412214006,-0.585749021077216,1.12166600271
226,-0.522213403687762,0.601521754592151,NA
"15567","LINC01792",0.104487577740536,-0.939216628356831,3.35247519753
738,-0.280156175069319,0.779357688030112,NA
"15568","AC005037.1",0.0852396197509296,0.359191432502738,3.3524751975
3738,0.10714215955023,0.91467620116095,NA
"15569","AC007272.1",0.203160351946096,0.359191432502738,3.35247519753

738,0.10714215955023,0.91467620116095,NA
"15570","CFLAR-
AS1",1.54349380336415,-0.0322573094016417,1.22928708804522,-0.02624066
39712912,0.979065381879857,NA
"15571","FLACC1",0.51816881923428,0.244204884354645,1.98849682537782,0
.122808787642014,0.902258515408228,NA
"15572","AC007279.2",0.263832551984778,-1.03705123952077,2.61674970071
281,-0.396312738370917,0.691874331103868,NA
"15573","MPP4",0.52106092669312,1.36427062910316,1.82704490379111,0.7
46708866471917,0.455239316522988,NA
"15574","AC069148.1",1.84069214403823,-0.576139789197547,1.14295342859
525,-0.504079846810255,0.614205268307231,NA
"15575","KIAA2012",0.472189900145349,-0.917036323712305,2.020509453381
5,-0.453863911489038,0.649926779721341,NA
"15576","KIAA2012-
AS1",0.48485213146133,1.19823590832951,2.23813098723957,0.535373450062
173,0.592391645396176,NA
"15577","AC079354.3",0.232794078501679,-0.90181358082427,3.12933488781
614,-0.288180592091764,0.773208504171299,NA
"15578","AC079354.2",0.157344574920455,0.946214072007857,3.34552927999
138,0.282829409883477,0.777307608562567,NA
"15579","AC064836.2",0.585991538155167,1.44490522145293,1.928133992325
48,0.74938008831547,0.453628148762142,NA
"15580","AC009498.1",0.255718859252789,1.41947394389201,3.326212722592
44,0.426753807491204,0.669558654609534,NA
"15581","AC007679.1",0,NA,NA,NA,NA,NA
"15582","AC007383.2",1.06668913514457,0.725352064777485,1.842687732482
51,0.393638082020699,0.693848261170883,NA
"15583","AC007383.3",0.523967452666877,2.26006213187112,2.432830589967
76,0.928984591525165,0.352897071716031,NA
"15584","GPR1-
AS",0.472930172974016,1.69172405687485,2.55922718335019,0.661029262224
498,0.508593547768755,NA
"15585","MDH1B",0.578521701825687,0.55915686389942,1.97123029411442,0.
283658822395798,0.776671853962445,NA
"15586","CPO",0.114130778193026,-0.939216628356831,3.35247519753738,-0
.280156175069319,0.779357688030112,NA
"15587","KLF7-
IT1",0.809484861094358,-0.107935738464258,1.817025977027,-0.0594024190
236737,0.95263158636165,NA
"15588","MYOSLID",0.452005165192231,-1.61777514371583,2.23501924951201
,-0.723830519164007,0.46916979030637,NA
"15589","AC007879.3",0.283673240731374,-1.61851549881525,2.89644275100
986,-0.558794230699344,0.576302160315398,NA
"15590","AC007879.2",0.32157802385943,0.28926759090872,2.4920059852094
5,0.116078208730469,0.907590558967189,NA
"15591","AC009226.1",0,NA,NA,NA,NA,NA
"15592","LINC01857",0.187579882450464,-1.57167522621382,3.317648721762
9,-0.473731656972615,0.635691275621751,NA
"15593","AC096772.1",0.0666094690818238,0.359191432502738,3.3524751975

3738,0.10714215955023,0.91467620116095,NA
"15594","AC083900.1",0,NA,NA,NA,NA,NA
"15595","IDH1-
AS1",0.988333869846794,1.35995678320391,1.5257841603984,0.891316621643
795,0.372759334009709,NA
"15596","AC019185.2",0,NA,NA,NA,NA,NA
"15597","MAP2",0.218618355933563,-1.71512321784709,3.15240431335981,-0
.544068287997971,0.586394481847521,NA
"15598","AC007038.1",1.20929750318527,-0.295584245455966,1.38440861686
088,-0.213509394449016,0.830929662342145,NA
"15599","AC006994.2",1.2138603823001,-1.09479862351383,1.5270831615618
,-0.716921416639901,0.473422586402475,NA
"15600","MYL1",0.199370397943956,-0.362148716949221,3.26311252083995,-
0.110982601622331,0.911630143386501,NA
"15601","LINC01878",0,NA,NA,NA,NA,NA
"15602","AC108066.2",0.515256781155562,-1.26199034484663,1.97147519137
654,-0.640124892449429,0.522091406986122,NA
"15603","AC079610.1",1.37804334402456,-0.403362708548819,1.26048243242
141,-0.320006608718816,0.748963320746317,NA
"15604","SNHG31",0.18074024727485,-0.362147980542956,3.35247519753738,
-0.108024059599002,0.913976607082588,NA
"15605","LINC00607",1.19980587688879,-0.855622459042342,1.219837608320
24,-0.701423249460693,0.483038916874097,NA
"15606","AC093382.1",0,NA,NA,NA,NA,NA
"15607","TMEM169",0.278042685404183,0.250550992867118,2.58936724201233
,0.0967614746961895,0.922915819060029,NA
"15608","AC098820.1",0.26719040510327,-0.362151043418806,2.96314189103
91,-0.122218596589652,0.902725898914264,NA
"15609","AC098820.3",0,NA,NA,NA,NA,NA
"15610","AC007563.4",0.582178532298435,2.47624018006168,2.109406563187
65,1.17390370508741,0.240433598244496,NA
"15611","LINC01921",0.114130778193026,-0.939216628356831,3.35247519753
738,-0.280156175069319,0.779357688030112,NA
"15612","AC007749.1",0,NA,NA,NA,NA,NA
"15613","AC007749.2",0.0852396197509296,0.359191432502738,3.3524751975
3738,0.10714215955023,0.91467620116095,NA
"15614","DIRC3-
AS1",0.268399484951693,0.250807602063781,2.61654731113631,0.0958544112
679779,0.923636202325112,NA
"15615","AC010136.1",0.821722305483364,-2.62534444144337,1.57251083154
767,-1.66952391600349,0.0950135947240108,NA
"15616","RUFY4",0.323087539291176,1.63986284743208,2.81636913523065,0.
582261333189118,0.560390666306499,NA
"15617","CXCR1",0.413681241493744,0.966624584743449,2.08484017904813,0
.463644453161286,0.642902500073556,NA
"15618","AC021016.2",1.82346982497706,-0.115554083542402,1.09958297055
501,-0.105089007957332,0.916305181385858,NA
"15619","CATIP",0,NA,NA,NA,NA,NA
"15620","CATIP-
AS1",0.0666094690818238,0.359191432502738,3.35247519753738,0.107142159

55023,0.91467620116095,NA
"15621","AC021016.3",0.0852396197509296,0.359191432502738,3.3524751975
3738,0.10714215955023,0.91467620116095,NA
"15622","VIL1",0.878085185334482,1.03471716976118,1.56580331695575,0.6
60821929904254,0.508726517330419,NA
"15623","PLCD4",1.2608615643758,-0.64454899372404,1.40635636002282,-0.
45831128727116,0.646728816045818,NA
"15624","AC009974.2",0.122207625915474,0.359191432502738,3.35247519753
738,0.10714215955023,0.914676201160951,NA
"15625","AC009974.1",0.354567657823409,-0.452272257696804,2.4327477477
3189,-0.185910050936629,0.852515300103562,NA
"15626","PRKAG3",0.189727197491466,-0.362148248616811,3.32022295329944
,-0.109073472989797,0.913144214822408,NA
"15627","WNT10A",0.170479239501859,1.00912327877525,3.3428978888652,0.
301870805607469,0.762750551898993,NA
"15628","AC097468.4",0,NA,NA,NA,NA,NA
"15629","AC097468.3",1.65456739225678,1.01207176187645,1.2856087316388
,0.787231555736508,0.431146327944805,NA
"15630","SLC23A3",0.821098298041682,2.94899355186191,1.69574594672269,
1.73905387039923,0.0820252876797928,NA
"15631","PTPRN",0,NA,NA,NA,NA,NA
"15632","AC053503.2",0,NA,NA,NA,NA,NA
"15633","DES",0.637263360046956,-2.36539064912893,2.33075410784087,-1.
01486065869048,0.310172253817243,NA
"15634","SPEG",1.05986865863289,-0.680664476502012,1.52640676744153,-0
.445926008073786,0.655650694669699,NA
"15635","SPEGNB",1.34985315511613,-0.91189546558065,1.33031851245015,-
0.685471529597178,0.493046420299363,NA
"15636","AC053503.5",0.505195898361709,1.26846994791545,1.994978496303
44,0.63583138879233,0.52488632630747,NA
"15637","ASIC4",0,NA,NA,NA,NA,NA
"15638","AC009955.4",0.0666094690818238,0.359191432502738,3.3524751975
3738,0.10714215955023,0.91467620116095,NA
"15639","INHA",0,NA,NA,NA,NA,NA
"15640","AC093843.1",0.32278856193692,-0.362152324877374,2.78414257136
106,-0.130076788668309,0.896505674038834,NA
"15641","AC093843.2",0.122207625915474,0.359191432502738,3.35247519753
738,0.10714215955023,0.914676201160951,NA
"15642","AC079834.2",0.244856896880082,-0.902701635500065,3.0767047150
267,-0.293398853354776,0.769217301797547,NA
"15643","PAX3",1.51528120369593,-0.640127059446733,1.30047404795191,-0
.492225939037274,0.622559629292382,NA
"15644","AC010980.1",0.322328328334613,1.70230134870348,2.798993086243
12,0.608183477504889,0.543065791670001,NA
"15645","MOGAT1",0.0786722874602274,0.359191432502738,3.35247519753738
,0.10714215955023,0.914676201160951,NA
"15646","SCG2",3.49490631121126,0.600175343762386,2.01683193686133,0.2
97583220888698,NA,NA
"15647","FAM124B",0.352862125737848,0.18604602110421,2.43752552439083,
0.076325773511933,0.939159921350957,NA

"15648","AC073052.2",0,NA,NA,NA,NA,NA
"15649","NYAP2",0.187579882450464,-1.57167522621382,3.3176487217629,-0
.473731656972615,0.635691275621751,NA
"15650","AC010735.2",0.157344574920455,0.946214072007857,3.34552927999
138,0.282829409883477,0.777307608562567,NA
"15651","AC010735.1",0,NA,NA,NA,NA,NA
"15652","COL4A3",1.63888938655098,-1.26905721615504,1.50664512891509,-
0.84230665323882,0.399616327892045,NA
"15653","AC064853.1",0,NA,NA,NA,NA,NA
"15654","SCYGR4",0,NA,NA,NA,NA,NA
"15655","DAW1",0.200580936021446,-0.939216628356831,3.35247519753738,-
0.280156175069319,0.779357688030112,NA
"15656","LINC01807",0.845829491593305,0.326483175296548,1.691464652564
72,0.193018030144177,0.846944847591829,NA
"15657","AC008273.1",0.671268002915638,-0.935017230770537,1.6890292442
9085,-0.553582617903759,0.579864524601113,NA
"15658","LINC01907",0,NA,NA,NA,NA,NA
"15659","GPR55",0.249276914129782,-1.90751516048253,3.31840217494316,-
0.57482940882993,0.565406675152336,NA
"15660","AC017104.1",1.21457969209197,1.07251416309392,1.3858045237545
5,0.773928894522698,0.438972841389263,NA
"15661","B3GNT7",1.39267492032664,0.604645385637112,1.24964441789655,0
.483853948353464,0.628489524972737,NA
"15662","AC017104.3",0.0830923047099275,-0.939216628356831,3.352475197
53738,-0.280156175069319,0.779357688030112,NA
"15663","LINC00471",0.584780512596648,1.22196114944416,1.8314533496861
7,0.66720844931898,0.504638994839217,NA
"15664","AC017104.6",0.149701773791751,-0.362147980542956,3.3524751975
3738,-0.108024059599003,0.913976607082588,NA
"15665","AC073476.3",0.0830923047099275,-0.939216628356831,3.352475197
53738,-0.280156175069319,0.779357688030112,NA
"15666","NPPC",0.511912232743864,0.298352888866755,2.25240707804775,0.
132459577034072,0.894620794090251,NA
"15667","CHRNG",0,NA,NA,NA,NA,NA
"15668","KCNJ13",0.104487577740536,-0.939216628356831,3.35247519753738
,-0.280156175069319,0.779357688030112,NA
"15669","SNORC",0.501193563453372,-0.473645919195038,1.83424481801214,
-0.258223937472181,0.796234084799633,NA
"15670","SAG",0,NA,NA,NA,NA,NA
"15671","HJURP",0,NA,NA,NA,NA,NA
"15672","AC097713.3",0,NA,NA,NA,NA,NA
"15673","AC010148.1",0.114130778193026,-0.939216628356831,3.3524751975
3738,-0.280156175069319,0.779357688030112,NA
"15674","AGAP1-
IT1",1.02953679399234,0.82566371176929,1.59919624183374,0.516299182158
239,0.605645467353569,NA
"15675","AC084030.1",0.452005165192231,-1.61777514371583,2.23501924951
201,-0.723830519164007,0.46916979030637,NA
"15676","AC105760.1",0.266252169910691,-1.00025133134918,2.61314057122
404,-0.382777467987741,0.701884769397932,NA

"15677","AC107079.1",0.0830923047099275,-0.939216628356831,3.352475197
53738,-0.280156175069319,0.779357688030112,NA
"15678","RBM44",0.785165781539001,-0.117815673282835,1.51988068938523,
-0.0775163959287423,0.938212748453205,NA
"15679","RAMP1",1.50757627208359,1.07626412656927,1.37221574381658,0.7
84325738440973,0.432849000594419,NA
"15680","ESPNL",1.38837317350013,-0.536548861129331,1.34210818591361,-
0.399780633752776,0.689318095944241,NA
"15681","ERFE",0.548084809317015,0.107694612450622,2.00600759589433,0.
0536860441959637,0.957185301884699,NA
"15682","LINC02610",0.525504906626783,1.43742242363353,1.9416217768550
5,0.74032050977601,0.459105538931258,NA
"15683","AC012485.1",0.0852396197509296,0.359191432502738,3.3524751975
3738,0.10714215955023,0.91467620116095,NA
"15684","LINC01940",0,NA,NA,NA,NA,NA
"15685","AC017028.1",0.104487577740536,-0.939216628356831,3.3524751975
3738,-0.280156175069319,0.779357688030112,NA
"15686","AC017028.2",0,NA,NA,NA,NA,NA
"15687","AC093802.1",0,NA,NA,NA,NA,NA
"15688","AC110619.1",0.157344574920455,0.946214072007857,3.34552927999
138,0.282829409883477,0.777307608562567,NA
"15689","GPR35",1.14679541608646,0.933235254990819,1.35198513038118,0.
69027035432535,0.490024187290461,NA
"15690","CROCC2",0.459993470756524,-0.661841380693949,2.09085089769128
,-0.31654164408603,0.751591421434787,NA
"15691","AC104809.2",0.416422401147476,-0.444123842470041,2.3432318269
6576,-0.189534743152211,0.849673727454178,NA
"15692","AC104809.1",0.0666094690818238,0.359191432502738,3.3524751975
3738,0.10714215955023,0.91467620116095,NA
"15693","AC005237.1",0,NA,NA,NA,NA,NA
"15694","ANO7",1.10523380248566,-1.12386759211035,1.39038610494992,-0.
808313308158982,0.41891024334342,NA
"15695","AC104841.1",0,NA,NA,NA,NA,NA
"15696","BOK-
AS1",0.0666094690818238,0.359191432502738,3.35247519753738,0.107142159
55023,0.91467620116095,NA
"15697","AC133528.1",0.0830923047099275,-0.939216628356831,3.352475197
53738,-0.280156175069319,0.779357688030112,NA
"15698","AC114730.2",0.968194747490915,0.805422459586286,1.57868789184
61,0.510184732363049,0.609922047399185,NA
"15699","GAL3ST2",0,NA,NA,NA,NA,NA
"15700","AC131097.1",0,NA,NA,NA,NA,NA
"15701","NEU4",0.232794078501679,-0.90181358082427,3.12933488781614,-0
.288180592091764,0.773208504171299,NA
"15702","AC131097.3",0,NA,NA,NA,NA,NA
"15703","RTP5",0.145281756542051,0.889415776427968,3.34729258829349,0.
265711990501974,0.790461028169891,NA
"15704","LINC01237",0.0786722874602274,0.359191432502738,3.35247519753
738,0.10714215955023,0.914676201160951,NA
"15705","FAM240C",0.199828407245471,1.19974298654701,3.33462853032117,

0.359783098968286,0.719009343087826,NA
"15706","LINC01238",0.949085425111855,1.41587452130931,1.5252720461645
5,0.928276712911425,0.353264050423963,NA
"15707","LINC01238.1",0.0852396197509296,0.359191432502738,3.352475197
53738,0.10714215955023,0.91467620116095,NA
"15708","CHL1-
AS1",0.281832639406324,0.978331721828331,2.92526312973549,0.3344422974
75233,0.738045826283596,NA
"15709","AC087430.1",0,NA,NA,NA,NA,NA
"15710","CNTN6",0,NA,NA,NA,NA,NA
"15711","AC087427.1",0.0852396197509296,0.359191432502738,3.3524751975
3738,0.10714215955023,0.91467620116095,NA
"15712","IL5RA",0.254189351532287,-0.999830626761722,2.64981024805783,
-0.377321594063025,0.705934626414087,NA
"15713","LRRN1",0.199370397943956,-0.362148716949221,3.26311252083995,
-0.110982601622331,0.911630143386501,NA
"15714","AC023480.1",0.0786722874602274,0.359191432502738,3.3524751975
3738,0.10714215955023,0.914676201160951,NA
"15715","AC023483.1",0.183159865200764,-0.362147980542956,3.3524751975
3738,-0.108024059599003,0.913976607082588,NA
"15716","ITPR1-
DT",0.157344574920455,0.946214072007857,3.34552927999138,0.28282940988
3477,0.777307608562567,NA
"15717","EGOT",0.646018768922974,-2.23608609327829,2.02826648363267,-1
.10246168899533,0.270261005232039,NA
"15718","AC026202.2",1.23892119809434,-0.326769861994436,1.18992098248
542,-0.274614757453812,0.783612227286045,NA
"15719","AC026202.3",0.149701773791751,-0.362147980542956,3.3524751975
3738,-0.108024059599003,0.913976607082588,NA
"15720","AC069277.1",0.436856796580246,-2.61279970672448,2.48118924953
286,-1.05304329656289,0.292321149013195,NA
"15721","GRM7",1.43375623002493,-1.01949601179087,1.3167190992859,-0.7
74269935283674,0.438771178851122,NA
"15722","AC066585.1",0.183159865200764,-0.362147980542956,3.3524751975
3738,-0.108024059599003,0.913976607082588,NA
"15723","GRM7-AS1",0,NA,NA,NA,NA,NA
"15724","AC077690.1",0,NA,NA,NA,NA,NA
"15725","AC023481.1",1.35608055131634,-0.542379521592342,1.22467138416
114,-0.442877598519094,0.657854271456562,NA
"15726","CAV3",0.133218938163648,0.832405827491147,3.34883738676897,0.
248565615870131,0.803696808386323,NA
"15727","OXTR",0.870593332007327,-0.539492963044058,1.50872760352138,-
0.357581422772989,0.720656582768885,NA
"15728","AC026191.1",0.530235807603324,0.911905109240407,2.21273602168
466,0.412116538215042,0.680254007362593,NA
"15729","LHFPL4",0.744699766971261,1.5406311510777,2.07164030033862,0.
743676955321766,0.457071934261849,NA
"15730","AC022382.2",0.145281756542051,0.889415776427968,3.34729258829
349,0.265711990501974,0.790461028169891,NA
"15731","CPNE9",0.0786722874602274,0.359191432502738,3.35247519753738,

0.10714215955023,0.914676201160951,NA
"15732","AC022382.1",1.25499589554656,-0.477250461871735,1.33007754075
21,-0.358814014408416,0.719734227898229,NA
"15733","AC018809.1",0.321256482131612,1.70014465421147,2.801781870833
04,0.606808357178063,0.543978101327833,NA
"15734","AC034193.1",0.0666094690818238,0.359191432502738,3.3524751975
3738,0.10714215955023,0.91467620116095,NA
"15735","GHRL",0.427575117031712,0.898247932584502,2.33176426451467,0.
385222445619502,0.700072614975807,NA
"15736","GHRLOS",1.14889349228456,1.75685303946984,1.5515560804097,1.1
3231681513306,0.257501263185385,NA
"15737","SLC6A1",0.275895370363181,-1.03718220655362,2.58196919069566,
-0.401702007247487,0.687903347118004,NA
"15738","AC022001.2",0,NA,NA,NA,NA,NA
"15739","AC022001.3",0.0666094690818238,0.359191432502738,3.3524751975
3738,0.10714215955023,0.91467620116095,NA
"15740","SYN2",0.228374061251979,0.170807695949971,2.75008424109352,0.
0621099868133684,0.95047524402806,NA
"15741","CAND2",1.49858758508771,-0.58935063896698,1.14829668840056,-0
.513238995566445,0.607784151264483,NA
"15742","AC034198.2",0.122207625915474,0.359191432502738,3.35247519753
738,0.10714215955023,0.914676201160951,NA
"15743","WNT7A",0,NA,NA,NA,NA,NA
"15744","AC090004.2",0.313462733221608,-2.14519230363698,3.25345188633
189,-0.659358852869214,0.509665358193341,NA
"15745","AC090946.1",0.594241350676839,2.48908177482304,2.389209575954
6,1.04180135550835,0.29750378557383,NA
"15746","AC090948.1",0.662540473282164,1.72564816261579,1.845903641570
63,0.934852786328266,0.349864171603807,NA
"15747","AC090948.2",0.408460282571498,0.305951066256654,2.39125610255
537,0.127945754505218,0.898191901718287,NA
"15748","AC090948.3",0.979346919630179,1.30801502971586,1.519062639979
69,0.861067210324747,0.389201027218908,NA
"15749","AC090948.4",0.200879913375701,0.981067669524494,3.25622950176
531,0.301289472683859,0.763193767774683,NA
"15750","AC010727.1",0.267489382457525,1.43658475709043,2.590121492191
77,0.554639912228514,0.579140983489215,NA
"15751","AC090644.1",0,NA,NA,NA,NA,NA
"15752","AC144521.1",0.451412434435241,-1.86834128961914,1.98539596371
557,-0.941042151673682,0.346683258546458,NA
"15753","SATB1-
AS1",0.459993470756524,-0.661841380693949,2.09085089769128,-0.31654164
408603,0.751591421434787,NA
"15754","KCNH8",0,NA,NA,NA,NA,NA
"15755","AC061958.1",0.524416834077708,2.21856618070798,2.168758653113
19,1.02296591532824,0.306323961763505,NA
"15756","EFHB",1.20443632933459,-0.595324007124685,1.35551156289201,-0
.439187701102712,0.660525535937108,NA
"15757","PP2D1",0,NA,NA,NA,NA,NA
"15758","SGO1",0.616093234674754,0.0443462516852922,1.96623614851321,0

.0225538787489107,0.982006133885267,NA
"15759","SGO1-AS1",0,NA,NA,NA,NA,NA
"15760","AC116096.1",0.0786722874602274,0.359191432502738,3.3524751975
3738,0.10714215955023,0.914676201160951,NA
"15761","UBE2E2-
AS1",0.282462702653884,-1.0372525382335,2.56395923547331,-0.4045511035
75567,0.685807506504499,NA
"15762","UBE2E1-
AS1",0.122207625915474,0.359191432502738,3.35247519753738,0.1071421595
5023,0.914676201160951,NA
"15763","LINC00691",0.669256807664318,0.805924249211452,2.147455715013
25,0.375292604907794,0.707442865345319,NA
"15764","THRB-
AS1",0.483342616029585,0.093245494467271,1.89783124080355,0.0491326586
171015,0.960813577014817,NA
"15765","AC012087.2",0.192803065653254,-0.362148402639638,3.3015497885
8522,-0.109690425960478,0.912654893573412,NA
"15766","RARB",0.703736108078967,0.936760944325022,1.90318778582288,0.
492206261149367,0.622573538706239,NA
"15767","RBMS3-
AS2",0.307046348712206,0.591373023504277,2.52180569687551,0.2345038018
73785,0.814593882002255,NA
"15768","AC025614.2",0.0786722874602274,0.359191432502738,3.3524751975
3738,0.10714215955023,0.914676201160951,NA
"15769","GADL1",0.0786722874602274,0.359191432502738,3.35247519753738,
0.10714215955023,0.914676201160951,NA
"15770","OSBPL10-
AS1",0.902345891330778,-0.00879266449981841,1.5689635158535,-0.0056041
2298372361,0.995528580199505,NA
"15771","ZNF860",0.904502750537266,-0.833355904239197,1.50204712053375
,-0.55481342285924,0.579022284987587,NA
"15772","AC097639.1",0.399951333965402,-1.22259966134345,2.35192678574
217,-0.519828962685013,0.603182790842112,NA
"15773","TRIM71",0.0786722874602274,0.359191432502738,3.35247519753738
,0.10714215955023,0.914676201160951,NA
"15774","CCR4",1.03676311341532,1.65723388944241,1.53280790741164,1.08
11751958149,0.279619189341341,NA
"15775","TMPPE",0.797574104352994,-0.222506679989089,1.48856626488696,
-0.149477174941881,0.881177118773767,NA
"15776","SUSD5",1.32688498145028,3.61022684804978,1.45581220337572,2.4
7987126339402,0.0131429825373507,NA
"15777","ARPP21",0,NA,NA,NA,NA,NA
"15778","DCLK3",0.422521275993144,-0.902686497936556,2.07376699750791,
-0.435288293728915,0.663353180875282,NA
"15779","LINC02033",0.313462733221608,-2.14519230363698,3.253451886331
89,-0.659358852869214,0.509665358193341,NA
"15780","AC011816.2",0.261832152660991,0.224500406754452,3.01038077252
586,0.074575418765409,0.940552532631568,NA
"15781","AC126118.1",0.203160351946096,0.359191432502738,3.35247519753
738,0.10714215955023,0.91467620116095,NA

"15782","AC097359.3",0.0666094690818238,0.359191432502738,3.3524751975
3738,0.10714215955023,0.91467620116095,NA
"15783","DLEC1",0.302626331462506,1.6366950852251,2.84830283044638,0.5
74621163076471,0.565547536063556,NA
"15784","SLC22A13",0.187579882450464,-1.57167522621382,3.3176487217629
,-0.473731656972615,0.635691275621751,NA
"15785","SLC22A14",0.240436879630382,0.224611920977265,3.0970535943405
2,0.0725243894350796,0.942184596610118,NA
"15786","ACVR2B-
AS1",0.427744459195935,-0.0478191855160199,1.93729569381596,-0.0246834
72775304,0.980307437879675,NA
"15787","SCN11A",0.508836364582075,0.357187296101515,2.02545770311269,
0.176348928715024,0.86001982474897,NA
"15788","AC092053.2",1.82396105326268,-1.95783349631872,1.170473071402
53,-1.67268563810077,0.0943891980064381,NA
"15789","AC092053.5",0,NA,NA,NA,NA,NA
"15790","CX3CR1",1.44695589410229,-0.978887318249062,1.23014690683777,
-0.795748306814347,0.426178343060751,NA
"15791","AC104850.2",0.197223082902954,-1.61249994371822,3.25614921967
676,-0.495216845092344,0.620447061165921,NA
"15792","CCR8",0.317291130139123,-0.362152203948855,2.80152312911717,-
0.129269753365548,0.897144203908295,NA
"15793","AC099332.2",0.314711714214473,-1.82631342394583,2.81227742011
208,-0.649407277847091,0.51607516109671,NA
"15794","AC099332.1",0.950298674950918,2.22036312681253,1.648321971970
08,1.34704454868046,0.177965885454871,NA
"15795","MYRIP",0,NA,NA,NA,NA,NA
"15796","AC099560.1",0.249769334282587,0.171289752160848,2.67665743248
737,0.0639939015287703,0.94897508273126,NA
"15797","AC099541.2",0.183159865200764,-0.362147980542956,3.3524751975
3738,-0.108024059599003,0.913976607082588,NA
"15798","VIPR1",0.755542713004645,-2.67300681305821,1.69994426736988,-
1.57240849854086,0.115855844596808,NA
"15799","VIPR1-AS1",0,NA,NA,NA,NA,NA
"15800","AC006059.5",0.627387160024802,-0.854857664199224,1.7176484056
0852,-0.497690715636516,0.618702053020933,NA
"15801","CCDC13",1.83224659936146,0.54169317986224,1.01754370318594,0.
532353724136066,0.59448102939516,NA
"15802","LINC02158",1.16989134503512,-1.6353594814041,1.53681117328832
,-1.0641251897622,0.287271991008704,NA
"15803","AC099329.1",0.279253223481674,-0.362151360256245,2.9199061145
6708,-0.124028426273541,0.901292767920445,NA
"15804","AC099329.2",0.504711214570601,1.0996636233872,2.0101950514143
9,0.547043244690841,0.584349022624697,NA
"15805","CYP8B1",0,NA,NA,NA,NA,NA
"15806","AC092042.4",0,NA,NA,NA,NA,NA
"15807","SNRK-
AS1",1.37923765577622,-2.26763697607009,1.38653664903044,-1.6354684729
4356,0.101950878521936,NA
"15808","AC104187.1",0.294871025467877,-1.1768104696752,2.895522041690

79,-0.406424282989752,0.684430880475328,NA
"15809","ZKSCAN7-AS1",0,NA,NA,NA,NA,NA
"15810","AC124045.1",1.26090146552024,-0.903525932536036,1.22018724483
03,-0.740481378054151,0.459007956472647,NA
"15811","KIF15",0.717347232608754,-0.911378680764643,1.69707319397841,
-0.537029683809995,0.591247110095375,NA
"15812","TGM4",0.166184609419855,-1.47576652541587,3.34432018311399,-0
.441275489370681,0.659013568361442,NA
"15813","CDCP1",0.355001704417153,-0.370457855519663,2.44239188938105,
-0.151678302376587,0.879440671339284,NA
"15814","SLC6A20",0.168331924460857,-0.362147980542956,3.3524751975373
8,-0.108024059599003,0.913976607082588,NA
"15815","CCR9",0,NA,NA,NA,NA,NA
"15816","XCR1",0.281832639406324,0.978331721828331,2.92526312973549,0.
334442297475233,0.738045826283596,NA
"15817","CCR3",0.470649734403622,1.96706297574743,2.03082446487806,0.9
68603150969794,0.332743230623977,NA
"15818","CCR5AS",0,NA,NA,NA,NA,NA
"15819","CCRL2",1.46698451356628,-1.84094270497248,1.24726327787719,-1
.4759856540519,0.139947743485328,NA
"15820","LINC02009",0,NA,NA,NA,NA,NA
"15821","LTF",0.0830923047099275,-0.939216628356831,3.35247519753738,-
0.280156175069319,0.779357688030112,NA
"15822","LRRC2-
AS1",0.104487577740536,-0.939216628356831,3.35247519753738,-0.28015617
5069319,0.779357688030112,NA
"15823","ALS2CL",0.731602434880427,-0.594062444673848,1.5417012478956,
-0.385329158606271,0.699993560847292,NA
"15824","TMIE",0,NA,NA,NA,NA,NA
"15825","PRSS45P",0.0786722874602274,0.359191432502738,3.3524751975373
8,0.10714215955023,0.914676201160951,NA
"15826","CSPG5",0,NA,NA,NA,NA,NA
"15827","AC112512.1",0.151849088832753,0.903480691260343,3.34685071118
547,0.269949504541936,0.787199101303289,NA
"15828","CDC25A",0.168331924460857,-0.362147980542956,3.35247519753738
,-0.108024059599003,0.913976607082588,NA
"15829","CAMP",0.567944436171258,0.631963736638957,2.17136994456222,0.
291043789300662,0.771017829359566,NA
"15830","AC134772.1",0,NA,NA,NA,NA,NA
"15831","PFKFB4",0.406038440365041,-0.0621573804740824,2.1240874049868
3,-0.0292630992152923,0.976654756850444,NA
"15832","COL7A1",1.74257393624474,-0.939726763622068,1.0450005708762,-
0.89925956962314,0.368514417180419,NA
"15833","CELSR3",0.149701773791751,-0.362147980542956,3.35247519753738
,-0.108024059599003,0.913976607082588,NA
"15834","PRKAR2A-
AS1",0.921417687049018,-1.18205277082303,1.50847875870482,-0.783605843
968226,0.433271425669415,NA
"15835","AC137630.2",0.283673240731374,-1.61851549881525,2.89644275100
986,-0.558794230699344,0.576302160315398,NA

"15836","AC137630.1",0.0852396197509296,0.359191432502738,3.35247519753738,0.10714215955023,0.91467620116095,NA
"15837","AC137630.4",0.797583786424787,0.783901596945459,1.6799001409807,0.466635830203473,0.640760447512393,NA
"15838","RHOA-IT1",0.302626331462506,1.6366950852251,2.84830283044638,0.574621163076471,0.565547536063556,NA
"15839","BSN",0.735073906720585,-1.16390881277811,1.76260996132187,-0.660332596727884,0.509040416268564,NA
"15840","MST1",1.8396632274164,-1.78783119260397,1.07649616931764,-1.66078732424773,0.0967561678343724,NA
"15841","AMIGO3",0.412472161645321,0.553901834992007,2.32279873413172,0.238463120740015,0.811521915767398,NA
"15842","MST1R",0.637178734423574,-0.389568044889178,2.42042256870754,-0.160950426560102,0.872132445348592,NA
"15843","RBM5-AS1",1.76570644622076,-0.264185723483516,1.33516181420145,-0.197867944299712,0.843148385027687,NA
"15844","SEMA3F-AS1",0.467132083286392,0.111196133431294,2.06620756220183,0.0538165358918727,0.957081334874633,NA
"15845","SEMA3F",0.114130778193026,-0.939216628356831,3.35247519753738,-0.280156175069319,0.779357688030112,NA
"15846","U73169.1",0.779686311919666,2.85403739831107,1.90916127592489,1.49491686967537,0.134936139008582,NA
"15847","U73166.1",0.575767375391718,-0.47237149214842,1.68276768476072,-0.280711055023373,0.778932028874993,NA
"15848","SEMA3B-AS1",0.814561128579933,-0.784467346746528,1.63510540617806,-0.479765612530243,0.631394067146207,NA
"15849","LSMEM2",0,NA,NA,NA,NA,NA
"15850","HYAL3",0.421611173498977,0.100795065206169,2.33019939303004,0.0432559829462071,0.965497478876789,NA
"15851","RASSF1-AS1",0.956272789003601,1.1889594075278,1.50465714590747,0.790186263203987,0.429418997211719,NA
"15852","ZMYND10",1.01116271986207,0.705410943712632,1.60297973312848,0.440062297191934,0.659891987967046,NA
"15853","CACNA2D2",1.57372382704778,0.142164992268135,1.23378489038602,0.115226724995518,0.908265416272699,NA
"15854","CYB561D2.1",1.66679104445797,-1.24587516844848,1.09928354172067,-1.13335197077394,0.257066475057051,NA
"15855","DOCK3",0.283673240731374,-1.61851549881525,2.89644275100986,-0.558794230699344,0.576302160315398,NA
"15856","AC099050.1",0,NA,NA,NA,NA,NA
"15857","GRM2",0.122207625915474,0.359191432502738,3.35247519753738,0.10714215955023,0.914676201160951,NA
"15858","GPR62",0.742286831246184,-0.555548805337303,1.72962137503903,-0.32119677367237,0.748061276957601,NA
"15859","TLR9",0.375803132835032,1.92177647131771,2.29881573598359,0.8

3598543425467,0.40316309736035,NA
"15860","AC006252.1",0.715279397271309,-1.73361003772187,1.80421238188126,-0.960868052526179,0.336618515959095,NA
"15861","SEMA3G",1.54722315375655,-2.06353313627369,1.25822045244893,-1.64004100573738,0.100996641232142,NA
"15862","TNNC1",0.792246152645975,-0.92654723116524,1.65463705988092,-0.559970070555487,0.575499852491146,NA
"15863","ITIH3",0,NA,NA,NA,NA,NA
"15864","CACNA1D",0.890106910681008,-0.471795600842239,1.61650762537237,-0.291861042556826,0.770392870766527,NA
"15865","IL17RB",0.461202550604947,-0.0952912860672431,2.06815863200518,-0.046075424095904,0.963250133930189,NA
"15866","AC115282.2",0,NA,NA,NA,NA,NA
"15867","CACNA2D3-AS1",0,NA,NA,NA,NA,NA
"15868","LRTM1",1.16597609405587,-1.47776504107873,1.60841628816877,-0.918770253664371,0.358215754472843,NA
"15869","WNT5A-AS1",1.00809938621803,-1.41683581800721,1.53402106709745,-0.923609100550381,0.355689873922645,NA
"15870","ERC2",0.0666094690818238,0.359191432502738,3.35247519753738,0.10714215955023,0.91467620116095,NA
"15871","ERC2-IT1",0,NA,NA,NA,NA,NA
"15872","SPATA12",0.450156827505484,-0.759200852052902,2.27830006555285,-0.333231282187878,0.73895970645481,NA
"15873","AC097358.2",0.286119533126631,1.51303924540838,2.55102271431352,0.593110848021414,0.553106971009519,NA
"15874","ASB14",0,NA,NA,NA,NA,NA
"15875","AC093928.1",0.236016862380682,1.34721548542771,3.32885593646028,0.404708257474268,0.68569197172232,NA
"15876","DNAH12",0.200879913375701,0.981067669524494,3.25622950176531,0.301289472683859,0.763193767774683,NA
"15877","AC092418.2",0,NA,NA,NA,NA,NA
"15878","DENND6A-AS1",1.42329822730948,-1.3379433528521,1.36449948350608,-0.980537822861063,0.326820709045861,NA
"15879","DENND6A-DT",0.0852396197509296,0.359191432502738,3.35247519753738,0.10714215955023,0.91467620116095,NA
"15880","FLNB-AS1",0.0786722874602274,0.359191432502738,3.35247519753738,0.10714215955023,0.914676201160951,NA
"15881","HTD2",0.557916427967931,0.114165072602,2.19641416520293,0.0519779349499196,0.95854627500195,NA
"15882","AC116036.2",1.43611698015324,-0.350182618355297,1.21822638346325,-0.28745282741273,0.773765616646991,NA
"15883","FAM3D-AS1",0.0786722874602274,0.359191432502738,3.35247519753738,0.10714215955023,0.914676201160951,NA
"15884","AC126121.3",0.600941806496934,1.47889537823892,2.16670338163398,0.682555531493027,0.494887741518523,NA

"15885","AC093418.1",0.570408009093698,2.44604900572949,2.34564861900671,1.04280282473225,0.297039622820572,NA
"15886","CADPS",0.557273344512294,2.41416305425686,2.42139322765754,0.997014044097382,0.318757693080667,NA
"15887","C3orf49",0.104487577740536,-0.939216628356831,3.35247519753738,-0.280156175069319,0.779357688030112,NA
"15888","SCAANT1",1.78428030391466,-1.73006359751777,1.4844842075998,-1.16543078643797,0.243844661512895,NA
"15889","AC012557.1",0.38374080122221,-1.17244511446869,2.37544818352895,-0.49356796018464,0.621611333031814,NA
"15890","LINC00994",0,NA,NA,NA,NA,NA
"15891","PRICKLE2-AS3",0.0786722874602274,0.359191432502738,3.35247519753738,0.10714215955023,0.914676201160951,NA
"15892","ADAMTS9-AS1",0.782312117019343,-0.274456927762136,1.60747795291168,-0.170737600018093,0.864430096364875,NA
"15893","MAGI1-IT1",0.0786722874602274,0.359191432502738,3.35247519753738,0.10714215955023,0.914676201160951,NA
"15894","MAGI1-AS1",0,NA,NA,NA,NA,NA
"15895","KBTBD8",1.49517511895146,0.479124868943141,1.30875205625706,0.366092925434175,0.714295736328253,NA
"15896","TAFA1",0.349344474620618,-1.43745544152659,2.38499104700468,-0.602708946573151,0.546702328818333,NA
"15897","AC104167.1",0,NA,NA,NA,NA,NA
"15898","AC109587.1",0.751901132928538,-0.0654893831078776,1.57158115199769,-0.0416710158585395,0.966760959877519,NA
"15899","LMOD3",0.765899999297487,-2.41040505212926,1.88788457364967,-1.27677564919631,0.201681464846194,NA
"15900","SAMMSON",0.236016862380682,1.34721548542771,3.32885593646028,0.404708257474268,0.68569197172232,NA
"15901","AC097634.3",0.0852396197509296,0.359191432502738,3.35247519753738,0.10714215955023,0.91467620116095,NA
"15902","AC097634.1",1.6941219059759,0.624817747471976,1.12717043979672,0.554324106995441,0.579357054945155,NA
"15903","GPR27",1.19947256671494,0.601568368400706,1.36361794791031,0.441156094581027,0.659099996086966,NA
"15904","PROK2",1.23519492659197,3.39377324329255,1.63695217708975,2.07322687295982,0.0381511705716145,NA
"15905","LINC00877",1.21704312710811,0.257374099511785,1.44780072563769,0.177769008506625,0.85890438726743,NA
"15906","PDZRN3-AS1",0,NA,NA,NA,NA,NA
"15907","AC117461.1",0,NA,NA,NA,NA,NA
"15908","CADM2",0.0786722874602274,0.359191432502738,3.35247519753738,0.10714215955023,0.914676201160951,NA
"15909","AC107204.1",1.17094139291604,1.0221859857148,1.32578031160999,0.771007064114182,0.440702744473875,NA
"15910","HTR1F",0,NA,NA,NA,NA,NA
"15911","STX19",0.698751492790938,-0.660529503563673,1.73866621789205,

-0.379905870814294,0.704015289226102,NA
"15912","AC109782.1",0.458252069610855,-2.67346529121449,2.40827692786756,-1.11011539423821,0.266949304409175,NA
"15913","EPHA6",0.509157906309894,-0.926037584524776,1.84728767954531,-0.501295815902756,0.616162949528196,NA
"15914","AC110491.3",0.200580936021446,-0.939216628356831,3.35247519753738,-0.280156175069319,0.779357688030112,NA
"15915","AC026100.1",0,NA,NA,NA,NA,NA
"15916","ST3GAL6-AS1",0.545505393392364,-1.10546865951885,1.96990658739496,-0.561178213521657,0.574676053430066,NA
"15917","AC107029.1",0,NA,NA,NA,NA,NA
"15918","AC107029.2",0.776035619902782,-0.924486761930243,1.68151337847005,-0.549794473102199,0.582460349916802,NA
"15919","AC069222.1",0.0786722874602274,0.359191432502738,3.35247519753738,0.10714215955023,0.914676201160951,NA
"15920","AC129803.1",0.0830923047099275,-0.939216628356831,3.35247519753738,-0.280156175069319,0.779357688030112,NA
"15921","IMPG2",1.41641609711662,0.0690694340744487,1.12189741377238,0.0615648393752893,0.950909377918071,NA
"15922","AC020651.1",0,NA,NA,NA,NA,NA
"15923","AC020651.2",0,NA,NA,NA,NA,NA
"15924","AC106712.1",0,NA,NA,NA,NA,NA
"15925","AC117435.1",0,NA,NA,NA,NA,NA
"15926","AC063944.3",0.0830923047099275,-0.939216628356831,3.35247519753738,-0.280156175069319,0.779357688030112,NA
"15927","LINC01990",0.303857975684492,-1.13129237031979,2.51681897225932,-0.44949294438298,0.653076096718193,NA
"15928","LINC00636",0.282462702653884,-1.0372525382335,2.56395923547331,-0.404551103575567,0.685807506504499,NA
"15929","LINC01215",0.26976982102792,0.904712707040654,2.95638786916641,0.306019624987755,0.759589703549634,NA
"15930","HHLA2",0,NA,NA,NA,NA,NA
"15931","MYH15",0.200580936021446,-0.939216628356831,3.35247519753738,-0.280156175069319,0.779357688030112,NA
"15932","MORC1",0,NA,NA,NA,NA,NA
"15933","DPPA4",0.0830923047099275,-0.939216628356831,3.35247519753738,-0.280156175069319,0.779357688030112,NA
"15934","LINC01205",0,NA,NA,NA,NA,NA
"15935","NECTIN3-AS1",0.432164476445635,-0.930105770766251,2.06396801207871,-0.450639624898789,0.652249302931621,NA
"15936","ZBED2",0.367072259157253,1.5178050344468,2.39032181156191,0.63497936851232,0.525441870944802,NA
"15937","PLCXD2",1.81322204387917,-0.805437746914488,1.29234280090496,-0.623238467649978,0.533127826483254,NA
"15938","GCSAM",0.75678988619372,0.388719104277147,2.10777114958043,0.184421873482102,0.853682511553393,NA
"15939","TBILA",0.145281756542051,0.889415776427968,3.34729258829349,0.265711990501974,0.790461028169891,NA

"15940","SLC9C1",0.307647929686632,-0.362152004845914,2.82990681512005
,-0.127973120143375,0.898170245124239,NA
"15941","AC112487.1",0.114130778193026,-0.939216628356831,3.3524751975
3738,-0.280156175069319,0.779357688030112,NA
"15942","BTLA",0.46101878987819,1.14409297605862,2.28797584627811,0.50
0045915222284,0.617042747507622,NA
"15943","CD200R1",1.70768561189536,0.575275571246752,1.10661532254897,
0.519851442072633,0.603167121743793,NA
"15944","AC074044.1",0.283972218085629,0.252983192935449,2.58123709711
829,0.0980085065482289,0.921925538642289,NA
"15945","SIDT1",1.54083096005181,-0.0668375224316077,1.19830963371073,
-0.0557765042951681,0.955519852655222,NA
"15946","SIDT1-AS1",0,NA,NA,NA,NA,NA
"15947","GRAMD1C",0.189727197491466,-0.362148248616811,3.3202229532994
4,-0.109073472989797,0.913144214822408,NA
"15948","AC128687.2",0.0786722874602274,0.359191432502738,3.3524751975
3738,0.10714215955023,0.914676201160951,NA
"15949","ZDHHC23",0.309193663753208,1.67003531445362,2.45970992724041,
0.678956203720844,0.497165612394039,NA
"15950","AC128687.3",0,NA,NA,NA,NA,NA
"15951","AC093010.2",1.74361721754487,0.907875404501296,1.418779851491
68,0.639898715467921,0.522238449078474,NA
"15952","ZNF80",0.576360106148707,0.11402539865629,1.98612499097244,0.
057410988318747,0.954217810028105,NA
"15953","ZBTB20-
AS1",0.624476658206847,0.709004843182086,1.95711181850513,0.3622709936
54126,0.717149531837747,NA
"15954","ZBTB20-
AS5",0.122207625915474,0.359191432502738,3.35247519753738,0.1071421595
5023,0.914676201160951,NA
"15955","ZBTB20-
AS4",1.1443211288991,0.760870729149349,1.56295729144043,0.486814792263
534,0.626389586648107,NA
"15956","AC026341.1",0.283673240731374,-1.61851549881525,2.89644275100
986,-0.558794230699344,0.576302160315398,NA
"15957","LSAMP-
AS1",0.0786722874602274,0.359191432502738,3.35247519753738,0.107142159
55023,0.914676201160951,NA
"15958","AC092691.1",0.166184609419855,-1.47576652541587,3.34432018311
399,-0.441275489370681,0.659013568361442,NA
"15959","UPK1B",0,NA,NA,NA,NA,NA
"15960","ARHGAP31-AS1",0,NA,NA,NA,NA,NA
"15961","AC073352.1",0.314711714214473,-1.82631342394583,2.81227742011
208,-0.649407277847091,0.51607516109671,NA
"15962","CD80",0.883899988869458,-0.240904580213554,1.58456906748417,-
0.15203160604166,0.879162007380565,NA
"15963","AC073352.2",0.545233090507453,-0.182909987841266,1.8627074576
2144,-0.0981957671844137,0.921776843519472,NA
"15964","PLA1A",1.40098400156727,-0.394793961630408,1.25621623466241,-
0.314272297027355,0.753314236857625,NA

"15965","AC092910.3",0.944670553802228,-1.56209756736786,1.52212522831077,-1.02626087414729,0.304768634417426,NA
"15966","GPR156",0.281832639406324,0.978331721828331,2.92526312973549,0.334442297475233,0.738045826283596,NA
"15967","AC126182.3",0.133218938163648,0.832405827491147,3.34883738676897,0.248565615870131,0.803696808386323,NA
"15968","HGD",0.0786722874602274,0.359191432502738,3.35247519753738,0.10714215955023,0.914676201160951,NA
"15969","AC072026.2",0.24915152696208 7,1.39724561189142,3.03826011163213,0.459883472959405,0.645599863589248,NA
"15970","POLQ",0.114130778193026,-0.939216628356831,3.35247519753738,-0.280156175069319,0.779357688030112,NA
"15971","FBXO40",0.122207625915474,0.359191432502738,3.35247519753738,0.10714215955023,0.914676201160951,NA
"15972","SLC15A2",0.207447245666404,1.02558430340912,3.22299417080721,0.318208550514461,0.750326747237409,NA
"15973","ILDR1",0,NA,NA,NA,NA,NA
"15974","AC096861.2",0.510685816124562,-2.81680479540165,2.07900976555508,-1.35487809728955,0.175456399023961,NA
"15975","AC112503.2",0.297130845374805,1.62930537364398,2.4846829288714,0.655739754441847,0.511991585412106,NA
"15976","ADCY5",1.06068408051304,0.0160380016113974,1.57215405960057,0.01020129135148,0.991860688302051,NA
"15977","AC112503.1",0,NA,NA,NA,NA,NA
"15978","AC020634.2",0,NA,NA,NA,NA,NA
"15979","AC117488.1",0.283673240731374,-1.61851549881525,2.89644275100986,-0.558794230699344,0.576302160315398,NA
"15980","AC092902.6",0.200879913375701,0.981067669524494,3.25622950176531,0.301289472683859,0.763193767774683,NA
"15981","AC079848.2",0.203160351946096,0.359191432502738,3.35247519753738,0.10714215955023,0.91467620116095,NA
"15982","AC016924.1",0.163911907211157,0.976662149432262,3.34440272959326,0.29202886984578,0.770264550640504,NA
"15983","AC063919.1",0.114130778193026,-0.939216628356831,3.35247519753738,-0.280156175069319,0.779357688030112,NA
"15984","CHST13",0.366765545441301,-2.20455412572815,2.64323073774586,-0.834037715378638,0.404259731170285,NA
"15985","KBTBD12",0.549322021863849,2.25425632411291,1.93858902917008,1.16283353005354,0.244897050238797,NA
"15986","AL449214.1",0,NA,NA,NA,NA,NA
"15987","GATA2",1.49153514023237,-0.266366219005806,1.61260657286498,-0.1651774360144,0.868804324756021,NA
"15988","GATA2-AS1",0.17109704682236,-0.362147980542956,3.35247519753738,-0.108024059599003,0.913976607082588,NA
"15989","AC112484.3",0,NA,NA,NA,NA,NA
"15990","KIAA1257",0.168331924460857,-0.362147980542956,3.35247519753738,-0.108024059599003,0.913976607082588,NA
"15991","EFCC1",1.39612853938962,-2.1235897657401,1.28448957462209,-1.65325574274503,0.0982788260581145,NA

"15992","AC108673.2",0.466833105932137,-1.69187726382196,2.01967559778713,-0.837697532056969,0.402200599869386,NA
"15993","AC137695.3",0.445736810255784,0.203771321783445,2.3096774264434,0.0882250133505555,0.929697837051532,NA
"15994","EFCAB12",0.114130778193026,-0.939216628356831,3.35247519753738,-0.280156175069319,0.779357688030112,NA
"15995","TRH",0.494699432713348,-0.00969594354598221,2.53709519052288,-0.00382167117032134,0.996950754999194,NA
"15996","ALG1L2",0.254189351532287,-0.999830626761722,2.64981024805783,-0.377321594063025,0.705934626414087,NA
"15997","LINC02021",0.631177114026942,-0.347087843091205,1.90107776965081,-0.182574247425427,0.855132092254945,NA
"15998","AC130888.1",0.32157802385943,0.28926759090872,2.49200598520945,0.116078208730469,0.907590558967189,NA
"15999","AC010210.1",1.55419242410195,0.142351519017933,1.19007618634624,0.119615467187004,0.90478776665545,NA
"16000","AC107027.3",1.4598020856964,0.869428991683201,1.24721511328044,0.697096260641374,0.485742554198179,NA
"16001","ACPP",0.500450995339886,-0.515419213533506,2.05214508147035,-0.25116119624652,0.801689484035114,NA
"16002","NPHP3-AS1",0.430017161404633,-1.79301758676743,2.24773276035005,-0.79770051778228,0.425044303313964,NA
"16003","TMEM108-AS1",0.200580936021446,-0.939216628356831,3.35247519753738,-0.280156175069319,0.779357688030112,NA
"16004","BFSP2",0.30693384384628,-1.17622046601614,2.85783338026898,-0.41157769173563,0.680648985462367,NA
"16005","AC022296.4",0,NA,NA,NA,NA,NA
"16006","AC080128.2",0.157344574920455,0.946214072007857,3.34552927999138,0.282829409883477,0.777307608562567,NA
"16007","SLCO2A1",1.42682819059747,0.568891684171834,1.27306864352574,0.446866464793525,0.654971478496241,NA
"16008","LINC02004",0.192803065653254,-0.362148402639638,3.30154978858522,-0.109690425960478,0.912654893573412,NA
"16009","AC010207.1",1.52653528073454,1.14994243241667,1.33431207139075,0.861824199205584,0.388784266244358,NA
"16010","EPHB1",0.244856896880082,-0.902701635500065,3.0767047150267,-0.293398853354776,0.769217301797547,NA
"16011","AC092969.1",0,NA,NA,NA,NA,NA
"16012","AC072039.2",0,NA,NA,NA,NA,NA
"16013","AC117382.2",0.348442108488148,1.43515432875047,2.41256076779889,0.59486763935892,0.551931950038862,NA
"16014","CLDN18",0.122207625915474,0.359191432502738,3.35247519753738,0.1071421595502 3,0.914676201160951,NA
"16015","AC016252.1",0.350581687167453,0.711640851901938,2.19448947702661,0.324285379060539,0.745721987930964,NA
"16016","NME9",0,NA,NA,NA,NA,NA
"16017","ESYT3",0.554220040724069,-0.104502023882428,2.00074353326423,-0.052231593977431,0.958344158930149,NA

"16018","FOXL2",0,NA,NA,NA,NA,NA
"16019","AC024933.1",0.406179844001103,0.900121711997641,2.36485200375239,0.380624965354867,0.703481553632706,NA
"16020","RASA2-IT1",0.26719040510327,-0.362151043418806,2.9631418910391,-0.122218596589652,0.902725898914264,NA
"16021","ATP1B3-AS1",0.200580936021446,-0.939216628356831,3.35247519753738,-0.280156175069319,0.779357688030112,NA
"16022","AC109992.2",0.104487577740536,-0.939216628356831,3.35247519753738,-0.280156175069319,0.779357688030112,NA
"16023","PLS1",0.0666094690818238,0.359191432502738,3.35247519753738,0.10714215955023,0.91467620116095,NA
"16024","AC072028.1",0,NA,NA,NA,NA,NA
"16025","AC021074.3",0,NA,NA,NA,NA,NA
"16026","PAQR9",0,NA,NA,NA,NA,NA
"16027","PAQR9-AS1",0.35500944077885,1.46214354848971,2.40471023689567,0.608033153456878,0.543165485527888,NA
"16028","AC018450.1",0.0786722874602274,0.359191432502738,3.35247519753738,0.10714215955023,0.914676201160951,NA
"16029","AC131210.1",0,NA,NA,NA,NA,NA
"16030","AC107021.2",0.504995773382571,-0.291862868521176,2.51202270073069,-0.116186397693094,0.907504816808058,NA
"16031","LNCSRLR",0,NA,NA,NA,NA,NA
"16032","PLSCR2",0.347071772411921,0.658784253785852,2.4422728853822,0.269742278894751,0.787358531872649,NA
"16033","AC092958.1",1.82100679954814,-0.432575356784805,1.24061125807126,-0.348679212743334,0.727330151842178,NA
"16034","AC093001.2",0,NA,NA,NA,NA,NA
"16035","TM4SF18",0.313462733221608,-2.14519230363698,3.25345188633189,-0.659358852869214,0.509665358193341,NA
"16036","TM4SF1-AS1",0,NA,NA,NA,NA,NA
"16037","WWTR1-AS1",0.104487577740536,-0.939216628356831,3.35247519753738,-0.280156175069319,0.779357688030112,NA
"16038","LINC01213",0,NA,NA,NA,NA,NA
"16039","AC018545.1",0,NA,NA,NA,NA,NA
"16040","ERICH6",0,NA,NA,NA,NA,NA
"16041","AC011317.1",0.104487577740536,-0.939216628356831,3.35247519753738,-0.280156175069319,0.779357688030112,NA
"16042","SIAH2-AS1",0.371492276406953,0.286980760168805,2.41816197757207,0.118677227923725,0.905531078509693,NA
"16043","MED12L",1.80015544013001,-0.993179903537919,1.15126836734213,-0.86268322114228,0.388311660098864,NA
"16044","P2RY13",1.07723546601054,-0.752426118964922,1.44130763696277,-0.522044079743094,0.601639640013536,NA
"16045","P2RY12",1.40501663898133,0.563226524510895,1.39167186357283,0.40471215898064,0.685689103540381,NA

"16046","LINC02066",1.32202715706553,0.916907452165561,1.3723656467146
2,0.66812183353656,0.504055825923708,NA
"16047","AADACL2",1.23707286040759,0.0497888594992764,1.2417678082185,
0.0400951443335496,0.968017272951076,NA
"16048","AC108718.1",0.114130778193026,-0.939216628356831,3.3524751975
3738,-0.280156175069319,0.779357688030112,NA
"16049","AC026347.1",0,NA,NA,NA,NA,NA
"16050","AC078788.1",0.0666094690818238,0.359191432502738,3.3524751975
3738,0.10714215955023,0.91467620116095,NA
"16051","LINC02006",0.508527843062334,0.906333497734048,2.051213637690
14,0.441852316638584,0.65859607770875,NA
"16052","ARHGEF26-
AS1",1.4637680640636,0.000898807587356174,1.16573328199186,0.000771023
356063409,0.999384812429131,NA
"16053","MME-AS1",0,NA,NA,NA,NA,NA
"16054","STRIT1",0.0786722874602274,0.359191432502738,3.35247519753738
,0.10714215955023,0.914676201160951,NA
"16055","PLCH1",0,NA,NA,NA,NA,NA
"16056","AC104472.4",0.283673240731374,-1.61851549881525,2.89644275100
986,-0.558794230699344,0.576302160315398,NA
"16057","AC104472.5",0.199370397943956,-0.362148716949221,3.2631125208
3995,-0.110982601622331,0.911630143386501,NA
"16058","LEKR1",0.85796139779569,0.279929430160949,1.99425519114604,0.
140367908482205,0.888369312108094,NA
"16059","LINC00880",0.192803065653254,-0.362148402639638,3.30154978858
522,-0.109690425960478,0.912654893573412,NA
"16060","LINC02029",0.499736909499534,-1.07557660766688,2.501382394006
14,-0.429992875237387,0.667200823944978,NA
"16061","LINC00881",0.332861638992515,-0.46854418161012,2.236949960888
52,-0.209456711058486,0.834091725397205,NA
"16062","AC084212.1",0,NA,NA,NA,NA,NA
"16063","AC080013.4",1.56584376023992,0.384897887069296,1.196232777822
46,0.321758351890289,0.747635768089205,NA
"16064","AC080013.6",0.358224488296156,1.80762754813311,2.720709431968
4,0.664395663459482,0.50643710764916,NA
"16065","AC080013.3",0.413063434173244,2.00860046950676,2.574312268059
58,0.780247406046342,0.435245263535554,NA
"16066","AC080013.5",1.19459759972956,-0.970430823360341,1.30629653268
907,-0.742887085034715,0.457550045438859,NA
"16067","IQCJ-
SCHIP1",0.803842459289441,-1.3786476913523,1.51358333238108,-0.9108502
06829044,0.362374304497249,NA
"16068","IQCJ",0,NA,NA,NA,NA,NA
"16069","SCHIP1",0.544147939597659,-2.16145041753665,2.07359241032852,
-1.04236995022286,0.297240192823922,NA
"16070","IL12A",0.585224103355878,-0.656792644305192,1.9218927555868,-
0.341742608892169,0.732544603266688,NA
"16071","C3orf80",1.2575169379324,-2.616225429963,1.36243687198248,-1.
92025442335258,0.0548257702478958,NA
"16072","AC069224.1",0.69748809323554,-0.480608317531789,1.80804513148

585,-0.265816549134936,0.790380497241165,NA
"16073","SPTSSB",0.767137211844321,-0.690999917674247,1.67295586942862
,-0.413041330199732,0.679576333082193,NA
"16074","WDR49",0,NA,NA,NA,NA,NA
"16075","AC069243.1",1.31863503102231,0.468550192227153,1.173935022308
68,0.399127876179802,0.689798983076684,NA
"16076","MECOM",0.0666094690818238,0.359191432502738,3.35247519753738,
0.10714215955023,0.91467620116095,NA
"16077","TERC",0.272819502201393,-1.00047660537212,2.59418636066544,-0
.385661038290051,0.699747721586005,NA
"16078","AC078802.1",0.30171066064349,-2.11338103047016,2.456886905897
93,-0.860186533371494,0.389686226749445,NA
"16079","AC078795.1",0.104487577740536,-0.939216628356831,3.3524751975
3738,-0.280156175069319,0.779357688030112,NA
"16080","AC078795.3",0.0852396197509296,0.359191432502738,3.3524751975
3738,0.10714215955023,0.91467620116095,NA
"16081","AC078795.2",1.28655501207147,-0.0762372702695293,1.1541518578
9968,-0.0660548001094637,0.947334196501395,NA
"16082","AC008040.1",0,NA,NA,NA,NA,NA
"16083","GPR160",1.23674769606502,-1.53167937716102,1.25311485094798,-
1.22229768165488,0.221595075655053,NA
"16084","SLC7A14-
AS1",1.08097644053367,1.71817093051187,1.7234493167066,0.9969373127810
82,0.318794938848258,NA
"16085","SLC7A14",0,NA,NA,NA,NA,NA
"16086","AC026316.5",1.24484850647499,1.3145684840688,1.32517151427184
,0.991998748774143,0.321198135780631,NA
"16087","GHSR",0,NA,NA,NA,NA,NA
"16088","LINC02068",0,NA,NA,NA,NA,NA
"16089","TBL1XR1-AS1",0,NA,NA,NA,NA,NA
"16090","LINC00578",0.782123836727225,2.74207015598941,1.7533490910656
,1.56390428464129,0.117840048155063,NA
"16091","LINC02015",0.522269519036706,1.17120614277035,2.2251922337678
9,0.526339309025522,0.598652475658343,NA
"16092","AC117453.1",1.11896670627429,2.5856340129555,1.92595100247254
,1.34252325715247,0.179426394670133,NA
"16093","LINC01014",0.157344574920455,0.946214072007857,3.345529279991
38,0.282829409883477,0.777307608562567,NA
"16094","KCNMB2-
AS1",1.24504054730842,1.83235650020537,1.50700492771276,1.215892839173
66,0.224025746272035,NA
"16095","AC007823.1",0.26976982102792,0.904712707040654,2.956387869166
41,0.306019624987755,0.759589703549634,NA
"16096","AC007620.3",0.249769334282587,0.171289752160848,2.67665743248
737,0.0639939015287703,0.94897508273126,NA
"16097","AC090425.3",0.323105933674099,-2.17962193225865,2.75047370576
267,-0.792453288207046,0.428096418402325,NA
"16098","PEX5L",0.114130778193026,-0.939216628356831,3.35247519753738,
-0.280156175069319,0.779357688030112,NA
"16099","AC007687.1",0,NA,NA,NA,NA,NA

"16100","SOX2-
OT",0.770079956476717,0.217936791541164,1.55510726315168,0.140142610548599,0.888547314422416,NA
"16101","LINC01206",0,NA,NA,NA,NA,NA
"16102","AC092953.2",0.390308133512912,-1.17188561924843,2.36426143555539,-0.495666681198962,0.620129598355301,NA
"16103","MCCC1-
AS1",0.0786722874602274,0.359191432502738,3.35247519753738,0.10714215955023,0.914676201160951,NA
"16104","LAMP3",0.638992316885705,-2.43777898928082,1.77753166151686,-1.37144054424355,0.170237665824936,NA
"16105","MCF2L2",0.83360276146922,1.38137630213628,1.68370813090207,0.820436913490578,0.411967079331041,NA
"16106","KLHL6-AS1",0,NA,NA,NA,NA,NA
"16107","YEATS2-
AS1",0.293304672737834,0.185328792052959,2.54859534931574,0.0727180139062551,0.942030513475199,NA
"16108","MAP6D1",0.992304505685663,0.747647082688859,1.51555024750172,0.493317251553555,0.62178844059954,NA
"16109","AC131235.3",0,NA,NA,NA,NA,NA
"16110","AC131235.2",0.247349716356674,0.113410451520479,3.07142756361078,0.0369243451690434,0.970545328342794,NA
"16111","CAMK2N2",0.192803065653254,-0.362148402639638,3.30154978858522,-0.109690425960478,0.912654893573412,NA
"16112","LINC02054",0.598661367926539,1.57424683788467,2.16418997288816,0.727406954845006,0.466976695271855,NA
"16113","AC107294.3",0.288399971697026,1.02047894340869,2.90825331250117,0.35089066658057,0.725670373838576,NA
"16114","AC107294.1",0.145281756542051,0.889415776427968,3.34729258829349,0.265711990501974,0.790461028169891,NA
"16115","AC099661.1",1.01144950297483,-2.23003683447906,1.42311003162297,-1.56701645334889,0.117110845819923,NA
"16116","LIPH",0.0786722874602274,0.359191432502738,3.35247519753738,0.10714215955023,0.914676201160951,NA
"16117","DGKG",0.104487577740536,-0.939216628356831,3.35247519753738,-0.280156175069319,0.779357688030112,NA
"16118","CRYGS",1.40828241209588,0.555218795095259,1.25455647509339,0.442561818553389,0.658082706965486,NA
"16119","AC068631.1",0.531601973688911,1.11941971931193,1.98896833035222,0.562814250096026,0.573561375754766,NA
"16120","AC112907.3",0.187579882450464,-1.57167522621382,3.3176487217629,-0.473731656972615,0.635691275621751,NA
"16121","LINC02043",0.580990558594508,0.295998671066575,1.80794430679408,0.163721122356612,0.869950688681686,NA
"16122","AC072022.1",0.88781679003882,-0.896802254090369,1.73831969513264,-0.515901796776191,0.605922999100606,NA
"16123","LPP-AS1",0,NA,NA,NA,NA,NA
"16124","TPRG1-
AS1",1.34627275057149,-0.591816991273822,1.21504786810436,-0.48707298437315,0.626206610576362,NA

"16125","TP63",0.717850310762537,-0.390795202556757,1.83382593529887,-0.213103760304855,0.831246031712163,NA
"16126","P3H2",0.189727197491466,-0.362148248616811,3.32022295329944,-0.109073472989797,0.913144214822408,NA
"16127","CLDN1",0.18074024727485,-0.362147980542956,3.35247519753738,-0.108024059599002,0.913976607082588,NA
"16128","OSTN",0.48941300860212,0.317611264138219,2.59417792041425,0.12243233651742,0.902556630302687,NA
"16129","OSTN-AS1",1.5947097042122,-0.743605326275874,1.25332205136318,-0.593307462728426,0.552975405341047,NA
"16130","UTS2B",0.402201471800537,0.0844247467069442,2.69562808697438,0.0313191375007908,0.975015028375246,NA
"16131","AC073365.1",0,NA,NA,NA,NA,NA
"16132","PLAAT1",0.349072171735707,-0.489203931913523,2.20641365119026,-0.221719046947348,0.824532601806914,NA
"16133","ATP13A4",0.383900599220947,0.186451415862013,2.38106138678372,0.0783060096211407,0.937584637157847,NA
"16134","OPA1-AS1",0,NA,NA,NA,NA,NA
"16135","LINC02028",0,NA,NA,NA,NA,NA
"16136","LRRC15",0.551790740726363,-1.29931731831596,2.10333029008533,-0.617742883483719,0.536744839042036,NA
"16137","GP5",0.0666094690818238,0.359191432502738,3.35247519753738,0.10714215955023,0.91467620116095,NA
"16138","AC108676.1",0.82183481034929,-1.23416712983882,1.52363144975768,-0.810016838412664,0.417930498137052,NA
"16139","AC046143.2",0.17109704682236,-0.362147980542956,3.35247519753738,-0.108024059599003,0.913976607082588,NA
"16140","XXYLT1-AS2",0.0786722874602274,0.359191432502738,3.35247519753738,0.10714215955023,0.914676201160951,NA
"16141","AC090018.2",0.362345528191601,-1.04303717464845,2.4060340982707,-0.433508891415179,0.664645108002679,NA
"16142","ACAP2-IT1",0.161764592170155,-0.362147980542956,3.35247519753738,-0.108024059599003,0.913976607082588,NA
"16143","AC069213.1",0.163911907211157,0.976662149432262,3.34440272959326,0.29202886984578,0.770264550640504,NA
"16144","AC233280.1",0.582138553122297,-1.54258047092986,1.91018337194181,-0.807556223966989,0.419346095811954,NA
"16145","MUC4",0.655216154335691,-0.785118697307469,1.91870634821834,-0.409191692119282,0.682398990760255,NA
"16146","AC124944.3",0.114130778193026,-0.939216628356831,3.35247519753738,-0.280156175069319,0.779357688030112,NA
"16147","TNK2-AS1",0.524110120361756,-1.03167501225757,2.17622541390957,-0.474066245924484,0.63545266779672,NA
"16148","ZDHHC19",0.403741287967542,-0.36215353881519,2.60324677651195,-0.139116099972832,0.889358405998339,NA
"16149","SLC51A",0.708766196287471,-1.77917235076349,1.82561126600383,

-0.974562539076575,0.329777303238125,NA
"16150","TM4SF19-
AS1",1.33670163781394,0.966164219099665,1.38733604659635,0.69641686415
4882,0.486167804809174,NA
"16151","TM4SF19",0.776942018099338,-2.10044040025591,1.92258767376733
,-1.09250695243462,0.274610336268956,NA
"16152","AC117490.2",0.347071772411921,0.658784253785852,2.44227288538
22,0.269742278894751,0.787358531872649,NA
"16153","LINC01063",0.469412521856787,-0.403068222503402,2.08259222247
51,-0.193541595975216,0.846534832951393,NA
"16154","NRROS",0.439496531774053,-0.102646575127407,2.09076969393339,
-0.0490951133571756,0.960843497689337,NA
"16155","NCBP2-AS1",0,NA,NA,NA,NA,NA
"16156","AC011322.1",0,NA,NA,NA,NA,NA
"16157","DLG1-AS1",0,NA,NA,NA,NA,NA
"16158","LINC02012",0,NA,NA,NA,NA,NA
"16159","BDH1",1.14819214806158,2.58482568718021,1.51557859611979,1.70
55042171999,0.0881004264300695,NA
"16160","AC024560.3",0.279253223481674,-0.362151360256245,2.9199061145
6708,-0.124028426273541,0.901292767920445,NA
"16161","AC024560.4",0.614068734716641,0.0739474386942598,1.9073989238
7724,0.0387687325228978,0.969074773916564,NA
"16162","AC024560.1",0.762003108754269,-0.543909218759389,1.5336190733
537,-0.354657312372868,0.722846334411073,NA
"16163","AC055764.2",0.335008954033517,0.621700837329771,2.21722287966
942,0.280396185259672,0.779173563438129,NA
"16164","AC135893.1",0.32278856193692,-0.362152324877374,2.78414257136
106,-0.130076788668309,0.896505674038834,NA
"16165","AC253576.2",0.611497055153688,0.343262446797434,1.92937848263
117,0.177913483480604,0.858790921498714,NA
"16166","ZNF732",0,NA,NA,NA,NA,NA
"16167","AC079140.6",1.35558409905895,2.05922794711723,1.3927051959108
8,1.47858136320833,0.139252236723808,NA
"16168","AC092574.1",0.0786722874602274,0.359191432502738,3.3524751975
3738,0.10714215955023,0.914676201160951,NA
"16169","PDE6B",0.869196321441507,-0.281127836166021,1.49067843786195,
-0.188590529671334,0.850413755849018,NA
"16170","AC107464.3",0.70793445964112,0.740482234887056,1.830401202989
23,0.404546409649303,0.68581095744978,NA
"16171","AC139887.4",1.7840775986915,2.46506567704584,1.28757996405337
,1.91449521262019,0.055556910254151,NA
"16172","CPLX1",0.432436779330546,-1.76941473546535,2.2416539110945,-0
.789334485001489,0.429916536278128,NA
"16173","SLC26A1",0.420248573784447,0.985237586720796,2.07917236844601
,0.473860465670372,0.635599412870079,NA
"16174","AC092535.2",1.53719982811922,1.28749244208507,1.2951667812801
5,0.994074632467416,0.32018653435034,NA
"16175","AC092535.5",0.274966817242395,0.284464995938123,2.96042306445
425,0.0960893053947896,0.923449645061639,NA
"16176","FAM53A",0.831672219651807,-0.0227225564734704,1.6988751633002

7,-0.0133750595478299,0.989328564660706,NA
"16177","AC016773.1",1.15272494876791,1.05329147569571,1.4378780719429
,0.732531844144807,0.463844013609785,NA
"16178","FGFR3",0.200580936021446,-0.939216628356831,3.35247519753738,
-0.280156175069319,0.779357688030112,NA
"16179","NAT8L",0.104487577740536,-0.939216628356831,3.35247519753738,
-0.280156175069319,0.779357688030112,NA
"16180","AL121750.1",0.0786722874602274,0.359191432502738,3.3524751975
3738,0.10714215955023,0.914676201160951,NA
"16181","HTT-AS",0,NA,NA,NA,NA,NA
"16182","MSANTD1",0.450156827505484,-0.759200852052902,2.2783000655528
5,-0.333231282187878,0.73895970645481,NA
"16183","HGFAC",0.0830923047099275,-0.939216628356831,3.35247519753738
,-0.280156175069319,0.779357688030112,NA
"16184","DOK7",0,NA,NA,NA,NA,NA
"16185","ADRA2C",0.358676929272824,-1.50882299437205,2.3731366179591,-
0.635792723838055,0.524911530586359,NA
"16186","NSG1",0.453832052361155,-1.82912497444579,2.18784610131088,-0
.836039140664346,0.403132884045081,NA
"16187","CYTL1",0.157344574920455,0.946214072007857,3.34552927999138,0
.282829409883477,0.777307608562567,NA
"16188","CRMP1",1.0673309650677,-2.49309570755715,1.81712529093254,-1.
37199989455746,0.170063470637879,NA
"16189","C4orf50",0,NA,NA,NA,NA,NA
"16190","JAKMIP1",0.909520722495477,0.742444637209101,1.4555049157582,
0.510094214846639,0.609985457989636,NA
"16191","PPP2R2C",0.254189351532287,-0.999830626761722,2.6498102480578
3,-0.377321594063025,0.705934626414087,NA
"16192","LINC002481",1.41763485905709,-0.640996653918542,1.15185710063
32,-0.556489736067236,0.577876109095032,NA
"16193","S100P",0.879418538494861,1.33606775379541,1.56593218592979,0.
853209203949086,0.393543300220814,NA
"16194","AC097382.1",0,NA,NA,NA,NA,NA
"16195","AC097382.2",0.0852396197509296,0.359191432502738,3.3524751975
3738,0.10714215955023,0.91467620116095,NA
"16196","AFAP1-
AS1",0.367072259157253,1.5178050344468,2.39032181156191,0.634979368512
32,0.525441870944802,NA
"16197","AC097381.1",0.236338404108501,-0.362150168278439,3.0794114327
3211,-0.117603696741858,0.90638167569981,NA
"16198","ABLIM2",0.163911907211157,0.976662149432262,3.34440272959326,
0.29202886984578,0.770264550640504,NA
"16199","LINC02517",1.407364225456,0.676835644290543,1.59813434787873,
0.423516111263696,0.671918746272871,NA
"16200","AC105345.1",1.38750654431185,0.313488531341511,1.238446394143
64,0.25313048091862,0.800167385344148,NA
"16201","GPR78",0.927288696085958,3.1596287099127,1.84900718387503,1.7
0882446399746,0.0874834697076103,NA
"16202","CPZ",1.53553719684908,2.95921385847915,1.35189586341802,2.188
93624764656,0.0286014740352122,NA

"16203","HMX1",0.406038440365041,-0.0621573804740824,2.12408740498683,-0.0292630992152923,0.976654756850444,NA
"16204","AC073648.7",0.977661007323793,3.14546663006439,2.48178430026545,1.26742143937648,0.205004637809083,NA
"16205","AC105916.1",0.415841438836516,-2.51633216722487,2.21011082787076,-1.13855474372257,0.254888913491687,NA
"16206","SLC2A9",1.69496186069484,-0.859245089032915,1.14553258643224,-0.750083497588694,0.453204417829474,NA
"16207","AC005674.2",1.52365737491642,1.7333667861007,1.38204245743577,1.254206610495,0.209766917756914,NA
"16208","CLNK",1.14876962842193,-0.434940360803242,1.33462608344255,-0.325889300530792,0.744508111122455,NA
"16209","HS3ST1",0.950298674950918,2.22036312681253,1.64832197197008,1.34704454868046,0.177965885454871,NA
"16210","AC006230.1",0.626757096777242,0.511245133907408,1.95553421897385,0.261435023200811,0.793757051285667,NA
"16211","AC025539.1",0,NA,NA,NA,NA,NA
"16212","AC005699.1",0.338518868789049,0.659927798105604,2.44910427084671,0.269456799353608,0.78757818224272,NA
"16213","AC007370.2",1.21445305316223,2.55887664432321,1.68646330284732,1.51730348357,0.129190079102941,NA
"16214","AC007370.1",0.104487577740536,-0.939216628356831,3.35247519753738,-0.280156175069319,0.779357688030112,NA
"16215","NKX3-2",0.992312104141053,-0.370664346712033,1.44504182403593,-0.256507694480971,0.797558839811249,NA
"16216","LINC01096",0,NA,NA,NA,NA,NA
"16217","LINC00504",1.52744990100336,0.525499485866418,1.20706941152257,0.435351505762675,0.663307304438276,NA
"16218","CPEB2-DT",0.114130778193026,-0.939216628356831,3.35247519753738,-0.280156175069319,0.779357688030112,NA
"16219","AC098829.1",0.358546030023975,0.325387423573685,2.74973617638295,0.11833405196047,0.905802977374664,NA
"16220","AC116651.1",0.339428971283217,-0.456359008767591,2.22549160404263,-0.205059865397205,0.837525352840848,NA
"16221","AC006427.2",0.800929385386679,2.94911902581165,1.90848897167193,1.54526385511574,0.122282454480847,NA
"16222","AC097515.1",0,NA,NA,NA,NA,NA
"16223","AC104656.1",0.0830923047099275,-0.939216628356831,3.35247519753738,-0.280156175069319,0.779357688030112,NA
"16224","AC024230.1",0.463202949928734,-1.02151734421452,2.0359565512643,-0.501738282960984,0.615851631501734,NA
"16225","SLIT2-IT1",0.207447245666404,1.02558430340912,3.22299417080721,0.318208550514461,0.750326747237409,NA
"16226","KCNIP4",0.960543666275791,-3.69867808171095,1.55507543392275,-2.37845573341794,0.0173853242013683,NA
"16227","KCNIP4-IT1",1.80739693532862,-1.20056936795472,1.17568848460667,-1.02116281963617,0.307177304410643,NA

"16228","STIM2-
AS1",0.490367957621803,1.28285419482131,2.01146260773488,0.63777183323
6283,0.523622217403963,NA
"16229","AC098595.1",0.145281756542051,0.889415776427968,3.34729258829
349,0.265711990501974,0.790461028169891,NA
"16230","LINC02506",0.203160351946096,0.359191432502738,3.352475197537
38,0.10714215955023,0.91467620116095,NA
"16231","AC104078.1",0.122207625915474,0.359191432502738,3.35247519753
738,0.10714215955023,0.914676201160951,NA
"16232","C4orf19",0.279552200835929,1.4935861496343,2.9278366319717,0.
510133022220051,0.609958271749706,NA
"16233","AC027607.1",0,NA,NA,NA,NA,NA
"16234","PTTG2",0,NA,NA,NA,NA,NA
"16235","LINC02278",0,NA,NA,NA,NA,NA
"16236","TLR10",0.247004211921084,0.251392038816408,2.68232836300611,0
.0937215749881831,0.925330331348742,NA
"16237","AC079921.1",1.14677068732721,-0.652935151144504,1.31914181990
035,-0.494969639575091,0.62062155161908,NA
"16238","KLB",0.66377211750415,-0.20505334607307,1.7152211670604,-0.11
9549216165806,0.904840250718262,NA
"16239","AC021148.2",0.528703727798015,2.32704649493668,2.116683493646
51,1.09938330502487,0.271600909719276,NA
"16240","AC108471.3",1.36125177699901,2.71410692000197,1.5077326914981
1,1.80012474048379,0.0718409438751158,NA
"16241","AC095057.3",1.48864595100569,0.420936674614836,1.149791863247
84,0.366098150517267,0.714291837537754,NA
"16242","LINC02265",0.415051577569971,1.80375159601878,2.2817131848951
8,0.790525122946879,0.429221156173938,NA
"16243","AC108050.1",0,NA,NA,NA,NA,NA
"16244","DCAF4L1",0.433239945283636,-0.0373922250043671,2.334073385599
01,-0.0160201582499819,0.987218309800208,NA
"16245","SHISA3",0.678230103599418,1.50295651860822,1.94578383015979,0
.772417005071314,0.439867485362442,NA
"16246","AC096734.1",0,NA,NA,NA,NA,NA
"16247","KCTD8",0.0852396197509296,0.359191432502738,3.35247519753738,
0.10714215955023,0.91467620116095,NA
"16248","YIPF7",0.133218938163648,0.832405827491147,3.34883738676897,0
.248565615870131,0.803696808386323,NA
"16249","AC096586.2",0.350581687167453,0.711640851901938,2.19448947702
661,0.324285379060539,0.745721987930964,NA
"16250","AC096586.1",0.676427666639543,0.349626092225164,1.77316930977
266,0.197175808479332,0.843689961018731,NA
"16251","GABRA2",0.104487577740536,-0.939216628356831,3.35247519753738
,-0.280156175069319,0.779357688030112,NA
"16252","AC107398.3",0.203160351946096,0.359191432502738,3.35247519753
738,0.10714215955023,0.91467620116095,NA
"16253","CORIN",0.104487577740536,-0.939216628356831,3.35247519753738,
-0.280156175069319,0.779357688030112,NA
"16254","AC107068.2",0.0786722874602274,0.359191432502738,3.3524751975
3738,0.10714215955023,0.914676201160951,NA

"16255","AC107068.1",1.57186831967917,0.975668581170016,1.30362536984705,0.748427120043303,0.454202569394583,NA
"16256","NIPAL1",0,NA,NA,NA,NA,NA
"16257","SLC10A4",0,NA,NA,NA,NA,NA
"16258","AC027271.1",0.433681728239077,1.86455413015681,2.05360812758522,0.907940568169295,0.363909622461608,NA
"16259","LRRC66",0.183159865200764,-0.362147980542956,3.35247519753738,-0.108024059599003,0.913976607082588,NA
"16260","RASL11B",0.874516759113582,-2.18895004042933,1.71558799085145,-1.27591825782305,0.20198441624309,NA
"16261","AC023154.1",0.200879913375701,0.981067669524494,3.25622950176531,0.301289472683859,0.763193767774683,NA
"16262","AC124017.1",1.59729106584695,-0.336088745491886,1.10279898744006,-0.304759751613529,0.760549137671981,NA
"16263","AC110792.3",0.627572866441406,0.75149837554784,1.76824661823499,0.424996359556429,0.670839328566559,NA
"16264","AC110792.2",1.09287123017521,0.879090298208926,1.73023405024611,0.508075943878162,0.611400085002609,NA
"16265","AC110792.4",1.09438200966577,-0.856801355203904,1.2929673879358,-0.662662773398926,0.507546556804154,NA
"16266","KDR",0.385718636172434,0.93196623084445,2.37298349846326,0.392740291471892,0.694511308616677,NA
"16267","AC069200.1",0.26719040510327,-0.362151043418806,2.9631418910391,-0.122218596589652,0.902725898914264,NA
"16268","AC024243.1",0.332749134126589,-2.20931931675378,2.72679285873797,-0.810226310250904,0.417810118610788,NA
"16269","NMU",0,NA,NA,NA,NA,NA
"16270","EXOC1L",0.22826155638605З,-1.75913219955101,3.32763566182624,-0.528643270575355,0.597052937379996,NA
"16271","AC110611.1",0.203160351946096,0.359191432502738,3.35247519753738,0.10714215955023,0.91467620116095,NA
"16272","AC110611.2",0.0852396197509296,0.359191432502738,3.35247519753738,0.10714215955023,0.91467620116095,NA
"16273","KIAA1211",0.279253223481674,-0.362151360256245,2.91990611456708,-0.124028426273541,0.901292767920445,NA
"16274","AC068620.1",0.908649006672988,-0.0367990197258061,1.61369774527606,-0.0228041588541172,0.981806490602623,NA
"16275","AC068620.2",0,NA,NA,NA,NA,NA
"16276","SPINK2",0.249276914129782,-1.90751516048253,3.31840217494316,-0.57482940882993,0.565406675152336,NA
"16277","AC069307.1",0.199370397943956,-0.362148716949221,3.26311252083995,-0.110982601622331,0.911630143386501,NA
"16278","IGFBP7-
AS1",0.259412534735078,0.171500712501937,2.64651507850536,0.0648024694417359,0.948331275255137,NA
"16279","LINC02271",0,NA,NA,NA,NA,NA
"16280","ADGRL3",0.133218938163648,0.832405827491147,3.34883738676897,0.248565615870131,0.803696808386323,NA
"16281","AC020741.1",1.00260946847543,-0.977908863642375,1.43781308224192,-0.68013629568425,0.496418164412525,NA

"16282","EPHA5",0.157344574920455,0.946214072007857,3.34552927999138,0
.282829409883477,0.777307608562567,NA
"16283","AC104806.2",0.0786722874602274,0.359191432502738,3.3524751975
3738,0.10714215955023,0.914676201160951,NA
"16284","STAP1",0.44316513069283,0.661259764145285,2.28988093900029,0.
288774736224485,0.772753766416315,NA
"16285","TMPRSS11F",0.0786722874602274,0.359191432502738,3.35247519753
738,0.10714215955023,0.914676201160951,NA
"16286","SULT1B1",0.163911907211157,0.976662149432262,3.34440272959326
,0.29202886984578,0.770264550640504,NA
"16287","AC009570.2",0,NA,NA,NA,NA,NA
"16288","AC053527.2",0.813154573848627,1.0282168254163,1.5770871834309
7,0.651972088936392,0.51441917810734,NA
"16289","ALB",0,NA,NA,NA,NA,NA
"16290","AFP",0.81478098646369,1.81008087918236,2.09466663928737,0.864
137923062627,0.387512128535974,NA
"16291","CXCL6",4.51897732148422,2.29451871233368,1.22860586001342,1.8
6757917002661,NA,NA
"16292","PF4V1",0,NA,NA,NA,NA,NA
"16293","CXCL5",1.16298993238153,-3.29688199932627,1.40886947807683,-2
.34009044175382,0.0192790705921045,NA
"16294","AC093677.2",0.723489368523149,-2.27697998790781,1.75267669847
204,-1.29914432587187,0.193894403651133,NA
"16295","EPGN",0.275895370363181,-1.03718220655362,2.58196919069566,-0
.401702007247487,0.687903347118004,NA
"16296","BTC",0.279552200835929,1.4935861496343,2.9278366319717,0.5101
33022220051,0.609958271749706,NA
"16297","PARM1",1.04737631322611,-1.8985712759404,1.48362771315096,-1.
27968172817976,0.200657093273935,NA
"16298","LINC02562",0.114130778193026,-0.939216628356831,3.35247519753
738,-0.280156175069319,0.779357688030112,NA
"16299","CDKL2",0.266252169910691,-1.00025133134918,2.61314057122404,-
0.382777467987741,0.701884769397932,NA
"16300","AC110615.1",0.0786722874602274,0.359191432502738,3.3524751975
3738,0.10714215955023,0.914676201160951,NA
"16301","PPEF2",0,NA,NA,NA,NA,NA
"16302","FAM47E",0.39217346359721,-0.555080789639082,2.37408693512403,
-0.233808114364643,0.815133949809328,NA
"16303","CCDC158",0,NA,NA,NA,NA,NA
"16304","SHROOM3",0.268399484951693,0.250807602063781,2.61654731113631
,0.0958544112679779,0.923636202325112,NA
"16305","AC107072.2",0.0786722874602274,0.359191432502738,3.3524751975
3738,0.10714215955023,0.914676201160951,NA
"16306","CXCL13",0.944595385523937,3.18438165036365,2.174906038888,1.4
6414676929757,0.143153850799841,NA
"16307","LINC01094",0.674898158898793,1.67631840927675,1.9053584606640
7,0.879791621306001,0.378972204464634,NA
"16308","AC112253.1",0.0666094690818238,0.359191432502738,3.3524751975
3738,0.10714215955023,0.91467620116095,NA
"16309","LINC00989",0.240436879630382,0.224611920977265,3.097053594340

52,0.0725243894350796,0.942184596610118,NA
"16310","PCAT4",0.228261556386053,-1.75913219955101,3.32763566182624,-0.528643270575355,0.597052937379996,NA
"16311","FGF5",0,NA,NA,NA,NA,NA
"16312","AC073840.1",0.168331924460857,-0.362147980542956,3.35247519753738,-0.108024059599003,0.913976607082588,NA
"16313","WDFY3-AS1",0.302947873190324,0.185474739119599,2.52471436804289,0.0734636525490903,0.941437166955118,NA
"16314","AC108021.1",0.281832639406324,0.978331721828331,2.92526312973549,0.334442297475233,0.738045826283596,NA
"16315","SLC10A6",0.941267769737775,0.81776547331695,1.99570011076807,0.409763705931862,0.681979292602052,NA
"16316","ABCG2",0.843551487181162,-1.70566610744416,1.8947448659495,-0.900208855607273,0.368009114346898,NA
"16317","PPM1K-DT",0.0830923047099275,-0.939216628356831,3.35247519753738,-0.280156175069319,0.779357688030112,NA
"16318","PIGY-DT",0.693086526612458,0.17400010720969,1.66788768641523,0.104323635594233,0.916912521759261,NA
"16319","TIGD2",1.81238146042607,0.0738682030937279,0.999996777628715,0.0738684411252714,0.941115067554971,NA
"16320","AC104785.1",0.0786722874602274,0.359191432502738,3.35247519753738,0.10714215955023,0.914676201160951,NA
"16321","AC093866.1",0,NA,NA,NA,NA,NA
"16322","SNCA",1.7961269164629,-3.90616500759586,1.43662002149299,-2.71899663735468,0.00654802737743941,NA
"16323","MMRN1",0,NA,NA,NA,NA,NA
"16324","CCSER1",0.26719040510327,-0.362151043418806,2.9631418910391,-0.122218596589652,0.902725898914264,NA
"16325","AC109925.2",0,NA,NA,NA,NA,NA
"16326","AC106881.1",0.726986400976911,1.72326924896156,1.62228298922469,1.06224947213749,0.28812244610574,NA
"16327","LINC02267",0,NA,NA,NA,NA,NA
"16328","AC114811.2",0,NA,NA,NA,NA,NA
"16329","ADH4",0,NA,NA,NA,NA,NA
"16330","ADH1A",0.512515759428386,-0.51770867776939,2.04100790055016,-0.25365344133643,0.799763306235634,NA
"16331","MTTP",0.0666094690818238,0.359191432502738,3.35247519753738,0.10714215955023,0.91467620116095,NA
"16332","AC097460.1",0.731989466911808,0.235203592647087,1.86175186559284,0.126334554563312,0.89946710479071,NA
"16333","AF213884.3",0.937800212072937,-0.80677300653029,1.46451483016274,-0.550880735322183,0.581715434408712,NA
"16334","AC018797.3",1.29721127571856,1.22905449179986,1.30331383785693,0.943022667372911,0.345669308780354,NA
"16335","AC105460.1",0.161764592170155,-0.362147980542956,3.35247519753738,-0.108024059599003,0.913976607082588,NA
"16336","LINC02503",0.352862125737848,0.18604602110421,2.4375255243908

3,0.076325773511933,0.939159921350957,NA
"16337","CXXC4",0,NA,NA,NA,NA,NA
"16338","CXXC4-AS1",0,NA,NA,NA,NA,NA
"16339","AC004069.1",0.218618355933563,-1.71512321784709,3.15240431335981,-0.544068287997971,0.586394481847521,NA
"16340","AC109361.1",0.461202550604947,-0.0952912860672431,2.06815863200518,-0.046075424095904,0.963250133930189,NA
"16341","AC092445.1",1.00088359629685,2.18134606274724,1.75168982193903,1.24528100547653,0.213028475231231,NA
"16342","AC096564.1",0.114130778193026,-0.939216628356831,3.35247519753738,-0.280156175069319,0.779357688030112,NA
"16343","AC096564.2",0,NA,NA,NA,NA,NA
"16344","LEF1-AS1",0.114130778193026,-0.939216628356831,3.35247519753738,-0.280156175069319,0.779357688030112,NA
"16345","RPL34-AS1",0.203160351946096,0.359191432502738,3.35247519753738,0.10714215955023,0.91467620116095,NA
"16346","AC107071.1",0.104487577740536,-0.939216628356831,3.35247519753738,-0.280156175069319,0.779357688030112,NA
"16347","COL25A1",1.09420960793571,1.45336545162101,1.5161508608136,0.958588943346379,0.337765864761205,NA
"16348","AC004067.1",0.756787661913176,-0.127298602215331,1.58935798606071,-0.0800943546587928,0.93616212654031,NA
"16349","AC126283.1",0.0852396197509296,0.359191432502738,3.35247519753738,0.10714215955023,0.91467620116095,NA
"16350","RRH",0.232794078501679,-0.90181358082427,3.12933488781614,-0.288180592091764,0.773208504171299,NA
"16351","LRIT3",0.0786722874602274,0.359191432502738,3.35247519753738,0.10714215955023,0.914676201160951,NA
"16352","AC109347.1",1.55402997598671,-0.822771385552788,1.10403666308523,-0.745239187305207,0.45612713684732,NA
"16353","AC109347.2",0.326022003816901,0.54804389107956,2.47324803026942,0.221588730435523,0.82463405622565 7,NA
"16354","AC017007.5",0.302947873190324,0.185474739119599,2.52471436804289,0.0734636525490903,0.941437166955118,NA
"16355","AC093879.1",0.331182781396546,-1.09385736146069,2.78562854043494,-0.392678831934247,0.694556707035699,NA
"16356","AC104779.1",0,NA,NA,NA,NA,NA
"16357","UGT8",0.599485221736414,2.52383390618827,2.73674222800149,0.922203735655183,0.356422314904502,NA
"16358","AC092661.1",0.666944264185789,1.54520203526093,1.7687128001251,0.873630832067046,0.382319329859843,NA
"16359","METTL14-DT",0.0786722874602274,0.359191432502738,3.35247519753738,0.10714215955023,0.914676201160951,NA
"16360","MYOZ2",0.697168775788265,0.918182779306542,1.87880552932411,0.488705597772459,0.625050140155535,NA
"16361","AC093752.3",0.0830923047099275,-0.939216628356831,3.35247519753738,-0.280156175069319,0.779357688030112,NA

"16362","AC093752.2",0,NA,NA,NA,NA,NA
"16363","AC073475.1",1.73476841545777,0.486675618546724,1.07215199397083,0.453924090318825,0.649883463837894,NA
"16364","TNIP3",0.649240442356926,0.0211878244423078,1.83888819554886,0.0115220841014664,0.990806910397629,NA
"16365","QRFPR",0,NA,NA,NA,NA,NA
"16366","AC079341.1",0.0786722874602274,0.359191432502738,3.35247519753738,0.10714215955023,0.914676201160951,NA
"16367","CCNA2",1.09610196029362,0.387782870958163,1.3616564403642,0.284787600941721,0.775806872067689,NA
"16368","TRPC3",0.104487577740536,-0.939216628356831,3.35247519753738,-0.280156175069319,0.779357688030112,NA
"16369","IL2",0.286252656656024,-0.362151520384174,2.89780959249269,-0.124974229266959,0.900543952427349,NA
"16370","IL21",0.247004211921084,0.251392038816408,2.68232836300611,0.0937215749881831,0.925330331348742,NA
"16371","IL21-AS1",0.236016862380682,1.34721548542771,3.32885593646028,0.404708257474268,0.68569197172232,NA
"16372","AC053545.1",0.0666094690818238,0.359191432502738,3.35247519753738,0.10714215955023,0.91467620116095,NA
"16373","AC021205.3",0.55804732721678,-1.38533782379472,1.77472455653642,-0.780593145393987,0.435041824597188,NA
"16374","AC108075.1",1.26808354556561,0.0412036721324134,1.32941619092843,0.0309938094733432,0.975274476627273,NA
"16375","LINC02516",0,NA,NA,NA,NA,NA
"16376","AC096773.1",0.192803065653254,-0.362148402639638,3.30154978858522,-0.109690425960478,0.912654893573412,NA
"16377","AC093772.2",1.28237830460839,-0.579827326842791,1.50104264684907,-0.386283046694204,0.699287054774997,NA
"16378","AC093591.2",0.157344574920455,0.946214072007857,3.34552927999138,0.282829409883477,0.777307608562567,NA
"16379","PLK4",1.05084486340674,1.31988091650656,1.51228802356227,0.872770858422532,0.382787986563292,NA
"16380","AC110609.1",1.16829943344169,-0.538830865948335,1.32461034901331,-0.406784430115397,0.684166323212691,NA
"16381","AC078850.1",0.421618909860674,1.82758340282461,2.27552689244673,0.803147354087971,0.421889570170481,NA
"16382","PCDH10",1.43392982021149,0.72583440559999,1.28601837885115,0.564404379856844,0.572478958792225,NA
"16383","AC015631.1",0.286119533126631,1.51303924540838,2.55102271431352,0.593110848021414,0.553106971009519,NA
"16384","LINC02510",0.890740734469875,1.84377340997136,1.66910016411727,1.10465114653348,0.269310779660701,NA
"16385","LINC02172",0,NA,NA,NA,NA,NA
"16386","AC116563.1",0,NA,NA,NA,NA,NA
"16387","SLC7A11",0.517258716740113,1.30006953745868,1.96150147758804,0.662793045181544,0.507463108493197,NA
"16388","LINC00499",0.0666094690818238,0.359191432502738,3.35247519753738,0.10714215955023,0.91467620116095,NA

"16389","AC109927.1",0.348442108488148,1.43515432875047,2.41256076779889,0.59486763935892,0.551931950038862,NA
"16390","MGARP",0.490527755640788,-1.00003351671662,1.85602482206413,-0.538803956083121,0.590022133603583,NA
"16391","AC112236.2",0.674748671117019,0.150305451525693,1.67495393316571,0.0897370659273067,0.928496160401128,NA
"16392","AC108053.1",0.406320703892193,1.06059202807511,3.08289175337242,0.344025062480676,0.730827438064171,NA
"16393","CLGN",0.203160351946096,0.359191432502738,3.35247519753738,0.10714215955023,0.91467620116095,NA
"16394","LINC02432",0.578215126016042,1.12728006508964,2.13323419125927,0.528437088486845,0.597196001223479,NA
"16395","AC139720.1",0,NA,NA,NA,NA,NA
"16396","AC097658.3",0.244415251830948,1.06920175933036,3.34018855838734,0.320102215979858,0.748890845867476,NA
"16397","SMARCA5-AS1",0.359914141819658,0.66011251553761,2.40654147005796,0.274299248008268,0.783854662360472,NA
"16398","GYPE",0.883885082825895,-0.649003958275878,1.60959442025597,-0.40320962231757,0.68679402158247,NA
"16399","SMAD1-AS1",0.203160351946096,0.359191432502738,3.35247519753738,0.10714215955023,0.91467620116095,NA
"16400","REELD1",0.208975155481072,-1.67232023626263,3.33344910552292,-0.501678646748169,0.615893587270659,NA
"16401","AC002460.2",0.48120303735028,0.782381850039811,2.0600111454287,0.379794959738915,0.704097624056885,NA
"16402","IQCM",0.170479239501859,1.00912327877525,3.3428978888652,0.301870805607469,0.762750551898993,NA
"16403","AC110813.1",0.605361823746633,0.603838341495348,2.16401074581818,0.279036664980628,0.780216687210599,NA
"16404","MAB21L2",0,NA,NA,NA,NA,NA
"16405","PRSS48",0.0786722874602274,0.359191432502738,3.35247519753738,0.10714215955023,0.914676201160951,NA
"16406","FAM160A1",0.510685816124562,-2.81680479540165,2.07900976555508,-1.35487809728955,0.175456399023961,NA
"16407","AC092611.1",0.122207625915474,0.359191432502738,3.35247519753738,0.10714215955023,0.914676201160951,NA
"16408","AC023424.3",0.463033607764511,-0.463667165626776,2.29239393697528,-0.202263301323579,0.839710888304177,NA
"16409","TIGD4",0.461042752585962,2.1801012561426,2.1445713904125,1.01656735042206,0.309359292687586,NA
"16410","AC093599.2",0.903183399401921,3.12063268504833,2.13964870993811,1.45847898795433,0.144708562685881,NA
"16411","MND1",0.927098879553324,0.38034008520707,1.58224779329056,0.240379595926683,0.810035994639324,NA
"16412","AC106865.2",0.0786722874602274,0.359191432502738,3.35247519753738,0.10714215955023,0.914676201160951,NA
"16413","DCHS2",0.0786722874602274,0.359191432502738,3.35247519753738,0.10714215955023,0.914676201160951,NA

"16414","AC079298.1",0.0786722874602274,0.359191432502738,3.3524751975
3738,0.10714215955023,0.914676201160951,NA
"16415","LRAT",1.64073926047825,1.06815897530805,1.42309372638465,0.75
0589336109079,0.452899840880215,NA
"16416","RBM46",0.145281756542051,0.889415776427968,3.34729258829349,0
.265711990501974,0.790461028169891,NA
"16417","AC104083.1",1.01167170661696,-2.32999149827103,1.699158839521
68,-1.37126173496937,0.170293379543193,NA
"16418","TDO2",0.342392334579079,-2.2379177893557,3.16281551721231,-0.
707571395541965,0.479211461452815,NA
"16419","GLRB",0.719210476318815,1.8248104040449,1.80283381009885,1.01
21900276237,0.311447203411511,NA
"16420","AC084740.1",0.0852396197509296,0.359191432502738,3.3524751975
3738,0.10714215955023,0.91467620116095,NA
"16421","RXFP1",0.0666094690818238,0.359191432502738,3.35247519753738,
0.10714215955023,0.91467620116095,NA
"16422","C4orf45",0,NA,NA,NA,NA,NA
"16423","NPY5R",1.3533052021507,2.82444902266585,1.56636394940821,1.80
318822054923,0.0713586597971838,NA
"16424","MARCH1",1.42949663618155,0.187372603036403,1.55264485936358,0
.120679627350975,0.903944796063066,NA
"16425","AC093788.1",0.97508864610956,0.138808164515042,1.396761315914
54,0.099378585971331,0.92083768450648,NA
"16426","FAM218A",0.350469182301527,-1.17415432878938,2.7379263077683,
-0.428848039283584,0.668033816139827,NA
"16427","GK3P",0.122207625915474,0.359191432502738,3.35247519753738,0.
10714215955023,0.914676201160951,NA
"16428","AC068989.1",0.669721154993322,-0.0825842961426588,1.804806841
16208,-0.0457579693622417,0.963503159287106,NA
"16429","AC080188.1",0,NA,NA,NA,NA,NA
"16430","AC096741.1",0,NA,NA,NA,NA,NA
"16431","AC084724.1",0.449412035111455,-0.926992265007577,2.2563340457
3974,-0.410839993642724,0.681189865182341,NA
"16432","MFAP3L",0.994336604099166,-0.581034435438493,1.42323358916917
,-0.408249524083871,0.68309049272267,NA
"16433","GALNTL6",0,NA,NA,NA,NA,NA
"16434","AC105285.1",1.51753404988195,1.36079979978015,1.2485552223212
9,1.0898995698806,0.275757386107105,NA
"16435","AC093849.4",1.50330441165481,0.763547380867659,1.221278808253
33,0.625203168766749,0.531837722445459,NA
"16436","AC093849.3",0.0666094690818238,0.359191432502738,3.3524751975
3738,0.10714215955023,0.91467620116095,NA
"16437","LINC02269",0.0830923047099275,-0.939216628356831,3.3524751975
3738,-0.280156175069319,0.779357688030112,NA
"16438","AC106895.1",0,NA,NA,NA,NA,NA
"16439","LINC02268",0.456007500100568,0.660887513635441,2.261677003467
18,0.292211271822763,0.77012509389369,NA
"16440","AC096751.2",1.46667793950684,0.593163194461031,1.194392259701
71,0.496623441455631,0.619454619407303,NA
"16441","GLRA3",0.515270085862356,0.965985478992931,2.50247739953968,0

.386011669544197,0.699488026314689,NA
"16442","GPM6A",0.236016862380682,1.34721548542771,3.32885593646028,0.404708257474268,0.68569197172232,NA
"16443","SPATA4",0.183159865200764,-0.362147980542956,3.35247519753738,-0.108024059599003,0.913976607082588,NA
"16444","AC093801.1",0.0830923047099275,-0.939216628356831,3.35247519753738,-0.280156175069319,0.779357688030112,NA
"16445","NEIL3",0.0786722874602274,0.359191432502738,3.35247519753738,0.10714215955023,0.914676201160951,NA
"16446","AC078881.1",0.355911806911321,-1.43801134465999,2.37199285548545,-0.606246069137377,0.544351365070765,NA
"16447","TENM3-AS1",1.05690932742136,-1.68553154490272,1.53592760029543,-1.09740299254894,0.272465265834085,NA
"16448","AC079766.1",0.505948427137684,-0.547777106418731,2.04582285238672,-0.267753928830972,0.788888735634182,NA
"16449","AC019193.2",0.0786722874602274,0.359191432502738,3.35247519753738,0.10714215955023,0.914676201160951,NA
"16450","CLDN22",0,NA,NA,NA,NA,NA
"16451","AC107214.1",1.63421840479532,0.482269473649531,1.10886169126655,0.434923018305989,0.663618305985681,NA
"16452","AC107214.2",0.0786722874602274,0.359191432502738,3.35247519753738,0.10714215955023,0.914676201160951,NA
"16453","AC074194.1",0,NA,NA,NA,NA,NA
"16454","ENPP6",0.78307684005706,0.230081171511078,1.82648351893562,0.125969475840196,0.899756086815008,NA
"16455","LINC02363",0.0830923047099275,-0.939216628356831,3.35247519753738,-0.280156175069319,0.779357688030112,NA
"16456","LINC02362",0.716448976592313,2.74549327902477,2.25190547915149,1.21918673072338,0.222773327111883,NA
"16457","AC099343.2",1.15625103362582,-0.786803505045453,1.29823801360287,-0.606054896560853,0.544478300179949,NA
"16458","AC099343.4",0.509157906309894,-0.926037584524776,1.84728767954531,-0.501295815902756,0.616162949528196,NA
"16459","LINC02427",1.48329300173398,1.35992563550027,1.66181956746696,0.818335312763911,0.413165747511511,NA
"16460","LINC02365",0,NA,NA,NA,NA,NA
"16461","AC084871.1",1.28131922513735,2.60488317183686,1.49533836506276,1.74200250103764,0.0815080066260243,NA
"16462","LINC01093",0,NA,NA,NA,NA,NA
"16463","C4orf47",0.58508903411639,0.589219493779528,1.79448093851505,0.328350934876529,0.742646336494855,NA
"16464","AC093797.1",0,NA,NA,NA,NA,NA
"16465","KLKB1",0.442572399935841,-0.155831311862669,2.08265704221862,-0.0748233188200129,0.940355288086137,NA
"16466","AC107050.1",0,NA,NA,NA,NA,NA
"16467","AC108865.1",0.122207625915474,0.359191432502738,3.35247519753738,0.10714215955023,0.914676201160951,NA
"16468","AC108865.2",1.095137188538,0.811330435123739,1.56170190249611,0.519516838538114,0.603400373692206,NA

"16469","AC097652.1",0,NA,NA,NA,NA,NA
"16470","FRG1-
DT",0.750850392870033,-1.28107216273539,1.52703532535575,-0.838927653777065,0.401509908535064,NA
"16471","AC021087.1",0,NA,NA,NA,NA,NA
"16472","AHRR",0,NA,NA,NA,NA,NA
"16473","SLC9A3",0.525948913883016,0.296454489025655,2.2469215128022,
0.13193807052741,0.895033275906466,NA
"16474","AC026740.1",0,NA,NA,NA,NA,NA
"16475","ZDHHC11",1.47111383354815,-0.449693287586776,1.1541738298341
8,-0.389623535001988,0.696814946422182,NA
"16476","AC122719.3",0,NA,NA,NA,NA,NA
"16477","AC116351.1",0.631521992087821,2.61703638364408,1.97754730466844,1.3233748580709,0.185710748542355,NA
"16478","SLC6A3",0,NA,NA,NA,NA,NA
"16479","AC026412.3",0.114130778193026,-0.939216628356831,3.35247519753738,-0.280156175069319,0.779357688030112,NA
"16480","AC126768.1",0,NA,NA,NA,NA,NA
"16481","AC091891.1",0,NA,NA,NA,NA,NA
"16482","AC094105.2",0.836052680130392,0.716961007479228,1.69367884498382,0.423315795437048,0.672064871411059,NA
"16483","AC091978.1",1.63224084841555,0.0998732781906507,1.13083387489086,0.0883182582413262,0.92962372768172,NA
"16484","LINC02145",0.17109704682236,-0.362147980542956,3.35247519753738,-0.108024059599003,0.913976607082588,NA
"16485","UBE2QL1",1.72357676915688,0.910546926836056,1.36217661570169,
0.668449976559765,0.50384640312285,NA
"16486","LINC01018",0,NA,NA,NA,NA,NA
"16487","LINC02102",0.189727197491466,-0.362148248616811,3.32022295329944,-0.109073472989797,0.913144214822408,NA
"16488","AC091965.4",0.122207625915474,0.359191432502738,3.35247519753738,0.10714215955023,0.914676201160951,NA
"16489","AC091932.1",0,NA,NA,NA,NA,NA
"16490","AC034229.1",0,NA,NA,NA,NA,NA
"16491","AC034229.4",0,NA,NA,NA,NA,NA
"16492","AC012640.1",0.414894491332807,1.9616904801836,2.59445883502614,0.756107768487234,0.449584584671079,NA
"16493","AC012640.4",0.357925510941901,0.224037573956491,2.72971566738288,0.082073593463779,0.93458819162738,NA
"16494","ROPN1L",1.04977496291383,1.22279461459827,1.45010048018154,0.
843248196459587,0.399089648501637,NA
"16495","LINC01513",0.0786722874602274,0.359191432502738,3.35247519753738,0.10714215955023,0.914676201160951,NA
"16496","DNAH5",1.00360037674581,-0.0707243930842106,1.52865784248491,
-0.0462656790281104,0.963098494170302,NA
"16497","AC016549.1",0,NA,NA,NA,NA,NA
"16498","AC010491.1",0.840933280536874,-0.480987134803662,1.57167621818942,-0.306034493133556,0.759578383183394,NA
"16499","AC010491.2",0,NA,NA,NA,NA,NA
"16500","AC016575.1",1.32043635414669,0.646057689271318,1.311936628688

58,0.492445805036422,0.622404225070348,NA
"16501","LINC02149",0.188817094997298,0.903560157643627,3.316755251044
27,0.272422922179483,0.785296849615702,NA
"16502","AC114964.2",0.333047345409119,1.75780413186539,3.235081698046
83,0.543356952291702,0.586884058677133,NA
"16503","AC010638.1",0,NA,NA,NA,NA,NA
"16504","AC024588.1",0,NA,NA,NA,NA,NA
"16505","AC022113.1",0.133218938163648,0.832405827491147,3.34883738676
897,0.248565615870131,0.803696808386323,NA
"16506","BASP1-
AS1",1.05539326408442,1.69265045533193,1.61068655496732,1.050887554820
54,0.293310236887033,NA
"16507","AC026790.1",0,NA,NA,NA,NA,NA
"16508","AC026785.2",0,NA,NA,NA,NA,NA
"16509","LINC02217",0,NA,NA,NA,NA,NA
"16510","H3.Y",0,NA,NA,NA,NA,NA
"16511","LINC02223",1.24686026479548,2.63549875396347,1.64024349821431
,1.6067728705114,0.108104201582015,NA
"16512","CDH18",0.487770369640982,0.820033188608036,2.2536398125648,0.
363870563537294,0.715954666931591,NA
"16513","LINC02241",0.170479239501859,1.00912327877525,3.3428978888652
,0.301870805607469,0.762750551898993,NA
"16514","AC091946.2",0.151849088832753,0.903480691260343,3.34685071118
547,0.269949504541936,0.787199101303289,NA
"16515","CDH12",1.32841798096133,-2.36987884656127,1.38256547768696,-1
.71411689703557,0.0865072591441746,NA
"16516","AC091938.1",0.187579882450464,-1.57167522621382,3.31764872176
29,-0.473731656972615,0.635691275621751,NA
"16517","C5orf17",0.625056084257043,-0.551981318130894,2.1060215724646
,-0.262096706580707,0.793246885822964,NA
"16518","CDH10",0,NA,NA,NA,NA,NA
"16519","LINC02211",0,NA,NA,NA,NA,NA
"16520","AC113370.1",1.13957183355988,0.999501118462008,1.445316681979
12,0.691544718831696,0.489223288242289,NA
"16521","PURPL",0.0830923047099275,-0.939216628356831,3.35247519753738
,-0.280156175069319,0.779357688030112,NA
"16522","CDH6",0,NA,NA,NA,NA,NA
"16523","AC010343.3",0.163911907211157,0.976662149432262,3.34440272959
326,0.29202886984578,0.770264550640504,NA
"16524","AC034223.1",0.0786722874602274,0.359191432502738,3.3524751975
3738,0.10714215955023,0.914676201160951,NA
"16525","AC034231.1",0.183159865200764,-0.362147980542956,3.3524751975
3738,-0.108024059599003,0.913976607082588,NA
"16526","SLC45A2",0,NA,NA,NA,NA,NA
"16527","AC139792.1",0.0852396197509296,0.359191432502738,3.3524751975
3738,0.10714215955023,0.91467620116095,NA
"16528","AC026801.2",0.83654913236753,-0.625737824206291,1.81153130763
766,-0.345419271291694,0.729779191338308,NA
"16529","PRLR",0.960220316744183,-0.165998606406586,1.74737133515396,-
0.0949990440308785,0.924315586240536,NA

"16530","UGT3A2",0.0852396197509296,0.359191432502738,3.35247519753738
,0.10714215955023,0.91467620116095,NA
"16531","AC008957.2",0.279552200835929,1.4935861496343,2.9278366319717
,0.510133022220051,0.609958271749706,NA
"16532","AC008957.1",0.161764592170155,-0.362147980542956,3.3524751975
3738,-0.108024059599003,0.913976607082588,NA
"16533","AC026741.1",0.777538996878664,1.96028279856398,1.798488994330
14,1.08996096431165,0.275730339710464,NA
"16534","GDNF",0,NA,NA,NA,NA,NA
"16535","AC008964.1",0.292686865417333,1.54246197535693,2.892423225138
22,0.533276721729829,0.593842039130769,NA
"16536","C9",0.539286277298825,0.0510100966562488,2.20714403351328,0.0
231113583353471,0.981561445465519,NA
"16537","AC108105.1",0.200879913375701,0.981067669524494,3.25622950176
531,0.301289472683859,0.763193767774683,NA
"16538","AC114977.1",0,NA,NA,NA,NA,NA
"16539","OXCT1-
AS1",1.34005865907808,0.0113106319628447,1.3142274814155,0.00860629694
842671,0.993133253307366,NA
"16540","AC008945.1",0.199370397943956,-0.362148716949221,3.2631125208
3995,-0.110982601622331,0.911630143386501,NA
"16541","AC008945.2",0,NA,NA,NA,NA,NA
"16542","AC008875.2",0.776026769685345,-0.0134583188036789,1.567486288
39132,-0.00858592442138101,0.993149507631608,NA
"16543","AC008875.1",0.686181426247863,1.713382878245,1.90427026578237
,0.899758248097759,0.368248917919897,NA
"16544","AC025171.5",0.0830923047099275,-0.939216628356831,3.352475197
53738,-0.280156175069319,0.779357688030112,NA
"16545","AC025171.4",1.22665979099756,0.799474215552866,1.417539007474
13,0.563987453846103,0.5727626705825,NA
"16546","NIM1K",1.00757323109508,-2.96274723376665,1.87395152760393,-1
.58101593884601,0.113874393397115,NA
"16547","AC114947.2",0.192803065653254,-0.362148402639638,3.3015497885
8522,-0.109690425960478,0.912654893573412,NA
"16548","AC114956.3",0.665270837008362,1.76818187867902,1.906513639537
51,0.927442553785218,0.353696805133449,NA
"16549","AC114956.1",0.188817094997298,0.903560157643627,3.31675525104
427,0.272422922179483,0.785296849615702,NA
"16550","LINC02224",0,NA,NA,NA,NA,NA
"16551","HCN1",0.472930172974016,1.69172405687485,2.55922718335019,0.6
61029262224498,0.508593547768755,NA
"16552","AC010478.1",0,NA,NA,NA,NA,NA
"16553","ISL1",0.199828407245471,1.19974298654701,3.33462853032117,0.3
59783098968286,0.719009343087826,NA
"16554","AC022126.1",0.286252656656024,-0.362151520384174,2.8978095924
9269,-0.124974229266959,0.900543952427349,NA
"16555","AC008966.2",0,NA,NA,NA,NA,NA
"16556","AC027329.1",0.705617802435895,0.707752882311892,1.72990841500
418,0.409127371237269,0.682446190445483,NA
"16557","AC025175.1",0.0666094690818238,0.359191432502738,3.3524751975

3738,0.10714215955023,0.91467620116095,NA
"16558","AC025175.2",0.62310221201459,-1.47776469604565,1.742487976652
82,-0.848077413357148,0.396394859517532,NA
"16559","AC016583.2",0.114130778193026,-0.939216628356831,3.3524751975
3738,-0.280156175069319,0.779357688030112,NA
"16560","ESM1",0.114130778193026,-0.939216628356831,3.35247519753738,-
0.280156175069319,0.779357688030112,NA
"16561","AC034238.1",0.0830923047099275,-0.939216628356831,3.352475197
53738,-0.280156175069319,0.779357688030112,NA
"16562","CDC20B",0,NA,NA,NA,NA,NA
"16563","CCNO",0.681477259655774,-3.10454492767072,1.9308808793671,-1.
60783866102,0.107870518727296,NA
"16564","IL31RA",0.916864546269925,0.337454736715292,1.50401082462192,
0.224369885635711,0.822469501142079,NA
"16565","AC008892.1",0.668197703078698,2.66942993809305,1.957804098468
46,1.36348163750463,0.172730767709534,NA
"16566","ANKRD55",0.459993470756524,-0.661841380693949,2.0908508976912
8,-0.31654164408603,0.751591421434787,NA
"16567","LINC01948",1.21918235621269,1.56301540273462,1.41370784206601
,1.10561415606949,0.268893559985736,NA
"16568","AC022431.1",0.133218938163648,0.832405827491147,3.34883738676
897,0.248565615870131,0.803696808386323,NA
"16569","AC008937.3",0.207447245666404,1.02558430340912,3.222994170807
21,0.318208550514461,0.750326747237409,NA
"16570","AC008780.1",0.200580936021446,-0.939216628356831,3.3524751975
3738,-0.280156175069319,0.779357688030112,NA
"16571","GAPT",1.2384170094955,-0.356910219569709,1.24918918532153,-0.
285713504218214,0.775097560801348,NA
"16572","RAB3C",0.104487577740536,-0.939216628356831,3.35247519753738,
-0.280156175069319,0.779357688030112,NA
"16573","AC092343.1",0,NA,NA,NA,NA,NA
"16574","PART1",0.151849088832753,0.903480691260343,3.34685071118547,0
.269949504541936,0.787199101303289,NA
"16575","DEPDC1B",0.376396977447761,-0.45703839455678,2.16690397315889
,-0.21091769650065,0.832951494936538,NA
"16576","ELOVL7",0.26719040510327,-0.362151043418806,2.9631418910391,-
0.122218596589652,0.902725898914264,NA
"16577","AC104113.1",1.47613940396175,-0.152537233106004,1.15166653875
71,-0.132449131734457,0.89462905544551,NA
"16578","C5orf64",0.0830923047099275,-0.939216628356831,3.352475197537
38,-0.280156175069319,0.779357688030112,NA
"16579","LRRC70",0.5746526283530,1.03087083243299,2.22323928551139,0.
463679658393529,0.642877273142636,NA
"16580","RGS7BP",0,NA,NA,NA,NA,NA
"16581","AC092354.2",0.555590376800296,0.731900435591399,1.99371836091
256,0.367103222772346,0.713542023450076,NA
"16582","AC092354.1",0.703470487394893,-0.0121369035330321,1.708389830
7119,-0.0071042939467595,0.994331641225798,NA
"16583","AC025176.1",0,NA,NA,NA,NA,NA
"16584","AC010359.2",0,NA,NA,NA,NA,NA

"16585","AC025442.2",0,NA,NA,NA,NA,NA
"16586","AC092353.2",0.0852396197509296,0.359191432502738,3.35247519753738,0.10714215955023,0.91467620116095,NA
"16587","MAST4-IT1",0.0786722874602274,0.359191432502738,3.35247519753738,0.10714215955023,0.914676201160951,NA
"16588","MAST4-AS1",0.915787131721828,-0.0731358953645438,1.4711940050327,-0.0497119313390068,0.960351948382931,NA
"16589","CD180",0,NA,NA,NA,NA,NA
"16590","AC112206.2",1.05437922837132,0.220298551046387,1.38861409579029,0.158646345096339,0.873947513052577,NA
"16591","LINC02242",0.157344574920455,0.946214072007857,3.34552927999138,0.282829409883477,0.777307608562567,NA
"16592","AC010280.2",0,NA,NA,NA,NA,NA
"16593","AC093523.1",0.367072259157253,1.5178050344468,2.39032181156191,0.63497936851232,0.525441870944802,NA
"16594","LINC02198",0.17109704682236,-0.362147980542956,3.35247519753738,-0.108024059599003,0.913976607082588,NA
"16595","AC010273.3",1.18890526170275,2.40116718268371,1.53304490521339,1.5662732216898,0.117284667238525,NA
"16596","AC010273.2",0.435522329564127,-0.493802246910433,2.1089806936729,-0.234142611353379,0.814874266126724,NA
"16597","MARVELD2",0.0666094690818238,0.359191432502738,3.35247519753738,0.10714215955023,0.91467620116095,NA
"16598","SERF1B",0.518614634274054,-1.05928759914261,1.98799423533696,-0.532842389738147,0.59414268756701,NA
"16599","SERF1A",1.69153240216104,-0.556741410846395,1.090102058204,-0.51072411675257,0.609544251066327,NA
"16600","LINC02197",0.207447245666404,1.02558430340912,3.22299417080721,0.318208550514461,0.750326747237409,NA
"16601","AC025774.1",0.401778221134326,-1.60644439392201,2.30579393600293,-0.696699027974185,0.485991167243569,NA
"16602","ZNF366",0.0830923047099275,-0.939216628356831,3.35247519753738,-0.280156175069319,0.779357688030112,NA
"16603","AC035140.1",0.133218938163648,0.832405827491147,3.34883738676897,0.248565615870131,0.803696808386323,NA
"16604","AC008972.1",0,NA,NA,NA,NA,NA
"16605","AC008972.2",0.0830923047099275,-0.939216628356831,3.35247519753738,-0.280156175069319,0.779357688030112,NA
"16606","TMEM171",0.401161872042892,-2.07999739171429,3.0419148688779,-0.683778962059367,0.494114753149597,NA
"16607","AC099522.2",1.28745737820394,-1.22738177417677,1.17432785022672,-1.04517811950027,0.295940654160557,NA
"16608","ARHGEF28",1.69578607888793,1.32356284392277,1.29838474571916,1.0193918623017,0.308016967958004,NA
"16609","FAM169A",0.789758901494231,1.93807653713637,1.71113859309813,1.13262394113112,0.257372210317327,NA
"16610","AC010501.2",0,NA,NA,NA,NA,NA
"16611","GCNT4",1.18678642893489,0.61152226888664,1.29052689635278,0.4

73854726015315,0.635603506111791,NA
"16612","ANKRD31",1.77565113641744,1.17660004669695,1.18712022691876,0.991138066740623,0.321618167331838,NA
"16613","AC008897.3",0.348442108488148,1.43515432875047,2.41256076779889,0.59486763935892,0.551931950038862,NA
"16614","AC010245.1",0.688969112982929,-1.21389670911671,1.76545085868465,-0.687584535783188,0.491714449239045,NA
"16615","ANKDD1B",0.230521376292981,1.33222568121774,2.70690437450901,0.492158383489031,0.622607381833459,NA
"16616","SV2C",0,NA,NA,NA,NA,NA
"16617","AC026725.1",0.166184609419855,-1.47576652541587,3.34432018311399,-0.441275489370681,0.659013568361442,NA
"16618","F2RL2",0.283673240731374,-1.61851549881525,2.89644275100986,-0.558794230699344,0.576302160315398,NA
"16619","F2RL1",0.340504440121218,0.631065267568559,2.80473121548499,0.225000265296165,0.82197906787763,NA
"16620","S100Z",0.309787508365938,-1.00169890791137,2.49878488750629,-0.400874406164283,0.688512594116363,NA
"16621","CRHBP",0.710017771153144,-3.27864734323822,2.18450562671039,-1.50086468221896,0.133390564634459,NA
"16622","ZBED3-AS1",1.71600500335296,-0.326089042628536,1.03732457794161,-0.314355843448349,0.7532507892558,NA
"16623","BHMT",0.327507556540876,0.329023874713084,2.82936419574862,0.116288979413634,0.907423519508415,NA
"16624","AC012636.1",0.247004211921084,0.251392038816408,2.68232836300611,0.0937215749881831,0.925330331348742,NA
"16625","AC010260.1",0,NA,NA,NA,NA,NA
"16626","ANKRD34B",0,NA,NA,NA,NA,NA
"16627","RASGRF2-AS1",0.104487577740536,-0.939216628356831,3.35247519753738,-0.280156175069319,0.779357688030112,NA
"16628","RASGRF2",0.511923028671397,-1.0585377389038,1.98791484679345,-0.532486459674689,0.594389117377751,NA
"16629","CKMT2",0.56908753823509,-2.02534562340146,2.10010400536034,-0.964402533508787,0.334844152539751,NA
"16630","AC010623.1",0.949880854703248,1.47580797441084,1.5244657866473,0.96808205689977,0.33300339028658,NA
"16631","AC026726.1",0.795622943872114,0.132382351154423,1.63885209335082,0.0807774854677413,0.935618913540561,NA
"16632","LINC01338",0.402530749890052,0.285225005632048,2.36559333107678,0.120572290209415,0.904029817895232,NA
"16633","AC104118.1",1.80691767342808,-0.289111199419285,1.10302253727443,-0.262108152507634,0.793238061660041,NA
"16634","VCAN-AS1",0.546256811723287,-2.16928648051396,2.07232406183912,-1.04678921625259,0.295196805069636,NA
"16635","AC113383.1",1.36477123594027,-0.629554101404111,1.24095935478423,-0.507312426452172,0.611935620879546,NA
"16636","LINC01949",0.286119533126631,1.51303924540838,2.5510227143135

2,0.593110848021414,0.553106971009519,NA
"16637","AC018754.1",0.200580936021446,-0.939216628356831,3.3524751975
3738,-0.280156175069319,0.779357688030112,NA
"16638","AC114971.1",0.293304672737834,0.185328792052959,2.54859534931
574,0.0727180139062551,0.942030513475199,NA
"16639","LINC02060",0,NA,NA,NA,NA,NA
"16640","MEF2C-
AS1",0.580952115679134,-0.513027987819039,1.95123517497162,-0.26292473
3214949,0.792608592454681,NA
"16641","AC093510.2",0.31135386109598,-2.13919741733647,2.780486611264
61,-0.769360804928863,0.441679149794276,NA
"16642","AC093510.1",0.151849088832753,0.903480691260343,3.34685071118
547,0.269949504541936,0.787199101303289,NA
"16643","ADGRV1",0.26976982102792,0.904712707040654,2.95638786916641,0
.306019624987755,0.759589703549634,NA
"16644","AC123595.1",0.852687577395536,-0.24433960683907,1.54906511839
162,-0.157733592951061,0.874666728492588,NA
"16645","AC123595.2",0.311466365961906,-0.380892033320124,2.5387834661
695,-0.150029350039376,0.8807414593427,NA
"16646","ARRDC3-
AS1",0.607263858176332,0.480884213635991,1.95123950582636,0.2464506341
73858,0.805333414238031,NA
"16647","AC106818.2",0,NA,NA,NA,NA,NA
"16648","AC108102.1",0.114130778193026,-0.939216628356831,3.3524751975
3738,-0.280156175069319,0.779357688030112,NA
"16649","AC099501.1",0,NA,NA,NA,NA,NA
"16650","MCTP1",0.895788103205562,-1.48780606509698,1.58281685097398,-
0.939973607294784,0.347231098787722,NA
"16651","SPATA9",0.421177126905233,-0.0731535521177196,2.1036781276087
3,-0.0347741183205027,0.972259858734036,NA
"16652","AC008840.1",0,NA,NA,NA,NA,NA
"16653","LINC01554",1.53374298469906,1.05884169363104,1.26704982859363
,0.835674864347136,0.403337839543408,NA
"16654","AC008592.5",0.901750101007953,2.03611427919459,1.794963421200
95,1.13434861966842,0.256648342309861,NA
"16655","AC020900.1",0.122207625915474,0.359191432502738,3.35247519753
738,0.10714215955023,0.914676201160951,NA
"16656","AC008906.2",0.553443061759294,-0.493413698128251,2.0102402922
3917,-0.245450108642806,0.806107934241846,NA
"16657","AC009126.1",1.13096103808884,-1.14385664834081,1.337023842105
68,-0.85552449576318,0.392260849384374,NA
"16658","LIX1-AS1",0,NA,NA,NA,NA,NA
"16659","LIX1",0,NA,NA,NA,NA,NA
"16660","AC008883.3",1.13714999135343,0.565585694513303,1.349219122690
02,0.419194840187011,0.675073742747704,NA
"16661","LINC01340",0,NA,NA,NA,NA,NA
"16662","LINC02234",0,NA,NA,NA,NA,NA
"16663","RGMB-
AS1",0.830116869316317,-1.89132378603401,1.82728765590046,-1.035044362
02301,0.300648194148763,NA

"16664","AC008522.1",1.26442720257389,-0.457815398840061,1.23018275825074,-0.372152345470239,0.709779424661399,NA
"16665","LINC02062",1.17979584597797,-0.179150027620466,1.28322965897922,-0.139608702438327,0.888969164414599,NA
"16666","AC113385.1",0,NA,NA,NA,NA,NA
"16667","AC021086.1",0.279552200835929,1.4935861496343,2.9278366319717,0.510133022220051,0.609958271749706,NA
"16668","SLCO4C1",1.61681955056419,-0.0824695060674277,1.15507945272121,-0.0713972583211836,0.943081591585751,NA
"16669","SLCO6A1",1.28529437477144,-1.30435951618244,1.6584043944787,-0.786514749071467,0.431565981759286,NA
"16670","AC024587.2",0,NA,NA,NA,NA,NA
"16671","AC012603.1",0.631982225690128,1.35385707411875,1.76239158496103,0.768193110811233,0.44237246577932,NA
"16672","TMEM232",1.08968000745937,-0.554020883355938,1.33531622461382,-0.414898638347754,0.678216106675311,NA
"16673","AC104126.1",0,NA,NA,NA,NA,NA
"16674","AC079465.1",0.0786722874602274,0.359191432502738,3.35247519753738,0.10714215955023,0.914676201160951,NA
"16675","TRIM36",0.197223082902954,-1.61249994371822,3.25614921967676,-0.495216845092344,0.620447061165921,NA
"16676","AC008494.3",1.12115252753621,-0.509112964906867,1.43836808932612,-0.353951793483814,0.723375010700887,NA
"16677","LINCADL",0.515569063216611,2.2834341274861,2.46915334125609,0.924784252696306,0.355078143441938,NA
"16678","ARL14EPL",1.2323440430677,0.277746114824391,1.48593045460076,0.186917304214628,0.85172547150709,NA
"16679","SEMA6A-AS1",0.575767375391718,-0.47237149214842,1.68276768476072,-0.280711055023373,0.778932028874993,NA
"16680","SEMA6A-AS2",0.842313298705142,-1.86842458866049,1.73185063360529,-1.07886012361868,0.280650087566213,NA
"16681","LINC02147",0,NA,NA,NA,NA,NA
"16682","AC008629.1",0.145281756542051,0.889415776427968,3.34729258829349,0.265711990501974,0.790461028169891,NA
[truncated: 551,402 more chars]
