# Supplementary material for: PPARγ agonist treatment reduces fibroadipose tissue in secondary lymphedema by exhausting fibroadipogenic PDGFRα+ mesenchymal cells
Source: JCI Insight. 2023 Dec 22;8(24):e165324. doi: 10.1172/jci.insight.165324 (PMC10807713; doi:10.1172/jci.insight.165324)
Supplement: Supplemental data set 7 [file jciinsight-8-165324-s189.pdf]

"","gene","baseMean","log2FoldChange","lfcSE","stat","pvalue","padj"
"1","PLP2",166.308611485797,-1.83566596364975,0.202089271049687,-9.083
44096702897,1.05195413533736e-19,1.41372116247988e-15
"2","CAPG",63.4295776627214,-2.66774645651428,0.325492174693665,-8.196
03868825731,2.48438406510372e-16,1.66938187254645e-12
"3","CTHRC1",289.890008158843,-1.81069809604709,0.250340937640029,-7.2
3292847393075,4.72688388251706e-13,2.11748641657156e-09
"4","CAV1",377.578594858749,-1.75282537711181,0.249102657205446,-7.036
55832810398,1.97046188210458e-12,6.62025930840086e-09
"5","PERP",28.5682946889083,-3.26367893917726,0.473470374747134,-6.893
10063152376,5.45892283138963e-12,1.46724927862091e-08
"6","PDGFD",204.976169154237,1.63906946745803,0.238912456065569,6.8605
4421125784,6.85987086794045e-12,1.53649674323753e-08
"7","FABP4",50.4737587823781,-2.96848775306448,0.47160949845991,-6.294
37651862056,3.08637849128628e-10,5.92540579205662e-07
"8","PTGFR",90.333391520575,-1.77898625644683,0.28944780839461,-6.1461
3828418249,7.93920752628843e-10,1.33368762432238e-06
"9","LMNA",893.472061683615,-1.00565098265054,0.165886432518553,-6.062
28591080265,1.3420037786098e-09,2.00390986452634e-06
"10","ADIRF",337.973677206483,-2.36702193253476,0.395752479568274,-5.9
8106658767353,2.2168122228608e-09,2.97917394630263e-06
"11","CDH13",21.2861065077619,-2.98823597702682,0.504778634090939,-5.9
1989394006022,3.22149300621124e-09,3.93578586458844e-06
"12","CXCL3",17.4307349156374,-3.1064613816822,0.555821634479828,-5.58
895370200805,2.28441843756044e-08,2.55835828186456e-05
"13","CD36",24.6521995665908,-3.33994507245854,0.625644446405927,-5.33
840760777974,9.37664682293703e-08,9.69328897334236e-05
"14","ABCA1",132.642699531574,1.86042166396116,0.349497184354625,5.323
13777404698,1.01992436758194e-07,9.79054541138119e-05
"15","SERTAD4-
AS1",10.1078392291928,-3.70781529461513,0.710414587803637,-5.219227417
72301,1.79671034805237e-07,0.000160973269116505
"16","VGLL3",138.956887850564,1.95061141613942,0.377784973640694,5.163
28481077867,2.42653501938717e-07,0.000203813775784651
"17","CD9",318.494009131793,-2.98568150769766,0.590253504050727,-5.058
30374103305,4.23002290531427e-07,0.000334395751908932
"18","GAL",35.9936605733649,2.81497990237657,0.563621536460875,4.99445
056704638,5.90035016545318e-07,0.000440526699297363
"19","NBL1",226.118269158008,-1.03382585221047,0.209937121297436,-4.92
445474064473,8.45959049271181e-07,0.000557783471964093
"20","SEMA3E",18.2655063363153,-2.67878719138425,0.542536636773264,-4.
93752312713171,7.91210260831438e-07,0.000557783471964093
"21","CRIP1",1936.28338888044,-1.22563556305186,0.249183230007165,-4.9
1861175014312,8.71601526247932e-07,0.000557783471964093
"22","MARCKS",2176.72982117135,1.32796709172254,0.272523426728338,4.87
285481349208,1.09997060638507e-06,0.000615937707467037
"23","C11orf96",228.155895610833,-1.74166640908974,0.357009979118009,-
4.87848102563551,1.06905946053453e-06,0.000615937707467037
"24","MAF",238.848492569429,1.06158673602566,0.217321931932017,4.88485
780789831,1.03503521540807e-06,0.000615937707467037

"25","CCL2",52.3162606851357,-2.18087881670599,0.45434205809086,-4.800
08129969305,1.58601232481248e-06,0.000852576785326195
"26","LDHA",556.635629373806,-0.874039017568116,0.183176603455556,-4.7
7156471448704,1.82800226886759e-06,0.00094486624966583
"27","C1QC",5.35377615494001,-4.0695167980633,0.856478550508027,-4.751
45208907968,2.01961016330628e-06,0.00100161586935293
"28","DBNDD2",225.147747595329,-1.50456854709812,0.317096665670868,-4.
74482613658194,2.08685499976799e-06,0.00100161586935293
"29","TNFAIP6",103.285921698135,-2.07533788645552,0.441687416108071,-4
.69865749117862,2.61877206780239e-06,0.00121357509721367
"30","FXYD5",59.8195983587789,-1.88457413053264,0.403048683967064,-4.6
7579775223046,2.92813081481381e-06,0.00131170500067609
"31","PXDNL",36.3591138918746,2.00116389929504,0.429897038300845,4.654
9841497038,3.24005631444346e-06,0.00140461667128405
"32","ANOS1",60.2681421091469,1.56450395748949,0.337616183646242,4.633
97204657935,3.58715453915992e-06,0.00150649280786782
"33","VEGFD",218.423195935126,1.3335228736035,0.288993178138324,4.6143
7492121429,3.94280131349021e-06,0.00160567596521197
"34","MS4A7",6.7178926594792,-2.96565129933564,0.650422463749873,-4.55
957698975801,5.12567631314427e-06,0.00202599894036311
"35","LAMC1",477.021741938192,0.992429367074837,0.21797076853006,4.553
03880317315,5.28765154331627e-06,0.00203030711687507
"36","PLAUR",32.0471341469978,-1.28064252042407,0.282063938302943,-4.5
4025611401848,5.61859363856478e-06,0.00209745221968533
"37","TAGLN2",434.636455328641,-0.843209901755094,0.186510473266304,-4
.5209788329213,6.15543435839514e-06,0.00223575357682358
"38","MAZ",98.4093556973776,-1.17770223083589,0.263990644983031,-4.461
15138251051,8.15204766020754e-06,0.0028830360133034
"39","ANGPTL1",104.027502804269,-1.06979896548433,0.241758465335402,-4
.42507344675666,9.64094764473749e-06,0.00316011452189334
"40","TUBA1C",38.5274732877966,-1.2940679173689,0.292230592738101,-4.4
2824245485023,9.50040719942467e-06,0.00316011452189334
"41","SLITRK2",4.50849215255144,-3.95866879912574,0.892687183965801,-4
.4345531897738,9.22634394337414e-06,0.00316011452189334
"42","RGCC",28.2482372483212,-1.66922862096227,0.380159731629124,-4.39
086121459791,1.12902589326012e-05,0.00361261404274351
"43","GALNT13",11.4584182774377,-2.75436608482121,0.628937471948509,-4
.37939573911524,1.19008842609149e-05,0.00363490871778262
"44","SCIN",25.8304686561201,2.87680962372141,0.65631596951553,4.38326
927477473,1.16911458671975e-05,0.00363490871778262
"45","LINC00702",8.30954106728534,-3.39451071214701,0.77853594325196,-
4.36012073889367,1.29990699166053e-05,0.00383737490124558
"46","MEG8",99.5034014741211,1.91510851198585,0.439937680896385,4.3531
3589889314,1.34203899366428e-05,0.00383737490124558
"47","KLHL13",23.9960576711407,1.64808720998142,0.378519596503176,4.35
403404528249,1.33654936039072e-05,0.00383737490124558
"48","S100A6",8604.28049464211,-0.919746284838584,0.212526348249832,-4
.3276812141777,1.50687354398145e-05,0.00421893199115973
"49","SH3BGRL3",1163.92675920873,-1.04195854618886,0.241469077360125,-
4.31508066200496,1.59544636630578e-05,0.00436070729894352

"50","IL7",6.98084045149015,-2.76722101427487,0.641841526698914,-4.31137734030252,1.62240765642664e-05,0.00436070729894352
"51","C1QA",12.7397036134859,-2.08408685892432,0.485395297108946,-4.29358683806233,1.75809505488761e-05,0.00454366143127587
"52","ADAMTSL3",55.4743148275964,1.40590825012198,0.327219263885921,4.29653264733254,1.7349042722825e-05,0.00454366143127587
"53","KCNAB1",54.500774913259,2.17714540239921,0.508774522378183,4.27919501987344,1.87570461263333e-05,0.00466807301651469
"54","IGFBP6",4068.42251555104,-0.908634114526557,0.212329566159946,-4.27935746754218,1.87433622631084e-05,0.00466807301651469
"55","MARCH2",82.1301591880251,-1.05875183826167,0.248758597853508,-4.25614168674951,2.0798493039578e-05,0.00508201723561615
"56","S100A4",4829.97415989524,-0.889017182353378,0.209482279348717,-4.24387774048164,2.19690074617037e-05,0.0052721694871042
"57","CYP26B1",26.3809251857275,-2.15419820079397,0.51140253146335,-4.21233386277899,2.52745601125757e-05,0.00595903181320886
"58","SDK1",75.7981381415416,1.08604327330514,0.259421944944904,4.18639708192689,2.83417353514372e-05,0.00646562911821283
"59","CRIP2",80.7137118819536,-1.73722896847644,0.415004645233419,-4.18604704412244,2.83854541241578e-05,0.00646562911821283
"60","FRMD6",178.4704918342,1.10792967490988,0.265604444501164,4.17135216615332,3.02797474107454e-05,0.00678215875755013
"61","S100A3",7.80789241619973,-2.34595564480196,0.563544297149929,-4.16285934693405,3.14286979174762e-05,0.00692410280840922
"62","IER3",46.6302324482718,-1.36792309566414,0.329193789575938,-4.15537333625361,3.24756819146736e-05,0.00703936595566611
"63","ADAMTSL1",63.1193119721897,1.46850319115458,0.35702086274348,4.11321394461393,3.90188459089814e-05,0.00832340111382225
"64","KCP",11.5315770160447,4.43003346886101,1.0783225411651,4.10826380766784,3.98644604456443e-05,0.00837091381139085
"65","SPI1",4.53384938948054,-3.64264774174423,0.893427815083422,-4.077159542435,4.55892126152892e-05,0.00942574505133649
"66","DPT",587.849580928478,-1.34153406297993,0.332929817367147,-4.02948006756788,5.59003552816062e-05,0.0113824981004471
"67","CXCL2",37.1869050152269,-2.23200137772559,0.555200814480234,-4.02016949455511,5.81562850511708e-05,0.0116651091761595
"68","ADM",269.093429521394,1.5553495048651,0.388045211246921,4.0081656976702,6.1192182026568e-05,0.0120935549155154
"69","CSF1",196.267284909723,0.709019792762302,0.17713149933209,4.00278773360923,6.26004633918919e-05,0.0121925743119367
"70","RNASE1",10.3087551187684,-2.43769863403415,0.610611341337016,-3.99222626408556,6.54558717709226e-05,0.0125665922961347
"71","ADAMTS5",864.832747492522,1.17752293364129,0.295975661746889,3.97844514204777,6.93674113457045e-05,0.0131299808602102
"72","NID1",316.213450114025,1.12285641407091,0.28265959833289,3.97246872454873,7.11315778655382e-05,0.013276906596319
"73","LAMA4",416.77077987726,0.854745167526323,0.215797275537567,3.96087098596166,7.46768742501728e-05,0.0137477056581928
"74","TXN",479.507731175691,-1.0604404448551,0.268763822713894,-3.94562197451688,7.95931017177988e-05,0.0142620225864733

"75","APBB1IP",137.082228176376,1.08530376709193,0.274899562217517,3.94800107478228,7.88064509378923e-05,0.0142620225864733
"76","LARP6",100.152455492711,-0.761876961592632,0.194074097176661,-3.92570143402039,8.64773665297642e-05,0.015291701694651З
"77","IL6ST",432.878560210377,0.86997624066509,0.223058510849987,3.90021540693497,9.61071404421963e-05,0.0167738163688659
"78","CLU",1208.64295824912,-1.57541992286272,0.405263475835696,-3.88739675988329,0.000101325060609079,0.0170213436190677
"79","VIM",10371.734176006,-0.939732036166579,0.241540354885497,-3.89057984373693,0.000100004963402174,0.0170213436190677
"80","SLITRK4",40.4519044266531,-1.30094758176769,0.334466794899522,-3.88961655269399,0.000100402739018529,0.0170213436190677
"81","GPX3",1059.64486983616,0.805694751823149,0.20815622447235,3.87062531454673,0.00010855653535655,0.0177913570567888
"82","ENOSF1",31.5427175102553,1.26668096851908,0.327066056229887,3.8728597614812,0.000107565727411647,0.0177913570567888
"83","S100A10",6471.29576454117,-0.832184603965385,0.215982984394029,-3.85301002437853,0.000116674611810073,0.0186983220735047
"84","FABP5",26.0407732902525,-1.93607356680143,0.502612297946309,-3.85202187593158,0.000117146554638862,0.0186983220735047
"85","MID1IP1",51.5308944040324,-1.33581800549457,0.346993122300924,-3.84969591511415,0.000118264556607478,0.0186983220735047
"86","ANTXR2",221.364880709862,0.776289376369614,0.202279799825104,3.83770093227703,0.000124191603999133,0.0193150527933454
"87","C16orf89",14.9549293938379,2.01174553432226,0.524434426303876,3.83602874529176,0.000125039779226211,0.0193150527933454
"88","VEGFC",29.4079945088526,1.72579940886093,0.452308601921722,3.81553523750938,0.000135888061599054,0.020752268861701
"89","TWIST1",115.41923327464,-1.03972492196686,0.273604570042215,-3.80010071398455,0.000144637292825525,0.0215975619809137
"90","STEAP4",70.3125456380954,1.49706826090974,0.393954039561609,3.80010892279637,0.000144632501659551,0.0215975619809137
"91","CRIM1",66.2742544425807,1.15040628580274,0.303014396172712,3.79654003352049,0.000146729676346585,0.0216692320925468
"92","NTM",195.748812250095,0.74091653870147,0.195593358847996,3.78804547897389,0.000151837060164933,0.0221797636038754
"93","HRCT1",60.6719009731518,-1.5840755304994,0.422023854316505,-3.75352130998593,0.000174367701362159,0.0249487527958758
"94","LMO2",7.14095490166545,-2.09314748762561,0.557678482734576,-3.75332302111041,0.000174505749148919,0.0249487527958758
"95","PTMA",1077.08641509349,-0.854995680574934,0.228082748524525,-3.74862056032703,0.000177809859399082,0.0251535442154132
"96","CTSO",101.914067054929,0.883503926493968,0.236111350877844,3.74189518296841,0.00018263769499805,0.0255673748237374
"97","PTX3",38.5369500594532,2.1476394179401,0.575912036701312,3.72911014369707,0.0001921571514945,0.0264631099204736
"98","CCNG2",81.7963385174111,0.803527529137156,0.215536171097969,3.72804028689887,0.000192974534727764,0.0264631099204736
"99","AQP1",52.0604019619583,-2.96634534631502,0.80017089037753,-3.707139789746,0.000209613252220074,0.0281699249658558

"100","FOXRED2",16.3646176709954,1.55951224546606,0.420482788415618,3.
70886107215545,0.000208193614798372,0.0281699249658558
"101","C1QB",8.65670800232811,-1.87302207210007,0.507749342976271,-3.6
888714835572,0.000225250957200067,0.0294695588327471
"102","COL6A6",69.2220323921193,1.50685501073817,0.408660434393909,3.6
8730340379784,0.000226643069882858,0.0294695588327471
"103","PDLIM4",63.6950638027736,-1.00316996548843,0.272024687499233,-3
.68779015871954,0.000226210075751589,0.0294695588327471
"104","TBC1D12",87.10143660888,0.90088281991555,0.244425062230426,3.68
572196195767,0.000228055221266887,0.0294695588327471
"105","TXNIP",2564.4269156483,0.941384549106059,0.256172359141249,3.67
480922712273,0.000238027201542868,0.0301778071842888
"106","TRIO",245.717570275131,1.07513485135248,0.292393135065588,3.677
01810479002,0.000235976298153217,0.0301778071842888
"107","ENO1",280.620154663649,-0.691978219806614,0.189206625919464,-3.
6572620881742,0.000254923688102433,0.0320179387327906
"108","TUSC1",61.4627653410456,-0.91686690684729,0.250878888425265,-3.
65461961587262,0.000257563560876039,0.0320499693945657
"109","PDLIM3",38.6228561000634,-2.06455731413795,0.567441665192463,-3
.6383604532066,0.000274379230904622,0.0338291971020846
"110","PODN",383.765334640767,0.841029801697203,0.231362266146123,3.63
512086783429,0.000277850337014177,0.0339457334466684
"111","SPP1",6.69900537625011,-2.50968708602812,0.691476593073173,-3.6
2946065155172,0.000284014018396972,0.0340791463681866
"112","ASPH",301.107198071677,0.824843848515594,0.227204034345842,3.63
041022088563,0.000282971121813295,0.0340791463681866
"113","CPB1",176.508628720722,1.20973452013217,0.334949826119186,3.611
68875394998,0.000304209544536993,0.0361793988409969
"114","KCNB2",11.773267217488,1.98572622507563,0.551922281930453,3.597
83667028294,0.000320875005625457,0.0378266596543904
"115","ITGBL1",147.69172004268,-1.02961239204501,0.286521075199733,-3.
59349618986062,0.000326270422091336,0.0381282452390041
"116","LUZP2",21.6407666251285,1.43549672415021,0.400780113058182,3.58
175637308086,0.000341291991610483,0.0395398540970111
"117","ADRA2B",2.9461159030805,4.26514113718428,1.19217606029577,3.577
61011920179,0.000346750150670329,0.0398288485030645
"118","MEX3B",18.8778821352277,1.44822882904627,0.406429553488326,3.56
329606598814,0.000366227230342087,0.0417095571912483
"119","SOD3",650.244505563226,-1.03185973832778,0.290110512689785,-3.5
5678161663567,0.000375425992986019,0.0423978984852025
"120","GSTM1",3.09098181661439,4.36047847873863,1.23816154908315,3.521
73630490103,0.00042873037367594,0.0477008675099436
"121","NFATC2",5.88895714755082,-2.51409279561611,0.713972891791514,-3
.52127206021464,0.000429481729942941,0.0477008675099436
"122","PPL",174.641995515755,-1.28458347404933,0.365227235250952,-3.51
7217091345,0.000436096953184946,0.0480385815889548
"123","MEG3",623.221702845844,1.39840241265878,0.399063010092278,3.504
21456585371,0.000457956210240129,0.050036369995261
"124","ITGA4",7.0200800219502,-2.14941030752182,0.615794512097172,-3.4
9046681205668,0.000482177497242057,0.0522579305277097

"125","MYADM",482.87605359484,-0.749014201625601,0.214768299310536,-3.48754543398694,0.000487476091139197,0.0524095295105573
"126","AIF1",14.1741207594227,-1.67878964516807,0.481714261622576,-3.48503205928207,0.00049207808015061,0.0524844231678099
"127","WASF1",32.2734200384986,1.16825491604068,0.335782842886642,3.47919776364237,0.000502917301065207,0.0532181544016954
"128","MT-CYB",10582.7161919789,-0.541615503614145,0.156413018004318,-3.46272650783575,0.000534731621663517,0.056142642683875
"129","XG",123.228468868495,-0.842042434944512,0.243344840191733,-3.46028473125241,0.000539604486176219,0.0562150751141257
"130","ABCA9",255.076034489788,0.830365850333348,0.240844421811014,3.44772714306383,0.000565324832237118,0.058441541695651
"131","NR4A2",19.5962337245311,-1.67747562942773,0.487177755502039,-3.44325168890169,0.000574764135558728,0.0587666744165761
"132","PLEKHA2",59.4112867394849,0.977406477519914,0.283956398733007,3.44210055445495,0.000577215642755268,0.0587666744165761
"133","GPC6",38.9605965536591,1.35020925729905,0.392742806179633,3.43789685273444,0.00058625096745897,0.0592377951254218
"134","FMO4",11.9501099054911,1.81319500787819,0.528444509780125,3.43119282028803,0.000600933290115019,0.0602682275063861
"135","ACTN1",10.9623602176705,-2.04475768215885,0.59847634979488,-3.41660565678103,0.000634070536801257,0.0631205477338673
"136","TPRG1",13.2356122064431,-1.35551107460387,0.397207642032353,-3.41260069335088,0.00064346150200141,0.0635844053338011
"137","CCDC85B",132.077818046973,-0.996944119113079,0.292557703843104,-3.40768370142709,0.000655167913422118,0.0642686247334295
"138","KCND2",20.6084126703808,1.39975922393437,0.411380785746595,3.40258775429683,0.000667509150602862,0.0650047498184917
"139","PHACTR2",133.69435373593,0.934849864213988,0.276384377283743,3.38242658069724,0.00071848480313158,0.0689694090663236
"140","FBN1",4185.58434565425,0.861099572759485,0.254441793333791,3.3842693901699,0.000713679428993448,0.0689694090663236
"141","TRIM47",50.9026074770959,-0.921428676064009,0.272654000772389,-3.37947975622487,0.00072623155527546,0.0692186232010419
"142","TET1",32.6086054365593,1.11812521390763,0.331235066112969,3.37562452861282,0.000736483538610377,0.0697014244745413
"143","INKA1",5.61580260701576,-2.14396965179841,0.636484560840504,-3.36845507920445,0.000755907137766955,0.0710394127583924
"144","SCARB2",189.659196152952,0.576155731117252,0.171399660053203,3.36147534329072,0.000775272780935459,0.0718544200206319
"145","SYNJ2",18.3058481939034,-1.77946802351955,0.529090588583058,-3.36325775191937,0.000770284075211997,0.0718544200206319
"146","MS4A4A",4.3334267364057,-2.48664406508982,0.740361014475985,-3.3586912553058,0.000783125090545252,0.0720850554235455
"147","SELENOM",545.177301486703,-0.716867509598465,0.213657234238613,-3.35522226594896,0.000793012409564708,0.0724985970893885
"148","PTGER3",2.7129068421123,-3.68316996101915,1.1000484205432,-3.34818894535608,0.000813415343824093,0.0738614108489999
"149","EPB41L2",336.121756203394,0.658002018823508,0.196710699530981,3

.34502403983306,0.00082275438771416,0.0742080282985946
"150","SMOC2",440.579252691866,1.04267426606489,0.313093751844405,3.33023019438299,0.000867742138531749,0.0777439106648545
"151","NR2F2-AS1",8.60820151202451,2.43267621011763,0.731640987688688,3.32495889521259,0.000884315716112004,0.0787040987339684
"152","TMCC3",16.3248971595684,1.66697863439897,0.501892161546496,3.3213880632494,0.000895709032335776,0.0791936426681611
"153","SCARA5",1142.43762798379,0.584022266904968,0.176540701169445,3.30814516446505,0.00093916114715477,0.0815541412541975
"154","KCNQ1OT1",395.801896543842,1.25492068110891,0.379599034316071,3.30591115272438,0.000946681005703907,0.0815541412541975
"155","KITLG",57.7556468539303,-0.933308516070538,0.281852036592394,-3.31134210472377,0.000928496216121188,0.0815541412541975
"156","SSBP4",112.974790969828,-0.798985076359368,0.241661520820289,-3.30621554332406,0.000945653131284838,0.0815541412541975
"157","ST6GALNAC4",24.4149852573324,-1.10826831076964,0.335451333623401,-3.30381250477857,0.000953795991218608,0.0816437218215725
"158","RPL22L1",104.753667789452,-0.836440290689914,0.253653816800526,-3.29756634944583,0.00097526629565567,0.0829531882741553
"159","SVEP1",323.095075092563,1.40016650903142,0.425086940501743,3.29383562661031,0.000988302743941767,0.0830112535989588
"160","FSTL3",62.3022063213881,-0.766023082334493,0.232454819461455,-3.2953633059069,0.000982945113222612,0.0830112535989588
"161","ANGPT2",15.5308920039769,-1.45303933524414,0.44268686259507,-3.28231862749731,0.00102957195312076,0.0859404812297509
"162","AC022424.1",7.27544315751386,2.62914825497868,0.802425897566647,3.2764997527517,0.00105102404727626,0.0871895813046024
"163","ROR1",32.8523023861305,1.25940317091043,0.385138092377195,3.27000417729909,0.00107545896188825,0.0875678049572921
"164","RGS2",20.6116164797651,-1.19290558254792,0.365508212027018,-3.26369023539132,0.00109971328435193,0.0875678049572921
"165","APBB2",38.7953502056225,1.07539496994286,0.329878454961688,3.2599733440238,0.0011142268507848,0.0875678049572921
"166","PRDM6",15.9265643036199,1.4911988347221,0.45727422335488,3.26106034095173,0.00110996417047831,0.0875678049572921
"167","PLAGL1",280.118162515119,0.935774724571941,0.286757602510101,3.26329525836713,0.00110124723089183,0.0875678049572921
"168","CCDC71L",99.3067169917555,-1.21815578534416,0.372336004921188,-3.27165723766631,0.00106919115440347,0.0875678049572921
"169","EGR3",7.33421639638987,-2.20777215060355,0.676584380303697,-3.26311427646697,0.00110195075932299,0.0875678049572921
"170","GDF10",77.8233300283539,-1.54303088980343,0.471855803255696,-3.27013227167468,0.00107497206219257,0.0875678049572921
"171","CADM1",2.26650013123825,-4.49278780638421,1.37516098085428,-3.26709953884322,0.0010865546950295,0.0875678049572921
"172","TYMP",224.266858411248,-0.790299791855259,0.24275796453988,-3.25550510094769,0.00113190863175503,0.0884402331520687
"173","ARHGEF10",132.190501235748,0.941332778201368,0.289505007427324,3.25152503083276,0.00114787667690374,0.0891694489069901

"174","FIBIN",53.0228267684697,-1.38028901765983,0.424853912583526,-3.
24885561078237,0.00115870277212973,0.0894931411186866
"175","PTGS1",21.7116540835729,1.33794414317618,0.413144826797093,3.23
843857261532,0.00120185898967957,0.0921637074375515
"176","PTMS",410.696253680577,-0.553142931512367,0.170869641133497,-3.
23722182502981,0.00120699549884732,0.0921637074375515
"177","CYP11A1",4.75984397978493,3.56658603134089,1.10241392035514,3.2
3525126586929,0.00121535726201696,0.0922778883855701
"178","A2M",10.6476141454503,-1.62083547332951,0.50143015731494,-3.232
4251935878,0.00122744268587824,0.0926719227838069
"179","ACSL3",99.7694677369361,0.814672098119888,0.252677018030346,3.2
241638138299,0.00126341104479106,0.0948546426309894
"180","NAV2",4.74850430191496,-2.62088372525687,0.814434925338634,-3.2
1803945743993,0.00129070060929956,0.0963651416020931
"181","APP",597.314874147628,0.580435385977326,0.18081432128901,3.2101
1843442185,0.00132680292280165,0.0984441242257748
"182","MT-
CO2",7223.3567564662,-0.602614502584631,0.187804304281379,-3.208736375
29501,0.00133319671174128,0.0984441242257748
"183","SLC12A7",17.8906537886719,1.30503472181531,0.407053300233879,3.
20605365701613,0.00134568892961336,0.0988235711752672
"184","CAV2",46.6875689673442,-0.918462569862286,0.287225009249737,-3.
19771099411367,0.00138523032082086,0.101174512399519
"185","GHR",30.081486490318,0.976008050966194,0.305620065260193,3.1935
3393938732,0.00140542815814728,0.102094859553196
"186","STXBP6",68.9858837389438,-0.80702002456844,0.253679704559418,-3
.18125577278656,0.00146638103696028,0.105949971804888
"187","NCKAP5",5.60623329710134,2.63226554738775,0.829478229916015,3.1
7339919536438,0.00150665210986643,0.108277527831524
"188","GSN",12999.3392516703,-0.903362030620932,0.284913160870867,-3.1
7065743070524,0.00152094392070492,0.108723219948688
"189","SNHG14",49.8383848686932,1.29442636410996,0.40856598974392,3.16
821859039535,0.00153376153267273,0.109059371627454
"190","CXXC5",64.1930223215877,-0.790645218510666,0.249780783992379,-3
.1653564612672,0.00154893064367642,0.109558310107197
"191","BBOX1-
AS1",4.79145707982154,-2.55246841532139,0.808660423682281,-3.156415648
11418,0.001597210773728,0.11238175700592
"192","FOLR2",3.79612290621289,-2.58446822791204,0.819962871551018,-3.
15193323695661,0.0016219335347322,0.113526899860761
"193","SASH1",229.239512569495,0.854606467984349,0.271556384994384,3.1
4706821569311,0.00164916481701038,0.114242917401044
"194","FREM1",28.8278835653083,1.55130900165228,0.492787803212069,3.14
802637472073,0.00164376861289879,0.114242917401044
"195","EHBP1",91.7570101491508,0.774334858781499,0.247102196451213,3.1
336623870698,0.00172639364102948,0.118396087494192
"196","ABCA6",253.098849967305,0.875841801969167,0.279499849438066,3.1
3360384175536,0.00172673808682652,0.118396087494192
"197","GTF2I",164.688984067221,0.658540560939281,0.210442911110154,3.1
2930740914611,0.00175218892266668,0.119531304221916

"198","SHTN1",10.4940523608267,-1.67167244839281,0.535714830915367,-3.
12045206128875,0.00180573686862208,0.12256210998693
"199","EMCN",6.52135580313739,-2.01530624843472,0.646682641457341,-3.1
1637597677448,0.00183088676606555,0.123644659543492
"200","MRC1",4.05793232493105,-2.92161430191003,0.942042446276536,-3.1
0136163551636,0.00192632874531661,0.12943966004155
"201","SYNE2",146.967795570918,0.915945540053273,0.295482079110501,3.0
9983449016797,0.00193628808524358,0.129461570037753
"202","REEP5",264.38534383986,-0.652088156821927,0.21070508499471,-3.0
9479079177562,0.00196951764579684,0.13103142396962 2
"203","KRT7",2.88264852362718,-3.44341536480328,1.11614181712459,-3.08
510559498096,0.00203479897238152,0.13451361107096
"204","SSC5D",121.116578396482,-0.957509988505823,0.310469323177885,-3
.08407277957447,0.00204187637908147,0.13451361107096
"205","CDKN2C",63.5115108999714,-1.09067559426168,0.354155364033927,-3
.07965290102791,0.00207241963108995,0.135859743523014
"206","SERTAD4",7.43719907977849,-2.35394746283725,0.767141301002053,-
3.06846660421292,0.00215160375566,0.140366033360751
"207","H19",356.727902237182,1.20400978961555,0.393381715508138,3.0606
6535924351,0.00220845773470962,0.143133080640539
"208","GNS",101.851543122368,0.72347979305999,0.236451682903065,3.0597
3628175269,0.002215319649768,0.143133080640539
"209","PEG10",35.0281943712049,1.66943185448753,0.547997539152457,3.04
642217384681,0.00231582422174874,0.148910821608045
"210","CCL4L2",11.7376106714219,-1.92249137822385,0.631940224706115,-3
.04220447292766,0.00234852302314687,0.150294290038432
"211","TSPAN9",13.1317209966438,1.4123539135421,0.464941236151843,3.03
770413059436,0.00238387885207187,0.151833876270113
"212","RASSF4",54.0267887415835,0.931824408343489,0.307374301522384,3.
03156250775776,0.00243291541244086,0.153502113745505
"213","MMP9",3.50918715126877,-3.12864396093614,1.03193985676437,-3.03
180843382283,0.00243093425505281,0.153502113745505
"214","ALPL",103.261247075088,2.50210411050655,0.828068515111927,3.021
61483602399,0.00251430249787271,0.156433848467182
"215","ALCAM",20.3517249988213,-1.1609220157,0.384009912418702,-3.0231
5637736506,0.00250152941391959,0.156433848467182
"216","ABCG1",16.1962249497038,2.29061775101637,0.757993836011496,3.02
194772858499,0.00251153913761422,0.156433848467182
"217","CCL26",27.2889110174519,-1.52392809521822,0.506440233071519,-3.
00909761054274,0.00262024917408125,0.162274325578239
"218","C6orf120",43.6257984832428,0.78279133532359,0.260552390766686,3
.00435291735453,0.00266146407368167,0.163754857500485
"219","MUC12",6.8259907717099,-1.73725011456223,0.578399558627706,-3.0
0354675007702,0.00266852547009497,0.163754857500485
"220","CAPS",31.132829331466,-1.39585378104513,0.465351744572305,-2.99
956709591371,0.00270363568645821,0.164407963757067
"221","DSTN",925.186004942005,-0.505652790548975,0.168528050939899,-3.
00040727777302,0.00269618828115982,0.164407963757067
"222","RDH10",23.242305823616,1.33013777437072,0.443713903867956,2.997
7374221894,0.00271991906740672,0.164653118679635

"223","TBC1D2B",101.305013877323,0.634619632181203,0.212022937392809,2.99316498481228,0.00276100448769227,0.166390759238101
"224","NRN1",48.9698986576678,-0.872044649870481,0.291640732811796,-2.99013324189264,0.00278855779118573,0.167301018552433
"225","AKR1C2",211.078057307416,-0.939831873506252,0.314792534659864,-2.98555959886962,0.00283059973438123,0.168320485974997
"226","LILRB5",2.90481977465879,-2.88179746286411,0.964809330339597,-2.98690878315797,0.00281813794162349,0.168320485974997
"227","SLC25A3",417.908140585433,-0.671140181269886,0.225425277007999,-2.97721795078939,0.00290877146591075,0.172206959164645
"228","DDX11",6.61029885051042,2.23893529303245,0.752819957992351,2.97406474053016,0.00293883062877377,0.17322344219338
"229","AC108734.4",17.7603868909239,1.2574681719385,0.42368358894531,2.96794165445199,0.00299801232945656,0.173665033170546
"230","GPR150",2.4682290127679,-3.24489481301363,1.09194392616019,-2.97166799070377,0.00296186787181457,0.173665033170546
"231","ABCB5",14.5391682618953,1.58929361086112,0.535437432924906,2.96821535651583,0.00299534386333533,0.173665033170546
"232","EGFR",325.631643149953,0.573239318236493,0.193110775868298,2.96844811305323,0.00299307630183646,0.173665033170546
"233","CKB",152.394628600564,-1.01770879014092,0.343106335227021,-2.96616146556296,0.00301542132317348,0.17392380756278з
"234","IFI44L",26.0946854338883,-1.1272196525503,0.38069847023261,-2.96092509082465,0.00306716529050237,0.175310162362871
"235","SBF2",56.1516064938513,0.85082771070638,0.287463198710954,2.95977959795088,0.00307859203196946,0.175310162362871
"236","MMP14",246.659903602798,0.864170261644793,0.291925115441811,2.96024636433533,0.00307393117570672,0.175310162362871
"237","SPG11",35.3069655868782,1.04793900004603,0.354331549915123,2.95750971172917,0.00310134979232428,0.175860927675299
"238","MEG9",3.27344238564505,3.62768315763182,1.23217597212167,2.94412749453745,0.00323866553592859,0.182318323283086
"239","RASL12",6.70673339553792,-1.73197726426867,0.588352536619036,-2.94377461890701,0.00324236023994774,0.182318323283086
"240","LIFR",33.9626361523428,0.970639579288837,0.330642846175856,2.93561342855303,0.0033288888137396,0.186101630752101
"241","LHFPL6",629.218540858686,0.413548078504241,0.140972286110594,2.93354169045544,0.00335118644556949,0.186101630752101
"242","TNFRSF12A",12.0766843290821,-1.40660508124983,0.479488204376876,-2.93355512901052,0.00335104137234508,0.186101630752101
"243","FAXDC2",70.8436189050681,0.682113410258368,0.232684946467048,2.93148921155055,0.00337341083057291,0.186564889514689
"244","TPBG",61.5995880876132,-0.653872300165105,0.223841385597979,-2.92114122872463,0.00348751695050962,0.187474961191595
"245","VPS13B",41.4923830511076,0.943985070975088,0.322984323617983,2.9226962485387,0.00347014862080389,0.187474961191595
"246","DUSP6",20.9237422364505,-1.15770609490117,0.396024504945304,-2.92331934121364,0.00346321129236945,0.187474961191595
"247","NLK",4.64864106207659,2.75984369820823,0.943888509153407,2.92390856700183,0.00345666264230103,0.187474961191595

"248","ABCA8",555.830613886562,0.733709692416755,0.25054197591615,2.92
84900852794,0.00340612695644831,0.187474961191595
"249","SEC11C",12.1820669611006,-1.25332981032664,0.428883086454515,-2
.92231111440567,0.00347444291186743,0.187474961191595
"250","RAD23A",134.113406122695,-0.542459877689009,0.18564329155306,-2
.92205483511352,0.00347730313555366,0.187474961191595
"251","NDFIP1",341.443143071576,-0.556982482214854,0.191137169095635,-
2.91404589097043,0.00356777455426638,0.191025188186398
"252","MLXIPL",19.9494561618152,1.40476793146033,0.483077269161577,2.9
0795701047667,0.00363798344745335,0.193608878949568
"253","SNAI2",104.079850666178,0.879768779993945,0.30259974838576,2.90
736785039357,0.00364484309652807,0.193608878949568
"254","LGALS3",1826.61424079527,-0.768574556865825,0.264653072635076,-
2.90408325591442,0.00368330199024403,0.19488147813736
"255","GEM",33.8367971185311,-1.22096448919223,0.421148532108884,-2.89
913034500751,0.00374199296502392,0.196630334336213
"256","ATP23",7.21764272466452,-1.63416104780201,0.563731884354569,-2.
89882671737293,0.00374561839348691,0.196630334336213
"257","ANXA1",1560.52546215978,-0.607323716769252,0.209983888761607,-2
.89223959205147,0.00382506126660519,0.198520081360504
"258","VPS51",67.7234892023451,-0.714424025820088,0.247020230557847,-2
.89216807954029,0.00382593206878268,0.198520081360504
"259","TYROBP",16.8762921440286,-1.3259583674948,0.458177907742784,-2.
89398145368279,0.00380390629263696,0.198520081360504
"260","CCL3",14.0350034089409,-1.72102343993081,0.595318979916832,-2.8
9092654188724,0.00384107891179533,0.198539459598529
"261","C4orf48",19.473636553374,1.26396267970189,0.437664740556044,2.8
8797008892251,0.00387736741758079,0.198574969747136
"262","CLTB",222.207234454906,-0.711196259002813,0.246335949267621,-2.
88709894401228,0.00388811939219503,0.198574969747136
"263","PDCD4",132.276789096059,0.508442300311726,0.17617123971406,2.88
606869734792,0.00390087000619421,0.198574969747136
"264","PLXNC1",54.8647306205772,1.38186275719027,0.478718386798694,2.8
8658801353154,0.00389443806778152,0.198574969747136
"265","LINC01894",10.6651840099737,1.51515101687529,0.525259922012391,
2.88457381456099,0.00391943862572137,0.198767304494602
"266","EMP3",747.766875371271,-0.671027998140371,0.232766834240225,-2.
88283337413887,0.00394115854626863,0.199117404899639
"267","MDGA2",2.72960232635111,3.38902487002659,1.17913397450234,2.874
16438107209,0.00405098061399861,0.203899357571263
"268","PLEKHG3",4.47280898783736,2.9357674024493,1.02356546789949,2.86
81774586182,0.00412843901402375,0.207022731005467
"269","SPHK1",2.77497490670795,-3.49735203906005,1.22069065615745,-2.8
6506005548464,0.00416930163064336,0.208294589643926
"270","LAMP2",210.945367009698,0.560540932011553,0.195781412466821,2.8
6309576046474,0.00419523751134662,0.208814062648101
"271","IRX1",22.858627427149,-0.9476354674846,0.331442610581325,-2.859
12383390452,0.00424812915020801,0.209172449535155
"272","PDE7B",8.04911490320189,2.02687055259867,0.708931731306137,2.85
904899314404,0.00424913153680313,0.209172449535155

"273","ANXA2",2446.12669829516,-0.558922961569266,0.195333638307977,-2
.86137588185415,0.0042180662812397,0.209172449535155
"274","ANXA5",991.737610615744,-0.524779993588145,0.183666154827222,-2
.85724930693853,0.00427330049114919,0.209594471899832
"275","GPC1",9.21619485444497,-1.39610453034491,0.488848044772337,-2.8
5590695365283,0.00429140877334878,0.209717245472852
"276","SEMA3C",861.804756610683,1.04156481497663,0.364877557528823,2.8
5455982009624,0.0043096514672203,0.209845674159325
"277","PTHLH",21.0988613123487,-1.70230778252296,0.596745977283742,-2.
85265062074067,0.00433562602735889,0.210348296684751
"278","KIAA0040",7.14674402518979,2.32647646169826,0.817963356976574,2
.84423066370305,0.00445188062803344,0.213768335930751
"279","DISP1",18.5742978147481,1.41929885702401,0.4988992656705,2.8448
6058546614,0.00444308649775131,0.213768335930751
"280","METTL7A",251.706201080068,0.629132754111497,0.221295384006952,2
.84295470931166,0.00446974215671935,0.213768335930751
"281","TUBB6",127.198563936538,-0.644225376983614,0.22660418051407,-2
.84295451006115,0.00446974495100387,0.213768335930751
"282","SREBF1",56.3816818978812,1.07253460425935,0.37795773885111,2.83
77103946054,0.00454383893839577,0.216541317351421
"283","ODF2L",144.607605208084,0.643780517944138,0.227137210447232,2.8
3432431294078,0.00459227010173997,0.216984831524673
"284","RALGPS2",52.8542490817795,0.826570880563901,0.291925848836369,2
.83144121652349,0.00463387503888542,0.216984831524673
"285","SPRED2",26.2248981977842,1.17906712444453,0.416312515256728,2.8
3216833805112,0.00462335014517878,0.216984831524673
"286","LINC00963",51.5608424819672,-0.634285626710487,0.22392481266272
9,-2.83258304056655,0.00461735713327426,0.216984831524673
"287","CNTN1",15.5532756266158,2.07871376026429,0.733680649166027,2.83
326780204325,0.00460747680110357,0.216984831524673
"288","AIF1L",52.3686803679145,1.32239183951851,0.467873044504837,2.82
639031046986,0.00470758639075584,0.21967101911586
"289","PTGDS",270.603893464292,3.24101039855957,1.1473410465884,2.8248
0122906495,0.00473099549578803,0.219999475667458
"290","DUSP2",15.5337161965551,-1.31405685059504,0.466027023556909,-2.
8197009704837,0.00480684203523175,0.222737808072383
"291","HLA-F-
AS1",17.836319472319,-1.11075776261544,0.394311270732098,-2.8169566661
2105,0.00484810663830597,0.222737808072383
"292","PLA2G7",11.4994781993183,1.95822307828048,0.695287438776711,2.8
1642234429804,0.00485617812078342,0.222737808072383
"293","SULT1A1",32.5454258217131,1.58254410982934,0.561842439993456,2.
81670446584236,0.0048519148700627,0.222737808072383
"294","TMEM245",39.3596628900802,0.827162153392291,0.293983450156448,2
.81363509732301,0.00489847981677564,0.223913844413768
"295","CCDC34",28.8527167387298,-1.008176982427,0.3588087756145,-2.809
78908807453,0.0049573975305268,0.225838865805931
"296","MFAP4",1415.16109524649,-0.647498711174234,0.230545291327431,-2
.80855318035807,0.00497646624158821,0.225941654799676
"297","KANK1",46.4495059580345,0.910494056352639,0.324552098879272,2.8

0538643717516,0.00502562872103676,0.227405469299707
"298","NT5E",234.115822152726,0.436302167898446,0.155862182490935,2.79
928178167158,0.00512164214499812,0.230972311364529
"299","NT5DC2",22.6412409322523,-0.975113356206861,0.34906088614213,-2
.79353372124832,0.00521355929603154,0.233020280655915
"300","PDE1C",9.04269653520425,1.79831593943912,0.644254202201691,2.79
131425653649,0.00524944774545705,0.233020280655915
"301","CD44",511.566099402053,-0.434252721365903,0.155514605148893,-2.
79235973335198,0.00523251481291734,0.233020280655915
"302","C3AR1",2.03564592921284,-3.55702942703109,1.27444171644444,-2.7
9104911674961,0.00525374990986995,0.233020280655915
"303","NID2",54.4712284457928,1.22682583833915,0.439306588745813,2.792
64156233497,0.00522795865740087,0.233020280655915
"304","RASSF1",40.2065212425266,-0.813425739951929,0.291671267105548,-
2.78884426300919,0.00528964947600629,0.233840787197528
"305","STX16",78.9575405717397,0.740736516546188,0.26621346174963,2.78
249083152241,0.00539433894316311,0.2376869542858
"306","NEGR1",325.184203249537,0.671026228325989,0.24166568246931,2.77
667156325021,0.0054918642720516,0.240325560235167
"307","CHL1",214.332774827874,0.51357469551042,0.185023595993687,2.775
72540276399,0.00550787056718741,0.240325560235167
"308","LINC02762",61.9809023388134,-0.88818216755857,0.319966068827018
,-2.77586361208427,0.00550552984284724,0.240325560235167
"309","DOCK4",45.8091481798694,0.917974827682017,0.331640030464471,2.7
6798559690267,0.00564039483161429,0.244679975369508
"310","ZFP82",11.5547054975441,1.35723392294051,0.490370438558495,2.76
77727208236,0.00564408009260715,0.244679975369508
"311","FAM43A",32.2884958934263,1.00772317589406,0.364452798063598,2.7
6503070150173,0.00569174385565027,0.245952879987408
"312","HLA-
DMB",4.60926982826533,-2.03125588157062,0.735303054828345,-2.762474422
25683,0.00573650562459927,0.247092625285223
"313","HLA-
DQB1",15.4851693961133,-1.67888168782338,0.607995375552411,-2.76133956
824587,0.00575647906028079,0.247160773454037
"314","RGL1",44.0260621585309,0.740467661454705,0.26863335175527,2.756
42490635074,0.00584370292237323,0.247334052395604
"315","SLC4A4",20.0031630496024,1.24095018031119,0.45028348730147,2.75
593090865523,0.00585253580339326,0.247334052395604
"316","HLA-
DRA",103.716042610377,-1.12142641499434,0.406809295902873,-2.756639109
00916,0.00583987662774785,0.247334052395604
"317","RAB8B",83.3564494602974,0.573238764148772,0.207924124299131,2.7
5696130057559,0.00583412558441946,0.247334052395604
"318","IL16",19.7604790079041,-1.38434271021013,0.502308933381769,-2.7
5595877001453,0.0058520373108129,0.247334052395604
"319","KIAA1522",10.510908273073,-1.51263494205613,0.549675200611022,-
2.75187045072195,0.00592559570913646,0.247463622184158
"320","LSM10",58.7987221327996,-0.742862515090207,0.269921722541579,-2
.75214053946984,0.00592071059746309,0.247463622184158

"321","TPP1",85.1317742537487,0.700070950983027,0.254416925592997,2.75166815003088,0.00592925711312589,0.247463622184158
"322","MEIS2",52.1065768562671,0.695469985916498,0.252478754897599,2.75456834456653,0.00587696142061751,0.247463622184158
"323","NATD1",17.2523935492211,1.06652895658578,0.388117971445526,2.74795045592336,0.00599690700228205,0.249512177101141
"324","CAMK2D",85.3004158072076,0.737944071398206,0.268805252403186,2.74527400339391,0.00604603940469583,0.250733372265247
"325","PRSS23",350.378319334803,-0.75437322457399,0.27488493248092,-2.74432366214307,0.0060635721397578,0.250733372265247
"326","MAN1C1",99.013130064616,0.659756584397449,0.240780422836738,2.74007569479517,0.0061425036002751,0.253218116208887
"327","ADAMTS16",65.7888570184122,1.96352483476524,0.717219600204796,2.73768987100265,0.0061872392398314 5,0.253988680179073
"328","NHSL1",79.7585228383911,0.658011154808522,0.240407527574202,2.73706552140064,0.00619899450098489,0.253988680179073
"329","GTF3A",115.743413077213,-0.754964677249166,0.276236780821227,-2.73303459084893,0.00627537414067218,0.256336635490861
"330","PFKP",41.0722145583549,-0.896269917833576,0.328230611325433,-2.73061039070772,0.00632171574223795,0.257447084424048
"331","DPYD",51.58812521765,0.650419834181039,0.238387232933305,2.72841723181966,0.00636390583038006,0.258166254287872
"332","TRIL",52.2619333540921,0.874352932319354,0.320546084312717,2.72769805999675,0.00637779570083887,0.258166254287872
"333","EBF1",487.073975122946,0.560234387548224,0.205984584578683,2.71978793313158,0.0065323795288163,0.260790778984155
"334","TEAD1",87.821679252561,0.937723651437144,0.344861169117536,2.71913377153096,0.0065453131409491,0.260790778984155
"335","ARHGAP20",89.3621665514336,0.905643171194223,0.332677133681519,2.72228860809298,0.00648314960531414,0.260790778984155
"336","ATP11A",50.2982067183536,0.828819572986577,0.304888000464532,2.71843946538984,0.00655906565195657,0.260790778984155
"337","C17orf49",93.2024505898718,-0.784995502866822,0.288450786543269,-2.72141917959052,0.00650022776851359,0.260790778984155
"338","CCL18",2.15816425158734,-3.82636134348244,1.40716984362718,-2.71918941470452,0.00654421210868009,0.260790778984155
"339","BMP8B",2.06677512088032,3.74833382467817,1.38124987219985,2.71372609700834,0.00665311501657375,0.263749889993318
"340","AFF3",32.2563713786428,-1.44348724844137,0.533348655668858,-2.70646083588817,0.00680046031782369,0.265248467491581
"341","PXDC1",104.953205501019,-0.609671604932444,0.22531825472443,-2.70582428253799,0.00681350881817081,0.265248467491581
"342","CRISPLD1",13.4455704387175,1.71670434056605,0.634236969585001,2.70672386330512,0.0067950751640515,0.265248467491581
"343","SLCO2B1",2.3933659089668,-3.20388344486212,1.18396194435658,-2.70606961662374,0.00680847713218912,0.265248467491581
"344","MGA",27.6691282914363,1.09304456117835,0.404073124036304,2.70506622727064,0.00682907729385275,0.265248467491581
"345","MSL1",53.1984492401521,0.759576690646748,0.280787346579214,2.70516709495832,0.00682700389294447,0.265248467491581

"346","FTX",191.125199391857,0.971435227130109,0.359026362967385,2.705
74901269397,0.00681505323677947,0.265248467491581
"347","LSAMP",21.7588321041848,2.93945844814369,1.08704809857537,2.704
0739521977,0.0068495043352424,0.265275183750209
"348","STAC",11.3868067508468,1.48552196532274,0.549856042888966,2.701
65615988823,0.00689950721313221,0.265680451109696
"349","TIAM1",13.2269500034412,1.49804898801647,0.554484350960225,2.70
169750583985,0.00689864938001051,0.265680451109696
"350","PLAT",66.5982644392572,0.845853746208783,0.313255123703222,2.70
020721835071,0.00692962999620354,0.26607799291137
"351","PRELP",454.518315194916,0.475315514638544,0.176567784640898,2.6
9197189966015,0.00710309298377474,0.271196590608932
"352","AL161431.1",13.3096198903709,-1.23293842005366,0.45800730457251
6,-2.69196235026084,0.00710329636835657,0.271196590608932
"353","TPPP3",642.200151018098,-0.685016149924259,0.254649516576716,-2
.69003514765318,0.00714444938711058,0.271995057544983
"354","GAB1",24.9475115735263,0.909790881542979,0.338665253003444,2.68
640161184096,0.00722262162342203,0.274194384172793
"355","PLPP3",715.624853063886,0.74975944605559,0.279247368271062,2.68
492931803679,0.00725451462464452,0.274283924299752
"356","IGSF3",16.5171190032051,1.09023979033022,0.406262660772818,2.68
358354237207,0.00728377747051328,0.274283924299752
"357","TMEM37",83.2937946967494,1.21444972092508,0.453117088270766,2.6
8021169883438,0.00735756126162192,0.274283924299752
"358","SP5",12.3397383800514,-1.45745787932093,0.543453299226306,-2.68
184567357647,0.00732172276124102,0.274283924299752
"359","SEPTIN11",151.085656969974,0.619021243412383,0.230893486843948,
2.68098183224526,0.00734065011359163,0.274283924299752
"360","PIM1",24.1235085482501,-1.27398854966301,0.475353385563489,-2.6
8008725372349,0.00736029719537419,0.274283924299752
"361","RUNX1T1",179.212677310471,0.710680688151729,0.265204688303936,2
.67974405994384,0.0073678470624459,0.274283924299752
"362","SMURF2",162.633681523153,0.84980298619389,0.317412727140461,2.6
772807563505,0.00742224109761652,0.275545574891902
"363","PCYOX1",169.876914919365,0.595714640194254,0.2227122347388,2.67
481775706178,0.00747698825798191,0.276052871425876
"364","RFX2",20.9140558635418,-1.06949688338284,0.399726380534339,-2.6
7557243020382,0.00746017514477418,0.276052871425876
"365","PINK1",73.6504951936809,-0.608880011957439,0.228234482663624,-2
.66778273314123,0.00763536160007254,0.27840794624723
"366","ITGB1BP1",232.398405072884,-0.69744500706599,0.261363278889643,
-2.66848889418959,0.00761932974412254,0.27840794624723
"367","KIAA1109",74.4846168293271,0.691503409729602,0.259344092816355,
2.66635496579158,0.00766786836606084,0.27840794624723
"368","TMEM173",56.1711856965307,-0.695114276750656,0.260949514905566,
-2.66378834619489,0.0077266160562702,0.27840794624723
"369","GARS-
DT",82.1432232866673,0.855350048691798,0.3211430045666,2.6634553346293
2,0.00773426788938497,0.27840794624723
"370","TMEM225B",8.57447151773506,-1.29823323442724,0.486869249001294,

-2.66649256877546,0.00766473009067384,0.27840794624723
"371","AL138762.1",5.53439923198632,2.08296480455523,0.783019023115866
,2.66017139183477,0.00781008971911642,0.27840794624723
"372","RRAS2",56.3833398267698,0.760818128586078,0.285870298392738,2.6
6141020198202,0.00778140937991048,0.27840794624723
"373","FZD10-
AS1",4.72509295561552,-2.26857209247139,0.852108944350242,-2.662302875
1344,0.00776080117616512,0.27840794624723
"374","TNFRSF19",7.13392078719632,2.02507272393172,0.761074914627093,2
.6608060323785,0.00779538501542693,0.27840794624723
"375","AC099518.5",7.76670374489091,-1.48279264186319,0.55593566003731
3,-2.6672018876495,0.00764857109530458,0.27840794624723
"376","ARSA",42.5134035235082,0.841000276671761,0.31540388629093,2.666
42331697846,0.00766630935443121,0.27840794624723
"377","MT-
ATP6",13823.181125143,-0.431910031201892,0.162167282259059,-2.66336109
963244,0.00773643442329358,0.27840794624723
"378","SESN1",52.3849425189305,0.789506972263544,0.297203513056978,2.6
5645235529966,0.0078967608674681,0.280012056194997
"379","PELI3",10.3535800355475,-1.24740049770849,0.469515610749241,-2.
65678173238568,0.00788905019912952,0.280012056194997
"380","ADD3",592.205321636768,0.431744597617483,0.163004743679321,2.64
866278043328,0.00808109216072601,0.285794204073676
"381","MS4A6A",9.37203700653416,-2.07293324548926,0.783467374631609,-2
.64584501232609,0.00814871423269729,0.287429319089813
"382","RHOBTB3",203.324075927529,0.626552179049497,0.237522664653432,2
.63786270654927,0.00834303518933778,0.29261848676772
"383","HLA-
DPA1",49.4897106118866,-0.840024384665904,0.318359229409776,-2.6386054
0881216,0.00832478154856918,0.29261848676772
"384","GPR183",14.3529657315141,-1.63214843826914,0.618911562080253,-2
.63712707641662,0.00836115030276097,0.29261848676772
"385","AC074387.1",3.73900699846247,-2.74944046928213,1.04371402724047
,-2.63428525201633,0.00843146203519362,0.294312774781733
"386","SPRY2",113.994406485479,0.697376992987919,0.264933887323857,2.6
3226799724356,0.0084816928965224,0.294568833101226
"387","PKM",213.729553824476,-0.548367466013316,0.208328077319567,-2.6
3223024504825,0.0084826354944694,0.294568833101226
"388","AC022893.2",3.33931946580042,3.01600949749133,1.14682601588873,
2.62987537403751,0.00854161746452518,0.295092023408108
"389","SESN3",99.43700179336,0.859783323970219,0.326889265370214,2.630
19748597888,0.00853352800443857,0.295092023408108
"390","FZD6",5.76853345988894,-1.69284454680408,0.644368774315004,-2.6
2713622118585,0.0086106855734745,0.295523350728777
"391","MEGF9",31.7343910106603,0.878879649850961,0.334650106557438,2.6
2626436576411,0.00863277403187414,0.295523350728777
"392","TPI1",216.562961156387,-0.577566805310439,0.219950177369278,-2.
62589833851669,0.00864206241806752,0.295523350728777
"393","RAB34",170.255381607128,-0.621657347145968,0.236657029033667,-2
.6268281558522,0.00861848463059194,0.295523350728777

"394","GPRC5A",44.1363753688305,-1.3935784447207,0.531084145971204,-2.
62402569403053,0.00868972294793138,0.296398951008248
"395","DEPTOR",22.9623455380582,1.13626054673474,0.433549051675568,2.6
2083504125623,0.00877146931996703,0.298429813141866
"396","DACT2",18.9460214436687,1.47742540753984,0.56458377349117,2.616
84001012641,0.00887479275358989,0.301182676301754
"397","QPCT",12.7548936958183,-1.53663137375354,0.588508227957972,-2.6
1106183525318,0.00902615715656152,0.304780260262057
"398","LVRN",43.8598097198924,0.81917080532358,0.313730915826058,2.611
06178575577,0.00902615846300309,0.304780260262057
"399","MSX1",153.883459221298,-0.726896506491314,0.278793582470374,-2.
60729282234664,0.00912612787585513,0.305850455171115
"400","BTN3A3",22.8104065492801,0.993291342849845,0.380926229701809,2.
60756877684007,0.0091187749599845,0.305850455171115
"401","ETHE1",35.3053297217525,-0.86060462238146,0.329898979049827,-2.
60869137837337,0.00908891728964796,0.305850455171115
"402","ZNF544",3.89165078442882,2.56069836040755,0.983365701852771,2.6
0401431083361,0.00921389094409246,0.307259256569872
"403","DDX17",388.307097707195,0.555118693113696,0.213176458054926,2.6
040337576613,0.00921336815542914,0.307259256569872
"404","AADAC",179.35590488848,-1.93224471388724,0.742610955402218,-2.6
0196095927602,0.0092692403536206,0.307578570647672
"405","HAS2",54.6808379824178,-1.02836725047006,0.395183323455099,-2.6
0225366161459,0.00926133228467484,0.307578570647672
"406","APOC1",16.1132204851123,-1.39178663702122,0.535150846236763,-2.
60073705723986,0.00930237237075762,0.307917690370965
"407","LTBP1",96.1620343156347,0.685344157443232,0.263671667446024,2.5
9923322092818,0.00934322709616452,0.308510144828882
"408","FBLL1",1.66677071391771,3.44863349627528,1.32815548043943,2.596
55857093951,0.00941628502143489,0.309402089005045
"409","PPP1R14B",395.877951288432,-0.64550352525739,0.248584149514104,
-2.59672037223261,0.00941185098921608,0.309402089005045
"410","SPOCK1",138.520313370525,-0.654786714695479,0.252355599939239,-
2.59469857159158,0.00946739077323338,0.309567553774899
"411","AC110048.2",3.69844374330221,2.98501882353574,1.15017130730372,
2.59528194155124,0.0094513354079853,0.309567553774899
"412","NT5DC1",21.202051726551,-0.920977822581567,0.355130049843285,-2
.59335368265226,0.00950449719398202,0.310026548033797
"413","YWHAQ",325.613281103005,-0.50762233373662,0.195886486488563,-2.
59141068297356,0.00955833495724502,0.311027756635389
"414","TKT",78.7260228669364,-0.576700664877006,0.222697732812658,-2.5
8961174679829,0.00960842318450262,0.311223406194582
"415","AC004540.2",3.21656414746581,3.16573985040167,1.2225145344876,2
.58953146248568,0.00961066400556229,0.311223406194582
"416","SPON1",20.3131419554183,1.90067146640342,0.735310673454518,2.58
485499397689,0.00974199593713621,0.313212161242042
"417","EMP1",384.25031852036,-0.785652452929039,0.303923230044194,-2.5
8503587506225,0.00973688656241099,0.313212161242042
"418","FLRT2",117.015276322317,0.854810397160409,0.330546307529669,2.5
8605338401393,0.00970818931836364,0.313212161242042

"419","MAP7D3",124.074838263785,0.746863985445366,0.28912893374251,2.5
8315200688459,0.00979021774622776,0.31401130379846
"420","SUCNR1",2.13763390954485,-2.88116385087604,1.11698824011964,-2.
57940392511871,0.00989709846686175,0.315609295096582
"421","TPMT",12.6449095839376,-1.09561398634722,0.424850473614913,-2.5
7882256085299,0.00991376951700275,0.315609295096582
"422","HYMAI",72.3279602226727,0.769892756728983,0.298493578325646,2.5
7926070318691,0.0099012031414045,0.315609295096582
"423","PCSK6",26.5133432201079,1.13661406393733,0.440869511498831,2.57
811899959506,0.00993397811041403,0.315609295096582
"424","ADAM33",99.0522579751572,0.592853279471938,0.230144660555063,2.
57600275427678,0.00999498501449898,0.316798593419462
"425","AL158071.1",4.84818548651561,2.33255408292956,0.905912819606265
,2.57481076815245,0.0100294941882618,0.317144405637764
"426","CLEC10A",5.01787595763319,-1.96877128898857,0.765753998109992,-
2.57102319262821,0.0101398536092325,0.319881438156046
"427","GBA",31.9818099414339,0.837105983004846,0.326243462039585,2.565
89351330288,0.0102910416030975,0.321023196109914
"428","LRRFIP1",53.5282465325761,-0.780433729922829,0.30492825756145,-
2.55940113967808,0.0104852673727314,0.321023196109914
"429","ZMAT3",38.8842789100216,0.859551627297843,0.335425521692851,2.5
625707398763,0.0103900419243688,0.321023196109914
"430","TMEM140",51.1207744212834,0.874407267630752,0.34160366722716,2.
55971276517148,0.0104758708011384,0.321023196109914
"431","PHLDA2",3.99959187842195,-1.99565783152671,0.77722700517002,-2.
56766403927275,0.0102386333189306,0.321023196109914
"432","TMC3-
AS1",4.19076144282729,2.36764324114659,0.924659797875864,2.56055605162
63,0.0104504803942345,0.321023196109914
"433","RGMA",40.079364898063,-0.76257799873484,0.297913199488109,-2.55
973216374818,0.0104752861156498,0.321023196109914
"434","AC093249.6",2.56358950547558,-2.39055782746074,0.93160417395453
,-2.56606603350987,0.0102859244567633,0.321023196109914
"435","C1QL1",1.68655839988689,-4.16967070943637,1.62879351390125,-2.5
5997502068219,0.0104679687090523,0.321023196109914
"436","AC006141.1",5.36326758675492,2.15772411856452,0.843332531599693
,2.55856858085576,0.0105104086479576,0.321023196109914
"437","ABCA10",94.9410261331767,0.906785558553316,0.353485614829057,2.
56526862908362,0.0103095953545852,0.321023196109914
"438","ACP5",2.04638692448343,-2.8833455657825,1.12486059165508,-2.563
29147555969,0.0103684962879591,0.321023196109914
"439","ZNF528-
AS1",11.6181758404078,1.24666048792787,0.487198898870978,2.55883272892
62,0.0105024262092949,0.321023196109914
"440","EYA2",4.65916068110236,2.86646658400777,1.12034083396996,2.5585
6655143984,0.0105104699969017,0.321023196109914
"441","ENTPD1",22.0207088263215,2.38462562848447,0.932908375129176,2.5
5611986349065,0.0105846651199045,0.321826503498635
"442","SOX9",10.0589289167192,-1.49316372887658,0.584106248567338,-2.5
5632212896015,0.0105785138607215,0.321826503498635

"443","PTPRD",55.0696439018548,0.785680666680633,0.307498512180953,2.5
5507144118566,0.0106166005507433,0.322068837023566
"444","EPB41L3",122.428908918548,0.478378696421151,0.187489180874344,2
.55150027425722,0.0107260239699556,0.324655486784308
"445","HLA-
DMA",8.31267705015771,-1.59780754480319,0.626777895917543,-2.549240417
07653,0.0107957847711459,0.326032700088607
"446","TWF2",89.2289796899888,-0.700931735562565,0.275865927656587,-2.
54084200073857,0.0110585880603642,0.329011361763791
"447","TRIM7",8.04097188515916,-1.3828224666814,0.544286638081121,-2.5
4061439310091,0.0110657887876504,0.329011361763791
"448","AKAP12",244.290320145761,0.853618415233453,0.335835066238647,2.
5417786915285,0.011028998164802,0.329011361763791
"449","CXCL12",877.007552071676,0.62552236442386,0.246101391420432,2.5
4172624060965,0.0110306532189097,0.329011361763791
"450","DDX21",70.8988879217894,-0.584152401080073,0.229790118298256,-2
.54211280017652,0.0110184607649546,0.329011361763791
"451","GALNT16",131.488694936307,0.520128477710868,0.204544051908891,2
.54286777276978,0.0109946827408541,0.329011361763791
"452","EHD4",39.2080750051681,0.797085311490253,0.313325358893144,2.54
395403648796,0.0109605506266446,0.329011361763791
"453","NUFIP2",88.5671460864749,0.619434313853458,0.244199010539795,2.
53659632970754,0.0111935942192734,0.332076628505111
"454","KCNE4",10.2553389377261,-1.51134955444616,0.596169269807013,-2.
5351014065777,0.011241477886909,0.332762602031211
"455","WDR5B",3.59913958677619,2.52595870338863,0.997487192781823,2.53
232194023881,0.0113309902896475,0.333203402596502
"456","HLA-
DQA1",12.1450167640815,-1.3658532438595,0.539745144684317,-2.530552163
94462,0.0113883149560268,0.333203402596502
"457","CATSPERZ",2.22219343348398,3.12872583400623,1.2367335909158,2.5
2983007576387,0.0114117779036191,0.333203402596502
"458","CD163",5.53561599982182,-1.87292453534407,0.740274100887238,-2.
53004195756588,0.0114048887439428,0.333203402596502
"459","CAB39L",126.527957351271,-0.778876862159987,0.307955331313013,-
2.52918778460217,0.011432684040572,0.333203402596502
"460","MYH10",42.5805281954949,0.972180240859636,0.384487108090661,2.5
2851193291609,0.0114547192499132,0.333203402596502
"461","TBC1D16",52.9405585823951,0.629781827440222,0.249065579316368,2
.52857833334032,0.0114525526873299,0.333203402596502
"462","GATD3B",50.3606564028658,-0.722715839655056,0.285808538237457,-
2.52867127102622,0.0114495208579935,0.333203402596502
"463","FCGR2A",5.98202126639956,-1.81820772439545,0.719983462434866,-2
.52534651038591,0.0115584259866277,0.335493924048143
"464","MYO9A",86.460392451565,0.652064487304255,0.258373238033432,2.52
373075581412,0.011611682436973,0.336313362651896
"465","HS6ST1",29.0744928166256,0.872711416755827,0.346296694251811,2.
52012632878681,0.01173127227379,0.33889973759776
"466","LNX1",11.3704001564843,-1.22257298740424,0.485239972875649,-2.5
1952241312474,0.0117514158583642,0.33889973759776

"467","ATP6AP1L",5.25861725404042,1.96067742720723,0.778635545491777,2.51809391256199,0.0117991856008545,0.339548726530799
"468","C5orf38",41.2369830979219,-0.914009429695326,0.363179383957805,-2.51668863946726,0.0118463465690089,0.3401774605575
"469","PXN",157.023356313504,0.583727060121886,0.232128726511901,2.51466963565132,0.0119143969480533,0.341402090799336
"470","TPGS1",83.55033893385,-0.718130540317344,0.285671262874234,-2.51383542429852,0.0119426150222131,0.341482560177705
"471","RBM3",304.241871760498,-0.741206842547887,0.295276366739476,-2.5102139081854,0.0120658047439662,0.344272505210533
"472","MAP1LC3C",2.2931256321222,3.10259089009599,1.2368116158711,2.5085395789324,0.0121231386033621,0.345175550191914
"473","ITGB3BP",21.1752841694734,-0.867093913820322,0.345855995855179,-2.50709521943174,0.0121727914788231,0.3458565426721
"474","SWT1",8.34416819076178,1.40550933910455,0.561422610544292,2.50347832934966,0.0122979211356961,0.348674603676413
"475","BOLA3",40.197382827112,-0.658504829518102,0.263620464303705,-2.49792758410238,0.0124921709880822,0.349754762310076
"476","UBE2E3",229.595890161421,-0.691034591948799,0.276394097373373,-2.50017854402766,0.0124130729015579,0.349754762310076
"477","NUMA1",137.225050720044,0.524248157908114,0.209687091898613,2.50014511223035,0.0124142444326588,0.349754762310076
"478","NEDD1",24.3826693177848,-0.85995276426826,0.344258057352109,-2.49798877877445,0.0124900147367102,0.349754762310076
"479","EMILIN2",185.051889067708,0.567560888125218,0.227115701094519,2.49899450099672,0.0124546243641159,0.349754762310076
"480","A4GALT",83.3074288263727,-0.739310600946743,0.295740193613741,-2.49986514147056,0.0124240591302442,0.349754762310076
"481","KHDRBS3",75.5128506756779,-0.763040845180216,0.305768721436813,-2.49548365050118,0.0125785551745371,0.350712039399595
"482","EIF4G2",246.909456342469,0.421245731439536,0.168778761296204,2.49584561590813,0.0125657277381907,0.350712039399595
"483","PCDHB9",3.21191135672923,2.86844347382489,1.15066757320507,2.49285157644195,0.0126721805584545,0.352107447401355
"484","NABP2",32.9727620104623,-0.70123729477418,0.281327147740429,-2.49260443013195,0.0126810033888128,0.352107447401355
"485","XRN1",61.6333930480052,0.641040741178825,0.257590459118988,2.48860436590437,0.0128245590265326,0.355359275788806
"486","CXCL14",968.411972473514,-1.02331891403271,0.411508587681705,-2.48674983867949,0.0128916014620959,0.356481958948779
"487","SAMD9L",38.7982776429099,0.891468367044036,0.358779786778806,2.48472294118855,0.0129652296843751,0.357781769462664
"488","WDR86-AS1",23.5480850068155,1.4676983808533,0.591189154890274,2.48262061086981,0.0130419908255444,0.358428046430453
"489","DHCR7",5.38211677164656,-1.50855751305868,0.607575819611596,-2.48291236149432,0.0130313143436892,0.358428046430453
"490","SOX13",15.147857166179,1.03912230503186,0.419398083821511,2.47765153231863,0.013225023773443,0.359052716144043
"491","CLIC1",607.052851899511,-0.586955957990784,0.236856862802709,-2

.47810407959212,0.0132082609807426,0.359052716144043
"492","LAMB1",88.5909134329543,1.1247770042985,0.45378464400499,2.4786
5814579246,0.0131877634091944,0.359052716144043
"493","ARC",5.41648592270609,-1.52297850285329,0.614627779734935,-2.47
788751675054,0.0132162803344714,0.359052716144043
"494","ZNF524",46.3160138426533,-0.831228585987136,0.335429487958401,-
2.4781023011615,0.0132083268187649,0.359052716144043
"495","MT-
ND4",8345.90583202803,-0.418781664522945,0.168821273445977,-2.48062140
496148,0.013115359233072,0.359052716144043
"496","AUP1",90.4198771864757,-0.608220003753649,0.245558275530851,-2.
47688660640246,0.0132534001429477,0.359097670405391
"497","ADAP2",2.55707878468398,-2.32471880616633,0.938848735037168,-2.
4761377625697,0.0132812320452651,0.359127721240075
"498","PARP14",58.5779272666466,0.606875243538239,0.24525159607692,2.4
7450068927543,0.0133422563324,0.360053379219123
"499","CCDC18-
AS1",54.1671253744345,0.673266785233987,0.272265102254468,2.4728354080
6023,0.0134045862617614,0.360364396999732
"500","HAND2",22.5814606485053,-1.14996151542603,0.46519801694302,-2.4
719828407327,0.013436596555987,0.360364396999732
"501","MRGPRF",65.345115691482,-0.632141632624152,0.255789743216725,-2
.47133299668139,0.0134610407987101,0.360364396999732
"502","SGCA",8.21614428248764,2.33798325969715,0.945841314028794,2.471
85571725404,0.0134413752872107,0.360364396999732
"503","C4orf46",8.07707596913246,1.49530176895079,0.605617568768633,2.
46905282485628,0.0135471218513854,0.360514397150037
"504","STK32A",1.97794057869588,-2.962872481152,1.19941253722617,-2.47
026972721504,0.0135011210226881,0.360514397150037
"505","SH2D3C",2.08898897833001,-2.8724548561638,1.16312680250159,-2.4
6959733881628,0.013526521273859,0.360514397150037
"506","MIR4435-2HG",68.9550177678375,0.782036855547036,0.3170449215816
44,2.46664369088672,0.0136385995287761,0.362231500132849
"507","URGCP",23.5333542576152,0.835115194824199,0.339027335828623,2.4
6326802168645,0.013767695518743,0.36493897450964
"508","RAI2",27.7658257133037,-0.831126429218052,0.337582632231826,-2.
46199404194259,0.0138166961963528,0.365516890123593
"509","EVA1B",127.297439194718,-0.579887036352765,0.23572578640329,-2.
46000679518646,0.0138934385204282,0.366824990719126
"510","MYLK",1.96463670679287,-2.62592046711786,1.06822843951226,-2.45
820123298423,0.0139634906973194,0.367951669571128
"511","RAB13",86.9744103314553,-0.78018660642711,0.318533311941293,-2.
44930930982471,0.0143130475985052,0.369630722456002
"512","DUSP10",4.21237409558614,-1.84282068211305,0.751040046477924,-2
.45369163835556,0.0141398174828111,0.369630722456002
"513","SLC19A3",4.31676128664404,2.46485414321413,1.00535462273002,2.4
5172607504491,0.0142172845760033,0.369630722456002
"514","NECTIN3",48.3591368096234,-0.657254567741951,0.26778208797674,-
2.45443813179559,0.0141104943018239,0.369630722456002
"515","AC093627.6",2.3747742880669,-3.23121523008495,1.3194616547147,-

2.44888907422143,0.0143297571545187,0.369630722456002
"516","PPP1R9A",6.78960097132876,1.78516550738286,0.726999650540659,2.45552457426253,0.014067913392352,0.369630722456002
"517","DLGAP2",4.79735811107144,2.1419166029652,0.874276937296899,2.44992920617077,0.0142884304386924,0.369630722456002
"518","FBLN5",482.72103105613,0.546208975879729,0.222846302935605,2.45105693334103,0.0142437422021417,0.369630722456002
"519","PSMD7",119.743002400928,-0.53595941331582,0.218629915209852,-2.45144591855775,0.0142283565831421,0.369630722456002
"520","MAP2K3",26.4643842065681,-0.825878952310681,0.336972093553889,-2.45088233746753,0.0142506528029801,0.369630722456002
"521","DYNLT3",14.5084035550766,-1.28301188813855,0.52302043660656l,-2.4530817504244,0.0141638145305024,0.369630722456002
"522","LENG8",71.9400006828074,0.652096057531255,0.26656808859795,2.44626452086234,0.0144345054201475,0.371619383795712
"523","TTC28",54.7981360915479,0.625310537996283,0.255711851182002,2.44537175381527,0.0144702901518486,0.371828354399031
"524","C1orf162",5.7589091373637,-1.50946980570942,0.61808499008431,-2.44217191798093,0.014599192504466,0.374337468973523
"525","GLI1",6.21961192334448,-1.65298007276542,0.677206457996865,-2.44088055163388,0.0146515000133993,0.374337468973523
"526","RAB20",3.61767166195834,-1.92972549148085,0.79046761896658,-2.44124546683352,0.0146367022009232,0.374337468973523
"527","CDA",25.1421985727634,-1.20873739059547,0.495695769024847,-2.43846622490515,0.0147497369684876,0.375419914999061
"528","QPRT",6.30855889295933,2.28251033391653,0.935998972915973,2.43858209246287,0.0147450091853619,0.375419914999061
"529","IGFBP5",4778.27512574745,0.937202492625993,0.384463230226025,2.43769083476465,0.0147814098747117,0.375514872034499
"530","TMEM63A",15.6639854546062,1.20439141050587,0.495036964240581,2.43293228083176,0.014977101721775,0.37692372666467
"531","IFT57",106.259055943677,-0.463754046079229,0.190522867933493,-2.43411224652106,0.0149283649277396,0.37692372666467
"532","GALNT4",12.9517038561044,1.16588428365354,0.47916612335439,2.43315256824042,0.0149679924384946,0.37692372666467
"533","AL161669.3",20.5986058357386,-0.894018757139994,0.367199988093143,-2.43469168335926,0.0149044833127399,0.37692372666467
"534","PYGO1",13.2205525802832,1.11906950235553,0.459747055741455,2.43409824680824,0.0149289423458125,0.37692372666467
"535","FLCN",38.710959869981,0.838153681872829,0.344718950451358,2.43141167834084,0.0150401147033993,0.377802058876604
"536","PELI1",9.74654751537319,-1.16794493478965,0.48090475672879,-2.42864084509008,0.0151555372562125,0.379991166392239
"537","DNASE1",19.7150482234686,1.20446081574481,0.49747380269019,2.42115425823721,0.0154713104627114,0.387186110443907
"538","TIGAR",2.4641065154554,-2.2937817778303,0.947879348737609,-2.41990901150571,0.0155243910477253,0.387792362993272
"539","PRSS12",8.58412227160584,2.01031539766753,0.832494988755091,2.41480780643947,0.0157435144690318,0.391809427683923
"540","DHRS4",24.9104335044201,-1.05503048893261,0.436832003643925,-2.

41518588411987,0.0157271812097936,0.391809427683923
"541","HDGF",82.7338487234582,-0.473847728469129,0.196365607190295,-2.41308921276591,0.0158179473015375,0.392479164180369
"542","SMARCE1",223.750645706806,0.449533952448425,0.186309181085106,2.41283843249291,0.0158288345104368,0.392479164180369
"543","AL583785.1",18.5325645695835,-1.58371090858699,0.65728747750695,-2.40946459925557,0.0159759458850314,0.393946305961351
"544","CPXM2",42.4654648517778,0.933821087865368,0.387518438364001,2.40974620925836,0.0159636208482275,0.393946305961351
"545","VAMP1",4.79693989219104,2.17734261851904,0.903484886322377,2.40993806479917,0.0159552288237382,0.393946305961351
"546","ATM",65.0144329011113,0.649723975684473,0.270115769627833,2.40535373621343,0.0161568181104437,0.397031766804947
"547","REXO2",244.411746335653,-0.574961868947011,0.239041734220334,-2.40527818634819,0.0161601589733095,0.397031766804947
"548","MUC20",7.20346963473103,2.21093649665072,0.920261461679514,2.40250905717129,0.0162830313310969,0.398877560742408
"549","GRIA1",5.51594959246865,1.87172342098149,0.779154886395901,2.40224819693993,0.016294648474409,0.398877560742408
"550","RND2",2.37327180498855,-2.26344747118107,0.942673682669561,-2.40109330810128,0.0163461678048345,0.399411180234857
"551","PTPRG",97.5315459143128,0.647645940366669,0.270285642425611,2.39615369338353,0.016568141469132,0.40408364986274
"552","EBF2",99.0183567513885,0.745447892306684,0.31124177203806,2.39507662299111,0.0166168923330217,0.40408364986274
"553","HOXB2",47.7831692665262,-0.648425417670084,0.270759331079164,-2.39484052160144,0.0166275956822751,0.40408364986274
"554","LINC01151",1.50034662534284,-3.74058057062314,1.56323665493522,-2.39284343724537,0.0167183733491812,0.40547986007784
"555","MIR497HG",23.4427608357535,0.985932422154738,0.412234420330244,2.39167903874912,0.0167715017648004,0.40547986007784
"556","MMP11",11.2129729795834,1.22755171920899,0.513278465226023,2.39159014526049,0.0167755638219569,0.40547986007784
"557","RPS6KB2",16.7258117273808,-0.930863769484174,0.389620740721511,-2.38915353366551,0.0168872434949118,0.407446436854793
"558","CD52",12.2056234124612,-1.25305600263079,0.525488466476678,-2.38455471921648,0.0170998047692867,0.407972773469743
"559","CACHD1",24.3346437199867,0.781914458890024,0.327645017948584,2.38646833022416,0.0170110724613005,0.407972773469743
"560","MGST3",580.838164617709,-0.609821222777821,0.255911168462215,-2.38294102771002,0.0171749454341024,0.407972773469743
"561","PXDN",68.6106968074523,0.687858283925539,0.288776472291367,2.38197481417915,0.0172200751452312,0.407972773469743
"562","RTKN",8.56196304317551,-1.31519319703575,0.551594179680627,-2.38434930150504,0.0171093538843202,0.407972773469743
"563","CEP19",7.64016906388174,-1.35207986829301,0.566244494217816,-2.38780223401679,0.0169494598442068,0.407972773469743
"564","CARD19",33.4650911150625,-0.70499710896065,0.295440658312932,-2.38625622142337,0.0170208877923364,0.407972773469743
"565","RNASE6",1.94233098856855,-3.18211592175039,1.33605880252381,-2.

38171846608801,0.0172320660473258,0.407972773469743
"566","PNMA8B",8.76434020692305,1.43082199313652,0.600810816670196,2.38148507556239,0.0172429894583536,0.407972773469743
"567","PFKL",55.8304533353267,-0.590969939487231,0.24787235370687,-2.38417044357477,0.0171176721425767,0.407972773469743
"568","YDJC",4.89978642429243,-1.7642140297264,0.740418411728283,-2.38272576935029,0.0171849906865904,0.407972773469743
"569","PNISR",423.835080568792,0.456833684535366,0.192140481367382,2.37760247754286,0.0174256002533468,0.411568790517974
"570","BASP1",519.004381216507,-0.541247259159615,0.22788365727797,-2.37510344368139,0.0175440326897022,0.412914632779173
"571","AC245060.5",24.4537620389568,0.962289621005911,0.40507259753903,2.37559792208159,0.0175205428792027,0.412914632779173
"572","ACTR3",118.882718580689,-0.416986103207037,0.175759839220381,-2.37247658541715,0.017669283010113,0.413077008208496
"573","PARP16",21.838188267853,-0.969813189448043,0.40858615410263,-2.3735830979834,0.0176164285508283,0.413077008208496
"574","DOK6",3.5633118923157,2.63197671575322,1.10942437112838,2.37238047427809,0.0176738804762174,0.413077008208496
"575","JUND",1385.68239391622,-0.485578673674801,0.204586445982001,-2.37346453399714,0.0176220853249172,0.413077008208496
"576","SNX18",67.3892753534153,0.56343279792962,0.237658880704108,2.37076265048605,0.0177514264135715,0.413451333746945
"577","SNAPC2",43.4986650121833,-0.688326363459254,0.290280985235891,-2.37124165366842,0.0177284357010694,0.413451333746945
"578","CWC22",58.8364094817032,0.609568323701751,0.257247529226632,2.36957892475899,0.0178083537852828,0.414059630658158
"579","FHIT",9.9469710654599,1.33793544219639,0.564979198172033,2.36811451912784,0.0178790008378862,0.414946466341857
"580","MXRA5",89.7647338623146,1.17119174802658,0.494693527896933,2.36750974488308,0.0179082484171647,0.414946466341857
"581","CCN3",35.4391536942759,-1.04013641665813,0.439636665061036,-2.36590007003563,0.0179862984887473,0.416037634062436
"582","CYB5R3",307.674646097519,-0.487271542832367,0.206038737260806,-2.36495112186392,0.0180324506540348,0.41638849542882
"583","SCX",2.42246827315115,-3.61578863091194,1.53126569222946,-2.36130715215561,0.0182106406671343,0.418854940190286
"584","AKR1C1",276.899604162405,-0.698508052243839,0.295770687299743,-2.36165408621426,0.0181936094486378,0.418854940190286
"585","AC009271.1",2.0875665987925,2.90108441187524,1.22882688966439,2.36085687599785,0.0182327658316331,0.418854940190286
"586","AZIN2",7.99724967586231,-1.30514402542892,0.553216829161338,-2.35919074878377,0.0183148389187993,0.419306848772987
"587","AP4E1",11.5691967053846,1.27881260251202,0.542009011972293,2.35939361572348,0.0183048284727843,0.419306848772987
"588","LRRC32",64.8708281033131,-0.686986048825642,0.291617560168403,-2.35577736961012,0.0184839918721296,0.422459807431206
"589","LINGO2",2.93245342364092,3.41871005444324,1.45265379824676,2.35342382236521,0.01860141884675,0.424421846997409
"590","OGA",107.611651061163,0.688341203921789,0.292581087915645,2.352

65105077553,0.0186401173022887,0.424583960043149
"591","AMPH",17.7398746587748,1.53280051685354,0.653354076830991,2.346
04875244399,0.0189736255097655,0.431449328639151
"592","FAM87B",2.40068209205883,-2.20598593630324,0.940695002426375,-2
.34505969587725,0.0190240334167552,0.431864839675291
"593","CREM",29.2145311067046,-0.742552309188338,0.316805307302196,-2.
34387585079194,0.0190845227661573,0.431961086713023
"594","SCD",1.52309138278469,3.35241384778167,1.4305954706239,2.343369
53850387,0.019110444420047,0.431961086713023
"595","NFAT5",77.5490430432807,0.733093041554506,0.312969438519617,2.3
4237900359257,0.0191612458253459,0.431961086713023
"596","ACTG1",2988.43053129148,-0.544661869846692,0.232460616510603,-2
.34302858704605,0.0191279174380019,0.431961086713023
"597","ICAM1",10.8614979222872,-1.26858936235949,0.54170645502899,-2.3
4183910969199,0.0191889849518323,0.431961086713023
"598","SNED1",81.2616698047066,1.03230060022576,0.441459982007102,2.33
837865786247,0.0193676138631501,0.433890458603082
"599","ZNF706",204.042475858951,-0.487422609081399,0.20845145892526,-2
.33830269931651,0.0193715510947131,0.433890458603082
"600","CEP152",27.8126534327288,1.03791048595845,0.443798573088869,2.3
3869721286961,0.0193511095129332,0.433890458603082
"601","ARL6IP5",613.493959008366,-0.396056010628704,0.169617605156305,
-2.33499353008629,0.0195437587290874,0.434262708950976
"602","FGF12",4.48911202705419,2.87344022558735,1.23061169709148,2.334
96905025254,0.019545037615234,0.434262708950976
"603","NDUFV1",93.799332708892,-0.675957853471843,0.289362141978172,-2
.33602726621658,0.0194898205119149,0.434262708950976
"604","NAALAD2",12.1141467137613,1.10747787778463,0.474030363226459,2.
33630156145832,0.0194755302027329,0.434262708950976
"605","NAA10",52.9092295238174,-0.691196707515175,0.296031051347605,-2
.33487907558575,0.0195497387391429,0.434262708950976
"606","EXOSC6",48.5614953854483,-0.60687680308296,0.260018569891881,-2
.33397485162428,0.019597038784264,0.434595056471492
"607","SMN2",4.50886953476605,-1.85027909139414,0.793203977565169,-2.3
3266491813844,0.0196657388230132,0.434946793585012
"608","SLC7A8",14.4072466492991,1.08152901904463,0.463788100583189,2.3
3194645935216,0.0197035079556856,0.434946793585012
"609","TMOD3",173.52185192825,-0.569181646211266,0.244092966376019,-2.
33182321744763,0.0197099931016647,0.434946793585012
"610","PRRX2",154.926892242387,-0.605539369520205,0.260048503767357,-2
.328563174745,0.0198822188345163,0.438028096585352
"611","GSTM2",14.9012037642737,1.04577545997418,0.449276721565958,2.32
768672351667,0.0199287446327094,0.438334532109626
"612","WDR34",26.1791278590135,-0.738683677314462,0.317543897568069,-2
.32624113696317,0.0200056902499709,0.439307959590456
"613","SRM",86.6441545945919,-0.63454476804025,0.272982246095602,-2.32
449097740234,0.0200991945851044,0.439923576594818
"614","CDKN1C",458.321384933645,0.686376931744747,0.295277608817275,2.
32451398700365,0.0200979628001133,0.439923576594818
"615","BBS9",17.4828318726442,0.979943061708284,0.422291892015635,2.32

053487229161,0.0203119607259179,0.443137078239627
"616","AOPEP",16.0876300280529,-1.00602650743811,0.43345963074652,-2.3
2092318656179,0.0202909899038262,0.443137078239627
"617","ZNRF1",34.1093642800253,-0.714318184519126,0.308651088957032,-2
.31432258001386,0.0206500341011571,0.449782509376743
"618","SOX4",323.455085820582,0.76546453337123,0.331308157458396,2.310
43068556908,0.0208643218787601,0.453714598266435
"619","AL157396.1",1.55237806858912,-3.24097192868936,1.4033059939526,
-2.30952617793694,0.0209144007586608,0.454068872044657
"620","BEX4",61.1461645570243,0.791068675397243,0.342754309961872,2.30
797586611016,0.0210004788401312,0.455202314729875
"621","CDK14",38.699594055021,0.678354039888781,0.294044296436041,2.30
697907801906,0.0210559864921343,0.455670535374869
"622","SMO",13.0428616871372,1.00210665060821,0.434688773283864,2.3053
4283882646,0.0211473801107877,0.45691260660591
"623","MIR99AHG",192.984340685008,0.437563212659708,0.189871603193869,
2.30452160986354,0.0211933807109161,0.45717149819262
"624","DENND6B",6.06970771570536,-1.29082391825058,0.560904703735959,-
2.30132482336647,0.0213732776585065,0.460313266751071
"625","RBMS3",221.457446585934,0.549441991491444,0.239255154653065,2.2
9646877321481,0.0216490926370613,0.460427921157268
"626","DDIT4L",8.94080871493443,-1.26971085952636,0.553056561455957,-2
.29580651965103,0.0216869465058822,0.460427921157268
"627","HEBP2",53.1023348955686,-0.737284441194891,0.320968163813854,-2
.29706408397089,0.0216151142636852,0.460427921157268
"628","ILK",139.668384611308,-0.461491364747056,0.200999785508083,-2.2
9597938913471,0.0216770598762328,0.460427921157268
"629","NAALADL1",18.6609323733529,-0.87368129624766,0.380150582691174,
-2.29825057760708,0.0215475315655297,0.460427921157268
"630","ARHGAP42",27.8171675172192,0.717998909765569,0.312646438585831,
2.29652035383239,0.0216461467470537,0.460427921157268
"631","LINC01089",11.1762371575145,1.24457706407061,0.541047805019079,
2.30030886832028,0.021430727608522,0.460427921157268
"632","LINC01578",170.709961477879,-0.466633321768232,0.20295841748450
2,-2.29915727345412,0.0214960102312466,0.460427921157268
"633","SNHG16",79.2490216132799,-0.528298268079593,0.230074670244423,-
2.29620352174513,0.0216642472826884,0.460427921157268
"634","ANTXR1",140.613389732708,0.53462062494986,0.233008163041976,2.2
9442873575871,0.0217658841428,0.460648373220613
"635","ACE",137.382633964382,0.630531155535539,0.274795857905853,2.294
54388556163,0.0217592772805112,0.460648373220613
"636","CSRNP1",17.4969745704411,-1.21284886070728,0.529181336008058,-2
.29193431094253,0.0219094340053324,0.462469889756714
"637","INSYN1",4.8219173461971,-1.67949490068097,0.732847749153002,-2.
29173781678673,0.0219207768267748,0.462469889756714
"638","DAB2",337.823504298436,0.42361047844107,0.184930544037054,2.290
64636481139,0.0219838750389571,0.462987038196178
"639","PFN1",331.060718620804,-0.573764267181096,0.250538632246618,-2.
29012293248376,0.0220141913391887,0.462987038196178
"640","COL24A1",2.07259854522764,2.95102929689516,1.28998049170871,2.2

876542055192,0.0221576665367492,0.463826916802761
"641","APEX1",107.289826947817,-0.580863409769261,0.253783907232138,-2
.28881104442112,0.0220903334941887,0.463826916802761
"642","LYL1",1.94268071883486,-2.5022278593805,1.09351835176503,-2.288
23581729718,0.0221237919291132,0.463826916802761
"643","EFHD2",36.9943022896456,-0.651226126369308,0.285217226727194,-2
.28326365080394,0.0224148427848983,0.467028018893408
"644","CSAD",76.1230173702393,0.663026469752853,0.290357062288127,2.28
348663031629,0.0224017195077602,0.467028018893408
"645","HERPUD1",198.454669707309,-0.530282101927571,0.23214366183985,-
2.2842842131671,0.0223548330837547,0.467028018893408
"646","LINC00648",5.93199724917617,1.48241201129967,0.649653992478027,
2.28184853547222,0.0224982841484909,0.468040929832151
"647","AC003681.1",17.3294944233894,1.36908320727192,0.600695391246363
,2.27916382782837,0.0226573284521345,0.470621077385217
"648","UFC1",121.632088707738,-0.594933612723389,0.261281952652904,-2.
27697935767389,0.0227874583252097,0.472265366365935
"649","LPIN2",51.5578300553291,0.567729692995259,0.249369988605887,2.2
7665604898639,0.0228067730315866,0.472265366365935
"650","RBP7",1.83480872348406,-3.29649952969508,1.4488793186993,-2.275
20642137016,0.0228935498490213,0.473332948339996
"651","PRXL2A",23.9905584309106,0.937314985874504,0.41220809892799,2.2
7388784527071,0.0229727306881745,0.473513079322665
"652","SF3B3",38.7848922114447,0.626920278471767,0.275641038165315,2.2
7440834878794,0.0229414459444971,0.473513079322665
"653","SEMA3B",56.2165305156614,0.821819745106289,0.361628562170449,2.
27255209094611,0.0230531855169649,0.473609518972094
"654","LINC01615",1.72140996689016,-2.79197137445336,1.22946111226667,
-2.27089034911077,0.0231536166355135,0.473609518972094
"655","ATP6V1C1",38.9129947642209,0.677474205116358,0.298308701917773,
2.27105076305518,0.023143905117109,0.473609518972094
"656","LYVE1",2.13927782178104,-3.17053556288191,1.39561957958447,-2.2
7177635600805,0.0231000216280453,0.473609518972094
"657","ZMYM2",43.8046664336522,0.659811206437855,0.290470589290563,2.2
7152500378561,0.0231152150943141,0.473609518972094
"658","FCER1G",10.5912703145446,-1.27150980247164,0.561237493413431,-2
.26554679149881,0.0234791460311762,0.47709770899671
"659","C1QTNF2",61.468730592321,-0.698543522839239,0.308436610618042,-
2.26478796223154,0.0235256944916965,0.47709770899671
"660","R3HDM4",19.8277359450868,-0.81296728068064,0.358686154963639,-2
.26651424770787,0.0234199158154391,0.47709770899671
"661","SLC7A4",7.6193879097526,1.59320407211734,0.703525167967124,2.26
460138834975,0.0235371516530113,0.47709770899671
"662","SDF2L1",34.0946534025887,-0.745205270295898,0.329006351159866,-
2.26501788694589,0.0235115818925631,0.47709770899671
"663","SLC2A11",11.5612353832719,1.09005372023042,0.48087875942758,2.2
6679531765549,0.0234027323042654,0.47709770899671
"664","LRRN4CL",258.931866111084,0.44853510171701,0.198336347248136,2.
26148715523057,0.0237291072032419,0.480264264615012
"665","CREB5",233.976080556502,0.60543002686373,0.267841869352686,2.26

040099080446,0.0237963750722348,0.480417527141051
"666","STK3",56.7543882145715,0.570119736566706,0.252304830339793,2.2596465386687,0.023843196777074,0.480417527141051
"667","DNAJC4",50.702401997104,-0.730297258342779,0.323289679125768,-2.2589563029591,0.0238861031522824,0.480417527141051
"668","CHMP4B",95.1056918962751,-0.467003986767571,0.20677751843802,-2.25848530485945,0.0239154197229975,0.480417527141051
"669","DUXAP8",103.639652411067,0.768722725818167,0.340281423260674,2.25907931867701,0.0238784513588939,0.480417527141051
"670","ATXN7",42.3154814290888,0.723786824649735,0.320637756182468,2.25733498533418,0.023987150850769,0.4811392840052
"671","ELMO1",20.9790846713458,1.00827502563101,0.447303440738315,2.25411864475435,0.0241887050257828,0.483019326658983
"672","ACADVL",132.443792311963,-0.530477308336927,0.23531301912462,-2.25434746581527,0.0241743174415698,0.483019326658983
"673","HOXB7",9.75849379671808,-1.30254850283159,0.577602906744233,-2.25509340002052,0.0241274668316897,0.483019326658983
"674","SUCO",35.1754823757596,0.652281310682162,0.289483924256103,2.25325572865006,0.0242430294147577,0.483385863953899
"675","TOR1B",12.7500489784277,0.975461122086449,0.43308051736764,2.2523782136761,0.0242983812958981,0.483771772200851
"676","BCHE",5.91632417996864,2.08491708849231,0.925903616506551,2.25176470998,0.0243371449115354,0.483826761044563
"677","WARS2",26.3559246209717,-0.784856336523514,0.34950222328404,-2.2456404687465,0.02472704614781,0.487000113155975
"678","TFPI",287.530449489743,0.399189749798797,0.177641161968504,2.2471692110952,0.0246292150626199,0.487000113155975
"679","EPHA4",5.19446672357524,1.7351682368995,0.772991823927133,2.24474332481823,0.024784615005738,0.487000113155975
"680","SLC9A9",27.568354159756,0.824626095212299,0.367233994442776,2.24550588369018,0.0247356749490879,0.487000113155975
"681","CCDC136",8.25808796709645,1.58527584878876,0.706393344589678,2.24418287761417,0.0248206372425699,0.487000113155975
"682","PKN3",1.93287728665222,-2.29486041132403,1.02256639565748,-2.2442165330971,0.0248184727878428,0.487000113155975
"683","BDNF",2.19414358880355,-2.29991708529284,1.02463362948137,-2.24462385297363,0.024792290150664,0.487000113155975
"684","UNKL",24.4035846684748,0.819692454582014,0.365247504768167,2.24421096347337,0.0248188309707432,0.487000113155975
"685","OLFM2",2.30161018484725,-2.28545710621439,1.01840768169516,-2.24414755239297,0.0248229092575224,0.487000113155975
"686","SLC25A26",26.5625446895716,-0.744970017203935,0.332097366816071,-2.24322771464951,0.0248821339750083,0.487450435116817
"687","ANKRD10",57.2979148628225,-0.543518880797422,0.242416199884614,-2.2420897656845,0.0249555713145822,0.48746791118702
"688","PDE4A",34.1595474204744,-0.69437999045043,0.309669214674691,-2.24232812803132,0.024940173121692,0.48746791118702
"689","WWC2",32.8353774670767,0.763289956835833,0.340825822071173,2.23953089058035,0.0251213937101839,0.489284652277045
"690","LDLR",3.65799679915626,-1.92332646935459,0.858716869464926,-2.2

3976788828317,0.0251059956057781,0.489284652277045
"691","FAM78B",7.50572044320949,-1.17566189583189,0.525303863887344,-2
.23806062862697,0.0252171016868374,0.489729233481804
"692","HSD11B1",37.7463479233256,-0.945059568371964,0.422161991203172,
-2.2386183220297,0.0251807610622616,0.489729233481804
"693","VEPH1",2.52555453027141,3.21783211276794,1.43819931078643,2.237
40345905769,0.0252599826702667,0.489798525885243
"694","DENND4C",42.2610021156715,0.615417153773505,0.275121837155222,2
.23688951824747,0.0252935617951007,0.489798525885243
"695","RAB3GAP2",40.4308012501819,0.67916449145739,0.304023913029236,2
.23391799904924,0.0254884690347738,0.490220585740877
"696","FSTL1",3006.01446987567,0.603648510060785,0.270429289585229,2.2
3218613259914,0.0256026635604786,0.490220585740877
"697","ERV3-1",14.7611653711894,1.0188908681794,0.456468228975891,2.23
211782004047,0.0256071769618346,0.490220585740877
"698","CASTOR3",8.35143097321388,1.30795683853995,0.585715033363064,2.
23309419092405,0.0255427336123266,0.490220585740877
"699","TPCN1",47.9348844532028,0.674067218329384,0.301731432622679,2.2
3399734151104,0.0254832479841255,0.490220585740877
"700","SPSB3",66.2311402008763,-0.606255610841742,0.271244692965806,-2
.23508745632184,0.0254116077188931,0.490220585740877
"701","ARHGAP28",30.1767549320398,0.913092509852663,0.408861319634818,
2.23325725864263,0.0255319843480935,0.490220585740877
"702","BCL2L1",28.8557720907829,0.743064014956847,0.332516341908936,2.
23466916149446,0.0254390766245405,0.490220585740877
"703","MAN2A1",45.9735988307974,0.634632745884195,0.284395247350196,2.
23151670710843,0.0256469220943866,0.49028305266922
"704","PBRM1",70.9806507528038,0.49523463714361,0.222106995727654,2.22
971201569384,0.0257665676737088,0.490498723821076
"705","NEK6",22.1308745549547,1.0625955496021,0.476682811089131,2.2291
4593285683,0.0258041965727734,0.490498723821076
"706","FGFR2",2.07516690808928,-3.38563664264606,1.51875303250209,-2.2
2922132182897,0.0257991825435289,0.490498723821076
"707","NPEPPS",113.872756891145,0.662062769401834,0.296833215695986,2.
23042009584235,0.0257195666813635,0.490498723821076
"708","PARP3",9.75916048815218,1.11920158170117,0.502557084973146,2.22
701383617142,0.0259463490900083,0.490761926512918
"709","IL17RD",48.1842438831259,0.755016295955645,0.338871489908377,2.
22803132880782,0.0258784259296959,0.490761926512918
"710","MTURN",116.726470927732,0.59266896430339,0.266060513048622,2.22
757205686919,0.0259090657654512,0.490761926512918
"711","PLAU",107.175628624105,0.799061356143018,0.358846767608169,2.22
674809492927,0.0259641141268461,0.490761926512918
"712","VMO1",2.01055894926987,-2.2616174177204,1.0175921176587,-2.2225
1860885477,0.0262482778564842,0.49543624454114
"713","SLC46A3",7.83514316241331,1.35973327867438,0.612274558354762,2.
22079010163039,0.0263651813250367,0.496944841272325
"714","C19orf25",31.5074374597081,-0.623852529219345,0.281033827651564
,-2.21984853009517,0.0264290513517357,0.497451009966352
"715","TRMT61B",15.2901204414217,-0.884587025884004,0.398664350320933,

-2.21887666948874,0.0264951161473608,0.497997015250883
"716","TUFM",140.94081435907,-0.576001102842307,0.259782764134819,-2.21724141230316,0.0266065990791048,0.498697468652845
"717","SERTAD1",55.7420738194934,-1.10160225270698,0.496776296695047,-2.21750164014611,0.0265888310987301,0.498697468652845
"718","PLOD1",30.1752166462398,0.712895390894874,0.321786513224429,2.21542967650004,0.026730586266359,0.498997167484648
"719","PHLDA3",65.7563559951452,-0.60013572753337,0.270895141004089,-2.21538018477899,0.0267339802506843,0.498997167484648
"720","ENPP4",2.68287848117954,2.8193826751317,1.27229942906908,2.21597417299369,0.0266932709940268,0.498997167484648
"721","NCEH1",10.3314295523277,-1.07399378654398,0.485052330343955,-2.2141812735595,0.0268163115340227,0.499839681977435
"722","CMKLR1",11.5795640416258,-1.81874790967837,0.821809327600565,-2.21310205250234,0.0268906106420775,0.500530355150803
"723","MXD4",140.900614001712,0.419310670647027,0.18979886665715,2.2092369571651,0.0271581635694854,0.501300776329361
"724","FOXP4",7.86983361878258,-1.27477395169693,0.57715128819125,-2.20873448223944,0.02719311451329,0.501300776329361
"725","AL139384.1",3.25294716758066,2.15005605585277,0.973354759573045,2.20891307584079,0.0271806875291301,0.501300776329361
"726","ANKFY1",40.1739340606531,0.65178900520626,0.295068231975948,2.20894333775446,0.027178582316059,0.501300776329361
"727","ZNF507",20.4654411003248,0.859237913422616,0.388890700028783,2.20945863030158,0.0271427568498048,0.501300776329361
"728","ERVK3-1",43.5274398922845,-0.594833823756035,0.269243913472629,-2.20927491390258,0.0271555249660457,0.501300776329361
"729","MTMR1",6.19163493414798,1.469853503459,0.665319646493374,2.20924409974362,0.0271576670285547,0.501300776329361
"730","RFX8",3.59271865273221,-1.79525842982618,0.81354328038604,-2.20671533169606,0.0273339534369408,0.502544538300049
"731","LINC00968",11.5755744771338,1.34406178784117,0.609083611027782,2.20669504729108,0.0273353714932164,0.502544538300049
"732","APOL1",57.6764040942923,0.820668122037342,0.372151110367057,2.20520132595576,0.0274399701940793,0.503778359888294
"733","CRKL",21.6402741323142,0.786382141155957,0.35673582516945,2.20438230666187,0.0274974689274993,0.504145272737603
"734","LINC01686",1.56865337928668,-2.7606800423919,1.2528574746082,-2.20350686198782,0.027559043860833,0.504585818045959
"735","MARC2",14.8945719787717,0.922883217228828,0.418973617823472,2.20272393766252,0.02761421202537,0.504908020964554
"736","PTCD1",6.88175831615422,1.50530768298253,0.684270220881988,2.19987314520609,0.0278158965273155,0.507904664987218
"737","FAM171A1",35.5921455149893,0.62711974404671,0.285298862107585,2.1981151253601,0.0279409029234315,0.50949497203256
"738","DLEU2",23.2304127224446,0.900560841229131,0.410114466134696,2.19587679926744,0.0281007627588142,0.510332636102303
"739","LMO7",7.08648518079603,1.787090198435,0.813758685909865,2.19609354637714,0.0280852484228702,0.510332636102303
"740","ZNF91",57.4271207875891,0.562647713952557,0.25619123404630 9,2.1

9620205213912,0.0280774845650809,0.510332636102303
"741","PLCB1",49.9906885695111,0.607740270144759,0.276926907501876,2.19458728524112,0.0281932164381249,0.511320695967559
"742","KCNC4",2.69419364087078,2.78983397932514,1.27206091333284,2.1931606812882,0.0282958042860231,0.511716930703288
"743","PEAR1",5.86948659214402,2.2267901428646,1.01563974789986,2.192499995662,0.0283434234075516,0.511716930703288
"744","IPPK",4.25632548149876,1.8513103182262,0.844503855364048,2.19218693492896,0.0283660114559611,0.511716930703288
"745","CDRT4",4.09626235832112,1.86228808470242,0.849518838535305,2.19216808412781,0.0283673720793176,0.511716930703288
"746","RTCA-AS1",12.9046705569468,1.12408305267603,0.513139036360652,2.19060132444492,0.0284806552301918,0.512362416335744
"747","XPO1",72.59276635172,0.526542943290243,0.240451131023214,2.1898127118371,0.028537822444836,0.512362416335744
"748","HIST1H2AC",16.3973660345989,1.05050363568265,0.479865074861819,2.18916460212311,0.0285848784465369,0.512362416335744
"749","SURF1",65.6654118862223,-0.587118635200709,0.268262668300576,-2.18859612080974,0.0286262080288612,0.512362416335744
"750","NOC4L",18.2478327036121,-0.763329188288172,0.348788199996927,-2.18851781194116,0.0286319052509966,0.512362416335744
"751","SLC43A2",6.1709487907831,1.46373536474791,0.66808548536491,2.19094022668134,0.0284561182334984,0.512362416335744
"752","ATP7A",20.0321134413524,0.787447982634403,0.360065817599538,2.18695567350466,0.0287457600517295,0.513715783690417
"753","CDC42BPA",63.8884868302056,0.607216850146438,0.278232720704576,2.18240632736785,0.0290795577646182,0.514210758945662
"754","KLHL24",64.6020684874287,0.603127504534025,0.276104705668017,2.18441588336858,0.0289317021330634,0.514210758945662
"755","FAM193A",24.9779258909924,0.804232674752152,0.367982763457739,2.18551724324043,0.0288509430581095,0.514210758945662
"756","EDN1",4.80170578004381,2.02297979905877,0.925893516965886,2.18489465796023,0.0288965712970711,0.514210758945662
"757","CISD1",35.7223582100607,-0.680120667771369,0.311141834608528,-2.18588628117811,0.028823926173593,0.514210758945662
"758","BMPR1A",40.8892279456238,0.610502989321885,0.279606447390194,2.18343673767265,0.0290036629805127,0.514210758945662
"759","NOVA2",9.31071028923172,1.51418406774665,0.693406080764613,2.18369020657703,0.0289850198994365,0.514210758945662
"760","GRWD1",22.1278219069737,-0.810573410443281,0.371373927795757,-2.18263413173439,0.0290627641522417,0.514210758945662
"761","CMTM8",5.27469426886363,1.84800554543712,0.847380340260091,2.180845433433,0.0291948508274561,0.515571091025208
"762","AL627171.2",28.8481472041133,1.09355635940688,0.501588017384122,2.18018836476594,0.0292435017256272,0.515752519279139
"763","IFI30",9.87267971016762,-1.07047899832908,0.491136397857205,-2.1795961427406,0.0292874109838179,0.515849955716289
"764","SLC52A2",34.6853324311221,-0.650974965638854,0.298759364164735,-2.17892740352704,0.0293370616569592,0.5160481303768

"765","RBM43",68.718183707169,0.511134530301909,0.234749386016163,2.17
736258644237,0.0294535248678894,0.516070300781702
"766","RNF130",123.580066641112,-0.45292799260566,0.207945417293862,-2
.17810999876759,0.0293978483730524,0.516070300781702
"767","PCSK5",79.2572106420445,0.644932312998345,0.296175373190206,2.1
7753524221666,0.0294406552847226,0.516070300781702
"768","REV3L",159.653436639995,-0.524226443510347,0.240894506254476,-2
.17616603907341,0.029542847324483,0.516289109484691
"769","SRRT",22.7131636379171,-0.73097360553089,0.335896297072166,-2.1
761883411708,0.029541180340837,0.516289109484691
"770","SH3GL1",86.185882811996,-0.495427242697499,0.227946164201339,-2
.17343969982272,0.0297472396061136,0.519185913073456
"771","FOLH1",3.33729832340337,2.11795716231597,0.974794029291487,2.17
27227482665,0.029801190593731,0.519218431061255
"772","SYCE1L",6.44685395387018,-1.56058251196449,0.718371748816872,-2
.17238848066437,0.0298263731512232,0.519218431061255
"773","KANK4",2.48922192597908,2.54233376145421,1.1710164040518,2.1710
4880226917,0.0299274835378564,0.519251474283573
"774","AL356599.1",16.4337121104466,0.927153226140333,0.42729752842389
1,2.1698071354641,0.0300214595965724,0.519251474283573
"775","SLC2A8",10.19718008532,-0.99911253368665,0.460411765421517,-2.1
7004127331964,0.030003719397517,0.519251474283573
"776","ALDOA",18.1135778776217,-0.81562937809907,0.375599663512029,-2.
17153916079839,0.0298904402665795,0.519251474283573
"777","EBP",36.3179729423796,-0.733842644990643,0.338127384616416,-2.1
7031414306517,0.0299830559240448,0.519251474283573
"778","CHST15",3.87996289370927,2.61073962744001,1.20365442040088,2.16
90109579546,0.0300818519864828,0.519377791637674
"779","ZNF609",20.7323296252203,0.893084933451463,0.411808055224161,2.
16869223931359,0.030106056974905,0.519377791637674
"780","KIAA0825",7.56965239953677,1.2233495682262,0.564241471381145,2.
16813125279801,0.0301487015817517,0.519446667380975
"781","NDUFAF2",74.5037369903443,-0.598755226138862,0.276348812222401,
-2.16666473549737,0.0302604273791299,0.520038214255916
"782","GLUD1",130.572377813419,-0.483715020923137,0.223249366222539,-2
.166702773261,0.0302575250056376,0.520038214255916
"783","TMEM176B",411.189446781961,0.710472709670768,0.328047888070546,
2.16575913306286,0.0303295977149193,0.520409404342278
"784","PKN1",62.0273046184874,-0.708455514993026,0.327175492537742,-2.
16536852897471,0.0303594741427447,0.520409404342278
"785","KDM5B",89.7854473576481,0.55562604345693,0.257289270376046,2.15
953833848122,0.0308084263237305,0.522111527572023
"786","DANCR",30.6533227146039,-0.721305877989479,0.333428022673012,-2
.16330310873976,0.0305178741495187,0.522111527572023
"787","KL",4.14555747734479,1.91029909694261,0.883761241056153,2.16155
564217737,0.030652443684283,0.522111527572023
"788","C15orf48",3.56245884126177,-1.81999679945998,0.842483104130185,
-2.16027691301776,0.0307512391131975,0.522111527572023
"789","TGFB1I1",88.3950057030689,-0.600358684853798,0.277792651073957,
-2.16117554777921,0.0306817815107758,0.522111527572023

"790","ABR",24.8485571450276,0.804175115249845,0.372247401868533,2.160
32431982925,0.0307475715583289,0.522111527572023
"791","ZSCAN30",16.5754233402341,0.869117292874797,0.402378824238393,2
.1599478911939,0.0307767037310013,0.522111527572023
"792","MYO5B",7.83181903651211,1.24689146740682,0.577061890086795,2.16
075864448313,0.0307139881806375,0.522111527572023
"793","HPS4",31.7806451848685,0.705946159716109,0.32666020241162,2.16
110243765644,0.0306874273260469,0.522111527572023
"794","CXCL1",6.36972364206269,-3.64156486514312,1.68669369504922,-2.1
5899595512323,0.0308504807414907,0.522165756530093
"795","RUFY3",107.786411809626,0.516864512211992,0.23954937349519,2.15
765336669674,0.0309547921713538,0.522614889435709
"796","EMC8",30.8017840881543,-0.750481517736914,0.347751924677719,-2.
15809450496191,0.0309204849087904,0.522614889435709
"797","GLIS3",11.3169205197522,-1.21798902426812,0.565593096490288,-2.
15347222557381,0.0312815846356385,0.525491519897932
"798","MRPL54",94.3154589272512,-0.627828459536017,0.291475476076441,-
2.15396666638042,0.0312427861041062,0.525491519897932
"799","SMS",66.9292770406018,-0.542041363542748,0.251569925043511,-2.1
54634992434,0.0311904085133807,0.525491519897932
"800","VSIG4",2.07925332596565,-2.59716016068521,1.20557012596491,-2.1
5430036357819,0.0312166243898964,0.525491519897932
"801","SLC66A1L",29.2756601915991,1.14663641340476,0.533393722358385,2
.14969986586069,0.0315789630786345,0.525550190579405
"802","TRIM23",28.7814468617905,0.764668340387646,0.355715031025196,2.
14966552912824,0.0315816809704062,0.525550190579405
"803","SLC25A27",7.59198511594393,1.41074768406288,0.656316325898274,2
.14949351158079,0.0315952998779295,0.525550190579405
"804","IGF2R",80.316240083354,0.629064047407409,0.292400553863201,2.15
137775594542,0.0314463954572777,0.525550190579405
"805","RECK",153.259962254085,0.490306981238257,0.228178403529868,2.14
878785044211,0.0316512209370566,0.525550190579405
"806","ECPAS",43.3777420750842,0.611252855437059,0.283917446582297,2.1
5292460113007,0.0313246047042851,0.525550190579405
"807","ALDH2",93.9472576661784,-0.684750925806774,0.318715055442424,-2
.14847373575195,0.031676140662945,0.525550190579405
"808","KCTD12",141.986695488212,0.771800914894373,0.359192232098924,2.
14871271125321,0.0316571804395834,0.525550190579405
"809","ATP8B3",2.00445038099025,-2.40379184636561,1.11813778040685,-2.
14981721258981,0.0315696761228667,0.525550190579405
"810","PTPRA",262.864280120968,0.402980707242688,0.187237432983112,2.1
5224434998014,0.0313781142907079,0.525550190579405
"811","SEC22C",50.3117080806775,-0.513190716859572,0.239100525432188,-
2.14633872481857,0.0318459641559093,0.526427510025881
"812","C3orf14",26.4766080023684,-0.676572346645975,0.315222658242235,
-2.14633158167856,0.0318465336446939,0.526427510025881
"813","DMRT3",5.31921113094013,1.86705782985395,0.869494261001353,2.14
729172301121,0.0317700643605466,0.526427510025881
"814","NDUFS6",173.483350723887,-0.539150570064033,0.251279763176036,-
2.14561874481841,0.031903408686347,0.526719790338841

"815","SAMD12",2.08810802220963,2.89576023210412,1.35026502294814,2.14
458656848089,0.0319859171522086,0.526787672314377
"816","AC005332.6",32.8459099979108,0.669055091793986,0.31192826273099
6,2.14490051634395,0.0319608019541883,0.526787672314377
"817","ISOC2",62.6975865215387,-0.613564187532139,0.286181159347554,-2
.14397128354279,0.032035187780197,0.526953351992739
"818","ZMYM1",18.2905279488623,0.889530352185175,0.415573653982225,2.1
4048783810348,0.0323153626289575,0.528418922050782
"819","CRABP2",200.016573205186,-0.786266239224297,0.367311375966211,-
2.14059866007696,0.0323064169464021,0.528418922050782
"820","HNRNPLL",23.9333965490778,-0.639378918276112,0.298716644707223,
-2.14041945638073,0.0323208835423575,0.528418922050782
"821","PRKG1",15.6183386078708,1.03242620437701,0.482179337373978,2.14
116641745738,0.0322606201631761,0.528418922050782
"822","PPIF",10.2619371972706,1.1018284117726,0.514692540210297,2.1407
5069229176,0.0322941481758522,0.528418922050782
"823","LPIN1",30.6380987606931,0.69255916609345,0.323888950157659,2.1
3826117180666,0.0324955520365103,0.528701844816781
"824","USP40",19.8250173731253,0.847194812667943,0.396121453882768,2.1
3872488945946,0.0324579556358593,0.528701844816781
"825","ZNF727",15.5630382626416,0.943440105761254,0.44108527843368,2.1
3890635641132,0.032443253157558,0.528701844816781
"826","MRC2",435.324206324314,0.5162213726639,0.241399157479051,2.1384
5556900379,0.0324797865387431,0.528701844816781
"827","RAVER2",5.75486661299384,-1.2230270849446,0.57274965812394,-2.1
3536065468928,0.0327315619414669,0.531897776216896
"828","POLR3GL",70.6282968623684,-0.518323929404585,0.242965239594298,
-2.1333254512871,0.032898037777817,0.532496627527742
"829","PRIMPOL",13.9494310095936,0.965933941288285,0.452857936052137,2
.13297342144199,0.0329269065760513,0.532496627527742
"830","KCNMA1",11.1231630806715,-1.17495200110133,0.550531709217587,-2
.13421312783448,0.0328253385689985,0.532496627527742
"831","CEBPG",25.4416937838329,-0.656651612330972,0.307806493878034,-2
.13332605189014,0.0328979885428798,0.532496627527742
"832","EFEMP1",1616.61669613105,0.624125331284444,0.292861254362675,2.
13112974825798,0.033078454563799,0.532995271301136
"833","FAM120B",16.0968829932527,0.893529305772055,0.419460176229845,2
.13018864818872,0.0331560418786926,0.532995271301136
"834","SHARPIN",63.6629489155578,-0.584197758154954,0.27419665351345,-
2.13057946065085,0.0331238031537269,0.532995271301136
"835","BDKRB2",84.1281411391859,0.881107027459642,0.413500888316987,2.
13084675838517,0.0331017688031105,0.532995271301136
"836","TRIP10",125.634713370487,-0.500875443058242,0.235120369785115,-
2.13029370239597,0.0331473731565371,0.532995271301136
"837","THEMIS2",2.38815289801284,-1.99518575348449,0.937624541997008,-
2.12791545455393,0.0333440939874364,0.534055237876433
"838","TTC13",5.67951417774428,1.47352235135941,0.692597521407284,2.12
753050049236,0.0333760298673903,0.534055237876433
"839","HMGA1",8.41526069369356,-1.14713227270314,0.539442400403186,-2.
12651484541399,0.0334604144870866,0.534055237876433

"840","CBWD1",26.6154811644384,-0.796409069205029,0.374395315712226,-2
.12718758964703,0.0334044998673121,0.534055237876433
"841","TJP2",4.48193183778024,-1.62040699233268,0.761839937698713,-2.1
2696514339671,0.0334229794636163,0.534055237876433
"842","SIGIRR",58.8968035837884,-0.643886708874896,0.302677570504649,-
2.12730235610572,0.0333949691276236,0.534055237876433
"843","FCGR2B",2.29781961057141,-2.43446948519746,1.14525985757384,-2.
12569179745348,0.0335289303678403,0.534513991949473
"844","AC012511.1",3.94452822965646,-1.52760130925116,0.71907376699487
2,-2.12440138879668,0.0336365939747355,0.534961167368604
"845","RHBDL1",1.87447539634951,2.76827508877339,1.3030557609232,2.124
44867809195,0.0336326432393282,0.534961167368604
"846","BMPER",15.7632705655482,1.27771686664269,0.602004297729981,2.12
243811457935,0.0338009643664705,0.536939905580374
"847","DOCK7",45.9965641214575,0.593548272479812,0.279831185950994,2.1
2109408200042,0.0339138858774279,0.538097653254727
"848","DYNLT1",155.072612917867,-0.569796952884515,0.268710310499098,-
2.12048786600776,0.0339649237455455,0.538271946009889
"849","SNHG18",14.8852863269452,-0.955056830245598,0.451065626446359,-
2.11733453903337,0.0342314655531657,0.539316137830004
"850","DYRK2",49.621182372135,-0.828124596504306,0.390897696288678,-2.
1185200229288,0.0341310509959943,0.539316137830004
"851","GLG1",228.760811624805,0.469979782657453,0.221967526028989,2.11
73357700805,0.0342313611484353,0.539316137830004
"852","CYGB",122.702873225629,-0.703537531522082,0.33220420982766,-2.1
1778632151309,0.0341931682908545,0.539316137830004
"853","SLC25A6",453.741909849818,-0.55345060116779,0.261179157548597,-
2.11904581652849,0.0340865951311045,0.539316137830004
"854","UBAP2L",40.5030203710349,0.595730278068377,0.281923279078331,2.
11309360481316,0.0345927569494791,0.542544689876551
"855","PAM",406.848536848197,0.49222935627191,0.232908603445935,2.1134
0134709181,0.0345664307692826,0.542544689876551
"856","FKTN",16.375037378817,0.981772528718324,0.464321633431237,2.114
42340401682,0.0344791204639092,0.542544689876551
"857","MPEG1",2.63004493087044,-2.31486239475544,1.09551600819502,-2.1
1303383742371,0.034597871807739,0.542544689876551
"858","NRF1",7.32016720352927,1.32767877460124,0.628497033426331,2.112
46625519174,0.034646477369106,0.542673670586732
"859","AZI2",60.6663200463376,0.477008818637531,0.226006934318686,2.11
059373056631,0.0348072470140391,0.544407568440269
"860","N4BP2L2",226.205524282302,0.369471268629082,0.175085440112612,2
.11023411422128,0.0348381954653346,0.544407568440269
"861","GAP43",6.6411951918242,-1.51124369189171,0.716503228024196,-2.1
0919313798356,0.0349279139888598,0.545175651679775
"862","ARPC2",373.016267711792,-0.407693926857236,0.193338859948668,-2
.10870141142593,0.0349703629382529,0.545204997131301
"863","AK1",64.1889204311186,-0.690304173622019,0.327567311099233,-2.1
0736587636151,0.0350858771155113,0.545739702031663
"864","ZNF317",9.0360618722369,1.14688396681536,0.544153310018573,2.10
764860876472,0.0350613956542309,0.545739702031663

"865","AC005162.3",2.73181890962848,2.19007301640745,1.04033248294163,
2.10516642738562,0.0352768235481291,0.546811109184899
"866","AL121839.2",7.97413935848451,1.63510057996779,0.776560329791422
,2.10556799934265,0.0352418948126463,0.546811109184899
"867","IL34",9.69208319040126,1.20139047721236,0.570496668699073,2.105
86764678563,0.0352158507068254,0.546811109184899
"868","SLC25A5",105.119294946558,-0.569468830295759,0.270736647304584,
-2.10340504680585,0.0354303776652881,0.548558577700238
"869","CYP7B1",42.245794544827,0.660638471665865,0.314256336040814,2.1
0222800911185,0.0355333073726024,0.548887491701614
"870","CWF19L1",6.95404045669439,-1.14022331474126,0.542367932388395,-
2.10230591937861,0.0355264863923606,0.548887491701614
"871","KIF9",10.6023465608948,-1.03823252400785,0.494033415179686,-2.1
0154311855652,0.0355933170779353,0.549032975280021
"872","EMX2",96.0056768380303,-0.664945561475731,0.316461659274784,-2.
10118838092281,0.0356244329521674,0.549032975280021
"873","AL391422.4",7.26109490156322,1.35184925946773,0.643988188614832
,2.09918331324594,0.0358007443990593,0.549259427194173
"874","RARG",71.4168903975722,-0.522987244066777,0.249109291983622,-2.
0994288888315,0.0357791103074219,0.549259427194173
"875","RILPL2",35.6157084678033,-0.607693609244895,0.28950676092875,-2
.09906534581572,0.0358111407590624,0.549259427194173
"876","NHLRC4",5.00185232897675,1.56718412079801,0.746672002664263,2.0
9889230506301,0.0358263953381848,0.549259427194173
"877","ZKSCAN2-
DT",2.8469709471323,2.23904048006589,1.06687091376151,2.09869858779036
,0.0358434792506355,0.549259427194173
"878","FKBP11",49.9279180407647,0.648905082497292,0.309363019854073,2.
0975521987191,0.0359447214939805,0.54998145734483
"879","PLEKHG4",22.7034757696471,-0.70036141086884,0.333949190491888,-
2.09720948817767,0.0359750349483058,0.54998145734483
"880","PIK3IP1",71.590820584586,0.621395843977298,0.29635770054522,2.0
9677643885782,0.0360133702257199,0.54998145734483
"881","HILPDA",71.6560276172723,0.853324043888855,0.407124271367858,2.
09597929649798,0.0360840275615859,0.550366727627514
"882","UBN1",46.4479464902686,0.560169471588752,0.267311484183001,2.09
556829666645,0.0361205040380584,0.550366727627514
"883","TUBB2A",83.719334820243,-0.74661320381431,0.356748543807328,-2.
09282761422438,0.0363645458091153,0.552222958996172
"884","SLC29A1",59.6648969316136,-0.678546892174666,0.324226764077738,
-2.09281579238158,0.0363656015113931,0.552222958996172
"885","TSHR",1.56467860503709,3.29751916229009,1.57495069421318,2.0937
2850490248,0.036284172177237,0.552222958996172
"886","UBE2H",92.0105612750031,0.455143282506226,0.217568693342532,2.0
9195208884978,0.0364428017887946,0.552770669570667
"887","ECM1",421.243202406173,-0.620090575610676,0.296599467204329,-2.
09066651891013,0.0365579683005247,0.553857755478733
"888","AL158151.1",5.66941408375469,1.91289731628011,0.915159954141041
,2.09023275944754,0.0365968961131866,0.553857755478733
"889","CLIC3",9.56976336068612,-1.19228182991773,0.570626322729382,-2.

08942662198772,0.0366693368601244,0.554329829092477
"890","MIR17HG",3.60066869027688,-2.01222737657773,0.963424632032722,-2.08861939966403,0.0367419974607617,0.554804161657501
"891","ZDHHC7",65.2360017509318,0.516354488537756,0.247447344456354,2.08672471176523,0.0369130261388121,0.556761120403474
"892","CUX1",48.7296086675065,0.662567943430952,0.317915945174312,2.08409786765388,0.0371512659874441,0.559726304490204
"893","AL512308.1",6.31501408041422,1.44612839168803,0.694323349252583,2.08278807452571,0.0372705450803355,0.56089457484281
"894","TRIB1",3.98103970597977,-1.71334943585695,0.823441917305321,-2.08071680570235,0.0374598349020852,0.563112663589623
"895","MAST4",41.1025511451493,0.708046370786828,0.340578168468166,2.07895407380761,0.0376215718839715,0.56447478442832
"896","RIPOR2",12.7775806913536,1.10916777740422,0.533557977807512,2.07881396874992,0.0376344524776973,0.56447478442832
"897","PRXL2B",2.21923882541281,2.59306164911785,1.24809029364914,2.07762343983649,0.037744055538851,0.564702900503267
"898","RCAN3",3.80244679513953,-1.61656598987372,0.77836757038329,-2.07686708874276,0.0378138280745119,0.564702900503267
"899","BBS10",15.1897680419296,0.899796021736303,0.433145364825574,2.07735345869082,0.037768948414766,0.564702900503267
"900","ACD",16.755575846703,-0.775854882300846,0.373659293823037,-2.07636982440021,0.0378597599042669,0.564702900503267
"901","RPS5",1509.38157936115,-0.536801234510897,0.258492251493625,-2.07666276806806,0.0378326952386438,0.564702900503267
"902","NT5DC3",4.37845189193502,1.8344207986485,0.884265067299619,2.07451460708549,0.0380315435901669,0.565888169552138
"903","LASP1",121.118210764955,0.437703245320235,0.210936329315853,2.07504912378002,0.0379819822173723,0.565888169552138
"904","CTXN1",12.9391635616762,-1.14875883622697,0.553846070904343,-2.07414820935938,0.0380655484243718,0.565888169552138
"905","RPL41",5138.01351203403,-0.546179976608045,0.263465695207257,-2.07305917447199,0.0381667728242645,0.566140463559924
"906","ZNF66",8.9226989980761,1.1660309521913,0.562442665449673,2.0731552277583,0.0381578356003389,0.566140463559924
"907","MCUB",105.021826547135,-0.447114740038036,0.215797860338349,-2.07191461183631,0.0382734050457624,0.567096240804852
"908","RRP9",9.5106230988613,-1.01904686311898,0.492234965858358,-2.07024476886148,0.038429428814848,0.568301304575262
"909","CCNI",666.390197119334,-0.417587036648065,0.201719302009817,-2.07013921071243,0.0384393099083945,0.568301304575262
"910","ZNF638",108.891963012784,0.572395684808729,0.276786731288933,2.06800261755039,0.038639776960924,0.570385557444759
"911","STAT1",61.4384333918755,0.597297538526022,0.28886594408398,2.06773263085791,0.0386651717264808,0.570385557444759
"912","PLEKHA6",3.41441985473202,-1.86501896373592,0.90304961107517,-2.06524529866685,0.038899796416925,0.570972145936714
"913","UBAC1",47.4226309502644,-0.513225457285896,0.24844751289069,-2.06572990534101,0.0388539898829978,0.570972145936714
"914","ZDHHC13",6.25216901771453,-1.18599903978606,0.574328940249933,-

2.0650170253826,0.0389213894090325,0.570972145936714
"915","FAM89B",109.374802691216,-0.602496400610927,0.291759276335395,-2.0650462538107,0.0389186240440925,0.570972145936714
"916","ADGRD1",128.450510093766,0.630161014563704,0.305286575801885,2.06416221515303,0.0390023387134387,0.570972145936714
"917","ZDHHC20",27.7923842762877,0.753645608452797,0.36466158269245,2.06669867137721,0.0387625564529979,0.570972145936714
"918","ZNF805",11.756332854095,0.95535431883674,0.462794178956435,2.06431792420335,0.0389875826464161,0.570972145936714
"919","PIAS2",27.9725641319505,0.73480020349303,0.356072404459475,2.06362580837589,0.0390532086558297,0.571094745512182
"920","SLC25A29",12.586657703101,0.972754015552938,0.471622836473354,2.06256767129193,0.039153721939371,0.571942249068704
"921","ADD1",180.177444303717,0.386102557130919,0.187320011732416,2.06119225362029,0.0392847022667195,0.572610752453843
"922","PPP1R10",66.8902523367932,0.505675366050908,0.245329557758776,2.06120848490715,0.0392831544028473,0.572610752453843
"923","KLF12",33.4890466769121,0.786488832144502,0.381785687992419,2.06002701746148,0.039395958044282,0.572724985270662
"924","MYL9",398.791092925952,0.62921648066017,0.305478776239501,2.05977151148089,0.0394203892682017,0.572724985270662
"925","USP51",13.8286358167101,0.947827520336352,0.460107193737042,2.06001456451483,0.0393971484844067,0.572724985270662
"926","RHNO1",5.88252207851115,1.63466196543873,0.793805009636968,2.05927393452242,0.0394680039898876,0.572797522267926
"927","DHRS3",148.307305406519,-0.584415938144848,0.284413196929875,-2.05481301308582,0.0398970688188479,0.574717684899559
"928","CFH",1023.83323275934,-0.506789042030823,0.246580666808884,-2.05526673518008,0.0398532484340133,0.574717684899559
"929","AC007038.2",2.83724441697173,2.19323957571198,1.06608546797594,2.05728306181308,0.0396590056964412,0.574717684899559
"930","CTDSP1",94.5561185780526,-0.441875837988197,0.215039571031324,-2.05485825640822,0.0398926973940679,0.574717684899559
"931","RARRES1",74.8926203037998,-0.864873037725235,0.420993046718774,-2.054364185979,0.0399404566610623,0.574717684899559
"932","CDK6",30.9969590955141,0.776063514637388,0.377881605817871,2.05372133146759,0.0400026707390783,0.574717684899559
"933","PALM2-AKAP2",25.8269038691112,-0.7514994665949,0.365507453390141,-2.05604416442024,0.0397782594146066,0.574717684899559
"934","TSSC4",47.6389840062184,-0.597549215214324,0.290988655358259,-2.05351378554134,0.0400227741351846,0.574717684899559
"935","BCL2A1",6.67531085750003,-1.48743990343167,0.724357773371255,-2.05346026247352,0.0400279598977593,0.574717684899559
"936","MXRA7",254.79441550975,-0.462853349854649,0.22522977331133,-2.0550273751546,0.0398763606832531,0.574717684899559
"937","GNAI1",34.5050898598315,-0.70415866670408,0.3429952789763,-2.05296897614948,0.0400755864604698,0.574787413492266
"938","POLD2",56.409910814834,-0.589682921326005,0.287398420880743,-2.05179596853351,0.0401894952762197,0.5753113067731

"939","MAOA",26.0014989368556,-1.00412648302211,0.489409256734547,-2.05171126047324,0.0401977317553345,0.5753113067731
"940","GBE1",29.9606460519104,-0.60668348099618,0.295799797742563,-2.05099356262637,0.0402675736480853,0.5756977896347
"941","BCAP29",97.0411874619703,-0.400628091298227,0.195391150201604,-2.05039015781861,0.0403263728591573,0.575701240096828
"942","MBNL3",24.3820103457231,0.765317263916844,0.373305078001413,2.05011211745142,0.0403534911951196,0.575701240096828
"943","TMEM65",31.7112243308558,0.677578075535043,0.330680473582362,2.04904168726576,0.0404580387407623,0.576154308708151
"944","XIST",631.827020882003,0.618142273453388,0.301693343424019,2.04890922165495,0.0404709924414387,0.576154308708151
"945","ATG101",36.0913741654076,-0.567244891300515,0.277022341698961,-2.04765033687044,0.0405942733237135,0.577297819256493
"946","PCNX2",34.9458533889739,0.778664792230966,0.380534227010491,2.04624114458303,0.0407326510996762,0.578653380685569
"947","SLC37A3",56.0110409599843,0.552721645645815,0.270251030189844,2.04521568431226,0.0408335988691845,0.579414337447572
"948","EXOSC8",50.7228154441234,-0.560762322824716,0.274235322629579,-2.04482164240439,0.0408724452638067,0.579414337447572
"949","PDZRN4",4.50692998923665,2.16989500485594,1.06254092271513,2.04217546681511,0.0411341291163202,0.58250954814987
"950","LINC01358",7.40050491894354,-1.11617157262238,0.547187146549936,-2.03983514536104,0.0413667480443184,0.584571742342371
"951","COX6A1",279.365676110115,-0.506170869932066,0.248113540575597,-2.04007757399214,0.0413425999602402,0.584571742342371
"952","DIP2A",33.2540223859804,0.660590023353324,0.3239871800585,2.03893877292937,0.0414561386107325,0.584748138756381
"953","SPIN4",7.1304753645109,1.54593246412708,0.758242136004038,2.03883745141651,0.041466253161309,0.584748138756381
"954","GRAMD2B",15.9501034935953,0.905111742891064,0.44445010616242,2.03647547911779,0.0417026329329786,0.584986343984304
"955","SFRP4",796.759644632103,-1.33252702822304,0.654190740258604,-2.03690903313044,0.041659158744918,0.584986343984304
"956","GPD1",2.10297219071594,-2.81283222439131,1.38150745267626,-2.03606011602925,0.0417443190625007,0.584986343984304
"957","BDKRB1",5.02884572597611,-1.35109028313065,0.663328171706116,-2.03683537163147,0.0416665423700209,0.584986343984304
"958","EIF4A1",442.513803832305,-0.446756611759143,0.219330267692775,-2.0369127182434,0.0416587893884749,0.584986343984304
"959","HSD17B10",60.2122034951617,-0.655707055715247,0.321971097598583,-2.03654011371154,0.0416961493296832,0.584986343984304
"960","ST3GAL5",141.503065000132,0.540374782719905,0.265581555633406,2.03468490660477,0.0418825878563641,0.586222192642365
"961","ADH1B",768.543461945587,0.825446303654148,0.406364599832498,2.03129481257568,0.0422250972898139,0.586222192642365
"962","CD83",12.3058796631476,-1.10785979756592,0.545331308106173,-2.03153529074517,0.0422007233526384,0.586222192642365
"963","UBAP1",28.4331510879638,0.624378092333733,0.307350820612565,2.03148340742776,0.0422059810377194,0.586222192642365

"964","FBXO10",3.47948804777157,1.79401597073827,0.882832949632941,2.03211261143365,0.0421422569482839,0.586222192642365
"965","GANAB",90.7928904724142,0.396868149887455,0.195289125895057,2.03220813278011,0.0421325899242927,0.586222192642365
"966","ECI1",70.2874233892884,-0.566931052222079,0.279079891533468,-2.03142924094942,0.0422114706821437,0.586222192642365
"967","KXD1",60.9409252381744,-0.486135697638383,0.239249914337063,-2.03191586916725,0.0421621737223745,0.586222192642365
"968","OXT",4.96469554797873,-1.38522667863445,0.681242442023528,-2.03338282112877,0.0420138615567693,0.586222192642365
"969","DGUOK",96.4580437273964,-0.484079856341777,0.238558346252658,-2.02918851486792,0.0424390929343773,0.586540806481392
"970","RSPO3",28.1029956433192,0.985438494324093,0.485415579989638,2.03009242996512,0.0423471448638517,0.586540806481392
"971","LERFS",6.18535807371676,2.0372550874421,1.00453118933175,2.02806553850991,0.042553559514797,0.586540806481392
"972","CTSH",211.344868365133,0.810808473452325,0.39935124301308,2.03031413483235,0.0423246183492911,0.586540806481392
"973","MT1F",19.4321911053878,-1.25395136826757,0.61821357964478,-2.02834652869981,0.0425248933196944,0.586540806481392
"974","MRPL4",53.3301526106334,-0.546147613099848,0.269228628727127,-2.02856440521185,0.0425026771296004,0.586540806481392
"975","RPL28",2783.70933167197,-0.487995259180613,0.240619103317745,-2.0280819454979,0.0425518852495062,0.586540806481392
"976","POLR2J",115.590040402304,-0.491442460336955,0.242507077095814,-2.02650770535158,0.0427127839217576,0.588132277791496
"977","CLIC4",103.75239275102,0.472614065754573,0.23364973141584,2.02274602624487,0.0430993389405923,0.588524533677252
"978","MRPL30",19.6547446687472,0.755203213753085,0.373382510300709,2.02259932621073,0.0431144737324566,0.588524533677252
"979","TMEM108",15.4273443861775,1.25958493708841,0.622203930555394,2.02439244632234,0.0429297883537635,0.588524533677252
"980","RWDD4",45.301685595773,-0.529053939165714,0.261529855520279,-2.02291986172374,0.0430814104442795,0.588524533677252
"981","ZKSCAN8",15.3394135901126,0.909917031068562,0.44979413424614,2.02296331096801,0.0430769302976522,0.588524533677252
"982","PLEKHG1",11.4445983381524,0.976840594070302,0.483112702959872,2.02197248816999,0.0431791941517799,0.588524533677252
"983","LANCL2",8.06550693738133,1.10370807724874,0.544945349801481,2.02535552904674,0.0428308704011466,0.588524533677252
"984","SERPINA3",6.82936844063441,-1.09111373029894,0.539177272194903,-2.02366417608293,0.0430047169493219,0.588524533677252
"985","ZNF444",13.239243312122,0.927548840572533,0.458728733654734,2.02199856368853,0.0431765002441201,0.588524533677252
"986","ZNF134",8.35129577310811,1.16245290795926,0.574543531654345,2.02326341506637,0.0430459966587885,0.588524533677252
"987","C14orf93",6.96231043432243,-1.17120222021566,0.579996699104994,-2.01932566516838,0.0434533820233856,0.591661601836149
"988","NBPF12",18.5100394818512,1.02628319988557,0.50850403541031,2.01824003040106,0.043566268969275,0.591999078542049

"989","RBMS3-
AS3",4.86076731636482,1.58791656445368,0.786722612590181,2.01839446209
087,0.0435501956893933,0.591999078542049
"990","AC092338.3",10.2411248510108,-0.954581989742581,0.4732246289555
91,-2.0171857746486,0.0436761300274757,0.592892435797218
"991","CFAP36",81.7006546746138,-0.431825539618153,0.21429027499499,-2
.01514296263911,0.0438896717794777,0.593494242122371
"992","ZNF385B",25.0535183523395,0.962796805361133,0.478067422244497,2
.01393519106753,0.044016338229208,0.593494242122371
"993","CARF",16.6485547266994,0.925549480755484,0.459657358538202,2.01
356393749229,0.0440553358380083,0.593494242122371
"994","SEPTIN2",270.577357120182,0.372738285901952,0.185204962258388,2
.0125718088586,0.0441596952067579,0.593494242122371
"995","FBLN2",1659.93184776947,0.385646819069427,0.191324704314286,2.0
1566661478244,0.0438348488752806,0.593494242122371
"996","ZFAND3",49.5507304816489,0.512705865907345,0.25437152415693,2.0
1557885697552,0.0438440324999219,0.593494242122371
"997","TMEM25",7.27879013220832,-1.21407474084125,0.60290164911639,-2.
01371938958965,0.0440390031270439,0.593494242122371
"998","MNT",21.5487310534107,0.97481907412095,0.484370325060813,2.0125
4912550343,0.0441620836462811,0.593494242122371
"999","CBX1",85.64178733577,0.424748752326165,0.211002449180647,2.0130
0389628427,0.0441142194600993,0.593494242122371
"1000","STXBP4",17.2784632134946,0.872987091091393,0.433390954422196,2
.01431774748339,0.0439761838267875,0.593494242122371
"1001","MAFB",114.623028312219,0.823393048757048,0.409298485109953,2.0
117178018283,0.0442496929690971,0.59407754626543
"1002","NCSTN",63.6292541581828,0.499650730286365,0.248643369503419,2.
00950755809113,0.0444833339503472,0.595429805735773
"1003","EIF3H",225.954486128555,-0.49005419841849,0.243862135275862,-2
.00955428305477,0.0444783839880374,0.595429805735773
"1004","DIO3OS",28.8324481816665,1.11917396450318,0.556846086566447,2.
00984435646139,0.0444476645075375,0.595429805735773
"1005","KLHL8",16.2362298814753,0.802675288035479,0.399679196430823,2.
0082988937214,0.0446115393867041,0.595958725465125
"1006","ZEB1",161.344898504393,0.526199625037937,0.261996365162989,2.0
084233791205,0.0445983205893988,0.595958725465125
"1007","IL33",102.940626679347,0.48565266267033,0.242017117402254,2.00
668724544443,0.0447829749500136,0.597654816636775
"1008","TWISTNB",44.1529833338994,-0.576032446398519,0.287180603512214
,-2.00581947162744,0.0448755124135889,0.598295646156965
"1009","ELL2",15.2224322192084,1.0501031733129,0.523749088673169,2.004
97374796997,0.044965853613864,0.598312976947246
"1010","NAMPT",117.358906155692,-0.670838594766131,0.334538146850086,-
2.00526786282088,0.0449344185398441,0.598312976947246
"1011","CAPN1",85.6072829445141,-0.492244897675808,0.245641569981691,-
2.0039152889004,0.0450791355018088,0.599227004954311
"1012","IVNS1ABP",25.2201376631324,-0.641189228886093,0.32032354729672
8,-2.00169245844463,0.0453178180054034,0.60180450214883
"1013","WDCP",6.86304703757051,1.29054477514487,0.645113081707427,2.00

049388508628,0.0454469595627659,0.602329082410268
"1014","LMCD1",72.1241522271194,-0.676515456253042,0.338133193678668,-2.00073659995636,0.0454207829764111,0.602329082410268
"1015","IL1B",22.6400546266912,-1.8572398067695,0.928791849552178,-1.99962974229908,0.0455402598446887,0.60297098724411
"1016","CITED4",47.4537050277822,0.949465131082621,0.475620917332919,1.99626445448788,0.0459051449501151,0.603639181002541
"1017","GBP1",40.2151144064534,0.590398779488735,0.2956559151688,1.99691177885501,0.0458347674117606,0.603639181002541
"1018","ATL2",15.9265274963514,0.814184619300916,0.407817000426067,1.99644600016747,0.0458853980039494,0.603639181002541
"1019","GPR146",4.65015409777327,1.67427543193412,0.838201468401722,1.9974618215912,0.0457750378478971,0.603639181002541
"1020","PRF1",2.1290982921083,2.5137188612424,1.25897222887172,1.99664361420837,0.0458639114163336,0.603639181002541
"1021","ZBTB16",162.604335031171,-0.799309826598014,0.400245383391494,-1.99704945957161,0.045819810399915,0.603639181002541
"1022","G6PD",30.7998795678377,-0.586542196699555,0.293713171541465,-1.99698976256756,0.045826295108112,0.603639181002541
"1023","TMEM131",43.9927918367433,0.563689272101841,0.282679396361482,1.99409394302304,0.0461417890517776,0.604781148963842
"1024","DNASE1L3",30.1212044636853,1.73467606021482,0.87026292565778,1.99327813362115,0.0462309994381278,0.604781148963842
"1025","DMGDH",4.63650045043068,1.578012209182,0.791839821256685,1.9928427023001,0.0462786741371568,0.604781148963842
"1026","NAT1",3.84324517342354,-1.46206449629924,0.733937226629765,-1.99208385029471,0.0463618585959472,0.604781148963842
"1027","PBX3",34.8622369864195,0.585467497815077,0.293496731675156,1.99480074096047,0.0460646165320484,0.604781148963842
"1028","TMTC1",27.7201702431711,0.707935536173934,0.354981056650173,1.99429102739866,0.0461202592359353,0.604781148963842
"1029","ZNF668",17.8953869834864,-0.883091217971197,0.443314018386171,-1.99202186564273,0.0463686588376701,0.604781148963842
"1030","CCL3L1",5.55281233453709,-1.86752939544919,0.937626047522106,-1.99176356116019,0.0463970060705202,0.604781148963842
"1031","CHRDL1",442.231289124157,0.40247806672771,0.202054891603105,1.99192439012215,0.0463793544270928,0.604781148963842
"1032","USF2",66.8848096979772,-0.438987128168171,0.220456736501346,-1.9912620278015,0.0464520877517147,0.604912410169858
"1033","CYB561A3",37.7583075962845,-0.600681131129734,0.301916487286359,-1.98956054546304,0.0466393660194512,0.606763252599617
"1034","UBR5-AS1",1.98686667680555,2.59417536572213,1.30422245288976,1.98905896764331,0.0466946946659294,0.60689555281956
"1035","TICAM1",11.2506947695459,-0.909816928144984,0.457558658842636,-1.9884159343554,0.0467657079434117,0.607231255122232
"1036","GABPB2",38.0878667712631,0.690460894127672,0.347449035865796,1.9872292706386,0.0468969955675323,0.608348188640991
"1037","TMTC4",5.87391801223462,1.3685868750307,0.688938054916926,1.98651658921023,0.0469759926725098,0.608785309089546

"1038","AGTR1",209.696629234888,0.527156655657536,0.26545047366127,1.9
8589457531057,0.047045031207481,0.609092653561982
"1039","HMCES",16.2904500494364,-0.782615118073779,0.39430517240056,-1
.98479546517018,0.0471672322452197,0.610087039599141
"1040","KANSL1L",21.5222676633963,0.757056196061429,0.381917924294569,
1.98224840444388,0.0474514455770526,0.610359669400555
"1041","ZIC1",9.44296813984434,-0.963373615442562,0.486116079680586,-1
.98177689591253,0.0475042164187566,0.610359669400555
"1042","SERPINE1",19.3194853585269,-2.32907334212638,1.1744710845096,-
1.98308274494376,0.0473581876620687,0.610359669400555
"1043","ZDHHC12",20.1255899018381,-0.723455860485056,0.364965049983013
,-1.9822606589829,0.0474500747167049,0.610359669400555
"1044","CREB3L1",53.5595635954646,0.961836219126622,0.485206655688182,
1.98232280586181,0.0474431231365819,0.610359669400555
"1045","AC007686.3",2.81139243553328,2.05199733004371,1.03544245917646
,1.98175892040951,0.0475062291980787,0.610359669400555
"1046","CISD3",53.8129023381486,-0.544603151243809,0.274605265120648,-
1.98322181114967,0.0473426586147298,0.610359669400555
"1047","DCHS1",26.9847315161042,-0.622108936987687,0.314241994590917,-
1.9797129209212,0.0477357958615488,0.61192698745525
"1048","HSPA8",285.573908009111,-0.447329559600944,0.225902678196911,-
1.98018705741516,0.0476825136284917,0.61192698745525
"1049","RPL6",1966.31538437627,-0.516840829771624,0.261155996244659,-1
.97905021214766,0.0478103532128888,0.61192698745525
"1050","IL17D",43.466617510849,-0.635532221221094,0.321098075527897,-1
.97924643483538,0.0477882671938642,0.61192698745525
"1051","INTU",23.30120370122,0.780418901730787,0.394590412950983,1.977
7948883612,0.047951850389059,0.612315675704692
"1052","ERBIN",46.2061254290445,0.540455197712717,0.27329278696039,1.9
775684668584,0.0479774095183452,0.612315675704692
"1053","MON2",46.1342495222097,0.566866688912002,0.286644616401261,1.9
7759405367122,0.047974520629569,0.612315675704692
"1054","STARD4-
AS1",3.88247501061044,1.71930251205601,0.869636734388382,1.97703528849
341,0.0480376414920373,0.612502717278453
"1055","KIF3C",3.25298130887891,1.91606374457929,0.969821626809045,1.9
756867568355,0.0481902657453771,0.613866333035187
"1056","SOS1",49.1835106656536,0.528269376322768,0.267611405930735,1.9
7401666975098,0.0483798475067879,0.615697699473222
"1057","TTC21B",17.9953184911908,0.771823384577999,0.39171805488878,1.
97035437847546,0.0487977702762818,0.617053170426568
"1058","IGF2BP2",13.2150950037039,-1.04096751779823,0.52825269528679З,
-1.9705862877483,0.0487712163341348,0.617053170426568
"1059","DNAH5",1.98898247357668,-2.17655298679285,1.10466158310624,-1.
97033464373091,0.0488000304929206,0.617053170426568
"1060","GANC",17.1062034528044,0.751426617914712,0.38134671996201,1.97
045517525251,0.0487862274108155,0.617053170426568
"1061","RASD1",190.258073826964,1.26804106689428,0.643588395694316,1.9
7026713871416,0.0488077624944893,0.617053170426568
"1062","RINL",10.1266205033044,-0.958281733448789,0.486170561680038,-1

.97108136316871,0.0487145700830518,0.617053170426568
"1063","ZNF217",47.7346802558117,0.513605307678591,0.260394580091126,1
.97241166655179,0.0485626311014161,0.617053170426568
"1064","DNAJB11",51.4911350840328,-0.502353479660469,0.255350192610991
,-1.9673119276858,0.0491472607885771,0.62076131366324
"1065","LGALS1",2719.27060449599,-0.563484253796418,0.286543024953196,
-1.96649090965819,0.0492419313603597,0.621373066245891
"1066","KIDINS220",163.230547126303,0.427423296976288,0.21743976893228
1,1.96570893666376,0.0493322419271598,0.621840108824219
"1067","EEF1D",731.252643821055,-0.478357866521441,0.243488880215967,-
1.96459840834273,0.0494607363449544,0.621840108824219
"1068","ELL",9.59910786782976,1.0092437601778,0.513722476577702,1.9645
6998903599,0.0494640283006987,0.621840108824219
"1069","POLR2I",90.3198936842608,-0.57834468199387,0.294328828979957,-
1.96496104033782,0.0494187469320476,0.621840108824219
"1070","NNMT",673.73357680242,-0.461713699945525,0.235207582744711,-1.
96300516572486,0.0496455738006312,0.623539127389423
"1071","TRIM24",12.8944679643136,-0.834012022654419,0.424993729869223,
-1.96241018170093,0.0497147483172506,0.623824932432802
"1072","SORT1",11.6483242531611,-0.874777675045994,0.446379136674273,-
1.95971899933202,0.0500286432310314,0.62431637054231
"1073","CYP2U1",37.5244270544259,0.532380095852728,0.27166619010695,1.
95968477212104,0.0500326461101323,0.62431637054231
"1074","ARHGAP18",42.167058513177,-0.658278749295822,0.335646202340256
,-1.961228057121,0.0498524253052118,0.62431637054231
"1075","PLXNA4",7.7035789079109,1.38682041998263,0.707415755200082,1.9
604036378726,0.049948630997868,0.62431637054231
"1076","SLC39A4",10.3173186423061,-0.872714577132494,0.445074407245148
,-1.96082848828421,0.0498990336119222,0.62431637054231
"1077","ARNTL2",3.15868687892733,-1.92092205718234,0.97940887462934,-1
.96130758760922,0.0498431527094261,0.62431637054231
"1078","AC007563.2",10.2140729479352,1.05042315080714,0.53677792461476
5,1.9569045272512,0.050358694197709,0.624320426836507
"1079","KDM3B",25.5862967325909,0.729406972195288,0.372738352148316,1.
95688736614109,0.0503607122514613,0.624320426836507
"1080","AC004951.1",6.0095659998185,1.38061725341044,0.705530589289691
,1.95684960279385,0.050365153255551,0.624320426836507
"1081","TCAF2",1.67294089785419,2.57353090299566,1.31502339057404,1.95
702291034705,0.0503447748319383,0.624320426836507
"1082","ARHGAP27",5.74976641239132,1.2858197036182,0.657402418096021,1
.95590960456491,0.0504758037091355,0.624320426836507
"1083","LSM7",138.711092914415,-0.511370501947928,0.261413345375349,-1
.95617596038825,0.0504444293783121,0.624320426836507
"1084","AC020910.5",3.59798051815789,-1.54336438856761,0.7881570580606
79,-1.95819395738862,0.0502072579133334,0.624320426836507
"1085","AL035681.1",5.77058541170884,1.39841253298472,0.71492418686749
,1.95602912682532,0.0504617230353614,0.624320426836507
"1086","UXT",143.119580415975,-0.568439158116871,0.290653894895681,-1.
95572523919176,0.050497529873598,0.624320426836507
"1087","MT-

CO3",11133.5975518602,-0.32939905638307,0.168328322928566,-1.956884323
75613,0.0503610700265533,0.624320426836507
"1088","TMOD2",63.6618274558617,0.527085360264615,0.269653571301255,1.
95467598563996,0.0506213262817773,0.625275738879416
"1089","GALNT8",8.11818135803635,1.29862934777312,0.664711237212394,1.
95367443044771,0.0507397320757078,0.626162772603707
"1090","TRPS1",13.756935808571,0.922139727458137,0.47211749829535,1.95
319963947038,0.0507959437919075,0.626281365705913
"1091","TLR3",21.2041071860597,0.676933276607435,0.34667177856177,1.95
266335037658,0.0508594991520222,0.626490200828622
"1092","RASSF8",67.1186533466193,-0.447230995878066,0.229143149537078,
-1.95175372592013,0.0509674505908124,0.627245026089677
"1093","CYP27C1",17.2455562856072,0.757492453939067,0.388544644666721,
1.94956349118855,0.0512281684191229,0.62784139711813
"1094","COX17",85.2198111103973,-0.445840619469048,0.228663347793779,-
1.94976861736115,0.0512037036262953,0.62784139711813
"1095","TAPBP",120.68826838865,0.41054387607301,0.210557356596762,1.94
979592595875,0.0512004473484528,0.62784139711813
"1096","RWDD2A",8.9774371351553,-0.928590687861982,0.476637496325836,-
1.94821157592517,0.0513896522680217,0.62784139711813
"1097","TNKS1BP1",84.8769182492556,0.531046689114207,0.272470418707087
,1.94900676423556,0.0512946169275667,0.62784139711813
"1098","PLCB3",14.0929154289495,-0.909903020476799,0.467044109113029,-
1.94821645905097,0.0513890682215555,0.62784139711813
"1099","AC020765.2",1.69192211451446,-2.46978076540167,1.2676713248745
8,-1.94828163810206,0.0513812730104421,0.62784139711813
"1100","RIPOR3",24.9291505767576,0.641251865773095,0.329133155379933,1
.9483052840206,0.0513784452770637,0.62784139711813
"1101","PALMD",8.41293257508099,-1.38901005298165,0.71342917464105,-1.
94694876850321,0.0515408770274823,0.628107764135524
"1102","PCDHGB6",6.12051208982733,1.22360074479271,0.628516725584747,1
.94680697423656,0.0515578805258182,0.628107764135524
"1103","HSDL1",17.2572991351575,0.730087442258764,0.37489969471515,1.9
4742074360313,0.0514843131448243,0.628107764135524
"1104","PLEKHM1",30.531203205492,0.64881462938155,0.333329000529469,1.
94646918915233,0.0515984055067801,0.628107764135524
"1105","EDEM3",49.7530148696752,0.579788652839094,0.298018997145975,1.
94547548442055,0.0517177775113938,0.628991142059385
"1106","CLTC",149.994243140508,0.351010189131947,0.180571407805991,1.9
4388576462271,0.0519092282673499,0.630748751071353
"1107","C12orf76",49.3136462489972,-0.505922938786946,0.26038169344501
7,-1.94300502502023,0.0520155511734577,0.630899812473012
"1108","LINC01697",15.3619523046273,1.15819983344001,0.596002249102329
,1.9432809778561,0.051982218561336,0.630899812473012
"1109","PLEKHO2",16.6989326021438,0.808426478003696,0.416162564550361,
1.94257376051388,0.0520676798370411,0.63096262338142
"1110","GPR135",4.96856250229367,1.43402177840275,0.738516933004004,1.
94175883357163,0.0521663026130754,0.631588234970379
"1111","PTPN12",93.8389950132558,0.410264904000137,0.211348947423095,1
.94117315937626,0.0522372775798021,0.631878283883853

"1112","AURKAIP1",148.092898053376,-0.538250308550796,0.277558157593849,-1.93923433278592,0.0524728108105952,0.632044628797166
"1113","TTC27",22.807146078842,0.794860582100595,0.409907625907249,1.93912123576947,0.052486577466262,0.632044628797166
"1114","PDLIM7",73.9655169373784,-0.548656056034106,0.282996450522509,-1.93873829520158,0.0525332130639508,0.632044628797166
"1115","EGR2",3.59361018152476,-1.80533290117914,0.93107304790505,-1.93898094810199,0.0525036580807244,0.632044628797166
"1116","VEGFB",279.515151017955,-0.487357078556782,0.251376682730675,-1.93875212793279,0.0525315278715184,0.632044628797166
"1117","FRMD6-AS1",9.00478477703087,1.173083024782,0.604911061818036,1.9392652884481,0.0524690432804162,0.632044628797166
"1118","SEC63",149.999264820864,0.383463750988355,0.197895686494689,1.93770646435311,0.0526590448267652,0.632649280946836
"1119","PLPP7",5.68892177002479,1.28998868241556,0.665938230167402,1.9370996047055,0.0527331689457544,0.632649280946836
"1120","CHMP2A",203.414830264333,-0.472473131363587,0.243947270408566,-1.93678384091892,0.0527717720024856,0.632649280946836
"1121","JAG1",96.3681451476326,0.666505586495696,0.344084195824916,1.93704213847369,0.0527401926051903,0.632649280946836
"1122","TRMT1L",18.5316072467376,0.760618550775589,0.393000225724576,1.93541504810394,0.0529393841736665,0.633124638642238
"1123","COL8A1",22.9014311137881,-1.24318392207654,0.64249820864421,-1.93492200499654,0.0529998674360085,0.633124638642238
"1124","PDS5B",70.5273644969126,0.448931924315856,0.231959920403535,1.93538574911933,0.052942976766416 7,0.633124638642238
"1125","APOL6",74.7879214489679,0.478283810460702,0.247142861882593,1.93525237515423,0.0529593334347537,0.633124638642238
"1126","NDUFA4L2",5.42818968268119,-1.85651809093674,0.959855457668235,-1.93416422869205,0.0530929389724745,0.633586685112543
"1127","SRPX2",152.078267028969,0.411336414019797,0.212704501112453,1.93383972538659,0.0531328368272815,0.633586685112543
"1128","ESCO2",1.60888401155816,-2.67471802714245,1.38467378093794,-1.93165932941308,0.0534015677522095,0.635663125794458
"1129","LRFN4",15.4999685597133,-0.867021828452732,0.448780924609448,-1.93194893300614,0.0533658092479001,0.635663125794458
"1130","ZNF510",9.37882634241638,0.999145304891613,0.517375372270784,1.93118064454116,0.0534607167462798,0.635804046330313
"1131","STAB1",3.90237385808046,-1.85253156633001,0.95975478091753,-1.93021342864162,0.0535803983564397,0.636663990726962
"1132","DDX60L",49.2465073980446,0.559248839909651,0.289877425973701,1.92925971393298,0.0536986283366495,0.637461073487344
"1133","AGA",15.8162411703911,-0.755399550726548,0.391620412508077,-1.9289074997105,0.05374234662260 3,0.637461073487344
"1134","SLC40A1",32.3822907076178,-0.65435706081561,0.339611130524894,-1.92678331774419,0.0540066393350846,0.638342327198066
"1135","MELTF",26.4963611497628,1.08211497368309,0.561566722739315,1.92695708250757,0.0539849786966447,0.638342327198066
"1136","MSANTD3",22.2896976145739,-0.671558523286103,0.348536863314964

,-1.92679338678512,0.0540053839813003,0.638342327198066
"1137","PHF19",27.7411577296055,-0.627723326085759,0.325625983381471,-1.92774335624925,0.0538870563986213,0.638342327198066
"1138","SKIL",53.0363441266129,-0.73001258489281,0.379272750242681,-1.92476940256242,0.0542582134829235,0.639066094517044
"1139","NCOA2",46.4958116716794,0.615568668981475,0.319653630770801,1.92573651516836,0.0541372819898337,0.639066094517044
"1140","DBX2",3.21772477620375,2.04735314106932,1.06389685937784,1.92439062398078,0.0543056388078328,0.639066094517044
"1141","STARD9",43.5451994620846,0.763465110850432,0.396496374564931,1.92552860461377,0.0541632609305029,0.639066094517044
"1142","XYLT1",24.3373894133749,-0.750806394356946,0.390148684311603,-1.92441093497907,0.0543030948730889,0.639066094517044
"1143","ZBED3-AS1",2.2722263978661,2.34311964997926,1.21797659541799,1.92378052155849,0.0543820999672651,0.639405985529375
"1144","BTN3A2",52.1371708793702,0.653628329453526,0.339834547340947,1.92337222500736,0.0544333199316576,0.639448764476877
"1145","CNST",24.2487325959656,-0.646403653128993,0.336232125898942,-1.92249224074227,0.0545438490029549,0.639826436979668
"1146","PDGFA",10.1174880277454,-1.21740134908056,0.633410061704102,-1.92197980847559,0.0546082984757556,0.639826436979668
"1147","C9orf92",3.97131895223672,-1.98142647437075,1.03087079375461,-1.9220900294925,0.0545944304327565,0.639826436979668
"1148","LAPTM5",8.33226559468007,-0.984541702326581,0.512899234956148,-1.91956165115114,0.0549132919246325,0.641951497720364
"1149","KCNK15-AS1",11.7009820148384,-0.927811354304095,0.483374882829669,-1.9194446945044,0.0549280791810809,0.641951497720364
"1150","TMSB4X",6867.75691018814,-0.500629402788545,0.260825174125718,-1.91940599471139,0.0549329728683994,0.641951497720364
"1151","HES1",43.524536238686,-0.570263294560856,0.297524819948852,-1.91669150378409,0.055277135282036,0.642137414670706
"1152","CCM2",47.0360767976407,-0.523343796760665,0.273036593774178,-1.91675331693271,0.0552692782231314,0.642137414670706
"1153","DCTN3",172.217066059455,-0.56679515664765,0.295562111869968,-1.91768543356805,0.0551509098426687,0.642137414670706
"1154","CREBZF",69.9359388621259,0.476897208802225,0.248578539625536,1.91849710566581,0.0550480087666504,0.642137414670706
"1155","NF1",74.5156694405759,0.511382223413535,0.266809082943623,1.91665972451766,0.055281175101567,0.642137414670706
"1156","CD37",3.80567772481599,-1.40305099768107,0.732035803778216,-1.91664258829907,0.0552833535809217,0.642137414670706
"1157","AL354822.1",5.9379377017931,1.60682415749693,0.837517760553181,1.91855532285742,0.0550406343416736,0.642137414670706
"1158","LUM",2923.95206151721,-0.536124205562399,0.279868375155492,-1.91562982157071,0.055412230905973,0.642662899882868
"1159","PIM3",17.8596713292627,-0.653187932083133,0.340994934912945,-1.91553558486108,0.0554242355059338,0.642662899882868
"1160","RENBP",10.6062656659418,1.06128752087677,0.554251823151179,1.9

148110597866,0.0555166035106107,0.643178995326808
"1161","MME",68.1919199095203,0.680515054961913,0.355489469839083,1.91430439632982,0.0555812730322633,0.643373581637026
"1162","PLD1",22.3599529799688,0.715499721718673,0.374016416850554,1.91301688771744,0.0557458905364804,0.644723771875869
"1163","COPRS",113.197735000399,-0.536922548393234,0.280747330494055,-1.9124760596952,0.0558151604374881,0.644969854788824
"1164","MVB12B",4.32776135873875,1.47797147155675,0.772968566381216,1.91207189507864,0.0558669731246405,0.64501396204643
"1165","ZFP36L2",1127.29183226316,-0.290004922108072,0.151783169510772,-1.91065269649341,0.056049227535315,0.645454643399399
"1166","ZNF665",6.80266012738827,1.23691581309339,0.647155791250691,1.91131073818088,0.0559646599181645,0.645454643399399
"1167","MKKS",23.062087773504,-0.654247606899343,0.342395659991989,-1.9107940997694,0.0560310462530307,0.645454643399399
"1168","COLGALT2",2.70452067655088,-1.8336024904833,0.960488397456474,-1.90903137959706,0.0562580437749823,0.647304666345879
"1169","C15orf61",110.641749555201,-0.534399901532494,0.280023500601953,-1.90841090259825,0.0563381288115866,0.647671610863055
"1170","SNHG8",121.379022597549,-0.55162225564427,0.289186408191917,-1.90749717143756,0.0564562369732384,0.647920895545133
"1171","PCNX4",53.851323115635,0.520754939064826,0.272978877925361,1.90767484657627,0.0564332546979463,0.647920895545133
"1172","UAP1",210.610838885623,-0.423051819704089,0.22266412076353,-1.89993549435297,0.0574415853319431,0.649176372156273
"1173","TGFBR2",380.385853210985,0.351380049459917,0.184999515975337,1.89935658808297,0.0575176072523219,0.649176372156273
"1174","SHPRH",71.1636461258705,0.497213263425662,0.261117055452535,1.90417765918796,0.0568870502947064,0.649176372156273
"1175","DPY19L1",19.5633943097589,0.698847557231164,0.367747195542254,1.90034775438788,0.0573874983313356,0.649176372156273
"1176","SEC61G",237.328153032489,-0.462681901087433,0.243185669666731,-1.90258703040153,0.0570944528111121,0.649176372156273
"1177","AKR1B1",72.4889935391479,-0.487093566827311,0.256167810829284,-1.90146281552884,0.057241418560382,0.649176372156273
"1178","KMT2C",107.237226612586,0.534492704983134,0.281266005127535,1.90031036541646,0.0573924018799929,0.649176372156273
"1179","C9orf16",132.748963277322,-0.581892434002678,0.306004242143588,-1.90158289939534,0.0572257053081684,0.649176372156273
"1180","FIBP",82.240959449502,-0.506575995658982,0.26584176290604,-1.90555460557199,0.0567080164391692,0.649176372156273
"1181","AGAP2-AS1",7.610680709349,-1.03431886583967,0.543274293843479,-1.90386123098559,0.0569282594316305,0.649176372156273
"1182","MRPL57",106.977834384298,-0.512628835709937,0.268920842249829,-1.90624434841574,0.0566185108448897,0.649176372156273
"1183","GOLGA8B",6.35333613986568,-1.21263574035287,0.637398493133982,-1.90247663497059,0.0571088706133277,0.649176372156273
"1184","SECISBP2L",51.850339587781,0.569984513949865,0.299654055541807,1.90214183125028,0.0571526149825488,0.649176372156273

"1185","AC007336.1",1.69285395398191,-2.23897390638344,1.1755150698540
8,-1.90467477942361,0.0568223593662928,0.649176372156273
"1186","RABEP1",53.8945620215526,0.504998419916703,0.265351283244309,1
.90313162891981,0.0570233717646855,0.649176372156273
"1187","NFE2L1",117.924234497651,0.46547884722589,0.244989149763177,1.
89999780674308,0.0574334074572823,0.649176372156273
"1188","ELOF1",54.2530268758872,-0.577957739195385,0.303491291872842,-
1.90436350126823,0.0568628592451415,0.649176372156273
"1189","HAS1",17.027065132082,-2.14752992243756,1.13064351891639,-1.89
938728388569,0.0575135741830259,0.649176372156273
"1190","NCOA6",25.6368142967951,0.76365029464543,0.402157931695924,1.8
9888159466374,0.0575800458077445,0.649176372156273
"1191","SCO2",31.9008507525188,-0.604242386626602,0.318153635015284,-1
.89921572512467,0.0575361180123843,0.649176372156273
"1192","LINC00632",72.3024741180274,0.489543728048737,0.25703056426463
4,1.90461289866178,0.0568304086564475,0.649176372156273
"1193","CLSTN1",90.5562109022205,0.48271608264144,0.254899325017798,1.
89375190619958,0.0582579480504455,0.652002502697625
"1194","MRPS18C",79.9205956580243,-0.46037108502378,0.243088654862365,
-1.8938402752051,0.0582462139622869,0.652002502697625
"1195","DDX60",18.495583677464,0.730459942470411,0.385768881066382,1.8
9351702099765,0.05828914685582,0.652002502697625
"1196","AC010359.3",4.0116817912024,1.88724936029085,0.996782375678469
,1.89334142169826,0.0583124800323659,0.652002502697625
"1197","EPB41L4A-
AS1",107.94296262667,-0.504602133839677,0.266471497780275,-1.893643928
31145,0.0582722885721311,0.652002502697625
"1198","GSDME",18.7969523356119,0.830250201250232,0.438557259033818,1.
89313979907515,0.0583392806891751,0.652002502697625
"1199","IFT22",50.4049699651238,-0.578223158950649,0.305520097693552,-
1.89258632514129,0.0584129037315233,0.652002502697625
"1200","ARL3",78.9619475180124,-0.438601401109932,0.231418560348657,-1
.89527322462439,0.0580562135276553,0.652002502697625
"1201","HSPA12A",32.6547343916556,0.655290732194661,0.345881758991162,
1.89455128858473,0.0581518734111877,0.652002502697625
"1202","ST5",60.763345787889,0.505291290291616,0.266712896591678,1.894
51390146007,0.0581568309437788,0.652002502697625
"1203","NAT14",14.8418360041588,-0.711420688166625,0.375266708614869,-
1.89577351743383,0.0579899991386945,0.652002502697625
"1204","HIC2",10.0215263409978,0.997467695991441,0.527004885514323,1.8
9271052965283,0.0583963753481461,0.652002502697625
"1205","STRAP",82.2811569532762,-0.470425354789114,0.248826328507033,-
1.89057708487555,0.0586808215698263,0.654449428279582
"1206","AP2M1",239.452573252369,-0.355628988338245,0.188314212483793,-
1.88848724505513,0.0589605686514225,0.654852133972287
"1207","MANBA",28.8328064005656,0.589330896467887,0.311874203167872,1.
88964297297352,0.0588057257669787,0.654852133972287
"1208","FAM53C",37.2381374994458,0.72128224906705,0.381908846126981,1.
88862409546604,0.0589422159674805,0.654852133972287
"1209","NUP98",20.6745883429323,0.771517952558111,0.408385308020533,1.

88919125493937,0.0588662061050034,0.654852133972287
"1210","IQSEC2",11.123485139828,0.9134849623913,0.483472003926899,1.88942680232095,0.0588346623244616,0.654852133972287
"1211","PRADC1",27.311415454954,-0.682021093608357,0.36129318849993,-1.88772198125315,0.0590632838204565,0.655451256204059
"1212","WDR46",38.3714729103497,-0.58960929820461,0.31248552244112,-1.88683716800258,0.0591822302525106,0.655773821864586
"1213","ETNK1",37.2544810807703,0.568852768173277,0.301493981812772,1.88677984466879,0.0591899431447089,0.655773821864586
"1214","TGFBI",23.4683891297775,-0.973472051241763,0.516433038296754,-1.88499181704634,0.0594309426085615,0.656819438911561
"1215","AHSA1",68.4705162799362,-0.447536585888038,0.237388417705813,-1.88525030080724,0.0593960525887678,0.656819438911561
"1216","BTBD3",7.80106544759273,1.10727654451632,0.587355397453356,1.88519003880313,0.0594041852068109,0.656819438911561
"1217","PRELID1",157.059406270951,-0.485451170253181,0.257689893636103,-1.88385801011937,0.0595841843015803,0.657971941519259
"1218","KAT6B",77.6442933642552,0.456317929120538,0.242388013604094,1.88259279960051,0.0597555729301971,0.658781906980245
"1219","ZFYVE26",7.40329897325947,1.16785223995111,0.620330939723735,1.88262774781346,0.0597508332708664,0.658781906980245
"1220","ARHGEF4",8.03545239254259,1.23557435069321,0.65651768481703,1.88201228888078,0.0598343471289601,0.659109664808274
"1221","SELENOW",341.411810359118,-0.442803460195576,0.235661140362158,-1.87898377948561,0.0602467080620398,0.663108525508397
"1222","PRDX1",516.500110304067,-0.423764348082913,0.225739759011345,-1.8772251283462,0.0604872448877466,0.663524293722261
"1223","CCT4",114.866406120471,-0.374875291471911,0.19961522841393,-1.87798944224113,0.0603826094055603,0.663524293722261
"1224","UPP1",15.293463169377,-0.925066186978665,0.492759192617629,-1.87731898427818,0.0604743878103533,0.663524293722261
"1225","SLC30A4",18.5851860642399,0.731418268796641,0.389689790828016,1.87692437936985,0.0605284589614295,0.663524293722261
"1226","ESS2",11.943201528569,-0.91145202192973,0.485614799483292,-1.87690330463474,0.0605313478758459,0.663524293722261
"1227","TMEM161B-AS1",42.4661468735689,0.568930347294032,0.303351729314142,1.87548081093965,0.0607266069524439,0.664580513708383
"1228","KCNK5",1.59717611534779,-2.20199239714679,1.17398294533402,-1.87565961319845,0.0607020349585143,0.664580513708383
"1229","C2CD5",17.0678291758744,0.751154538098794,0.400652610405538,1.87482751538416,0.0608164565166463,0.665022261291464
"1230","AC099791.2",3.04862743998736,1.94594631853828,1.03881138540733,1.87324315643233,0.0610348153747943,0.666113192770861
"1231","ZNF783",11.4669475942539,0.842736482951662,0.449850717075293,1.87336921108122,0.0610174185770039,0.666113192770861
"1232","TUBB3",12.4714493883494,-1.34623453223142,0.718748795610534,-1.87302509646347,0.0610649195247936,0.666113192770861
"1233","NMB",24.3700731808112,-0.631857651885144,0.337649945111965,-1.87133941833048,0.0612980501584961,0.668113946536926

"1234","SEC14L2",4.94157974021524,-1.35431448388854,0.724192218949135,
-1.8701036112398,0.0614694311097553,0.669438966518639
"1235","KLHL23",4.22862409509654,1.54445262043892,0.826550061117171,1.
86855302914312,0.0616850258404548,0.671038624669347
"1236","SGK1",27.9867311423136,-0.901907827710196,0.483035607349699,-1
.8671663413361,0.0618783623522039,0.671038624669347
"1237","DHX37",6.53974445935544,1.16963971005548,0.626039709767564,1.8
6831552664566,0.0617181036769845,0.671038624669347
"1238","ZSCAN29",11.2656631610255,1.01252833407748,0.542353337653513,1
.8669163878629,0.0619132649659417,0.671038624669347
"1239","POLR2E",148.555221554037,-0.491385399441483,0.263209635090113,
-1.86689746092787,0.0619159085192343,0.671038624669347
"1240","RTF2",122.186256188881,-0.442536880814944,0.237020947365615,-1
.86707920010256,0.0618905285946521,0.671038624669347
"1241","ADAM23",5.11999076980017,1.60941397149547,0.862569014447488,1.
86583791504076,0.0620640459274709,0.672102105736729
"1242","ARHGAP35",35.4243063253962,0.540859011394386,0.290060698506444
,1.86464079476927,0.0622317706447439,0.673375817789625
"1243","CAMK2N1",137.772214646426,-0.492430482247174,0.264967010164397
,-1.85845959442894,0.0631037761785925,0.67380694967305
"1244","ZMYM4",54.7097866066033,0.435160727090688,0.234820768240434,1.
85316115925966,0.063859262921155,0.67380694967305
"1245","RRNAD1",18.4651662901805,-0.693821180009971,0.374563712163556,
-1.85234489481728,0.0639763128047333,0.67380694967305
"1246","PRG4",518.55911329373,-1.35852001327478,0.730852553478507,-1.8
588154434282,0.0630533026794841,0.67380694967305
"1247","COL6A3",1958.7515029334,0.378580098629581,0.204183912290219,1.
85411325693222,0.0637229584807286,0.67380694967305
"1248","LRRC2",19.8195723736994,-0.876261992427143,0.472891103557755,-
1.85298895630445,0.0638839415790882,0.67380694967305
"1249","IMPDH2",101.907627975731,-0.459702226176448,0.247875690420872,
-1.85456760764201,0.0636579973672126,0.67380694967305
"1250","GNAI2",327.448714955,-0.321600485062498,0.173500164650826,-1.8
5360334216241,0.0637959291062538,0.67380694967305
"1251","PRRC2A",47.3222146527792,0.507218065059746,0.272676921136052,1
.86014299613816,0.0628652974456581,0.67380694967305
"1252","SNX13",38.7879596295383,0.490815331505175,0.264780377530712,1.
85366958111632,0.0637864461766408,0.67380694967305
"1253","R3HCC1",49.6665592036872,-0.636140449105334,0.342582793249858,
-1.85689550566941,0.0633260221234395,0.67380694967305
"1254","UBR5",53.401321267123,0.571273163949765,0.30804516072961,1.854
51108076717,0.0636660763562382,0.67380694967305
"1255","NUDT2",42.2278694561022,-0.509995097775347,0.274526627851474,-
1.85772543001281,0.0632080153323064,0.67380694967305
"1256","TRMT112",227.22504370988,-0.468288569585542,0.251703412210779,
-1.86047763704289,0.0628179795081296,0.67380694967305
"1257","CFL1",495.159880883851,-0.472377110155656,0.25424986156833,-1.
8579247486776,0.0631797013291149,0.67380694967305
"1258","CEP295",15.5713496395159,0.792090187380228,0.425513817565685,1
.86149110717881,0.0626748553008944,0.67380694967305

"1259","AC022075.1",2.02202231025217,2.20143909971113,1.18748082258903
,1.85387339132887,0.0637572755134154,0.67380694967305
"1260","CSRP2",5.23773398394974,-1.29397497303569,0.69654579803049,-1.
85770264739871,0.0632112523603179,0.67380694967305
"1261","N4BP2L1",39.0750383528651,0.574324761462573,0.309893013906343,
1.85330012517206,0.0638393532537857,0.67380694967305
"1262","PRRT2",12.7051336024208,-0.864945665731658,0.464840207445733,-
1.86073762957056,0.0627812371169098,0.67380694967305
"1263","RBL2",68.0776462852337,0.439063278951674,0.236513101036364,1.8
5640151445212,0.0633963490370911,0.67380694967305
"1264","HP",1.97795177624581,2.92168788464399,1.56996291444841,1.86099
165639877,0.0627453549679435,0.67380694967305
"1265","CMC2",24.4299350279733,-0.714395612917983,0.383895084341906,-1
.86091367682563,0.0627563680422884,0.67380694967305
"1266","CAMKK1",6.66284277076959,-1.51817323147609,0.818471666360765,-
1.85488795015528,0.0636122290600513,0.67380694967305
"1267","SPIRE1",13.759383453368,-0.782553063707779,0.421273617365685,-
1.85758858720195,0.0632274604645706,0.67380694967305
"1268","ARID3A",9.54734709217468,1.03441831143907,0.55788407740128,1.8
5418145693917,0.0637132040452361,0.67380694967305
"1269","NFIC",424.656567529337,-0.333574997254854,0.179345336044434,-1
.85995914146442,0.0628913068789327,0.67380694967305
"1270","SIN3B",22.7575311427378,0.678307611121892,0.364061856425967,1.
86316583061161,0.0624389386452374,0.67380694967305
"1271","ZNF790-
AS1",5.86381302332361,1.41899901086054,0.765844017099035,1.85285642921
86,0.0639029395929954,0.67380694967305
"1272","ZNF667",8.39640899665539,1.06863976786196,0.574254415706812,1.
86091693617477,0.0627559076916011,0.67380694967305
"1273","CLDN5",1.44961337588877,-2.62289525045192,1.41521280815757,-1.
85335748470691,0.0638311368394223,0.67380694967305
"1274","RRP7A",35.7712608668249,-0.675553740875517,0.363130516548236,-
1.86036069702167,0.0628345113788132,0.67380694967305
"1275","EFNB1",13.4757662617391,1.02868642947697,0.554089381881859,1.8
5653517846387,0.0633773136340243,0.67380694967305
"1276","GPRASP1",9.59383715514266,0.997675573665352,0.53850563181053,1
.8526743542329,0.0639290480024534,0.67380694967305
"1277","MEST",179.899556536408,0.723561643954274,0.390954309508934,1.8
507575600410,0.0642044391396002,0.674000171375346
"1278","ERLIN2",38.3939647258204,0.564647260486849,0.30518258171897,1.
85019491383295,0.0642854617740546,0.674000171375346
"1279","LMO7DN",3.40194015944805,1.80802270478641,0.977243681670772,1.
85012473213976,0.0642955740533666,0.674000171375346
"1280","HSD3B7",143.911646150257,-0.584884709099247,0.315990263402877,
-1.85095800991038,0.0641755941713009,0.674000171375346
"1281","NAE1",52.53018175784,-0.482006352095625,0.260398372379165,-1.8
510344273342,0.0641646004328622,0.674000171375346
"1282","ZNF112",3.52158847313325,1.61122593395933,0.870441953659442,1.
85104351552168,0.0641632930706856,0.674000171375346
"1283","PTGS2",6.86998602688655,-1.50532626025418,0.814210339366307,-1

.84881742158392,0.0644841808643112,0.674500763174518
"1284","ATP5F1A",149.556233684281,-0.344661383089449,0.186435265190749,-1.8486919989993,0.0645022996697272,0.674500763174518
"1285","EIF3D",144.818384763845,-0.454777456876546,0.246038062991823,-1.84840285013811,0.0645440867209189,0.674500763174518
"1286","MT-ND5",1915.06724523833,-0.391989398021247,0.211969859832041,-1.84926950620173,0.0644189066438509,0.674500763174518
"1287","DPY19L3",27.6303777128436,0.613419639318934,0.331946253929627,1.84794867258535,0.0646097684015269,0.67466253111742
"1288","PCDHGA6",6.20556167724137,1.30218118518356,0.705339422737689,1.84617666786482,0.0648665575987725,0.676731390472346
"1289","ZNF680",22.7764336804129,0.652769433226016,0.353740331862998,1.84533505068014,0.0649888147511983,0.676731390472346
"1290","TRADD",56.1746607338477,-0.556646692136721,0.301730405888728,-1.84484785514789,0.0650596738217331,0.676731390472346
"1291","FKBP8",224.971262042454,-0.429410741646492,0.232710041100621,-1.84526090758938,0.0649995942192358,0.676731390472346
"1292","SRSF6",74.8775538031354,0.456971303207782,0.247660188849621,1.84515446479472,0.0650150722345045,0.676731390472346
"1293","GULP1",65.2053982588261,0.474087027762514,0.257092017224998,1.84403636052071,0.0651778413247224,0.677436202291527
"1294","ZFAS1",389.351332516669,-0.407595316570737,0.22114381780319,-1.84312326982381,0.0653110145435881,0.677771987993267
"1295","CD99L2",32.5661886497357,0.555775230598139,0.301522694454694,1.84322852249401,0.0652956521307891,0.677771987993267
"1296","MMAA",7.79137006293513,1.21396803953645,0.65911289817148,1.84182109454125,0.0655013234137765,0.678656882043474
"1297","WDR11",48.8996487469207,0.620367320755807,0.336779919001388,1.84205555543604,0.0654670239723073,0.678656882043474
"1298","ALDH16A1",36.9385735125699,-0.527163772051236,0.286268106216164,-1.8415036834497,0.0655477813001287,0.678656882043474
"1299","RPSA",1066.11120522587,-0.449587072177227,0.244301049571295,-1.84029938866891,0.0657242950876216,0.679960586360698
"1300","TLR5",19.4021743955513,0.871516658276127,0.473850206481547,1.83922397068759,0.065882250363372,0.680262479229046
"1301","MAP9",16.1403922319514,0.803538034817755,0.43677487716892,1.83970754001721,0.0658111859828717,0.680262479229046
"1302","HCFC1R1",186.322639691232,-0.51162710647001,0.278199274485877,-1.83906700481343,0.0659053313458009,0.680262479229046
"1303","FGFR1OP",9.88206330582159,1.05695112270774,0.575028189347997,1.83808575351093,0.0660497701313426,0.680365280149574
"1304","CIZ1",76.4595055190134,-0.450106464749186,0.244985096095241,-1.83728100983825,0.0661684218435518,0.680365280149574
"1305","SPOCK2",1.87212214861514,2.35155715313546,1.27926234915705,1.83821336935695,0.066030970518199,0.680365280149574
"1306","NME2",784.011651177103,-0.483975096984689,0.263335285871672,-1.83786648789839,0.0660820813462121,0.680365280149574
"1307","C20orf194",26.3647028873809,0.712225779426694,0.387622255974331,1.83742230599334,0.0661475763837294,0.680365280149574

"1308","DPY30",72.6972860188329,-0.510710794550481,0.278780409422654,-1.83194649727414,0.0669593921549183,0.680409411564099
"1309","NIFK",70.5107359521298,-0.434717975418121,0.236679786297202,-1.83673469635569,0.0662490705451787,0.680409411564099
"1310","NR2C2",24.121523405412,0.788262277166416,0.430357379549036,1.83164577773111,0.0670042118735771,0.680409411564099
"1311","MAPKAPK3",9.04472355313887,-0.865360500761315,0.472500223487303,-1.83144992900637,0.0670334147563708,0.680409411564099
"1312","RBPJ",410.119340407438,0.437584714722241,0.238623749782957,1.8337852586771,0.0666858767925591,0.680409411564099
"1313","HSP90AB1",523.747238808928,-0.290830658322055,0.158559577631469,-1.8342042951074,0.0666236741113068,0.680409411564099
"1314","MBLAC1",3.44538436616338,-1.54182432644778,0.840894680631975,-1.83355224139249,0.0667204870724279,0.680409411564099
"1315","MKLN1-AS",6.71179333396473,1.16327225174151,0.634203039155147,1.83422686414616,0.0666203252705218,0.680409411564099
"1316","OPLAH",4.45878187733965,1.54005044627745,0.840554027637861,1.83218495853898,0.0669238690572727,0.680409411564099
"1317","DENND1A",10.9076312674848,0.891524833024466,0.485536288921329,1.83616519169985,0.066333229004235,0.680409411564099
"1318","ITGB1",545.123812562064,0.333976123482642,0.182335338092724,1.83165878307585,0.0670022730255111,0.680409411564099
"1319","C11orf80",5.28541060250858,-1.18304466284919,0.644411010229613,-1.83585420495478,0.0663792221850176,0.680409411564099
"1320","ATG14",27.5536734759535,0.632893260645214,0.345549122584522,1.83155800226466,0.0670172987240129,0.680409411564099
"1321","RHOJ",16.7297821147377,0.865631282358682,0.472174016270153,1.83328868707467,0.0667596508720592,0.680409411564099
"1322","GOLGA6L10",3.38498062407848,-1.44351142424982,0.787464446813116,-1.83311313938265,0.0667857475131347,0.680409411564099
"1323","ZNF101",4.73506785129695,-1.26618202099572,0.690573723320124,-1.83352186484623,0.066725000019491,0.680409411564099
"1324","PCBP3",2.37553973433107,2.26031894369377,1.23139568218059,1.83557484925651,0.0664205597072676,0.680409411564099
"1325","AC253572.1",1.89840681508929,-1.88511830605457,1.03120770495208,-1.82806848416845,0.0675392741502377,0.68152068777536
"1326","LYPLAL1-AS1",2.05902005289795,2.85009454608788,1.55903684448873,1.82811237345871,0.0675326883116698,0.68152068777536
"1327","SLC26A6",3.68790678320553,-1.31536965860227,0.720229775679834,-1.82631946500779,0.0678021548891774,0.68152068777536
"1328","BICC1",125.213955838161,0.517328496807157,0.282827653893258,1.82912982406735,0.0673801620847146,0.68152068777536
"1329","HERC4",51.464940785444,0.529444991206639,0.289770955084711,1.82711545762708,0.0676824115896518,0.68152068777536
"1330","ACIN1",61.6643772630138,0.472170152951894,0.258056911426793,1.82971326108366,0.0672928268841723,0.68152068777536
"1331","FLVCR2",4.95117924311199,1.29369682846048,0.708281392144883,1.82652945963014,0.0677705479270787,0.68152068777536

"1332","ATG2B",20.5046718769825,0.697444172203139,0.381328723152092,1.82898410179546,0.0674019899313972,0.68152068777536
"1333","APOBR",1.45893177403671,2.52273927391832,1.38010988649804,1.82792638368793,0.0675606007638208,0.68152068777536
"1334","DDX19B",30.5335896020252,-0.571500800745897,0.312801584392859,-1.82703934142522,0.0676938544259161,0.68152068777536
"1335","BORCS6",12.0767280983812,-0.857791362025816,0.469552707767364,-1.82682656885199,0.0677258497735073,0.68152068777536
"1336","TLK2",32.6961029815193,0.619802860081135,0.339045524961465,1.82808152430733,0.0675373173477768,0.68152068777536
"1337","RNF215",13.1505343107852,-0.772895037702926,0.422706532441108,-1.82844356163482,0.0674830086704148,0.68152068777536
"1338","CRPPA",2.41606506650077,1.96626958565615,1.07705459479296,1.82559880916168,0.0679107153225443,0.681899484160907
"1339","GCHFR",7.38846048541612,-0.965319012250097,0.528828816660819,-1.82539033773803,0.0679421463458253,0.681899484160907
"1340","TUG1",74.5079773333351,0.530482447048981,0.29066584761247,1.82505943304439,0.0679920610741585,0.681899484160907
"1341","RETREG1",24.1644221958676,-0.7624030125533,0.418014384322012,-1.82386788863704,0.0681720473362404,0.682871040236381
"1342","MT-ND2",5574.58611506904,-0.352980354285847,0.193546938742857,-1.82374547785956,0.068190560011699,0.682871040236381
"1343","BTBD8",15.588342854027,0.842508371319359,0.463151208973745,1.8190784240555,0.0688994662534138,0.683855189792931
"1344","RPS7",1604.14258249282,-0.525794025311542,0.288887148950432,-1.82006720348006,0.068748771331684,0.683855189792931
"1345","PPARG",21.0684736833051,0.88793469064845,0.487295780297072,1.82216782198921,0.0684295254214854,0.683855189792931
"1346","LST1",6.04454966958522,-1.14115974434148,0.627273727879542,-1.81923727014536,0.0688752390342828,0.683855189792931
"1347","AL365203.2",1.52096032607048,-2.24184193285206,1.23232476159311,-1.81919734368875,0.0688813279621591,0.683855189792931
"1348","SHOC2",46.1952716517407,0.482251497774543,0.264757518696643,1.82148367362176,0.0685333662228761,0.683855189792931
"1349","TUB",9.20592195598629,0.931963964634913,0.511593409141216,1.82168876295602,0.0685022239504838,0.683855189792931
"1350","NHLRC3",72.3432973782018,0.451868720460886,0.24822413631059,1.82040605388787,0.0686971911923732,0.683855189792931
"1351","RPS15",2401.26573919087,-0.441512815377771,0.242530341563397,-1.82044362998747,0.0686914732812865,0.683855189792931
"1352","TGFB1",40.1216175034692,-0.489769188675433,0.268783881147128,-1.82216726161247,0.0684296104231369,0.683855189792931
"1353","REM1",4.8759926624187,-1.32644533689099,0.728378156277402,-1.8210943387844,0.0685925177008941,0.683855189792931
"1354","USP9X",64.34944914379,0.481527972149859,0.264480068588538,1.82065882967911,0.0686587341297306,0.683855189792931
"1355","GGCX",36.920839989846,0.521285735779142,0.286657895104339,1.81849425633088,0.0689886237959569,0.684234771360786
"1356","SLC39A1",118.120255075465,-0.427227297840327,0.235067383134622

,-1.81746736677481,0.069145580704038,0.685285736785816
"1357","PDZRN3",21.6902380043114,0.859391746001778,0.47324972605463,1.81593712302028,0.0693800178849781,0.685667506980053
"1358","IGSF10",21.8263828722612,1.73619687711637,0.955949137201658,1.81620214878663,0.0693393686000199,0.685667506980053
"1359","ERAP1",21.6409487156938,0.706411772784929,0.38889116929652,1.81647676408488,0.0692972691217949,0.685667506980053
"1360","EML1",56.9510669487671,-0.519014146303847,0.285819015112605,-1.81588389456653,0.0693881843509839,0.685667506980053
"1361","ZBTB44",64.6291049280562,0.449613752101898,0.247836327829793,1.81415596349007,0.0696537176688044,0.687280698789326
"1362","CRNDE",117.419740418868,-0.47144547973624,0.259829647336204,-1.8144406712996,0.0696099089486654,0.687280698789326
"1363","HMCN2",79.5838762859888,-0.811013172250635,0.447529349609594,-1.81220108347783,0.069955131727071,0.689748360440284
"1364","ATF7IP",56.4524395084186,0.528413620328401,0.2916443124488,1.81184270624571,0.0700105042302753,0.689788245125125
"1365","AC016831.1",5.32015047000449,-1.2340378382021,0.68129136846015,-1.81132169778,0.0700910688769499,0.689911116303522
"1366","SDCBP2-AS1",3.52321499114003,1.62346908504851,0.896400350771596,1.81109822597802,0.0701256481040711,0.689911116303522
"1367","TUT4",72.4153622250537,0.486106732732155,0.268546199077386,1.81014192121213,0.0702737814514233,0.690862727816882
"1368","PRXL2C",39.8434498369602,-0.512683123697701,0.28332604752724,-1.80951637935234,0.0703708178506276,0.691053082992552
"1369","SUPT20H",39.0166029655485,0.596744052227613,0.329810513771368,1.8093542422401,0.0703959870985046,0.691053082992552
"1370","BANF1",117.816864622686,-0.477908617766922,0.264205908768596,-1.80884909044747,0.0704744513570161,0.691318358968569
"1371","IPO8",24.4067988576907,0.701559700982006,0.388054105676221,1.8078914530732,0.0706233962507449,0.692274122694209
"1372","CLEC7A",3.2898134422958,-1.77357786107421,0.981937753097043,-1.80620192622223,0.0708868041679005,0.694349680183976
"1373","TMEM192",21.4745202657551,0.688256564754782,0.381334292750167,1.80486407291383,0.0710959550068953,0.695384672007035
"1374","TRPM7",42.515644736417,0.577434081025344,0.31991870968981,1.80494001612228,0.0710840690363787,0.695384672007035
"1375","RABGGTB",27.9986597931852,-0.570680212524089,0.31659118529417,-1.80257770598959,0.0714545608859209,0.695891935006755
"1376","RHOC",199.689674098607,-0.458966629755847,0.254555575776882,-1.80301149701836,0.071386409097854,0.695891935006755
"1377","CBWD2",14.1161591304129,-1.02507371150948,0.568782869455997,-1.80222324995457,0.0715102881348559,0.695891935006755
"1378","TMED9",205.341468336984,-0.442183166617907,0.245351785730942,-1.80224148481566,0.0715074203981894,0.695891935006755
"1379","DLC1",171.357853339231,0.457395593513863,0.253724041592333,1.80272862848676,0.0714308437962819,0.695891935006755
"1380","SCAI",13.5318771802551,0.776633101265167,0.430844900571819,1.80258162562541,0.071453944843425,0.695891935006755

"1381","RTRAF",196.884255077854,-0.502866981448466,0.278933433250115,-1.80282075041737,0.0714163702384036,0.695891935006755
"1382","ITM2C",84.7751301171327,0.607372034638267,0.337451205016077,1.7998810660917,0.0718794199379145,0.698186353713097
"1383","PSMC5",174.138903607957,-0.419149142448345,0.232894454270663,-1.79973861447651,0.0719019207931339,0.698186353713097
"1384","CCBE1",8.020052833584,-1.56029924542843,0.866860091425236,-1.79994356743669,0.0718695493980446,0.698186353713097
"1385","MPG",140.465453609876,-0.454824659875988,0.252770514324252,-1.79935805049059,0.0719620608357567,0.698265801856848
"1386","RPRD2",38.8511128373714,0.549050274476363,0.305671589052247,1.79620970394641,0.07246117198853,0.698706487341851
"1387","MCFD2",91.7983554099594,0.419333094201488,0.233508544392362,1.79579336290533,0.0725273866985551,0.698706487341851
"1388","KAT2B",17.7377423489967,0.689612848057555,0.383847828896048,1.79657873809236,0.0724025223309005,0.698706487341851
"1389","TNIP2",40.4607773243452,-0.524336419370687,0.29164774730911,-1.79784148586259,0.072202131002313,0.698706487341851
"1390","KDM2A",50.5934080187364,0.552205949591918,0.307378550773949,1.79650124643219,0.0724148346583968,0.698706487341851
"1391","ANGPTL5",130.653567344916,-0.790678919969613,0.439645012142345,-1.79844851671742,0.0721059602212608,0.698706487341851
"1392","TFCP2",18.2163483990284,0.72572844746453,0.403766492218231,1.79739641959264,0.0722727087501571,0.698706487341851
"1393","LCP1",9.06108420305566,-1.04416842777773,0.580953938624575,-1.79733427791165,0.0722825675506224,0.698706487341851
"1394","ADCY9",19.0034348232841,0.728423277362737,0.405083223633308,1.79820647922494,0.0721442931927262,0.698706487341851
"1395","DLGAP1-AS1",19.3162936328482,0.673708982691602,0.375131905354378,1.79592557464598,0.0725063544318889,0.698706487341851
"1396","GALNT17",11.8023865136501,1.00724301394491,0.561096478240042,1.79513337368338,0.0726324526055355,0.699217428772057
"1397","ZNF189",10.2267501247201,0.92201256631082,0.513808917438961,1.79446587051567,0.07273884137091,0.699239834895322
"1398","IMPA2",65.5216311412906,-0.716231007183557,0.399126262244198,-1.79449731810769,0.0727338262929534,0.699239834895322
"1399","AC073316.1",3.27882859924549,1.87170776546098,1.04337289770107,1.79390107753904,0.0728289594462393,0.699605708361694
"1400","GOLGA6L4",10.7531023943529,0.935801733319044,0.521881347397462,1.79313121265157,0.0729519458301548,0.70028657143675
"1401","TCEANC2",4.87737042942846,1.35831888732752,0.760151203921173,1.78690618434957,0.0739526528045433,0.700443205278933
"1402","GSTM5",66.4637081740253,0.747452027949303,0.419733565129007,1.78077735508136,0.0749488302099193,0.700443205278933
"1403","C1orf54",19.3988151343156,0.880481491783451,0.493877313012671,1.78279396235571,0.0746198489592684,0.700443205278933
"1404","RCOR3",30.9525225106358,0.560858179802971,0.314246050625556,1.78477399695715,0.0742979827500918,0.700443205278933
"1405","GALNT2",42.9895874118301,0.517893750646606,0.289235601775294,1

.7905601781656,0.0733639021725948,0.700443205278933
"1406","TBCE",5.66730627788166,1.22618450758564,0.686053449096495,1.78730171708994,0.0738887366613047,0.700443205278933
"1407","HEATR1",19.8679210718476,0.622091432863251,0.349176868850537,1.78159405263908,0.07481545499889,0.700443205278933
"1408","SOCS5",35.5530111421481,-0.49837239760887,0.279756475002521,-1.7814508050417,0.0748388347930095,0.700443205278933
"1409","IMP4",41.0828784281544,-0.460922305662284,0.258158935658248,-1.78542069243908,0.0741931047319844,0.700443205278933
"1410","OLA1",43.0169645936849,-0.51133047275944,0.287016048517992,-1.78153965745016,0.0748243322693498,0.700443205278933
"1411","NABP1",33.0724392616703,0.72003185741704,0.403750581948008,1.78335806710926,0.0745280346089832,0.700443205278933
"1412","COL4A4",4.17710320674397,-2.00755344112943,1.12223771201675,-1.78888431535747,0.0736334482813881,0.700443205278933
"1413","LZTFL1",16.1029248403947,0.690781781622538,0.387909292820721,1.78078173018092,0.0749481151936395,0.700443205278933
"1414","ITIH4",5.22146720804727,1.65956029982456,0.926001149445801,1.79217952463427,0.0731042134095135,0.700443205278933
"1415","LINC00882",51.3778931704058,0.650002140083928,0.364424019540594,1.78364241990235,0.0744817880161934,0.700443205278933
"1416","COX7A2",261.220056833954,-0.387132833842909,0.216596675505435,-1.78734430221296,0.0738818578088438,0.700443205278933
"1417","RBAK",9.88243605144418,0.943040789778292,0.529464148417818,1.78112303278013,0.0748923537622394,0.700443205278933
"1418","TMEM176A",251.120351538509,0.570720675630338,0.318390057143993,1.79252040955609,0.073049642857138,0.700443205278933
"1419","MATN2",31.5739616414193,-0.893128326080857,0.499250933084173,-1.78893671878281,0.0736250074552561,0.700443205278933
"1420","RGS22",5.49160491660441,-1.19364233515861,0.669363107923927,-1.7832508559678,0.074545477312453,0.700443205278933
"1421","FBXL6",9.34390655515199,-0.861889041563125,0.482630848536595,-1.78581423913638,0.0741293404285222,0.700443205278933
"1422","ACO1",35.6038185379077,0.536256819936783,0.299458996503411,1.79075207690638,0.0733330887064072,0.700443205278933
"1423","VDAC2",178.90558832652,-0.424085946467109,0.236797990180142,-1.79091869041831,0.0733063439135537,0.700443205278933
"1424","DKK3",30.5617720110397,-2.70456302734961,1.51374925357176,-1.7866651434959,0.0739916259793765,0.700443205278933
"1425","APLP2",404.894751964693,0.32302297233715,0.180727401263138,1.78734917936894,0.0738810700263742,0.700443205278933
"1426","PRR4",2.20211984852045,2.1273858408692,1.19444956604643,1.78105957869008,0.0749027182174199,0.700443205278933
"1427","PDE3A",5.88639066866143,1.20513784111413,0.674283261257529,1.78728719865678,0.0738910819699275,0.700443205278933
"1428","DYNLL1",669.071653669599,-0.452839069901016,0.253803233411209,-1.7842131631449,0.0743890341912407,0.700443205278933
"1429","NR2F2",83.1111661439441,0.378974556006881,0.212435330542602,1.78395258001061,0.074431370909396,0.700443205278933
"1430","GPATCH8",66.4345520348429,0.475665329176977,0.266522331088578,

1.78471097425188,0.0743082099501185,0.700443205278933
"1431","APCDD1",13.2433306306853,1.33081238776551,0.746388445517409,1.78300239742185,0.074585913066068,0.700443205278933
"1432","RPL18A",2705.68143441661,-0.431027847364278,0.241604494066211,-1.7840224745412,0.0744200132733275,0.700443205278933
"1433","AC006942.1",2.52573701418705,-1.69464758750636,0.94974468841886,-1.78431909982841,0.0743718283759547,0.700443205278933
"1434","ID1",30.961543556013,-1.05627538489888,0.59203845672261,-1.78413306248074,0.0744020459822267,0.700443205278933
"1435","ADAMTS1",119.251712717929,1.29461078396331,0.72575246751181,1.78381864604855,0.0744531387040234,0.700443205278933
"1436","PSMG1",16.0716835557651,-0.723578511956521,0.405822972138379,-1.78299051959482,0.0745878465893035,0.700443205278933
"1437","IFT27",33.8063529547753,0.540838786780963,0.303473192371771,1.78216330264325,0.0747226052504875,0.700443205278933
"1438","ODF3B",28.3103719010451,-0.759554602258029,0.424650172186349,-1.78865958854413,0.0736696548880093,0.700443205278933
"1439","NAV3",33.981183880529,0.538709624033209,0.302598517593857,1.78027846374402,0.0750303998522888,0.700718237397435
"1440","IL6R",35.5231551550362,0.670053412310696,0.376903340737128,1.77778581373209,0.0754390392985877,0.701089663384344
"1441","CLIP4",32.7454682201611,0.623628950023372,0.351405102646724,1.77467243738439,0.0759519889131432,0.701089663384344
"1442","NOA1",31.98859628916,-0.640226838569471,0.360337569355914,-1.77674184713475,0.0756107237413905,0.701089663384344
"1443","AFF1",78.2715941217506,0.504405961261987,0.284082189259631,1.77556348244344,0.0758048934433679,0.701089663384344
"1444","STOX2",4.97793021013616,1.40773844041907,0.793191944857872,1.77477652104916,0.0759347945740961,0.701089663384344
"1445","CYSTM1",152.67918050067,-0.469548711166685,0.264339673879564,-1.77630812762755,0.0756821443295428,0.701089663384344
"1446","SUN1",138.407555020391,0.375504199355681,0.211144265744882,1.77842480368086,0.07533411195295,0.701089663384344
"1447","CYCS",57.5721161821009,-0.495595186610221,0.278983879588945,-1.77642947449305,0.0756621565974179,0.701089663384344
"1448","RPS6KA4",14.9542300895401,-0.691728803523489,0.388973840104241,-1.77834273723424,0.0753475812590452,0.701089663384344
"1449","LINC02384",20.8127690054408,0.733912701371734,0.413444689561597,1.77511701057269,0.0758785688146957,0.701089663384344
"1450","DHRS1",29.3848389996335,-0.514765579233929,0.289826481422832,-1.7761164428689,0.0757137265952142,0.701089663384344
"1451","VASH1",12.3608992503401,0.928754497791637,0.52270133178224,1.77683591244141,0.0755952412688655,0.701089663384344
"1452","MBP",150.922884064072,0.338276014766796,0.190236789002178,1.778184001849,0.0753736395741706,0.701089663384344
"1453","MAFF",36.8591692649532,-0.785269810354242,0.442257015912798,-1.77559604958099,0.07579952160267,0.701089663384344
"1454","MED14",15.7289777603268,0.741708709964983,0.417948378082672,1.77464191479233,0.0759570317648341,0.701089663384344
"1455","PABPC5",14.1643516080385,0.742869553461497,0.417601884245574,1

.77889416089092,0.075257115603522,0.701089663384344
"1456","NXT2",12.0748753843166,-0.830948170326684,0.46705051621259,-1.77913981781889,0.0752168420952662,0.701089663384344
"1457","ESR1",10.0256159158929,0.962404211428302,0.542431901445573,1.77423969509077,0.0760235108265946,0.701221662318878
"1458","NR1H2",71.4934646849702,-0.424725495363338,0.239538451999495,-1.77309944110449,0.0762122305947848,0.702480224254673
"1459","NRBP2",36.5637593959504,0.537390741892471,0.303396541933002,1.77124873760473,0.0765193478358124,0.703062332190553
"1460","UROS",54.3779542485424,-0.444450394745902,0.250927228246596,-1.77123223275365,0.0765220912866612,0.703062332190553
"1461","ROBO3",48.3013067782252,-0.540306655169596,0.304869560104038,-1.77225517360675,0.0763522086522312,0.703062332190553
"1462","BNIP2",64.0233074323948,0.382101501045133,0.215747907699659,1.77105541888756,0.0765514864750149,0.703062332190553
"1463","POFUT2",27.3648043819669,0.661921189759473,0.373791871919471,1.77082820544069,0.0765892740774588,0.703062332190553
"1464","RPGR",12.6648108864292,-0.873923740304149,0.493252976704548,-1.77175563367683,0.0764351301731179,0.703062332190553
"1465","KDM4A",22.6372072085546,0.6507354738597,0.367850002459876,1.76902397582743,0.0768898735510321,0.703082581998028
"1466","UBR3",31.5248073232246,0.566000270695448,0.320161198783523,1.76786029302117,0.0770842623095754,0.703082581998028
"1467","BMP2K",22.9921582138891,0.621507535311357,0.351486241563867,1.76822720726161,0.0770229275052905,0.703082581998028
"1468","FBXL7",36.732337192063,0.623440145644458,0.352893034879406,1.76665471977226,0.0772860709022194,0.703082581998028
"1469","C7",5.85581311089931,-1.42581245655256,0.805616617169395,-1.76983992902515,0.0767538101464961,0.703082581998028
"1470","ZNF608",3.90911559104236,1.6739440509941,0.947028414512504,1.76757531806032,0.0771319272847098,0.703082581998028
"1471","RREB1",29.8588482564644,0.565751359340254,0.320279714732626,1.76642894731111,0.0773239122102155,0.703082581998028
"1472","BEND6",23.103870811814,-0.732692108448817,0.414531723731737,-1.76751757827581,0.0771415877794913,0.703082581998028
"1473","SERGEF",17.3313532857557,-0.869358541763834,0.492096451498506,-1.76664257406553,0.0772881062377359,0.703082581998028
"1474","CPT1A",44.2603717806555,0.609108948323417,0.344206132544828,1.76960516019885,0.0767929386433725,0.703082581998028
"1475","MTHFSD",7.8126769597321,0.984753335709085,0.557260549329606,1.76713269384269,0.0772060083311734,0.703082581998028
"1476","CDK12",61.293583674326,0.465306453166817,0.263067576321847,1.76877158208788,0.0769320009907641,0.703082581998028
"1477","NFATC1",9.15007582425952,-0.841218247209981,0.475324957502439,-1.76977504322538,0.0767646229201049,0.703082581998028
"1478","BCL3",17.9393842334492,-0.776048746716665,0.439193946376821,-1.7669841606852,0.0772308809890463,0.703082581998028
"1479","MOV10",10.1834522468908,0.902884776545928,0.511429714752445,1.76541321417541,0.0774943441502989,0.704155842485373
"1480","PDPN",70.832604844839,0.605588144670973,0.343547761272206,1.76

274804536171,0.0779429935418585,0.705264525950677
"1481","ARHGAP30",2.10982232307072,-1.73438629800681,0.984182373703896
,-1.76226108529009,0.0780251955414772,0.705264525950677
"1482","OSMR",45.1306252462008,0.472807171097869,0.268209367271138,1.7
6282870321938,0.0779293847852924,0.705264525950677
"1483","MAP3K1",13.1976652943493,0.762681739555759,0.432541259845567,1
.76325777528845,0.0778570233981909,0.705264525950677
"1484","FAM92A",52.7559135323759,-0.452843629951274,0.256782322669184,
-1.76353117007465,0.0778109449437674,0.705264525950677
"1485","AGAP9",6.45449821093068,1.32513436483128,0.751978992478388,1.7
6219598962981,0.0780361894552166,0.705264525950677
"1486","ATP2B1",154.003230271055,0.513210761957134,0.291172072438104,1
.7625686339346,0.077973271139367,0.705264525950677
"1487","KIF22",25.0565135207303,-0.560916171520083,0.317937296125336,-
1.76423520724338,0.0776923874656252,0.705264525950677
"1488","PPP1CB",154.947214575187,-0.446510652138674,0.253493967310869,
-1.76142516082484,0.0781664695560031,0.705493072104181
"1489","GPAM",13.5109838194444,0.819230153332463,0.465092879353293,1.7
6143344630775,0.0781650682607529,0.705493072104181
"1490","FMO3",2.77051729478635,2.02917164840598,1.15281582989036,1.760
18718323721,0.0783760743749086,0.705677612684375
"1491","MTX3",21.8350752910571,0.716338739161792,0.407220592297888,1.7
590926213225,0.0785617779914706,0.705677612684375
"1492","RACK1",1778.61281485445,-0.464227932781795,0.264034607674537,-
1.7582086563214,0.0787120129037784,0.705677612684375
"1493","GTF2H4",11.8210158473737,-1.0048471558784,0.571087540631496,-1
.75953261870722,0.0784870849451394,0.705677612684375
"1494","GJA1",172.259921712692,0.555877448480106,0.315934504391303,1.7
5947052554798,0.0784976222476577,0.705677612684375
"1495","PIK3C2A",35.7195091590379,0.561739241389862,0.31940247521849,1
.7587191239066,0.0786252275230179,0.705677612684375
"1496","GPALPP1",31.6722140447049,0.509723646568961,0.28960354574035,1
.76007391506858,0.0783952748890002,0.705677612684375
"1497","PNP",3.99869684153089,-1.2060728579328,0.685541120226321,-1.75
930053259917,0.078526476197365,0.705677612684375
"1498","MBTPS1",108.345939149518,0.345290575585374,0.196367717445325,1
.75838768244335,0.0786815675270274,0.705677612684375
"1499","MN1",28.3686672101023,0.647275642112736,0.367756809532799,1.76
006432874768,0.0783969000780813,0.705677612684375
"1500","PRR5L",9.41752734190549,1.14671909459214,0.652543474184019,1.7
5730681549772,0.0788655267280168,0.706582542465212
"1501","NUDT16",33.1488242544649,-0.579313306214249,0.330300278897093,
-1.75389893144697,0.0794478267373902,0.708153246949587
"1502","SLC25A37",73.88438685354,0.392643522609637,0.223656274788379,1
.75556676413909,0.0791624113901283,0.708153246949587
"1503","C8orf82",31.2133459192927,-0.540031724408334,0.307815543286412
,-1.75440043944062,0.0793619160999573,0.708153246949587
"1504","TMEM80",30.0380686955935,0.637944386660246,0.363504933140839,1
.75498137301228,0.0792624939421673,0.708153246949587
"1505","CALCOCO1",91.3329383059161,0.396826352923312,0.226233370190236

,1.7540575583064,0.0794206450508958,0.708153246949587
"1506","JMJD8",46.7363875865197,-0.572160501898469,0.326237856341775,-1.75381394518195,0.0794623927673173,0.708153246949587
"1507","MRPL45",29.4565597919886,-0.534209357837887,0.304504060157735,-1.75435873518784,0.0793690573503635,0.708153246949587
"1508","TAF1",28.4772270768321,0.589897752262857,0.33626216575428,1.75427928663827,0.079382663211813 7,0.708153246949587
"1509","JMY",21.6108325860469,0.683111009577226,0.389625817255532,1.75324883342937,0.0795593040561648,0.708245668517751
"1510","NR4A1",44.1798215481028,-1.55423264971835,0.886542780783704,-1.75313891603111,0.0795781650019945,0.708245668517751
"1511","TTLL3",57.7524382848512,0.553502807852641,0.316270206072599,1.75009468873456,0.0801019761010289,0.710661219094879
"1512","RICTOR",50.1012919210367,0.501345817768814,0.286347047280587,1.75083285310623,0.0799747057575016,0.710661219094879
"1513","TRMT9B",9.08046296769965,1.1497911124719,0.657129106346167,1.7497187407587,0.0801668582593821,0.710661219094879
"1514","ECM2",257.813029473011,0.410270719569825,0.234441584245056,1.74999124362246,0.0801198246927647,0.710661219094879
"1515","MMP2",1907.58260225536,-0.331444574571884,0.189339831356729,-1.75052746269442,0.080027339591482,0.710661219094879
"1516","PAK3",33.5603080335814,0.577935237710262,0.330286605853456,1.74979919702431,0.0801529693008288,0.710661219094879
"1517","ATAD3A",12.5847347426061,-0.707434775411911,0.404818841561381,-1.74753421229937,0.0805447157272769,0.710773355159575
"1518","ASAP3",47.4319277488701,0.502990938492871,0.288105492930088,1.74585681577035,0.0808358354100743,0.710773355159575
"1519","ESRRG",1.49226689750966,2.39668494342322,1.37221253846199,1.74658434917777,0.0807094639772459,0.710773355159575
"1520","CLASP1",23.1298854540901,0.58617635634564,0.33530290821078,1.74819943994388,0.0804294985588128,0.710773355159575
"1521","LRP1B",14.1506240565455,0.894778357566198,0.512096530025291,1.74728455496859,0.0805879908603644,0.710773355159575
"1522","LY75",1.89833328036428,-1.9233463807481,1.10278906538765,-1.74407458426513,0.081146085211367,0.710773355159575
"1523","DNAJC10",81.9352111560266,0.397488584556833,0.228053338828109,1.74296323219557,0.0813400375194825,0.710773355159575
"1524","IFRD2",20.7752647292661,-0.653409396825176,0.373666340065965,-1.74864398198089,0.0803525787029737,0.710773355159575
"1525","AC008549.2",2.34563349470403,-1.86364788533727,1.06879920074902,-1.74368383137938,0.081214236268444,0.710773355159575
"1526","MDGA1",10.0594821431991,0.842195349110431,0.481924218614375,1.74756801293761,0.0805388582390185,0.710773355159575
"1527","LAMA2",255.838608579278,0.459642030431523,0.26371560160211,1.74294591461078,0.0813430627453997,0.710773355159575
"1528","ANKMY2",29.3459090929479,0.543104691923522,0.311174556042133,1.74533772565256,0.0809260987376285,0.710773355159575
"1529","RGS10",79.7200873270551,-0.491121202345261,0.281585564804768,-1.74412776693923,0.0811368132321869,0.710773355159575
"1530","ZDHHC5",23.3514895720604,0.60225086865192,0.344967463111754,1.

74581933965412,0.0808423493024211,0.710773355159575
"1531","ARL2",200.308130412062,-0.418814453471063,0.240241362132645,-1
.7433070215437,0.0812799995172757,0.710773355159575
"1532","IQSEC3",1.45486618780467,2.51199746931465,1.43926287026456,1.7
4533611698945,0.0809263785913119,0.710773355159575
"1533","ETFA",67.3941135884353,-0.446549873412121,0.255436390144296,-1
.7481842472009,0.0804321284413025,0.710773355159575
"1534","NLRP1",11.5380741198489,0.937472694374897,0.537028587113695,1.
74566627712209,0.0808689582149419,0.710773355159575
"1535","PSMB3",150.967426794058,-0.495793020532562,0.28400986448785,-1
.74568943732506,0.0808649315097413,0.710773355159575
"1536","TXNL4A",97.5572391664981,-0.449430343074197,0.257722439893607,
-1.74385413726384,0.0811845275766772,0.710773355159575
"1537","PRKCSH",98.5580477376915,0.363601694759484,0.20839558401022,1.
74476679285897,0.0810254713438753,0.710773355159575
"1538","HOMER3",84.0339006898055,-0.522751901979378,0.29965255516525,-
1.74452676264047,0.0810672789082285,0.710773355159575
"1539","PSMG3",44.788223076696,-0.538850255688484,0.309464720327414,-1
.74123323368939,0.0816427040374156,0.711079908981742
"1540","ADAM9",122.837729062754,0.392308678163935,0.225203973074938,1.
74201490678582,0.0815058358788829,0.711079908981742
"1541","KDM5A",92.8935852992314,0.418225334569411,0.240110281686626,1.
74180518898082,0.0815425384270485,0.711079908981742
"1542","PITPNA",35.1665667341481,-0.499675672891542,0.286951298537067,
-1.74132570732032,0.0816265025171114,0.711079908981742
"1543","KLHL4",11.7917862821021,-1.07321026746291,0.616189862996776,-1
.74168763868245,0.0815631166767177,0.711079908981742
"1544","STK32C",12.4290983430488,-0.696790025778036,0.400312073759828,
-1.74061706216756,0.0817507248441412,0.711559579780061
"1545","ERCC6",22.3955772326889,0.659528529403523,0.379066459478529,1.
73987572076627,0.081880842773997,0.712230838860677
"1546","TNFSF12",115.989410244041,-0.501226854130259,0.288451262268316
,-1.73764832987286,0.0822727983676095,0.714583284696778
"1547","PLD6",3.03457917415733,1.65674287805735,0.953557961904067,1.73
743279826343,0.0823108062140496,0.714583284696778
"1548","CD93",1.75276165167834,-2.24723527152082,1.29328559524569,-1.7
3761718199135,0.0822782902500605,0.714583284696778
"1549","SDC2",613.681798529269,-0.362715818248698,0.20884211584531,-1.
7367944046179,0.082423467059941,0.715099402077823
"1550","GTPBP10",13.2946210684526,0.848170031220001,0.48856079848787,1
.73605830399235,0.0825535259410825,0.715765700078844
"1551","PPP1R11",29.2468931863974,-0.536352006365372,0.309515608482004
,-1.73287547272937,0.0831178046056623,0.71603857442019
"1552","FGL2",231.957429413418,-0.586216585261355,0.338054467166433,-1
.73408915484837,0.0829022654918538,0.71603857442019
"1553","TSPYL5",17.6708086468912,0.66753084712583,0.38464922890139,1.7
3542749333565,0.0826651138526157,0.71603857442019
"1554","FAU",1779.96007480944,-0.449395401835956,0.259249643411045,-1.
73344655723762,0.0830163286052087,0.71603857442019
"1555","KNTC1",3.55294076128841,1.65265759917231,0.953331014814193,1.7

3356113825209,0.0829959808015821,0.71603857442019
"1556","GABPB1-
AS1",73.0240150259205,0.687533508925731,0.396511374947646,1.7339565832
5442,0.0829257869666466,0.71603857442019
"1557","VPS13C",120.46658966304,0.39215761388291,0.226164763122533,1.7
3394656386169,0.0829275648741358,0.71603857442019
"1558","CORO2B",24.6668039481885,0.661379871304855,0.381188230581041,1
.73504798481506,0.0827323062900215,0.71603857442019
"1559","SEPTIN1",26.6119733310905,-0.65456281172864,0.377529267048286,
-1.73380680349987,0.0829523681001657,0.71603857442019
"1560","SLC39A6",63.1907757324415,0.442758908423532,0.255482497378809,
1.73303029744165,0.0830902838798474,0.71603857442019
"1561","DCBLD2",54.1787537390322,0.479157336800195,0.276633768461146,1
.73209995101337,0.0832557679247936,0.716715796878083
"1562","FIS1",240.80346100206,-0.456236339523963,0.263486658756345,-1.
73153487799874,0.0833564097418293,0.716715796878083
"1563","SLC25A25",4.62436184435053,-1.12064558932779,0.647182568319963
,-1.7315756699642,0.0833491412272317,0.716715796878083
"1564","MRPL20",167.426370311977,-0.36786281359444,0.212587839285043,-
1.73040384074463,0.0835581481213093,0.717532238084521
"1565","OSR2",215.364577317394,0.605277887427999,0.349746591169225,1.7
3061840404081,0.0835198470042894,0.717532238084521
"1566","SLC35E2B",38.8317777405403,0.479824958920456,0.278104801904444
,1.72533863361814,0.0844664634288349,0.717656023522754
"1567","WLS",48.5073487580218,0.507992521160689,0.293917095772964,1.72
835309162515,0.0839249399585156,0.717656023522754
"1568","FPGT",11.5630515608319,0.776135932077196,0.452193482850487,1.7
1638018129911,0.0860924815173525,0.717656023522754
"1569","NBPF14",30.386968422706,0.893788581608325,0.521955755159143,1.
71238380413262,0.0868259617520073,0.717656023522754
"1570","DSTYK",36.4408259904234,0.555348399365396,0.322629244500316,1.
72132070738197,0.0851926439114998,0.717656023522754
"1571","IL10",2.20463046017031,-1.75917816115809,1.0200456960097,-1.72
460720930424,0.084598283335769,0.717656023522754
"1572","AHCTF1",30.0561861394591,0.555698880513284,0.324918681799879,1
.71027063582495,0.0872158391194177,0.717656023522754
"1573","PUM2",50.5537506211037,0.447888186261691,0.259252856663541,1.7
2761138305589,0.0840579206297789,0.717656023522754
"1574","IQSEC1",63.9563960896911,0.587408567316869,0.343557529470325,1
.70978225458338,0.0873061456499672,0.717656023522754
"1575","LINC01985",3.58885930086631,-1.40661388371508,0.82307811992578
6,-1.70896765405684,0.0874569412345017,0.717656023522754
"1576","RPL14",2161.61262084792,-0.434157252990213,0.25105574892968,-1
.72932607534839,0.0837507523502644,0.717656023522754
"1577","TUSC2",30.1597695169532,-0.545012703287341,0.315052106835499,-
1.72991289841434,0.0836458384260314,0.717656023522754
"1578","ACKR4",125.959560672033,0.575198553779899,0.333107137061087,1.
72676742640437,0.0842094407190834,0.717656023522754
"1579","RNF7",171.749100770788,-0.436543742752557,0.255115112547645,-1
.71116378952669,0.0870508811509325,0.717656023522754

"1580","ATP1B3",67.9991159204263,-0.497887681301682,0.290045373123131,
-1.7165854981259,0.0860549340374111,0.717656023522754
"1581","TADA2B",12.3438662899614,0.830493664239638,0.48298253851575,1.
71951074420169,0.0855214129724088,0.717656023522754
"1582","FGFBP2",42.8706173058803,-2.17411420204788,1.27067686067759,-1
.71098905577656,0.0870831331741049,0.717656023522754
"1583","MGST2",25.0009592569262,-0.668229778515779,0.389576832237076,-
1.71527083548215,0.0862955830319385,0.717656023522754
"1584","TTC37",83.3973659542082,0.402465694438119,0.234899301401474,1.
71335415659773,0.0866474047261354,0.717656023522754
"1585","VDAC1",126.627848430329,-0.394818394790859,0.229198433564577,-
1.72260511841421,0.0849599587742689,0.717656023522754
"1586","NQO2",89.4389512236091,-0.456502063009526,0.266372381038548,-1
.71377400776195,0.0865702387985341,0.717656023522754
"1587","COA1",37.6930798952049,0.442968911903565,0.258769055605783,1.7
1183107990469,0.0869278026649703,0.717656023522754
"1588","SGCE",250.602711633869,0.472737562268463,0.27526537356908,1.71
738841009665,0.0859082278763649,0.717656023522754
"1589","STMP1",134.54336519032,-0.337976682195323,0.195598840538645,-1
.72790739078306,0.084004829070091,0.717656023522754
"1590","DENND2A",50.1092567778608,0.411020017767139,0.240376796075187,
1.70989889406204,0.0872845709991498,0.717656023522754
"1591","PDGFRL",518.445275483863,-0.33619898998814,0.196096144176124,-
1.71445997268658,0.0864442822929733,0.717656023522754
"1592","AC026904.2",1.74939856712481,2.1547726127513,1.24638091541575,
1.72882349697448,0.0838406894429683,0.717656023522754
"1593","MTFR1",16.7155020711938,-0.637099237621127,0.371928580138584,-
1.71296122869541,0.0867196726343075,0.717656023522754
"1594","PDE7A",45.661452965258,0.567282628194123,0.329646919168357,1.7
2087950836998,0.0852726907480797,0.717656023522754
"1595","EFR3A",42.3639864523992,0.464313509045681,0.269400765563075,1.
72350478691186,0.0847972802697299,0.717656023522754
"1596","JRK",8.72683646446248,1.02856426365181,0.601864825778911,1.708
96224467127,0.087457943300194,0.717656023522754
"1597","FRMPD1",2.19043185808707,1.89905248004148,1.10528270480547,1.7
1815995291061,0.0857674438777412,0.717656023522754
"1598","KLF4",491.370077126887,0.529798039420546,0.307076747886131,1.7
2529520084993,0.0844742863935249,0.717656023522754
"1599","PTPA",26.9007534698172,-0.502248846122893,0.291836140605299,-1
.72099605306312,0.0852515401103565,0.717656023522754
"1600","AGPAT2",41.2508301554184,-0.547051055681956,0.31981654940352,-
1.7105151584627,0.08717065278915,0.717656023522754
"1601","ZEB1-
AS1",19.9945398372904,0.651129909785586,0.379641538557578,1.7151176666
7976,0.0863236559007341,0.717656023522754
"1602","ZNF248",15.8887491946381,0.758534018444096,0.441828009775445,1
.71680835452151,0.0860141939693625,0.717656023522754
"1603","CDH23",7.09472255182222,1.04175846448734,0.609476262708873,1.7
0926831482682,0.0874012596690159,0.717656023522754
"1604","CHST3",14.7749864496172,0.682566800808547,0.399420525641673,1.

7088926507019,0.0874708361135703,0.717656023522754
"1605","PPP1R3C",3.40103595267411,-1.49022642491168,0.868133409819171,
-1.71658688406208,0.0860546806281792,0.717656023522754
"1606","TCIRG1",38.8740263692192,-0.524474612134421,0.303676807839588,
-1.72708155049978,0.0841530185202576,0.717656023522754
"1607","SRSF8",47.3841235451035,-0.405432378336304,0.235642785906537,-
1.72053804565488,0.0853346841403146,0.717656023522754
"1608","GUCY1A2",31.1930453882105,0.695154906767505,0.404005942006671,
1.72065515500766,0.0853134185464451,0.717656023522754
"1609","YBX3",579.421061548793,-0.318714275291119,0.184404965879849,-1
.7283388967886,0.0839274833463484,0.717656023522754
"1610","ETV6",19.0776372502718,-0.609184386645155,0.355786777969269,-1
.71221761000285,0.0868565733315982,0.717656023522754
"1611","BHLHE41",22.1944016076028,-0.839962127194192,0.487105258017493
,-1.72439552513315,0.0846364648536406,0.717656023522754
"1612","SCAF11",192.516465825174,0.355612781855294,0.207513900016034,1
.71368174289923,0.0865871917240574,0.717656023522754
"1613","TUBA1A",802.234030488193,-0.442544511332088,0.257252578572498,
-1.72027240227399,0.0853829375417673,0.717656023522754
"1614","LYZ",28.2321345849151,-0.894336994017679,0.517088940867131,-1.
72956124824082,0.0837086946740939,0.717656023522754
"1615","ZNF84",24.2512763762705,0.586700544700971,0.340994032450491,1.
72055956664331,0.0853307758765902,0.717656023522754
"1616","LINC00640",3.72185024277538,1.60593784107179,0.934514881808778
,1.71847219593064,0.0857105216799486,0.717656023522754
"1617","AREL1",6.36272535591547,1.09462177542324,0.640324990782798,1.7
09478454191,0.0873623594097088,0.717656023522754
"1618","CYP46A1",7.97001195531382,1.07121372989157,0.622013891063202,1
.72217010790636,0.0850387080676429,0.717656023522754
"1619","USP3-
AS1",1.83335087976049,-1.7574138694416,1.02756529514039,-1.71026977823
487,0.0872159976302269,0.717656023522754
"1620","PIF1",2.42957381309362,1.79883352218389,1.04672864334764,1.718
52899375226,0.0857001706660764,0.717656023522754
"1621","IREB2",45.5729940429944,0.456031665101985,0.26627211857259,1.7
1265270861494,0.0867764502257471,0.717656023522754
"1622","TLNRD1",57.6351529112593,0.609249663394988,0.354708703820754,1
.71760562070352,0.0858685744216841,0.717656023522754
"1623","LINC02254",4.7125607594665,1.35658159525415,0.789004020993962,
1.71935954590595,0.0855489235957198,0.717656023522754
"1624","ARHGDIA",137.146441114307,-0.318530503431596,0.185270127977495
,-1.71927610192124,0.0855641093432135,0.717656023522754
"1625","RAB31",41.7121232372271,-0.528607214120294,0.309068546026162,-
1.71032355416571,0.0872060585221162,0.717656023522754
"1626","RNF165",3.93412533643704,1.64315072838663,0.953732962908896,1.
72286247019816,0.0849133985440558,0.717656023522754
"1627","ZNF532",38.8671946116685,0.580704063509061,0.336829189601559,1
.72403129371295,0.084702193980144,0.717656023522754
"1628","ATP9B",20.0838797376239,0.64194052713437,0.374115372102392,1.7
1588920157677,0.0861823234979093,0.717656023522754

"1629","RNF126",23.9276263810879,-0.592647349369993,0.344949015627732,
-1.71807230205166,0.0857834282025937,0.717656023522754
"1630","CDKN2D",15.0283349437191,-0.73419412003958,0.429033232422123,-
1.71127564150371,0.0870302407873812,0.717656023522754
"1631","ECSIT",32.0381333131939,-0.505460874857349,0.294822747727217,-
1.71445683467079,0.0864448581565591,0.717656023522754
"1632","EIF3K",339.93755861413,-0.480288794209639,0.280106538298445,-1
.7146647026779,0.0864067185748381,0.717656023522754
"1633","MRPS12",57.0530930573085,-0.540798495286947,0.31529077323066,-
1.7152373022071,0.0863017283998822,0.717656023522754
"1634","RPS16",2191.4890436,-0.403624181213109,0.235647141505386,-1.71
28329188915,0.0867432821098765,0.717656023522754
"1635","REPS2",9.98657555783931,0.878242357657197,0.508448832769803,1.
72729742120347,0.0841142620961448,0.717656023522754
"1636","RBM41",15.6061118238212,0.836550625614721,0.48878014389892,1.7
1150697518457,0.0869875646585294,0.717656023522754
"1637","ALG13",38.9895136393255,-0.447922853422294,0.259570676229649,-
1.72562964325756,0.0844140628682153,0.717656023522754
"1638","CMC4",26.3162326941781,-0.69306181546882,0.404839681427244,-1.
71194141104316,0.0869074661592354,0.717656023522754
"1639","RSAD2",3.06225386048888,-1.51616314115973,0.887822483850814,-1
.70773230993607,0.0876860240984487,0.718014790448936
"1640","CCNT2",32.0286479616699,0.563149349544848,0.329808363987353,1.
70750475438653,0.0877282748654776,0.718014790448936
"1641","VHL",23.0880649249952,0.622795842499773,0.364619310833726,1.70
807147069559,0.0876230817983723,0.718014790448936
"1642","CRAT",42.1270562926582,-0.4521577845716,0.264749679183074,-1.7
078690556559,0.0876606420998951,0.718014790448936
"1643","NCAPD3",7.38995378714753,1.05246958301471,0.616493193150651,1.
70718767815742,0.0877871745255639,0.71805954866041
"1644","AL360012.1",3.09746442553484,1.75850817671494,1.03046639398146
,1.70651676462783,0.0879119076800216,0.718173595130737
"1645","AC002451.1",9.67369158045734,0.902851007724563,0.5291242923457
09,1.70631176981509,0.0879500478669061,0.718173595130737
"1646","FAM110B",28.9446191870829,0.662217742705536,0.388112831869366,
1.70625057542141,0.0879614359390724,0.718173595130737
"1647","TBCE.1",5.81636610398081,1.13932491797545,0.667878285131601,1.
7058870505888,0.0880291112121376,0.718289754450465
"1648","NEURL4",4.87560629553546,1.26815027966195,0.743777292135736,1.
70501344027389,0.0881919177888924,0.718885016110293
"1649","NDUFV2",179.025950462555,-0.459269721955445,0.269424100609432,
-1.70463488944228,0.0882625401132512,0.718885016110293
"1650","MID1",16.1428020782104,0.947707614537454,0.555920135127787,1.7
0475497225804,0.0882401325649263,0.718885016110293
"1651","WDR6",25.2168368170398,0.604705728884281,0.354889071670825,1.7
0392885314082,0.088394379839949,0.719087209848108
"1652","MGP",4209.85455006406,-0.537173163778217,0.315230179296463,-1.
70406642212079,0.0883686788338724,0.719087209848108
"1653","MLH3",49.8403212106414,0.428977770511826,0.251854027357616,1.7
0327937580567,0.0885157983247973,0.719639330724108

"1654","CEP104",16.6602790844669,0.659389774352002,0.387628499642557,1
.70108692977953,0.0889266648440225,0.720660533983894
"1655","FABP3",25.1986446367867,-0.821511669812448,0.483539986189961,-
1.69895291656337,0.0893280553409991,0.720660533983894
"1656","ENSA",133.257831193851,-0.359713431545599,0.211537304019204,-1
.70047279941198,0.0890420285540891,0.720660533983894
"1657","ENPP1",33.9090125949879,0.846682287101204,0.497404433340885,1.
70220092614444,0.0887177092263095,0.720660533983894
"1658","TP53INP1",24.6979436983783,0.695195128830551,0.40859809034279,
1.70141551138265,0.0888649906239538,0.720660533983894
"1659","PSMB7",155.318259275854,-0.401940613356994,0.236587030873181,-
1.69891228557007,0.0893357118322161,0.720660533983894
"1660","CD248",1064.70157092207,0.447343738316331,0.263345868393524,1.
69869282949165,0.0893770752019698,0.720660533983894
"1661","NOVA1",1651.85152511967,-0.358400125324923,0.210620530300159,-
1.70163907960046,0.0888230469477614,0.720660533983894
"1662","EFL1",10.7053389744267,0.78530503447585,0.462321373944568,1.69
861286700971,0.0893921504688705,0.720660533983894
"1663","TRPV1",9.87820154327325,0.928667413542089,0.546065102382436,1.
70065329113762,0.0890081108796023,0.720660533983894
"1664","NT5M",2.5935906865816,-1.58058198165608,0.929962990980039,-1.6
9961815361102,0.0892027734336207,0.720660533983894
"1665","ACLY",52.768866452578,0.440172592720484,0.258877036821711,1.70
03153239259,0.0890716295636949,0.720660533983894
"1666","AGPAT3",20.9323931362174,0.666870455494282,0.392585847754697,1
.69866147572128,0.0893829860608056,0.720660533983894
"1667","LINC01278",22.9779597167392,0.749159630566097,0.44034354211208
8,1.70130718159914,0.0888853201346265,0.720660533983894
"1668","AL353622.1",2.30265816981223,2.05912020665108,1.21294665140926
,1.6976181139201,0.0895798617738499,0.72077180515540З
"1669","CMPK1",116.873705864835,-0.391449956405788,0.230804722806287,-
1.69602229818464,0.0898816573108516,0.720771805155403
"1670","GLMP",48.3498907914839,0.488251676958386,0.287570720390984,1.6
9784905881431,0.0895362538830658,0.720771805155403
"1671","CSNK2B",136.651482299373,-0.449677840375269,0.265275312445342,
-1.69513640839805,0.0900495469049722,0.720771805155403
"1672","DPAGT1",15.2554867171133,0.669361509790199,0.394167157799957,1
.69816661927452,0.0894763188273893,0.720771805155403
"1673","CIB1",148.15201301847,-0.411836551897247,0.242709468780122,-1.
69682935720296,0.0897289267692874,0.720771805155403
"1674","ZNF426",26.8009658568023,0.530055158769497,0.312594571404922,1
.69566335201287,0.0899496526833654,0.720771805155403
"1675","QTRT1",27.9888666070659,-0.530999499900075,0.312922340047924,-
1.69690505260427,0.0897146126429995,0.720771805155403
"1676","C19orf53",195.559347924819,-0.398934222139397,0.23530787367343
7,-1.69537132740846,0.0900050016057476,0.720771805155403
"1677","PNMA8A",1.79919095903972,2.24530666291177,1.32395146454991,1.6
9591312297471,0.0899023340515951,0.720771805155403
"1678","HM13",145.993453536286,-0.394180852501678,0.232407367863892,-1
.69607726349075,0.089871248856376,0.720771805155403

"1679","CA5B",28.8343625375857,-0.599328093900743,0.35352939319996,-1.69527090371735,0.0900240417566011,0.720771805155403
"1680","SS18L1",6.90676147585197,1.27535725220479,0.752953453444682,1.69380623247051,0.0903021094058314,0.722363123991052
"1681","HSD17B14",3.9048549319411,-1.59442817425287,0.941516395547303,-1.6934683047405,0.0903663629647216,0.722447086188515
"1682","FHL3",25.929831343178,-0.659005771756512,0.389727096381852,-1.69094163037312,0.090847951243056,0.72402154534881
"1683","EXTL2",37.9012612352442,0.43441454251042,0.257923266725809,1.68427822749405,0.0921279111145163,0.72402154534881
"1684","SPAG17",19.3307109284617,-1.74409129384545,1.03272257791932,-1.68882847255975,0.0912523057358973,0.72402154534881
"1685","RNASEH1",39.4054549307656,-0.47949632104655,0.284985084303486,-1.68253128832499,0.0924658630154292,0.72402154534881
"1686","PREPL",42.9069068275381,0.491511032736684,0.290685246812835,1.69087023894665,0.0908615884926931,0.72402154534881
"1687","COBLL1",51.3729671134739,-0.51772743967499,0.307930392803565,-1.6813132180988,0.0927020917691685,0.72402154534881
"1688","PPP1R1C",1.85318247338731,2.15815914268449,1.27790617003343,1.68882441707597,0.0912530831448479,0.72402154534881
"1689","INO80D",42.3500322008536,0.575143116505215,0.341003480605805,1.68661948987574,0.0916765418908176,0.72402154534881
"1690","WDFY1",45.8162063174653,0.467721418918963,0.277646843554761,1.68459116239408,0.0920674776067242,0.72402154534881
"1691","AC018816.1",1.58063217362272,-2.49195383198751,1.48197310220517,-1.68151083732862,0.0926637332155319,0.72402154534881
"1692","RBM5",85.1433893357468,0.433712410863598,0.25690977907505,1.68818957544197,0.0913748436828622,0.72402154534881
"1693","NLGN1",7.53667121500552,1.07079447191604,0.634974403091704,1.6863584842197,0.0917267726804817,0.72402154534881
"1694","DGKQ",5.56573468348019,1.19285405976965,0.707503063150301,1.68600550569805,0.0917947389133757,0.72402154534881
"1695","ZCCHC9",37.3873762393049,0.447770554261311,0.266329599581907,1.6812646996963,0.0927115113022892,0.72402154534881
"1696","ARSI",3.89698857522326,-1.24322423660078,0.73786808776752,-1.68488684794901,0.0920104045313122,0.72402154534881
"1697","POLR1C",14.6387928028603,-0.730188337090694,0.432172507905181,-1.68957609226475,0.0911090830091915,0.72402154534881
"1698","ENPP5",1.85570935862002,2.01213329332402,1.19209619048587,1.68789507875528,0.0914313715057481,0.72402154534881
"1699","RADIL",17.6934865987919,0.643851864424383,0.381628715336887,1.68711587611015,0.0915810728802226,0.72402154534881
"1700","LRWD1",8.3181240695869,-0.893302805418758,0.531181857533963,-1.6817268751722,0.0926218141287004,0.72402154534881
"1701","ZNF212",6.72582255039854,1.03600564485783,0.615258987773624,1.68385292282641,0.0922100963778328,0.72402154534881
"1702","SFRP1",198.685645877878,1.45975199740181,0.866367783166113,1.68491029533346,0.0920058799456786,0.72402154534881
"1703","AL391834.2",3.01119326687704,-1.44824601163998,0.856628969608957,-1.69063394190496,0.0909067378931495,0.72402154534881

"1704","FCN1",2.70753007844299,-1.44910024278525,0.857687534966564,-1.68954331700966,0.0911153580278745,0.72402154534881
"1705","HSPB2",128.046749823347,-0.432273782563893,0.256625705272683,-1.68445238992941,0.0920942732011974,0.72402154534881
"1706","AC026356.2",4.28336359037664,1.32071138407945,0.780993446712258,1.69106589772198,0.0908242175172257,0.72402154534881
"1707","FAIM2",5.56938612499134,1.41083287519211,0.839167186061095,1.68122979380821,0.092718288529303,0.72402154534881
"1708","C12orf10",65.5805297690148,-0.454490878196396,0.270149870807629,-1.68236570625656,0.0924979470688999,0.72402154534881
"1709","POLE",10.7545980084386,0.885090012287719,0.523455450760573,1.69086024608531,0.090863497468717,0.72402154534881
"1710","POLR3K",18.5498075007967,-0.603923068826585,0.358669696217083,-1.68378615532957,0.0922230037794488,0.72402154534881
"1711","AC009065.4",2.84645421244119,-1.51898199943266,0.898820246893442,-1.68997305599496,0.0910331095148576,0.72402154534881
"1712","THOC6",12.6991826164196,-0.794546357937182,0.470768479405888,-1.68776456516354,0.0914564322154018,0.72402154534881
"1713","APPBP2",48.9523491068477,0.457151827153527,0.270333112450472,1.69106855985791,0.0908237091324864,0.72402154534881
"1714","CEP131",20.23377338876,0.868689741187897,0.515170296847229,1.68621860868951,0.0917537009615453,0.72402154534881
"1715","CRTC1",13.5443382760058,0.806168342359047,0.478632986902265,1.68431421239185,0.0921209601452395,0.72402154534881
"1716","RABAC1",441.054890152721,-0.447943643175222,0.266280510440292,-1.68222466764298,0.09252528250289,0.72402154534881
"1717","RRBP1",378.556916280768,0.370150879175511,0.220014806459196,1.68239076784207,0.0924930904300824,0.72402154534881
"1718","GRK3",4.89675435149233,-1.10732114334195,0.658108709893354,-1.68258089688767,0.0924562523390952,0.72402154534881
"1719","DMD",20.5230886928763,0.674432378071569,0.400751464693384,1.68291930907247,0.0923907130801869,0.72402154534881
"1720","RRAGB",22.9434903263714,0.592805375925518,0.352481021234438,1.68180792784086,0.092606090933472,0.72402154534881
"1721","MT-ND1",8142.00000395468,-0.29580275288205,0.175282362196926,-1.68757853998865,0.0914921615850147,0.72402154534881
"1722","LUC7L3",222.241776239419,0.448958574112464,0.26710319341778,1.68084315416717,0.0927933839773047,0.724187158693959
"1723","LYPLA2",19.0804766720319,-0.6594767313514,0.392510320894249,-1.68015131385316,0.0929278791591718,0.724650149151394
"1724","FUCA1",28.6659916538854,-0.573916500449429,0.341834642174923,-1.67893018916364,0.0931656501013017,0.724650149151394
"1725","NIFK-AS1",17.9442976777952,0.636854339655697,0.379328966488226,1.67889719984109,0.0931720803743419,0.724650149151394
"1726","AC106707.1",8.11350388153111,-0.988304889611157,0.588755413877196,-1.67863405807645,0.0932233846733557,0.724650149151394
"1727","BCL7A",12.3460505488408,0.913523874250902,0.544352454506446,1.67818454144599,0.0933110785981852,0.724650149151394

"1728","ATXN3",71.1412095273697,0.436754998881566,0.260275846089121,1.67804660111264,0.0933380019481408,0.724650149151394
"1729","ZNF99",2.45787148261882,2.00032607254423,1.19173286029506,1.67850206970794,0.0932491267551705,0.724650149151394
"1730","ZNF543",2.61134447915518,1.74049752761516,1.03682429518233,1.67868127290467,0.0932141776107054,0.724650149151394
"1731","AC008969.1",8.62878747045891,1.00456377895322,0.598226694004092,1.67923596359335,0.0931060655497662,0.724650149151394
"1732","PTGIR",4.80020185022712,-1.75965691134416,1.0489132677807,-1.67760001269433,0.0934252103383155,0.72490843056387
"1733","MICOS10",232.177661747326,-0.392222295174377,0.23407665999948,-1.67561471175831,0.0938136852568289,0.725948589142988
"1734","MKLN1",69.4110812320925,0.480344402274437,0.286700813932365,1.675420434585,0.0938517700452733,0.725948589142988
"1735","RBM33",58.0256716419778,0.434061010625282,0.258913077620267,1.67647387538259,0.093645409195647,0.725948589142988
"1736","MEAK7",14.1963082017948,0.701475408773806,0.418726474695521,1.67525927106445,0.0938833728648347,0.725948589142988
"1737","TNFSF13",13.7009908144807,-0.81164694496599,0.484204343175491,-1.67624879125015,0.0936894708216729,0.725948589142988
"1738","CD320",26.8308579747546,-0.685719215723573,0.409255137615933,-1.67552989002935,0.0938303116125078,0.725948589142988
"1739","NPAS2",10.5160841062306,1.13256972302796,0.676173458036964,1.67496921029108,0.0939402728601281,0.725970860820737
"1740","TBC1D9",22.8452407471228,0.647937650006643,0.387239971533733,1.67321996084332,0.0942840022272923,0.726044701962193
"1741","ZNF398",11.6728711300187,0.772602077164519,0.461591726908725,1.67377799931257,0.0941742377281501,0.726044701962193
"1742","B4GALT1-AS1",7.76162853235592,-0.922387821146781,0.551496110472492,-1.67251917761764,0.0944219894359132,0.726044701962193
"1743","ORAI1",20.6242550218433,0.819673793276055,0.489620083917398,1.67410165595727,0.0941106223906302,0.726044701962193
"1744","PGAM5",6.0113178558142,-0.935085387244723,0.559017094009598,-1.67273129438269,0.0943802056669318,0.726044701962193
"1745","DZIP1",24.4772612895736,0.571196433619732,0.341573159424333,1.67225210137235,0.0944746204711697,0.726044701962193
"1746","WDR76",2.43618745865251,-1.56950528392492,0.938601837380588,-1.67217367516031,0.0944900798967093,0.726044701962193
"1747","SNHG9",54.5394746159743,-0.656204088780716,0.392046214572072,-1.67379269175441,0.0941713491535654,0.726044701962193
"1748","ZSWIM7",42.9815536985954,0.533157146961789,0.318644221887282,1.67320513080067,0.0942869206507759,0.726044701962193
"1749","YIF1B",28.0432423972444,-0.598134912331734,0.357293839008002,-1.67407004272004,0.0941168345146178,0.726044701962193
"1750","ZNF281",17.5734403860479,0.642334870916294,0.384349804564496,1.67122465860004,0.0946773114139336,0.727067650338202
"1751","NELFE",61.6800564433124,-0.476276118755021,0.285284411575009,-1.66947824497518,0.0950226383107757,0.728104766881486
"1752","STRBP",6.32042143483243,1.10510028336848,0.661956397645934,1.6

6944573282842,0.0950290766508019,0.728104766881486
"1753","UBE2N",61.9176819796508,-0.440115250252661,0.263554972978429,-1.6699182158429,0.094935545783409,0.728104766881486
"1754","PLA2G4C",14.8891335443741,0.879374308113464,0.526654898904939,1.66973536169877,0.0949717341139474,0.728104766881486
"1755","CARS",24.2152057568368,-0.564475361927369,0.338219279507302,-1.66896270002604,0.0951247722078768,0.728260456380983
"1756","TMEM265",27.5535632493555,-0.541800242765658,0.324665314929594,-1.66879619673309,0.0951577767248311,0.728260456380983
"1757","GALC",32.7265225492629,0.489524538741945,0.293395832131755,1.66847816202827,0.0952208435037694,0.728328352787226
"1758","LINC00629",1.70416368589849,2.31974742987166,1.39062992209819,1.66812707896548,0.0952905026968594,0.728446567544422
"1759","BOLA3-AS1",6.45697591192238,-0.984528264206693,0.590666129205211,-1.66681009038296,0.0955521731940589,0.728718284092293
"1760","CASP2",14.1074898776741,0.678168657543046,0.406981295225933,1.66633863889633,0.0956459849157202,0.728718284092293
"1761","DCLK1",360.143019836357,0.351937250650743,0.211089630845546,1.66724082675692,0.0954665275845202,0.728718284092293
"1762","FOPNL",28.2987032609516,0.569041261104841,0.341324169370777,1.66715782874051,0.0954830257395383,0.728718284092293
"1763","C1QBP",114.444947191816,-0.467063507785075,0.280259699474844,-1.66653824527846,0.0956062572645757,0.728718284092293
"1764","CPD",32.4786075488272,0.483249858127696,0.290011720147029,1.66631147831784,0.0956513917061392,0.728718284092293
"1765","CH25H",6.05857803176051,-1.35596027686199,0.814048004179311,-1.66570063423841,0.0957730556058615,0.728985718068582
"1766","CARS2",36.2962903878305,-0.517375811603809,0.310626038111943,-1.66559060775632,0.0957949831169816,0.728985718068582
"1767","NEXN-AS1",2.47561498536941,1.74470816399379,1.04803585541576,1.66474091032091,0.0959644572869964,0.729862106100705
"1768","DDT",107.986090053685,-0.420254052955485,0.252514609136385,-1.66427619531709,0.0960572472637434,0.730154607453308
"1769","ANPEP",122.666065096295,0.441391662242991,0.265278525961453,1.66388010730702,0.0961363911108601,0.730343109179677
"1770","NBPF26",17.0314762130219,0.797749026163639,0.480050224729129,1.66180325530266,0.0965522293360389,0.733087802286456
"1771","HERC5",6.70217259760846,1.11581418200792,0.671916104244068,1.66064509387411,0.0967847467027786,0.733864279080036
"1772","ADRA2A",3.48072101940122,-1.47668890363475,0.889378387392064,-1.66036067951332,0.0968419153673793,0.733864279080036
"1773","BRF1",12.3644291405401,0.770301815718281,0.463979530070695,1.660206465576,0.0968729244056838,0.733864279080036
"1774","PRPF31",55.4260929573537,-0.46786075634136,0.281696791455541,-1.6608664725072,0.096740267208836,0.733864279080036
"1775","BRCA1",13.0357087354822,0.760707329012589,0.458381928138562,1.65954912773664,0.0970051896893485,0.734452250273326
"1776","S100A13",1155.90814400327,-0.442560401295128,0.267201513520586

,-1.65627954521684,0.0976652208444882,0.735060640909764
"1777","ARF1",245.334741979167,-0.300265523806636,0.181010474313005,-1
.65882955086574,0.0971501438288797,0.735060640909764
"1778","AC104794.5",1.83086375452114,-1.87832038905293,1.1347594174714
7,-1.65525869195984,0.0978720342820379,0.735060640909764
"1779","AP3B1",45.8605110347556,0.447276231606505,0.269806187407008,1.
65776862237699,0.0973641768988305,0.735060640909764
"1780","PBX2",18.6648793031317,0.740495283047477,0.447033248929659,1.6
5646578821701,0.097627527794844,0.735060640909764
"1781","MROH1",12.3692151593476,0.743962789654073,0.44862875554587,1.6
5830384356182,0.0972561536269424,0.735060640909764
"1782","SETX",87.3771650251544,0.424101230433147,0.256093993124622,1.6
5603739962291,0.0977142452076431,0.735060640909764
"1783","COMTD1",23.8635049219895,-0.577146788460161,0.348749130177167,
-1.65490531307409,0.0979437063605652,0.735060640909764
"1784","FKBP2",21.3711850227315,0.729844948260607,0.441044414382083,1.
65481054619667,0.0979629340465394,0.735060640909764
"1785","C1RL-
AS1",12.2572043257121,0.928472191177086,0.561162243149094,1.6545521415
8163,0.0980153782655181,0.735060640909764
"1786","DDX47",4.2131737693832,-1.170552162162,0.7069021719115,-1.6558
8989350077,0.0977441186697259,0.735060640909764
"1787","SGSM2",23.9498381512966,0.614809355956199,0.37152525241614,1.6
5482521566948,0.0979599574927266,0.735060640909764
"1788","RNF112",5.75343218877777,-1.02383055584406,0.618527766597645,-
1.65527016107275,0.0978697088262096,0.735060640909764
"1789","AC009283.1",4.70687800746406,1.26143323698392,0.76161571382072
4,1.65625946798788,0.0976692848968473,0.735060640909764
"1790","TCF4",486.928420810385,0.365297573378558,0.220424583550665,1.6
5724515611751,0.0974699204357855,0.735060640909764
"1791","LTBP4",898.59553893297,0.413239687353048,0.249312546136309,1.6
5751661421449,0.0974150727089811,0.735060640909764
"1792","NKRF",5.87907982869159,1.08031706316798,0.652376169217678,1.65
597260314319,0.0977273671214456,0.735060640909764
"1793","ENPEP",5.45231022098422,1.25088749262978,0.756301364631461,1.6
5395376912922,0.0981369063815044,0.735561564339675
"1794","ILF2",77.1294199674765,-0.500375492887611,0.302647430420386,-1
.6533280728423,0.0982641126100902,0.736104464530101
"1795","F2R",17.5257144222737,-0.755106325107448,0.456811729766086,-1.
6529924165785,0.0983324069484921,0.736205691911301
"1796","ZNF704",73.6372960888034,0.481459192641462,0.291424178624348,1
.65209075964172,0.098516050373033,0.737169933721153
"1797","HINT1",387.201846237511,-0.397926520500934,0.240983836333148,-
1.65125813646199,0.0986858765848352,0.737316558262801
"1798","EXT2",64.1586989989863,0.398359696358386,0.24121374298426,1.65
148009988958,0.0986405809214721,0.737316558262801
"1799","EIF6",84.7616903808574,-0.472309563126585,0.286042307483239,-1
.65118778156361,0.0987002372434541,0.737316558262801
"1800","BCL7B",28.9704470787622,-0.644489354092022,0.390537001166423,-
1.65026451313734,0.098888847040202,0.738148396117722

"1801","CYP3A5",16.4994980542308,-0.801463551666141,0.485750773994019,-1.64994806920474,0.0989535579200748,0.738148396117722
"1802","PSMB6",177.702299828455,-0.461301634704177,0.279639516795528,-1.64962963743597,0.0990187094150289,0.738148396117722
"1803","KRT222",144.687519170535,0.601004334534382,0.364399677435419,1.64929985329336,0.0990862197035769,0.738148396117722
"1804","CDC42EP4",43.8623100137325,-0.42296605501026,0.256430163945482,-1.64943955306359,0.0990576172054407,0.738148396117722
"1805","NDUFS7",108.605017045844,-0.447287255888009,0.271267515228904,-1.64887880331183,0.0991724664559273,0.738381593740281
"1806","FASTKD1",4.61783372585846,-1.1634990137298,0.706047172804721,-1.64790549207624,0.0993720662399897,0.739414402316352
"1807","THAP9",4.70044791188544,-1.16122664163368,0.704999861154208,-1.64713031252595,0.0995312637689025,0.739414402316352
"1808","VPS50",15.931438462441,0.743493638048851,0.45136508629006,1.64721122796605,0.0995146367746472,0.739414402316352
"1809","AXL",287.959665578928,-0.26835278612967,0.16288456597455,-1.64750284671906,0.0994547315719332,0.739414402316352
"1810","SEMA4C",9.43258269648,0.806205033135747,0.489889743958844,1.64568669395021,0.0998282799016875,0.739984695862536
"1811","HEBP1",135.857950755525,-0.494452095948799,0.300358330201439,-1.64620736710444,0.0997210730292894,0.739984695862536
"1812","ATP8B4",10.5281094233185,0.8874218692912,0.539060002289761,1.64623950120897,0.0997144596103194,0.739984695862536
"1813","TXLNG",33.7807724557976,0.463619622803972,0.281683398854685,1.64588905377113,0.099786602994463,0.739984695862536
"1814","MR1",35.1916270762847,0.500885418630059,0.304665487905336,1.6440504045069,0.10016579120072,0.741281338096863
"1815","RHOU",50.8236347633228,0.521702654687774,0.31739208753305,1.64371663686622,0.10023474779269,0.741281338096863
"1816","CCDC121",13.2132091932141,0.76511202668919,0.46571707285334,1.64286875291371,0.100410091315229,0.741281338096863
"1817","BHLHE40",32.5512911485928,-0.604529982160787,0.367813177910466,-1.64357891034547,0.10026321320172 7,0.741281338096863
"1818","CD47",224.494384212418,-0.350442652151579,0.213351371057581,-1.64256105041387,0.100473785033061,0.741281338096863
"1819","NEPRO",21.1173661233288,0.570422149144273,0.347471401918627,1.64163768872656,0.100665112138312,0.741281338096863
"1820","KLF3-AS1",23.4437263186598,0.643092533282587,0.391614627558934,1.64215656930692,0.100557560688467,0.741281338096863
"1821","SPRY1",331.770987848152,0.474910183338316,0.289176576433121,1.64228441043236,0.10053107636832,0.741281338096863
"1822","KCNMB4",10.6258278328494,0.955158188003125,0.58147548837206,1.64264566108754,0.100456267606644,0.741281338096863
"1823","MIR22HG",37.9068997687613,-0.507547114906929,0.308977132955122,-1.64266886048376,0.100451464929685,0.741281338096863
"1824","CSTB",340.400181758506,-0.383277877242003,0.233464358617475,-1.64169760006063,0.100652689282035,0.741281338096863
"1825","CHM",37.2309391629399,0.490908285112232,0.298752670867522,1.64

31929585323,0.100343016315105,0.741281338096863
"1826","PON2",31.0737127337753,0.535869113848832,0.326532170420843,1.64109133001563,0.100778458205692,0.741678783885892
"1827","AEBP2",27.9741082542225,0.558502987054573,0.340375093203847,1.64084563825618,0.100829461876592,0.741678783885892
"1828","ALDH6A1",34.0073986507872,0.447161728313968,0.272581003308856,1.64047282417292,0.100906894420778,0.741842316258662
"1829","RTTN",5.26392297016259,1.22621468251114,0.747616299577634,1.64016579521325,0.100970699130609,0.741905536148857
"1830","CABLES1",17.4714654401871,0.629359982839499,0.383926683545387,1.63927127186798,0.101156776261569,0.742866620862963
"1831","CCT7",121.555377284966,-0.381182589076826,0.232820625610547,-1.63723719957034,0.101580917109148,0.745573973254963
"1832","LRRC8C",20.4725038959868,0.645101381152018,0.394353988843726,1.63584342849809,0.101872360281479,0.746913468848073
"1833","FAM71F2",1.60374783607939,2.62719345263562,1.6063139047611,1.63554174862625,0.101935530310208,0.746913468848073
"1834","HOXC10",50.403867351912,-0.400415871770167,0.244846365697472,-1.6353760066217,0.101970249000566,0.746913468848073
"1835","CALM1",859.363244512872,-0.285621047312012,0.174659497902866,-1.6353021206488,0.101985729245942,0.746913468848073
"1836","RPL5",1967.82809956853,-0.426274724739114,0.260884137665887,-1.63396183667189,0.102266864170661,0.747137607629759
"1837","INTS3",27.784052072739,0.643355688868817,0.393702318813667,1.63411709335984,0.102234266332865,0.747137607629759
"1838","SELENOP",1022.51373942416,0.420943838625065,0.257767391230234,1.63303758716743,0.102461091663193,0.747137607629759
"1839","MLIP",2.18536591100241,-2.34619483243406,1.43661675217752,-1.63313899053304,0.102439767811361,0.747137607629759
"1840","CYB5R2",5.64575655331576,-0.973592237107117,0.595849299338637,-1.63395717371449,0.102267843337431,0.747137607629759
"1841","C1RL",49.5035319919112,0.519129765998859,0.317662643066811,1.63421723431822,0.102213245031683,0.747137607629759
"1842","TBX3",23.2232188831395,-0.544528000180709,0.333407376419334,-1.63322121432564,0.102422479774833,0.747137607629759
"1843","BTBD7",68.2145902233401,0.471020044280935,0.288306619477512,1.63374689465871,0.102312007245734,0.747137607629759
"1844","ZNF438",25.5682604891751,0.578408311766963,0.35436919244375,1.63221951597493,0.102633250938114,0.747811533245174
"1845","ZNF720",33.0801819993377,0.493590804783216,0.302432428298706,1.63206970747101,0.102664802354144,0.747811533245174
"1846","CPED1",84.0440141028683,0.370042622038383,0.226840014073559,1.63129341862228,0.102828421428072,0.748584060645598
"1847","PARVA",132.992743238237,0.367181936373864,0.225121608094896,1.63103817301752,0.102882265050407,0.748584060645598
"1848","GID4",13.6541388278626,0.672381008765778,0.412565188460613,1.62975701191515,0.103152862600737,0.750009190981727
"1849","LRRC59",48.6131506902852,-0.42000837212299,0.257781582909157,-1.62931877205131,0.103245554231431,0.750009190981727
"1850","TSPO",449.441688887929,-0.463963763181457,0.284717136544758,-1

.62956037283875,0.103194445328045,0.750009190981727
"1851","STK40",25.1144099242384,-0.532550636123989,0.326947525064421,-1.62885660632866,0.103343378123885,0.750314240198211
"1852","ZNF219",14.9910779696876,-0.667245601597595,0.409899383239465,-1.62782777647603,0.103561409056194,0.751491239906149
"1853","CLPTM1L",52.1081079328002,-0.405650748730659,0.249289629504797,-1.62722673035564,0.103688952604661,0.752010703752854
"1854","NKTR",170.155060476745,0.451825540419565,0.27789886404212,1.62586321457969,0.103978757022296,0.752242886348959
"1855","EPS8L2",28.1911125501973,-0.618412401736075,0.380185752703515,-1.62660593496343,0.103820818022883,0.752242886348959
"1856","MRPL52",62.8735494543959,-0.446477986006745,0.274521640167863,-1.62638539436722,0.103867695923623,0.752242886348959
"1857","CDH11",68.377658614104,0.621106865913067,0.381926220579138,1.62624829730529,0.103896845621938,0.752242886348959
"1858","TEKT3",8.69618626701707,0.979627867260689,0.602566309903385,1.62575944117712,0.104000839559221,0.752242886348959
"1859","AC084824.4",2.18556805870636,-2.11309199626702,1.3000227154149,-1.62542698001445,0.104071610966068,0.752349854638508
"1860","TPD52L2",52.5344942224497,0.475506756577366,0.292722209729757,1.62443005953104,0.104284056018689,0.753480338083417
"1861","AK2",47.3773445372886,-0.422776960502115,0.261253673005238,-1.61826226456015,0.105606089325528,0.754232535665574
"1862","UQCRH",248.002774374611,-0.414532433532433,0.255564360969276,-1.62202754703449,0.104797449981403,0.754232535665574
"1863","GBP4",14.1581343624061,0.701286657625153,0.432344597117036,1.62205486619118,0.104791600880395,0.754232535665574
"1864","JTB",240.294292208562,-0.354904719545316,0.219372183141573,-1.61782006479954,0.105701380936796,0.754232535665574
"1865","ATAD2B",32.1320572008929,0.565125459174097,0.349263180878423,1.61805048488868,0.105651718170083,0.754232535665574
"1866","PLEK",4.44652031238236,-1.51533425931974,0.938453043761857,-1.61471505622211,0.106372415495067,0.754232535665574
"1867","RIF1",81.3574049888338,0.451106123762525,0.278132911248656,1.62190846720483,0.1048229483034,0.754232535665574
"1868","CD302",123.554722495884,0.399424133895332,0.24673009116805,1.61887077496065,0.105475070124238,0.754232535665574
"1869","ATF2",39.804052746971,0.443630730244048,0.274897509166894,1.6138041104429,0.106569922702951,0.754232535665574
"1870","TSEN2",6.19886387917215,1.06601366449772,0.662084923089394,1.61008599852044,0.107379083299192,0.754232535665574
"1871","TBC1D5",68.4614971202769,0.391399281993653,0.242899523203577,1.6113629077222,0.107100646841437,0.754232535665574
"1872","DLG1",41.1370447424802,0.486937884281143,0.302454545229452,1.60995393179407,0.10740791379768,0.754232535665574
"1873","LIMCH1",50.8786252180628,0.523873117101521,0.324244584727475,1.61567267975141,0.106165100817945,0.754232535665574
"1874","C6orf132",39.8677050238137,-0.494370656131132,0.306824997290261,-1.6112463472572,0.10712603968788,0.754232535665574
"1875","BMP5",6.37351618565624,1.39314487697782,0.860558360866459,1.61

88848314426,0.105472045128704,0.754232535665574
"1876","MMS22L",10.9385191445907,0.849407680884947,0.527031448888996,1
.6116831029259,0.10703091644844,0.754232535665574
"1877","ADCY1",6.35626058672149,1.21982970612758,0.756239028958485,1.6
1302135887851,0.106739867510164,0.754232535665574
"1878","VOPP1",24.7929772763424,-0.519461256966656,0.321991481888865,-
1.61327639451638,0.106684472623321,0.754232535665574
"1879","WNT2",43.5298129063077,-0.645322172209687,0.398414699452783,-1
.61972480708173,0.105291404869271,0.754232535665574
"1880","RPL8",2417.34082903318,-0.40843190164863,0.253072553063798,-1.
61389252490635,0.106550740344583,0.754232535665574
"1881","OLFM1",33.3993059494112,0.718428077282628,0.445441507785443,1.
61284492963928,0.10677820207137,0.754232535665574
"1882","WAC-
AS1",57.5576607228905,0.478879492775184,0.296725802453866,1.6138788363
3625,0.106553710030924,0.754232535665574
"1883","UBXN1",166.330343397766,-0.448043654468159,0.277016884055409,-
1.61738753215685,0.105794655313196,0.754232535665574
"1884","NEAT1",2545.41169825046,0.633891387128329,0.391283242997487,1.
62003203171264,0.105225396285962,0.754232535665574
"1885","FKBP4",26.8120446958827,-0.506769322455708,0.314009961298066,-
1.61386384164631,0.106556963150586,0.754232535665574
"1886","DIP2B",15.0684247197316,0.694225617919698,0.43037834697707,1.6
1305888829181,0.106731714522804,0.754232535665574
"1887","LRP1",959.134514990819,0.300763671747018,0.185282969135507,1.6
2326668851606,0.104532407271444,0.754232535665574
"1888","GNPTAB",41.5584087570412,0.469323446012297,0.291148428614548,1
.61197313770714,0.10696778528261,0.754232535665574
"1889","MEDAG",387.445168658058,0.341321900828217,0.212013804953222,1.
60990413291024,0.107418786610902,0.754232535665574
"1890","SERP2",7.66341343887498,-0.877362843527864,0.544449293641333,-
1.61146842097998,0.107077664755558,0.754232535665574
"1891","SPTSSA",42.5366491290084,-0.438496405923274,0.270556217167312,
-1.62072197236594,0.105077279145632,0.754232535665574
"1892","PTGER2",3.39678634781131,-1.27925737602867,0.790528412461727,-
1.61823073764678,0.10561288094453,0.754232535665574
"1893","TC2N",25.0107724625202,0.814590127389339,0.503154039971937,1.6
1896767724408,0.105454217871214,0.754232535665574
"1894","DYNC1H1",243.12504361886,0.28213168436311,0.174332777703716,1.
61835133977273,0.105586902357637,0.754232535665574
"1895","WDR61",68.1560478920487,-0.353707661082517,0.218402665330066,-
1.61952080826472,0.105335253076136,0.754232535665574
"1896","ERI2",6.83097392623948,0.948333543013719,0.588826108828787,1.6
1054941143832,0.107277967580154,0.754232535665574
"1897","MINK1",20.67090108538,0.580228444329598,0.35989193085378,1.612
22965725602,0.106911973887079,0.754232535665574
"1898","THRA",65.7755012869318,0.434078207833738,0.268892289803853,1.6
1432002438739,0.106458028874241,0.754232535665574
"1899","HLF",28.5615717421952,-0.709443542652304,0.438572781608365,-1.
61761872237165,0.10574479171308,0.754232535665574

"1900","MYO15B",9.10138295496234,0.823232207613076,0.508156044237894,1
.62003820863278,0.105224069483956,0.754232535665574
"1901","RNF213",133.829267986363,0.414961781697421,0.257065680990629,1
.61422473858947,0.106478687888742,0.754232535665574
"1902","JUNB",1193.44425838536,-0.645688788094449,0.400366710260169,-1
.61274344631416,0.106800257319898,0.754232535665574
"1903","UQCC1",13.0550121541872,0.76695599564938,0.475580335734929,1.6
1267390180079,0.106815373429583,0.754232535665574
"1904","CCN5",2734.65819280831,-0.375501024636329,0.233141507735285,-1
.61061420715647,0.107263835303743,0.754232535665574
"1905","SON",376.34426707272,0.318595104553508,0.196535011701027,1.621
06029758281,0.10500470764929,0.754232535665574
"1906","BRWD1",57.4214465752779,0.473062436894496,0.291579949705881,1.
62241072258802,0.104715434793773,0.754232535665574
"1907","ARVCF",5.01168567247483,1.14436962687431,0.70829418246208,1.61
566995072076,0.106165691166715,0.754232535665574
"1908","RNF185",15.9076278579107,0.70214456353443,0.435254096231814,1.
61318312593311,0.106704728335594,0.754232535665574
"1909","CERK",23.9485808214998,0.588041764550711,0.362578173719337,1.6
2183442681771,0.104838804904212,0.754232535665574
"1910","NBDY",79.207319097299,-0.479626672791434,0.297720388521993,-1.
6109970673238,0.1071803616481,0.754232535665574
"1911","FAM133A",1.56590348558773,-1.927394169657,1.19672724489455,-1.
6105542661284,0.107276908696392,0.754232535665574
"1912","LINC00894",2.95797679053927,1.65247353455931,1.02417004226723,
1.61347575730802,0.106641185997822,0.754232535665574
"1913","UBL4A",33.7787468725223,-0.567071387357034,0.350403291893962,-
1.61833921220306,0.105589514496249,0.754232535665574
"1914","MT-
ND3",9848.3300244782,-0.580871938202812,0.358228090723791,-1.621514206
2957,0.104907405742154,0.754232535665574
"1915","AL021155.5",5.30540181248583,1.34036740050254,0.83292780762061
,1.60922397864409,0.107567375013917,0.754372840173063
"1916","FBXL15",39.1750803714176,-0.545710224717089,0.33919793482668,-
1.60882531609731,0.107654543582296,0.754372840173063
"1917","CREBBP",80.9485742295206,0.431646389706121,0.268284848422955,1
.60891079851675,0.107635847926826,0.754372840173063
"1918","GDPD3",3.75928244492987,1.34411103057235,0.835481931659614,1.6
0878527666348,0.107663301395337,0.754372840173063
"1919","ALMS1",13.0610078830944,0.739380775381624,0.459666671149269,1.
60851508666706,0.107722414719531,0.754393711003529
"1920","TRAPPC3L",1.63144590776772,-1.90227153316093,1.18324355020924,
-1.60767538756035,0.107906291740434,0.755287840989423
"1921","AL356488.3",1.90558348954921,1.93204566494719,1.20288538758657
,1.6061760204965,0.108235240511316,0.755337231459857
"1922","PCOLCE2",1344.75134773785,-0.352011322171261,0.219119956481717
,-1.60647769296464,0.108168992360368,0.755337231459857
"1923","PCDHB10",4.87018886855855,1.22044700873688,0.760242483803214,1
.60533913157738,0.108419191865917,0.755337231459857
"1924","TUBE1",22.673116090054,0.524140646378304,0.326470848046636,1.6

```
0547457610497,0.108389403877726,0.755337231459857
"1925","HSF1",37.0982660515939,-0.444219703683868,0.276666965542424,-1
.60561165230892,0.108359263633169,0.755337231459857
"1926","ACACB",21.8492213021443,0.537375092250842,0.334400499180181,1.
60698053252993,0.108058638666452,0.755337231459857
"1927","SHC2",29.8679802225483,0.473729469859061,0.29503024885419,1.60
569796384908,0.108340288899694,0.755337231459857
"1928","PNMA6A",5.76932092111614,1.28493122489036,0.799898856631663,1.
60636712283993,0.108193270154341,0.755337231459857
"1929","AC244090.1",9.47595278915127,0.926060796776771,0.5766889177746
59,1.60582381286305,0.108312626958074,0.755337231459857
"1930","GPR157",3.46963960864718,1.62827631781506,1.01471359467849,1.6
0466591396262,0.108567347122629,0.755407204268143
"1931","ECHDC2",67.6776550082766,0.359459112524804,0.224229455035887,1
.60308605516289,0.108915656283512,0.755407204268143
"1932","PRKACB",28.014036136954,0.52421326603934,0.327124434560473,1.6
024888716848,0.109047546415671,0.755407204268143
"1933","SPR",22.1092522948071,-0.569390188577097,0.355259146102617,-1.
60274603714953,0.108990735015747,0.755407204268143
"1934","GFPT2",164.952123389359,0.434415962516868,0.270973985107786,1.
60316482906678,0.108898268204984,0.755407204268143
"1935","EIF3M",120.191352845638,-0.357735051108756,0.223209900658923,-
1.60268451378147,0.109004324246079,0.755407204268143
"1936","SLC7A1",12.4725089505879,-0.712745923162046,0.444110054343916,
-1.6048858074492,0.108518937473977,0.755407204268143
"1937","COX5A",99.8483473178049,-0.508790573289766,0.317357059460753,-
1.60321177084982,0.108887907601772,0.755407204268143
"1938","LRRC37A3",4.14844784433559,1.50774749770447,0.939811331003861,
1.60430870320958,0.108646023649201,0.755407204268143
"1939","BPTF",189.36191890282,0.370857694529967,0.231298889045566,1.60
336997752249,0.108852995271543,0.755407204268143
"1940","ZNF133",6.9115248900029,0.957709610081322,0.597195583037077,1.
60367832128099,0.108784976816616,0.755407204268143
"1941","CD74",80.0373425661371,-0.555717962458464,0.346950356910907,-1
.60172183538398,0.10921713442691,0.756192204823926
"1942","DDI2",34.6477896230325,0.655331531579233,0.409347013670002,1.6
009192926653,0.109394795917341,0.75672942862221
"1943","PURB",38.5900823948865,0.498133167483272,0.311165462064756,1.6
0086265415795,0.109407342794327,0.75672942862221
"1944","CCNL2",73.995512305851,0.47513454547927,0.298972253817896,1.58
922622220547,0.112009328382931,0.757500090203998
"1945","UBR4",27.0725380042777,0.548689581991991,0.343811533878943,1.5
9590219618748,0.110510631136197,0.757500090203998
"1946","NCDN",9.58080022846095,0.831156236630712,0.525118100303353,1.5
8279868119298,0.113467357419085,0.757500090203998
"1947","NPR1",89.7116100008961,-0.329870844068121,0.209152112045117,-1
.57718151082769,0.114753766021033,0.757500090203998
"1948","RCSD1",1.75266786318165,-1.8292601388033,1.15656892015188,-1.5
8162657402474,0.113734843823485,0.757500090203998
"1949","DYRK3",6.41039736235509,-0.934943767011766,0.590947645566153,-
```

1.58210930194341,0.113624620474243,0.757500090203998
"1950","DNMT3A",19.1717067396275,0.634958561674386,0.399959387001429,1
.58755759287159,0.112386413979517,0.757500090203998
"1951","LINC01119",1.98248019912714,-2.0086977675596,1.26620504325065,
-1.58639217105217,0.112650375271869,0.757500090203998
"1952","NBEAL1",78.2928703575217,0.348457081181937,0.2189097586155,1.5
9178413692364,0.111433215658551,0.757500090203998
"1953","CNTN4",34.8437712371358,0.819230345892865,0.515996930148532,1.
58766515462998,0.11236207646687,0.757500090203998
"1954","AC006059.1",3.81698262959743,-1.17422747590304,0.7429332731275
45,-1.5805288555187,0.113985804076496,0.757500090203998
"1955","TASOR",62.4445336389217,0.394520192572712,0.247194759717077,1.
59598930424032,0.110491181340543,0.757500090203998
"1956","PSMD6",44.7963145377363,-0.402655220283827,0.254822115816088,-
1.58014236321009,0.114074267600181,0.757500090203998
"1957","CCDC80",3674.05999358117,-0.372368744545908,0.234217533298362,
-1.58984145764852,0.111870546323338,0.757500090203998
"1958","SEL1L3",3.99260297615629,1.372787329242,0.86864579864078,1.580
3764104887,0.114020690490097,0.757500090203998
"1959","SLAIN2",52.8989636426397,0.462448595440407,0.291429089262808,1
.58683059611587,0.112551017248626,0.757500090203998
"1960","CDS1",16.8491675303976,0.844734280144525,0.532825006480419,1.5
8538782878158,0.112878244821446,0.757500090203998
"1961","WDFY3",47.0460575488661,0.498491956392001,0.313495050932827,1.
5901110875872,0.111809767173514,0.757500090203998
"1962","SLC10A7",11.6873701023517,0.720461724901122,0.456273996610495,
1.57901114298248,0.114333501189405,0.757500090203998
"1963","ARL15",7.51163225119443,0.862066555453352,0.545768767547402,1.
57954541687562,0.114211007766453,0.757500090203998
"1964","FCHO2",48.9934191168516,0.451455628406124,0.285721032490968,1.
58005738839123,0.114093724578786,0.757500090203998
"1965","PCDHB7",3.55244867775654,1.45891679097507,0.92239217138331,1.5
8166649310037,0.113725725736109,0.757500090203998
"1966","NHP2",98.5120424487898,-0.385190776786437,0.243444280576703,-1
.58225436996896,0.113591512911373,0.757500090203998
"1967","IRF4",16.6152731780973,0.69611484311635,0.437179918653448,1.59
228457990578,0.111320776120834,0.757500090203998
"1968","CHST12",74.9820157075207,-0.423042215054336,0.264850879344834,
-1.59728454026969,0.110202295312274,0.757500090203998
"1969","POLM",11.1545618526062,0.749772954117297,0.471665896669998,1.5
8962723277379,0.111918854811613,0.757500090203998
"1970","CCT6A",68.6915834974753,-0.36364298693726,0.229782480197679,-1
.58255314602063,0.113523349895535,0.757500090203998
"1971","POM121C",25.8781970568596,0.59152973539725,0.372007724839965,1
.59010067775265,0.111812113242786,0.757500090203998
"1972","MDH2",106.918593007509,-0.471780164892286,0.296813408974164,-1
.58948400115358,0.111951163240765,0.757500090203998
"1973","KLHDC10",32.8835264610736,0.558917156421473,0.350899455875393,
1.59281283302972,0.111202185364436,0.757500090203998
"1974","PNMA2",4.30124881645684,1.44647426821997,0.915075633463113,1.5

8071553358466,0.11394309502189,0.757500090203998
"1975","BAG4",24.9471700118875,0.481931198015555,0.304921635748757,1.5
8050837170717,0.113990491223095,0.757500090203998
"1976","PABPC1",323.35511216391,-0.334866062835249,0.209908688821983,-
1.59466963832188,0.110786130968761,0.757500090203998
"1977","ROR2",2.78748380323626,1.67070370349914,1.05917266189583,1.577
36671612041,0.114711169323177,0.757500090203998
"1978","RC3H2",30.6399502544639,0.583271788228609,0.366015968073361,1.
59356924043189,0.111032548515903,0.757500090203998
"1979","PRRC2B",82.1915227780842,0.417669489677377,0.264866443187908,1
.57690602346732,0.114817150364279,0.757500090203998
"1980","NTNG2",4.43611184216119,1.33214992010509,0.833405353678761,1.5
9844175973289,0.109944697164693,0.757500090203998
"1981","EGFL7",3.42674380747707,-1.27937485227309,0.809487208925319,-1
.58047568654185,0.113997970622051,0.757500090203998
"1982","FAM107B",77.9178041393828,0.466780251123282,0.292004466621761,
1.59853805156998,0.109923283972097,0.757500090203998
"1983","EIF3F",304.399608729733,-0.40137632752921,0.250861825822686,-1
.59998966049506,0.109600877151125,0.757500090203998
"1984","PTPMT1",54.7876484266653,-0.49241542960524,0.310556838148332,-
1.58558875258141,0.112832629310053,0.757500090203998
"1985","TRPT1",19.9334549711815,-0.589707190795612,0.372166492267251,-
1.58452521397909,0.113074248485124,0.757500090203998
"1986","RPS25",1836.84665601027,-0.371846668344041,0.235614575909109,-
1.57819891621427,0.114519919166454,0.757500090203998
"1987","RECQL",90.098314152769,-0.329995398219626,0.208476593519065,-1
.5828894392859,0.113446666216054,0.757500090203998
"1988","TDG",40.4662430521003,0.437307028025504,0.277073506046366,1.57
830690586607,0.114495120176409,0.757500090203998
"1989","CKAP4",110.003506149044,0.343133888516446,0.21668943922,1.5835
2843475712,0.113301070928298,0.757500090203998
"1990","ARPC3",322.071787813047,-0.432255954537121,0.271037267280975,-
1.59482110660825,0.11075224576271,0.757500090203998
"1991","POLR1D",125.354236520563,-0.353099701206538,0.222032694124723,
-1.59030498908503,0.111766074600482,0.757500090203998
"1992","F10",544.111785074101,0.404329300534981,0.256397748985941,1.57
696119460531,0.114804454341241,0.757500090203998
"1993","GMPR2",43.934962573385,-0.475823206444904,0.298621844224677,-1
.5933971866,0.111071116468087,0.757500090203998
"1994","EGLN3",2.12753988522515,1.95012799153394,1.22932486182328,1.58
63406428156,0.112662057391069,0.757500090203998
"1995","BRMS1L",19.4839061970161,-0.546526105258042,0.343741303603303,
-1.58993434751375,0.111849604434096,0.757500090203998
"1996","TNFAIP2",319.229976141471,0.433243133240105,0.271494685581733,
1.5957702166869,0.110540105140401,0.757500090203998
"1997","LCMT2",3.55853204003577,1.30871679256702,0.819443976481962,1.5
9707903179129,0.110248091511976,0.757500090203998
"1998","PATL2",3.22412392981682,1.44052618081092,0.912140304812159,1.5
7928135968904,0.114271535461016,0.757500090203998
"1999","AC022467.1",4.23811235198329,1.49052921339289,0.94297041368558

9,1.58067442176385,0.113952499688672,0.757500090203998
"2000","MCRIP2",11.3809327090425,-0.716679930255711,0.453342739734462,-1.5808788085489,0.113905750565482,0.757500090203998
"2001","EMP2",141.924918461529,-0.43604052622992,0.275304981130487,-1.58384539371356,0.113228906385879,0.757500090203998
"2002","ZC3H7A",42.9631947777192,0.419640350981152,0.263025701612029,1.59543477465991,0.110615044866479,0.757500090203998
"2003","PPP4C",74.3766885959784,-0.495527246551139,0.312396108187558,-1.58621453201213,0.112690652373683,0.757500090203998
"2004","ZNF423",38.8981009311891,0.467558632949764,0.295907983764427,1.58008116915827,0.11408827915093,0.757500090203998
"2005","IRX3",33.9224369527752,0.684883839618849,0.428797366267237,1.59722025715991,0.110216618760828,0.757500090203998
"2006","POLR2C",57.7963878964719,-0.409861652637878,0.258660617886739,-1.58455375227375,0.113067759716242,0.757500090203998
"2007","WWOX",16.6753795729314,0.696468327599519,0.441506032765262,1.57748315065451,0.114684396076946,0.757500090203998
"2008","WSB1",327.710888985927,0.467321824063557,0.292807495462332,1.59600362458507,0.11048798410186,0.757500090203998
"2009","ZNF207",94.4846397980103,0.40889690525452,0.256909907036659,1.59159648598594,0.111475400170931,0.757500090203998
"2010","CCL8",3.14644294059774,2.31778511890165,1.4659597995714,1.58107003996924,0.113862024127645,0.757500090203998
"2011","MRPS23",41.350895637638,-0.469410620745252,0.294177011207144,-1.59567404271001,0.110561586872432,0.757500090203998
"2012","PIGN",15.3563090693642,0.658166957680155,0.41563811836751,1.58350961712852,0.113305356425234,0.757500090203998
"2013","THOP1",13.2637371870286,-0.665679887755195,0.420072613650845,-1.58467813926211,0.113039481199793,0.757500090203998
"2014","TNFSF9",21.0945585810411,-0.778633044455028,0.490542864518069,-1.58728849357536,0.112447320067421,0.757500090203998
"2015","ZNF429",23.9069172335257,0.556752824553303,0.350716554387286,1.58747232655148,0.112405709757416,0.757500090203998
"2016","ZNF676",5.75664968479132,1.04650974761693,0.661547085000123,1.58191271845259,0.113669497072517,0.757500090203998
"2017","FAM98C",27.6227070114312,-0.513720032977738,0.325767180072469,-1.57695453809514,0.114805986083683,0.757500090203998
"2018","XRCC1",17.6816575194482,-0.552855789137081,0.348919998976401,-1.58447721758268,0.113085162115806,0.757500090203998
"2019","AC074212.1",5.02161921523339,1.19889743453995,0.758864016904677,1.57985806130342,0.114139375444748,0.757500090203998
"2020","ZNF581",32.9976077252816,-0.590714225585605,0.371494276442825,-1.59010316724629,0.111811552180909,0.757500090203998
"2021","PSMF1",74.3706885205434,-0.355382480464695,0.224983070947769,-1.57959654016457,0.114199292104731,0.757500090203998
"2022","NXT1",29.045393399073,-0.512178302553008,0.32237064293976,-1.58878704922494,0.112108478098274,0.757500090203998
"2023","YWHAB",395.2533957617,-0.311722609713711,0.195716946824042,-1.59272160521676,0.111222658524396,0.757500090203998
"2024","PSMA7",381.354366565582,-0.352532908996564,0.223309529162303,-

1.5786738269478,0.114410891155684,0.757500090203998
"2025","DIDO1",41.2474926769059,0.492375542883857,0.31045143049068,1.5
8599862820938,0.112739620742701,0.757500090203998
"2026","IFNAR1",100.950343833238,0.348647317333329,0.219218484846404,1
.59041021370807,0.111742369568866,0.757500090203998
"2027","C2CD2",30.3809123088138,0.54763760949986,0.342832090386546,1.5
9739308208398,0.11017811355558,0.757500090203998
"2028","DGCR2",24.802386222253,0.512624886861454,0.322526352469966,1.5
8940465774557,0.11196906371808,0.757500090203998
"2029","TANGO2",16.1222652456282,-0.580267316366207,0.364007178983588,
-1.59410953923074,0.110911502716248,0.757500090203998
"2030","CYTH4",1.90260228884703,-1.95724266311122,1.23735550208237,-1.
58179493267484,0.113696392207571,0.757500090203998
"2031","SH3KBP1",70.599014435001,0.357247552091003,0.223391607891928,1
.59919862461365,0.109776475856109,0.757500090203998
"2032","GPR34",2.19843397104759,-1.90360369380929,1.20153248916343,-1.
58431312592693,0.11312248026445,0.757500090203998
"2033","OGT",89.5507848120849,0.399957748519479,0.252818378485014,1.58
199633632721,0.113650406857653,0.757500090203998
"2034","PRRG3",83.6723296320649,0.510206745930743,0.321125809248131,1.
58880641554573,0.112104104410077,0.757500090203998
"2035","HAUS7",11.1994706854183,-0.716341422429204,0.448323931947698,-
1.5978210650436,0.11008280516418,0.757500090203998
"2036","BGN",69.8087508742545,-1.32293276175232,0.82919498057224,-1.59
544231784826,0.110613359232872,0.757500090203998
"2037","CCNQ",13.151953035942,-0.847001993850022,0.53625647383482,-1.5
7947182957631,0.114227873051151,0.757500090203998
"2038","AL360178.1",2.01949031007298,1.9245034046527,1.22089704040585,
1.57630278472374,0.114956040189022,0.758044270902978
"2039","ENAH",93.7165104167819,-0.341609859634233,0.216829310150821,-1
.57547823860444,0.115146097623557,0.758181482588431
"2040","CNPPD1",37.6427277399797,0.429541175321723,0.272567226885971,1
.57590910774252,0.115046751713312,0.758181482588431
"2041","HMG20A",23.9967677773427,0.503962359160131,0.319853193388741,1
.57560521381954,0.11511681381678,0.758181482588431
"2042","SND1",50.9901391899971,-0.373013944248644,0.237052631373008,-1
.57354905570189,0.115591738799469,0.760743084096993
"2043","TMEM115",58.5131438888435,-0.437320677079812,0.277998564849372
,-1.57310408173066,0.115694719974254,0.761048135944199
"2044","NECAB2",1.76681399637366,2.14923261152398,1.36649057366263,1.5
7281188245839,0.11576238343118,0.761120680494922
"2045","IRX2",15.4468195224835,-0.741660427708507,0.471724096989082,-1
.57223349928989,0.115896409112276,0.761629262620964
"2046","TPPP",8.59726524640247,-1.19727216957809,0.761742527820269,-1.
57175439974986,0.116007520651712,0.761986837750908
"2047","AC015712.1",2.61555210512337,-1.42248809039089,0.9053874957262
84,-1.57113732750396,0.116150753802664,0.762554948878358
"2048","PREX2",4.61130024018016,1.37714839677385,0.8770215142712,1.570
25611614357,0.116355539101108,0.762781507307213
"2049","SCN2B",3.59549818810548,1.33399397989155,0.849429389318624,1.5

7045894180989,0.116308379194802,0.762781507307213
"2050","LINC01852",3.18064636958371,1.38276305903758,0.880425983481932
,1.57056139298501,0.116284563521646,0.762781507307213
"2051","STPG1",2.37360822607377,1.73398974236954,1.10465995436208,1.56
970453714951,0.116483865192876,0.763250445795741
"2052","VAMP8",8.54069164088978,-0.844466099241026,0.538094590356106,-
1.56936366649248,0.116563225085226,0.763398236803291
"2053","BZW2",43.6800593415387,-0.419688416414181,0.26755914423921,-1.
56858184610937,0.116745405221006,0.764218948253823
"2054","ACVR2A",22.9143662103812,0.623846869129302,0.398044459652443,1
.56727936792292,0.117049405792789,0.764798886817575
"2055","CCT5",99.3212810531834,-0.378441197001411,0.241445284112069,-1
.56739941470861,0.117021360696061,0.764798886817575
"2056","VSTM4",28.2406102789072,0.650088946122298,0.414611144826988,1.
5679485566978,0.116893138477213,0.764798886817575
"2057","PCF11",63.6176436155764,0.400176410668293,0.255340421849219,1.
56722702880393,0.117061634807929,0.764798886817575
"2058","PRKG2",4.267143785894,1.51055214823058,0.964147492417444,1.566
72310005507,0.117179428900645,0.765196474730693
"2059","IRX5",15.1845911902528,-0.754226153132146,0.481623298132781,-1
.56600844696722,0.117346639659067,0.765916216793689
"2060","DANT2",3.08368333962759,1.8803942211079,1.20107635923277,1.565
59090240446,0.11744442118111,0.766182318569384
"2061","ZNF697",4.73460733068834,1.21481894056453,0.776451670806816,1.
56457766302725,0.117681969745671,0.766838327417817
"2062","NBPF19",26.6862538960174,0.866193275563764,0.554164837730614,1
.56306069347697,0.118038319776922,0.766838327417817
"2063","TTN",1.72102268742089,2.15443791113973,1.37825075089753,1.5631
6832023255,0.118013009400626,0.766838327417817
"2064","AC147067.1",11.8167189440802,-0.830675054733553,0.531285989105
016,-1.5635177131866,0.117930872679272,0.766838327417817
"2065","SAP30L-
AS1",1.87434424474647,1.95158653170791,1.2480333616343,1.5637294576423
1,0.117881116759763,0.766838327417817
"2066","FBXL18",6.11916634278511,1.02372267037266,0.65508513505177,1.5
6273225508584,0.118115584325834,0.766838327417817
"2067","P4HA1",78.3476940495861,0.441136013748167,0.282277713110028,1.
56277308926694,0.118105975998116,0.766838327417817
"2068","DYNLRB1",281.266026895537,-0.414116961552129,0.264995030120457
,-1.56273482323003,0.118114980020612,0.766838327417817
"2069","MIR155HG",2.49296019879582,-1.53814925913157,0.983904033951033
,-1.5633122805228,0.117979161193603,0.766838327417817
"2070","PLXNB2",28.3485938934336,0.484660269343742,0.309797869265979,1
.5644402929306,0.117714204435494,0.766838327417817
"2071","RBM8A",117.018037666173,-0.382997460811612,0.245194620878067,-
1.56201412347489,0.118284661716229,0.767441541585519
"2072","RPS10",1318.8271842717,-0.562845977804597,0.360426647898094,-1
.56161033343943,0.118379813654794,0.767441541585519
"2073","RAD9A",12.7174637853545,-0.7311482991975,0.468147514066358,-1.
56179041269855,0.118337371091749,0.767441541585519

"2074","SLC35F5",46.4316433875493,0.406122226187659,0.260529859489926,
1.55883178604855,0.119036198833936,0.769155693889224
"2075","CTNNA1",141.088418272608,0.307589081227419,0.197211694818345,1
.55968986276775,0.118833188873547,0.769155693889224
"2076","USP49",6.19908456110977,1.13675552870867,0.729704948826942,1.5
5782899723525,0.119273790167806,0.769155693889224
"2077","TMEM243",86.14997416983,-0.355457122985699,0.228169526235249,-
1.55786414097741,0.119265457261838,0.769155693889224
"2078","NIPAL2",45.8478523618171,0.49971484540467,0.320307189819772,1.
5601112347364,0.118733597079058,0.769155693889224
"2079","LARP4",24.4487867301128,0.527537130580237,0.338623410995423,1.
55788735642784,0.11925995291427,0.769155693889224
"2080","FNDC3A",82.9188049103978,0.322086035509338,0.206545519883651,1
.55939492510305,0.118902936712759,0.769155693889224
"2081","FAM177A1",73.9333275711435,-0.361930107322113,0.232271471447181,-1.55822023715219,0.119181049296628,0.769155693889224
"2082","APH1B",36.6077739594193,0.444974132754749,0.285541930930528,1.
5583495261262,0.119150414614505,0.769155693889224
"2083","COTL1",121.396500446067,-0.404474104380453,0.259426577303579,-
1.55910820157466,0.118970772803771,0.769155693889224
"2084","UBE2M",96.6979593328737,-0.456205345159662,0.292766849156383,-
1.55825479037067,0.119172861398115,0.769155693889224
"2085","VWA1",10.5777828989606,0.819697885429271,0.532210319956896,1.5
4017660817937,0.123517310137653,0.769776438214376
"2086","RNF207",3.87745307091839,1.28027338178588,0.823352660864283,1.
55495141102959,0.119957641814302,0.769776438214376
"2087","PHF13",13.9313022645052,0.63072818853827,0.409191159319177,1.5
4140228637318,0.12321890792889,0.769776438214376
"2088","CSF3R",4.15969872803085,1.5927852215441,1.02730616475867,1.550
44842149688,0.121033924171623,0.769776438214376
"2089","AKR1A1",128.870700428135,-0.435334937474215,0.280874169457856,
-1.5499287040688,0.121158629256582,0.769776438214376
"2090","MYSM1",38.7169960134251,0.523083408574297,0.337677520367962,1.
54906198080435,0.121366821212983,0.769776438214376
"2091","AK4",6.74539767500313,1.03273607607811,0.668586440402938,1.544
65602900308,0.122429485988203,0.769776438214376
"2092","SRSF11",229.085725505425,0.342817517124323,0.222116343542932,1
.54341419301305,0.122730312198911,0.769776438214376
"2093","AL160006.1",3.10464210557363,1.5002294030278,0.966153903383934
,1.55278511816107,0.120474479185316,0.769776438214376
"2094","CERS2",99.4899695254057,-0.385300695761932,0.249453254474297,-
1.544580753512,0.122447704537342,0.769776438214376
"2095","MSTO1",8.10711650297191,-0.742292196915295,0.478193925500348,-
1.55228278179948,0.120594575947284,0.769776438214376
"2096","Z97200.1",37.3673854212285,0.62573167673703,0.405337025753878,
1.54373185023807,0.122653307017602,0.769776438214376
"2097","SRGAP2",36.4346127354347,0.501287719434255,0.323374039324248,1
.55017923047191,0.121098503439569,0.769776438214376
"2098","SPTBN1",585.083139285029,0.319716447886714,0.207311000852101,1
.54220686105705,0.123023333345474,0.769776438214376

"2099","PCBP1",251.7208388,-0.236192278956678,0.15278893758664,-1.5458729060325,0.122135265148871,0.769776438214376
"2100","LONRF2",3.05150462998871,1.65019101623053,1.06959527217693,1.5428181660451,0.122874900559163,0.769776438214376
"2101","CFLAR",158.597698659283,0.441213265691742,0.286470389328289,1.54017058002501,0.123518779138076,0.769776438214376
"2102","FAM117B",13.7327606807691,0.694738708959224,0.449956920234754,1.54401161025985,0.122585519988685,0.769776438214376
"2103","OBSL1",25.8362550650286,0.479129735788323,0.309944679137627,1.54585565760131,0.122139431670944,0.769776438214376
"2104","RBSN",29.6632301501509,0.57550264452795,0.370045836411394,1.5552198887279,0.119893709124211,0.769776438214376
"2105","METTL6",13.5548711341092,0.718006143894065,0.461729854855845,1.55503512788496,0.11993770342569,0.769776438214376
"2106","AC084198.2",2.794356713643,1.53591976179734,0.993870864697322,1.54539167647811,0.122251552404447,0.769776438214376
"2107","SLC35A5",27.968287767407,0.506725202149297,0.328488146050858,1.54259813707507,0.122928310327096,0.769776438214376
"2108","PIK3R4",14.053112911905,0.67976733986927,0.441592801384919,1.53935330860782,0.123718066241268,0.769776438214376
"2109","CEP70",5.64589498116724,-0.906078882056319,0.588067570590493,-1.54077342021513,0.123371940569044,0.769776438214376
"2110","TFRC",11.2854447417551,-0.781035512423136,0.50680943839951,-1.54108320257338,0.123296537452836,0.769776438214376
"2111","LINC02447",2.24571083839597,1.72809243029002,1.11233222504252,1.55357580350957,0.120285634669036,0.769776438214376
"2112","CXCL8",66.0138776555521,-1.69839509504589,1.09861425139332,-1.54594307591759,0.122118316093177,0.769776438214376
"2113","MCC",30.6398950969075,0.612284707256278,0.394487433097657,1.55210193249605,0.120637835678153,0.769776438214376
"2114","LTC4S",10.4266997844356,-0.795969846566133,0.516180427451093,-1.54203802437191,0.123064353754436,0.769776438214376
"2115","PPP1R3G",8.77143719453256,-0.747200920377827,0.480597722312876,-1.55473254592619,0.120009779990813,0.769776438214376
"2116","HIST1H4H",3.19069475894992,1.46739053071381,0.947554335157359,1.54860832383836,0.12147590375106,0.769776438214376
"2117","ITPR3",27.4945675295518,0.599432031310798,0.388232259173827,1.5440036657088,0.122587444582893,0.769776438214376
"2118","PHIP",117.682442788869,0.391583572083427,0.25220928331436,1.55261363474614,0.120515466291269,0.769776438214376
"2119","MPDZ",44.7413294766973,0.498066329699315,0.321624086755854,1.54859772700233,0.121478452693265,0.769776438214376
"2120","OGN",596.791975095312,0.477821080970265,0.310011149375088,1.54130289163291,0.123243085465033,0.769776438214376
"2121","TMEM38B",12.6352958584209,-0.747280647132455,0.480996407057677,-1.55360962403789,0.12027756226406,0.769776438214376
"2122","KIF5B",128.467164434826,-0.332352850712248,0.215038871940061,-1.54554777800772,0.122213821596931,0.769776438214376
"2123","NUDT13",1.99410881616184,1.89774354872655,1.22423611497927,1.550145045965,0.121106706274741,0.769776438214376

"2124","PLCE1",19.0665093922138,0.622755195585474,0.404499060182179,1.53957142769379,0.123664854576325,0.769776438214376
"2125","FAM53B",11.674468081755,0.712236782942035,0.461585703501869,1.54302175639014,0.122825497245076,0.769776438214376
"2126","RPS13",2229.9475065288,-0.428672537969597,0.277059510789204,-1.5472218829396,0.12180975095446,0.769776438214376
"2127","FTH1",7120.74312213748,-0.419517469556354,0.272304162691278,-1.5406208462262,0.123409091338097,0.769776438214376
"2128","GPR137",18.81241509252,-0.595247935248239,0.387021495127955,-1.53802293345862,0.124043007502009,0.769776438214376
"2129","MRE11",30.83589507842,0.488356064707213,0.316633071761547,1.54234067209186,0.122990830323152,0.769776438214376
"2130","C11orf65",1.5292368471422,1.93114102733173,1.24751417803369,1.54799124638051,0.121624404071002,0.769776438214376
"2131","BACE1",66.5983497586033,0.358467400158409,0.231499497824118,1.54845865121813,0.12151190958736,0.769776438214376
"2132","CREBL2",66.5861316465321,0.334707959698138,0.21744967131784,1.53924334614838,0.123744899121435,0.769776438214376
"2133","LINC02381",76.4797435740404,0.443754976606018,0.285148862648657,1.55622215177055,0.1196552756052,0.769776438214376
"2134","PHLDA1",29.2268876469714,0.698829353307137,0.44923275962646,1.55560639408448,0.119801717331529,0.769776438214376
"2135","FGD6",2.55546550014528,1.55749956519319,1.0124954442279,1.53827809702482,0.123980632790876,0.769776438214376
"2136","SCYL2",15.9131179602294,0.65515159556128,0.425775773728418,1.53872445541998,0.123871579379359,0.769776438214376
"2137","EID3",7.43810756947871,-0.81386753358227,0.528498964001385,-1.53996050894839,0.12356997986055,0.769776438214376
"2138","PXN-AS1",7.66161988469337,1.03424208282037,0.671277074490433,1.54070818462773,0.123387823939466,0.769776438214376
"2139","ACADS",45.4917262718787,-0.513141278891544,0.329706456690982,-1.55635799201981,0.119622988471543,0.769776438214376
"2140","PIP4P1",23.9433477552972,-0.474977668668247,0.305534564826584,-1.5545791650049,0.120046329060882,0.769776438214376
"2141","SLC12A6",25.0877926728043,0.546795457182861,0.354503669571256,1.54242537981106,0.12297025808135,0.769776438214376
"2142","HACD3",63.4220716418792,0.429231038158402,0.279097508550232,1.53792500831711,0.12406695179495,0.769776438214376
"2143","CIB2",6.00795537252559,-0.951995483449738,0.615943091114687,-1.54559000203556,0.122203617324588,0.769776438214376
"2144","MESD",152.067525554655,0.279084873806494,0.180683341196975,1.54460766530903,0.122441190966951,0.769776438214376
"2145","B3GNT9",15.8357972721428,0.714622299669154,0.463644971454329,1.54131359912645,0.123240480714461,0.769776438214376
"2146","HSF4",7.22817953872939,1.11074354090833,0.718511898866569,1.54589442799833,0.122130066469784,0.769776438214376
"2147","NQO1",44.8624929727464,0.577858211554795,0.375071989768603,1.5406594662302,0.123399686794043,0.769776438214376
"2148","IFI35",49.6865570835971,-0.431614183118031,0.277622407591561,-

1.5546806429005,0.120022146963636,0.769776438214376
"2149","PNPO",10.445424748223,0.754690014881915,0.487534522366047,1.54797246196913,0.121628926784209,0.769776438214376
"2150","GDPD1",3.08046824671476,-1.32944951481866,0.858978486461859,-1.5477099086552,0.12169215537887,0.769776438214376
"2151","C17orf58",584.56676982872,0.472123281316894,0.306566787782326,1.54003401585732,0.123552062102975,0.769776438214376
"2152","NAT9",15.8447738926885,0.628499207717757,0.405336062803938,1.5505632619265 9,0.121006382007739,0.769776438214376
"2153","MTCL1",2.56046922293646,-1.51316853801278,0.973752678384555,-1.55395571339851,0.120194980789784,0.769776438214376
"2154","PTPRS",18.3321480943458,0.678602860997845,0.439447175220999,1.54421941762756,0.122535186339494,0.769776438214376
"2155","ZNF559",15.6546170633505,0.650267511969894,0.419949847999559,1.54844087947039,0.12151618538343,0.769776438214376
"2156","BLVRB",247.693957667234,-0.45418302075324,0.29242979098191,-1.55313526446195,0.120390822859558,0.769776438214376
"2157","ZNF160",23.5721860010079,0.641183272278677,0.412639969703571,1.55385643504018,0.12021866536992,0.769776438214376
"2158","RNF24",33.4328120893826,-0.643992501039994,0.414411603253219,-1.55399244612003,0.120186218485823,0.769776438214376
"2159","AL162457.2",1.87496478627781,2.58493772274906,1.67202044936911,1.54599647613426,0.122105418868116,0.769776438214376
"2160","C21orf62-AS1",9.90236484792686,0.724467746065837,0.470884479075852,1.53852543088203,0.123920195414378,0.769776438214376
"2161","DEPDC5",5.98396784889604,1.04130865705185,0.676968160898355,1.53819443394502,0.124001081525872,0.769776438214376
"2162","MCAT",17.2885963941607,-0.621688673562832,0.401362803192507,-1.54894441791271,0.121395082039698,0.769776438214376
"2163","ASMTL",38.8148195110312,-0.493506217048891,0.320779132089098,-1.53846110198907,0.123935912317397,0.769776438214376
"2164","DOCK11",65.8808635996191,-0.418517723222149,0.271075577201244,-1.54391527094839,0.12260886010357,0.769776438214376
"2165","NDUFA1",230.614092685217,-0.384137779053655,0.246819295601859,-1.55635230267127,0.119624340605055,0.769776438214376
"2166","IRAK1",28.3343345295817,-0.54795411035933,0.352715507555957,-1.55352996571153,0.120296576076047,0.769776438214376
"2167","LAMTOR4",262.882041223541,-0.415308553317892,0.27012330871421,-1.53747766268215,0.124176380964721,0.769872051725572
"2168","WASL",98.0709638058735,0.321769730086002,0.2092955993049,1.53739367265554,0.124196934901484,0.769872051725572
"2169","AP001160.3",3.67037618698532,-1.10360578439477,0.717978247710671,-1.53710197755113,0.124268338788514,0.769959522811822
"2170","CITED2",150.802188127613,0.430631929592096,0.280244813675571,1.53662765046072,0.124384517437018,0.770324207297736
"2171","AL513534.2",5.40479312798518,1.06208582119209,0.691295405477723,1.53637043263455,0.12444755414968,0.77035959475705
"2172","ARHGEF19",2.15236277117778,1.75013461556507,1.13984126223193,1.53541960056623,0.124680792154046,0.770383984256656

"2173","HFE",10.8203256809697,0.803401529232743,0.523173376136368,1.53
563152461209,0.124628777930578,0.770383984256656
"2174","NUP43",12.5450899034974,0.688765113104074,0.448396314448972,1.
53606327908936,0.124522861316587,0.770383984256656
"2175","ZNF709",5.16310629172748,1.0217971201464,0.665318171957377,1.5
358022119556,0.124586897068022,0.770383984256656
"2176","UBXN10",1.5335943706349,-2.03745945343545,1.32955931873673,-1.
53243215607057,0.125415827411647,0.770626929745664
"2177","MRPL53",7.13935806659804,-0.872189072205774,0.56869608424417,-
1.53366463453844,0.12511217839706,0.770626929745664
"2178","TWIST2",128.95070747285,-0.360144420541622,0.23485653325892,-1
.53346562492505,0.125161170091279,0.770626929745664
"2179","SEC24B-
AS1",2.79746432298815,1.49105678622642,0.973785495508733,1.53119633954
647,0.125720875207442,0.770626929745664
"2180","C1QTNF3",142.832358748366,-0.705035522423042,0.460230901112641
,-1.5319169588973,0.12554292801345,0.770626929745664
"2181","NDST1",35.5809724234778,0.470196990398044,0.306825735626547,1.
53245616583592,0.125409906581117,0.770626929745664
"2182","TMEM170B",6.58228528451099,0.909211679217198,0.593844334922577
,1.53106062607423,0.125754409791775,0.770626929745664
"2183","ARMC2",6.23215986108794,0.99462066361102,0.649425302342426,1.5
3153974756373,0.125636050750678,0.770626929745664
"2184","CD164",280.997156740352,0.259635408630093,0.169336541641712,1.
53325092217508,0.125214041851638,0.770626929745664
"2185","BAG1",99.7111405875463,-0.374439321984889,0.244448404594075,-1
.53177241065113,0.125578606500903,0.770626929745664
"2186","SLC11A2",19.2852468159817,0.609826972441521,0.397791384834776,
1.53303212611006,0.125267939522757,0.770626929745664
"2187","USP3",30.3433695270547,0.44772682009608,0.29176227950689,1.534
56033059787,0.124891863191374,0.770626929745664
"2188","UACA",115.441701647924,0.401540997395238,0.262240627359783,1.5
3119294076559,0.125721714955753,0.770626929745664
"2189","MRPL38",49.1698604262588,-0.403107946240728,0.26287191688191,-
1.53347664909301,0.125158455797686,0.770626929745664
"2190","MFSD11",31.5874998331038,-0.447025442418976,0.291519835890864,
-1.53343061906198,0.125169789294441,0.770626929745664
"2191","ZNF519",19.2175876723485,0.578906301914084,0.378163191877272,1
.53083725319825,0.125809620050747,0.770626929745664
"2192","MICOS13",139.970107737024,-0.436983083785168,0.285325490916736
,-1.53152486439667,0.125639726084234,0.770626929745664
"2193","ZNF43",28.2049169051347,0.482349834695237,0.314884648229664,1.
5318302667567,0.125564325072695,0.770626929745664
"2194","ARSD",15.1495791382867,0.680984610693441,0.44386387492456,1.53
42194964823,0.124975662800645,0.770626929745664
"2195","CRAMP1",9.66854629461339,0.823135693401789,0.53794162080467,1.
53015803493791,0.125977615967423,0.770701756496863
"2196","ABHD8",16.9791379366403,-0.625950873882608,0.408993915685799,-
1.53046500174218,0.125901670040943,0.770701756496863
"2197","MBOAT7",25.0269852349681,-0.555513109569895,0.363058558249791,

-1.53009231416518,0.125993880424407,0.770701756496863
"2198","NPC2",277.845981813517,-0.344164294987201,0.224965234106006,-1
.5298554745798,0.126052506621658,0.770709570740886
"2199","RRN3",20.0079260559955,0.525792275063647,0.343746770936056,1.5
2959189589436,0.126117776673997,0.770757981228669
"2200","CYC1",84.2002888175368,-0.446297344834328,0.291922697953242,-1
.52882029374027,0.126309000023329,0.770874955183252
"2201","RBM26",63.8699290645799,0.40017693609106,0.261718100533722,1.5
2903805764668,0.126255009533561,0.770874955183252
"2202","CHP1",58.9717875637868,-0.388225144079328,0.253867150806796,-1
.52924528772446,0.126203647400756,0.770874955183252
"2203","TET2",24.7448550654195,0.582176816930524,0.381093132124987,1.5
2764972090756,0.12659952981188,0.770897635315749
"2204","PPP6C",33.6651597751167,-0.42937689726789,0.281035756485725,-1
.5278372497405,0.12655295121319,0.770897635315749
"2205","COL5A1",384.106736777909,0.555313148877006,0.363508067846074,1
.52765013488545,0.126599426972941,0.770897635315749
"2206","GAPDH",808.863230725068,-0.485917787353825,0.317894478085027,-
1.52855057527567,0.126375896597917,0.770897635315749
"2207","GPRC5B",72.4809636603107,0.399676813756828,0.261527288386913,1
.52824134040472,0.126452628122775,0.770897635315749
"2208","CYP1B1",62.8313510669425,0.703227499134228,0.460645901214349,1
.5266118666863,0.126857554452949,0.772118964806697
"2209","FAM162A",89.7880287593509,-0.445397624178915,0.29187599286583,
-1.52598238658037,0.127014251054252,0.772722734231823
"2210","MMP23B",110.408615841358,0.975396811751224,0.643563094622145,1
.51561955603422,0.129615579207292,0.774494655437953
"2211","FAM76A",40.6752710896724,-0.452986217047156,0.299287090037068,
-1.51355080832605,0.130139805513073,0.774494655437953
"2212","ADAMTSL4",210.020628589576,0.290996732823025,0.191656560396159
,1.51832388216468,0.128932769992889,0.774494655437953
"2213","MTX1",49.9662563833,-0.430688498464296,0.284964338123296,-1.51
137683157374,0.130692468766847,0.774494655437953
"2214","CAPN2",211.846057614025,-0.346414923092665,0.22734576982846,-1
.52373595230756,0.127574684588867,0.774494655437953
"2215","CENPA",2.46274065966546,2.27674392753551,1.49613263925543,1.52
175272953644,0.12807104989614,0.774494655437953
"2216","ORMDL1",94.2668457866764,0.328028199343124,0.216382664772154,1
.51596339609058,0.129528608327195,0.774494655437953
"2217","PHLDB2",65.9064442453724,0.485194038517872,0.318362442004185,1
.52403039587029,0.127501118362823,0.774494655437953
"2218","ACAD9",23.2057035627543,-0.534968070989865,0.354076866882097,-
1.51088117024036,0.130818729127543,0.774494655437953
"2219","ATP11B",29.5426229907016,0.502036187209827,0.330520556729706,1
.51892575813487,0.128781184558497,0.774494655437953
"2220","ADGRA3",15.2312031304552,0.617414991195606,0.408646606296597,1
.51087756923028,0.130819646762881,0.774494655437953
"2221","ANKH",38.7324239024748,-0.403572424942941,0.265583298522474,-1
.51957004521047,0.128619071177528,0.774494655437953
"2222","PELO",13.5873407833701,-0.750381051780072,0.495440602768108,-1

.51457318513575,0.129880527151462,0.774494655437953
"2223","RASA1",29.8446668910884,0.459154777425289,0.301846651944227,1.52115246091955,0.128221582081445,0.774494655437953
"2224","ARHGEF37",1.77807683843378,-1.76196020286093,1.16264470645716,-1.51547604618612,0.129651891987112,0.774494655437953
"2225","RPS14",3420.85382629704,-0.415908886278959,0.273181423003501,-1.52246401569418,0.127892855236335,0.774494655437953
"2226","PDSS2",16.236863700638,0.574031677142168,0.377610581377874,1.52016841013185,0.128468654658935,0.774494655437953
"2227","ABRACL",22.868722001935,-0.648520576495633,0.426038242335053,-1.52221212100864,0.127955938981961,0.774494655437953
"2228","SCAF8",24.74524569306,0.476089702001881,0.313279052257822,1.5196984878838,0.128586771825173,0.774494655437953
"2229","HOXA9",13.1424809084334,-0.609630878440171,0.402405190846257,-1.51496772981014,0.129780576531537,0.774494655437953
"2230","FKBP14",53.6105854669409,0.506543598571111,0.332423245985112,1.52379114484008,0.12756089231411,0.774494655437953
"2231","GARS",48.46145041834,-0.399085830435224,0.262780896337782,-1.5187018386688,0.128837563597115,0.774494655437953
"2232","CHCHD2",651.12817308547,-0.437125894841359,0.288633540390335,-1.51446673262646,0.129907505166018,0.774494655437953
"2233","PAXIP1-AS2",21.6024384588639,0.570728554546382,0.375664288745668,1.51925155423218,0.128699188990435,0.774494655437953
"2234","AC004908.2",3.72033037429357,-1.23287211717652,0.811760189219007,-1.51876395707785,0.128821921337098,0.774494655437953
"2235","SORBS3",104.183412450271,-0.339336114826115,0.223712943522823,-1.51683719986232,0.129307792368204,0.774494655437953
"2236","AC016877.3",1.60822411129875,1.98173084904719,1.31117316103631,1.51141810093252,0.130681960441813,0.774494655437953
"2237","LINC01230",2.56566568075471,1.6297563855805,1.0736970742611,1.51789217336004,0.12904158333571,0.774494655437953
"2238","PTPRD-AS1",10.211830443007,0.900663836518756,0.594943042223565,1.51386565200019,0.130059917278284,0.774494655437953
"2239","GALNT12",59.2526056124402,-0.47886385938711,0.314496100324352,-1.52263846481161,0.127849180901855,0.774494655437953
"2240","DPCD",36.4192921513349,-0.44012233798276,0.290441662141107,-1.51535538923108,0.129682428334331,0.774494655437953
"2241","NHLRC2",31.6433019597055,0.531703504642337,0.350842107069884,1.51550653108017,0.129644177631114,0.774494655437953
"2242","HSD17B12",91.8447076378399,-0.296355621364302,0.195004567349048,-1.51973682151681,0.1285771333258,0.774494655437953
"2243","NDUFS3",93.1712221274578,-0.476245692601423,0.314655623881753,-1.51354578292996,0.130141080962618,0.774494655437953
"2244","COX8A",204.955871208906,-0.366059440715515,0.241879980998654,-1.51339287858449,0.130179892846056,0.774494655437953
"2245","SAC3D1",17.2134904970597,-0.650541777254191,0.428616370793107,-1.51777165218966,0.129071973749283,0.774494655437953
"2246","TMEM117",4.12630590649345,1.12017802168812,0.741373886404711,1

.51094885081591,0.130801483204292,0.774494655437953
"2247","NCKAP5L",35.536000451049,0.523631388722755,0.344859613172387,1
.51839000196583,0.128916110619715,0.774494655437953
"2248","IGDCC4",24.8299575903824,0.655960165709139,0.433721450525082,1
.5123996401723,0.130432226410985,0.774494655437953
"2249","USP31",8.13325793129747,0.862491251326488,0.568630625609231,1.
51678649105896,0.129320598820019,0.774494655437953
"2250","UBFD1",25.7121946305639,0.511296137760039,0.338139190684644,1.
51208777877772,0.130511533456822,0.774494655437953
"2251","CMIP",22.2321611803863,0.541239245221052,0.358179754340317,1.5
1108274173086,0.130767371188862,0.774494655437953
"2252","FBXO31",13.3247101821734,0.691375233626299,0.455836994028042,1
.51671593724086,0.129338418746932,0.774494655437953
"2253","RPL13",5793.32811206863,-0.389637605415975,0.257900262345155,-
1.51080732478865,0.130837547956265,0.774494655437953
"2254","SCARF1",2.19200433605856,1.8662000419832,1.23538235674285,1.51
062546085208,0.130883903247585,0.774494655437953
"2255","WDR81",18.6614967059569,0.621191487427191,0.410834665373818,1.
51202305886717,0.130527996560617,0.774494655437953
"2256","TXNDC17",75.4478783283481,-0.467199914387239,0.306615680941624
,-1.52373131391212,0.127575843748226,0.774494655437953
"2257","AC005670.3",26.1313173141533,0.573397452033108,0.3777727056023
62,1.5178371638015,0.129055453762898,0.774494655437953
"2258","SPATA20",23.6502051706279,-0.586292238038863,0.387189399712343
,-1.51422595369202,0.129968541263736,0.774494655437953
"2259","MSI2",13.4159934174467,0.795677489033133,0.524675740205632,1.5
1651282508562,0.129389729860854,0.774494655437953
"2260","WDR7",12.3680583585737,0.714450838133846,0.472821428999774,1.5
1103734795867,0.130778935599542,0.774494655437953
"2261","AC012615.1",4.62416292146236,-0.997330046687987,0.659645447813
238,-1.51191833430246,0.13055463924657,0.774494655437953
"2262","ZNF136",17.4223324909938,0.616151157553025,0.406983347449421,1
.51394685166965,0.130039319900937,0.774494655437953
"2263","NR2C2AP",18.3981508969792,-0.591248177657108,0.390863319167603
,-1.51267245776926,0.13036287895721,0.774494655437953
"2264","RPL18",2035.82997678722,-0.408834589854582,0.269235556738041,-
1.51850147435158,0.128888028113806,0.774494655437953
"2265","KMT5C",9.77779038209368,0.711206528427454,0.469563327608325,1.
5146125913408,0.129870541630587,0.774494655437953
"2266","PI3",1.66548679549901,-4.87907382831156,3.2302756568534,-1.510
42026953955,0.130936219745147,0.774494655437953
"2267","SPECC1L",49.5245323897129,0.414404631985641,0.272538890804328,
1.52053393467124,0.128376836670846,0.774494655437953
"2268","SLC35E4",7.49524275823233,-0.870085690944419,0.575025113040459
,-1.51312642041635,0.130247549691016,0.774494655437953
"2269","LINC00899",12.6510660374127,0.723724267396748,0.47584276476562
6,1.52093153660373,0.128277018926704,0.774494655437953
"2270","TSPAN6",32.377424876374,0.519080655875366,0.341590069121545,1.
51960113246344,0.128611253118406,0.774494655437953
"2271","AL683813.1",8.5276817772361,0.933144521662338,0.61702196030448

4,1.51233599725017,0.130448407910055,0.774494655437953
"2272","RPL10",7243.41600975895,-0.419386592951234,0.275996591068077,-1.51953540921739,0.128627782135811,0.774494655437953
"2273","ROBO1",53.4917822165873,0.4242834770132,0.281067546720837,1.50954274857854,0.131160139433139,0.774717295582257
"2274","SFMBT2",8.63003848371641,1.07836641784259,0.714428219917221,1.50941184541609,0.131193567826019,0.774717295582257
"2275","PHF21A",38.1233029149319,0.470440677213302,0.311675187914797,1.5093940597604,0.131198110211376,0.774717295582257
"2276","SRRM2",397.00125617835,0.352852661133624,0.233774910602524,1.50936924849717,0.131204447112524,0.774717295582257
"2277","PIGC",50.0480631277317,-0.417204418023296,0.276468245660935,-1.50905004307425,0.131285994690589,0.774858358650342
"2278","FZD7",38.6680623017413,0.514700105453891,0.341180769430195,1.50858474911552,0.131404933965339,0.775035760433509
"2279","PSMA2",182.234517258213,-0.431562562786572,0.286090764072273,-1.50848128280558,0.131431393558149,0.775035760433509
"2280","CNKSR2",13.9346325877844,-0.979437575515772,0.649416382313905,-1.50818119497692,0.131508158824981,0.775148309846018
"2281","RPIA",21.6571376385009,0.511436250848678,0.339396803562027,1.50689766515497,0.131836890027439,0.775979580342173
"2282","GRPEL1",25.4319656502017,-0.470404170301114,0.312155562901423,-1.50695430806615,0.131822369511684,0.775979580342173
"2283","BBS1",18.8575318119182,0.572364997325599,0.379771468927354,1.50713006151361,0.131777322676886,0.775979580342173
"2284","CLDND2",3.68066756461493,1.31370781985785,0.871893935385161,1.50672893403888,0.131880151908737,0.775979580342173
"2285","GASAL1",3.12474298432667,-1.22072291530965,0.810873444152134,-1.50544197015364,0.132210485552823,0.776902805135281
"2286","CLPX",34.7386696071754,0.436618786763195,0.289965911011018,1.50575902264113,0.132129046122221,0.776902805135281
"2287","TANC2",24.710772994514,0.707911739886425,0.470178236250369,1.50562421930024,0.132163667522699,0.776902805135281
"2288","BX255925.3",2.16997041358448,1.81344738556323,1.20497082530946,1.50497202710074,0.132331268622977,0.777272691881202
"2289","GSR",13.4966184352438,0.654701234961453,0.435165349508042,1.50448843342329,0.132455649341373,0.777490762066806
"2290","NKAP",59.5360970780231,-0.339831423696225,0.225894991506291,-1.50437785906714,0.132484101877594,0.777490762066806
"2291","WDR35",12.7926663568313,-0.621199971653143,0.413269709280687,-1.50313453346089,0.132804355223602,0.777672213442259
"2292","LRIG1",9.27026787272602,-0.734494533104239,0.488597348389486,-1.50327163159047,0.132769012383159,0.777672213442259
"2293","USP47",62.3153507968407,0.408479513602832,0.271731777319354,1.50324528707132,0.132775803231686,0.777672213442259
"2294","C12orf57",389.539308761132,-0.411452160991966,0.273651937976992,-1.5035601941418,0.132694646979705,0.777672213442259
"2295","AL592183.1",4.67804873693655,-1.09100032826459,0.725388714988056,-1.50402164483983,0.13257579363779,0.777672213442259
"2296","HOTAIR",4.85962592157365,1.23866102361423,0.824276299894818,1.

50272551057489,0.132909841455499,0.777697153095896
"2297","ATP11C",31.0714599592781,0.471997661107594,0.314106151886319,1
.50266926729413,0.132924351563455,0.777697153095896
"2298","PIK3CA",43.7161572089283,0.46159891388431,0.307242057533659,1.
50239494420044,0.132995141289136,0.77777271705165
"2299","AC008280.3",2.19052071987218,1.66899264791518,1.11188714667117
,1.50104500525246,0.133343921497414,0.777821369897411
"2300","DDX56",26.7469997815484,-0.516111684990657,0.344045975423062,-
1.50012417484614,0.133582239878207,0.777821369897411
"2301","RPL30",3443.04075558733,-0.428026330144916,0.285275346781875,-
1.50039719510775,0.133511545673413,0.777821369897411
"2302","FRMD4A",26.7019697723733,0.5725206868419,0.38157321684201,1.50
042157460688,0.13350523440252,0.777821369897411
"2303","SHMT2",34.1339018499285,-0.441428206850658,0.294153981441256,-
1.50067051510847,0.133440802856855,0.777821369897411
"2304","RNASEH2B",45.3435431920234,-0.433178292891328,0.28854501168275
1,-1.50125032612797,0.1332908276797,0.777821369897411
"2305","ERBB2",25.2077086538072,0.48174969896141,0.320981947689537,1.5
0086228346827,0.133391185190793,0.777821369897411
"2306","LDLRAD4",13.9422532649808,-0.712274141044931,0.47476738638405,
-1.50025920371193,0.133547272706425,0.777821369897411
"2307","SPATC1L",35.9099738941721,0.453007403900414,0.301617467359628,
1.50192695358813,0.133115974737448,0.777821369897411
"2308","C21orf58",5.17422969050532,1.12497867459742,0.749177249611033,
1.50161884277919,0.133195574190451,0.777821369897411
"2309","HHIP",1.76005033832565,2.12802383326274,1.41932412413285,1.499
32196394032,0.133790127070234,0.778694464138968
"2310","SP3",76.5746668681208,0.354711123066884,0.236798258174993,1.49
794650433938,0.134147149879479,0.778747505509269
"2311","CYP4V2",31.099422822543,0.46406644621912,0.309829549733579,1.4
9781209254627,0.134182078117784,0.778747505509269
"2312","ASAP1",47.3011926435075,0.528434312150724,0.352819692808341,1.
49774608085094,0.134199234486864,0.778747505509269
"2313","SRGAP1",104.798595888998,0.374673623135172,0.250161926742915,1
.4977244060013,0.134204868127053,0.778747505509269
"2314","ZDHHC17",39.5915165124402,0.452545604389573,0.302053231837266,
1.49823129399055,0.134073167694674,0.778747505509269
"2315","DHPS",55.783749252155,-0.476319547266573,0.31777451141368,-1.4
9892307330621,0.133893589833426,0.778747505509269
"2316","ACSS2",13.8571533556733,-0.581343001723522,0.387994825554886,-
1.49832668745549,0.134048393594608,0.778747505509269
"2317","EIF3G",247.72121348723,-0.421488112078407,0.28148693936841,-1.
49736294346063,0.134298844983086,0.778956485855717
"2318","SHPK",14.0961835972477,0.678574122894926,0.453320551509433,1.4
9689688816327,0.134420090063749,0.779323378070201
"2319","GLUL",303.763874697436,0.37743434984176,0.252387454545035,1.49
545606584186,0.134795457739926,0.779823733268763
"2320","MTR",48.3518427593891,0.440159081919695,0.294286575041107,1.49
568182598276,0.134736588510932,0.779823733268763
"2321","IRF1",99.4006898120523,-1.04526512966226,0.69883675369599,-1.4

9572146017519,0.134726255547319,0.779823733268763
"2322","AC005520.2",5.43578853442332,0.978338968572682,0.653928752568545,1.49609413063716,0.134629127213074,0.779823733268763
"2323","FKBP1A",330.798335031558,-0.391171453637938,0.261574067895713,-1.49545196427459,0.134796527448719,0.779823733268763
"2324","IFT172",9.29525507958068,0.790825808536003,0.529857248587561,1.49252616746134,0.135561262594848,0.780799761883442
"2325","DUSP19",3.98488863788605,-1.10644092954846,0.741190219068753,-1.49278943661536,0.135492313263809,0.780799761883442
"2326","USP19",15.0763589890588,0.595545801997761,0.399024341744631,1.4925049419138,0.135566822676734,0.780799761883442
"2327","CDKN2AIPNL",27.0010633435809,-0.518729021246446,0.347495414029786,-1.4927650849573,0.135498689746303,0.780799761883442
"2328","DPYSL3",182.354991185274,-0.459221499177155,0.307714597971541,-1.49236176055458,0.135604333970977,0.780799761883442
"2329","SEMA3A",10.0420192671408,1.11317994651347,0.745093969276569,1.49401282578396,0.135172267003763,0.780799761883442
"2330","FBP1",7.17564403521681,1.08517248238807,0.726617138279499,1.49345841877274,0.135317230897513,0.780799761883442
"2331","UEVLD",16.652353039992,0.586941468585005,0.39320191297514,1.49272282055788,0.135509757231008,0.780799761883442
"2332","MLST8",23.9995158682087,-0.549825227412635,0.368332194229753,-1.49274279041075,0.135504527777332,0.780799761883442
"2333","PIN1",93.1146013972208,-0.408651166028065,0.27366766046558,-1.49323878946033,0.135374691802583,0.780799761883442
"2334","KANK2",129.711343041389,0.420429523741874,0.2814380256105,1.49386182918912,0.135211737030424,0.780799761883442
"2335","CAMTA1",66.61034920542,-0.478615407254618,0.321299119222496,-1.48962564358349,0.136322695489112,0.780941450753088
"2336","FBXO44",9.29214052197088,0.729135179738754,0.489897664425455,1.48834181643604,0.136660772364515,0.780941450753088
"2337","CLCN6",13.7935075825379,0.697521664463161,0.468272795617448,1.48956264594323,0.136339269909376,0.780941450753088
"2338","AC010969.2",4.94666557970953,1.02853033626088,0.690617672572278,1.4892904961873,0.136410889266182,0.780941450753088
"2339","METTL5",83.4789084426609,-0.436858506385949,0.293647696161288,-1.48769601157028,0.136831079913968,0.780941450753088
"2340","TTC14",96.0481418308604,0.351890564266728,0.236371112256628,1.48872068548157,0.136560935586645,0.780941450753088
"2341","FRAS1",5.59430455096416,1.44849767887333,0.973293130101128,1.48824401824642,0.136686552565916,0.780941450753088
"2342","MTRF1L",21.7270530826504,-0.519863494897062,0.349458196112556,-1.48762713446166,0.136849253406021,0.780941450753088
"2343","OSGIN2",19.3514322152409,-0.536444545140052,0.360315450137258,-1.48881915814517,0.136534996014348,0.780941450753088
"2344","AL356481.1",2.85001701292804,-1.1752518672501,0.787810570382525,-1.49179499670771,0.135752895980099,0.780941450753088
"2345","C11orf95",22.4031083339211,0.568027737241363,0.381289192049177,1.48975567387208,0.136288489972641,0.780941450753088
"2346","MTERF2",16.6927319698086,0.591826507116333,0.397044567869467,1

.49057953441364,0.136071920755164,0.780941450753088
"2347","WSCD2",18.1330271373744,0.663611797779983,0.445657220650766,1.48906326887501,0.136470709002687,0.780941450753088
"2348","UBAC2",48.9449851136305,-0.413915846149933,0.278097503858644,-1.48838389560061,0.136649681193734,0.780941450753088
"2349","MOK",19.4652068759715,-0.557170085691191,0.373602481533857,-1.49134471324623,0.135871015340308,0.780941450753088
"2350","RPLP1",5654.18199686614,-0.377988842327097,0.253834165408757,-1.48911728142825,0.13645648785386,0.780941450753088
"2351","RPS2",3286.28899353101,-0.371943913161736,0.249607699013103,-1.49011394533232,0.136194278048829,0.780941450753088
"2352","IL4R",52.1905850810916,0.428038414853322,0.287665282543393,1.48797383913978,0.136757792936207,0.780941450753088
"2353","DDX19A",25.3732787876157,0.480002516264152,0.321949363277993,1.49092550262224,0.13598105492671,0.780941450753088
"2354","ABHD15",9.56054078825197,0.784208108311698,0.526416198976543,1.48971120158603,0.136300188020748,0.780941450753088
"2355","GPX4",1727.87524614974,-0.463719879034689,0.310930136798991,-1.49139573220101,0.135857627949188,0.780941450753088
"2356","ZBTB10",34.3443137987501,0.48122196293542,0.32355598009228,1.48729120320438,0.136937916862221,0.781115732050673
"2357","PIK3C3",26.1980471625307,0.496961156516303,0.334226480388441,1.4868994100609,0.137041380107848,0.781374250008216
"2358","AC092828.1",3.46565138778586,1.24173836795993,0.835663919016158,1.48593033599183,0.137297548537108,0.782502864626887
"2359","ANKRD33B",12.2482295373865,0.631449398207612,0.425097752757406,1.48542163328717,0.137432168519438,0.782938072375044
"2360","SSNA1",85.6986312456448,-0.421685323877774,0.28393281353738,-1.48515882551299,0.137501756230486,0.783002585585382
"2361","VPS45",19.6909484134583,0.54071844816532,0.364512446804938,1.48340187805624,0.137967668756176,0.783564808754025
"2362","PLXND1",38.8708651551045,0.443116320155803,0.298611340384645,1.48392328163096,0.137829274617313,0.783564808754025
"2363","U2SURP",132.519047852397,0.276871826651654,0.186603906485585,1.48374078477849,0.137877701874673,0.783564808754025
"2364","NSD1",55.2821214266258,0.385259852307815,0.259740746074852,1.48324765416971,0.138008624326273,0.783564808754025
"2365","MICU3",28.6278286869038,0.537480834163109,0.362047346142986,1.48455951932551,0.137660545490813,0.783564808754025
"2366","ARNTL",16.9104949974526,0.985845471534634,0.664284288666846,1.4840716367282,0.137789916858701,0.783564808754025
"2367","TNMD",16.6779297594377,-1.06149772588078,0.715576320176893,-1.48341650771559,0.137963784202236,0.783564808754025
"2368","GAS2L1",26.3785714761504,-0.507621806927948,0.342440907137375,-1.48236322339933,0.138243674017199,0.78456787800555
"2369","ZBTB37",24.2113420590287,0.63846445805871,0.430849486116072,1.48187355128168,0.138373943756922,0.784975698670021
"2370","BAZ2B",84.0156422936657,0.517474565096199,0.349267952814777,1.48159761273782,0.138447394620316,0.785060985781616
"2371","ING5",22.6663319316718,0.531624124025703,0.359104280049647,1.4

8041711993019,0.138761963973576,0.785513069977203
"2372","AC097534.2",7.82521425366707,-0.808586842650186,0.546488867440438,-1.4796035030637,0.138979091094848,0.785513069977203
"2373","NFKBIL1",41.4495778914724,-0.526755290022007,0.356025198453881,-1.47954496566411,0.138994722851833,0.785513069977203
"2374","MARS",15.4464623033139,-0.564486189472007,0.381231620232218,-1.48069089633269,0.138688960991583,0.785513069977203
"2375","APPL2",66.9400330272444,0.329192637379038,0.222444546361925,1.47988630318421,0.13890359155796,0.785513069977203
"2376","SRSF5",297.806152281194,0.33388225503815,0.225516659610299,1.48052146398014,0.138734136950618,0.785513069977203
"2377","TNRC6C",42.2523799912945,0.589388612508312,0.398216213809402,1.48007186063602,0.138854070194824,0.785513069977203
"2378","LINC00205",9.33641927194904,0.889011435593404,0.600344531701725,1.48083540142096,0.138650440374109,0.785513069977203
"2379","EXOC3L4",1.86167944761518,1.75847170009248,1.18902722458179,1.47891626342785,0.139162696114134,0.786131766741426
"2380","HSPA14.1",14.8678395619058,-0.622870294237537,0.421377553868324,-1.47817625433408,0.139360607944352,0.786918995867291
"2381","COX7C",561.774948569138,-0.337336288155513,0.228323276176743,-1.47745027928902,0.139554976919925,0.787661779853157
"2382","NORAD",262.200253959642,0.279233167031879,0.189022634707228,1.47724724853389,0.139609372692181,0.787661779853157
"2383","RPP21",70.1678635647571,-0.384313720268864,0.2603472065954,-1.47615841665672,0.139901369773294,0.788647025328564
"2384","RBM12B",23.9422309012271,0.592067407041585,0.401064008808805,1.47624168221945,0.139879023481192,0.788647025328564
"2385","PTCH2",11.6206049440388,0.731315774375781,0.497096634062656,1.47117426323913,0.141243995484198,0.790354450877438
"2386","SNRNP27",63.8175787891312,0.33739923026616,0.229000150708526,1.4733581144914,0.14065449812482,0.790354450877438
"2387","PCNP",126.850075322057,-0.280751044825303,0.190377572845169,-1.47470650365752,0.140291466823234,0.790354450877438
"2388","CEP63",41.2884058833894,-0.437548220634944,0.296924753357864,-1.47359967697808,0.140589408393111,0.790354450877438
"2389","GK5",35.2922480769152,0.705491690177697,0.479205918690234,1.47220988443955,0.140964209032789,0.790354450877438
"2390","LINC02482",7.95793512833099,0.843842638440561,0.573696410039016,1.47088708186822,0.141321656779409,0.790354450877438
"2391","GUCY1A1",3.10379542235281,1.79981102131937,1.22220225851748,1.47259670711337,0.140859813247611,0.790354450877438
"2392","TMEM14B",138.982864141101,-0.371288769380937,0.251818638405301,-1.47442926279091,0.140366050383631,0.790354450877438
"2393","LSM2",91.4928314384445,-0.422114133176743,0.286912365700868,-1.47123018607304,0.141228876314894,0.790354450877438
"2394","ANKIB1",56.3372462649583,0.389687951983743,0.264899638287498,1.47107770513756,0.141270103639603,0.790354450877438
"2395","KAT6A",46.0965477997777,0.467790715113395,0.317477162269941,1.47346256898834,0.140626349706346,0.790354450877438
"2396","MIR1915HG",1.61131260140949,-1.72866281909144,1.17471943950124

,-1.47155376931993,0.141141417509306,0.790354450877438
"2397","AL157893.2",3.30009166806555,-1.18663762393812,0.806090289102285,-1.47209021120902,0.140996518513769,0.790354450877438
"2398","ADAMTS15",13.4902779272784,1.63742373320833,1.11221680777923,1.47221631767802,0.140962472342792,0.790354450877438
"2399","EZH1",59.0384334333322,0.368979407198667,0.25043278713866,1.47336701162204,0.140652100355509,0.790354450877438
"2400","FAM174C",55.2614868205253,-0.441911744607401,0.299975537149064,-1.47315927427711,0.140708093598483,0.790354450877438
"2401","ATF5",19.3378570012354,-0.621808223188082,0.42212161608572,-1.47305468256762,0.140736291592811,0.790354450877438
"2402","CD99",1315.74322916378,-0.35943432604572,0.244249964063102,-1.47158394648877,0.141133263279106,0.790354450877438
"2403","PLXNA3",13.4763308539355,0.739544022552085,0.502774227361057,1.47092667504811,0.141310947806245,0.790354450877438
"2404","TASOR2",42.401712362005,0.440911018558175,0.30005058789932,1.46945560628637,0.141709254470361,0.791691852584542
"2405","KDM4D",2.05403591938251,-1.35928636122119,0.92490181866003,-1.46965476096748,0.141655280913157,0.791691852584542
"2406","SPATA33",15.8796797162787,-0.560641794152242,0.381557349952194,-1.46935131566064,0.141737524913938,0.791691852584542
"2407","NME1",47.0793561879683,-0.475583558842225,0.323764966453701,-1.4689160598549,0.141855558082875,0.792021954746885
"2408","TSN",46.832858647829,-0.382006350853341,0.260313005432053,-1.46748853450218,0.142243205983249,0.792539985575824
"2409","NMNAT3",13.3995371334484,0.833208378112342,0.567621327735822,1.46789476962734,0.14213280915757,0.792539985575824
"2410","SRFBP1",34.7647656235494,-0.409329362071298,0.278919188595582,-1.46755540245316,0.142225029691798,0.792539985575824
"2411","BCL2",17.7275890917793,0.588170216618383,0.400704603548806,1.46783992848923,0.142147708721978,0.792539985575824
"2412","PMEPA1",55.6011357158237,-0.449517451940445,0.306239968093868,-1.46786017102333,0.142142208971066,0.792539985575824
"2413","NEMP2",3.85636800878797,1.16380558705755,0.793193682517537,1.46724011134799,0.142310748898764,0.792587714235597
"2414","STX11",5.50717428580504,-0.878213898252163,0.598687342854095,-1.46689905630123,0.14240351728932,0.792775836309514
"2415","RNF181",96.3385413771907,-0.461146257952929,0.314511086140756,-1.46623212431548,0.142585059723529,0.793016836617082
"2416","THOC3",18.6032685890516,-0.566711618147763,0.386472671583304,-1.46636918938163,0.142547735386221,0.793016836617082
"2417","DPY19L4",39.4702803072277,0.405089212896963,0.276346699334083,1.46587317262378,0.142682841799249,0.793016836617082
"2418","GLIPR1",55.8604416014488,0.33696892900621,0.229848621813664,1.46604720249046,0.142635427875046,0.793016836617082
"2419","JARID2",11.8087381376001,0.665980960770743,0.454480351793537,1.46536799257119,0.142820545147228,0.793454033168082
"2420","UPK3BL1",43.2931291575555,-0.530100823818002,0.361932600211364,-1.464639613863,0.143019268111899,0.793724060422946
"2421","CNOT2",76.7160611259405,0.312981724946282,0.213706436246325,1.

46454047170358,0.143046333383762,0.793724060422946
"2422","PRAC2",2.24005787243843,-1.61946007918509,1.10550059449162,-1.46491108847374,0.142945177127535,0.793724060422946
"2423","AC007920.2",5.97280198662585,-1.16280702492901,0.7944867449056,-1.46359524861194,0.143304571558193,0.794828781333288
"2424","SLC27A3",80.1144742274818,-0.449682908845104,0.307635269396671,-1.46174042308937,0.143812355539947,0.79567486459504
"2425","OXSM",5.00559416375674,-0.998038186943684,0.682461229026616,-1.46241008938657,0.143628866274733,0.79567486459504
"2426","SUFU",10.3144470153782,0.730104657097283,0.499209947821012,1.46252024881334,0.143598699681936,0.79567486459504
"2427","ATG2A",11.0744588728642,0.719742388820224,0.49224387451401,1.46216626774853,0.143695652861131,0.79567486459504
"2428","CPB2-AS1",2.7904152416391,1.37459402233632,0.940353035119309,1.461785064757,0.143800118087007,0.79567486459504
"2429","PSMG2",159.643018010366,-0.396729091214548,0.271391022305558,-1.46183572265656,0.143786232394192,0.79567486459504
"2430","WNT4",3.40500463253765,-1.16531157554418,0.800110801135002,-1.45643775073542,0.145271646099417,0.796657879971451
"2431","KHDRBS1",137.99479529145,0.260964595689633,0.178836095499828,1.45923894703844,0.144499352687155,0.796657879971451
"2432","WDR47",14.0892029156285,-0.596669446902004,0.410821222617144,-1.45238223843674,0.146395352168184,0.796657879971451
"2433","KIRREL1",56.5499740391143,0.413682335964978,0.284210950958668,1.45554678512419,0.145517947938832,0.796657879971451
"2434","SCYL3",10.0672159550414,0.739315816089315,0.506774449313035,1.45886560992075,0.144602100101697,0.796657879971451
"2435","FMOD",52.6577904241774,-0.622830556248487,0.426667207740133,-1.45975726502945,0.144356797352825,0.796657879971451
"2436","CDC42EP3",1.57849523549394,-1.60913491751399,1.10441652611418,-1.45700003528164,0.145116370480051,0.796657879971451
"2437","CYP1B1-AS1",10.3711180974298,0.75844470698395,0.522352983412964,1.45197736218218,0.146507899858396,0.796657879971451
"2438","RND3",148.138613423,-0.516191658364765,0.355230904293985,-1.4531158526049,0.146191590297471,0.796657879971451
"2439","SACM1L",22.48018338513,0.488091753933945,0.334963618743465,1.4571485576998,0.145075377050193,0.796657879971451
"2440","C3orf18",7.49505902138063,0.824739776124541,0.564869288002395,1.46005419951428,0.144275178701774,0.796657879971451
"2441","PARP15",2.91985310177161,1.58830265657639,1.09099327779415,1.45583175341624,0.145439135491542,0.796657879971451
"2442","RPL35A",2419.12693726377,-0.390609726342988,0.268074367491732,-1.45709464876397,0.145090255341832,0.796657879971451
"2443","MTREX",47.5068167433009,0.416388274823379,0.285761715586281,1.4571170738148,0.145084066124881,0.796657879971451
"2444","BTF3",854.862634156585,-0.369545481433046,0.253831171102867,-1.45587115966654,0.145428239648368,0.796657879971451
"2445","CDC23",13.8555008287404,0.65737697390618,0.451660977042361,1.4

5546550913236,0.145540432080479,0.796657879971451
"2446","F13A1",2.47234012061055,-1.37767989997291,0.94351897883473,-1.46015070271758,0.144248660396317,0.796657879971451
"2447","TRERF1",35.0224011836769,0.419686725964696,0.289109690098645,1.4516522868005,0.146598328534072,0.796657879971451
"2448","RHBDD2",24.2957358845733,-0.61640666388439,0.424485140293593,-1.4521277787440 5,0.146466079266851,0.796657879971451
"2449","BMT2",9.57882695940572,-0.660212714610981,0.45440291725576,-1.45292358288136,0.146244972355382,0.796657879971451
"2450","SCARA3",86.5016410888966,-0.537373354831618,0.369186631040334,-1.45556016835536,0.145514245864831,0.796657879971451
"2451","PPP1R16A",7.20220390938982,-0.831149119544289,0.570808337930391,-1.45609141337674,0.145367350924079,0.796657879971451
"2452","STOML2",93.0261988057365,-0.377574375818626,0.259938434509855,-1.45255308831334,0.146347879091273,0.796657879971451
"2453","RAB5B",48.4248672492924,0.343326059628295,0.235660959745528,1.45686438686759,0.145153818328686,0.796657879971451
"2454","STAT2",66.4016147989774,0.440729550896804,0.301761864063624,1.46052103788661,0.144146929944653,0.796657879971451
"2455","EP400",31.9018066743004,0.532056972279348,0.366000322055293,1.45370629537033,0.146027752074798,0.796657879971451
"2456","UBL3",179.742785118429,-0.319477407168358,0.218697288255334,-1.46082015793155,0.144064802328284,0.796657879971451
"2457","DPP8",35.2213558972913,0.481678785293703,0.331250887702615,1.45412073801335,0.145912834976159,0.796657879971451
"2458","AC015871.7",2.53548101162071,-1.24458639951076,0.852804098631706,-1.45940480528606,0.144453724212168,0.796657879971451
"2459","VPS4A",42.9245074150842,-0.375287097772847,0.257351868641124,-1.45826451447369,0.144767647306521,0.796657879971451
"2460","AC134312.5",1.67713749280741,-1.57684061769205,1.08606564593815,-1.45188334019162,0.146534045603665,0.796657879971451
"2461","MED9",8.65708711675673,0.745091946969767,0.512677092465367,1.45333575055355,0.146130555777617,0.796657879971451
"2462","RPL19",3162.93010183721,-0.39701231347269,0.272896065607607,-1.45481142276176,0.145721474994573,0.796657879971451
"2463","MRPL10",41.3094366469951,-0.432355429921422,0.297012414888404,-1.45568133939408,0.145480730772688,0.796657879971451
"2464","TMEM241",7.62886762016811,-0.80097871940821,0.549997937011175,-1.45633040691194,0.145301303697206,0.796657879971451
"2465","PHLPP1",17.4420281524037,0.582718738149703,0.400464608042533,1.45510670967411,0.145639721939734,0.796657879971451
"2466","ALKBH7",121.072310380316,-0.489635361034196,0.336544655168873,-1.45488972566955,0.145699792650551,0.796657879971451
"2467","FLT3LG",24.1608421673624,-0.549706407031331,0.377567319750587,-1.45591627843865,0.145415765058312,0.796657879971451
"2468","ZNF551",3.90644452897023,1.16542026552245,0.80242575832817,1.45237145421474,0.146398349113479,0.796657879971451
"2469","SDCBP2",5.89450338775357,-0.837218246213808,0.575191267173388,-1.45554756129744,0.145517733231553,0.796657879971451
"2470","OSER1",39.5644815700074,-0.397980898527207,0.27383519197806,-1

.45335921089023,0.146124045315811,0.796657879971451
"2471","CTSZ",223.513761223111,-0.247088559381246,0.170187806635637,-1
.45185818106375,0.146541042488752,0.796657879971451
"2472","C1QTNF6",25.5310664103192,0.72857572365268,0.501252793624746,1
.45350955230409,0.146082329443066,0.796657879971451
"2473","NR0B1",4.43836387223693,-1.47680300160421,1.01437504887276,-1.
45587473119073,0.145427252151468,0.796657879971451
"2474","ZSWIM6",14.3739152845025,0.602011453876894,0.414875946807263,1
.45106376619267,0.14676210489214,0.796863732670736
"2475","LMO3",86.3700732947988,0.404971510246752,0.279095656449606,1.4
5101330274509,0.146776156003557,0.796863732670736
"2476","CNEP1R1",15.0466975354491,-0.53425387425415,0.368228214939676,
-1.45087707182263,0.146814093466236,0.796863732670736
"2477","ATG4D",7.69187479460006,-0.773217722433592,0.533018101323944,-
1.45064064524831,0.146879951123763,0.796899339181369
"2478","SP4",6.46139626108157,-0.81956773195291,0.565064263050682,-1.4
5039738936632,0.146947734694218,0.796945361806132
"2479","KLF6",562.020332620087,-0.398559135848988,0.274955761228909,-1
.44953913337781,0.147187079954118,0.797599664315883
"2480","COLEC12",142.561619572248,0.438705925875347,0.302649687168352,
1.44955023737167,0.147183981437146,0.797599664315883
"2481","MRPL24",49.854071845332,-0.396528274557825,0.273621358419102,-
1.44918611927388,0.147285612868882,0.797811911062031
"2482","DLEU2L",5.42840201667999,1.10707610541075,0.764279980376183,1.
44852165938697,0.147471212973079,0.797895490612007
"2483","ANK2",161.015905278887,-0.441269152644135,0.305138402305224,-1
.44612788593794,0.148141335558371,0.797895490612007
"2484","POLR3G",12.9402679305103,0.699106361257533,0.483392646106282,1
.44624947625666,0.148107241090182,0.797895490612007
"2485","CDO1",57.204035272127,-0.616306495569727,0.425682489903491,-1.
44780795590031,0.147670767118852,0.797895490612007
"2486","ISOC1",11.7540246520039,-0.653578656954533,0.451371483768561,-
1.44798393442518,0.14762154371076,0.797895490612007
"2487","SMAD5-
AS1",2.38670633223154,1.50053820703077,1.03704228577605,1.446940233404
16,0.147913663455805,0.797895490612007
"2488","AC004889.1",21.0283521496956,0.641141370286426,0.4425408388748
73,1.44877334240266,0.147400890650552,0.797895490612007
"2489","CLN8",33.4633340882022,0.47693049744187,0.329472218150716,1.44
755906922537,0.147740405268621,0.797895490612007
"2490","GRK5",67.4832831386165,0.411497980835472,0.284620409931526,1.4
4577818904298,0.148239425548554,0.797895490612007
"2491","FANCF",9.07370238103159,-0.661353944571052,0.45745815007476,-1
.44571463960795,0.148257256493136,0.797895490612007
"2492","RPLP0",1983.54341499057,-0.279621051852521,0.193412482197312,-
1.4457239195517,0.148254652588727,0.797895490612007
"2493","PNN",142.310660750633,0.32974014067962,0.227693867748143,1.448
17312798408,0.147568637880542,0.797895490612007
"2494","AC087190.1",5.63837336588007,-0.971519271768349,0.671434208414
625,-1.44693144852163,0.147916124115131,0.797895490612007

"2495","TXNL4B",5.96108237349825,0.913580704354657,0.632098464832248,1
.44531391101719,0.148369732200268,0.797895490612007
"2496","ZNF682",5.77877496325043,-0.861778704321429,0.595806757076952,
-1.44640639617675,0.148063248904896,0.797895490612007
"2497","ZNF587",14.4774904710856,0.681742729374053,0.471075935870559,1
.44720347074018,0.147839944776209,0.797895490612007
"2498","RBX1",212.907015596005,-0.372311546695918,0.25759226333859,-1.
44535220844943,0.148358980136965,0.797895490612007
"2499","PGK1",139.48751923296,-0.322460277729274,0.222623636794473,-1.
44845481087424,0.14748989531171,0.797895490612007
"2500","IL17RE",5.66777465367254,-1.01731343308381,0.704227836491528,-
1.44457997876381,0.148575899827332,0.79851063533842
"2501","CUTA",167.712032716063,-0.419982289290882,0.290749062446677,-1
.44448372681479,0.148602954013051,0.79851063533842
"2502","PCNX1",27.1907362295695,0.469586429940901,0.325234145517788,1.
4438411108197,0.148783674841742,0.798922446759808
"2503","BTBD2",46.7429174163246,-0.436850232066416,0.302572186515354,-
1.44378846283761,0.148798488298222,0.798922446759808
"2504","NUDC",136.422718532526,-0.351366356547402,0.2435355304828,-1.4
4277246055568,0.149084579407072,0.799722104273548
"2505","ARHGAP29",173.457760887673,0.324106283536043,0.224697379975034
,1.44241238403427,0.149186072299745,0.799722104273548
"2506","RIOK2",21.6436879320394,-0.527124008500864,0.365285503914691,-
1.44304661108033,0.149007341373233,0.799722104273548
"2507","TSG101",67.5958853095906,-0.344699147273171,0.239008667696624,
-1.44220354263763,0.149244961494014,0.799722104273548
"2508","OSCAR",1.79135691248842,-1.70367916382791,1.18125026350055,-1.
44226775347264,0.149226853404346,0.799722104273548
"2509","GGA3",19.2091569457396,0.561544161271708,0.389467927103898,1.4
4182388893324,0.149352061861489,0.799977026447409
"2510","MSRA",11.6370802104597,-0.612340084245718,0.424892751946266,-1
.44116387356769,0.149538391895534,0.80065595565103
"2511","LMOD1",51.1318721494891,0.464641132582505,0.322475761421737,1.
44085599033548,0.149625371567389,0.800802615887752
"2512","PTPN13",48.6955990700955,0.448434058690777,0.311406366816977,1
.44002854943019,0.14985932203188,0.800956753419529
"2513","ZDHHC11",3.24663736256685,-1.22945674688249,0.853565497655416,
-1.4403777451872,0.149760556490415,0.800956753419529
"2514","AL136366.1",2.60440871704237,-1.390997248392,0.966295153161111
,-1.43951591171862,0.150004405033203,0.800956753419529
"2515","GOLGA1",20.1681162419327,0.569960623006791,0.395964753212953,1
.43942262128635,0.15003081893138,0.800956753419529
"2516","LARP4B",24.8578677924919,0.477929385370319,0.332092903609941,1
.4391436256996,0.150109833848554,0.800956753419529
"2517","CAVIN3",374.137932587331,-0.387046764042381,0.268995676186608,
-1.43885868178,0.150190566159477,0.800956753419529
"2518","CASC4",113.972119949004,0.280759688572116,0.195115568337984,1.
4389404749383,0.150167388560517,0.800956753419529
"2519","TOB1",38.4930022061779,0.514644778806058,0.3573975518803,1.439
97846683187,0.149873491325536,0.800956753419529

"2520","PRNP",125.634002028994,0.313823699052877,0.218090825545891,1.4
3895873779817,0.150162213815091,0.800956753419529
"2521","TRIM61",3.69284978748461,1.42697332313088,0.992888025544447,1.
43719461451699,0.150662702826866,0.801263179730173
"2522","NPR3",8.17158105516231,1.31080212257114,0.91198892837083,1.437
30047788272,0.150632633137895,0.801263179730173
"2523","RXRB",21.341380048295,0.526010232607697,0.365960195729078,1.43
73427458682,0.150620628514068,0.801263179730173
"2524","IRF7",14.6442614359404,-0.666127096541858,0.463177733219426,-1
.43816735729454,0.15038657467733,0.801263179730173
"2525","FBRSL1",19.1813099250605,0.526175044949542,0.365935849730547,1
.43788875929206,0.150465619589078,0.801263179730173
"2526","MMP25-
AS1",3.26721331551175,-1.31598943710497,0.915250886463122,-1.437845574
98814,0.150477874843119,0.801263179730173
"2527","SCML2",1.87796554192103,1.61288047481887,1.12224958197656,1.43
718518654129,0.150665380993984,0.801263179730173
"2528","RASSF2",26.8284453489848,-0.653846685106464,0.455019989421994,
-1.43696255177061,0.150728634504356,0.8012824838228
"2529","DCLK2",1.78291947309522,1.73626222470745,1.20997361477682,1.43
495874910273,0.151298852832009,0.801776531233977
"2530","BHMT2",50.5792475421912,0.384281078655034,0.267630980432922,1.
43586171538668,0.151041694330838,0.801776531233977
"2531","OAS2",7.13156180730771,0.836050283285368,0.582410373415789,1.4
3550033008856,0.151144574340104,0.801776531233977
"2532","NIPA2",36.1730163219915,-0.425311880959418,0.296166342674687,-
1.43605744365958,0.150985996231306,0.801776531233977
"2533","HDGFL3",45.6299236130875,0.40823072956037,0.284295096998144,1.
4359400984078,0.151019387113423,0.801776531233977
"2534","AC027796.1",3.7662686541566,1.39404532483543,0.971259256462949
,1.43529682271668,0.151202532788239,0.801776531233977
"2535","HMG20B",60.5911253179348,-0.414323887907783,0.288721472629576,
-1.43502969881063,0.151278634782956,0.801776531233977
"2536","PHETA2",23.7265162103512,-0.514111298214303,0.358149861773689,
-1.43546418158096,0.151154868118343,0.801776531233977
"2537","RPH3AL",2.39039582914631,1.44810503533527,1.00977572702594,1.4
3408580398374,0.151547778489851,0.802779107262555
"2538","AC005899.6",3.084783217567,1.39604755829455,0.973725730497919,
1.43371743661398,0.151652914248295,0.803019509291897
"2539","NR2F1-
AS1",4.45276368391239,1.08024473839608,0.753580260810005,1.43348332563
137,0.151719760745943,0.803057055795482
"2540","RPL29",2513.12633332706,-0.408421900025281,0.284978681881573,-
1.43316649978403,0.15181026082683,0.803219722540068
"2541","DDAH2",523.678360130043,-0.358917768388648,0.250925514679822,-
1.43037573857973,0.152609207650472,0.804358287124644
"2542","ZKSCAN1",102.038989778795,0.374901635988923,0.262101077854297,
1.43037044737883,0.152610725463176,0.804358287124644
"2543","BSCL2",72.9199042508231,-0.404685878211884,0.28290513147937,-1
.43046496221435,0.152583615045922,0.804358287124644

"2544","MDP1",44.6001684158365,-0.543843423403048,0.380169434736904,-1
.4305290581275,0.152565232007696,0.804358287124644
"2545","NPAS3",3.48237367605407,1.29000117787858,0.900756149308624,1.4
3213141411104,0.152106215601842,0.804358287124644
"2546","TOP3A",12.7291979504617,0.662058340781239,0.462443041983985,1.
43165380527915,0.152242922948967,0.804358287124644
"2547","ACACA",11.7710396778699,0.632185315841105,0.441932354939035,1.
43050244856663,0.152572863562272,0.804358287124644
"2548","PLXDC1",99.4235999631074,0.357782426423893,0.250012112549774,1
.4310603705357,0.152412913756481,0.804358287124644
"2549","MAPT",2.57219955478408,1.55717711428991,1.08868818534362,1.430
324251933,0.152623977391758,0.804358287124644
"2550","NAPB",7.95723575805359,0.883768764105136,0.617629437344389,1.4
3090453703933,0.152457576575363,0.804358287124644
"2551","CAMLG",190.169044987055,-0.339789351907952,0.23765221171308,-1
.42977567706453,0.152781412124507,0.804408220191963
"2552","ADAM22",7.43037771307804,0.911071612314428,0.637633700116478,1
.42883227807439,0.153052445794393,0.804408220191963
"2553","ZNF618",26.4124948978482,0.470166761320237,0.32898230929415,1.
42915514919026,0.152959645449472,0.804408220191963
"2554","NDUFA8",66.1431540459624,-0.455639909101516,0.318783763218398,
-1.42930714068194,0.152915974532764,0.804408220191963
"2555","KBTBD7",20.2032133862935,0.502488690053611,0.351636747664784,1
.42899936764469,0.153004415187472,0.804408220191963
"2556","TOMM40",29.4600513812772,-0.423872208931737,0.296644113236254,
-1.42889135505668,0.153035462554314,0.804408220191963
"2557","PCIF1",20.8209801709484,-0.47376427379195,0.331445990150374,-1
.42938604741305,0.152893306417098,0.804408220191963
"2558","KMT2B",27.8238862494943,-0.466435435716225,0.326497200398932,-
1.42860470211172,0.153117881919141,0.804437535227261
"2559","DEGS1",90.032397691546,-0.317346844532033,0.222449948612764,-1
.42659886644642,0.153695551148729,0.804870518332088
"2560","AC104825.1",3.84489049386184,1.18884171342551,0.83252705823223
5,1.42799168107504,0.153294252976205,0.804870518332088
"2561","AC004982.2",3.3521884845079,-1.23359431060388,0.86476129424509
2,-1.42651425175171,0.153719956069609,0.804870518332088
"2562","AC004540.1",14.4885797089922,0.884597323422927,0.6201402727251
89,1.42644714805505,0.153739312490399,0.804870518332088
"2563","PHB2",157.383691210249,-0.348834183111314,0.244388712525972,-1
.42737436400318,0.153472016125473,0.804870518332088
"2564","DERA",70.212733725662,-0.384267914647839,0.269257663812579,-1.
4271382630554,0.153540045379292,0.804870518332088
"2565","AC025159.1",11.0856618669037,0.691721951832784,0.4845595966281
8,1.42752709191222,0.153428021880736,0.804870518332088
"2566","LGI4",24.6811950705032,0.839824152493919,0.588717182947751,1.4
2653242816671,0.153714713304235,0.804870518332088
"2567","COX7A1",180.261710633363,-0.340384197555925,0.238424050536168,
-1.42764203858826,0.153394917085712,0.804870518332088
"2568","ACAP2",89.0576402136749,0.313030586451396,0.219485719012357,1.
42620024601133,0.153810548659289,0.804929892302255

"2569","AGRN",2.98063680893285,1.44509239485527,1.013612791426,1.42568
484442886,0.153959333160173,0.805394892308119
"2570","ZNF487",10.0459780028249,-0.698887641853422,0.490395101130739,
-1.42515216861251,0.154113219259627,0.80588620763818
"2571","LINC00377",1.61157262079707,2.04053254819551,1.43248439179738,
1.42447105174752,0.154310159139599,0.806288580356558
"2572","GOLGA8A",25.325121526199,0.76647711079783,0.538070382707238,1.
42449228842775,0.154304015824471,0.806288580356558
"2573","OTUD7B",17.1881602084979,0.626791501652687,0.440302636344614,1
.42354701042969,0.15457764443932,0.806821376884354
"2574","DCAF8",60.6571267961864,0.398953770012914,0.280198342313189,1.
42382630360813,0.154496759402266,0.806821376884354
"2575","HIBCH",40.8760982104812,0.434976308235794,0.305650873089588,1.
42311488869295,0.154702852959123,0.806821376884354
"2576","PARD3B",39.0278962230793,0.475877904884617,0.334443683275199,1
.42289398389695,0.154766890510911,0.806821376884354
"2577","LSM12",42.364510837571,-0.386605470296765,0.27160209656 1109,-1
.42342594255262,0.154612716440657,0.806821376884354
"2578","SPIN2B",20.3482336940021,-0.603946030663792,0.424454680973147,
-1.42287518017029,0.154772342407014,0.806821376884354
"2579","YBX1",852.285903108296,-0.288575380768453,0.202891609773077,-1
.42231303251627,0.154935397185732,0.807071332127101
"2580","FAM204A",113.823213549336,-0.319411979732346,0.22457493133369,
-1.42229579158923,0.154940400095835,0.807071332127101
"2581","TALDO1",148.142535082849,-0.412511897301455,0.290201663722434,
-1.42146634175056,0.155181231814983,0.808012620829739
"2582","LPL",1.70007708278546,-1.56033096131498,1.09824963005608,-1.42
074344357876,0.15539135784268,0.808480239275175
"2583","AC135050.6",3.23382008388369,1.30690593402517,0.91975351707569
4,1.4209306186514,0.155336930638236,0.808480239275175
"2584","IER5",31.1782502609667,0.549412867466692,0.387134104440651,1.4
1917971360469,0.155846628714233,0.808502378907764
"2585","CTDSPL",63.2386766984348,-0.334673092359517,0.235866691359009,
-1.41890781793397,0.155925892747665,0.808502378907764
"2586","DDIT4",258.285424237225,1.13261335457035,0.7982505382863,1.418
86951558074,0.155937061249269,0.808502378907764
"2587","ANXA11",218.690935491381,-0.291160157854922,0.205163361834176,
-1.41916254077691,0.155851634095853,0.808502378907764
"2588","MED19",39.1562552366498,-0.371590203738985,0.261684440133624,-
1.41999349884632,0.155609573731388,0.808502378907764
"2589","GLRX5",100.243293076661,-0.403512373184142,0.284351684802207,-
1.41906095427155,0.155881246113393,0.808502378907764
"2590","GLYR1",36.930672246186,0.429509890790583,0.302662588838471,1.4
1910466185766,0.155868505021891,0.808502378907764
"2591","FAM171A2",2.43841059102594,1.44364377151282,1.01664287864509,1
.42001070566373,0.155604564355529,0.808502378907764
"2592","CBR3-
AS1",6.72692118396222,0.860429840383484,0.605850546757117,1.4202014754
1177,0.155549034257094,0.808502378907764
"2593","OSBPL11",30.8959613020453,0.458452985898378,0.323237884078806,

1.4183145246261,0.15609895800411,0.808717770476959
"2594","MORC2-
AS1",2.65359503050718,-1.34659861469364,0.94934121752543,-1.41845586163815,0.1560577163996,0.808717770476959
"2595","CYB5RL",7.03338238215419,0.90137562263122,0.63595820905903,1.4173504010034,0.156380506234343,0.809240517244257
"2596","AFAP1",42.9712182133274,0.499187900017135,0.352132895362796,1.41761223274194,0.156304006743396,0.809240517244257
"2597","PDLIM1",211.256442972159,-0.347611419962892,0.245235547018515,-1.41745935362563,0.156348670050996,0.809240517244257
"2598","IGF1",112.884605191845,-0.584848592894377,0.412697889821177,-1.41713492440631,0.156443483422765,0.809254801277343
"2599","HSPB11",52.5811969414852,-0.376095014900468,0.266481726768892,-1.411335101512,0.158145829073719,0.809954953095162
"2600","SNX7",95.3022248717811,-0.353319795356955,0.250087592273726,-1.41278418551145,0.157719189559714,0.809954953095162
"2601","PYHIN1",5.96805745814707,0.964249972304287,0.682618377042476,1.41257546637114,0.157780586869878,0.809954953095162
"2602","CD84",2.38617415398848,-1.66239149031683,1.17704204664439,-1.41234673396427,0.157847892121128,0.809954953095162
"2603","ITSN2",40.4364456936712,0.468296384116367,0.331137961597748,1.41420325793161,0.157302231686874,0.809954953095162
"2604","ZKSCAN7",2.61647119205689,1.36870755992962,0.968923189373986,1.41260687631383,0.157771346090524,0.809954953095162
"2605","RFT1",22.3046291915577,-0.450285414315663,0.319041965003563,-1.41136735510839,0.158136323464624,0.809954953095162
"2606","TLR1",1.92044508452081,1.59067485512733,1.12651538751449,1.4120311828469,0.157940779753951,0.809954953095162
"2607","PCDHGA8",3.90205863132021,1.10939640391097,0.785550210764365,1.41225397015868,0.157875194369131,0.809954953095162
"2608","ZNF623",10.1828183323189,0.679308555735702,0.480581645238434,1.41351331759388,0.157504848416898,0.809954953095162
"2609","PTPN20",2.804279083757,-1.32114171961685,0.935221661191857,-1.41265089811241,0.157758395603515,0.809954953095162
"2610","ARHGAP19",8.40889199315294,0.798200592762557,0.564049201939722,1.41512582593434,0.15703160728498,0.809954953095162
"2611","LRRC23",13.9302615252209,-0.57240288375551,0.405340404465789,-1.4121535318195,0.157904759419393,0.809954953095162
"2612","AC008124.1",23.9102897897596,-0.446042334568258,0.315907989585508,-1.41193749215869,0.157968367110413,0.809954953095162
"2613","AC139768.1",1.5859614897801,1.72671591210526,1.22253793357376,1.41240272770733,0.157831413770921,0.809954953095162
"2614","HELB",7.21743126705417,-0.939401536260455,0.663626292005096,-1.41555804460689,0.156904942525788,0.809954953095162
"2615","SLC7A7",2.62983230412692,-1.25084655394662,0.884070949044477,-1.41487123324046,0.157106253753819,0.809954953095162
"2616","FBXL8",9.92935998074877,-0.667912150746793,0.472412166725883,-1.41383350766736,0.157410792713892,0.809954953095162
"2617","STX8",83.1240610328252,-0.465859691586706,0.329815153918496,-1.41248722519836,0.157806549544417,0.809954953095162

"2618","KANSL1",71.5304124780536,0.335754123958263,0.23783082388048,1.41173510851139,0.158027971720394,0.809954953095162
"2619","GCDH",17.5187325215179,-0.55050605335189,0.389527618748441,-1.41326577848491,0.157577592111356,0.809954953095162
"2620","LSR",2.40449020169001,1.57325475929215,1.11228119498869,1.41443977150773,0.157232819560295,0.809954953095162
"2621","ZNF787",22.7341342096648,-0.554476506172801,0.391828072583484,-1.41510153296804,0.157038728811778,0.809954953095162
"2622","TOP1",102.345614188484,0.296652867240424,0.209846349767836,1.41366703575557,0.157459688456544,0.809954953095162
"2623","DRG1",33.1376596941412,-0.399404708842829,0.282920925009355,-1.41171851756678,0.158032858724172,0.809954953095162
"2624","NCAPH2",19.4900531383495,-0.517231998941815,0.366288041904875,-1.41209086775522,0.1579232073507,0.809954953095162
"2625","BRCA2",2.79435984530972,1.39622380541138,0.990028032385276,1.41028714313013,0.15845491330515,0.811228792345871
"2626","MTMR11",17.1065373912702,-0.652287192198035,0.462924301752019,-1.40905800306732,0.15881801770498,0.811541954348755
"2627","PPIL6",7.37496177210154,-0.786517345684689,0.557961151056755,-1.40962743408759,0.158649721952398,0.811541954348755
"2628","ZBTB43",25.4449503683427,0.50051466527761,0.355209032713626,1.40907077011505,0.1588142429145,0.811541954348755
"2629","CBX4",10.7766727030917,0.683710995951164,0.485139111705878,1.40930916401907,0.158743770254545,0.811541954348755
"2630","TOB2",63.611088770625,0.378484946413842,0.268536777929896,1.40943430293428,0.15870678686989,0.811541954348755
"2631","PRRC2C",304.711754568914,0.248792042994742,0.176605651595721,1.40874338248397,0.158911061982845,0.811708765483637
"2632","CAVIN1",711.435021777011,-0.287072914612701,0.203830076942526,-1.40839329955042,0.159014642198822,0.811929246394364
"2633","HLA-DPB1",57.7481360590532,-0.436948173284128,0.310477102595812,-1.40734427637635,0.159325326140292,0.81320663045932
"2634","SLC25A33",5.21173140112831,-1.00787608115082,0.717395399740385,-1.40491015347402,0.160047996932441,0.814112426485645
"2635","AGO3",53.6082059194607,0.413800170171035,0.294522979400975,1.40498432758169,0.160025938710505,0.814112426485645
"2636","MZT2A",112.92312538013,-0.402614984045608,0.286516740794829,-1.40520579331144,0.15996009193607,0.814112426485645
"2637","PDE10A",13.5278595638574,0.662563630281831,0.471483313177296,1.40527482471619,0.159939571525533,0.814112426485645
"2638","CNTFR",5.2966288744958,1.08404396969991,0.77094955930503,1.40611529848609,0.159689890272225,0.814112426485645
"2639","RAD23B",104.972964667868,0.32744910012708,0.232940871724511,1.40571767291375,0.159807976943936,0.814112426485645
"2640","ADCY7",22.4015285010365,-0.470788728728235,0.335020941076881,-1.40525164550892,0.159946461600088,0.814112426485645
"2641","SLFN11",36.3745700192913,0.426321569462901,0.303102708363639,1.40652510749404,0.159568254438128,0.814112426485645
"2642","LINC01436",13.8074042122152,-0.952594034155332,0.6776191248209

83,-1.40579567379683,0.159784807070328,0.814112426485645
"2643","FLOT1",96.1420123321899,-0.27152328976138,0.193364578903708,-1.40420386867542,0.160258150181603,0.814256816745015
"2644","BTBD6",65.2778274338441,-0.361235515002353,0.257249545253456,-1.40422217130236,0.160252701649994,0.814256816745015
"2645","BEX3",189.945308073862,0.44084767058306,0.313919298583098,1.40433440241764,0.160219294492649,0.814256816745015
"2646","CZIB",137.758299277551,-0.384648589926422,0.274028074898042,-1.40368314476368,0.160413223798937,0.814736702431562
"2647","TRAF5",27.8207645624134,0.464930624256615,0.331424120602277,1.40282675688096,0.16066850606431,0.815109117779637
"2648","CDK9",27.4159026009382,-0.407081510056419,0.290171005550149,-1.40290208969919,0.160646037656494,0.815109117779637
"2649","TMEM250",26.8887607164087,-0.457834169855249,0.326333130664433,-1.4029656410401,0.160627084982495,0.815109117779637
"2650","B4GALT2",37.6407535295647,-0.457513725684837,0.326203646223976,-1.40254019530702,0.160753996252764,0.815197162784946
"2651","SETD9",7.0531698922855,-0.761241438242992,0.542906730687536,-1.40215877832083,0.16086783806129,0.815197162784946
"2652","AL662884.5",7.90315999511063,-0.878381206001257,0.626437614320625,-1.40218464843281,0.160860114664891,0.815197162784946
"2653","SMG6",49.6585120001071,0.38042557246172,0.271353930027068,1.40195342821743,0.160929154276776,0.815200491641761
"2654","SLC14A2-AS1",1.94060845098016,-1.64077043876763,1.17055129657456,-1.40170742074192,0.161002633765625,0.815265408883284
"2655","AL354953.1",1.71637503373017,-1.46974632389437,1.04871197477054,-1.40147758321913,0.161071306381501,0.815305945936341
"2656","GMDS-DT",21.6975949356843,0.528039490790458,0.377054667448662,1.40043218232368,0.161383938488502,0.815966421876214
"2657","PYCARD",58.9593848109395,-0.47226635384054,0.337139169548547,-1.40080535427829,0.161272287090041,0.815966421876214
"2658","RPS6KA6",4.20843565927358,-1.13245284162469,0.808560767754753,-1.40057851776476,0.16134014861647,0.815966421876214
"2659","AC118549.1",28.89306373346,0.411316764087406,0.294121710977624,1.39845767495449,0.161975674117085,0.817473119117125
"2660","SNRPG",110.749629877702,-0.306192751500044,0.219362705798369,-1.39582865914084,0.162766097908695,0.817473119117125
"2661","TRIP12",63.7255755637219,0.373184629866559,0.266897351609552,1.39823279480306,0.162043171683991,0.817473119117125
"2662","RPL32",3453.18623780753,-0.381309463327505,0.273138863002413,-1.39602786339539,0.162706104692242,0.817473119117125
"2663","AC092902.4",3.58797597751223,1.40334271429388,1.00451598010256,1.39703373773168,0.16240342595267,0.817473119117125
"2664","ADH1C",60.1307296450437,-0.597771922061047,0.428131901661239,-1.39623307616548,0.162644319368989,0.817473119117125
"2665","FBXW7",30.9221956641878,-0.425515183772374,0.304397115356214,-1.39789492838795,0.162144621876834,0.817473119117125
"2666","ECI2",109.229906131533,-0.334751259783523,0.239249855723024,-1

.3991701636429,0.161761961433411,0.817473119117125
"2667","GET4",3.52264167766262,-1.42050144874563,1.01766466170319,-1.3
9584433085083,0.162761377544,0.817473119117125
"2668","SGMS1",39.5876759572506,0.368670458713659,0.263781418525022,1.
39763619733013,0.162222342722165,0.817473119117125
"2669","CCDC6",60.0318464219288,0.361663092100668,0.258792419276695,1.
39750265139716,0.16226246990520‌5,0.817473119117125
"2670","DDB2",21.7428429687947,-0.519987848957355,0.372456468711846,-1
.39610368630662,0.162683273918997,0.817473119117125
"2671","KLC2",7.68394852422926,-0.746001080046373,0.53432205562267,-1.
39616374094276,0.162665192791319,0.817473119117125
"2672","MTFMT",11.279869898475,-0.613197943802239,0.439287135638713,-1
.39589324169638,0.162746646118879,0.817473119117125
"2673","NEO1",19.8414203229364,0.537550396749636,0.385067309494187,1.3
9599073589432,0.162717284906465,0.817473119117125
"2674","AC009041.2",3.61734119220495,1.22533806999688,0.87712668274402
3,1.39699098671073,0.162416281558332,0.817473119117125
"2675","SLC44A2",32.783982572969,0.46686477408955,0.334354683356872,1.
39631594031313,0.162619375703842,0.817473119117125
"2676","CFAP410",38.0927357011626,-0.468163282516897,0.335410260083755
,-1.39579296828902,0.162776848482582,0.817473119117125
"2677","OTUB1",46.7386286862144,-0.326530294194379,0.233978771072915,-
1.39555521510378,0.162848476669122,0.81752733580737
"2678","AC018926.2",4.14628279667561,1.14311166610544,0.81928780445262
5,1.39525043567463,0.16294033269915,0.817683021338266
"2679","AL034417.3",2.11975659426838,-1.91956938385794,1.3777767773743
,-1.393236854751,0.16354817842277,0.819090107054019
"2680","AGL",53.3751929337054,-0.380069385910143,0.273518856036169,-1.
38955460481995,0.164664168639305,0.819090107054019
"2681","ADAR",84.8914506720068,0.298397983158399,0.214784370948281,1.3
8929095185539,0.164744294169457,0.819090107054019
"2682","CAPN7",32.2985519667697,0.442887708349981,0.31800828264523,1.3
9269236846911,0.163712837345996,0.819090107054019
"2683","SH3BP5",319.481240470027,0.240708001427831,0.17330974829283,1.
38888899094771,0.164866508708684,0.819090107054019
"2684","C3orf67",3.68643307674202,-1.16694004595246,0.840109888659346,
-1.38903262740387,0.164822828806992,0.819090107054019
"2685","DCK",12.2707310093744,-0.632824408995065,0.455474535293757,-1.
38937385069602,0.164719097609796,0.819090107054019
"2686","ZNF391",2.14133464039925,1.52494493451123,1.09843511506616,1.3
8828858764169,0.165049185944065,0.819090107054019
"2687","AUXG01000058.1",44.5614376837994,1.28523296711343,0.9240163827
18706,1.39092010829064,0.164249654432457,0.819090107054019
"2688","BNIP3L",171.896677362566,0.274158126297902,0.197102223677305,1
.39094385229642,0.164242453627129,0.819090107054019
"2689","LRRC24",2.7615394375549,1.38831903249658,0.998397116488144,1.3
9054791882812,0.164362558797914,0.819090107054019
"2690","USP54",13.2986643794203,0.660693998989003,0.474791342129009,1.
39154601266819,0.164059916928983,0.819090107054019
"2691","IFIT3",70.9012579992513,0.391278243800178,0.281480909338633,1.

39007027055413,0.164507539928489,0.819090107054019
"2692","PSMC3",126.788661213904,-0.357620925394628,0.256688503009494,-1.39320975112548,0.163556371917909,0.819090107054019
"2693","BUD13",11.1973486394611,-0.585406793758484,0.421262188550646,-1.38964950966185,0.164635333740016,0.819090107054019
"2694","JAML",2.01153000160996,-1.5489439565877,1.11492309903887,-1.38928322314156,0.164746643409298,0.819090107054019
"2695","MGAT4C",3.57923388252083,1.38072928823578,0.993296215287321,1.39004786989588,0.164514341589732,0.819090107054019
"2696","SLC8B1",27.3173768264147,0.494674507877392,0.355215258020124,1.39260489719551,0.163739301310516,0.819090107054019
"2697","C16orf72",23.2730186317316,-0.444650873606939,0.32026097917358,-1.38840165528233,0.165014772620954,0.819090107054019
"2698","MIR193BHG",11.8628241865127,-0.612695889525911,0.440943149384381,-1.38951220895783,0.16467705098559,0.819090107054019
"2699","CIAO2B",136.552870572484,-0.386447415544396,0.278348981127073,-1.38835577547156,0.165028735971608,0.819090107054019
"2700","RFWD3",9.56155052015076,0.810633301882506,0.581755540426086,1.39342601067243,0.163491004719243,0.819090107054019
"2701","TLCD2",18.5177982862747,0.630689146698516,0.452725416687364,1.3930941878928,0.163591310450015,0.819090107054019
"2702","DRC3",5.23093941454474,-0.868585588435652,0.624892437649994,-1.38997615605992,0.16453611796434,0.819090107054019
"2703","FAM20A",37.1027987209452,0.439375727033424,0.3163749074883,1.38878184278781,0.164899098180602,0.819090107054019
"2704","CRLF1",13.2680723896452,-0.858036527020348,0.615870079674067,-1.39321028141916,0.163556211605808,0.819090107054019
"2705","PTPRH",2.89391127363585,1.41519299766654,1.01663892582483,1.39203109552229,0.163912981875454,0.819090107054019
"2706","ZGPAT",22.0401335943033,-0.457903101468031,0.329002197644483,-1.39179344316368,0.163984956073516,0.819090107054019
"2707","Z82217.1",3.8080658272895,-1.08423307981334,0.780121586303703,-1.38982576414857,0.164581792500086,0.819090107054019
"2708","BCLAF3",33.4713094498042,0.430066556233414,0.309023445234134,1.39169555859288,0.164014607825286,0.819090107054019
"2709","TMEM128",35.8387113426503,0.438166032548214,0.315739763070445,1.38774422419027,0.165214944296614,0.819610054042892
"2710","TXNDC16",10.4265978599557,0.758749612134205,0.546908957413058,1.38734171720861,0.165337587989813,0.819915809961293
"2711","SLC6A6",9.94441844823846,-0.760149718720698,0.548014809289983,-1.38709703795334,0.165412175124839,0.819983113796647
"2712","EIF5",278.953656721491,-0.252727003520263,0.182302505098121,-1.38630570865846,0.165653574448085,0.820876986359816
"2713","FANK1",2.27050935730026,1.43632964039682,1.03630142091965,1.38601531504432,0.165742227056969,0.821013560419687
"2714","HS2ST1",23.8440245293606,0.508276314279995,0.367011533092458,1.38490556413101,0.166081345435765,0.821133902908767
"2715","VGLL4",102.502521441233,0.321722694912251,0.232311364121123,1.3848771287164,0.166090041599584,0.821133902908767
"2716","ASIC1",9.84606822488423,0.80745336116103,0.58305955244823,1.38

485572832241,0.166096586528038,0.821133902908767
"2717","MVK",5.00408032319833,-0.867339696775848,0.626346237906874,-1.3847607669431,0.16612563111067,0.821133902908767
"2718","AC092329.4",3.11031856935559,1.25037823403452,0.902972115740258,1.38473626398691,0.166133126126121,0.821133902908767
"2719","ZNF256",1.51841495942126,1.74846307842869,1.2622445501776,1.38520152705961,0.165990853911307,0.821133902908767
"2720","AP2S1",176.418498477987,-0.380685334054428,0.275010887561303,-1.38425550141017,0.166280234238727,0.82155884850524
"2721","STX17",43.2777049994084,0.409525419456573,0.296095148427494,1.38308723270707,0.166638119939667,0.822420012438189
"2722","CHD8",46.1489853631108,0.421120427630735,0.304475159928945,1.38310273891969,0.166633366000678,0.822420012438189
"2723","EIF5A",165.113694742227,-0.360303301883161,0.260432477749023,-1.38348068181567,0.166517526711758,0.822420012438189
"2724","MT2A",1374.7644512636,-0.447062405374063,0.323365686682849,-1.38252889463975,0.166809364758736,0.822962941627259
"2725","MKNK1",35.2294283779719,0.506821603049228,0.367013923527902,1.38093290351884,0.167299591891406,0.823646393435779
"2726","TADA1",5.84359805428339,0.851904965231869,0.617145901649508,1.38039475423056,0.167465134458263,0.823646393435779
"2727","MAT2A",56.2470129724913,0.375847144073119,0.272394270973383,1.37979092853185,0.167651026505436,0.823646393435779
"2728","LIMD1",17.2646449849587,0.560239694706231,0.405831307055024,1.38047431276728,0.167440653344373,0.823646393435779
"2729","GPBP1",111.022290638338,0.309401150379009,0.224254760397979,1.37968598673189,0.167683349389113,0.823646393435779
"2730","HOXC-AS1",8.81647944152377,0.774481077308138,0.560611613638561,1.38149310229499,0.167127397218212,0.823646393435779
"2731","SRP54",48.3762779143379,0.380689275227841,0.275646401916907,1.38107834015043,0.167254874573383,0.823646393435779
"2732","CHMP6",28.9029124889711,-0.495983255757993,0.359386038516658,-1.38008492985741,0.167560496750324,0.823646393435779
"2733","ADGRE5",44.4109137334866,-0.425747551824262,0.308320176033011,-1.38086179536522,0.167321458744027,0.823646393435779
"2734","NFS1",10.697995486391,-0.67040829268777,0.485813895399337,-1.37996936488755,0.167596077475568,0.823646393435779
"2735","TNRC6B",217.624801439087,0.318333271403235,0.230558990111486,1.38070205481602,0.167370589253573,0.823646393435779
"2736","ARMCX5",9.88304489902593,0.75624742958205,0.547790674169714,1.38054089863485,0.167420166142767,0.823646393435779
"2737","AC108463.3",7.5139849262779,0.83306589922548,0.604126811898168,1.37895866036467,0.167907500164807,0.824446070410976
"2738","FHOD3",4.53698139687745,1.15287605052558,0.836190133896745,1.37872477058901,0.167979629178431,0.824498990697201
"2739","BAIAP2",14.5271779002189,-0.567110440615473,0.411489962890839,-1.37818778526542,0.168145317520592,0.82501092448311
"2740","ZNF329",6.11063277545044,0.853632208795868,0.619552843497535,1.37781985468244,0.168258914359075,0.825266989077232

"2741","STIP1",33.9593262809574,-0.387104062881523,0.281059469901566,-1.37730304201135,0.168418575125714,0.825447567875447
"2742","ZPR1",23.9782360953235,-0.455230316321978,0.330512149685064,-1.37734820567339,0.168404618022959,0.825447567875447
"2743","PIP4K2A",39.348203491365,0.420762282893877,0.305647562760197,1.3766256766261,0.168628008167929,0.825871647874926
"2744","RELA",47.1530117610236,-0.346841479769143,0.2519396344861,-1.37668485737316,0.168609702420901,0.825871647874926
"2745","CLIP2",6.40879255157205,0.917297259776396,0.666450663613578,1.37639184692637,0.168700350794551,0.82592495968232
"2746","PGAM1",144.654564424262,-0.351266772376311,0.255296624938236,-1.37591624041756,0.168847566593449,0.826043846905481
"2747","CENPX",62.0950861009838,-0.468602205031107,0.340543400715213,-1.37604253686004,0.168808464309797,0.826043846905481
"2748","LAP3",50.3098613744406,-0.365675033521689,0.265931272987876,-1.3750734519229,0.169108673954652,0.826537774816818
"2749","PCDH18",189.13988094309,0.27056553370924,0.196820556607721,1.37468127502809,0.169230278916215,0.826537774816818
"2750","HIGD2A",197.058458658973,-0.397598318915956,0.289308239502992,-1.37430693159309,0.16934641530711,0.826537774816818
"2751","PKIA",11.3234388077211,-0.612248174931591,0.445491634441295,-1.37432025115226,0.169342282018793,0.826537774816818
"2752","SNHG7",144.428158062222,-0.451712881512346,0.328696075443715,-1.37425699683992,0.16936191159347,0.826537774816818
"2753","ALKBH8",7.523657262722,0.73753436735643,0.536700198103758,1.3742017796197,0.169379048429609,0.826537774816818
"2754","SNHG19",29.3170008022152,-0.426804230892347,0.310405702752604,-1.37498836879461,0.169135050690502,0.826537774816818
"2755","OXA1L",54.2765894611759,-0.35214060231342,0.25641351438255,-1.37333089935366,0.169649500852715,0.827557038823826
"2756","ABCC1",19.6542327850595,0.599293551472709,0.436456683245051,1.37308826850117,0.169724907658368,0.827624468077216
"2757","RFC2",21.924254201585,-0.489324176348331,0.356726168904852,-1.37170810274602,0.170154324974462,0.828679450544268
"2758","GNG10",104.720898187936,-0.298451445146845,0.217510211169037,-1.3721261339538,0.170024175127777,0.828679450544268
"2759","MRPL41",110.190054372102,-0.397632168596724,0.289826117529795,-1.37196803374991,0.170073389272765,0.828679450544268
"2760","NDUFB11",190.030518571615,-0.386712242002075,0.281942376415169,-1.37160027846481,0.170187907098905,0.828679450544268
"2761","TCEA3",48.6608221225195,0.438069131489458,0.319458288459495,1.37128741784079,0.170285376387277,0.828853738960018
"2762","NMI",39.137841439262,-0.351562834777129,0.256872784326494,-1.36862624703083,0.171116135190387,0.830274905629976
"2763","AC135507.1",2.60524276149444,-1.25906987324074,0.919824296137642,-1.3688156298193,0.171056913939351,0.830274905629976
"2764","IL1RAP",3.54326167986062,-0.995915957291348,0.727409880535395,-1.36912624359505,0.170959816189657,0.830274905629976
"2765","GM2A",20.2915450747454,0.529539411315297,0.386918631625711,1.36860664757946,0.171122264945081,0.830274905629976

"2766","HLA-
C",802.15258096631,-0.421691492322182,0.308169851778861,-1.36837360918
998,0.171195160614678,0.830274905629976
"2767","SRGN",45.1262722408504,-0.499173191626432,0.364766837365117,-1
.3684719675511,0.171164390751616,0.830274905629976
"2768","DHRS7",145.668524764431,-0.303219918034829,0.221463377089207,-
1.36916506024691,0.170947685020964,0.830274905629976
"2769","DAPK2",1.98589274826631,1.69379302735186,1.23740201066448,1.36
883002674474,0.171052412553336,0.830274905629976
"2770","DACT3",2.81654818693851,-1.22533632622617,0.894483452474093,-1
.3698814917559,0.170723898108392,0.830274905629976
"2771","ZNF470",3.53325254419884,1.19474885228007,0.872400550618657,1.
36949575677459,0.170844360276472,0.830274905629976
"2772","NDUFS1",70.8770903356023,0.361161274227283,0.264116210835869,1
.36743319572959,0.171489563282959,0.83065100329787
"2773","NOL7",156.316964084521,-0.29934090327408,0.218921291864234,-1.
36734486045204,0.171517236687238,0.83065100329787
"2774","ZNF318",16.5957804161162,0.572217233400611,0.41853503081299,1.
36719077561823,0.171565515911312,0.83065100329787
"2775","PODXL",2.71040521782382,-1.37835589567135,1.00820467844757,-1.
36713896011051,0.171581753490207,0.83065100329787
"2776","AHCY",25.5418835774735,-0.430343439440284,0.314628727475805,-1
.36778177534147,0.171380393951089,0.83065100329787
"2777","GSAP",6.09105704612113,-0.874256888409887,0.639613452817716,-1
.36685193933693,0.17167171887343,0.830787263212109
"2778","LINC01128",13.3894377877863,-0.532186764530185,0.3898428990031
55,-1.36513135391464,0.17221176904121,0.831145717196005
"2779","RBBP4",76.9439063288545,0.294503023250333,0.215873021331313,1.
36424191144452,0.172491441380592,0.831145717196005
"2780","ERI3",51.177998389325,-0.407819114361723,0.300711844323311,-1.
3561790865918,0.175042194413295,0.831145717196005
"2781","MPC2",124.272993039339,-0.319281043276274,0.234760599609859,-1
.36002823219432,0.173820989999629,0.831145717196005
"2782","SIPA1L2",45.3197818796613,0.526762712839663,0.387984628944807,
1.35768964423227,0.174562184166444,0.831145717196005
"2783","LGALS8",33.7398860895242,0.43281088381304,0.318572523272211,1.
35859451834525,0.174275113172155,0.831145717196005
"2784","MPV17",47.7118573573816,0.383179847691656,0.282070496846793,1.
35845418778335,0.174319609881317,0.831145717196005
"2785","STRN",46.4462067241898,0.375310381140407,0.275603105781683,1.3
6177848967242,0.173267801968613,0.831145717196005
"2786","DPP4",158.755758226747,0.411040978346016,0.303041617984321,1.3
5638458202557,0.174976836256104,0.831145717196005
"2787","ARPC4",96.682844807477,-0.337641353890317,0.248178983356829,-1
.3604752075435,0.173679593255168,0.831145717196005
"2788","TMEM43",133.063632058397,-0.303178744454023,0.223415157131196,
-1.35701958786971,0.174774986711728,0.831145717196005
"2789","CMTM7",10.4825097301791,-0.702351732904346,0.515592475476564,-
1.3622226202102,0.173127639194174,0.831145717196005
"2790","PDCD6IP",96.8249237158794,0.29178085472596,0.214696830387992,1

.35903662014323,0.174134984800009,0.831145717196005
"2791","CACNA2D3",2.02493976413212,1.80675833455259,1.32646684724966,1
.36208329540899,0.173171599461667,0.831145717196005
"2792","PRDM5",22.4688802013335,0.487955902017927,0.358161930536368,1.
36238907716179,0.173075129029555,0.831145717196005
"2793","ZFYVE16",67.3812131536279,0.329252717607761,0.24245907529625,1
.35797233906572,0.174472461436368,0.831145717196005
"2794","PWWP2A",32.7702730152732,0.371819182546677,0.27361222971701,1.
35892749725127,0.174169564514583,0.831145717196005
"2795","NEU1",93.6492242638645,0.484930573740078,0.357607056537835,1.3
5604307821809,0.175085462116369,0.831145717196005
"2796","SLC39A7",101.927633520385,-0.285501027481773,0.209757522988129
,-1.36110030007329,0.173481994854359,0.831145717196005
"2797","UBE3D",9.65238379093069,0.672209306029153,0.494607853679818,1.
35907527757193,0.174122735962047,0.831145717196005
"2798","RPS12",4066.09918788819,-0.351542016849741,0.257493798310256,-
1.36524459678895,0.172176185813817,0.831145717196005
"2799","NOD1",9.0581594933007,0.727212109529039,0.532607664918826,1.36
538048065807,0.17213349558327,0.831145717196005
"2800","AASS",46.658006231066,0.494097198735376,0.36242740359365,1.363
2997776552,0.172788052082883,0.831145717196005
"2801","FGFR1",525.383875594491,0.225782383486335,0.165865948127754,1.
36123409316318,0.173439723270635,0.831145717196005
"2802","MAF1",125.46732143116,-0.326479672568627,0.239664696590591,-1.
36223514440401,0.173123687923716,0.831145717196005
"2803","PGM5",3.73529367676338,1.3186771414555,0.966979570221691,1.363
70734404779,0.172659691735946,0.831145717196005
"2804","AKR1C3",180.984934990274,-0.452309427923659,0.333133131102538,
-1.35774375375603,0.174545008020794,0.831145717196005
"2805","RIC8A",51.7081879305268,-0.347716297974823,0.2558725590932,-1.
3589432927357,0.174164558800648,0.831145717196005
"2806","RPLP2",2026.49127008736,-0.261417372350816,0.192339107992428,-
1.35914830363624,0.174099598971929,0.831145717196005
"2807","POLA2",3.91290315376222,-0.966090842382391,0.709244008768529,-
1.36214170361457,0.173153169276784,0.831145717196005
"2808","PPP6R3",39.5470348908792,0.365294667349362,0.268498906348592,1
.36050709597715,0.173669508913179,0.831145717196005
"2809","MLF2",94.8092556717402,-0.33361050841416,0.245480252544109,-1.
35901159036903,0.1741429159772,0.831145717196005
"2810","PRICKLE1",38.0601746092281,0.51079262578439,0.37648671914271,1
.35673477924402,0.174865497429353,0.831145717196005
"2811","PTGES3",281.26714526864,-0.31788714067193,0.234121217295982,-1
.35778868888269,0.174530745090115,0.831145717196005
"2812","CAND1",54.7473574493074,0.331218389812487,0.243435702219517,1.
36059906904622,0.173640425962673,0.831145717196005
"2813","TUBGCP3",19.5291445655972,0.535709141553532,0.392978271027861,
1.36320295814918,0.172818555233711,0.831145717196005
"2814","CIPC",7.47547670658069,-0.793806550455657,0.581343826442939,-1
.36546827255862,0.172105918474643,0.831145717196005
"2815","ST20-

AS1",3.30164286258542,-1.15564977145929,0.851541474278919,-1.35712681808938,0.174740918562164,0.831145717196005
"2816","ZNF500",12.2455297855305,0.621961652086164,0.458520247865562,1.3564540606035,0.174954742601756,0.831145717196005
"2817","TAOK2",14.1002882461006,0.573264249340949,0.421501491710832,1.36005271775938,0.173813241979347,0.831145717196005
"2818","KAT8",33.3154369144676,-0.402205714524498,0.295654396346534,-1.36039145534327,0.1737060809867,0.831145717196005
"2819","POLR2A",61.7178786681332,0.359702119202348,0.264627600884875,1.35927665141337,0.174058939860396,0.831145717196005
"2820","AC006449.6",3.79599734782974,-1.09019116466265,0.799451745410552,-1.36367350615114,0.172670346043089,0.831145717196005
"2821","ELP2",41.4979167082977,0.381586432002662,0.281027093759539,1.35782791224097,0.174518295852307,0.831145717196005
"2822","SETBP1",41.4048368437684,-0.438251328559161,0.322010082242801,-1.36098635641077,0.173518001132462,0.831145717196005
"2823","AC027097.2",6.56647897137709,0.865844012762301,0.634072380958751,1.36552866638521,0.172086949542254,0.831145717196005
"2824","AC010618.3",5.46793699831868,0.926969586225477,0.680328018666152,1.36253330862793,0.173029639698785,0.831145717196005
"2825","COX6B1",345.819703925079,-0.353101127662004,0.260146469347425,-1.35731662454522,0.174680627242087,0.831145717196005
"2826","HIF3A",9.04835653559329,-0.84791196309351,0.624351272994232,-1.35806876636471,0.174441864861114,0.831145717196005
"2827","GNAS",1173.72662248905,-0.283363997976398,0.208823132611928,-1.35695693495316,0.174794894485755,0.831145717196005
"2828","UFD1",69.2155553278481,-0.406252681544683,0.29904022706676,-1.35852184680822,0.174298155163801,0.831145717196005
"2829","TRIOBP",278.988203027298,0.277276902797691,0.204352604601428,1.35685524213648,0.174827210672725,0.831145717196005
"2830","WDR13",84.6338762533577,-0.339375130577102,0.248419748212367,-1.36613587695524,0.171896319347677,0.831145717196005
"2831","TBC1D8B",28.548858414677,0.470667201200375,0.344581938003809,1.36590792868305,0.171967863835003,0.831145717196005
"2832","KHDC4",21.6210642966013,0.564455605097355,0.41643587110069,1.35544424548594,0.175276060885917,0.831328346516041
"2833","DAAM1",75.1666349783432,-0.308346374269635,0.227537554844275,-1.35514497587295,0.175371371558373,0.831328346516041
"2834","AC005332.7",3.85429967684002,1.07248598203087,0.791179589878244,1.35555314589943,0.175241388134469,0.831328346516041
"2835","SRRD",17.9453132377923,-0.472378553754754,0.348542871507125,-1.35529540946327,0.175323456995282,0.831328346516041
"2836","SNX3",345.358565290618,-0.284443825901721,0.209934748365897,-1.3549154111732,0.175444508978747,0.831381789903167
"2837","FKBP15",31.3068641645737,0.431529862626393,0.318555939015258,1.35464390951356,0.175531036535475,0.831498625308513
"2838","CUL3",58.7730563251862,0.317627640188545,0.234573903356281,1.35406213412461,0.17571655548839,0.832084139960702
"2839","AC010680.3",1.59153086143098,1.73524305348636,1.28182935671502,1.35372391371447,0.175824475818978,0.832301912832421

"2840","NDUFS5",291.709724713413,-0.298473249696738,0.220605258300988,-1.35297432162523,0.176063833959744,0.832510606447417
"2841","MRPL36",69.9154339260072,-0.413385605569766,0.305575520327371,-1.35280995390892,0.176116351970385,0.832510606447417
"2842","SNCAIP",13.6985151454048,0.704965711863649,0.521020097120458,1.35304898171838,0.176039982769919,0.832510606447417
"2843","ID4",36.724262767522,-0.675921815786334,0.499521278176225,-1.35313918609064,0.17601116896106,0.832510606447417
"2844","SNU13",189.092458401461,-0.337269773246243,0.249438255614094,-1.3521172701273,0.176337803412944,0.833264324917915
"2845","PRKAB1",10.118357352487,0.619921989366317,0.458555863445054,1.35190069255455,0.17640708597835,0.833298709477343
"2846","Z83843.1",27.3866094047427,0.596826895019029,0.441541867838569,1.35168811496996,0.176475108688321,0.833327120752755
"2847","TMEM109",189.304118218157,-0.283242981223268,0.209647643301868,-1.35104300130592,0.176681658324084,0.834009415601463
"2848","SLC45A1",3.36714398033192,-1.05050950269975,0.779454398349593,-1.34774979129514,0.17773887161702,0.834637400967263
"2849","UBE4B",23.1966637209541,0.44110879187761,0.326704617846257,1.35017617683381,0.17695947794128,0.834637400967263
"2850","SF3B4",34.3422220735479,-0.380566841506883,0.282484583460531,-1.34721278182622,0.177911712509678,0.834637400967263
"2851","PLA2R1",40.8038862041978,0.456473436859614,0.338687896761291,1.34777014834203,0.177732321995169,0.834637400967263
"2852","LNPK",70.429116057788,0.367784144994554,0.272925812973504,1.34756086640386,0.177799664379355,0.834637400967263
"2853","SATB1",21.0432956363439,0.474512196181599,0.352234663629493,1.34714792488659,0.177932595711824,0.834637400967263
"2854","HLTF",44.4894357258594,0.342004847677259,0.253770937951799,1.3476911518616,0.177757739115986,0.834637400967263
"2855","LINC02511",67.65178701103,-0.33569861597647,0.248995697886961,-1.34821050654807,0.177590686305829,0.834637400967263
"2856","SMARCA5",79.5308950302472,0.288560112187185,0.214125928560728,1.34761873130814,0.177781042809629,0.834637400967263
"2857","MAMDC2",46.9616501913522,-0.685249286044073,0.508361185613067,-1.34795752594228,0.177672044065955,0.834637400967263
"2858","DKK1",39.9580081896904,-1.37810712606227,1.02106908495633,-1.34967079736942,0.177121603567844,0.834637400967263
"2859","TCF7L2",224.847202419259,0.292364356132057,0.216698276211034,1.34917711965246,0.177280082086517,0.834637400967263
"2860","PDHX",13.2528136505014,0.596866168513959,0.442460939018023,1.34896917643988,0.177346866818776,0.834637400967263
"2861","KMT5B",51.5382957275402,0.353688973318045,0.2620171522644,1.34986954197998,0.177057833142535,0.834637400967263
"2862","ABCA5",11.4416257023548,0.713916908162602,0.529061658852968,1.34940208993864,0.177207849898616,0.834637400967263
"2863","TMEM94",12.2952539144506,0.606712623938116,0.449608692427638,1.34942369699795,0.177200913580175,0.834637400967263
"2864","SF3A2",29.1312125999784,-0.478006031575987,0.354801985685656,-1.34724734037843,0.177900585791271,0.834637400967263

"2865","IDH3B",59.5931461794776,-0.442589854463415,0.327789615298667,-1.35022536958698,0.176943702823056,0.834637400967263
"2866","FBXO21",39.3641094519699,0.387089377890791,0.28739139675789,1.34690663067027,0.17801030574758,0.834710571856847
"2867","LINC00294",9.99016258482949,0.659713086240844,0.489869673084394,1.34671142650463,0.178073190730065,0.834714199588887
"2868","IGFBP3",117.678724365427,-0.437216273993314,0.324716763281067,-1.34645427472086,0.178156057362037,0.834811455679362
"2869","SDHB",56.694325114573,-0.385969565691275,0.290253168043341,-1.32976865780029,0.183594505523618,0.835043123442507
"2870","STMN1",14.221063701421,-0.603097617677792,0.449472922239574,-1.34178854350726,0.179664567898673,0.835043123442507
"2871","TMEM200B",15.2599834461707,-0.504469469322752,0.381636028051347,-1.32186018154156,0.186214713808994,0.835043123442507
"2872","EIF3I",174.783655784291,-0.359005863488256,0.271237318036149,-1.3235858033385,0.18564064262101,0.835043123442507
"2873","RNF19B",11.8503954607319,-0.694692616373581,0.520279378177408,-1.33522996588325,0.181801092108394,0.835043123442507
"2874","TRAPPC3",60.4097268492916,-0.355318812738918,0.267094449621907,-1.33031148060882,0.183415666103093,0.835043123442507
"2875","MRPS15",93.0891484585393,-0.360163797365486,0.272210794527183,-1.32310622725698,0.185800054114208,0.835043123442507
"2876","LRRC41",46.3724568477979,0.360814758279482,0.268419233579731,1.3442209541676,0.178876947651665,0.835043123442507
"2877","KYAT3",32.2343639764888,-0.420740344469024,0.316756567352998,-1.32827662575389,0.184086738624517,0.835043123442507
"2878","ALG14",21.9843358117721,-0.568465224957872,0.427265305833745,-1.3304736359265,0.183362267152406,0.835043123442507
"2879","SCNM1",30.9545605490166,-0.486578416517552,0.365278554051609,-1.33207496339576,0.182835556510094,0.835043123442507
"2880","TPM3",165.408659114567,-0.266910837301001,0.200955665439839,-1.32820757611786,0.184109542276933,0.835043123442507
"2881","ADAM15",36.7484171777864,-0.373992314269814,0.281709567553493,-1.32758115926893,0.184316512095026,0.835043123442507
"2882","DCAF6",57.5020276154471,0.335412634435445,0.251009123273747,1.33625674661095,0.181465368843417,0.835043123442507
"2883","PRDX6",296.605384051429,-0.319988385418892,0.24158554232151,-1.32453449963922,0.185325593123857,0.835043123442507
"2884","DESI2",78.4249045595865,-0.299366464581036,0.224462007899634,-1.33370661423868,0.182300026261789,0.835043123442507
"2885","NCOA1",79.1614006580103,0.322824246046638,0.240400818117895,1.34285835037517,0.179317844971104,0.835043123442507
"2886","RHOQ",148.274440312111,-0.32201037372133,0.242962186391022,-1.32535181093197,0.185054492101519,0.835043123442507
"2887","POLE4",66.0319478516195,-0.362798620540975,0.274050022065667,-1.32384087330612,0.185555898359029,0.835043123442507
"2888","PTPN18",36.3117005770628,0.360577719175901,0.270114958184948,1.33490467021457,0.181907549071106,0.835043123442507
"2889","MYO1B",50.7445590941654,0.351235199224451,0.26240538004863,1.33852133351595,0.180726550753913,0.835043123442507

"2890","METTL21A",18.7051886250398,-0.521448671790727,0.394195555184305,-1.32281722848682,0.185896166406173,0.835043123442507
"2891","AC079610.1",1.87799647009688,-1.48259099346283,1.11118845549194,-1.33423901781491,0.182125536036714,0.835043123442507
"2892","SH3BP4",16.2256378236318,-0.606703762324248,0.454327964229871,-1.33538723145221,0.181749641616662,0.835043123442507
"2893","WDR48",35.4870268962407,0.453201387915298,0.337807096598648,1.34159818570583,0.179726314786393,0.835043123442507
"2894","GNL3",58.1813062913246,-0.3949549960323,0.294386354904748,-1.34162127235854,0.179718825264516,0.835043123442507
"2895","ZBTB20-AS2",5.48935350799002,0.976192994609263,0.730873031823232,1.33565332431826,0.18166261214538,0.835043123442507
"2896","AC046134.2",6.23503697232465,0.85559304494462,0.636940853538342,1.34328492228379,0.179179732425905,0.835043123442507
"2897","GYG1",43.4309302124862,-0.423388527018329,0.315085199375806,-1.34372711843361,0.179036644686833,0.835043123442507
"2898","ATP13A3",39.9650590753622,0.386835830574631,0.291916304890109,1.32516006846639,0.185118066455021,0.835043123442507
"2899","KLF3",126.498865193694,0.293116416806247,0.220613405501616,1.32864281814507,0.183965838487679,0.835043123442507
"2900","NUP54",47.1704639655633,0.348303530374133,0.262546968361931,1.3266332212756,0.184630040831996,0.835043123442507
"2901","METAP1",14.6974156430923,0.562738328432385,0.419925021956126,1.34009239509233,0.180215308209727,0.835043123442507
"2902","HMGB2",59.1562085790265,0.347932975817656,0.263015774568819,1.3228597272846,0.185882030280629,0.835043123442507
"2903","ARSB",7.89192024414393,-0.732281874898687,0.553767165453109,-1.32236420030341,0.18604690390316,0.835043123442507
"2904","CCDC112",21.2637027112014,-0.508189162875121,0.381501257729242,-1.33207729353226,0.182834790896674,0.835043123442507
"2905","CDC42SE2",22.0315827920626,-0.434938217946076,0.326414759603422,-1.33247105147606,0.182705447897807,0.835043123442507
"2906","UBE2D2",158.399325002486,-0.306521426455006,0.230261406547526,-1.3311888911429,0.18312686574754,0.835043123442507
"2907","TUBB2B",5.73292448759418,-0.992334686509795,0.740204826989458,-1.34062174458628,0.180043293670759,0.835043123442507
"2908","HMGN4",55.3705712741536,-0.33778506330702,0.252219290406665,-1.33925150119324,0.180488811512585,0.835043123442507
"2909","TRIM39",5.76198440218559,0.864176579922208,0.643839314513717,1.34222399974893,0.179523377044616,0.835043123442507
"2910","MAP3K7",29.8959832704612,0.406893412543476,0.307291024647387,1.32413048187913,0.185459713699966,0.835043123442507
"2911","ZBTB24",15.0943893905626,-0.542618586576444,0.410490967916783,-1.32187704233835,0.186209098303865,0.835043123442507
"2912","TRMT11",38.6464926184081,0.389547015472882,0.291808421981195,1.33494096170393,0.181895669948626,0.835043123442507
"2913","EZR",10.5655492142405,-0.628696879449143,0.475023689793988,-1.32350637022293,0.185667039264978,0.835043123442507
"2914","AL356417.2",4.84175482054143,-0.938519774379931,0.705795973170

536,-1.3297324015097,0.183606455189419,0.835043123442507
"2915","MRPL18",111.260472522622,-0.361530805713157,0.271488459466284,
-1.33166178195525,0.182971353193414,0.835043123442507
"2916","ZDHHC4",36.6563423399138,-0.423820029588662,0.319943827161009,
-1.32467012521976,0.185280585989899,0.835043123442507
"2917","CCZ1B",17.2533027234593,-0.657101269881281,0.496001440323994,-
1.32479710029079,0.185238456841945,0.835043123442507
"2918","RP9",25.8695508563378,0.407788490579591,0.306967066925855,1.32
844377953446,0.184031544723149,0.835043123442507
"2919","ZNF107",12.9852627723679,0.634911488309021,0.476918352252383,1
.33127921228124,0.183097155615891,0.835043123442507
"2920","AC006001.2",1.49616845766193,1.6905402996881,1.25939687967518,
1.34234118487265,0.179485395426557,0.835043123442507
"2921","RCC1L",37.1531362066387,-0.379149975703327,0.286375141939555,-
1.32396259373432,0.185515468136379,0.835043123442507
"2922","ARPC1B",301.978222332512,-0.362182574263561,0.273415627001425,
-1.32465937750396,0.185284152307375,0.835043123442507
"2923","WDR86",34.4733484079716,0.668703545425128,0.49856541803797,1.3
4125537237763,0.179837553872725,0.835043123442507
"2924","PRKDC",120.788219097914,0.28138011103943,0.209844804886349,1.3
4089624564127,0.179954141346739,0.835043123442507
"2925","GDF6",4.13821001088038,1.31660356795047,0.991627427541575,1.32
771999985375,0.184270623940132,0.835043123442507
"2926","NDUFB9",136.75618717304,-0.384740697649934,0.290102295100761,-
1.32622424623115,0.184765430384593,0.835043123442507
"2927","FOCAD",16.0830973579769,0.506245691463962,0.3797549573991,1.33
308514240652,0.182503864547684,0.835043123442507
"2928","NPR2",17.4071410493586,0.589465349491978,0.445963203665105,1.3
2178023802752,0.186241340779336,0.835043123442507
"2929","ZBTB6",8.58432033057355,0.682530433269332,0.516302572070322,1.
32195822796786,0.186182061101431,0.835043123442507
"2930","PTGES2",19.4217809889256,-0.575549141601084,0.433060061266013,
-1.32902844912208,0.183838584183991,0.835043123442507
"2931","ATP5F1C",207.239642397683,-0.401436189767527,0.29996829364842,
-1.33826207058415,0.180811021493977,0.835043123442507
"2932","PPA1",224.399650357047,-0.291715937137197,0.217929954241555,-1
.33857659977231,0.180708548184202,0.835043123442507
"2933","MCU",21.0773203301649,0.567584113492531,0.424920961035233,1.33
574044478702,0.181634124872463,0.835043123442507
"2934","PAPSS2",79.5024517246619,0.301728624544525,0.225918486127737,1
.33556412189272,0.181691783626215,0.835043123442507
"2935","MARCH5",37.4197676744827,-0.372810429166691,0.27813649124946,-
1.34038661195419,0.180119686029142,0.835043123442507
"2936","FRAT1",4.48667656340073,-0.964728568024121,0.728408595726316,-
1.32443325584614,0.185359195987059,0.835043123442507
"2937","DNMBP",32.2538624828049,0.414861726203854,0.308682512446051,1.
34397547472457,0.178956317761979,0.835043123442507
"2938","WBP1L",49.00652914938,0.401845885188617,0.303096919219134,1.32
579996597752,0.1849059642947,0.835043123442507
"2939","DUSP5",3.16998178473879,-1.12132584027831,0.837421846443958,-1

.3390214800819,0.180563680254459,0.835043123442507
"2940","TMEM9B-
AS1",4.57911921418367,1.06300001597468,0.790523189910954,1.34467910561159,0.178728885000013,0.835043123442507
"2941","RBM4B",12.3730834496922,0.588416681825563,0.444674340087733,1.32325306135153,0.185751235591557,0.835043123442507
"2942","PPP1CA",78.8987193362052,-0.324275875905571,0.241129050641557,-1.34482292798313,0.178682424150749,0.835043123442507
"2943","MRPL21",79.1323540270072,-0.419596456619447,0.311997243768198,-1.3448723185875,0.178666470921112,0.835043123442507
"2944","MRPL48",22.261900207246,-0.492834177682071,0.371265318104953,-1.32744469695592,0.184361622450978,0.835043123442507
"2945","PGM2L1",9.08584796181687,-0.824618531212216,0.614957812416717,-1.34093512524307,0.179941516700583,0.835043123442507
"2946","NOX4",50.3917851650753,0.433252389007964,0.324498249947529,1.33514553338276,0.181828719228109,0.835043123442507
"2947","CEP57",56.191409940042,0.336681731894198,0.251993749566798,1.33607175762489,0.181525820107176,0.835043123442507
"2948","CUL5",55.1236724783234,0.319679652199886,0.237969615426853,1.343363318155,0.179154358551843,0.835043123442507
"2949","HINFP",6.74935560277961,0.843992461692288,0.629636784845408,1.3404433826075,0.180101239578221,0.835043123442507
"2950","CBL",11.3327765082646,-0.64578525410293,0.481618106891144,-1.34086581227498,0.179964023863996,0.835043123442507
"2951","THY1",726.541144181501,-0.423137014318681,0.315979431322536,-1.33912834942336,0.180528892869686,0.835043123442507
"2952","SENP1",10.4862297492907,0.637994643299212,0.476907119510932,1.33777546444154,0.180969642317722,0.835043123442507
"2953","ADCY6",15.3597126057049,0.602247827996403,0.448967762792154,1.34140550370698,0.179788831641654,0.835043123442507
"2954","DAZAP2",119.083318839046,0.256610721043508,0.19182945870856,1.33770236735833,0.180993478970018,0.835043123442507
"2955","ATP5F1B",186.881680253087,-0.313038494323095,0.232658667242997,-1.34548391440816,0.178469012464095,0.835043123442507
"2956","MSRB3",30.5241968302808,-0.397200636600405,0.300532832023548,-1.3216547221346,0.186283152323881,0.835043123442507
"2957","CCT2",77.1794649800606,-0.315536994338483,0.238590552168649,-1.32250414557675,0.186000329829898,0.835043123442507
"2958","TMTC2",2.75277835023441,1.46867917308125,1.10945980375127,1.32377862462019,0.185576577178188,0.835043123442507
"2959","IKBIP",85.2035899797797,-0.311170293125938,0.233424675589289,-1.33306511978812,0.182510434631236,0.835043123442507
"2960","ANAPC7",27.9831862495832,0.398813080763411,0.300163089183119,1.32865463854588,0.183961936906712,0.835043123442507
"2961","ESD",256.523804844302,-0.358249625192596,0.270238665984184,-1.32567863258162,0.184946167969225,0.835043123442507
"2962","SUGT1",93.8141156272248,-0.29865204384751,0.223298907969234,-1.33745411727968,0.181074449680634,0.835043123442507
"2963","ARGLU1",337.117660067816,0.268471597822082,0.201948377030647,1.32940705822726,0.183713710459618,0.835043123442507

"2964","ZFHX2-
AS1",5.59006812295423,0.958617214836379,0.715819134937599,1.3391891443
6388,0.180509105533566,0.835043123442507
"2965","PCK2",15.4848215771496,0.557718896796221,0.420653838074998,1.3
2583812701784,0.184893321016786,0.835043123442507
"2966","TOGARAM1",32.9552403683856,0.38291857870854,0.284973423876298,
1.34369925974135,0.179045656813767,0.835043123442507
"2967","RIN3",9.68386405669461,-0.606803170359121,0.458573906793024,-1
.32323963786496,0.185755698158276,0.835043123442507
"2968","THBS1",67.6760113567628,-0.57919172013915,0.436643419957666,-1
.32646386883674,0.184686095369879,0.835043123442507
"2969","PEAK1",35.4631292037927,0.574093867894999,0.432959291554035,1.
32597655043823,0.184847464800701,0.835043123442507
"2970","RAMAC",24.1396479816264,-0.507627518030482,0.379261692589868,-
1.33846240722083,0.180745747020885,0.835043123442507
"2971","UBE2Q2L",1.89578172051761,1.71934840693937,1.29722301812746,1.
32540695232285,0.185036212349054,0.835043123442507
"2972","AP3S2",28.8875722827118,0.415935130242367,0.3143897397448,1.32
299206259083,0.185838017454844,0.835043123442507
"2973","NTAN1",147.757635770553,-0.376157743860576,0.281341340022074,-
1.33701554073448,0.181217564110307,0.835043123442507
"2974","RIPOR1",29.0867852577014,0.440137004491044,0.329976887536912,1
.33384191776707,0.182255670067612,0.835043123442507
"2975","CTDNEP1",122.510452236528,-0.268782734381078,0.201852842921519
,-1.33157765078187,0.182999012998564,0.835043123442507
"2976","ZBTB4",57.6300474346187,0.363306756802373,0.27481658035218,1.3
219972257O156,0.186169074740773,0.835043123442507
"2977","TVP23B",13.641974246788,-0.700984770943999,0.526970831871664,-
1.33021550444127,0.183447277195163,0.835043123442507
"2978","FBXL20",41.3909729347712,0.424837082559625,0.318410314117748,1
.334244098646,0.182123871439422,0.835043123442507
"2979","ZNF397",41.1072480034981,0.361667589470722,0.272097450621866,1
.3291840428646,0.183787258292242,0.835043123442507
"2980","NDUFA11",300.207137511187,-0.322179869478033,0.243150504207117
,-1.32502241987374,0.185163715343258,0.835043123442507
"2981","C3",2420.92692666352,0.344085740569934,0.259611031686783,1.325
38951959895,0.185041991273185,0.835043123442507
"2982","CALR",399.318930778861,-0.250446859327606,0.189034582137973,-1
.32487324009746,0.185213197760675,0.835043123442507
"2983","TMEM221",4.96799769156026,1.14465615045695,0.865232793083051,1
.32294587029953,0.185853379473369,0.835043123442507
"2984","ZFP36",1174.56226037929,-0.508122025712232,0.379983701724324,-
1.33722057921545,0.181150646379321,0.835043123442507
"2985","HNRNPUL1",116.032770139563,-0.270858453442679,0.20465441069386
7,-1.32349189311069,0.185671850494202,0.835043123442507
"2986","BFSP1",1.53486783849632,-1.51714422764748,1.14637439263232,-1.
323428225018,0.185693010627806,0.835043123442507
"2987","COMMD7",59.2913673545228,-0.353657455297597,0.264352041742542,
-1.3378275914435,0.180952645344716,0.835043123442507
"2988","MANBAL",65.2939712786436,-0.363518410577406,0.272684673301341,

-1.3331090676141,0.182496014125473,0.835043123442507
"2989","SYS1",49.0979547592151,-0.354275378784654,0.267340231101943,-1
.32518542878629,0.185109657030998,0.835043123442507
"2990","ACOT8",15.5924050965276,0.53068888985715,0.399545788326086,1.3
2823046910466,0.184101981632933,0.835043123442507
"2991","N6AMT1",16.0347965546403,0.601184549947954,0.452143437832389,1
.32963236806019,0.183639428077504,0.835043123442507
"2992","IFNAR2",18.4949706002953,-0.578424677181351,0.437467851999887,
-1.32221070539716,0.186097997233989,0.835043123442507
"2993","AC245060.6",3.69409712924981,1.21991155542967,0.92045933721749
9,1.32532911135151,0.185062017557621,0.835043123442507
"2994","EMID1",2.77465871000989,1.35679811792834,1.01698267584443,1.33
414083657006,0.182157704689893,0.835043123442507
"2995","HMOX1",19.4240474942335,0.570518495603701,0.427617924193625,1.
3341781607484,0.182145475090499,0.835043123442507
"2996","APOBEC3G",17.4668941500918,-0.533244490239296,0.40168701356117
4,-1.32751239705709,0.184339241802563,0.835043123442507
"2997","TCEAL9",186.482776771789,0.327678144299784,0.245721933083389,1
.33353233953512,0.182357170067859,0.835043123442507
"2998","FAM199X",16.7697087350471,0.53525197231091,0.404055069505177,1
.32470054878015,0.185270491089882,0.835043123442507
"2999","AL645728.1",1.76103030560825,-1.3430606951355,1.02704220329484
,-1.30769766892426,0.19097587431788,0.835324492793159
"3000","PEX10",14.3974627362525,0.600479244020541,0.455936078829157,1.
31702506536129,0.187830222859285,0.835324492793159
"3001","DNAJC11",12.8044202065934,0.590606252401463,0.451070238662261,
1.30934431443099,0.190417745045624,0.835324492793159
"3002","MIR34AHG",3.35155351927596,1.15874419098837,0.885895238406734,
1.30799234576805,0.190875905463685,0.835324492793159
"3003","PGD",62.5627356425665,0.38875949036197,0.296486266230798,1.311
22259153597,0.189782571925873,0.835324492793159
"3004","WDTC1",22.4083118521934,0.455396714650255,0.345960042200538,1.
3163274919081,0.188064147325256,0.835324492793159
"3005","KIAA0319L",24.1070925643093,0.454125609523065,0.34577193445686
2,1.31336746643826,0.189059154669858,0.835324492793159
"3006","CCT3",99.5166148658958,-0.308079985497065,0.233937910783122,-1
.31693056702844,0.18786189937433,0.835324492793159
"3007","SOAT1",30.9568928261373,0.425995702347891,0.324203078485219,1.
31397796818673,0.18885361854428,0.835324492793159
"3008","RPS6KC1",21.2563634670704,0.496248213241331,0.379387199546178,
1.30802571577254,0.190864587148091,0.835324492793159
"3009","LYST",55.4863436950607,0.399077118904863,0.303070940058503,1.3
167779095806,0.187913079567951,0.835324492793159
"3010","HNRNPU",274.489033183815,0.287747951972943,0.218368526079193,1
.31771715063273,0.187598351086214,0.835324492793159
"3011","LCLAT1",5.5779149950981,0.935924305936902,0.713383883865022,1.
31195044786572,0.189536854366571,0.835324492793159
"3012","GPATCH11",35.4844988412012,0.425973313152848,0.325764214728747
,1.30761235855062,0.191004822977143,0.835324492793159
"3013","RPS27A",2930.97648723229,-0.360414242218169,0.275511227525047,

-1.30816535302687,0.190817230855603,0.835324492793159
"3014","SLC1A4",6.21109588467009,-0.765728404773237,0.582406250828437,
-1.31476680355686,0.188588287326339,0.835324492793159
"3015","INPP5D",1.47255487563566,1.60731943379514,1.22932330710712,1.3
074830880556,0.191048694928433,0.835324492793159
"3016","GLB1",40.1819414298385,0.359520122874132,0.27531177967584,1.30
586538395648,0.191598340219711,0.835324492793159
"3017","PLCD1",19.0066233921094,-0.438707234239523,0.334678831397101,-
1.31083054284658,0.189915021150512,0.835324492793159
"3018","CDHR4",3.06867098726578,-1.29982964859484,0.989606984717976,-1
.31348067330515,0.189021029142029,0.835324492793159
"3019","PIGX",21.2837246086075,-0.445932380903209,0.34027426359603,-1.
31050869434138,0.190023804923719,0.835324492793159
"3020","FGF2",35.7604392958655,0.359364741809475,0.275211940199774,1.3
0577452979916,0.191629244086711,0.835324492793159
"3021","DCTD",57.4609046657484,-0.327443029136816,0.248409350668026,-1
.31815903167997,0.187450416680739,0.835324492793159
"3022","MRPS36",85.8801649589646,-0.405927153119485,0.309764883380339,
-1.31043631766702,0.190048274330225,0.835324492793159
"3023","AC113383.1",1.95964113237124,-1.28374585691999,0.9729127752200
63,-1.31948710060839,0.187006319775168,0.835324492793159
"3024","SLC25A46",31.2796825374938,0.401763530518159,0.305923525546149
,1.31328092470467,0.189088303803541,0.835324492793159
"3025","U91328.1",2.48755355889827,-1.13156246611259,0.865164681737887
,-1.30791569512475,0.190901905418125,0.835324492793159
"3026","MTCH1",252.921893215464,-0.277665716425695,0.212225021163544,-
1.3083552302333,0.190752850166186,0.835324492793159
"3027","LCA5",36.0463157529256,0.374166878551949,0.286012449254169,1.3
082188538564,0.190799088985782,0.835324492793159
"3028","ARID1B",122.894141068935,0.318355335866585,0.24128423472326,1.
31942037668446,0.187028613230033,0.835324492793159
"3029","ITGB8",7.8743169365874,0.737081185285011,0.56374938793675,1.30
746250205722,0.19105568210987,0.835324492793159
"3030","SCRN1",56.3364937446615,0.320150719440322,0.244173447605927,1.
31116107250537,0.18980335093301,0.835324492793159
"3031","ING3",31.0069481258137,-0.398836603701779,0.303622525807646,-1
.31359359006997,0.188983006960936,0.835324492793159
"3032","AC004918.1",2.19302035430015,1.45236300251791,1.10995857793697
,1.30848396632724,0.19070920937674,0.835324492793159
"3033","FAM86B1",2.69914674497,1.34717237842637,1.02940756244298,1.308
68708136286,0.190640369504981,0.835324492793159
"3034","RBPMS",52.0502750175197,0.345904649437902,0.262324026782141,1.
31861596393217,0.187297533975975,0.835324492793159
"3035","RIPK2",19.8790421796489,-0.462622587123903,0.350537295031873,-
1.31975282995734,0.186917555121297,0.835324492793159
"3036","DCAF10",41.1145971402485,0.381826932945894,0.290499957641613,1
.31437861831619,0.18871882222267,0.835324492793159
"3037","OSTF1",49.7390356046734,-0.3287790011074,0.251679801162988,-1.
30633844904575,0.191437487279251,0.835324492793159
"3038","ANKRD26",37.4455849849326,0.459028902368287,0.348895911003876,

1.31566145629946,0.188287696423413,0.835324492793159
"3039","SWAP70",97.5705724639047,0.331926519783949,0.251901191924153,1
.31768538786387,0.187608988050622,0.835324492793159
"3040","SNX15",31.6414922596684,-0.398871713472896,0.304780913989484,-
1.30871617993329,0.190630508898113,0.835324492793159
"3041","MALAT1",51632.4804103682,0.204107059188324,0.155108787126368,1
.31589617177547,0.188208893842777,0.835324492793159
"3042","GRK2",26.6149268292749,-0.406143953740534,0.310340418923085,-1
.30870466421969,0.190634411172916,0.835324492793159
"3043","CORO1B",76.9015638887437,-0.370703587573864,0.28204824289118,-
1.31432688172033,0.18873622469794,0.835324492793159
"3044","RIMKLB",28.7482915574022,0.437624095558408,0.334746459800457,1
.30733001872305,0.191100653342689,0.835324492793159
"3045","ARHGDIB",15.9590958911272,-0.614876564741052,0.47074157776271,
-1.30618707542973,0.191488946954965,0.835324492793159
"3046","ATP5MC2",337.256097255892,-0.348866434314156,0.266135917362836
,-1.31085814260286,0.189905694656726,0.835324492793159
"3047","DRAM1",42.885883526161,0.398717441337385,0.303273841940464,1.3
1471095161467,0.188607064534252,0.835324492793159
"3048","KCTD10",33.7028449607674,0.454889890211386,0.346909957348573,1
.31126213178804,0.189769217477048,0.835324492793159
"3049","RB1",21.5370073166228,0.494677596154719,0.377090722806139,1.31
182648163167,0.189578687646711,0.835324492793159
"3050","RCBTB2",13.3797118692703,-0.552972684557303,0.42185364230278,-
1.31081642803599,0.189919790949604,0.835324492793159
"3051","DOCK9",38.6734421903653,0.388576889095088,0.297311659018777,1.
3069682177198,0.191223505737002,0.835324492793159
"3052","RNF31",12.045503473409,-0.565672857643353,0.430639765821334,-1
.31356391708155,0.188992998126187,0.835324492793159
"3053","RPS15A",2355.00494138142,-0.337705834055059,0.257561214708788,
-1.31116726731114,0.18980125846535,0.835324492793159
"3054","VPS35L",28.2249557793446,0.410469425355172,0.314191714725275,1
.30642982013094,0.191406430473605,0.835324492793159
"3055","AC008915.2",9.04633457338335,-0.667430624721017,0.508428392576
254,-1.31273279475815,0.18927300281538,0.835324492793159
"3056","RANBP10",8.56947147828876,0.779958514274538,0.59490413577302,1
.31106587998595,0.189835506986921,0.835324492793159
"3057","NIP7",18.8903406869416,-0.562750421608089,0.42907007691357,-1.
31155830221516,0.189669209962635,0.835324492793159
"3058","AARS",27.740179507445,0.402075425970896,0.306630696636124,1.31
126932294074,0.189766788788979,0.835324492793159
"3059","AC040169.1",2.29908105955325,1.43285397385769,1.09609282528422
,1.30723780030778,0.191131961318396,0.835324492793159
"3060","CTU2",11.0998385308974,-0.584270473049209,0.446029960963535,-1
.30993548457381,0.190217661663502,0.835324492793159
"3061","APRT",250.595970120901,-0.375014157258952,0.285488365077891,-1
.31358823382044,0.188984810430095,0.835324492793159
"3062","METTL16",22.2520067684607,-0.417786710117855,0.319294773894536
,-1.30846711025672,0.190714923068689,0.835324492793159
"3063","TBKBP1",3.33728412339688,1.10394833069823,0.842704192603602,1.

31000692815766,0.190193491850258,0.835324492793159
"3064","CALCOCO2",109.466114595384,0.256140117217,0.19599346668356,1.30688089532366,0.191253165451816,0.835324492793159
"3065","TRAPPC8",23.059587941896,0.490589394461473,0.37344204586015,1.31369619436263,0.188948462163199,0.835324492793159
"3066","RNF125",2.99530047934327,1.24257844336663,0.951464439153571,1.3059641456196,0.191564750788656,0.835324492793159
"3067","SERPINB2",1.59300200151115,-1.9170816684345,1.4594590480089,-1.313556328319,0.188995553394138,0.835324492793159
"3068","NCLN",18.9628286882641,0.48283258412794,0.366892929241873,1.31600405907423,0.188172680289566,0.835324492793159
"3069","PET100",142.060865416398,-0.392646841503368,0.297607836964717,-1.31934308420083,0.187054440251478,0.835324492793159
"3070","DDX39A",21.1617132290391,-0.453653734198281,0.347311078321986,-1.30618849358357,0.191488464804335,0.835324492793159
"3071","NFKBIB",9.94739006388668,0.613788171994116,0.467368107793561,1.3132863833859,0.189086465103365,0.835324492793159
"3072","TMEM160",93.4754854774708,-0.321816268696552,0.244821517247097,-1.31449340039726,0.188680217561694,0.835324492793159
"3073","PPP1R15A",234.563138216808,-0.320467239813372,0.244776908235918,-1.30922169955878,0.190459263830589,0.835324492793159
"3074","POLD1",5.67794497460309,-0.896357739439851,0.684254079353816,-1.3099779255775,0.190203303332582,0.835324492793159
"3075","ZNF816",2.9077978757605,-1.10775860943112,0.844539482808891,-1.31167178323834,0.189630901253461,0.835324492793159
"3076","TBC1D20",39.3704782965316,0.375466448418468,0.286146587715564,1.31214721592868,0.189470467564708,0.835324492793159
"3077","SEC23B",12.8208003288348,-0.547330966666775,0.414812992652772,-1.31946437638449,0.187013912055784,0.835324492793159
"3078","SAMHD1",155.550578152918,-0.341532150857045,0.25941076188694,-1.31656893635699,0.187983156966473,0.835324492793159
"3079","C22orf39",36.6607277486293,-0.380962313702577,0.289380057439098,-1.31647742789861,0.188013849622614,0.835324492793159
"3080","DNAL4",20.8355826687728,-0.46022570283885,0.351362651494305,-1.30983102751975,0.190253004212654,0.835324492793159
"3081","TUBGCP6",10.6189210072805,0.606675414384955,0.459259368060928,1.32098647643584,0.186505873468545,0.835324492793159
"3082","PRAF2",39.3211317531734,-0.395771599138067,0.299879040118926,-1.31977079485486,0.186911555221654,0.835324492793159
"3083","RPS4X",3777.95364315333,-0.36249194327332,0.274706115866505,-1.31956269750279,0.186981064100473,0.835324492793159
"3084","LIAS",20.1789640904776,0.495630177497179,0.379895101706589,1.30465008701265,0.19201202469107,0.835436586559195
"3085","ARSJ",2.73461521006024,1.5418104047264,1.18182312972325,1.30460334203093,0.19202794968981,0.835436586559195
"3086","ZDHHC6",26.1746572587188,-0.438545309798013,0.336012818568387,-1.30514458247895,0.191843620311204,0.835436586559195
"3087","RDH5",18.0062844847015,0.46955956964529,0.359727368469872,1.30532067004687,0.19178367851907,0.835436586559195
"3088","SPART",110.62136275535,0.285357430563006,0.21859491611338,1.30

541659264847,0.191751031398902,0.835436586559195
"3089","SUCLA2",60.7053848180153,-0.315400604910023,0.241727492608426,
-1.30477754725624,0.191968606692161,0.835436586559195
"3090","APBA1",9.27384308389756,0.647722016647934,0.496583184823973,1.
30435753050627,0.19211170831286,0.835530500976222
"3091","FNDC10",7.08073725713707,0.85163126587917,0.654187911034279,1.
30181443514101,0.192979827972547,0.83624682273638
"3092","ZNF644",71.5464607786515,0.280811894846167,0.215994246588863,1
.30008969813294,0.193570228078117,0.83624682273638
"3093","FDPS",97.7702205809838,-0.385830603264099,0.297595700301668,-1
.29649253289946,0.194805850967478,0.83624682273638
"3094","OST4",507.205021312044,-0.313128416270868,0.241311004630578,-1
.29761349570541,0.194420182285015,0.83624682273638
"3095","THADA",23.2959245732131,0.43441016392071,0.335121351883232,1.2
9627718878406,0.194880004601789,0.83624682273638
"3096","GPAT2",7.94843097299769,-0.887470742726081,0.684605977201899,-
1.29632339225743,0.194864092715389,0.83624682273638
"3097","NCKAP1",93.3546465367433,0.32004874191318,0.246222147723281,1.
29983734149241,0.193656724184497,0.83624682273638
"3098","FBXL2",9.01415959686631,0.70233539194197,0.540650936255929,1.2
9905516636247,0.193924997702139,0.83624682273638
"3099","TMEM131L",8.57961951196411,0.738288709307085,0.567458035746597
,1.3010454743772,0.193242889683414,0.83624682273638
"3100","NIPBL-
DT",16.3535867334403,0.550592570834055,0.423313915991225,1.30067203093
193,0.193370739782789,0.83624682273638
"3101","CPLANE1",40.1299999683382,0.43025636816616,0.330333802436017,1
.30248967860168,0.192749044025364,0.83624682273638
"3102","HNRNPA0",183.789786535042,-0.250906270567288,0.193298216669863
,-1.29802682554395,0.194278117102752,0.83624682273638
"3103","RNF145",51.8863169971038,0.365425477080711,0.280443489950673,1
.30302713443262,0.192565497991,0.83624682273638
"3104","MRPS18B",77.8507708245643,-0.320533694267012,0.246615700151093
,-1.29972947411958,0.193693704754409,0.83624682273638
"3105","SLC35A1",31.6759061104519,-0.388741216532166,0.299948282554146
,-1.29602747921049,0.194966017885794,0.83624682273638
"3106","CCN2",445.429756944098,-0.516063848883076,0.397511197884378,-1
.29823726131404,0.194205817710452,0.83624682273638
"3107","QKI",219.979226492085,0.236894341239498,0.182753625949797,1.29
624974611761,0.194889455956916,0.83624682273638
"3108","ZNF117",20.3843334577131,0.674314853418731,0.519521664104319,1
.29795329051635,0.194303386180145,0.83624682273638
"3109","ELN",300.202605556434,-0.415111245976173,0.318442797381344,-1.
30356613303791,0.192381554134862,0.83624682273638
"3110","AKAP9",236.850382270761,0.33671073886977,0.258902792771876,1.3
0052957430418,0.193419526837765,0.83624682273638
"3111","NDUFA5",125.092194071687,-0.327971481720811,0.251938720295358,
-1.30179069472258,0.19298794563878,0.83624682273638
"3112","EXOC4",37.3166585528092,0.372633574530347,0.286382869141171,1.
3011727120684,0.193199343453383,0.83624682273638

"3113","TMEM70",39.5562047817243,-0.360545765255176,0.27676724332781,-1.30270389270069,0.192675872566902,0.83624682273638
"3114","AL162231.1",5.74749116658852,-0.845079058032073,0.649015334447851,-1.30209413118262,0.192884209156647,0.83624682273638
"3115","NINJ1",14.4523222991875,-0.547004535275178,0.421614400483089,-1.29740477234273,0.194491951342135,0.83624682273638
"3116","TOR1A",41.132118760996,-0.368644090347644,0.284472345814916,-1.29588726556743,0.195014327141589,0.83624682273638
"3117","UBTD1",11.0878054532756,-0.615393083022277,0.473807540565975,-1.29882500875181,0.194003989996763,0.83624682273638
"3118","AP2A2",69.4903000461578,0.351014081522976,0.269604554862093,1.30195901809791,0.192930395499245,0.83624682273638
"3119","FADS3",16.1098860273708,-0.490430358982773,0.378032516685325,-1.2973232125188,0.194520000785012,0.83624682273638
"3120","DRAP1",160.439468127441,-0.375082525896039,0.289266159045862,-1.29666922371162,0.19474502303846,0.83624682273638
"3121","CCND1",46.7762711577957,-0.53830061794684,0.415081337543848,-1.29685574671248,0.194680825380009,0.83624682273638
"3122","CERS5",32.1223487674224,-0.35510147458593,0.272742775278288,-1.30196473297454,0.192928441791465,0.83624682273638
"3123","BTG1",311.669601519974,-0.356485220825066,0.274652221713656,-1.29795134589054,0.194304054450885,0.83624682273638
"3124","MAB21L1",6.57808583305765,0.825826210139536,0.635467085552498,1.29955780388143,0.193752569788786,0.83624682273638
"3125","RIPK3",2.30715835356079,-1.18996093542437,0.915866895045521,-1.29927278937757,0.193850329120834,0.83624682273638
"3126","PRPF39",16.0196939567244,-0.526867019514652,0.405399043829617,-1.29962570838249,0.193729284038374,0.83624682273638
"3127","FGF7",315.24687500114,0.229669796725134,0.177204109547569,1.29607488963726,0.194949685067277,0.83624682273638
"3128","EDC3",8.63103655812069,0.669000937529294,0.513980470244243,1.30160769962988,0.193050526380153,0.83624682273638
"3129","DEXI",66.5218712622989,-0.368793471909175,0.284110484082241,-1.29806357938703,0.19426548816576,0.83624682273638
"3130","COPS3",38.3248381779482,-0.408964224038647,0.315328136781424,-1.29694808783312,0.194649049074283,0.83624682273638
"3131","COPE",189.903898401556,-0.320945553122884,0.247043112517982,-1.29914794973093,0.193893160240892,0.83624682273638
"3132","MRPS6",93.5290158139098,-0.389876993323156,0.30065523021989,-1.29675772823913,0.194714559532625,0.83624682273638
"3133","ZNRF3",10.9107495115973,0.664396026132581,0.512499527607246,1.29638368494603,0.194843330116354,0.83624682273638
"3134","AL022238.2",2.52354238095126,-1.44024115789167,1.11030734222084,-1.29715539393884,0.194577725028976,0.83624682273638
"3135","C8orf58",9.31378778895687,-0.610513821954178,0.471470706066383,-1.294913584447,0.195350041744146,0.836353818197627
"3136","LINC01235",2.80363893411114,4.18964830145476,3.23585944616271,1.29475595932423,0.195404428990154,0.836353818197627
"3137","NR1H3",25.357285878019,0.467102949959118,0.360822157962971,1.29455173317558,0.195474912043958,0.836353818197627

"3138","DIXDC1",67.2384388779454,-0.403150968566018,0.311368813267838,-1.29476990432959,0.195399616933707,0.836353818197627
"3139","TTYH2",1.6326696744904,1.73722080723344,1.34125188344688,1.29522338695171,0.195243179296481,0.836353818197627
"3140","KCNK6",6.97207010207502,-1.04740982209517,0.80873638819668,-1.29511895023134,0.195279198629082,0.836353818197627
"3141","MORF4L2-AS1",1.56684146722839,1.62958329104564,1.25868046945212,1.29467591703792,0.19543205116927,0.836353818197627
"3142","LIMD1-AS1",2.92046577479769,1.40733433666998,1.0877363135054,1.2938193928036,0.19572781249108,0.836370770132789
"3143","GLTP",59.8518422396096,-0.289524971232481,0.22373202465779,-1.29407031324784,0.195641134678437,0.836370770132789
"3144","MBIP",36.7559180185872,0.375676142428181,0.29030056128254,1.2940937515534,0.195633039600881,0.836370770132789
"3145","IQCH-AS1",12.9700633953109,0.712041979560273,0.550271045301702,1.29398409318425,0.195670915313528,0.836370770132789
"3146","AL031280.1",2.41293608289067,1.47147808186975,1.1400224355313,1.29074484502051,0.19679216878955,0.836631929562881
"3147","MIGA1",50.4094603010569,0.3519391796296,0.272335921707746,1.29229804655472,0.196253948208722,0.836631929562881
"3148","CLCC1",34.9874454396091,0.382823455855501,0.296286866644278,1.29207028374673,0.196332805790411,0.836631929562881
"3149","AC007878.1",4.41276284172069,1.07677739952262,0.832690642872736,1.29313017834306,0.195966039166056,0.836631929562881
"3150","HMBOX1",25.5673866245206,0.506059284749779,0.39166259340472,1.29207969632895,0.196329546442703,0.836631929562881
"3151","SNCG",8.91632658470963,-0.924152298478853,0.715555219358862,-1.29151779412202,0.196524188931062,0.836631929562881
"3152","PPP1R14B-AS1",3.68720064143471,-1.13578148969663,0.879357992313838,-1.29160307818215,0.19649463750399,0.836631929562881
"3153","AP003068.2",2.77954557563724,-1.29568958730814,1.00359958914188,-1.29104236522855,0.196688987488134,0.836631929562881
"3154","KHNYN",25.0556625150419,0.440131285427168,0.340836783035115,1.29132566475908,0.196590774809338,0.836631929562881
"3155","CCNDBP1",106.497536719075,-0.334714256747249,0.258841096291221,-1.29312640667641,0.195967343425291,0.836631929562881
"3156","LEO1",33.9857989063169,-0.395594667878506,0.306489967736296,-1.29072631903853,0.196798594991289,0.836631929562881
"3157","CIAO3",13.8009780685164,-0.507866917231554,0.393207931067005,-1.29159886438051,0.196496097534532,0.836631929562881
"3158","CDC6",2.26861838121671,-1.19145802160899,0.92319144462143,-1.29058607350674,0.196847247658146,0.836631929562881
"3159","SEH1L",9.76719098500021,0.649871638370271,0.503380467535429,1.29101480943841,0.196698542290776,0.836631929562881
"3160","MBD2",83.1022592042073,-0.303309296813846,0.234596550141217,-1.29289751546332,0.196046506940153,0.836631929562881

"3161","RBM12",24.5066245524152,0.473935479667536,0.367063111039047,1.29115529568189,0.196649833091249,0.836631929562881
"3162","WDR4",4.56391299850935,-1.00526239498574,0.778688565414035,-1.29096848166922,0.196714606929129,0.836631929562881
"3163","ASH1L",117.414112756881,0.321350763626989,0.249220106089516,1.28942551493644,0.197250195812487,0.836746213956233
"3164","FAM20C",50.1055372571936,-0.340525045753298,0.264235592448526,-1.28871755162823,0.197496298338128,0.836746213956233
"3165","SMC5",55.7450866778474,0.3539634261552,0.274663592695594,1.28871621710524,0.19749676245771,0.836746213956233
"3166","AP000873.2",12.3796174196664,-0.601792629950876,0.466737795448976,-1.28935911301544,0.197273268933892,0.836746213956233
"3167","RASSF8-AS1",43.2733848695005,0.355552066350151,0.275746060370475,1.28941848116507,0.197252639790714,0.836746213956233
"3168","AC009120.2",11.9626619590607,0.659784873360655,0.511732604958575,1.28931568355717,0.197288360727964,0.836746213956233
"3169","WDR83OS",272.234709245587,-0.347388969265577,0.269393400458496,-1.28952293810589,0.197216347104869,0.836746213956233
"3170","XBP1",100.963631791964,-0.449035644456851,0.348327456773501,-1.28911929199091,0.197356617608065,0.836746213956233
"3171","TFE3",26.8151413876383,0.395670670599132,0.30681496365612,1.28960682322718,0.1971872054638,0.836746213956233
"3172","IGBP1",119.029012797983,-0.407790114306093,0.31639693763175,-1.28885607224402,0.197448128065055,0.836746213956233
"3173","MFAP2",136.226609808253,0.391433594317416,0.304976285188071,1.28348862953731,0.199320940639091,0.836812412758196
"3174","EIF4G3",53.7416428520181,0.37763804089176,0.293273342186421,1.28766575944605,0.197862337612758,0.836812412758196
"3175","PSMA5",84.1176619697583,-0.37332083117825,0.290051081288602,-1.28708650048712,0.198064140140976,0.836812412758196
"3176","NUDT17",1.95840925201487,1.43831836982625,1.12044923488958,1.28369793564811,0.199247667021794,0.836812412758196
"3177","RO60",73.3277325779321,-0.267020914792241,0.208048808280415,-1.28345322907277,0.199333335535112,0.836812412758196
"3178","ACP1",73.3883417384737,-0.371947006144854,0.289094324025991,-1.28659394264487,0.19823585609403,0.836812412758196
"3179","EIF4E2",83.2412024052995,-0.334304882193546,0.259911258835698,-1.28622701337027,0.198363846052233,0.836812412758196
"3180","EIF2A",83.7112990161623,-0.305496091964452,0.237845056212407,-1.28443322232281,0.198990414825262,0.836812412758196
"3181","AL031963.3",3.6897203900564,-1.10915526487442,0.864222037804124,-1.28341469709873,0.199346827518284,0.836812412758196
"3182","SNHG32",139.710227788069,0.320352276351212,0.248861596017828,1.28727084241742,0.197999902680818,0.836812412758196
"3183","PLPBP",57.5121780446255,-0.349511857567912,0.271898867607216,-1.2854480073555,0.198635774338835,0.836812412758196
"3184","AC108863.2",9.57462244665121,0.660317712301356,0.514229272664028,1.28409203326854,0.199109755251884,0.836812412758196
"3185","MPP7",7.3214767624537,0.738594337740952,0.574896162779509,1.28

474389908952,0.198881792408595,0.836812412758196
"3186","GFRA1",76.5392343474541,0.412201091654058,0.320584056695011,1.2857816321359,0.198519282307802,0.836812412758196
"3187","PNPLA2",32.3217678141273,0.388031510738083,0.302320525467723,1.28351030793478,0.199313350579361,0.836812412758196
"3188","AP000487.1",2.43608103516158,-1.259272425419,0.977372663133399,-1.28842607627458,0.19759768640920 1,0.836812412758196
"3189","HDAC7",145.046041773712,0.328972898743851,0.255718471644291,1.28646513733845,0.19828077826826,0.836812412758196
"3190","PTGDR",1.66618901064853,-1.46123653281768,1.13707587775022,-1.28508269448898,0.19876338823828,0.836812412758196
"3191","TMEM30B",6.75610003800195,-0.709151506186759,0.55193634681154,-1.28484291763612,0.198847181504889,0.836812412758196
"3192","PLLP",2.11707056477089,-1.35295849709149,1.05320926316214,-1.28460558068905,0.198930147539269,0.836812412758196
"3193","BAIAP2-DT",4.32620769441509,0.954720355813274,0.741621538992803,1.28734173108009,0.197975204232404,0.836812412758196
"3194","CYBC1",47.4699607505355,-0.347365877303267,0.270519228608755,-1.28407092941128,0.199117138634291,0.836812412758196
"3195","KDM4B",22.1157405468155,0.497270773499072,0.387185274448612,1.2843225357866,0.199029124810678,0.836812412758196
"3196","MAST3",3.08125513610135,1.13231743951917,0.880796256442849,1.2855611399759,0.198596266132498,0.836812412758196
"3197","HCST",8.04577008675063,-0.750919684453936,0.584411275028508,-1.28491649038993,0.198821467764742,0.836812412758196
"3198","LRP5L",3.24096372977936,-1.02527705881188,0.798926240442848,-1.28331879328881,0.199380411165395,0.836812412758196
"3199","RTCB",60.416045793776,-0.324518886428747,0.252406428522118,-1.28569976735085,0.198547862474278,0.836812412758196
"3200","AR",90.9092589946374,0.359361256971311,0.279447937708761,1.2859685418249,0.198454040738463,0.836812412758196
"3201","TCEAL7",28.5253688444839,-0.443197066896931,0.345069263488722,-1.28437132422667,0.199012061535048,0.836812412758196
"3202","ZNF449",10.9745904323487,0.614722804199982,0.478692791029999,1.28416975504747,0.199082565283823,0.836812412758196
"3203","RERE",87.9138696830427,0.347212034876666,0.271869176031234,1.27712909549105,0.201556674322707,0.837125475205355
"3204","ACADM",43.5758690057895,-0.328349941052922,0.256231748635126,-1.28145689518161,0.200033230997362,0.837125475205355
"3205","ANKRD35",71.8569402758153,-0.421366858127678,0.330291635015604,-1.27574183980703,0.202046793064925,0.837125475205355
"3206","NBPF9",10.6552225156565,0.746738672106374,0.584598820887545,1.27735234048653,0.201477882712188,0.837125475205355
"3207","NENF",324.83045971327,-0.291300464608634,0.228712604453069,-1.27365286799665,0.202786466922545,0.837125475205355
"3208","AL392172.1",7.68653725612733,-0.770686311655921,0.606724605920602,-1.27024073877231,0.203998890258994,0.837125475205355
"3209","CNIH4",106.940868459851,-0.329975822159974,0.258909547113667,-1.2744830225017,0.20249228571348,0.837125475205355

"3210","CYS1",51.7692515713803,-0.519728386211239,0.408311750608988,-1.27287148958137,0.203063647704096,0.837125475205355
"3211","SLC66A3",120.992978696887,-0.354666265159691,0.278725482196349,-1.27245726642907,0.203210698221873,0.837125475205355
"3212","CALM2",571.249895325886,-0.231237450461908,0.180751301992534,-1.27931277901091,0.200786934312342,0.837125475205355
"3213","TIA1",59.0978673316768,0.363871311833899,0.284187523131789,1.28039158026356,0.200407453653818,0.837125475205355
"3214","TMSB10",4621.4185842858,-0.318569740720515,0.250845693439211,-1.26998289806285,0.204090722057117,0.837125475205355
"3215","DOCK10",4.9273432045697,-0.92427220718578,0.725246010286876,-1.27442577287696,0.202512563230358,0.837125475205355
"3216","TCTA",22.5941584293861,0.502009097629089,0.394101399717691,1.27380693899767,0.202731845385508,0.837125475205355
"3217","EOGT",3.88363052914378,1.03073982775693,0.810970231315227,1.27099588610704,0.203730112170431,0.837125475205355
"3218","IFT122",17.422876478236,-0.488594588567026,0.381471517089812,-1.28081538641322,0.200258518326353,0.837125475205355
"3219","CHST2",2.43707877204184,-1.27583595286255,0.997500224384543,-1.27903324898973,0.200885347670321,0.837125475205355
"3220","PLSCR4",169.028851565043,0.246267312194575,0.194003902515995,1.26939359982344,0.20430071773221,0.837125475205355
"3221","USP46",25.535487214658,0.414303568314024,0.325229666020407,1.2738800041938,0.202705945923601,0.837125475205355
"3222","CLOCK",27.8343351150887,0.43876158930172,0.345376162565292,1.27038758564808,0.203946603142874,0.837125475205355
"3223","HERC6",3.46313301959894,1.12598989632654,0.879833408381968,1.27977624582051,0.200623840373342,0.837125475205355
"3224","MTRR",12.0451863503448,0.620426508697794,0.486528881138903,1.27521002914658,0.202234912442725,0.837125475205355
"3225","AC025171.2",12.2088182014466,0.581391069915572,0.455554073294563,1.27622845233488,0.201874773575197,0.837125475205355
"3226","NAIP",16.6668119446539,-0.472297536723952,0.370470444023955,-1.27485888373166,0.202359194376374,0.837125475205355
"3227","EEF1E1",24.3826444894332,-0.491057121803225,0.383325284304463,-1.28104547732675,0.200177692886134,0.837125475205355
"3228","AL121944.1",6.0256992889674,0.797681382495647,0.628481389870506,1.26922037048703,0.204362477669938,0.837125475205355
"3229","YIPF3",141.068699909607,-0.288979503587367,0.225659108529355,-1.28060199063392,0.200333500457704,0.837125475205355
"3230","SLC17A5",41.1783326621722,0.396053844556198,0.312097236423338,1.26900785503585,0.204438262491552,0.837125475205355
"3231","TRIM73",12.6825039658207,0.719726420394666,0.565120175760336,1.27358118019113,0.202811885470504,0.837125475205355
"3232","PDAP1",165.857228261129,-0.273350777232338,0.214695293377128,-1.27320339879169,0.202945874832612,0.837125475205355
"3233","ADCK2",7.57049673416297,0.701043600642998,0.551533958075999,1.27107966858207,0.203700307533329,0.837125475205355
"3234","CKS2",6.77927669763222,-0.720299656006987,0.56305577940052,-1.27926873741334,0.2008024375799,0.837125475205355

"3235","TRUB2",25.3771981847933,-0.408771034916906,0.320873822228572,-1.27393076841812,0.202687952919133,0.837125475205355
"3236","SET",177.883537813804,-0.268103699821131,0.209110974085029,-1.28211205076264,0.199803342205808,0.837125475205355
"3237","CCNY",93.6393756548696,-0.290068595342437,0.226722821087357,-1.27939743317975,0.200757137296196,0.837125475205355
"3238","HTRA1",689.223542682998,-0.255351222891071,0.199670936341629,-1.27886024661183,0.200946273766755,0.837125475205355
"3239","PEX16",26.1688233309906,-0.498060021184422,0.391218975224301,-1.27309781152324,0.20298333545142,0.837125475205355
"3240","CELF1",60.3709573641548,0.397004261337495,0.312024593796319,1.27234926102219,0.203249053222143,0.837125475205355
"3241","SDHAF2",48.2709844162333,-0.324093603150402,0.253761530231591,-1.27715813683273,0.201546423262558,0.837125475205355
"3242","PACS1",44.7136023178005,0.38895853840333,0.303974580712111,1.27957586944319,0.200694340938542,0.837125475205355
"3243","AP002387.2",1.69315118854495,-1.43320802132459,1.12424617224965,-1.27481690104998,0.202374057155075,0.837125475205355
"3244","CD4",3.20766872473322,-1.036466499594,0.81385750467578,-1.27352330554094,0.202832407942032,0.837125475205355
"3245","FGD4",33.3411323585154,0.396926756401027,0.311552332814465,1.27402915848941,0.202653082597954,0.837125475205355
"3246","PA2G4",141.172966277884,-0.2709536824919,0.212809594950409,-1.27322117480201,0.202939568693021,0.837125475205355
"3247","RFXAP",17.4713898136383,-0.443021932345133,0.347455932038157,-1.27504495245308,0.202293331593725,0.837125475205355
"3248","TRIM13",31.4159778196831,0.414584501085972,0.323973614960021,1.27968600509993,0.200655588494381,0.837125475205355
"3249","BMP4",56.1322251636909,-0.415194027798269,0.326765626702347,-1.27061720655359,0.203864862594111,0.837125475205355
"3250","EIF2S1",49.4238939428379,-0.316265758717159,0.249209021499978,-1.26907828943579,0.204413142719502,0.837125475205355
"3251","AC123768.3",1.74386081611553,1.472251250548,1.15171170393644,1.27831578468465,0.201138104429062,0.837125475205355
"3252","RMDN3",20.298000865542,-0.459585308251835,0.361653343833142,-1.27078960028606,0.20380350947069,0.837125475205355
"3253","AC090517.2",2.81282027406002,-1.22130149351585,0.960766258983785,-1.27117442155769,0.203666604093184,0.837125475205355
"3254","AC026401.3",2.97393199886088,1.16410196577453,0.915977930722034,1.27088429396646,0.203769814700446,0.837125475205355
"3255","AC106739.1",2.54595732572407,-1.29491159633134,1.01253223676312,-1.27888431529937,0.20093779671079,0.837125475205355
"3256","LGALS9",7.92295478293865,-0.677957831783801,0.528487185915274,-1.28282737945606,0.199552559581785,0.837125475205355
"3257","ARHGAP23",22.9988913007999,0.488804418575269,0.384802225391727,1.27027440674925,0.203986901389903,0.837125475205355
"3258","AC067852.3",2.10462671158702,1.38253133319093,1.08558027965026,1.27354131159819,0.20282602279452,0.837125475205355
"3259","AC015912.3",2.70920389898696,-1.21701141519616,0.958792365522951,-1.26931696471359,0.204328038101616,0.837125475205355

"3260","GAA",85.3520948212956,0.31265941423665,0.244623798997538,1.278
12345126648,0.201205901322295,0.837125475205355
"3261","TGIF1",34.3697472304923,0.419738298989535,0.328039550261431,1.
27953564945149,0.200708494147142,0.837125475205355
"3262","PTPRM",19.4487737151872,0.489623575012566,0.384134352841473,1.
27461543439367,0.202445391783708,0.837125475205355
"3263","LMNB2",11.4032110803148,-0.539719697526442,0.425222298193128,-
1.26926480530264,0.204346634419473,0.837125475205355
"3264","TNFSF14",7.04067249297334,0.984631231165674,0.77573689610354,1
.26928503222084,0.204339422800014,0.837125475205355
"3265","ZNF177",2.99313545311761,1.18514521906496,0.926301453831679,1.
27943793477011,0.200742882476356,0.837125475205355
"3266","ZNF441",12.9605675684312,0.650440709922136,0.511970622313171,1
.27046490867647,0.203919074994917,0.837125475205355
"3267","ZNF443",2.41341656822175,1.42962424524223,1.12498792893675,1.2
707907422557,0.203803103100468,0.837125475205355
"3268","GADD45GIP1",170.627023644027,-0.346832551717264,0.272064002695
674,-1.27481970521923,0.202373064393626,0.837125475205355
"3269","ZNF253",12.5261391069863,0.637222805671075,0.501920023820631,1
.26957040052022,0.204237698530752,0.837125475205355
"3270","ZNF431",34.4762089410646,0.408610433788527,0.319781339741274,1
.27778073016744,0.201326750689559,0.837125475205355
"3271","CATSPERG",1.70390873262023,1.63320117348127,1.27786339010981,1
.27807180808343,0.201224108211954,0.837125475205355
"3272","KCNK15",3.55140361954771,-0.978030443043857,0.764330664691988,
-1.27959074288611,0.200689107234815,0.837125475205355
"3273","RCAN1",18.6882586125761,0.479444486005655,0.374690702967559,1.
27957401186751,0.200694994593846,0.837125475205355
"3274","ICOSLG",1.79168705437094,-1.38477860821745,1.08573987609698,-1
.27542391939722,0.202159236902617,0.837125475205355
"3275","SLC19A1",1.45581333594141,1.62818269305751,1.27932701862453,1.
27268686532397,0.20312918031857,0.837125475205355
"3276","ZNF70",4.76498300564228,0.882989946987459,0.690905562128855,1.
27801829278483,0.201242976386952,0.837125475205355
"3277","TIMP3",2114.31092775555,-0.259445028883623,0.202948195321664,-
1.2783805663924,0.201115272843836,0.837125475205355
"3278","FBLN1",2138.9855320408,-0.335127624464734,0.262095105997044,-1
.27864892093221,0.201020714438415,0.837125475205355
"3279","MOSPD2",25.4045395789448,0.393061373798547,0.307024532365713,1
.28022790481886,0.20046499464598,0.837125475205355
"3280","MAP7D2",1.69555555497361,-1.56647206146019,1.22801958882533,-1
.27560836627908,0.202093995162379,0.837125475205355
"3281","LPAR4",5.96271796978784,0.845771092040555,0.660039273972988,1.
28139510085451,0.200054924090449,0.837125475205355
"3282","CUL4B",26.1529373468905,0.414408922766122,0.325308703714114,1.
27389435952599,0.202700857664107,0.837125475205355
"3283","PUM1",99.9439915916036,0.303603285521438,0.242991998920753,1.2
4943737600369,0.211505145127486,0.837411576766515
"3284","ACOT11",1.55713724270233,-1.44639723865222,1.14220397342813,-1
.26632131589518,0.205398068470451,0.837411576766515

"3285","FNBP1L",14.3442151871174,0.602264652893496,0.47691678436085,1.26282964375144,0.206650408876308,0.837411576766515
"3286","MAGI3",35.7466534572281,-0.378599174169812,0.30219598495302,-1.25282661921756,0.210268811885522,0.837411576766515
"3287","LMX1A",6.1591130790566,0.753675920429156,0.601743257091987,1.25248752112555,0.210392272624788,0.837411576766515
"3288","CREG1",113.919854606214,0.270905400729127,0.216442763812153,1.25162604634009,0.210706159093969,0.837411576766515
"3289","SNRPE",143.763099118597,-0.284918078896034,0.225207414992374,-1.26513631403159,0.205822467276,0.837411576766515
"3290","RBM34",3.86825209324173,1.01379653435603,0.799595954605145,1.26788602233069,0.204838656399334,0.837411576766515
"3291","TTC32",18.9394517932609,-0.724822619194463,0.577134175111619,-1.25589966848572,0.209152348787493,0.837411576766515
"3292","ASXL2",25.5430359271871,0.423391474466971,0.33759626746766,1.25413553189693,0.209792747389672,0.837411576766515
"3293","LGALSL",19.7803388647632,-0.473735417147217,0.374393452503345,-1.26534108430485,0.20574908496603,0.837411576766515
"3294","EXOC6B",20.5683427998054,0.450773904490504,0.359462015337301,1.25402375009643,0.209833373035421,0.837411576766515
"3295","MALL",6.28701516006585,0.788301010138265,0.626621078010547,1.25801866199751,0.208385007182229,0.837411576766515
"3296","GCA",10.1567910163375,-0.616017088485847,0.491953192332083,-1.25218638294762,0.21050195667379,0.837411576766515
"3297","OSBPL6",2.08934871732031,-1.1441045392337,0.908953390889393,-1.25870539754983,0.208136761118475,0.837411576766515
"3298","RFTN2",35.0005542238908,0.347674498601642,0.275118838718673,1.26372479696733,0.206328821625322,0.837411576766515
"3299","NGLY1",28.331989542778,-0.400116846258895,0.31719337646945,-1.26142875589784,0.20715441334428,0.837411576766515
"3300","KLHDC8B",30.570808536273,-0.410676478023456,0.324340958909855,-1.26618753118257,0.205445950506363,0.837411576766515
"3301","THOC7",116.336397348396,-0.35239522473083,0.278996278150489,-1.26308217108455,0.206559650630702,0.837411576766515
"3302","BOC",70.7985381351385,0.477594663569365,0.382284813111751,1.24931634003926,0.211549393788401,0.837411576766515
"3303","NDUFB4",236.192950841202,-0.334334387516268,0.264100522084009,-1.26593610977383,0.205535957091236,0.837411576766515
"3304","NPHP3",50.2575284194025,0.470634482389884,0.376427751920153,1.25026510396533,0.211202721385221,0.837411576766515
"3305","YEATS2",14.486086770968,0.574632064499265,0.45360498051073,1.26681162947608,0.205222652787931,0.837411576766515
"3306","FAM114A1",237.132041096185,-0.211533947039468,0.168819257206851,-1.2530202450795,0.210198339013964,0.837411576766515
"3307","PTCD2",7.30720204136526,0.745894553819368,0.593824409902335,1.25608604392339,0.209084775500495,0.837411576766515
"3308","DELE1",16.4554408425263,0.500673486264098,0.396841311536948,1.26164658695692,0.20707598465428,0.837411576766515
"3309","CREBRF",110.442804220929,0.301532173382283,0.239619985787316,1.25837655983303,0.208255604953375,0.837411576766515

"3310","RAB24",13.4251796391008,-0.585149990413694,0.467571236598392,-1.2514670377731,0.210764132382143,0.837411576766515
"3311","MYLIP",56.4606124118759,0.360529637557612,0.285420791589804,1.26315127762574,0.206534818805451,0.837411576766515
"3312","ZKSCAN4",6.97230420029524,0.721363659616737,0.570500037404591,1.26444103824862,0.20607177102753,0.837411576766515
"3313","MRPL2",45.8422297753286,-0.377012772931686,0.297626330536873,-1.26673191935543,0.205251162643742,0.837411576766515
"3314","AL445250.1",3.02401703838361,1.20046763931885,0.948450959848879,1.26571398009879,0.205615501340355,0.837411576766515
"3315","KHDC1",2.04814578477643,-1.27918813379262,1.02378504784106,-1.2494694433076,0.211493422995506,0.837411576766515
"3316","TRAF3IP2-AS1",12.3000167809225,0.565979060420725,0.446350211142299,1.2680156663806,0.204792355953356,0.837411576766515
"3317","NDUFA4",414.417503566449,-0.362745353165554,0.287041985910626,-1.26373621620114,0.206324721570434,0.837411576766515
"3318","PHKG1",13.1091715164911,-0.709125351426542,0.56251618995568,-1.26063100776248,0.207441821516819,0.837411576766515
"3319","AC107959.1",8.81853146094891,0.767411841073233,0.605118128412807,1.26820170317176,0.204725928974228,0.837411576766515
"3320","SMIM19",156.751025458207,-0.396312735841664,0.315892346622952,-1.2545816322505,0.209630674712262,0.837411576766515
"3321","ANGPT1",43.90168763746,0.48100279223181,0.383820453164882,1.25319739546339,0.210133877601478,0.837411576766515
"3322","TAF2",19.542770380722,0.452857558845762,0.359759410672511,1.25877891004775,0.208110200006929,0.837411576766515
"3323","PLPP6",4.2636946292456,0.925552353900637,0.733957424037163,1.26104365674183,0.207293118585691,0.837411576766515
"3324","WHRN",1.74252495557083,1.46868729245309,1.17425032271,1.25074463600195,0.211027659744868,0.837411576766515
"3325","ANAPC16",153.681141140439,-0.311116344206165,0.246566594344952,-1.26179438472881,0.207022783276778,0.837411576766515
"3326","MICU1",65.8436419476641,0.32690872103607,0.260168478903437,1.25652701055074,0.208924959236636,0.837411576766515
"3327","FUT11",33.0767366176936,0.418956526068901,0.335106584706567,1.25021872200976,0.211219659505536,0.837411576766515
"3328","MRPL23",24.6929585644275,-0.564929987257815,0.446229001304084,-1.26600912447831,0.205509815536963,0.837411576766515
"3329","TCP11L1",7.85452196754091,0.66665203732241,0.531068517858254,1.25530325166129,0.209368695210917,0.837411576766515
"3330","AP001462.1",3.48472282180592,-1.07461107360385,0.857885563385684,-1.2526275292043,0.210341291340163,0.837411576766515
"3331","LAMTOR1",190.78122557581,-0.316495689246604,0.250122623024594,-1.26536210687141,0.205741552309472,0.837411576766515
"3332","STARD10",14.5689959501938,-0.56135325257073,0.44766944626693,-1.25394586843439,0.209861681481775,0.837411576766515
"3333","TMEM123",115.61508289982,0.227106635292862,0.181772402372849,1.24940107699641,0.211518414715996,0.837411576766515
"3334","CRYAB",299.998136760671,-0.342577467062916,0.271768051334404,-

1.26055091973038,0.207470691150052,0.837411576766515
"3335","KCNA1",3.73173984069752,-1.11058371682386,0.887606365059175,-1
.25121197925369,0.210857148833406,0.837411576766515
"3336","HOXC-
AS3",5.83378489082737,0.820754397816114,0.656743760815865,1.2497330721
4445,0.211397071859016,0.837411576766515
"3337","HOXC5",15.567318140778,-0.55213696426987,0.437463083394514,-1.
26213384678208,0.206900627864663,0.837411576766515
"3338","ARHGEF25",13.1819941065744,0.641195001495453,0.509232702883812
,1.25913948154612,0.207979955983997,0.837411576766515
"3339","THAP2",11.652800759668,0.557868056197854,0.440195589217249,1.2
6731859624002,0.205041393760174,0.837411576766515
"3340","CDK17",11.7427542813365,0.568428174530028,0.45496527885333,1.2
4938803234098,0.211523183511581,0.837411576766515
"3341","UTP20",19.4070740514631,-0.433250246600334,0.343025785567179,-
1.26302530255553,0.206580086637248,0.837411576766515
"3342","GLT8D2",79.7298446944212,0.37771753734814,0.302108970531907,1.
25026918824394,0.211201229903904,0.837411576766515
"3343","PPP1CC",153.466357017844,-0.209368393223326,0.166897311682702,
-1.25447432982844,0.209669650461651,0.837411576766515
"3344","SDSL",5.34043748935518,0.909617022773797,0.721449194860128,1.2
6081923613506,0.207373981604093,0.837411576766515
"3345","RAB35",27.4377198256692,-0.392394382307032,0.312589909590315,-
1.25530085990718,0.209369563130734,0.837411576766515
"3346","STX2",55.8268269701603,0.378173162825997,0.299800502657121,1.2
6141603991408,0.20715899231931,0.837411576766515
"3347","VPS36",86.6848121578769,0.316148027953532,0.25111649374827,1.2
5896958512989,0.208041317841214,0.837411576766515
"3348","COMMD6",405.63343825216,-0.363217164639912,0.28925909959709,-1
.25568103180103,0.209231639049756,0.837411576766515
"3349","CLN5",73.2743320920824,-0.351311941137575,0.279904614697406,-1
.25511307313516,0.209437715335656,0.837411576766515
"3350","MBNL2",129.128592593113,0.278157934331852,0.221539360139765,1.
25556891631522,0.209272307030679,0.837411576766515
"3351","UBAC2-
AS1",2.81141370355649,-1.14142805791098,0.908377444331587,-1.256557023
77207,0.208914084985404,0.837411576766515
"3352","ING1",21.3886561061377,-0.41076842913903,0.327856876727525,-1.
25288947189115,0.21024593389171,0.837411576766515
"3353","HECTD1",91.4504797770257,0.279678100175968,0.223378535612048,1
.25203659075686,0.210556531133299,0.837411576766515
"3354","RPL36AL",505.375949929884,-0.331229505211194,0.264788609776952
,-1.25092051916511,0.210963476800687,0.837411576766515
"3355","FBXO34",19.3054231969896,0.434402121249367,0.346788955787817,1
.25264116402587,0.210336326956397,0.837411576766515
"3356","L3HYPDH",20.5059921654243,-0.430744032739882,0.342971862035486
,-1.25591653549502,0.209146232742355,0.837411576766515
"3357","PRKCH",1.83167241900231,1.53345007307497,1.22587121008986,1.25
090634354857,0.210968649216138,0.837411576766515
"3358","DNAL1",15.6427387750096,-0.533123469692691,0.425471586895647,-

1.25301779510707,0.210199230609294,0.837411576766515
"3359","PGF",2.70326921703725,-1.06911953130201,0.844429907620832,-1.2660843980695,0.205482867760031,0.837411576766515
"3360","FOS",2643.57200412052,-0.413443745312835,0.330326793911973,-1.25162037392284,0.21070822701479,0.837411576766515
"3361","SERPINA1",2.11710563178811,1.42235070131358,1.12566422217034,1.26356570041039,0.206385951106138,0.837411576766515
"3362","WDR20",22.1500706844322,0.447377947323737,0.353997429456263,1.26378868911818,0.206305882026722,0.837411576766515
"3363","SNRNP25",23.9599211382489,-0.565399435883304,0.448948043568675,-1.25938723641373,0.207890497346688,0.837411576766515
"3364","FLYWCH1",20.0527857369599,0.440629544968307,0.351197786556913,1.2546478418562,0.209606627835304,0.837411576766515
"3365","ZNF174",7.72372961396361,0.704270894590275,0.559949036599638,1.25774105955615,0.208485417784326,0.837411576766515
"3366","ITGAX",3.3048248341309,-1.00693879676049,0.802668851389197,-1.25448844192441,0.20966452418717,0.837411576766515
"3367","ARL2BP",97.7762308461531,-0.298616174477602,0.236937339730369,-1.26031707293338,0.20755500346224,0.837411576766515
"3368","PLD2",13.6110410181626,-0.492187320688277,0.38949712327181,-1.26364815368561,0.206356341785851,0.837411576766515
"3369","SOX15",4.11249105125892,-0.89872216451252,0.708634747787832,-1.26824456085182,0.204710628274651,0.837411576766515
"3370","POLDIP2",33.12817727225,-0.414970301816618,0.331291151501157,-1.25258492397485,0.210356804279486,0.837411576766515
"3371","CCT6B",3.94085312438585,0.951800268367434,0.751124472928977,1.26716716425965,0.205095523997158,0.837411576766515
"3372","PCGF2",28.2845745778057,-0.420119286026567,0.333376119339813,-1.26019610180397,0.207598628761135,0.837411576766515
"3373","TTC25",2.00956814220881,-1.34589716325306,1.06252231841825,-1.26670013412675,0.205262532044196,0.837411576766515
"3374","STAT5B",29.7766094847709,0.410868487049828,0.325998206679438,1.26033971546919,0.207546838720718,0.837411576766515
"3375","HOXB6",2.89256979721608,-1.09121775237745,0.866956058170044,-1.25867711759322,0.208146979723827,0.837411576766515
"3376","SMCHD1",79.0909000993708,0.302782324969701,0.24180516893852,1.25217474175122,0.210506197596169,0.837411576766515
"3377","ME2",29.158658410018,-0.373440259427792,0.296105128755202,-1.26117457336082,0.207245957402131,0.837411576766515
"3378","NACC1",8.63445945007418,0.670234531984182,0.533615740395567,1.25602466577792,0.20910702734613,0.837411576766515
"3379","ZNF737",20.8186569022972,0.51076091465108,0.404908588891217,1.26142277211191,0.207156568072576,0.837411576766515
"3380","TBCB",123.07951965662,-0.3286405932931,0.261908307279332,-1.25479255204608,0.209554077035694,0.837411576766515
"3381","SAMD4B",128.874016078623,0.297161586132353,0.23685842966778,1.25459577921358,0.209625536464009,0.837411576766515
"3382","FBL",77.5332886080415,-0.299004440910527,0.238011438434545,-1.25626080358636,0.20902142806095,0.837411576766515
"3383","PSMC4",64.2984486830849,-0.362768756537551,0.286651518297394,-

1.26553928160669,0.205678076278543,0.837411576766515
"3384","SNRPB",121.148520576485,-0.341386236880367,0.273043541526742,-1.25029962243927,0.211190116300132,0.837411576766515
"3385","MCM8",4.5987429449485,-0.878767081344,0.701620659604645,-1.25248176391952,0.210394369193476,0.837411576766515
"3386","MACROD2",1.963865087842,1.60430507626708,1.28393629778757,1.24952077375767,0.211474660240671,0.837411576766515
"3387","TOMM34",7.23899582177435,-0.674429419823243,0.53450915965175,-1.26177336280384,0.207030349736715,0.837411576766515
"3388","RPS21",1383.20461703145,-0.361118296252644,0.287214272998088,-1.25731319855071,0.208640246554981,0.837411576766515
"3389","PCMTD2",60.0520837984164,0.32062434560651,0.254346917309387,1.26057885426031,0.207460621154291,0.837411576766515
"3390","EVA1C",7.3111956398318,-0.701024843109484,0.55973583050747,-1.25242088303319,0.21041654076177,0.837411576766515
"3391","COL18A1",101.665004772213,0.494855952456606,0.395908424700084,1.24992528974719,0.211326840126762,0.837411576766515
"3392","ASCC2",35.7031227201534,0.368384127010664,0.291189766625258,1.26509983946225,0.205835540446291,0.837411576766515
"3393","TRABD",16.9410398614584,-0.478730799461986,0.378472089387126,-1.26490384069592,0.205905800413883,0.837411576766515
"3394","STAG2",128.773443160627,0.284061944873386,0.225999507914065,1.25691399727029,0.208784779446424,0.837411576766515
"3395","GPC3",947.864846524745,0.439112474351112,0.34895441306418,1.2583663020486,0.208259312966519,0.837411576766515
"3396","KCNJ8",23.5624738995254,-0.514663250939217,0.412159530709331,-1.24869913854346,0.211775136077264,0.838058319712117
"3397","SDCCAG8",64.1142079472266,0.318940486652618,0.255570056128217,1.24795718044765,0.212046738639685,0.838145087936581
"3398","STIMATE",18.2582732457959,-0.508429661416962,0.407354195986532,-1.24812673203389,0.21198465009478,0.838145087936581
"3399","SNX25",2.29117309440985,1.36459285414837,1.09334050691199,1.24809503125655,0.211996257687901,0.838145087936581
"3400","HLA-DQA2",1.9059574826407,-1.88402702504988,1.50934954778751,-1.24823771127874,0.21194401742494,0.838145087936581
"3401","CD109",103.808449765575,-0.387740051416547,0.310757882603624,-1.24772394562591,0.212132168985743,0.838145087936581
"3402","ERMARD",9.03641816258638,-0.604298395015171,0.4843830562992,-1.24756303334009,0.212191123188469,0.838145087936581
"3403","PDLIM2",232.429439292122,-0.354839316851354,0.284452410602127,-1.24744703727499,0.212233628562258,0.838145087936581
"3404","SPAST",21.6875104849086,0.428046670022167,0.34332867541748,1.24675478825552,0.212487422853212,0.838782002746822
"3405","ZNF12",35.8638879223691,0.373947393559278,0.299998735418282,1.24649656618683,0.212582149070293,0.838782002746822
"3406","PHF14",104.615771357543,0.229430803603019,0.184044741729617,1.24660341527214,0.212542948839705,0.838782002746822
"3407","ZNF141",31.1957100797111,0.424224377987507,0.340568627410589,1.24563551614536,0.212898237178405,0.839037562018446

"3408","LY6E-
DT",2.95593787396859,1.39282865930973,1.11846841966048,1.2452999430529
6,0.213021516601454,0.839037562018446
"3409","C12orf65",32.6436656875648,0.385023393198984,0.309144283634148
,1.24544885214386,0.212966805538088,0.839037562018446
"3410","IRF8",3.85496491567203,-0.991937958900481,0.796487380875046,-1
.24539067751545,0.212988178416915,0.839037562018446
"3411","AC008760.2",11.3248712318622,-0.95564795552178,0.7673508493660
39,-1.24538593566594,0.212989920601583,0.839037562018446
"3412","SUMO3",168.974245989577,-0.270085499242496,0.216741135300591,-
1.24612016481285,0.212720282822917,0.839037562018446
"3413","AOC3",2.94664273144897,1.19894008099102,0.963012905855867,1.24
498859122295,0.213135943947076,0.839242294375844
"3414","DDHD1",36.885351880176,0.451707689968619,0.362911846929085,1.2
4467606607752,0.213250847122134,0.839448779869468
"3415","PCDHGA12",8.78579255875156,0.916532863804202,0.736469753651465
,1.24449491545304,0.213317469531027,0.839465145835278
"3416","C6orf62",76.4823881361795,0.286935027640368,0.230735483814588,
1.24356697503423,0.213658977050422,0.839504475879705
"3417","RARRES2",138.55608631358,0.442240270934521,0.355510639928799,1
.24395790523483,0.213515055958584,0.839504475879705
"3418","EMSY",17.2356755944249,0.558243347997359,0.448914346196853,1.2
4354089533277,0.213668580791735,0.839504475879705
"3419","DENND5B",17.8935106127758,0.504749437217795,0.405982168660001,
1.2432798191206,0.213764738184415,0.839504475879705
"3420","RDH11",63.8066236127529,-0.292423411392446,0.235184766927663,-
1.24337734629891,0.213728814142722,0.839504475879705
"3421","LOXL1",262.230745034407,0.448294886875089,0.360470763124313,1.
24363730081624,0.213633081426785,0.839504475879705
"3422","HDAC8",27.2609947808933,0.467316478578454,0.375850798922752,1.
24335635288752,0.213736546677563,0.839504475879705
"3423","PRR13",83.715726538115,-0.28624511525195,0.230304121959176,-1.
24290053003346,0.213904490367992,0.839562630273202
"3424","ADRM1",66.3596859242541,-0.341197640936079,0.274482211041965,-
1.24305921189156,0.213846014715349,0.839562630273202
"3425","NOP16",8.92849927229581,-0.591400391883997,0.47622411897289,-1
.24185308623074,0.214290771956306,0.840238342777439
"3426","PRPH2",2.42395424251439,1.54817723184834,1.24649790409004,1.24
202152829012,0.214228619127055,0.840238342777439
"3427","ZC3HAV1",68.111889628145,0.313912518976968,0.252831828079391,1
.24158624078926,0.214389260911068,0.840238342777439
"3428","PRIMA1",3.79413083508945,1.00283837999076,0.807684172915047,1.
24162192800111,0.214376087365313,0.840238342777439
"3429","ELP5",36.1754095205742,-0.412144591772069,0.331900839017868,-1
.2417702618398,0.214321337798739,0.840238342777439
"3430","EFCAB13",11.8553177318239,0.695471929981091,0.560329715179281,
1.24118338032201,0.214538012956324,0.840412665862689
"3431","ARHGEF9",19.0719605883866,0.455132565812031,0.366709079673831,
1.24112707058371,0.214558810668568,0.840412665862689
"3432","PTP4A2",214.728019028413,-0.214461912755002,0.172852400035824,

-1.24072279419061,0.214708170757178,0.840476830372363
"3433","FH",33.1438944369719,-0.409683097544397,0.330236475560421,-1.2405749451182,0.214762812374336,0.840476830372363
"3434","LINC00174",6.97712492295954,0.722453454150922,0.582222350377951,1.24085489621266,0.214659357365888,0.840476830372363
"3435","YARS",16.323702302283,-0.494148189474644,0.398392371340995,-1.24035555151654,0.214843913676461,0.840549448587469
"3436","PITHD1",73.0325554388103,-0.27229833746846,0.220452073974628,-1.23518156377063,0.216762939080408,0.840664595664442
"3437","PPIH",29.6103057203576,-0.417416875032686,0.337846091080853,-1.23552376674617,0.216635636776599,0.840664595664442
"3438","SERBP1",268.82876851445,-0.198797171882745,0.161539202037714,-1.23064351795135,0.218456221507993,0.840664595664442
"3439","IFI44",22.7914615740804,-0.408596680637763,0.329895926101297,-1.23856237167445,0.215507610352144,0.840664595664442
"3440","GSTM3",151.820192820984,0.4448802352456,0.361523781027658,1.23056976772315,0.21848381821301,0.840664595664442
"3441","PSMD4",118.035751008863,-0.309613181423313,0.251953038700247,-1.22885273787733,0.219127024228926,0.840664595664442
"3442","POGZ",53.5497076195003,0.392460283699032,0.317235006758185,1.23712791885603,0.21603959633205,0.840664595664442
"3443","DDR2",274.020495987705,0.360797422231808,0.293084195744495,1.23103677192592,0.218309111513569,0.840664595664442
"3444","RGS16",13.4679762701553,-1.64040641987374,1.32813127567709,-1.23512370344373,0.216784468914011,0.840664595664442
"3445","ADIPOR1",36.6983707701405,-0.356190300657218,0.287880685881515,-1.23728446584228,0.215981492869223,0.840664595664442
"3446","SH3YL1",39.1734890073305,0.358451493102047,0.290689516993528,1.23310773917598,0.217535570442612,0.840664595664442
"3447","TTC7A",25.3797214306718,0.476799860668634,0.387528255798368,1.23036153760286,0.218561749663686,0.840664595664442
"3448","RTN4",732.430317175583,-0.214834591601998,0.174823981765467,-1.22886225008996,0.219123457175836,0.840664595664442
"3449","PNO1",14.3886002193232,-0.493847489922883,0.399150514478266,-1.23724628181526,0.215995664089005,0.840664595664442
"3450","LBX2-AS1",7.10944973494577,0.726713274686991,0.590588411337742,1.2304902377629,0.218513580451131,0.840664595664442
"3451","AC079922.2",5.40150847118295,-0.878338881095658,0.709299174363812,-1.23831933384592,0.21559767771621,0.840664595664442
"3452","FAP",89.4876078827526,-0.563411713848409,0.45659058816233,-1.23395384936866,0.217220101841739,0.840664595664442
"3453","ERICH2",2.02022526990525,-1.2797835755417,1.03583188176067,-1.23551282604506,0.216639705974071,0.840664595664442
"3454","STK36",5.89488338090307,0.890783212510221,0.72173903034737,1.234217875236,0.217121728310953,0.840664595664442
"3455","SCAP",24.1483619734289,0.405620122669599,0.327841620940816,1.23724413485262,0.21599646091011,0.840664595664442
"3456","CD96",1.95006381367043,-1.54040025563106,1.25242370794792,-1.22993540113911,0.218721296201899,0.840664595664442

"3457","ARHGEF26",8.8049879745891,-0.727220799167972,0.590551510164293,-1.23142653375936,0.218163378127683,0.840664595664442
"3458","YTHDC1",78.3620903596617,0.334485376217494,0.271649204391012,1.23131366045172,0.218205574679536,0.840664595664442
"3459","AC097376.3",16.2163362285117,0.481512516957757,0.390847365472107,1.23197073716011,0.217960015451977,0.840664595664442
"3460","LMNB1",3.70295665523542,1.14257787337459,0.929040469971599,1.22984725671801,0.218754308129055,0.840664595664442
"3461","ANKHD1",78.4576294692472,0.322620731667563,0.261469153597124,1.23387683491209,0.217248802735496,0.840664595664442
"3462","PCDHB13",3.64721299651629,1.02613821204942,0.832444530538168,1.23268058640018,0.217694957629696,0.840664595664442
"3463","RANBP17",3.60287331851822,-1.03257995801891,0.836908225911973,-1.23380309339619,0.217276286462079,0.840664595664442
"3464","SFXN1",24.4160785774702,0.416701098605999,0.337480404922612,1.23474161026194,0.216926684458734,0.840664595664442
"3465","ZNF454",2.3644280368782,1.23488630802033,0.997878504685534,1.23751168325796,0.215897179622672,0.840664595664442
"3466","ME1",7.56947485180549,-0.688964512272392,0.558566027074393,-1.23345223102985,0.217407088376269,0.840664595664442
"3467","TBL2",24.5281001197613,-0.413972476019339,0.334183924586666,-1.23875640197642,0.21543572414212,0.840664595664442
"3468","COPS6",141.441740716765,-0.310097906569643,0.250163924048159,-1.23957883915327,0.2151312115117,0.840664595664442
"3469","CHPF2",26.8674536964354,0.390630690900141,0.317429170814002,1.23060741361112,0.218469731130567,0.840664595664442
"3470","ZNF251",11.7389545707218,0.644235713264688,0.522771898052441,1.23234572413849,0.217819966362711,0.840664595664442
"3471","SUSD1",4.36793536659694,0.993610392561385,0.803255903632793,1.23697863665576,0.21609501390352,0.840664595664442
"3472","RALGDS",33.8932419696318,-0.391326800244637,0.317485229520333,-1.23258269632217,0.217731496018725,0.840664595664442
"3473","RPL7A",2959.88411677436,-0.334596646568612,0.271531122904315,-1.23225891378397,0.217852382295386,0.840664595664442
"3474","CCDC186",151.808256974786,-0.273351707027788,0.22171728143039,-1.23288408221625,0.217619015017592,0.840664595664442
"3475","TRIM3",10.0919074444639,-0.555201433689199,0.45118246007688,-1.23054746763559,0.218492163208333,0.840664595664442
"3476","YIF1A",114.875414264878,-0.325305226160833,0.264703835225197,-1.22894035851078,0.219094168313706,0.840664595664442
"3477","ME3",14.7033788147369,-0.51484153214698,0.415608947607133,-1.23876431224876,0.215432793834972,0.840664595664442
"3478","YARS2",18.7450814346786,-0.47211750071101,0.381481774790875,-1.23758861342674,0.21586863862484,0.840664595664442
"3479","PAWR",5.29018602405716,0.965619005740676,0.782279224235532,1.23436616469562,0.217066491122824,0.840664595664442
"3480","TGDS",11.3768990379876,0.594170301581847,0.479545006921265,1.23902927359517,0.215334657275772,0.840664595664442
"3481","TOX4",60.6394702489044,0.284704503734699,0.231353371153222,1.23060451773639,0.218470814743042,0.840664595664442

"3482","SOS2",35.6447914681479,0.38878986196199,0.315713243157227,1.23
14651678022,0.218148936526216,0.840664595664442
"3483","TRIM69",36.9889333823688,0.402408146887037,0.326175926624731,1
.23371504160702,0.217309106954324,0.840664595664442
"3484","SPG21",58.4203151474314,-0.288735292204036,0.233167444638185,-
1.23831735022905,0.215598412936118,0.840664595664442
"3485","RPP25",17.5556530482472,-0.458747655243905,0.373011291027538,-
1.22984924660106,0.2187535628368,0.840664595664442
"3486","RHOT2",29.2519384562044,-0.353727421701662,0.286011410310251,-
1.23675982478446,0.21617626125528,0.840664595664442
"3487","STUB1",170.658015663686,-0.306933848333218,0.247962053867418,-
1.23782588321893,0.215780628937685,0.840664595664442
"3488","BBS2",31.557630831192,0.388457302315036,0.315799742451115,1.23
007479138514,0.218669098947064,0.840664595664442
"3489","AC025280.2",3.23431085338479,2.63531212430769,2.14086253468765
,1.23095812160223,0.218338527639812,0.840664595664442
"3490","AP2B1",120.72416431715,0.309497922461045,0.250568522457759,1.2
3518277326004,0.216762489045694,0.840664595664442
"3491","KANSL1-
AS1",8.78328661364493,0.633655965348852,0.515323321180308,1.2296279623
0047,0.218836453997761,0.840664595664442
"3492","MBTD1",18.9095871576295,0.446633842219642,0.361607893257322,1.
23513300054492,0.216781009357947,0.840664595664442
"3493","SLC16A3",1.4895602152442,-1.42011351122654,1.15164743841012,-1
.23311480915291,0.217532933067473,0.840664595664442
"3494","UQCR11",258.791283856206,-0.301075898124121,0.244088588072791,
-1.23346978447978,0.217400543083525,0.840664595664442
"3495","MATK",1.71717802622681,-1.30778557446519,1.05929666350685,-1.2
345791500331,0.216987172685489,0.840664595664442
"3496","POP4",34.1705936824942,-0.416486406638343,0.338869768112493,-1
.2290456270507,0.219054699478922,0.840664595664442
"3497","ZNF571",8.7374896131621,0.662345003204236,0.534917893957218,1.
2382180717574,0.215635212424116,0.840664595664442
"3498","A1BG",2.21447767575393,-1.26853389761255,1.02574585683341,-1.2
3669414715323,0.216200652402016,0.840664595664442
"3499","ATP5PO",244.881208567025,-0.358515430372003,0.289168512324625,
-1.23981490062627,0.215043865556141,0.840664595664442
"3500","YBEY",39.9717511808966,0.439789839598162,0.35773750172738,1.22
936465278195,0.218935116827824,0.840664595664442
"3501","LINC01638",6.69736927959585,-0.859321855937573,0.6948379862512
93,-1.2367226215908,0.216190077414007,0.840664595664442
"3502","SMARCA1",117.483687168614,0.264432490508651,0.214048984190297,
1.23538306667955,0.216687971836768,0.840664595664442
"3503","SLC6A8",5.80936200033989,-0.725062099916209,0.585017360073455,
-1.23938561383062,0.215202726534453,0.840664595664442
"3504","RSRP1",219.054211308147,0.247752942809452,0.202387772726802,1.
22414975703047,0.220895738686466,0.841691932609061
"3505","MIR29B2CHG",5.72422766317568,0.882775394105342,0.7210093881253
31,1.22436047108986,0.22081627418482,0.841691932609061
"3506","ORC2",14.4029926840057,0.580605403167085,0.472944853817396,1.2

2763869504172,0.219582629209893,0.841691932609061
"3507","LMCD1-
AS1",4.02212989787076,0.904150032610434,0.736884678455623,1.2269898656
3999,0.21982639997678,0.841691932609061
"3508","SFMBT1",9.3245117956151,0.590836612634352,0.482009858960175,1.
22577702852167,0.22028259395893,0.841691932609061
"3509","UMPS",20.0504833272537,0.431511453995985,0.352587481160741,1.2
2384224356299,0.221011744978106,0.841691932609061
"3510","MLF1",24.3925691209131,-0.37516312654976,0.305962524944947,-1.
22617345577621,0.220133408002325,0.841691932609061
"3511","TNFSF10",185.480673038226,-0.411306247369685,0.335906556925105
,-1.22446626566266,0.220776384657327,0.841691932609061
"3512","ETV5",5.62208734409797,0.889103186781927,0.726464495687559,1.2
2387699888959,0.220998631700104,0.841691932609061
"3513","TCERG1",38.9331467242751,0.399962125995751,0.326241152573336,1
.22597079749417,0.22020966456192,0.841691932609061
"3514","CENPQ",14.8322161390425,0.488419548302933,0.398867020232622,1.
22451725394113,0.220757161524569,0.841691932609061
"3515","AC105446.1",3.44291144636109,-0.963398424110734,0.786226567986
773,-1.22534452960249,0.22044543733682,0.841691932609061
"3516","NDUFB2",230.646858122701,-0.320193906955401,0.260854231394278,
-1.22748212763868,0.219641435155981,0.841691932609061
"3517","ASAH1",179.263196754252,0.273098549630369,0.222784166008067,1.
22584362490322,0.220257526929505,0.841691932609061
"3518","PIWIL2",2.56042284622635,1.16440279276186,0.95145570658996,1.2
2381187552609,0.221023203376544,0.841691932609061
"3519","YTHDF3",41.1649708985896,0.342040715173641,0.278581189052418,1
.22779544569064,0.219523765759303,0.841691932609061
"3520","RANBP6",12.2233058426788,0.555626041397752,0.452866594498098,1
.22690886929635,0.219856844626928,0.841691932609061
"3521","GRHPR",120.805573413334,-0.314020792369392,0.256277784933234,-
1.22531413501643,0.220456884674243,0.841691932609061
"3522","GDI2",182.809273603515,-0.209108808121211,0.170454289613665,-1
.22677351561616,0.219907727694009,0.841691932609061
"3523","ARHGEF7",34.8135048793677,0.422800142688858,0.345287140629281,
1.22448852835443,0.220767991233998,0.841691932609061
"3524","KTN1-
AS1",1.94828168996248,1.40342740294557,1.1445801698567,1.2261503736529
7,0.220142092421832,0.841691932609061
"3525","SERPINA5",12.739302466091,-0.502966381256822,0.410440689604507
,-1.22543011449832,0.220413206282103,0.841691932609061
"3526","SHMT1",8.92984638276486,-0.594685783151007,0.485678085853345,-
1.22444433972378,0.220784651342582,0.841691932609061
"3527","HAUS1",32.9485343804212,-0.375819045421683,0.30618870534111,-1
.22740989091352,0.219668570724804,0.841691932609061
"3528","MAN2B1",43.2370162630641,-0.339968367109132,0.277503430150983,
-1.22509608953,0.220539018386718,0.841691932609061
"3529","ZNF583",7.14033139019831,0.768236021212104,0.62656660908495,1.
22610431209229,0.22015942336098,0.841691932609061
"3530","ZNF146",28.6699875013869,0.38488860634165,0.314554289570415,1.

22359992886217,0.221103186466636,0.841757568825987
"3531","AC005261.1",33.9970715334559,0.49512717439454,0.40470271251382
6,1.22343428666203,0.221165709913279,0.841757568825987
"3532","STAMBPL1",11.0524016907209,0.537323367579778,0.439457456266742
,1.22269712327656,0.221444113930155,0.841870877551919
"3533","CD82",3.93371697265652,-0.892241245198527,0.729735091208575,-1
.22269198226553,0.221446056413873,0.841870877551919
"3534","CES1",44.4191271652757,0.548046438674953,0.448202876475173,1.2
2276421558243,0.221418764839107,0.841870877551919
"3535","ZNF331",28.4478535352061,-0.399989921621873,0.327036014718314,
-1.22307606385919,0.221300968335219,0.841870877551919
"3536","FARSB",14.1273787945434,-0.504601916103467,0.41292122837628,-1
.22202948510955,0.221696477047815,0.842414865032627
"3537","RNPEPL1",41.5572570636135,-0.337083651901085,0.275849981077635
,-1.22198178366456,0.221714515783942,0.842414865032627
"3538","CYP4X1",13.3355655641133,0.613670352879496,0.502277361014844,1
.22177585635073,0.221792401142534,0.842472605696586
"3539","FBXL3",75.3345907035883,0.293279417614277,0.240080128648352,1.
22158972200422,0.22186281731332,0.842501950232752
"3540","MSH3",22.0130635960528,0.502332875871636,0.411424097349988,1.2
209612395268,0.222100695780969,0.8427723422199
"3541","P4HA2",107.628923353888,-0.26825131028996,0.21975732414804,-1.
22067062533603,0.222210753996869,0.8427723422199
"3542","OMD",151.865276630158,-0.257303265327245,0.210727885708767,-1.
22102143464224,0.222077904229106,0.8427723422199
"3543","TAFA2",1.83640667835615,1.42641175217858,1.16860680273687,1.22
060880429407,0.222234171219979,0.8427723422199
"3544","SMTN",22.0641783856055,0.516727309802543,0.423347995911936,1.2
205734166509,0.222247576518143,0.8427723422199
"3545","ALDH5A1",4.1495423094195,0.91206204180809,0.747689892731122,1.
21984000409121,0.222525533074971,0.843112669578386
"3546","RPS6",2697.72275918434,-0.322843891748893,0.264653666797022,-1
.21987311060572,0.222512980651992,0.843112669578386
"3547","CCDC124",66.1936932544196,-0.3467233908892,0.284160138850169,-
1.22016899446976,0.222400818008611,0.843112669578386
"3548","C8orf88",3.43106730245482,-0.961875721796008,0.788739938592438
,-1.21950933981173,0.222650933133654,0.84312387254181
"3549","SPPL3",58.2473458936874,0.274280252200607,0.224911807099955,1.
21950134916088,0.222653964108258,0.84312387254181
"3550","ZNF593",37.1796675538426,-0.392287472271447,0.324309679789058,
-1.20960765810815,0.22642947817736,0.843649863695554
"3551","COL8A2",33.75819284361,-0.445993803079533,0.36648815042699,-1.
21693921770707,0.223627342832956,0.843649863695554
"3552","GADD45A",42.3470455163992,0.530959962079042,0.438316664105178,
1.2113615692960l,0.22575686139896,0.843649863695554
"3553","AL049597.2",6.96245427343906,0.74823549344575,0.61430944286164
5,1.21801073081383,0.223219894937354,0.843649863695554
"3554","VCAM1",4.41184497817021,0.967119921678356,0.800507616957597,1.
20813331590021,0.226995986612656,0.843649863695554
"3555","ZNF687",6.25160884244072,0.765498938954833,0.628093120687594,1

.2187666346621,0.222932778639975,0.843649863695554
"3556","PBX1",140.818543391952,0.296712737583489,0.245701938412145,1.2
0761252231465,0.227196340238103,0.843649863695554
"3557","PLA2G4A",9.77512090261693,0.576683479961551,0.476979112027114,
1.20903298576473,0.226650173004386,0.843649863695554
"3558","VASH2",28.8903091903783,0.390265401719757,0.321413751740317,1.
21421500980166,0.224665630252914,0.843649863695554
"3559","OSR1",30.5866022609702,-0.511225849496295,0.41963314975386,-1.
21826850380186,0.223121954854595,0.843649863695554
"3560","AL133243.3",8.09555755859279,0.725726451348012,0.5972769724699
62,1.21505848174066,0.224343787177454,0.843649863695554
"3561","VAMP5",176.734393892566,-0.328468846111378,0.269522015758626,-
1.21870877667204,0.222954745597873,0.843649863695554
"3562","PDCL3",16.930684462243,-0.490050105778095,0.406121774174241,-1
.2066580443132,0.227563863077341,0.843649863695554
"3563","AC024060.2",1.69440650341447,1.41637140340456,1.17106438273635
,1.2094735560952,0.226480964449338,0.843649863695554
"3564","JAGN1",64.4258055255846,-0.37088554306958,0.304478341495846,-1
.21810156100919,0.223185380764792,0.843649863695554
"3565","HYAL1",2.80013991650731,-1.17905623053667,0.971296287800474,-1
.21389965692825,0.224786043950033,0.843649863695554
"3566","ADAMTS9-
AS2",1.77527168151407,1.39381451940671,1.149572592525,1.212463248054,0
.2253351027338,0.843649863695554
"3567","ARL13B",33.0478192104228,-0.387975077845038,0.321167013920842,
-1.20801658024775,0.227040884830639,0.843649863695554
"3568","USO1",82.5999246578527,0.265743580194584,0.220062179937704,1.2
0758405769593,0.227207294446381,0.843649863695554
"3569","TSLP",2.23368289550698,1.27485960517917,1.05598657477533,1.207
26876234237,0.227328656615865,0.843649863695554
"3570","MCUR1",37.0719071804548,-0.371913660390191,0.307203275401733,-
1.210643538562,0.226032050207263,0.843649863695554
"3571","BTN3A1",21.101460266963,0.494339148685737,0.409603597720903,1.
20687208666212,0.227481408993888,0.843649863695554
"3572","UBR2",48.4667594082173,0.318994851253296,0.2641692330132,1.207
53975629462,0.227224343966819,0.843649863695554
"3573","MRPS17",37.0520146684824,-0.389889929498279,0.321687908785792,
-1.21201300655009,0.225507401869423,0.843649863695554
"3574","METTL27",20.8104849898346,0.678325040653193,0.559524011213172,
1.21232516756954,0.225387933595717,0.843649863695554
"3575","GNG11",287.127329077682,-0.319298930916874,0.263066295727096,-
1.21375841794691,0.224839989302372,0.843649863695554
"3576","SYPL1",142.063657829819,-0.309103976327551,0.255148700224026,-
1.21146600416209,0.225716856154695,0.843649863695554
"3577","CBLL1",18.0269475223636,0.462433444155876,0.381184711995837,1.
21314792960775,0.225073267925158,0.843649863695554
"3578","TMEM168",23.2834565137204,0.391407852823045,0.321726615829148,
1.21658524214515,0.223762060517664,0.843649863695554
"3579","POLR3D",19.1989525699132,0.479469583946465,0.397326884397875,1
.2067383375607,0.227532929757589,0.843649863695554

"3580","MMP16",14.6034703766788,0.834484086695176,0.691562131279211,1.20666538688519,0.227561034195318,0.843649863695554
"3581","ZNF517",1.63676213208661,1.40818442365193,1.16158292239127,1.21229780199677,0.225398404965383,0.843649863695554
"3582","GNE",13.8277695648697,0.526860110980078,0.436580599850908,1.20678772982583,0.2275139026634,0.843649863695554
"3583","DOLPP1",3.19635066618766,-0.956858138265993,0.785969027133089,-1.2174247396952,0.223442654956891,0.843649863695554
"3584","TPRN",6.47710083038796,-0.657700493352936,0.544250405049087,-1.20845200527433,0.226873446313078,0.843649863695554
"3585","ABI1",67.058042057229,0.273820824671124,0.225581207362354,1.21384590442094,0.224806573296493,0.843649863695554
"3586","ARHGAP12",95.3534441889271,0.249100429223386,0.206002081881609,1.20921316400359,0.226580961581493,0.843649863695554
"3587","ZNF37A",52.1000649026588,0.352218044579676,0.290317706542559,1.21321585505169,0.225047303841466,0.843649863695554
"3588","BUB3",68.2154698776658,-0.264520134435397,0.217687336372996,-1.21513790761882,0.224313497678822,0.843649863695554
"3589","QSER1",36.5628805223485,0.354183453301925,0.291267507300621,1.2160074310533,0.223982091254475,0.843649863695554
"3590","NUDT22",34.1402729009759,-0.389199288533804,0.321421368732638,-1.21086936462381,0.225945475464352,0.843649863695554
"3591","LTO1",11.4138056749491,0.642873363321578,0.532680223910079,1.20686545973612,0.22748396152019,0.843649863695554
"3592","BIRC2",71.0142943284895,0.249209897174588,0.205130421882126,1.21488511985702,0.224409909846066,0.843649863695554
"3593","LAYN",2.23664798595162,-1.3333931868458,1.09864398097399,-1.21367177169049,0.224873087879472,0.843649863695554
"3594","SLC37A4",5.90018688560428,-0.711179801073832,0.589233314676839,-1.20695789487713,0.227448359657138,0.843649863695554
"3595","LRRK2",43.9425829005241,0.398437389110456,0.330023990896042,1.20729825740446,0.227317301540948,0.843649863695554
"3596","SLC35E3",46.2859586982511,0.380336475128581,0.315155565483748,1.20682138214752,0.227500939625386,0.843649863695554
"3597","GIT2",30.8714803929541,0.461407983608257,0.380849375221987,1.21152354087312,0.225694818070592,0.843649863695554
"3598","MAPKAPK5-AS1",33.4636266740405,-0.355862112044624,0.29348128849375,-1.21255468745907,0.225300122044941,0.843649863695554
"3599","SFSWAP",60.7910128629496,0.336279935190756,0.277230812306586,1.21299624811858,0.22513125496634,0.843649863695554
"3600","LPAR6",17.880577866466,0.451872166590761,0.372961285983138,1.21157928067416,0.225673469715548,0.843649863695554
"3601","KPNA3",34.8171099467607,-0.354406589278566,0.29206662231786,-1.21344433837038,0.224959983198875,0.843649863695554
"3602","CLEC14A",9.75343402414476,0.858262594626859,0.707014028815609,1.21392583406672,0.224776046770335,0.843649863695554
"3603","GMFB",29.908983905724,-0.35903717410319,0.294851213438429,-1.21768932173028,0.22334205644734,0.843649863695554
"3604","MARK3",72.212196941597,0.292276511066544,0.241910705139195,1.2

081999880839,0.226970346368672,0.843649863695554
"3605","TSR3",71.2423807109405,-0.282967019335727,0.234211927529178,-1
.20816656231342,0.226983200720374,0.843649863695554
"3606","NUBP2",53.5187280289612,-0.340296818835847,0.280912003553143,-
1.21140006312144,0.225742115208784,0.843649863695554
"3607","PGP",31.035622850526,-0.429900570516789,0.35523249310279,-1.21
019495362546,0.22620409404583,0.843649863695554
"3608","CES2",27.1852507534741,0.423235603492917,0.347710832219751,1.2
1720569011619,0.223525965788703,0.843649863695554
"3609","SUMO2",686.990152073352,-0.293118703941835,0.242032723030382,-
1.21107055389796,0.225868365643202,0.843649863695554
"3610","MIF4GD",23.0203316049272,-0.43136561052487,0.356067103330386,-
1.21147279962175,0.225714253233673,0.843649863695554
"3611","SIRT7",18.6842687795904,-0.458538236104873,0.377146446983373,-
1.21580950787822,0.224057496051636,0.843649863695554
"3612","AC130371.2",3.00921422674545,1.0538385180864,0.87245664084786,
1.20789786992999,0.227086549026921,0.843649863695554
"3613","CDC34",26.5388490703132,-0.422240136044952,0.349291542461178,-
1.20884729435406,0.226721517966286,0.843649863695554
"3614","ZNF724",2.58996042102676,1.16805103143655,0.962574221368969,1.
21346593904764,0.224951729209619,0.843649863695554
"3615","TIMM50",30.4461871246216,-0.344918136377034,0.284103952293414,
-1.21405610021508,0.224726302202606,0.843649863695554
"3616","ERCC1",73.123471578709,-0.308303842647267,0.254538889205136,-1
.21122490795032,0.225809219094545,0.843649863695554
"3617","CENPB",23.7111134119238,-0.432185583606141,0.356071675975553,-
1.21376007350782,0.224839356917587,0.843649863695554
"3618","MAVS",51.2701548633547,0.384207729076411,0.317656877856776,1.2
0950546283982,0.226468713620093,0.843649863695554
"3619","PYGB",32.9459912743469,-0.344986515477862,0.284812649817298,-1
.21127525655607,0.225789928528041,0.843649863695554
"3620","CBFA2T2",21.1198338786352,0.546720996401937,0.451591136190792,
1.21065484370126,0.226027715602905,0.843649863695554
"3621","PLCG1-
AS1",1.45179091244229,1.55491727595244,1.28787786060549,1.207348401207
39,0.227297997995657,0.843649863695554
"3622","ZMYND8",35.4561264417262,0.377557143439792,0.310731202576957,1
.21506028460816,0.224343099611525,0.843649863695554
"3623","RHBDD3",14.1954501794784,0.534649132541134,0.442883130661619,1
.20720139361015,0.227354593897454,0.843649863695554
"3624","PLCXD1",3.23814300620056,1.21033875460798,1.00276143935134,1.2
0700568162145,0.22742995590776,0.843649863695554
"3625","EIF2S3",128.324379315123,-0.232374437166601,0.191261034874874,
-1.21495963523686,0.224381486922123,0.843649863695554
"3626","SLC66A2",42.4678278465477,-0.318580811478299,0.264059942212093
,-1.20647156403002,0.227635716974263,0.843683508112829
"3627","TYK2",16.3698375564087,0.455912162124692,0.377972203268213,1.2
0620553094263,0.227738251850105,0.843830815167786
"3628","HSPA14",6.45904546320699,0.722893271555013,0.59970715199928,1.
20541045599516,0.228044886994021,0.844665353250744

"3629","CHCHD10",24.3431387292932,0.528655623456628,0.438610742948164,
1.20529565669828,0.228089185724157,0.844665353250744
"3630","SMCR8",11.0919148053274,0.637359230274423,0.528921112228632,1.
20501756412952,0.228196521440697,0.844830041774524
"3631","MROH6",5.14471856352019,-0.781400663369248,0.648631750873297,-
1.2046907391092,0.228322712462837,0.845064426545875
"3632","ADAMTS14",2.26656351283227,1.46768201572194,1.21873131716142,1
.20427037120894,0.228485094567331,0.845199886014412
"3633","MIF",437.225027725525,-0.318569050850205,0.26450118173764,-1.2
0441447088201,0.228429421664277,0.845199886014412
"3634","PYGO2",8.77532097093333,0.723334172276851,0.600794431307285,1.
20396284416773,0.228603939941203,0.84540681036594
"3635","EVI5",65.2680637372455,0.297204085618868,0.247400048621224,1.2
013097300312,0.229631077490059,0.845691476782645
"3636","TTF2",15.082592830985,0.478891125417369,0.398640805237304,1.20
130984868018,0.229631031482514,0.845691476782645
"3637","VIT",280.188953748289,0.27513615499319,0.228977748292817,1.201
58468254891,0.22952447899714,0.845691476782645
"3638","C2orf74",36.000833052947,-0.450688438806591,0.374474872051994,
-1.20352117710054,0.228774701398069,0.845691476782645
"3639","GCFC2",17.3754688817141,-0.42061905004555,0.349776938190884,-1
.20253511343851,0.229156270216982,0.845691476782645
"3640","HRH1",33.1283036867159,-0.443252747331526,0.368707503895268,-1
.20217989232308,0.229293838077069,0.845691476782645
"3641","SHROOM1",5.11327382307491,0.911095570760915,0.757791239939354,
1.20230417394879,0.22924570036715,0.845691476782645
"3642","GINM1",185.927045671474,-0.203472517303756,0.169345653888293,-
1.20152193240208,0.229548803991351,0.845691476782645
"3643","PLAA",18.1968905624619,-0.417327079283785,0.34729463441369,-1.
20165138741151,0.229498622974726,0.845691476782645
"3644","INVS",11.4804596439894,0.582463929314962,0.48448906741337,1.20
222306031521,0.2292771171049,0.845691476782645
"3645","PLXDC2",231.455575262485,0.430121211966035,0.357801984627598,1
.20212081107853,0.229316724398998,0.845691476782645
"3646","AL355075.4",5.74861974939421,1.03556904411408,0.86206352679203
4,1.20126766987546,0.229647387226387,0.845691476782645
"3647","NGRN",95.6234331271728,-0.301708952263364,0.250726413909494,-1
.20333931937571,0.228845039322323,0.845691476782645
"3648","C5AR1",7.55001402034722,-0.812539135013779,0.676460034321515,-
1.20116354815957,0.22968776622194,0.845691476782645
"3649","LSM14B",16.9951902740984,0.459584181774824,0.382472671146236,1
.20161312544892,0.229513453756441,0.845691476782645
"3650","ARHGAP4",2.38113272356599,-1.18402547557893,0.985515952246602,
-1.20142700164295,0.229585607234659,0.845691476782645
"3651","MARCKSL1",10.9976623108042,0.617323850632687,0.514673646805303
,1.19944717291153,0.230354114287078,0.845855075744427
"3652","TRIM33",35.0164957950248,0.364155938446713,0.303970155970962,1
.19799898540533,0.230917411970328,0.845855075744427
"3653","RAB10",96.398936466545,-0.225476136926415,0.18823729735593,-1.
1978292298793,0.23098350536834,0.845855075744427

"3654","RANBP2",43.9329989663517,0.369709555763923,0.308438538392883,1
.19864903293308,0.230664443737375,0.845855075744427
"3655","TRAK1",25.8636127080189,0.454770939370674,0.379618213646398,1.
19796923072368,0.230928995823631,0.845855075744427
"3656","AC104162.2",3.64166009631605,1.06757488639203,0.89005133575047
1,1.19945315906062,0.230351787899628,0.845855075744427
"3657","TBCK",18.6086882034271,0.454844747997456,0.379170285791358,1.1
9957909425355,0.230302849781171,0.845855075744427
"3658","CPE",1103.57738889769,0.336475197913635,0.280467689737892,1.19
96932631637,0.23025849040113,0.845855075744427
"3659","SMAD5",102.430566944544,0.256490179650742,0.213753866300584,1.
19993235252204,0.23016561390353,0.845855075744427
"3660","FAM135A",11.526748796241,0.567243491031058,0.473193540990391,1
.19875577727418,0.23062292264453,0.845855075744427
"3661","FKBP9",266.610171437842,0.316934090726259,0.264549404987726,1.
19801475547059,0.230911272662799,0.845855075744427
"3662","SEC61B",329.201815041065,-0.317320466881242,0.264242267854447,
-1.200869450061,0.22980184642238,0.845855075744427
"3663","POLR2L",415.623959411161,-0.337293065511907,0.280972396741251,
-1.20044911679535,0.229964963005571,0.845855075744427
"3664","BTBD10",17.7919186930304,-0.484457391538452,0.403795371782998,
-1.19975964409716,0.230232701434917,0.845855075744427
"3665","CPSF7",34.1207944635275,0.364537993708218,0.303825073015495,1.
19982853979147,0.230205937657081,0.845855075744427
"3666","EPS8",127.155904043063,-0.271880867167677,0.226740120097545,-1
.19908583911269,0.230494569793048,0.845855075744427
"3667","FAN1",19.1019047795109,0.475167297517668,0.396750263953748,1.1
9764834629842,0.231053946205655,0.845855075744427
"3668","RPL23A",2267.83503541767,-0.300415697985317,0.250827943170907,
-1.19769629407128,0.231035272591731,0.845855075744427
"3669","UQCRFS1",94.8718328507295,-0.296141422733293,0.247168037759309
,-1.19813801743117,0.230863290612673,0.845855075744427
"3670","PDCD2L",5.96263223410199,-0.684907876006119,0.571567414687023,
-1.19829762580353,0.23080117056189,0.845855075744427
"3671","USP25",30.2495212270463,0.396826730751621,0.330874104719385,1.
19932846086027,0.23040025260995,0.845855075744427
"3672","DTX1",25.3044867667508,0.509973171038204,0.425895555552975,1.1
9741369542132,0.23114534818696,0.845959241362897
"3673","SMARCA2",104.948361940124,0.264189717550934,0.220696569481639,
1.19707215282707,0.231278432883012,0.846192130693134
"3674","CCDC40",2.75799255591792,-1.11803517568088,0.934087829593319,-
1.19692724844478,0.231334912431474,0.846192130693134
"3675","SMDT1",157.087886502415,-0.300778372593117,0.25141159354753,-1
.19635840316272,0.231556726635361,0.846773020204792
"3676","BDH2",140.650045493616,0.287609389805627,0.240459921897213,1.1
9608035940629,0.231665201311185,0.846870257407065
"3677","IGHMBP2",6.81175285379832,-0.732443361611783,0.61242761940611,
-1.19596722682438,0.231709348648395,0.846870257407065
"3678","SASS6",13.8649162219352,-0.480706818961829,0.40266053377815,-1
.19382650802991,0.232545840186071,0.846967352457585

"3679","BRK1",269.842779384074,-0.282089203055432,0.236264211807566,-1.19395654931095,0.232494965162706,0.846967352457585
"3680","PCCB",13.0147683442167,-0.492372949177786,0.412114668001761,-1.1947474511528,0.232185716967702,0.846967352457585
"3681","PCDHB6",1.53804216862798,-1.56267170798829,1.30801700278719,-1.19468761083264,0.232209104740175,0.846967352457585
"3682","AL662844.4",10.167242731862,-0.718087126711477,0.601456786587564,-1.19391308357435,0.232511969040742,0.846967352457585
"3683","MED20",7.66279325976029,0.677981398762671,0.567284278749615,1.19513518029629,0.232034218836725,0.846967352457585
"3684","TRA2A",76.3479904153135,0.31849426871304,0.266384214756181,1.19561990189454,0.231844921380452,0.846967352457585
"3685","EIF3E",432.134594563008,-0.28585132868246,0.239446070161275,-1.19380254806408,0.232555214716012,0.846967352457585
"3686","GRASP",26.0963880578592,0.403213083312832,0.337326628954343,1.19531945806569,0.231962240246896,0.846967352457585
"3687","TEDC1",8.74699975936071,-0.722635492634285,0.605061991477857,-1.19431645486317,0.232354203334716,0.846967352457585
"3688","ATP6V0D1",71.9489280084965,-0.344262723488507,0.287999918706991,-1.19535701618985,0.231947572050974,0.846967352457585
"3689","RAB5C",142.918987158768,-0.285707127525069,0.239274070583127,-1.1940580390879,0.232455265606379,0.846967352457585
"3690","UPF3B",27.9017181663684,-0.374936181929213,0.31390721673681,-1.19441720973103,0.232314808169507,0.846967352457585
"3691","E2F6",8.10629303223131,0.654690149941504,0.548781072952569,1.19298966784536,0.232873419544458,0.846978588703105
"3692","SURF2",23.4422215457422,-0.45943539787162,0.384985977297891,-1.19338216185501,0.232719737839679,0.846978588703105
"3693","HEXIM1",46.0462045051714,0.384455431417836,0.322140597476111,1.19343986579135,0.232697149832367,0.846978588703105
"3694","PAF1",43.2385863742621,0.370007051041925,0.310130330131099,1.19306954236148,0.232842138707168,0.846978588703105
"3695","SNRPD2",228.829872208811,-0.300368369529623,0.251744539844867,-1.1931475046677,0.232811609613206,0.846978588703105
"3696","AC097662.1",2.89098463390196,1.11583086419178,0.936669672384901,1.19127467995276,0.233545770486115,0.847534530342804
"3697","DLD",31.598758175606,0.360422456124752,0.302337247670969,1.19212058355773,0.233213967144754,0.847534530342804
"3698","PPDPFL",8.86537762763706,0.919079645740729,0.771490377482379,1.19130409473153,0.233534227007304,0.847534530342804
"3699","SULF1",56.8381479249322,0.463830997331038,0.389275636296876,1.19152331685434,0.233448208642796,0.847534530342804
"3700","AUH",22.1164347255102,0.390573586035344,0.327696426113221,1.19187624554806,0.233309773698221,0.847534530342804
"3701","SWI5",62.909945023541,-0.329518237214282,0.276578088576988,-1.19141121738774,0.233492191418461,0.847534530342804
"3702","RBBP6",90.1968038440426,0.305655964177939,0.256401073625017,1.19210095284139,0.233221663447918,0.847534530342804
"3703","AC068473.5",3.20549552421694,-1.18627465463905,0.994946936985904,-1.19229941873358,0.233143862371707,0.847534530342804

"3704","CD70",49.249869383107,-0.620356946497455,0.520804120383216,-1.1911521476462,0.233593861179384,0.847534530342804
"3705","SLC26A2",35.8980046692107,0.39318554630205,0.330138559209273,1.19097129170183,0.233664855186366,0.84756328983794
"3706","IDS",115.410481701863,-0.317277838870668,0.266497736940107,-1.19054609061079,0.23383182578363,0.847940071966057
"3707","BOLA1",27.7912325167885,0.44285204984094,0.372645749363373,1.18839957411968,0.234676024141334,0.848042145777497
"3708","GPATCH4",18.906498479326,-0.412125430862005,0.347259148351646,-1.18679502849173,0.235308480306413,0.848042145777497
"3709","CEP350",67.7729359013101,0.352559669314376,0.297072220923995,1.18678100637547,0.235314012650238,0.848042145777497
"3710","MZT2B",365.86534524199,-0.316633560236053,0.267086542591134,-1.18550922545269,0.235816169266352,0.848042145777497
"3711","MBD5",36.7711120898938,0.371259366072606,0.312732339536775,1.18714734338803,0.23516950674369,0.848042145777497
"3712","SLC25A12",20.5897482326233,-0.399446510174249,0.336234942989922,-1.18799820929445,0.234834115193929,0.848042145777497
"3713","AC018809.2",2.04586028716232,1.64234007113981,1.38250849996006,1.18794211477706,0.234856215913616,0.848042145777497
"3714","AC108471.3",2.01106440193253,1.32002638795752,1.11080804451303,1.18834788285695,0.234696380256288,0.848042145777497
"3715","ATPSCKMT",9.4754483125882,0.613064766419722,0.51642668550698,1.18712836424762,0.235176991743619,0.848042145777497
"3716","OTULINL",4.90940967335413,0.892612031409645,0.752766821492595,1.18577493843281,0.235711191163996,0.848042145777497
"3717","RFPL4B",1.96628276205114,1.51552981664414,1.27714938518879,1.18665039048671,0.235365550807053,0.848042145777497
"3718","FAM185A",6.0884601802128,0.704846405519208,0.592382281482896,1.18985058728425,0.234105122589894,0.848042145777497
"3719","LSMEM1",2.42351051945751,-1.14375780145982,0.964668993737448,-1.18564793611591,0.235761363217619,0.848042145777497
"3720","TNKS",31.2628203508475,0.368265461963904,0.309956515474401,1.18811976383287,0.234786228886421,0.848042145777497
"3721","MTUS1",93.7385485058145,0.401320147948219,0.338015745876353,1.18728240575817,0.235116245669913,0.848042145777497
"3722","EMX2OS",109.608481044598,-0.330055013258659,0.277979998784759,-1.18733367401092,0.235096030563593,0.848042145777497
"3723","MUS81",20.8968177322617,-0.388523884571189,0.326922147398429,-1.18842937886886,0.234664287544286,0.848042145777497
"3724","AIP",106.061952297446,-0.312353712513918,0.263372460932585,-1.18597711927775,0.235631335545206,0.848042145777497
"3725","PPFIBP1",87.5442842519655,0.273191160819754,0.229692851699069,1.18937598100647,0.234291748165431,0.848042145777497
"3726","TFDP1",25.4302743155144,0.412964801963813,0.348225381378127,1.18591241204037,0.235656890959645,0.848042145777497
"3727","STARD5",10.7819330566472,0.597016279980244,0.50264888200758,1.1877401927082,0.234935783529149,0.848042145777497
"3728","DCUN1D3",29.0467741241227,0.394602823851224,0.331749711892647,1.18945943193137,0.234258925800502,0.848042145777497

"3729","MT1G",13.1678421504165,-0.717534675038936,0.603413996577081,-1
.18912501053873,0.234390477839602,0.848042145777497
"3730","CDK10",25.58250587702,-0.436057597688855,0.366638070462966,-1.
18934074996148,0.234305605984685,0.848042145777497
"3731","ATP5F1D",280.551991447186,-0.322870127260311,0.271565075401233
,-1.18892360066284,0.234469732107399,0.848042145777497
"3732","ZNF266",7.71414327719109,0.659814617469694,0.556416591030182,1
.1858284388107,0.23569005819057,0.848042145777497
"3733","EPN1",73.0393551567203,-0.270047043879052,0.227492976800205,-1
.18705661896639,0.235205288196057,0.848042145777497
"3734","KIAA1755",2.3183623472016,-1.21675324702367,1.02242742464243,-
1.19006319441128,0.234021554985674,0.848042145777497
"3735","FBXO7",63.8682066046528,-0.262647387668805,0.221338761565926,-
1.18663078175115,0.23537328867249,0.848042145777497
"3736","ARMCX4",12.2248613057748,0.536526739842421,0.452329845109278,1
.18614048054424,0.235566826527944,0.848042145777497
"3737","XIAP",66.3324390644696,0.315485675781408,0.266066833113096,1.1
8573845559813,0.23572560287359,0.848042145777497
"3738","FOSL2",36.3429572617769,0.392997126023846,0.332367289228819,1.
18241818241411,0.237039812135662,0.848299143848238
"3739","TMEM237",51.2010354009271,-0.374671240503112,0.316770825496223
,-1.18278329425125,0.236895043028886,0.848299143848238
"3740","FNDC3B",162.026535144763,0.319879209253913,0.270037274657332,1
.18457427649508,0.236185812616892,0.848299143848238
"3741","AC007566.1",5.36744010139216,0.870624916740481,0.7361705827959
84,1.182640188411,0.236951777933004,0.848299143848238
"3742","RBPMS-
AS1",2.35750502932951,1.24838980702641,1.05599107674086,1.182197306893
31,0.237127420995733,0.848299143848238
"3743","ARFGEF1",43.074961269068,0.315901097727126,0.266932204552216,1
.18345067526437,0.236630583859102,0.848299143848238
"3744","AC083843.3",1.80100565286443,-1.20126668158317,1.0141598471101
5,-1.18449442166951,0.236217403170556,0.848299143848238
"3745","RIC1",19.0012432608333,0.424953197282889,0.358956311138152,1.1
8385771219756,0.236469392221753,0.848299143848238
"3746","AL137026.1",10.7872356733648,0.579959314437194,0.4898747045725
6,1.18389316497417,0.23645535616228,0.848299143848238
"3747","ENTPD1-
AS1",17.0510900307566,0.447252956728792,0.3781473178095,1.182747928293
13,0.236909063116407,0.848299143848238
"3748","GAB2",9.53857645476548,0.602040626335839,0.509141354474683,1.1
8246263251785,0.237022184052147,0.848299143848238
"3749","KMT2A",145.130092112316,0.296182301734061,0.250535666189704,1.
18219615689286,0.237127877195909,0.848299143848238
"3750","APOLD1",4.64578061005662,-1.15531264098882,0.976247354943854,-
1.18342204476985,0.236641924810925,0.848299143848238
"3751","AC008033.3",1.92690709797252,1.39975589634749,1.18286234331365
,1.18336330872301,0.236665192209036,0.848299143848238
"3752","RUVBL2",31.0521837870196,-0.346893637619157,0.29302463373813,-
1.18383779955227,0.236477276067822,0.848299143848238

"3753","CDS2",31.0852389924104,0.392372334149574,0.331916920740371,1.1
8214019723475,0.237150076898417,0.848299143848238
"3754","ACTR5",4.96312735393145,-0.775131347905899,0.655332491171856,-
1.18280622180021,0.23688595419944,0.848299143848238
"3755","AP1S2",37.6085003776912,-0.39936613052912,0.337453275033341,-1
.18347089827373,0.236622573464976,0.848299143848238
"3756","MED12",10.7277103327914,0.563607150505453,0.475907777469149,1.
1842780832511,0.236303001621296,0.848299143848238
"3757","DCAF12L2",5.48795291371391,-0.691482680916498,0.58467262060289
,-1.18268353357041,0.236934592550938,0.848299143848238
"3758","TNKS2",42.8196198702202,0.334354023169436,0.282959242982303,1.
1816331555225,0.237351291873964,0.848520737662389
"3759","RIOX1",4.13848903075527,-0.803141000788352,0.679759930334346,-
1.18150683049723,0.237401441596144,0.848520737662389
"3760","CDC37",113.72247521823,-0.261365488304258,0.221209062223707,-1
.18153155967879,0.237391623778823,0.848520737662389
"3761","AGO1",17.7425645888144,0.47077376942115,0.399072572859131,1.17
966956748824,0.238131662212749,0.848796264976192
"3762","ETV3",10.5330013463299,-0.584435780059924,0.495747304081826,-1
.17889855425913,0.238438573500459,0.848796264976192
"3763","CSRP1",64.1220663683947,-0.398045933675988,0.337279148568356,-
1.18016763077578,0.237933550346247,0.848796264976192
"3764","VPS8",25.521986142303,0.380924915172967,0.322551639012521,1.18
097342905822,0.237613278962299,0.848796264976192
"3765","AL023806.1",1.69535497649164,-1.29853977218608,1.1004950350728
1,-1.17995968250795,0.23801625061631,0.848796264976192
"3766","WTAP",86.5905645205391,-0.263719718227953,0.223711699419098,-1
.17883740060418,0.238462928409289,0.848796264976192
"3767","TTYH3",14.6890947831936,0.533881933007776,0.452898861744412,1.
17881049855468,0.238473642911478,0.848796264976192
"3768","ELOC",95.5176136864489,-0.320120383558662,0.271571136331811,-1
.17877174976185,0.238489076311489,0.848796264976192
"3769","C9orf72",6.24561228515402,-0.693510371338636,0.587829331014896
,-1.17978184270132,0.238086992960547,0.848796264976192
"3770","RIN1",5.26916286893767,-0.848315279683313,0.71850530334859,-1.
18066669199203,0.237735158304856,0.848796264976192
"3771","MPI",15.6101830455253,0.46937123222903,0.397973912335744,1.179
4020102329,0.238238135048792,0.848796264976192
"3772","CD2BP2",56.3917646536489,-0.326864202375253,0.27683529107398,-
1.1807172456488,0.23771506820464,0.848796264976192
"3773","PHB",138.104102332289,-0.334107683219709,0.28289214937006,-1.1
8104261275435,0.237585795515073,0.848796264976192
"3774","ZNF526",6.52078058894768,0.821429159833363,0.696246370491018,1
.17979668497814,0.238081088329629,0.848796264976192
"3775","NKG7",9.43949848974728,0.720408132419063,0.610825506554124,1.1
7940086766044,0.238238589800837,0.848796264976192
"3776","ADARB1",11.0054760549819,0.747165265971907,0.633699307413185,1
.17905331003422,0.238376948677889,0.848796264976192
"3777","SMYD2",12.3358757097474,-0.504853096238035,0.429177644731501,-
1.17632663871362,0.239464374745779,0.848895207651947

"3778","BMPR2",83.5141556495945,0.305327452328956,0.259374479419801,1.17716844391147,0.239128281075492,0.848895207651947
"3779","LMLN",11.0720992241868,0.565960263465178,0.481016231176332,1.17659286066317,0.239358048704458,0.848895207651947
"3780","ANKRD37",7.47037660831927,-0.614355818584736,0.521888257940183,-1.1771788486093,0.239124129046334,0.848895207651947
"3781","ANKS1A",26.0447747969224,0.464320136486244,0.394629416391027,1.1765978844977,0.239356042561798,0.848895207651947
"3782","CDC5L",87.621047982413,-0.243583014018041,0.206909148708834,-1.17724622394931,0.239097243924407,0.848895207651947
"3783","ANK1",5.27669277507071,-0.721436894929714,0.612724510371777,-1.17742457289978,0.239026086735741,0.848895207651947
"3784","ZDHHC21",28.0708409196491,0.528001285737768,0.448699076223319,1.17673807172043,0.23930006708297,0.848895207651947
"3785","NYNRIN",23.0156627557393,0.425765017307661,0.361445023665644,1.1779523563216,0.238815600638281,0.848895207651947
"3786","PRTG",3.96221407511175,0.926577704158955,0.786528317401518,1.17806019651031,0.238772608827483,0.848895207651947
"3787","PHKB",59.3347045066771,0.299528032380314,0.254619547603367,1.17637485102638,0.239445116822987,0.848895207651947
"3788","SPG7",66.8554444718605,0.398813122678273,0.338527867617584,1.17808062740876,0.238764464415185,0.848895207651947
"3789","TCF25",223.80600408301,-0.195521411035418,0.166192034245717,-1.17647883620184,0.23940358471005,0.848895207651947
"3790","SPATA22",4.32142696368103,-1.01944860545187,0.865845081366339,-1.17740301052833,0.239034688839201,0.848895207651947
"3791","SOGA1",32.777246278786,0.38369833862403,0.326117520828855,1.1765646250739,0.239369324101416,0.848895207651947
"3792","DOK2",2.72622196884032,-1.22004013024844,1.03763447847209,-1.17578989091124,0.23967884702021,0.849431441219569
"3793","ISG15",82.442312071052,-0.497771022793618,0.424785649096314,-1.17181694780079,0.241270556876725,0.849597674485153
"3794","STXBP3",53.4331830803549,-0.28027900172614,0.238774566238096,-1.17382268196294,0.240466056858093,0.849597674485153
"3795","DENND2C",7.68862857407401,0.727041803622064,0.620499324649036,1.171704423745,0.241315746347992,0.849597674485153
"3796","LINC00623",48.5469819083712,-0.34965031656774,0.298136619865071,-1.17278553948181,0.24088181844698,0.849597674485153
"3797","PRRX1",844.140810737936,0.208612531570496,0.177654898600113,1.17425713118143,0.240292048581685,0.849597674485153
"3798","RAB3GAP1",55.2359088178824,0.295427910311058,0.25151271867577,1.17460425805305,0.240153078974147,0.849597674485153
"3799","MAN2B2",56.9237628265163,0.269994200904276,0.230406522531465,1.17181665665478,0.241270673792782,0.849597674485153
"3800","ATP8A1",4.72267514744161,0.931073500870151,0.794018337752631,1.17260956907549,0.240952410297486,0.849597674485153
"3801","OCIAD2",1.94628960931443,-1.33845473409626,1.1420226550352,-1.17200366226974,0.241195585806236,0.849597674485153
"3802","LYRM7",30.6186608276866,0.401804560942255,0.342521057862053,1.1730798785051,0.240763774696536,0.849597674485153

"3803","TCP1",90.9420244785354,-0.258945155258065,0.220890318547332,-1.17227933284264,0.241084926365917,0.849597674485153
"3804","FSBP",1.59901180986002,1.47847647171768,1.26160195700702,1.1719040728386,0.241235571700942,0.849597674485153
"3805","PTDSS1",19.0184188760938,-0.420756845433685,0.359139086591068,-1.17157073998124,0.241369441266784,0.849597674485153
"3806","METTL15",14.7041584424195,0.472747297888911,0.402638397692077,1.17412373136466,0.240345469255605,0.849597674485153
"3807","TBCEL",9.19965418470225,0.571479097029211,0.487594321724071,1.17203804795867,0.241181780781138,0.849597674485153
"3808","KMT5A",25.0138442157373,0.398813412886421,0.340107997691799,1.17260815856445,0.24095297619344,0.849597674485153
"3809","EIF1",2586.69498818596,-0.245449475311896,0.209482052749302,-1.17169691670741,0.24131876137366,0.849597674485153
"3810","SMAD2",49.4611005572909,0.321542516830631,0.273697486066632,1.17480990217189,0.240070777634732,0.849597674485153
"3811","SOCS6",16.1481596327488,0.488648654915814,0.416315657939672,1.17374555964125,0.240496955641808,0.849597674485153
"3812","PGPEP1",17.5539693526584,0.50659152840524,0.431636428195082,1.17365332329245,0.240533913479813,0.849597674485153
"3813","UBE2S",31.6583444580683,-0.38843966330207,0.3313576746942,-1.17226698811352,0.241089881011538,0.849597674485153
"3814","ISM1",63.0991621532028,0.426684199582081,0.363308958726546,1.17443897083539,0.240219243391096,0.849597674485153
"3815","KIAA1671",29.0106255646965,0.451563110488456,0.38510988302993,1.17255653616493,0.240973687704679,0.849597674485153
"3816","KDELR3",86.5755599142616,-0.34525831115444,0.29469020962039,-1.17159749419293,0.241358694597388,0.849597674485153
"3817","TAB1",13.3765423439394,0.47166154910681,0.401617972987212,1.17440348995992,0.240233448048064,0.849597674485153
"3818","KIAA0930",73.0370683864878,-0.317167419301833,0.270235800324276,-1.17366913977068,0.240527575750025,0.849597674485153
"3819","NOL9",23.4165820120192,0.402547370354924,0.347299650865731,1.1590779586201,0.246424409367826,0.849677243399363
"3820","PRDM2",78.1757970009707,0.269697317440344,0.231700668705678,1.16399024200889,0.244427966266995,0.849677243399363
"3821","MRTO4",32.1499794023264,-0.373595999586838,0.320913510314067,-1.16416413637809,0.244357501076367,0.849677243399363
"3822","TMEM35B",184.045714759372,0.305648753695377,0.262688112687222,1.16354238708665,0.244609510980464,0.849677243399363
"3823","MAGOH",75.1172043842778,-0.371156767076639,0.319986352761781,-1.1599143647009,0.246083673627882,0.849677243399363
"3824","CAPZA1",90.1629258078367,0.254432517446063,0.217821739663821,1.16807678535093,0.242775793443795,0.849677243399363
"3825","GATAD2B",38.9117263987464,0.352319765842698,0.30108500874555,1.17016708108655,0.241933737459226,0.849677243399363
"3826","Z99127.4",3.96364215179239,1.07284894863173,0.920830684165952,1.16508818296326,0.243983299696494,0.849677243399363
"3827","TAF5L",10.580055142227,-0.520723515380124,0.447901110683493,-1.16258589889519,0.244997554696578,0.849677243399363

"3828","GGPS1",45.2141478316156,-0.283935419233358,0.242946253898225,-1.16871700912213,0.242517666690377,0.849677243399363
"3829","LBH",16.1827007077496,-0.640816248658082,0.553581273516864,-1.15758295902428,0.247034266773549,0.849677243399363
"3830","CNNM4",4.46663290209731,0.884490506403314,0.757107592320423,1.1682494210532,0.242706170808309,0.849677243399363
"3831","RPL15",2428.17142052108,-0.295644935952625,0.252874602503981,-1.16913653259414,0.242348627052865,0.849677243399363
"3832","CNOT10",16.3787601349909,0.448497309113128,0.384237972432388,1.16723838165695,0.243114114820159,0.849677243399363
"3833","QRICH1",29.5652311321149,0.413261406781028,0.357083467955329,1.15732439014154,0.247139852296995,0.849677243399363
"3834","RNF123",7.07536226558509,0.668221330732671,0.575165674731648,1.16178930713214,0.245321058533619,0.849677243399363
"3835","RBM5-AS1",2.07172285193517,1.42053162188469,1.21638771431769,1.16782799198323,0.242876154571753,0.849677243399363
"3836","SELENOK",157.233130293773,-0.258250260490194,0.221478607917033,-1.16602801019471,0.243603120814388,0.849677243399363
"3837","EBLN2",2.91308926755572,1.11816092953801,0.956169450633446,1.16941712454549,0.242235613699715,0.849677243399363
"3838","PDIA5",17.5214532874076,0.464831272342877,0.400457633154867,1.16075018643262,0.245743506002021,0.849677243399363
"3839","ITGB5",236.47767228376,-0.257248712459857,0.221752838413484,-1.1600695364277,0.246020495990258,0.849677243399363
"3840","TSC22D2",42.7658069998856,-0.467069289359624,0.402267387600779,-1.16109161159034,0.245604645689816,0.849677243399363
"3841","AC007620.2",1.74436533884949,1.39392816076407,1.20248343151216,1.15920778967504,0.24637149702824,0.849677243399363
"3842","ZNF595",15.6043914108661,0.508040361079414,0.434007092161233,1.17058078140962,0.241767326075988,0.849677243399363
"3843","PYURF",144.439940337231,-0.276723464597396,0.238212377256032,-1.1616670291652,0.24537074334845,0.849677243399363
"3844","LNPEP",70.0345443293755,0.294236759651315,0.253390182184204,1.1612003161094,0.245560446253332,0.849677243399363
"3845","APBB3",7.4212110260756,0.651793505142166,0.562133301811607,1.15949989627302,0.2462524787858,0.849677243399363
"3846","PCDHGC3",19.713514795063,0.418477481905217,0.361326031500223,1.15817141701001,0.246794090195899,0.849677243399363
"3847","LYRM4",40.5727018635418,-0.367921079569478,0.31566866664324,-1.16552929843142,0.243804807819451,0.849677243399363
"3848","SSR1",114.275468376944,0.222110859530867,0.191082203666142,1.16238380796015,0.245079597483288,0.849677243399363
"3849","TMEM14C",258.111134576812,-0.272225187629291,0.2340029308461,-1.16334093186349,0.244691204733732,0.849677243399363
"3850","NUP153",21.7309792905665,0.425824396517309,0.366253003635514,1.16265093334519,0.244971156782732,0.849677243399363
"3851","PTPRK",9.2517820938402,0.734207540094574,0.632880437971044,1.16010465175441,0.246006200483031,0.849677243399363
"3852","SF3B5",218.410721749107,-0.336814684391055,0.287658018100085,-

1.17088578519604,0.241644689584065,0.849677243399363
"3853","SOD2",236.250815481531,-0.34849490416775,0.299460594185452,-1.16374211143097,0.244528538030871,0.849677243399363
"3854","ETV1",7.21974200209438,0.76428517257565,0.658121787421019,1.16131267370839,0.245514767331738,0.849677243399363
"3855","LIMK1",12.3380514773121,-0.498919436795017,0.426393167103654,-1.17009247634996,0.241963755867309,0.849677243399363
"3856","SRI",272.529865530158,-0.278607068237931,0.239907717554937,-1.16130931958924,0.245516130860778,0.849677243399363
"3857","RBM48",14.9194856731273,-0.462883745577543,0.396457039800472,-1.16755082924118,0.242987994005879,0.849677243399363
"3858","PDK4",560.974697969675,0.385272418616372,0.332903223702782,1.15731056711048,0.24714549776383,0.849677243399363
"3859","GIGYF1",34.8672518936938,0.353506540953293,0.303174620477121,1.16601627272416,0.24360786628765,0.849677243399363
"3860","BRAF",31.5740797456682,0.418242996374089,0.361230499105927,1.15782858149927,0.246933997126694,0.849677243399363
"3861","AC016065.1",5.67652593668983,-0.758609137948116,0.653234534526023,-1.16131205233745,0.245515019933371,0.849677243399363
"3862","KCTD9",38.8359373698878,0.370652318650851,0.317880803403572,1.16601038717107,0.243610245848189,0.849677243399363
"3863","GSDMD",53.7821668416317,-0.273440258498876,0.234141432180759,-1.16784225650323,0.24287039959764,0.849677243399363
"3864","GLIPR2",161.373101003816,-0.243263192660595,0.207874584535022,-1.17024018691237,0.241904324703893,0.849677243399363
"3865","GKAP1",9.95770836092663,-0.631202699994613,0.538990821209949,-1.17108246588998,0.241565631066445,0.849677243399363
"3866","EDF1",439.215505681958,-0.331354615402245,0.28314034021196,-1.17028401941663,0.241886690726037,0.849677243399363
"3867","TMEM254",13.6919778103612,0.545219820417976,0.470126813819435,1.15972925685405,0.246159054529039,0.849677243399363
"3868","CNNM2",5.04865492096013,0.825911284066505,0.710441593097907,1.16253227864248,0.245019321013416,0.849677243399363
"3869","ATG13",22.9960159315188,0.389276864362261,0.336254962604776,1.15768362598058,0.246993168388259,0.849677243399363
"3870","TTC9C",24.0311486019466,0.366314912982402,0.315166313337553,1.16229082068827,0.245117353968459,0.849677243399363
"3871","CFAP300",7.99240342137094,0.595195105814171,0.508702618606613,1.1700256378559,0.241990651629148,0.849677243399363
"3872","PHLDB1",101.867243120058,0.366064836290764,0.315512950032593,1.16022127222654,0.245958728275621,0.849677243399363
"3873","SPSB2",12.9136033980208,-0.529498368451381,0.453910006689449,-1.16652719844894,0.243401358526157,0.849677243399363
"3874","LINC01252",3.68247705890656,1.36504676045218,1.16942516283871,1.16728013371852,0.243097258757579,0.849677243399363
"3875","C12orf81",9.45947101944575,0.703009167974639,0.602385449528073,1.1670420799928,0.243193376364144,0.849677243399363
"3876","STAT6",72.0080464706656,0.28780740935457,0.248562749197479,1.15788632964432,0.24691042693306,0.849677243399363
"3877","EXOC5",39.8474283984037,0.325466229121522,0.279455766706853,1.

16464309524496,0.244163491895638,0.849677243399363
"3878","ATP6V1D",60.0603601912639,-0.273383161237461,0.235198906625253
,-1.16234877602151,0.245093821347421,0.849677243399363
"3879","VRK1",9.04019374737716,-0.551565538959569,0.476018918614761,-1
.15870507954737,0.246576419630799,0.849677243399363
"3880","ADSSL1",2.48850612405562,1.27262086134057,1.09166666189959,1.1
6575957273084,0.243711666642158,0.849677243399363
"3881","ZNF280D",69.26964820862,0.308274516567843,0.265433996690444,1.
16139801386241,0.245480076320452,0.849677243399363
"3882","CCP110",19.0079689402148,0.485295341870565,0.416151079435974,1
.1661518276687,0.24355306521901,0.849677243399363
"3883","TMEM219",143.723042859638,-0.297073725498903,0.254386339286622
,-1.16780534022381,0.242885293547277,0.849677243399363
"3884","NLRC5",8.22103380687605,0.776524409453252,0.66592157059628,1.1
6608988766941,0.243578104751692,0.849677243399363
"3885","AC104564.3",1.53717001614387,1.46074621551868,1.25866672498759
,1.16055043524971,0.245824771909181,0.849677243399363
"3886","COASY",25.075646881926,-0.35274698066733,0.304042018104916,-1.
16019155137172,0.245970826009717,0.849677243399363
"3887","RPL27",1256.43393585383,-0.280339098107636,0.241995369244085,-
1.1584481925556,0.246681182038766,0.849677243399363
"3888","SNHG25",28.6519347805701,-0.558805515879416,0.477949916010673,
-1.16917170013047,0.242334460675477,0.849677243399363
"3889","ASPSCR1",18.0575330725679,-0.441001498382481,0.377004703821829
,-1.16975065274224,0.242101327569636,0.849677243399363
"3890","AP005329.3",6.34472431417682,-0.675383793548466,0.578231055526
333,-1.16801715697145,0.242799844367669,0.849677243399363
"3891","RPL17",2383.16273395403,-0.315239567246147,0.270851213835279,-
1.16388463903235,0.244470765493179,0.849677243399363
"3892","S1PR2",50.6486063766427,-0.288374994436702,0.247583361324401,-
1.16475918613469,0.244116483900785,0.849677243399363
"3893","RNASEH2A",4.00603753453815,-1.03972927849016,0.893090803235209
,-1.16419212326871,0.244346161607085,0.849677243399363
"3894","GIPC1",55.8770070851806,-0.349260008045014,0.301571046466064,-
1.1581350800675,0.246808916221125,0.849677243399363
"3895","PSMD8",203.785367378134,-0.302681979820691,0.25985177867767,-1
.16482550691388,0.244089631874047,0.849677243399363
"3896","MEIS3",21.1911716701938,-0.422801753912109,0.364056982690982,-
1.16136147365422,0.245494929596364,0.849677243399363
"3897","NOP53",451.349037608036,-0.318483539330679,0.272747893714961,-
1.16768468857001,0.242933975186338,0.849677243399363
"3898","ZNF837",1.83331113811769,1.37488079209113,1.18539590600729,1.1
5984945208902,0.246110105949351,0.849677243399363
"3899","DDX27",61.9815782760206,-0.326454039994243,0.281892887367015,-
1.15807831493531,0.246832078507142,0.849677243399363
"3900","MED15",35.2758327844746,-0.342157861284673,0.294647018745138,-
1.16124664265017,0.245541611500945,0.849677243399363
"3901","CASTOR1",10.973369415909,-0.54459862623246,0.466214759941746,-
1.16812823836918,0.242755041373136,0.849677243399363
"3902","NCF4",2.1062596238352,-1.10217302048428,0.95138536482474,-1.15

849272149285,0.246663020229441,0.849677243399363
"3903","ANKRD54",15.0343808977237,-0.472701462999474,0.407692941547389
,-1.15945461602878,0.246270925490018,0.849677243399363
"3904","MIR222HG",12.6206954826987,0.555727655404278,0.475293356946022
,1.16923084929081,0.242310635204159,0.849677243399363
"3905","FGD1",9.23734326507344,0.593578849491541,0.507014630999755,1.1
7073317651819,0.241706045292347,0.849677243399363
"3906","COX7B",182.251784504017,-0.284847841319752,0.24573186563374,-1
.15918153547214,0.246382196224271,0.849677243399363
"3907","MORF4L2",179.743662117325,-0.240008173117701,0.205927837443623
,-1.16549649672016,0.243818077465525,0.849677243399363
"3908","PLP1",45.6553284632592,0.396495984560232,0.341054717127715,1.1
6255827774332,0.245008766908881,0.849677243399363
"3909","ACSL4",51.8577044065092,0.310924232392473,0.266712305477855,1.
16576635575702,0.243708923427829,0.849677243399363
"3910","P3H1",67.0094485647385,0.30755985911131,0.266077935945217,1.15
590140166539,0.247721489261134,0.849887021974354
"3911","SELENBP1",148.930957582867,0.352430867359665,0.305068478788374
,1.15525166270668,0.247987384069322,0.849887021974354
"3912","MAP3K2-
DT",2.75701242997152,-0.914851117213202,0.790870528522872,-1.156764709
54341,0.247368503764258,0.849887021974354
"3913","IKZF2",4.22368638222719,0.84886323615958,0.734606765083376,1.1
5553419394829,0.247871738350071,0.849887021974354
"3914","DIRC3",7.73176370064898,0.98818781132296,0.855997658774862,1.1
5442817067665,0.248324671309344,0.849887021974354
"3915","SEC61A1",79.0064773371327,0.272204021486602,0.235721793654147,
1.15476815811941,0.24818537980758,0.849887021974354
"3916","TNIK",3.61070526740227,0.966314866243921,0.835635620493466,1.1
5638304847906,0.247524512191919,0.849887021974354
"3917","CLINT1",106.719087862206,0.276799554747842,0.239814282961551,1
.15422464137476,0.24840808261889,0.849887021974354
"3918","ZKSCAN5",3.44605468440131,0.961929460910629,0.832546590438324,
1.15540616219951,0.247924139642301,0.849887021974354
"3919","CTNND1",54.7581571563505,0.29589305013054,0.256294971489937,1.
15450197251395,0.248294430343996,0.849887021974354
"3920","ANAPC5",127.81902267723,-0.233092468679364,0.201931876762584,-
1.154312396915,0.248372115838795,0.849887021974354
"3921","GEMIN2",16.0761337109499,0.483813415840118,0.418258353482461,1
.1567334204131,0.247381290971161,0.849887021974354
"3922","SLC25A39",29.8511146224757,-0.378914811909928,0.32805210874523
2,-1.15504458532287,0.248072168982132,0.849887021974354
"3923","WDR45B",57.7810084079506,0.349218218162131,0.302048868294828,1
.15616463035796,0.247613824138982,0.849887021974354
"3924","UBA52",1565.17456862529,-0.279401937690846,0.241853432356733,-
1.15525314223669,0.247986778369603,0.849887021974354
"3925","TMEM238",4.82715391987776,-0.806026324665843,0.697031804864063
,-1.15636950716049,0.247530048629428,0.849887021974354
"3926","ATP9A",22.6704352133896,0.447860469535479,0.38787756178126,1.1
5464392288834,0.248236272147568,0.849887021974354

"3927","TAFA5",18.5869806307191,-0.459387279990899,0.397622941420834,-1.15533394111859,0.247953701964506,0.849887021974354
"3928","RPL39",2589.57178178342,-0.294867923631163,0.254977939143047,-1.15644484625682,0.247499246954295,0.849887021974354
"3929","C19orf12",36.6680190858031,0.342298149856153,0.296625644454847,1.15397355641736,0.248511010405426,0.850022771402015
"3930","CERCAM",152.720470635243,0.379092837380742,0.32859297932902,1.15368514006246,0.248629278339587,0.850183342549831
"3931","AC009318.1",4.49319471312506,-0.903964080212162,0.783636292146954,-1.15355055562261,0.248684479467474,0.850183342549831
"3932","FAM133B",117.782722371531,-0.227224888576991,0.19703221602888,-1.15323723783163,0.248813023034556,0.850260928312893
"3933","ZNF740",15.922972927375,0.469235940125815,0.406935642782751,1.15309619210801,0.248870904431126,0.850260928312893
"3934","BORA",2.09471640036172,-1.12458349523811,0.97532665966692,-1.15303266253601,0.248896978345332,0.850260928312893
"3935","FZD1",43.9689571320975,0.346973120872683,0.301284385805145,1.15164654134146,0.249466348075435,0.85090818573243
"3936","ATP6V1F",230.297898041249,-0.295326585084897,0.256329345057266,-1.15213724366564,0.249264680865316,0.85090818573243
"3937","ARRDC1",18.0920236620439,-0.426086902527807,0.36981967058385,-1.15214775313364,0.249260362965871,0.85090818573243
"3938","AKT1",55.9298278230299,-0.285635322131608,0.247986938787582,-1.15181599292322,0.249396694542397,0.85090818573243
"3939","CDIP1",18.728960008604,0.441782265330029,0.383574049342939,1.15175222642617,0.249422904339015,0.85090818573243
"3940","TRIR",249.944571648278,-0.272825678973944,0.236819536149224,-1.15204042457896,0.249304462225201,0.85090818573243
"3941","RXRG",2.52536026476277,-1.06377148579721,0.924571844090908,-1.15055578708778,0.24991503098798,0.851066633518156
"3942","RPP40",4.02794815730491,0.909312580419034,0.790108777502402,1.15087011600282,0.249785673675692,0.851066633518156
"3943","PSMB9",93.1648939284247,0.34654762736344,0.301266596073098,1.15030219705923,0.250019426231839,0.851066633518156
"3944","MRPS33",55.6514067354748,-0.332477991635533,0.288929946211017,-1.15072181335164,0.249846699559422,0.851066633518156
"3945","TENM4",1.90795295097198,1.69412644631727,1.47267149351731,1.15037634243265,0.249988899703062,0.851066633518156
"3946","SIN3A",20.6651028672711,0.42525614056529,0.369556349122513,1.15072069949558,0.249847157945661,0.851066633518156
"3947","AC079414.3",2.01176635425713,1.43044922727245,1.24311129419316,1.15070085353933,0.249855325269311,0.851066633518156
"3948","PIGW",3.30114381556163,-0.876250399983272,0.761459306134431,-1.15075144912416,0.24983450373768,0.851066633518156
"3949","BIVM",20.3717530997667,0.459839358938217,0.399987275823644,1.14963496774073,0.250294249595066,0.851786381440389
"3950","EXOC3",35.4368813870029,0.310557921880398,0.270254849582117,1.14912987633931,0.250502430633976,0.852136671072687
"3951","MED29",57.5911827686568,0.282914934063069,0.246210474773748,1.14907757000611,0.250523996384269,0.852136671072687

"3952","BPGM",21.1488764752593,-0.395010533494433,0.343996299079667,-1
.14829878853712,0.25084523906998,0.852362107534523
"3953","PTPRN2",16.9363216897938,0.471581501352484,0.410602703943321,1
.14851046236067,0.250757896447144,0.852362107534523
"3954","C11orf54",38.330449622605,0.361111678746978,0.314504227418795,
1.14819340175711,0.250888732561267,0.852362107534523
"3955","AC138150.2",3.56284898424759,-1.04792016076107,0.9127046285903
56,-1.14814818281304,0.250907396190682,0.852362107534523
"3956","RBMX",172.536026620579,0.215915564996014,0.188029684732086,1.1
4830573323388,0.250842373154269,0.852362107534523
"3957","SPAG7",86.8711051146345,-0.329183141495277,0.286761315480125,-
1.1479342705069,0.250995699312843,0.852411736238469
"3958","APMAP",44.5279259984801,0.353356074413823,0.307853613664062,1.
14780551122396,0.251048861673626,0.852411736238469
"3959","ZNF436",29.609059007557,0.377783613125988,0.329537527142937,1.
14640543795221,0.251627433697194,0.852498370872913
"3960","FAM107A",3.31919172165868,-0.958582258800211,0.83584896289286,
-1.14683669102438,0.251449121878301,0.852498370872913
"3961","SENP2",21.655127177738,0.42200309315132,0.368210390521293,1.14
609229944291,0.251756963504712,0.852498370872913
"3962","LRBA",22.4794437675752,0.396619676723091,0.345835362090427,1.1
4684534954926,0.25144554270857,0.852498370872913
"3963","IMPDH1",11.9167085651179,-0.488926525484186,0.426302859304783,
-1.14689947489803,0.251423169747994,0.852498370872913
"3964","UBE2R2",113.494842322682,-0.234166125283315,0.204150634520983,
-1.14702619383359,0.251370795328008,0.852498370872913
"3965","TMEM179B",130.209540213789,-0.300098883435626,0.26170532346665
5,-1.14670530755888,0.251503436151469,0.852498370872913
"3966","CMAS",33.4523071331616,0.333931243745274,0.291259472903208,1.1
4650775274955,0.251585121237675,0.852498370872913
"3967","PPTC7",31.3807447722521,-0.388765617982574,0.339220563934209,-
1.1460555736178,0.251772158195892,0.852498370872913
"3968","SMARCA4",29.5472040460716,0.438892849399433,0.382872073389673,
1.14631721638455,0.251663921862348,0.852498370872913
"3969","NINL",7.8430164398269,0.68101666180844,0.593581667545808,1.147
30069852753,0.251257365428233,0.852498370872913
"3970","GIN1",8.32007146660804,0.634734762269295,0.55400665517243,1.14
571685437919,0.251912327723378,0.852543382592416
"3971","ZNF277",31.8428257270904,-0.350648493914119,0.306021147814727,
-1.14583092187606,0.251865117989521,0.852543382592416
"3972","CEP170",72.4666215201446,0.270637546178917,0.236306094546111,1
.14528381800202,0.252091607014616,0.852935323934902
"3973","MAGI1",4.43728472550539,0.816678050073824,0.713211309630133,1.
14507164853758,0.252179478727568,0.853017874306515
"3974","AKR7A2",119.495218089145,-0.344052479992764,0.300620371439768,
-1.14447493476568,0.252426727068819,0.853304818048836
"3975","ADAM17",36.5100307907133,-0.355383308328285,0.31047768657493,-
1.14463397434043,0.252360812514812,0.853304818048836
"3976","SLC41A2",30.2572853027719,-0.327351772356973,0.286044831486671
,-1.14440722685188,0.252454792511509,0.853304818048836

"3977","AC004160.1",6.87066475814158,-0.764793227528021,0.668456575284525,-1.1441180411794,0.252574686642036,0.85349540200712
"3978","GATA6",5.42833958517468,0.745501259666722,0.651716365964471,1.14390446304576,0.252663259946369,0.853580078034001
"3979","ZBTB20",405.373789507344,0.257310345873949,0.225006666565333,1.14356765424653,0.25280298239861,0.853658135052793
"3980","RRP12",4.33947860257523,-0.760489827801687,0.665029771705511,-1.14354253021088,0.25281340706229,0.853658135052793
"3981","TMEM165",108.878119564065,-0.249781672790075,0.218503371114782,-1.1431479135343,0.252977183834547,0.853782112896151
"3982","LINC02175",3.59212073676611,0.966108507773184,0.845129225692798,1.14314885629616,0.252976792474391,0.853782112896151
"3983","ZBTB38",125.720804357905,0.262892950569414,0.230075125523411,1.14263960509136,0.253188254716943,0.853816934936494
"3984","CDK7",8.8751582654636,0.576298429109043,0.504296496647509,1.1427769832632,0.25313119747399,0.853816934936494
"3985","PPM1N",1.48669099457762,-1.41618981541278,1.23954140076558,-1.14251110494421,0.253241632759645,0.853816934936494
"3986","AL162458.1",3.81547831700004,1.00907788818254,0.883079187592531,1.14268108948815,0.25317102406843,0.853816934936494
"3987","KIAA1217",30.6832141612801,0.556561788270851,0.487286525257503,1.1421653573875,0.253385292736503,0.854087020086749
"3988","TAF1A",8.3797216347858,0.626176259418031,0.549134074696975,1.14029758536396,0.254162343455736,0.854301857614404
"3989","LANCL1",20.9405134827615,0.398967547214683,0.349902373433451,1.14022532428053,0.254192439576158,0.854301857614404
"3990","ENTPD3-AS1",15.5640360270972,-0.509887554480438,0.447080760751119,-1.14048198724499,0.254085552894861,0.854301857614404
"3991","ATR",26.6707066908807,0.53252397469512,0.466628690604584,1.1412156719407,0.253780184165674,0.854301857614404
"3992","ACTA2",69.0040616018016,0.278077251949485,0.243757431439788,1.14079497107835,0.253955253854782,0.854301857614404
"3993","HNRNPUL2",67.9311599909574,0.316989495915749,0.27794017175395,1.14049543078058,0.254079955228639,0.854301857614404
"3994","SPCS2",298.913770578971,-0.286940948851312,0.251425569394848,-1.14125603669486,0.253763391268795,0.854301857614404
"3995","AL136038.5",5.39600248922836,0.824006920390893,0.722088738893198,1.14114356866154,0.253810183125498,0.854301857614404
"3996","WIZ",17.8337982920839,0.407745733787312,0.35744319865203,1.14072875165895,0.253982817938191,0.854301857614404
"3997","IFNGR2",113.368070700312,-0.294331098683151,0.257908313198208,-1.14122377457818,0.253776813173743,0.854301857614404
"3998","ZC3H12B",2.63549777121218,1.06914140276206,0.936996909362355,1.14102980712032,0.253857519250535,0.854301857614404
"3999","ATRX",213.122675373538,0.239732722339857,0.210258901007017,1.14017870916132,0.254211855688667,0.854301857614404
"4000","MACF1",169.739613318692,0.322194854004702,0.282715770979345,1.13964230891188,0.254435351189437,0.854328261115519
"4001","AC097448.1",11.3337162920447,-0.49002815386335,0.4300384169060

93,-1.13949855314986,0.254495271410376,0.854328261115519
"4002","AL359643.3",3.20822051540569,-0.978711512089207,0.858973149413488,-1.13939709612283,0.254537566597703,0.854328261115519
"4003","LONP2",55.6132325148118,0.307037631133669,0.269373249265415,1.13982227994415,0.254360349570254,0.854328261115519
"4004","ANKRD11",189.595509187269,0.242415952018429,0.21274021999676,1.13949281439176,0.254497663641056,0.854328261115519
"4005","FAM229A",4.3989019176525,0.827542712619468,0.726569507542612,1.13897253329329,0.254714610481286,0.854709026281649
"4006","AMDHD2",25.9559251477811,0.367735284465742,0.322955644392628,1.13865569730893,0.254846787766925,0.85493908657007
"4007","PCBP1-AS1",24.5639547056321,0.460250290376734,0.404290143777235,1.13841580721377,0.254946896577489,0.854968304891569
"4008","ARL6IP4",321.701960788743,-0.2574487830828,0.226163586493854,-1.13832994547863,0.254982734281227,0.854968304891569
"4009","AGBL5",4.18116028595038,0.834273227016516,0.733063435789864,1.13806416509862,0.255093690168303,0.855022469731833
"4010","ZIC4",4.15973770530324,-0.759921735662307,0.667866891892736,-1.13783411767678,0.255189755643603,0.855022469731833
"4011","RAD50",94.4714947615481,0.256017045511982,0.22499944899526,1.13785632211648,0.255180482197759,0.855022469731833
"4012","HIST2H2BE",1.46415684190342,1.49376282071633,1.31441426406858,1.13644751243995,0.255769320640875,0.855318364202351
"4013","AMT",6.52744970943009,0.732151426439285,0.644199103995611,1.13652971868193,0.2557349350813,0.855318364202351
"4014","AC097382.3",9.81389678229866,0.652266567560937,0.573668483072514,1.13700959144114,0.255534276069327,0.855318364202351
"4015","TMX2",49.8288936616563,-0.321842318656122,0.283103462183653,-1.13683639251059,0.255606686656787,0.855318364202351
"4016","AC026124.1",2.94162892265141,-0.910141651752468,0.800510052064146,-1.13695218368043,0.255558275386437,0.855318364202351
"4017","P2RX4",17.7451895135329,0.422686710347287,0.372000319274835,1.13625362250027,0.255850434433708,0.855318364202351
"4018","RSL24D1",166.264106295107,-0.296178759256117,0.260437106352577,-1.1372371756241,0.255439149904796,0.855318364202351
"4019","GOSR1",58.9313115071178,-0.278916198212013,0.2453347508218,-1.13688010882162,0.255588408502553,0.855318364202351
"4020","OCRL",18.1286445799923,0.397213977549457,0.349582463372241,1.13625258463408,0.255850868672777,0.855318364202351
"4021","CD58",37.7873215812424,-0.291843804969248,0.258189970577868,-1.13034524275307,0.258330777536138,0.855399196295873
"4022","HIST2H2AC",32.614714421865,0.443967624034865,0.391635650085978,1.13362413237252,0.256952245844178,0.855399196295873
"4023","HSD17B7",17.7430069307897,-0.4400140820956,0.387829345831187,-1.13455592472656,0.256561429182111,0.855399196295873
"4024","PGBD2",4.47777833088149,-0.763726284422529,0.675181181821664,-1.13114272877388,0.257995022451366,0.855399196295873
"4025","FAM136A",43.9637514914302,-0.286755100915684,0.253361095262129,-1.13180399942227,0.25771684593181,0.855399196295873

"4026","TPRKB",48.5739282180174,-0.358675785804971,0.316572005432521,-1.13299906387782,0.257214646432202,0.855399196295873
"4027","MARCH7",65.8069215793555,0.266722379727037,0.235909537311394,1.13061295769052,0.25821803126585,0.855399196295873
"4028","HSPE1",158.460865037112,-0.301506231424098,0.265806536933602,-1.13430705994794,0.256665768778973,0.855399196295873
"4029","CLASP2",31.9439757406631,0.359973132613499,0.317484290090455,1.13382974795679,0.25686597009176,0.855399196295873
"4030","WNT5A",16.2693340037644,0.633380368853024,0.560062757152885,1.13090963604303,0.258093127114134,0.855399196295873
"4031","QTRT2",9.83971187635068,0.572834390088408,0.506084036164978,1.13189579033011,0.257678248729118,0.855399196295873
"4032","MRPL3",63.868471510358,-0.273580750847015,0.241716775149935,-1.13182360089537,0.257708603362295,0.855399196295873
"4033","AC026304.1",4.60353531827534,-0.836564984744079,0.739846586434287,-1.13072764013946,0.258169743995096,0.855399196295873
"4034","FRYL",24.6382276740175,0.411720420236115,0.363109634710884,1.13387357667867,0.25684758227699,0.855399196295873
"4035","EREG",3.73979163419062,-1.13242371998181,0.997090922020359,-1.13572764025093,0.256070568941608,0.855399196295873
"4036","SAP30",24.8922274767064,-0.41270508356906,0.36380696334327,-1.13440677379133,0.256623958995864,0.855399196295873
"4037","AGPAT1",32.5105269223735,0.335640446108677,0.296546345235559,1.13183133598246,0.25770535074927,0.855399196295873
"4038","BAZ1B",64.504087374436,0.297759247935134,0.263084519716069,1.13180071657765,0.257718226410987,0.855399196295873
"4039","LINC01003",37.8411115872274,-0.438925311246142,0.387324066081618,-1.13322499086243,0.257119781977058,0.855399196295873
"4040","ZNF782",3.85364848018641,1.11533390045573,0.983202466760779,1.13438883461131,0.256631480503543,0.855399196295873
"4041","STKLD1",2.17175113002656,-1.03276119248152,0.911618330362974,-1.13288769881395,0.257261416431811,0.855399196295873
"4042","TUBB4B",175.047045917901,-0.295028509794066,0.260999284939485,-1.13038052905957,0.258315915003003,0.855399196295873
"4043","ALOX5",1.70945583184592,-1.37015360267726,1.20950128208832,-1.13282525861532,0.257287642031564,0.855399196295873
"4044","CKAP5",32.3075767003652,0.312705214544559,0.276627259758821,1.13042082265209,0.258298944134358,0.855399196295873
"4045","POLR2G",83.0608723524082,-0.318346317145711,0.281681951570358,-1.13016228186063,0.258407849863057,0.855399196295873
"4046","TMEM135",25.1479834261231,0.351069222268649,0.309268568854658,1.13515972078506,0.256308402738815,0.855399196295873
"4047","LPCAT3",22.0366314664475,0.435352919949993,0.384042159534216,1.13360710313162,0.256959392170207,0.855399196295873
"4048","PFKM",18.1724663652098,0.418013885101793,0.369844664288219,1.13024176219029,0.258374366794076,0.855399196295873
"4049","ALG5",73.7529789166653,-0.333981208490865,0.29482626189774,-1.13280684814539,0.257295374994267,0.855399196295873
"4050","SEC11A",398.225284136252,-0.306787522367309,0.270254862958676,-1.13517854594246,0.256300516665743,0.855399196295873

"4051","NDE1",23.8899886908472,0.37615681672023,0.332251920970801,1.13
214339174065,0.257574154690455,0.855399196295873
"4052","ZKSCAN2",2.69424633801355,1.15619697203456,1.01946872047762,1.
13411716201835,0.256745405619508,0.855399196295873
"4053","EDC4",11.0764690759152,0.525158590952503,0.463940625136679,1.1
3195215615746,0.257654549422677,0.855399196295873
"4054","COG1",13.3478962147916,-0.467241667774176,0.41305558658649,-1.
13118350882379,0.257977861498242,0.855399196295873
"4055","AC132872.1",4.52522426824203,-0.824125646786201,0.728714627850
735,-1.13093056635472,0.258084316854251,0.855399196295873
"4056","TBXA2R",6.43763152996129,-0.631306015774338,0.558613176662502,
-1.13013090658933,0.25842106830577,0.855399196295873
"4057","MAP2K2",186.646696063687,-0.295083264243692,0.260005429115443,
-1.13491193336842,0.256412219420879,0.855399196295873
"4058","CCDC130",27.4289127041751,-0.334569839586358,0.295325699812936
,-1.13288426912483,0.257262856892437,0.855399196295873
"4059","CCDC8",22.7107929240965,0.442647220189787,0.391229366852897,1.
13142636441252,0.257875680043602,0.855399196295873
"4060","POLR2F",111.428694536611,-0.287538828658058,0.253586369779275,
-1.1338891317713,0.256841056544081,0.855399196295873
"4061","STX5",49.2028681537534,-0.30704314258804,0.271736805360156,-1.
12992843270196,0.258506382090778,0.855448876062924
"4062","PEMT",26.2821394821947,-0.428630067855656,0.379388084305978,-1
.12979317376232,0.25856338526435,0.855448876062924
"4063","PLAAT3",112.124052551671,-0.337750095855121,0.29900224312465,-
1.12959050850437,0.258648812285888,0.855520893012566
"4064","SRGAP3",2.11843587363769,1.17030482602553,1.0365608772578,1.12
9026622268,0.258886603314199,0.855873548787316
"4065","GAS1",501.848717071657,-0.273673442390805,0.242460585542086,-1
.12873373533655,0.259010173593125,0.855873548787316
"4066","NIPSNAP3B",1.54499443804126,-1.30493190622478,1.15583753841651
,-1.12899249492496,0.25890099968673,0.855873548787316
"4067","NOMO1",16.2659162284489,-0.439691695604335,0.38949992066573,-1
.12886209284155,0.258956013970506,0.855873548787316
"4068","JDP2",203.533622633162,-0.249391821452502,0.221009123299501,-1
.12842319687653,0.259141235755143,0.856096132574573
"4069","C5orf58",1.61589232999155,-1.43446108906232,1.27209186335036,-
1.12763954427342,0.25947217906179,0.856953077637793
"4070","INHBA",2.40614349399791,1.45910895914737,1.29410186283454,1.12
750703870513,0.259528166231551,0.856953077637793
"4071","FAM198B-
AS1",4.45888950895678,0.942104038603802,0.836193540367998,1.1266578765
8344,0.259887158677538,0.857087725895949
"4072","SLC35A4",21.8633606110943,0.388914218353208,0.345101906301446,
1.12695470888964,0.259761630558265,0.857087725895949
"4073","GALNT10",50.6039364293461,0.43181100962837,0.383293317060653,1
.12658110749173,0.259919630574779,0.857087725895949
"4074","KIAA0513",7.50923030539195,0.68977484056194,0.61196772339037,1
.12714251781207,0.259682229282539,0.857087725895949
"4075","FKRP",7.64709851106651,0.609433356234474,0.540930857152974,1.1

2663817967798,0.259895489834173,0.857087725895949
"4076","LGALS2",2.14202533543198,-1.12525029033958,0.998885722959672,-1.126505529587,0.259951601365569,0.857087725895949
"4077","LRIG2",20.6652985304148,0.445627423872087,0.395669624386422,1.12626139689934,0.260054892409656,0.857217978683681
"4078","KIAA1841",13.0191755815939,0.561117808592902,0.498396614426771,1.12584594748556,0.260230731808524,0.857377005338258
"4079","SENP5",26.5561667123024,0.356915208917047,0.316983975954656,1.12597240236555,0.260177200939582,0.857377005338258
"4080","TMEM220",27.4042527023452,-0.386084098070654,0.343007789815069,-1.12558405241703,0.260341621444842,0.857424788483315
"4081","TAOK1",96.5097510250869,0.311018864809679,0.276335856989256,1.12551034164839,0.260372837398646,0.857424788483315
"4082","AC108047.1",1.9803191634164,-1.2826917471233,1.14043054335193,-1.12474341782642,0.260697777506914,0.858074315923443
"4083","FBXL5",48.289131691318,0.284765335187194,0.253166370054528,1.12481501838439,0.260667428998517,0.858074315923443
"4084","KLF15",1.81910791112273,1.49840105657239,1.33326668564161,1.12385696928391,0.261073708753385,0.858302504596444
"4085","RPL39L",4.38319735014229,-0.801032002541948,0.712698891969036,-1.12394169763456,0.261037760372125,0.858302504596444
"4086","GNB3",1.53560950720722,1.47820289305161,1.31532961333833,1.12382696934794,0.261086437889,0.858302504596444
"4087","LIMA1",505.328787828372,-0.214316752937941,0.190623906363143,-1.1242910557591,0.260889571536716,0.858302504596444
"4088","MIEN1",60.21805527075,-0.367975261531618,0.327372472216707,-1.12402627820226,0.26100187810617,0.858302504596444
"4089","HSD11B1L",9.34995513832069,-0.642272323546108,0.571716115328705,-1.12341126360736,0.261262868268015,0.858672459440904
"4090","ITIH5",276.016309324052,-0.350918759707997,0.312440748889003,-1.12315298486454,0.261372526295579,0.858822831512538
"4091","TMEM54",61.9371334172803,-0.285909439129148,0.254687898233773,-1.12258745355352,0.261612746386594,0.859017025935813
"4092","WDR66",1.73371787731158,-1.31308224700207,1.16971870014164,-1.12256241337603,0.261623386201003,0.859017025935813
"4093","SULF2",35.5518505827371,0.555226985734834,0.494502428718296,1.1227993099527,0.261522738516146,0.859017025935813
"4094","KDELR1",296.749251809499,-0.234743088101259,0.209150354332573,-1.12236524222183,0.261707176587394,0.859082253580359
"4095","SERINC5",19.2285802508441,-0.495014543660551,0.441188165247642,-1.12200322368733,0.261861069245365,0.859377511499013
"4096","TMEM259",76.3827655822216,-0.263393279738311,0.234786281067719,-1.12184271815413,0.261929319522046,0.859391632093938
"4097","LATS1",33.5122829066572,0.318236249519934,0.283723804676636,1.12164099125427,0.262015115407931,0.859463298991257
"4098","ELP3",11.7351027018059,0.526936636640732,0.469945554764981,1.12127166923465,0.262172241001388,0.859768849882296
"4099","UBTD2",54.2179476806917,0.265911020863447,0.237273057784842,1.12069622799135,0.262417188346741,0.860239254339598
"4100","JCAD",1.55801078073854,-1.20040586374062,1.0711846232487,-1.12

063395766456,0.262443704352433,0.860239254339598
"4101","Z99289.1",1.58292590492821,1.50832467672103,1.34643543584823,1
.12023542797714,0.262613450435591,0.860585749915607
"4102","RPL22",1452.8962953102,-0.303630345331293,0.27159341861281,-1.
1179591423169,0.263584443797741,0.860619859134559
"4103","PIGV",18.9111951435806,-0.465192197312019,0.416015706062563,-1
.11820825640189,0.263478058856971,0.860619859134559
"4104","RLF",25.5296934305687,0.404770554121892,0.361598056537903,1.11
93936106774,0.262972255549548,0.860619859134559
"4105","ORC4",29.3237921859197,0.345290899838331,0.308517959081311,1.1
1919222098617,0.26305814337808,0.860619859134559
"4106","TFEB",12.5370957098004,-0.51281873278401,0.458183634429678,-1.
1192427975354,0.26303657188354,0.860619859134559
"4107","TBP",11.5476387699801,0.497567780067328,0.445059969936839,1.11
79791796102,0.263575885713795,0.860619859134559
"4108","AGBL3",5.29705774938089,-0.954807801525048,0.853305031109863,-
1.11895250433853,0.263160401964126,0.860619859134559
"4109","PTPRE",10.3862189903588,0.636738757359975,0.56922501697593,1.1
1860641814852,0.263308084177057,0.860619859134559
"4110","GSTP1",771.14844137034,-0.308639276922691,0.275800104411223,-1
.11906874575546,0.263110812149277,0.860619859134559
"4111","TNS2",97.2130914164773,0.276734716244736,0.247472015518986,1.1
1824650421334,0.263461727634704,0.860619859134559
"4112","NAA16",16.4477896070034,0.503782718088737,0.450035164129871,1.
11942967626271,0.262956876494393,0.860619859134559
"4113","DENND4A",14.885198750204,0.451905867494583,0.404157303678215,1
.11814351338405,0.263505704713289,0.860619859134559
"4114","NLGN2",14.6397262799871,0.43663708610024,0.390196087060863,1.1
190196431471,0.263131759053164,0.860619859134559
"4115","SMARCB1",55.5801874739101,-0.330466584004374,0.29538373123306,
-1.11877042999241,0.263238089766012,0.860619859134559
"4116","HEPH",11.7354224410039,-0.550251921603667,0.491706070154686,-1
.11906676570123,0.263111656807298,0.860619859134559
"4117","ZNF462",22.1871234473122,0.492277993130989,0.440436115238701,1
.11770578319671,0.263692671987933,0.860764104650432
"4118","ZNF486",7.3591783838079,0.620469170668292,0.555245909188843,1.
11746734266756,0.263794555356199,0.860887573927137
"4119","HTRA2",43.8500501840607,-0.350613191646816,0.313863434147215,-
1.1170883687023,0.263956543193644,0.860998054363927
"4120","GTF3C1",35.5563626370386,0.312001682567831,0.279276926636544,1
.11717672607403,0.2639187697723,0.860998054363927
"4121","SDC3",11.7187430011087,0.574549467982843,0.515663173791422,1.1
1419526773351,0.265195425151325,0.861016327369776
"4122","TMEM167B",42.8596341707057,0.313401903820473,0.2807068383198,1
.11647406132452,0.264219267161281,0.861016327369776
"4123","ACP6",1.84594406982218,-1.26506792460172,1.13602729236055,-1.1
1358937686526,0.265455385855717,0.861016327369776
"4124","TMEM214",42.898498949519,-0.302087702954127,0.27135110372317,-
1.11327243121264,0.26559144297274,0.861016327369776
"4125","DCTN1",50.6469860532903,0.346521222297344,0.310807527318091,1.

11490614557318,0.264890642893208,0.861016327369776
"4126","AC008063.1",3.35179088941409,0.903385343338881,0.8109890157199
8,1.11393043041017,0.26530903343184,0.861016327369776
"4127","ABI2",48.6731069488195,0.308315981455053,0.276471430087307,1.1
1518206911177,0.264772408296237,0.861016327369776
"4128","SMC4",23.3177672145078,0.35022590590469,0.314614928706502,1.11
318908910203,0.265627227701654,0.861016327369776
"4129","SPATA18",1.71777427075157,1.29418633221021,1.16084996216007,1.
11486098496487,0.264909997900769,0.861016327369776
"4130","SEMA5A",4.89914101003343,1.83572478953098,1.64828172575024,1.1
1372028267523,0.265399205162304,0.861016327369776
"4131","THG1L",17.0037915229998,-0.423939664461998,0.380909406281917,-
1.11296717138099,0.265722529041808,0.861016327369776
"4132","HIST1H2BD",1.47852896555176,1.66574111415888,1.49230389296712,
1.11622111421751,0.264327498729656,0.861016327369776
"4133","MRPS10",68.003768148036,-0.252686161685492,0.226960104489739,-
1.11335056993207,0.265557895447409,0.861016327369776
"4134","LRRC1",1.53726555130412,-1.31334970183193,1.17785951757875,-1.
11503085234791,0.26483720092012,0.861016327369776
"4135","UBXN2B",8.39621946258819,-0.55834496935398,0.501380315597651,-
1.11361565658681,0.265444106761278,0.861016327369776
"4136","NET1",13.7605276446962,0.615843206777667,0.552267529458375,1.1
1511753620866,0.26480005770447,0.861016327369776
"4137","CAPN5",17.704443450421,0.449536789981814,0.403146698738453,1.1
1507000153673,0.264820425417875,0.861016327369776
"4138","OSBPL8",189.336639943771,0.235823149448665,0.211696110883127,1
.11397015497775,0.265291990495157,0.861016327369776
"4139","NUP58",20.1425041537153,0.435561403482633,0.390223896160728,1.
11618331877664,0.264343673351106,0.861016327369776
"4140","CFL2",72.6664592056423,0.252013634355868,0.226250853363561,1.1
1386821578485,0.265335726739976,0.861016327369776
"4141","LTBP2",144.197469412751,0.379523943600675,0.340602927648826,1.
11427093777649,0.265162970840077,0.861016327369776
"4142","AL928654.1",1.56543402865013,1.30592631676124,1.17331697017655
,1.1130209056507,0.265699450996407,0.861016327369776
"4143","RGS11",3.25465872544973,1.0306288195097,0.925279946565008,1.11
385621544678,0.265340875721596,0.861016327369776
"4144","GABARAPL2",276.682218736802,-0.254198571425698,0.2278635060620
42,-1.11557386182097,0.264604585683862,0.861016327369776
"4145","MLX",40.7131267256177,-0.317761981126962,0.285528882960992,-1.
11288909840471,0.26575606264825,0.861016327369776
"4146","MIB1",44.3664225682679,0.341840497452478,0.306077923768242,1.1
1684140183634,0.264062143107158,0.861016327369776
"4147","DYM",35.1998361426872,0.30937289624969,0.277591004365675,1.114
49179326484,0.265068263233228,0.861016327369776
"4148","ZNF224",29.4702767799297,0.354604658552167,0.318250395323493,1
.11423163572733,0.265179826851281,0.861016327369776
"4149","SAMD9",29.9188273832934,-0.315467283182166,0.283619435347505,-
1.11229078076275,0.266013146486571,0.861038667901814
"4150","IFITM1",76.6919596330856,-0.875194654735567,0.786657757505975,

-1.11254817788906,0.265902527667404,0.861038667901814
"4151","AP001160.2",2.22740297171184,1.11579100185941,1.00341062287165
,1.1119983947012,0.266138840553445,0.861038667901814
"4152","DPP3",9.63898426131516,-0.528601560878074,0.475433996721982,-1
.11182953790152,0.266211449150386,0.861038667901814
"4153","VSIG10L2",2.08176029856251,-1.30305626901541,1.17198533656985,
-1.11183666583165,0.266208383857532,0.861038667901814
"4154","PDCD5",172.661775033507,-0.295738524662296,0.265820313575763,-
1.11255050708533,0.265901526818159,0.861038667901814
"4155","IDH3G",90.0994667156629,-0.314985845535064,0.283214076407085,-
1.11218287428027,0.266059529727923,0.861038667901814
"4156","SLC45A4",2.26410838095716,1.14469622602344,1.03001424288969,1.
11134019158027,0.266421945572558,0.861512157495093
"4157","TPM4",299.086654422397,-0.295861904113908,0.266303037871388,-1
.11099710494777,0.266569595446469,0.861782245178038
"4158","SLC16A1",29.9308008523976,0.328704954819675,0.295934471853364,
1.11073560562606,0.266682171419327,0.861938841198733
"4159","SCRN3",12.0907246136465,0.483345482645346,0.43554898047878,1.1
097385238143,0.267111717193648,0.862633723738017
"4160","RARS2",57.0305833447158,-0.254169872911611,0.229073698800339,-
1.10955502199816,0.267190822126261,0.862633723738017
"4161","PHF2",23.7921480107648,0.370211043307248,0.333626333438649,1.1
0965774041732,0.267146539742648,0.862633723738017
"4162","PCBP2",327.586910206288,-0.214920603637591,0.193710866124654,-
1.10949172825074,0.267218110865493,0.862633723738017
"4163","TBC1D15",52.2239916781419,0.299556184931992,0.269882098969282,
1.10995203489242,0.267019695983532,0.862633723738017
"4164","COP1",24.3829075429139,-0.353740278709873,0.319162419880105,-1
.10833938044071,0.267715274154205,0.862787906320949
"4165","KIF9-
AS1",1.7531721403201,1.25470517846828,1.13164554873476,1.108743970116,
0.267540647597497,0.862787906320949
"4166","ZBTB22",12.1341379227812,-0.531031527742888,0.478822317466234,
-1.10903671022046,0.267414345712401,0.862787906320949
"4167","CSTF2T",24.3925205785922,-0.43801808281783,0.395048781782716,-
1.10876960774618,0.267529584676786,0.862787906320949
"4168","RASA3",43.8721808937707,0.287450311003456,0.259338035648502,1.
10840012451184,0.267689051167631,0.862787906320949
"4169","DOHH",13.3133705793154,-0.464255491933697,0.418757985126403,-1
.1086486907075,0.267581764471132,0.862787906320949
"4170","APOL3",60.5252417480888,0.26378535635709,0.237962494805071,1.1
0851651884542,0.267638809105065,0.862787906320949
"4171","GASK1A",20.5778110241177,-0.479917964099551,0.43310528137997,-
1.10808615071704,0.267824611493233,0.86284545180632
"4172","SLC39A11",12.652215687301,-0.470362405700671,0.424514561090548
,-1.10800064076093,0.267861539172257,0.86284545180632
"4173","CLCA2",2.33525015418515,1.24458211425619,1.12408079897303,1.10
719986978984,0.268207523842399,0.862937091365702
"4174","ZFAND2B",30.4459399090526,-0.422705564705042,0.382117628638929
,-1.10621843386468,0.26863198598135,0.862937091365702

"4175","LARP1B",31.6099398435084,-0.375320923843586,0.339133575161197,
-1.10670529647555,0.268421364690632,0.862937091365702
"4176","AFF4",91.810040804117,0.247576297731846,0.223741800252684,1.10
652679764015,0.268498571772486,0.862937091365702
"4177","AIG1",49.8286850132106,-0.341502193170775,0.308692641153175,-1
.1062855009923,0.268602965380202,0.862937091365702
"4178","TMEM68",16.3839892536689,0.481361155061504,0.434689400740801,1
.10736805231773,0.26813483269359,0.862937091365702
"4179","KYAT1",2.63616139779608,1.00238181200483,0.905426385983658,1.1
0708261601614,0.268258210786948,0.862937091365702
"4180","MED22",14.253479695522,0.468382663098729,0.423169534564121,1.1
0684400657807,0.268361378178164,0.862937091365702
"4181","C10orf55",1.48420839708484,1.41079294270753,1.2746643561771,1.
10679563280383,0.268382296834387,0.862937091365702
"4182","CCNJ",2.98101627941879,1.00055607426801,0.903258673440729,1.10
771820264581,0.267983535612263,0.862937091365702
"4183","NOP10",152.266202656402,-0.301847634536284,0.272613483683303,-
1.1072366284235,0.268191635023479,0.862937091365702
"4184","PDXDC1",37.1374762135037,0.371215996650749,0.335617528130844,1
.10606856178866,0.268696844874295,0.862937091365702
"4185","SSH2",25.3637684204727,0.407061228502139,0.368046755936986,1.1
0600412022605,0.268724736019456,0.862937091365702
"4186","HSPA12B",30.6880992724746,0.323683934938969,0.292734397900063,
1.10572565868898,0.268845280644124,0.863117947103771
"4187","BBS7",25.4543001074034,-0.34187573378771,0.30928623309547,-1.1
0537003333796,0.268999283080138,0.863199943962266
"4188","RSF1",129.063442340542,0.228301512364819,0.206536315731921,1.1
0538193516122,0.268994128053845,0.863199943962266
"4189","CHEK2",15.4190161679186,-0.426852119355928,0.386234294225178,-
1.10516369400142,0.269088665436455,0.863280633755197
"4190","MAP7D1",56.9624284172811,-0.2561938688387,0.231857506788669,-1
.10496257976333,0.269175803994658,0.863354088277854
"4191","FBXO6",11.2043538895582,0.520615567378063,0.471596209565894,1.
10394349406941,0.269617650048647,0.86341834504067
"4192","NME7",22.3435459234157,-0.409722587694315,0.370873035505942,-1
.10475162243967,0.269267228165644,0.86341834504067
"4193","TARBP1",20.8198123257406,0.467911166646161,0.42443399274719,1.
10243565464105,0.270272317967181,0.86341834504067
"4194","LRCH3",44.5866276113109,0.33514306776798,0.303534139415878,1.1
0413632026015,0.269534008046961,0.86341834504067
"4195","CENPW",15.6780510612983,0.565329066544854,0.512571371979374,1.
10292750912277,0.270058646802855,0.86341834504067
"4196","CYHR1",55.4279611523894,-0.302383137895419,0.273858528214222,-
1.10415819389376,0.269524521069816,0.86341834504067
"4197","RCL1",8.1095806533463,-0.540088500145788,0.489757921704429,-1.
1027662365648,0.27012869397308,0.86341834504067
"4198","TM9SF3",182.359814873127,0.192171455420994,0.174143261562159,1
.10352507296069,0.269799209349568,0.86341834504067
"4199","CUTC",141.478760638959,-0.292833847347268,0.265623062813433,-1
.10244134769633,0.270269844130189,0.86341834504067

"4200","CCDC82",78.7827305002968,0.238780411777507,0.216322807307874,1
.10381524143994,0.26967329190915,0.86341834504067
"4201","CARD16",6.97021880256888,-0.569810563043288,0.516642877853583,
-1.10290993540953,0.270066279169805,0.86341834504067
"4202","LINC02328",4.8027727597778,0.859449289313383,0.779463501897686
,1.10261646276056,0.270193757929136,0.86341834504067
"4203","PDPR",20.4414749193796,0.432743355127837,0.392652074887334,1.1
0210382882099,0.270416535030596,0.86341834504067
"4204","MED1",33.9848500446456,0.436571504969744,0.395321123436152,1.1
0434651499278,0.269442852396244,0.86341834504067
"4205","RUNDC1",12.6290164773644,-0.485784677969428,0.44077036940804,-
1.10212643971926,0.270406706280468,0.86341834504067
"4206","RELCH",13.5161611403403,0.558500267421375,0.506719947112129,1.
10218725472394,0.270380271770948,0.86341834504067
"4207","KIF3B",27.4541632629916,-0.360883795476256,0.327066227981699,-
1.10339669645271,0.269854930684156,0.86341834504067
"4208","ZNF280C",4.59594836484527,0.787380823169151,0.713453095537282,
1.10361960456026,0.269758183314795,0.86341834504067
"4209","MT-
ND4L",491.857888637481,-0.35082927204543,0.317770803114653,-1.10403243
031377,0.269579070060716,0.86341834504067
"4210","COA7",7.14473540264875,-0.571035926750413,0.520118962373454,-1
.09789484341161,0.272250409923153,0.863808533967927
"4211","AC245297.2",2.33886281972635,-1.01498993937841,0.9262697157761
75,-1.09578227819733,0.273174065731356,0.863808533967927
"4212","NHEJ1",10.8141483323524,-0.498362391338333,0.454002759160355,-
1.09770784710652,0.272332082117758,0.863808533967927
"4213","IRS1",22.2976917072093,-0.380481136952874,0.3470492492699,-1.0
9633182539166,0.272933586729445,0.863808533967927
"4214","TADA3",70.4747930137252,-0.313729485941798,0.284925373526363,-
1.1010935321728,0.270855951705191,0.863808533967927
"4215","POMGNT2",25.8224912790761,0.378945960191756,0.345669421724415,
1.09626694285348,0.272961971450451,0.863808533967927
"4216","ZNF35",5.14263458307471,0.768042053924186,0.699372196559146,1.
09818785719949,0.272122467427521,0.863808533967927
"4217","NDUFB5",118.819264838287,-0.334686589478829,0.305255828484131,
-1.09641342850306,0.272897889982235,0.863808533967927
"4218","UBE2D3-
AS1",8.69785005284287,-0.568576374990452,0.517920990266965,-1.09780523
60793,0.272289544580164,0.863808533967927
"4219","ELOVL6",17.8412820145401,0.47202194929549,0.429021291708361,1.
10022965856986,0.271232071292954,0.863808533967927
"4220","SRP19",70.3470224418962,-0.269630804342483,0.244928174328411,-
1.10085662901708,0.270959060739699,0.863808533967927
"4221","DST",501.965478993646,0.320258866568572,0.290946155767093,1.10
07496068274,0.271005649582538,0.863808533967927
"4222","ZNF292",108.696033853258,0.305034945356276,0.278248785309518,1
.09626694332901,0.272961971242409,0.863808533967927
"4223","RWDD1",237.333941393133,-0.257403830844581,0.233740730550438,-
1.10123652920233,0.270793727113114,0.863808533967927

"4224","DLL1",1.77437573722259,1.27875128093896,1.16262122878158,1.09988640262408,0.271381619856454,0.863808533967927
"4225","AIMP2",17.2034667867948,-0.434422015851498,0.396259708712443,-1.09630630190249,0.272944752468473,0.863808533967927
"4226","SARAF",206.2252152006,-0.22766633439288,0.206739276997158,-1.10122439093182,0.270799008652985,0.863808533967927
"4227","RMRP",2.84278904163003,1.17348640545852,1.06900113817388,1.09774102529314,0.272317590044779,0.863808533967927
"4228","SNX30",9.47219381216057,0.595587017178314,0.541921100500597,1.09902902217342,0.271755407103077,0.863808533967927
"4229","CFAP70",2.38562435949177,1.15437603131618,1.04984109111802,1.09957215533147,0.271518579544778,0.863808533967927
"4230","LCOR",34.0474330857373,0.370161820303326,0.33660451344527,1.09969357366776,0.271465655681618,0.863808533967927
"4231","HPS6",7.11385537272765,-0.575577078967668,0.524475885184288,-1.09743287580405,0.27245220857958,0.863808533967927
"4232","LGR4",53.518510976716,-0.429520716323641,0.391042377335355,-1.09839940942075,0.27203012016421,0.863808533967927
"4233","BRMS1",29.0769294345271,-0.368973520039131,0.335561895794095,-1.099569184296,0.271519874649238,0.863808533967927
"4234","AVIL",5.28579926915197,0.929537652933085,0.845552326799581,1.09932599494036,0.271625897699726,0.863808533967927
"4235","HELLPAR",27.2862512085728,-0.52749135051752,0.479619214990263,-1.09981279738392,0.271413695285834,0.863808533967927
"4236","AC000403.1",2.66323150838404,-1.0760743306128,0.981674887926426,-1.09616161505952,0.27300805440395,0.863808533967927
"4237","RCOR1",25.1834110292513,0.349639235143876,0.318308056754332,1.09843036556792,0.272016608915039,0.863808533967927
"4238","ATP5MPL",228.156667646815,-0.262262904630488,0.238712358481227,-1.09865658526897,0.271917886065983,0.863808533967927
"4239","GLIS2",32.9838923365138,-0.399014699451813,0.363951772598167,-1.0963394864197,0.272930235327008,0.863808533967927
"4240","DDX28",4.23055586636367,-0.788539651170244,0.717685751037584,-1.09872552162311,0.271887806944066,0.863808533967927
"4241","TMEM231",4.92540158686457,-0.6984793342262,0.63713754122042,-1.09627716001208,0.272957501528428,0.863808533967927
"4242","MLYCD",18.9475453264942,0.436005560314899,0.397595485084709,1.09660591397814,0.272813701121697,0.863808533967927
"4243","RAMP2-AS1",48.4374341264711,0.428089935880629,0.39059714938103,1.09598837717841,0.273083860911029,0.863808533967927
"4244","STAP2",3.16602814249863,-0.982728528293255,0.89298114695852,-1.10050310876149,0.271112975869511,0.863808533967927
"4245","C20orf27",22.5748951363592,-0.39953333854059,0.363409801403696,-1.09940165894636,0.271592907491383,0.863808533967927
"4246","SMIM26",131.298245333369,-0.283527661167709,0.258159965057242,-1.0982634782463,0.272089454689745,0.863808533967927
"4247","PTGIS",411.985284036166,-0.319295173901488,0.290697876579757,-1.09837463437365,0.272040933915629,0.863808533967927
"4248","SLC2A4RG",123.084998713435,-0.326196891858838,0.29658501690239

1,-1.0998427879659,0.271400625783382,0.863808533967927
"4249","NHS",3.09141064204483,1.0506441570698,0.95868550039959,1.09592160998772,0.273113081155594,0.863808533967927
"4250","NUDT11",5.57328594262878,-0.677487332435014,0.618009313200014,-1.09624129922416,0.272973190545815,0.863808533967927
"4251","ZC3H12A",7.61763764974422,-0.615621037824251,0.562187424417047,-1.0950459065544,0.27349652524069,0.864106677613592
"4252","ANKRD36B",20.0168967602102,-0.421151559524781,0.384487089146773,-1.09535943185861,0.273359199705448,0.864106677613592
"4253","G3BP2",60.1627629623512,0.259742344653282,0.237329061373724,1.09443969124482,0.273762184092012,0.864106677613592
"4254","FRG1",102.167121442869,-0.313914397302129,0.28666542509917,-1.09505496588412,0.273492556550737,0.864106677613592
"4255","DERL1",60.2373915926702,-0.260544071696492,0.237997384557434,-1.09473502064313,0.273632741271135,0.864106677613592
"4256","PPFIBP2",5.27441825264733,0.770759372634385,0.704120719766251,1.09464094862917,0.273673968475988,0.864106677613592
"4257","AMBRA1",8.86930775080135,0.5962902844066,0.544583058616237,1.09494828194206,0.273539294900425,0.864106677613592
"4258","MRPL34",102.587994881996,-0.285799830522467,0.261149223459896,-1.09439280245976,0.273782739287051,0.864106677613592
"4259","SAMD11",1.82629471858571,-1.21585741596081,1.11295761899604,-1.09245616832885,0.274632646248568,0.86423016728202
"4260","TNFRSF1B",25.3045340970373,-0.334991711482643,0.309189568478638,-1.08345088461737,0.278608340209008,0.86423016728202
"4261","HP1BP3",279.671378622436,0.205364851047121,0.189534721366623,1.08352100114615,0.278577234346452,0.86423016728202
"4262","EBNA1BP2",49.7730488673868,-0.323960503866265,0.297612706362429,-1.08853048589851,0.276360985754308,0.86423016728202
"4263","ELOVL1",55.8387238707089,-0.299510259415184,0.274341623593065,-1.09174195112096,0.274946540327931,0.86423016728202
"4264","FUBP1",41.2394104435022,0.307779968529468,0.282609563104728,1.08906423812492,0.276125558836481,0.86423016728202
"4265","RPAP2",35.8554162890347,0.348425683033709,0.319810046158987,1.08947697928318,0.275943601166006,0.86423016728202
"4266","AC239809.3",4.73411229595413,0.841892371225155,0.773638828765961,1.08822403933379,0.276496214737741,0.86423016728202
"4267","SNAPIN",43.106916552458,-0.314188528347942,0.287294431610453,-1.09361161852923,0.274125351373078,0.86423016728202
"4268","PBXIP1",56.6813916116162,0.281747065245427,0.260237829983791,1.08265222340263,0.278962817810959,0.86423016728202
"4269","USF1",6.70689777841085,0.63857964346248,0.591792247093104,1.07906050915536,0.280560754280406,0.86423016728202
"4270","POU2F1",35.1631156180713,0.398833475109934,0.367039580217799,1.0866225241247,0.277203666492343,0.86423016728202
"4271","AL513217.1",1.64649549953792,-1.25266761609646,1.15200274721149,-1.08738249030103,0.276867806174155,0.86423016728202
"4272","NUCKS1",613.87887834428,0.15242418926436,0.140407328280795,1.08558571073679,0.277662324484264,0.86423016728202
"4273","IRF2BP2",235.564148159855,0.225912481509381,0.209655105689049,

1.07754342908512,0.281237559726155,0.86423016728202
"4274","NLRP3",5.79083080449016,-0.676323122191118,0.621748071893543,-1.08777679057591,0.276693657976148,0.86423016728202
"4275","THUMPD2",21.0299811612218,0.415476292095229,0.38362084955763,1.08303887177752,0.278791169600134,0.86423016728202
"4276","MSH2",14.2594886632714,0.546020144710432,0.5044234933266,1.08246374709772,0.279046515808042,0.86423016728202
"4277","ETAA1",18.7371881249457,0.410364767249449,0.376129194202541,1.09102078108958,0.275263738569137,0.86423016728202
"4278","SUCLG1",90.6872119433157,-0.295662052340936,0.270989370736156,-1.09104667661966,0.275252344400301,0.86423016728202
"4279","ACTR1B",27.3040449503445,-0.386966158037893,0.358998656451498,-1.07790419569488,0.281076512833835,0.86423016728202
"4280","MRPS9",25.2574954015755,-0.346115250694589,0.317199428479538,-1.09115975509053,0.275202593053044,0.86423016728202
"4281","CHCHD5",60.9344395753147,-0.290206953270242,0.267286518978547,-1.08575230198398,0.277588594228394,0.86423016728202
"4282","AC009404.1",5.70893263453245,0.747671330668671,0.688383689689168,1.08612586536772,0.277423310346156,0.86423016728202
"4283","RALB",46.3187027188114,-0.258146357626685,0.238903111068381,-1.08054832970675,0.2798980774199,0.86423016728202
"4284","AC007405.3",5.0952336837333,-0.755752672946986,0.69260115762583,-1.09118020469044,0.275193596459571,0.86423016728202
"4285","SATB2-AS1",3.1746536914737,-0.858649835037188,0.794305509373833,-1.08100702425453,0.279693989073519,0.86423016728202
"4286","SGO2",6.81886173286161,0.664616373481282,0.612774106711043,1.08460257410107,0.278097714455346,0.86423016728202
"4287","KCTD6",13.8123359295355,0.446174995977042,0.411966500920907,1.08303707942191,0.278791965130513,0.86423016728202
"4288","LINC02614",11.9940807324035,0.502325936630595,0.464159577897043,1.08222680420917,0.279151760931167,0.86423016728202
"4289","RPN1",95.1783479771395,0.243165812531646,0.224517483728655,1.08305958401676,0.278781976661723,0.86423016728202
"4290","SRPRB",47.8093905980321,-0.279932264894483,0.257136099478686,-1.08865408420682,0.276306456958583,0.86423016728202
"4291","CRIPAK",6.38006542614249,-0.619722835631795,0.566947393320755,-1.09308701818333,0.274355595233607,0.86423016728202
"4292","MFSD10",100.180432582425,-0.27695813713175,0.256883423610022,-1.07814717368531,0.280968082289437,0.86423016728202
"4293","PCDH7",15.4889279627265,0.75360613714894,0.697553054589372,1.08035673013082,0.279983356354308,0.86423016728202
"4294","AASDH",25.3892447188937,0.345374464227938,0.319509811357311,1.08095104422851,0.279718891014716,0.86423016728202
"4295","MOB1B",11.4592033156007,0.513132508306502,0.469364693537954,1.09324905637584,0.274284463573156,0.86423016728202
"4296","HPF1",68.5268201290406,-0.296014565543569,0.274476597065062,-1.07846923456793,0.280824404249776,0.86423016728202
"4297","GOLPH3",65.7249432522594,0.246552832301451,0.227586832581569,1.0833352242076,0.278659655911044,0.86423016728202

"4298","C6",4.04235639814829,-0.952496439757584,0.88003186459405,-1.08234312651504,0.279100089599661,0.86423016728202
"4299","PRR16",2.62169426043539,-1.09003555665642,1.0052456687545,-1.0843474292279,0.278210783330439,0.86423016728202
"4300","NME5",1.91750807597507,1.25094731596068,1.15968058532749,1.07869988666527,0.280721536228104,0.86423016728202
"4301","HBEGF",4.42713993146432,-0.891742015831376,0.824043036415249,-1.0821546647741,0.279183809133711,0.86423016728202
"4302","SLC22A23",6.44846332384092,0.630621861169225,0.57849712625457,1.09010370587687,0.275667464048243,0.86423016728202
"4303","HIST1H1E",17.5540317432843,0.523189979389286,0.485400247555271,1.07785272468307,0.28109948576074,0.86423016728202
"4304","HLA-E",673.486046053145,-0.200244207304493,0.184366128615351,-1.08612253676091,0.277424782798982,0.86423016728202
"4305","COL21A1",6.6225365161377,1.10961408375669,1.0210864811712,1.08669941696217,0.277169671767995,0.86423016728202
"4306","AL021368.2",16.319995634466,0.54577414464905,0.501868473930736,1.08748441673261,0.276822781791334,0.86423016728202
"4307","SMIM8",29.3739521799737,0.323520744337706,0.296000877621022,1.09297224703475,0.274405985190735,0.86423016728202
"4308","TOMM7",625.386177354892,-0.270244518520582,0.2479247492542,-1.09002638636733,0.275701520995952,0.86423016728202
"4309","GLI3",78.1643659903228,0.267386385693162,0.247612653175405,1.07985752046261,0.280205632194129,0.86423016728202
"4310","AC002456.1",3.33418793242628,-0.863217319599728,0.798884647983807,-1.08052811100862,0.279907075714323,0.86423016728202
"4311","RBM28",37.5679856681302,-0.351833228229377,0.322282626616155,-1.09169157494927,0.274968689590014,0.86423016728202
"4312","MKRN1",55.3170180931891,-0.240330225872095,0.221203910027129,-1.08646463727798,0.277273477994785,0.86423016728202
"4313","ESYT2",100.265694390524,-0.231869603018233,0.215250520569459,-1.07720809410731,0.281387310042316,0.86423016728202
"4314","LRRCC1",21.5871264910213,0.430643633075177,0.397289440756987,1.08395438915929,0.278385022451358,0.86423016728202
"4315","NFIB",498.888738463725,0.230206619064316,0.210645190065153,1.09286435162898,0.274453362136577,0.86423016728202
"4316","PRPF4",16.849042626795,-0.403778214130523,0.371913949743652,-1.08567644319025,0.277622166297107,0.86423016728202
"4317","URM1",57.222944008329,-0.287074589044333,0.263915293594715,-1.08775276011546,0.276704269226106,0.86423016728202
"4318","IER5L",75.4352966002061,-0.332915440547681,0.304694388039816,-1.09262084769404,0.274560305411776,0.86423016728202
"4319","MRPS2",29.705233203482,-0.352328247165836,0.324504178944989,-1.08574332790199,0.27759256565403,0.86423016728202
"4320","PAXX",46.8383098523757,-0.349572529088486,0.319886943689409,-1.09280024078726,0.274481515896716,0.86423016728202
"4321","CELF2",324.073184882991,0.239776999904255,0.21937712856639,1.09298996422816,0.274398206093103,0.86423016728202
"4322","EIF5AL1",4.12287913536843,-0.835271943116938,0.77341315117956,

-1.07998156204486,0.28015039080973,0.86423016728202
"4323","ARMH3",11.6301518305624,0.468468424918031,0.43454848369481,1.0
7805789801592,0.28100791885009,0.86423016728202
"4324","TAF5",4.96837915615667,-0.68264551048498,0.630425659612335,-1.
08283268625956,0.278882694405557,0.86423016728202
"4325","DOCK1",78.1869365999649,0.258770442509698,0.237800420493794,1.
08818328400076,0.276514202681561,0.86423016728202
"4326","MOB2",42.630864647664,-0.300912981034528,0.278476153064645,-1.
08057001550389,0.27988842641426,0.86423016728202
"4327","SLC22A18",10.5354766028576,-0.568434925648566,0.52351467762209
1,-1.08580513583786,0.277565213710867,0.86423016728202
"4328","LRRC4C",4.65421233334999,-0.76569151176711,0.702645353643963,-
1.08972685551279,0.275833482541168,0.86423016728202
"4329","TMEM258",309.606891921582,-0.254708352532416,0.232824956496092
,-1.09399076613461,0.273959028034974,0.86423016728202
"4330","EML3",13.4268585242425,-0.438517150295778,0.407067850890483,-1
.07725812622269,0.281364963800235,0.86423016728202
"4331","CLCF1",2.97125966289398,-0.876485832175869,0.806140572955203,-
1.08726177738802,0.276921135641831,0.86423016728202
"4332","FDXACB1",1.85284788314825,1.21044423290571,1.1156320011991,1.0
8498522057874,0.277928201174521,0.86423016728202
"4333","ADIPOR2",24.1767930471719,0.363910675877501,0.335333291852659,
1.08522083765366,0.277823857263618,0.86423016728202
"4334","RAD51AP1",1.86024099149285,-1.10663443814331,1.0245485372274,-
1.08011909434573,0.280089150042637,0.86423016728202
"4335","MFAP5",5413.0714986046,0.334299558770897,0.310202983959673,1.0
7768002262143,0.281176576749175,0.86423016728202
"4336","KMT2D",13.293037903163,0.500471193511211,0.463622985343624,1.0
7947882079288,0.280374330244348,0.86423016728202
"4337","TUBA1B",526.28356336597,-0.332160758978597,0.307633516316378,-
1.079728772586,0.280262977332854,0.86423016728202
"4338","TMBIM4",377.359191943556,-0.271951596315532,0.249829480019866,
-1.08854886258382,0.276352877909446,0.86423016728202
"4339","POP5",35.2078007697999,-0.30573020567325,0.28311714769312,-1.0
7987173565566,0.280199301143708,0.86423016728202
"4340","CHAMP1",19.2045358854636,0.40273913163916,0.368127782498249,1.
09401993217145,0.273946236422177,0.86423016728202
"4341","NOP9",4.71121650450094,0.76796922486648,0.707366599917555,1.08
567357429088,0.277623436011508,0.86423016728202
"4342","ARMH4",19.9103006525127,0.388028231229192,0.358807952066824,1.
08143710024834,0.27950272596769,0.86423016728202
"4343","SLC39A9",24.7064214448131,0.330876325112809,0.306480051512714,
1.0796015058066,0.2803196706165,0.86423016728202
"4344","ELMSAN1",47.5381911686241,0.301094185418083,0.27754329988827,1
.08485481558839,0.277985962985232,0.86423016728202
"4345","GSKIP",13.1809097162536,0.48560682664801,0.44774398870917,1.08
456358743754,0.278114989587934,0.86423016728202
"4346","RASGRP1",2.0821465139769,1.24036517905726,1.1505427824338,1.07
806958419525,0.281002704028783,0.86423016728202
"4347","TLN2",74.7521412013692,0.273003265130618,0.252940465623564,1.0

7931826747292,0.280445872204871,0.86423016728202
"4348","C15orf39",8.97789802894236,-0.49979769943453,0.462877836428607
,-1.07976157011704,0.280248368343196,0.86423016728202
"4349","METRN",26.455899988721,-0.383854448403697,0.354048112457032,-1
.08418724715072,0.278281784898902,0.86423016728202
"4350","AC093525.4",2.3788764811196,1.09592272188624,1.01078195944831,
1.08423257028093,0.278261693926353,0.86423016728202
"4351","PAM16",31.1991482746238,-0.338299472957673,0.311047591556298,-
1.08761322106698,0.276765891659516,0.86423016728202
"4352","AC137932.3",2.54980154504829,1.06044197218894,0.97932375805596
8,1.0828308447188,0.278883511949129,0.86423016728202
"4353","FAM222B",13.2649224009747,0.467146452706439,0.430078162009628,
1.086189660325,0.277395090839836,0.86423016728202
"4354","ZNHIT3",45.8304046154653,-0.330292223062891,0.306153798043488,
-1.07884411421208,0.280657225505802,0.86423016728202
"4355","FAM215B",10.6364734541186,-0.517284460999732,0.476378444975391
,-1.08586873830207,0.277537069549107,0.86423016728202
"4356","ITGB3",23.9384009038571,0.469765688304764,0.432326698660478,1.
08659883777774,0.277214138922092,0.86423016728202
"4357","SNF8",115.584836535105,-0.29537851621912,0.271622414947163,-1.
08746001789498,0.276833559136148,0.86423016728202
"4358","ATP5PD",259.122953797147,-0.258783164195513,0.23977623379701,-
1.07926945092729,0.280467627133494,0.86423016728202
"4359","AFMID",18.0350666843136,-0.456206989276525,0.422964457598654,-
1.07859414917888,0.280768690680341,0.86423016728202
"4360","INO80C",12.5941218408186,-0.435000491955981,0.4038413362518,-1
.07715692502749,0.281410165341627,0.86423016728202
"4361","ADNP2",13.1892765035358,0.488865215637507,0.451673019994462,1.
08234318632426,0.279100063033584,0.86423016728202
"4362","AP3D1",42.5494357143648,0.279939617128473,0.259685951794451,1.
07799291873152,0.281036916296589,0.86423016728202
"4363","DOT1L",6.79417945106351,-0.586161410900715,0.53707411836544,-1
.09139761320965,0.275097962205023,0.86423016728202
"4364","NDUFA13",432.975547528435,-0.292421955795161,0.270872331782939
,-1.07955638684238,0.280339771543779,0.86423016728202
"4365","SUPT5H",47.5336723038973,0.320218184461181,0.294198532673774,1
.0884424934106,0.27639981053322,0.86423016728202
"4366","SERTAD3",21.3898406969682,-0.464320452454569,0.429813650397262
,-1.0802831692884,0.280016102193368,0.86423016728202
"4367","WFDC3",7.26260397817888,-0.760519148881752,0.697660286322234,-
1.09009952808248,0.275669304169241,0.86423016728202
"4368","JAM2",13.8486688210698,-0.489892627530541,0.449809878842773,-1
.08911042325457,0.27610519397544,0.86423016728202
"4369","NIPSNAP1",12.7727481613759,0.515753531893532,0.474442523248443
,1.08707273614987,0.277004665780432,0.86423016728202
"4370","XPNPEP3",18.5636593381065,0.448746455886299,0.412689391545788,
1.08737095035434,0.276872904076871,0.86423016728202
"4371","CHST7",57.1736615263696,0.516211233140055,0.476179370533267,1.
08406887211842,0.278334263193453,0.86423016728202
"4372","GPR173",4.21846828540964,0.828442835054556,0.768069644133657,1

.07860379769207,0.280764387628057,0.86423016728202
"4373","TCEAL8",145.436353125675,0.254596826761387,0.234912208749223,1
.08379563632292,0.278455420435645,0.86423016728202
"4374","ZBTB33",12.0704930310879,0.513404731482438,0.472180761111428,1
.08730548503072,0.276901825339034,0.86423016728202
"4375","VAMP7",27.1998543346543,0.351907694419621,0.326662611024028,1.
07728182700939,0.281354378549547,0.86423016728202
"4376","AC009509.1",1.72022658108114,3.50099029542444,3.24188963899614
,1.07992272571886,0.28017659239156,0.86423016728202
"4377","NIBAN1",139.064428790046,0.270248756108409,0.250974559691257,1
.0767974110239,0.281570782216087,0.864525872104636
"4378","SPSB1",62.243047810408,-0.276898627639871,0.258156308325245,-1
.07260066366851,0.283450326859802,0.864629320095251
"4379","SPEN",57.4390254156465,0.373150365667917,0.347079352683125,1.0
7511542470979,0.282323054443659,0.864629320095251
"4380","RPS8",3367.59517377883,-0.259510356076203,0.242046687227053,-1
.07215000151094,0.283652663366101,0.864629320095251
"4381","WARS2-
AS1",9.31015189861128,0.570363546168236,0.532351069926138,1.0714049024
9672,0.283987409458742,0.864629320095251
"4382","DDX59",20.7319052286393,-0.386085647728598,0.360434574491955,-
1.07116707178494,0.284094314496211,0.864629320095251
"4383","CENPF",6.87955022018846,-0.591651713462007,0.550721179947277,-
1.07432169853836,0.282678523139119,0.864629320095251
"4384","HSPD1",116.052705571275,-0.204051396295222,0.190502104327901,-
1.0711241065558,0.284113630295456,0.864629320095251
"4385","DUSP28",6.9131650438456,-0.632195268411589,0.58865411888493,-1
.07396728932966,0.282837342530533,0.864629320095251
"4386","TOP2B",62.6198559064896,0.245569912772566,0.228652488889996,1.
07398749064441,0.282828288204852,0.864629320095251
"4387","MAP4",127.93105398492,-0.260057294859992,0.242345461881597,-1.
07308506146919,0.283232952893714,0.864629320095251
"4388","TMCC1",19.0323621566124,0.377245026248995,0.351306488776134,1.
07383449580799,0.282896866110572,0.864629320095251
"4389","PDGFRA",582.76078448683,0.251857085090859,0.234536865801782,1.
0738486004316,0.282890543428657,0.864629320095251
"4390","SLC9B1",2.42173267921459,1.01667264966771,0.946837855136954,1.
07375581167554,0.282932139616632,0.864629320095251
"4391","HADH",32.608814008273,-0.342393593968194,0.318956797095284,-1.
07347953417625,0.283056016350177,0.864629320095251
"4392","SREK1",90.5746395381643,0.285027966815685,0.265156158415177,1.
07494379357161,0.282399893414617,0.864629320095251
"4393","LRP11",48.7668463767994,0.270049222405452,0.25141975868901,1.0
7409705511445,0.282779184308409,0.864629320095251
"4394","AC004130.2",9.33238054664558,-0.595744931001891,0.556120906884
664,-1.07125073635371,0.284056704120388,0.864629320095251
"4395","ZNF138",20.5465959293613,0.395993833971548,0.367842549724441,1
.07653079902854,0.281689934247295,0.864629320095251
"4396","CAPZA2",104.81324532187,-0.240399674197484,0.223497191380435,-
1.07562727170149,0.282093985592868,0.864629320095251

"4397","RB1CC1",128.480393218665,0.27240782833306,0.254129565942806,1.07192497387088,0.283753731986856,0.864629320095251
"4398","KLHL9",33.3497623079288,0.384352460395696,0.357396963889918,1.07542172774047,0.28218595832159,0.864629320095251
"4399","TSC1",30.813544636212,0.416925318498544,0.389236357778851,1.07113662474312,0.284108002425175,0.864629320095251
"4400","PSMA1",148.968368807806,-0.260787032417918,0.242933650049694,-1.07349077562772,0.283050975214303,0.864629320095251
"4401","TRPV4",1.71774084416371,-1.23982890369893,1.15590822290097,-1.07260150861056,0.283449947592916,0.864629320095251
"4402","SRSF9",181.817801094997,-0.283283412803191,0.264241251335637,-1.07206354560957,0.283691491172655,0.864629320095251
"4403","USPL1",10.3793471846903,0.522491728972339,0.487397354671727,1.0720036207916,0.283718405839886,0.864629320095251
"4404","TPP2",39.4395617745239,0.330892764525413,0.308567766926108,1.07235038779877,0.283562682652819,0.864629320095251
"4405","CCNB1IP1",32.7258808396738,-0.319904898614187,0.298022490913568,-1.07342535670224,0.283080312643108,0.864629320095251
"4406","KTN1",184.674908626983,-0.241849526086342,0.225209588719349,-1.07388645155659,0.282873576372048,0.864629320095251
"4407","CDC42BPB",80.2379576474238,0.298725221692391,0.277593016271285,1.07612657445407,0.281870652197262,0.864629320095251
"4408","C16orf91",19.1242322969206,-0.41731421087612,0.389123475783804,-1.07244676008183,0.283519414886246,0.864629320095251
"4409","GALNS",17.7915016194825,0.552304488391475,0.514148383280288,1.07421224368683,0.282727566068983,0.864629320095251
"4410","KRT10",331.982088944229,-0.261905493322832,0.243929977599169,-1.07369129411884,0.282961064576829,0.864629320095251
"4411","TIMP2",1691.0684125806,0.175653093407321,0.163434394657362,1.0747621011818,0.282481252239613,0.864629320095251
"4412","TNPO2",24.2143973756736,0.388631411784532,0.362064833875473,1.07337519533365,0.283102809143332,0.864629320095251
"4413","CEBPA",12.1703499604962,-0.499940100231556,0.466554586344842,-1.07155757303399,0.28391879832133,0.864629320095251
"4414","ZNF607",2.50720504279105,1.08693294790569,1.01359404143903,1.07235530544609,0.283560474693813,0.864629320095251
"4415","EIF2S2",180.255172369336,-0.188124021232602,0.175526861971848,-1.071767700506,0.283824384016087,0.864629320095251
"4416","TMSB15B-AS1",3.04662926626723,-0.894523810308462,0.832089172641374,-1.07503358981213,0.282359690020888,0.864629320095251
"4417","LDLRAD2",2.97993450932942,-1.0172090451915,0.953307696373894,-1.0670311894687,0.285957752101772,0.864731200290855
"4418","GALE",3.09656555588782,-0.942408043602242,0.883465273282153,-1.0667176991588,0.286099332332516,0.864731200290855
"4419","FGGY",6.78176747795873,-0.694169699171002,0.649022958507561,-1.0695610841984,0.284816919828742,0.864731200290855
"4420","RMDN2",9.11219015214904,0.55748076085083,0.52253236114998,1.06688274698229,0.286024786617562,0.864731200290855
"4421","PTCD3",33.7194534478231,0.32003419972164,0.299402514303238,1.0

6890952624902,0.285110438989602,0.864731200290855
"4422","ZNF445",17.0062797996102,0.426998364823672,0.398826833352812,1
.07063599817001,0.284333130702899,0.864731200290855
"4423","TEX264",81.4592723842891,-0.335038124570268,0.313601779496608,
-1.06835530432279,0.285360270373293,0.864731200290855
"4424","FNIP2",35.4721869810686,-0.32609086377254,0.305603178965066,-1
.06704015605092,0.285953703260786,0.864731200290855
"4425","RFESD",2.16930432704871,1.22272787193433,1.14366238818249,1.06
913358747199,0.285009478986001,0.864731200290855
"4426","HRH2",3.63747544958394,0.978680233638491,0.915986521706285,1.0
6844392406061,0.285320312553727,0.864731200290855
"4427","PAK1IP1",31.0647287248774,-0.32399541288424,0.303602590027285,
-1.06716946273457,0.285895319412227,0.864731200290855
"4428","MFSD4B",15.148826051391,0.481665451408661,0.45078150392566,1.0
6851201128273,0.285289615225414,0.864731200290855
"4429","FAM120AOS",57.2385735002271,-0.257212066229407,0.2404963682605
28,-1.06950499123866,0.28484218100035,0.864731200290855
"4430","TMOD1",3.058074540636,-0.892506384439596,0.836907391419654,-1.
06643386543119,0.286227559738733,0.864731200290855
"4431","DHTKD1",11.0675064144988,0.534340088332225,0.501097830262198,1
.06633885852715,0.286270489626759,0.864731200290855
"4432","AP006621.3",10.2842297304711,0.551886582006175,0.5173040040419
58,1.06685155671328,0.286038873049256,0.864731200290855
"4433","IFT46",13.4889153054891,-0.435786817574556,0.40701573961429,-1
.0706878755783,0.284309796188757,0.864731200290855
"4434","PARPBP",2.09069751731986,1.19984780616722,1.12253298563586,1.0
688753217239,0.285125853373444,0.864731200290855
"4435","ZNF664",25.6456242299276,0.367019429336103,0.34378817936928,1.
0675743127918,0.285712575818273,0.864731200290855
"4436","KLF5",3.28529112751381,-0.929161674104436,0.869325746433712,-1
.06883027210018,0.285146155997228,0.864731200290855
"4437","EXD2",9.16060789392623,0.520596992895992,0.486361382816002,1.0
7039130015169,0.284443213610046,0.864731200290855
"4438","NUMB",59.9075878011989,0.290996518031388,0.271929317136841,1.0
7011822445371,0.284566096901921,0.864731200290855
"4439","TRIP11",111.151229311418,0.217093642671781,0.202936805788679,1
.06975982906641,0.28472742831223,0.864731200290855
"4440","SUPT4H1",87.4268578461585,-0.250471596017454,0.234874930408432
,-1.06640413083636,0.286240995166545,0.864731200290855
"4441","MBD1",21.7527805741453,-0.398710878441261,0.373697640194515,-1
.06693442921857,0.286001446449909,0.864731200290855
"4442","ZCCHC2",14.6142247001797,-0.462154243199848,0.432095027924941,
-1.06956621421742,0.284814609627097,0.864731200290855
"4443","SLC27A1",27.9731126648356,0.343472287363387,0.321822573941552,
1.06727220268199,0.285848936574799,0.864731200290855
"4444","AKT2",70.7102564776333,0.251897526262546,0.235881863984058,1.0
6789696336964,0.285566992451965,0.864731200290855
"4445","ZNF586",4.87308442335765,0.780528075743361,0.730956642249554,1
.06781720095086,0.285602977435388,0.864731200290855
"4446","BLCAP",42.0369957427677,0.302197615654167,0.282285791024205,1.

07053782111284,0.284377294396344,0.864731200290855
"4447","RPL3",2760.46234999753,-0.290008090524862,0.271859144778102,-1.06675863621058,0.286080841426352,0.864731200290855
"4448","DLG3",4.19674389132058,-0.744145518650383,0.697637189148118,-1.0666654963722,0.286122913043383,0.864731200290855
"4449","FGF16",16.7381494851054,-0.429927530724212,0.402613888416865,-1.06784078516205,0.285592337049628,0.864731200290855
"4450","PCDHB15",1.71927453605772,-1.19990980885674,1.12547481552526,-1.0661365250512,0.28636193067171,0.864813030628564
"4451","NEK1",37.7796409319649,0.290601399600606,0.27268909147844,1.06568765924977,0.286564858078738,0.865077498899486
"4452","BAD",120.145586994314,-0.292728204299738,0.274692432853149,-1.06565805711958,0.286578244296489,0.865077498899486
"4453","HERC3",20.2138843142401,-0.380385304022407,0.357009842735232,-1.06547567738773,0.2866607265553,0.865132159033613
"4454","PLEKHA8",13.0925699084966,0.518749562609454,0.486989144020327,1.06521791908323,0.286777326533746,0.865289737603731
"4455","DHX57",16.7580383728701,0.422496136443083,0.396794485781637,1.06477320522944,0.2869785733155,0.865702591871382
"4456","DEF6",1.70839285736947,-1.12984308197204,1.06142885793627,-1.06445483700979,0.287122703330947,0.865748712152703
"4457","ATP5MG",367.341537172363,-0.291383904833849,0.273735781861043,-1.06447137766507,0.287115213927854,0.865748712152703
"4458","TRIM25",47.0199526959784,0.313511309111691,0.29459468882267,1.06421236025883,0.287232508925121,0.86588552881218
"4459","ZBTB14",14.1305127378815,0.416779300895338,0.391722664528481,1.06396524540396,0.287344444041523,0.865978498879776
"4460","ATP6V1E1",73.164806192614,0.220685134351475,0.207438177532107,1.0638597821142,0.287392224496153,0.865978498879776
"4461","MARK1",7.84390115746075,0.584432537939941,0.549574218564686,1.06342786505942,0.287587961731471,0.866374045664479
"4462","PSMB5",163.070568976657,-0.308780641416917,0.290454794072802,-1.06309363012105,0.28773949286312,0.866636271758734
"4463","PILRA",2.38678881308797,1.09688115621564,1.03261742283525,1.06223382635163,0.288129547077152,0.866645699008469
"4464","VWCE",3.42866913333024,0.942867412497577,0.887539879613675,1.062338080975,0.288082232466913,0.866645699008469
"4465","POLD4",124.359985997338,-0.278571732982197,0.262177489146877,-1.06253108872416,0.287994652230289,0.866645699008469
"4466","EPSTI1",32.6726483177432,0.4442274551993,0.418178149010971,1.06229236570571,0.288102979104566,0.866645699008469
"4467","FUS",260.643059552841,0.302782202630815,0.284939383988708,1.06261969964396,0.287954449676867,0.866645699008469
"4468","XRN2",95.7178690718388,-0.203646879927502,0.191701018700652,-1.06231506388343,0.288092678025012,0.866645699008469
"4469","PNRC1",467.448075762667,0.223609327821394,0.210547333482657,1.06203827957675,0.288218307579577,0.866718692226882
"4470","SRP9",177.372674717272,-0.217088420636581,0.204516636311866,-1.06147071725522,0.288476033810895,0.867235340470253
"4471","TGFBR1",47.1625206505401,0.345718632009712,0.325731299823727,1

.06136141106735,0.288525686851234,0.867235340470253
"4472","PSMA3",110.881973380633,-0.294349905783836,0.277365772998947,-1.06123370090424,0.288583707313265,0.867235340470253
"4473","PCBP4",16.2463535962039,-0.437035843158599,0.411993990715351,-1.06078208179631,0.288788947085982,0.867344220402517
"4474","DACT1",3.15258201369163,-0.855475694651288,0.806498687105038,-1.06072794454515,0.288813556537039,0.867344220402517
"4475","TSIX",14.1835515807162,0.71630333863468,0.675182408945104,1.06090343756707,0.288733786898443,0.867344220402517
"4476","THNSL1",2.60344016385294,1.06378361953537,1.00316712442102,1.06042512123723,0.288951238560686,0.867370045793402
"4477","MYDGF",247.571406156923,-0.285693608934434,0.269391365190315,-1.06051509383978,0.288910326887482,0.867370045793402
"4478","CUL7",11.7852608922582,0.462594384651255,0.436352803270217,1.06013845031904,0.289081617463289,0.867567632221784
"4479","AL133330.1",5.46486405094593,-0.861080577644129,0.81237088895885,-1.05995991405811,0.289162836384207,0.867617628525867
"4480","ADSL",36.6092348708508,-0.346722144501994,0.3271818001618,-1.05972320077257,0.289270544638994,0.867747064598981
"4481","ATP5MD",240.399958114329,-0.280969751228252,0.265256967572737,-1.05923608265335,0.289492276037178,0.868218410547564
"4482","ANXA4",297.900509333231,-0.277143696790131,0.262200799974871,-1.05699027926952,0.290516023863076,0.868483298778516
"4483","MASP1",18.8006152513288,-0.713120882814421,0.674598455031045,-1.05710423363126,0.29046401926034,0.868483298778516
"4484","RNF4",28.5783964564906,0.314144421493775,0.297048875235806,1.05755129099344,0.290260059124282,0.868483298778516
"4485","EXOC1",65.4659642547586,0.30833874614285,0.291446309464407,1.0579607156786,0.290073352648725,0.868483298778516
"4486","GTF3C6",83.1475307809806,-0.282322240032324,0.26711891969519,-1.0569159247667,0.290549959915783,0.868483298778516
"4487","PTAR1",34.2719471763255,0.374424358837269,0.353955855828421,1.05782784116099,0.290133937428428,0.868483298778516
"4488","ZFAND5",166.530810713764,0.33909761258361,0.320794429605948,1.05705580050173,0.290486121603762,0.868483298778516
"4489","RABEPK",25.9829419554366,-0.375793316297574,0.355528019009571,-1.05700056311865,0.290511330433323,0.868483298778516
"4490","AC022613.3",1.70344468741217,1.34857432680649,1.27444791527542,1.05816354724473,0.289980887035942,0.868483298778516
"4491","BMF",3.40297848218995,0.8458722902622,0.799028738213137,1.05862561608713,0.289770316013989,0.868483298778516
"4492","ELL3",2.61864808532742,0.967895120298112,0.915135669477203,1.05765205376712,0.290214101627911,0.868483298778516
"4493","ORAI3",95.2099956626062,0.353096310554669,0.333995576101503,1.05718858517863,0.290425528333946,0.868483298778516
"4494","AC087500.1",3.69054547703299,0.855912547151172,0.809376104915228,1.05749668411673,0.290284967147696,0.868483298778516
"4495","PRKCA",9.31267356707555,0.595514284101622,0.56252002192623,1.05865437831423,0.289757212082576,0.868483298778516
"4496","ARMCX6",53.1892744293568,0.323580436407239,0.306056869338564,1

.05725591817804,0.290394805699784,0.868483298778516
"4497","DVL1",26.5604636790544,-0.332038634568888,0.3142020129822,-1.05676800545418,0.290617479528693,0.868486002253401
"4498","OPN3",6.08087686451421,-0.63990684489234,0.605610623953129,-1.05663081125516,0.290680112964938,0.868486002253401
"4499","SGMS1-AS1",4.29210833381688,0.867455934213281,0.821298538072489,1.05620050931677,0.290876618140398,0.868879944696335
"4500","RPS9",2448.8952912717,-0.302456154335252,0.28659637175939,-1.05533839273156,0.291270588073801,0.86986342958307
"4501","MTHFR",22.4608202627182,0.360838720819135,0.342062381826498,1.0548915636159,0.291474921084773,0.869867947538383
"4502","TRIM45",1.64234342230199,1.2681691310137,1.2018904834729,1.05514533017125,0.291358862923354,0.869867947538383
"4503","GMPPA",24.5012348313279,-0.375381712482932,0.355985484814856,-1.05448600714201,0.291660463695807,0.869867947538383
"4504","SUCLG2",76.4373801339145,-0.240094850988044,0.227634067478085,-1.0547404158262,0.291544062116683,0.869867947538383
"4505","RPL35",1699.18088007287,-0.254273834081282,0.241126982188426,-1.05452252491006,0.291643753518361,0.869867947538383
"4506","ITGA5",67.4882445156173,-0.331756744745717,0.3145576633883,-1.05467703813716,0.291573056881485,0.869867947538383
"4507","CSNK1G1",24.7105465617933,0.414010059294161,0.392688384153887,1.05429667899705,0.291747108740046,0.869933302497777
"4508","WNT9A",6.34548333519619,-0.724504037891697,0.687383815965896,-1.05400217616942,0.291881920811379,0.870087330559728
"4509","RPL10A",1888.38465017619,-0.288996294172387,0.27428938242663,-1.05361823201338,0.29205773853374,0.870087330559728
"4510","LINC00824",2.44758896621435,-1.02746362771041,0.975144566764221,-1.05365262006206,0.292041988477691,0.870087330559728
"4511","LRP6",66.9402523006728,0.32386775674406,0.307344733454439,1.05376055448847,0.291992557181923,0.870087330559728
"4512","SLC2A5",3.78071698474917,1.13016166938477,1.07290607902632,1.05336496034249,0.292173756967084,0.870240053165037
"4513","CEP192",16.63608716406,0.473945722957898,0.449996915530397,1.05321993685062,0.29224020312063,0.870245089682727
"4514","NIT2",44.0226423618839,-0.361642150384255,0.343662313005533,-1.05231832731811,0.292653525870248,0.870388638806761
"4515","ARHGAP31",5.37153627333525,-0.652266517105496,0.61953272113476,-1.05283626651838,0.292416040261486,0.870388638806761
"4516","DBR1",14.8073696525284,-0.412397761094186,0.391741004974375,-1.05273064564983,0.292464459058935,0.870388638806761
"4517","AL353135.2",3.01196401521835,-0.919626284289939,0.873938284826517,-1.05227829042012,0.292671888989425,0.870388638806761
"4518","AP003071.4",2.15682399807019,-1.03184648410869,0.980593659779597,-1.05226713819525,0.292677004149695,0.870388638806761
"4519","CNTROB",7.35175302674475,0.612332458948484,0.581686673247189,1.05268435243706,0.292485682525139,0.870388638806761
"4520","PSMA3-AS1",52.0175863072926,0.315194418229921,0.299728268432018,1.0516005710

0657,0.292982845705041,0.870965550441048
"4521","HSCB",25.6650934316773,-0.382832182506759,0.364060543759698,-1
.05156185988518,0.29300061414867,0.870965550441048
"4522","COPA",133.268534604563,0.218902428158531,0.208340916018688,1.0
5069341318868,0.293399422191754,0.87107808578178
"4523","NOL8",39.7175456374133,0.345995454281054,0.329321080804411,1.0
5063257243027,0.293427375127176,0.87107808578178
"4524","ZCCHC8",24.2535918278282,0.381620523950346,0.363065444501181,1
.05110670742752,0.293209583833938,0.87107808578178
"4525","SCPEP1",234.64057626176,0.231636493469102,0.220463360146808,1.
05068022783856,0.293405479973133,0.87107808578178
"4526","HDHD5",15.4905910483985,-0.533063568060817,0.507222058554546,-
1.050947132662,0.293282871506428,0.87107808578178
"4527","PNPLA4",12.186760015304,-0.467585213313521,0.444940761883281,-
1.05089318257647,0.293307651864732,0.87107808578178
"4528","SYTL4",12.2466313362883,0.46063149900818,0.438514786830151,1.0
5043549919468,0.293517931556172,0.871154479280785
"4529","LOXL3",6.3223133562633,-0.656992404291736,0.62652935762863,-1.
04862189822741,0.294352173156536,0.871344073435553
"4530","DNAJC1",91.5708015302846,-0.212978282412278,0.203106259225262,
-1.04860521396373,0.294359855152721,0.871344073435553
"4531","AL353801.3",1.57058360467282,-1.15807801851285,1.1040205063424
7,-1.04896422834524,0.294194582524961,0.871344073435553
"4532","EIF4EBP2",113.35564176971,0.260468857883671,0.24819507549143,1
.04945215922572,0.293970062817109,0.871344073435553
"4533","EI24",86.2423240671813,-0.277730001959019,0.264808192469365,-1
.04879686451222,0.294271620888884,0.871344073435553
"4534","ACVR1B",14.6682328811245,0.437836923777353,0.417044933341894,1
.04985551621224,0.293784546275531,0.871344073435553
"4535","NIN",50.9240987399298,0.30795914528313,0.293568464764704,1.049
01984458705,0.294168985099494,0.871344073435553
"4536","NUTF2",45.4619822787612,-0.283784604467471,0.27030612146463,-1
.04986377271003,0.293780749673319,0.871344073435553
"4537","KCNJ12",16.0944989173279,0.455929446216147,0.434288710687069,1
.04983029721137,0.293796142983897,0.871344073435553
"4538","ZNF528",8.87731495290292,0.558571214869682,0.532521460565761,1
.04891775493188,0.294215973100695,0.871344073435553
"4539","RPS6KA3",44.3336637049011,0.322337026553101,0.307065918447701,
1.04973234471152,0.293841188385344,0.871344073435553
"4540","AC007325.4",7.05673937734138,0.71070180494174,0.67748632036656
4,1.04902753543009,0.294165445499582,0.871344073435553
"4541","FKBP1B",6.1760896739985,-0.640257666527212,0.611203043594918,-
1.04753677724084,0.294852079251753,0.871649162574638
"4542","PPM1G",88.7006442807194,-0.225447389970717,0.215185683168417,-
1.04768768373065,0.294782523867777,0.871649162574638
"4543","CD24",12.1990585373812,-0.546276316037456,0.52120398466661,-1.
04810464253623,0.294590397582229,0.871649162574638
"4544","AC009061.2",7.44717478863479,0.599505091319798,0.5722785144260
89,1.04757574538861,0.294834117112358,0.871649162574638
"4545","XYLT2",13.8820567442297,-0.401859309328932,0.383443229626471,-

1.04802817804451,0.294625624606387,0.871649162574638
"4546","RBM42",62.0199855802443,-0.264501101974809,0.252486369429979,-1.04758566797865,0.294829543469744,0.871649162574638
"4547","ZNF148",81.9809812255079,0.247110112933775,0.235959738785954,1.04725540977961,0.294981795695414,0.871840851627594
"4548","AL645933.2",2.78191940026557,-0.874942467464724,0.835838214966216,-1.04678447551012,0.295198992048462,0.872280936693645
"4549","WDR45",83.2912369046382,-0.280872395545468,0.268353404905003,-1.04665113395858,0.295260509042294,0.872280936693645
"4550","ZNF26",11.4393471360265,0.501504377297321,0.479235683598147,1.0464671026414,0.295345425812154,0.87234003900869
"4551","C1orf21",245.367136148071,0.233601911290334,0.223373920823255,1.04578865083884,0.295658622166297,0.872881419879803
"4552","IQGAP2",2.01996264731135,-1.14551317624006,1.09531170943893,-1.04583304128725,0.295638123233934,0.872881419879803
"4553","CBR1",115.095698674372,-0.266238655621355,0.254678219804646,-1.04539232222361,0.29584168374992,0.873230043469181
"4554","KPNB1",112.864412087586,0.254268134665477,0.243320281504415,1.04499359072484,0.296025931762561,0.873582015142087
"4555","MIOS",7.76457595150785,-0.525881925661068,0.503421124080924,-1.04461632717767,0.296200330444561,0.873763478309448
"4556","ACVRL1",113.795970694181,0.269771803518252,0.258293577052365,1.04443868328774,0.296282474191246,0.873763478309448
"4557","TBCD",29.7134051876205,0.309077273364308,0.295922267419082,1.04445426178962,0.29627526997597,0.873763478309448
"4558","CD55",812.79239647151,-0.257323576603123,0.246412287104768,-1.04428062263679,0.296355575329015,0.873787313919842
"4559","CLHC1",5.19815290572872,0.781804177078672,0.749030556135706,1.04375471824814,0.296598886612581,0.874312883787338
"4560","EYA3",13.5796659094115,0.455937849072762,0.438992931790248,1.03859952189529,0.29899102825472,0.874447842349716
"4561","AL512353.1",1.53057677053346,1.23053540445247,1.18605855826559,1.03749970511736,0.299503033070391,0.874447842349716
"4562","AL445426.1",14.7006612611407,0.440397735853814,0.423495235337019,1.03991190244052,0.298380832135369,0.874447842349716
"4563","RIT1",20.953453892077,0.393619804433424,0.378343062464303,1.04037801531134,0.298164311534463,0.874447842349716
"4564","FMO1",2.53234940465755,1.2629562993238,1.21575214986465,1.03882711576073,0.298885148007017,0.874447842349716
"4565","ODC1",14.900557301317,-0.459430849087866,0.442323429922418,-1.03867626720214,0.298955322333143,0.874447842349716
"4566","LINC01121",2.12966354968701,1.31327949880201,1.2660300830324,1.03732092657422,0.299586316234439,0.874447842349716
"4567","PGAP1",11.5109405042641,0.504845232717799,0.486520451128458,1.03766497697443,0.299426055673888,0.874447842349716
"4568","CHPF",45.5644288008197,-0.357965720429397,0.345096045512803,-1.03729302344647,0.299599316172012,0.874447842349716
"4569","DVL3",43.3972562176698,0.311764969391356,0.299684525102091,1.04031053750656,0.298195650093775,0.874447842349716
"4570","AREG",15.2797815548016,-0.491671431920709,0.471634793686932,-1

.04248337591284,0.297187628907639,0.874447842349716
"4571","C5orf56",36.5490798125421,0.291270972323406,0.280807542724661,
1.03726192500821,0.299613805233281,0.874447842349716
"4572","PCDHGA10",15.4502703145895,0.453655140449985,0.435195143009755
,1.04241774692742,0.297218042040288,0.874447842349716
"4573","MGAT4B",54.7590916451271,-0.282102515635285,0.270895669246554,
-1.04136960336022,0.297704044304722,0.874447842349716
"4574","TRAF3IP2",18.5410639382382,-0.383983214318471,0.37021457931387
1,-1.0371909583629,0.299646871025623,0.874447842349716
"4575","FAM184A",10.6859011013775,-0.594860642805963,0.573188271306277
,-1.03781021452218,0.299358420399942,0.874447842349716
"4576","AC027644.3",26.2989753557067,-0.396990469899367,0.381486817953
232,-1.04064007251762,0.298042625780209,0.874447842349716
"4577","BUD23",45.0316426957506,-0.310969191442001,0.298666808670548,-
1.04119099415906,0.29778691458546,0.874447842349716
"4578","DENND11",13.6905458138563,0.491873818910055,0.474419184795522,
1.0367915857409,0.29983299780843,0.874447842349716
"4579","PLIN2",196.668977684951,0.223814817392472,0.214976343219818,1.
0411137060026,0.29782277916865,0.874447842349716
"4580","AL157392.3",6.33179024147631,0.676937200112483,0.6504850659645
13,1.04066524434154,0.298030939039339,0.874447842349716
"4581","BORCS7",106.36109012796,-0.231603543077955,0.222843729289412,-
1.03930922272965,0.298660946925481,0.874447842349716
"4582","FADS2",5.9155781938237,0.85164517918964,0.821121983623173,1.03
717254704567,0.299655449886978,0.874447842349716
"4583","RAB30",66.815181612731,0.2540893471676,0.244530833037577,1.039
08919791949,0.298763254269725,0.874447842349716
"4584","NDUFA9",54.0533833691599,-0.308101374058342,0.295646275961143,
-1.04212837809882,0.297352163281655,0.874447842349716
"4585","MDM1",19.5609610527544,0.378533940219278,0.364859089841527,1.0
3747981277838,0.299512299061516,0.874447842349716
"4586","CHFR",10.6129446312858,0.519258615940435,0.500611503629651,1.0
372486692287,0.299619981370423,0.874447842349716
"4587","ZFP36L1",757.760425064109,0.236381304212643,0.227070080689307,
1.04100594624827,0.297872788525203,0.874447842349716
"4588","DMXL2",15.4197989831743,0.492210957549452,0.472213762393556,1.
04234776016382,0.29725047690377,0.874447842349716
"4589","SNUPN",41.1018406908464,0.314630467391069,0.303007673207668,1.
03835808532623,0.299103375706734,0.874447842349716
"4590","PRC1",3.48679571154953,-0.809802866360489,0.780596972121585,-1
.03741481876304,0.299542575063195,0.874447842349716
"4591","ZFP1",10.7359454298598,0.501997827103083,0.483186408518627,1.0
3893201102682,0.298836357505137,0.874447842349716
"4592","TAX1BP3",201.744246070719,-0.281855766472115,0.271575151092041
,-1.0378555082773,0.299337329760994,0.874447842349716
"4593","RPL26",3083.74439657146,-0.289946011186103,0.2785610507265,-1.
04087061141502,0.297935602925496,0.874447842349716
"4594","UBL5",333.271258337327,-0.242882284532514,0.232978796973466,-1
.0425081066934,0.297176168954681,0.874447842349716
"4595","ZNF844",12.9831248985099,0.567623555576563,0.544670880664278,1

.04214044797896,0.297346568134266,0.874447842349716
"4596","KIAA0355",28.8398950712874,0.366473715771722,0.353438533096153
,1.03688104565561,0.299791298501046,0.874447842349716
"4597","NUMBL",54.6853009205292,-0.274068497304113,0.262726086093283,-
1.04317200236753,0.296868637928856,0.874447842349716
"4598","ZNF576",25.5863826809043,-0.371940741693403,0.358058117762571,
-1.03877198488776,0.29891079345942,0.874447842349716
"4599","SLC27A5",10.8220641235837,-0.516321271254403,0.497907468415811
,-1.03698237927055,0.299744069262997,0.874447842349716
"4600","CPXM1",130.436838914307,0.76124394922625,0.7298172480535,1.043
06105570479,0.296920016037031,0.874447842349716
"4601","GPCPD1",31.8698924293014,0.345302579800829,0.331948431085708,1
.04022958828708,0.298233248053398,0.874447842349716
"4602","ZSWIM1",2.73306791329185,0.98304170844173,0.948035625952087,1.
03692485971135,0.299770877181028,0.874447842349716
"4603","PTTG1IP",172.355344035997,0.206350837994349,0.198539380649249,
1.03934462432368,0.298644488043516,0.874447842349716
"4604","THOC5",19.9989913379476,-0.380535559658012,0.365390837312391,-
1.04144800799335,0.297667671349184,0.874447842349716
"4605","PQBP1",39.7204782619451,-0.310849213330143,0.298767504411441,-
1.04043849729408,0.298136223898103,0.874447842349716
"4606","YIPF6",56.7383299701259,-0.256969816977519,0.247597352468129,-
1.03785365399089,0.299338193173699,0.874447842349716
"4607","PJA1",19.072145224937,0.390375962669902,0.376379415164669,1.03
71873352827,0.299648559208167,0.874447842349716
"4608","EMD",47.2560401226967,-0.263199430179997,0.253768648908247,-1.
0371629092574,0.299659940736331,0.874447842349716
"4609","MANF",86.0429952499005,-0.249757928200542,0.240962913097921,-1
.03649945541224,0.29996919335748,0.874655237476932
"4610","SLF2",35.3468534194628,0.321586650931722,0.310308824252088,1.0
3634388002602,0.300041741771876,0.874676999495063
"4611","ATP13A2",6.90013877961401,-0.53328523365316,0.514977317515961,
-1.03555091751518,0.300411700364731,0.874684859851553
"4612","HMGCL",47.5699703507352,0.329878650838211,0.318938653251753,1.
03430125974045,0.300995348130532,0.874684859851553
"4613","C1orf216",18.5451230273712,-0.362013278134141,0.35012726901720
6,-1.03394768179668,0.301160622326735,0.874684859851553
"4614","GON4L",58.2204422837497,0.244194344431195,0.236318402704191,1.
03332766994393,0.301450582569927,0.874684859851553
"4615","CENPL",7.78152961976464,0.624472364870859,0.60413667797315,1.0
3366073876848,0.301294793549086,0.874684859851553
"4616","URB2",2.8152252298705,-0.818751693441802,0.790930896300248,-1.
0351747507547,0.30058730816583,0.874684859851553
"4617","FAM228B",23.2323765525275,-0.332589562998206,0.321063049970091
,-1.03590108867772,0.300248289684669,0.874684859851553
"4618","SRP72",123.19961996304,-0.195509451630915,0.188872628750039,-1
.03513914602025,0.300603933244402,0.874684859851553
"4619","LINC01091",10.3212162527957,0.51056888196042,0.492946795208665
,1.03574845586387,0.300319509942318,0.874684859851553
"4620","CEP120",18.0297721992845,0.442188751251207,0.427129611784008,1

.03525660373745,0.300549090508487,0.874684859851553
"4621","NCS1",15.4979504448392,-0.435861245954012,0.421825975922403,-1
.03327265467926,0.301476320472683,0.874684859851553
"4622","CAMSAP1",9.93560244342791,0.560758817396103,0.541226891431697,
1.03608824002211,0.300160978044172,0.874684859851553
"4623","C11orf24",27.4485607875517,-0.341152364682771,0.33011720456129
4,-1.03342800668672,0.301403645595799,0.874684859851553
"4624","NR2C1",24.4650839200874,0.348846645290252,0.33707243735942,1.0
3493079417311,0.300701232192304,0.874684859851553
"4625","AC007541.1",8.18470514474441,0.555966377365709,0.5373280977370
6,1.03468696259723,0.300815126587861,0.874684859851553
"4626","SNX33",42.9788690828901,0.28656778424085,0.276671955498683,1.0
3576737195619,0.300310682860947,0.874684859851553
"4627","ASPA",45.0452369686743,0.379154688384705,0.366613253881605,1.0
3420889553314,0.301038516429316,0.874684859851553
"4628","AC132938.3",1.69783819524278,-1.15191979600916,1.1135209423258
8,-1.03448417737261,0.300909870009112,0.874684859851553
"4629","ZBTB7C",6.37307758864891,-0.602646101454607,0.58246597822163,-
1.03464601193462,0.300834257572797,0.874684859851553
"4630","CSNK2A1",79.5600583904254,0.262576330195422,0.253986979671485,
1.03381807419832,0.30122122040351,0.874684859851553
"4631","RAE1",19.7666588956908,-0.354235159082243,0.34279736380086,-1.
03336605379506,0.30143262624159,0.874684859851553
"4632","GABRE",34.3779531967755,0.439640097202901,0.424966012421911,1.
03453001970055,0.300888450245396,0.874684859851553
"4633","GEMIN6",22.0570488285945,-0.372712948809821,0.360777884374432,
-1.03308147464772,0.301565771990813,0.874755538481446
"4634","FAAP20",66.9303256026113,-0.25487563427634,0.246921261913471,-
1.03221420586153,0.301971781595362,0.874800339051535
"4635","ZNF2",4.65270030186298,-0.638205226000501,0.618214445612195,-1
.03233632039851,0.301914592000719,0.874800339051535
"4636","GCC2",156.158942426436,0.19387753297995,0.187796941974344,1.03
237854110764,0.301894820559116,0.874800339051535
"4637","AC068587.4",5.65728249582535,0.757775845245245,0.7338919220087
29,1.03254419693181,0.301817254286942,0.874800339051535
"4638","FNBP1",55.7957452750626,0.273872209292109,0.265233699486105,1.
03256942772634,0.301805441446465,0.874800339051535
"4639","RBM7",51.1039437139783,-0.28830801586292,0.279204668408631,-1.
0326045674887,0.301788989825464,0.874800339051535
"4640","AC092747.4",7.23520923034005,0.663485418306313,0.6429557397817
85,1.03193015825863,0.30210483678383,0.87499717705558
"4641","TIMM10",47.9447925887723,-0.341954066759901,0.331536899988254,
-1.0314208366309,0.302343513785544,0.875238885479884
"4642","NCAM2",11.4435115487023,0.64524719896104,0.625642581411392,1.0
3133517144153,0.302383670309034,0.875238885479884
"4643","MSN",127.361422380311,-0.205217477487065,0.198942059146939,-1.
03154394986678,0.30228580928137,0.875238885479884
"4644","FASTK",46.5053764888338,-0.302713886013876,0.29362706543141,-1
.03094680856179,0.302565764290727,0.875577370004971
"4645","AMFR",51.0616122600917,-0.292545993668695,0.28384299758682,-1.

03066130274788,0.302699677585936,0.875776311534421
"4646","TAB2",35.8098761277921,0.322091441225493,0.312699362280224,1.0
3003549120402,0.302993345333048,0.875902109999345
"4647","IGSF9B",2.65890894219576,-1.15094432585457,1.11736743386718,-1
.03005000053669,0.30298653455276,0.875902109999345
"4648","FLOT2",22.4197057122954,0.383777433941255,0.372594716592437,1.
03001308620554,0.303003862592972,0.875902109999345
"4649","SKA2",32.2534505601324,-0.355838162254854,0.345357181058064,-1
.03034823588923,0.302846563381138,0.875902109999345
"4650","RPL11",3577.37176671651,-0.279103650799189,0.271222797543346,-
1.02905675086027,0.30345300778657,0.875983580264266
"4651","ZMYM6",13.9620631104067,0.447239855714331,0.434778778529377,1.
02866073000873,0.303639129411808,0.875983580264266
"4652","PSMB2",119.837286640447,-0.278837120726361,0.271190796176277,-
1.02819536893543,0.303857936425956,0.875983580264266
"4653","SPATA6",51.3464530027631,0.251951259910654,0.244976157925624,1
.02847257481746,0.303727585042306,0.875983580264266
"4654","ATP5PB",119.150531087595,-0.257129899470161,0.250030929565693,
-1.02839236696356,0.303765297610503,0.875983580264266
"4655","AL390728.6",13.2678835968985,0.48833349326141,0.47501431330198
6,1.02803953393917,0.303931231516686,0.875983580264266
"4656","N4BP2",17.9372518036951,0.440426992234641,0.428541805880694,1.
02773401845713,0.304074961078414,0.875983580264266
"4657","GEMIN5",16.9780281631245,0.407462402589782,0.396407482360846,1
.0278877688259,0.304002623674684,0.875983580264266
"4658","MRPL11",91.1589569343837,-0.282714883799028,0.274557218285099,
-1.02971207810482,0.303145184081518,0.875983580264266
"4659","RERG",53.3891832839138,-0.395963055240873,0.384809838985463,-1
.02898370864117,0.303487330418122,0.875983580264266
"4660","USP12",26.8420428633258,0.364602603766495,0.354495820278042,1.
02851030367728,0.303709846556098,0.875983580264266
"4661","ERH",220.316624039499,-0.265197397523495,0.257943815957258,-1.
02812078102868,0.303893016473399,0.875983580264266
"4662","MRPL28",48.6581797005193,-0.346539015032908,0.336707735865703,
-1.02919825747968,0.303386520986438,0.875983580264266
"4663","MYL12B",778.647681320971,-0.285093817076865,0.277381547260024,
-1.02780383155629,0.304042113572289,0.875983580264266
"4664","COLGALT1",46.093497656221,0.295692193492259,0.287421138802454,
1.02877677934291,0.303584580765049,0.875983580264266
"4665","RRP1B",38.7291310072952,-0.32290889557518,0.314132056354654,-1
.0279399667846,0.303978067862471,0.875983580264266
"4666","GLMN",7.77041771919222,0.564905987276356,0.551173052343803,1.0
249158315599,0.305402903872108,0.876090057080099
"4667","PSME4",18.8546165270423,0.443165480996067,0.432269382855793,1.
02520673120148,0.30526565283103,0.876090057080099
"4668","TMEM182",1.77826912898706,1.12492742801701,1.09779909854039,1.
02471156107952,0.305499306367896,0.876090057080099
"4669","POLR2H",46.8961491715006,-0.34682347575324,0.338662029909328,-
1.02409908735885,0.305788475442549,0.876090057080099
"4670","ANXA2R",11.5804152261566,-0.488153666659328,0.475624420037552,

-1.02634273198333,0.304730061709121,0.876090057080099
"4671","WDR41",40.3548839756022,-0.289872479994311,0.282910785047173,-1.02460738619766,0.305548477965494,0.876090057080099
"4672","CETN3",37.5247881707653,0.318897312435934,0.31140014037942,1.02407568618106,0.305799527510037,0.876090057080099
"4673","HSD17B4",47.0175506381865,0.25529046983017,0.248928675113583,1.02555669696826,0.305100587695247,0.876090057080099
"4674","ALDH7A1",43.1381053899576,0.25340783087642,0.247362472486534,1.02443927055352,0.305627841319303,0.876090057080099
"4675","SLIT3",414.470854150405,-0.324779739642191,0.316655383594777,-1.0256567753726,0.305053395525628,0.876090057080099
"4676","FAM66D",3.17924220633294,0.899147852335034,0.875664671211556,1.02681755002287,0.304506383844838,0.876090057080099
"4677","AL162231.2",10.2050147423364,-0.451885695728631,0.440923688642399,-1.02486146099336,0.305428561302918,0.876090057080099
"4678","ZSWIM8",30.53875608652,0.298426381421678,0.290591447170787,1.02696202633344,0.304438345401933,0.876090057080099
"4679","CTR9",41.1268066963961,0.271618015802735,0.265070042899709,1.02470280244193,0.305503440331305,0.876090057080099
"4680","CTTN",166.316598148152,-0.194958586165076,0.190177113647896,-1.02514220783702,0.305296092440358,0.876090057080099
"4681","FMNL3",5.09989661119471,-0.791819713348432,0.773083896163953,-1.02423516681365,0.30572421215986,0.876090057080099
"4682","SPRYD3",84.35713252658,-0.23175003063692,0.22560068624438,-1.02725764932239,0.304299158688399,0.876090057080099
"4683","MMAB",16.4826932574916,-0.430702660866025,0.420075825910489,-1.02529742084659,0.305222872393891,0.876090057080099
"4684","HERC2",61.3957483543994,0.331333099228965,0.322788831147729,1.02647014783893,0.304670027788383,0.876090057080099
"4685","GATM",2.82716828298101,1.32777351954689,1.29238638240663,1.02738123646456,0.304240983290995,0.876090057080099
"4686","SQOR",54.1822067754434,-0.280550925799072,0.273922872287553,-1.02419678742475,0.305742335851841,0.876090057080099
"4687","ZNF688",34.8929124223846,-0.361010621565893,0.351864987462037,-1.025991884472,0.304895409431335,0.876090057080099
"4688","JUP",15.8605495824512,-0.452824036479266,0.440949216183366,-1.02693013131689,0.30445336490107,0.876090057080099
"4689","MED13",57.8121519141105,0.271672540312972,0.265215183674131,1.02434761294352,0.30567111643133,0.876090057080099
"4690","MIEF1",16.5333709401042,0.418724169384824,0.408029928611467,1.02620945186484,0.304792867068257,0.876090057080099
"4691","DDX3X",193.305052604624,0.282492001168398,0.275854890960631,1.02406015055471,0.305806864927654,0.876090057080099
"4692","UVSSA",13.9970525342704,0.499310366778383,0.487712373605851,1.02378039557779,0.305939012123632,0.876095116967715
"4693","SBF2-AS1",5.93975539718758,0.66009351152582,0.64468843319059,1.02389538502962,0.305884690246724,0.876095116967715
"4694","AC093157.1",7.83234636457446,0.567797234305248,0.554740849851404,1.02353600687121,0.306054484416027,0.876239074577542

"4695","ACBD5",26.7470485878351,0.338533867949915,0.330809131910575,1.02335103627498,0.306141901191179,0.876302664559799
"4696","ATF6B",36.4719896848701,0.376631516969579,0.368125535653026,1.02310619745915,0.306257637030358,0.876447270879681
"4697","FARP1",51.8110809635652,0.308211276547732,0.30143848870959,1.02246822516639,0.306559344125306,0.877123914349582
"4698","DIPK2A",25.8939389761431,0.375832154702341,0.367723192969237,1.02205180931784,0.3067563798928,0.877207731148407
"4699","DDX23",34.3334284871708,0.293362921735124,0.28708871801203,1.02185458128254,0.306849731686038,0.877207731148407
"4700","SLC23A2",17.5038665631345,0.400051981412491,0.391463658129,1.02193900533331,0.306809769868284,0.877207731148407
"4701","AL022323.4",14.6592055610828,0.58497383178812,0.572461833375469,1.0218564761582,0.306848834715704,0.877207731148407
"4702","ARID1A",48.5832421185179,0.299106734210954,0.293369764963926,1.01955542094712,0.307939356349662,0.877208279932743
"4703","PEF1",52.2987946384623,-0.254472584147838,0.249763345115114,-1.01885480445721,0.308271902871164,0.877208279932743
"4704","SHISAL2A",1.69844537575948,-1.17580152098219,1.15502400445468,-1.01798881793572,0.308683270175578,0.877208279932743
"4705","ZRANB2",152.903653874181,0.244824397259955,0.240103283887028,1.01966284382495,0.307888389244365,0.877208279932743
"4706","AL359504.2",6.10054277187761,0.643038555257984,0.629950877648496,1.02077571136712,0.307360714385332,0.877208279932743
"4707","PLEKHO1",29.7068660006779,-0.36502761208736,0.357982677795971,-1.0196795396212,0.30788046837519,0.877208279932743
"4708","MLLT11",2.48465289257848,-0.861112349848686,0.845929435790442,-1.01794820397053,0.308702571827426,0.877208279932743
"4709","CD247",2.30627349967507,1.02613823576284,1.0068307348817,1.01917651121706,0.308119175775695,0.877208279932743
"4710","MPZL1",154.036942940151,0.241837192083183,0.237237774972697,1.01938737248322,0.30801909864258,0.877208279932743
"4711","SOWAHC",23.7331687973597,0.341385175749852,0.334482645719384,1.02063643695362,0.307426719642129,0.877208279932743
"4712","ZFR",81.3536448065131,0.210001901335875,0.205715565939454,1.02083622295107,0.307332039517521,0.877208279932743
"4713","CMYA5",24.5945773880767,0.535199427416884,0.524698825242047,1.02001262756781,0.307722471984429,0.877208279932743
"4714","BCLAF1",151.602985653452,0.272130267498671,0.266570646768234,1.0208560874869,0.30732262661616,0.877208279932743
"4715","CDK13",62.3458815141129,0.298279267621427,0.29290119629234,1.01836138396553,0.308506246645359,0.877208279932743
"4716","LSM8",104.850748503725,-0.226809131514242,0.222156107133772,-1.02094484117723,0.307280572604792,0.877208279932743
"4717","FAM180A",28.8640013029201,0.371399093276875,0.364715265400673,1.01832615333186,0.308522983490931,0.877208279932743
"4718","TPK1",12.1682686447098,0.430891091297964,0.42198008761778,1.02111711889177,0.307198953422584,0.877208279932743
"4719","AC106845.1",1.57789057459938,1.41841846316841,1.39076720485343,1.01988201779455,0.307784418813683,0.877208279932743

"4720","BMP1",68.5728192457291,0.393403245933209,0.38616858793119,1.01
873445491975,0.308329050489736,0.877208279932743
"4721","SLC39A13",59.6892898457803,-0.243731244289254,0.23902619402736
1,-1.01968424540682,0.307878235866779,0.877208279932743
"4722","ZNF641",10.5197644282534,-0.511244583726128,0.501459228787739,
-1.01951375979667,0.307959124109263,0.877208279932743
"4723","NDUFA12",143.862150589189,-0.27613093957089,0.270925723071189,
-1.01921270686554,0.308101995383828,0.877208279932743
"4724","UNC119B",11.1768814896169,0.510524037301594,0.501431491162003,
1.01813317731306,0.308614670320891,0.877208279932743
"4725","DAD1",456.955193960302,-0.27377636157696,0.268765512362238,-1.
0186439441976,0.308372033852362,0.877208279932743
"4726","FANCM",8.19483265912527,0.533224616018401,0.523866548570058,1.
017863456779,0.308742850218162,0.877208279932743
"4727","C14orf132",63.8264669213568,-0.28223726025381,0.27661316357755
7,-1.02033199217099,0.307571035501355,0.877208279932743
"4728","TM6SF2",2.44669890934571,0.953652959584488,0.935578310021901,1
.01931922680226,0.308051438998293,0.877208279932743
"4729","PPP6R2",34.0081441840989,0.366826593056127,0.3600060241995,1.0
189457075664,0.308228742446197,0.877208279932743
"4730","JPX",131.676372890143,0.204575771256167,0.200311721244604,1.02
128707189509,0.307118449675147,0.877208279932743
"4731","NRP2",3.7431827661412,-0.739803188669725,0.727174537150388,-1.
01736674054736,0.308978997882035,0.877508189462525
"4732","CUEDC1",45.7644416494445,-0.286207394385933,0.281295620127142,
-1.01746125395259,0.30893405534562,0.877508189462525
"4733","SIX5",25.5623701152721,0.370551824330195,0.364311007127812,1.0
1713046567433,0.309091369020659,0.877641856807234
"4734","KIAA0895L",2.67949301023805,0.891217041750723,0.87678902146679
6,1.01645552114668,0.309412518010578,0.878368151572488
"4735","RAB4A",86.2914227040895,-0.272369812381844,0.268640461561155,-
1.01388231243729,0.310638912231313,0.878856303642893
"4736","ZNF513",6.19812144207106,-0.575267992483143,0.566363914257683,
-1.01572147871944,0.309762036882792,0.878856303642893
"4737","RPL24",2021.2258721086,-0.254858313566451,0.251603477578019,-1
.0129363712289,0.311090554226903,0.878856303642893
"4738","PSMD2",94.6875309935098,-0.210556109332141,0.207351556565598,-
1.01545468391759,0.309889137483253,0.878856303642893
"4739","PPP3CA",85.3742647358378,-0.322472595225251,0.317874074815103,
-1.01446648460659,0.310360214012815,0.878856303642893
"4740","HSPA1B",93.0715030045878,0.437685974733158,0.431323068164386,1
.01475206646343,0.310224028045498,0.878856303642893
"4741","USP42",16.2306389688217,0.462318619285805,0.45527493415985,1.0
1547127811671,0.309881231029964,0.878856303642893
"4742","AVL9",20.9868987875075,0.397032467137792,0.391757419870434,1.0
1346508578983,0.3108380647699,0.878856303642893
"4743","NSUN5",23.7462506815551,-0.318460072004237,0.314002849950471,-
1.01419484585719,0.310489787521481,0.878856303642893
"4744","ZFP37",8.81655318471046,0.544641458980938,0.53718903869052,1.0
1387299396239,0.310643359248948,0.878856303642893

"4745","NUP214",26.3483989005562,0.355039423165211,0.350693267275418,1
.01239304057235,0.311350164569076,0.878856303642893
"4746","API5",35.5077079022463,0.297406628565004,0.293742594058991,1.0
1247362343807,0.311311652022845,0.878856303642893
"4747","RAB30-
DT",26.0593111050146,0.375579739850461,0.370019912538278,1.01502575165
224,0.310093552293838,0.878856303642893
"4748","AL096870.8",17.0909794460047,-0.457632891413921,0.450947595105
963,-1.01482499603172,0.310189256307749,0.878856303642893
"4749","MRPS34",92.7989716306822,-0.283671063738024,0.280036420385929,
-1.01297918087613,0.311070105300159,0.878856303642893
"4750","NAA60",28.39258707443,0.318993808250326,0.314824229871447,1.01
324414699778,0.310943558398877,0.878856303642893
"4751","USP7",55.5987185595099,0.282561595849929,0.278622011016259,1.0
1413953197488,0.310516176990168,0.878856303642893
"4752","C16orf74",7.47730753441577,0.69870513474576,0.688443188574643,
1.01490601743386,0.310150629521009,0.878856303642893
"4753","SP2",12.0601919419681,0.445615918609279,0.439538434070881,1.01
382696953736,0.310665323913143,0.878856303642893
"4754","CNDP2",32.991908556869,0.282145702465866,0.278081925192241,1.0
1461359730884,0.310290055168556,0.878856303642893
"4755","ZNF490",4.68792341658888,0.875386718686488,0.863775846324658,1
.0134419970311,0.310849088061706,0.878856303642893
"4756","ATP13A1",16.0516428226257,0.394128521829546,0.38930198991682,1
.01239791225767,0.311347836180832,0.878856303642893
"4757","GPI",59.4418445964058,-0.216772985943253,0.214090194969652,-1.
01253112490267,0.311284172570518,0.878856303642893
"4758","ZNF565",9.76077068305317,0.536543601711377,0.528131766149966,1
.01592753191639,0.309663897051886,0.878856303642893
"4759","LENG1",26.5829484023355,-0.317746294131908,0.313454481000443,-
1.01369198206313,0.310729751151573,0.878856303642893
"4760","ATRN",26.9730235418112,0.333263382879053,0.328636174695157,1.0
1408003299755,0.310544564760962,0.878856303642893
"4761","OPHN1",44.4969380108892,0.336988927494336,0.332690987101539,1.
0129187160441,0.311098987854839,0.878856303642893
"4762","VANGL2",4.09307425316697,0.742317247387886,0.734000956125566,1
.0113300823288,0.311858472707085,0.878950709938273
"4763","PUS10",5.4416807828315,0.745847536653888,0.737427318236561,1.0
1141837060968,0.311816232306884,0.878950709938273
"4764","GPD2",20.10217373556,0.374825155919903,0.370443989690854,1.011
82679798019,0.311620874531328,0.878950709938273
"4765","ARCN1",71.4450717126149,0.235117795071693,0.232512770620275,1.
01120379084757,0.311918901809518,0.878950709938273
"4766","BRI3BP",7.29416798524049,0.567314111809449,0.56102577370164,1.
01120864388514,0.311916579541148,0.878950709938273
"4767","DUT",182.712417963996,-0.270660534681099,0.267691212881382,-1.
01109233944497,0.311972236506106,0.878950709938273
"4768","RAB40C",8.03095491511068,0.536864430623553,0.530694760924471,1
.01162564651728,0.311717078618608,0.878950709938273
"4769","PET117",15.5118017516665,0.408503515291207,0.403911224511165,1

.01136955474709,0.311839587166584,0.878950709938273
"4770","COL6A2",3340.17184898508,-0.242411530413489,0.239550754789271,-1.01194225260002,0.311565665251502,0.878950709938273
"4771","KLHL17",2.5698842540118,-0.879244992102345,0.873025665893188,-1.00712387556532,0.313875249013569,0.879327129181194
"4772","LRRC8D",4.30312460385493,0.841085097975863,0.835502707897671,1.00668147454871,0.314087867389638,0.879327129181194
"4773","TRMT13",29.3970450512802,0.313435077231843,0.311451452436428,1.00636896948111,0.314238114722434,0.879327129181194
"4774","CCDC88A",82.1758768351785,0.29492833124952,0.292949996976575,1.00675314658939,0.314053415305308,0.879327129181194
"4775","ATIC",25.1854271035553,-0.301800982005316,0.299833889867981,-1.00656060640177,0.314145973216236,0.879327129181194
"4776","CYP27A1",65.4293053108757,-0.240263524964537,0.238325417497818,-1.0081321895376,0.313391006228702,0.879327129181194
"4777","RETREG2",63.8282117089503,-0.219595247662152,0.217542687995912,-1.00943520412085,0.312765962168089,0.879327129181194
"4778","MRPL14",74.0138030199256,-0.311903224215084,0.310030381305171,-1.00604083671422,0.314395926461466,0.879327129181194
"4779","SEM1",271.051943065396,-0.254499767518814,0.252259682323517,-1.00888007617652,0.313032151463818,0.879327129181194
"4780","SND1-IT1",5.01758112253627,0.750753361719214,0.744140762290797,1.00888622121446,0.313029204041606,0.879327129181194
"4781","UBN2",35.7115001655391,0.329103090716223,0.326386582105645,1.00832297882178,0.313299435028725,0.879327129181194
"4782","ADAM32",5.69406660348799,0.668462421982964,0.66252735310241,1.00895822467218,0.312994669474656,0.879327129181194
"4783","ZFHX4-AS1",5.5298800662704,-0.628823264100666,0.623735004756921,-1.00815772612558,0.313378748670214,0.879327129181194
"4784","ALDH1B1",6.93678743003562,-0.554811846473353,0.550338993958814,-1.00812744974214,0.313393281364548,0.879327129181194
"4785","NRBF2",68.5645478300487,-0.273126049837591,0.270836790068114,-1.00845254357394,0.31323725918985,0.879327129181194
"4786","CD151",356.231833996688,-0.259127113007657,0.257136343146576,-1.00774207891705,0.313578298502,0.879327129181194
"4787","TNNT3",119.023564495349,-0.376132481394001,0.372544397362193,-1.0096312924237,0.312671971543372,0.879327129181194
"4788","RNF141",18.7062108904938,0.359969184834754,0.357565413911035,1.00672260467652,0.314068096215232,0.879327129181194
"4789","USP35",2.89290224436139,-0.90856968841796,0.899865193109983,-1.00967311034433,0.312651929447919,0.879327129181194
"4790","AP003486.1",3.91037610272799,0.772736370828991,0.765739152169689,1.00913786194617,0.312908522112458,0.879327129181194
"4791","VPS18",11.7387201135755,0.444343084263211,0.440622499807484,1.00844392752833,0.313241393626,0.879327129181194
"4792","ADAL",12.250767890092,0.486367625383044,0.483422887952157,1.00609143154839,0.314371590051199,0.879327129181194
"4793","TMEM97",3.74194534389484,0.830026946255996,0.824861767654594,1

.00626187175106,0.314289616435321,0.879327129181194
"4794","ASB16-
AS1",7.76044630305547,0.543408875376121,0.538670803382888,1.0087958581
818,0.313072547846271,0.879327129181194
"4795","PPM1D",8.46449959973669,0.54166840209652,0.537092161086972,1.0
0852040178782,0.313204698471512,0.879327129181194
"4796","MCRIP1",149.031578566765,-0.285024899898135,0.282770539291932,
-1.00797240268328,0.313467711060168,0.879327129181194
"4797","ALPK2",13.1342754525496,0.542679193238399,0.539075740839668,1.
00668450112987,0.314086412489908,0.879327129181194
"4798","RPL36",1587.83183696144,-0.243523932370173,0.241009423344998,-
1.01043323945709,0.31228776947984,0.879327129181194
"4799","EID2",21.7841650790324,-0.359672270849601,0.357160295978932,-1
.00703318621625,0.313918826688817,0.879327129181194
"4800","AL117332.1",2.20206922863904,-0.983163011986512,0.975856028088
483,-1.00748776836717,0.313700432756664,0.879327129181194
"4801","ROMO1",127.042639239181,-0.264646784931889,0.262089820422947,-
1.0097560618906,0.312612175723529,0.879327129181194
"4802","CHD6",59.3878515582827,0.254920303518499,0.252417363426989,1.0
0991587923084,0.312535594199785,0.879327129181194
"4803","MRPL40",104.338906516013,-0.286666503285356,0.284080419489193,
-1.00910335108915,0.312925071025439,0.879327129181194
"4804","AKAP17A",24.8342468142223,0.341306520648566,0.338902562643096,
1.00709336036506,0.313889911608551,0.879327129181194
"4805","TAB3",8.01029857396774,0.583192147881665,0.578979677255102,1.0
072756795999,0.313802313666345,0.879327129181194
"4806","TMEM164",4.2925858074206,-0.684072130184279,0.680091568234651,
-1.00585297941564,0.31448629775621,0.879396869651625
"4807","SLC4A11",1.91309640670898,-1.47046697507589,1.46219470830808,-
1.00565743175023,0.314580386746828,0.879425914875893
"4808","SH3BP1",2.294391328913,1.02431243542816,1.01864935446077,1.005
55940171423,0.314627561479522,0.879425914875893
"4809","ODR4",37.286539498854,0.282768646129258,0.281501331799644,1.00
450198342406,0.315136715692073,0.879600669471718
"4810","DIRC3-
AS1",1.87158995295775,1.18958096327521,1.18472835613856,1.004095966059
65,0.315332359629285,0.879600669471718
"4811","PHC3",47.2626223362422,0.280843342963514,0.279743637312404,1.0
0393111944091,0.315411815557713,0.879600669471718
"4812","POLH",9.61082301835705,-0.510647554671458,0.508625753067822,-1
.00397502798756,0.315390650391106,0.879600669471718
"4813","PARP12",6.7943398789991,0.585035950370238,0.582232532187805,1.
00481494596652,0.314985965690996,0.879600669471718
"4814","FBXO25",31.2149857330902,0.366452442049246,0.365164314392979,1
.00352752885618,0.315606401383483,0.879600669471718
"4815","RNF139",63.3931589017031,0.246502861628508,0.245611480621372,1
.00362923184568,0.315557359212389,0.879600669471718
"4816","ERCC6L2",34.4037578197689,0.307924769691515,0.306615726400385,
1.00426932860391,0.31524881321927,0.879600669471718
"4817","POMT1",18.3614580882137,0.370232366116941,0.368321793831992,1.

00518723658753,0.314806699851813,0.879600669471718
"4818","CACUL1",43.7220380884335,-0.280990666604839,0.27992296394272,-
1.0038142732096,0.315468143304971,0.879600669471718
"4819","IQCD",3.08203234471131,-0.841781890465329,0.838626604319997,-1
.00376244460774,0.31549313029446,0.879600669471718
"4820","ZNF738",10.6071597742782,0.508040293252743,0.505739870307172,1
.00454862881223,0.315114244207255,0.879600669471718
"4821","ZNF350",9.82426042890105,0.490338064612688,0.488192084738382,1
.00439576949605,0.315187888337679,0.879600669471718
"4822","CRNKL1",34.3807816620367,0.349808901666271,0.34805759953515,1.
00503164457107,0.314881612553337,0.879600669471718
"4823","SPPL2A",166.955974720414,0.187590512105701,0.187041292780407,1
.00293635334278,0.315891571081278,0.880212901464087
"4824","PER2",36.0294005853716,-0.422648767491529,0.421688066982955,-1
.00227822550315,0.316209236014019,0.880367914396602
"4825","ARL10",12.706304770967,0.4965543244818,0.49541857292889,1.0022
925090317,0.316202339419842,0.880367914396602
"4826","DBN1",349.9308019905,-0.180110724935429,0.179648686408172,-1.0
0257190039346,0.316067459214847,0.880367914396602
"4827","CLBA1",4.23102622072286,-0.645176845867073,0.643579599686838,-
1.00248181605044,0.316110944602811,0.880367914396602
"4828","VPS72",31.6694620333567,-0.283489148998253,0.283442745013645,-
1.00016371554899,0.317231285608394,0.880559869706351
"4829","ZCWPW2",10.9459373280706,0.524238074304747,0.523818788394323,1
.00080044076256,0.31692329643235,0.880559869706351
"4830","PCGF3",25.3257402478915,0.304783161400945,0.304182769791651,1.
00197378572661,0.316356253814507,0.880559869706351
"4831","CDKL3",7.9247250751107,0.536647686734672,0.536186211217144,1.0
0086066278445,0.316894176705352,0.880559869706351
"4832","AC132217.1",5.25599952660816,1.15912375201026,1.15740133688901
,1.00148817447013,0.316590854436892,0.880559869706351
"4833","SPATA13",25.162413414587,-0.324967043710199,0.324841707672277,
-1.00038583727077,0.31712382123728,0.880559869706351
"4834","DICER1",73.0119606127875,0.282948071203619,0.282869448843788,1
.00027794574548,0.317176017089261,0.880559869706351
"4835","ZWILCH",7.15278160958726,0.5684949095231,0.567893720095358,1.0
0105863017404,0.316798464018512,0.880559869706351
"4836","MIR762HG",3.80009368287812,-0.737919787935058,0.73780788977203
9,-1.00015166300682,0.317237117413358,0.880559869706351
"4837","DYNLRB2",2.31933108325059,-1.01874062098126,1.01806825855461,-
1.00066042961362,0.316991004138413,0.880559869706351
"4838","KSR1",10.0057370400889,0.484873030435002,0.484343504318366,1.0
0109328629767,0.316781710529289,0.880559869706351
"4839","PIP4K2B",36.0088675640457,0.328203329814114,0.327943305992705,
1.00079289260265,0.316926946389678,0.880559869706351
"4840","PLIN3",96.4429215165372,-0.244113271041241,0.244088310840612,-
1.00010225889369,0.317261023075984,0.880559869706351
"4841","IFT52",40.4683594999978,-0.304809860912489,0.304449956041256,-
1.00118214788372,0.316738755650713,0.880559869706351
"4842","RTL8C",140.366954243663,-0.197541374631598,0.197216547485759,-

1.00164705827163,0.316514084514405,0.880559869706351
"4843","PAN3",24.7508226800827,-0.327828537142582,0.327932361565383,-0
.999683396837369,0.317463749510763,0.880940600800154
"4844","HS1BP3",27.2389711371655,0.310391013576039,0.310641605598401,0
.999193308243823,0.317701056903142,0.881100004517985
"4845","TRIM38",61.242637498377,0.298907334497505,0.29919958808098,0.9
99023215287999,0.317783445338096,0.881100004517985
"4846","AC087854.1",4.86492558445309,0.803587590013979,0.8043082214617
33,0.999104035706057,0.317744296364073,0.881100004517985
"4847","NPTN-
IT1",2.91034179384222,0.9447117935543,0.945311872015271,0.999365205834
46,0.317617808576622,0.881100004517985
"4848","REXO4",38.1019986837592,0.325638081359132,0.326020540662494,0.
99882688586865,0.317878559414779,0.881181922437131
"4849","MUTYH",8.18927398507742,-0.529392584926921,0.532049559626086,-
0.995006151868573,0.319733272330357,0.881276096337479
"4850","ARNT",52.9143464043608,0.258657802022967,0.259836389805432,0.9
95464115771672,0.319510588597101,0.881276096337479
"4851","EFNA3",1.71001195742657,-1.28817798387797,1.29792437364947,-0.
992490787622629,0.320958171164208,0.881276096337479
"4852","CD48",6.30381941112911,-0.621774051845368,0.627060035319404,-0
.991570211500811,0.321407226482816,0.881276096337479
"4853","SLC19A2",16.321619250479,0.479396002433316,0.483801428774542,0
.990894143590306,0.321737272325635,0.881276096337479
"4854","RHOB",532.453522389933,0.349990186479391,0.352871279219443,0.9
91835286945357,0.321277881132977,0.881276096337479
"4855","TTL",23.5131224041778,0.372064643252151,0.374493239772517,0.99
3514979010458,0.320459054445353,0.881276096337479
"4856","GYPC",435.897130268201,-0.272612678780751,0.273544020582793,-0
.996595276328623,0.3189609997239,0.881276096337479
"4857","CCDC74A",10.2000943715599,-0.512182872168012,0.517231993546632
,-0.990238188198687,0.322057710953263,0.881276096337479
"4858","MAP3K20",127.54205086578,-0.188787017210746,0.190318398401057,
-0.991953583031507,0.321220168733353,0.881276096337479
"4859","PLEKHA3",56.9287897723971,0.244079206445312,0.246938729500639,
0.98842011108946,0.322946942135678,0.881276096337479
"4860","PID1",77.1145521345334,0.320105603779598,0.323697378084763,0.9
88903912887965,0.322710155923483,0.881276096337479
"4861","HDAC4",41.9714389240949,0.282978963773188,0.283656206751523,0.
997612451403439,0.318467320916159,0.881276096337479
"4862","CAPN10-
DT",2.97493600288439,1.05381387456707,1.0639713997275,0.99045319717894
8,0.321952654679445,0.881276096337479
"4863","SH3BP5-
AS1",4.65347150076561,-0.676865714680839,0.681264999044616,-0.99354247
7053795,0.320445660887536,0.881276096337479
"4864","ZNF620",2.46850702353722,0.978911365306258,0.983137734427905,0
.995701142399843,0.319395374887096,0.881276096337479
"4865","DALRD3",47.3236960184162,-0.283412539331875,0.284263414769699,
-0.997006736028575,0.31876124033133,0.881276096337479

"4866","WDR82",68.9373307293186,0.243281108042004,0.246172241686425,0.
988255647246761,0.323027461162171,0.881276096337479
"4867","TOMM70",39.7424635977024,0.306147115574329,0.308651974180483,0
.991884521027917,0.321253860774722,0.881276096337479
"4868","TMEM45A",27.5439004724066,0.570696487599195,0.574862378818126,
0.992753237344395,0.320830223802198,0.881276096337479
"4869","TIPARP-
AS1",1.77552410306291,1.15920379528938,1.16874397029335,0.991837241306
515,0.321276927615547,0.881276096337479
"4870","LPP",276.382816036086,0.226155143568051,0.228030407306926,0.99
1776255802802,0.321306682833976,0.881276096337479
"4871","WFS1",18.0122449286718,0.410723840805454,0.412475694121759,0.9
95752832612269,0.319370252961882,0.881276096337479
"4872","MRPL1",42.9005770607926,-0.288660043193848,0.289746576376792,-
0.996250056872005,0.319128663376211,0.881276096337479
"4873","CISD2",35.7163070726601,-0.268700831837294,0.270029786402004,-
0.995078488997758,0.319698091843497,0.881276096337479
"4874","IL15",1.55311906557131,-1.2552978114991,1.26477752588471,-0.99
2504836469974,0.320951321339603,0.881276096337479
"4875","NIPBL",98.9255528359149,0.238877562528194,0.24155263155969,0.9
88925523128343,0.322699581906314,0.881276096337479
"4876","RPS23",3477.71046073884,-0.257691044502702,0.260540507215766,-
0.989063264121522,0.322632189742137,0.881276096337479
"4877","PAQR8",6.0933959448129,-0.565943677828901,0.56780895468678,-0.
996714956954301,0.318902887578867,0.881276096337479
"4878","DOP1A",14.2876334218797,0.424112744107834,0.428934395821128,0.
988758999603976,0.322781068680352,0.881276096337479
"4879","CCDC170",1.68429921621699,-1.13799768630579,1.13987097503491,-
0.998356578270565,0.318106481278741,0.881276096337479
"4880","TP53TG1",53.8256418770801,-0.345008124134042,0.348464160183334
,-0.990082090372009,0.322133996462456,0.881276096337479
"4881","LRRC17",167.865790071824,0.387565707124287,0.390644496891331,0
.992118691568562,0.321139629546719,0.881276096337479
"4882","INTS10",45.2997146355828,-0.256288249145461,0.258695904640926,
-0.990693105486897,0.321835458665791,0.881276096337479
"4883","AF117829.1",31.6456623566083,0.386088415971923,0.3880317114896
98,0.994991915711439,0.319740196250571,0.881276096337479
"4884","HGH1",10.4647327817563,-0.523404707156125,0.527808105625065,-0
.991657198095271,0.321364777039547,0.881276096337479
"4885","RGP1",11.6991653399111,-0.442860068610869,0.444039165877531,-0
.997344609761321,0.318597267419903,0.881276096337479
"4886","POLR1E",23.6314758182209,-0.436603269783242,0.437436854005985,
-0.998094389589928,0.318233590406176,0.881276096337479
"4887","BRD3",59.6100668367549,0.281507197537768,0.283046492710349,0.9
94561687877348,0.319949488818506,0.881276096337479
"4888","BEND7",17.2835197797321,-0.457677586527843,0.458983276403953,-
0.99715525609922,0.318689155510266,0.881276096337479
"4889","CPEB3",3.65530717920841,0.803529257219615,0.810441283188658,0.
99147128100157,0.321455508999802,0.881276096337479
"4890","SMC3",121.8496143991,0.203548534592584,0.204215662248509,0.996

733219927505,0.318894020416061,0.881276096337479
"4891","SLC25A22",2.1003999831682,1.10073418751697,1.11106741445664,0.990699730002684,0.321832222962737,0.881276096337479
"4892","IMMP1L",14.7486174019379,0.379581964623751,0.382535014891388,0.992280313820488,0.321060803730916,0.881276096337479
"4893","LRTOMT",7.74058192383198,-0.510354445606301,0.516410463898124,-0.988272859062326,0.323019033902675,0.881276096337479
"4894","SERPINH1",238.983328368159,0.279108809447912,0.280611333784056,0.994645532253164,0.319908694089238,0.881276096337479
"4895","PKNOX2",3.99916790498497,0.776574756888599,0.781357195647258,0.993879318210287,0.32028162430835,0.881276096337479
"4896","C1S",2172.67966332392,0.223063002309041,0.22440316807175,0.993863337399774,0.320289405482134,0.881276096337479
"4897","CLEC2B",214.37920780476,-0.277037402994392,0.279489416613812,-0.991226810484893,0.321574841879644,0.881276096337479
"4898","SLC48A1",24.5541183261868,0.316142129215296,0.319386922867747,0.989840555701792,0.322252058468513,0.881276096337479
"4899","PFDN5",792.224334527369,-0.257537955047314,0.258514428995188,-0.996222748758475,0.31914192864193 2,0.881276096337479
"4900","MDM2",37.6070453300413,0.267453926642221,0.270139518072595,0.990058502178668,0.322145525113778,0.881276096337479
"4901","PRDM4",17.025748265513,-0.385100198338247,0.38882091063122,-0.990430781392563,0.321963606289038,0.881276096337479
"4902","GCN1",19.3567820500097,0.410818885850898,0.412526901304002,0.995859626492953,0.319318354235405,0.881276096337479
"4903","TPT1-AS1",61.8402084850255,0.283060496785673,0.284353287269665,0.99545357644216,0.319515712177122,0.881276096337479
"4904","CDADC1",24.9990641296546,-0.329888196809202,0.332738578629127,-0.991433569766187,0.321473915019383,0.881276096337479
"4905","RBM26-AS1",5.36033282854469,0.650252881596189,0.652895629940213,0.995952265227651,0.319273338969421,0.881276096337479
"4906","LAMP1",388.179109499737,0.158557438566081,0.15916655309707,0.996173099692512,0.319166047235807,0.881276096337479
"4907","HAUS4",30.2399477986179,-0.385642794221902,0.387903135741998,-0.994172922794832,0.320138688077206,0.881276096337479
"4908","IRF9",56.0743914023285,-0.303318094926954,0.305664867186762,-0.992322401061639,0.321040279169443,0.881276096337479
"4909","GPATCH2L",47.4525362863831,0.325483921583682,0.327181137431183,0.994812610956652,0.319827411595502,0.881276096337479
"4910","AL049840.2",8.29522873074793,0.561832226614668,0.568345129779038,0.988540584192388,0.322887968619529,0.881276096337479
"4911","UBAP1L",3.0333992892062,0.869606940441902,0.876324248714391,0.992334677167334,0.321034292678453,0.881276096337479
"4912","AC104041.1",2.29916060692158,1.0079468489433,1.01661394187265,0.991474548427511,0.321453914273892,0.881276096337479
"4913","ITGAM",2.29299237853732,-0.980703501278926,0.990491495074314,-0.990118043573253,0.322116424972436,0.881276096337479
"4914","NCBP3",47.0041982439524,0.252119521627169,0.254017426209337,0.

992528447317613,0.320939809566529,0.881276096337479
"4915","CD68",89.9452436157077,-0.242112996400288,0.244726466349539,-0
.989320852835267,0.322506184644549,0.881276096337479
"4916","MRPL58",24.6502534409641,-0.348761308445315,0.352455889966551,
-0.989517605957483,0.322409960222039,0.881276096337479
"4917","EIF4A3",74.4970918041013,-0.236750107134795,0.238988705060781,
-0.990633038806515,0.32186479882711,0.881276096337479
"4918","VMAC",12.0631946315474,-0.434006304524461,0.437024452685623,-0
.993093868906843,0.320664211567732,0.881276096337479
"4919","SYMPK",27.4046757301116,0.317568960151696,0.320434664604556,0.
991056821344855,0.321657835367951,0.881276096337479
"4920","ZNF784",2.86353434021168,0.893855880335669,0.903541487561466,0
.989280395688373,0.322525973008729,0.881276096337479
"4921","ZSCAN22",2.63961146178967,0.980963742778995,0.992606048539658,
0.988270970363528,0.323019958641516,0.881276096337479
"4922","AAR2",17.3828730929771,0.386109521509908,0.387097728087133,0.9
9744713929966,0.318547519914581,0.881276096337479
"4923","ZNF512B",11.1470513070374,0.485225820815602,0.490127995947662,
0.989998173594263,0.322175011734347,0.881276096337479
"4924","DNAJC28",1.48981825548815,-1.18373176735058,1.18648120313083,-
0.997682697565719,0.318433245915969,0.881276096337479
"4925","MTMR3",35.8606050564254,0.275572861126768,0.278835836941484,0.
988297860667741,0.323006792856925,0.881276096337479
"4926","UPRT",7.95544478442769,0.534462458073585,0.536261840663321,0.9
96644582080444,0.318937057975028,0.881276096337479
"4927","METTL9",116.990261214959,-0.239192702036343,0.24209732454049,-
0.988002252773092,0.323151544906519,0.881428094670004
"4928","TUBG1",12.1530168913436,-0.437072423069092,0.442437409815207,-
0.987874020986707,0.323214350065762,0.881428094670004
"4929","LZIC",75.9776480264747,0.207508432985906,0.210322124671294,0.9
8662198905709,0.323827986354011,0.881472147576734
"4930","TNPO1",72.015573516436,0.256765280005882,0.260242067716582,0.9
86640177965052,0.323819066316467,0.881472147576734
"4931","SEC24A",19.7276802982546,0.354247299466513,0.358935008436574,0
.986939950520626,0.323672077666771,0.881472147576734
"4932","MTCH2",29.329402741922,-0.349362459581419,0.353890341663858,-0
.987205409276924,0.3235419505369,0.881472147576734
"4933","CTSC",23.8788459041113,0.403190826197736,0.408343700786828,0.9
8738103568351,0.323455877711678,0.881472147576734
"4934","RAP2A",34.4945903360609,-0.289284561094003,0.293242494641333,-
0.986502864967879,0.323886410055355,0.881472147576734
"4935","TRIM16L",3.60115797411417,-0.792958463615075,0.803783757487963
,-0.986532081829172,0.323872080183937,0.881472147576734
"4936","CCL13",2.30593509512037,-1.21391041861172,1.23024603502173,-0.
986721667093425,0.323779105130066,0.881472147576734
"4937","GMFG",6.14304635491876,-0.602653333922599,0.610421248023859,-0
.987274502441703,0.323508086855662,0.881472147576734
"4938","GYS1",6.51884967686623,0.584884882744185,0.592286117486876,0.9
87503953707214,0.323395645639769,0.881472147576734
"4939","PLEKHA5",116.337716246565,-0.193726610266886,0.196439154856799

,-0.986191426083611,0.324039185845436,0.88170937812853
"4940","PTGES",230.438398056442,-0.28227612655905,0.286287294592399,-0
.985989011356373,0.324138505192383,0.881801087303733
"4941","PTPN4",13.2513615916669,0.485690230933539,0.492759242242377,0.
985654228875202,0.324302817268849,0.882069532741564
"4942","AC073332.1",15.8109682840125,-0.437435931141727,0.444069568464
619,-0.985061716014841,0.32459375697234,0.882325141589209
"4943","TIMM23B",9.77716498120504,-0.452728749181528,0.4595034884713,-
0.98525639204109,0.324498147089825,0.882325141589209
"4944","HSPA2",2.56985691633157,-1.01212912254393,1.02746999133741,-0.
985069278010244,0.324590042764545,0.882325141589209
"4945","VPS37C",10.3637332357287,0.453193519056276,0.460133867475702,0
.984916675537272,0.32466500162153,0.88234033504383
"4946","FILIP1",122.093058548686,0.22428924313782,0.227797574191081,0.
984598909511133,0.32482112553942,0.882459001525157
"4947","ITGAE",52.5954998762484,-0.284837484474775,0.289304193021722,-
0.984560512240443,0.324839994087726,0.882459001525157
"4948","FIZ1",4.37417492401985,0.700619900175037,0.711769637690354,0.9
84335188065204,0.324950733522419,0.882581428417096
"4949","GTF2E2",29.3726187344028,-0.292628918183628,0.297385342315713,
-0.984005855517129,0.325112633846744,0.882842733131217
"4950","AC016831.7",2.31760736249102,-0.974735996865178,0.991173792778
285,-0.983415828754883,0.325402823020042,0.883132131735929
"4951","COQ4",45.0612052236064,-0.238873414824988,0.24296919765671,-0.
983142789821826,0.325537167056257,0.883132131735929
"4952","AL353796.2",1.766137152045,-1.2348419725132,1.25638368565658,-
0.982854192242936,0.325679205661378,0.883132131735929
"4953","ATP2A2",49.9762767939109,0.299882520409872,0.305093082721275,0
.982921401347656,0.325646123865353,0.883132131735929
"4954","SNHG21",6.52437045349136,0.580284005864931,0.590353171385463,0
.982943827511079,0.325635085701909,0.883132131735929
"4955","GABARAP",711.801429169802,-0.259805554410549,0.264307091993854
,-0.982968532742174,0.325622926062433,0.883132131735929
"4956","PIP5K1C",26.0085156612916,-0.303359132093252,0.30860009608804,
-0.983016972252358,0.325599085532315,0.883132131735929
"4957","CRYBG3",52.927636773673,0.314662723115745,0.320409680661945,0.
982063720626896,0.326068457134639,0.883474595853308
"4958","PSMB1",374.618972126305,-0.274139221834168,0.279111211849356,-
0.982186347935488,0.326008052030922,0.883474595853308
"4959","ABL1",85.2558017787088,0.21019034551672,0.214007491766559,0.98
2163492416412,0.326019309901418,0.883474595853308
"4960","HPRT1",9.59260319056655,-0.456335948241927,0.464616973369703,-
0.982176662493158,0.326012822727657,0.883474595853308
"4961","OGDH",55.1433525070046,0.25266250126893,0.257369269312038,0.98
1712004484103,0.326241749683053,0.88374620453696
"4962","RABL6",51.2416702331083,-0.234164566194362,0.238563707570444,-
0.981559888463828,0.326316716652007,0.88374620453696
"4963","FAM227A",1.49380472223677,1.1775575962953,1.19980199851111,0.9
81459939020431,0.326365980587613,0.88374620453696
"4964","ABHD17C",2.14742991505493,-0.952124956103094,0.970331849845812

,-0.981236425718057,0.326476165221711,0.883866475506562
"4965","KLC4",6.18490227352369,0.619666066377786,0.631622310663196,0.981070579547995,0.326557937494427,0.883909792948157
"4966","RPS10-NUDT3",1.52667971621639,-1.16090171656362,1.18361306002924,-0.980811851243805,0.326685532892594,0.884077099585897
"4967","PARP9",29.2669495453717,0.299014570529059,0.304989772754883,0.980408516089403,0.326884507724899,0.884259440280781
"4968","ZNF785",11.8033148075828,0.484886360280653,0.494531100846717,0.980497201188053,0.326840750505179,0.884259440280781
"4969","NASP",82.4068474838665,0.21455994048292,0.219034676766708,0.979570649041319,0.327298099025366,0.884374869955662
"4970","HDAC4-AS1",2.59631939862917,0.971105688456421,0.990947182915546,0.979977243185911,0.327097351976029,0.884374869955662
"4971","LAMB2",402.848704840691,0.22241762442708,0.227193013304204,0.978980916676649,0.327589408716533,0.884374869955662
"4972","GMPPB",13.4552288051476,-0.472022490049325,0.482218434583074,-0.978856170145041,0.327651051232178,0.884374869955662
"4973","NMD3",37.8811368703989,-0.251849047203217,0.257152444920724,-0.979376444508851,0.327394011526338,0.884374869955662
"4974","AC058791.1",14.0136552568548,-0.593342497460262,0.605646450110959,-0.979684595446001,0.327241832391436,0.884374869955662
"4975","FAM118B",8.32798217296555,-0.550664383113574,0.56202443348468,-0.979787266007865,0.327191139137961,0.884374869955662
"4976","PTPN6",2.57779011784034,-0.84628751884796,0.864387071839086,-0.979060824044237,0.327549927075745,0.884374869955662
"4977","LRRC57",18.3971207501923,-0.365471561158717,0.373238590471966,-0.979190176172759,0.327486021690187,0.884374869955662
"4978","LCMT1",33.1706104291178,-0.335696809106456,0.342933253818547,-0.978898387276493,0.327630189126638,0.884374869955662
"4979","SLC37A1",5.49928200412402,0.620729218392729,0.633785455769419,0.97939959451919,0.327382577388497,0.884374869955662
"4980","PPIA",657.585934203267,-0.225439273251645,0.230351233114674,-0.978676216330111,0.327739987253621,0.884437286887833
"4981","PPCS",101.442857361017,-0.211007949447402,0.215987516666475,-0.976945115644057,0.328596324700848,0.884439416714339
"4982","GREB1",2.20433237085552,-1.00387922508952,1.02739624800801,-0.977110075139865,0.328514660383847,0.884439416714339
"4983","ACYP2",20.1601120016574,0.33821168516631,0.346106657420961,0.977189192737634,0.328475497350744,0.884439416714339
"4984","RBM45",10.5183981846695,-0.440968801550047,0.451254930338038,-0.977205503815137,0.328467423780475,0.884439416714339
"4985","ANKRD44",9.27838668525631,-0.520389883322738,0.532500639510013,-0.977256823206035,0.328442022821077,0.884439416714339
"4986","CC2D2A",29.7823784712961,-0.40647219514685,0.415810117533909,-0.977542820645057,0.32830048934098,0.884439416714339
"4987","LIFR-AS1",4.71228617241763,0.738275208727081,0.754575980710835,0.978397441211423,0.327877793255852,0.884439416714339

"4988","MOB3B",8.21743684627633,-0.780202799788179,0.798028132685347,-
0.977663277562425,0.328240889855371,0.884439416714339
"4989","WASHC2C",36.334118860503,-0.274492027207722,0.280600985229677,
-0.978229021480607,0.327961065852172,0.884439416714339
"4990","AXIN2",3.50455696634627,0.851840068228539,0.870900182196424,0.
978114467814423,0.328017713024973,0.884439416714339
"4991","ABHD17A",109.859216661292,-0.246623040699329,0.252301210915423
,-0.977494478938519,0.328324409748589,0.884439416714339
"4992","AC006504.5",4.77764740314156,0.701299148136959,0.7178094977206
26,0.976998981434357,0.328569656628874,0.884439416714339
"4993","NAGA",27.2798140775509,0.30462195209302,0.311730842031923,0.97
7195423165171,0.328472413431716,0.884439416714339
"4994","ALOX12-
AS1",16.1874393115839,-0.383964096207192,0.393200504516569,-0.97650967
3300819,0.328811956612402,0.88484258808852
"4995","EMC1-
AS1",6.81374487492866,-0.600593656124562,0.615795981376664,-0.97531272
4162123,0.329405160714011,0.885180270900149
"4996","BCL9",8.36479934053886,0.506361352209673,0.519086879577264,0.9
75484783244851,0.32931984614775,0.885180270900149
"4997","TUBA4A",4.03053087900955,0.767430846987101,0.787356985783234,0
.974692370607074,0.329712878544549,0.885180270900149
"4998","AEBP1",629.009993053433,0.172335717782788,0.176823212933548,0.
974621572154972,0.329748008973581,0.885180270900149
"4999","EIF4H",129.770721158253,-0.202376418628236,0.207665772617832,-
0.974529485899781,0.329793706108866,0.885180270900149
"5000","ARHGAP21",203.53064525027,0.21426534516356,0.21974553483183,0.
975061201255064,0.329529902751924,0.885180270900149
"5001","WNT5B",6.97831255020974,-0.50310462209731,0.516028383708401,-0
.974955328003056,0.329582419426606,0.885180270900149
"5002","KCTD13",9.05641960239699,0.56109593282235,0.574864060999913,0.
976049767046464,0.329039802683631,0.885180270900149
"5003","UBALD2",29.3383758421897,-0.298457707808153,0.30614369592546,-
0.974894181328565,0.329612752692256,0.885180270900149
"5004","GAREM1",12.3660110790837,0.456437446684953,0.468211503552399,0
.974853123474937,0.329633121434126,0.885180270900149
"5005","EMC10",56.9620427774573,0.259888158977259,0.26661864286271,0.9
74756139281241,0.32968123838992,0.885180270900149
"5006","ZNF28",6.50236327677058,0.651978810964438,0.668809635409573,0.
974834656150209,0.329642283313067,0.885180270900149
"5007","LINC01260",1.70658189702533,-1.17782791056138,1.2081215007944,
-0.974925046683547,0.329597440971058,0.885180270900149
"5008","LINC00857",2.62815367154058,-0.818074846145527,0.8397093849197
97,-0.974235683007954,0.329939531075417,0.885218079082157
"5009","XPNPEP2",34.0671084174573,0.388411709920359,0.398652998076197,
0.97431026931878,0.329902507246849,0.885218079082157
"5010","CCDC85C",1.84852991318699,1.19876365999555,1.23064622474076,0.
974092826919507,0.330010450785873,0.88523162636953
"5011","C1orf198",8.77226926934516,-0.512838314677557,0.52660810603384
5,-0.973851919105471,0.330130069810957,0.885317905794473

"5012","ELOVL5",34.4375538826552,0.311558429776113,0.320007803143026,0
.97359635207665,0.330256998321652,0.885317905794473
"5013","LINC02185",2.52374179544099,-1.08605902049424,1.11562594270508
,-0.973497459068455,0.330306122453567,0.885317905794473
"5014","ACSS1",6.36225177623685,0.669523202142882,0.687627561978663,0.
973671270849461,0.330219786304703,0.885317905794473
"5015","EXOSC10",25.7883213620235,0.335375453536851,0.344971427113983,
0.97218328005478,0.330959377008181,0.885832118624368
"5016","LINC01359",6.84043182427858,0.675441205133615,0.69470290206961
6,0.97227347564172,0.330914515724741,0.885832118624368
"5017","GTDC1",16.8148199307447,0.395229700735967,0.406420050210957,0.
972466049671562,0.33081874684175,0.885832118624368
"5018","NLN",10.0995576227998,0.463587304798833,0.476507091969589,0.97
2886474538388,0.330609727899804,0.885832118624368
"5019","DPM2",48.2447945180991,-0.295529704483174,0.303882570227686,-0
.97251285015046,0.330795475230463,0.885832118624368
"5020","RAD51-
AS1",16.0279813545232,0.390509061428118,0.401628951477908,0.9723130267
1565,0.330894845138972,0.885832118624368
"5021","SEMA4B",2.14428424450544,0.992006161962299,1.02030723783703,0.
972262202182623,0.33092012267801,0.885832118624368
"5022","FIGN",9.77220328890756,0.493053593720404,0.507821586018454,0.9
70918935498907,0.331588648001145,0.885948212740112
"5023","GTF3C3",16.7850664127914,0.354120391315515,0.364728590471019,0
.970914813281284,0.331590700914916,0.885948212740112
"5024","YME1L1",97.9096390670141,0.207091381380418,0.213277329666605,0
.970995753295218,0.331550393321321,0.885948212740112
"5025","MAP4K2",4.50505266274975,-0.79519426266533,0.818287774468401,-
0.971778251461628,0.331160877738837,0.885948212740112
"5026","CEP290",52.4013179168348,0.26724868800978,0.275155033865821,0.
971265850582635,0.33141590957483,0.885948212740112
"5027","SSH1",14.4621733828039,0.511580164871036,0.526839946882291,0.9
71035260136293,0.331530720325224,0.885948212740112
"5028","EIF2B1",53.8826399391775,-0.225207985310458,0.231828101411916,
-0.971443858353931,0.331327297269976,0.885948212740112
"5029","RNF40",23.7839034735607,0.33512180838538,0.344837307153186,0.9
7182584782368,0.331137194552329,0.885948212740112
"5030","OPA3",6.65389865655942,0.566964730858593,0.583955473939957,0.9
7090404347659,0.331596064445477,0.885948212740112
"5031","FER",54.3751973023601,0.26875198889131,0.276927259622791,0.970
478634921616,0.331807969394805,0.8863381635255
"5032","AHNAK",1693.25212644682,0.168891990805767,0.174085660856507,0.
970166008933837,0.331963750755742,0.886401916631514
"5033","AC130650.2",3.15903914951145,0.880224130520756,0.9072503903058
55,0.970210803903884,0.331941426545899,0.886401916631514
"5034","ANGEL1",2.95445598285889,0.889374622245789,0.916916498523828,0
.969962503322408,0.332065182884825,0.886496621531419
"5035","ZSCAN12",3.22663858563279,-0.810928791535578,0.836293586903354
,-0.969669986993805,0.332211015189268,0.886709798039437
"5036","PLEKHM2",71.7374321430378,0.22660836708068,0.234245652336978,0

.96739625610933,0.333345980938974,0.887232699551082
"5037","ZBTB17",11.6041953750739,-0.442002175346408,0.456503598496856,-0.968233715575962,0.33292766014555,0.887232699551082
"5038","TGFBR3",570.063727934492,0.218634840124172,0.226488703569921,0.965323376742612,0.334382866562122,0.887232699551082
"5039","GNAI3",108.998360079217,-0.223962648619657,0.231429382162219,-0.967736449569191,0.333176009438478,0.887232699551082
"5040","SSR2",443.592239049358,-0.264574801591379,0.273573559872836,-0.967106622856246,0.333490735040551,0.887232699551082
"5041","IARS2",68.7966931430855,0.220327497631132,0.22807484195712,0.966031569902636,0.334028384042048,0.887232699551082
"5042","TAF1A-AS1",1.64117800619834,1.37492935626377,1.42604053039237,0.964158680598979,0.33496637652639,0.887232699551082
"5043","ERCC3",19.9337740156362,0.37344915198284,0.386382997620716,0.966525841671293,0.333781122442673,0.887232699551082
"5044","UGGT1",35.3898641244194,0.284384354781569,0.295123399215979,0.963611680866584,0.335240648341019,0.887232699551082
"5045","ITPRID2",22.0688392096966,0.341437462921572,0.352947152280663,0.967389765621516,0.333349224337541,0.887232699551082
"5046","DCP1A",41.3474204438239,0.292795639468271,0.303221776108879,0.965615475331614,0.334236628710086,0.887232699551082
"5047","CDKN2AIP",26.4653604259044,0.317684396549706,0.329198395593801,0.965024133780098,0.334532723978606,0.887232699551082
"5048","TRIM52-AS1",24.1901757448559,-0.327514641646253,0.339667619595124,-0.964220970007807,0.334935153087903,0.887232699551082
"5049","RANBP9",26.72786856594,0.322818283485617,0.33409106865391,0.966258346223643,0.333914923540894,0.887232699551082
"5050","RRP36",38.9563254998442,-0.265797860703773,0.275066681480955,-0.966303367869643,0.333892401313712,0.887232699551082
"5051","BUD31",133.86831763837,-0.259776344504992,0.269588977022242,-0.963601506910126,0.335245751046982,0.887232699551082
"5052","PRKRIP1",28.1669372511967,0.28618617372766,0.296065016989258,0.966632858680646,0.333727602271454,0.887232699551082
"5053","THEM6",3.64612752674083,0.788133844830185,0.817032959374062,0.9646291937035,0.334730571660237,0.887232699551082
"5054","CDC14B",38.7832463730722,0.284008361519923,0.294443258212433,0.964560585438905,0.334764949081817,0.887232699551082
"5055","RUFY2",42.8501410685953,0.320389049555437,0.331657277116839,0.966024482684779,0.334031930312354,0.887232699551082
"5056","PPP3CB",60.5382291624978,0.244084009316842,0.251918511827791,0.968900647855904,0.33259476220276,0.887232699551082
"5057","GOT1",11.6325462085966,-0.446202554426524,0.462549937156712,-0.964658123551644,0.334716076514405,0.887232699551082
"5058","SH3PXD2A",56.1429223387313,0.33852115228189,0.350909355908079,0.964696855704711,0.334696670609398,0.887232699551082
"5059","APBB1",17.5863183014478,-0.336678836598379,0.348716247801681,-0.965480784795125,0.334304055800785,0.887232699551082
"5060","FBXO3",36.6769526965303,-0.255065504235841,0.263995945690849,-

0.966172050742529,0.33395809587935,0.887232699551082
"5061","PRDX5",400.439459346794,-0.253787965805855,0.262448448168282,-
0.96700120567345,0.333543430940792,0.887232699551082
"5062","ANAPC15",16.3689734806855,-0.413691844941625,0.429162978829372
,-0.963950446215219,0.335070770540417,0.887232699551082
"5063","AC021054.1",6.13419712159088,0.59441789120202,0.61670902695541
,0.963854695198094,0.335118780372027,0.887232699551082
"5064","SP1",43.0262685517965,0.320129717798849,0.331792497722667,0.96
4849175301227,0.334620361212826,0.887232699551082
"5065","FAM124A",1.49830256008287,1.16010026020807,1.19958072877903,0.
967088110350733,0.333499988675729,0.887232699551082
"5066","AL049828.1",1.69513433931029,1.09518299329224,1.13454583471635
,0.965305199473097,0.334391968293855,0.887232699551082
"5067","TMEM251",28.0126745186409,-0.337748685396075,0.350114674211956
,-0.964680175591856,0.33470502772918,0.887232699551082
"5068","ZNF710",4.95490714745252,-0.814510467359027,0.84175693727831,-
0.967631428132472,0.333228475527269,0.887232699551082
"5069","RNPS1",81.8029369758751,-0.210241336410066,0.217318856320708,-
0.967432554954193,0.333327842212259,0.887232699551082
"5070","RPGRIP1L",10.9134841239932,0.474876145362528,0.492458546201604
,0.964296688574722,0.334897200621126,0.887232699551082
"5071","TTC19",31.6971207430506,0.274948703773845,0.284492943623908,0.
966451751918739,0.333818178639027,0.887232699551082
"5072","SS18",57.4597422666206,0.21108538727674,0.218770350904489,0.96
4872005754089,0.334608924530192,0.887232699551082
"5073","TLE5",334.367285967117,-0.203360757777347,0.209900182583282,-0
.968845073284583,0.332622493928286,0.887232699551082
"5074","PPAN",11.1739340218122,-0.417215065983763,0.432906593806591,-0
.963753086584219,0.335169732065195,0.887232699551082
"5075","KLF2",550.716686583899,0.278453748274963,0.288491644828259,0.9
65205590063894,0.334441847603919,0.887232699551082
"5076","EML2",5.28558506568546,-0.621514977747651,0.641420176077728,-0
.968966990636625,0.332561659093174,0.887232699551082
"5077","CTNNBL1",47.0170188904312,-0.332293105901416,0.344674562768553
,-0.964077833978566,0.335006904823522,0.887232699551082
"5078","SLC9A8",5.82311617716432,-0.607992385872588,0.628127048167814,
-0.967944920770476,0.33307187822628,0.887232699551082
"5079","SLA",3.20684648825086,0.881232189511873,0.915020630267925,0.96
3073574913651,0.335510601812538,0.887442206730838
"5080","TESK1",33.7436217307418,0.345458234273271,0.358687564014391,0.
96311740057821,0.335488610405613,0.887442206730838
"5081","CDCA4",10.0532806429053,-0.468484222530729,0.48652594417421,-0
.96291724653224,0.335589053843747,0.887442206730838
"5082","ARHGEF18",13.3785939303261,-0.414956463445861,0.43089243620730
7,-0.96301635530734,0.335539315601306,0.887442206730838
"5083","DDX20",9.01923271043135,-0.45594749207223,0.473639155710448,-0
.962647379497835,0.335724512046848,0.887600908023377
"5084","DYNLL2",32.4828924162284,-0.292707244338429,0.304142073819271,
-0.962403000225361,0.33584720718051,0.887600908023377
"5085","AURKC",2.6088922957102,-1.00172856195298,1.04072915914525,-0.9

62525699554442,0.33578560010546,0.887600908023377
"5086","GRAMD1A",30.5730309183536,-0.283171634768749,0.294303154867779
,-0.962176687830509,0.335960857242773,0.887726692977905
"5087","NPEPL1",44.5115876484222,-0.259503412948524,0.269873005343616,
-0.961576029503623,0.336262617196694,0.88834938323302
"5088","LINC02802",309.679038145476,0.213343203672588,0.22248167322640
4,0.958924843465571,0.337596608296355,0.88903814429634
"5089","CIAO1",63.6839525561367,0.230093393412091,0.239470351518962,0.
960842926703065,0.336631151115734,0.88903814429634
"5090","CCDC39",2.89481489998082,-0.778123899957487,0.810586164453849,
-0.959952111299315,0.337079317328754,0.88903814429634
"5091","SLC30A9",30.324613266964,0.329075737347065,0.343087756708648,0
.959159080766961,0.337478610893487,0.88903814429634
"5092","HPGD",29.5328695574418,-0.360703463923482,0.375741084120804,-0
.959978770401142,0.337065899659387,0.88903814429634
"5093","FAM50B",18.5709295676154,-0.36533674386571,0.380969261316152,-
0.958966459927934,0.337575642002687,0.88903814429634
"5094","GOPC",85.3531187058024,0.196714016455242,0.205094398905872,0.9
59138901426187,0.337488775224183,0.88903814429634
"5095","LUC7L2",70.398648130485,-0.200439740899182,0.208995323337123,-
0.959063282846094,0.337526866040681,0.88903814429634
"5096","PDIA4",79.8021625165483,0.240352134625105,0.250425100869751,0.
959776531147789,0.337167696341773,0.88903814429634
"5097","PLGRKT",35.4844116777681,-0.33863270897877,0.35336874757729,-0
.958298410089882,0.337912305312997,0.88903814429634
"5098","APTX",24.8316425881784,0.321287728129677,0.334894076143424,0.9
59371189331161,0.337371783745761,0.88903814429634
"5099","TOMM5",149.192697066815,-0.24749524587197,0.258151746465644,-0
.958720013559575,0.337699813402542,0.88903814429634
"5100","PWWP2B",1.56394128171685,-1.06343913578186,1.10929955841102,-0
.958658216095522,0.337730954504929,0.88903814429634
"5101","SIX1",31.9697488297366,0.338447886122191,0.352726914256049,0.9
5951817806709,0.337297766541644,0.88903814429634
"5102","COX16",122.328979348633,-0.246883797076737,0.257120410498975,-
0.960187472467191,0.336960870653261,0.88903814429634
"5103","SRP14",942.728231670556,-0.199043464126123,0.207705204714184,-
0.95829791265953,0.337912556073049,0.88903814429634
"5104","TCF12",45.903114540545,0.262490731816266,0.273785833543282,0.9
5874475468348,0.337687346322798,0.88903814429634
"5105","ELOB",405.981361520094,-0.238292385112929,0.248421129859985,-0
.959227523227333,0.337444137902423,0.88903814429634
"5106","SPC24",2.11158404636342,-1.0700561694943,1.11486715602203,-0.9
59805985595969,0.337152869280761,0.88903814429634
"5107","ZNF257",2.10834257151203,1.00695127049528,1.0503478650673,0.95
8683598058022,0.337718163752066,0.88903814429634
"5108","AC010642.2",42.5759492199482,-0.271120604365099,0.282909105461
552,-0.958331135799886,0.337895808189157,0.88903814429634
"5109","MIR503HG",5.26009418225163,0.971707867312356,1.01421483597136,
0.958088792284041,0.338017986517617,0.889141460326923
"5110","FHDC1",13.4463092264724,0.444564860765974,0.464190292568625,0.

957721149888654,0.338203388879811,0.889455057369038
"5111","NOC2L",29.3546166437526,-0.273834282633833,0.286926757489352,-0.954369975912739,0.339896398478844,0.88949685586072
"5112","SLC22A15",2.03267183514843,1.07179748247731,1.12128350042478,0.95586663147302,0.339139619361925,0.88949685586072
"5113","ZNF687-AS1",1.55770465031143,1.2218370181372,1.27947491952247,0.954951909954763,0.339602016845086,0.88949685586072
"5114","TBX19",3.32121065766716,0.829728497005943,0.869315052589361,0.954462360377282,0.339849653241845,0.88949685586072
"5115","SPATA17",2.55658944301752,-0.890901528177588,0.9319723614226,-0.955931275491562,0.339106956692335,0.88949685586072
"5116","MSX2",2.81657050975737,-0.767914224468437,0.803753826355281,-0.955409727815091,0.339370536522948,0.88949685586072
"5117","GRK6",10.3517329611392,-0.43354214932868,0.453159333340607,-0.956710184324545,0.338713556268169,0.88949685586072
"5118","ADTRP",1.4880078486712,-1.07069039606816,1.12054524154658,-0.955508404632007,0.339320657144079,0.88949685586072
"5119","FAM3C",167.612771750431,-0.198648354318323,0.207587617660191,-0.956937396157698,0.338598854489355,0.88949685586072
"5120","AC055822.1",1.75513009323304,1.2032894386782,1.25960925333456,0.955287868434381,0.33943214076589,0.88949685586072
"5121","PDCL",45.6171184004385,0.252152060028559,0.263595921457678,0.956585589921747,0.338776464985304,0.88949685586072
"5122","ZNF215",3.12617630994721,-0.8155388465263,0.854842866123969,-0.9540219364807,0.340072538538015,0.88949685586072
"5123","MEN1",10.9176957242678,-0.402209384050602,0.421085814781954,-0.955172009911741,0.339490718012806,0.88949685586072
"5124","UGGT2",37.3152514035018,0.287672909870093,0.30044517897199,0.957488853222413,0.338320569940545,0.88949685586072
"5125","PSMC1",122.744493622945,-0.248249639578561,0.259923027477803,-0.955089058431968,0.339532661674705,0.88949685586072
"5126","SEMA6D",2.4220075070187,0.929988345959766,0.974766406088533,0.954062778683101,0.340051865586479,0.88949685586072
"5127","UNC45A",33.6496382570053,-0.273999774618246,0.286491466829911,-0.956397681404309,0.338871355690503,0.88949685586072
"5128","BCL7C",141.301573226232,-0.251283597630754,0.26324973862417,-0.954544528492395,0.339808080796868,0.88949685586072
"5129","RFLNB",11.8409732112296,-0.574680728041207,0.602322061180078,-0.954108715386058,0.340028614932327,0.88949685586072
"5130","GPS2",78.0253577027484,-0.207152826424535,0.217131972488141,-0.954041102518191,0.340062837233046,0.88949685586072
"5131","TMUB2",35.5291178198899,0.289767368155036,0.303550444541144,0.954593786192792,0.339783160733971,0.88949685586072
"5132","WDR18",26.0293775502165,-0.341253230336212,0.357124199812236,-0.955558963844039,0.339295102182177,0.88949685586072
"5133","PLD3",264.139861361246,0.203223955366379,0.212494283282183,0.956373753812973,0.338883439954006,0.88949685586072
"5134","KPTN",6.36578799702226,0.581559773941007,0.608208640698136,0.95618466267342,0.338978947265419,0.88949685586072

"5135","HMGXB4",47.547581086346,0.244239798103173,0.255525483727235,0.955833424285348,0.339156398735322,0.88949685586072
"5136","APOL2",26.8097322614547,0.343573707786553,0.36007635904222,0.954169023205069,0.339998091949956,0.88949685586072
"5137","PRPS2",16.8435303825036,-0.349265476860698,0.364993731362186,-0.956908151702255,0.338613616367371,0.88949685586072
"5138","UBE2A",70.7427644815338,-0.221476559348335,0.231576063402627,-0.956387962098085,0.33887626424389,0.88949685586072
"5139","CASZ1",2.07717430656149,1.0660201043778,1.12319919894256,0.949092650156267,0.342573492205695,0.889814729093396
"5140","MAD2L2",22.8505520484444,-0.354811475320614,0.372890701589915,-0.95151601744904,0.341342491431005,0.889814729093396
"5141","OTUD3",9.94028210336846,-0.436760991572229,0.458632495552884,-0.952311482084825,0.340939036356158,0.889814729093396
"5142","MYCBP",7.14986583777848,-0.526397763668969,0.559356997215267,-0.941076568791695,0.346665622121824,0.889814729093396
"5143","CRYZ",18.1644404533823,0.364830233247841,0.38744650438474,0.941627370795837,0.346383451399562,0.889814729093396
"5144","USP33",95.2810434516912,0.219924522962799,0.232823731536359,0.944596676256154,0.344864824553193,0.889814729093396
"5145","PRPF3",17.670477186014,0.418030308650694,0.442513238010591,0.944673001264403,0.344825844821739,0.889814729093396
"5146","ATP1B1",11.6620837178948,0.549831983163034,0.583732674182518,0.941924287402689,0.346231404487508,0.889814729093396
"5147","RNF187",86.3977669979356,-0.260966194847693,0.276214628235575,-0.944794982491379,0.344763553965497,0.889814729093396
"5148","TOMM20",238.897506633018,-0.223093493477855,0.236289757684602,-0.944152195439798,0.345091879945149,0.889814729093396
"5149","SMC6",35.8060027850592,-0.280066681771456,0.297397862014246,-0.94172392455881,0.346334002880123,0.889814729093396
"5150","KCNS3",9.21167950963625,-0.609161711553583,0.644128734143928,-0.945714232673042,0.344294361140424,0.889814729093396
"5151","ADCY3",85.2544084559506,0.238268598954751,0.252418924318363,0.943941107419645,0.345199744013793,0.889814729093396
"5152","EMILIN1",109.262496274527,0.264843076294503,0.277921183986642,0.952943106011062,0.340618897947689,0.889814729093396
"5153","C2orf68",36.454759282242,0.289656290082766,0.306831923603493,0.944022664529124,0.345158066520603,0.889814729093396
"5154","MRPL35",28.371929637482,-0.284863307034152,0.301070474424366,-0.946168193937977,0.34406280597706,0.889814729093396
"5155","INHBB",12.9033650569664,0.56431851694677,0.599272219494305,0.941673080429075,0.346360041356002,0.889814729093396
"5156","ZEB2",166.533522306898,0.213472298235683,0.22448455916789,0.950944238779598,0.341632682986962,0.889814729093396
"5157","HOXD-AS2",30.8581303980819,-0.380724180431548,0.401519383664753,-0.948208718982872,0.343023209335593,0.889814729093396
"5158","CAVIN2",14.6615802098393,-0.437308775906738,0.461005409971695,-0.948597926288084,0.342825146103152,0.889814729093396
"5159","AC064836.4",1.90062959440461,-0.988833959135102,1.046195117563

99,-0.945171643925797,0.344571253581853,0.889814729093396
"5160","ITPR1",4.49703108701104,0.691447099674714,0.728258369166827,0.94945300864276,0.342390261045479,0.889814729093396
"5161","UBE2E2",61.737688838121,-0.199132558261023,0.20975802712555,-0.949344160935653,0.342445600135658,0.889814729093396
"5162","OSBPL10",26.8055923675278,0.336408421712637,0.356606748817173,0.943359661107,0.345496968965129,0.889814729093396
"5163","ANO10",21.2256271173659,0.359309524218464,0.378757553218612,0.948653092631732,0.342797078485374,0.889814729093396
"5164","ZNF654",12.5329488735994,0.405393645813387,0.425949063305219,0.951742076077527,0.341227804725638,0.889814729093396
"5165","ASTE1",12.8291150761003,-0.422180357973934,0.444992534323332,-0.94873582231197,0.342754989903539,0.889814729093396
"5166","AC092944.1",6.58692450680025,0.563710113393257,0.599109148902767,0.940913879258326,0.346748994438732,0.889814729093396
"5167","PRKCI",29.621944069771,0.322939260306063,0.341294800755802,0.946217931216383,0.344037442183366,0.889814729093396
"5168","TMEM44",21.4979332655331,0.371419454243852,0.391268533419984,0.949269932333591,0.342483341861225,0.889814729093396
"5169","IQCG",10.2231659968357,-0.41700334499142,0.442976051007155,-0.941367697064697,0.346516461598178,0.889814729093396
"5170","ZNF718",6.00008299787752,0.58009212717159,0.614385923953569,0.944181994663389,0.34507665454288,0.889814729093396
"5171","CCDC110",1.89194076432696,-1.06533141578067,1.13044369197308,-0.942401132710323,0.345987307534243,0.889814729093396
"5172","RAD1",22.420696404585,-0.358366887247796,0.375946395824998,-0.953239321423407,0.340468827602778,0.889814729093396
"5173","ZNF131",35.540375985592,0.278630719930144,0.295959078603121,0.941450153329425,0.346474222288629,0.889814729093396
"5174","CHD1",44.5561408756813,-0.28210067666884,0.297388851421692,-0.948591970816102,0.342828176225255,0.889814729093396
"5175","H2AFY",74.3805078614075,-0.227780693508647,0.239931726863596,-0.949356287666545,0.342439434520563,0.889814729093396
"5176","CCNG1",67.6255466163734,0.217712635656785,0.230208363459009,0.945719922532486,0.344291458259118,0.889814729093396
"5177","HCG11",47.3075166960594,0.242347906254644,0.256812489606863,0.943676480165111,0.345334996510374,0.889814729093396
"5178","ZSCAN31",2.20346578045341,0.962437419060729,1.01921149433562,0.944296080263594,0.345018368421314,0.889814729093396
"5179","CMTR1",14.4445361263345,-0.396105454521201,0.419853365681243,-0.943437606790386,0.345457115060909,0.889814729093396
"5180","CCDC167",29.2742874123,-0.307367302658678,0.325980032662146,-0.942902238976219,0.345730909583665,0.889814729093396
"5181","MPP6",7.19111348710767,0.521354608964258,0.551232132347189,0.945798654269827,0.344251292106128,0.889814729093396
"5182","HGF",6.6598457736682,-0.623963436788147,0.654618486918172,-0.953171120671608,0.340503376102948,0.889814729093396
"5183","AC100810.1",99.3782270753416,0.259849725771402,0.274573875842066,0.946374541184926,0.343957585880806,0.889814729093396
"5184","MCPH1-

AS1",1.46334751317912,1.33326943735344,1.40571046275729,0.948466610071
495,0.342891963280738,0.889814729093396
"5185","UBE2V2",79.9114394409901,-0.21325270734818,0.225069871307145,-
0.947495575972325,0.343386309515744,0.889814729093396
"5186","EXOSC4",17.2137304111897,-0.41713567596107,0.4388508043641,-0.
950518198469534,0.341849011400913,0.889814729093396
"5187","FAM214B",10.4257642640759,-0.464493180858072,0.488981417324509
,-0.949919903704264,0.342152952544002,0.889814729093396
"5188","TMEM8B",16.564914384126,0.347232456926388,0.366733760099473,0.
946824357899869,0.343728287624088,0.889814729093396
"5189","ABHD17B",8.18859406664157,0.493566558655185,0.520190381045781,
0.948819079781769,0.342712636144701,0.889814729093396
"5190","CARNMT1",8.657116127994,0.500813238051252,0.525447281025754,0.
953117955189696,0.340530309734298,0.889814729093396
"5191","ZNF79",1.63032399696245,1.14223409919914,1.20990706348023,0.94
4067634346697,0.345135087318064,0.889814729093396
"5192","ST6GALNAC6",70.9941328093852,-0.228139522522106,0.241168377819
039,-0.945976104268908,0.344160774347988,0.889814729093396
"5193","PROSER2",9.18376328518737,0.465544888618997,0.489527992610014,
0.951007696489128,0.341600468872489,0.889814729093396
"5194","MARCH8",20.5356880545352,0.340292346342802,0.359289808045774,0
.947124963532079,0.343575105532221,0.889814729093396
"5195","NUTM2B-
AS1",37.2385043597092,0.316257924494689,0.334353090985782,0.9458800681
70621,0.344209760753435,0.889814729093396
"5196","AL133482.1",7.04769668314394,0.720264470006624,0.7637609484324
64,0.943049617141186,0.345655524556164,0.889814729093396
"5197","FAM111A",38.0947856971015,0.280631598520738,0.296423863860674,
0.946724041936925,0.343779416160752,0.889814729093396
"5198","TM7SF2",1.95418566926674,-0.926160238862864,0.974927137926421,
-0.949978929535925,0.342122959008562,0.889814729093396
"5199","CTSW",2.09719170765733,1.08530807781754,1.1520236926614,0.9420
88330935514,0.346147418284065,0.889814729093396
"5200","MCRS1",27.2271255915649,-0.293657943584692,0.311780557756582,-
0.941873815666085,0.346257247370513,0.889814729093396
"5201","TBK1",28.3968556809009,0.317144385616204,0.336160359779971,0.9
43431836590686,0.345460065284026,0.889814729093396
"5202","WASHC3",98.125509679982,-0.294899498209676,0.31170847581369,-0
.946074685456867,0.344110494363492,0.889814729093396
"5203","HCFC2",33.2502459661834,0.280165757199883,0.294416328843112,0.
951597210320414,0.341301296875282,0.889814729093396
"5204","CDK2AP1",225.296251508125,-0.22460241346702,0.236259545415424,
-0.950659636088326,0.341777184593711,0.889814729093396
"5205","NCOR2",84.9269423002114,0.307022860336595,0.323872951468562,0.
947973144853369,0.343143125884899,0.889814729093396
"5206","CDK8",5.88403718517866,0.567631867481701,0.601826190542631,0.9
43182394521416,0.345587616909826,0.889814729093396
"5207","AKAP11",44.2755276826557,0.274479054715412,0.291261531954225,0
.942380041997955,0.345998101544875,0.889814729093396
"5208","MYCBP2",181.221333313461,0.217259986410116,0.228667034796263,0

.950115029058254,0.342053807462511,0.889814729093396
"5209","DICER1-
AS1",3.42384776680764,0.747674402222193,0.791577569830578,0.9445371252
52574,0.344895239631449,0.889814729093396
"5210","RPL4",703.478780841943,-0.187980672374123,0.19905274142401,-0.
944376204162382,0.344977437023385,0.889814729093396
"5211","TTC23",15.7479821852133,0.374075881248579,0.394266018276618,0.
948790572628366,0.342727137591166,0.889814729093396
"5212","MARF1",40.2614418667672,0.347443806061163,0.365100786047482,0.
951638066361154,0.341280569086172,0.889814729093396
"5213","SMG1",60.7082652056123,0.280168688739198,0.296831105133371,0.9
43865666010014,0.345238299105397,0.889814729093396
"5214","ITPRIPL2",71.96271759109,-0.343078807794305,0.360015067366801,
-0.952956803457229,0.340611957534962,0.889814729093396
"5215","SHISA6",11.0731676640903,0.623742411642848,0.658309471119362,0
.947491170956818,0.343388553113191,0.889814729093396
"5216","SUZ12",39.9014513397196,-0.249968622097809,0.265299507130857,-
0.942212915512554,0.346083642754491,0.889814729093396
"5217","MMP28",24.2962526064783,-0.366356622866351,0.384691375012613,-
0.952339061031298,0.340925053953183,0.889814729093396
"5218","NOL11",29.9312342150656,-0.284791907228906,0.299789305847616,-
0.949973536993566,0.342125699118875,0.889814729093396
"5219","NDUFV2-
AS1",4.44830353834085,-0.652266610630438,0.693152325497152,-0.94101481
9740539,0.346697264704215,0.889814729093396
"5220","PWWP3A",79.2548271566632,0.279827898678654,0.296134179670848,0
.944936173830664,0.344691462248644,0.889814729093396
"5221","OAZ1",963.418987232053,-0.211794392721989,0.222535390488476,-0
.951733529921199,0.341232140012814,0.889814729093396
"5222","LONP1",20.6208761936762,-0.323602239486145,0.339875744054286,-
0.952119252836291,0.341036505929084,0.889814729093396
"5223","TMED1",69.9004202794764,-0.312875305370039,0.328623615665028,-
0.952077971441221,0.341057439930094,0.889814729093396
"5224","UBA2",73.5525662740706,0.211758893112091,0.223738170485396,0.9
4645849947143,0.343914779951909,0.889814729093396
"5225","ZNF30",2.33825547479039,-0.889409135605259,0.939733958615813,-
0.946447797752589,0.34392023600818,0.889814729093396
"5226","GRIN2D",2.9792129592676,0.916439562521686,0.962056597069403,0.
952583834790308,0.340800970841877,0.889814729093396
"5227","SNRNP70",101.439618032806,0.242081659851736,0.256145445306537,
0.945094532374097,0.34461061647078,0.889814729093396
"5228","ZNF473",3.37018561429351,-0.794550736231593,0.844155346354522,
-0.941237581048149,0.346583121913661,0.889814729093396
"5229","PPP1R12C",33.1449343058892,-0.263460265057022,0.27747875762520
1,-0.949479042330462,0.342377026147056,0.889814729093396
"5230","RALY-
AS1",3.98893962065741,0.752196524621056,0.792478690983349,0.9491694012
46224,0.342534461393788,0.889814729093396
"5231","MMP24OS",87.9247356177534,-0.251319323320493,0.264583250786838
,-0.94986860495931,0.342179020984,0.889814729093396

"5232","ADA",54.4625006222193,-0.249840398345332,0.264975739312911,-0.942880276485593,0.345742144457907,0.889814729093396
"5233","SDC4",24.7961636292237,-0.33395300043803,0.354509282233987,-0.942014827746062,0.34618504848173,0.889814729093396
"5234","ELMO2",16.5891771160225,0.38275716327139,0.401732767666139,0.952765604595843,0.34070884502954,0.889814729093396
"5235","SNAP29",41.4010248186014,-0.243453017928489,0.256333423350171,-0.949751361904582,0.342238605057537,0.889814729093396
"5236","TOP3B",10.0481437904986,-0.452620833045035,0.479645234737368,-0.943657520735861,0.34534468807942,0.889814729093396
"5237","PARVB",20.413570325129,-0.331678851317692,0.351222809138699,-0.944354531333161,0.344988508310825,0.889814729093396
"5238","RPL7L1",123.610882479435,-0.18386844449491,0.195470379348668,-0.940646071837495,0.346886263549194,0.889997040060638
"5239","PIGA",4.57463961043406,0.672747248614651,0.715346845822571,0.940449031883358,0.346987281710902,0.890086291069442
"5240","GXYLT2",79.0150202657356,0.255654690304919,0.272015269750704,0.939854187374191,0.347292359308732,0.890165800851519
"5241","COL15A1",13.4363163611953,-0.533926742564834,0.568311780864416,-0.939496171894798,0.34747605676577,0.890165800851519
"5242","BAZ2A",37.9332977592693,0.297235654386797,0.316209108522597,0.939997129670146,0.347219032993662,0.890165800851519
"5243","GTF2A1",41.6825641160445,0.256162935268094,0.272634411662642,0.939584015480297,0.347430978570748,0.890165800851519
"5244","RABEP2",18.3771049040293,-0.330983616105299,0.352242707788679,-0.939646467582419,0.347398932642127,0.890165800851519
"5245","MAPK3",88.0722439125786,-0.245673554042778,0.26146184447224,-0.939615317633323,0.347414916321321,0.890165800851519
"5246","CDKL5",19.5833949566274,0.394149067709841,0.419537502217578,0.939484707866305,0.347481939970762,0.890165800851519
"5247","MAST2",21.2689444417719,-0.37123961451122,0.396122102207822,-0.937184803478731,0.348663505307115,0.890347158806245
"5248","KPNA1",24.6880354664846,0.313613593254022,0.3345376591072,0.937453780513023,0.348525187924158,0.890347158806245
"5249","PGM2",12.6492037246118,-0.417514800202768,0.445122114469295,-0.937978111243827,0.348255659002851,0.890347158806245
"5250","MFSD8",17.9584620024191,0.409030485566817,0.435715151757835,0.938756625553732,0.347855713074925,0.890347158806245
"5251","USP38",12.0564743108327,0.462981076042277,0.493702473199156,0.937773459067753,0.348360843401657,0.890347158806245
"5252","RNF5",42.7855415718799,-0.260914963260856,0.27820771288209,-0.937842306950839,0.348325455630513,0.890347158806245
"5253","EEF1A1",7790.64525821977,-0.200972598406308,0.214329408285474,-0.937680927755023,0.348408408040605,0.890347158806245
"5254","LTV1",33.5057477332852,-0.285142341696568,0.303647942375286,-0.939055734960829,0.347702129381,0.890347158806245
"5255","HOTAIRM1",11.9376153322747,-0.548669916129246,0.585099312556089,-0.937738097370691,0.348379020181161,0.890347158806245
"5256","AQP7",1.49994153407551,-1.12534932639466,1.19924246639268,-0.938383486185003,0.348047369394211,0.890347158806245

"5257","NANS",57.9460855287067,-0.298151973527671,0.317676049406061,-0
.938540925842874,0.347966495162635,0.890347158806245
"5258","NPM3",11.9032803995478,-0.410811953047042,0.437646119832379,-0
.938685240039111,0.34789237376716,0.890347158806245
"5259","WNT10B",26.0075138340387,0.326097927512012,0.347965961867465,0
.937154673870711,0.348679001175479,0.890347158806245
"5260","KATNB1",8.38213084829875,-0.442790467998445,0.472432190936624,
-0.937257190541116,0.348626277922509,0.890347158806245
"5261","SNTB2",160.725096745021,-0.197497795840832,0.210670912573311,-
0.937470642854436,0.348516517881032,0.890347158806245
"5262","ZNF778",4.19610800539127,0.777369899207217,0.828079338382459,0
.938762583698446,0.3478526533256,0.890347158806245
"5263","ZNF699",9.17957027132685,0.516395108815125,0.550133446489033,0
.938672447768396,0.347898943615835,0.890347158806245
"5264","PIFO",6.00009716831233,0.709447412616318,0.757561849312385,0.9
36487777546165,0.349022102647081,0.891053958486726
"5265","SPOPL",23.5218477091406,0.375377569478653,0.401109687933418,0.
935847676511277,0.349351620215054,0.891387207911547
"5266","TSPAN17",10.64448617659,-0.452317569641165,0.483288271059324,-
0.9359167120893,0.349316071894912,0.891387207911547
"5267","FBXL4",29.7554933726696,0.303770912448517,0.324537251818432,0.
936012463119233,0.349266770854863,0.891387207911547
"5268","AL133243.2",4.28342345082684,0.745654406619327,0.7969483738857
71,0.935637026252598,0.349460104029851,0.89144113215909
"5269","RAB8A",72.2892653502879,-0.209134852510598,0.223542371255192,-
0.935549047530917,0.349505418955744,0.89144113215909
"5270","FOXF2",11.9183104038704,0.510960322081046,0.546542916738664,0.
934895149918059,0.349842336842805,0.89184568626885
"5271","STK17A",45.545875831759,0.372998476257839,0.399045756032992,0.
934726082457072,0.349929481635214,0.89184568626885
"5272","GLIPR1L2",4.51573343556256,0.673457924295604,0.720429846089643
,0.934800144595628,0.349891305062064,0.89184568626885
"5273","C17orf80",13.1707377748826,0.475288193843847,0.508323543677536
,0.935011174979836,0.349782540401043,0.89184568626885
"5274","RASA4",1.90255431040785,1.07905464101151,1.15462382690543,0.93
4550817215978,0.350019835574927,0.891906820305545
"5275","SNX19",16.8234721346575,0.368457695760464,0.39434201756891,0.9
34360730900501,0.350117846907582,0.891987439732889
"5276","AC018647.2",8.11849910657625,-0.463981606713043,0.496672947320
191,-0.934179341187123,0.350211390375546,0.892056648077514
"5277","DIS3L2",14.2906566230031,-0.379699982404281,0.406788591620661,
-0.933408630983337,0.350609025762531,0.892892162951259
"5278","IGF2",24.1156240833332,1.09518048815813,1.17346708787871,0.933
28607122497,0.350672284846845,0.892892162951259
"5279","AMOTL2",104.608080172066,0.204098726843381,0.21879415726823,0.
932834447645538,0.350905452320492,0.893055533461808
"5280","HMGCLL1",7.96178698877173,-0.614671261135579,0.658970081402512
,-0.932775672952178,0.350935804167855,0.893055533461808
"5281","TCEA1",173.3584785931,-0.179334548598371,0.19222213209516,-0.9
32954736500326,0.350843339124584,0.893055533461808

"5282","KCNAB2",6.89805278490893,-0.620210132988302,0.66516514076976,-0.932415267989791,0.351121957329697,0.893206634853272
"5283","RESF1",41.8276767516087,0.272525400705377,0.292282731043153,0.932403360721096,0.351128108633815,0.893206634853272
"5284","NIT1",22.5849608855325,0.314789477406824,0.337851505774143,0.931739157667876,0.35147134459173,0.893498071856873
"5285","CARMIL1",58.7904212354959,0.262873654134448,0.28223198298142,0.931409868426407,0.351641588068383,0.893498071856873
"5286","CDK2AP2",16.3474742564906,0.366820057821254,0.393580850182391,0.932006873940297,0.351332973005142,0.893498071856873
"5287","PSMB10",70.1649142089468,-0.293025326761124,0.314592526020642,-0.931444018927192,0.351623929734938,0.893498071856873
"5288","ALYREF",25.3484512611847,-0.342489533983327,0.367560454905034,-0.9317910276061,0.351444532446883,0.893498071856873
"5289","TRAPPC10",19.0485949053035,0.376791266107842,0.404504091708043,0.93148938127379,0.351600474916955,0.893498071856873
"5290","POLR3C",18.6804632072147,-0.349730921923489,0.375671965486863,-0.930947619341903,0.351880660271019,0.893598675998153
"5291","PIK3C2B",11.3902510220984,0.443274354562748,0.476025614539245,0.931198534330557,0.351750875911408,0.893598675998153
"5292","PIAS1",54.009192577854,0.260719160614217,0.280052660171044,0.930964770893375,0.351871787762523,0.893598675998153
"5293","NDC1",7.69484143670458,0.518290797990388,0.557198832925862,0.930172081066346,0.352281994738827,0.893773405190691
"5294","SLC4A5",1.81937528037774,1.039827277426,1.11745665248102,0.930530303003109,0.352096581936022,0.893773405190691
"5295","MOXD1",14.3270762152477,-0.648679968134871,0.697264691190901,-0.930320976137413,0.352204920339369,0.893773405190691
"5296","NUCB2",193.870150705255,-0.206834640403644,0.222335993881766,-0.930279604271519,0.352226335099594,0.893773405190691
"5297","SLC35F2",9.16957793560717,-0.480695143427968,0.516681935689076,-0.930350202367509,0.352189792857363,0.893773405190691
"5298","BBS4",24.9277073067466,0.300199864690703,0.322818541777718,0.929933773436754,0.352405375084274,0.893917673793422
"5299","SRGAP2B",40.3487293832351,0.313712539241271,0.337435906509841,0.929695190076406,0.352528925578214,0.894062319465111
"5300","TMEM50A",317.762873022654,-0.223354043054462,0.241296961450953,-0.925639683613929,0.354633263083244,0.894400252150159
"5301","CCN1",259.026531535011,0.413945783185796,0.446713519600854,0.926647090411912,0.354109796792569,0.894400252150159
"5302","RTCA",32.5964658317597,0.262460268465846,0.283677445618246,0.925206682871245,0.35485840795183,0.894400252150159
"5303","TNS1",96.0048058084324,-0.256793502775864,0.276969133774119,-0.927155669935737,0.353845715454655,0.894400252150159
"5304","LXN",51.6010302402101,0.319757589809013,0.345360020111163,0.925867417155267,0.354514886006374,0.894400252150159
"5305","RELL1",34.2488495208968,0.252714333328875,0.272283531038839,0.928129337696989,0.353340483079476,0.894400252150159
"5306","JCHAIN",13.963545798233,-0.454107563089924,0.490772713190366,-0.925290976627259,0.354814571147658,0.894400252150159

"5307","STK38",66.9387562651074,0.257149818616801,0.276985170635966,0.928388397206891,0.353206134980156,0.894400252150159
"5308","MYBL1",2.99304344995314,0.868526167796803,0.937695502571111,0.926234758954641,0.354323992429085,0.894400252150159
"5309","AC087672.2",3.29321923831871,-0.899869692224855,0.97205203521074,-0.925742305585281,0.354579916560679,0.894400252150159
"5310","RBIS",189.484604358595,-0.239116125124522,0.257906996185819,-0.927140902188793,0.35385338189355,0.894400252150159
"5311","TNFRSF11B",19.9278667118344,0.339239183901933,0.365418117823783,0.928358960202203,0.353221399365343,0.894400252150159
"5312","AQP3",14.661493970545,-0.588975479112071,0.635159257562622,-0.927287876385873,0.353777087273396,0.894400252150159
"5313","PI4K2A",19.6128006870332,0.353339164397026,0.381411113907751,0.92639976003029,0.354238268774169,0.894400252150159
"5314","TSPAN18",16.4955623714438,0.419423948483759,0.452937349308228,0.926008749608189,0.35444143321414,0.894400252150159
"5315","ZFPL1",35.5073821046042,-0.296031227784395,0.319837558752828,-0.925567431601022,0.354670825269562,0.894400252150159
"5316","NFRKB",16.0731074740664,0.362428987439322,0.3913895253239,0.926005843256505,0.354442943595943,0.894400252150159
"5317","WNK1",93.1694496190068,0.258987127152412,0.279348078586595,0.927112613277305,0.353868067921211,0.894400252150159
"5318","ANKRD13A",26.6976113861551,0.281364808911243,0.302964602626006,0.928705223225609,0.353041873123113,0.894400252150159
"5319","CTDSPL2",34.3009228594811,0.27500009362376,0.29667212911036,0.926949540047497,0.353952733971748,0.894400252150159
"5320","ANP32A",68.0225811472043,-0.206213213054061,0.222649938077924,-0.926176826430958,0.354354093447653,0.894400252150159
"5321","NDUFAB1",133.297822160814,-0.272046337306464,0.293575548427513,-0.926665516810353,0.354100226660276,0.894400252150159
"5322","PALB2",7.29561957096022,0.530885828379544,0.573573298600808,0.92557625969445,0.354666235608869,0.894400252150159
"5323","MON1B",20.523121930034,0.386185464345207,0.416152223082656,0.927990871908673,0.353412304599112,0.894400252150159
"5324","AC009113.1",6.35069531972726,0.632642314621316,0.681129677497092,0.928813316351228,0.352985842135579,0.894400252150159
"5325","TEN1",22.7839600784343,-0.399587906778843,0.43125680898667,-0.926566023891332,0.354151902312667,0.894400252150159
"5326","RANBP3",32.1608420208864,-0.253866859368658,0.273897017397209,-0.926869747546382,0.353994166133472,0.894400252150159
"5327","BCKDHA",26.1937596638241,-0.329442916354857,0.355042328583412,-0.927897576802479,0.353460701513513,0.894400252150159
"5328","ZNF525",4.93669706089542,-0.610076737521803,0.65923697357144,-0.925428581799183,0.354743017191391,0.894400252150159
"5329","LENG8-AS1",5.97631782028212,0.631835429103817,0.6805275599702,0.928449435804605,0.353174485104541,0.894400252150159
"5330","CST7",4.51502095509723,0.705703618800761,0.761169854524655,0.927130277960716,0.353858897356024,0.894400252150159
"5331","ZNF337-

AS1",6.04774911058232,0.588787387983377,0.634181861962126,0.928420415812113,0.353189532394869,0.894400252150159
"5332","CDC42EP1",31.4597472319135,-0.330085160930875,0.356200428251294,-0.92668378460793,0.354090739061937,0.894400252150159
"5333","SNRNP40",34.5982817812703,-0.305466133034473,0.330337266680344,-0.924709876376324,0.355116840659197,0.894808372780667
"5334","TBC1D1",10.0691175077074,-0.471270818663251,0.509680644972836,-0.924639425317727,0.35515349805879,0.894808372780667
"5335","Z93241.1",2.84171796938288,0.945839430820631,1.02340640348687,0.92420706729804,0.355378516750127,0.895207476402053
"5336","C11orf53",1.52290275666683,-1.04909869807723,1.1355128638642,-0.923898558495494,0.355539133681642,0.895444231174586
"5337","KDM2B",9.81862322169946,0.459741972838868,0.497822957205878,0.923504965337986,0.355744113997551,0.895792607834568
"5338","LINC02035",2.68848129736768,0.871929932457428,0.944304336346656,0.923356908251389,0.355821240288412,0.895818967447728
"5339","TXLNA",43.3213706503688,0.272818038592215,0.295711174290762,0.922582784524614,0.356224670570364,0.895892341248312
"5340","TGFB2",10.8231046778404,0.547135934767915,0.592801728820892,0.922966159117301,0.356024841002312,0.895892341248312
"5341","TTN-
AS1",15.2693798556951,0.382097802476808,0.414642670592634,0.921511049334815,0.356783675151497,0.895892341248312
"5342","CEP97",14.417748013808,0.406243711844695,0.440270636106121,0.922713618690608,0.356156466829952,0.895892341248312
"5343","SRD5A3-
AS1",1.64534807134494,-1.0942235691662,1.18630703053281,-0.922378053070076,0.356331413426462,0.895892341248312
"5344","TACC2",31.1566306498475,-0.353274006339851,0.383132534518069,-0.922067364454506,0.356493438720483,0.895892341248312
"5345","CCND2-
AS1",3.46709731489447,-0.816299567834211,0.885360335619844,-0.921996993758159,0.356530143754381,0.895892341248312
"5346","LATS2",59.5027827288023,0.236586893350876,0.256734537973904,0.921523435132533,0.356777211708778,0.895892341248312
"5347","MAP4K5",60.0061245513536,0.236815285052053,0.256700270918958,0.922536171092771,0.356248972102249,0.895892341248312
"5348","GOLGA8R",2.79237011217619,0.94378407182668,1.02367788590982,0.921954146726403,0.356552493737,0.895892341248312
"5349","LINC02693",11.9329051450492,0.448324082066601,0.486451077826002,0.921622137359024,0.356725707272181,0.895892341248312
"5350","ACBD4",18.4455342515542,-0.34527236080801,0.374563677828667,-0.92179883220269,0.356633516712487,0.895892341248312
"5351","CDR2L",13.1422173454067,0.388892348817979,0.421596744564982,0.922427304839016,0.356305732704793,0.895892341248312
"5352","ZNF493",30.4221451674468,0.310215840761689,0.336451742863592,0.922021797602813,0.356517205910485,0.895892341248312
"5353","DUSP16",16.6792512141475,0.342567241658085,0.37189114095363,0.921149239478116,0.356972515670703,0.89619907306157
"5354","SNRPC",71.1805539549209,-0.253083842702836,0.274893789222858,-

0.920660460966834,0.357227725283628,0.896672282421868
"5355","PARP10",19.3659192621414,-0.320316157999709,0.348002282570491,
-0.920442692598794,0.357341467315997,0.896790285576039
"5356","SMIM4",24.2867568432031,0.324973976955626,0.353136463392166,0.
92025041490755,0.357441914332882,0.896874885496566
"5357","PMVK",80.2341559320807,-0.274802251114393,0.29874782304385,-0.
919846873910301,0.357652784364313,0.896907705377579
"5358","RAD18",11.3770672131492,0.410202639567121,0.446176284708904,0.
919373471036784,0.357900260456044,0.896907705377579
"5359","AC096733.2",3.50009499355036,0.922079107212169,1.0031097861719
5,0.919220527925453,0.357980236024318,0.896907705377579
"5360","CACNA2D1",41.305085999763,0.316205074528733,0.343978449600407,
0.919258386378748,0.357960438392028,0.896907705377579
"5361","AC022034.1",5.32984895806412,0.593558649811883,0.6451056510526
9,0.920095257022331,0.357522982699904,0.896907705377579
"5362","TIMM10B",22.8131259734471,-0.310661936303135,0.33796845389336,
-0.919203945588245,0.357988907779249,0.896907705377579
"5363","ST3GAL4",56.463779172853,-0.313087646352401,0.340606161689107,
-0.919207229839184,0.357987190265428,0.896907705377579
"5364","LPIN3",28.8576579709041,-0.332961911498726,0.362216407290266,-
0.919234757998975,0.357972794494143,0.896907705377579
"5365","TRABD2A",1.78666919528992,1.13453169360451,1.23541463934274,0.
918340820542724,0.358440462640113,0.896921073002706
"5366","PPP2R3A",17.0972867083532,-0.348039125775828,0.37900341289132,
-0.91830076969684,0.358461424443599,0.896921073002706
"5367","LAMTOR3",43.0977004781578,0.228401295855776,0.248527239109444,
0.919019165360763,0.358085547783751,0.896921073002706
"5368","TCOF1",18.0656827346837,-0.34193032255551,0.37229827228653,-0.
918431129039331,0.358393199826864,0.896921073002706
"5369","MAFK",15.6495195465127,-0.367187464185997,0.399711222358534,-0
.918631861320713,0.358288160935019,0.896921073002706
"5370","CCZ1",52.7222243228168,-0.323807676649882,0.352448055935287,-0
.918738722478118,0.358232250685828,0.896921073002706
"5371","ZNF282",11.7953883627586,0.399010008887239,0.434317327889976,0
.918706170038692,0.358249281690558,0.896921073002706
"5372","ATP6V0A2",13.0173192939123,0.439895972850802,0.479195731934566
,0.917988086151963,0.358625103197936,0.897163581883295
"5373","AC079866.2",4.90173905127234,0.631873490072322,0.6884382448781
18,0.917836123680476,0.35870466713381,0.897195611690168
"5374","ARL4A",54.4228996272987,-0.364325241073395,0.397101808275681,-
0.917460544074047,0.358901359293224,0.897353556751933
"5375","VCPIP1",26.7725573622311,0.324239439893745,0.353366998136649,0
.917571368021073,0.358843313411512,0.897353556751933
"5376","NMRK1",16.5172389327521,-0.431356281163872,0.470449938474096,-
0.916901557183695,0.359194227917276,0.897417964116058
"5377","SLTM",100.494666355339,-0.179841188408202,0.19612317736746,-0.
916980801668576,0.359152700415424,0.897417964116058
"5378","RSPRY1",35.5089549697284,-0.255598207557529,0.278694303870762,
-0.917127490614435,0.359075837092011,0.897417964116058
"5379","RNMT",78.7759719806236,0.184589390846617,0.201262886691557,0.9

17155635999138,0.359061090415867,0.897417964116058
"5380","ARHGEF10L",26.547280201505,-0.27501356986355,0.300018374821819,-0.91664837911906,0.35932692427981,0.897453231063818
"5381","HPS3",23.629530574235,0.306538140390389,0.334422340748634,0.916619803880854,0.359341903144163,0.897453231063818
"5382","ARMC9",6.44100161873564,0.574804282181415,0.627390367746369,0.916182829274465,0.359571009892304,0.897730147288759
"5383","SETDB2",13.915207158982,0.405124477049166,0.44220151918831,0.916153517049871,0.35958638163966,0.897730147288759
"5384","PCGF1",12.4200713921917,-0.397125089405874,0.434156314825382,-0.914705316599157,0.360346352522721,0.897991580728455
"5385","TRANK1",20.7150713568811,0.311667406732569,0.340685750104592,0.914823724317457,0.360284177988888,0.897991580728455
"5386","CD200",13.8278540770408,0.372176709771528,0.407288962544223,0.913790315962999,0.360827035935237,0.897991580728455
"5387","FOXC1",13.0147839334865,-0.464690204078753,0.508511101342031,-0.913825092219956,0.360808759340414,0.897991580728455
"5388","SMPDL3A",23.2400564463818,-0.384216425962982,0.419824695756427,-0.915183003397914,0.360095565886469,0.897991580728455
"5389","FBXO30",13.4866657383986,0.392477015050439,0.429254612826847,0.914322183903372,0.360547577252081,0.897991580728455
"5390","EIF4EBP1",36.4394381752613,-0.343712828709065,0.375733521387335,-0.91477818492202,0.360308089405782,0.897991580728455
"5391","ECD",16.3224672148761,0.370001869963917,0.404165973370627,0.915470114612096,0.359944884597287,0.897991580728455
"5392","GHITM",138.726552824192,-0.186025930196416,0.203477627891675,-0.914232842813808,0.360594510131012,0.897991580728455
"5393","CEP126",134.66474247005,0.220236368658204,0.241010447553929,0.913804239166534,0.360819718552734,0.897991580728455
"5394","THSD1",14.3982326907954,0.500204726816812,0.547118679653757,0.914252694010312,0.360584081520809,0.897991580728455
"5395","RAD51B",9.2656861391685,0.496241769459208,0.543024911729281,0.91384715275568,0.36079716576946,0.897991580728455
"5396","EMC4",106.691300942908,-0.241402542547081,0.263719841873404,-0.915374970772822,0.359994813461446,0.897991580728455
"5397","CILP",1060.93438195322,-0.255557559978787,0.27948248650485,-0.914395614461347,0.360509005402721,0.897991580728455
"5398","ITFG1",66.6849441966127,-0.22041768707267,0.241012123793151,-0.914550204378283,0.360427810358799,0.897991580728455
"5399","ZNF254",33.2139295508883,0.253160074003382,0.276554865664211,0.915406327765592,0.359978357695585,0.897991580728455
"5400","B9D2",8.71645015392087,-0.499268063511961,0.545448764806081,-0.91533448368787,0.360016061286552,0.897991580728455
"5401","TMEM267",14.0226605476317,-0.396841301661507,0.434419441218902,-0.91349802519897,0.360980671738175,0.89820759997951
"5402","ZC3H8",20.5688851553495,0.341274215560658,0.374058384538261,0.912355476223125,0.361581619493586,0.898359862290022
"5403","FARS2",17.2706463711916,-0.388573921181821,0.425737734636841,-0.912707259818721,0.361396524698635,0.898359862290022
"5404","HOXA3",8.73040940932604,0.547251025127275,0.599769990366278,0.

912434823211261,0.361539865016453,0.898359862290022
"5405","CALU",321.216491499837,-0.168597869054794,0.184820709387239,-0
.912223903986567,0.361650862935302,0.898359862290022
"5406","AL158152.1",11.8502270675658,0.468739895510941,0.5138883035314
99,0.912143538371485,0.361693161598739,0.898359862290022
"5407","UVRAG",35.8012753195815,0.250542064334329,0.274482125697349,0.
912780982360152,0.36135774230628,0.898359862290022
"5408","BLOC1S1",291.752121536011,-0.246300585783406,0.269964027489823
,-0.912345945026659,0.361586635263816,0.898359862290022
"5409","COG8",17.6028860945105,0.353126707801857,0.387153255880109,0.9
12110908118544,0.361710336695536,0.898359862290022
"5410","SNPH",2.46411409431303,-0.827172047773976,0.906645999517645,-0
.912342908052371,0.361588233473716,0.898359862290022
"5411","DNTTIP1",35.1431352795729,-0.252291943669538,0.276384581448113
,-0.912829298753421,0.3613323264576,0.898359862290022
"5412","ABHD10",15.3962916545036,0.360650886220859,0.39545911640305,0.
911980205441224,0.361779137827881,0.898364714203416
"5413","AC019080.1",5.58829436666749,-0.566308923686209,0.621292108597
915,-0.911501877859372,0.362030996624019,0.898738141449069
"5414","USB1",14.1124290144008,-0.402509616920631,0.441619144275091,-0
.911440597941792,0.362063270913406,0.898738141449069
"5415","FAM155A",3.3112293498373,-0.779374372433044,0.855661489065738,
-0.910844279417099,0.362377428062259,0.899185793155224
"5416","KREMEN1",76.4070327764028,0.248362576050801,0.27264256125333,0
.910945726555257,0.362323970845323,0.899185793155224
"5417","TMEM141",50.2147371247177,-0.27155214225812,0.298246061743489,
-0.910496992552654,0.362560467068682,0.899473900117412
"5418","RC3H1",35.110018930473,0.279295000908215,0.30712701570064,0.90
9379463968895,0.363149858040879,0.899733192266196
"5419","ZNF512",18.3600580664056,0.317994750369332,0.349694809463853,0
.909349357678134,0.36316574456129,0.899733192266196
"5420","MTA3",15.4121146218217,0.363471278944003,0.399758609414091,0.9
09226894391911,0.363230370604805,0.899733192266196
"5421","STK16",29.3546896216628,-0.346276210904225,0.381039117973414,-
0.908768141040009,0.363472526837661,0.899733192266196
"5422","ZNF827",16.5642345932561,0.43478669258724,0.478415194568834,0.
908806194960188,0.363452435963402,0.899733192266196
"5423","PARP8",9.19501999695215,0.470850333423146,0.517720902077266,0.
909467497900782,0.363103406694832,0.899733192266196
"5424","LUCAT1",2.03742591992699,-0.856973257915406,0.941750495789617,
-0.909979088672389,0.362833538022594,0.899733192266196
"5425","ARMT1",23.723594299741,0.355655506462941,0.391391135967287,0.9
08695864008192,0.363510687992948,0.899733192266196
"5426","AP4M1",21.6472053007355,-0.322241191970866,0.35413323887477,-0
.909943367628414,0.362852377111553,0.899733192266196
"5427","SUOX",8.65972843752466,0.456479318989907,0.502510353076821,0.9
08397839357796,0.363668066874812,0.899733192266196
"5428","NEDD8",285.037060384791,-0.241585193227403,0.265902391713012,-
0.908548402558732,0.363588553131604,0.899733192266196
"5429","LRR1",7.33495713311946,-0.510958547541734,0.561989619037471,-0

.909195704391944,0.363246831269609,0.899733192266196
"5430","AC009118.3",2.57530050366833,0.938339903406048,1.0330450630562
2,0.908324270608301,0.363706923130309,0.899733192266196
"5431","SLC46A1",6.1709805398658,0.584473036745672,0.642942543948193,0
.909059514333162,0.363318711663616,0.899733192266196
"5432","AC099850.1",3.58717102259291,-0.706555912724056,0.776742830540
33,-0.909639439133994,0.363012692155814,0.899733192266196
"5433","NPB",5.19491548450497,0.7437593789321,0.818875758993864,0.9082
68892763344,0.36373617334491,0.899733192266196
"5434","DDX49",32.1150212731526,-0.276536020367521,0.304565675950086,-
0.907968435723667,0.363894898428783,0.899960165620981
"5435","TMEM201",3.53085286562083,-0.706802029678617,0.779374005973493
,-0.906884274124298,0.364467998067811,0.900234315405913
"5436","MED10",108.314924730609,-0.19881227081024,0.21922866740343,-0.
906871684095863,0.364474656605668,0.900234315405913
"5437","FRK",6.37109324154757,-0.626348063276254,0.690440083426581,-0.
907172220024879,0.364315731740111,0.900234315405913
"5438","ECHDC1",79.4068255102948,0.206585594326397,0.227777554691926,0
.906962034103004,0.364426874526867,0.900234315405913
"5439","UST",39.6759829654023,0.271865077980882,0.299748806814426,0.90
6976347529527,0.364419305154865,0.900234315405913
"5440","AL669970.3",3.44676076055672,-0.874443586048736,0.963668798822
39,-0.907410914535484,0.364189539786514,0.900234315405913
"5441","TSPAN3",113.215464267667,0.169426510523525,0.186783813363874,0
.907072767560779,0.364368317892377,0.900234315405913
"5442","IP6K1",24.5717522598446,0.307561990481,0.339200512757181,0.906
726195609176,0.364551607189138,0.900258921171413
"5443","LINC01871",3.21961049166951,0.799424603140882,0.88180027862635
7,0.906582388912604,0.364627678230408,0.900281346268317
"5444","VAMP3",73.9871784987995,-0.232290445574079,0.258223676289079,-
0.899570670328588,0.368348771419297,0.900394360641411
"5445","SLC66A1",8.8238896199412,0.469115205155569,0.526861840568386,0
.890395107471593,0.37325376852235,0.900394360641411
"5446","SYF2",149.654111435381,-0.20264699389069,0.225678132928771,-0.
897946962166024,0.369213826102981,0.900394360641411
"5447","AL031432.4",1.77506959573576,1.00106812619415,1.1203312519572,
0.893546551027029,0.371564554741779,0.900394360641411
"5448","RPA2",54.3623153736402,-0.254848432937996,0.282853051186303,-0
.900992341674044,0.367592391568087,0.900394360641411
"5449","JAK1",193.062366395494,-0.153455271665542,0.17164618248500‌1,-0
.894020883213946,0.371310717857844,0.900394360641411
"5450","MAN1A2",72.4220966739584,0.206616431565682,0.232123373910256,0
.890114718242742,0.373404290707454,0.900394360641411
"5451","PFDN2",94.3890132779207,-0.26636257287882,0.294917315075444,-0
.903177125462033,0.366431896092753,0.900394360641411
"5452","Z99572.1",1.90565071872105,0.934914261644552,1.03819805362458,
0.900516292031715,0.367845559141584,0.900394360641411
"5453","VAMP4",31.3693277893897,0.263678651031412,0.291564385469915,0.
904358228136952,0.365805480284347,0.900394360641411
"5454","RNASEL",16.992940911471,0.359259274709291,0.400294155748617,0.

897488183501994,0.369458475977707,0.900394360641411
"5455","CD46",133.644695575562,0.183813561097075,0.205152958562758,0.895982989398832,0.370261848415136,0.900394360641411
"5456","CD34",648.585792167322,-0.181833108360678,0.204725834847717,-0.888178614564851,0.37444468030654,0.900394360641411
"5457","LINC01354",12.5391670110302,0.52361691525266,0.588409548752198,0.889885142691958,0.373527562409807,0.900394360641411
"5458","GPR137B",21.4990016431607,0.32572428013108,0.364111580643175,0.894572700916882,0.371015550351858,0.900394360641411
"5459","ZNF496",12.1513837980685,-0.381433436114471,0.425606758956292,-0.896210946108689,0.370140110621297,0.900394360641411
"5460","MRPL33",143.430613564613,-0.213167045781012,0.237701779249768,-0.896783551447565,0.36983442667814,0.900394360641411
"5461","SLC30A6",14.7274741267536,0.404284583911721,0.451752109680502,0.894925724193403,0.370826794467406,0.900394360641411
"5462","AC019171.1",1.73268503750146,1.07709777711262,1.19642817223595,0.900261129006751,0.367981301869309,0.900394360641411
"5463","GNLY",9.50437440977478,0.566316126281488,0.626410755400004,0.904065138408836,0.365960862511663,0.900394360641411
"5464","POLR1A",11.7391550106075,-0.367518725111457,0.412589290474083,-0.89076166928415,0.373057042723188,0.900394360641411
"5465","KRCC1",71.2934451769563,-0.18351482530598,0.205710412787648,-0.892102751723219,0.372337860118228,0.900394360641411
"5466","STARD7",26.8121504723435,0.316000357421352,0.353105902728887,0.894916666584233,0.370831636680519,0.900394360641411
"5467","COX5B",320.655679499384,-0.226005150416205,0.251167142870676,-0.899819728938724,0.368216193416631,0.900394360641411
"5468","DCAF17",8.79915316529558,0.456393639614796,0.505928629885989,0.902090952468226,0.367008554638606,0.900394360641411
"5469","KCTD18",19.0699602455788,-0.299725677976654,0.336443615244301,-0.890864514575539,0.373001859416162,0.900394360641411
"5470","ZDBF2",11.451758471054,0.415559888307863,0.462127525210577,0.899232063960063,0.368529064817652,0.900394360641411
"5471","CREB1",61.3230893111122,0.267300018778752,0.296462807656593,0.901630868612626,0.367252987545635,0.900394360641411
"5472","ZNF621",24.9804584535045,0.378846999553897,0.425293025667448,0.890790529563332,0.373041556762605,0.900394360641411
"5473","ZBTB11",24.61137992438,0.328391754600464,0.367375585177592,0.893885625093233,0.371383089706645,0.900394360641411
"5474","AC108673.3",4.61389201591969,-0.761167939020273,0.855021191927623,-0.890232834234481,0.373340877567562,0.900394360641411
"5475","SHOX2",45.275955487368,-0.227132717923886,0.251095964727448,-0.904565384674449,0.365695680584583,0.900394360641411
"5476","ALG3",23.090679034796,-0.398218588267317,0.44559558082525,-0.893677148973985,0.371494655061545,0.900394360641411
"5477","UBA6",30.1274281978108,0.293824526810739,0.325454713962371,0.902812324435131,0.366625509027217,0.900394360641411
"5478","UBA6-AS1",8.71093215005983,0.45356036806524,0.505116298556925,0.897932554069279,0.369221507881246,0.900394360641411

"5479","SEC31A",243.274004711858,0.182535454820209,0.202197731718605,0
.902757183617866,0.366654779777056,0.900394360641411
"5480","FASTKD3",6.19939914484529,-0.524842939127075,0.586781345998698
,-0.894443803822351,0.371084484419726,0.900394360641411
"5481","ANKRA2",24.288768204696,0.31068658248256,0.343250932101885,0.9
05129610515788,0.365396726874015,0.900394360641411
"5482","KCTD16",8.49547327883392,0.730228235014828,0.809902672237621,0
.901624676700144,0.367256277870587,0.900394360641411
"5483","TRIM52",22.1021740075077,0.349809431080709,0.393476902478928,0
.88902151276705,0.373991518251602,0.900394360641411
"5484","SERPINB9P1",1.48305993681179,1.18598730260006,1.3199116572339,
0.898535364923968,0.368900199935715,0.900394360641411
"5485","NKAPL",3.41961991221619,0.777681310286824,0.859735745517384,0.
904558539460075,0.365699308442078,0.900394360641411
"5486","ZNF76",14.1593472412382,0.352514362905138,0.395989795504612,0.
890210724889835,0.373352746943028,0.900394360641411
"5487","TMEM63B",14.6684451033217,-0.342562852693261,0.380432011200118
,-0.900457486773015,0.367876839851561,0.900394360641411
"5488","HACE1",6.11341323866698,0.52742159514199,0.587833570942279,0.8
97229456113828,0.369596490259782,0.900394360641411
"5489","HSF2",12.6833221541405,0.383852445712237,0.431742734559275,0.8
89076792697102,0.373961810311207,0.900394360641411
"5490","EYA4",3.18711858357683,1.16589526536173,1.28894789315789,0.904
532504029558,0.365713107021818,0.900394360641411
"5491","EPM2A",2.82612801181157,0.804005796012847,0.900388095906985,0.
892954715491824,0.371881423945327,0.900394360641411
"5492","MAP3K4",33.1783218768686,0.279308940238901,0.313270289403786,0
.891590903083978,0.372612247858953,0.900394360641411
"5493","PDCD2",63.6103529912626,-0.211752292043058,0.233827472120915,-
0.905592016722307,0.365151835856772,0.900394360641411
"5494","GPER1",5.46388370214715,-0.628907426968763,0.69989403921118,-0
.898575201008394,0.368878972786314,0.900394360641411
"5495","HOXA13",2.05750936313514,-0.944487542449803,1.05697504155322,-
0.893576012033245,0.371548785673281,0.900394360641411
"5496","GRB10",11.5198690470853,-0.442051710790651,0.497191455842807,-
0.889097561102117,0.373950649557563,0.900394360641411
"5497","TAF6",22.386228328648,-0.321934743986383,0.359623422123548,-0.
895199600975331,0.370680398015443,0.900394360641411
"5498","ARMC10",51.8602708622293,-0.219014383178271,0.244203603824738,
-0.896851560534113,0.369798130621426,0.900394360641411
"5499","SLC35B4",23.8015440008962,-0.276143284109959,0.305476575413174
,-0.903975317048317,0.366008489766402,0.900394360641411
"5500","PSD3",57.406365570622,-0.309570381896113,0.343105889519404,-0.
902259014934822,0.366919291865023,0.900394360641411
"5501","GINS4",1.57159453104492,1.02027964836007,1.13922304352354,0.89
5592530506068,0.370470426501527,0.900394360641411
"5502","COX6C",475.322946929177,-0.229389976582709,0.255080420673986,-
0.899284923462974,0.368500915778149,0.900394360641411
"5503","POLR2K",97.3298719276982,-0.266073160157114,0.297827639871501,
-0.893379675143356,0.371653883261302,0.900394360641411

"5504","MRPL13",57.8776843215998,-0.295770593093645,0.332933760952566,
-0.888376691650037,0.374338158861521,0.900394360641411
"5505","STOM",143.28084511352,0.171823451256414,0.191026188568129,0.89
9475891469897,0.368399231571305,0.900394360641411
"5506","RABGAP1",36.0796952950663,0.266153049419047,0.298212738186481,
0.892493898944767,0.372128261468524,0.900394360641411
"5507","NR6A1",1.5223428694608,1.21075654178182,1.36322053722443,0.888
158965274222,0.374455248279251,0.900394360641411
"5508","NMT2",37.9474035610355,0.238798577206257,0.264700123340374,0.9
02147585700929,0.366978473600119,0.900394360641411
"5509","CUL2",28.9039833120543,0.26887199370631,0.300087398401262,0.89
5978955260187,0.370264003026799,0.900394360641411
"5510","CAMK2G",31.0906297308677,0.297012332270803,0.333957661421256,0
.889371218515481,0.373803607778958,0.900394360641411
"5511","PIDD1",4.05106011494789,0.73326947989858,0.817624977128242,0.8
96828619979364,0.36981037361558,0.900394360641411
"5512","STIM1",52.6323345990509,0.22032410971492,0.247895860665413,0.8
88776880434856,0.374123003432758,0.900394360641411
"5513","DDB1",79.0726472313169,0.20934635649118,0.23117403771576,0.905
579011206185,0.365158722196297,0.900394360641411
"5514","AP001107.9",2.31788043271136,-0.811626216046312,0.902645305803
98,-0.899164057939017,0.368565281734175,0.900394360641411
"5515","KCTD21-
AS1",4.90190186415665,-0.577628440866808,0.643024680459332,-0.89829902
0893242,0.369026154396778,0.900394360641411
"5516","ARHGAP32",30.5958049527513,-0.382937059139523,0.42992357938723
9,-0.890709599332318,0.373084983627076,0.900394360641411
"5517","VPS26B",34.7729246579557,0.27658483791036,0.307107498288993,0.
900612454763606,0.367794410167653,0.900394360641411
"5518","ACAD8",13.9148115695915,-0.382636594881236,0.425028615269717,-
0.900260785120127,0.367981484832644,0.900394360641411
"5519","FOXJ2",17.5787530100407,0.333713351225804,0.3691517236812,0.90
4000522869018,0.365995124136012,0.900394360641411
"5520","GOLT1B",38.8760537375915,-0.239249799729291,0.266969540386446,
-0.89616890145209,0.370162562241105,0.900394360641411
"5521","ALG10",4.24394759939925,-0.60654356976121,0.675359599604265,-0
.898104609924286,0.369129781583092,0.900394360641411
"5522","SPATS2",34.3809358763409,0.249916835095053,0.279233133150194,0
.895011391648239,0.370780998442997,0.900394360641411
"5523","BIN2",1.58068843204532,-1.03301463628139,1.14800474088619,-0.8
99834817305692,0.36820816258463,0.900394360641411
"5524","ESYT1",21.981079322143,0.331263945646013,0.367164613461088,0.9
0222187406173,0.366939017277961,0.900394360641411
"5525","LRIG3",46.1574982076947,0.241039571736448,0.268198551796692,0.
898735545444586,0.368793538958306,0.900394360641411
"5526","RXYLT1",25.0251217632168,-0.337895173381776,0.373602075465748,
-0.904425311236674,0.365769921808225,0.900394360641411
"5527","C12orf49",19.3434251473135,0.32952897597931,0.369407039071251,
0.89204844825859,0.372366964747397,0.900394360641411
"5528","OGFOD2",8.68355904497452,0.475090411140515,0.529346346834254,0

.897503900767021,0.369450092868557,0.900394360641411
"5529","SMAD9",11.3056961630306,-0.536699743630759,0.599334179723152,-0.895493302048408,0.370523444684758,0.900394360641411
"5530","ANKRD10-IT1",4.39859633924103,0.75964979172507,0.84683500884811,0.897045804422243,0.369694475971221,0.900394360641411
"5531","HNRNPC",185.014663441204,0.152061167304311,0.169279724319964,0.898283405854868,0.369034477037039,0.900394360641411
"5532","PRMT5",16.8148777387998,-0.375547432634223,0.416927949634893,-0.900748997430114,0.367721790709615,0.900394360641411
"5533","PSME1",220.10501998986,-0.21137297839949,0.236201391720531,-0.894884559569327,0.370848801460911,0.900394360641411
"5534","TMEM260",19.3342114619695,0.340106554000544,0.382693862460509,0.888717033019153,0.37415517462246,0.900394360641411
"5535","ARID4A",62.0539554247581,0.25002219633574,0.276847994476804,0.903102790425626,0.366471343177586,0.900394360641411
"5536","ZBTB25",18.7690025716801,-0.334789615704363,0.370670905134192,-0.903199067062363,0.366420252935209,0.900394360641411
"5537","UBE3A",65.4562625768436,0.211878676021101,0.236078991018998,0.897490603067049,0.369457185447901,0.900394360641411
"5538","SPRED1",39.0271341779081,0.260305683738414,0.289152689309682,0.900236080666803,0.367994628874311,0.900394360641411
"5539","MFAP1",38.4159414911578,-0.241485585958188,0.267026528025949,-0.904350544282707,0.365809553372597,0.900394360641411
"5540","AC090517.5",7.06151773651351,0.513623005810681,0.573003828395301,0.896369239362821,0.370055590465716,0.900394360641411
"5541","ADAM10",42.0687468796681,0.342512077463761,0.385615561835983,0.888221615935316,0.374421553534448,0.900394360641411
"5542","RAB11A",95.4619825187242,-0.213233569051016,0.237604698747132,-0.897429933731857,0.369489545657355,0.900394360641411
"5543","LOXL1-AS1",5.80631443709973,0.577853674567292,0.646390212939958,0.893970334017059,0.371337763925757,0.900394360641411
"5544","UBL7",21.6151161801997,-0.307850840954993,0.344909241866957,-0.892556080227452,0.372094947984129,0.900394360641411
"5545","KIF7",10.3856105278257,-0.40993716884467,0.453947171155766,-0.90305038756152,0.366499153193288,0.900394360641411
"5546","LINS1",13.1972566239184,0.380298117109743,0.427320331822447,0.889960268185315,0.373487220640297,0.900394360641411
"5547","CHSY1",18.400038036458,0.340884437302117,0.378038382661472,0.901719118842421,0.367206094187406,0.900394360641411
"5548","AC108134.2",7.0231405747014,0.816663656069619,0.9092859521747,0.898137328654907,0.369112340199924,0.900394360641411
"5549","TRAP1",27.0072120569309,-0.282059014171093,0.312955291430386,-0.90127574735011,0.367441725379336,0.900394360641411
"5550","EEF2KMT",7.81049494053363,0.572029860108269,0.6410180803217,0.892377107087512,0.372190837444644,0.900394360641411
"5551","CARHSP1",193.803099958173,0.21572958302151,0.241345276820896,0.89386287506096,0.371395263306051,0.900394360641411
"5552","TMEM159",141.340385013637,-0.186393037273681,0.206327275731415

,-0.903385345504766,0.366321414870726,0.900394360641411
"5553","DCTPP1",39.3610517442146,-0.289576917933205,0.323436959711434,
-0.895311773248058,0.370620448481131,0.900394360641411
"5554","FBXL19-
AS1",1.99949480328627,0.981990472406358,1.09759680967621,0.89467322039
3238,0.370961798128566,0.900394360641411
"5555","PRPF8",81.1144089641613,0.208072197902566,0.231997924063428,0.
896870947197265,0.369787784476739,0.900394360641411
"5556","CLDN7",1.90150416493481,-1.08161413632469,1.21656568571632,-0.
889071711477567,0.373964540944159,0.900394360641411
"5557","CHRNB1",18.7481394104321,-0.431606032314799,0.484241496694758,
-0.891303275867047,0.372766491732764,0.900394360641411
"5558","NDEL1",30.5318799800179,-0.275023783981142,0.304250001836858,-
0.903940122664692,0.366027152438445,0.900394360641411
"5559","SYNRG",50.7100853352967,0.243761932267889,0.274163017198898,0.
889113107808579,0.373942295032527,0.900394360641411
"5560","ARL4D",72.057986107743,0.321065889965404,0.361293498757267,0.8
8865670450691,0.374187606159143,0.900394360641411
"5561","AC008105.3",18.4981028031448,0.404107060767337,0.4490851240654
73,0.899845127598619,0.368202674960664,0.900394360641411
"5562","ATP5MC1",133.588841440864,-0.258743032797747,0.291179722383553
,-0.888602512151997,0.374216740489091,0.900394360641411
"5563","MKS1",6.61083363512519,0.521788462148488,0.585164252838582,0.8
91695724093426,0.372556046048461,0.900394360641411
"5564","SLC25A19",7.33816362397002,-0.504979551880923,0.55997488434809
5,-0.901789644492369,0.36716862178832,0.900394360641411
"5565","METTL23",44.9986561766773,-0.270722482665204,0.300308861467677
,-0.901480167258876,0.367333074066405,0.900394360641411
"5566","AC022966.1",2.41333552111432,0.97882325914636,1.08694791848964
,0.900524526056851,0.367841179288769,0.900394360641411
"5567","CACTIN",6.92724643561138,0.534283844941831,0.598804086306916,0
.89225150121635,0.37225814316537,0.900394360641411
"5568","HDGFL2",47.3888497466392,-0.239044545500075,0.266703000079707,
-0.896294925173823,0.37009526878596,0.900394360641411
"5569","FEM1A",7.19613069340393,0.542311724384448,0.599686945264705,0.
904324712529915,0.36582324657915,0.900394360641411
"5570","GPR108",52.7336603672743,-0.238848289676369,0.267387571444417,
-0.893266236669565,0.371714614384476,0.900394360641411
"5571","XAB2",29.1990994986204,-0.280299699383449,0.314975436441312,-0
.889909710262998,0.373514369530265,0.900394360641411
"5572","ICAM3",14.9517793261428,-0.378737473406448,0.422722377770247,-
0.89594848374054,0.370280277952442,0.900394360641411
"5573","ZNF433",3.18032407488598,0.786705167438453,0.88181028765332,0.
892147867238019,0.372313680959328,0.900394360641411
"5574","ZFP14",23.7124642205716,0.34196877761645,0.384049091727794,0.8
90429856448742,0.37323511674223,0.900394360641411
"5575","ZNF234",13.4081076127874,0.390478369322777,0.435993684980482,0
.89560556213161,0.370463463999067,0.900394360641411
"5576","CLEC11A",73.9872104389899,0.320489140758871,0.358171968007335,
0.894791243831534,0.370898691930045,0.900394360641411

"5577","ZNF808",7.86278007418624,0.506433346925469,0.564909514494662,0.89648578034395,0.369993371461473,0.900394360641411
"5578","LILRB2",1.95212234929939,-1.11364236995751,1.2420963897911,-0.896582889307647,0.369941531804985,0.900394360641411
"5579","SLX4IP",10.1372488145062,0.478135010230087,0.529273507503481,0.903379828107007,0.366324342125149,0.900394360641411
"5580","NAA20",69.4884926672446,-0.23483447373833,0.264122767730047,-0.889111059060037,0.373943395987145,0.900394360641411
"5581","NANP",5.58681125470201,-0.535839273213688,0.601912817294059,-0.890227384794015,0.373343803071523,0.900394360641411
"5582","PKIG",80.7581279689074,-0.260510938910312,0.291394022082747,-0.894016071600585,0.371313292232299,0.900394360641411
"5583","TCEA2",56.5526370975454,-0.234723764495053,0.262449621304017,-0.894357413543963,0.371130690323337,0.900394360641411
"5584","PCNT",18.5057947404857,-0.398695942683152,0.448459963508443,-0.889033526123555,0.373985062039686,0.900394360641411
"5585","KLHL22",16.3181072705703,-0.349046875090765,0.390736722004504,-0.893304507700562,0.371694124680632,0.900394360641411
"5586","RBFOX2",110.54757807201,-0.179155539473245,0.200187714904445,-0.894937731612355,0.370820375341522,0.900394360641411
"5587","PPP1R3F",6.94815598304454,-0.51994554512322,0.58479444328063,-0.889108217592468,0.373944922935709,0.900394360641411
"5588","RAB9B",11.567355673287,-0.392913961042712,0.438192087103244,-0.896670598595579,0.369894713854451,0.900394360641411
"5589","ZNF75D",8.46867269495641,0.48807469883608,0.539366973838808,0.904902825922641,0.365516869802826,0.900394360641411
"5590","KANTR",14.4773614360889,0.536701849637014,0.604435573322758,0.887938886003364,0.374573626055389,0.90051788203191
"5591","PPP1R15B",32.9568278617665,-0.376655748775408,0.42473374631768,-0.886804385196387,0.375184226276028,0.900524807041322
"5592","MAP4K3-DT",23.9519967433438,0.360614026803522,0.407768346645564,0.884360028849838,0.376501892956695,0.900524807041322
"5593","TGOLN2",204.432655877518,0.156333569851997,0.176758760995509,0.884445947524884,0.376455528852916,0.900524807041322
"5594","COPS7B",19.6573381150924,0.310602620465138,0.351291048731636,0.884174594219217,0.376601970641483,0.900524807041322
"5595","SS18L2",51.1959837304234,-0.291990458332847,0.329780200289872,-0.885409306186944,0.375935914986335,0.900524807041322
"5596","GAK",28.6155298560395,0.286013532661676,0.323276358909675,0.884733834624726,0.376300202656629,0.900524807041322
"5597","RPL34",3423.47938126282,-0.225245424133294,0.254652224841299,-0.884521720843666,0.37641464238884,0.900524807041322
"5598","AC093849.2",9.59580334566171,-0.498483394678615,0.564167304015376,-0.883573704343967,0.37692637915639,0.900524807041322
"5599","SENP6",87.0649877030306,0.241854463458744,0.273411613698254,0.884580066615832,0.376383161507966,0.900524807041322
"5600","L3MBTL3",7.73011900219799,-0.516042960871824,0.582069300868946,-0.886566187396321,0.375312504902769,0.900524807041322
"5601","HIPK2",16.7831262076883,0.404884236039354,0.458616724070115,0.

882837922799897,0.37732384764117,0.900524807041322
"5602","ZFP41",3.83129865321509,0.671610433172461,0.759598974642648,0.884164480986061,0.376607429149751,0.900524807041322
"5603","MAMDC4",1.48270403779423,1.06493568096843,1.20458060919194,0.88407174483974,0.37665748475803,0.900524807041322
"5604","RPP38",22.5221912661819,-0.289811850291165,0.327831908633835,-0.88402575423146,0.376682310342653,0.900524807041322
"5605","CASP7",28.1519877464877,0.301321997869003,0.341294054715652,0.8828808873335,0.37730063114058,0.900524807041322
"5606","BCCIP",25.5780261877981,-0.300399282889627,0.33963967053936,-0.884464651648563,0.376445436055159,0.900524807041322
"5607","CDC42EP2",62.4247413329787,0.347447548338568,0.391432783000743,0.88763017158404,0.374739718512189,0.900524807041322
"5608","SF3B2",142.152499386595,-0.173185393616574,0.19558772721773,-0.885461455481727,0.375907799475839,0.900524807041322
"5609","CCDC77",7.29210293969281,0.492403994304922,0.557366779145515,0.883446973750058,0.376994820488525,0.900524807041322
"5610","SOX5",27.2646740462018,-0.308655658439902,0.34955835842292,-0.882987492653425,0.377243029233781,0.900524807041322
"5611","AC009779.2",6.17238380169544,0.527308597844855,0.595566214059472,0.885390382121641,0.375946117933475,0.900524807041322
"5612","DCTN2",141.210940477603,-0.201336923694947,0.228032021871206,-0.882932677800243,0.377272646591296,0.900524807041322
"5613","METTL1",5.30857979695846,-0.556049485589676,0.629205877425557,-0.883732186140401,0.376840801252215,0.900524807041322
"5614","PPP1R3E",5.28318994858773,0.64578733586599,0.729577849875295,0.88515205879177,0.376074625059795,0.900524807041322
"5615","YLPM1",32.4191254005667,0.303676866332615,0.343013111976973,0.885321451947881,0.375983283218046,0.900524807041322
"5616","ZSCAN2",3.46810905952296,-0.705851555662741,0.798983064772456,-0.883437442899697,0.376999967969805,0.900524807041322
"5617","ABHD2",56.2694955577134,-0.317531070066868,0.35941007015098,-0.883478501126806,0.376977793294498,0.900524807041322
"5618","FES",24.321524051828,-0.312219181141781,0.352757157571755,-0.885082483629753,0.37611214603035,0.900524807041322
"5619","IGF1R",42.0746192135204,0.306834897006266,0.345755294281286,0.887433691056206,0.374845451349927,0.900524807041322
"5620","GOT2",13.2079280328075,0.410052716525421,0.462298164809701,0.886987549894803,0.375085603507028,0.900524807041322
"5621","TMEM107",24.4268391851016,0.28295173373549,0.319260074282919,0.886273469587515,0.375470181743308,0.900524807041322
"5622","STRADA",21.308893252142,0.306680953470968,0.346198864280949,0.885851991767624,0.375697288958279,0.900524807041322
"5623","TEX2",7.51633425356035,0.531464047556265,0.600843588570109,0.884529780572421,0.376410293607526,0.900524807041322
"5624","FASN",9.89401308364751,0.433831714441259,0.490868699761927,0.883803988014857,0.37680203321152,0.900524807041322
"5625","ZNF561-AS1",8.35245164388685,0.494112026375505,0.55936679486884,0.88334171943718,0.377051669299829,0.900524807041322

"5626","OCEL1",28.9586002228999,-0.32965508230661,0.372684998709426,-0
.884540787657607,0.376404354573571,0.900524807041322
"5627","MRPL39",19.2694320059177,-0.370952655135827,0.419598302658056,
-0.884066147994237,0.376660505863556,0.900524807041322
"5628","SF3A1",38.7003542739568,0.240792031714183,0.272684921464521,0.
883041241961422,0.37721398899962,0.900524807041322
"5629","PES1",16.6171155267301,-0.327956264732679,0.370464696761754,-0
.885256456551346,0.376018329054789,0.900524807041322
"5630","GK",6.88880911226884,0.630271656140935,0.713814429012357,0.882
962896971679,0.377256318503729,0.900524807041322
"5631","ZXDA",2.73058685504878,-0.747296838937319,0.845345758812499,-0
.884013234995212,0.376689068361486,0.900524807041322
"5632","AC083862.3",3.8753608120454,0.672993686022186,0.76251768762636
8,0.882594196755147,0.377455565164239,0.900582454325004
"5633","NR4A3",6.08698585206891,-0.804103220417621,0.911118416948067,-
0.882545238313907,0.37748202732441,0.900582454325004
"5634","RPL37",3257.33824114475,-0.229922030618268,0.260570341172509,-
0.882379896283165,0.3775714035575,0.90063579914967
"5635","MRPL19",52.1676866066234,0.228892147206034,0.259527842905352,0
.881956034634438,0.377800583068629,0.901022544074411
"5636","DDX54",29.1727695251858,-0.247326743331609,0.280559782596678,-
0.881547387307313,0.378021617416375,0.901281727943107
"5637","NUPR1",780.073903929863,0.20480950870422,0.232340157359207,0.8
815071446628,0.378043388675891,0.901281727943107
"5638","FLG-
AS1",2.35476426652581,0.902527487821716,1.02468090274276,0.88078882450
7144,0.378432129603579,0.901762083288552
"5639","SMG7",22.5344560242975,0.321535128722065,0.365022104295398,0.8
80864815961555,0.37839099286538,0.901762083288552
"5640","TMEM64",28.9512407958362,0.358938909566948,0.407531831817939,0
.880762879222893,0.378446175291869,0.901762083288552
"5641","DNAJC16",17.0596441525236,0.39834963471747,0.452499437983342,0
.880331777853244,0.378679602457876,0.902122423766306
"5642","SPRYD4",9.766909642245,-0.43906895837649,0.498808415410458,-0.
880235667265537,0.378731655248865,0.902122423766306
"5643","PIP5K1A",22.5685341671681,-0.29521362314811,0.335438257871114,
-0.88008334237635,0.378814162314921,0.902159051453166
"5644","SRSF2",169.896048454104,-0.201778501158934,0.229312216294153,-
0.879929139492946,0.378897697867043,0.902198115101912
"5645","LINC02613",2.07201481227282,1.05484650047153,1.19990283530093,
0.879109932436302,0.379341672843835,0.902199112464907
"5646","MFF",74.358226420177,-0.206727635613559,0.235154765605717,-0.8
79113102730728,0.379339954064105,0.902199112464907
"5647","CCDC66",63.8140815549702,-0.203117199907212,0.231009088541778,
-0.879260643766741,0.3792599697761,0.902199112464907
"5648","GCLC",8.5591341474131,-0.42666702935629,0.485366647411345,-0.8
79061286208842,0.37936804706743,0.902199112464907
"5649","STON2",25.206241242472,0.413467126623219,0.470121603378598,0.8
79489739785998,0.37913579382794,0.902199112464907
"5650","ZNF18",5.52031256818221,-0.508236538618494,0.577847926621689,-

0.879533377561518,0.379112143865124,0.902199112464907
"5651","DOCK6",7.4192423534746,0.50534420902608,0.574645275196584,0.87
9402008227082,0.379183343638743,0.902199112464907
"5652","SIGMAR1",35.1459793640663,0.246721437049427,0.280743779249714,
0.878813549168527,0.379502378620013,0.90235889353757
"5653","UQCRQ",206.381884650104,-0.2002837438235,0.227935963131262,-0.
878684263211956,0.379572493531992,0.902365954462487
"5654","RPS27",4526.55770380345,-0.249083014996808,0.283560101148918,-
0.878413479144573,0.379719372116693,0.902555472563891
"5655","RBM38",5.2521007386653,0.555185977975048,0.632187059350229,0.8
78198896614042,0.379835790701887,0.902577275374701
"5656","RTL6",9.13149165182181,-0.416048131761857,0.473778520414263,-0
.878148995437936,0.379862866993029,0.902577275374701
"5657","KPNA5",23.6762661401135,0.297515653352299,0.33887772573407,0.8
77943962554124,0.379974129931197,0.902682045632908
"5658","C5orf30",6.51597077753873,0.499599046309491,0.569139868377445,
0.877814179023853,0.380044568483169,0.902689811920345
"5659","GSTM4",16.3796479723197,0.350572696437382,0.399888723506462,0.
876675624567134,0.380662850098805,0.90270669672135
"5660","YY1AP1",26.5657695928892,0.2704630523048,0.308634411130663,0.8
76321766305888,0.380855135379379,0.90270669672135
"5661","PLCL2",19.5266559171675,0.352273669848349,0.401601142109124,0.
877172978140156,0.380392690817041,0.90270669672135
"5662","TECPR1",9.69460917524187,0.423363914118996,0.482948390217278,0
.876623512356104,0.380691163938279,0.90270669672135
"5663","ABCB8",15.7691635044498,-0.356995622750782,0.407520489595523,-
0.87601883062398,0.381019796897664,0.90270669672135
"5664","SQLE",16.030349211419,-0.425331279759313,0.485841820541634,-0.
875452177593808,0.381327920030293,0.90270669672135
"5665","TIGD5",10.74259589428,-0.435270203667662,0.497190848859125,-0.
875459000636177,0.381324209024769,0.90270669672135
"5666","CUEDC2",84.8909651588766,-0.240616330420483,0.274747950002934,
-0.87577115832134,0.381154452326715,0.90270669672135
"5667","PITPNM1",4.65007784853595,0.678342441499339,0.7743416591824,0.
876024728174352,0.381016590850896,0.90270669672135
"5668","MSANTD2",13.0775599793985,0.428231249467848,0.488579076672323,
0.876482988965676,0.380767520181651,0.90270669672135
"5669","CACNA1C",5.54316651086158,0.584791009582476,0.667594292901611,
0.87596765850223,0.38104761595918,0.90270669672135
"5670","SUDS3",46.8347046407223,0.227478112715948,0.259721046960408,0.
875855520290677,0.381108582806705,0.90270669672135
"5671","HYKK",8.02532423322644,-0.527051000181467,0.601000570251146,-0
.876955906982289,0.380510587991882,0.90270669672135
"5672","RARA-
AS1",19.500329635695,-0.436679011462767,0.497993958403376,-0.876876122
880705,0.3805539265209,0.90270669672135
"5673","ZNF98",1.83790837391543,1.10836369200269,1.2646638791834,0.876
409700827672,0.380807346514278,0.90270669672135
"5674","AP000525.1",10.8547611649468,0.53918821284668,0.61503084230706
5,0.876684835550866,0.380657845680465,0.90270669672135

"5675","SREBF2",18.0831844908461,0.327800493472758,0.373739437131573,0
.877082964507591,0.380441576913988,0.90270669672135
"5676","MAGEH1",79.1455818037788,-0.243358257565551,0.277465537652741,
-0.877075616756857,0.380445567622752,0.90270669672135
"5677","ZNF185",5.77111229331149,0.58873167551758,0.672451121037656,0.
87550106929573,0.381301328661664,0.90270669672135
"5678","PARK7",454.902380089788,-0.224931640326016,0.257132791314574,-
0.874768399534218,0.381699934602053,0.902951139080617
"5679","RPS6KA2",8.37896992611991,0.475648379540929,0.543571239988079,
0.875043314564178,0.381550338264263,0.902951139080617
"5680","CEP95",27.9645034514061,0.31784164829349,0.363337189050988,0.8
74784244144318,0.381691311704023,0.902951139080617
"5681","ZNF382",1.76881114013464,0.970083784390402,1.10870993238855,0.
874966261283963,0.381592263548664,0.902951139080617
"5682","MCOLN3",31.5581276744239,0.45249221645748,0.519118579249309,0.
871654829060102,0.383396709651758,0.903447470668511
"5683","NGF-
AS1",3.02271950061221,0.72258701326433,0.828226970523864,0.87245047430
33,0.382962675101634,0.903447470668511
"5684","AC245297.3",103.057920096362,-0.189393461534587,0.217244140089
565,-0.871800093003678,0.38331744386046,0.903447470668511
"5685","POLR2D",16.0653644005324,0.326750335255835,0.375014747504871,0
.871299962014403,0.383590391222154,0.903447470668511
"5686","PARL",46.4771048940804,-0.249274433523817,0.286021711047851,-0
.871522768710778,0.383468779383011,0.903447470668511
"5687","HAUS3",16.8240734931135,0.35883778823412,0.411308409240228,0.8
7242998239926,0.382973849916288,0.903447470668511
"5688","CEP135",11.7703945941797,0.413764925668074,0.474655327052785,0
.871716595360281,0.38336300457175,0.903447470668511
"5689","ADH5",293.207472505923,0.190301011283165,0.218201273381639,0.8
72135200376781,0.383134624995263,0.903447470668511
"5690","AL590062.1",6.18291150912462,-0.502433859860167,0.575914986609
142,-0.872409768008269,0.382984873590973,0.903447470668511
"5691","HLA-
DRB1",62.9518192207724,-0.363673949640109,0.417263001095759,-0.8715700
85737481,0.383442955951878,0.903447470668511
"5692","NUDT18",14.279689067113,-0.433086640725465,0.496142152769213,-
0.87290837577133,0.382713020596772,0.903447470668511
"5693","ZFAT",2.90086495201628,0.81654548039394,0.93463800461577,0.873
648916865542,0.38230947803151,0.903447470668511
"5694","GLIDR",4.52621480677294,0.606441041062254,0.694200148416429,0.
873582413437441,0.382345707034254,0.903447470668511
"5695","MAP3K8",39.9543527545716,-0.275543013304095,0.315462866469633,
-0.873456252990143,0.382414441112638,0.903447470668511
"5696","DLG5",25.0626503691144,0.332906001459608,0.381402128106896,0.8
72847781715323,0.382746051625353,0.903447470668511
"5697","TLCD5",16.5824021516005,0.355568889322966,0.407813909403754,0.
871890048681339,0.383268363016657,0.903447470668511
"5698","NACA",1863.48186994188,-0.23877187476934,0.273422553732755,-0.
873270589823826,0.382515606928544,0.903447470668511

"5699","ARHGAP11A",1.76542997819411,-0.893500363685319,1.02506023425058,-0.871656448890102,0.383395825708074,0.903447470668511
"5700","NPIPB5",13.816188267326,0.525174670859083,0.601690803531226,0.872831473868169,0.382754941656489,0.903447470668511
"5701","AKTIP",24.891257596444,-0.292110159508011,0.334412510720245,-0.873502486132696,0.382389251733195,0.903447470668511
"5702","CMTM3",180.490011798153,0.238538920054013,0.273081688771916,0.873507561516678,0.382386486554342,0.903447470668511
"5703","RPRD1A",19.7752309397095,0.32000384075998,0.367247474581514,0.871357498440612,0.383558984560789,0.903447470668511
"5704","CSNK1G2",38.8613534048916,-0.243970243897888,0.279477202787558,-0.872952217442007,0.382689122716908,0.903447470668511
"5705","C19orf44",6.71204106213685,0.551319074779018,0.630636803099115,0.874225976139819,0.381995202432912,0.903447470668511
"5706","MORC2",11.8923971590603,0.384352625010399,0.440977719888117,0.871591936907642,0.383431030959566,0.903447470668511
"5707","THEM4",20.9823524749723,-0.379783928365941,0.436225396340638,-0.870613979726612,0.383964960745152,0.903589524498733
"5708","TTC33",21.5451575337615,0.346933900288651,0.398433654539006,0.870744467331854,0.383893692865133,0.903589524498733
"5709","RAB2A",286.218177696459,-0.145783545601372,0.167389641645842,-0.870923338911358,0.383796012465695,0.903589524498733
"5710","TMEM134",37.6522836016625,0.245845587803471,0.282364371639434,0.870667876319058,0.383935523282869,0.903589524498733
"5711","SGTA",25.8727004977808,-0.289810162214495,0.332895556840057,-0.870573836927892,0.383986887001433,0.903589524498733
"5712","SHE",19.7153296599306,0.320386793996671,0.368087511101094,0.87040930304391,0.384076764479769,0.903611063535501
"5713","PFKFB2",1.82941617713124,0.972262569966999,1.11733397114627,0.870162901222413,0.384211386812275,0.903611063535501
"5714","C4orf33",9.97338915028991,0.444713031880553,0.511154375502403,0.870017069585786,0.384291075938151,0.903611063535501
"5715","HCP5",1.81035706533862,0.971516932433348,1.11676088213527,0.869941764593136,0.384332230014802,0.903611063535501
"5716","TRAFD1",20.6168952842448,0.320964738234346,0.368813405938366,0.870263209163235,0.384156579800982,0.903611063535501
"5717","BIRC6",59.0349486314854,0.241311433127082,0.277669790608992,0.8690590092564,0.384814855644044,0.903657884601407
"5718","SF3B1",191.082415816638,0.182808670722628,0.210477783865405,0.868541407864354,0.385098014247253,0.903657884601407
"5719","ALS2",15.6630192431886,0.387857293938698,0.446862072770921,0.867957514348122,0.385417591395901,0.903657884601407
"5720","AC012063.1",11.2462620257358,-0.388675443983344,0.448852831609613,-0.865930694008391,0.386528169611792,0.903657884601407
"5721","FGFRL1",7.16859323557647,-0.509921725717539,0.588193802225654,-0.86692808354671,0.385981414997913,0.903657884601407
"5722","WDR19",20.2570385970889,0.349062904430231,0.402158831847737,0.867972743073788,0.385409254337823,0.903657884601407
"5723","UBLCP1",32.1296553151692,-0.244971695597251,0.282703348131703,-0.866532700147317,0.386198101969571,0.903657884601407

"5724","PSMG4",42.1945408218751,-0.241844534114769,0.278421264300072,-0.868628101099773,0.385050579037853,0.903657884601407
"5725","HCG18",38.8119939550607,0.299904468618626,0.345699966256344,0.867528197547582,0.385652668819647,0.903657884601407
"5726","HBS1L",34.4038276517046,-0.245038511598666,0.282880825521575,-0.866225242191177,0.386366653367489,0.903657884601407
"5727","PSMG3-AS1",9.30119982414055,0.440987054936635,0.509334979213967,0.865809482822465,0.386594648039834,0.903657884601407
"5728","DMTF1",37.4079585481527,0.290083950194437,0.333929800807246,0.868697401349578,0.385012663171168,0.903657884601407
"5729","MAP11",2.5603668304104,-0.754990879466622,0.87220733933261,-0.865609408932881,0.386704394080003,0.903657884601407
"5730","FDFT1",35.5511543659849,-0.25381031795287З,0.292276452141765,-0.868391264821313,0.385180175204652,0.903657884601407
"5731","MSC-AS1",15.0926206613225,0.3550469740695,0.409620306378143,0.866770930398497,0.386067532736169,0.903657884601407
"5732","LY6K",3.28655746900702,0.929835969781674,1.07298615057165,0.866587112318539,0.386168277371958,0.903657884601407
"5733","ASS1",13.3691761673241,-0.535571491682244,0.617730531521559,-0.866998576811566,0.385942789481283,0.903657884601407
"5734","PLEKHA1",38.6270646630124,-0.262600228749279,0.30322141702066,-0.866034567510073,0.386471205610178,0.903657884601407
"5735","AP003307.1",7.21237400917047,-0.482067118384288,0.55691208161145,-0.865607219346733,0.386705595233477,0.903657884601407
"5736","SPA17",8.39860630967006,-0.473171256766466,0.545446447880828,-0.867493515825127,0.385671663024442,0.903657884601407
"5737","NECAP1",31.0495658711651,-0.245009428987531,0.282375492344822,-0.867672427776905,0.38557368410617,0.903657884601407
"5738","ATL1",33.3047630080129,-0.262857690786694,0.303382520233523,-0.866423321239353,0.386258059389937,0.903657884601407
"5739","LACTB",17.2230290870476,0.34655789375807,0.398454174613486,0.869755961508605,0.384433782681977,0.903657884601407
"5740","CLK3",24.4264229054814,0.344372385591784,0.397541841101883,0.866254441638829,0.386350644020979,0.903657884601407
"5741","SETD1A",15.1165987982139,0.351743917228718,0.405114438654024,0.868258160329639,0.385253021320593,0.903657884601407
"5742","AC007906.2",1.76054245205918,-0.969958077806598,1.11782220312658,-0.867721248597134,0.385546950628863,0.903657884601407
"5743","MAP1LC3B",177.649088532875,0.184138318831992,0.212114954151643,0.868106256668496,0.385336166230268,0.903657884601407
"5744","ALDH3A2",114.658682240862,0.166906694808163,0.192640806985291,0.866414013833044,0.38626316162379,0.903657884601407
"5745","NXPH3",21.8085444108266,0.39031411135198,0.44990860212032,0.867540895000708,0.385645714924295,0.903657884601407
"5746","L3MBTL4",6.28173559846353,-0.512769464029501,0.590191949791536,-0.868818126391962,0.384946616990536,0.903657884601407
"5747","ZNF420",13.2521495779008,0.378635146838328,0.436045467350677,0.868338682979181,0.385208951461638,0.903657884601407

"5748","AC093227.1",3.0906568698787,0.747584159848902,0.86214941974566
,0.867116700106862,0.385878071238109,0.903657884601407
"5749","LIPE-
AS1",6.82205021954263,0.593562201779967,0.683656758263713,0.8682166812
5894,0.385275723922002,0.903657884601407
"5750","KCNB1",3.25787734103132,0.807139024123602,0.92885498359456,0.8
68961289307044,0.384868304490506,0.903657884601407
"5751","RWDD2B",21.4798772564193,0.280916424303905,0.324470641324649,0
.865768388650098,0.38661718777427,0.903657884601407
"5752","HMGCS1",17.6269451075875,0.352185702883797,0.40716004278697,0.
864981004700562,0.387049214716346,0.90375376511216
"5753","SLC22A4",4.67342269283137,0.620692093425683,0.717731400140599,
0.864797183603913,0.38715011725208,0.90375376511216
"5754","EEPD1",3.33433548993071,-0.722180046405258,0.834886800382871,-
0.865003550270616,0.387036840172783,0.90375376511216
"5755","DPH7",18.4877790165343,-0.325025139495571,0.375726242612035,-0
.865058392610556,0.387006739970899,0.90375376511216
"5756","PLAC9",2609.57472900903,-0.210019839057676,0.242826826840431,-
0.864895538068727,0.387096126813135,0.90375376511216
"5757","FAM227B",2.57971432736253,-0.91591961087982,1.05911110731781,-
0.864800307117332,0.387148402568304,0.90375376511216
"5758","SELENOT",69.3201715340232,-0.223544423842419,0.258546928833344
,-0.864618368708272,0.387248287190868,0.903825934622799
"5759","MXD3",3.15428762666398,0.753241356363252,0.871532475676031,0.8
64272276003229,0.387438336291137,0.904112485052368
"5760","SP140L",15.9687104643632,0.360023564588112,0.416670048659457,0
.8640495417091,0.387560675937541,0.90424095901469
"5761","WARS",29.5599001207112,0.265959350282568,0.307879479172532,0.8
63842406766993,0.387674468572316,0.904349450293934
"5762","ELOA",30.7094252066395,-0.256256938428617,0.297811926103632,-0
.860465669663764,0.389532399792976,0.904514008385798
"5763","GNG5",233.437898141727,-0.227980360598292,0.264106858628255,-0
.863212571541684,0.388020602930778,0.904514008385798
"5764","MTF2",26.3557987182196,0.333588407138435,0.387302190154088,0.8
61312989233851,0.389065683603894,0.904514008385798
"5765","TATDN3",14.6110874156265,0.351762421034046,0.407392217568644,0
.863449044592453,0.387890623936375,0.904514008385798
"5766","ZNF669",5.28279914686411,-0.518123568272508,0.602035105541791,
-0.860620192249806,0.389447261079235,0.904514008385798
"5767","STK39",12.8911993033315,-0.372640338896302,0.431889361010181,-
0.862814351399404,0.388239547261964,0.904514008385798
"5768","BOK",8.69062041469098,-0.506867665048733,0.587562640770857,-0.
862661493221803,0.388323609784135,0.904514008385798
"5769","COQ2",10.78476014539,-0.399868513385095,0.464799225139515,-0.8
60303743546624,0.389621629845055,0.904514008385798
"5770","MARCH6",79.9539065697762,0.21663999934742,0.251822245949676,0.
860289362166652,0.389629555364639,0.904514008385798
"5771","FAM172A",68.0655596991176,0.188713966259962,0.219126258468089,
0.86121100756524,0.38912178728054,0.904514008385798
"5772","SLC35B2",46.4800763483931,0.242705155551003,0.281707778903005,

0.861549356202084,0.388935549906746,0.904514008385798
"5773","TRBC2",5.11345662742463,0.628119717795736,0.729034845396066,0.
861577086146678,0.388920284703862,0.904514008385798
"5774","CHRAC1",39.8130338310356,-0.253228830253721,0.29418453292180l,
-0.860782270701611,0.389357971406837,0.904514008385798
"5775","CREB3",43.1179498809976,-0.237604483955649,0.275316843777531,-
0.863021966602395,0.388125389524603,0.904514008385798
"5776","AL139339.1",4.14298506246538,-0.698786160413844,0.810693291079
167,-0.861961198030446,0.388708870450934,0.904514008385798
"5777","SLC43A1",30.0611048788951,0.311144597304506,0.360371293245932,
0.863400063034901,0.387917544798185,0.904514008385798
"5778","RBP5",1.54735398443412,-1.02993960956028,1.1942237056418,-0.86
2434403784309,0.38844851536549,0.904514008385798
"5779","IRAK3",37.7102792471934,0.24666651461209,0.286341079538472,0.8
61442986139712,0.388994109503839,0.904514008385798
"5780","ZNF140",9.30365906159828,0.410871698632343,0.477494795591875,0
.860473668876434,0.389527992116202,0.904514008385798
"5781","RNASE4",425.52049572107,-0.199145985055343,0.230811987031116,-
0.862806077001955,0.388244097383834,0.904514008385798
"5782","EFS",42.6699437044164,0.234485027660241,0.272497532935703,0.86
050330487054,0.389511662538131,0.904514008385798
"5783","SELENOS",210.144699233386,-0.164229728108223,0.190865403008041
,-0.860447862839257,0.389542211708405,0.904514008385798
"5784","MAP2K6",9.95348464935248,0.410744194825135,0.476757072517239,0
.861537706523029,0.388941963108564,0.904514008385798
"5785","FAAP24",6.71243362545862,0.546725952164307,0.633794712037613,0
.862623088802074,0.388344731576823,0.904514008385798
"5786","FCGRT",703.71360808404,0.241062364597184,0.280160411461148,0.8
604440696669,0.389544301841445,0.904514008385798
"5787","NSFL1C",60.906638071411,-0.198036999378447,0.230019412129139,-
0.860957766761284,0.389261303965985,0.904514008385798
"5788","IL10RB",79.3776591501485,0.192596499383599,0.223291876408966,0
.862532495498638,0.388394559160526,0.904514008385798
"5789","EP300",58.0781757181012,0.247135600550504,0.286741608173693,0.
861875617300727,0.38875596781874,0.904514008385798
"5790","HNRNPA2B1",473.312859465597,0.139551460198005,0.16226534865337
2,0.860020092743967,0.389777966745566,0.904645504994942
"5791","TMEM248",96.1844844163641,0.165721945743679,0.192712858164607,
0.859942337641667,0.389820828887991,0.904645504994942
"5792","EXPH5",2.96222505361235,-0.709472980585937,0.825249529299332,-
0.859707222357711,0.389950452542223,0.904790077989457
"5793","TIMM13",149.893503033508,-0.234481973812316,0.272797251574677,
-0.859546686994857,0.390038973881443,0.904839249092475
"5794","SDF4",202.350588921254,0.163187612300638,0.193777408055333,0.8
42139514292809,0.399709865564036,0.905373927055604
"5795","TPRG1L",41.2458548832229,-0.226184300945709,0.266533988331218,
-0.848613350822011,0.396096475536334,0.905373927055604
"5796","THAP3",25.6438836709645,-0.278435843359977,0.327990688692829,-
0.848913865419939,0.395929222952694,0.905373927055604
"5797","ERRFI1",197.068015095231,0.295272260452564,0.347178322770176,0

.850491638120006,0.395051807427328,0.905373927055604
"5798","H6PD",90.4564106350667,0.199260139302916,0.232983446446826,0.855254492719476,0.392410274636949,0.905373927055604
"5799","USP48",59.4334954507654,0.22965523008761,0.268921089833132,0.853987428914978,0.393111954787189,0.905373927055604
"5800","KDM4A-AS1",4.01883351511451,0.729239313953671,0.864113343913443,0.843916274514467,0.398716193259666,0.905373927055604
"5801","CYP4B1",103.354190713334,0.306843657944562,0.359056981259184,0.854582069031177,0.39278255775662,0.905373927055604
"5802","EPS15",85.4436162180272,0.192123863898859,0.227651673382803,0.843937850506362,0.398704135801372,0.905373927055604
"5803","C1orf52",45.966881219137,0.246358188144708,0.291120772513988,0.846240500178908,0.397418592774011,0.905373927055604
"5804","GPR89A",12.892644506566,-0.385513297767523,0.457468713704801,-0.842709645967813,0.399390851113977,0.905373927055604
"5805","ADAMTSL4-AS1",4.52536437054152,0.596715897062568,0.702861958405322,0.8489802157118,0.39589230118572,0.905373927055604
"5806","EEF1AKNMT",21.8702400948491,-0.291203551463835,0.339364718904923,-0.858084341835839,0.390845892871688,0.905373927055604
"5807","CAMSAP2",38.6065792895787,0.240678263885599,0.285784277515555,0.842167616699975,0.399694137406645,0.905373927055604
"5808","ETNK2",5.70909519672776,0.541919332662987,0.636741334754846,0.851082383196676,0.394723590761588,0.905373927055604
"5809","FLVCR1-DT",3.71890346170582,-0.688859199892617,0.804579706892266,-0.856172724705392,0.391902247017779,0.905373927055604
"5810","LYPLAL1",47.7125342225275,0.272945988995092,0.324705340399979,0.840595934328863,0.400574337156146,0.905373927055604
"5811","CHML",8.9596980853054,0.502808786460691,0.588431262020147,0.854490267451962,0.392833399722629,0.905373927055604
"5812","TAF1B",31.3699492707468,-0.286316009185429,0.33444997475635,-0.856080223638867,0.391953406740242,0.905373927055604
"5813","NBAS",36.0658041385565,0.25468993784464,0.298585184950371,0.852989199336774,0.393665293753684,0.905373927055604
"5814","MGAT4A",3.09870776018109,-0.693675149712755,0.822732329010682,-0.843135884239391,0.399152451631574,0.905373927055604
"5815","ZEB2-AS1",1.52179681514151,1.04216984289616,1.23882448815324,0.841257056881206,0.400203942459916,0.905373927055604
"5816","CCDC174",39.4898797724901,0.223730184258455,0.264689237027954,0.845256069988318,0.397967882777789,0.905373927055604
"5817","STT3B",70.6126134700633,0.1984025539057,0.233859172701257,0.848384742039382,0.396223737224177,0.905373927055604
"5818","GOLGA4",213.738815815208,0.191840862900344,0.226047787223804,0.848673925351929,0.396062759110185,0.905373927055604
"5819","MYL3",2.15505099431583,0.886981941148648,1.05038719483162,0.844433315174631,0.398427312322877,0.905373927055604
"5820","NDUFAF3",127.343681648211,-0.247813795430611,0.289438288040146

,-0.856188713347551,0.391893404562889,0.905373927055604
"5821","UBA7",25.1061762543263,0.279705635940032,0.329868796313234,0.847929962052039,0.396476977046922,0.905373927055604
"5822","ARHGEF3",33.3820222768023,0.325542239001694,0.383288346882099,0.84934029862857 5,0.395691963092789,0.905373927055604
"5823","ST3GAL6",1.92237263070582,-1.07435275771291,1.26136248256114,-0.851739902340747,0.394358468574952,0.905373927055604
"5824","ADPRH",3.34960794827616,0.761813146686689,0.895082747336431,0.851109183987377,0.394708704213061,0.905373927055604
"5825","CCDC14",47.3708672518083,0.26519090851702,0.311274099410534,0.851953018317995,0.394240168552296,0.905373927055604
"5826","H1FX-AS1",6.43555403833011,-0.530981657474417,0.623999876576294,-0.850932311698135,0.394806954525698,0.905373927055604
"5827","DNAJC13",50.2777021128829,0.243910405444337,0.28765169229335,0.847936626062311,0.396473265552302,0.905373927055604
"5828","IL20RB",3.97085490028928,-0.693852944526778,0.817038765161863,-0.849228915581893,0.395753926351441,0.905373927055604
"5829","FAIM",33.0835399723765,-0.267815813740518,0.312457646710129,-0.857126770813113,0.391374827519757,0.905373927055604
"5830","PIK3CB",5.13973987443897,-0.527467869385402,0.616432137123749,-0.855678731882067,0.392175507423556,0.905373927055604
"5831","TMEM129",27.9262509381596,0.283002704214714,0.335277078517105,0.844086048072254,0.398621323480753,0.905373927055604
"5832","BLOC1S4",45.3794612573659,-0.24932714758201,0.292404867907827,-0.852677827718667,0.393837989828701,0.905373927055604
"5833","FAM184B",1.80599738214817,1.12359183074242,1.32463561434435,0.848227103797571,0.396311505488113,0.905373927055604
"5834","RASGEF1B",5.39453524023826,-0.613126681033516,0.725687177158993,-0.844891160174357,0.398171610452945,0.905373927055604
"5835","NFKB1",53.4772837600803,0.196757972571717,0.233915655359369,0.841149226499758,0.400264340504791,0.905373927055604
"5836","FAM241A",8.93614192340998,-0.421243259482382,0.492405937547909,-0.855479650753392,0.392285665147631,0.905373927055604
"5837","LARP7",103.82241207788,-0.185636284997402,0.219160875972224,-0.84703204517639,0.396977260132854,0.905373927055604
"5838","TRIM2",34.1856883029169,0.260131542122994,0.306462871728482,0.848819110307964,0.395981954678647,0.905373927055604
"5839","GASK1B",61.8234709454374,0.621502854502051,0.738593274587071,0.841468337021505,0.400085615932432,0.905373927055604
"5840","ZNF622",31.5798367261358,-0.248468906010245,0.294824402336321,-0.842769133223932,0.399357574132379,0.905373927055604
"5841","FBXO4",17.7567354717028,-0.326705419105364,0.388815048460782,-0.840259193667286,0.40076307566097,0.905373927055604
"5842","GZMA",6.97485171858131,0.507357225214732,0.593598350378108,0.854714681891482,0.392709120544587,0.905373927055604
"5843","CKMT2-AS1",7.87543991636187,-0.440237956673374,0.51573978218464,-0.85360480591308,0.393323994782882,0.905373927055604
"5844","SLF1",7.71113665146767,0.461299501658475,0.547847844748313,0.8

42021203661759,0.399776084907249,0.905373927055604
"5845","FAM174A",27.7217257629771,-0.304181037681918,0.355548294445591
,-0.855526639935736,0.392259662893183,0.905373927055604
"5846","DMXL1",36.2333110110565,0.293068099289393,0.342966054283101,0.
854510513881587,0.392822186410542,0.905373927055604
"5847","TXNDC15",111.649026763902,-0.19384329276969,0.22738105499036,-
0.852504148940234,0.39393433723187,0.905373927055604
"5848","FBXO38",14.0540827269904,0.32529357944593,0.386541043636696,0.
841549907315067,0.400039938470946,0.905373927055604
"5849","ERGIC1",125.52862978695,0.17653074208836,0.209181741547431,0.8
43910853702938,0.398719222643151,0.905373927055604
"5850","BNIP1",11.1375122754034,-0.378570721720931,0.448692247907319,-
0.843720219118046,0.398825766291483,0.905373927055604
"5851","EXOC2",11.956803536944,0.397657957524252,0.469770204610693,0.8
46494634230364,0.397276865939169,0.905373927055604
"5852","ZSCAN9",3.19304082549915,0.811757383096077,0.96286304601051,0.
843066297392429,0.399191366425764,0.905373927055604
"5853","PFDN6",42.6086145703734,-0.257194564392641,0.302514835985007,-
0.85018826780908,0.395220423409761,0.905373927055604
"5854","SRF",10.248667139748,-0.367595620188278,0.436495332945246,-0.8
42152464054844,0.399702617886148,0.905373927055604
"5855","B3GAT2",4.31011608621251,-0.600418472200992,0.702241789459899,
-0.855002480930079,0.392549774295262,0.905373927055604
"5856","HMGN3",170.70640989322,-0.219766365048187,0.259508169956809,-0
.846857211026396,0.397074714949902,0.905373927055604
"5857","MAP3K5",7.20949301697633,0.514856702519835,0.601898142362606,0
.85538842253091,0.392336150803715,0.905373927055604
"5858","RGS17",1.83629779924996,-0.888220135705889,1.0447301663904,-0.
850190952918245,0.395218930810837,0.905373927055604
"5859","ZNF786",2.86262258026618,-0.677286196305101,0.796057616803145,
-0.850800472238413,0.394880199292828,0.905373927055604
"5860","LRRC61",1.56316943895592,-1.02527962816489,1.21453188354504,-0
.844176791120749,0.39857062167177,0.905373927055604
"5861","UBE3C",33.5355296982049,0.297381907328487,0.348622073477736,0.
853020878345154,0.393647726192014,0.905373927055604
"5862","HTRA4",1.53234299160045,-1.25106042722635,1.48612821780726,-0.
84182536354249,0.399885712581781,0.905373927055604
"5863","HGSNAT",98.0611150265309,-0.212036564109397,0.251035490483688,
-0.844647757577427,0.398307536066536,0.905373927055604
"5864","AC090578.1",2.38389331168135,0.932821006755722,1.1076515180959
1,0.842161087233709,0.399697791739159,0.905373927055604
"5865","MTDH",341.84947134317,-0.122509254773144,0.143100314136501,-0.
856107518088916,0.391938310530889,0.905373927055604
"5866","KLF10",49.2355059544046,-0.28287086186448,0.330284928040693,-0
.856444959636877,0.391751705088968,0.905373927055604
"5867","PUF60",73.7203911549633,-0.26117511298606,0.307257793303354,-0
.850019490728437,0.395314250079297,0.905373927055604
"5868","PLEC",109.041790969385,-0.257406615371951,0.306352302638428,-0
.84023071853896,0.400779038027665,0.905373927055604
"5869","KDM4C",12.3011761469406,0.391930676149148,0.458498894756374,0.

854812695584364,0.392654848711881,0.905373927055604
"5870","DMAC1",79.0142266750853,0.247116470122261,0.291012608951053,0.849160697926406,0.395791879260155,0.905373927055604
"5871","FAM122A",19.4194006022171,0.350981874484255,0.414537404598636,0.84668324399841,0.397171700744935,0.905373927055604
"5872","ENG",66.1381104646549,0.18106251300592,0.214629151544044,0.843606340067762,0.398889420262112,0.905373927055604
"5873","GTF3C5",21.9734139929352,0.294653028030402,0.346814456205773,0.849598460381297,0.395548368231622,0.905373927055604
"5874","FBXW5",78.7372563275259,-0.175589126281917,0.20533716009328,-0.855125911949648,0.392481446016993,0.905373927055604
"5875","CAMK1D",108.208964182424,-0.264734720915889,0.309386685480133,-0.855675868872801,0.392177091481841,0.905373927055604
"5876","NRP1",44.0186714211934,0.269853958714066,0.315726554467515,0.854707831494202,0.392712913894948,0.905373927055604
"5877","CSGALNACT2",40.0330353075147,0.276459232199118,0.324043420560277,0.853154900417715,0.393573409641413,0.905373927055604
"5878","ABLIM1",383.557009714426,0.243895371481551,0.289602279485161,0.842173521269015,0.399690832826337,0.905373927055604
"5879","HTATIP2",35.8406649450329,0.332905200974612,0.392404212059295,0.848373158961678,0.3962301859363,0.905373927055604
"5880","CAPRIN1",91.9136078888379,0.173064800700979,0.201927920261276,0.857062264975789,0.391410474301242,0.905373927055604
"5881","TP53I11",46.4291488109249,0.287976088647579,0.337335681100635,0.853678115839955,0.393283362910073,0.905373927055604
"5882","SLC35C1",19.1487638282035,-0.336551202055739,0.3933401141223,-0.855623898942318,0.392205846306771,0.905373927055604
"5883","TMEM216",25.2187199598949,-0.301523448273544,0.35545800810691,-0.84826742230226,0.396289056232083,0.905373927055604
"5884","PYGM",3.96131234401818,0.742548840309735,0.878674421785966,0.845078474915036,0.398067025542328,0.905373927055604
"5885","RAB6A",75.7641918138266,0.193181556489918,0.2276287970307,0.848669232583358,0.396065371093033,0.905373927055604
"5886","IFFO1",31.8557677468396,0.312749059137265,0.366644977260175,0.853002437055982,0.393657952732309,0.905373927055604
"5887","KRAS",88.8570511222289,-0.181400334276364,0.211405477917711,-0.858068277431172,0.390854762803466,0.905373927055604
"5888","BICD1",15.5670630273612,0.366421995444442,0.435668833015959,0.841056251161808,0.400316422328423,0.905373927055604
"5889","RASSF3",3.98144030629138,-0.600873173142164,0.71505399310573,-0.840318603819499,0.400729773208066,0.905373927055604
"5890","LLPH",53.5943440227165,-0.221951953577925,0.258923861998294,-0.857209342796638,0.391329200022311,0.905373927055604
"5891","GPN3",29.4190277862518,-0.267760370329146,0.317835839597324,-0.842448638480733,0.399536877587989,0.905373927055604
"5892","ZMYM5",29.9863894654314,0.254804576905772,0.300926318372352,0.846734105158888,0.397143344410719,0.905373927055604
"5893","MRPS31",44.5988672491813,-0.224575836727174,0.264087727712312,-0.850383464133627,0.395111926524574,0.905373927055604
"5894","ALG11",9.16524633288469,0.451811389929353,0.527367701822366,0.

856729352912738,0.391594477255388,0.905373927055604
"5895","PPP2R3C",35.4785343971904,-0.236767534362556,0.280647281228916
,-0.843648060033873,0.398866099709949,0.905373927055604
"5896","AL122035.2",1.81867501565038,1.01903577809567,1.19981453495151
,0.849327748923171,0.395698944298898,0.905373927055604
"5897","GON7",19.9688332404732,-0.30901977390227,0.364887468442083,-0.
846890618693086,0.397056091969031,0.905373927055604
"5898","BAG5",53.5533326469711,-0.216317644682741,0.254105547788566,-0
.851290523033887,0.394607988027646,0.905373927055604
"5899","IGHA1",26.3920609856862,0.457851101723396,0.543345913743411,0.
842651228512985,0.399423531275641,0.905373927055604
"5900","GOLGA8N",4.74272906805552,0.638042640108243,0.754451621380347,
0.845703849029946,0.397717975399067,0.905373927055604
"5901","CDAN1",4.56357570023852,0.577862866866435,0.675190120524716,0.
855852076771141,0.392079605635971,0.905373927055604
"5902","MAP1A",132.165324471119,-0.217951861644257,0.256132405456193,-
0.850934348803177,0.39480582285551,0.905373927055604
"5903","FAM214A",28.1140552332894,0.316087849725806,0.373134502568458,
0.847115041761151,0.396931001814099,0.905373927055604
"5904","AC103691.1",1.57504655583158,1.07324434518153,1.27697300515307
,0.840459697151454,0.400650689784868,0.905373927055604
"5905","LRRC49",8.23580260071614,0.428845510385536,0.50673551044435,0.
846290622122548,0.397390638087613,0.905373927055604
"5906","PSMA4",182.700480192539,-0.200535548064197,0.235748053911895,-
0.850635009437416,0.394972135479377,0.905373927055604
"5907","DET1",6.94344820748231,0.517963145078828,0.60392046040019,0.85
7667820586172,0.391075913832792,0.905373927055604
"5908","MESP2",1.98802509310348,0.951072103892895,1.12817381398622,0.8
43019127108112,0.399217746587143,0.905373927055604
"5909","SLX4",4.17305443376033,0.659357789852576,0.772958399471986,0.8
53031405445607,0.39364188850432,0.905373927055604
"5910","CDIPT",110.751257702999,-0.177628315700472,0.2107764049555,-0.
84273339673847,0.399377564808455,0.905373927055604
"5911","ZNF821",7.08735448770168,0.548060043334861,0.639693904409591,0
.856753580981354,0.391581084452055,0.905373927055604
"5912","LRRC75A",37.5530973239658,-0.3698094498084,0.436790801005899,-
0.846651186235504,0.397189574355004,0.905373927055604
"5913","AC104984.3",2.77770297142616,-0.744879342094873,0.886351907618
564,-0.840387814018704,0.40069097942015,0.905373927055604
"5914","UTP6",29.086818584826,-0.256645581681524,0.304568329263901,-0.
842653542808606,0.399422236571121,0.905373927055604
"5915","CCDC43",27.627695195485,0.26549078415134,0.312438757194106,0.8
49737038181856,0.395471301406065,0.905373927055604
"5916","AC015813.1",5.95820745439602,0.542017779116385,0.6372841954054
4,0.850511879980883,0.395040558364657,0.905373927055604
"5917","MAP3K3",31.9091570850167,0.281796492477025,0.328550107004181,0
.857697156292324,0.391059710702752,0.905373927055604
"5918","SLC26A11",19.2987974954494,0.314541502609622,0.371485771783383
,0.846712112551741,0.397155605674341,0.905373927055604
"5919","LINC01915",24.4249780958459,-0.445207400355183,0.5212436522862

68,-0.854125318173993,0.393035557025793,0.905373927055604
"5920","CNN2",57.2189953397594,0.273520837929174,0.320098057451816,0.854490777315468,0.392833117336701,0.905373927055604
"5921","DNMT1",50.9657567551543,-0.216186066359005,0.252419951344052,-0.85645395781073,0.39174672982981,0.905373927055604
"5922","NDUFB7",280.264325794823,-0.22788566663611,0.266180331201075,-0.856132628612454,0.391924422534748,0.905373927055604
"5923","AC024075.1",2.80608123119308,0.865710889101752,1.0183279288163,0.850129771171114,0.395252941376198,0.905373927055604
"5924","MYO9B",31.3955934170293,-0.321105543193788,0.377806972520347,-0.849919579439459,0.39536979917428,0.905373927055604
"5925","MAP1S",20.0031367211052,0.284494326262653,0.332013955670623,0.856874602418485,0.391514190326791,0.905373927055604
"5926","ZNF90",4.13935696895058,0.596918911613048,0.704723175873585,0.847026083501651,0.396980583009832,0.905373927055604
"5927","ZNF302",56.910769566676,0.224935144914917,0.262075106902493,0.858285044976082,0.390735085304817,0.905373927055604
"5928","ZNF790",9.70333292076343,0.436289985489822,0.513528575516343,0.849592420540845,0.395551727354989,0.905373927055604
"5929","ZNF569",7.64629074244015,-0.44776764267333,0.524735607143634,-0.853320484787998,0.393481603222117,0.905373927055604
"5930","ZNF780B",8.96558456325753,-0.437016458737793,0.515078581918166,-0.848446186813538,0.396189529788518,0.905373927055604
"5931","PAFAH1B3",3.71646540758374,-0.629156828876342,0.739578532004734,-0.850696446219067,0.394937997816344,0.905373927055604
"5932","DBP",31.0247458362602,-0.481593531984405,0.568985366692629,-0.846407588272066,0.397325406761472,0.905373927055604
"5933","PRMT1",88.2475705604024,-0.27152748442799,0.320670733912997,-0.84674856702595,0.397135281791009,0.905373927055604
"5934","ZNF880",11.4908887185144,0.400169063263074,0.476102134860688,0.840510961750186,0.400621958077951,0.905373927055604
"5935","HSPBP1",15.5895567587858,-0.328062646673559,0.390349505552415,-0.840433104197971,0.400665594533245,0.905373927055604
"5936","ZNF582",4.59664682293299,0.647179483260076,0.766818077972629,0.843980471836472,0.398680318078805,0.905373927055604
"5937","ZNF582-AS1",5.87996854887428,0.592121379686959,0.695705151486598,0.851109666820348,0.394708436025712,0.905373927055604
"5938","ZSCAN18",43.5565186681266,0.256050689331265,0.300014308143014,0.853461592935788,0.393403377609851,0.905373927055604
"5939","TRIM28",65.440113778349,-0.19136175140002,0.22389798979751,-0.85468275786256,0.392726798398776,0.905373927055604
"5940","AP5S1",12.2289500267457,0.404143953767309,0.478389856190522,0.844800424878481,0.398222277354685,0.905373927055604
"5941","ABHD12",38.5919315704873,-0.265920539149546,0.31506966322863,-0.844005533330518,0.39866631357302,0.905373927055604
"5942","SNTA1",21.6882241308619,-0.343440492767384,0.40653003200712,-0.844809646834084,0.398217127605598,0.905373927055604
"5943","RBM39",316.528805156883,0.13194114282027,0.155537519162948,0.848291418882947,0.396275695352426,0.905373927055604

"5944","CNBD2",5.24695144264949,-0.632916264662227,0.746194360577496,-0.848192237974567,0.396330919321833,0.905373927055604
"5945","SPATA2",7.40958743628644,0.480819104788493,0.57086295113286,0.842267139309571,0.399638440286547,0.905373927055604
"5946","RAB36",1.66332041124032,0.95720172060128,1.1150092241814,0.858469777507017,0.390633111929071,0.905373927055604
"5947","TFIP11",11.7740520157447,0.376575657722281,0.444157168951007,0.847843250198262,0.396525272788711,0.905373927055604
"5948","USP11",39.5518391741583,0.228232062568552,0.271606516400305,0.840304075150297,0.400737917122535,0.905373927055604
"5949","SLC35A2",15.6034996587409,-0.313717537290998,0.373068806994187,-0.840910661544228,0.400397985178168,0.905373927055604
"5950","COQ3",8.34377128277243,-0.466967979995915,0.555848473827731,-0.840099419146085,0.400852645763969,0.905388017886047
"5951","ACTR8",8.62402005147824,0.460894412234641,0.54871933576823,0.839945637398343,0.400938867654664,0.905430590221985
"5952","GATAD1",95.7326181141148,0.203507877450701,0.242372447153434,0.839649390187783,0.401104998049451,0.905562101831845
"5953","C17orf100",2.32078033567881,0.807701996479883,0.96200639954106,0.839601479642141,0.401131869350768,0.905562101831845
"5954","NRDC",74.5138076770434,0.219861595261551,0.262479131808879,0.837634572113872,0.402235969909602,0.906052943469205
"5955","USP34",77.5087961660556,0.213283903025116,0.255233407107276,0.835642580814162,0.403356006478384,0.906052943469205
"5956","ACTL6A",35.1932904249258,-0.245148627292518,0.293435694680948,-0.835442421410484,0.403468653257978,0.906052943469205
"5957","METTL14",24.7386379966835,0.280797111935487,0.335076872007736,0.838008037537741,0.4020261893976,0.906052943469205
"5958","HOMER1",2.63160100231996,-0.749234956412388,0.894938701896149,-0.837191368330533,0.402485008552148,0.906052943469205
"5959","TENT2",31.1336644180577,0.247574074077778,0.296312588543552,0.83551655801957,0.403426928064001,0.906052943469205
"5960","ZNF354C",10.9650217216609,0.369166102805332,0.440802979560149,0.837485497883206,0.40231972522325,0.906052943469205
"5961","CDKAL1",14.2984171525019,0.332247675418662,0.397148203354413,0.836583604338168,0.402826664744707,0.906052943469205
"5962","PREP",11.6779354745813,0.378271530236027,0.452064660380885,0.836764214033709,0.40272511636254,0.906052943469205
"5963","EIF3B",69.6518862526156,-0.206972177543576,0.247304375896007,-0.836912718562687,0.402641630709795,0.906052943469205
"5964","IMMP2L",20.1444278960665,-0.29293723515503,0.350665643262019,-0.835374781601134,0.403506724210372,0.906052943469205
"5965","ZER1",20.5171566744645,0.288245238904907,0.344919735345793,0.835687869863209,0.403330521078789,0.906052943469205
"5966","FUBP3",38.1664549211717,-0.232200102162611,0.277897869634767,-0.835559129934263,0.403402969120948,0.906052943469205
"5967","SEC16A",28.4197086614705,0.293829588107548,0.350668750357245,0.837912097408759,0.40208007398292,0.906052943469205
"5968","AL450326.1",11.0496050697333,-0.377208951574583,0.451331588854786,-0.835769001969743,0.403284868211791,0.906052943469205

"5969","NCOA4",73.9091366699161,0.170838033454207,0.204473778474338,0.835500936740638,0.403435719738841,0.906052943469205
"5970","ZFYVE27",9.99844059889336,0.410775650098213,0.491016509065748,0.836582156636222,0.402827478781775,0.906052943469205
"5971","MTA2",8.69281742672364,-0.401569993789923,0.48061076272926 3,-0.835540992693362,0.403413176429901,0.906052943469205
"5972","MRPL49",17.0314565559097,-0.338078535350174,0.40398247240076,-0.836864365280662,0.402668812615804,0.906052943469205
"5973","GDPD5",27.3072856775668,0.291424945448362,0.348258024390479,0.836807553705082,0.402700750775901,0.906052943469205
"5974","ZFC3H1",54.2917905496889,0.235293455185788,0.280943390354821,0.837511980220008,0.402304845721365,0.906052943469205
"5975","ZNF268",16.4332144947852,0.321003862214466,0.383585144935008,0.836851651981608,0.40267595960802,0.906052943469205
"5976","KLF13",89.7645280349187,-0.238921352829567,0.285171230087618,-0.837817169551635,0.402133394291341,0.906052943469205
"5977","SGF29",23.2114488763375,-0.311672661053252,0.372474690500747,-0.836761984107553,0.402726370052131,0.906052943469205
"5978","BECN1",37.6137582041398,-0.225846328676385,0.26993794313873,-0.836660182152736,0.402783606748373,0.906052943469205
"5979","SLC38A10",73.1121964480528,0.188735016630923,0.225894400257346,0.835501085533376,0.403435635997566,0.906052943469205
"5980","NDC80",1.82146631449849,0.923390320918805,1.1044589281846,0.836056730906766,0.403122988695453,0.906052943469205
"5981","ZNF460",8.42493376894791,0.482494725179176,0.575335843740537,0.838631436627074,0.401676164055355,0.906052943469205
"5982","RANBP1",112.818978450452,-0.215295224841525,0.256992057861778,-0.837750499501119,0.402170844924063,0.906052943469205
"5983","ZNF41",4.45535029355168,0.654576078186797,0.782497861295443,0.836521236113198,0.402861735044126,0.906052943469205
"5984","LINC00891",15.5318309974923,0.339216289479212,0.405269167624443,0.837014795543389,0.402584251518115,0.906052943469205
"5985","SMIM10",38.6363149804339,-0.277761236043841,0.33134177874425,-0.838292222298457,0.401866603005516,0.906052943469205
"5986","BCAS2",52.2273639733689,-0.232900371775411,0.279320817203132,-0.833809574622709,0.404388299063013,0.906282581531217
"5987","RUSC1",31.9509866652094,-0.235383850752075,0.283848616775366,-0.829258403391672,0.406958203224279,0.906282581531217
"5988","TRAPPC12",41.9216505605706,-0.206888927274865,0.248748815952603,-0.831718239472088,0.405568002897344,0.906282581531217
"5989","GEN1",3.20334646955522,0.712620762451572,0.855428545906964,0.833057028388057,0.404812567152629,0.906282581531217
"5990","AC009236.1",7.54915634143186,0.546743770081593,0.658470330865243,0.830324077568022,0.406355578419647,0.906282581531217
"5991","ZC3H6",70.098450776514,0.184780611807337,0.221663388059206,0.83360907466587,0.404501310277758,0.906282581531217
"5992","AC009962.1",1.47362537182914,-1.2141507393362,1.46179396808052,-0.830589512508732,0.406205561337517,0.906282581531217
"5993","EFHD1",113.566686299104,0.217103032870921,0.261871049087176,0.82904556890765,0.407078622176627,0.906282581531217

"5994","SETD5",83.6491437787858,0.215988586009287,0.260339547121059,0.829641859632072,0.406741302361946,0.906282581531217
"5995","MUSTN1",28.2468030611424,-0.677607891250384,0.817260016131031,-0.829121549905537,0.407035630688126,0.906282581531217
"5996","GFM2",8.2806607012534,0.452730163419162,0.542214638396903,0.83496484852879,0.403737500371993,0.906282581531217
"5997","TMEM167A",115.787614111296,-0.151458430015647,0.182703708087005,-0.828983886542257,0.407113525219574,0.906282581531217
"5998","WRNIP1",19.2653852860637,-0.30628730344109,0.366944720705124,-0.834696035011828,0.403888874676429,0.906282581531217
"5999","ABCC10",6.83982379732426,-0.441638755476368,0.53074533193857,-0.832110484821909,0.405346584208505,0.906282581531217
"6000","DSE",131.922818581061,0.223960036391429,0.269604716125869,0.830697769718795,0.406144386607975,0.906282581531217
"6001","SRRM3",4.45108728506752,-0.6402742739031,0.772262401643008,-0.829089015004356,0.407054039220227,0.906282581531217
"6002","ZNHIT1",189.690340410235,-0.217266491066441,0.260442598677504,-0.834220254941757,0.404156879279613,0.906282581531217
"6003","AC016831.6",1.97362489423797,0.939660191911009,1.12874580182364,0.83248167159768,0.405137119422498,0.906282581531217
"6004","SMARCD3",17.2249501111252,0.342097954935105,0.411491130372994,0.831361673883499,0.405769343396021,0.906282581531217
"6005","PAXIP1",9.59413788911792,0.444018858675917,0.534316650006174,0.831003223782726,0.405971808182098,0.906282581531217
"6006","AP3M2",15.3961820979297,-0.327023863257222,0.392554506449964,-0.833066129375602,0.404807434637696,0.906282581531217
"6007","YTHDF3-AS1",4.83774768056186,-0.644036701733405,0.776722224613103,-0.829172490917469,0.407006808814749,0.906282581531217
"6008","ERMP1",5.54847304627126,0.520049304728375,0.624854047817645,0.832273242919192,0.405254730055051,0.906282581531217
"6009","CDK5RAP2",36.1322695533393,0.269967305783252,0.325602616790884,0.829131253440254,0.407030140439314,0.906282581531217
"6010","RPS24",2971.77240689116,-0.213842533048974,0.257254951150049,-0.831247492392268,0.405833830431483,0.906282581531217
"6011","AL158212.2",6.58901001258898,0.708211521420755,0.849179441571126,0.833995133125743,0.404283726387244,0.906282581531217
"6012","RBM14",19.0726170656576,0.35116245007254,0.421098302921416,0.833920364048753,0.404325861030157,0.906282581531217
"6013","COLCA2",4.52302889760137,-0.59836990659542,0.721059709987051,-0.829847928413814,0.406624768678765,0.906282581531217
"6014","INTS13",10.86985992766,0.373637213091052,0.449588171715065,0.831065487478732,0.405936635203846,0.906282581531217
"6015","MTIF3",86.0399561349992,-0.194271550761483,0.23435009558309,-0.828980036377255,0.407115703899394,0.906282581531217
"6016","MGAT2",35.6704134585036,-0.235296226340491,0.282159939467382,-0.833910819461638,0.404331239883616,0.906282581531217
"6017","ENTPD5",14.3557598055378,0.373883964944867,0.450106395668322,0.830656859229296,0.406167503942399,0.906282581531217
"6018","NEDD4",25.327757249793,0.255455204324362,0.307931901564685,0.8

29583433955123,0.40677434621443,0.906282581531217
"6019","MSRB1",18.4948617289465,-0.358519657994099,0.43145114371335,-0
.83096235394915,0.405994896682493,0.906282581531217
"6020","PRMT7",15.8101292128259,0.310120206305448,0.373138568318968,0.
831112708886071,0.405909960880298,0.906282581531217
"6021","DHODH",5.02751273758418,-0.515025265000833,0.620916881856505,-
0.829459272327945,0.406844573714368,0.906282581531217
"6022","SNHG29",580.589473935518,-0.249899430601558,0.300611212155732,
-0.831304424108098,0.405801675964926,0.906282581531217
"6023","TMEM199",15.5330628280617,-0.376893908331368,0.45245247624616 9
,-0.833002200492563,0.404843488245999,0.906282581531217
"6024","CORO6",4.62290996213797,0.651302882584132,0.780486660717422,0.
834483041626176,0.404008839661604,0.906282581531217
"6025","SNHG30",20.1905942677185,-0.383489485599068,0.460341120653991,
-0.833055028962561,0.404813694736708,0.906282581531217
"6026","NR1D1",9.97458588045402,-1.11776182466743,1.34381587052746,-0.
831781979348622,0.405532017439897,0.906282581531217
"6027","NKIRAS2",18.704531097223,0.346503134134294,0.41794525926403,0.
829063439418979,0.40706851045908,0.906282581531217
"6028","DCAKD",13.6422472244095,-0.37285195110855,0.447194577659542,-0
.833757763924431,0.404417500201627,0.906282581531217
"6029","ZNF652",61.6029111890009,0.19152726122004,0.229492446707154,0.
834568910515996,0.403960472870651,0.906282581531217
"6030","KCTD2",17.4008711894573,0.32825056910837,0.393762123119487,0.8
33626572581139,0.404491446876584,0.906282581531217
"6031","ADAT3",1.5642791228372,-1.13275615129252,1.36008237140393,-0.8
32858490859822,0.404924542358829,0.906282581531217
"6032","GTF2F1",42.0352284586274,-0.208969044716759,0.251564697407204,
-0.830677145364735,0.406156040735773,0.906282581531217
"6033","GDF15",7.18960755283904,0.597537395313581,0.719984611931895,0.
829930786590343,0.40657791727814,0.906282581531217
"6034","CLIP3",20.6968728117076,0.305936275174483,0.366618850849822,0.
834480481473671,0.404010281754663,0.906282581531217
"6035","CU638689.4",2.53963538405936,0.852949295124274,1.0261816146721
5,0.831187465190342,0.405867734845867,0.906282581531217
"6036","LARGE1",11.9588637375697,0.399182273655859,0.481137621549308,0
.829663397284243,0.406729121700429,0.906282581531217
"6037","PMM1",63.2846338938324,-0.239004633267491,0.287297644012265,-0
.83190599800842,0.405462006012803,0.906282581531217
"6038","LINC00847",11.8237767955924,-0.398947194423775,0.4813523332305
68,-0.82880494573749,0.40721478919465,0.906353022853081
"6039","CROCC",35.5716336082406,-0.233755117718106,0.284530881717962,-
0.821545683571225,0.411335513665985,0.906467667417708
"6040","PPP1R8",24.187367885239,0.277596386297487,0.337019073677466,0.
823681530153251,0.410120536714759,0.906467667417708
"6041","ARPC5",224.580261002477,-0.149842555078257,0.180904558140268,-
0.828296183460863,0.40750278372622,0.906467667417708
"6042","YOD1",4.29152775337279,-0.707953469075989,0.862087939537537,-0
.821207949453239,0.411527829244572,0.906467667417708
"6043","RNF103",36.4509734572807,0.240213056852633,0.29206188132598,0.

822473154531669,0.410807659324249,0.906467667417708
"6044","IL1R1",125.36435178393,0.20636848565872,0.249569933575238,0.82
6896424190079,0.408295770348679,0.906467667417708
"6045","HAT1",47.0600512762897,-0.220618326784531,0.267424921743431,-0
.824972950711727,0.409386947642328,0.906467667417708
"6046","AC012459.1",1.83344220012827,1.02314607732853,1.24591135110695
,0.821202950289758,0.411530676313579,0.906467667417708
"6047","TTLL4",4.85055149913615,-0.556842759122343,0.677111114179482,-
0.822380178764488,0.41086055675277,0.906467667417708
"6048","B4GALT4",26.5554275664534,-0.266130292450778,0.323990283565646
,-0.821414424907762,0.411410249808748,0.906467667417708
"6049","LINC00884",4.46805206816129,0.602910559400557,0.73060646335896
,0.825219307024301,0.409247094100017,0.906467667417708
"6050","AC139887.2",11.0074368250116,0.504681983823626,0.6095144168599
39,0.828006639159771,0.407666739968649,0.906467667417708
"6051","EIF4E",36.2240451326984,-0.218003102461299,0.265116563087903,-
0.82229152310268,0.410911000077266,0.906467667417708
"6052","SPATA5",7.66761387018212,-0.462634321226181,0.563471542634183,
-0.82104292093156,0.411621820662028,0.906467667417708
"6053","ARHGAP10",85.9293976763924,0.168377878448714,0.205102921359498
,0.820943345578128,0.411678539617314,0.906467667417708
"6054","TRAPPC11",29.2055769494061,0.293781354737271,0.35539676638012,
0.826629228311699,0.408447245603531,0.906467667417708
"6055","CCNB1",4.90398234866098,-0.545733326718452,0.66415740257665,-0
.821692756267169,0.41125178289279,0.906467667417708
"6056","MRPL22",65.0019877652619,-0.267620004029179,0.324766192470727,
-0.824038986303358,0.409917406245019,0.906467667417708
"6057","MICB",1.65750940164889,1.01854950958681,1.23967159824802,0.821
628495019401,0.411288366575976,0.906467667417708
"6058","TJAP1",13.064677873453,-0.32027915467629,0.389482333553309,-0.
822320108217317,0.410894735308629,0.906467667417708
"6059","RAC1",551.357021238282,-0.159330979712086,0.194093166389537,-0
.820899481810269,0.411703526257998,0.906467667417708
"6060","AC073349.1",2.28866920555289,0.826798512635834,0.9991930318423
53,0.827466251552364,0.407972842925482,0.906467667417708
"6061","ARF5",119.887016830355,-0.348815202907432,0.423405600392166,-0
.823832284184131,0.41003486090664,0.906467667417708
"6062","RHEB",155.993705124872,-0.222499521186187,0.269278754553574,-0
.826279524186967,0.408645545927011,0.906467667417708
"6063","PTK2B",7.75987584601535,0.505673356778586,0.614065744575658,0.
82348406704892,0.410232774094135,0.906467667417708
"6064","AC103706.1",1.7594275674101,-0.861404394817171,1.0442062562160
4,-0.824937017652723,0.409407348787186,0.906467667417708
"6065","TPM2",71.2697845048774,-0.296030431137449,0.357956827691859,-0
.827000376124359,0.408236848295324,0.906467667417708
"6066","TUT7",30.3408401962043,0.288797249046187,0.35119016461115,0.82
2338659073648,0.410884180180809,0.906467667417708
"6067","SPIN1",52.0937237858557,0.198965934876108,0.24110485283372,0.8
25225757746682,0.409243432483743,0.906467667417708
"6068","ZNF483",11.8783840495475,0.378833603150329,0.461554635663957,0

.820777376887067,0.411773087078497,0.906467667417708
"6069","ANGPTL2",641.082141128012,-0.160664474720476,0.194649661876223
,-0.825403307520985,0.409142657757119,0.906467667417708
"6070","AL391121.1",6.7399773810536,0.552933684439335,0.66968983098115
6,0.825656384283507,0.408999040512805,0.906467667417708
"6071","RNH1",485.629870211803,-0.209588847521227,0.254482336011108,-0
.823588979912849,0.410173139694495,0.906467667417708
"6072","NAP1L4",89.9598160882549,0.179764687626186,0.217990260050183,0
.82464550289908,0.409572879863922,0.906467667417708
"6073","CCDC86",7.69464899901946,-0.461066962390547,0.558193213683798,
-0.825998867574427,0.408804734192819,0.906467667417708
"6074","NAA40",6.37734565537511,0.529696931969729,0.645377162417472,0.
820755618289274,0.411785483264016,0.906467667417708
"6075","COA4",62.8312575442722,-0.229895407132979,0.279167991655386,-0
.82350202747014 7,0.41022256469516,0.906467667417708
"6076","M6PR",55.7714194977994,0.194453801583488,0.234706155269155,0.8
28498943116737,0.407387993229213,0.906467667417708
"6077","ORMDL2",27.3741635734886,-0.317456947732948,0.384042747165906,
-0.826618781569666,0.408453168616028,0.906467667417708
"6078","PRIM1",8.73867486283629,-0.458843539494861,0.555901168928252,-
0.825404883352713,0.409141763403557,0.906467667417708
"6079","NEMP1",7.88519929572722,0.5128426132413,0.623534774887956,0.82
2476362017585,0.410805834536442,0.906467667417708
"6080","ARHGEF40",39.2477968813596,0.27487558172444,0.334895011835738,
0.820781355379706,0.411770820498068,0.906467667417708
"6081","PRORP",14.0691329452862,0.357440042336556,0.434516047198676,0.
822616436472189,0.410726148732004,0.906467667417708
"6082","CEP128",5.49197595156305,-0.549213073858082,0.665010563438261,
-0.825871202734768,0.408877157827764,0.906467667417708
"6083","USP8",95.8832732118388,0.191206794319432,0.232156379820594,0.8
23612060401669,0.410160021011989,0.906467667417708
"6084","PPCDC",9.21652522750929,0.485139370983933,0.589854649884797,0.
822472741511293,0.410807894298209,0.906467667417708
"6085","PEX11A",5.35497831965223,-0.501523981521883,0.607066524334359,
-0.826143365542677,0.408722770420457,0.906467667417708
"6086","METTL26",114.826326073964,-0.238945702470418,0.28946327024555,
-0.825478487366368,0.409099991196438,0.906467667417708
"6087","HAGHL",3.67646282787338,0.656344334422755,0.796001613082248,0.
82455151300 6716,0.40962625871104,0.906467667417708
"6088","TRAPPC1",132.18523376244,-0.228992715101127,0.276921462667739,
-0.826922958210292,0.408280729849322,0.906467667417708
"6089","AC005838.2",4.4495512976333,0.689101519829635,0.83792712622513
8,0.822388365601717,0.4108558987892,0.906467667417708
"6090","LLGL1",8.32732879183052,0.527501431562627,0.641762455291868,0.
821957450475541,0.411101113855515,0.906467667417708
"6091","HOXB5",2.04601051030235,-0.787102843917041,0.955631504974915,-
0.823646813462583,0.41014026824515,0.906467667417708
"6092","C17orf67",5.27662356924799,0.580162655850169,0.706774497226196
,0.820859635042114,0.411726225427189,0.906467667417708
"6093","DLGAP1-

AS2",2.9791207657316,-0.654108465546691,0.795820867649545,-0.821929270
940833,0.411117152630037,0.906467667417708
"6094","RBFADN",1.55362362081237,-0.883157029510332,1.07429858322399,-
0.822077812724991,0.411032612187554,0.906467667417708
"6095","YIPF2",47.7410250466992,-0.233287297841137,0.28409037789433,-0
.821172823839569,0.411547833848037,0.906467667417708
"6096","TMEM205",59.4015375262504,-0.23620269059042,0.285918726239607,
-0.826118294862843,0.408736990593628,0.906467667417708
"6097","USE1",51.2615382175771,-0.252732552714958,0.307179983581512,-0
.822750720174752,0.410649765800829,0.906467667417708
"6098","SNHG17",17.9992638886911,-0.377579571723743,0.459546158806519,
-0.821635791069062,0.411284212865533,0.906467667417708
"6099","ADA2",2.96972860050965,-0.688033399543749,0.831798427164698,-0
.827163621707013,0.408144327628975,0.906467667417708
"6100","Z95114.1",3.08521702468915,-0.800468519488931,0.97103725688243
,-0.824343776529111,0.409744251325026,0.906467667417708
"6101","KCTD17",16.1570297540388,-0.322342229457951,0.389424164693482,
-0.827740696861143,0.407817366068199,0.906467667417708
"6102","GPM6B",3.53447708193965,-0.859087214073292,1.04186458154963,-0
.82456705918107,0.409617429424381,0.906467667417708
"6103","ZRSR2",16.962977540129,-0.313102918739563,0.378510187264199,-0
.827198129071802,0.408124771918877,0.906467667417708
"6104","CXorf40A",7.26947777032078,-0.500501348346928,0.60652978292224
4,-0.825188411911985,0.409264631328029,0.906467667417708
"6105","CLIC2",38.8625712686944,-0.216376616715976,0.26349713929133,-0
.821172545925608,0.41154799212682,0.906467667417708
"6106","NXPE3",10.0180016018498,-0.394572700241337,0.480936621343035,-
0.82042556696863,0.411973545386123,0.906584653093845
"6107","CDK16",27.8158396752223,-0.240650297433264,0.293293741692365,-
0.82050948664865,0.411925723408481,0.906584653093845
"6108","PABPC4",75.0325523518774,-0.188402205809421,0.230784559574287,
-0.81635533224993,0.414296936077507,0.906699027876652
"6109","MIA3",89.394220762018,-0.169789713929462,0.207392196866888,-0.
818689017689704,0.4129638640544,0.906699027876652
"6110","MGAT5",8.17808394245559,0.458014400026738,0.560006835146334,0.
817872874546354,0.413429780606408,0.906699027876652
"6111","APPL1",115.442480298053,0.157795694935216,0.19306725321304,0.8
17309472783021,0.41375159467904,0.906699027876652
"6112","UBA5",27.9046535809696,-0.26198661453243,0.32061756162495,-0.8
1713120517988,0.413853451683788,0.906699027876652
"6113","NCK1",76.4155551976869,0.182170749201671,0.222366294144889,0.8
19237240527887,0.412651071506942,0.906699027876652
"6114","AADACL2",3.12379097441587,-0.775712559623905,0.949747861081378
,-0.816756311238945,0.414067703761528,0.906699027876652
"6115","GNL1",51.5709256526485,-0.196651280045145,0.239989632334883,-0
.819415731137651,0.412549262691588,0.906699027876652
"6116","BCKDHB",14.5654563686232,0.342376294949971,0.417578612161934,0
.819908599191377,0.412268214231512,0.906699027876652
"6117","MANEA-
DT",4.60682929014635,0.651392862754032,0.797503630281675,0.81678984022

1746,0.414048539258989,0.906699027876652
"6118","FOXO3",88.1268350270355,0.240512703550562,0.294494283198712,0.816697359752395,0.414101400536317,0.906699027876652
"6119","ZC2HC1B",2.42275059459911,0.945433002121751,1.15434024270306,0.819024553720729,0.412772404846239,0.906699027876652
"6120","PMS2",12.9609288531128,0.372503171303284,0.455963424524911,0.816958447251359,0.413952174775775,0.906699027876652
"6121","KCTD7",16.7925021619891,0.38160083936546,0.467586893002194,0.816106792291267,0.414439059479032,0.906699027876652
"6122","SLC12A9",13.9297691391402,-0.316301212910981,0.385675275645242,-0.820123126590895,0.412145919604861,0.906699027876652
"6123","FLNC",70.8997147792978,0.219106413527267,0.267379437951327,0.819458725794585,0.412524741301236,0.906699027876652
"6124","AC246817.1",2.4487936373971,0.964254988862935,1.18029272930604,0.816962576249941,0.41394981508328,0.906699027876652
"6125","PEX2",58.4389529226448,0.211117240255543,0.258559413850707,0.816513454727444,0.41420653140126,0.906699027876652
"6126","YWHAZ",278.021742206284,-0.153296080516422,0.187189832614646,-0.818933797713261,0.412824185669379,0.906699027876652
"6127","CEP78",12.8895051130669,0.429070758153014,0.524053052821704,0.818754429237138,0.412926535639649,0.906699027876652
"6128","GVQW3",14.0053539848786,0.376693067955675,0.460686132889251,0.817678330348685,0.413540887145594,0.906699027876652
"6129","C12orf4",11.1932544062048,0.387764711202109,0.474139453086778,0.817828401913518,0.413455177905201,0.906699027876652
"6130","LTBR",81.024709953024,0.178942624161956,0.218703581886836,0.818197043771084,0.413244682918827,0.906699027876652
"6131","LTA4H",26.0502060733537,0.268289632209447,0.328455228356823,0.816822534844798,0.414029852165364,0.906699027876652
"6132","THUMPD1",67.1830813631177,0.175744049622671,0.214876388835416,0.817884415198739,0.413423190155954,0.906699027876652
"6133","CMTM4",4.66068796893689,0.673911664619654,0.825872057763069,0.816000079291931,0.414500090362582,0.906699027876652
"6134","TVP23C",3.48786437584685,0.675332851916734,0.824989590040081,0.818595604198987,0.413017175806984,0.906699027876652
"6135","PIGL",12.6709785173782,0.366582785123785,0.448099899000735,0.81808272204762,0.413309953983377,0.906699027876652
"6136","ABI3",1.56242081072782,-1.05977946961558,1.2987566076944,-0.815995440051646,0.414502743739392,0.906699027876652
"6137","BAHCC1",8.28413267902518,-0.41430994791782,0.505497010657475,-0.819609095964676,0.41243898661057,0.906699027876652
"6138","MIDN",51.0203947420262,-0.285343364188872,0.348452709603308,-0.818886914421524,0.412850936430357,0.906699027876652
"6139","SCAMP4",30.3024773495835,0.291547722563682,0.356256323146686,0.818365046797049,0.413148774026425,0.906699027876652
"6140","CD40",41.3190949108051,-0.256779804548168,0.314442265741954,-0.816619877554544,0.414145691966218,0.906699027876652
"6141","VAPB",44.3564527605446,0.207395361972589,0.253114000106669,0.819375308695634,0.412572317843726,0.906699027876652
"6142","HMGN1",109.019935069371,0.212415218896385,0.260324867108979,0.

815962075599416,0.414521826569994,0.906699027876652
"6143","MBTPS2",12.0486429365465,0.389380700581668,0.475997533879807,0
.81803092005092,0.413339531924789,0.906699027876652
"6144","TIMP1",1426.26402686127,-0.216568390940233,0.264977999544959,-
0.817307064405881,0.413752970657904,0.906699027876652
"6145","TRMT10C",41.1098436567033,-0.244966035254267,0.300435567262422
,-0.815369623132191,0.4148607668407,0.906963925947401
"6146","LIN54",8.33653391184626,-0.47435907278519,0.581602740787395,-0
.81560666674814,0.414725135246079,0.906963925947401
"6147","MRNIP",31.8682788080477,0.236401626429806,0.289956098820566,0.
815301445258094,0.414899781705261,0.906963925947401
"6148","PRPSAP2",13.4872256461069,-0.325805757599299,0.399625080225898
,-0.815278554126607,0.414912881667135,0.906963925947401
"6149","REX1BD",175.232584606447,-0.208145402328114,0.255470213230039,
-0.814754094798083,0.415213082207867,0.907472534036677
"6150","MOB4",67.5300467366626,-0.195736506240573,0.240405900958182,-0
.814191770919222,0.41553509895198,0.907585924722194
"6151","MAN1A1",121.411869777379,0.206365710819046,0.253398364998389,0
.814392432328276,0.415420172567438,0.907585924722194
"6152","MEOX2",29.6584754173608,0.401481471334656,0.493038949043015,0.
814299706167086,0.415473278016042,0.907585924722194
"6153","FUT10",12.9180679331087,0.361404471286413,0.443844031745071,0.
814260067586064,0.41549598075764,0.907585924722194
"6154","NCF2",3.28961836744126,-0.747081609407155,0.917818837583426,-0
.813975022973146,0.415659259801396,0.907650077053305
"6155","RAB33A",1.750397080055,-0.94683001619928,1.16331803470217,-0.8
13904700137899,0.415699547902604,0.907650077053305
"6156","TDRD10",5.82133494404394,0.520026169727084,0.639279027014091,0
.813457266314513,0.415955937696634,0.907709637994356
"6157","ABT1",24.9805134360345,-0.315699626849834,0.3880645198009,-0.8
13523552763356,0.415917948158526,0.907709637994356
"6158","MLEC",108.249309798291,0.197186593956368,0.242375006378691,0.8
13559933024941,0.415897099076348,0.907709637994356
"6159","MRPL12",34.9704666947659,-0.279351554162363,0.343442944506521,
-0.813385625271038,0.415996998318866,0.907709637994356
"6160","EIF5B",128.758417189074,-0.207151245573101,0.254726049543314,-
0.813231492988224,0.416085346397724,0.907755027636203
"6161","ATF7",54.0981523309831,0.197579358955848,0.243053892591387,0.8
12903495802108,0.416273390082195,0.908017868741215
"6162","TTC4",21.8130058208451,-0.314499085170591,0.388447389978481,-0
.809631093641829,0.418152231845982,0.908099702730471
"6163","S100A9",10.6792150929144,-0.488038173539328,0.602762753606878,
-0.809668763736564,0.418130575280144,0.908099702730471
"6164","KLHDC9",3.82316548895664,-0.621758139331372,0.766202657936732,
-0.811480008442769,0.417090068924908,0.908099702730471
"6165","TIPRL",64.2237784157357,-0.189390657963989,0.233082332182472,-
0.812548322262889,0.416477070758833,0.908099702730471
"6166","SLC30A1",29.1336478614655,0.241379215871114,0.297812058068349,
0.810508538293355,0.417647959767271,0.908099702730471
"6167","COX7A2L",150.899769887216,-0.197601056095512,0.243886344172668

,-0.810217795366243,0.417815011556555,0.908099702730471
"6168","CCDC85A",5.92801083921284,0.743105427214382,0.915871897780738,
0.811363935300353,0.417156703685315,0.908099702730471
"6169","PJVK",5.39940758966555,0.507445464135928,0.625109630950297,0.8
11770350369591,0.416923418189659,0.908099702730471
"6170","ANKRD28",92.6767276257541,-0.258678857469898,0.31838416099038,
-0.812474014615678,0.416519691257108,0.908099702730471
"6171","FLNB",63.0135064296049,0.296736305435822,0.365153256013825,0.8
12634970519305,0.416427375330224,0.908099702730471
"6172","RASA2",27.870156934033,0.259678737538949,0.320514809825085,0.8
10192632535963,0.417829471182089,0.908099702730471
"6173","EIF2B5",23.2608626598701,-0.258031907399157,0.318339442679003,
-0.810555880941663,0.41762076188913,0.908099702730471
"6174","SERPINB1",102.998692866505,-0.179020091547866,0.22053206723088
3,-0.81176444675705,0.416926806359655,0.908099702730471
"6175","TCF19",6.74633349066589,-0.449645052179505,0.556037496227274,-
0.808659587222724,0.418710979763574,0.908099702730471
"6176","HLA-
B",1246.84760388348,0.328643114743177,0.406261956197979,0.808943859323
669,0.418547439304833,0.908099702730471
"6177","MANEA",17.6544277260481,-0.297716898819834,0.368240181295368,-
0.808485640465817,0.418811069091708,0.908099702730471
"6178","RSBN1L",84.9700515795822,0.191631913665143,0.236818026516957,0
.809194791813795,0.418403110220662,0.908099702730471
"6179","ZNF746",7.18152538767933,0.488289116774848,0.603111505117643,0
.809616650704753,0.418160535276555,0.908099702730471
"6180","WASHC5",32.0098672464205,0.251977961612023,0.310747853899705,0
.810875951192732,0.417436912110457,0.908099702730471
"6181","FAM49B",21.5953278366078,0.294254694954973,0.362892911309035,0
.810858205781784,0.417447103904411,0.908099702730471
"6182","REEP3",69.6243930954849,-0.183182139216799,0.226565809344392,-
0.808516252945973,0.418793453585812,0.908099702730471
"6183","SEC24C",18.907440339155,0.337518945639457,0.416433802636789,0.
810498435771407,0.417653763700244,0.908099702730471
"6184","MMS19",17.506582493679,-0.30737656779763,0.379355059457739,-0.
810260889197058,0.417790248705179,0.908099702730471
"6185","BLOC1S2",43.821632229806,-0.264156963201839,0.325571860999672,
-0.811363004132918,0.417157238271207,0.908099702730471
"6186","TIAL1",81.9610257420577,0.172888817989466,0.213699368326149,0.
809028212594446,0.418498918482679,0.908099702730471
"6187","DCUN1D2",5.99595118614824,0.502509638757231,0.621047972453159,
0.809131759616414,0.418439361755084,0.908099702730471
"6188","AC015871.1",4.46642584533336,0.666634257605286,0.8235825411274
25,0.809432235769244,0.418266566333133,0.908099702730471
"6189","AATF",34.126231036035,-0.240496661012557,0.29743087469412,-0.8
08580014633266,0.418756764251218,0.908099702730471
"6190","ICAM2",14.6737664767984,-0.407725410396675,0.504062017377329,-
0.808879455980633,0.418584486963453,0.908099702730471
"6191","SNRPD1",87.6811172649614,-0.197926687080706,0.24398463024554,-
0.811226046827284,0.417235870250907,0.908099702730471

"6192","SBNO2",10.4949854977432,0.377151572013548,0.464826214871836,0.811381888427996,0.417146396822274,0.908099702730471
"6193","AP1M1",38.5765874636996,0.240030341341873,0.296259823424062,0.810202134625244,0.417824010845437,0.908099702730471
"6194","BACH1",31.5907871926797,0.252489757927007,0.311308321857476,0.811060097656506,0.417331159181103,0.908099702730471
"6195","GART",19.873366448279,-0.26950594473596,0.332983787014368,-0.809366567520992,0.418304326715838,0.908099702730471
"6196","DUSP18",15.2180649090674,-0.319456868646185,0.394301111060965,-0.810185058283523,0.417833823725313,0.908099702730471
"6197","LAGE3",23.6633023325487,-0.284600655277805,0.350391411180433,-0.812236391066248,0.416656001823003,0.908099702730471
"6198","F8A1",26.471357547402,-0.324564485513479,0.40086416417736,-0.809662011518389,0.418134457086872,0.908099702730471
"6199","UTP11",32.1305329967688,0.254942767520095,0.315595147105925,0.807815867442749,0.419196589335137,0.908365157855534
"6200","THUMPD3-AS1",50.0286085365676,-0.234358604331922,0.290474477650691,-0.80681306745906,0.419774189375873,0.908365157855534
"6201","THRB-IT1",3.21665028825546,-0.665781960932664,0.824845997386268,-0.807159109751835,0.419574820576435,0.908365157855534
"6202","ARF4-AS1",1.97263085307938,0.845126294786158,1.04718481855917,0.807045976801857,0.419639994866374,0.908365157855534
"6203","TBL1XR1",118.837207865164,0.173691129490719,0.215329413909241,0.806629834435569,0.419879779790057,0.908365157855534
"6204","SPEF2",13.1298340250137,-0.338341830802038,0.418881026344908,-0.807727754475675,0.419247322544131,0.908365157855534
"6205","KMT2E",152.866090158482,0.170553536865196,0.211228776095605,0.807435142208093,0.419415827163166,0.908365157855534
"6206","UTP23",42.6301411184786,-0.207082804177257,0.256626107766972,-0.806943634765711,0.419698957790281,0.908365157855534
"6207","BCO2",4.38335925661764,0.556840673229035,0.690233231260385,0.806742776223956,0.419814693769477,0.908365157855534
"6208","HOXC8",45.182590935044,-0.225469232534181,0.279371277624029,-0.807059460269971,0.419632226919026,0.908365157855534
"6209","POGLUT2",13.8638785138681,-0.360447315810376,0.446048826576908,-0.808089371238883,0.419039135795184,0.908365157855534
"6210","WRAP53",7.34088899548313,-0.432869066599715,0.536129484117127,-0.807396495480086,0.419438085366048,0.908365157855534
"6211","ZNF529",9.96695475394255,0.414689982436072,0.513724684544718,0.807222224105476,0.419538463877417,0.908365157855534
"6212","IGLC3",8.96327729564347,0.684996003823596,0.849084112514392,0.806746933227991,0.419812298287034,0.908365157855534
"6213","HIGD1A",64.6022935793354,-0.219803642840927,0.272758694715337,-0.805853844807123,0.420327126907026,0.908747789334543
"6214","LINC00271",7.77570931567382,0.490200682764351,0.608161608769168,0.806036875225397,0.420221587221922,0.908747789334543
"6215","VSIR",272.551730522751,0.267287729963396,0.331618516653827,0.8

06009666349286,0.420237275520775,0.908747789334543
"6216","KRT25",3.40022794391549,0.997791640633271,1.23815417640139,0.805870270157535,0.420317655024103,0.908747789334543
"6217","AGGF1",51.311757759489,0.225403678679709,0.279849402563225,0.805446345838758,0.420562156399918,0.908869230390972
"6218","REC8",2.22635162051294,0.81507308378893,1.01195865391916,0.805441092511315,0.420565186817814,0.908869230390972
"6219","PSEN1",34.1314231296464,0.240402931830681,0.298530577910849,0.805287463391685,0.420653814497496,0.908869230390972
"6220","NDUFB10",252.385167899271,-0.205014858973455,0.254575421764585,-0.805320708308752,0.420634634782743,0.908869230390972
"6221","RNF150",1.72030398363122,1.23397389320681,1.53266971793358,0.805114029962383,0.420753880336428,0.908939302015956
"6222","AGK",11.6693539432144,0.375047052678474,0.465907710300303,0.804981425262818,0.420830398660616,0.908958490453233
"6223","IMPACT",28.2490673563861,0.245843270390802,0.305449008532014,0.804858629505209,0.42090126410928,0.90896546494691
"6224","WRAP73",21.8880973076501,-0.288784247802378,0.360555054844535,-0.800943556114881,0.423164323053366,0.909300949544838
"6225","IQCC",1.92919931728974,0.883300535034914,1.1039964383435,0.800093645555837,0.423656542546221,0.909300949544838
"6226","USP24",20.5325017690113,-0.2737133177378,0.341672445420352,-0.801098600155059,0.42307456655141,0.909300949544838
"6227","AC092807.3",2.21274057773101,-0.752484068374546,0.937741788820206,-0.802442716476636,0.422296911853736,0.909300949544838
"6228","WNT2B",10.5029082903258,0.412217150102404,0.514682825943366,0.800914911716449,0.423180906793997,0.909300949544838
"6229","C1orf112",4.46209858822156,-0.533240285246778,0.665411357765194,-0.801369377038713,0.422917837916745,0.909300949544838
"6230","RGS1",18.6521159458644,-0.439547680071065,0.546851649099096,-0.803778649648753,0.421524822282427,0.909300949544838
"6231","ZBTB41",34.1152077959838,0.222942966136289,0.278087908149377,0.801699605063493,0.422726744332947,0.909300949544838
"6232","RAB7B",36.1015016788171,0.214018834473943,0.267353374519251,0.800509194465139,0.423415838589602,0.909300949544838
"6233","QARS",52.0688606777902,-0.215676400980164,0.269426186088821,-0.800502742925895,0.42341957498926,0.909300949544838
"6234","SLC33A1",30.398615088795,0.23465924141909,0.292863103791897,0.80125914934588,0.422981634812561,0.909300949544838
"6235","SGMS2",58.8604424142993,0.232675952118287,0.290519847902355,0.80089520147515,0.423192318304692,0.909300949544838
"6236","GALNT7",12.5888381500316,-0.37077403650584,0.461728044281668,-0.803013897677952,0.421966701867292,0.909300949544838
"6237","CCDC125",9.6514426259635,0.395761277488039,0.491975774893888,0.804432449084305,0.421147267225298,0.909300949544838
"6238","AHI1",116.407525440134,-0.182071417386644,0.226529552460922,-0.803742449533391,0.421545732866431,0.909300949544838
"6239","AL139393.3",11.3170440474282,0.375609283144867,0.467901579360861,0.80275275765886,0.422117652758859,0.909300949544838
"6240","SLC4A2",23.2847345734329,-0.254587109312601,0.316746586305392,

-0.803756442278245,0.42153765004473,0.909300949544838
"6241","LMBR1",16.2836212592419,0.303821898664801,0.379603741037112,0.800365923251263,0.423498818450515,0.909300949544838
"6242","PPP3CC",15.328154239151,-0.317369753679392,0.395222028393741,-0.803016357588276,0.42196528007693,0.909300949544838
"6243","SNHG6",269.272851683715,-0.213758207163863,0.267193660715983,-0.800012270467299,0.423703687900238,0.909300949544838
"6244","RNF38",24.1178833494884,0.297490337855708,0.37153436019852 7,0.800707470761913,0.423301016567934,0.909300949544838
"6245","AL512625.1",4.63710571720044,0.541820160380957,0.67466809379681,0.803091424305796,0.421921894018446,0.909300949544838
"6246","CTNNAL1",19.5391532073436,-0.336515406318524,0.420814549204526,-0.799676263462482,0.42389838893507,0.909300949544838
"6247","DNAJC25",14.8848104513717,0.31872159411923,0.398251164773349,0.800302980408405,0.423535276712108,0.909300949544838
"6248","NACC2",11.5333257511187,0.399305094131463,0.497597141186019,0.802466616226376,0.422283091949495,0.909300949544838
"6249","CTBP2",58.2132002655365,0.227747717492438,0.284258978327101,0.801197973878471,0.423017043999098,0.909300949544838
"6250","APIP",24.0518066444327,-0.28877475578603,0.359773667502009,-0.802656730802617,0.422173168638467,0.909300949544838
"6251","SLC3A2",73.8846224858071,-0.22962402832991,0.287089918078495,-0.799833131956682,0.423807484144938,0.909300949544838
"6252","KLRK1",1.93691109526371,1.07361786780162,1.3362372283898,0.803463520542198,0.421706873422792,0.909300949544838
"6253","POU6F1",5.80916103150768,-0.467969008200583,0.584828627711341,-0.800181430980774,0.42360568675059,0.909300949544838
"6254","TBC1D4",30.1471790454755,0.25339894534616,0.315541724074656,0.803060026655007,0.421940040494978,0.909300949544838
"6255","SUPT6H",47.2293243168847,0.217447151096563,0.27056151881799,0.803688388676007,0.421576961637215,0.909300949544838
"6256","EVI2A",3.64999286980208,-0.662730657619489,0.826266098448267,-0.802078965679581,0.422507281546887,0.909300949544838
"6257","FDXR",7.29544145603732,0.524860673590608,0.652607142228267,0.804252113757933,0.421251387158244,0.909300949544838
"6258","GRB2",58.9965057144062,0.182784060154445,0.228566681023993,0.799696873295624,0.423886444952005,0.909300949544838
"6259","PIK3R2",22.832209030259,0.28220361251269,0.351405309015787,0.803071567993902,0.421933370049897,0.909300949544838
"6260","LRFN3",12.0679039437849,-0.371933803276127,0.465006494026835,-0.799846471078881,0.423799754692459,0.909300949544838
"6261","GZF1",6.49466826043569,-0.448964605358712,0.559897683697631,-0.801869017199172,0.422628729970356,0.909300949544838
"6262","RRP1",18.2849452414383,-0.292000341753227,0.363409998953097,-0.803501121582826,0.421685148753393,0.909300949544838
"6263","DMC1",1.48417733780715,-1.17867568715912,1.46894429551536,-0.802396449448474,0.422323666271946,0.909300949544838
"6264","NDUFA6",109.197501239483,-0.204830892353464,0.255434156928548,-0.80189311725746,0.42261478782513,0.909300949544838
"6265","FOXO4",3.45802376664807,0.642679803604738,0.802932810130618,0.

800415421435063,0.423470149002945,0.909300949544838
"6266","TMC6",2.57214926258862,-0.677461620869561,0.847311244614663,-0
.799542818740302,0.423975728701336,0.909321707312041
"6267","MRPS28",34.1279203937562,-0.273507877877446,0.342139516974254,
-0.799404524494105,0.424055887784948,0.909348504219229
"6268","TIMM22",26.7774147637379,0.309181789521858,0.387041365118009,0
.798833968114979,0.424386691385022,0.909912690734416
"6269","STXBP5",24.1278285433137,0.278055737561935,0.348231444016192,0
.798479696017934,0.42459217114374,0.910208037645673
"6270","PSMB8-
AS1",10.185095538103,-0.403022998162436,0.505091439843322,-0.797920864
165611,0.424916414854299,0.910378750484862
"6271","TAF3",33.4992870767975,-0.248469164296754,0.311456003042676,-0
.797766496292923,0.425006007202379,0.910378750484862
"6272","MIR100HG",71.7466521476051,0.182285508015685,0.228474486557791
,0.797837477444453,0.424964809643152,0.910378750484862
"6273","NSF",26.6771466827236,0.309176340827567,0.387556196808193,0.79
7758733762636,0.425010512727288,0.910378750484862
"6274","SIPA1L3",8.05193204770978,0.437855082409129,0.548713387930706,
0.797966829386755,0.424889739595356,0.910378750484862
"6275","PPFIA1",56.9867098263122,0.202065012505091,0.253417630059242,0
.797359727726337,0.425242141234675,0.910729742797259
"6276","AC093425.1",36.1459591696574,0.34259709355083,0.43015009953918
1,0.796459407815677,0.425765060122523,0.910733273937106
"6277","ITPKB",9.29973958551646,0.429238772765313,0.539181125524505,0.
796093840168751,0.425977494208569,0.910733273937106
"6278","CHMP3",128.077776377171,-0.166280787647381,0.208907928257785,-
0.795952499429299,0.426059644941853,0.910733273937106
"6279","OSTC",271.915705217828,-0.218565471380749,0.274312649871334,-0
.796775035650987,0.425581696180886,0.910733273937106
"6280","HOXA10-
AS",7.4479194108699,0.452060943736093,0.567487470748336,0.796600748101
042,0.425682942805618,0.910733273937106
"6281","NRDE2",28.1947788424114,0.299438314861623,0.376007570992058,0.
796362461722739,0.425821390195015,0.910733273937106
"6282","PGBD4",2.59454702622525,0.750596712729012,0.942943128251341,0.
7960148287214,0.426023416476554,0.910733273937106
"6283","AC037198.1",8.57968810154239,-0.633138012981913,0.795558968092
924,-0.795840457307196,0.426124773161435,0.910733273937106
"6284","OIP5-
AS1",81.7208669146224,0.184519552012652,0.231613538264405,0.7966699761
82518,0.425642725343629,0.910733273937106
"6285","AC022306.2",1.50369217165506,1.13318268959786,1.42305989548242
,0.796300066634727,0.425857646868506,0.910733273937106
"6286","CD276",151.901154527401,0.199943388714241,0.251211888694079,0.
795915311785776,0.426081260855774,0.910733273937106
"6287","TEPSIN",8.69424881448333,0.407051156305778,0.511058692108549,0
.796486123005458,0.425749538151221,0.910733273937106
"6288","DTD1",64.6329175711257,-0.211767317751856,0.265896518294433,-0
.796427569304842,0.425783559260252,0.910733273937106

"6289","BARD1",7.32294581550934,0.561568038004627,0.706425767949311,0.794942743431922,0.426646808941211,0.911425270034335
"6290","ASB1",21.9911372415187,-0.26466073297172,0.332934305418581,-0.794933801246392,0.42665201084798,0.911425270034335
"6291","AC005736.1",4.83900655677403,0.55731994800147,0.701089023197702,0.794934636773382,0.426651524798177,0.911425270034335
"6292","CCDC50",126.619500076438,0.191035048506719,0.240462640329593,0.79444793688065,0.426934706633649,0.911739317090356
"6293","ARID2",24.8650750919937,0.298630427440561,0.375890729581083,0.794460740687523,0.42692725545418,0.911739317090356
"6294","SLC9A1",12.0229967070677,0.355995708893901,0.448511595780766,0.793726878508427,0.427354449091632,0.912048586304185
"6295","AC105942.1",4.15644057894893,-0.571174208922752,0.723955407082565,-0.788963247369753,0.430133487610382,0.912048586304185
"6296","AL359258.2",2.15216966110199,-0.82068207461965,1.03425293874505,-0.793502289309866,0.427485236025746,0.912048586304185
"6297","FOXN2",14.3061274539887,-0.348836405779541,0.441647633726985,-0.789852314696613,0.429614022692111,0.912048586304185
"6298","SH3RF3",12.9759711647779,0.446581490611872,0.564904284144138,0.790543642784491,0.429210345015353,0.912048586304185
"6299","CLK1",85.288771585139,0.184998514346997,0.233383476708321,0.79268042860723,0.42796403584887,0.912048586304185
"6300","HES6",2.93597088563811,-0.668686703085562,0.844671631360564,-0.791652848585039,0.4285631226293,0.912048586304185
"6301","MYLK-AS1",1.60610926393632,-0.867654699221485,1.09958185788889,-0.789076950475805,0.430067032744596,0.912048586304185
"6302","EVC",11.5774740185841,-0.429386816164951,0.543779593556189,-0.789633927519905,0.429741588334628,0.912048586304185
"6303","TMEM184C",17.5062769151141,0.304966245482948,0.385972830673944,0.790123607795007,0.42945558396528,0.912048586304185
"6304","FBXO8",29.0507896432829,0.239241823444625,0.302697353561129,0.790366419230388,0.429313807700944,0.912048586304185
"6305","PPIP5K2",47.9835902993298,0.20824293134505,0.263440180037725,0.790475208888902,0.429250294841584,0.912048586304185
"6306","MBOAT1",28.934132017266,-0.308896416945528,0.390828018166514,-0.79036405423196,0.429315188479348,0.912048586304185
"6307","CDC40",78.8047020040302,-0.170185690414228,0.214847171018315,-0.792124418523157,0.428288133308871,0.912048586304185
"6308","PCMT1",117.803590238081,-0.214516991622415,0.271615852431426,-0.789780823549588,0.429655780112319,0.912048586304185
"6309","INMT",38.4084438359531,0.374277425681969,0.472297594065927,0.79246100421533,0.428091920947458,0.912048586304185
"6310","PRKAG2",51.2156388787495,0.250776907815567,0.317297042536766,0.790353751206202,0.429321203818095,0.912048586304185
"6311","SLC25A32",15.9770152666161,-0.295853997710697,0.373240275259255,-0.792663646776047,0.427973815863614,0.912048586304185
"6312","EBAG9",43.698504944785,-0.194847288091812,0.245671154688798,-0.793122368552521,0.427706531571049,0.912048586304185
"6313","CALHM2",141.263556742117,-0.202140513753278,0.2550836641822,-0

.792447899011263,0.428099559636727,0.912048586304185
"6314","HRAS",35.844721584376,-0.285153617444453,0.361248991628135,-0.789354777598898,0.429904679113038,0.912048586304185
"6315","AASDHPPT",38.5138814169004,0.213095584764363,0.269452265400764,0.790847256182537,0.429033129928619,0.912048586304185
"6316","PPP2R1B",11.7410463270994,-0.36607032766487,0.461376827927267,-0.79343024076315,0.42752719760593 1,0.912048586304185
"6317","TBRG1",55.1666940295836,-0.187140017373991,0.235794542228515,-0.793657120327358,0.427395069469452,0.912048586304185
"6318","C1R",2116.0459242211,0.200165968218549,0.252852570467744,0.791631138446679,0.428575785061072,0.912048586304185
"6319","TMEM19",18.9143826051276,-0.313816171115191,0.397715988628926,-0.78904590232098,0.430085178543358,0.912048586304185
"6320","METAP2",109.44069585835,0.180711598714184,0.228926260312713,0.789387807529518,0.429885379804515,0.912048586304185
"6321","PCID2",63.707433033534,-0.192719121379456,0.243513403518757,-0.791410733843285,0.428704348299873,0.912048586304185
"6322","RTN1",1.80743762605594,1.02275993286253,1.29533713315269,0.789570457517314,0.429778666943765,0.912048586304185
"6323","PLA2G4B",3.84850603737197,0.609144238915622,0.770379139258176,0.79070707898735,0.429114944199159,0.912048586304185
"6324","VPS39",23.2793928045816,0.308197171931134,0.390025651248126,0.790197185607839,0.429412619411493,0.912048586304185
"6325","GINS3",2.55727341128263,-0.680231038924049,0.861782928165975,-0.789329907441657,0.429919211017802,0.912048586304185
"6326","RRAD",2.52799296595678,-0.907805812219267,1.14814805625094,-0.790669641669327,0.429136795984361,0.912048586304185
"6327","AC067852.2",5.39664664425597,0.505571025096624,0.639146106408664,0.791010099298589,0.428938098105146,0.912048586304185
"6328","SEC14L1",30.0379590680298,0.219575311953043,0.277739998917441,0.790578644807704,0.42918991263219,0.912048586304185
"6329","SDHAF1",40.6470805794823,-0.29655215751535,0.374193257527212,-0.792510692135556,0.428062959846384,0.912048586304185
"6330","MEGF8",17.9196905781477,-0.307250620420315,0.388505143660001,-0.790853417089387,0.42902953432996,0.912048586304185
"6331","ZNF230",7.97150621139764,-0.456162310815611,0.576103197923202,-0.79180659378395,0.428473457015318,0.912048586304185
"6332","IRF3",52.5075093467607,0.199167767974997,0.251777735642985,0.791045989298326,0.428917155100956,0.912048586304185
"6333","AP000254.2",1.81569011103874,-0.836999300810343,1.05844755513439,-0.790780135255802,0.42907230385436,0.912048586304185
"6334","PHF5A",48.2330831193712,-0.257867997643719,0.326483882030603,-0.7898337769077,0.429624850243297,0.912048586304185
"6335","HCCS",13.1428362312942,-0.353175539077125,0.446709972111057,-0.790614853319911,0.42916877655769,0.912048586304185
"6336","SLC16A2",9.42338887995366,-0.386462450171549,0.48974495482786,-0.789109609730204,0.43004794583377,0.912048586304185
"6337","CXorf56",12.7865339274245,0.350365344470444,0.443174141473714,0.790581651053361,0.429188157766766,0.912048586304185
"6338","RTL8A",78.0720016503793,-0.193429451671556,0.245127056934203,-

0.789098739611908,0.43005429855672,0.912048586304185
"6339","IQCH",1.89602145858282,-0.828629424067966,1.05058189934835,-0.788733771809644,0.430267624949372,0.912189085296515
"6340","GREM2",1.477430450699,1.00259701482462,1.27167888806321,0.788404230215382,0.43046029722492,0.912342063520396
"6341","RNF144A",10.4756685291462,0.434236881096391,0.550797724331385,0.788378132141907,0.430475558061078,0.912342063520396
"6342","AL109811.2",4.20630888828216,0.608495145580419,0.772229896874768,0.78797149403696,0.430713380059868,0.912356991824911
"6343","ZNF655",50.6025419175506,0.254070268837731,0.322441347631029,0.787958091306779,0.430721219934326,0.912356991824911
"6344","STAM",44.7479304121077,0.212659148527268,0.269905656369262,0.787901785342082,0.430754156792102,0.912356991824911
"6345","ZNF10",4.65796819468458,0.563227366228841,0.714653896141125,0.788112076727024,0.430631151755652,0.912356991824911
"6346","ACTRT3",2.08525830035562,-0.856496141006113,1.08766329788385,-0.787464413548298,0.431010052490117,0.912611327464106
"6347","TM9SF4",36.4964874119167,0.206140892909407,0.261744171359823,0.787566316523713,0.430950423637498,0.912611327464106
"6348","TMLHE",12.5649038204796,-0.426762579926684,0.542076115980427,-0.787274272644939,0.43112132684301,0.912703136648268
"6349","ARRB1",34.9466051176105,-0.217787923984235,0.276679591127198,-0.787148495835789,0.431194943163405,0.912715205728934
"6350","TRIM11",14.2401070486705,-0.312187845278717,0.396704379480929,-0.786953362317809,0.431309167924524,0.912749077495638
"6351","CCAR2",14.2852594154229,0.310412025571659,0.39460129781349,0.786647249493783,0.431488391674506,0.912749077495638
"6352","LINC02550",1.60805024439837,1.06847749974524,1.35803875945333,0.786779826648946,0.431410764750127,0.912749077495638
"6353","MTMR10",11.3729861118801,-0.359040174323434,0.456479848544743,-0.786541126553678,0.431550534891531,0.912749077495638
"6354","TLE2",21.497252971008,-0.332855048738075,0.423081343398618,-0.786740077130905,0.431434038142567,0.912749077495638
"6355","TBC1D22B",4.34060325450726,-0.532102762356549,0.676824108029993,-0.786175841025109,0.43176447757193,0.912770617286325
"6356","CD2AP",27.676961500837,0.280424557874896,0.356665216760442,0.786240274344572,0.431726735404284,0.912770617286325
"6357","AC005921.2",12.4214739281644,-0.386844114946674,0.492001564882194,-0.786266025473518,0.431711652075073,0.912770617286325
"6358","ZNF175",6.93607136111454,0.487351324453244,0.620082004967528,0.785946569242507,0.431898790271576,0.912910953516784
"6359","RNF44",11.9117322474849,0.370479632397575,0.471554924352933,0.785655314502221,0.432069448922204,0.913128058510064
"6360","FAM20B",27.383027795069,0.263851955012564,0.336423113302537,0.784286051045751,0.432872282452069,0.913531815934887
"6361","INSIG2",45.1000828567699,-0.193461847790211,0.246640064938682,-0.784389380688448,0.432811667589904,0.913531815934887
"6362","ABCB6",24.2657772995449,-0.274604513587439,0.349863340407728,-0.784890789836445,0.432517602527928,0.913531815934887
"6363","CSTA",5.36616271325039,-0.511600756399826,0.652277435725691,-0

.784329992698037,0.432846504958209,0.913531815934887
"6364","VARS2",5.3065353837577,0.49781053139028,0.634137330646497,0.785020069521481,0.432441801694152,0.913531815934887
"6365","SMPD2",6.56073606254297,-0.465275173787236,0.593049179922183,-0.784547368985971,0.432718998562955,0.913531815934887
"6366","DNM1L",41.0397713309262,-0.224034740501775,0.285477092646327,-0.784773091336363,0.432586619630018,0.913531815934887
"6367","AL161772.1",20.9633505085198,-0.265399052414026,0.338209394986162,-0.784718154931459,0.432618835914133,0.913531815934887
"6368","ZNF469",6.90433093576614,0.515482446117819,0.656827726481255,0.784806160481915,0.432567227603876,0.913531815934887
"6369","UCP2",11.2594657560438,-0.434604922870627,0.554365459061591,-0.783968257341122,0.433058736234585,0.913781811313643
"6370","ZNF771",9.65225569518995,-0.373511213220619,0.47664291759107,-0.783629000737757,0.433257833855777,0.914058403326182
"6371","ELOA-AS1",3.94733393737333,0.649341861741621,0.829189563230088,0.783104238808946,0.433565902214805,0.91405930869696
"6372","DYNC1I2",217.591981039228,0.177295488431483,0.226387210358386,0.78315152234445,0.433538138605179,0.91405930869696
"6373","ERICH6-AS1",3.68967005428588,-0.628602975272097,0.802763272591404,-0.783048996801885,0.433598340125242,0.91405930869696
"6374","UTP3",23.7582647239635,-0.290253555904673,0.370604060937354,-0.783190435556876,0.433515290593434,0.91405930869696
"6375","FANCG",10.1543897075028,-0.423603043000907,0.540753018602479,-0.78335770384725,0.433417086434583,0.91405930869696
"6376","SPTY2D1OS",3.020267287936,0.782526122174671,0.999509595496252,0.782910064796477,0.433679926707712,0.914087913272419
"6377","KIF1B",74.5299940916708,0.21102115872576,0.269663650451172,0.782534681158185,0.433900411787442,0.914409225970116
"6378","ECE1",70.2345463836061,-0.225715946036676,0.288484929929666,-0.782418499613505,0.433968665228276,0.914409672625085
"6379","NDUFB1",192.48230035009,-0.218703626633903,0.279649552096138,-0.782063210881586,0.434177426019264,0.914600319108925
"6380","ZFP30",7.29169486914241,0.450218337116857,0.575702573744306,0.782032871919759,0.434195255295405,0.914600319108925
"6381","MYOF",217.931461609282,0.175925834538007,0.225001377035741,0.781887812669081,0.434280508008675,0.914636537710167
"6382","AL139260.1",3.41158163425801,0.63358025232276,0.810642861127988,0.78157753889444,0.434462891343859,0.914877279343485
"6383","SH3D19",200.659187529308,0.162794214970924,0.208504053824583,0.780772421374043,0.434936357160401,0.914921587686988
"6384","HSPA9",70.6254552827094,-0.164018923526711,0.210024204425954,-0.780952481048618,0.434830443537425,0.914921587686988
"6385","TTC1",77.0537763895019,-0.194626662960055,0.249210404233728,-0.780973264573335,0.434818219335697,0.914921587686988
"6386","LMAN2",160.445791266987,-0.196512062335225,0.251644306432118,-0.78091201474584,0.434854245086293,0.914921587686988
"6387","ZBTB9",1.76543456786631,0.830165620524024,1.06330779773788,0.7

80738768482791,0.434956153914137,0.914921587686988
"6388","CALML4",4.27603431514546,-0.604543256314046,0.774444166824783,
-0.780615675359361,0.435028569485814,0.914921587686988
"6389","IDH2",18.0697751925536,0.29799101449681,0.381686360319505,0.78
0722198842435,0.434965901413047,0.914921587686988
"6390","LINC00649",2.8245032438313,0.742588359071481,0.951190770261063
,0.78069340272054,0.434982841740749,0.914921587686988
"6391","NELFB",40.8228347770062,-0.208747646074715,0.267550336497391,-
0.780218215411405,0.435262442316639,0.915127027893197
"6392","RPS27L",351.579246303051,-0.190556992998135,0.2442236601160З,-
0.780256068996765,0.435240165437088,0.915127027893197
"6393","ACAP3",33.2428580314902,-0.241773905066024,0.310276552289158,-
0.779220676787416,0.435849732056635,0.915325610960309
"6394","MEAF6",103.041570643011,-0.175765475632326,0.226783458305353,-
0.775036578707016,0.438318045435119,0.915325610960309
"6395","USP1",58.6572894344104,-0.180842022783784,0.233067718533576,-0
.775920508947412,0.437795922381839,0.915325610960309
"6396","HIPK1",37.9320214025839,0.246185887279112,0.317494725310572,0.
77540150324795,0.438102447133953,0.915325610960309
"6397","CDC73",44.6580976265187,0.208807542338048,0.268684905563883,0.
777146531174977,0.43707232385685,0.915325610960309
"6398","GUK1",471.871097218456,-0.227195528425269,0.292655475033382,-0
.776324203056014,0.437557585981342,0.915325610960309
"6399","TANK",69.589445949457,0.167223485794822,0.215000218411712,0.77
7782864734582,0.436697031307644,0.915325610960309
"6400","HDLBP",283.378926977219,0.144326577042323,0.185268864599115,0.
779011505007156,0.435972937537124,0.915325610960309
"6401","GRIP2",1.68030217137482,0.98714307260776,1.27305527045158,0.77
5412580678919,0.438095903514802,0.915325610960309
"6402","GSTCD",7.75853052913726,0.463309011332775,0.597408806274376,0.
775530937051483,0.438025991983632,0.915325610960309
"6403","PGRMC2",162.105287866406,0.143484720167107,0.185202343676528,0
.774745704178102,0.438489938489654,0.915325610960309
"6404","SORBS2",19.5047690396967,-0.404692664317373,0.521812855174198,
-0.775551350076022,0.438013934930997,0.915325610960309
"6405","MAPK14",44.1732215721039,0.230698339467067,0.296675705462818,0
.777611159994295,0.436798279889632,0.915325610960309
"6406","TPD52L1",3.98593360356643,-0.559326595489435,0.717621085510073
,-0.779417727242331,0.435733684596136,0.915325610960309
"6407","ACTR3B",4.13863830761173,-0.533652737087735,0.686899650394284,
-0.776900580428911,0.437217428787428,0.915325610960309
"6408","SH3GLB2",64.3019886633803,-0.191428340649787,0.246446642338011
,-0.776753697407797,0.437304099406013,0.915325610960309
"6409","SGPL1",13.2197566736847,0.336484070526533,0.432951383337523,0.
777186731527808,0.437048609267732,0.915325610960309
"6410","RNLS",5.14958915143643,0.535720758014504,0.687250315310822,0.7
79513295344527,0.435677408801296,0.915325610960309
"6411","LHPP",10.8667777960258,0.408489043516451,0.524632179982181,0.7
78619877130535,0.436203666522691,0.915325610960309
"6412","IPO7",35.6674801803669,-0.269637151548559,0.347938426030711,-0

.77495651924562,0.438365352916315,0.915325610960309
"6413","MED21",49.7290355134366,-0.228245227116489,0.294515577446692,-0.774985245586208,0.438348378060059,0.915325610960309
"6414","TMEM106C",91.1377252907081,0.294828689610234,0.379567935109453,0.776748145296353,0.437307375709975,0.915325610960309
"6415","RAB3IP",2.00788514037592,1.07012216636645,1.37663630375831,0.77734559479867,0.43695490148899,0.915325610960309
"6416","APAF1",17.20418668112,0.38926292761586,0.501862623469115,0.775636418040237,0.4379636911777,0.915325610960309
"6417","GTF2H3",24.5285202019907,-0.240191594422734,0.309288325684709,-0.776594441096321,0.437398082251254,0.915325610960309
"6418","PPM1A",45.4509984681921,0.225054256794656,0.29001108873137,0.776019488700025,0.437737478954178,0.915325610960309
"6419","ACOT4",4.02446563245154,0.640239331545034,0.825582381909492,0.775500235438918,0.438044126400458,0.915325610960309
"6420","AC027237.3",2.23498625074131,0.766199552247518,0.985746263435689,0.777278677757327,0.436994372055084,0.915325610960309
"6421","SNN",8.27540555731212,0.387787530271296,0.499315345932235,0.776638517983633,0.437372069740191,0.915325610960309
"6422","GFOD2",14.9516670900644,-0.289980334931926,0.373616634776625,-0.776144068385223,0.437663926211486,0.915325610960309
"6423","GCSH",71.3146925989349,0.232472202183833,0.300041267325714,0.774800760761584,0.438457399686727,0.915325610960309
"6424","SMYD4",27.0382233880557,0.247547502568742,0.317960316088343,0.778548422690467,0.436245771756555,0.915325610960309
"6425","AC004148.1",10.7790074911842,-0.445296451484455,0.573112086002885,-0.776979690988776,0.437170752464796,0.915325610960309
"6426","ZNF287",7.78600971293211,0.408863642297438,0.524907542296643,0.778925066514619,0.436023857036608,0.915325610960309
"6427","CCDC144NL-AS1",5.77328975015086,-0.527776760534909,0.678776467705179,-0.777541334512122,0.436839457524023,0.915325610960309
"6428","TP53I13",79.3369618228535,-0.223710425149519,0.288060570229328,-0.776608978352785,0.437389502814156,0.915325610960309
"6429","PSMD3",70.54957088336,-0.186637915688077,0.240130281297272,-0.777236068186776,0.437019506093114,0.915325610960309
"6430","MED24",18.797245695902,0.293509335283498,0.376846469981932,0.778856533530939,0.436064231130987,0.915325610960309
"6431","RBBP8",9.92133578550281,-0.463041454882896,0.596281832403756,-0.776547984056907,0.437425500401866,0.915325610960309
"6432","ZNF85",8.51769897811787,0.440521479010623,0.567132150303498,0.776752788172705,0.437304635945164,0.915325610960309
"6433","NOSIP",65.3248973386899,-0.2193255730406,0.281818270641967,-0.77825178807956,0.436420591673659,0.915325610960309
"6434","ETFB",181.527680973778,-0.235016133786392,0.301915941896713,-0.778415781260042,0.436323938238115,0.915325610960309
"6435","ZNF417",3.89146666464982,-0.56161370216421,0.724393349840167,-0.775288318546998,0.438169310403939,0.915325610960309
"6436","DYRK1A",30.1259464065895,0.253996229146514,0.326793083433046,0.77723869329858,0.437017957598625,0.915325610960309

"6437","CSNK1E",83.5360538154472,-0.162658488635823,0.20938334384219,-0.776845405422587,0.437249984501165,0.915325610960309
"6438","FAM3A",41.5537759431772,-0.239078988645975,0.307322464388692,-0.777941791927046,0.436603329270106,0.915325610960309
"6439","TFAM",43.6989173821923,-0.197283943781388,0.254719906227759,-0.774513255375436,0.438627332560751,0.915366490087728
"6440","COA3",89.3337805498325,-0.246157099175382,0.317848847531709,-0.77444704011653,0.438666475099812,0.915366490087728
"6441","ILF3-DT",52.1208145943581,0.229918526710999,0.296911619958509,0.774366886493458,0.438713859859741,0.915366490087728
"6442","SMIM15",67.4499261545794,-0.19359649324084,0.250046221132391,-0.774242827442442,0.438787206178167,0.915377408231665
"6443","SCCPDH",76.0964086812822,-0.202909132019824,0.262142565993922,-0.774041145322918,0.438906459922858,0.915484078054213
"6444","SNRPD3",150.198505164739,-0.162292412661565,0.209724975887823,-0.773834456170692,0.439028693621067,0.915596929480682
"6445","CAMK1",37.0454395826459,-0.244080214046821,0.315613138871085,-0.773352512889259,0.439313785536215,0.91604933496062
"6446","NUDT4",38.509172303269,0.284666704230242,0.368161605207542,0.773211275167512,0.439397354369566,0.916081452896772
"6447","DCAF13",32.3048642233744,-0.225140168522429,0.291242770342609,-0.773032643033787,0.4395030621435,0.916159710275554
"6448","CIP2A",3.98813433275506,-0.560155352055519,0.724897513340097,-0.772737306649738,0.439677863119258,0.916179520699044
"6449","CCNK",38.9087246305687,0.21768927998582,0.281862052774057,0.772325603405443,0.439921604812183,0.916179520699044
"6450","SAXO2",1.98014603088757,-0.78959901126664,1.02184533153391,-0.772718714760246,0.439688868450293,0.916179520699044
"6451","TMEM204",37.1693298713539,0.285323822867061,0.36942679474345,0.772341982029771,0.439911906653959,0.916179520699044
"6452","ZNF358",70.8023966613321,-0.21873476035802,0.283186775669806,-0.772404572355681,0.439874846620058,0.916179520699044
"6453","ATP5F1E",766.290246909036,-0.190423877278625,0.246474518723702,-0.772590522804067,0.439764755030911,0.916179520699044
"6454","TMEM44-AS1",9.52832034794273,0.429094358339212,0.555688248036234,0.772185411254607,0.440004620811351,0.916210427499805
"6455","HNMT",114.647283328997,-0.207376617031776,0.268727518862561,-0.77169847699088,0.440293032978423,0.916293102887566
"6456","TNK2",2.99685335727211,0.647833009597386,0.839935100775903,0.771289363903164,0.440535435298591,0.916293102887566
"6457","HPSE",6.87513912396239,0.502638181203459,0.652228419986312,0.770647469201062,0.440915916497885,0.916293102887566
"6458","NDUFC1",161.858432980242,-0.180558088217028,0.234175177275441,-0.771038546090874,0.440684084407026,0.916293102887566
"6459","GATB",9.7876543505016,0.366154551950441,0.474631390952024,0.771450348482012,0.440440041698019,0.916293102887566
"6460","PENK",18.5191835950378,-0.728306598824055,0.944512356038965,-0.771092716963895,0.440651977187599,0.916293102887566

"6461","ALAD",12.9848268049257,0.333604754075432,0.432824244418188,0.7
7076263258744,0.440847639878847,0.916293102887566
"6462","SIK3",34.2488655049647,0.257449496826319,0.334010255468655,0.7
70783209830151,0.440835440940324,0.916293102887566
"6463","CLMP",127.769935853044,-0.151735226057215,0.196717881441788,-0
.771334181443573,0.440508876865926,0.916293102887566
"6464","SARNP",69.6576094720262,-0.199304423416466,0.258558297925897,-
0.77082973169009,0.440807861801479,0.916293102887566
"6465","TEX26-
AS1",4.84063087969521,-0.554868471530361,0.719245741839485,-0.77145882
0334861,0.440435021914554,0.916293102887566
"6466","IER3IP1",116.420960296851,-0.213638364137155,0.277225839361283
,-0.770629334658591,0.440926668429434,0.916293102887566
"6467","FAM32A",84.9600870274264,-0.20643694812269,0.267883344539108,-
0.770622557657939,0.440930686537234,0.916293102887566
"6468","C1orf50",28.0174795698549,-0.259468644876825,0.337367600825082
,-0.769097697118087,0.441835315194114,0.916525865261004
"6469","AC119674.1",4.52740788546589,0.61844554835807,0.80401595319284
7,0.769195618447802,0.441777191130444,0.916525865261004
"6470","PHGDH",75.4170268657184,0.22357107093862,0.290400687712596,0.7
69871010635775,0.441376411622782,0.916525865261004
"6471","SFT2D3",16.2535758059637,0.296766325510865,0.385679234970506,0
.769464100221937,0.441617848393393,0.916525865261004
"6472","GOLGB1",174.823870398341,0.201117658547121,0.261701951868499,0
.768498886275709,0.442190852077222,0.916525865261004
"6473","PDGFC",10.9847635745472,-0.545154790947276,0.707846538422098,-
0.770159577473548,0.441205238817761,0.916525865261004
"6474","WDR36",27.3524520196053,-0.245769440523308,0.319812415600489,-
0.7684799855623,0.442202076817646,0.916525865261004
"6475","CNPY3",42.9420817515592,0.207360858087687,0.269608000571404,0.
769119824516369,0.441822180448101,0.916525865261004
"6476","RALA",104.898054120386,-0.162923996296404,0.211783904598918,-0
.769293571222768,0.441719052781414,0.916525865261004
"6477","POP1",6.83538219945531,-0.420452829990997,0.546682235386873,-0
.76909912701563,0.441834466405192,0.916525865261004
"6478","RRM1",25.8827647383157,-0.227967368430839,0.29595820259284,-0.
770268796180187,0.441140462112293,0.916525865261004
"6479","PCF11-
AS1",1.63028015451659,1.01893866387343,1.32538272002224,0.768788251484
317,0.442019024457067,0.916525865261004
"6480","PSMD9",102.162784513084,-0.20168482277198,0.261933264787279,-0
.769985526411744,0.441308478313866,0.916525865261004
"6481","SPATA7",22.2321931272871,0.314597726315649,0.409326203038481,0
.768574608662601,0.442145883762269,0.916525865261004
"6482","DERL2",69.5561713390322,-0.223824188382914,0.2911421109766,-0.
7687798499232,0.442024012839647,0.916525865261004
"6483","ZNF264",36.2583775440993,0.232196847700823,0.302070924919253,0
.76868320829915,0.442081395610819,0.916525865261004
"6484","APEX2",10.9523983356692,-0.361348751394979,0.470004009080206,-
0.76882057262051,0.441999834253406,0.916525865261004

"6485","ZBTB18",2.52511858100243,0.689327015290416,0.897436447056925,0
.768106775193956,0.442423752081751,0.916563002751404
"6486","XPC",51.8384227918932,0.219662881598515,0.286101791536611,0.76
7778769992099,0.44261862936857,0.916563002751404
"6487","NDUFS8",91.103532165005,-0.229978716933243,0.299463174209234,-
0.767969943351223,0.442505041831805,0.916563002751404
"6488","KLRD1",5.29290986298842,0.543343021349163,0.707383543346423,0.
768102433905611,0.442426331044966,0.916563002751404
"6489","MTMR6",28.4947817818868,0.267794201704242,0.348798925070575,0.
767760971883893,0.442629205138523,0.916563002751404
"6490","AC004825.2",1.94934016509784,-0.746781578671755,0.972473179480
919,-0.767919973968195,0.442534730023952,0.916563002751404
"6491","PACRGL",16.4242643150671,0.280391552012273,0.365316218482655,0
.767531080817824,0.442765821129768,0.916704647999223
"6492","KIAA1549",1.53313907509531,1.05891634517112,1.38000781988718,0
.767326336786763,0.44288751343919,0.91681535630149
"6493","CCDC126",17.393047988501,0.412544082327565,0.537791957762841,0
.767107198931917,0.443017782090686,0.916927917418822
"6494","TARBP2",8.36807058220418,-0.405875736943757,0.529169382330974,
-0.767005330421588,0.443078346284532,0.916927917418822
"6495","KRTCAP2",397.990710185558,-0.198168963771209,0.258481934360703
,-0.766664657866072,0.443280921744599,0.917051272501242
"6496","MFSD1",36.0778372712919,-0.235771795946303,0.307847269358379,-
0.765872623907637,0.443752096515781,0.917051272501242
"6497","ZBTB49",2.62216518750073,0.737504315759721,0.962673603334186,0
.766100070891527,0.443616760826718,0.917051272501242
"6498","UBE2D3",215.748561360657,-0.120975979983717,0.157928957600969,
-0.766015186963883,0.443667265772509,0.917051272501242
"6499","CARD6",42.5147981263598,0.244678128791076,0.319397257493011,0.
766062084288341,0.443639362006066,0.917051272501242
"6500","AC037459.2",1.54871730246392,0.979880969457208,1.2792687620916
6,0.765969590201717,0.443694396669437,0.917051272501242
"6501","TSPAN31",64.9776253614536,0.185389873099821,0.241974170340027,
0.766155630740697,0.443583705143537,0.917051272501242
"6502","UBC",1715.09533378644,-0.170540018908631,0.222629134307308,-0.
766027408942914,0.443659993635308,0.917051272501242
"6503","DNAAF2",12.2371219103874,0.334685519734288,0.436756438219746,0
.76629785034994,0.443499097099528,0.917051272501242
"6504","PRKAB2",25.4873326144895,0.265233262156692,0.346723001499267,0
.764971637329497,0.444288433934313,0.917189233103835
"6505","SNRNP200",70.5140982481026,0.185046911768814,0.241957323152605
,0.764791531654128,0.444395691220466,0.917189233103835
"6506","COPS9",200.575664067346,-0.19021519103728,0.248687711476868,-0
.764875714636882,0.444345556378869,0.917189233103835
"6507","CCDC51",31.9791834388992,0.296579363565429,0.387926030069586,0
.764525555328755,0.444554113576267,0.917189233103835
"6508","AC072022.2",5.42581625781356,0.552028306323482,0.7220779703956
66,0.764499581701677,0.444569585864899,0.917189233103835
"6509","EXOSC2",10.0547269003107,0.364507085959468,0.476524817592144,0
.764927811737704,0.44431453177402,0.917189233103835

"6510","TRAPPC4",59.2328038886358,-0.225437350995128,0.294659455940482,-0.765077605521216,0.44422533421745,0.917189233103835
"6511","LBHD2",7.8113737385257,0.60893114813586,0.79586071586283,0.765122760803301,0.444198447651229,0.917189233103835
"6512","EEF2",811.102628509523,-0.131883576448949,0.172253274595442,-0.765637557594731,0.443891990639005,0.917189233103835
"6513","SBF1",19.4262507866676,-0.256006900252828,0.334855325543163,-0.764529875215704,0.444551540282843,0.917189233103835
"6514","ARMCX2",27.5427149830302,0.319532625872846,0.417598735988439,0.765166650029546,0.444172315816007,0.917189233103835
"6515","CENPK",1.8972461450162,-0.931256844091304,1.2195777149175,-0.763589587363279,0.445111856361068,0.917521817740238
"6516","FCHSD1",9.73145933265569,-0.377236587196121,0.494272485117603,-0.763215834493325,0.445334686906999,0.917521817740238
"6517","AC034213.1",2.63422258181681,-0.739017643330788,0.967469175408481,-0.76386686223751,0.444946586842814,0.917521817740238
"6518","NUDT1",79.2719693013802,-0.214273992895542,0.280583275139149,-0.763673432742126,0.445061876683195,0.917521817740238
"6519","AGO2",58.5854151940133,0.247731103361766,0.324596061052033,0.763198119406798,0.44534525017632,0.917521817740238
"6520","ARFIP2",25.4782804455982,-0.268576467197846,0.351686045627094,-0.763682467750305,0.445056491165348,0.917521817740238
"6521","SLC1A2",4.60089072277022,0.688207381477745,0.901649082951948,0.763276306148499,0.445298629543129,0.917521817740238
"6522","TTC3",293.584730651223,0.159445963641485,0.20884919610749,0.763450214859442,0.445194942472714,0.917521817740238
"6523","TBC1D10A",22.3529256662853,-0.277680380160795,0.363437757185714,-0.764038338534272,0.444844396017202,0.917521817740238
"6524","PLEKHB2",31.2548570693263,0.228087973902023,0.299444431673732,0.761703841434402,0.446236782119233,0.917861920772624
"6525","BRPF1",8.07002061819811,0.412903505825312,0.542810243394812,0.760677439030913,0.446849753463706,0.917861920772624
"6526","ALAS1",11.3020600359782,0.356045564533449,0.467329694282951,0.761872333149618,0.446136204004902,0.917861920772624
"6527","ATP2C1",42.6270623519051,0.203170831364606,0.267066086026459,0.760751147356379,0.446805718617206,0.917861920772624
"6528","CHSY3",3.87067104680648,0.636702973933242,0.837068718727046,0.760634055112578,0.446875673049514,0.917861920772624
"6529","FKBP5",63.5479789280597,-0.531978292016584,0.699096847899912,-0.760950780445724,0.446686466166513,0.917861920772624
"6530","MAPK13",2.72511453054641,-0.631833110104209,0.82946771814088 2,-0.761733213102448,0.446219248298524,0.917861920772624
"6531","KIF13B",19.116613664952,-0.437144733320664,0.574281211335432,-0.761203265390015,0.446535668173101,0.917861920772624
"6532","RPS20",1617.35968986296,-0.162171753993265,0.212769851864083,-0.762193292764333,0.445944648627106,0.917861920772624
"6533","DDX31",3.57021527391084,0.666281743314928,0.875956910934106,0.760633011736177,0.446876296422002,0.917861920772624
"6534","SUPV3L1",11.1284597413927,-0.335357103298677,0.440520568208638,-0.761274563551925,0.446493090208684,0.917861920772624

"6535","ANXA7",167.732689620541,-0.123956622902431,0.162866168270014,-0.761094978896568,0.44660033931587,0.917861920772624
"6536","LDB1",12.6107096453211,-0.335495742832133,0.439931292612965,-0.762609408481631,0.445696371859384,0.917861920772624
"6537","ZNRD2",40.2424574851966,-0.233448095387498,0.306277316808459,-0.762211507597519,0.445933779040494,0.917861920772624
"6538","UBE4A",38.7469370577027,0.212363602491424,0.279058686167288,0.760999793298382,0.446657190709612,0.917861920772624
"6539","HECTD4",24.75227821537,0.275363275800213,0.361896321870476,0.760889954274712,0.44672279925611,0.917861920772624
"6540","PLCB2",1.91199686058309,-0.779972362839426,1.02376574064381,-0.76186605184593,0.446139953285071,0.917861920772624
"6541","WIPF2",49.3524552420315,0.232178722768954,0.304985316057266,0.761278365038922,0.446490820094742,0.917861920772624
"6542","KAT7",21.1221784453811,0.276415963648939,0.362376975256608,0.762785669407395,0.445591228966587,0.917861920772624
"6543","AL732509.1",3.28896004274958,0.647544419552278,0.850472447456404,0.761393765887368,0.446421909905534,0.917861920772624
"6544","SHISA4",34.4538730869766,-0.256452318450232,0.33724584841291,-0.760431357886552,0.446996785204385,0.917969100911022
"6545","SMAP2",21.6808610080539,0.288900438666972,0.380054373204228,0.76015554361672,0.447161614980949,0.918004233570432
"6546","TUBD1",8.73288872354848,0.372397068111031,0.489957567779952,0.760059834973874,0.447218819643249,0.918004233570432
"6547","PIGP",75.4224266468335,0.251780201561656,0.331256113133655,0.760077147497252,0.447208471710907,0.918004233570432
"6548","BOLA2B",5.00745829404108,-0.476678296240281,0.627378713270604,-0.759793544405256,0.447378002317127,0.918119853581835
"6549","DHX40",48.9359875364029,0.241508862851994,0.317884805700989,0.759737044743069,0.447411780720845,0.918119853581835
"6550","MPHOSPH10",66.989130917577,-0.209877802743369,0.276736557718338,-0.758402881331576,0.448209833494669,0.918651253472951
"6551","SLC4A7",44.9417158020274,-0.293107602850117,0.386506159944819,-0.758351698435967,0.448240465518633,0.918651253472951
"6552","ESRG",5.42369050679829,0.522191671265053,0.687948558648886,0.759056276374246,0.447818893037452,0.918651253472951
"6553","WEE2-AS1",2.75449870539821,0.717447147160765,0.945864738871924,0.758509243104274,0.448146181727963,0.918651253472951
"6554","ZC3H3",8.81082403924054,0.427274040511371,0.563481121560998,0.75827569755612,0.448285952844379,0.918651253472951
"6555","RBM18",20.2519178271286,0.25022270297143,0.329745642065278,0.758835511530109,0.447950959763186,0.918651253472951
"6556","ALDH18A1",21.1231308155064,0.285472842569586,0.376390874379525,0.758447831765802,0.448182932465158,0.918651253472951
"6557","ISLR",726.253916410434,0.156147959375847,0.205832429306751,0.758616899687563,0.448081760316304,0.918651253472951
"6558","EWSR1",71.9236238281943,0.218564348127407,0.288024061120043,0.758840588794814,0.447947922175346,0.918651253472951
"6559","HSPG2",467.481762074257,0.141029704518088,0.186037776728082,0.

758070253248733,0.448408926543376,0.918763159600005
"6560","BCAR1",13.5645589478703,0.306771592987939,0.404814796171809,0.757807263689394,0.448566373307945,0.918945654098395
"6561","H3F3A",1287.64491502715,-0.167767707737296,0.221640170794239,-0.756937278725722,0.449087440074164,0.919032451219232
"6562","GPD1L",9.52987710794558,0.410193019723848,0.541792284820222,0.757103840745828,0.448987653233643,0.919032451219232
"6563","TXNDC5",116.25031538855,0.162815710181551,0.21506762422864,0.757044258825586,0.449023347150041,0.919032451219232
"6564","ST8SIA1",33.68406316328,0.321304316752192,0.42446998674707,0.756954147016403,0.449077333755792,0.919032451219232
"6565","MIEF2",9.60708813682954,-0.370918969351979,0.489586066220504,-0.757617495561897,0.448680003309191,0.919032451219232
"6566","KDSR",172.743364740474,-0.128630792098921,0.169882686662488,-0.757174227850986,0.448945488297876,0.919032451219232
"6567","RPS19",1927.2222883831,-0.187166149442751,0.247209382592499,-0.757115880796791,0.448980440560414,0.919032451219232
"6568","MAP3K6",19.9636293342217,-0.285608364036868,0.378069597579001,-0.755438590846193,0.449985865557459,0.919252612394694
"6569","AOX1",116.702385351055,0.169602281353136,0.224342651346366,0.755996598664087,0.449651235012247,0.919252612394694
"6570","SCOC",65.9884945058614,-0.175088108157941,0.231683219447458,-0.755722009455451,0.449815885207768,0.919252612394694
"6571","GSTA4",47.2246089764321,0.268200472207622,0.355215806670777,0.755035297334607,0.450227803640212,0.919252612394694
"6572","AKAP7",8.76191246218559,-0.403351679946298,0.533749785002122,-0.755694318349364,0.449832491347903,0.919252612394694
"6573","C12orf29",25.6933291797614,-0.248053367542272,0.328576859555537,-0.754932553308262,0.450289452146311,0.919252612394694
"6574","VSIG10",17.9042747015166,0.304597964371116,0.403449462727284,0.754984186401091,0.450258470642411,0.919252612394694
"6575","ABCA3",8.13710689062252,0.436367169757072,0.576807484088027,0.756521338219109,0.449336683805447,0.919252612394694
"6576","HMOX2",48.8210943286596,-0.216351425760641,0.286145719642952,-0.756088282678493,0.449596266715276,0.919252612394694
"6577","AC015908.2",2.15684146534327,-0.776940078537931,1.02865901848123,-0.755294091218927,0.450072543238263,0.919252612394694
"6578","LRRC37A2",4.17899300497365,0.625235444971198,0.827748015837511,0.755345144909333,0.450041917749859,0.919252612394694
"6579","ZNF563",7.01111270236026,-0.419019625963382,0.554239654873586,-0.756026066122884,0.449633567656249,0.919252612394694
"6580","ZNF304",4.4991370260412,-0.48500503847415,0.642135448812126,-0.75530020865746,0.450068873518782,0.919252612394694
"6581","H1F0",78.6951158855833,-0.160683731441464,0.212780196944462,-0.75516299800871,0.450151187303464,0.919252612394694
"6582","PARVG",2.42293692597322,-0.772960543337686,1.02256952486575,-0.755900234205751,0.449709013532645,0.919252612394694
"6583","FAM122C",5.1583301500753,-0.48957802048946,0.647484601626675,-0.756123032516131,0.449575433769518,0.919252612394694
"6584","MDK",50.0425271756003,0.324506094346403,0.430220250090285,0.75

4278986817341,0.450681717282637,0.91931643708006
"6585","CLP1",7.74935870923057,0.462127426365388,0.612352631166242,0.754675333859927,0.450443810018934,0.91931643708006
"6586","IGHA2",3.24515439788577,-0.763237296725725,1.01187443349703,-0.75428064141119,0.450680723965012,0.91931643708006
"6587","EXOC7",64.7186675368458,0.178059067571289,0.23603815440705,0.754365615248055,0.45062971250244,0.91931643708006
"6588","ZNF236",12.5782506407387,0.365105579609574,0.484098656847385,0.754196638320101,0.450731155883661,0.91931643708006
"6589","GGT5",45.3692274708672,0.341160741640114,0.452114704996764,0.754588908234153,0.450495680924916,0.91931643708006
"6590","RNF135",25.4883246851776,-0.265575705517665,0.352263609201664,-0.753911839260204,0.4509021610257 9,0.919525666468224
"6591","AL031293.1",1.76907109143996,0.902029907379924,1.19694040924997,0.753613045736466,0.451081608495039,0.919684880879689
"6592","BMERB1",61.8633279281927,-0.210366143789733,0.279165337277407,-0.753553954231402,0.4511171020729 9,0.919684880879689
"6593","AL022069.3",2.22868263698042,0.765617054932188,1.01686707100036,0.752917541305571,0.451499466519143,0.919766762248106
"6594","LRRN3",4.69017889867957,0.703766460569963,0.934579572688703,0.753030005294562,0.451431883485629,0.919766762248106
"6595","PTCH1",8.9981275465147,0.454791958135456,0.603697341739612,0.753344311281758,0.451243037802381,0.919766762248106
"6596","BCAT1",27.4678839035907,-0.267198776766699,0.354773534376974,-0.753153070552269,0.45135793638509,0.919766762248106
"6597","ZNF432",6.98644971465047,0.448701536939459,0.595852452371079,0.753041353029492,0.451425064604243,0.919766762248106
"6598","EHMT2",28.9134827151823,0.233099713474596,0.309662319786542,0.752754528336797,0.451597436132214,0.919768279579442
"6599","TJP1",60.6486056505502,-0.225548679383181,0.299657380554592,-0.752688550389602,0.451637091818196,0.919768279579442
"6600","LINC01140",66.9503473027649,-0.207365288024992,0.275799387120622,-0.751870010263292,0.452129234657057,0.919795807889792
"6601","EIF2B4",23.5970351058808,-0.258997823015542,0.344365038496114,-0.75210254834991,0.451989391569725,0.919795807889792
"6602","LINC01886",10.3656038772005,-0.35099566659217,0.466920514325601,-0.751724663670288,0.452216655192628,0.919795807889792
"6603","FRZB",2.75804730787833,0.930808298956519,1.23727535469262,0.752304889470428,0.451867728145081,0.919795807889792
"6604","AC080013.1",2.88894983704139,0.803573048100357,1.06869310839225,0.751921240803412,0.452098423681604,0.919795807889792
"6605","COMMD8",47.2082671154629,-0.198877404991141,0.264583824671632,-0.751661237182442,0.452254806844835,0.919795807889792
"6606","ZNF451",60.7758339274078,0.227081057910869,0.30181011271516,0.752397114424001,0.451812281380027,0.919795807889792
"6607","MYO1C",97.8258992975876,-0.160575634616683,0.213648899858663,-0.75158652688083,0.452299748154386,0.919795807889792
"6608","MRGBP",16.9221682990582,-0.295695158537996,0.393458670942988,-0.75152787414575,0.452335031947588,0.919795807889792
"6609","AL118516.1",14.5635253220713,-0.357714127202144,0.475931546323

806,-0.751608356212573,0.452286616655378,0.919795807889792
"6610","LRRC20",3.82580128580263,0.54624821615231,0.727133142370771,0.
751235481264549,0.452510950270769,0.920014320830388
"6611","GUCY1B1",7.96564816773779,0.598472198178137,0.797728381104976,
0.750220516598846,0.453121904132245,0.920560131463831
"6612","PMPCB",52.8177372591303,-0.184026052986848,0.245274086399713,-
0.750287385382199,0.453081638415997,0.920560131463831
"6613","ITPK1",14.4342236013618,-0.311142652397333,0.414726618880243,-
0.750235548510039,0.453112852338417,0.920560131463831
"6614","ATP2A3",2.09330792744802,0.800384273978316,1.06670290527588,0.
750334765209354,0.453053109395189,0.920560131463831
"6615","AC027097.1",7.45782945818629,0.528512919442282,0.7044138761946
65,0.75028749049831,0.453081575120854,0.920560131463831
"6616","NEXN",70.3952735144417,0.193054041919526,0.257595365542756,0.7
49446875772627,0.453587906664225,0.920658910605006
"6617","SENP7",42.3861443029707,0.218915657328107,0.29200469932513,0.7
49699089891556,0.453435955797566,0.920658910605006
"6618","FAM168A",25.7656938239143,0.24871169974911,0.331808737936843,0
.749563442167248,0.453517675603263,0.920658910605006
"6619","NTN4",68.419762126663,-0.202666664382712,0.270458886412237,-0.
749343706436049,0.453650071138206,0.920658910605006
"6620","KIAA0556",7.83349298580865,0.452012450402861,0.602680441894982
,0.750003515928968,0.453252587200886,0.920658910605006
"6621","GINS2",1.90952374435811,0.791812832923887,1.05635837244153,0.7
49568379047156,0.453514701276681,0.920658910605006
"6622","ARHGEF6",39.9384559031157,0.211136541799212,0.281571986246091,
0.749849246773721,0.453345504765345,0.920658910605006
"6623","GOLIM4",198.79937844868,0.190682261321164,0.254524600090693,0.
749170261944107,0.453754590595644,0.920731985960269
"6624","GPR153",10.2659377022896,0.375177894226476,0.507318988377243,0
.7395305573453,0.459584891542675,0.921039760975479
"6625","ACOT7",5.57814480609778,-0.451622935942001,0.610378331548846,-
0.739906567122066,0.459356689357264,0.921039760975479
"6626","NBPF3",2.26900934340031,0.715715862952311,0.961401895326492,0.
744450230888357,0.456604139902725,0.921039760975479
"6627","PNRC2",185.202937807946,0.119048702412491,0.160423004185319,0.
742092463715282,0.458031315599248,0.921039760975479
"6628","ERMAP",10.6853368521838,0.386242477563189,0.519438114293681,0.
743577467526409,0.457132139111575,0.921039760975479
"6629","PRKAA2",5.68478988352731,0.521495660668801,0.698146257974209,0
.746971934193288,0.455080502341479,0.921039760975479
"6630","SYDE2",19.1250726914984,0.326036814020246,0.438717350154161,0.
743159152255274,0.457385330532581,0.921039760975479
"6631","GBP3",33.2354635529841,-0.225498627579213,0.30278925791767,-0.
744737871911321,0.456430199670063,0.921039760975479
"6632","NOTCH2",129.427166025147,0.172980277725499,0.233515673339027,0
.740765171142751,0.45883583634669,0.921039760975479
"6633","TOMM40L",6.86636585301413,-0.442491507330399,0.595449423766766
,-0.743121900313939,0.457407881631251,0.921039760975479
"6634","KIAA1614",7.76977950131435,0.395411815635229,0.52936996591089,

0.746947959079699,0.455094974843332,0.921039760975479
"6635","GPATCH2",47.3907351850827,-0.185451333082473,0.250965996022881
,-0.738950041126546,0.459937334318509,0.921039760975479
"6636","C1orf131",42.8897590407338,0.207627031748524,0.278111722076669
,0.746559800493724,0.455329321615084,0.921039760975479
"6637","MSH6",26.4524586539019,0.243666070445469,0.327618412763065,0.7
43749621367249,0.457027963357723,0.921039760975479
"6638","MOB1A",93.8067856816078,0.161130138064034,0.217338746125617,0.
741377876409134,0.458464354813669,0.921039760975479
"6639","C2orf49",47.0291463859702,0.215900601760717,0.289271269581677,
0.746360335310646,0.45544977309678,0.921039760975479
"6640","FAM168B",33.5918018148537,0.210405494312482,0.281974586404891,
0.746186019793849,0.455555052078045,0.921039760975479
"6641","TYW5",10.6245528815124,0.359327895377295,0.483610743777613,0.7
43010572036693,0.457475279825147,0.921039760975479
"6642","SUMO1",195.777062541801,-0.191095780349659,0.25600663099642,-0
.746448557234174,0.45539649611658,0.921039760975479
"6643","CCL20",3.08136558646069,-0.608267554315962,0.819328259598064,-
0.742397869462428,0.457846310032903,0.921039760975479
"6644","TATDN2",13.6393004238249,0.318061366342552,0.426693468648598,0
.745409502868418,0.456024200848168,0.921039760975479
"6645","LSM3",146.356651416333,-0.206354328083734,0.277164497121937,-0
.744519338611214,0.456562346172322,0.921039760975479
"6646","ARF4",204.435967878057,-0.176321368305776,0.238079648729625,-0
.740598237802405,0.458937076811445,0.921039760975479
"6647","HCLS1",3.7431448104928,-0.585675709761888,0.788736474244747,-0
.742549291032471,0.457754598976848,0.921039760975479
"6648","RNF212",28.8973152045367,-0.249887141679621,0.338084953096043,
-0.739125297920708,0.45983091654557,0.921039760975479
"6649","ANKRD17",77.7810606778163,0.207104991059546,0.279343172196064,
0.741399868238711,0.458451024363312,0.921039760975479
"6650","SCOC-
AS1",3.16242157910347,0.647373419878219,0.867368999557713,0.7463644887
10487,0.455447264791194,0.921039760975479
"6651","AC107223.1",2.47987126057402,0.725632061015141,0.9709885906296
01,0.747312654358412,0.454874855702422,0.921039760975479
"6652","AC099343.3",8.13219007233412,0.376012480524022,0.5088445052955
66,0.738953602939296,0.459935171411045,0.921039760975479
"6653","AC025181.2",12.6771681890225,-0.310975843533868,0.417651456981
769,-0.744582206850633,0.45652432771278,0.921039760975479
"6654","NSA2",219.761321284302,-0.172667612481485,0.232240126050384,-0
.743487421480409,0.457186634144629,0.921039760975479
"6655","DIAPH1",75.3906474563844,-0.250299691715312,0.338497323583959,
-0.739443635964905,0.459637653588367,0.921039760975479
"6656","TAP1",22.9836055955286,0.268612794073205,0.36029860452658,0.74
5528266550333,0.455952429713546,0.921039760975479
"6657","CDKN1A",90.6804764887796,-0.544542503729759,0.732047365564693,
-0.743862391075889,0.456959730057261,0.921039760975479
"6658","SAYSD1",32.9680876756437,0.234843793240578,0.316905164515722,0
.741053853128122,0.458660788231725,0.921039760975479

"6659","C7orf26",14.9810818360634,0.299189322678791,0.402523667315593,
0.743283804090497,0.457309874955721,0.921039760975479
"6660","TYW1B",6.45400097245965,-0.442971852047053,0.598619119960259,-
0.73998948125222,0.459306376905132,0.921039760975479
"6661","STYXL1",10.0946197918953,0.355358442869861,0.475746177606005,0
.746949654242214,0.455093951555301,0.921039760975479
"6662","PNPLA8",51.7791001042384,-0.190492277960586,0.256136999592213,
-0.743712459597256,0.457050449995619,0.921039760975479
"6663","PTPRZ1",6.23283081968927,-0.554292886825222,0.740269138608469,
-0.748772112622662,0.453994570896258,0.921039760975479
"6664","TRBC1",3.32182893722584,0.591846558758456,0.798833586421848,0.
740888426348556,0.458761093499047,0.921039760975479
"6665","NCAPG2",8.15820845284208,0.391432685834829,0.528077089783866,0
.741241560006015,0.458546988487836,0.921039760975479
"6666","XPO7",24.1402665331245,0.265614336900885,0.356083013169618,0.7
4593374880919,0.455707437017576,0.921039760975479
"6667","EXTL3",7.91681784300989,0.400721641139084,0.538536040853128,0.
744094379466741,0.456819379389804,0.921039760975479
"6668","STAU2",21.7781690365951,0.253079697462703,0.339932826570726,0.
744499141244446,0.456574560551527,0.921039760975479
"6669","LRRC69",1.79630434237395,0.83299778693137,1.12532180337008,0.7
40230736165188,0.459160000522982,0.921039760975479
"6670","ZFPM2",30.266682044353,-0.22912498841211,0.306115203073,-0.748
492678939144,0.454163039332475,0.921039760975479
"6671","DOCK8",10.9395095958586,0.50975073741369,0.688017746926161,0.7
40897658077993,0.458755495586327,0.921039760975479
"6672","AL158206.1",9.18368235763469,0.399971497627531,0.5411090316306
34,0.739169879353548,0.45980384842868,0.921039760975479
"6673","FAM219A",11.1314297648136,-0.315208747242253,0.422263619776045
,-0.74647384354217,0.455381226430687,0.921039760975479
"6674","MFSD14C",27.6814261467383,0.224152357524597,0.302776472702713,
0.740322903968451,0.459104086532477,0.921039760975479
"6675","NUP188",12.4230219904422,0.332540759717145,0.448862034654875,0
.740852943762178,0.458782609696317,0.921039760975479
"6676","PHPT1",263.165975999202,-0.200826266830349,0.268821679838837,-
0.747061274785379,0.455026574456709,0.921039760975479
"6677","LINC00710",4.16930027790071,-0.609549320947613,0.8206019389899
34,-0.742807556240847,0.457598200041314,0.921039760975479
"6678","ZCCHC24",121.718509577704,0.164169335105981,0.221983782490502,
0.739555535382431,0.45956973028082,0.921039760975479
"6679","BTRC",12.5856658020367,0.375182885110228,0.503724469777997,0.7
44817668428099,0.45638195228948,0.921039760975479
"6680","TRUB1",15.5660623363274,-0.277252826025912,0.372508355453541,-
0.744286193764291,0.456703351944676,0.921039760975479
"6681","TMEM86A",2.40939797126672,0.765573376363182,1.02946418108925,0
.743661984968871,0.457080993257681,0.921039760975479
"6682","SELENOH",170.495139771467,-0.202148295889565,0.273245919497912
,-0.73980353031808,0.459419216587476,0.921039760975479
"6683","MYL6B",75.4579678101978,-0.250189252314617,0.334700134341811,-
0.747502694633257,0.454760176859275,0.921039760975479

"6684","RHOF",5.06228994112357,0.54502802803025,0.735764305639449,0.74
0764432105154,0.458836284525014,0.921039760975479
"6685","POMP",228.824006988258,-0.1999013451373,0.269808613573043,-0.7
40900531269298,0.458753753355672,0.921039760975479
"6686","DHRS4-
AS1",39.9671797351422,0.200283886361107,0.268492774663914,0.7459563357
4055,0.455693792154425,0.921039760975479
"6687","AL049838.1",2.51250710711255,0.701106725606943,0.9426737997380
72,0.743742666659188,0.45703217161382,0.921039760975479
"6688","EIF2AK4",76.7377429290032,0.18326977429562,0.2448552115483,0.7
48482228075706,0.454169340761985,0.921039760975479
"6689","ARPP19",60.1698742924087,-0.180907133487002,0.243754569516366,
-0.742169198493143,0.457984828046461,0.921039760975479
"6690","C16orf87",19.9259805662314,-0.319958694836297,0.43218739791168
9,-0.740323980713745,0.459103433342902,0.921039760975479
"6691","MT1M",243.416341559923,-0.29564465476295,0.39528947862087,-0.7
47919362271993,0.454508797892639,0.921039760975479
"6692","ARRB2",9.52556574698061,0.426392499152532,0.570092978270502,0.
747935013067666,0.454499357166811,0.921039760975479
"6693","ALDH3A1",1.4896524007193,0.887685740241334,1.19125220097903,0.
745170283430992,0.456168784957945,0.921039760975479
"6694","CDK5RAP3",47.9922487079696,-0.218275856276497,0.29481502156666
5,-0.740382410355368,0.459067988756046,0.921039760975479
"6695","HOXB-
AS1",25.7823442213734,0.288133616980335,0.385739702191401,0.7469638601
97532,0.455085376154659,0.921039760975479
"6696","TRIM37",18.554426820165,0.311397393733122,0.421287120293661,0.
739157165583559,0.459811567645789,0.921039760975479
"6697","VMP1",281.337670499052,0.234628611353515,0.315474954697553,0.7
43731341774673,0.457039024286549,0.921039760975479
"6698","PITPNC1",14.2100742819382,-0.332308351359491,0.447397981850266
,-0.742757823772899,0.457628314444952,0.921039760975479
"6699","SLC16A5",5.76587383421771,0.479614598300407,0.646438155449779,
0.741934234941161,0.458127182297327,0.921039760975479
"6700","MAFG-
DT",2.39002277134723,-0.747208005605068,1.00893341325105,-0.7405919912
96402,0.458940865387939,0.921039760975479
"6701","GPS1",39.4861531771824,0.189578402243143,0.253493763129635,0.7
47862195513638,0.454543282426186,0.921039760975479
"6702","SECTM1",9.83928689432786,-0.395903591593224,0.530617345365315,
-0.746118827534096,0.455595636933808,0.921039760975479
"6703","FN3K",6.8527425931389,0.420080808031556,0.564725490572334,0.74
3867268335657,0.456956779114368,0.921039760975479
"6704","ZNF564",8.15506298763098,-0.383100479978946,0.51719318749793,-
0.740729942388267,0.458857200572109,0.921039760975479
"6705","ITPA",74.5535809757591,-0.220834801889716,0.297444082810581,-0
.742441402104976,0.457819942689566,0.921039760975479
"6706","CRLS1",32.5245750022211,-0.207917360390345,0.280159858941427,-
0.742138296242553,0.458003548965633,0.921039760975479
"6707","TMEM189",28.3914246991104,0.25176151462929,0.337187332715409,0

```
.746651757650043,0.455273797283649,0.921039760975479
"6708","TST",13.3908015924958,0.349198568668095,0.46945853642986,0.743
83261048714,0.456977748771399,0.921039760975479
"6709","LINC00685",7.05638436281073,0.541353126239092,0.73222723366202
7,0.739323943923347,0.459710313265006,0.921039760975479
"6710","ARHGAP6",49.5864464745351,0.236080015843139,0.316297417892772,
0.746386162163271,0.455434175967384,0.921039760975479
"6711","FUNDC2",102.659834449741,-0.167615288519489,0.226629701285719,
-0.739599829892426,0.459542844923134,0.921039760975479
"6712","LRRC34",8.05773329787557,0.396617087751224,0.53701399721638,0.
73856005580319,0.46017418697368,0.921205859476611
"6713","ZCCHC10",34.7301276943117,0.213315562036772,0.288996392017358,
0.738125346644327,0.460438282499707,0.921205859476611
"6714","TTI2",12.9452467977673,-0.295671884199621,0.400439887726316,-0
.738367713262622,0.46029102890888,0.921205859476611
"6715","UBE2D1",32.2191233396412,0.231785775411967,0.313826987805217,0
.738578211622225,0.460163158752137,0.921205859476611
"6716","PAN2",10.6576591500257,-0.337921106297769,0.457873036751092,-0
.738023598627995,0.460500108933989,0.921205859476611
"6717","ZBTB45",11.3149650530536,-0.346823622700854,0.469861897163075,
-0.738139493316867,0.460429686745771,0.921205859476611
"6718","ZC3H7B",30.8177564899451,0.2313905609995,0.313397307876967,0.7
38329766030853,0.460314082597273,0.921205859476611
"6719","CCDC47",71.5330767697943,-0.170029280691617,0.230425811718089,
-0.737891642537152,0.460580297997811,0.921229144931178
"6720","PDP2",1.97206038438039,0.750527980087203,1.01754520414312,0.73
758686791633,0.460765537870913,0.921325407446393
"6721","ZNF761",12.358110631641,-0.329701423261917,0.446965831137103,-
0.73764346241667,0.460731136982339,0.921325407446393
"6722","TERF2",22.2908045810905,0.253904138634019,0.344437264917809,0.
737156412778408,0.461027236425155,0.921711548693493
"6723","S100A2",2.11570696863848,-0.704959673321698,0.956699143560243,
-0.736866629459156,0.461203459228644,0.921808522624659
"6724","RHOBTB1",3.32926046842943,0.671067432150133,0.910723298508788,
0.736851064696527,0.461212925524831,0.921808522624659
"6725","BYSL",5.31295352057641,-0.49617313949255,0.673625039743999,-0.
736571698227122,0.461382851218667,0.921837501758826
"6726","GRINA",135.718243191799,0.162019596227479,0.220056557424079,0.
736263432110524,0.461570395813315,0.921837501758826
"6727","RDX",134.777418465199,0.150390252867564,0.204255597424903,0.73
6284609888631,0.461557510201061,0.921837501758826
"6728","FBXL19",3.37697319640124,0.620601852801699,0.842791027891285,0
.736365044552601,0.461508571586494,0.921837501758826
"6729","URB1",11.1526302246966,0.381057507233435,0.517290564272792,0.7
36641132762835,0.461340614138251,0.921837501758826
"6730","GLOD4",62.1210405391905,-0.185499209528314,0.252010230531066,-
0.736078091502111,0.461683174819941,0.921925733492599
"6731","TSPAN5",6.72316736224345,-0.53269433041009,0.723887376538826,-
0.735880121238056,0.461803655916064,0.922029316870596
"6732","MIB2",23.771141335628,-0.239962132236207,0.326373601582377,-0.
```

735237565393719,0.462194824631313,0.922134270539747
"6733","TBC1D14",17.1274523505988,-0.283393400663559,0.385375915517733
,-0.735368738035522,0.462114955645371,0.922134270539747
"6734","C7orf25",13.0849366941656,-0.317172882083077,0.43144418440962,
-0.735142327893677,0.462252818093924,0.922134270539747
"6735","AP001330.4",1.98411691529823,-0.929033719659185,1.262904817067
37,-0.735632414338656,0.461954430544256,0.922134270539747
"6736","AC073611.1",4.90735663398508,0.500974567469747,0.6813505566845
96,0.735266981959262,0.462176912670995,0.922134270539747
"6737","RPL21",2761.5278598785,-0.179170284122779,0.243730121096029,-0
.735117528014464,0.462267920278762,0.922134270539747
"6738","PRPS1",20.8824997787343,0.292015504601835,0.397341704753462,0.
734922866410464,0.462386471366574,0.922233865938763
"6739","DAB2IP",12.6791441642581,-0.406022924069445,0.552664624136686,
-0.734664218292767,0.46254401719501,0.922411195590406
"6740","CCDC74B",5.37748650814914,-0.483980752636339,0.659897344338219
,-0.733418245714721,0.463303374210923,0.922921028640273
"6741","AC093323.1",16.4088207780494,0.287644733918876,0.3920458093708
89,0.733701845660475,0.463130473400679,0.922921028640273
"6742","ATG5",39.7154901335856,-0.197854953455126,0.269700961389266,-0
.733608632449617,0.463187298214953,0.922921028640273
"6743","SMC2",30.4530561981547,-0.224242797669694,0.305489890434872,-0
.734043268503844,0.462922367747424,0.922921028640273
"6744","EXOSC5",12.6973294377153,-0.321682073668755,0.438604396378772,
-0.733421908956325,0.463301140633374,0.922921028640273
"6745","DLGAP4",164.125734672129,0.131601488126283,0.179416781930906,0
.733495979082732,0.463255979360056,0.922921028640273
"6746","ETS2",6.86588166088861,-0.477944452352865,0.651240072542466,-0
.73389902204106,0.463010283166865,0.922921028640273
"6747","HUWE1",57.3200037322336,0.226733166194146,0.309177388375212,0.
733343299733766,0.463349072121134,0.922921028640273
"6748","VEZF1",55.4749130865129,0.222143744777134,0.303036063973966,0.
733060421469238,0.463521578256994,0.923113457417524
"6749","TTPAL",13.2023389449789,-0.299822998995095,0.409058004170217,-
0.732959619267915,0.463583058569155,0.923113457417524
"6750","CBR4",34.051909391888,0.238013447663185,0.325030175129274,0.73
2281079959792,0.463997024948654,0.923664052478886
"6751","TOLLIP-
AS1",3.08575303587309,0.636895762705106,0.869709186054458,0.7323088831
50311,0.463980058607347,0.923664052478886
"6752","AC239868.1",1.65376169664167,-0.852215192014808,1.165640519267
88,-0.731113218807864,0.464710000793249,0.92387353518992
"6753","SLC4A1AP",29.4037983680696,0.238317568540543,0.325952952114557
,0.731141003615718,0.46469303117451,0.92387353518992
"6754","BBS5",12.6386597368524,0.332351973051878,0.454594620873854,0.7
31095261120794,0.464720968664622,0.92387353518992
"6755","DENND6A",25.0593328054347,0.237529055192014,0.324623455607573,
0.731706385009823,0.46434779841041,0.92387353518992
"6756","PXYLP1",25.0304879183107,0.29465256231759,0.402944390666404,0.
73124870116763,0.464627257968998,0.92387353518992

"6757","RPS3A",2861.74539633823,-0.208303452267988,0.284838254510338,-0.731304341918819,0.464593278997781,0.92387353518992
"6758","AP1G1",29.3601856758393,0.230933131522013,0.315618829112604,0.73168363298003,0.464361688480701,0.92387353518992
"6759","SAT2",180.75179339141,-0.199642329532735,0.272894543507847,-0.731573181956986,0.46442912190309,0.92387353518992
"6760","SYNGR1",14.1124556796485,0.311253606106483,0.425518482110286,0.731469064664064,0.464492693396333,0.92387353518992
"6761","PBDC1",71.0429834316006,-0.207862698935962,0.284374088654169,-0.730948096993275,0.46481085633438,0.923915559573693
"6762","DYRK1B",10.8570203403459,0.362650249206774,0.49632761040914,0.730667086821604,0.464982523864949,0.924120103256588
"6763","TRIP6",90.231163059139,-0.218870663914412,0.299688650313972,-0.730326836485499,0.465190428059186,0.924259929433383
"6764","MIAT",1.71474149032128,0.86205933902201,1.18021187653556,0.730427608941312,0.465128847374203,0.924259929433383
"6765","MPHOSPH8",218.326171019573,0.131876897291797,0.180699853677565,0.729811865410327,0.46550519067306,0.924748596815262
"6766","ZBTB8OS",46.2634190456391,-0.193528058713543,0.266319081818185,-0.726677402881944,0.467423599388953,0.924921224107227
"6767","SF3A3",48.049319102392,-0.192660341547741,0.265203868154995,-0.726461280101477,0.467556035959336,0.924921224107227
"6768","NBPF10",7.71874260191935,0.497049001798862,0.682616090254917,0.728153070070825,0.466519890177905,0.924921224107227
"6769","NME6",27.5958305611495,0.240608840807117,0.331094791455403,0.726706813325169,0.467405578749085,0.924921224107227
"6770","COMMD2",62.0479317063462,-0.171695829985167,0.236163495153992,-0.727021040543168,0.467213066571926,0.924921224107227
"6771","TSTD3",24.6619287059182,-0.241144838066465,0.331550197113597,-0.727325274319903,0.467026718802995,0.924921224107227
"6772","HIBADH",66.1128943886732,-0.203889295959958,0.27967907304358,-0.729011626580257,0.465994550522845,0.924921224107227
"6773","POM121",25.7227044761072,0.256640888030213,0.352620694801784,0.727810057133704,0.466729867226132,0.924921224107227
"6774","FBXO32",11.5562217266356,-0.379749091963336,0.521242063236758,-0.728546521370909,0.466279101611366,0.924921224107227
"6775","TLR4",35.0159226659873,0.346956017626354,0.476176347770557,0.728629255213517,0.466228478043729,0.924921224107227
"6776","SLC27A4",8.08578126488002,-0.374888489116211,0.514370241606457,-0.728830050403726,0.466105627241695,0.924921224107227
"6777","PTER",22.2974707983584,-0.27237046323074,0.374589508036188,-0.727117170629424,0.467154180991606,0.924921224107227
"6778","IFIT1",83.0357337087995,0.218581944207272,0.300389509733986,0.727661709627744,0.466820695118552,0.924921224107227
"6779","TRAF6",12.9095123382421,0.350823510299572,0.481764787389034,0.728204965333581,0.466488126805461,0.924921224107227
"6780","FGF14-AS2",11.8709601852675,-0.354419961437682,0.486872718318251,-0.727951984374712,0.466642979411828,0.924921224107227
"6781","ZNF770",29.2296519538527,0.215456161236502,0.296552466343781,0

.726536399757111,0.467510001482338,0.924921224107227
"6782","DNAJC17",20.5085867409877,-0.263869848230315,0.36198895087873,
-0.728944481840591,0.466035623677158,0.924921224107227
"6783","MEF2A",81.1896693439518,0.241710192860674,0.33182697275177,0.7
28422378856737,0.466355068233664,0.924921224107227
"6784","GFER",29.0450263699721,-0.247397600503582,0.339527968647365,-0
.728651608552843,0.46621480090233,0.924921224107227
"6785","ZFP90",29.0784991921748,0.241118218828971,0.330569205246748,0.
729403147667649,0.465755092935055,0.924921224107227
"6786","COG4",29.0313714577071,0.249525064141651,0.342438782279645,0.7
2867057428642,0.466203196680192,0.924921224107227
"6787","RNF167",46.9892637562075,-0.20782315672681,0.28609377437816,-0
.726416215027832,0.467583653733214,0.924921224107227
"6788","DLG4",10.0160874491669,-0.405518920655596,0.557295480527565,-0
.727655139553098,0.466824717967922,0.924921224107227
"6789","DUS1L",32.4698335227747,-0.219423092753367,0.3013281793866,-0.
728186435135396,0.466499468388587,0.924921224107227
"6790","ZNF765",13.2386269029413,0.33104583550574,0.455458887675853,0.
726840214261761,0.467323844924057,0.924921224107227
"6791","RPRD1B",14.4788803420332,0.300162415797958,0.41321397184137,0.
726409163902107,0.467587975041632,0.924921224107227
"6792","THAP7",42.3204085320235,-0.214333001950881,0.293808420007481,-
0.729499181628026,0.465696368199396,0.924921224107227
"6793","BCAP31",113.453293710227,-0.163416334943898,0.224778414806513,
-0.727010798988708,0.467219340395469,0.924921224107227
"6794","MT-
CO1",8331.51851877202,-0.164759249092816,0.226786641505819,-0.72649450
602049,0.46753567429676,0.924921224107227
"6795","TANC1",4.13300349340881,-0.581510868817882,0.800966370718711,-
0.726011590594109,0.467831665115282,0.925130922231353
"6796","LMTK2",9.65507968808276,-0.375852118616532,0.517678782434761,-
0.726033462002855,0.467818257343265,0.925130922231353
"6797","PYCR3",4.08656414400604,-0.51563675953705,0.711092568962175,-0
.725133100869851,0.468370379336416,0.926059957025466
"6798","P2RX7",4.03437287412548,0.557050606140993,0.768449408325742,0.
724902121214025,0.468512079459421,0.926203859349097
"6799","PPARD",11.2310371220957,0.344556222757427,0.47561729075065,0.7
24440068639276,0.468795608124086,0.926215545311042
"6800","EPHB4",10.3428290584919,-0.334434412429474,0.461501899834812,-
0.724665299426026,0.468657388217091,0.926215545311042
"6801","RCE1",12.5543340420047,-0.377856517752223,0.521618163826203,-0
.724392944794232,0.468824529996703,0.926215545311042
"6802","MIER2",5.06289863866679,0.47957173882051,0.66193815763884,0.72
4496289096192,0.468761104572305,0.926215545311042
"6803","STK26",2.41953015377905,-0.669904176198257,0.924859267284503,-
0.724330933251256,0.468862590576012,0.926215545311042
"6804","ANKZF1",15.6323558609518,0.305469728740298,0.422079067708145,0
.723726316016983,0.469233773687441,0.926812563872064
"6805","PIK3CD",1.68040089822315,0.908311327889175,1.26255302805792,0.
719424299576831,0.471879529213559,0.927004608544132

"6806","ATP6V0B",108.217947066666,-0.187240235140257,0.260803881280869,-0.717935002426636,0.472797364509796,0.927004608544132
"6807","MINDY1",10.999589875447,0.332493685732305,0.463829242082647,0.71684502736259,0.473469724908619,0.927004608544132
"6808","HMCN1",106.971974896509,0.303482598909641,0.421143133428077,0.720616281783612,0.471145633506776,0.927004608544132
"6809","LBX2",5.44582704501243,-0.524707839211209,0.728436471681178,-0.72032065884925,0.471327587889103,0.927004608544132
"6810","PRPF40A",107.571937617953,0.158993953151509,0.221250324186556,0.718615684456341,0.472377746757249,0.927004608544132
"6811","RAPGEF4",2.19921961786704,-0.694140422472418,0.965255838140567,-0.719125847308611,0.47206338291574,0.927004608544132
"6812","MTERF4",29.1706438214354,-0.213671724927962,0.297994008353546,-0.717033627986432,0.473353347396148,0.927004608544132
"6813","DTYMK",46.2862964094853,-0.220213044428192,0.306475284322831,-0.718534432278157,0.472427825229454,0.927004608544132
"6814","SMARCC1",30.5638960746532,0.235925875460195,0.329048248615494,0.716994776458707,0.473377319752887,0.927004608544132
"6815","PPM1M",19.0609486681919,-0.263399277299284,0.366819101501092,-0.718063143989519,0.472718353838323,0.927004608544132
"6816","DZIP3",24.3441755448502,0.225865959113562,0.313206629422882,0.721140416247719,0.470823126743598,0.927004608544132
"6817","AC083799.1",3.54016765089322,0.605422901822781,0.843968078055642,0.717352844929362,0.473156407907052,0.927004608544132
"6818","AC046143.1",2.33353447056318,-0.682750393371368,0.944110946783053,-0.723167542647143,0.46957695704746,0.927004608544132
"6819","TRMT44",8.61247052378054,0.382803289410251,0.531556106967971,0.720155942885851,0.471428986520945,0.927004608544132
"6820","ANAPC4",17.2884705765161,0.275678403896839,0.383597596420964,0.718665618525686,0.472346972147953,0.927004608544132
"6821","HNRNPD",155.293944773805,-0.134197271709956,0.186934666650864,-0.717883280368722,0.472829257818277,0.927004608544132
"6822","HSD17B11",100.834213417138,-0.175541341814358,0.244083031956589,-0.719186993078563,0.47202571245119,0.927004608544132
"6823","LINC01060",3.10639280405846,-0.908819555313182,1.26367979195798,-0.719185003271306,0.472026938298364,0.927004608544132
"6824","GTF2H2",17.931061023649,0.314468455590761,0.43686612021291,0.719827977132909,0.471630917030301,0.927004608544132
"6825","FOXD1",2.87799984001348,-0.686383638528744,0.953395137702235,-0.71993616433055,0.47156430024433,0.927004608544132
"6826","ARRDC3",72.9685376604973,-0.261254221034124,0.36330156054384,-0.71911119964099,0.472072407243707,0.927004608544132
"6827","LOX",256.395620247194,0.242567347955656,0.337376834566893,0.718980448871219,0.472152966111057,0.927004608544132
"6828","PCDHB3",3.07973602131755,0.572543169861574,0.795212517693324,0.719987622330634,0.471532616554693,0.927004608544132
"6829","RPS18",4816.02963567086,-0.198451212688703,0.275658539774,-0.719916795798906,0.47157622612933,0.927004608544132
"6830","ETV7",1.68345956222465,0.844556040968852,1.17479191800182,0.718898409179846,0.472203516713704,0.927004608544132

"6831","CREB3L2",66.8835458282407,-0.183923712045776,0.256536341841767
,-0.716949928908011,0.47340499263493,0.927004608544132
"6832","CNOT7",42.4276413509691,0.180052167626793,0.249614617861135,0.
721320606820226,0.470712281286999,0.927004608544132
"6833","POLB",4.34519206528401,0.481797646952896,0.670769321628291,0.7
18276211236574,0.47258699502219,0.927004608544132
"6834","BX664615.2",2.40113060919483,0.724953311117758,1.0065121717573
8,0.720262835820439,0.471363182194536,0.927004608544132
"6835","FAM225B",3.461641779962,0.628884333273041,0.875018093869863,0.
718710090315655,0.4723195648977,0.927004608544132
"6836","FAM225A",2.97833263048313,0.618378250059308,0.861998307490328,
0.717377568709723,0.473141156560838,0.927004608544132
"6837","CNTRL",21.6505415785531,0.269041203006244,0.374054677988351,0.
719256351646598,0.471982984280958,0.927004608544132
"6838","ZBTB26",5.05993776345236,0.47397807422401,0.658999312538456,0.
719239102690795,0.471993610257382,0.927004608544132
"6839","MTPAP",14.0191292412478,0.283200735563571,0.392428649700272,0.
721661723168972,0.47050248069615,0.927004608544132
"6840","SFR1",14.4554700743811,-0.290815743782167,0.403139322325095,-0
.721377766140239,0.47067712235209,0.927004608544132
"6841","BAG3",34.7037403395689,-0.194061744497949,0.270523702794305,-0
.717355789875113,0.473154591245627,0.927004608544132
"6842","AC048341.1",9.26698490695939,0.360295048780738,0.4983795006438
02,0.722933122881884,0.46972097257607,0.927004608544132
"6843","TBX5-
AS1",2.56021800642011,-0.777526290892497,1.0835936259785,-0.7175441717
74158,0.473038391263638,0.927004608544132
"6844","MED13L",129.222324797147,0.222712972166651,0.308430505179308,0
.722084775749325,0.470242357688782,0.927004608544132
"6845","GATC",24.1014430956882,-0.231123112478669,0.321673489812965,-0
.718502207356454,0.472447687351011,0.927004608544132
"6846","NUDT15",14.4835698217769,-0.290083574957781,0.402622269786493,
-0.720485667898126,0.47122602089695,0.927004608544132
"6847","OLFM4",5.94471127357349,0.533517408620945,0.744027799931185,0.
717066497609753,0.473333066544365,0.927004608544132
"6848","PCCA-
DT",6.17841536272695,-0.39700031071651,0.551099339833944,-0.7203788537
21969,0.471291766183378,0.927004608544132
"6849","AL359232.1",1.80112966393698,0.772681491668695,1.0733544654129
3,0.719875415407564,0.471601706046386,0.927004608544132
"6850","CYFIP1",73.9915838920592,0.156884830015837,0.218111093843102,0
.719288630631011,0.471963099621157,0.927004608544132
"6851","NSMCE3",44.8634129783138,-0.225343694149833,0.313358790441848,
-0.719123576626299,0.472064781861377,0.927004608544132
"6852","MAPK8IP3",8.38216478450666,0.459239714500621,0.636621348184592
,0.721370271057045,0.470681732526279,0.927004608544132
"6853","KLHL36",40.7177976432943,0.192650764279411,0.268426390440274,0
.717704261356061,0.472939655232772,0.927004608544132
"6854","RETREG3",36.3135069189782,0.191375544580611,0.266450104157842,
0.718241582924068,0.472608342473452,0.927004608544132

"6855","WIPI1",46.5903757102593,-0.186175796500517,0.259000137637622,-0.718825087116378,0.472248698276762,0.927004608544132
"6856","USP14",62.4299572425735,0.187509707002646,0.260866095101481,0.718796771691246,0.472266147075133,0.927004608544132
"6857","CYB5A",270.544769829697,-0.216055444822499,0.30055649124828,-0.718851367758427,0.472232503689397,0.927004608544132
"6858","MOB3A",23.4598508512144,-0.23457436268717,0.326222184296366,-0.719063184476944,0.472101989724797,0.927004608544132
"6859","COQ8B",14.3765315290388,-0.285478143255231,0.397081519355177,-0.718940895861435,0.472177337216201,0.927004608544132
"6860","ZNF649",5.8763002395143,0.481151390689279,0.671196415095831,0.716856317864245,0.473462757572114,0.927004608544132
"6861","PEG3",13.6522916393898,0.314508894141985,0.437337224063734,0.719145036911267,0.472051560476463,0.927004608544132
"6862","ADNP-AS1",1.8172433044565,0.827361235618648,1.15322100155738,0.717435109576856,0.473105662401841,0.927004608544132
"6863","ZBED1",48.1584143999948,0.223304987667315,0.309479074488812,0.721551167994683,0.470570471021932,0.927004608544132
"6864","SH3BGRL",241.118188363221,-0.173114984555392,0.239322467912804,-0.723354501836682,0.469462116370644,0.927004608544132
"6865","MRPL17",47.8256915234517,-0.199781896606184,0.278858217070458,-0.716428221857655,0.4737269738645,0.927373168501824
"6866","PPT2",3.91550246819256,0.545104715618941,0.761298336440329,0.716019843374065,0.473979096246323,0.927485800190116
"6867","MAGI2",22.7662447267481,0.300212379860281,0.419545341935116,0.715565994549189,0.474259377352306,0.927485800190116
"6868","ARPC1A",103.512804162951,-0.188117099665794,0.262897629864284,-0.715552664978024,0.47426761060395,0.927485800190116
"6869","AC012531.1",2.59052799224063,-0.793758042513307,1.10899272093742,-0.715746846239309,0.474147678783356,0.927485800190116
"6870","G2E3",20.5003152260967,-0.248660594347335,0.347437036253982,-0.715699733765748,0.474176775243748,0.927485800190116
"6871","ISYNA1",5.63605663701176,-0.409137087033009,0.571555469440181,-0.715830936643375,0.474095747351117,0.927485800190116
"6872","ZNF180",6.53118945749948,0.430567316630203,0.601317346311928,0.716040073134444,0.473966605176147,0.927485800190116
"6873","LPGAT1",30.315063507637,0.220286539820528,0.308206926529944,0.714735850685452,0.47477228060074,0.927527937053837
"6874","SLC35D2",24.6102717656722,0.228150736453785,0.319094914195055,0.714993333658466,0.474613162524078,0.927527937053837
"6875","AC007262.2",2.01748391698108,-0.762137094780385,1.06556280871035,-0.715243708348643,0.474458465278408,0.927527937053837
"6876","TECPR2",14.923341323413,0.287027491082089,0.401541285203135,0.714814395577991,0.474723738705245,0.927527937053837
"6877","SNAPC5",33.8318771824158,-0.231453429818872,0.323821495836673,-0.714756224631891,0.474759688941276,0.927527937053837
"6878","CXorf38",18.5471770051066,-0.249539396622045,0.348823075132052,-0.7153752558588,0.4743771980452,0.927527937053837
"6879","C1GALT1C1",69.2493292707247,-0.228799416783578,0.3199307488147

01,-0.715152943664366,0.47451454221383,0.927527937053837
"6880","CPT2",14.6788647791629,0.305642992006564,0.428467504897509,0.7
1333995813679,0.475635417104907,0.927784592350695
"6881","AL078459.1",1.62127394900363,-0.80894746938337,1.1323726082348
4,-0.714382760144977,0.474990529392132,0.927784592350695
"6882","CDC42SE1",25.8552269508868,0.222437735678717,0.311894028950257
,0.713183693921224,0.475732095088075,0.927784592350695
"6883","SCLY",4.287172425327,-0.468792987058324,0.6570038362157 5,-0.71
3531582644184,0.475516877026964,0.927784592350695
"6884","BCL6",65.0171183252451,0.231550348117914,0.324448340757447,0.7
13674009173059,0.475428781615204,0.927784592350695
"6885","TICAM2",16.5922439982858,0.288361810142526,0.404194609124325,0
.713423196730042,0.475583923217413,0.927784592350695
"6886","SMAGP",2.73862405884217,-0.624709705656065,0.875047109824939,-
0.71391551225287,0.475279424618419,0.927784592350695
"6887","AL049840.4",4.71307778406683,0.596053126103576,0.8346190924358
7,0.714161863184762,0.475127096008582,0.927784592350695
"6888","ZZEF1",21.1158172762703,-0.272321369898799,0.381328109119395,-
0.714139250126809,0.475141077447571,0.927784592350695
"6889","MYOM1",4.97361823701246,0.458501910576274,0.642854178721594,0.
713228482838939,0.475704383853219,0.927784592350695
"6890","RBFA",37.9135837815782,0.184512087511263,0.258520776817636,0.7
1372247052089,0.475398808746774,0.927784592350695
"6891","RALGAPA2",11.8528523389276,0.378716742104469,0.530772739268183
,0.713519580200436,0.47552430133543,0.927784592350695
"6892","ALDH4A1",2.50502888434048,0.672830415636701,0.94848596007064,0
.709373089282831,0.478092982918274,0.927811061001975
"6893","PAQR7",7.190046098568,-0.444850164450549,0.626621500417683,-0.
709918450219196,0.477754708340504,0.927811061001975
"6894","SFPQ",186.154691131391,0.181241161073107,0.255460942854953,0.7
09467204840048,0.478034595902411,0.927811061001975
"6895","BTBD19",5.00212802622479,0.499520714250098,0.701007505130683,0
.71257541551852,0.476108529070489,0.927811061001975
"6896","SETDB1",11.5544781653005,0.330558576323994,0.464862904170133,0
.711088308743634,0.477029514348539,0.927811061001975
"6897","LAPTM4A",1786.82816486316,-0.174171496341245,0.244486091314462
,-0.71239838391143,0.476218116003532,0.927811061001975
"6898","RAPH1",17.9334675112967,-0.254444519176974,0.358010270865653,-
0.710718490175543,0.477258699305921,0.927811061001975
"6899","AC069224.1",1.85465917141335,0.781008351818661,1.1002387219631
9,0.709853540170888,0.477794963654644,0.927811061001975
"6900","PPAT",8.31006400081353,0.405060064288227,0.57084678772653,0.70
9577548647388,0.477966146237363,0.927811061001975
"6901","PAIP1",79.7566366651203,-0.167695797091456,0.235738088850976,-
0.711364879170914,0.476858156764097,0.927811061001975
"6902","UQCC2",45.1796807582787,-0.241257293835519,0.339703636821367,-
0.710199325779912,0.477580538910633,0.927811061001975
"6903","UFL1",81.1244836404321,-0.142252735991301,0.199827925056757,-0
.711876160205823,0.476541465879888,0.927811061001975
"6904","GUSB",120.111158348339,-0.214039377673472,0.300441675043092,-0

.712415738072198,0.476207372738727,0.927811061001975
"6905","CEP41",10.5467073918411,0.389001233052706,0.54814629031162,0.709666816921373,0.477910774296863,0.927811061001975
"6906","RHOBTB2",7.54909532976706,0.377853325995502,0.531917381462387,0.710360930407424,0.477480344490691,0.927811061001975
"6907","DCTN6",69.6059060324743,-0.201305129866426,0.283404042418878,-0.710311427276299,0.477511035072512,0.927811061001975
"6908","WAC",119.878636537612,0.153666280341188,0.21657279301551,0.709536402064054,0.477991670104761,0.927811061001975
"6909","AL022345.4",2.06549022202601,-0.681336558212291,0.958287669475601,-0.710993765144798,0.477088099428765,0.927811061001975
"6910","AP002360.1",5.96948107494469,0.486029284172854,0.683235563314732,0.711364147695815,0.476858609927191,0.927811061001975
"6911","TMEM126A",39.4511929169501,-0.235718852959568,0.331983019409919,-0.710032860652164,0.477683758851351,0.927811061001975
"6912","PUS3",15.1857532187994,-0.293238845255368,0.412030309879733,-0.711692412485288,0.476655267179786,0.927811061001975
"6913","HOXC11",4.98721630966504,0.480390462070477,0.675807872721999,0.710838807094055,0.477184129569084,0.927811061001975
"6914","FRS2",34.4884669833486,0.200221640717576,0.282160022263088,0.709603150409765,0.477950265440939,0.927811061001975
"6915","RAN",174.812945544414,-0.1847713186786,0.260369092041292,-0.709651507519552,0.477920270268988,0.927811061001975
"6916","NPTN",124.698841069146,-0.156596631619928,0.220447139199489,-0.710359101000714,0.477481478653406,0.927811061001975
"6917","NME4",68.1423479090052,-0.216385903877358,0.30457534444816,-0.710451150500752,0.477424413286302,0.927811061001975
"6918","ZNF629",10.4359822765799,0.424143531217202,0.595197136813957,0.712610167259219,0.476087018508561,0.927811061001975
"6919","TK2",81.07125186884,0.180708382153799,0.254079962816959,0.711226419235514,0.476943939597137,0.927811061001975
"6920","AC073508.3",5.04610993653953,0.499045475141663,0.702059562039111,0.710830678941542,0.477189167015478,0.927811061001975
"6921","HSPB6",85.3712396139082,0.283834681847588,0.399503203788693,0.710469100512433,0.477413285751849,0.927811061001975
"6922","ADNP",47.2451839169402,0.178267204498247,0.250157515395463,0.712619823619662,0.476081041528745,0.927811061001975
"6923","BMP7",19.798995608216,-0.489201394268072,0.688594283957497,-0.710434875318058,0.477434502686864,0.927811061001975
"6924","SUN2",50.2305504026341,-0.209753548926842,0.294587437264096,-0.712024758675636,0.476449444609203,0.927811061001975
"6925","GPKOW",12.9143980837922,-0.314112079679952,0.441649758620181,-0.711224388894296,0.476945197557596,0.927811061001975
"6926","LINC01703",2.22200179209604,-0.660953482932871,0.932426645849753,-0.708853061926948,0.478415665584378,0.927852761286374
"6927","ZXDC",13.0168712745659,0.30053704686916,0.424288305319431,0.70833214845009,0.478739017372652,0.927852761286374
"6928","PDZD2",3.11021049690215,0.643604939721068,0.908485741299884,0.708437029292482,0.478673904051411,0.927852761286374
"6929","FNIP1",82.0096710935669,0.174490639321802,0.246415727855335,0.

708114862799025,0.478873930521787,0.927852761286374
"6930","FAM126A",64.5508496332977,-0.195167184622596,0.275600564854604
,-0.708152338967662,0.478850660002869,0.927852761286374
"6931","TBC1D10C",1.99541026964797,0.788703561100614,1.11282280309971,
0.708741372753794,0.478484985460163,0.927852761286374
"6932","AP1G2",4.80977645130487,-0.445195080888925,0.628243389438643,-
0.708634724014719,0.478551182112582,0.927852761286374
"6933","LSM4",61.8207806732461,-0.230012885900478,0.324739832752923,-0
.708298960280251,0.47875962263851,0.927852761286374
"6934","AC011484.1",1.68081616200316,0.766339649046094,1.0808490466130
8,0.709016352882465,0.478314328943761,0.927852761286374
"6935","Z95331.1",7.57582214126351,0.511890583793301,0.722401430725196
,0.708595750259561,0.478575374290276,0.927852761286374
"6936","AL050309.1",11.4336162505559,0.385714353672405,0.5443250225254
11,0.708610366436715,0.478566301512838,0.927852761286374
"6937","KIT",1.6369384952419,0.963525795876946,1.36101448807699,0.7079
46759066711,0.478978320750384,0.927921241540206
"6938","SERP1",181.40165872141,-0.153261703555765,0.216565831846186,-0
.707691062109084,0.479137129052046,0.927961360041858
"6939","TTC12",5.92792909675052,-0.503280765389331,0.711048322205184,-
0.707801072968571,0.479068799969453,0.927961360041858
"6940","MAML1",25.6500035654535,0.221302228460409,0.312983892505147,0.
707072260777028,0.479521573193754,0.928438326199519
"6941","ATP5PF",320.383529871873,-0.184713191687559,0.261234834523684,
-0.707077186028237,0.479518512607159,0.928438326199519
"6942","ZNF580",40.1262575750674,0.2067635533696,0.29252809445817,0.70
6816053865097,0.479680796712443,0.928612824404857
"6943","INPP5B",8.3488274760656,0.387226217216248,0.550056223000714,0.
703975704708545,0.48144790379401,0.928640895499594
"6944","UHMK1",36.746303041715,0.205537139008128,0.291111467304349,0.7
06042743390952,0.480161556625149,0.928640895499594
"6945","USP39",17.9048727625381,0.285761702008161,0.404706850426637,0.
706095539788651,0.480128725244211,0.928640895499594
"6946","RHOH",2.10939793645636,-0.69812374166132,0.992061784658874,-0.
703709942724357,0.481613427164712,0.928640895499594
"6947","CENPE",4.72628160003418,0.496488084361475,0.702846249471964,0.
706396434119807,0.479941637805876,0.928640895499594
"6948","ANAPC10",27.8728826374576,-0.238230384839941,0.338387245921683
,-0.704017032885091,0.481422166329026,0.928640895499594
"6949","AC107214.1",1.54013273184283,-0.811254411927077,1.150128250852
86,-0.705359955574959,0.480586257707937,0.928640895499594
"6950","CCDC127",43.2650146522966,0.191419539385875,0.271813820445767,
0.704230340723486,0.48128933900936,0.928640895499594
"6951","PDE4D",5.91097598874661,0.477330268355267,0.678759189715947,0.
703239492867897,0.481906511289097,0.928640895499594
"6952","FAF2",47.5846906672702,0.187459155359576,0.266166389723284,0.7
0429311362139,0.481250253969739,0.928640895499594
"6953","AL357054.4",1.86413721896515,-0.817868040951783,1.162885851143
98,-0.703308962050926,0.481863226790435,0.928640895499594
"6954","WAKMAR2",1.5425493115426,-0.764775793366259,1.0825579517119,-0

.706452520307928,0.479906769423919,0.928640895499594
"6955","ATXN7L1",9.1690680540005,-0.35479009787739,0.502991644438803,-0.705359824164149,0.480586339466515,0.928640895499594
"6956","RBM17",80.7773876132513,-0.15038267841347,0.213097060671747,-0.705700388073952,0.480374479593122,0.928640895499594
"6957","EDRF1-DT",8.85640222276566,-0.3897708697168,0.553240502792671,-0.704523381331081,0.481106894429252,0.928640895499594
"6958","LSP1",350.32427433739,0.155514535943127,0.220734094058105,0.704533373544865,0.481100674026236,0.928640895499594
"6959","OAS1",18.0498918300906,-0.300687960263626,0.427470511296304,-0.70341217070574,0.481798923981317,0.928640895499594
"6960","SIRT4",2.32173136636121,0.753596338179082,1.06845874540726,0.70531159150355,0.480616348420831,0.928640895499594
"6961","SLC15A4",42.6787531569903,-0.174791860131028,0.247974845100333,-0.704877384076217,0.480886545573643,0.928640895499594
"6962","CKAP2",11.9186843130677,0.344713726360122,0.489349282637938,0.704432883812298,0.48116323339253,0.928640895499594
"6963","ALG1",13.370517857926,-0.309363186989604,0.438903292270204,-0.704855015758574,0.480900467096285,0.928640895499594
"6964","YPEL3",185.665710734039,-0.170233326841798,0.241122201152579,-0.706004366367227,0.480185422102618,0.928640895499594
"6965","RANGRF",34.9995186761074,-0.224082115217973,0.318545374225606,-0.703454306196485,0.481772673354094,0.928640895499594
"6966","CENPV",10.053921378451,-0.384593825264507,0.546708404477854,-0.703471580305816,0.481761911720103,0.928640895499594
"6967","DNAJC7",75.4360568189984,-0.147490631038863,0.209474113952009,-0.704099557965684,0.481370775390695,0.928640895499594
"6968","ACOX1",30.3249172762408,0.219428143627999,0.310751993700639,0.706119825700566,0.480113623487989,0.928640895499594
"6969","USP36",24.4758542676063,0.253906129842994,0.360251890477057,0.704801658380651,0.480933676378312,0.928640895499594
"6970","CLPTM1",62.3350754375067,-0.16454884118794,0.23396641861357,-0.703301106898234,0.481868121032239,0.928640895499594
"6971","ZNF274",7.565884319202,-0.375767687461185,0.533949940360935,-0.703750780845066,0.481587990123871,0.928640895499594
"6972","U2AF1",7.26620566506247,0.432406845368034,0.613631122875665,0.704669025491474,0.481016231629116,0.928640895499594
"6973","SAMM50",38.7352604904475,-0.210462317447182,0.29901430165949,-0.703853682847756,0.481523898289846,0.928640895499594
"6974","PDK3",11.4029464599076,-0.328502556148859,0.465962438350898,-0.704997933549049,0.480811522153959,0.928640895499594
"6975","LINC02104",3.76202038190426,-0.614076133888084,0.873648449530246,-0.702886995585316,0.482126176055533,0.928664709762119
"6976","UHRF2",23.6375351419584,0.268469636614153,0.381920576403825,0.702946249039711,0.482089247436327,0.928664709762119
"6977","SMG9",24.4263150640775,-0.252325093697915,0.358976982221128,-0.702900481631674,0.48211777099145,0.928664709762119
"6978","ZNF684",11.0310364490603,-0.318465931070542,0.453264106759377,-0.702605669236467,0.482301528483182,0.928869338103394

"6979","ORC3",21.8394745507455,0.230401285544477,0.328161433154575,0.70209738947584,0.482618430240619,0.92894716184527
"6980","TTC17",70.3181539250454,0.156987240333408,0.223569815699425,0.702184415379524,0.48256416339071,0.92894716184527
"6981","RAMP2",472.539058323307,-0.251269321467282,0.357855871175193,-0.702152295677356,0.482584191926859,0.92894716184527
"6982","SNX21",140.751853625624,-0.182100202993742,0.25926586014156,-0.702368614573145,0.482449313010344,0.92894716184527
"6983","TRNAU1AP",38.1329635865808,-0.189521822038688,0.27032554541435,-0.70108735653596,0.483248500237823,0.929071140638773
"6984","AL604028.1",2.07180692762539,-0.697210201091549,1.00051776202433,-0.696849398936106,0.485897047596397,0.929071140638773
"6985","ADGRL2",17.2167801494711,0.262896780881589,0.375079244432194,0.700909967117935,0.48335920385365,0.929071140638773
"6986","AC118553.1",1.86571848437945,-0.759283426734416,1.08773651014646,-0.698039846646484,0.48515227505466,0.929071140638773
"6987","TMEM79",4.20900665520394,0.519472558484579,0.745280209008005,0.697016440535857,0.485792505086585,0.929071140638773
"6988","FCGR3A",2.95063196527424,-0.627243813527691,0.894307354404707,-0.701373873801155,0.483069722158816,0.929071140638773
"6989","LBR",21.7569554045819,0.24494268954605,0.351208027973442,0.69742907347372,0.485534312107898,0.929071140638773
"6990","SLC5A6",5.2694863658958,0.533080012358996,0.760106010470969,0.701323243094334,0.483101311570574,0.929071140638773
"6991","PRKD3",46.6720449516043,0.213718058980616,0.305534941977615,0.699488109599832,0.484247041295151,0.929071140638773
"6992","AC092683.1",13.6727661157787,0.329562960401037,0.471764166721716,0.698575652091523,0.484817264209699,0.929071140638773
"6993","PAX8-AS1",50.1171984906128,-0.516267746697702,0.740732031720465,-0.696969652437725,0.485821786057583,0.929071140638773
"6994","CDK15",2.21172739567946,0.700229658552785,0.998572077518095,0.701230962008444,0.483158890290889,0.929071140638773
"6995","IDH1-AS1",2.35360738313604,0.664271404016283,0.946611095672542,0.701736338241774,0.482843606815932,0.929071140638773
"6996","RAP2B",43.9872574442748,-0.181688924197313,0.260068697527543,-0.698618964622111,0.484790188648213,0.929071140638773
"6997","ATP5ME",296.461213066123,-0.200174613964046,0.286434965678415,-0.698848387765565,0.484646785144988,0.929071140638773
"6998","FAM200B",91.7579283028102,-0.184246637849737,0.264504586944694,-0.696572562230315,0.486070331857371,0.929071140638773
"6999","NAAA",47.4231191326186,-0.233899455355824,0.334653688453206,-0.698929859213339,0.484595866036342,0.929071140638773
"7000","ALPK1",20.9924936641622,-0.260406837595278,0.373515922702454,-0.697177340422834,0.485691817858434,0.929071140638773
"7001","USP53",202.491101794229,0.20939620289312,0.299860150310568,0.698312872438191,0.484981550784978,0.929071140638773
"7002","FAM193B",11.6268743558239,0.355552335694858,0.507445921960896,0.700670397194083,0.483508734376514,0.929071140638773

"7003","AL451165.2",7.31179741502544,0.390607855437411,0.560172008020021,0.697299846913183,0.485615163855323,0.929071140638773
"7004","TAF8",11.3586633313929,0.318328316455021,0.456183254610118,0.697807982292301,0.485297286441815,0.929071140638773
"7005","CUL9",9.86295074937063,-0.329607005604349,0.472886089685791,-0.697011421552612,0.485795646025616,0.929071140638773
"7006","AL391840.3",10.6863704346164,0.330473488767268,0.474158218626072,0.696968808691861,0.485822314100167,0.929071140638773
"7007","QRSL1",16.7939781635236,-0.259942086390625,0.37146549250534,-0.699774519128148,0.484068130136655,0.929071140638773
"7008","UTRN",180.070899221468,0.210796679375435,0.300908586960063,0.700533944561085,0.483593914191243,0.929071140638773
"7009","CPSF4",14.8292257228694,-0.273683313162655,0.392384140191365,-0.697488214047542,0.485497312712032,0.929071140638773
"7010","ADGRA2",47.5233058722244,0.168952050726152,0.241453905014259,0.699727969676754,0.484097205696796,0.929071140638773
"7011","GLI4",22.883493611251,-0.243767004116136,0.349474397014963,-0.697524643287959,0.485474522690259,0.929071140638773
"7012","LINC01504",13.6214599310238,0.338895540261175,0.482889231198537,0.701808030425594,0.48279889006032,0.929071140638773
"7013","OLFML2A",127.857902870272,0.178413237477767,0.25511991142459,0.699330900836029,0.484345259977641,0.929071140638773
"7014","STAM-AS1",1.73438492225319,-0.721078559917829,1.03157044120834,-0.699010490328884,0.484545474984395,0.929071140638773
"7015","C10orf25",1.98767789530525,-0.728174036782565,1.0393252090548,-0.700621932806571,0.483538987074592,0.929071140638773
"7016","SLK",91.5442684187868,-0.178644664987154,0.255513545613946,-0.699159273759469,0.484452499060821,0.929071140638773
"7017","CYP2R1",4.3756651511068,-0.530134514138383,0.756683270869781,-0.700602926676325,0.483550851462857,0.929071140638773
"7018","AC022081.1",7.51764911455159,-2.15871789000672,3.08942106382448,-0.698745119363685,0.484711331370268,0.929071140638773
"7019","ADAMTS7",17.1316910071941,-0.328748291886504,0.469921808283757,-0.699580836835718,0.484189113539396,0.929071140638773
"7020","MLLT6",43.078960312325,0.205596594293688,0.293480580953127,0.700545820190144,0.483586500570821,0.929071140638773
"7021","RAPGEFL1",2.20232980061242,-0.626227339290586,0.895798806394646,-0.699071415166298,0.484507401410179,0.929071140638773
"7022","WDR83",14.023611334592,-0.281440601219584,0.402700205524774,-0.698883679120122,0.48462472792905 5,0.929071140638773
"7023","ZNF83",57.8085862147092,0.199217753469023,0.284293634006391,0.700746445361998,0.483461265254856,0.929071140638773
"7024","ZNF471",8.7108664421883,0.417550426341693,0.597353890136267,0.699000095649233,0.484551971062009,0.929071140638773
"7025","CST3",5075.30631905653,-0.199680508662438,0.286588307624016,-0.696750367514661,0.485959031875909,0.929071140638773
"7026","MATN4",16.0499592969691,0.317316931499224,0.455284620781254,0.696963870544799,0.48582540455347,0.929071140638773
"7027","PRR14L",33.327334550733,0.25614465944578,0.366145245182962,0.6

99571175143309,0.484195149132842,0.929071140638773
"7028","PDHA1",37.9624164509143,-0.198914225143115,0.285210324413463,-0.697429959985439,0.485533757479117,0.929071140638773
"7029","YY2",2.8150698101386,0.636979913322738,0.91340882772594,0.697365619849098,0.485574011522255,0.929071140638773
"7030","DNASE1L1",1.83179047207517,-0.712152207968466,1.0223371949817,-0.696592290160403,0.486057982172926,0.929071140638773
"7031","VBP1",51.9487623423503,-0.194715570566982,0.278369951950429,-0.699484873287099,0.484249063124274,0.929071140638773
"7032","AAGAB",13.8479561374457,-0.317459088542709,0.455859244313878,-0.696397171939601,0.486180133642281,0.929148864621532
"7033","AC124016.1",2.86314300911216,-0.584892697685056,0.841416035790192,-0.695129012053794,0.486974454666893,0.929833483444472
"7034","HEXB",149.43160015827,-0.139142989419888,0.200053720350223,-0.695528127026574,0.486724390572686,0.929833483444472
"7035","ZNF25",22.1346354293185,0.229398609579143,0.330045227095581,0.695052043617976,0.487022686953318,0.929833483444472
"7036","STT3A",52.8390198172648,0.184495973966809,0.265319802268685,0.695372046825112,0.486822173815619,0.929833483444472
"7037","CCR10",4.30888809916614,-0.491672082317537,0.707000284472408,-0.695434065750685,0.486783318086579,0.929833483444472
"7038","ZNF236-DT",2.16244283433311,0.71961225376436,1.03525651585551,0.695105264002798,0.486989336112762,0.929833483444472
"7039","CLCN5",11.0944577767885,0.406108656403015,0.583778498356596,0.695655385640714,0.486644671724338,0.929833483444472
"7040","PDHB",52.8800567361007,-0.199433648442061,0.287210874697886,-0.69438056150152,0.48744358079758,0.929857240763508
"7041","RBM47",7.01161079107872,0.511165952357938,0.735756715623871,0.694748605759588,0.487212861488044,0.929857240763508
"7042","FEN1",4.56532144219182,-0.442423933843664,0.637127148317519,-0.694404460729677,0.487428597076786,0.929857240763508
"7043","CLEC2D",12.900145029528,0.330067130892919,0.475306404705926,0.694430219380555,0.487412447861598,0.929857240763508
"7044","NUP88",14.6129072139714,-0.288375888277436,0.415305870204701,-0.694369882456267,0.48745027614993,0.929857240763508
"7045","TGIF2",5.31557539423597,0.479787958639277,0.690520614723492,0.694820615647225,0.487167726882579,0.929857240763508
"7046","SHLD2",31.9608729768016,-0.196858655945821,0.283571873717929,-0.694210795184989,0.487550023654352,0.929915521982803
"7047","CEBPZ",61.5406150992567,0.196334719204518,0.282921820301273,0.693953965782662,0.487711078591417,0.930074422490518
"7048","SIAH1",29.2806359505913,0.220350829164107,0.317573731242018,0.693857229004185,0.487771748620669,0.930074422490518
"7049","SNAPC4",16.3472944389126,0.284653410861044,0.4103510086284,0.693682737158362,0.487881194290886,0.930130178609474
"7050","CDK2",4.65689104740507,-0.439204273611915,0.633253263384075,-0.693568117225055,0.487953094002782,0.930130178609474
"7051","AL110292.1",5.46738274049898,-0.563603069673841,0.812717589997367,-0.693479600553086,0.488008623363003,0.930130178609474

"7052","DNAJA2",80.0804659056465,-0.151936513244621,0.219134923176265,
-0.69334687069668,0.488091895466167,0.930156974357603
"7053","FBXO28",18.4025398859708,0.241264956248376,0.348209881254942,0
.692872228033453,0.488389739642563,0.93044933167332
"7054","RITA1",11.366493006702,-0.319904001479369,0.461774250027201,-0
.692771416900195,0.488453012497602,0.93044933167332
"7055","KLHL11",2.72138656256089,0.637837292726582,0.92042639444507,0.
692980228050867,0.488321959677095,0.93044933167332
"7056","KANSL3",16.8329043067046,0.256022524438704,0.369945905772222,0
.692053947466412,0.488903450893076,0.930779639687214
"7057","RBM15B",32.4881726649738,0.190047517661927,0.274568739753165,0
.692167352455266,0.488832238600296,0.930779639687214
"7058","AMER1",1.63686602384811,0.742436273351386,1.07269087329146,0.6
92125095716795,0.488858772928035,0.930779639687214
"7059","ZCCHC18",2.15980406735889,-0.677679086795302,0.978859004053115
,-0.692315322216243,0.488739329876421,0.930779639687214
"7060","FXR1",115.873298412314,0.166558977029494,0.240724388643166,0.6
91907363305802,0.488995506219626,0.930823032306736
"7061","HDDC2",77.4899251652706,-0.177534774833818,0.256685094854338,-
0.691644269156197,0.489160753613417,0.930923252608554
"7062","GCH1",2.98260216832635,-0.600180525739343,0.867810798948302,-0
.691602969756427,0.489186696176919,0.930923252608554
"7063","APH1A",99.2037133052967,-0.148132299665612,0.214371511618366,-
0.691007394346896,0.489560894235389,0.931503448623728
"7064","LINC00115",1.79884781924778,0.834494056045086,1.21008961364438
,0.689613435761897,0.49043731583374,0.93163114846732
"7065","AC254633.1",3.03972584785463,-0.682316871139932,0.992138566879
858,-0.687723362358271,0.491627005101365,0.93163114846732
"7066","DDOST",159.421480931854,-0.185038608926063,0.268334977890514,-
0.689580651694102,0.490457938262646,0.93163114846732
"7067","CDC42",395.399166413598,-0.14614024253752,0.211833402322614,-0
.689882902956703,0.490267828358109,0.93163114846732
"7068","MMACHC",2.46257652255891,0.64973161004914,0.941862104688653,0.
689837298702998,0.490296509968462,0.93163114846732
"7069","EFCAB7",15.165624098727,0.289194592512221,0.421385804134327,0.
686294103111344,0.492527666772076,0.93163114846732
"7070","DNAJB4",34.8449866560456,-0.222404533226574,0.323823922954572,
-0.68680698818467,0.492204365503951,0.93163114846732
"7071","DBT",22.7965682091019,0.244911237285384,0.355592267062413,0.68
8741741513736,0.490985803331514,0.93163114846732
"7072","ATP1A1-
AS1",5.69441646407822,-0.503903590799269,0.732369831723865,-0.68804525
933731,0.4914242806274,0.93163114846732
"7073","C1orf43",99.9416941329068,-0.180545815677858,0.261673774549752
,-0.689965266823219,0.490216030031011,0.93163114846732
"7074","AL359541.1",2.02404699317815,0.690992048047819,1.0088916149206
4,0.684902161767071,0.493405661779437,0.93163114846732
"7075","RPL37A",2770.49474943303,-0.180320196623533,0.262759532954325,
-0.686255583560038,0.492551952479503,0.93163114846732
"7076","ABHD6",7.08727603713787,0.385904191563667,0.559529088162642,0.

689694601635248,0.490386261410078,0.93163114846732
"7077","FILIP1L",260.246933555997,0.196174029320573,0.28663956637856,0
.6843927089308,0.493727218793609,0.93163114846732
"7078","STAG1",83.1225367242018,0.162268570239912,0.236577259670253,0.
685900963034595,0.492775562872692,0.93163114846732
"7079","KPNA4",63.4970757946982,-0.152267163615374,0.221860404524598,-
0.686319688011257,0.492511536425147,0.93163114846732
"7080","MRPL47",48.9288627952102,-0.213129338018257,0.311255545323895,
-0.684740693684584,0.493507565244513,0.93163114846732
"7081","STK32B",17.5997405662118,0.342684937945903,0.498392212630513,0
.687580843483112,0.49171677506043,0.93163114846732
"7082","TAPT1",19.2928819035156,0.274067114373632,0.399511347028715,0.
686005832905501,0.492709430202958,0.93163114846732
"7083","RAPGEF2",16.0526183882979,-0.281671419927029,0.409728685833039
,-0.687458383233162,0.491793917495932,0.93163114846732
"7084","MAP1B",320.882291533228,-0.205818365040262,0.300222104950962,-
0.685553667255381,0.492994607278577,0.93163114846732
"7085","AC008771.1",7.87496519977772,-0.382599639392076,0.558741185625
949,-0.684752886013683,0.493499870199969,0.93163114846732
"7086","AP3S1",251.046603927853,0.150538014016962,0.218678447110871,0.
688398952918478,0.491201583035878,0.93163114846732
"7087","GRPEL2",8.68239683119994,0.363679348687819,0.528450127932351,0
.688199944450341,0.491326878913116,0.93163114846732
"7088","MCM3",24.7677003074399,0.233405268753227,0.338040708425279,0.6
90464973406653,0.489901829543026,0.93163114846732
"7089","SAMD5",1.52214767752352,-0.792240204465099,1.15451120545322,-0
.686212659282156,0.492579016023884,0.93163114846732
"7090","TMEM242",55.5710319332241,-0.177916365247382,0.259533692556857
,-0.685523191592573,0.493013831203683,0.93163114846732
"7091","HOXA7",19.5874108931193,0.289579304467993,0.420402948600131,0.
688813685613391,0.490940522187374,0.93163114846732
"7092","KRIT1",52.8045562741927,0.169264213565844,0.246058158149128,0.
687903277985438,0.491513692195766,0.93163114846732
"7093","DNAJC2",36.5461949536135,-0.23228397285058,0.338002884150993,-
0.687224824823724,0.491941062952669,0.93163114846732
"7094","ZNF775",7.68905592794132,0.385531853528429,0.563215249511778,0
.684519557775871,0.493647143710845,0.93163114846732
"7095","DNAJB6",122.866159487853,-0.150083500972852,0.218959499733973,
-0.685439550031846,0.493066594026362,0.93163114846732
"7096","FRA10AC1",50.8110444818876,-0.166614659454555,0.24126539648415
1,-0.690586639785701,0.489825345785132,0.93163114846732
"7097","MARVELD1",87.8734020173097,0.177964690220123,0.259043777634364
,0.687006234410761,0.492078799734951,0.93163114846732
"7098","ERLIN1",36.4706682663362,0.19425084928529,0.283690356144006,0.
684728419836328,0.493515311803798,0.93163114846732
"7099","HIF1AN",14.0072810532673,0.360145011664817,0.525640044221426,0
.685155203877705,0.493245988069859,0.93163114846732
"7100","ACSL5",14.0583372409215,-0.301306310049388,0.436958178789493,-
0.689554114501525,0.49047463151024,0.93163114846732
"7101","C11orf49",23.0167569493528,-0.237673198041176,0.34731378121456

8,-0.684318362519406,0.493774154222463,0.93163114846732
"7102","MRPL16",57.466346899001,-0.193968413288126,0.282881796849324,-0.685687150776415,0.492910411144501,0.93163114846732
"7103","PLBD1-AS1",2.95260105363272,0.620045409849679,0.900580004469558,0.688495643665646,0.491140712666073,0.93163114846732
"7104","CACNB3",5.87634272228386,0.421907215832055,0.613884254874349,0.687274860826024,0.491909537499193,0.93163114846732
"7105","DDIT3",55.7256952718791,-0.216400749598729,0.315329666392157,-0.686268285742593,0.492543943969238,0.93163114846732
"7106","ACAD10",9.95946848464017,-0.323890650241601,0.470955836463681,-0.687730409444832,0.491622566502346,0.93163114846732
"7107","ARL6IP1",112.853883454189,0.160068045816989,0.232057063671582,0.689778812523133,0.490333294652178,0.93163114846732
"7108","KLHDC4",10.9083713468534,0.318175315956255,0.462616889422271,0.687772805600766,0.491595863788386,0.93163114846732
"7109","NXN",6.84251246438718,0.438336828889509,0.639935076617503,0.684970780483578,0.493362359512172,0.93163114846732
"7110","NBR2",5.48947127621815,0.442778535438634,0.643713038451349,0.687850810826941,0.491546735159783,0.93163114846732
"7111","TACO1",15.6049100299619,0.272647544360747,0.397126022897864,0.686551695532851,0.492365277068886,0.93163114846732
"7112","C18orf32",149.382224404842,-0.188898110437809,0.275968739019811,-0.684490972088867,0.493665188213144,0.93163114846732
"7113","ZNF57",2.84952133370497,-0.59342504955885,0.863214841887237,-0.687459275215254,0.491793355578522,0.93163114846732
"7114","UBXN6",88.0915930829283,-0.154730274422433,0.226059562658701,-0.684466839635717,0.49368042191955,0.93163114846732
"7115","SUGP1",12.9651028018543,0.293308715987209,0.426655860417785,0.687459714487452,0.491793078852581,0.93163114846732
"7116","ZNF573",8.37990073433567,0.363122160336458,0.525728924175631,0.690702268104901,0.489752663719198,0.93163114846732
"7117","ZNF225",5.2071758953936,0.465515989131433,0.680283142536833,0.684297405041501,0.493787385261754,0.93163114846732
"7118","CARD8-AS1",2.44993257633801,0.741594431547772,1.07786270357955,0.688023093372617,0.49143823888288,0.93163114846732
"7119","ZNF841",7.39947476680937,0.407422539377929,0.59280466819403,0.687279573251567,0.491906568465887,0.93163114846732
"7120","SOX12",5.79744606705819,0.40893075731147,0.592599527334558,0.690062577590624,0.490154835447204,0.93163114846732
"7121","L3MBTL2",13.4442734789875,-0.299277837417159,0.437058607619629,-0.684754475028254,0.493498867316918,0.93163114846732
"7122","RAP2C",29.5668147757103,0.201142411871818,0.292630488000395,0.687359725387009,0.491856070602874,0.93163114846732
"7123","CETN2",66.1214754489056,0.189984864433342,0.276458808902019,0.687208576163242,0.491951300742138,0.93163114846732
"7124","TP53I3",100.561549746022,-0.223746804838144,0.32709358130705,-0.684045232389039,0.493946603755118,0.931774741889349
"7125","CCS",82.3512871349286,0.1666794809227,0.243713122133256,0.6839

16727436466,0.494027750655864,0.931774741889349
"7126","PHC1",18.0616042712513,0.249483085653691,0.364822713326206,0.6
83847459438787,0.494071494211139,0.931774741889349
"7127","AIDA",120.174202404563,0.137372019841764,0.201315832631614,0.6
82370671228524,0.495004596175778,0.931837187953736
"7128","PPP1R7",79.5129544961326,-0.179567875965846,0.2629071230971,-0
.683008789760045,0.494601288370022,0.931837187953736
"7129","TRAM1L1",4.37933987332303,0.481432435167633,0.705216882837532,
0.682672872536072,0.494813575048229,0.931837187953736
"7130","AC025171.1",11.2905235092232,0.300975855227178,0.4413590171380
38,0.681929774945657,0.49528335691298,0.931837187953736
"7131","SUMO4",2.80754683803446,0.635841118685772,0.930229098160162,0.
683531744968374,0.494270897856606,0.931837187953736
"7132","AC002074.1",3.24514291396954,-0.604063638812022,0.885563413922
821,-0.682123526463422,0.495160845404517,0.931837187953736
"7133","SNRPF",115.14466216394,-0.170801100495319,0.25019872442523,-0.
68266175572115,0.494820601277159,0.931837187953736
"7134","POSTN",252.537883809332,-0.292430617421745,0.428454689293432,-
0.682524021160779,0.494907658921561,0.931837187953736
"7135","EAPP",104.764493461686,0.156583888318374,0.229340264827888,0.6
82757946738592,0.494759806842097,0.931837187953736
"7136","ADAT1",10.119373073,0.346690319896278,0.507308444978379,0.6833
91580266426,0.494359438960805,0.931837187953736
"7137","CHMP1A",40.6252893216515,-0.199670581501095,0.292667943628494,
-0.682242745910541,0.495085469502677,0.931837187953736
"7138","SOCS3",355.45546976284,-0.437807346857185,0.640573546324197,-0
.683461484429781,0.49431527990839,0.931837187953736
"7139","MYL12A",490.944875486368,-0.194332995360435,0.284934538169912,
-0.682026814329369,0.495221995673087,0.931837187953736
"7140","SLC35E1",41.5527584176087,-0.197122773296434,0.289057556930398
,-0.681949904336523,0.495270628093741,0.931837187953736
"7141","LINC00662",31.1125889145151,-0.194209454183112,0.2842690144380
87,-0.683188966504157,0.49448744370988,0.931837187953736
"7142","URI1",87.0563634390957,-0.148841475954585,0.217833694790218,-0
.6832803166559,0.494429729477581,0.931837187953736
"7143","RTL10",8.53467326870696,0.353827070236085,0.518405583766015,0.
682529435091475,0.494904236793251,0.931837187953736
"7144","UBE2G1",27.2170918997146,0.240224588762518,0.352348971351694,0
.68178030388725,0.495377880394403,0.931884565316404
"7145","ENOX1",1.57817187054332,0.793529163229424,1.1641586556147,0.68
163317723255,0.495470930714137,0.931929158553853
"7146","OAF",297.323382362401,0.215688010469685,0.316485835866966,0.68
150920523454,0.495549344105443,0.931946212627072
"7147","CCDC30",10.0945654964741,-0.34792305731804,0.511875554430112,-
0.679702428269687,0.496692898167446,0.932036766570291
"7148","MOB3C",11.6453217112173,0.297165874349898,0.438907412274612,0.
677058226950084,0.498369011493588,0.932036766570291
"7149","GTF2B",33.8825731835223,0.210910954419406,0.310030011930865,0.
680292056584633,0.496319553184883,0.932036766570291
"7150","MUC1",1.94724112237187,0.76846429633161,1.12889716973421,0.680

721253391518,0.496047885503279,0.932036766570291
"7151","METTL18",11.4861345965233,-0.364702057402956,0.538202903234772
,-0.677629301534756,0.498006762727558,0.932036766570291
"7152","AL136454.1",2.12914132108281,0.788846760292235,1.1594904151737
1,0.680339181737912,0.49628972061115,0.932036766570291
"7153","VPS54",18.2573763200786,0.265134817430963,0.38956707900008,0.6
80588354928494,0.496131997446287,0.932036766570291
"7154","GALNT15",280.013371796229,-0.432543164506597,0.639048201663652
,-0.676855303528819,0.498497765298027,0.932036766570291
"7155","PTPN23",30.7424345122526,0.19577329923227,0.288425123141043,0.
678766457998649,0.497285849678982,0.932036766570291
"7156","RHOA",679.550167628332,-0.122416691186725,0.180555124258959,-0
.678001755359487,0.497770580237632,0.932036766570291
"7157","GNB4",54.3009909288581,0.194307198407931,0.286202256920617,0.6
78915674874728,0.497191293244935,0.932036766570291
"7158","NAF1",10.7628956326816,-0.30232170794217,0.444843953460897,-0.
679612941999324,0.496749572792035,0.932036766570291
"7159","BDP1",83.719769853103,0.198886624002037,0.292069162945322,0.68
0957284214458,0.495898519454961,0.932036766570291
"7160","VPS37D",5.37174494203069,-0.430081917675055,0.633939468998333,
-0.678427418874256,0.497500729146398,0.932036766570291
"7161","REEP4",8.99616705977594,-0.334256743938165,0.492766348781188,-
0.678327050467059,0.497564351081298,0.932036766570291
"7162","LRRC6",8.15772434603006,0.376129989839739,0.553330974853193,0.
679755891018992,0.496659040079058,0.932036766570291
"7163","TSNARE1",1.4899093315744,-0.841806453984717,1.23713487142095,-
0.680448408198078,0.496220578488682,0.932036766570291
"7164","C8orf31",4.95735007153732,0.463027107242401,0.680203857532183,
0.680718143708106,0.49604985354681,0.932036766570291
"7165","ZBTB34",5.83995160785392,0.412386891513168,0.608382225442767,0
.677841781477166,0.497872016460123,0.932036766570291
"7166","TUBGCP2",53.5941221543213,0.167606372260845,0.246119173769792,
0.680996810177884,0.495873508809922,0.932036766570291
"7167","ZNF408",5.12289595375062,0.42434968798513,0.626303509390271,0.
677546399824982,0.498059340932858,0.932036766570291
"7168","JRKL",17.8846902026039,-0.265134760227446,0.391476675187613,-0
.677268345809828,0.498235710863031,0.932036766570291
"7169","SDHD",143.419330435647,-0.188205675198979,0.278111990742992,-0
.676726216284947,0.498579679654277,0.932036766570291
"7170","AC092821.3",12.9016135337467,-0.304643766466028,0.449825042656
558,-0.677249458293552,0.498247692437712,0.932036766570291
"7171","PRPF40B",6.3032307947003,0.395209418214545,0.582061810920722,0
.678981872370893,0.497149347979325,0.932036766570291
"7172","UHRF1BP1L",32.7054577900503,-0.196345215582729,0.2897027092647
16,-0.677747253662438,0.497931959817347,0.932036766570291
"7173","DDX51",13.4310069460904,-0.282823854412619,0.417893889573699,-
0.676783895311639,0.498543077629412,0.932036766570291
"7174","TMCO3",66.5805576949727,0.173325334862937,0.255089820729636,0.
679467861034879,0.496841464705278,0.932036766570291
"7175","PSMA6",144.517409053494,-0.170603037797217,0.250800347009656,-

0.680234456735615,0.496356018062658,0.932036766570291
"7176","SEC23A",94.1621275387875,-0.148570534625605,0.219459760911774,
-0.676983033282957,0.498416719402722,0.932036766570291
"7177","DHRS7B",45.1950018028267,-0.192538577879898,0.283681381200166,
-0.678714186547344,0.497318975557082,0.932036766570291
"7178","PROCA1",1.8943855044501,0.682382004654081,1.0058234455171,0.67
8431197538114,0.497498333996169,0.932036766570291
"7179","KRT27",12.6799725226507,0.520027362007975,0.768055881195333,0.
677069696020882,0.498361734958789,0.932036766570291
"7180","MPP2",3.2254038451224,0.551499306248703,0.812997913657899,0.67
8352671001771,0.497548110220613,0.932036766570291
"7181","H3F3B",1498.50105434369,-0.143746149327771,0.211595568963922,-
0.67934385408742,0.496920015861118,0.932036766570291
"7182","DSG3",1.58435588113825,-0.992180516697695,1.46356514938418,-0.
677920294231637,0.497822231726395,0.932036766570291
"7183","RPS28",2514.02337321331,-0.189507647117776,0.279725745939923,-
0.677476599377723,0.498103612300908,0.932036766570291
"7184","ZNF440",3.4023610381989,0.53412682843262,0.787167124983928,0.6
78543109182215,0.497427400277125,0.932036766570291
"7185","ZNF772",1.61207683273403,0.791276929838809,1.16780304389304,0.
677577382570415,0.498039690604673,0.932036766570291
"7186","GSS",18.2857959841002,0.238861541316199,0.350989060692313,0.68
0538421468331,0.49616360248962,0.932036766570291
"7187","CCDC117",19.2947460806973,-0.276473680271488,0.408020103749647
,-0.677598181390412,0.498026499505456,0.932036766570291
"7188","TCN2",25.1692237147012,0.254674243097662,0.37491360402502,0.67
9287815548743,0.496955515172204,0.932036766570291
"7189","TSPYL2",16.8252065495767,0.307001937620097,0.451953044301827,0
.679278392945336,0.496961484338687,0.932036766570291
"7190","SEMA4F",3.07858155199915,0.632466513674268,0.934829645122436,0
.676558041322526,0.498686408476092,0.932106626357469
"7191","AC005229.4",2.53640411676259,0.701492277944475,1.0374396035151
2,0.676176497954806,0.498928592247382,0.932299965407754
"7192","HSPA5",219.767131397739,-0.159410611308902,0.235718106461798,-
0.676276479994279,0.498865122835987,0.932299965407754
"7193","WDR1",68.6232394115487,-0.156592025043779,0.231700577300525,-0
.675837871740271,0.499143586796495,0.932572037113596
"7194","CCND3",17.6701825157585,-0.247948067460222,0.367107402467802,-
0.675410154612636,0.499415215697434,0.932949830936588
"7195","MRPS21",165.824567674438,-0.202084714382416,0.299600328955515,
-0.674514327427263,0.499984379992888,0.9331051357762
"7196","ELF3-
AS1",2.09828690614236,0.820431067437432,1.21579772611793,0.67480885168
0525,0.499797215874777,0.9331051357762
"7197","RABIF",13.5447057790105,-0.291630644850878,0.432047969897893,-
0.674995984635225,0.499678316046259,0.9331051357762
"7198","PDCD6",158.00786295383,-0.15822314207097,0.2343731624917,-0.67
509069890446,0.499618142576249,0.9331051357762
"7199","USP20",5.30094547228835,0.465879274201158,0.690678016154522,0.
674524544439719,0.499977886668503,0.9331051357762

"7200","SLC38A2",143.524844694265,-0.187069756746205,0.277244465226324
,-0.674746587252856,0.4998367805405,0.9331051357762
"7201","RPS6KA5",42.9082443261587,-0.273166513404422,0.404852661237662
,-0.674730684909748,0.499846885627874,0.9331051357762
"7202","C1orf174",29.8204057773492,-0.207582190783834,0.30817327089364
9,-0.673589212269713,0.500572513490033,0.93318502065899
"7203","ACY1",8.99366438427792,0.356581490291813,0.529473705994529,0.6
73464019562658,0.500652131777016,0.93318502065899
"7204","ADAM8",1.46501840299223,-0.906851106218767,1.3458236548,-0.673
826101201596,0.500421878589441,0.93318502065899
"7205","NDRG2",26.6989045604273,-0.299317797661746,0.444230024477511,-
0.673790111359075,0.500444762560501,0.93318502065899
"7206","RFX7",14.3121888199168,0.279742131187561,0.415375301620008,0.6
73468379310317,0.500649359013545,0.93318502065899
"7207","RCCD1",8.15862178027522,0.353361556023141,0.524516915496472,0.
673689533327392,0.500508717568358,0.93318502065899
"7208","BCAT2",15.4418340005744,-0.254436908523241,0.377612950586607,-
0.673803449081879,0.50043628176785,0.93318502065899
"7209","LINC01426",4.45029125340623,0.496423130733544,0.73696207081072
7,0.673607435708913,0.500560924564694,0.93318502065899
"7210","SLC25A17",14.1061240748136,-0.300538581368683,0.44574289882140
6,-0.674241995023006,0.500157474735221,0.93318502065899
"7211","ZNF703",43.1248794062402,0.211526082093306,0.314173737598565,0
.673277415579475,0.500770817998834,0.933276802535894
"7212","LINC01355",5.87280854950265,0.475706826259193,0.70730944754244
9,0.672558281120152,0.501228350429673,0.933999972465942
"7213","RFK",30.1993374282136,-0.187855460800717,0.279466617945891,-0.
672192844288432,0.50146093586769,0.934093182094025
"7214","LETMD1",54.7885754707037,-0.183095934028965,0.272402543670114,
-0.672152071570589,0.50148688956086,0.934093182094025
"7215","ARHGAP17",26.2990743016302,0.219018698440316,0.325804478394135
,0.672239680436075,0.50143112340295,0.934093182094025
"7216","ZNF383",12.4505171311336,0.338576718982556,0.504004791356213,0
.67177281801526,0.501728335808987,0.934413401460223
"7217","ASIP",2.62478905034384,-0.721283426806424,1.07421857970573,-0.
671449405580021,0.501934280223557,0.934667423018482
"7218","NAGPA",15.04164385446,0.28111235899485,0.418944499036667,0.671
001432507761,0.502219616951735,0.935069192603819
"7219","ATP10D",27.1258097519687,-0.212559373576734,0.316855994892135,
-0.670839046769792,0.502323069834105,0.935132253151481
"7220","PDIK1L",6.0314530977755,0.433266752920028,0.646156338172804,0.
670529293491443,0.502520439041008,0.935158318594466
"7221","SLC20A2",14.8643602320617,0.264262586535151,0.394190773058739,
0.670392623562937,0.502607535692293,0.935158318594466
"7222","EEF1AKMT3",8.93480936766139,0.339843723541463,0.50690219597505
2,0.670432533612833,0.502582101096057,0.935158318594466
"7223","PRDX2",232.821544140945,-0.18130090521145,0.270444873056365,-0
.670380263314011,0.502615412992621,0.935158318594466
"7224","MARC1",1.63426387529818,0.873330258796144,1.30305505048979,0.6
70217469682403,0.502719168962291,0.935221886999478

"7225","ADGRL1",5.79465687076724,0.423708295925573,0.633048468795286,0
.669314147038228,0.503295104247146,0.936163724010712
"7226","NFIA",643.962083638648,0.16077271295511,0.240540384709386,0.66
8381374501213,0.503890181561676,0.936431695124446
"7227","SPSB4",21.9055774513006,0.284954258126169,0.426210921378433,0.
668575683618295,0.503766188308709,0.936431695124446
"7228","ARFIP1",45.8016636116734,0.174082113681625,0.260473708609043,0
.668328925062118,0.503923653552184,0.936431695124446
"7229","PHAX",74.5288287221546,-0.136311536856512,0.2039274971505,-0.6
68431372724166,0.503858274971692,0.936431695124446
"7230","NT5C3A",37.3661139783628,-0.180650415130223,0.270112104309647,
-0.66879792592757,0.503624389970519,0.936431695124446
"7231","FERMT2",214.847803256178,0.145719530961455,0.218005678085892,0
.668420805553709,0.503865018369997,0.936431695124446
"7232","HCFC1",23.9340856060657,0.245267040617563,0.36698834335158,0.6
683237902807,0.503926930511198,0.936431695124446
"7233","ARMC8",25.1374068466867,0.205412354987385,0.307651078053344,0.
667679620325492,0.50433812162172,0.936852926478081
"7234","ZFAND1",36.049531056223,-0.198252429487378,0.296957009178033,-
0.667613234778106,0.504380507368466,0.936852926478081
"7235","RNF208",4.48855779468801,0.525774797222394,0.787639986044418,0
.667531875651554,0.504432455985966,0.936852926478081
"7236","MIS18BP1",37.494502358725,0.204196324441424,0.305780669814999,
0.667786896290616,0.504269632192012,0.936852926478081
"7237","IFFO2",6.55418860001147,-0.372308493755585,0.557902175505992,-
0.66733651543466,0.50455720696273,0.93695513394668
"7238","NRBP1",97.4806165023625,-0.152762712511365,0.229053504886284,-
0.666930255388172,0.504816684108825,0.937307463075227
"7239","ESRRA",39.9373569803116,-0.206193604637391,0.309228352656968,-
0.666800449783224,0.504899605406111,0.937331923891798
"7240","UBXN11",13.1299825791925,-0.310113089538913,0.466137631723265,
-0.665282243770913,0.505869985377469,0.937672531255002
"7241","F3",136.286432968736,-0.279636672515523,0.419986465221147,-0.6
65823057817536,0.505524204949194,0.937672531255002
"7242","ZNF184",7.09776210592967,-0.383311360117944,0.575586070005929,
-0.665949681711366,0.505443263378493,0.937672531255002
"7243","NAPRT",34.4380013068937,0.23112303269329,0.346977817537171,0.6
66103194532113,0.505345142799034,0.937672531255002
"7244","HINT2",70.4669336349715,-0.203371036168177,0.305360037184702,-
0.666004098123568,0.505408480967019,0.937672531255002
"7245","ITGA11",268.062182259627,-0.199547442196792,0.299854880611462,
-0.665480054184464,0.505743496842119,0.937672531255002
"7246","ZNF205",21.2374415002422,-0.264388365622664,0.39688518125266,-
0.666158320117153,0.50530991070248,0.937672531255002
"7247","ZNF653",2.07875054799097,-0.659611295338095,0.99107925023013,-
0.665548486848991,0.505699741912332,0.937672531255002
"7248","PGLS",196.66168821617,-0.197180939449697,0.296355785538721,-0.
665352083784219,0.505825324750498,0.937672531255002
"7249","ALKBH6",11.3145011043333,0.31041920372113,0.465990305267178,0.
666149488975203,0.505315554812164,0.937672531255002

"7250","RBM10",24.7446918667943,0.214794862181432,0.322741236151562,0.665532749216347,0.505709804167568,0.937672531255002
"7251","IKBKG",20.8436843077161,-0.234515060227204,0.352546344231082,-0.665203494702778,0.5059203455711,0.937672531255002
"7252","SUPT7L",20.7515994031111,0.224092525291079,0.336948412115216,0.665064791029353,0.506009053275495,0.937707620927933
"7253","PLCL1",3.3389549195159,-0.513386701282652,0.772233863796592,-0.664807288764377,0.506173760119933,0.937883518854513
"7254","BRD9",43.2554850743992,0.235594730544583,0.354441245983508,0.664693325662063,0.506246663637783,0.937889290409176
"7255","SNHG3",17.1242668605578,-0.261529454537255,0.393665159834095,-0.664344933769281,0.506469568216667,0.938083037623095
"7256","METTL8",19.439918828281,0.240465077513123,0.362048199322502,0.664179736187347,0.506575281298146,0.938083037623095
"7257","BBS12",3.87508656883053,-0.49121146729352,0.739944753661787,-0.663848841231251,0.50678706217255,0.938083037623095
"7258","WIPI2",74.0401325027455,-0.138305859783896,0.208274528149352,-0.664055566529566,0.506654747465081,0.938083037623095
"7259","CLDN15",5.72130481129074,-0.398651743883504,0.600533145476074,-0.663829710127775,0.506799307970089,0.938083037623095
"7260","CDNF",3.15342911860416,0.566725860783672,0.853483363073339,0.664015123555459,0.506680631596975,0.938083037623095
"7261","FAAP100",13.1830376729436,-0.270914026893088,0.408146670757079,-0.663766352401121,0.506839864289106,0.938083037623095
"7262","GDAP1",3.35214539481865,-0.560672358627556,0.844922415687373,-0.66357851113635,0.506960114584807,0.938176394919475
"7263","AC108488.1",5.84632846221849,-0.412613608646913,0.62207619525781,-0.663284677652568,0.50714824795558,0.938395333095833
"7264","PCED1B",2.95070232592308,0.546106216129443,0.823671804036032,0.663014338300153,0.507321371079668,0.938457247892588
"7265","GTF2F2",40.7293266121147,-0.206501305462044,0.311418233868103,-0.663099597275043,0.507266768574503,0.938457247892588
"7266","MOCS1",7.17578876203343,-0.373463241828681,0.563374026379304,-0.662904614593004,0.507391646104224,0.938458069363429
"7267","AC068491.3",7.95511436321654,0.401581010853606,0.60598401094582,0.662692420261747,0.507527565253811,0.938580287525246
"7268","HES4",4.63928333044345,-0.594506251293658,0.904379474686144,-0.657363715048902,0.510947076870851,0.938862577973696
"7269","FCER1A",2.60775585300277,-0.696975380863874,1.06198254982571,-0.656296453249875,0.511633399156645,0.938862577973696
"7270","DUSP12",23.7802614067268,-0.218226323381949,0.330239140364297,-0.660813019138849,0.508732232528302,0.938862577973696
"7271","RASSF5",25.3062593329969,0.250419694727159,0.381417648089243,0.656549837118619,0.511470412460348,0.938862577973696
"7272","AL162595.1",1.72783214079527,-0.68089992293251,1.03927725119174,-0.655166773016268,0.512360384658833,0.938862577973696
"7273","ATRAID",290.975104967367,-0.184866342786938,0.281879737064481,-0.655834096881711,0.511930875223174,0.938862577973696
"7274","CXCR4",25.855354266913,0.281781651980094,0.426825586425271,0.660179850838039,0.509138420944999,0.938862577973696

"7275","DYNC1LI1",57.0741425682994,-0.155813277132273,0.237822728527631,-0.655165627343183,0.512361122209556,0.938862577973696
"7276","UBP1",18.1875220500923,0.251493833420539,0.381108724696294,0.659900488032528,0.509317691016502,0.938862577973696
"7277","LRRFIP2",46.7547220730734,-0.16168965725214,0.246206700830652,-0.656723219581887,0.511358901508811,0.938862577973696
"7278","USP46-AS1",2.97323983370041,-0.536269450933608,0.812406775872127,-0.660099677723536,0.509189865499756,0.938862577973696
"7279","ABHD18",10.3310967262296,0.342431891226825,0.521175124914519,0.65703805660907,0.511156446498699,0.938862577973696
"7280","AC008875.3",18.3339793061743,-0.255974493137159,0.390766474906366,-0.65505745649878,0.512430761919567,0.938862577973696
"7281","TRAPPC13",10.5856895925826,0.303818319619689,0.461968371068297,0.657660434451631,0.510756351508217,0.938862577973696
"7282","PRRC1",61.3040418559738,-0.178180477707627,0.270452412919381,-0.65882376786463,0.510008942867111,0.938862577973696
"7283","SKP1",446.87386428766,-0.13806266061506,0.210408392891855,-0.656165178192395,0.511717851213815,0.938862577973696
"7284","SIL1",76.1519612596565,0.164424293039926,0.248336791017265,0.662102028323685,0.507905836006427,0.938862577973696
"7285","MFAP3",3.67455765680576,0.509362150700152,0.776816033774715,0.655704991341454,0.512013956719612,0.938862577973696
"7286","B4GALT7",29.5491771088543,-0.209265925452454,0.31864490952564,-0.656737073766482,0.51134999173613,0.938862577973696
"7287","PSORS1C1",19.0805263839849,-0.263490098766263,0.401977157661051,-0.655485252692989,0.512155378254971,0.938862577973696
"7288","CFB",266.829910455023,-0.428561475210966,0.651593511484518,-0.657712926322086,0.510722614646075,0.938862577973696
"7289","IFNGR1",123.899838004527,-0.177870825686,0.268767603020365,-0.661801585038964,0.508098390182418,0.938862577973696
"7290","AL355312.2",2.2567893315179,0.637658479866909,0.969946062739677,0.657416432070254,0.510913188694203,0.938862577973696
"7291","SFT2D1",97.3478122022236,-0.184262085646461,0.280866202195845,-0.656049336680163,0.511792380582888,0.938862577973696
"7292","TNS3",8.50130761705964,-0.444403669726437,0.676381042993255,-0.657031527317463,0.511160644714992,0.938862577973696
"7293","SLC39A14",26.7139299800322,-0.245235075478468,0.373832365180936,-0.656002792480992,0.511822327486367,0.938862577973696
"7294","RFX3",9.36346790234875,-0.33134989947028,0.504689822152465,-0.656541671589685,0.511475664434256,0.938862577973696
"7295","NFX1",24.6653818533747,0.248571822329236,0.37636647017611,0.660451559919575,0.508964094062651,0.938862577973696
"7296","ANP32B",103.144350642214,-0.16395994817145,0.247666926183009,-0.662017939570561,0.507959724650029,0.938862577973696
"7297","TBC1D2",3.12484503560364,-0.500085207743014,0.760860482506555,-0.657262690389109,0.511012022014342,0.938862577973696
"7298","MASTL",4.78148841168539,0.485638150521772,0.740352304930403,0.655955478611529,0.511852770538952,0.938862577973696
"7299","CCDC7",9.0147185122931,0.375773133620142,0.567866319429063,0.6

61728158130504,0.508145455329181,0.938862577973696
"7300","AL137026.3",5.08565706682058,0.528762151244728,0.800571995220637,0.660480449480376,0.508945560537033,0.938862577973696
"7301","RTKN2",2.83660278400447,-0.661422004603617,1.00513778398458,-0.658041131417427,0.510511701534349,0.938862577973696
"7302","AVPI1",21.9551899189521,-0.278080738902752,0.420051426813471,-0.662015936982491,0.507961008053888,0.938862577973696
"7303","HPS1",44.1321046471498,-0.174600146985806,0.265831314822391,-0.656808048000141,0.511304348727038,0.938862577973696
"7304","GAS2",1.47996478692356,0.822243231297659,1.25297099093796,0.656234850802204,0.51167302842515,0.938862577973696
"7305","DTX4",7.86313709274544,0.389455189380006,0.588388758674907,0.661901138725163,0.508034581957901,0.938862577973696
"7306","SLC25A45",18.4331678868598,0.281502248772368,0.429394762069561,0.655579139846997,0.512094950955018,0.938862577973696
"7307","NDUFC2",293.309436732298,-0.187344955738598,0.285093915091243,-0.657134178674559,0.511094643835126,0.938862577973696
"7308","SC5D",21.1313007790066,0.225462341377699,0.343175700923852,0.656988069874236,0.511188587523058,0.938862577973696
"7309","RPUSD4",15.7553124920841,-0.263434415013043,0.401195926770327,-0.656622855405636,0.511423449193061,0.938862577973696
"7310","FOXRED1",14.4361663880502,0.2748412328995,0.418322046813979,0.657008720895166,0.51117530897295,0.938862577973696
"7311","CHD4",91.6443593389224,0.184684200079548,0.28172095919297,0.655557188959609,0.512109078570187,0.938862577973696
"7312","ZCRB1",127.884792921778,-0.15347697387357,0.233167190054943,-0.658227145240312,0.51039218444574,0.938862577973696
"7313","BCDIN3D",10.5492108487932,-0.318702601505645,0.484764316882096,-0.657438244538036,0.510899167290225,0.938862577973696
"7314","ISCU",238.413333928235,-0.169180123754708,0.25801970296355,-0.655686840235638,0.512025637812775,0.938862577973696
"7315","TM9SF2",113.896982254923,0.132827026376639,0.202217101738708,0.656853575857643,0.51127507121597,0.938862577973696
"7316","UPF3A",118.586060732122,-0.149472237467172,0.226623308024902,-0.65956250824274,0.509534620408917,0.938862577973696
"7317","EVL",64.8360602608316,-0.148509113635985,0.226456614579543,-0.655794991511813,0.511956039416497,0.938862577973696
"7318","PACS2",39.4078053674964,0.180809362493881,0.273637139710164,0.660763238080158,0.508764161785275,0.938862577973696
"7319","AC090825.1",2.50225755445095,0.596549531415136,0.903437418187786,0.660310852091738,0.509054367438494,0.938862577973696
"7320","SEPHS2",45.3360043200253,-0.180242065001699,0.272510337238483,-0.661413680039459,0.508347055224198,0.938862577973696
"7321","TENT4B",25.7416182112163,-0.207683603034879,0.316590423324398,-0.656000901271969,0.511823544324445,0.938862577973696
"7322","CFAP20",43.5475137376363,-0.219557657734001,0.333279690163109,-0.658778990182533,0.510037700662274,0.938862577973696
"7323","RARA",32.5824185177346,-0.220435426283599,0.336464073841983,-0.655152937330016,0.51236929170479,0.938862577973696
"7324","ARL17B",8.05886579577669,-0.346241952927576,0.527647446882971,

-0.656199428184422,0.511695816758658,0.938862577973696
"7325","GALNT1",100.404077862464,-0.118486697907483,0.180825313648958,
-0.655255038780162,0.512303563413741,0.938862577973696
"7326","ZNF516",14.2926485465411,0.30679459043584,0.464317537581576,0.
660743059660845,0.508777104395262,0.938862577973696
"7327","KRI1",13.7934584116772,-0.281540520542857,0.425915641140038,-0
.661024140342122,0.508596832393855,0.938862577973696
"7328","EPS15L1",25.8302887488372,0.221792343625955,0.335707881879277,
0.660670647303161,0.508823551718887,0.938862577973696
"7329","TMEM147-
AS1",1.58224239228649,-0.69033493097308,1.05085254639682,-0.6569284466
59916,0.511226926098115,0.938862577973696
"7330","TEAD2",11.9365446618816,0.352822225952178,0.5360711188254,0.65
8163093593357,0.510433337081652,0.938862577973696
"7331","ZNF587B",3.6291728263323,0.509784316682571,0.770932091570574,0
.661257096774915,0.50844745041534,0.938862577973696
"7332","ZNF324B",2.75465938719298,-0.537659149033144,0.820349012214928
,-0.655402933419123,0.512208363338879,0.938862577973696
"7333","RALGAPB",17.9800618759058,0.281826108710086,0.427343297438329,
0.659484097210526,0.509584954761014,0.938862577973696
"7334","MIS18A",17.9808575066388,-0.233025857716219,0.355472579396677,
-0.655538208071407,0.51212129485452,0.938862577973696
"7335","CBR3",105.79401076694,-0.206089913192093,0.311733168328427,-0.
661109994477605,0.508541776244018,0.938862577973696
"7336","GLYCTK",2.30409367691597,-0.570862375037794,0.872449754735589,
-0.65432120524901,0.512904886922841,0.938996823975373
"7337","SUCLG2-
AS1",18.3433476295197,0.257758104933987,0.39387128548221,0.65442217910
965,0.512839849088383,0.938996823975373
"7338","SBNO1",46.2237097236165,0.177796286276971,0.271666171970383,0.
65446604922292,0.512811593439582,0.938996823975373
"7339","CNOT1",46.2348233552235,0.184664615521868,0.282235489609733,0.
654292682246364,0.512923259528478,0.938996823975373
"7340","CCDC137",22.2259446870202,0.229911741455282,0.351286988128652,
0.654484080608988,0.512799980107542,0.938996823975373
"7341","IRF2BP1",17.520821321546,-0.248285732926733,0.379383977640462,
-0.654444435083736,0.512825514467651,0.938996823975373
"7342","ANKRD36C",67.0083738740704,-0.281075608093957,0.42976405288307
,-0.654023076635571,0.513096938320504,0.939059880347877
"7343","NECAB1",1.67756755140631,0.796148128354695,1.21731041850259,0.
654022274231444,0.513097455271558,0.939059880347877
"7344","KHK",3.06102419333969,-0.579608386771838,0.886723681002812,-0.
653651638260464,0.513336267510489,0.939369022204992
"7345","SMARCD1",27.441969214268,0.217866401080553,0.333369274520641,0
.653528737445368,0.513415469088967,0.9393860434427
"7346","CNBP",315.100568214844,-0.126353531602716,0.193414568820095,-0
.653278252892337,0.513576909779865,0.939491445568235
"7347","BAG2",61.7779288214862,-0.165581625999352,0.25355621253195,-0.
653037148433062,0.513732329827388,0.939491445568235
"7348","NCBP1",10.0119964180863,0.368780951767024,0.564743986788861,0.

653005539490408,0.513752707305674,0.939491445568235
"7349","DTX3",11.0737445788577,0.294246060103976,0.450453878620439,0.653221282065845,0.513613631938074,0.939491445568235
"7350","SPAG1",4.09157406973045,-0.510818617968622,0.782449277215776,-0.652845664049037,0.513855781364403,0.939552087857988
"7351","CAPZB",248.724930396523,-0.144787634840428,0.221936156629518,-0.652384167768231,0.514153375180356,0.939771003684134
"7352","TMEM234",13.2278897644355,-0.254766694909139,0.395600473230753,-0.643999975097436,0.519575450141604,0.939771003684134
"7353","CTPS1",3.09842334861556,-0.533499196065036,0.826158631808805,-0.645758787143559,0.518435582135793,0.939771003684134
"7354","PARS2",2.45407869992084,-0.703223836576845,1.08469508979369,-0.648314759782494,0.516781389547349,0.939771003684134
"7355","CCDC18",10.7337679158345,0.293262581658739,0.455405927031512,0.643958640526096,0.519602254197116,0.939771003684134
"7356","YPEL5",79.5968831360914,-0.146309080850341,0.226813870958914,-0.645062315773639,0.518886803447439,0.939771003684134
"7357","RFTN1",102.688475018091,0.162328203729514,0.249326611082688,0.651066498776893,0.515003561214796,0.939771003684134
"7358","SNRK",23.0827418989411,0.231477810497467,0.357368714157865,0.647728246281831,0.517160731630368,0.939771003684134
"7359","LARS2",5.0346653313408,-0.421211117840692,0.648473340361795,-0.649542689921056,0.515987662539987,0.939771003684134
"7360","P4HTM",54.55698243164,-0.189886549577217,0.291515414064232,-0.651377390066163,0.514802902218098,0.939771003684134
"7361","AC121247.1",2.29525045153949,0.678399854346591,1.05120813940412,0.645352550952606,0.51869874479682,0.939771003684134
"7362","CLDND1",61.6780375476984,-0.167996177424624,0.260019553247375,-0.646090554831454,0.518220711899644,0.939771003684134
"7363","TXNRD3",9.4219351192234,-0.30241858314359,0.466382426683636,-0.648434773355496,0.516703785578349,0.939771003684134
"7364","CLDN11",27.6065663582426,0.394364581553254,0.609670584244467,0.646848628988668,0.517729915989198,0.939771003684134
"7365","NCBP2",50.6180832534797,-0.155747440495057,0.240500324828124,-0.647597630507834,0.517245230232938,0.939771003684134
"7366","AC124016.2",1.65184867136767,0.814572789632857,1.25585391312286,0.64862065652788,0.516583600494628,0.939771003684134
"7367","FAM13A",74.6386184653322,0.186821039009131,0.28950812212985,0.64530500089161,0.51872955257217,0.939771003684134
"7368","NPY1R",2.92348461891712,1.46728907467612,2.27593840424678,0.644696302825351,0.51912401270454,0.939771003684134
"7369","PJA2",173.758159864244,0.123216300996129,0.191215032596073,0.644386057535622,0.519325123347748,0.939771003684134
"7370","TMCO6",5.63812714958813,0.401171460087605,0.622274364607617,0.644685821728441,0.519130806221064,0.939771003684134
"7371","PCYOX1L",8.83344396372651,0.320571430998997,0.494413808205602,0.648386889036253,0.516734748129,0.939771003684134
"7372","ZNF346",15.341654888939,0.286076103809345,0.438736399178908,0.652045520601288,0.514371807300433,0.939771003684134
"7373","PEX6",11.9752733909684,-0.29304958063164,0.452373028931745,-0.

647805156120074,0.517110980081234,0.939771003684134
"7374","TCF21",1.75577726017774,0.87602699921483,1.34820958538564,0.64
9770635597621,0.51584038937961,0.939771003684134
"7375","PHF10",45.7388358339257,0.187029220026516,0.290431763297278,0.
643969577924843,0.519595161598764,0.939771003684134
"7376","MINDY4",14.7927373633573,-0.274216245590776,0.421936688469003,
-0.649899032449084,0.515757443208145,0.939771003684134
"7377","TRIM4",30.6617574756729,0.191461783392154,0.293776324661287,0.
651726389500249,0.514577695435805,0.939771003684134
"7378","NAPEPLD",15.2962678445916,0.25949773473033,0.402471130157405,0
.644761115235375,0.519082004363664,0.939771003684134
"7379","RINT1",11.7756987741814,0.283036069982519,0.43542979471743,0.6
50015394941437,0.515682277380359,0.939771003684134
"7380","CHMP7",16.8987309916887,-0.232830186690363,0.36140180216822,-0
.64424190829571,0.51941857904929,0.939771003684134
"7381","AC104051.2",3.07547021286873,0.634545907683818,0.9730230285887
42,0.652138632940839,0.514311743741014,0.939771003684134
"7382","HAS2-
AS1",15.4619049513914,-0.278854999342769,0.429681530841531,-0.64898065
038223,0.516350883183428,0.939771003684134
"7383","TOP1MT",4.82788866499776,-0.450795857446399,0.694875005481049,
-0.648743808441234,0.5165039830686,0.939771003684134
"7384","TSTA3",33.5922354778038,-0.239453266214557,0.370202959943922,-
0.646816184967374,0.517750916121725,0.939771003684134
"7385","CDC37L1",26.6592137827446,0.201768522686983,0.312619778762632,
0.645411891357595,0.518660299361127,0.939771003684134
"7386","NDUFB6",102.349826552524,-0.197168865000572,0.303186384820347,
-0.650322293058787,0.51548405992064,0.939771003684134
"7387","CLTA",313.337311023328,-0.154543069045214,0.239895845020001,-0
.644209027598327,0.519439897676351,0.939771003684134
"7388","FAM24B",5.70438173670943,-0.41955726439475,0.650487604223645,-
0.644988869381286,0.518934398814415,0.939771003684134
"7389","LDLRAD3",2.45148602053142,0.688907220109573,1.06016012356981,0
.649814310870189,0.515812173762722,0.939771003684134
"7390","TAF6L",5.00895309443037,-0.414317792537455,0.640785976297793,-
0.646577496797322,0.517905426028559,0.939771003684134
"7391","ATL3",132.425773345459,-0.118589022128483,0.183221707568895,-0
.647243297216248,0.517474493272251,0.939771003684134
"7392","HIKESHI",88.480714022312,-0.199344790575466,0.306970369900553,
-0.649394241665885,0.516083585028143,0.939771003684134
"7393","KLRB1",5.79701995543882,-0.391258256617936,0.601478608938149,-
0.650494050501088,0.51537314354841,0.939771003684134
"7394","TWF1",42.3563971947516,-0.201575028883285,0.312645578397029,-0
.644739739857458,0.519095858679342,0.939771003684134
"7395","VPS33A",15.6738838451625,0.254539404749634,0.39362711185155,0.
6466510996977,0.517857778152556,0.939771003684134
"7396","STK24",74.0826480355602,-0.138768760877699,0.212854195238894,-
0.651942803955325,0.51443807049713,0.939771003684134
"7397","HEATR5A",36.3973574381505,0.235197825443397,0.362735409569013,
0.64840051243646,0.516725938984149,0.939771003684134

"7398","AC004846.1",3.58417429018562,-0.507871164151481,0.780075645918664,-0.651053736658298,0.51501179915475,0.939771003684134
"7399","ZNF410",18.71953549998,0.246171547729222,0.377912472227582,0.651398315271731,0.514789397892883,0.939771003684134
"7400","IFI27L1",42.8961728725604,0.222491313674993,0.345536604350423,0.643900851237616,0.519639729769834,0.939771003684134
"7401","TMEM87A",98.8811586319619,-0.131980858720627,0.203482561407819,-0.648610169871566,0.516590380387437,0.939771003684134
"7402","CPPED1",9.01361410507655,-0.307144021696148,0.473422496869453,-0.648773608620554,0.516484718277638,0.939771003684134
"7403","ZNF764",5.06710035734459,-0.405162405523176,0.626450994842286,-0.64675834001218,0.517788358681177,0.939771003684134
"7404","AC026471.1",3.53418154797989,0.561183581056917,0.871468590672038,0.643951585936284,0.519606828928557,0.939771003684134
"7405","PSKH1",15.7778938661277,0.266673047742208,0.412892780724924,0.645865125745247,0.518366706640125,0.939771003684134
"7406","RPA1",32.2702953172352,0.208735012783858,0.320300872540207,0.651684184087434,0.514604927557889,0.939771003684134
"7407","EMC6",44.1284186744836,-0.212278443994889,0.326559693049609,-0.650044841763863,0.51566325673329,0.939771003684134
"7408","ARHGAP44",5.38823449399893,0.486132841388556,0.745907497458027,0.651733416067334,0.514573161769382,0.939771003684134
"7409","SARM1",5.43280767941172,-0.409375694318568,0.635125422113713,-0.644558822659241,0.51921312666095,0.939771003684134
"7410","DHX58",10.4284503415301,-0.319067291818269,0.48990298972125,-0.651286680246257,0.51486144498565,0.939771003684134
"7411","HOXB4",14.0294332091015,-0.30158206652712,0.467547673236344,-0.645029552686217,0.518908034588396,0.939771003684134
"7412","SLC35B1",32.8726630660719,-0.230692608665082,0.356174156602884,-0.647696090208734,0.517181533532944,0.939771003684134
"7413","PPP1R9B",18.7131822593169,0.234998444203751,0.36332878124247,0.646792812284593,0.517766044896664,0.939771003684134
"7414","RSAD1",24.0857837381918,0.211687140918686,0.32658967688175,0.648174623704755,0.516872012905049,0.939771003684134
"7415","ANAPC11",200.189271647959,-0.155507101624105,0.23913629650263,-0.650286484730243,0.515507185544377,0.939771003684134
"7416","CCDC57",7.4233310929444,0.389100466822744,0.60173957449611,0.646626021146395,0.517874012851871,0.939771003684134
"7417","ZBTB7A",144.845307813736,-0.151184929305391,0.233750861380955,-0.64677806281534,0.517775592116978,0.939771003684134
"7418","AC008569.2",1.49158515051262,0.933623687824177,1.43096054743917,0.652445442674836,0.51411385717051,0.939771003684134
"7419","RAB3A",2.58382315016522,-0.553974251281338,0.85766464058223,-0.645910097104237,0.518337580121147,0.939771003684134
"7420","ZNF529-AS1",4.93034105790799,0.42971642107035,0.664567909134303,0.646610248800786,0.517884223276998,0.939771003684134
"7421","ZNF45",4.81461778678606,-0.429586265690234,0.666876185859055,-0.644176947384694,0.5194606977346,0.939771003684134
"7422","RTN2",35.4759328046456,0.225674447242574,0.348060092626264,0.6

48377829069005,0.516740606516005,0.939771003684134
"7423","JOSD2",90.1420976747682,-0.168206483796367,0.258378089701603,-0.651009085138089,0.515040622223141,0.939771003684134
"7424","AC022137.4",3.9987673041697,-0.493727875492507,0.758064650467196,-0.651300486295229,0.514852534543993,0.939771003684134
"7425","ZNF135",6.36009140348187,0.384122053452564,0.592617270138908,0.648178972851276,0.516869200270264,0.939771003684134
"7426","TMEM230",222.580784940385,-0.172482887173262,0.267367123278914,-0.645116292003224,0.51885182674137,0.939771003684134
"7427","ESF1",57.8524446645593,-0.175008785015011,0.271635059708282,-0.64427907503162,0.519394482057227,0.939771003684134
"7428","OSER1-DT",21.7801317365979,-0.221765440033232,0.342790732670273,-0.646941176926583,0.517670014647884,0.939771003684134
"7429","DGCR6L",72.2641554856625,-0.218387681628999,0.338108415901223,-0.645910220977168,0.518337499893758,0.939771003684134
"7430","CLCN4",5.46147663807944,0.463672029810057,0.717821291481979,0.645943545158409,0.518315917428853,0.939771003684134
"7431","CYBB",1.83960943661294,-0.760111149745919,1.16844153397385,-0.650534175347905,0.515347233778724,0.939771003684134
"7432","CREB3L4",6.02524532387048,-0.385975997877212,0.599945962389423,-0.64335127173783,0.519996194330713,0.939926903571572
"7433","EXOG",23.5225056084349,-0.222884567821964,0.346352361092175,-0.643519700917089,0.519886935529128,0.939926903571572
"7434","KIF23",2.34185908987895,0.610044495604117,0.948250837101154,0.643336627541559,0.52000569447538,0.939926903571572
"7435","ZNF850",2.57929663920581,0.685923564440191,1.06612179710893,0.643381990969749,0.519976266102266,0.939926903571572
"7436","TARS2",13.3325188709773,-0.250687288341388,0.390798385932672,-0.641474728057288,0.521214295158082,0.94015087898416
"7437","NES",10.5579634751417,-0.407299425702045,0.634335078935584,-0.642088762276075,0.520815552041517,0.94015087898416
"7438","RABGAP1L",69.3402317266785,-0.194235602926974,0.303190108323586,-0.640639643558795,0.521756836553832,0.94015087898416
"7439","HSD11B1-AS1",3.32592850084052,0.597712828933888,0.933979749200039,0.639963371203534,0.522196412943861,0.94015087898416
"7440","UTP25",15.7071468736786,-0.237829782620071,0.370309173521121,-0.642246532427602,0.520713124233358,0.94015087898416
"7441","DDX18",112.392426779435,-0.140243472023357,0.218443465126399,-0.64201266878002,0.520864957300807,0.94015087898416
"7442","TMA7",516.668417702723,-0.157550963859407,0.246019965910069,-0.640399096376588,0.52191317018242,0.94015087898416
"7443","AC012020.1",1.64083489954405,-0.804446325618443,1.25405387328663,-0.641476688326112,0.521213021943685,0.94015087898416
"7444","P2RY1",4.16214738999636,0.607624392582693,0.946912228518794,0.641690300623922,0.521074288192736,0.94015087898416
"7445","UBXN7",51.5633516400888,0.205599839722158,0.32082866901909,0.640839985873969,0.521626650791614,0.94015087898416
"7446","CENPC",55.7720386733164,0.175970168605684,0.27432226083485,0.6

41472434902476,0.521215784587322,0.94015087898416
"7447","EMB",3.90336276332166,-0.524672800744759,0.818666397046999,-0.640887182663536,0.521595983966562,0.94015087898416
"7448","PANK3",30.0442656407796,0.210643068361515,0.328822798293962,0.640597517734167,0.521784212740302,0.94015087898416
"7449","MRS2",13.8365225821582,0.258630333365484,0.403748979685855,0.640572103901579,0.52180072871066,0.94015087898416
"7450","LEMD2",19.3532666171096,-0.217261274875878,0.339097671129199,-0.640704119708036,0.521714937058029,0.94015087898416
"7451","TBCC",17.2644263421361,-0.261526626232966,0.408957850295989,-0.639495307508299,0.522500765336302,0.94015087898416
"7452","AC009812.1",3.49286511865273,0.487896402851489,0.762121196517288,0.640182171918403,0.52205417185775,0.94015087898416
"7453","VPS28",255.87480963987,-0.170662185979361,0.266595381751159,-0.640154322473063,0.52207227551553,0.94015087898416
"7454","IFIT5",23.6661618194617,0.199312593602835,0.311579530423681,0.639684491891405,0.52237773965335,0.94015087898416
"7455","CTSF",242.824517725981,-0.149024207245187,0.233012589717371,-0.639554315180754,0.522462391336869,0.94015087898416
"7456","ROBO4",10.523256026728,0.372374128327142,0.581885638228504,0.639943837522439,0.522209112646141,0.94015087898416
"7457","DGKA",9.05396911046326,-0.3150327458046,0.492085043645138,-0.640199798536821,0.522042713761591,0.94015087898416
"7458","MBD6",17.0573106860634,0.23566418307728,0.36772845784601,0.640864687105524,0.521610600678588,0.94015087898416
"7459","UBE3B",19.3262251834015,0.244474786088277,0.381565721730196,0.640714750213188,0.521708029044838,0.94015087898416
"7460","SNW1",98.2685663309092,-0.122506031208928,0.191239764532671,-0.640588695077583,0.521789946387905,0.94015087898416
"7461","AC009093.1",4.57806517835306,-0.626342896083289,0.97660120967323,-0.641349703317347,0.521295503296503,0.94015087898416
"7462","CCL5",10.2492296755773,0.406648842000483,0.633352905538836,0.642057277142384,0.520835994116053,0.94015087898416
"7463","DUSP14",23.4537874830123,-0.260489126472629,0.40553757567234,-0.642330432736757,0.520658658557743,0.94015087898416
"7464","NT5C3B",44.41305480215,-0.194583085116895,0.30283005604267,-0.642548786800334,0.520516923119309,0.94015087898416
"7465","CD300A",1.46608291601616,-0.756076437604351,1.1816638559682,-0.639840538225533,0.522276274686399,0.94015087898416
"7466","TSEN34",46.8202220045591,-0.185847219333185,0.290620608845438,-0.639483965268358,0.522508141612671,0.94015087898416
"7467","MTG2",18.6786896928774,0.268211630299612,0.418179468065763,0.641379242123464,0.521276316174704,0.94015087898416
"7468","ZBTB21",16.9684448626552,-0.251842077831119,0.391995040152073,-0.642462409048385,0.520572989259439,0.94015087898416
"7469","ITGB2",7.83049156064523,-0.351811598628077,0.5475575751471,-0.642510695854337,0.52054164683739,0.94015087898416
"7470","AL691432.2",4.3150774643524,0.432981780526494,0.711622236054128,0.608443298409748,0.542893501752829,0.940215282864022
"7471","MIIP",12.6666108353028,0.263535416769392,0.441746124969302,0.5

96576635024713,0.550790074631953,0.940215282864022
"7472","VPS13D",33.5131938570152,-0.169580383618025,0.275024521124356,-0.616600959524429,0.537497961488292,0.940215282864022
"7473","NBPF1",26.4457850264351,0.2620794992289,0.420920919922051,0.622633579907202,0.533525339183882,0.940215282864022
"7474","PLA2G2A",4475.69061032567,-0.23880580543968,0.401265959378825,-0.59513098447065,0.551755918717976,0.940215282864022
"7475","TMEM222",41.7888306718393,-0.206278992186763,0.326960698430129,-0.630898432677666,0.528106936721068,0.940215282864022
"7476","AL020997.2",2.46432819308856,-0.581944951523144,0.923851395186652,-0.62991186088491,0.528752252254572,0.940215282864022
"7477","SRSF4",99.5130601338718,0.150672716181809,0.247931412249932,0.607719347921596,0.543373628530568,0.940215282864022
"7478","MECR",23.463461349326,-0.209849981447506,0.328893842167175,-0.638047766612914,0.523442586603381,0.940215282864022
"7479","FNDC5",39.8491004186498,0.228614095932692,0.38402788395518,0.595305980331819,0.551638959132854,0.940215282864022
"7480","MTF1",15.0404536085999,0.252806259878826,0.409302619651666,0.617651213896395,0.536805277431812,0.940215282864022
"7481","SCMH1",14.0058268507653,0.261817492422387,0.41748653222946,0.627127996259496,0.530575336882682,0.940215282864022
"7482","ST3GAL3",14.4546482195627,-0.243723327360513,0.383068110643721,-0.636240189638735,0.524619881801283,0.940215282864022
"7483","FAF1",24.8186953906978,-0.235023880142854,0.372081437317102,-0.631646345589009,0.527617995264686,0.940215282864022
"7484","PIGK",45.1269379632658,0.183853543830433,0.308000118996539,0.596926859734425,0.550556213544368,0.940215282864022
"7485","TTLL7",27.2640058909449,0.25155060618275,0.42205111717228,0.59601928758802,0.551162341810886,0.940215282864022
"7486","PKN2",88.249506157229,0.144853652914053,0.241939491175856,0.59871851515454,0.549360608241455,0.940215282864022
"7487","HFM1",1.78041691264976,0.727650526214921,1.18841989700221,0.612284031974237,0.5403498525587,0.940215282864022
"7488","DNTTIP2",40.4692733405915,-0.183992258936353,0.293743918965463,-0.626369592890145,0.531072549134367,0.940215282864022
"7489","CYB561D1",8.0464535916399,0.33046958572145,0.524164312267882,0.630469450107391,0.528387484435421,0.940215282864022
"7490","AHCYL1",157.370417045271,0.109082503919344,0.176178699271866,0.619158299897631,0.535812079836905,0.940215282864022
"7491","SLC16A4",19.8445373280247,0.232645037578886,0.391158495770397,0.594759004583769,0.552004574280463,0.940215282864022
"7492","PTGFRN",80.6376126439593,-0.207977189072085,0.33437560788536,-0.621986724412594,0.533950597419095,0.940215282864022
"7493","AC253572.2",3.18172490903633,0.571415563949431,0.95611251281119,0.597644687516262,0.550077039841583,0.940215282864022
"7494","NBPF15",8.59619067851782,-0.319889403073799,0.520034884993832,-0.615130662008556,0.538468434209324,0.940215282864022
"7495","PSMB4",138.575905492272,-0.15373150538594,0.25788912458332,-0.596114728119418,0.55109858576265,0.940215282864022
"7496","TUFT1",7.36317672939474,-0.426519954373481,0.680948358269261,-

0.626361675146041,0.531077741285166,0.940215282864022
"7497","S100A16",222.38811238925,-0.151813032884355,0.246686611498243,-0.615408481077765,0.538284991869421,0.940215282864022
"7498","HAX1",76.8254596875736,-0.174834964373461,0.283523443114901,-0.616650822424611,0.53746506479498,0.940215282864022
"7499","AL353807.5",19.0752099740663,-0.222200778914708,0.354788552603481,-0.626290722415286,0.531124270495048,0.940215282864022
"7500","DUSP23",24.0847844009729,-0.223546020115023,0.35071819374685,-0.637394991479625,0.523867589960804,0.940215282864022
"7501","AL590560.3",1.67243239216255,0.842576669722136,1.4091802464257,0.59791972805415,0.549893495669438,0.940215282864022
"7502","SDHC",81.2525315493963,-0.156084852211565,0.255659891447602,-0.61051755646057,0.541519017100119,0.940215282864022
"7503","CACYBP",111.563040695472,-0.136334083915212,0.22175721952806,-0.614789832797128,0.5386935246088,0.940215282864022
"7504","STX6",14.5617346421556,0.262187043034566,0.432659412364541,0.605989458548189,0.544521753207594,0.940215282864022
"7505","PPP1R12B",78.5072677918788,0.211737862111197,0.346107630903509,0.611768834909703,0.540690711912457,0.940215282864022
"7506","INTS7",6.22218090093032,-0.377152171355258,0.592579386024544,-0.636458473328731,0.524477639214537,0.940215282864022
"7507","AC092803.1",3.61930427752397,0.470362923592939,0.769393804027596,0.611342229597769,0.540973039432567,0.940215282864022
"7508","LINC01139",3.90511510721848,-0.5020380815727,0.838268468419409,-0.598898921391275,0.549240290765203,0.940215282864022
"7509","ADI1",212.351209548343,0.180346384785965,0.287974955220388,0.626257184927576,0.531146264347144,0.940215282864022
"7510","LINC00954",1.70436476057186,0.753275358960242,1.23799772842658,0.608462634190458,0.542880681086274,0.940215282864022
"7511","KLHL29",12.4869858217274,-0.324614177714412,0.537812953983992,-0.603581924365612,0.546121633310647,0.940215282864022
"7512","PTRHD1",44.9304865980569,-0.209561127984957,0.333606715209035,-0.628168194557021,0.529893763312342,0.940215282864022
"7513","POMC",2.46143181311456,-0.675637842502985,1.0784805283813,-0.626471989732678,0.531005403559559,0.940215282864022
"7514","GPN1",19.0899608934595,-0.243670046852159,0.392354819606007,-0.621045122108724,0.534569935072447,0.940215282864022
"7515","ASB3",21.7991854465331,0.208099986927334,0.331617607023953,0.627529969819434,0.530311897279454,0.940215282864022
"7516","MTIF2",20.6216786872141,-0.219291929700895,0.354120922011029,-0.619257197387691,0.535746936932312,0.940215282864022
"7517","TMEM17",6.62149192301807,0.336647421153192,0.556845124752787,0.604562033837771,0.545470039475438,0.940215282864022
"7518","STAMBP",37.6748859415742,-0.164802526880442,0.268424921745721,-0.61396134833024,0.539240868907126,0.940215282864022
"7519","KDM3A",21.1000354724759,0.278050939555438,0.456340004695564,0.609306518592277,0.542321287153057,0.940215282864022
"7520","KCNIP3",6.40378799341181,0.408131337544616,0.679437248215189,0.600690260383479,0.54804630729996,0.940215282864022
"7521","REV1",48.1038452956518,0.178876268447128,0.287511207059638,0.6

22154072797669,0.533840562183966,0.940215282864022
"7522","RGPD8",1.49672145102693,0.740774425630907,1.19520354885905,0.619789345788049,0.535396483969735,0.940215282864022
"7523","C2orf76",25.6203854074597,-0.205918143562788,0.343019768054344,-0.60030984432992,0.548299759189109,0.940215282864022
"7524","PDK1",5.4145746119991,0.422655354549593,0.703887386819358,0.600458770058997,0.548200530645816,0.940215282864022
"7525","HOXD4",1.58206267153349,0.753161409856293,1.25504758501244,0.600105859610755,0.548435687687239,0.940215282864022
"7526","MTX2",42.9904312465982,0.176026260110187,0.295469257531736,0.595751522783316,0.551341233035639,0.940215282864022
"7527","FAM171B",26.2378169935572,-0.234133303216857,0.368861215711293,-0.634746330717818,0.525593871454735,0.940215282864022
"7528","COL3A1",6294.97881522268,0.254596041829456,0.416120025054155,0.611833188744814,0.540648128919885,0.940215282864022
"7529","COL5A2",411.592397896018,0.204881929079,0.329974685509385,0.620901960290445,0.534664131302889,0.940215282864022
"7530","C2orf88",13.6876734256582,0.376197939025601,0.612879594860082,0.613820303662558,0.539334078841313,0.940215282864022
"7531","SLC39A10",44.2232957754544,0.16390518767122,0.275636713616986,0.59464207623291,0.552082748165591,0.940215282864022
"7532","COQ10B",46.7948718474368,-0.175177228787446,0.282502034955581,-0.620091918328977,0.535197272583205,0.940215282864022
"7533","CASP8",18.1848122779252,-0.222954348722065,0.351868474451204,-0.633629793262379,0.526322452584715,0.940215282864022
"7534","CYP20A1",41.7018559124153,0.18287284838924,0.294038860384441,0.621934284979011,0.53398507985431,0.940215282864022
"7535","ACADL",7.74645801747033,0.35559565514236,0.558169179930018,0.637075044499849,0.524075962952816,0.940215282864022
"7536","FN1",1656.9169198452,-0.121149190671933,0.200560486242999,-0.604053135995834,0.545808315466823,0.940215282864022
"7537","AGFG1",55.9374154610897,0.145051481199795,0.240839249811179,0.602275091429315,0.546991038330472,0.940215282864022
"7538","FARP2",14.6032773075636,0.268226274931115,0.419887222879649,0.638805518042629,0.52294945812413,0.940215282864022
"7539","THAP4",28.0488765785066,-0.204883049091563,0.325692511378679,-0.629068959013762,0.529303911247126,0.940215282864022
"7540","BHLHE40-AS1",1.75243400162424,-0.611636970102542,1.01169969566318,-0.604563758123507,0.545468893480274,0.940215282864022
"7541","FGD5",36.0074944447042,0.225911791714875,0.356668007453225,0.633395165795748,0.526475621084786,0.940215282864022
"7542","FGD5-AS1",123.633880050303,0.111201790752398,0.186886513194107,0.595023091029044,0.551828036049337,0.940215282864022
"7543","BTD",45.4677510349107,0.207991783636237,0.344059474082684,0.604522762207829,0.545496140518186,0.940215282864022
"7544","AC096921.2",3.59181928144679,-0.462833068372479,0.750330448931828,-0.616838979454145,0.537340938644916,0.940215282864022
"7545","DAG1",51.9671468579813,0.160173977809715,0.266597912949975,0.6

00807320797632,0.547968327543993,0.940215282864022
"7546","DNAH1",1.52054660009931,0.740422248891172,1.23797496581088,0.598091455271223,0.549778911395023,0.940215282864022
"7547","LRTM1",1.86798698005821,0.827774408205754,1.36296337812532,0.607334299285659,0.543629080364808,0.940215282864022
"7548","MTRNR2L12",9.92380775103492,-0.319825624015032,0.52222682958284,-0.612426642787601,0.540255518862807,0.940215282864022
"7549","H1FX",260.056483025694,0.167460072601178,0.268002109876444,0.624846097959384,0.532072072240559,0.940215282864022
"7550","PLSCR1",66.0506954955199,0.147398361324688,0.245620524199119,0.60010604490525,0.548435564205721,0.940215282864022
"7551","TIPARP",42.6681746083637,0.226187878607989,0.375226529641685,0.602803535304348,0.54663939463618,0.940215282864022
"7552","CCNL1",225.422927501292,0.192781229257679,0.307847399932962,0.626223347345664,0.53116845546889,0.940215282864022
"7553","EIF5A2",18.1496524947565,-0.226115252135708,0.362344566852138,-0.624033786680121,0.53260539651039,0.940215282864022
"7554","DNAJC19",99.4416619599679,-0.163034461501082,0.270192676604502,-0.6034007418333,0.546242128850772,0.940215282864022
"7555","EIF4A2",321.526552318916,0.126968316858024,0.209936829397397,0.604792961875598,0.545316570666823,0.940215282864022
"7556","SLBP",52.8631046485663,-0.154580000808307,0.246265894384326,-0.627695528829774,0.530203414932993,0.940215282864022
"7557","LETM1",11.1871806200382,0.283768105572669,0.454395980797265,0.624495192661653,0.532302426484869,0.940215282864022
"7558","DHX15",41.0146924046662,0.162628571537485,0.272502705715497,0.596796171658109,0.550643474245225,0.940215282864022
"7559","RPL9",2668.69488645549,-0.139234280841575,0.231460571271866,-0.601546432191402,0.547476095391526,0.940215282864022
"7560","CXCL9",4.65108520305155,-1.04132007244287,1.63991433599793,-0.6349844315552,0.525438568789745,0.940215282864022
"7561","HNRNPDL",192.185249420981,0.162000239564126,0.256169748149432,0.63239410872835,0.527129382580484,0.940215282864022
"7562","GPAT3",1.7694776123701,-0.656878643164705,1.09166348775414,-0.6017226467069,0.54735877264224,0.940215282864022
"7563","ARHGAP24",74.4496176439939,0.152074125498253,0.241576260120529,0.629507739801832,0.52901670325236,0.940215282864022
"7564","DNAJB14",75.0669617589637,0.143447569068166,0.236043326379171,0.607717113923982,0.543375110453549,0.940215282864022
"7565","TIFA",10.2727259475742,-0.309398107233942,0.504472549442626,-0.613310095020602,0.539671319655597,0.940215282864022
"7566","NAA15",26.6554748025884,0.208395565889017,0.32688714662492,0.63751532613222,0.52378923018435,0.940215282864022
"7567","LSM6",79.4094799100709,-0.175050519449116,0.277226048862301,-0.631436043501327,0.527755455065393,0.940215282864022
"7568","MIR4453HG",1.85761345615341,0.633054346579739,1.01330367163774,0.624742971232476,0.532139765264584,0.940215282864022
"7569","PALLD",117.974181868277,-0.161859000368685,0.253650878182846,-0.638117248117738,0.523397359587551,0.940215282864022
"7570","OTULIN",25.3159740582583,0.242979992473926,0.386459101468185,0

.628734040810083,0.529523188391709,0.940215282864022
"7571","MTMR12",16.019374374766,0.227419932200042,0.369692886106256,0.615159070533691,0.538449674783718,0.940215282864022
"7572","BRIX1",35.7818896115479,0.186799464913762,0.298538989856986,0.625712122236522,0.53150378068596,0.940215282864022
"7573","LMBRD2",15.4081181758229,0.248437474270528,0.407923600132132,0.609029421661447,0.542504937417685,0.940215282864022
"7574","MRPS30",30.4416147131252,-0.172097627038305,0.275689226054513,-0.624245022198567,0.532466683467404,0.940215282864022
"7575","AC093297.2",5.10041961407649,0.470348380928521,0.748748775518889,0.628179165438455,0.529886577186635,0.940215282864022
"7576","GZMK",3.04098137173634,0.539584118842625,0.882939233305358,0.611122598802917,0.541118419874716,0.940215282864022
"7577","SREK1IP1",77.2993924385113,-0.14816959972079,0.248829163084239,-0.595467178702958,0.551531231967663,0.940215282864022
"7578","CWC27",38.6006781153711,0.17201645512503,0.272458749170528,0.63134861937345,0.527812603489564,0.940215282864022
"7579","SLC30A5",34.950081052678,-0.191885900869275,0.30750515266163,-0.624008733539566,0.532621849489667,0.940215282864022
"7580","RAD17",27.3900303401365,0.201184324746019,0.316807667673442,0.635036160025632,0.525404831696402,0.940215282864022
"7581","MRPS27",24.5023367091116,0.202428906065205,0.32783270774825,0.61747623492362,0.536920651772795,0.940215282864022
"7582","POC5",10.9337056732997,-0.272899181236415,0.45465240215993,-0.60023697211308,0.548348316852031,0.940215282864022
"7583","MEF2C",13.6508777770859,-0.276088509517012,0.457006991550629,-0.60412316358715,0.545761760358929,0.940215282864022
"7584","MBLAC2",10.752152988411,0.275809854404049,0.447358519133065,0.616529791225482,0.537544916018729,0.940215282864022
"7585","ARSK",16.8203129279542,-0.220440607258823,0.347526538680001,-0.634313017060842,0.525876563050538,0.940215282864022
"7586","TMEM232",1.9324103719367,0.652844354294602,1.08803272080275,0.600022721571215,0.548491092830278,0.940215282864022
"7587","CSNK1G3",74.5828830410461,0.180737990391909,0.303812927723418,0.594898945697425,0.551911022076713,0.940215282864022
"7588","BRD8",28.4286264733958,0.212467280296838,0.352537432829421,0.602680057523545,0.546721550731382,0.940215282864022
"7589","AC011379.2",6.53669675439904,0.455810442339952,0.753267654382507,0.605110865557614,0.545105335153925,0.940215282864022
"7590","GNPDA1",33.1852914912021,0.170229498693676,0.270585290697126,0.629115863080003,0.529273205974445,0.940215282864022
"7591","TNIP1",30.0598654646623,-0.193776983907741,0.311497312887695,-0.622082361197138,0.533887712732197,0.940215282864022
"7592","ZBED8",5.73136002785561,0.381210921902371,0.631754393935186,0.603416336414878,0.546231757147757,0.940215282864022
"7593","SLU7",73.7116374130983,-0.1309653275559,0.21374005836125,-0.612731785328472,0.540053701930104,0.940215282864022
"7594","NUDCD2",79.6010323406933,-0.168081536901216,0.274406409483414,-0.612527736570147,0.540188652695474,0.940215282864022
"7595","LCP2",3.88417121480495,0.466007365496534,0.730256897180734,0.6

38141683146883,0.523381454774621,0.940215282864022
"7596","SH3PXD2B",70.1811976497724,0.178145390641594,0.292063508750038,0.609954291804593,0.541892086438764,0.940215282864022
"7597","ATP6V0E1",461.463211994257,-0.15687247777019,0.260773264871002,-0.601566567216125,0.547462688962656,0.940215282864022
"7598","RMND5B",25.3048313983587,0.200226277822056,0.33389817584229,0.599662688533613,0.548731059204676,0.940215282864022
"7599","CLK4",36.773017863007,0.186939551898064,0.310497400248003,0.602064789427384,0.547131011244081,0.940215282864022
"7600","ADAMTS2",138.724009285669,0.165073379282199,0.25914694282851,0.636987561884654,0.524132945458145,0.940215282864022
"7601","SIRT5",7.28403143564278,-0.333715656361822,0.53876494718281,-0.619408627281365,0.535647199127733,0.940215282864022
"7602","NHLRC1",1.48194338850802,0.777986641943321,1.21734791881016,0.639083231607056,0.522768787805366,0.940215282864022
"7603","HIST1H4C",200.718520944997,-0.229549892948007,0.36781095569458,-0.62409748647786,0.532563564532211,0.940215282864022
"7604","BTN2A2",5.62211700307097,-0.391573076672845,0.637100461143248,-0.614617474880155,0.53880737116949,0.940215282864022
"7605","PGBD1",6.70076026673883,-0.327303337826305,0.537361914656095,-0.609092920244962,0.54246285001421,0.940215282864022
"7606","TRIM26",13.8263771478278,-0.239281503745817,0.399465077938238,-0.599004811586589,0.549169675973143,0.940215282864022
"7607","NRM",12.099824912576,-0.277476692082925,0.438547344486415,-0.632717756865863,0.52691797201073,0.940215282864022
"7608","B3GALT4",30.0544760323811,0.20680690958538,0.345853463720061,0.5979610768125,0.549865904806137,0.940215282864022
"7609","TREM1",1.5588585996228,-0.739778901823258,1.16204421883828,-0.636618546721766,0.524373341398153,0.940215282864022
"7610","VEGFA",88.7096917059562,0.198648405466827,0.327245746005822,0.60703128426089,0.543830150859132,0.940215282864022
"7611","RUNX2",2.1681249019307,-0.614485223586738,1.02795343863432,-0.597775347104344,0.549989842365079,0.940215282864022
"7612","KIAA1586",28.798023711333,-0.191280807805862,0.313835031526655,-0.609494761867003,0.542196543704449,0.940215282864022
"7613","PHF3",132.062097184255,0.163866933666126,0.26424036862361,0.620143449389227,0.535163348661726,0.940215282864022
"7614","MTO1",13.0189760941868,-0.252390388400677,0.39778556074734,-0.634488561944025,0.525762029107985,0.940215282864022
"7615","HMGN3-AS1",13.8243257005031,-0.274783840254589,0.447397170585711,-0.614183232081812,0.5390942524766,0.940215282864022
"7616","PM20D2",9.33280249920829,0.315732077725917,0.517106691637961,0.610574341488059,0.54148141356144,0.940215282864022
"7617","MDN1",18.4312093919722,0.260127345067882,0.41187762527826,0.631564642270001,0.527671396854782,0.940215282864022
"7618","FIG4",14.9429033394896,0.238531346331467,0.385814513387134,0.618253948606956,0.536407953025145,0.940215282864022
"7619","RPF2",40.0098654116987,-0.187242077027161,0.304308955033636,-0.615302553309562,0.53835493166932,0.940215282864022

"7620","TIAM2",5.80494532020081,-0.354497160117253,0.589582708742837,-0.60126790501225,0.547661562768254,0.940215282864022
"7621","BX322234.1",11.9670390547565,-0.357156438823944,0.578745301936966,-0.617121966482665,0.537154280796325,0.940215282864022
"7622","AL135910.1",3.49891918706394,0.486207523386042,0.789538425602328,0.615812362793008,0.538018367023641,0.940215282864022
"7623","MAD1L1",19.5617655514931,-0.212510119093057,0.352159967023601,-0.603447691369232,0.546210903772085,0.940215282864022
"7624","ACTB",3322.80511496432,-0.148679356033025,0.241081401276794,-0.616718482826145,0.537420427944369,0.940215282864022
"7625","KDELR2",302.335977952379,-0.132579310290781,0.213334421659561,-0.621462346579732,0.534295461517195,0.940215282864022
"7626","OSBPL3",1.94958695278711,-0.754100635023507,1.21931245359146,-0.618463817705065,0.536269641470059,0.940215282864022
"7627","HOXA10",86.6314952746143,-0.16189949278234,0.254200076918286,-0.636897890610724,0.524191356859569,0.940215282864022
"7628","AC018645.3",4.19596669143349,-0.45069308061562,0.757063439295683,-0.595317455872538,0.551631289808648,0.940215282864022
"7629","SNHG15",20.9985506724046,-0.213064997158169,0.356111356510308,-0.598310032137383,0.549633083908477,0.940215282864022
"7630","RABGEF1",39.8148910663819,-0.224002134283518,0.371504799204938,-0.602958924791572,0.546536014737075,0.940215282864022
"7631","HIP1",14.963586901494,0.259977106880654,0.415725414517391,0.625357742880494,0.531736289848911,0.940215282864022
"7632","SEMA3D",74.5928538845028,0.246505423775739,0.394054592982847,0.62556160533439,0.531602528897095,0.940215282864022
"7633","MTERF1",8.80532432101131,0.304464412696096,0.49263310943125,0.61803481509313,0.536552389328616,0.940215282864022
"7634","COL1A2",8096.30680585475,0.200482774969932,0.325104992341197,0.616670859238992,0.537451845934267,0.940215282864022
"7635","POP7",48.2256587818123,-0.190897226947256,0.303719837558875,-0.628530650093776,0.52965637459495,0.940215282864022
"7636","POLR2J3",69.5713308443789,-0.214254549003231,0.342848688708657,-0.62492451060619,0.532020604611345,0.940215282864022
"7637","RELN",1.45826556540138,-0.985091665926522,1.65325163183464,-0.595851016843304,0.551274758628782,0.940215282864022
"7638","SRPK2",88.9958136776585,0.175161183705127,0.275714168702935,0.635299899635742,0.525232839049792,0.940215282864022
"7639","PUS7",7.12959824383221,-0.33283363973824,0.550535669383209,-0.604563261289009,0.545469223686404,0.940215282864022
"7640","HBP1",97.3149710216917,0.154883632098903,0.254255077871372,0.609166327750822,0.542414196920982,0.940215282864022
"7641","DUS4L",5.54804365690282,0.382809962659918,0.642063349498925,0.596218368419049,0.551029356224932,0.940215282864022
"7642","TES",25.5847555405574,-0.355894217563166,0.59413497449771,-0.599012400951558,0.549164615039842,0.940215282864022
"7643","ST7-OT4",3.2970974546901,-0.486816540602935,0.804185867509449,-0.605353265048786,0.544944296749324,0.940215282864022
"7644","CHCHD3",52.9892138086188,-0.160786676685096,0.266563104885905,

-0.603184288215417,0.54638609871553,0.940215282864022
"7645","WDR60",59.7314721918298,-0.170935533587362,0.285960983197753,-0.597758238469735,0.550001259660661,0.940215282864022
"7646","DEFB1",32.1315963894021,1.22402637255302,1.97559655195973,0.619573045589102,0.535538917225123,0.940215282864022
"7647","FAM66B",3.9248091082922,-0.437244094955247,0.707996363808856,-0.617579577108243,0.536852510441307,0.940215282864022
"7648","LOXL2",43.2295268262717,-0.234064836707423,0.387976275303776,-0.603296777680945,0.546311276214606,0.940215282864022
"7649","UBXN8",10.9884024902764,0.286235206757766,0.473743545714271,0.604198641537594,0.545711583998117,0.940215282864022
"7650","RAB11FIP1",2.85654789836227,-0.574362422879425,0.922566505204423,-0.62257020999495,0.533566992511745,0.940215282864022
"7651","NSD3",157.630367547507,0.10772385975299,0.174224431811922,0.618305128808113,0.536374221710819,0.940215282864022
"7652","RRS1",7.36776852509198,-0.401560515097114,0.629789032181826,-0.637611159575069,0.523726829457899,0.940215282864022
"7653","TMEM67",14.8854746619139,-0.265943244228733,0.434110871501358,-0.612615950641771,0.540130308900716,0.940215282864022
"7654","VIRMA",40.9005854777593,0.180977452899056,0.303703558893606,0.595901653435865,0.551240928600054,0.940215282864022
"7655","RNF19A",97.545023345983,0.161825207970441,0.257271254805714,0.629006175185208,0.529345013463649,0.940215282864022
"7656","AC021546.1",2.08056256117368,-0.792860033347585,1.25203873850746,-0.633255193279996,0.526567008185881,0.940215282864022
"7657","AC091563.1",2.75617887844995,-0.585820107207503,0.941714414410732,-0.622078305527556,0.533890379407458,0.940215282864022
"7658","NDRG1",246.709965079219,0.130946210493786,0.217304089811681,0.602594321198769,0.546778599098369,0.940215282864022
"7659","PTK2",51.8839248094578,0.194070584616685,0.306825453062884,0.632511360056265,0.527052787706966,0.940215282864022
"7660","MFSD3",5.97670528449378,-0.393741079545585,0.625054152350328,-0.629931147669431,0.528739632944098,0.940215282864022
"7661","CDKN2B",10.6225836758889,-0.365978656057848,0.587605178847389,-0.622830889230299,0.533395657405144,0.940215282864022
"7662","TRPM3",2.10410179021855,0.603765440128757,0.988291120264569,0.610918612692915,0.541253462093225,0.940215282864022
"7663","HNRNPK",351.832094165215,0.0987679800017954,0.155978979402723,0.633213400805667,0.526594295777876,0.940215282864022
"7664","RMI1",4.13196478031668,0.421589772057228,0.702651805211221,0.599998134112095,0.548507479020356,0.940215282864022
"7665","NAA35",21.4590558947332,-0.213935344724624,0.341509080968241,-0.626441159684767,0.531025619562656,0.940215282864022
"7666","CCDC180",7.41140294985237,0.397034863039446,0.65546164780051 2,0.60573317198916,0.544691952637845,0.940215282864022
"7667","MRPL50",30.8964789363374,-0.214484999884147,0.341120895658672,-0.628765351562522,0.529502686705762,0.940215282864022
"7668","ABITRAM",12.7264824501074,0.281915303612168,0.44571450422484,0.632501973662397,0.527058919195114,0.940215282864022
"7669","INIP",26.6115321824965,-0.186923650709445,0.309012213262394,-0

.60490699942245,0.545240792163889,0.940215282864022
"7670","COL27A1",2.42320447076554,0.659324092857018,1.06758512546854,0.617584562699535,0.536849223173219,0.940215282864022
"7671","AKNA",12.6515191591472,0.321021226940324,0.531780304680097,0.60367265224958,0.54606129963380l,0.940215282864022
"7672","TRIM32",7.46902829500935,0.317702237082012,0.5298927707039,0.59955948570497,0.548799854694037,0.940215282864022
"7673","NAIF1",4.98598216762096,0.459730169193933,0.729019879436633,0.630614036957675,0.528292918476532,0.940215282864022
"7674","GPR107",32.4872714922691,0.181997462583141,0.294826467107682,0.617303678223259,0.537034441170072,0.940215282864022
"7675","TTF1",34.5919054362469,0.168624159589567,0.274980500710336,0.613222243591719,0.539729398880755,0.940215282864022
"7676","CACFD1",2.41943211011471,0.557206584432452,0.924328788464467,0.602822925550232,0.546626493867437,0.940215282864022
"7677","CCDC183",3.29024508711371,-0.556158157772088,0.896505174544459,-0.620362462553202,0.535019180037088,0.940215282864022
"7678","EXD3",8.63958148195471,-0.330856794519237,0.518897122692235,-0.637615396290168,0.523724070862797,0.940215282864022
"7679","ZMYND19",11.3213683114693,-0.26262919001382,0.426878592326749,-0.615231578098893,0.538401796266681,0.940215282864022
"7680","IDI1",31.1858216263259,0.171312844067438,0.283598738316734,0.604067722882845,0.545798617782559,0.940215282864022
"7681","WDR37",8.16981849612464,0.375442269655518,0.591580871611335,0.634642341685079,0.525661706274979,0.940215282864022
"7682","PFKFB3",10.6567629809927,-0.299942566493195,0.486384521908803,-0.616677860792277,0.537447226847406,0.940215282864022
"7683","RSU1",74.0126641265889,0.149503475630735,0.250760679293959,0.59619983504462,0.551041735814709,0.940215282864022
"7684","ZNF22",106.353793742218,0.128655340539551,0.203673738068599,0.631673684391354,0.527600127138164,0.940215282864022
"7685","WDFY4",1.716686256963,0.643744278059945,1.07396157214244,0.599410905155334,0.548898906660876,0.940215282864022
"7686","ADO",14.3143128789146,-0.268746469768857,0.432958164663109,-0.62072156550731,0.534782837642982,0.940215282864022
"7687","SIRT1",21.2072620944761,0.211857001598298,0.340202878138468,0.622737240665168,0.533457206045267,0.940215282864022
"7688","HK1",25.1643286464019,-0.191922714777643,0.305120618501001,-0.629006049215955,0.529345095932624,0.940215282864022
"7689","PSAP",992.292074787478,0.0899325058739929,0.148050277690443,0.607445708828943,0.543555162072205,0.940215282864022
"7690","DNAJB12",58.1097134979792,-0.159461371378512,0.253191499794139,-0.629805390418577,0.528821918459691,0.940215282864022
"7691","VCL",106.75179119339,0.138963288961594,0.227049432502496,0.612039798690385,0.540511426259523,0.940215282864022
"7692","TSPAN14",59.876675014531,0.191991753998715,0.311893402719853,0.615568499764534,0.538179346680356,0.940215282864022
"7693","GRID1",5.16368324954142,0.458691294992259,0.724751951788272,0.632894183810711,0.526802746193735,0.940215282864022
"7694","NUTM2A-

AS1",55.9523552235062,-0.191991603429486,0.309611823006922,-0.620104237509023,0.535189162523456,0.940215282864022
"7695","LOXL4",17.1468890618732,-0.3206749953749,0.50237761273615,-0.638314660616295,0.523268870460665,0.940215282864022
"7696","NT5C2",19.0812538480159,0.24273965260889,0.399044897279963,0.608301607822812,0.542987454864042,0.940215282864022
"7697","GSTO1",188.464378089619,-0.151158672741336,0.253876296702118,-0.595402858419254,0.55157421529277,0.940215282864022
"7698","FAM160B1",40.8710562123446,0.172997839176924,0.288826092958714,0.598968872253703,0.549193642265267,0.940215282864022
"7699","ENO4",1.7034079640625,-0.610876889185192,0.993012039267272,-0.61517571291074,0.538438685226654,0.940215282864022
"7700","PDZD8",42.976758502375,0.172813679856335,0.288683131157373,0.598627564982755,0.549421270136149,0.940215282864022
"7701","DHX32",19.6934417655158,-0.23046929181175,0.364445731974379,-0.632383017804011,0.527136628062806,0.940215282864022
"7702","PSMD13",111.622854725943,-0.152946678432482,0.252564459341462,-0.6055748256555,0.544797123352408,0.940215282864022
"7703","GTF2H1",30.0465211616218,-0.184349358845208,0.30895267326782,-0.59669125661004З,0.550713531166559,0.940215282864022
"7704","DNAJC24",17.0439874619734,0.238572049752055,0.382730063009355,0.623342853906472,0.533059242361463,0.940215282864022
"7705","NAT10",14.4065346436293,0.286018018393363,0.451323473303946,0.633731758509141,0.526255895165534,0.940215282864022
"7706","ZBTB3",4.32791616706422,-0.442178222553549,0.707666683888692,-0.624839677521259,0.532076286528696,0.940215282864022
"7707","FRMD8",10.2412451864821,0.307908762738567,0.508350048913915,0.605702238834069,0.544712497076247,0.940215282864022
"7708","PCNX3",11.9528201943502,0.256891915532005,0.425345430920013,0.603960679620687,0.54586978449174,0.940215282864022
"7709","SSH3",13.4273307118595,-0.230669577012963,0.386435113881619,-0.596916710533782,0.550562989943006,0.940215282864022
"7710","FADD",21.3966281379181,-0.212251866328612,0.345892313447168,-0.61363568393095,0.5394560977261,0.940215282864022
"7711","FAM86C1",1.78440497831835,-0.677851541349738,1.0717392813213,-0.632478022559783,0.52707456497476,0.940215282864022
"7712","INPPL1",15.4753435709762,0.235967473565892,0.373544872348659,0.631697798666727,0.527584366757884,0.940215282864022
"7713","NPAT",20.5910011193202,0.213122522298949,0.34520071681531,0.617387253031036,0.536979327759781,0.940215282864022
"7714","USP28",17.550007296905,0.254180950327322,0.413267711394696,0.61505155936212,0.538520670968248,0.940215282864022
"7715","BACE1-
AS",5.93674318059477,0.411184874434819,0.652697420963422,0.629977783316325,0.528709119952819,0.940215282864022
"7716","NLRX1",7.71656516599988,0.326326827417973,0.538639133488094,0.605835720298972,0.544623847323175,0.940215282864022
"7717","SIAE",8.5391056037002,0.31362644746619,0.500878200382626,0.626153119114802,0.531214513547323,0.940215282864022
"7718","AP000866.1",3.60984745145289,0.512165608080677,0.8190505506818

02,0.625316236774806,0.531763525478115,0.940215282864022
"7719","TIRAP",5.06390596803134,-0.414784210399446,0.694292265944774,-0.597420179864771,0.550226884018008,0.940215282864022
"7720","AC007406.5",4.694468804188,-0.438154898296345,0.719073191058755,-0.609332824174978,0.54230385433364,0.940215282864022
"7721","DCP1B",9.79302033500411,0.296282014213987,0.483289125620887,0.613053343241171,0.539841068933863,0.940215282864022
"7722","NOP2",5.63450199105178,0.374317508162627,0.593036086337246,0.631188416331483,0.527917335069859,0.940215282864022
"7723","AMN1",4.50493743141283,0.441903251253666,0.701617891910217,0.629834638410581,0.528802780324641,0.940215282864022
"7724","GXYLT1",19.9471046505778,-0.199307127383809,0.335217946853575,-0.594559835636924,0.552137734382235,0.940215282864022
"7725","MAP3K12",27.0718812835414,0.221333852559309,0.368850752463206,0.600063443225286,0.548463954617807,0.940215282864022
"7726","NCKAP1L",1.57207263000863,-0.710766190176453,1.12573689596357,-0.631378604294633,0.527793002240926,0.940215282864022
"7727","RAP1B",453.554827373675,0.132786956954148,0.211877419831836,0.626715942923691,0.530845451360725,0.940215282864022
"7728","TSPAN8",24.5968963414547,-0.567712689610608,0.921804969805688,-0.615870719085274,0.537979848262113,0.940215282864022
"7729","LINC02823",1.97172889974254,0.670993473470419,1.10064799488429,0.609634939225923,0.542103661463581,0.940215282864022
"7730","USP44",9.16085881038836,0.387260634923959,0.64496181260389,0.600439634341265,0.548213280191677,0.940215282864022
"7731","ALKBH2",21.8144173196634,0.230037530170777,0.379778838144413,0.60571445026988,0.544704386732748,0.940215282864022
"7732","VPS29",140.596059594048,-0.159805951135443,0.26384171868657,-0.605688713411105,0.544721480187074,0.940215282864022
"7733","PHETA1",8.14919875972353,-0.301131866186543,0.497235468926292,-0.605612199863365,0.544772299183454,0.940215282864022
"7734","LINC00173",8.25358455368042,0.477936312572882,0.789003622699189,0.60574666430283,0.544682991758273,0.940215282864022
"7735","RFC5",10.1271627353082,-0.276514014297376,0.454769913894267,-0.608030579528762,0.543167192618378,0.940215282864022
"7736","TMEM233",11.3735741910821,-0.460763263701942,0.753530794105125,-0.611472374196907,0.540886901891291,0.940215282864022
"7737","TMED2",191.53494964643,-0.103035381190469,0.170908950106272,-0.602867088741702,0.546597111661057,0.940215282864022
"7738","XPO4",10.6298821111823,-0.278953097896895,0.454864771768546,-0.613265997303563,0.539700472589226,0.940215282864022
"7739","LINC00539",3.59238872918376,0.479661060020001,0.76461309273522,0.627325198296211,0.530446089170245,0.940215282864022
"7740","AL162377.1",4.81927553894156,0.39338806007258,0.636804201235857,0.617753556445018,0.536737802507626,0.940215282864022
"7741","HNRNPA1L2",4.33936998859881,-0.418883320080223,0.684637825870933,-0.611831985104473,0.540648925354433,0.940215282864022
"7742","DIS3",36.5405952479983,0.200989873589046,0.315290407397476,0.637475384196083,0.523815238993298,0.940215282864022
"7743","GGACT",7.36129601960771,-0.37813978254036,0.635880166513336,-0

.594671452977972,0.552063107470566,0.940215282864022
"7744","METTL3",19.2346876807671,0.215846726396071,0.359291475096242,0
.600756603919568,0.548002111902186,0.940215282864022
"7745","NGDN",23.1762336140422,-0.243592317064391,0.387917495307888,-0
.627948777796301,0.530037495657159,0.940215282864022
"7746","AL136295.7",2.36387587226224,0.550449732506887,0.9129186087322
77,0.602955977939007,0.546537975174212,0.940215282864022
"7747","LTB4R",4.98987031410632,-0.405287447783114,0.675127935021466,-
0.600312069400932,0.548298276569015,0.940215282864022
"7748","SCFD1",79.8192463935627,0.135041054303129,0.220244953211203,0.
613140289183527,0.539783582363166,0.940215282864022
"7749","ERO1A",26.0005317426641,0.194199453955608,0.321894591278977,0.
60330138876829,0.54630820925274,0.940215282864022
"7750","AL139317.5",5.59908024737057,-0.441660563912997,0.727816888736
074,-0.60682923239111,0.543964246183454,0.940215282864022
"7751","AL138995.1",3.28782182250684,-0.476264608418355,0.778825376155
028,-0.61151655172,0.540857664101583,0.940215282864022
"7752","HIF1A",48.8726104763761,0.161857782308593,0.259575761658308,0.
623547365418711,0.532924886627643,0.940215282864022
"7753","PPP2R5E",73.5128474151716,-0.130268930790388,0.207512735329928
,-0.627763547057826,0.53015884933617,0.940215282864022
"7754","SUSD6",12.578875905255,0.294467290355077,0.483024666394609,0.6
09631993647528,0.54210561313738,0.940215282864022
"7755","IRF2BPL",50.3164504602667,-0.158650369243655,0.256571130291606
,-0.618348483180244,0.536345648997505,0.940215282864022
"7756","TTC8",15.6654404623532,-0.257987532456422,0.411360947212198,-0
.627156112423431,0.530556908358176,0.940215282864022
"7757","FOXN3-
AS1",4.41944838226618,0.474662939015803,0.759670578471865,0.6248273297
23133,0.53208439150273,0.940215282864022
"7758","SIVA1",142.186303787539,-0.185096310556446,0.30103663547332,-0
.614863072281614,0.538645151814961,0.940215282864022
"7759","NUDT14",5.34618430641846,0.409060144400274,0.661259606150788,0
.618607488791618,0.536174967221615,0.940215282864022
"7760","INO80",29.8747541906087,0.228354079704088,0.36051640477375,0.6
33408290664044,0.526467052363215,0.940215282864022
"7761","NDUFAF1",15.1287313125624,-0.256151914044786,0.401992892488802
,-0.637205082057318,0.523991267812127,0.940215282864022
"7762","SMAD3",95.9394649694892,0.147982967633356,0.245942342166537,0.
601697805793649,0.547375310845784,0.940215282864022
"7763","PARP6",15.2812010022893,-0.239700827504303,0.394711981113978,-
0.607280343575602,0.543664880836957,0.940215282864022
"7764","SCAMP2",71.0631577326305,-0.160884000805969,0.26540929438621,-
0.606173198184457,0.544399748321557,0.940215282864022
"7765","ZNF592",15.1413677521774,0.275506276000659,0.452541208122992,0
.608798206782931,0.542658202366838,0.940215282864022
"7766","MAN2A2",13.4504050582521,0.291875161159049,0.474926422873553,0
.614569219781566,0.538839246986934,0.940215282864022
"7767","NPRL3",30.3948146450453,-0.179914834486935,0.299219665110081,-
0.601280114462881,0.547653432013754,0.940215282864022

"7768","WDR90",8.17860427512228,0.356550424476329,0.577449775752678,0.617457031672725,0.536933314410036,0.940215282864022
"7769","RPUSD1",7.41748943340605,-0.341756362542286,0.559800864363403,-0.610496310917502,0.541533086418495,0.940215282864022
"7770","JPT2",8.4710544883416,0.361510799768594,0.572713807706851,0.631224173232499,0.527893958336193,0.940215282864022
"7771","E4F1",11.5358811124879,0.259976604111407,0.43656599688461,0.595503557232199,0.551506922011619,0.940215282864022
"7772","AC093525.6",2.1080575746663,0.633409555653791,1.00411273354893,0.630815180896147,0.528161376113647,0.940215282864022
"7773","PARN",18.755054825928,0.229348498887506,0.365913489999227,0.626783393222234,0.530801230701516,0.940215282864022
"7774","GGA2",29.698957011163,0.194580005307007,0.322136207556056,0.604030223063787,0.545823548662024,0.940215282864022
"7775","SULT1A3",1.65025905404404,0.702470566781032,1.17937252239222,0.595630772672366,0.55142191442121,0.940215282864022
"7776","PHKG2",12.1460205620867,-0.315642250630773,0.49925745654456,-0.632223407969473,0.527240903650204,0.940215282864022
"7777","BCKDK",31.5607755101661,-0.213796036627289,0.358661406310472,-0.596094346549845,0.551112200725232,0.940215282864022
"7778","MT1A",38.0363175239708,-0.565975325288537,0.913651482094612,-0.61946522977339,0.535609920856881,0.940215282864022
"7779","CPNE2",10.7035135649815,-0.31063522308657,0.495276887602381,-0.627195071811941,0.530531373262193,0.940215282864022
"7780","AC009107.2",19.1577364229865,-0.289969951740757,0.473349948107668,-0.612591071151444,0.540146763596574,0.940215282864022
"7781","CSNK2A2",62.7209288559317,0.165997842420849,0.277219722465442,0.598795211771204,0.549309455742197,0.940215282864022
"7782","CENPT",28.3003661233852,-0.194166067924745,0.326119636625557,-0.595382939628629,0.551587526759633,0.940215282864022
"7783","LCAT",14.0418636808865,0.260083770266154,0.425024605159866,0.611926385222635,0.540586463696003,0.940215282864022
"7784","CMTR2",19.837905057404,0.229883432973157,0.371062320542053,0.619527826585411,0.53556869626273,0.940215282864022
"7785","IST1",56.2474292941658,0.161103687622732,0.258313949226609,0.623673975428258,0.53284171759425,0.940215282864022
"7786","AC116667.1",3.2488860127558,-0.589662930282027,0.932593756219074,-0.632282734416582,0.52720214353074,0.940215282864022
"7787","RILP",23.6688366527788,-0.207718815302292,0.345850983392122,-0.600602066430392,0.548105061294441,0.940215282864022
"7788","SLC25A11",55.3282071096522,-0.177189485509609,0.281974576518434,-0.628388160724926,0.529749690953668,0.940215282864022
"7789","ZNF594",4.1174393357693,0.46094800422534,0.744702374808481,0.618969429691807,0.535936498059015,0.940215282864022
"7790","TP53",15.1964059249565,0.265617676069077,0.420318741899279,0.631943450508154,0.527423829628788,0.940215282864022
"7791","KDM6B",23.8099385061299,-0.239516197221559,0.380664340865003,-0.629205763474702,0.529214356122696,0.940215282864022
"7792","ELAC2",20.3875025923339,-0.225204651429734,0.354329763703483,-0.635579266827255,0.525050686608032,0.940215282864022

"7793","TNFAIP1",11.0750318110668,-0.299800857048853,0.483483048051082
,-0.620085560925764,0.535201457863254,0.940215282864022
"7794","NSRP1",81.5367302113467,-0.133838062040679,0.221706740174652,-
0.603671597603421,0.546062000950261,0.940215282864022
"7795","ATAD5",5.76475091308753,0.455444411133259,0.740564400428457,0.
614996360707806,0.538557123830696,0.940215282864022
"7796","ZNF830",27.8603413503491,-0.202488398456972,0.322699008843971,
-0.627483794209227,0.530342155803908,0.940215282864022
"7797","KAT2A",7.12594642879986,-0.336985012404226,0.547096004858117,-
0.615952244965889,0.53792603845743,0.940215282864022
"7798","HSD17B1",2.80332503371833,-0.496836112791787,0.828646039662221
,-0.59957580077775,0.548788978706739,0.940215282864022
"7799","CNTD1",2.05314503566911,-0.572647810634013,0.950304228143661,-
0.602594194232548,0.546778683583014,0.940215282864022
"7800","DHX8",20.4447132629413,0.247635945212019,0.393627059092663,0.6
29113114791541,0.529275005088709,0.940215282864022
"7801","AC002558.3",8.47498808558053,0.320040418454709,0.5236964567899
37,0.611118166459319,0.541121353981456,0.940215282864022
"7802","SPAG9",169.147451884258,0.132746374198953,0.220277150572198,0.
602633427271632,0.546752577818033,0.940215282864022
"7803","RAD51C",22.408556349011,-0.207424521486508,0.344349581774641,-
0.60236611996891,0.546930457005889,0.940215282864022
"7804","DCAF7",54.6007491557144,0.166976513537028,0.267777593197675,0.
62356417332411,0.532913845280207,0.940215282864022
"7805","DDX42",72.472862332366,0.147306152404291,0.2364928645139,0.622
877788330198,0.533364835261937,0.940215282864022
"7806","CBX8",2.1281221306379,0.688691008546394,1.10849640920694,0.621
28393274554,0.534412823393376,0.940215282864022
"7807","RPTOR",6.31780652064982,0.333675280548725,0.560999849280011,0.
594786756140781,0.551986021434382,0.940215282864022
"7808","AATK",3.38180077173943,0.485533655099743,0.793535939366472,0.6
11860951738839,0.54062975863858,0.940215282864022
"7809","NPLOC4",33.9255644643718,0.217234682041313,0.352128252260911,0
.616919206699586,0.537288017556017,0.940215282864022
"7810","YES1",43.6106695040877,0.156365224483334,0.248442477599936,0.6
29382004212368,0.529098996530496,0.940215282864022
"7811","LINC00667",52.4876647580125,-0.140134110089646,0.2263740329839
34,-0.619037918097217,0.535891379650245,0.940215282864022
"7812","RAB12",28.3747269520665,0.216968468064483,0.342692032840254,0.
633129595299413,0.526649017146621,0.940215282864022
"7813","GNAL",14.0764618032078,-0.276825751349702,0.465307284980079,-0
.594931049406531,0.551889561485376,0.940215282864022
"7814","TTC39C",20.3747240992921,-0.222025181682747,0.364668496475948,
-0.608841136068333,0.542629744285122,0.940215282864022
"7815","OSBPL1A",157.985928663905,-0.112372808783861,0.183506644446825
,-0.612363705535599,0.540297149357851,0.940215282864022
"7816","KIAA1328",25.1218994733516,0.200801830985669,0.319485976207832
,0.628515321295489,0.529666413031457,0.940215282864022
"7817","PSTPIP2",7.81780250945188,-0.310240312899382,0.512181798769928
,-0.60572303358781,0.544698686074821,0.940215282864022

"7818","AC016588.2",2.22801428506163,0.628816113365028,1.0432289418225
9,0.602759459746627,0.546668719661635,0.940215282864022
"7819","TCF3",31.4955736531364,0.196801158361862,0.312915766055766,0.6
28926950030346,0.529396881516003,0.940215282864022
"7820","DPP9",22.2232150692471,0.226413327547126,0.355775829018965,0.6
3639322595762,0.524520155001806,0.940215282864022
"7821","SAFB",51.0571381152007,0.158444258616211,0.264705167109421,0.5
98568816568338,0.549460455876508,0.940215282864022
"7822","COL5A3",40.6390893391305,0.260710666130397,0.429212439700157,0
.607416379433286,0.543574621130592,0.940215282864022
"7823","STX10",59.8220366317056,-0.196732276519095,0.315630657813144,-
0.623299009931925,0.533088048296801,0.940215282864022
"7824","MRI1",26.7136840575583,0.208390303862694,0.350483739050881,0.5
94579093532329,0.552124858276171,0.940215282864022
"7825","PRKACA",47.1671391314269,0.189649797658256,0.315067155139156,0
.601934522735296,0.547217723105175,0.940215282864022
"7826","NOTCH3",8.07338898520674,0.453527147057948,0.753268810113259,0
.602078754581326,0.54712171576036,0.940215282864022
"7827","MPV17L2",2.27076699231448,0.583805551342895,0.941719562842727,
0.619935673397913,0.535300138353787,0.940215282864022
"7828","ZNF506",40.7418484164705,0.19063202195919,0.312481826736083,0.
6100579478505,0.541823421886927,0.940215282864022
"7829","ZNF675",14.2852947310339,0.237411011861725,0.389737336411306,0
.60915644892481,0.542420744291548,0.940215282864022
"7830","TMEM147",95.158921595533,-0.176716368578523,0.290816549316535,
-0.607655819429239,0.543415770959714,0.940215282864022
"7831","LINC00665",7.56223403888417,0.387746225682763,0.62050892975183
5,0.624884199229554,0.532047063433637,0.940215282864022
"7832","ZNF540",3.25736144743392,0.549570100658245,0.876478978280604,0
.627020287168029,0.530645936989168,0.940215282864022
"7833","ZNF781",3.38185598332421,0.474406850918163,0.78722852784198,0.
602629140255688,0.546755430379418,0.940215282864022
"7834","PLEKHG2",4.46994163316196,-0.42509293623343,0.692472791391213,
-0.613876734968021,0.539296785018271,0.940215282864022
"7835","ZNF221",3.15126026465492,0.551529882321147,0.866183387738858,0
.636735696075744,0.52429701801009,0.940215282864022
"7836","PPP1R37",16.3885053220553,-0.26594079871603,0.421052310192867,
-0.631609879053302,0.527641829586176,0.940215282864022
"7837","STRN4",15.9882151990582,-0.26679193993014,0.42639867199364,-0.
625686610801919,0.531520517078897,0.940215282864022
"7838","ZC3H4",22.9581639047161,0.223391810763791,0.367118523767738,0.
608500514959366,0.542855564528506,0.940215282864022
"7839","C5AR2",1.8108850611704,-0.7532236566331,1.19185927670314,-0.63
1973649369604,0.527404095943941,0.940215282864022
"7840","NAPA-
AS1",4.78972967055948,0.440393247787314,0.720710808931501,0.6110540348
91227,0.541163808453709,0.940215282864022
"7841","CA11",6.42871539980904,-0.36964307652191,0.607751367361721,-0.
608214306660549,0.543045347115659,0.940215282864022
"7842","PLEKHA4",27.0029659968801,-0.25097974099346,0.392725048207728,

-0.639072404825848,0.522775830713869,0.940215282864022
"7843","AP2A1",71.9270543339849,-0.160920834618616,0.25641547160309,-0.627578490535503,0.530280102969752,0.940215282864022
"7844","PNKP",23.9082161444443,-0.200975752026482,0.327520078555914,-0.6136287976988,0.539460649242544,0.940215282864022
"7845","CTU1",19.2269336661635,-0.256243539516483,0.40538015900399,-0.632106761579224,0.527317117206625,0.940215282864022
"7846","SPACA6",6.78975949680415,-0.372811516587925,0.588757817000585,-0.633217098478974,0.526591881424116,0.940215282864022
"7847","ZNF611",9.79602721541749,-0.280678274175207,0.468762828715037,-0.598763931313829,0.549330317833468,0.940215282864022
"7848","ZNF888",1.48496045577793,-0.711233494758739,1.19260046364102,-0.59637197573053,0.550926757644061,0.940215282864022
"7849","ZNF865",10.4717485599151,-0.280281105950322,0.468145905278024,-0.598704597840855,0.549369890589503,0.940215282864022
"7850","ZNF667-AS1",37.1286496960333,-0.195336202130287,0.325773009035793,-0.59960830612835,0.548767310240355,0.940215282864022
"7851","ZNF549",2.57093403482311,0.603373716725841,0.987900439950537,0.610763688652726,0.541356035645515,0.940215282864022
"7852","ZCCHC3",17.0456091534704,-0.237106637867107,0.388661900353818,-0.610058865176282,0.541822814245047,0.940215282864022
"7853","LZTS3",11.3572933087017,0.294613078059811,0.491293608087891,0.599668046173941,0.54872748789276,0.940215282864022
"7854","FAM182A",8.8856661268993,-0.330956098018244,0.53325581287207,-0.620632893312016,0.534841192051373,0.940215282864022
"7855","NOL4L",6.21368474848513,0.392976895519654,0.616794282627961,0.637127980248628,0.52404148436909,0.940215282864022
"7856","RALY",97.1669606773482,-0.152192499962517,0.253740854897893,-0.59979501536621,0.548642855746921,0.940215282864022
"7857","CPNE1",110.501681491287,0.150046201895862,0.243752749765978,0.615567217354134,0.53818019329317,0.940215282864022
"7858","TTI1",9.97798022449532,0.285906968761851,0.464580583329307,0.615408777338402,0.538284796266957,0.940215282864022
"7859","ZNFX1",29.9718474199842,0.18976547498139,0.311492242220569,0.609214128828982,0.542382516448689,0.940215282864022
"7860","B4GALT5",23.5056998984613,0.222067353343739,0.358230086823648,0.619901458620533,0.535322665402923,0.940215282864022
"7861","APCDD1L",10.8541063563838,0.418381678312999,0.682413991588132,0.613090709554957,0.539816362848354,0.940215282864022
"7862","APCDD1L-DT",6.4191375068702,0.604595991038346,0.980819214378395,0.616419399391065,0.537617753039368,0.940215282864022
"7863","PRELID3B",18.9598795375233,0.263416516187748,0.416300749737144,0.632755324976166,0.5268934349062,0.940215282864022
"7864","LAMA5",1.70490681397021,0.645669405133702,1.07940550136308,0.598171312188373,0.549725631206275,0.940215282864022
"7865","OGFR",32.926018085778,0.177550509466883,0.296134718072006,0.599559925370556,0.548799561601867,0.940215282864022
"7866","PPDPF",300.967827596123,-0.225859410610822,0.359756559804196,-

0.627811792323536,0.530127240153438,0.940215282864022
"7867","DNAJC5",13.4955138551755,0.245684934330672,0.406380280976946,0.604569035042846,0.545465386340408,0.940215282864022
"7868","LINC02246",2.56193580771372,0.63922626390285,1.04692064737304,0.610577569089707,0.541479276254737,0.940215282864022
"7869","LINC01684",4.20530640078151,0.42082537096375,0.693073503909516,0.607187215482834,0.543726675568005,0.940215282864022
"7870","URB1-AS1",6.53198922862309,0.422401713461347,0.701919406452262,0.601780930372488,0.547319970404632,0.940215282864022
"7871","PAXBP1",31.5399852704218,0.220746468078007,0.347282453730401,0.635639565739117,0.525011374874385,0.940215282864022
"7872","DOP1B",8.7508215629176,0.309834963302853,0.508293880700044,0.609558712129557,0.542154168946257,0.940215282864022
"7873","MORC3",57.67506832946,0.170958111914936,0.269313779117314,0.634791552349299,0.525564373574991,0.940215282864022
"7874","PRDM15",2.10259093078468,0.663220708762565,1.05801480426345,0.62685390231782,0.530755006682481,0.940215282864022
"7875","MCM3AP-AS1",1.88812718451166,-0.619780912665394,1.03563417859666,-0.598455444474837,0.549536080015706,0.940215282864022
"7876","AC006946.2",2.41919761617573,0.616844866832499,1.01294257418663,0.608963313964574,0.542548755893127,0.940215282864022
"7877","ZNF74",4.95989123235503,-0.397812616203121,0.640989263152665,-0.620622901304938,0.534847767906553,0.940215282864022
"7878","LIMK2",5.97799010048758,0.395271088338177,0.650081404892635,0.608033217629811,0.543165442966903,0.940215282864022
"7879","NOL12",14.3411091853698,-0.241400212929256,0.381688719510181,-0.632453097484891,0.527090847242152,0.940215282864022
"7880","EIF3L",129.674715282075,-0.14951260216505,0.242646276934327,-0.616175133836963,0.537778938149252,0.940215282864022
"7881","RPS19BP1",150.37258744674,-0.156798326352246,0.249320628214775,-0.628902339429266,0.529412994398715,0.940215282864022
"7882","MSL3",25.1658624315624,-0.192647741491548,0.318269426349858,-0.605297667768378,0.544981230783955,0.940215282864022
"7883","PIR",15.8374352108798,0.266092549577218,0.443938699646147,0.59939029823107,0.548912645066695,0.940215282864022
"7884","RBBP7",105.317018915813,-0.128145970746172,0.204073422862872,-0.627940517429749,0.53004290712497,0.940215282864022
"7885","WAS",1.47171805864368,-0.644329756351128,1.08078869059313,-0.596166264468886,0.551064160031778,0.940215282864022
"7886","MAGED1",49.8228692002755,0.197461370347782,0.318642602297612,0.619695448518066,0.535458312792272,0.940215282864022
"7887","RPL36A",1000.72818591296,-0.175722885578049,0.281507112070278,-0.624221833280644,0.532481910151937,0.940215282864022
"7888","ARMCX5-GPRASP2",6.31492445496111,-0.366244137827143,0.573234731124902,-0.638907794558145,0.522882917023242,0.940215282864022
"7889","SLC25A53",6.04172833222287,-0.38402183452748,0.60198405974423,-0.637926915690496,0.523521255861903,0.940215282864022

"7890","IL13RA1",230.854753470089,-0.199238561257815,0.322120533595628
,-0.618521765855287,0.536231454642435,0.940215282864022
"7891","TMEM255A",12.6696394872465,0.317441141744285,0.508520922988972
,0.624244013163583,0.532467346031784,0.940215282864022
"7892","RAP2C-
AS1",9.24723267623199,0.312241286480679,0.518827501524407,0.6018210013
21748,0.547293294037149,0.940215282864022
"7893","NEK3",11.8842047876032,-0.247754057807342,0.416802512265966,-0
.594415941642041,0.552233948644567,0.940259981734998
"7894","LRIF1",13.3829006810993,0.264948129772602,0.445964768017474,0.
594101033923425,0.552444539401318,0.940335183474191
"7895","SPRR2F",1.50865913735218,1.00645949287914,1.70135379927674,0.5
91563902409357,0.554142652042826,0.940335183474191
"7896","EFNA4",14.3982851549035,0.267573803953074,0.451882517890658,0.
592131346886534,0.553762637417289,0.940335183474191
"7897","CMPK2",1.75985777889629,-0.6473776026297,1.09019116431214,-0.5
93820261823681,0.552632335600316,0.940335183474191
"7898","SFXN5",10.9133088590483,-0.268522472311278,0.453038872914052,-
0.592713977465286,0.553372585573652,0.940335183474191
"7899","SLC35G2",11.2040531969612,0.260557632256271,0.439495440613305,
0.592856280585457,0.553277338815928,0.940335183474191
"7900","PAPSS1",45.2083413044914,0.179486797502817,0.30330400824451,0.
591771927254332,0.5540033240652,0.940335183474191
"7901","NDUFS4",182.783561986113,-0.166751315041792,0.281645617673807,
-0.592060747896734,0.553809910242861,0.940335183474191
"7902","PCDHB16",10.906099219378,0.35699253873952,0.603053789048717,0.
591974621870224,0.553867582591117,0.940335183474191
"7903","DCTN4",30.116399519384,0.176286904460386,0.297374280736674,0.5
92811537109655,0.553307285789422,0.940335183474191
"7904","KIAA1324L",4.04236431635074,0.456335265053991,0.76844328874016
9,0.593843777075774,0.552616606073272,0.940335183474191
"7905","CASD1",29.3343881584497,0.203098009348466,0.342177191870751,0.
593546309261843,0.55281560069015,0.940335183474191
"7906","PLOD3",42.0148483429866,0.162586045064888,0.274338371742476,0.
592647845914567,0.553416851678945,0.940335183474191
"7907","SH2D4A",41.1011953208606,-0.184170340161741,0.310594168801392,
-0.592961358136468,0.553207013146624,0.940335183474191
"7908","C9orf85",23.12314670067,0.20781864960701,0.350444655608348,0.5
93014178647557,0.553171663408529,0.940335183474191
"7909","FPGS",16.7097266707781,-0.216560296889629,0.364907060744614,-0
.593466995260998,0.552868664664164,0.940335183474191
"7910","PARD3",27.3035478236671,-0.206587684849942,0.34799168983133,-0
.593656948963562,0.552741582846734,0.940335183474191
"7911","NOLC1",23.924769920369,0.207292937656796,0.350217591303766,0.5
91897559700242,0.55391918802516,0.940335183474191
"7912","FAM111A-
DT",8.13170229142815,-0.288409343470599,0.48756744722935,-0.5915270699
66451,0.554167322949296,0.940335183474191
"7913","C2CD3",6.8254004358223,0.343444307495705,0.579827364473736,0.5
92321660788505,0.553635213768142,0.940335183474191

"7914","PTPN21",25.8967836101963,0.210744984585586,0.356195332803534,0
.591655659626022,0.554081194060923,0.940335183474191
"7915","NPIPB2",5.76059432104071,-0.349300596147256,0.589422928216937,
-0.592614537754622,0.553439147632184,0.940335183474191
"7916","ZNF48",3.19741168511189,0.543017226965826,0.917603725722934,0.
591777487104262,0.553999600501803,0.940335183474191
"7917","CRISPLD2",55.5571831941142,-0.14958148852976,0.252727919372178
,-0.591867684826226,0.553939194659214,0.940335183474191
"7918","VPS25",59.0920704331817,-0.16837598166685,0.284319214695971,-0
.592207536331647,0.553711623460341,0.940335183474191
"7919","UBTF",87.4495776261906,0.124506178909473,0.210306045135172,0.5
92023775776147,0.553834667428071,0.940335183474191
"7920","ZNF570",12.3427379423153,0.25539497656362,0.429996979674517,0.
593945977846028,0.552548245769714,0.940335183474191
"7921","CCDC150",3.46382629495859,0.545004236502417,0.921612838973235,
0.591359205791452,0.55427976762444,0.940407246194274
"7922","LMF2",77.4488350769978,-0.154007296347527,0.260492587767711,-0
.591215656719032,0.554375933558431,0.940451675219863
"7923","ICA1L",13.7845405717869,0.25579862238495,0.432824226617081,0.5
90998855087784,0.554521188083053,0.940526289709387
"7924","SLC23A3",1.80567645753273,-0.636887253799156,1.0781294607834,-
0.590733559341173,0.554698958413126,0.940526289709387
"7925","SLC9B2",9.21363900765336,-0.334450267669259,0.566012471402905,
-0.590888513181165,0.554595123000725,0.940526289709387
"7926","GNG2",29.4966223978303,0.195862696887517,0.331559191378556,0.5
90732219104405,0.55469985655455,0.940526289709387
"7927","TSPAN11",1.50890199589808,0.864035021693397,1.46350124786237,0
.59038898870461,0.554929891140635,0.940797629246751
"7928","ATP6V1A",30.9200847618299,0.176999779313968,0.300022858634825,
0.589954312545913,0.555221279995071,0.940935533651167
"7929","ITCH",33.9998319615205,0.177871477368061,0.301446183078953,0.5
90060472988221,0.55515010754248,0.940935533651167
"7930","GABPA",49.398970383731,0.183737362405464,0.311395200638764,0.5
90045582040329,0.555160090514704,0.940935533651167
"7931","POLK",53.7120845384238,0.148803439424571,0.252530123875314,0.5
89250253162043,0.55569341072272,0.941232493726673
"7932","UFM1",86.8503199163752,0.133968104397921,0.227279125534955,0.5
89443065141137,0.555564094671622,0.941232493726673
"7933","DMAC2L",17.1509025002468,0.229180483966412,0.389048517064058,0
.589079443602342,0.555807982360774,0.941232493726673
"7934","CCDC32",39.6947453748352,-0.177301302745626,0.300967469814802,
-0.589104539619272,0.555791148319451,0.941232493726673
"7935","HAPLN3",2.23174964967062,-0.624966259293463,1.06003246537185,-
0.589572753391312,0.55547712300851,0.941232493726673
"7936","EEF2K",69.5606774084325,0.204983183802714,0.347979759477227,0.
58906639889246,0.555816732659787,0.941232493726673
"7937","DENND4B",11.5056323602853,0.274570803310817,0.466290987024188,
0.588840039699446,0.555968583509302,0.941252430559525
"7938","MITF",42.8680946877461,0.193776922967409,0.329037476380068,0.5
88920523884577,0.555914589162018,0.941252430559525

"7939","GMCL1",21.3505115952014,0.204507332585188,0.347372841681525,0.58872573801461,0.556045269367485,0.941263682457442
"7940","TNPO3",32.1964963878173,0.178827067787573,0.303895177748126,0.588449836923007,0.556230394741188,0.941458472912698
"7941","PLEKHM3",14.2088456273199,0.235764700903773,0.400924218616879,0.588053028368107,0.556496699857165,0.941555166405549
"7942","ADAMTS19",2.16092246767916,-0.680221074190274,1.1566230726659,-0.588109549485669,0.556458763756739,0.941555166405549
"7943","WFDC10B",1.46561861319842,-1.73567447489691,2.95156868655443,-0.588051527583823,0.556497707177563,0.941555166405549
"7944","UBQLN2",37.7396646434933,0.154752651932189,0.263227632209347,0.587904281299439,0.556596542615848,0.941603843934338
"7945","ARL14EP",44.2544097182204,0.153293805388158,0.260909721168591,0.587535813926626,0.556843904747666,0.94190374271918
"7946","MEGF6",31.6331179028727,0.216284131862285,0.369049671589066,0.586056968784178,0.557837232168026,0.941946856457898
"7947","PLA2G5",37.4701110327024,-0.490434304672884,0.835106132171681,-0.587271827830455,0.55702115869886,0.941946856457898
"7948","GPATCH3",6.99404100302382,-0.310259899011804,0.529321831851781,-0.586146046397501,0.557777375118715,0.941946856457898
"7949","S100A8",7.33695614281379,-0.433986354488019,0.739736978205692,-0.586676571908974,0.557420945223093,0.941946856457898
"7950","G0S2",63.9865630035972,-0.25480490396917,0.43462219127064,-0.586267588463983,0.557695708130467,0.941946856457898
"7951","AK6",55.2510536725216,-0.18148055169741,0.309674229188359,-0.586036985296005,0.557850660804257,0.941946856457898
"7952","WDR55",24.0731485318512,-0.20134182762631,0.343535840392091,-0.586086818180342,0.55781717406675,0.941946856457898
"7953","SLC25A13",13.3722749360917,0.26005427102033,0.443570781774888,0.586274573766465,0.557691014717387,0.941946856457898
"7954","ZC3H12C",8.61317926928516,0.300462954657443,0.512525596842253,0.586239900033561,0.557714312129747,0.941946856457898
"7955","WBP11",47.3678612105299,-0.137025594046549,0.233377293565668,-0.587141927789956,0.557108390409912,0.941946856457898
"7956","CRYL1",78.7586559837298,-0.169954004984718,0.289773670058515,-0.586506030552739,0.557535510159805,0.941946856457898
"7957","CTSG",6.39365350859301,-0.503838269255244,0.858707972877689,-0.586739945556565,0.557378375491331,0.941946856457898
"7958","ZNF823",7.49988390761534,0.317625782926524,0.541634294297788,0.586421107877439,0.557592563123741,0.941946856457898
"7959","NR2F6",34.9099600999503,0.215114625605003,0.36681552217202,0.586438175601859,0.557581096414321,0.941946856457898
"7960","CERS3-AS1",1.74045327900223,-0.641329070748116,1.09490142937168,-0.585741376843531,0.558049324090298,0.942163927945919
"7961","C1QTNF4",51.2653203403611,-0.279066544723865,0.476665220808506,-0.585456065476144,0.558241099858401,0.942250959683126
"7962","RASL11A",7.75186130471796,-0.329992454877653,0.563561819478284,-0.585547926548932,0.55817935073924,0.942250959683126
"7963","BROX",37.3955616135159,0.160296298550655,0.274514392171116,0.5

83926756199855,0.559269590920999,0.942739366813288
"7964","SLC20A1",34.0076618846893,-0.45122837927679,0.771537037228922,
-0.584843445620495,0.55865298854094,0.942739366813288
"7965","C1QTNF7",70.0279530353873,-0.285727386480587,0.488902970181185
,-0.584425548436939,0.558934042120268,0.942739366813288
"7966","ENOPH1",20.9570192962555,0.230016696030237,0.393560219034884,0
.584451082465348,0.558916867433761,0.942739366813288
"7967","SPRY4",1.63512716714746,-0.730732199912461,1.25391706011978,-0
.582759596430294,0.560055147518843,0.942739366813288
"7968","MED30",43.4563728521847,-0.187945273485182,0.322200765976117,-
0.583317277089009,0.559679733963795,0.942739366813288
"7969","ANKRD2",3.32282517017873,-0.466374628343163,0.799091695085434,
-0.583630928980311,0.559468646751879,0.942739366813288
"7970","AAMDC",103.900692982871,-0.155697294574672,0.267068422325731,-
0.582986536629087,0.55990236359184,0.942739366813288
"7971","MAML2",50.8651481099348,-0.177966925992237,0.305145422092649,-
0.583220042338377,0.559745180636035,0.942739366813288
"7972","MYL6",1569.34253221557,-0.150288937181475,0.257225210895528,-0
.584269856979589,0.559038768782505,0.942739366813288
"7973","METTL25",10.8758052126639,0.282588520072534,0.484332383773,0.5
83459891471927,0.559583749927262,0.942739366813288
"7974","SUPT16H",98.2616376102141,-0.122150345616656,0.209212504731766
,-0.583857765926882,0.559316009931492,0.942739366813288
"7975","AL121603.2",7.15030092051054,-0.310585159449032,0.531167742556
052,-0.584721425202617,0.558735045349027,0.942739366813288
"7976","RBM25",188.623537011598,0.177306266342051,0.303965548351608,0.
58331040245704,0.559684361012517,0.942739366813288
"7977","RPAP1",10.9431182977126,0.254374115878366,0.436239437547857,0.
583106647368304,0.559821509101245,0.942739366813288
"7978","B2M",5867.34503080634,0.16399087516626,0.280810387766935,0.583
991484326313,0.559226041328405,0.942739366813288
"7979","PARD6A",5.11765185811172,-0.428831478363111,0.735898321892129,
-0.582731969357542,0.560073748391792,0.942739366813288
"7980","CENPBD1",6.19282194073012,-0.340272123006407,0.583335551154889
,-0.583321421663287,0.559676944420213,0.942739366813288
"7981","GET3",90.0923732245759,-0.182172357835353,0.311754283166923,-0
.584345966267967,0.558987572286529,0.942739366813288
"7982","PPP2R3B",11.0736224942296,0.260551703584258,0.447088243267604,
0.582774670342439,0.560044998617489,0.942739366813288
"7983","ZNF674",3.60827835192311,-0.429919597000717,0.735945189467591,
-0.584173391107749,0.559103661829264,0.942739366813288
"7984","LDOC1",9.01760203632475,-0.294587688855564,0.505009245714854,-
0.583331278298811,0.559670310348718,0.942739366813288
"7985","KLF9",218.091036478613,-0.182042946523081,0.312485895235226,-0
.582563723031552,0.560187032457707,0.942811963581606
"7986","FAM71A",1.69099251885701,0.689253765231648,1.18349754744979,0.
582387151301543,0.560305934148653,0.942872586993908
"7987","MALSU1",52.4468772751878,-0.176773549439604,0.303577166860442,
-0.582301861723576,0.560363371703277,0.942872586993908
"7988","ADPRHL2",36.2057646183595,-0.193748489973475,0.333132830969498

,-0.581595303619941,0.560839307039831,0.943296424305596
"7989","SRGAP2C",46.1779129290755,0.167897536580133,0.289548831768892,
0.579859140009044,0.562009612004892,0.943296424305596
"7990","FAM161A",5.28647643842032,0.385864293758179,0.664582656465137,
0.580611440886166,0.561502359720562,0.943296424305596
"7991","MCM6",18.9357909511381,0.213493060036647,0.368642793174931,0.5
79132602045305,0.562499703274265,0.943296424305596
"7992","RBMS1",228.600091056994,0.125092655984728,0.215481755978198,0.
580525508606791,0.561560289889836,0.943296424305596
"7993","MBNL1",196.215628664404,-0.123800160501469,0.213557562061679,-
0.579703941674113,0.562114284795895,0.943296424305596
"7994","TMEM41A",22.0713657053841,-0.198451578160282,0.342417714211793
,-0.579559905704924,0.562211437607641,0.943296424305596
"7995","APOD",2283.49017627518,0.239845274024703,0.413353924896181,0.5
80241917589007,0.561751489725275,0.943296424305596
"7996","LSM11",7.06883449128571,-0.34947607802327,0.60113565913334,-0.
581359752517611,0.560998017013712,0.943296424305596
"7997","HIVEP1",46.5086729265808,0.185216337538078,0.318437858144279,0
.581640445069694,0.560808894048635,0.943296424305596
"7998","POMZP3",2.9569560428159,0.580345312334055,1.00098307014553,0.5
79775352493907,0.562066120932701,0.943296424305596
"7999","MDFIC",50.7650223890156,-0.148196339303689,0.255765004231356,-
0.579423833800326,0.562303226077547,0.943296424305596
"8000","PIP4P2",71.8446052180875,-0.149200525208781,0.257296138026718,
-0.579878603515175,0.561996485598915,0.943296424305596
"8001","C8orf37",5.78254949709417,0.384387653864884,0.662434737754267,
0.580264940766851,0.561735966100119,0.943296424305596
"8002","ALDH1A1",130.367783785559,0.356138710562226,0.613598649583696,
0.580409866944545,0.561638252828627,0.943296424305596
"8003","TTLL11",3.28320463509317,0.498034811063656,0.857214540911676,0
.580992023926681,0.5612458291833,0.943296424305596
"8004","ARID5B",334.379291084914,-0.161797957671527,0.278517649226173,
-0.580925331378686,0.5612907789413,0.943296424305596
"8005","EHD1",36.3834676363285,0.190542570484615,0.328314905680002,0.5
80365274887431,0.561668317162036,0.943296424305596
"8006","NARS2",9.52755123495695,0.312211133360888,0.538377295214888,0.
579911404392105,0.561974364656283,0.943296424305596
"8007","PDE8A",32.3530943946298,0.189913336559968,0.327105615702465,0.
580587209278341,0.561518694862735,0.943296424305596
"8008","ZNF597",3.22847764460158,-0.475709539258443,0.819230646541481,
-0.580678397795212,0.561457223562779,0.943296424305596
"8009","ORMDL3",15.0290400666382,0.238755912557059,0.41198087522067,0.
579531543616374,0.562230568900559,0.943296424305596
"8010","MRPL27",97.5160685043789,-0.162356021124557,0.280256516959645,
-0.579312206138404,0.562378530877838,0.943296424305596
"8011","AC027307.2",3.38870054841685,0.486602501174757,0.8386605870615
95,0.580213865635036,0.561770404337067,0.943296424305596
"8012","AL031846.2",6.62340070362525,-0.354891576286278,0.609992449329
203,-0.581796670887557,0.560703646738292,0.943296424305596
"8013","TTLL12",6.27725077866843,0.334016997866314,0.575799607969015,0

.580092437097123,0.56185228359778,0.943296424305596
"8014","ZXDB",7.02712156166447,0.326975738044178,0.564611119444334,0.579116717300879,0.562510420744478,0.943296424305596
"8015","GPR1",15.6367838131013,0.309797972768943,0.535951434187305,0.578033666872649,0.563241389559238,0.943625292881102
"8016","CNOT9",19.0740725853817,-0.198495559719782,0.343413445318504,-0.578007537054015,0.563259030667545,0.943625292881102
"8017","RHBDD1",10.0295839364283,0.272913273651558,0.472016264849838,0.578186164280545,0.563138438810326,0.943625292881102
"8018","C5orf22",7.37745445625249,-0.299608672936532,0.518518500153219,-0.57781674684317,0.563387847549517,0.943625292881102
"8019","ATG10",19.1641669739543,-0.208172611356295,0.360055227383721,-0.578168557276463,0.563150324806311,0.943625292881102
"8020","AC019205.1",4.56229286158714,0.478131388294568,0.827142229409777,0.578052203471401,0.563228875047392,0.943625292881102
"8021","ATP6V1H",32.0372018661516,0.167177324580208,0.289084590464799,0.578298982700584,0.563062281122237,0.943625292881102
"8022","ABCD4",44.849298839353,0.15051716629085,0.260496359854701,0.577809096352845,0.563393013269563,0.943625292881102
"8023","TP53BP1",40.6100279133472,0.162982435941349,0.281795632126891,0.578371051074201,0.563013634214105,0.943625292881102
"8024","SHKBP1",18.4202928920753,-0.223067178316945,0.386072392710191,-0.577785882981777,0.563408687408138,0.943625292881102
"8025","AC060780.1",16.0732964596101,0.263253190567652,0.455862001739375,0.577484391248206,0.563612279830787,0.943848651544665
"8026","PRMT9",9.0856317450796,0.328614632055598,0.569742234689853,0.576777728676695,0.564089616375815,0.94389168558897
"8027","PPID",35.0361986946596,0.186026705630346,0.322430212971896,0.57695184305374,0.563971987496405,0.94389168558897
"8028","HLA-
A",1129.77966826406,0.146835100500482,0.254566669396099,0.576804107343727,0.564071794612256,0.94389168558897
"8029","CCDC146",42.174091425801,-0.290206985484073,0.502958836104787,-0.576999477196999,0.563939808691902,0.94389168558897
"8030","ATP6V0A1",29.4125109690913,0.189870919471993,0.32911217401662,0.576918553801067,0.563994476267858,0.94389168558897
"8031","LINC01560",6.80434556795536,-0.422683518739743,0.73291130612566,-0.576718513150175,0.564129624127584,0.94389168558897
"8032","SH2D1A",2.17221926136175,0.70167110440926,1.21576734124206,0.577142583623292,0.563843139790406,0.94389168558897
"8033","LINC-
PINT",68.5774683766307,-0.200685491545228,0.348127317402116,-0.576471542201381,0.564296499697802,0.9439358550459
"8034","WDR59",15.7821770353868,0.233110754141288,0.404310195219138,0.576564125509972,0.56423393938393,0.9439358550459
"8035","PAIP2B",4.3350817968351,0.448596896937711,0.780005125184909,0.575120447870603,0.565209839726167,0.943936254017641
"8036","SERPINE2",11.1077346156731,-0.368833376374169,0.640863506050105,-0.575525635165958,0.564935858452547,0.943936254017641
"8037","AC108519.1",2.5653219915372,0.587660869683434,1.02179260662919

,0.575127345677397,0.565205175003401,0.943936254017641
"8038","SLIT2",49.7232901711149,0.198039296341614,0.344051126810696,0.575610079168784,0.564878766789815,0.943936254017641
"8039","H2AFX",15.334422643611,0.25231205397787,0.438627806852117,0.575230411835101,0.56513547750922,0.943936254017641
"8040","ING4",56.5215802749208,-0.179562076330827,0.312025922715509,-0.575471661995673,0.56497235057363,0.943936254017641
"8041","FRY",2.95934238276049,-0.519168582595931,0.902040427213928,-0.575549129432538,0.564919973958374,0.943936254017641
"8042","SENP8",1.71991894075218,-0.58573342505847,1.01820589076934,-0.575260298892889,0.565115267447716,0.943936254017641
"8043","INPP5K",14.5545195745637,-0.235972478044415,0.409605630221427,-0.576096763896658,0.564549778684312,0.943936254017641
"8044","EVI2B",3.20572522333506,-0.548933796578169,0.954363479234521,-0.57518315455497,0.565167434275786,0.943936254017641
"8045","ARFGEF2",20.8461786889094,0.198896683945774,0.345534647797766,0.575620086765319,0.564872000947643,0.943936254017641
"8046","ZNF275",11.1045859023998,-0.277593363358933,0.481840549431416,-0.576110424260681,0.564540545910964,0.943936254017641
"8047","ATP6AP1",71.8566739406324,0.156228987003441,0.271407754577391,0.575624625194313,0.564868932661904,0.943936254017641
"8048","AL391684.1",1.60381608211164,-0.708318111055293,1.23213488207748,-0.574870593600118,0.565378819081729,0.94396347541143
"8049","CCDC102A",20.5553654379365,-0.227667802991691,0.396056088372707,-0.574837276020979,0.565401353983201,0.94396347541143
"8050","RAB5IF",74.6777347298941,-0.159416386980277,0.27734970099866,-0.574784780391909,0.565436861154998,0.94396347541143
"8051","AVEN",20.2041421244304,-0.218982370322785,0.381115662524628,-0.574582447942911,0.56557372548589,0.944074685977502
"8052","NIPAL3",5.78650443001596,0.350661478502087,0.617719197083415,0.567671330529711,0.570258162210148,0.944095275637103
"8053","UCHL5",26.7075419102544,0.184458319560321,0.323898052912373,0.56949499356893,0.569020267298824,0.944095275637103
"8054","PCAT6",2.55153386850827,0.525604290147851,0.925451125978995,0.567943865854435,0.57007308480409,0.944095275637103
"8055","TP53BP2",27.096673622096,0.222990971256999,0.391234315354131,0.569967823643372,0.568699521862459,0.944095275637103
"8056","AGT",1.62958935768361,0.773030991721901,1.35121992671978,0.572098572878886,0.567255195923149,0.944095275637103
"8057","MCEE",39.0059352348351,-0.172598676344616,0.300508661511894,-0.574355080070744,0.565727543588664,0.944095275637103
"8058","TMEM87B",22.8111935316313,0.206754904216432,0.362477925875486,0.570393090053859,0.568411115177006,0.944095275637103
"8059","LINC01960",1.77938484238517,-0.618719277395163,1.08386897665914,-0.570843239099128,0.568105909824663,0.944095275637103
"8060","AC009950.1",4.09137273144666,-0.411721531819369,0.722770724435368,-0.569643343179137,0.568919624676695,0.944095275637103
"8061","DGKD",9.02041686259939,0.342030979894327,0.600758813517646,0.56933160562659,0.569131121992875,0.944095275637103
"8062","NDUFA10",104.159295794632,-0.129082872896957,0.225821928928093

,-0.571613543067644,0.567583818306553,0.944095275637103
"8063","IL17RC",25.4607106543352,0.183867216897965,0.321921410940721,0.571155600867513,0.567894171645823,0.944095275637103
"8064","MKRN2",22.4488442852094,0.213170061458196,0.371869229103468,0.573239313110481,0.566482669348972,0.944095275637103
"8065","ACAA1",63.3565738908637,-0.158422718011188,0.278977522229659,-0.567869112697803,0.570123846456591,0.944095275637103
"8066","EPHB3",2.64615998881859,0.534069593874503,0.937960874717987,0.569394319390006,0.569088571029683,0.944095275637103
"8067","NFXL1",10.4447382458885,0.287134431040393,0.505263985971302,0.568285963402707,0.569840808578448,0.944095275637103
"8068","AP1AR",13.6723051955091,0.252763659805411,0.441455025888839,0.572569446449248,0.566936252043414,0.944095275637103
"8069","HHIP-AS1",6.39377111453115,-0.452744813400401,0.796940559962615,-0.568103615433551,0.569964612961903,0.944095275637103
"8070","SH3RF1",21.4318226074699,0.203451780684008,0.35626877321319,0.571062624571542,0.567957192797846,0.944095275637103
"8071","NUDT12",16.6014278612651,0.219276660508754,0.385553888551884,0.568731549647556,0.569538333660566,0.944095275637103
"8072","EFNA5",44.4605741239921,0.162399528060003,0.283816387206689,0.572199264666616,0.56718698561502,0.944095275637103
"8073","IGIP",13.009056846748,0.277547705155085,0.484462437864917,0.572898296054221,0.566713558380268,0.944095275637103
"8074","SNHG5",238.011235742948,0.22902084354576,0.4029153848316,0.568409279386242,0.569757090983437,0.944095275637103
"8075","LINC01625",1.83593164048234,0.748410278294987,1.30815880255567,0.572109652767588,0.567247690028044,0.944095275637103
"8076","HOXA11-AS",5.71946570068599,0.354564935882565,0.620927174679194,0.571024993495821,0.567982700828301,0.944095275637103
"8077","CHN2",1.58461678835484,0.706916251022486,1.23407370534432,0.572831467003221,0.566758810949275,0.944095275637103
"8078","DBF4",5.56359249913091,-0.387730393972164,0.676658786037265,-0.573007255610823,0.566639781310292,0.944095275637103
"8079","ALKBH4",23.1443760411526,-0.207669394709786,0.362874631716688,-0.572289646502272,0.567125762788468,0.944095275637103
"8080","KDM7A-DT",11.1329208841836,-0.278553639407541,0.486223422973052,-0.572892267723967,0.566717640327802,0.944095275637103
"8081","ZC2HC1A",60.7376109346132,-0.127316893720359,0.223226175356249,-0.570349303871614,0.568440806810034,0.944095275637103
"8082","E2F5",2.60485256770104,-0.6033628490853,1.05810162550534,-0.570231473557315,0.568520711828357,0.944095275637103
"8083","CAAP1",18.7772771222492,-0.195230642213597,0.341921889468121,-0.570980239133825,0.568013038055175,0.944095275637103
"8084","B4GALT1",114.160078653701,-0.160492474128043,0.282848634248406,-0.567414704173872,0.570432462090468,0.944095275637103
"8085","HDHD3",7.87270332634178,-0.332317949878679,0.581447328469592,-0.571535775653767,0.567636516561933,0.944095275637103

"8086","AL135925.1",15.8890831857099,-0.280752283594497,0.490891329676211,-0.571923492272066,0.567373807822589,0.944095275637103
"8087","ARFGAP2",51.0673865364474,-0.164651132383214,0.288979913720494,-0.569766701994615,0.568835942664347,0.944095275637103
"8088","PAK1",20.8525916470247,0.235167739334007,0.413626872368982,0.568550437710009,0.569661267661819,0.944095275637103
"8089","AQP11",1.64486940314616,-0.695869850325626,1.22155430209332,-0.569659366868216,0.568908754472251,0.944095275637103
"8090","SINHCAF",4.26368677180315,0.424626769536846,0.74389268732947,0.57081723852029,0.568123536331993,0.944095275637103
"8091","AC073896.2",5.02673293598113,-0.368770389861952,0.650033273458455,-0.567310020762377,0.570503570050697,0.944095275637103
"8092","ATXN7L3B",77.2659751932428,-0.126236343684152,0.220559362710839,-0.572346338566693,0.567087362347287,0.944095275637103
"8093","PITPNM2",10.3123796027446,-0.299731162281388,0.525189847271981,-0.570710122898026,0.568196155716497,0.944095275637103
"8094","NBEA",5.53603555147416,0.37871894119358,0.664340392245419,0.570067612347849,0.568631840994276,0.944095275637103
"8095","DLEU1",2.13558032369139,-0.528347547313976,0.929446604871671,-0.568453899927823,0.569726800131889,0.944095275637103
"8096","GPR180",12.7424305322506,0.265450801233779,0.465086979167714,0.570755177254822,0.568165610427788,0.944095275637103
"8097","EFCAB11",10.3521234845101,0.261692732910442,0.459860034162226,0.569070398533751,0.569308365991557,0.944095275637103
"8098","PPP4R3A",44.2349702695237,0.16722997379962,0.294083645524454,0.56864764955355,0.569595281255097,0.944095275637103
"8099","AC123768.4",2.29631231302911,-0.514364094659835,0.905898679314487,-0.56779428693843,0.570174659568507,0.944095275637103
"8100","SNX1",50.037850462874,0.135514433863658,0.237562191475189,0.570437715792039,0.568380854999839,0.944095275637103
"8101","DNAJA4",5.82702511473471,0.400393731945618,0.698255354852256,0.573420209617065,0.566360209866528,0.944095275637103
"8102","FURIN",20.8546737020037,-0.197643522752323,0.348398876257102,-0.56729093065866,0.570516537778616,0.944095275637103
"8103","ITGAL",1.49096565302029,0.740310371742498,1.29000964550279,0.573879718127189,0.566049198654536,0.944095275637103
"8104","DYNC1LI2",107.182473242525,-0.156865805231398,0.274406568642526,-0.571654702026285,0.567555928326885,0.944095275637103
"8105","PLA2G15",16.9003898317951,0.215281910068897,0.375870592434646,0.572755396144295,0.566810323616851,0.944095275637103
"8106","LINC02193",2.57832182536723,0.569226121746416,0.998073690107073,0.570324743942854,0.56845746134733,0.944095275637103
"8107","MED31",31.7681565321335,-0.21019772297682,0.366906886820238,-0.572891189910544,0.566718370146277,0.944095275637103
"8108","TMEM98",150.50619920419,-0.124403844177776,0.219255620935967,-0.56739181256433,0.570448011238784,0.944095275637103
"8109","TADA2A",11.0362180867428,0.260301599776247,0.458666686193775,0.567517998606676,0.570362301757193,0.944095275637103
"8110","HDAC5",26.5571957290318,-0.193302955030618,0.340238235952823,-0.568140010746533,0.569939901493291,0.944095275637103

"8111","PEX11G",12.6327728364566,0.280398995805428,0.49435074753496,0.
567206577927948,0.57057383947649,0.944095275637103
"8112","COMP",4.98380950298545,-0.642512988740463,1.13034055191512,-0.
568424257319497,0.569746923061692,0.944095275637103
"8113","GEMIN7",11.9753949662558,0.274077993733911,0.47933424282048,0.
571788888106953,0.567465006213812,0.944095275637103
"8114","AC008440.2",1.51787944739614,-0.687479295514231,1.206051579956
8,-0.570024787446369,0.568660886159965,0.944095275637103
"8115","MZF1",12.6431120054514,0.256993887828022,0.448547839545989,0.5
72946439978724,0.566680959316175,0.944095275637103
"8116","SMOX",4.60331474719554,-0.406338701935571,0.71053248939106,-0.
571879130092715,0.567403863764706,0.944095275637103
"8117","DOK5",10.7304606685004,0.289721154043073,0.509383701109753,0.5
68768010071546,0.569513586818456,0.944095275637103
"8118","RAB22A",77.105783754906,-0.134249499763382,0.23640253386209,-0
.567885198056715,0.570112923399445,0.944095275637103
"8119","AL008721.2",2.09567855336668,0.587370943489976,1.0318194203690
9,0.569257499805408,0.569181404366514,0.944095275637103
"8120","DESI1",24.6864368452434,-0.187856353166042,0.330647551549366,-
0.568146814593894,0.569935281915012,0.944095275637103
"8121","ARMCX3",117.831146160835,0.110345931439182,0.193612726897733,0
.569931187929949,0.568724370699915,0.944095275637103
"8122","TMSB15B",3.67348174697608,-0.429899252855491,0.751487088973368
,-0.572064722286567,0.56727812776547,0.944095275637103
"8123","SZRD1",57.0465843629436,0.13108676562445,0.231354445179835,0.5
66605778949069,0.570982047836529,0.944213504675434
"8124","PDE1A",17.5188429407143,0.266504965790774,0.470211840048704,0.
56677638266865,0.570866118284821,0.944213504675434
"8125","LINC01088",12.3875925537378,-0.354830306693037,0.6262620679931
,-0.566584381886827,0.57099658847364,0.944213504675434
"8126","LINC00324",5.85906443236235,-0.399386939374523,0.7046070785874
15,-0.566822207030913,0.570834981378818,0.944213504675434
"8127","SWSAP1",3.71820676809656,-0.442921089479858,0.781443249062538,
-0.566798791865194,0.570850891500593,0.944213504675434
"8128","SLC30A7",34.8688155349623,0.152608779748307,0.270309333781415,
0.564570884820846,0.57236567335512,0.944247039933031
"8129","EIF2D",35.7595198334857,-0.151130716348593,0.268250893462629,-
0.56339315182802,0.573167199367808,0.944247039933031
"8130","RN7SL832P",2.69680414249566,0.535137060430444,0.94949802695382
4,0.563599970973366,0.573026406586093,0.944247039933031
"8131","CRIPT",52.1237096492901,0.131190544213761,0.232148024542689,0.
565115918915075,0.571994921510813,0.944247039933031
"8132","MDH1",132.981143608915,-0.15389385613672,0.272666014717037,-0.
564404244865008,0.572479050641309,0.944247039933031
"8133","SPAG16",37.1410045397703,-0.173054432825757,0.307012307061779,
-0.563672624338586,0.572976951474625,0.944247039933031
"8134","RPUSD3",19.6082874001787,-0.187662102072942,0.332772003044473,
-0.563935969240363,0.572797709700221,0.944247039933031
"8135","SERPINI1",9.47480771544703,-0.34177766643922,0.604832132602576
,-0.565078553231886,0.572020335351273,0.944247039933031

"8136","OCIAD1",202.035238411813,-0.122181077178393,0.215951952314593,-0.565778988653934,0.571544031516425,0.944247039933031
"8137","ZDHHC11B",2.63940472390987,-0.678091239527218,1.19852399613433,-0.565771934241037,0.571548827654733,0.944247039933031
"8138","PTGER4",37.2059885418294,0.247852512703509,0.439559321286155,0.563865900917059,0.572845398059514,0.944247039933031
"8139","KIF2A",36.0493961150535,-0.183228204960634,0.323906844491984,-0.565681794245531,0.571610113516239,0.944247039933031
"8140","LARP1",70.0834362356817,0.126116677324386,0.223251998706369,0.564907270954649,0.572136837893069,0.944247039933031
"8141","CDHR3",4.47254451353362,-0.416377798693477,0.738822991140528,-0.563569087164857,0.573047429793386,0.944247039933031
"8142","GIMAP4",1.92472977502605,0.631388098926257,1.11909454871173,0.564195491482905,0.57262109580210,0.944247039933031
"8143","MRPL15",38.8804665706907,-0.184399037329117,0.325916680918929,-0.565785822343304,0.571539385461384,0.944247039933031
"8144","ARPC5L",34.0757011195498,-0.168142245868326,0.298614127986276,-0.56307532065614,0.573383595912108,0.944247039933031
"8145","UNC5B",2.32679871450046,0.539174071106066,0.95318353244133,0.565656091146595,0.571627589534164,0.944247039933031
"8146","PSTK",4.28789234221005,0.431718634448704,0.76495142476964,0.564373920316199,0.57249968378511,0.944247039933031
"8147","ENO2",11.0246572171352,0.293440846286095,0.52077088594742,0.563473984825876,0.573112170097798,0.944247039933031
"8148","RCBTB1",6.72863484740032,0.312168002565828,0.552467963196519,0.565042723490533,0.572044705042501,0.944247039933031
"8149","L2HGDH",3.31611962579045,0.456414412490599,0.808351903128515,0.564623415525053,0.572329935106078,0.944247039933031
"8150","JKAMP",44.3284134704707,0.169423317915888,0.300519271109007,0.563768563961522,0.572911648521527,0.944247039933031
"8151","IGHG2",9.88749005875095,1.01120916485765,1.79138506899249,0.564484533426627,0.572424423159926,0.944247039933031
"8152","NUSAP1",1.570693606731,-0.657251429904562,1.16548722788482,-0.563928470582532,0.572802813181556,0.944247039933031
"8153","SLCO3A1",56.4896795805145,-0.159356639341921,0.282974850522444,-0.563147710998726,0.573334305269477,0.944247039933031
"8154","STX4",91.4674505397026,-0.142252172682825,0.252510075365002,-0.563352462182747,0.573194900902941,0.944247039933031
"8155","ATXN1L",19.3984783572205,0.189070904166999,0.33574925630922,0.563131267200387,0.573345501688652,0.944247039933031
"8156","AC005726.5",1.46298283520663,-0.697174160750279,1.23322162937066,-0.565327548711633,0.571850994061636,0.944247039933031
"8157","C18orf54",5.59123979744927,-0.409858037138702,0.726052693044894,-0.564501779367905,0.572412689528337,0.944247039933031
"8158","MEX3D",30.3131872491485,-0.183370481650441,0.32433363667383,-0.565376084734776,0.571817987592508,0.944247039933031
"8159","APBA3",7.42945270985185,-0.326784738879172,0.580390622789355,-0.563042761284883,0.573405766269325,0.944247039933031
"8160","FP565260.6",1.7775236110769,-0.710105145773069,1.25786397308138,-0.56453254164958,0.572391760059592,0.944247039933031

"8161","TTC38",8.42101887728927,0.287438176144516,0.507678391780612,0.566181623638473,0.571270320683385,0.944247039933031
"8162","ZMIZ2",15.5037173962029,0.225114883656569,0.40007498252103,0.56268173090462,0.573651626645986,0.944326461809697
"8163","RNF214",27.1221714361962,0.165849753956217,0.294758912017475,0.562662390158315,0.573664799033735,0.944326461809697
"8164","VPS53",27.3702553272713,0.236230215333735,0.419732882305921,0.562810838254886,0.573563699271186,0.944326461809697
"8165","PFDN4",59.9732689034702,-0.157818271079252,0.280599548788017,-0.56243237653414,0.573821465230915,0.944468667634815
"8166","PUDP",7.47310411357785,-0.286568639180207,0.509665687214051,-0.562267867681374,0.573933527445186,0.944537432688691
"8167","SETMAR",13.5788120166843,-0.255578688759737,0.454648838807989,-0.562145257930979,0.574017055042814,0.944559226487129
"8168","LYSMD1",3.81616534012675,0.417693571031575,0.743661987358903,0.561671267500176,0.574340014025448,0.944639225948705
"8169","CLSTN2",10.1551727814299,-0.407316079526405,0.725198816281509,-0.5616612581015 2,0.57434683497484,0.944639225948705
"8170","PEX3",18.6262040463491,0.220659432030373,0.392676672446001,0.561936696305067,0.574159150374412,0.944639225948705
"8171","UBR7",19.6492872811658,-0.196793552287723,0.350345317878191,-0.561713093468955,0.574311511947251,0.944639225948705
"8172","AP002748.4",3.49113231528455,-0.533423528811926,0.949949567935064,-0.56152826088594,0.574437470160687,0.944672682512173
"8173","MMGT1",29.4666381372188,-0.173285607368839,0.308739612576243,-0.561267813750484,0.574614979757314,0.944848979928857
"8174","AL137186.2",4.79800323576317,-0.394832982887223,0.703933929091952,-0.560894945632955,0.57486915584130 1,0.945151282768687
"8175","EIF2AK2",113.13882342175,-0.134370983573567,0.239808300454209,-0.560326658080898,0.575256647354368,0.945210427932641
"8176","DUSP11",36.9971816890901,-0.1767108212005,0.315487752589657,-0.560119433321841,0.575397975959595,0.945210427932641
"8177","TTC30B",3.5421952353905,0.422610810651484,0.754229637154324,0.560321140715149,0.575260410020328,0.945210427932641
"8178","DCAF1",9.7492341226443,0.34419972970829,0.614103858578997,0.560491071501732,0.575144528028548,0.945210427932641
"8179","AL035530.2",2.29347091703905,-0.538341575339674,0.961471974871022,-0.559913954238646,0.575538130201117,0.945210427932641
"8180","BOP1",8.71065513306276,-0.268727312482462,0.479922494015141,-0.559938981468087,0.575521058635801,0.945210427932641
"8181","TMEM86B",1.66213345695612,0.670761699420564,1.19662579241157,0.560544243383534,0.575108270452852,0.945210427932641
"8182","NUP50",26.4942509612094,-0.186099166994041,0.331951339348025,-0.560621828969128,0.575055367263131,0.945210427932641
"8183","SEPTIN6",25.0138939110284,0.193177569167021,0.344922414587921,0.560060932536988,0.575437876836898,0.945210427932641
"8184","FSIP2",2.41841089691601,0.51978813760637,0.929597609886432,0.55915390925959,0.576056685739205,0.945652933611341
"8185","PNKD",107.901151749219,-0.161578522365806,0.289741956373727,-0.557663530639631,0.577074165380356,0.945652933611341

"8186","ILKAP",22.8041373840173,-0.193206914418002,0.345777492977376,-0.558760816831545,0.57632496722412,0.945652933611341
"8187","SFRP2",230.709576247343,0.428314037589376,0.767965933546679,0.557725309000756,0.577031972551519,0.945652933611341
"8188","IL11RA",76.0329475275229,-0.181747996548542,0.325491339487435,-0.558380437509485,0.576584628251931,0.945652933611341
"8189","AC104389.5",1.62831375526159,0.649182454516972,1.1615865387163 4,0.558875669508342,0.576246575374087,0.945652933611341
"8190","SLC38A1",5.15078563690985,-0.369076723626588,0.66142929495009,-0.557998755792088,0.576845233783576,0.945652933611341
"8191","AC068888.1",32.551041270926,-0.169411116817069,0.3031648527123 95,-0.558808566696829,0.576292375293619,0.945652933611341
"8192","ARL1",160.462951641124,-0.110081506680437,0.197012779450786,-0 .558753127524581,0.576330215683741,0.945652933611341
"8193","MICU2",37.2507737945553,-0.153193747760541,0.274407746083381,-0.558270493260756,0.576659690548669,0.945652933611341
"8194","COX4I1",510.8314794236,-0.150822380202286,0.27018277862203,-0.558223514361282,0.576691765883267,0.945652933611341
"8195","CXCL16",9.58023735814704,-0.284155317737435,0.509025983875564,-0.558233423712411,0.576685000100216,0.945652933611341
"8196","MPRIP",47.6861792080153,0.165304089110685,0.295762715573695,0.55890780144496,0.576224644857746,0.945652933611341
"8197","PEX12",6.90937610936856,-0.310585456768508,0.5566259595611,-0.557978749344362,0.576858895363818,0.945652933611341
"8198","MADCAM1",1.74111043369578,0.633398271462268,1.13576886449756,0 .557682369416305,0.57706129888823,0.945652933611341
"8199","GATAD2A",25.6675048774914,0.182406674777619,0.326644126490787,0.558426311647652,0.576553309939048,0.945652933611341
"8200","CCDC9",9.90733461510958,0.295510117367351,0.529556630411658,0.55803307974377,0.57682179572408,0.945652933611341
"8201","PRR34-AS1",6.72835223117746,-0.390448283400373,0.699943448926429,-0.55782832 7415939,0.576961617195256,0.945652933611341
"8202","TFB2M",9.71996539449549,-0.258059050759195,0.463161059312331,-0.557169143585478,0.577411869752701,0.945657590601452
"8203","AC093702.1",1.94249181332187,0.600524335491485,1.0800939335778 ,0.555992693619023,0.578215849539358,0.945657590601452
"8204","MRPS22",44.4318458169766,-0.147390963995173,0.264779668468814,-0.556655142169773,0.577763070887184,0.945657590601452
"8205","MAEA",40.3594794803385,0.145456378267285,0.261669046276441,0.555879192962002,0.578293443134434,0.945657590601452
"8206","ENC1",2.86566992276579,0.613283037005602,1.10291204623013,0.556057973164648,0.578171224045281,0.945657590601452
"8207","HCG25",6.46872077634409,0.338134385692437,0.607995508550705,0.55614619012311,0.578110920970016,0.945657590601452
"8208","DNAAF5",17.0356694337224,0.222778255704168,0.400685321124526,0 .555993054796565,0.57821560263174,0.945657590601452
"8209","PSMC2",67.8054999324198,-0.155590425246569,0.279237399555887,-0.557197658673328,0.57739238922628,0.945657590601452
"8210","PHYHIP",4.32805097164741,-0.394536392313847,0.709738265100343,

-0.555889983271604,0.578286066236014,0.945657590601452
"8211","HOOK3",126.984493545091,0.141782500314336,0.255093587539967,0.555805818882539,0.578343607199444,0.945657590601452
"8212","GBGT1",2.527230375687,-0.501686334165153,0.902094366147158,-0.55613509294804,0.578118506579917,0.945657590601452
"8213","NADSYN1",15.9095870286513,0.224259866708427,0.402862322693241,0.556666270524357,0.577755466164998,0.945657590601452
"8214","ZC3H14",45.6442573922856,0.142257009472182,0.255841179625622,0.556036403835965,0.57818596879026,0.945657590601452
"8215","ZNF106",163.243313238019,0.140178787906025,0.251786688889059,0.556736293425701,0.577707616082637,0.945657590601452
"8216","RNASEK",386.08216694715,-0.153983710352041,0.27675393549728,-0.556392125283994,0.577942820996098,0.945657590601452
"8217","AFG3L2",35.0509190660559,0.174990477958846,0.314814547345377,0.555852578714755,0.578311638408415,0.945657590601452
"8218","MCM5",12.7486933304785,-0.247207943422114,0.443870558257222,-0.556937014233906,0.577570464048979,0.945657590601452
"8219","PHF6",27.7472815863071,-0.171195700506106,0.30745147180628,-0.556821860374678,0.577649146522725,0.945657590601452
"8220","CHORDC1",31.0096233541939,0.166904900748338,0.300355707405258,0.555690791396017,0.578422252798406,0.945671125955935
"8221","TEAD3",7.91289436063122,0.309727204041825,0.55770034656354,0.555364912269311,0.57864508735175,0.945690299029572
"8222","TOLLIP",31.8040584551412,0.17474868441223,0.314652686903981,0.555370068921597,0.57864156094403,0.945690299029572
"8223","ALDH3B1",4.78812276839035,-0.351891566425307,0.633610461372929,-0.555375246902989,0.578638019960435,0.945690299029572
"8224","SIAH2",19.1252862345731,-0.203406686090816,0.366739540017979,-0.554635276253128,0.579144155113449,0.946160746483058
"8225","C5orf63",4.29767892728401,0.391648646729119,0.705901090692958,0.554820855064343,0.579017200876432,0.946160746483058
"8226","KLF16",4.10672837663585,-0.387606761091077,0.698790486609081,-0.554682366916527,0.579111939211011,0.946160746483058
"8227","SESN2",13.8561627016971,0.224892212759529,0.406244384396621,0.553588483674803,0.579860509292919,0.946212906129755
"8228","ZNF326",44.3845603238287,0.156130754269722,0.282654605047871,0.55237293672707,0.580692868584889,0.946212906129755
"8229","UBE2Q1",65.392716197148,0.172897800142822,0.313809385092816,0.550964401818904,0.581658077722198,0.946212906129755
"8230","RAB11FIP5",13.5755196935151,-0.260600798777867,0.472469215709312,-0.551572018055462,0.581241612145248,0.946212906129755
"8231","ATP5MC3",276.41528891384,-0.173799984491136,0.315690999099869,-0.550538295316283,0.581950218101941,0.946212906129755
"8232","EIF1B",167.686321313894,-0.13030387897273,0.235524937038854,-0.553248758330985,0.580093083661288,0.946212906129755
"8233","EIF4E3",21.6927098135535,-0.193420925141831,0.349068303106591,-0.55410624058515,0.579506139480248,0.946212906129755
"8234","TRA2B",131.366199496086,0.124652038456544,0.22672446175249,0.549795277902673,0.58245979785178,0.946212906129755
"8235","CPEB2",11.5722218897279,0.243252473704275,0.439448139196599,0.

553540798122368,0.579893151959044,0.946212906129755
"8236","OTUD4",21.5758894270127,0.197647757137025,0.35958378444494,0.549657035959276,0.582554630580192,0.946212906129755
"8237","FAT1",51.1322792016173,-0.214260239520889,0.388695605633784,-0.551228870137416,0.581476791637924,0.946212906129755
"8238","SUB1",249.860066397908,-0.138595325160365,0.251906541807866,-0.550185494055469,0.582192151974286,0.946212906129755
"8239","DHX29",73.2996039116419,-0.12213821182606,0.22126026440574,-0.552011506154842,0.580940470057249,0.946212906129755
"8240","HSPA4",41.2754160819222,0.170768886328276,0.308885032462598,0.552855814886252,0.580362145415284,0.946212906129755
"8241","EFHC1",59.8058172595923,-0.143645464217506,0.260205122176039,-0.552047027422943,0.580916133683089,0.946212906129755
"8242","AK9",9.81829805737104,0.315322020616254,0.56870074992514,0.554460356624746,0.579263829380187,0.946212906129755
"8243","YKT6",23.0304134992689,-0.17689109277255,0.321579860411113,-0.550068939474037,0.582272089735181,0.946212906129755
"8244","AC009275.1",4.13172172778801,-0.387163653485471,0.700743782133321,-0.552503872823249,0.580603181754482,0.946212906129755
"8245","FMC1",54.6969587646617,-0.185189598022804,0.334449817880323,-0.553714154178642,0.579774486939301,0.946212906129755
"8246","AC026979.2",5.93514787475297,0.360172716476955,0.652572390757646,0.551927604627572,0.58099795466777,0.946212906129755
"8247","LY6E",317.174832990888,-0.167654383120234,0.304357196019405,-0.550847442784118,0.58173825849318,0.946212906129755
"8248","TEK",6.27640435208081,-0.342971575650333,0.620450782908456,-0.552778052825725,0.580415398675336,0.946212906129755
"8249","TLE1",8.94540328039369,0.321057559141156,0.583749514733697,0.549991993205544,0.582324865350699,0.946212906129755
"8250","TSTD2",18.8891540137576,0.220254121836861,0.400713011559288,0.549655527729907,0.5825556652517,0.946212906129755
"8251","MAN1B1-DT",3.75475753651922,-0.429658708868675,0.778773518421477,-0.551712017300698,0.58114567519376,0.946212906129755
"8252","LPXN",4.38707037808478,0.40934021793524,0.742430231296826,0.551351764353982,0.581392559873703,0.946212906129755
"8253","TMEM223",38.5392443673666,-0.185930220623885,0.338105278126318,-0.549918125071153,0.582375531848515,0.946212906129755
"8254","MRPL51",204.902051308371,-0.149784368436891,0.270450166878883,-0.553833521958851,0.579692784387749,0.946212906129755
"8255","TM7SF3",50.6376435213513,-0.139793084277963,0.253297661885781,-0.551892517432709,0.581021995230917,0.946212906129755
"8256","RACGAP1",3.83312911067053,-0.393680339108695,0.714311739444203,-0.551132394121106,0.581542920351218,0.946212906129755
"8257","RAB21",68.2982410175528,0.124481818948173,0.226133272161252,0.550479890723054,0.581990265874458,0.946212906129755
"8258","CHST11",3.32093387091143,-0.441866378244523,0.801361020214611,-0.551394898302125,0.581362997194766,0.946212906129755
"8259","NRAV",4.73097158676258,-0.364762685895142,0.658109899747431,-0.554258013798502,0.579402280177927,0.946212906129755

"8260","DTWD1",97.644957811097,0.119648434449844,0.216822704819375,0.551826131629148,0.581067481819314,0.946212906129755
"8261","CGNL1",1.51678390444138,0.759668050078606,1.37253595226955,0.553477705864433,0.579936342454916,0.946212906129755
"8262","RSL1D1",123.402754096211,-0.116227889783112,0.210182102836727,-0.552986615960352,0.580272574974607,0.946212906129755
"8263","ZNF768",12.730044821892,-0.24417637675628,0.442815049368326,-0.551418424248671,0.581346873532247,0.946212906129755
"8264","MPHOSPH6",15.4373552224433,-0.221629824756411,0.401299143494216,-0.552280831767101,0.580755961277071,0.946212906129755
"8265","LRRC37B",5.65229717286389,0.349425122766717,0.635207939653832,0.550095647351547,0.582253771959405,0.946212906129755
"8266","ZNF554",4.40529922680632,0.415164214721821,0.752668302833811,0.551589874528686,0.581229375269866,0.946212906129755
"8267","BST2",110.353311275337,0.252710584673232,0.459352091506345,0.550145714683746,0.582219433671092,0.946212906129755
"8268","SLC25A42",7.61378891147537,0.292301113776472,0.53169925176521,0.5497489658036,0.582491566731973,0.946212906129755
"8269","ARHGEF1",28.8617093456134,-0.179683246684088,0.324572011670387,-0.553600557729425,0.57985224425677,0.946212906129755
"8270","ZNF578",1.45811727692818,0.706653349461171,1.28216974320606,0.551138687529927,0.581538606477296,0.946212906129755
"8271","BTG3",31.6941995349526,-0.200906416277575,0.364368406497156,-0.551382646505997,0.581371394126817,0.946212906129755
"8272","AP000692.2",4.03718219397686,-0.491990393994062,0.893419160225208,-0.550682608899996,0.581851268434779,0.946212906129755
"8273","FAM104B",29.5869874948325,-0.187095663854495,0.337485841536853,-0.554380779360975,0.579318277372964,0.946212906129755
"8274","WDR44",16.4409751152219,-0.201479093816317,0.364107258585212,-0.553350940047698,0.580023125941949,0.946212906129755
"8275","AAMP",57.633005546815,-0.133742509067353,0.243465764016024,-0.54932778580955,0.582780522235891,0.946223251687157
"8276","TFDP2",83.7868264167477,0.128050191009706,0.233017343759118,0.549530729961793,0.582641281656987,0.946223251687157
"8277","EDIL3",65.9531722223447,-0.229456510663913,0.417774167892147,-0.54923575534031,0.582843669727382,0.946223251687157
"8278","PAPLN",23.6344067792671,-0.304620098885592,0.554551701683273,-0.549308744272094,0.582793587487331,0.946223251687157
"8279","TDRD7",10.7181309986842,-0.262232638555804,0.477863578985425,-0.548760462374142,0.583169846852958,0.946295529081974
"8280","ARIH1",38.7031345705434,0.182576740862223,0.332698780551985,0.548774902508832,0.583159935834402,0.946295529081974
"8281","ZNF232",4.61470515669946,-0.418204286037146,0.761665665555535,-0.549065429819685,0.582960548456402,0.946295529081974
"8282","TRPC4AP",41.8703207897413,0.138164525510685,0.251690436197545,0.548946267478528,0.583042325103156,0.946295529081974
"8283","KCMF1",47.5998501207385,-0.144799337194782,0.263922666443446,-0.548643051944196,0.583250434677865,0.946312035691878
"8284","CCDC9B",2.38853636294704,0.578757529468073,1.05549626419352,0.548327406833873,0.583467111974606,0.946320844536172

"8285","ULK2",19.5987461446861,0.198797606982476,0.362552690809211,0.548327490105683,0.58346705480701,0.946320844536172
"8286","RGS19",6.25724801734708,-0.327511818884635,0.597113865207902,-0.548491398320826,0.583354533928103,0.946320844536172
"8287","SOBP",85.1713073645165,-0.144099764597459,0.262871857301806,-0.548174939974713,0.583571787593828,0.946376403218711
"8288","IL18R1",1.56262079445264,0.689601853280071,1.26088800853873,0.546917607757458,0.584435338291456,0.946633350110249
"8289","ELOVL4",5.48177600370319,-0.318475641232329,0.582333927327545,-0.546895219885061,0.584450719933696,0.946633350110249
"8290","DAGLB",6.78890505840949,-0.291781052909869,0.533511951893983,-0.546906309922464,0.584443100472958,0.946633350110249
"8291","ORAI2",17.5621321119392,-0.223621993571231,0.408708259627681,-0.547143318745118,0.584280273529733,0.946633350110249
"8292","INTS8",15.6176747833552,-0.214965118628535,0.393268849677419,-0.546611100281299,0.584645941358365,0.946633350110249
"8293","ENTR1",21.5411257061189,-0.182917567342608,0.334296611583506,-0.54717146690826,0.584260936920685,0.946633350110249
"8294","ATE1",32.1190586493715,0.175808096543262,0.321603732031134,0.546660623099493,0.58461191155189,0.946633350110249
"8295","PRG2",5.20926089786424,-0.382422886418047,0.699593028525453,-0.546636216807489,0.58462868231984,0.946633350110249
"8296","NECTIN1",10.4879881471154,0.261798380139893,0.478348937016322,0.547295833399041,0.584175505915756,0.946633350110249
"8297","PPIB",757.021410315769,0.121519863303454,0.222063263323292,0.547230827309506,0.584220159787713,0.946633350110249
"8298","CLN6",6.7481644942546,-0.304822991300496,0.557034348987174,-0.547224766039544,0.584224323465884,0.946633350110249
"8299","LMF1",20.7702614433591,0.217009561858904,0.396870249261802,0.546802291838586,0.584514568387499,0.946633350110249
"8300","NUP85",19.2336265570196,0.190398861695519,0.347640255849935,0.547689338307551,0.583905234115892,0.946633350110249
"8301","LRP5",22.722723704812,-0.224317240434456,0.410525346424337,-0.546415081037631,0.584780645819713,0.946737392985318
"8302","RGS9",46.2960001460484,-0.187799868229276,0.343847152533635,-0.546172527082089,0.584947348916763,0.946893209117367
"8303","PRKCE",20.3961935781507,0.212175634594335,0.388667287345086,0.54590556371149,0.585130853709561,0.946943000932186
"8304","TBC1D13",12.2520291284239,0.251225791957635,0.460531436735236,0.54551279656087З,0.585400881891852,0.946943000932186
"8305","IKZF5",14.7500991676419,0.23121886144503,0.423710542346288,0.545700043630401,0.585272141947326,0.946943000932186
"8306","EID1",875.422698932756,-0.107946332350487,0.197783981960943,-0.545778941652635,0.585217900300172,0.946943000932186
"8307","ZNF747",9.20785326607771,-0.260707578862712,0.477530669330826,-0.545949392586757,0.585100724862273,0.946943000932186
"8308","RNF113A",12.1626229294648,-0.230391324487178,0.422321529646167,-0.545535352365783,0.585385373165652,0.946943000932186
"8309","EPHB2",11.3849617743542,0.270034134755882,0.495388639368503,0.545095533680602,0.585687814323289,0.946980562012348

"8310","BRAT1",19.9308426548833,-0.212560364045797,0.38996954035488,-0
.545069145278278,0.585705962604854,0.946980562012348
"8311","NAP1L1",231.279702784857,-0.095967802730034,0.176061966772028,
-0.54507969261923,0.585698708776547,0.946980562012348
"8312","CERS4",42.7294367352427,-0.158573773776977,0.2907606385113,-0.
54537565534619,0.585495180374994,0.946980562012348
"8313","FBXL12",25.3620462194144,0.20994965566492,0.385255951744415,0.
544961485252287,0.58578000709677,0.946986348535245
"8314","NDUFAF4",16.8971296886027,0.23764084099671,0.43629028577499,0.
544685152855476,0.585970077946882,0.947179682166003
"8315","SMUG1",21.304787469008,-0.218680084543863,0.401621809031637,-0
.544492554005296,0.586102570927293,0.947244397991279
"8316","RALGAPA1",51.4229691361959,0.156212993630213,0.286933633530236
,0.544422038323898,0.586151083688926,0.947244397991279
"8317","ZRANB3",4.53384152806756,0.420940245746829,0.773400881899631,0
.544271742634834,0.586254489017283,0.947297592629947
"8318","NME3",136.498059706068,-0.147929921435469,0.271875657534903,-0
.544108739917174,0.586366646503511,0.947364914926747
"8319","PINK1-
AS",1.66753312654316,-0.600073202343367,1.1151611868444,-0.53810445469
4493,0.590504937203972,0.947656755384479
"8320","KDM1A",38.5729143887844,0.151773631955672,0.279490104820477,0.
54303758644035,0.587103923809287,0.947656755384479
"8321","NSUN4",12.8910348235719,-0.230509265143815,0.428394727532018,-
0.538076802372845,0.590524026878249,0.947656755384479
"8322","CYP4Z1",1.47596953668541,0.665039037639622,1.23091619584858,0.
540279703754449,0.58900415322131,0.947656755384479
"8323","CLK2",9.92639024892946,0.297787094579042,0.548726976516493,0.5
42687178366,0.587345203610919,0.947656755384479
"8324","DAP3",37.9281846884013,-0.178814993118469,0.333839667099774,-0
.535631354631764,0.592213354428581,0.947656755384479
"8325","HLX",9.02376429364675,0.301931146663069,0.557978650895462,0.54
1115948035861,0.58842766648607,0.947656755384479
"8326","AC074117.1",2.3661673499409,0.570416073281809,1.05890637863529
,0.538684141290147,0.5901048181921,0.947656755384479
"8327","LINC01914",6.04275896476374,-0.325921590056226,0.6045312664846
06,-0.53913107249437,0.589796416658545,0.947656755384479
"8328","UGP2",210.995784476037,0.0980803899247632,0.182794556732521,0.
536560779915793,0.591571041542803,0.947656755384479
"8329","CYTOR",134.760033519589,-0.17348839105677,0.321195682752653,-0
.540133010412754,0.589105306956433,0.947656755384479
"8330","ANAPC1",19.6905237122281,-0.19069765001043,0.355548733267639,-
0.536347431919783,0.591718455071647,0.947656755384479
"8331","AMMECR1L",11.4484761923655,0.26029304877694,0.481559895989129,
0.540520610094192,0.588838051445624,0.947656755384479
"8332","INPP1",30.0265222411083,-0.156256322798245,0.287868094189904,-
0.542805284614708,0.587263874294821,0.947656755384479
"8333","SP100",119.691732597926,0.111270221317645,0.205890977212809,0.
540432722326808,0.588898646408189,0.947656755384479
"8334","CMTM6",78.0657219426289,-0.128173300688862,0.236678861124756,-

0.541549423044168,0.588128941835739,0.947656755384479
"8335","ITGA9-
AS1",10.5966493853023,0.248859417197889,0.464483626753958,0.5357765115
14608,0.592113017440351,0.947656755384479
"8336","NISCH",36.7956936233858,0.160114597479424,0.296919473657843,0.
539252597705776,0.589712571942298,0.947656755384479
"8337","CCDC191",14.8339075753503,-0.217734167931713,0.406325950758231
,-0.535860846508591,0.592054726032161,0.947656755384479
"8338","PLXNA1",39.8100772292089,0.166108531820778,0.308800723298282,0
.537914970038225,0.590635752905315,0.947656755384479
"8339","NELFA",21.617307186922,0.174386358720383,0.322127067904953,0.5
41358911110931,0.588260222456203,0.947656755384479
"8340","PAICS",17.1136518868341,0.217939160690414,0.401040229939661,0.
543434659219112,0.586830567669637,0.947656755384479
"8341","APC",61.834285747456,0.137296577123577,0.253680573959821,0.541
218332095553,0.58835710328737,0.947656755384479
"8342","PCDHGA5",10.2820088971014,0.287658195179603,0.532649830629631,
0.540051228101528,0.589161704176887,0.947656755384479
"8343","AL512274.1",1.79000731971799,-0.631093637266335,1.176015585140
1,-0.53663713750116,0.591518286107678,0.947656755384479
"8344","FBXO9",64.1317619377197,-0.119198467690027,0.220945773195001,-
0.539491957534873,0.589547444855654,0.947656755384479
"8345","NACAD",5.87289647439213,-0.341189566165772,0.634602311756113,-
0.537643118919012,0.59082345574183,0.947656755384479
"8346","ZNF92",14.6016028819982,0.215693930179966,0.403107520624505,0.
535077911336923,0.592595983349747,0.947656755384479
"8347","MAGI2-
AS3",136.202115937463,0.114997401268266,0.214321025986217,0.5365661196
287,0.591567352266303,0.947656755384479
"8348","TRRAP",26.5156777035579,0.17974217050988,0.334829615516997,0.5
36816823184376,0.591394149846739,0.947656755384479
"8349","DDHD2",27.5146431308032,0.187112410665112,0.347056399701414,0.
53914121977319,0.589789415467189,0.947656755384479
"8350","RRM2B",16.6783909647994,0.210313053192506,0.392906276172099,0.
535275372135785,0.592459453713676,0.947656755384479
"8351","ATAD2",10.1385840276663,-0.261584009951679,0.484439145065863,-
0.539972899828554,0.589215721822058,0.947656755384479
"8352","SNAPC3",29.8151797892575,0.164277194110906,0.303759373984682,0
.540813578708488,0.588636082555516,0.947656755384479
"8353","DDX58",13.8808803375307,0.221224118998207,0.412113676204449,0.
536803633977092,0.591403261233299,0.947656755384479
"8354","AL161457.2",3.48886526761917,0.480102100344088,0.8972943249867
14,0.535055317942858,0.592611605941749,0.947656755384479
"8355","TRIM14",1.6677352742471,-0.615924153141991,1.1479281971591,-0.
536552856412348,0.591576516012982,0.947656755384479
"8356","GARNL3",7.58522748614662,0.299775407754217,0.552780699053411,0
.542304404382347,0.58760882198252,0.947656755384479
"8357","SEPHS1",30.6370542754311,-0.160601406485802,0.298517707456201,
-0.537996247707904,0.59057963910138,0.947656755384479
"8358","SAR1A",125.483797361523,-0.11435477571699,0.213063423710086,-0

.536717066335103,0.591463065613575,0.947656755384479
"8359","AP3M1",19.3492659280102,-0.186714791327593,0.344543218759561,-0.541919797463469,0.587873757827117,0.947656755384479
"8360","FGFBP3",5.94682686936349,-0.338469979441666,0.623146813791715,-0.543162497104252,0.5870179254161,0.947656755384479
"8361","FRAT2",6.62061989418247,-0.296017064742226,0.549309932809582,-0.538888971528648,0.589963467398207,0.947656755384479
"8362","SLC25A28",22.8255818845944,-0.198104986790539,0.367618940589895,-0.538886778990909,0.589964980358566,0.947656755384479
"8363","ITPRIP",7.75985089415357,0.316900668834928,0.58534427030971,0.541391937888542,0.58823746293329,0.947656755384479
"8364","BBIP1",42.1580864737959,-0.139517108002597,0.258504337401621,-0.539708963512821,0.589397757440801,0.947656755384479
"8365","EBF3",67.3799027860212,0.148219369815716,0.276736764308086,0.535596960477235,0.592237129889808,0.947656755384479
"8366","TRIM5",19.6749538222452,0.202603486920096,0.375159028405545,0.540046944308329,0.589164658356596,0.947656755384479
"8367","INCENP",3.41052040462248,0.45978796600196,0.851371615725319,0.540055549785093,0.589158723874235,0.947656755384479
"8368","RAB1B",80.308423832192,-0.135694649119153,0.251142604821229,-0.540309157085254,0.588983844374073,0.947656755384479
"8369","MMP27",16.4338478360278,-0.251910245275397,0.467618976259447,-0.538708346035192,0.590088113986567,0.947656755384479
"8370","KBTBD3",16.0336561119664,-0.212910760892754,0.396743190052329,-0.536646289668316,0.591511963022932,0.947656755384479
"8371","IL10RA",1.55533808723934,0.636515749876636,1.18087603374011,0.539019957802551,0.589873083581947,0.947656755384479
"8372","AP004609.3",2.39284677260423,-0.540700259806866,1.00563867230355,-0.537668523196629,0.59080591389408,0.947656755384479
"8373","ZNF202",4.44428468671213,-0.353331550608706,0.659077944823983,-0.536099794240677,0.591889582328265,0.947656755384479
"8374","ZNF384",16.5570476828481,0.203116167899795,0.37910948003743,0.535771798372705,0.592116275189238,0.947656755384479
"8375","SAP18",297.885934382746,-0.141760155277824,0.262606458945075,-0.53981975861254,0.589321339430048,0.947656755384479
"8376","COL4A2",95.0854429446853,-0.156881262785981,0.290314930040303,-0.540383034259389,0.588932905538991,0.947656755384479
"8377","DCAF4",6.81507913702016,0.314862640328894,0.585837808488984,0.537457015860755,0.590951968631211,0.947656755384479
"8378","ACOT2",21.231403953444,-0.291693284100073,0.54239623330696,-0.537786338082836,0.590724564947542,0.947656755384479
"8379","MOAP1",20.2194734505897,0.190394353477468,0.350782494274819,0.542770396427776,0.587287898157532,0.947656755384479
"8380","IDH3A",16.5491731316213,-0.19329314904689,0.358300759024291,-0.539471782234728,0.589561362360572,0.947656755384479
"8381","RHBDF1",49.4924422510559,0.167006559724225,0.311909274682996,0.535433131618031,0.592350384990866,0.947656755384479
"8382","VASN",201.400690378818,-0.153572628738014,0.286410165574677,-0.536198247118336,0.591821544843818,0.947656755384479
"8383","VAC14",11.4668820549637,0.241028747021159,0.445261660272396,0.

541319337653517,0.588287493984807,0.947656755384479
"8384","DHX38",28.6982993990124,0.178744134827469,0.332549919568613,0.
537495648952007,0.590925289612541,0.947656755384479
"8385","RCVRN",1.9748004395539,-0.589092780547165,1.08375587767788,-0.
543565938308348,0.586740204400619,0.947656755384479
"8386","TEFM",16.5387902618165,-0.212055666710121,0.391956888461293,-0
.541017833728065,0.588495290635673,0.947656755384479
"8387","ASXL3",1.72057705795318,0.664199417966925,1.23956795358112,0.5
35831388709305,0.592075086635462,0.947656755384479
"8388","ELAVL1",51.2362379489463,0.128082146561112,0.23927164440993,0.
535300147566487,0.592442324342221,0.947656755384479
"8389","ZNF227",9.47738649474601,-0.255985366595731,0.472340201751632,
-0.541951258111065,0.58785208413736,0.947656755384479
"8390","VASP",16.1217900522644,-0.228707431236221,0.424506546770549,-0
.5387606692432,0.590052005380475,0.947656755384479
"8391","PPP5D1",5.42067574841058,0.356095268419519,0.664553757757524,0
.535841178027688,0.592068320431491,0.947656755384479
"8392","AC020915.2",12.0526587405246,0.278164152114154,0.5179642203555
2,0.537033526993868,0.591244455457289,0.947656755384479
"8393","TRIB3",2.79971257258405,0.623718607346557,1.15856355427081,0.5
38355107967398,0.590331912635616,0.947656755384479
"8394","SLC5A3",36.0700563020847,-0.222888796914225,0.411318430607377,
-0.541888669041876,0.587895203005321,0.947656755384479
"8395","CLIC6",10.2280904924475,0.330015434481655,0.614711067021093,0.
536862685880895,0.591362467416981,0.947656755384479
"8396","LZTR1",21.6511569013468,0.190012340910749,0.352692453582584,0.
538747962936659,0.590060773996526,0.947656755384479
"8397","LIF",2.6758513354041,-0.496555155447886,0.922088693486606,-0.5
38511272240319,0.590224125104896,0.947656755384479
"8398","TMEM184B",25.3698646720017,0.209477014385769,0.388488118456659
,0.539210864975628,0.589741364269465,0.947656755384479
"8399","ST13",271.647015427205,0.0953812651242816,0.177384361870682,0.
537709548453977,0.590777586146402,0.947656755384479
"8400","BX537318.1",2.24433682771954,-0.492902737147335,0.912660277571
143,-0.540072521244262,0.589147020149707,0.947656755384479
"8401","SRPX",300.037876442856,0.185325300929011,0.342450526948301,0.5
41173940015543,0.588387697874246,0.947656755384479
"8402","GRIPAP1",19.0037053074699,0.201982256085923,0.377166024418896,
0.535526115845456,0.592286103655519,0.947656755384479
"8403","LONRF3",5.75796385918011,-0.363959530455987,0.67832802399036З,
-0.536553875977203,0.591575811578665,0.947656755384479
"8404","INTS6L",4.31474262285549,0.409402276727941,0.764879191530851,0
.535250901398627,0.592476372647451,0.947656755384479
"8405","HEXIM2",11.6719335638579,-0.262958122445384,0.4916040379589,-0
.534898215110609,0.592720242626743,0.947717708585462
"8406","KCTD5",10.4275639774045,0.255228832867336,0.477708590491606,0.
534277251754428,0.59314972842965,0.948272284892785
"8407","MT-
ATP8",95.3592264099223,-0.179851116591757,0.336678338815607,-0.5341927
17073665,0.59320820738847,0.948272284892785

"8408","CD53",7.01358284215264,-0.329669633660004,0.618706026922747,-0
.532837275401502,0.594146228170559,0.948273479302222
"8409","LINC01138",4.50673948632046,0.399971496262864,0.75042112221222
1,0.532996053047866,0.594036312511701,0.948273479302222
"8410","BABAM2",29.5345603611367,-0.201212242912262,0.377176781785062,
-0.533469324278091,0.593708740229375,0.948273479302222
"8411","MREG",3.28325171354083,-0.412995962529799,0.773926448720294,-0
.53363722510414,0.593592548393581,0.948273479302222
"8412","ACKR2",17.3628000854548,0.243989767611123,0.458320195546956,0.
53235657075933,0.594479058197873,0.948273479302222
"8413","LINC02427",1.81768747463505,0.726423341641178,1.36437595881027
,0.532421681099258,0.594433972146134,0.948273479302222
"8414","TMUB1",18.6895058678765,-0.224001997169474,0.419923155396078,-
0.533435687675266,0.59373201890432,0.948273479302222
"8415","CENPP",5.56878648442839,0.344265565544352,0.646139142422528,0.
532804071045161,0.594169215445395,0.948273479302222
"8416","AL499604.1",3.96225226759769,0.461913914429466,0.8674805641670
57,0.53247753726101,0.594395295451982,0.948273479302222
"8417","DIP2C",31.0698426576363,0.194277417365799,0.364482833969165,0.
533022132346115,0.594018259705698,0.948273479302222
"8418","CD27-
AS1",41.8812137576062,0.16062513527155,0.301212699079268,0.53326149847
7789,0.593852575622912,0.948273479302222
"8419","SLC16A7",30.3927709260757,0.186795309485996,0.350640528391618,
0.532725952538411,0.594223298253709,0.948273479302222
"8420","CHPT1",129.156023905266,-0.159386397436258,0.299247606270668,-
0.532623800813611,0.594294023075046,0.948273479302222
"8421","DCTN5",25.035731177872,0.184232870976297,0.345253823853972,0.5
33615729203973,0.593607423547008,0.948273479302222
"8422","CIAPIN1",26.5047568966531,-0.192135403628447,0.360208832018182
,-0.533400035062852,0.593756693242503,0.948273479302222
"8423","ANGPTL4",126.283607580324,-0.197633881242971,0.370052253754256
,-0.534070200189122,0.593292966164497,0.948273479302222
"8424","CCDC106",14.468618752498,-0.267780072737681,0.502082918103872,
-0.533338345285593,0.593799388401155,0.948273479302222
"8425","PREX1",2.35062331414726,0.558487291459226,1.04900868960399,0.5
32395295667246,0.594452242711012,0.948273479302222
"8426","HENMT1",4.09197391362467,0.451205404870811,0.848515817014457,0
.531758390148104,0.59489334512283,0.948390301218428
"8427","PRRT3",7.124666334359,-0.30046065119523,0.565204254400467,-0.5
3159658451959,0.595005430845798,0.948390301218428
"8428","ZFHX4",220.903862167772,0.145904439518387,0.274476196926979,0.
531574107889593,0.595021001581871,0.948390301218428
"8429","IMPA1",17.1748460483176,-0.20459755505021,0.384880415141515,-0
.531587337264181,0.595011836880293,0.948390301218428
"8430","BAMBI",2.86847006755654,0.518924021099768,0.976269617653648,0.
531537611860689,0.59504628468441,0.948390301218428
"8431","TMBIM6",524.456700933828,0.088623185814153,0.166617060942767,0
.531897425826009,0.594797040265154,0.948390301218428
"8432","RBPMS2",1.95546878344062,0.593632759591157,1.11648384855638,0.

53169847495665,0.594934848349314,0.948390301218428
"8433","MFSD14A",37.2672681314646,0.152552698347265,0.287308529491878,0.530971700064258,0.595438390253486,0.948495907837108
"8434","AC024560.5",5.33536477322037,0.351796775906132,0.662325672470733,0.53115376698867,0.595312227954041,0.948495907837108
"8435","TRIM6",5.09626980230541,0.435292931122306,0.819746532603411,0.531009176385133,0.59541242024784,0.948495907837108
"8436","SLC25A1",40.5461127779011,-0.145038439182379,0.27317512464933,-0.530935748152628,0.595463304380092,0.948495907837108
"8437","SERPIND1",1.56584087750122,-0.818938856666328,1.54245330200661,-0.530932674331828,0.59546543451311,0.948495907837108
"8438","RBM27",62.7314141154231,0.128289158568021,0.241940604899689,0.530250631642466,0.595938170531753,0.949056681221122
"8439","NPDC1",138.878407336326,-0.143607441121285,0.270896553278182,-0.530119115150997,0.596029346640841,0.949056681221122
"8440","SIPA1L1",8.20128275932499,0.310160111394926,0.584995886328909,0.530191952872196,0.595978849830566,0.949056681221122
"8441","FBLIM1",3.00799381462224,-0.462472967605702,0.877491501743604,-0.527039825100019,0.59816593279891,0.949079874609401
"8442","RCC2",16.1635361085567,0.2114306530724,0.401216037463542,0.52697458060014,0.598211240730203,0.949079874609401
"8443","TNNI3K",1.92782618333925,0.547428572999956,1.04008495225036,0.526330634642416,0.598658501568959,0.949079874609401
"8444","AL445524.1",3.98418254634749,-0.394771847738881,0.746950124206295,-0.528511656863788,0.59714425856372,0.949079874609401
"8445","EPM2AIP1",45.9036252768361,0.138296935549005,0.261109523678127,0.529651058302591,0.596353886803957,0.949079874609401
"8446","EIF1B-AS1",7.47941973971538,0.303735610969125,0.575260528405868,0.527996613657504,0.597501686303966,0.949079874609401
"8447","CTBP1",44.9792541570938,-0.147661125003644,0.279328533933406,-0.52862886195095,0.597062934604914,0.949079874609401
"8448","SPARCL1",69.5228416072314,-0.49239186570372,0.935339989830494,-0.526430892570897,0.598588856183618,0.949079874609401
"8449","NSUN2",11.2284761024358,-0.262822833842496,0.497172114221485,-0.528635509362883,0.597058322380549,0.949079874609401
"8450","CENPH",6.30818870142807,-0.301003222676931,0.571976831489583,-0.526250725738378,0.598714013889813,0.949079874609401
"8451","DDX41",22.0633705965784,-0.195364582902464,0.370080200743585,-0.527897959712318,0.597570160892198,0.949079874609401
"8452","GMNN",16.6847308545089,0.199584211837648,0.377512414695071,0.528682512332367,0.597025710415319,0.949079874609401
"8453","TAF11",47.0483654156523,0.144239365418008,0.273772629903117,0.526858238053422,0.598292036695051,0.949079874609401
"8454","AL121933.2",2.43485008480558,0.484198629724744,0.917793563447641,0.52756812534823,0.597799121086273,0.949079874609401
"8455","ASL",11.3450032713584,-0.22565714607143,0.428817431367617,-0.526231280644882,0.598727522653359,0.949079874609401
"8456","THAP1",16.9763288867276,-0.201344916176017,0.380896241454579,-0.528608303949428,0.597077198621992,0.949079874609401

"8457","MSC",7.00190248790034,-0.333649244559146,0.630613083526683,-0.529087095201424,0.59674503310208,0.949079874609401
"8458","SNTB1",9.90814573876501,0.293698821290996,0.557293314188797,0.527009411046869,0.598187053126076,0.949079874609401
"8459","RFX3-AS1",1.95639759957708,-0.501420412376881,0.949624817508565,-0.528019490573558,0.597485808204537,0.949079874609401
"8460","PTPDC1",9.43308115810005,0.2580357789863,0.489215595746092,0.527447982505085,0.597882530207269,0.949079874609401
"8461","MALRD1",1.45508720493025,0.777346778785095,1.46955419726445,0.528967751058188,0.596827821250748,0.949079874609401
"8462","MAPK8",22.2633399629975,0.18982346359477,0.360442456516968,0.526640133987197,0.598443515938758,0.949079874609401
"8463","SERPING1",1622.5954852406,0.0935039949876215,0.177097319519843,0.527980859569954,0.597512620798768,0.949079874609401
"8464","XRRA1",43.5334139425963,0.209006506208063,0.396648076638283,0.526931853494561,0.598240912684822,0.949079874609401
"8465","SORL1",2.28348610850961,0.515696371091262,0.974655084766929,0.529106531275709,0.596731550936188,0.949079874609401
"8466","RPS26",713.478299265504,-0.325441542764505,0.618111652995726,-0.526509314599114,0.598534381933113,0.949079874609401
"8467","LIG4",6.59941116481108,0.304531081407949,0.575489128572125,0.529169129855739,0.596688129301154,0.949079874609401
"8468","PELI2",18.6282688898838,-0.261325807555518,0.496314324883706,-0.526532873329317,0.598518017784574,0.949079874609401
"8469","COQ6",14.9781741766698,0.203550920071342,0.385207975239637,0.528418239380204,0.597209080856237,0.949079874609401
"8470","HYPK",4.87706900142065,-0.382294634595281,0.724841672520716,-0.527418123278991,0.597903260783202,0.949079874609401
"8471","CHD2",73.8288469646445,0.137578179797066,0.261115400168682,0.526886501938184,0.598272407927291,0.949079874609401
"8472","TXNDC11",17.2603698128271,0.207764521694455,0.393092083327352,0.52853906376292,0.597125241552729,0.949079874609401
"8473","G6PC3",31.8344290011661,0.167876925500816,0.317224321244992,0.529205720551185,0.596662748755356,0.949079874609401
"8474","AC007114.1",2.3343589102787,0.599802858660386,1.13674672220061,0.527648637067753,0.597743228815423,0.949079874609401
"8475","RNFT1",24.2309627948521,0.184379498861018,0.349689515676897,0.527266305093857,0.59800866971724,0.949079874609401
"8476","CANT1",20.4664844418956,0.177192954140938,0.335269575763894,0.528508898361009,0.597146172640011,0.949079874609401
"8477","BBC3",9.69541715614816,0.292712735562456,0.554382731513224,0.527997570854123,0.597501021942083,0.949079874609401
"8478","TMEM35A",20.8192403648903,0.241219143782673,0.455793297059162,0.529229247860929,0.59664642967597,0.949079874609401
"8479","TAOK3",68.8714008945648,0.147344161662047,0.280119237386574,0.526005150651997,0.598884628298976,0.949216950077832
"8480","TSR2",45.4813432291932,-0.129276143946266,0.24587279051757,-0.52578466968279,0.599037827233324,0.949347801909038
"8481","ATAD3B",6.4045792595017,-0.302971986990873,0.579798083615539,-

0.522547410128682,0.601289245745086,0.949428992477035
"8482","S100PBP",26.7714211649285,0.189688084044882,0.370919809547396,
0.511399173520398,0.609071573528126,0.949428992477035
"8483","HYI",72.0858475464398,-0.156624453250086,0.305628617445175,-0.
512466583003085,0.608324502007639,0.949428992477035
"8484","EIF2B3",34.3525981887348,0.164810251812748,0.319912974875785,0
.51517213978814,0.606432735634775,0.949428992477035
"8485","LRRC42",15.2980681606226,-0.197196458237807,0.377158139761601,
-0.522848209937754,0.601079887452738,0.949428992477035
"8486","OMA1",17.9373468316809,0.228003579332135,0.446281221762466,0.5
10896645912406,0.609423429924137,0.949428992477035
"8487","PTBP2",19.5913550075173,0.186003998310582,0.365131284550398,0.
509416766464196,0.610460126489159,0.949428992477035
"8488","RNF115",97.9871194124628,-0.101956157806837,0.197074267532231,
-0.517348911572954,0.604912616083397,0.949428992477035
"8489","S100A11",1890.89670760673,-0.132026048785965,0.25690214671402,
-0.513915708664495,0.607310924266838,0.949428992477035
"8490","MRPL55",72.4224405772044,0.138727088978632,0.264503873847725,0
.524480367567308,0.599944471273175,0.949428992477035
"8491","NUP133",22.0166833280732,0.179360037172269,0.345243161481859,0
.519518001174639,0.603399563148679,0.949428992477035
"8492","NOL10",20.9260301397733,0.179068457812383,0.345026721285644,0.
518998810136025,0.603761570999334,0.949428992477035
"8493","GTF3C2",13.2662264783728,0.219412551479846,0.425404041432368,0
.515774487569668,0.606011923181035,0.949428992477035
"8494","APLF",5.59207620258023,0.388597526046776,0.758846085906969,0.5
1209004469243,0.608587991609183,0.949428992477035
"8495","MTHFD2",4.83290797005522,0.354731286554829,0.67958054811468,0.
521985638845814,0.601680329707109,0.949428992477035
"8496","TTC31",12.8268768202011,0.276235941397056,0.535577689056565,0.
515771935689952,0.606013705700647,0.949428992477035
"8497","AC012360.3",3.415596177593,0.399418012656319,0.783854530365697
,0.509556297990618,0.610362347379156,0.949428992477035
"8498","TMEM177",4.91081822000489,-0.325619801626577,0.632424092822887
,-0.514875706542333,0.606639877909861,0.949428992477035
"8499","PSMD14",52.1692374607044,-0.140127686324176,0.270013996270829,
-0.518964528726228,0.603785477272009,0.949428992477035
"8500","SESTD1",220.342567481367,0.130079534106992,0.250769903091236,0
.518720677814619,0.603955539912492,0.949428992477035
"8501","BZW1",244.175847822277,0.0977888897848849,0.190305931419246,0.
513850982234784,0.607356180491183,0.949428992477035
"8502","HTR2B",2.80377325802057,0.581862894933632,1.12815863471192,0.5
15763366099852,0.606019691682719,0.949428992477035
"8503","TRAF3IP1",18.3909539601777,0.194073286824387,0.372495278595245
,0.521008716019908,0.602360699608843,0.949428992477035
"8504","CHL1-
AS2",7.56425569401107,0.366302023017836,0.718669813383006,0.5096944607
89353,0.610265534278358,0.949428992477035
"8505","TAMM41",12.1063415580935,-0.228033217460536,0.441578946103982,
-0.516404188814833,0.605572140795771,0.949428992477035

"8506","RAF1",39.7993990812731,0.159911746149323,0.309363068946008,0.5
16906386706526,0.605221509039596,0.949428992477035
"8507","PTH1R",16.95086932593,-0.234240139201313,0.453276654301748,-0.
516770799859854,0.605316166050889,0.949428992477035
"8508","AC099778.1",5.13970176518608,0.336283642220069,0.6492824896649
97,0.517931174138976,0.604506290600519,0.949428992477035
"8509","APEH",23.4720971823272,-0.173314940684,0.333258746294112,-0.52
0061191525468,0.603020926195558,0.949428992477035
"8510","PRKCD",6.76715749319609,-0.278302418741076,0.531626528442885,-
0.523492346321041,0.600631675807255,0.949428992477035
"8511","SLMAP",32.1218157151082,0.18402451116181,0.354601151938825,0.5
18961966580293,0.603787264011391,0.949428992477035
"8512","PXK",79.2376781210443,-0.130560213782882,0.254141713963044,-0.
5137299648568,0.607440798953756,0.949428992477035
"8513","FRMD4B",8.26930000592191,0.296558363825134,0.56949388893321,0.
520740203868832,0.602547763404511,0.949428992477035
"8514","NSUN3",16.7983551632836,0.18560446167439,0.360047274460133,0.5
15500254661534,0.606203491685595,0.949428992477035
"8515","CMSS1",28.8842901689101,-0.166467185414735,0.321848833050073,-
0.51722165290199,0.605001438442459,0.949428992477035
"8516","CHCHD6",15.1192925415862,-0.243589761376064,0.474004466653133,
-0.513897607539462,0.607323580283323,0.949428992477035
"8517","TOPBP1",26.3990652538194,0.173685427029915,0.340011333825155,0
.510822463110096,0.609475378393501,0.949428992477035
"8518","IFT80",26.2926561165295,0.166380614170011,0.323294345776549,0.
514641274564722,0.606803717192845,0.949428992477035
"8519","SLC49A3",19.9340734413675,-0.224506036364403,0.436841114925949
,-0.513930645933958,0.607300480456118,0.949428992477035
"8520","NOP14",21.1321617239542,-0.207082343426716,0.396020359906754,-
0.522908325914038,0.601038050355476,0.949428992477035
"8521","MTHFD2L",9.15007011904423,-0.248576534375275,0.480477046176412
,-0.517353610028661,0.60490933682806,0.949428992477035
"8522","SPCS3",69.8020640300945,-0.114489203225086,0.221143183931171,-
0.517715270214794,0.604656942475961,0.949428992477035
"8523","LPCAT1",27.5758226539722,0.172467188986314,0.33053966163459,0.
52177456748587,0.601827299268512,0.949428992477035
"8524","RANBP3L",1.6979704605713,-0.670313189016254,1.31156699397494,-
0.511078116554877,0.609296358606429,0.949428992477035
"8525","NUP155",10.1525205048736,0.264944640967178,0.506181662505899,0
.523418093922141,0.600683335407478,0.949428992477035
"8526","MOCS2",46.7003049536186,-0.125163961393183,0.244149260815632,-
0.512653452134349,0.608193755773633,0.949428992477035
"8527","OCLN",7.06720612269233,-0.388924015763516,0.753181632983238,-0
.516374800887068,0.605592662099687,0.949428992477035
"8528","CCNH",37.356838480014,-0.132264945880679,0.258609228996701,-0.
511447121952349,0.609038006041843,0.949428992477035
"8529","EGR1",411.796452695508,0.172065478727001,0.328903116267913,0.5
23149432816084,0.600870267695144,0.949428992477035
"8530","MALINC1",5.28850528496197,-0.316615958558738,0.608007505562308
,-0.520743503430801,0.602545464546849,0.949428992477035

"8531","PCDHB2",11.2718967249238,0.234166538069753,0.45675948772027,0.512669237016838,0.608182712180004,0.949428992477035
"8532","LARS",57.3884882006817,0.127424244424967,0.246425896249038,0.517089503840912,0.605093680320557,0.949428992477035
"8533","CPEB4",15.4127242511723,0.262735943547743,0.506787136984464,0.518434514954544,0.604155138517057,0.949428992477035
"8534","UIMC1",18.6927015594311,-0.181093809257631,0.34692151223581,-0.522002247973994,0.601668765413157,0.949428992477035
"8535","PHACTR1",16.0104090502996,0.211784293187306,0.40357889103902,0.524765536279819,0.59974619229833,0.949428992477035
"8536","PSMB8",123.518573441402,-0.158005483720262,0.309211097031404,-0.510995514835016,0.609354197406705,0.949428992477035
"8537","DAXX",40.226993217144,0.146839786365023,0.279744065145967,0.524907601841004,0.599647424602681,0.949428992477035
"8538","BICRAL",28.8695624439773,0.166591598699726,0.325971811297695,0.511061364590161,0.609308088352977,0.949428992477035
"8539","AL035587.2",3.43378741254063,0.461155337071548,0.895107189417198,0.515195657596946,0.606416303162758,0.949428992477035
"8540","PTK7",17.9703083132145,0.214874053522087,0.417177658037598,0.515066062101344,0.606506857200317,0.949428992477035
"8541","AL590428.1",1.84103020834382,0.667611876839334,1.28167780634924,0.520889004656306,0.602444095440789,0.949428992477035
"8542","CEP162",30.722824277457,0.172193046929049,0.336179830706647,0.51220516878452,0.608507426016413,0.949428992477035
"8543","RNF146",68.285784508389,0.114744221355682,0.223456578806795,0.513496724815128,0.607603901228542,0.949428992477035
"8544","MTHFD1L",9.19154551136485,-0.294591227191726,0.562689787615203,-0.523541094357275,0.600597761441836,0.949428992477035
"8545","TNRC18",52.8043656905316,0.13277798205997,0.258843005366148,0.512967240015422,0.607974236806709,0.949428992477035
"8546","ZNF316",11.9420528319729,-0.250339173760059,0.483064799527867,-0.518231040648652,0.604297079867575,0.949428992477035
"8547","C1GALT1",60.0823767993291,0.132110381619848,0.256228264617881,0.515596442167953,0.606136295729026,0.949428992477035
"8548","UMAD1",13.3418387378482,0.237480693487455,0.454432881451421,0.522586949978095,0.601261723917294,0.949428992477035
"8549","CBX3",140.156339430794,0.100481354013109,0.192553682396028,0.521835535746584,0.601784845240192,0.949428992477035
"8550","JAZF1",56.4870513001012,0.119341782071878,0.233882205864847,0.510264479636565,0.609866184155549,0.949428992477035
"8551","HUS1",11.9509357974885,0.247411482063579,0.479198933183766,0.516302238863083,0.605643332788475,0.949428992477035
"8552","POR",44.1031147591269,-0.139698566989185,0.270582362550406,-0.516288518114929,0.605652914318978,0.949428992477035
"8553","ZSCAN25",6.67255237070955,0.321674731445575,0.620926713758243,0.518055874095986,0.604419286042413,0.949428992477035
"8554","GIMAP7",1.51635287343268,0.60533990267902,1.18786287392602,0.509604194193141,0.610328784896493,0.949428992477035
"8555","CCDC25",77.4034619819531,0.125106474316869,0.242642174465408,0.515600697168599,0.606133323290647,0.949428992477035

"8556","FNTA",94.5542103254383,-0.114730829947561,0.223832544603426,-0.512574389710998,0.608249071643256,0.949428992477035
"8557","AC027031.2",1.53684723566074,-0.642609594411221,1.24037804988445,-0.518075593542696,0.604405528077999,0.949428992477035
"8558","EXT1",66.9802361725434,0.143816743731915,0.278424016547745,0.516538571331373,0.605478306760653,0.949428992477035
"8559","MYC",134.673994688653,-0.350967415860523,0.679688676135261,-0.516364959699106,0.605599534175056,0.949428992477035
"8560","C8orf33",47.4176484562777,-0.164983226576522,0.315967883375668,-0.522151887128246,0.601564581975191,0.949428992477035
"8561","IDNK",10.5024369445494,-0.258287077122961,0.49589005571326,-0.520855528654342,0.602467417123378,0.949428992477035
"8562","CDC26",84.5456222514991,-0.133440501138058,0.256656853924629,-0.519917933605016,0.603120775362758,0.949428992477035
"8563","PAPPA",2.00032718349335,-0.7200490060045,1.39148029469817,-0.51746978289814,0.604828257295934,0.949428992477035
"8564","BRD3OS",14.6290104330038,-0.205042612331835,0.393383574731284,-0.521228199402828,0.602207812018485,0.949428992477035
"8565","TMEM203",60.5502178901098,-0.126946731027878,0.2489482332746,-0.509932243173828,0.610098931947041,0.949428992477035
"8566","HNRNPH3",135.075274680388,0.0954395963127432,0.187113835189301,0.510061675643537,0.610008253520543,0.949428992477035
"8567","ZNF503",193.875008286289,0.121456130849919,0.23741221216103,0.511583333242936,0.608942652448215,0.949428992477035
"8568","ATAD1",41.1445709108367,0.132211507423309,0.257704238415852,0.513035828343506,0.607926258654936,0.949428992477035
"8569","FAS",29.4247630363797,-0.162091116822618,0.309528043813189,-0.523671828974715,0.600506812685486,0.949428992477035
"8570","IFIT2",31.4272155723019,0.161605847951474,0.315569389637684,0.512108757243596,0.608574895957622,0.949428992477035
"8571","BTAF1",21.6418666492572,0.197920135549875,0.383416321979585,0.516201643498142,0.60571358254175,0.949428992477035
"8572","EEF1AKMT2",34.8131305885583,-0.143744839212716,0.276045683040259,-0.52072844476163,0.602555956194932,0.949428992477035
"8573","ZRANB1",29.6707804179193,0.158937371712883,0.303958897266548,0.522890999875905,0.601050108099161,0.949428992477035
"8574","DUSP8",3.4678950724914,-0.393503357785039,0.761944040724664,-0.516446532491794,0.605542573165934,0.949428992477035
"8575","CD81",1969.77193327511,-0.123251783531747,0.238350984368995,-0.517102053755059,0.60508492003546,0.949428992477035
"8576","C11orf74",33.4145395197272,0.20037215216729,0.389660890872653,0.514221870505282,0.607096878653689,0.949428992477035
"8577","NRXN2",5.75995587124741,0.448299164031782,0.873089724477939,0.513462879545216,0.607627570488227,0.949428992477035
"8578","SSSCA1-AS1",2.03311349804376,0.508955062059326,0.999102466327269,0.509412276730995,0.610463272862122,0.949428992477035
"8579","POLD3",27.0916015776082,0.190640013453768,0.372648166453506,0.511581783074607,0.608943737593428,0.949428992477035
"8580","JAM3",53.1657089935258,-0.133343001567372,0.254597695727226,-0

.523740017310429,0.60045937826101,0.949428992477035
"8581","THYN1",60.6707749635246,0.165160318900789,0.320994467935211,0.514526994696136,0.606883592012798,0.949428992477035
"8582","SMIM10L1",44.9813093749718,-0.154533608007638,0.301298500682901,-0.512892057734718,0.608026829426886,0.949428992477035
"8583","CPM",3.51025034914137,-0.451065818980992,0.8629665545049,-0.522692121295219,0.601188521882586,0.949428992477035
"8584","ATP2B1-AS1",24.9221389669671,0.204403097010531,0.400936698451562,0.509813887828044,0.610181855147161,0.949428992477035
"8585","WASHC4",59.2173644021228,0.128745440234668,0.246122267457206,0.523095458061525,0.600907826075424,0.949428992477035
"8586","RILPL1",27.9776550876798,-0.173793862540238,0.34012549497185,-0.510969818815322,0.609372190592308,0.949428992477035
"8587","INTS6-AS1",5.86096068458922,0.317428592863103,0.609417986932828,0.520871716407167,0.602456139571972,0.949428992477035
"8588","MIA2",18.1866048376466,-0.175047818436052,0.33951401464056,-0.515583483707949,0.606145348229359,0.949428992477035
"8589","ZBTB1",43.6525670690668,0.147799601059016,0.286453233348659,0.51596415698028,0.605879443314928,0.949428992477035
"8590","GPHN",10.2672267693356,0.271821366031036,0.531342823860285,0.511574361833313,0.608948932605346,0.949428992477035
"8591","LGMN",139.796846249691,-0.120065548616675,0.233852285757321,-0.513424738303703,0.607654244574058,0.949428992477035
"8592","WDR25",5.86719365082056,0.315399454949651,0.607467641894935,0.51920371258919,0.603618690356823,0.949428992477035
"8593","DPH6",16.2540258176484,0.209751277913339,0.409257864906692,0.512516180870859,0.608289798777043,0.949428992477035
"8594","HAUS2",10.7497067960251,0.245856265777028,0.468031680843363,0.525298341629375,0.599375810067033,0.949428992477035
"8595","EIF3J-DT",42.1582740777933,-0.142097807425453,0.277950917394986,-0.511233453579586,0.609187596211731,0.949428992477035
"8596","TEX9",2.98507406716661,-0.428039736160281,0.823606106688906,-0.519714136022016,0.603262832812599,0.949428992477035
"8597","ROGDI",8.2819232675431,-0.277742258377773,0.541672961708023,-0.512748979572446,0.608126923179688,0.949428992477035
"8598","NUBP1",27.1071297407883,-0.202282864693826,0.386207633422633,-0.523767132464896,0.600440516389183,0.949428992477035
"8599","MYH11",1.7128303014302,0.613558854440291,1.20415378512602,0.509535295258055,0.610377064959768,0.949428992477035
"8600","GDE1",45.5024296746357,-0.145636832135105,0.2779984133939,-0.52387648676523,0.600364449956285,0.949428992477035
"8601","ZNF319",6.06577453190442,0.321871017419934,0.621433796432138,0.517949006423378,0.6044938484714,0.949428992477035
"8602","ZCCHC14",33.0928449479351,0.15767456614055,0.308605470596189,0.510926024208,0.609402857539851,0.949428992477035
"8603","MVD",16.1689615145962,-0.223076714075809,0.428756349720145,-0.520287837652817,0.602862971576007,0.949428992477035

"8604","CRK",74.6384007873074,0.11150661641968,0.21593094729704,0.516399422201804,0.605575469254095,0.949428992477035
"8605","CLUH",8.32984950998126,0.275214688720826,0.534163977888092,0.515225099620034,0.606395731577016,0.949428992477035
"8606","MED11",43.4833609597404,0.141415953532208,0.271372146390505,0.521114474765255,0.602287028068678,0.949428992477035
"8607","ZNF624",11.5171263049185,0.247986913966886,0.484589133035278,0.511746750104758,0.608828262585804,0.949428992477035
"8608","AC079915.1",1.62599990333109,0.623882013483548,1.21356171219665,0.514091708079904,0.607187874434561,0.949428992477035
"8609","SKAP1",1.9660714179655,0.669391330267711,1.30132573464121,0.514391833227113,0.60697806788989,0.949428992477035
"8610","ACSF2",9.24943114262193,0.25448581199468,0.497249849082604,0.511786604790712,0.60880036622985,0.949428992477035
"8611","AKAP1",9.92004392673753,-0.246520431996262,0.473652085982346,-0.520467320406672,0.602737899352499,0.949428992477035
"8612","POLG2",13.500588806669,0.248667473444988,0.482407631735489,0.515471682216951,0.606223452848537,0.949428992477035
"8613","UNK",21.6428432557709,0.168930884407146,0.326479979431308,0.517431067906231,0.604855276775973,0.949428992477035
"8614","ZNF24",51.6651563909979,-0.116703978417018,0.225721226642686,-0.517027043281837,0.605137280851194,0.949428992477035
"8615","LMAN1",197.017088712123,-0.0859391976517446,0.168591834817032,-0.509747092704769,0.610228655963508,0.949428992477035
"8616","ZNF121",10.942582674471,0.221429067832993,0.429565968504699,0.515471625007395,0.606223492816334,0.949428992477035
"8617","DNM2",30.4856347914896,-0.159779110942417,0.310205949572937,-0.515074295520075,0.606501103970928,0.949428992477035
"8618","ZNF69",4.51644493035792,0.369714106508939,0.713635884942319,0.518071070009074,0.604408684067644,0.949428992477035
"8619","ZNF791",51.7211914910206,0.150577291748611,0.292979774110329,0.513951149719664,0.607286144790062,0.949428992477035
"8620","AC005253.1",3.59477134140324,-0.429750820691835,0.842629189917126,-0.510011789093257,0.610043202572621,0.949428992477035
"8621","MEF2B",7.07852105863391,-0.297273480090401,0.568826286105306,-0.522608549133341,0.601246690002301,0.949428992477035
"8622","ITPKC",10.4506241019044,-0.250540603312964,0.477188041915424,-0.52503537663538,0.599558598512961,0.949428992477035
"8623","AC011462.5",2.32636411420626,0.653123994011696,1.2434784099848,0.525239512618222,0.599416700251454,0.949428992477035
"8624","INAFM1",34.9562108572192,-0.146645040390155,0.287439699670984,-0.510176710308324,0.609927667002362,0.949428992477035
"8625","LIG1",3.04379708563998,0.485291410288232,0.948921655011235,0.511413568997418,0.609061495531751,0.949428992477035
"8626","MED25",23.8362535910091,-0.168790124739772,0.322486485769057,-0.523402164705432,0.60069441809843,0.949428992477035
"8627","CDC25B",13.7975727213236,0.228811947676137,0.441363646480612,0.518420466888608,0.604164937806196,0.949428992477035
"8628","NCOA5",12.3817652771194,-0.243337956727623,0.468903997012199,-0.518950485127327,0.603795270752401,0.949428992477035

"8629","DPM1",45.722040079793,-0.184448669978005,0.358220784536875,-0.514902199816433,0.606621363594733,0.949428992477035
"8630","TXNRD2",27.79899701372,0.192089408295293,0.366337179474565,0.524351387349778,0.600034161488484,0.949428992477035
"8631","AC245452.1",9.79109331436863,-0.234456667654139,0.459345404879177,-0.510414744903802,0.60976092898821,0.949428992477035
"8632","SUSD2",1.45114650774784,0.709183388690504,1.37654858336647,0.515189508935555,0.606420599364629,0.949428992477035
"8633","ASPHD2",2.95630826514059,-0.430792839620622,0.828364385244656,-0.520052343261218,0.603027093121188,0.949428992477035
"8634","CCDC157",1.54094641698609,0.602997348131393,1.18077612896701,0.510678809757883,0.609575981049766,0.949428992477035
"8635","RAC2",3.48995726533162,0.536115232776707,1.02802696716498,0.521499192044707,0.602019068270883,0.949428992477035
"8636","GGA1",28.6188896780184,0.17888510818336,0.348791113418541,0.512871748451635,0.60804103682468,0.949428992477035
"8637","MICALL1",2.90047226397288,0.447881202620247,0.855731559990056,0.523389838076618,0.600702994366197,0.949428992477035
"8638","APOBEC3F",6.82393554232209,0.297686857506708,0.571916111815155,0.520507905542136,0.602709619295752,0.949428992477035
"8639","ACO2",33.7457014409839,-0.154256411491766,0.302111503640694,-0.51059429923339,0.609635168520623,0.949428992477035
"8640","TBC1D22A",29.8528106115481,0.161744217124036,0.308162493471318,0.524866654932781,0.599675891209357,0.949428992477035
"8641","CCDC22",15.7077536163316,-0.20928866583015,0.409435759676365,-0.511163621847736,0.609236489258437,0.949428992477035
"8642","TWNK",3.07607503016143,-0.391628951608098,0.768965802790584,-0.509293066332563,0.610546817290014,0.949449048549004
"8643","AKAP8",15.8686362662903,0.206449985087103,0.405963020101536,0.508543820162407,0.611072016287549,0.950155828634545
"8644","HOXD3",1.54794047090245,-0.616308359367499,1.21218252302256,-0.508428679396187,0.611152744244649,0.950171417156853
"8645","ACOX3",12.8897180654516,-0.223874337454434,0.440722007912502,-0.507971767769946,0.611473142542128,0.950490362653274
"8646","C12orf45",39.1464501705361,-0.145653433795122,0.286756383220998,-0.507934408151847,0.611499343366337,0.950490362653274
"8647","DTNB",1.92444259092061,-0.59468654153351,1.17395488474207,-0.506566776340955,0.612458824762034,0.950573035330976
"8648","MAP4K4",64.4590654581436,0.136895418427929,0.269753403075881,0.507483564125492,0.611815565801241,0.950573035330976
"8649","ZC3H15",104.327888777032,-0.103297746065634,0.203620149505184,-0.507306110503587,0.611940051790768,0.950573035330976
"8650","ABI3BP",660.390948421021,-0.131522719809398,0.259108777991984,-0.507596542381388,0.611736315947389,0.950573035330976
"8651","TMEM150C",8.16408725174839,-0.34114614794186,0.67377802214357,-0.506318307706938,0.612633212855471,0.950573035330976
"8652","FAM149A",18.453233157839,0.219232902790972,0.43304948119636,0.506253701506142,0.612678560411406,0.950573035330976
"8653","TAGAP",3.46878864947272,-0.448859207213947,0.886347115547566,-0.506414698418296,0.61256555828106,0.950573035330976

"8654","C7orf50",51.8563140955693,0.169566909775039,0.334375986762668,0.507114495322277,0.612074484879985,0.950573035330976
"8655","STARD3NL",24.9809497504721,-0.152917267723716,0.302482823641392,-0.505540334101772,0.61317937668608,0.950573035330976
"8656","SDHAF3",28.7358056809833,-0.180023624020231,0.355980526065027,-0.50571200062597 2,0.613058842487541,0.950573035330976
"8657","GBA2",12.2152829168574,0.298874547214925,0.589502004387317,0.506994963529517,0.612158352431083,0.950573035330976
"8658","INTS5",10.9374931279544,0.2367723854971,0.468287492595531,0.50561330217206,0.613128141494073,0.950573035330976
"8659","FDX1",60.1876509430651,-0.13743376662064,0.271705546474752,-0.50581877478681,0.612983877191212,0.950573035330976
"8660","RAD52",3.8113296611603,0.372109130947788,0.735780160400486,0.505734118660183,0.6130433132577,0.950573035330976
"8661","RSRC2",160.457125195478,0.0897323226520347,0.176900286547454,0.507248034490683,0.6119807952922,0.950573035330976
"8662","VCPKMT",7.02430277679888,0.299561139973994,0.591916465584089,0.506086850749108,0.61279568101627,0.950573035330976
"8663","UBL7-AS1",12.935740102017,0.227744792157294,0.450296852220519,0.505765898727099,0.613021000549772,0.950573035330976
"8664","CENPN",2.2255734339671,0.557048755969236,1.09708573007609,0.50775316887095,0.611626456072331,0.950573035330976
"8665","FAM106A",7.28497410430023,-0.400734723490559,0.791036548730861,-0.506594447669324,0.61243940495572,0.950573035330976
"8666","STXBP2",2.24543618620084,-0.44878225409346,0.887590923368013,-0.505618345431622,0.613124600393489,0.950573035330976
"8667","SAMD1",38.8633010563653,-0.135605122011822,0.267549538844172,-0.506841172657754,0.612266264787899,0.950573035330976
"8668","ZNF813",3.64137686211616,0.384836134747122,0.760185853371326,0.506239537397892,0.612688502498863,0.950573035330976
"8669","KRBOX4",16.38784553871,0.201982200559527,0.399011199091215,0.506206845871897,0.612711449645239,0.950573035330976
"8670","BRD2",114.487867276146,0.139246725040063,0.275499863525129,0.50543300914326,0.613254739312926,0.950580212413657
"8671","KAZN",38.0045067980656,0.195817695821983,0.387653725850663,0.505135595929803,0.613463601573496,0.950587180436399
"8672","MED8",39.9557127775078,-0.160199268210604,0.31764551710093,-0.504333477370314,0.614027056069542,0.950587180436399
"8673","FMO5",7.55650961112681,-0.308507196243578,0.610532974413817,-0.505308000013891,0.613342524760612,0.950587180436399
"8674","ANKAR",20.153989051148,0.186885677870184,0.370636531906326,0.504228973083034,0.614100482720892,0.950587180436399
"8675","PRDM1",4.31254055472761,0.430379836339956,0.85232129910933,0.504950230376385,0.613593792796268,0.950587180436399
"8676","EPHA1-AS1",4.08770681982269,-0.468023788634969,0.927865704719947,-0.50440897454684,0.613974012760111,0.950587180436399
"8677","MSR1",6.75672479042747,-0.281466916332493,0.557479166354149,-0.504892260231455,0.613634510543638,0.950587180436399

"8678","ANKRD46",17.5458059466211,0.194470033496426,0.385601638461131,
0.504328856776964,0.614030302502402,0.950587180436399
"8679","TNFSF15",1.44946973640452,-1.61100189593164,3.19467627456885,-
0.504277040135798,0.614066709437132,0.950587180436399
"8680","DNM1",140.07402925125,-0.123991266461852,0.245907943976772,-0.
50421822270843,0.614108036353063,0.950587180436399
"8681","ABHD13",23.018276640319,-0.162658005073241,0.322570104108988,-
0.504256293442132,0.614081286556084,0.950587180436399
"8682","CHTF18",3.51767521619635,-0.408610842264111,0.810338659073024,
-0.504247005482295,0.614087812546337,0.950587180436399
"8683","DNM3",2.26043913110358,-0.545468391066404,1.08447015420663,-0.
502981468831158,0.614977300966774,0.950602252390497
"8684","LIPT1",13.0506053244116,0.217067757166221,0.431092070343232,0.
503529923418431,0.614591747463741,0.950602252390497
"8685","NICN1",5.91528378088815,0.310740920209482,0.61749470967345,0.5
03228473607185,0.61480364797427,0.950602252390497
"8686","AC004522.5",1.490681439592,0.570894435274369,1.13480639177857,
0.503076506627367,0.614910483519503,0.950602252390497
"8687","TOPORS",55.5294169697403,-0.150092293745671,0.298232779492085,
-0.503272289522603,0.614772846107699,0.950602252390497
"8688","FJX1",6.03076411205894,-0.358742695933975,0.712059566490192,-0
.503809951886822,0.614394933642371,0.950602252390497
"8689","PPHLN1",59.3861922353778,-0.109982593672811,0.218369223452184,
-0.50365427844686,0.61450434296521,0.950602252390497
"8690","SPTLC2",25.0160141678066,-0.159529043209411,0.316683269828096,
-0.503749513815516,0.614437409287829,0.950602252390497
"8691","CAPN3",5.74621627176219,0.400296946984874,0.795890361728716,0.
502954887046764,0.6149959901765,0.950602252390497
"8692","CLEC16A",7.66159462997743,0.259461562769605,0.516037957112805,
0.502795500201718,0.615108057652189,0.950602252390497
"8693","MOSMO",45.8820090733829,0.125030008215291,0.248112713480873,0.
503924230488613,0.614314622612765,0.950602252390497
"8694","CYB5D2",62.2896982413852,0.146981807348947,0.291845782971666,0
.503628340462323,0.614522573352658,0.950602252390497
"8695","ARSG",15.7937609366362,-0.233164238354254,0.463665289884524,-0
.502871885045189,0.615054349109072,0.950602252390497
"8696","MIRLET7BHG",16.8770077418382,-0.241272132189833,0.479777518622
266,-0.502883363277779,0.615046278587442,0.950602252390497
"8697","GCC1",5.61968315115161,0.322552610610039,0.64192889539853,0.50
247404801709,0.615334103216918,0.950842245962074
"8698","KTI12",7.08171862641566,-0.280117798803227,0.558046564122789,-
0.501961335867291,0.615694718578637,0.950962110444581
"8699","KIFAP3",75.0020353385409,-0.120585108913017,0.240213010916431,
-0.501992412704772,0.615672858085915,0.950962110444581
"8700","CRCP",40.1374821412955,-0.137355410418448,0.273482820187478,-0
.502245114791079,0.61549511160166,0.950962110444581
"8701","NFATC4",29.6705691981033,0.21455580796991,0.427331744636425,0.
502082540468542,0.615609461114283,0.950962110444581
"8702","MED4",89.3872386460632,-0.119451777799891,0.238099654603407,-0
.5016881607781 3,0.615886893796299,0.951149616838481

"8703","MAP4K3",21.8068622679815,0.163351988722259,0.325703476350325,0.50153590791447,0.61599401319044,0.951205738626487
"8704","ZNF829",7.85654490332112,0.262805562059957,0.524133989734797,0.501409119055478,0.616083223310012,0.951234195549546
"8705","TMCC1-AS1",2.06616432581821,0.510286582884917,1.01821174782574,0.501159590796873,0.616258810865977,0.951290872813236
"8706","AC091132.4",4.69520381238276,-0.374887370286931,0.748045610334342,-0.501155765247221,0.616261502992189,0.951290872813236
"8707","ZBED5",31.7986291633914,0.150925464816063,0.301499454932111,0.500582877836537,0.616664715123925,0.951585383689336
"8708","NXF1",18.5951258470287,0.194244291142018,0.387973894058434,0.500663302651912,0.616608103197037,0.951585383689336
"8709","SUGP2",43.8533187243039,0.181896705992585,0.363291968470002,0.500690138454311,0.616589213682826,0.951585383689336
"8710","ZNF672",9.99910613423319,-0.254032680403976,0.507898207401842,-0.500164554042203,0.616959214673707,0.951821247388354
"8711","ZNF862",6.02148069790057,0.31081946661095,0.621329720846989,0.500248831147567,0.616899878714179,0.951821247388354
"8712","NVL",12.8617257641271,0.224233494780437,0.448618406897862,0.499831240387532,0.617193911281143,0.951964762275598
"8713","FAM102A",29.6058976102871,0.164537996648698,0.329138098661701,0.499905654549628,0.617141510542406,0.951964762275598
"8714","MAPK10",15.7695061075823,0.261134777875114,0.522564081384418,0.499718191849878,0.617273521186699,0.951978293691537
"8715","OSGEPL1",11.0711564400155,0.222172354090956,0.444685908394198,0.499616358191427,0.617345237313065,0.951979649368936
"8716","ZNF852",2.45116965683161,-0.463033195145036,0.927031912564587,-0.49947924000165,0.61744180825598,0.952019327805429
"8717","C1GALT1C1L",9.66956629106551,0.241682967736785,0.483985902778503,0.49935951925317,0.617526131767022,0.952040115270966
"8718","RER1",175.395889156142,-0.13347466641778,0.268984010859464,-0.49621784577938,0.619740721497706,0.952073096457026
"8719","LINC01772",1.69128506931513,-0.571551736488139,1.16375670632467,-0.491126481490442,0.623336992787146,0.952073096457026
"8720","GPN2",13.4153923617948,0.202900916412166,0.410207006148325,0.494630548408527,0.620860934450705,0.952073096457026
"8721","PDE4B",10.7055543641395,0.268037626452571,0.545628772296916,0.491245403581307,0.623252889520223,0.952073096457026
"8722","AL355488.1",2.8934965147701,-0.402778109194809,0.819331914664519,-0.491593335967792,0.623006855378394,0.952073096457026
"8723","GPR89B",6.76629418797339,0.269134234160455,0.548024883517775,0.491098565511991,0.623356736046236,0.952073096457026
"8724","NOTCH2NLC",7.44369212317358,0.28417431408884,0.577149454953576,0.492375608518418,0.622453839077884,0.952073096457026
"8725","AC096642.1",6.78780027114622,0.308979670688057,0.623482915892959,0.495570388236754,0.62019754923014,0.952073096457026
"8726","PYCR2",27.4568795168831,0.153953579186673,0.309315969767011,0.497722698581121,0.61867950707105,0.952073096457026
"8727","AC104695.4",5.57637128996973,-0.353827522931444,0.714488204917

044,-0.495218144255476,0.620446144205734,0.952073096457026
"8728","FAM98A",27.9107036226676,0.147229733739087,0.298835155207892,0
.492678760089866,0.622239587687488,0.952073096457026
"8729","UXS1",43.6460752779773,-0.134557025693062,0.272591651923603,-0
.493621227002113,0.621573706689007,0.952073096457026
"8730","AGPS",51.4838005663387,-0.122599552505325,0.249611372588568,-0
.491161725661452,0.623312067133594,0.952073096457026
"8731","C3orf33",7.18744354342909,0.290627407594942,0.58497543259466,0
.496819851571994,0.619316094886889,0.952073096457026
"8732","DCUN1D4",26.0592268803995,0.169546217178127,0.344616807905558,
0.491984758980738,0.622730117918471,0.952073096457026
"8733","TENT4A",15.1803259110983,0.237593259544796,0.482676059799044,0
.492241648868425,0.622548524816077,0.952073096457026
"8734","NNT",31.9530097145191,0.140077064665088,0.283294209314626,0.49
445791710313,0.62098281967104,0.952073096457026
"8735","AC008966.1",2.169290328527,-0.602840070797975,1.21382163025274
,-0.496646340593266,0.619438468361683,0.952073096457026
"8736","IPO11",10.1222980150605,-0.222735193181591,0.448791675952907,-
0.496299742433198,0.619682947986623,0.952073096457026
"8737","MIR3936HG",3.60949102535927,-0.415145192550466,0.8350619693669
39,-0.49714297594607,0.619088230492757,0.952073096457026
"8738","SPDL1",11.7237094097647,0.229810612150512,0.467309318827136,0.
491774083870821,0.622879059349264,0.952073096457026
"8739","AC008443.5",2.70925205662182,0.426995915144429,0.8616939649284
38,0.495530817811739,0.620225473758169,0.952073096457026
"8740","HLA-
F",36.6798235886759,-0.226934041931486,0.461237695593516,-0.4920110478
81655,0.622711533481461,0.952073096457026
"8741","NUDT3",31.9323071060258,0.173389293639557,0.348119533623121,0.
498074014505805,0.618431875226423,0.952073096457026
"8742","C6orf226",32.6941546095744,-0.178315704449964,0.35925973218724
1,-0.496342029106196,0.619653118021964,0.952073096457026
"8743","PPP2R5D",23.1285248749219,0.168805755358377,0.341870687711036,
0.493770777742311,0.62146807308823,0.952073096457026
"8744","KLHDC3",62.1153082212102,0.134001938165204,0.271539076468953,0
.493490439415724,0.62166609352453,0.952073096457026
"8745","CEP57L1",36.1706247585431,0.143875834296832,0.291601335650747,
0.493399092208389,0.621730623670239,0.952073096457026
"8746","RNASET2",47.7375602086562,0.153551655064313,0.309412089922542,
0.496269086003566,0.619704574109009,0.952073096457026
"8747","GGCT",31.8529921136466,-0.160450775727138,0.326502127955737,-0
.49142336906573,0.623127039166718,0.952073096457026
"8748","DNAJC30",18.748374906671,-0.221454559810989,0.445137575299367,
-0.497496891072507,0.618838694737179,0.952073096457026
"8749","LRP12",15.6272714000879,0.200906423275509,0.408661459472011,0.
491620676770155,0.62298752360366,0.952073096457026
"8750","CD72",4.15177491889871,0.415523476799291,0.845384419354869,0.4
91520150225131,0.623058603868807,0.952073096457026
"8751","AL590399.1",2.84088326966407,0.450767855046109,0.9139985923243
37,0.493182220226169,0.621883839571475,0.952073096457026

"8752","AL117336.2",5.10167559548588,-0.372377391623071,0.754997086309733,-0.493216991661747,0.621859273114996,0.952073096457026
"8753","RAB11FIP2",51.3763236122392,0.130274903873265,0.262803292651795,0.495712601462244,0.620097195030899,0.952073096457026
"8754","TMEM138",21.1110836836517,-0.158729918132209,0.322053958610315,-0.492867464871848,0.622106237351555,0.952073096457026
"8755","WDR74",41.0556812039014,-0.140717391127741,0.285124942710458,-0.4935288712031,0.62163894513653,0.952073096457026
"8756","SCYL1",33.1155330649631,0.147583891492065,0.299307734047245,0.493084122807094,0.621953148902345,0.952073096457026
"8757","IRAK4",16.132784137335,0.188018897673734,0.380307426365618,0.49438660577976,0.621033171608021,0.952073096457026
"8758","ZC3H10",7.39661705600649,-0.252038390562441,0.50680597981982,-0.497307452157621,0.618972257407693,0.952073096457026
"8759","YEATS4",24.7472022382923,-0.190053841032298,0.385275598089199,-0.493293221722018,0.621805417093148,0.952073096457026
"8760","AC027288.3",5.77458147238684,-0.408746900230752,0.82779730555657,-0.493776553133295,0.621463993857647,0.952073096457026
"8761","ITM2B",2672.9461063237,-0.0997034337817745,0.200859640373772,-0.49638361194036,0.619623785171497,0.952073096457026
"8762","FKBP3",87.2168413133745,-0.134390254203474,0.2730258173621,-0.492225444106039,0.622559979135359,0.952073096457026
"8763","CDKL1",8.58926179151774,0.256384157285828,0.521818190764982,0.491328515991308,0.623194114247321,0.952073096457026
"8764","LINC01550",5.46554807194947,0.392351718085099,0.78938511851915,0.497034601844449,0.619164650853343,0.952073096457026
"8765","AL049840.5",5.67781304925497,0.316295792889022,0.640734829161227,0.493645387286154,0.621556640796933,0.952073096457026
"8766","PPIP5K1",1.66506153094266,0.630255936005622,1.26505978246431,0.49820249188374,0.618341326272767,0.952073096457026
"8767","ALDH1A3",85.2489973555865,-0.151584621331704,0.306935398950796,-0.493864903982628,0.621401592002608,0.952073096457026
"8768","SLC9A3R2",7.91680157552844,-0.256073957458698,0.516212225436424,-0.496063333723266,0.619849727509044,0.952073096457026
"8769","MT1E",457.620669830292,-0.19595859403358,0.396761192705894,-0.493895566492154,0.621379935829968,0.952073096457026
"8770","ATMIN",44.7643797431193,0.129322461854477,0.260948265065877,0.495586593847747,0.620186113219935,0.952073096457026
"8771","ZC3H18",33.6590648469424,0.155821850429551,0.31412772882875,0.496046149795643,0.619861851036716,0.952073096457026
"8772","SPATA2L",7.3370372702733,-0.265544507295865,0.536354738988399,-0.495091192438607,0.620535750645858,0.952073096457026
"8773","B9D1",12.2874805513193,-0.205216300894503,0.411244773910334,-0.499012544142987,0.617770546949124,0.952073096457026
"8774","MAPK7",16.9927843053504,0.185340983036223,0.375116886511443,0.494088615311028,0.621243597753471,0.952073096457026
"8775","GIT1",21.949376700798,-0.159471710342123,0.321807590319802,-0.495549872467723,0.620212026973615,0.952073096457026
"8776","MILR1",4.47186520145617,-0.383424472537389,0.77678787433612,-0.493602546081299,0.621586902309923,0.952073096457026

"8777","RECQL5",6.37332253932472,0.293224298868879,0.593034269835817,0
.494447477630691,0.620990190726902,0.952073096457026
"8778","AC027601.6",3.6385716445848,-0.370302409998499,0.7516111003545
79,-0.492678207950635,0.622239977881018,0.952073096457026
"8779","AC144831.1",12.4877454178343,0.216022857323781,0.4354606639412
73,0.49607892333741,0.619838728883685,0.952073096457026
"8780","PRELID3A",2.24961715627217,0.549702924635364,1.10871936928763,
0.495799874939098,0.620035613123039,0.952073096457026
"8781","CTIF",21.1241859983274,0.181673809525173,0.366960920266684,0.4
95076722047526,0.620545964644062,0.952073096457026
"8782","PIAS4",10.6617481719768,-0.235766544490006,0.477097319516246,-
0.494168662966002,0.621187069010405,0.952073096457026
"8783","SIRT6",15.9153186099362,-0.217614122815245,0.439071701036784,-
0.495623203001675,0.62016027913204,0.952073096457026
"8784","CLPP",53.4165676952987,-0.15676725909127,0.317288351582098,-0.
494084508017957,0.621246498337406,0.952073096457026
"8785","TRMT1",12.5966186022681,-0.202687346655263,0.412313585619316,-
0.491585418779777,0.623012453408021,0.952073096457026
"8786","CACNA1A",7.29926605300178,-0.336742968945507,0.684279822372534
,-0.492112960130772,0.62263949083329,0.952073096457026
"8787","ZNF708",25.186290304503,0.170388329103597,0.343143344628191,0.
496551460988465,0.619505389319102,0.952073096457026
"8788","LRP3",15.1467490858394,-0.200758807570915,0.407967588821537,-0
.492094992523379,0.622652192027648,0.952073096457026
"8789","ZNF574",4.17602016990952,0.347116814642337,0.701632395435909,0
.494727462557769,0.62079251340144,0.952073096457026
"8790","PTOV1",66.1595711397997,-0.125985744354771,0.25348178573829,-0
.497020896344978,0.619174315625412,0.952073096457026
"8791","CSE1L",26.1451257682283,0.158310808520397,0.318856410505079,0.
496495611518763,0.619544782820622,0.952073096457026
"8792","CR381653.1",1.57506480209059,0.605417290621414,1.2197396769635
,0.496349591683844,0.619647783276589,0.952073096457026
"8793","MX1",18.8731255715245,0.278952863193704,0.560091097255449,0.49
8049093371819,0.618449439943695,0.952073096457026
"8794","BCL2L13",27.1824579867236,0.162530152551298,0.330394640340598,
0.491927327827561,0.622770718612766,0.952073096457026
"8795","CBX7",35.7272163003761,-0.135745996043683,0.272972382858316,-0
.49728838728034,0.618985699671962,0.952073096457026
"8796","SGSM3",31.5354459479196,0.161593106829854,0.324682389411024,0.
497695939477916,0.618698370519146,0.952073096457026
"8797","CHADL",8.93567620635942,-0.272912099157763,0.552830474827142,-
0.49366326855099,0.621544010292724,0.952073096457026
"8798","ZNF674-
AS1",3.30490212459893,0.418534640411394,0.841096876970403,0.4976057477
69435,0.618761951727792,0.952073096457026
"8799","USP27X",4.55017143894346,-0.339639267814243,0.680536081956654,
-0.499076061974148,0.617725800740789,0.952073096457026
"8800","NR1D2",48.1195496787318,-0.436928198613418,0.890126512860018,-
0.490860784732215,0.623524914775859,0.952113547286987
"8801","Z68871.1",1.77718772664799,-0.534740553669908,1.0893872458876,

-0.490863607673521,0.623522918037244,0.952113547286987
"8802","SMYD5",7.54274847221393,0.266701684679284,0.543462314581162,0.490745498857317,0.623606461788595,0.952129884114625
"8803","SAR1B",63.2070499212205,-0.108396110151003,0.22100209713096,-0.490475482170519,0.623797475092274,0.952313332700792
"8804","RBMXL1",12.9022979518344,-0.199738991116039,0.407856185333338,-0.489728973836191,0.624325696386748,0.952424530598374
"8805","SLC25A44",8.19368686808579,-0.24493401869678,0.500532482360105,-0.489346900208893,0.624596122328632,0.952424530598374
"8806","ERO1B",11.5024872035394,0.240329364436396,0.491659623499489,0.488812489270121,0.624974455137756,0.952424530598374
"8807","EN1",26.3232299078397,0.176324773414517,0.360322268793355,0.489352972840099,0.624591823816464,0.952424530598374
"8808","CNOT6L",35.2226599503456,0.160880041716496,0.329287427066146,0.488570253501295,0.625145976963186,0.952424530598374
"8809","AZIN1",84.88232362055,0.118954770735082,0.243437110524002,0.488646823317245,0.625091757364269,0.952424530598374
"8810","AL354733.3",1.82631181866739,0.587293962518874,1.20080851398144,0.489082110661943,0.624783566036755,0.952424530598374
"8811","WASHC2A",38.5581706717467,0.169740207409387,0.347292072992671,0.488753474695545,0.625016240190496,0.952424530598374
"8812","BDNF-
AS",9.7377935858941,0.230481107093932,0.471558908152323,0.488764188544355,0.625008654199657,0.952424530598374
"8813","FLI1",5.15773755073019,0.335420681698145,0.685411110319047,0.489371527027061,0.624578690313845,0.952424530598374
"8814","CDKN1B",56.099212191535,0.123355057887367,0.252475909773451,0.488581496737867,0.625138015425095,0.952424530598374
"8815","ASB8",54.9121202536244,-0.119270773553941,0.243664852328943,-0.489486983510151,0.624496967611466,0.952424530598374
"8816","COA8",68.8756311825838,-0.141820593082369,0.290092532838571,-0.488880536477953,0.624926276058439,0.952424530598374
"8817","AC116407.2",3.43800093191132,-0.448130505205864,0.916323971994146,-0.489052473690742,0.624804547468589,0.952424530598374
"8818","STARD3",25.4234525217717,-0.15893234070556,0.324796777471422,-0.489328563980423,0.62460910170598,0.952424530598374
"8819","TASP1",34.9397678680358,0.168275657564444,0.343604788802835,0.489736066108795,0.624320677061547,0.952424530598374
"8820","TMEM50B",68.507795719532,0.118769307717634,0.242752609250977,0.489260684299549,0.62465715164045,0.952424530598374
"8821","NHSL2",9.93601032924108,-0.296328025665961,0.604453144684889,-0.49024151544524,0.623963006644168,0.952424530598374
"8822","RUBCN",10.914740560277,0.23919124965543,0.489814261304079,0.488330513322762,0.62531575170402,0.95243910384132
"8823","CHID1",110.642646534361,0.153259106495739,0.314068999925241,0.487979095460616,0.625564648190418,0.95243910384132
"8824","AL139383.1",1.58560051681103,0.573658610771825,1.17587623650441,0.487856283648667,0.625651641395281,0.95243910384132
"8825","IGHG4",32.8670247421148,0.403405025126511,0.826845967633813,0.487884129472067,0.625631916472232,0.95243910384132

"8826","KATNAL2",3.81258285732704,0.364999537560354,0.747308858002365,
0.488418588448204,0.625253377989522,0.95243910384132
"8827","EHD2",134.169644042991,0.100053762789594,0.204992851681407,0.4
88084154978703,0.625490233881919,0.95243910384132
"8828","HDAC10",13.8893366803061,0.19260374116241,0.394685226096084,0.
487993287885373,0.625554595385014,0.95243910384132
"8829","SKI",94.7387668004258,-0.121649377273131,0.249524523124907,-0.
487524736044623,0.625886517703701,0.952500491153829
"8830","IPMK",24.8616135285496,0.184433400266281,0.378305410991193,0.4
87525144784606,0.625886228119073,0.952500491153829
"8831","NFKBIA",373.227206808548,-0.128739094998973,0.264080616205018,
-0.487499222203522,0.625904593896827,0.952500491153829
"8832","CDC14A",11.3196436822439,0.229664958287716,0.47225945538423,0.
486310979418847,0.626746695905311,0.952540150755303
"8833","VARS",19.4429953928692,-0.176908580536325,0.363605920663227,-0
.486539328660103,0.626584828073802,0.952540150755303
"8834","DAAM2",6.16329752082289,0.281462781878872,0.578159053001732,0.
486825866372846,0.626381738155253,0.952540150755303
"8835","ZFAND2A",19.6545173585632,-0.19035260491385,0.391494648599199,
-0.486220196355038,0.62681105345712,0.952540150755303
"8836","MFHAS1",11.4641888068251,-0.231045248973842,0.475340761684192,
-0.486062352732426,0.626922958064637,0.952540150755303
"8837","PINX1",11.2913276818841,-0.219208673298535,0.450672189373259,-
0.486403817380841,0.626680884542076,0.952540150755303
"8838","RNF169",34.7979111500006,0.141272220935413,0.2899584804625,0.4
87215344452335,0.626105732406967,0.952540150755303
"8839","TELO2",17.72903375997,-0.186641887349993,0.38303470823212,-0.4
87271475244189,0.62606595933445,0.952540150755303
"8840","CHD3",53.4846112831782,0.14094409496908,0.289952433684001,0.48
6093850561313,0.626900626719431,0.952540150755303
"8841","SOCS7",7.96671136780504,0.27318319078269,0.561389563222626,0.4
86619646461713,0.626527898205281,0.952540150755303
"8842","ZNF599",10.6251335022451,0.223959459123279,0.459824771055684,0
.487053924061342,0.626220117487634,0.952540150755303
"8843","CAPNS1",196.732010297193,-0.106242056581832,0.218326781191835,
-0.486619442662336,0.626528042657014,0.952540150755303
"8844","MAP3K10",6.79768566994399,0.28924943083127,0.594043259703545,0
.486916442710955,0.626317546071524,0.952540150755303
"8845","SNRPA",35.3313185106905,-0.150551194113951,0.309277653553311,-
0.486783291273259,0.626411912403814,0.952540150755303
"8846","KPNA6",42.764800262863,0.145506291299264,0.300683477724827,0.4
83918479326706,0.628443725113069,0.952844854583325
"8847","DNAH14",2.72596747652423,-0.495275777116186,1.02136633897053,-
0.48491492055181,0.62773669800844,0.952844854583325
"8848","WBP1",51.1360366453347,-0.116799234485119,0.240561737419445,-0
.485527065684044,0.627302518384766,0.952844854583325
"8849","BIN1",216.76562056,0.131352337089163,0.270636065931816,0.48534
6757598288,0.627430393116201,0.952844854583325
"8850","AC006460.2",1.67768956299065,-0.542641568952285,1.121994937355
38,-0.483639944250842,0.628641421307166,0.952844854583325

"8851","TRAK2",22.4350352491038,-0.172902986094807,0.357355478350289,-0.483840311873777,0.628499203436208,0.952844854583325
"8852","EAF1",11.2028359262802,-0.230817076318242,0.475679879962666,-0.485236155744821,0.627508837633998,0.952844854583325
"8853","SLC12A2",9.09191497021042,0.260464893016148,0.538461371686155,0.483720665422145,0.628584124997659,0.952844854583325
"8854","SLC22A5",6.81413035146172,-0.294572231670093,0.608761636483377,-0.483887640114352,0.628465612588396,0.952844854583325
"8855","ZNRD1",36.4971364938951,0.14218686432797,0.293861904363229,0.483856063738767,0.628488023588081,0.952844854583325
"8856","SPTAN1",195.429557014798,0.0962483414159575,0.198618168489833,0.48458981445539,0.627967340255192,0.952844854583325
"8857","MSS51",2.57614908590493,-0.461968582250591,0.95308204597048,-0.484710192793726,0.627881935187779,0.952844854583325
"8858","HPS5",13.1032495078928,-0.20913160317593,0.430907276139517,-0.485328549217208,0.627443307146178,0.952844854583325
"8859","RCN1",165.215990995524,-0.117350072036918,0.242668956735819,-0.483580898090195,0.628683333997347,0.952844854583325
"8860","CD3D",4.9826150841324,-0.347717078604347,0.718580278007641,-0.483894547688448,0.628460710056149,0.952844854583325
"8861","FAM66C",5.76466752307475,0.31548277953906,0.651770128113976,0.484039948949442,0.628357517807978,0.952844854583325
"8862","IGHG3",15.4224475648565,0.797573010131593,1.64867716590791,0.483765425168835,0.628552355259563,0.952844854583325
"8863","ERVK13-1",6.54357465628707,0.354384952237215,0.731731760014572,0.484309922846806,0.628165934788137,0.952844854583325
"8864","NAGLU",77.75416120783,0.153110907462692,0.315956117441966,0.484595483392768,0.62796331819073,0.952844854583325
"8865","SLC25A10",1.84202962859796,-0.501923123609763,1.03659170113929,-0.484205230524334,0.628240225187812,0.952844854583325
"8866","UBE2L3",145.396844978552,0.117758152554529,0.243033516685815,0.484534619588138,0.628006501137199,0.952844854583325
"8867","LINC00893",4.47457609711039,0.403906468657956,0.831793751974838,0.485584879303319,0.627261519259908,0.952844854583325
"8868","AC004241.1",2.07381849485698,-0.475439448536389,0.983557460190037,-0.483387567864645,0.628820573814325,0.952945386952042
"8869","DHDDS",17.8005231905847,-0.178352516300954,0.369952032306827,-0.482096327972146,0.629737518264508,0.953045773216406
"8870","PRPF38A",35.7593035466508,0.142486385402477,0.29547075493523,0.482235155332754,0.629638905777496,0.953045773216406
"8871","WWTR1",37.1473485491073,0.146071149536275,0.302985134333491,0.482106654696452,0.629730182711137,0.953045773216406
"8872","EIF4EBP3",57.825268858603,0.165884742432565,0.343621112175705,0.482754803342067,0.629269845636907,0.953045773216406
"8873","KIF13A",35.8808195753632,-0.136420358335815,0.282908687623695,-0.482206324173656,0.629659384715464,0.953045773216406
"8874","AC007032.1",4.9795234219395,0.358061139216849,0.742717640425527,0.482095913342928,0.62973781279572,0.953045773216406
"8875","GADD45G",26.2287347251709,0.231767426112886,0.480083997438216,0.48276432322182,0.629263085369362,0.953045773216406

"8876","NDUFAF8",92.6367270919393,-0.140896446146883,0.291852286001502
,-0.482766292761393,0.629261686761619,0.953045773216406
"8877","ZNF414",21.1833482177337,-0.166628679142536,0.345218126336499,
-0.48267650633187,0.629325447174964,0.953045773216406
"8878","CCT8",91.6692018826339,-0.129048774655626,0.267502701116363,-0
.482420454511559,0.629507293502165,0.953045773216406
"8879","DONSON",4.18167580325437,-0.420955533964928,0.87163622205953,-
0.482948646822273,0.62913219941525,0.953045773216406
"8880","NF2",25.7952319034048,-0.147926147022178,0.306744752714944,-0.
482245077423199,0.629631858125495,0.953045773216406
"8881","TAX1BP1",143.86348738525,-0.094018331533477,0.195146348748284,
-0.481783708158175,0.629959603966886,0.953274081489808
"8882","ZNF562",13.3992379614088,0.234778652664688,0.487494213086455,0
.481602953147366,0.630088027929285,0.953361068153756
"8883","AC010226.1",26.9699516529374,0.187832782879232,0.3905726293932
06,0.480916400033072,0.630575916375557,0.953464485640287
"8884","SERAC1",5.45103141642285,0.2916842227653,0.606504308547541,0.4
80926876618283,0.630568470139902,0.953464485640287
"8885","RAB3IL1",57.0889621741522,0.149707647284731,0.311302212919432,
0.480907751604955,0.63058206327635,0.953464485640287
"8886","HSP90B1",544.175679745964,0.10166778397491,0.211240891430134,0
.481288368395926,0.630311562750564,0.953464485640287
"8887","KIAA0586",24.0183891369367,0.151850059518601,0.315621672616986
,0.48111417146843,0.630435356607947,0.953464485640287
"8888","TPST2",8.7938762109347,0.238515244427098,0.495814604593263,0.4
8105731904118,0.630475761295835,0.953464485640287
"8889","FAAH",2.81020265301184,0.438426844742652,0.912073179599103,0.4
80692618256093,0.630734978289904,0.953588409634156
"8890","MMP17",18.4037111550836,-0.188159314152644,0.391569815225789,-
0.480525584037028,0.630853715744095,0.953660639582102
"8891","YPEL4",11.0512110351838,0.233189266006121,0.485382302748112,0.
48042391468717,0.63092599278563,0.953662627043761
"8892","ADGRG2",13.6866717553093,0.305319375377239,0.635794382106878,0
.480217164495037,0.631072982997525,0.953777532445314
"8893","SELENON",67.4973001855607,0.124321994654687,0.259440870215413,
0.479192019944517,0.631802030777943,0.953796609515238
"8894","SPATA1",2.29318302939989,-0.458542417833378,0.957603652995808,
-0.478843638909328,0.632049869051946,0.953796609515238
"8895","OLFML3",393.551982815865,0.118129767053508,0.247166121880897,0
.47793672593379,0.632695240820131,0.953796609515238
"8896","PLEKHH2",59.4675730520532,-0.196168239366763,0.40870327940447,
-0.479977160086906,0.631243633785575,0.953796609515238
"8897","RPE",30.2302194685093,-0.150251655371098,0.314163989621534,-0.
478258681245113,0.632466100930651,0.953796609515238
"8898","CBLB",126.321159844975,0.164815633173088,0.344098401331143,0.4
7897820081552,0.631954136807484,0.953796609515238
"8899","PRPF4B",141.758116731752,0.109501902877417,0.228885067496426,0
.478414359115529,0.632355315566575,0.953796609515238
"8900","PRR3",7.48610857122564,0.260111342952488,0.543852765972183,0.4
78275296600757,0.632454276517592,0.953796609515238

"8901","ILRUN",24.326672151927,0.159642865999161,0.333136974320601,0.479210890129313,0.631788607709096,0.953796609515238
"8902","AARS2",6.24211624085668,0.323523087019012,0.677128854071696,0.47778659124273,0.632802105738936,0.953796609515238
"8903","CYP39A1",2.45703927242298,0.513171341512593,1.07444367514635,0.477615861476121,0.632923639416972,0.953796609515238
"8904","LSM5",96.3683265894107,-0.124993186277792,0.260573792334093,-0.479684411690691,0.631451813845342,0.953796609515238
"8905","OR2A1-AS1",3.59510621368273,-0.40570723610048,0.849428822008291,-0.477623581386458,0.632918143798695,0.953796609515238
"8906","AC004263.2",1.94409406627029,0.67959348087587,1.42274279577252,0.477664327589769,0.63288913789344,0.953796609515238
"8907","FNTB",6.82584313701233,-0.285310156304628,0.596200791450116,-0.478547094194021,0.63226086354526,0.953796609515238
"8908","SORD",11.3861907738763,-0.216547009291856,0.452511306414871,-0.478544969422977,0.632262375448902,0.953796609515238
"8909","AC105020.1",1.62394464517173,0.566078875307148,1.18078146609549,0.479410366406759,0.631646720189795,0.953796609515238
"8910","LINC00921",5.44073651614572,-0.318117292061921,0.663538910739537,-0.479425225729971,0.63163615129293,0.953796609515238
"8911","TMEM170A",27.1035655018001,0.170586333089657,0.355900431761364,0.47930914903761,0.631718714430989,0.953796609515238
"8912","FLII",71.222717599109,-0.105576518508919,0.220122878135222,-0.479625377440606,0.631493797989363,0.953796609515238
"8913","AC008736.1",1.8512894762651,0.506658782201712,1.05814740574018,0.478816825948084,0.632068945506918,0.953796609515238
"8914","GSK3A",24.9886611613871,-0.152755556770584,0.319646733384408,-0.477888684026938,0.632729435909905,0.953796609515238
"8915","BLOC1S3",6.71821689042554,0.273292452396971,0.572213568481487,0.477605683350399,0.632930885010558,0.953796609515238
"8916","VRK3",21.434629203724,0.175158837622107,0.36644506019094,0.477994811912156,0.632653897647024,0.953796609515238
"8917","ZFP64",10.1126709171497,-0.233415349233873,0.486234886570132,-0.48004648716255,0.631194338004362,0.953796609515238
"8918","UTP14A",16.2355467326168,0.184995852027082,0.38601905987088,0.479240201478552,0.631767757689824,0.953796609515238
"8919","NSMCE4A",40.3914282333306,-0.146883082901241,0.307824700954735,-0.47716470590461,0.633244841390814,0.954055764960891
"8920","ELF1",92.2673718146848,0.116254559166205,0.243601044777939,0.477233417747366,0.633195917260222,0.954055764960891
"8921","NFIA-AS2",1.80530435238403,-0.57186331469928,1.20076824149904,-0.476247867769523,0.63389780089955,0.954079115136942
"8922","HEATR5B",13.2863672719132,0.213651383639108,0.449027959703991,0.475808641804735,0.634210712648416,0.954079115136942
"8923","FBXO11",49.0738002772311,0.13424114829565,0.282273153329457,0.475571788220927,0.634379478154866,0.954079115136942
"8924","CAPN10",9.61114445238896,0.23287342191912,0.490036801580204,0.475216190229349,0.634632888419937,0.954079115136942

"8925","KIAA1143",42.0585906661303,0.138007611694414,0.290306178051561
,0.475386409688818,0.634511579357257,0.954079115136942
"8926","IRF2",58.2935216721563,-0.115827354655252,0.243449806007666,-0
.475775095304881,0.63423461441156,0.954079115136942
"8927","TMEM14A",48.0837908703571,-0.119102579739687,0.250266997930792
,-0.475902059498166,0.634144154880958,0.954079115136942
"8928","ZNF707",1.87618422279539,0.512867545707287,1.07747901138897,0.
475988432522831,0.634082618896241,0.954079115136942
"8929","TMEM72-
AS1",3.11469146612878,0.395958583144929,0.832993826064842,0.4753439590
48871,0.634541831427078,0.954079115136942
"8930","MACROD1",23.9726479493637,-0.15464126435712,0.325197837039335,
-0.475529805994421,0.634409393773827,0.954079115136942
"8931","PARP11",9.65767569567518,-0.236967110105975,0.497125115857645,
-0.47667499095707,0.633593574134289,0.954079115136942
"8932","HMGB1",900.08340104105,-0.0956839430754738,0.20087860920064,-0
.47632718812735,0.633841298757076,0.954079115136942
"8933","CAMTA2",17.5949660290407,0.172616382029981,0.362478601559162,0
.476211233676941,0.633923897124162,0.954079115136942
"8934","ZFP3",2.6886482748988,0.411466604726705,0.863706934808063,0.47
6396087775008,0.63379222131474,0.954079115136942
"8935","VAMP2",173.310777372363,-0.118153524293724,0.247743331498847,-
0.476919090329881,0.633419737435739,0.954079115136942
"8936","TMEM38A",2.93545461678841,-0.414741535355675,0.872864803389667
,-0.475149798393835,0.634680206066245,0.954079115136942
"8937","CARD8",17.827270224793,0.190100956244154,0.399259402831949,0.4
76133949246446,0.633978952031221,0.954079115136942
"8938","ZNF577",8.67812804236523,0.253576305267468,0.531719506902974,0
.476898631657198,0.633434306414141,0.954079115136942
"8939","PXMP4",24.3196546375062,-0.156552388438213,0.328320313646552,-
0.476828212971151,0.633484453877907,0.954079115136942
"8940","BCOR",4.01163702204848,0.358675344802296,0.754161868691196,0.4
75594643129804,0.634363192496354,0.954079115136942
"8941","NFATC2IP",43.4575651764087,-0.165290488004933,0.34809170601229
3,-0.474847533422975,0.634895650019443,0.954189514718328
"8942","ZNF20",3.57539173942954,-0.368555370863178,0.776105602130323,-
0.474877864367342,0.634874029781592,0.954189514718328
"8943","TARDBP",43.6215893386234,-0.156525574617494,0.330513205002898,
-0.47358342192749,0.635796999637211,0.954420488674633
"8944","RNF11",59.3288261254108,0.11864774518631,0.250238042517441,0.4
74139519286085,0.635400418888135,0.954420488674633
"8945","PLXNB1",8.06798964906111,0.252436472226419,0.533312057990219,0
.473337267448486,0.63597257802525,0.954420488674633
"8946","CNOT8",35.8625741141484,-0.148179747650178,0.312793467648128,-
0.47373031401305,0.635692233412388,0.954420488674633
"8947","GMPR",2.73894871838322,0.445449051409555,0.939030546978552,0.4
74371204262569,0.635235223629405,0.954420488674633
"8948","LSM1",53.6296736459467,-0.145925099065979,0.30786790950138,-0.
473986065330154,0.635509844113404,0.954420488674633
"8949","ZNF169",4.94877935064198,-0.357445596540579,0.753925034566104,

-0.474112915943022,0.635419388677858,0.954420488674633
"8950","DGKZ",21.2884950572604,0.162464814116299,0.343189382129065,0.4
73396971399309,0.635929990191127,0.954420488674633
"8951","SLC22A17",22.9080654623658,0.179085689309297,0.37770308437293,
0.474144100799755,0.635397152016441,0.954420488674633
"8952","ANKDD1A",10.5982965382775,0.239369286019874,0.50558046272268,0
.473454382969644,0.635889038685866,0.954420488674633
"8953","MPV17L",7.22552270581283,-0.283223260750711,0.598183939832391,
-0.473471856884138,0.635876574814697,0.954420488674633
"8954","NAA38",162.532061965137,-0.139890149819713,0.295314322643857,-
0.473699170996246,0.635714444594464,0.954420488674633
"8955","ELANE",29.1655560255896,-0.213628429059367,0.450335546789541,-
0.474376119278907,0.635231719337277,0.954420488674633
"8956","QSOX1",42.0747372125581,-0.131318921930302,0.277556822400357,-
0.473124460766752,0.636124386406611,0.954541718280309
"8957","PCDHB5",3.49893842750694,0.429773217535861,0.908867470548096,0
.472866761615623,0.636308239869823,0.954604424604883
"8958","DGCR8",19.919481593468,0.17424357890111,0.368436332170641,0.47
2927243289375,0.636265087675667,0.954604424604883
"8959","AC008467.1",1.81817967357478,-0.508562200257625,1.075955203683
57,-0.472661128006579,0.636454963648213,0.954717965896678
"8960","CAPRIN2",17.3995909792319,0.199442985976797,0.422159392636942,
0.472435268420804,0.636616135508393,0.954746595814897
"8961","AL049775.1",3.12971870019592,0.388649541679164,0.8224925068991
4,0.472526543912725,0.636550999868662,0.954746595814897
"8962","AK3",132.958183070447,0.119975922919167,0.254134127918948,0.47
20968564971,0.636857656137316,0.954816437177987
"8963","ISCA1",66.8394737641298,-0.134075564961407,0.283964309205569,-
0.472156396472864,0.636815160355481,0.954816437177987
"8964","ARFGAP3",60.8047643083493,-0.120220285833619,0.254665489929405
,-0.472071366508847,0.636875849606628,0.954816437177987
"8965","AL080250.1",1.64541371764193,0.630718090608666,1.3364778090589
3,0.471925599013712,0.636979895305626,0.954865902176498
"8966","AL050341.2",8.17364745267763,0.249363067061866,0.5287697645795
38,0.471591009482459,0.637218745175642,0.954885937844824
"8967","EPC2",36.22188799916,0.128830532604393,0.27371344905279,0.4706
76662218179,0.637871652706724,0.954885937844824
"8968","ITGAV",120.466866388867,0.0987588609549646,0.209739549804805,0
.470864274510339,0.637737661552176,0.954885937844824
"8969","MLLT3",24.3174703517152,0.16234086784789,0.344571531709105,0.4
71138364340968,0.637541930129875,0.954885937844824
"8970","TMEFF1",2.49021424634238,-0.447785591260999,0.951493833359529,
-0.470613235274432,0.637916954384317,0.954885937844824
"8971","NSMF",13.2917225132835,-0.218258767192187,0.462835221235605,-0
.471569053473315,0.637234419996398,0.954885937844824
"8972","PICALM",77.2195428757857,0.106133111817348,0.225360939981259,0
.470947236136725,0.63767841481557,0.954885937844824
"8973","PLBD1",116.844916623815,0.102307892454116,0.217307830013211,0.
470797082865798,0.637785647911311,0.954885937844824
"8974","C15orf65",3.72417084804637,-0.361538101695623,0.7670184253045,

-0.471355171881426,0.637387122702993,0.954885937844824
"8975","AC087477.2",4.97913707720877,0.330402475914104,0.701844700618488,0.470762941748997,0.637810031106336,0.954885937844824
"8976","WDR24",7.07388357165516,-0.264424272107263,0.561296355746617,-0.471095650987321,0.637572430677335,0.954885937844824
"8977","TMEM100",45.8656422546535,-0.211904504877417,0.449456329212098,-0.471468507850112,0.637306203540103,0.954885937844824
"8978","FTSJ1",13.8908232346272,0.213188233566395,0.452186014916481,0.471461360001969,0.637311306804474,0.954885937844824
"8979","IAH1",123.377083800795,-0.135200221534494,0.28748772148305,-0.470281724857813,0.638153752384985,0.955084798812923
"8980","PEBP1",535.263038796129,-0.10144945934377,0.215745131634642,-0.470228266914368,0.638191940869116,0.955084798812923
"8981","AL606970.4",6.90628157097651,0.291748121559765,0.621008898447536,0.46979700659541,0.638500053252655,0.955144171446062
"8982","TRIM21",9.32744699253388,-0.242843966135708,0.516671865790946,-0.470015849932048,0.63834369361303,0.955144171446062
"8983","ZNF527",4.25612576699505,0.329483635116718,0.701361651613895,0.469777089121635,0.638514284728748,0.955144171446062
"8984","GEMIN8",15.5737461084119,-0.191856399792388,0.40840077105281,-0.46977482265228,0.638515904179732,0.955144171446062
"8985","MINDY3",25.0074296487201,0.166838735585069,0.35537472391995,0.469472712478702,0.63873178514453,0.955177744647863
"8986","SEC23IP",24.1974171871862,0.156113971914155,0.332608650211848,0.469362332623403,0.638810667682811,0.955177744647863
"8987","NINJ2",41.8216562241074,0.176067741011194,0.375214058533667,0.469246119666371,0.638893723241286,0.955177744647863
"8988","H2AFJ",293.573027598938,0.129416568526651,0.275662225428052,0.469475164127734,0.638730033129397,0.955177744647863
"8989","CHURC1",191.392426212493,-0.0961584488550658,0.204908719347817,-0.469274558745566,0.638873397863518,0.955177744647863
"8990","UBE2E1",59.8047850545388,-0.117286439211639,0.250071174607076,-0.469012229801875,0.63906089435056,0.955215143941405
"8991","SEPTIN7",410.392739235121,-0.0746397281897824,0.159130884518613,-0.469046146608027,0.639036651418684,0.955215143941405
"8992","THBS3",91.9794020583016,-0.122893867686793,0.262571461395936,-0.468039698729784,0.639756200570474,0.955330456043958
"8993","WDR26",41.8633396540815,0.133961736850074,0.286068122479395,0.468286139990043,0.639579978659992,0.955330456043958
"8994","CPSF3",11.2529153524454,0.241201332869523,0.51627792390203,0.467192807793374,0.640361938883212,0.955330456043958
"8995","RBKS",4.46339816905623,-0.339191327501727,0.725601023251252,-0.467462581546382,0.640168957309031,0.955330456043958
"8996","CALCRL",6.58973657013489,-0.34822098583832,0.745288598374765,-0.46722972362341,0.640335529855217,0.955330456043958
"8997","XYLB",3.29244047477598,0.381605206648973,0.814467748463115,0.468533232124973,0.639403311747144,0.955330456043958
"8998","XXYLT1",17.5314690743451,0.171134792134905,0.365691661694694,0.467975647412438,0.639802004855753,0.955330456043958
"8999","HEIH",29.6463494494198,-0.166462662476803,0.356190455447867,-0

.467341726682418,0.640255407343802,0.955330456043958
"9000","RNF8",35.5763645531311,-0.137389198515431,0.293863129855885,-0
.467527854150363,0.640122268468972,0.955330456043958
"9001","AUTS2",61.2247870650841,0.134663795777335,0.287752210214492,0.
467985269954855,0.639795123509516,0.955330456043958
"9002","FAM120A",97.5987228728336,-0.0979267371430435,0.20914258727015
1,-0.468229538618793,0.639620450610623,0.955330456043958
"9003","ATP6V1G1",230.810361018923,-0.105321738935567,0.22504245792821
9,-0.468008303433837,0.639778651757469,0.955330456043958
"9004","PXMP2",18.9973369468138,-0.201388373317124,0.431397712332558,-
0.466827633897783,0.640623203313218,0.955330456043958
"9005","AL136419.3",2.47925673231081,-0.431727142887911,0.923628314808
183,-0.467425192543574,0.640195701930116,0.955330456043958
"9006","SEL1L",87.818845093665,0.0926914303440832,0.198465146757053,0.
467041351384228,0.640470293281308,0.955330456043958
"9007","NOB1",47.3403801441445,-0.137764491556592,0.295099700611653,-0
.466840499231439,0.640613998026107,0.955330456043958
"9008","AC005332.2",1.55452440209607,-0.500635680084815,1.070425064533
4,-0.467698017051797,0.64000055934848,0.955330456043958
"9009","B4GALT6",2.75129437614966,0.436743348367157,0.934957382225027,
0.46712647728155,0.640409391875322,0.955330456043958
"9010","ZNF284",4.12982686533661,0.322803566728048,0.691499257621526,0
.466816938948511,0.64063085570862,0.955330456043958
"9011","UBA1",89.4151860917637,-0.0950407245428142,0.203152642393609,-
0.467829132926917,0.639906785244505,0.955330456043958
"9012","NONO",86.5754764840266,-0.107613066125874,0.229966265928294,-0
.467951530592877,0.639819251596634,0.955330456043958
"9013","DXO",7.85509531491106,-0.241539801750322,0.51772302854666,-0.4
66542511018616,0.640827226043094,0.955517262930561
"9014","CLDN4",5.60383678559097,0.427358729060406,0.916336338772534,0.
466377585366601,0.640945252750959,0.955587225617944
"9015","TOR1AIP2",71.353207295451,0.108849895890019,0.233508736563677,
0.466149136395744,0.641108754019327,0.955613692927471
"9016","B3GNT2",4.74608918244058,0.316927988563863,0.67977711431804,0.
466223386884403,0.641055610923805,0.955613692927471
"9017","CACNB4",38.2474751536219,0.237800839541914,0.510345348133669,0
.465960629231854,0.641243681989286,0.955613692927471
"9018","PMP22",1151.50294529707,0.0808384759182056,0.173489733973196,0
.465955385756111,0.641247435286847,0.955613692927471
"9019","EGLN1",38.1862660996495,0.124892286586246,0.268125752929335,0.
465797429831971,0.641360504966848,0.955633545280845
"9020","ATF4",170.157009650382,-0.0918388958790378,0.197189998441846,-
0.465738103376083,0.641402974807145,0.955633545280845
"9021","HSPB1",376.623483669652,0.117132290347224,0.251585519443916,0.
465576439399704,0.641518710636451,0.955700027961786
"9022","AL450306.1",2.03038035014752,-0.516578180118499,1.110013795898
97,-0.465379963768953,0.641659379982807,0.955725637483649
"9023","LTN1",53.6515503940843,0.121602321058743,0.261311561657698,0.4
65353772666343,0.641678132823496,0.955725637483649
"9024","ARHGAP26",13.4742438915235,0.222260504697596,0.47834843610923,

0.464641436910319,0.642188253139824,0.956273455284885
"9025","AC022098.1",3.88493288505319,-0.318352960024851,0.685139540417124,-0.464654192678811,0.642179116949806,0.956273455284885
"9026","THOC2",103.191042061125,0.110313003976011,0.237585464369336,0.464308724731261,0.642426573991596,0.956522349642484
"9027","AC027279.1",9.13779469702825,0.283878455663909,0.611933456778975,0.463904126370465,0.64271643633379,0.95684792155642
"9028","TACC1",290.571956707769,-0.102317373653767,0.220692989964987,-0.463618593730593,0.642921030367636,0.95690437532746
"9029","ALKBH1",4.39556816940283,-0.306769979181464,0.661884976795195,-0.46347929011295,0.643020856069768,0.95690437532746
"9030","DAZAP1",37.1043872551665,0.134104571507675,0.28935913499291,0.46345373375187,0.643039170591732,0.95690437532746
"9031","ZNF726",1.7324978049966,0.519459310968817,1.1205874474436,0.463559816017804,0.642963149998388,0.95690437532746
"9032","SNX11",10.453596175197,-0.218676762256801,0.472000354727065,-0.463297876933272,0.643150867366808,0.956964626499394
"9033","DCAF15",10.6746330697436,0.230954484847797,0.49872253584115,0.46309213690989,0.643298325926947,0.957078069537501
"9034","TLCD4",9.88263408851439,-0.257789366869806,0.558948955611599,-0.461203772333261,0.644652415485024,0.957139427171467
"9035","HOXD8",22.8600226052807,-0.200337488193491,0.434432534853929,-0.461147524922026,0.644692766928798,0.957139427171467
"9036","UQCRC1",65.6773895902657,-0.11334755883521,0.245129349649701,-0.462398970164887,0.64379523770008,0.957139427171467
"9037","PPA2",22.299854629324,-0.152372289542865,0.329411918333013,-0.462558520389744,0.643680846504571,0.957139427171467
"9038","RUFY1",42.2883664229037,-0.117518304577338,0.254751475363791,-0.461305687865082,0.644579304762804,0.957139427171467
"9039","PTN",2.2051460761908,0.650050703171687,1.40544695226623,0.46252240408185,0.643706739728582,0.957139427171467
"9040","PTEN",111.322999420476,0.116605461809066,0.25201018431353,0.462701386956628,0.643578423995294,0.957139427171467
"9041","TAF10",101.427744083948,-0.134557395736251,0.291602187918071,-0.461441653428391,0.644481773062403,0.957139427171467
"9042","CLPB",8.04024745226689,-0.238748583289164,0.5173235860167,-0.461507245643844,0.644434724216552,0.957139427171467
"9043","AP001372.2",4.93574849338552,0.303164679936837,0.65681996802532,0.461564347454721,0.644393766639221,0.957139427171467
"9044","GDF11",6.99782815894196,-0.27005648039081,0.58459240256067,-0.461956876633857,0.644112245345893,0.957139427171467
"9045","UCHL3",34.6186662027395,0.16741930757561,0.36168453292095,0.462887661309536,0.64344489216708,0.957139427171467
"9046","MED6",40.5689981095068,0.123870918367865,0.268501580396698,0.46134148702161,0.644553624486106,0.957139427171467
"9047","RNF166",14.4228487351101,-0.206532232635562,0.446710786912908,-0.462339927054029,0.643837571412956,0.957139427171467
"9048","SGSH",21.4793607908148,0.186714844235654,0.404573954502033,0.461509798537256,0.644432893072426,0.957139427171467
"9049","PARD6G",1.51837843249429,-0.574062924222239,1.24281726020684,-

0.461904531424596,0.644149784296587,0.957139427171467
"9050","C21orf91",10.2858051698341,0.221429247281298,0.479732372294128
,0.46156828279568,0.644390943965845,0.957139427171467
"9051","NUP50-
DT",3.58741544823411,-0.342336550213844,0.740633270580031,-0.462221404
050267,0.643922555498182,0.957139427171467
"9052","TMEM187",17.4510630349471,0.206179112694592,0.446390670846197,
0.461880424838964,0.644167072448088,0.957139427171467
"9053","SLC9A7",2.57302836036173,0.506005645996026,1.09759713224905,0.
461012179358731,0.644789867044586,0.957177844163503
"9054","GLB1L",12.9805072940474,-0.216352205141476,0.46962834163303,-0
.460688135620516,0.645022368320136,0.957222889245885
"9055","RBCK1",86.6110046280346,-0.120832020336061,0.262295083458026,-
0.460672074912901,0.645033892775559,0.957222889245885
"9056","ZMYM3",11.7122016767494,0.224882375405449,0.487965147192322,0.
460857454060782,0.644900878063649,0.957222889245885
"9057","XCL1",1.47123711421814,-0.623804326493843,1.3546580487824,-0.4
60488406690186,0.645165691053953,0.957273135680204
"9058","JMJD4",14.5525640787171,-0.195681813907215,0.426429188205417,-
0.458884662024945,0.646316993678593,0.957273135680204
"9059","LRRC58",84.6048138205149,-0.0990455225348518,0.215701598385509
,-0.459178435747307,0.646106034746003,0.957273135680204
"9060","EAF2",3.57257230782489,-0.343720858140729,0.748991719425318,-0
.458911426156296,0.646297773176079,0.957273135680204
"9061","PFN2",36.2268878923026,-0.123120177195324,0.268538842045703,-0
.458481820571677,0.646606320393915,0.957273135680204
"9062","ABCC5",22.5931057904392,-0.162379877120429,0.353492498722426,-
0.459358763502178,0.645976555464686,0.957273135680204
"9063","PDS5A",47.6286340806775,0.139884833888976,0.304770769929279,0.
458983759897432,0.646245828309226,0.957273135680204
"9064","FAM114A2",23.2447363411604,0.165007688560196,0.359265800839457
,0.459291388644956,0.646024930834426,0.957273135680204
"9065","NOM1",22.3001251652218,0.171371149948246,0.373236587639283,0.4
5914884988143,0.646127279070241,0.957273135680204
"9066","PVT1",3.82363519874033,-0.326489589471221,0.711922103789355,-0
.458602967562619,0.64651930520464,0.957273135680204
"9067","CCDC107",144.662598029567,-0.122788814583094,0.267771202704346
,-0.458558699901232,0.646551100390045,0.957273135680204
"9068","RPAP3",27.1535109579354,0.155918953553666,0.340059622873842,0.
458504753478214,0.646589848204877,0.957273135680204
"9069","AC004812.2",6.77227048290887,0.278071440017132,0.6057195962847
81,0.459076182647385,0.646179459477155,0.957273135680204
"9070","DHRS12",32.2611314310268,0.16126939169574,0.351447193621095,0.
45887232740179,0.646325851794669,0.957273135680204
"9071","TMX1",48.2757762295059,-0.113227182711796,0.245977078786524,-0
.460315990702785,0.645289424997309,0.957273135680204
"9072","SLC38A6",20.9624555679493,0.169682015927757,0.370181590125631,
0.45837507983628,0.646682992133419,0.957273135680204
"9073","SHF",8.62703641783591,-0.245436234873611,0.534805822795521,-0.
458925883773431,0.646287390622112,0.957273135680204

"9074","HSBP1L1",8.86647209420295,-0.262438194936132,0.57121749704243,-0.459436547891036,0.6459207078789,0.957273135680204
"9075","PARD6G-AS1",2.00848772366317,-0.464391498786485,1.01319653424945,-0.458342960214026,0.646706064353045,0.957273135680204
"9076","CARM1",37.3201377839241,0.158306070118512,0.344917749012376,0.458967596106026,0.646257435826779,0.957273135680204
"9077","SCAND1",144.966431876327,-0.136096217625083,0.296150738713934,-0.459550491807299,0.645838902101926,0.957273135680204
"9078","NDUFV3",59.82505945177,-0.117322949068367,0.255777128387061,-0.458692103583339,0.646455285341813,0.957273135680204
"9079","UBE2G2",64.5084772038438,0.107019402327545,0.233114018263497,0.459086086391328,0.646172347759731,0.957273135680204
"9080","C1orf167",10.4113389543681,-0.254743176394596,0.556538802600002,-0.457727610733525,0.647148148764905,0.957312493971549
"9081","COL16A1",88.5061332729838,0.121408087563027,0.265311454345011,0.457605902703124,0.647235602000469,0.957312493971549
"9082","CEBPZOS",54.9596393903474,0.118468382740428,0.258937211386501,0.457517797871074,0.647298912716377,0.957312493971549
"9083","HDAC9",1.85342212867865,-0.47309242785331,1.0342974761826,-0.457404604330473,0.6473802555205,0.957312493971549
"9084","WAPL",42.9349902504272,0.116956120875679,0.255671325782095,0.457447156101339,0.64734967659991,0.957312493971549
"9085","DNAJC14",12.6547234144162,-0.193539876426966,0.422557091546208,-0.458020656377511,0.646937600974145,0.957312493971549
"9086","SDR39U1",21.5476182745075,-0.162391082887832,0.354769563261064,-0.45773679510475,0.647141549537897,0.957312493971549
"9087","AL606834.1",2.42589671434071,0.41532835335836,0.90793197162798,0.457444353031923,0.647351690947517,0.957312493971549
"9088","TPSAB1",2.4383033634219,0.532541580430519,1.16449741711253,0.457314522647033,0.647444992760429,0.957312493971549
"9089","YWHAE",283.737027362026,-0.0786685574595157,0.171867602877778,-0.457727670266396,0.647148105988787,0.957312493971549
"9090","IL17RA",28.6742497020286,0.167578788827384,0.366602321297271,0.45711327804577,0.647589626911878,0.957421011668728
"9091","AL513477.2",1.72194237678515,0.601667249562466,1.33438620098056,0.450894388086701,0.652065668382422,0.957570068113219
"9092","RNPC3",38.3340639569948,-0.138877843667898,0.308227523795186,-0.450569248190115,0.652300034523666,0.957570068113219
"9093","RAP1A",133.471601689442,0.0872295899806679,0.192087785138748,0.454113154137629,0.649747386033239,0.957570068113219
"9094","DEDD",19.0601850227303,-0.162143756602319,0.357164911783603,-0.453974484202855,0.649847191932386,0.957570068113219
"9095","ATF6",44.3687332492312,0.125233266416582,0.278540619699656,0.44960503983806,0.652995253452832,0.957570068113219
"9096","GAS5",277.815666215751,-0.115422353027786,0.254895130430616,-0.45282290341441,0.650676269975349,0.957570068113219
"9097","TOR3A",31.3599461963634,-0.15161183564208,0.335441015318575,-0.451977631590732,0.651285096809754,0.957570068113219
"9098","FAM72A",4.86646441463217,-0.343441864200541,0.762458949957566,

-0.450439809539458,0.65239334557127,0.957570068113219
"9099","ATF3",119.863966772842,-0.311841071744741,0.686058406372273,-0.454540122019186,0.649440120873946,0.957570068113219
"9100","PEX13",16.7840518533965,-0.175680687224458,0.389841367793159,-0.450646600741637,0.652244274429259,0.957570068113219
"9101","EIF2AK3-DT",1.90084720982177,0.45651243884736,1.00877626599592,0.452540820234965,0.650879421108582,0.957570068113219
"9102","UNC50",75.3008434102566,-0.135517586980001,0.297476292357479,-0.455557603955707,0.648708135858059,0.957570068113219
"9103","RABL2A",12.6805080037802,-0.201189219451287,0.442299352795426,-0.454871159498038,0.649201932551398,0.957570068113219
"9104","PDE6D",14.7293690463927,-0.17857430678985,0.396710224482735,-0.450137898569894,0.652611011356132,0.957570068113219
"9105","NAA80",7.71110657430336,0.262872749066404,0.579036507438618,0.453983031621319,0.649841039856334,0.957570068113219
"9106","SPCS1",226.872621400392,-0.11920438443355,0.261303107972774,-0.456191988523806,0.648251925933716,0.957570068113219
"9107","FYTTD1",51.5879616063303,-0.118418370777125,0.262778783750731,-0.450639009310034,0.652249746676538,0.957570068113219
"9108","RAB28",32.1366949741666,-0.137002058746937,0.304497553044602,-0.449928274881307,0.652762159088782,0.957570068113219
"9109","PRDM8",28.9027013988638,0.150222386466113,0.329008228773158,0.456591578351334,0.647964633536745,0.957570068113219
"9110","AMACR",20.8665024677126,-0.17334566021006,0.382649672308662,-0.453014004073763,0.650538657527584,0.957570068113219
"9111","CCDC152",16.8009019708967,0.242248398976066,0.531585664210943,0.455709051777472,0.648599212016503,0.957570068113219
"9112","TAF9",54.1581017948327,-0.144072696891343,0.318584592613977,-0.452227446748855,0.651105137355889,0.957570068113219
"9113","TCF7",4.82262813394162,0.397812741670938,0.875480777790984,0.454393462155389,0.649545657142126,0.957570068113219
"9114","PFDN1",94.2629022182164,0.11475374946581,0.253558421207619,0.452573213381255,0.650856090840268,0.957570068113219
"9115","RGS14",1.75970707584807,-0.481101101862365,1.05990147032629,-0.453911156208001,0.649892773530884,0.957570068113219
"9116","MGAT1",127.321700745732,0.0941866638625806,0.206946466654043,0.45512574041689,0.649018780701761,0.957570068113219
"9117","AC008443.1",9.39013352941504,0.215606811917557,0.478427551616838,0.450657181403784,0.652236647440623,0.957570068113219
"9118","SLC35B3",32.2093154028874,0.129692952831301,0.28801245229454,0.450303283063151,0.652491772058137,0.957570068113219
"9119","PI16",630.972594889518,-0.176718816212619,0.389379658350352,-0.453847067824003,0.649938903768737,0.957570068113219
"9120","UFSP1",1.59682295869929,0.5413411304782,1.19757032611793,0.452032852411284,0.651245315613531,0.957570068113219
"9121","AC078846.1",4.85030642144885,-0.304829359103046,0.676662024261963,-0.450489828264744,0.652357286920106,0.957570068113219
"9122","AGAP3",35.1561262380305,-0.127842700269273,0.283733252628429,-0.450573554861733,0.652296929980075,0.957570068113219

"9123","SLC7A2",5.88328992099251,-0.314021450704867,0.693054106146939,
-0.45309803075936,0.650478153302195,0.957570068113219
"9124","LYN",4.34735825550191,0.338645135213995,0.74832279510003,0.452
538847448484,0.650880841964569,0.957570068113219
"9125","JAK2",14.7450196432186,-0.213990472863211,0.474323237912102,-0
.45114903879718,0.651882135986664,0.957570068113219
"9126","CEMIP2",5.05210480318812,0.318879374369603,0.702008770553595,0
.45423844793013,0.649657212844792,0.957570068113219
"9127","GNAQ",67.1407786042934,-0.114301522302502,0.250493565449414,-0
.456305223239698,0.648170508449155,0.957570068113219
"9128","AL161729.1",2.35944182800567,-0.452387317160056,0.999085857252
771,-0.452801242131486,0.650691869114508,0.957570068113219
"9129","TEX10",9.34553025909882,0.227558649648982,0.506192418768294,0.
44954969930742,0.653035164545807,0.957570068113219
"9130","RALGPS1",4.86897561894519,-0.283914658678183,0.622763122616219
,-0.455895104201837,0.648465410248084,0.957570068113219
"9131","AL445222.1",1.9552538160867,-0.492936231851105,1.0792364293255
,-0.456745360383342,0.647854083103713,0.957570068113219
"9132","PANX1",7.52973326693601,0.266815718879681,0.585571719616608,0.
455649939949923,0.648641725350927,0.957570068113219
"9133","FAM234B",4.61047340439791,-0.307893724857454,0.683993571272305
,-0.450141255399136,0.652608591053278,0.957570068113219
"9134","DDX55",23.9924191374912,0.156076933553792,0.344537849269908,0.
453003737860807,0.650546049971133,0.957570068113219
"9135","ULK1",23.3469069918758,0.172471719056945,0.380592242671254,0.4
53166669521223,0.650428731009046,0.957570068113219
"9136","AL133453.1",9.71091602047871,-0.278500182861751,0.618510909253
731,-0.450275296191263,0.652511949474239,0.957570068113219
"9137","MAX",48.7252485665469,0.113677428807393,0.252980162521036,0.44
9353133757833,0.653176933878554,0.957570068113219
"9138","SLIRP",112.753936383585,-0.143326059355736,0.31754727094354,-0
.45135345969079,0.651734820585664,0.957570068113219
"9139","ZFYVE19",18.5178439417224,-0.155986252013283,0.347115738979289
,-0.449378217397945,0.653158842061539,0.957570068113219
"9140","TLE3",31.5787198771968,0.138949625185222,0.307655306925937,0.4
51640592758153,0.651527921819782,0.957570068113219
"9141","ANTKMT",52.6980335475073,-0.138122721384786,0.304856363485375,
-0.453074752337955,0.650494914923379,0.957570068113219
"9142","PKD1",56.8494004392091,-0.117109903566342,0.259570841647822,-0
.451167407028072,0.651868898411796,0.957570068113219
"9143","FCSK",4.83268757558861,0.30987122720733,0.680387727441371,0.45
5433298852427,0.648797543811917,0.957570068113219
"9144","NPIPB15",10.2417817611584,0.234977778218879,0.519471547521526,
0.452340035445622,0.651024038432785,0.957570068113219
"9145","NUDT7",14.5788881813703,-0.192655678334105,0.423014644351173,-
0.455435008945385,0.648796313772465,0.957570068113219
"9146","GRN",298.23045227263,0.118908990290767,0.261223835819956,0.455
199618049873,0.648965635265754,0.957570068113219
"9147","ANKRD40",28.4281609588191,0.135025596743626,0.29786893897657,0
.453305394001646,0.650328849256947,0.957570068113219

"9148","JMJD6",11.4462932412017,0.192829255037155,0.424569728562583,0.454175703223107,0.649702369191424,0.957570068113219
"9149","AC139530.1",9.69278204770655,-0.250950766609548,0.553773896656791,-0.453164672666177,0.650430168792343,0.957570068113219
"9150","GAPLINC",2.1637673635585,0.561855306839735,1.24050413246322,0.452924977947539,0.650602764161188,0.957570068113219
"9151","ESCO1",34.5680958583186,-0.122392604448119,0.272221153836845,-0.449607250292808,0.652993659313372,0.957570068113219
"9152","AC011815.2",1.79961529592238,0.585661993506291,1.28210135647032,0.456798513277177,0.647815874493212,0.957570068113219
"9153","FZR1",16.6655740986287,0.167650481468451,0.370164468988369,0.452908086847522,0.650614927529186,0.957570068113219
"9154","PLPPR2",25.7101231377831,-0.142987476847782,0.31714880968628,-0.450852951298236,0.652095534785651,0.957570068113219
"9155","SYDE1",28.460561956082,-0.137005631488118,0.30371380370227,-0.451101101820265,0.65191668361488,0.957570068113219
"9156","MED26",6.94328505076642,0.264656527080477,0.581748280805857,0.454933062653603,0.649157395928431,0.957570068113219
"9157","ARRDC2",13.2069738163722,0.214135477525476,0.470801040828467,0.454832209267554,0.649229956187754,0.957570068113219
"9158","ARMC6",9.58978624310954,-0.217367877014266,0.482138670453601,-0.450840993131216,0.652104153980098,0.957570068113219
"9159","RPS11",2320.47694550904,-0.0949218334195901,0.210495549617948,-0.450944609479272,0.652029471046137,0.957570068113219
"9160","ZNF614",4.27035109628832,0.321970245089819,0.705902171869847,0.456111707712925,0.648309651606918,0.957570068113219
"9161","ERGIC3",118.624733643285,-0.0882290059847196,0.195708842105195,-0.45081767913836,0.65212095834779,0.957570068113219
"9162","TTC28-AS1",14.0934431119206,-0.199246052137854,0.440667169222749,-0.452146350020322,0.651163554813288,0.957570068113219
"9163","SFI1",8.39576597027241,0.262953410264365,0.576264670255288,0.456306665733777,0.648169471301086,0.957570068113219
"9164","YWHAH",234.044346873453,-0.0939877728944865,0.20688091431607,-0.454308572664626,0.6496067467352,0.957570068113219
"9165","CASK",25.5828988939644,0.150646488548285,0.33522151964369,0.449393847711234,0.65314756865067,0.957570068113219
"9166","ZNF182",7.36948518683955,0.263465520094104,0.580866372618239,0.453573373350123,0.650135921668856,0.957570068113219
"9167","RLIM",27.3303318901165,0.142653837665321,0.317452394660486,0.449370803511779,0.653164189377111,0.957570068113219
"9168","ADCK1",3.19729834649395,-0.396338613135763,0.882894850158137,-0.448908058603778,0.653497982521715,0.957638848544281
"9169","AC138969.1",2.36081998320857,-0.42358515233872,0.943511540982163,-0.448945385339731,0.653471054945546,0.957638848544281
"9170","VPS35",106.391339047189,0.08817199309439,0.196420980520105,0.448892948507427,0.653508883101392,0.957638848544281
"9171","TRO",3.99272866097561,0.329591456947988,0.734205107241709,0.44890924034322,0.653497130005785,0.957638848544281
"9172","ARIH2OS",5.9392115516382,0.331579887344994,0.740342472512601,0

.447873652607915,0.654244384317863,0.957649307383978
"9173","GPX8",169.235902414603,-0.100723134007496,0.224709642669704,-0.448236812674508,0.653982297559146,0.957649307383978
"9174","SMN1",5.20557497085668,0.301376152003619,0.673116562916618,0.447732485882913,0.654346273573148,0.957649307383978
"9175","ASH2L",28.5638271943551,0.132225762042834,0.294924500588,0.448337665332015,0.653909521391229,0.957649307383978
"9176","H2AFY2",6.5967943809206,-0.29454578608802,0.658026330050482,-0.447620061138622,0.654427422468558,0.957649307383978
"9177","BNIP3",69.713503995853,0.119619988989499,0.267087322559225,0.447868464303371,0.654248128942462,0.957649307383978
"9178","SLC25A15",2.44571639827424,-0.41348488075632,0.921840371679576,-0.448542820925664,0.653761489459594,0.957649307383978
"9179","TSC22D1-AS1",2.01871046275321,0.487094496216787,1.08823776961129,0.447599329685804,0.654442387009037,0.957649307383978
"9180","ARHGAP5",67.3779846334418,0.134651612395622,0.300676926587541,0.447828218559425,0.654277176338343,0.957649307383978
"9181","TTBK2",25.0476274941616,0.168049602840084,0.374986847286122,0.448147992539746,0.654046393677072,0.957649307383978
"9182","REXO5",4.49025591332282,-0.306907212093989,0.685144279408006,-0.44794537635076,0.654192619070961,0.957649307383978
"9183","MRM3",10.3750360668121,-0.211138686207286,0.470966141497746,-0.448309692785626,0.653929706297884,0.957649307383978
"9184","ZNF44",19.6978207934786,0.183705524481121,0.409793735311625,0.448287781513846,0.653945517583523,0.957649307383978
"9185","ZNF721",46.7245799371889,-0.137657284938164,0.307636801324446,-0.447466897151182,0.654537983780298,0.957684917150073
"9186","GNRH1",2.71156329708236,-0.402835463336052,0.901038194398793,-0.447079231313651,0.654817853937242,0.957885832052094
"9187","SERPINF1",1567.12745850827,-0.127972372148187,0.286225510729999,-0.447103306137187,0.654800472027873,0.957885832052094
"9188","PIGBOS1",40.1024396402735,0.127418273122604,0.285292150960841,0.446623829970329,0.655146686797652,0.958262551575278
"9189","FKBPL",5.11788347848188,-0.281569484010779,0.63254882448051,-0.445134783456475,0.656222353208728,0.959105188120946
"9190","MICALL2",7.50730739205363,-0.240355270978414,0.539265292233548,-0.445708771619443,0.655807627594718,0.959105188120946
"9191","MRPL43",113.441115306235,-0.116729457771998,0.261963779585289,-0.445593883081054,0.655890629911554,0.959105188120946
"9192","PAMR1",137.651269493338,-0.123446572249958,0.277193500435687,-0.445344396805579,0.656070888274871,0.959105188120946
"9193","C14orf28",19.6968716098379,-0.174364572273383,0.391626758901313,-0.445231507577655,0.656152459373911,0.959105188120946
"9194","CCDC159",6.11081760323736,-0.25430149263418,0.571202852773179,-0.445203470885257,0.656172718663552,0.959105188120946
"9195","ZNF585B",7.43043234909736,0.246507648858288,0.553499373479395,0.4453621100033,0.656058089507298,0.959105188120946
"9196","WDPCP",10.1093349856394,0.228912844374803,0.514622520490174,0.444816997430983,0.656452009809459,0.959127914745523

"9197","SUV39H2",2.10530279415678,-0.416162800960181,0.935220755344056
,-0.444988842027014,0.656327817667366,0.959127914745523
"9198","PISD",9.62897483626924,0.238755349618214,0.536665059877712,0.4
44887076629544,0.65640136240472,0.959127914745523
"9199","TBC1D19",10.0509111640183,0.225303220988484,0.50679742931949,0
.444562675250767,0.656635825947677,0.959292190989328
"9200","HECTD3",19.2110490984088,0.172263917785219,0.388111961640561,0
.443851091466117,0.657150246909003,0.959400441653714
"9201","ASH1L-
AS1",3.81360028959787,-0.350772238553099,0.789668271825473,-0.44420201
6300111,0.656896534619802,0.959400441653714
"9202","PROX1",1.94497448142658,0.524943045161533,1.18425467130007,0.4
4326871397117,0.657571382477203,0.959400441653714
"9203","MOGS",19.0996482790647,-0.165782792886018,0.374599991764359,-0
.442559520904376,0.65808436920385,0.959400441653714
"9204","ELMOD3",25.6638506408287,0.14146861387854,0.319704482741848,0.
442498061538822,0.658128832721082,0.959400441653714
"9205","TXNDC9",37.0737347568154,-0.13563682052012,0.306616441642704,-
0.44236642951514,0.658224067560124,0.959400441653714
"9206","STAM2",45.7322288199238,0.110913513520045,0.250096683059086,0.
443482545083741,0.657416741805082,0.959400441653714
"9207","AP001816.1",12.4296902877779,-0.213642728165436,0.481203438562
486,-0.443975896771764,0.657060010407343,0.959400441653714
"9208","PDE5A",14.8139078240469,0.179824965950561,0.405044512764774,0.
443963466442496,0.657068997536888,0.959400441653714
"9209","HIST1H1C",27.5538746628254,0.138196973515959,0.312505848995622
,0.44222203827582,0.658328539979492,0.959400441653714
"9210","RTN4IP1",1.62821189556705,0.534568479002329,1.20573910028269,0
.443353358016671,0.657510166937541,0.959400441653714
"9211","ACAT2",15.3446843425938,-0.186544493846705,0.420227019637928,-
0.443913611284286,0.657105043320584,0.959400441653714
"9212","CLDN12",10.9560963327191,0.204551703233487,0.462155341684669,0
.442603784450151,0.658052346958404,0.959400441653714
"9213","ZDHHC2",19.2601797569446,0.186265582051771,0.420514637424212,0
.44294672640340
7,0.657804268544729,0.959400441653714
"9214","SPACA9",9.39559507410492,0.258120613838407,0.582748360009119,0
.442936662806512,0.65781154784412,0.959400441653714
"9215","ALKBH3",22.1279716725032,-0.145428542358829,0.328687594809103,
-0.442452178468407,0.658162028168619,0.959400441653714
"9216","B3GAT3",47.3394695865909,-0.135921080966242,0.307049900290384,
-0.442667725466442,0.658006090250954,0.959400441653714
"9217","RPS3",2123.5872808565,-0.117941797781411,0.266621790513188,-0.
442356183845284,0.65823148046251,0.959400441653714
"9218","VWA5A",22.4821640562457,0.16076322478851,0.363561597298686,0.4
42189785673193,0.658351876845788,0.959400441653714
"9219","PDIA3",344.335723943077,0.0832397338910091,0.188208251473019,0
.442274625259679,0.658290490572936,0.959400441653714
"9220","MYO1E",38.5326007805252,-0.159965359250805,0.361651533772934,-
0.442319040049309,0.658258354861519,0.959400441653714
"9221","CCDC113",3.64275032272148,-0.349606730464448,0.788506800865939

,-0.443378205591264,0.657492197324176,0.959400441653714
"9222","NAPA",53.9665609090005,-0.129676252193994,0.2929591563807,-0.442642769033238,0.658024144272246,0.959400441653714
"9223","CAB39",39.5228323250063,-0.11232644934616,0.254215254287234,-0.441855661498754,0.658593657107248,0.959440667519167
"9224","CAHM",4.45160396940462,0.315190946652999,0.713116907942531,0.441990567244269,0.658496031778026,0.959440667519167
"9225","TSPAN15",8.19887557294482,-0.573666895921666,1.29803279400953,-0.441951003525611,0.658524661690517,0.959440667519167
"9226","AGO4",16.5840665440159,0.195454287593711,0.442664156967189,0.441540803603392,0.658821528497262,0.959598719847433
"9227","ZNF789",4.91225834625459,-0.288454736129589,0.653339131447543,-0.441508432979496,0.658844957811761,0.959598719847433
"9228","S1PR3",34.6505090605552,-0.194344209852055,0.440310817278806,-0.44137959419924,0.658938212477828,0.959630541557167
"9229","SDE2",16.0105371664406,0.176140280468013,0.399607450212594,0.440783274621894,0.659369902999497,0.959936343975834
"9230","PREB",23.3797655124079,0.171714884036059,0.389580250713964,0.440768965370719,0.659380263215815,0.959936343975834
"9231","PPP1R21",20.7857893600054,0.164499579405897,0.37360769783629,0.440300294556507,0.659719627424719,0.959936343975834
"9232","ANKRD36",22.9349365827672,0.184418707655776,0.418619096163669,0.440540599666464,0.659545613909874,0.959936343975834
"9233","TLE4",18.9449648070728,0.213955792559344,0.485890061857466,0.440337865198213,0.659692419966336,0.959936343975834
"9234","EIF3C",7.91258763801379,0.256667825241007,0.582610908201172,0.44054757922998,0.659540560033835,0.959936343975834
"9235","ZNF521",69.5820790196532,0.0998078796777925,0.22659341473165,0.440471228151061,0.659595846411476,0.959936343975834
"9236","TCEANC",4.81038738080882,-0.341016900952659,0.774215257846814,-0.440467812402804,0.659598319823358,0.959936343975834
"9237","MNDA",3.65324615468703,0.412314069520646,0.937675954467638,0.439719145570643,0.660140534259094,0.959983550604044
"9238","ROCK2",150.388035919385,0.0972544174858456,0.221262974259129,0.439542213565056,0.66026870157792,0.959983550604044
"9239","ADAP1",2.8883010776839,-0.500965619049511,1.13890680414055,-0.439865331586594,0.660034646470769,0.959983550604044
"9240","KDM7A",27.413882997116,0.185572146359104,0.421954395845436,0.439791949524043,0.660087798886608,0.959983550604044
"9241","ARHGEF12",66.4477011320121,0.133415609525349,0.303585373610216,0.439466526133257,0.660323531645493,0.959983550604044
"9242","SMAD6",12.0971573095677,0.203553764232544,0.463164264081301,0.439485038070237,0.660310120918186,0.959983550604044
"9243","SETD6",6.75684218657945,0.290880989173054,0.661580558382997,0.439675842174097,0.660171901773312,0.959983550604044
"9244","RAB3D",9.61586338534525,0.220977164407363,0.502244091625517,0.439979619654994,0.659951868255054,0.959983550604044
"9245","ZNF362",20.022070365263,0.150383274259352,0.342328964741715,0.439294625194264,0.660448068211549,0.960060745126555
"9246","NOTCH2NLA",14.0170187701921,0.208701697427005,0.47559053507902

6,0.438826431632678,0.660787306701333,0.960449990780793
"9247","DPYSL2",471.79995959448,-0.116561257842371,0.265743452252724,-0.43862325432395,0.660934544350423,0.960456243677047
"9248","ENDOD1",21.2390128836959,-0.148979292083884,0.339576978383379,-0.438720235962779,0.660864262480677,0.960456243677047
"9249","GORAB",14.0604463366292,0.183396261544638,0.41851412138872,0.4382080607844,0.661235465800965,0.960789644815566
"9250","TCEAL1",36.1060261160706,-0.1259542039453,0.287499416038489,-0.438102468800972,0.661312004852092,0.960796976562947
"9251","ZNF799",4.53373131646369,0.315068929079296,0.71967267616231,0.437794763529743,0.661535067254159,0.961017162342303
"9252","ACAA2",153.217294225302,-0.100975631505445,0.230813580436098,-0.437476994701361,0.661765456494139,0.961247943128484
"9253","JUN",2396.27226851539,0.140343271521899,0.323156079226668,0.43428943641645,0.664078273839491,0.961375268633655
"9254","WDR77",10.5149944987024,-0.203825976518907,0.467738035366415,-0.435769514359111,0.663003964477158,0.961375268633655
"9255","HIST2H2BF",1.65533686714243,0.484706756403744,1.11446843115633,0.434921925873513,0.663619098960912,0.961375268633655
"9256","MRPL9",44.0104482180235,-0.112196601376554,0.257296832020663,-0.436059008171323,0.662793917344729,0.961375268633655
"9257","CHTOP",47.7390550974238,-0.106703918241947,0.24476652438245,-0.435941632587063,0.66287907799045,0.961375268633655
"9258","NSL1",50.5525815825608,-0.107681351161096,0.248047626844186,-0.434115627434473,0.664204477836096,0.961375268633655
"9259","NTPCR",63.5349376157395,-0.132570663231129,0.30508149934997,-0.434541797892019,0.663895049249595,0.961375268633655
"9260","DOK1",14.0321628840819,0.181066641166947,0.417596005981152,0.433592847090397,0.664584129922845,0.961375268633655
"9261","NCK2",27.2861027942714,-0.144904960654139,0.333801033337871,-0.43410578812519,0.664211622516696,0.961375268633655
"9262","EPB41L5",9.47507264975763,0.209697591038742,0.48204825821315,0.435013688911658,0.663552491291164,0.961375268633655
"9263","ZDHHC3",62.1271981937182,0.107427238170343,0.246209456278915,0.436324582304612,0.662601248857933,0.961375268633655
"9264","CYB561D2",30.267385322132,-0.145952139841373,0.336000205998404,-0.434381102260592,0.664011718423758,0.961375268633655
"9265","ABHD14B",63.315183580026,-0.117460192540574,0.268884765696611,-0.43684212542226,0.662225846289239,0.961375268633655
"9266","ZFYVE28",8.48275616857613,-0.230585876354973,0.529691256725693,-0.435321280891719,0.663329240125994,0.961375268633655
"9267","SRD5A3",20.7580861058979,0.157496223093991,0.3629650948796,0.433915616999574,0.664349718687056,0.961375268633655
"9268","POLR2B",49.3379563509322,0.109050891291337,0.249891751307499,0.436392520844541,0.662551964451754,0.961375268633655
"9269","PPM1K",21.9862442712439,0.196618186478209,0.450003038934957,0.43692635263876,0.662164759670681,0.961375268633655
"9270","E2F3",9.10721776301903,0.268974681694912,0.6207848453114,0.433281649393339,0.664810167919533,0.961375268633655
"9271","SKIV2L",11.3169326053278,-0.186073823272276,0.428976356510809,

-0.433762421746867,0.664460972452646,0.961375268633655
"9272","CYB5R4",14.7479563384615,0.175221485847207,0.40274800247397,0.435064816636879,0.663515380566976,0.961375268633655
"9273","MPC1",97.3847078059284,-0.112826666267558,0.258644520573111,-0.436222913277088,0.662675004977188,0.961375268633655
"9274","RPL12",2945.32845344068,-0.109610999452499,0.2530167910723,-0.433216305479021,0.664857634249661,0.961375268633655
"9275","CDC123",57.4984915660846,-0.126898380604559,0.290916740662926,-0.436201712955363,0.662690385230183,0.961375268633655
"9276","ZMIZ1",76.6143367676074,-0.141848192451869,0.325080535245984,-0.43634784944763,0.662584370095091,0.961375268633655
"9277","FADS1",7.64909365433713,0.287731562983823,0.661977312443142,0.434654719391969,0.663813070200949,0.961375268633655
"9278","SART1",28.4633383880409,-0.143750100057089,0.331749376455163,-0.433309330052402,0.664790060877629,0.961375268633655
"9279","PROSER1",9.09263657801441,0.22360565530527,0.513308518726772,0.435616490176141,0.66311500446841,0.961375268633655
"9280","WDFY2",45.348564672958,0.156637612944982,0.361107240422794,0.433770346896357,0.664455216853232,0.961375268633655
"9281","IGHM",7.63433909976936,0.303276739694539,0.697537958553045,0.434781700373193,0.663720889019228,0.961375268633655
"9282","NIPA1",5.03965968545775,0.296140642776607,0.677665013425014,0.437001522743325,0.662110243710183,0.961375268633655
"9283","CPEB1",12.0127443836128,0.204867673597204,0.471464869457827,0.434534335151624,0.66390046721129,0.961375268633655
"9284","AC027020.2",1.78478475625375,0.538124081247213,1.2320972085829
7,0.436754565710044,0.662289352215958,0.961375268633655
"9285","CHD9",184.84898330811,0.0861675950679058,0.197621991518278,0.436022298965326,0.662820550862515,0.961375268633655
"9286","MPP3",2.78746431390645,-0.380056505781378,0.877174083670409,-0.43327375130725,0.664815905079608,0.961375268633655
"9287","SMARCD2",68.2733710778153,-0.104156613465569,0.239251315728693,-0.435343952648021,0.663312786081091,0.961375268633655
"9288","AP001269.4",1.45603018643943,0.589592546714191,1.3580536777497
1,0.434145245047415,0.664182971593513,0.961375268633655
"9289","U2AF1L4",13.4373444293461,0.19009944926536,0.437896275268272,0.434119813302587,0.664201438334155,0.961375268633655
"9290","ZNF575",4.62575223126484,0.290127811822314,0.668390404287287,0.434069385139784,0.664238056314701,0.961375268633655
"9291","ZNF613",2.46249616171923,-0.386858815753813,0.892987625758268,-0.433218562715599,0.664855994552958,0.961375268633655
"9292","AC011468.1",3.01492499578573,-0.343266785741628,0.78607656846259,-0.436683650821688,0.662340787647255,0.961375268633655
"9293","AL031595.3",2.22691413906127,0.45572190147871,1.04717182688695,0.435193050249917,0.663422306671305,0.961375268633655
"9294","PRKX",5.91351807016599,0.281969893296586,0.646544523912939,0.436118291730448,0.662750906425873,0.961375268633655
"9295","CTSS",54.3509969952147,-0.118674253246,0.274048338387203,-0.43304131652251,0.664984754212403,0.9613955355992
"9296","AC073475.1",1.90518399812279,0.518530601903489,1.1975300905265

8,0.433000060712864,0.665014725718444,0.9613955355992
"9297","PCOLCE",945.725523475192,0.163412906259244,0.377752843313027,0
.432592127768131,0.665311109514241,0.961619167700628
"9298","ZBTB42",1.93077026653058,-0.489504125129041,1.13156550432989,-
0.432590179937418,0.665312524836702,0.961619167700628
"9299","ZBTB48",8.6258281509774,-0.223462209897633,0.517693259943349,-
0.431649834348019,0.665995932957878,0.961821777738884
"9300","PHC2",44.9994125662537,-0.131409187356268,0.305368462583425,-0
.430329924198925,0.666955662381774,0.961821777738884
"9301","SIX2",7.05252652048562,0.260684937430438,0.604301243025891,0.4
31382427951201,0.666190324648203,0.961821777738884
"9302","PTPRG-
AS1",3.04219034437878,0.398442905345303,0.923597057725788,0.4314033939
50177,0.666175082557064,0.961821777738884
"9303","PI4K2B",10.6985494305021,0.192167923098176,0.446478153510264,0
.430408344926462,0.66689862606933,0.961821777738884
"9304","TMA16",51.044874606709,0.119893606173303,0.278392516486824,0.4
3066389745781,0.666712773083956,0.961821777738884
"9305","DROSHA",13.4793779500013,0.172893909270255,0.401162015385315,0
.43098275170492,0.666480912011155,0.961821777738884
"9306","OSMR-
AS1",6.39140255423913,0.282120419073469,0.654386372791805,0.4311220875
0598,0.666379601273204,0.961821777738884
"9307","LINC00997",2.8433093480408,0.395222626130498,0.914723587923588
,0.432067819555904,0.665692122606589,0.961821777738884
"9308","AC007938.3",4.20430673233183,0.363561997548138,0.8438930480940
02,0.430815253626358,0.666602707528487,0.961821777738884
"9309","NSMAF",18.7654319209145,-0.145447160318776,0.337582762867302,-
0.430848894900326,0.666578244713371,0.961821777738884
"9310","ADK",90.1665960360456,0.0865561975303867,0.200296003504931,0.4
32141410790833,0.665638638882984,0.961821777738884
"9311","LIN7C",37.0171339026015,-0.122509006280888,0.28456314414546,-0
.430516069285013,0.666820280021889,0.961821777738884
"9312","NCAPD2",10.5480833922782,0.202449586309984,0.469196413803465,0
.431481529598361,0.666118279863656,0.961821777738884
"9313","CPNE8",25.4010120518719,0.144345216787688,0.33532587363011,0.4
30462508678681,0.666859233262755,0.961821777738884
"9314","FEM1B",37.4463749506434,0.150043253903637,0.347922384160495,0.
431254960113238,0.666282995577206,0.961821777738884
"9315","AC012645.1",1.56151022268712,-0.519584878219928,1.207344940054
77,-0.430353299195804,0.666938661271748,0.961821777738884
"9316","ZNF286A",11.8272420803716,0.185688974793754,0.430414044593019,
0.431419413763168,0.666163436391197,0.961821777738884
"9317","RNF138",9.82709740888853,0.198506393684146,0.461128894683719,0
.430479191333908,0.666847100303662,0.961821777738884
"9318","NDUFA7",50.1775573633866,-0.134110650502347,0.31130617130466,-
0.430799845503542,0.666613911921122,0.961821777738884
"9319","ZNF585A",29.2406888945893,-0.151973451321142,0.352726025873263
,-0.430854091202637,0.666574466166835,0.961821777738884
"9320","AP002495.1",7.05933813843002,0.281594134880723,0.6552491250863

75,0.429751256582911,0.667376593593779,0.96219871226622
"9321","TRMT5",17.057007282377,0.166650143144601,0.38785131938837,0.42
9675328699159,0.667431832409086,0.96219871226622
"9322","RNF227",2.03833905751202,0.432381505369587,1.00627316513781,0.
429686014046067,0.667424058530123,0.96219871226622
"9323","ADAM12",12.0914559302068,0.541747338998797,1.26145666868016,0.
429461710774115,0.667587252717091,0.962216333040003
"9324","FP565260.1",3.71490608162045,-0.321514366568888,0.748554646517
178,-0.429513553973391,0.667549532259134,0.962216333040003
"9325","SMPD1",27.3940695559909,0.172118262159321,0.400982364011252,0.
429241476950571,0.667747501430379,0.962225760289911
"9326","DDX5",770.683384833819,0.0943560186987072,0.219764629799108,0.
429350340793969,0.667668286949894,0.962225760289911
"9327","ZNF283",5.44445508865995,0.314233618887176,0.732316560788562,0
.429095333510972,0.667853848127984,0.962225760289911
"9328","CHKB",44.1274880747204,-0.119613260396391,0.278780361574984,-0
.429059133579673,0.667880191382119,0.962225760289911
"9329","PATJ",4.94346636029372,-0.332392797982969,0.77674705608887,-0.
427929266519085,0.668702618792164,0.962360197312875
"9330","VANGL1",22.4219149418524,0.158158968418713,0.369605237693922,0
.427913222781999,0.668714299854331,0.962360197312875
"9331","IGKC",189.299140061261,0.328948921332802,0.769142105044137,0.4
27682893935348,0.668882005630552,0.962360197312875
"9332","NPRL2",17.1077675952822,0.184960568155944,0.432563929402905,0.
427591289017688,0.668948709093014,0.962360197312875
"9333","PDE8B",1.92446738642403,0.437156491258444,1.02269492357402,0.4
27455423099892,0.669047646662266,0.962360197312875
"9334","ATG12",106.209863406412,0.0848016478750871,0.197838360047721,0
.428641077769913,0.668184446749976,0.962360197312875
"9335","PCDHB4",15.9164134254354,0.191513201854798,0.447597520884696,0
.427869219374281,0.668746338097165,0.962360197312875
"9336","PCDHGA1",1.59224566741393,0.507703021351814,1.18410501541098,0
.428765197971566,0.668094108051709,0.962360197312875
"9337","SNX8",11.1706892688813,-0.207386024950823,0.483907943112109,-0
.428565035773295,0.668239794949614,0.962360197312875
"9338","GPSM1",12.4108808285907,0.221286291982676,0.51738343759219,0.4
27702697659792,0.668867585602073,0.962360197312875
"9339","AGAP4",5.44360036486188,0.268386249055992,0.627042600765384,0.
428019162858142,0.66863716889631,0.962360197312875
"9340","RIC8B",11.4938402062318,-0.199475815137714,0.466617867848342,-
0.427492877753552,0.669020371607539,0.962360197312875
"9341","STRN3",45.5462873622815,0.108314428682201,0.253035747695424,0.
428059788661078,0.66860759171519,0.962360197312875
"9342","IFT20",73.1125309260359,-0.105503619122942,0.246709737771192,-
0.427642703024516,0.668911270909194,0.962360197312875
"9343","APLN",3.00251573508623,0.378837024555386,0.884260938594098,0.4
28422208898773,0.668343758304855,0.962360197312875
"9344","ZBTB47",24.3872266976597,-0.136872490744514,0.320611408337596,
-0.426910855899395,0.669444258185836,0.962577250473478
"9345","CEP85L",28.5981404565538,-0.145395768324827,0.340621094178363,

-0.426854856642237,0.669485048007709,0.962577250473478
"9346","WNT11",195.552198822445,-0.250568154615818,0.586738354232075,-0.427052625430909,0.669340997712351,0.962577250473478
"9347","SNX5",79.507772613755,-0.0907915679118202,0.21267677669454,-0.426899301873617,0.669452674050732,0.962577250473478
"9348","WDR12",11.1486410848056,0.198090580268807,0.464489321899057,0.426469610666004,0.669765687343121,0.962774742989005
"9349","ZNF484",5.86897481896318,0.25490211011681,0.597666174134337,0.426495795058189,0.66974661139245,0.962774742989005
"9350","ETFDH",24.2367884899635,-0.132537151012737,0.311307739882525,-0.425743192452431,0.670294985129856,0.963432546006431
"9351","LDAH",9.57583232392186,0.20877603985842,0.490851970062914,0.425334016346436,0.670593199541546,0.963462047187522
"9352","ZNF777",6.32934391851283,0.265932184583289,0.625308645320362,0.425281477512669,0.670631494486212,0.963462047187522
"9353","LMX1B",3.42588814713926,0.361060975930478,0.849109289848734,0.425223207715463,0.670673967664206,0.963462047187522
"9354","SH2B3",8.42708179521726,-0.232756039295819,0.547088532276885,-0.425444924475257,0.670512362706858,0.963462047187522
"9355","PIK3R5",1.75247193751836,0.468902759529709,1.10218482330633,0.425430245104532,0.670523061738474,0.963462047187522
"9356","CCDC122",9.94133939852686,0.217656095352237,0.512162445057224,0.424974727164774,0.670855098325207,0.963619246087266
"9357","DPH2",4.36808939126469,-0.314323635034611,0.74080776325943,-0.424298516597126,0.671348120927566,0.963969631082197
"9358","UGDH-AS1",1.75290291582796,-0.488819579368895,1.15199572867061,-0.424324124823787,0.671329447486459,0.963969631082197
"9359","COL25A1",1.73193596689985,0.537096254517585,1.26651095337426,0.424075491085682,0.671510759030419,0.963969631082197
"9360","AP000787.1",3.1658592573772,0.384466432761935,0.90568395626264,0.424503967530196,0.671198312447955,0.963969631082197
"9361","GNA11",82.471254679518,0.128109724424201,0.302019052735653,0.424177624768364,0.671436277621604,0.963969631082197
"9362","HDX",7.22409285225642,0.253225203910921,0.597297482444015,0.423951567441365,0.671601135190313,0.963969631082197
"9363","AIFM1",18.2195365700604,-0.164362620304182,0.387668242958385,-0.42397752018554,0.671582207744413,0.963969631082197
"9364","ZNF330",31.7380666990749,0.155832028627599,0.367860428690065,0.423617264793905,0.671844962137462,0.964065680301307
"9365","AS3MT",3.73393025352297,-0.315203086253665,0.744030496500866,-0.423642697088422,0.671826411627663,0.964065680301307
"9366","AC245140.2",1.70452395424925,0.476997874312865,1.12615099888315,0.423564757111544,0.671883262274131,0.964065680301307
"9367","CEP85",2.48537173880806,-0.363148111155612,0.857915187240817,-0.423291388888394,0.672082676194708,0.96414593140272
"9368","AP001267.3",1.70518647876654,0.462445120486524,1.0924354002231,0.423315758892548,0.672064898070443,0.96414593140272
"9369","FBXL14",5.1341636581751,-0.262294496326467,0.619969749719138,-0.423076281456141,0.672239606839499,0.964165216255712

"9370","ANKRD27",14.8904400028709,-0.168487719923009,0.398218045431595
,-0.423104180877588,0.672219252137789,0.964165216255712
"9371","AAK1",77.3641123934224,0.130558755758856,0.309071144426077,0.4
22422986142216,0.672716302984437,0.964667571741586
"9372","COA5",62.8762262621958,-0.114723684427107,0.271599936451926,-0
.422399526030132,0.672733423793596,0.964667571741586
"9373","AP5M1",22.5806495802682,-0.144442835043311,0.342084761362725,-
0.422242822123705,0.672847788113103,0.964728627382053
"9374","ZNF385D",23.7398725617219,-0.188477440764369,0.446605340918652
,-0.422022361794144,0.673008695417566,0.964856396172037
"9375","SLC35E2A",23.1358424015088,-0.231006262054308,0.56074013129837
8,-0.411966701080266,0.68036383019298,0.964879202878059
"9376","PER3",57.4631550234671,-0.379079255163423,0.908486882825091,-0
.417264423218322,0.676485007842345,0.964879202878059
"9377","AL020997.5",2.91428055794883,0.36455736275713,0.88956705222155
1,0.409814371886534,0.681942122701879,0.964879202878059
"9378","ZCCHC17",67.503717289647,-0.0945743190449147,0.232837546241499
,-0.406181565523038,0.684609197979146,0.964879202878059
"9379","C1orf122",134.450340544281,-0.129384303080651,0.31300768879915
8,-0.413358226365075,0.679344175637689,0.964879202878059
"9380","TMEM53",4.54758173018592,0.280133878389091,0.685839857670475,0
.408453774239068,0.682940561210425,0.964879202878059
"9381","GPBP1L1",40.2717759019647,0.117592462769501,0.286649678192493,
0.410230576608339,0.681636812997505,0.964879202878059
"9382","SARS",109.538265043178,-0.0825186442021366,0.200461004113709,-
0.411644372265684,0.680600103432896,0.964879202878059
"9383","BGLAP",7.87188660446508,-0.230971523030068,0.549330455508624,-
0.420460072282379,0.674149391715167,0.964879202878059
"9384","AL592148.3",4.59368026657033,-0.315091828928031,0.776903186690
343,-0.405574123425007,0.685055545159723,0.964879202878059
"9385","PARP1",64.2086854122983,0.1146092060901,0.273614969029231,0.41
8870380143038,0.675310865245764,0.964879202878059
"9386","ARV1",19.7737423905814,-0.146401904740171,0.354135868839838,-0
.413406033169667,0.679309155115444,0.964879202878059
"9387","FAM89A",3.84080405453751,0.322830354286809,0.777198009529367,0
.415377227332709,0.677865774755332,0.964879202878059
"9388","DISC1",10.8613224578227,-0.253831397603118,0.604985902982018,-
0.419565805338547,0.674802672814932,0.964879202878059
"9389","COX20",68.8366813711673,-0.112779029910973,0.274766244331692,-
0.410454458062281,0.681472604845408,0.964879202878059
"9390","AC104794.3",15.0792467762109,0.173274338234888,0.4269317667438
72,0.405859558206265,0.684845794574983,0.964879202878059
"9391","AC010896.1",1.69476511099129,-0.44397231451431,1.0714400599345
8,-0.414369717090303,0.67860336324964,0.964879202878059
"9392","FEZ2",88.3724914220034,0.0805666257228282,0.198154984589063,0.
40658389638751,0.684313626714003,0.964879202878059
"9393","CNRIP1",70.8170373030616,0.101156720661625,0.249462896376865,0
.405498060556498,0.685111443746678,0.964879202878059
"9394","ATOH8",29.9162083142864,0.170781902646568,0.411138764468788,0.
415387497861523,0.677858257388604,0.964879202878059

"9395","CCDC138",3.65104895433395,0.302931835865244,0.741800653556989,
0.408373643798592,0.682999380130967,0.964879202878059
"9396","PPIG",182.362714351271,0.0780207379424723,0.186276117309814,0.
418844557580661,0.675329738324068,0.964879202878059
"9397","METAP1D",11.0858722115906,0.222428435464436,0.542154665497019,
0.410267493060352,0.681609735207964,0.964879202878059
"9398","CIR1",126.474764588122,0.0902197107056699,0.219242471799214,0.
411506538697915,0.680701147668333,0.964879202878059
"9399","WIPF1",70.5327805704732,0.0992810588773338,0.238094870570824,0
.416981090937873,0.676692238672209,0.964879202878059
"9400","FAM126B",33.3134453744939,0.163024825057555,0.391439112053927,
0.416475564238189,0.677062044561517,0.964879202878059
"9401","STRADB",13.8891976369175,0.186624265370177,0.455549994488838,0
.409668022451815,0.6820494906825,0.964879202878059
"9402","THUMPD3",31.995524195489,-0.126540563641892,0.304442299034206,
-0.4156471161968,0.677668244113231,0.964879202878059
"9403","PRRT3-
AS1",1.56859976818866,-0.506550910263574,1.23743032134201,-0.409357118
156127,0.68227760421232,0.964879202878059
"9404","RAB5A",51.0274261603561,0.0967360479773092,0.231785415239944,0
.4173517469905,0.676421143680383,0.964879202878059
"9405","TTC21A",3.8802395746348,-0.387807720024358,0.930571802958352,-
0.416741318393154,0.676867628793449,0.964879202878059
"9406","ZNF662",13.2774477688881,0.168365907418246,0.403330194694376,0
.417439382503524,0.676357053866985,0.964879202878059
"9407","FYCO1",23.9163863624722,0.153416065471454,0.370934002385415,0.
413593966810431,0.679171492421408,0.964879202878059
"9408","CCDC12",61.1851701275048,-0.0983801566171582,0.23937809824039,
-0.410982280084631,0.681085528094411,0.964879202878059
"9409","IP6K2",85.9577809910427,0.0979726828025109,0.234322080648803,0
.418111184960628,0.675865827589137,0.964879202878059
"9410","CFAP44",9.04735371971812,0.234900952529898,0.56880671119584,0.
412971485579081,0.679627505187767,0.964879202878059
"9411","EIF4G1",80.4768203729724,-0.0953099434894809,0.233617021631014
,-0.407975167323287,0.683291906242399,0.964879202878059
"9412","RTP4",9.16365550128133,-0.214055884518405,0.509365993564739,-0
.420239841730226,0.674310252059889,0.964879202878059
"9413","BOD1L1",182.748997907271,0.091716778304064,0.224001959645523,0
.409446321135776,0.682212152136614,0.964879202878059
"9414","TMEM33",28.6608984483392,-0.131610502746377,0.317272220357227,
-0.414818866266301,0.678274507402505,0.964879202878059
"9415","GNPDA2",39.2892663360529,-0.108977445866358,0.26286034024279,-
0.414583066299394,0.678447146633563,0.964879202878059
"9416","REST",59.5071188635709,-0.0916761604179932,0.222578690949998,-
0.411882018115509,0.680425901402402,0.964879202878059
"9417","DKK2",16.9542030723938,-0.20856188167812,0.512010286757225,-0.
407339241168434,0.683758845361442,0.964879202878059
"9418","FST",75.2250663227376,-0.172190164989118,0.413018052625784,-0.
416907115547154,0.67674634872215,0.964879202878059
"9419","AC011379.1",1.49353192649962,0.454309677812665,1.1166001242951

4,0.406868732975872,0.684104401523452,0.964879202878059
"9420","TAF7",133.895869519997,0.0871078270026848,0.214565585512832,0.405972965303308,0.684762464576132,0.964879202878059
"9421","RBM22",45.5568360601035,-0.10205148465017,0.245454349818563,-0.415765639213371,0.677581504565439,0.964879202878059
"9422","FAM153CP",4.68616259867322,-0.303447948196977,0.745301375963234,-0.407147977963677,0.683899307149705,0.964879202878059
"9423","CNOT6",14.2404522194907,-0.194815565085751,0.469938375840577,-0.414555556858461,0.678467288570226,0.964879202878059
"9424","GABBR1",15.3413143575447,-0.188440660813309,0.460643281951341,-0.409081534881072,0.682479826649931,0.964879202878059
"9425","MDC1",11.2555194165291,0.214953610542353,0.525865138994239,0.408761856611126,0.682714434465661,0.964879202878059
"9426","TUBB",571.205809153125,0.108016692838733,0.263687953103839,0.409638330334328,0.682071274812801,0.964879202878059
"9427","HCG27",2.64928848492875,0.37179171089334,0.889620031863828,0.417921918995468,0.676004206129527,0.964879202878059
"9428","TOMM6",162.100807856684,-0.107012602621847,0.255801459933022,-0.418342423260082,0.6756967765639,0.964879202878059
"9429","MEA1",46.3613034852613,-0.114691296967175,0.274959779053791,-0.417120268869353,0.676590440086723,0.964879202878059
"9430","TNFRSF21",11.7373357510129,0.237426948120629,0.578235410735285,0.410606032962798,0.68136143924324,0.964879202878059
"9431","ICK",48.2035607159176,0.109008434907988,0.265682699009223,0.410295571802379,0.681589140050822,0.964879202878059
"9432","AP5Z1",17.8959206751319,-0.145826404008169,0.358454522558398,-0.406819819059227,0.684140329255034,0.964879202878059
"9433","RNF216",51.5474290849038,0.111748397288211,0.272477904759487,0.410119115481493,0.681718570929842,0.964879202878059
"9434","NUP42",26.1297570767352,-0.134295344949299,0.325100709074707,-0.413088440598996,0.679541818182177,0.964879202878059
"9435","C7orf31",2.16208178030473,-0.406266640910102,0.991644030037258,-0.409689998229342,0.682033367922945,0.964879202878059
"9436","KBTBD2",22.0072249355279,0.154792493056226,0.369078253836509,0.419402908318717,0.674921698975374,0.964879202878059
"9437","LINC02604",1.97677688003999,0.437216033825914,1.07115529373135,0.408172406358446,0.683147104877228,0.964879202878059
"9438","SBDS",168.543215752657,-0.0771192276461837,0.183876896821451,-0.419406836743978,0.674918828444252,0.964879202878059
"9439","GTF2IRD2",27.6347602063757,-0.163721520773876,0.39762913866196,-0.411744273381992,0.680526870633549,0.964879202878059
"9440","AC006333.2",11.6468885814762,-0.201069979409806,0.480505356290974,-0.418455230055847,0.675614313008646,0.964879202878059
"9441","CYREN",18.5569082480717,-0.15621600633862,0.385089560838001,-0.405661493390459,0.684991339117446,0.964879202878059
"9442","FAM131B",2.8985346698651,-0.336607930850988,0.83011289330548,-0.405496569882956,0.685112539259594,0.964879202878059
"9443","AC093673.1",18.0992522043619,-0.157361405816559,0.383185078392636,-0.410666841403767,0.681316844048297,0.964879202878059
"9444","ZNF425",2.9579235750433,-0.325319139456784,0.792358961731239,-

0.410570404537343,0.681387568642575,0.964879202878059
"9445","ERICH1",73.379147367691,0.0944815776977139,0.22633383931881,0.417443445408217,0.676354082630652,0.964879202878059
"9446","AC145124.1",11.8743402431767,-0.191175435208722,0.464910656112796,-0.411208976811105,0.680919306474906,0.964879202878059
"9447","CSGALNACT1",1.87707637981325,0.539870363924497,1.29646853710943,0.416416093774383,0.677105553868361,0.964879202878059
"9448","AC023632.2",1.94825024083821,0.421285076983525,0.99970945709219,0.421407513948,0.673457533283674,0.964879202878059
"9449","AP003117.1",3.33036358455802,0.386888864011059,0.945508377978629,0.409186077058541,0.682403111131558,0.964879202878059
"9450","NCALD",61.5518286797232,0.115592875124243,0.275859379462301,0.419028257620075,0.675195480904834,0.964879202878059
"9451","TRMT12",6.00313414494703,-0.238407320128374,0.570241926027457,-0.418081009562412,0.675887889076561,0.964879202878059
"9452","PTP4A3",4.26773390682034,0.278434774081589,0.664035856393686,0.419306836220775,0.674991901082205,0.964879202878059
"9453","DMRT2",13.477602915507,-0.218053742757348,0.530281600831426,-0.41120367445422,0.680923194163321,0.964879202878059
"9454","SMU1",49.0163988458157,0.107249270679405,0.255974572000412,0.418984080493872,0.675227766872907,0.964879202878059
"9455","VPS13A",23.4918834736926,0.198730559599385,0.47763899428228,0.416068541258876,0.677359849015008,0.964879202878059
"9456","PSMD5",28.3043136789019,-0.132510195272461,0.325147760810628,-0.407538390982915,0.683612603354734,0.964879202878059
"9457","AJM1",2.32132356176085,-0.401993291414095,0.976922430192145,-0.411489468345024,0.680713662150582,0.964879202878059
"9458","ARL5B",21.0602291443744,-0.155699000918374,0.380380022621169,-0.409324863712516,0.682301271277907,0.964879202878059
"9459","PARG",15.0615773722684,0.171936978985651,0.422793273388401,0.406669144964612,0.684251005286709,0.964879202878059
"9460","EXOSC1",35.3582333267579,0.118053034831096,0.286946065336185,0.411411930994021,0.6807705068272,0.964879202878059
"9461","PGGHG",5.72592986304213,0.252793366704409,0.622486726581383,0.406102420998946,0.684667346973673,0.964879202878059
"9462","TRIM68",4.14773085972916,-0.294774513372226,0.726482921927091,-0.405755599306172,0.684922185536248,0.964879202878059
"9463","DNHD1",2.8779635000477,0.406987008148312,0.985744806566038,0.412872586735811,0.679699966579025,0.964879202878059
"9464","AMOTL1",21.1345493878437,0.155796163391863,0.373234263664174,0.417421921187933,0.676369823531128,0.964879202878059
"9465","IL18",14.300962412532,-0.216650567739029,0.527927807592701,-0.410379155299537,0.681527834746525,0.964879202878059
"9466","TRIM29",3.74438642283459,-0.345288449743731,0.825420033924585,-0.418318474900597,0.675714283694994,0.964879202878059
"9467","TMEM218",31.2975607449348,-0.127298749562867,0.309581902312787,-0.411195708185326,0.68092903504813,0.964879202878059
"9468","LOH12CR2",4.7491240555761,0.299406609563527,0.720685208491005,0.415447141187253,0.677814602942974,0.964879202878059
"9469","ETFBKMT",9.80279461647584,0.191938451687136,0.473278284580813,

0.405550936817517,0.685072584810264,0.964879202878059
"9470","PRKAG1",53.1573562819987,-0.123973105880989,0.303860048257938,
-0.407994096597231,0.683278008970882,0.964879202878059
"9471","USP15",59.584869418864,-0.0983474723481196,0.235208187979583,-
0.418129458812279,0.675852467557822,0.964879202878059
"9472","MRPL42",53.1776366601743,-0.119044582732391,0.282982399502145,
-0.420678398875082,0.673989936758638,0.964879202878059
"9473","TCTN1",47.5787997758137,-0.128881383988572,0.306030993942465,-
0.421138337422131,0.673654068296997,0.964879202878059
"9474","ATXN2",71.1038683955703,0.105853778504463,0.255200000337735,0.
414787532775763,0.678297447017098,0.964879202878059
"9475","PLBD2",74.7495465081611,-0.0864026628244235,0.210852069915303,
-0.409778584858714,0.681968376943113,0.964879202878059
"9476","ZNF605",24.4898026600554,0.165801452079352,0.397589567773172,0
.417016605863116,0.676666261512869,0.964879202878059
"9477","CUL4A",34.8510783429717,-0.12025169628643 2,0.291414185250739,-
0.412648739741219,0.679863986144383,0.964879202878059
"9478","CDC16",40.5326396714352,-0.1022935049824,0.250916353858728,-0.
407679704448412,0.683508839606738,0.964879202878059
"9479","OSGEP",28.293580560257,0.138546739256125,0.339012689680088,0.4
08677148300454,0.682776605956437,0.964879202878059
"9480","RABGGTA",10.874883155475,-0.195536739908556,0.474813848040113,
-0.411817685426977,0.680473057643839,0.964879202878059
"9481","AL136418.1",1.55255649315825,0.537106715154402,1.3165690855697
5,0.407959385528157,0.683303492817011,0.964879202878059
"9482","FUT8",43.5864296988518,0.118042221906656,0.284975041739038,0.4
14219509141268,0.678713355432051,0.964879202878059
"9483","KLC1",74.3965471289983,-0.104337565987755,0.252266731342601,-0
.41360018196793,0.679166939958232,0.964879202878059
"9484","RNF111",15.657073315492,0.15861419370682,0.380600702809385,0.4
16746980591515,0.676863486775799,0.964879202878059
"9485","SPESP1",6.22739776724017,0.246659955870723,0.58922450771282,0.
418617950614746,0.675495368558962,0.964879202878059
"9486","PTPN9",25.3580224598072,0.140661986979679,0.334781481739032,0.
42016059624626 2,0.674368138025298,0.964879202878059
"9487","RPS17",734.897042721586,0.0931480427373846,0.224513845005326,0
.41488774438465,0.678224081975262,0.964879202878059
"9488","HDDC3",26.1841098627937,0.142334875583376,0.346260589398164,0.
411062881371422,0.681026426761317,0.964879202878059
"9489","LITAF",77.15632847349,-0.11252083857522,0.269822193077621,-0.4
17018471652741,0.676664896803911,0.964879202878059
"9490","SNX29",55.5110899722723,0.132031694194584,0.320658347902897,0.
411751931793043,0.680521256737704,0.964879202878059
"9491","EARS2",6.04415855791373,0.252562070209674,0.600488193580091,0.
420594564405849,0.674051163581315,0.964879202878059
"9492","PRKCB",1.8991852917519,-0.466735754618258,1.10702589980066,-0.
421612317021944,0.673308014467735,0.964879202878059
"9493","TNRC6A",121.050294232771,0.112509244503074,0.269360475809816,0
.417690250081499,0.676173601752049,0.964879202878059
"9494","AC133552.5",3.52591104867495,-0.330164910366469,0.798172137248

766,-0.413651260121056,0.679129526802402,0.964879202878059
"9495","PAGR1",40.7597040612868,-0.11861946091528,0.2812400290328,-0.421773036134542,0.673190688690204,0.964879202878059
"9496","CKLF",89.5283875028682,0.126968883362602,0.302437872686276,0.419818067872436,0.674618364803969,0.964879202878059
"9497","ZDHHC1",33.335383814559,-0.128004075710975,0.3140901908469,-0.407539233765405,0.683611984498116,0.964879202878059
"9498","C16orf95",4.72674845329631,-0.308972265796232,0.747592939639572,-0.413289437892757,0.679394567340234,0.964879202878059
"9499","PITPNA-AS1",27.2673500335239,-0.15159173178484,0.361068967920216,-0.419841485293016,0.674601256561874,0.964879202878059
"9500","RPAIN",48.2467686648708,0.141338615333385,0.33722171529369,0.419126672225992,0.675123558709571,0.964879202878059
"9501","CRLF3",8.33421769073273,-0.198635578033472,0.4891552087235,-0.406078836514552,0.684684675306592,0.964879202878059
"9502","LIG3",10.6146548432285,0.185375652597893,0.45602530359438,0.406502996953823,0.6843730553789,0.964879202878059
"9503","RAD51D",12.4016485710592,-0.180137132271752,0.44169225708911,-0.407834027834021,0.683395529754331,0.964879202878059
"9504","KRT24",3.05420980089646,0.378002968477314,0.923108359588404,0.409489270193435,0.682180639412097,0.964879202878059
"9505","STAT5A",24.5199143061096,0.157391539614754,0.374175874035273,0.42063518932254,0.674021493718817,0.964879202878059
"9506","VAT1",304.168330523083,0.0638715189278304,0.154026380874117,0.414679086565251,0.678376844056648,0.964879202878059
"9507","UTP18",17.936440780423,-0.151930609607983,0.370782770614081,-0.409756390126649,0.681984659736288,0.964879202878059
"9508","TSEN54",4.79546276652024,-0.27361725283342,0.651284419083596,-0.420119451373363,0.674398193629859,0.964879202878059
"9509","PYCR1",15.8764479757316,0.22463887399529,0.532972000851231,0.421483443101157,0.673402098835334,0.964879202878059
"9510","RIOK3",63.2656213924801,0.110925058632875,0.269483633813306,0.411620761762927,0.680617411619563,0.964879202878059
"9511","ZADH2",38.6036002165343,0.12397738526545,0.299953879871476,0.413321492352663,0.679371085337846,0.964879202878059
"9512","STK11",25.4803697886816,0.142464285915293,0.351386843470393,0.405434319931495,0.685158288047125,0.964879202878059
"9513","DAPK3",34.582450456168,-0.126071993500215,0.306380674582775,-0.411488073364608,0.68071468483149,0.964879202878059
"9514","TRAPPC5",194.91486366404,-0.119718791618457,0.290897312113502,-0.411550009687768,0.680669278956674,0.964879202878059
"9515","ADAMTS10",14.8462782730914,-0.188379579484084,0.460116540588817,-0.409417099509208,0.682233593041818,0.964879202878059
"9516","ILF3",78.2737184212791,0.0972256987666474,0.235202649736197,0.413369912608108,0.679335614902601,0.964879202878059
"9517","ZNF763",3.96454638748364,0.315313326142356,0.757403945305295,0.416308005915205,0.677184635005357,0.964879202878059
"9518","PBX4",4.12275785625894,-0.289734253734168,0.697327800210624,-0.415492188389241,0.677781632483298,0.964879202878059

"9519","NFKBID",2.41393807995538,-0.354600121295884,0.869380681531164,
-0.407876697549062,0.683364201353284,0.964879202878059
"9520","C19orf47",2.91669164924083,0.346003027391112,0.846844590498763
,0.408579131605869,0.682848547794344,0.964879202878059
"9521","RELB",8.11115929657316,-0.213339838602748,0.52328775602667,-0.
407691248544854,0.683500363263802,0.964879202878059
"9522","FOSB",739.79633922011,0.253292565963684,0.60898596799981,0.415
925126806473,0.677464792363944,0.964879202878059
"9523","TRPM4",3.74299857552088,-0.298709785652386,0.731336491480943,-
0.408443704275585,0.682947952857148,0.964879202878059
"9524","BCL2L12",12.6683759647856,-0.16858720463861,0.402231184432564,
-0.419130120098568,0.675121039030005,0.964879202878059
"9525","AKT1S1",29.4409972516683,0.140647036092875,0.336364428859859,0
.41813885186853,0.675845600324674,0.964879202878059
"9526","ZNF468",6.87823011321957,0.250799691954967,0.618229385607106,0
.405674168510576,0.684982024671906,0.964879202878059
"9527","CNOT3",23.4313914910396,-0.13710196200673,0.334545604218551,-0
.409815463954397,0.681941321540168,0.964879202878059
"9528","PCED1A",23.083291002767,-0.1432603888862,0.342169322730817,-0.
418682737958078,0.675448012969555,0.964879202878059
"9529","TRMT6",12.0379546854648,-0.172825571167065,0.415803739122862,-
0.415642176599085,0.677671859187156,0.964879202878059
"9530","SPTLC3",7.82496403261898,-0.247137613486944,0.60047276483395,-
0.411571728078768,0.680653357353359,0.964879202878059
"9531","ENTPD6",21.5480466621664,-0.144928161200203,0.356293239397009,
-0.406766520311976,0.684179478499858,0.964879202878059
"9532","NEURL2",3.46073987689192,0.339039096288798,0.815841788597966,0
.415569661945659,0.677724930321164,0.964879202878059
"9533","HLCS",5.05240584115543,-0.257189072777075,0.610058708261153,-0
.421580856554182,0.673330981701293,0.964879202878059
"9534","SH3BGR",3.82438642868438,-0.301957660405286,0.71778188466972,-
0.420681640000192,0.67398756971129,0.964879202878059
"9535","THAP7-
AS1",9.0179271327081,-0.236516329116958,0.563214487332786,-0.419940066
238402,0.674529237379419,0.964879202878059
"9536","SERHL2",1.81854265588913,0.418925099820784,1.02737610142381,0.
407762161529949,0.683448295726114,0.964879202878059
"9537","FAM118A",16.1257721639966,0.188401862931667,0.462214945411355,
0.407606601218823,0.683562517149897,0.964879202878059
"9538","ATXN10",91.701587666089,0.117313243234249,0.279939575758502,0.
419066303563498,0.675167676267914,0.964879202878059
"9539","SCML1",22.9814199240732,0.146446799807632,0.354426358764434,0.
413193872820747,0.679464576893778,0.964879202878059
"9540","INE1",2.2269494367945,-0.442040697065279,1.08562726035555,-0.4
07175384413714,0.683879179454883,0.964879202878059
"9541","ABCB7",27.1677336807807,-0.150441957074872,0.358801374189497,-
0.419290359226489,0.675003941487631,0.964879202878059
"9542","CDR1",1.67438286387122,0.460519096382747,1.12922287010758,0.40
7819491239027,0.683406202747812,0.964879202878059
"9543","MPP1",10.7427681141197,-0.202407061745321,0.484175973840533,-0

.418044415008469,0.675914644036156,0.964879202878059
"9544","TOR1AIP1",72.6011878286002,0.0870037617163778,0.21529951223409
5,0.404105707502852,0.686134988097209,0.964926556475481
"9545","LIN9",4.04910467888487,0.283769488946955,0.70357314036239,0.40
3326211118284,0.686708261868585,0.964926556475481
"9546","HPCAL1",15.0817655942972,0.196451534655763,0.485330419698629,0
.404778943750839,0.685640007710312,0.964926556475481
"9547","RPL31",824.646089540653,0.0904298055734115,0.223181850959029,0
.405184405384345,0.685341967081036,0.964926556475481
"9548","MRFAP1",346.015404710084,0.0762937772824361,0.188801378806506,
0.404095445513806,0.686142534014753,0.964926556475481
"9549","SEPTIN8",49.9069366202258,0.125229670142401,0.310668517085778,
0.403097395632865,0.686876576444565,0.964926556475481
"9550","AC106791.1",5.80862971589782,0.271828548789048,0.6734152050692
79,0.403656684231065,0.686465196291326,0.964926556475481
"9551","IK",97.5346031648216,-0.0861505753440314,0.213198130013558,-0.
404086918297795,0.686148804330663,0.964926556475481
"9552","KIAA1191",24.1273587117269,0.143432480902169,0.355718706776268
,0.403218830412487,0.686787248198766,0.964926556475481
"9553","MAK16",12.4470417048898,-0.168839017043737,0.418624534035501,-
0.403318495015438,0.686713937510166,0.964926556475481
"9554","ENY2",172.404056282908,-0.0913052350592381,0.225839154621192,-
0.404293202444844,0.685997123514945,0.964926556475481
"9555","VLDLR",3.44894837990693,0.439891308777029,1.08652025123287,0.4
04862503278597,0.685578582028198,0.964926556475481
"9556","AL441992.1",2.00864596699563,-0.405053762191421,1.005227405305
24,-0.402947392852292,0.686986925541886,0.964926556475481
"9557","MAPK8IP1",13.2200269423325,-0.196571764233805,0.48697568686308
6,-0.403658271935599,0.686464028600376,0.964926556475481
"9558","NUDT8",15.3599196641376,0.165113281239763,0.409454961665018,0.
403251387083802,0.68676330003445,0.964926556475481
"9559","WBP4",68.9618742713161,0.0862391425759548,0.213619668821404,0.
403704130110111,0.686430302134652,0.964926556475481
"9560","ZC3H13",103.205269091491,-0.119323657487592,0.295642398696036,
-0.403608068443102,0.686500951552822,0.964926556475481
"9561","ISG20",11.6822715850736,-0.170631306497492,0.423456261897104,-
0.402949069009054,0.686985692445318,0.964926556475481
"9562","CDR2",18.2903923573605,-0.14208745747054,0.352081917508851,-0.
403563632224787,0.686533633496448,0.964926556475481
"9563","COX11",63.6601331359244,-0.0914259069665453,0.225806431239101,
-0.404886195954873,0.685561165615671,0.964926556475481
"9564","ZNF77",4.17028347156587,0.289794939228762,0.718717758386506,0.
403211046126564,0.686792974240989,0.964926556475481
"9565","AC104532.2",5.10715280816286,0.281069308114703,0.6942806458089
83,0.404835292199739,0.685598585013623,0.964926556475481
"9566","PAK4",10.142741442171,0.189363207431647,0.469625515567926,0.40
322171848488,0.68678512376641,0.964926556475481
"9567","ZNF428",108.142605428331,-0.125857712712242,0.312146434046281,
-0.403200866595776,0.686800462228297,0.964926556475481
"9568","PRPF6",77.293765204764,-0.0992844348832127,0.245602274496366,-

0.404248841289463,0.68602974122276,0.964926556475481
"9569","CDK11B",27.3792981444194,-0.13120018651157,0.326219172221714,-0.402184168447343,0.687548492558507,0.964970209425607
"9570","FBXO42",20.4187296139656,0.144041366715117,0.358230885952546,0.402090864756417,0.68761715559573,0.964970209425607
"9571","DNPEP",43.2204105233651,-0.106955515499028,0.266249613330706,-0.401711439731479,0.687896404465011,0.964970209425607
"9572","PAQR3",14.7488095598249,-0.200939802553019,0.49970616149803,-0.402115919384779,0.687598717413167,0.964970209425607
"9573","RAP1GDS1",13.6650069656702,-0.166547207546344,0.413908905657801,-0.402376477697816,0.687406978555591,0.964970209425607
"9574","MYO1G",1.93480462593685,0.477698629606408,1.18628615091142,0.402684149384527,0.687180595934913,0.964970209425607
"9575","TYW1",13.6818746150194,-0.159405752239388,0.396708551376817,-0.401820811994484,0.687815904402786,0.964970209425607
"9576","TMEM120A",35.2463767348651,-0.110503248660349,0.274383599669197,-0.402732702659979,0.687144873344724,0.964970209425607
"9577","YWHAG",75.8245303897621,0.0933972219477067,0.232651416319838,0.401447037912328,0.688091023832739,0.964970209425607
"9578","AC067750.1",2.6345174044553,0.36127012922501З,0.89995995870888,0.401429114405607,0.68810421761211,0.964970209425607
"9579","PLAAT4",102.906151201009,0.139359695086316,0.346767697149517,0.401881998328777,0.687770871642105,0.964970209425607
"9580","AC091057.6",1.74388571477454,0.529376892717878,1.3166716034024,0.402056892052596,0.687642157058122,0.964970209425607
"9581","HEXA",177.670244828012,0.128282491249147,0.319632254149412,0.401344012013199,0.688166864136842,0.964970209425607
"9582","MRPL46",24.009708936042,-0.138786443601075,0.344958386416427,-0.402328075113196,0.687442595382197,0.964970209425607
"9583","RAB4B",11.2721074930424,0.196486773009548,0.489073797051405,0.40175281152692,0.687865953622086,0.964970209425607
"9584","ZNF222",3.33802568102577,0.31647318078313,0.787990309047731,0.401620650849857,0.687963229492281,0.964970209425607
"9585","IBA57",10.3540025542634,0.194037630031261,0.483889803694775,0.400995492258099,0.688423442716919,0.964996132546319
"9586","TRNT1",16.0460448824761,0.158570697468953,0.395522777958882,0.400914198386414,0.688483296036041,0.964996132546319
"9587","FLYWCH2",65.6024585700744,-0.121410285416799,0.302720601576001,-0.40106383505028,0.688373126252162,0.964996132546319
"9588","CDC42EP5",52.7802433775548,0.12234221968055 9,0.305221271506738,0.400831236553767,0.688544379417119,0.964996132546319
"9589","GPR82",2.77676278564712,-0.366582270707613,0.914053342085582,-0.401051288616469,0.688382363292552,0.964996132546319
"9590","AL390208.1",1.52668658410995,0.51530307364396,1.28648879110152,0.400549990958528,0.6887514708608,0.965085081524168
"9591","POLR3E",14.190514828272,-0.167206164736679,0.417362990147269,-0.400625279873713,0.688696030584669,0.965085081524168
"9592","SNX14",50.8182300535414,0.0967549242484865,0.241650104205837,0.400392644424729,0.688867341061255,0.965146809478962
"9593","C1orf109",15.996350486412,-0.158185746700537,0.395356309329173

,-0.400109326619679,0.689075995081021,0.965338507025315
"9594","IDH1",39.928654726283,-0.110333464459717,0.276212483590216,-0.399451404315982,0.689560623914794,0.965413725950406
"9595","ING2",33.8022287777803,-0.135150771181583,0.338587015108887,-0.399161117085719,0.689774491439836,0.965413725950406
"9596","WDR70",32.9863918425729,0.149809966174892,0.375242884566557,0.399234661965509,0.689720305308357,0.965413725950406
"9597","TDP2",30.4100823039683,0.122913281069793,0.307987905379142,0.399084765742614,0.689830746990175,0.965413725950406
"9598","ZNF853",10.249627264819,-0.184375431700391,0.461744259641448,-0.399302054872457,0.689670653202165,0.965413725950406
"9599","MINPP1",16.8202011335208,0.152800932228582,0.382211412075128,0.399781187586693,0.689317687987513,0.965413725950406
"9600","MAP3K11",7.96161963118313,0.203345680322964,0.509290013495653,0.399272860127855,0.689692162431709,0.965413725950406
"9601","TTC7B",11.3660070376765,-0.197598761264374,0.495158947797513,-0.399061275461751,0.689848054937253,0.965413725950406
"9602","ZNF14",4.84973211683849,0.257163353001956,0.644163830044735,0.399220417240901,0.689730800360991,0.965413725950406
"9603","NDUFAF5",9.65313680794296,0.198852393645603,0.497471732992587,0.399726015485118,0.689358328466753,0.965413725950406
"9604","WDR33",74.94884175186,-0.0948555151477134,0.23820646037524,-0.398207147691503,0.690477497622286,0.965566431236159
"9605","SEMA6A",8.50380339239986,0.27101974178331,0.680628319579674,0.398190516008326,0.690489756335831,0.965566431236159
"9606","ASCC3",28.727038223881,0.144754511724854,0.363672101502002,0.398035788632131,0.690603805122551,0.965566431236159
"9607","FUT4",13.6968779314792,0.187036463142384,0.469512209451976,0.398363363885034,0.69036235932651,0.965566431236159
"9608","TEX30",15.8451291404703,0.167305333485038,0.420192840650824,0.398163217692866,0.690509877279259,0.965566431236159
"9609","DHRS4L2",62.3859736111449,0.111374347107472,0.279277007693639,0.398795260760052,0.690044069379241,0.965566431236159
"9610","ZNF213",7.99275288817676,-0.213463863249996,0.535828572548998,-0.398380889310408,0.690349442754011,0.965566431236159
"9611","UBE2O",11.6435728145517,0.194592334795368,0.488691780927284,0.398190316248276,0.69048990357345,0.965566431236159
"9612","ACTN4",92.3708608784177,-0.082575628323362,0.207434719393527,-0.398080073407127,0.690571162315475,0.965566431236159
"9613","ANKRD23",1.83244165945698,-0.401602267117696,1.01085107762769,-0.397291229149397,0.691152714434363,0.965649136137669
"9614","NPM1",399.828868873863,-0.104377050254422,0.262541522804564,-0.39756397060331,0.690951623224136,0.965649136137669
"9615","TBC1D7",8.79575388400445,0.214450245919067,0.539547207092304,0.397463360203029,0.691025800342498,0.965649136137669
"9616","PPP1R35",31.1879310908654,-0.122990769198254,0.309587306137932,-0.397273294995685,0.691165937979629,0.965649136137669
"9617","MRRF",15.6960714769314,0.156240987615724,0.392916103592381,0.397644652859053,0.690892140690055,0.965649136137669
"9618","POLR2M",35.3109376256761,0.116478351718545,0.292850498594061,0

.397739980904056,0.690821863090576,0.965649136137669
"9619","INE2",1.65000903046622,-0.468539602508792,1.17824969899821,-0.
397657307196564,0.690882811524826,0.965649136137669
"9620","DPP7",208.654598099072,-0.10669196106796,0.268716012969029,-0.
397043554975107,0.691335342516273,0.965785412481933
"9621","SMIM1",2.54514586356824,0.411395550331901,1.03668645129621,0.3
9683700873829,0.69148765772978,0.965786116695749
"9622","MDM4",73.5874231156423,0.110999255266413,0.280114866823773,0.3
96263349121862,0.691910762076544,0.965786116695749
"9623","SSR3",262.076249337933,0.104590360150637,0.263579283292571,0.3
96807969291513,0.691509073546388,0.965786116695749
"9624","AC146944.4",4.17281375953814,0.277267481718857,0.6991652477815
38,0.396569312617627,0.691685085817467,0.965786116695749
"9625","COX15",23.8273509253545,0.13174405131619,0.332421321037017,0.3
9631649048624,0.691871563461637,0.965786116695749
"9626","CD69",12.88883576302,-0.251762374612644,0.635017486931513,-0.3
96465262443704,0.691761829332767,0.965786116695749
"9627","ZNF385A",81.7605217071432,-0.143144383096896,0.361096755836458
,-0.396415588850447,0.691798467833159,0.965786116695749
"9628","AC005332.5",8.08292633936987,-0.19408603674631,0.4892866055158
98,-0.396671469356224,0.691609741909916,0.965786116695749
"9629","CTNNBIP1",22.0147847192981,0.147963531094169,0.38471687252663,
0.384603696017848,0.700531055363733,0.965884611183634
"9630","TMCO4",15.9375380743398,0.145144845793897,0.372334204938279,0.
389824098535232,0.696666622671415,0.965884611183634
"9631","RUNX3",3.81761730692492,0.337334106514161,0.883023235500633,0.
382021778082553,0.702445210975796,0.965884611183634
"9632","THRAP3",162.896976495918,-0.0747428401550828,0.194043126314489
,-0.385186744692753,0.700099063331905,0.965884611183634
"9633","PGM1",42.0686074390541,-0.111841359773682,0.284530007900119,-0
.393074040236004,0.694264796997348,0.965884611183634
"9634","ST7L",15.2861400295448,0.154347451615177,0.397020642151771,0.3
88764298950919,0.697450512682784,0.965884611183634
"9635","PI4KB",37.7050027570774,0.124886963412977,0.320799579162362,0.
389299025076868,0.69705495741384,0.965884611183634
"9636","FLAD1",10.0030115718376,-0.202860495167884,0.514745866196509,-
0.3940983473395,0.693508431114718,0.965884611183634
"9637","IGSF8",24.0592726713901,-0.139601083077279,0.360170127054184,-
0.387597617323432,0.698313832450943,0.965884611183634
"9638","RNPEP",38.5535543046821,-0.138114906260947,0.350042569457633,-
0.394566027997527,0.693163189187046,0.965884611183634
"9639","ARL8A",49.1835432464345,-0.117088101092107,0.304451307775635,-
0.384587282437936,0.700543217904967,0.965884611183634
"9640","AL591895.1",8.16107074779109,0.217823422112898,0.5596046107337
75,0.389245224100781,0.697094752118857,0.965884611183634
"9641","AC082651.1",2.21796208484537,-0.372984996267926,0.948822331426
97,-0.393103096242454,0.694243337344497,0.965884611183634
"9642","CENPO",1.83211999939102,0.433711255778941,1.10525387076711,0.3
92408719164149,0.694756244672813,0.965884611183634
"9643","DNAJC27-

AS1",3.04080183177246,0.314505463496635,0.814307359738812,0.386224513060415,0.699330400764369,0.965884611183634
"9644","CRIM1-
DT",9.81232860571566,0.223034437796082,0.582638301261814,0.382800851425419,0.701867430145515,0.965884611183634
"9645","AC007390.2",2.44012271349498,0.363493987905915,0.951277668662339,0.382111343386259,0.702378778287549,0.965884611183634
"9646","REL",18.7587430592026,0.141972679591519,0.370487563735226,0.383204980378185,0.701567785564667,0.965884611183634
"9647","WDR54",14.0529738030373,-0.167392094278814,0.426210690409413,-0.392744945271125,0.694507871031675,0.965884611183634
"9648","LIMS1",195.425697441357,-0.0621748005548888,0.160692182462331,-0.386918639115898,0.698816441803954,0.965884611183634
"9649","FBLN7",24.5370962086008,0.174268705627418,0.444890099990081,0.391711808447306,0.695271164035555,0.965884611183634
"9650","AC009951.6",12.8217081622095,-0.183032823090911,0.468235863464698,-0.390898769984352,0.695872063192098,0.965884611183634
"9651","CYBRD1",702.468408826904,0.0860572204833146,0.217476370959197,0.395708371000272,0.69232017937612,0.965884611183634
"9652","PPIL3",38.9093147254882,0.124261402391047,0.319306863791575,0.389159822358714,0.697157922519333,0.965884611183634
"9653","XRCC5",184.667418423137,-0.0796515321122259,0.204952959445315,-0.388633237245243,0.697547476077051,0.965884611183634
"9654","GIGYF2",52.9137892413882,-0.103283223643066,0.261755196404751,-0.394579458446966,0.693153275768007,0.965884611183634
"9655","CAND2",1.51527592120667,0.473199951673615,1.21814192437444,0.38846044307737,0.697675321950903,0.965884611183634
"9656","TCAIM",12.1944582250131,-0.173816529070006,0.442913253466498,-0.392439214021292,0.69473371646029,0.965884611183634
"9657","ZNF502",6.57442612927279,-0.220937483599926,0.571211295178711,-0.386787665903566,0.698913409101429,0.965884611183634
"9658","SETD2",55.4916583247639,0.0992752502749573,0.253451026006005,0.391694016155236,0.695284311887503,0.965884611183634
"9659","RABL3",23.143561022499,-0.121982042817182,0.316101717200999,-0.38589490717514,0.699574502538959,0.965884611183634
"9660","SNX4",32.3810666999692,0.112551968955538,0.291810083686407,0.38570280894231,0.699716782281438,0.965884611183634
"9661","DZIP1L",10.81837842557,-0.211223304426417,0.533740230726949,-0.395741771495719,0.692295536688334,0.965884611183634
"9662","SLC25A36",116.940517232088,0.0768474399118343,0.19479085414454,0.394512567077771,0.693202650822031,0.965884611183634
"9663","PIGZ",9.46728241841735,0.198413128457979,0.510291554404438,0.388823069371679,0.697407034161053,0.965884611183634
"9664","KIAA0232",38.2579258521839,0.127994598063589,0.333861594370018,0.383376226022968,0.701440828132821,0.965884611183634
"9665","UBE2K",91.9769546279231,-0.0807841126599984,0.205614465099521,-0.392891193821882,0.694399845975113,0.965884611183634
"9666","SCFD2",7.89760462250894,0.208671027859367,0.530345125345523,0.393462705485257,0.693977764033189,0.965884611183634
"9667","SMAD1",16.4831672738197,0.149419392785727,0.387999671501678,0.

385101853842886,0.700161954572423,0.965884611183634
"9668","TENM3",4.20284500300545,0.327371852382697,0.85485293934839,0.382956924301197,0.701751703191045,0.965884611183634
"9669","ACSL1",17.6473908423884,0.154623827458546,0.40228342945296,0.384365390512876,0.700707648416429,0.965884611183634
"9670","DCP2",38.2716849336989,0.12178770470033,0.318233117676501,0.38269656118547,0.701942469458521,0.965884611183634
"9671","LEAP2",1.55707349381383,-0.520357952538954,1.33221883545417,-0.39059495233871,0.696096657289373,0.965884611183634
"9672","HDAC3",36.7646199604324,-0.1183236905796,0.309516271715336,-0.382285848572197,0.702249350225299,0.965884611183634
"9673","AC008393.1",2.51497983470185,-0.365191042223469,0.949784801321263,-0.384498721937269,0.700608842984223,0.965884611183634
"9674","SNRNP48",25.7078437828048,0.120097786202901,0.312530640345292,0.384275238006149,0.70077445892289,0.965884611183634
"9675","DDX39B",141.448241767397,-0.076442494694047,0.196634069621425,-0.388755086243294,0.697457328359735,0.965884611183634
"9676","RCAN2",15.4685177887998,-0.203529374469261,0.531999062211117,-0.382574686548023,0.702035142101563,0.965884611183634
"9677","TENT5A",191.534554076014,-0.0740773481861467,0.191369077662421,-0.387091525396913,0.698688451280888,0.965884611183634
"9678","LYRM2",54.431466040948,-0.092071356662239,0.234402742896249,-0.392791293841585,0.694473635443305,0.965884611183634
"9679","SLC18B1",13.4782032134249,-0.181861884497869,0.464762264456801,-0.391300883066363,0.695574846209828,0.965884611183634
"9680","PEX7",9.77804330637487,-0.206010546924241,0.537382229278127,-0.383359433379436,0.701453277429211,0.965884611183634
"9681","WDR27",21.7029651748068,0.138440050152481,0.358090542033112,0.386606273839199,0.699047712601269,0.965884611183634
"9682","HERPUD2",34.8658386579472,0.109771648297742,0.279121278616101,0.393275814878738,0.694115779038378,0.965884611183634
"9683","ZNF713",3.74388553014613,0.30435229533203,0.77980638282496,0.390292131528176,0.696320541009522,0.965884611183634
"9684","AC005076.1",2.64127322950707,-0.348891370614172,0.908078543356159,-0.38420836299546,0.700824020363861,0.965884611183634
"9685","CYP51A1",36.7622328063728,0.106918487838996,0.270852373926601,0.394748202827161,0.693028724938291,0.965884611183634
"9686","FAM200A",11.7785149210078,0.17432094034903,0.443704481358401,0.392876222064169,0.694410904431847,0.965884611183634
"9687","MOSPD3",30.7096771389406,-0.152267018472678,0.397486281345288,-0.383074902503131,0.701664227763145,0.965884611183634
"9688","AC002467.1",2.39683435064598,-0.355171884830695,0.928005287998149,-0.382726143292627,0.701922828154745,0.965884611183634
"9689","THAP5",28.552587241274,-0.136822988942653,0.355894827215686,-0.384447815701837,0.700646566521088,0.965884611183634
"9690","ST7-AS1",7.95691730690215,-0.235259797632874,0.615322674790189,-0.382335654562206,0.702212411400888,0.965884611183634
"9691","POT1",9.75852917204939,-0.208184644406179,0.532793963827325,-0.390741371975547,0.695988414737021,0.965884611183634

"9692","GIMAP2",3.02773042578785,-0.321608261673747,0.832410443423676,-0.386357792858752,0.699231704323605,0.965884611183634
"9693","LONRF1",21.8843968116204,-0.125892992756966,0.325323655463468,-0.386977677899302,0.698772733470434,0.965884611183634
"9694","AC019270.1",3.924345795559,-0.312718402172732,0.816110705872404,-0.383181350180234,0.701585305083385,0.965884611183634
"9695","CEBPD",818.64469055007,-0.136317414485548,0.34695680945144,-0.392894477848912,0.694397420332026,0.965884611183634
"9696","ENPP2",35.9679760708496,-0.184074450836622,0.466305350422194,-0.394750887310129,0.693026743578857,0.965884611183634
"9697","WASHC1",26.2816809466863,0.141909629905186,0.371068817922586,0.382434801985415,0.702138880386519,0.965884611183634
"9698","CBWD3",4.22891307981189,-0.278012746601858,0.709919919943912,-0.391611418121388,0.695345350008213,0.965884611183634
"9699","UBQLN1",47.6836227684371,0.104129405538824,0.264437020639873,0.39377771420528,0.693745159313679,0.965884611183634
"9700","HABP4",19.856645880933,-0.140218715816827,0.359727146810304,-0.389791866029976,0.696690458954976,0.965884611183634
"9701","VAV2",5.58646404530014,-0.255947471719282,0.66197214703168,-0.38664386842704,0.699019876625567,0.965884611183634
"9702","AL359532.1",1.74449202721651,0.481838482125939,1.22429352706132,0.393564510042374,0.693902587656957,0.965884611183634
"9703","AL442003.1",1.65147768967462,-0.471559398376956,1.22081403096974,-0.38626636524023,0.699299407814358,0.965884611183634
"9704","BET1L",20.8602612888392,-0.144736848518708,0.370475390738811,-0.390678712100338,0.696034736077515,0.965884611183634
"9705","DEAF1",36.6982746241082,-0.119641029265478,0.304506610731689,-0.392901254189511,0.694392415211456,0.965884611183634
"9706","TRIM66",6.92251666946344,-0.239253520052151,0.613215077821423,-0.390162487364385,0.696416398581646,0.965884611183634
"9707","COMMD9",48.2938336340228,-0.121926617456984,0.314610284906069,-0.38754809777877,0.698350484514113,0.965884611183634
"9708","FAM180B",92.4659449960113,-0.137270240458883,0.353810651886298,-0.387976562398683,0.698033378230928,0.965884611183634
"9709","EFEMP2",228.159511289314,0.113341421214039,0.290557672903359,0.390082354671588,0.696475650302054,0.965884611183634
"9710","RHOD",12.3665524067144,-0.204489634716745,0.529534569702541,-0.386168621307604,0.699371791272717,0.965884611183634
"9711","ANKRD13D",33.3788808889564,-0.106569288453138,0.274958155251585,-0.38758366106882,0.698324162150132,0.965884611183634
"9712","ALG8",21.1556032945891,-0.134982137327908,0.347761547658253,-0.388145665433246,0.697908239729743,0.965884611183634
"9713","DLG2",5.89341497426134,0.246304533398376,0.637885917033026,0.386126306948431,0.699403127667794,0.965884611183634
"9714","SMCO4",17.8288921167507,0.144363231736309,0.374865352683547,0.38510689425645,0.700158220333261,0.965884611183634
"9715","C11orf52",2.95526116837069,-0.31158289168171,0.792883784154874,-0.392974226372686,0.694338517558668,0.965884611183634
"9716","C11orf71",17.8162929115286,-0.141656582768284,0.366596595844961,-0.38640997863546,0.699193061063595,0.965884611183634

"9717","AC079630.1",2.53601629771486,0.352860825641685,0.904124030811319,0.390279224549583,0.696330084080445,0.965884611183634
"9718","HOXC9",75.1763505593254,-0.102897931274381,0.265254818582321,-0.387921063316882,0.698074450081381,0.965884611183634
"9719","CBX5",88.1349501539309,0.111835966813993,0.289252866324296,0.386637367626316,0.699024689952529,0.965884611183634
"9720","PYM1",21.9748299324589,0.134669937057232,0.349672624767262,0.385131484475993,0.700140002535109,0.965884611183634
"9721","PTPN11",30.7538628746015,-0.120944097153619,0.311048959452283,-0.38882656083012,0.697404451201593,0.965884611183634
"9722","RBM19",15.4013217276863,0.170678791335749,0.444389287994591,0.384074944978931,0.700922900999529,0.965884611183634
"9723","RNF34",20.1156962210239,0.13777774797989,0.360276964566843,0.382421751958355,0.702148558559687,0.965884611183634
"9724","AL356966.1",1.46931507044487,0.456878876668071,1.16354602524084,0.392660768682101,0.694570050056578,0.965884611183634
"9725","COL4A1",129.916040176074,0.136091105334083,0.355150375815553,0.383192908135233,0.701576735951729,0.965884611183634
"9726","WDR89",7.2165822834843,-0.212267154394593,0.553055190732906,-0.383808267151958,0.701120560203498,0.965884611183634
"9727","DLST",43.4618321163589,0.119883464096757,0.313098989081669,0.382893168861323,0.701798976571846,0.965884611183634
"9728","HHIPL1",4.48752309248198,0.286821604603991,0.746252381414545,0.384349332407237,0.700719548638995,0.965884611183634
"9729","GPR176",7.64854651750694,-0.207722632455045,0.532083994452389,-0.390394438887096,0.696244899415912,0.965884611183634
"9730","RAB27A",8.76685195178257,0.199117002296362,0.506228807248191,0.393334001236995,0.694072808479457,0.965884611183634
"9731","INTS14",14.1527590499627,-0.154902182168602,0.404300513387458,-0.383136249001378,0.7016187437094,0.965884611183634
"9732","COMMD4",70.836197940577,-0.115690443631137,0.295315165538402,-0.391752463576385,0.69524112172864,0.965884611183634
"9733","GOLGA6L9",3.69758777632524,0.372085513630347,0.97271593208571,0.38252227426377,0.702074008015968,0.965884611183634
"9734","FANCI",3.53799559064018,-0.283348527228115,0.739534397606446,-0.383144486781402,0.701612636064372,0.965884611183634
"9735","ARPIN",27.2668338108054,0.124860728552703,0.317826236799375,0.392858468231245,0.694424017872442,0.965884611183634
"9736","AXIN1",12.041367529994,-0.17140373846988,0.440305796726854,-0.389283401999387,0.697066513173329,0.965884611183634
"9737","GSPT1",77.0667709711897,-0.0829782224555553,0.210200215869077,-0.394758026829232,0.693021474062802,0.965884611183634
"9738","NSMCE1-DT",3.02397275870458,0.317984647556744,0.826866312417183,0.384565972493398,0.700559008790359,0.965884611183634
"9739","ZFHX3",108.315658219473,-0.127057826923328,0.329643071145802,-0.385440611512595,0.699910998805786,0.965884611183634
"9740","TERF2IP",148.138004826899,0.0754508987629134,0.19359397605248,0.389737843611725,0.696730409784635,0.965884611183634
"9741","GAS8",6.59828923196754,0.237614297190011,0.613609758021095,0.3

87240088808761,0.698578474215411,0.965884611183634
"9742","FXR2",15.4543426830549,-0.148102957886923,0.376188216813435,-0
.393693771541952,0.693807140365085,0.965884611183634
"9743","TOM1L2",18.4318769557264,-0.153325415806755,0.387188326688288,
-0.395997000008194,0.692107241358993,0.965884611183634
"9744","FOXO3B",1.6702067793896,0.440199365704117,1.1524082026799,0.38
1982152400897,0.702474602999393,0.965884611183634
"9745","RPL23",1404.04086699214,-0.0852089804086225,0.215340056030549,
-0.395694985778839,0.69233005500466,0.965884611183634
"9746","OSBPL7",2.03806490209859,-0.375626576067299,0.953247324366902,
-0.394049441803333,0.693544536864782,0.965884611183634
"9747","PSMD12",62.560488268167,0.0929103374343128,0.236318664184175,0
.393157001606539,0.694203525557596,0.965884611183634
"9748","AC110285.2",1.6836894455163,0.437661009790553,1.11655886179914
,0.391973074384397,0.695078108634438,0.965884611183634
"9749","AC036176.1",18.9514978778732,0.161835796202384,0.4202632754660
32,0.385081937085565,0.700176710165715,0.965884611183634
"9750","DSEL",48.6963045756376,-0.115912302829496,0.303264492321953,-0
.382215213993601,0.70230173786706,0.965884611183634
"9751","PALM",193.0053814912,-0.0725423920283512,0.186830413883604,-0.
388279351955755,0.69780931573298,0.965884611183634
"9752","RAB11B-
AS1",20.755833706806,-0.141912677316466,0.369142235132937,-0.384439015
127489,0.700653088170125,0.965884611183634
"9753","MYO1F",4.52279175218332,-0.306510270498468,0.786578067888465,-
0.389675587219565,0.696776450871962,0.965884611183634
"9754","AC011447.3",2.41977179978049,-0.383921105237159,0.975704300873
579,-0.393481001255628,0.693964253515049,0.965884611183634
"9755","ZNF714",9.91401289667352,0.219644387351263,0.564760626754544,0
.388915899844988,0.697338359941259,0.965884611183634
"9756","ZNF345",9.55059382265047,0.20902821122855,0.536360022521641,0.
389716239934934,0.6967463864395,0.965884611183634
"9757","ZNF546",8.20376216141538,0.208081574065237,0.539142131173744,0
.385949385206144,0.699534154684689,0.965884611183634
"9758","DMWD",29.6418977592835,0.116694911733918,0.29999306809174,0.38
8992027303218,0.69728204414483,0.965884611183634
"9759","ZNF749",10.8599787266329,0.17358469546748,0.449436353766659,0.
386227535918474,0.699328162219288,0.965884611183634
"9760","ZNF550",4.8024630565389,0.262898003576121,0.66804634858892,0.3
93532580683102,0.69392616519211,0.965884611183634
"9761","NRSN2-
AS1",3.69599048148273,0.29220858415192,0.751353837137146,0.38890941885
0259,0.69734315437748,0.965884611183634
"9762","DZANK1",3.30920361832694,-0.323599407944628,0.831420467032061,
-0.38921270377164,0.697118806675883,0.965884611183634
"9763","RBBP9",13.7065271115295,0.173504220561244,0.440808440650461,0.
39360457868098,0.693873000268668,0.965884611183634
"9764","RIN2",45.336887701072,-0.123194893068587,0.318321820465497,-0.
387013660855649,0.698746094606096,0.965884611183634
"9765","SRC",15.4788118152067,-0.143327458959282,0.369740425283607,-0.

3876434632468,0.698279900058479,0.965884611183634
"9766","SAMSN1",5.69488026598123,-0.239279728177222,0.618386319667373,
-0.386942143716777,0.698799040463036,0.965884611183634
"9767","AF165147.1",3.07855283049134,-0.327352158998828,0.833990012142
642,-0.392513284610942,0.694678997602644,0.965884611183634
"9768","SCAF4",16.8407820480714,0.163627802241801,0.414464092922049,0.
394793674617733,0.692995163466153,0.965884611183634
"9769","CBX6",82.4452382789274,-0.0917613272648804,0.23650803254361,-0
.387983977871705,0.698027890509832,0.965884611183634
"9770","ITM2A",952.795647817592,-0.130442609066426,0.339523201040646,-
0.384193506265895,0.700835030941345,0.965884611183634
"9771","ZMAT1",12.6582621993722,0.220406472700572,0.561731407876676,0.
392369857924984,0.69478495397826,0.965884611183634
"9772","MORC4",59.5585402960954,-0.1114271946510З,0.290675954167871,-0
.383338191733185,0.701469025127147,0.965884611183634
"9773","AMMECR1",24.3768814883026,0.145353055987639,0.374356601243242,
0.388274323211933,0.697813036758888,0.965884611183634
"9774","ENOX2",19.8114853477126,-0.178781021925973,0.463956323232663,-
0.385340199009032,0.6999853821837,0.965884611183634
"9775","COA6",42.5898497535966,0.143114613126691,0.375236494220117,0.3
81398438934189,0.70290761918937,0.966314953483232
"9776","HAGH",36.5956514447036,-0.11922101088819,0.312615406676659,-0.
381366395711589,0.702931392607491,0.966314953483232
"9777","MORN1",3.86237602288395,0.286743730510865,0.752583330129174,0.
381012598912665,0.703193899846926,0.966478095729479
"9778","CEP89",33.8518628868745,0.101938631175687,0.267501070958682,0.
381077469373693,0.703145765145406,0.966478095729479
"9779","CHRD",19.0578049607361,-0.139264842298858,0.36570563838116,-0.
380811307463922,0.703343268217328,0.966584536411972
"9780","PCYT1A",29.6862983327102,0.123963864058416,0.325810522568906,0
.380478393027344,0.703590332585397,0.966630868490185
"9781","AC104564.5",2.01056075717564,-0.420186563890098,1.104278793724
43,-0.380507681826365,0.703568595358226,0.966630868490185
"9782","KIF16B",19.8573733705309,0.135524516761478,0.356198107915879,0
.380475116935445,0.703592764013021,0.966630868490185
"9783","ATP5IF1",232.559018295226,-0.105333437436353,0.277236984242562
,-0.379940063639539,0.703989906883714,0.96668790499342
"9784","CCDC93",31.3458292461288,0.111347512848406,0.292820525698947,0
.380258564807317,0.703753489920136,0.96668790499342
"9785","TATDN1",64.3416431935076,-0.106589879993865,0.280372410209106,
-0.380172499549327,0.703817371595131,0.96668790499342
"9786","TRDMT1",9.9491069530061,-0.212321060829986,0.558610452306886,-
0.380087876897338,0.703880184538189,0.96668790499342
"9787","EPC1",105.942715917082,-0.0759166797231729,0.199815107513,-0.3
79934633912672,0.703993937508044,0.96668790499342
"9788","SLC35D1",9.87549533058472,0.210897905569761,0.555240932868044,
0.379831336426132,0.70407061944284,0.966694427328599
"9789","PPWD1",34.2398358439608,0.108717724489814,0.286417842567682,0.
37957734586358,0.704259179783022,0.966854542558386
"9790","TCEAL3",34.8519886572039,-0.118929166466727,0.313410899509702,

-0.379467231844134,0.704340933103992,0.966868008170025
"9791","SYNC",32.0421036834481,0.1210436239923,0.319960636089987,0.378307861465362,0.705201906041706,0.966916440025532
"9792","LRRC8B",2.98383424885352,0.306093347523877,0.808778213983135,0.378463888160889,0.705086015304204,0.966916440025532
"9793","AL451085.2",6.59923257610971,0.239367853616854,0.632723900470038,0.378313279202876,0.705197881836211,0.966916440025532
"9794","SCD5",9.52464692132567,0.198327905115697,0.526960281943797,0.376362150832555,0.706647680207383,0.966916440025532
"9795","FAM13A-AS1",3.97236890250793,-0.270867667032561,0.714788970971069,-0.37894774266673,0.704726669975174,0.966916440025532
"9796","RAI14",6.30543736801138,0.25569639141897,0.678144798765215,0.377052794454148,0.706134371045668,0.966916440025532
"9797","ZKSCAN3",4.1219205424136,0.288618195968055,0.76648942418683,0.376545568484849,0.706511345124884,0.966916440025532
"9798","KMT2E-AS1",23.6936251487764,0.188632635979969,0.498907674479845,0.378091269445063,0.705362793754112,0.966916440025532
"9799","MINCR",10.6295805381998,-0.170411318150436,0.450358303747494,-0.378390532010667,0.705140500630283,0.966916440025532
"9800","NTRK2",405.178339975601,0.135595103080382,0.359548104042032,0.377126458340414,0.706079629495435,0.966916440025532
"9801","CTSL",315.800883655303,0.124850778031517,0.329439635615957,0.378979225733061,0.704703290656584,0.966916440025532
"9802","LPAR1",88.7861148197206,0.104887453467865,0.278347598511937,0.376821837258879,0.706306011201306,0.966916440025532
"9803","AFAP1L2",12.3418113077261,-0.194899780967555,0.514691395442278,-0.37867308972608,0.704930639020877,0.966916440025532
"9804","AC136475.1",2.98293722284,0.345615617591788,0.91524251184236,0.377621901430335,0.705711492368577,0.966916440025532
"9805","IFITM3",1241.34332415087,-0.0762539476285202,0.202630278627413,-0.376320598012563,0.706678567894246,0.966916440025532
"9806","IFITM10",2.01263724075904,-0.436168208362791,1.15681834424745,-0.377041227373113,0.706142966982628,0.966916440025532
"9807","LRP4",4.67924664147706,-0.270462470875887,0.713030573590992,-0.379313988618712,0.704454713062736,0.966916440025532
"9808","PPP1R32",2.85945200373543,0.334240046678888,0.88493212316846,0.377701337682438,0.705652473963994,0.966916440025532
"9809","RBM4",114.145419173504,-0.085361913274105,0.225290669338404,-0.378896798188677,0.704764501924951,0.966916440025532
"9810","EED",18.9905237266834,0.145898837668444,0.387118806608915,0.376883879516187,0.706259901870591,0.966916440025532
"9811","PSME2",83.0273902724999,-0.117009430438401,0.310876550148616,-0.376385515029887,0.706630312981877,0.966916440025532
"9812","CPSF2",27.68184118189,0.120509285095083,0.31902128788881,0.37774684533618,0.705618664139406,0.966916440025532
"9813","CCDC144A",21.6265987685658,-0.229886737323454,0.609314939724585,-0.37728721607805,0.705960171519468,0.966916440025532
"9814","C17orf75",11.0619162180379,-0.175895142642928,0.46675195836511

8,-0.376849286843984,0.706285610746802,0.966916440025532
"9815","CWC25",45.0719601673712,0.100180820734762,0.26510006319381,0.377898139773441,0.70550626440411,0.966916440025532
"9816","AC006441.4",1.65001496952504,-0.418677381103476,1.109610854675 5,-0.377319110875061,0.70593647157507,0.966916440025532
"9817","AC018521.5",12.7291891910072,-0.179931814455226,0.477772033776 533,-0.376605999796516,0.706466428348567,0.966916440025532
"9818","COL1A1",7675.96075974919,0.156037772999969,0.413190137795908,0.377641571583306,0.705696877956179,0.966916440025532
"9819","PHLDB3",2.28808703196812,-0.359448704461977,0.951181668924467,-0.377897005593492,0.705507106985714,0.966916440025532
"9820","TAF9B",11.9384713056433,0.17241612882354,0.456843283189139,0.377407603806571,0.705870716965625,0.966916440025532
"9821","PLS3",176.884638438346,0.0847953775116204,0.224818579390573,0.377172463866107,0.706045442363902,0.966916440025532
"9822","GDI1",48.5209324611827,-0.0900154484010769,0.238994788276095,-0.376641888513015,0.706439753828848,0.966916440025532
"9823","MICAL1",12.7836593557594,0.184395316838768,0.49012599540856,0.376220234319667,0.706753173783706,0.966920075585791
"9824","ABL2",74.3853321620653,0.137422254859338,0.365695649611579,0.375783127322678,0.707078132457933,0.966970898758743
"9825","TMEM42",34.1305654673937,0.134326831929147,0.357365099725322,0.375881226321188,0.707005198022554,0.966970898758743
"9826","SIRT2",60.9014946860724,-0.100908234004423,0.268509903533662,-0.375808239012579,0.707059462215915,0.966970898758743
"9827","TBC1D17",35.7389892006505,-0.116963547761716,0.311105151737942,-0.375961462252607,0.706945546381723,0.966970898758743
"9828","RNF139-
AS1",3.44062070233676,-0.306066482909571,0.815085284139945,-0.37550240 3079849,0.707286859576542,0.967143264610826
"9829","VPS16",22.1656484852086,0.12917342726493,0.344077068626952,0.375420041156478,0.707348102378139,0.967143264610826
"9830","ERGIC2",98.8288489383076,-0.0862844442685124,0.229948336920564,-0.375234043542221,0.707486413727522,0.967233968879366
"9831","AL021368.3",1.59175068323671,0.537977220859215,1.4346703980462 4,0.374983147064191,0.707673000394212,0.967390647166902
"9832","TMBIM1",91.2135679503376,0.0748574822423545,0.199854177956789,0.374560507104033,0.707987348924166,0.967678978268557
"9833","CEP44",28.1985974139739,0.113635205438109,0.303426972068507,0.374505946730578,0.70802793312856,0.967678978268557
"9834","NTNG1",2.31082432076462,-0.402918416262926,1.07720030014279,-0.37404224284891,0.708372888224918,0.967691430471672
"9835","YIPF4",102.969724101196,0.0780615647281711,0.208596428503743,0.374222920728341,0.708238472580139,0.967691430471672
"9836","ANXA6",148.726201936774,0.0708164086010512,0.189343758741684,0.374009732729896,0.708397075152936,0.967691430471672
"9837","DDX6",90.8111815640355,0.0902503323524867,0.241181992729886,0.374200127177667,0.708255429383741,0.967691430471672
"9838","PARP2",14.6274691589946,0.153665762791591,0.410836700857539,0.37403124519023,0.70838107024691,0.967691430471672

"9839","EML4",27.4482093028426,0.11768726626524,0.314837838211665,0.37
3802802527563,0.708551034293615,0.967705015230884
"9840","ZBTB11-
AS1",3.56598194647454,-0.330278587134377,0.88354929109599,-0.373808898
340789,0.708546498747858,0.967705015230884
"9841","MISP3",3.90566579294359,-0.26125407070284,0.699161054096215,-0
.373667939843354,0.708651380542041,0.967743715385071
"9842","LINC00653",2.44923974224248,-0.409747114751202,1.0973127318255
7,-0.373409605910172,0.708843611220316,0.967907873520609
"9843","NFE2L2",193.514945291177,-0.0770264231999982,0.206373035202899
,-0.373238796067851,0.708970723917366,0.967944480538362
"9844","NDUFA3",114.106262236921,-0.129315954760838,0.346524331515994,
-0.373180013637424,0.709014470304311,0.967944480538362
"9845","PPP1R26",2.22803736421124,0.364600593366053,0.97739590314708,0
.373032659736029,0.70912413655437,0.967995862991791
"9846","DNAJC8",201.616794833518,-0.0777899140374796,0.20893973939487,
-0.372307892518552,0.709663622959363,0.968020647795763
"9847","ISG20L2",21.7828864811603,-0.151021597248926,0.404996766668106
,-0.372895809740347,0.709225990806467,0.968020647795763
"9848","RETSAT",20.3806745638191,0.130782985456708,0.351007274120892,0
.372593376545423,0.709451103196347,0.968020647795763
"9849","ATRIP",4.34919233740512,-0.256241148537271,0.687870306684814,-
0.372513751570734,0.709510375276067,0.968020647795763
"9850","ZHX2",27.4971819517631,0.120133183221695,0.323155486998873,0.3
71750405160577,0.710078692311231,0.968020647795763
"9851","TBC1D31",3.31916208044018,-0.275293162649476,0.740416220429187
,-0.371808659850673,0.710035315576004,0.968020647795763
"9852","BICD2",39.0913699346458,0.104479635357623,0.280497185610059,0.
372480155657848,0.709535384283976,0.968020647795763
"9853","AC145207.5",1.61622407223909,-0.413849717354369,1.112989269656
41,-0.37183621499076,0.710014798207406,0.968020647795763
"9854","PTBP1",70.6559954793413,0.0881258263676699,0.236822114049802,0
.372118231953362,0.709804822396629,0.968020647795763
"9855","SAFB2",41.4654154488108,0.113209373935733,0.304451442953852,0.
371847059870537,0.710006723241844,0.968020647795763
"9856","PATZ1",18.3646589761034,-0.135577565023292,0.364496168261692,-
0.371958821048438,0.709923509125354,0.968020647795763
"9857","HMGN5",7.22662845489017,-0.22389368879853,0.601282812098868,-0
.372360034734728,0.709624805611019,0.968020647795763
"9858","CXorf40B",9.83376919133249,-0.171519340647298,0.46018031367741
3,-0.372722029929194,0.709355338547647,0.968020647795763
"9859","LINC02018",2.18146672022048,-0.342009514640338,0.9218505247308
72,-0.371003221742685,0.710635132091251,0.968091793225983
"9860","CAST",504.599360002207,-0.0781654327717709,0.210516186844715,-
0.371303670009132,0.710411364766138,0.968091793225983
"9861","RAD21",101.070498961575,0.079635892328892,0.214563568736836,0.
371152907260627,0.710523646458562,0.968091793225983
"9862","AARSD1",31.6019556990619,-0.118133895394571,0.318166674064655,
-0.371295629065678,0.710417353160684,0.968091793225983
"9863","AC138150.1",8.89218911217018,0.175815817953694,0.4738815161508

49,0.371012187564892,0.710628454180981,0.968091793225983
"9864","ZNF419",3.97760157655922,0.261638043435018,0.704769396514433,0.37123922339562,0.710459361097942,0.968091793225983
"9865","DKC1",28.1733075321145,0.118013126296135,0.317953019595326,0.371165294943059,0.710514420401825,0.968091793225983
"9866","PRMT3",9.81270484912624,0.168620489464757,0.454706962323853,0.370833313400337,0.710761687166983,0.968166056541363
"9867","TRIM35",8.17283271940918,0.18957599434559,0.51141157228527,0.370691639804824,0.710867217879892,0.968211669310618
"9868","OGFOD3",39.9550262105425,-0.123712900488618,0.333960085033723,-0.370442175675353,0.7110530537497,0.968366638563256
"9869","DEK",316.443853646652,-0.0762251864947919,0.205830670641357,-0.370329583328268,0.711136933945886,0.968382739416229
"9870","CAP1",164.919689679142,-0.068408227184534,0.185313437069761,-0.369148769059751,0.712016839525251,0.968569852303411
"9871","BEND5",18.3390066508009,0.155330469502133,0.420136477324635,0.369714314003996,0.711595365322004,0.968569852303411
"9872","LRRC40",18.898874588661,0.14047159982504,0.379983590810228,0.369678068270044,0.711622374931251,0.968569852303411
"9873","EPHX1",317.281597397065,-0.104226490147015,0.281758808009286,-0.369913866698287,0.711446669055073,0.968569852303411
"9874","AC019131.2",1.89259614421213,0.371502521758868,1.0060778259438,0.369258234481374,0.71193525319315,0.968569852303411
"9875","FAT4",53.1522428717409,-0.13368761304357,0.361374956309147,-0.369941554359412,0.71142603852009,0.968569852303411
"9876","ABCF1",62.769479509585,0.0904922682703762,0.245228155887824,0.369012554625949,0.712118366928254,0.968569852303411
"9877","NUB1",77.94915281221,0.0767744358822407,0.207908860487059,0.369269667980406,0.71192673181338,0.968569852303411
"9878","PYGL",22.0346996969747,0.166167153937508,0.450167693851695,0.36912278736788,0.712036204580522,0.968569852303411
"9879","LINC00519",4.71056174436804,0.306818157077959,0.831555639846218,0.368968884793685,0.712150917310052,0.968569852303411
"9880","CATSPER2",3.12451253063432,-0.307194507591535,0.831187075007085,-0.369585279690395,0.711691520807947,0.968569852303411
"9881","CXXC1",18.0704978815257,-0.128784090014725,0.348953721610434,-0.369057792020048,0.712084648678868,0.968569852303411
"9882","ZNF100",14.4142516177903,0.164168935331819,0.445037445702377,0.368887914752255,0.7122112717064,0.968569852303411
"9883","SCNN1D",1.55047116410446,0.435814017540042,1.18288123564688,0.368434297887656,0.712549427279645,0.968596925316505
"9884","PABPC4L",2.29263706951333,-0.351310858056885,0.953741815720939,-0.368350063157634,0.712612227561057,0.968596925316505
"9885","ABLIM3",7.38585384966102,-0.206606920218385,0.561022336585405,-0.368268617388521,0.712672950416061,0.968596925316505
"9886","HLA-DRB5",9.20558093761588,0.647510941510518,1.75828352594588,0.368263099753599,0.712677064219817,0.968596925316505
"9887","LINC00310",2.31687452518372,-0.452668521176772,1.22926401430539,-0.368243531014416,0.712691654225448,0.968596925316505

"9888","YPEL1",8.15475176245878,-0.200204700191713,0.543761945450305,-0.368184463563219,0.712735694207524,0.968596925316505
"9889","MAMLD1",2.07721146483889,0.374449577143861,1.01699399476341,0.368192515464139,0.712729690750535,0.968596925316505
"9890","FLVCR1",5.3820663686986,0.237232078256467,0.647109210312148,0.366602846128604,0.713915284920147,0.969024089894138
"9891","PSMD1",74.8233628143367,-0.0849349623318148,0.231361170049463,-0.367109840919531,0.713537087029475,0.969024089894138
"9892","LHFPL2",34.6981791764671,0.10469881783826,0.285460485714053,0.366771665704855,0.713789344439064,0.969024089894138
"9893","HOXA5",8.02709660898865,0.210075494942308,0.572576341053302,0.366895171665418,0.713697213143364,0.969024089894138
"9894","GNA14",2.34883712602596,-0.325550622927574,0.887935491710946,-0.366637696056362,0.713889286013113,0.969024089894138
"9895","COX14",87.431559388142,-0.106638093660349,0.290060543642943,-0.367640811539049,0.713141079584599,0.969024089894138
"9896","CPSF6",42.440819650072,0.096204263002913,0.26196487069193,0.367241083694954,0.713439196619025,0.969024089894138
"9897","C1QTNF1",34.8881369714223,-0.138209102275468,0.376809560955105,-0.366787673659733,0.713777402807294,0.969024089894138
"9898","GZMM",2.94897418631525,0.298087864047927,0.811850923315386,0.367170690439833,0.713491700443604,0.969024089894138
"9899","APOE",74.3518906747958,0.312284333414708,0.850516800628576,0.367170093740551,0.71349214550617,0.969024089894138
"9900","ZNF600",3.61495369444619,0.300888291239729,0.818657274041789,0.367538774503541,0.713217174645469,0.969024089894138
"9901","DHRSX",31.8845525665796,-0.1283129104977,0.349620948538444,-0.367005784504902,0.713614703226383,0.969024089894138
"9902","TNFRSF14",49.2922369253449,0.0992034556910869,0.270952621181154,0.366128422226118,0.714269249900033,0.969055502481513
"9903","EXOSC7",50.7677241386255,-0.117802868369951,0.321787866150876,-0.366088596748755,0.714298966261022,0.969055502481513
"9904","TRAIP",3.21164928325007,-0.298899593635003,0.816143203311605,-0.366234249604947,0.71419028736289,0.969055502481513
"9905","STEAP2",21.8013110321735,-0.163489562090154,0.446514559591532,-0.366146094406672,0.714256063683469,0.969055502481513
"9906","FOXN3",172.636064874734,0.0897514341726064,0.245073391599215,0.366222679610126,0.714198920104141,0.969055502481513
"9907","HHAT",2.18254142673223,0.387342839622663,1.05898553149779,0.36576782977839,0.714538327010389,0.969282383838965
"9908","CPTP",13.1978725759259,-0.153544605807082,0.422809545123495,-0.363153120779794,0.716490504656401,0.969287934922405
"9909","AJAP1",1.9477667595031,-0.436319109705727,1.20173983294036,-0.363072852996945,0.716550463084502,0.969287934922405
"9910","ID3",280.777333284031,-0.147817255913278,0.409057205163648,-0.361360841582395,0.717829716916367,0.969287934922405
"9911","PMF1",66.5497773386827,0.125335512356286,0.343797779140108,0.364561727739398,0.715438588189428,0.969287934922405
"9912","CD1C",1.50999339616781,-0.43847628718716,1.20086197149725,-0.365134626288867,0.715010914631747,0.969287934922405

"9913","RNF2",17.2862820144127,-0.130181625431652,0.359001650411014,-0.362621245007117,0.716887837781721,0.969287934922405
"9914","SF3B6",199.602857842706,-0.0910137043810112,0.250999113733304,-0.362605680264341,0.716899466440826,0.969287934922405
"9915","AC007098.1",5.21518304668279,-0.228007363237511,0.631023411868098,-0.361329483105091,0.717853156076254,0.969287934922405
"9916","ANKRD39",19.0187211105039,0.137983735354503,0.380204073654939,0.362920191853947,0.716664502726231,0.969287934922405
"9917","HACL1",16.8024265419106,-0.153157176475943,0.42119436723972,-0.363625889585495,0.716137391289115,0.969287934922405
"9918","OPA1",32.1660288652,0.101122978190439,0.27725681730786,0.364726751076255,0.715315387723239,0.969287934922405
"9919","CLCN3",54.4242620965658,0.099847264439003,0.274587472461452,0.363626437666489,0.716136981959912,0.969287934922405
"9920","SLC9A3-AS1",1.54361938664853,-0.501458778687128,1.38149850703142,-0.36298177387442,0.716618499483336,0.969287934922405
"9921","SKP2",7.46520985103866,0.195905535540577,0.536393697364914,0.365227139138625,0.714941861401526,0.969287934922405
"9922","C5orf34",2.56990095570378,-0.341897902995879,0.940946895166388,-0.363355152933919,0.716339598415092,0.969287934922405
"9923","AC011346.1",5.32579176162384,0.255245395725331,0.704502115606553,0.362306074135169,0.717123319583086,0.969287934922405
"9924","RAB23",55.3846539877169,0.092900389181901,0.256854727887348,0.36168455977437,0.717587766534514,0.969287934922405
"9925","COL12A1",476.460595621549,-0.133336019926573,0.365836220141578,-0.364469160202267,0.715507699016247,0.969287934922405
"9926","KLHL7",26.459920266041,-0.111781886428626,0.308915593425531,-0.361852521554801,0.717462241293255,0.969287934922405
"9927","ABHD11",15.0924922912308,0.174800940959908,0.482967698415624,0.361930914910755,0.717403657094316,0.969287934922405
"9928","OTUD6B-AS1",81.2081332719334,0.0892711224424211,0.247114783086386,0.361253670571436,0.717909823894832,0.969287934922405
"9929","AZIN1-AS1",11.9844187589498,0.152503237833856,0.421206214593326,0.362063123833768,0.717304859708292,0.969287934922405
"9930","ZNF572",2.16897139240346,0.346923917931754,0.956623904889121,0.362654451931101,0.716863028596643,0.969287934922405
"9931","TRAPPC9",10.9920769630723,0.163899335295633,0.450499474040678,0.363816929297532,0.715994719995674,0.969287934922405
"9932","LURAP1L",54.0040747882835,0.115596653484744,0.319734587321227,0.361539408211124,0.717696250837914,0.969287934922405
"9933","UGCG",32.1845347256934,-0.11782297985692,0.322878771228133,-0.36491398740387,0.715175612819507,0.969287934922405
"9934","VIM-AS1",2.86917053205414,0.318791201802879,0.874986454206288,0.364338442350012,0.715605296799678,0.969287934922405
"9935","AC016394.1",3.63991785497568,-0.278839504739967,0.768637501033646,-0.362771142918463,0.716775850103053,0.969287934922405

"9936","CHCHD1",72.8504601675987,-0.101834814519703,0.280944724925413,-0.362472776617318,0.716998763475453,0.969287934922405
"9937","DAGLA",3.65216506837196,0.271987548405142,0.74978527476675,0.362753921100617,0.716788716092844,0.969287934922405
"9938","PAAF1",18.5340892158788,0.133862680716113,0.370643103848918,0.361163284372555,0.717977387162216,0.969287934922405
"9939","PEX5",7.43646502712182,-0.198573046824945,0.545097392721626,-0.364289115076273,0.715642127197446,0.969287934922405
"9940","A2M-AS1",4.90413798999801,0.2438210604257,0.675166848276149,0.361127121463722,0.718004419387793,0.969287934922405
"9941","GIHCG",24.6617048937997,-0.142064774973207,0.391608427611946,-0.362772517025559,0.71677482354544,0.969287934922405
"9942","SALL2",4.79699415829181,-0.244668720598054,0.674305470646326,-0.362845522168962,0.716720284146814,0.969287934922405
"9943","MNAT1",44.0749640140124,-0.0944198395650206,0.259227393581,-0.364235578118088,0.715682101521177,0.969287934922405
"9944","NEIL1",6.37772611309511,-0.226159722300215,0.626014817191418,-0.361268960557305,0.717898394918958,0.969287934922405
"9945","NUDT21",49.3509806720621,0.09025241639994,0.249438209027255,0.361822740597365,0.71748449735899,0.969287934922405
"9946","NUP93",12.9646220229911,-0.176244784867187,0.48374471153322,-0.364334287621631,0.715608398918002,0.969287934922405
"9947","KIAA0753",8.88244601134362,-0.177995170372492,0.489680279049716,-0.363492625673865,0.716236920625613,0.969287934922405
"9948","MPPE1",31.5171728397492,0.115623990465286,0.316333452883487,0.365513003482033,0.714728501936083,0.969287934922405
"9949","SERPINB8",8.20511825553976,-0.198919880328631,0.548981089501822,-0.362343774917899,0.717095149801508,0.969287934922405
"9950","AC027575.2",2.86262793676179,-0.299678084928344,0.8230993226582,-0.364084961169125,0.715794566530778,0.969287934922405
"9951","FBXO27",2.65332044698461,0.341462472361805,0.940032751105102,0.363245293273434,0.716421655803273,0.969287934922405
"9952","RTEL1",3.50590411082369,-0.288891924338764,0.791584846039169,-0.364953833798467,0.715145868082931,0.969287934922405
"9953","CFAP298",58.2954151720218,-0.082880399604031,0.227496407043436,-0.364315202517491,0.715622648827137,0.969287934922405
"9954","APOL4",6.17780028714003,0.213475684451616,0.590263293747646,0.361661798578454,0.717604777566188,0.969287934922405
"9955","FAM122B",13.2840636477745,0.173765932361803,0.480064941024303,0.36196338768468,0.717379390327586,0.969287934922405
"9956","UCKL1",24.707877234055,0.126330592794121,0.3500865896637,0.360855275591894,0.718207638868345,0.969464891397318
"9957","UNC93B1",15.2429177366707,0.150903618878421,0.418663321164639,0.360441460356825,0.718517026388159,0.96978510772627
"9958","FZD3",3.86152176866282,-0.266549374766882,0.739921685556973,-0.360239982108699,0.718667677606686,0.9698910342796
"9959","NOP58",37.0056736682677,-0.107795521111889,0.299332483217757,-0.360119690162294,0.718757628650478,0.969915028761299
"9960","CHI3L2",28.2165829764386,-0.194111408427927,0.540477078187908,

-0.359148271521036,0.719484171769421,0.969915409572347
"9961","AC244033.2",2.5721738212194,0.316634898740962,0.88277457780584
1,0.358681487552537,0.719833378902436,0.969915409572347
"9962","PIGF",41.0454476027635,-0.111160664300774,0.31227333610013,-0.
355972321201098,0.721861293737824,0.969915409572347
"9963","AC005041.4",2.93086154967839,-0.307307510890847,0.863089957040
544,-0.35605501881238,0.721799362388787,0.969915409572347
"9964","CNNM3",22.7903213165188,0.135832479145484,0.379816143178997,0.
357626924460316,0.720622526378749,0.969915409572347
"9965","OGG1",22.6408469395864,0.126177630964911,0.352401520003786,0.3
58050756885371,0.720305329555078,0.969915409572347
"9966","DPH3",25.5993332126611,-0.111187235789627,0.311821191721349,-0
.356573699099279,0.72141096991755,0.969915409572347
"9967","CLEC3B",927.445347747821,0.138425309906127,0.387475488342768,0
.357249204325599,0.720905253216617,0.969915409572347
"9968","NCBP2AS2",52.5840034392018,-0.110314259699985,0.30831007031937
9,-0.357802972785515,0.72049076570671,0.969915409572347
"9969","CXCL10",1.91325596677262,-0.467036773045795,1.2985302571304,-0
.359665683938618,0.719097157246501,0.969915409572347
"9970","SHLD3",1.76970246275049,-0.413432780324557,1.14909180326071,-0
.359790905436263,0.719003504797966,0.969915409572347
"9971","RNF14",21.8121189750488,0.122913507095906,0.343484362096432,0.
357843094648364,0.72046073827424,0.969915409572347
"9972","HMGXB3",22.0396858092834,0.119078833500289,0.334051406467949,0
.356468588949689,0.721489671544491,0.969915409572347
"9973","KDM1B",15.5037886824106,0.138812650944741,0.386447151783138,0.
359202158184461,0.719443862226206,0.969915409572347
"9974","CYP21A2",1.78611685397102,0.392937960555516,1.10059727384222,0
.357022473064791,0.721074981883517,0.969915409572347
"9975","ZNF736",9.34175945534027,0.186656318314309,0.520344039211387,0
.358717126071432,0.719806715202289,0.969915409572347
"9976","SLC25A40",5.82208888017015,0.212822942656956,0.593862628400094
,0.358370660956248,0.720065944740489,0.969915409572347
"9977","STEAP1",26.9333116365393,0.16030342637138,0.450299843173806,0.
355992632023873,0.721846083012824,0.969915409572347
"9978","COPG2",8.10841117186641,-0.179152730573604,0.500241009634743,-
0.358132834220079,0.720243908315972,0.969915409572347
"9979","AC073111.4",8.22500958057172,0.182396712669951,0.5067993379301
81,0.359899271800309,0.71892246161391,0.969915409572347
"9980","INSIG1",33.1233806134126,0.0995186461751619,0.277921478498926,
0.358081882381561,0.720282037083521,0.969915409572347
"9981","LEPROTL1",36.7735572510225,0.103569340828884,0.289098032711116
,0.358249898339421,0.720156308409291,0.969915409572347
"9982","AC087623.3",1.5185090965974,-0.401754171328035,1.1272989009045
1,-0.356386554626887,0.721551097106666,0.969915409572347
"9983","SPIDR",16.0005974010849,0.14944869934765,0.417040788458769,0.3
58355114136336,0.720077577820657,0.969915409572347
"9984","CCDC171",11.3895636024962,0.169329589799863,0.472021228759272,
0.358732996490334,0.719794841527552,0.969915409572347
"9985","TMEM268",3.6204191368009,-0.277529428039699,0.777218831135078,

-0.357080164455595,0.721031793404022,0.969915409572347
"9986","RAB14",150.070749770624,0.0633798163052274,0.177533198168809,0
.357002616744177,0.721089846773331,0.969915409572347
"9987","SURF4",107.801704416136,-0.0710483118627201,0.199437152560739,
-0.356244114752301,0.721657757331774,0.969915409572347
"9988","MSRB2",88.409916181997,-0.0987304166891807,0.275261030489523,-
0.358679238080302,0.719835061903373,0.969915409572347
"9989","R3HCC1L",13.2678847784262,-0.146030337043795,0.408557699411856
,-0.357428919474567,0.720770730089206,0.969915409572347
"9990","PCGF6",13.6571263448205,0.145850648904257,0.406678155758131,0.
358639004429343,0.719865163981155,0.969915409572347
"9991","RBMS2",27.3265169914863,0.114399614133306,0.321204133045708,0.
356158599357211,0.721721794608521,0.969915409572347
"9992","AL161891.1",1.78261454852087,-0.414288412773114,1.158957602777
91,-0.357466409280291,0.720742668736544,0.969915409572347
"9993","PAPOLA",124.335243079624,0.0716289800565274,0.199867017451883,
0.358383193834229,0.720056566922932,0.969915409572347
"9994","TPM1",425.152303956788,-0.0880271636688599,0.246779142644811,-
0.356704228426457,0.721313239690035,0.969915409572347
"9995","AC012291.3",1.977849043826,0.375274839586222,1.04598248334395,
0.358777365359396,0.719761646699453,0.969915409572347
"9996","THAP11",32.6325720196871,-0.115358235795906,0.323217829665165,
-0.356905545450295,0.721162518051709,0.969915409572347
"9997","POLRMT",7.42425946389488,-0.190595111185833,0.530901973325503,
-0.359002453865388,0.719593253562469,0.969915409572347
"9998","ZNF625",4.23557181903499,0.258701551029534,0.72056939596868,0.
359023783797749,0.719577296926371,0.969915409572347
"9999","CHERP",21.4088500408164,0.121357351029587,0.340097628798311,0.
356830923692079,0.721218384461899,0.969915409572347
"10000","EIF1AX",276.009672708199,-0.0629005998540218,0.17500615882646
4,-0.359419350014956,0.71928140130123,0.969915409572347
"10001","ATG4A",6.78045627373326,0.205552427872345,0.576494692307483,0
.356555629418893,0.721424499449991,0.969915409572347
"10002","VMA21",32.6701409795832,-0.105276565568484,0.294404244029951,
-0.357591874788918,0.72064875975871,0.969915409572347
"10003","RRAS",310.051836230556,-0.0932961688057509,0.262283727859672,
-0.355707041252924,0.722059971332176,0.970085369862352
"10004","ZNF436-
AS1",3.38692144359234,0.299457024657426,0.842679328983282,0.3553629647
21978,0.722317690418795,0.970143864891313
"10005","STK19",34.9325717164159,0.115846393319324,0.326036198429128,0
.355317580923476,0.722351685997317,0.970143864891313
"10006","GALK2",27.4819943826557,0.121190681189753,0.34105531115221,0.
355340254870469,0.722334701586999,0.970143864891313
"10007","CRELD2",51.1867353407629,-0.111756267571036,0.31457297560706,
-0.355263408610896,0.722392265493516,0.970143864891313
"10008","CADM3",86.9617872257424,-0.120026454403775,0.338122453142045,
-0.354979248755632,0.722605137274199,0.970272487604504
"10009","CSTF1",13.5060001018948,-0.145648256874367,0.410342887882225,
-0.354942808016135,0.72263243756481,0.970272487604504

"10010","AL355001.2",8.1010017227063,-0.181195176667351,0.510796602232694,-0.35473058331897,0.722791436850722,0.970389022960724
"10011","GPAA1",87.3401691782997,-0.092928918739293,0.262186032074538,-0.354438861612863,0.723010015033858,0.970585515137351
"10012","MICA",22.4940038151676,-0.125976699725313,0.355844583817309,-0.354021686585482,0.723322630834046,0.97090819374538
"10013","RBBP5",16.0720586049446,-0.13283364149906,0.375409365637375,-0.353836781012465,0.723461207119776,0.970917657395639
"10014","ZNF696",6.5285085185527,0.215164470661389,0.60811934576744,0.353819479940823,0.723474173759947,0.970917657395639
"10015","CDV3",134.819165862119,0.0668957913110463,0.189141902980226,0.353680439167623,0.723578383578434,0.970924312062287
"10016","SURF6",21.2825531591893,-0.128735524351505,0.364547182845076,-0.353138168142737,0.723984859824107,0.970924312062287
"10017","VTI1A",20.8263881887247,0.119296678533884,0.33773844059828,0.353222091990944,0.723921946978618,0.970924312062287
"10018","SMARCC2",79.8171991396501,0.0860660297476868,0.24365095339562,0.353234939359913,0.72391231621521 5,0.970924312062287
"10019","DEDD2",10.6257210323971,-0.183026374263045,0.518013954187723,-0.35332325081868,0.723846116546567,0.970924312062287
"10020","MARK4",13.3640322498359,0.139171805253908,0.393959802142391,0.353263973880274,0.723890551250858,0.970924312062287
"10021","EDEM2",30.0299866899737,-0.138492430892762,0.392161561808354,-0.353151467099782,0.723974890242372,0.970924312062287
"10022","OTUD6B",15.6064460266685,0.151835015419707,0.43052084909769,0.352677496892268,0.724330231561441,0.971141188836178
"10023","TAF1D",98.3775350803336,-0.0744822928109903,0.211308957396619,-0.352480527700442,0.72447791927424,0.971141188836178
"10024","ADORA2B",2.10584798040134,-0.374082626351648,1.06140630175618,-0.352440555263991,0.72450789190204,0.971141188836178
"10025","KPNA2",42.9382263211769,0.180865542937824,0.512907197989754,0.352628201839813,0.724367192082749,0.971141188836178
"10026","BCORL1",15.04683130265,0.143565752607696,0.407328466631156,0.352456958864351,0.72449559189999,0.971141188836178
"10027","PCBD1",163.912389649704,-0.124095031805197,0.352405180866557,-0.352137365007093,0.724735247523799,0.971189134632269
"10028","ERC1",54.2989326057089,0.120677849227217,0.342772574009553,0.352063900024433,0.724790340932557,0.971189134632269
"10029","TRMT61A",15.0274735939462,-0.139287083769247,0.395766243389651,-0.351942809918005,0.724881152839937,0.971189134632269
"10030","MGRN1",22.3753704772102,0.119053753796633,0.338306408929445,0.351911021057427,0.724904993637644,0.971189134632269
"10031","AL022322.1",2.5748699122379,0.314995037793395,0.894229954063293,0.35225283649031,0.724648655166974,0.971189134632269
"10032","KDM5C",28.8673013864358,-0.112375495182549,0.319635226612191,-0.35157418778154,0.725157625992987,0.971430755155478
"10033","MAN1B1",60.6070366527292,0.0944562328437173,0.268792610438038,0.351409336327315,0.725281279110736,0.971499562440863
"10034","IFI16",280.020432202757,0.0738616935352551,0.210494449192099,0.350896158158776,0.725666253805312,0.971902971442036

"10035","ACAD11",18.5666570364606,0.154871363276616,0.441694001928719,
0.350630442343224,0.725865615068832,0.971902971442036
"10036","INO80E",30.2466106437613,0.104065325692014,0.296687672268173,
0.350757161214136,0.725770538116012,0.971902971442036
"10037","MOCS3",5.68554066950624,0.210650123416516,0.600789295539909,0
.350622298000853,0.725871725899525,0.971902971442036
"10038","ICMT",42.164127989148,-0.0898798510938068,0.256707993954678,-
0.350124862530285,0.726244992778198,0.972115895729485
"10039","UBIAD1",12.6777650566791,0.164962735609147,0.475013313295677,
0.34728023613617,0.728380797204142,0.972115895729485
"10040","ZMPSTE24",50.1069844792724,-0.0807309412130466,0.235596106644
992,-0.342666703464231,0.731849214531984,0.972115895729485
"10041","EXO5",5.0990758265612,-0.229712959692802,0.66372670857503,-0.
346095699818948,0.729270795017533,0.972115895729485
"10042","GCLM",25.0310430807933,0.114885578100598,0.330791678835219,0.
347304921650787,0.728362253706457,0.972115895729485
"10043","GDAP2",10.4683283899861,0.163596791904,0.46826460247069,0.349
368265379915,0.72681285467104,0.972115895729485
"10044","NAXE",52.4319752736721,-0.0958009307515144,0.278819712384375,
-0.343594539755659,0.731151231626962,0.972115895729485
"10045","POGK",16.6304828303385,-0.132686929061407,0.38568868124702,-0
.344025986535045,0.730826743139969,0.972115895729485
"10046","COLEC11",1.73068067790614,-0.466204719116953,1.33219625615679
,-0.3499519811457,0.726374735174674,0.972115895729485
"10047","SNX17",89.8205402354163,-0.0965117631563122,0.277737498852646
,-0.347492735244644,0.728221175326525,0.972115895729485
"10048","GALM",23.7048718860322,-0.138951582659908,0.400518478467334,-
0.346929268261567,0.728644457754649,0.972115895729485
"10049","MORN2",38.2437616378497,0.113210730532736,0.329344745510598,0
.343745367357297,0.731037789637342,0.972115895729485
"10050","HAAO",9.28717774537772,-0.194158481545791,0.557806457114326,-
0.348074998181666,0.727783860241567,0.972115895729485
"10051","ZNF514",15.176070447659,0.136446323052616,0.392408154900295,0
.347715309553862,0.728053997949402,0.972115895729485
"10052","ARL6IP6",26.2838088633515,-0.116408907871788,0.33867898892053
,-0.343714584252239,0.731060942061762,0.972115895729485
"10053","ASNSD1",34.6186177837238,-0.102132269000347,0.297559722383327
,-0.343232841401756,0.73142329982122,0.972115895729485
"10054","FASTKD2",25.4484915163566,0.110754493470984,0.32289383993172,
0.343005904028409,0.731594018540316,0.972115895729485
"10055","SEC13",67.1802521076152,0.0937034366941524,0.27239984098921,0
.343992259150636,0.730852107565183,0.972115895729485
"10056","OXNAD1",6.32712427501112,0.204782311678806,0.588299649143727,
0.348091847372112,0.727771206787538,0.972115895729485
"10057","VILL",5.63413273022569,-0.23262727209089,0.667707292808292,-0
.348397081470369,0.727541994049836,0.972115895729485
"10058","NBEAL2",3.62000658300976,-0.252096933961901,0.724687369982386
,-0.34786991522707,0.727937879982851,0.972115895729485
"10059","SHISA5",88.2320063241766,-0.0839211815875655,0.24485732379411
6,-0.342735027432258,0.731797808920806,0.972115895729485

"10060","RAB43",5.42682302309544,0.226683494586071,0.659116329114807,0
.343920313566662,0.730906214682668,0.972115895729485
"10061","ANAPC13",61.9066220317374,-0.0896967833268434,0.2582175301956
01,-0.347369070019754,0.728314066873031,0.972115895729485
"10062","MSL2",17.053197923738,-0.134198649392783,0.38560221214078З,-0
.348023546461884,0.72782250006297,0.972115895729485
"10063","ABCF3",15.7439576501095,-0.135751943107357,0.39422736347428,-
0.344349367103771,0.730583562163306,0.972115895729485
"10064","NAP1L5",11.1831070263666,0.150987619419107,0.437099411107084,
0.345430846124194,0.729770490820512,0.972115895729485
"10065","CMBL",129.559824094181,-0.0782900760664405,0.228356538717081,
-0.342841402774268,0.73171776599551,0.972115895729485
"10066","TARS",51.6510175368555,0.0824984711121195,0.238666090292194,0
.345664819879181,0.729594625945633,0.972115895729485
"10067","AC099522.2",2.72064870988019,0.311480575744306,0.896888916510
348,0.347290026680481,0.728373442631707,0.972115895729485
"10068","PCDHB12",7.58303093286731,0.23785714409154,0.679740612697071,
0.349923396731838,0.726396187693229,0.972115895729485
"10069","ATOX1",132.381262733775,-0.102827545457593,0.297525374845514,
-0.345609329997431,0.729636333273715,0.972115895729485
"10070","LY6G5B",5.30520740809387,-0.247994743205827,0.720401629918008
,-0.344245116760844,0.730661955074254,0.972115895729485
"10071","OARD1",31.0318360085777,0.11477910577781,0.330116433086803,0.
347692796461391,0.728070907128573,0.972115895729485
"10072","MCM9",8.06128412023574,0.186853248274872,0.54359377363814,0.3
43736917780181,0.731044144663721,0.972115895729485
"10073","TFB1M",10.2060013032949,-0.16559462821541,0.478914947645168,-
0.345770431742924,0.729515248122821,0.972115895729485
"10074","THBS2",286.872714129211,0.0786132082313858,0.229506179957968,
0.342531988662716,0.731950574827665,0.972115895729485
"10075","AHR",84.3854279330797,0.0927516895597884,0.267864758287361,0.
346263129770456,0.729144974853663,0.972115895729485
"10076","SNX10",4.15547464741128,-0.250898188394719,0.719351730595888,
-0.348783741976797,0.727251669896761,0.972115895729485
"10077","FIGNL1",7.83644463248847,0.218739997767441,0.632398344381071,
0.345889580058154,0.729425699789745,0.972115895729485
"10078","COG5",24.4958726407407,-0.110573447520665,0.320223836731648,-
0.345300489336547,0.729868478831664,0.972115895729485
"10079","ADHFE1",2.63357228838553,0.296603970089872,0.846771983880039,
0.350276078727578,0.726131515906262,0.972115895729485
"10080","IFT74",33.6674517004937,-0.117118881628658,0.334557100466376,
-0.350071427165627,0.726285093610029,0.972115895729485
"10081","SPAG8",3.16586575752047,0.286918922620395,0.826372361361068,0
.347202951158516,0.728438853890452,0.972115895729485
"10082","GLE1",11.9638804006551,0.15991542051997,0.463232557687599,0.3
45216280388944,0.729931780276405,0.972115895729485
"10083","OTUD1",27.4137868781485,0.115873613260485,0.338341200026659,0
.342475622984592,0.731992986119689,0.972115895729485
"10084","BMS1",29.8066487521562,0.10992533877715,0.316103446477485,0.3
47751155522373,0.728027074959814,0.972115895729485

"10085","ZNF503-
AS2",3.26329970062863,-0.268723900283551,0.776776640838884,-0.34594745
2788155,0.729382205697152,0.972115895729485
"10086","MADD",14.4153119348812,-0.149315576908577,0.435285208806469,-
0.343029291801559,0.731576423950078,0.972115895729485
"10087","ZDHHC24",28.9984618518117,0.11068289246952,0.320888981010998,
0.344925812412756,0.730150144619007,0.972115895729485
"10088","C11orf1",11.729688742639,-0.155833865855483,0.452906536674637
,-0.344075108740222,0.730789801682313,0.972115895729485
"10089","DLAT",15.3408482401651,0.141144808015203,0.411684036636236,0.
342847415625976,0.731713252870646,0.972115895729485
"10090","ITFG2-
AS1",1.94502189511744,-0.367257113598738,1.0554697813494,-0.3479560666
61809,0.727873177890565,0.972115895729485
"10091","CCND2",36.9633370901011,-0.120014887830603,0.348855684275885,
-0.344024458365114,0.730827892382282,0.972115895729485
"10092","LMBR1L",17.0308730168516,-0.160978202989202,0.464507732907547
,-0.346556562108391,0.728924484084443,0.972115895729485
"10093","HOMEZ",4.50037980634995,0.246954291326093,0.718644910359452,0
.343638823243835,0.731117924069716,0.972115895729485
"10094","MAPK1IP1L",53.3717296979445,-0.0803594223049286,0.23425564701
9077,-0.343041558773541,0.731567195580014,0.972115895729485
"10095","MAP2K1",29.4221163221086,-0.106184063740546,0.308178447416857
,-0.344553827922028,0.730429822364744,0.972115895729485
"10096","AC116913.1",2.56527111728218,-0.285572911654323,0.83398351746
2807,-0.342420330467813,0.732034590727166,0.972115895729485
"10097","AC015712.2",7.08076404395758,0.237260154016513,0.688432861320
065,0.344638042933581,0.730366501894746,0.972115895729485
"10098","TBC1D24",16.8592702967346,0.126773963957179,0.362610564270375
,0.349614645707487,0.726627918102142,0.972115895729485
"10099","ATXN2L",34.9400537912244,-0.0976704813642649,0.28182100720348
4,-0.346569201258101,0.728914987284929,0.972115895729485
"10100","MIS12",10.1742884637443,-0.163697289444962,0.473045125590399,
-0.346050050173659,0.729305101051862,0.972115895729485
"10101","RAI1",9.55130160848312,-0.16928435027689,0.48845292842698,-0.
346572495372391,0.728912512161418,0.972115895729485
"10102","EPN2",70.416865836201,0.103110617096724,0.299655870544777,0.3
44096769768828,0.730773512099542,0.972115895729485
"10103","TRAF4",2.51100981836592,-0.327208184311402,0.938334693843211,
-0.348711591352579,0.727305841243815,0.972115895729485
"10104","COIL",32.346374306442,0.098755690716217,0.287917364061508,0.3
43000121017778,0.73159836911319,0.972115895729485
"10105","SMG8",8.26435832760924,0.181414684289967,0.52396003866221,0.3
46237634368378,0.729164133653068,0.972115895729485
"10106","ABHD3",6.52777281218119,0.201259226836732,0.587041014267558,0
.342836738737651,0.731721285562835,0.972115895729485
"10107","LINC00909",20.059336829262,-0.141774746388644,0.4094842499133
51,-0.346227593414505,0.729171679084357,0.972115895729485
"10108","MFSD12",17.1733709982384,-0.140424610269474,0.407797519532235
,-0.344348858302396,0.730583944758771,0.972115895729485

"10109","DUS3L",10.1931403952988,-0.16695785394767,0.486374692261625,-0.343270027417179,0.731395327076456,0.972115895729485
"10110","AC024075.2",12.3568663746388,-0.144505602395617,0.419632677130425,-0.344362129717327,0.730573965280982,0.972115895729485
"10111","ZNF681",5.65880699429452,0.218365008853263,0.624324894424802,0.349761816008387,0.726517457585361,0.972115895729485
"10112","AC012313.1",2.858640979353,-0.295421143584471,0.847534092568514,-0.348565498632835,0.727415533164559,0.972115895729485
"10113","FASTKD5",5.83854975971787,0.207387100982817,0.598717768486189,0.346385412123611,0.729053086789967,0.972115895729485
"10114","MGME1",11.7572386337623,-0.167964300458014,0.48386594261567,-0.347129825980388,0.728493787158369,0.972115895729485
"10115","ZNF337",12.0522675022299,0.15285661600292,0.445044111313412,0.343463967092634,0.7312494440141,0.972115895729485
"10116","LINC01637",5.57052645997942,0.23653950989678,0.679826528946872,0.347940981743102,0.727884506938525,0.972115895729485
"10117","BCR",12.0428348341552,0.153703253785966,0.447502535569131,0.343469012059312,0.731245649277196,0.972115895729485
"10118","ZFX",55.1552010839646,-0.0855008998081931,0.24654953045592,-0.346789951901692,0.728749126459427,0.972115895729485
"10119","MOSPD1",7.63322640338069,-0.181940406285191,0.527849402768764,-0.344682413830245,0.730333140576757,0.972115895729485
"10120","F8A3",2.94447970606685,-0.337662925507926,0.982573002908395,-0.343651743441404,0.73110820631693,0.972115895729485
"10121","TRAM2-AS1",9.74662232501393,-0.180813960014793,0.52834725590081,-0.342225606351476,0.732181116290561,0.972118358212691
"10122","IFT43",46.0033973410345,0.0993357940034985,0.290189161208826,0.342313936157024,0.732114648867541,0.972118358212691
"10123","ZNF223",3.51987977022779,-0.256838079798153,0.751101071447492,-0.341948759709775,0.732389454035582,0.97229891067709
"10124","COLCA1",7.42214600041526,0.197249952081335,0.577306945183817,0.341672577693534,0.732597311277143,0.972478789633893
"10125","LINC01963",1.49266771337285,0.393880686226972,1.1539368381956,0.341336434707187,0.732850322185381,0.972527509269195
"10126","CFAP97",93.6974876673278,-0.0708290722975207,0.207446149212787,-0.3414335362035,0.732777232037771,0.972527509269195
"10127","MTHFD1",11.1471731559038,0.152713243081231,0.44748197151877,0.341272392634985,0.73289852919997,0.972527509269195
"10128","RPA4",1.78629185286019,0.427047825775514,1.25146162384039,0.341239249882089,0.732923477481837,0.972527509269195
"10129","AL445423.1",2.17855501268271,-0.329388557789045,0.966409551658597,-0.340837440217487,0.733225963050783,0.97271271174229
"10130","ABHD5",46.9683983505107,0.14278863487611,0.419279028043314,0.340557541221353,0.733436697765112,0.97271271174229
"10131","RRAGD",8.76480645353864,-0.213783275933575,0.627453158971844,-0.340715913015536,0.733317457874313,0.97271271174229
"10132","TAPBPL",24.7109920808708,0.18284296519276,0.537019997756671,0.340477013810588,0.733497330217752,0.97271271174229
"10133","OBI1",21.2062195088035,0.11472706749504,0.336505400938533,0.3

40936778949342,0.73315117619235,0.97271271174229
"10134","LENG9",11.7715176224248,0.168632858121358,0.495184790893656,0
.34054530999837,0.733445907056924,0.97271271174229
"10135","AC009163.7",2.28495443247481,-0.346994350493966,1.02025624512
191,-0.340105098256473,0.733777382543753,0.97298808524968
"10136","HSPBAP1",4.98226305037308,0.239199385873593,0.704125743719612
,0.339711178020562,0.734074043021504,0.973165798243461
"10137","CNPY2",155.142195527752,0.0816876755692544,0.240497395646098,
0.339661372838569,0.734111554024348,0.973165798243461
"10138","TMEM143",5.38022296012944,0.201967018533518,0.594652582917383
,0.339638680357969,0.734128645181354,0.973165798243461
"10139","LINC02308",4.60889941967901,0.296618478377529,0.8738894961632
74,0.339423324893826,0.734290849696672,0.973284813992857
"10140","MSMO1",24.3437146180387,-0.116518802073464,0.343690364397576,
-0.339022603318249,0.734592702469905,0.973316747798635
"10141","CTTNBP2",3.69893177128078,0.3406082076314,1.00461870462039,0.
339042271525398,0.734577885981644,0.973316747798635
"10142","HSBP1",208.522020687843,-0.0790512437145699,0.233167502028355
,-0.339031996427,0.734585626424442,0.973316747798635
"10143","AC100793.4",17.9264348301037,0.169908245193226,0.501194275717
563,0.339006755314527,0.734604641187704,0.973316747798635
"10144","TAF13",14.5622256027757,0.134640881840121,0.397739965008146,0
.338514843076841,0.734975243488002,0.97350825428477
"10145","MAPRE3",9.3762474637489,0.165914032279116,0.490245781888835,0
.338430310690031,0.735038935652024,0.97350825428477
"10146","SPINDOC",4.44108875647429,-0.2312673094409,0.683285168211536,
-0.338463821842102,0.735013685966023,0.97350825428477
"10147","TYMS",1.61082351388165,-0.396876724724854,1.17250358371528,-0
.338486577130349,0.734996540676272,0.97350825428477
"10148","AL079343.1",16.027163068086,-0.161104842088504,0.476411910431
458,-0.338162918602518,0.735240418129815,0.973679146555635
"10149","AGAP5",4.22959386914175,-0.259347429129574,0.76819358689269,-
0.337606865710274,0.735659467589613,0.974042126594759
"10150","MRPS26",74.9772825824263,-0.0967056636987098,0.28637946307663
5,-0.337683654616083,0.735601593680663,0.974042126594759
"10151","TIMM17A",28.8596267470961,-0.0966203155596202,0.2874956071453
87,-0.33607579788431,0.736813708533852,0.97416274196761
"10152","FAM49A",17.4980203061029,0.134036950204548,0.399134412007984,
0.335819077914701,0.737007302727484,0.97416274196761
"10153","TGFBRAP1",14.8874880732018,-0.133601981706307,0.3968077561914
62,-0.336691961338184,0.736349123850861,0.97416274196761
"10154","CHROMR",14.1892740318766,-0.173802059518812,0.516953436769556
,-0.336204476373929,0.736716677533796,0.97416274196761
"10155","AC064836.3",2.40252229513867,0.343766037033564,1.021665146514
64,0.336476230207427,0.736511773270732,0.97416274196761
"10156","WDFY3-
AS2",48.2909078818027,0.127105193367577,0.376737765229987,0.3373837323
95353,0.735827646192087,0.97416274196761
"10157","AC025171.4",1.51572205217829,0.40592708406528,1.2087097490395
7,0.335835037640614,0.736995266908015,0.97416274196761

"10158","AL137003.2",1.56014016935655,0.455745322581756,1.352627363328
41,0.336933389740323,0.736167114108762,0.97416274196761
"10159","EZH2",3.46121321007079,-0.292717821617772,0.868207713694532,-
0.337151832448198,0.736002445741104,0.97416274196761
"10160","AC048341.2",2.70872714331425,0.298991394792263,0.889229528580
007,0.33623646671936,0.736692555620408,0.97416274196761
"10161","EEA1",294.859736790132,0.0590494312700163,0.175810136380564,0
.335870459381229,0.736968554294069,0.97416274196761
"10162","ICE2",42.047338656853,0.100918364728946,0.300718488976096,0.3
35590821410944,0.737179446454815,0.97416274196761
"10163","IQGAP1",227.346244975371,0.0646771539331932,0.192655307330769
,0.335714363799744,0.737086273113661,0.97416274196761
"10164","AC018557.1",1.72120270697215,-0.433371367534024,1.28719099289
526,-0.336679925454768,0.736358197934582,0.97416274196761
"10165","MTSS2",3.41199554246336,0.258587206095318,0.768502463934589,0
.336481947984135,0.736507462228892,0.97416274196761
"10166","TAF1C",11.0440857437153,-0.171957700082798,0.510791822104143,
-0.336649281843315,0.736381300907912,0.97416274196761
"10167","VPS9D1",5.26519943435323,-0.215797453465768,0.641483505528281
,-0.336403744766676,0.736566425960913,0.97416274196761
"10168","RBL1",9.95739494449888,0.169930425067624,0.505539343760716,0.
336136894516476,0.73676763762742,0.97416274196761
"10169","ARFRP1",45.7262497665235,-0.0947256735674099,0.28120273286803
6,-0.336859007738958,0.736223188157616,0.97416274196761
"10170","BRD1",43.4546358833733,0.0981874258789893,0.292604914794349,0
.335563146463204,0.737200318908445,0.97416274196761
"10171","DNAJA3",11.8044682051027,-0.146287071576223,0.436141170959782
,-0.335412204388548,0.737314162876318,0.974217386185708
"10172","ANXA3",10.3868434665854,0.209624000282112,0.625691908964609,0
.335027506794831,0.73760433666676,0.974217659013718
"10173","FUOM",23.5503820109703,0.156222422989996,0.466195309817836,0.
335100803676123,0.737549046630624,0.974217659013718
"10174","IGFBP4",1233.94688532569,-0.104597980842166,0.312098645770499
,-0.335143975341317,0.737516481584087,0.974217659013718
"10175","POLI",18.333843678144,0.117638386906798,0.351024292312014,0.3
35128905558003,0.737527848898317,0.974217659013718
"10176","NFKB2",11.7556299539288,0.162850240314768,0.48644762540832,0.
334774458356279,0.737795229127756,0.97427467361075
"10177","ABAT",5.04175841443006,0.279283810888002,0.83446372407033,0.3
34686581132271,0.737861524960801,0.97427467361075
"10178","SETD4",16.4097072417719,0.128802461564255,0.384850297152548,0
.334681985481747,0.737864992038858,0.97427467361075
"10179","EVC2",27.2897254813559,-0.122238673041726,0.366193152375103,-
0.333809281383048,0.738523479248923,0.974282618791232
"10180","AC108471.2",3.18936135830186,0.284692777244243,0.852418636594
185,0.333982347431686,0.738392879310614,0.974282618791232
"10181","LINC00265",2.17224207401239,-0.315840445230558,0.945748286813
603,-0.333958252564941,0.73841106144321,0.974282618791232
"10182","LRCH4",9.72793567069268,-0.172699030255592,0.516821048016212,
-0.334156340804012,0.738261587200195,0.974282618791232

"10183","TRIM22",66.3376571140685,0.0814991225642902,0.24401087989646,
0.333997904515046,0.73838113991888,0.974282618791232
"10184","CDT1",2.60643750957095,-0.317763170259904,0.951344292600798,-
0.334014901578059,0.738368313986688,0.974282618791232
"10185","XAF1",65.1241106259097,0.113475268495617,0.339918555914252,0.
333830755989215,0.738507273567701,0.974282618791232
"10186","ATPAF2",8.7748106541195,0.172582270531651,0.51695477420774,0.
333844040411741,0.738497248617024,0.974282618791232
"10187","BICRA",5.24144901743116,0.225479121381676,0.674873589025158,0
.334105712608158,0.738299789492705,0.974282618791232
"10188","NR3C2",6.62727302826099,0.205924631861217,0.618256999498456,0
.333072867801363,0.739079279542048,0.974408334557013
"10189","CGAS",7.72520378615287,0.17579903675021,0.527907909283092,0.3
33010802942786,0.739126128565274,0.974408334557013
"10190","LINC00476",11.945617275229,0.153231842457685,0.45960646141892
7,0.333397929142723,0.738833926138364,0.974408334557013
"10191","YAP1",55.3567849293435,0.0833406999973855,0.250203223026251,0
.333092032106403,0.739064813758228,0.974408334557013
"10192","RAB11FIP3",11.6224249464689,-0.146294596855149,0.439047780537
008,-0.333208829062326,0.738976653960599,0.974408334557013
"10193","PIGQ",14.5578853933443,0.13006213299642,0.390564596988398,0.3
33010554462219,0.739126316130233,0.974408334557013
"10194","ZSWIM9",5.5488282330688,0.20513719392917,0.615462259721639,0.
333305886248734,0.738903396597045,0.974408334557013
"10195","YRDC",11.2507927694455,-0.14951656629596,0.45010947502863,-0.
332178224611801,0.739754685350955,0.974409504173633
"10196","HIVEP3",2.13228992053056,-0.334920611974899,1.01224677181298,
-0.330868540459793,0.740743785895156,0.974409504173633
"10197","YIPF1",10.3641068134848,0.164341493441495,0.496504423813605,0
.330997037607848,0.740646723200994,0.974409504173633
"10198","ABCD3",22.2911899783096,0.10907800156914,0.33004884021786,0.3
3049048588427,0.741029380327797,0.974409504173633
"10199","DPH5",21.8665337590629,-0.112987201790332,0.342469199125652,-
0.329919309762153,0.741460932583273,0.974409504173633
"10200","ATP8B2",49.0658574414633,0.105991106063723,0.318625059037163,
0.332651507022118,0.739397358436383,0.974409504173633
"10201","ACBD3",103.153622903934,-0.0780176009301986,0.234557540665075
,-0.332616042566715,0.739424132053639,0.974409504173633
"10202","OBSCN",2.47792931810816,-0.307623073388775,0.928116419015859,
-0.331448800049208,0.740305508876214,0.974409504173633
"10203","MAP10",2.42987178260353,-0.294041663726254,0.891478412988312,
-0.329835988670327,0.741523892654518,0.974409504173633
"10204","CCDC142",8.95764073969282,0.196361341876688,0.59181372215808,
0.331795858265412,0.740043411920783,0.974409504173633
"10205","FMNL2",45.1476137578004,0.103184243856509,0.311854857995071,0
.330872651847996,0.740740680218223,0.974409504173633
"10206","TTC30A",2.1406393411052,-0.353647420781169,1.06819594228598,-
0.331069803564645,0.740591759922209,0.974409504173633
"10207","SLC25A20",14.9258010390023,0.139532645159235,0.41935418466061
,0.332732211250405,0.739336432578549,0.974409504173633

"10208","PDCD10",69.4491832410624,-0.0762618142293893,0.231746886272618,-0.329073738404743,0.74209995350972,0.974409504173633
"10209","AC106897.1",2.46514277534258,-0.361072471920878,1.09317478366745,-0.330297110137806,0.741175476305048,0.974409504173633
"10210","IL7R",7.10973088488899,0.217346617297945,0.659068066520637,0.329778710786831,0.741567174651527,0.974409504173633
"10211","HMGCR",12.4334654356608,-0.145170132305281,0.440911686030188,-0.329249908552756,0.741966802330135,0.974409504173633
"10212","PGGT1B",34.5387882512825,0.10054173197348,0.302370557720072,0.332511646410229,0.739502946960404,0.974409504173633
"10213","BLOC1S5",35.5933417541979,-0.0946309092651079,0.286745886477662,-0.330016623525233,0.741387401394987,0.974409504173633
"10214","SYNGAP1",17.3091747057505,0.14855275146133,0.447083541378083,0.332270678100638,0.739684878804181,0.974409504173633
"10215","CPVL",43.6195998158495,-0.13747365803698,0.416518795181749,-0.330053912637947,0.741359226018905,0.974409504173633
"10216","AL391988.1",1.73639886895747,0.371391694513862,1.12036843614355,0.331490679791228,0.740273879864898,0.974409504173633
"10217","MMP19",29.6715110751549,0.111105470492332,0.336916144198009,0.32977188064646,0.741572335897407,0.974409504173633
"10218","IFT81",16.2169624224559,0.136708134618825,0.411791475495684,0.331983886879315,0.739901426077826,0.974409504173633
"10219","TM2D3",70.0630252506954,-0.0848848914188239,0.257851613331573,-0.329200544150445,0.742004111665972,0.974409504173633
"10220","PDPK1",40.3880741484782,-0.0939627412234689,0.284494204874073,-0.330279983260327,0.741188416164108,0.974409504173633
"10221","HEATR3",6.11105799131749,0.212241517093389,0.644615506919619,0.329252887675031,0.741964550745784,0.974409504173633
"10222","KRBA2",8.90407849357101,0.176870463879658,0.532681876529426,0.332037697681813,0.739860793615935,0.974409504173633
"10223","DHRS13",1.60181786073025,0.362594475600685,1.09647837148624,0.330690039156176,0.74087862699962,0.974409504173633
"10224","MYO1D",17.4678992314827,-0.192500334716699,0.581169848171254,-0.331229046590136,0.740471481387818,0.974409504173633
"10225","USP32",17.2543549286408,0.130882064919762,0.397514948632665,0.329250674395913,0.741966223514988,0.974409504173633
"10226","THOC1",23.5252151742923,0.105897478589881,0.321746089862647,0.329133692456337,0.742054638764479,0.974409504173633
"10227","LINC00526",5.85736843504162,-0.193866374306427,0.583287774733224,-0.332368314071207,0.739611161578442,0.974409504173633
"10228","ATP8B1",7.91851389090886,-0.178841492636186,0.540488380562354,-0.330888690798698,0.740728564691615,0.974409504173633
"10229","AC022150.4",1.70657434751456,0.386725190093741,1.17286911080048,0.329725786562663,0.741607167535288,0.974409504173633
"10230","RDH13",11.0487639332817,0.159100665238095,0.481449136958203,0.330462042664139,0.741050868683445,0.974409504173633
"10231","AL121890.5",1.49484664677125,-0.450396472454704,1.36699370312252,-0.329479551680375,0.741793247327443,0.974409504173633
"10232","THBD",7.08559983909615,0.227094776095999,0.688631410430201,0.329776964362006,0.741568494349203,0.974409504173633

"10233","GNB1L",5.8821260380212,0.200355926986844,0.60355378957508,0.331960349595188,0.739919199276429,0.974409504173633
"10234","HNRNPH2",61.0553318832205,0.085101325085426,0.258566427900174,0.32912751193779,0.742059310110914,0.974409504173633
"10235","ABCD1",6.58904746961121,0.19213316040852,0.57952279538366,0.331536847107667,0.740239013239793,0.974409504173633
"10236","PAX8",1.56903823502478,0.401511077991396,1.22297346434407,0.328307268879905,0.742679348732738,0.974514722762186
"10237","AP002026.1",1.90410445512248,0.33817591187772,1.03041510316197,0.328193861716489,0.742765088692239,0.974514722762186
"10238","SYNCRIP",98.9897487476696,0.0737795959250428,0.224442481829275,0.328723846411413,0.742364428304087,0.974514722762186
"10239","PCGF5",23.6363150323677,0.11391872227005,0.347310733658285,0.328002308106414,0.74290991746787,0.974514722762186
"10240","IPO5",93.8978350358651,-0.0726135440952724,0.221408925616396,-0.327961232335682,0.742940974990516,0.974514722762186
"10241","LINC00346",2.05832997462653,-0.310548965345739,0.945566153799118,-0.328426481952646,0.742589222735225,0.974514722762186
"10242","KLHDC1",14.1614138371636,0.142527411968409,0.43433738626151,0.328149075987197,0.74279894921911,0.974514722762186
"10243","GTF2A2",125.844736774436,-0.102565904288336,0.312784265097884,-0.327912608571402,0.742977740116181,0.974514722762186
"10244","AC015871.4",2.02842088071171,0.384718883068922,1.17278693819846,0.328038171758544,0.742882801187057,0.974514722762186
"10245","ACAP1",4.58526244515084,-0.235998208470763,0.718398637379546,-0.328505924414887,0.742529165583473,0.974514722762186
"10246","FAM210B",80.1924429077011,0.0753271055315122,0.229236140348279,0.328600479039071,0.742457685936959,0.974514722762186
"10247","WDR3",12.838977883145,-0.15687729359927,0.479189106628901,-0.327380759347605,0.743379917199477,0.974591805506767
"10248","AL596325.2",1.8262776456007,-0.399628662912905,1.22002790136524,-0.327556986578513,0.743246648823198,0.974591805506767
"10249","LINC01473",8.23277227209018,0.198169078450787,0.605374718585408,0.327349445503528,0.743403598482606,0.974591805506767
"10250","CBWD5",9.01581009145526,-0.187260757683839,0.572208786698619,-0.327259493452115,0.743471626613243,0.974591805506767
"10251","ARRDC1-AS1",9.91606022181134,0.155036485098054,0.473265775967257,0.327588625611458,0.74322272324142,0.974591805506767
"10252","CTSA",166.090927578848,0.0715226300681964,0.218510018142256,0.327319683904072,0.74342610609639,0.974591805506767
"10253","DNAJA1",195.987111986237,-0.0656142372961769,0.200569735452561,-0.327139272274187,0.743562549534134,0.974615927356796
"10254","MBD4",49.8360694275337,0.0758085651652472,0.231810477035162,0.327028209142369,0.743646549429645,0.974630971112249
"10255","AC025449.2",1.89612061510708,0.33962801836498,1.03971811919349,0.326653938308229,0.743929642521263,0.974663000111655
"10256","BNC2",55.2447746310159,0.1158403213461,0.354672181898484,0.32661236843,0.743961087517327,0.974663000111655
"10257","INPP5F",7.54406585446725,0.187633305116099,0.574128306091901,

0.326814238429247,0.743808389568924,0.974663000111655
"10258","AL078587.2",1.7994650219247,-0.382196400976518,1.16983811195981,-0.326708795917266,0.743888146846842,0.974663000111655
"10259","AL357568.2",3.45399318223175,0.263808355526174,0.808370003763587,0.326346047352008,0.744162552775219,0.974831908250918
"10260","ZFYVE1",18.603255175055,0.113761245670593,0.348962016644664,0.325998934681857,0.744425161141562,0.974985843541707
"10261","NFIX",272.111088853219,0.0630709393025444,0.193459808138267,0.326015723418206,0.744412458925591,0.974985843541707
"10262","EDA2R",5.45889474011641,0.262939485900577,0.806835232594344,0.325889940446833,0.744507626948813,0.974998830497476
"10263","TAF12",54.867357453645,-0.0935875743248824,0.28736980795294,-0.325669474436258,0.744674442042007,0.975027262919187
"10264","KLHL28",28.6689831900254,0.103358550181576,0.317341686076,0.325701143961346,0.74465047863277,0.975027262919187
"10265","FGF14",18.1768848093538,-0.112718341616922,0.346438280315244,-0.325363413980561,0.744906041826739,0.97523548914852
"10266","CCHCR1",9.51972853595194,0.16250000210937,0.499733393567226,0.325173390854278,0.745049846315663,0.975328743876505
"10267","SGPP1",20.0339535464633,0.117212524350344,0.360785797258064,0.324881204418653,0.745270982636731,0.975523204018216
"10268","NBPF11",11.8824184069994,0.164496465543126,0.506625246210052,0.324690620480694,0.745415234153222,0.975527846448551
"10269","TGFB3",14.6405818805063,0.133332833593358,0.410653256895666,0.324684709921182,0.745419707952986,0.975527846448551
"10270","TGS1",41.9895295520579,0.092450977595106,0.28484095112602,0.324570526919086,0.74550613663119,0.975545956201223
"10271","TPRA1",32.3429079181903,-0.101258365537148,0.312102804878627,-0.32443913977808,0.745605591622491,0.975581106592801
"10272","RNF170",48.3209918443924,0.0779979910676969,0.240558009101498,0.324237764350583,0.745758033271669,0.975685573319506
"10273","HDAC1",8.29945805973774,0.169008875980445,0.522356717523914,0.323550689233181,0.746278225556693,0.97570124265555
"10274","GTF2IRD1",2.86923275349956,-0.31857410152178,0.98423749439447,-0.323676047027426,0.74618330713124,0.97570124265555
"10275","CHCHD7",17.4318177464505,-0.137666165442027,0.424795755659817,-0.324076132136009,0.745880396420936,0.97570124265555
"10276","GBF1",26.7257288564113,-0.099812061725464,0.308479668391939,-0.323561232562814,0.74627024220915,0.97570124265555
"10277","OXLD1",32.1641673258077,0.119703846424802,0.36946532385089,0.323992100739372,0.74594401465264,0.97570124265555
"10278","ZNF335",13.0405888573255,-0.129298830409409,0.399500485545473,-0.323651247214045,0.746202084750528,0.97570124265555
"10279","PPM1F",18.7576655738342,-0.127151152432062,0.392682988654842,-0.323801020430311,0.74608868359284,0.97570124265555
"10280","BTG2",103.139364352804,0.15132042052901,0.468019774762353,0.323320570387964,0.746452476983149,0.975739211961535
"10281","GJC2",8.56359392942571,0.1828878990327,0.565577947947002,0.32336462144001,0.746419119482129,0.975739211961535
"10282","SPIN3",11.1140609534729,0.156880194095196,0.485521090918937,0

.323117156040062,0.746606517929196,0.975845652057037
"10283","ZNF155",3.17915710678496,0.253489091814494,0.784793491575109,
0.323001011776654,0.746694475814997,0.975865706552343
"10284","ENPP6",1.79170137146533,0.521512468546402,1.61637445274192,0.
322643350160442,0.746965359330293,0.976101313941102
"10285","ZSCAN21",11.184019981092,-0.139305480509074,0.431988068320671
,-0.322475296715061,0.74709264939342,0.976101313941102
"10286","AL117335.1",3.84854640027314,0.250573335237371,0.776953264003
17,0.322507603541451,0.747068178440211,0.976101313941102
"10287","CEPT1",27.1006315647558,0.111204154962819,0.345017293332665,0
.322314727730462,0.747214276886119,0.976165321966808
"10288","B3GALT6",12.902046321115,0.16172154213445,0.502646087012319,0
.321740378196731,0.747649385600235,0.976226319244255
"10289","MRM2",20.771507660931,-0.128621006956107,0.400041334274217,-0
.321519292973712,0.747816894087742,0.976226319244255
"10290","HOXA4",3.09022510639514,0.274073095694882,0.852698023985776,0
.321418706254037,0.747893109044465,0.976226319244255
"10291","FAM13C",6.55538898601247,0.264470008752541,0.82306566462924,0
.321323097436794,0.747965554508973,0.976226319244255
"10292","GLCE",27.1556857956988,0.113646357393969,0.353185604170335,0.
321775168783943,0.747623027082335,0.976226319244255
"10293","LUC7L",41.2380228646894,-0.0830045400393538,0.258421375506555
,-0.321198429799583,0.748060021993998,0.976226319244255
"10294","NMRAL1",28.4810807863057,-0.110517727436733,0.343600299744584
,-0.321646190410446,0.747720746912563,0.976226319244255
"10295","CEP76",4.88355397105686,-0.216331770475208,0.672089464730058,
-0.321879425028775,0.747544040815188,0.976226319244255
"10296","SHFL",43.7662463567137,-0.0961304815697016,0.298548181401271,
-0.321993190909761,0.747457852913616,0.976226319244255
"10297","UPF1",42.2231028986962,0.092501336158237,0.287673802022048,0
.321548688012731,0.747794621815378,0.976226319244255
"10298","STS",15.5519176800818,0.123812109018642,0.3853745579402,0.321
277329983611,0.748000234571085,0.976226319244255
"10299","ZNF692",8.42089635684254,-0.188020685815693,0.586181489233369
,-0.320755072053869,0.748396009037846,0.9762855722636
"10300","ELMOD2",21.7895499348728,0.104235556952106,0.324835888083811,
0.320886825550545,0.748296158111877,0.9762855722636
"10301","NCOA7",166.350117445838,0.0678308842489219,0.211292957670119,
0.321027662241459,0.748189428049105,0.9762855722636
"10302","TAGLN",776.259675707029,0.127202770913483,0.39647018346907,0.
320838177036347,0.748333026464937,0.9762855722636
"10303","BNC2-
AS1",3.76960093111232,-0.246621728233461,0.769212357540828,-0.32061592
0708699,0.748501471091563,0.976328377171651
"10304","SH3BP2",12.2407647104716,0.15080640141908,0.470584839809347,0
.320465915306957,0.748615164646179,0.976381909712733
"10305","AKIRIN1",34.1158550629903,-0.092269638445501,0.28814316509775
6,-0.320221506604876,0.748800420975711,0.976434005190431
"10306","INPP5E",10.1114953759254,-0.162002907399115,0.505812324226301
,-0.320282641683192,0.748754080593528,0.976434005190431

"10307","SSB",178.26922025442,-0.0667970380299974,0.208662899260375,-0
.320119380430186,0.748877834627606,0.976440207583234
"10308","EMC1",27.5961537082747,0.0973148163277218,0.309468398568564,0
.314458008565166,0.753173204335197,0.976495747501424
"10309","BSDC1",63.3080732204153,0.087573166470771,0.27831929680794З,0
.314649999030437,0.753027412146006,0.976495747501424
"10310","SNIP1",20.6892235802613,0.128415510843767,0.409036556449473,0
.313946293598895,0.753561829349495,0.976495747501424
"10311","SVBP",142.983902590038,0.0818893741976279,0.256787929156352,0
.318898845699898,0.749803219658176,0.976495747501424
"10312","TYW3",45.5566017405414,-0.0749450797750903,0.237453959903422,
-0.315619414414366,0.752291399821958,0.976495747501424
"10313","SLC16A1-
AS1",28.4805897453864,0.12937983729927,0.40479392183055,0.319619021733
803,0.749257152937003,0.976495747501424
"10314","LIX1L",41.0062832998893,0.0830887656904163,0.264997937848966,
0.313544951952691,0.753866674374738,0.976495747501424
"10315","NPL",2.93495150691806,0.273967840841265,0.869270232825936,0.3
15169932772936,0.752632633239005,0.976495747501424
"10316","AL096803.2",3.86506466394932,0.244043754170436,0.769418019143
433,0.317179670996166,0.751107274349728,0.976495747501424
"10317","LAMB3",4.54308109904143,-0.269011452177071,0.841255708051535,
-0.319773702100803,0.749139884386984,0.976495747501424
"10318","SMYD3",13.2493447121931,-0.128217264159495,0.407052105985538,
-0.314989806646646,0.752769393338339,0.976495747501424
"10319","MEIS1-
AS2",2.5813167086037,-0.3119745058489,0.978718536392106,-0.31875814572
6907,0.749909918746495,0.976495747501424
"10320","BCL2L11",11.8498890287426,0.146452088693712,0.462202071174638
,0.316857274831158,0.751351902524268,0.976495747501424
"10321","MAP3K2",77.596662661541,0.0888514093160828,0.280703860396473,
0.316530770865021,0.751599673091883,0.976495747501424
"10322","EEFSEC",13.801308371658,-0.12270914996425,0.391116956315957,-
0.313740296815773,0.753718292479553,0.976495747501424
"10323","CFI",10.2923354863305,0.466566955652217,1.47147622440643,0.31
7074070184466,0.751187399515064,0.976495747501424
"10324","NUDT6",10.1952796863174,-0.152495150719856,0.481437238365246,
-0.316749803645568,0.751433455203004,0.976495747501424
"10325","SETD7",125.200613127112,0.0762233901544859,0.240196746763428,
0.317337312772012,0.750987667825725,0.976495747501424
"10326","WWC2-
AS2",2.11872752351399,-0.325622785090575,1.03309012236702,-0.315193009
826196,0.752615112632095,0.976495747501424
"10327","EGFLAM",2.07959855498659,-0.303670671905516,0.967432623660465
,-0.313893354925866,0.753602037509094,0.976495747501424
"10328","C5orf24",86.8494308445821,0.0705558704571854,0.22372243362770
7,0.315372353648701,0.752478955197842,0.976495747501424
"10329","PHYKPL",89.2216447136895,0.0827999194389324,0.262794456220291
,0.315074833121762,0.752704836332448,0.976495747501424
"10330","CDYL",39.0481482701196,-0.0832016483319796,0.261112657640694,

-0.318642723350738,0.749997452274712,0.976495747501424
"10331","RNF144B",1.61974536691575,0.36456796695551,1.1612600801466,0.313941702800536,0.753565316141697,0.976495747501424
"10332","ZSCAN16",2.67012385491783,-0.326104899680722,1.02583170594004,-0.317893176621879,0.750565968608771,0.976495747501424
"10333","PPIL1",4.3058588299469,-0.281415366949692,0.897253952510244,-0.313640710260877,0.75379393623908,0.976495747501424
"10334","DCBLD1",4.09962653496243,0.234423900645221,0.742352083793594,0.315785333890705,0.752165450799657,0.976495747501424
"10335","PAPOLB",2.11902427057709,-0.318784539071878,1.00836805453118,-0.316139070093895,0.751896952647257,0.976495747501424
"10336","THSD7A",1.8734429638003,0.349041531318961,1.09686214991477,0.318218229470389,0.750319405813599,0.976495747501424
"10337","IL6",7.1033070174357,0.303969011456875,0.958679103200549,0.317070655281913,0.751189990634931,0.976495747501424
"10338","CFAP69",5.82051464773064,-0.187234596855514,0.590247424267996,-0.317213746570289,0.751081419903234,0.976495747501424
"10339","CDK5",8.37793428647322,-0.162472211390059,0.5157496908759,-0.315021442114937,0.752745373663462,0.976495747501424
"10340","ENTPD4",26.0997099728697,-0.10701143197322,0.34139193566435,-0.31345623839935,0.753934063254317,0.976495747501424
"10341","DECR1",113.239187304349,0.0957578352473377,0.30484735182546,0.314117326832361,0.753431930178379,0.976495747501424
"10342","TRIM8",121.303455238907,-0.075094108012568,0.235944703813414,-0.318269945452782,0.750280179967508,0.976495747501424
"10343","USP5",25.217399214477,-0.113763642977285,0.362036085994261,-0.31423288279359,0.753344169760809,0.976495747501424
"10344","SLC2A3",83.558946927826,-0.144487499840949,0.460603069444622,-0.313692003866076,0.753754974448199,0.976495747501424
"10345","AICDA",1.89277635656669,-0.311093196985337,0.974048117004668,-0.319381754919861,0.749437044402811,0.976495747501424
"10346","ATF1",35.941586059087,-0.0855730808607,0.271323036243285,-0.315391874002065,0.752464135853284,0.976495747501424
"10347","SCARB1",4.71659222059377,0.212553782875094,0.675575720580949,0.314626142414224,0.753045527713982,0.976495747501424
"10348","CENPJ",9.47460634308057,0.161007857844631,0.507593647234694,0.317198331227708,0.751093116080942,0.976495747501424
"10349","ARF6",96.7307570440162,-0.0701987779185466,0.22356747062282,-0.313993702764472,0.753525821547249,0.976495747501424
"10350","LINC01588",3.06751772481246,-0.292892286888474,0.925103698915238,-0.316604816553988,0.751543480586923,0.976495747501424
"10351","NEK9",28.7320709542085,0.0981805086893529,0.307884454203291,0.318887515588968,0.749811811611307,0.976495747501424
"10352","ULK3",10.4694904942805,-0.162702069221795,0.511310510855546,-0.318205993750363,0.750328686529778,0.976495747501424
"10353","IL32",95.4580039215566,0.102235319018741,0.323516460643273,0.316012727190013,0.751992847885737,0.976495747501424
"10354","TIGD7",2.36094618729627,-0.300282816664224,0.952247973513355,-0.315340987869283,0.752502767472831,0.976495747501424
"10355","AC027682.6",2.60210389807851,0.274535505536388,0.863628895108

8,0.317885965941195,0.750571438425509,0.976495747501424
"10356","SNAI3-
AS1",2.05258252518168,0.349884632510785,1.09379118056239,0.319882477321573,0.749057421593657,0.976495747501424
"10357","TRPV3",3.01112043439323,0.29205443863193,0.920501767103781,0.317277433970426,0.751033098576626,0.976495747501424
"10358","AC004687.1",1.46915178516434,-0.394683138004806,1.23691185061778,-0.319087522532571,0.74966014508404,0.976495747501424
"10359","GALK1",42.3849714063461,0.0999738698234477,0.313212568536623,0.319188563506697,0.749583528755473,0.976495747501424
"10360","PTPN2",29.6127435430326,-0.0969961781275948,0.305888815263896,-0.317096190797021,0.751170615163027,0.976495747501424
"10361","ADAMTSL5",4.30523292293812,0.218082521616665,0.687176741003437,0.317360161664107,0.750970332330148,0.976495747501424
"10362","MKNK2",57.3130510811693,-0.0828312496195477,0.263732424281571,-0.314073060395159,0.753465549720768,0.976495747501424
"10363","ZNF555",7.59290392306101,0.188137671463448,0.593367342642451,0.317067789112916,0.751192165395326,0.976495747501424
"10364","PNPLA6",12.212962098357,-0.135273200623816,0.430048236644335,-0.31455355259529,0.753100649770619,0.976495747501424
"10365","CC2D1A",13.3918874054935,0.138581165289931,0.433521459929522,0.319663910784164,0.749223120394082,0.976495747501424
"10366","FXYD1",353.529793935445,0.0840568747896005,0.26424991491038,0.318096128122154,0.750412020531969,0.976495747501424
"10367","IGFLR1",2.8177681486273,-0.272023291489487,0.852764935640171,-0.318989770944649,0.749734269520995,0.976495747501424
"10368","BAX",65.4803878810811,-0.0966585839521775,0.303653171771359,-0.31831903282393,0.750242948483363,0.976495747501424
"10369","NUP62",26.6780337854412,0.0972536713995606,0.309819147040605,0.313904651563754,0.753593457392481,0.976495747501424
"10370","PCNA",38.0754653513373,-0.0927382397819557,0.293106248158172,-0.316398030968997,0.751700411318095,0.976495747501424
"10371","UBE2V1",135.448556223921,-0.0574716889233777,0.182895075633074,-0.314233112753008,0.753343995118434,0.976495747501424
"10372","BACE2",55.9123215760938,-0.0730093788857855,0.230331198375227,-0.316975639430519,0.751262086777627,0.976495747501424
"10373","LINC01315",13.1914578810988,0.187010690195384,0.596108084818151,0.313719432697233,0.75373414021,0.976495747501424
"10374","MAGED2",178.628230318297,-0.0889777217438914,0.282564556850288,-0.314893427313443,0.752842572179846,0.976495747501424
"10375","SNX12",15.9831583807973,0.127472280137004,0.402023801151358,0.31707645112537,0.751185592934198,0.976495747501424
"10376","RTL5",9.5693767665293,-0.167085142885073,0.527838436134929,-0.316545994847488,0.751588119662008,0.976495747501424
"10377","RGPD5",6.05044514280033,-0.197849108437464,0.63315522660219,-0.312481205437117,0.754674844360963,0.976514079854321
"10378","ZNF660",8.30989753701166,0.175956462680868,0.562236470684175,0.312958109008386,0.754312488720932,0.976514079854321
"10379","CADPS2",15.8863678268003,-0.127357299341275,0.4073317825436,-0.312662318039577,0.75453722700598,0.976514079854321

"10380","COL14A1",678.444051428455,0.0943614171099272,0.30127181565303
2,0.313210238088123,0.754120940616979,0.976514079854321
"10381","CNTLN",57.9651834473004,-0.08550670334873,0.273573717002214,-
0.312554525652909,0.754619131483963,0.976514079854321
"10382","RPP25L",19.5570846973793,0.135121333470875,0.4314948403022,0.
313147043371925,0.754168949636978,0.976514079854321
"10383","ACTR10",96.5799876696216,-0.077411611165577З,0.24745396801288
8,-0.312832369540122,0.754408021458019,0.976514079854321
"10384","EID2B",9.80168323988001,-0.142361867952458,0.454466126683311,
-0.313250778427457,0.754090142625289,0.976514079854321
"10385","PDZD11",51.6274076002725,-0.101109807630636,0.323564904494338
,-0.312486942267885,0.754670485144085,0.976514079854321
"10386","ZDHHC9",35.7600860530941,-0.116318389385304,0.372063820240458
,-0.312630207662034,0.754561625320926,0.976514079854321
"10387","ELP6",34.4678036742984,-0.0905703800045781,0.290295631554394,
-0.311993602933731,0.755045384980024,0.97689948288693
"10388","PMAIP1",1.65010565526105,0.35359079481681,1.13417930601462,0.
311759166246199,0.755223559009774,0.977035946239156
"10389","MIR4458HG",25.6248083112452,0.122917197405543,0.3948107819827
63,0.311331916489831,0.755548306224901,0.977283065227283
"10390","LTBP3",225.447399130195,-0.0576439682392069,0.185161936277669
,-0.311316512443271,0.755560015455872,0.977283065227283
"10391","DNAJC27",8.67493648952149,0.175073721229614,0.565262829296,0.
309720915928007,0.756773195772859,0.977318116397535
"10392","MXD1",12.0436230891633,-0.13510123914494,0.437285339078685,-0
.30895442191038,0.757356197775847,0.977318116397535
"10393","NCKIPSD",10.059456662742,-0.145138346782318,0.466907581185163
,-0.310850268084981,0.755914452972266,0.977318116397535
"10394","C3orf62",5.67740931072181,0.183829820104553,0.593555463902507
,0.30970959124175,0.756781808421075,0.977318116397535
"10395","PCDHGA3",2.92574937862257,0.262206294675399,0.845594809006596
,0.310085033496645,0.756496293255393,0.977318116397535
"10396","AL358852.1",1.86237456108226,-0.318086021221468,1.02890737773
971,-0.309149324908365,0.75720793976029,0.977318116397535
"10397","AGPAT4",22.0895527109393,-0.117787233727437,0.38132416247147,
-0.308890034568029,0.757405177638242,0.977318116397535
"10398","SKAP2",63.4636429508325,-0.113597508322649,0.367620274583823,
-0.3090077348189,0.757315643043335,0.977318116397535
"10399","VDAC3",121.922885616339,-0.0780219707755863,0.250787662296626
,-0.311107691905926,0.755718753824967,0.977318116397535
"10400","ZCCHC7",43.3569979589428,-0.0773955989039757,0.25011341405716
5,-0.309442015318245,0.75698531399767,0.977318116397535
"10401","KIF20B",16.3754136628328,-0.122367629866569,0.393917919766602
,-0.310642455512235,0.756072448002126,0.977318116397535
"10402","MARK2",21.558332462149,-0.114377733359571,0.36823600155007,-0
.310609861279462,0.7560972295591,0.977318116397535
"10403","DCUN1D5",37.8649168830005,0.0981210576597274,0.31656455775621
7,0.309955916591551,0.756594479932413,0.977318116397535
"10404","PRDM10",3.93943497534606,-0.216424784295399,0.698371759379525
,-0.30989910658427,0.756637682227064,0.977318116397535

"10405","TAS2R14",3.38200686307536,0.258143336626796,0.831147749755896
,0.310586579465097,0.756114930991742,0.977318116397535
"10406","VPS37B",10.7854835420213,0.140748333726747,0.452988738411412,
0.310710447726225,0.756020753991982,0.977318116397535
"10407","BCL2L2",14.1023491190889,-0.125518023885204,0.405494415377532
,-0.309543162927019,0.756908383934505,0.977318116397535
"10408","CNIH1",73.8677806438711,0.0675401682546845,0.217660559782284,
0.31030044360008,0.756332493849046,0.977318116397535
"10409","ZNF200",5.56059454611826,-0.190350635268669,0.615509932710941
,-0.309256805053157,0.757126186024201,0.977318116397535
"10410","COX10-
AS1",7.24546005770983,-0.166829457312046,0.538613047583115,-0.30973898
248594,0.75675945586023,0.977318116397535
"10411","SP2-
AS1",1.86532290676349,-0.314661068296417,1.01829591879005,-0.309007492
311569,0.757315827515344,0.977318116397535
"10412","CLASRP",15.9747864287479,-0.11911921423257,0.385379192050442,
-0.309096123220319,0.757248408116639,0.977318116397535
"10413","NUCB1",261.027944024474,0.059764565945063,0.193319607524905,0
.309149013440675,0.757208176679134,0.977318116397535
"10414","PPP2R1A",99.8503157581498,0.065162777626313,0.210846684407682
,0.309052892196861,0.757281292684795,0.977318116397535
"10415","ACOT9",72.854591147044,-0.0629722551923958,0.202470779132817,
-0.311018979934319,0.755786192812126,0.977318116397535
"10416","SHC3",11.8859062607707,-0.169367773465957,0.548540356382121,-
0.308760825881648,0.757503470449584,0.97735110785061
"10417","C2orf27A",27.8951469220839,0.102910252084218,0.33386479059492
5,0.308239308196705,0.757900243984524,0.977483168803972
"10418","ANKMY1",4.09703355779907,-0.215961479851457,0.700242541907981
,-0.308409539447599,0.757770724091535,0.977483168803972
"10419","VPS13B-
DT",6.3080117822159,0.17197114595041,0.557940580963486,0.3082248393788
45,0.757911252850908,0.977483168803972
"10420","PPRC1",7.84052838910035,0.169431637691833,0.549837998201976,0
.308148287760923,0.757969499375414,0.977483168803972
"10421","RPL38",1269.69028568255,-0.0738545426705595,0.239519493729592
,-0.308344600769481,0.757820131671329,0.977483168803972
"10422","IKBKE",6.47623087439007,-0.174318975180832,0.566050980881407,
-0.307956316778035,0.758115572122498,0.977577736879126
"10423","C1orf159",4.19005536605258,0.249395461289613,0.82264484634220
6,0.303162977800835,0.761765661562634,0.977629134279952
"10424","SSU72",111.524901201948,-0.0788947886468995,0.261904756023628
,-0.301234654325185,0.763235566020892,0.977629134279952
"10425","LEPROT",359.86603158167,-0.0443031167851811,0.1466435498713,-
0.302114322955652,0.762564914170955,0.977629134279952
"10426","WDR78",3.37112765071984,0.222086314486516,0.767424705103043,0
.289391666713018,0.772281671319642,0.977629134279952
"10427","AC092802.1",3.73725687576574,-0.290291057886768,1.00423608369
916,-0.289066547795679,0.772530452301878,0.977629134279952
"10428","KCND3",3.426864342476,-0.25648103534639,0.847369274716727,-0.

302679177778933,0.762134367604577,0.977629134279952
"10429","PHTF1",11.4692037298832,0.141269277193483,0.483388627524251,0.292247829488697,0.770097144360589,0.977629134279952
"10430","SIKE1",46.2881086100568,0.0747078450223378,0.244818537444447,0.305155997589807,0.76024734382469,0.977629134279952
"10431","GOLPH3L",25.0537517860127,-0.0952330639941853,0.327715452859614,-0.290596806354997,0.771359702884296,0.977629134279952
"10432","RFX5",8.98816657127132,-0.144997341900181,0.498035682011925,-0.291138460831626,0.770945425836665,0.977629134279952
"10433","ARHGEF2",33.6002549416115,-0.0831537919682125,0.279887728057676,-0.297096955787491,0.766392488433218,0.977629134279952
"10434","ARHGEF11",19.0347876978977,0.117200243629625,0.382611942720575,0.306316218977036,0.75936389141071,0.977629134279952
"10435","AL592295.5",13.5843773481438,-0.134642093723171,0.455230607412233,-0.295766786175795,0.767408189535024,0.977629134279952
"10436","UCK2",5.19840709120709,0.187126202577986,0.630042160078129,0.297005842521366,0.766462048614486,0.977629134279952
"10437","ZC3H11A",2.30947639799466,-0.27165307983663,0.938105702820512,-0.289576194899867,0.772140480769282,0.977629134279952
"10438","COQ8A",25.3017403635753,0.0972281179751647,0.327391119159629,0.296978483181636,0.766482936397322,0.977629134279952
"10439","COA6-
AS1",4.61409266144461,-0.196783624517387,0.667170217851874,-0.294952651140488,0.76803005027921,0.977629134279952
"10440","CATSPERE",1.57642179558611,-0.352855787094282,1.21424028444651,-0.290597990870583,0.771358796852495,0.977629134279952
"10441","EFCAB2",16.6843374616306,0.110011155943657,0.367471026893957,0.299373686338009,0.76465493727431,0.977629134279952
"10442","ZNF124",4.41527643600664,-0.21123933253058,0.723219742939946,-0.29208181136189,0.770224073057251,0.977629134279952
"10443","EIPR1",11.3588403893905,0.144419730897647,0.469320691763712,0.307720783319644,0.758294803814219,0.977629134279952
"10444","WDR43",37.8086481122244,-0.0921914778466532,0.308418763478913,-0.298916566575744,0.765003706393784,0.977629134279952
"10445","MEMO1",6.95497732416117,0.162817508876033,0.542099544237373,0.300346146029482,0.763913137579895,0.977629134279952
"10446","LHCGR",4.86070515771367,0.27079218303418,0.922791757933346,0.293448853120055,0.769179088705545,0.977629134279952
"10447","AC016727.1",3.45742687295796,-0.221336681666305,0.753187233247253,-0.293866746402545,0.7688597292514,0.977629134279952
"10448","SERTAD2",33.5514816696827,-0.0897494116137343,0.303094437519664,-0.296110388393094,0.767145780769325,0.977629134279952
"10449","C1D",73.4904789451178,0.0803421462417363,0.26727188982318,0.300600808767763,0.76371891466129,0.977629134279952
"10450","NFU1",49.8743157611671,-0.0959944452464127,0.323575023682512,-0.296668278515221,0.76671977736131,0.977629134279952
"10451","FAHD2A",35.0285814998953,-0.0887619710647922,0.306458508954449,-0.289637808940673,0.772093338863767,0.977629134279952
"10452","RNF149",53.0269929457788,0.0746364994402558,0.256629339526205,0.290833852349273,0.771178393453355,0.977629134279952

"10453","WDSUB1",12.2543623416126,-0.129361818920087,0.433800376266465
,-0.298205870712814,0.765546041347445,0.977629134279952
"10454","IFIH1",11.3963178085943,-0.144529506323526,0.475595151749917,
-0.303891883236698,0.761210261780443,0.977629134279952
"10455","FKBP7",70.4299930723735,0.0917659999202459,0.298941626954811,
0.306969627666198,0.758866490437736,0.977629134279952
"10456","HECW2",24.9908750885553,0.111955964295157,0.375883932316333,0
.297847166824195,0.76581981343551,0.977629134279952
"10457","CCNYL1",9.68604447977719,-0.148546508609967,0.490873041444245
,-0.302616962163809,0.76218178630788,0.977629134279952
"10458","PAX3",1.46093235161021,-0.446980645559638,1.53747903241433,-0
.290723083785889,0.771263115477927,0.977629134279952
"10459","FBXO36",8.42616413620503,-0.149605403642137,0.516733157058182
,-0.28952158691317,0.772182262931852,0.977629134279952
"10460","ARL4C",119.46823490035,0.196181887822679,0.637749697228214,0.
30761580707184,0.758374690792972,0.977629134279952
"10461","STK25",59.7710155117697,-0.0734899991536114,0.251797116915139
,-0.291861956379663,0.770392172043934,0.977629134279952
"10462","HDAC11",4.04077913638734,0.216072671044072,0.741895281432601,
0.291244164037322,0.770864587794195,0.977629134279952
"10463","MRPS25",45.849561744897,0.0777662434876607,0.268945837524338,
0.289152062004393,0.772465014565695,0.977629134279952
"10464","NKIRAS1",15.5627062679963,0.134186422173008,0.443329601553129
,0.302678688052656,0.762134740854412,0.977629134279952
"10465","MYD88",20.6952004311909,0.10056853346746,0.348207937149275,0.
288817464331223,0.772721066778735,0.977629134279952
"10466","ACVR2B",10.0718759028704,0.179676147413861,0.592625179869421,
0.303186826205142,0.761747487990714,0.977629134279952
"10467","HEMK1",14.8589474615127,0.117490912109223,0.388835608291373,0
.302160886513205,0.762529419474503,0.977629134279952
"10468","PDE12",14.2049865235263,0.122890231329076,0.40650689916766,0.
302307861393493,0.762417386019966,0.977629134279952
"10469","UBA3",30.4557418521183,0.0913020097383122,0.300752404180325,0
.303578652969203,0.761448916945508,0.977629134279952
"10470","ROBO2",12.1420714048617,0.170974722186818,0.571171582363986,0
.299340386437262,0.764680342521783,0.977629134279952
"10471","POGLUT1",22.1627818441327,-0.0948848188259165,0.3177350910438
43,-0.298628705171327,0.765223360755958,0.977629134279952
"10472","IQCB1",12.359779103602,0.126853885278121,0.424683657497894,0.
298702064556723,0.76516738164564,0.977629134279952
"10473","MAP3K13",30.4799764767519,-0.0913110124510882,0.3092023180330
84,-0.295311539162905,0.76775590242076,0.977629134279952
"10474","NSD2",13.2308686161629,0.132054009115412,0.451882879873347,0.
292230608852505,0.770110310063844,0.977629134279952
"10475","LYAR",29.8631042695065,-0.0871322129940809,0.292884414011404,
-0.297496926520263,0.766087154120977,0.977629134279952
"10476","SEPSECS-
AS1",7.00363345813247,0.177533419802039,0.579378337517572,0.3064205344
00209,0.759284475631423,0.977629134279952
"10477","RFC1",119.83860260433,0.0553428072695954,0.19141384955033,0.2

89126452446398,0.772484611507688,0.977629134279952
"10478","UGDH",169.545634125541,-0.0824998882787935,0.270022789415835,
-0.305529353493729,0.759963017190387,0.977629134279952
"10479","SGCB",81.3477490644332,0.0653533078696261,0.22650770859596,0.
288525756031562,0.77294431791827,0.977629134279952
"10480","AC124852.1",17.1739837521458,-0.12747498168651 2,0.42117489059
4814,-0.302665198075988,0.762145022398332,0.977629134279952
"10481","DNAJC21",64.0596397372515,0.0676244669563138,0.23238290294267
9,0.29100448483938,0.771047889472889,0.977629134279952
"10482","DDX46",111.208685410326,-0.0656807345572026,0.225298877607816
,-0.291527127230234,0.770648199884365,0.977629134279952
"10483","ETF1",39.1369903998029,-0.0875505640131646,0.287569746712743,
-0.304449842217304,0.760785200168823,0.977629134279952
"10484","HARS",35.3587372663981,-0.0845045035465372,0.283749776388896,
-0.297813463051751,0.765845538530445,0.977629134279952
"10485","PCDHGA2",1.93410231986076,0.31536967165731,1.04330453482573,0
.302279594433075,0.762438932480195,0.977629134279952
"10486","TIGD6",3.91493776238453,0.229580075946581,0.765021656458657,0
.30009617898835,0.764103793692074,0.977629134279952
"10487","SLC36A1",9.28546090082304,0.182325211850728,0.619550831759379
,0.294286122307297,0.768539276183655,0.977629134279952
"10488","GMDS",9.58773410145794,-0.149884814555431,0.496555649687235,-
0.301848976343012,0.762767193413151,0.977629134279952
"10489","SERPINB6",135.727103190312,0.067179251347853,0.22706840389934
8,0.295854686051483,0.767341057923632,0.977629134279952
"10490","LINC01011",5.20793369379758,0.212313052725041,0.7340488622683
17,0.289235585855911,0.772401101465505,0.977629134279952
"10491","HIST1H2BC",2.35924189460285,0.302999483335295,1.0213453978194
9,0.296667008028998,0.76672074742115,0.977629134279952
"10492","SMAP1",69.1318750474666,-0.0794747272300906,0.265109003791627
,-0.299781320488673,0.76434396449458,0.977629134279952
"10493","ANKRD6",7.36538190468455,0.163806490119398,0.538487516372838,
0.304197377169989,0.76097752293374,0.977629134279952
"10494","FYN",330.985389207216,0.0614799134103688,0.206768643090567,0.
297336735838808,0.766209437988556,0.977629134279952
"10495","TBPL1",19.022236667276,-0.113622297141755,0.388255211309807,-
0.292648479226954,0.769790854088268,0.977629134279952
"10496","HIVEP2",40.956565784207,-0.0957941331104552,0.320346258171503
,-0.299033095180309,0.764914793919778,0.977629134279952
"10497","RAET1G",2.99850951361664,0.240902928200818,0.819797679353255,
0.293856562744687,0.768867511268532,0.977629134279952
"10498","PON3",6.01741026158783,-0.17400878016986,0.595794540934004,-0
.292061722984359,0.770239431987873,0.977629134279952
"10499","ZC3HAV1L",2.54477792710726,0.296836821977261,1.0285595850211,
0.28859467774166,0.772891568836134,0.977629134279952
"10500","SSBP1",171.947940001874,-0.0705620040572496,0.243978781230654
,-0.289213691868316,0.772417854765635,0.977629134279952
"10501","TCAF1",76.7808782741157,0.0718381563896906,0.241112813446173,
0.297944167142855,0.765745777395897,0.977629134279952
"10502","ATP6V0E2",11.9022419006933,0.132921483376031,0.44799565943755

5,0.296702614357715,0.76669356090546,0.977629134279952
"10503","PAG1",2.01785310352118,0.327181201394244,1.1105095552909,0.29
4622589995221,0.768282203443726,0.977629134279952
"10504","MTERF3",10.9731788639659,-0.139321690594526,0.4604292048555,
-0.302590906342709,0.762201645466235,0.977629134279952
"10505","WDYHV1",10.1975359581107,-0.159504515880596,0.541365379938482
,-0.294633757147015,0.768273671797286,0.977629134279952
"10506","AC090198.1",1.68432984581876,-0.329312429564963,1.13234295658
225,-0.2908239307276,0.771185981956124,0.977629134279952
"10507","CPSF1",17.7453493129124,-0.115774441067188,0.397324400158325,
-0.291385178008335,0.770756749212139,0.977629134279952
"10508","KIAA2026",63.6920616500463,0.078148306632031,0.26830086079574
5,0.291271173712426,0.770843932156965,0.977629134279952
"10509","XPA",66.0339719542149,0.0730614772670922,0.243819818607273,0.
299653562554627,0.764441423390378,0.977629134279952
"10510","ERP44",105.439193479366,-0.0784574096774523,0.255825529742865
,-0.306683268696097,0.75908446613445,0.977629134279952
"10511","AL359091.5",2.32635909416476,0.30306628531805,1.0211240049316
5,0.296796749321681,0.76662168737885,0.977629134279952
"10512","ENDOG",32.6000100524348,-0.089290711854865,0.306794439184012,
-0.291044101361007,0.771017590696704,0.977629134279952
"10513","MED27",16.8284575974865,-0.10841214198903,0.371163541220661,-
0.292087260598093,0.770219906761168,0.977629134279952
"10514","IL15RA",55.9614709388822,-0.114825310094549,0.377781558015061
,-0.30394630880836,0.761168796380238,0.977629134279952
"10515","MLLT10",33.1926458756437,0.0921192503782135,0.306460560325183
,0.300590882821811,0.763726484576361,0.977629134279952
"10516","ZNF485",1.83737919811796,-0.324386649059194,1.07891486556229,
-0.300660097856874,0.763673698950657,0.977629134279952
"10517","KIF1BP",31.3361638627396,0.093417076113633,0.303818224710879,
0.307476867796627,0.758480427619729,0.977629134279952
"10518","VPS26A",73.7794385815679,0.0641888962765706,0.208816088735182
,0.307394399853807,0.758543190257632,0.977629134279952
"10519","RPP30",38.5058593207542,0.0946645156164773,0.320428824583257,
0.295430711452367,0.76766487535824,0.977629134279952
"10520","SEC31B",6.7266585830821,0.175637814703207,0.590178117951973,0
.29760136704611,0.766007431076974,0.977629134279952
"10521","LRRC27",5.03857416119216,-0.181128117375531,0.601256663787237
,-0.301249247259279,0.763224439041487,0.977629134279952
"10522","AP006623.1",7.26611484610369,0.174331204761942,0.598121683387
095,0.29146444545318,0.770696132420198,0.977629134279952
"10523","FAM160A2",11.9429436590227,-0.130207795061699,0.4375871912331
65,-0.297558515583511,0.766040140771871,0.977629134279952
"10524","OLFML1",131.798373532738,0.055520427533979,0.183229411137127,
0.30301045661511 2,0.76188189277026,0.977629134279952
"10525","TMEM9B",66.9315686280196,-0.0672930935199345,0.23001823865043
2,-0.292555468273985,0.769861956264869,0.977629134279952
"10526","DENND5A",40.5458354247087,0.0883279253708482,0.30111020359381
2,0.293340857654892,0.769261626608149,0.977629134279952
"10527","ZNF143",15.9538656573393,0.119272954416341,0.411211844158121,

0.290052332175718,0.771776202266606,0.977629134279952
"10528","WEE1",25.6413811220165,-0.0964220394086509,0.334376395530323,
-0.288363774170497,0.773068294515444,0.977629134279952
"10529","TRIM44",67.8804005726643,-0.0720505085404865,0.23993702939410
5,-0.300289241399839,0.763956538905782,0.977629134279952
"10530","ACP2",27.8316469485241,-0.103218585064821,0.345697851560441,-
0.298580348703075,0.765260261300502,0.977629134279952
"10531","FNBP4",81.50613318335,0.0910186539031077,0.297017898796998,0.
306441646351138,0.759268403321421,0.977629134279952
"10532","SLC43A3",95.6961182310232,0.077929650468591,0.265104173481296
,0.293958595390009,0.768789542321792,0.977629134279952
"10533","ZFP91",95.8018698674037,-0.0735993543936307,0.249068436648703
,-0.29549851994068,0.767613082805932,0.977629134279952
"10534","BEST1",3.50057553983117,0.227312585736648,0.787797633064051,0
.28854184906921,0.772932001052746,0.977629134279952
"10535","ROM1",5.73957086696543,-0.195457606612395,0.673262081864751,-
0.290314294948901,0.771575803768724,0.977629134279952
"10536","PLAAT5",4.26004817934507,-0.203526271224881,0.666390873028393
,-0.305415754420469,0.760049524356401,0.977629134279952
"10537","FCHSD2",11.8332861448363,0.13333450805311,0.459202011657436,0
.290361332634094,0.771539822096021,0.977629134279952
"10538","AP001528.1",34.2474567844682,-0.131778062686804,0.43386952866
8947,-0.303727397245622,0.761335583464435,0.977629134279952
"10539","SLC36A4",19.3952025376794,-0.103874086932239,0.34960381606694
1,-0.297119431077233,0.766375330022401,0.977629134279952
"10540","CCDC84",20.3134821589314,0.128465223727828,0.423038968797918,
0.303672316743933,0.761377550633676,0.977629134279952
"10541","SRPRA",66.1835845794128,-0.07220182708578,0.240239518578552,-
0.300541008044736,0.763764521376241,0.977629134279952
"10542","ATN1",62.1595161878439,0.0751337537261905,0.259703925725985,0
.28930542161101,0.772347663788219,0.977629134279952
"10543","EMG1",36.9934840710875,-0.0934902584813163,0.316335214667952,
-0.295541735938095,0.767580074695185,0.977629134279952
"10544","CLEC12A",3.28138312797828,0.29867681883011,0.987891108653333,
0.302337794331663,0.762394569870701,0.977629134279952
"10545","ITPR2",18.7744665383123,0.115738604639862,0.3906247231736,0.2
96291037852272,0.767007829613567,0.977629134279952
"10546","MRPS35",37.0486930268613,-0.0847816149170884,0.28703366178871
4,-0.295371680062725,0.767709964751866,0.977629134279952
"10547","ANO6",94.6955985667325,0.0712343219709701,0.235458452765275,0
.302534570895114,0.762244583607239,0.977629134279952
"10548","CAPS2",6.64359024019845,-0.170285029854079,0.563302175779808,
-0.302297837955879,0.762425026353457,0.977629134279952
"10549","SOCS2",5.96560157769674,0.182273214825285,0.619419878426861,0
.294264393464743,0.768555878632605,0.977629134279952
"10550","VEZT",39.7405481402354,0.0828680522619731,0.286271161289475,0
.289473979456064,0.772218689324145,0.977629134279952
"10551","C12orf75",89.4320820118143,-0.119596504648933,0.4068037741777
28,-0.293990646696122,0.76876505057872,0.977629134279952
"10552","PWP1",59.5685991135581,-0.067287007184169,0.225656623956985,-

0.298183168764393,0.765563367194152,0.977629134279952
"10553","NAA25",15.2919059830417,-0.132083981427984,0.457682714687772,
-0.288592898943302,0.772892930222431,0.977629134279952
"10554","KATNAL1",28.0483213466224,-0.0943107704548478,0.3221917637430
82,-0.29271626735329,0.769739034708132,0.977629134279952
"10555","DGKH",7.81782331331612,0.15947147554709,0.547286757325966,0.2
91385591579569,0.770756432945486,0.977629134279952
"10556","MZT1",22.953883656842,0.116148512280144,0.388442385949862,0.2
99010912509266,0.764931719277739,0.977629134279952
"10557","PCCA",18.0499498761962,0.0993781607193813,0.343834079443395,0
.289029408836543,0.772558872520368,0.977629134279952
"10558","MCF2L",19.9636303838683,-0.132489877894412,0.452355851924232,
-0.292888612650476,0.769607293162187,0.977629134279952
"10559","LINC01551",1.54917391184456,0.368605657726157,1.2359542000530
6,0.298235693288905,0.765523281303875,0.977629134279952
"10560","SYNJ2BP",35.9284098676955,-0.0906136820107478,0.2947397356998
75,-0.307436259978929,0.758511332202423,0.977629134279952
"10561","PNMA1",42.3388011466331,-0.0799908699969941,0.264731937416971
,-0.302157989615749,0.762531627721101,0.977629134279952
"10562","ISCA2",34.0174689278423,0.0943004515611436,0.316181603547103,
0.298247749088587,0.765514080595938,0.977629134279952
"10563","ACYP1",31.3780453190369,0.10613352796727,0.356940862851299,0.
297342050219353,0.766205381086075,0.977629134279952
"10564","IFI27L2",287.500149415744,-0.0867730293201323,0.2836113963617
21,-0.305957484195949,0.759637017103031,0.977629134279952
"10565","BCL11B",3.03115801182509,-0.265321559434659,0.868931814344807
,-0.305342208737883,0.760105531947164,0.977629134279952
"10566","AL118558.3",3.51137108994851,0.239009413619761,0.821647718317
111,0.290890375877021,0.771135162133279,0.977629134279952
"10567","TRAF3",12.1987726265436,0.135690651444898,0.442189593186214,0
.306860798028226,0.758949329016346,0.977629134279952
"10568","STOML1",20.8163537461468,0.12502967465991,0.429976673722486,0
.290782459377333,0.771217701348222,0.977629134279952
"10569","SNRPA1",39.4715909898772,-0.0925015806852103,0.31126426327971
6,-0.297180215006193,0.766328926050065,0.977629134279952
"10570","DECR2",9.83693035249579,-0.167333901084027,0.565973986837423,
-0.295656523048107,0.767492403059771,0.977629134279952
"10571","GNPTG",74.6551155290425,0.070010408250118,0.229601579038765,0
.30492128383097,0.760426105068732,0.977629134279952
"10572","SOCS1",82.3791207728046,0.133441517218001,0.454295797741615,0
.293732669070157,0.768962188613577,0.977629134279952
"10573","KNOP1",53.1840797836546,0.0859966372033914,0.286052769233935,
0.300632073703377,0.763695070944716,0.977629134279952
"10574","LYRM1",48.576768672628,0.0800993714269498,0.275688041151576,0
.290543511036485,0.771400468608414,0.977629134279952
"10575","CRYM",3.77721664587955,-0.220254949342693,0.725038848097391,-
0.303783652311424,0.761292722091029,0.977629134279952
"10576","AC008870.2",1.45689658335484,0.37960241766815,1.2696990943857
7,0.298970377585239,0.764962647678138,0.977629134279952
"10577","FBRS",18.9324217154873,0.104787286506752,0.361638728903592,0.

289756815660878,0.77200228726691,0.977629134279952
"10578","BRD7",92.3415718828109,-0.0640737869519184,0.214417538290067,
-0.298827173667289,0.765071916163458,0.977629134279952
"10579","IRX6",2.17972136684763,-0.31066882468097,1.01585431134899,-0.
305820255139165,0.759741505509604,0.977629134279952
"10580","C16orf70",9.33170547609655,0.153146007655665,0.52594899062485
4,0.29118034331375,0.770913395310192,0.977629134279952
"10581","SLC12A4",38.8564241871641,0.0830466125531404,0.27899826296860
6,0.297659962716274,0.765962704072473,0.977629134279952
"10582","SLC7A6",13.4404226600176,-0.13110342314733,0.438574064264888,
-0.298931090161653,0.764992624585594,0.977629134279952
"10583","AC009063.3",1.71875214988974,-0.395585451714962,1.32840253222
549,-0.297790347517805,0.765863182084665,0.977629134279952
"10584","CTNS",11.0653049826568,0.130691238605568,0.443477187522503,0.
294696643441071,0.768225627516765,0.977629134279952
"10585","AC012146.1",6.81662288815536,0.166297919218472,0.558038070168
365,0.298004613140997,0.76569964272939,0.977629134279952
"10586","AC016876.1",22.6758294871043,0.116208487654567,0.382679198818
588,0.303670771793522,0.761378727778961,0.977629134279952
"10587","TRIM16",10.34869361582,0.148281653690966,0.496657899989862,0.
298558935021456,0.765276602128766,0.977629134279952
"10588","PIGS",20.1439795243133,0.111131431627902,0.372287533332749,0.
29850967781017,0.765314190813237,0.977629134279952
"10589","NEK8",3.3827709786812,-0.219374135265409,0.759343012119504,-0
.288899919751792,0.772657965138359,0.977629134279952
"10590","PLEKHH3",39.3017804994793,-0.0749269373144577,0.2573543165546
07,-0.291143114743752,0.770941866638264,0.977629134279952
"10591","AC100793.2",2.41142069160861,-0.268684808939231,0.90847263912
7262,-0.295754431522943,0.767417625271369,0.977629134279952
"10592","TMEM106A",8.44060025728851,0.189395634059289,0.64847495206215
9,0.292063145163908,0.770238344631936,0.977629134279952
"10593","UBE2Z",60.5321670639162,0.0759985013645417,0.253481859490972,
0.299818304620132,0.764315752206666,0.977629134279952
"10594","TRIM65",16.5193090577367,0.116634227252072,0.398161995958363,
0.292931591754098,0.769574440771485,0.977629134279952
"10595","LRRC45",11.312218346691,-0.136108958088541,0.452876812070512,
-0.300543005207671,0.763762998236932,0.977629134279952
"10596","AP005205.2",1.62849679526309,-0.314492030125799,1.08207574551
53,-0.290637722386092,0.771328406536882,0.977629134279952
"10597","FECH",19.3693837899358,-0.108879570159424,0.363108960683277,-
0.299853713206474,0.76428874207145,0.977629134279952
"10598","CIRBP",517.910643404555,-0.0604932728968649,0.209245142892466
,-0.289102399514015,0.772503017421014,0.977629134279952
"10599","FDX2",34.9284850050196,0.106970090110563,0.370804551634053,0.
288481060006329,0.772978526485475,0.977629134279952
"10600","IER2",198.360412519629,-0.124122491380597,0.423653473579321,-
0.292981172399988,0.769536542804083,0.977629134279952
"10601","SMIM7",124.534653976844,-0.0745622207781704,0.250783772418184
,-0.297316768382594,0.766224680842623,0.977629134279952
"10602","BABAM1",52.5841068241271,-0.0812416175048914,0.27615532933516

,-0.294188121230466,0.768614157122626,0.977629134279952
"10603","DDA1",28.5244826357941,-0.102149983934471,0.345292853643736,-0.295835789407523,0.767355489675689,0.977629134279952
"10604","ZNF626",29.1509481217886,0.0937739039928266,0.318521078493698,0.294404076603935,0.768449152196182,0.977629134279952
"10605","ZNF430",32.3032549984015,-0.0938845580808134,0.320126693464095,-0.293273132161793,0.769313388635587,0.977629134279952
"10606","GPATCH1",12.1503857174859,0.156721984185453,0.520016500780784,0.301378867690047,0.763125606790112,0.977629134279952
"10607","ZNF235",1.75899399544545,0.293358370138712,1.01700091078305,0.28845438290988,0.77299894428856,0.977629134279952
"10608","DMPK",7.73312027251977,0.151992957829809,0.526560944983888,0.288652167005018,0.77284757035501,0.977629134279952
"10609","CCDC61",14.5533643799657,0.141754058519495,0.468944894873893,0.302282976249513,0.762436354684551,0.977629134279952
"10610","SCAF1",27.7095528214002,-0.0853003192637086,0.29165095662204,-0.292473991005118,0.769924243112206,0.977629134279952
"10611","ZNF17",4.58540065634313,-0.195851384088366,0.67189486871687,-0.291491114469124,0.770675738609875,0.977629134279952
"10612","NRSN2",13.8120021231571,-0.134158186026037,0.44636212177636,-0.300559074081233,0.763750743319674,0.977629134279952
"10613","PHF20",70.7192190784959,0.0725390532171136,0.241237586323999,0.300695485817408,0.763646711371082,0.977629134279952
"10614","MROH8",1.7742640715097,0.33527688479092,1.10176245400624,0.304309593752976,0.76089203678705,0.977629134279952
"10615","HIRA",8.4083665447084,0.146493568447714,0.505494975466748,0.289802224665932,0.771967545834345,0.977629134279952
"10616","JOSD1",39.2527817183294,-0.0822041817655001,0.274378213810826,-0.299601708983268,0.764480980446883,0.977629134279952
"10617","XRCC6",154.505191556379,-0.0642471791283753,0.217320759437589,-0.295632958833028,0.767510400598152,0.977629134279952
"10618","TCF20",13.3752328372815,0.123519592544504,0.408117035995588,0.302657281245763,0.762151056322878,0.977629134279952
"10619","PPARA",19.9185836972376,0.101829568333004,0.333689219916929,0.305162894858737,0.760242090962908,0.977629134279952
"10620","RAB9A",82.601348239654,0.0752221765830693,0.246875450409089,0.304696868232224,0.760597035056328,0.977629134279952
"10621","FUNDC1",26.8335753943323,-0.107319296158764,0.367107581347327,-0.292337455317293,0.770028623742317,0.977629134279952
"10622","NLGN3",1.45726840185783,0.382294959623453,1.2880530181231,0.296800639604509,0.766618717129884,0.977629134279952
"10623","TIMM8A",8.62052868670199,-0.158491461851978,0.526272583980533,-0.301158499751606,0.763293633983624,0.977629134279952
"10624","TMSB15B.1",3.55067836519966,0.24245378668819,0.812189573228713,0.298518713709114,0.765307295384434,0.977629134279952
"10625","TSC22D3",761.253457190498,-0.101446086805812,0.346803206914668,-0.292517729891618,0.769890805904244,0.977629134279952
"10626","AMOT",16.1806569593797,0.119635705144938,0.393372650857702,0.304128171808809,0.761030244757547,0.977629134279952
"10627","BRCC3",17.7615073907117,-0.109801606477696,0.374666735206789,

-0.293064732360329,0.769472673307614,0.977629134279952
"10628","IGHG1",22.0008855506123,0.301133147320361,1.04484498516169,0.288208443928896,0.773187185579708,0.977687484663689
"10629","KCTD21",7.27823403037164,0.16220676250126,0.56318698221647,0.288015823559843,0.773334626211322,0.977781921314701
"10630","AL162253.2",7.03536326190477,0.160770908455466,0.558694415394267,0.287761796118922,0.773529083191654,0.977807875127721
"10631","SRP68",35.3127014918032,-0.0867416694778605,0.301323548819788,-0.287868869916097,0.773447116898479,0.977807875127721
"10632","CCDC97",21.4739838380243,-0.096031518408173,0.333785984974847,-0.28770386634241,0.773573430192569,0.977807875127721
"10633","H2AFZ",159.266230324583,-0.0799743262732564,0.278366965618166,-0.287298193216492,0.773884005977907,0.978016471350112
"10634","ZNF846",10.4692541073002,-0.144601945629973,0.503222192163753,-0.287352083993383,0.773842746117363,0.978016471350112
"10635","MYORG",1.88196146865148,0.317964710413027,1.10741096497288,0.287124401392228,0.774017068838448,0.978092655206385
"10636","C1orf53",1.85506383697946,-0.350637078773501,1.2235375820968,-0.286576468025287,0.774436634793397,0.978162963814705
"10637","AC064807.1",7.32381188215698,-0.159795315479281,0.556921037795068,-0.28692634078241,0.77416872107135,0.978162963814705
"10638","FGFR1OP2",83.4730364614805,-0.066106996934341,0.230541875757291,-0.28674615714473,0.774306692644106,0.978162963814705
"10639","NPFF",2.66737935929554,-0.251276993925437,0.876227231653634,-0.286771495849566,0.774287289669255,0.978162963814705
"10640","IQCK",27.7612125645135,-0.0928683449431322,0.323983826437607,-0.286645003129552,0.774384152183465,0.978162963814705
"10641","MRPL20-AS1",11.3956300822391,-0.113879294894314,0.437384252863037,-0.260364414468242,0.794582689719157,0.978328522364823
"10642","NMNAT1",12.5291225769127,0.116015078235832,0.424395201924573,0.273365668861758,0.784572140335399,0.978328522364823
"10643","CASP9",8.67425845069326,-0.144289841875391,0.532745022257194,-0.270842215031964,0.786512389993609,0.978328522364823
"10644","AL450998.2",6.92333780944974,0.150914071577348,0.566914858106661,0.266202357231135,0.790083367698964,0.978328522364823
"10645","ZBTB40",13.1891971826607,0.128685863458457,0.480235287871838,0.267964197359858,0.788726877565876,0.978328522364823
"10646","MACO1",41.2716400843277,0.0736743184685249,0.264886738658922,0.278135171437898,0.780908597590918,0.978328522364823
"10647","SYTL1",1.53783281588301,-0.315182908024515,1.14342307401559,-0.275648546183018,0.782818019117665,0.978328522364823
"10648","PHACTR4",40.5476758336735,0.088390070267814,0.324882633247639,0.272067698369212,0.785569965975378,0.978328522364823
"10649","RCC1",4.74499358966748,0.179207366842708,0.643141672241834,0.278643687040268,0.780518282554626,0.978328522364823
"10650","SNHG12",13.6626878176557,0.120918434658523,0.456536740716948,0.264860248637672,0.79111712196652,0.978328522364823
"10651","EPB41",1.76365091269529,0.302933149200202,1.08940123545317,0.278073072933672,0.78095626555682,0.978328522364823

"10652","OSCP1",11.6012175334093,-0.129230592952131,0.489855253566604,
-0.263813834824086,0.791923373484421,0.978328522364823
"10653","GNL2",26.0430364337466,-0.082839147755505,0.333324409118018,-
0.248524096914171,0.803728928147944,0.978328522364823
"10654","FOXJ3",32.1569087293304,0.090621669942271,0.331423426590196,0
.273431696952202,0.784521390138812,0.978328522364823
"10655","ZNF691",6.40030943926695,0.16420580384327,0.588005928909325,0
.279258755345972,0.780046256082634,0.978328522364823
"10656","SZT2",18.4363800246548,0.11164486240173,0.453530966399135,0.2
46168113476679,0.805552097918149,0.978328522364823
"10657","DMAP1",33.980031439219,-0.0813762848316473,0.293725369369675,
-0.277048880749654,0.78174257312718,0.978328522364823
"10658","PLK3",30.058316932566,-0.118225387933624,0.476079233148658,-0
.248331327438321,0.803878062192763,0.978328522364823
"10659","TOE1",7.16349300891569,-0.145004965343644,0.549103840286168,-
0.264075671494255,0.791721610000159,0.978328522364823
"10660","ALG6",13.0732353328285,-0.120735587834483,0.441758932461059,-
0.273306500361769,0.784617618924461,0.978328522364823
"10661","AL513166.1",3.05034463293684,0.241145640977273,0.899626549563
103,0.268050827417647,0.788660195137425,0.978328522364823
"10662","DDAH1",4.46521907450563,-0.204092630424955,0.810429494703466,
-0.251832678547357,0.801170397329028,0.978328522364823
"10663","ZNHIT6",41.8500881681954,-0.08571334877799,0.338639004059697,
-0.253111271148435,0.800182229297072,0.978328522364823
"10664","CNN3",189.021952633022,0.0571305310407625,0.21976559972631,0.
259961209178831,0.794893695696567,0.978328522364823
"10665","FRRS1",9.57980284701832,0.134248947821461,0.518626212018393,0
.258854922312913,0.795747179741525,0.978328522364823
"10666","AMY2B",2.71762436773105,-0.23965239696359,0.861588906003973,-
0.278151674532454,0.780895929647303,0.978328522364823
"10667","PRMT6",8.50558901061335,0.129563876883365,0.483398324317385,0
.268027153520494,0.78867841767675,0.978328522364823
"10668","AC245407.2",9.39281447609591,-0.150779282440105,0.55582564870
4758,-0.27127082528751,0.786182743505382,0.978328522364823
"10669","AL365436.2",1.95172553313628,-0.257687187519503,1.04231953873
821,-0.247224750129359,0.804734291862809,0.978328522364823
"10670","ZBTB7B",9.95835279396374,-0.13837384035345,0.487690275881159,
-0.283733031386439,0.776614979167711,0.978328522364823
"10671","HCN3",1.97256333789238,0.276931678371299,0.976025318516316,0.
283734113365288,0.776614149933044,0.978328522364823
"10672","UBQLN4",7.71237135671195,0.157171422153243,0.578361294150524,
0.271753009308984,0.785811938954693,0.978328522364823
"10673","RGS5",1.7113087493497,-0.29005821974514,1.12222874725916,-0.2
58466217741753,0.796047117683793,0.978328522364823
"10674","XPR1",11.9634493131413,0.119507046538853,0.438430241941225,0.
27257938688197,0.785176558995712,0.978328522364823
"10675","NEK7",41.032208929466,0.0666764879156587,0.265977208667506,0.
25068496751919,0.802057683825833,0.978328522364823
"10676","PTPRC",11.6703354222267,-0.157474129358155,0.639198662252774,
-0.246361794317838,0.805402178627375,0.978328522364823

"10677","CYB5R1",41.357052726699,-0.0845291276718962,0.315489109305497
,-0.267930414010089,0.788752882306126,0.978328522364823
"10678","ATP2B4",153.780040201934,-0.0595373406413903,0.22855116812458
5,-0.260498955791537,0.79447892067467,0.978328522364823
"10679","SLC41A1",9.06728481344796,0.121083711328965,0.481853535388609
,0.251287377670296,0.801591933303383,0.978328522364823
"10680","PPP2R5A",32.1816394304924,-0.0846661225398559,0.3392643733299
64,-0.249557953017103,0.802929218461817,0.978328522364823
"10681","EPRS",101.10529481849,-0.0561642327371037,0.205065424757202,-
0.273884458111855,0.784173415704376,0.978328522364823
"10682","CNIH3",6.30181704646725,0.160453708504838,0.615858661607383,0
.260536578451387,0.794449903716653,0.978328522364823
"10683","HIST3H2A",23.321747278126,0.125508108220983,0.44368722788725,
0.282875188493993,0.777272514817954,0.978328522364823
"10684","ABCB10",11.291396941205,-0.124934925990981,0.438787576065102,
-0.284727583017176,0.77585285675535,0.978328522364823
"10685","COG2",18.1307718180155,-0.092301258882332,0.36775328740929,-0
.25098690356398,0.8018242346168,0.978328522364823
"10686","EXOC8",8.83521393706546,0.131204993154167,0.483870591087739,0
.27115719692577,0.786270131967825,0.978328522364823
"10687","KCNK1",13.0234170882234,0.159723164110958,0.591332804558557,0
.27010705795393,0.787077892399492,0.978328522364823
"10688","ARID4B",134.418660898221,-0.0566749013734727,0.21966068687493
7,-0.258011126978494,0.796398319808677,0.978328522364823
"10689","ADSS",62.0528521437119,-0.0712776659234627,0.256481327978547,
-0.277905867398755,0.781084619724397,0.978328522364823
"10690","TMEM18",53.5888583249348,-0.0623193297115876,0.25188360361249
3,-0.247413205217844,0.804588455523586,0.978328522364823
"10691","GRHL1",3.56607766805722,-0.236830380124344,0.889731516884974,
-0.266181848827282,0.790099161448704,0.978328522364823
"10692","DDX1",102.709103122079,0.0644959860798484,0.240266303011378,0
.268435420495875,0.788364178017414,0.978328522364823
"10693","SLC35F6",89.9654405430862,0.0658526425873454,0.26329136339819
8,0.250113189196225,0.802499816566113,0.978328522364823
"10694","CAD",11.272860818557,-0.13249671847847,0.480152600687545,-0.2
75947101585504,0.782588696013881,0.978328522364823
"10695","FNDC4",20.4571966361607,0.103826895217929,0.379393046603905,0
.273665783143166,0.784341475308561,0.978328522364823
"10696","SRBD1",26.4742480198475,0.100236507660632,0.350496944567344,0
.28598396994406,0.774890399177965,0.978328522364823
"10697","AC015982.2",1.52018321987748,0.320910245762844,1.272174788403
71,0.252253266365634,0.800845308290149,0.978328522364823
"10698","CEP68",35.3200144400052,0.0853754236279568,0.343964608880065,
0.248209907135318,0.803972001377524,0.978328522364823
"10699","AC017083.1",1.49595210748833,0.33649289361825,1.3682963970657
7,0.245921055072453,0.805743344660533,0.978328522364823
"10700","INO80B",12.5761196156208,0.118490211632922,0.42248125527189,0
.280462648115991,0.779122578433717,0.978328522364823
"10701","RMND5A",21.9028553103327,0.10284008867833,0.398322321644849,0
.258183092159278,0.796265606184345,0.978328522364823

"10702","PLGLB2",3.30120401522975,0.210652251031434,0.80851802943798,0
.260541191861688,0.794446345584479,0.978328522364823
"10703","TBC1D8",5.6384374725186,0.18486890756856,0.645749027630186,0.
28628600223682,0.77465907826731,0.978328522364823
"10704","SEPTIN10",133.28583573167,0.0487129362946441,0.19372686307765
5,0.251451634124265,0.801464951438227,0.978328522364823
"10705","THORLNC",3.04231141790187,-0.228537716314917,0.80526461746788
7,-0.283804492780947,0.776560211300407,0.978328522364823
"10706","IWS1",46.4317633398768,0.0785080768276065,0.299427276566421,0
.262194138516273,0.793171772152439,0.978328522364823
"10707","LINC01117",4.61121547950417,0.170709981132967,0.6657820897131
94,0.256405186878045,0.7976379831533,0.978328522364823
"10708","NIF3L1",17.2791630163397,0.106675639145416,0.390535032842809,
0.273152547593222,0.784735954839486,0.978328522364823
"10709","CASP10",6.75193135401041,-0.148380461131487,0.546841910670645
,-0.271340689577932,0.78612901412932,0.978328522364823
"10710","SMARCAL1",17.2902138386684,0.102120896845572,0.39812122322539
1,0.256507040790834,0.797559344501095,0.978328522364823
"10711","NCL",267.150502706769,-0.058917582872631,0.230374185424639,-0
.255747330214237,0.798145945933133,0.978328522364823
"10712","COPS8",75.7425677903488,0.0771138007102884,0.281873891006722,
0.273575535622948,0.78441083671672,0.978328522364823
"10713","UBE2F",29.6302832902203,0.0857407052458549,0.338006187387322,
0.253666081998684,0.799753539758795,0.978328522364823
"10714","ARL8B",51.7870174395675,0.0659453703442134,0.233903114248268,
0.281934554638798,0.777993693058533,0.978328522364823
"10715","ZNF589",14.8233043989777,0.120641952749121,0.423016821989861,
0.285194220366046,0.775495348060755,0.978328522364823
"10716","MON1A",7.28165847592895,0.148200240606914,0.543071701564967,0
.272892585232937,0.784935786404396,0.978328522364823
"10717","CISH",1.47545994927174,-0.341126611175564,1.25357547571287,-0
.272122913844956,0.785527511429659,0.978328522364823
"10718","RYBP",44.3301810407672,0.0770908350958543,0.297448285142364,0
.25917391004274,0.79550106027679,0.978328522364823
"10719","PROS1",152.729444325246,0.0565292359447384,0.206183907058369,
0.274169001602707,0.78395474883453,0.978328522364823
"10720","CPOX",11.7211416631324,0.113941553720282,0.450991743572961,0.
25264665117278,0.800541276753095,0.978328522364823
"10721","DUBR",17.8822763310669,0.0890085630863645,0.353266224900421,0
.251958882034234,0.801072846020181,0.978328522364823
"10722","MAATS1",2.20438555037334,-0.227728555220606,0.909742215552129
,-0.250322070722415,0.80233828970599,0.978328522364823
"10723","AC083798.2",4.02679137365491,0.198306642561912,0.749805692304
151,0.264477376735453,0.791412095113717,0.978328522364823
"10724","HEG1",113.039917419127,0.0720767563321691,0.253940036854381,0
.283833763375803,0.776537778696724,0.978328522364823
"10725","SLC41A3",34.9814663033543,-0.089812141927813,0.33734867021852
6,-0.266229423313378,0.790062523940203,0.978328522364823
"10726","ISY1",38.624249845153,0.0702696654872567,0.265653572768434,0.
264516169517169,0.791382206919301,0.978328522364823

"10727","MRAS",14.3426160352227,-0.108503439284748,0.422625960062378,-0.256736333160257,0.797382321581788,0.978328522364823
"10728","AC084036.1",1.89760285428447,-0.307115981301989,1.08583191626461,-0.282839338853204,0.77729999700576,0.978328522364823
"10729","LINC00886",2.09400217631143,0.284745734395382,1.00431478588735,0.283522395962534,0.776776416187443,0.978328522364823
"10730","GFM1",25.3550052029308,0.0866853397712617,0.318755447975368,0.271949359052085,0.785660957866479,0.978328522364823
"10731","PPM1L",26.1845755534152,-0.119457051990986,0.452721129090285,-0.263864538929357,0.791884301333204,0.978328522364823
"10732","MYNN",13.1404586853324,-0.102573340747057,0.397627414724037,-0.257963452591027,0.796435113420183,0.978328522364823
"10733","MYL5",14.301029451727,0.124501919996481,0.505960208867246,0.246070575935642,0.805627599878531,0.978328522364823
"10734","TACC3",1.591108077121,-0.286755018136018,1.14929161601714,-0.249505881831598,0.802969491704404,0.978328522364823
"10735","LRPAP1",220.679488043528,-0.0631854287951722,0.232491325508507,-0.271775424984018,0.785794702247568,0.978328522364823
"10736","HELQ",17.6349875500766,0.0962081873117352,0.367457680116205,0.261821136195358,0.793459343007672,0.978328522364823
"10737","AC098487.1",10.5597951195596,0.123289097212323,0.460506638481907,0.267724907546945,0.788911076017281,0.978328522364823
"10738","CASP6",16.7928820196443,0.110800659518457,0.42001094556886,0.263804219121931,0.791930783321477,0.978328522364823
"10739","SEC24D",60.8435665407169,0.0681891637643483,0.256479092641985,0.265866363850298,0.790342130796649,0.978328522364823
"10740","NDNF",18.5198646183236,-0.117736204419289,0.473595248350942,-0.24860089882499,0.80366951316042,0.978328522364823
"10741","JADE1",24.6511582651159,0.0844044935373039,0.325569789527169,0.259251614407732,0.795441109463209,0.978328522364823
"10742","RAB33B",19.3426150694947,-0.0953521495228428,0.345509949732013,-0.275975119086442,0.782567176487462,0.978328522364823
"10743","KLHL2",11.1655114289605,0.1298671406607,0.466702739036575,0.27826522066013,0.780808772058793,0.978328522364823
"10744","SLC25A4",40.2696200047443,0.0790709574337214,0.293923233310565,0.269019078699959,0.787915001021032,0.978328522364823
"10745","FYB1",6.94106667764314,0.194903998870398,0.783965292731071,0.24861304534467,0.803660116553889,0.978328522364823
"10746","MCCC2",16.3348325195181,-0.105096156990479,0.408825486809781,-0.257068505710306,0.797125889515828,0.978328522364823
"10747","SCAMP1",62.1011794605659,0.0596828325784312,0.233436462173546,0.255670566726035,0.798205224287901,0.978328522364823
"10748","XRCC4",15.2336577794064,0.105009424852658,0.385964244803205,0.272070343993134,0.785567931770955,0.978328522364823
"10749","TMEM161B",16.1524530250085,0.0993323729727174,0.363553504815379,0.273226283496182,0.784679277059338,0.978328522364823
"10750","NR2F1",2.25776793336875,0.263991951402927,0.989877902853977,0.266691427944594,0.789706754466549,0.978328522364823
"10751","FBXL17",19.7208933878907,-0.100198838651568,0.362046228487251,-0.276757029261793,0.781966678440586,0.978328522364823

"10752","LINC01184",26.3826106395269,0.0726210443207987,0.293491047670787,0.247438703487334,0.804568724159947,0.978328522364823
"10753","SPARC",1759.96990404495,0.0949267099982728,0.36281131174018,0.261642090327803,0.793597390661585,0.978328522364823
"10754","CYFIP2",1.61950538456901,0.318152228255091,1.13081947438086,0.281346612313414,0.778444562036121,0.978328522364823
"10755","MAT2B",38.3234399845062,0.0919261624011361,0.345948777228321,0.265721888476184,0.790453404666609,0.978328522364823
"10756","DUSP1",822.214168244896,-0.0973718149405175,0.374979718599769,-0.259672217217824,0.795116625101832,0.978328522364823
"10757","ZFP62",13.9562920461931,0.114079061020453,0.436371043693756,0.261426743751845,0.793763435395154,0.978328522364823
"10758","RIPK1",23.9087854493999,-0.087135300793596,0.332759387194435,-0.261856777439856,0.793431863725416,0.978328522364823
"10759","SMIM13",16.1275198631136,-0.0948128496094803,0.381486058664408,-0.248535555772136,0.803720063349861,0.978328522364823
"10760","ACOT13",77.1218856016474,-0.0973813224194195,0.365005663726671,-0.266794003756452,0.789627771283122,0.978328522364823
"10761","APOM",5.50521355026012,0.179731471669939,0.710173804796857,0.253080964766577,0.800205648074097,0.978328522364823
"10762","C6orf47",20.5664281760475,-0.0901681910417364,0.332052168263923,-0.271548267590496,0.785969381545029,0.978328522364823
"10763","EGFL8",8.67064025671712,-0.150250314451287,0.531221911740651,-0.282839075592651,0.777300198821252,0.978328522364823
"10764","GPSM3",3.27688626583909,0.239687255208991,0.840196723795186,0.285275160472322,0.775433341593804,0.978328522364823
"10765","NOTCH4",3.0858498443378,-0.2244339681912,0.886443043814633,-0.253184871557447,0.800125356493553,0.978328522364823
"10766","PHF1",27.2028636908992,-0.0853675996930353,0.326700147876366,-0.261302604997109,0.79385915784246,0.978328522364823
"10767","SRPK1",24.7087001427428,0.091396687434484,0.325336731074156,0.280929506891896,0.778764468398576,0.978328522364823
"10768","C6orf89",96.5525130971974,0.0556381227854675,0.213764119812238,0.260278117928948,0.794649250435756,0.978328522364823
"10769","DNPH1",59.6708213163546,0.10242715669563,0.380184491930306,0.269414347164939,0.787610846758795,0.978328522364823
"10770","AL359711.2",2.14778978595739,-0.298670171231723,1.08423484568623,-0.275466309185709,0.78295800628004,0.978328522364823
"10771","Z97989.1",4.562593663544,0.196900778652318,0.793070263740267,0.248276587403111,0.803920412537719,0.978328522364823
"10772","TSPYL1",58.7888701603163,-0.0697773762525403,0.279499857868169,-0.249650846997754,0.802857373081185,0.978328522364823
"10773","CALHM5",14.727423550407,-0.107963544726538,0.386238472820728,-0.279525610015161,0.779841487014183,0.978328522364823
"10774","ZUP1",4.55146905089275,0.18685346852959,0.717988572587028,0.260245741594921,0.79467422276136,0.978328522364823
"10775","MED23",19.0524579829794,-0.0841824694320451,0.335774758391622,-0.250711130983411,0.80203745420651,0.978328522364823
"10776","REPS1",15.2550756951227,0.12084292284987 5,0.428254013237691,0.282175809483435,0.777808706178527,0.978328522364823

"10777","VTA1",30.8630826572276,0.0746460108580621,0.280061288499524,0
.266534554839731,0.789827550642875,0.978328522364823
"10778","PPIL4",75.90510986973,0.0556505901158907,0.216456771879847,0.
257097939845383,0.797103167874938,0.978328522364823
"10779","KATNA1",32.8362428268292,0.0685828737337929,0.272016600950242
,0.252127530063278,0.800942491263391,0.978328522364823
"10780","RMND1",20.3211828498414,-0.0861190855112432,0.345706131212935
,-0.249110668674253,0.803275177141967,0.978328522364823
"10781","FBXO5",3.7589294505221,-0.19165039762428,0.721742465466054,-0
.265538480544477,0.790594670259433,0.978328522364823
"10782","SYTL3",1.95826244433681,-0.266645299118941,1.00031647099021,-
0.266560940314209,0.789807232820588,0.978328522364823
"10783","GPNMB",989.451733909348,0.0487584600115135,0.178940850388778,
0.27248367214964,0.785250144213416,0.978328522364823
"10784","FAM221A",2.97227559017391,-0.234762013766377,0.84592689726653
5,-0.277520450673657,0.78138050491043,0.978328522364823
"10785","AC007036.3",2.44772403825914,-0.260289880331202,1.01057823067
846,-0.257565295223561,0.796742416572668,0.978328522364823
"10786","EPDR1",50.9242844279663,0.0836062496038028,0.300070245772347,
0.27862258560191,0.780534729032816,0.978328522364823
"10787","SUGCT",2.42695416851404,0.235954282403347,0.911368431880893,0
.258901092192081,0.795711555464108,0.978328522364823
"10788","BLVRA",35.3209748544751,-0.0937769949318281,0.376017916201639
,-0.249395017873405,0.803055238587033,0.978328522364823
"10789","TMED4",117.78419240054,0.0519542487148279,0.186124569389599,0
.279136972003284,0.780139710752982,0.978328522364823
"10790","H2AFV",131.197231615155,-0.0592488502743342,0.231448457453937
,-0.255991553912715,0.797957359079702,0.978328522364823
"10791","TBRG4",17.2785288745543,-0.122662761133086,0.497921128850399,
-0.246349781171749,0.805411477234504,0.978328522364823
"10792","IKZF1",2.78568910629834,-0.264944014575647,0.977596605697785,
-0.27101568584778,0.786378968028651,0.978328522364823
"10793","CASTOR2",13.436788683991,-0.11096798118489,0.440367989677846,
-0.251989208539135,0.801049405052038,0.978328522364823
"10794","DTX2",13.5956224236532,0.108548391512374,0.427270108304799,0.
25405098414921,0.799456170187903,0.978328522364823
"10795","ATP5MF",198.430728750141,-0.0740546713650495,0.27272405229721
6,-0.271537001380224,0.785978045306262,0.978328522364823
"10796","AGFG2",3.91842473461853,0.204307206427787,0.730816862467134,0
.279560060694379,0.779815052638767,0.978328522364823
"10797","AP1S1",45.5256259227479,-0.0798909233283041,0.319300249159822
,-0.250206266792844,0.802427839163311,0.978328522364823
"10798","FOXP2",19.4583089086555,0.112271255545071,0.421082135575315,0
.266625548936378,0.789757482319278,0.978328522364823
"10799","ST7",21.5290423955823,-0.0904572138855181,0.361848653292827,-
0.24998632180155,0.80259792649871,0.978328522364823
"10800","WDR91",7.73930395819626,0.141950267779414,0.513337344797545,0
.276524334763523,0.782145371581613,0.978328522364823
"10801","MTPN",181.920874302518,-0.0472230454925206,0.186297080756465,
-0.25348247702417,0.799895400412029,0.978328522364823

"10802","CUL1",33.8551116458769,0.0726073199435987,0.268228816757646,0
.270691720678177,0.786628145103529,0.978328522364823
"10803","PRKAG2-
AS1",22.4237366564706,-0.115527632055503,0.42130542124554,-0.274213495
079079,0.783920557890759,0.978328522364823
"10804","GALNT11",91.8338389591977,0.0538012269682995,0.21292198500430
3,0.252680468704123,0.800515141932258,0.978328522364823
"10805","PPP2R2A",75.5185824872906,-0.0842955817907367,0.2966582813757
62,-0.28415044205007,0.776295092193497,0.978328522364823
"10806","INTS9",7.12603957678242,0.141174364544587,0.546861247694649,0
.258153901267868,0.796288133754146,0.978328522364823
"10807","AC044849.1",3.80166944408966,0.209701000833142,0.773436766726
032,0.271128823783241,0.786291953395183,0.978328522364823
"10808","IMPAD1",59.8644111762744,0.0576272986280207,0.219220026629011
,0.262874243353433,0.7926475091417,0.978328522364823
"10809","BHLHE22",5.00537146458622,0.176701334995438,0.687494718422476
,0.257022098149199,0.797161714119275,0.978328522364823
"10810","RPL7",1703.21188970881,-0.0609631445283164,0.228034919860129,
-0.267341267581779,0.789206415707826,0.978328522364823
"10811","EMC2",62.7290609410062,-0.075451844851747,0.282308969911164,-
0.267266905743342,0.789263665602106,0.978328522364823
"10812","AC016405.3",1.50880850270714,0.330125002092644,1.313654870513
44,0.25130268954403,0.801580095919622,0.978328522364823
"10813","ASAP1-
IT2",1.96301024545991,0.290431489189226,1.11919282189207,0.25950085053
1039,0.795248825863611,0.978328522364823
"10814","DENND3",4.25911752285098,0.196220363384172,0.759064910754462,
0.258502745422841,0.796018930373337,0.978328522364823
"10815","ADCK5",6.96711167829192,-0.170509909886795,0.622667031947727,
-0.273838024398744,0.78420910084698,0.978328522364823
"10816","ZNF34",9.81463864575738,0.12636905602826,0.461613853078173,0.
273754903986514,0.784272981509935,0.978328522364823
"10817","ZNF16",5.03950190713855,0.174918585002041,0.646370784352903,0
.27061647778087,0.786686021135174,0.978328522364823
"10818","CDC37L1-
DT",1.82693252870417,-0.285712377880967,1.01886027463389,-0.2804235134
04362,0.77915259934931,0.978328522364823
"10819","RRAGA",142.147948890129,-0.0687089268527521,0.279308991630839
,-0.245996115096661,0.805685239821374,0.978328522364823
"10820","SIT1",1.46623970115341,0.313939940789155,1.24004410029362,0.2
53168367733713,0.800138109302316,0.978328522364823
"10821","AL162390.1",3.62166102818668,0.23334754758716,0.8199110906023
54,0.284601038163455,0.775949815791548,0.978328522364823
"10822","PRUNE2",1.84780494616807,-0.300959636516696,1.15954688551906,
-0.259549346624284,0.795211412975314,0.978328522364823
"10823","CDK20",5.03502155754067,-0.17247233890875,0.66198934958994,-0
.260536425571779,0.794450021626432,0.978328522364823
"10824","NFIL3",36.3544582314868,-0.113677101553951,0.442190888770966,
-0.25707698742936,0.797119342046605,0.978328522364823
"10825","FANCC",3.36175242067811,0.204966232915175,0.818239256431414,0

.250496699228406,0.802203256987162,0.978328522364823
"10826","AL589843.1",1.48843498125259,-0.375221278307326,1.34072968422
73,-0.279863482342137,0.779582244955002,0.978328522364823
"10827","NIPSNAP3A",19.5806761552179,-0.100037644829798,0.361462603676
828,-0.27675793792278,0.78196598067489,0.978328522364823
"10828","ASTN2",2.90301195732361,-0.207994447909324,0.797075303173245,
-0.260947048643052,0.794133341814558,0.978328522364823
"10829","TRAF1",2.27954313973713,-0.253760950859275,0.928633157071164,
-0.27326285834938,0.784651163885602,0.978328522364823
"10830","AL596244.1",2.48469756260775,-0.237345012350513,0.92367271144
1705,-0.256957913133598,0.797211262842711,0.978328522364823
"10831","GAPVD1",31.070175477878,-0.0891249231041679,0.326741845069185
,-0.272768622841364,0.785031080571517,0.978328522364823
"10832","TOR2A",13.4685975397107,0.127820165218419,0.514847486877491,0
.248268018153566,0.803927042301934,0.978328522364823
"10833","GOLGA2",111.014658439517,-0.0619101727694162,0.24996345962294
6,-0.247676891905736,0.804384412478038,0.978328522364823
"10834","PHYHD1",56.3191156075728,0.0864870110222912,0.320640268962786
,0.26973221829579,0.787366272295031,0.978328522364823
"10835","AL157938.3",3.08893869966917,-0.210878016584031,0.76743321409
8125,-0.274783541694702,0.783482543372815,0.978328522364823
"10836","GTF3C4",17.9167880457785,0.103533600789211,0.362287419096581,
0.285777521746098,0.775048525507711,0.978328522364823
"10837","RXRA",49.7926756065922,0.0645814830133128,0.241855161613708,0
.267025448547022,0.789449567168666,0.978328522364823
"10838","QSOX2",4.81713771492315,-0.160621156774062,0.64111676350598,-
0.250533390978107,0.802174885584782,0.978328522364823
"10839","PMPCA",26.6560920973523,0.0864724620855865,0.302907766870941,
0.285474562038647,0.775280590470361,0.978328522364823
"10840","GTPBP4",29.4769981530759,0.0880601648704543,0.317721900456767
,0.277161142319293,0.781656375155694,0.978328522364823
"10841","OPTN",203.182887663936,-0.0471745878069266,0.185292352791242,
-0.254595438485664,0.799035582679445,0.978328522364823
"10842","ZNF33B",4.87738296831508,-0.170850950483307,0.669358558880517
,-0.25524578451503,0.798533270927924,0.978328522364823
"10843","AIFM2",18.9084846330618,-0.10036612338056,0.399442168425052,-
0.251265718329865,0.801608677897814,0.978328522364823
"10844","MRPS16",61.3904192286816,0.0796583532460391,0.311763857604104
,0.255508620717652,0.798330286164075,0.978328522364823
"10845","ZMIZ1-
AS1",2.14804493144864,-0.294935599492344,1.03860757211396,-0.283972125
190691,0.776431742522001,0.978328522364823
"10846","TMEM254-
AS1",3.50272184454018,0.225903167659602,0.899795770620544,0.2510604906
53126,0.801767341646424,0.978328522364823
"10847","SLC16A12",2.5903830576146,0.279566685898529,0.993116867615094
,0.281504317381991,0.778323617128336,0.978328522364823
"10848","IDE",15.5364549669685,0.10908024792086,0.408642804388182,0.26
6932995637045,0.789520751243695,0.978328522364823
"10849","LGI1",5.71445711765136,-0.206349348245048,0.819126019788629,-

0.251914044066498,0.801107503997722,0.978328522364823
"10850","PYROXD2",17.0977807681351,0.112636773259601,0.428904167589609
,0.262615245481541,0.792847148212343,0.978328522364823
"10851","CHUK",8.74672063573385,-0.13331721085974,0.506521326263672,-0
.263201575031693,0.792395216892197,0.978328522364823
"10852","NDUFB8",206.429081421295,-0.0730578099468361,0.28082844163369
9,-0.260151035706454,0.794747271964138,0.978328522364823
"10853","KAZALD1",39.6362589618768,0.126254062091004,0.502390582333249
,0.251306586012507,0.80157708362458,0.978328522364823
"10854","PDCD11",19.1384105601989,0.105883969568321,0.384533256469385,
0.275357118758728,0.783041885359817,0.978328522364823
"10855","EIF3A",124.947222720179,0.061919020199273,0.242800481491608,0
.255020170548604,0.79870752033604,0.978328522364823
"10856","DENND10",46.7131291136282,-0.0654525851590709,0.2484642980511
78,-0.263428531472917,0.792220302031208,0.978328522364823
"10857","MGMT",144.14954016184,-0.0829645921942738,0.309587975298619,-
0.267983897353405,0.788711713600558,0.978328522364823
"10858","C10orf143",3.56268930170105,0.19054520839458,0.75757268628689
1,0.251520694771222,0.801411564235834,0.978328522364823
"10859","SSRP1",51.2774706214174,-0.062772632369659,0.238760308274801,
-0.262910668960148,0.792619432880166,0.978328522364823
"10860","AP000662.1",1.86045949641905,0.288812038993292,1.171156586728
87,0.246604119607922,0.805214616015722,0.978328522364823
"10861","STX3",4.2757560487608,-0.224835791666339,0.813669280592122,-0
.276323313450794,0.782299751183456,0.978328522364823
"10862","TMEM132A",5.20279383962059,-0.194167995317937,0.6906291847773
95,-0.281146524933669,0.778598017900852,0.978328522364823
"10863","EIF1AD",12.3598128708486,-0.114183057802718,0.417313394734052
,-0.273614648471769,0.784380775599876,0.978328522364823
"10864","PC",3.12598847462429,-0.22261195265896,0.780787820313887,-0.2
85111968792581,0.775558360680715,0.978328522364823
"10865","AP002807.1",2.17805215360345,0.252383837532696,0.990707444674
994,0.254751126469522,0.798915325416376,0.978328522364823
"10866","ARHGEF17",7.97434236964233,-0.143944095213736,0.5361401290319
65,-0.268482225856953,0.788328154523774,0.978328522364823
"10867","PPME1",19.5937545566628,-0.091054348114983,0.339484557716006,
-0.26821351971819,0.788534968911978,0.978328522364823
"10868","P4HA3",1.80070185970355,0.313246405338281,1.09821508657641,0.
285232291166934,0.775466182664287,0.978328522364823
"10869","AP003119.2",2.01829485962837,-0.295764721783916,1.09207527780
259,-0.270828145088164,0.786523211913284,0.978328522364823
"10870","ACER3",18.6959825299371,0.100059130425486,0.372670700942404,0
.268492076711311,0.788320572924523,0.978328522364823
"10871","PRCP",61.1509396558707,-0.0710502397877364,0.278744017637997,
-0.254894222985653,0.798804798321947,0.978328522364823
"10872","FZD4",52.4024691305666,0.0763286805430674,0.284448668932703,0
.26833903223898,0.788438364145013,0.978328522364823
"10873","MED17",23.9873572105403,-0.0925840058394648,0.36654876474004,
-0.252583052367197,0.800590427704598,0.978328522364823
"10874","DYNC2H1",49.3258473345457,-0.0735249888217492,0.2834345632595

97,-0.259407278971859,0.795321014087138,0.978328522364823
"10875","ALG9",13.4091707072639,0.117536313155826,0.420868895664682,0.279270609842051,0.780037159293785,0.978328522364823
"10876","PIH1D2",2.44789137301281,0.239414126806668,0.959061689034933,0.249633709222168,0.802870627528459,0.978328522364823
"10877","ZW10",9.63154405250847,0.129660138107491,0.486181958030224,0.266690558886248,0.789707423649083,0.978328522364823
"10878","HYOU1",14.1847196048062,-0.124616516749883,0.503393975958934,-0.247552657960392,0.804480544122875,0.978328522364823
"10879","CCDC15-DT",1.61822704771192,0.338931429150627,1.19415288166265,0.283825826956698,0.77654386104682,0.978328522364823
"10880","DYRK4",2.90332193168273,-0.283383038963724,1.12201863141344,-0.252565359459975,0.800604101422922,0.978328522364823
"10881","PYROXD1",21.494849723133,0.122966820998321,0.439606866666587,0.279719973281453,0.779692353330762,0.978328522364823
"10882","AC010198.2",1.97694395888526,-0.379201208769704,1.40204826957485,-0.270462306468728,0.786804611739955,0.978328522364823
"10883","PCED1B-AS1",2.60175915539188,0.237142565564481,0.915487417361781,0.259034216164183,0.795608840548735,0.978328522364823
"10884","RHEBL1",1.79251743314528,0.271115667930502,1.08518195976455,0.249834293217817,0.802715498346774,0.978328522364823
"10885","AC011603.2",3.1171132621747,-0.229242345761865,0.924467911324929,-0.247972204284862,0.804155913017601,0.978328522364823
"10886","AC008147.2",8.43752549124194,0.177368345984458,0.625706408399716,0.283468961806048,0.776817371188278,0.978328522364823
"10887","AAAS",18.3847577603354,-0.0957122194332002,0.361785935659029,-0.264554837541849,0.791352415150106,0.978328522364823
"10888","HOXC6",57.7118581340776,-0.055330098608286,0.224396238901259,-0.246573199618702,0.805238547825783,0.978328522364823
"10889","ANKRD52",9.36524121533601,0.141756002198011,0.55123198215336,0.257162150940968,0.79705360080299,0.978328522364823
"10890","NAB2",12.7035070697007,0.111755941060033,0.416461815647832,0.268346188920561,0.788432855871835,0.978328522364823
"10891","KRR1",84.8899917716217,-0.055488245385761,0.219776770817389,-0.252475478547575,0.800673565584903,0.978328522364823
"10892","PPP1R12A",125.151541949411,-0.0598744005729975,0.221409530055794,-0.270423773348461,0.786834252696338,0.978328522364823
"10893","POC1B",9.59798145724772,0.156425676790111,0.571758614323894,0.273586917400597,0.784402088945692,0.978328522364823
"10894","DCN",20684.4558854753,0.0480829797386862,0.178576871696667,0.269256479194912,0.787732320353903,0.978328522364823
"10895","TMPO",42.5228133035255,-0.0724345253969468,0.294302414931254,-0.246122769376075,0.805587197700875,0.978328522364823
"10896","TXNRD1",64.0720345424654,-0.0669915559283588,0.256124098784851,-0.261558971788253,0.793661478781141,0.978328522364823
"10897","TCP11L2",30.8615092617159,-0.0995005748069889,0.386401011850176,-0.257505989258562,0.796788192403231,0.978328522364823
"10898","HVCN1",5.62850859897004,-0.188087873352754,0.671242820631911,

-0.280208394893054,0.779317626369654,0.978328522364823
"10899","RNF10",58.2810947260904,-0.059085822237925,0.233841946880064,
-0.2526741802583,0.800520001743925,0.978328522364823
"10900","SBNO1-
AS1",2.92388196465398,0.247200983762547,0.911000870415965,0.2713509852
62697,0.786121096283168,0.978328522364823
"10901","TMED2-
DT",1.88933850462335,-0.291130512772168,1.09638187948938,-0.2655375086
16758,0.790595418881949,0.978328522364823
"10902","TCTN2",8.08209721785487,-0.144906580308116,0.549408441384238,
-0.263750189099795,0.791972419076677,0.978328522364823
"10903","PARP4",49.6132401126013,-0.0626853953784931,0.239564151305282
,-0.261664339330185,0.793580235919946,0.978328522364823
"10904","WASF3",23.6192784388864,-0.0938788088211168,0.345806827505648
,-0.271477603546111,0.786023722918959,0.978328522364823
"10905","STARD13",11.0006411057672,0.131079238932495,0.520924489183962
,0.251628098993451,0.801328537447832,0.978328522364823
"10906","MTRF1",5.807832477759,-0.16875643566578,0.608822238403902,-0.
277185071472084,0.781638001944702,0.978328522364823
"10907","TPT1",7082.02687692038,-0.0677173932076875,0.262655760427555,
-0.257818039465254,0.796547341548834,0.978328522364823
"10908","AL161421.1",2.82000661802601,-0.256109734132879,0.95098996386
6105,-0.269308556203584,0.787692248440735,0.978328522364823
"10909","PIBF1",33.6846130777803,0.083038486830318,0.308660791227864,0
.269028296402624,0.787907907742763,0.978328522364823
"10910","ERCC5",45.9304527257626,0.0688317596095663,0.267971274452579,
0.25686245568738,0.79728495436661,0.978328522364823
"10911","GAS6",155.756267079591,0.0872738938353032,0.327740171430141,0
.266289888891164,0.790015959564174,0.978328522364823
"10912","METTL17",21.758842114636,0.0967496579416498,0.381963732278738
,0.25329540415907,0.80003994728889,0.978328522364823
"10913","TRAC",114.20496391301,-0.192996080481576,0.72754144338561,-0.
265271596877656,0.790800243338367,0.978328522364823
"10914","ABHD4",14.7707898944946,0.113278235487512,0.433002772608436,0
.261610877928372,0.793621456657162,0.978328522364823
"10915","ZFHX2",1.87584518517571,0.278721061931169,1.04430954462346,0.
266895063217743,0.789549957800575,0.978328522364823
"10916","EMC9",9.17469903008241,-0.136065705193807,0.536188130969757,-
0.253764858516575,0.799677223746162,0.978328522364823
"10917","AL121821.2",1.74495705215662,0.290235115861611,1.152691809298
08,0.251788998169725,0.801204161603491,0.978328522364823
"10918","PSMC6",90.4067910512118,0.0630479747177817,0.228876982373072,
0.275466646161092,0.782957747422561,0.978328522364823
"10919","MPP5",14.6331427074167,0.110209499201728,0.417339136265369,0.
264076597723272,0.791720896300615,0.978328522364823
"10920","LINC01220",2.23103504517245,-0.309760922114013,1.207479743722
34,-0.256535087834353,0.797537690497842,0.978328522364823
"10921","POMT2",8.98900752000665,0.12232709687205,0.485764841912255,0.
25182369393079,0.801177342272065,0.978328522364823
"10922","DIO2",2.32015474932411,0.29421255824967,1.15178358311854,0.25

5440833297058,0.798382636147458,0.978328522364823
"10923","DGLUCY",22.7163247269405,-0.0998941368854468,0.366221697021924,-0.272769575636221,0.785030348112949,0.978328522364823
"10924","IFI27",834.904600351256,-0.16009995587007,0.572983942220625,-0.279414385069144,0.779926832844871,0.978328522364823
"10925","GSC",31.6165413318572,-0.0984926574715589,0.349763150173711,-0.281598154129851,0.77825165574408,0.978328522364823
"10926","HSP90AA1",829.074237870069,0.0555112094775487,0.207430701643473,0.267613275362487,0.78899701131622,0.978328522364823
"10927","KATNBL1",56.3900292086749,0.0667994285492958,0.2474496760226,0.269951570044468,0.787197512238667,0.978328522364823
"10928","AQR",25.373298185731,0.0768754802094866,0.307622501945538,0.249902005618226,0.802663132168438,0.978328522364823
"10929","SRP14-AS1",4.876516013196,-0.167782508314087,0.637225033614113,-0.263301815627808,0.792317960382754,0.978328522364823
"10930","TYRO3",2.89177169959091,0.23729803191613,0.879059531622141,0.269945348841437,0.787202298434294,0.978328522364823
"10931","TMEM62",5.38806832837236,-0.161634428734666,0.64759074640813,-0.249593481116235,0.802901740383456,0.978328522364823
"10932","GNB5",20.7375233450533,0.0876601568270313,0.355106209279239,0.246856164540056,0.805019542178932,0.978328522364823
"10933","LIPC",7.29592722841829,0.172872876358833,0.66378976044038,0.260433177282131,0.794529653875321,0.978328522364823
"10934","ADPGK",42.2487818412867,-0.0668760808126166,0.252293967934611,-0.26507205606259,0.790953953597984,0.978328522364823
"10935","CSK",25.0274181456124,-0.0991356117308896,0.372498931561531,-0.266136633775858,0.790133982366905,0.978328522364823
"10936","MORF4L1",473.743995646361,-0.0462332249561475,0.162198925816058,-0.285040265979186,0.775613293141014,0.978328522364823
"10937","TMED3",98.0148047164929,-0.0675305784674815,0.268185640808239,-0.251805347459926,0.801191523804756,0.978328522364823
"10938","MTHFS",23.0247134846008,-0.092631427823295,0.37636643951123,-0.246120318122921,0.805589095168418,0.978328522364823
"10939","ZFAND6",73.9815248584152,0.0564346396140984,0.226149487712819,0.24954573271359,0.802938669923236,0.978328522364823
"10940","AC027807.2",2.25851057330997,0.258552704401715,0.99522896611322,0.259792181704146,0.795024082047289,0.978328522364823
"10941","AKAP13",239.719009037383,-0.069900848430625,0.250616534222854,-0.27891554979556,0.780309634899322,0.978328522364823
"10942","AEN",2.78240018131117,0.247099381164115,0.93518146817356,0.264226131048884,0.791605676690943,0.978328522364823
"10943","ADAMTS17",3.26916179062796,-0.25663268584879,0.949091408205954,-0.270398281587963,0.786853861975242,0.978328522364823
"10944","SPATA41",3.35125953738523,0.209435138023836,0.785079036207339,0.266769494999641,0.789646642783566,0.978328522364823
"10945","TMEM8A",15.8441671241045,0.103263917111054,0.406208421864233,0.254214121502305,0.799330141511431,0.978328522364823
"10946","CLCN7",25.4301891135992,-0.0863726041516843,0.343936204852692,-0.251129723864569,0.801713815783707,0.978328522364823

"10947","FAHD1",26.7440510142597,-0.0911734958483543,0.359684380075914
,-0.253481943889561,0.799895812343077,0.978328522364823
"10948","TBL3",13.6947121512677,-0.118998791440206,0.462583428085551,-
0.25724828045115,0.79698711532941,0.978328522364823
"10949","TRAF7",34.8287761984341,0.0737545574823877,0.270070621703861,
0.273093596841723,0.784781268698125,0.978328522364823
"10950","BFAR",64.5255139007731,-0.069288114485638,0.242704617221599,-
0.285483297676101,0.775273898753612,0.978328522364823
"10951","COG7",17.1412952119295,-0.102426039726253,0.382667170989816,-
0.267663514121987,0.788958336821853,0.978328522364823
"10952","CTF1",9.01210418467984,-0.143623255027396,0.530540836391567,-
0.270711027645371,0.786613294580942,0.978328522364823
"10953","ZNF646",7.04669506199292,0.142642926496903,0.546159287137288,
0.261174587444206,0.793957874453566,0.978328522364823
"10954","VKORC1",341.010745934577,-0.0705342863496641,0.26686011323982
2,-0.264311835490665,0.791539641073613,0.978328522364823
"10955","ZNF267",15.2851570854849,-0.0931620534403045,0.37639738002606
9,-0.247509835041499,0.804513680982138,0.978328522364823
"10956","HSD11B2",11.310678841927,-0.180389326077291,0.640251382477075
,-0.281747655708889,0.778137010125987,0.978328522364823
"10957","DUS2",5.25863331444035,-0.183100103421545,0.646311462671641,-
0.283300102190155,0.77694679895561,0.978328522364823
"10958","ZNF23",10.8465772078338,-0.11530526879058,0.444084211935526,-
0.259647304929003,0.795135843311701,0.978328522364823
"10959","AC004943.2",2.06821879178201,0.308103940952614,1.134171120298
23,0.271655604201593,0.785886840586621,0.978328522364823
"10960","TRAPPC2L",118.431248108503,0.0889757285920198,0.3240951584550
81,0.274535815394945,0.783672883798329,0.978328522364823
"10961","TLCD3A",11.9163054284135,-0.122085539041923,0.457342915637275
,-0.266945293930716,0.78951128207689,0.978328522364823
"10962","HIC1",144.011980903709,0.0507156513256836,0.1892671073247,0.2
67958083380424,0.788731583785324,0.978328522364823
"10963","CTC1",5.54593372198481,-0.166337566009043,0.65991802157547,-0
.252057923212846,0.800996292489875,0.978328522364823
"10964","NTN1",9.33411076736444,-0.164335045390546,0.646912004422309,-
0.254029982852608,0.799472394721926,0.978328522364823
"10965","ADPRM",7.59975417470625,-0.174513632436733,0.61721884034184,-
0.282741907781169,0.777374688674265,0.978328522364823
"10966","SMCR5",3.23849468446184,-0.204348349069884,0.782837263522206,
-0.261035541602175,0.794065098913837,0.978328522364823
"10967","ALKBH5",47.2170818137878,-0.0633418411162763,0.24447414374329
2,-0.259094234451182,0.79556253319345,0.978328522364823
"10968","RSKR",2.17166279990734,0.271519092059516,1.02906579083713,0.2
63850080798663,0.791895442592173,0.978328522364823
"10969","ANKRD13B",17.5254818808666,-0.110363774165877,0.4485602738090
99,-0.246040009804448,0.805651260913854,0.978328522364823
"10970","AC005562.1",18.1344028524291,-0.0994438691956787,0.3619377917
11256,-0.274754036392564,0.783505213083089,0.978328522364823
"10971","RHOT1",24.748029532433,0.0955110468200596,0.355953962343873,0
.268324156840794,0.788449813305929,0.978328522364823

"10972","PSMD11",69.0133922949771,0.0577130367888816,0.234384726656212
,0.246232071569805,0.805502590155504,0.978328522364823
"10973","SLFN13",2.04811396265005,-0.260523124761518,1.01240612140064,
-0.257330649483915,0.796923534038244,0.978328522364823
"10974","PGAP3",8.30869258391021,0.129444720941305,0.503400358787403,0
.257140700600836,0.797070159073764,0.978328522364823
"10975","CASC3",37.3472640091075,-0.0733720319580009,0.280230286897931
,-0.261827630304377,0.793454336051876,0.978328522364823
"10976","GHDC",21.6741834180655,0.10023189282698,0.357412428400025,0.2
80437625730233,0.779141773501548,0.978328522364823
"10977","PSME3",18.6452194540943,-0.0976481446669714,0.365035112724912
,-0.267503429842812,0.789081573733103,0.978328522364823
"10978","FZD2",2.98576253107419,0.240975331703418,0.962328808606485,0.
250408518947247,0.802271442270911,0.978328522364823
"10979","EFTUD2",18.4841882213482,-0.119119360939715,0.419626387302408
,-0.283870043791767,0.776509974115864,0.978328522364823
"10980","ARL17A",9.33787675438982,0.118167294216072,0.476836720579434,
0.247815004835365,0.804277544812293,0.978328522364823
"10981","MTMR4",13.0386364880053,-0.112453289268187,0.414640985678766,
-0.271206400602441,0.786232290436451,0.978328522364823
"10982","BCAS3",13.5330791266257,0.105892841250354,0.405103112373374,0
.261397254219945,0.793786174267094,0.978328522364823
"10983","LIMD2",6.55559211652318,-0.160287700783462,0.577508536096266,
-0.277550357726217,0.78135754407353,0.978328522364823
"10984","AMZ2",23.5279220312254,-0.0813607306311989,0.32892060199283,-
0.247356748523075,0.80463214393421,0.978328522364823
"10985","FAM104A",30.2750222848183,0.0848191756486113,0.29874308548780
9,0.283920129934095,0.776471589574623,0.978328522364823
"10986","PRPSAP1",36.536965643864,0.0757107587608272,0.273947399313527
,0.276369693417596,0.782264131707406,0.978328522364823
"10987","AC015802.6",2.43462832549251,0.382660137082211,1.392981815166
88,0.274705766375255,0.783542300620568,0.978328522364823
"10988","SNHG20",8.39885025442475,0.156983161290618,0.555773886163366,
0.282458685445457,0.77759182153078,0.978328522364823
"10989","SEPTIN9",39.1770030092205,-0.0710799546382825,0.2763074739142
77,-0.257249482365919,0.79698618755245,0.978328522364823
"10990","CEP295NL",1.84244166121643,0.342775598798966,1.19796855804107
,0.286130713947515,0.774778008180486,0.978328522364823
"10991","RNF213-
AS1",3.4125030690535,0.209682064240034,0.84954915652373,0.246815693512
112,0.8050508645013,0.978328522364823
"10992","P4HB",349.920198171243,-0.0600548112252482,0.22067424414538,-
0.272142367396914,0.785512553968738,0.978328522364823
"10993","FOXK2",16.685757455278,0.0989874324273674,0.378194266578921,0
.26173699914277,0.793524213415537,0.978328522364823
"10994","B3GNTL1",5.145990606905,0.200685627259139,0.759334640002235,0
.264291416046223,0.791555374202013,0.978328522364823
"10995","AC139099.2",2.51104482536178,-0.221155180042921,0.88590660680
3031,-0.249637126921317,0.802867984257136,0.978328522364823
"10996","AC120049.1",2.38276259390323,-0.261391987325438,0.97583745245

6601,-0.267864270496487,0.788803796970517,0.978328522364823
"10997","SMAD7",22.2226805009758,-0.0912586273874503,0.370085702908451
,-0.246587821875479,0.805227230300006,0.978328522364823
"10998","NARS",92.2397365598845,0.0576211657414768,0.228134006483187,0
.252575960198741,0.800595908781434,0.978328522364823
"10999","VPS4B",121.744301737327,0.055575225470025,0.224827149428522,0
.247190900259551,0.804760487370819,0.978328522364823
"11000","BSG",253.494973211844,0.0650901593780061,0.25001317401004,0.2
60346918260364,0.794596184463148,0.978328522364823
"11001","CFD",39230.6884849706,0.068968886538684,0.274482349542448,0.2
51268930966426,0.801606194238576,0.978328522364823
"11002","MED16",25.6045339757551,0.0955164972417942,0.37551396661036,0
.254362036395051,0.799215877176438,0.978328522364823
"11003","GAMT",42.370744957303,-0.106884058840946,0.388785594114708,-0
.274917745047444,0.783379433678386,0.978328522364823
"11004","EVI5L",3.86909370392551,0.185925573944748,0.721069277020812,0
.257847033384813,0.79652496404913,0.978328522364823
"11005","ZNF558",5.97963770146306,0.159443498619121,0.570965227719165,
0.279252554934124,0.780051014106861,0.978328522364823
"11006","TIMM29",19.2438792003246,0.0866199735064216,0.351890803842526
,0.246155831753946,0.805561604867003,0.978328522364823
"11007","ZNF627",2.65944451614651,-0.242526760580747,0.936013065626002
,-0.259106170081661,0.795553324294679,0.978328522364823
"11008","HOOK2",15.9413029879183,0.0948049885997908,0.368455463936692,
0.257303793481212,0.796944264245648,0.978328522364823
"11009","DNASE2",79.6569941682464,-0.06514639680073,0.261977120895181,
-0.24867208471535,0.803614443814591,0.978328522364823
"11010","FARSA",15.3118447697409,0.115274254737087,0.432314862336188,0
.266644209533201,0.789743113281363,0.978328522364823
"11011","ZNF333",15.374854788496,0.109832011398012,0.407772764794976,0
.269346118427586,0.787663345628355,0.978328522364823
"11012","TSHZ3",15.3965179452643,0.110118005909048,0.392983206989893,0
.280210461796859,0.779316040708064,0.978328522364823
"11013","PEPD",92.4211468286811,-0.076943806582484,0.297083984176952,-
0.258996818006366,0.795637695614728,0.978328522364823
"11014","HAUS5",5.89011146936482,-0.168693825001504,0.607407506664221,
-0.277727593338354,0.781221477138338,0.978328522364823
"11015","ZNF567",17.956536078479,-0.100138869154778,0.3707245597447,-0
.270116631128347,0.787070527736443,0.978328522364823
"11016","ZNF780A",10.2414376617842,-0.129740200110089,0.48757124259747
5,-0.266094857069326,0.790166155726763,0.978328522364823
"11017","TMEM91",35.2166052671339,-0.0861133517466139,0.32230077571028
4,-0.267183197300218,0.78932811264712,0.978328522364823
"11018","FBXO46",8.28711288156864,-0.139522928566889,0.508025509219152
,-0.274637643257992,0.783594642823786,0.978328522364823
"11019","DHX34",3.60586841661768,0.221757751452268,0.826531247062154,0
.268299295689655,0.788468948312189,0.978328522364823
"11020","SPHK2",9.84676767640645,-0.129007128905943,0.46969198565141,-
0.274663253466043,0.783574965178449,0.978328522364823
"11021","LIN7B",3.47950038395255,-0.236479855713627,0.883838507900424,

-0.267560027765015,0.789038002623427,0.978328522364823
"11022","ZNF615",7.98144129985227,-0.125206432358755,0.49984329546734,
-0.25049137098396,0.802207377002262,0.978328522364823
"11023","ZNF836",11.2262286493716,-0.135537495422422,0.504735645973382
,-0.268531649198341,0.788290116607086,0.978328522364823
"11024","ZNF701",11.6811542548171,0.114358351607521,0.452045006189969,
0.252980013143786,0.800283658138769,0.978328522364823
"11025","ZNF677",36.5199758651802,0.0754969367956271,0.265040280152564
,0.28485080362943,0.775758448119652,0.978328522364823
"11026","ZNF845",7.40588286713419,0.166778380108228,0.61719408845084,0
.270220313559455,0.786990765846103,0.978328522364823
"11027","ZNF579",9.35363472920784,0.117317470756327,0.477190210435984,
0.245850539660358,0.805797932439632,0.978328522364823
"11028","ZNF71",7.51392574635095,-0.143213731063931,0.51101825806011,-
0.280251691216647,0.779284411025757,0.978328522364823
"11029","ZNF773",5.08236102559865,0.199279211703808,0.741745126028906,
0.268662650701451,0.788189295752046,0.978328522364823
"11030","ZNF8",7.41055444369794,0.150048351956186,0.540316018011832,0.
277704800439399,0.781238975277513,0.978328522364823
"11031","C20orf96",10.0702292293847,-0.115748038107915,0.4506553132502
71,-0.256843833201706,0.797299330819271,0.978328522364823
"11032","ACTL10",2.48806593907467,-0.265809825370223,0.966624189992886
,-0.274987764761173,0.783325638385768,0.978328522364823
"11033","TP53INP2",4.33547820262993,-0.268451623214981,0.9390936823216
95,-0.285862452563088,0.774983472715906,0.978328522364823
"11034","STK4",43.0388885574896,0.0655776045214678,0.247632242530987,0
.264818522221564,0.791149267486878,0.978328522364823
"11035","ZSWIM3",2.56223592959912,-0.234829931885755,0.842969408335711
,-0.278574678468326,0.780571247370239,0.978328522364823
"11036","GMEB2",6.74850209215655,0.147098728645952,0.566864308813423,0
.259495484825749,0.795252965329746,0.978328522364823
"11037","AL118506.1",1.54122608178242,0.314699725063855,1.138666557621
86,0.276375663232892,0.782259546965986,0.978328522364823
"11038","SOD1",425.546233476031,-0.0689539350355295,0.247673911292119,
-0.278406129558805,0.780700614737542,0.978328522364823
"11039","SYNJ1",11.9501216687922,-0.115544349871265,0.419538044567505,
-0.275408515073711,0.783002402870062,0.978328522364823
"11040","ITSN1",52.1455280817313,0.077967853194071,0.288532999500192,0
.270221615306151,0.786989764439176,0.978328522364823
"11041","MX2",48.680890397733,0.0885037171825133,0.322777790667769,0.2
74193949340241,0.783935577732461,0.978328522364823
"11042","PKNOX1",14.6877210306018,0.104254212035816,0.412731277534695,
0.252595860092169,0.800580529469849,0.978328522364823
"11043","MICAL3",8.74392340677303,0.154328935694277,0.581267321367612,
0.265504235351079,0.79062104756671,0.978328522364823
"11044","GP1BB",14.7931322459915,-0.159469531595933,0.558983879937072,
-0.285284669772382,0.775426056818425,0.978328522364823
"11045","COMT",305.688543556594,0.0763417473713866,0.267796516395447,0
.285073713425962,0.77558766847618,0.978328522364823
"11046","ZDHHC8",26.607309223159,-0.0851063783093478,0.309213673011189

,-0.275234848060125,0.783135815576915,0.978328522364823
"11047","GUCD1",28.7818423390072,0.08911869961457,0.35233982033678,0.252933941810457,0.800319260285979,0.978328522364823
"11048","PITPNB",62.9445559206502,-0.0630780466331656,0.246272558216883,-0.25613104070497,0.797849654202284,0.978328522364823
"11049","AP1B1",17.7740800814111,-0.11502103485534,0.418173222680149,-0.275055954367783,0.78327325013768,0.978328522364823
"11050","TXN2",118.277737530934,-0.0801163571134381,0.288207760193969,-0.277981262751281,0.781026742267112,0.978328522364823
"11051","TOMM22",64.6024416799119,-0.0639586069213495,0.259786292001557,-0.246197004578541,0.805529734212197,0.978328522364823
"11052","TEF",16.9499474391579,-0.199034145869558,0.715715457552101,-0.278091165657226,0.780942377160997,0.978328522364823
"11053","TRMU",16.3136292949398,0.100625840044031,0.376190662165917,0.267486278007748,0.789094777960922,0.978328522364823
"11054","SELENOO",17.6752468883806,-0.0949552351166639,0.351579537397937,-0.270081802312597,0.787097321708443,0.978328522364823
"11055","MAOB",6.66227756559218,0.154610999740407,0.584159213195776,0.264672706083968,0.79126160524413,0.978328522364823
"11056","TIMM17B",24.8755953555317,-0.102438832800576,0.392728568125143,-0.260838760189032,0.794216852503117,0.978328522364823
"11057","SMC1A",53.2776779583129,-0.0950044600391236,0.338759371866673,-0.28044821170738,0.779133652814628,0.978328522364823
"11058","PHF8",11.8884059265269,0.118310294429826,0.458235789985859,0.258186499211414,0.796262976861357,0.978328522364823
"11059","IL2RG",3.13677629103351,0.256424314156985,0.913679264298241,0.280650250231884,0.778978670175523,0.978328522364823
"11060","CSTF2",5.07520670747259,-0.206343862608798,0.761198557026783,-0.271077579829855,0.786331364901123,0.978328522364823
"11061","NRK",3.27132476110603,0.673348232591061,2.60429142352711,0.258553334894877,0.795979892442244,0.978328522364823
"11062","PSMD10",23.7668576907735,0.0952400180909113,0.366654004174383,0.259754474263465,0.795053170026313,0.978328522364823
"11063","SLC25A43",27.8330630490385,-0.0897796376352958,0.316070349866163,-0.284049540468798,0.776372415687591,0.978328522364823
"11064","RBMX2",40.4671907297351,-0.0711880110535337,0.288605405419413,-0.24666208503642,0.805169751756197,0.978328522364823
"11065","HTATSF1",62.6661886165465,-0.0722648500239884,0.256427982252588,-0.281813433109674,0.778086570109609,0.978328522364823
"11066","MTM1",7.12460131777374,0.157538805121234,0.601281332021253,0.262005149223001,0.793317472325124,0.978328522364823
"11067","HMGB3",2.5189676091663,-0.288974465007874,1.02883427738869,-0.280875619483953,0.778805801032434,0.978328522364823
"11068","NSDHL",9.83220333565424,-0.121534322878254,0.483527761084807,-0.251349214377245,0.801544128536699,0.978328522364823
"11069","MT-ND6",78.7952480981133,0.0938740283429626,0.351941323651352,0.266732043196945,0.789675480539709,0.978328522364823
"11070","RNF220",25.8306731456837,0.0781818441276111,0.318300981770552,0.245622378205446,0.805974564525019,0.97835049935405

"11071","THRB",35.0085119988948,-0.0815506159763113,0.332395249963166,
-0.245342302530943,0.806191399740044,0.97835049935405
"11072","FGF10",5.44486915471977,0.182240836604592,0.743241605212972,0
.245197302366263,0.806303665047504,0.97835049935405
"11073","STK10",8.48070547564277,0.132623542772557,0.541060599204207,0
.245117724276393,0.806365279492886,0.97835049935405
"11074","MTRES1",26.540941421931,-0.082178971253446,0.335068855352445,
-0.245259951621004,0.806255158834489,0.97835049935405
"11075","GOLGA3",62.0883022895067,0.0627309068769134,0.255966253546649
,0.245074911273337,0.806398428554566,0.97835049935405
"11076","GOSR2",28.4249197315044,0.0748673842700727,0.304903233666671,
0.245544736832539,0.80603467315629,0.97835049935405
"11077","ZNF439",3.84602044324976,0.205057258197646,0.835970809135573,
0.245292366619455,0.806230061800258,0.97835049935405
"11078","HACD2",21.9021712761413,0.0832972649540531,0.340280989234033,
0.244789652050659,0.806619306673864,0.978530137424631
"11079","OGFRL1",60.6520552235429,0.0600719332477046,0.245737213114941
,0.244455988111196,0.806877684459192,0.978755230747096
"11080","CRY2",24.8886561510923,0.0795025919003341,0.325440050241671,0
.244292587348409,0.807004224033635,0.978820376063901
"11081","SH3BP5L",10.186433825507,0.112202091785354,0.460473941411579,
0.24366653939504,0.807489090045861,0.978888559888767
"11082","DHX30",30.7444638365218,0.0718663006865018,0.294675058759881,
0.243883214917971,0.80732126928695l,0.978888559888767
"11083","KCNMB3",1.70394626269432,-0.266352817301851,1.09526587658647,
-0.243185534211996,0.807861672574322,0.978888559888767
"11084","RGS12",21.6144263822541,0.0939310802046669,0.385965983263663,
0.243366214323867,0.80772171417923,0.978888559888767
"11085","AC091729.3",5.93914965192569,-0.157150132489601,0.64546764889
2763,-0.243467093601325,0.807643573768771,0.978888559888767
"11086","MXI1",45.5404718797259,0.0779800047852362,0.320138634558797,0
.243581987199718,0.807554580297133,0.978888559888767
"11087","SYVN1",27.8303873950757,-0.0735356757866401,0.302319384003534
,-0.243238375299747,0.807820740184166,0.978888559888767
"11088","EIF4B",178.355812611296,-0.0573782240401723,0.235087007186842
,-0.244072289348468,0.807174833528432,0.978888559888767
"11089","LACC1",5.19014176244447,-0.147988235302668,0.608276041738931,
-0.243291244678322,0.80777978640556,0.978888559888767
"11090","AF001548.2",3.11138733996874,0.217606499843326,0.894133786362
757,0.243371297631562,0.807717776637342,0.978888559888767
"11091","SAE1",29.1311510938912,-0.0829582833419265,0.341005688729369,
-0.243275364851066,0.807792087212214,0.978888559888767
"11092","C1orf220",4.01406701395045,0.194999821906434,0.80307816628262
5,0.242815494298731,0.8081483320142,0.979018799302051
"11093","GSK3B",33.6140405974416,0.0714553765381835,0.29453396954614,0
.242604873890411,0.808311505184197,0.979018799302051
"11094","Z99289.3",1.66721204904043,-0.289484318385778,1.1924802042338
7,-0.242758175236765,0.808192737772906,0.979018799302051
"11095","YAE1",18.211816893885,0.0948502129104499,0.391162994358781,0.
242482582141836,0.808406251644829,0.979018799302051

"11096","SEMA4D",3.13335632871694,0.20404505798314,0.840719799204181,0
.242702810349283,0.808235630196066,0.979018799302051
"11097","PHF12",19.0414622270812,0.101265326365928,0.417596837115652,0
.242495434269496,0.808396294229209,0.979018799302051
"11098","SENP3",5.69127683407697,-0.168555029631551,0.696258579698142,
-0.242086825995921,0.808712885975004,0.979075057972828
"11099","METTL2A",17.0318754276389,0.0899577308186371,0.37145927392064
3,0.24217387243872,0.808645439326316,0.979075057972828
"11100","ZNF792",1.79653347571571,0.265205854186,1.09568134843572,0.24
2046517050443,0.808744119246698,0.979075057972828
"11101","SLC10A3",30.3731975576324,-0.0800016259093583,0.3304022193164
95,-0.242134045209678,0.808676298689237,0.979075057972828
"11102","PHF7",7.33678028492807,-0.132043805819817,0.545805613565853,-
0.241924601978989,0.808838586647314,0.979101221937781
"11103","ZNF689",9.48991483763822,-0.116048256065449,0.480388523090429
,-0.241571666448001,0.80911207881135,0.979344071615395
"11104","ERF",12.0376574949408,0.109061919194938,0.45173619298327,0.24
1428340011219,0.809223150109875,0.979390302082728
"11105","AC008608.2",4.62907664844845,-0.162603558706394,0.67426617169
7694,-0.241156334889209,0.809433951949907,0.979513207361552
"11106","FAM157C",2.04674796928528,0.281514596773228,1.168124241499,0.
24099713606831,0.80955733620577,0.979513207361552
"11107","SDF2",99.1552162707416,-0.0595676315600101,0.247249509941965,
-0.240921130941744,0.809616244316699,0.979513207361552
"11108","CD7",1.86124354150943,0.271542193588255,1.12648053333445,0.24
1053605058289,0.809513570362893,0.979513207361552
"11109","GMEB1",17.5996864785199,0.0913049119018004,0.379444532233469,
0.240627823424851,0.809843583720984,0.97961187413378
"11110","NPIPA5",1.57379974518337,0.322354378103891,1.33927218336322,0
.240693700734069,0.809792521551145,0.97961187413378
"11111","CENPS",10.0854437444461,0.119911900705242,0.512972956373143,0
.233758718106801,0.815172299864771,0.979737011813886
"11112","NECAP2",53.81098839171,-0.0640203769809297,0.274495575668714,
-0.233229176189693,0.815583451155245,0.979737011813886
"11113","TRIM62",2.46827895628391,-0.203407779965453,0.863806583652386
,-0.23547838580414,0.813837452874311,0.979737011813886
"11114","POMGNT1",15.626642438117,0.0922304490207039,0.387145179577058
,0.238232203023843,0.811701001614087,0.979737011813886
"11115","ZFYVE9",25.750587814799,-0.0909674975087813,0.383764708877579
,-0.237039767869327,0.81262593896288,0.979737011813886
"11116","KCNT2",9.98085966488742,0.122675487944153,0.515713071576105,0
.237875467397474,0.811977683603271,0.979737011813886
"11117","ID2",162.913706948534,-0.0667978401446683,0.280243466405106,-
0.238356458409306,0.811604635409962,0.979737011813886
"11118","CGREF1",16.796922254461,0.126101133349145,0.534846230338226,0
.235770818220783,0.813610513535192,0.979737011813886
"11119","MFSD9",11.710970848796,-0.112862863699734,0.480330727566843,-
0.2349690686487,0.814232740811358,0.979737011813886
"11120","CERS6",2.70743872896751,0.228757996852658,0.972775383897869,0
.23516014142549,0.814084441093381,0.979737011813886

"11121","GPR155",7.27005724522719,0.125140045696724,0.524618751025294,0.2385352133376,0.811466007118387,0.979737011813886
"11122","PRKAR2A",47.9684504859108,0.0693687778662427,0.29499885707736,0.23514931058886,0.814092847188768,0.979737011813886
"11123","RBM6",79.2069365547344,0.0689376804887216,0.290826716908935,0.237040397187125,0.81262545075025,0.979737011813886
"11124","DHFR2",12.7085202745273,0.106339574860996,0.44916993874342,0.236746865025045,0.812853175249616,0.979737011813886
"11125","LDB2",32.513849524548,-0.0727585683371431,0.311988458725202,-0.233209166244283,0.815598988438033,0.979737011813886
"11126","TLR6",1.62406920703905,0.262200584072149,1.1061068721271,0.237048146683985,0.812619438845833,0.979737011813886
"11127","FIP1L1",71.3050528119329,-0.054940173702353,0.228722300925961,-0.240204708854068,0.810171563397657,0.979737011813886
"11128","C4orf3",267.788033380606,-0.0612806799458093,0.259782953981929,-0.235891843581361,0.81351659755511,0.979737011813886
"11129","NNT-AS1",44.3292381350909,0.0603797615219209,0.251591318444406,0.239991434900259,0.810336896561173,0.979737011813886
"11130","PLPP1",268.928618553965,0.0783751530881991,0.332985015281573,0.235371411599182,0.813920473072982,0.979737011813886
"11131","AC034236.2",4.04098446548971,0.173563610964773,0.723563929159146,0.239873221936963,0.810428540666702,0.979737011813886
"11132","COMMD10",33.0077830931615,0.0749544447421042,0.318553684620304,0.235296116042246,0.813978909461401,0.979737011813886
"11133","G3BP1",86.5884432113335,0.0516916994307451,0.22049281005495,0.234437120275544,0.814645643649535,0.979737011813886
"11134","SAP30L",44.9183474920697,0.058025177062411,0.246738337925015,0.235168873837697,0.814077663655624,0.979737011813886
"11135","PTTG1",4.45543957100395,-0.166698006299269,0.696545470774049,-0.239321068463805,0.810856629735288,0.979737011813886
"11136","FBXW11",48.1394692886937,0.0622247115007587,0.264717886958839,0.235060472171396,0.81416179781438,0.979737011813886
"11137","NFKBIE",3.6558096335616,0.1942189539269,0.8296605072689,0.234094490728786,0.814911622882405,0.979737011813886
"11138","NUS1",24.3635264235173,0.0747690390277399,0.318737394620374,0.23457881092613,0.814535657128991,0.979737011813886
"11139","RNF217",18.1958245147122,0.0894246795934909,0.375105970458294,0.238398443736405,0.81157207435487,0.979737011813886
"11140","GLCCI1",5.2336162842641,-0.149179894694741,0.631404955229655,-0.236266588437654,0.813225811626802,0.979737011813886
"11141","HOXA6",3.55813618177999,-0.17711251579424,0.742602372172592,-0.23850249128086,0.811491383335326,0.979737011813886
"11142","TMEM60",36.9400726155527,-0.0797275037838086,0.33432346257501,-0.238474150661564,0.811513361872499,0.979737011813886
"11143","SMURF1",12.5561013383394,0.0959180065741497,0.410915454276969,0.23342516222052,0.815431276142959,0.979737011813886
"11144","ORC5",7.43647324836129,-0.135261517594373,0.567814846174404,-0.23821412649860,0.811715021092218,0.979737011813886
"11145","SMIM30",45.1296959357947,0.086093511893542,0.365326405232982,

0.235661892106148,0.813695042761822,0.979737011813886
"11146","GSTK1",136.876416651875,-0.0632106200567338,0.263832346415253
,-0.239586316521042,0.810650973695735,0.979737011813886
"11147","ARHGEF35",5.76065214868529,-0.14357608767492,0.60619225650893
,-0.236849095535757,0.81277386223946,0.979737011813886
"11148","FAM160B2",24.0390967856001,-0.0830241686389108,0.354240855463
512,-0.234372087122126,0.814696126469542,0.979737011813886
"11149","TRAM1",226.665371112725,0.0426689161209604,0.179237328706814,
0.238058201541018,0.811835953174007,0.979737011813886
"11150","MTAP",42.8136850969344,0.0681467550032276,0.292179260442693,0
.233236112994384,0.8155780648958,0.979737011813886
"11151","PTGR1",66.9291560872845,0.0756036589455501,0.324249870713867,
0.233164808297692,0.815633431667071,0.979737011813886
"11152","LRRC8A",32.4170127858949,0.0723547582073332,0.309879607841902
,0.233493125640742,0.815378506997098,0.979737011813886
"11153","TRAF2",6.36813278059968,0.130021686506381,0.556487822022298,0
.23364695750911,0.815259069713946,0.979737011813886
"11154","USP6NL",8.84797769503696,0.11327803504619,0.479034464171359,0
.236471576720768,0.813066760405632,0.979737011813886
"11155","LIPA",52.396834799928,-0.05739684463015,0.242837822564626,-0.
236358751795656,0.813154300758604,0.979737011813886
"11156","ABRAXAS2",18.6123173130404,-0.0954712604954831,0.404022865920
023,-0.236301626835106,0.813198624649371,0.979737011813886
"11157","TPCN2",6.83341256419851,0.128822435742714,0.546560990621686,0
.235696359515495,0.813668295006862,0.979737011813886
"11158","PTS",23.9466147536343,0.0820498207458024,0.347264434240667,0.
236274759680511,0.813219471379633,0.979737011813886
"11159","VPS11",25.3261044079993,-0.0742017239861652,0.314722449582163
,-0.235768767320788,0.813612105061772,0.979737011813886
"11160","SSPN",434.92144234482,0.0683927375638172,0.291828416972493,0.
234359416650859,0.81470596217505,0.979737011813886
"11161","PUS7L",47.0979954600538,-0.0609581645106153,0.258482314934734
,-0.235831083940915,0.813563746851788,0.979737011813886
"11162","ZBTB39",4.61945629380197,0.169674869493981,0.713427256163901,
0.237830652008339,0.812012443808209,0.979737011813886
"11163","MARCH9",16.7395964992181,0.0939481316317673,0.398350316805481
,0.235842994641431,0.813554504131074,0.979737011813886
"11164","FAM216A",10.4368430059208,0.109585373244733,0.462787598165244
,0.236794100963795,0.812816528177645,0.979737011813886
"11165","FBXW8",11.5282001283108,-0.102383932543667,0.43702981258899,-
0.234272192867435,0.814773672165488,0.979737011813886
"11166","IPO4",5.47218297457442,-0.137187569994299,0.584959691975427,-
0.234524826028632,0.814577562152013,0.979737011813886
"11167","LRFN5",4.79316231650803,0.196805917663748,0.839725412918872,0
.234369371982746,0.814698234148068,0.979737011813886
"11168","ERG28",22.0676046285309,-0.0862841303685209,0.366544559025958
,-0.235398748238984,0.813899257542168,0.979737011813886
"11169","SETD3",22.8055085626968,-0.0762079691734153,0.326213668637041
,-0.233613660309885,0.81528492177613,0.979737011813886
"11170","ZNF75A",18.4767463030278,0.0969703398947104,0.411153941267501

,0.235849228626561,0.813549666560006,0.979737011813886
"11171","ARMC5",5.07678724609582,0.157348572399988,0.656461775442822,0
.239691903300611,0.810569112230893,0.979737011813886
"11172","PHLPP2",10.1047147923146,-0.124845253305638,0.522898056177757
,-0.238756392055123,0.811294486447868,0.979737011813886
"11173","DDX52",33.4329100336839,-0.0784996453090446,0.336070616245298
,-0.233580805683255,0.815310430420505,0.979737011813886
"11174","NMT1",101.917745332424,0.0562108668567709,0.239833229895452,0
.234374806532332,0.81469401547705l,0.979737011813886
"11175","ERN1",13.2913577778822,-0.106568461243163,0.448954089511324,-
0.237370510109754,0.812369365546784,0.979737011813886
"11176","WBP2",53.675869418984,-0.0602829808913215,0.252108673911489,-
0.239115060803048,0.811016363644073,0.979737011813886
"11177","ZNF396",5.48852011983823,0.139111132609995,0.593435409596023,
0.234416636352546,0.814661544474638,0.979737011813886
"11178","CTDP1",9.48778370020695,-0.135760389066539,0.573667149080855,
-0.236653587858497,0.812925543704238,0.979737011813886
"11179","MCOLN1",38.0201620700613,-0.0819139171174125,0.35007831691121
9,-0.233987405561556,0.814994756198521,0.979737011813886
"11180","ZNF875",12.2713501251363,-0.109456154463007,0.457037463899108
,-0.239490551888695,0.810725221837519,0.979737011813886
"11181","C19orf54",3.90900838782928,-0.177756966439252,0.7522634907552
59,-0.236296149718482,0.813202874436371,0.979737011813886
"11182","ZNF628",4.52024559625851,0.164447044741823,0.691385246156655,
0.237851538857633,0.811996243274373,0.979737011813886
"11183","SLC2A10",39.9254363455036,0.0685728027329972,0.28759777933664
9,0.238433004911101,0.811545271228082,0.979737011813886
"11184","TSHZ2",311.228736322871,-0.0618804442110131,0.265258922568264
,-0.233283177100625,0.815541520996765,0.979737011813886
"11185","COL6A1",2303.95483275792,0.0610333001729234,0.260003140395544
,0.23474062690194l,0.81441005291652,0.979737011813886
"11186","LINC00630",4.78772952101903,0.162962866595966,0.6852110394401
41,0.237828723146545,0.812013939901521,0.979737011813886
"11187","TCEAL4",171.429778703378,0.0495363488677602,0.210354205782383
,0.235490175646913,0.813828303178784,0.979737011813886
"11188","GPC4",31.5559599160162,0.0785543239146207,0.333450951096965,0
.235579846619714,0.813758713421581,0.979737011813886
"11189","GUCA1B",2.50956307431489,-0.199245259340113,0.855618720698738
,-0.232866876939533,0.815864779782796,0.97983974758722
"11190","AP001505.1",2.42499172718022,-0.222306799638964,0.95432339901
792,-0.23294702809104,0.815802539646563,0.97983974758722
"11191","SSX2IP",4.83547398884702,-0.160439851236956,0.693139193837819
,-0.231468444813547,0.816950897225294,0.97989166213483
"11192","TMEM183A",42.3160876572855,0.0575267021775061,0.2479329310477
51,0.232025257533969,0.816518396376906,0.97989166213483
"11193","EEF1B2",683.594189535578,-0.0627111568576544,0.27064262575841
,-0.231712047139365,0.816761673753414,0.97989166213483
"11194","RUVBL1",17.9323425308678,-0.085864775285841,0.369334178890429
,-0.232485321406754,0.816161086748269,0.97989166213483
"11195","DTNBP1",29.3603748418107,-0.0790103833187883,0.34098741450111

9,-0.23171055575287,0.816762832188239,0.97989166213483
"11196","SIPA1",20.3074132700123,0.0835708510382253,0.359990226128274,
0.232147555607376,0.816423409581495,0.97989166213483
"11197","DCPS",29.1097990639656,-0.0803218767838466,0.346448074233476,
-0.2318439118519,0.816659249388517,0.97989166213483
"11198","CCDC59",76.0512930837654,0.0516565961162788,0.223312116132646
,0.231320167534461,0.817066080092186,0.97989166213483
"11199","MTA1",27.9477358827446,0.0657314889479222,0.284171577774598,0
.231309159989462,0.817074630990618,0.97989166213483
"11200","HNRNPA1P48",17.160000834068,-0.0981401843124967,0.42171619526
1858,-0.232716185470558,0.815981800055722,0.97989166213483
"11201","AC105429.1",1.61672175036006,0.278331342277104,1.199964499091
01,0.23194964725035,0.816577122923049,0.97989166213483
"11202","MAP3K14",6.63101946371429,0.13855507928279,0.598956314609443,
0.231327520727679,0.817060367985186,0.97989166213483
"11203","HDHD2",28.3158715227642,0.0709391073042387,0.305877733288426,
0.231919814958701,0.816600293962673,0.97989166213483
"11204","ZNF81",3.12710302750123,-0.195663030554101,0.844727918048459,
-0.23162846447189,0.816826597228186,0.97989166213483
"11205","CHIC1",13.2318383388254,0.0958436158267267,0.413919696167733,
0.231551232555718,0.816886588825741,0.97989166213483
"11206","F8",6.02870972940466,0.140840250634061,0.607816615152274,0.23
1715038916429,0.816759349891056,0.97989166213483
"11207","SAV1",105.734872356178,-0.0516077534396156,0.223388717946479,
-0.231022201631419,0.817297554113017,0.98004539390013
"11208","SAP30BP",47.9223345825433,-0.0566178180423314,0.2451450434932
48,-0.23095640538166,0.817348669903464,0.98004539390013
"11209","FAM131A",7.75767925774195,-0.138112166748233,0.59857326021688
8,-0.230735610705679,0.817520206546779,0.980076186956482
"11210","CPQ",350.839369774613,0.0510834064931738,0.221381189497726,0.
230748631394893,0.817510090456486,0.980076186956482
"11211","LCK",1.79442114032844,-0.278333939805822,1.21564606507809,-0.
228959684731874,0.818900250728766,0.980340532297193
"11212","CC2D1B",4.53890840905144,0.156366878368305,0.686622134509823,
0.2277335240291,0.819853408735251,0.980340532297193
"11213","PRUNE1",11.1521267884935,0.10485880069392,0.461944677395156,0
.226994282703297,0.820428187884279,0.980340532297193
"11214","PRCC",25.9304877570658,-0.0708745357533152,0.311732230088623,
-0.227357099819823,0.820146076170431,0.980340532297193
"11215","MRPS14",50.3436638117844,-0.0615719488413661,0.27021149072825
4,-0.227865767941333,0.819750595885301,0.980340532297193
"11216","VRK2",14.0285595409628,-0.10211156244686,0.450326937067398,-0
.226749843373411,0.820618266987279,0.980340532297193
"11217","LINC00342",94.4636228842328,0.0800099486753875,0.350598280227
527,0.228209757969901,0.8194831758195,0.980340532297193
"11218","ARID5A",8.37581970537168,-0.116170622475667,0.50547678961667,
-0.22982385118764,0.818228650185808,0.980340532297193
"11219","FAHD2B",5.94402727789477,0.156762544913862,0.68634329740269,0
.22840529036263,0.81933332352607,0.980340532297193
"11220","EMC3",86.1831544897969,0.0615119087801819,0.270078962685153,0

.227755276340756,0.819836497217283,0.980340532297193
"11221","TRPC1",13.8794922159154,0.0974145301519221,0.429747719484565,
0.226678410926206,0.82067381574809,0.980340532297193
"11222","GMPS",35.3478572219367,0.0621577827117772,0.272648337498503,0
.227977853384558,0.819663457635346,0.980340532297193
"11223","TBCCD1",8.29305912173148,0.118517087912789,0.515199263674254,
0.230041260283562,0.818059708268923,0.980340532297193
"11224","ABRAXAS1",58.7871136822661,-0.0582863349673607,0.254883061339
323,-0.228678730791626,0.819118627148874,0.980340532297193
"11225","HSPA4L",1.69184690331027,-0.260989385508574,1.13854988443462,
-0.229229644723188,0.818690432783067,0.980340532297193
"11226","ERCC8",5.89774096286368,0.149382957253379,0.65678103755438,0.
227447122726972,0.820076081650747,0.980340532297193
"11227","KLHL3",3.46447571861386,0.207202520070872,0.907083575287267,0
.228427154581927,0.819314181115482,0.980340532297193
"11228","MRPS18A",50.1855905610106,-0.0753082676965977,0.3271417205115
99,-0.230200744737869,0.817935783180086,0.980340532297193
"11229","ZNF395",29.0250195563527,-0.0738294597502372,0.32216606329840
3,-0.229165850041298,0.818740014029911,0.980340532297193
"11230","SLC44A1",142.613155230814,-0.0394001545911532,0.1736220594252
57,-0.226930579683135,0.820477723139777,0.980340532297193
"11231","ABCA2",16.3283913838898,-0.0884412647177824,0.386580061887642
,-0.228778650109191,0.819040961483237,0.980340532297193
"11232","AL121749.1",2.03818136556465,-0.301142234831485,1.32808169713
547,-0.22674978164447,0.820618314989813,0.980340532297193
"11233","PAOX",5.22491149400612,0.153614585234044,0.667955201319739,0.
2299773771213,0.818109349031836,0.980340532297193
"11234","CD59",597.765795290947,0.0524363727185991,0.231592477262954,0
.226416563000283,0.820877446978221,0.980340532297193
"11235","C11orf98",1.86177018498716,-0.259254711014146,1.1294935677992
7,-0.229531817095057,0.818455594174484,0.980340532297193
"11236","ZNHIT2",7.91664279020076,0.122338267039835,0.532353309366602,
0.229806530526491,0.818242109901631,0.980340532297193
"11237","CHKA",4.70029148666177,0.160888942462451,0.71025622775076,0.2
26522395969627,0.820795142417271,0.980340532297193
"11238","CCDC15",4.82456032014206,-0.149645182057562,0.660733518210749
,-0.226483412651439,0.820825458873663,0.980340532297193
"11239","HIST4H4",1.68390788968249,0.249679062576198,1.09554252998837,
0.227904490918165,0.819720491345883,0.980340532297193
"11240","HOXC-
AS2",4.5255308969624,-0.155031237483819,0.679168253559048,-0.228266319
386114,0.819439206618723,0.980340532297193
"11241","COPZ1",104.610804483371,-0.056525335730409,0.247044354706986,
-0.228806425459317,0.819019372469201,0.980340532297193
"11242","COQ5",25.1093927348609,0.0963679073583216,0.421159678259188,0
.228815606842152,0.819012236063576,0.980340532297193
"11243","TMED10",182.630785887376,-0.0454966495093901,0.19973972033278
8,-0.227779679643026,0.819817524764049,0.980340532297193
"11244","EMC7",148.997850875077,-0.050581167220678,0.223386195559816,-
0.226429243283898,0.820867585626035,0.980340532297193

"11245","DEF8",31.5351479137618,0.0769236570859303,0.339061577814326,0
.226872232418073,0.820523094406338,0.980340532297193
"11246","AC124283.1",1.98379593904682,0.23607137111383,1.0287428545857
6,0.229475587666548,0.818499292637548,0.980340532297193
"11247","TIMM21",17.6018774062955,0.0861459450131239,0.378702765258749
,0.227476408719288,0.820053311544557,0.980340532297193
"11248","ARHGAP45",2.85100440105607,0.205235715288155,0.90148004578496
9,0.227665289151736,0.819906459059105,0.980340532297193
"11249","SCN1B",22.6163924353523,0.085760127231142,0.373314400607107,0
.229726276542436,0.818304475184317,0.980340532297193
"11250","OSBPL2",25.3173776308698,0.0799725694342821,0.35300912823502,
0.226545329958265,0.820777307291299,0.980340532297193
"11251","AJ011932.1",2.23599776839951,-0.232386919202872,1.01346377286
611,-0.229299680387857,0.818636001877369,0.980340532297193
"11252","GSPT2",20.1519306680553,-0.0885576648908076,0.387163901431053
,-0.228734302354834,0.819075432054649,0.980340532297193
"11253","FMR1",37.4485801173932,0.0786367061245094,0.342305257939818,0
.229726842636857,0.818304035268938,0.980340532297193
"11254","HNRNPAB",89.5082449629272,-0.055300436622491,0.24470994149085
6,-0.225983612621465,0.821214165900207,0.980394170341378
"11255","ARF3",80.16684298846,-0.0495953660673712,0.219407719580747,-0
.226042028795249,0.82116873190811,0.980394170341378
"11256","TMEM220-
AS1",5.71550510828206,0.144977384896484,0.640783200216415,0.2262502900
31824,0.821006758716173,0.980394170341378
"11257","TP53RK",13.3068287925034,-0.107156439088857,0.473917762385674
,-0.226107665915364,0.821117682440273,0.980394170341378
"11258","CCDC28B",27.8843128232365,-0.0899021302588648,0.3982434635362
83,-0.225746656230238,0.821398468180367,0.980440004785146
"11259","FAM161B",8.33149748492167,0.121328075425054,0.537276370761056
,0.22582060560972,0.821340950043537,0.980440004785146
"11260","CTBS",50.1278277142167,-0.0567051457239491,0.251541984507479,
-0.225430143739138,0.821644663766951,0.980509542243742
"11261","C9orf64",16.6820637981165,0.0873542314252337,0.38732749462368
2,0.225530675301285,0.821566464547284,0.980509542243742
"11262","SLC16A8",3.06403794459717,0.191975785806394,0.851747966121779
,0.225390365979396,0.821675605681153,0.980509542243742
"11263","SIMC1",8.02412262174372,0.113056869145293,0.502091109427061,0
.22517201962469,0.821845455634955,0.980623152335271
"11264","REXO1",13.7021202287225,0.0972418354374579,0.432031606663408,
0.225080373606133,0.821916748858136,0.980623152335271
"11265","RRAGC",65.3328693412094,0.0532858642555182,0.237294087985472,
0.224556223494201,0.822324523681796,0.980632781318716
"11266","USP37",14.1290944787409,0.098701915347326,0.439758754077797,0
.224445595299883,0.822410595607818,0.980632781318716
"11267","ADAMTS9",1.52599655034984,0.26187310195313,1.16667712730234,0
.224460646244646,0.822398885413054,0.980632781318716
"11268","CBLN3",4.66446735885645,0.157854518347154,0.703156166547262,0
.22449425299400 2,0.822372738255023,0.980632781318716
"11269","ANKRD9",25.0269067063276,-0.0825624686507018,0.36769990805986

6,-0.22453763746185,0.822338984001631,0.980632781318716
"11270","IQCN",6.60003171206338,0.139745424384719,0.622337301519177,0.224549330473344,0.822329886588385,0.980632781318716
"11271","B3GNT8",9.23528074593787,-0.103687799080482,0.462039141291767,-0.224413452917845,0.822435603708851,0.980632781318716
"11272","BOD1",72.0482218388983,-0.0654701132684383,0.291880175761373,-0.224304761697703,0.822520171304389,0.980646609488971
"11273","SPRTN",14.6017736112387,-0.0888594878399627,0.397798085051876,-0.223378370030049,0.823241037257896,0.980765944625965
"11274","TSGA10",5.92253295717851,0.1337619173141,0.598770379390813,0.22339434600982,0.823228604381968,0.980765944625965
"11275","NUDT9",34.2338794627796,-0.0672496218636611,0.301245914007682,-0.223238287182033,0.823350054860491,0.980765944625965
"11276","EXOSC9",20.4980220191769,-0.073357860185089,0.328446709373416,-0.223347831144465,0.823264803446381,0.980765944625965
"11277","SERINC1",221.608594361868,0.0418733290247034,0.187290613653888,0.223574092731017,0.823088724397228,0.980765944625965
"11278","NFYB",61.4700198701262,-0.0689647140688784,0.308758180663136,-0.223361576754855,0.823254106219753,0.980765944625965
"11279","CCDC102B",17.1983209519313,0.117223161974495,0.523142077765415,0.224075192871523,0.822698794931985,0.980765944625965
"11280","ZNF181",15.0500618507444,0.0860786538347866,0.385202479182104,0.223463395192981,0.823174869225708,0.980765944625965
"11281","BMP2",1.73355439059155,-0.292662610417658,1.30697946848023,-0.223922882857502,0.822817309853385,0.980765944625965
"11282","SMIM11A",2.89338254429808,-0.186035483319889,0.833146193571653,-0.223292724320524,0.823307689480546,0.980765944625965
"11283","HMGN2",274.106277460484,0.0452764783405946,0.202963698386731,0.223076730964588,0.823475788058665,0.980828181477801
"11284","RPL27A",2232.96053291099,0.0480929864511082,0.215679477331203,0.222983600693984,0.823548269945346,0.980828181477801
"11285","GLT8D1",146.96225590511,-0.0431538144874776,0.193637912037629,-0.222858292745336,0.823645797609173,0.980857410196693
"11286","PPM1B",30.6120501364235,0.0752590579860239,0.338521546118477,0.222316891934803,0.82406720325198,0.981026967718522
"11287","MTBP",3.62103562994336,-0.175643178799396,0.790116943416518,-0.222300230697374,0.824080172525813,0.981026967718522
"11288","ZWINT",3.7179529822816,-0.181940774500122,0.818191984057557,-0.222369294792948,0.824026412656322,0.981026967718522
"11289","MAGT1",67.9708231203728,0.0580412696513797,0.261027077733597,0.222357274790535,0.824035769032565,0.981026967718522
"11290","TCIM",5.80926421654323,0.346407947012614,1.56258664768073,0.22168879244346,0.824556155376778,0.981506658291277
"11291","FAM13B",25.5740818184718,0.0722111967903456,0.326188907182525,0.221378456471969,0.824797765546834,0.98153434087502
"11292","ATP7B",1.55649896226828,0.24640976426445,1.11285196694984,0.221421870637315,0.82476396471511,0.98153434087502
"11293","ZFP28",2.64955399831608,0.200431764398095,0.905768397515931,0.221283680185552,0.82487155635408,0.98153434087502
"11294","MAPK1",68.66651629482,-0.0511476392183085,0.231123937906345,-

0.221299618211915,0.824859147236396,0.98153434087502
"11295","ZBTB8A",39.0387936260452,0.0655617211847511,0.296470468512182
,0.221140815521251,0.824982790677261,0.981548471554628
"11296","PDRG1",20.0048063427393,0.07482772662247 5,0.338463225901715,0
.221080817341745,0.825029506263939,0.981548471554628
"11297","YTHDC2",24.0895011652024,0.0777686886290248,0.353098451977792
,0.220246472884328,0.82567920497449,0.981663158564995
"11298","CSPP1",23.9192167385663,-0.0744621302436598,0.33824267089139,
-0.220144105554233,0.825758925994,0.981663158564995
"11299","NOL6",7.40100558366985,-0.117922153985456,0.534186756989031,-
0.220750800057511,0.825286474318366,0.981663158564995
"11300","DNAJC9",23.8691655331854,0.0799305122837728,0.36209813955463,
0.220742675955433,0.825292800389027,0.981663158564995
"11301","CASC2",2.82587509060027,0.183229366738317,0.831897270649498,0
.220254799724564,0.825672720326492,0.981663158564995
"11302","CASP4",106.25919352276,0.0521995019165974,0.236545078462623,0
.220674647960793,0.825345772830006,0.981663158564995
"11303","GZMH",3.14170892559651,0.178471905609877,0.809833633995226,0.
220380950009949,0.825574480411305,0.981663158564995
"11304","COQ7",22.5568580474379,-0.081648626657897,0.37036686218619,-0
.220453380132186,0.825518076469305,0.981663158564995
"11305","MAGEE1",5.89113038829703,0.130052562215853,0.590845107223074,
0.220112785273098,0.825783317774929,0.981663158564995
"11306","HNRNPF",165.443441733996,-0.054209140562566,0.246624494814016
,-0.219804365350839,0.826023519716925,0.981700110341359
"11307","ARID3B",3.67062236483495,0.179609776435313,0.817182578698384,
0.219791489830091,0.826033547714867,0.981700110341359
"11308","TRAPPC2B",23.3404521277531,-0.091906313274632,0.4178769741939
82,-0.219936294532391,0.825920769348306,0.981700110341359
"11309","C11orf58",279.997176655173,-0.0339309720387942,0.154568634227
312,-0.219520423457289,0.826244672209993,0.981864192221249
"11310","RSRC1",82.6128222281317,0.046332713377098,0.211240959206748,0
.219335840696267,0.826388444715225,0.981948214723953
"11311","RNF121",18.1559259839955,0.0851359944652643,0.388665549247276
,0.21904692769952 5,0.826613492286117,0.982055552280959
"11312","HOXB3",19.8670421019485,-0.0996097801484156,0.454772214748096
,-0.219032247173655,0.82662492800076,0.982055552280959
"11313","PKP4",2.61233547110698,-0.222940888732632,1.01927034566922,-0
.218725963803309,0.826863522470763,0.982239408031619
"11314","RNF122",2.62126757955454,0.194854913433278,0.891189252770817,
0.218645941731736,0.826925862227081,0.982239408031619
"11315","MRFAP1L1",41.2456413507034,0.059094983569953,0.27061208085558
9,0.218375260199447,0.827136739891388,0.982403062076922
"11316","MEIS1",13.4281635087202,-0.0946905876641022,0.434301431713186
,-0.218029646576519,0.827406012408521,0.982421718134283
"11317","HTRA3",420.194340048669,-0.0644893906133302,0.2959777108132,-
0.217885969981136,0.827517958871946,0.982421718134283
"11318","GREB1L",2.94504233923992,-0.206815024632607,0.948041148596024
,-0.218149839739429,0.82731236589323,0.982421718134283
"11319","ZNF93",4.90439214472889,-0.145480213566666,0.667656441940981,

-0.217896817027231,0.827509507209922,0.982421718134283
"11320","LIPE",1.72959781106749,-0.239328364523654,1.09835720632484,-0
.217896657977471,0.827509631136126,0.982421718134283
"11321","KANSL2",12.5262142724049,-0.0885136829887611,0.40683296755512
6,-0.217567626145655,0.827766010823511,0.982482512244698
"11322","OAS3",8.40059230822907,-0.10923780283044,0.502520234111194,-0
.217379909136692,0.827912287048097,0.982482512244698
"11323","CSNK1D",44.005077606224,-0.0638169713495618,0.293580558711254
,-0.217374650520806,0.827916384847338,0.982482512244698
"11324","METRNL",459.821124282351,0.0571488239387599,0.262519433267923
,0.217693689291317,0.827667780984126,0.982482512244698
"11325","DHX35",6.24175278153504,-0.121412033870429,0.558598546222409,
-0.217351145454088,0.827934701329802,0.982482512244698
"11326","RAPGEF6",15.5263547738457,0.0879160094624166,0.40479320118044
2,0.217187465614638,0.828062252547586,0.982503337071438
"11327","ZDHHC16",12.2897676374918,0.0951026555389162,0.43816581593478
8,0.217047181866579,0.828171575440528,0.982503337071438
"11328","AC240274.1",24.8139416006508,-0.11478833932286,0.528770546090
144,-0.217085350482611,0.828141830371437,0.982503337071438
"11329","IMP3",88.9933891644203,0.059011723650189,0.272144740355982,0.
216839478775148,0.828333444228499,0.982608628915773
"11330","SHB",1.73883165068677,0.238145856255953,1.09910527514434,0.21
6672471365109,0.828463603029717,0.982621340873534
"11331","RCN2",91.7075876088849,-0.0515677041916208,0.238036177479289,
-0.216638095678156,0.828490394630405,0.982621340873534
"11332","RBM15",8.22745326804601,-0.120734924637114,0.558167206846115,
-0.216306015753449,0.828749220170544,0.98282791676268
"11333","FBF1",4.1170386483472,0.15631224995134,0.723521106008405,0.21
6043801145898,0.828953605305488,0.98282791676268
"11334","ZNF616",10.5228804682508,0.0980634451395807,0.453904242837125
,0.216044345667791,0.828953180861828,0.98282791676268
"11335","GET1",40.0593279500121,0.0700849237792138,0.324408184391731,0
.216039320680592,0.828957097738297,0.98282791676268
"11336","NRAS",18.3773412988829,0.0781032964573868,0.361821676930795,0
.215861296978969,0.829095866376746,0.982905729378713
"11337","YTHDF2",66.2889235063022,-0.0558026256792378,0.25890779188997
2,-0.215530885617194,0.829353434610412,0.983037644005056
"11338","ZNF32",55.9733698034208,-0.0687193037349799,0.318724330535731
,-0.215607335716958,0.829293837215492,0.983037644005056
"11339","TBC1D32",9.68938217214707,0.117259285021352,0.545334757569,0.
21502257722224,0.829749716159672,0.983365542447337
"11340","ENGASE",11.778920997113,0.106481286720065,0.49528867840708,0.
214988331779609,0.82977641575659,0.983365542447337
"11341","DCAF11",15.74930446499,0.0830300693961299,0.386382007718882,0
.214891138141556,0.8298521942375,0.983368630487414
"11342","ZCCHC4",6.4928328597747,-0.146016103526397,0.679919080830459,
-0.214755119606367,0.829958245790281,0.983407588183352
"11343","TMEM240",14.8291909724804,0.0903170518299665,0.42266119574190
3,0.213686642492533,0.830791428505899,0.983516670595121
"11344","ATG4C",16.4622388013912,0.0860752706652686,0.402988874160465,

0.21359217632146,0.830865101001883,0.983516670595121
"11345","HHLA3",9.69675441857859,-0.116402209634283,0.544989533092607,
-0.213586138019469,0.830869810217358,0.983516670595121
"11346","DHX9",64.3468813426482,-0.0506768572497565,0.237663592287833,
-0.213229366609851,0.831148063691404,0.983516670595121
"11347","TMF1",99.1918011506556,0.0514289878957861,0.23997471286808,0.
214310029924102,0.830305296991047,0.983516670595121
"11348","SH3BGRL2",6.61336137978143,-0.138877664757082,0.6511272544877
45,-0.213288053602272,0.831102291024679,0.983516670595121
"11349","ARMC1",35.1732619182533,-0.0572677939236396,0.268305104861789
,-0.213442804053765,0.830981596824861,0.983516670595121
"11350","TMEM71",2.66652729751425,0.192420001753781,0.897973517217786,
0.214282490590547,0.830326771411868,0.983516670595121
"11351","TRIM34",14.0589301834858,0.0906801211618065,0.425168963614161
,0.21328019898484,0.831108417166475,0.983516670595121
"11352","RRP8",19.934080355857,-0.0736136986328703,0.343721753406216,-
0.214166539950913,0.830417187946465,0.983516670595121
"11353","CORO1A",6.19002490610896,-0.141486109437859,0.659958917564446
,-0.214386237797965,0.830245872831221,0.983516670595121
"11354","PRKAR1A",145.428381135846,0.0380428124837559,0.17787534408561
7,0.213873444233196,0.830645749510919,0.983516670595121
"11355","MAPRE2",34.608021985334,-0.0595130999459536,0.278421281153087
,-0.213751979372694,0.830740474298638,0.983516670595121
"11356","SARS2",5.87705065489726,-0.136371483964671,0.63751764783392,-
0.213910131630108,0.830617139202517,0.983516670595121
"11357","TFPT",11.5058336232281,0.104261257660034,0.487700047397669,0.
213781520457839,0.830717436355765,0.983516670595121
"11358","HMBS",6.21794238252528,-0.134325525577695,0.630277827707456,-
0.213121134319899,0.831232480501496,0.983529961741469
"11359","PPP3R1",28.4551985638701,0.0645972535594552,0.304212196639802
,0.21234274717769,0.831839648302757,0.984056378328433
"11360","PAXIP1-
AS1",17.0615324939531,-0.0787224052770311,0.370861247572951,-0.2122691
59401849,0.831897054408016,0.984056378328433
"11361","AC080038.1",11.9005987559563,0.0972879434416493,0.45792226969
6714,0.212455147695883,0.831751965960361,0.984056378328433
"11362","PUSL1",10.2928098795383,-0.108731102873183,0.523439776839151,
-0.207724188501241,0.835444329922677,0.984359685657801
"11363","DFFB",5.52906715147684,-0.15396810920494,0.732640835382103,-0
.210154965119626,0.833546727245078,0.984359685657801
"11364","EFCAB14",84.9640718114657,-0.0512486774252722,0.2432690349055
75,-0.210666669702391,0.833147384622101,0.984359685657801
"11365","SH3GLB1",170.824736935436,0.0390457318186718,0.18576520815482
3,0.21018861500766,0.833520465001946,0.984359685657801
"11366","PRPF38B",71.9923847244939,0.0549185462127907,0.26064833769493
4,0.210699775407999,0.833121549869656,0.984359685657801
"11367","RSBN1",45.4430860056959,-0.0576063956383558,0.273607314101147
,-0.210544063222885,0.833243064805735,0.984359685657801
"11368","TBX15",43.89669251274,-0.0594163761764414,0.284128360407481,-
0.209118076390649,0.834356063740629,0.984359685657801

"11369","AKT3",57.8741115894638,0.0690812879546163,0.33226824836633,0.207908183506157,0.835300659280551,0.984359685657801
"11370","AC159540.2",3.15195006095209,-0.185018079304207,0.897894494300928,-0.2060577055306,0.83674583585654,0.984359685657801
"11371","CYTIP",5.35993449183871,0.149017173600468,0.716630082965097,0.207941554705465,0.835274602305112,0.984359685657801
"11372","SATB2",4.29226068103502,0.146930959013359,0.713796716407253,0.205844262989756,0.836912564634566,0.984359685657801
"11373","ARL6",7.59503984186016,0.107300121405105,0.52125139120213,0.205851002445568,0.836907300055437,0.984359685657801
"11374","BBX",121.483006394544,0.0422913938276367,0.204948701816893,0.206351118366297,0.836516650950651,0.984359685657801
"11375","SEC22A",13.6193786018362,-0.0844446806431444,0.408003527991861,-0.206970466796623,0.836032923177136,0.984359685657801
"11376","MBNL1-AS1",3.89962770736287,-0.165216934685159,0.785194419353524,-0.210415319585673,0.833343536989935,0.984359685657801
"11377","ZNF639",26.1997722457495,-0.0727814347705421,0.350536778630955,-0.207628526326951,0.835519028935709,0.984359685657801
"11378","RNF168",45.7190320055219,-0.072848096017017,0.353809525372395,-0.205896367375475,0.836871863072244,0.984359685657801
"11379","ANKRD50",19.780425060261,0.0846313421835237,0.410114797193484,0.206360128341325,0.836509613474352,0.984359685657801
"11380","ADAMTS12",10.2096921489029,-0.0912959306691131,0.437975968470352,-0.208449634777834,0.834877904421642,0.984359685657801
"11381","FEM1C",31.7819711602159,0.0761001704642022,0.361629608484864,0.21043678028202,0.833326788765122,0.984359685657801
"11382","ADAM19",7.22378413121332,-0.13243473025552,0.626235060484786,-0.211477668070833,0.832514556451239,0.984359685657801
"11383","FGF18",52.9780549709409,-0.0826761200316397,0.400045508746738,-0.206666787212903,0.836270097359072,0.984359685657801
"11384","ATAT1",10.2325737629389,-0.0967729158456939,0.46814502722554,-0.206715676163898,0.836231914013737,0.984359685657801
"11385","RGL2",23.3348965264623,0.0722940982037719,0.348119414882552,0.207670400193458,0.835486331017296,0.984359685657801
"11386","PHTF2",30.6939010989605,0.0744926685277037,0.357505278740894,0.208368024075228,0.834941621455846,0.984359685657801
"11387","GAL3ST4",12.02279162527,-0.0956222574982882,0.463774356527161,-0.206182718282071,0.836648186641408,0.984359685657801
"11388","WRN",23.943255968099,0.0814186865415121,0.386397110050392,0.210712462448008,0.833111649311709,0.984359685657801
"11389","HACD4",14.0733907709353,0.0883421127550109,0.422802967802863,0.20894392774509,0.834492011526633,0.984359685657801
"11390","C5",5.51865092279936,0.125359315721733,0.604180326329404,0.207486590110162,0.835629864350987,0.984359685657801
"11391","KIN",49.3859515763703,0.0510075267387372,0.247039624027831,0.206475082446655,0.836419826716879,0.984359685657801
"11392","ZNF239",3.56416845787203,0.17312090659436,0.821815401689778,0.210656683043902,0.833155177962138,0.984359685657801
"11393","ACADSB",16.889316088241,0.0834476348766966,0.400503032357603,

0.208357061332278,0.834950180629862,0.984359685657801
"11394","ZBED5-
AS1",10.3154405568191,0.113040880686071,0.533917300764219,0.2117198310
00552,0.832325615905543,0.984359685657801
"11395","TKFC",7.13125408337385,0.122252566975107,0.591684257442387,0.
206617913925166,0.83630826885667,0.984359685657801
"11396","TUT1",6.75542725081462,0.127791372654442,0.620823224622446,0.
205841804214329,0.836914485328226,0.984359685657801
"11397","GSEC",7.6547053707495,0.118270931201602,0.561736059703706,0.2
10545378311632,0.833242038517889,0.984359685657801
"11398","ETFRF1",54.6003588523224,0.0652152189833744,0.310277512266726
,0.210183517674053,0.833524443232504,0.984359685657801
"11399","AC011611.4",1.51827727516786,0.231964235149736,1.125100201528
45,0.206172067905251,0.836656505702196,0.984359685657801
"11400","ANKS1B",3.47565344755975,-0.167124256078802,0.795216723669593
,-0.210161898139659,0.833541316315665,0.984359685657801
"11401","TMEM119",51.1486752602033,0.0812260071223759,0.38810574144636
8,0.209288341934,0.834223152054591,0.984359685657801
"11402","UNG",15.3466336335129,-0.0853233070675933,0.414362416946832,-
0.20591468622151,0.836857553331558,0.984359685657801
"11403","LINC01232",2.85631735195174,0.181920880221434,0.8690125235104
91,0.209342069647674,0.834181212425935,0.984359685657801
"11404","TRAPPC6B",32.5131681920114,-0.0597528573025719,0.284917352082
184,-0.209719965687931,0.833886241690353,0.984359685657801
"11405","MNS1",20.3085877059842,0.0887352340046134,0.425561279269284,0
.208513411175419,0.834828112161528,0.984359685657801
"11406","DIS3L",22.7660608883286,0.0698597153794465,0.332209566107401,
0.210288090731443,0.833442829703801,0.984359685657801
"11407","MAN2C1",26.9055059162627,0.0641874662843867,0.308002622282465
,0.208399090269826,0.834917366595318,0.984359685657801
"11408","ZSCAN32",7.2809610573568,-0.113501903409393,0.540437897009737
,-0.210018401813425,0.833653310746423,0.984359685657801
"11409","PRR14",16.2609981907808,0.0824503557374234,0.395183259926092,
0.208638280257224,0.834730624819139,0.984359685657801
"11410","TANGO6",8.70039372497377,-0.116514143707277,0.559676802920744
,-0.208181120066497,0.835087549647108,0.984359685657801
"11411","CYB5B",46.7036878994116,-0.0541932966296496,0.256813099108242
,-0.211022322528837,0.832869852692008,0.984359685657801
"11412","C16orf46",2.36034779489821,0.236641587818272,1.14447532625818
,0.206768623480912,0.8361905614306,0.984359685657801
"11413","PHF23",13.9522348278119,0.0950439756272695,0.453235030600892,
0.2097013011136,0.833900809972049,0.984359685657801
"11414","MPDU1",29.9785704584451,0.081720561253911,0.393078155537049,0
.207899014744941,0.835307818485114,0.984359685657801
"11415","PFAS",7.35022472484088,-0.114382066972385,0.554451460999634,-
0.206297710472551,0.83655836684949,0.984359685657801
"11416","GAS7",307.011042003557,0.0564681064268802,0.267547230608327,0
.211058459840857,0.83284165428381,0.984359685657801
"11417","TRPV2",22.9986640783616,-0.0729632847037443,0.350530643610107
,-0.208150944956758,0.835111109862535,0.984359685657801

"11418","NLE1",11.8392093632144,0.0902038724820225,0.43179783361188,0.208903022341474,0.834523944729483,0.984359685657801
"11419","FKBP10",138.053927065599,-0.0537441639019497,0.2567592403174,-0.209317350509031,0.834200508023627,0.984359685657801
"11420","TMC8",1.67473728759588,-0.271620084875354,1.29982857783318,-0.208966081764524,0.834474716888921,0.984359685657801
"11421","VAPA",186.980554771114,-0.0456903215182213,0.217814959026476,-0.209766683254603,0.833849777424251,0.984359685657801
"11422","STK35",12.1285977646751,0.0981473683501273,0.463362703103688,0.21181542599937,0.832251033368805,0.984359685657801
"11423","CDK5RAP1",14.7105680384634,0.0808107664433129,0.390236135192427,0.20708171067619,0.835946045280095,0.984359685657801
"11424","SLC13A3",1.51864909367084,0.241440966915255,1.1631261743438,0.20757934284427,0.835557435054856,0.984359685657801
"11425","RNF114",47.05995270772,-0.0581229392321991,0.281358625931877,-0.206579553193695,0.836338230003821,0.984359685657801
"11426","PIM2",1.48502310319013,0.255093825783174,1.23069477837448,0.207276272123382,0.8357941038504,0.984359685657801
"11427","DIPK1A",25.7586384368135,0.070537911398896,0.343909752546861,0.205105876982318,0.837489404879653,0.984429850406023
"11428","TENT5C",2.27970221914087,-0.18821169432686,0.916399085175537,-0.205381800758572,0.837273838091748,0.984429850406023
"11429","B4GALT3",14.2755995400951,-0.0862297712941098,0.422298336871388,-0.204191595763663,0.838203778615189,0.984429850406023
"11430","HADHB",107.661055954366,-0.0543691020590482,0.265100196805882,-0.205088878522635,0.837502685407799,0.984429850406023
"11431","SLC1A3",8.80767611480015,0.151679211068522,0.742534085323538,0.204272388387978,0.838140645908145,0.984429850406023
"11432","KCTD20",60.5619784587602,0.0476656020629036,0.233458555171848,0.204171579952669,0.838219419465445,0.984429850406023
"11433","AL513550.1",3.46530399356492,0.152104820832873,0.742990123118913,0.204719842296651,0.837791016820209,0.984429850406023
"11434","NUDCD3",36.0856169206026,0.0658615055477701,0.322507549434106,0.204216942094333,0.838183972456615,0.984429850406023
"11435","FZD8",6.94305530716082,-0.121037401942447,0.591598704203462,-0.204593757698327,0.837889532876286,0.984429850406023
"11436","SFXN3",59.0426115118062,-0.059539946082851,0.290338445262346,-0.205070830454615,0.837516786022865,0.984429850406023
"11437","POLL",19.4084990777994,-0.0704995908372581,0.34436703636577,-0.204722239332968,0.837789143923262,0.984429850406023
"11438","HOXC4",17.4470128582489,0.0793514206055048,0.386263285148217,0.20543350521926,0.837233445089701,0.984429850406023
"11439","NPIPB4",1.83442108862484,0.2625436720817,1.28240758942782,0.204727166500036,0.83778529414044,0.984429850406023
"11440","MYBBP1A",7.45536698248415,-0.10812935672221,0.525951027978826,-0.205588260066227,0.8371125487457,0.984429850406023
"11441","PVR",5.20404755217074,-0.125641413266714,0.614534722824194,-0.20444965695235,0.83800212894056,0.984429850406023
"11442","PPP5C",17.930016418512,0.0851745976799025,0.41495851824549,0.205260511436261,0.837368594459086,0.984429850406023

"11443","RP2",13.8048317295797,-0.0843299325666414,0.412772427440807,-0.204301273438945,0.838118074899344,0.984429850406023
"11444","PACSIN2",24.698881564998,-0.0670456930805803,0.328556372603527,-0.204061459984175,0.838305471078169,0.984444881668954
"11445","KLHL21",18.6469486998165,0.0825515937831615,0.406952584114594,0.202853101333092,0.839249851795907,0.984565908517513
"11446","SRSF10",150.898589376825,-0.0386061300140996,0.189983698675658,-0.203207592457753,0.838972778717905,0.984565908517513
"11447","GPX7",37.8926479377907,0.0688129697929921,0.339382861766151,0.202759118226798,0.839323313080981,0.984565908517513
"11448","NDUFS2",70.3247898699798,-0.0520394161794256,0.257403212880521,-0.20217081052356 9,0.839783191762311,0.984565908517513
"11449","DNM3OS",32.49123632419,-0.0807671030346378,0.40009953961798,-0.201867523046278,0.840020292213841,0.984565908517513
"11450","NPHP1",16.7762153142486,0.0924927435633306,0.456168441683972,0.202760066483092,0.839322571875611,0.984565908517513
"11451","LINC01116",46.1538746665029,0.0860434868221212,0.425773349376301,0.202087535417994,0.839848292130026,0.984565908517513
"11452","KRBOX1",4.91159081615299,-0.157727051388627,0.781257346667783,-0.2018887272694,0.840003714958025,0.984565908517513
"11453","NCK1-DT",4.74848847779849,-0.125968002900235,0.622052093232483,-0.20250394504043,0.839522774604429,0.984565908517513
"11454","LYSMD3",32.0133035745619,-0.0581855194532457,0.287806249245407,-0.202169062019331,0.839784558645368,0.984565908517513
"11455","ZNF596",3.32468653474177,0.178921645861959,0.879223419347736,0.203499636070539,0.838744530103782,0.984565908517513
"11456","DOLK",14.1320390699951,-0.083144418648661,0.411646604618888,-0.201980090970599,0.839932288514979,0.984565908517513
"11457","DNLZ",6.00826844417696,-0.128264100319431,0.629354489880672,-0.203802630126228,0.838507737435991,0.984565908517513
"11458","OAT",114.67419194661,-0.0437768472013513,0.21656691818662,-0.202140047833289,0.839807240387728,0.984565908517513
"11459","KLHL42",28.5051532333047,-0.074506177370229,0.365960384817992,-0.203590826934134,0.838673262060327,0.984565908517513
"11460","ERCC4",16.7748500747983,0.0756165955082693,0.371219701318818,0.203697689642088,0.838589747744659,0.984565908517513
"11461","DHX33",15.611248315593,0.0876204420371489,0.431525450302698,0.203048144612761,0.839097401952065,0.984565908517513
"11462","LINC00672",2.93956254159418,0.161019330886213,0.797595463752004,0.201880951188909,0.840009794214033,0.984565908517513
"11463","CHAF1A",8.13125320124852,0.11403575631554,0.562309146166976,0.202799042293502,0.83929210652229,0.984565908517513
"11464","DSN1",11.6136523956532,-0.0842439130065276,0.416821598603153,-0.202110239221875,0.839830543307991,0.984565908517513
"11465","TNFRSF6B",2.39379535090745,-0.210788905628003,1.03872399199956,-0.20293062185098,0.839189259440203,0.984565908517513
"11466","ZMAT5",28.1945334021218,0.0752675273417549,0.370881516264548,0.2029422444662,0.839180174938632,0.984565908517513
"11467","STXBP1",38.0053702870746,-0.0623172962171036,0.30958478053915

,-0.201293151777605,0.840469357066235,0.985006339026173
"11468","PTH2R",2.49866776485523,-0.195605814601598,0.975947727677045,-0.200426528034632,0.841147014016246,0.985163925460446
"11469","SUMF1",43.1059107178023,0.052096918998992,0.259541473716618,0.200726759592475,0.840912234420142,0.985163925460446
"11470","CSNK1A1",183.380876961994,0.036807354532523,0.183228308678571,0.20088246624l025,0.84079047815995,0.985163925460446
"11471","C8orf76",22.5118098569847,-0.0708427542506145,0.352474138418095,-0.200987098141603,0.840708662412593,0.985163925460446
"11472","STN1",16.193844050071,0.0738145715831094,0.368389101005088,0.200371214516713,0.841190270456032,0.985163925460446
"11473","AC023355.1",1.6972266074098,0.246657765725756,1.22901687730634,0.200695181880953,0.840936927369223,0.985163925460446
"11474","ZNF561",15.9778528698207,0.0772940659970585,0.385596538929588,0.200453215196449,0.84112614421505,0.985163925460446
"11475","TNNC2",18.2393097579844,0.0906903278835768,0.452523299908205,0.20041029467869,0.841159708823239,0.985163925460446
"11476","MYOC",265.07855249069,-0.169746335549625,0.84786360622022,-0.200204766785963,0.841320439290869,0.985230514432728
"11477","EEF1G",1016.15532248111,-0.051666883403062,0.258500639268631,-0.199871395093032,0.841581162433594,0.985302749861872
"11478","CDON",63.5803310391927,-0.0474483375517045,0.237426098642816,-0.199844658287064,0.841602073492405,0.985302749861872
"11479","AC005632.3",3.01407535389197,0.19467798199988,0.973902330843289,0.199894769562068,0.841562881175561,0.985302749861872
"11480","LINC01409",3.34260038103318,-0.153924240440686,0.780375271345677,-0.197243872393931,0.843636699719677,0.985309006224134
"11481","NADK",30.782389756832,-0.0547359244212998,0.279301509909067,-0.195974323372331,0.844630263273326,0.985309006224134
"11482","PIK3CD-AS2",1.71897671484527,0.281700115265319,1.42938134848411,0.197078348310664,0.843766226686438,0.985309006224134
"11483","SMIM12",29.9229878978833,-0.0593965712963698,0.309406704245794,-0.19196924462627З,0.847766296569744,0.985309006224134
"11484","PLPPR4",1.86705475015388,0.270041571561909,1.40882010295924,0.191679243499354,0.847993465785259,0.985309006224134
"11485","LINC02591",14.3484764084651,0.0962692373039179,0.483024021520548,0.199305278857281,0.842023950080625,0.985309006224134
"11486","LAMTOR2",115.238442854489,-0.0584562137967808,0.307604067103799,-0.190037194069528,0.849279984916333,0.985309006224134
"11487","TMCO1",181.339903131026,-0.0382063530706923,0.19976426823964 9,-0.191257192326596,0.848324097519333,0.985309006224134
"11488","CCSAP",3.14551388251938,-0.16444633467776,0.843333650496476,-0.194995580433616,0.845396407516049,0.985309006224134
"11489","LRPPRC",41.9782907068223,0.0654650517449138,0.333055307515602,0.196559100748896,0.844172579019405,0.985309006224134
"11490","GLS",88.217852502945,0.0552689014953968,0.28147689203019,0.196353246253227,0.844333687949255,0.985309006224134
"11491","MAIP1",16.1743240251227,-0.0780459483088177,0.412509123622957,-0.189198114270446,0.849937545568059,0.985309006224134

"11492","KLF7",58.0145029713592,-0.0479423180368586,0.252752388248453,
-0.189680969462222,0.849559134242561,0.985309006224134
"11493","PIKFYVE",20.5193002383846,0.074848462232422,0.387538313911936
,0.193138225423127,0.846850716650129,0.985309006224134
"11494","BCS1L",14.4712063766774,0.0806752578173753,0.418008866047598,
0.192998915501924,0.846959817426638,0.985309006224134
"11495","HYAL2",27.4238913226856,0.0740348277460311,0.374442106920455,
0.197720358842439,0.84326386010925,0.985309006224134
"11496","AC012467.2",1.94844631075256,0.200214547061705,1.046134105613
2,0.191385163706471,0.848223842874831,0.985309006224134
"11497","AC092903.2",5.16262992087893,-0.122972254075216,0.62934961736
206,-0.195395771575516,0.845083126609619,0.985309006224134
"11498","COPG1",51.6582539844969,-0.0547775941777829,0.288691738871895
,-0.189744238584153,0.849509553097773,0.985309006224134
"11499","ECT2",6.47199949563955,-0.116661401323021,0.606719819573854,-
0.192282166428915,0.847521186879023,0.985309006224134
"11500","PIGG",18.5917643220143,-0.0694823509269031,0.361610129047672,
-0.192147136779299,0.847626952951459,0.985309006224134
"11501","CD38",1.69094378598118,0.206141597130037,1.05227151783988,0.1
95901526968256,0.844687242079164,0.985309006224134
"11502","CHIC2",50.0182592792996,-0.0486997355160101,0.24760562962903,
-0.196682666662198,0.844075875143305,0.985309006224134
"11503","GPRIN3",1.71217761481295,-0.229926495526735,1.15977217219664,
-0.198251433375271,0.842848347981134,0.985309006224134
"11504","SMARCAD1",24.5909356005279,0.0634280627852084,0.3272616779704
43,0.193814513139962,0.846321122648467,0.985309006224134
"11505","AADAT",5.50065903991218,0.126518874589277,0.659140616771763,0
.191945195562248,0.847785134660763,0.985309006224134
"11506","GTF2H2C",15.6526306621832,-0.0806771366674977,0.4173281240737
58,-0.193318235732512,0.846709745650177,0.985309006224134
"11507","VCAN",969.360025981576,0.0545127568189581,0.277107456029156,0
.196720642598741,0.844046155280989,0.985309006224134
"11508","ZMAT2",86.9884852616263,-0.0382741843777858,0.200447499622487
,-0.190943685752476,0.848569713505507,0.985309006224134
"11509","MED7",18.9448975391905,0.0713981066360859,0.368263476738504,0
.193877783559797,0.84627157977619,0.985309006224134
"11510","LINC02202",9.78572445341654,-0.105370584133493,0.538145714753
227,-0.195803072002183,0.844764305507837,0.985309006224134
"11511","RPL26L1",46.5903352661478,-0.0645750167453605,0.3254291309053
72,-0.198430351227953,0.842708372696565,0.985309006224134
"11512","ZNF879",2.23384931186393,0.197663777511371,1.01216860698753,0
.195287401868418,0.845167959054492,0.985309006224134
"11513","TBC1D9B",53.1061559777717,-0.0459081364401582,0.2418084306936
42,-0.189853332691784,0.849424062385653,0.985309006224134
"11514","BTN2A1",17.6431490046956,-0.0749152613777106,0.39369001678110
2,-0.190289969733636,0.849081913046638,0.985309006224134
"11515","DHX16",21.3085744626168,0.0703982703702262,0.365497542969417,
0.192609421662013,0.847264865678635,0.985309006224134
"11516","GPANK1",29.9062602527328,0.0710887125780057,0.360590928464628
,0.197145038785908,0.843714039119769,0.985309006224134

"11517","NFYA",6.35566902140098,-0.112370108156572,0.585842940759435,-0.191809272312654,0.847891607571698,0.985309006224134
"11518","PGM3",48.0690012035493,-0.0538679500208122,0.272014745958371,-0.198033197909999,0.843019089879954,0.985309006224134
"11519","RPA3",52.6081966455441,-0.0516101165364336,0.268150105625228,-0.192467261633545,0.847376209903109,0.985309006224134
"11520","UBE2D4",32.2686909015449,-0.0679876356795669,0.353209135949866,-0.192485495871253,0.847361928101833,0.985309006224134
"11521","MCM7",13.2345189897624,0.0811573666073622,0.425272646881878,0.190836084103721,0.848654017153956,0.985309006224134
"11522","PRKAR2B",10.3277352434446,0.10061952704276,0.507883010890683,0.198115559853641,0.842954651114599,0.985309006224134
"11523","ZYX",157.761072285072,0.0510291805811161,0.256943379143515,0.198600877559931,0.842574967091956,0.985309006224134
"11524","PPP1R3B",27.4459972948386,-0.0747985839582244,0.379709530854285,-0.196988955715543,0.843836180510432,0.985309006224134
"11525","TNFRSF10B",8.99509056773834,0.0957501090851706,0.493866182312955,0.193878650764744,0.846270900729695,0.985309006224134
"11526","AC067838.1",2.10872668915431,0.220719124976001,1.14509302190304,0.192752135201372,0.847153090993495,0.985309006224134
"11527","PCMTD1",161.172015184595,0.0371335432749299,0.188004352547814,0.197514274386206,0.843425112117558,0.985309006224134
"11528","COPS5",75.5851523963696,0.0494181454977891,0.25329762956525,0.195099123440707,0.845315348785048,0.985309006224134
"11529","COMMD5",44.4053911726156,-0.0527375076437122,0.273731950272249,-0.192661132875649,0.847224364578872,0.985309006224134
"11530","CDKN2A",9.13101907449987,0.2379694036775,1.20613067770486,0.197299851563622,0.843592895497687,0.985309006224134
"11531","VCP",134.363920306416,-0.0382794465960854,0.201291623115221,-0.190169098960338,0.849176624694917,0.985309006224134
"11532","EBLN3P",65.9978253120546,-0.0453425505673367,0.23700125930013,-0.191317762197696,0.848276645893405,0.985309006224134
"11533","TRMO",8.30886269236941,0.0942104355128938,0.495440528448194,0.190154882580917,0.849187764476594,0.985309006224134
"11534","HSDL2",36.3699744004578,-0.0536230767570532,0.273821956749703,-0.195831909878831,0.844741733149237,0.985309006224134
"11535","LINC01503",4.20175276825544,-0.182672832255709,0.944344329442981,-0.193438798286064,0.846615332557589,0.985309006224134
"11536","DCLRE1C",21.0392868386234,0.0798404113601863,0.421691253790308,0.189333809137735,0.849831198647377,0.985309006224134
"11537","BMI1",65.2087885826869,0.0412088433670334,0.21341278678158,0.193094537532135,0.846884930572515,0.985309006224134
"11538","ZFAND4",4.92343419397451,-0.130786559730978,0.68973272420861,-0.189619188912692,0.849607549434782,0.985309006224134
"11539","DDX50",54.5004997574616,-0.0525069615224586,0.267656813598499,-0.19617270644648,0.844474990078361,0.985309006224134
"11540","ENTPD7",1.91770281442077,0.20687556159881,1.08726522047965,0.190271479030245,0.849096401809249,0.985309006224134
"11541","SCUBE2",1.99695470929565,0.243566038241961,1.23584538267718,0.197084555767267,0.843761369111423,0.985309006224134

"11542","KBTBD4",9.83628798448197,-0.105780435864613,0.546993603872434,-0.193385142194976,0.846657350618543,0.985309006224134
"11543","IL18BP",2.64901007855739,0.17345537308459,0.905015912038477,0.191660025837442,0.848008520177177,0.985309006224134
"11544","ARAP1",73.7272929038095,0.0503968506820274,0.26652237675179,0.189090504505599,0.850021883544653,0.985309006224134
"11545","NEU3",3.15154091674056,0.165916387970964,0.877102858908744,0.189164117167957,0.84996419023991,0.985309006224134
"11546","CCDC90B",89.8965934454939,0.0535711250906895,0.278162753194404,0.192589138824239,0.847280751651413,0.985309006224134
"11547","TMEM126B",86.5984342632087,-0.055049858031482,0.2793564770421 4,-0.197059537027229,0.843780947279997,0.985309006224134
"11548","PGR",7.46073955520145,-0.12305441474289,0.617642983652845,-0.199232271716462,0.842081056234274,0.985309006224134
"11549","SIDT2",41.2312181134099,-0.0513663014184221,0.270267520989697,-0.190057248574757,0.84926427010755,0.985309006224134
"11550","AP003396.5",1.98638861970237,0.202088414090415,1.05818714500785,0.190976062262517,0.848544347523845,0.985309006224134
"11551","JHY",8.39364429103986,0.100099013682845,0.522775746120995,0.191476009408588,0.848152674517612,0.985309006224134
"11552","COPS7A",44.887985737284,-0.0533764897117467,0.274908148966019,-0.194161176787613,0.846049680782366,0.985309006224134
"11553","CSRNP2",12.1701882320527,0.0876010680457816,0.446733731095924,0.196092351994283,0.844537882279217,0.985309006224134
"11554","C12orf66",5.06416267986379,-0.128303111098478,0.654388959846123,-0.19606551908921,0.844558884204391,0.985309006224134
"11555","NUAK1",36.6073873344926,0.0960334027625124,0.484704158233076,0.19812787064297,0.842945019425602,0.985309006224134
"11556","AC156455.1",1.4754181462151,0.251720814727411,1.31970649931721,0.190739997762871,0.848729300261921,0.985309006224134
"11557","SNRNP35",35.7706571692966,-0.0693146399908476,0.348969763610296,-0.198626492088447,0.842554928807629,0.985309006224134
"11558","TMEM132C",5.3186282610068,0.164926120832961,0.871464973804164,0.189251577275696,0.849895645160653,0.985309006224134
"11559","TM9SF1",68.3812306349963,-0.0526699859217009,0.264292662645992,-0.199286599160151,0.842038561252466,0.985309006224134
"11560","SOCS4",11.2315903199518,0.103829318356005,0.540462700119574,0.192111903990847,0.847654550554157,0.985309006224134
"11561","ZFYVE21",98.2642683815323,0.0505421078186469,0.253738209977557,0.199189975459815,0.842114140727771,0.985309006224134
"11562","TUBGCP4",26.2729183958795,0.0653852593675101,0.335335424028599,0.194984647258542,0.845404966656981,0.985309006224134
"11563","AC011939.2",2.73784608653394,0.196316350105547,0.995065915516034,0.197289794620026,0.84360076511413,0.985309006224134
"11564","FAH",5.31337035827032,0.119554422897655,0.613171792015571,0.194977043064335,0.845410919684083,0.985309006224134
"11565","WHAMM",17.6586500007834,0.0823576030348525,0.425585445085152,0.193516023599854,0.846554858214437,0.985309006224134
"11566","GDPGP1",2.59109754357402,0.192255976378042,0.964106328584035,0.199413664943372,0.841939172064019,0.985309006224134

"11567","NTHL1",19.4334917738538,-0.0885049709025546,0.445698660433462
,-0.198575806390083,0.842594580404691,0.985309006224134
"11568","C16orf58",27.2146197237186,0.0636720515533927,0.3253574791296
25,0.19569874872317,0.844845963848647,0.985309006224134
"11569","FAM192A",35.5888667734799,-0.0548114540005314,0.2858748266155
18,-0.191732355903617,0.847951859818307,0.985309006224134
"11570","KCTD11",7.18920962536233,-0.100968331796964,0.529149365490215
,-0.190812534951118,0.848672467654977,0.985309006224134
"11571","NCOR1",129.913902133852,-0.0356811491411223,0.184282334869891
,-0.193622189377589,0.846471722369095,0.985309006224134
"11572","USP22",81.9550565180916,0.0429028170394828,0.217851524010834,
0.19693604272123,0.843877587946591,0.985309006224134
"11573","DHRS11",3.41707221551205,0.152054180175782,0.782581068340439,
0.194298311481303,0.845942307685249,0.985309006224134
"11574","HELZ",50.0898697709018,0.0503604764407474,0.254242001789878,0
.198080868173656,0.842981793243272,0.985309006224134
"11575","MRPS7",56.1444385939517,-0.053355653365562,0.282267490004388,
-0.189025145491366,0.850073108795184,0.985309006224134
"11576","HGS",40.944917806618,0.0522948404542935,0.269553825238615,0.1
94005187676342,0.84617181990089,0.985309006224134
"11577","ROCK1",143.113846723048,0.0561130837207067,0.296439657071341,
0.189290070954314,0.849865476883076,0.985309006224134
"11578","RAB27B",2.81448195935887,-0.208300785696653,1.06659876983694,
-0.19529441772045,0.845162466949844,0.985309006224134
"11579","KEAP1",35.7742389657312,-0.0562104857910536,0.288484070015631
,-0.194847797966827,0.845512101980324,0.985309006224134
"11580","AC020916.1",74.3645102987592,0.134673205756769,0.712378381916
485,0.18904729449322,0.850055749402352,0.985309006224134
"11581","TECR",73.5572709384567,-0.0578606646026103,0.306181157870703,
-0.188975262243421,0.850112205310576,0.985309006224134
"11582","TMEM161A",20.4683054822591,0.0690927412613017,0.3603857852975
55,0.191718830431269,0.847962455049834,0.985309006224134
"11583","AC011445.2",1.49868618662724,0.229693183469288,1.152687723980
67,0.199267484758205,0.842053512504691,0.985309006224134
"11584","CD3EAP",4.97460808156606,0.119990251610505,0.628597288235016,
0.190885728997361,0.848615121199662,0.985309006224134
"11585","ZNF814",3.95626645035966,0.16364291893618,0.829501369357045,0
.197278660387289,0.843609477733761,0.985309006224134
"11586","MZF1-
AS1",2.92981873637351,-0.172177696357267,0.864911273363021,-0.19906977
9363368,0.8422081606632,0.985309006224134
"11587","ZNF343",5.45092336416142,0.126024554163875,0.666038164692683,
0.189215214449496,0.849924143647786,0.985309006224134
"11588","AL050403.2",7.97471467058318,0.120151074503123,0.613723057212
031,0.195774092387754,0.844786988937677,0.985309006224134
"11589","MAPRE1",41.7557818576782,-0.056137492415479,0.288842601647884
,-0.19435322938932,0.845899309114214,0.985309006224134
"11590","PLCG1",11.1426750443131,0.0932428198486109,0.480855864016638,
0.193910123232655,0.846246256956503,0.985309006224134
"11591","PIGT",309.005219104507,-0.0447178911203388,0.227951567809071,

-0.196172772796166,0.844474938147886,0.985309006224134
"11592","PICK1",4.49025418999269,0.128063703931677,0.675427520810083,0.189603917498182,0.849619517179911,0.985309006224134
"11593","CCDC134",1.54829987464955,-0.223622144309438,1.16614348312221,-0.191762118080631,0.847928545597592,0.985309006224134
"11594","PRRG1",20.0790803689042,0.0693086813596685,0.36420977315572,0.190298796100771,0.849074996987088,0.985309006224134
"11595","TENM1",18.94550315531,0.114231305181076,0.600120597505661,0.19034724962927,0.849037030555728,0.985309006224134
"11596","PIH1D1",165.998220500748,0.0519082410180081,0.275026405442587,0.188739117374837,0.850297291312564,0.985364825621737
"11597","FLNA",269.813858556629,-0.0349444671888092,0.185159086849761,-0.188726720267114,0.850307008165435,0.985364825621737
"11598","SPIN2A",7.47848470994788,-0.112851006283788,0.598450858807036,-0.188571884596754,0.85042837027651,0.985420492166409
"11599","UQCR10",282.432146502416,-0.053816429416357,0.285611226089303,-0.188425469661091,0.850543135371464,0.985459635139221
"11600","FAM50A",85.2226328916503,-0.0462900260010402,0.245776845768663,-0.188341687990457,0.850608807769548,0.985459635139221
"11601","ASAP2",97.0858058094835,-0.05691859368491,0.302386429306224,-0.188231309902036,0.850695329394637,0.98547491869102
"11602","CWC15",130.453001318454,-0.0416400909425204,0.221534278964601,-0.187962292504511,0.850906210500103,0.985566376127079
"11603","REEP6",2.25575501841981,0.191903732510791,1.021071460845,0.187943488648658,0.850920951127502,0.985566376127079
"11604","LINC01137",1.66838765812825,-0.218072349075477,1.16163819555132,-0.187728287439773,0.851089654322927,0.985619766491783
"11605","ACVR1",51.7845336289526,-0.0485285382110534,0.258567849402121,-0.187682027457259,0.851125919911302,0.985619766491783
"11606","HOXA11",7.16964729785821,0.0997891387142396,0.531913624901873,0.187604028252987,0.851187068227073,0.985619766491783
"11607","SEC16B",5.52851496167603,-0.109858209636131,0.58687226812209,-0.187192708879672,0.851509541269397,0.985719255147007
"11608","LAMC2",12.326943266497,0.0949583495244007,0.507682067537235,0.18704294596231,0.851626961075412,0.985719255147007
"11609","GTPBP8",23.1894175333698,-0.0678504032786851,0.362315955792819,-0.18726860408401,0.851450037807963,0.985719255147007
"11610","RAB18",73.0125248852668,0.0444372703025336,0.237807498912135,0.186862359285618,0.851768552251658,0.985719255147007
"11611","PRDX3",113.127797936246,0.040692119400822,0.217940374091049,0.186712166437881,0.851886316416976,0.985719255147007
"11612","TMEM41B",30.8516611175256,0.0532975681574045,0.285544387028409,0.186652480590003,0.851933116194098,0.985719255147007
"11613","SGCG",13.5794055513055,0.118997911503983,0.636943439640728,0.186826496825377,0.851796671217785,0.985719255147007
"11614","HYDIN",1.75523613677974,0.216510965421074,1.15773657582306,0.187012287546631,0.851650998841506,0.985719255147007
"11615","CYB561",50.8556962574511,-0.0474074798783609,0.253755689314757,-0.186823318154483,0.851799163552795,0.985719255147007
"11616","GBP2",99.4690081403622,-0.0416797192114912,0.224624403776328,

-0.185552943094261,0.852795358562516,0.985751613325846
"11617","DPM3",75.1996775270044,-0.0525306579536531,0.282111016830192,
-0.186205624097525,0.852283513699834,0.985751613325846
"11618","ZNF678",11.6845774423525,0.0881880650880386,0.475421748035971
,0.185494385674099,0.852841283439215,0.985751613325846
"11619","LFNG",2.52753232836334,-0.191510751033334,1.02967607095524,-0
.185991261169803,0.85245161433734,0.985751613325846
"11620","TNFSF13B",34.7854745010965,-0.0715325658480948,0.385025008196
711,-0.185786804299082,0.852611953017898,0.985751613325846
"11621","SNRPN",69.3261456275098,-0.0520590575226687,0.279263590081558
,-0.186415484766435,0.852118950175246,0.985751613325846
"11622","CFDP1",178.747714817551,0.0389859393722154,0.209825719340865,
0.185801528500336,0.852600405836238,0.985751613325846
"11623","GEMIN4",10.3011151618362,0.0836319590305359,0.450701500337734
,0.185559531015242,0.852790191879464,0.985751613325846
"11624","CASKIN2",7.10957108286231,0.100620290644425,0.540404859753206
,0.186194274215774,0.852292413962259,0.985751613325846
"11625","SNRPB2",129.62277550349,-0.0389654321311141,0.209974575389065
,-0.185572143955593,0.852780299995629,0.985751613325846
"11626","HUNK",26.4540347679652,0.0807718975395961,0.434399506187607,0
.18593920202274,0.852492439459424,0.985751613325846
"11627","PI4KA",25.5235988285682,-0.0589044534172287,0.316588431582963
,-0.186060031071579,0.852397685138608,0.985751613325846
"11628","PIGB",6.03040236114326,0.10963911939898,0.591696149008696,0.1
85296320725874,0.852996623697474,0.985846373053866
"11629","C16orf86",14.4467716787454,0.102398218400989,0.55353618787090
1,0.184989203316317,0.853237503927334,0.986039970356818
"11630","MED18",6.14406999665619,0.102548954349669,0.559879204769563,0
.183162642005746,0.854670405806718,0.986069707924975
"11631","HIST2H2AA4",3.23671219471267,0.174240529861708,0.949526202373
535,0.183502603115278,0.854403676464475,0.986069707924975
"11632","SFT2D2",92.0215677375531,0.0589319116782436,0.321156878053948
,0.183498830961808,0.85440663595887,0.986069707924975
"11633","ARHGAP15",3.35690601714824,0.140641120196591,0.76775406051606
9,0.183185120638834,0.854652768832092,0.986069707924975
"11634","ZNF619",5.25177546939785,-0.120362195774541,0.655806647720786
,-0.183533052299565,0.854379787217127,0.986069707924975
"11635","CCDC36",7.82195549000205,-0.105234073002016,0.5747471141200З,
-0.183096305169161,0.85472245481765,0.986069707924975
"11636","LPP-
AS2",6.61577483403334,0.112514065483096,0.612925823471783,0.1835688123
65882,0.854351731428475,0.986069707924975
"11637","LINC02615",2.29308116035708,-0.197794542539414,1.081372172997
43,-0.182910701309385,0.854868086122897,0.986069707924975
"11638","CASP3",25.1511893556787,0.0603583114649701,0.326572782524338,
0.184823459562102,0.853367506783009,0.986069707924975
"11639","PIK3R1",423.429214975706,0.0460688093042121,0.252418669783784
,0.182509516208423,0.855182886926148,0.986069707924975
"11640","PCDHGA9",2.61852182854061,0.164200300328217,0.892029840272475
,0.184074896281566,0.853954699819624,0.986069707924975

"11641","NR3C1",226.670680210871,-0.0356455350026174,0.194734688467623
,-0.183046663556011,0.854761404885717,0.986069707924975
"11642","TNXB",2587.97507484972,-0.0257073962706165,0.13967471769931,-
0.184051893528499,0.853972745076765,0.986069707924975
"11643","MAD2L1BP",12.1405173814128,0.0936345163136576,0.5131334799638
98,0.182475944310329,0.855209231075116,0.986069707924975
"11644","ASF1A",22.1380382071306,0.0733433703981258,0.402032999934131,
0.182431219352994,0.855244327373577,0.986069707924975
"11645","VPS41",31.4979399493956,0.0620420948705126,0.337210354599044,
0.183986327894003,0.854024180588563,0.986069707924975
"11646","RIDA",20.8265367607179,0.0677737010066535,0.367792542268812,0
.184271547727901,0.853800433321876,0.986069707924975
"11647","SVIL",75.1466814686951,0.0503262401659516,0.275090169861548,0
.182944523940207,0.854841547330651,0.986069707924975
"11648","ZNF195",16.6466436094137,0.0735519254441444,0.401718877450473
,0.183093027419934,0.854725026611868,0.986069707924975
"11649","POGLUT3",96.5249653789275,0.0411469803123363,0.22285251887813
,0.184637716995418,0.853513200649545,0.986069707924975
"11650","SIK2",32.0875098862396,-0.0533927796100897,0.292131269524737,
-0.182769820214568,0.854978629677379,0.986069707924975
"11651","HSPH1",42.2761436438098,0.0605519271182004,0.329932960160683,
0.183527972133219,0.85438377290905,0.986069707924975
"11652","SPRYD7",18.0456891150025,0.0641243377050806,0.348863503611801
,0.183809246427896,0.854163102131603,0.986069707924975
"11653","AC010542.2",2.55132645413618,-0.177627982679914,0.96845592369
3415,-0.183413595120046,0.854473509445783,0.986069707924975
"11654","MYPOP",10.6739526737818,-0.086757364634137,0.474504484366464,
-0.182837818171458,0.85492527414415,0.986069707924975
"11655","PTPN1",30.0940204497244,0.055712893348528,0.304948836182519,0
.182695871366378,0.855036655412098,0.986069707924975
"11656","ATP6AP2",294.938959384272,-0.0337356103433103,0.1838774998690
94,-0.183467854236257,0.854430939248397,0.986069707924975
"11657","TPR",175.35112600052,0.0448865024777919,0.247486330167066,0.1
81369623314108,0.856077460522513,0.98608472063739
"11658","PLXNA2",11.5949261568306,0.0827947174867407,0.45713457262566,
0.18111672676864,0.856275955658193,0.98608472063739
"11659","B3GALNT2",18.7420820845975,-0.0779631224652522,0.430463701524
633,-0.181114277903385,0.856277877784435,0.98608472063739
"11660","ATP6V1E2",9.60550051251118,-0.0923674086313473,0.508404092704
594,-0.181681087852723,0.855833008660265,0.98608472063739
"11661","ARL5A",97.1455247807493,-0.0368903479668735,0.203523333667041
,-0.181258567763169,0.856164625427248,0.98608472063739
"11662","ABTB1",36.1074037645219,0.0529510494816999,0.292376463847326,
0.181105718240542,0.856284596312103,0.98608472063739
"11663","TYSND1",14.4242862238696,-0.0728434995729078,0.40095503156775
6,-0.18167498556655,0.855837797885616,0.98608472063739
"11664","POLR3B",6.55131270186814,0.106036691197441,0.582143532567938,
0.182148706058271,0.855466026114926,0.98608472063739
"11665","PCDH9",28.036615809341,-0.0828972762770687,0.456362068673858,
-0.181648042130144,0.85585894382541,0.98608472063739

"11666","DGKE",4.30669583530068,0.130657343491877,0.719234293450985,0.181661726479927,0.855848203963546,0.98608472063739
"11667","TWSG1",44.7078291934574,-0.0447562288118027,0.246550664016338,-0.18152954075531,0.855951948147362,0.98608472063739
"11668","LINC00663",2.94596400606777,-0.142972469247971,0.785579077057351,-0.181996279462435,0.855585645701027,0.98608472063739
"11669","PDXP",2.72481264301633,-0.163594512129942,0.900921802668053,-0.181585695501498,0.855907875547659,0.98608472063739
"11670","KLHL15",6.95089742545018,0.112320970649893,0.619153625048615,0.181410503154325,0.856045375333986,0.98608472063739
"11671","PTPRF",4.69247364779297,-0.141890465510454,0.785552226086573,-0.180625120518488,0.856661836871136,0.986134640231855
"11672","DENND1B",17.8113582009819,0.0723700132201276,0.401641247594312,0.180185709644112,0.857006776871721,0.986134640231855
"11673","DBI",465.289700741115,-0.0500542712743278,0.276696540354201,-0.180899519778068,0.856446445795203,0.986134640231855
"11674","RIOX2",20.1031477589073,-0.0677198461814668,0.376930789595297,-0.179661221770119,0.857418538544523,0.986134640231855
"11675","ADAMTS6",14.8576439846784,0.112702032397003,0.627274777676383,0.179669319424074,0.857412180993607,0.986134640231855
"11676","DOCK2",1.84528648354843,-0.204628013905091,1.13608329933975,-0.180117086505904,0.857060648887406,0.986134640231855
"11677","SMNDC1",36.0939912804197,0.0506265632469336,0.28126000041904,0.179999157973074,0.857153229242405,0.986134640231855
"11678","CASP1",13.8280239054536,0.0880477284845787,0.48847796977395,0.180249128789419,0.856956990799013,0.986134640231855
"11679","IKZF4",10.1971742418782,-0.0899143863334077,0.497310189272211,-0.180801415842681,0.856523451744538,0.986134640231855
"11680","NUP107",31.3714833999643,-0.0556004266902287,0.309183544903932,-0.179829837669739,0.857286158387162,0.986134640231855
"11681","AACS",16.3131969376429,-0.0738174605747111,0.4096877770647,-0.180179797170403,0.857011418383103,0.986134640231855
"11682","LYSMD4",8.38456539205331,-0.0976576451995789,0.543604384772676,-0.179648376531064,0.857428623492018,0.986134640231855
"11683","MRM1",2.45392119095992,-0.163307139010336,0.90673171773316,-0.180105245925008,0.857069944302906,0.986134640231855
"11684","RFX1",19.6102513777651,-0.0771666011301859,0.42880629487018,-0.179956782475752,0.857186496807936,0.986134640231855
"11685","NECTIN2",30.0790879316767,0.0646419717799796,0.357748816481979,0.180690945159943,0.856610166514566,0.986134640231855
"11686","CDC7",2.3875219243748,-0.155458699390617,0.867282427866998,-0.179248067752224,0.857742922254081,0.986143900029284
"11687","TMEM185B",17.03698287854,0.066014370495258,0.369214049665894,0.178797016405511,0.85809708809751,0.986143900029284
"11688","PODXL2",11.4398693039021,0.0829688425862406,0.463855150974665,0.178867998796401,0.858041350777266,0.986143900029284
"11689","PDLIM5",83.5507173591996,0.0510810461902891,0.285125390821653,0.179152919503547,0.857817630347073,0.986143900029284
"11690","SECISBP2",59.4139257815837,-0.0425260463106103,0.237213751304537,-0.179273107384129,0.857723261955261,0.986143900029284

"11691","DOK4",11.9993696030234,0.0788450060763579,0.439626216184274,0.179345551229159,0.857666381918045,0.986143900029284
"11692","AC015922.3",13.9215790913067,0.07219784821991,0.403215852907179,0.179055083522547,0.857894450110133,0.986143900029284
"11693","GADD45B",356.831762188191,0.111764444509866,0.624210676844106,0.179049235548047,0.857899041919626,0.986143900029284
"11694","PSENEN",79.8767508008273,-0.0565150643451135,0.315822258656733,-0.178945792438714,0.857980265888373,0.986143900029284
"11695","PPT1",64.1234140413358,-0.0440604656441271,0.247010543323129,-0.178374837978025,0.858428608784574,0.986425085727888
"11696","PARTICL",3.98955901097415,0.124428143705825,0.69786425316879,0.178298491634776,0.858488563336065,0.986425085727888
"11697","DTX3L",23.0808767255396,0.07101698422519,0.39872975001582,0.178108064979782,0.858638108365688,0.986477876874913
"11698","SLC38A9",14.5145883917775,-0.075412775928402,0.42354105829868,-0.17805304692614,0.858681315848109,0.986477876874913
"11699","PECR",3.71721112526413,0.135397867771093,0.761759577122852,0.177743571380471,0.858924365029621,0.986496183452201
"11700","RNGTT",12.8861247968707,-0.0794140644272779,0.446781280640795,-0.177747071930539,0.858921615768847,0.986496183452201
"11701","PROSER3",6.27790274043089,0.108589693905868,0.610835353077575,0.177772444503679,0.858901688717466,0.986496183452201
"11702","EFCAB6",1.56404434472932,0.230877232559767,1.29955350289251,0.177658889800141,0.858990872740357,0.986496183452201
"11703","ZNF670",4.9114955279172,-0.119949573687526,0.678416026931816,-0.176808284188105,0.859658981735435,0.986531852384077
"11704","LINC01943",3.42396470444048,-0.155853383350845,0.882190936024081,-0.176666271423345,0.859770535599507,0.986531852384077
"11705","PLOD2",23.9924738725391,0.0691132715869655,0.39185887294188,0.176372863699877,0.86000102209075,0.986531852384077
"11706","CALHM6",2.94314609855867,0.146838290899624,0.833429157071825,0.176185689753795,0.860148062851401,0.986531852384077
"11707","HINT3",17.1364982663652,-0.0653242713300671,0.369465426217955,-0.176807535142764,0.859659570118171,0.986531852384077
"11708","ST3GAL1",21.1761884365589,-0.0591272192565711,0.336583686150492,-0.175668701988528,0.860554225079669,0.986531852384077
"11709","ODF2",11.8544144668041,0.0856494704982584,0.485856411475907,0.176285561896934,0.860069604341532,0.986531852384077
"11710","PRPF18",39.1561135998202,0.0481021253532054,0.273182870542228,0.176080313006923,0.860230847217114,0.986531852384077
"11711","ZNF518A",36.9801232525804,-0.0512710643483279,0.291680068835655,-0.175778429266678,0.860468016710867,0.986531852384077
"11712","RNF41",24.5410839488228,0.0554055521771448,0.314083043086711,0.176404149783561,0.859976444733487,0.986531852384077
"11713","PIP4K2C",3.6029229938968,0.148577474706375,0.844354993855434,0.175965649267912,0.860320929223578,0.986531852384077
"11714","LINC00645",4.74499701847257,-0.145586668904239,0.826785179534792,-0.176087661593253,0.860225074090427,0.986531852384077
"11715","RPUSD2",8.41424894346794,0.094528860987969,0.536895888677487,0.17606553333984,0.860242458301597,0.986531852384077

"11716","THAP10",4.09047512697154,-0.126873750026587,0.720767257244358
,-0.176025962266448,0.860273545962224,0.986531852384077
"11717","CRTC3",29.7483703599884,-0.0513117805204339,0.290551343198816
,-0.176601422507701,0.859821476649357,0.986531852384077
"11718","UQCRC2",90.7561785955189,-0.0379370022161625,0.21566723558621
5,-0.175905264946918,0.860368369021248,0.986531852384077
"11719","N4BP1",41.4192752329697,0.0456512209276078,0.258214383352343,
0.176795809493366,0.859668780745208,0.986531852384077
"11720","ACSF3",17.9931681494489,0.0647826177485877,0.367412869747191,
0.176321035768734,0.860041736771716,0.986531852384077
"11721","LSM14A",82.4531992762996,0.0394234358343618,0.223906765721199
,0.176070766362864,0.860238347172221,0.986531852384077
"11722","ZNF568",10.2715555293519,-0.0766697693622707,0.43664677915032
2,-0.175587621444188,0.860617927914007,0.986531852384077
"11723","ZNF584",7.03322621203202,-0.095285348172712,0.538610851719973
,-0.176909447458091,0.859579517673852,0.986531852384077
"11724","AC011043.1",6.96087315973258,0.0991318024791437,0.56464939742
1386,0.175563461028833,0.860636910287292,0.986531852384077
"11725","RAVER1",8.60785055350591,-0.0839029743183675,0.47819424271398
7,-0.17545793492238,0.860719821061597,0.98654274415751
"11726","HYLS1",5.19797136686694,0.108622535914488,0.619476638500786,0
.175345653352431,0.860808041219627,0.986559719081577
"11727","CDK11A",29.2926294792976,0.056051764883715,0.349852990924828,
0.160215194203552,0.87271156075998,0.986582224452921
"11728","TXNDC12",82.7779347938846,-0.0447175077723198,0.2822773699063
69,-0.158416906701209,0.874128292374166,0.986582224452921
"11729","RPF1",33.8768073975399,-0.0501159467218331,0.317649402524732,
-0.157771260778402,0.874637045564249,0.986582224452921
"11730","SELENOF",245.713539957441,0.0335550288772983,0.19534430079833
4,0.171773779629943,0.863615379924658,0.986582224452921
"11731","SLC35A3",43.8761516047072,0.0490254877227368,0.31417300032107
1,0.156046151873761,0.875996642842501,0.986582224452921
"11732","INKA2",1.66994643091544,0.17763875047867,1.0980724251985,0.16
1773255026014,0.871484414985667,0.986582224452921
"11733","LINC01719",2.47304809063171,-0.146232373821781,0.928881179254
848,-0.157428503330306,0.874907151119988,0.986582224452921
"11734","PEA15",68.8682715241992,-0.0367222567280794,0.231119324151127
,-0.158888733613927,0.873756537205954,0.986582224452921
"11735","PPOX",9.89210087559058,0.0865536570664557,0.510100399741027,0
.169679649556044,0.865262079277713,0.986582224452921
"11736","GPR161",11.2232613510281,0.0859414575376221,0.495896766460366
,0.173305137984784,0.862411585275354,0.986582224452921
"11737","NAV1",179.691977259889,0.0413817212308017,0.265741710232293,0
.155721588434983,0.876252479649008,0.986582224452921
"11738","PSEN2",5.85972143659018,0.0956478580030033,0.614624087480116,
0.155620093568327,0.876332485531567,0.986582224452921
"11739","NDUFAF7",18.0370043141615,0.055657871511048,0.354616324861749
,0.156952366850981,0.875282388468869,0.986582224452921
"11740","PKDCC",135.201744084454,0.0486274363321172,0.298641474318895,
0.162828811513943,0.870653222688178,0.986582224452921

"11741","TEX261",36.0687480908532,-0.0456435483963921,0.272157036236976,-0.16771033748563,0.866811163247309,0.986582224452921
"11742","TCF7L1",74.9708441051996,-0.0379218599579141,0.233963474932875,-0.162084530368657,0.871239288087811,0.986582224452921
"11743","MRPS5",57.4342890588323,-0.0406938086109936,0.254158071567409,-0.16011220245744,0.872792689014533,0.986582224452921
"11744","STARD7-AS1",3.13047950533684,-0.1424770794594,0.865602065256047,-0.164598821072885,0.869259759207634,0.986582224452921
"11745","CNOT11",13.7588753752997,0.0623569662315251,0.389035886684725,0.160285897434596,0.872655867462842,0.986582224452921
"11746","POLR1B",5.47953381760768,0.103791294779151,0.614875182157621,0.168800592040393,0.865953492627355,0.986582224452921
"11747","GALNT5",7.03185913599381,-0.257577212718886,1.61453977283545,-0.159535997225097,0.87324659979767,0.986582224452921
"11748","TLK1",63.1912703226477,-0.0406806261194355,0.235996270074976,-0.172378258802612,0.863140163291423,0.986582224452921
"11749","NAB1",21.8257790336024,-0.0602181918484692,0.376047856013106,-0.160134384189576,0.872775215994372,0.986582224452921
"11750","NDUFB3",103.530343464832,-0.04679264030486,0.291957856565585,-0.160271899702581,0.872666893497928,0.986582224452921
"11751","C2CD6",4.20967716299411,-0.119656032588898,0.708400119731066,-0.16891023766953,0.865867246411274,0.986582224452921
"11752","MRPL44",22.9793275691343,-0.0591753360447064,0.349480598952937,-0.169323665525351,0.865542062339723,0.986582224452921
"11753","EDEM1",18.0102106449898,-0.064112983740091,0.374080981096433,-0.1713879800897,0.863918705444255,0.986582224452921
"11754","CRELD1",27.2924923025477,-0.0777945540487092,0.454753234240929,-0.171069820269807,0.86416886596879,0.986582224452921
"11755","TIMP4",8.80204104033107,0.0897914838229759,0.541704143429025,0.165757424808659,0.8683478526101,0.986582224452921
"11756","COLQ",2.31383638007003,-0.147881132726083,0.935759248823123,-0.158033311358738,0.874430549858158,0.986582224452921
"11757","CTNNB1",107.854575257612,0.0473395465389751,0.274336065137763,0.172560419699841,0.862996965596605,0.986582224452921
"11758","CCDC71",8.92408654732068,-0.0797087402235134,0.485064830831462,-0.164325952237936,0.869474552455513,0.986582224452921
"11759","DUSP7",6.54952229108629,0.0887616703609085,0.569855133075251,0.15576181595821,0.876220769641443,0.986582224452921
"11760","ACOX2",18.8927239849922,-0.0765776581501551,0.462088576577989,-0.165720734144205,0.868376728190648,0.986582224452921
"11761","FOXP1",202.856380590268,0.0481952990395869,0.29199607808406,0.165054610855809,0.868900998211931,0.986582224452921
"11762","CNTN3",11.6938304316655,-0.114641458121447,0.674833868023596,-0.169881008576526,0.865103716976772,0.986582224452921
"11763","TBC1D23",40.3655953995069,0.0409647560555802,0.260589308654517,0.157200447965769,0.875086875265333,0.986582224452921
"11764","AC091607.2",7.18041732474264,0.100559813032424,0.622289567056552,0.161596495194472,0.871623617467068,0.986582224452921
"11765","TMEM175",24.167307078843,-0.0599784760787793,0.35756629861777

,-0.16774085340435,0.866787155149496,0.986582224452921
"11766","NOP14-
AS1",9.63514301716482,-0.073892594730672,0.458213769583331,-0.16126227
4588267,0.871886834836941,0.986582224452921
"11767","HTT",41.8757524386969,-0.0597202684056109,0.342562561711464,-
0.174333903002256,0.861603056441797,0.986582224452921
"11768","STX18",28.8818947894282,0.0494272347874307,0.310637550596408,
0.159115453661455,0.873577913070193,0.986582224452921
"11769","SMIM20",77.4870845976208,-0.050329062881306,0.304689986713448
,-0.165181217224047,0.86880134866556,0.986582224452921
"11770","KLHL5",11.6795911741187,0.0835654274520765,0.502403273920694,
0.16633137519177,0.867896176110549,0.986582224452921
"11771","TMEM154",4.19952147649651,-0.131328979351283,0.74988978959424
8,-0.175131040819136,0.860976668129041,0.986582224452921
"11772","CCL28",3.34502682579616,0.145585830287568,0.860275111662082,0
.169231712406849,0.865614386630494,0.986582224452921
"11773","MIER3",9.47817233339832,0.0832626563530071,0.475662194240699,
0.175045772737772,0.861043667341418,0.986582224452921
"11774","DIMT1",26.4178829489006,-0.053612094773969,0.337530025444227,
-0.158836520405584,0.873797674926539,0.986582224452921
"11775","SSBP2",78.3810035768069,-0.0411734500048435,0.239716180288269
,-0.171759161001692,0.863626873099994,0.986582224452921
"11776","AC114980.1",2.59421366240772,-0.149242054450405,0.88028191855
1387,-0.169538929864652,0.865372753936804,0.986582224452921
"11777","LINC01023",6.47937526859662,0.0998744290420599,0.619944619181
016,0.161102179052703,0.872012924028433,0.986582224452921
"11778","UBE2B",229.226081740627,-0.0335630801874792,0.195350379744467
,-0.171809649059204,0.863587179477602,0.986582224452921
"11779","PURA",120.561107354254,-0.038077858184472,0.223691311192944,-
0.170225021174953,0.864833174826779,0.986582224452921
"11780","CD14",27.9461879462127,0.0790486831185305,0.502378406091618,0
.157348887133725,0.874969893716877,0.986582224452921
"11781","HARS2",11.3176465408909,-0.0681728549404726,0.438627920839907
,-0.155422971729506,0.876487875390963,0.986582224452921
"11782","ZNF300",3.92587082371313,0.124530925280289,0.753026931982113,
0.165373800047893,0.86864977425952,0.986582224452921
"11783","ZNF354B",10.0754785628455,0.0842410796262273,0.49473664137548
4,0.170274591734336,0.864794192310478,0.986582224452921
"11784","ZFP2",1.59403844253854,0.228983311585012,1.34413774854185,0.1
70357027643497,0.864729365060305,0.986582224452921
"11785","MAPK9",32.9529318992066,-0.0502642795086839,0.291608597179102
,-0.172368990471883,0.863147449298028,0.986582224452921
"11786","FAM8A1",52.5475937016885,-0.0468173712389847,0.28835525856238
5,-0.162360039738467,0.871022336930301,0.986582224452921
"11787","BTBD9",6.10687830504328,0.0941793346492407,0.601231538728418,
0.156644035754389,0.875525395408248,0.986582224452921
"11788","FRS3",5.51566764132965,0.106257021107345,0.615090745079545,0.
17275015427781,0.862847818975063,0.986582224452921
"11789","MMUT",28.5252281880497,-0.0538638813779638,0.309992120192308,
-0.1737588727886,0.862054967391428,0.986582224452921

"11790","SIM1",11.4441963919258,0.116457610112454,0.724112496387042,0.16082806289564,0.872228821949141,0.986582224452921
"11791","AFG1L",3.24730432039799,0.124783026018676,0.768048420614322,0.162467655253907,0.870937597217939,0.986582224452921
"11792","AL080317.2",1.98041086139156,0.185320854747812,1.15201546480064,0.160866638001151,0.872198439052314,0.986582224452921
"11793","RNF217-AS1",1.46824403283148,0.199693233458405,1.19574953710869,0.167002559700973,0.867368034861157,0.986582224452921
"11794","AL355312.3",2.47970343472244,0.167705241805668,1.03210129622965,0.162489130106036,0.870920687447026,0.986582224452921
"11795","PRKN",4.24439501762887,-0.121533870587669,0.699207408533519,-0.173816623085513,0.862009579922721,0.986582224452921
"11796","SNHG26",7.30964515041246,0.0950416331373456,0.554402968968235,0.171430599143835,0.863885196261665,0.986582224452921
"11797","ZNF273",15.4314635654371,0.065669650626872,0.388897961248325,0.16886087655507,0.865906073199984,0.986582224452921
"11798","VKORC1L1",52.9554488091632,0.0500552474455625,0.290311498262152,0.172419100673589,0.863108056878186,0.986582224452921
"11799","IFRD1",64.1199020345363,-0.0667770768333125,0.400580896460889,-0.166700602608074,0.867605631359409,0.986582224452921
"11800","ZNF800",39.7944219324149,-0.0452307038212397,0.281841225740578,-0.160482923328159,0.872500672488448,0.986582224452921
"11801","AHCYL2",7.04121088085168,0.101524727028243,0.588692592554611,0.172457965859023,0.863077504577327,0.986582224452921
"11802","ARHGEF5",8.21402352629429,-0.0956352021863591,0.548146909670872,-0.174470019805059,0.8614960901253,0.986582224452921
"11803","REPIN1",26.8867232405165,-0.0518655599981595,0.310336771307929,-0.167126698455905,0.867270359114719,0.986582224452921
"11804","AC021097.2",2.48235013046344,0.15079258594028,0.930888081329894,0.161987879063672,0.871315398911829,0.986582224452921
"11805","AC021097.1",2.50182876915652,-0.143540954156866,0.85900157711485,-0.16710208453747,0.867289725853724,0.986582224452921
"11806","MCPH1",23.932497903006,-0.0561198523943034,0.350213758660181,-0.16024456780054,0.872688422950601,0.986582224452921
"11807","MTMR9",23.745790742256,0.0514741142943035,0.325376424999014,0.15819865958162,0.874300259953223,0.986582224452921
"11808","PPP2CB",80.9013544576449,0.0351429165589364,0.220609730446126,0.159299032222509,0.873433283197857,0.986582224452921
"11809","AC062004.1",26.0558255228545,0.0760276111978732,0.469792923441791,0.161832176272219,0.871438014017254,0.986582224452921
"11810","NDUFAF6",8.60220734376335,0.0804468332430156,0.492230917059229,0.163433117374331,0.870177429520604,0.986582224452921
"11811","LAPTM4B",42.1602964515509,0.0530900778202988,0.332850474620016,0.159501283214052,0.873273947401973,0.986582224452921
"11812","MTSS1",77.1754518441416,-0.0559243187005522,0.339921432822717,-0.164521307868572,0.869320774037048,0.986582224452921
"11813","ZNF250",6.87348677514519,-0.0938151098906779,0.592978136398058,-0.158210065653586,0.874291272404184,0.986582224452921
"11814","PDCD1LG2",8.25852506351562,-0.0982054431074547,0.572921902952

327,-0.171411570410193,0.863900157551488,0.986582224452921
"11815","TTC39B",15.680359329271,-0.0622649878883175,0.3677226258035,-0.169325963427636,0.86554025497495,0.986582224452921
"11816","HAUS6",15.0003486872188,-0.0722788665346546,0.448327682931138,-0.161218834541066,0.871921047339572,0.986582224452921
"11817","PIGO",7.02097873266424,0.0998365676936233,0.628501019114404,0.158848696592885,0.873788081526711,0.986582224452921
"11818","ZNF658",4.8917556314,0.108016329809745,0.685176427219262,0.157647469350516,0.874734596107991,0.986582224452921
"11819","SPTLC1",74.867318192648,-0.0348923500299179,0.20189988441837,-0.172820059458851,0.862792869118649,0.986582224452921
"11820","AL691447.2",3.08359005846674,-0.175862416893004,1.04879406692211,-0.16768059854601,0.866834560182703,0.986582224452921
"11821","PTRH1",20.0635595218675,0.0596064196991655,0.374020254700599,0.15936682291947,0.873379876288364,0.986582224452921
"11822","RAPGEF1",33.9542018845588,-0.0492957447408741,0.291900237796555,-0.168878741288425,0.865892021003641,0.986582224452921
"11823","ANAPC2",9.59187610784126,-0.0890475641483068,0.56601758580577,-0.157322963776012,0.87499032313149,0.986582224452921
"11824","NUDT5",52.2944697911016,-0.0425784611988072,0.270933778252906,-0.157154495365513,0.875123090022941,0.986582224452921
"11825","NDST2",15.3858249583752,-0.0604874688369199,0.373808229923334,-0.161814170997052,0.871452193273337,0.986582224452921
"11826","AC010997.5",1.95042780997048,0.180654027502902,1.15701119133247,0.156138530773286,0.87592382763483,0.986582224452921
"11827","ADIRF-AS1",3.80870377800642,0.138118266433768,0.889022968225481,0.155359615409551,0.876537819775867,0.986582224452921
"11828","EXOC6",6.16382398675838,0.10274509529593,0.63263133976346,0.162409113867716,0.870983694292673,0.986582224452921
"11829","PTDSS2",17.480953751166,-0.0646166928831994,0.401702606595184,-0.160857041558401,0.872205997477537,0.986582224452921
"11830","CTSD",294.460090721538,-0.0423545455499706,0.24714706076601,-0.171373859024244,0.863929808169814,0.986582224452921
"11831","RHOG",44.2311694347423,0.0505716862232116,0.319003599784038,0.158530142786627,0.874039070466723,0.986582224452921
"11832","PTPRJ",2.96198149713984,0.147613137405304,0.904380177859959,0.163220226425796,0.870345041372435,0.986582224452921
"11833","UBE2L6",86.0317154302815,-0.0568655418524367,0.357640252439742,-0.159002073912298,0.873667239874062,0.986582224452921
"11834","PRPF19",30.3847976644992,0.0474902471355506,0.286444171018145,0.165792332120951,0.868320380692063,0.986582224452921
"11835","AP5B1",7.36180241851942,0.0959090643405434,0.591651924673645,0.162103866041587,0.871224061804329,0.986582224452921
"11836","CLNS1A",64.3838295959654,-0.0429361189248914,0.259311081214587,-0.165577647988597,0.868489338779958,0.986582224452921
"11837","FAM76B",21.3755443275683,-0.0570269201832469,0.345820917186004,-0.164903038969659,0.869020300397459,0.986582224452921
"11838","NKAPD1",46.9047231232472,-0.0402252609451816,0.250754859429429,-0.160416675619809,0.872552854480287,0.986582224452921

"11839","PCSK7",70.0867384227915,-0.049694166569667,0.316339334243274
,-0.157091329437554,0.875172870848631,0.986582224452921
"11840","RNF26",16.3657146242709,0.0598342589794282,0.370586172602914,
0.161458422906515,0.871732355373694,0.986582224452921
"11841","ETS1",32.6254825339969,-0.0641487761938509,0.386865589018154,
-0.165816702272894,0.868301201572187,0.986582224452921
"11842","GABARAPL1",96.2143797048239,-0.0350845865810781,0.21607333814
452,-0.162373511153013,0.871011729049868,0.986582224452921
"11843","BORCS5",17.3462908813733,0.0641500893569486,0.37670030075575,
0.170294765436206,0.864778327712572,0.986582224452921
"11844","CCDC91",32.9196604185699,0.0491823668520107,0.311649267166048
,0.157813195902065,0.87460400013684,0.986582224452921
"11845","ALG10B",10.4054707182181,-0.0769195495991429,0.48914880864811
2,-0.157251838784458,0.875046375015251,0.986582224452921
"11846","SLC2A13",16.2804950614392,-0.0636932737779561,0.4090562905142
47,-0.155707845729212,0.876263312608642,0.986582224452921
"11847","TSFM",20.0422551985152,0.0637849539390852,0.389701477646053,0
.163676448763733,0.869985858650677,0.986582224452921
"11848","NUP37",22.483149959997,0.0637454805011523,0.380380261083828,0
.167583565770528,0.866910900957953,0.986582224452921
"11849","BRAP",16.7680067517453,0.0634263970132057,0.382204751125832,0
.165948740371163,0.868197289964441,0.986582224452921
"11850","TBX5",6.64351983879189,-0.109198134992976,0.686159398383339,-
0.159143976239717,0.873555441667541,0.986582224452921
"11851","HSPB8",55.290337239066,-0.0569343013756034,0.344321425374304,
-0.165352188913924,0.868666783297196,0.986582224452921
"11852","CAMKK2",57.5942925356627,0.0474546886763685,0.284974413920103
,0.166522629254966,0.867745676214981,0.986582224452921
"11853","TMEM120B",9.6543813000054,0.0813689598549839,0.47161436421825
7,0.172532827726442,0.863018655511262,0.986582224452921
"11854","B3GLCT",11.079238240251,-0.0858032637455437,0.511360125235235
,-0.167794201212057,0.86674518458363,0.986582224452921
"11855","KBTBD6",15.9325990819837,0.064644599209266,0.389003477760635,
0.166180003277615,0.868015295705206,0.986582224452921
"11856","AL354811.1",1.81886665547153,0.175599400175813,1.114577908983
67,0.157547892130694,0.874813066670474,0.986582224452921
"11857","CHMP4A",84.1039946460968,-0.0399160012623921,0.2308081920256,
-0.172940140954636,0.862698479081768,0.986582224452921
"11858","COCH",5.87210378419189,0.115801438072899,0.702597965013908,0.
164818920405793,0.869086511380359,0.986582224452921
"11859","AP4S1",10.073139862019,0.0780867123708072,0.461307863144724,0
.169272450372929,0.865582344679945,0.986582224452921
"11860","BAZ1A",37.5351798773113,0.0502636207467399,0.317976853329563,
0.158073206336956,0.874399113388037,0.986582224452921
"11861","GNPNAT1",22.6614166008273,-0.0597251503424796,0.3594689718852
2,-0.166148277080087,0.86804026248534,0.986582224452921
"11862","SMOC1",2.03962078013307,-0.174028585096142,1.06008212359657,-
0.164165191754871,0.869601102256807,0.986582224452921
"11863","FCF1",29.4963390171947,0.0569326694133035,0.332708717092828,0
.17111865871978,0.864130464721531,0.986582224452921

"11864","EIF2B2",18.4179097338777,0.0698213430736116,0.422556432800914,0.165235546435304,0.868758587780454,0.986582224452921
"11865","TTLL5",10.612132545186,0.0740271782457188,0.463955815188463,0.159556526337855,0.873230427089257,0.986582224452921
"11866","GSTZ1",26.2165411604896,-0.0630087634119946,0.367189034372219,-0.171597617340944,0.863753880755341,0.986582224452921
"11867","LPCAT4",15.3129793338684,0.069753264533137,0.409973758666753,0.17014080306012,0.864899405094444,0.986582224452921
"11868","C15orf41",8.84326234424588,0.084975924185643,0.539589954280447,0.157482405874216,0.874864672953646,0.986582224452921
"11869","EIF3J",59.1519262351646,0.0462724968604327,0.280034850238534,0.165238350944597,0.868756380446114,0.986582224452921
"11870","GABPB1-IT1",57.4755976278978,-0.0380102762598855,0.240856490776729,-0.157812962139021,0.874604184344578,0.986582224452921
"11871","MAPK6",40.1860981691815,0.0520147435919008,0.308272020245787,0.168730018217123,0.866009006174495,0.986582224452921
"11872","PDCD7",79.9981149013254,-0.0393436149322955,0.242586001016184,-0.16218419351276,0.871160806753899,0.986582224452921
"11873","UBE2Q2",67.0480891922483,0.0362043625987316,0.230482749197964,0.157080574249986,0.875181347019946,0.986582224452921
"11874","SYNM",6.5600575519803,0.143095648474581,0.835436764997739,0.171282441077354,0.86400168642547,0.986582224452921
"11875","LRRK1",26.0363635334704,-0.0525213117610999,0.307809305797864,-0.170629382451453,0.86451519286656,0.986582224452921
"11876","EME2",2.74838936096815,0.152311939832708,0.879785752622811,0.173123899061376,0.862554039852347,0.986582224452921
"11877","TSC2",34.7747024808828,0.0475532964787799,0.283206868621564,0.167910110055711,0.86665399638157,0.986582224452921
"11878","ATP6V0C",201.854008405903,-0.0444849785878214,0.278830179202778,-0.159541476876755,0.873242282956708,0.986582224452921
"11879","NUDT16L1",27.1399698170583,0.0560215887352076,0.327896129523039,0.170851631633155,0.864340429708022,0.986582224452921
"11880","NPIPA1",22.281938947379,0.0728580806497622,0.440633202058779,0.165348594498431,0.868669612286921,0.986582224452921
"11881","SPNS1",45.5711326574593,0.0424631318055972,0.264390406306687,0.160607687694768,0.87240239960438,0.986582224452921
"11882","LAT",3.00196685385866,-0.135322385225346,0.805449230274374,-0.168008584699061,0.866576525467553,0.986582224452921
"11883","FTO",30.4449437564479,0.0503854102725467,0.307745516048844,0.163724271012773,0.869948209863688,0.986582224452921
"11884","C17orf97",9.39954942580107,-0.0918655250333564,0.58609202217431,-0.156742493597762,0.875447795928114,0.986582224452921
"11885","SRR",7.98056733370634,0.106232400329584,0.630659011680768,0.168446653995261,0.86623190783809,0.986582224452921
"11886","PELP1",19.3552401012756,0.0647387971398273,0.383369741185645,0.16886778006947,0.865900642968944,0.986582224452921
"11887","AC040977.1",3.0254465903834,-0.166889939081869,0.955540515132808,-0.174655010895768,0.861350720407428,0.986582224452921
"11888","SCO1",28.4119021164516,-0.0495018229100265,0.298684877963514,

-0.165732605036916,0.868367385769933,0.986582224452921
"11889","UBB",817.024044819238,-0.044500340227667,0.264482664941578,-0.168254279491236,0.866383240688769,0.986582224452921
"11890","AC007952.4",12.8150947016041,-0.0797127804153057,0.464982688582861,-0.171431716432817,0.863884317797774,0.986582224452921
"11891","SLFN5",127.354874442697,0.0395932637565984,0.253076183598299,0.156448003892155,0.875679901345756,0.986582224452921
"11892","SCRN2",33.8913667702688,0.0521627553162699,0.308042702734559,0.169336117535687,0.865532268491816,0.986582224452921
"11893","MMD",11.0641842239079,-0.0828351980373047,0.52676889073726,-0.157251499649817,0.875046642280553,0.986582224452921
"11894","PCTP",12.0456198291592,-0.0704534720732469,0.449278380148937,-0.156814739337983,0.875390856260393,0.986582224452921
"11895","FTSJ3",25.0029875681686,0.0673957053362219,0.390239462522884,0.17270345982057,0.862884524116589,0.986582224452921
"11896","CEP112",19.3569267881127,0.059454829323562,0.349707148853244,0.170013193949639,0.864999760437808,0.986582224452921
"11897","SLC9A3R1",7.82108884351641,0.104452827240627,0.645889715694838,0.161719291548493,0.871526912058299,0.986582224452921
"11898","ARMC7",6.15567530883454,0.0963525532493683,0.596352185239878,0.161569883760233,0.871644574935386,0.986582224452921
"11899","AC087741.1",3.57269573820708,0.13074774925169,0.831444832728262,0.157253667477445,0.875044933858732,0.986582224452921
"11900","NARF",34.3966739873029,-0.0448468150707542,0.288628605071432,-0.155378968968288,0.876522563130719,0.986582224452921
"11901","PIEZO2",14.9713584828911,-0.0979715620588199,0.621298889941383,-0.157688294064171,0.87470242506726,0.986582224452921
"11902","CHMP1B",54.2695127285167,0.039639446220323,0.253776353293614,0.156198344352679,0.875876681733732,0.986582224452921
"11903","EPG5",20.5011074420114,0.060870279761073,0.370845885219043,0.164139018894923,0.869621705709836,0.986582224452921
"11904","SMAD4",66.5231554317283,-0.0435339254668829,0.261691575340143,-0.166355853872247,0.86787691330424,0.986582224452921
"11905","ZNF557",8.48580046319045,-0.0860681799155985,0.538512066758793,-0.15982590777144,0.873018214968093,0.986582224452921
"11906","RAB11B",93.6351817882438,-0.0364423191883819,0.227945793764483,-0.159872742490851,0.872981320650681,0.986582224452921
"11907","WTIP",37.9394732516454,-0.049825632507927,0.319590378270468,-0.15590467015174,0.876108164077129,0.986582224452921
"11908","ZNF260",15.6913962294723,0.0649318359758218,0.372489057189232,0.17431877453204,0.861614945187633,0.986582224452921
"11909","HNRNPL",48.5410773654385,-0.0480902674325226,0.300629482261517,-0.159965240503887,0.872908455624836,0.986582224452921
"11910","CNFN",2.26330058514103,-0.166048504519807,1.04749078270662,-0.158520253601423,0.874046862371024,0.986582224452921
"11911","CALM3",184.53337250583,0.035397450932304,0.202706776777417,0.174623914873711,0.861375155958406,0.986582224452921
"11912","SLC1A5",26.4524551591469,-0.0529659822995785,0.32029014268068,-0.16536875551735,0.868653744550605,0.986582224452921
"11913","PTOV1-

AS1",1.80933898915426,-0.206648366320264,1.18785533248461,-0.173967621030098,0.861890908842296,0.986582224452921
"11914","ZNF776",3.65357952773108,0.115808760222275,0.735120946908647,0.157537015792133,0.874821637705432,0.986582224452921
"11915","PANK2",26.4859448961347,0.0498489234557084,0.303793858621318,0.164087989408125,0.869661876722772,0.986582224452921
"11916","SHLD1",5.29675619421325,-0.104088257789668,0.625014983914908,-0.166537219856219,0.867734194909399,0.986582224452921
"11917","KAT14",7.93868944901529,-0.0878863623286129,0.507392877775806,-0.17321165940261,0.862485059286442,0.986582224452921
"11918","AL035071.1",4.64349715260547,0.110223960029862,0.683971646262837,0.161152820635352,0.871973039015328,0.986582224452921
"11919","NDRG3",23.1106533045034,0.0539633137061522,0.344047209181984,0.156848572713195,0.875364191087959,0.986582224452921
"11920","PLTP",592.927258634437,0.0555759671001593,0.31931843887198,0.17404559315925,0.861829630838994,0.986582224452921
"11921","NCOA3",42.914890453112,-0.042312878216912,0.271204865945413,-0.156018138057407,0.876018724194731,0.986582224452921
"11922","MHENCR",6.75734997338504,-0.106374639929314,0.637115529435198,-0.16696287410168,0.867399260996553,0.986582224452921
"11923","MAP3K7CL",3.42469024606981,0.128075668137669,0.751112498774925,0.170514627764232,0.864605431526501,0.986582224452921
"11924","LSS",19.0009852428156,-0.0635976616088007,0.364237389732707,-0.174604978515444,0.861390036394506,0.986582224452921
"11925","CABIN1",33.2434363609735,-0.0555364853181705,0.34839580447581,-0.159406297678383,0.87334877752478,0.986582224452921
"11926","MPST",63.7753477036806,0.0476076511418088,0.30190829577803,0.157689112248876,0.874701780316454,0.986582224452921
"11927","AL022311.1",6.60305994288582,-0.0946003110704566,0.588025113817135,-0.16087801158077,0.872189480919775,0.986582224452921
"11928","MRTFA",26.7844935494105,0.0549703953791101,0.329517454008144,0.166820891307783,0.867510980140303,0.986582224452921
"11929","AL022328.4",1.87725692059241,-0.194895799519032,1.20590218100819,-0.161618249463726,0.87160648525905,0.986582224452921
"11930","TMEM47",52.0332579327123,0.0421812970511809,0.253734990943578,0.166241545536621,0.867966865692751,0.986582224452921
"11931","HDAC6",16.0121096416736,0.064100173826269,0.374990707238774,0.17093803283358,0.8642724908812,0.986582224452921
"11932","FAM120C",6.90661973622201,0.0843433892413533,0.541412476956186,0.155783977708698,0.876203300361587,0.986582224452921
"11933","WNK3",1.92427648142284,0.189951673610396,1.16423504832938,0.163155776733373,0.870395784614223,0.986582224452921
"11934","ZDHHC15",5.79440135072075,-0.0925326264951922,0.584849329749971,-0.158216179429924,0.87428645498863,0.986582224452921
"11935","ZNF711",1.65918943632097,-0.172749230332569,1.06666704191803,-0.161952346462247,0.871343380370867,0.986582224452921
"11936","TRMT2B",6.24003452842474,-0.0893688806168928,0.562562005005523,-0.158860498614753,0.873778782942834,0.986582224452921
"11937","LRCH2",10.4836867214566,-0.0838065491057469,0.52886123142578,-0.158466047662085,0.874089572627596,0.986582224452921

"11938","CCDC160",1.61423837988725,-0.235276905187481,1.36878086328316
,-0.171887927058788,0.863525638132212,0.986582224452921
"11939","MECP2",52.3474150045728,-0.0577217047590467,0.330801290791042
,-0.174490566892944,0.861479943578876,0.986582224452921
"11940","TAZ",23.2669534540782,-0.0578408517485356,0.331359752985243,-
0.174556056453577,0.861428480213412,0.986582224452921
"11941","AGTRAP",113.092277142772,-0.0446303594307316,0.28807198490914
7,-0.154927801968689,0.876878235469694,0.98658471831259
"11942","SH2D2A",2.63369041203522,-0.149849663970762,0.973781264777641
,-0.153884316109716,0.87770095072948,0.98658471831259
"11943","KLF11",33.6493799111774,0.0516025452377072,0.3355373257683,0.
153790774601752,0.877774708099673,0.98658471831259
"11944","ACKR3",882.749050126487,-0.052712993405144,0.343627702836414,
-0.153401466092617,0.878081688796554,0.98658471831259
"11945","NFKBIZ",138.955837650079,0.068979301902902,0.447356048979982,
0.154193292032559,0.877457331122468,0.98658471831259
"11946","MAD2L1",3.86260502593835,-0.123834842211731,0.802286278135337
,-0.154352437012317,0.877331853906364,0.98658471831259
"11947","HIST1H4J",1.78133410154939,-0.178239776939364,1.1576291411976
9,-0.153969670074957,0.877633650153553,0.98658471831259
"11948","RAB32",209.624039285483,-0.0428342042035222,0.278719693379897
,-0.153682015375709,0.87786046595686,0.98658471831259
"11949","EPHB6",16.643836921021,0.0694293650763105,0.44892440135473,0.
154657142420398,0.877091618761665,0.98658471831259
"11950","TLN1",219.925453683822,0.0271456564558047,0.176753409824196,0
.153579251923935,0.877941497396531,0.98658471831259
"11951","POLE3",43.721804439293,-0.0424156678974085,0.273986005630549,
-0.154809614453824,0.876971411356279,0.98658471831259
"11952","MMRN2",1.75996093512062,0.193952447546654,1.26358001328469,0.
153494393317028,0.878008411401856,0.98658471831259
"11953","ZNF511",45.9503946660597,-0.0453181541239856,0.29223617224964
9,-0.155073732916511,0.876763189753989,0.98658471831259
"11954","ACSS3",14.8148832007207,-0.0670257111158592,0.434666819013874
,-0.154200201588702,0.877451883246889,0.98658471831259
"11955","TCHP",18.6783082427389,0.0620004451273151,0.400914153067068,0
.154647683682405,0.877099076028213,0.98658471831259
"11956","MAPKBP1",7.22018464541513,-0.0853449760754413,0.5526210717584
22,-0.1544367025381,0.877265416340995,0.98658471831259
"11957","TMEM99",29.8269148975589,0.0584360940716422,0.380548441821069
,0.153557570205789,0.877958594113288,0.98658471831259
"11958","BORCS8",19.7687046614746,-0.0548896419919307,0.35538647351462
9,-0.154450566024909,0.877254486018963,0.98658471831259
"11959","THAP8",7.80628400105727,0.085342905786956,0.550322443082626,0
.155078003558983,0.876759823001379,0.98658471831259
"11960","LINC00654",3.9827154703146,-0.105077650744135,0.6821462939009
92,-0.154039759042342,0.877578386506311,0.98658471831259
"11961","TMEM191B",2.17448061298371,-0.188616189868844,1.2158774971755
,-0.155127626185206,0.876720703264888,0.98658471831259
"11962","ZBTB2",6.74921611112409,0.0844937108215468,0.551810647477245,
0.15312084173771,0.878302980357697,0.986722538825388

"11963","PORCN",7.97949747174405,-0.0828316586033946,0.541172228921657
,-0.153059699254053,0.878351196664046,0.986722538825388
"11964","OSBPL5",16.1296177048716,-0.0561276271907416,0.36735166867605
8,-0.152789906720791,0.878563957545968,0.986879055956224
"11965","RDH14",72.8946050277898,-0.0350780738385963,0.230242033212545
,-0.152353040620582,0.878908492777423,0.98688148377984
"11966","EPB41L4A",18.4103403746343,0.074073988258666,0.48659740426972
9,0.152228490346828,0.879006723777415,0.98688148377984
"11967","ASPN",459.254706406184,0.0443731466341311,0.291286230929258,0
.152335201332972,0.878922562251282,0.98688148377984
"11968","C12orf73",12.9641810140554,0.0741002034405317,0.4853738685379
68,0.15266623986935,0.878661485298867,0.98688148377984
"11969","LRRC29",4.61052395825722,0.103672688839364,0.680812176790182,
0.152277959140138,0.87896770823237,0.98688148377984
"11970","FPR1",2.37510510093948,0.153601865743018,1.00857463366582,0.1
52295983475937,0.878953492691123,0.98688148377984
"11971","SLC25A30",3.66285109949954,0.114101800714264,0.75063278543688
,0.152007483456581,0.87918103329977,0.986994729472526
"11972","USP4",25.2565097814664,0.0546763127193532,0.3601570266258,0.1
51812428127805,0.879334879527774,0.987054062656668
"11973","SRSF1",61.8827920840586,0.0364800662158643,0.240389114964274,
0.151754234884079,0.879380779238655,0.987054062656668
"11974","MEF2D",36.2709246548814,-0.0439700392630632,0.290165543998592
,-0.151534322983836,0.879554237614933,0.987077844155524
"11975","TCTE3",4.47984348286063,0.110649969720534,0.730613517702241,0
.151448018739819,0.879622312791618,0.987077844155524
"11976","ERBB3",1.4931041222662,-0.206555787666416,1.36231963080739,-0
.151620649805949,0.879486145519703,0.987077844155524
"11977","TPGS2",54.7860019822551,-0.0385901268181148,0.255969864018284
,-0.150760430201886,0.880164701537938,0.98760402638126
"11978","FAM43B",8.8134022661994,-0.0750078936771583,0.498074659764592
,-0.150595683210645,0.880294666854412,0.987667392541029
"11979","LINC02227",1.62787710555777,0.172109753935131,1.1442385253163
1,0.150414227564619,0.880437816996561,0.987688551506465
"11980","AC011365.1",1.84147439189579,0.202456723809569,1.346477354184
97,0.15036028877895,0.880480369993605,0.987688551506465
"11981","AGER",2.06390525630021,0.142154798251871,0.945855501552322,0.
150292299424774,0.880534008155291,0.987688551506465
"11982","SNHG1",14.3681032079529,-0.0602475381192647,0.401362603793879
,-0.150107502666603,0.880679800783529,0.987718678385643
"11983","C12orf43",23.2283012758908,-0.0533266269773747,0.355340429948
848,-0.150071938014628,0.880707859446028,0.987718678385643
"11984","AFDN",22.4551919754994,0.0629927227464841,0.42096109856642,0.
149640246951572,0.881048453195363,0.987858193966348
"11985","COPS2",79.749828982087,0.0362358645235941,0.241887845144409,0
.149804404193857,0.880918934527807,0.987858193966348
"11986","SLC38A7",11.5975492457154,0.0645578529869495,0.43191090511521
9,0.14947030098656,0.881182542467732,0.987858193966348
"11987","PDXK",41.1239988343442,0.0376255458533577,0.251547958492693,0
.149576033448312,0.881099117969003,0.987858193966348

"11988","CHKB-
DT",5.09432488261307,0.0935757811294307,0.62614091624739,0.14944843676
7641,0.881199793829048,0.987858193966348
"11989","GNB1",167.467318092238,0.0275792911706456,0.186085507145193,0
.148207625589707,0.882178913931456,0.987884544981655
"11990","RNASEH1-
AS1",9.24948112255424,-0.0769175613631787,0.51819145501869,-0.14843463
8622909,0.881999765175969,0.987884544981655
"11991","ATG3",50.9220628020945,0.0387213284176865,0.260313091345002,0
.148749062974969,0.881751645203914,0.987884544981655
"11992","BAG6",48.1813864027765,-0.0401262088448554,0.26960069362198,-
0.148835703297998,0.881683277237236,0.987884544981655
"11993","METTL2B",27.4680953261628,-0.0517593183835405,0.3489205437628
08,-0.148341275137774,0.882073442824106,0.987884544981655
"11994","COMMD3",94.5668813780405,0.0370424312643073,0.248957540325476
,0.148790156007646,0.881719218540319,0.987884544981655
"11995","DEPP1",140.072162522097,0.0600703962156925,0.403362372424983,
0.14892414444747,0.881613489145879,0.987884544981655
"11996","CAT",129.491727606762,0.0429307794556864,0.288781732796295,0.
148661686596256,0.881820594885245,0.987884544981655
"11997","CD3E",3.06908017009598,-0.128086777456307,0.863798551679692,-
0.148283158390619,0.882119306079473,0.987884544981655
"11998","AC023794.2",4.70588527512411,-0.106820440592652,0.71855657824
9906,-0.14865974903858,0.88182212384391,0.987884544981655
"11999","CDK4",84.6236812076995,0.03962299531993,0.266938416878564,0.1
48434967822392,0.881999505390519,0.987884544981655
"12000","PUS1",3.52956821160694,0.115013881276114,0.772371437409013,0.
148910065423882,0.881624598711404,0.987884544981655
"12001","XPO6",28.3312115982371,0.0450277774237242,0.30346329003033,0.
148379652178765,0.882043157518735,0.987884544981655
"12002","LINC01266",2.07725512376321,0.142380206565354,0.9634881894489
71,0.147775767388267,0.882519734165571,0.98801921921452
"12003","SGCD",51.2994863435476,-0.0507880011648702,0.34347402358014,-
0.147865624991056,0.882448817221248,0.98801921921452
"12004","HAUS8",5.21776260892697,-0.0989011178855377,0.668429911666106
,-0.147960341330357,0.882374066710537,0.98801921921452
"12005","AC092958.1",3.39583480811303,0.125767652677138,0.852931785408
716,0.147453354217385,0.882774195138509,0.988221774965966
"12006","ORC6",1.76995375231052,-0.166862896654812,1.13363551363689,-0
.147192721688374,0.882979905277629,0.988343578144755
"12007","POLDIP3",32.6597520960429,-0.0415401030027478,0.2823376940068
78,-0.147129143166183,0.883030087267213,0.988343578144755
"12008","DNAJC3-
DT",3.06091785090807,-0.133934688886533,0.911025252780051,-0.147015341
756799,0.883119910928645,0.98836179904814
"12009","NPIPB11",2.29102304340774,0.154407018535417,1.05186643132293,
0.14679337027727,0.883295117748431,0.988393262899348
"12010","GNL3L",27.8337723569249,0.056972153490832,0.388029714587549,0
.14682420275826,0.883270780672421,0.988393262899348
"12011","POU5F2",7.39627209578995,0.0872595031915968,0.597093181613535

,0.146140511864152,0.883810465865659,0.988497837339435
"12012","ALOX5AP",3.90629247957386,0.102620020836693,0.702294655765585
,0.14612103337854,0.88382584239227,0.988497837339435
"12013","GNA13",42.4205602101074,-0.0394459922273091,0.269280752120876
,-0.146486490091213,0.88353735422064,0.988497837339435
"12014","NRIP1",41.9290070907343,-0.040519253344779,0.277281706210294,
-0.146130279918462,0.883818543068478,0.988497837339435
"12015","DGCR6",6.06512893314239,-0.0911530149950345,0.623840515686773
,-0.146115894532252,0.883829899060246,0.988497837339435
"12016","CTPS2",9.46876920500584,0.0722592624457925,0.493421959925707,
0.146445169275953,0.883569971718942,0.988497837339435
"12017","INTS12",13.0528317949696,-0.0666286914753216,0.45823621278784
8,-0.145402501190295,0.884393089987125,0.988586601697387
"12018","FOXK1",27.8298941608905,0.048315410555711,0.332590574643586,0
.145269933182825,0.884497752720394,0.988586601697387
"12019","SLC25A51",6.15362984347863,-0.0874787337285567,0.602051417869
307,-0.145301100756724,0.884473145670994,0.988586601697387
"12020","NIBAN2",11.3176115905722,-0.0794057720447881,0.54600586151277
3,-0.145430255684042,0.884371178008649,0.988586601697387
"12021","ANK3",1.57477703542193,-0.174720760415522,1.20222564700275,-0
.145331087263955,0.884449471188378,0.988586601697387
"12022","CHEK1",4.0470003441186,-0.123621848737552,0.848336048611033,-
0.145722734451703,0.884140273449624,0.988586601697387
"12023","COX10",6.88951171491444,-0.0808159977142594,0.555816509371964
,-0.145400498818533,0.884394670849031,0.988586601697387
"12024","PRCD",5.96153226117578,-0.102729536946139,0.70462031052266,-0
.145794175120979,0.884083874350327,0.988586601697387
"12025","ITFG2",22.8501851419295,0.0510002055081537,0.351532175060138,
0.145079765456544,0.884647894077357,0.988589975761317
"12026","NUBPL",12.6107084414409,-0.0615267157818424,0.423882843564553
,-0.145150285546937,0.884592216522483,0.988589975761317
"12027","ZNF33A",30.7508807738807,-0.0462553809291273,0.31915215111308
2,-0.144932066939878,0.884764507972391,0.988638082866962
"12028","PAFAH2",7.16988434378144,0.0812950476395867,0.562059121012362
,0.144637894129644,0.884996776624071,0.988776365104256
"12029","BCL10",34.199588794823,0.042824783681823,0.297965914556154,0.
143723767014137,0.885718602683637,0.988776365104256
"12030","DRAM2",52.9454930422711,-0.0362411303215351,0.25544216346382,
-0.141876070223106,0.887177896286921,0.988776365104256
"12031","TSEN15",15.6265268212914,0.0564814617425084,0.392286188563058
,0.143980245517691,0.885516068881022,0.988776365104256
"12032","AC092164.1",6.51915180925317,-0.0792143165746947,0.5591341345
17445,-0.141673190178346,0.887338152443662,0.988776365104256
"12033","INPP4A",10.4094532951542,-0.0688390948417526,0.48472866762135
3,-0.142015728468378,0.887067582086627,0.988776365104256
"12034","PMS1",20.3715258084868,0.0488894030321593,0.342472285344106,0
.142754334071257,0.886484203726115,0.988776365104256
"12035","STK17B",16.1635952724996,-0.0727857264796148,0.50937817132083
8,-0.142891334135655,0.886376002698741,0.988776365104256
"12036","MLH1",20.4532293837098,0.0508768314665148,0.353604386252013,0

.143880657154109,0.885594710100735,0.988776365104256
"12037","CHMP2B",81.0663766583585,0.0326175618311668,0.23019307936581,
0.141696535452018,0.887319711638179,0.988776365104256
"12038","SPON2",542.560883258251,-0.0321621398341048,0.226232856344569
,-0.142163876431457,0.886950564362059,0.988776365104256
"12039","UTP15",5.50304661360054,-0.0932108781312592,0.658134154994268
,-0.141628993760506,0.887373064068819,0.988776365104256
"12040","AC011374.2",1.97276762015692,0.145528181629434,1.013986163756
89,0.143520875166819,0.885878825912834,0.988776365104256
"12041","AL662884.3",1.63935040659149,0.179765322018851,1.269459303373
09,0.141607786512884,0.887389816171027,0.988776365104256
"12042","LMBRD1",98.9595776378678,0.031324989603886,0.220956485695958,
0.141769948527286,0.887261721872097,0.988776365104256
"12043","NUP205",10.6918439445898,0.0684340826563424,0.483240673846445
,0.141614906112162,0.887384192226992,0.988776365104256
"12044","RUSC2",19.4565127616316,0.0534304625517359,0.371421005930128,
0.143854175446898,0.8856156219078 1,0.988776365104256
"12045","JMJD1C",130.369721987699,0.0299298395271979,0.21081634010397,
0.1419711560898 8,0.887102788981111,0.988776365104256
"12046","EDRF1",18.5645718707578,0.0557397764348176,0.389274651812132,
0.143188815853641,0.886141062458921,0.988776365104256
"12047","P3H3",54.7061750445909,0.0387663725754111,0.272198438004841,0
.142419526208749,0.886748639909354,0.988776365104256
"12048","R3HDM2",68.4856738915235,-0.0366322431031806,0.25751736153422
,-0.142251547176996,0.886881316975864,0.988776365104256
"12049","DENR",80.5921042836811,0.0332736789740575,0.231899454289224,0
.143483213774875,0.885908567541775,0.988776365104256
"12050","ANG",88.7848056489829,-0.0403151508388381,0.279933282427274,-
0.144016997511941,0.885487047485479,0.988776365104256
"12051","VTI1B",90.082762708493,-0.0305017333471984,0.213976676258978,
-0.142547000357562,0.886647957460193,0.988776365104256
"12052","BBOF1",13.7104789011629,-0.0661253953483141,0.463689939883694
,-0.142606922558855,0.886600629955172,0.988776365104256
"12053","GOLGA5",33.5684569653305,0.0465523806659809,0.327766181923675
,0.142029236795458,0.887056912153791,0.988776365104256
"12054","TRIP4",19.6822101617343,-0.0498318782989639,0.346230127907939
,-0.143927042398269,0.885558081261655,0.988776365104256
"12055","ZNF263",13.6867581435911,-0.06329308791731,0.441336308095331,
-0.143412374545985,0.885964510520781,0.988776365104256
"12056","PLSCR3",29.965503407985,-0.0459839661388047,0.323470649002952
,-0.142158079196808,0.886955143381112,0.988776365104256
"12057","KCTD1",4.85775116943616,0.100081826217064,0.693886091347404,0
.144233797830884,0.885315853159414,0.988776365104256
"12058","MAMSTR",2.86549918820796,-0.135219816703739,0.945086565800624
,-0.14307664672938,0.886229648378862,0.988776365104256
"12059","CEBPB-
AS1",1.4734186590865,0.161669626715114,1.13562167794808,0.142362223136
872,0.886793899984446,0.988776365104256
"12060","CEBPB",176.159199496405,0.0512746963900207,0.358489497201559,
0.143029842687948,0.886266612438759,0.988776365104256

"12061","TAF4",9.19473980122857,0.0730323242596369,0.50921691003154,0.143420854297854,0.885957813882353,0.988776365104256
"12062","MTOR",9.49141326715176,0.0703253281233846,0.499331250807853,0.140839028219458,0.887997110281572,0.988857384141683
"12063","C2orf42",6.03304187292645,0.0883689815945854,0.62587400897763,0.141192924337818,0.887717535640643,0.988857384141683
"12064","SAP130",9.70765239786271,-0.0707218352828387,0.500635675170651,-0.141264074436429,0.887661329410178,0.988857384141683
"12065","GORASP1",11.9537181550085,0.0671853629619368,0.477193668150166,0.140792653897483,0.88803374659787,0.988857384141683
"12066","SUMF2",99.0898853044046,-0.0281596553239816,0.199230016887459,-0.141342433052588,0.887599429342364,0.988857384141683
"12067","SRCAP",17.9124203631012,0.0548537372952571,0.389411129914533,0.140863301229465,0.887977934383997,0.988857384141683
"12068","HSPA13",22.389806441372,0.0517299676039976,0.367439800715447,0.140784878239302,0.88803988949218,0.988857384141683
"12069","MIATNB",5.91625710679905,-0.0878028594281918,0.623730135416599,-0.14077059042457,0.888051177111836,0.988857384141683
"12070","PRKAA1",65.7179043742547,-0.0333115159669559,0.236798243108514,-0.140674675325571,0.888126952279229,0.988859826982648
"12071","NEK11",22.1022661964227,0.0517512524125419,0.368636526368029,0.140385579590873,0.888355350840996,0.988914082048491
"12072","TBX18",23.3306190062942,-0.0462055612978574,0.32925521095766,-0.140333576387343,0.888396436682151,0.988914082048491
"12073","ANKS6",9.9282267171768,-0.0695298649024071,0.494876293609073,-0.140499486033841,0.888265358548291,0.988914082048491
"12074","AL357873.1",1.91696646097551,0.137520618989709,0.999619399433709,0.137572979343553,0.890577911796181,0.989088050175043
"12075","LMO4",116.777631124838,0.0290773417902619,0.211805284810051,0.137283362954512,0.890806820361037,0.989088050175043
"12076","MCL1",277.038959510755,0.0499627076240828,0.362418710346367,0.137859073490805,0.890351796114334,0.989088050175043
"12077","TNFSF4",6.17114381689812,-0.112325167811682,0.811052128116932,-0.138493154653911,0.889850679280365,0.989088050175043
"12078","KLHL20",36.6203525922182,0.0418562909547274,0.303760323671189,0.137793805487367,0.890403380163829,0.989088050175043
"12079","OXSR1",25.4243262830194,-0.0535549295553962,0.386375164175543,-0.138608623226788,0.889759428744127,0.989088050175043
"12080","FBXO45",12.8951266481345,-0.0574278802945475,0.414668145259246,-0.138491178912825,0.889852240648066,0.989088050175043
"12081","QDPR",46.9980709526363,-0.037172937096003,0.267226089716691,-0.139106691024867,0.889365840962837,0.989088050175043
"12082","SRD5A1",7.11674208269193,-0.0848761790197568,0.610447618984054,-0.139039249855726,0.889419133358535,0.989088050175043
"12083","OXCT1",10.1666090658974,0.0699244113398878,0.509692156343488,0.137189498542655,0.890881011375351,0.989088050175043
"12084","GTPBP2",13.645372457945,-0.061545810672112,0.44022403558491,-0.139805657340673,0.888813543287914,0.989088050175043
"12085","TSC22D4",46.1222961760539,0.0368149805450894,0.26301970182001,0.139970429174476,0.888683354448749,0.989088050175043

"12086","PLEKHF2",14.6264794332202,0.0591036039490767,0.425706820487891,0.138836403610681,0.889579426626829,0.989088050175043
"12087","LRRC14",7.26098851210691,-0.0735165793563836,0.534487961921528,-0.137545809436167,0.89059938610686,0.989088050175043
"12088","ZNF7",20.6761955507368,-0.0463514201137341,0.337567502023917,-0.137310078238663,0.890785704609204,0.989088050175043
"12089","ACCS",18.1747514727781,0.0716644118315501,0.518263966677723,0.138277820646007,0.890020853668132,0.989088050175043
"12090","TMEM116",8.45557622536605,-0.0715063474689645,0.519452044556952,-0.137657264454418,0.890511295787798,0.989088050175043
"12091","SETD1B",11.4501994283182,-0.0646532905906493,0.466631723891011,-0.138553140047011,0.889803274863827,0.989088050175043
"12092","RTF1",156.5677777127,-0.0285962727787222,0.207399243872873,-0.137880313566864,0.890335009288564,0.989088050175043
"12093","AC048382.6",2.48378910912764,-0.12465688460712,0.891859420622341,-0.13977189871486,0.888840216883864,0.989088050175043
"12094","PMM2",28.3564373056132,0.0580247910431854,0.416821250952356,0.139207852072345,0.889285903864849,0.989088050175043
"12095","PRSS53",3.14802756933379,0.106377286089131,0.768431778737127,0.13843426187287,0.889897220625868,0.989088050175043
"12096","TSR1",16.5010333931546,0.0580379267474879,0.422688072988946,0.137306752795473,0.890788333034191,0.989088050175043
"12097","PCYT2",5.00469083546591,0.102446920519278,0.737578091140973,0.138896371448345,0.889532038339757,0.989088050175043
"12098","RALBP1",130.753910947331,0.0269885342342936,0.194499640442009,0.138758787280845,0.889640761839016,0.989088050175043
"12099","LAMA3",3.77603975426074,0.118183974443118,0.851330887348001,0.13882260845871,0.889590328003218,0.989088050175043
"12100","ZNF766",27.4033907656884,0.0431595868918876,0.31349040769092,0.137674346114093,0.890497795134663,0.989088050175043
"12101","ARFGAP1",19.1086999797129,-0.0596071700862415,0.434595488488782,-0.137155519707564,0.890907868693273,0.989088050175043
"12102","LRRC75B",20.031392096929,-0.0506707641998303,0.3667873234594,-0.138147533894909,0.890123819280662,0.989088050175043
"12103","AL022322.2",1.74047530878434,0.153399027414948,1.09928032218714,0.139544958932535,0.889019531359778,0.989088050175043
"12104","ALG12",17.7133599036806,0.0482212429672647,0.349383478151118,0.138018097542688,0.890226114658176,0.989088050175043
"12105","NAP1L3",10.4967628051546,0.0606213570858902,0.434736776575539,0.139443820611199,0.889099446745993,0.989088050175043
"12106","CSMD2",2.48070388159157,0.131867203357841,0.972127605979294,0.135648038947523,0.892099526816868,0.989091778737887
"12107","ATPAF1",24.1419589076798,-0.0503294967290492,0.368870913907957,-0.136442031158921,0.891471848282368,0.989091778737887
"12108","CHD1L",2.1654518524298,-0.129412630581167,0.9532217868655,-0.135763399834489,0.892008325805383,0.989091778737887
"12109","SLC50A1",18.8939358215529,-0.053284699703759,0.39384644645946,-0.135293082323757,0.892380154298372,0.989091778737887
"12110","RAB29",28.6683745401957,-0.0519542848917296,0.387530487436604,-0.134065026045799,0.893351155104459,0.989091778737887

"12111","IMMT",36.5750031632317,-0.0381373837718394,0.281559863405402,
-0.135450356135908,0.89225581250052,0.989091778737887
"12112","AC009506.1",2.555616961956,0.130590204354518,0.97502676167866
3,0.133934994901767,0.893453977649272,0.989091778737887
"12113","CHCHD4",10.0759349724559,0.0644447761989176,0.471776765250847
,0.136600148514418,0.891346859104565,0.989091778737887
"12114","RYK",55.3087752055345,0.0395698367718177,0.292963088570861,0.
1350676529417,0.892558384989306,0.989091778737887
"12115","RNF13",140.789660837365,0.025075705663479,0.186068908992647,0
.134765694060527,0.89279713051587,0.989091778737887
"12116","ZGRF1",3.25961817061688,-0.112315063838792,0.83596099043561,-
0.134354431754365,0.893122313047547,0.989091778737887
"12117","BRPF3",11.5366911871874,0.0633083565007421,0.466193087387199,
0.135798574053418,0.891980518359939,0.989091778737887
"12118","TMEM209",10.4485639314537,-0.0580520480707994,0.4309837321108
01,-0.134696610905664,0.892851752876124,0.989091778737887
"12119","AC083862.2",3.58533572132666,-0.118448861210007,0.88048713804
7673,-0.134526509351001,0.892986250179443,0.989091778737887
"12120","AC009403.1",4.5661660848578,0.0888486141524729,0.661764338019
507,0.134260202685407,0.893196821914731,0.989091778737887
"12121","OXR1",70.3072368882368,0.0296257399461418,0.220487345900285,0
.134364808216885,0.893114108222385,0.989091778737887
"12122","EHMT1",41.3229577648346,-0.0365410953542087,0.271275268298689
,-0.134701167501841,0.892848150071355,0.989091778737887
"12123","ARHGAP1",87.6000913488637,0.0346119760260333,0.25755681693858
7,0.134385788881241,0.893097518530105,0.989091778737887
"12124","CSKMT",23.4807256344791,-0.0598423695103585,0.445044989201744
,-0.134463640670789,0.89303596048637,0.989091778737887
"12125","AP000766.1",6.04835478504498,0.0942867753623531,0.69529168894
0078,0.135607511008921,0.892131567382594,0.989091778737887
"12126","AMIGO2",5.79912103459736,0.0819771155816225,0.612594995867325
,0.133819433940295,0.893545359353715,0.989091778737887
"12127","ALDH1L2",33.6355418519266,0.0485754748655875,0.36086193504818
,0.134609583743162,0.892920563847598,0.989091778737887
"12128","LINC01048",8.48095411133825,-0.0892846993536513,0.66594291577
0188,-0.134072601779073,0.893345164624279,0.989091778737887
"12129","AL365295.1",8.27392886174501,0.100684060813774,0.734924266033
132,0.136999233073677,0.891031401314517,0.989091778737887
"12130","SAMD4A",32.8062621712036,-0.0490009777877063,0.3615542882412,
-0.135528686510881,0.892193884935224,0.989091778737887
"12131","BLOC1S6",110.830880055702,0.0278037151342833,0.20610870148031
3,0.134898308196556,0.892692277188544,0.989091778737887
"12132","UBALD1",11.0185698181859,-0.0615469352312863,0.45532113905198
,-0.135172584693592,0.89247542237465,0.989091778737887
"12133","ZFPM1",29.8787908885155,-0.0539085936088145,0.397425023501732
,-0.135644688736062,0.892102175419377,0.989091778737887
"12134","NEDD4L",1.53481822831924,0.148757991227445,1.1064615991,0.134
444784480948,0.893050870170049,0.989091778737887
"12135","ZNF208",18.2600104306642,-0.0723408716943499,0.53936891873548
5,-0.134121320642554,0.893306640527135,0.989091778737887

"12136","ECH1",151.747693601518,-0.0380090439480999,0.278392629258514,
-0.13653035301019,0.891402030973997,0.989091778737887
"12137","TRAPPC6A",34.9577692933407,0.0523621050338314,0.3895157219724
83,0.134428733116787,0.893063562093809,0.989091778737887
"12138","FAM217B",27.0304413524247,0.0414164551316645,0.30807480123316
4,0.134436360799009,0.893057530829732,0.989091778737887
"12139","POLR3H",19.2329444159563,0.0493462688300646,0.368806369067904
,0.133799936684334,0.893560777264431,0.989091778737887
"12140","TTLL1",7.7444741201497,0.0789797343607273,0.577935464416803,0
.136658397387719,0.891300815005256,0.989091778737887
"12141","ELF4",11.4503893940159,0.0587932537258052,0.436609341827802,0
.134658716828357,0.89288171501176,0.989091778737887
"12142","OSTM1",33.7578805812865,0.035923046769203,0.26906414118582,0.
133511089998403,0.893789194239036,0.989194274661202
"12143","ZNF19",3.00174788149763,0.111632311812328,0.83680129603939,0.
133403607691202,0.893874192386758,0.989194274661202
"12144","TSPAN10",6.10800243412261,0.0793120813018349,0.59447091038865
2,0.133416252865901,0.893864192385305,0.989194274661202
"12145","ERLEC1",141.380848361199,0.026000695362491,0.19546376515496,0
.13302053882917,0.894177137208531,0.989285135996168
"12146","SYNE3",103.382319544111,0.0397444370357513,0.298737073542695,
0.133041529008856,0.894160537009025,0.989285135996168
"12147","OFD1",36.8720470913317,0.039902929759995,0.299704333802281,0.
133140983494485,0.894081883510214,0.989285135996168
"12148","BCL9L",44.6731351948394,-0.0456720341974685,0.343904551298426
,-0.132804390128109,0.894348082297107,0.989392811820121
"12149","SLC25A21-
AS1",9.21520416309745,-0.0692720523735877,0.522008664718926,-0.1327028
78429972,0.89442836634362,0.98940018234356
"12150","CES4A",2.38538054766494,-0.148274137201961,1.11869868682146,-
0.132541620857042,0.894555904710268,0.989459819209983
"12151","TSKU",55.6692517959709,0.0434151208145727,0.328325357195495,0
.132232006645536,0.894800785752976,0.989649227202226
"12152","LRRC47",25.5581550170174,0.0418686767815152,0.317386668629774
,0.131916935775126,0.895049992885768,0.989665185590573
"12153","BX284668.5",25.1310578057934,0.0460493376514085,0.35064791305
764,0.131326427269621,0.895517086893607,0.989665185590573
"12154","SRRM1",185.213657573452,0.0231766674094203,0.178211673615613,
0.130051342536687,0.896525806108493,0.989665185590573
"12155","MRPL37",34.1930349116197,-0.0440005063730965,0.33931098687588
9,-0.12967604373268,0.896822736836202,0.989665185590573
"12156","AL603840.1",1.96065797335597,0.138129103704493,1.058118371390
75,0.130542203442647,0.896137466275943,0.989665185590573
"12157","RWDD3",28.7074878547452,-0.0477902755404087,0.363418767149246
,-0.131501947230982,0.895378246284489,0.989665185590573
"12158","AMPD2",11.8140356116591,0.0557391997942362,0.430062375022544,
0.129607245440419,0.896877170571292,0.989665185590573
"12159","CKS1B",119.257969601153,-0.0370672946227045,0.285983192009939
,-0.129613542537899,0.896872188239414,0.989665185590573
"12160","SELENOI",7.57622464622944,0.0654946749887573,0.50479398576912

6,0.129745355204593,0.896767897560521,0.989665185590573
"12161","NEK4",9.40021267551624,-0.0680641111127096,0.523907858140329,
-0.12991618670182,0.896632737689717,0.989665185590573
"12162","SLC49A4",8.5378807405633,-0.070310432919502,0.542223519284607
,-0.129670570196342,0.896827067522243,0.989665185590573
"12163","MAML3",6.16546264850287,-0.0794392404511346,0.612827520480793
,-0.129627403790239,0.896861221080434,0.989665185590573
"12164","HSD17B8",9.31853363031805,0.0673426631140786,0.51537335551661
3,0.130667723492562,0.89603816630042,0.989665185590573
"12165","CRYBG1",7.23146681863667,0.0780374792238503,0.594299459686943
,0.131310028895126,0.895530058574942,0.989665185590573
"12166","KIAA0408",30.0493872455885,-0.0544823024941044,0.413474368405
1,-0.13176706141244,0.895168540519376,0.989665185590573
"12167","SNX9",278.492669713128,-0.0257188725924367,0.198056663433119,
-0.129856133828699,0.896680250474719,0.989665185590573
"12168","CTSB",211.956104307152,0.0266501170428,0.202405803210338,0.13
1666763601167,0.895247875396736,0.989665185590573
"12169","TIMM23",16.1410185101786,-0.0609507382405526,0.46417187569183
7,-0.131310709313672,0.895529520339786,0.989665185590573
"12170","FBXW4",17.3698540253803,0.0543857686780092,0.415181862676961,
0.130992640977491,0.895781128892012,0.989665185590573
"12171","PACSIN3",3.88159827282525,0.0984529625409116,0.75280153692736
7,0.130782095561012,0.895947687005595,0.989665185590573
"12172","RTN3",200.084903075253,-0.0265944688022697,0.203267636039928,
-0.130834742413424,0.895906038741908,0.989665185590573
"12173","EEF1AKMT1",33.322336122192,-0.049406289056943,0.3766784432903
64,-0.131163038227962,0.895646334523003,0.989665185590573
"12174","RNF6",20.8585337317411,-0.0444470369540269,0.341047039324821,
-0.130325239128361,0.896309112420285,0.989665185590573
"12175","TMEM121",9.39171831855906,-0.0870410827163884,0.6683084154624
72,-0.130240889838497,0.896375844660565,0.989665185590573
"12176","GABPB1",10.2222733670705,0.0663985443448961,0.50340461850531,
0.131898957427217,0.895064213276962,0.989665185590573
"12177","CNTNAP1",12.4214207660499,0.0701667113630563,0.53454532358975
8,0.131264288109097,0.895566241266165,0.989665185590573
"12178","PDE9A",3.53431175830105,0.102993687731471,0.784191302092455,0
.131337452298505,0.895508365730181,0.989665185590573
"12179","PHKA2",5.05707696037128,-0.08457330671239,0.646506921161996,-
0.130815779296504,0.895921040192473,0.989665185590573
"12180","NOL3",12.5254369668802,-0.0624856220286099,0.482542876461582,
-0.129492372754123,0.896968059801055,0.98968421639297
"12181","ZNF322",48.9574329646627,-0.033753516654663,0.260909590428889
,-0.129368631483336,0.897065967518779,0.989710987397165
"12182","HOXD9",2.56808835240061,0.114386849985801,0.893388295367257,0
.128037103887486,0.898119610022519,0.990060654506819
"12183","BPHL",26.5837308593894,0.040159572866884,0.31261790705024,0.1
28462164070563,0.897783238785614,0.990060654506819
"12184","CCDC28A",24.3384305130955,0.0421774603580155,0.32913792314963
7,0.12814524669295,0.898034029510081,0.990060654506819
"12185","NCF1",1.49386551319098,-0.145894361782175,1.13314291954089,-0

.128751951114239,0.897553926428879,0.990060654506819
"12186","SAXO1",1.49441884179918,0.143200488174938,1.11581517351658,0.128337104185123,0.897882202980786,0.990060654506819
"12187","MCMBP",40.1960152717969,-0.0336846110229936,0.261448023018155,-0.128838652647431,0.897485320010025,0.990060654506819
"12188","ABCC9",90.1800214137774,0.0403527893557223,0.314655689143172,0.12824427063628,0.897955666392975,0.990060654506819
"12189","TMEM202-AS1",2.34122552612858,0.115053553901989,0.895810395784194,0.128435162667732,0.897804605791241,0.990060654506819
"12190","CIC",18.3978621201971,0.0463782909424166,0.361118263879704,0.128429646410424,0.897808970977296,0.990060654506819
"12191","PIGU",12.8599127783163,-0.0632787500714591,0.494124117756167,-0.128062460012699,0.898099543947069,0.990060654506819
"12192","MRPS11",48.1991134942782,-0.0378716009667816,0.296245881480117,-0.127838404968082,0.89827685663018,0.990071571906257
"12193","RPN2",190.919332590989,0.0276142687729673,0.215918454817276,0.12789211925556,0.89823434775373,0.990071571906257
"12194","STIM2",13.7616627307663,0.0556922656135252,0.437509629772986,0.127293805264178,0.898707863106457,0.990083818400332
"12195","INTS6",64.3772652913936,-0.0400251250607948,0.313378671478022,-0.127721280047618,0.898369548972701,0.990083818400332
"12196","CGRRF1",22.6321729717812,0.041111058785224,0.323002137658983,0.127277977425115,0.898720390004225,0.990083818400332
"12197","AC009093.2",1.75919827770998,0.136907330005203,1.0757587294252,0.127265832254372,0.89873000228184,0.990083818400332
"12198","AC005523.2",4.10478943364509,0.0861327903043614,0.676399872333231,0.127340045182516,0.898671266800476,0.990083818400332
"12199","SLC17A7",27.485211674935,0.041798486141507,0.328264313362395,0.127331800747292,0.898677791793494,0.990083818400332
"12200","FGR",2.31746816493538,0.121838584453096,0.958582602410299,0.127102853887333,0.89885899270882,0.990102379858324
"12201","FHL2",14.1847691870076,0.0769453712202872,0.606479342456084,0.126872204597569,0.899041546351003,0.990102379858324
"12202","TDP1",5.66900749233225,0.0872290876948424,0.686803328513798,0.127007374707401,0.898934561649294,0.990102379858324
"12203","ZNF341",2.33325529736841,-0.115245666629476,0.907709729554818,-0.12696312805416,0.898969581874439,0.990102379858324
"12204","SMIM3",20.5740848963451,-0.0549744072931109,0.434685821191963,-0.12646929026202,0.899360456651649,0.990154271386757
"12205","SUPT3H",9.63492108598334,-0.0623336785163494,0.493019606573963,-0.126432453568148,0.899389614035914,0.990154271386757
"12206","VWA8",7.25042394250756,0.0685732889277901,0.542666018576593,0.126363705447518,0.899444030666138,0.990154271386757
"12207","ZNF566",19.446287509403,0.0512292770887393,0.405763043802362,0.126254171914414,0.899530731405679,0.990154271386757
"12208","FBXO17",66.4661715223246,0.0314651830478456,0.249105058895965,0.126312902625501,0.899484243245587,0.990154271386757
"12209","GLA",35.3067364163802,-0.0471583039594094,0.372563927800501,-0.12657775066367,0.899274607672955,0.990154271386757

"12210","GAR1",21.4984876799801,-0.0426455771927377,0.340799276738879,
-0.125134001459202,0.900417465573249,0.990186755393921
"12211","SNX24",25.0554749811335,-0.0411140568858869,0.330827471158383
,-0.124276429469195,0.901096409166394,0.990186755393921
"12212","PPP1R18",39.1370112850443,-0.0380442214192397,0.3061603851132
1,-0.124262390789625,0.90110752425535,0.990186755393921
"12213","RING1",31.6767560947313,-0.0400592787486928,0.320997812437489
,-0.124796111364447,0.900684966007357,0.990186755393921
"12214","CCNC",37.3319529035754,-0.0381980782562692,0.306343744010264,
-0.12469025074979,0.900768775936697,0.990186755393921
"12215","EXOSC3",22.886505370138,0.0462796763010532,0.371222762723159,
0.124668207201417,0.900786227969948,0.990186755393921
"12216","IFITM2",354.713245739471,-0.0260475665472182,0.20833269684272
2,-0.125028701408702,0.900500828182072,0.990186755393921
"12217","SAAL1",8.40262801148763,0.0707244536555724,0.564862160719184,
0.125206569980764,0.90036001608552,0.990186755393921
"12218","RNASEH2C",138.449532385032,0.0291916780161024,0.2342805389298
07,0.124601378114673,0.90083913732395,0.990186755393921
"12219","AC074032.1",9.18806228295631,0.0600503265413725,0.48239975783
1734,0.124482497278364,0.900933257733527,0.990186755393921
"12220","TIMM9",55.3638721458298,-0.0391842349477335,0.315095619585476
,-0.124356647671847,0.901032896979881,0.990186755393921
"12221","TARSL2",14.4897617115024,0.060399955507218,0.482491832860204,
0.125183373880482,0.900378379418987,0.990186755393921
"12222","CAPN15",14.8413716899937,-0.0513578759250872,0.41101581480137
1,-0.124953527517929,0.900560341566241,0.990186755393921
"12223","MT1X",225.347587140399,0.0371228571593055,0.296339547129677,0
.125271356856939,0.900308727444611,0.990186755393921
"12224","COQ9",28.1909989511542,0.040831655428138,0.324002458539237,0.
126022671593997,0.899713978310848,0.990186755393921
"12225","BANP",13.0543811269359,0.0527570508151634,0.42336132839138,0.
124614713903183,0.900828579192131,0.990186755393921
"12226","CACNB1",4.69394412823528,-0.0867295597471451,0.69054742739510
8,-0.125595370146128,0.900052228023,0.990186755393921
"12227","MLLT1",34.2887461199523,0.0352297424687598,0.28223699793335,0
.124823261042053,0.900663471765252,0.990186755393921
"12228","ZNF480",21.1638891545866,0.0480036823789562,0.381850369105148
,0.125713332401514,0.89995884786865,0.990186755393921
"12229","PLA2G6",6.00587194517974,0.0733060737100558,0.586298126441469
,0.125032079080631,0.900498154172544,0.990186755393921
"12230","SYP",4.47340848359456,0.0933834738786623,0.749329696742961,0.
124622678487938,0.900822273526437,0.990186755393921
"12231","CLCN2",2.98730740659099,-0.0997028900810653,0.803961951460543
,-0.124014438618565,0.901303842951751,0.990251331651717
"12232","COPB1",123.272172528456,-0.0331067033735454,0.267078870778511
,-0.123958526846554,0.901348112510162,0.990251331651717
"12233","RPS29",2457.13828281129,-0.0343805028534293,0.277465797271656
,-0.123908976138665,0.901387345791759,0.990251331651717
"12234","LAS1L",18.3344712191357,0.0494184533212339,0.39917592037556,0
.123801188395179,0.901472690851175,0.990264140293357

"12235","MAGEF1",42.9177653843157,-0.0318274115377597,0.25735567299747
6,-0.123670914913431,0.90157584137192,0.990279503306257
"12236","KLHDC2",79.4228559380106,-0.02781844023771,0.225073019266325,
-0.123597401094055,0.901634050335245,0.990279503306257
"12237","IPP",20.8517407380778,-0.0414980820870063,0.338722782724715,-
0.122513406843178,0.902492428849731,0.99033598319412
"12238","RCHY1",17.5370423778011,0.0457386474687541,0.377934608753487,
0.121022648916992,0.903673095805719,0.99033598319412
"12239","KIF3A",27.1961760497884,-0.0421177826173373,0.343180758471786
,-0.12272769255739,0.902322734142368,0.99033598319412
"12240","HSPA1A",284.26182037981,-0.0469821702650198,0.386060935324566
,-0.121696255606699,0.903139578790098,0.99033598319412
"12241","CYTH3",36.0821093607344,0.0405763430332687,0.331086938245195,
0.122554949610301,0.90245953042538,0.99033598319412
"12242","MRPS24",192.228501148614,0.0373085984440425,0.304765063050931
,0.122417570014604,0.902568324304036,0.99033598319412
"12243","PSPH",4.40080271916274,0.091967866451311,0.747236330417967,0.
123077348768453,0.902045847955175,0.99033598319412
"12244","AC124242.1",1.95901810541279,0.130617391821166,1.069158855898
58,0.122168367310944,0.902765677993295,0.99033598319412
"12245","GAS1RR",18.5381012357858,-0.0456841448303854,0.37647777011468
7,-0.121346194800475,0.903416832167985,0.99033598319412
"12246","GATD1",22.2334746713621,0.0414422659658864,0.337702549242556,
0.122718250302932,0.90233021145127,0.99033598319412
"12247","ZNF214",5.14687113253914,-0.0757596855859177,0.61769843821844
4,-0.122648335981588,0.902385576780049,0.99033598319412
"12248","POC1B-
AS1",7.93706356120875,0.0748889897038382,0.616972368871679,0.121381432
106588,0.903388923162505,0.99033598319412
"12249","ERP29",197.69230527656,0.0316774633396249,0.260613703474365,0
.121549492284241,0.903255816093759,0.99033598319412
"12250","ANKLE2",47.6130730242617,-0.0323063664217944,0.26511996950096
,-0.12185565079313,0.903013339337261,0.99033598319412
"12251","LNX2",2.56940131805588,0.113237179807218,0.934301314775647,0.
121199850643911,0.903532742414883,0.99033598319412
"12252","NDFIP2",45.3480710268537,-0.0352473968323976,0.28644027099048
2,-0.123053217030257,0.902064957043053,0.99033598319412
"12253","AL356019.2",2.64706434134731,-0.102021633895904,0.84272179781
0983,-0.121062056494694,0.903641882603698,0.99033598319412
"12254","SLC24A1",3.39938014132679,0.09021339866725,0.74544161961778,0
.121020072253956,0.903675136684984,0.99033598319412
"12255","LRRC28",14.7515560366955,-0.047843003017449,0.39223505222671,
-0.12197533786398,0.902918549941242,0.99033598319412
"12256","NSMCE1",75.2782676698857,-0.0371529313248693,0.30347430803365
1,-0.122425293810208,0.902562207614372,0.99033598319412
"12257","TMX3",53.8958223545148,0.0297108849914788,0.242138821063818,0
.122701865239726,0.902343186781862,0.99033598319412
"12258","LPAR2",2.0202633174567,-0.13800495070902,1.13987540348329,-0.
121070206697414,0.903635427165263,0.99033598319412
"12259","AVP",1.82618569684989,0.141209184245999,1.15097677247745,0.12

26863891806,0.902355442296133,0.99033598319412
"12260","MAP1LC3A",76.9435668062877,-0.0328251476166348,0.270010972116
141,-0.121569680518448,0.903239826785733,0.99033598319412
"12261","STAU1",105.463074018491,-0.0238243516745627,0.196678219536598
,-0.121133655423037,0.903585172272152,0.99033598319412
"12262","MED14OS",4.95430636283375,-0.0817263573037923,0.6709459042055
02,-0.121807670024558,0.903051339386155,0.99033598319412
"12263","KLF8",19.6517740266336,-0.0562824992821749,0.464732624465597,
-0.121107269684144,0.903606071186966,0.99033598319412
"12264","TMEM59",666.749852075684,-0.0293254376180012,0.24506148981453
1,-0.119665630206506,0.904748027785179,0.99047717197517
"12265","DYNC2LI1",45.2521754147561,0.0357369939248627,0.2977941436270
7,0.120005697525121,0.904478634606462,0.99047717197517
"12266","SPICE1",17.7398659535402,-0.0439361413495299,0.36661313523553
2,-0.119843336549584,0.904607251710115,0.99047717197517
"12267","HOPX",1.6320946027458,0.151740010421265,1.25806094104283,0.12
0614197191024,0.903996623211963,0.99047717197517
"12268","ATXN1",45.3306926351409,0.0352429800531987,0.294555835609195,
0.119647875861328,0.904762092653262,0.99047717197517
"12269","XPO5",6.2069193476776,0.0709227265128837,0.590954237505107,0.
12001390634291,0.904472131913534,0.99047717197517
"12270","KLF14",2.51671257061317,0.115763771929206,0.963330528922647,0
.120170355297129,0.904348200614495,0.99047717197517
"12271","TUSC3",24.5443321585218,0.0439739465423768,0.364936894293227,
0.120497398947676,0.904089139972863,0.99047717197517
"12272","ABCC2",37.7178381077002,-0.0671917012996912,0.559363990459602
,-0.120121606763573,0.904386816596601,0.99047717197517
"12273","EHBP1L1",25.4283978688062,-0.0378265875927434,0.3150953707756
26,-0.120048058781794,0.904445077803681,0.99047717197517
"12274","CTDSP2",127.194343951734,0.0255748570222113,0.213624650115171
,0.119718660783871,0.904706017510609,0.99047717197517
"12275","USP16",99.3172827696012,-0.0233793297788233,0.193960028625103
,-0.120536844341327,0.904057894836264,0.99047717197517
"12276","CRYZL1",24.3575183995984,0.0389267311952572,0.325145219894527
,0.119721062508268,0.904704114895709,0.99047717197517
"12277","CRBN",94.6436589619803,-0.0271108980190805,0.227439441522443,
-0.119200512618236,0.905116500535312,0.990703750667377
"12278","LINC00513",5.39673781500994,0.0967213641156623,0.810981053562
229,0.119264640882564,0.905065695998831,0.990703750667377
"12279","IPO13",6.45799828786217,-0.0640278921946754,0.566516017200698
,-0.113020444701729,0.910014346536794,0.990730869809033
"12280","TM2D1",102.88262071341,-0.0256460139191574,0.222777721435532,
-0.115119293589592,0.908350567497866,0.990730869809033
"12281","SLC25A24",40.667262985798,-0.0314816130438858,0.2682768984253
26,-0.117347461628898,0.906584715865864,0.990730869809033
"12282","SEMA6C",11.9377220226367,0.051496205848263,0.458722416999849,
0.112260059547689,0.910617209430295,0.990730869809033
"12283","SHC1",37.6499070399757,-0.0309076338203931,0.270369872831004,
-0.114316116277171,0.908987207307058,0.990730869809033
"12284","GLRX2",30.3034558279528,0.0350079277665267,0.30815723713909,0

.113604107083571,0.909551631450738,0.990730869809033
"12285","PTPN14",27.8722239742682,0.0424395448787074,0.364309031325779
,0.116493254982626,0.90726163144817,0.990730869809033
"12286","RRP15",35.6025388128873,-0.0328067491375482,0.292170385340021
,-0.112286360232467,0.910596356364322,0.990730869809033
"12287","COMMD1",87.8414570786311,0.0379098272354057,0.324569763684824
,0.116800242896989,0.907018351266194,0.990730869809033
"12288","WDR92",18.6827979270855,-0.0438904939437584,0.380523263999033
,-0.115342472054139,0.908173675156265,0.990730869809033
"12289","LIMS2",67.5040040437062,0.0281051207946564,0.243951026141117,
0.115208044988499,0.908280222160603,0.990730869809033
"12290","ATG16L1",8.40967267907883,-0.0562471089050462,0.5007980338293
43,-0.112314955541965,0.910573684025456,0.990730869809033
"12291","ATG4B",27.1520349716172,-0.039511187172895,0.346871680727786,
-0.113907215169583,0.90931134591948,0.990730869809033
"12292","CRTAP",176.737204313064,-0.0246024149494751,0.212100318859825
,-0.115994238394967,0.907657108067926,0.990730869809033
"12293","ZNF717",8.36783025597801,0.0603912673854067,0.531729364631998
,0.113575197087719,0.909574549957858,0.990730869809033
"12294","THAP9-
AS1",13.11848182743,0.0489854252585916,0.429439711152943,0.11406822421
4937,0.909183711179354,0.990730869809033
"12295","AIMP1",75.5300623394161,-0.0246425496480784,0.211875923712819
,-0.116306511925722,0.907409624869788,0.990730869809033
"12296","SEC24B",20.7383130997591,0.0379831984260069,0.338552077516029
,0.1121930744147,0.910670320317805,0.990730869809033
"12297","SCRG1",1.71118248333636,0.13403849984241,1.167127076723,0.114
844820684613,0.908568122294379,0.990730869809033
"12298","ICE1",44.4804455674797,0.0355797961401633,0.312535174160566,0
.113842533838716,0.909362620622618,0.990730869809033
"12299","DAP",56.7035418032832,-0.0280183120494388,0.248252744686368,-
0.112862043418034,0.910139928968275,0.990730869809033
"12300","PLK2",8.98935971222976,0.067283446852579,0.575200144989558,0.
116973974083057,0.906880677545208,0.990730869809033
"12301","SGTB",17.1511180277597,0.0480247758679336,0.427944947674987,0
.1122218550046,0.910647500837104,0.990730869809033
"12302","TMED7",87.0168265412988,0.0215567189656511,0.188959433349951,
0.11408120030572,0.909173424902928,0.990730869809033
"12303","PPP2CA",44.6904454679084,-0.0304361756240893,0.26881433697916
,-0.113223781015998,0.909853142371637,0.990730869809033
"12304","GLO1",114.630378595042,0.0271216500882684,0.230510590778269,0
.117659019469336,0.906337838889247,0.990730869809033
"12305","Z94721.3",1.52540354518469,-0.144696935772545,1.2461487375699
2,-0.116115301015121,0.907561162385274,0.990730869809033
"12306","COX19",28.4305416855759,-0.041525475164042,0.351928917227167,
-0.117993927555654,0.906072469380125,0.990730869809033
"12307","ZNF394",25.085163212171,-0.0356189975044972,0.305449105842126
,-0.116611889913036,0.907167615224299,0.990730869809033
"12308","ZC3HC1",7.0024770802059,-0.0638972535348888,0.562559223446526
,-0.113583158664472,0.909568238380661,0.990730869809033

"12309","PEBP4",2.36330187354821,0.108506212771224,0.912996907117799,0
.118846199724556,0.905397205947924,0.990730869809033
"12310","TM2D2",58.007532377362,-0.0320974041586914,0.276180437443329,
-0.116218963427768,0.90747900795002,0.990730869809033
"12311","LACTB2",20.8335568522187,0.0419808679110157,0.363743771662634
,0.115413296890626,0.90811753999616,0.990730869809033
"12312","MAPKAP1",41.8524593083922,-0.0289256803334259,0.246092477966508,-0.117539880017635,0.906432243366592,0.990730869809033
"12313","PITRM1",18.2061261046186,0.0471732165898654,0.409844167931215
,0.115100372973424,0.908365564279804,0.990730869809033
"12314","AL135791.1",1.8168568109395,0.120801400096491,1.05591128290931,0.114404876670748,0.908916848355569,0.990730869809033
"12315","PRDM11",5.64549246041938,-0.0678034072185692,0.59009852464506
,-0.114901841619334,0.908522925363559,0.990730869809033
"12316","UQCC3",58.7318637898065,-0.0340790021004947,0.292811888651382
,-0.11638530886657,0.907347178092158,0.990730869809033
"12317","C11orf68",46.1810049460445,-0.0333906112046057,0.28072552432017,-0.118943980193705,0.905319737906632,0.990730869809033
"12318","ATG16L2",4.95335945161103,-0.0753887169348879,0.671237666029389,-0.112313001415488,0.910575233389746,0.990730869809033
"12319","MSANTD4",19.5857395413443,0.043152636679477,0.366164651663178
,0.117850361807102,0.906186224599197,0.990730869809033
"12320","TULP3",63.0144718285618,-0.0278896654828655,0.242777164952613
,-0.114877630638405,0.908542115858652,0.990730869809033
"12321","MANSC1",26.8849765044184,0.037003073770715,0.313614492723061,
0.117989042691948,0.906076339900789,0.990730869809033
"12322","CEP83",14.4920015337274,-0.0458577223827,0.398674033431747,-0
.115025606227627,0.908424825913649,0.990730869809033
"12323","ACTR6",27.6486435960386,0.0356942614597714,0.311783038279861,
0.114484295414851,0.908853894986572,0.990730869809033
"12324","MAPKAPK5",22.692918813217,-0.0390844163707727,0.34256582386881,-0.114093157132162,0.909163946620979,0.990730869809033
"12325","TINF2",40.8737733271243,-0.0340402917114578,0.288626676490683
,-0.117938827156736,0.906116128298201,0.990730869809033
"12326","CIDEB",7.53256161913615,0.0619740175571705,0.529996258012396,
0.116932934186341,0.906913199463671,0.990730869809033
"12327","STYX",11.5464438408767,-0.0569072388810201,0.47866547764674,-
0.118887284624729,0.905364655710664,0.990730869809033
"12328","AL133467.1",9.75395922704426,-0.0663156733917621,0.576045618532987,-0.115122259866585,0.908348216382406,0.990730869809033
"12329","AC078909.1",2.20839174739673,-0.117949364626288,1.00836765835092,-0.116970594653126,0.906883355556398,0.990730869809033
"12330","MVP",110.159285999266,-0.0230983775321071,0.200103049850491,-
0.115432411196957,0.908102390238936,0.990730869809033
"12331","LPCAT2",11.7618421228964,0.0687687597893212,0.594658860975698
,0.115644051240551,0.907934649254881,0.990730869809033
"12332","FHOD1",8.88401820653602,0.0573686087430055,0.488457896775887,
0.117448421085364,0.906504715074735,0.990730869809033
"12333","ATXN7L3",12.1312574154783,0.0561167771394061,0.48943931795242
,0.114655229118437,0.908718401805625,0.990730869809033

"12334","AC103702.1",4.68214120892404,-0.0899482893947362,0.7681732564
40136,-0.117093752796829,0.906785760227502,0.990730869809033
"12335","HEATR6",12.7676002780258,0.0473382025696398,0.408366985796033
,0.115920738493008,0.907715359556666,0.990730869809033
"12336","RFNG",68.5541938380805,0.0398224573519779,0.341287463966547,0
.116683035729321,0.907111233963197,0.990730869809033
"12337","HNRNPM",136.102454698317,-0.0273673340934121,0.23585961318032
8,-0.116032302963578,0.907626940633885,0.990730869809033
"12338","BISPR",1.7636104485428,0.14028358382152,1.24567510293857,0.11
2616510910902,0.910334594511488,0.990730869809033
"12339","AC011477.2",1.95921399862421,0.141522292181933,1.235215540251
28,0.11457295311646,0.90878361878959,0.990730869809033
"12340","PPP1R13L",2.33489612781905,-0.0996954223164283,0.881798601226
063,-0.113059174938371,0.909983641084083,0.990730869809033
"12341","FTL",6944.32626711804,0.0329117897720099,0.290869056444615,0.
113149848850549,0.909911755048932,0.990730869809033
"12342","RPL13A",3553.72814713452,-0.0244055092472754,0.21702722864558
2,-0.112453674129208,0.910463699370221,0.990730869809033
"12343","U2AF2",31.2963722678656,0.0361001129514785,0.32025511949286,0
.112722984752421,0.910250178191618,0.990730869809033
"12344","ZNF548",10.6308625694912,-0.0508949513461623,0.44980915977764
1,-0.113147876693578,0.909913318561862,0.990730869809033
"12345","ZNF552",4.05163176861425,-0.0901217682931239,0.76940254407864
,-0.117132142318355,0.906755339157166,0.990730869809033
"12346","ZHX3",49.9900638356576,0.0365757199214663,0.321142862166051,0
.113892364522037,0.909323118410453,0.990730869809033
"12347","MTFP1",2.26934138409503,-0.119048931125238,1.02054118319656,-
0.116652745705322,0.907135237984396,0.990730869809033
"12348","CBY1",64.5034250338737,-0.0339306306020731,0.288180997792175,
-0.117740693737699,0.906273122076903,0.990730869809033
"12349","ZBED4",6.88817876642352,-0.0670033049401148,0.571459967980846
,-0.117249341501312,0.906662467666427,0.990730869809033
"12350","TBL1X",28.1821617755903,-0.0391791471817127,0.34456261343793,
-0.113706901601414,0.9094701413228,0.990730869809033
"12351","ARAF",21.0226926330141,0.0394947970277329,0.345753387747525,0
.114228228637264,0.909056875144027,0.990730869809033
"12352","CCDC120",3.63128034854815,-0.0896499168679545,0.7761745896939
38,-0.11550225691272,0.908047031693101,0.990730869809033
"12353","TMEM185A",33.8621937133545,0.032955464119521,0.28373845899969
9,0.116147328901775,0.907535779569838,0.990730869809033
"12354","SERPINB9",4.96450185273838,-0.0766454231443613,0.683966251366
64,-0.112060241263681,0.910775641682272,0.990737092931265
"12355","DSCC1",9.97811048970508,0.0534199836232606,0.47696454804117,0
.111999904065511,0.910823482637531,0.990737092931265
"12356","AC012640.2",1.76713244678829,-0.140944884258622,1.25992007973
129,-0.111868114911449,0.910927978488106,0.990770565142575
"12357","CCDC149",15.4622286749848,-0.0481397440532906,0.4313626452831
84,-0.111599241565499,0.911141173409847,0.990838522914069
"12358","FICD",6.94713588558652,0.0699887440520527,0.626961647824589,0
.111631619405904,0.9111155000494,0.990838522914069

"12359","ZNF606",6.25432103076636,0.0762899725930358,0.684151392932628,0.111510366537467,0.911211645561052,0.990838522914069
"12360","SLC25A34",2.09616364119444,-0.114003419289619,1.02782777825175,-0.1109168497893,0.911682283848846,0.990846824143231
"12361","NFYC",26.9434069529405,0.0328660656474288,0.29713273120865,0.110610721052976,0.911925045488664,0.990846824143231
"12362","PHF20L1",66.9179440375985,0.031071795954923,0.279755335896849,0.111067750880647,0.911562621550024,0.990846824143231
"12363","UAP1L1",7.75282203504248,-0.0718201570826119,0.64805495721768,-0.110824176688595,0.911755773218583,0.990846824143231
"12364","OGFOD1",15.1050289203473,0.0442471047166311,0.397938542761181,0.111190799487813,0.911465047337131,0.990846824143231
"12365","LDHD",2.95174689096356,0.0943156638887401,0.849913670345831,0.110970875254145,0.911639442236405,0.990846824143231
"12366","USP10",26.6178618210228,0.0336366825731492,0.304208995153743,0.110570966371818,0.911956571755907,0.990846824143231
"12367","TMEM256",90.1429879858155,-0.0304974782352077,0.275370392218262,-0.110750752793477,0.911813998587113,0.990846824143231
"12368","LINC00910",4.99602729498781,0.0692321665256529,0.625903942889223,0.110611488092041,0.911924437212515,0.990846824143231
"12369","C18orf21",19.2718470061565,-0.0421884552465493,0.37959261987164,-0.111141400116829,0.911504219540102,0.990846824143231
"12370","JMJD7",4.89933904874631,0.0808372851893717,0.731830022113248,0.110459099444902,0.912045285237879,0.990852443893928
"12371","CDPF1",6.77909503512192,0.0611899148296019,0.554364434576082,0.110378500158282,0.912109203319576,0.990852443893928
"12372","ANKS3",23.0691773460834,0.0373913776935811,0.340090505263699,0.109945373701594,0.912452697629526,0.991065368418588
"12373","FITM2",9.23951717624296,-0.0546445571524402,0.496962707318858,-0.109957057838909,0.912443431218456,0.991065368418588
"12374","CLSTN3",14.1056986057656,0.0645992562281523,0.588224178457261,0.109820810830281,0.912551486216205,0.991092566935475
"12375","DHFR",15.4787531997635,0.0433302816744205,0.395671183112612,0.109510835066521,0.912797328316233,0.991199361283278
"12376","AC005332.3",4.56830033093951,-0.0805773403244856,0.735461117203801,-0.109560299572108,0.91275809740529,0.991199361283278
"12377","AP4B1",7.79319724179717,-0.0625502428084207,0.573243368583555,-0.109116382738065,0.91311018093121,0.991269236529628
"12378","LMAN2L",16.1950932505825,-0.0404755848262428,0.370840339342079,-0.109145582430574,0.913087021269629,0.991269236529628
"12379","TIMM44",34.5491729974838,-0.0358594361686789,0.328551070788756,-0.109144176832511,0.913088136114454,0.991269236529628
"12380","FAM207A",31.9547441797827,-0.0380851458396102,0.349518163346833,-0.108964711518633,0.913230479758414,0.991269236529628
"12381","KDM6A",19.0790162217284,0.0426067457328436,0.390979773614267,0.108974296391298,0.913222877407255,0.991269236529628
"12382","SPATS2L",90.8288590164295,-0.0231610662775606,0.212754621338111,-0.108862811683666,0.913311303266419,0.991276902325747
"12383","RAB1A",142.331875691561,-0.020705935143128,0.190724867377446,-0.108564422814099,0.913547980394999,0.991378243127571

"12384","FBXW2",37.446832468476,-0.0316479415762753,0.291527331239729,
-0.108559089268548,0.913552210945148,0.991378243127571
"12385","IFT88",20.7897473945537,0.0401996708725991,0.370850098435463,
0.108398706221713,0.913679427374923,0.991436239361453
"12386","KLHL26",4.65144260445088,0.0728073403625553,0.673501327295806
,0.108102742209709,0.91391419293511,0.991610918686819
"12387","PANK4",9.1484878974743,-0.0544794402250113,0.516541591817521,
-0.105469609975294,0.916003183193484,0.991658891040246
"12388","KIAA2013",46.6334499251824,-0.0285901633032005,0.268685636698
838,-0.10640748666162,0.915259053306408,0.991658891040246
"12389","EPHA2",5.42023859044833,-0.0691465439448227,0.647155089240306
,-0.1068469445647,0.914910404236238,0.991658891040246
"12390","TMEM69",12.9008191825375,0.0421959674644306,0.394597487822441
,0.106934201982091,0.914841179488825,0.991658891040246
"12391","FMO2",2.0842315250121,0.108701590546916,1.0103061041973,0.107
592728674326,0.914318765184638,0.991658891040246
"12392","SRSF7",200.659820131954,0.0214611636242128,0.202406845472936,
0.106029831027046,0.915558683851837,0.991658891040246
"12393","AC010913.1",1.9051797436938,-0.106697253372498,0.996970446524
294,-0.10702148067124,0.914771938511845,0.991658891040246
"12394","EIF2AK3",12.6359098264727,0.04788190069446,0.454904732002117,
0.105256985311459,0.916171894060372,0.991658891040246
"12395","SMPD4",21.2090345986369,0.0395806870324536,0.367625271721715,
0.107665849105214,0.914260760461813,0.991658891040246
"12396","WDR75",21.1749399325127,0.0365727677626386,0.342783117196044,
0.106693608663702,0.915032053211644,0.991658891040246
"12397","STK11IP",5.61086934002336,-0.0635996718316235,0.6024695843158
23,-0.105564950476045,0.9159275347851,0.991658891040246
"12398","ESPNL",1.56629891416758,0.11751726736557,1.11927770856438,0.1
04993842427454,0.916380694745658,0.991658891040246
"12399","PCDHGB7",9.19141064093654,-0.0566580028924586,0.5357649042266
37,-0.105751613152494,0.915779428534104,0.991658891040246
"12400","UBE2J1",58.7764444496671,0.0253242925942762,0.237790771408723
,0.106498214561691,0.915187071927419,0.991658891040246
"12401","TNFAIP3",44.689983878739,0.0425744540805427,0.398117640441765
,0.106939381116849,0.914837070697451,0.991658891040246
"12402","CDCA7L",6.47499749082356,-0.062902536441125,0.586222764834462
,-0.107301422282513,0.914549856240238,0.991658891040246
"12403","AC120053.1",4.01981802639785,-0.0797011570385195,0.7462220431
19147,-0.106806221785375,0.914942711442593,0.991658891040246
"12404","AGTPBP1",4.31677172797726,-0.075260637160124,0.71691989430061
6,-0.104977749618099,0.916393464381935,0.991658891040246
"12405","FSD1L",5.36318992978257,0.0702572495282902,0.665642163133894,
0.105548075857325,0.915940923982274,0.991658891040246
"12406","WDR31",4.65316070404848,-0.0715008637576732,0.669051093858918
,-0.10686906338539,0.914892856443153,0.991658891040246
"12407","ASB13",11.9405358596523,-0.0499874170972895,0.468925986638175
,-0.106599801507396,0.91510647606277,0.991658891040246
"12408","SPTY2D1",42.732285152153,0.0333622823475204,0.316449674673403
,0.105426818283041,0.916037136728129,0.991658891040246

"12409","FAR2",12.5526743274927,0.0504096436548454,0.467443231930086,0
.107841209822854,0.914121652778866,0.991658891040246
"12410","TMED8",16.0217641254112,-0.0488342812888904,0.461714501913232
,-0.105767267622163,0.915767007732749,0.991658891040246
"12411","FAM98B",17.0378190632773,-0.0384034202914542,0.36092823156699
6,-0.106401818790187,0.915263550085175,0.991658891040246
"12412","SCAPER",27.3645188281522,0.036606466703571,0.346975120552544,
0.105501704690748,0.915977717392241,0.991658891040246
"12413","BTBD1",26.0493014518021,-0.0355015565437265,0.334927588617649
,-0.105997707415661,0.915584171157334,0.991658891040246
"12414","UBE2I",184.965207777811,0.0234219433714523,0.222605702074169,
0.105217176169407,0.916203481760597,0.991658891040246
"12415","CDK3",2.8261382957574,-0.096477627278009,0.904350270257424,-0
.106681703374232,0.915041498351554,0.991658891040246
"12416","CYTH1",26.1537717660098,0.0348815889236596,0.330410664938511,
0.105570408661449,0.915923203982986,0.991658891040246
"12417","ELAC1",13.920597802579,0.0440299342750977,0.418971099265195,0
.105090624036643,0.916303899041734,0.991658891040246
"12418","MALT1",22.5272432975852,0.0456468231090132,0.431518706387803,
0.105781794469857,0.915755481644331,0.991658891040246
"12419","AP000547.3",2.80765116869841,-0.0951512794813891,0.8888231096
58506,-0.10705311152176,0.914746844905597,0.991658891040246
"12420","LINC01004",3.02270027813246,0.101202758067823,0.9696428246097
99,0.10437117204322,0.916874799089985,0.991677262858455
"12421","CRADD",9.48758158594912,0.0491337333035966,0.469565513146415,
0.104636588352425,0.916664180748941,0.991677262858455
"12422","CEP170B",5.67448654463338,0.0616274537234543,0.59019594461653
4,0.104418632973656,0.916837136535358,0.991677262858455
"12423","SPECC1",3.03174348723375,-0.094316670417526,0.904235586865982
,-0.104305417512289,0.916926978813837,0.991677262858455
"12424","CEP250",8.48469533187079,0.0543448109109188,0.519290507706829
,0.104652039858968,0.916651919547278,0.991677262858455
"12425","KCNJ15",7.66666964642989,-0.0630406383560574,0.60290844492303
9,-0.104560881319393,0.916724256678945,0.991677262858455
"12426","RGN",6.29734658871082,-0.071630159285672,0.683319650452827,-0
.104826722366617,0.916513305482192,0.991677262858455
"12427","NAA50",51.3260192123147,-0.0244623695123088,0.234864552776191
,-0.104155221480441,0.917046168709909,0.991726358839017
"12428","LINC02767",8.1828415173694,0.0564067869117658,0.5431518054562
39,0.103850868845746,0.91728769717609,0.991907737556282
"12429","PPP4R3B",63.8206919898246,-0.0250249027439097,0.2416915906436
05,-0.103540643169547,0.917533894232809,0.992063233364789
"12430","NEDD9",28.8807664504442,-0.0410581571852612,0.396812435569976
,-0.103469935679525,0.917590009251125,0.992063233364789
"12431","MEPCE",18.230991788737,-0.0384069931415614,0.371573456284976,
-0.103363123742901,0.917674778330306,0.992063233364789
"12432","WSB2",34.0677815981428,0.0314071086448474,0.304487446555612,0
.103147466340985,0.917845933207934,0.992063233364789
"12433","RBM23",40.4170943563132,0.0277280469159469,0.268913022107947,
0.103111581204187,0.917874413546974,0.992063233364789

"12434","ERAL1",17.0115188978382,0.0451740130091239,0.437356247724751,
0.103288825172,0.917733744402798,0.992063233364789
"12435","ELK4",41.1165273087669,-0.0291172876204268,0.283070463030115,
-0.102862330844208,0.918072234697304,0.992197246650347
"12436","FBXO22",30.346400982129,-0.0300873910962998,0.2931830113826,-
0.102623241893904,0.918261995864106,0.992280881640307
"12437","PLEKHF1",51.5396752549865,0.0370422038108198,0.36110984705491
1,0.10257876962626,0.918297293322457,0.992280881640307
"12438","AC007364.1",3.31143300534124,0.0906899935545297,0.88976942386
7043,0.101925275382447,0.918815987583135,0.992425285752799
"12439","ZNF142",5.80960235725553,0.0597384997590934,0.585432881384611
,0.102041586078673,0.918723666423566,0.992425285752799
"12440","SMIM29",22.0632906832616,-0.0395590919616445,0.38758682628620
3,-0.10206510974765,0.918704994736624,0.992425285752799
"12441","TMEM181",28.5412792279601,0.0348507848113826,0.34217104805519
6,0.101851939284357,0.918874198386433,0.992425285752799
"12442","SCRIB",16.2213875817197,-0.0427391904025884,0.418896138774973
,-0.102028131669047,0.918734345752489,0.992425285752799
"12443","SIX4",1.49348808405377,0.130107386336815,1.2785817975145,0.10
1759141722288,0.91894785742301,0.992425285752799
"12444","TMEM208",86.8108431396362,-0.0291326410974538,0.2858569593547
86,-0.10191335261947,0.918825451291417,0.992425285752799
"12445","PEX14",9.31120641235439,-0.0459080263491444,0.458317727059987
,-0.100166377250199,0.920212238798748,0.99273547123168
"12446","ANP32E",49.0707555602795,-0.0249818767968287,0.25074819966956
3,-0.0996293366403024,0.920638602422831,0.99273547123168
"12447","TCHH",1.45857976628769,-0.139664511831724,1.3948399083109,-0.
100129420587667,0.920241578447169,0.99273547123168
"12448","UCHL1",14.2003568128072,0.0433475971001116,0.432374710715278,
0.100254700438889,0.920142120047108,0.99273547123168
"12449","TBCA",237.358068986362,0.0221029154563671,0.219566812913227,0
.100666012149578,0.919815592653498,0.99273547123168
"12450","SYNE1",162.446928262938,0.0275859910174522,0.275383799745926,
0.100172889773848,0.920207068560821,0.99273547123168
"12451","DNAJB9",28.6200003065728,0.0340977385212094,0.341203929280187
,0.0999336044961231,0.920397037349705,0.99273547123168
"12452","LYPLA1",31.4943576385864,-0.0301064974510529,0.30178616263934
9,-0.0997610267738874,0.920534049772847,0.99273547123168
"12453","IARS",32.6955943064713,-0.0343361626656602,0.34190578736442,-
0.100425801301406,0.920006286830447,0.99273547123168
"12454","SPOUT1",10.0134823779666,0.050203673939716,0.503609540479062,
0.099687694343438,0.920592270365549,0.99273547123168
"12455","CCAR1",61.388718765143,0.0262300123911366,0.259794212355886,0
.100964575589562,0.919578581021178,0.99273547123168
"12456","TNFRSF1A",195.200182622895,-0.0259925517271384,0.260367462257
654,-0.0998302610539593,0.920479083203047,0.99273547123168
"12457","DNAAF4",10.6634636869881,0.0431498760597698,0.431010131992985
,0.100113368240893,0.920254322332259,0.99273547123168
"12458","MESP1",5.32099747974564,0.0634985107143988,0.627497925255875,
0.101193180341602,0.91939711022936,0.99273547123168

"12459","LIN37",17.3121784867237,-0.0430840373073307,0.431145820629013
,-0.099929154466751,0.920400570274931,0.99273547123168
"12460","TCFL5",8.83731303830123,-0.0510715534686029,0.510379488790873
,-0.100065842358977,0.920292053033811,0.99273547123168
"12461","PEX26",24.7192445354941,0.033530062922992,0.335151521838232,0
.100044489546361,0.920309005046995,0.99273547123168
"12462","TBX1",7.05311099574097,-0.0723437647529803,0.726020979243747,
-0.0996441794675638,0.920626818199378,0.99273547123168
"12463","GTPBP1",13.8794750034377,0.0391656062338795,0.38984538613677,
0.100464460082488,0.919975596796363,0.99273547123168
"12464","SQSTM1",419.424613145001,0.019063721283921,0.191670334264612,
0.0994609904399837,0.92077225939406,0.992799935333511
"12465","AL356512.1",2.17205661993953,0.0999728673727876,1.02316481217
906,0.0977094463988388,0.922163014312192,0.992802370845449
"12466","ACTR2",164.718108414079,-0.0200399892144663,0.202846977347759
,-0.0987936299396265,0.921302126322914,0.992802370845449
"12467","TIGD1",3.82524844122331,0.0852215522892276,0.872852410096582,
0.0976356956839906,0.922221578850765,0.992802370845449
"12468","ZNF197",14.0354463776784,-0.0398248573618972,0.40236987983219
6,-0.0989757418684166,0.921157530698862,0.992802370845449
"12469","SMIM14",370.713050814259,-0.0223146227177191,0.2250309742609,
-0.0991624499294376,0.921009288483101,0.992802370845449
"12470","UFSP2",56.6043901002697,0.02862617742974,0.289245645344453,0.
0989683955160327,0.921163363601919,0.992802370845449
"12471","EPB41L4A-
DT",2.73971623672862,0.0884427172114774,0.905555514384328,0.0976668087
230501,0.922196872316408,0.992802370845449
"12472","TRIM27",27.9317439141799,-0.0288507401321089,0.29225191502652
1,-0.0987187376667517,0.921361591029772,0.992802370845449
"12473","AL009176.1",3.09698158818493,-0.0896083954679521,0.9178006578
20585,-0.0976338322536583,0.922223058583412,0.992802370845449
"12474","AC072061.1",1.90973112696175,0.105507074062621,1.078688987219
36,0.0978104674402924,0.922082795426571,0.992802370845449
"12475","BET1",37.828919665404,0.0326935761671727,0.333414259331612,0.
0980569224385087,0.921887093532197,0.992802370845449
"12476","CLYBL",6.97517698067485,-0.0530188363269617,0.542299577868794
,-0.0977666929694519,0.922117555803149,0.992802370845449
"12477","MIPOL1",10.9970053002065,-0.0469197038835721,0.48054477010046
7,-0.0976385693964845,0.922219296863003,0.992802370845449
"12478","ZNF839",7.17326030211367,-0.0548192312806408,0.55605087278805
4,-0.0985867192434671,0.921466415155152,0.992802370845449
"12479","AC091057.2",1.53448587560665,0.115921557176818,1.187752873386
4,0.0975973704414747,0.92225201262256,0.992802370845449
"12480","ANKRD12",271.949199958818,0.0180384763886651,0.18437163869862
3,0.0978375877981492,0.922061259801682,0.992802370845449
"12481","ZNF433-
AS1",6.34003766775869,0.0601835644196352,0.609797526544222,0.098694340
0061017,0.921380962946184,0.992802370845449
"12482","MAU2",28.8084492585346,0.0312299268965575,0.319401282181398,0
.097776460643076,0.922109799488304,0.992802370845449

"12483","TSKS",7.93723952356584,-0.0527325449046879,0.539540459614248,
-0.0977360343696741,0.922141901233966,0.992802370845449
"12484","GCNA",2.78378829674866,-0.0947457749033543,0.968347468620118,
-0.0978427454747888,0.922057164220989,0.992802370845449
"12485","NPHP4",7.5832878709266,-0.0513502537235222,0.530239457755938,
-0.0968435165893634,0.922850665086338,0.992836889286918
"12486","TRIT1",11.6335760621171,-0.0416630614089745,0.435329370162887
,-0.095704687679091,0.923755117763506,0.992836889286918
"12487","ATG7",21.799895013531,-0.0344633029536693,0.358284110267754,-
0.0961898726904583,0.923369773940594,0.992836889286918
"12488","ZNF518B",12.0869378596822,-0.0419401483962882,0.4342832030257
76,-0.0965732685585791,0.923065285845041,0.992836889286918
"12489","SDAD1",37.2280241531941,-0.0272046987139156,0.281907832059836
,-0.096502103241109,0.92312180357934,0.992836889286918
"12490","IBTK",44.0291892100684,0.0255933314380327,0.26455981331305,0.
0967393010961513,0.922933428422555,0.992836889286918
"12491","UBAP2",27.2114520232562,0.0330139531937369,0.345394410551576,
0.0955833452574274,0.923851493188126,0.992836889286918
"12492","GLRX3",52.6798367564109,-0.0265345875067808,0.272698443819407
,-0.0973037731170667,0.922485159707556,0.992836889286918
"12493","MTG1",34.9032894496189,-0.0251589979410118,0.262684422882893,
-0.0957765126112097,0.923698071808245,0.992836889286918
"12494","BIRC3",6.54150112983008,-0.0671124448060694,0.703254028711907
,-0.0954312980317991,0.923972257280128,0.992836889286918
"12495","MAGOHB",27.0013477802939,-0.0355412515197599,0.36557958397364
7,-0.0972189177892437,0.922552544983967,0.992836889286918
"12496","CORO1C",43.2456350839548,0.0283031983872562,0.29377932782436,
0.0963416949615243,0.923249197289032,0.992836889286918
"12497","KNSTRN",2.87148391682928,0.0777329011153291,0.809375895986367
,0.0960405437088016,0.923488372149259,0.992836889286918
"12498","ASB7",11.1018705448259,-0.0452947394740467,0.467540697169188,
-0.0968787097001226,0.922822716462661,0.992836889286918
"12499","AC009054.2",1.65411217264816,0.108500357247858,1.133911420023
42,0.095686801748251,0.923769323476778,0.992836889286918
"12500","KARS",38.9971681323071,0.025792203985752,0.269738563196509,0.
0956192680798184,0.9238229616049,0.992836889286918
"12501","FAM117A",17.4945374287643,-0.0343852405017823,0.3603663721721
64,-0.0954174505643242,0.923983255771373,0.992836889286918
"12502","AP001033.2",4.24922522962187,-0.0729871153934042,0.7551633848
33974,-0.0966507604304077,0.923003744159575,0.992836889286918
"12503","MPND",10.6339703479957,0.0470230601654995,0.489128681239327,0
.0961363787671479,0.923412259024115,0.992836889286918
"12504","BRD4",101.44240305511,0.0232089542401319,0.24117213981409,0.0
962339773492195,0.923334746012189,0.992836889286918
"12505","DMAC2",22.4671150115101,-0.0341464064955445,0.351986308072969
,-0.0970106101072141,0.922717968547126,0.992836889286918
"12506","PRKD2",10.8411487830662,0.0419044484616652,0.437076921263973,
0.0958743105000435,0.92362039782367,0.992836889286918
"12507","SUV39H1",1.94529555892932,0.11001055912088,1.13525900719648,0
.0969034893566279,0.922803037745215,0.992836889286918

"12508","EPB41L1",16.2885881162925,0.0436066891610313,0.45851202429176
,0.0951047886440673,0.924231594531585,0.993024336337541
"12509","USF3",24.5708795080561,0.0337484221712128,0.355631314242332,0
.0948972174824183,0.924396466673968,0.993042695094441
"12510","C15orf40",50.6251602119557,0.0289087496910249,0.3045733555262
51,0.0949155570127776,0.924381899598238,0.993042695094441
"12511","HNRNPA1",419.646425514946,0.0221626806813774,0.23449422337076
6,0.0945126935870624,0.924701899557468,0.993291409811591
"12512","MXRA8",832.558963381231,0.0174955044292759,0.200608832572268,
0.0872120345098626,0.930502973650734,0.993363403513347
"12513","DFFA",45.4621989467159,-0.0204355140398325,0.236614140527365,
-0.0863664107068408,0.931175147503565,0.993363403513347
"12514","DNALI1",9.59113779600327,0.0453349002138531,0.48654732390247,
0.0931767538052283,0.925763141809885,0.993363403513347
"12515","PPIE",49.1949947117374,0.0266543037282728,0.285147799240814,0
.0934753969668992,0.925525894479061,0.993363403513347
"12516","DHCR24",8.23772523252863,-0.0519264173263927,0.57457250406487
,-0.0903740032094021,0.927990014574133,0.993363403513347
"12517","LINC02609",24.3606131345667,-0.0360806146241446,0.39725078427
7658,-0.0908257857558467,0.927631020658368,0.993363403513347
"12518","DR1",42.732901107099,0.0239698131149118,0.275430134155729,0.0
87026836001028,0.930650180983269,0.993363403513347
"12519","FAM189B",10.8049177610178,-0.0440051203946699,0.4829057407705
84,-0.0911256932345635,0.927392717324585,0.993363403513347
"12520","OLFML2B",77.1171041472499,-0.0285688495734038,0.3089490111019
52,-0.0924710827573298,0.926323765377579,0.993363403513347
"12521","EPAS1",18.3518592899476,0.0367365237259764,0.406590826780307,
0.0903525640676252,0.928007050837172,0.993363403513347
"12522","PNPT1",26.9919562762178,-0.0318642208111506,0.350426001168109
,-0.090929955839277,0.927548247466926,0.993363403513347
"12523","AFTPH",22.8774633805026,-0.0324524131576294,0.34923930683611,
-0.0929231404438061,0.92596462186744,0.993363403513347
"12524","NAGK",64.9707486419825,0.0233101511176501,0.271831485774241,0
.085752211710344,0.931663396006417,0.993363403513347
"12525","THNSL2",20.2502604560022,0.0465082437159175,0.544556330131138
,0.0854057535328212,0.931938819219903,0.993363403513347
"12526","MTLN",70.7468012019219,-0.0295347912770688,0.338524175487117,
-0.0872457372787923,0.930476184842258,0.993363403513347
"12527","CMC1",56.1330303124645,0.0262703409756776,0.294643172603682,0
.0891598496701405,0.928954873570939,0.993363403513347
"12528","SUSD5",1.75441640142398,-0.11720838778536,1.26790904653264,-0
.0924422679259925,0.926346658230458,0.993363403513347
"12529","CCR1",3.61663974677324,0.0728776241050334,0.847598140885347,0
.0859813402008056,0.931481250649354,0.993363403513347
"12530","FOXP1-
IT1",2.70389057098757,0.0878160498083662,0.987149571267003,0.088959213
8460382,0.929114324192234,0.993363403513347
"12531","GTF2E1",8.35289941901822,-0.0446622647863605,0.52149502289528
9,-0.0856427440829636,0.931750418400013,0.993363403513347
"12532","MFN1",34.7888754948153,0.0255234582861375,0.278038411420367,0

.0917983172028289,0.926858281095763,0.993363403513347
"12533","SORCS2",12.6510037071245,0.0536511702771714,0.590270730874514
,0.0908924794520721,0.927578025985569,0.993363403513347
"12534","COX18",9.94945034333869,-0.0415046843952769,0.476577179156188
,-0.0870891142306977,0.930600678094695,0.993363403513347
"12535","THAP6",17.9358148201801,-0.0336482754261895,0.369423468196236
,-0.0910832102532147,0.927426473460092,0.993363403513347
"12536","COPS4",51.4783754714856,0.0265209661747778,0.299636647663979,
0.0885104221447545,0.929471001177208,0.993363403513347
"12537","TRMT10A",16.4183435756719,-0.0384854549549752,0.4445375295424
49,-0.0865741414332043,0.931010020854847,0.993363403513347
"12538","ZBED3",15.8169228511366,-0.0361421525398338,0.386246881647579
,-0.0935726714107451,0.925448619394988,0.993363403513347
"12539","RGMB",8.9302907445046,0.0467173345640767,0.520688958073078,0.
08972215338878,0.928508011081922,0.993363403513347
"12540","PCDHGB2",2.32375141256886,-0.0934946673849082,1.0762280727380
1,-0.0868725410098718,0.93077282608333,0.993363403513347
"12541","ABHD16A",35.2703166904405,0.0242651301608753,0.28316256983423
8,0.085693282749482,0.931710242076293,0.993363403513347
"12542","TMEM30A",90.6418295766713,0.0198080552766066,0.21679664182003
9,0.0913669838716833,0.927200995044338,0.993363403513347
"12543","BVES",1.90341929035803,0.0958663857292557,1.02113325562767,0.
0938823461099876,0.925202617654839,0.993363403513347
"12544","AGPAT5",6.66521225399563,-0.052481855299484,0.593299467512048
,-0.0884576140267955,0.92951297133616,0.993363403513347
"12545","CLDN23",1.8971272477245,-0.101988669996809,1.12474011542279,-
0.0906775428370591,0.927748815323837,0.993363403513347
"12546","C8orf48",4.44832446321069,-0.0638099509245105,0.6965382962710
43,-0.091610111412568,0.927007817490706,0.993363403513347
"12547","AC026979.4",3.40889383000258,0.081577803366437,0.890299302113
931,0.0916296386762724,0.926992302244381,0.993363403513347
"12548","PLPP5",31.5207974306855,-0.0298488167221704,0.348040786825704
,-0.0857624101887759,0.93165528866479,0.993363403513347
"12549","GOLGA7",71.5791132907769,-0.0209129482849134,0.23792470963947
6,-0.0878973365843443,0.929958273510328,0.993363403513347
"12550","FAM91A1",23.9872563191011,-0.0321788328516055,0.3475926304660
62,-0.0925762804822968,0.926240188240285,0.993363403513347
"12551","DCAF12",20.385829839992,0.0309412530159097,0.345785833862285,
0.0894809734404348,0.928699673915531,0.993363403513347
"12552","UNC13B",8.05900244247403,0.0449976236899576,0.508027943422571
,0.0885731272709328,0.929421165455723,0.993363403513347
"12553","ZBTB5",4.20086370527231,0.0684754309476789,0.763622583551345,
0.0896718253528115,0.928548005830872,0.993363403513347
"12554","TRMT10B",18.9630785868749,-0.0369785635268352,0.3932280736993
4,-0.0940384626635503,0.925078603311122,0.993363403513347
"12555","ELP1",16.6709784246539,0.0370096297795227,0.393563947277639,0
.0940371444984372,0.925079650414639,0.993363403513347
"12556","WDR5",11.3497614999612,-0.0403322090010677,0.431899013964126,
-0.0933834245901236,0.925598958232817,0.993363403513347
"12557","PDSS1",2.17372445621999,0.0828117439804935,0.937307137483576,

0.0883507024205761,0.929597941771991,0.993363403513347
"12558","FAM149B1",17.867494471575,0.0298130972772184,0.345380869799446,0.0863194805622271,0.931212453023618,0.993363403513347
"12559","HECTD2",14.9472216429647,-0.0440573837562476,0.473394012241556,-0.0930670490478589,0.925850294839625,0.993363403513347
"12560","XPNPEP1",26.5770615728095,-0.0270425764679705,0.295906036648045,-0.0913890665236251,0.927183449044476,0.993363403513347
"12561","TSPAN4",409.763222317043,0.0197252163223358,0.22607117380658 5,0.0872522400366341,0.930471016102881,0.993363403513347
"12562","RTN4RL2",4.2485293188183,-0.063190053397587,0.715553441014677,-0.0883093417984009,0.929630814284546,0.993363403513347
"12563","THAP12",76.1184191487088,0.0199503508748376,0.22877968649704,0.0872033316432388,0.930509891179048,0.993363403513347
"12564","MTMR2",18.9193607002976,0.0318817113883123,0.360566046386848,0.0884212801171708,0.929541848448884,0.993363403513347
"12565","CEP164",33.4563088780837,-0.0315469047086262,0.343840126269085,-0.0917487585027758,0.926897657048084,0.993363403513347
"12566","LDHB",135.22408791569,-0.0253063899336969,0.288091364529423,-0.0878415428210876,0.930002618965253,0.993363403513347
"12567","STK38L",12.1651249148331,0.0364796129884065,0.423699062650922,0.0860979317730079,0.931388567727156,0.993363403513347
"12568","AC023157.3",3.37213253331074,-0.0735204160259749,0.821186909328854,-0.0895294544893101,0.928661146270655,0.993363403513347
"12569","RASSF9",5.74899219707937,-0.0543234891830853,0.630573522234135,-0.0861493343244353,0.931347706246866,0.993363403513347
"12570","RFC3",9.8490123569256,0.0470410042405217,0.520414790162254,0.0903913669053398,0.927976216821604,0.993363403513347
"12571","COG6",25.9940779360687,0.0310870641077751,0.342080967862601,0.0908763334657701,0.927590855523359,0.993363403513347
"12572","ARHGAP5-AS1",2.6762826899074,-0.0880239129285886,0.953158534646595,-0.0923497086045874,0.926420195319306,0.993363403513347
"12573","SNX6",178.335424013016,0.0159119891515652,0.177757251304886,0.0895152745373704,0.928672414989602,0.993363403513347
"12574","NEMF",76.8993409263405,-0.0245704450867949,0.266447463269086,-0.0922149709565112,0.926527243623279,0.993363403513347
"12575","PIGH",31.0748888378328,-0.0279800774055479,0.304125785990203,-0.092001660807707,0.926696720056286,0.993363403513347
"12576","PTGR2",10.5115116163015,0.0425297989940038,0.473643148329148,0.0897929150754834,0.928451778398375,0.993363403513347
"12577","LIN52",6.39223723315418,0.051996954039628,0.574595431481462,0.0904931560377462,0.92789533233143,0.993363403513347
"12578","AC127502.2",12.1026681065652,-0.0487062729462907,0.521393847483738,-0.0934155114820564,0.925573468021394,0.993363403513347
"12579","TIPIN",7.47225815403234,-0.0455108904166645,0.504892408093764,-0.0901397796581894,0.928176138272607,0.993363403513347
"12580","ARRDC4",8.68099703534431,0.0492467040383298,0.56264625889772,0.0875269376798293,0.930252674420179,0.993363403513347
"12581","FAM234A",48.9202287574325,0.026829022303742,0.289880947183488,0.0925518650481014,0.926259585660427,0.993363403513347

"12582","CLUAP1",39.8810667024042,0.0226111844852292,0.259332303826223
,0.0871900035268297,0.930520485126442,0.993363403513347
"12583","TFAP4",8.06808788380423,-0.0465256961456675,0.518078890306838
,-0.0898042692264726,0.928442755549204,0.993363403513347
"12584","KIFC3",2.61936644918197,-0.0798534649425185,0.902233333475044
,-0.0885064450400593,0.929474162042659,0.993363403513347
"12585","NFATC3",27.6920960854968,0.0337132054933819,0.382124474749406
,0.0882257162813002,0.929697278372479,0.993363403513347
"12586","MLKL",5.71510442085996,-0.053568043247557,0.626535909602867,-
0.0854987598101302,0.931864881390881,0.993363403513347
"12587","AKAP10",17.7789036212299,0.0360358169564632,0.395594857708094
,0.091092733523482,0.927418906446807,0.993363403513347
"12588","STAT3",150.313913489992,-0.0159953630665243,0.187214884721385
,-0.0854385221043122,0.931912768900057,0.993363403513347
"12589","MAFG",45.7749238555295,-0.0258348550614142,0.288540564820589,
-0.0895363016894279,0.928655704848752,0.993363403513347
"12590","RAB40B",32.0230650300766,-0.0280377796994344,0.30882032918670
3,-0.0907899417543967,0.927659502360275,0.993363403513347
"12591","PPP4R1",28.5498337706268,0.0308395727947984,0.355703950617813
,0.0867001132296507,0.930909886413659,0.993363403513347
"12592","NAPG",36.5376427212452,0.0274819808722502,0.301403151442093,0
.0911801377681687,0.92734945696764,0.993363403513347
"12593","TAF4B",7.14542104271611,-0.053872758009157,0.601849440058558,
-0.0895120181617438,0.928675002812926,0.993363403513347
"12594","PRX",2.2200375480973,0.0847121241161695,0.954553620807721,0.0
887452755608304,0.929284349767983,0.993363403513347
"12595","SIRPA",60.5192533485543,0.023245954367328,0.256301675926267,0
.0906976292032343,0.927732854490363,0.993363403513347
"12596","NOP56",55.7685527099823,0.0222343622256803,0.257323337677031,
0.0864063183168676,0.931143424427155,0.993363403513347
"12597","KIZ",34.124606437274,-0.0244429127410937,0.28079536966201,-0.
087048845465348 4,0.930632686363153,0.993363403513347
"12598","PROCR",493.425428414979,0.0282716340008288,0.328413483425978,
0.0860854849986725,0.931398462080805,0.993363403513347
"12599","NELFCD",44.4240503908125,0.025433525695196,0.292848101801596,
0.0868488664899291,0.930791644492809,0.993363403513347
"12600","APOBEC3D",6.37606945136348,-0.0553718636612434,0.601115413988
991,-0.092115195139976,0.926606515805889,0.993363403513347
"12601","RANGAP1",16.9249469307683,-0.0361618205547534,0.4184759770057
94,-0.0864131337083962,0.931138006795037,0.993363403513347
"12602","PRR5",19.6360930867928,0.0309963134640538,0.343294621647028,0
.0902907051539068,0.928056206195234,0.993363403513347
"12603","RABL2B",22.1379982393247,-0.0342884343588186,0.36381669635854
9,-0.0942464562567151,0.924913382220298,0.993363403513347
"12604","TRAPPC2",23.4196633151187,0.0331859871463353,0.35796871096473
8,0.0927063906141349,0.926136819903149,0.993363403513347
"12605","CFP",1.51864996174557,0.0965183397339666,1.1028542219266,0.08
75168610819266,0.930260683647578,0.993363403513347
"12606","DIAPH2",64.6054227843098,-0.0215211408181892,0.24106328083904
9,-0.0892758977778879,0.928862648357354,0.993363403513347

"12607","MCTS1",74.8175853933929,-0.0255434614186041,0.281405751294148,-0.0907709288141164,0.927674610111199,0.993363403513347
"12608","RTL8B",14.1211789426869,-0.0359961994676592,0.414810162239582,-0.0867775255874009,0.930848352204637,0.993363403513347
"12609","IDUA",22.7922064173851,0.0344446683578686,0.405469372534051,0.0849501113798082,0.932301052617188,0.993513111261786
"12610","AP003469.4",4.22686444284297,0.0671891554938286,0.790040937149348,0.0850451569462499,0.932225490673095,0.993513111261786
"12611","PRH1",23.244788799991,-0.0298351846621895,0.351002796156251,-0.0849998489724514,0.932261510778762,0.993513111261786
"12612","MFN2",13.148247464964,-0.03720025580169,0.440855320248076,-0.0843820049188856,0.932752714248259,0.993517559863769
"12613","OSBPL9",53.1073790265602,-0.0201846022287846,0.239806732382337,-0.0841702900842799,0.932921039420952,0.993517559863769
"12614","LRRC8C-DT",4.99859657110247,-0.0530366655895743,0.630578269213772,-0.0841079817985203,0.932970578575843,0.993517559863769
"12615","EIF2AK1",63.1925196673561,0.019664059813522,0.233615671233318,0.084172691453918,0.932919130180518,0.993517559863769
"12616","LRP10",192.806662907089,-0.0175730357925083,0.208243330947189,-0.0843870279666477,0.932748720679937,0.993517559863769
"12617","PABPN1",97.8772969882324,0.0164068858911123,0.193385950288561,0.0848401130828314,0.932388502856713,0.993517559863769
"12618","EPOR",5.53697203224977,-0.0509439624083901,0.605072021072256,-0.0841948737244727,0.932901493893554,0.993517559863769
"12619","ANO8",6.67209214728715,-0.0491213230725003,0.581928436211166,-0.0844112781157777,0.932729440650695,0.993517559863769
"12620","NDUFA6-DT",4.745318953516,-0.0548899950292184,0.647928475369431,-0.0847161332088725,0.932487069630729,0.993517559863769
"12621","AC016949.1",2.70998135121649,0.0714452869831519,0.852574408970547,0.0837994739596038,0.933215866269633,0.993595690370955
"12622","MBD3",29.4073520271314,0.0251091360234516,0.29973011986764,0.0837724818397953,0.933237327403385,0.993595690370955
"12623","ZCCHC12",3.96537848401241,-0.152526600223413,1.82150162781596,-0.08373673561099,0.933265748906359,0.993595690370955
"12624","IKBKB",20.4289825318462,0.0367178571930526,0.44079947149361,0.08329832399444,0.933614332923525,0.993809348131426
"12625","CCSER2",84.8816199725308,0.0170773756617532,0.204975701542138,0.0833141466684651,0.933601751987439,0.993809348131426
"12626","BTF3L4",73.434537275029,-0.0232012053303711,0.279268011822307,-0.0830786353903419,0.933789013598301,0.993811957539176
"12627","LY96",66.8250766787117,0.0295233537263867,0.355336397356532,0.0830856448875515,0.933783440097871,0.993811957539176
"12628","SART3",22.9166145542942,-0.0276653246053459,0.333251995701353,-0.0830162308469369,0.93383863381239,0.993811957539176
"12629","HTR2A",3.27354840966171,-0.0642721844599387,0.775741199548991,-0.0828526117954107,0.933968734732314,0.993871710037815
"12630","TMED5",57.2280523541597,-0.0229412173985903,0.279318905075456,-0.0821327055982584,0.93454118566710,0.993980696230852

"12631","TPST1",142.958058790554,-0.0211411703288109,0.256490420343957
,-0.0824247950487209,0.934308919533919,0.993980696230852
"12632","CSTF3",25.8825835231624,-0.0263581045609987,0.319264109749579
,-0.0825589339862637,0.934202255683668,0.993980696230852
"12633","AC087239.1",5.95337805770217,0.0566232227540768,0.68885917653
218,0.0821985460644171,0.934488829607168,0.993980696230852
"12634","ZNF891",10.5407747545639,0.0417641104203028,0.508867162274233
,0.0820727166470133,0.934588888873654,0.993980696230852
"12635","GTPBP6",42.9905129578182,-0.0240444076653853,0.29140276846377
8,-0.0825126260541143,0.934239078432793,0.993980696230852
"12636","ELK1",9.925861971237,-0.0418294261501102,0.5089586641547 9,-0.
0821862934970857,0.934498572775383,0.993980696230852
"12637","SLC31A2",30.6756995133634,0.0257808288560729,0.31488133521644
7,0.0818747444600086,0.934746317997655,0.994017004500661
"12638","KLRG1",2.2287737095956,-0.0760913452728999,0.929714689451963,
-0.0818437593126052,0.934770957874794,0.994017004500661
"12639","KCTD3",39.9159946951295,0.0215959129318372,0.264272465657586,
0.0817183616844092,0.934870676681276,0.994044388315505
"12640","AKNAD1",5.66034508891499,0.0494506670944269,0.610097792965812
,0.0810536731399025,0.935399267886397,0.994208669110626
"12641","AC093690.1",2.83740068116055,-0.0789018577697306,0.9943575107
75026,-0.0793495869591537,0.936754565917217,0.994208669110626
"12642","TET3",2.5215662976249,-0.0721704056094586,0.899750612540503,-
0.0802115659645741,0.936068991493853,0.994208669110626
"12643","ITGA9",15.4041820420405,-0.0324742317345697,0.411255776203885
,-0.078963588145374,0.937061585046146,0.994208669110626
"12644","ADRA1B",9.86573740535876,-0.0453662307263048,0.56257926159588
1,-0.0806397139446865,0.935728481872499,0.994208669110626
"12645","UHRF1BP1",9.50478454250796,0.0406055295188218,0.5035811471533
36,0.0806335379081572,0.935733393641892,0.994208669110626
"12646","GNA12",46.0788137992464,-0.0201672063393535,0.254132288207705
,-0.0793571194025949,0.936748574789546,0.994208669110626
"12647","PILRB",16.409703521462,0.0319328847695725,0.394049659474694,0
.081037716952077,0.935411957339073,0.994208669110626
"12648","MYOM2",3.28055943337238,0.0781804914255899,0.98170710086852,0
.0796372883077074,0.936525737603979,0.994208669110626
"12649","NSMCE2",27.2614198414247,0.0294628830780949,0.370767908733601
,0.0794644908150996,0.936663174492614,0.994208669110626
"12650","GALT",23.5853965202568,0.0314676252054641,0.394612179585746,0
.0797431676804757,0.93644152590671,0.994208669110626
"12651","PRTFDC1",26.3221693534934,0.0307678472721591,0.37833772704683
9,0.0813237620057646,0.935184477226678,0.994208669110626
"12652","ALG1L9P",1.80239459514668,-0.0953281244483981,1.2055419070653
8,-0.0790749155128529,0.936973035543894,0.994208669110626
"12653","ACAT1",92.0241253037859,-0.0185003225539083,0.232230315795095
,-0.0796636842634782,0.936504743379916,0.994208669110626
"12654","LEMD3",22.4443286917211,-0.0319092290943769,0.403188357315262
,-0.0791422383990774,0.936919487471209,0.994208669110626
"12655","LRCH1",10.5915045539939,-0.0395115171060681,0.497503693407777
,-0.0794195452810089,0.936698922857596,0.994208669110626

"12656","FUT8-
AS1",1.61831756221008,0.0918836937373794,1.15293216011843,0.0796956637309355,0.936479308326535,0.994208669110626
"12657","CINP",45.5357188302654,-0.0218755848461823,0.27250948980943,-0.0802745800209755,0.936018875159457,0.994208669110626
"12658","NDN",215.814622300836,-0.019913621854516,0.251320622624154,-0.0792359244004283,0.936844970867348,0.994208669110626
"12659","CORO7",10.3693287400619,0.040825127832314,0.501679071500174,0.0813769801284203,0.935142155580059,0.994208669110626
"12660","GAN",2.06024628480048,0.0984093033817895,1.21757282020723,0.0808241624226142,0.935581792095675,0.994208669110626
"12661","INSR",45.5424863831304,-0.029887057526625,0.374870299101763,-0.0797263949644402,0.936454866124286,0.994208669110626
"12662","IRGQ",15.7423786402323,0.0299081754866382,0.372999881014727,0.0801828016815301,0.936091868385406,0.994208669110626
"12663","SULT2B1",1.8307438628052,0.0967809565288569,1.22450231605582,0.0790369730296573,0.937003214810099,0.994208669110626
"12664","ZSCAN5A",2.92821691547813,0.0764823584957695,0.950345108600844,0.0804785101786565,0.935856687181216,0.994208669110626
"12665","AP001347.1",7.29580342124694,-0.0494282596779461,0.626311853262317,-0.0789195660603986,0.937096600314331,0.994208669110626
"12666","TOM1",26.9237053794102,-0.0260597063597306,0.324536435773411,-0.0802982453961664,0.936000053680587,0.994208669110626
"12667","POLA1",4.87453131741522,0.0542268889889422,0.668517354614122,0.0811151552232219,0.935350373279093,0.994208669110626
"12668","PEX19",29.9743125238537,-0.0254934920400646,0.323641847428028,-0.078770691252261,0.937215016581922,0.994211006395975
"12669","AL121832.2",2.38065734109555,0.0865830782862292,1.09973606353732,0.078730780190779,0.937246762410194,0.994211006395975
"12670","LAMTOR5",164.448493148859,-0.0194874960789262,0.252541076707869,-0.0771656489825957,0.938491768278496,0.99437871520733
"12671","CAMKMT",6.19967641392082,0.0461780442158447,0.597255170586142,0.0773171108263926,0.938371279181422,0.99437871520733
"12672","MFSD6",3.31930954016434,-0.0667830166163706,0.86219327515856,-0.0774571300200514,0.938259894058275,0.99437871520733
"12673","TULP4",24.5129809287182,-0.0319563818152839,0.409355108654593,-0.0780651838456672,0.937776201913272,0.99437871520733
"12674","FNDC1",250.979995790499,-0.0256681703744798,0.327329551675956,-0.0784169050519775,0.937496426651049,0.99437871520733
"12675","MPLKIP",69.6781799708216,0.0201014196299058,0.260583757625282,0.0771399561242474,0.938512207290707,0.99437871520733
"12676","CPNE3",149.798696337816,0.0147977498460905,0.190766325497684,0.0775700313327583,0.938170082060897,0.99437871520733
"12677","FERMT3",6.82060346454572,-0.0439315851629656,0.56469745712831,-0.0777966760933785,0.937989790530505,0.99437871520733
"12678","DDX10",16.6951439646157,-0.030237001132065,0.386402731620372,-0.0782525553203693,0.937627157045952,0.99437871520733
"12679","CD63",3330.74756407207,-0.0215630744191324,0.279576395506931,-0.0771276644440387,0.938521985500922,0.99437871520733
"12680","COG3",16.726730551508,0.0303847544338664,0.393773336877069,0.

0771630569881679,0.938493830242869,0.99437871520733
"12681","SPATA5L1",6.90475700157667,-0.0468321039264749,0.607692681715
329,-0.0770654400416381,0.938571486059424,0.99437871520733
"12682","MAP2K4",17.9850821846669,-0.030626986026833,0.39243642134602З
,-0.0780431793812233,0.93779370553441,0.99437871520733
"12683","GTPBP3",12.8455652732597,-0.0369233545629209,0.47193290737879
1,-0.0782385673590777,0.937638283711348,0.99437871520733
"12684","STMN3",58.1337102244824,0.0213058215954169,0.27654182660926l,
0.0770437581057889,0.93858873445978,0.99437871520733
"12685","WWC3",32.1654485572501,-0.0241769108143001,0.312813342682824,
-0.0772886175728579,0.93839394568275,0.99437871520733
"12686","UBE2J2",44.7305974812491,-0.0211556652482428,0.27781408749020
7,-0.0761504408914778,0.93929941057898,0.994446919069619
"12687","LEPR",89.8243868097467,-0.0310171591510059,0.417674669532216,
-0.0742615279632452,0.940802288708751,0.994446919069619
"12688","SMG5",11.4434011296606,0.0377633851268611,0.502965240989309,0
.0750815007664989,0.940149866524056,0.994446919069619
"12689","PAPOLG",13.6728720032489,0.032803855518003,0.431405961811743,
0.0760394116489238,0.939387742985142,0.994446919069619
"12690","AC012306.2",8.00534022616141,-0.0471990910877278,0.6362238374
15579,-0.0741862978279098,0.940862148555306,0.994446919069619
"12691","NOSTRIN",13.1351446258053,-0.0393315629679149,0.5174416512372
76,-0.0760115906283687,0.93940987688977,0.994446919069619
"12692","PHOSPHO2",6.81286051252097,-0.0435462173592761,0.571506405300
934,-0.076195501844544,0.939263561295783,0.994446919069619
"12693","PRKRA",45.8217572002299,0.0193746646120768,0.256412521179952,
0.0755605245910729,0.939768743452777,0.994446919069619
"12694","SLC11A1",2.33220834425927,0.0814085554732096,1.09693172211116
,0.0742147882427269,0.940839478985329,0.994446919069619
"12695","LNP1",2.46371035925093,-0.0678232845975281,0.89534013225885,-
0.0757514179850477,0.939616867838555,0.994446919069619
"12696","TFG",121.677059226499,0.0189841113873148,0.25125427560116,0.0
755573665040833,0.939771256058952,0.994446919069619
"12697","STBD1",5.95375461951695,0.0470743146910566,0.634589332112241,
0.074180753298151,0.940866560293951,0.994446919069619
"12698","CCDC69",44.4971025221388,-0.0274749171807127,0.36848151695629
4,-0.0745625381909497,0.940562781309362,0.994446919069619
"12699","RARS",38.1259570767629,-0.0206613668937087,0.27164732497135,-
0.0760595264315147,0.939371740054294,0.994446919069619
"12700","IQCE",11.4979850741221,-0.0355130586584461,0.476689689609046,
-0.0744993219542297,0.940613080671492,0.994446919069619
"12701","ZNRF2",12.650656118623,-0.0345736979898232,0.458314907453809,
-0.0754365555811812,0.939867374909662,0.994446919069619
"12702","DOCK5",1.54374812246567,0.0847005487697577,1.14769386375609,0
.0738006461867416,0.941169012846677,0.994446919069619
"12703","BRF2",14.942091456294,-0.0297847703274001,0.403379214931826,-
0.0738381384683739,0.941139179729694,0.994446919069619
"12704","ZHX1",58.6638135878393,0.0172996414909104,0.231193499378537,0
.074827542891184,0.940351927186725,0.994446919069619
"12705","ASCC1",18.4158068232636,-0.0292247092204011,0.382039678903711

,-0.0764965285916464,0.939024075667427,0.994446919069619
"12706","SLC15A3",9.87641306727147,0.0339411456023225,0.45971220525933
6,0.073831290999062,0.941144628347731,0.994446919069619
"12707","CS",22.7601178939122,-0.0259325457574107,0.343118127321137,-0
.0755790606572628,0.939753995981804,0.994446919069619
"12708","OS9",215.066216387885,0.0137441388533443,0.183406309050373,0.
074938201005776,0.940263881986276,0.994446919069619
"12709","SNHG10",8.72176807937389,0.0356516510957532,0.479243516201021
,0.0743915147321448,0.940698860359174,0.994446919069619
"12710","HIRIP3",22.4995204620936,-0.0271973572128253,0.36172969985929
4,-0.0751869620421119,0.940065957773938,0.994446919069619
"12711","ENKD1",16.9388632194408,0.0287232702642072,0.383407832789651,
0.074915710655201,0.940281776389178,0.994446919069619
"12712","DVL2",25.5114079243334,0.025768282964148,0.336748635059145,0.
076520823787815,0.939004747557662,0.994446919069619
"12713","DBF4B",4.48382005578947,0.0543022425637643,0.719549515336783,
0.0754669990130538,0.939843153609191,0.994446919069619
"12714","FMNL1",55.5605129958445,0.0202575337627796,0.272375466994513,
0.0743735622973257,0.940713144758944,0.994446919069619
"12715","NPC1",12.1348901610501,-0.0324061022019197,0.430157253961002,
-0.0753354776736082,0.939947794572005,0.994446919069619
"12716","ERCC2",6.25493066127734,0.0444642899593115,0.601665956389534,
0.0739019542108249,0.941088400867873,0.994446919069619
"12717","PPP1R3D",8.5589813705643,-0.0378578190767868,0.49967540163617
,-0.075764824429665,0.939606201695399,0.994446919069619
"12718","LIME1",4.85149490621964,-0.0461227296144612,0.621849541542873
,-0.0741702397979195,0.940874925808233,0.994446919069619
"12719","APOOL",33.7482843968045,0.0245792453496819,0.324055457051666,
0.0758488857842722,0.939539322880311,0.994446919069619
"12720","CALD1",919.466028120382,-0.0124801377344983,0.169496120989959
,-0.0736308162193146,0.941304149888284,0.994449743670589
"12721","OAZ2",67.7612770539426,0.0195988318423401,0.26624760428063,0.
0736112983825483,0.941319680722789,0.994449743670589
"12722","ANKRD65",32.7586197583462,0.0272273260192519,0.37261158178473
2,0.0730716041858889,0.941749137811993,0.99456066158631
"12723","BCAR3",1.66478089557798,0.0898359636583321,1.23039043990051,0
.0730141918735944,0.941794824172445,0.99456066158631
"12724","IPO9",30.8834112096369,0.0212363588020041,0.29102894097858,0.
072969920897042,0.941830053328225,0.99456066158631
"12725","FBXO48",7.05035778703833,-0.0438227787533365,0.60032757867366
,-0.0729981102153542,0.941807621330011,0.99456066158631
"12726","GORASP2",56.5638650439562,-0.0171762119260589,0.2373168275953
79,-0.0723767130215649,0.942302116499454,0.99456066158631
"12727","DNER",5.00362687679448,-0.049568166363387,0.687840525121469,-
0.0720634573757246,0.942551407375105,0.99456066158631
"12728","ARIH2",46.095896942968,0.0187232210345354,0.260887973854852,0
.0717672829371289,0.942787110091296,0.99456066158631
"12729","TCTEX1D2",25.0453359186548,-0.0316346750021265,0.442315522853
284,-0.0715206077282975,0.942983423959091,0.99456066158631
"12730","EXOC3-

AS1",6.29411779809926,0.0514063746129765,0.709877700656661,0.0724158183380375,0.942270996631177,0.99456066158631
"12731","AC006480.2",2.902493750336,0.0628918522014435,0.881573491921489,0.0713404529262371,0.943126800450624,0.99456066158631
"12732","CNOT4",66.4259044275073,-0.0168670217911061,0.229964899691613,-0.0733460706991593,0.941530731282783,0.99456066158631
"12733","CDKN2B-AS1",1.46093895136634,0.0899332449644501,1.25686887420589,0.0715534029126719,0.942957324057013,0.99456066158631
"12734","LRSAM1",11.6568032185213,0.032925104045227,0.456889768757593,0.072063561709336,0.942551324344801,0.99456066158631
"12735","AL450384.2",2.01677170465318,-0.0744785780720435,1.03725758054381,-0.0718033586536876,0.942758399903115,0.99456066158631
"12736","TCTN3",37.2513239217759,0.0188052089461795,0.263379577657556,0.0713996472825617,0.943079690322604,0.99456066158631
"12737","PAFAH1B2",60.6221710290103,0.0170058002901793,0.23764873400866,0.0715585562074134,0.942953222835809,0.99456066158631
"12738","USP30",8.71149690626153,0.0347057506714912,0.476701091985144,0.0728040091684388,0.941962080570727,0.99456066158631
"12739","MIPEP",6.45222375537937,0.0429422660255058,0.594977465042757,0.0721746092054425,0.94246295138235,0.99456066158631
"12740","RAB2B",24.0155865622534,-0.0225058978484349,0.313192264775713,-0.0718596861405632,0.942713572868842,0.99456066158631
"12741","PLEKHJ1",76.968405808334,0.0229507440936471,0.317410098894995,0.0723062819158745,0.942358165538725,0.99456066158631
"12742","SLC25A23",19.2518060587871,-0.0273854101542031,0.380501214571318,-0.0719719388676753,0.942624239460982,0.99456066158631
"12743","SLPI",108.26083694253,0.0322201543618039,0.442534310565467,0.0728082627551143,0.941958695682684,0.99456066158631
"12744","ARMCX1",89.7935348530081,-0.0168962489173733,0.234857611610984,-0.071942522115741,0.942647649946746,0.99456066158631
"12745","UBXN2A",53.7617807993906,0.0173367420944077,0.243941983109811,0.0710691200973124,0.94334274459898,0.994568883762161
"12746","AC012358.3",3.71178221691611,0.0561004205876058,0.788899624078413,0.0711122415011186,0.943308425528625,0.994568883762161
"12747","WWP1",28.7901978820967,-0.0215746580245135,0.303647354654776,-0.0710516910283716,0.943356615917573,0.994568883762161
"12748","PDE4DIP",150.347559032163,0.0223748745762629,0.31583943150017,0.0708425622158291,0.943523057149917,0.994588310066495
"12749","CP",75.9141189124966,0.0469155615691561,0.661864481596285,0.0708839390444477,0.943490126004602,0.994588310066495
"12750","HDAC2",60.0846292046808,-0.0162336334574534,0.23063288825768,-0.0703873310527851,0.94388537459367,0.994892199934457
"12751","TIMMDC1",83.0265563568451,0.0169294065036278,0.242022302644835,0.0699497786717262,0.944233632478305,0.995103182785127
"12752","POLE2",1.52043883038198,-0.0813059620301878,1.16184832735238,-0.069979841702288,0.944209704287239,0.995103182785127
"12753","ZMYND11",62.5315252159279,0.0165026303721898,0.236852820799321,0.069674620367608,0.944452642689677,0.995255944884072
"12754","FBH1",46.5595713978376,0.0174162334513345,0.251665630202751,0

.0692038616369799,0.944827349337605,0.995368457314782
"12755","B4GAT1",51.7010610199773,0.0213629955868666,0.308759469913916
,0.0691897663667539,0.944838568843306,0.995368457314782
"12756","EGLN2",54.7359182072587,-0.018133217425348,0.262160898302975,
-0.0691682762102523,0.944855674526726,0.995368457314782
"12757","UBOX5",7.14457673194581,-0.0369647427050626,0.53352077432046,
-0.0692845423913328,0.944763129553278,0.995368457314782
"12758","SFXN4",11.2481675099817,0.033521956558694,0.485925899616141,0
.0689857375068396,0.945000972272122,0.995443491641719
"12759","ANKRD49",25.6674735283414,0.0229388497012712,0.33298109241816
4,0.0688893460427001,0.94507769899888,0.995446288646912
"12760","SNAP47",17.5553681905772,-0.0320578355258892,0.46740780674027
4,-0.0685864357924662,0.945318816109709,0.995479633421903
"12761","USP13",12.0928501383754,0.0349006522637409,0.507943001816458,
0.0687097807016387,0.945220632731529,0.995479633421903
"12762","SGK3",26.4798101052974,0.023352751113411,0.340566047263867,0.
0685704030129508,0.945331578371183,0.995479633421903
"12763","PCDHB14",14.5762082315905,0.0282738801681444,0.41509603830095
3,0.0681140689366091,0.945694830975874,0.995485163807447
"12764","BAK1",9.01890030311539,-0.03243433269317,0.475128916809346,-0
.068264278484622,0.945575259409885,0.995485163807447
"12765","AL080276.2",3.28097862515139,0.0514861379861626,0.75785292243
3214,0.0679368469291609,0.945835906889581,0.995485163807447
"12766","CCNF",1.84228332345125,0.0678876428224101,0.999635136652616,0
.0679124215758752,0.945855350595825,0.995485163807447
"12767","FUZ",20.1213226489028,-0.0256396047522982,0.376398901868383,-
0.0681181709750677,0.945691565606853,0.995485163807447
"12768","PPP6R1",18.2606164331721,0.0250128672384978,0.365828187358119
,0.0683732640153614,0.945488504236942,0.995485163807447
"12769","PIN4",72.0429135407722,0.0197455818719593,0.290226588034656,0
.0680350549743617,0.945757729087931,0.995485163807447
"12770","MYO18A",7.23445877469,0.0415800595085469,0.613659215533649,0.
0677575736761129,0.945978617406209,0.995536933384655
"12771","UROD",64.8994542771581,-0.02113058505991,0.314677042612885,-0
.067150068795787,0.946462234677299,0.995585598150975
"12772","RAD54L2",13.5214425443366,-0.0290817083263301,0.4319959942866
22,-0.0673193934919566,0.946327438135699,0.995585598150975
"12773","ZNF213-
AS1",6.92371377545657,-0.0399808967757323,0.592714713352511,-0.0674538
624991987,0.94622039071602,0.995585598150975
"12774","BOLA2",25.4508905911544,0.0230398498674334,0.343155525999815,
0.0671411302507941,0.946469350545194,0.995585598150975
"12775","C19orf48",19.5941313222672,-0.0244308320316684,0.361940001973
806,-0.0674996736985057,0.946183921785564,0.995585598150975
"12776","SLC9A6",9.50330928404708,0.0312837317517397,0.465525604649685
,0.0672008831292561,0.946421782080279,0.995585598150975
"12777","SNX2",148.254223492531,0.014309566344656,0.213714591311364,0.
066956431270564,0.946616388131375,0.995609491594617
"12778","TMEM101",33.4897340495663,-0.0221328782706263,0.3307043538282
34,-0.0669264798434495,0.946640232427712,0.995609491594617

"12779","CCDC115",66.0221603775671,0.0180678321503913,0.27144031195587
9,0.0665628182498115,0.946929746807004,0.99571748269279
"12780","YAF2",26.7802601558804,0.0223920680932291,0.336630180347205,0
.066518302280959,0.946965186866326,0.99571748269279
"12781","YY1",175.95951574032,-0.0115085375776621,0.17288219738406,-0.
0665686678663377,0.946925089817285,0.99571748269279
"12782","BLZF1",25.4808528337759,0.0223485405648974,0.338783180649827,
0.0659670899896219,0.947404026745245,0.995736823774035
"12783","FSCN1",47.1080365239841,0.0242385739917286,0.365757058715381,
0.0662696000368653,0.947163185660467,0.995736823774035
"12784","ZCWPW1",12.5011804239626,-0.0306161957206432,0.46432635678226
8,-0.0659368034431864,0.94742813941503,0.995736823774035
"12785","EPHX2",7.80947745905957,-0.0342612767619068,0.518712518004194
,-0.0660506071720247,0.9473375346913,0.995736823774035
"12786","HELLS",4.00094033027772,0.0493099399756502,0.745266801369483,
0.0661641440153238,0.947247143126882,0.995736823774035
"12787","LZTS2",53.5574568311118,0.0163582318062472,0.247860672374102,
0.0659976899504141,0.947379664599175,0.995736823774035
"12788","SYT11",15.7511026904495,-0.0262779258675423,0.400882484141185
,-0.0655501971452752,0.947735940699478,0.995926139526089
"12789","MATR3.1",12.458566931608,-0.0319437653379332,0.48750951448470
2,-0.0655243936555736,0.947756484738384,0.995926139526089
"12790","OLMALINC",4.64768089834184,-0.0414300220490929,0.633913362522
429,-0.0653559689674896,0.947890580761321,0.995989172388693
"12791","SKIDA1",2.01394846099306,0.0663837518547538,1.01871767811672,
0.0651640324701893,0.948043398167659,0.99607186521579
"12792","PML",37.493794047888,-0.0179158016279916,0.276014675054412,-0
.0649088735026853,0.948246555461893,0.996207431117291
"12793","SEC62",403.038486743975,0.0111913689775156,0.172697644696779,
0.064803251932968,0.948330652227014,0.996217903171956
"12794","SCLT1",15.285850905612,-0.0267320334577908,0.41472956234374,-
0.0644565420095,0.948606709569148,0.996352135201233
"12795","EIF4ENIF1",13.2706045622057,0.0267196086166981,0.414217105341
924,0.0645062897502552,0.948567099047887,0.996352135201233
"12796","ANGEL2",27.891372408032,-0.0199767804779227,0.314241794032483
,-0.0635713671996719,0.949311531275743,0.996371000394099
"12797","PPP4R2",55.8779340098468,-0.0168937507016393,0.26487150626259
7,-0.0637809288738317,0.949144663889425,0.996371000394099
"12798","GLRX",118.173135413531,0.0204032515082801,0.320639355407744,0
.0636330230964134,0.949262436388432,0.996371000394099
"12799","GTF2H5",140.485458221584,0.0174804824110848,0.272883968308305
,0.0640582974494687,0.948923807425638,0.996371000394099
"12800","NBN",56.6111764183191,-0.0159528551822404,0.248209353830631,-
0.064271772743609,0.948753829058426,0.996371000394099
"12801","AC132192.2",2.26611526408383,-0.0646733751249666,1.0112012339
8821,-0.0639569780486647,0.949004483178449,0.996371000394099
"12802","TDRD3",32.123435822932,0.0202495786801471,0.318400140468675,0
.0635978949328991,0.949290407951015,0.996371000394099
"12803","DDX24",211.130109413816,0.00996067046854671,0.156912909696674
,0.0634789737045952,0.949385102023658,0.996371000394099

"12804","TBC1D10B",20.156365844016,0.0217947581024749,0.344218408744123,0.0633166546263253,0.949514353898893,0.996371000394099
"12805","NBR1",68.8125796686688,0.0146906799538072,0.230522275758955,0.0637278107091415,0.949186960006703,0.996371000394099
"12806","JPT1",46.8986442880972,0.0206176064226807,0.324077245564863,0.0636194200760515,0.949273268081204,0.996371000394099
"12807","NUDT19",10.1676676950593,-0.0302465765010408,0.477228702946725,-0.063379625563757,0.949464211072402,0.996371000394099
"12808","LSG1",17.7747115743942,0.0228878875465345,0.374148122302446,0.0611733326514811,0.951221167408022,0.996400405899605
"12809","WDR53",5.40445711263335,-0.0410172592875949,0.673868380613623,-0.0608683542181408,0.951464052372872,0.996400405899605
"12810","SDHA",77.5754457740193,-0.0125931492092185,0.201161709499941,-0.0626021186662377,0.950083342277177,0.996400405899605
"12811","MRPS30-DT",9.22168162152524,0.0330075909261859,0.544627418073296,0.0606058193745651,0.951673138855383,0.996400405899605
"12812","C5orf15",102.209448680549,0.0128202664994719,0.205701560545614,0.0623245952313963,0.950304342384729,0.996400405899605
"12813","JADE2",21.8763451280701,-0.0239592537478604,0.387712371092215,-0.0617964644263617,0.950724919087357,0.996400405899605
"12814","DUSP22",61.8294096882204,-0.0145498147640689,0.235107192637898,-0.0618858768241852,0.950653714600612,0.996400405899605
"12815","USP45",7.94010486374691,-0.0313010220813108,0.50108359171907,-0.0624666674355194,0.950191205614003,0.996400405899605
"12816","AC093627.5",2.05954767566691,0.0649209217692963,1.05013179805502,0.0618216893246525,0.950704830939134,0.996400405899605
"12817","ABCF2",14.9826932206373,0.0255483824177108,0.421251360232758,0.0606487831958437,0.951638921629146,0.996400405899605
"12818","AC107375.1",3.36002591343874,0.0491134505966734,0.815714392430681,0.0602091259543972,0.951989077442587,0.996400405899605
"12819","SLC29A3",6.95756550473698,0.0339202563586851,0.543205042745246,0.0624446639656715,0.950208727635368,0.996400405899605
"12820","LRMDA",25.6952779334799,0.0213892621379895,0.348571717937443,0.0613625863410646,0.951070447959422,0.996400405899605
"12821","POLR3A",13.5015698226493,-0.0253354879561543,0.407483992041639,-0.0621754190372352,0.950423137374598,0.996400405899605
"12822","DPF2",38.6433918282679,-0.0181806761367317,0.295223951501468,-0.0615826596868827,0.950895186294637,0.996400405899605
"12823","RELA-DT",1.64895751535171,-0.0651264576238598,1.06782102508357,-0.0609900499184898,0.951367133319155,0.996400405899605
"12824","TIMELESS",1.69997923554382,0.0719626410503269,1.18149062854717,0.0609083468895699,0.951432201933572,0.996400405899605
"12825","SACS",36.7571430481512,0.0210188787732245,0.339488758146338,0.0619133278167762,0.950631853798085,0.996400405899605
"12826","FOXO1",72.7381032711403,0.0176860196113978,0.293495333237597,0.060259968757596,0.951948584280222,0.996400405899605
"12827","TSC22D1",95.7947269577997,-0.0195995006654833,0.323483088836531,-0.0605889499076341,0.951686574052626,0.996400405899605

"12828","TTC5",22.1618224789427,0.0227109877400396,0.365194866225698,0
.0621886829208703,0.95041257476711,0.996400405899605
"12829","AL109628.2",2.0712209193852,0.0720896601973638,1.170782320015
63,0.0615739228077865,0.95090214411144,0.996400405899605
"12830","OTUD7A",5.89137029646588,-0.0407028955424939,0.67446922879719
6,-0.0603480393243155,0.95187844178996,0.996400405899605
"12831","MRTFB",18.9051969495301,-0.0255017669002179,0.41203705272361,
-0.0618919263004345,0.950648897051926,0.996400405899605
"12832","HEXD",23.8869493315338,0.019474428183823,0.32199995256934,0.0
60479599541647,0.951773663310186,0.996400405899605
"12833","ANKRD29",4.47256032992628,-0.0454608307665248,0.7536115500116
94,-0.0603239570383699,0.95189762173067,0.996400405899605
"12834","AKAP8L",30.1609249397862,0.0172746514486615,0.285349513141603
,0.0605385699049329,0.951726697825054,0.996400405899605
"12835","ZNF324",9.48201100844046,-0.0351392051757381,0.56419728768163
2,-0.0622817690601282,0.95033844647062,0.996400405899605
"12836","DDRGK1",61.492243766587,0.0167520961741041,0.271910458452847,
0.0616088703223201,0.950874312868165,0.996400405899605
"12837","GGT7",17.6815191462529,0.0240565312967786,0.394127532861991,0
.0610374289816547,0.951329400594919,0.996400405899605
"12838","IGLC1",12.4128617229625,0.114925170755186,1.82268854893931,0.
0630525554253718,0.949724654363309,0.996400405899605
"12839","GRAMD4",9.24917955655408,-0.0301773251830569,0.49401354068699
1,-0.0610860284134951,0.951290696081455,0.996400405899605
"12840","SSR4",392.37097848983,-0.0167706028350879,0.271129243871542,-
0.0618546439167352,0.950678587204386,0.996400405899605
"12841","C1orf35",39.8155031167258,-0.0151632665537401,0.2527906665778
93,-0.0599834905260149,0.952168783663247,0.996441147682348
"12842","PAK2",113.461448824574,0.0128204412491322,0.214917230578219,0
.0596529241263707,0.952432066026188,0.996441147682348
"12843","CRY1",12.8374217406669,0.0282651966472782,0.474234857116654,0
.0596016851631922,0.952472876190747,0.996441147682348
"12844","TEP1",6.65032547434986,-0.0359324643822868,0.599825889546019,
-0.0599048240640003,0.952231437750015,0.996441147682348
"12845","DCAF5",56.4843547364013,-0.0169909724113454,0.284797878503161
,-0.0596597576521514,0.952426623354998,0.996441147682348
"12846","ZNF416",2.1934972771091,0.0577939701694147,0.965361815260215,
0.0598676778548944,0.952261023037366,0.996441147682348
"12847","NAALADL2",29.8696907781967,0.0211685628587442,0.3561213787612
8,0.0594419883815348,0.952600070272708,0.996496640802905
"12848","DCUN1D1",36.1422499252918,-0.0171050868539192,0.2889838676679
25,-0.0591904558270182,0.952800411462135,0.99657174584137
"12849","ZNF835",3.96537309388856,0.0404495704601806,0.683666579148015
,0.0591656396464324,0.952820177268826,0.99657174584137
"12850","AC012510.1",1.5692549564914,-0.0664711636056279,1.12529999287
541,-0.0590697272073897,0.952896570712651,0.996574086677612
"12851","ANKRD42",10.3918437071604,-0.0264453182991051,0.4488775999862
32,-0.0589143194044797,0.953020352627429,0.996625983889193
"12852","CCDC13",1.81896928880807,0.0603960353869916,1.03824994473143,
0.0581709979311528,0.95361242188872,0.996702235010305

"12853","POLR2J2",3.1455567161105,-0.0542218478795109,0.929389727144229,-0.058341346257528,0.953476734032247,0.996702235010305
"12854","ERI1",8.45586969710166,0.0357780186284842,0.610157391971798,0.0586373599652102,0.953240952915207,0.996702235010305
"12855","AKIP1",28.7287058890128,-0.0186323148468042,0.318818252981939,-0.0584418071190537,0.953396714394778,0.996702235010305
"12856","AC006115.2",7.94098444625772,-0.0341976691675685,0.585860962754078,-0.0583716467586583,0.953452598861149,0.996702235010305
"12857","GID8",46.0381630543279,-0.0147447772671339,0.25319295696655 9,-0.0582353373639903,0.95356117332721,0.996702235010305
"12858","OTUD5",34.2782425747543,0.0175846483232377,0.301517391047819,0.0583205110064412,0.953493329899683,0.996702235010305
"12859","WASF2",418.490442703454,0.00818725769373931,0.147127497353929,0.0556473659987847,0.955622730327219,0.996827521523841
"12860","IFI6",221.964563525939,0.0178553691208435,0.33005177185162,0.054098691913312,0.956856534425159,0.996827521523841
"12861","ATP1A1",98.627561686546,-0.0111793137979597,0.204670583093018,-0.0546210091798043,0.956440400837533,0.996827521523841
"12862","CD2",2.40240774273398,0.0513544982423637,0.948135065370772,0.0541636947287476,0.956804745613361,0.996827521523841
"12863","TSNAX",69.4619172778352,-0.0155286933860431,0.287996980238172,-0.0539196396198354,0.956999189272328,0.996827521523841
"12864","GLI2",17.3124813814607,-0.0233131961388814,0.421251122589755,-0.0553427513630284,0.955865403672555,0.996827521523841
"12865","UBXN4",263.110218025457,0.00944929105087961,0.164607636633281,0.0574049372443825,0.954222630137614,0.996827521523841
"12866","MMADHC",50.4311775940744,-0.0135026863765868,0.237808464676985,-0.056779670963048,0.954720708029729,0.996827521523841
"12867","ATG9A",23.4720127270613,0.0184202684739629,0.335663537829893,0.0548771802652509,0.956236311987841,0.996827521523841
"12868","COPB2",92.5850102832127,-0.0126780304225593,0.232614343300351,-0.0545023588944792,0.956534929259017,0.996827521523841
"12869","GRSF1",35.9423747309934,-0.0154313615127156,0.269997383911409,-0.0571537445628693,0.954422724383174,0.996827521523841
"12870","ELF2",90.6941260811532,0.0115254707620373,0.202980068671823,0.0567812930474055,0.954719415878306,0.996827521523841
"12871","ABCE1",45.3131740855771,-0.0167963828193131,0.31123518412551,-0.0539668510374447,0.956961574777673,0.996827521523841
"12872","DTWD2",2.15724673182317,0.0558271082174124,1.02689117041665,0.0543651652927944,0.956644231861855,0.996827521523841
"12873","C6orf136",8.14319072368959,-0.0334610112681731,0.585604388355524,-0.057139276845478,0.954434249117839,0.996827521523841
"12874","VPS52",16.4192932381609,0.0217283203434358,0.381307515766176,0.056983719032593,0.954558164395651,0.996827521523841
"12875","SRSF3",266.188418813492,-0.0121680022160632,0.220671099858486,-0.0551408962200597,0.956026215216079,0.996827521523841
"12876","PRIM2",12.9548554687826,-0.023296768842676,0.4195735854164,-0.0555248701358439,0.955720317005977,0.996827521523841
"12877","AMD1",64.9852778769474,-0.0207578990662485,0.361833520953847,-0.0573686457007288,0.95425153895887,0.996827521523841

"12878","HECA",24.4720163951003,-0.0180002976802844,0.321002161911305,
-0.0560753160449369,0.95528180793274,0.996827521523841
"12879","FUCA2",91.5574046326231,0.0137261829839436,0.254073698468032,
0.0540244152256107,0.956915712106402,0.996827521523841
"12880","MRPL32",55.836929458212,-0.0151449005000588,0.270847211005153
,-0.0559167674049659,0.955408113270497,0.996827521523841
"12881","SAP25",1.68649589355537,-0.0613352324878222,1.1089937588437,-
0.0553071033977447,0.955893803137186,0.996827521523841
"12882","SMIM27",16.7317478924225,0.0232869059295811,0.41677691613579,
0.0558737900973238,0.955442350675274,0.996827521523841
"12883","PTBP3",43.0599939183861,0.0165824870479761,0.301472918176312,
0.0550048977808285,0.95613456183735,0.996827521523841
"12884","UPF2",58.7484405173986,0.0163086972182355,0.291470652265568,0
.0559531365901502,0.955379140218888,0.996827521523841
"12885","SLC35G1",3.71892305356068,0.0446747883462893,0.83097959582008
8,0.0537615948345881,0.957125108126387,0.996827521523841
"12886","NUP160",27.6686402357271,-0.0172317064097918,0.30971498324751
7,-0.0556373031395146,0.955630746907713,0.996827521523841
"12887","KIF21A",18.3725638334745,-0.0208774344642455,0.38864792167403
1,-0.0537181168352058,0.957159748494486,0.996827521523841
"12888","AC026124.2",1.83057545849202,0.0566523246956538,1.03786591284
698,0.0545853987440924,0.9564687715291,0.996827521523841
"12889","AL135999.1",2.60887092656257,-0.0526952786352524,0.9746890652
15244,-0.0540636809376902,0.956884428320544,0.996827521523841
"12890","GZMB",4.88065685801883,-0.0401543747418283,0.708291159034054,
-0.0566919044938943,0.954790622923537,0.996827521523841
"12891","CHST14",30.2686943198494,0.0207970337780738,0.368529829356526
,0.0564324299457297,0.954997322749082,0.996827521523841
"12892","HERC1",47.1433499977478,0.0144925001859861,0.270158675468696,
0.0536444005021981,0.95721848093349,0.996827521523841
"12893","IFT140",10.9333112117141,-0.024383341466583,0.453846832574185
,-0.0537259262740305,0.957153526448886,0.996827521523841
"12894","FN3KRP",30.1250831462489,-0.0201337147425531,0.36421682705467
4,-0.0552794743322794,0.955915814268376,0.996827521523841
"12895","TXNL1",108.722838045811,-0.0148086406548733,0.268034916163181
,-0.0552489237852048,0.95594015288315,0.996827521523841
"12896","ZNF700",5.38952418527892,-0.035674379742882,0.625675311898229
,-0.0570174003424395,0.954531334220437,0.996827521523841
"12897","ARHGAP33",4.85390024609741,-0.0391461680232677,0.689710084402
228,-0.0567574244723356,0.95473842958284,0.996827521523841
"12898","PINLYP",7.68445774888305,0.0284183085162328,0.524084093385287
,0.0542247110242681,0.956756133093426,0.996827521523841
"12899","RCN3",162.816273666516,-0.0161546402584948,0.286504258257108,
-0.0563853408559034,0.955034834678559,0.996827521523841
"12900","VSIG10L",1.52007815680148,-0.069702708877519,1.21634918985655
,-0.0573048508263812,0.954302356304392,0.996827521523841
"12901","NECAB3",18.4440777466215,0.0229003706875326,0.407212541952504
,0.0562368992313689,0.955153086325571,0.996827521523841
"12902","HELZ2",15.9445717361667,0.0240303487535246,0.445342149617835,
0.0539592957328335,0.956967594267774,0.996827521523841

"12903","PRMT2",122.765373345636,-0.0102297229444406,0.188753936654062
,-0.0541960773151402,0.956778945942395,0.996827521523841
"12904","Z95115.1",1.62082620707642,-0.0642297301440222,1.148861470075
46,-0.0559072889264912,0.95541566419023l,0.996827521523841
"12905","GCAT",3.70624970762512,-0.0386275731191285,0.71287488831031,-
0.0541856274537674,0.956787271491862,0.996827521523841
"12906","RSPH3",4.46762741281919,0.0413344252265724,0.775272114138073,
0.0533160221718111,0.957480114511159,0.996827581300534
"12907","LINC01006",5.2169243031324,-0.053791326065971,1.0097173463625
,-0.053273647580438,0.957513876561479,0.996827581300534
"12908","SLC47A1",1.79839050699538,0.0747996419408639,1.40410953264505
,0.0532719422536481,0.957515235286003,0.996827581300534
"12909","INTS2",8.1592734361816,-0.0283110718030507,0.52942831905418,-
0.0534747968405396,0.957353611118064,0.996827581300534
"12910","B3GALNT1",13.9832969376457,0.0216970286978826,0.4117332711145
94,0.0526968069380137,0.957973483187024,0.997082395150831
"12911","DCAF16",19.1899424919152,0.0237436173748036,0.450667958099345
,0.0526853905366167,0.957982579521357,0.997082395150831
"12912","C18orf25",12.2865016998982,-0.0218966307667635,0.414536741726
089,-0.0528219300310708,0.957873788252505,0.997082395150831
"12913","LURAP1",6.91267380078203,0.0293977867307945,0.560216932687895
,0.0524757196997693,0.958149641545506,0.997101830008522
"12914","CDK19",21.410322929458,0.020274321320499,0.386012225771342,0.
052522484954942,0.958112379655609,0.997101830008522
"12915","PUM3",37.8985214256319,-0.0162549786667568,0.311067998789483,
-0.0522553870215285,0.958325200717377,0.997207307196348
"12916","AC010883.3",1.59325160694501,-0.073214135204532,1.40469305224
711,-0.0521210915704396,0.958432207163002,0.997241439459863
"12917","SSBP3",55.3635239476298,0.0145705783887297,0.291836524595508,
0.0499271926600853,0.960180407711791,0.997399988617524
"12918","AC103591.3",28.107187813539,-0.0214102973716709,0.44655359749
9649,-0.0479456385337658,0.961759566901312,0.997399988617524
"12919","STRIP1",15.0821252448191,0.0218249713682439,0.446648248433146
,0.0488638910928374,0.96102776520303,0.997399988617524
"12920","WARS2-
IT1",2.34468920259826,-0.0424476768615401,0.871777520042497,-0.0486909
514017645,0.961165587059337,0.997399988617524
"12921","CTSK",728.69487529246,-0.010438831654143,0.210934150053315,-0
.0494885804479952,0.960529937532274,0.997399988617524
"12922","STON1",3.55116238224706,0.038951601422816,0.804310390265126,0
.0484285692367797,0.961374691052945,0.997399988617524
"12923","TMEM127",33.8730121095145,0.0146642184157945,0.30084068573027
9,0.0487441330623142,0.961123204558617,0.997399988617524
"12924","C3orf38",25.7740923012147,-0.0158633441283523,0.3077252200175
82,-0.0515503543305482,0.958886978206479,0.997399988617524
"12925","TMEM39A",21.6855404652927,0.0186532269360946,0.37251022344409
3,0.0500744026932568,0.960063097832953,0.997399988617524
"12926","POPDC2",2.85292837536552,0.044409750905641,0.919657810442944,
0.0482894293957569,0.961485578847265,0.997399988617524
"12927","AC093827.4",7.07925581097551,-0.0283646069689321,0.5616468750

5076,-0.0505025634948447,0.959721906639887,0.997399988617524
"12928","NADK2",21.7207845882741,-0.0178236998988219,0.350729178567432
,-0.0508189822461408,0.959469764775027,0.997399988617524
"12929","YIPF5",92.8284624041824,0.0100162685155562,0.203888535891494,
0.0491261976636424,0.960818725892155,0.997399988617524
"12930","RIOK1",26.1406184256667,0.0147898590139946,0.308064904197763,
0.048008905956065,0.961709144866463,0.997399988617524
"12931","HULC",2.93219189114083,0.0420621713537886,0.867071317204202,0
.048510624811595,0.961309297036964,0.997399988617524
"12932","ZSCAN26",15.8604361630695,-0.0195140751668818,0.4071783278785
54,-0.0479251321369493,0.961775909851514,0.997399988617524
"12933","IRAK1BP1",13.2249455075086,0.0214966333557966,0.4320875514061
45,0.0497506426321239,0.960321099411275,0.997399988617524
"12934","ABCB4",1.83383253937347,-0.0620636308292362,1.22815612180849,
-0.0505339913445581,0.95969686282134,0.997399988617524
"12935","BRI3",493.54720830146,-0.0128135041362475,0.264862127653322,-
0.0483780155727629,0.961414979817453,0.997399988617524
"12936","GNB2",216.632896389044,-0.0101809284307254,0.201135057594472,
-0.0506173739798868,0.959630418137774,0.997399988617524
"12937","TSPAN12",10.2154413025391,0.0267757687771983,0.52826942453356
5,0.0506858196475027,0.959575876406938,0.997399988617524
"12938","AC091182.1",1.72015661027888,-0.0599190064269671,1.2399637463
8912,-0.0483231921912687,0.961458671446525,0.997399988617524
"12939","SDCBP",589.42920932275,-0.0137871946411986,0.269405304093335,
-0.0511764038484635,0.959184954201904,0.997399988617524
"12940","MFSD14B",38.97853221723,0.0138053584708955,0.283668412789609,
0.0486672391019252,0.961184484334006,0.997399988617524
"12941","ALG2",26.4095260216681,-0.0150771703385137,0.306272569239799,
-0.0492279487383961,0.960737638390283,0.997399988617524
"12942","ANKRD16",16.3435292448578,0.0176872241790603,0.36018014668801
5,0.0491066049633791,0.960834339760225,0.997399988617524
"12943","LINC00863",8.0896826372608,-0.0294430864851776,0.602816830638
962,-0.0488425090154982,0.961044805286049,0.997399988617524
"12944","ACTR1A",45.3879692162979,-0.0125036938455415,0.24824381869319
5,-0.0503686009640177,0.959828657414968,0.997399988617524
"12945","TIMM8B",110.279106183697,0.013738745095414,0.279398283607823,
0.0491726180920222,0.960781732457902,0.997399988617524
"12946","CACNA2D4",5.48233820707343,-0.0352159313991892,0.726699667969
77,-0.0484600901188992,0.961349570522159,0.997399988617524
"12947","FBXO33",22.1525742167197,-0.0169728741882332,0.35158410912522
7,-0.0482754303954841,0.961496735421803,0.997399988617524
"12948","SERF2",1987.08824393159,-0.0138332898032811,0.287096003967016
,-0.0481834982449647,0.961570001404433,0.997399988617524
"12949","MAP2K5",13.8784467985293,0.0207367883346464,0.425534673862221
,0.0487311366343804,0.961133561899312,0.997399988617524
"12950","WDR73",28.5012900238011,0.014547092059526,0.299211461305113,0
.0486180977027882,0.961223647138516,0.997399988617524
"12951","CCDC189",1.91399381945333,0.0522735256573866,1.01763889629803
,0.0513674603511592,0.959032713406102,0.997399988617524
"12952","LYRM9",23.8108347242923,0.0179894690045626,0.369909727709998,

0.0486320517060477,0.961212526609207,0.997399988617524
"12953","UNC119",5.48907738485683,0.0317770386594674,0.624351738947672
,0.0508960521404598,0.95940835137013,0.997399988617524
"12954","TAF15",34.4811026437979,0.0182840637139981,0.353698678292917,
0.0516938988922544,0.958772598720323,0.997399988617524
"12955","PSMC3IP",5.04491694482594,-0.0321962608030196,0.6379447948222
05,-0.0504687256081347,0.959748870981995,0.997399988617524
"12956","PDK2",32.0173066412601,0.0175872613672168,0.348290918087273,0
.0504958942478363,0.95972722114833,0.997399988617524
"12957","POLR3F",12.6623918527093,-0.0207482514569513,0.42785385015928
,-0.0484937822792227,0.961322719636131,0.997399988617524
"12958","LINC01597",1.78358110078122,-0.0535707691920635,1.06228562999
028,-0.050429722176091,0.959779951640993,0.997399988617524
"12959","PRICKLE3",4.23140567320417,-0.0353738202521745,0.716907126526
804,-0.0493422633745459,0.960646539215225,0.997399988617524
"12960","LINC02606",1.51818964736151,0.0550515190852618,1.234113173808
7,0.0446081609479644,0.964419637641109,0.997484768676216
"12961","ZYG11B",55.4540205692489,0.0132095777444392,0.284375246475575
,0.0464512221374856,0.962950611181693,0.997484768676216
"12962","SNX27",71.1085696074102,0.0103071411104652,0.224002998438573,
0.0460134068843353,0.963299564079876,0.997484768676216
"12963","CRTC2",14.2658243680755,0.0164653549537832,0.379884242008478,
0.0433430849006255,0.965428046690136,0.997484768676216
"12964","ACBD6",66.4861950977383,0.0104649027898911,0.242154151019461,
0.0432158719800348,0.965529452898198,0.997484768676216
"12965","FANCL",11.6580382767019,0.019627989991695,0.432631265112401,0
.045368866225134,0.96381329657728,0.997484768676216
"12966","D2HGDH",23.6802241252678,0.0170980881206784,0.3877537370714,0
.0440952245871714,0.964828499311457,0.997484768676216
"12967","AC093495.1",4.1736758242487,-0.0408807399908506,0.91038932817
4995,-0.0449046783894115,0.964183287792595,0.997484768676216
"12968","SHQ1",8.24239061451061,0.021691985931097,0.497197422776506,0.
0436285164351057,0.965200520505812,0.997484768676216
"12969","CGGBP1",69.6054275251105,-0.010787273144418,0.234127779795888
,-0.0460742982051184,0.96325103130804,0.997484768676216
"12970","RAB6B",6.0040223053434,0.0296506756879871,0.670496536978121,0
.0442219669345655,0.964727472097976,0.997484768676216
"12971","CTBP1-
DT",13.3666844903476,0.0193454619166526,0.406441256229636,0.0475971905
414113,0.962037271116883,0.997484768676216
"12972","BST1",53.2541791959936,0.0190984095635784,0.411281881795346,0
.0464363017408137,0.962962503103345,0.997484768676216
"12973","SCAMP1-
AS1",11.1295864942126,-0.0188565577258659,0.436458320007112,-0.0432035
703330357,0.965539259033717,0.997484768676216
"12974","DNAJC18",25.7896067888651,-0.0166241867808588,0.3698368290066
25,-0.0449500576389621,0.964147116913316,0.997484768676216
"12975","PDGFRB",382.715538811973,0.00868537558208479,0.19164916472896
,0.0453191413297735,0.96385293053735,0.997484768676216
"12976","SDHAF4",12.2996676773302,-0.0201441968180455,0.42825061952975

9,-0.047038336664089,0.962482673091353,0.997484768676216
"12977","MYO6",30.8729948294827,0.0140757145542802,0.314795315839558,0
.0447138627737845,0.964335383854744,0.997484768676216
"12978","ADAT2",9.00204673264351,-0.022675393920847,0.500598927183259,
-0.0452965291964079,0.963870953900772,0.997484768676216
"12979","INTS1",31.2039996938479,0.0138039876838879,0.297218735408954,
0.0464438678971381,0.962956472690377,0.997484768676216
"12980","TMEM106B",88.2640275956428,-0.00923632813910653,0.20593246084
1124,-0.0448512492949439,0.964225875134436,0.997484768676216
"12981","GTF2IRD2B",21.5015233348536,0.0150744846261334,0.337681684713
436,0.0446411082049828,0.964393375694905,0.997484768676216
"12982","BIN3",24.8999534615607,-0.0154200156638917,0.329254546804827,
-0.0468331138127979,0.962646236973843,0.997484768676216
"12983","TERF1",69.0642949824769,0.00995427541829232,0.217343700987907
,0.0457996959334294,0.963469901167993,0.997484768676216
"12984","PSIP1",146.623185377981,-0.00775118746266342,0.17464834157743
,-0.0443816837460603,0.964600161514607,0.997484768676216
"12985","KIF27",10.2612920297648,-0.0243160111068355,0.537706237357448
,-0.0452217389672403,0.96393056678344,0.997484768676216
"12986","PHRF1",16.4387528808955,-0.016800802211413,0.368814664446239
,-0.0455515516075425,0.963667685271528,0.997484768676216
"12987","FAR1",35.44424534221,0.01204940298538 9,0.27277857254838,0.044
172835398396,0.964766635122717,0.997484768676216
"12988","OSBP",33.7091564962243,0.0132291827723646,0.302175298888468,0
.0437798285333952,0.965079906163857,0.997484768676216
"12989","KAT5",33.6806181700016,-0.0144162816697878,0.316746024244705,
-0.0455136941471139,0.96369785985922,0.997484768676216
"12990","PIWIL4",4.81672143940764,-0.0301714098025174,0.67103581130967
7,-0.0449624435745555,0.964137244348123,0.997484768676216
"12991","VDR",5.89980687887099,0.0338123340119124,0.753037761062308,0.
0449012463388468,0.964186023413478,0.997484768676216
"12992","MFSD5",19.3883056903725,-0.0160745098596588,0.35207608080227,
-0.0456563530900199,0.963584152694132,0.997484768676216
"12993","AC025164.1",2.63458272256577,0.0415544352920251,0.94231116911
5854,0.0440984216827383,0.964825950877223,0.997484768676216
"12994","TRIAP1",28.8664450057545,-0.0168107418749919,0.38943964659785
6,-0.0431664881114454,0.965568818789262,0.997484768676216
"12995","CCDC92",33.3855606517601,-0.0144701339490239,0.30960332871247
2,-0.0467376562429093,0.962722317783872,0.997484768676216
"12996","TUBGCP5",17.5522044739775,0.0174692719129432,0.40013525776503
4,0.0436584169326099,0.965176686070653,0.997484768676216
"12997","LINC02256",13.1886966366861,0.0224424203833406,0.507908354424
35,0.0441859642351744,0.964756170044507,0.997484768676216
"12998","SEC14L5",2.15601460668162,-0.0418837320391344,0.9488409120965
41,-0.044141996308516,0.964791217178717,0.997484768676216
"12999","KIF1C",8.6106959075038,0.0241966763674777,0.51609896887516,0.
0468837913398945,0.962605846524696,0.997484768676216
"13000","P3H4",41.1675389683154,-0.0152791183888983,0.326066695571202,
-0.046858874568997,0.96262570540531,0.997484768676216
"13001","AC005332.4",6.37282223801209,0.0291461427905349,0.64390355041

9999,0.0452647648417589,0.963896272220077,0.997484768676216
"13002","NT5C",105.63883480883,0.0130067637869313,0.294314243771353,0.044193456695341,0.964750197760196,0.997484768676216
"13003","PGS1",13.6302871164615,-0.0179411272516501,0.412786434734453,-0.0434634613494304,0.965332090605941,0.997484768676216
"13004","BID",16.2684166209371,-0.0180973083147474,0.392564277165264,-0.0461002423486656,0.963230352849027,0.997484768676216
"13005","Z93930.2",5.27060181380225,0.0313802775438659,0.665145778170127,0.0471780451350015,0.96237132547835,0.997484768676216
"13006","SAT1",354.137453917099,-0.014411376337367,0.331501673280559,-0.0434730123524,0.965324477204244,0.997484768676216
"13007","APOO",5.32850206552797,-0.0297193421738686,0.659176255156638,-0.0450855775543773,0.964039097177292,0.997484768676216
"13008","BRWD3",16.1498720333833,-0.0204479141122904,0.466416961585208,-0.043840417043999,0.965031609897099,0.997484768676216
"13009","SLC25A14",5.33946835504341,-0.0272002149343474,0.594497416696182,-0.0457532937409686,0.963506885990018,0.997484768676216
"13010","CPS1",2.17637753798317,0.0402635396960758,0.940220560136781,0.0428235048276527,0.965842227030754,0.997505381554625
"13011","ULK4",6.24510176875785,0.029350129048034,0.683331957056116,0.0429514948700457,0.965740199626645,0.997505381554625
"13012","MCCC1",8.27114669020416,-0.0223902343156634,0.520237941907379,-0.0430384493556406,0.965670884081971,0.997505381554625
"13013","DBNL",64.7372903455008,-0.00983573309219964,0.230466537494117,-0.0426774888846964,0.965958624484271,0.997505381554625
"13014","PGAP2",9.79256099043461,0.0224373133218516,0.526881691559191,0.0425851072096531,0.966032267445674,0.997505381554625
"13015","CCPG1",169.881395016548,-0.00806985702124609,0.189509854481711,-0.042582783060629,0.966034120167679,0.997505381554625
"13016","AC016747.1",13.0208796301288,-0.0207366809768045,0.495446992246482,-0.0418544895848073,0.966614696630731,0.997534869722294
"13017","ABHD14A",99.2532895354177,-0.0125056211438208,0.29825296361317,-0.0419295788123013,0.966554836643914,0.997534869722294
"13018","AC008267.5",5.60286934154835,0.0279650747315749,0.671981523135015,0.0416158387824544,0.966804946657703,0.997534869722294
"13019","LINC01239",10.655804476871,0.0242641449236947,0.577964535690379,0.0419820653783042,0.966512995265968,0.997534869722294
"13020","ASB6",9.39876951584864,0.0195235745935701,0.465198411602752,0.0419682744107086,0.966523989176656,0.997534869722294
"13021","LINC00545",2.68939317334162,0.0465059871411049,1.10223949144477,0.0421922708286807,0.966345424060493,0.997534869722294
"13022","PHF11",49.0864093454767,-0.0106613857870084,0.255224186910952,-0.0417726310192072,0.966679953244954,0.997534869722294
"13023","C14orf119",69.8933607745314,0.0132312084916347,0.314767435633272,0.0420348708087136,0.966470899787899,0.997534869722294
"13024","IVD",28.0684022226877,0.0132024678880898,0.316795122142975,0.041675098400446,0.966757705307604,0.997534869722294
"13025","MAP2K7",25.6216711893399,-0.0139216783216868,0.333236354064254,-0.0417771895289746,0.966676319252693,0.997534869722294
"13026","POFUT1",22.0388260583432,0.014016545462227,0.33818250293027,0

.0414466902952607,0.966939791280696,0.997597409413579
"13027","TRIQK",39.7668860682022,0.0110553757525489,0.271809308135433,0.0406732787349591,0.967556364442098,0.997773939666771
"13028","C10orf88",10.2346940408299,-0.020432667185521,0.50001930963675,-0.0408637562424635,0.967404511627518,0.997773939666771
"13029","SYTL2",12.9370341649345,0.0202230569614209,0.496416506271764,0.0407380832545277,0.967504700736361,0.997773939666771
"13030","NAXD",17.8434742175275,-0.0145919343003163,0.355054882475904,-0.0410976866409001,0.96721801884031,0.997773939666771
"13031","SNAP23",49.6732629580209,0.0105636416274071,0.25860013580848,0.0408493274544549,0.967416014530043,0.997773939666771
"13032","TSHZ1",33.3167615834922,0.0128038086708535,0.311956385865794,0.0410435857413791,0.967261148721733,0.997773939666771
"13033","ADD3-AS1",6.32272670908403,0.024628791761999,0.607858619715572,0.0405173028121625,0.967680712718814,0.997825604099451
"13034","VPS26C",22.2038328227975,-0.0153609987332118,0.380810160546725,-0.0403376808831944,0.967823913212538,0.997826642349333
"13035","AL136169.1",4.47574413205394,-0.0306720749785793,0.760531781786506,-0.0403297741305826,0.967830216758952,0.997826642349333
"13036","CROT",16.900108913087,0.0153641188646049,0.383122250123932,0.0401023925382433,0.96801149436805,0.997936980117538
"13037","LRIG2-DT",3.90522751589888,-0.0317199204661195,0.797643787452434,-0.0397670250368635,0.968278865634017,0.997946002841305
"13038","MAPKAPK2",22.266393823907,0.0139138620235594,0.350308816151825,0.0397188462922642,0.968317276363614,0.997946002841305
"13039","BPNT1",10.0294511928717,0.0191126866492525,0.478728473934248,0.0399238559849641,0.968153831936851,0.997946002841305
"13040","ZNF3",18.640235482518,-0.0139971482449392,0.350857908542878,-0.0398940651019374,0.968177582700717,0.997946002841305
"13041","CSDE1",205.635166803031,-0.00687742710052402,0.191464029778995,-0.0359202044815549,0.971345985415763,0.997977315310808
"13042","C1orf56",27.8964824525427,0.0150048572813333,0.396351303468482,0.0378574692451503,0.969801323347183,0.997977315310808
"13043","RASAL2",9.77594629981749,-0.0220884016518501,0.603208923088516,-0.0366181613142497,0.970789462606515,0.997977315310808
"13044","LINC00467",23.3614345678028,0.0133280945063358,0.357454027978312,0.0372861779785134,0.970256826205409,0.997977315310808
"13045","AC231981.1",3.25304477049629,0.0358380178754011,0.973978717438295,0.0367954835498462,0.970648075216758,0.997977315310808
"13046","CCDC58",11.7761703897227,-0.017077381582523,0.471652196147506,-0.0362075735510457,0.971116847130308,0.997977315310808
"13047","MB21D2",6.85239898783006,0.021336399507688,0.594956660994484,0.0358621071189012,0.971392310557384,0.997977315310808
"13048","PLRG1",45.6354555146054,-0.0108523646120745,0.280087895168194,-0.038746282146744,0.969092673276319,0.997977315310808
"13049","MARCH3",41.2597380794592,-0.0125599307583969,0.34883651840694,-0.0360052061514527,0.971278207738886,0.997977315310808
"13050","PRELID2",11.7189710121976,-0.0193387201331163,0.5003623160194

01,-0.0386494336483296,0.969169889359783,0.997977315310808
"13051","AKIRIN2",48.797398990914,-0.0106259113108688,0.28128508128078
,-0.0377763060254944,0.969866035937199,0.997977315310808
"13052","TSPYL4",23.827774675166,-0.0147455193989035,0.383159785501211
,-0.0384839953379107,0.969301791901438,0.997977315310808
"13053","NIPSNAP2",66.3433169663343,0.00881811442946049,0.231432206224
558,0.0381023651518246,0.969606065562678,0.997977315310808
"13054","PEX1",23.7645356695007,-0.0124126012522678,0.341470704281019,
-0.0363504133638727,0.971002952425883,0.997977315310808
"13055","VIPR2",2.96484003389652,-0.0410409674640016,1.14294885767071,
-0.0359079649002332,0.971355744892708,0.997977315310808
"13056","VPS37A",31.7606842838454,0.0106268797968634,0.29495355918368,
0.0360289932634635,0.971259240675793,0.997977315310808
"13057","AKR1E2",1.47155294625204,-0.0496881656826618,1.26153690586505
,-0.0393870091724269,0.968581837052182,0.997977315310808
"13058","NSUN6",16.6459148256244,0.0167675776439591,0.448964159780746,
0.0373472520660616,0.970208130051306,0.997977315310808
"13059","HIPK3",64.1573460934053,0.00977400784001028,0.271240863167831
,0.0360344224165169,0.971254911649459,0.997977315310808
"13060","RASGRP2",1.67398220845257,-0.0475972678291377,1.2424243072061
,-0.0383099940600584,0.969440522529411,0.997977315310808
"13061","KCNE3",3.33644920354782,0.0322275201328821,0.827640657835113,
0.0389390248386065,0.968939002827527,0.997977315310808
"13062","AP002840.2",1.67521616075051,-0.0444432902185214,1.1725693070
1452,-0.0379024846997561,0.969765431970334,0.997977315310808
"13063","TMTC3",36.4730040886439,0.0117152911854049,0.325299593132302,
0.0360138513319326,0.971271314353584,0.997977315310808
"13064","NEK5",2.29089594981099,0.0345056488443833,0.883968145111462,0
.0390349460387313,0.968862526926127,0.997977315310808
"13065","ATP10A",6.67728829439003,0.0242165414937231,0.673972029787647
,0.0359310778836819,0.971337315292942,0.997977315310808
"13066","CA12",53.7657817494489,-0.0150993734125856,0.395563878156248,
-0.0381717700892327,0.96955072869129,0.997977315310808
"13067","CELF6",2.6622065716836,0.03592281979718,0.980249342856228,0.0
366466145159648,0.97076677546357,0.997977315310808
"13068","CTCF",56.775799161053,0.00928273794457526,0.246306032885647,0
.0376878220797985,0.969936585672312,0.997977315310808
"13069","UTP4",10.9142825738774,0.0174873534211507,0.449452264640465,0
.0389081439719511,0.968963623536442,0.997977315310808
"13070","AC233723.1",3.23821842996232,-0.0352735893347543,0.9382827207
99592,-0.0375937748322751,0.97001157137926,0.997977315310808
"13071","CYB5D1",4.34593019728818,0.0317521454467416,0.823944990016434
,0.0385367297956485,0.969259747080598,0.997977315310808
"13072","KIAA0100",32.1083327926549,-0.0110939479947552,0.300504212352
829,-0.0369177786490711,0.970550564097883,0.997977315310808
"13073","MYO19",10.521245811982,0.0194034749530033,0.506233842892294,0
.0383290750419695,0.969425309282106,0.997977315310808
"13074","COPZ2",251.272523060842,0.0107074189061918,0.27136401692554,0
.0394577697791443,0.968525422111546,0.997977315310808
"13075","DCXR",16.9813047410774,0.0138719843520984,0.374510019957914,0

.0370403556990472,0.970452828609375,0.997977315310808
"13076","KHSRP",66.0942852775918,0.00815962023834128,0.227448369175768
,0.0358746042801285,0.971382345677552,0.997977315310808
"13077","DNAJB1",148.835242882912,0.0131266093495525,0.351201022837191
,0.0373763414568348,0.970184936269322,0.997977315310808
"13078","LINC01534",6.47913661178061,0.0232131864331682,0.600856930604
56,0.0386334670548143,0.969182619350722,0.997977315310808
"13079","ASXL1",46.9597063498941,0.0113496966546982,0.30172039423472,0
.0376166042189009,0.969993369039112,0.997977315310808
"13080","SLC35C2",48.5683914534377,0.0100121264737039,0.25649197740375
8,0.0390348523764672,0.968862601600888,0.997977315310808
"13081","IGLC2",54.5602090634881,0.0327042877015474,0.879870811387535,
0.0371694199628848,0.970349921090499,0.997977315310808
"13082","SCP2",258.454009216547,0.00766021601330426,0.215509526932445,
0.035544674624549,0.971645423665535,0.998008472075904
"13083","ELK3",48.1488350085564,0.00925000765183488,0.259123350828992,
0.0356973141256552,0.971523712205963,0.998008472075904
"13084","ETV2",1.6417030804541,-0.0408881747167784,1.15006745211699,-0
.0355528492189856,0.971638905402756,0.998008472075904
"13085","MARCO",1.75401433703817,0.0411689875540577,1.16720223706277,0
.0352715118655516,0.971863239428631,0.998155909413937
"13086","PEX11B",12.5753227713033,0.0168402928155246,0.480450730722645
,0.0350510296658206,0.972039050059119,0.998183907216665
"13087","PIEZO1",23.0008089869285,-0.011883534385634,0.338652311731998
,-0.0350906637100955,0.972007446108954,0.998183907216665
"13088","AL592435.1",1.83989883154844,-0.0375478810278481,1.0773018576
5043,-0.0348536306339796,0.972196455520913,0.998239919171528
"13089","ALKAL2",1.56810932098453,0.0498092745896193,1.43288613452506,
0.0347615022502321,0.972269918819566,0.998239919171528
"13090","SLC39A8",14.0092453981771,0.0176382871406938,0.50865866905590
6,0.0346760769327519,0.972338037294278,0.998239919171528
"13091","KLHL35",3.68386638217513,0.0248944887509234,0.719301377046777
,0.0346092605204403,0.972391317097534,0.998239919171528
"13092","CRACR2A",5.44667340733448,-0.0218082938967606,0.6335252415382
62,-0.0344237174256985,0.972539270906527,0.998239919171528
"13093","RFXANK",32.2595201330447,0.0118753167406316,0.344888620943964
,0.0344323240010897,0.972532407921415,0.998239919171528
"13094","KLHL12",11.3422138382401,0.0149388589898219,0.443391365412529
,0.0336922641150733,0.973122547813838,0.998304879394669
"13095","GFPT1",57.8812933861131,0.0089753500088819,0.264891426804727,
0.0338831275785244,0.97297034770462,0.998304879394669
"13096","NUP35",12.9587366316429,0.0139564210496835,0.41306417002181,0
.0337875372946208,0.973046574070686,0.998304879394669
"13097","PPP2R5B",8.59922276925457,-0.0196642197606455,0.5819421081438
83,-0.0337906803536953,0.973044067703549,0.998304879394669
"13098","INTS4",9.83188376426837,-0.0169529834816979,0.495656879677851
,-0.0342030629993804,0.97271522405132,0.998304879394669
"13099","SLFN12L",1.65968271635218,0.0385740674476584,1.13591906985379
,0.0339584645344702,0.97291027208269,0.998304879394669
"13100","CDC27",52.4043065991124,0.00814283326809058,0.241048863100463

,0.0337808407944942,0.973051914056425,0.998304879394669
"13101","MUL1",20.4934904351413,-0.0117195522541729,0.35859813552868,-0.0326815760960241,0.973928516188146,0.998312162490252
"13102","ZDHHC18",14.0963932427978,-0.013355692908703,0.398363274084264,-0.0335261058428926,0.973255048077925,0.998312162490252
"13103","DCLRE1B",4.04638920902826,-0.0228218941375889,0.693654639829406,-0.0329009464179489,0.973753578073397,0.998312162490252
"13104","TMCC2",1.76695239834933,-0.038825625530137,1.16166853528279,-0.0334222924620106,0.973337832777664,0.998312162490252
"13105","LCORL",14.1031385918823,-0.0138597003499756,0.419116181917401,-0.0330688743311445,0.97361966385373,0.998312162490252
"13106","ELP4",15.2985685434199,-0.0124762154230827,0.381943132682733,-0.0326651125664989,0.973941645174392,0.998312162490252
"13107","PRSS36",4.71337784841286,0.0263181934976397,0.805856134266891,0.0326586748906268,0.973946778957489,0.998312162490252
"13108","PER1",56.6241662149896,0.0117833825344143,0.357008723242563,0.0330058672723475,0.97366990878531,0.998312162490252
"13109","YJU2",23.1935737439057,0.0112488102102071,0.338172726178744,0.033263505124483,0.973464456336228,0.998312162490252
"13110","YTHDF1",23.7829661061418,-0.0111014539337867,0.335593091668977,-0.0330801026879808,0.973610709820013,0.998312162490252
"13111","MYH9",94.2220167160475,0.00754687736108992,0.229641314497472,0.0328637613732742,0.973783231410776,0.998312162490252
"13112","FAM174B",4.74922066255603,0.021728412912659,0.669429369158207,0.0324581112119147,0.974106720824349,0.998364378667168
"13113","PRDX4",246.29136370552,-0.0101384762627586,0.31283403129437,-0.0324084826091649,0.974146297898845,0.998364378667168
"13114","STX12",80.6329636956047,0.00741557202653473,0.230914452505437,0.0321139363347564,0.974381189555047,0.998501526505129
"13115","SULT1A2",1.95180725945298,-0.0365593844195492,1.14054399241854,-0.0320543395630223,0.974428716430893,0.998501526505129
"13116","INTS11",44.026449378625,-0.00814749176323864,0.26186524218084,-0.031113299708604,0.975179183171375,0.99868040164261
"13117","SCAMP3",23.3425514822975,-0.0102567531742222,0.329179680347429,-0.0311585246191284,0.975143116400121,0.99868040164261
"13118","MITD1",54.4103213262984,0.00716327112518637,0.230404451010485,0.0310899858651619,0.975197775932433,0.99868040164261
"13119","CHMP5",138.990850472887,0.00787173411922904,0.249645874788897,0.0315316010163815,0.974845590701812,0.99868040164261
"13120","PHYH",86.5092129735533,0.00951433938706795,0.302645281483603,0.0314372632556091,0.974920824047104,0.99868040164261
"13121","DCLRE1A",1.99405513698157,0.0319021708932022,1.02409799452868,0.0311514826351011,0.975148732364268,0.99868040164261
"13122","ZNF530",2.02227101020353,0.0308489732036694,0.990927851088031,0.0311314019177052,0.975164746691503,0.99868040164261
"13123","GAB3",3.85660941000422,-0.0237669155561565,0.75428543534121,-0.0315091800034628,0.974863471197201,0.99868040164261
"13124","CCL4",54.6552453730032,0.0153741306947618,0.503728380646446,0.0305206759941377,0.975651804002441,0.99891700396075
"13125","MVB12A",79.4701196158088,0.00893519849904054,0.29216367622557

3,0.0305828521001422,0.975602217794957,0.99891700396075
"13126","TRMT2A",12.8621369601841,0.014196855943738,0.464143905969289,
0.0305871859161658,0.975598761527012,0.99891700396075
"13127","USP18",12.976332809671,-0.0166697830474499,0.549318180187026,
-0.0303463159398335,0.975790858786593,0.998983267405578
"13128","TMEM144",2.48881589918533,0.0313866360927909,1.04607504429881
,0.0300041916340996,0.976063710236512,0.998998299969307
"13129","LINC00472",2.75630470606449,-0.024341310507198,0.812593990770
937,-0.0299550707778488,0.976102885400474,0.998998299969307
"13130","ILVBL",34.4774854907913,-0.00987221277340614,0.32792463878815
8,-0.0301051266226557,0.975983212132009,0.998998299969307
"13131","EDA",7.83863351814721,0.0157532754013201,0.523042506133274,0.
0301185376266648,0.975972516549028,0.998998299969307
"13132","ENDOV",29.6586834702598,-0.00865058859150397,0.29106429526977
2,-0.0297205419286697,0.976289929067342,0.99911364276089
"13133","ANKRD13C",26.2993987220787,0.0103358556584486,0.3524940527086
76,0.0293220710506309,0.976607724316104,0.999199096173246
"13134","NBPF20",5.31655408689591,-0.0228738985183124,0.78829561011920
3,-0.0290169046036594,0.976851108346897,0.999199096173246
"13135","MBOAT2",16.7157031470585,-0.0131547901718576,0.44997892021167
5,-0.0292342364963885,0.976677776119818,0.999199096173246
"13136","HNRNPA3",182.972702707352,0.00655581791648401,0.2279427939276
87,0.0287608035486474,0.977055362174714,0.999199096173246
"13137","AC079781.5",4.28237971156798,-0.0210664833958185,0.7321593630
10492,-0.0287730847410014,0.977045567254615,0.999199096173246
"13138","NEIL2",8.4030665499333,-0.0156024834113672,0.543951393045904,
-0.0286835985914104,0.977116937414153,0.999199096173246
"13139","UBE2W",26.3079472305762,0.00889482415200862,0.306490967833894
,0.0290214886750897,0.976847452326793,0.999199096173246
"13140","SLC25A16",9.83004004257612,0.0150467024223831,0.5150244302783
08,0.0292155119986293,0.976692709728779,0.999199096173246
"13141","GMIP",1.95878179556238,0.0295726337140363,1.01943932260258,0.
0290087237742985,0.976857632957736,0.999199096173246
"13142","SNHG11",12.614821100028,0.0137414944666103,0.474712671038068,
0.0289469721475126,0.976906882945011,0.999199096173246
"13143","LINC01133",230.44152077445,0.00688388618908312,0.249478053856
287,0.0275931533161976,0.977986642450017,0.999316214431921
"13144","AL137796.1",2.11132448934617,0.0260408945001753,0.93514258353
3023,0.027846977518435,0.977784197832807,0.999316214431921
"13145","CHST10",6.73605857197327,-0.0164060205814804,0.59250094659068
4,-0.0276894419762238,0.977909844558817,0.999316214431921
"13146","TNFAIP8",5.95992280853025,0.0171945192225705,0.60964838623809
2,0.0282039936637433,0.977499452009629,0.999316214431921
"13147","HNRNPH1",133.241388852374,-0.00708475552901578,0.251356209278
006,-0.0281861170223961,0.977513709837423,0.999316214431921
"13148","LINC00900",1.73235200842348,0.0303262594261882,1.099407433121
09,0.02758418627396,0.97799379439226,0.999316214431921
"13149","VPS33B",6.71875564468076,0.0158364139962844,0.57708413789782,
0.02744212317803 9,0.978107101449713,0.999316214431921
"13150","OSGIN1",4.66928858630812,-0.0199496376252503,0.72752575959741

4,-0.0274212113620412,0.978123780388235,0.999316214431921
"13151","AC127024.5",1.5678135405305,0.0317481224952053,1.120442580717
14,0.0283353409104507,0.977394693937785,0.999316214431921
"13152","SLFN12",13.1582886147533,-0.0121879807087295,0.44321639755693
7,-0.0274989390643288,0.978061786032655,0.999316214431921
"13153","YJEFN3",6.0759698627023,0.0183567148305556,0.663638996702816,
0.027606934218122,0.977932773803983,0.999316214431921
"13154","PAXBP1-
AS1",2.12272269449381,0.0309693996534702,1.11246359295398,0.0278385736
392826,0.977790900559685,0.999316214431921
"13155","TESK2",2.58772190936695,0.0236089451711934,0.873703316086708,
0.0270216957364168,0.978442429652975,0.999400545519715
"13156","LINC01341",4.06502199259756,0.0250342313151139,0.932937333475
248,0.0268337758784504,0.978592313655576,0.999400545519715
"13157","STX18-
AS1",6.75067898542709,0.0156483401561855,0.577518436661419,0.027095827
8780624,0.978383302411464,0.999400545519715
"13158","SEPSECS",7.91271698293192,-0.014296074170093,0.53361808035947
7,-0.026790835423834,0.978626562868258,0.999400545519715
"13159","AC048382.5",3.18292558752046,0.0257957450089231,0.95185958537
7418,0.0271003679588885,0.978379681280823,0.999400545519715
"13160","Z69706.1",1.49735624069517,-0.0319709517048215,1.194805272525
92,-0.0267582947949603,0.97865251722892,0.999400545519715
"13161","BAHD1",7.12595164623996,0.0145720514284879,0.547874499947775,
0.0265974259248732,0.978780826349084,0.999455628394905
"13162","GPSM2",60.2839177522921,-0.00879514270344172,0.33339021775801
5,-0.0263809261189106,0.978953507607527,0.999542829796282
"13163","PIP5KL1",2.19380341906817,-0.0285791748775949,1.0898007197585
6,-0.0262242209602562,0.979078496991195,0.999542829796282
"13164","MYO5A",19.9373362211523,-0.00972647684749715,0.37108928850577
3,-0.0262106106232862,0.979089352737425,0.999542829796282
"13165","ERAP2",53.0138678817378,-0.0219220625136518,0.849186923138088
,-0.0258153557436341,0.979404613818691,0.999712790909114
"13166","PGRMC1",236.063360872785,-0.00591561749620477,0.2289630372587
62,-0.0258365610756606,0.979387700053175,0.999712790909114
"13167","LINC00339",9.50497504345889,0.00791540992402829,0.53329765963
1289,0.0148423863879336,0.988157923851717,0.999751247167862
"13168","LUZP1",49.0752803422733,0.00607949131684601,0.267919165940777
,0.0226915132984173,0.981896345500964,0.999751247167862
"13169","HNRNPR",151.831810115382,-0.00282202127357506,0.1929818928068
63,-0.0146232438314788,0.98833275533865,0.999751247167862
"13170","LDLRAP1",9.09938795117755,-0.00415886618198484,0.562025670598
944,-0.00739977976015365,0.994095883857774,0.999751247167862
"13171","MTFR1L",33.5797353998993,0.00845620097368811,0.33471368922975
3,0.0252639830571245,0.979844402112154,0.999751247167862
"13172","AHDC1",42.7413731089005,0.00290799028811558,0.340047793831263
,0.0085517104973737,0.993176805391154,0.999751247167862
"13173","XKR8",6.79959785133903,0.00754014365573911,0.668705083033899,
0.0112757385087155,0.991003452972898,0.999751247167862
"13174","RAB42",11.6753685352108,0.00360525168981501,0.488405059782229

,0.0073816837430442,0.994110321996105,0.999751247167862
"13175","PTPRU",7.91871459725191,-0.00529008253272842,0.596078367579376,-0.00887481046193807,0.992919018704839,0.999751247167862
"13176","TMEM39B",14.2870936693157,-0.00900853605287271,0.424236421410316,-0.0212347068715247,0.98305842843878,0.999751247167862
"13177","MIER1",98.9070682759252,0.00305125211294196,0.208573982842439,0.01462911796973,0.988328073880347,0.999751247167862
"13178","GNG12",137.633325674589,0.00238327126063284,0.223067537959402,0.010684079281256,0.991475500273369,0.999751247167862
"13179","FAM102B",35.9767053484032,0.00970363937641785,0.387446812527057,0.0250450876421656,0.980019000136586,0.999751247167862
"13180","CTTNBP2NL",37.1778773352043,0.00227266569642605,0.288299545395579,0.0078830013183257,0.99371034009709,0.999751247167862
"13181","SEC22B",84.6585408913759,-0.000848590761850744,0.244676043275669,-0.00346822169628868,0.997232765002734,0.999751247167862
"13182","PIAS3",15.3904625036055,0.00136433770734595,0.408198811858329,0.00334233630209453,0.997333206432768,0.999751247167862
"13183","AC242426.2",4.97570239054205,-0.0153034922100163,0.655485489755101,-0.0233468054582473,0.981373636518479,0.999751247167862
"13184","PIGM",23.8611388739523,-0.00146910571595557,0.369836915763277,-0.00397230685564096,0.996830566024315,0.999751247167862
"13185","USP21",5.62570070306568,-0.00336114891494082,0.597992457100725,-0.00562072125664735,0.995515336902382,0.999751247167862
"13186","ALDH9A1",107.599092207476,0.00126859437351079,0.271277032968401,0.00467637956530791,0.99626880254369,0.999751247167862
"13187","AL645568.1",2.70153333955686,-0.0228911290352881,0.910380947145735,-0.0251445607545472,0.979939657063126,0.999751247167862
"13188","DARS2",6.5569964441932,-0.00301028891356041,0.573984936218823,-0.00524454340803952,0.99581547896898,0.999751247167862
"13189","TMEM9",85.78533185032,-0.00260457812638255,0.242020080116946,-0.0107618265605234,0.991413470485861,0.999751247167862
"13190","UBE2T",2.16115721512621,-0.00685699192779441,0.934233521664297,-0.00733969801852,0.994143820849982,0.999751247167862
"13191","PACC1",5.38287398281564,-0.00418295173286846,0.682111751391647,-0.00613235547450162,0.995107118912331,0.999751247167862
"13192","GNPAT",29.2841451056977,0.00230040576514593,0.296551776056943,0.00775718087321187,0.993810727218038,0.999751247167862
"13193","PDIA6",241.170649064134,-0.00302917399103176,0.210259989319419,-0.0144068017925652,0.98850543290815,0.999751247167862
"13194","PFN4",2.32364751274645,-0.0208452030593388,0.913325537371863,-0.0228234098428057,0.981791134532666,0.999751247167862
"13195","GAREM2",5.54689257760256,-0.00105349328409659,0.643795409564676,-0.00163637899314776,0.998694359048439,0.999751247167862
"13196","HADHA",184.753702806083,0.00232414235197974,0.160394620488815,0.0144901515081786,0.988438936398151,0.999751247167862
"13197","ARHGEF33",1.63216797593231,0.0255812093791435,1.12777072372022,0.0226829876331227,0.981903146247238,0.999751247167862
"13198","C2orf81",8.03516897495988,0.00510217762902966,0.561817074205285,0.00908156384575337,0.992754060020909,0.999751247167862
"13199","TMEM150A",16.1999776014298,0.00972460253274793,0.403103163472

761,0.0241243518134906,0.980753419034898,0.999751247167862
"13200","R3HDM1",16.7801263254218,0.00753802009027354,0.46525987512165
,0.0162017412060359,0.987073446365059,0.999751247167862
"13201","DARS",57.3191571068841,-0.00449766925950218,0.232626418597352
,-0.0193343012656146,0.984574420584931,0.999751247167862
"13202","AC009961.1",1.5642260770778,-0.0250997931132309,1.38893136206
398,-0.0180712984088228,0.985581974763682,0.999751247167862
"13203","C2orf69",19.953530358396,0.00712160016024582,0.35224485649690
8,0.0202177548625422,0.983869664448139,0.999751247167862
"13204","RNF25",7.79705658619247,-0.00346392213452547,0.55709696383680
9,-0.00621780831593288,0.995038938709421,0.999751247167862
"13205","DNAJB2",36.550124215902,-0.00669499936595388,0.31492635956058
6,-0.0212589361376271,0.983039100644478,0.999751247167862
"13206","SP110",45.7025154706701,0.00254880664120094,0.313307758978028
,0.00813515327394011,0.993509158397798,0.999751247167862
"13207","AGAP1",63.4485482514259,0.000311493603356044,0.34840249958352
3,0.000894062481550506,0.999286641444615,0.999751247167862
"13208","MTMR14",20.3298539572887,-0.00857509170928062,0.4047418077921
93,-0.0211865726351732,0.983096825364615,0.999751247167862
"13209","SLC25A38",11.1920878655063,-0.00238720987245247,0.46113421544
5184,-0.00517682226235984,0.995869511892012,0.999751247167862
"13210","ZNF501",4.09098029143534,0.015558621561666,0.817035934895726,
0.019042762866546,0.98480699175134,0.999751247167862
"13211","KLHL18",7.73340087825812,0.000941972651471319,0.5437633366855
13,0.00173232100790957,0.998617808504746,0.999751247167862
"13212","BAP1",22.5251653113215,0.00613853567992096,0.324365550067784,
0.0189247461040119,0.984901138538052,0.999751247167862
"13213","RPP14",18.2476746290379,0.00199462442570287,0.348903473625344
,0.00571683739624993,0.995438648550781,0.999751247167862
"13214","PSMD6-
AS2",2.11970845560948,0.0152208446600327,1.09032588197225,0.0139599040
174119,0.988861969876606,0.999751247167862
"13215","AC092040.1",10.4802820777268,0.00085654670388336,0.5471199432
30824,0.00156555562355363,0.998750867849152,0.999751247167862
"13216","PRICKLE2",18.1522677823821,-0.00581692582157094,0.37348498431
0149,-0.015574724730407,0.987573669980943,0.999751247167862
"13217","MCM2",3.45106295637336,-0.016875125567355,0.741901359592997,-
0.0227457806205027,0.981853057615201,0.999751247167862
"13218","MGLL",117.749314303244,-0.00176413023409444,0.242883108051539
,-0.00726328911156762,0.994204784712064,0.999751247167862
"13219","RAB7A",171.381340042679,-0.00331317903023225,0.24500201857404
5,-0.0135230682976228,0.989210481444261,0.999751247167862
"13220","DHX36",118.641631213542,-0.00420741987988615,0.21309022024888
4,-0.0197447816937445,0.984246967106915,0.999751247167862
"13221","RFC4",12.8171837168628,0.00609625359814074,0.47146540653673,0
.0129304367056797,0.989683291674627,0.999751247167862
"13222","PPP1R2",60.9620872634064,-0.000363288610163498,0.243664562614
538,-0.00149093740290088,0.998810404505826,0.999751247167862
"13223","MELTF-
AS1",4.33780871283876,-0.0107492200542815,0.675000537286868,-0.0159247

5777499,0.987294418657226,0.999751247167862
"13224","POLN",2.13467269779469,-0.00760564635811631,1.16975718614059,
-0.00650190180340743,0.994812269486879,0.999751247167862
"13225","GRK4",11.9867039337074,-0.00795928363882527,0.545887933425037
,-0.0145804351982773,0.988366908045041,0.999751247167862
"13226","MED28",56.1053158667996,-0.00549360947511209,0.24219428443673
7,-0.0226826553231361,0.981903411324044,0.999751247167862
"13227","GUF1",15.1376108225041,-0.00929596589422479,0.421590118536595
,-0.0220497717700134,0.982408253042744,0.999751247167862
"13228","IGFBP7",455.81778565927,0.00960015188312397,0.409696958739894
,0.0234323240100468,0.981305421248233,0.999751247167862
"13229","AC098818.2",4.16630638289436,0.00156095675414031,0.9198244119
63535,0.00169701601070595,0.998645977775522,0.999751247167862
"13230","PKD2",76.3559917318737,-0.0042836844316657,0.309644720573074,
-0.0138341917270144,0.988962264086877,0.999751247167862
"13231","PLA2G12A",37.4624924244462,0.000599373791378337,0.28764434356
5039,0.00208373223665639,0.998337423422659,0.999751247167862
"13232","SYNPO2",15.6748138418801,-0.00636083035683325,0.4543665560867
54,-0.013999336596461,0.988830510304811,0.999751247167862
"13233","HAND2-
AS1",22.1352775464279,0.00593757452062562,0.429273906256726,0.01383166
88577728,0.988964276852713,0.999751247167862
"13234","C5orf51",16.1480474921784,0.00800055187576504,0.3687544462544
31,0.021696150262131,0.982690334697052,0.999751247167862
"13235","CERT1",76.7572767784995,-0.000144483576124471,0.2462644870989
82,-0.000586700818402608,0.999531880502042,0.999751247167862
"13236","FAM151B",2.13180326348082,-0.0261432342709616,1.1200765479435
8,-0.02334057821223,0.981378603788346,0.999751247167862
"13237","NREP",25.9312968809876,0.00617187742346253,0.37275136785219,0
.0165576251511166,0.986789530150406,0.999751247167862
"13238","PPIC",546.983359261893,-0.00581894932444308,0.332605126304588
,-0.0174950680679356,0.986041667357074,0.999751247167862
"13239","PCBD2",14.0239473326326,-0.00382412707542507,0.39648318788085
3,-0.00964511785698782,0.992304428692621,0.999751247167862
"13240","PAIP2",150.508490100768,-0.00173092834202414,0.21478580628485
1,-0.00805885813389628,0.993570031116602,0.999751247167862
"13241","SRA1",39.5074944293128,-0.00236362544348619,0.334804044174369
,-0.00705972787549482,0.994367198914285,0.999751247167862
"13242","NDUFA2",159.655685946459,0.00435271421130407,0.25263287127389
7,0.0172294056167575,0.986253603378456,0.999751247167862
"13243","PCDHB11",6.44420986153066,-0.0056930346627113,0.7239432940120
02,-0.00786392347273669,0.993725561543732,0.999751247167862
"13244","AFAP1L1",10.6367537223371,-0.00061085179954968,0.492305617493
702,-0.00124079794713595,0.999009986728938,0.999751247167862
"13245","ZNF354A",11.398512534807,0.00351114367029369,0.43336426216797
1,0.00810206096074617,0.993535561373427,0.999751247167862
"13246","CANX",200.784642476344,0.00413426073638951,0.175683873748898,
0.0235323860304818,0.981225605316162,0.999751247167862
"13247","TRIM41",9.38541816845604,-0.0015579669867285,0.48956814305493
3,-0.00318232917895085,0.997460872966439,0.999751247167862

"13248","AL138724.1",2.7208401974446,0.0069562815965146,0.831776312695919,0.00836316385828323,0.99332723846305,0.999751247167862
"13249","ZSCAN16-AS1",50.7572447253992,0.00644774064222147,0.276274723809455,0.0233381489023528,0.981380541569339,0.999751247167862
"13250","LTB",3.32158030236033,-0.00775259968068407,0.816422897729862,-0.00949581363070643,0.992423550773975,0.999751247167862
"13251","C2",117.183750620455,0.00619063490135334,0.341112840445324,0.0181483490720298,0.98552050730956,0.999751247167862
"13252","TAP2",18.890036311818,0.00954463779385203,0.405928984611063,0.0235130728666669,0.981241010728748,0.999751247167862
"13253","KIFC1",2.63332505497521,-0.0147947120650818,1.06118251554125,-0.0139417224166532,0.988876475283514,0.999751247167862
"13254","AL354740.1",6.99068029513388,-0.00042919970250566,0.559382612634566,-0.00076727394239915,0.999387804027521,0.999751247167862
"13255","TDRD6",2.28598959213684,0.012300353715308,1.04184597395959,0.0118063072879765,0.990580148532447,0.999751247167862
"13256","TRAM2",108.421284664667,-0.00344392745293084,0.29307490027798,-0.011751014671213,0.990624262597454,0.999751247167862
"13257","CASP8AP2",41.2159703852015,-0.00154306948674071,0.301465965519898,-0.0051185528823447,0.9959160035147,0.999751247167862
"13258","BACH2",10.9918480905051,0.0141532280572773,0.622195313572624,0.0227472431060432,0.981851891022407,0.999751247167862
"13259","FAM229B",59.8078034192814,-0.00645771434806294,0.316388010565369,-0.0204107429245607,0.983715714021813,0.999751247167862
"13260","STX7",69.8035077245097,0.000329324089454988,0.233236791684915,0.00141197315859103,0.998873408790834,0.999751247167862
"13261","ZDHHC14",21.0076609214011,0.00076955797931681,0.421573930920202,0.00182544014910276,0.998543510297255,0.999751247167862
"13262","APTR",9.74173921726887,0.00690434333674881,0.500447741062061,0.0137963323045405,0.988992468652966,0.999751247167862
"13263","CNPY4",59.7974185783894,0.00504030630927569,0.346347300977047,0.014552751804495,0.98838899385426,0.999751247167862
"13264","TRIM56",81.0416416395857,-0.0075443644060177,0.340471228044323,-0.0221585960415884,0.982321445046222,0.999751247167862
"13265","AC090114.2",9.02962320923762,0.00272535788517036,0.523046645723136,0.00521054461863995,0.995842605707475,0.999751247167862
"13266","AC016831.5",12.8006460911542,0.0129777215514775,0.587867719585568,0.0220759213665047,0.982387393760879,0.999751247167862
"13267","TTC26",6.92347634845489,-0.00652316647383296,0.551460758815729,-0.0118288860441159,0.990562134549442,0.999751247167862
"13268","KRBA1",2.86504578170512,-0.0099356554779827,0.899456565815329,-0.0110462871200193,0.99118651729064,0.999751247167862
"13269","PCM1",144.533761871172,0.00532841990364497,0.24456034590987,0.0217877509283892,0.982617265212178,0.999751247167862
"13270","ATP6V1B2",41.252332983079,0.003689130699425,0.254835850532875,0.0144764980739987,0.98844982911996,0.999751247167862
"13271","IDO1",2.54044875493455,-0.0200908358507219,0.901672331885517,-0.0222817481919506,0.982223208101434,0.999751247167862
"13272","GPAT4",27.2473750027315,-0.006445072543834,0.312645726082248,

-0.0206146190597165,0.983553078621192,0.999751247167862
"13273","MCM4",10.0203687443249,-0.00269884383207884,0.480262082760003
,-0.00561952302494701,0.995516292937858,0.999751247167862
"13274","CHD7",3.6507894380799,0.0114285695446645,0.82891250778229,0.
0137874256177421,0.988999574484997,0.999751247167862
"13275","GGH",7.06762396258954,0.00920472680448916,0.59248010082021,0.
015535925665261l,0.987604623410896,0.999751247167862
"13276","SNX16",16.3522128848621,-0.00263220916418244,0.44394666211644
1,-0.0059291112847517,0.995269281364196,0.999751247167862
"13277","RMDN1",31.2753946433465,0.00700166431037363,0.315298757627286
,0.0222064443357252,0.982283277022787,0.999751247167862
"13278","PDP1",37.855377335654,-0.000380795983773567,0.303524657869604
,-0.00125458006096216,0.998998990201661,0.999751247167862
"13279","UQCRB",472.224653512376,-0.00428333678895776,0.26159686617488
7,-0.0163738077278578,0.986936175353666,0.999751247167862
"13280","NUDCD1",12.8198659250943,0.0108969168335733,0.430440002665373
,0.0253157623968437,0.979803101386051,0.999751247167862
"13281","LYNX1",2.2275149745722,-0.0155782301203694,1.00590475378133,-
0.0154867844712025,0.98764382769433,0.999751247167862
"13282","DGAT1",18.2629510091083,0.00445298210927495,0.394182568105401
,0.0112967504643287,0.990986688925653,0.999751247167862
"13283","FXN",9.31751227709164,0.00849125718274967,0.499882948928545,0
.0169864909394288,0.986447392887197,0.999751247167862
"13284","C9orf40",8.76112404333492,0.011634879285937,0.58678499446188,
0.0198281813538993,0.984180436830458,0.999751247167862
"13285","GOLM1",58.1494608460658,0.0042606089346818,0.248530111863297,
0.017143230261874,0.986322351209861,0.999751247167862
"13286","RNF20",28.4175768559061,0.00434626597800582,0.327837430410364
,0.0132573817839088,0.989422449607344,0.999751247167862
"13287","SLC31A1",14.4520739661931,0.00799718176936154,0.4336231938574
58,0.0184426983672612,0.985285689855323,0.999751247167862
"13288","MIGA2",6.54750199086692,-0.00473826589540451,0.56260656342511
1,-0.00842198830130645,0.993280305001087,0.999751247167862
"13289","NTMT1",33.7253501800178,0.00309464559918772,0.322376282195519
,0.00959948287172951,0.992340838457183,0.999751247167862
"13290","C9orf78",72.9399388111456,-0.00285404688544352,0.209124705220
446,-0.0136475835432019,0.98911114181983,0.999751247167862
"13291","LAMC3",1.61258369273422,0.00128522320038495,1.2701871382413,0
.00101183767469451,0.999192670479082,0.999751247167862
"13292","UCK1",24.6803191063951,-0.00225662842668849,0.305443866259691
,-0.00738802993270747,0.994105258607423,0.999751247167862
"13293","C9orf116",3.76178812157226,0.00912438444757867,0.721911007113
201,0.0126392094838192,0.989915638388305,0.999751247167862
"13294","NOTCH1",1.59337275727322,0.00273695237237127,1.28358347496234
,0.0021322745468l245,0.998298692348898,0.999751247167862
"13295","PNPLA7",5.51438275869923,0.00478473949824962,0.77103793523272
4,0.0062055824747526,0.995048693331147,0.999751247167862
"13296","SEC61A2",6.74567631964776,-0.00854262811020959,0.597745226564
112,-0.0142914200407978,0.988597484749346,0.999751247167862
"13297","PBLD",9.37042947988769,0.0110446430664599,0.498024981659455,0

.0221768856446887,0.982306855639406,0.999751247167862
"13298","SAMD8",31.3341200758121,-0.00537536517840473,0.330612692807374,-0.0162587985741267,0.987027927167752,0.999751247167862
"13299","AL157394.3",3.01493857512416,0.0188860244495405,0.822605484312149,0.0229587874257035,0.981683147146007,0.999751247167862
"13300","NOC3L",33.441269418736,-0.00429497337571039,0.323314769630235,-0.0132841855032556,0.989401065216631,0.999751247167862
"13301","SORBS1",3.9829016083101,0.00976159998664124,0.873996446067146,0.0111689241192764,0.991088673158548,0.999751247167862
"13302","MORN4",11.9393383487745,0.00313024434485221,0.549443891083639,0.00569711374655307,0.995454385490082,0.999751247167862
"13303","RPARP-AS1",2.8522039467991,-0.000592063967103787,0.859575296491974,-0.000688786624650649,0.999450427829959,0.999751247167862
"13304","AL158212.3",3.56992220597536,-0.000891600145027126,0.777610396086405,-0.00114658979549967,0.99908515390505,0.999751247167862
"13305","PPP2R2D",33.2124866063349,0.00627633363054627,0.292731204904308,0.0214406032749326,0.982894184271739,0.999751247167862
"13306","INPP5A",10.4277901412669,0.0110918811706154,0.486382336504181,0.0228048601648182,0.981805931183123,0.999751247167862
"13307","ECHS1",121.678393102124,-0.00391004736914099,0.262373594384223,-0.0149025948221568,0.988109889784273,0.999751247167862
"13308","SIRT3",9.8691798926821,0.00783424432282594,0.510142051057855,0.0153569859739664,0.987747379596376,0.999751247167862
"13309","HARBI1",1.57090024285582,0.00818858326654005,1.24447303249066,0.00657996039508514,0.994749989074067,0.999751247167862
"13310","PATL1",16.0949492154809,-0.000436865052256531,0.383234467480445,-0.00113994196589017,0.999090458102192,0.999751247167862
"13311","LRRC10B",3.34040692613346,-0.000514223904278847,0.803140889530957,-0.000640266123891612,0.999489141579845,0.999751247167862
"13312","SF1",167.20681431336,0.00390805300244534,0.184138942807668,0.0212233921997005,0.983067454206677,0.999751247167862
"13313","CCDC89",8.66562856352803,0.0110289326312906,0.537041477216649,0.0205364633816569,0.983615424631461,0.999751247167862
"13314","CWF19L2",73.7156114829463,0.00290948720051438,0.288455125627061,0.0100864465285183,0.991952316498826,0.999751247167862
"13315","AP002433.1",1.95154365712702,0.00842025450499443,1.01393063368796,0.00830456662934381,0.993373990663744,0.999751247167862
"13316","C2CD2L",3.1068124483789,0.0125956923432604,0.852395205258209,0.014776822142547,0.988210230814432,0.999751247167862
"13317","FEZ1",92.4690320561593,0.00798968223846008,0.314347274476449,0.0254167377521152,0.979722560625102,0.999751247167862
"13318","AC010168.2",1.99573823764421,-0.0233174010683507,1.10351576488683,-0.0211301023603791,0.983141872040742,0.999751247167862
"13319","MGST1",666.917791520953,0.00301737804675126,0.268225615719522,0.011249403002234,0.991024464334279,0.999751247167862
"13320","AC008014.1",3.3954728806974,-0.0158846333898455,0.805234591464235,-0.0197267151190822,0.98426137934085,0.999751247167862
"13321","CCNT1",25.3800738680841,-0.00743142686349949,0.313579132982981,-0.0236987289071394,0.981092919907355,0.999751247167862

"13322","COQ10A",13.435626685892,0.00171728402447916,0.474799105702951
,0.00361686448826959,0.997114165958235,0.999751247167862
"13323","XPOT",25.3653016181878,-0.00152690093119329,0.303528288552791
,-0.00503050617941901,0.995986253715064,0.999751247167862
"13324","HMGA2-
AS1",4.07648699711421,-0.00903447885068173,0.736442932250049,-0.012267
7242934205,0.990212017701135,0.999751247167862
"13325","AC121761.2",2.57212055943138,0.00335561943381934,0.9862931057
6413,0.0034022537663584,0.997285399484957,0.999751247167862
"13326","TMEM263",75.3498567935117,-0.00494007912509547,0.224202225875
468,-0.0220340324713788,0.982420808135835,0.999751247167862
"13327","MLXIP",37.9938051965929,0.00453527348740577,0.334053739628409
,0.0135764787200127,0.989167870004687,0.999751247167862
"13328","CLIP1",126.735061116615,0.00533027985872788,0.267238197424224
,0.0199458008252705,0.984086608626261,0.999751247167862
"13329","MPHOSPH9",9.20599035900745,0.000946954919470019,0.48282177236
9587,0.0019612929110934,0.998435115670292,0.999751247167862
"13330","PSPC1",26.7759852554883,-0.00212500867813306,0.30241367268621
7,-0.00702682738930909,0.994393449053141,0.999751247167862
"13331","DNAJC15",120.782945640703,-0.00432358292247346,0.274454245780
195,-0.0157533832649692,0.987431138581759,0.999751247167862
"13332","NUFIP1",13.6911224542632,-0.00291706225288977,0.4020229128801
91,-0.00725596019388851,0.994210632188238,0.999751247167862
"13333","EBPL",64.7985710919048,-0.0040580304496575,0.369976285390939,
-0.0109683528644803,0.991248696062938,0.999751247167862
"13334","DNAJC3",104.51733263663,-0.00417674133338333,0.21630033910814
5,-0.0193099157893371,0.984593873748262,0.999751247167862
"13335","IRS2",37.5251259370668,-0.00689752014686255,0.371462780282212
,-0.018568536372937,0.985185302844132,0.999751247167862
"13336","GRTP1",6.35653270633291,-0.00737663098972059,0.57044964796713
2,-0.0129312569759805,0.989682637248333,0.999751247167862
"13337","ADPRHL1",2.37918969362264,0.0227994005344728,0.95568330136346
8,0.0238566484335816,0.980966953969737,0.999751247167862
"13338","AL442125.1",3.48806100002705,-0.01192102868087,0.999005388431
005,-0.0119328972785548,0.990479151448342,0.999751247167862
"13339","LINC00641",2.69378584139532,-0.0163406872886679,0.84680497474
0148,-0.0192968720970046,0.98460427917017,0.999751247167862
"13340","PRKD1",7.72638790931908,0.00115635104322074,0.56563798737912,
0.00204433059487162,0.998368861317341,0.999751247167862
"13341","DTD2",10.1559184281263,0.00964578244034887,0.548769170644418,
0.01757712159562 8,0.985976208179788,0.999751247167862
"13342","SNAPC1",26.0514192723464,0.0010202138744607 8,0.33984873749317
2,0.00300196458573361,0.9976047824025,0.999751247167862
"13343","VIPAS39",18.420640976455,-0.0017845611238169,0.36680620224822
1,-0.00486513344888666,0.996118200448285,0.999751247167862
"13344","GPR65",2.34728550652209,-0.0114894943799954,1.08660226970518,
-0.0105737809503313,0.991563500637627,0.999751247167862
"13345","PPP2R5C",64.975499170798,-0.00235682219575991,0.2402124489323
96,-0.00981140738639735,0.992171755122837,0.999751247167862
"13346","INF2",20.0900728744856,0.00366518536733545,0.351638485858259,

0.0104231633189685,0.9916836694976,0.999751247167862
"13347","AHNAK2",250.174386629837,-0.00367271936705274,0.293551130601434,-0.0125113446489816,0.990017651699434,0.999751247167862
"13348","UBR1",26.336331157368,0.00406683371279129,0.324692494793584,0.0125251854539375,0.990006609200103,0.999751247167862
"13349","LYSMD2",41.6122139738654,0.0032443253682835,0.334571626816026,0.00969695308343493,0.992263072099802,0.999751247167862
"13350","LINC00926",1.74283816807387,-0.00541396738605521,1.04009238488852,-0.00520527547813505,0.995846809816319,0.999751247167862
"13351","MINDY2",87.6532649475922,-0.0036215398309198,0.263310416668789,-0.0137538798378616,0.989026337607165,0.999751247167862
"13352","RORA",73.7707883262783,0.00708161370517146,0.330685485057574,0.0214149517446722,0.982914646533535,0.999751247167862
"13353","CIAO2A",29.4105625145831,-0.00459726796798236,0.298107138562544,-0.0154215292869206,0.987695887581244,0.999751247167862
"13354","FAM219B",26.8284095115137,0.00405962193984645,0.322726164577223,0.0125791534292381,0.989963552377922,0.999751247167862
"13355","ALPK3",1.80866423152493,-0.012466933709016,1.20845959172882,-0.0103163844238936,0.991768862148759,0.999751247167862
"13356","MFGE8",258.024688682677,-0.00218387495637068,0.210051254535074,-0.010396866998983,0.99170464978849,0.999751247167862
"13357","POLG",16.3314769558611,-0.00786741026130923,0.377667335261294,-0.0208315878201806,0.98337999976547,0.999751247167862
"13358","TPSB2",5.55543363241117,-0.0102481987056489,0.863423532376055,-0.0118692603587568,0.990529922768495,0.999751247167862
"13359","ZNF598",13.4991181785964,0.00936915919865504,0.413825275349622,0.0226403744690062,0.981937137903612,0.999751247167862
"13360","METTL22",6.46358703112957,0.00897979070238361,0.555195788739802,0.0161740972905543,0.987095500128651,0.999751247167862
"13361","TMEM186",11.4160477804829,0.00426326249184715,0.446065297722599,0.00955748522383018,0.992374346194871,0.999751247167862
"13362","ATF7IP2",4.69267343658781,-0.0171293579368492,0.825438230836998,-0.0207518349610242,0.983443619587694,0.999751247167862
"13363","CIITA",2.24870446983876,0.00921075248328099,1.2404610950157,0.00742526510528279,0.994075550053002,0.999751247167862
"13364","IGSF6",2.26835932001074,0.00692357790564251,0.953684199268424,0.00725982239294058,0.994207550680407,0.999751247167862
"13365","SDR42E2",6.06746502865487,-0.0156075044781092,0.681272667420587,-0.0229093360477852,0.98172259326183,0.999751247167862
"13366","CLN3",47.7549414119913,-0.00295427219554064,0.301807742399525,-0.00978858982229108,0.992189960030731,0.999751247167862
"13367","SH2B1",19.6317924291309,-0.00492302374906008,0.367448100962782,-0.0133978750636099,0.989310362143914,0.999751247167862
"13368","AC009133.1",3.5577003955142,0.0209279703877587,0.831520116247303,0.0251683272344725,0.979920700155087,0.999751247167862
"13369","CYLD",31.1131959743214,0.00327474475256453,0.348860898961147,0.0093869641519477,0.992510396221705,0.999751247167862
"13370","CBFB",27.8175405683777,0.00714135879343549,0.31384581038549,0.0227543543903419,0.981846218506691,0.999751247167862
"13371","SLC7A6OS",30.672372151102,0.00277064871235401,0.3035050750776

69,0.00912883816405699,0.992716342135743,0.999751247167862
"13372","WWP2",13.1544542580079,-0.00942477007053611,0.444702880163368
,-0.0211934091073883,0.983091371873474,0.999751247167862
"13373","ST3GAL2",23.9761224339755,-0.00513470001394442,0.345213376645
851,-0.0148739891363249,0.988132711289887,0.999751247167862
"13374","AC092376.2",7.75537592928038,-0.0102419575453627,0.5719874411
38636,-0.0179059133273527,0.985713911618177,0.999751247167862
"13375","GSE1",24.4914350588146,-0.00434845791338182,0.380292309714134
,-0.0114345144571831,0.990876776262163,0.999751247167862
"13376","CYBA",348.648968759924,-0.000440317952181699,0.30974116958551
1,-0.00142156740988265,0.998865753693538,0.999751247167862
"13377","ZNF276",14.5792752903033,-0.00453305183725315,0.4334922384562
73,-0.0104570542102345,0.991656629952348,0.999751247167862
"13378","PAFAH1B1",114.861653834759,0.000281301040232287,0.22819397550
6277,0.00123272772477093,0.999016425829841,0.999751247167862
"13379","SLC25A35",4.78656001537789,-0.016028219278388,0.7604409466458
62,-0.0210775331721482,0.983183806845086,0.999751247167862
"13380","DRG2",29.4531953523615,-0.000223042641538685,0.36023602222294
1,-0.000619156963155254,0.999505984249949,0.999751247167862
"13381","TMEM11",38.6835066770601,-0.00172174647761213,0.3116261680958
43,-0.00552503818319454,0.995591679763893,0.999751247167862
"13382","BLMH",20.2335362590363,-0.00467087716020113,0.337686908310884
,-0.0138319758487676,0.988964031932768,0.999751247167862
"13383","OMG",1.64838856614299,-0.0101681654850592,1.44268261548017,-0
.0070480959401281,0.994376479625031,0.999751247167862
"13384","RFFL",11.7917186086473,-0.00956726452791475,0.463570656326083
,-0.0206382013126926,0.983534266707813,0.999751247167862
"13385","GGNBP2",93.7969731582676,0.00340983769101834,0.24265097898375
6,0.0140524373950563,0.988788146164709,0.999751247167862
"13386","CNP",49.0404996587732,0.000999646073179815,0.249165722912605,
0.0040119726802488,0.996798917527466,0.999751247167862
"13387","TUBG2",11.3578017974523,-0.0037254709918715,0.480235078536538
,-0.0077575986394506,0.993810393898836,0.999751247167862
"13388","DUSP3",23.8252835798168,-0.00679181080641415,0.36502423748402
3,-0.0186064652945448,0.985155045170567,0.999751247167862
"13389","PLCD3",31.0671503314861,0.00141226875751504,0.326699407369676
,0.00432283844309822,0.996550884689641,0.999751247167862
"13390","AC142472.1",8.97112549994324,0.0040655870668452,0.56480793651
5576,0.00719817623655698,0.994256735911622,0.999751247167862
"13391","SPOP",131.422426045439,-0.00095714430364948,0.245770343729061
,-0.00389446622862131,0.996892673378352,0.999751247167862
"13392","YPEL2",52.4438733304439,-0.000148585447372264,0.2982218149602
9,-0.000498238022567359,0.999602463590636,0.999751247167862
"13393","AC091271.1",7.52110873743201,-0.00687734256217048,0.677971840
239707,-0.0101439944168461,0.991906402276207,0.999751247167862
"13394","PTRH2",15.5216979786401,0.00023073500335428,0.413105087979798
,0.000558538275291258,0.999554350956699,0.999751247167862
"13395","RPS6KB1",37.552199980396,-0.000721012288996444,0.282419163080
128,-0.00255298642320485,0.997963013761743,0.999751247167862
"13396","TMEM104",5.05135875244643,0.00654434070854077,0.6469422202665

96,0.0101158040139101,0.991928893809459,0.999751247167862
"13397","LGALS3BP",594.170634424435,-0.00114484786273173,0.279615469819614,-0.00409436524906972,0.996733178308888,0.999751247167862
"13398","ARL16",56.1076650351881,0.000789290552477503,0.275320548856337,0.00286680582236292,0.997712623028681,0.999751247167862
"13399","METTL4",13.5410784782804,-0.00258908481518248,0.437465829017737,-0.00591837040391448,0.995277851196823,0.999751247167862
"13400","FAM210A",17.9712127429828,-0.00757754310294521,0.362222780524468,-0.0209195652796148,0.983309819202561,0.999751247167862
"13401","RMC1",6.99591896210873,0.0109147918567538,0.572074880620216,0.0190793062700471,0.984777839630189,0.999751247167862
"13402","CFAP53",1.64732976624692,-0.0015696916489109,1.0927871669448,-0.00143641112962501,0.998853910130824,0.999751247167862
"13403","MEX3C",17.2400432356133,0.0031743572608552 6,0.422055786159437,0.00752117934394603,0.993999023699833,0.999751247167862
"13404","ZNF407",16.4412839400429,-0.00568769501681658,0.427308036628793,-0.0133105266675748,0.989380049866353,0.999751247167862
"13405","LINC01002",1.83466794809167,0.0174938377130278,1.12534529404423,0.015545306676637,0.98759713935055,0.999751247167862
"13406","SPPL2B",31.8372606863382,0.00748577779531153,0.31460724679935,0.02379404121001,0.98101689313089,0.999751247167862
"13407","SLC39A3",32.8938775713325,-0.00465237728044636,0.342569786494314,-0.0135808161252527,0.989164409575043,0.999751247167862
"13408","FBXW9",8.60777650503799,0.0146229729999873,0.581031336323301,0.0251672708265957,0.979921542779713,0.999751247167862
"13409","CYP4F12",8.26718467438114,-0.00262405229821704,0.638882934641354,-0.00410725057117089,0.996722897395768,0.999751247167862
"13410","AC008764.8",3.32311833204329,-0.00869509993281358,0.823843883278473,-0.0105543053839419,0.991579039024304,0.999751247167862
"13411","AC092279.1",8.95454019465007,-0.00984585772959291,0.54017922155829,-0.0182270204714463,0.985457746995764,0.999751247167862
"13412","ZNF226",29.2676287599478,-0.00047910445820313,0.357991930133588,-0.00133831077707352,0.998932182812174,0.999751247167862
"13413","AC010331.1",2.06126302813186,-0.00663437229556019,1.11951307887768,-0.00592612308041207,0.995271665564416,0.999751247167862
"13414","CYTH2",40.5833042099205,-0.000406219813011069,0.278325317226719,-0.00145951441664996,0.998835476394127,0.999751247167862
"13415","PRR12",25.4429240002827,-0.00572376582495674,0.368054759417162,-0.0155513973899446,0.98759228025182,0.999751247167862
"13416","ZNF415",5.20013125507883,-0.00854681821473414,0.664077731935986,-0.0128702075129332,0.989731343619094,0.999751247167862
"13417","ZNF347",14.7441413996005,0.0014316471164334,0.409636142449134,0.0034949238313637 6,0.997211459910541,0.999751247167862
"13418","ZNF211",2.18942785806309,0.0149488173688693,1.01137721978192,0.0147806546128186,0.988207173279491,0.999751247167862
"13419","ZNF446",7.85477955345399,-0.00697096929949354,0.548874710710308,-0.0127004745590707,0.989866759853922,0.999751247167862
"13420","SRXN1",5.46297809241433,0.00776692320773341,0.622677816038209,0.012473422061429,0.990047907185624,0.999751247167862
"13421","AL353194.1",3.84839207612647,0.0047625903963102 2,0.7343234434

528,0.00648568480112312,0.994825208509806,0.999751247167862
"13422","TMX4",110.476537565624,0.00103000737188986,0.214227142032555,
0.00480801527816364,0.996163773621803,0.999751247167862
"13423","PLAGL2",6.5048068803001,-0.00321583623180289,0.57209587874063
4,-0.00562114909634024,0.99551499554109,0.999751247167862
"13424","L3MBTL1",4.43722602631502,0.0189202427208991,0.77583141341811
6,0.0243870541894421,0.98054387451633,0.999751247167862
"13425","SERINC3",165.388372521663,0.000282861447798832,0.176217041539
331,0.00160518781457183,0.998719245975569,0.999751247167862
"13426","RUNX1",46.7336282508853,0.00670689371811145,0.283378504224869
,0.0236676163439318,0.981117737180295,0.999751247167862
"13427","MCM3AP",25.9011337261914,-0.00495156750204138,0.3730224945832
16,-0.013274179369729,0.989409048252203,0.999751247167862
"13428","SEPTIN5",3.5127486139907,-0.00323516036833168,0.7981122190436
01,-0.00405351564747181,0.996765771304841,0.999751247167862
"13429","SCARF2",5.73425323568912,-0.00229729619831845,0.6930177870624
01,-0.00331491664601618,0.997355084031815,0.999751247167862
"13430","PPIL2",22.2356826477311,0.00314504659896419,0.325822232139273
,0.00965264579496169,0.992298422546733,0.999751247167862
"13431","APOBEC3C",56.9468104917121,-0.0028282902511802,0.230035616037
709,-0.0122950102244888,0.99019024831983,0.999751247167862
"13432","SYAP1",82.0520939269557,0.00157490365541547,0.214299989444973
,0.00734906081654224,0.994136350619469,0.999751247167862
"13433","TBC1D25",7.1533093617278,-0.00576904108202895,0.5420657776342
4,-0.0106426956285767,0.991508517773681,0.999751247167862
"13434","ZC4H2",7.12322525964204,-0.00759431191835668,0.59680272029356
1,-0.0127249954802839,0.98984719657038,0.999751247167862
"13435","GPRASP2",5.22524240271204,-0.00746546267346738,0.627886811054
341,-0.0118898224043462,0.990513517787386,0.999751247167862
"13436","GRIA3",8.75691811134999,-0.00251440444310764,0.53828462945502
4,-0.00467114293353184,0.9962729807257,0.999751247167862
"13437","FHL1",654.434472896511,0.00121190316136503,0.253667762605597,
0.00477752138827866,0.996188103946297,0.999751247167862
"13438","NAA30",17.9746343586336,-0.000110464198485405,0.3686024149808
91,-0.000299683870739512,0.999760886869994,0.999835284911881
"13439","E2F4",24.2423450583649,2.00111650204962e-05,0.34546979429944,
5.7924499770163e-05,0.999953782935967,0.999953782935967
"13440","AL627309.1",0.314808297382012,-2.74994603383303,2.64513346523
022,-1.0396246805617,0.298514305856315,NA
"13441","AL627309.5",0.828756440966117,1.21932373882118,1.758892620891
24,0.693233756477608,0.488162867152544,NA
"13442","AL627309.4",0,NA,NA,NA,NA,NA
"13443","FAM41C",0.30194018855455,0.0910421727158873,2.45620633524313,
0.0370661745349156,0.970432242295643,NA
"13444","LINC02593",0,NA,NA,NA,NA,NA
"13445","PLEKHN1",0.136922337397099,0.183394507878855,3.3377988902691,
0.0549447446979257,0.956182484581931,NA
"13446","AL645608.7",0.110901857212745,-1.2169087618042,3.340133369026
82,-0.36432939267894,0.715612053721651,NA
"13447","AL390719.2",0,NA,NA,NA,NA,NA

"13448","TTLL10",0,NA,NA,NA,NA,NA
"13449","TNFRSF18",0.613989881264282,0.77162442883918,1.81742550988748
,0.424570044076773,0.671150134245904,NA
"13450","TNFRSF4",0.561745576692298,-0.841651897618886,1.7255490263392
5,-0.487758901527387,0.625720625296203,NA
"13451","C1QTNF12",0.305234002012195,-0.332214363285073,2.419253729817
26,-0.137321009032883,0.890777064935366,NA
"13452","AL162741.1",0,NA,NA,NA,NA,NA
"13453","LINC01786",0.173995588670669,0.483768689799832,3.284683762686
88,0.14728014163656,0.882910906219075,NA
"13454","TAS1R3",0.102366282257309,-0.49131726092088,3.34845351515744,
-0.146729604785264,0.883345450266286,NA
"13455","AL391244.2",0.265168140257861,-0.115484819744169,2.9325971244
9375,-0.0393797084432814,0.968587657675463,NA
"13456","ATAD3C",0.175910378850688,0.313649592621519,3.2560472709057,0
.0963283289601242,0.923259812444105,NA
"13457","AL691432.4",0.164130935857893,0.400527358383716,3.33018851502
426,0.120271677286953,0.904267939474822,NA
"13458","FO704657.1",0.324169996682799,-0.762130878718679,2.3944812359
1694,-0.318286427676611,0.750267678567961,NA
"13459","AL109917.1",0.0367720482966892,-0.560503011376585,3.349520014
37583,-0.167338307868279,0.867103863749685,NA
"13460","CALML6",0,NA,NA,NA,NA,NA
"13461","TMEM52",0.549030436971896,-0.937606412493812,1.90566837742939
,-0.492009220281326,0.622712825460876,NA
"13462","CFAP74",0.176496091173365,-1.14849506633421,3.26459492750587,
-0.351803238024283,0.724985830023263,NA
"13463","PRKCZ",0.367402586358926,0.387883044574848,2.30191642888907,0
.168504399076749,0.866186483192033,NA
"13464","AL590822.2",0,NA,NA,NA,NA,NA
"13465","PRKCZ-
AS1",0.296698427056759,0.680778658728797,2.47341973472073,0.2752378212
12606,0.783133531518291,NA
"13466","AL590822.1",0.0732428936164972,-0.49131726092088,3.3484535151
5744,-0.146729604785264,0.883345450266286,NA
"13467","AL590822.3",0.469950650192765,-0.190361419678693,1.9963658853
6408,-0.0953539734746451,0.924033673278506,NA
"13468","PLCH2",0.147372702532553,-1.1477220656059,3.3390638510896,-0.
343725701810518,0.731052580354999,NA
"13469","AL139246.1",0,NA,NA,NA,NA,NA
"13470","HES5",0,NA,NA,NA,NA,NA
"13471","AL139246.5",0.348127452817314,-2.77495368732187,2.61435422383
524,-1.06142987894389,0.288494584202949,NA
"13472","TNFRSF14-
AS1",0.621770377076403,0.823271544833337,1.67168843357348,0.4924790578
78908,0.622380722982169,NA
"13473","AL139246.3",0.110901857212745,-1.2169087618042,3.340133369026
82,-0.36432939267894,0.715612053721651,NA
"13474","MMEL1",0.305687010103899,-0.848207973241147,2.41217665093934,
-0.351635927207421,0.725111317827357,NA

"13475","TTC34",0.507700627949628,1.70885637406036,2.13053233476397,0.802079530161024,0.422506955040145,NA
"13476","PRDM16-DT",0.743948294094023,-0.999183256166749,1.55899268937706,-0.640915934356306,0.521577302575688,NA
"13477","PRDM16",1.42296657440286,-0.197457824314228,1.63209678185345,-0.120984139243256,0.903703597956916,NA
"13478","AL354743.1",0.22180371442549,-1.25279977890202,3.08716608771214,-0.405808998708733,0.684882946245175,NA
"13479","AL513320.1",0.598638615611309,1.05488723322738,1.70766214849292,0.617737667932944,0.536748277598737,NA
"13480","TP73",0,NA,NA,NA,NA,NA
"13481","AL365330.1",0.157560096502761,-0.491317252697356,3.34845351503297,-0.146729602334801,0.883345452200538,NA
"13482","LINC01134",0.110014941913186,-0.033834469553869,3.34232334787728,-0.0101230389858472,0.991923121432614,NA
"13483","AL365255.1",0.204732564514618,0.248608670877116,3.33613854356166,0.0745198880774602,0.940596716765987,NA
"13484","CHD5",0.0741298089160557,-1.76673671430743,3.34309696236258,-0.528473069790613,0.597171033704837,NA
"13485","AL031847.1",0.0735440965933784,-0.185659846583073,3.34418846206699,-0.0555171601986567,0.955726459171656,NA
"13486","LINC00337",0,NA,NA,NA,NA,NA
"13487","AL031848.2",1.10762685550911,-0.208693223082807,1.33438708909041,-0.156396314674375,0.875720641895614,NA
"13488","HES2",0,NA,NA,NA,NA,NA
"13489","ESPN",0,NA,NA,NA,NA,NA
"13490","TNFRSF25",0.553643908944716,-0.523182484095715,1.78974621283222,-0.292322162966219,0.770040314903036,NA
"13491","PLEKHG5",0.0741298089160557,-1.76673671430743,3.34309696236258,-0.528473069790613,0.597171033704837,NA
"13492","CAMTA1-DT",0,NA,NA,NA,NA,NA
"13493","Z98884.2",0.491616284178886,0.465433277934102,2.25880697513037,0.206052700854281,0.836749745131845,NA
"13494","Z98884.1",0.0367720482966892,-0.560503011376585,3.34952001437583,-0.167338307868279,0.867103863749685,NA
"13495","UTS2",0.323868793705918,-0.88601961950788,2.43915923736479,-0.363247960992131,0.716419663167488,NA
"13496","TNFRSF9",0.360880824349351,-0.842801168501668,2.31268383976649,-0.364425588145575,0.715540230675511,NA
"13497","AL034417.4",1.16931155472061,-0.373102766570344,1.37935514814461,-0.27049071957444,0.786782755632875,NA
"13498","AL034417.2",1.11482403819042,-1.25505687440984,1.37396294956879,-0.913457582537964,0.36100193270805,NA
"13499","RERE-AS1",0.597428959154319,1.19061388306553,1.64164706971668,0.725255692912736,0.468295181887322,NA
"13500","ENO1-AS1",0.239791573166398,-1.32959277084231,2.9363559394555,-0.45280367852436,0.650690114564834,NA

"13501","CA6",0,NA,NA,NA,NA,NA
"13502","LNCTAM34A",1.1886588974565,1.93880898444308,1.45243185035793,
1.33487088152555,0.181918609481066,NA
"13503","BX323043.1",0.127358887561203,0.15032497835859,3.338664945584
14,0.0450254759937551,0.964087002635848,NA
"13504","PIK3CD-AS1",0,NA,NA,NA,NA,NA
"13505","AL139424.3",0.173694385693788,0.450698267773282,2.85372304708
896,0.15793342953621,0.874509255668894,NA
"13506","AL139424.2",0.931682642936558,-0.711831658511954,1.6912238861
1745,-0.420897353895652,0.673830037537251,NA
"13507","CORT",0.751729752413601,-0.78276965222799,1.72292049051832,-0
.45432720577402,0.649593337464,NA
"13508","AL354956.1",0,NA,NA,NA,NA,NA
"13509","AL139423.2",0,NA,NA,NA,NA,NA
"13510","AL139423.1",0.0732428936164972,-0.49131726092088,3.3484535151
5744,-0.146729604785264,0.883345450266286,NA
"13511","C1orf127",0,NA,NA,NA,NA,NA
"13512","MASP2",0,NA,NA,NA,NA,NA
"13513","AL109811.1",0.733329524645388,-0.285975400432883,1.7042276699
7008,-0.167803519137735,0.866737853887688,NA
"13514","ANGPTL7",0.102366282257309,-0.49131726092088,3.34845351515744
,-0.146729604785264,0.883345450266286,NA
"13515","DISP3",0.0636794437806017,-0.49131726092088,3.34845351515744,
-0.146729604785264,0.883345450266286,NA
"13516","FBXO2",0.607315150147646,1.94639957338797,1.65157411469582,1.
17851179433534,0.238592633148811,NA
"13517","DRAXIN",0.127358887561203,0.15032497835859,3.33866494558414,0
.0450254759937551,0.964087002635848,NA
"13518","AL096840.2",0.0732428936164972,-0.49131726092088,3.3484535151
5744,-0.146729604785264,0.883345450266286,NA
"13519","TNFRSF8",0.102366282257309,-0.49131726092088,3.34845351515744
,-0.146729604785264,0.883345450266286,NA
"13520","AADACL4",0,NA,NA,NA,NA,NA
"13521","KAZN-AS1",0,NA,NA,NA,NA,NA
"13522","TMEM51-
AS1",0.137809252696657,-1.1477220656059,3.3390638510896,-0.34372570181
0518,0.731052580354999,NA
"13523","TMEM51",0.249738984789862,-0.445595840703958,2.59717004458552
,-0.171569759797946,0.863775783061595,NA
"13524","FHAD1",0.690936495622832,-0.826167963559151,1.62357962262604,
-0.508855834383333,0.610853279384017,NA
"13525","AL031283.1",0.139138330553998,-0.0338344975521838,3.342323348
20666,-0.0101230473617515,0.991923114749952,NA
"13526","CTRC",0.0367720482966892,-0.560503011376585,3.34952001437583,
-0.167338307868279,0.867103863749685,NA
"13527","CELA2A",0,NA,NA,NA,NA,NA
"13528","AL121992.3",0,NA,NA,NA,NA,NA
"13529","AGMAT",0.254717775122407,0.857533257909012,3.31322339204986,0
.25882144257664,0.795773012681309,NA
"13530","AL121992.1",0.293907567111104,-0.687518690041658,2.7928624230

7872,-0.246169909538104,0.805550707638064,NA
"13531","AL121992.2",0.175910378850688,0.313649592621519,3.25604727090
57,0.0963283289601242,0.923259812444105,NA
"13532","AL450998.3",0,NA,NA,NA,NA,NA
"13533","UQCRHL",0.569569173311715,0.533000189388187,1.88718372668121,
0.282431531097144,0.777612640368161,NA
"13534","HSPB7",0.478939233239905,-1.24939567869091,1.94574902256357,-
0.642115537102931,0.520798168511421,NA
"13535","CLCNKB",0,NA,NA,NA,NA,NA
"13536","FAM131C",0.662935750199327,-3.62221842009454,1.84206392208931
,-1.96639127266883,0.0492534307831359,NA
"13537","AL451042.2",0.0735440965933784,-0.185659846583073,3.344188462
06699,-0.0555171601986567,0.955726459171656,NA
"13538","AL109627.1",0.558593886807792,-0.872560702958852,1.7680433303
0825,-0.493517714187878,0.621646826449378,NA
"13539","CPLANE2",1.02897853864114,-0.342313957521402,1.38737580285384
,-0.246734847773228,0.80511343553876,NA
"13540","SPATA21",0.366610439396214,-2.24370892473058,2.2580699376172,
-0.99364013813417,0.320398095757749,NA
"13541","AL137802.2",0.376069997470606,-0.337572100309193,2.3348194320
0206,-0.144581673290141,0.885041167775182,NA
"13542","AL137798.1",0.339138869770174,-1.32417497355052,2.65062070458
238,-0.499571655522532,0.617376720220205,NA
"13543","BX284668.2",0,NA,NA,NA,NA,NA
"13544","BX284668.6",0.284918984063964,0.682792286303301,2.83389871436
466,0.24093743465224,0.809603607962577,NA
"13545","PADI2",0,NA,NA,NA,NA,NA
"13546","PADI4",0.102366282257309,-0.49131726092088,3.34845351515744,-
0.146729604785264,0.883345450266286,NA
"13547","PADI6",0,NA,NA,NA,NA,NA
"13548","IGSF21",0.0741298089160557,-1.76673671430743,3.34309696236258
,-0.528473069790613,0.597171033704837,NA
"13549","PAX7",0,NA,NA,NA,NA,NA
"13550","AL137127.1",0.952183696890315,-0.722157455224644,1.5122246782
1152,-0.477546402746663,0.632973086325242,NA
"13551","AKR7A3",0.517314023205809,0.743379026496226,1.80777043385341,
0.411213178717418,0.680916225642943,NA
"13552","HTR6",0.0741298089160557,-1.76673671430743,3.34309696236258,-
0.528473069790613,0.597171033704837,NA
"13553","PLA2G2D",0,NA,NA,NA,NA,NA
"13554","Z98257.1",0,NA,NA,NA,NA,NA
"13555","PLA2G2C",0,NA,NA,NA,NA,NA
"13556","UBXN10-
AS1",0.2915937151806,-0.850240888675731,2.45302809421104,-0.3466087040
26764,0.72888530597779,NA
"13557","VWA5B1",0.102366282257309,-0.49131726092088,3.34845351515744,
-0.146729604785264,0.883345450266286,NA
"13558","KIF17",0.146485787232994,0.248608662259703,3.33613854377696,0
.0745198854896011,0.940596718825075,NA
"13559","AL031005.1",0,NA,NA,NA,NA,NA

"13560","AL031728.1",0,NA,NA,NA,NA,NA
"13561","LINC02596",0,NA,NA,NA,NA,NA
"13562","RAP1GAP",0,NA,NA,NA,NA,NA
"13563","LINC01635",0,NA,NA,NA,NA,NA
"13564","CDC42-
AS1",0.716499713286486,-0.959181927297487,1.64302490162467,-0.58379025
5612695,0.559361434985281,NA
"13565","CDC42-
IT1",1.18265223224349,0.711689481063255,1.34117619875746,0.53064577325
6788,0.59566426994654,NA
"13566","AL445253.1",0.183559038506565,0.529394638499161,3.22699253814
936,0.164052018168831,0.869690193908075,NA
"13567","TEX46",0,NA,NA,NA,NA,NA
"13568","AL109936.6",0,NA,NA,NA,NA,NA
"13569","AL109936.2",0.175910378850688,0.313649592621519,3.25604727090
57,0.0963283289601242,0.923259812444105,NA
"13570","E2F2",0,NA,NA,NA,NA,NA
"13571","MDS2",0,NA,NA,NA,NA,NA
"13572","CNR2",0,NA,NA,NA,NA,NA
"13573","MYOM3",0,NA,NA,NA,NA,NA
"13574","IL22RA1",0.836778369762935,-0.307867163899165,1.4822118261862
6,-0.207707939216292,0.835457018276747,NA
"13575","IFNLR1",0.0741298089160557,-1.76673671430743,3.34309696236258
,-0.528473069790613,0.597171033704837,NA
"13576","RCAN3AS",0,NA,NA,NA,NA,NA
"13577","NCMAP-
DT",0.157560096502761,-0.491317252697356,3.34845351503297,-0.146729602
334801,0.883345452200538,NA
"13578","AL031432.1",0,NA,NA,NA,NA,NA
"13579","AL031432.5",0.0367720482966892,-0.560503011376585,3.349520014
37583,-0.167338307868279,0.867103863749685,NA
"13580","AL031432.3",0.148259617832111,-2.17639404086898,3.33507601540
8,-0.652577041966681,0.514028990213041,NA
"13581","RHD",0.127358887561203,0.15032497835859,3.33866494558414,0.04
50254759937551,0.964087002635848,NA
"13582","RHCE",0.457570059671173,-1.88394161052829,2.17090636577639,-0
.867813389019444,0.385496499136352,NA
"13583","AL606491.1",0.295046608041987,-0.778299399754659,2.4673923307
6565,-0.315433986743871,0.752432165265782,NA
"13584","AL020996.1",0.231104193096139,0.317741310460635,2.97787872987
845,0.106700554079852,0.915026543025481,NA
"13585","AUNIP",0,NA,NA,NA,NA,NA
"13586","SLC30A2",0,NA,NA,NA,NA,NA
"13587","TRIM63",0.369869407396491,-2.18301008743594,2.60759739189274,
-0.837172983154194,0.40249534128038,NA
"13588","FAM110D",0.102366282257309,-0.49131726092088,3.34845351515744
,-0.146729604785264,0.883345450266286,NA
"13589","CNKSR1",0.0636794437806017,-0.49131726092088,3.34845351515744
,-0.146729604785264,0.883345450266286,NA
"13590","CRYBG2",0.0741298089160557,-1.76673671430743,3.34309696236258

,-0.528473069790613,0.597171033704837,NA
"13591","ZNF683",0.284295039929652,-0.0383820061561763,2.5093626766356
7,-0.0152955196606477,0.987796416859156,NA
"13592","AL513365.2",0,NA,NA,NA,NA,NA
"13593","RPS6KA1",1.09056741920689,-0.98087767122347,1.4345708981398,-
0.68374290353678,0.494137526417476,NA
"13594","AL512408.1",0,NA,NA,NA,NA,NA
"13595","SFN",0.358074864440778,-1.83062655359436,2.30107188171825,-0.
79555383216773,0.426291410012607,NA
"13596","TRNP1",0.330679615407794,-0.405387626957155,2.37965677974227,
-0.170355502696091,0.864730564264229,NA
"13597","TENT5B",0.341129980543248,-1.35584050242381,2.13008704224124,
-0.636518825539269,0.524438314730738,NA
"13598","FCN3",0,NA,NA,NA,NA,NA
"13599","GPR3",0,NA,NA,NA,NA,NA
"13600","BX293535.1",0,NA,NA,NA,NA,NA
"13601","LINC02574",0.324108776052662,-1.32491523663082,2.684712726775
92,-0.49350354077619,0.62165683860654,NA
"13602","AL445490.1",0,NA,NA,NA,NA,NA
"13603","AL020997.3",0.264582427935183,0.930632542201311,2.84339986125
834,0.327295697971042,0.743444245943564,NA
"13604","SMPDL3B",0,NA,NA,NA,NA,NA
"13605","PTAFR",0.845000683663113,2.39708665476758,2.01282646773975,1.
19090577016275,0.233690579080291,NA
"13606","LINC01715",1.1024722601383,-1.50457435842221,1.42344726519598
,-1.05699339568793,0.290514601560383,NA
"13607","MATN1",0.139138330553998,-0.0338344975521838,3.34232334820666
,-0.0101230473617515,0.991923114749952,NA
"13608","MATN1-
AS1",0.995629312311402,-0.398966450517033,1.37978566984055,-0.28915103
2104237,0.772465802662932,NA
"13609","AL137857.1",0,NA,NA,NA,NA,NA
"13610","LINC01778",0.0367720482966892,-0.560503011376585,3.3495200143
7583,-0.167338307868279,0.867103863749685,NA
"13611","NKAIN1",0.312832716247786,1.10977866355563,2.12048700769892,0
.523360275034139,0.600723563116301,NA
"13612","SERINC2",1.20222348748822,-1.2992372444041,1.26308362032017,-
1.02862330213321,0.30365672366297,NA
"13613","LINC01226",0,NA,NA,NA,NA,NA
"13614","TINAGL1",0.240175534953966,-0.492654690648343,2.6203058694694
2,-0.188014191926418,0.850865526027581,NA
"13615","AC114488.2",0.157560096502761,-0.491317252697356,3.3484535150
3297,-0.146729602334801,0.883345452200538,NA
"13616","AC114488.3",0,NA,NA,NA,NA,NA
"13617","ADGRB2",1.41249213935743,-0.676642663751363,1.17240198587631,
-0.577142201994486,0.563843397571409,NA
"13618","SPOCD1",0.26891496180721,-1.3277650763353,2.82969623342791,-0
.469225304345422,0.63890860009957,NA
"13619","AL354919.1",0.420798011374483,-2.43995659027234,2.44810148298
057,-0.996672975869322,0.318923271132377,NA

"13620","AL136115.2",0.611959732530476,-1.71936074095806,2.05647600660277,-0.836071383978063,0.403114745755657,NA
"13621","AL136115.1",0,NA,NA,NA,NA,NA
"13622","AL445248.1",0.248852069490303,0.780083555267702,2.96292468503169,0.263281601185674,0.792333539707393,NA
"13623","AL049795.2",0.583723259189223,-2.6394486315839,1.96882725623174,-1.34061971319703,0.180043953546716,NA
"13624","AL049795.1",0.409465129029236,1.3798722604301 6,3.00126968086102,0.459762836118845,0.645686461413841,NA
"13625","DCDC2B",0.0741298089160557,-1.76673671430743,3.34309696236258,-0.528473069790613,0.597171033704837,NA
"13626","FAM167B",1.25693384627437,0.0912348946029476,1.22380461203392,0.0745502130861545,0.940572588026208,NA
"13627","TSSK3",1.15692704501322,-0.536731193342104,1.44755022890786,-0.37078588543836,0.710797014986351,NA
"13628","HPCA",0.415259789459303,-1.69558844674257,2.00739027031345,-0.844673042316684,0.398293414769668,NA
"13629","AL031602.2",0,NA,NA,NA,NA,NA
"13630","AL020995.1",0.157560096502761,-0.491317252697356,3.34845351503297,-0.146729602334801,0.883345452200538,NA
"13631","AL662907.3",0.0732428936164972,-0.49131726092088,3.34845351515744,-0.146729604785264,0.883345450266286,NA
"13632","AL513327.1",0.323605822540671,0.859187086673796,2.43516471689191,0.352825039190945,0.724219610906701,NA
"13633","ZSCAN20",1.14719334231871,1.13915425241417,1.42605766840547,0.798813594746067,0.424398506475081,NA
"13634","GJA4",0,NA,NA,NA,NA,NA
"13635","DLGAP3",0,NA,NA,NA,NA,NA
"13636","AL590434.1",0,NA,NA,NA,NA,NA
"13637","AC004865.2",0.30194018855455,0.0910421727158873,2.45620633524313,0.0370661745349156,0.970432242295643,NA
"13638","TFAP2E",0.389178518320742,0.428257360405757,2.26996748318886,0.18866233264458,0.850357475548766,NA
"13639","CLSPN",0.166548679549901,-1.95393031097672,3.33920037562282,-0.585149164824313,0.558447422910535,NA
"13640","AL139286.3",0.0732428936164972,-0.49131726092088,3.34845351515744,-0.146729604785264,0.883345450266286,NA
"13641","AL139286.2",0,NA,NA,NA,NA,NA
"13642","TEKT2",0,NA,NA,NA,NA,NA
"13643","SH3D21",0.663467945856715,-0.425907344531702,1.78445035298135,-0.238677049109336,0.811356014632332,NA
"13644","AL591845.1",0.146485787232994,0.248608662259703,3.33613854377696,0.0745198854896011,0.940596718825075,NA
"13645","CDCA8",0.818246305838478,0.10426076005577,1.59046275134426,0.0655537264029913,0.947733130802872,NA
"13646","MANEAL",1.30380221245502,0.106877440413504,1.27678842640452,0.0837080272684445,0.93328857471127,NA
"13647","AL929472.2",0.0367720482966892,-0.560503011376585,3.34952001437583,-0.167338307868279,0.867103863749685,NA
"13648","POU3F1",0.907699331010911,-1.84001942036754,1.90805789542884,

-0.964341503879784,0.334874739121864,NA
"13649","GJA9",0.369941320673256,1.11554984681986,2.1030042091201,0.530455356190946,0.595796254308461,NA
"13650","RHBDL2",0.137809252696657,-1.1477220656059,3.3390638510896,-0.343725701810518,0.731052580354999,NA
"13651","AL356055.1",0.551870078345599,0.911103995633706,1.772473224836,0.514029765227064,0.607231180454691,NA
"13652","AL442071.2",0,NA,NA,NA,NA,NA
"13653","BMP8A",0.444071129589312,0.414615036158139,1.88057031848348,0.22047302995428,0.825502774601196,NA
"13654","PABPC4-AS1",0,NA,NA,NA,NA,NA
"13655","HEYL",0.230802990119258,0.237440402410633,3.03060926850593,0.0783474151148786,0.937551701540005,NA
"13656","HPCAL4",0,NA,NA,NA,NA,NA
"13657","OXCT2",0,NA,NA,NA,NA,NA
"13658","AL033527.4",0.0735440965933784,-0.185659846583073,3.34418846206699,-0.0555171601986567,0.955726459171656,NA
"13659","MYCL",0.157560096502761,-0.491317252697356,3.34845351503297,-0.146729602334801,0.883345452200538,NA
"13660","MFSD2A",0,NA,NA,NA,NA,NA
"13661","COL9A2",0.304932799035314,-0.455323059556486,2.47402355059449,-0.184041521935826,0.853980881434662,NA
"13662","ZFP69B",1.26602392189115,0.110320370407536,1.29665838293504,0.085080520713421,0.932197376366626,NA
"13663","AL031985.3",0.305819714334872,-1.57468193089132,2.7692160162842,-0.568638171103845,0.569601714969488,NA
"13664","ZFP69",1.29653078045157,0.520065395880507,1.34960042691753,0.385347681808556,0.699979838990852,NA
"13665","AL603839.3",0.751848211306496,-0.0637118578675504,1.6302672165907,-0.03908062262381,0.968826110071849,NA
"13666","AL603839.2",0,NA,NA,NA,NA,NA
"13667","RIMS3",0.100451492077291,-0.0338344573480013,3.34232334773369,-0.010123035334371,0.991923124345921,NA
"13668","NFYC-AS1",1.01658331243123,0.172721451785379,1.37987214475861,0.125172069340956,0.900387328745588,NA
"13669","KCNQ4",0.0636794437806017,-0.49131726092088,3.34845351515744,-0.146729604785264,0.883345450266286,NA
"13670","SLFNL1-AS1",0.136922337397099,0.183394507878855,3.3377988902691,0.0549447446979257,0.956182484581931,NA
"13671","SLFNL1",0,NA,NA,NA,NA,NA
"13672","FOXO6",1.36813894659186,-0.271278754884181,1.20431327874038,-0.225255969250723,0.821780150876501,NA
"13673","AC093151.2",0.239288619654408,0.726512763451421,2.6183991094885,0.277464486150602,0.781423471618519,NA
"13674","EDN2",0,NA,NA,NA,NA,NA
"13675","RIMKLA",0,NA,NA,NA,NA,NA
"13676","ZMYND12",0.531183537811889,0.512344079929104,1.7415912785189,0.294181583387818,0.768619152652684,NA

"13677","AC098484.4",0.0367720482966892,-0.560503011376585,3.34952001437583,-0.167338307868279,0.867103863749685,NA
"13678","AC098484.2",0.305184056591909,1.0238829631905,2.42729878959865,0.42181991256206,0.673156470155355,NA
"13679","TMEM269",0.44495804488887,-0.476413877250727,2.0578524524919,-0.231510221577755,0.816918445445258,NA
"13680","SLC2A1",0.669578502831224,-0.517405507217267,1.55389427099576,-0.332973431252632,0.739154338670139,NA
"13681","SLC2A1-AS1",0.184144750829242,-0.691295663073562,2.81313162320765,-0.245738826214366,0.805884414604706,NA
"13682","CFAP57",0.548156898935687,-1.20160317576281,2.10764641676969,-0.570116109705183,0.568598949450693,NA
"13683","TIE1",0.20332072784659,-1.39924864619322,3.10758170858468,-0.450269301794319,0.652516271227926,NA
"13684","AL139289.2",0.100451492077291,-0.0338344573480013,3.34232334773369,-0.010123035334371,0.991923124345921,NA
"13685","CDC20",0.4722356896538,-1.63083635579888,2.18683090224777,-0.745753297213148,0.455816456863918,NA
"13686","AL139289.1",0,NA,NA,NA,NA,NA
"13687","SZT2-AS1",0.100451492077291,-0.0338344573480013,3.34232334773369,-0.010123035334371,0.991923124345921,NA
"13688","HYI-AS1",0.0741298089160557,-1.76673671430743,3.34309696236258,-0.528473069790613,0.597171033704837,NA
"13689","AL451062.1",0.139138330553998,-0.0338344975521838,3.34232334820666,-0.0101230473617515,0.991923114749952,NA
"13690","ARTN",0,NA,NA,NA,NA,NA
"13691","AL357079.1",0.611836342716288,-0.783542964837656,2.0887035488451,-0.375133639846066,0.707561079830245,NA
"13692","CCDC24",1.31868306466883,-0.969715368614809,1.29753600065024,-0.747351416938608,0.454851463297004,NA
"13693","SLC6A9",1.3666905417669,-1.25461788848893,1.45721830437723,-0.860967697647142,0.389255834232356,NA
"13694","AL139220.2",0.633179235544979,-0.795356793553292,1.75094130824894,-0.454245262137715,0.649652308847731,NA
"13695","ERI3-IT1",0,NA,NA,NA,NA,NA
"13696","ARMH1",0.857854036997383,-0.472932976795809,1.55548249496944,-0.304042622353715,0.761095419324381,NA
"13697","KIF2C",0.102366282257309,-0.49131726092088,3.34845351515744,-0.146729604785264,0.883345450266286,NA
"13698","AL592166.1",0,NA,NA,NA,NA,NA
"13699","BEST4",0.146786990209876,0.2964989704902,3.33426311477484,0.0889248869341921,0.929141604986601,NA
"13700","TCTEX1D4",0.239589822631289,0.773110999527443,2.56583771895994,0.301309390619147,0.763178580793342,NA
"13701","ZSWIM5",0.636739741765339,2.02740964397481,1.75931341064908,1.15238685256586,0.24916214116659,NA
"13702","LINC01144",0.200601781177701,0.585305641041376,3.160438969187

92,0.185197577535177,0.853074069074656,NA
"13703","HPDL",0.110316144890068,0.0897481123376931,3.34029447074234,0.0268683234738127,0.978564758597723,NA
"13704","CCDC163",0.957539269154236,-0.0571797315269176,1.61988587988651,-0.0352986171661202,0.971841625986735,NA
"13705","CCDC17",1.31552367814173,-0.617553411547165,1.37672018667576,-0.448568574445264,0.653742907729562,NA
"13706","PIK3R3",0.935971829184921,1.26896643899763,1.71737872665147,0.738897262033663,0.459969385040497,NA
"13707","TSPAN1",0.157560096502761,-0.491317252697356,3.34845351503297,-0.146729602334801,0.883345452200538,NA
"13708","RAD54L",0.166548679549901,-1.95393031097672,3.33920037562282,-0.585149164824313,0.558447422910535,NA
"13709","TMEM275",0,NA,NA,NA,NA,NA
"13710","MKNK1-AS1",0,NA,NA,NA,NA,NA
"13711","TEX38",0,NA,NA,NA,NA,NA
"13712","CYP4A22-AS1",0.0367720482966892,-0.560503011376585,3.34952001437583,-0.167338307868279,0.867103863749685,NA
"13713","CYP4A22",0.0732428936164972,-0.49131726092088,3.34845351515744,-0.146729604785264,0.883345450266286,NA
"13714","LINC00853",0.831060702316601,-0.951515862035261,1.70055401743941,-0.559532865335261,0.575798107986255,NA
"13715","PDZK1IP1",0,NA,NA,NA,NA,NA
"13716","TAL1",0.0741298089160557,-1.76673671430743,3.34309696236258,-0.528473069790613,0.597171033704837,NA
"13717","STIL",0.360640842002607,-0.556886351045847,2.12807129898026,-0.261685946007871,0.7935635765282,NA
"13718","FOXD2-AS1",0.624762987557167,0.624188168454204,1.74249994666725,0.358214167895983,0.720183045362805,NA
"13719","FOXD2",0.183257835529684,0.495279120319891,3.246926734798,0.152537818304269,0.878762764724423,NA
"13720","TRABD2B",0.962896012228992,1.08669348983828,1.60514715071883,0.677005525226535,0.49840244874956,NA
"13721","SLC5A9",0,NA,NA,NA,NA,NA
"13722","AL109659.2",0.285925320763874,1.00190796089301,2.43754991556965,0.411030746280684,0.681049989757148,NA
"13723","AGBL4",0.0741298089160557,-1.76673671430743,3.34309696236258,-0.528473069790613,0.597171033704837,NA
"13724","DMRTA2",0,NA,NA,NA,NA,NA
"13725","LINC01562",0.283945055524722,-1.32691080208876,2.78374542960066,-0.476663845759455,0.633601511730444,NA
"13726","TTC39A",0.110014941913186,-0.033834469553869,3.34232334787728,-0.0101230389858472,0.991923121432614,NA
"13727","TTC39A-AS1",0,NA,NA,NA,NA,NA
"13728","AC104170.2",0.0732428936164972,-0.49131726092088,3.34845351515744,-0.146729604785264,0.883345450266286,NA
"13729","AC104170.1",0,NA,NA,NA,NA,NA
"13730","AL050343.2",0.603984268870604,-0.679866372342693,1.6575115976

9222,-0.410172920231317,0.681679104101936,NA
"13731","AL050343.3",0,NA,NA,NA,NA,NA
"13732","RAB3B",0.157560096502761,-0.491317252697356,3.34845351503297,
-0.146729602334801,0.883345452200538,NA
"13733","AL513218.1",0.0367720482966892,-0.560503011376585,3.349520014
37583,-0.167338307868279,0.867103863749685,NA
"13734","AL513218.2",0.239791573166398,-1.32959277084231,2.93635593945
55,-0.45280367852436,0.650690114564834,NA
"13735","ORC1",0,NA,NA,NA,NA,NA
"13736","AL445183.1",0.218350821564103,-1.39752731860778,3.03720335813
394,-0.460136235153652,0.645418436617112,NA
"13737","SLC1A7",5.91729342100151,1.66392502128168,0.969444995678184,1
.71636867351888,NA,NA
"13738","AL445183.2",0.856899462850056,-0.703442805857689,1.6883820683
2245,-0.416637216809949,0.676943783041121,NA
"13739","AL606760.2",0.367988298681603,-0.416045891399803,2.6943195694
901,-0.154415940896922,0.877281785385367,NA
"13740","AL606760.3",0,NA,NA,NA,NA,NA
"13741","AL606760.1",1.0281106297927,-0.242399201332363,1.384547443627
71,-0.175074680501552,0.861020953015848,NA
"13742","LRP8",1.17773726137628,1.28524011899083,1.34001874041572,0.95
9121003480971,0.337497790580929,NA
"13743","LINC01771",0.166548679549901,-1.95393031097672,3.339200375622
82,-0.585149164824313,0.558447422910535,NA
"13744","LINC02812",0,NA,NA,NA,NA,NA
"13745","GLIS1",0.0741298089160557,-1.76673671430743,3.34309696236258,
-0.528473069790613,0.597171033704837,NA
"13746","DIO1",0,NA,NA,NA,NA,NA
"13747","SSBP3-
AS1",0.102366282257309,-0.49131726092088,3.34845351515744,-0.146729604
785264,0.883345450266286,NA
"13748","FAM151A",0,NA,NA,NA,NA,NA
"13749","MROH7",0.166548679549901,-1.95393031097672,3.33920037562282,-
0.585149164824313,0.558447422910535,NA
"13750","TTC22",0.137809252696657,-1.1477220656059,3.3390638510896,-0.
343725701810518,0.731052580354999,NA
"13751","LEXM",0,NA,NA,NA,NA,NA
"13752","TMEM61",0.146786990209876,0.2964989704902,3.33426311477484,0.
0889248869341921,0.929141604986601,NA
"13753","MIR4422HG",0.0741298089160557,-1.76673671430743,3.34309696236
258,-0.528473069790613,0.597171033704837,NA
"13754","LINC01767",0,NA,NA,NA,NA,NA
"13755","DAB1",0.102366282257309,-0.49131726092088,3.34845351515744,-0
.146729604785264,0.883345450266286,NA
"13756","TACSTD2",0.20332072784659,-1.39924864619322,3.10758170858468,
-0.450269301794319,0.652516271227926,NA
"13757","AL136985.3",0,NA,NA,NA,NA,NA
"13758","LINC01135",1.16863051535375,0.874474164822472,1.4237614895715
,0.614199900213384,0.539083239308965,NA
"13759","AL136985.2",0,NA,NA,NA,NA,NA

"13760","LINC02777",0.185031666128801,-1.62793589934517,3.24336249302777,-0.501928447049853,0.61571785405042,NA
"13761","AC093424.1",0.388435152786078,-1.25011571613562,2.31504211076261,-0.539996966069793,0.589199124740611,NA
"13762","HOOK1",0,NA,NA,NA,NA,NA
"13763","AC099792.1",0,NA,NA,NA,NA,NA
"13764","NFIA-AS1",0,NA,NA,NA,NA,NA
"13765","AC099791.4",0.239791573166398,-1.32959277084231,2.9363559394555,-0.45280367852436,0.650690114564834,NA
"13766","L1TD1",0.157560096502761,-0.491317252697356,3.34845351503297,-0.146729602334801,0.883345452200538,NA
"13767","ANGPTL3",0,NA,NA,NA,NA,NA
"13768","AC103923.1",0.0367720482966892,-0.560503011376585,3.34952001437583,-0.167338307868279,0.867103863749685,NA
"13769","ROR1-AS1",0.766579298533352,-2.32431954880001,2.20246395971388,-1.05532693897155,0.291275824625406,NA
"13770","AL357078.2",0.647474616246589,0.428089454549083,1.76082444236764,0.243118759740446,0.807913398959812,NA
"13771","DNAJC6",0.174581300993347,-0.691169037761009,2.86286045881777,-0.241426030958711,0.809224939549254,NA
"13772","AC119800.1",0.2915937151806,-0.850240888675731,2.45302809421104,-0.346608704026764,0.72888530597779,NA
"13773","SGIP1",0.675619678258394,-2.54149630812262,1.81208675282763,-1.40252463308217,0.160758639929975,NA
"13774","AL139147.1",0,NA,NA,NA,NA,NA
"13775","TCTEX1D1",0,NA,NA,NA,NA,NA
"13776","IL23R",0.0367720482966892,-0.560503011376585,3.34952001437583,-0.167338307868279,0.867103863749685,NA
"13777","IL12RB2",0,NA,NA,NA,NA,NA
"13778","GNG12-AS1",0.737594734912848,-0.247909245551211,1.68640584279927,-0.147004498715271,0.883128469439671,NA
"13779","DIRAS3",0.867371795841989,1.44327444757979,1.5449170288783,0.93420838828328,0.350196409595084,NA
"13780","DEPDC1",0.0741298089160557,-1.76673671430743,3.34309696236258,-0.528473069790613,0.597171033704837,NA
"13781","LRRC7",0,NA,NA,NA,NA,NA
"13782","AL353771.1",0.0367720482966892,-0.560503011376585,3.34952001437583,-0.167338307868279,0.867103863749685,NA
"13783","CTH",1.38161645829805,0.29956838105716,1.36633433552119,0.219249691140118,0.826455549060452,NA
"13784","AL354872.2",0.0636794437806017,-0.49131726092088,3.34845351515744,-0.146729604785264,0.883345450266286,NA
"13785","AL031429.1",0.518955149573953,-0.431642602861939,1.92601140244629,-0.224112174161428,0.822670019793585,NA
"13786","AL031429.2",0,NA,NA,NA,NA,NA
"13787","ZRANB2-AS1",0.175609175873806,0.247512349757584,3.30210845793575,0.0749558510601773,0.940249838776562,NA

"13788","ZRANB2-
AS2",1.4369406732362,1.93723728050273,1.63971353654258,1.1814486111930
9,0.237424556574731,NA
"13789","NEGR1-IT1",0,NA,NA,NA,NA,NA
"13790","LRRIQ3",0.0732428936164972,-0.49131726092088,3.34845351515744
,-0.146729604785264,0.883345450266286,NA
"13791","AC105271.1",0.0732428936164972,-0.49131726092088,3.3484535151
5744,-0.146729604785264,0.883345450266286,NA
"13792","ERICH3",0,NA,NA,NA,NA,NA
"13793","AC135803.1",1.14409770082892,-0.131775123596941,1.31230904694
804,-0.100414703307428,0.920015097213366,NA
"13794","LHX8",0,NA,NA,NA,NA,NA
"13795","SLC44A5",0.200601781177701,0.585305641041376,3.16043896918792
,0.185197577535177,0.853074069074656,NA
"13796","ST6GALNAC3",0,NA,NA,NA,NA,NA
"13797","AC093433.2",0,NA,NA,NA,NA,NA
"13798","AK5",0.763391926381543,1.31072073073822,1.56304442936788,0.83
8569081026249,0.40171116716649,NA
"13799","AC093575.1",0,NA,NA,NA,NA,NA
"13800","GIPC2",1.18432661040084,-0.965569457546071,1.33293036550805,-
0.724396024377493,0.468822639897634,NA
"13801","AC096531.2",0,NA,NA,NA,NA,NA
"13802","ADGRL4",0.700232719864486,-1.13383713355962,1.70984134762151,
-0.663124175314189,0.507251028605289,NA
"13803","LINC01781",0,NA,NA,NA,NA,NA
"13804","LINC01725",0,NA,NA,NA,NA,NA
"13805","TTLL7-
IT1",0.942186339846565,-0.668916056721527,1.40199059260099,-0.47711879
1132933,0.633277534470056,NA
"13806","SAMD13",1.14585699017245,0.416214516581488,1.29636892289182,0
.321061782052778,0.748163571808622,NA
"13807","DNASE2B",0,NA,NA,NA,NA,NA
"13808","AL359762.1",0.0741298089160557,-1.76673671430743,3.3430969623
6258,-0.528473069790613,0.597171033704837,NA
"13809","LPAR3",0,NA,NA,NA,NA,NA
"13810","MCOLN2",0.460407169315448,-0.672874675686778,2.2400938489751,
-0.300377895325517,0.763888922622206,NA
"13811","WDR63",0.330931741039118,0.249707170562266,2.3504963061479,0.
106235934048966,0.91539516100748,NA
"13812","AL360219.1",0.346668202458428,-1.94732862277873,3.00338753971
666,-0.648377406187962,0.516740879961632,NA
"13813","AC092807.2",0.175609175873806,0.247512349757584,3.30210845793
575,0.0749558510601773,0.940249838776562,NA
"13814","LINC02795",0.307098846771927,1.01615615877409,3.2570492212097
2,0.311986737000149,0.755050602967235,NA
"13815","CLCA4-
AS1",0.339740111731699,1.20312752727266,2.09157395552813,0.57522590778
7166,0.565138523241819,NA
"13816","AL121989.1",0,NA,NA,NA,NA,NA
"13817","LINC02801",0,NA,NA,NA,NA,NA

"13818","PKN2-
AS1",0.147673905509434,-0.842065770106804,3.33478677621422,-0.25250962
8535456,0.800647172695484,NA
"13819","GBP7",0,NA,NA,NA,NA,NA
"13820","AC099063.4",0.881662778453834,1.45371527483805,1.553406489398
03,0.935824128944759,0.349363746030788,NA
"13821","GBP5",1.36056488194871,-0.340766090632414,1.32143689479029,-0
.257875417264245,0.796503057540291,NA
"13822","GBP6",0,NA,NA,NA,NA,NA
"13823","AC099568.1",0.102366282257309,-0.49131726092088,3.34845351515
744,-0.146729604785264,0.883345450266286,NA
"13824","AC099568.2",0.173694385693788,0.450698267773282,2.85372304708
896,0.15793342953621,0.874509255668894,NA
"13825","LINC01763",0,NA,NA,NA,NA,NA
"13826","BRDT",0,NA,NA,NA,NA,NA
"13827","EPHX4",0.0636794437806017,-0.49131726092088,3.34845351515744,
-0.146729604785264,0.883345450266286,NA
"13828","AC104836.1",0.185031666128801,-1.62793589934517,3.24336249302
777,-0.501928447049853,0.61571785405042,NA
"13829","GFI1",0.184144750829242,-0.691295663073562,2.81313162320765,-
0.245738826214366,0.805884414604706,NA
"13830","AC093577.1",0.0741298089160557,-1.76673671430743,3.3430969623
6258,-0.528473069790613,0.597171033704837,NA
"13831","AL049796.1",0.247238482287166,0.868764518721955,2.52920099602
734,0.343493664634222,0.731227106130206,NA
"13832","ABCA4",0.0367720482966892,-0.560503011376585,3.34952001437583
,-0.167338307868279,0.867103863749685,NA
"13833","ARHGAP29-
AS1",0.267000171627192,-0.853137625449662,2.51498162517089,-0.33922221
0178849,0.734442338867411,NA
"13834","AC118469.2",0,NA,NA,NA,NA,NA
"13835","SLC44A3",0.686709517167008,0.479701814182684,1.79275208050155
,0.267578445118011,0.789023824427067,NA
"13836","AC092802.4",0,NA,NA,NA,NA,NA
"13837","DPYD-
AS1",0.419911096074925,-1.32028169514223,2.49077535946509,-0.530068554
807674,0.59606440017536,NA
"13838","DPYD-
IT1",0.276060667951097,0.926641171611055,2.22442592371387,0.4165754236
77831,0.676988988618009,NA
"13839","PLPPR5",0.506087040746491,1.76156984108242,2.03719669053394,0
.864702877865331,0.387201889506756,NA
"13840","LRRC39",0.175609175873806,0.247512349757584,3.30210845793575,
0.0749558510601773,0.940249838776562,NA
"13841","AL445928.2",0.0732428936164972,-0.49131726092088,3.3484535151
5744,-0.146729604785264,0.883345450266286,NA
"13842","GPR88",0,NA,NA,NA,NA,NA
"13843","AC104506.1",1.38940382316936,-0.537601795107908,1.16319361087
133,-0.462177396852447,0.643954110992234,NA
"13844","AC093157.2",0.0732428936164972,-0.49131726092088,3.3484535151

5744,-0.146729604785264,0.883345450266286,NA
"13845","AL109741.1",0,NA,NA,NA,NA,NA
"13846","S1PR1",1.22535564612215,-0.705506050109639,1.17029625034721,-0.602843980659023,0.546612485598553,NA
"13847","COL11A1",0.622219039777449,1.91340053936603,2.34335276501477,0.81652262003923,0.414201291599348,NA
"13848","VAV3",0.576916629990711,0.563807479882612,2.12821214517239,0.264920713454975,0.79107054124159,NA
"13849","VAV3-AS1",0.0741298089160557,-1.76673671430743,3.34309696236258,-0.528473069790613,0.597171033704837,NA
"13850","LINC02785",0,NA,NA,NA,NA,NA
"13851","AL160171.1",0.157560096502761,-0.491317252697356,3.34845351503297,-0.146729602334801,0.883345452200538,NA
"13852","FNDC7",0,NA,NA,NA,NA,NA
"13853","SPATA42",0,NA,NA,NA,NA,NA
"13854","AL356488.2",0.723835456356831,1.04133493034795,1.77543563674429,0.586523616399577,0.557523695947376,NA
"13855","KIAA1324",0.0367720482966892,-0.560503011376585,3.34952001437583,-0.167338307868279,0.867103863749685,NA
"13856","CELSR2",0.230802990119258,0.237440402410633,3.03060926850593,0.0783474151148786,0.937551701540005,NA
"13857","PSRC1",1.16226659876915,1.07593044418158,1.36328709058069,0.789217804243479,0.429984717683037,NA
"13858","SYPL2",0.925538251609625,1.64362090453078,1.59604155517153,1.02981084621831,0.303098808218626,NA
"13859","ATXN7L2",1.11039898169582,0.130501884686154,1.35577798013758,0.0962560880896669,0.92331718571385,NA
"13860","AMIGO1",1.3858151539908,1.66733716022945,1.42364966750419,1.17117096873452,0.241530062067347,NA
"13861","GNAT2",0,NA,NA,NA,NA,NA
"13862","AC000032.1",0.5040727981247,0.407656716514595,2.13663545532543,0.190793761986181,0.848687176125093,NA
"13863","ALX3",0.567585001584359,-0.99041573415116,1.89861833041983,-0.521650780613793,0.601913499934438,NA
"13864","UBL4B",0,NA,NA,NA,NA,NA
"13865","RBM15-AS1",0.0367720482966892,-0.560503011376585,3.34952001437583,-0.167338307868279,0.867103863749685,NA
"13866","LAMTOR5-AS1",1.38219936619913,-0.135797673116805,1.34718910293887,-0.100800750852694,0.919708630784128,NA
"13867","KCNA2",1.40641458735575,1.3730141923871,1.34639677586652,1.01976937036518,0.307837852921823,NA
"13868","AL365361.1",0.729695247376305,1.35463753355188,1.63984102163635,0.82607857449506,0.408759520721588,NA
"13869","KCNA3",0.146485787232994,0.248608662259703,3.33613854377696,0.0745198854896011,0.940596718825075,NA
"13870","AL360270.2",0.304932799035314,-0.455323059556486,2.47402355059449,-0.184041521935826,0.853980881434662,NA

"13871","AL360270.1",0.0732428936164972,-0.49131726092088,3.3484535151
5744,-0.146729604785264,0.883345450266286,NA
"13872","AL360270.3",0.174581300993347,-0.691169037761009,2.8628604588
1777,-0.241426030958711,0.809224939549254,NA
"13873","AL355816.1",0,NA,NA,NA,NA,NA
"13874","AL355816.2",0.450493735074624,-2.14067227781541,2.19393815434
663,-0.975721340902118,0.329202573686981,NA
"13875","DENND2D",0.926380434954286,0.326120242491401,1.37616594807436
,0.236977410280886,0.812674315139959,NA
"13876","OVGP1",0.185031666128801,-1.62793589934517,3.24336249302777,-
0.501928447049853,0.61571785405042,NA
"13877","AL390195.2",0.800111876882448,-0.772568132303377,1.6274475371
3692,-0.474711543490067,0.634992588998819,NA
"13878","AL390195.1",0,NA,NA,NA,NA,NA
"13879","TMIGD3",0,NA,NA,NA,NA,NA
"13880","ADORA3",0,NA,NA,NA,NA,NA
"13881","LINC01160",0,NA,NA,NA,NA,NA
"13882","INKA2-
AS1",1.26124414765865,-2.62907438997051,1.85179564539386,-1.4197432619
0368,0.15568243839379,NA
"13883","AL049557.1",0,NA,NA,NA,NA,NA
"13884","LINC01750",0.488140689144711,1.66600694741326,2.1453672357574
8,0.776560264203452,0.437418252773092,NA
"13885","AL354760.1",0.460407169315448,-0.672874675686778,2.2400938489
751,-0.300377895325517,0.763888922622206,NA
"13886","AL603832.1",0.184144750829242,-0.691295663073562,2.8131316232
0765,-0.245738826214366,0.805884414604706,NA
"13887","PPM1J",0.75330999339481,0.327296450740656,1.52768762796144,0.
214243045993247,0.830357529453228,NA
"13888","TAFA3",0,NA,NA,NA,NA,NA
"13889","AL390242.1",0.383440250755117,-1.38194811510753,2.56276654272
887,-0.53924073537967,0.589720755955717,NA
"13890","AL137856.1",0,NA,NA,NA,NA,NA
"13891","PTPN22",1.42152950383262,-0.82442204491025,1.17161666877241,-
0.703661928755296,0.48164333479645,NA
"13892","AP4B1-
AS1",0.221502511448609,-1.56478135306832,3.04955054434762,-0.513118681
036017,0.607868304703499,NA
"13893","BCL2L15",0,NA,NA,NA,NA,NA
"13894","HIPK1-
AS1",0.978255388980819,-1.09282441315499,1.95846484734954,-0.558000525
071432,0.576844025622827,NA
"13895","AMPD1",0,NA,NA,NA,NA,NA
"13896","TSPAN2",0.944079100244473,-2.21694988823577,1.4478763769833,-
1.5311734644465,0.125726527120187,NA
"13897","LINC01765",0,NA,NA,NA,NA,NA
"13898","NGF",0.6990901076886,1.34216450332723,1.64503803440635,0.8158
86608853747,0.414564991764409,NA
"13899","CASQ2",0,NA,NA,NA,NA,NA
"13900","MAB21L3",0.417113788685114,1.41442366572219,2.25597241774124,

0.626968510163952,0.530679876972655,NA
"13901","LINC01779",0.200902984154582,0.617734931975277,3.113565194884
92,0.198401155366881,0.842731213560209,NA
"13902","LINC01762",0,NA,NA,NA,NA,NA
"13903","AL390066.1",0,NA,NA,NA,NA,NA
"13904","AL157904.1",0.157560096502761,-0.491317252697356,3.3484535150
3297,-0.146729602334801,0.883345452200538,NA
"13905","CD101",0.397735631456727,0.15200363794564,2.11198752656295,0.
07197184454636,0.94262431452384,NA
"13906","AL445231.1",0,NA,NA,NA,NA,NA
"13907","AL358072.1",0.312390553690004,-0.679283711575989,2.4487373888
1309,-0.277401617126955,0.781471740045957,NA
"13908","AL157902.1",0,NA,NA,NA,NA,NA
"13909","AL139420.1",0.184144750829242,-0.691295663073562,2.8131316232
0765,-0.245738826214366,0.805884414604706,NA
"13910","AL139420.2",0.391744004281009,-0.194185375615089,2.2961989252
5906,-0.0845681850465898,0.932604693084977,NA
"13911","AL359915.1",0.137809252696657,-1.1477220656059,3.339063851089
6,-0.343725701810518,0.731052580354999,NA
"13912","HAO2",0.102366282257309,-0.49131726092088,3.34845351515744,-0
.146729604785264,0.883345450266286,NA
"13913","HSD3B2",0,NA,NA,NA,NA,NA
"13914","LINC00622",1.0392504141216,0.722497414602125,1.51115588585224
,0.478109122537455,0.632572539471116,NA
"13915","ADAM30",0,NA,NA,NA,NA,NA
"13916","AC245008.1",0,NA,NA,NA,NA,NA
"13917","FCGR1B",0.0741298089160557,-1.76673671430743,3.34309696236258
,-0.528473069790613,0.597171033704837,NA
"13918","AC244453.2",0.0367720482966892,-0.560503011376585,3.349520014
37583,-0.167338307868279,0.867103863749685,NA
"13919","FAM72B",0.276864824439969,-0.72952978945291,2.87172568327462,
-0.2540388149543,0.799465571478648,NA
"13920","LINC02798",0.857858291426379,0.669158790608668,1.408900057723
1,0.474951212430273,0.634821747745524,NA
"13921","AL592494.1",0,NA,NA,NA,NA,NA
"13922","AL592494.2",0,NA,NA,NA,NA,NA
"13923","AC239800.2",0.518200938505368,-0.0507451875318495,1.833766198
13857,-0.0276726594608191,0.977923229939557,NA
"13924","HIST2H3PS2",1.39285027664749,1.79796740903276,1.3351673577427
,1.346623251839,0.178101601601545,NA
"13925","FAM72C",0.315634421727363,0.0162048499507083,2.43727149812695
,0.00664876685390272,0.994695090663815,NA
"13926","AC246785.3",0.741645287697441,-3.0538344465633,1.684681494108
04,-1.81270730238546,0.0698769776073902,NA
"13927","FP700111.1",0,NA,NA,NA,NA,NA
"13928","FAM72D",1.01402851911759,2.66317701448645,1.44769035308092,1.
83960403467411,0.065826391604083,NA
"13929","AC245014.3",0.554036126082213,1.84022205000134,1.871306379835
12,0.983388968172858,0.325416037700512,NA
"13930","PDZK1",0.267575038415947,0.563240138104268,2.52089568762832,0

.223428577734674,0.82320196461372,NA
"13931","CD160",0.378426950208406,1.30388489209699,2.05276066214983,0.635186028327066,0.525307094470031,NA
"13932","ITGA10",0.102366282257309,-0.49131726092088,3.34845351515744,-0.146729604785264,0.883345450266286,NA
"13933","LIX1L-AS1",0.391941315496387,1.42320456477131,2.54196858586189,0.559882829664771,0.575559361218714,NA
"13934","ANKRD34A",0.0732428936164972,-0.49131726092088,3.34845351515744,-0.146729604785264,0.883345450266286,NA
"13935","AC239799.2",0.98290216306484,1.92365053555857,1.51222598602488,1.27206552019066,0.203349840696296,NA
"13936","AC239799.1",0.746739154039678,0.116041688778838,1.68912031666764,0.0686994808089036,0.945228831483675,NA
"13937","LINC00624",0.292971574465989,1.01027116194909,3.29385652095093,0.306713773208745,0.759061245268142,NA
"13938","AC239803.1",0.368026530493238,1.19074263094412,2.05006005643351,0.580833047894051,0.561352979471559,NA
"13939","LINC02804",0.0636794437806017,-0.49131726092088,3.34845351515744,-0.146729604785264,0.883345450266286,NA
"13940","LINC02805",0.137809252696657,-1.1477220656059,3.3390638510896,-0.343725701810518,0.731052580354999,NA
"13941","LINC01731",0.315120193005521,0.245400022010969,3.2393007694085,0.0757570968180826,0.939612349767913,NA
"13942","AC245100.6",0.276947583250656,-0.158224666587883,2.24258642930609,-0.0705545456443527,0.94375228752887,NA
"13943","PPIAL4G",0.102366282257309,-0.49131726092088,3.34845351515744,-0.146729604785264,0.883345450266286,NA
"13944","NUDT4B",0.110014941913186,-0.033834469553869,3.34232334787728,-0.0101230389858472,0.991923121432614,NA
"13945","AC239802.2",0,NA,NA,NA,NA,NA
"13946","AC239804.1",0,NA,NA,NA,NA,NA
"13947","FCGR1A",0.620042299394207,-1.60600615194282,1.79928316144149,-0.892581104719604,0.372081541689668,NA
"13948","AC243772.2",0.110901857212745,-1.2169087618042,3.34013336902682,-0.36432939267894,0.715612053721651,NA
"13949","HIST2H3D",0.201488696477259,-0.508277411998464,3.16327545678924,-0.160680730762022,0.872344866840293,NA
"13950","HIST2H4A",0.0735440965933784,-0.185659846583073,3.34418846206699,-0.0555171601986567,0.955726459171656,NA
"13951","HIST2H4B",0.533196186765512,0.495828603525761,1.93337758626408,0.256457200625701,0.7975978244884,NA
"13952","HIST2H2AB",0.184144750829242,-0.691295663073562,2.81313162320765,-0.245738826214366,0.805884414604706,NA
"13953","SV2A",0,NA,NA,NA,NA,NA
"13954","AC242988.2",0,NA,NA,NA,NA,NA
"13955","CIART",0.445756106464212,1.57689858612009,2.91444898602858,0.541062339289345,0.588464615248459,NA
"13956","FALEC",0.55897784859536,-0.231121109873379,1.72648937694543,-0.133867669827362,0.893507215871836,NA

"13957","AL356356.1",0.358161877680461,1.11569273694527,2.33981371918157,0.476829726998748,0.633483375671316,NA
"13958","HORMAD1",0.139138330553998,-0.0338344975521838,3.34232334820666,-0.0101230473617515,0.991923114749952,NA
"13959","ANXA9",0.237675032451271,0.812476888782201,2.93416062775353,0.276902662075544,0.781854848453636,NA
"13960","BNIPL",0.2915937151806,-0.850240888675731,2.45302809421104,-0.346608704026764,0.72888530597779,NA
"13961","AL592424.1",0,NA,NA,NA,NA,NA
"13962","TNFAIP8L2",0.379062607951369,-0.896733663230212,2.35597047097777,-0.380621775305211,0.70348392106999,NA
"13963","TMOD4",0.0367720482966892,-0.560503011376585,3.34952001437583,-0.167338307868279,0.867103863749685,NA
"13964","AL391069.2",1.23154362575632,-0.996861546210566,1.47070582183972,-0.677811654382098,0.497891120676297,NA
"13965","CGN",0,NA,NA,NA,NA,NA
"13966","AL391335.1",0.148259617832111,-2.17639404086898,3.335076015408,-0.652577041966681,0.514028990213041,NA
"13967","OAZ3",0.553622370764166,-0.732705490110449,2.0831738585821,-0.351725559099115,0.725044090476922,NA
"13968","AL589765.5",0,NA,NA,NA,NA,NA
"13969","TDRKH",1.38779667418385,-0.0797629343493284,1.24906554002428,-0.0638580857396627,0.949083226859262,NA
"13970","TDRKH-AS1",0,NA,NA,NA,NA,NA
"13971","LINGO4",0,NA,NA,NA,NA,NA
"13972","RORC",0,NA,NA,NA,NA,NA
"13973","C2CD4D",0.0367720482966892,-0.560503011376585,3.34952001437583,-0.167338307868279,0.867103863749685,NA
"13974","C2CD4D-AS1",0,NA,NA,NA,NA,NA
"13975","AL450992.2",0.0732428936164972,-0.49131726092088,3.34845351515744,-0.146729604785264,0.883345450266286,NA
"13976","AL450992.1",0.449034484715737,-1.31903840108013,2.44559733198216,-0.539352240792252,0.589643828608869,NA
"13977","AL589986.2",0,NA,NA,NA,NA,NA
"13978","HRNR",0.175609175873806,0.247512349757584,3.30210845793575,0.0749558510601773,0.940249838776562,NA
"13979","FLG",0,NA,NA,NA,NA,NA
"13980","FLG2",0.102366282257309,-0.49131726092088,3.34845351515744,-0.146729604785264,0.883345450266286,NA
"13981","SPRR2D",0,NA,NA,NA,NA,NA
"13982","SPRR2A",0.661415708886232,0.237048193568644,1.8719596899314,0.126631035296135,0.899232432055055,NA
"13983","SPRR2B",0.292971574465989,1.01027116194909,3.29385652095093,0.306713773208745,0.759061245268142,NA
"13984","SPRR2E",0.175609175873806,0.247512349757584,3.30210845793575,0.0749558510601773,0.940249838776562,NA
"13985","LOR",0,NA,NA,NA,NA,NA
"13986","S100A12",0.308937660828617,-0.6869866595118,2.75278474113728,-0.24956061737976,0.802927157786805,NA
"13987","S100A7",0.0735440965933784,-0.185659846583073,3.3441884620669

9,-0.0555171601986567,0.955726459171656,NA
"13988","BX470102.1",0.477165402640788,0.339129872826896,1.93186602242931,0.175545233929029,0.860651231022044,NA
"13989","S100A5",0.358140339499911,0.413822999339114,2.07222666238332,0.199699679022161,0.841715464820706,NA
"13990","BX470102.2",1.44512255878328,0.4079169343486,1.12863199550206,0.361425988253277,0.717781023333633,NA
"13991","S100A14",0.313133919224668,1.13737532363375,2.37252941477638,0.479393560539247,0.631658673683647,NA
"13992","S100A1",0.837397571337755,0.641253001727093,1.85197800103244,0.346253033982912,0.729152561423669,NA
"13993","AL162258.2",1.36631356884339,-0.378433615598915,1.36937439114273,-0.276355113726871,0.782275328754288,NA
"13994","AL358472.5",0.257387644445739,-0.166363093190703,2.56504828799876,-0.0648576847340752,0.948287312309793,NA
"13995","AL358472.4",0.920170425538005,-0.564417241060794,1.51479651030335,-0.372602681100556,0.709444177088056,NA
"13996","AL358472.3",0.157560096502761,-0.491317252697356,3.34845351503297,-0.146729602334801,0.883345452200538,NA
"13997","AL358472.2",0.95901648644867,1.77665616901365,1.52646103457694,1.16390535281895,0.24446237010638,NA
"13998","NUP210L",0,NA,NA,NA,NA,NA
"13999","C1orf189",0,NA,NA,NA,NA,NA
"14000","IL6R-AS1",0.684493524010108,0.49756416922913,1.79855816279139,0.276646137735631,0.78205183396175,NA
"14001","AL162591.2",0,NA,NA,NA,NA,NA
"14002","UBE2Q1-AS1",0,NA,NA,NA,NA,NA
"14003","CHRNB2",0,NA,NA,NA,NA,NA
"14004","KCNN3",0.34054426822057,-0.292421495304613,2.34583666707645,-0.124655522444813,0.900796270603264,NA
"14005","AL451085.1",0.173995588670669,0.483768689799832,3.28468376268688,0.14728014163656,0.882910906219075,NA
"14006","DCST2",0.424823239295199,-1.69637615105059,1.99462087517732,-0.850475482414561,0.395060785819042,NA
"14007","DCST1-AS1",0.416588867316644,-0.981167612923955,2.01295263683305,-0.487427073528969,0.625955711422229,NA
"14008","EFNA1",0.295369349199418,-0.498711181181302,2.48533021027214,-0.200661939858163,0.840962921935508,NA
"14009","TRIM46",0,NA,NA,NA,NA,NA
"14010","AC234582.1",0.842469560785176,-2.60138736875647,1.6216317186373,-1.6041788889912,0.108674626715026,NA
"14011","AC234582.2",0.181578773267413,-1.95393031097672,3.33920037562282,-0.585149164824313,0.558447422910535,NA
"14012","RUSC1-AS1",1.17714729462461,0.74939851709042,1.35036974736704,0.554958016907298,0.578923377028338,NA
"14013","ASH1L-IT1",0,NA,NA,NA,NA,NA
"14014","AL355388.2",0.593982566436195,0.921364700268173,2.14818106048

106,0.428904582215172,0.667992665420688,NA
"14015","MEX3A",1.11547673286857,0.718034284323787,1.53163495870355,0.468802491248676,0.639210819079317,NA
"14016","SEMA4A",0.946584286852094,0.480405121074772,1.44696659351535,0.332008439744034,0.739882886153884,NA
"14017","PAQR6",0.343044770723266,-1.83025924174448,2.32925281575478,-0.785770968855264,0.432001677398361,NA
"14018","TSACC",0,NA,NA,NA,NA,NA
"14019","C1orf61",0,NA,NA,NA,NA,NA
"14020","IQGAP3",0.0741298089160557,-1.76673671430743,3.34309696236258,-0.528473069790613,0.597171033704837,NA
"14021","TTC24",0,NA,NA,NA,NA,NA
"14022","HAPLN2",0,NA,NA,NA,NA,NA
"14023","BCAN",0,NA,NA,NA,NA,NA
"14024","NTRK1",0.537376915522716,-0.725192510625201,1.82038269295815,-0.398373657050514,0.690354773056456,NA
"14025","INSRR",0,NA,NA,NA,NA,NA
"14026","LRRC71",0.102366282257309,-0.49131726092088,3.34845351515744,-0.146729604785264,0.883345450266286,NA
"14027","FCRL5",0,NA,NA,NA,NA,NA
"14028","FCRL3",0.0732428936164972,-0.49131726092088,3.34845351515744,-0.146729604785264,0.883345450266286,NA
"14029","AL356276.1",0,NA,NA,NA,NA,NA
"14030","FCRL2",0,NA,NA,NA,NA,NA
"14031","FCRL1",0,NA,NA,NA,NA,NA
"14032","KIRREL1-IT1",0.0367720482966892,-0.560503011376585,3.34952001437583,-0.167338307868279,0.867103863749685,NA
"14033","CD1D",0.110901857212745,-1.2169087618042,3.34013336902682,-0.36432939267894,0.715612053721651,NA
"14034","AL138899.1",0,NA,NA,NA,NA,NA
"14035","CD1A",0.0732428936164972,-0.49131726092088,3.34845351515744,-0.146729604785264,0.883345450266286,NA
"14036","CD1B",0,NA,NA,NA,NA,NA
"14037","CD1E",0.461907183215397,1.4579683082444,2.17918867913612,0.669041796244265,0.503468816460873,NA
"14038","SPTA1",0.0367720482966892,-0.560503011376585,3.34952001437583,-0.167338307868279,0.867103863749685,NA
"14039","AIM2",0,NA,NA,NA,NA,NA
"14040","CADM3-AS1",1.3504222199334,-0.2659009864743,1.18876696060984,-0.22367797498165,0.823007885164788,NA
"14041","ACKR1",1.28188938375887,-1.31437150298774,1.19439275973674,-1.10045166656691,0.271135377688243,NA
"14042","FCRL6",0.47173273614181,0.207096797661373,1.97948491349532,0.104621558997228,0.916676106980748,NA
"14043","SLAMF8",0.267000171627192,-0.853137625449662,2.51498162517089,-0.339222210178849,0.734442338867411,NA
"14044","AL590560.2",1.10308896930939,1.03440602152663,1.51234613851885,0.683974386009076,0.493991340169444,NA

"14045","VSIG8",0.696474717538012,-1.12275345075283,1.86717744948977,-0.60131052410666,0.54763318129262,NA
"14046","CFAP45",0.211052146313155,-0.488988616138086,2.71532944071237,-0.180084452665824,0.85708626804187,NA
"14047","IGSF9",0.173995588670669,0.483768689799832,3.28468376268688,0.14728014163656,0.882910906219075,NA
"14048","SLAMF9",0.230228123330503,-1.33023934114211,2.97714568949567,-0.446817011957333,0.655007187193824,NA
"14049","KCNJ10",0.257387644445739,-0.166363093190703,2.56504828799876,-0.0648576847340752,0.948287312309793,NA
"14050","AL121987.1",1.09660572575716,0.995259465996943,1.47144793566405,0.676381027064877,0.498798760095482,NA
"14051","KCNJ9",0.102366282257309,-0.49131726092088,3.34845351515744,-0.146729604785264,0.883345450266286,NA
"14052","ATP1A2",0.0367720482966892,-0.560503011376585,3.34952001437583,-0.167338307868279,0.867103863749685,NA
"14053","ATP1A4",0,NA,NA,NA,NA,NA
"14054","AL121987.2",0,NA,NA,NA,NA,NA
"14055","AL139011.1",0.304648289689518,0.883629640495721,2.74575758756345,0.321816333859189,0.747591839419236,NA
"14056","NHLH1",0.0741298089160557,-1.76673671430743,3.34309696236258,-0.528473069790613,0.597171033704837,NA
"14057","SLAMF6",0.398238584968717,-1.83124581126634,2.25547900778347,-0.811909933520493,0.41684331409865,NA
"14058","SLAMF1",0.44915381168336,-0.252395733607446,2.01296812629867,-0.125384863431264,0.900218870775263,NA
"14059","AL121985.1",0,NA,NA,NA,NA,NA
"14060","SLAMF7",0.544785592831849,-0.310826770021341,1.89313192731556,-0.164186534248613,0.869584301366373,NA
"14061","LY9",0.110014941913186,-0.033834469553869,3.34232334787728,-0.0101230389858472,0.991923121432614,NA
"14062","CD244",0.137809252696657,-1.1477220656059,3.3390638510896,-0.343725701810518,0.731052580354999,NA
"14063","ITLN1",0,NA,NA,NA,NA,NA
"14064","F11R",0.322981878406359,0.0998814290120277,2.43523825703497,0.0410150541629708,0.967283894474422,NA
"14065","TSTD1",1.3300641956106,-0.479335148306833,1.27825844013552,-0.374990794706596,0.707667312736854,NA
"14066","NECTIN4",0,NA,NA,NA,NA,NA
"14067","AL590714.1",0.282030265344704,-0.851485042625668,2.47907279984832,-0.343469156161032,0.731245540886465,NA
"14068","ADAMTS4",0.925729739174036,0.743221478304304,1.70442639258034,0.436053725487751,0.662797750047699,NA
"14069","NR1I3",0,NA,NA,NA,NA,NA
"14070","MPZ",0.767125746550407,-3.81952008441798,1.97283432494663,-1.93605719249704,0.0528606966414986,NA
"14071","CFAP126",0.181578773267413,-1.95393031097672,3.33920037562282,-0.585149164824313,0.558447422910535,NA
"14072","AL592295.4",0.946837646462186,-0.191460138324906,1.49826855526722,-0.127787596991087,0.898317065754193,NA

"14073","HSPA6",0.833480977738036,-1.6309810961804,1.63080585669513,-1
.00010745576155,0.317258508359934,NA
"14074","FCGR3B",0.996324301647751,1.86783906293285,1.60296950188834,1
.16523680627267,0.243923150877476,NA
"14075","FCRLA",0,NA,NA,NA,NA,NA
"14076","FCRLB",1.20578399370859,0.476564660123821,1.22654343730509,0.
38854283152899,0.697614363825303,NA
"14077","NOS1AP",0.176496091173365,-1.14849506633421,3.26459492750587,
-0.351803238024283,0.724985830023263,NA
"14078","C1orf226",0,NA,NA,NA,NA,NA
"14079","SH2D1B",0.449034484715737,-1.31903840108013,2.44559733198216,
-0.539352240792252,0.589643828608869,NA
"14080","AL596325.1",0.744019872127586,-1.46371255289236,1.87968997997
858,-0.77869891763164,0.436157093856249,NA
"14081","RGS4",0.148259617832111,-2.17639404086898,3.335076015408,-0.6
52577041966681,0.514028990213041,NA
"14082","AL499616.1",0,NA,NA,NA,NA,NA
"14083","NUF2",0.951810518711037,-1.60060175936291,1.49326935844498,-1
.07187745486835,0.28377507772764,NA
"14084","AL391001.1",0.925751277354586,1.08879899638273,1.511326086005
74,0.7204262577511,0.47126258786378,NA
"14085","AL357568.1",0.210165231013596,0.627110932648478,3.11655680733
581,0.20121915672205,0.840527212935289,NA
"14086","AL356441.1",0.166548679549901,-1.95393031097672,3.33920037562
282,-0.585149164824313,0.558447422910535,NA
"14087","AL451074.5",0.100451492077291,-0.0338344573480013,3.342323347
73369,-0.010123035334371,0.991923124345921,NA
"14088","TMCO1-
AS1",0.410347646239029,-1.32074922904993,2.50843327198929,-0.526523565
046849,0.598524483393891,NA
"14089","AL358115.1",0.442609347500998,0.132555327879018,1.84961014356
03,0.0716666311225257,0.942867212359173,NA
"14090","AL596087.2",0.911202107366953,-1.17448012535019,1.43348537618
26,-0.819317828325432,0.412605103389786,NA
"14091","LINC01675",0,NA,NA,NA,NA,NA
"14092","ILDR2",0.0636794437806017,-0.49131726092088,3.34845351515744,
-0.146729604785264,0.883345450266286,NA
"14093","GPA33",0,NA,NA,NA,NA,NA
"14094","DUSP27",0,NA,NA,NA,NA,NA
"14095","AL451050.2",0.127358887561203,0.15032497835859,3.338664945584
14,0.0450254759937551,0.964087002635848,NA
"14096","AL136984.1",0.413787161837068,0.110504566484034,1.99593571726
468,0.0553647923268164,0.955847844449542,NA
"14097","AL359962.3",0.0741298089160557,-1.76673671430743,3.3430969623
6258,-0.528473069790613,0.597171033704837,NA
"14098","AL359962.2",0,NA,NA,NA,NA,NA
"14099","AL031733.2",0,NA,NA,NA,NA,NA
"14100","ADCY10",0,NA,NA,NA,NA,NA
"14101","Z99943.1",0.0732428936164972,-0.49131726092088,3.348453515157
44,-0.146729604785264,0.883345450266286,NA

"14102","AL023755.1",0.0732428936164972,-0.49131726092088,3.3484535151
5744,-0.146729604785264,0.883345450266286,NA
"14103","XCL2",0.98326415699593,0.058538655199643,1.4341242166l049,0.0
40818399495406,0.967440670906418,NA
"14104","CCDC181",0.296698427056759,0.680778658728797,2.47341973472073
,0.275237821212606,0.783133531518291,NA
"14105","F5",0.898068969431473,-1.63602317498088,2.18806848288518,-0.7
47701997344993,0.454639926145324,NA
"14106","SELP",0.110901857212745,-1.2169087618042,3.34013336902682,-0.
36432939267894,0.715612053721651,NA
"14107","SELL",0.68047103052446,0.152574534120054,1.65372764977901,0.0
922609803013471,0.926490689307805,NA
"14108","SELE",0.0741298089160557,-1.76673671430743,3.34309696236258,-
0.528473069790613,0.597171033704837,NA
"14109","LINC01681",0.0741298089160557,-1.76673671430743,3.34309696236
258,-0.528473069790613,0.597171033704837,NA
"14110","LINC01142",1.08432415800141,-0.680491211750987,1.290282149925
63,-0.527397214469882,0.597917777484226,NA
"14111","GORAB-
AS1",1.10962236437195,0.255512743244976,1.43013153018299,0.17866380668
6566,0.85820168993174,NA
"14112","BX284613.2",0.175609175873806,0.247512349757584,3.30210845793
575,0.0749558510601773,0.940249838776562,NA
"14113","MROH9",0,NA,NA,NA,NA,NA
"14114","AL021026.1",0,NA,NA,NA,NA,NA
"14115","MYOCOS",0.157560096502761,-0.491317252697356,3.34845351503297
,-0.146729602334801,0.883345452200538,NA
"14116","FASLG",0.303471016947,-0.658169734815469,2.43860095812758,-0.
269896447232116,0.787239920484044,NA
"14117","ANKRD45",1.21580255368968,-1.1152997134452,1.4065806484716,-0
.792915581951942,0.427827008279763,NA
"14118","GAS5-
AS1",0.660589584540881,-2.51207830616547,1.81754013645839,-1.382130856
85163,0.166931525491156,NA
"14119","SERPINC1",0,NA,NA,NA,NA,NA
"14120","RC3H1-
IT1",0.642601725799973,-2.22125767423893,1.97237400569085,-1.126184824
90135,0.260087295404371,NA
"14121","RABGAP1L-
DT",0.137809252696657,-1.1477220656059,3.3390638510896,-0.343725701810
518,0.731052580354999,NA
"14122","GPR52",0.0732428936164972,-0.49131726092088,3.34845351515744,
-0.146729604785264,0.883345450266286,NA
"14123","RABGAP1L-
IT1",0.642671107347311,-0.59925969674612,1.55496275012508,-0.385385242
635501,0.699952014498215,NA
"14124","Z99127.1",0,NA,NA,NA,NA,NA
"14125","TNN",0.0735440965933784,-0.185659846583073,3.34418846206699,-
0.0555171601986567,0.955726459171656,NA
"14126","TNR",0,NA,NA,NA,NA,NA

"14127","LINC02803",0.0367720482966892,-0.560503011376585,3.3495200143
7583,-0.167338307868279,0.867103863749685,NA
"14128","AL590723.1",0.230802990119258,0.237440402410633,3.03060926850
593,0.0783474151148786,0.937551701540005,NA
"14129","AL591043.2",0.137809252696657,-1.1477220656059,3.339063851089
6,-0.343725701810518,0.731052580354999,NA
"14130","PAPPA2",0.59758192826138,-0.718462918015111,1.89174986834824,
-0.379787481440361,0.704103175696231,NA
"14131","ASTN1",0.305687010103899,-0.848207973241147,2.41217665093934,
-0.351635927207421,0.725111317827357,NA
"14132","RASAL2-
AS1",0.555141485547965,0.596584589444265,2.0675653139599,0.28854449502
3308,0.772929975967525,NA
"14133","CLEC20A",0,NA,NA,NA,NA,NA
"14134","TEX35",0.310917926067768,1.15052592799326,2.34028283809482,0.
491618324616644,0.622989186723195,NA
"14135","AL449106.1",0,NA,NA,NA,NA,NA
"14136","AXDND1",0.157560096502761,-0.491317252697356,3.34845351503297
,-0.146729602334801,0.883345452200538,NA
"14137","AL160286.2",0,NA,NA,NA,NA,NA
"14138","AL353708.3",0.35783913652303,0.365859147993896,2.059710929392
24,0.177626453680007,0.859016347873581,NA
"14139","AL353708.1",0.562046779669179,-0.748876317552344,1.7026098667
9994,-0.439840231256181,0.660052827058607,NA
"14140","LHX4",0.459500285057311,0.597439447676033,2.00042882720096,0.
298655687996649,0.765202770547793,NA
"14141","AL162431.1",0.623231823921515,-1.64163340961381,1.90624581423
546,-0.861186630472537,0.389135262284184,NA
"14142","KIAA1614-
AS1",0.314222585059335,-1.49515354645625,2.42031350762541,-0.617752015
078063,0.536738818706479,NA
"14143","LINC01732",0,NA,NA,NA,NA,NA
"14144","TEDDM1",0,NA,NA,NA,NA,NA
"14145","RGSL1",0,NA,NA,NA,NA,NA
"14146","AL355999.1",0.0735440965933784,-0.185659846583073,3.344188462
06699,-0.0555171601986567,0.955726459171656,NA
"14147","SHCBP1L",0.100451492077291,-0.0338344573480013,3.342323347733
69,-0.010123035334371,0.991923124345921,NA
"14148","NMNAT2",0.671459315628603,-0.399765179987685,1.8472449820271,
-0.216411566347305,0.828666951243084,NA
"14149","SMG7-
AS1",0.59537931236783,-1.76813548456826,1.85517800914211,-0.9530813085
61812,0.340548875709377,NA
"14150","AL445228.2",0.539653637708192,-2.45445954386727,2.05825028093
578,-1.19249809734089,0.2330659963461,NA
"14151","AL078645.1",0.807497565373079,0.73622771688706,1.640970066662
85,0.448653958925822,0.653681302487175,NA
"14152","AL713852.1",0.498165585353468,0.197948396422649,1.94525111178
797,0.101759816623729,0.918947321710412,NA
"14153","AL713852.2",0.660960169065101,-0.430945428932687,1.6008397100

7608,-0.269199612066224,0.787776078782502,NA
"14154","AL078644.2",0,NA,NA,NA,NA,NA
"14155","OCLM",0,NA,NA,NA,NA,NA
"14156","AL096803.4",0.0367720482966892,-0.560503011376585,3.349520014
37583,-0.167338307868279,0.867103863749685,NA
"14157","PACERR",0,NA,NA,NA,NA,NA
"14158","ERVMER61-1",0,NA,NA,NA,NA,NA
"14159","BRINP3",0.551821296917551,0.369348724302321,1.9043823254568,0
.193946729795304,0.846217593240801,NA
"14160","RGS18",0.421370346433812,-2.14315573922607,2.22923659272606,-
0.961385501305303,0.336358371562453,NA
"14161","AL390957.1",0.666056228422498,-2.54235346198754,1.81764172829
87,-1.39870989007673,0.161899997234295,NA
"14162","AL136987.1",0,NA,NA,NA,NA,NA
"14163","RGS13",0.295632320364664,-1.87186550711609,2.77313524931022,-
0.674999716505601,0.499675945057307,NA
"14164","B3GALT2",0.35886701140349,1.25799451591014,2.32360437602023,0
.54139789410484,0.588233358409003,NA
"14165","LINC01031",0,NA,NA,NA,NA,NA
"14166","AL138778.1",0.102366282257309,-0.49131726092088,3.34845351515
744,-0.146729604785264,0.883345450266286,NA
"14167","AL136456.1",0,NA,NA,NA,NA,NA
"14168","CFHR3",0.0367720482966892,-0.560503011376585,3.34952001437583
,-0.167338307868279,0.867103863749685,NA
"14169","CFHR1",0.0636794437806017,-0.49131726092088,3.34845351515744,
-0.146729604785264,0.883345450266286,NA
"14170","ASPM",0.294159692742428,0.0483394037151892,2.44071044902073,0
.0198054643206793,0.984198558841866,NA
"14171","CRB1",0.574673885187874,1.80636074759547,1.91367040448354,0.9
43924692237149,0.345208132924303,NA
"14172","LHX9",0.659516353912334,2.12096571216169,1.77045900056737,1.1
9797505137481,0.230926729742069,NA
"14173","AL450352.1",0,NA,NA,NA,NA,NA
"14174","AL157402.2",0,NA,NA,NA,NA,NA
"14175","MIR181A1HG",0.666776797351647,0.221687977593299,1.63259037077
278,0.135789100292417,0.891988007954322,NA
"14176","NR5A2",0.0741298089160557,-1.76673671430743,3.34309696236258,
-0.528473069790613,0.597171033704837,NA
"14177","LINC00862",0,NA,NA,NA,NA,NA
"14178","AL359834.1",0,NA,NA,NA,NA,NA
"14179","KIF14",0.166548679549901,-1.95393031097672,3.33920037562282,-
0.585149164824313,0.558447422910535,NA
"14180","DDX59-
AS1",0.0367720482966892,-0.560503011376585,3.34952001437583,-0.1673383
07868279,0.867103863749685,NA
"14181","INAVA",0.166045726037911,0.183394512381981,3.33779889015228,0
.0549447460489789,0.956182483505573,NA
"14182","KIF21B",0.780529141472017,-0.0205601731117668,1.9393358108989
7,-0.0106016570189751,0.991541259999462,NA
"14183","AL358473.1",0,NA,NA,NA,NA,NA

"14184","ASCL5",0,NA,NA,NA,NA,NA
"14185","IGFN1",0,NA,NA,NA,NA,NA
"14186","PKP1",0,NA,NA,NA,NA,NA
"14187","LAD1",0,NA,NA,NA,NA,NA
"14188","TNNI1",0,NA,NA,NA,NA,NA
"14189","AC096677.1",0.405384291112604,0.287714103939607,1.89638327060
822,0.151717275931953,0.879409930689183,NA
"14190","IPO9-
AS1",1.37268382165565,-0.322255005891454,1.30288420497316,-0.247339713
430706,0.804645326478433,NA
"14191","AL645504.1",0.0732428936164972,-0.49131726092088,3.3484535151
5744,-0.146729604785264,0.883345450266286,NA
"14192","ELF3",0.0367720482966892,-0.560503011376585,3.34952001437583,
-0.167338307868279,0.867103863749685,NA
"14193","GPR37L1",0,NA,NA,NA,NA,NA
"14194","PTPN7",1.20858540327065,0.715469512386657,1.46802600886532,0.
487368417225568,0.625997270892098,NA
"14195","LGR6",0.0732428936164972,-0.49131726092088,3.34845351515744,-
0.146729604785264,0.883345450266286,NA
"14196","SYT2",0.102366282257309,-0.49131726092088,3.34845351515744,-0
.146729604785264,0.883345450266286,NA
"14197","AC104463.2",0.147673905509434,-0.842065770106804,3.3347867762
1422,-0.252509628535456,0.800647172695484,NA
"14198","AC098934.4",0,NA,NA,NA,NA,NA
"14199","PPFIA4",0,NA,NA,NA,NA,NA
"14200","ADORA1",0.383440250755117,-1.38194811510753,2.56276654272887,
-0.53924073537967,0.589720755955717,NA
"14201","CHI3L1",40.9687303223521,0.904666151685198,1.47717900193448,0
.612428250401928,NA,NA
"14202","CHIT1",0.221502511448609,-1.56478135306832,3.04955054434762,-
0.513118681036017,0.607868304703499,NA
"14203","LINC01353",0.181578773267413,-1.95393031097672,3.339200375622
82,-0.585149164824313,0.558447422910535,NA
"14204","LINC01136",0,NA,NA,NA,NA,NA
"14205","LAX1",0.246937279310285,0.827316883439734,2.55507596510077,0.
323793458488075,0.746094409004131,NA
"14206","ZBED6",1.07852296492099,0.25513288602365,1.30132400564392,0.1
96056389428862,0.84456602995022,NA
"14207","LINC00303",0,NA,NA,NA,NA,NA
"14208","AL592146.2",0.102366282257309,-0.49131726092088,3.34845351515
744,-0.146729604785264,0.883345450266286,NA
"14209","LINC00628",0,NA,NA,NA,NA,NA
"14210","AL512306.3",0.204732564514618,0.248608670877116,3.33613854356
166,0.0745198880774602,0.940596716765987,NA
"14211","LRRN2",0.614197693543855,1.97076569868583,2.71444873187221,0.
726027968605822,0.467821624925937,NA
"14212","AL512306.2",0.0367720482966892,-0.560503011376585,3.349520014
37583,-0.167338307868279,0.867103863749685,NA
"14213","NFASC",0,NA,NA,NA,NA,NA
"14214","CNTN2",0.351519554724494,1.2182323152282,2.30159582767501,0.5

29298976205921,0.596598065696855,NA
"14215","TMEM81",1.19964502949434,1.27531545909025,1.42814713224714,0.892986044850706,0.37186464598334,NA
"14216","NUAK2",0.211052146313155,-0.488988616138086,2.71532944071237,-0.180084452665824,0.85708626804187,NA
"14217","CDK18",0.348275686687135,0.335441884772111,2.06969612118201,0.162073012235506,0.871248358307384,NA
"14218","MFSD4A",0.0735440965933784,-0.185659846583073,3.34418846206699,-0.0555171601986567,0.955726459171656,NA
"14219","SLC45A3",0.22061559614905,-0.418038608046788,3.07273464003886,-0.136047741513241,0.891783539523794,NA
"14220","PM20D1",0.268461953715506,-0.692213851544516,2.51693598492032,-0.275022430324715,0.783299005639148,NA
"14221","RHEX",0.350190476867153,0.252842669947774,1.94464010584612,0.130020289712045,0.896550374083493,NA
"14222","C1orf147",0,NA,NA,NA,NA,NA
"14223","AC244034.2",0.296519235664223,-2.72668234943778,3.16193663701917,-0.862345664209225,0.388497331277209,NA
"14224","AL591846.2",0.488239711910554,0.128337068229332,1.95822837784094,0.0655373345017247,0.947746181583193,NA
"14225","IL19",0.346668202458428,-1.94732862277873,3.00338753971666,-0.648377406187962,0.516740879961632,NA
"14226","IL20",0.166548679549901,-1.95393031097672,3.33920037562282,-0.585149164824313,0.558447422910535,NA
"14227","IL24",0.0732428936164972,-0.49131726092088,3.34845351515744,-0.146729604785264,0.883345450266286,NA
"14228","FCMR",0.489964465164114,-0.897553445004759,1.99159980432532,-0.450669578825761,0.652227710900958,NA
"14229","PIGR",0,NA,NA,NA,NA,NA
"14230","C4BPB",0.0367720482966892,-0.560503011376585,3.34952001437583,-0.167338307868279,0.867103863749685,NA
"14231","AL445493.3",0,NA,NA,NA,NA,NA
"14232","CR2",0,NA,NA,NA,NA,NA
"14233","CR1",0.45985543437541,0.096685612506136,2.17458742061307,0.0444615891684308,0.964536469084358,NA
"14234","CR1L",0.157560096502761,-0.491317252697356,3.34845351503297,-0.146729602334801,0.883345452200538,NA
"14235","AL356275.1",0.401504765332282,1.47227555468942,2.19982361369066,0.669269820328625,0.503323374784758,NA
"14236","TRAF3IP3",0.829270669687959,0.629381670485555,1.49557624614655,0.420828875897969,0.673880044300358,NA
"14237","C1orf74",0.633621303670034,0.53960897461696,1.91612533387807,0.281614654885251,0.778239001866131,NA
"14238","IRF6",0.0636794437806017,-0.49131726092088,3.34845351515744,-0.146729604785264,0.883345450266286,NA
"14239","KCNH1",0,NA,NA,NA,NA,NA
"14240","AL590101.1",0.563781357494638,0.894895764358641,1.93140036143012,0.463340373249183,0.643120411040619,NA
"14241","RD3",0,NA,NA,NA,NA,NA
"14242","NEK2",0,NA,NA,NA,NA,NA

"14243","DTL",0,NA,NA,NA,NA,NA
"14244","AL360091.2",0,NA,NA,NA,NA,NA
"14245","AL360091.3",0,NA,NA,NA,NA,NA
"14246","LINC01740",0,NA,NA,NA,NA,NA
"14247","AC092803.4",0.221239540283362,0.178834610992208,3.06205963446949,0.0584033730039328,0.953427328091912,NA
"14248","AL590648.2",0,NA,NA,NA,NA,NA
"14249","LINC02773",0.0636794437806017,-0.49131726092088,3.34845351515744,-0.146729604785264,0.883345450266286,NA
"14250","BATF3",1.31704997941301,-1.56765691475342,1.43780570610026,-1.09031206935835,0.275575700412871,NA
"14251","PROX1-AS1",0.0367720482966892,-0.560503011376585,3.34952001437583,-0.167338307868279,0.867103863749685,NA
"14252","AC011700.1",0,NA,NA,NA,NA,NA
"14253","KCNK2",0.166548679549901,-1.95393031097672,3.33920037562282,-0.585149164824313,0.558447422910535,NA
"14254","TGFB2-AS1",0.100451492077291,-0.0338344573480013,3.34232334773369,-0.010123035334371,0.991923124345921,NA
"14255","LYPLAL1-DT",0.301053273254991,1.08368293634553,2.41489596894672,0.448749325138916,0.653612498174957,NA
"14256","AL360093.1",0,NA,NA,NA,NA,NA
"14257","AC096642.2",0.419911096074925,-1.32028169514223,2.49077535946509,-0.530068554807674,0.59606440017536,NA
"14258","ZC3H11B",1.24142446025405,-1.46087009276537,1.19786131832979,-1.21956529559057,0.222629709088847,NA
"14259","SLC30A10",0.204732564514618,0.248608670877116,3.33613854356166,0.0745198880774602,0.940596716765987,NA
"14260","C1orf115",0,NA,NA,NA,NA,NA
"14261","LINC01352",0.136922337397099,0.183394507878855,3.3377988902691,0.0549447446979257,0.956182484581931,NA
"14262","HLX-AS1",0.89158735414609,-0.632341157236998,1.88265245787255,-0.335877795496871,0.736963021945447,NA
"14263","LINC02817",1.10270037661966,0.601167850946711,1.50034973789094,0.400685144112987,0.688651949724982,NA
"14264","LINC01655",0.102366282257309,-0.49131726092088,3.34845351515744,-0.146729604785264,0.883345450266286,NA
"14265","LINC02257",0,NA,NA,NA,NA,NA
"14266","AL356108.1",0.0735440965933784,-0.185659846583073,3.34418846206699,-0.0555171601986567,0.955726459171656,NA
"14267","AL513314.2",0.731407857345284,0.0756603792637646,1.75076323114501,0.043215654702939,0.965529626098428,NA
"14268","HHIPL2",0,NA,NA,NA,NA,NA
"14269","FAM177B",0,NA,NA,NA,NA,NA
"14270","AL392172.2",0,NA,NA,NA,NA,NA
"14271","AL929091.1",0.24871109909402,-1.11566793947822,2.59977760371317,-0.429139760987536,0.66782151812021,NA

"14272","SUSD4",0.181578773267413,-1.95393031097672,3.33920037562282,-0.585149164824313,0.558447422910535,NA
"14273","CAPN8",0.181578773267413,-1.95393031097672,3.33920037562282,-0.585149164824313,0.558447422910535,NA
"14274","AC138393.3",0.176496091173365,-1.14849506633421,3.26459492750587,-0.351803238024283,0.724985830023263,NA
"14275","AC092809.2",0.0732428936164972,-0.49131726092088,3.34845351515744,-0.146729604785264,0.883345450266286,NA
"14276","AC092809.4",0.504228298961188,-1.31726387959561,2.38522289744417,-0.552260286033266,0.580770035719828,NA
"14277","AC096537.1",0.157560096502761,-0.491317252697356,3.34845351503297,-0.146729602334801,0.883345452200538,NA
"14278","AC099066.2",0.346668202458428,-1.94732862277873,3.00338753971666,-0.648377406187962,0.516740879961632,NA
"14279","LEFTY1",0.255708582183469,-2.44071869332471,2.85324394739347,-0.855418862994307,0.392319304627377,NA
"14280","AL117348.1",0,NA,NA,NA,NA,NA
"14281","LEFTY2",0.245258217048015,-1.32928540753819,2.91755618878102,-0.455616043540048,0.648666104227452,NA
"14282","AL512343.2",0.84736969447929,0.143228951569712,1.46418994606155,0.0978212915304989,0.922074200269624,NA
"14283","ACBD3-AS1",0,NA,NA,NA,NA,NA
"14284","MIXL1",0,NA,NA,NA,NA,NA
"14285","STUM",0.598588670191023,1.97044879234018,2.03126042026431,0.970062121371807,0.332015528332663,NA
"14286","ITPKB-IT1",0.166548679549901,-1.95393031097672,3.33920037562282,-0.585149164824313,0.558447422910535,NA
"14287","ITPKB-AS1",0.378799636786123,0.955583479781293,2.66241880823434,0.358915538316458,0.719658275181294,NA
"14288","AL359732.1",0.461415075237329,0.524484459998416,2.02654392241427,0.258807348904426,0.795783887415333,NA
"14289","AL353689.3",0,NA,NA,NA,NA,NA
"14290","AL627308.3",0.102366282257309,-0.49131726092088,3.34845351515744,-0.146729604785264,0.883345450266286,NA
"14291","AL731702.1",0.166548679549901,-1.95393031097672,3.33920037562282,-0.585149164824313,0.558447422910535,NA
"14292","PRSS38",0,NA,NA,NA,NA,NA
"14293","LINC02809",0.0732428936164972,-0.49131726092088,3.34845351515744,-0.146729604785264,0.883345450266286,NA
"14294","IBA57-DT",0,NA,NA,NA,NA,NA
"14295","OBSCN-AS1",0,NA,NA,NA,NA,NA
"14296","AL353593.1",1.2907016510373,1.43720309407998,1.45596588878654,0.987113163260852,0.323587165406894,NA
"14297","AL353593.3",0,NA,NA,NA,NA,NA
"14298","AL670729.3",0.332594405587812,-0.658132278841225,2.38474260669479,-0.27597623198144,0.782566321704692,NA
"14299","TRIM17",1.35046335059936,0.646052663822738,1.40590344573876,0.459528473154324,0.64585471001016,NA

"14300","HIST3H2BB",0.971812620073736,-0.566556573050422,1.45225879332
286,-0.390120945147872,0.696447115498593,NA
"14301","AL117350.1",0.110901857212745,-1.2169087618042,3.340133369026
82,-0.36432939267894,0.715612053721651,NA
"14302","ACTA1",0.386311337782032,-0.61003754468642,2.606628632716,-0.
234033163385759,0.814959232819682,NA
"14303","LINC01736",0.100451492077291,-0.0338344573480013,3.3423233477
3369,-0.010123035334371,0.991923124345921,NA
"14304","AL136988.2",0,NA,NA,NA,NA,NA
"14305","AL136988.1",0.0636794437806017,-0.49131726092088,3.3484535151
5744,-0.146729604785264,0.883345450266286,NA
"14306","PGBD5",0,NA,NA,NA,NA,NA
"14307","AL158214.2",0,NA,NA,NA,NA,NA
"14308","AL118511.1",0,NA,NA,NA,NA,NA
"14309","TRIM67",0,NA,NA,NA,NA,NA
"14310","AL136171.2",1.28928044090762,1.14382138817249,1.5732698418061
9,0.727034458919857,0.467204846745152,NA
"14311","DISC1-
IT1",0.0732428936164972,-0.49131726092088,3.34845351515744,-0.14672960
4785264,0.883345450266286,NA
"14312","MAP3K21",0.773482887769902,-0.0870515388710107,1.562200690257
68,-0.0557236592032564,0.955561951464249,NA
"14313","SLC35F3",0,NA,NA,NA,NA,NA
"14314","AL160408.2",1.25158040857104,-0.515827303281796,1.42690720342
58,-0.361500244755488,0.717725522047138,NA
"14315","LINC01132",0,NA,NA,NA,NA,NA
"14316","AL391832.4",0,NA,NA,NA,NA,NA
"14317","AL391832.2",1.1222913214995,0.860651980388175,1.5003388075647
6,0.573638418235094,0.566212508524202,NA
"14318","AL391832.3",0.137809252696657,-1.1477220656059,3.339063851089
6,-0.343725701810518,0.731052580354999,NA
"14319","LINC01348",0,NA,NA,NA,NA,NA
"14320","AL732292.2",0.19433214479945,-0.0333008172506769,3.1614299744
8539,-0.0105334666652225,0.991595664992027,NA
"14321","GNG4",1.29368420350321,3.02098460182292,1.51640569064613,1.99
220078139888,0.0463490325485792,NA
"14322","LINC02768",0.345709709125306,-0.403701136108005,2.35390943449
03,-0.171502407948596,0.863828737224812,NA
"14323","EDARADD",0.102366282257309,-0.49131726092088,3.34845351515744
,-0.146729604785264,0.883345450266286,NA
"14324","LGALS8-
AS1",0.200902984154582,0.617734931975277,3.11356519488492,0.1984011553
66881,0.842731213560209,NA
"14325","AL359921.2",0.805697512488487,-3.02971535761365,1.62427389098
083,-1.86527369210136,0.0621430507510178,NA
"14326","AL359921.1",0.338411033874358,0.245695297616098,2.39493458792
811,0.10258956501549,0.918288725051033,NA
"14327","ACTN2",0.254821666883911,-1.3286579469836,2.88029438084422,-0
.461292413657444,0.64458882703139,NA
"14328","RYR2",0,NA,NA,NA,NA,NA

"14329","AC093426.1",0,NA,NA,NA,NA,NA
"14330","CHRM3",0,NA,NA,NA,NA,NA
"14331","CHRM3-
AS2",0.0367720482966892,-0.560503011376585,3.34952001437583,-0.1673383
07868279,0.867103863749685,NA
"14332","FMN2",0,NA,NA,NA,NA,NA
"14333","AL358176.4",0,NA,NA,NA,NA,NA
"14334","RGS7",0,NA,NA,NA,NA,NA
"14335","AL359764.2",0.20332072784659,-1.39924864619322,3.107581708584
68,-0.450269301794319,0.652516271227926,NA
"14336","KMO",0.0732428936164972,-0.49131726092088,3.34845351515744,-0
.146729604785264,0.883345450266286,NA
"14337","AL133390.1",0.0367720482966892,-0.560503011376585,3.349520014
37583,-0.167338307868279,0.867103863749685,NA
"14338","EXO1",0.420798011374483,-2.43995659027234,2.44810148298057,-0
.996672975869322,0.318923271132377,NA
"14339","PLD5",0.91695307570363,1.54026319037134,1.70516290681871,0.90
3293863719437,0.366369952400655,NA
"14340","AL591686.2",0,NA,NA,NA,NA,NA
"14341","AL591686.1",0,NA,NA,NA,NA,NA
"14342","AL591721.1",0,NA,NA,NA,NA,NA
"14343","AKT3-
IT1",0.313418428570464,0.0405212521647926,2.20326788522023,0.018391432
3068083,0.985326587316889,NA
"14344","C1orf100",0.0741298089160557,-1.76673671430743,3.343096962362
58,-0.528473069790613,0.597171033704837,NA
"14345","AL451007.3",0,NA,NA,NA,NA,NA
"14346","AL451007.2",0.211052146313155,-0.488988616138086,2.7153294407
1237,-0.180084452665824,0.85708626804187,NA
"14347","BX323046.2",0.330679615407794,-0.405387626957155,2.3796567797
4227,-0.170355502696091,0.864730564264229,NA
"14348","BX323046.1",0.100451492077291,-0.0338344573480013,3.342323347
73369,-0.010123035334371,0.991923124345921,NA
"14349","KIF26B",0.166548679549901,-1.95393031097672,3.33920037562282,
-0.585149164824313,0.558447422910535,NA
"14350","SMYD3-IT1",0,NA,NA,NA,NA,NA
"14351","AL591623.1",0.314305343870022,-0.928034973562832,2.4529818242
0738,-0.378329331430209,0.705185958556623,NA
"14352","AL591848.3",0,NA,NA,NA,NA,NA
"14353","AL591848.4",1.40482281765663,-0.466521670853123,1.30516578185
36,-0.357442462359508,0.720760593112245,NA
"14354","ZNF695",0,NA,NA,NA,NA,NA
"14355","C1orf229",0.760839326083063,-0.0456151411064137,1.62757616825
996,-0.0280264248125363,0.977641075469044,NA
"14356","AL390728.5",0.986206822056407,0.432655159371057,1.45777604852
654,0.296791238824624,0.766625894674757,NA
"14357","AC104335.1",0.264582427935183,0.930632542201311,2.84339986125
834,0.327295697971042,0.743444245943564,NA
"14358","AC104335.2",0.246937279310285,0.827316883439734,2.55507596510
077,0.323793458488075,0.746094409004131,NA

"14359","OR2B11",0,NA,NA,NA,NA,NA
"14360","GCSAML",0.305819714334872,-1.57468193089132,2.7692160162842,-0.568638171103845,0.569601714969488,NA
"14361","OR2C3",0.474027090019631,-0.682441326031679,2.44835278820398,-0.278734882211273,0.780448290849661,NA
"14362","AL390860.1",0.0741298089160557,-1.76673671430743,3.34309696236258,-0.528473069790613,0.597171033704837,NA
"14363","TRIM58",0.0741298089160557,-1.76673671430743,3.34309696236258,-0.528473069790613,0.597171033704837,NA
"14364","OR2L2",0.174581300993347,-0.691169037761009,2.86286045881777,-0.241426030958711,0.809224939549254,NA
"14365","FAM110C",0,NA,NA,NA,NA,NA
"14366","AC079779.2",0.0636794437806017,-0.49131726092088,3.34845351515744,-0.146729604785264,0.883345450266286,NA
"14367","AC093326.1",0.490154502090573,0.00282707757597508,2.01155956810223,0.00140541578822955,0.998878640810213,NA
"14368","AC116609.3",0.239589822631289,0.773110999527443,2.56583771895994,0.301309390619147,0.763178580793342,NA
"14369","LINC01115",0,NA,NA,NA,NA,NA
"14370","TPO",0,NA,NA,NA,NA,NA
"14371","AC144450.1",0,NA,NA,NA,NA,NA
"14372","MYT1L",0,NA,NA,NA,NA,NA
"14373","EIPR1-IT1",0,NA,NA,NA,NA,NA
"14374","TRAPPC12-AS1",0,NA,NA,NA,NA,NA
"14375","AC114810.1",0,NA,NA,NA,NA,NA
"14376","AC108488.3",0.592646988435591,0.743846339116681,1.79967671292345,0.41332220046808,0.679370566600192,NA
"14377","SOX11",0,NA,NA,NA,NA,NA
"14378","SILC1",0.813940536985803,-0.625163446715791,1.51638046652981,-0.41227347655464,0.680138986989358,NA
"14379","LINC00487",0,NA,NA,NA,NA,NA
"14380","NRIR",0,NA,NA,NA,NA,NA
"14381","AC017076.1",0,NA,NA,NA,NA,NA
"14382","RNF144A-AS1",0.773505192773856,-2.83777569479574,1.92773075216902,-1.47208093848312,0.140999022209843,NA
"14383","AC010904.2",0,NA,NA,NA,NA,NA
"14384","AC013460.1",0.0732428936164972,-0.49131726092088,3.34845351515744,-0.146729604785264,0.883345450266286,NA
"14385","AC092580.4",0,NA,NA,NA,NA,NA
"14386","LINC00298",0,NA,NA,NA,NA,NA
"14387","LINC00299",0.456683144371614,-0.835921186995024,2.20212795909758,-0.379597009130015,0.704244581299108,NA
"14388","U91324.1",0.729924537076421,-0.67096834001036,1.62441572407304,-0.413052108562445,0.679568436377672,NA
"14389","LINC01814",0.102366282257309,-0.49131726092088,3.34845351515744,-0.146729604785264,0.883345450266286,NA
"14390","AC092617.1",0,NA,NA,NA,NA,NA
"14391","ID2-AS1",1.24204064929119,2.01496813476035,1.35972762339166,1.481891005298

75,0.138369298758973,NA
"14392","AC073195.1",1.22770986442489,0.19210344334861,1.5542301550077
9,0.12360038359161,0.901631688754128,NA
"14393","AC082651.4",0,NA,NA,NA,NA,NA
"14394","AC082651.3",0.494927820290539,-2.84339218695741,2.30196208233
711,-1.2352037458717,0.216754685526326,NA
"14395","AC010969.1",0,NA,NA,NA,NA,NA
"14396","RRM2",0.516355194629486,-1.10861473456796,2.15669169458585,-0
.514034869866205,0.607227611603326,NA
"14397","AC007240.3",0,NA,NA,NA,NA,NA
"14398","ODC1-
DT",1.32371223009759,0.749134978313319,1.29522337401908,0.578382843716
562,0.563005674249853,NA
"14399","ATP6V1C2",0.0741298089160557,-1.76673671430743,3.343096962362
58,-0.528473069790613,0.597171033704837,NA
"14400","AC092687.3",0.147673905509434,-0.842065770106804,3.3347867762
1422,-0.252509628535456,0.800647172695484,NA
"14401","C2orf50",0.181578773267413,-1.95393031097672,3.33920037562282
,-0.585149164824313,0.558447422910535,NA
"14402","LINC00570",0.211353349290036,-0.402177768036322,2.70013642896
566,-0.148947202712414,0.881595294233763,NA
"14403","AC099344.3",0.346668202458428,-1.94732862277873,3.00338753971
666,-0.648377406187962,0.516740879961632,NA
"14404","MIR3681HG",0,NA,NA,NA,NA,NA
"14405","AC009486.1",0,NA,NA,NA,NA,NA
"14406","TRIB2",1.07622249025383,-0.445572892544818,1.31408391577753,-
0.339074915380253,0.734553294873804,NA
"14407","LINC00276",0.136922337397099,0.183394507878855,3.337798890269
1,0.0549447446979257,0.956182484581931,NA
"14408","LRATD1",0.363157546534827,-2.82913804218295,2.97971220862415,
-0.949466876027358,0.342383211161164,NA
"14409","MYCN",0,NA,NA,NA,NA,NA
"14410","AC079148.1",0.675033965935717,-1.31327469130271,1.86634484177
677,-0.70366132876734,0.481643708531971,NA
"14411","NT5C1B",0,NA,NA,NA,NA,NA
"14412","AC130814.1",0,NA,NA,NA,NA,NA
"14413","LINC01376",1.09113531494649,1.1560249932527,1.3470700863737,0
.858177317532684,0.390794558911682,NA
"14414","AC010096.1",0,NA,NA,NA,NA,NA
"14415","AC013400.1",0,NA,NA,NA,NA,NA
"14416","AC079145.1",0.864233483220832,0.972381902530792,1.62018175278
197,0.600168407563622,0.548394006083834,NA
"14417","MATN3",1.02168918829963,0.0018719104130348,1.4441600862164,0.
00129619315123095,0.998965787786414,NA
"14418","LAPTM4A-
DT",0.342459058400589,-0.481809408085001,2.32728154585689,-0.207026695
563644,0.835989010065959,NA
"14419","SDC1",0.593325370512683,1.95891447360725,2.28231283891516,0.8
58302350232745,0.390725532021288,NA
"14420","AC012065.3",0.0732428936164972,-0.49131726092088,3.3484535151

5744,-0.146729604785264,0.883345450266286,NA
"14421","GDF7",0.0636794437806017,-0.49131726092088,3.34845351515744,-0.146729604785264,0.883345450266286,NA
"14422","AC012065.4",0.0367720482966892,-0.560503011376585,3.34952001437583,-0.167338307868279,0.867103863749685,NA
"14423","APOB",0.388592805998064,1.38085751013908,2.5417271917706‌4,0.543275263612038,0.586940293032918,NA
"14424","TDRD15",0.0636794437806017,-0.49131726092088,3.34845351515744,-0.146729604785264,0.883345450266286,NA
"14425","AC018742.1",0,NA,NA,NA,NA,NA
"14426","AC011752.1",0,NA,NA,NA,NA,NA
"14427","AC018467.1",0.312531513270905,1.06986046542867,2.41843726213171,0.442376770396623,0.658216585804703,NA
"14428","AC009242.1",0.481296185977706,0.238065932153633,1.9749859083959,0.120540572538562,0.904054941697304,NA
"14429","MFSD2B",0.276563621463087,-0.851854214261183,2.4869613794202,-0.342528123400042,0.731953483145556,NA
"14430","FAM228A",0.898373656542882,-0.133389081421452,1.94865766743681,-0.0684517776777629,0.945426005823995,NA
"14431","AC009228.1",0.2028177743346,0.512115644193803,2.71757760068597,0.188445637785848,0.850527326709045,NA
"14432","AC012073.1",0.0636794437806017,-0.49131726092088,3.34845351515744,-0.146729604785264,0.883345450266286,NA
"14433","EFR3B",0.315120193005521,0.245400022010969,3.2393007694085,0.0757570968180826,0.939612349767913,NA
"14434","AC012074.1",0,NA,NA,NA,NA,NA
"14435","ADGRF3",0.449034484715737,-1.31903840108013,2.44559733198216,-0.539352240792252,0.589643828608869,NA
"14436","OTOF",0,NA,NA,NA,NA,NA
"14437","KCNK3",0.0741298089160557,-1.76673671430743,3.34309696236258,-0.528473069790613,0.597171033704837,NA
"14438","DPYSL5",0,NA,NA,NA,NA,NA
"14439","AC013472.1",0.0741298089160557,-1.76673671430743,3.34309696236258,-0.528473069790613,0.597171033704837,NA
"14440","AGBL5-AS1",0.181578773267413,-1.95393031097672,3.33920037562282,-0.585149164824313,0.558447422910535,NA
"14441","AC013472.3",0.157560096502761,-0.491317252697356,3.34845351503297,-0.146729602334801,0.883345452200538,NA
"14442","AC013403.2",0.176496091173365,-1.14849506633421,3.26459492750587,-0.351803238024283,0.724985830023263,NA
"14443","ABHD1",0.676465300656428,0.894539740442854,1.87269024621053,0.477676296041479,0.632880618046741,NA
"14444","TCF23",0.146485787232994,0.248608662259703,3.33613854377696,0.0745198854896011,0.940596718825075,NA
"14445","SLC30A3",0,NA,NA,NA,NA,NA
"14446","TRIM54",0,NA,NA,NA,NA,NA
"14447","UCN",1.01771713989673,1.21028307751342,1.45218722723946,0.833420825367066,0.404607433639008,NA
"14448","GTF3C2-

AS1",0.147372702532553,-1.1477220656059,3.3390638510896,-0.343725701810518,0.731052580354999,NA
"14449","KRTCAP3",0.593217662720982,-1.12715711634127,1.9118224632257,-0.589572064363909,0.555477585067182,NA
"14450","GCKR",0,NA,NA,NA,NA,NA
"14451","LINC01460",0,NA,NA,NA,NA,NA
"14452","AC104695.2",0.346668202458428,-1.94732862277873,3.00338753971666,-0.648377406187962,0.516740879961632,NA
"14453","AC104695.3",0.961707893952552,1.69867120845384,1.63103368258797,1.0414691165412,0.297657879319582,NA
"14454","PLB1",0.445411052980575,-1.13988653355172,2.24235716978756,-0.508342983406036,0.611212830829659,NA
"14455","SPDYA",0.443496262800557,-0.827986380539087,1.85605283041527,-0.446100653478615,0.655524540798626,NA
"14456","AC097724.1",0.110014941913186,-0.033834469553869,3.34232334787728,-0.0101230389858472,0.991923121432614,NA
"14457","TOGARAM2",0.229725169818512,0.686619208900669,3.0259364803766,0.226911309392461,0.820492707794461,NA
"14458","PCARE",0,NA,NA,NA,NA,NA
"14459","ALK",0.0735440965933784,-0.185659846583073,3.34418846206699,-0.0555171601986567,0.955726459171656,NA
"14460","AC016907.2",0.166045726037911,0.183394512381981,3.33779889015228,0.0549447460489789,0.956182483505573,NA
"14461","LINC01936",0,NA,NA,NA,NA,NA
"14462","GALNT14",0,NA,NA,NA,NA,NA
"14463","CAPN14",0,NA,NA,NA,NA,NA
"14464","EHD3",0.515852241117495,0.448717276770201,1.77649169607135,0.252586194330389,0.800587999488245,NA
"14465","AL121652.1",0,NA,NA,NA,NA,NA
"14466","AL121655.1",0.110014941913186,-0.033834469553869,3.34232334787728,-0.0101230389858472,0.991923121432614,NA
"14467","AL121658.1",0.8849366229529,1.6346050654326,1.69866672256246,0.962287094767343,0.33590540974946,NA
"14468","NLRC4",0.148259617832111,-2.17639404086898,3.335076015408,-0.652577041966681,0.514028990213041,NA
"14469","AL133245.1",0.769143079608956,0.496882389244959,1.49063059675927,0.333337038918438,0.73887988351392,NA
"14470","AL133243.1",0.146485787232994,0.248608662259703,3.33613854377696,0.0745198854896011,0.940596718825075,NA
"14471","LINC00486",0.164130935857893,0.400527358383716,3.33018851502426,0.12027167728695З,0.904267939474822,NA
"14472","AC019127.1",0,NA,NA,NA,NA,NA
"14473","RASGRP3",0.19433214479945,-0.0333008172506769,3.16142997448539,-0.0105334666652225,0.991595664992027,NA
"14474","AC020594.1",0.175609175873806,0.247512349757584,3.30210845793575,0.0749558510601773,0.940249838776562,NA
"14475","AC017050.1",0,NA,NA,NA,NA,NA
"14476","AC007378.1",0.212381224170495,0.56712693135589,2.68810250756675,0.210976675837131,0.832905471698532,NA
"14477","AC006369.1",0,NA,NA,NA,NA,NA

"14478","LINC00211",0,NA,NA,NA,NA,NA
"14479","RMDN2-
AS1",0.102366282257309,-0.49131726092088,3.34845351515744,-0.146729604
785264,0.883345450266286,NA
"14480","AC009229.3",0.148259617832111,-2.17639404086898,3.33507601540
8,-0.652577041966681,0.514028990213041,NA
"14481","AC009229.1",0.191038331341805,0.541704852733846,3.32505412972
637,0.162916100490197,0.870584493834308,NA
"14482","AC074366.1",0,NA,NA,NA,NA,NA
"14483","SOS1-
IT1",0.488814578699309,1.51681181378356,1.97202506526045,0.76916457123
39,0.441795619636595,NA
"14484","CDKL4",0.0636794437806017,-0.49131726092088,3.34845351515744,
-0.146729604785264,0.883345450266286,NA
"14485","AC007684.2",0.605313346727945,-0.0205378770009734,1.597629075
92986,-0.012855222348166,0.989743299061651,NA
"14486","TMEM178A",0.496535304519246,-0.437790965907973,1.917388402226
42,-0.228326699691947,0.819392269350643,NA
"14487","SLC8A1-
AS1",0.0636794437806017,-0.49131726092088,3.34845351515744,-0.14672960
4785264,0.883345450266286,NA
"14488","SLC8A1",0.888279308800256,-0.245774289112232,1.47825773401392
,-0.16625943058311,0.867952791342538,NA
"14489","AC007877.1",0,NA,NA,NA,NA,NA
"14490","AC009413.2",0.102366282257309,-0.49131726092088,3.34845351515
744,-0.146729604785264,0.883345450266286,NA
"14491","LINC01913",0.102366282257309,-0.49131726092088,3.348453515157
44,-0.146729604785264,0.883345450266286,NA
"14492","C2orf91",0.102366282257309,-0.49131726092088,3.34845351515744
,-0.146729604785264,0.883345450266286,NA
"14493","AC083949.1",0,NA,NA,NA,NA,NA
"14494","OXER1",1.43503946180431,0.591036916999982,1.22034255086736,0.
484320502124508,0.628158427868685,NA
"14495","LINC01819",0.13722354037398,0.244427849343087,3.3362431208708
4,0.0732643996518113,0.941595720500819,NA
"14496","LINC02580",1.42189768916497,1.10243892805431,1.25425050910616
,0.87896231259253,0.379421710461169,NA
"14497","AC010883.2",0,NA,NA,NA,NA,NA
"14498","LINC01126",0.65963109120776,-1.08107649763112,1.5817294180325
2,-0.68347751853528,0.494305151325512,NA
"14499","AC010883.1",0.110316144890068,0.0897481123376931,3.3402944707
4234,0.0268683234738127,0.978564758597723,NA
"14500","ABCG5",0,NA,NA,NA,NA,NA
"14501","ABCG8",0,NA,NA,NA,NA,NA
"14502","AC019129.2",0.363157546534827,-2.82913804218295,2.97971220862
415,-0.949466876027358,0.342383211161164,NA
"14503","SLC3A1",0.723752401628635,1.44647438605713,2.08121584858443,0
.695014112563561,0.487046457405675,NA
"14504","LINC01833",0,NA,NA,NA,NA,NA
"14505","SIX3",0.110014941913186,-0.033834469553869,3.34232334787728,-

0.0101230389858472,0.991923121432614,NA
"14506","AC093833.1",0.147088193186757,0.338238253117599,3.33259753179
347,0.10149387974118,0.919158415731812,NA
"14507","U51244.1",0,NA,NA,NA,NA,NA
"14508","AC018682.1",0,NA,NA,NA,NA,NA
"14509","LINC01118",0.807927776859269,0.604395460715779,1.651409336107
69,0.365987673377404,0.714374273647065,NA
"14510","AC016722.2",0.0741298089160557,-1.76673671430743,3.3430969623
6258,-0.528473069790613,0.597171033704837,NA
"14511","STPG4",0,NA,NA,NA,NA,NA
"14512","AC073283.1",0,NA,NA,NA,NA,NA
"14513","EPCAM-DT",0,NA,NA,NA,NA,NA
"14514","KCNK12",0.250625900089421,-1.56858798056831,2.93131916770576,
-0.535113336633345,0.592571488297769,NA
"14515","AC079807.1",1.0899256584895,1.23956905877391,1.36620346670807
,0.907309261746141,0.36424327779063,NA
"14516","AC092650.1",0,NA,NA,NA,NA,NA
"14517","AC093635.1",1.0356335150368,1.73537049428451,1.5219842687749,
1.14020264853419,0.254201884340879,NA
"14518","STON1-GTF2A1L",0,NA,NA,NA,NA,NA
"14519","GTF2A1L",0.102366282257309,-0.49131726092088,3.34845351515744
,-0.146729604785264,0.883345450266286,NA
"14520","NRXN1",0.181578773267413,-1.95393031097672,3.33920037562282,-
0.585149164824313,0.558447422910535,NA
"14521","CHAC2",0.735222682212129,-1.0835592159384,1.53837287380408,-0
.70435408371378,0.481212293086667,NA
"14522","GPR75",0,NA,NA,NA,NA,NA
"14523","TSPYL6",0,NA,NA,NA,NA,NA
"14524","C2orf73",0.258274559745298,-1.11669114604128,2.56884985789992
,-0.434704715266695,0.663776775348859,NA
"14525","AC093110.1",1.4249559595956,-0.366006375332799,1.304569898914
85,-0.28055712126827 9,0.779050107799055,NA
"14526","EML6",0.0732428936164972,-0.49131726092088,3.34845351515744,-
0.146729604785264,0.883345450266286,NA
"14527","AC012358.1",0,NA,NA,NA,NA,NA
"14528","AC011306.1",0,NA,NA,NA,NA,NA
"14529","AC007743.1",0.100451492077291,-0.0338344573480013,3.342323347
73369,-0.010123035334371,0.991923124345921,NA
"14530","AC132153.1",0.728868254956225,-0.231985699924583,2.0053371550
8154,-0.115684137870148,0.907902877985331,NA
"14531","AC068193.1",0.724639612845702,-0.165231823708136,1.5511053260
4746,-0.106525212010703,0.915165652921492,NA
"14532","AC007381.1",0,NA,NA,NA,NA,NA
"14533","BCL11A",0.239791573166398,-1.32959277084231,2.9363559394555,-
0.45280367852436,0.650690114564834,NA
"14534","LINC01185",0,NA,NA,NA,NA,NA
"14535","AC016747.3",0.347624499305324,-0.658125241455401,2.3594506545
9191,-0.278931555603663,0.780297351347269,NA
"14536","AC016727.3",0.0636794437806017,-0.49131726092088,3.3484535151
5744,-0.146729604785264,0.883345450266286,NA

"14537","AC107081.2",0.383440250755117,-1.38194811510753,2.56276654272
887,-0.53924073537967,0.589720755955717,NA
"14538","AC107081.4",0,NA,NA,NA,NA,NA
"14539","AC092155.1",0.551585568999803,1.86515242622154,1.828434312744
81,1.02008172414004,0.307689703551198,NA
"14540","AC092567.2",0.346668202458428,-1.94732862277873,3.00338753971
666,-0.648377406187962,0.516740879961632,NA
"14541","OTX1",0,NA,NA,NA,NA,NA
"14542","AC009501.3",0,NA,NA,NA,NA,NA
"14543","AC012368.2",0,NA,NA,NA,NA,NA
"14544","AC012368.1",0.28518195522921,-0.923093880701459,2.51322729499
66,-0.367294228635499,0.713399558597788,NA
"14545","LINC00309",0.110901857212745,-1.2169087618042,3.3401333690268
2,-0.36432939267894,0.715612053721651,NA
"14546","LGALSL-
DT",0.531699868587228,-2.29242442442076,2.05619654395917,-1.1148858464
7007,0.264899342595533,NA
"14547","AC008074.2",0.377399075327947,0.41478541520833,2.038770241574
8,0.203448827508851,0.838784238896543,NA
"14548","AC007365.1",0.793603980857023,-0.41249155160606,1.48173242351
928,-0.278384642907623,0.780717106953655,NA
"14549","LINC01800",0,NA,NA,NA,NA,NA
"14550","LINC02245",0.90608211063714,1.59599546596734,1.54293735948939
,1.03438772556231,0.30095494029983,NA
"14551","LINC02576",0,NA,NA,NA,NA,NA
"14552","AC012370.1",0.110014941913186,-0.033834469553869,3.3423233478
7728,-0.0101230389858472,0.991923121432614,NA
"14553","AC007389.1",0.447840263464867,1.63379520094101,2.082254240419
67,0.784628106033644,0.432671646175622,NA
"14554","AC007389.5",0.662788629189429,1.32800748458859,1.692588019146
83,0.784601727984575,0.432687116607061,NA
"14555","LINC01873",1.28468977235891,1.62267398535559,1.54292623293546
,1.05168604351771,0.292943616298136,NA
"14556","LINC01829",0.0367720482966892,-0.560503011376585,3.3495200143
7583,-0.167338307868279,0.867103863749685,NA
"14557","LINC02831",0,NA,NA,NA,NA,NA
"14558","AC017083.2",0.184144750829242,-0.691295663073562,2.8131316232
0765,-0.245738826214366,0.805884414604706,NA
"14559","PROKR1",0,NA,NA,NA,NA,NA
"14560","ARHGAP25",0.756027776071462,0.43253236333288,1.52239331688996
,0.284113414407574,0.776323467177135,NA
"14561","LINC01888",0,NA,NA,NA,NA,NA
"14562","AC114802.1",0.240678488465957,-2.44080205522383,2.91280317905
203,-0.837956396359809,0.402055193025598,NA
"14563","ASPRV1",0.947033723698795,1.91289206637776,1.46101124174195,1
.30929318798193,0.190435056230354,NA
"14564","LINC01816",0.247238482287166,0.868764518721955,2.529200996027
34,0.343493664634222,0.731227106130206,NA
"14565","TGFA",0.0741298089160557,-1.76673671430743,3.34309696236258,-
0.528473069790613,0.597171033704837,NA

"14566","ADD2",0.102366282257309,-0.49131726092088,3.34845351515744,-0.146729604785264,0.883345450266286,NA
"14567","CLEC4F",0,NA,NA,NA,NA,NA
"14568","VAX2",0.783656569233559,-0.97520637586154,1.74930088897947,-0.557483496410082,0.577197131827647,NA
"14569","ATP6V1B1",0.185031666128801,-1.62793589934517,3.24336249302777,-0.501928447049853,0.61571785405042,NA
"14570","ANKRD53",1.05733433895002,0.545069718365305,1.31674881257204,0.41395117516803,0.678909864324222,NA
"14571","AC007881.3",0.249738984789862,-0.445595840703958,2.59717004458552,-0.171569759797946,0.863775783061595,NA
"14572","AC007881.4",0,NA,NA,NA,NA,NA
"14573","DYSF",0.328951475799966,-1.82996535219318,2.35245146173024,-0.777897177460669,0.436629632387116,NA
"14574","FBXO41",0,NA,NA,NA,NA,NA
"14575","EGR4",0,NA,NA,NA,NA,NA
"14576","ALMS1-IT1",1.27160722145181,-0.0798265305200111,1.26565586869293,-0.0630712759246713,0.949709747236806,NA
"14577","AC074008.1",0.552494022479911,1.72600669414969,1.89161611977036,0.912450827686552,0.361531443405949,NA
"14578","AC092653.1",0.0741298089160557,-1.76673671430743,3.34309696236258,-0.528473069790613,0.597171033704837,NA
"14579","AC073046.4",0.0367720482966892,-0.560503011376585,3.34952001437583,-0.167338307868279,0.867103863749685,NA
"14580","ACTG2",0.659487245525356,2.13727513987099,2.27044513583479,0.941346305241225,0.346527420377656,NA
"14581","DGUOK-AS1",0.815805381745536,-0.00306706951333592,2.0247957581779,-0.00151475500723883,0.99879140082851,NA
"14582","AC073046.1",0,NA,NA,NA,NA,NA
"14583","AC073263.2",0.662940434304252,1.03314221570373,1.63551459378106,0.631692446910708,0.527587864487294,NA
"14584","DCTN1-AS1",0,NA,NA,NA,NA,NA
"14585","AC005041.5",0.230228123330503,-1.33023934114211,2.97714568949567,-0.446817011957333,0.655007187193824,NA
"14586","AC005041.3",0.102366282257309,-0.49131726092088,3.34845351515744,-0.146729604785264,0.883345450266286,NA
"14587","TLX2",0,NA,NA,NA,NA,NA
"14588","M1AP",0,NA,NA,NA,NA,NA
"14589","AC019069.1",0.240092776143279,-1.01655735204591,2.97416330481592,-0.341796077706914,0.732504361553979,NA
"14590","HK2",1.09223873627435,0.600558111278173,1.61166586447466,0.372631898780044,0.709422428239571,NA
"14591","TACR1",0,NA,NA,NA,NA,NA
"14592","AC007681.1",0.20332072784659,-1.39924864619322,3.10758170858468,-0.450269301794319,0.652516271227926,NA
"14593","EVA1A",0.201488696477259,-0.508277411998464,3.16327545678924,-0.160680730762022,0.872344866840293,NA
"14594","AC005034.3",0,NA,NA,NA,NA,NA

"14595","AC005034.5",0.284295039929652,-0.0383820061561763,2.50936267663567,-0.0152955196606477,0.987796416859156,NA
"14596","AC005034.6",0.221239540283362,0.178834610992208,3.06205963446949,0.0584033730039328,0.953427328091912,NA
"14597","AC005034.4",0.357489152118101,-0.479767574744153,2.30314372505503,-0.2083098720783,0.834987023916724,NA
"14598","LRRTM4",1.0607970563833,2.74576510987146,1.55750898103411,1.76292088412127,0.0779138342066405,NA
"14599","AC079117.2",0,NA,NA,NA,NA,NA
"14600","AC013716.1",0,NA,NA,NA,NA,NA
"14601","CTNNA2",0.0367720482966892,-0.560503011376585,3.34952001437583,-0.167338307868279,0.867103863749685,NA
"14602","DNAH6",0.137809252696657,-1.1477220656059,3.3390638510896,-0.343725701810518,0.731052580354999,NA
"14603","AC022210.2",0.230228123330503,-1.33023934114211,2.97714568949567,-0.446817011957333,0.655007187193824,NA
"14604","TCF7L1-IT1",0.362292661017379,1.07435604480197,2.68613932098777,0.399962889641516,0.689183850256067,NA
"14605","AC062037.3",0,NA,NA,NA,NA,NA
"14606","SH2D6",0.102366282257309,-0.49131726092088,3.34845351515744,-0.146729604785264,0.883345450266286,NA
"14607","AC105053.1",0,NA,NA,NA,NA,NA
"14608","ST3GAL5-AS1",0.598456834034779,1.99982267803697,1.76571187018934,1.13258720847956,0.257387642837788,NA
"14609","AC009309.1",1.15194289755452,0.200370257389413,1.67225516268863,0.119820384986739,0.904625433396445,NA
"14610","REEP1",0.204732564514618,0.248608670877116,3.33613854356166,0.0745198880774602,0.940596716765987,NA
"14611","AC015971.1",0.564859177795383,0.800196169254057,1.97978987527063,0.404182372709969,0.686078615091201,NA
"14612","CD8A",0.774560708070647,-0.191234974106846,1.58289233745202,-0.120813633108286,0.903838651545768,NA
"14613","CD8B",1.14490854967404,-1.19221692104711,1.49768158516308,-0.796041650547026,0.426007827031077,NA
"14614","RGPD1",0.0741298089160557,-1.76673671430743,3.34309696236258,-0.528473069790613,0.597171033704837,NA
"14615","PLGLB1",0.918233076215414,2.6070966569098,1.48198509552518,1.75919222452498,0.0785448644946162,NA
"14616","AC068279.2",0.35665101824659,1.26236994571574,2.66861061896275,0.473043889110509,0.636181867222591,NA
"14617","AC133644.2",0.146786990209876,0.2964989704902,3.33426311477484,0.0889248869341921,0.929141604986601,NA
"14618","RGPD2",1.06436410397689,1.57077886537429,1.81210071298987,0.866827574270l9,0.386036491330544,NA
"14619","AC108479.3",0.0636794437806017,-0.49131726092088,3.34845351515744,-0.146729604785264,0.883345450266286,NA
"14620","AC062029.1",0.0367720482966892,-0.560503011376585,3.34952001437583,-0.167338307868279,0.867103863749685,NA

"14621","AC233263.6",1.15830606829085,-0.655094801264694,1.25989223118694,-0.519960981621049,0.603090770595666,NA
"14622","AC233266.2",1.19235883467545,-1.41903391748292,1.24717939177666,-1.13779455212249,0.25520628035326,NA
"14623","TEKT4",0.687798183001675,-2.00722251528739,1.70477991194464,-1.17740859170364,0.239032462261329,NA
"14624","MAL",0.239791573166398,-1.32959277084231,2.9363559394555,-0.45280367852436,0.650690114564834,NA
"14625","AC092835.1",1.24870596106566,0.816240775229781,1.4912991955432,0.547335355419722,0.584148358293364,NA
"14626","PROM2",0.379231106697278,-0.172944401134216,2.2735351625237,-0.0760684963157737,0.939364603796265,NA
"14627","AC009237.14",1.08777436624608,1.76829921724813,1.41950912776793,1.24571176236721,0.212870233801562,NA
"14628","AC009237.15",0.0636794437806017,-0.49131726092088,3.34845351515744,-0.146729604785264,0.883345450266286,NA
"14629","ASTL",0,NA,NA,NA,NA,NA
"14630","AC021188.1",0,NA,NA,NA,NA,NA
"14631","ITPRIPL1",0.340817932032444,1.02566256958608,2.35874002368957,0.434834936993916,0.663682243685771,NA
"14632","NCAPH",0.369869407396491,-2.18301008743594,2.60759739189274,-0.837172983154194,0.40249534128038,NA
"14633","NEURL3",0,NA,NA,NA,NA,NA
"14634","AC013270.1",0,NA,NA,NA,NA,NA
"14635","FER1L5",0.102366282257309,-0.49131726092088,3.34845351515744,-0.146729604785264,0.883345450266286,NA
"14636","CNNM3-DT",0.0732428936164972,-0.49131726092088,3.34845351515744,-0.146729604785264,0.883345450266286,NA
"14637","FAM178B",0.726905551422888,-0.966860334868509,1.65044488374982,-0.585818008458298,0.557997820607112,NA
"14638","C2orf92",1.39096999668164,0.251900641238667,1.21052191161106,0.208092591156335,0.835156671942924,NA
"14639","ZAP70",1.18991151169057,0.365585241693449,1.30101661579134,0.280999671530776,0.778710651766735,NA
"14640","LINC02611",0,NA,NA,NA,NA,NA
"14641","KIAA1211L",0.0741298089160557,-1.76673671430743,3.34309696236258,-0.528473069790613,0.597171033704837,NA
"14642","C2orf15",0.391744004281009,-0.194185375615089,2.29619892525906,-0.0845681850465898,0.932604693084977,NA
"14643","AC079447.1",0.110901857212745,-1.2169087618042,3.34013336902682,-0.36432939267894,0.715612053721651,NA
"14644","LYG2",0.728317388090916,0.190850722285734,1.66431961312364,0.114671918050368,0.908705173213979,NA
"14645","LYG1",0.412256333444618,0.613398341644278,2.20887265970322,0.277697466601218,0.781244605498498,NA
"14646","AC018690.1",0,NA,NA,NA,NA,NA
"14647","AC092667.1",0,NA,NA,NA,NA,NA
"14648","AC092168.2",0.229725169818512,0.686619208900669,3.0259364803766,0.226911309392461,0.820492707794461,NA

"14649","AC016738.2",0,NA,NA,NA,NA,NA
"14650","AC016738.1",0,NA,NA,NA,NA,NA
"14651","TBC1D8-
AS1",0.902563508724742,0.200841871658326,1.45889375173703,0.1376672368
49287,0.890503414006256,NA
"14652","LINC01127",0.2028177743346,0.512115644193803,2.71757760068597
,0.188445637785848,0.850527326709045,NA
"14653","IL1R2",1.38972201398028,-0.398060537315542,1.22516963084679,-
0.324902386815135,0.745254950395205,NA
"14654","AC007271.1",0,NA,NA,NA,NA,NA
"14655","IL1RL2",0.313335669759777,-0.504482984843873,2.39455653806486
,-0.210679086847357,0.833137694613702,NA
"14656","IL1RL1",0.625425020472899,-0.546521225040392,1.6640428102802,
-0.32842978658005,0.742586724461814,NA
"14657","IL18RAP",0.461678046402575,-0.742187863026571,2.0287788035205
6,-0.365829858700537,0.714492037979809,NA
"14658","AC007278.2",0.0732428936164972,-0.49131726092088,3.3484535151
5744,-0.146729604785264,0.883345450266286,NA
"14659","AC007278.1",0,NA,NA,NA,NA,NA
"14660","PANTR1",0.166548679549901,-1.95393031097672,3.33920037562282,
-0.585149164824313,0.558447422910535,NA
"14661","POU3F3",0.671497547440238,0.919532008250066,1.63721943211563,
0.561642495937053,0.574359620638868,NA
"14662","AC010884.1",0.0367720482966892,-0.560503011376585,3.349520014
37583,-0.167338307868279,0.867103863749685,NA
"14663","LINC01918",0,NA,NA,NA,NA,NA
"14664","AC012360.2",0.304357932246558,-1.82931526615362,2.40596782312
181,-0.760324077726043,0.447060892953357,NA
"14665","AC108058.1",0.200601781177701,0.585305641041376,3.16043896918
792,0.185197577535177,0.853074069074656,NA
"14666","AC010978.1",0.157560096502761,-0.491317252697356,3.3484535150
3297,-0.146729602334801,0.883345452200538,NA
"14667","ECRG4",0.955368966988626,-2.40986901469587,1.67085525717733,-
1.44229669466822,0.149218692249592,NA
"14668","RGPD3",0,NA,NA,NA,NA,NA
"14669","ST6GAL2",0.26891496180721,-1.3277650763353,2.82969623342791,-
0.469225304345422,0.63890860009957,NA
"14670","LINC01885",0.19433214479945,-0.0333008172506769,3.16142997448
539,-0.0105334666652225,0.991595664992027,NA
"14671","SULT1C2",0.175609175873806,0.247512349757584,3.30210845793575
,0.0749558510601773,0.940249838776562,NA
"14672","SULT1C4",0.221502511448609,-1.56478135306832,3.04955054434762
,-0.513118681036017,0.607868304703499,NA
"14673","GCC2-
AS1",0.647452586464479,-1.33397004022798,1.84382574695282,-0.723479451
587308,0.469385373979188,NA
"14674","SH3RF3-
AS1",0.794031793151346,1.63394676588273,1.7976248927855,0.908947563220
964,0.363377805534311,NA
"14675","AC011753.2",0.419911096074925,-1.32028169514223,2.49077535946

509,-0.530068554807674,0.59606440017536,NA
"14676","LINC01123",0.136922337397099,0.183394507878855,3.337798890269
1,0.0549447446979257,0.956182484581931,NA
"14677","LINC01106",0.741602128435578,0.499612622651161,1.694533042784
,0.294837934721138,0.768117686132141,NA
"14678","RGPD6",0,NA,NA,NA,NA,NA
"14679","BUB1",0,NA,NA,NA,NA,NA
"14680","ACOXL",0,NA,NA,NA,NA,NA
"14681","ACOXL-
AS1",0.673089596462825,0.243385557596731,1.65750506398866,0.1468385001
55796,0.883259495310794,NA
"14682","AC017002.3",1.20106544010622,-1.04890176250994,1.466220641191
25,-0.715377844945455,0.474375598639674,NA
"14683","AC017002.1",0.0636794437806017,-0.49131726092088,3.3484535151
5744,-0.146729604785264,0.883345450266286,NA
"14684","MERTK",0.656764402270611,-0.511284320327422,1.6512171025755,-
0.30964088218923,0.756834063664084,NA
"14685","AC093675.1",0.371281244064519,-0.609248717377829,2.6341163094
4316,-0.23129150189523,0.817088348224649,NA
"14686","AC092645.2",0.19433214479945,-0.0333008172506769,3.1614299744
8539,-0.0105334666652225,0.991595664992027,NA
"14687","AC012442.1",0,NA,NA,NA,NA,NA
"14688","CKAP2L",0.181578773267413,-1.95393031097672,3.33920037562282,
-0.585149164824313,0.558447422910535,NA
"14689","IL1A",0.221502511448609,-1.56478135306832,3.04955054434762,-0
.513118681036017,0.607868304703499,NA
"14690","AC079753.2",0,NA,NA,NA,NA,NA
"14691","IL1RN",1.20003088031866,-0.902379355321145,1.40572229114571,-
0.641932877500061,0.520916766014409,NA
"14692","PSD4",0.862965196317699,-2.41434913916911,1.67507284058162,-1
.4413397917256,0.149488710819277,NA
"14693","AC016745.2",0.0636794437806017,-0.49131726092088,3.3484535151
5744,-0.146729604785264,0.883345450266286,NA
"14694","PGM5P4-
AS1",0.395820841276709,0.260603393041236,2.05990848793743,0.1265121215
66224,0.89932655451841,NA
"14695","AC104653.1",0.102366282257309,-0.49131726092088,3.34845351515
744,-0.146729604785264,0.883345450266286,NA
"14696","AC110769.2",1.41122209717596,2.40618917492741,1.4231815895042
5,1.69071128566634,0.0908919577940024,NA
"14697","LINC01191",0.0636794437806017,-0.49131726092088,3.34845351515
744,-0.146729604785264,0.883345450266286,NA
"14698","AC009303.2",0.102366282257309,-0.49131726092088,3.34845351515
744,-0.146729604785264,0.883345450266286,NA
"14699","LINC01956",0.13722354037398,0.244427849343087,3.3362431208708
4,0.0732643996518113,0.941595720500819,NA
"14700","STEAP3",1.08309327416088,0.554846181217083,1.54354425708155,0
.359462437614945,0.719249172998874,NA
"14701","SCTR",0.102366282257309,-0.49131726092088,3.34845351515744,-0
.146729604785264,0.883345450266286,NA

"14702","AC013275.1",0.633144725330813,2.07508842962452,1.81578636613144,1.14280427936329,0.253119861657979,NA
"14703","CFAP221",0,NA,NA,NA,NA,NA
"14704","AC073257.2",0,NA,NA,NA,NA,NA
"14705","AC073257.1",0.181578773267413,-1.95393031097672,3.33920037562282,-0.585149164824313,0.558447422910535,NA
"14706","AC017033.1",0.350994633356024,-1.19800308996477,2.37879327969663,-0.503617989923678,0.614529848245348,NA
"14707","TFCP2L1",0,NA,NA,NA,NA,NA
"14708","AC012447.1",0,NA,NA,NA,NA,NA
"14709","AC011246.1",0.358441542476793,0.426942177942248,2.33556389537268,0.182800470065548,0.854954579767651,NA
"14710","AC012508.2",0.0735440965933784,-0.185659846583073,3.34418846206699,-0.0555171601986567,0.955726459171656,NA
"14711","AC012508.1",0.0636794437806017,-0.49131726092088,3.34845351515744,-0.146729604785264,0.883345450266286,NA
"14712","AC110926.2",0.147088193186757,0.338238253117599,3.33259753179347,0.10149387974118,0.919158415731812,NA
"14713","AC068282.1",0.102366282257309,-0.49131726092088,3.34845351515744,-0.146729604785264,0.883345450266286,NA
"14714","AC068282.2",0.0367720482966892,-0.560503011376585,3.34952001437583,-0.167338307868279,0.867103863749685,NA
"14715","PROC",0.346668202458428,-1.94732862277873,3.00338753971666,-0.648377406187962,0.516740879961632,NA
"14716","AC010976.1",0.166548679549901,-1.95393031097672,3.33920037562282,-0.585149164824313,0.558447422910535,NA
"14717","MYO7B",0.184144750829242,-0.691295663073562,2.81313162320765,-0.245738826214366,0.805884414604706,NA
"14718","AC010976.2",0.0741298089160557,-1.76673671430743,3.34309696236258,-0.528473069790613,0.597171033704837,NA
"14719","AC012306.3",1.43195291173573,0.217686320754616,1.2916811679211,0.168529453057655,0.86616677484204,NA
"14720","LINC02572",0.102366282257309,-0.49131726092088,3.34845351515744,-0.146729604785264,0.883345450266286,NA
"14721","RAB6C",0.0735440965933784,-0.185659846583073,3.34418846206699,-0.0555171601986567,0.955726459171656,NA
"14722","AC073869.3",0,NA,NA,NA,NA,NA
"14723","AC093787.2",0.0732428936164972,-0.49131726092088,3.34845351515744,-0.146729604785264,0.883345450266286,NA
"14724","AC097532.2",0.276563621463087,-0.851854214261183,2.4869613794202,-0.342528123400042,0.731953483145556,NA
"14725","AC097532.3",0.452172797336894,0.156831999275383,1.99183356568934,0.0787375019564477,0.937241415814801,NA
"14726","GPR39",0.453493619844306,-1.08036622926776,2.19402823773957,-0.492412180793454,0.622427990044312,NA
"14727","LYPD1",0,NA,NA,NA,NA,NA
"14728","AC092623.1",0,NA,NA,NA,NA,NA
"14729","TMEM163",0,NA,NA,NA,NA,NA
"14730","AC110620.2",0,NA,NA,NA,NA,NA
"14731","CCNT2-

AS1",0.860446971160996,0.519943866007532,1.77780270297237,0.292464324156004,0.769931633217317,NA
"14732","MAP3K19",0,NA,NA,NA,NA,NA
"14733","AC011893.1",0.0636794437806017,-0.49131726092088,3.34845351515744,-0.146729604785264,0.883345450266286,NA
"14734","DARS-
AS1",0.148259617832111,-2.17639404086898,3.335076015408,-0.652577041966681,0.514028990213041,NA
"14735","AC068492.1",0,NA,NA,NA,NA,NA
"14736","THSD7B",0,NA,NA,NA,NA,NA
"14737","AC114763.1",0.221502511448609,-1.56478135306832,3.04955054434762,-0.513118681036017,0.607868304703499,NA
"14738","AC092620.1",1.08624964082806,1.35467706943077,1.72311635040311,0.786178524226676,0.431762905833853,NA
"14739","LINC02631",0,NA,NA,NA,NA,NA
"14740","KYNU",1.21153734342222,-1.39833652121111,1.43810283467926,-0.972348073789164,0.3308774152339,NA
"14741","AC079793.1",0.147372702532553,-1.1477220656059,3.3390638510896,-0.343725701810518,0.731052580354999,NA
"14742","AC092652.3",0.0741298089160557,-1.76673671430743,3.34309696236258,-0.528473069790613,0.597171033704837,NA
"14743","AC092652.1",0,NA,NA,NA,NA,NA
"14744","AC092652.2",0,NA,NA,NA,NA,NA
"14745","AC016910.1",0.575937832455809,-0.835691092715063,1.92194248794687,-0.434815868818113,0.663696085483548,NA
"14746","AC009951.5",0,NA,NA,NA,NA,NA
"14747","LINC01412",0.22061559614905,-0.418038608046788,3.07273464003886,-0.136047741513241,0.891783539523794,NA
"14748","TEX41",1.43414185385812,-0.213898597890334,1.1116389138208,-0.192417335549316,0.84741531431217,NA
"14749","AC018678.1",0,NA,NA,NA,NA,NA
"14750","AC105402.3",0,NA,NA,NA,NA,NA
"14751","KIF5C",0.810646723528158,-0.429541604895048,1.50112993443236,-0.286145519479948,0.774766668908921,NA
"14752","LYPD6B",0.240678488465957,-2.44080205522383,2.91280317905203,-0.837956396359809,0.402055193025598,NA
"14753","LYPD6",0.204732564514618,0.248608670877116,3.33613854356166,0.0745198880774602,0.940596716765987,NA
"14754","LINC02612",0,NA,NA,NA,NA,NA
"14755","NEB",0.811746511685383,2.34850180291558,1.73038626599113,1.35721246121334,0.174713712416997,NA
"14756","AC079790.2",0.0367720482966892,-0.560503011376585,3.34952001437583,-0.167338307868279,0.867103863749685,NA
"14757","AC012066.2",0.0367720482966892,-0.560503011376585,3.34952001437583,-0.167338307868279,0.867103863749685,NA
"14758","AC012443.2",0.346668202458428,-1.94732862277873,3.00338753971666,-0.648377406187962,0.516740879961632,NA
"14759","RPRM",0.102366282257309,-0.49131726092088,3.34845351515744,-0.146729604785264,0.883345450266286,NA
"14760","AC073225.1",0.0741298089160557,-1.76673671430743,3.3430969623

6258,-0.528473069790613,0.597171033704837,NA
"14761","LINC01876",0,NA,NA,NA,NA,NA
"14762","AC074099.1",0,NA,NA,NA,NA,NA
"14763","ERMN",0.102366282257309,-0.49131726092088,3.34845351515744,-0.146729604785264,0.883345450266286,NA
"14764","ACVR1C",0.0741298089160557,-1.76673671430743,3.34309696236258,-0.528473069790613,0.597171033704837,NA
"14765","AC019186.1",0.102366282257309,-0.49131726092088,3.34845351515744,-0.146729604785264,0.883345450266286,NA
"14766","UPP2",0.255122869860792,-1.01416582893919,2.91937932186279,-0.347390906465717,0.728297664063611,NA
"14767","AC013731.1",1.17368747482479,-1.41831558258621,1.61602991729181,-0.87765428561067,0.380131359869547,NA
"14768","AC091488.1",0.691237698599714,-0.752125874464154,1.63525908331111,-0.459942942460978,0.645557175831708,NA
"14769","CCDC148",0.0367720482966892,-0.560503011376585,3.34952001437583,-0.167338307868279,0.867103863749685,NA
"14770","PKP4-AS1",0.0367720482966892,-0.560503011376585,3.34952001437583,-0.167338307868279,0.867103863749685,NA
"14771","DAPL1",0.0732428936164972,-0.49131726092088,3.34845351515744,-0.146729604785264,0.883345450266286,NA
"14772","AC008277.1",0,NA,NA,NA,NA,NA
"14773","AC009961.4",0.630678733042283,0.323590816789748,1.84513861572274,0.175374800588083,0.860785139932414,NA
"14774","LINC02478",1.44863047560629,-0.0844022786790816,1.4954848915579,-0.0564380684522707,0.954992831030387,NA
"14775","LINC01806",0,NA,NA,NA,NA,NA
"14776","TBR1",0,NA,NA,NA,NA,NA
"14777","SLC4A10",0,NA,NA,NA,NA,NA
"14778","AC007750.1",0.146485787232994,0.248608662259703,3.33613854377696,0.0745198854896011,0.940596718825075,NA
"14779","GRB14",0,NA,NA,NA,NA,NA
"14780","AC019197.1",0.175609175873806,0.247512349757584,3.30210845793575,0.0749558510601773,0.940249838776562,NA
"14781","SLC38A11",0.384899501114004,-2.20157759821794,2.28647392189998,-0.96287019813832,0.335612666980918,NA
"14782","SCN3A",0,NA,NA,NA,NA,NA
"14783","SCN2A",0.320717103821412,-0.846710013286843,2.38334198606247,-0.35526165285482,0.722393580711595,NA
"14784","CSRNP3",0.332091452075822,1.13393387627575,2.70064835955158,0.419874683894066,0.674577002697029,NA
"14785","GALNT3",1.27016388710729,-1.80956374374049,1.25197181185926,-1.44537099525681,0.148353705929337,NA
"14786","AC009495.3",0,NA,NA,NA,NA,NA
"14787","TTC21B-AS1",0.293907567111104,-0.687518690041658,2.79286242307872,-0.246169909538104,0.805550707638064,NA
"14788","SCN9A",0.611327533046235,-3.59348320528558,2.13935106894165,-1.67970711186888,0.0930143153929888,NA

"14789","SCN7A",0.716563975801164,2.19540632171719,2.62525426221469,0.836264263357531,0.403006252788843,NA
"14790","B3GALT1",0.139138330553998,-0.0338344975521838,3.34232334820666,-0.0101230473617515,0.991923114749952,NA
"14791","CERS6-AS1",0.0732428936164972,-0.49131726092088,3.34845351515744,-0.146729604785264,0.883345450266286,NA
"14792","SPC25",0,NA,NA,NA,NA,NA
"14793","ABCB11",0.139138330553998,-0.0338344975521838,3.34232334820666,-0.0101230473617515,0.991923114749952,NA
"14794","DHRS9",0.786590978944108,-1.99435197092999,1.67105443147791,-1.19346918530125,0.23268567340708,NA
"14795","KLHL41",0.2028177743346,0.512115644193803,2.71757760068597,0.188445637785848,0.850527326709045,NA
"14796","CCDC173",0.599404540288545,-1.25916399994897,1.67100049055958,-0.753538976836146,0.451126098563798,NA
"14797","MYO3B",0.870999625666917,2.49062022465191,1.86588926907997,1.33481673640793,0.18193633441696,NA
"14798","AC007405.1",0.558090933295802,0.5948207362968,1.71168924693572,0.347505096127498,0.72821189062752,NA
"14799","LINC01124",1.07422884775133,-0.829526838861423,1.27849271078568,-0.648831887630905,0.516447043978386,NA
"14800","AC007405.2",0,NA,NA,NA,NA,NA
"14801","AC007405.4",0,NA,NA,NA,NA,NA
"14802","AC010092.1",0,NA,NA,NA,NA,NA
"14803","DLX1",0,NA,NA,NA,NA,NA
"14804","DLX2",0,NA,NA,NA,NA,NA
"14805","ITGA6",0.949399369595019,-0.864004644840149,1.7163704881567,-0.50339052716296,0.614689730384326,NA
"14806","ITGA6-AS1",0.20332072784659,-1.39924864619322,3.10758170858468,-0.450269301794319,0.652516271227926,NA
"14807","AC078883.1",0,NA,NA,NA,NA,NA
"14808","MAP3K20-AS1",0.343044770723266,-1.83025924174448,2.32925281575478,-0.785770968855264,0.432001677398361,NA
"14809","CDCA7",0,NA,NA,NA,NA,NA
"14810","AC016737.1",0.315120193005521,0.245400022010969,3.2393007694085,0.0757570968180826,0.939612349767913,NA
"14811","AC016737.2",0.0636794437806017,-0.49131726092088,3.34845351515744,-0.146729604785264,0.883345450266286,NA
"14812","LINC01305",0.0636794437806017,-0.49131726092088,3.34845351515744,-0.146729604785264,0.883345450266286,NA
"14813","SP9",0,NA,NA,NA,NA,NA
"14814","AC010894.2",0.460506621757221,0.768899685425682,1.78929804430786,0.429721413864905,0.667398304459194,NA
"14815","AC010894.3",0,NA,NA,NA,NA,NA
"14816","CHRNA1",0,NA,NA,NA,NA,NA
"14817","CHN1",1.24368177565934,1.07791859782507,1.33108008702945,0.809807470135511,0.418050838560777,NA

"14818","EVX2",0,NA,NA,NA,NA,NA
"14819","HOXD13",0,NA,NA,NA,NA,NA
"14820","HOXD11",0.102366282257309,-0.49131726092088,3.34845351515744,
-0.146729604785264,0.883345450266286,NA
"14821","HOXD10",0.204732564514618,0.248608670877116,3.33613854356166,
0.0745198880774602,0.940596716765987,NA
"14822","HAGLR",0.258011588580052,0.510354618063005,2.53828617896562,0
.201062678547531,0.84064956422761,NA
"14823","HAGLROS",0,NA,NA,NA,NA,NA
"14824","HOXD1",0,NA,NA,NA,NA,NA
"14825","AC017048.3",1.17857539524779,0.398484070617791,1.234603648276
17,0.322762751571468,0.746874924359664,NA
"14826","AC092162.2",0.341654901911717,1.15701766215399,2.372227232111
99,0.487734752595306,0.625737732511514,NA
"14827","AC074286.1",0.0636794437806017,-0.49131726092088,3.3484535151
5744,-0.146729604785264,0.883345450266286,NA
"14828","AC079305.3",0.647391857435902,-0.0578061610754079,1.613478205
31245,-0.0358270479793768,0.971420265738874,NA
"14829","AC019080.4",0,NA,NA,NA,NA,NA
"14830","AC019080.5",0.0732428936164972,-0.49131726092088,3.3484535151
5744,-0.146729604785264,0.883345450266286,NA
"14831","AC019080.6",0.921860476364412,1.42979908373295,1.477526381132
92,0.967697837406212,0.33319529849166,NA
"14832","PDE11A",0.137809252696657,-1.1477220656059,3.3390638510896,-0
.343725701810518,0.731052580354999,NA
"14833","AC009948.3",1.24221387278169,-0.335611917045508,1.46658779302
594,-0.228838613440969,0.81899435380994,NA
"14834","AC009948.2",0.790233846806322,-0.0201953278961975,1.547348615
05125,-0.0130515694393332,0.989586649891972,NA
"14835","AC010680.2",0.900031508545552,-0.514696850013739,1.5120504356
0475,-0.340396615016273,0.733557867488138,NA
"14836","AC010680.4",0.821169001940377,0.889014726698567,1.50961654169
426,0.588901023634,0.555927671041319,NA
"14837","AC010680.5",0.0636794437806017,-0.49131726092088,3.3484535151
5744,-0.146729604785264,0.883345450266286,NA
"14838","AC010680.1",0.0636794437806017,-0.49131726092088,3.3484535151
5744,-0.146729604785264,0.883345450266286,NA
"14839","CCDC141",0.411806896597916,-2.14409022170048,2.24320078708113
,-0.955817345486216,0.339164523438083,NA
"14840","SCHLAP1",0,NA,NA,NA,NA,NA
"14841","AC104076.1",0.0367720482966892,-0.560503011376585,3.349520014
37583,-0.167338307868279,0.867103863749685,NA
"14842","AC068196.1",0.110901857212745,-1.2169087618042,3.340133369026
82,-0.36432939267894,0.715612053721651,NA
"14843","LINC01934",0.212381224170495,0.56712693135589,2.6881025075667
5,0.210976675837131,0.832905471698532,NA
"14844","CERKL",0,NA,NA,NA,NA,NA
"14845","AC012500.1",0,NA,NA,NA,NA,NA
"14846","AC021851.1",0.653832429856761,1.14945050875657,1.815842521711
42,0.633012221606758,0.526725661969349,NA

"14847","AC021851.2",0,NA,NA,NA,NA,NA
"14848","AC096667.1",0.102366282257309,-0.49131726092088,3.34845351515744,-0.146729604785264,0.883345450266286,NA
"14849","ZNF804A",0,NA,NA,NA,NA,NA
"14850","AC009315.1",1.12172015043241,-0.153410153983729,1.33658332424424,-0.114777845272365,0.908621209895304,NA
"14851","FSIP2-AS1",0.110901857212745,-1.2169087618042,3.34013336902682,-0.36432939267894,0.715612053721651,NA
"14852","FSIP2-AS2",0.305184056591909,1.0238829631905,2.42729878959865,0.42181991256206,0.673156470155355,NA
"14853","AC097500.1",0.497864382376587,0.152168235041596,1.77396993023166,0.0857783621065801,0.931642607606228,NA
"14854","AC017071.1",0.764552505492976,1.4830419665261,1.50803840631092,0.983424533698735,0.325398540497804,NA
"14855","AC007319.1",0.421217377326751,0.576856084266614,2.24713630275234,0.256707207106248,0.797404807487813,NA
"14856","DIRC1",0.139138330553998,-0.0338344975521838,3.34232334820666,-0.0101230473617515,0.991923114749952,NA
"14857","AC133106.1",0.157560096502761,-0.491317252697356,3.34845351503297,-0.146729602334801,0.883345452200538,NA
"14858","OSGEPL1-AS1",0,NA,NA,NA,NA,NA
"14859","AC013468.1",0,NA,NA,NA,NA,NA
"14860","MSTN",0.603797273828686,1.89161435727128,2.11036040594963,0.896346591766198,0.370067682339367,NA
"14861","AC093388.1",0.258274559745298,-1.11669114604128,2.56884985789992,-0.434704715266695,0.663776775348859,NA
"14862","AC006460.1",0,NA,NA,NA,NA,NA
"14863","AC005540.1",0,NA,NA,NA,NA,NA
"14864","AC067945.3",0,NA,NA,NA,NA,NA
"14865","STAT4",1.31954718336288,-0.734561173693022,1.1958643645242,-0.614251244107675,0.539049315464419,NA
"14866","AC067945.4",0,NA,NA,NA,NA,NA
"14867","AC092614.1",0.166045726037911,0.183394512381981,3.33779889015228,0.0549447460489789,0.956182483505573,NA
"14868","AC098617.1",0,NA,NA,NA,NA,NA
"14869","TMEFF2",0.166548679549901,-1.95393031097672,3.33920037562282,-0.585149164824313,0.558447422910535,NA
"14870","AC096647.1",0.35616007426076,-1.35549659837783,2.1075333522812,-0.643167329670316,0.520115529757457,NA
"14871","LINC01821",0,NA,NA,NA,NA,NA
"14872","LINC01825",0.166548679549901,-1.95393031097672,3.33920037562282,-0.585149164824313,0.558447422910535,NA
"14873","LINC01827",0,NA,NA,NA,NA,NA
"14874","DNAH7",0.414756835947313,-0.22104055406692,2.04612302872437,-0.108028965494184,0.913972715517691,NA
"14875","AC114760.2",1.21147392630586,-0.106706669472013,1.43801652532671,-0.074204063439236,0.940848012610569,NA
"14876","HECW2-

AS1",0.284918984063964,0.682792286303301,2.83389871436466,0.2409374346
5224,0.809603607962577,NA
"14877","C2orf66",0.181578773267413,-1.95393031097672,3.33920037562282
,-0.585149164824313,0.558447422910535,NA
"14878","AC013264.1",0.0741298089160557,-1.76673671430743,3.3430969623
6258,-0.528473069790613,0.597171033704837,NA
"14879","ANKRD44-
IT1",0.137809252696657,-1.1477220656059,3.3390638510896,-0.34372570181
0518,0.731052580354999,NA
"14880","AC011997.1",0,NA,NA,NA,NA,NA
"14881","MARS2",0.55897784859536,-0.231121109873379,1.72648937694543,-
0.133867669827362,0.893507215871836,NA
"14882","AC011997.2",0.22061559614905,-0.418038608046788,3.07273464003
886,-0.136047741513241,0.891783539523794,NA
"14883","FTCDNL1",0.351820757701375,1.24278979418629,2.61670261372257,
0.474944989036516,0.634826183670586,NA
"14884","AC097717.1",0,NA,NA,NA,NA,NA
"14885","LINC01792",0,NA,NA,NA,NA,NA
"14886","AC005037.1",0.146485787232994,0.248608662259703,3.33613854377
696,0.0745198854896011,0.940596718825075,NA
"14887","AC007272.1",0.2915937151806,-0.850240888675731,2.453028094211
04,-0.346608704026764,0.72888530597779,NA
"14888","CFLAR-
AS1",0.321954003525899,-0.649223595204747,2.18296399927226,-0.29740462
7571129,0.766157611143954,NA
"14889","FLACC1",0.683009764838791,0.775454766889393,1.88081566602947,
0.41229705860885,0.680121704305204,NA
"14890","AC007279.2",0,NA,NA,NA,NA,NA
"14891","MPP4",0.787093870530467,-1.85760727489908,1.6121101481541,-1.
15228309742115,0.249204760349102,NA
"14892","AC069148.1",0.95424022079689,0.868127000789923,1.601827711887
39,0.541960283460842,0.587845866513121,NA
"14893","KIAA2012",0.81821906259098,-0.0927928706710356,1.589768950103
55,-0.0583687778434668,0.953454884028094,NA
"14894","KIAA2012-
AS1",0.218350821564103,-1.39752731860778,3.03720335813394,-0.460136235
153652,0.645418436617112,NA
"14895","AC079354.3",0.618088256440531,1.8791334134612,1.8738084874885
3,1.00284176638553,0.315937213385383,NA
"14896","AC079354.2",0.136922337397099,0.183394507878855,3.33779889026
91,0.0549447446979257,0.956182484581931,NA
"14897","AC064836.2",0.409465129029236,1.37987226043016,3.001269680861
02,0.459762836118845,0.645686461413841,NA
"14898","CD28",0.830207530407803,-0.543134453229988,1.44796289827667,-
0.375102465592464,0.707584263413726,NA
"14899","CTLA4",0.883806858333968,-0.617990721645881,1.6477150255529,-
0.375059225692568,0.707616420392767,NA
"14900","ICOS",0.943214214727025,-0.232585312070562,1.41124338353487,-
0.164808788323942,0.869094486555858,NA
"14901","AC009498.1",0.157560096502761,-0.491317252697356,3.3484535150

3297,-0.146729602334801,0.883345452200538,NA
"14902","AC007679.1",0,NA,NA,NA,NA,NA
"14903","AC007383.2",0.877418230231296,0.693334796240419,1.46560948929
162,0.473069259790021,0.63616376722974,NA
"14904","AC007383.3",0.100451492077291,-0.0338344573480013,3.342323347
73369,-0.010123035334371,0.991923124345921,NA
"14905","GPR1-
AS",0.110316144890068,0.0897481123376931,3.34029447074234,0.0268683234
738127,0.978564758597723,NA
"14906","MDH1B",1.23524067312324,0.390681353506566,1.35657956270681,0.
287990003864596,0.773354390434451,NA
"14907","AC008269.1",1.19390552794995,1.33267931590691,1.3313659086551
2,1.00098651110356,0.31683332973684,NA
"14908","CPO",0.318802313641394,-0.548249818839563,2.39386723732325,-0
.229022650166933,0.818851311656768,NA
"14909","KLF7-
IT1",1.29322447923962,1.35093834413365,1.45037153579548,0.931442951541
868,0.351624481643552,NA
"14910","MYOSLID",0.166548679549901,-1.95393031097672,3.33920037562282
,-0.585149164824313,0.558447422910535,NA
"14911","AC007879.3",0,NA,NA,NA,NA,NA
"14912","AC007879.2",0,NA,NA,NA,NA,NA
"14913","AC009226.1",0.0741298089160557,-1.76673671430743,3.3430969623
6258,-0.528473069790613,0.597171033704837,NA
"14914","LINC01857",0,NA,NA,NA,NA,NA
"14915","AC096772.1",0,NA,NA,NA,NA,NA
"14916","FZD5",1.40667028423208,0.340005486990508,1.1430367503278,0.29
7458053639135,0.766116827760188,NA
"14917","AC083900.1",0.204732564514618,0.248608670877116,3.33613854356
166,0.0745198880774602,0.940596716765987,NA
"14918","AC019185.2",0.102366282257309,-0.49131726092088,3.34845351515
744,-0.146729604785264,0.883345450266286,NA
"14919","MAP2",0.62846713797515,1.19188536032583,1.67774295796072,0.71
0409991393766,0.477449929091295,NA
"14920","AC007038.1",1.26684980877356,-0.507235657466626,1.43128885254
235,-0.354390839113741,0.723045998995779,NA
"14921","AC006994.2",0.868139289741196,0.0455298162281891,1.6132261705
6466,0.0282228351231451,0.977484424682112,NA
"14922","MYL1",0.0636794437806017,-0.49131726092088,3.34845351515744,-
0.146729604785264,0.883345450266286,NA
"14923","LINC01878",0,NA,NA,NA,NA,NA
"14924","AC108066.2",0.0732428936164972,-0.49131726092088,3.3484535151
5744,-0.146729604785264,0.883345450266286,NA
"14925","SNHG31",0,NA,NA,NA,NA,NA
"14926","LINC00607",1.23060351059437,-2.389884065872,1.30700540111878,
-1.82851889045469,0.0674717132242924,NA
"14927","AC093382.1",0,NA,NA,NA,NA,NA
"14928","TMEM169",0.157560096502761,-0.491317252697356,3.3484535150329
7,-0.146729602334801,0.883345452200538,NA
"14929","AC098820.1",0.136922337397099,0.183394507878855,3.33779889026

91,0.0549447446979257,0.956182484581931,NA
"14930","AC098820.3",0.0367720482966892,-0.560503011376585,3.34952001437583,-0.167338307868279,0.867103863749685,NA
"14931","IGFBP2",0.797813124914862,-1.22769254594918,1.53946753583461,-0.797478684916598,0.425173077233046,NA
"14932","AC007563.4",0.483594937945292,0.701364060710641,2.53840308705191,0.276301295207296,0.78231666119359,NA
"14933","LINC01921",0,NA,NA,NA,NA,NA
"14934","AC007749.1",0.0367720482966892,-0.560503011376585,3.34952001437583,-0.167338307868279,0.867103863749685,NA
"14935","AC007749.2",0,NA,NA,NA,NA,NA
"14936","AC010136.1",0.453584634004922,1.5389431092149,2.49049807065504,0.617925838750051,0.536624225607653,NA
"14937","RUFY4",0,NA,NA,NA,NA,NA
"14938","CXCR2",0.0741298089160557,-1.76673671430743,3.34309696236258,-0.528473069790613,0.597171033704837,NA
"14939","CXCR1",0.360377870837361,1.07706477854197,2.11045352449717,0.510347546648102,0.609807997840901,NA
"14940","AC021016.2",1.07982450361332,1.70360818606963,1.47118802124027,1.15798127871747,0.246871676420779,NA
"14941","GPBAR1",0.347974483710253,0.280695632154393,2.35407473614482,0.119238199129598,0.905086643984856,NA
"14942","CATIP",0,NA,NA,NA,NA,NA
"14943","CATIP-AS1",0.268461953715506,-0.692213851544516,2.51693598492032,-0.275022430324715,0.783299005639148,NA
"14944","AC021016.3",0.304347086712636,0.835009903170014,2.40703490964789,0.346903943861854,0.728663483658821,NA
"14945","VIL1",0.347624499305324,-0.658125241455401,2.35945065459191,-0.278931555603663,0.780297351347269,NA
"14946","PLCD4",1.41049610176468,0.390016464999854,1.64166115282761,0.237574279155042,0.812211301659979,NA
"14947","AC009974.2",0.292971574465989,1.01027116194909,3.29385652095093,0.306713773208745,0.759061245268142,NA
"14948","AC009974.1",1.04909837330329,0.176279897162548,1.37740078895362,0.12798010468432,0.898164717716673,NA
"14949","PRKAG3",0.2028177743346,0.512115644193803,2.71757760068597,0.188445637785848,0.850527326709045,NA
"14950","WNT10A",0.0732428936164972,-0.49131726092088,3.34845351515744,-0.146729604785264,0.883345450266286,NA
"14951","AC097468.4",0,NA,NA,NA,NA,NA
"14952","AC097468.3",1.21220913092332,0.0027316342333161,1.3281105253657,0.00205678230926146,0.998358926307562,NA
"14953","PTPRN",0.110014941913186,-0.033834469553869,3.34232334787728,-0.0101230389858472,0.991923121432614,NA
"14954","AC053503.2",0,NA,NA,NA,NA,NA
"14955","DES",0.127358887561203,0.15032497835859,3.33866494558414,0.0450254759937551,0.964087002635848,NA
"14956","SPEG",0,NA,NA,NA,NA,NA
"14957","SPEGNB",0.721844407509389,0.0466605941563659,1.75126692582894

,0.0266439076009385,0.978743752476106,NA
"14958","AC053503.5",0.471196969239419,0.0966590483646935,2.16270796978202,0.0446935276122535,0.964351592761972,NA
"14959","ASIC4",0.166548679549901,-1.95393031097672,3.33920037562282,-0.585149164824313,0.558447422910535,NA
"14960","TMEM198",0.958979021460439,-1.42982393813961,1.58857882596098,-0.900064834538293,0.368085748671521,NA
"14961","AC009955.4",0,NA,NA,NA,NA,NA
"14962","INHA",0.350190476867153,0.252842669947774,1.94464010584612,0.130020289712045,0.896550374083493,NA
"14963","SLC4A3",0.988688122423928,-2.39073269605454,1.70101373564929,-1.40547524452645,0.159880005618965,NA
"14964","AC093843.1",0,NA,NA,NA,NA,NA
"14965","AC093843.2",0,NA,NA,NA,NA,NA
"14966","AC079834.2",0.633179235544979,-0.795356793553292,1.75094130824894,-0.454245262137715,0.649652308847731,NA
"14967","AC010980.1",0.0636794437806017,-0.49131726092088,3.34845351515744,-0.146729604785264,0.883345450266286,NA
"14968","SGPP2",0.304932799035314,-0.455323059556486,2.47402355059449,-0.184041521935826,0.853980881434662,NA
"14969","MOGAT1",0.0367720482966892,-0.560503011376585,3.34952001437583,-0.167338307868279,0.867103863749685,NA
"14970","SCG2",0.303854978734568,0.0104934003039746,2.46246497721938,0.00426133991794828,0.996599952961392,NA
"14971","AP1S3",1.12112214256842,0.574154143916693,1.50904624333428,0.380474850557317,0.703592961711863,NA
"14972","FAM124B",0.0741298089160557,-1.76673671430743,3.34309696236258,-0.528473069790613,0.597171033704837,NA
"14973","AC073052.2",0.346668202458428,-1.94732862277873,3.00338753971666,-0.648377406187962,0.516740879961632,NA
"14974","NYAP2",0,NA,NA,NA,NA,NA
"14975","AC010735.2",0.183257835529684,0.495279120319891,3.246926734798,0.152537818304269,0.878762764724423,NA
"14976","AC010735.1",0,NA,NA,NA,NA,NA
"14977","COL4A3",0.810277241070545,-2.31801956905785,1.7830125755674,-1.30005789124634,0.193581128435081,NA
"14978","AC064853.1",0,NA,NA,NA,NA,NA
"14979","SCYGR4",0,NA,NA,NA,NA,NA
"14980","DAW1",0,NA,NA,NA,NA,NA
"14981","LINC01807",0.789632604844798,-1.98816382959966,1.83313307531808,-1.08457146748862,0.278111497849704,NA
"14982","AC008273.1",0.255122869860792,-1.01416582893919,2.91937932186279,-0.347390906465717,0.728297664063611,NA
"14983","SLC16A14",0.597751700311751,1.9295375788579,1.67654902348381,1.15089839415992,0.249774038521653,NA
"14984","SP140",0.622546994400267,0.650510654983293,1.89612223187703,0.343074219608371,0.731542625217565,NA
"14985","LINC01907",0.0636794437806017,-0.49131726092088,3.34845351515744,-0.146729604785264,0.883345450266286,NA
"14986","GPR55",0.346668202458428,-1.94732862277873,3.00338753971666,-

0.648377406187962,0.516740879961632,NA
"14987","AC017104.1",0.0636794437806017,-0.49131726092088,3.3484535151
5744,-0.146729604785264,0.883345450266286,NA
"14988","B3GNT7",0.295632320364664,-1.87186550711609,2.77313524931022,
-0.674999716505601,0.499675945057307,NA
"14989","AC017104.3",0,NA,NA,NA,NA,NA
"14990","LINC00471",0.268461953715506,-0.692213851544516,2.51693598492
032,-0.275022430324715,0.783299005639148,NA
"14991","AC017104.6",0.315120193005521,0.245400022010969,3.23930076940
85,0.0757570968180826,0.939612349767913,NA
"14992","NMUR1",0.981847044936883,1.5790364720041,1.46616325195809,1.0
7698543794169,0.281486771344861,NA
"14993","AC073476.3",0.366647507215611,1.28945761217035,2.315246384711
76,0.556941853223489,0.577567157768564,NA
"14994","NPPC",0.409465129029236,1.37987226043016,3.00126968086102,0.4
59762836118845,0.645686461413841,NA
"14995","CHRNG",0.102366282257309,-0.49131726092088,3.34845351515744,-
0.146729604785264,0.883345450266286,NA
"14996","KCNJ13",0.204732564514618,0.248608670877116,3.33613854356166,
0.0745198880774602,0.940596716765987,NA
"14997","SNORC",0,NA,NA,NA,NA,NA
"14998","SAG",0.146485787232994,0.248608662259703,3.33613854377696,0.0
745198854896011,0.940596718825075,NA
"14999","HJURP",0.239791573166398,-1.32959277084231,2.9363559394555,-0
.45280367852436,0.650690114564834,NA
"15000","AC097713.3",0.212381224170495,0.56712693135589,2.688102507566
75,0.210976675837131,0.832905471698532,NA
"15001","AC010148.1",0.157560096502761,-0.491317252697356,3.3484535150
3297,-0.146729602334801,0.883345452200538,NA
"15002","AGAP1-
IT1",0.0735440965933784,-0.185659846583073,3.34418846206699,-0.0555171
601986567,0.955726459171656,NA
"15003","IQCA1",1.31289518692612,0.217594737629225,1.33702541228413,0.
162745401568317,0.870718898216525,NA
"15004","AC084030.1",0.929711316231328,2.59249965629503,1.478760523257
21,1.7531571985602,0.0795750276143245,NA
"15005","AC105760.1",0.146485787232994,0.248608662259703,3.33613854377
696,0.0745198854896011,0.940596718825075,NA
"15006","AC107079.1",0,NA,NA,NA,NA,NA
"15007","AC112715.1",0.776944708371894,-1.16715325844081,1.61429912471
699,-0.723009286550556,0.469674178831745,NA
"15008","MLPH",0.614388943014768,0.879636915537126,1.7657928072355,0.4
98154093692494,0.618375435921,NA
"15009","AC012076.1",0.387990400044302,1.3346877833647,2.2587233549962
9,0.59090360951569,0.5545850073729,NA
"15010","RBM44",1.12613544311513,-0.943535897548541,1.57431869520485,-
0.599329665856359,0.548953068976623,NA
"15011","RAMP1",0.127358887561203,0.15032497835859,3.33866494558414,0.
0450254759937551,0.964087002635848,NA
"15012","ERFE",0.375910918066863,-0.84151360567691,2.29073989540971,-0

.367354498589374,0.713354607339421,NA
"15013","LINC02610",0.559350629605804,-0.152689664103771,2.08638941352
69,-0.073183684269975,0.941659949636156,NA
"15014","AC012485.1",0,NA,NA,NA,NA,NA
"15015","AC012485.3",0.597176833522995,0.774288198723821,1.83872601738
517,0.42110036590711,0.673681794395866,NA
"15016","LINC01940",0.102366282257309,-0.49131726092088,3.348453515157
44,-0.146729604785264,0.883345450266286,NA
"15017","AC017028.1",0.102366282257309,-0.49131726092088,3.34845351515
744,-0.146729604785264,0.883345450266286,NA
"15018","AC017028.2",0,NA,NA,NA,NA,NA
"15019","AC093802.1",0.397351669669159,-0.620585281597984,2.3000689652
3181,-0.26981159738201,0.787305200194518,NA
"15020","AC110619.1",0,NA,NA,NA,NA,NA
"15021","GPR35",0.851880041514011,-0.406000034409928,1.55850217337265,
-0.260506556453065,0.794473058544246,NA
"15022","CROCC2",0.139138330553998,-0.0338344975521838,3.3423233482066
6,-0.0101230473617515,0.991923114749952,NA
"15023","AC104809.2",0.0636794437806017,-0.49131726092088,3.3484535151
5744,-0.146729604785264,0.883345450266286,NA
"15024","AC104809.1",0.110901857212745,-1.2169087618042,3.340133369026
82,-0.36432939267894,0.715612053721651,NA
"15025","AC005237.1",0,NA,NA,NA,NA,NA
"15026","PASK",0.938246891186764,1.83036698513985,1.4276182966585,1.28
211230510567,0.199803252996386,NA
"15027","ANO7",0.157560096502761,-0.491317252697356,3.34845351503297,-
0.146729602334801,0.883345452200538,NA
"15028","AC104841.1",0.100451492077291,-0.0338344573480013,3.342323347
73369,-0.010123035334371,0.991923124345921,NA
"15029","BOK-
AS1",0.0741298089160557,-1.76673671430743,3.34309696236258,-0.52847306
9790613,0.597171033704837,NA
"15030","AC133528.1",0,NA,NA,NA,NA,NA
"15031","AC114730.2",0.845595863880173,1.41809171733226,1.618296714789
2,0.876286594647743,0.380874250787752,NA
"15032","GAL3ST2",0,NA,NA,NA,NA,NA
"15033","AC131097.1",0,NA,NA,NA,NA,NA
"15034","NEU4",0.20332072784659,-1.39924864619322,3.10758170858468,-0.
450269301794319,0.652516271227926,NA
"15035","AC131097.3",0,NA,NA,NA,NA,NA
"15036","PDCD1",0.110014941913186,-0.033834469553869,3.34232334787728,
-0.0101230389858472,0.991923121432614,NA
"15037","RTP5",0,NA,NA,NA,NA,NA
"15038","LINC01237",0.0636794437806017,-0.49131726092088,3.34845351515
744,-0.146729604785264,0.883345450266286,NA
"15039","FAM240C",0.0636794437806017,-0.49131726092088,3.3484535151574
4,-0.146729604785264,0.883345450266286,NA
"15040","LINC01238",0.323605822540671,0.859187086673796,2.435164716891
91,0.352825039190945,0.724219610906701,NA
"15041","LINC01238.1",0.378487741162614,-2.15786196901571,2.2407871508

2696,-0.962992834111599,0.335551119403315,NA
"15042","CHL1-AS1",0,NA,NA,NA,NA,NA
"15043","AC087430.1",0,NA,NA,NA,NA,NA
"15044","CNTN6",0.0367720482966892,-0.560503011376585,3.34952001437583
,-0.167338307868279,0.867103863749685,NA
"15045","AC087427.1",0,NA,NA,NA,NA,NA
"15046","IL5RA",0,NA,NA,NA,NA,NA
"15047","LRRN1",0.0367720482966892,-0.560503011376585,3.34952001437583
,-0.167338307868279,0.867103863749685,NA
"15048","AC023480.1",0.0735440965933784,-0.185659846583073,3.344188462
06699,-0.0555171601986567,0.955726459171656,NA
"15049","AC023483.1",0,NA,NA,NA,NA,NA
"15050","ITPR1-
DT",0.0367720482966892,-0.560503011376585,3.34952001437583,-0.16733830
7868279,0.867103863749685,NA
"15051","EGOT",0.230228123330503,-1.33023934114211,2.97714568949567,-0
.446817011957333,0.655007187193824,NA
"15052","AC026202.2",0.456025854015374,0.234746545218556,2.14609518203
59,0.109383100611532,0.912898637074469,NA
"15053","AC026202.3",0,NA,NA,NA,NA,NA
"15054","AC069277.1",0.0636794437806017,-0.49131726092088,3.3484535151
5744,-0.146729604785264,0.883345450266286,NA
"15055","GRM7",0,NA,NA,NA,NA,NA
"15056","AC066585.1",0,NA,NA,NA,NA,NA
"15057","GRM7-AS1",0,NA,NA,NA,NA,NA
"15058","AC077690.1",0,NA,NA,NA,NA,NA
"15059","AC023481.1",1.15165130613279,-0.195320180220165,1.42480374619
278,-0.137085672845879,0.890963076963971,NA
"15060","CAV3",0.2028177743346,0.512115644193803,2.71757760068597,0.18
8445637785848,0.850527326709045,NA
"15061","OXTR",0.452031837755993,-1.37640832031325,1.9625872045661,-0.
701323394502388,0.483101217102684,NA
"15062","AC026191.1",0.100451492077291,-0.0338344573480013,3.342323347
73369,-0.010123035334371,0.991923124345921,NA
"15063","LHFPL4",0.0636794437806017,-0.49131726092088,3.34845351515744
,-0.146729604785264,0.883345450266286,NA
"15064","AC022382.2",0,NA,NA,NA,NA,NA
"15065","CPNE9",0.0636794437806017,-0.49131726092088,3.34845351515744,
-0.146729604785264,0.883345450266286,NA
"15066","AC022382.1",0.44074024831227,-2.50975333340643,2.137041643671
35,-1.17440544073572,0.240232667045727,NA
"15067","CIDEC",0.100451492077291,-0.0338344573480013,3.34232334773369
,-0.010123035334371,0.991923124345921,NA
"15068","AC018809.1",0.576425686004881,-1.96709542127708,1.86916787839
861,-1.05239098318037,0.29262020387937,NA
"15069","AC022007.1",0.249153272467185,0.812295408448132,2.54252578312
747,0.31948364647416,0.7493597903733,NA
"15070","FANCD2",1.15746793441934,0.221625653350636,1.41003186036711,0
.157177762843554,0.875104753137217,NA
"15071","AC034193.1",0.0367720482966892,-0.560503011376585,3.349520014

37583,-0.167338307868279,0.867103863749685,NA
"15072","IRAK2",1.05072650176202,-2.38741121572182,1.69833688904076,-1.40573476977837,0.159802898166177,NA
"15073","LINC00852",1.3582959148604,-2.22489920211906,1.23934755700907,-1.79521812871317,0.072618953198622,NA
"15074","GHRL",0.102366282257309,-0.49131726092088,3.34845351515744,-0.146729604785264,0.883345450266286,NA
"15075","GHRLOS",0.929756139147889,0.802611436152667,1.57049685987624,0.511055740802893,0.609312026158339,NA
"15076","SLC6A1",0,NA,NA,NA,NA,NA
"15077","AC083855.2",1.21050474379718,0.9439090966457,1.39318804371689,0.677517368098743,0.498077754231589,NA
"15078","AC022001.2",0,NA,NA,NA,NA,NA
"15079","AC022001.3",0.102366282257309,-0.49131726092088,3.34845351515744,-0.146729604785264,0.883345450266286,NA
"15080","SYN2",0.0735440965933784,-0.185659846583073,3.34418846206699,-0.0555171601986567,0.955726459171656,NA
"15081","AC034198.2",0.173694385693788,0.450698267773282,2.85372304708896,0.15793342953621,0.874509255668894,NA
"15082","NUP210",0.800844159420247,-0.447302391581119,1.70640019585906,-0.262132173136521,0.793219543198501,NA
"15083","WNT7A",0,NA,NA,NA,NA,NA
"15084","AC090004.2",0,NA,NA,NA,NA,NA
"15085","EAF1-AS1",0.656764402270611,-0.511284320327422,1.6512171025755,-0.30964088218923,0.756834063664084,NA
"15086","AC090946.1",0.204732564514618,0.248608670877116,3.33613854356166,0.0745198880774602,0.940596716765987,NA
"15087","AC090948.1",0.601559312815308,-1.91635145920392,1.85621788557072,-1.03239575165213,0.301886761318908,NA
"15088","AC090948.2",0.102366282257309,-0.49131726092088,3.34845351515744,-0.146729604785264,0.883345450266286,NA
"15089","AC090948.3",0.204732564514618,0.248608670877116,3.33613854356166,0.0745198880774602,0.940596716765987,NA
"15090","AC090948.4",0.139138330553998,-0.0338344975521838,3.34232334820666,-0.0101230473617515,0.991923114749952,NA
"15091","AC010727.1",0.0636794437806017,-0.49131726092088,3.34845351515744,-0.146729604785264,0.883345450266286,NA
"15092","AC090644.1",0,NA,NA,NA,NA,NA
"15093","AC144521.1",0.283408124630093,0.975610017042562,2.84250964510619,0.343221356776123,0.731431939068564,NA
"15094","SATB1-AS1",0.0741298089160557,-1.76673671430743,3.34309696236258,-0.528473069790613,0.597171033704837,NA
"15095","KCNH8",0,NA,NA,NA,NA,NA
"15096","AC061958.1",0.512992334867705,0.228494235795889,1.95556874188788,0.116842855432074,0.906984582595472,NA
"15097","EFHB",0.868342142342912,-1.38478066885235,1.80767735837599,-0.766055215791626,0.443643448678214,NA
"15098","PP2D1",0,NA,NA,NA,NA,NA

"15099","SGO1",0.0741298089160557,-1.76673671430743,3.34309696236258,-0.528473069790613,0.597171033704837,NA
"15100","SGO1-AS1",0.148259617832111,-2.17639404086898,3.335076015408,-0.652577041966681,0.514028990213041,NA
"15101","AC116096.1",0.100451492077291,-0.0338344573480013,3.34232334773369,-0.010123035334371,0.991923124345921,NA
"15102","UBE2E2-AS1",0.664735803083919,0.00920784038461028,1.61449027427806,0.00570324921203239,0.995449490176433,NA
"15103","UBE2E1-AS1",0.0732428936164972,-0.49131726092088,3.34845351515744,-0.146729604785264,0.883345450266286,NA
"15104","LINC00691",0,NA,NA,NA,NA,NA
"15105","THRB-AS1",0.887244651232438,-0.239970010944999,1.48713494205968,-0.161363978585992,0.871806735762753,NA
"15106","AC012087.2",0,NA,NA,NA,NA,NA
"15107","RARB",1.00483106413372,0.548599637201854,1.47813200161699,0.37114387389064,0.710530374328428,NA
"15108","NEK10",0.725778653776585,-0.239620990939981,1.59104737134402,-0.150605818064087,0.880286671595952,NA
"15109","LINC02084",0.258011588580052,0.510354618063005,2.53828617896562,0.201062678547531,0.84064956422761,NA
"15110","EOMES",0.430780827162646,0.604773573905575,2.27500885169993,0.265833503660295,0.790367439097745,NA
"15111","RBMS3-AS2",0.183257835529684,0.495279120319891,3.246926734798,0.152537818304269,0.878762764724423,NA
"15112","AC025614.2",0,NA,NA,NA,NA,NA
"15113","GADL1",0,NA,NA,NA,NA,NA
"15114","OSBPL10-AS1",0.936517883504208,1.56753896462633,1.48983475269883,1.05215626215374,0.292727862623442,NA
"15115","ZNF860",1.02746197414032,-0.0618722906463059,1.56407451095266,-0.0395584035242796,0.968445190640688,NA
"15116","AC097639.1",0.0741298089160557,-1.76673671430743,3.34309696236258,-0.528473069790613,0.597171033704837,NA
"15117","TRIM71",0,NA,NA,NA,NA,NA
"15118","CCR4",0.0367720482966892,-0.560503011376585,3.34952001437583,-0.167338307868279,0.867103863749685,NA
"15119","TMPPE",0.254821666883911,-1.3286579469836,2.88029438084422,-0.461292413657444,0.64458882703139,NA
"15120","AC112220.2",0.781413466587581,2.37377146068518,1.72059080094205,1.37962580026901,0.167701889395658,NA
"15121","ARPP21",0,NA,NA,NA,NA,NA
"15122","DCLK3",0.148259617832111,-2.17639404086898,3.335076015408,-0.652577041966681,0.514028990213041,NA
"15123","LINC02033",0.218350821564103,-1.39752731860778,3.03720335813394,-0.460136235153652,0.645418436617112,NA

"15124","AC011816.2",0.0741298089160557,-1.76673671430743,3.3430969623
6258,-0.528473069790613,0.597171033704837,NA
"15125","AC126118.1",0.395770895856423,1.36763722394447,2.564983235450
5,0.533195385077935,0.593898335781326,NA
"15126","AC097359.3",0.221239540283362,0.178834610992208,3.06205963446
949,0.0584033730039328,0.953427328091912,NA
"15127","AC097359.2",0.66521721841536,-1.46619896226912,1.963956580500
34,-0.746553654406958,0.455333032798311,NA
"15128","C3orf35",1.39332685498671,0.872697799809835,1.28430367510101,
0.679510474608892,0.496814473023829,NA
"15129","DLEC1",0,NA,NA,NA,NA,NA
"15130","SLC22A13",0.102366282257309,-0.49131726092088,3.3484535151574
4,-0.146729604785264,0.883345450266286,NA
"15131","SLC22A14",0.444825340657898,0.0964063451783711,2.19019770977,
0.0440171883790779,0.964890702800851,NA
"15132","ACVR2B-
AS1",1.17161360524103,-1.84689488209717,1.60682750421757,-1.1494045734
5265,0.250389195084717,NA
"15133","SCN11A",0.377399075327947,0.41478541520833,2.0387702415748,0.
203448827508851,0.838784238896543,NA
"15134","AC092053.3",0.524912737441401,1.7831979140026,1.8830766646151
,0.946959806528686,0.343659260551436,NA
"15135","AC092053.2",0.0732428936164972,-0.49131726092088,3.3484535151
5744,-0.146729604785264,0.883345450266286,NA
"15136","AC092053.4",0.613668374085619,-1.64348018088634,1.91394329632
625,-0.858688020716677,0.390512661310397,NA
"15137","AC092053.5",0,NA,NA,NA,NA,NA
"15138","CX3CR1",0.267000171627192,-0.853137625449662,2.51498162517089
,-0.339222210178849,0.734442338867411,NA
"15139","AC104850.2",0,NA,NA,NA,NA,NA
"15140","CCR8",0,NA,NA,NA,NA,NA
"15141","AC099332.2",0.692377911583734,-1.22808724775035,1.87015329861
707,-0.656677315521931,0.511388423474679,NA
"15142","AC099332.1",0.52896754465499,0.511322216823306,1.850699560410
38,0.27628591250647,0.782328475168257,NA
"15143","MYRIP",0.175609175873806,0.247512349757584,3.30210845793575,0
.0749558510601773,0.940249838776562,NA
"15144","AC099560.1",0.346668202458428,-1.94732862277873,3.00338753971
666,-0.648377406187962,0.516740879961632,NA
"15145","AC099541.2",0.148259617832111,-2.17639404086898,3.33507601540
8,-0.652577041966681,0.514028990213041,NA
"15146","VIPR1",0.456683144371614,-0.835921186995024,2.20212795909758,
-0.379597009130015,0.704244581299108,NA
"15147","VIPR1-AS1",0,NA,NA,NA,NA,NA
"15148","AC006059.5",0.22061559614905,-0.418038608046788,3.07273464003
886,-0.136047741513241,0.891783539523794,NA
"15149","LINC02158",1.23322275800512,-0.240315184819425,1.387629607235
31,-0.17318395598248,0.862506834357434,NA
"15150","AC099329.1",0.0732428936164972,-0.49131726092088,3.3484535151
5744,-0.146729604785264,0.883345450266286,NA

"15151","AC099329.2",1.09081867676349,-0.265879775819961,1.34010847670296,-0.198401681984804,0.842730801568854,NA
"15152","CYP8B1",0.102366282257309,-0.49131726092088,3.34845351515744,-0.146729604785264,0.883345450266286,NA
"15153","AC092042.4",0,NA,NA,NA,NA,NA
"15154","SNRK-AS1",0.342157855423707,-0.617755040995007,2.37281219406507,-0.260347212704043,0.794595957359595,NA
"15155","AC104187.1",0,NA,NA,NA,NA,NA
"15156","ZKSCAN7-AS1",0.0367720482966892,-0.560503011376585,3.34952001437583,-0.167338307868279,0.867103863749685,NA
"15157","AC124045.1",0.184144750829242,-0.691295663073562,2.81313162320765,-0.245738826214366,0.805884414604706,NA
"15158","KIF15",0.227810379638494,0.737818182751471,3.02320406785502,0.24405172994986,0.807190756195641,NA
"15159","TGM4",0,NA,NA,NA,NA,NA
"15160","CDCP1",0.0732428936164972,-0.49131726092088,3.34845351515744,-0.146729604785264,0.883345450266286,NA
"15161","TMEM158",0.862940973520428,0.220061045343961,1.49423705780966,0.147273181449896,0.882916399741917,NA
"15162","SLC6A20",0,NA,NA,NA,NA,NA
"15163","CCR9",0,NA,NA,NA,NA,NA
"15164","CXCR6",0.32866696645417,-0.463602958677204,2.72219798872936,-0.170304643746211,0.864770559430071,NA
"15165","XCR1",0,NA,NA,NA,NA,NA
"15166","CCR3",0.110901857212745,-1.2169087618042,3.34013336902682,-0.36432939267894,0.715612053721651,NA
"15167","CCR2",0.388938106298068,-3.01478160288299,2.45633321092062,-1.22735042195398,0.21969091191595,NA
"15168","CCR5AS",0,NA,NA,NA,NA,NA
"15169","CCR5",0.433441573061322,-1.54362562381143,2.22689915984128,-0.693172664325516,0.488201200749473,NA
"15170","CCRL2",1.1800705820126,-3.12294254696957,1.50302153443404,-2.07777631618932,0.0377299661816734,NA
"15171","LINC02009",0.166548679549901,-1.95393031097672,3.33920037562282,-0.585149164824313,0.558447422910535,NA
"15172","LTF",0,NA,NA,NA,NA,NA
"15173","LRRC2-AS1",0,NA,NA,NA,NA,NA
"15174","ALS2CL",0.701004897868618,1.30104841396355,1.64132437686187,0.79268207570955,0.427963075967488,NA
"15175","TMIE",0,NA,NA,NA,NA,NA
"15176","PRSS45P",0,NA,NA,NA,NA,NA
"15177","CSPG5",0,NA,NA,NA,NA,NA
"15178","AC112512.1",0.166045726037911,0.183394512381981,3.33779889015228,0.0549447460489789,0.956182483505573,NA
"15179","CDC25A",0.102366282257309,-0.49131726092088,3.34845351515744,-0.146729604785264,0.883345450266286,NA
"15180","CAMP",0.663727897162038,-1.01796282270642,1.82688704898461,-0.557211691479347,0.577382802609908,NA

"15181","AC134772.1",0,NA,NA,NA,NA,NA
"15182","PFKFB4",0.457570059671173,-1.88394161052829,2.17090636577639,
-0.867813389019444,0.385496499136352,NA
"15183","COL7A1",0.666327852106506,-0.486849085527285,1.65002992963514
,-0.29505469978653,0.76795209426991,NA
"15184","CELSR3",0.0741298089160557,-1.76673671430743,3.34309696236258
,-0.528473069790613,0.597171033704837,NA
"15185","PRKAR2A-
AS1",0.646888903923912,1.13196818603525,1.5882611459604,0.712709108898
314,0.476025778730941,NA
"15186","AC137630.2",0.139138330553998,-0.0338344975521838,3.342323348
20666,-0.0101230473617515,0.991923114749952,NA
"15187","AC137630.1",0.347388771387576,1.2723569470996,2.2549773752801
4,0.564243775147205,0.572588239944582,NA
"15188","AC137630.4",0.763847371769947,1.42019389963552,1.601315260330
14,0.886892128501113,0.375136979978486,NA
"15189","AC137630.3",0.426022050218266,0.00097441000183233,2.303955265
48958,0.000422929219341971,0.999662551315635,NA
"15190","RHOA-
IT1",0.0732428936164972,-0.49131726092088,3.34845351515744,-0.14672960
4785264,0.883345450266286,NA
"15191","BSN",0.296698427056759,0.680778658728797,2.47341973472073,0.2
75237821212606,0.783133531518291,NA
"15192","MST1",0.712936145976236,0.989336676627397,1.87426646128465,0.
527852734423521,0.597601552446119,NA
"15193","AMIGO3",0.183559038506565,0.529394638499161,3.22699253814936,
0.164052018168831,0.869690193908075,NA
"15194","MST1R",0.268461953715506,-0.692213851544516,2.51693598492032,
-0.275022430324715,0.783299005639148,NA
"15195","SEMA3F-
AS1",0.102366282257309,-0.49131726092088,3.34845351515744,-0.146729604
785264,0.883345450266286,NA
"15196","SEMA3F",0.110901857212745,-1.2169087618042,3.34013336902682,-
0.36432939267894,0.715612053721651,NA
"15197","U73169.1",0.497704138980607,1.6938748139119,2.42780167284217,
0.69769900600197,0.485365449927194,NA
"15198","U73166.1",0.98326415699593,0.058538655199643,1.43412421661049
,0.040818399495406,0.967440670906418,NA
"15199","SEMA3B-
AS1",0.664413061926488,-0.399280157498269,1.53604425067701,-0.25994053
0568887,0.794909646692208,NA
"15200","LSMEM2",0,NA,NA,NA,NA,NA
"15201","HYAL3",0.928849787721278,-0.336147060837668,1.46487744604158,
-0.229471114970068,0.818502768598953,NA
"15202","RASSF1-
AS1",0.621700995529065,-0.6467763142453,1.84500904742647,-0.3505545488
50297,0.725922559849992,NA
"15203","ZMYND10",0.42881725555458,0.112992871480689,1.9821718950858,0
.0570045775347844,0.95454154872733,NA
"15204","CACNA2D2",0.89570274160258,0.688567419622748,1.95062684455983

,0.352998022939711,0.724089922717569,NA
"15205","CYB561D2.1",0.210165231013596,0.627110932648478,3.11655680733
581,0.20121915672205,0.840527212935289,NA
"15206","LINC02019",0.956713144808885,-1.94657150468949,1.488532273310
73,-1.30771199227009,0.19097101424484,NA
"15207","DOCK3",0.20332072784659,-1.39924864619322,3.10758170858468,-0
.450269301794319,0.652516271227926,NA
"15208","AC099050.1",0,NA,NA,NA,NA,NA
"15209","GRM2",0.0741298089160557,-1.76673671430743,3.34309696236258,-
0.528473069790613,0.597171033704837,NA
"15210","GPR62",0.139138330553998,-0.0338344975521838,3.34232334820666
,-0.0101230473617515,0.991923114749952,NA
"15211","POC1A",1.11849658659684,0.651956633783168,1.33204901843204,0.
489438920611638,0.624530987053828,NA
"15212","TLR9",0.848099194029805,0.768704357439611,1.82580428287941,0.
42102232131218,0.673738782509469,NA
"15213","AC006252.1",0.268461953715506,-0.692213851544516,2.5169359849
2032,-0.275022430324715,0.783299005639148,NA
"15214","SEMA3G",0.19433214479945,-0.0333008172506769,3.16142997448539
,-0.0105334666652225,0.991595664992027,NA
"15215","TNNC1",0.294745405065106,-0.847819422426427,2.82820104587465,
-0.29977339258222,0.764350012112202,NA
"15216","ITIH3",0,NA,NA,NA,NA,NA
"15217","CACNA1D",0.294783636876741,0.78572505329692,2.42199479408685,
0.324412362576179,0.745625861450258,NA
"15218","CHDH",0.954364782664215,0.497068815839909,1.45910554973167,0.
340666798184216,0.733354436290647,NA
"15219","IL17RB",0,NA,NA,NA,NA,NA
"15220","AC115282.2",0,NA,NA,NA,NA,NA
"15221","CACNA2D3-
AS1",0.239791573166398,-1.32959277084231,2.9363559394555,-0.4528036785
2436,0.650690114564834,NA
"15222","WNT5A-
AS1",0.127358887561203,0.15032497835859,3.33866494558414,0.04502547599
37551,0.964087002635848,NA
"15223","ERC2",0,NA,NA,NA,NA,NA
"15224","ERC2-IT1",0,NA,NA,NA,NA,NA
"15225","SPATA12",0.499214998414478,1.59351582292497,1.97275322154641,
0.807762372668101,0.419227389837337,NA
"15226","AC097358.2",0.258011588580052,0.510354618063005,2.53828617896
562,0.201062678547531,0.84064956422761,NA
"15227","HESX1",0.887940078967586,-0.0705363532616509,1.65578695677887
,-0.0425998966671841,0.966020477864563,NA
"15228","ASB14",0,NA,NA,NA,NA,NA
"15229","AC093928.1",0,NA,NA,NA,NA,NA
"15230","DNAH12",0.0367720482966892,-0.560503011376585,3.3495200143758
3,-0.167338307868279,0.867103863749685,NA
"15231","AC092418.2",0.239791573166398,-1.32959277084231,2.93635593945
55,-0.45280367852436,0.650690114564834,NA
"15232","DENND6A-

AS1",0.441269424109735,1.48700001704137,1.94523108125461,0.76443360964719,0.444608886288397,NA
"15233","DENND6A-DT",0.0732428936164972,-0.49131726092088,3.34845351515744,-0.146729604785264,0.883345450266286,NA
"15234","FLNB-AS1",0,NA,NA,NA,NA,NA
"15235","AC137936.1",0.564284311006628,-0.562768415413309,1.81953571495959,-0.309292316048772,0.757099175524869,NA
"15236","HTD2",0.0732428936164972,-0.49131726092088,3.34845351515744,-0.146729604785264,0.883345450266286,NA
"15237","AC116036.2",0.635863671999703,0.862218094010156,1.7890355219988,0.481945765418252,0.62984447400838,NA
"15238","FAM3D-AS1",0.231104193096139,0.317741310460635,2.97787872987845,0.106700554079852,0.915026543025481,NA
"15239","AC126121.3",0,NA,NA,NA,NA,NA
"15240","AC093418.1",0,NA,NA,NA,NA,NA
"15241","CADPS",0.102366282257309,-0.49131726092088,3.34845351515744,-0.146729604785264,0.883345450266286,NA
"15242","C3orf49",0,NA,NA,NA,NA,NA
"15243","SCAANT1",0.411806896597916,-2.14409022170048,2.24320078708113,-0.955817345486216,0.339164523438083,NA
"15244","AC012557.1",0.255708582183469,-2.44071869332471,2.85324394739347,-0.855418862994307,0.392319304627377,NA
"15245","PSMD6-AS1",0.609027760116626,0.540802307110845,2.09777355041637,0.257798229462616,0.796562630999997,NA
"15246","AC012557.2",0.604055846904167,-0.835453338111786,1.89011052726181,-0.442012954301726,0.658479831816476,NA
"15247","LINC00994",0,NA,NA,NA,NA,NA
"15248","PRICKLE2-AS3",0,NA,NA,NA,NA,NA
"15249","ADAMTS9-AS1",0.449034484715737,-1.31903840108013,2.44559733198216,-0.539352240792252,0.589643828608869,NA
"15250","MAGI1-IT1",0.0636794437806017,-0.49131726092088,3.34845351515744,-0.146729604785264,0.883345450266286,NA
"15251","MAGI1-AS1",0,NA,NA,NA,NA,NA
"15252","KBTBD8",0.212381224170495,0.56712693135589,2.68810250756675,0.210976675837131,0.832905471698532,NA
"15253","TAFA1",0.424035776437413,0.644399003506104,2.00295959778104,0.321723415799298,0.747662236989494,NA
"15254","AC104167.1",0,NA,NA,NA,NA,NA
"15255","AC109587.1",0.230228123330503,-1.33023934114211,2.97714568949567,-0.446817011957333,0.655007187193824,NA
"15256","LMOD3",0.42175430822138,-1.18743696082707,2.04384528905385,-0.5809818224435,0.561252704714215,NA
"15257","SAMMSON",0.295369349199418,-0.498711181181302,2.48533021027214,-0.200661939858163,0.840962921935508,NA
"15258","AC097634.3",0.102366282257309,-0.49131726092088,3.34845351515

744,-0.146729604785264,0.883345450266286,NA
"15259","AC097634.1",1.08836007856876,1.03456901882393,1.3790609945513
5,0.750198158682969,0.453135367628723,NA
"15260","GPR27",0.166045726037911,0.183394512381981,3.33779889015228,0
.0549447460489789,0.956182483505573,NA
"15261","PROK2",0.0636794437806017,-0.49131726092088,3.34845351515744,
-0.146729604785264,0.883345450266286,NA
"15262","LINC00877",0.514676132367215,-3.24724171037767,2.307062948094
82,-1.40752193738764,0.159272676891155,NA
"15263","PDZRN3-
AS1",0.166548679549901,-1.95393031097672,3.33920037562282,-0.585149164
824313,0.558447422910535,NA
"15264","LINC00960",0.22061559614905,-0.418038608046788,3.072734640038
86,-0.136047741513241,0.891783539523794,NA
"15265","AC117461.1",0,NA,NA,NA,NA,NA
"15266","CADM2",0.0741298089160557,-1.76673671430743,3.34309696236258,
-0.528473069790613,0.597171033704837,NA
"15267","AC107204.1",0.62625114481825,1.20354333052438,1.6330277419069
8,0.737001154137729,0.461121647506765,NA
"15268","HTR1F",0,NA,NA,NA,NA,NA
"15269","EPHA3",0.534848873855013,1.70942421438182,2.40548055542316,0.
71063730302368,0.477309020891269,NA
"15270","STX19",0.388291603021183,1.36359484651523,2.22743782542675,0.
612180879281775,0.540418090770536,NA
"15271","AC109782.1",0,NA,NA,NA,NA,NA
"15272","EPHA6",0.26891496180721,-1.3277650763353,2.82969623342791,-0.
469225304345422,0.63890860009957,NA
"15273","AC110491.3",0,NA,NA,NA,NA,NA
"15274","AC026100.1",0,NA,NA,NA,NA,NA
"15275","ST3GAL6-
AS1",0.222389426748167,-2.46578197109637,3.33045300506323,-0.740374347
678133,0.459072879645795,NA
"15276","AC107029.1",0,NA,NA,NA,NA,NA
"15277","AC107029.2",1.11856720212294,0.68241804895881,1.6152274939568
5,0.42249036220098,0.672667133973305,NA
"15278","AC069222.1",0.0636794437806017,-0.49131726092088,3.3484535151
5744,-0.146729604785264,0.883345450266286,NA
"15279","AC129803.1",0,NA,NA,NA,NA,NA
"15280","IMPG2",0.315634421727363,0.0162048499507083,2.43727149812695,
0.00664876685390272,0.994695090663815,NA
"15281","AC020651.1",0,NA,NA,NA,NA,NA
"15282","AC020651.2",0,NA,NA,NA,NA,NA
"15283","AC106712.1",0,NA,NA,NA,NA,NA
"15284","AC117435.1",0,NA,NA,NA,NA,NA
"15285","AC063944.3",0.157560096502761,-0.491317252697356,3.3484535150
3297,-0.146729602334801,0.883345452200538,NA
"15286","LINC01990",0.231104193096139,0.317741310460635,2.977878729878
45,0.106700554079852,0.915026543025481,NA
"15287","LINC00636",0,NA,NA,NA,NA,NA
"15288","LINC01215",0,NA,NA,NA,NA,NA

"15289","HHLA2",0,NA,NA,NA,NA,NA
"15290","MYH15",0.0732428936164972,-0.49131726092088,3.34845351515744,
-0.146729604785264,0.883345450266286,NA
"15291","TRAT1",0.442609347500998,0.132555327879018,1.8496101435603,0.
071666311225257,0.942867212359173,NA
"15292","MORC1",0,NA,NA,NA,NA,NA
"15293","DPPA4",0,NA,NA,NA,NA,NA
"15294","LINC01205",0,NA,NA,NA,NA,NA
"15295","NECTIN3-
AS1",0.377399075327947,0.41478541520833,2.0387702415748,0.203448827508
851,0.838784238896543,NA
"15296","ZBED2",0,NA,NA,NA,NA,NA
"15297","PLCXD2",0.184144750829242,-0.691295663073562,2.81313162320765
,-0.245738826214366,0.805884414604706,NA
"15298","C3orf52",0.737213792985203,-1.18133608747588,1.56243559614055
,-0.756086260703458,0.449597478930489,NA
"15299","GCSAM",0.285483158206092,-0.82351996307896,2.498184683591,-0.
329647350929714,0.741666439733092,NA
"15300","TBILA",0.102366282257309,-0.49131726092088,3.34845351515744,-
0.146729604785264,0.883345450266286,NA
"15301","SLC9C1",0.166548679549901,-1.95393031097672,3.33920037562282,
-0.585149164824313,0.558447422910535,NA
"15302","AC112487.1",0.0367720482966892,-0.560503011376585,3.349520014
37583,-0.167338307868279,0.867103863749685,NA
"15303","BTLA",0,NA,NA,NA,NA,NA
"15304","CD200R1",1.06022662891428,-0.391310651231563,1.45609645703865
,-0.268739511960214,0.788130143747803,NA
"15305","AC074044.1",0,NA,NA,NA,NA,NA
"15306","AC078785.1",0.799847242062504,-0.705637906440701,1.5118197186
591,-0.466747389077952,0.640680620608315,NA
"15307","AC026329.1",1.39515547605923,0.354985442680561,1.467600917956
52,0.241881453150656,0.808872021718136,NA
"15308","SIDT1",0.268461953715506,-0.692213851544516,2.51693598492032,
-0.275022430324715,0.783299005639148,NA
"15309","SIDT1-AS1",0,NA,NA,NA,NA,NA
"15310","GRAMD1C",0.369941320673256,1.11554984681986,2.1030042091201,0
.530455356190946,0.595796254308461,NA
"15311","AC128687.2",0,NA,NA,NA,NA,NA
"15312","ZDHHC23",0.220029883826373,0.698180369696046,3.02638332375644
,0.230697930501891,0.817549481385233,NA
"15313","AC128687.3",0.0732428936164972,-0.49131726092088,3.3484535151
5744,-0.146729604785264,0.883345450266286,NA
"15314","AC093010.2",0.0732428936164972,-0.49131726092088,3.3484535151
5744,-0.146729604785264,0.883345450266286,NA
"15315","ZNF80",0,NA,NA,NA,NA,NA
"15316","TIGIT",0.459017300503899,1.59668131033552,2.20764158307741,0.
723252054398149,0.469525043331563,NA
"15317","ZBTB20-
AS1",0.553784868525617,0.880999667320216,1.96102554376352,0.4492545597
49097,0.653248033306958,NA

"15318","ZBTB20-
AS5",0.175609175873806,0.247512349757584,3.30210845793575,0.0749558510
601773,0.940249838776562,NA
"15319","ZBTB20-
AS4",0.989617716177105,-0.383429239032083,1.42306108480676,-0.26943976
1318573,0.787591291985263,NA
"15320","AC026341.1",0.164130935857893,0.400527358383716,3.33018851502
426,0.120271677286953,0.904267939474822,NA
"15321","LSAMP-
AS1",0.102366282257309,-0.49131726092088,3.34845351515744,-0.146729604
785264,0.883345450266286,NA
"15322","AC092691.1",0,NA,NA,NA,NA,NA
"15323","UPK1B",0.102366282257309,-0.49131726092088,3.34845351515744,-
0.146729604785264,0.883345450266286,NA
"15324","ARHGAP31-AS1",0,NA,NA,NA,NA,NA
"15325","AC073352.1",0.0636794437806017,-0.49131726092088,3.3484535151
5744,-0.146729604785264,0.883345450266286,NA
"15326","CD80",0.420798011374483,-2.43995659027234,2.44810148298057,-0
.996672975869322,0.318923271132377,NA
"15327","AC073352.2",0.278862373430674,-0.427610315239008,2.8803333160
6682,-0.148458622081601,0.881980838831896,NA
"15328","PLA1A",0.176496091173365,-1.14849506633421,3.26459492750587,-
0.351803238024283,0.724985830023263,NA
"15329","AC092910.3",0.471279728050106,0.590529255541241,1.81969176558
07,0.324521584760149,0.745543183459673,NA
"15330","GPR156",0.640072816057542,0.194998657021937,2.0722717109108,0
.0940989813233669,0.925030529578473,NA
"15331","AC126182.3",0,NA,NA,NA,NA,NA
"15332","HGD",0,NA,NA,NA,NA,NA
"15333","AC072026.2",0,NA,NA,NA,NA,NA
"15334","POLQ",0.0636794437806017,-0.49131726092088,3.34845351515744,-
0.146729604785264,0.883345450266286,NA
"15335","FBXO40",0,NA,NA,NA,NA,NA
"15336","SLC15A2",0.258274559745298,-1.11669114604128,2.56884985789992
,-0.434704715266695,0.663776775348859,NA
"15337","ILDR1",0,NA,NA,NA,NA,NA
"15338","CD86",1.35432778564329,-2.15581333153801,1.30098863297444,-1.
65705777659962,0.0975077946011232,NA
"15339","AC096861.2",0.0636794437806017,-0.49131726092088,3.3484535151
5744,-0.146729604785264,0.883345450266286,NA
"15340","AC112503.2",0.423083386078721,-0.153038729122502,2.2490708169
9273,-0.0680453136318456,0.94574956278821,NA
"15341","ADCY5",0.580943521566125,1.90993949357142,2.03274915040211,0.
939584450542559,0.34743075532157,NA
"15342","AC112503.1",0.351218351747613,1.19734761786025,2.664153967506
07,0.449428836495172,0.653122333171162,NA
"15343","AC020634.2",0,NA,NA,NA,NA,NA
"15344","KALRN",1.40794169415074,-1.14811520899405,1.28482380928516,-0
.893597394986653,0.371537340660343,NA
"15345","AC117488.1",0.110901857212745,-1.2169087618042,3.340133369026

82,-0.36432939267894,0.715612053721651,NA
"15346","SLC12A8",0.757015848697457,-2.32697631296323,2.20970511914233
,-1.05307096987964,0.292308466645741,NA
"15347","AC092902.6",0,NA,NA,NA,NA,NA
"15348","ALG1L",0.902242336789282,0.557240947081173,1.61256717747204,0
.345561384893582,0.729672370436813,NA
"15349","AC079848.1",0.827066686057219,-2.09251515774436,1.54956539098
138,-1.35038841853529,0.176891423823814,NA
"15350","AC079848.2",0.19433214479945,-0.0333008172506769,3.1614299744
8539,-0.0105334666652225,0.991595664992027,NA
"15351","AC016924.1",0,NA,NA,NA,NA,NA
"15352","AC063919.1",0,NA,NA,NA,NA,NA
"15353","CHST13",0,NA,NA,NA,NA,NA
"15354","KBTBD12",0,NA,NA,NA,NA,NA
"15355","AL449214.1",0,NA,NA,NA,NA,NA
"15356","GATA2",1.16915519925928,-0.0106866707417187,1.64944834650539,
-0.00647893628458269,0.994830592933833,NA
"15357","GATA2-AS1",0,NA,NA,NA,NA,NA
"15358","AC112484.3",0.0367720482966892,-0.560503011376585,3.349520014
37583,-0.167338307868279,0.867103863749685,NA
"15359","KIAA1257",0.546498202800829,-2.44298980326156,2.0355678694483
1,-1.20015148594563,0.2300805128744,NA
"15360","EFCC1",0.0732428936164972,-0.49131726092088,3.34845351515744,
-0.146729604785264,0.883345450266286,NA
"15361","AC108673.2",0.254821666883911,-1.3286579469836,2.880294380844
22,-0.461292413657444,0.64458882703139,NA
"15362","AC137695.3",0,NA,NA,NA,NA,NA
"15363","EFCAB12",0.19433214479945,-0.0333008172506769,3.1614299744853
9,-0.0105334666652225,0.991595664992027,NA
"15364","TRH",0.399064709314068,1.24599958031216,2.29353613743834,0.54
3265728397819,0.586946857221725,NA
"15365","ALG1L2",0.841665404296305,-1.35310394335246,1.56923108457087,
-0.862271947488526,0.388537885911407,NA
"15366","LINC02021",0.45985543437541,0.096685612506136,2.1745874206130
7,0.0444615891684308,0.964536469084358,NA
"15367","AC130888.1",0.181578773267413,-1.95393031097672,3.33920037562
282,-0.585149164824313,0.558447422910535,NA
"15368","AC010210.1",0.913599586182873,-0.721050004883116,1.7294801983
134,-0.416917178691197,0.676738987835287,NA
"15369","AC107027.3",1.12129795683321,-1.55715007350365,1.435399934207
75,-1.0848196634223,0.278001534744491,NA
"15370","ACPP",0.102366282257309,-0.49131726092088,3.34845351515744,-0
.146729604785264,0.883345450266286,NA
"15371","NPHP3-
AS1",0.0367720482966892,-0.560503011376585,3.34952001437583,-0.1673383
07868279,0.867103863749685,NA
"15372","TMEM108-
AS1",0.419911096074925,-1.32028169514223,2.49077535946509,-0.530068554
807674,0.59606440017536,NA
"15373","BFSP2",0.0367720482966892,-0.560503011376585,3.34952001437583

,-0.167338307868279,0.867103863749685,NA
"15374","AC022296.4",0.147673905509434,-0.842065770106804,3.3347867762
1422,-0.252509628535456,0.800647172695484,NA
"15375","TF",1.29581402704896,1.13815359824471,1.60585139418174,0.7087
5399950981,0.478477148389991,NA
"15376","AC080128.2",0,NA,NA,NA,NA,NA
"15377","SLCO2A1",0.218350821564103,-1.39752731860778,3.03720335813394
,-0.460136235153652,0.645418436617112,NA
"15378","LINC02004",0,NA,NA,NA,NA,NA
"15379","AC010207.1",0.239288619654408,0.726512763451421,2.61839910948
85,0.277464486150602,0.781423471618519,NA
"15380","EPHB1",0,NA,NA,NA,NA,NA
"15381","AC092969.1",0,NA,NA,NA,NA,NA
"15382","AC072039.2",0,NA,NA,NA,NA,NA
"15383","AC117382.2",0.0367720482966892,-0.560503011376585,3.349520014
37583,-0.167338307868279,0.867103863749685,NA
"15384","AC096992.2",1.12540594356461,-0.852104171126825,1.34591042834
317,-0.633106151184052,0.526664325659117,NA
"15385","CLDN18",0.181578773267413,-1.95393031097672,3.33920037562282,
-0.585149164824313,0.558447422910535,NA
"15386","AC016252.1",0.563157413360326,0.294149429492247,1.92152833884
387,0.153080973902903,0.878334419653114,NA
"15387","NME9",0.102366282257309,-0.49131726092088,3.34845351515744,-0
.146729604785264,0.883345450266286,NA
"15388","ESYT3",0,NA,NA,NA,NA,NA
"15389","FOXL2",0.164130935857893,0.400527358383716,3.33018851502426,0
.120271677286953,0.904267939474822,NA
"15390","AC024933.1",0.810277241070545,-2.31801956905785,1.78301257556
74,-1.30005789124634,0.193581128435081,NA
"15391","RBP1",0.68363892243849,-0.403778980636972,1.7851589278737,-0.
226186573269368,0.821056313010949,NA
"15392","RASA2-
IT1",0.102366282257309,-0.49131726092088,3.34845351515744,-0.146729604
785264,0.883345450266286,NA
"15393","ATP1B3-
AS1",0.176496091173365,-1.14849506633421,3.26459492750587,-0.351803238
024283,0.724985830023263,NA
"15394","AC109992.2",0.0732428936164972,-0.49131726092088,3.3484535151
5744,-0.146729604785264,0.883345450266286,NA
"15395","PLS1",0.0367720482966892,-0.560503011376585,3.34952001437583,
-0.167338307868279,0.867103863749685,NA
"15396","AC072028.1",0,NA,NA,NA,NA,NA
"15397","AC021074.3",0.267575038415947,0.563240138104268,2.52089568762
832,0.223428577734674,0.82320196461372,NA
"15398","PAQR9",0.0367720482966892,-0.560503011376585,3.34952001437583
,-0.167338307868279,0.867103863749685,NA
"15399","PAQR9-
AS1",0.110901857212745,-1.2169087618042,3.34013336902682,-0.3643293926
7894,0.715612053721651,NA
"15400","AC018450.1",0,NA,NA,NA,NA,NA

"15401","AC131210.1",0.0636794437806017,-0.49131726092088,3.3484535151
5744,-0.146729604785264,0.883345450266286,NA
"15402","AC107021.2",0.0732428936164972,-0.49131726092088,3.3484535151
5744,-0.146729604785264,0.883345450266286,NA
"15403","LNCSRLR",0.175609175873806,0.247512349757584,3.30210845793575
,0.0749558510601773,0.940249838776562,NA
"15404","PLSCR2",0,NA,NA,NA,NA,NA
"15405","CPA3",1.19818498355548,0.723729306931742,1.59592148551621,0.4
53486787100711,0.65019825553653,NA
"15406","AC093001.2",0,NA,NA,NA,NA,NA
"15407","TM4SF18",0.204732564514618,0.248608670877116,3.33613854356166
,0.0745198880774602,0.940596716765987,NA
"15408","TM4SF1",1.36479280311634,1.50345203205164,1.66236999665423,0.
904402771391185,0.365781869175255,NA
"15409","TM4SF1-AS1",0,NA,NA,NA,NA,NA
"15410","WWTR1-
AS1",0.433045897665103,0.0925424035774023,2.00313886296437,0.046198696
1006049,0.9631518817278,NA
"15411","LINC01213",0.339138869770174,-1.32417497355052,2.650620704582
38,-0.499571655522532,0.617376720220205,NA
"15412","AC018545.1",0,NA,NA,NA,NA,NA
"15413","ERICH6",0,NA,NA,NA,NA,NA
"15414","AC011317.1",0.166548679549901,-1.95393031097672,3.33920037562
282,-0.585149164824313,0.558447422910535,NA
"15415","SIAH2-
AS1",0.175910378850688,0.313649592621519,3.2560472709057,0.09632832896
01242,0.923259812444105,NA
"15416","MED12L",0.221239540283362,0.178834610992208,3.06205963446949,
0.0584033730039328,0.953427328091912,NA
"15417","GPR171",0.614624670932516,-0.696771334178063,1.74778816940561
,-0.398658914378064,0.690144545045934,NA
"15418","P2RY14",1.29999925408412,-2.69942671580541,1.34480413960055,-
2.00730101604775,0.0447176210431102,NA
"15419","P2RY13",0.0732428936164972,-0.49131726092088,3.34845351515744
,-0.146729604785264,0.883345450266286,NA
"15420","P2RY12",0.824336893854408,0.553538983551241,1.48337541747017,
0.373161761366705,0.709028053995379,NA
"15421","LINC02066",0.485433752001982,-0.679593966196398,1.98358409430
652,-0.342609102456021,0.731892553333912,NA
"15422","AC108718.1",0,NA,NA,NA,NA,NA
"15423","AC026347.1",0,NA,NA,NA,NA,NA
"15424","AC078788.1",0,NA,NA,NA,NA,NA
"15425","LINC02006",0.0741298089160557,-1.76673671430743,3.34309696236
258,-0.528473069790613,0.597171033704837,NA
"15426","ARHGEF26-
AS1",0.283945055524722,-1.32691080208876,2.78374542960066,-0.476663845
759455,0.633601511730444,NA
"15427","MME-AS1",0,NA,NA,NA,NA,NA
"15428","STRIT1",0.0636794437806017,-0.49131726092088,3.34845351515744
,-0.146729604785264,0.883345450266286,NA

"15429","PLCH1",0,NA,NA,NA,NA,NA
"15430","AC104472.4",0.340243065243689,-0.348902370642203,2.1288113109
4097,-0.163895394978892,0.869813492355055,NA
"15431","AC104472.5",0.0732428936164972,-0.49131726092088,3.3484535151
5744,-0.146729604785264,0.883345450266286,NA
"15432","LEKR1",0.200902984154582,0.617734931975277,3.11356519488492,0
.198401155366881,0.842731213560209,NA
"15433","LINC00880",0.139138330553998,-0.0338344975521838,3.3423233482
0666,-0.0101230473617515,0.991923114749952,NA
"15434","LINC02029",0.227810379638494,0.737818182751471,3.023204067855
02,0.24405172994986,0.807190756195641,NA
"15435","LINC00881",0,NA,NA,NA,NA,NA
"15436","AC084212.1",0,NA,NA,NA,NA,NA
"15437","AC080013.4",0.127358887561203,0.15032497835859,3.338664945584
14,0.0450254759937551,0.964087002635848,NA
"15438","AC080013.6",0.211939061612713,-1.56342887923112,3.09537917223
915,-0.505084770632529,0.613499297433191,NA
"15439","AC080013.3",0.716499713286486,-0.959181927297487,1.6430249016
2467,-0.583790255612695,0.559361434985281,NA
"15440","AC080013.5",0.564284311006628,-0.562768415413309,1.8195357149
5959,-0.309292316048772,0.757099175524869,NA
"15441","IQCJ-
SCHIP1",0.494927820290539,-2.84339218695741,2.30196208233711,-1.235203
7458717,0.216754685526326,NA
"15442","IQCJ",0,NA,NA,NA,NA,NA
"15443","SCHIP1",0.240678488465957,-2.44080205522383,2.91280317905203,
-0.837956396359809,0.402055193025598,NA
"15444","IL12A",0,NA,NA,NA,NA,NA
"15445","C3orf80",0.934014893382812,-3.8086178370845,1.79306069530902,
-2.12408751530194,0.0336628262737273,NA
"15446","TRIM59",0.534575210043139,0.399813787548105,1.75850844383736,
0.22735960634664,0.820144127278845,NA
"15447","SPTSSB",0.240678488465957,-2.44080205522383,2.91280317905203,
-0.837956396359809,0.402055193025598,NA
"15448","WDR49",0.147372702532553,-1.1477220656059,3.3390638510896,-0.
343725701810518,0.731052580354999,NA
"15449","AC069243.1",1.04440041982022,-0.830476328251785,1.38610278272
341,-0.599144838754358,0.549076303201084,NA
"15450","MECOM",0.166045726037911,0.183394512381981,3.33779889015228,0
.0549447460489789,0.956182483505573,NA
"15451","TERC",0.333169272376567,0.908589184944016,2.42247183547103,0.
375066975656849,0.707610656801772,NA
"15452","AC078802.1",0.418952602741803,0.00787819335423566,2.014321020
63106,0.00391109126775012,0.996879408617331,NA
"15453","AC078795.1",0.580034638410087,-0.737467811554113,1.8992728749
8437,-0.388289550842019,0.697801769075372,NA
"15454","AC078795.3",0,NA,NA,NA,NA,NA
"15455","AC078795.2",0.392630919580568,-1.18563504912561,2.07059770158
235,-0.572605218396384,0.566912025605628,NA
"15456","AC008040.1",0,NA,NA,NA,NA,NA

"15457","GPR160",0.0367720482966892,-0.560503011376585,3.34952001437583,-0.167338307868279,0.867103863749685,NA
"15458","SLC7A14-AS1",0.987334149378639,1.91138418433158,1.51355431827328,1.26284478941737,0.206644964712667,NA
"15459","SLC7A14",0.175609175873806,0.247512349757584,3.30210845793575,0.0749558510601773,0.940249838776562,NA
"15460","AC026316.5",0.247238482287166,0.868764518721955,2.52920099602734,0.343493664634222,0.731227106130206,NA
"15461","GHSR",0,NA,NA,NA,NA,NA
"15462","LINC02068",0,NA,NA,NA,NA,NA
"15463","TBL1XR1-AS1",0,NA,NA,NA,NA,NA
"15464","LINC00578",0,NA,NA,NA,NA,NA
"15465","LINC02015",0,NA,NA,NA,NA,NA
"15466","AC117453.1",0.146786990209876,0.2964989704902,3.33426311477484,0.0889248869341921,0.929141604986601,NA
"15467","KCNMB2",0.873074167641354,0.0506404104446442,1.79850628963237,0.0281569270769664,0.977536990804087,NA
"15468","LINC01014",0,NA,NA,NA,NA,NA
"15469","KCNMB2-AS1",0.843588846995085,1.24897022127995,1.68436814599811,0.741506673732451,0.458386286804816,NA
"15470","AC007823.1",0.100451492077291,-0.0338344573480013,3.34232334773369,-0.010123035334371,0.991923124345921,NA
"15471","AC007620.3",0.320717103821412,-0.846710013286843,2.38334198606247,-0.35526165285482,0.722393580711595,NA
"15472","AC090425.3",0,NA,NA,NA,NA,NA
"15473","PEX5L",0.204732564514618,0.248608670877116,3.33613854356166,0.0745198880774602,0.940596716765987,NA
"15474","AC007687.1",0,NA,NA,NA,NA,NA
"15475","SOX2-OT",0.495363879873892,0.94051273243175,1.89606569869077,0.496033831043499,0.619870542171166,NA
"15476","LINC01206",0,NA,NA,NA,NA,NA
"15477","AC092953.2",0,NA,NA,NA,NA,NA
"15478","MCCC1-AS1",0,NA,NA,NA,NA,NA
"15479","LAMP3",0.231689905418816,-1.15178064054131,2.99600284051573,-0.384439101647526,0.700653024054507,NA
"15480","MCF2L2",0.110014941913186,-0.033834469553869,3.34232334787728,-0.0101230389858472,0.991923121432614,NA
"15481","B3GNT5",0.67323314622772,-1.57673979068499,1.7905907562486,-0.880569602620067,0.378550817236465,NA
"15482","KLHL6",0.479777071193906,-1.209115034652,2.16911110989743,-0.557424204382583,0.577237631994869,NA
"15483","KLHL6-AS1",0,NA,NA,NA,NA,NA
"15484","YEATS2-AS1",0.0367720482966892,-0.560503011376585,3.34952001437583,-0.167338307868279,0.867103863749685,NA
"15485","MAP6D1",0.920220604950169,-1.18440851837074,1.64523614620384,-0.719901833608265,0.471585438989308,NA

"15486","AC131235.3",0.166548679549901,-1.95393031097672,3.33920037562
282,-0.585149164824313,0.558447422910535,NA
"15487","AC131235.2",0.0735440965933784,-0.185659846583073,3.344188462
06699,-0.0555171601986567,0.955726459171656,NA
"15488","EEF1AKMT4",1.12205977743392,0.312153458607963,1.4333252612332
9,0.217782709236126,0.827598417252703,NA
"15489","CAMK2N2",0,NA,NA,NA,NA,NA
"15490","THPO",1.22314790831518,0.205882095284602,1.33054941948464,0.1
54734647409299,0.877030514259762,NA
"15491","LINC02054",0.328064560500408,-0.607303666148901,2.70536905215
305,-0.224480895006019,0.822383131186076,NA
"15492","AC107294.2",1.1721964486868,1.10139733409126,1.50981031296473
,0.729493847428095,0.46569962995302,NA
"15493","AC107294.3",0.175609175873806,0.247512349757584,3.30210845793
575,0.0749558510601773,0.940249838776562,NA
"15494","AC107294.1",0.136922337397099,0.183394507878855,3.33779889026
91,0.0549447446979257,0.956182484581931,NA
"15495","C3orf70",1.33857779348303,-0.280335163117765,1.33883535628684
,-0.209387331908573,0.834145881260398,NA
"15496","EHHADH",1.18490147718959,-0.254163238609828,1.40044778202792,
-0.18148712281281,0.855985239825332,NA
"15497","AC099661.1",0.796050475092869,0.552370053495436,1.52261803979
711,0.362776506686496,0.716771842982397,NA
"15498","LIPH",0.292971574465989,1.01027116194909,3.29385652095093,0.3
06713773208745,0.759061245268142,NA
"15499","DGKG",0,NA,NA,NA,NA,NA
"15500","CRYGS",1.18923376424525,2.87859622981102,1.43529540181372,2.0
0557754603928,0.0449013395462408,NA
"15501","AC068631.1",0.241504612811307,0.71883124973489,2.978478094155
1,0.241341795041404,0.809290220283093,NA
"15502","AC112907.3",0.136922337397099,0.183394507878855,3.33779889026
91,0.0549447446979257,0.956182484581931,NA
"15503","LINC02043",0.304932799035314,-0.455323059556486,2.47402355059
449,-0.184041521935826,0.853980881434662,NA
"15504","ST6GAL1",1.01998712794706,0.524270196211572,1.6531770271465,0
.317128890374492,0.751145804075721,NA
"15505","AC072022.1",0,NA,NA,NA,NA,NA
"15506","LPP-AS1",0,NA,NA,NA,NA,NA
"15507","TPRG1-
AS1",0.404497375813046,1.3317642143449,2.02661760121176,0.657136409724
56,0.511093209405098,NA
"15508","TP63",0.147673905509434,-0.842065770106804,3.33478677621422,-
0.252509628535456,0.800647172695484,NA
"15509","P3H2",0,NA,NA,NA,NA,NA
"15510","CLDN1",0,NA,NA,NA,NA,NA
"15511","OSTN",0,NA,NA,NA,NA,NA
"15512","OSTN-
AS1",0.315120193005521,0.245400022010969,3.2393007694085,0.07575709681
80826,0.939612349767913,NA
"15513","UTS2B",0.738553563489171,1.28013288946246,1.58133166453243,0.

809528398231983,0.418211274869234,NA
"15514","AC073365.1",0,NA,NA,NA,NA,NA
"15515","PLAAT1",0.758475099056344,-2.77443545551218,1.78976731475304,
-1.55016545035912,0.12110181003125,NA
"15516","ATP13A4",0.544823824643484,0.913711225072656,1.75049109824222
,0.52197421968622,0.601688280484707,NA
"15517","OPA1-
AS1",0.157560096502761,-0.491317252697356,3.34845351503297,-0.14672960
2334801,0.883345452200538,NA
"15518","LINC02028",0.444071129589312,0.414615036158139,1.880570318483
48,0.22047302995428,0.825502774601196,NA
"15519","LINC00887",0.703997508349382,1.18610919523398,1.7189933062204
2,0.690002218706659,0.490192792086464,NA
"15520","LRRC15",0.528246975725841,-2.84780569868221,2.33554953918413,
-1.21933003385448,0.222718953697351,NA
"15521","GP5",0.166548679549901,-1.95393031097672,3.33920037562282,-0.
585149164824313,0.558447422910535,NA
"15522","AC108676.1",0.286226523740755,1.02083909497203,2.805216817595
13,0.363907377343895,0.715927175510104,NA
"15523","AC046143.2",0,NA,NA,NA,NA,NA
"15524","XXYLT1-
AS2",0.0636794437806017,-0.49131726092088,3.34845351515744,-0.14672960
4785264,0.883345450266286,NA
"15525","AC090018.2",0.212381224170495,0.56712693135589,2.688102507566
75,0.210976675837131,0.832905471698532,NA
"15526","ACAP2-
IT1",0.157560096502761,-0.491317252697356,3.34845351503297,-0.14672960
2334801,0.883345452200538,NA
"15527","AC069213.1",0,NA,NA,NA,NA,NA
"15528","AC233280.1",0.147673905509434,-0.842065770106804,3.3347867762
1422,-0.252509628535456,0.800647172695484,NA
"15529","MUC4",0.0636794437806017,-0.49131726092088,3.34845351515744,-
0.146729604785264,0.883345450266286,NA
"15530","AC124944.3",0,NA,NA,NA,NA,NA
"15531","TNK2-
AS1",0.0367720482966892,-0.560503011376585,3.34952001437583,-0.1673383
07868279,0.867103863749685,NA
"15532","ZDHHC19",0,NA,NA,NA,NA,NA
"15533","SLC51A",0.0732428936164972,-0.49131726092088,3.34845351515744
,-0.146729604785264,0.883345450266286,NA
"15534","TM4SF19-
AS1",0.584042031932817,0.0221458054576762,1.89666294929803,0.011676194
4792946,0.99068395637802,NA
"15535","TM4SF19",0.753895370474285,-3.61289105696522,1.85660557003495
,-1.94596586118031,0.0516588405630632,NA
"15536","AC117490.2",0.183257835529684,0.495279120319891,3.24692673479
8,0.152537818304269,0.878762764724423,NA
"15537","LINC01063",0,NA,NA,NA,NA,NA
"15538","NRROS",0.166548679549901,-1.95393031097672,3.33920037562282,-
0.585149164824313,0.558447422910535,NA

"15539","NCBP2-
AS1",0.100451492077291,-0.0338344573480013,3.34232334773369,-0.0101230
35334371,0.991923124345921,NA
"15540","AC011322.1",0,NA,NA,NA,NA,NA
"15541","DLG1-AS1",0,NA,NA,NA,NA,NA
"15542","LINC02012",0,NA,NA,NA,NA,NA
"15543","BDH1",0.229725169818512,0.686619208900669,3.0259364803766,0.2
26911309392461,0.820492707794461,NA
"15544","AC024560.3",0.175910378850688,0.313649592621519,3.25604727090
57,0.0963283289601242,0.923259812444105,NA
"15545","AC024560.4",1.25260575172207,-0.699386813330429,1.28466328764
824,-0.544412547672904,0.58615761312618,NA
"15546","AC024560.1",0.579577375889387,-1.82723002832079,1.86426404980
884,-0.980134776781299,0.327019594671503,NA
"15547","AC055764.2",0.304045883735755,0.789679836645197,2.79279246778
834,0.282756361510298,0.777363608165881,NA
"15548","AC135893.1",0,NA,NA,NA,NA,NA
"15549","AC253576.2",0.240175534953966,-0.492654690648343,2.6203058694
6942,-0.188014191926418,0.850865526027581,NA
"15550","ZNF732",0.102366282257309,-0.49131726092088,3.34845351515744,
-0.146729604785264,0.883345450266286,NA
"15551","AC079140.6",0.605899059050622,-0.809322703223874,1.6058545045
5205,-0.503982584306186,0.614273615361514,NA
"15552","AC092574.1",0.231104193096139,0.317741310460635,2.97787872987
845,0.106700554079852,0.915026543025481,NA
"15553","PDE6B",0.240092776143279,-1.01655735204591,2.97416330481592,-
0.341796077706914,0.732504361553979,NA
"15554","AC107464.3",0.313636872736658,-0.466923955273698,2.7580636353
7793,-0.16929411971661,0.865565301011883,NA
"15555","AC139887.4",1.30024919476118,0.897591901566792,1.285550517145
53,0.698215970197597,0.485042140471398,NA
"15556","CPLX1",0.305819714334872,-1.57468193089132,2.7692160162842,-0
.568638171103845,0.569601714969488,NA
"15557","SLC26A1",0.110901857212745,-1.2169087618042,3.34013336902682,
-0.36432939267894,0.715612053721651,NA
"15558","AC092535.2",1.1754359186344,0.977428348981928,1.4178556644104
,0.689370839018637,0.490589929476111,NA
"15559","AC092535.5",0.369366453884501,-0.298623700666588,2.0854036368
725,-0.143197074842756,0.886134539952422,NA
"15560","CTBP1-
AS",1.24158744961706,0.363543278317595,1.42009053651955,0.256000071100
107,0.797950782413982,NA
"15561","FAM53A",0.560892638775378,-0.336572841678827,1.77222748901493
,-0.189915145637373,0.849375623958259,NA
"15562","AC016773.1",1.16481568701584,-0.550286683267027,1.26131444059
23,-0.43628033229257,0.662633349767777,NA
"15563","FGFR3",0,NA,NA,NA,NA,NA
"15564","NAT8L",0,NA,NA,NA,NA,NA
"15565","AL121750.1",0.255122869860792,-1.01416582893919,2.91937932186
279,-0.347390906465717,0.728297664063611,NA

"15566","HTT-AS",0,NA,NA,NA,NA,NA
"15567","MSANTD1",0.305184056591909,1.0238829631905,2.42729878959865,0.42181991256206,0.673156470155355,NA
"15568","HGFAC",0,NA,NA,NA,NA,NA
"15569","DOK7",0.0636794437806017,-0.49131726092088,3.34845351515744,-0.146729604785264,0.883345450266286,NA
"15570","ADRA2C",0.136922337397099,0.183394507878855,3.3377988902691,0.0549447446979257,0.956182484581931,NA
"15571","NSG1",0.267575038415947,0.563240138104268,2.52089568762832,0.223428577734674,0.82320196461372,NA
"15572","CYTL1",0.102366282257309,-0.49131726092088,3.34845351515744,-0.146729604785264,0.883345450266286,NA
"15573","CRMP1",0.420798011374483,-2.43995659027234,2.44810148298057,-0.996672975869322,0.318923271132377,NA
"15574","C4orf50",0,NA,NA,NA,NA,NA
"15575","JAKMIP1",0.0741298089160557,-1.76673671430743,3.34309696236258,-0.528473069790613,0.597171033704837,NA
"15576","PPP2R2C",0.147372702532553,-1.1477220656059,3.3390638510896,-0.343725701810518,0.731052580354999,NA
"15577","LINC002481",0.146786990209876,0.2964989704902,3.33426311477484,0.0889248869341921,0.929141604986601,NA
"15578","S100P",0.175609175873806,0.247512349757584,3.30210845793575,0.0749558510601773,0.940249838776562,NA
"15579","AC097382.1",0,NA,NA,NA,NA,NA
"15580","AC097382.2",0,NA,NA,NA,NA,NA
"15581","CCDC96",1.06863677392766,-0.0802344894231313,1.31773231002347,-0.0608883069898334,0.951448161838215,NA
"15582","AFAP1-AS1",0.312531513270905,1.06986046542867,2.41843726213171,0.442376770396623,0.658216585804703,NA
"15583","AC097381.1",0,NA,NA,NA,NA,NA
"15584","ABLIM2",0.0741298089160557,-1.76673671430743,3.34309696236258,-0.528473069790613,0.597171033704837,NA
"15585","SH3TC1",0.620567220762677,0.343642684451145,1.76318417373173,0.194898916160207,0.845472082701713,NA
"15586","LINC02517",0,NA,NA,NA,NA,NA
"15587","AC105345.1",0.72522532516838,-0.901384327517214,1.57121765116929,-0.573685209586596,0.566180838738378,NA
"15588","GPR78",0.43271231097375,1.56912079812923,2.15131947485791,0.729376002247583,0.465771693096376,NA
"15589","CPZ",1.33477199811177,2.22529179386829,1.36767223465429,1.62706512385314,0.103723267210803,NA
"15590","HMX1",0.350107718056466,-0.253434672451047,2.32882288692438,-0.10882522405375,0.91334111673156,NA
"15591","AC073648.7",0.293858489765547,0.00709152447587447,2.85449073404838,0.0024843396376407,0.998017785798355,NA
"15592","AC105916.1",0.213268139470054,-0.691656524248949,2.68459372570788,-0.257639179301356,0.796685389475746,NA
"15593","SLC2A9",1.14262478719153,-1.21245043510819,1.52700221066484,-0.794006993991384,0.42719135973559,NA

"15594","AC005674.2",0.379816819019955,-1.35490224425385,2.07036443936
748,-0.654426930105015,0.512836789051267,NA
"15595","CLNK",0.181578773267413,-1.95393031097672,3.33920037562282,-0
.585149164824313,0.558447422910535,NA
"15596","HS3ST1",1.34095616171575,-0.760500235677543,1.39767972380701,
-0.544116239739163,0.586361485873165,NA
"15597","AC006230.1",0.0735440965933784,-0.185659846583073,3.344188462
06699,-0.0555171601986567,0.955726459171656,NA
"15598","AC025539.1",0.0741298089160557,-1.76673671430743,3.3430969623
6258,-0.528473069790613,0.597171033704837,NA
"15599","AC005699.1",0.442910550477879,0.19113381161474,1.971070242506
09,0.0969695587163478,0.922750569118878,NA
"15600","AC007370.2",0.554036126082213,1.84022205000134,1.871306379835
12,0.983388968172858,0.325416037700512,NA
"15601","AC007370.1",0.0741298089160557,-1.76673671430743,3.3430969623
6258,-0.528473069790613,0.597171033704837,NA
"15602","NKX3-2",0.55631291019332,-1.05727564308755,1.89681546477565,-
0.557395098638445,0.577257513530341,NA
"15603","LINC01096",0.157560096502761,-0.491317252697356,3.34845351503
297,-0.146729602334801,0.883345452200538,NA
"15604","LINC00504",1.15497393205991,0.524569308378929,1.5626259985384
2,0.335697287047303,0.737099151848369,NA
"15605","CPEB2-
DT",0.0367720482966892,-0.560503011376585,3.34952001437583,-0.16733830
7868279,0.867103863749685,NA
"15606","AC098829.1",0.175609175873806,0.247512349757584,3.30210845793
575,0.0749558510601773,0.940249838776562,NA
"15607","AC116651.1",0.212381224170495,0.56712693135589,2.688102507566
75,0.210976675837131,0.832905471698532,NA
"15608","TAPT1-
AS1",1.0965029979879,0.952041456148997,1.36788462442732,0.695995436418
901,0.486431687713241,NA
"15609","AC006427.2",0.35904879298002,0.00556896818116425,2.3635192802
9378,0.00235621863870391,0.998120011265845,NA
"15610","AC097515.1",0,NA,NA,NA,NA,NA
"15611","AC104656.1",0,NA,NA,NA,NA,NA
"15612","AC006160.1",0.983067141698061,-1.98816137528047,1.49066976329
227,-1.33373697128561,0.182290073695697,NA
"15613","NCAPG",0.651648844860529,0.148947877124253,1.66412434717374,0
.0895052568500776,0.928680375991539,NA
"15614","AC024230.1",1.16328452338019,-1.98363297730324,1.402363202587
84,-1.4144930312224,0.157217192074556,NA
"15615","SLIT2-IT1",0,NA,NA,NA,NA,NA
"15616","KCNIP4",0.513216882008329,-2.79767777251513,2.35085107316122,
-1.19007018541292,0.234018807453478,NA
"15617","KCNIP4-
IT1",0.955368966988626,-2.40986901469587,1.67085525717733,-1.442296694
66822,0.149218692249592,NA
"15618","PPARGC1A",0.9745496281109,1.31160663871987,1.55950425832165,0
.841040754920043,0.400325103225584,NA

"15619","LGI2",1.3495757329317,0.259935633150991,1.6994993115985,0.152948360365326,0.878438998475506,NA
"15620","STIM2-AS1",0.617190648494345,0.0555545770979446,1.58013324070079,0.0351581598734713,0.971953625172634,NA
"15621","AC098595.1",0.230228123330503,-1.33023934114211,2.97714568949567,-0.446817011957333,0.655007187193824,NA
"15622","LINC02506",0,NA,NA,NA,NA,NA
"15623","ARAP2",0.643606804074918,-0.474116015638896,2.02006600081224,-0.23470323021538,0.814439080413756,NA
"15624","AC104078.1",0,NA,NA,NA,NA,NA
"15625","DTHD1",0.0735440965933784,-0.185659846583073,3.34418846206699,-0.0555171601986567,0.955726459171656,NA
"15626","C4orf19",0.147372702532553,-1.1477220656059,3.3390638510896,-0.343725701810518,0.731052580354999,NA
"15627","AC027607.1",0.181578773267413,-1.95393031097672,3.33920037562282,-0.585149164824313,0.558447422910535,NA
"15628","PTTG2",0,NA,NA,NA,NA,NA
"15629","LINC02278",0.0636794437806017,-0.49131726092088,3.34845351515744,-0.146729604785264,0.883345450266286,NA
"15630","TLR10",0.245258217048015,-1.32928540753819,2.91755618878102,-0.455616043540048,0.648666104227452,NA
"15631","TMEM156",0.29327277744287,1.03801187260161,2.7639650260909,0.375551739187407,0.707250175067299,NA
"15632","AC079921.1",0.254821666883911,-1.3286579469836,2.88029438084422,-0.461292413657444,0.64458882703139,NA
"15633","KLB",0.157560096502761,-0.491317252697356,3.34845351503297,-0.146729602334801,0.883345452200538,NA
"15634","AC021148.2",1.00591972996838,-0.968663542030306,1.45829951300949,-0.66424183330575,0.506535542807752,NA
"15635","AC095057.3",0.664101013415685,1.23152800601869,1.6679162669569,0.73836320828359,0.460293765738307,NA
"15636","LINC02265",0.0741298089160557,-1.76673671430743,3.34309696236258,-0.528473069790613,0.597171033704837,NA
"15637","NSUN7",1.00518221253088,0.00323515205229786,1.44815832467421,0.00223397676702627,0.998217545910999,NA
"15638","AC108050.1",0,NA,NA,NA,NA,NA
"15639","DCAF4L1",0.446746913525274,-0.784159373907405,2.24466915739519,-0.349342962780929,0.726831848062333,NA
"15640","SHISA3",0.720504484118126,1.19611616565322,1.73665865095979,0.688745692765918,0.490983316381091,NA
"15641","AC096734.1",0,NA,NA,NA,NA,NA
"15642","KCTD8",1.2264432909948,0.805194308772694,1.43827732356704,0.559832443701295,0.575593731799694,NA
"15643","YIPF7",0,NA,NA,NA,NA,NA
"15644","AC096586.2",0.747843349513193,0.343616125049579,1.46883814320252,0.233937365147933,0.815033604734797,NA
"15645","AC096586.1",0.511322396030091,0.371910992164034,2.16389538257213,0.171871059552777,0.863538899119222,NA
"15646","GABRA2",0,NA,NA,NA,NA,NA

"15647","AC107398.3",0,NA,NA,NA,NA,NA
"15648","CORIN",0,NA,NA,NA,NA,NA
"15649","AC107068.2",0,NA,NA,NA,NA,NA
"15650","AC107068.1",1.23089710213486,0.76182281759256,1.5288659989055
6,0.498292733397113,0.618277728855311,NA
"15651","NIPAL1",0.0636794437806017,-0.49131726092088,3.34845351515744
,-0.146729604785264,0.883345450266286,NA
"15652","TXK",0.173694385693788,0.450698267773282,2.85372304708896,0.1
5793342953621,0.874509255668894,NA
"15653","TEC",1.25262244535316,-0.167683913625739,1.26571868065299,-0.
132481187319784,0.894603702204422,NA
"15654","SLC10A4",0.102366282257309,-0.49131726092088,3.34845351515744
,-0.146729604785264,0.883345450266286,NA
"15655","AC027271.1",0,NA,NA,NA,NA,NA
"15656","LRRC66",0.0367720482966892,-0.560503011376585,3.3495200143758
3,-0.167338307868279,0.867103863749685,NA
"15657","RASL11B",0.100451492077291,-0.0338344573480013,3.342323347733
69,-0.010123035334371,0.991923124345921,NA
"15658","AC023154.1",0,NA,NA,NA,NA,NA
"15659","AC124017.1",0.258274559745298,-1.11669114604128,2.56884985789
992,-0.434704715266695,0.663776775348859,NA
"15660","AC110792.3",0.387465478675832,-1.00097401054312,2.05547168079
657,-0.486980200162721,0.626272362417671,NA
"15661","AC110792.2",0,NA,NA,NA,NA,NA
"15662","AC110792.4",0.176496091173365,-1.14849506633421,3.26459492750
587,-0.351803238024283,0.724985830023263,NA
"15663","KDR",0.594492397068271,-0.866007131154221,1.76856098090702,-0
.489667667953458,0.624369084357414,NA
"15664","AC069200.1",0.147372702532553,-1.1477220656059,3.339063851089
6,-0.343725701810518,0.731052580354999,NA
"15665","AC024243.1",0.175910378850688,0.313649592621519,3.25604727090
57,0.0963283289601242,0.923259812444105,NA
"15666","NMU",0,NA,NA,NA,NA,NA
"15667","EXOC1L",0,NA,NA,NA,NA,NA
"15668","AC110611.1",0,NA,NA,NA,NA,NA
"15669","AC110611.2",0,NA,NA,NA,NA,NA
"15670","KIAA1211",0.0367720482966892,-0.560503011376585,3.34952001437
583,-0.167338307868279,0.867103863749685,NA
"15671","AC068620.1",0.458038502968997,0.249855005656417,2.20593739351
949,0.113264776412255,0.909820641845179,NA
"15672","AC068620.2",0.496698966272935,-0.613403124794402,2.4974353581
6898,-0.245613213886795,0.805981659325098,NA
"15673","ARL9",0.574510895824863,-1.64176158481446,1.8687957430978,-0.
87851312315866,0.379665319187486,NA
"15674","SPINK2",0.0741298089160557,-1.76673671430743,3.34309696236258
,-0.528473069790613,0.597171033704837,NA
"15675","AC069307.1",0.415400749040204,-0.0604383114512968,2.270638850
93632,-0.026617315838833,0.978764962110375,NA
"15676","IGFBP7-
AS1",0.0735440965933784,-0.185659846583073,3.34418846206699,-0.0555171

601986567,0.955726459171656,NA
"15677","LINC02271",0,NA,NA,NA,NA,NA
"15678","ADGRL3",0.556837831561789,0.938100894454578,2.18196238318678,
0.429934494601356,0.667243292270749,NA
"15679","AC020741.1",0.311503638390445,0.135186863550907,2.16550210957
302,0.0624274910438958,0.950222402963504,NA
"15680","EPHA5",0,NA,NA,NA,NA,NA
"15681","AC104806.2",0.157560096502761,-0.491317252697356,3.3484535150
3297,-0.146729602334801,0.883345452200538,NA
"15682","STAP1",0.157560096502761,-0.491317252697356,3.34845351503297,
-0.146729602334801,0.883345452200538,NA
"15683","TMPRSS11F",0.102366282257309,-0.49131726092088,3.348453515157
44,-0.146729604785264,0.883345450266286,NA
"15684","SULT1B1",0,NA,NA,NA,NA,NA
"15685","AC009570.2",0,NA,NA,NA,NA,NA
"15686","AC009570.1",0.166548679549901,-1.95393031097672,3.33920037562
282,-0.585149164824313,0.558447422910535,NA
"15687","ADAMTS3",1.37226203328618,-0.0608101659468651,1.2890307443347
2,-0.047175109060916 8,0.962373665521048,NA
"15688","AC053527.2",0.315120193005521,0.245400022010969,3.23930076940
85,0.0757570968180826,0.939612349767913,NA
"15689","ALB",0.348127452817314,-2.77495368732187,2.61435422383524,-1.
06142987894389,0.288494584202949,NA
"15690","AFP",0.552407009240228,-0.726782636071104,1.81053645231014,-0
.40141839461104,0.688112108669356,NA
"15691","CXCL6",0.26891496180721,-1.3277650763353,2.82969623342791,-0.
469225304345422,0.63890860009957,NA
"15692","PF4V1",0,NA,NA,NA,NA,NA
"15693","CXCL5",0.343044770723266,-1.83025924174448,2.32925281575478,-
0.785770968855264,0.432001677398361,NA
"15694","AC093677.2",0.221239540283362,0.178834610992208,3.06205963446
949,0.0584033730039328,0.953427328091912,NA
"15695","EPGN",0,NA,NA,NA,NA,NA
"15696","BTC",0,NA,NA,NA,NA,NA
"15697","PARM1",0.147372702532553,-1.1477220656059,3.3390638510896,-0.
343725701810518,0.731052580354999,NA
"15698","LINC02562",0,NA,NA,NA,NA,NA
"15699","CDKL2",0,NA,NA,NA,NA,NA
"15700","AC110615.1",0,NA,NA,NA,NA,NA
"15701","PPEF2",0.102366282257309,-0.49131726092088,3.34845351515744,-
0.146729604785264,0.883345450266286,NA
"15702","ART3",1.12094655355878,1.99952460153282,1.46351001734407,1.36
625276071665,0.171859642520696,NA
"15703","CXCL11",0.258274559745298,-1.11669114604128,2.56884985789992,
-0.434704715266695,0.663776775348859,NA
"15704","FAM47E",0.320717103821412,-0.846710013286843,2.38334198606247
,-0.35526165285482,0.722393580711595,NA
"15705","CCDC158",0,NA,NA,NA,NA,NA
"15706","SHROOM3",0,NA,NA,NA,NA,NA
"15707","AC107072.2",0.0741298089160557,-1.76673671430743,3.3430969623

6258,-0.528473069790613,0.597171033704837,NA
"15708","CXCL13",0.157560096502761,-0.491317252697356,3.34845351503297,-0.146729602334801,0.883345452200538,NA
"15709","LINC01094",0.536026299484825,-1.50692115267787,2.08705932246187,-0.722030819373319,0.470275529510899,NA
"15710","AC112253.1",0.750623025210659,-1.99356467296405,1.61636307661465,-1.23336439801595,0.217439841490706,NA
"15711","LINC00989",0.0636794437806017,-0.49131726092088,3.34845351515744,-0.146729604785264,0.883345450266286,NA
"15712","PCAT4",0.502295877088825,-1.69701434412636,2.15327580306091,-0.788108212479808,0.430633411873012,NA
"15713","FGF5",0,NA,NA,NA,NA,NA
"15714","AC073840.1",0,NA,NA,NA,NA,NA
"15715","PLAC8",0.968278733307684,1.75462743589747,1.46519146323112,1.19754139983048,0.231095601160156,NA
"15716","WDFY3-AS1",0.147372702532553,-1.1477220656059,3.3390638510896,-0.343725701810518,0.731052580354999,NA
"15717","AC108021.1",0.110316144890068,0.0897481123376931,3.34029447074234,0.0268683234738127,0.978564758597723,NA
"15718","SLC10A6",0.304357932246558,-1.82931526615362,2.40596782312181,-0.760324077726043,0.447060892953357,NA
"15719","C4orf36",1.22912868995698,1.97683377921035,1.44860559161377,1.36464596758054,0.1723643496848,NA
"15720","HSD17B13",0.514907125047723,0.174515920715263,1.96830023232382,0.0886632627732944,0.929349529567923,NA
"15721","ABCG2",0.601805343533102,2.01142938477639,2.29293202806947,0.877230271178123,0.380361577173655,NA
"15722","PPM1K-DT",0.0732428936164972,-0.49131726092088,3.34845351515744,-0.146729604785264,0.883345450266286,NA
"15723","PIGY-DT",0.257990050399502,-0.0838183463325425,2.93675595546608,-0.0285411343685315,0.977230560898511,NA
"15724","TIGD2",0.368026530493238,1.19074263094412,2.05006005643351,0.580833047894051,0.561352979471559,NA
"15725","AC104785.1",0,NA,NA,NA,NA,NA
"15726","AC093866.1",0,NA,NA,NA,NA,NA
"15727","SNCA",1.1908710745832,-1.07469257392632,1.48066128111413,-0.725819326563102,0.467949537388632,NA
"15728","MMRN1",0,NA,NA,NA,NA,NA
"15729","CCSER1",0.166548679549901,-1.95393031097672,3.33920037562282,-0.585149164824313,0.558447422910535,NA
"15730","HPGDS",0.43432848836088,-2.41395349369949,2.44018179751814,-0.98925149599701,0.322540108885353,NA
"15731","AC109925.2",0,NA,NA,NA,NA,NA
"15732","UNC5C",0.787581539188804,1.25182698431009,1.72852701411027,0.724216037175707,0.468933114403784,NA
"15733","AC106881.1",0.137809252696657,-1.1477220656059,3.3390638510896,-0.343725701810518,0.731052580354999,NA

"15734","LINC02267",0.147372702532553,-1.1477220656059,3.3390638510896
,-0.343725701810518,0.731052580354999,NA
"15735","AC114811.2",0.0741298089160557,-1.76673671430743,3.3430969623
6258,-0.528473069790613,0.597171033704837,NA
"15736","ADH4",0,NA,NA,NA,NA,NA
"15737","ADH1A",0.20332072784659,-1.39924864619322,3.10758170858468,-0
.450269301794319,0.652516271227926,NA
"15738","MTTP",0,NA,NA,NA,NA,NA
"15739","DAPP1",0.821793967036713,-1.86723594436668,1.55250174172762,-
1.2027271172583 8,0.229081936613638,NA
"15740","AC097460.1",0.604662159346134,-1.0215129207689,2.090501756855
65,-0.488644851609869,0.625093153518007,NA
"15741","BANK1",1.35948184818258,0.610118065133531,1.39676606670424,0.
436807622748986,0.662250870341911,NA
"15742","AF213884.3",0.89053963674322,-0.732213686561753,1.84419162977
659,-0.397037745285969,0.691339626635887,NA
"15743","AC018797.3",0.136922337397099,0.183394507878855,3.33779889026
91,0.0549447446979257,0.956182484581931,NA
"15744","AC105460.1",0,NA,NA,NA,NA,NA
"15745","LINC02503",0.221239540283362,0.178834610992208,3.062059634469
49,0.0584033730039328,0.953427328091912,NA
"15746","CXXC4",0.222389426748167,-2.46578197109637,3.33045300506323,-
0.740374347678133,0.459072879645795,NA
"15747","CXXC4-AS1",0,NA,NA,NA,NA,NA
"15748","AC004069.1",0.0636794437806017,-0.49131726092088,3.3484535151
5744,-0.146729604785264,0.883345450266286,NA
"15749","AC109361.1",0.277975458131115,0.892787139500793,2.87631492378
557,0.310392694526571,0.7562623489228,NA
"15750","AC092445.1",0.433620764453858,1.39289160892939,1.857665923467
68,0.749807374583961,0.453370726579907,NA
"15751","AC096564.1",0.102366282257309,-0.49131726092088,3.34845351515
744,-0.146729604785264,0.883345450266286,NA
"15752","AC096564.2",0,NA,NA,NA,NA,NA
"15753","LEF1",0,NA,NA,NA,NA,NA
"15754","LEF1-AS1",0,NA,NA,NA,NA,NA
"15755","RPL34-
AS1",0.0367720482966892,-0.560503011376585,3.34952001437583,-0.1673383
07868279,0.867103863749685,NA
"15756","AC107071.1",0,NA,NA,NA,NA,NA
"15757","AC004067.1",0.539417909790444,-0.269724932656213,1.7350159982
7088,-0.155459622807526,0.87645898322482,NA
"15758","AC126283.1",0.0732428936164972,-0.49131726092088,3.3484535151
5744,-0.146729604785264,0.883345450266286,NA
"15759","RRH",0,NA,NA,NA,NA,NA
"15760","LRIT3",0.110014941913186,-0.033834469553869,3.34232334787728,
-0.0101230389858472,0.991923121432614,NA
"15761","AC109347.1",0.239589822631289,0.773110999527443,2.56583771895
994,0.301309390619147,0.763178580793342,NA
"15762","AC109347.2",0.147372702532553,-1.1477220656059,3.339063851089
6,-0.343725701810518,0.731052580354999,NA

"15763","AC017007.5",0.210165231013596,0.627110932648478,3.11655680733
581,0.20121915672205,0.840527212935289,NA
"15764","AC093879.1",0.322094963106801,1.10762670259855,2.749425975580
69,0.402857437310932,0.687053104266605,NA
"15765","AC104779.1",0,NA,NA,NA,NA,NA
"15766","UGT8",0.147088193186757,0.338238253117599,3.33259753179347,0.
10149387974118,0.919158415731812,NA
"15767","AC092661.1",0,NA,NA,NA,NA,NA
"15768","METTL14-
DT",0.343044770723266,-1.83025924174448,2.32925281575478,-0.7857709688
55264,0.432001677398361,NA
"15769","MYOZ2",0.176496091173365,-1.14849506633421,3.26459492750587,-
0.351803238024283,0.724985830023263,NA
"15770","AC093752.3",0.412682966363553,-0.472696900163557,2.2250668041
592,-0.212441666596243,0.831762482299565,NA
"15771","AC093752.2",0.304932799035314,-0.455323059556486,2.4740235505
9449,-0.184041521935826,0.853980881434662,NA
"15772","TNIP3",0.0636794437806017,-0.49131726092088,3.34845351515744,
-0.146729604785264,0.883345450266286,NA
"15773","QRFPR",0,NA,NA,NA,NA,NA
"15774","AC079341.1",0,NA,NA,NA,NA,NA
"15775","CCNA2",0.97126065114182,-0.200745382967751,1.40401703226532,-
0.142979307483085,0.886306523508095,NA
"15776","TRPC3",0.0367720482966892,-0.560503011376585,3.34952001437583
,-0.167338307868279,0.867103863749685,NA
"15777","IL2",0,NA,NA,NA,NA,NA
"15778","IL21",0,NA,NA,NA,NA,NA
"15779","IL21-AS1",0,NA,NA,NA,NA,NA
"15780","AC053545.1",0,NA,NA,NA,NA,NA
"15781","AC021205.3",0.176496091173365,-1.14849506633421,3.26459492750
587,-0.351803238024283,0.724985830023263,NA
"15782","AC108075.1",0.102366282257309,-0.49131726092088,3.34845351515
744,-0.146729604785264,0.883345450266286,NA
"15783","LINC02516",0.100451492077291,-0.0338344573480013,3.3423233477
3369,-0.010123035334371,0.991923124345921,NA
"15784","AC096773.1",0,NA,NA,NA,NA,NA
"15785","AC093772.1",1.28493100965956,-1.13898142609725,1.276076922663
4,-0.892564864914252,0.372090241756693,NA
"15786","AC093772.2",0.255708582183469,-2.44071869332471,2.85324394739
347,-0.855418862994307,0.392319304627377,NA
"15787","AC093591.2",0.184144750829242,-0.691295663073562,2.8131316232
0765,-0.245738826214366,0.805884414604706,NA
"15788","PLK4",1.1336177564006,1.19088691751896,1.3160904484563,0.9048
67077271019,0.365535810498379,NA
"15789","AC110609.1",0.54336204255517,0.618435795056071,1.853582298815
02,0.333643558989225,0.738648544554647,NA
"15790","AC078850.1",0,NA,NA,NA,NA,NA
"15791","PCDH10",0.276947583250656,-0.158224666587883,2.24258642930609
,-0.0705545456443527,0.94375228752887,NA
"15792","AC015631.1",0,NA,NA,NA,NA,NA

"15793","LINC02510",0.249738984789862,-0.445595840703958,2.59717004458
552,-0.171569759797946,0.863775783061595,NA
"15794","LINC02172",0,NA,NA,NA,NA,NA
"15795","AC116563.1",0.0741298089160557,-1.76673671430743,3.3430969623
6258,-0.528473069790613,0.597171033704837,NA
"15796","SLC7A11",0.470906611796459,-2.39770178202472,2.12513181341507
,-1.12826026455819,0.259210019400695,NA
"15797","LINC00499",0,NA,NA,NA,NA,NA
"15798","AC109927.1",0.0741298089160557,-1.76673671430743,3.3430969623
6258,-0.528473069790613,0.597171033704837,NA
"15799","NOCT",1.08404312546789,-2.70445954899802,1.65181014425974,-1.
63727021437444,0.10157402163782,NA
"15800","MGARP",0.5062887912816,0.423556559455169,1.77954156544367,0.2
38014423309954,0.811869907446422,NA
"15801","AC112236.2",0.323605822540671,0.859187086673796,2.43516471689
191,0.352825039190945,0.724219610906701,NA
"15802","AC108053.1",0.0732428936164972,-0.49131726092088,3.3484535151
5744,-0.146729604785264,0.883345450266286,NA
"15803","CLGN",0.0732428936164972,-0.49131726092088,3.34845351515744,-
0.146729604785264,0.883345450266286,NA
"15804","LINC02432",0.110901857212745,-1.2169087618042,3.3401333690268
2,-0.36432939267894,0.715612053721651,NA
"15805","INPP4B",0.72809250570709,-0.168322532710984,1.48140550239662,
-0.11362353686325,0.909536228462667,NA
"15806","AC139720.1",0,NA,NA,NA,NA,NA
"15807","AC104596.1",0.431082030139527,0.647039988012108,1.97537317568
419,0.327553292702783,0.743249442165126,NA
"15808","AC097658.3",0.0732428936164972,-0.49131726092088,3.3484535151
5744,-0.146729604785264,0.883345450266286,NA
"15809","SMARCA5-
AS1",0.352105267047171,0.203443411411541,2.39121938749498,0.0850793584
542931,0.932198300364955,NA
"15810","GYPE",1.20072371786981,1.04251332823475,1.50427794880858,0.69
3032380791354,0.488289230841609,NA
"15811","SMAD1-AS1",0,NA,NA,NA,NA,NA
"15812","REELD1",0.0636794437806017,-0.49131726092088,3.34845351515744
,-0.146729604785264,0.883345450266286,NA
"15813","EDNRA",0.526402058694722,0.95832950091286,1.88643549320433,0.
508010745326375,0.611445807565065,NA
"15814","AC002460.2",0,NA,NA,NA,NA,NA
"15815","IQCM",0,NA,NA,NA,NA,NA
"15816","AC110813.1",0.0732428936164972,-0.49131726092088,3.3484535151
5744,-0.146729604785264,0.883345450266286,NA
"15817","MAB21L2",0,NA,NA,NA,NA,NA
"15818","AC095055.1",0.993402434294888,-2.22992626871497,1.86151663757
401,-1.19790832040109,0.230952710228252,NA
"15819","PRSS48",0,NA,NA,NA,NA,NA
"15820","FAM160A1",0,NA,NA,NA,NA,NA
"15821","AC092611.1",0,NA,NA,NA,NA,NA
"15822","LINC02273",0,NA,NA,NA,NA,NA

"15823","AC023424.3",0.157560096502761,-0.491317252697356,3.3484535150
3297,-0.146729602334801,0.883345452200538,NA
"15824","TIGD4",0.166045726037911,0.183394512381981,3.33779889015228,0
.0549447460489789,0.956182483505573,NA
"15825","AC093599.2",0.523299150238264,1.85138259165284,1.801539452878
68,1.0276669704316,0.304106509818544,NA
"15826","MND1",0.173694385693788,0.450698267773282,2.85372304708896,0.
15793342953621,0.874509255668894,NA
"15827","AC106865.2",0,NA,NA,NA,NA,NA
"15828","TLR2",1.33980098068097,-1.15446742674751,1.18111148510867,-0.
97744153816377,0.328350607160521,NA
"15829","RNF175",0.211052146313155,-0.488988616138086,2.71532944071237
,-0.180084452665824,0.85708626804187,NA
"15830","AC079298.3",0.633549820069198,0.828286870039255,1.66604030973
295,0.497158961401132,0.619076958644025,NA
"15831","DCHS2",0,NA,NA,NA,NA,NA
"15832","AC079298.1",0,NA,NA,NA,NA,NA
"15833","LRAT",0.544216574140257,1.06369979840237,2.14398635331058,0.4
96131795223363,0.619801427884106,NA
"15834","RBM46",0.166548679549901,-1.95393031097672,3.33920037562282,-
0.585149164824313,0.558447422910535,NA
"15835","AC104083.1",0.627815950593339,0.679011500974866,2.10328994577
788,0.32283304655067,0.746821684370925,NA
"15836","TDO2",0.0741298089160557,-1.76673671430743,3.34309696236258,-
0.52847306979061З,0.597171033704837,NA
"15837","GLRB",1.08363899323696,-1.26884292978654,1.54362085733149,-0.
821991309433349,0.411081843049606,NA
"15838","AC084740.1",0.148259617832111,-2.17639404086898,3.33507601540
8,-0.652577041966681,0.514028990213041,NA
"15839","RXFP1",0.230802990119258,0.237440402410633,3.03060926850593,0
.0783474151148786,0.937551701540005,NA
"15840","C4orf45",0,NA,NA,NA,NA,NA
"15841","NPY5R",0.358161877680461,1.11569273694527,2.33981371918157,0.
476829726998748,0.633483375671316,NA
"15842","MARCH1",1.27620429571106,-1.01346586072852,1.31985272541336,-
0.767862838947515,0.442568676915557,NA
"15843","AC093788.1",0.740906606267703,-0.169425839817779,1.5602290139
0724,-0.108590366098558,0.913527402305145,NA
"15844","FAM218A",0.237675032451271,0.812476888782201,2.93416062775353
,0.276902662075544,0.781854848453636,NA
"15845","GK3P",0,NA,NA,NA,NA,NA
"15846","TLL1",0.744019872127586,-1.46371255289236,1.87968997997858,-0
.77869891763164,0.436157093856249,NA
"15847","AC068989.1",0.331517453361795,-0.597214710352191,2.4127145192
6306,-0.247528128829184,0.804499524999222,NA
"15848","AC080188.1",0.0636794437806017,-0.49131726092088,3.3484535151
5744,-0.146729604785264,0.883345450266286,NA
"15849","AC096741.1",0,NA,NA,NA,NA,NA
"15850","AC084724.1",0.157560096502761,-0.491317252697356,3.3484535150
3297,-0.146729602334801,0.883345452200538,NA

"15851","MFAP3L",0.596526514215913,-0.343584943176733,1.7567314660373,-0.195581937148178,0.844937399297048,NA
"15852","GALNTL6",0,NA,NA,NA,NA,NA
"15853","AC105285.1",0.999476758355661,-0.0155223299295409,1.62032047406154,-0.00957979003414807,0.99235655034579,NA
"15854","AC093849.4",1.1213807156439,-1.18748882211853,1.40091015152616,-0.847655233866977,0.396630004095337,NA
"15855","AC093849.3",0.102366282257309,-0.49131726092088,3.34845351515744,-0.146729604785264,0.883345450266286,NA
"15856","LINC02269",0.0732428936164972,-0.49131726092088,3.34845351515744,-0.146729604785264,0.883345450266286,NA
"15857","AC106895.1",0,NA,NA,NA,NA,NA
"15858","LINC02268",0.460725065923415,0.308594529727252,2.49227218462806,0.123820556851942,0.901457355052409,NA
"15859","AC096751.2",0.294762098696191,0.110405081667536,2.82766066957157,0.0390446713976694,0.968854773123445,NA
"15860","GLRA3",0.230802990119258,0.237440402410633,3.03060926850593,0.0783474151148786,0.937551701540005,NA
"15861","GPM6A",0,NA,NA,NA,NA,NA
"15862","WDR17",1.3805735453803,-0.509094407349485,1.40637490801724,-0.361990536411963,0.717359102417329,NA
"15863","SPATA4",0,NA,NA,NA,NA,NA
"15864","AC093801.1",0,NA,NA,NA,NA,NA
"15865","AC097518.2",1.44577815231575,2.4543660373209,1.39969099698865,1.75350562559974,0.0795152547376875,NA
"15866","NEIL3",0.0636794437806017,-0.49131726092088,3.34845351515744,-0.146729604785264,0.883345450266286,NA
"15867","AC078881.1",0.533006149839054,-0.317111715655266,1.9028375312166,-0.166652018605349,0.867643861050434,NA
"15868","TENM3-AS1",0.444372332566193,0.4999343759006,2.00236462425676,0.24967199771928,0.802841015078245,NA
"15869","AC079766.1",0.166548679549901,-1.95393031097672,3.33920037562282,-0.585149164824313,0.558447422910535,NA
"15870","AC019193.2",0.181578773267413,-1.95393031097672,3.33920037562282,-0.585149164824313,0.558447422910535,NA
"15871","CLDN22",0,NA,NA,NA,NA,NA
"15872","AC107214.2",0.157560096502761,-0.491317252697356,3.34845351503297,-0.146729602334801,0.883345452200538,NA
"15873","AC074194.1",0.102366282257309,-0.49131726092088,3.34845351515744,-0.146729604785264,0.883345450266286,NA
"15874","LINC02363",0.0735440965933784,-0.185659846583073,3.34418846206699,-0.0555171601986567,0.955726459171656,NA
"15875","LINC02362",0.525716893930272,0.514252381493716,1.87791064429509,0.273842838612138,0.784205401016425,NA
"15876","AC099343.2",0.358074864440778,-1.83062655359436,2.30107188171825,-0.79555383216773,0.426291410012607,NA
"15877","AC099343.4",0.377589980329133,1.23398568878864,2.26050004314689,0.545890584045636,0.585141151197391,NA
"15878","LINC02365",0,NA,NA,NA,NA,NA

"15879","CENPU",1.16687871106568,0.662166707931555,1.27436759172575,0.519604164631061,0.603339494807357,NA
"15880","AC084871.1",0.166045726037911,0.183394512381981,3.33779889015228,0.0549447460489789,0.956182483505573,NA
"15881","MIR3945HG",0.312390553690004,-0.679283711575989,2.44873738881309,-0.277401617126955,0.781471740045957,NA
"15882","LINC01093",0,NA,NA,NA,NA,NA
"15883","LRP2BP",0.45447154930448,0.602231321607586,2.26441176068791,0.265954863891288,0.790273970827156,NA
"15884","C4orf47",0.605614549704826,0.0209147303800328,1.71602279492623,0.012187909415814,0.990275695998931,NA
"15885","AC093797.1",0,NA,NA,NA,NA,NA
"15886","KLKB1",0.562221716632719,0.485831605671953,2.09047545008442,0.232402444932961,0.816225450125791,NA
"15887","AC107050.1",0,NA,NA,NA,NA,NA
"15888","AC108865.1",0.102366282257309,-0.49131726092088,3.34845351515744,-0.146729604785264,0.883345450266286,NA
"15889","AC108865.2",0.392045207257891,-0.10443549837766,2.03486961329477,-0.0513229435907505,0.959068185852561,NA
"15890","AC110772.2",1.03057874858093,0.160288312322443,1.38992036132556,0.115321939862495,0.908189948881641,NA
"15891","AC097652.1",0.127358887561203,0.15032497835859,3.33866494558414,0.0450254759937551,0.964087002635848,NA
"15892","TRIML2",0.239791573166398,-1.32959277084231,2.9363559394555,-0.45280367852436,0.650690114564834,NA
"15893","FRG1-DT",0.480401015328219,-0.940822955840685,1.99300848454414,-0.472061691225504,0.636882755393714,NA
"15894","AC021087.1",0,NA,NA,NA,NA,NA
"15895","AHRR",0.0636794437806017,-0.49131726092088,3.34845351515744,-0.146729604785264,0.883345450266286,NA
"15896","SLC9A3",0.102366282257309,-0.49131726092088,3.34845351515744,-0.146729604785264,0.883345450266286,NA
"15897","CEP72",1.21983905682025,-0.254316424809221,1.25419136968747,-0.202773221819086,0.839312289010313,NA
"15898","AC026740.1",0,NA,NA,NA,NA,NA
"15899","TRIP13",1.16355002627487,-2.37470047968666,1.47526322489004,-1.60967916750155,0.107467915180119,NA
"15900","AC122719.3",0,NA,NA,NA,NA,NA
"15901","AC116351.1",0.26841200829522,0.85362770346921,2.90278708422675,0.294071758865015,0.768703070410226,NA
"15902","AC116351.2",0.388291603021183,1.36359484651523,2.22743782542675,0.612180879281775,0.540418090770536,NA
"15903","NKD2",0.127358887561203,0.15032497835859,3.33866494558414,0.0450254759937551,0.964087002635848,NA
"15904","AC026748.3",1.04697757815917,0.0914911341265784,1.33194732811337,0.0686897538629928,0.945236574173387,NA
"15905","SLC6A3",0,NA,NA,NA,NA,NA
"15906","AC026412.3",0,NA,NA,NA,NA,NA
"15907","AC126768.1",0,NA,NA,NA,NA,NA

"15908","AC091891.1",0,NA,NA,NA,NA,NA
"15909","AC094105.2",0.276563621463087,-0.851854214261183,2.4869613794
202,-0.342528123400042,0.731953483145556,NA
"15910","AC091978.1",0.638414119922684,-0.934929691757625,1.7479398060
5056,-0.534875221973509,0.592736143158158,NA
"15911","LINC02145",0.322981878406359,0.0998814290120277,2.43523825703
497,0.0410150541629708,0.967283894474422,NA
"15912","UBE2QL1",0.296698427056759,0.680778658728797,2.47341973472073
,0.275237821212606,0.783133531518291,NA
"15913","LINC01018",0,NA,NA,NA,NA,NA
"15914","LINC02102",0,NA,NA,NA,NA,NA
"15915","ADCY2",1.34144457685291,-0.592642600868122,1.21742202581459,-
0.4868012803297,0.626399162944294,NA
"15916","C5orf49",0.680138677811876,0.13375394463524,1.85511103585925,
0.0721002366164 5,0.942522137924628,NA
"15917","AC091965.4",0.181578773267413,-1.95393031097672,3.33920037562
282,-0.585149164824313,0.558447422910535,NA
"15918","AC091932.1",0,NA,NA,NA,NA,NA
"15919","AC034229.1",0,NA,NA,NA,NA,NA
"15920","AC034229.4",0,NA,NA,NA,NA,NA
"15921","AC012640.1",0.166548679549901,-1.95393031097672,3.33920037562
282,-0.585149164824313,0.558447422910535,NA
"15922","AC012640.4",0.771210838896035,-1.52784650142979,1.79049479124
251,-0.853309659934586,0.393487604545887,NA
"15923","ROPN1L",1.05593803192083,1.22971956869475,1.48829667755679,0.
826259701602959,0.408656788073611,NA
"15924","LINC01513",0.56218773925008,0.622315868804543,1.8657119112687
8,0.333554106100624,0.738716054617014,NA
"15925","CTNND2",0.68008262941716,2.20914336105829,1.88435550061511,1.
1723601838067,0.241052478023862,NA
"15926","AC016549.1",0,NA,NA,NA,NA,NA
"15927","AC010491.1",0.986042873657004,0.452518606938701,1.54967408545
705,0.292008888311015,0.770279828118535,NA
"15928","AC010491.2",0.230228123330503,-1.33023934114211,2.97714568949
567,-0.446817011957333,0.655007187193824,NA
"15929","AC016575.1",0.100451492077291,-0.0338344573480013,3.342323347
73369,-0.010123035334371,0.991923124345921,NA
"15930","LINC02149",0.229725169818512,0.686619208900669,3.025936480376
6,0.226911309392461,0.820492707794461,NA
"15931","AC114964.2",0.110014941913186,-0.033834469553869,3.3423233478
7728,-0.0101230389858472,0.991923121432614,NA
"15932","AC010638.1",0,NA,NA,NA,NA,NA
"15933","AC024588.1",0,NA,NA,NA,NA,NA
"15934","AC022113.1",0,NA,NA,NA,NA,NA
"15935","MYO10",0.508001830926509,1.73048819659133,2.06855152069878,0.
836570024616427,0.402834300607182,NA
"15936","BASP1-
AS1",0.700232719864486,-1.13383713355962,1.70984134762151,-0.663124175
314189,0.507251028605289,NA
"15937","AC026790.1",0.102366282257309,-0.49131726092088,3.34845351515

744,-0.146729604785264,0.883345450266286,NA
"15938","AC026785.2",0,NA,NA,NA,NA,NA
"15939","LINC02217",0,NA,NA,NA,NA,NA
"15940","H3.Y",0.384160819684265,1.41778543227635,2.17630311956775,0.651465055363217,0.514746327627056,NA
"15941","LINC02223",0.383440250755117,-1.38194811510753,2.56276654272887,-0.53924073537967,0.589720755955717,NA
"15942","CDH18",0.596673880011005,1.99425618319448,1.9887154666663,1.00278607805945,0.315964087439908,NA
"15943","LINC02241",0.77471251318547,-0.642818534131801,1.62185937260514,-0.396346653100547,0.691849315007915,NA
"15944","AC091946.2",0.240092776143279,-1.01655735204591,2.97416330481592,-0.341796077706914,0.732504361553979,NA
"15945","CDH12",0.348127452817314,-2.77495368732187,2.61435422383524,-1.06142987894389,0.288494584202949,NA
"15946","AC091938.1",0,NA,NA,NA,NA,NA
"15947","C5orf17",1.28331845495041,-1.32482497847237,1.33470516272872,-0.99259747805564,0.320906154287848,NA
"15948","CDH10",0.166548679549901,-1.95393031097672,3.33920037562282,-0.585149164824313,0.558447422910535,NA
"15949","LINC02211",0.248852069490303,0.780083555267702,2.96292468503169,0.263281601185674,0.792333539707393,NA
"15950","AC113370.1",0.241504612811307,0.71883124973489,2.9784780941551,0.241341795041404,0.809290220283093,NA
"15951","PURPL",0,NA,NA,NA,NA,NA
"15952","CDH6",0.278862373430674,-0.427610315239008,2.88033331606682,-0.148458622081601,0.881980838831896,NA
"15953","AC010343.3",0.139138330553998,-0.0338344975521838,3.34232334820666,-0.0101230473617515,0.991923114749952,NA
"15954","AC034223.1",0,NA,NA,NA,NA,NA
"15955","AC034231.1",0.136922337397099,0.183394507878855,3.3377988902691,0.0549447446979257,0.956182484581931,NA
"15956","SLC45A2",0,NA,NA,NA,NA,NA
"15957","AC139792.1",0,NA,NA,NA,NA,NA
"15958","AC026801.2",0.328951475799966,-1.82996535219318,2.35245146173024,-0.777897177460669,0.436629632387116,NA
"15959","PRLR",0,NA,NA,NA,NA,NA
"15960","UGT3A2",0,NA,NA,NA,NA,NA
"15961","AC008957.2",0,NA,NA,NA,NA,NA
"15962","AC008957.1",0.102366282257309,-0.49131726092088,3.34845351515744,-0.146729604785264,0.883345450266286,NA
"15963","AC026741.1",0.221502511448609,-1.56478135306832,3.04955054434762,-0.513118681036017,0.607868304703499,NA
"15964","GDNF",0,NA,NA,NA,NA,NA
"15965","AC010457.1",0.240092776143279,-1.01655735204591,2.97416330481592,-0.341796077706914,0.732504361553979,NA
"15966","AC008964.1",0.0367720482966892,-0.560503011376585,3.34952001437583,-0.167338307868279,0.867103863749685,NA
"15967","C9",0.137809252696657,-1.1477220656059,3.3390638510896,-0.343725701810518,0.731052580354999,NA

"15968","LINC00603",0.471481478585215,-1.16313012954609,2.0184214737257,-0.576257310322371,0.564441273080995,NA
"15969","AC108105.1",0,NA,NA,NA,NA,NA
"15970","AC114977.1",0,NA,NA,NA,NA,NA
"15971","PLCXD3",0,NA,NA,NA,NA,NA
"15972","OXCT1-AS1",0.447119694535718,-0.836861397497281,2.2163164323279,-0.377591117085338,0.70573436455466,NA
"15973","AC008945.1",0.434123717965848,-0.25264285781757,2.02558456078757,-0.124725900220793,0.900740552099731,NA
"15974","AC008945.2",0,NA,NA,NA,NA,NA
"15975","AC008875.2",1.02879579455715,0.166987054546669,1.3855839979741,0.120517453139489,0.904073254805122,NA
"15976","AC008875.1",0.565373406517225,0.281869699900563,1.9439868468566,0.144995682638667,0.884714280523853,NA
"15977","AC025171.3",0.0636794437806017,-0.49131726092088,3.34845351515744,-0.146729604785264,0.883345450266286,NA
"15978","AC025171.5",0.164130935857893,0.400527358383716,3.33018851502426,0.120271677286953,0.904267939474822,NA
"15979","NIM1K",0.275032793070637,-0.0248595051845633,2.48079203608562,-0.0100207936912715,0.992004697236763,NA
"15980","AC114947.2",0,NA,NA,NA,NA,NA
"15981","AC114956.3",0.602747369166116,-0.854317133709453,1.87224090283809,-0.456307269227167,0.648169037391706,NA
"15982","AC114956.1",0,NA,NA,NA,NA,NA
"15983","FGF10-AS1",0.980377007498641,-0.0965629579891203,1.57233315874735,-0.0614138024450559,0.951029660347509,NA
"15984","LINC02224",0.201488696477259,-0.508277411998464,3.16327545678924,-0.160680730762022,0.872344866840293,NA
"15985","HCN1",0.444322387145907,1.52738617063694,2.17375882393448,0.702647485001298,0.482275462268703,NA
"15986","AC010478.1",0,NA,NA,NA,NA,NA
"15987","ISL1",0.0367720482966892,-0.560503011376585,3.34952001437583,-0.167338307868279,0.867103863749685,NA
"15988","AC022126.1",0.230802990119258,0.237440402410633,3.03060926850593,0.0783474151148786,0.937551701540005,NA
"15989","ITGA1",0.413427758089972,-0.841109711978644,2.58757795462954,-0.325056762241223,0.745138112174142,NA
"15990","ITGA2",0,NA,NA,NA,NA,NA
"15991","AC008966.2",0.181578773267413,-1.95393031097672,3.33920037562282,-0.585149164824313,0.558447422910535,NA
"15992","AC027329.1",0.110901857212745,-1.2169087618042,3.34013336902682,-0.36432939267894,0.715612053721651,NA
"15993","AC025175.1",0.557720348771582,-1.19625710293857,1.92843665418425,-0.620324810951389,0.535043963361683,NA
"15994","AC025175.2",0.549485976793028,-0.291403672406866,1.9366598080864,-0.150467145127981,0.880396069990334,NA
"15995","AC016583.2",0.49404090499098,-1.83318908307749,2.12601175584889,-0.862266672813165,0.388540787828544,NA

"15996","ESM1",0,NA,NA,NA,NA,NA
"15997","AC034238.1",0,NA,NA,NA,NA,NA
"15998","CDC20B",0,NA,NA,NA,NA,NA
"15999","CCNO",0.90438855123001,-2.05271796594251,1.7634836626379,-1.16401303251767,0.244418730322247,NA
"16000","IL31RA",0.75935116882198,-1.28126392433083,1.82249662800327,-0.703026773626782,0.482039064442702,NA
"16001","AC008914.1",1.36305762935424,2.18539708530016,1.54872892614461,1.41109076508337,0.158217852667716,NA
"16002","AC008892.1",0.110316144890068,0.0897481123376931,3.34029447074234,0.0268683234738127,0.978564758597723,NA
"16003","AC016596.1",1.21313028639534,1.38175192069465,1.37598963365184,1.0041877401558,0.315288130292481,NA
"16004","ANKRD55",0.601346716746277,1.89770692311957,2.03268141729175,0.933597811725946,0.350511394536293,NA
"16005","LINC01948",1.40272103243469,1.25961158347587,1.49585960571594,0.842065377434271,0.399751359745321,NA
"16006","AC022431.1",0.248852069490303,0.780083555267702,2.96292468503169,0.263281601185674,0.792333539707393,NA
"16007","AC008937.3",0,NA,NA,NA,NA,NA
"16008","AC008780.1",0.157560096502761,-0.491317252697356,3.34845351503297,-0.146729602334801,0.883345452200538,NA
"16009","GAPT",0.146485787232994,0.248608662259703,3.33613854377696,0.0745198854896011,0.940596718825075,NA
"16010","RAB3C",0,NA,NA,NA,NA,NA
"16011","AC092343.1",0,NA,NA,NA,NA,NA
"16012","PART1",0,NA,NA,NA,NA,NA
"16013","DEPDC1B",0,NA,NA,NA,NA,NA
"16014","ELOVL7",0.0741298089160557,-1.76673671430743,3.34309696236258,-0.528473069790613,0.597171033704837,NA
"16015","AC104113.1",0.970993425547578,-0.24600486773854,1.75946307756205,-0.139818147294918,0.88880367467188,NA
"16016","C5orf64",0,NA,NA,NA,NA,NA
"16017","LRRC70",0.185031666128801,-1.62793589934517,3.24336249302777,-0.501928447049853,0.61571785405042,NA
"16018","RNF180",0.785977289057625,2.34259985464183,1.72933111486773,1.35462771386092,0.175536199094579,NA
"16019","RGS7BP",0,NA,NA,NA,NA,NA
"16020","AC092354.2",0.239791573166398,-1.32959277084231,2.9363559394555,-0.45280367852436,0.650690114564834,NA
"16021","AC092354.1",0,NA,NA,NA,NA,NA
"16022","AC025176.1",0,NA,NA,NA,NA,NA
"16023","AC010359.2",0.338411033874358,0.245695297616098,2.39493458792811,0.10258956501549,0.918288725051033,NA
"16024","AC025442.2",0,NA,NA,NA,NA,NA
"16025","AC092353.2",0.584343234909699,0.0719463550787189,2.04103652185779,0.0352499106744214,0.971880463974407,NA
"16026","MAST4-IT1",0.212381224170495,0.56712693135589,2.68810250756675,0.210976675837131,0.832905471698532,NA

"16027","MAST4-
AS1",0.917867562171637,0.0742721214021116,1.61656761208592,0.045944333
4425557,0.96335461848779,NA
"16028","CD180",0.240678488465957,-2.44080205522383,2.91280317905203,-
0.837956396359809,0.402055193025598,NA
"16029","AC112206.2",1.25446572748615,-0.21997603812315,1.507456358212
73,-0.145925311153922,0.883980350055462,NA
"16030","LINC02242",0.100451492077291,-0.0338344573480013,3.3423233477
3369,-0.010123035334371,0.991923124345921,NA
"16031","AC010280.2",0,NA,NA,NA,NA,NA
"16032","AC093523.1",0.0367720482966892,-0.560503011376585,3.349520014
37583,-0.167338307868279,0.867103863749685,NA
"16033","LINC02198",0,NA,NA,NA,NA,NA
"16034","AC010273.3",0.796247786308246,1.63069590024526,1.770104620623
26,0.921242666250477,0.35692374712247,NA
"16035","AC010273.2",0.239791573166398,-1.32959277084231,2.93635593945
55,-0.45280367852436,0.650690114564834,NA
"16036","MARVELD2",0,NA,NA,NA,NA,NA
"16037","SERF1B",0.0636794437806017,-0.49131726092088,3.34845351515744
,-0.146729604785264,0.883345450266286,NA
"16038","SERF1A",0.753698209611374,2.27138383289795,1.86850541858557,1
.21561533100469,0.224131491217702,NA
"16039","LINC02197",0,NA,NA,NA,NA,NA
"16040","AC025774.1",0.947484579415001,-0.818483776904533,1.4483198652
1234,-0.565126389939099,0.571987799860291,NA
"16041","ZNF366",0,NA,NA,NA,NA,NA
"16042","AC035140.1",0,NA,NA,NA,NA,NA
"16043","AC008972.1",0,NA,NA,NA,NA,NA
"16044","AC008972.2",0.494997201837877,-0.654157138962558,1.8378698426
1605,-0.355932244925147,0.721891307084456,NA
"16045","TMEM171",0,NA,NA,NA,NA,NA
"16046","ARHGEF28",0.517626071716612,-1.18981814542056,1.7704527979758
9,-0.672041721067541,0.501557136252948,NA
"16047","FAM169A",0,NA,NA,NA,NA,NA
"16048","AC010501.2",0,NA,NA,NA,NA,NA
"16049","GCNT4",0.230802990119258,0.237440402410633,3.03060926850593,0
.0783474151148786,0.937551701540005,NA
"16050","ANKRD31",0.345709709125306,-0.403701136108005,2.3539094344903
,-0.171502407948596,0.863828737224812,NA
"16051","AC008897.3",0,NA,NA,NA,NA,NA
"16052","AC010245.1",0.346668202458428,-1.94732862277873,3.00338753971
666,-0.648377406187962,0.516740879961632,NA
"16053","AC010245.2",1.26247206818955,0.0789846224080144,1.28430676990
417,0.0614998100601059,0.950961165671692,NA
"16054","ANKDD1B",0.366024727073537,-1.19582861061534,2.35129598193766
,-0.508582764484578,0.611044712557475,NA
"16055","SV2C",0,NA,NA,NA,NA,NA
"16056","AC026725.1",0,NA,NA,NA,NA,NA
"16057","F2RL2",0,NA,NA,NA,NA,NA
"16058","F2RL1",0.147372702532553,-1.1477220656059,3.3390638510896,-0.

343725701810518,0.731052580354999,NA
"16059","S100Z",0.0367720482966892,-0.560503011376585,3.34952001437583
,-0.167338307868279,0.867103863749685,NA
"16060","CRHBP",0.759936881144657,-2.53750849446348,1.78686782882139,-
1.42008740295986,0.155582237160479,NA
"16061","BHMT",0.693152488779732,-0.855253687027318,1.64248627197806,-
0.520706749041701,0.602571072141372,NA
"16062","THBS4",16.6764987345601,-0.293802007186401,2.24879152604543,-
0.130648841292577,NA,NA
"16063","AC012636.1",0.102366282257309,-0.49131726092088,3.34845351515
744,-0.146729604785264,0.883345450266286,NA
"16064","AC010260.1",0,NA,NA,NA,NA,NA
"16065","ANKRD34B",0,NA,NA,NA,NA,NA
"16066","RASGRF2-AS1",0,NA,NA,NA,NA,NA
"16067","RASGRF2",0.387788219833263,-0.658138061478555,2.3041734338657
6,-0.285628699561206,0.775162519724375,NA
"16068","CKMT2",0.527429503899252,-1.62619138582155,2.13506096060678,-
0.761660400253579,0.446262715648008,NA
"16069","AC010623.1",0.747214474457588,1.20060645836436,1.755127323066
29,0.684056616625876,0.493939415305784,NA
"16070","AC026726.1",0.449755053644885,1.60864175400859,1.890377182215
69,0.850963378706844,0.394789696125851,NA
"16071","LINC01338",0,NA,NA,NA,NA,NA
"16072","AC104118.1",0.379231106697278,-0.172944401134216,2.2735351625
237,-0.0760684963157737,0.939364603796265,NA
"16073","VCAN-
AS1",0.403968200015581,-3.04670923771917,2.42900297968504,-1.254304446
39233,0.209731368411591,NA
"16074","LINC01949",0.136922337397099,0.183394507878855,3.337798890269
1,0.0549447446979257,0.956182484581931,NA
"16075","AC018754.1",0.0367720482966892,-0.560503011376585,3.349520014
37583,-0.167338307868279,0.867103863749685,NA
"16076","AC114971.1",0.181578773267413,-1.95393031097672,3.33920037562
282,-0.585149164824313,0.558447422910535,NA
"16077","LINC02060",0.102366282257309,-0.49131726092088,3.348453515157
44,-0.146729604785264,0.883345450266286,NA
"16078","MEF2C-
AS1",0.62140419064195,0.471445136693775,2.14148238531929,0.22014896780
1799,0.82575513936116,NA
"16079","AC093510.2",0.268461953715506,-0.692213851544516,2.5169359849
2032,-0.275022430324715,0.783299005639148,NA
"16080","AC093510.1",0.284596242906533,0.0204368619746971,2.2035438942
8378,0.00927454271626377,0.992600091644834,NA
"16081","ADGRV1",0,NA,NA,NA,NA,NA
"16082","AC123595.1",0.348127452817314,-2.77495368732187,2.61435422383
524,-1.06142987894389,0.288494584202949,NA
"16083","AC123595.2",0.20332072784659,-1.39924864619322,3.107581708584
68,-0.450269301794319,0.652516271227926,NA
"16084","ARRDC3-
AS1",0.137809252696657,-1.1477220656059,3.3390638510896,-0.34372570181

0518,0.731052580354999,NA
"16085","AC106818.2",0,NA,NA,NA,NA,NA
"16086","AC108102.1",0,NA,NA,NA,NA,NA
"16087","AC099501.1",0,NA,NA,NA,NA,NA
"16088","MCTP1",0.546810098424338,0.763868934858005,2.49056758607195,0
.30670476044489,0.759068106006984,NA
"16089","SPATA9",0.925801222774872,0.555880726233655,1.5746634979172,0
.35301556616313,0.724076770774702,NA
"16090","AC008840.1",0.139138330553998,-0.0338344975521838,3.342323348
20666,-0.0101230473617515,0.991923114749952,NA
"16091","LINC01554",1.34763083062728,3.06745358329985,1.51065207309912
,2.03054934880335,0.042300730313473,NA
"16092","AC008592.5",0.102366282257309,-0.49131726092088,3.34845351515
744,-0.146729604785264,0.883345450266286,NA
"16093","PCSK1",0.175609175873806,0.247512349757584,3.30210845793575,0
.0749558510601773,0.940249838776562,NA
"16094","AC020900.1",0.0741298089160557,-1.76673671430743,3.3430969623
6258,-0.528473069790613,0.597171033704837,NA
"16095","AC008906.1",1.22612025391986,1.1440391726001,1.53767121713992
,0.744007665519048,0.456871837630043,NA
"16096","AC008906.2",0,NA,NA,NA,NA,NA
"16097","AC009126.1",0.730883365652744,0.727646159019324,1.64646791521
122,0.441943722253449,0.658529930769104,NA
"16098","LIX1-AS1",0,NA,NA,NA,NA,NA
"16099","LIX1",0,NA,NA,NA,NA,NA
"16100","AC008883.3",1.03407549194907,1.45972588003887,1.6076832546164
5,0.907968578914584,0.363894822773742,NA
"16101","LINC01340",0.245258217048015,-1.32928540753819,2.917556188781
02,-0.455616043540048,0.648666104227452,NA
"16102","LINC02234",0.0367720482966892,-0.560503011376585,3.3495200143
7583,-0.167338307868279,0.867103863749685,NA
"16103","RGMB-
AS1",0.0735440965933784,-0.185659846583073,3.34418846206699,-0.0555171
601986567,0.955726459171656,NA
"16104","AC008522.1",0.459017300503899,1.59668131033552,2.207641583077
41,0.723252054398149,0.469525043331563,NA
"16105","LINC02062",1.04688363857136,-0.773203440314082,1.650376525981
15,-0.468501234804229,0.639426188165659,NA
"16106","AC113385.1",0,NA,NA,NA,NA,NA
"16107","AC021086.1",0.346668202458428,-1.94732862277873,3.00338753971
666,-0.648377406187962,0.516740879961632,NA
"16108","ST8SIA4",0.9356426818133,0.306270417936807,1.44667204173823,0
.211706875574102,0.832335723753086,NA
"16109","SLCO4C1",0.979790131183725,1.34606071402487,1.74146784889551,
0.772946060921294,0.439554303431798,NA
"16110","SLCO6A1",0.100451492077291,-0.0338344573480013,3.342323347733
69,-0.010123035334371,0.991923124345921,NA
"16111","AC024587.2",0.0367720482966892,-0.560503011376585,3.349520014
37583,-0.167338307868279,0.867103863749685,NA
"16112","AC012603.1",0.100451492077291,-0.0338344573480013,3.342323347

73369,-0.010123035334371,0.991923124345921,NA
"16113","CAMK4",0.66284098186248,-0.414923810414891,2.06222189632338,-0.201202310553794,0.84054038487774,NA
"16114","STARD4",1.27210064514379,0.0710581788245259,1.64798489565951,0.0431182221461375,0.965607293635224,NA
"16115","AC104126.1",0,NA,NA,NA,NA,NA
"16116","AC079465.1",0,NA,NA,NA,NA,NA
"16117","TRIM36",0,NA,NA,NA,NA,NA
"16118","AC008494.3",1.09942363326626,0.249697259512692,1.32676653147282,0.18819984796836,0.850719991662422,NA
"16119","LINCADL",0.110316144890068,0.0897481123376931,3.34029447074234,0.0268683234738127,0.978564758597723,NA
"16120","ARL14EPL",0.479967108120365,-0.652752146076172,1.84937444539975,-0.352958346374834,0.724119668059795,NA
"16121","SEMA6A-AS1",0.239288619654408,0.726512763451421,2.6183991094885,0.277464486150602,0.781423471618519,NA
"16122","SEMA6A-AS2",0.2028177743346,0.512115644193803,2.71757760068597,0.188445637785848,0.850527326709045,NA
"16123","LINC02147",0,NA,NA,NA,NA,NA
"16124","AC008629.1",0.213268139470054,-0.691656524248949,2.68459372570788,-0.257639179301356,0.796685389475746,NA
"16125","AC114284.1",0,NA,NA,NA,NA,NA
"16126","AC010255.1",0.346668202458428,-1.94732862277873,3.00338753971666,-0.648377406187962,0.516740879961632,NA
"16127","ZNF474",0,NA,NA,NA,NA,NA
"16128","AC010255.2",0.0636794437806017,-0.49131726092088,3.34845351515744,-0.146729604785264,0.883345450266286,NA
"16129","AC113349.2",0.771289691218517,-0.737575966870104,1.60534889473083,-0.459449014037396,0.645911757628658,NA
"16130","LINC02201",0,NA,NA,NA,NA,NA
"16131","AC106786.1",0.898554974435112,1.1354213120362,1.61637979980457,0.702447105670014,0.482400377395617,NA
"16132","LINC01170",0,NA,NA,NA,NA,NA
"16133","AC093535.1",0,NA,NA,NA,NA,NA
"16134","AC011416.3",0.589808706738178,0.00328920541581905,2.09527234191916,0.00156982238061058,0.998747463473755,NA
"16135","MEGF10",0.0367720482966892,-0.560503011376585,3.34952001437583,-0.167338307868279,0.867103863749685,NA
"16136","FBN2",0,NA,NA,NA,NA,NA
"16137","MEIKIN",0,NA,NA,NA,NA,NA
"16138","ACSL6",0.443005022897217,-1.454952451486,2.48495927977389,-0.585503538560351,0.558209187981723,NA
"16139","CSF2",0.593038471328446,-3.54908917470203,2.50705948925658,-1.41563819682414,0.156881461864222,NA
"16140","AC116366.2",0,NA,NA,NA,NA,NA
"16141","AC116366.1",0.249738984789862,-0.445595840703958,2.59717004458552,-0.171569759797946,0.863775783061595,NA
"16142","IL13",0.0636794437806017,-0.49131726092088,3.34845351515744,-

0.146729604785264,0.883345450266286,NA
"16143","IL4",0.0741298089160557,-1.76673671430743,3.34309696236258,-0
.528473069790613,0.597171033704837,NA
"16144","CCNI2",0.0636794437806017,-0.49131726092088,3.34845351515744,
-0.146729604785264,0.883345450266286,NA
"16145","GDF9",0.0741298089160557,-1.76673671430743,3.34309696236258,-
0.528473069790613,0.597171033704837,NA
"16146","AC010240.3",0,NA,NA,NA,NA,NA
"16147","AC113410.4",0.368913445792797,0.12177558220634,2.075635828735
16,0.0586690499944525,0.953215711385443,NA
"16148","AC104109.2",0,NA,NA,NA,NA,NA
"16149","AC109454.2",0.986290744859332,-0.217834545532166,1.3994891570
4524,-0.155652899799583,0.876306625056021,NA
"16150","LINC01843",0.586760978601707,-1.80705765983095,2.106090930662
78,-0.858015023720879,0.390884167617079,NA
"16151","CATSPER3",0.487265783371313,-1.64064677236868,1.9799875017399
6,-0.828614711419605,0.407322460726528,NA
"16152","PITX1",0.873091799333699,0.889129804280967,1.64375427565962,0
.540914063280029,0.588566817120445,NA
"16153","C5orf66",0.387548237486519,-0.408455828168204,2.1129050769419
8,-0.193314802745122,0.846712434070688,NA
"16154","AC026691.1",0.56236952082661,-0.410391542406868,1.76762200428
619,-0.232171551050924,0.816404773052515,NA
"16155","DCANP1",0,NA,NA,NA,NA,NA
"16156","TIFAB",0,NA,NA,NA,NA,NA
"16157","AC034206.1",0,NA,NA,NA,NA,NA
"16158","SLC25A48",0.870788792937117,-1.27618063163971,1.6536437191888
1,-0.771738565466654,0.440269284396621,NA
"16159","SLC25A48-AS1",0,NA,NA,NA,NA,NA
"16160","AC114296.1",1.02489209009029,-0.616032774141882,1.74967834489
581,-0.352083442044638,0.724775685695617,NA
"16161","AC002428.2",0,NA,NA,NA,NA,NA
"16162","MYOT",0,NA,NA,NA,NA,NA
"16163","PKD2L2",0.333470475353448,0.961753938299967,2.36516696187672,
0.406632577658209,0.684277866430116,NA
"16164","KIF20A",0,NA,NA,NA,NA,NA
"16165","CDC25C",0,NA,NA,NA,NA,NA
"16166","AC104116.1",0,NA,NA,NA,NA,NA
"16167","REEP2",0.893235786806595,-1.07073815484881,1.43123652913304,-
0.748121035939044,0.454387154662315,NA
"16168","AC034243.1",0.286226523740755,1.02083909497203,2.805216817595
13,0.363907377343895,0.715927175510104,NA
"16169","LRRTM2",1.12607465216092,1.46382365073531,1.64643713995782,0.
889085659700803,0.373957045238136,NA
"16170","AC011405.1",0.486225898964693,1.70255919977209,1.901184314444
13,0.895525587307345,0.370506194017731,NA
"16171","SNHG4",1.14216417688995,-0.390692518306874,1.27277000189841,-
0.306962387331674,0.75887200152535,NA
"16172","AC135457.1",0.110901857212745,-1.2169087618042,3.340133369026
82,-0.36432939267894,0.715612053721651,NA

"16173","SLC23A1",0.0741298089160557,-1.76673671430743,3.3430969623625
8,-0.528473069790613,0.597171033704837,NA
"16174","MZB1",1.23235136428351,0.693241797781419,1.43751150812655,0.4
82251302937318,0.629627436165581,NA
"16175","PROB1",0.377288777352252,1.21524412320682,2.63990646084813,0.
460336054034427,0.645275026087781,NA
"16176","SPATA24",0.983302388807565,1.0003986984893,1.40983137957269,0
.709587481867881,0.477959984617429,NA
"16177","ECSCR",0.174581300993347,-0.691169037761009,2.86286045881777,
-0.241426030958711,0.809224939549254,NA
"16178","SMIM33",0.19433214479945,-0.0333008172506769,3.16142997448539
,-0.0105334666652225,0.991595664992027,NA
"16179","AC113361.1",0,NA,NA,NA,NA,NA
"16180","PSD2",0.0732428936164972,-0.49131726092088,3.34845351515744,-
0.146729604785264,0.883345450266286,NA
"16181","NRG2",0.564574668449587,1.78724841384001,2.06460633656391,0.8
65660626041909,0.386676298274566,NA
"16182","SLC4A9",0.0367720482966892,-0.560503011376585,3.3495200143758
3,-0.167338307868279,0.867103863749685,NA
"16183","AC008438.1",0,NA,NA,NA,NA,NA
"16184","DND1",0.359311764145266,-1.24452834018846,2.3561397552602,-0.
528206502780656,0.59735601650281,NA
"16185","PCDHA3",0.636739741765339,2.02740964397481,1.75931341064908,1
.15238685256586,0.24916214116659,NA
"16186","PCDHA4",0.578104748267151,-0.180600602845237,1.72158947317208
,-0.104903407960828,0.916452454730232,NA
"16187","PCDHA5",0.26891496180721,-1.3277650763353,2.82969623342791,-0
.469225304345422,0.63890860009957,NA
"16188","PCDHA7",0.35718794914122,-0.618669716037269,2.34864996752757,
-0.263415036123303,0.792230702576974,NA
"16189","PCDHA9",0.139138330553998,-0.0338344975521838,3.3423233482066
6,-0.0101230473617515,0.991923114749952,NA
"16190","PCDHA10",0.560892638775378,-0.336572841678827,1.7722274890149
3,-0.189915145637373,0.849375623958259,NA
"16191","PCDHA11",0.320717103821412,-0.846710013286843,2.3833419860624
7,-0.35526165285482,0.722393580711595,NA
"16192","PCDHA12",0.201488696477259,-0.508277411998464,3.1632754567892
4,-0.160680730762022,0.872344866840293,NA
"16193","PCDHA13",0,NA,NA,NA,NA,NA
"16194","PCDHAC1",0,NA,NA,NA,NA,NA
"16195","AC244517.5",0.183559038506565,0.529394638499161,3.22699253814
936,0.164052018168831,0.869690193908075,NA
"16196","AC244517.1",0.798484912415962,0.133944322812225,1.61639135859
166,0.0828662700405233,0.933957874375381,NA
"16197","PCDHB8",0.343870895068617,1.1594692588655,2.70210858682761,0.
429097951325032,0.667851943119762,NA
"16198","SLC25A2",0,NA,NA,NA,NA,NA
"16199","AC005618.4",0.379313865507965,0.344076933198593,2.08729762576
917,0.164843254239701,0.869067357760439,NA
"16200","AC005618.1",0.449034484715737,-1.31903840108013,2.44559733198

216,-0.539352240792252,0.589643828608869,NA
"16201","PCDHGB1",0.110316144890068,0.0897481123376931,3.3402944707423
4,0.0268683234738127,0.978564758597723,NA
"16202","PCDHGA4",0.489914519743828,0.0238794503936408,2.0021647161426
1,0.0119268161111376,0.990484003172671,NA
"16203","PCDHGB3",0.755224783574828,0.252165741780809,1.55554114985335
,0.162108049539276,0.871220767427114,NA
"16204","PCDHGA7",1.05133633431667,2.73668040346611,1.47670048766849,1
.85323999437892,0.0638479675723308,NA
"16205","PCDHGB4",0.110014941913186,-0.033834469553869,3.3423233478772
8,-0.0101230389858472,0.991923121432614,NA
"16206","PCDHGB5",1.08288424933685,1.06120323339748,1.46757822377459,0
.723098241855945,0.469619529371872,NA
"16207","PCDHGA11",0.416085913804654,0.519041316743244,2.0061371489160
1,0.258726736117568,0.795846089327329,NA
"16208","AC008781.3",0.102366282257309,-0.49131726092088,3.34845351515
744,-0.146729604785264,0.883345450266286,NA
"16209","PCDHGC4",0.305184056591909,1.0238829631905,2.42729878959865,0
.42181991256206,0.673156470155355,NA
"16210","PCDHGC5",0.102366282257309,-0.49131726092088,3.34845351515744
,-0.146729604785264,0.883345450266286,NA
"16211","AC008781.2",0,NA,NA,NA,NA,NA
"16212","RELL2",0.945689116202607,-0.218914829559114,1.3666732148917,-
0.160180815116408,0.872738641570946,NA
"16213","ARAP3",0.410347646239029,-1.32074922904993,2.50843327198929,-
0.526523565046849,0.598524483393891,NA
"16214","AC005753.3",0,NA,NA,NA,NA,NA
"16215","PCDH1",0.110901857212745,-1.2169087618042,3.34013336902682,-0
.36432939267894,0.715612053721651,NA
"16216","PCDH12",0.0741298089160557,-1.76673671430743,3.34309696236258
,-0.528473069790613,0.597171033704837,NA
"16217","AC005740.4",0.517937967340121,1.58740079885281,1.958528054827
07,0.810507051425909,0.417648813974657,NA
"16218","FGF1",0.204732564514618,0.248608670877116,3.33613854356166,0.
0745198880774602,0.940596716765987,NA
"16219","ARHGAP26-AS1",0,NA,NA,NA,NA,NA
"16220","ARHGAP26-IT1",0,NA,NA,NA,NA,NA
"16221","HMHB1",0,NA,NA,NA,NA,NA
"16222","AC132803.1",0,NA,NA,NA,NA,NA
"16223","AC137770.1",0.693336404916855,-3.03393243374782,2.49756523051
135,-1.21475603387009,0.224459154109302,NA
"16224","AC008700.1",0,NA,NA,NA,NA,NA
"16225","SH3RF2",0.389250001921577,-0.440140233367592,2.67442347393031
,-0.164573874578197,0.869279395855967,NA
"16226","PLAC8L1",0.176496091173365,-1.14849506633421,3.26459492750587
,-0.351803238024283,0.724985830023263,NA
"16227","POU4F3",0.0741298089160557,-1.76673671430743,3.34309696236258
,-0.528473069790613,0.597171033704837,NA
"16228","PPP2R2B",0.248852069490303,0.780083555267702,2.96292468503169
,0.263281601185674,0.792333539707393,NA

"16229","JAKMIP2",0.484477455155085,-1.83301316746489,2.13697299351441
,-0.857761503317066,0.391024171032547,NA
"16230","AC011370.1",0,NA,NA,NA,NA,NA
"16231","SCGB3A2",0,NA,NA,NA,NA,NA
"16232","SPINK6",0,NA,NA,NA,NA,NA
"16233","SPINK9",0.494927820290539,-2.84339218695741,2.30196208233711,
-1.2352037458717,0.216754685526326,NA
"16234","AC114939.1",0.332594405587812,-0.658132278841225,2.3847426066
9479,-0.27597623198144,0.782566321704692,NA
"16235","ADRB2",0.876855077051191,-1.59896315920462,1.48363110125935,-
1.07773634419458,0.281151434269656,NA
"16236","SH3TC2",0,NA,NA,NA,NA,NA
"16237","AC012613.2",0.102366282257309,-0.49131726092088,3.34845351515
744,-0.146729604785264,0.883345450266286,NA
"16238","CARMN",0.68999137955306,-0.858020681613839,1.62984033220208,-
0.526444624458744,0.598579317447457,NA
"16239","PPARGC1B",1.03702295417242,-2.0690809146913,1.57650575480263,
-1.31244742265488,0.189369215019084,NA
"16240","PDE6A",0.0741298089160557,-1.76673671430743,3.34309696236258,
-0.528473069790613,0.597171033704837,NA
"16241","CSF1R",1.10755078905468,-0.918328391314986,1.46875580342939,-
0.625242391669727,0.531811983178562,NA
"16242","CDX1",0.360942044979488,-0.457661975100024,2.33474335741298,-
0.196022390918006,0.844592640550718,NA
"16243","SLC6A7",0.148259617832111,-2.17639404086898,3.335076015408,-0
.652577041966681,0.514028990213041,NA
"16244","CAMK2A",0.549988930305018,-2.20911919048621,2.11123153428697,
-1.04636519235788,0.295392456866928,NA
"16245","NDST1-
AS1",1.12624223695159,0.883610210675794,1.6589759216245,0.532623891135
53,0.594293960538899,NA
"16246","SYNPO",0.926009850430066,-0.742761546745857,1.50564395431451,
-0.493318187621601,0.621787779294224,NA
"16247","MYOZ3",0.329252678776848,-1.49385501129296,2.38810932116682,-
0.625538788384727,0.531617499010726,NA
"16248","IRGM",0.0741298089160557,-1.76673671430743,3.34309696236258,-
0.528473069790613,0.597171033704837,NA
"16249","AC011337.1",0.751622984452944,1.07446178821609,2.058364919521
19,0.521997716744041,0.601671920323481,NA
"16250","AC011374.1",0.147673905509434,-0.842065770106804,3.3347867762
1422,-0.252509628535456,0.800647172695484,NA
"16251","CLMAT3",0.166045726037911,0.183394512381981,3.33779889015228,
0.0549447460489789,0.956182483505573,NA
"16252","AC091982.3",0.606610016424617,1.90935095832213,2.050444434976
05,0.931188831919962,0.351755893864898,NA
"16253","LINC01933",0,NA,NA,NA,NA,NA
"16254","NMUR2",0,NA,NA,NA,NA,NA
"16255","AC008625.1",0.0741298089160557,-1.76673671430743,3.3430969623
6258,-0.528473069790613,0.597171033704837,NA
"16256","PPP1R2B",0.0636794437806017,-0.49131726092088,3.3484535151574

4,-0.146729604785264,0.883345450266286,NA
"16257","TIMD4",0.0741298089160557,-1.76673671430743,3.34309696236258,
-0.528473069790613,0.597171033704837,NA
"16258","HAVCR1",0,NA,NA,NA,NA,NA
"16259","HAVCR2",0.728584613685158,0.361934530267251,1.64022956392691,
0.220660899076063,0.825356478989446,NA
"16260","ITK",1.1430984474258,0.870257071930921,1.51468780753636,0.574
545505417645,0.565598716344425,NA
"16261","AC010609.1",0,NA,NA,NA,NA,NA
"16262","AC016571.1",0,NA,NA,NA,NA,NA
"16263","AC008676.1",0.0367720482966892,-0.560503011376585,3.349520014
37583,-0.167338307868279,0.867103863749685,NA
"16264","NIPAL4",0.166548679549901,-1.95393031097672,3.33920037562282,
-0.585149164824313,0.558447422910535,NA
"16265","AC106801.1",0,NA,NA,NA,NA,NA
"16266","SOX30",0.315120193005521,0.245400022010969,3.2393007694085,0.
0757570968180826,0.939612349767913,NA
"16267","AC025437.4",0,NA,NA,NA,NA,NA
"16268","AC025437.2",0,NA,NA,NA,NA,NA
"16269","AC091979.2",0.698909490104308,0.978972665201945,1.82597330552
07,0.536137446392065,0.591863561764118,NA
"16270","AC136424.2",0.333169272376567,0.908589184944016,2.42247183547
103,0.375066975656849,0.707610656801772,NA
"16271","AC136424.1",0,NA,NA,NA,NA,NA
"16272","AC008691.1",0.293907567111104,-0.687518690041658,2.7928624230
7872,-0.246169909538104,0.805550707638064,NA
"16273","AC008703.1",0,NA,NA,NA,NA,NA
"16274","CCNJL",0.740978184301266,-0.154637782468743,1.68232347011982,
-0.0919191732239988,0.926762257921432,NA
"16275","MIR3142HG",0.175609175873806,0.247512349757584,3.302108457935
75,0.0749558510601773,0.940249838776562,NA
"16276","GABRB2",0.638403274388762,1.03436891356428,1.72452527673885,0
.599799218669796,0.548640054117621,NA
"16277","GABRG2",0,NA,NA,NA,NA,NA
"16278","HMMR",0.332594405587812,-0.658132278841225,2.38474260669479,-
0.27597623198144,0.782566321704692,NA
"16279","AC109466.1",0.102366282257309,-0.49131726092088,3.34845351515
744,-0.146729604785264,0.883345450266286,NA
"16280","TENM2",0,NA,NA,NA,NA,NA
"16281","WWC1",0,NA,NA,NA,NA,NA
"16282","AC011365.2",0.284295039929652,-0.0383820061561763,2.509362676
63567,-0.0152955196606477,0.987796416859156,NA
"16283","AC011389.1",0.175609175873806,0.247512349757584,3.30210845793
575,0.0749558510601773,0.940249838776562,NA
"16284","INSYN2B",0,NA,NA,NA,NA,NA
"16285","LINC01366",0.787816103114314,1.25666392586735,1.5861230516847
4,0.792286528168516,0.428193625088343,NA
"16286","AC034199.1",0,NA,NA,NA,NA,NA
"16287","KCNIP1",0.0636794437806017,-0.49131726092088,3.34845351515744
,-0.146729604785264,0.883345450266286,NA

"16288","KCNMB1",0.690285308241022,-1.96443912467929,1.83782583168906,
-1.06889297712932,0.285117896835469,NA
"16289","AC027306.1",0,NA,NA,NA,NA,NA
"16290","AC008619.1",0,NA,NA,NA,NA,NA
"16291","GABRP",0,NA,NA,NA,NA,NA
"16292","NEURL1B",0.146485787232994,0.248608662259703,3.33613854377696
,0.0745198854896011,0.940596718825075,NA
"16293","AC022217.3",0,NA,NA,NA,NA,NA
"16294","AC008429.1",0.388363086622019,1.03128946935905,2.649486690202
21,0.389241234225767,0.697097703323401,NA
"16295","AC008378.1",0.102366282257309,-0.49131726092088,3.34845351515
744,-0.146729604785264,0.883345450266286,NA
"16296","STC2",0,NA,NA,NA,NA,NA
"16297","LINC01484",0,NA,NA,NA,NA,NA
"16298","LINC01485",0,NA,NA,NA,NA,NA
"16299","FAM153B",0,NA,NA,NA,NA,NA
"16300","AC138956.2",0,NA,NA,NA,NA,NA
"16301","GPRIN1",0.474059403747572,-2.50656086699194,2.12839038554275,
-1.17767909685081,0.238924563324123,NA
"16302","HK3",0.314808297382012,-2.74994603383303,2.64513346523022,-1.
0396246805617,0.298514305856315,NA
"16303","ZNF346-IT1",0,NA,NA,NA,NA,NA
"16304","FGFR4",0,NA,NA,NA,NA,NA
"16305","AC146507.3",1.29244833282165,0.767096200867831,1.431880285127
14,0.535726491128912,0.592147592218049,NA
"16306","F12",0.547269983636128,-0.224588255521411,2.13136262294802,-0
.105373085322651,0.916079771948164,NA
"16307","PRR7-
AS1",0.157560096502761,-0.491317252697356,3.34845351503297,-0.14672960
2334801,0.883345452200538,NA
"16308","PRR7",0.320180172926782,1.1465069317774,2.3818635835708,0.481
348696745511,0.630268692547473,NA
"16309","DOK3",0.0636794437806017,-0.49131726092088,3.34845351515744,-
0.146729604785264,0.883345450266286,NA
"16310","AC139795.3",1.3121223243688,-0.396067488174509,1.428377765451
34,-0.277284831614107,0.781561405722056,NA
"16311","AC139795.2",0.350408921033347,-0.237495051401567,2.6669933487
2584,-0.0890497351690174,0.929042384138531,NA
"16312","FAM153A",0.0741298089160557,-1.76673671430743,3.3430969623625
8,-0.528473069790613,0.597171033704837,NA
"16313","AC106795.2",0.0741298089160557,-1.76673671430743,3.3430969623
6258,-0.528473069790613,0.597171033704837,NA
"16314","N4BP3",1.07367204233085,0.859655885484139,1.39888258730708,0.
614530406829225,0.538864886302172,NA
"16315","COL23A1",0.157560096502761,-0.491317252697356,3.3484535150329
7,-0.146729602334801,0.883345452200538,NA
"16316","AC104117.3",0.0636794437806017,-0.49131726092088,3.3484535151
5744,-0.146729604785264,0.883345450266286,NA
"16317","GRM6",0,NA,NA,NA,NA,NA
"16318","AC136604.2",0.166548679549901,-1.95393031097672,3.33920037562

282,-0.585149164824313,0.558447422910535,NA
"16319","AC136604.3",0,NA,NA,NA,NA,NA
"16320","AC010285.3",0,NA,NA,NA,NA,NA
"16321","AC008610.1",0.213268139470054,-0.691656524248949,2.6845937257
0788,-0.257639179301356,0.796685389475746,NA
"16322","SCGB3A1",0,NA,NA,NA,NA,NA
"16323","FLT4",0,NA,NA,NA,NA,NA
"16324","AC022413.2",0,NA,NA,NA,NA,NA
"16325","BTNL8",0.102366282257309,-0.49131726092088,3.34845351515744,-
0.146729604785264,0.883345450266286,NA
"16326","BTNL9",1.0623651773869,-2.14296153990783,1.6348922671193,-1.3
1076620949694,0.189936761948993,NA
"16327","LINC01962",0,NA,NA,NA,NA,NA
"16328","AC008443.6",0.0367720482966892,-0.560503011376585,3.349520014
37583,-0.167338307868279,0.867103863749685,NA
"16329","AC008443.8",1.17145049296815,2.92476448718169,1.7666934275904
3,1.65550199118063,0.0978227128525834,NA
"16330","AC138035.1",0.0732428936164972,-0.49131726092088,3.3484535151
5744,-0.146729604785264,0.883345450266286,NA
"16331","AL365272.1",0.914342083642808,0.3867455236075,1.5567185270875
7,0.248436385176872,0.803796784510588,NA
"16332","AL031770.1",0.0732428936164972,-0.49131726092088,3.3484535151
5744,-0.146729604785264,0.883345450266286,NA
"16333","HUS1B",0.173694385693788,0.450698267773282,2.85372304708896,0
.15793342953621,0.874509255668894,NA
"16334","AL357054.2",0,NA,NA,NA,NA,NA
"16335","LINC01394",0,NA,NA,NA,NA,NA
"16336","AL034346.1",0,NA,NA,NA,NA,NA
"16337","AL512329.2",0.9866069858735,0.534218836804054,1.4908854258779
2,0.358323200114103,0.720101458044286,NA
"16338","AL031768.2",0.307098846771927,1.01615615877409,3.257049221209
72,0.311986737000149,0.755050602967235,NA
"16339","AL031768.1",0.181578773267413,-1.95393031097672,3.33920037562
282,-0.585149164824313,0.558447422910535,NA
"16340","LINC01600",0.35616007426076,-1.35549659837783,2.1075333522812
,-0.643167329670316,0.520115529757457,NA
"16341","MYLK4",0.469950650192765,-0.190361419678693,1.99636588536408,
-0.0953539734746451,0.924033673278506,NA
"16342","AL133351.1",0.35783913652303,0.365859147993896,2.059710929392
24,0.17762645368007,0.859016347873581,NA
"16343","AL031963.2",0,NA,NA,NA,NA,NA
"16344","AL031963.1",0.0732428936164972,-0.49131726092088,3.3484535151
5744,-0.146729604785264,0.883345450266286,NA
"16345","AL445309.1",0.304932799035314,-0.455323059556486,2.4740235505
9449,-0.184041521935826,0.853980881434662,NA
"16346","AL033523.1",0.164130935857893,0.400527358383716,3.33018851502
426,0.120271677286953,0.904267939474822,NA
"16347","AL590004.3",0.840776019192951,-2.6700474204422,1.728596023757
6,-1.54463355448318,0.122434925161597,NA
"16348","AL138831.2",0,NA,NA,NA,NA,NA

"16349","AL136309.2",0,NA,NA,NA,NA,NA
"16350","AL159166.1",0,NA,NA,NA,NA,NA
"16351","AL034376.1",0.0367720482966892,-0.560503011376585,3.34952001437583,-0.167338307868279,0.867103863749685,NA
"16352","AL359643.2",0.181578773267413,-1.95393031097672,3.33920037562282,-0.585149164824313,0.558447422910535,NA
"16353","AL022097.1",0,NA,NA,NA,NA,NA
"16354","LY86-AS1",0.157560096502761,-0.491317252697356,3.34845351503297,-0.146729602334801,0.883345452200538,NA
"16355","LY86",1.13520937574725,-0.915003399499482,1.4886193136807,-0.614665812199549,0.538775441987329,NA
"16356","AL031123.2",0.0741298089160557,-1.76673671430743,3.34309696236258,-0.528473069790613,0.597171033704837,NA
"16357","AL031123.1",0.873857724010936,-1.75153018599546,1.50031450973439,-1.16744200941278,0.243031914377877,NA
"16358","AL031123.5",0.102366282257309,-0.49131726092088,3.34845351515744,-0.146729604785264,0.883345450266286,NA
"16359","AL355336.1",0.488441892121593,1.69208569905443,1.86924825828432,0.905222562896759,0.365347490921305,NA
"16360","CAGE1",0,NA,NA,NA,NA,NA
"16361","DSP",0,NA,NA,NA,NA,NA
"16362","BMP6",0.102366282257309,-0.49131726092088,3.34845351515744,-0.146729604785264,0.883345450266286,NA
"16363","AL355499.1",0.0636794437806017,-0.49131726092088,3.34845351515744,-0.146729604785264,0.883345450266286,NA
"16364","TFAP2A",0.56788620456124,-0.904326179501734,2.11372307093889,-0.427835695193526,0.668770746971703,NA
"16365","TFAP2A-AS2",0.397351669669159,-0.620585281597984,2.30006896523181,-0.26981159738201,0.787305200194518,NA
"16366","TFAP2A-AS1",0,NA,NA,NA,NA,NA
"16367","GCNT2",0,NA,NA,NA,NA,NA
"16368","AL358777.1",0,NA,NA,NA,NA,NA
"16369","C6orf52",1.10615819630072,-0.293608504457005,1.42370976947197,-0.206227779532551,0.836612989283125,NA
"16370","AL024498.1",0.0732428936164972,-0.49131726092088,3.34845351515744,-0.146729604785264,0.883345450266286,NA
"16371","MAK",0.245258217048015,-1.32928540753819,2.91755618878102,-0.455616043540048,0.648666104227452,NA
"16372","GCM2",0,NA,NA,NA,NA,NA
"16373","AL357497.1",0.678320512426694,1.40668154255209,1.73434444803439,0.811073915649423,0.417323224323724,NA
"16374","SYCP2L",0.459619706457662,1.66297134018077,1.89126930185179,0.879288496118723,0.379244871747527,NA
"16375","ELOVL2",1.44416456511262,2.4760883612602,1.33740086032759,1.85141825066099,0.0641094054784577,NA
"16376","ELOVL2-AS1",0,NA,NA,NA,NA,NA
"16377","ERVFRD-1",0,NA,NA,NA,NA,NA
"16378","AL022098.1",0.200902984154582,0.617734931975277,3.11356519488

492,0.198401155366881,0.842731213560209,NA
"16379","AL022724.3",0,NA,NA,NA,NA,NA
"16380","AL157373.2",0,NA,NA,NA,NA,NA
"16381","AL008729.1",0.249153272467185,0.812295408448132,2.54252578312747,0.31948364647416,0.7493597903733,NA
"16382","AL008729.2",0.383440250755117,-1.38194811510753,2.56276654272887,-0.53924073537967,0.589720755955717,NA
"16383","GFOD1",0.878327551786133,0.454147376388525,1.79671911163451,0.252764816407712,0.800449957422267,NA
"16384","AL441883.1",0.857217949578491,-1.59732646757231,1.61062382837268,-0.991743968662252,0.32132243666559,NA
"16385","Z93020.1",0,NA,NA,NA,NA,NA
"16386","AL023583.1",0.527059254618235,-0.22159230441492,2.22451514615702,-0.0996137539444199,0.920650974071801,NA
"16387","AL133259.1",0,NA,NA,NA,NA,NA
"16388","AL138720.1",0.0741298089160557,-1.76673671430743,3.34309696236258,-0.528473069790613,0.597171033704837,NA
"16389","AL136162.1",0,NA,NA,NA,NA,NA
"16390","JARID2-AS1",0,NA,NA,NA,NA,NA
"16391","AL021978.1",0,NA,NA,NA,NA,NA
"16392","AL137003.1",0,NA,NA,NA,NA,NA
"16393","RBM24",0.181578773267413,-1.95393031097672,3.33920037562282,-0.585149164824313,0.558447422910535,NA
"16394","CAP2",1.05870917778518,-0.584067641880837,1.5342121464989,-0.380695488048174,0.703429217233842,NA
"16395","AL138824.1",0.469063734893206,0.614695798253097,1.82376653477466,0.337047416175474,0.736081156204608,NA
"16396","AL022068.1",0.49491444302719,-1.28398319070718,1.96637317447474,-0.652970253751633,0.513775455617391,NA
"16397","AL132775.1",0,NA,NA,NA,NA,NA
"16398","E2F3-
IT1",0.0732428936164972,-0.49131726092088,3.34845351515744,-0.146729604785264,0.883345450266286,NA
"16399","LINC00581",0.0735440965933784,-0.185659846583073,3.34418846206699,-0.0555171601986567,0.955726459171656,NA
"16400","AL512380.1",0,NA,NA,NA,NA,NA
"16401","AL512380.2",0,NA,NA,NA,NA,NA
"16402","CASC15",1.06120098712945,2.76183906385089,1.45195869357599,1.90214713136834,0.0571519222692471,NA
"16403","NBAT1",0.0741298089160557,-1.76673671430743,3.34309696236258,-0.528473069790613,0.597171033704837,NA
"16404","PRL",0,NA,NA,NA,NA,NA
"16405","GPLD1",1.36598995961123,-0.546795613638179,1.24272209732663,-0.439998302769747,0.659938336574203,NA
"16406","KIAA0319",0.0741298089160557,-1.76673671430743,3.34309696236258,-0.528473069790613,0.597171033704837,NA
"16407","AL031775.1",0.321368291203222,0.229925664674723,2.12751149802765,0.108072583808774,0.913938115704468,NA
"16408","AL031775.2",0.25992637876007,0.233924006470616,2.95011957287767,0.0792930593801107,0.936799526733212,NA

"16409","LINC02828",0,NA,NA,NA,NA,NA
"16410","ARMH2",0.157560096502761,-0.491317252697356,3.34845351503297,
-0.146729602334801,0.883345452200538,NA
"16411","AL022170.1",0,NA,NA,NA,NA,NA
"16412","SLC17A3",0,NA,NA,NA,NA,NA
"16413","U91328.3",0.136922337397099,0.183394507878855,3.3377988902691
,0.0549447446979257,0.956182484581931,NA
"16414","HIST1H1A",0.420798011374483,-2.43995659027234,2.4481014829805
7,-0.996672975869322,0.318923271132377,NA
"16415","HIST1H3A",0.294783636876741,0.78572505329692,2.42199479408685
,0.324412362576179,0.745625861450258,NA
"16416","HIST1H4A",0.127358887561203,0.15032497835859,3.33866494558414
,0.0450254759937551,0.964087002635848,NA
"16417","HIST1H4B",0.20332072784659,-1.39924864619322,3.10758170858468
,-0.450269301794319,0.652516271227926,NA
"16418","HIST1H3B",0.0732428936164972,-0.49131726092088,3.348453515157
44,-0.146729604785264,0.883345450266286,NA
"16419","HIST1H2AB",0.100451492077291,-0.0338344573480013,3.3423233477
3369,-0.010123035334371,0.991923124345921,NA
"16420","HIST1H2BB",0.421370346433812,-2.14315573922607,2.229236592726
06,-0.961385501305303,0.336358371562453,NA
"16421","HIST1H3C",0,NA,NA,NA,NA,NA
"16422","AL353759.1",0.0732428936164972,-0.49131726092088,3.3484535151
5744,-0.146729604785264,0.883345450266286,NA
"16423","HIST1H2BE",0.394611184819719,0.536249437905654,2.000949529821
44,0.267997483151666,0.788701256051203,NA
"16424","HIST1H4D",0.181578773267413,-1.95393031097672,3.3392003756228
2,-0.585149164824313,0.558447422910535,NA
"16425","HIST1H3D",0.425063651317872,1.50781002770324,2.17054505398985
,0.694668845934169,0.487262856261903,NA
"16426","HIST1H2AD",0,NA,NA,NA,NA,NA
"16427","HIST1H2BF",0.183257835529684,0.495279120319891,3.246926734798
,0.152537818304269,0.878762764724423,NA
"16428","HIST1H4E",1.32430184890848,0.177723723636305,1.34973130017021
,0.131673410562453,0.895242617663753,NA
"16429","HIST1H2BG",0.175910378850688,0.313649592621519,3.256047270905
7,0.0963283289601242,0.923259812444105,NA
"16430","HIST1H2AE",0,NA,NA,NA,NA,NA
"16431","HIST1H3E",0,NA,NA,NA,NA,NA
"16432","HIST1H1D",1.27427992648298,0.240588062915259,1.6855941708568,
0.14273190253913,0.886501920086029,NA
"16433","HIST1H4F",0,NA,NA,NA,NA,NA
"16434","HIST1H3F",0,NA,NA,NA,NA,NA
"16435","HIST1H2BH",0.0732428936164972,-0.49131726092088,3.34845351515
744,-0.146729604785264,0.883345450266286,NA
"16436","HIST1H3G",0,NA,NA,NA,NA,NA
"16437","HIST1H2BI",0,NA,NA,NA,NA,NA
"16438","AL513548.3",0,NA,NA,NA,NA,NA
"16439","AL513548.4",0.799580016468261,-1.01845122696069,1.81760497599
703,-0.56032594563185,0.575257133221046,NA

"16440","AL513548.1",1.3850003048553,0.802835313627099,1.29922565164691,0.617933699669045,0.536619043594775,NA
"16441","LINC00240",0.673210587085147,2.09375617462403,1.61142137425711,1.29932257823581,0.193833249044789,NA
"16442","AL133255.1",0,NA,NA,NA,NA,NA
"16443","AL021807.1",0,NA,NA,NA,NA,NA
"16444","HIST1H2BJ",1.08021617127001,-1.63075583437364,1.68866481578122,-0.965707237536781,0.334190696885445,NA
"16445","HIST1H2AG",0.529553256977667,-0.380232544145317,1.92531335763675,-0.197491251300535,0.84344312707558,NA
"16446","HIST1H4I",0.127358887561203,0.15032497835859,3.33866494558414,0.0450254759937551,0.964087002635848,NA
"16447","HIST1H2BK",1.11289880770622,1.8110535079092,1.44763455561815,1.25104329741277,0.210918681168273,NA
"16448","HIST1H2AH",0.229725169818512,0.686619208900669,3.0259364803766,0.226911309392461,0.820492707794461,NA
"16449","PRSS16",0,NA,NA,NA,NA,NA
"16450","AL031118.1",0.756378924468629,-0.174079951099796,1.50200127402814,-0.115898670733441,0.907732849198627,NA
"16451","AL021918.4",0,NA,NA,NA,NA,NA
"16452","AL021918.5",0.240175534953966,-0.492654690648343,2.62030586946942,-0.188014191926418,0.850865526027581,NA
"16453","AL009179.1",0,NA,NA,NA,NA,NA
"16454","AL009179.2",0.181578773267413,-1.95393031097672,3.33920037562282,-0.585149164824313,0.558447422910535,NA
"16455","HIST1H2BL",0.0636794437806017,-0.49131726092088,3.34845351515744,-0.146729604785264,0.883345450266286,NA
"16456","HIST1H2AI",0,NA,NA,NA,NA,NA
"16457","HIST1H3H",0.330176661895803,1.16963308605726,2.36427179681943,0.494711770292537,0.620803591863057,NA
"16458","HIST1H2AJ",0,NA,NA,NA,NA,NA
"16459","HIST1H2BM",0,NA,NA,NA,NA,NA
"16460","HIST1H2BN",1.36576301327874,1.85141175086438,1.41268329176285,1.31056391879177,0.19000513597329,NA
"16461","HIST1H2AK",0.23737382947439,0.788402968215764,2.58205902180894,0.305338863889883,0.760108079194951,NA
"16462","HIST1H2AL",0.0732428936164972,-0.49131726092088,3.34845351515744,-0.146729604785264,0.883345450266286,NA
"16463","HIST1H1B",0.0732428936164972,-0.49131726092088,3.34845351515744,-0.146729604785264,0.883345450266286,NA
"16464","HIST1H3I",0,NA,NA,NA,NA,NA
"16465","HIST1H2AM",0,NA,NA,NA,NA,NA
"16466","HIST1H2BO",0,NA,NA,NA,NA,NA
"16467","ZNF165",1.31441132175101,-1.57699490566885,1.32196256686666,-1.19291948591757,0.232900906984515,NA
"16468","AL358933.1",0.0367720482966892,-0.560503011376585,3.34952001437583,-0.167338307868279,0.867103863749685,NA
"16469","ZSCAN23",1.23832746988128,0.717005098638197,1.38232470064586,0.518695136029323,0.603973354104636,NA
"16470","Z98745.2",0,NA,NA,NA,NA,NA

"16471","HCG14",0,NA,NA,NA,NA,NA
"16472","LINC01556",0.166548679549901,-1.95393031097672,3.339200375622
82,-0.585149164824313,0.558447422910535,NA
"16473","ZNF311",0.822426166520953,-0.4299219121476,1.47559768973747,-
0.291354422101385,0.77078026901024,NA
"16474","LINC02829",0,NA,NA,NA,NA,NA
"16475","UBD",0,NA,NA,NA,NA,NA
"16476","AL645939.4",0.513134458440843,0.24772197215915,1.914632407826
25,0.129383567909204,0.897054149297952,NA
"16477","HLA-G",0,NA,NA,NA,NA,NA
"16478","AL645929.2",0.383440250755117,-1.38194811510753,2.56276654272
887,-0.53924073537967,0.589720755955717,NA
"16479","HCG9",0,NA,NA,NA,NA,NA
"16480","HCG17",0.320782578880545,1.2086714770253,2.35194331823262,0.5
13903318866357,0.607319587002391,NA
"16481","AL662873.1",0.102366282257309,-0.49131726092088,3.34845351515
744,-0.146729604785264,0.883345450266286,NA
"16482","LINC02569",0.994192764125303,-0.987425317609957,1.57654442875
772,-0.626322544165801,0.531103402248864,NA
"16483","MDC1-AS1",0,NA,NA,NA,NA,NA
"16484","AL662797.1",0.176496091173365,-1.14849506633421,3.26459492750
587,-0.351803238024283,0.724985830023263,NA
"16485","IER3-
AS1",1.04499072181509,1.69765211042726,1.47319512075576,1.152360665949
23,0.249172897282036,NA
"16486","HCG20",0.694943889145562,-0.285380991974866,1.66234010281326,
-0.171674250950153,0.863693630009993,NA
"16487","LINC00243",0,NA,NA,NA,NA,NA
"16488","DDR1",0.74842906183587,-0.375321543988061,1.48173969300029,-0
.253297894199009,0.800038023247622,NA
"16489","CDSN",0,NA,NA,NA,NA,NA
"16490","POU5F1",0.644301388181533,0.0901178682340261,1.82938583561038
,0.0492612692630572,0.960711084674426,NA
"16491","PSORS1C3",0.308937660828617,-0.6869866595118,2.75278474113728
,-0.24956061737976,0.802927157786805,NA
"16492","AL645933.3",0.102366282257309,-0.49131726092088,3.34845351515
744,-0.146729604785264,0.883345450266286,NA
"16493","ATP6V1G2",1.28088773116388,0.890453704662083,1.25245106052589
,0.710968861560301,0.47710353189029,NA
"16494","LTA",0.0636794437806017,-0.49131726092088,3.34845351515744,-0
.146729604785264,0.883345450266286,NA
"16495","TNF",1.01677304478898,-1.47508745847686,1.49456301907674,-0.9
8696906028632,0.323657806477649,NA
"16496","NCR3",0.285483158206092,-0.82351996307896,2.498184683591,-0.3
29647350929714,0.741666439733092,NA
"16497","C6orf47-
AS1",0.874915178184269,-3.71920342119683,2.0507521858061,-1.8135801326
6479,0.0697423914222069,NA
"16498","LY6G5C",0.611567945068909,-0.628255791691975,2.02021862208001
,-0.310984061242403,0.75581273856971,NA

"16499","MPIG6B",0,NA,NA,NA,NA,NA
"16500","LY6G6C",0,NA,NA,NA,NA,NA
"16501","MSH5",1.17641101116588,0.89289479571188,1.41054973152859,0.63
3011921348043,0.526725858045085,NA
"16502","SAPCD1",0.692124613899272,-1.3363774304418,1.74326520577892,-
0.766594449319422,0.443322676705234,NA
"16503","SAPCD1-AS1",0,NA,NA,NA,NA,NA
"16504","VWA7",0,NA,NA,NA,NA,NA
"16505","HSPA1L",0.864117082270708,2.51037494779809,1.51429844535962,1
.65778083936546,0.0973617100844376,NA
"16506","SLC44A4",0,NA,NA,NA,NA,NA
"16507","ZBTB12",0.0367720482966892,-0.560503011376585,3.3495200143758
3,-0.167338307868279,0.867103863749685,NA
"16508","C2-AS1",0,NA,NA,NA,NA,NA
"16509","C4A",0.204732564514618,0.248608670877116,3.33613854356166,0.0
745198880774602,0.940596716765987,NA
"16510","C4B",0.102366282257309,-0.49131726092088,3.34845351515744,-0.
146729604785264,0.883345450266286,NA
"16511","PRRT1",0.247824194609844,-0.209546153089553,2.30954823409246,
-0.0907303644913461,0.927706842755531,NA
"16512","AL662884.1",0.0732428936164972,-0.49131726092088,3.3484535151
5744,-0.146729604785264,0.883345450266286,NA
"16513","TSBP1-
AS1",1.05485805924459,-0.716599739135089,1.56358367898226,-0.458305972
854312,0.646732633594592,NA
"16514","HLA-
DQB2",0.0367720482966892,-0.560503011376585,3.34952001437583,-0.167338
307868279,0.867103863749685,NA
"16515","HLA-
DOB",0.284897445883414,0.0578870307512366,2.4766822950744,0.0233728124
379788,0.981352891611699,NA
"16516","AL645941.3",0.0636794437806017,-0.49131726092088,3.3484535151
5744,-0.146729604785264,0.883345450266286,NA
"16517","HLA-
DOA",1.32766041183181,0.211681008227268,1.50184844132541,0.14094698399
8236,0.887911824742081,NA
"16518","COL11A2",0.147673905509434,-0.842065770106804,3.3347867762142
2,-0.252509628535456,0.800647172695484,NA
"16519","AL645940.1",0.284295039929652,-0.0383820061561763,2.509362676
63567,-0.0152955196606477,0.987796416859156,NA
"16520","SYNGAP1-
AS1",0.102366282257309,-0.49131726092088,3.34845351515744,-0.146729604
785264,0.883345450266286,NA
"16521","LINC00336",0,NA,NA,NA,NA,NA
"16522","IP6K3",0,NA,NA,NA,NA,NA
"16523","AL138889.3",0.0636794437806017,-0.49131726092088,3.3484535151
5744,-0.146729604785264,0.883345450266286,NA
"16524","PACSIN1",0.0636794437806017,-0.49131726092088,3.3484535151574
4,-0.146729604785264,0.883345450266286,NA
"16525","SPDEF",0.0367720482966892,-0.560503011376585,3.34952001437583

,-0.167338307868279,0.867103863749685,NA
"16526","AL591002.1",0,NA,NA,NA,NA,NA
"16527","TCP11",0.0636794437806017,-0.49131726092088,3.34845351515744,
-0.146729604785264,0.883345450266286,NA
"16528","SCUBE3",0.102366282257309,-0.49131726092088,3.34845351515744,
-0.146729604785264,0.883345450266286,NA
"16529","Z97832.2",1.09255852997997,-1.14897871694454,1.35142735676199
,-0.850196432087544,0.395215885059876,NA
"16530","FANCE",1.1922044978312,-1.41099425810506,1.25515109740567,-1.
12416286853551,0.26094393853909,NA
"16531","TULP1",0,NA,NA,NA,NA,NA
"16532","AL033519.4",0,NA,NA,NA,NA,NA
"16533","AL033519.5",0.181578773267413,-1.95393031097672,3.33920037562
282,-0.585149164824313,0.558447422910535,NA
"16534","ARMC12",0.100451492077291,-0.0338344573480013,3.3423233477336
9,-0.010123035334371,0.991923124345921,NA
"16535","LHFPL5",0.647151875089158,0.015537902063889,1.80833951488034,
0.00859235886625921,0.993144373876753,NA
"16536","SLC26A8",0.0367720482966892,-0.560503011376585,3.349520014375
83,-0.167338307868279,0.867103863749685,NA
"16537","Z84485.1",0.916914843891995,0.375133586491293,1.5271951612450
4,0.245635656798092,0.805964284579058,NA
"16538","PNPLA1",0,NA,NA,NA,NA,NA
"16539","Z84484.1",0.0741298089160557,-1.76673671430743,3.343096962362
58,-0.528473069790613,0.597171033704837,NA
"16540","PXT1",0,NA,NA,NA,NA,NA
"16541","DINOL",0.958174926897199,-1.71054000531297,1.49907061589022,-
1.14106699656518,0.253842044063567,NA
"16542","RAB44",0.204732564514618,0.248608670877116,3.33613854356166,0
.0745198880774602,0.940596716765987,NA
"16543","CPNE5",0,NA,NA,NA,NA,NA
"16544","Z85996.3",0,NA,NA,NA,NA,NA
"16545","FGD2",0.982033757434705,-0.268587146564852,1.59067647769454,-
0.168850894780396,0.865913924769027,NA
"16546","TMEM217",0.451730634779111,-1.53364922056465,1.97887618406504
,-0.775010196653231,0.438333634380283,NA
"16547","AL096712.2",0,NA,NA,NA,NA,NA
"16548","AL353597.1",0.0741298089160557,-1.76673671430743,3.3430969623
6258,-0.528473069790613,0.597171033704837,NA
"16549","AL121574.1",0,NA,NA,NA,NA,NA
"16550","BTBD9-AS1",0,NA,NA,NA,NA,NA
"16551","DNAH8",0.102366282257309,-0.49131726092088,3.34845351515744,-
0.146729604785264,0.883345450266286,NA
"16552","KCNK17",0.157560096502761,-0.491317252697356,3.34845351503297
,-0.146729602334801,0.883345452200538,NA
"16553","KIF6",0,NA,NA,NA,NA,NA
"16554","AL590999.1",0,NA,NA,NA,NA,NA
"16555","LINC00951",0,NA,NA,NA,NA,NA
"16556","LRFN2",0,NA,NA,NA,NA,NA
"16557","AL033380.1",0,NA,NA,NA,NA,NA

"16558","UNC5CL",0.847190646116969,0.459349470477703,1.52281041927486,0.301645867839831,0.762922038197996,NA
"16559","APOBEC2",0,NA,NA,NA,NA,NA
"16560","TREM2",1.23025778061844,-1.96202326621743,1.31686513666406,-1.48991966723921,0.136245359641469,NA
"16561","TREML2",0.0741298089160557,-1.76673671430743,3.34309696236258,-0.528473069790613,0.597171033704837,NA
"16562","MDFI",0.358463080657343,1.15922610032579,2.28060712253717,0.508297149855495,0.611244968548103,NA
"16563","PRICKLE4",0.805194558976497,-1.49699299647297,1.56631088752564,-0.955744487505817,0.339201340528079,NA
"16564","PTCRA",0.166548679549901,-1.95393031097672,3.33920037562282,-0.585149164824313,0.558447422910535,NA
"16565","AL035587.1",0.278276661107997,0.9214909476517,2.82327194492209,0.326391139652375,0.744128440342072,NA
"16566","GNMT",0.420798011374483,-2.43995659027234,2.44810148298057,-0.996672975869322,0.318923271132377,NA
"16567","AL136304.1",0.333470475353448,0.961753938299967,2.36516696187672,0.406632577658209,0.684277866430116,NA
"16568","AL355385.1",0.0636794437806017,-0.49131726092088,3.34845351515744,-0.146729604785264,0.883345450266286,NA
"16569","CRIP3",0.556624805816829,1.71586907345465,2.12173245392154,0.808711329405956,0.418681209919594,NA
"16570","AL359813.1",0,NA,NA,NA,NA,NA
"16571","DLK2",0.184144750829242,-0.691295663073562,2.81313162320765,-0.245738826214366,0.805884414604706,NA
"16572","LRRC73",0.185031666128801,-1.62793589934517,3.24336249302777,-0.501928447049853,0.61571785405042,NA
"16573","AL355802.3",0.110014941913186,-0.033834469553869,3.34232334787728,-0.0101230389858472,0.991923121432614,NA
"16574","POLH-AS1",0.221239540283362,0.178834610992208,3.06205963446949,0.0584033730039328,0.953427328091912,NA
"16575","RSPH9",0.184144750829242,-0.691295663073562,2.81313162320765,-0.245738826214366,0.805884414604706,NA
"16576","AL136131.2",0,NA,NA,NA,NA,NA
"16577","AL136131.3",0.277975458131115,0.892787139500793,2.87631492378557,0.310392694526571,0.7562623489228,NA
"16578","AL109615.3",0.597294102547848,-2.24646449491057,1.99684973332026,-1.12500427920295,0.260587220951647,NA
"16579","C6orf223",0.0367720482966892,-0.560503011376585,3.34952001437583,-0.167338307868279,0.867103863749685,NA
"16580","AL109615.2",0.254821666883911,-1.3286579469836,2.88029438084422,-0.461292413657444,0.64458882703139,NA
"16581","AL109615.4",0.81231068582751,0.964529768403027,1.58744757243649,0.607597872931714,0.543454211920704,NA
"16582","CAPN11",0.22061559614905,-0.418038608046788,3.07273464003886,-0.136047741513241,0.891783539523794,NA
"16583","MYMX",0.314222585059335,-1.49515354645625,2.42031350762541,-0.617752015078063,0.536738818706479,NA

"16584","TCTE1",0.0732428936164972,-0.49131726092088,3.34845351515744,
-0.146729604785264,0.883345450266286,NA
"16585","AL096865.1",0.0367720482966892,-0.560503011376585,3.349520014
37583,-0.167338307868279,0.867103863749685,NA
"16586","CLIC5",0.212682427147377,0.619345584110054,3.06492592002866,0
.202075221480154,0.839857918651898,NA
"16587","AL035701.1",0,NA,NA,NA,NA,NA
"16588","ANKRD66",0,NA,NA,NA,NA,NA
"16589","ADGRF5",0.148259617832111,-2.17639404086898,3.335076015408,-0
.652577041966681,0.514028990213041,NA
"16590","ADGRF1",0,NA,NA,NA,NA,NA
"16591","AL355353.1",0.480268311097246,-0.54211782218741,1.80191088075
471,-0.300857177775823,0.763523405444014,NA
"16592","PTCHD4",0,NA,NA,NA,NA,NA
"16593","AL353138.1",0,NA,NA,NA,NA,NA
"16594","C6orf141",0.0741298089160557,-1.76673671430743,3.343096962362
58,-0.528473069790613,0.597171033704837,NA
"16595","RHAG",0,NA,NA,NA,NA,NA
"16596","AL132799.1",0.157560096502761,-0.491317252697356,3.3484535150
3297,-0.146729602334801,0.883345452200538,NA
"16597","IL17A",0.100451492077291,-0.0338344573480013,3.34232334773369
,-0.010123035334371,0.991923124345921,NA
"16598","IL17F",0,NA,NA,NA,NA,NA
"16599","AL034374.1",0.0741298089160557,-1.76673671430743,3.3430969623
6258,-0.528473069790613,0.597171033704837,NA
"16600","AL033397.2",0,NA,NA,NA,NA,NA
"16601","AL590652.1",0.0741298089160557,-1.76673671430743,3.3430969623
6258,-0.528473069790613,0.597171033704837,NA
"16602","AL033384.1",0.157560096502761,-0.491317252697356,3.3484535150
3297,-0.146729602334801,0.883345452200538,NA
"16603","GFRAL",0,NA,NA,NA,NA,NA
"16604","ZNF451-
AS1",0.257387644445739,-0.166363093190703,2.56504828799876,-0.06485768
47340752,0.948287312309793,NA
"16605","AL021368.5",0.602971149483336,-0.378673307211633,1.9480919544
6888,-0.194381639092017,0.845877065609819,NA
"16606","AL021368.1",0.708960863475807,0.173590143266778,2.06972765177
477,0.0838710074332372,0.933158991036506,NA
"16607","KHDRBS2",0.0636794437806017,-0.49131726092088,3.3484535151574
4,-0.146729604785264,0.883345450266286,NA
"16608","AL355347.1",0.488190634564997,0.693562944803306,1.98736294819
994,0.348986552975391,0.727099404850652,NA
"16609","AL135905.1",0.528246975725841,-2.84780569868221,2.33554953918
413,-1.21933003385448,0.222718953697351,NA
"16610","AL135905.2",0.894411895556874,1.05037044911165,1.483389493822
53,0.708088100587095,0.478890548675301,NA
"16611","EYS",0.523164293631792,-1.83363477485289,2.09884711853435,-0.
873639036717138,0.382314860296463,NA
"16612","AL646090.2",0.267876241392828,0.623390345045958,2.84586722702
171,0.219051099477453,0.826610242595754,NA

"16613","AL646090.1",0.0367720482966892,-0.560503011376585,3.349520014
37583,-0.167338307868279,0.867103863749685,NA
"16614","AL391807.1",0.276947583250656,-0.158224666587883,2.2425864293
0609,-0.0705545456443527,0.94375228752887,NA
"16615","ADGRB3",0.0741298089160557,-1.76673671430743,3.34309696236258
,-0.528473069790613,0.597171033704837,NA
"16616","COL19A1",0,NA,NA,NA,NA,NA
"16617","COL9A1",0,NA,NA,NA,NA,NA
"16618","AL365226.2",0,NA,NA,NA,NA,NA
"16619","AL583856.2",0.255708582183469,-2.44071869332471,2.85324394739
347,-0.855418862994307,0.392319304627377,NA
"16620","AL589935.1",0.230802990119258,0.237440402410633,3.03060926850
593,0.0783474151148786,0.937551701540005,NA
"16621","RIMS1",0,NA,NA,NA,NA,NA
"16622","KCNQ5",0,NA,NA,NA,NA,NA
"16623","KCNQ5-IT1",0,NA,NA,NA,NA,NA
"16624","KCNQ5-AS1",0,NA,NA,NA,NA,NA
"16625","OOEP",0,NA,NA,NA,NA,NA
"16626","DDX43",0.377589980329133,1.23398568878864,2.26050004314689,0.
545890584045636,0.585141151197391,NA
"16627","AL603910.1",0.479815303005541,-0.0795097315647441,1.962540305
80471,-0.0405136808296746,0.967683600271782,NA
"16628","AL356277.3",0.324108776052662,-1.32491523663082,2.68471272677
592,-0.49350354077619,0.62165683860654,NA
"16629","IMPG1",0.347991177341339,1.29761695082272,2.60744442995824,0.
497658525686586,0.618724745258831,NA
"16630","LINC02540",0.0367720482966892,-0.560503011376585,3.3495200143
7583,-0.167338307868279,0.867103863749685,NA
"16631","AL450327.1",0,NA,NA,NA,NA,NA
"16632","AL356776.2",1.0892538709884,0.30947696377267,1.43478056366979
,0.215696373096322,0.829224428526569,NA
"16633","LCAL1",0,NA,NA,NA,NA,NA
"16634","AL391840.1",0.127358887561203,0.15032497835859,3.338664945584
14,0.0450254759937551,0.964087002635848,NA
"16635","AL451064.1",0.533684040314584,-0.507233685761732,1.9666513560
8311,-0.257917441336408,0.796470623906511,NA
"16636","TTK",0.139138330553998,-0.0338344975521838,3.34232334820666,-
0.0101230473617515,0.991923114749952,NA
"16637","AL359715.3",0.164130935857893,0.400527358383716,3.33018851502
426,0.120271677286953,0.904267939474822,NA
"16638","AL359715.1",1.41043507271698,1.74707017250982,1.3067201189677
5,1.33698880666958,0.181226290570682,NA
"16639","AL359715.2",0.55049221906021,-0.531780950456365,1.75928305801
347,-0.302271398587125,0.762445179789401,NA
"16640","AL122017.1",1.11395083539742,0.119561207071816,1.254770768842
09,0.0952852983514651,0.924088219735273,NA
"16641","AL078599.2",0,NA,NA,NA,NA,NA
"16642","LINC02542",0.0367720482966892,-0.560503011376585,3.3495200143
7583,-0.167338307868279,0.867103863749685,NA
"16643","PRSS35",0.100451492077291,-0.0338344573480013,3.3423233477336

9,-0.010123035334371,0.991923124345921,NA
"16644","RIPPLY2",0.0732428936164972,-0.49131726092088,3.3484535151574
4,-0.146729604785264,0.883345450266286,NA
"16645","MRAP2",1.25487754298626,-0.756661174165814,2.92097528332972,-
0.259044018100444,0.795601277775453,NA
"16646","LINC01611",0.102366282257309,-0.49131726092088,3.348453515157
44,-0.146729604785264,0.883345450266286,NA
"16647","AL035694.1",0.0367720482966892,-0.560503011376585,3.349520014
37583,-0.167338307868279,0.867103863749685,NA
"16648","LINC02535",0.102366282257309,-0.49131726092088,3.348453515157
44,-0.146729604785264,0.883345450266286,NA
"16649","AL139274.2",0.693503575251267,-0.388030707855641,1.9746660195
2481,-0.196504474183952,0.844215331045033,NA
"16650","GJB7",0.19433214479945,-0.0333008172506769,3.16142997448539,-
0.0105334666652225,0.991595664992027,NA
"16651","C6orf163",1.06407417620986,1.87154660199594,1.60428186505343,
1.1665946257727,0.243374114703624,NA
"16652","CFAP206",0.348127452817314,-2.77495368732187,2.61435422383524
,-1.06142987894389,0.288494584202949,NA
"16653","CNR1",0,NA,NA,NA,NA,NA
"16654","AL353135.1",0.200601781177701,0.585305641041376,3.16043896918
792,0.185197577535177,0.853074069074656,NA
"16655","SRSF12",0.433620764453858,1.39289160892939,1.85766592346768,0
.749807374583961,0.453370726579907,NA
"16656","GABRR2",0.110014941913186,-0.033834469553869,3.34232334787728
,-0.0101230389858472,0.991923121432614,NA
"16657","AL158136.1",0,NA,NA,NA,NA,NA
"16658","AL132996.1",0.0636794437806017,-0.49131726092088,3.3484535151
5744,-0.146729604785264,0.883345450266286,NA
"16659","EPHA7",0.127358887561203,0.15032497835859,3.33866494558414,0.
0450254759937551,0.964087002635848,NA
"16660","FHL5",0,NA,NA,NA,NA,NA
"16661","GPR63",0.0636794437806017,-0.49131726092088,3.34845351515744,
-0.146729604785264,0.883345450266286,NA
"16662","KLHL32",0.0636794437806017,-0.49131726092088,3.34845351515744
,-0.146729604785264,0.883345450266286,NA
"16663","FAXC",0.581513114129486,0.662959259834617,2.08920353696585,0.
317326315078632,0.750996011840308,NA
"16664","PRDM13",0,NA,NA,NA,NA,NA
"16665","AL133338.1",0.946591561141013,0.814554010659553,1.48345726001
443,0.54909166082185,0.582942547821622,NA
"16666","GRIK2",0.102366282257309,-0.49131726092088,3.34845351515744,-
0.146729604785264,0.883345450266286,NA
"16667","AL590608.1",0,NA,NA,NA,NA,NA
"16668","BVES-
AS1",0.258011588580052,0.510354618063005,2.53828617896562,0.2010626785
47531,0.84064956422761,NA
"16669","AL133406.2",0.102366282257309,-0.49131726092088,3.34845351515
744,-0.146729604785264,0.883345450266286,NA
"16670","AL096794.1",0.295369349199418,-0.498711181181302,2.4853302102

7214,-0.200661939858163,0.840962921935508,NA
"16671","AL022067.1",0.43170597427384,1.4611282008878,1.95084655668148
,0.748971361116827,0.453874467024349,NA
"16672","LINC02526",0,NA,NA,NA,NA,NA
"16673","BEND3",0.646587700947031,1.11975734209536,1.73163360917371,0.
646647960725179,0.517859810164411,NA
"16674","SCML4",0.431082030139527,0.647039988012108,1.97537317568419,0
.327553292702783,0.743249442165126,NA
"16675","AL024507.2",0.780243419581765,2.31619083194188,2.272646993728
95,1.01915996559654,0.308127029423744,NA
"16676","Z98200.1",0.176496091173365,-1.14849506633421,3.2645949275058
7,-0.351803238024283,0.724985830023263,NA
"16677","Z98742.4",0,NA,NA,NA,NA,NA
"16678","AL139106.1",0.175910378850688,0.313649592621519,3.25604727090
57,0.0963283289601242,0.923259812444105,NA
"16679","LINC00222",0,NA,NA,NA,NA,NA
"16680","Z95118.2",0.230802990119258,0.237440402410633,3.0306092685059
3,0.0783474151148786,0.937551701540005,NA
"16681","AL109947.1",0.415701952017086,0.0145756929821296,2.0108927506
5524,0.00724836915214907,0.99421668880397,NA
"16682","AL512303.1",0,NA,NA,NA,NA,NA
"16683","METTL24",0.181578773267413,-1.95393031097672,3.33920037562282
,-0.585149164824313,0.558447422910535,NA
"16684","DDO",0.892027794004303,0.261637597099114,1.62483967772604,0.1
61023638630781,0.87207478264716,NA
"16685","SLC22A16",0.139138330553998,-0.0338344975521838,3.34232334820
666,-0.0101230473617515,0.991923114749952,NA
"16686","SLC16A10",0.147372702532553,-1.1477220656059,3.3390638510896,
-0.343725701810518,0.731052580354999,NA
"16687","AL080317.1",0.803048438968496,0.972068673375818,1.56358451393
838,0.62169244112514,0.534144122917399,NA
"16688","REV3L-IT1",0,NA,NA,NA,NA,NA
"16689","AL008730.1",0.175609175873806,0.247512349757584,3.30210845793
575,0.0749558510601773,0.940249838776562,NA
"16690","CCN6",0.378487741162614,-2.15786196901571,2.24078715082696,-0
.962992834111599,0.335551119403315,NA
"16691","Z99289.2",1.26889720540429,-0.614488030616154,1.5767738651192
2,-0.389712211883784,0.69674936533541,NA
"16692","AL360015.1",0.452343733379502,1.42893663417479,2.186308449918
92,0.653584188556644,0.513379733668147,NA
"16693","LINC02518",0.549501411999148,1.84085799564531,2.0674141636728
3,0.890415683510154,0.37324272410608,NA
"16694","LINC02541",0.175910378850688,0.313649592621519,3.256047270905
7,0.0963283289601242,0.923259812444105,NA
"16695","LINC01268",0,NA,NA,NA,NA,NA
"16696","FO393415.1",0.610292137060489,0.788506114306944,2.01694188448
499,0.390941415006751,0.695840540428241,NA
"16697","HDAC2-
AS2",0.433921967430739,1.44756788574182,1.97425683550236,0.73322166584
9507,0.463423243076131,NA

"16698","HS3ST5",0,NA,NA,NA,NA,NA
"16699","AL590550.1",0,NA,NA,NA,NA,NA
"16700","AL357141.1",0.666266631476369,-0.748461589724698,1.9158378861
2358,-0.390670627794664,0.696040712486657,NA
"16701","COL10A1",0,NA,NA,NA,NA,NA
"16702","RSPH4A",0.0367720482966892,-0.560503011376585,3.3495200143758
3,-0.167338307868279,0.867103863749685,NA
"16703","FAM162B",0.95951943996066,0.799781641587764,1.45709620663469,
0.548887326688566,0.583082775934765,NA
"16704","SLC35F1",0.157560096502761,-0.491317252697356,3.3484535150329
7,-0.146729602334801,0.883345452200538,NA
"16705","PLN",0,NA,NA,NA,NA,NA
"16706","AL137009.1",0.174581300993347,-0.691169037761009,2.8628604588
1777,-0.241426030958711,0.809224939549254,NA
"16707","AL078600.1",0.293858489765547,0.00709152447587447,2.854490734
04838,0.0024843396376407,0.998017785798355,NA
"16708","AL096854.1",0,NA,NA,NA,NA,NA
"16709","PKIB",0.90447829890476,0.155023806268515,1.50907334180256,0.1
02727814463505,0.918178997674864,NA
"16710","AL512283.1",0,NA,NA,NA,NA,NA
"16711","FABP7",0,NA,NA,NA,NA,NA
"16712","TRDN",0,NA,NA,NA,NA,NA
"16713","NKAIN2",0.572457892030975,1.81329414718264,1.89100236074295,0
.958906337097444,0.33760593203778,NA
"16714","HEY2",0.766579298533352,-2.32431954880001,2.20246395971388,-1
.05532693897155,0.291275824625406,NA
"16715","SOGA3",0.836598157408376,0.979989775769094,1.54821397471805,0
.63298083583541,0.526746157789968,NA
"16716","C6orf58",0.514238653914358,0.55193752547049,1.87024392202627,
0.295115262223393,0.767905831111884,NA
"16717","THEMIS",0.38805119099851,-2.15897864366634,2.22490304067693,-
0.970369766320007,0.331862213197398,NA
"16718","AL356124.1",1.06710906797541,2.7548408685391,1.7339708536635,
1.58874692888795,0.112117539299867,NA
"16719","AL356124.2",0,NA,NA,NA,NA,NA
"16720","TMEM244",0.239791573166398,-1.32959277084231,2.9363559394555,
-0.45280367852436,0.650690114564834,NA
"16721","AL355581.1",0,NA,NA,NA,NA,NA
"16722","SAMD3",1.38022761044852,-0.96354883015049,1.20961774967624,-0
.796572992094726,0.425699068043052,NA
"16723","TMEM200A",1.01031367605298,0.0598405103755355,1.3383905095981
,0.0447107999843071,0.964337825165636,NA
"16724","ARG1",0,NA,NA,NA,NA,NA
"16725","ENPP3",0.531114156264551,-1.1770449047866,2.15095114535975,-0
.547220659718769,0.584227144235434,NA
"16726","AL133346.1",0.382481757421995,-0.164533352554252,2.2580773059
4307,-0.0728643577087526,0.941914056949521,NA
"16727","AL021408.1",0.42343337048365,0.56090709918418,1.8519433954396
8,0.302874861383661,0.761985229794635,NA
"16728","LINC01013",0.661025644124234,1.04967562974343,1.7416815778891

8,0.602679412281309,0.546721980060166,NA
"16729","VNN1",0.267000171627192,-0.853137625449662,2.51498162517089,-0.339222210178849,0.734442338867411,NA
"16730","VNN3",0.127358887561203,0.15032497835859,3.33866494558414,0.0450254759937551,0.964087002635848,NA
"16731","VNN2",0.45447154930448,0.602231321607586,2.26441176068791,0.265954863891288,0.790273970827156,NA
"16732","LINC00326",0,NA,NA,NA,NA,NA
"16733","AL591115.1",0.406340252716299,-2.11986182578706,2.53128067841097,-0.837466126876856,0.402330609325434,NA
"16734","TARID",0.110014941913186,-0.033834469553869,3.34232334787728,-0.0101230389858472,0.991923121432614,NA
"16735","AL450270.1",0,NA,NA,NA,NA,NA
"16736","SLC2A12",1.07127417316469,1.10871639587372,1.39912934569988,0.792433093681636,0.428108189381265,NA
"16737","AL355881.1",0.388938106298068,-3.01478160288299,2.45633321092062,-1.22735042195398,0.21969091191595,NA
"16738","LINC01010",0,NA,NA,NA,NA,NA
"16739","Z84486.1",0,NA,NA,NA,NA,NA
"16740","CT69",0,NA,NA,NA,NA,NA
"16741","AL078590.2",0.239791573166398,-1.32959277084231,2.9363559394555,-0.45280367852436,0.650690114564834,NA
"16742","ALDH8A1",0.249454475444066,0.842861112043892,2.9219716440684,0.288456294144709,0.772997481484846,NA
"16743","MYB",0,NA,NA,NA,NA,NA
"16744","AL049552.1",0.827210177367547,-0.12865975018352,1.69625427869521,-0.0758493297847344,0.93953896963678,NA
"16745","AL360178.2",0,NA,NA,NA,NA,NA
"16746","AL138828.1",0.0732428936164972,-0.49131726092088,3.34845351515744,-0.146729604785264,0.883345450266286,NA
"16747","MTFR2",0.245258217048015,-1.32928540753819,2.91755618878102,-0.455616043540048,0.648666104227452,NA
"16748","AL023284.4",0,NA,NA,NA,NA,NA
"16749","MAP7",0.830842167188743,2.30357745048355,1.98091883602543,1.16288330879095,0.244876850357486,NA
"16750","AL024508.2",0.0741298089160557,-1.76673671430743,3.34309696236258,-0.528473069790613,0.597171033704837,NA
"16751","AL024508.1",0,NA,NA,NA,NA,NA
"16752","IL20RA",1.44487296488138,0.752931151709829,1.3190828316235,0.570798992799517,0.568135905764011,NA
"16753","AL356234.2",0,NA,NA,NA,NA,NA
"16754","AL591468.2",0,NA,NA,NA,NA,NA
"16755","ARFGEF3",0.0367720482966892,-0.560503011376585,3.34952001437583,-0.167338307868279,0.867103863749685,NA
"16756","AL590617.2",0.78939687692705,-0.0754600031032702,1.54035327538908,-0.04898876401208,0.960928250208854,NA
"16757","GVQW2",1.06820071434431,-2.73207737794216,1.48773865992193,-1.83639603617313,0.0662991053910306,NA
"16758","ECT2L",0.0735440965933784,-0.185659846583073,3.34418846206699,-0.0555171601986567,0.955726459171656,NA

"16759","AL031772.1",0,NA,NA,NA,NA,NA
"16760","TXLNB",0.886391713315518,0.146202588866025,1.70185071636733,0.0859079985453135,0.931539553100682,NA
"16761","AL592429.2",0,NA,NA,NA,NA,NA
"16762","AL357146.1",0.20332072784659,-1.39924864619322,3.10758170858468,-0.450269301794319,0.652516271227926,NA
"16763","AL035446.1",0.350693430379143,-1.35559418604359,2.11385182547938,-0.641291016571691,0.521333624728689,NA
"16764","ADGRG6",0.615130156173933,-1.35126317223882,1.88247763308033,-0.717811010602938,0.472873823417767,NA
"16765","AL023584.2",0.147372702532553,-1.1477220656059,3.3390638510896,-0.343725701810518,0.731052580354999,NA
"16766","AL355304.1",0.176496091173365,-1.14849506633421,3.26459492750587,-0.351803238024283,0.724985830023263,NA
"16767","LINC01277",0.572819885962064,-1.19338725373427,1.74372011350564,-0.684391517016476,0.493727971238932,NA
"16768","AL023581.2",0.516739156417054,-0.410819930877521,1.68858336223313,-0.243292655882986,0.807778693262736,NA
"16769","PHACTR2-AS1",0,NA,NA,NA,NA,NA
"16770","AL023806.2",0.110316144890068,0.0897481123376931,3.34029447074234,0.0268683234738127,0.978564758597723,NA
"16771","AL023806.4",0,NA,NA,NA,NA,NA
"16772","GRM1",0.146485787232994,0.248608662259703,3.33613854377696,0.0745198854896011,0.940596718825075,NA
"16773","ADGB",0,NA,NA,NA,NA,NA
"16774","STXBP5-AS1",1.17128756553077,-0.358276459793481,1.25323095975824,-0.285882228653684,0.774968325438417,NA
"16775","AL031056.1",0.166548679549901,-1.95393031097672,3.33920037562282,-0.585149164824313,0.558447422910535,NA
"16776","ZC3H12D",0.850921548180889,0.162921714324658,1.49521326437832,0.108962191686012,0.913232478393738,NA
"16777","AL078581.2",0.608694173425273,1.9224179294909,1.98541848980931,0.968268372314564,0.332910356005393,NA
"16778","RAET1E",0.62522326993779,0.580594438077531,1.65943108630712,0.349875594635015,0.726432063505799,NA
"16779","RAET1E-AS1",0.490132963910023,-0.440547237339647,1.95453452545336,-0.225397521303677,0.821670039750708,NA
"16780","ULBP2",0.544823824643484,0.913711225072656,1.75049109824222,0.52197421968622,0.601688280484707,NA
"16781","ULBP1",0.0636794437806017,-0.49131726092088,3.34845351515744,-0.146729604785264,0.883345450266286,NA
"16782","ULBP3",0.221239540283362,0.178834610992208,3.06205963446949,0.0584033730039328,0.953427328091912,NA
"16783","PPP1R14C",0.102366282257309,-0.49131726092088,3.34845351515744,-0.146729604785264,0.883345450266286,NA
"16784","AL450344.3",0.175910378850688,0.313649592621519,3.2560472709057,0.0963283289601242,0.923259812444105,NA
"16785","AL450344.1",0.110014941913186,-0.033834469553869,3.3423233478

7728,-0.0101230389858472,0.991923121432614,NA
"16786","AL450344.2",0,NA,NA,NA,NA,NA
"16787","AL078582.2",0,NA,NA,NA,NA,NA
"16788","NANOGP11",0,NA,NA,NA,NA,NA
"16789","MYCT1",0,NA,NA,NA,NA,NA
"16790","AL590867.1",0,NA,NA,NA,NA,NA
"16791","OPRM1",0.303471016947,-0.658169734815469,2.43860095812758,-0.269896447232116,0.787239920484044,NA
"16792","IPCEF1",0.468881953316676,1.64606492969255,2.13708382773394,0.770238821861262,0.441158239088729,NA
"16793","CNKSR3",1.11588072554035,-0.146425648646206,1.25484396828779,-0.116688331256037,0.907107037410838,NA
"16794","AL357075.4",0.147088193186757,0.338238253117599,3.33259753179347,0.10149387974118,0.919158415731812,NA
"16795","AL596202.1",0.423049408696082,0.0560619112126286,2.24397805604634,0.0249832706971314,0.980068307494171,NA
"16796","CLDN20",0.146485787232994,0.248608662259703,3.33613854377696,0.0745198854896011,0.940596718825075,NA
"16797","AL355297.3",0,NA,NA,NA,NA,NA
"16798","AL355297.4",0.238260744773948,-0.25241805562962,2.61915068421643,-0.0963740105335467,0.923223532616171,NA
"16799","AL049820.1",0,NA,NA,NA,NA,NA
"16800","AL390955.2",0.255708582183469,-2.44071869332471,2.85324394739347,-0.855418862994307,0.392319304627377,NA
"16801","AL117344.2",0.183559038506565,0.529394638499161,3.22699253814936,0.164052018168831,0.869690193908075,NA
"16802","AL391863.2",0,NA,NA,NA,NA,NA
"16803","AL035634.1",0,NA,NA,NA,NA,NA
"16804","AL139330.1",0.567283798607478,-1.15461009573386,2.09357149411343,-0.551502587315656,0.581289193532241,NA
"16805","AL360169.2",0.0732428936164972,-0.49131726092088,3.34845351515744,-0.146729604785264,0.883345450266286,NA
"16806","AL627422.2",0,NA,NA,NA,NA,NA
"16807","C6orf99",0,NA,NA,NA,NA,NA
"16808","AL356417.3",0,NA,NA,NA,NA,NA
"16809","AL356417.1",0,NA,NA,NA,NA,NA
"16810","FNDC1-IT1",0.406724214503868,-1.18623546580274,2.0615465963257,-0.575410455391583,0.565013734706149,NA
"16811","AL078604.2",0.139138330553998,-0.0338344975521838,3.34232334820666,-0.0101230473617515,0.991923114749952,NA
"16812","SOD2-OT1",0.809089614395666,0.0522506115298861,1.62158012393017,0.0322220350131378,0.974294983902018,NA
"16813","PNLDC1",0,NA,NA,NA,NA,NA
"16814","MAS1",0.276563621463087,-0.851854214261183,2.4869613794202,-0.342528123400042,0.731953483145556,NA
"16815","AIRN",0.331254482196549,0.971729354124435,2.13678976678249,0.454761328994773,0.649280953898856,NA
"16816","SLC22A1",0,NA,NA,NA,NA,NA

"16817","SLC22A3",0.52833838705529,1.68885914123054,2.16848120633059,0
.778821202738644,0.436085046019879,NA
"16818","PLG",0,NA,NA,NA,NA,NA
"16819","PACRG",1.43924733255769,-0.998465530951236,1.1624836941137,-0
.858907127908134,0.390391756556374,NA
"16820","AL445307.1",0,NA,NA,NA,NA,NA
"16821","AL078602.1",0.19433214479945,-0.0333008172506769,3.1614299744
8539,-0.0105334666652225,0.991595664992027,NA
"16822","Z97205.2",0.626875088952562,1.98570154698629,1.78576587553399
,1.11196074143399,0.266155030315921,NA
"16823","TBXT",0,NA,NA,NA,NA,NA
"16824","AL022069.1",0.348861399009812,-0.508963646938794,2.3910732565
5586,-0.212859913657315,0.831436230075967,NA
"16825","RAMACL",0.100451492077291,-0.0338344573480013,3.3423233477336
9,-0.010123035334371,0.991923124345921,NA
"16826","CCR6",0.558440917700731,1.07497546584909,1.82557864702058,0.5
88840950568462,0.555967972421171,NA
"16827","AL121935.1",0,NA,NA,NA,NA,NA
"16828","AL121935.2",0,NA,NA,NA,NA,NA
"16829","GPR31",0,NA,NA,NA,NA,NA
"16830","AL356123.2",0.229725169818512,0.686619208900669,3.02593648037
66,0.226911309392461,0.820492707794461,NA
"16831","LINC01558",0,NA,NA,NA,NA,NA
"16832","AFDN-
DT",1.40300020562947,-0.0394677998463679,1.36328960506682,-0.028950415
0106341,0.976904137088473,NA
"16833","KIF25-AS1",0,NA,NA,NA,NA,NA
"16834","KIF25",0.100451492077291,-0.0338344573480013,3.34232334773369
,-0.010123035334371,0.991923124345921,NA
"16835","AL606970.1",0.102366282257309,-0.49131726092088,3.34845351515
744,-0.146729604785264,0.883345450266286,NA
"16836","AL138918.1",0,NA,NA,NA,NA,NA
"16837","AL513210.1",0.268461953715506,-0.692213851544516,2.5169359849
2032,-0.275022430324715,0.783299005639148,NA
"16838","AL109924.2",0,NA,NA,NA,NA,NA
"16839","AL136129.1",0.102366282257309,-0.49131726092088,3.34845351515
744,-0.146729604785264,0.883345450266286,NA
"16840","LINC02544",0.351892241302211,0.700193822812102,2.702055693558
24,0.2591337493455,0.795532045707916,NA
"16841","LINC02519",0.185031666128801,-1.62793589934517,3.243362493027
77,-0.501928447049853,0.61571785405042,NA
"16842","AL354892.2",0.276563621463087,-0.851854214261183,2.4869613794
202,-0.342528123400042,0.731953483145556,NA
"16843","AL354892.1",0,NA,NA,NA,NA,NA
"16844","AL354892.3",0.894735657676328,-1.57586677345221,1.68571132225
669,-0.934837864968821,0.349871862478045,NA
"16845","LINC00242",0.73363072762227,-0.197588120095833,1.571244321725
36,-0.125752639079621,0.899927732598936,NA
"16846","LINC00574",0.376512160028388,1.35661765944732,2.0003101394003
5,0.67820366088531,0.497642571708043,NA

"16847","AL603783.1",0,NA,NA,NA,NA,NA
"16848","AL596442.2",0,NA,NA,NA,NA,NA
"16849","LINC01624",0,NA,NA,NA,NA,NA
"16850","AL008628.1",0,NA,NA,NA,NA,NA
"16851","AL031259.1",0,NA,NA,NA,NA,NA
"16852","AL672310.1",0.28739794838611,-1.11963306374531,2.48645079881355,-0.450293673327284,0.652498700264967,NA
"16853","AC215522.2",0,NA,NA,NA,NA,NA
"16854","AC093627.4",0.319388025964071,-1.829717069326,2.37252256485154,-0.771211661559265,0.440581483117291,NA
"16855","AC093627.7",0,NA,NA,NA,NA,NA
"16856","AC147651.1",0.457570059671173,-1.88394161052829,2.17090636577639,-0.867813389019444,0.385496499136352,NA
"16857","PRKAR1B",0.782520395275305,2.29074302359578,1.70244674508154,1.34555928413847,0.178444690004174,NA
"16858","AC147651.4",0.213268139470054,-0.691656524248949,2.68459372570788,-0.257639179301356,0.796685389475746,NA
"16859","AC073957.3",0.634676421797991,1.1661900311261,1.90640078210864,0.61172343301087,0.540720755399789,NA
"16860","AC102953.2",1.17904178060284,-0.528164019609347,1.29556280463388,-0.407671490506092,0.683514870782302,NA
"16861","TMEM184A",0.926150810010967,0.421960606216918,1.65057228100487,0.255645033588004,0.798224941763546,NA
"16862","ELFN1",0.102366282257309,-0.49131726092088,3.34845351515744,-0.146729604785264,0.883345450266286,NA
"16863","ELFN1-AS1",0,NA,NA,NA,NA,NA
"16864","AC104129.1",0,NA,NA,NA,NA,NA
"16865","AMZ1",0.0741298089160557,-1.76673671430743,3.34309696236258,-0.52847306979061З,0.597171033704837,NA
"16866","CARD11",0.716454022295196,1.11672160732877,1.63775558681119,0.681860966509108,0.495326869243607,NA
"16867","AC024028.1",0.0732428936164972,-0.49131726092088,3.34845351515744,-0.146729604785264,0.883345450266286,NA
"16868","AC073316.3",0.255122869860792,-1.01416582893919,2.91937932186279,-0.347390906465717,0.728297664063611,NA
"16869","AC011284.1",0.102366282257309,-0.49131726092088,3.34845351515744,-0.146729604785264,0.883345450266286,NA
"16870","AC072054.1",0.431404771296958,1.43806046454093,2.21134919284444,0.650309082434495,0.51549259150238,NA
"16871","AC092428.1",1.21116236939662,2.08555242112847,1.39789028925316,1.49192854200503,0.135717879332121,NA
"16872","RBAKDN",0.8685594844425,-1.51233561415887,1.57554855574617,-0.959878772788847,0.337116230767259,NA
"16873","SLC29A4",0.559350629605804,-0.152689664103771,2.0863894135269,-0.073183684269975,0.941659949636156,NA
"16874","AC092171.1",0,NA,NA,NA,NA,NA
"16875","AC092171.4",0.570987854592733,-0.354990731700412,2.13627608730322,-0.166172684237899,0.868021055376994,NA
"16876","AC092171.3",0.872265674988348,-1.36254795164175,1.64637037140674,-0.827607186879533,0.407892996808158,NA

"16877","AC092171.5",0.631535577447408,-0.634810198265546,1.9036891109
6379,-0.333463166128085,0.738784689045945,NA
"16878","AC092171.2",0,NA,NA,NA,NA,NA
"16879","AC006483.2",0.166045726037911,0.183394512381981,3.33779889015
228,0.0549447460489789,0.956182483505573,NA
"16880","OCM",0,NA,NA,NA,NA,NA
"16881","ANKRD61",0,NA,NA,NA,NA,NA
"16882","FAM220A",0.514676132367215,-3.24724171037767,2.30706294809482
,-1.40752193738764,0.159272676891155,NA
"16883","AC073343.2",0.221502511448609,-1.56478135306832,3.04955054434
762,-0.513118681036017,0.607868304703499,NA
"16884","AC005532.1",0.486762829859323,0.207145125259014,1.96832722248
05,0.10523917105509,0.916186029274287,NA
"16885","COL28A1",1.18986230058659,-1.86914353594582,1.30381615914166,
-1.433594393535,0.151688044356144,NA
"16886","AC004982.1",0.450040726982919,-1.35364553278111,1.99848628192
982,-0.677335413818291,0.498193166520516,NA
"16887","AC004948.1",0,NA,NA,NA,NA,NA
"16888","AC006042.4",0.345709709125306,-0.403701136108005,2.3539094344
903,-0.171502407948596,0.863828737224812,NA
"16889","ICA1",0.350693430379143,-1.35559418604359,2.11385182547938,-0
.641291016571691,0.521333624728689,NA
"16890","AC007009.1",0,NA,NA,NA,NA,NA
"16891","AC007029.1",0,NA,NA,NA,NA,NA
"16892","AC004160.2",0.320717103821412,-0.846710013286843,2.3833419860
6247,-0.35526165285482,0.722393580711595,NA
"16893","AC013470.2",0,NA,NA,NA,NA,NA
"16894","AC011287.1",0.157560096502761,-0.491317252697356,3.3484535150
3297,-0.146729602334801,0.883345452200538,NA
"16895","AGMO",1.39880869326397,1.01014834747194,1.29718124808035,0.77
8725678440713,0.436141326358843,NA
"16896","AC005550.2",0.55491176617192,0.123444081731382,2.178027449671
51,0.0566770091671712,0.954802488596476,NA
"16897","LINC02587",0.46901378947292,1.61641286689612,2.15855671867238
,0.748839654253001,0.453953855851458,NA
"16898","AC006041.2",0,NA,NA,NA,NA,NA
"16899","AC005014.3",0.0636794437806017,-0.49131726092088,3.3484535151
5744,-0.146729604785264,0.883345450266286,NA
"16900","AC005014.2",0.823674922864307,0.395745742426588,1.80024170797
268,0.219829226638824,0.826004156778864,NA
"16901","TSPAN13",0.627641013629799,-0.579225087378908,1.5651766331126
9,-0.370070109101357,0.711330253020717,NA
"16902","AC019117.1",0.0741298089160557,-1.76673671430743,3.3430969623
6258,-0.528473069790613,0.597171033704837,NA
"16903","AC003986.2",0.313335669759777,-0.504482984843873,2.3945565380
6486,-0.210679086847357,0.833137694613702,NA
"16904","AC003986.3",0.157560096502761,-0.491317252697356,3.3484535150
3297,-0.146729602334801,0.883345452200538,NA
"16905","TMEM196",0,NA,NA,NA,NA,NA
"16906","AC005062.1",0.935258720025732,-0.0955139599571659,1.599110819

34803,-0.0597294188754898,0.952371140683232,NA
"16907","MACC1",0.350693430379143,-1.35559418604359,2.11385182547938,-0.641291016571691,0.521333624728689,NA
"16908","AC005083.1",0.387465478675832,-1.00097401054312,2.05547168079657,-0.486980200162721,0.626272362417671,NA
"16909","DNAH11",0.419911096074925,-1.32028169514223,2.49077535946509,-0.530068554807674,0.59606440017536,NA
"16910","RAPGEF5",0,NA,NA,NA,NA,NA
"16911","STEAP1B",0.341420337986207,1.11072068185056,2.66384239543665,0.416961860714173,0.676706304653868,NA
"16912","AC002480.2",0.191038331341805,0.541704852733846,3.32505412972637,0.162916100490197,0.870584493834308,NA
"16913","AC073072.1",0,NA,NA,NA,NA,NA
"16914","KLHL7-DT",0.622273330588393,-0.546005430617734,1.70314797724217,-0.320586019484846,0.748524133682017,NA
"16915","AC005082.1",0.770402775918959,-0.0136893115112987,1.54548553071317,-0.00885761221263695,0.99293274038309,NA
"16916","IGF2BP3",0,NA,NA,NA,NA,NA
"16917","STK31",0,NA,NA,NA,NA,NA
"16918","AC005165.1",0.533497389742394,0.545763554943214,1.87737296391281,0.290705984071346,0.771276194544064,NA
"16919","NFE2L3",0.266497218115202,0.892674172735432,2.50488901813024,0.356372743971613,0.721561438409243,NA
"16920","AC004947.1",0,NA,NA,NA,NA,NA
"16921","HOXA1",0.911925208025528,1.13948004911727,1.46086428630342,0.780004042675734,0.435388495615613,NA
"16922","HOXA2",0.660829902130827,0.680205585747471,1.85512671605547,0.366662600382244,0.713870706974289,NA
"16923","HOXA-AS2",0.888252065552757,-0.500190878499314,1.62388523009651,-0.308021077616174,0.758066293951732,NA
"16924","HOXA-AS3",0.582684538774841,-1.01411259568,1.84507923117045,-0.549630920205356,0.58257254656153,NA
"16925","AC005091.1",0.231689905418816,-1.15178064054131,2.99600284051573,-0.384439101647526,0.700653024054507,NA
"16926","JAZF1-AS1",1.04806575586334,0.0146927205748172,1.77624575362275,0.00827178364528139,0.993400146802185,NA
"16927","AC005013.1",0.0367720482966892,-0.560503011376585,3.34952001437583,-0.167338307868279,0.867103863749685,NA
"16928","AC005162.2",0,NA,NA,NA,NA,NA
"16929","AC004593.3",0,NA,NA,NA,NA,NA
"16930","AC004593.2",0.0741298089160557,-1.76673671430743,3.34309696236258,-0.528473069790613,0.597171033704837,NA
"16931","AC004593.1",0,NA,NA,NA,NA,NA
"16932","PRR15",0,NA,NA,NA,NA,NA
"16933","WIPF3",0.49021572272071,0.0673020408128115,1.94685255239111,0.0345696651398441,0.972422890746892,NA

"16934","AC007285.1",1.22066078307583,0.24218339389444,1.27100760834791,0.1905444092575,0.848882547018573,NA
"16935","AC007285.2",0.0732428936164972,-0.49131726092088,3.34845351515744,-0.146729604785264,0.883345450266286,NA
"16936","AC007036.5",0.638353328968476,1.9612554894762,1.72288048435494,1.1383584103981,0.254970852962081,NA
"16937","AC007036.2",0.899494242407721,-1.63134503573134,1.92840181744935,-0.845957010084694,0.397576727011425,NA
"16938","AC007036.1",0.620304249597431,1.86911509061695,1.87174338444371,0.998595804398935,0.317990533426145,NA
"16939","LINC01176",1.2170437895583,0.698429940943446,1.34237099374929,0.52029576338856,0.602857448295259,NA
"16940","AC006027.1",0.379816819019955,-1.35490224425385,2.07036443936748,-0.654426930105015,0.512836789051267,NA
"16941","ADCYAP1R1",0.531665358373062,0.963484317971597,1.94670986607269,0.494929591082486,0.620649821928629,NA
"16942","PPP1R17",0,NA,NA,NA,NA,NA
"16943","AC018648.1",0.662120748176533,-0.113481616785815,1.75376708635006,-0.0647073477824204,0.948407012401465,NA
"16944","AC008080.1",0.0636794437806017,-0.49131726092088,3.34845351515744,-0.146729604785264,0.883345450266286,NA
"16945","AC008080.4",0.346668202458428,-1.94732862277873,3.00338753971666,-0.648377406187962,0.516740879961632,NA
"16946","AC007350.1",0.102366282257309,-0.49131726092088,3.34845351515744,-0.146729604785264,0.883345450266286,NA
"16947","AC005400.1",0.20332072784659,-1.39924864619322,3.10758170858468,-0.450269301794319,0.652516271227926,NA
"16948","AC018647.3",0.499717951926468,0.0474299692155556,2.00863141950114,0.0236130774193183,0.981161240788159,NA
"16949","AC018647.1",0.623533026898396,-1.50191485641434,2.10525127914818,-0.713413582164899,0.475589870919251,NA
"16950","SEPTIN7-AS1",0.908133176293134,-0.81618108936012,1.49623153877514,-0.545491167782941,0.585415753400047,NA
"16951","AC083864.5",0,NA,NA,NA,NA,NA
"16952","AC006960.3",0,NA,NA,NA,NA,NA
"16953","KIAA0895",0.0735440965933784,-0.185659846583073,3.34418846206699,-0.0555171601986567,0.955726459171656,NA
"16954","ANLN",0.744067715494375,-0.0964293825174966,1.63960171998609,-0.0588126868507522,0.953101303307843,NA
"16955","AOAH",0.689055386907944,0.2109688147905,1.85650068265608,0.11363788699968,0.909524852391517,NA
"16956","AC007349.3",0,NA,NA,NA,NA,NA
"16957","AC007349.2",0,NA,NA,NA,NA,NA
"16958","ELMO1-AS1",0,NA,NA,NA,NA,NA
"16959","GPR141",0,NA,NA,NA,NA,NA
"16960","NME8",0,NA,NA,NA,NA,NA
"16961","TRGC2",0.301053273254991,1.08368293634553,2.41489596894672,0.448749325138916,0.653612498174957,NA
"16962","TRGC1",0.285624117786993,0.964238783217236,2.46507094134902,0

.39116066277977,0.695678483017121,NA
"16963","TRGV10",0,NA,NA,NA,NA,NA
"16964","TRGV9",0,NA,NA,NA,NA,NA
"16965","AC006033.2",0.183257835529684,0.495279120319891,3.24692673479
8,0.152537818304269,0.878762764724423,NA
"16966","TRGV8",0,NA,NA,NA,NA,NA
"16967","TRGV7",0,NA,NA,NA,NA,NA
"16968","TRG-
AS1",0.220029883826373,0.698180369696046,3.02638332375644,0.2306979305
01891,0.817549481385233,NA
"16969","TRGV5",0,NA,NA,NA,NA,NA
"16970","TRGV4",0,NA,NA,NA,NA,NA
"16971","TRGV3",0,NA,NA,NA,NA,NA
"16972","TRGV2",0,NA,NA,NA,NA,NA
"16973","POU6F2",0,NA,NA,NA,NA,NA
"16974","AC004837.4",0.505534580213014,0.753238353957723,1.95498054151
835,0.385291995475676,0.700021091325341,NA
"16975","AC004988.1",0,NA,NA,NA,NA,NA
"16976","INHBA-AS1",0,NA,NA,NA,NA,NA
"16977","AC010132.4",0.20332072784659,-1.39924864619322,3.107581708584
68,-0.450269301794319,0.652516271227926,NA
"16978","HECW1",0.531183537811889,0.512344079929104,1.7415912785189,0.
294181583387818,0.768619152652684,NA
"16979","LUARIS",0.342157855423707,-0.617755040995007,2.37281219406507
,-0.260347212704043,0.794595957359595,NA
"16980","SPDYE1",0.110901857212745,-1.2169087618042,3.34013336902682,-
0.36432939267894,0.715612053721651,NA
"16981","LINC00957",1.15079397012611,-0.841539640826559,1.574729946851
39,-0.534402512957338,0.593063080746974,NA
"16982","PGAM2",0.19433214479945,-0.0333008172506769,3.16142997448539,
-0.0105334666652225,0.991595664992027,NA
"16983","GCK",0.65593827792526,-2.37369409921183,1.75707758874562,-1.3
5093300057763,0.176716895841299,NA
"16984","CAMK2B",0,NA,NA,NA,NA,NA
"16985","NPC1L1",0,NA,NA,NA,NA,NA
"16986","AC004938.2",0.914721647340611,-0.0392825580879249,1.609320928
78658,-0.0244093998811932,0.980526050539741,NA
"16987","LINC01952",0,NA,NA,NA,NA,NA
"16988","AC004854.2",0.927709512811627,0.8821469079491,1.4276963801421
7,0.61788130881249,0.536653580756241,NA
"16989","AC004847.1",0.183257835529684,0.495279120319891,3.24692673479
8,0.152537818304269,0.878762764724423,NA
"16990","RAMP3",0.383440250755117,-1.38194811510753,2.56276654272887,-
0.53924073537967,0.589720755955717,NA
"16991","AC073115.2",0,NA,NA,NA,NA,NA
"16992","AC073115.1",0.157560096502761,-0.491317252697356,3.3484535150
3297,-0.146729602334801,0.883345452200538,NA
"16993","AC087175.1",0.0732428936164972,-0.49131726092088,3.3484535151
5744,-0.146729604785264,0.883345450266286,NA
"16994","PKD1L1",0,NA,NA,NA,NA,NA

"16995","SUN3",0,NA,NA,NA,NA,NA
"16996","C7orf57",0.148259617832111,-2.17639404086898,3.335076015408,-0.652577041966681,0.514028990213041,NA
"16997","ABCA13",0,NA,NA,NA,NA,NA
"16998","VWC2",0.247824194609844,-0.209546153089553,2.30954823409246,-0.0907303644913461,0.927706842755531,NA
"16999","COBL",0.513216882008329,-2.79767777251513,2.35085107316122,-1.19007018541292,0.234018807453478,NA
"17000","AC006459.1",0.147372702532553,-1.1477220656059,3.3390638510896,-0.343725701810518,0.731052580354999,NA
"17001","EGFR-AS1",0.212381224170495,0.56712693135589,2.68810250756675,0.210976675837131,0.832905471698532,NA
"17002","AC237221.1",1.1172727289125,-2.13627074015795,1.54456862956367,-1.38308567147413,0.166638598592575,NA
"17003","LINC02848",0.414372874159745,-0.868429051303812,1.88336171393898,-0.461105822039636,0.644722684919384,NA
"17004","AC093582.1",0.157560096502761,-0.491317252697356,3.34845351503297,-0.146729602334801,0.883345452200538,NA
"17005","SPDYE21P",0,NA,NA,NA,NA,NA
"17006","AC092100.1",0.363157546534827,-2.82913804218295,2.97971220862415,-0.949466876027358,0.342383211161164,NA
"17007","CT66",0,NA,NA,NA,NA,NA
"17008","CALN1",0.407227168015858,-3.00441642259496,2.45146029940343,-1.22556193274926,0.220363570475393,NA
"17009","AC211476.2",0.249153272467185,0.812295408448132,2.54252578312747,0.31948364647416,0.7493597903733,NA
"17010","AC211476.6",0.369941320673256,1.11554984681986,2.1030042091201,0.530455356190946,0.595796254308461,NA
"17011","FKBP6",0.268461953715506,-0.692213851544516,2.51693598492032,-0.275022430324715,0.783299005639148,NA
"17012","FZD9",0.20332072784659,-1.39924864619322,3.10758170858468,-0.450269301794319,0.652516271227926,NA
"17013","AC005089.1",0.61655585882611,1.21542550919917,1.82246530172976,0.666912839462882,0.504827809183121,NA
"17014","STX1A",0.666426812946718,-0.539822547347074,1.81110630561987,-0.298062320070337,0.765655599398932,NA
"17015","CLDN3",0,NA,NA,NA,NA,NA
"17016","ELN-AS1",0.0636794437806017,-0.49131726092088,3.34845351515744,-0.146729604785264,0.883345450266286,NA
"17017","LAT2",0.781558180344714,-0.803657375605259,1.57627548149641,-0.509845763027616,0.610159521962216,NA
"17018","AC005081.1",0,NA,NA,NA,NA,NA
"17019","AC211433.1",0.313418428570464,0.0405212521647926,2.20326788522023,0.0183914323068083,0.985326587316889,NA
"17020","AC211486.5",0.102366282257309,-0.49131726092088,3.34845351515744,-0.146729604785264,0.883345450266286,NA
"17021","SPDYE5",0.102366282257309,-0.49131726092088,3.34845351515744,-0.146729604785264,0.883345450266286,NA

"17022","SSC4D",1.14594057773218,-2.01588168685661,1.33070282296952,-1
.51489998522591,0.129797734125466,NA
"17023","ZP3",0.137809252696657,-1.1477220656059,3.3390638510896,-0.34
3725701810518,0.731052580354999,NA
"17024","UPK3B",0,NA,NA,NA,NA,NA
"17025","SPDYE16",0.920132336756585,0.603702244374645,1.45033532110691
,0.416250115120892,0.677226991547968,NA
"17026","AC004921.1",0,NA,NA,NA,NA,NA
"17027","AC004990.1",0,NA,NA,NA,NA,NA
"17028","AC006043.1",0.2028177743346,0.512115644193803,2.7175776006859
7,0.188445637785848,0.850527326709045,NA
"17029","AC074024.1",0.110901857212745,-1.2169087618042,3.340133369026
82,-0.36432939267894,0.715612053721651,NA
"17030","AC006145.1",0.239791573166398,-1.32959277084231,2.93635593945
55,-0.45280367852436,0.650690114564834,NA
"17031","GRM3",0,NA,NA,NA,NA,NA
"17032","AC005009.1",0,NA,NA,NA,NA,NA
"17033","AC005009.2",0.0732428936164972,-0.49131726092088,3.3484535151
5744,-0.146729604785264,0.883345450266286,NA
"17034","ABCB1",0.184144750829242,-0.691295663073562,2.81313162320765,
-0.245738826214366,0.805884414604706,NA
"17035","RUNDC3B",0.200601781177701,0.585305641041376,3.16043896918792
,0.185197577535177,0.853074069074656,NA
"17036","AC003991.2",0,NA,NA,NA,NA,NA
"17037","AC003991.1",0.503673736374214,0.286368654269739,2.18167416911
85,0.131260963861274,0.895568870880352,NA
"17038","AC002064.1",0,NA,NA,NA,NA,NA
"17039","FAM237B",0,NA,NA,NA,NA,NA
"17040","AC002458.1",0,NA,NA,NA,NA,NA
"17041","AC079760.2",0.674288407385893,2.03842018916823,1.770382245206
94,1.15140117038959,0.249567232566303,NA
"17042","AC003086.1",0.166045726037911,0.183394512381981,3.33779889015
228,0.0549447460489789,0.956182483505573,NA
"17043","CYP51A1-
AS1",0.137809252696657,-1.1477220656059,3.3390638510896,-0.34372570181
0518,0.731052580354999,NA
"17044","LRRD1",0.19433214479945,-0.0333008172506769,3.16142997448539,
-0.0105334666652225,0.991595664992027,NA
"17045","AC000120.1",0.176496091173365,-1.14849506633421,3.26459492750
587,-0.351803238024283,0.724985830023263,NA
"17046","AC000065.2",0,NA,NA,NA,NA,NA
"17047","AC000065.1",0.175609175873806,0.247512349757584,3.30210845793
575,0.0749558510601773,0.940249838776562,NA
"17048","AC002454.1",0.876225267405392,1.52770604746929,1.734948009710
61,0.880548603715284,0.378562187332606,NA
"17049","CALCR",0,NA,NA,NA,NA,NA
"17050","TFPI2",0.148259617832111,-2.17639404086898,3.335076015408,-0.
652577041966681,0.514028990213041,NA
"17051","AC003092.2",0,NA,NA,NA,NA,NA
"17052","AC002074.2",0,NA,NA,NA,NA,NA

"17053","AC002429.2",0.691862072409955,2.22001778593415,1.89228531284468,1.17319400561049,0.24071801533969,NA
"17054","PON1",0.176496091173365,-1.14849506633421,3.26459492750587,-0.351803238024283,0.724985830023263,NA
"17055","AC004012.1",0,NA,NA,NA,NA,NA
"17056","ASB4",0.0732428936164972,-0.49131726092088,3.34845351515744,-0.146729604785264,0.883345450266286,NA
"17057","DYNC1I1",1.1702716417219,-0.0521867257839291,1.42194061547272,-0.0367010585505921,0.970723364611889,NA
"17058","DLX5",0.552494022479911,1.72600669414969,1.89161611977036,0.912450827686552,0.361531443405949,NA
"17059","ASNS",1.43024469985652,1.66565509215504,1.30708452655668,1.27432852146369,0.202547012562318,NA
"17060","BAIAP2L1",0.0367720482966892,-0.560503011376585,3.34952001437583,-0.167338307868279,0.867103863749685,NA
"17061","AC093799.1",0.941874291335762,0.713106331536905,1.41107028343863,0.505365565348836,0.613302099834489,NA
"17062","NPTX2",0,NA,NA,NA,NA,NA
"17063","TMEM130",0.0741298089160557,-1.76673671430743,3.34309696236258,-0.528473069790613,0.597171033704837,NA
"17064","AC004893.3",0.239791573166398,-1.32959277084231,2.9363559394555,-0.45280367852436,0.650690114564834,NA
"17065","AC004834.1",0.239791573166398,-1.32959277084231,2.9363559394555,-0.45280367852436,0.650690114564834,NA
"17066","AC069294.1",0.0636794437806017,-0.49131726092088,3.34845351515744,-0.146729604785264,0.883345450266286,NA
"17067","AC073842.2",0.102366282257309,-0.49131726092088,3.34845351515744,-0.146729604785264,0.883345450266286,NA
"17068","GPC2",1.35475299969272,0.806710985332998,1.26022572483356,0.64013213620087,0.522086698032997,NA
"17069","STAG3",1.00944013801677,-0.0551825463948682,1.42578085595665,-0.0387033857021755,0.969126874033589,NA
"17070","PVRIG",0,NA,NA,NA,NA,NA
"17071","SPDYE3",0.571692988315762,-0.248410645511036,1.74985390735746,-0.14196079139325,0.887110975894532,NA
"17072","C7orf61",0.599260990523651,0.807596515397756,1.82169188581637,0.44332223340603,0.657532676344118,NA
"17073","AC092849.3",0.584998328779714,0.670695755005217,1.73083645755267,0.387498051637735,0.698387527054083,NA
"17074","NYAP1",0.800159290573307,-2.19634487640846,1.55555958862057,-1.41193233128159,0.157969886844075,NA
"17075","FBXO24",0.0636794437806017,-0.49131726092088,3.34845351515744,-0.146729604785264,0.883345450266286,NA
"17076","PCOLCE-AS1",0.922877658598244,-0.0758431049235753,1.40064895190461,-0.0541485465151304,0.956816814427922,NA
"17077","TFR2",0.514523163260154,-0.380375479542462,1.93844303618663,-0.196227318751006,0.844432246428642,NA
"17078","SLC12A9-AS1",0.51220931132965,-0.454948451162437,1.95636429738911,-0.232547921

555098,0.816112471156129,NA
"17079","ACHE",0.0732428936164972,-0.49131726092088,3.34845351515744,-0.146729604785264,0.883345450266286,NA
"17080","VGF",0,NA,NA,NA,NA,NA
"17081","AC006329.1",0.110901857212745,-1.2169087618042,3.34013336902682,-0.36432939267894,0.715612053721651,NA
"17082","AC005096.1",0,NA,NA,NA,NA,NA
"17083","AC005072.1",0.601882483648668,1.89352721428938,1.84894886366599,1.02411010466509,0.305783272205891,NA
"17084","SH2B2",0.342459058400589,-0.481809408085001,2.32728154585689,-0.207026695563644,0.835989010065959,NA
"17085","SPDYE6",0.240175534953966,-0.492654690648343,2.62030586946942,-0.188014191926418,0.850865526027581,NA
"17086","RASA4B",0,NA,NA,NA,NA,NA
"17087","UPK3BL2",0,NA,NA,NA,NA,NA
"17088","AC105052.4",0,NA,NA,NA,NA,NA
"17089","FBXL13",1.16637542231049,-0.00516312926752137,1.32587737416421,-0.00389412276589759,0.996892947419878,NA
"17090","NFE4",0.212381224170495,0.56712693135589,2.68810250756675,0.210976675837131,0.832905471698532,NA
"17091","SLC26A5",0,NA,NA,NA,NA,NA
"17092","AC005064.1",0,NA,NA,NA,NA,NA
"17093","LHFPL3-AS2",0,NA,NA,NA,NA,NA
"17094","AC007384.1",0.331254482196549,0.971729354124435,2.13678976678249,0.454761328994773,0.649280953898856,NA
"17095","AC005070.3",0.900982734912006,1.17235846454736,1.5114765987876,0.775637853399608,0.437962843438794,NA
"17096","AC004884.2",0.451418586268308,0.527440182692286,2.26177993606217,0.23319695001388,0.81560847410807,NA
"17097","EFCAB10",0,NA,NA,NA,NA,NA
"17098","AC073073.2",0.405837299204309,-0.106576764313145,2.06782133802848,-0.0515406057347092,0.958894746134285,NA
"17099","AC004917.1",0,NA,NA,NA,NA,NA
"17100","AC005050.3",0.0732428936164972,-0.49131726092088,3.34845351515744,-0.146729604785264,0.883345450266286,NA
"17101","AC005050.1",0.136922337397099,0.183394507878855,3.3377988902691,0.0549447446979257,0.956182484581931,NA
"17102","PIK3CG",0.623651189873782,0.854713238262306,1.83508819496307,0.465761395342368,0.641386300721785,NA
"17103","AC006387.1",0.60097360049263,-0.944993068809743,2.05973848681605,-0.458792742310951,0.64638300707464,NA
"17104","AC004492.1",0.304045883735755,0.789679836645197,2.79279246778834,0.282756361510298,0.777363608165881,NA
"17105","GPR22",0,NA,NA,NA,NA,NA
"17106","AC004839.2",0.531385288346998,-1.12239656744153,1.95315593576039,-0.574657940460121,0.565522658000296,NA
"17107","SLC26A4-AS1",0.254821666883911,-1.3286579469836,2.88029438084422,-0.461292413657444,0.64458882703139,NA
"17108","SLC26A4",0.637325454088016,1.12624392663817,1.67704877285066,

0.67156301287754,0.501861931624065,NA
"17109","AC005046.1",0.614544443851256,-0.147231125828971,2.0438889461
7273,-0.0720347972450598,0.942574215574421,NA
"17110","NRCAM",0.0367720482966892,-0.560503011376585,3.34952001437583
,-0.167338307868279,0.867103863749685,NA
"17111","AC073114.1",0.0732428936164972,-0.49131726092088,3.3484535151
5744,-0.146729604785264,0.883345450266286,NA
"17112","AC073326.1",0,NA,NA,NA,NA,NA
"17113","AC003077.1",0.219728680849491,0.668957297716141,3.32079168991
007,0.201445125193702,0.840350533531288,NA
"17114","DOCK4-
AS1",0.0732428936164972,-0.49131726092088,3.34845351515744,-0.14672960
4785264,0.883345450266286,NA
"17115","GPR85",0.0367720482966892,-0.560503011376585,3.34952001437583
,-0.167338307868279,0.867103863749685,NA
"17116","AC003992.1",0.254821666883911,-1.3286579469836,2.880294380844
22,-0.461292413657444,0.64458882703139,NA
"17117","LINC01393",0.139138330553998,-0.0338344975521838,3.3423233482
0666,-0.0101230473617515,0.991923114749952,NA
"17118","TFEC",1.02608134913363,-1.98457465985625,1.61790858126303,-1.
22662966427125,0.219961814522581,NA
"17119","AC073130.2",0,NA,NA,NA,NA,NA
"17120","AC002066.1",0.0367720482966892,-0.560503011376585,3.349520014
37583,-0.167338307868279,0.867103863749685,NA
"17121","AC073130.1",0.181578773267413,-1.95393031097672,3.33920037562
282,-0.585149164824313,0.558447422910535,NA
"17122","MET",0.426475058309971,-0.620974212993122,2.29052207896558,-0
.271105971296099,0.786309529077914,NA
"17123","AC002542.6",0.0367720482966892,-0.560503011376585,3.349520014
37583,-0.167338307868279,0.867103863749685,NA
"17124","ANKRD7",0.0735440965933784,-0.185659846583073,3.3441884620669
9,-0.0555171601986567,0.955726459171656,NA
"17125","AC006364.1",0.0367720482966892,-0.560503011376585,3.349520014
37583,-0.167338307868279,0.867103863749685,NA
"17126","WNT16",0.945883215975629,-0.621113559521011,1.93946524315621,
-0.320249904819245,0.748778895019716,NA
"17127","RNF148",0,NA,NA,NA,NA,NA
"17128","IQUB",1.09232787254266,-1.15756245808825,1.33475269162622,-0.
867248641153073,0.385805790262862,NA
"17129","ASB15",0,NA,NA,NA,NA,NA
"17130","LMOD2",0,NA,NA,NA,NA,NA
"17131","POT1-
AS1",0.573847760842524,-0.522058165304654,1.81851766503382,-0.28707896
2906277,0.774051859688179,NA
"17132","AC000123.1",0,NA,NA,NA,NA,NA
"17133","FSCN3",0.635892079239439,-0.368042790395005,1.83420938676258,
-0.200654730616448,0.840968559434568,NA
"17134","LRRC4",0.626452895353359,0.0125198611885614,1.74695305816911,
0.00716668437655836,0.9942818621324,NA
"17135","LEP",0.0367720482966892,-0.560503011376585,3.34952001437583,-

0.167338307868279,0.867103863749685,NA
"17136","AC018635.2",0,NA,NA,NA,NA,NA
"17137","PRRT4",0.860758957746168,0.0665461619971396,1.58987714945311,
0.0418561660692024,0.966613360160888,NA
"17138","AC018638.7",1.08580537621678,1.10638951171038,1.4058514914013
8,0.786988894970346,0.431288366968836,NA
"17139","AC018638.6",0.173694385693788,0.450698267773282,2.85372304708
896,0.15793342953621,0.874509255668894,NA
"17140","FAM71F1",0,NA,NA,NA,NA,NA
"17141","ATP6V1FNB",0.763257069775072,-1.61048054119122,1.649282346579
87,-0.976473521668917,0.328829863095248,NA
"17142","IRF5",0.313921382082454,-1.82957220103905,2.3844409020376,-0.
767296098416871,0.44290548763277,NA
"17143","TSPAN33",0.448727178764426,0.745648140103718,1.93474365662073
,0.385398932593523,0.69994187328447,NA
"17144","STRIP2",0,NA,NA,NA,NA,NA
"17145","SMKR1",0.747591653557798,2.30067895366779,1.86043251284414,1.
23663660884459,0.21622202242152,NA
"17146","AC073320.1",0.147372702532553,-1.1477220656059,3.339063851089
6,-0.343725701810518,0.731052580354999,NA
"17147","AC087071.1",0.854553632434813,2.49863991317623,1.503452425583
34,1.66193480462594,0.0965258472248093,NA
"17148","CPA2",0.0741298089160557,-1.76673671430743,3.34309696236258,-
0.528473069790613,0.597171033704837,NA
"17149","CPA4",0.562221716632719,0.485831605671953,2.09047545008442,0.
232402444932961,0.816225450125791,NA
"17150","CPA5",0.318501110664512,-0.658145771751813,2.40922756837849,-
0.273177088121556,0.784717091409864,NA
"17151","CPA1",0,NA,NA,NA,NA,NA
"17152","AC007938.1",0.399064709314068,1.24599958031216,2.293536137438
34,0.543265728397819,0.586946857221725,NA
"17153","MESTIT1",0.303269266411891,1.0727654749473,2.37822581631485,0
.451078054736446,0.651933293649195,NA
"17154","AC007938.2",0,NA,NA,NA,NA,NA
"17155","AC008264.2",0.55098316304604,1.81321979478368,1.9052645414267
6,0.951689256456666,0.341254599584123,NA
"17156","LRGUK",0.139138330553998,-0.0338344975521838,3.34232334820666
,-0.0101230473617515,0.991923114749952,NA
"17157","AKR1B10",0.499143085137713,-1.45226630715515,2.18273763738816
,-0.665341671064469,0.50583198324578,NA
"17158","AKR1B15",0,NA,NA,NA,NA,NA
"17159","AC083870.1",0.200601781177701,0.585305641041376,3.16043896918
792,0.185197577535177,0.853074069074656,NA
"17160","AC009542.1",0,NA,NA,NA,NA,NA
"17161","SLC13A4",0.794524577621711,-0.0640583721992239,1.708422058008
11,-0.0374956363381958,0.970089819399639,NA
"17162","AC091736.1",0.453192416867425,-1.22293739884947,2.25158379024
741,-0.543145409087838,0.587029689815395,NA
"17163","AC015987.1",0.88036734273593,-1.66507747246486,1.756238423125
31,-0.948093066715723,0.343082077567075,NA

"17164","AC024084.1",0,NA,NA,NA,NA,NA
"17165","AC078845.1",0.348127452817314,-2.77495368732187,2.61435422383524,-1.06142987894389,0.288494584202949,NA
"17166","DGKI",0,NA,NA,NA,NA,NA
"17167","SVOPL",0,NA,NA,NA,NA,NA
"17168","AC083880.1",0.697431014384909,-0.32739354879967,1.59879847679181,-0.204774744004402,0.837748120228527,NA
"17169","CLEC2L",0.293404613599114,1.00496856842666,2.81535074830612,0.356960342874262,0.721121494297541,NA
"17170","TBXAS1",1.34694243239586,-1.13288673774669,1.2128791145019,-0.934047527244246,0.350279377347498,NA
"17171","RAB19",0,NA,NA,NA,NA,NA
"17172","AC006452.1",0.166045726037911,0.183394512381981,3.33779889015228,0.0549447460489789,0.956182483505573,NA
"17173","NDUFB2-AS1",0.313719631547345,0.11156219962684,2.15239999387258,0.0518315368632379,0.958662926533518,NA
"17174","TMEM178B",0.388363086622019,1.03128946935905,2.64948669020221,0.389241234225767,0.697097703323401,NA
"17175","AC073878.1",0,NA,NA,NA,NA,NA
"17176","AC004918.4",0.331517453361795,-0.597214710352191,2.41271451926306,-0.247528128829184,0.804499524999222,NA
"17177","TAS2R4",1.20719583037661,1.27371629280744,1.32470793491695,0.961507256984379,0.336297178398474,NA
"17178","MGAM",0.29448243389986,0.720695510657489,2.47922205459795,0.29069421567983,0.771285195876597,NA
"17179","CLEC5A",0.102366282257309,-0.49131726092088,3.34845351515744,-0.146729604785264,0.883345450266286,NA
"17180","TRBV3-1",0,NA,NA,NA,NA,NA
"17181","TRBV4-1",0,NA,NA,NA,NA,NA
"17182","TRBV5-1",0,NA,NA,NA,NA,NA
"17183","TRBV9",0,NA,NA,NA,NA,NA
"17184","TRBV5-4",0,NA,NA,NA,NA,NA
"17185","TRBV14",0,NA,NA,NA,NA,NA
"17186","TRBV18",0,NA,NA,NA,NA,NA
"17187","TRBV20-1",0,NA,NA,NA,NA,NA
"17188","MTRNR2L6",0,NA,NA,NA,NA,NA
"17189","TRBV28",0.0732428936164972,-0.49131726092088,3.34845351515744,-0.146729604785264,0.883345450266286,NA
"17190","AC244472.1",0,NA,NA,NA,NA,NA
"17191","KEL",0,NA,NA,NA,NA,NA
"17192","AC073342.1",0,NA,NA,NA,NA,NA
"17193","CLCN1",0,NA,NA,NA,NA,NA
"17194","AC093673.2",0.419911096074925,-1.32028169514223,2.49077535946509,-0.530068554807674,0.59606440017536,NA
"17195","EPHA1",0.388352393975391,-1.81412735618453,2.26011214383589,-0.80267139006012 5,0.422164693424515,NA
"17196","CTAGE6",0.147372702532553,-1.1477220656059,3.3390638510896,-0.343725701810518,0.731052580354999,NA
"17197","OR2A42",0.355273158961201,-0.347538517327666,2.10983370624232

,-0.164723179983053,0.869161871219592,NA
"17198","OR2A7",0,NA,NA,NA,NA,NA
"17199","OR2A1",0.467202891054406,0.157272410674589,1.97987186999164,0.0794356508915161,0.93668611291076,NA
"17200","CNTNAP2",0.498137178113732,1.68974842586392,2.41614157540217,0.699358201136314,0.484328202904778,NA
"17201","AC005518.1",0,NA,NA,NA,NA,NA
"17202","AC006974.1",0.0367720482966892,-0.560503011376585,3.34952001437583,-0.167338307868279,0.867103863749685,NA
"17203","AC073140.2",0.0367720482966892,-0.560503011376585,3.34952001437583,-0.167338307868279,0.867103863749685,NA
"17204","AC073140.1",0.724772317076675,-0.459711507526458,1.54269870402737,-0.297991763606423,0.765709449909464,NA
"17205","GHET1",0.346668202458428,-1.94732862277873,3.00338753971666,-0.648377406187962,0.516740879961632,NA
"17206","AC073314.1",0,NA,NA,NA,NA,NA
"17207","ZNF467",0.342157855423707,-0.617755040995007,2.37281219406507,-0.260347212704043,0.794595957359595,NA
"17208","SSPO",0.181578773267413,-1.95393031097672,3.33920037562282,-0.585149164824313,0.558447422910535,NA
"17209","AC004877.1",0,NA,NA,NA,NA,NA
"17210","ATP6V0E2-AS1",0.110901857212745,-1.2169087618042,3.34013336902682,-0.36432939267894,0.715612053721651,NA
"17211","AC092681.3",0.166548679549901,-1.95393031097672,3.33920037562282,-0.585149164824313,0.558447422910535,NA
"17212","AC092666.1",0,NA,NA,NA,NA,NA
"17213","AC006008.1",0,NA,NA,NA,NA,NA
"17214","ACTR3C",0.0741298089160557,-1.76673671430743,3.34309696236258,-0.528473069790613,0.597171033704837,NA
"17215","ZBED6CL",0.110014941913186,-0.033834469553869,3.34232334787728,-0.0101230389858472,0.991923121432614,NA
"17216","LINC00996",0.0367720482966892,-0.560503011376585,3.34952001437583,-0.167338307868279,0.867103863749685,NA
"17217","GIMAP8",0.230228123330503,-1.33023934114211,2.97714568949567,-0.446817011957333,0.655007187193824,NA
"17218","GIMAP6",0.176496091173365,-1.14849506633421,3.26459492750587,-0.351803238024283,0.724985830023263,NA
"17219","GIMAP1",1.05088289654904,0.92686073886755,1.59159531022103,0.582346990416072,0.560332979806479,NA
"17220","GIMAP5",0.341129980543248,-1.35584050242381,2.13008704224124,-0.636518825539269,0.524438314730738,NA
"17221","AOC1",0,NA,NA,NA,NA,NA
"17222","KCNH2",0,NA,NA,NA,NA,NA
"17223","NOS3",0.328064560500408,-0.607303666148901,2.70536905215305,-0.224480895006019,0.822383131186076,NA
"17224","ATG9B",0.250625900089421,-1.56858798056831,2.93131916770576,-0.535113336633345,0.592571488297769,NA
"17225","ASIC3",0,NA,NA,NA,NA,NA
"17226","CRYGN",1.0298736148579,0.088345242967629,1.27713149781594,0.0

691747428661109,0.944850527210773,NA
"17227","AC093583.1",0.441505152027483,-0.621699711546511,2.2729866775
2869,-0.27351665440576,0.78445609189319,NA
"17228","AC006017.1",0,NA,NA,NA,NA,NA
"17229","XRCC2",0.735751858009595,2.19210720619606,1.70532180566041,1.
28545075710631,0.198634814001933,NA
"17230","DPP6",0,NA,NA,NA,NA,NA
"17231","AC093726.2",0.78248216346367,0.83461598887174,1.5307414770036
8,0.545236410857204,0.585590932288622,NA
"17232","AC099552.5",0.230228123330503,-1.33023934114211,2.97714568949
567,-0.446817011957333,0.655007187193824,NA
"17233","AC144652.1",1.21083571827992,-0.203229426869903,1.34993302382
551,-0.150547785173801,0.880332453178585,NA
"17234","EN2",0.110014941913186,-0.033834469553869,3.34232334787728,-0
.0101230389858472,0.991923121432614,NA
"17235","AC009403.2",0,NA,NA,NA,NA,NA
"17236","RNF32",0.0367720482966892,-0.560503011376585,3.34952001437583
,-0.167338307868279,0.867103863749685,NA
"17237","AC005534.1",0.204732564514618,0.248608670877116,3.33613854356
166,0.0745198880774602,0.940596716765987,NA
"17238","AC005481.1",0,NA,NA,NA,NA,NA
"17239","AC011899.2",0.100451492077291,-0.0338344573480013,3.342323347
73369,-0.010123035334371,0.991923124345921,NA
"17240","AC011899.3",0,NA,NA,NA,NA,NA
"17241","LINC00689",0,NA,NA,NA,NA,NA
[truncated: 466,260 more chars]
