# Supplementary material for: PPARγ agonist treatment reduces fibroadipose tissue in secondary lymphedema by exhausting fibroadipogenic PDGFRα+ mesenchymal cells
Source: JCI Insight. 2023 Dec 22;8(24):e165324. doi: 10.1172/jci.insight.165324 (PMC10807713; doi:10.1172/jci.insight.165324)
Supplement: Supplemental data set 8 [file jciinsight-8-165324-s190.pdf]

"","gene","baseMean","log2FoldChange","lfcSE","stat","pvalue","padj"
"1","SUCNR1",50.163180468349,-3.16798248015853,0.39383013192353,-8.044
03275261237,8.69292706875363e-16,1.23091847293551e-11
"2","PDGFD",109.089729228488,2.12828786481056,0.289188803603667,7.3595
0990594839,1.84586660258335e-13,1.30687355462901e-09
"3","FXYD5",120.965823748865,-1.70882347842476,0.242786694676586,-7.03
837366665032,1.94496529583265e-12,9.18023619633009e-09
"4","CAV1",1122.90518993686,-1.46895738719143,0.211796165189862,-6.935
7128627641,4.04177802213665e-12,1.43078941983637e-08
"5","EMCN",25.9464298769868,-2.10825090827209,0.331491932521181,-6.359
88602267834,2.01903509692517e-10,5.71790739449207e-07
"6","TNFRSF12A",77.5706468567418,-2.09164205101278,0.330884693184184,-
6.32136237818801,2.59267221117327e-10,6.11870641836892e-07
"7","PERP",41.1037443353063,-1.7122342904704,0.275186996875165,-6.2220
7557011544,4.90620605580925e-10,9.92455396432271e-07
"8","CD36",83.6810664943765,-2.78305178923199,0.476910958021741,-5.835
57945654316,5.36039437705118e-09,9.48789804738058e-06
"9","GPX3",593.393831350248,1.76714957677118,0.308117650824268,5.73530
78346656,9.73353952599238e-09,1.53141021875613e-05
"10","LMNA",1718.66318170161,-0.95145413355534,0.16692333950275,-5.699
94667246437,1.19844911830991e-08,1.64477778317367e-05
"11","NFATC2",29.5544304175887,-1.96502103024403,0.345406036901873,-5.
689017620738,1.27772285416034e-08,1.64477778317367e-05
"12","CAPG",79.5046726772635,-1.82772123375535,0.325050979809549,-5.62
287563269686,1.87804611498196e-08,2.21609441567872e-05
"13","PLP2",275.213606717266,-1.05000719480473,0.1887847813903,-5.5619
271165397,2.66811728299889e-08,2.90619544055879e-05
"14","MAF",247.611280231439,0.980400074560718,0.180872959423404,5.4203
7946239219,5.94726613270799e-08,6.01523488851037e-05
"15","TUBA1C",115.738791555887,-1.29725823292163,0.245042309597956,-5.
29401732725282,1.19658087815503e-07,0.000112957234897835
"16","CCN3",132.859566768407,-1.83292581632918,0.350614960343739,-5.22
774560027958,1.71589385132113e-07,0.00015185660584192
"17","FBLN2",1310.07474863417,0.833469804365557,0.164620949173607,5.06
296318026081,4.12789600585379e-07,0.00034382945554641
"18","PHACTR2",193.269173625934,0.810402039342799,0.163523196447231,4.
95588428400318,7.20020608063136e-07,0.000566416211676334
"19","TXNIP",2435.11451563142,0.990074193102858,0.201334829190135,4.91
7550515653,8.7633846987037e-07,0.000653102775440234
"20","CD9",446.882099956144,-1.6649181491229,0.342593170951094,-4.8597
5287978105,1.17532367819369e-06,0.000832129164161132
"21","KLHL13",14.6334429503364,2.35502046106185,0.489963451313074,4.80
652272072648,1.53578036691421e-06,0.00103555476169072
"22","AIF1",13.0293591720675,-2.36297867623404,0.495195642140433,-4.77
18083019073,1.82579237650986e-06,0.00112405304571216
"23","IL33",91.850378418861,0.948720216637621,0.198793052846172,4.7724
0126379945,1.82042358762161e-06,0.00112405304571216
"24","ECM2",273.293807429314,0.787470751133752,0.166626394062043,4.725
96646867685,2.29023441725511e-06,0.00135123830618051
"25","RBMS3",252.616773807388,0.818751969538475,0.175147992282838,4.67

462948828048,2.94484784610262e-06,0.00141458048929465
"26","ADH1B",706.00079681095,1.00506754503357,0.214294077614984,4.6901321595987,2.73028643569018e-06,0.00141458048929465
"27","LIFR",170.560847459582,1.13805389536174,0.243641105517308,4.67102582277888,2.99699256206494e-06,0.00141458048929465
"28","RASL12",59.7292838712306,-1.21753998059386,0.260613012774417,-4.67183110939953,2.98526383899422e-06,0.00141458048929465
"29","KLK1",9.87886110842481,-4.3231243705875,0.923964755235073,-4.67888449867075,2.88439859630926e-06,0.00141458048929465
"30","VEGFD",58.9757560817065,2.06897560657201,0.442859550121353,4.67185500686407,2.98491645430064e-06,0.00141458048929465
"31","C1QA",12.8284966534057,-2.35207344021177,0.513681106963177,-4.57885915664089,4.67518855872534e-06,0.00200608090883487
"32","IL1B",26.8798913023691,-3.01910058006395,0.657721224567457,-4.59024350635701,4.42729203213108e-06,0.00200608090883487
"33","LUZP2",8.0048646679061,4.04943647981203,0.883670580750867,4.58251815554521,4.59409807152386e-06,0.00200608090883487
"34","MME",131.904538068425,2.04110111552406,0.447641955557377,4.55967339563291,5.12332390363076e-06,0.00207275047072604
"35","ANXA5",1334.21339968893,-0.737525174418168,0.161593977113227,-4.564063510247,5.01728897471693e-06,0.00207275047072604
"36","ZNRF1",50.9798656858077,-1.11441268166886,0.245035726903668,-4.54795998832843,5.41684306226319e-06,0.00213062493782352
"37","GSTM1",3.80470655887117,5.71905490883847,1.26291313270577,4.52846261609894,5.9414390124586e-06,0.00227380476801118
"38","CLCF1",11.5138939240751,-2.42504771786183,0.536349652425278,-4.52139328681615,6.14339279707588e-06,0.00228922215806827
"39","ID1",125.220347282412,-1.30422903247629,0.289656813572653,-4.50266995756051,6.71050522469499e-06,0.00243642958927387
"40","BCL7A",17.215027780788,1.67286856268251,0.374780952974112,4.46359013020084,8.05976722269678e-06,0.00285315759683466
"41","FAM43B",19.2956276106982,-1.79524374366792,0.40535191041018,-4.42885230724901,9.47358673429511e-06,0.00325797837782601
"42","VGLL3",113.039855865453,1.72586053892445,0.39006296073668,4.42456914049198,9.66349518846697e-06,0.00325797837782601
"43","SCIN",29.2935341725351,2.60005336272362,0.590077092935814,4.4062943534845,1.05154045692743e-05,0.00346274717909126
"44","AK1",96.5625259706216,-0.893657937797118,0.204272673455381,-4.37482861843641,1.2152791880822e-05,0.0039109893871009
"45","SPP1",7.92035806753327,-3.36553397690373,0.775398765357051,-4.34039119904194,1.42229278667565e-05,0.00447548130207272
"46","S100A6",9682.69742000452,-0.822652855269378,0.190309421350946,-4.32271218854866,1.54122735115239e-05,0.00460873277298837
"47","CXXC5",193.605688861556,-1.09740750721857,0.254297272711024,-4.31545134369422,1.5927713317175e-05,0.00460873277298837
"48","FZD8",37.9948838157282,1.66174848226091,0.384727294943284,4.31928928387025,1.56532486245088e-05,0.00460873277298837
"49","AP1S2",29.3194407643646,-1.23628852203495,0.286498482365588,-4.31516604146397,1.5948298437601e-05,0.00460873277298837
"50","CPB1",79.4220317793764,2.26806060820187,0.52876320972613,4.28936

916654356,1.7918132802261e-05,0.00507441520960032
"51","MAN1C1",78.8961028161676,1.11045328693978,0.259393688910627,4.28095722607338,1.86091127246658e-05,0.00516676541531896
"52","IGSF10",83.3749328352489,1.72687252904292,0.404032195106071,4.27409634667744,1.91914041491025e-05,0.00522596697598639
"53","RGCC",78.6342659284082,-1.79814701808536,0.421265073819627,-4.26844552239161,1.96839885782077e-05,0.00525896751447963
"54","LDHA",764.468957220844,-0.684630331257206,0.160940110382351,-4.25394471043115,2.10037183290532e-05,0.00540750275526169
"55","ABCA8",788.911071099935,1.12013534840526,0.263156723256769,4.25653327242685,2.07621150466879e-05,0.00540750275526169
"56","ATP11A",43.1791323183435,1.26310829272204,0.298330466788546,4.2339232272154,2.2964937139376e-05,0.00570497385778182
"57","EMILIN2",70.6970689079073,1.02710632490979,0.242505750374851,4.23538956631811,2.28155812150628e-05,0.00570497385778182
"58","AGTR1",155.108049335364,1.20411868170705,0.285752816944794,4.21384710947461,2.51057266917043e-05,0.00612926017162989
"59","FBLN5",634.284710208609,0.805281161114773,0.191656758278523,4.2016841375586,2.64936621658028e-05,0.00635847891979267
"60","TNFAIP6",221.232172262964,-1.38722102416262,0.331246275903331,-4.18788413659768,2.81567188680799e-05,0.00653605146183626
"61","PCSK5",62.9758115020686,1.48740022769098,0.354857954604525,4.19153694708246,2.77071054856198e-05,0.00653605146183626
"62","S100A10",6463.64831405717,-0.902234860745348,0.216654829924783,-4.16438840093518,3.12188279452316e-05,0.00701680323340443
"63","ACADVL",240.6869432291,-0.568734184141922,0.136457858660549,-4.16783752672462,3.07502982403657e-05,0.00701680323340443
"64","MARCKS",1181.77427143127,1.13848360107251,0.274932279413555,4.14096010661592,3.45855114273025e-05,0.00763387367403978
"65","ABCA1",128.365894057087,1.49633382440327,0.361612480897849,4.13794850412248,3.50424992099284e-05,0.00763387367403978
"66","ANTXR2",199.799595700637,0.738670033940764,0.178929890599381,4.12826516277609,3.6551047412431e-05,0.00784186108121247
"67","CRIP2",132.485221720281,-1.2985391352279,0.3151212344577,-4.12076049861441,3.77623825491033e-05,0.00794207134024701
"68","ABCA6",312.372469764966,0.99682227279658,0.242037132052853,4.11846837029496,3.81398906170054e-05,0.00794207134024701
"69","DBNDD2",215.997899044223,-1.53426443481141,0.374639073960825,-4.09531344018815,4.21597608098564e-05,0.00865191613141402
"70","TMCC3",9.31933339105029,2.55247075810279,0.624133491253417,4.08962312369552,4.3207466506807e-05,0.00874025322480552
"71","PRELP",211.643002397354,0.99237804519819,0.243910968932392,4.06860769543029,4.72949000625247e-05,0.0094323349983852
"72","NEGR1",365.066808765743,0.92055692898976 4,0.226891184667099,4.05726176775193,4.96514217499523e-05,0.00956564633438355
"73","LHFPL6",886.99946737483,0.751840466490792,0.185379910156621,4.05567391771627,4.99899596570892e-05,0.00956564633438355
"74","C16orf89",29.9661930049695,1.47388143329856,0.363321774406166,4.05668346112081,4.97744673447733e-05,0.00956564633438355
"75","TUSC1",108.35126069075,-1.07209848945804,0.265591591298552,-4.03

664319422258,5.42214363842299e-05,0.0102370071893426
"76","CRIP1",1928.93280434899,-0.978181920662152,0.242735887451563,-4.02981994517538,5.58195930046132e-05,0.0104000715387542
"77","VEGFC",37.392608014763,1.69951397947931,0.422700160888456,4.02061351457063,5.80467702437898e-05,0.0106745748915852
"78","CTSO",116.048344181525,0.734897217545479,0.183073084584785,4.01422863012468,5.96405158896819e-05,0.010827047499973
"79","SNAI2",219.686981643991,0.883716108387519,0.220322546333025,4.01101077985797,6.04593494086572e-05,0.0108367644003365
"80","STEAP4",73.8318592730876,1.28851497924849,0.322927733642156,3.99010318722367,6.6044556315376e-05,0.0116898864678216
"81","TAGLN2",493.468145717134,-0.857619222193715,0.215354823496754,-3.98235436879659,6.8235936603431e-05,0.0118727315155287
"82","BATF3",9.05308281616709,-2.20091513233287,0.552916632168753,-3.98055512220718,6.87545186633721e-05,0.0118727315155287
"83","PLAGL1",388.168956509901,0.900040219928262,0.226530933859604,3.97314487956896,7.09298762137364e-05,0.0119567505617441
"84","TBC1D2B",106.625660430401,0.884526189990551,0.222497148816518,3.97544954933321,7.02464349607947e-05,0.0119567505617441
"85","ENPEP",22.8439350365739,2.00762820004999,0.507066932616084,3.95929624062081,7.51709472015354e-05,0.0123769838648109
"86","TYROBP",19.1384294316872,-2.05877002165424,0.519811925708164,-3.96060559566709,7.47599241489665e-05,0.0123769838648109
"87","WNT5A",27.1158580399569,2.11215950502266,0.53443053652966,3.95216844968858,7.74461907476297e-05,0.0126050351837521
"88","MAN2A1",35.8682773667824,1.07474480392553,0.272161614440692,3.9489213279918,7.8504144749787e-05,0.012632030564283
"89","TUBB3",43.1742537423261,-1.42885509741075,0.363206133217258,-3.9340059727352,8.35417057546738e-05,0.0132915792526537
"90","TPP1",84.9248137561802,0.718493421571824,0.183050089701271,3.92511919958287,8.66868016981046e-05,0.0136387234671685
"91","PLAUR",54.3982986563202,-1.16957983894522,0.299394757521952,-3.90648068999492,9.3650123509391e-05,0.0145723708669558
"92","GNS",95.2609468461481,0.667175826076438,0.170923717230038,3.90335429680901,9.48686708164857e-05,0.0146015258561026
"93","DDIT4L",14.2757170284624,-2.1578082154596,0.554960143955203,-3.88822195424862,0.00010098126322595,0.0152122076223582
"94","ADD3",630.301781430812,0.509175764690467,0.130953671172087,3.88821298504384,0.000100984994103225,0.0152122076223582
"95","OLFM4",9.92663696827414,2.18843030816864,0.564361083787888,3.87771299445437,0.000105443002703415,0.0155773832444678
"96","RNASE1",11.8891284393059,-2.34610663697075,0.605083153926708,-3.87732929225612,0.000105609377928595,0.0155773832444678
"97","SCARA5",691.137362862494,0.774251167869445,0.200347672102813,3.86453787929275,0.000111299717536838,0.0162474639208416
"98","DIO2",23.4044175206546,-2.02880319815202,0.526653487392749,-3.85225436974854,0.000117035353176805,0.0169104142957506
"99","FAXDC2",52.7751493507898,0.992629477373241,0.25925910624148,3.82871595819147,0.000128813569883963,0.0184242439349183
"100","ENO1",376.772968751553,-0.557475527445236,0.145827348646429,-3.

82284621245418,0.000131920062655162,0.018679880871971
"101","VIM",19420.6990000631,-0.776280855168574,0.20341050315701,-3.81632631118056,0.000135453346208293,0.0189902909139547
"102","TRIL",40.3784431443425,1.38771574527257,0.363944838685691,3.81298372106062,0.000137299159115495,0.019023215059139
"103","ANGPT1",62.1613941930241,1.54433517276115,0.405225128278345,3.811054806306,0.00013837508129176,0.019023215059139
"104","IGF2R",81.0868186370249,0.937624856055632,0.247095026378834,3.79459218502485,0.000147886329276044,0.0201352925245075
"105","CCT6A",101.273251578507,-0.687238879506595,0.181485633578194,-3.78673984247076,0.000152636762225198,0.0205841576486552
"106","NDUFA4L2",16.9173022474639,-2.16257671598313,0.571946233870991,-3.78108393396804,0.000156147014261647,0.0208588841692917
"107","UBE2E3",270.437300073632,-0.854147279079846,0.226232764216101,-3.77552421303552,0.000159671512574214,0.0211303609163633
"108","RECK",95.2887924764599,1.00464071709936,0.267086419819945,3.76148183713959,0.000168909688654977,0.0221459369569859
"109","XBP1",156.541905886136,-0.655391438712277,0.174529271125914,-3.75519495660671,0.000173206601388147,0.0225009676665702
"110","TXN",725.972815096399,-1.01343223847036,0.270052507797507,-3.75272293057265,0.000174924155852337,0.0225175095169917
"111","TET1",41.1451180549714,0.961161918203654,0.257206325798872,3.73692954564132,0.000186281092052931,0.0237634257970226
"112","AIF1L",10.7080675936801,2.32192193550647,0.623959566926663,3.7212698684038,0.000198223461809384,0.0250611091001864
"113","ABCA10",168.770111376769,1.30371485579506,0.350590643083455,3.71862421748865,0.000200310783584738,0.0251008911111494
"114","OLMALINC",9.98034055461187,1.99014973195198,0.535859536612944,3.71393918736857,0.000204057849613205,0.0253461328993244
"115","CXCL12",2453.4883203032,0.948424501992819,0.256007550412622,3.70467394599959,0.00021166281877732,0.0260621349033639
"116","IFRD2",32.9305647334732,-0.974759060513912,0.264728889724885,-3.68210308110654,0.000231317839049392,0.028236729318443
"117","ARHGAP20",67.6604639745469,1.00116223115753,0.2722568443923,3.67727112018875,0.000235742439225493,0.0285308798242135
"118","FGFBP2",70.8398577948671,-3.42971988530902,0.937751588402999,-3.65738637793178,0.000254800147575471,0.0305760177090565
"119","AKAP12",305.551957411283,0.849530884691342,0.232438377296963,3.65486497785167,0.000257317364888813,0.0306186040909713
"120","KRT222",91.0082330488047,1.41886291267785,0.390318130654681,3.63514477356713,0.000277824572788904,0.0327832995890906
"121","DPYD",76.6299366756368,0.773275777555379,0.213113390473097,3.62847109624956,0.000285104662170035,0.0333643141845264
"122","GEM",139.321402083742,-1.00741248242,0.27936811079337,-3.60603964267461,0.000310905661159186,0.036085443950935
"123","TWIST1",209.431371671319,-1.26283379430046,0.350985168098701,-3.59796911402631,0.000320711692511733,0.036677554273186
"124","PTGDR",6.52018735729244,-2.19187707022387,0.60926374043186,-3.59758332027148,0.000321187622166318,0.036677554273186
"125","CYP4B1",32.7143801080221,2.08254978983814,0.579680839676357,3.5

9257999798796,0.000327420090433228,0.0370506191559777
"126","AQP3",40.2613540709725,-1.57148929692328,0.43764545915758,-3.59078167964595,0.000329687712828615,0.0370506191559777
"127","PLAT",45.8446672684478,0.914304975154809,0.255093849450242,3.58419059152248,0.000338125123575184,0.0371151298436016
"128","AL117339.4",2.08872593065635,4.81295757783375,1.34234145687553,3.58549425198899,0.000336440413945589,0.0371151298436016
"129","RASSF4",38.4726025494428,0.983959716126847,0.274318487708846,3.58692454287367,0.000334601097775818,0.0371151298436016
"130","TMEM37",40.0097994777831,1.4343987174163,0.401369639739568,3.57375988464653,0.000351891644468165,0.038329120658994
"131","FOLR2",3.59273862277591,-3.8328855622374,1.07317812020425,-3.57152786669552,0.000354904777235836,0.0383622263027439
"132","TGFBR2",382.837787445329,0.529991169077883,0.14887368803467,3.5600056401804,0.000370846828763062,0.0391879932483952
"133","SVEP1",444.860428069704,1.60410903675681,0.450567831909592,3.56019432181452,0.000370580463343043,0.0391879932483952
"134","ADGRD1",46.0791668336108,1.11071974540398,0.311748057524865,3.56287623481146,0.000366813643872989,0.0391879932483952
"135","TUBB2A",153.326750392322,-0.951539530990031,0.267931409284051,-3.55142957495231,0.00038314451291785,0.0401876022438278
"136","PKM",349.14492530209,-0.781267360770696,0.221218527580617,-3.53165428463483,0.000412968874625752,0.0429973475345636
"137","PLEKHA2",43.2940006939186,1.05063853346194,0.298609408409721,3.51843747676015,0.000434096093451482,0.0445420339367608
"138","VASN",148.043164422669,-0.783240817871942,0.222565780413993,-3.51914304353095,0.000432943208048503,0.0445420339367608
"139","CACHD1",29.1376403965278,1.06612003999095,0.304169500138521,3.50501953517833,0.000456573780285744,0.0464474986959015
"140","LAMC1",479.237449958023,1.03583355846654,0.29581884477987,3.50158070300539,0.000462506872607494,0.0464474986959015
"141","CTNNAL1",45.6504634503775,-1.06644345616326,0.30443600046789,-3.50301362034791,0.000460025939725378,0.0464474986959015
"142","RAB13",305.024451147431,-1.15783804134628,0.331350403999802,-3.49430097977779,0.000475304841957361,0.0473965955078608
"143","EXOC4",39.4511999281255,0.799570057404856,0.229267659310525,3.48749605508861,0.000487566116068956,0.0482792741506043
"144","IL6ST",576.184057533709,0.692915729687448,0.19915582855073,3.47926412563389,0.000502792768852243,0.0493317388220133
"145","PTGER2",30.0031015047755,-1.33909027945459,0.385016900191281,-3.4780038974635,0.000505162579745193,0.0493317388220133
"146","IGFBP2",3.85590971432813,-3.9391572848526,1.13735171357064,-3.46344691606951,0.000533301810845433,0.0517229701477488
"147","SNX7",92.8644186089656,-0.67068375057904,0.19425941900866,-3.45251599125364,0.0005553845555905,0.0534982673956564
"148","DKK3",33.1981765851641,-1.60636972321538,0.466103302737315,-3.44638133602904,0.000568148021362129,0.0543579458276199
"149","ANOS1",29.6659040912758,1.92954475840704,0.560185643136127,3.44447377766545,0.000572172132818901,0.0543755530249372
"150","KCND2",28.9040600331509,1.18619891339352,0.344880198185095,3.43

945207534616,0.000582892977137383,0.055025097041769
"151","LRRFIP1",114.73588629404,-1.09726570265782,0.319460142583699,-3
.43474993087857,0.000593100866722373,0.0550328221615733
"152","PLXNA4",18.0271462959471,1.40960517247257,0.410403618927719,3.4
3468017205968,0.000593253551273306,0.0550328221615733
"153","EGR3",65.9889801765932,-1.08367451444333,0.316439540596423,-3.4
2458629664558,0.000615736257443487,0.0550328221615733
"154","CTHRC1",342.27106202429,-1.07502186077592,0.313601594342291,-3.
42798595469689,0.000608076918777036,0.0550328221615733
"155","ABCC9",79.5081826055223,1.08065646213346,0.314995871032283,3.43
07004043958,0.000602025098179374,0.0550328221615733
"156","TMOD2",47.2923358712763,0.915932711004683,0.267517918077862,3.4
2381817855692,0.000617479194949501,0.0550328221615733
"157","TRIM47",128.375496141706,-0.795394635945147,0.232242504858819,-
3.42484523420323,0.000615149734455909,0.0550328221615733
"158","PIM3",59.5397453834479,-1.08204317064716,0.316053318229643,-3.4
2360958811687,0.000617953299695632,0.0550328221615733
"159","PLP1",36.7995689855323,1.11523216445136,0.325667184641648,3.424
45360492344,0.000616037020884847,0.0550328221615733
"160","MLF1",64.8631636707017,-0.790574162946387,0.231180250778754,-3.
419730536165,0.000626831939890824,0.0554746266803379
"161","NAA10",82.010424445968,-0.77074892221168,0.225537984068157,-3.4
1737967285706,0.000632270364971041,0.0556083749564593
"162","TKT",127.685271931781,-0.529688997350736,0.155597998903917,-3.4
0421471408397,0.000663545726892355,0.0579988116839244
"163","PTPRT",10.8500664956385,-2.58445405041886,0.7615069177729,-3.39
386811872614,0.000689128705671234,0.0598654139405195
"164","ADAMTSL1",29.5819026593358,1.51078223488818,0.44554129614974,3.
39089159174244,0.000696656517784241,0.0601503432428344
"165","A4GALT",89.7665758759455,-0.762071589163971,0.224953407700065,-
3.38768635227814,0.000704848167852486,0.060488788222977
"166","PODN",483.873668076833,0.713893827262799,0.210869829989993,3.38
547163098996,0.000710560537853009,0.0606116699758952
"167","S100A3",15.6957233905884,-1.23908026523937,0.366740259249999,-3
.37863169910319,0.000728475304321791,0.0617677264023746
"168","ABCA9",292.145676678057,1.00420386214699,0.29789203820724,3.371
03290235764,0.000748869132301189,0.0631189697225288
"169","REEP5",334.827972919681,-0.620507207522086,0.184386752978395,-3
.36524830281486,0.000764748044056611,0.0640759307919622
"170","PLXNC1",28.401827458568,1.58805475913444,0.472823278482459,3.35
866449771963,0.000783200915193574,0.0652360291714177
"171","KIAA1109",79.9654652331271,0.816422534109158,0.243334939888056,
3.35513894751303,0.000793251302461715,0.0655555869478805
"172","AMPH",24.1122845901679,2.22350162613163,0.662924673219245,3.354
0788508203,0.000796296677615498,0.0655555869478805
"173","ADM",189.729171943113,1.45890308080339,0.435408906665252,3.3506
505229233,0.000806219794680034,0.0655590496104621
"174","SYNE2",87.8709998328552,0.850059433372439,0.253803883633753,3.3
4927669822066,0.000810228367360937,0.0655590496104621
"175","HPS4",35.5145312888128,0.973489088312125,0.290509907005153,3.35

096692001921,0.000805299215990755,0.0655590496104621
"176","CRABP2",248.211457774171,-1.24678147206953,0.37309067819355,-3.
34176527300618,0.000832474268616962,0.0669763388841828
"177","PCSK6",13.6050747800046,2.53891877676631,0.760671353692991,3.33
77341797349,0.000844645125405686,0.0675716100324549
"178","NTM",44.0906631370863,1.10389482361781,0.331050807096273,3.3345
1784425579,0.00085447417819317,0.0679739009169398
"179","SH3BGRL3",1769.95991020194,-1.02259704870966,0.307252159050813,
-3.32820134403202,0.000874086688298302,0.0682738628051793
"180","PID1",151.041188903649,0.862455256429752,0.259321445286398,3.32
581540056298,0.000881602947672932,0.0682738628051793
"181","MAST4",40.6048378570377,0.957812197018116,0.287799379012621,3.3
2805512056409,0.000874545610740176,0.0682738628051793
"182","CAPN1",97.2194063988446,-0.660224032870781,0.198529085176611,-3
.32557837700934,0.000882352887948292,0.0682738628051793
"183","DTNA",6.12359972764243,-2.39737920837645,0.720208465251243,-3.3
2872956101693,0.000872430738424998,0.0682738628051793
"184","CCDC71L",285.659125590599,-1.09786229741894,0.330684698350292,-
3.31996703474918,0.000900280781967134,0.0691582363757564
"185","GALC",30.6835776130144,0.91163520727317,0.274675387625474,3.318
95484030846,0.00090355040462676,0.0691582363757564
"186","EFHD2",57.5293366614551,-1.08948757273865,0.32858963510396,-3.3
1564801912864,0.000914309069408882,0.0692052388065133
"187","ANKRA2",23.3302148360039,1.07447098359101,0.324017008407191,3.3
1609438922024,0.000912849916280013,0.0692052388065133
"188","SEMA3E",19.4005721039438,-2.17871657088949,0.657962043897674,-3
.31131041842944,0.000928601368166492,0.0692052388065133
"189","MYH10",68.6121146543358,0.867141109216108,0.261785189905086,3.3
124146921012,0.000924943299034772,0.0692052388065133
"190","APP",478.299434460521,0.558022418322853,0.168457097879547,3.312
54916145985,0.000924498762594221,0.0692052388065133
"191","OGN",763.761099928515,0.862211718948522,0.260738449751047,3.306
80695452383,0.000943658993004302,0.0696336270279758
"192","TUBB6",197.620495719523,-0.80466941385789,0.243348764654433,-3.
30665090903814,0.000944184773260689,0.0696336270279758
"193","TRIO",110.434460599222,0.943120932500551,0.286822889748211,3.28
816480905159,0.00100842787185778,0.0736048384819903
"194","LVRN",15.0482459202058,1.35537108492055,0.412183277032386,3.288
27286414644,0.00100804087614804,0.0736048384819903
"195","C6orf120",49.359123325882,0.725456325425422,0.22079163616481,3.
28570564549784,0.00101727255121712,0.0738696375653047
"196","CAV2",87.0269703867485,-0.657697019089294,0.200454679963055,-3.
28102601151798,0.00103430205175926,0.0747230461883222
"197","STARD13",10.2087966287654,1.80841865975456,0.552104014852279,3.
27550354843629,0.00105473789334161,0.0758126323335902
"198","ASAP3",38.3405671798305,0.789630785493436,0.242672047856554,3.2
5390086113336,0.00113831997764472,0.0791094026958155
"199","PLPP3",745.787280912884,0.855562519249139,0.26307482602141,3.25
216415492188,0.00114529855597755,0.0791094026958155
"200","S100A4",4642.98675047458,-0.73581836016624,0.225747758236282,-3

.25947139371406,0.00111620036322072,0.0791094026958155
"201","DOCK4",22.9841597866067,1.22468782410162,0.376438518122042,3.25
335417377394,0.0011405124719318,0.0791094026958155
"202","KCP",8.91969145081628,2.23438213024112,0.686440197547818,3.2550
280974556,0.00113381148075015,0.0791094026958155
"203","H19",686.121798913655,0.771012958255616,0.23679576638599,3.2560
2509716674,0.00112983764102589,0.0791094026958155
"204","HSDL1",17.5909870666601,1.20983016024298,0.370932063212341,3.26
159499334088,0.00110787305743315,0.0791094026958155
"205","KDELR3",115.238235783255,-0.830458063502627,0.255047962280824,-
3.25608585960095,0.00112959587098548,0.0791094026958155
"206","ZNF528-
AS1",11.5212396407768,1.76939227455912,0.544976904542661,3.24672891605
191,0.00116739524476911,0.0802442556598575
"207","TFPI",411.845389075198,0.508554794617134,0.156895319799705,3.24
136370202989,0.00118959304930278,0.0813254766230468
"208","PTMA",1912.87910039366,-0.696537642569456,0.214969901522671,-3.
24016356539103,0.00119461152101651,0.0813254766230468
"209","SPG11",42.5950807773647,0.755252554903356,0.233256906780169,3.2
3785719929458,0.00120431073712756,0.0815934930034749
"210","GFRA1",85.1724655871156,1.24175535315362,0.383711627195107,3.23
616816678379,0.00121145990082235,0.0816870104554499
"211","SFRP1",325.010775158959,1.46186579730873,0.452223132448986,3.23
262056364098,0.00122660364676036,0.0818725491015325
"212","DIO3OS",103.916130941446,1.09745006431973,0.339528523384658,3.2
3227649146994,0.00122808165941904,0.0818725491015325
"213","ABCG1",6.95280661794979,2.43177638422436,0.752529728019317,3.23
146886253235,0.00123155741233237,0.0818725491015325
"214","MMP23B",95.116001756732,1.21669253773422,0.377184894919886,3.22
571914761498,0.00125656595708261,0.0831447380948117
"215","HS3ST3A1",5.12302826983463,-2.4144366383317,0.749771753451029,-
3.2202288592743,0.00128088295913321,0.0843595474480289
"216","AQP7",7.17172377303757,-2.47881882285275,0.770270248722282,-3.2
1811575478164,0.00129035731424772,0.0845900906006837
"217","IL1RN",3.35195650131588,-4.36710302080949,1.3593076942783,-3.21
274060258163,0.0013147497450886,0.085124820744264
"218","DLGAP2",3.10411611122748,3.29166795584534,1.02493501754561,3.21
158697819481,0.00132004004512529,0.085124820744264
"219","CCDC85B",203.620370991263,-0.868152366953253,0.270364957155142,
-3.21103879766151,0.00132256077427529,0.085124820744264
"220","FRMD6",223.310274397439,0.902304061131044,0.280952324092372,3.2
1159137603141,0.00132001984024562,0.085124820744264
"221","BCHE",15.522350074995,2.00792076684097,0.627213179135778,3.2013
3701528345,0.00136791437139401,0.0868594955109381
"222","TFRC",20.3650593070712,-1.00087312410984,0.312508779787679,-3.2
0270401615544,0.00136143828646032,0.0868594955109381
"223","PLIN4",4.96112934203782,-3.62762365483124,1.13305801191116,-3.2
0162217353059,0.00136656111049407,0.0868594955109381
"224","FCGR2A",7.80202008384938,-1.8491108690743,0.578863027135367,-3.
19438413302199,0.0014012952310713,0.0877979666901312

"225","APBB2",46.0682704226462,1.03916325139701,0.325242868112123,3.19
503778031737,0.00139812537030323,0.0877979666901312
"226","LINC00702",16.0729909830474,-1.42106255339938,0.444671704601607
,-3.19575664179609,0.00139464688876705,0.0877979666901312
"227","SASH1",239.821593089279,0.859502876673137,0.269425438543459,3.1
9013260707562,0.00142207525344229,0.0887074255010696
"228","SEPTIN11",221.689649736753,0.660218372791266,0.207048774955148,
3.18870938953531,0.00142909468142031,0.0887543012671562
"229","KANK4",1.89791751268856,4.74118569943028,1.48956398939021,3.182
93523017511,0.00145790222902041,0.0901480155586418
"230","TMEM35A",5.9318529762101,1.90418520562446,0.599379338927315,3.1
7692833562182,0.00148843811115193,0.0916360158865711
"231","IGFBP6",2513.55587427105,-0.636624454132901,0.200490582637444,-
3.17533345336294,0.00149664407406618,0.091742338046654
"232","CDA",20.9847593175972,-2.20243303250893,0.693892516544246,-3.17
402620722527,0.00150340115714599,0.0917593120051173
"233","GAS6",364.024776606568,0.847362685019967,0.267457894114276,3.16
820966465068,0.00153380862515179,0.0930475684522079
"234","MAP7D3",180.730244927722,0.702342078679926,0.221735120693641,3.
16748233876001,0.00153765049560852,0.0930475684522079
"235","FBN1",1508.7454934904,0.904353856657929,0.286083705693686,3.161
15122483149,0.00157146888867006,0.0946893594194384
"236","SEC14L2",17.0063422387928,-1.49640059247052,0.474882577973748,-
3.15109600115346,0.00162659016685389,0.0975954100112335
"237","DESI2",108.066948870946,-0.572283818690931,0.181856038774469,-3
.14690577529106,0.00165008127070252,0.0982188233776403
"238","MAZ",146.43711135209,-0.584859029118926,0.185860170542162,-3.14
676903293948,0.00165085310479367,0.0982188233776403
"239","LINC00882",17.9291066078247,1.5525269455855,0.494017523428857,3
.14265561838735,0.0016742269077295,0.0984001589572935
"240","ARHGAP12",76.3482348260502,0.614480385316304,0.1955346971558,3.
14256443615576,0.00167474846812908,0.0984001589572935
"241","HSD3B7",92.644434985208,-0.973957874066561,0.309922936211645,-3
.14258081693395,0.00167465475941769,0.0984001589572935
"242","CHPF",58.6424752732427,-0.793010316324731,0.253671399643016,-3.
12613214355544,0.0017712194310473,0.103638293981941
"243","GPR183",18.4640079635769,-1.60484758182609,0.514126960752889,-3
.12150053262321,0.00179931922486044,0.104849219028905
"244","S100A16",349.1343585017,-0.834649233920276,0.267711282097441,-3
.11772155204308,0.00182254904675812,0.105767600418422
"245","MS4A4A",5.03668454454601,-2.696041591851,0.867070826078756,-3.1
0936720595662,0.00187488543048218,0.108360725288276
"246","ROBO2",13.4152303017091,1.20127393341243,0.386752155528129,3.10
605620742315,0.0018960067233176,0.109135996756818
"247","ATOH8",28.5153463979158,0.970547427096096,0.313055000989585,3.1
0024572049045,0.00193360158899828,0.110849386640549
"248","HRCT1",36.0347810398635,-1.61191593487079,0.5208098755542,-3.09
501799126895,0.00196800959112079,0.112366999234961
"249","PTPRD",33.6722666295085,1.13311981213296,0.366924164248713,3.08
815805155011,0.00201401325826075,0.114531838301093

"250","RFTN2",32.4304589306708,1.06869074868611,0.346331373524177,3.08
574628342616,0.00203041995011013,0.115002985974238
"251","PPDPFL",23.4020647919676,1.59326868289406,0.516705866456133,3.0
8351189008481,0.0020457293521455,0.115408476599125
"252","SEMA3B",8.62292112538396,1.60745589063478,0.521839772815272,3.0
8036292818909,0.00206748495185351,0.116172963961292
"253","GLUL",323.669530161892,0.880960238981723,0.286246891207537,3.07
762377877845,0.00208658162196555,0.116782591964554
"254","PGP",38.5618752990565,-0.871412247341419,0.283657044777963,-3.0
7206277222387,0.00212585014914212,0.118511961070285
"255","SERPINE1",118.071022825093,-2.13101134237316,0.695014359266234,
-3.06614002137019,0.00216841741507402,0.120410943519404
"256","IL17RD",23.4363948258095,1.14218683773825,0.372796376555288,3.0
6383567429565,0.00218518903053062,0.120523147025101
"257","TCEAL3",52.5564182029275,-0.869411710386984,0.28379457662888,-3
.06352475341317,0.00218746107241885,0.120523147025101
"258","C11orf96",1208.84789525567,-1.0692957112396,0.350041922596982,-
3.05476470734257,0.00225237151898811,0.122667618111045
"259","ATP8B4",34.0047639921391,1.44062465550529,0.471368035253241,3.0
5626293630903,0.00224114626489373,0.122667618111045
"260","FTX",167.209197994343,0.958119673744388,0.313572508105442,3.055
49641304081,0.00224688290454757,0.122667618111045
"261","TGFBR3",260.997679757063,0.563555858952879,0.184827664973635,3.
04908823596981,0.00229537053780772,0.124530447568419
"262","SLC7A4",1.57121388964836,4.46535274111846,1.46846994163383,3.04
081998174936,0.00235934846361148,0.127512878796712
"263","CSPG4",8.12864461220865,-2.12070263599648,0.697743118471122,-3.
03937449163714,0.00237069959569154,0.127639187357385
"264","SPART",154.398638456517,0.460171253544823,0.15175537393367,3.03
232262302591,0.00242679676494274,0.129673366760714
"265","TUG1",81.6254958057476,0.718415385729288,0.236889881789876,3.03
269764120416,0.00242378319819029,0.129673366760714
"266","RIPOR3",35.1221359870923,1.1295515796998,0.373209322197908,3.02
658993898553,0.00247329229986246,0.131660973556588
"267","ZNF98",2.23148634826911,4.19442077345845,1.38776681372144,3.022
42475608037,0.00250758414784534,0.132986485144157
"268","FMO1",94.7858063222821,-1.09583005137657,0.36283377362519,-3.02
019858964002,0.0025260899537142,0.133152136028648
"269","DEPP1",318.36734004016,1.26733561179502,0.419676976192503,3.019
78827452687,0.00252951444856682,0.133152136028648
"270","NID1",228.600752471396,1.04431579237332,0.346195913187398,3.016
54569737229,0.00255672677186603,0.134086115146752
"271","SRM",165.648596959854,-0.752245489733145,0.249928080896134,-3.0
0984782116486,0.00261378613392728,0.136572736739521
"272","QPCT",12.464702106586,-1.80331204152287,0.601394824050379,-2.99
854932135533,0.00271268245512145,0.141219057222499
"273","NINL",11.0730287637936,1.49683103676121,0.500115687321468,2.992
9695762554,0.00276277288792997,0.143299868472851
"274","ANXA1",2226.68125234611,-0.590422468662155,0.197639208837522,-2
.98737518802526,0.00281384164170169,0.14541604980473

"275","LARP6",147.810026819651,-0.578043286323203,0.193655838442164,-2
.98489986655289,0.00283671167712717,0.146064863084075
"276","JTB",278.880673176755,-0.538071025721443,0.180813115989004,-2.9
7584067825127,0.00292186614916359,0.149363265964464
"277","CORO6",5.92758208192751,1.96973299025682,0.661676221526549,2.97
688344567145,0.00291194690346058,0.149363265964464
"278","FILIP1",75.1335452712939,0.687036692010907,0.231060493803839,2.
97340614442802,0.00294514461999455,0.1500116828026
"279","AC074387.1",2.62399700994776,-3.97167534478113,1.33653708468246
,-2.97161626886302,0.00296236682665795,0.150348079804575
"280","FCGR2B",5.16676745566055,-2.20198835653009,0.742861818396927,-2
.96419643868885,0.00303474493951654,0.152434822524993
"281","BHLHE40",64.6955804687315,-1.08296116056431,0.365360210252653,-
2.96409168315132,0.00303577824520113,0.152434822524993
"282","THOC6",27.1373156163007,-0.91977091097434,0.310238012888622,-2.
96472667037275,0.00302951966866093,0.152434822524993
"283","SET",300.17258922165,-0.457480061455445,0.154580361538399,-2.95
949664564476,0.0030814205647386,0.153097948058591
"284","CX3CL1",8.45939668516577,-1.67511807408292,0.566007946017926,-2
.9595310204883,0.00308107681019482,0.153097948058591
"285","LILRB5",3.84171465394703,-3.41756926665957,1.15417428231402,-2.
96105130657359,0.00306590860734916,0.153097948058591
"286","CALR",545.105794291889,-0.554887335017893,0.187593186347132,-2.
95792904754601,0.00309713403684673,0.15334062224388
"287","RNPEPL1",41.6694797346698,-0.730954325007898,0.247301435785652,
-2.95572212383535,0.00311937990488549,0.153412092688743
"288","GASAL1",12.9670281909374,-1.8510160478543,0.626454306343881,-2.
95475029720399,0.00312922207126594,0.153412092688743
"289","PARP16",57.7872624729524,-1.4233519793309,0.481746364480232,-2.
95456714212383,0.00313108014032815,0.153412092688743
"290","CCDC18-
AS1",64.1230722698801,0.655861277663371,0.222258570267216,2.9508930831
0967,0.00316856577401794,0.15368870390735
"291","AC002451.1",11.7342183639728,1.31791386072585,0.446418080035398
,2.95219642676961,0.00315522147491065,0.15368870390735
"292","EBF3",83.1750211405743,0.741932772101743,0.251432506281659,2.95
08227996209,0.00316928683198773,0.15368870390735
"293","PNISR",468.760108861617,0.524795447806691,0.177949159756755,2.9
4913136158693,0.00318668494114612,0.153754794479741
"294","CDKN1B",59.7904106469868,0.735265798494668,0.249362605935671,2.
94858082564451,0.00319236649555394,0.153754794479741
"295","YIF1B",30.4565648941153,-0.748277016141328,0.254001218234445,-2
.94595837509198,0.00321955723044541,0.154538747061379
"296","NRXN3",3.17601680337123,-3.62082807709346,1.22969988332018,-2.9
4448110974626,0.00323496693686432,0.154592922534573
"297","AP000525.1",5.32623687446817,2.48282074706627,0.843418404896231
,2.94375926900925,0.00324252104468701,0.154592922534573
"298","BZW2",62.0307588143464,-0.713847537155901,0.242669437656961,-2.
94164582095007,0.00326473091908578,0.155129496020989
"299","FABP4",368.537177145426,-2.26076365670958,0.769158052702316,-2.

93927060734363,0.00328985698631248,0.155281249753949
"300","Z83843.1",22.0059211195832,1.00169075187275,0.340744537611215,2
.93971184070944,0.00328517614298941,0.155281249753949
"301","PLXNB2",48.3892552050265,0.6954717818719,0.236878621205152,2.93
598374700762,0.00332491742699192,0.156414720153507
"302","ISM1",36.8902684459437,1.12995275730384,0.385086005682703,2.934
28673239006,0.00334315212024806,0.156751768287128
"303","EGR2",34.9563110245386,-1.352946375534,0.461852877160359,-2.929
38821525247,0.00339629948745626,0.158718154265283
"304","NR4A1",112.393884491375,-1.37017884347203,0.46808263117357,-2.9
2721573547119,0.00342011550732496,0.15930538020961
"305","CYP2U1",35.1373704174886,0.759063199008124,0.259416319782909,2.
92604258530589,0.00343303940096887,0.159383075140063
"306","FHDC1",10.3781687346474,1.34524810096112,0.460768336051671,2.91
9575838237,0.00350508097977138,0.161359420953324
"307","KCTD12",383.563134602459,0.981437185527879,0.336205700054156,2.
91915688927876,0.00350979531452146,0.161359420953324
"308","MYADM",576.615243691797,-0.690620800658008,0.236567547082596,-2
.91933872238563,0.00350774848084118,0.161359420953324
"309","CD52",17.2578290506166,-1.20488827268888,0.413206456115557,-2.9
159473547817,0.00354610330601521,0.161976847784437
"310","MAP3K21",2.96037206228511,2.87686360297547,0.986592238006737,2.
915960102005,0.00354595842912894,0.161976847784437
"311","IL6R",24.8870440953403,1.05420906953458,0.362256712402447,2.910
11604048184,0.00361294587307637,0.162763228788397
"312","NLGN1",4.62670929228093,2.19401908805698,0.75410409213371,2.909
43798202855,0.00362079216584357,0.162763228788397
"313","ZNF727",16.4421659160019,1.02017631286666,0.350487357264528,2.9
1073641237418,0.00360578066227389,0.162763228788397
"314","CCDC34",58.4954153422744,-0.917894062785134,0.315342876990596,-
2.91078102522832,0.0036052658883641,0.162763228788397
"315","VSIG4",3.25497462533724,-3.05098201692603,1.04761613936637,-2.9
1230910089963,0.00358767419998506,0.162763228788397
"316","CEBPG",31.9863949109599,-0.781534285637557,0.269015001491494,-2
.90516990243858,0.00367053793699153,0.164477269581646
"317","TBC1D12",37.4302238715899,0.750944299491677,0.259060362350261,2
.89872326541552,0.00374685437790294,0.167367375366264
"318","STAB1",3.38765871977062,-3.15698639609303,1.0900957159368,-2.89
60634831777,0.00377875948765782,0.168261743224009
"319","FKBP1B",14.3341183536621,-1.20437010131922,0.416552233386866,-2
.89128230456679,0.00383673302353214,0.17030764769033
"320","SMOC2",282.476506858699,1.81477337144961,0.629519400397494,2.88
279180959906,0.00394167858698139,0.174419277473926
"321","GPC1",69.9336563002578,-0.92734425859134,0.32200585016701,-2.87
989879100137,0.00397802849078312,0.174934420588475
"322","GALNT16",46.4557172328613,0.834520947892742,0.289752167071704,2
.8801197807305,0.00397524112177033,0.174934420588475
"323","LAMA2",302.092312941438,0.720765473342627,0.250553789667717,2.8
7668956952718,0.00401870727704956,0.17617614564403
"324","IL7",12.8885531108943,-1.36178171916358,0.473719125233424,-2.87

466063037367,0.00404461975443357,0.176410135936292
"325","TMEM64",40.0691036987419,1.02965590462684,0.358225684188132,2.8
7432183139076,0.00404896145334004,0.176410135936292
"326","HPGD",30.2495048634998,1.02306111038495,0.356630909134909,2.868
68323574818,0.00412184370584817,0.177394687975572
"327","PDE7B",21.6668328608824,1.10559705197664,0.385227618823689,2.86
998386915412,0.00410492739492779,0.177394687975572
"328","ST6GALNAC4",34.8175633577974,-0.94415843293941,0.32896480321429
5,-2.87008951630721,0.00410355609637593,0.177394687975572
"329","HMGA2",1.96718027892155,-3.5321116257427,1.23116100785048,-2.86
892746214365,0.00411866243025707,0.177394687975572
"330","TYMP",385.83148584569,-0.649365950969559,0.226438507883236,-2.8
6773639801763,0.00413419823671883,0.177394687975572
"331","PTMS",795.069221030543,-0.563621466710061,0.196660945058659,-2.
86595524364,0.00415753019640919,0.177856880909831
"332","KHDRBS3",151.837917659591,-0.821060045845047,0.286788689259992,
-2.86294430914849,0.00419724328735568,0.179014954665531
"333","RGS2",50.4649862904128,-1.21999858286874,0.42644215973869,-2.86
087703808723,0.0042247087212424,0.179107411655067
"334","ARC",22.095258024534,-1.06313712509408,0.371606003605591,-2.860
92558994946,0.00422406180404067,0.179107411655067
"335","ZNF668",13.1087267668922,-1.26142991848504,0.44125210453614,-2.
85875105300881,0.00425312415250603,0.179773844774583
"336","RHOBTB3",265.694842000057,0.894624816851733,0.313882348057013,2
.8501915523113,0.00436929063972852,0.182874537455785
"337","PDE7A",14.8008866788772,1.0868357541908,0.381237633088838,2.850
80920628195,0.00436081275533294,0.182874537455785
"338","TMEM245",52.0813546172524,0.887557373601397,0.311401292919277,2
.85020452317606,0.00436911244887811,0.182874537455785
"339","ADAMTSL3",42.4771038689235,0.98482659314124,0.345608007332754,2
.84954796256512,0.00437814040942875,0.182874537455785
"340","F13A1",3.23450619958563,-3.14874967840502,1.10619604909738,-2.8
4646621272448,0.00442074202259993,0.183068678596214
"341","MUC12",12.7482665396965,-1.48424608980038,0.521168851733301,-2.
84791787702599,0.00440062786405142,0.183068678596214
"342","COTL1",149.645566205171,-0.572217618881413,0.201031602464617,-2
.84640629565756,0.00442157401694247,0.183068678596214
"343","ABRACL",51.7583522238446,-1.11589988125459,0.392350907474884,-2
.84413737803326,0.00445318429857695,0.183305493220493
"344","ANGPTL4",380.511452651404,-1.09901518101689,0.386329091758267,-
2.84476422941651,0.00444443067217653,0.183305493220493
"345","RSPO3",30.3034632732639,1.03885952876594,0.36602428622564,2.838
22568026409,0.00453650950027216,0.186194129054649
"346","TMEM70",65.421131846947,-0.700008484480066,0.246987582529007,-2
.83418493072564,0.00459427366841758,0.187336507312643
"347","AKR1C1",332.07538062754,-0.862738011958409,0.304477127503816,-2
.83350680240372,0.00460403280683614,0.187336507312643
"348","NRBF2",68.8314717487465,-0.609153829153408,0.214862290400784,-2
.83508952649229,0.00458128453709656,0.187336507312643
"349","HIST1H2AC",23.1359716263255,1.09361880435222,0.386491440739431,

2.82960678834161,0.00466052438623889,0.187480185537337
"350","AL137026.1",17.0715618745065,1.04779384371067,0.370224483399025
,2.8301581626663,0.00465249983893094,0.187480185537337
"351","PDE1B",9.31030188036931,-1.57417556299015,0.555836934974657,-2.
83208161232022,0.00462460433942639,0.187480185537337
"352","ZNF83",58.401294899001,0.593438005694814,0.20960490338891,2.831
22196141435,0.00463705295385513,0.187480185537337
"353","ADIRF",397.771864748215,-1.07718595351847,0.380842963274972,-2.
82842551233047,0.00467775854456623,0.187640399408096
"354","ILF2",122.659168910891,-0.79486032848844,0.281169534774761,-2.8
26978851479,0.00469894310767768,0.187957724307107
"355","NET1",24.5179541881718,1.06514493021075,0.377060414222205,2.824
86543279256,0.00473004765786036,0.188668943198036
"356","STAC",7.87556620505345,1.75972924640065,0.623441549042013,2.822
60502063852,0.00476352176642545,0.189470416327484
"357","MLF2",134.247307108888,-0.513307244173818,0.181978147503018,-2.
82070815214395,0.00479177749984768,0.189529523457662
"358","EMP3",839.131056370995,-0.646030350628909,0.22901842662934,-2.8
2086625140645,0.00478941667394825,0.189529523457662
"359","AL359541.1",3.79024731913319,2.33348555505031,0.827637974364654
,2.81945201564928,0.00481057228188107,0.189742906717092
"360","EIF2AK1",57.9073394448259,-0.586475817539971,0.208329596331225,
-2.81513442097554,0.00487568352465565,0.191437464421489
"361","RFX2",25.5469455289903,-1.14681596873996,0.407421833344546,-2.8
1481225325038,0.00488057377515238,0.191437464421489
"362","AC093627.6",3.64419971313602,-3.86602550871201,1.37516874698033
,-2.81130989720443,0.00493402379727088,0.192664439721194
"363","THBD",32.258829760629,-1.25515226027438,0.446517486898881,-2.81
098119805244,0.00493906720471705,0.192664439721194
"364","EBF1",516.859491043489,0.378328292765978,0.134797251640806,2.80
664693204657,0.00500600765154013,0.19473919875222
"365","LURAP1L",37.2557567523258,0.836510073183913,0.298152998576187,2
.80564031614178,0.00502167121651011,0.194813327193927
"366","MEGF6",39.9867196521645,1.13537656496462,0.405361059185854,2.80
090191012664,0.00509600062426952,0.195252960623917
"367","SH3RF3",17.72237156699,1.12580644492362,0.401617389664825,2.803
1815202603,0.00506011807956419,0.195252960623917
"368","FAM43A",65.0400446274848,0.956818720161581,0.341730058816761,2.
79992554203327,0.00511143952059744,0.195252960623917
"369","CHIC2",62.8692695994421,-0.632493620780612,0.225918481017914,-2
.79965418469,0.00511573788075374,0.195252960623917
"370","CCNG2",106.258053743144,0.711809184787172,0.253983272650833,2.8
0258293137966,0.00506951807668936,0.195252960623917
"371","MAP1A",207.771032985237,-0.652033224900304,0.232771035279135,-2
.80117852342924,0.00509163432399017,0.195252960623917
"372","BOC",226.733930954032,0.771538803599195,0.27580577953675,2.7973
9897001103,0.00515158757512383,0.195566970680304
"373","WASHC2C",37.5071581223604,0.776722534269914,0.277599551044877,2
.79799636327347,0.00514206916092211,0.195566970680304
"374","SLN",2.63539405625541,-4.00978976467814,1.4345805203704,-2.7950

9564485292,0.00518843625270115,0.19643919074398
"375","RTN4RL2",6.45417072777018,-1.74728822838025,0.626083065117436,-2.79082493319398,0.00525739000233552,0.198519046488189
"376","S1PR2",59.6551877197451,-0.629833432110133,0.225829241999675,-2.78898085355588,0.00528741907863238,0.19912195253573
"377","LINC01140",167.921673264767,-0.902919212759415,0.324755798311296,-2.78030205297187,0.00543083578877477,0.203980463578384
"378","IMPDH1",24.0939744431453,-0.870210063798253,0.313244232078804,-2.77805614495507,0.00546851682721123,0.204852376384421
"379","UAP1",204.504545579776,-0.646798660743818,0.23294115485752,-2.77666117496256,0.00549203978360073,0.205063736763344
"380","DUSP6",40.1627370727004,-0.93918243942141,0.338321499014992,-2.77600578785504,0.00550312287924227,0.205063736763344
"381","SHOX2",92.6068730946755,-0.529549708077892,0.19085459307873,-2.7746238617346,0.00552655848634311,0.205171601387782
"382","SGMS1",48.2290796703536,0.641571666798336,0.231405918924835,2.77249462666826,0.00556284378057013,0.205171601387782
"383","CD163",7.10241831146805,-1.78199901919221,0.642757451227871,-2.77242841104062,0.00556397563085511,0.205171601387782
"384","TPCN1",46.8657847181717,0.95882170003711,0.345793230651441,2.77281801679802,0.00555731892799544,0.205171601387782
"385","PTP4A2",293.586004999421,-0.49630498182339,0.179072524923972,-2.77153059652286,0.00557934285875961,0.205203882805289
"386","DLK1",35.6022506279077,8.93774109210138,3.22722698343798,2.76948015679392,0.00561458241987413,0.205964992397455
"387","LRP1B",16.9228391589204,1.64341071348912,0.594786855091683,2.76302460187322,0.00572684497301285,0.209000321695521
"388","GRID1",5.81661695757015,1.73728191275189,0.628583455705247,2.76380470561817,0.00571317221402157,0.209000321695521
"389","ST3GAL4",73.6502083253621,-0.674270647807204,0.244133616594521,-2.76189185747038,0.00574675094743424,0.209187643742079
"390","HSD11B1",100.660355735673,-1.13443369526922,0.41099461618585,-2.76021546412722,0.0057763250986229,0.209725034350001
"391","PFKP",54.2369100542218,-0.811262498965461,0.294067930892327,-2.75875882318669,0.00580213383160821,0.210123312162589
"392","HAS2",154.601461678923,-0.844633536577718,0.30630167276256,-2.75752178876433,0.00582413316811717,0.210381953215661
"393","AC046134.2",5.53572572347221,1.7054699609953,0.619396595288738,2.75343773919241,0.00589729860835237,0.2124828200872
"394","C1QB",11.258284689342,-1.57515405109215,0.572362981669433,-2.75201943790606,0.00592290051980504,0.212863632894516
"395","GTF3A",171.293819446657,-0.657621194238706,0.239175474843115,-2.7495343938172,0.00596799993127616,0.213941465890811
"396","DHRS4",30.7811634768549,-1.14550057184877,0.417277493043575,-2.74517698880334,0.00604782712015186,0.21516892467676
"397","ALKBH5",84.5327779848006,-0.56244677726632,0.20478779972865,-2.7464857672751,0.00602374996064862,0.21516892467676
"398","GATD3B",62.668349576471,-0.635263542137598,0.231341448460433,-2.74599967435688,0.0060326823503811,0.21516892467676
"399","BICRAL",40.2690632523549,0.796443782426472,0.29078310467637,2.7

389616852495,0.0061633555748274,0.218729611377333
"400","HMGA1",53.5644200269426,-1.30655821799982,0.477578173805629,-2.73579968612967,0.00622288939650513,0.220196422937523
"401","HRAS",42.9681014302973,-0.751722153309112,0.274840831895994,-2.73511817048196,0.00623578853092844,0.220196422937523
"402","POLD4",162.972413115877,-0.548410961844973,0.200636653357164,-2.73335381481228,0.00626929461708485,0.220828885019705
"403","AL355596.1",2.2500629130849,-3.72120365121992,1.36210756721173,-2.73194550914751,0.00629615532909308,0.221224713300144
"404","GRIA1",5.94074034011128,2.03037739230223,0.744967803075319,2.72545656862026,0.00642126238595447,0.224250882723316
"405","BEND6",28.5524480073559,-0.914786820819922,0.335799431352723,-2.72420598550398,0.00644562918562074,0.224250882723316
"406","ASPH",262.886515555451,0.535287729596087,0.196362984408286,2.7260113773943,0.00641047886224144,0.224250882723316
"407","AC009120.2",10.3199686612381,1.34107863806628,0.492236679120803,2.72445897461689,0.00644069315549766,0.224250882723316
"408","FH",47.5001662865323,-0.678446934012237,0.249213021729996,-2.72235748077115,0.00648179846782032,0.224297134519113
"409","TXNL4A",104.722827724599,-0.612697782509371,0.224990252775822,-2.72321922816743,0.00646491419108539,0.224297134519113
"410","CLCN4",12.8418217303012,1.31782706105879,0.484190558346688,2.72171160370955,0.00649447917746019,0.224297134519113
"411","PRDM6",9.54178428542165,1.29505832793734,0.476069842523686,2.72031162711783,0.00652204200012998,0.224701009055573
"412","CNOT1",51.7058299049442,0.644097819918951,0.237072436169443,2.71688193839032,0.00659001104180394,0.226491641630931
"413","TCEA3",49.2362224934764,0.859203592191069,0.31703982833564,2.71008092800712,0.0067266793158149,0.22896581517293
"414","MUC20",6.40601206921653,2.3847928376831,0.879955173760663,2.7101299120627,0.00672568592606747,0.22896581517293
"415","AASS",62.0832602869758,0.833004448437505,0.307280923332469,2.71088891364798,0.00671031032314877,0.22896581517293
"416","SDC4",49.6133281018812,-0.846716307345412,0.31236367422427,-2.71067469496305,0.00671464669079424,0.22896581517293
"417","ANXA4",251.053067532727,-0.418139845114679,0.154407686720546,-2.70802480106738,0.00676849641880711,0.2298367129264
"418","UGDH",181.482816448577,-0.764192038914387,0.282403825274087,-2.70602580603397,0.006809375421674,0.230294170319927
"419","TPBG",97.0954989304863,-0.576019586198659,0.212885156003462,-2.70577618943658,0.00681449557655716,0.230294170319927
"420","EFHD1",40.2127120942511,0.905560790761132,0.334780728851661,2.70493703107499,0.00683173383216885,0.230327026341693
"421","ST3GAL5",62.6142714093471,0.645991331341962,0.239081214813488,2.70197443929634,0.00689290611937402,0.231136775008156
"422","ZBTB20",410.629040189841,0.576709400408019,0.213549060409307,2.70059441752002,0.00692156877057591,0.231136775008156
"423","PLD1",23.9253978797316,1.04246041499653,0.385905052830146,2.70133911787717,0.00690608828887952,0.231136775008156
"424","SPON1",36.7669223738361,0.995841440318325,0.368885799105328,2.6

9959278110888,0.00694243947639779,0.231136775008156
"425","ATG101",57.9001570392906,-0.586899267757247,0.217189118449743,-2.7022498730435,0.00688719822192934,0.231136775008156
"426","BEX5",4.20119524414803,-2.6790080282771,0.992572997244915,-2.69905390909608,0.00695369111253352,0.231136775008156
"427","CDKN1C",364.326600667896,0.705608538259325,0.261524909234289,2.69805480604221,0.0069745957083145,0.231288700772209
"428","CXCL8",76.0036685430908,-2.25470304709091,0.836677710780012,-2.69482862760729,0.0070424841618713,0.231372565503707
"429","GAB1",62.1170213079161,0.642664676820823,0.238350036781673,2.69630617850292,0.0070113187588835,0.231372565503707
"430","PDE1C",6.07406866229684,2.14792594411585,0.796883669487794,2.69540715459316,0.00703026673084338,0.231372565503707
"431","AC024132.3",6.35653995728559,-2.56387223516011,0.950830960891401,-2.6964543021995,0.00700820128455084,0.231372565503707
"432","SMAD5",118.15377759633,0.525408611068736,0.195169299019767,2.69206588181435,0.00710109161624681,0.232758002976979
"433","PBXIP1",38.6862284775635,0.722745489253947,0.269070081770446,2.68608640729722,0.00722943901550993,0.233229772987588
"434","GHR",71.6169725241426,0.723961361651799,0.269243279744511,2.68887439767773,0.00716933891419371,0.233229772987588
"435","BHLHE41",78.888628162629,-1.0018907989687,0.372948148941069,-2.68640775349985,0.0072224888460609,0.233229772987588
"436","SNHG14",55.1725257763307,0.920212357637374,0.342278156744993,2.68849279307928,0.00717753850242306,0.233229772987588
"437","ANKFY1",52.0507700232121,0.572190025982519,0.212877382349032,2.68788548444457,0.00719060518193476,0.233229772987588
"438","CCL3L1",7.18000937425438,-2.27304007072412,0.846247007014776,-2.68602435445237,0.00723078180378186,0.233229772987588
"439","AL592183.1",8.18376257384348,-1.50470346098465,0.559306438953803,-2.69030241060559,0.00713872956531628,0.233229772987588
"440","FREM1",21.3964149483705,0.931097263040753,0.346804813401003,2.6847876011575,0.00725759117033333,0.233345436167613
"441","LMO3",68.45004929647,0.822796930824302,0.306517458563899,2.68433953054187,0.00726732608403372,0.233345436167613
"442","CLPTM1L",63.1740722933889,-0.572154631711634,0.213281233897139,-2.68262997759836,0.00730457606256898,0.233482611841934
"443","CMC4",44.6601887629974,-0.736457501628646,0.274522004707432,-2.68269023611975,0.007303260163651,0.233482611841934
"444","ESR1",12.8504482951824,1.66709725799686,0.622050281188284,2.68000402606082,0.00736212747015636,0.234703513826429
"445","C17orf49",106.459887813363,-0.700280857933729,0.261359552628317,-2.67937732097976,0.00737592257434753,0.234703513826429
"446","TTF2",18.1394518134071,0.977832266876358,0.366372760586473,2.6689546059895,0.00760877328617196,0.24110385596673
"447","NR4A3",8.78858703362776,-1.81942523441673,0.681726008675669,-2.66885113852583,0.00761111748708534,0.24110385596673
"448","SERP1",247.330334480479,-0.399959346901913,0.150027192128509,-2.66591236713488,0.00767797040952012,0.242138220487316
"449","RGS3",8.11874480836491,-1.62933191527236,0.611029748700736,-2.6

6653451609662,0.00766377363804337,0.242138220487316
"450","EBF2",73.822180318064,0.792725551827514,0.297543642661543,2.664
23286593033,0.00771641259609012,0.242809783023636
"451","PRKG2",3.49633346940433,2.65391273233786,0.997212347278944,2.66
133160061595,0.00778322631341255,0.244369145449937
"452","CHST2",13.0629426560346,-2.03785379885266,0.766130711804447,-2.
65992965358739,0.00781569735819213,0.244467568005564
"453","PRRC2B",104.735492739593,0.633619478963137,0.238260156721112,2.
65935978420765,0.00782893098254761,0.244467568005564
"454","RPS6KA4",17.9704076085901,-0.875534213259875,0.329276558375226,
-2.65896308434493,0.00783815507588462,0.244467568005564
"455","LINC02254",3.48322158157448,2.11174642655315,0.794780110341488,
2.65701972039261,0.00788348313288817,0.244863780926019
"456","NXPH3",22.5193895813689,0.860728278294457,0.323955230790735,2.6
5693588646038,0.00788544379253281,0.244863780926019
"457","LINC01354",11.457888733677,1.25720011738521,0.474860497381378,2
.64751463707352,0.00810858494333939,0.251241931723601
"458","PDLIM4",126.864519154386,-0.763030819470606,0.288385204066712,-
2.64587367420589,0.00814802384998171,0.251912702436116
"459","RNF152",15.9573033603894,1.01537271008657,0.38404298787486,2.64
390378719121,0.00819559482872209,0.252831422167113
"460","ACKR4",35.7734785501935,1.25964494090656,0.476783081010623,2.64
196652749617,0.00824262014897702,0.253178961625845
"461","VPS13B",31.2342206949071,0.82426921499683,0.311906976548351,2.6
4267642910211,0.00822535994069574,0.253178961625845
"462","MTF1",23.0690332939737,0.856920097755392,0.325175161480511,2.63
525693000002,0.00840736174245665,0.253506782389124
"463","SLC1A3",38.453889721085,0.696399598027056,0.26395969350492,2.63
82800676122,0.00833277316551011,0.253506782389124
"464","PAM",259.916166313444,0.6061813064338,0.229586155944544,2.64032
168638348,0.00828273663187436,0.253506782389124
"465","UBTD2",71.7878259833511,0.582423314157253,0.220741759080055,2.6
3848270750633,0.00832779475349507,0.253506782389124
"466","KDM7A-
DT",19.4258165964074,-0.968115959670402,0.36716276062868,-2.6367487759
7263,0.00837047975753153,0.253506782389124
"467","DENND2A",32.8483540110293,0.787799579374783,0.299142006284539,2
.63353043980537,0.00845022607963746,0.253506782389124
"468","RUNX1T1",239.850197330635,0.708492768192107,0.268439272379321,2
.63930371257662,0.00830765170153557,0.253506782389124
"469","MSANTD3",33.1306019205066,-0.8671884246886,0.328991690471008,-2
.63589765275552,0.00839150377957333,0.253506782389124
"470","NID2",111.963912254124,0.802847729889053,0.304828054185269,2.63
377244602656,0.00844420592989204,0.253506782389124
"471","AC009271.1",3.95367949919033,2.50753428703271,0.951962493324405
,2.63406836363479,0.00843684989253967,0.253506782389124
"472","RBM3",503.212451036503,-0.618914258425665,0.234976383359617,-2.
63394239700445,0.00843998052019347,0.253506782389124
"473","PKN1",73.0183965757341,-0.704941380152825,0.267933030333863,-2.
6310357452921,0.00851250821334263,0.254835341016769

"474","GLIS3",28.9502972735726,-0.833708102064277,0.317258760151528,-2.62784895731826,0.00859266594940395,0.256692299248017
"475","SYCE1L",22.0219271078367,-1.19708126870758,0.456211987693584,-2.62395838118924,0.00869144048712814,0.259096415363652
"476","ACP5",7.45664425084567,-1.80187595119975,0.687079249965601,-2.62251545406146,0.00872833092021814,0.259649508046825
"477","CYP26B1",108.397080028911,-0.993250935252851,0.379051536669154,-2.62035855066269,0.00878373607673289,0.260749901145782
"478","USP53",272.686394278713,0.675957649883815,0.258313404007769,2.61681213361845,0.00887551752688294,0.262532712444275
"479","STX16",90.9386497364466,0.576586942381128,0.220356790483695,2.61660619178328,0.00888087353536777,0.262532712444275
"480","RGS16",292.229032805386,-1.03513065481963,0.395924391300698,-2.6144654827125,0.00893671899906879,0.262539296736129
"481","SGCG",4.81557546979459,2.01830371924742,0.771691241720734,2.61542908631043,0.00891154241831103,0.262539296736129
"482","ALYREF",43.1808573193663,-0.837068607129477,0.320105744901272,-2.61497527133619,0.00892339157913629,0.262539296736129
"483","GRK5",37.0885457075968,0.886275240959225,0.339103317418863,2.61358469656165,0.00895978731596621,0.262672025660624
"484","NBL1",280.72493479843,-0.67308483053376,0.257719181259908,-2.61169862190024,0.0090093636875406,0.263019964223622
"485","FABP3",8.83628284228961,-1.92741188385628,0.738184772006659,-2.61101550309262,0.00902738012801414,0.263019964223622
"486","ETS2",19.5171085858726,-0.921820979828293,0.352873364396216,-2.61232802709713,0.00899279230637607,0.263019964223622
"487","ABHD17A",118.412085746491,-0.502336432865571,0.192455031845058,-2.61014964404773,0.00905026234989326,0.263145205081085
"488","DDX39A",30.7389136761833,-0.782541116486018,0.300080823356981,-2.60776782645352,0.0091134744884854,0.264440161387199
"489","C1QC",4.73033539132083,-1.91325454783296,0.734075135154084,-2.60634702935601,0.00915136907432736,0.264996699575614
"490","RABGAP1",42.974753234069,0.623402238894987,0.239326946704332,2.60481424043381,0.00919240829337463,0.265641839661601
"491","RPL39L",17.7675556267376,-0.891853645577659,0.342679586349527,-2.60258760983791,0.00925231721078901,0.266274000677453
"492","PARVA",174.201622050445,0.375230786983904,0.144213756266579,2.60190703507015,0.0092706979049424,0.266274000677453
"493","MEG8",65.1181277908072,1.13759572182783,0.436987507640167,2.603268290142,0.00923396620200363,0.266274000677453
"494","RICTOR",64.465040665334,0.707403035696784,0.272044862724333,2.60031756752417,0.00931375258834126,0.266969102532211
"495","MATR3.1",11.9220773296321,1.07240347432058,0.412855180399899,2.59752941281208,0.00938970782077931,0.268602550994414
"496","ADAMTS5",330.627991037573,0.866795650863068,0.334254022408285,2.59322429276347,0.00950807398131451,0.271440176563334
"497","PCDHGA8",3.62976506131852,2.19373852715346,0.846589695573475,2.59126533032915,0.00956237338376271,0.27244106059171
"498","FAM174C",86.0297761939504,-0.713698042817631,0.275764971054169,-2.58806635262401,0.00965163870441184,0.274432136655565

"499","GPR1",5.96100409789988,2.78378891928135,1.07659304248541,2.5857
3928069859,0.00971704005517791,0.275186574362638
"500","STARD5",12.4729698351896,1.11818956817678,0.432348618052815,2.5
8631465786293,0.00970083267432599,0.275186574362638
"501","XPO1",79.450119446353,0.499517626911849,0.193675342991045,2.579
14930830893,0.0099043967157285,0.279375014929712
"502","MSL1",71.7941260955495,0.555709957987522,0.215410614098263,2.57
977054804749,0.00988659812934299,0.279375014929712
"503","MEGF9",22.5417544244093,0.893683772822286,0.346747322308613,2.5
7733431615915,0.00995656008042834,0.280288053158778
"504","DCN",17717.7448131647,0.40993698160365,0.15927138126148,2.57382
700116504,0.0100580551006095,0.282583452826647
"505","VWA1",44.3222942795978,0.752659358259833,0.292904459029335,2.56
96411749896,0.0101803902020823,0.284467711306532
"506","YARS",24.4730287910144,-0.788315904301552,0.306832294796914,-2.
5692077322673,0.0101931333962554,0.284467711306532
"507","NR4A2",47.1301722810016,-0.96032072482006,0.374169572253779,-2.
56653879960267,0.0102719132793105,0.284467711306532
"508","KIF9",12.62875534127,-1.05821922040922,0.412549933360808,-2.565
06942514452,0.0103155162737862,0.284467711306532
"509","GBE1",36.0676494254509,-0.657604774777486,0.256128476672614,-2.
56748013075502,0.0102440660061591,0.284467711306532
"510","UST",44.9187012577289,0.655595687926127,0.255411116542363,2.566
82519070147,0.0102634338869695,0.284467711306532
"511","ANXA7",195.129975557369,-0.477351748852281,0.1861056585468,-2.5
6495021473107,0.0103190610005287,0.284467711306532
"512","SLC25A3",647.640077684969,-0.53207888712018,0.207296608908501,-
2.56675152537124,0.0102656143552957,0.284467711306532
"513","SLC39A6",79.0474448487589,0.490985458282579,0.191438500534488,2
.56471638104022,0.010326017204206,0.284467711306532
"514","CECR2",1.63493475013566,4.47620432139659,1.74176283327695,2.569
92756756387,0.0101719780585878,0.284467711306532
"515","EGFR",285.613884311813,0.671421933585172,0.261915742186544,2.56
350354499489,0.0103621642698275,0.284909215651955
"516","FAM89B",114.228969892292,-0.543906206930187,0.212298313982917,-
2.56199023311111,0.0104074245213969,0.285046675479653
"517","AKT1",66.4891975079702,-0.509113152156424,0.198676200322408,-2.
56252712368288,0.0103913470621624,0.285046675479653
"518","DNASE1L3",48.8842901915409,1.48999882306249,0.581974989031396,2
.56024545924621,0.01045982560536,0.285928823497872
"519","MEG3",601.92867178541,0.990858530825976,0.387541128879276,2.556
78289860852,0.0105645128914845,0.288234108946861
"520","HECTD4",29.5502607782595,0.841863864297116,0.329700501927387,2.
55342002628351,0.0106670772261685,0.290472718312587
"521","FBXO38",17.3436829441295,0.84285353915482,0.330249871375072,2.5
5216916707765,0.0107054525574822,0.29095817315537
"522","SLITRK5",1.69133059404342,4.51908439101777,1.77236075491857,2.5
4975426333303,0.0107798872296743,0.291860809124642
"523","AVEN",32.129577243793,-0.705109479646782,0.276493927930034,-2.5
5018070351696,0.0107667096916645,0.291860809124642

"524","CLEC7A",4.08047624155622,-2.1511856382875,0.844362542884056,-2
.54762433738624,0.0108459195023632,0.293088206399737
"525","CAMK2N1",182.075527688364,-0.675955177434949,0.265484312459848,
-2.54612097856886,0.0108927430434638,0.293792840943709
"526","BRINP3",1.56900179172347,3.64005712551126,1.43025360602923,2.54
504313792087,0.0109264239068367,0.294140993385566
"527","GSTM2",19.3354446108213,0.873916080003869,0.343872755616558,2.5
4139377351065,0.0110411491319024,0.294298567983725
"528","EVA1A",5.50637915323767,-1.57094154334036,0.617675413647518,-2.
54331240750476,0.010980700217556,0.294298567983725
"529","RPL22L1",178.94854401998,-0.614666444585916,0.241946780670042,-
2.54050267948874,0.0110693245480223,0.294298567983725
"530","TRIM2",39.2462154610977,0.642346450022395,0.252843164064104,2.5
4049363920925,0.011069610718746,0.294298567983725
"531","CLIC1",688.495048982749,-0.580056671983599,0.228465364594871,-2
.53892607753561,0.0111193314880856,0.294298567983725
"532","DEPTOR",73.6995699366286,1.05263531866578,0.41403474313129,2.54
238402967063,0.0110099130801193,0.294298567983725
"533","APBB1IP",59.4153202866886,0.907987788904848,0.356940576184461,2
.54380658710994,0.0109651781881144,0.294298567983725
"534","NMB",33.7195005432745,-1.32088962779666,0.520116968403285,-2.53
960110521232,0.0110978963262495,0.294298567983725
"535","POLDIP2",43.7985302836896,-0.654630562526633,0.257795934521657,
-2.53933625346461,0.0111063021797078,0.294298567983725
"536","UBTD1",21.3010743920124,-0.960014523048595,0.378905857457553,-2
.53364920112416,0.0112881673596336,0.297944895680382
"537","CCL3",22.0631904017821,-1.6451719811549,0.649416639797309,-2.53
33074028845,0.011299181425167,0.297944895680382
"538","AC026904.2",1.98165141094592,3.21655404880103,1.27023672204293,
2.53224772436733,0.0113333890532557,0.298291429357064
"539","CREG1",150.858640116335,0.448046032386872,0.177347168380266,2.5
263782696895l,0.0115245319473918,0.29950689364746
"540","VIT",216.030728060357,0.538027612715039,0.213080439056325,2.524
99767269964,0.0115699055500456,0.29950689364746
"541","SLC6A6",13.6597887476147,-1.02750717279897,0.406347552622721,-2
.52864122391546,0.0114505009821229,0.29950689364746
"542","ELOVL2",15.2059973602979,1.05217736089836,0.416246318810465,2.5
2777577446267,0.0114787635624146,0.29950689364746
"543","ARHGAP21",239.086106668035,0.415681086559129,0.164466764849456,
2.52744733526936,0.0114895054443678,0.29950689364746
"544","CMKLR1",57.0070846657597,-0.849435411631053,0.336496040063049,-
2.52435485265115,0.011591085997091,0.29950689364746
"545","SLC12A6",20.560848286321,1.04695771060354,0.414721612836445,2.5
2448311879138,0.0115868569767466,0.29950689364746
"546","CDH13",15.8972016206203,-1.2760930870395,0.504548617869719,-2.5
2917764878111,0.0114330142268943,0.29950689364746
"547","TMEM221",9.5559140494383,1.58252450504901,0.626294636875917,2.5
2680513590689,0.0115105348983062,0.29950689364746
"548","RTCB",77.9291167404795,-0.500039020558525,0.198078166652676,-2.
52445299251648,0.0115878501371209,0.29950689364746

"549","C3",2822.47853529583,0.562979829214627,0.223133009331603,2.5230
6832996623,0.0116335793297153,0.300057346646208
"550","TNK2",7.87338611180152,1.60007355730004,0.6348400588016,2.52043
571465942,0.0117209645888165,0.301213899414959
"551","H2AFY",117.666890874141,-0.46108066487181,0.182912528743048,-2.
52077136563743,0.0117097909264641,0.301213899414959
"552","AREG",26.6663555352339,-1.37454380586029,0.546548750396707,-2.5
1495187732585,0.0119048632203064,0.304326846979541
"553","BCL7B",44.9845916850622,-0.610707630704766,0.242835630865562,-2
.51490124627907,0.0119065729679849,0.304326846979541
"554","ANP32B",189.975697746905,-0.612634622842701,0.243550968919789,-
2.51542675260087,0.0118888378639034,0.304326846979541
"555","CLSTN1",86.4673147411282,0.460690712681074,0.183397633795782,2.
51197740748425,0.012005677443713,0.304636922686187
"556","SUSD1",8.65848235958806,1.58392832727741,0.630640515698356,2.51
161840676127,0.0120178961577645,0.304636922686187
"557","CALHM2",158.818269977671,-0.435598338196756,0.173400592020822,-
2.51209256623789,0.0120017603091585,0.304636922686187
"558","GANC",15.3685937490019,0.871603225031216,0.346873336881273,2.51
274206564209,0.0119796887409293,0.304636922686187
"559","SLC47A1",4.27515854280755,2.99460414252284,1.19241737773957,2.5
1137244259189,0.0120262739958742,0.304636922686187
"560","KANK1",36.4932401661357,0.894668553205531,0.356390467567135,2.5
1036050238071,0.0120607963969747,0.304965851752075
"561","SEC61G",300.292457788175,-0.575043674936119,0.229474239623785,-
2.50591820623911,0.0122133869420598,0.305916253310393
"562","PPP1R14B",390.264246794864,-0.76798453130097,0.306396476199005,
-2.50650575629389,0.0121931072312691,0.305916253310393
"563","CFL2",80.1916248308338,0.52491254566826,0.209322028663873,2.507
67943067836,0.0121526863335769,0.305916253310393
"564","SNX33",39.1413903569558,0.646218272300947,0.257920387113479,2.5
0549512403076,0.0122280084303448,0.305916253310393
"565","COPS3",50.3201991900435,-0.63169527518245,0.252109809831427,-2.
50563544355864,0.0122231573466606,0.305916253310393
"566","TCF4",711.302656568649,0.625342243678831,0.249292480352513,2.50
846813668251,0.0121255903539024,0.305916253310393
"567","ASS1",58.6831612159394,-1.07840367505759,0.430837917881432,-2.5
0303798783645,0.0123132327030334,0.307173428867128
"568","IGF2",72.0318624062562,1.27658151697366,0.510189032215202,2.502
17357952765,0.0123433390554658,0.307173428867128
"569","FLRT2",182.948774468348,0.590317809545775,0.235881656183971,2.5
0260159732544,0.0123284235449683,0.307173428867128
"570","BCL2A1",9.19523484079747,-1.86478385634119,0.745578664610937,-2
.50112287925283,0.0123800215299559,0.307545798007325
"571","CHL1",135.465992514075,0.879593484913813,0.3518772825242,2.4997
166017767,0.0124292691501292,0.30768959994026
"572","PXDNL",4.72250752294707,2.27929895225637,0.911624748052867,2.50
026006547618,0.0124102166031589,0.30768959994026
"573","PSD",5.91126703589289,-1.78134081815359,0.712934480231348,-2.49
860382341943,0.01246836136056,0.30811866817719

"574","APCDD1",22.5189349998275,2.64992711147557,1.06114120635365,2.49724268137827,0.0125163266746656,0.308765131904642
"575","LGALS1",3635.21510301356,-0.652591660436025,0.261550652469663,-2.49508710559121,0.0125926213557845,0.310106988518101
"576","AC005838.2",8.68270001235473,1.31898445822221,0.528939580065472,2.49363917530797,0.0126441004501261,0.3108341360656
"577","APCDD1L",8.18168350719548,2.15591435758097,0.865923790667963,2.48972759590994,0.0127841036712444,0.313731209679066
"578","TNIK",3.59534841791853,2.21803689189285,0.891500139759358,2.48798266312281,0.0128469995533963,0.314729262415383
"579","FCER1G",13.4653951220849,-1.26378956540468,0.508705230620589,-2.48432587151292,0.012979696966419,0.315253017228976
"580","GALNT2",66.620550137121,0.518305070958642,0.208626819144175,2.48436453704669,0.0129782875556769,0.315253017228976
"581","AC025171.2",16.0239820546802,0.985165980080492,0.396294833839257,2.48594202083414,0.0129209014569745,0.315253017228976
"582","PPP1R14B-AS1",3.64803616865175,-2.12741756327576,0.85605726614449,-2.48513463691187,0.0129502445647843,0.315253017228976
"583","USB1",18.7415567710908,-0.855449765037777,0.344199946870694,-2.48532799849369,0.0129432117767284,0.315253017228976
"584","FOXJ3",38.0167214471981,0.635939808309345,0.25610405167151,2.48313060320119,0.0130233329422706,0.315771223394781
"585","SERTAD4-AS1",13.9855404528138,-1.42968077075878,0.57606728296935,-2.48179477124522,0.01307225400817,0.315805695516282
"586","EPB41L2",402.678671717335,0.442307749206118,0.178275462693025,2.48103548589709,0.013100133069318,0.315805695516282
"587","ARID2",30.354466180508,0.755525471931451,0.304506373758945,2.48114830111748,0.0130959874531927,0.315805695516282
"588","RNF126",33.6105980992696,-0.69393073031424,0.279736411873712,-2.48065929517791,0.0131139653222863,0.315805695516282
"589","ZFHX4",247.538765509534,0.5839228139615,0.235512805680073,2.47936757525923,0.0131615592681604,0.316413716871225
"590","SMARCE1",183.335031154928,0.392192077141934,0.158272725142398,2.47795112385333,0.0132139245077567,0.31713418818616
"591","ARSI",9.36319364193031,-1.91590122729105,0.774284194882387,-2.47441603477658,0.0133454186804636,0.319636271078661
"592","UXT",205.182222979992,-0.580495678251718,0.234644482873363,-2.47393704357843,0.0133633243275824,0.319636271078661
"593","CAMK1",53.748831090111,-0.569433226777253,0.230911349367971,-2.46602528778188,0.0136621688599345,0.324047422205482
"594","R3HCC1",56.0809126286871,-0.6126602126724,0.248375573174804,-2.46666854087627,0.013637653166869,0.324047422205482
"595","FOLH1",5.29105509827294,1.96294121781643,0.795521886908495,2.4674886387409,0.0136064539159352,0.324047422205482
"596","LRP6",88.796921884484,0.722986520275413,0.293128321017488,2.46645058985031,0.0136459553706776,0.324047422205482
"597","EFNB3",2.59635289047845,-3.28745961558692,1.33241032937183,-2.46730270932139,0.0136135217571247,0.324047422205482

"598","KCNIP4",3.73766813230466,-2.10621577319655,0.854622192524331,-2
.46449927420599,0.0137204841422398,0.324886380358052
"599","ANKRD33B",18.8567458352154,0.83218339267586,0.337926754119057,2
.46261470135823,0.0137928047666268,0.325510192492393
"600","MS4A6A",9.17395477816863,-1.67078996012187,0.678360245758762,-2
.46298330506241,0.0137786331475873,0.325510192492393
"601","SCARB2",212.338402030667,0.388399304197374,0.157955226310388,2.
45892024765395,0.0139355571030669,0.32724293296754
"602","MPG",174.152582258115,-0.597545334306887,0.242985970439998,-2.4
5917627764621,0.0139256222997679,0.32724293296754
"603","PLTP",643.241835085764,-0.60195040706004,0.244775134315022,-2.4
591975355035,0.0139247977063916,0.32724293296754
"604","TIMP1",4166.34767128423,-0.810007493023598,0.329778753076413,-2
.45621491823614,0.014040915720682,0.329171136763008
"605","MS4A7",5.91359388830074,-1.75684322108409,0.715663631466592,-2.
4548449073538,0.0140945382566291,0.329882085477468
"606","RBPJ",401.996261834854,0.524496256635174,0.213729165152817,2.45
402285766735,0.0141268001507771,0.330007372782561
"607","NIBAN2",25.1408565851995,-0.782049099420192,0.318745547077375,-
2.45352164631292,0.0141465024914558,0.330007372782561
"608","CRISPLD1",49.8632683716271,0.798195211122865,0.325427094930869,
2.45276199663837,0.0141764101247619,0.330161130537218
"609","PGM1",50.112669876601,-0.636124349536707,0.259548521830349,-2.4
5088796904227,0.0142504298559929,0.331340043942297
"610","OGA",110.889357320698,0.663302616036648,0.270716481222911,2.450
17448897202,0.0142787001509578,0.331453105143544
"611","ZBTB4",65.3865701195176,0.604855424875095,0.246959042632907,2.4
4921351502883,0.0143168551117215,0.331794874602252
"612","TBC1D4",36.5561208467012,0.686032206316088,0.28050800818597,2.4
4567779277541,0.0144580144067426,0.334518764705024
"613","AMOTL2",102.020891128651,0.652032769695683,0.266743027319457,2.
44442291987185,0.0145084079433978,0.334591297196274
"614","ZNF706",269.381190903408,-0.554040452092469,0.226639666778357,-
2.44458730445582,0.0145017977379285,0.334591297196274
"615","FOXS1",14.501033060121,-1.51166941053441,0.618902076860104,-2.4
4250175763444,0.0145858585907509,0.335830500235825
"616","KIAA0040",34.93672147707,1.35176520874563,0.555246576760752,2.4
3453136916517,0.0149110873265252,0.33674800086698
"617","G0S2",92.4896028363936,-0.763595965660294,0.313580172183408,-2.
43509007710373,0.0148880829622721,0.33674800086698
"618","FBXL7",49.2275202630686,0.637117816577791,0.261440620143946,2.4
3695037223749,0.014811712002247,0.33674800086698
"619","SNX18",59.6821080494752,0.50202744208815,0.205913040717099,2.43
805560026613,0.0147665025833286,0.33674800086698
"620","PCDHB14",13.0669996555026,0.938745712933591,0.3851345042425,2.4
374490018233,0.0147913004495675,0.33674800086698
"621","LRRK2",43.4406134333493,0.891376483516331,0.3660480087287,2.435
13545289351,0.01488621602315,0.33674800086698
"622","RAB20",6.48553667357436,-1.47439604850584,0.604081377427491,-2.
44072421961529,0.0146578435255337,0.33674800086698

"623","OXA1L",74.9747920005999,-0.5038317531646,0.206461454493636,-2.4
4031872390074,0.0146743086826701,0.33674800086698
"624","PSMC5",216.552930503999,-0.510233788848611,0.20956425863446,-2.
43473668732131,0.0149026298741761,0.33674800086698
"625","PSMC4",80.7099581376497,-0.559078334804008,0.229394016946422,-2
.437196672547,0.0148016265244751,0.33674800086698
"626","TGFB1",77.6794265214873,-0.626680915549825,0.257334246403865,-2
.4352798910655,0.0148802746398248,0.33674800086698
"627","CIC",28.6386246918914,-0.712111470685592,0.292300512449107,-2.4
3623066110628,0.0148412173641367,0.33674800086698
"628","GPC3",1017.20415894203,0.530051880164621,0.217871270702358,2.43
286725439237,0.0149797916162272,0.337760906506014
"629","ITPRIPL2",137.94666219654,-0.535570134298649,0.220287284856659,
-2.43123489695352,0.0150474555638147,0.3387471713571
"630","GATAD2B",45.3505666068824,0.663364684768458,0.272924853838008,2
.43057631227016,0.015074831128621,0.338824775843292
"631","ATXN7L1",13.252599818074,0.986903312878262,0.406332427058353,2.
42880766377165,0.015148566209521,0.339942468346779
"632","AC103591.3",49.0493439885458,-0.96468048051266,0.3976339556708,
-2.42605156515184,0.0152641013054204,0.340377440133469
"633","PLEKHO1",55.3329611020397,-0.922503685624635,0.380113131249656,
-2.42691875071987,0.0152276656923209,0.340377440133469
"634","LINC01119",4.61477073659358,-1.98599239879434,0.818602811546424
,-2.42607571191039,0.0152630857187882,0.340377440133469
"635","SERP2",13.5944559273103,-0.963739486852128,0.396978810510805,-2
.42768495782446,0.0151955365292087,0.340377440133469
"636","SCUBE2",4.43593359462434,2.16347013580438,0.892308763409122,2.4
2457569007695,0.0153262880805268,0.341226791226823
"637","ENPP5",1.90261943116468,3.06463084887817,1.2645693305756,2.4234
58148778,0.0153735245461283,0.341271655138169
"638","MDM2",40.204554507546,0.592868442297039,0.244701932931671,2.422
81879507093,0.0154006064712775,0.341271655138169
"639","TNFRSF6B",10.5683667534393,-1.28011539750317,0.528310966071649,
-2.42303393212088,0.015391488951237,0.341271655138169
"640","LYVE1",3.26622159482238,-3.18558360711938,1.3153612968759,-2.42
183164023863,0.0154425031272371,0.3415541698682
"641","CFD",30852.0065123013,0.585421189651707,0.24177146075858,2.4213
8252304426,0.0154615976614065,0.3415541698682
"642","AL139384.1",3.50755749194538,2.00880248706014,0.829822546876646
,2.42076151656886,0.0154880344189298,0.341605245127797
"643","YWHAQ",384.005492853757,-0.375966827499954,0.155422759805401,-2
.41899466957534,0.0155634682727394,0.342202966990668
"644","DHRS4-
AS1",43.5461520469176,0.603271163502337,0.249379764135517,2.4190862702
6574,0.0155595495371021,0.342202966990668
"645","GTPBP6",66.0086371486296,-0.596905721047799,0.246827097768061,-
2.41831519490903,0.015592563753159,0.342311167046094
"646","KCNE3",5.1545536786986,1.47263514660767,0.6092928714134,2.41695
778122521,0.015650832296956,0.343058491215011
"647","AC246817.1",3.85454440385321,2.21807761072138,0.918409616754266

,2.41512890354987,0.0157296418618099,0.343721803646956
"648","TUFM",187.505213294637,-0.469500883921106,0.194398874575929,-2.41514198549398,0.01572907690048,0.343721803646956
"649","AXL",231.72912244456,-0.4752223992326,0.196858483854649,-2.41403057631735,0.0157771383052611,0.344228472114788
"650","SAMD9L",50.5296614468885,0.782582943472046,0.324294686305828,2.41318460190256,0.0158138078699412,0.344497722212873
"651","MBNL2",131.369193857074,0.566728906454749,0.234940133922835,2.41222688091634,0.0158554116414208,0.344873469804176
"652","SENP5",33.7432153047428,-0.700609496048042,0.290657888329143,-2.41042656738312,0.0159338785607445,0.345362764680996
"653","MYH11",5.92256047242406,2.96708463113526,1.23113858111659,2.41003301873964,0.0159510768433172,0.345362764680996
"654","ARHGEF6",24.9003671655876,0.810349787079264,0.336178687417831,2.41047341014838,0.0159318325935224,0.345362764680996
"655","UBXN11",18.6714579413542,-0.845325689070885,0.351341474076393,-2.40599459910925,0.016128503153703,0.345799249663994
"656","RFTN1",142.611943034719,0.585672986545542,0.243539680369907,2.40483598260446,0.0161797256828841,0.345799249663994
"657","KCNAB1",5.49564409363895,2.60214554427569,1.08265756402931,2.40347976195845,0.016239865891706,0.345799249663994
"658","HMBOX1",29.0166338134524,0.807495679885653,0.335532914741761,2.4066064591819,0.0161015103251734,0.345799249663994
"659","CCND1",134.382407779643,-0.821732640972057,0.341304713847598,-2.40762171640847,0.016056808826788,0.345799249663994
"660","ITGA7",24.2385908994729,-1.12875980704947,0.469195354414787,-2.40573525809379,0.0161399562428842,0.345799249663994
"661","SLC25A29",13.5531070291818,0.922487323863522,0.383703528160326,2.4041669053355,0.0162093707189032,0.345799249663994
"662","ANXA2",2774.66285407721,-0.507913979742351,0.210870199372669,-2.40865699019291,0.0160113383849028,0.345799249663994
"663","NR2F2-AS1",24.28563474378,0.819588981344642,0.340928226858366,2.40399273740725,0.0162170954630209,0.345799249663994
"664","CARHSP1",165.607457420099,0.54138106946633,0.224919682274227,2.40699730673755,0.0160842884803362,0.345799249663994
"665","EBP",31.8519346761409,-0.672401700049154,0.279698051192509,-2.40402711846697,0.0162155703285191,0.345799249663994
"666","CISD1",65.5765165006088,-0.664716249330464,0.276685213603379,-2.40242780115932,0.0162866492037349,0.34627470379112
"667","BRCA1",11.7248311754755,1.05043435672471,0.43773316260425,2.39971390441442,0.0164078902017599,0.348329423173793
"668","KIRREL1",60.7198569934641,0.709155016118294,0.296652856998069,2.39052144413667,0.0168244666022838,0.354348822767393
"669","HERC5",10.6074386903752,1.09518733099784,0.458273997970457,2.38980901348987,0.0168571362488119,0.354348822767393
"670","PIK3R1",460.168815779603,0.709189983510925,0.296915902163418,2.38852139054714,0.0169163235438975,0.354348822767393
"671","ST8SIA4",4.11879682240407,-1.77361375939454,0.742068817645015,-2.39009336765177,0.0168440900628608,0.354348822767393

"672","STXBP5",27.8303483981147,0.74950551776637,0.313801114043358,2.3
8847309402098,0.0169185471021861,0.354348822767393
"673","GAPDH",1170.627460918,-0.677461525684932,0.283622186935816,-2.3
8860553542746,0.0169124501503079,0.354348822767393
"674","ALDH6A1",34.4865857949879,0.674010732787635,0.282183560999943,2
.38855421059688,0.0169148126645412,0.354348822767393
"675","SCPEP1",303.647709149166,0.433835236200705,0.181675251452645,2.
38797102374611,0.0169416774727066,0.354348822767393
"676","ARVCF",5.13698998961718,1.55375623123854,0.650219884894398,2.38
958584216613,0.0168673815738045,0.354348822767393
"677","MXRA5",258.165874451695,0.752345166437604,0.314900136144176,2.3
8915478300443,0.0168871860658962,0.354348822767393
"678","FADS1",12.9702770053625,1.42187562705368,0.595861958922472,2.38
625004627739,0.0170211736215395,0.355143480006536
"679","ICAM2",47.0699967153596,-0.703237868772917,0.29479415251557,-2.
38552177094413,0.0170549128816698,0.355143480006536
"680","SERPINB8",10.531238604527,-1.03483634284313,0.433782427978115,-
2.38561148653845,0.0170507534053466,0.355143480006536
"681","A1BG",16.8041449382563,-1.04802609215171,0.439514973090897,-2.3
8450600392849,0.0171020689410572,0.355602490756785
"682","SLC39A8",13.892009249002,1.04565851193718,0.438931315289409,2.3
8228277526228,0.0172056796793156,0.356280744459771
"683","GPD1",15.6702963211414,-1.84961040548266,0.776433870242357,-2.3
8218665667602,0.0172101715544127,0.356280744459771
"684","GPC4",51.6891141175054,0.664393031642986,0.278791221342383,2.38
312034519568,0.0171665813325338,0.356280744459771
"685","AMPD2",15.7901510147077,-0.892994746442994,0.375164981727859,-2
.38027212009559,0.0172998574519259,0.357614571560979
"686","NT5E",129.171103886077,0.568863811913634,0.239123486507579,2.37
895415553672,0.0173618350790198,0.357850923899447
"687","AL121839.2",9.52020846492687,1.45986060296659,0.613518156439152
,2.37949046437287,0.0173365915591212,0.357850923899447
"688","CCNQ",25.8277250143687,-0.759313626071345,0.319255659350554,-2.
37838736395771,0.0173885484190895,0.357880589555678
"689","SNTB1",7.61652219728923,1.26860259754181,0.533560150036324,2.37
761871357043,0.0174248331047259,0.358106874837328
"690","SGCD",70.9664881310936,0.61627707991698,0.259382236198497,2.375
94173351703,0.0175042266399151,0.359217172784344
"691","IER3",177.708986323071,-0.910433786359359,0.383511890459314,-2.
37393887649474,0.0175994636519499,0.360066681152693
"692","AL356218.2",3.60697038730245,-2.65458105440359,1.1186875662199,
-2.37294230718372,0.0176470201011324,0.360066681152693
"693","TGFB1I1",144.063104985801,-0.632707964281618,0.266573806466352,
-2.37348137339023,0.0176212818082218,0.360066681152693
"694","RAB31",67.2638753373109,-0.573807682972008,0.241813414392875,-2
.37293569677545,0.0176473359265515,0.360066681152693
"695","PCDH18",209.649924322471,0.374953107424504,0.158054565165653,2.
37230166070512,0.0176776512974307,0.360166248016717
"696","SAA1",1.98411802038777,-2.80675739823204,1.18428505633548,-2.37
000153233121,0.017788011538593,0.361894027854134

"697","PALMD",19.6181521351136,-1.28831670319769,0.543729052346287,-2.
36940935496894,0.0178165217682557,0.361954014689384
"698","ADCY3",94.9625965990132,0.52029692986556,0.219653552787082,2.36
871620451276,0.0178499441770375,0.362113480726147
"699","WDFY3-
AS2",37.5691713545314,0.617604803444654,0.260959637321085,2.3666679252
9587,0.0179490295576276,0.363602658849796
"700","ARL17B",8.22960356899483,1.18269419094949,0.499884827313203,2.3
6593336370355,0.0179846811291871,0.363804406841841
"701","GRHL1",9.5747412952273,-1.43613515085816,0.607207221230078,-2.3
6514833922568,0.0180228504435809,0.364052026782641
"702","ADAMTS19",3.89124151695507,-1.91067390973387,0.808108641496879,
-2.3643775250252,0.0180603978482018,0.364052026782641
"703","ZNF674-
AS1",2.69567422987383,-2.35542954365587,0.996333474448719,-2.364097567
79391,0.0180740518946466,0.364052026782641
"704","SWAP70",85.1037476371118,0.715057410291002,0.302615958259335,2.
36292036416075,0.0181315653368925,0.364691711889769
"705","NT5DC2",31.666034344183,-0.654965983599687,0.277271089073708,-2
.36218635627595,0.0181675070782169,0.364896312379505
"706","HLA-
DMA",15.3215538513673,-1.05753134845214,0.447972752021314,-2.360704626
96763,0.0182402521962173,0.365838485975122
"707","SNCG",42.867515550032,-1.09800741629978,0.465399370730789,-2.35
927997619687,0.0183104354120359,0.366208708240717
"708","PNP",7.31846047362674,-1.47260035894618,0.624066479570759,-2.35
968507707552,0.0182904547301511,0.366208708240717
"709","CYP7B1",121.952580329883,0.596359503740423,0.253008479791405,2.
35707318676471,0.0184196162062568,0.367872729873901
"710","PLSCR4",211.289320017871,0.554971418928721,0.235561655419584,2.
35594973188741,0.0184754176468448,0.367949245962479
"711","FGF12",16.1451339658353,1.35092237859944,0.57333939213548,2.356
23506273963,0.0184612314085234,0.367949245962479
"712","ARHGAP5-
AS1",3.88586260307251,-2.00341631973379,0.850764618126246,-2.354842076
23277,0.0185305791324606,0.368529495106239
"713","SPI1",3.41144028266954,-1.9097847903757,0.811440219015342,-2.35
35742321143,0.0185938948833534,0.368564506525844
"714","SLC43A1",28.354017840898,0.671131085091407,0.28519402528418,2.3
5324384661517,0.018610425294207,0.368564506525844
"715","NUAK1",56.6327855842043,0.857229068652817,0.364273729206887,2.3
5325525812475,0.0186098541197707,0.368564506525844
"716","EDIL3",32.202092554579,-1.14998620199991,0.48925333088451,-2.35
049233067229,0.0187485940234955,0.369429634065961
"717","SEMA3D",42.930516883465,0.79502731778297,0.338360908092488,2.34
964293678292,0.0187914276245339,0.369429634065961
"718","SHTN1",24.9671308086341,-0.796576832968484,0.33907529739791,-2.
34926235877835,0.0188106473277936,0.369429634065961
"719","SSBP4",149.089972809869,-0.542410836934072,0.230753265665305,-2
.35060958019467,0.0187426880276097,0.369429634065961

"720","ADAM33",59.4478561526284,0.627537035604661,0.267035508582399,2.35001344553779,0.018772732935333,0.369429634065961
"721","MT-CYB",13161.9378187484,-0.343981429519976,0.146287384786543,-2.35140870159037,0.0187024786739644,0.369429634065961
"722","ODF3B",50.6215074997188,-0.613865230195138,0.261561837425766,-2.34692199839497,0.018929217083644,0.371243371058724
"723","STXBP6",58.1597772423907,-0.838672317594536,0.357474192357651,-2.34610591624317,0.0189707156932236,0.371542647601724
"724","HSPA8",463.678883890892,-0.526482762333378,0.224461556465574,-2.3455364500875,0.0189997207315267,0.371596748008864
"725","ATP6V0D1",107.588848180639,-0.615842986017925,0.262752694197443,-2.34381226003778,0.0190877767311648,0.372804025535577
"726","PDE10A",5.81006358088847,1.61892749165232,0.692139818919514,2.33901799520738,0.0193345021850255,0.375572332462152
"727","SCX",3.20244673440461,-2.49558195018853,1.06715211072648,-2.33854379811855,0.01935905640377,0.375572332462152
"728","RRP12",7.8521255177247,-1.16829409615155,0.499558929550989,-2.33865121218357,0.0193534920512089,0.375572332462152
"729","STX4",104.927571502399,-0.437817587236968,0.187214481439134,-2.33858825381149,0.0193567533035251,0.375572332462152
"730","IQCN",11.1241902031518,1.72224638656556,0.736640882764926,2.33797285334102,0.0193886564286605,0.375572332462152
"731","C5AR1",9.17253545974525,-1.13410704987634,0.484990810241334,-2.3384093593691,0.0193660226811965,0.375572332462152
"732","PXK",103.048006473783,0.527666575614087,0.225787180501146,2.33700856905562,0.0194387385237532,0.376028056688996
"733","FBLL1",2.18463720684047,2.69710744199658,1.1546385098298,2.33588904149244,0.019497025252321,0.376641033523691
"734","CLIP4",31.0506180090057,0.775146779831801,0.332003859636226,2.33475231487104,0.0195563635886655,0.376894762181084
"735","PPM1K",23.5454444752084,0.793574063866508,0.339916044449474,2.3346178470386,0.0195633933759249,0.376894762181084
"736","SH3BP5",314.846708204771,0.384169937919751,0.164692846698322,2.33264495466192,0.0196667874414793,0.377858494126659
"737","CYCS",116.07567412542,-0.449168530139865,0.192539542007629,-2.33286381309699,0.0196552941644984,0.377858494126659
"738","DSE",135.731758759778,0.51726605183899,0.221805658143248,2.33206878566158,0.0196970728322939,0.377927576294419
"739","RELB",28.6214330460959,-0.685727281926761,0.294323461083681,-2.32984241012237,0.0198144817408694,0.379665847700555
"740","EPHB3",9.46536357461863,1.22695323048563,0.52693950965731,2.32845176343593,0.0198881277598299,0.380562012269178
"741","DMRT2",10.6427316655251,1.05139575672136,0.451740820303825,2.32743137096671,0.0199423177102663,0.381083965961363
"742","FAM66D",3.05839303607452,1.93115891061264,0.829985488752239,2.32673816203203,0.0199792054200475,0.38127432445805
"743","EHBP1",53.798068746247,0.552261027252809,0.237578965035774,2.32453671632777,0.0200967460840298,0.382492415873568
"744","TWISTNB",56.0853337564959,-0.517088447429482,0.222448518336509,

-2.32453086806924,0.0200970591391197,0.382492415873568
"745","PPP1R9A",3.88161823814271,1.92339669725464,0.827703412496053,2.32377524148942,0.0201375433625447,0.382748475186085
"746","CLDN11",9.79073242178783,1.49461976512197,0.643404907830528,2.32298471294168,0.0201799736855624,0.383029219335877
"747","PGK1",186.314217487185,-0.450196486282824,0.19384192771801,-2.32249282486369,0.0202064143251342,0.383029219335877
"748","ENAH",163.159535209529,-0.459954390489472,0.19816364970908,-2.32108356484513,0.02028233422504,0.383954348431239
"749","LMO2",14.3443565520777,-1.24284927798915,0.535698116562454,-2.32005534379037,0.0203378836409345,0.384491899006185
"750","SMYD2",18.1030575061916,-0.794139066416284,0.342656355002065,-2.31759620046007,0.0204712768996793,0.384749311886261
"751","USP49",9.179826320644,1.38397062047591,0.597348591356165,2.31685592048333,0.0205115816324709,0.384749311886261
"752","NRBP2",33.0809317062634,0.675554643650436,0.291580959145448,2.31686817146881,0.0205109140616806,0.384749311886261
"753","PPP1CA",98.8471768691148,-0.528181793374245,0.227774666328936,-2.31887857366668,0.0204016212115931,0.384749311886261
"754","HERPUD1",236.792494772452,-0.396275617540308,0.170908704694157,-2.31863917200384,0.0204146092659785,0.384749311886261
"755","LGALS2",4.84357758479477,-1.62544639629621,0.701590602639049,-2.31680183597393,0.0205145289882858,0.384749311886261
"756","CLTB",188.527972670605,-0.439072259304042,0.189688482753031,-2.31470173060376,0.0206292610012522,0.386389333039327
"757","RNF38",28.4175098203773,0.666301837666701,0.287975165558034,2.31374756352879,0.0206815732079316,0.386857432793014
"758","HMBS",11.4031603324541,-0.975651772803161,0.422327018445708,-2.31018080821316,0.0208781461047245,0.390019193724141
"759","RIN1",13.1500180952026,-1.00792569064348,0.436458437692269,-2.30932799918542,0.0209253870837384,0.3902904591824
"760","PCGF2",42.1710758420282,-0.561370748272062,0.243130879268112,-2.30892410689228,0.020947793006965,0.3902904591824
"761","PRRX1",1173.92070079529,0.305157707454911,0.132245755504639,2.30750473835969,0.0210266983903941,0.390988860325943
"762","PPL",226.075618316266,-0.791813365864603,0.343217857825219,-2.307028459655,0.021053233594434,0.390988860325943
"763","C16orf74",7.74876945265839,1.30851477175256,0.567251845879581,2.30676159321011,0.0210681144370547,0.390988860325943
"764","DEXI",79.6004901753553,-0.501530110069877,0.217550592087385,-2.30534932246208,0.0211470172805518,0.391427143389037
"765","MID1",32.2879402610536,0.768475937056558,0.333308564846359,2.30559912977572,0.0211330419617626,0.391427143389037
"766","H6PD",118.603249468458,0.540793273565693,0.234707387250364,2.30411696837145,0.021216078475747,0.392192782267072
"767","MPC2",160.556683966399,-0.481460642463134,0.209056384247826,-2.30301812688191,0.0212778232196704,0.392821351747761
"768","RUBCN",9.23438014794751,1.11721021682578,0.48552555161856,2.30103279446657,0.0213897774785919,0.394374022261538
"769","NLRP3",7.28159445867971,-1.42343200487673,0.619304197951863,-2.

29843752001721,0.0215369001157106,0.39657022839852
"770","SBF2",66.9681887005328,0.524611531012772,0.228308865385723,2.29
781498027443,0.0215723217954894,0.396706593018351
"771","MAT2B",44.0990181151326,0.614419869278621,0.267716102439009,2.2
9504263539246,0.0217306810397747,0.397650955394466
"772","PM20D2",18.0531869866351,0.955965393325517,0.416640682328977,2.
29446003203953,0.0217640883072536,0.397650955394466
"773","TM9SF3",198.531438643926,0.363952803973948,0.158607864655077,2.
29467060013343,0.0217520088924538,0.397650955394466
"774","CCL18",1.65221934201092,-3.31574956797913,1.44488581375269,-2.2
9481771944829,0.0217435727327388,0.397650955394466
"775","TIMP2",1171.66161964388,0.303128079235069,0.13206294067679,2.29
53303756649,0.0217141980923685,0.397650955394466
"776","MIR4435-2HG",57.5459193222966,0.586263273441111,0.2555879258035
16,2.29378313391769,0.0218029586898142,0.39784780289661
"777","TSC1",29.7219725141388,0.642960770412597,0.280440274593258,2.29
268342910136,0.0218662372451969,0.398488956746446
"778","NDUFAB1",169.076676196291,-0.471510398878341,0.20574089406376,-
2.29176800763886,0.0219190336975356,0.398937682721214
"779","SDF2L1",60.7648334437307,-0.634599411450702,0.276992094281638,-
2.29103799188384,0.0219612164321186,0.399192329497818
"780","WFS1",19.4017832843798,0.793487781146196,0.346479687475133,2.29
014227912897,0.0220130701678224,0.399621889200469
"781","LAMA4",486.816869502836,0.411667116302489,0.179843448401928,2.2
8903037592152,0.0220775875396147,0.40027994822144
"782","CRIM1",51.0052251780841,0.669794661306478,0.292879135716533,2.2
8693197850307,0.022199793728255,0.400444686868906
"783","ANGPT2",28.0907251459494,-0.995428537605758,0.435262826645651,-
2.28695968658068,0.0221981762440082,0.400444686868906
"784","ITGAM",2.44821818122673,-2.60671242510354,1.13972849284003,-2.2
8713456009861,0.0221879702077171,0.400444686868906
"785","C20orf194",29.8127194160603,0.777045485139127,0.339635519448234
,2.28788050908663,0.022144480661069,0.400444686868906
"786","VPS13D",32.6693308073051,0.629328333003685,0.275474722070893,2.
28452298008573,0.022340813620432,0.401538218340717
"787","DUSP2",18.5472011577264,-0.961493798523484,0.420887565067797,-2
.28444334859027,0.0223454884217857,0.401538218340717
"788","PIP4K2B",38.4640914678055,0.541323941350226,0.236915231300197,2
.28488450649385,0.0223196007485123,0.401538218340717
"789","RSRP1",248.879521364992,0.463325446314754,0.202917853673029,2.2
8331533144112,0.0224118005683225,0.401746327844945
"790","ZMYM1",24.7065328708051,0.664825820103804,0.29117124901113,2.28
328113562611,0.0224138134885245,0.401746327844945
"791","LRRN4CL",141.063893399356,0.637101705463103,0.279128064508812,2
.28247097469122,0.0224615492166057,0.402092967012815
"792","ITGB1BP1",242.317280288913,-0.601419991288313,0.264691510459115
,-2.27215444214714,0.0230771838217017,0.405604964146049
"793","ACKR3",221.024303610303,0.964756455877939,0.423862291405195,2.2
7610824421196,0.0228395317727883,0.405604964146049
"794","PTPRG",86.7015718271017,0.534825186948387,0.234790625250678,2.2

7788135227875,0.0227336475991125,0.405604964146049
"795","SHISA3",2.13909550276472,-2.96092331184591,1.30199767480242,-2.27413870942222,0.0229576479333116,0.405604964146049
"796","SAYSD1",41.6916164332944,0.559282187178609,0.246159573719467,2.27203101926065,0.0230846368655682,0.405604964146049
"797","ZDHHC14",35.9452592345944,0.720480073857058,0.316795188248139,2.27427720048804,0.0229493250931991,0.405604964146049
"798","SLC43A3",91.8779084363972,0.562435762269103,0.247414712587686,2.27325107867129,0.0230110537883106,0.405604964146049
"799","FADS2",12.4661610541874,1.77699664301644,0.781050911653547,2.27513548285143,0.0228978036834135,0.405604964146049
"800","OLR1",3.07256229690249,-2.07507974205511,0.912588238362654,-2.27384011191967,0.0229756015443032,0.405604964146049
"801","WASF3",40.4512350806782,0.696248864261072,0.30646075375061,2.27190221175159,0.0230924172956055,0.405604964146049
"802","AL161431.1",4.15509698946981,-1.60957995835522,0.707464116206763,-2.27514006927358,0.0228975286374542,0.405604964146049
"803","PRTG",8.13474574346744,1.13341173624805,0.49889529574342,2.27184290154332,0.0230960006080216,0.405604964146049
"804","MEX3B",18.4670688342725,0.795801099664526,0.350245297992722,2.27212500560411,0.0230789611940197,0.405604964146049
"805","EEF2K",47.293982317299,0.619347209052024,0.272715500495707,2.27103779552777,0.0231446900444921,0.405604964146049
"806","ZNF773",6.53270621478697,1.22223084933702,0.537528640378544,2.27379670128142,0.0229782126874339,0.405604964146049
"807","PARVG",2.94599982922144,-2.32827368231063,1.02500932305787,-2.27146585883217,0.0231187914852356,0.405604964146049
"808","PNMA6A",4.33791414506603,1.63785868968086,0.720970990687631,2.27174007114869,0.023102214400083,0.405604964146049
"809","SRP19",89.719924887671,-0.559834442399225,0.246765755874789,-2.26868772944043,0.0232873223247491,0.406594924930268
"810","NOP16",18.5490267785857,-0.882403057768874,0.388851506439148,-2.26925456930681,0.0232528495282482,0.406594924930268
"811","JUNB",3625.4759641752,-0.492090425038688,0.216885195050001,-2.2688981833234,0.0232745182371785,0.406594924930268
"812","TRABD2B",4.67843260597468,1.49522303986031,0.659574378821007,2.26695136723326,0.0233931967721964,0.40690314902302
"813","PLA2G4A",11.7080827972074,0.982482096225509,0.43343009210728,2.26676023219526,0.0234048766933726,0.40690314902302
"814","BHMT2",51.4565005668806,0.587220501266008,0.258941339695916,2.26777424553222,0.023342969907787,0.40690314902302
"815","GLRX3",84.3347784345323,-0.545097745684461,0.240508738045217,-2.26643634703193,0.0234246802941972,0.40690314902302
"816","EYA2",3.4854278119033,3.24950884167262,1.43400042558954,2.26604454481714,0.0234486560453944,0.40690314902302
"817","PDLIM3",67.8304591052973,-1.19501143219564,0.527578498588743,-2.26508744270714,0.0235073140620911,0.406910552535155
"818","AC004160.1",3.35191576166982,1.82607457938129,0.806414745471997,2.26443599851648,0.0235473119889743,0.406910552535155
"819","MRC1",4.19395175438136,-2.03915933202994,0.900209674546962,-2.2

6520486247403,0.0235001108981281,0.406910552535155
"820","LRRC20",4.85541019399086,1.43736060793494,0.634830604529882,2.2641640111213,0.0235640291722335,0.406910552535155
"821","ST8SIA1",26.3104276929643,1.02739235702005,0.453913709308445,2.26340896067079,0.023610490889428,0.407216261868819
"822","FCHO1",2.22797990332596,-3.03300399153723,1.34047636035108,-2.26263146538657,0.0236584167903623,0.40754644981938
"823","TNFRSF19",6.68648430340936,1.56567609013696,0.692430153314979,2.26113216277679,0.0237510742732525,0.40864545772692
"824","LINC01871",3.98901918617371,2.17735511921443,0.963476787507665,2.25989369691702,0.0238278492097933,0.408972539164452
"825","HNRNPAB",152.156143442978,-0.361507304600979,0.159952721073876,-2.26008849473722,0.0238157590609185,0.408972539164452
"826","STARD7",45.1626369165758,0.488150215105977,0.216096473852928,2.25894576807489,0.0238867585394081,0.409487289246997
"827","SERPINA3",102.022960437202,-0.891716961228293,0.394880560557819,-2.25819412322711,0.0239335594930511,0.409793473303027
"828","RHOB",695.538379664814,0.690429781893851,0.306004556571494,2.25627287916722,0.0240535467974813,0.410359304400404
"829","SIGIRR",87.1391834046334,-0.531847254601733,0.235694589967195,-2.25651023502813,0.0240386950465622,0.410359304400404
"830","SNHG16",143.1869792867,-0.404442504347448,0.179226531249486,-2.25659951976897,0.0240331104103749,0.410359304400404
"831","MAP4K3",33.5238785717208,0.599349565616145,0.265822459382651,2.25469874520039,0.0241522444816235,0.410559161896505
"832","FZD6",5.51274091851228,-1.38094680052004,0.612312237314292,-2.25529838596255,0.0241146058789966,0.410559161896505
"833","LYZ",34.566485003474,-1.2054844960142,0.534577681363492,-2.25502211940367,0.0241319404177403,0.410559161896505
"834","NFKBIZ",321.108913062293,0.713912321472946,0.317020065276586,2.251946799803,0.024325634159239,0.411355012662712
"835","ATP8A1",3.34263604094735,2.00327383257869,0.889854855175014,2.25123661564412,0.0243705549367646,0.411355012662712
"836","DDIT4",213.64539669618,1.53779819147254,0.683098808070884,2.25120901003383,0.0243723025056163,0.411355012662712
"837","GPR137",34.3279592773956,-0.611797454505434,0.271719675311151,-2.25157583382526,0.0243490896475578,0.411355012662712
"838","AL590807.1",9.49740460804719,1.85419567529454,0.823036415030423,2.25287197678368,0.0242672222487327,0.411355012662712
"839","F10",280.815378608298,0.502861111743802,0.223375466337971,2.251192219037,0.0243733655101706,0.411355012662712
"840","PXDN",80.229723867571,0.566600391420076,0.251851621012141,2.24973891032753,0.0244655239696099,0.411740026389376
"841","PGM3",66.9321885806561,-0.454463491811298,0.202032496755306,-2.24945738487668,0.0244834111737185,0.411740026389376
"842","DMTF1",36.222108111463,0.6328614801667,0.28131951954095,2.24961808977702,0.0244731991236541,0.411740026389376
"843","MAP2K3",49.2876802437863,-0.704199126068478,0.313197700229984,-2.24841729537407,0.0245495933574625,0.412363276324637
"844","PDZRN3",29.2751577753555,0.798504640378774,0.355237215481035,2.

24780683323818,0.0245885099636423,0.412527607920824
"845","ECHDC3",4.40723656953829,-1.7884030357166,0.796036321351981,-2.
24663496846374,0.0246633654625447,0.413293792839802
"846","PRELID1",223.510994919493,-0.515609902354597,0.229709864169383,
-2.24461367481545,0.0247929441140061,0.414974100064215
"847","EFEMP1",1854.23788042363,0.559383742428221,0.249591864311676,2.
24119381443337,0.025013523357877,0.418171771838888
"848","RBP4",1.67870879092327,-3.327553904642,1.48536693440605,-2.2402
2349465628,0.0250764171114864,0.418728851767273
"849","RAB2A",330.436455729572,-0.318240415732771,0.142134859620397,-2
.23900327184128,0.0251557032725393,0.419064421575479
"850","SPHK1",27.5164210749547,-1.4905040260571,0.665638343723409,-2.2
3920998559009,0.0251422564291357,0.419064421575479
"851","HARS",40.8661240457182,-0.577932622808432,0.258183858670543,-2.
23845373519615,0.0251914812125037,0.419167301961283
"852","SPATA18",3.4378918674799,2.18167832953201,0.976534909434309,2.2
3410172893442,0.0254763802846236,0.423410263885294
"853","KXD1",77.9571952218955,-0.503035022479607,0.225235894085917,-2.
23336970566389,0.0255245742306886,0.423435871664712
"854","BGN",540.622426853232,-0.643115482294039,0.287983203141468,-2.2
3317011297397,0.0255377284181966,0.423435871664712
"855","RNF19B",22.0074363495054,-0.886107953146305,0.396900609945489,-
2.23256888737965,0.0255773877325175,0.423597438938535
"856","DHRS9",6.69584938801448,-1.74723010378578,0.783010137940124,-2.
23142717970708,0.0256528461416241,0.424350819352099
"857","ARF1",321.630222599281,-0.307964266498739,0.138068667908039,-2.
23051522959473,0.0257132575407505,0.42485382354379
"858","DHRS3",158.981486359421,0.766926005168566,0.343994464941891,2.2
294719343758,0.0257825206619214,0.424936669816621
"859","ACVR2A",63.8142975062981,0.918024563243964,0.411839332190545,2.
22908423622643,0.0258083005679586,0.424936669816621
"860","PLS3",177.824309346387,0.472583268364751,0.211947006163667,2.22
972372631591,0.0257657897415403,0.424936669816621
"861","TMEFF2",6.13084983548273,-2.36503213504259,1.06178724269091,-2.
22740680990746,0.0259200977104692,0.425199456040803
"862","TMEM158",8.58340610399398,-1.16214855984831,0.522045169375445,-
2.22614560582691,0.0260044300092751,0.425199456040803
"863","FSTL1",1584.88674982935,0.622923626318003,0.279583919899213,2.2
2803810227198,0.0258779742798382,0.425199456040803
"864","HSPA5",369.963810417228,-0.412941551649125,0.185482163462149,-2
.22631407754414,0.0259931511864241,0.425199456040803
"865","NPAS3",3.89302673919637,1.90451140559692,0.855427879145776,2.22
63845404463,0.0259884350996323,0.425199456040803
"866","CDIP1",22.5029780578072,0.687076727526678,0.308588116835986,2.2
2651712765679,0.0259795630343053,0.425199456040803
"867","GLRX5",153.903320333283,-0.593305203131194,0.266662164371776,-2
.22493207661818,0.02608579828124,0.426037951167657
"868","HPRT1",26.7385786519191,-0.684644918476988,0.307840681081507,-2
.22402353084618,0.0261468611092816,0.426543264179063
"869","MYSM1",46.0053533426933,0.754275865516856,0.339465932079614,2.2

2194863825085,0.0262867768596539,0.426906645655038
"870","CMPK2",3.5612656248249,-1.70297512208349,0.766448240144549,-2.2
2190492832538,0.0262897312861012,0.426906645655038
"871","FNDC4",61.5989749623968,-0.732362658031351,0.329478746461296,-2
.22279180644321,0.0262298418454357,0.426906645655038
"872","TSPAN5",15.0907537454232,-1.27616457561452,0.574142812731883,-2
.22273021156929,0.0262339974339073,0.426906645655038
"873","PRKAG2",43.3365185716017,0.601470075296071,0.270832493147449,2.
22081947518983,0.0263631909628529,0.427119890199081
"874","TAB3",10.8664730029429,1.01156879993268,0.455485312331782,2.220
85931762349,0.0263604914337483,0.427119890199081
"875","RBM33",58.4330837022936,0.490721491349842,0.221075654449636,2.2
1969937201582,0.0264391815119394,0.427861497381785
"876","MLLT6",60.7926342764039,0.684081492111176,0.308255116970033,2.2
1920563342239,0.0264727379988525,0.427915490940356
"877","KIDINS220",156.852996264725,0.427072523953562,0.192571929122276
,2.21772989396802,0.0265732546967652,0.428561829733708
"878","GNG10",155.994402862379,-0.47558517675858,0.214431370098472,-2.
21788993159061,0.0265623381774477,0.428561829733708
"879","ZNF431",29.7419182264546,0.678448804059514,0.306052511273857,2.
21677254414826,0.0266386386049027,0.429127557048262
"880","CPED1",86.8792599483202,0.444900901451509,0.200778198373999,2.2
158825263626,0.026699548543157,0.429620008376253
"881","CCNI",1019.07700212396,-0.287731070668038,0.129884686004957,-2.
21528095049679,0.0267407865423429,0.429795161679428
"882","STMP1",148.025099882552,-0.416800770596527,0.18834832433926,-2.
21292529178954,0.0269027966793287,0.431090375098968
"883","KCNMA1",9.88250489255623,-1.28520741149166,0.580706952188611,-2
.21317724309632,0.0268854283821472,0.431090375098968
"884","EGLN3",15.979351504706,-1.05594944529499,0.477204541979273,-2.2
1278163220177,0.0269127042081559,0.431090375098968
"885","ZHX2",28.8695234959966,0.663099202594423,0.299891319282018,2.21
113169991708,0.0270267183473945,0.431939426409827
"886","FBLN1",2265.40151989146,0.561062158945414,0.253728176531953,2.2
1127257766249,0.0270169671151438,0.431939426409827
"887","C5orf56",55.854450675229,0.47419341896007,0.21469947692589,2.20
863797969919,0.0271998314388731,0.433090926923405
"888","WNT2",38.1019831146943,-0.962964995992122,0.436000380954448,-2.
20863338211792,0.0272001514828757,0.433090926923405
"889","KLF10",123.698448794181,-0.537198150327807,0.243225114170979,-2
.20864589645202,0.0271992803503545,0.433090926923405
"890","PTGS1",12.0957945479555,1.08039972209709,0.489237817360966,2.20
833239737057,0.0272211105199033,0.433090926923405
"891","CSF1",183.282380380223,0.432918525689154,0.196337463504646,2.20
497157272743,0.0274560893978816,0.435124597907
"892","PBX1",137.848079589677,0.49542368123928,0.224974907017857,2.202
12861872617,0.0276562244432416,0.435124597907
"893","PCYOX1",182.033811681241,0.6749883285302,0.306277678206128,2.20
384434309289,0.0275352928495767,0.435124597907
"894","MAP3K2-

DT",6.85392297953615,-1.17622545387198,0.533746256940199,-2.2037165386
6935,0.027544285302743,0.435124597907
"895","MAP3K13",39.3785724892851,-0.626839697875487,0.284491411184762,
-2.20336949809844,0.0275687162202694,0.435124597907
"896","CNTN1",32.8947717218825,0.94690984738357,0.429553519074259,2.20
440481880879,0.0274958870883565,0.435124597907
"897","FZD10-
AS1",25.8513750357665,-0.955243248307888,0.433711519792542,-2.20248530
351421,0.0276310461275067,0.435124597907
"898","COPE",245.140046201346,-0.536292038628431,0.243279046367268,-2.
20443168713681,0.0274939992604235,0.435124597907
"899","KCNB1",1.55661721496329,3.61596090841261,1.64126742614468,2.203
15157104315,0.0275840673729037,0.435124597907
"900","RTL5",11.1330968172273,0.937112922983941,0.425415844199526,2.20
281622267087,0.0276077042846982,0.435124597907
"901","HRH2",4.01529205884121,1.86816357236907,0.848649146727828,2.201
33794934247,0.0277121082093452,0.435519924799476
"902","IARS",41.4197962783798,-0.565324255173273,0.256887035593694,-2.
20067257916206,0.0277592113735662,0.435776533314521
"903","SEC61B",469.403440513052,-0.480546726992231,0.218467685779015,-
2.19962382664828,0.027833595266138,0.43597755416871
"904","MRGPRF",93.317598471791,-0.408316931032977,0.185611073302497,-2
.19985221661602,0.0278173818423738,0.43597755416871
"905","WIPF1",77.9088293599686,0.561840940973559,0.255582371602948,2.1
9827735946668,0.0279293467974328,0.436372505577165
"906","DEFB1",3.56329711507688,3.08082103988297,1.40211410405104,2.197
26841844165,0.0280012817694017,0.436372505577165
"907","PPP1R1A",4.66420623597945,-1.90808463975824,0.868453718011232,-
2.19710572962688,0.0280128960148053,0.436372505577165
"908","KL",3.44830883195857,2.05920820308163,0.936955842248082,2.19776
440919657,0.027965898935681,0.436372505577165
"909","SYNGR1",31.9223791989065,0.619276232918695,0.281652780650185,2.
19872224051586,0.0278976785368801,0.436372505577165
"910","NUDC",188.116170307883,-0.369324499617282,0.168330286165099,-2.
19404664502885,0.0282320563835253,0.437521413383108
"911","RNF144A",19.2356704330584,0.966917105258948,0.440652130003236,2
.19428669334209,0.028214805468644,0.437521413383108
"912","ALCAM",20.0759062803084,-0.909158471987676,0.414457251421486,-2
.19361217319636,0.0282633025299572,0.437521413383108
"913","PAMR1",42.7477653157734,0.804305717344976,0.366420517456294,2.1
950346092203,0.0281611152112479,0.437521413383108
"914","HSD11B2",16.2860871614898,-1.27782084429155,0.582551280722664,-
2.19349074764095,0.0282720404834424,0.437521413383108
"915","LRRC59",76.4641671411067,-0.522493288681945,0.238089052868496,-
2.19452882182926,0.0281974142661008,0.437521413383108
"916","PAQR4",3.80693962970359,-1.65477296132339,0.754648524772558,-2.
19277306852501,0.0283237332314702,0.437842863054168
"917","TBC1D5",75.0575509705258,0.438562756253562,0.200430808849027,2.
18810051594367,0.0286622813466673,0.439059518631253
"918","TMOD1",3.15748042231943,-2.06610622303683,0.944070116514646,-2.

18850929278915,0.028632525105606,0.439059518631253
"919","BICC1",192.840015898519,0.464078662638268,0.212069784125642,2.18832996200591,0.0286455759191342,0.439059518631253
"920","RIMKLB",36.1596122206126,0.605324362662236,0.2766771251197,2.18783667930752,0.0286815010405303,0.439059518631253
"921","DERA",63.9528164906378,-0.582792999047188,0.266116105337274,-2.18999522147883,0.0285245833793672,0.439059518631253
"922","SREBF1",28.3527463246278,0.830689967420215,0.379253453407686,2.19032934296645,0.028500360250477,0.439059518631253
"923","MRPL38",60.6602095038907,-0.459294318716593,0.209826145444469,-2.18892797055239,0.0286020757261967,0.439059518631253
"924","MARCH2",56.2056606784633,-0.675151375004552,0.308563272423963,-2.18804840155085,0.0286660768418813,0.439059518631253
"925","ZC3H7B",39.5213186942487,0.557608802819203,0.254543542312193,2.19062246778708,0.0284791238897274,0.439059518631253
"926","NTF3",1.94532239548013,2.6137763632043,1.19583826428919,2.18572731886776,0.0288355609676941,0.440747722076366
"927","INSR",58.7161855587483,0.643865796086431,0.294611372217987,2.18547502507821,0.0288540351952536,0.440747722076366
"928","FOXP4",11.3136086268262,-1.03360243790964,0.473131747364493,-2.18459751151167,0.0289183705574995,0.440966455655893
"929","RTRAF",243.742051906207,-0.456621050367169,0.209034380558208,-2.18443037527033,0.0289306382277065,0.440966455655893
"930","ARL3",99.315562655074,-0.606498982237692,0.278036373116734,-2.18136560853156,0.0291563852585978,0.442502052799297
"931","AP3S2",28.3659533736737,0.630661950094132,0.289098221083755,2.18147987120067,0.0291479416820759,0.442502052799297
"932","AATF",49.0901555710839,-0.475125156700165,0.217706574553756,-2.18241069510213,0.0290792356991398,0.442502052799297
"933","DUXAP8",50.553131654461,0.803084047214873,0.368083564976239,2.18179816658403,0.0291244319588707,0.442502052799297
"934","HIGD1A",77.611502705494,-0.513439840089194,0.235445248037689,-2.18071863572717,0.0292042337985226,0.442753694418716
"935","MIIP",17.551525401491,-0.733664012255359,0.336619819236638,-2.17950331599342,0.0292942985994258,0.443005780474657
"936","AFF3",30.1815029516819,-1.16961673219806,0.536711447877361,-2.17922821811194,0.02931471866559,0.443005780474657
"937","AFF1",87.3259881329856,0.591815209733853,0.271540924610679,2.17946967140356,0.0292967953253069,0.443005780474657
"938","ARPC5L",58.9908891494363,-0.667403281177071,0.306333421962825,-2.17868255086467,0.0293552588934966,0.443145486068136
"939","APOL1",116.144814347688,0.474545471560548,0.217908996275925,2.17772317651199,0.0294266523549815,0.443750156918571
"940","MPZL1",231.939123397869,0.404712555628992,0.18606382125407,2.17512761428433,0.0296205546456091,0.446198993384921
"941","STX3",8.56867409070282,-1.16123240499942,0.534000924876259,-2.1745887523856,0.0296609479466012,0.44624822665444
"942","ARFGAP3",83.0282488377312,-0.426460656475772,0.196142085547947,-2.17424350967005,0.0296868523664182,0.44624822665444
"943","GLT8D2",141.268787243778,0.542501758357887,0.249813536761569,2.

17162674765568,0.0298838278224977,0.448280136411753
"944","CABLES1",15.3276585302534,0.924824697688032,0.425871178452425,2
.17160668408873,0.0298853424274502,0.448280136411753
"945","TJAP1",17.615221627688,0.73817486087566,0.340017404915645,2.170
99139692215,0.0299318227029899,0.448502232247975
"946","SYNPO2",51.2237740950715,0.704474014841223,0.324649172805551,2.
16995475070306,0.0300102740087406,0.449053219437798
"947","NOL7",197.099130071569,-0.384228202609737,0.177124425813752,-2.
16925588238042,0.0300632626435429,0.449053219437798
"948","RAB34",262.771535074362,-0.515274339205377,0.237535744041887,-2
.16924969033087,0.0300637324877848,0.449053219437798
"949","LAPTM5",12.5978823879106,-1.08956796907752,0.502622811738151,-2
.16776466095842,0.0301765968916773,0.449357895541071
"950","SPRED2",36.1655496592981,0.682669156179455,0.314967841568025,2.
16742494338748,0.0302024670379577,0.449357895541071
"951","AC010864.1",1.65675949827156,2.82059516837894,1.30165141964467,
2.16693588299463,0.0302397433924256,0.449357895541071
"952","TPGS1",98.6599467697271,-0.641621361889379,0.296101625208622,-2
.16689577923565,0.0302428018679831,0.449357895541071
"953","SLITRK2",22.0091754857002,-0.960294806878705,0.443165109297863,
-2.16690074812109,0.0302424229061706,0.449357895541071
"954","GTPBP4",43.263923063772,-0.603479821406674,0.278701564370764,-2
.16532627927359,0.0303627072421235,0.45019469586227
"955","ATP2B1-
AS1",27.517099792031,-0.718354113169474,0.3317000423128,-2.16567386654
733,0.0303361174027609,0.45019469586227
"956","NDE1",22.6114463640037,0.645491225761347,0.29825910444995,2.164
19621775423,0.030449293452044,0.451006271214375
"957","CSNK2B",178.635104484917,-0.440825625499448,0.203735035424697,-
2.16372026824214,0.0304858245108505,0.451075522542992
"958","UBE3B",24.5037637764114,0.653817632975072,0.302240112309756,2.1
6323911468441,0.0305227932608507,0.45115109871988
"959","PIM1",97.4251313346643,-1.53151497613585,0.708404282129526,-2.1
6192224520719,0.0306241700240455,0.45141081892635
"960","AKR1C2",203.903879991806,-0.737135575446065,0.34098724170934,-2
.16176878569083,0.0306360026121626,0.45141081892635
"961","TLN2",71.6874118779862,0.474084149031689,0.219281412500923,2.16
198967174061,0.0306189723019227,0.45141081892635
"962","HMGB2",76.1411773436078,0.478928098644009,0.221827638608601,2.1
5901003882137,0.0308493881202196,0.453140389815673
"963","RNF144B",1.67807084175713,-2.63496402330263,1.22014796577368,-2
.15954465951335,0.0308079365043606,0.453140389815673
"964","DPY19L3",27.5937466620489,0.606478893686301,0.280876127841328,2
.15923972730396,0.0308315734463494,0.453140389815673
"965","RUFY3",114.589670626163,0.554547671561898,0.256984282108256,2.1
5790501665114,0.0309352173877126,0.453930236487058
"966","DIP2B",16.6741987461642,0.719407948580184,0.333544638875222,2.1
5685657849627,0.031016841093026,0.454656801115164
"967","STRAP",121.841008778834,-0.611334106958513,0.28362682936265,-2.
15541706097504,0.0311292124699608,0.455361207205212

"968","FGD6",9.55734184827776,1.15024237626107,0.533557223507451,2.155
79946364461,0.0310993273904887,0.455361207205212
"969","AC092807.3",6.05677637010629,-1.24770409171955,0.57925448474960
4,-2.15398261829411,0.0312415350530164,0.455773973643644
"970","GEN1",3.34291127410683,-1.69426055771065,0.786536868193406,-2.1
5407646637365,0.0312341757567377,0.455773973643644
"971","GALNT13",8.50134726639677,-1.23766966234765,0.574747741304601,-
2.15341370378994,0.0312861795467247,0.455773973643644
"972","RDH5",22.0332491641538,-0.754921227639053,0.35052119292451,-2.1
5371065395648,0.0312628701174903,0.455773973643644
"973","LDOC1",18.0131405127507,-0.80313458940494,0.37317131489261,-2.1
5218736637371,0.0313826002650556,0.456708756169772
"974","STARD9",42.105893271362,0.809559520531775,0.376590354880701,2.1
4970858929256,0.0315782726155453,0.459084538230104
"975","ZFP28",4.91719275187995,-1.44135988879454,0.670685091464636,-2.
14908592294323,0.0316275894377482,0.459329914295912
"976","ODC1",29.3338082485737,-0.765321749625409,0.356517015692231,-2.
14666261619918,0.0318201509991611,0.459364669468865
"977","VHL",27.6226101896807,0.75356622993667,0.350987542260332,2.1469
8853721065,0.0317941941972625,0.459364669468865
"978","LRP11",60.784595800487,0.515346891648848,0.24007516583691,2.146
6064174207,0.0318246285839659,0.459364669468865
"979","GTF2I",183.81527446498,0.331941636022779,0.154626790066649,2.14
672784631759,0.0318149545281623,0.459364669468865
"980","VPS11",27.08143346901,0.606867858122501,0.282543229972205,2.147
87612565412,0.0317235974459622,0.459364669468865
"981","MT-
CO2",9747.50409173865,-0.432342380203884,0.201308536296877,-2.14766044
280553,0.0317407399910117,0.459364669468865
"982","DYRK3",27.305113136607,-0.878996184381903,0.409635898550439,-2.
14579871415657,0.0318890412803423,0.459825686893735
"983","CAMK2D",163.320417106608,0.400683346244328,0.186927337620675,2.
14352459808431,0.0320709980864088,0.459905910679136
"984","MDFI",6.7786908799623,1.46665866738052,0.684042773721011,2.1441
0373696705,0.0320245757180146,0.459905910679136
"985","TMEM176B",399.322728187234,0.622990614541053,0.290587553643202,
2.14389985644737,0.0320409117126958,0.459905910679136
"986","POLI",21.9910777867776,0.655077444286114,0.30540245887966,2.144
9645385607,0.0319556823812039,0.459905910679136
"987","MIR155HG",3.57034456548154,-1.80520314809747,0.842415352347485,
-2.14288966015051,0.0321219594393831,0.459905910679136
"988","SELENOM",1031.38677780813,-0.432198863168708,0.201628458109875,
-2.14354098236066,0.0320696839702041,0.459905910679136
"989","MORF4L2",253.717061216704,-0.374025616231592,0.174527202412911,
-2.14307919373331,0.0321067398442294,0.459905910679136
"990","HIST1H4H",6.74452464846861,1.25864323089412,0.588073625698079,2
.14028171965718,0.0323320064017237,0.462445667321623
"991","DDX11",7.083653142789,1.36172710843511,0.63671350364571,2.13868
105613922,0.0324615078734383,0.463829416233992
"992","LEPR",159.429509098103,0.656634870917733,0.307924220907443,2.13

24560600743,0.0329693730197887,0.4645390640379
"993","PLPPR5",1.65569095314486,3.66725699700314,1.71556822663326,2.13763401540725,0.0325464587443606,0.4645390640379
"994","TWF2",103.443242588441,-0.57211843629929,0.268243453694415,-2.13283279953236,0.0329384445722647,0.4645390640379
"995","EREG",7.78143089495786,-1.54595855005987,0.724495765539614,-2.13384069803143,0.0328558231741544,0.4645390640379
"996","ZNF608",3.94935952506702,1.54918315585605,0.726618920710532,2.13204351235606,0.0330032696625796,0.4645390640379
"997","ZBED8",4.68326985720999,1.3976133029287,0.654722149072537,2.13466629303518,0.0327882781702806,0.4645390640379
"998","CALU",349.845195168973,-0.365915697546233,0.171572538030607,-2.13271716876367,0.0329479346439731,0.4645390640379
"999","KLF6",997.55442129064,-0.512736711112833,0.240483989424519,-2.13210331523449,0.0329983541604006,0.4645390640379
"1000","FAM204A",129.23332069141,-0.396083865023479,0.185581052350762,-2.13429043539881,0.032819013726567,0.4645390640379
"1001","CORO1C",35.5389180110175,-0.60606025657824,0.283754791778692,-2.13585910841965,0.0326908995074746,0.4645390640379
"1002","EVL",98.566078360166,-0.400733150435572,0.187885096052254,-2.13286289788585,0.0329359747180082,0.4645390640379
"1003","ERN1",17.7983102757697,-0.731771948655642,0.342471600113441,-2.13673761098219,0.0326193390790392,0.4645390640379
"1004","ZNF44",15.0517622875538,0.85771161774125,0.402183318737547,2.13263847052037,0.0329543949206157,0.4645390640379
"1005","SSC5D",136.766657184002,-0.524397754734069,0.245672648234331,-2.13453861674451,0.0327987160650436,0.4645390640379
"1006","COL9A3",1.571666567381,2.88886165400683,1.35309338255481,2.13500538192885,0.0327605705095922,0.4645390640379
"1007","PGBD1",8.04959319693976,1.02830221269073,0.482561580772687,2.13092432896169,0.0330953767198458,0.464911244397834
"1008","MAOA",11.2937613827737,-1.01435045636208,0.476008101367274,-2.13095208558107,0.0330930897352624,0.464911244397834
"1009","SLC23A2",21.5589326125436,0.753769838939823,0.35384449300944,2.1302291086376,0.0331527029879431,0.46525497949383
"1010","TNFAIP8L3",13.1912290266438,-1.08911167385633,0.511385671919605,-2.12972661077519,0.033194190697543,0.465375980472484
"1011","PFN1",451.571507464737,-0.553604096920976,0.260011800751436,-2.12914988981675,0.0332418612380375,0.465583338408121
"1012","RALGPS2",42.2288322211038,0.79402717828348,0.373456907944785,2.12615474875852,0.0334903765291074,0.468137938452281
"1013","AL139317.5",11.7412435431715,-1.23887348318341,0.582660467045637,-2.12623569514693,0.0334836393409441,0.468137938452281
"1014","MAPKAPK2",41.8126098195054,-0.53078788216272,0.249719614315859,-2.12553540744842,0.0335419628514148,0.468396641002006
"1015","SLIT3",852.015388680619,0.509684481662177,0.239957129400846,2.1240647566289,0.0336647290332966,0.469222447428522
"1016","CYB561",47.918874468769,-0.583935954913632,0.274969761543249,-2.12363698334076,0.033700510525057,0.469222447428522
"1017","GAS2L1",33.7153418581007,-0.644299244187368,0.303376095474437,

-2.12376404666879,0.0336898788015098,0.469222447428522
"1018","VMA21",40.5570174168936,-0.509260398695459,0.239908551004696,-2.12272716650893,0.0337767211587132,0.469821583111375
"1019","NOX4",30.9268763483969,0.704518529595584,0.331996291593794,2.12206746711972,0.0338320728816139,0.470129687932927
"1020","NMNAT3",13.6595024592344,0.860490087861037,0.405807446394758,2.12043937464858,0.0339690091114048,0.470966331085494
"1021","MAN2B2",47.7434455957617,0.517742454901933,0.244199035425396,2.12016584750232,0.0339920614667638,0.470966331085494
"1022","TMEM200A",6.70770065810422,-1.12150031183166,0.528890190564888,-2.12047856405472,0.0339657073965514,0.470966331085494
"1023","OLFML3",326.863462375122,0.587715987190982,0.277334010221995,2.11916305079402,0.0340766897213139,0.471172714506457
"1024","MYLIP",84.6721425357318,0.674192017071953,0.318365246143776,2.11766838635233,0.0342031620325327,0.471172714506457
"1025","AC093627.5",1.96732550025318,2.75770180570661,1.30348511577238,2.11563735737215,0.0343756620583181,0.471172714506457
"1026","CAPZA2",143.720055289002,-0.405651097756167,0.191663738169469,-2.11647284786594,0.0343046121105109,0.471172714506457
"1027","ATL1",44.6145669490433,-0.531660306260889,0.251107759963153,-2.11725956353919,0.0342378247148769,0.471172714506457
"1028","ZDHHC7",44.7426442544362,0.543963198594241,0.256789414818554,2.11832407102373,0.0341476314066112,0.471172714506457
"1029","LSM12",57.5389775804474,-0.479666079248358,0.226762660941963,-2.1152780499922,0.0344062561299207,0.471172714506457
"1030","DLX3",5.81206529171762,-1.77408504875117,0.837705452352345,-2.11779097744845,0.0341927738014148,0.471172714506457
"1031","POLR2I",136.82009440096,-0.54036105467831,0.255362753359753,-2.1160527428879,0.03434032205443,0.471172714506457
"1032","REM1",15.660322071583,-0.876084377748332,0.413554181256173,-2.11842708272764,0.0341389142446784,0.471172714506457
"1033","PRR5",27.7421668141071,-0.675905446928064,0.31946580966298,-2.11573641523989,0.0343672316319315,0.471172714506457
"1034","ADGRG2",2.79723745276665,2.05580863988213,0.971307856583563,2.11653661189681,0.0342991947876079,0.471172714506457
"1035","LPP",300.146518866105,0.421455849226585,0.199290071184551,2.11478598367452,0.0344481920174966,0.471291206732127
"1036","ZNF721",50.4188578531213,0.525827352848282,0.248753607775022,2.11384814697382,0.0345282392037494,0.471930373672869
"1037","SMARCAL1",25.4189971989509,0.628540419574499,0.297921071323249,2.10975483131411,0.0348794789026627,0.476271380194507
"1038","RECQL",98.6856268111824,-0.420849979782194,0.19954432568254,-2.10905511014999,0.0349398249814657,0.476635762752943
"1039","GLI3",76.3333017130782,0.526873193333336,0.249946186709849,2.10794651548319,0.0350356160462549,0.477482505500452
"1040","AC005332.6",23.4659014699629,0.70555242769276,0.334908030232888,2.10670501750028,0.0351431570177879,0.478487599396035
"1041","EEF1AKMT2",46.9521210011787,-0.552432231349246,0.262285134208299,-2.10622776245687,0.0351845726907897,0.478591305765208
"1042","MBD2",127.31808036942,-0.47390431916293,0.225091588478209,-2.1

0538440093157,0.0352578605412295,0.47912793211498
"1043","PINK1",114.882314004795,-0.517074608744598,0.245677921840146,-2.10468488528261,0.0353187470402065,0.479495165953331
"1044","DNMT3A",22.627109392072,0.697990503931352,0.331789626496765,2.10371406514772,0.0354033968007989,0.479724496362978
"1045","PPP1CC",176.007162702687,-0.319236973998943,0.15174721406133,-2.10374191034519,0.0354009664552867,0.479724496362978
"1046","MIGA1",54.7613897517394,0.499993220844584,0.237810895346849,2.10248239516251,0.0355110402124034,0.480723068267335
"1047","HDAC3",41.9662158864336,-0.530923492777126,0.252759508827604,-2.1005084842891,0.0356841350656857,0.480944212590462
"1048","ETS1",49.6297633095405,0.468424816295979,0.222966142748775,2.10087868284007,0.0356516171435783,0.480944212590462
"1049","SHMT1",8.66571550717892,-1.09263885822193,0.519979833811193,-2.10131006468738,0.0356137568255975,0.480944212590462
"1050","MMP28",26.6510150070586,-0.950124089375738,0.452362534360273,-2.10035981587068,0.0356972010898712,0.480944212590462
"1051","SLC2A10",42.0035876989214,0.572448232943543,0.272485426383781,2.10083981569451,0.035655030012152,0.480944212590462
"1052","LGI4",21.8584574765587,1.04128251622515,0.495906649689849,2.09975509882031,0.0357503899352237,0.481202967188943
"1053","CCL4L2",14.9276150289745,-1.58874452155158,0.757445821105992,-2.09750252398483,0.0359491139765421,0.48341828481276
"1054","DEAF1",35.071071205482,-0.633311529230303,0.30206284763029,-2.09662172689786,0.0360270743961019,0.483548221278486
"1055","NR1D1",16.5426268817402,-1.3361138932074,0.637213635248474,-2.0968068153256,0.0360106800424907,0.483548221278486
"1056","GPR137C",1.96735967807433,2.54299739568174,1.21345841538515,2.095660933609,0.0361122797120357,0.484232841593207
"1057","CFAP410",46.0367556137384,-0.519292071016788,0.24797154436699,-2.09415992606092,0.0362457362639808,0.485562559600727
"1058","CITED1",3.03225164583747,-2.5420966861183,1.21421759155225,-2.09360884227389,0.0362948392399935,0.485760797389705
"1059","AL513534.2",6.42749293270907,1.17500996111036,0.561756675680924,2.09167066806298,0.0364679861256556,0.487157248621965
"1060","NME1",91.9311545009746,-0.573562148158034,0.274207832920538,-2.0917059226541,0.0364648303811324,0.487157248621965
"1061","RTCA-AS1",12.8275753096982,0.949239072373102,0.45421351627404,2.08985210338919,0.0366310872419852,0.487900419216421
"1062","PRR16",16.8303910153922,-0.899521408538464,0.430571971733225,-2.08913135919537,0.0366959001741164,0.487900419216421
"1063","RERGL",2.02588067577273,3.19786315550188,1.53018626830977,2.08985221062937,0.0366310776056712,0.487900419216421
"1064","KRT27",6.10802944129889,1.86398990387622,0.891818031040346,2.09010116301617,0.0366087132362671,0.487900419216421
"1065","FOXRED2",15.3874889712996,0.86437697458377,0.413736900210708,2.08919478572871,0.036690192624703,0.487900419216421
"1066","FAT3",2.3327809434863,2.89128776612542,1.38509473176354,2.08742961749928,0.0368493170486832,0.489021864488616

"1067","UBE3A",93.2813502132428,0.403753641389271,0.193411359181987,2.08753841086121,0.0368394926965478,0.489021864488616
"1068","CASC2",2.15562353666846,-2.31965554778195,1.11252271945305,-2.08504105778834,0.0370655737932601,0.491431203101651
"1069","ITSN2",51.3470406818083,0.65636528633166,0.315148925666034,2.08271465607729,0.0372772407250225,0.491934509474668
"1070","AC026329.1",4.27664716901202,1.64787482979239,0.790789026282272,2.08383623826892,0.0371750658131912,0.491934509474668
"1071","RDH10",38.1667172652487,0.93527508270457,0.448929400766928,2.08334557974326,0.0372197348941697,0.491934509474668
"1072","C11orf80",5.22391708460226,-1.23758602767287,0.593865028280341,-2.08395168723195,0.0371645620858502,0.491934509474668
"1073","SPIN4",4.43856683463159,1.36297264438393,0.65441128532126,2.08274624071439,0.0372743601313128,0.491934509474668
"1074","LAMB1",238.055907494405,0.683380691581113,0.328202365065573,2.0821930745215,0.0373248376083975,0.492104004222448
"1075","FLG-AS1",5.25279371846768,1.31375405793407,0.631209469945436,2.08132818103574,0.037403877403181,0.492687352585156
"1076","SPTSSA",56.5080381371263,-0.560575768436226,0.269417436308686,-2.08069594944087,0.0374617450741925,0.492990994656659
"1077","ELP5",49.0335806207174,-0.703670423635685,0.338262790752583,-2.08024779216811,0.0375028106951606,0.493073165685677
"1078","NFATC1",23.0378393509413,-0.923763705369939,0.44415676142832,-2.07981457357375,0.0375425438609797,0.493137681884483
"1079","EN1",22.662220001512,0.782086974742168,0.376283693064381,2.07845035317104,0.0376678990745458,0.494325719087645
"1080","AKIRIN1",69.6252190484872,-0.522208195458122,0.251468904060125,-2.07663129328016,0.0378356023619682,0.495955909876039
"1081","KCTD17",24.0222428155803,-0.615854137806208,0.29660509798046,-2.07634373785032,0.0378621708033897,0.495955909876039
"1082","SYNPO",19.5114364251331,-0.905601673601191,0.436442257823689,-2.07496331385727,0.0379899349682714,0.497169574076454
"1083","ANKH",81.9023977870881,-0.547810966245912,0.264089173915345,-2.07434086798846,0.0380476648383369,0.497465313121745
"1084","KIF22",36.057802122645,-0.498539004480218,0.240449107321478,-2.07336600261808,0.038138230408659,0.498189430430454
"1085","NIPAL2",31.6723519970089,0.70670966499547,0.341008296579978,2.07241193860432,0.0382270409364991,0.498429926022862
"1086","COL14A1",584.587252925191,0.522338971232883,0.252039580002433,2.07244818939883,0.0382236632643434,0.498429926022862
"1087","XIST",676.112587497747,0.608426404399243,0.293646960774438,2.07196561065926,0.0382686483974302,0.498513395867168
"1088","PSMD6",51.2836083525331,-0.47415978582914,0.229045487376733,-2.07015554534477,0.0384377807139325,0.499675722590105
"1089","STMN2",5.77832202058429,-1.61398148209349,0.779747026141938,-2.06987834256862,0.0384637385327129,0.499675722590105
"1090","SKA2",43.0814587846033,0.48220990235568,0.232897665056716,2.0704797630249,0.0384074392200864,0.499675722590105
"1091","SLC25A13",20.0632886012932,0.674253067947523,0.325834579098938

,2.06931096697011,0.0385169151720401,0.499907899941418
"1092","IGSF3",7.5329147160419,1.24404888405447,0.601464031838505,2.06
836787937554,0.0386054432402867,0.500313278609948
"1093","CSF1R",2.28194084933585,-2.64220661529325,1.27853387438082,-2.
06659101353324,0.0387727083063301,0.500313278609948
"1094","CDK19",23.7256996693168,0.721913263795327,0.349320618811046,2.
06662082030097,0.0387698973799202,0.500313278609948
"1095","ZFAND1",58.2280754690737,-0.560257262327756,0.271178529542982,
-2.06600892508695,0.0388276368600605,0.500313278609948
"1096","CTBP2",59.0049535805307,0.51949856327884,0.251327077023926,2.0
6702186421953,0.0387320937781594,0.500313278609948
"1097","TEDC1",8.77035557965134,-1.03742451744953,0.501774088335503,-2
.06751313303343,0.0386858279939797,0.500313278609948
"1098","WDR45",104.012052332918,-0.565579632894458,0.273759145244476,-
2.06597530244835,0.0388308116661252,0.500313278609948
"1099","OPHN1",31.118133642086,0.583201822317109,0.282169267987511,2.0
6685095962655,0.0387481999259727,0.500313278609948
"1100","SIPA1L2",21.0105980884197,0.926902774329815,0.448748755249472,
2.06552723208006,0.0388731416137714,0.500403350228185
"1101","LRRC8D",6.75864112946183,1.29015405935696,0.625090772171856,2.
06394673668652,0.0390227667683139,0.501361221250922
"1102","AL158151.1",2.70060215471841,2.16071374489233,1.04693818219463
,2.06384080897972,0.0390328123885913,0.501361221250922
"1103","MIEF1",18.3016755742637,0.717142327236889,0.347516682303177,2.
06361985986977,0.0390537730960288,0.501361221250922
"1104","CFLAR",194.225075166967,0.530606664553367,0.257275331693423,2.
062407853333,0.0391689222547577,0.501478323001691
"1105","PCDHGA10",9.75683330467571,0.968158410074453,0.469324086918374
,2.06287816257519,0.0391242053761127,0.501478323001691
"1106","GLI1",25.2343854000869,-0.670206891279526,0.324963675215351,-2
.06240556220749,0.0391691402005558,0.501478323001691
"1107","FAM234A",55.1630206867578,0.492467921852515,0.238936257391553,
2.06108494051403,0.0392949372673311,0.502634427918164
"1108","AGPAT2",73.6041765298495,-0.505650901651208,0.24545676360653,-
2.06004061253644,0.0393946584572063,0.50345520194408
"1109","PTGER3",32.2902098465564,-1.14952658962357,0.558561654961444,-
2.05801200174208,0.039588981323724,0.504868558544289
"1110","WBP1L",61.0606697366416,0.459300262128308,0.22315975529482,2.0
5816797711361,0.0395740114529163,0.504868558544289
"1111","AC008969.1",8.2183021142376,0.993918949998581,0.48300779027311
8,2.0577700194785,0.0396122152925639,0.504868558544289
"1112","CMPK1",174.628709414058,-0.408408170318019,0.198590245881841,-
2.0565369084694,0.039730792489068,0.505582004985369
"1113","ZDHHC11",2.54962255318882,-2.58275105817128,1.25592978951179,-
2.05644541577062,0.0397396025105025,0.505582004985369
"1114","PRADC1",39.0537535028331,-0.590710295113996,0.287491422197223,
-2.05470580860935,0.0399074285733162,0.506352319532399
"1115","SDC2",516.318014452277,-0.567607791410826,0.276218932320026,-2
.0549199384827,0.0398867383063126,0.506352319532399
"1116","BBS2",32.1690018122392,0.533787182266471,0.259721920522578,2.0

5522576297162,0.0398572038432528,0.506352319532399
"1117","NFE2L1",131.958378551107,0.456821969961167,0.222547296841081,2
.05269610750374,0.0401020598077322,0.508366308753346
"1118","YY1AP1",40.012771776161,0.463873309825888,0.226166840541003,2.
05102263760805,0.040264742252861,0.509296281059268
"1119","CCDC93",40.271807612181,0.549946757715844,0.268065313767198,2.
05154016380294,0.0402143724859244,0.509296281059268
"1120","SLC66A2",49.6971673217056,-0.511287164119423,0.249307204936651
,-2.05083188129016,0.0402833216657048,0.509296281059268
"1121","BSDC1",57.5566137278245,0.474091259683412,0.231860804816688,2.
04472360068894,0.0408821155138596,0.509366787981454
"1122","AKR1A1",160.00691532065,-0.533808147656025,0.261089443174702,-
2.04454129268964,0.0409001024422092,0.509366787981454
"1123","SORT1",8.02227869612402,-1.09995021639124,0.537160677562089,-2
.04771172265136,0.0405882545226815,0.509366787981454
"1124","AC112715.1",4.31702811652942,-1.75885375287457,0.8602695300856
49,-2.0445380097322,0.0409004264078328,0.509366787981454
"1125","UBE2E2",68.4721758247591,-0.44685966981251,0.218359674925833,-
2.04643861081167,0.0407132365497628,0.509366787981454
"1126","VEPH1",1.92671372464044,2.60048293780708,1.27146023072848,2.04
527273048654,0.0408279775985563,0.509366787981454
"1127","CHN2",1.99646675377862,2.17736711834232,1.06354452977564,2.047
27405142279,0.0406311841894683,0.509366787981454
"1128","AC022034.1",11.4487651193263,0.992628429935302,0.4850744949504
57,2.04634224282743,0.0407227103090063,0.509366787981454
"1129","PLEC",163.282348834188,-0.416370255370924,0.203573545280654,-2
.04530630341433,0.0408246696637633,0.509366787981454
"1130","FZD4",79.8979798737305,0.500447554006643,0.244414807472675,2.0
4753369561127,0.0406057119417792,0.509366787981454
"1131","CLEC14A",39.1567179160711,1.1898262100377,0.581098700811928,2.
04754580998932,0.0406045237981145,0.509366787981454
"1132","MARF1",45.6767497394786,0.580399980208403,0.283438844189308,2.
04770796983901,0.0405886224596489,0.509366787981454
"1133","CDYL2",6.99809169779523,1.08382340414769,0.528737832867742,2.0
4983138480806,0.0403808878065949,0.509366787981454
"1134","PPP5D1",5.23243429814898,1.36912162384544,0.668923880611885,2.
04675249834565,0.0406823917945021,0.509366787981454
"1135","MX2",87.7477080892275,0.632163860785908,0.309155841351163,2.04
480645755565,0.0408739428798443,0.509366787981454
"1136","PRR14L",39.3472661094349,0.629687029384842,0.307348067553431,2
.04877497489186,0.0404841239069819,0.509366787981454
"1137","USP51",17.0810398993818,0.720276021492041,0.351345543364854,2.
0500502570601,0.0403595267772188,0.509366787981454
"1138","ACBD3",123.385817790916,-0.442327122859599,0.216513253003471,-
2.04295633973274,0.0410567603307204,0.509521232500439
"1139","PMS2",17.1400874314922,0.814776711740751,0.398744011009615,2.0
4335786681221,0.0410170251018526,0.509521232500439
"1140","SULT1A1",83.6632335978259,0.973573563003532,0.476381504967011,
2.04368463689822,0.0409847118981257,0.509521232500439
"1141","SLC43A2",17.0529261029147,0.751087580079228,0.367594616542966,

2.04324967308502,0.0410277287741857,0.509521232500439
"1142","FBLN7",29.3634447961344,0.67799975250925,0.332078693965235,2.0
4168398885665,0.041182887992954,0.509856887142541
"1143","DALRD3",60.2858805358041,-0.555920329051511,0.272367116451979,
-2.04106992170447,0.0412438775742338,0.509856887142541
"1144","STK38",52.6350826131045,0.513503272039931,0.251478607967456,2.
04193619564804,0.0411578607792478,0.509856887142541
"1145","ZNF483",15.073078770358,0.872223029436538,0.427390979431079,2.
04080823277458,0.0412698919423632,0.509856887142541
"1146","SLC35B1",51.7013891530379,-0.557207797899022,0.273073202044337
,-2.04050706450702,0.0412998481322383,0.509856887142541
"1147","PNPLA4",22.8192090869043,-0.704876431287393,0.345438285520156,
-2.04052781881429,0.0412977831807428,0.509856887142541
"1148","MYLK-
AS1",3.58022388320237,1.53997575701393,0.755464971566927,2.03844759846
355,0.0415051902261024,0.510167963195842
"1149","RRAS2",40.2894254411064,0.681438114388902,0.334154197316899,2.
03929239812197,0.0414208538891978,0.510167963195842
"1150","FIBP",110.376222529939,-0.452632807811198,0.22196184035928,-2.
03923704668578,0.0414263751760035,0.510167963195842
"1151","C12orf76",51.152409608285,-0.474069902350984,0.232483115423595
,-2.03915842011669,0.0414342192208188,0.510167963195842
"1152","IGDCC4",27.4073520453146,0.888754193739843,0.435935549425568,2
.03872841962752,0.0414771397343807,0.510167963195842
"1153","CPEB1",11.8058138216688,1.01071647557312,0.496317621053988,2.0
3643077073658,0.0417071182041951,0.512205371874591
"1154","PBX3",41.2904170994661,0.47285590269734,0.232410440993602,2.03
457254620655,0.0418939021083439,0.514053426216768
"1155","C1RL",77.8780013017549,0.480218792240849,0.236192203622877,2.0
3316953258797,0.0420353980433759,0.51534306172658З
"1156","ZSCAN26",16.4995108111442,0.726343907266785,0.357394941777274,
2.03232844778043,0.0421204163841753,0.515938664359794
"1157","TRIM69",37.5653564059703,0.546579534989698,0.26904843397875,2.
03152840143596,0.0422014214607128,0.516038106980737
"1158","PITPNA",39.0406792393768,-0.547967908474552,0.269692463826008,
-2.03182506734068,0.0421713685328891,0.516038106980737
"1159","DPP6",1.58416098654627,3.63568844494539,1.7906556861512,2.0303
6712923849,0.0423192353082155,0.516811341156339
"1160","UBAC2",59.2250639224529,-0.503014323012156,0.247811453150976,-
2.02982677602758,0.0423741502176914,0.516811341156339
"1161","SPINT1",1.65758419524138,-2.54459143537003,1.25340166187041,-2
.03014844544949,0.0423414524048823,0.516811341156339
"1162","TLR5",15.8393873340573,0.885307621929134,0.436280626487974,2.0
2921598663638,0.0424362959645227,0.517123881977317
"1163","KCNC4",4.20474247659191,1.4814709802575,0.730870485331519,2.02
699522007037,0.0426629015127997,0.517657828124459
"1164","PCDHB2",9.07724655044019,1.20842232594894,0.596139506895683,2.
02707975561231,0.0426542568663628,0.517657828124459
"1165","KAT6B",103.395664693826,0.430143714452916,0.212077127079506,2.
02824189659858,0.0425355658197184,0.517657828124459

"1166","TUBGCP4",25.4355476673801,0.582590297720118,0.287267215551988,
2.02804311170929,0.0425558481672008,0.517657828124459
"1167","ZNF561",19.6829848514489,0.704076258669841,0.347312480140268,2
.02721266562459,0.0426406684176852,0.517657828124459
"1168","WSB1",436.920977689558,0.46779703295404,0.230947390390548,2.02
555669567412,0.0428102329802222,0.519000769691735
"1169","LMAN1",248.314762802894,-0.321619507782007,0.158831330541048,-
2.02491225557598,0.0428763749332161,0.519357971817228
"1170","AXIN2",4.758432327749,1.7121113107738,0.8459700149087,2.023843
96680842,0.0429862087855632,0.520243347353483
"1171","KCNQ1OT1",350.49743622014,0.863726004767456,0.426933491443156,
2.02309264107582,0.043063597117662,0.520734872063274
"1172","MGAT4B",58.8512930560962,-0.526244907112579,0.2602720234589,-2
.02190346898993,0.0431863253301579,0.521328530839758
"1173","TERF2",29.8029252625886,0.631118886438907,0.312116826251273,2.
02205979734914,0.0431701746453793,0.521328530839758
"1174","KIF5C",2.38158705235243,-2.03726175354763,1.00876497393174,-2.
01956035964178,0.0434290104319316,0.522043266465965
"1175","STK16",39.0412744439415,-0.570330688753936,0.282349318170177,-
2.01994710824903,0.0433888741391483,0.522043266465965
"1176","DZIP3",31.510077020762,0.554735234686591,0.274552605257293,2.0
2050617646382,0.043330910126872,0.522043266465965
"1177","PRDM10",6.38214162300886,1.25386422866906,0.620510192503478,2.
02069884397916,0.0433109495856697,0.522043266465965
"1178","NTN4",40.5490974880282,0.769254457289715,0.380903501854045,2.0
1955207433214,0.0434298706141883,0.522043266465965
"1179","BCL2",31.1991188315258,0.541941917081685,0.26847671684645,2.01
858069275943,0.0435308194283239,0.522812894915239
"1180","TUFT1",5.29386555204991,1.35636951372342,0.672137801133414,2.0
1799320234065,0.0435919692855702,0.522939330945399
"1181","CACNA2D3",2.99283797889472,1.63124133121796,0.808437649228723,
2.01777011841819,0.0436152083224941,0.522939330945399
"1182","GABPB1-
AS1",81.4620801259418,0.666042453226943,0.330230593008529,2.0169011209
9863,0.0437058330764766,0.523582568834948
"1183","EIF6",121.953411797638,-0.48233652038047,0.239204293111322,-2.
01642083470467,0.0437559886827817,0.523740321004386
"1184","CLSTN2",23.0474264168619,0.848955188220299,0.421244707102729,2
.01534921129173,0.04386807200768,0.523753709636382
"1185","MIPEP",10.644344432113,-0.941801635797232,0.467264742727008,-2
.0155632335981,0.0438456676157965,0.523753709636382
"1186","SNHG9",83.9355186317529,-0.684115198720149,0.339375727730336,-
2.01580473446157,0.0438203983026212,0.523753709636382
"1187","NFAT5",90.3056781549871,0.546184850311599,0.271062130534143,2.
01498028970447,0.043906714337369,0.523773441463475
"1188","AL021155.5",17.8058712298697,0.965571242687746,0.4800376797581
12,2.01144885787776,0.044278067101682,0.524437481583898
"1189","GABPB2",41.5639728737544,0.714316615566807,0.355832969460707,2
.00744921598865,0.0447018528701959,0.524437481583898
"1190","SLC50A1",27.0501536645445,-0.667252679950922,0.33179541315042,

-2.01103648062917,0.0443216035602011,0.524437481583898
"1191","VANGL2",9.26286645867763,0.900682844156816,0.447398217501426,2
.01315697945074,0.0440981174587596,0.524437481583898
"1192","PCNX2",38.4283991862493,0.587810916157346,0.293108403441099,2.
0054386338175,0.0449161749954858,0.524437481583898
"1193","CNRIP1",69.9124906576924,0.39994619844385,0.199416460292504,2.
00558267786525,0.0449007915602788,0.524437481583898
"1194","RBM43",80.613552091704,0.428459486325765,0.21341703875212,2.00
761611552212,0.0446841007140017,0.524437481583898
"1195","INPP1",38.3042403992308,-0.486997219902476,0.241951038656487,-
2.01279243357122,0.044136470257076,0.524437481583898
"1196","NABP1",132.928566317156,0.85448686619413,0.426060586127322,2.0
0555248247905,0.0449040159610514,0.524437481583898
"1197","SEMA3G",2.6413755760681,-2.08757068842503,1.04118664989986,-2.
00499179337904,0.044963924381859,0.524437481583898
"1198","COL6A6",27.2419437625627,2.08550490830236,1.03791985548914,2.0
0931208442826,0.0445040471340387,0.524437481583898
"1199","DANCR",43.7985417597093,-0.616208784108734,0.306600847502674,-
2.00980783036929,0.044451531724209,0.524437481583898
"1200","RFESD",4.6075183202578,1.33690601553542,0.666805763013489,2.00
494070341196,0.044969386583601,0.524437481583898
"1201","POLR2J",135.188064681565,-0.411671864168159,0.205172326882889,
-2.00646875932318,0.0448062586491483,0.524437481583898
"1202","BAG1",126.525514165123,-0.340659029185291,0.169494178590777,-2
.00985681052663,0.0444463459925963,0.524437481583898
"1203","AL138756.1",2.65591301342076,2.30937066403327,1.14954720577934
,2.00893939146033,0.0445435617444725,0.524437481583898
"1204","USP20",10.1336113330637,0.938458071299176,0.467356869127349,2.
00801172143135,0.0446420461112883,0.524437481583898
"1205","AL157938.3",3.18452052317447,-1.61269995540337,0.8008668356998
52,-2.01369301800852,0.0440417735202672,0.524437481583898
"1206","TEAD1",101.030935052542,0.522591409214556,0.260477699802955,2.
00628080488227,0.0448262968073628,0.524437481583898
"1207","VEGFB",296.041761139024,-0.432987193937878,0.215423801989077,-
2.00993200352035,0.044438385993054,0.524437481583898
"1208","SIX4",2.8376302029398,-1.82782465010477,0.910893528644314,-2.0
0662820914441,0.044789265342957,0.524437481583898
"1209","CORO2B",15.8768738201805,1.15463559635113,0.574612915514562,2.
00941462535203,0.0444931804665232,0.524437481583898
"1210","GLYR1",39.6098445352757,0.511481909731104,0.255068012214594,2.
00527657423693,0.0449334877440196,0.524437481583898
"1211","GALNS",21.5987847185377,0.693926070521428,0.345466701048742,2.
00866268272704,0.0445729188115126,0.524437481583898
"1212","AKAP10",21.3973593418546,0.640757327718302,0.319633909424846,2
.00466004646156,0.0449994025511607,0.524437481583898
"1213","STAT5B",34.9019195873353,0.551655223747313,0.274573019824524,2
.0091384947431,0.0445224482240426,0.524437481583898
"1214","SERTAD3",42.6382678683367,-0.586052989882316,0.291640853845724
,-2.0095023799112,0.0444838825464346,0.524437481583898
"1215","ATP11C",40.7502158409658,0.505538434968262,0.251900992063313,2

.00689338627614,0.0447610162250962,0.524437481583898
"1216","MRPL57",124.7774895607,-0.393257074054209,0.196325816445839,-2
.00308385913525,0.0451682881715106,0.525540641338201
"1217","ZNF528",10.692362366706,0.96883482850755,0.483609573827795,2.0
0334087855038,0.0451407125973803,0.525540641338201
"1218","BHLHE40-
AS1",5.29998077751921,1.91480706462146,0.957413005806321,1.99998021022
164,0.04550240087717,0.526322465589311
"1219","BRK1",375.704140184152,-0.372554507029517,0.186108164141493,-2
.00181710860504,0.0453044052166083,0.526322465589311
"1220","SND1",75.6852393672782,-0.447494416137895,0.223559525598623,-2
.00167903800852,0.0453192622903576,0.526322465589311
"1221","HK1",29.2875091440526,-0.669972180056202,0.334864542739642,-2.
00072594899098,0.0454219314070683,0.526322465589311
"1222","NHLRC2",36.21178109398,0.527657034816146,0.263875257295636,1.9
9964574255243,0.0455385308542898,0.526322465589311
"1223","ZNF268",22.0585068072417,0.599054536101232,0.299467249315385,2
.00040083672167,0.0454569981183137,0.526322465589311
"1224","TUBGCP3",19.571501698027,0.685152714785988,0.342676659117971,1
.99941459844254,0.045563513703829,0.526322465589311
"1225","MBP",128.376576342806,0.411352239088619,0.205742521843481,1.99
935451069058,0.0455700100856282,0.526322465589311
"1226","TMSB4X",7472.81650664157,-0.549770538717032,0.274918536587126,
-1.99975798482691,0.0455264034879832,0.526322465589311
"1227","SERPINE2",122.796762946998,-0.437532375241751,0.21891436148524
7,-1.99864628466249,0.0456466387110124,0.526348863312651
"1228","SHMT2",46.8062494684842,-0.44332105064693,0.22178582748626,-1.
99887006158856,0.045622414775233,0.526348863312651
"1229","MTUS1",86.7841870851959,0.610121153737534,0.305408667052409,1.
99772049570825,0.0457469709050904,0.526847239507904
"1230","SPTLC2",30.2481214966373,-0.696954460421021,0.348902713781062,
-1.99756101885285,0.045764272923356,0.526847239507904
"1231","ZNF737",17.9560289034416,0.858495121848145,0.429931489629105,1
.99681842934733,0.0458449108206347,0.527346821462378
"1232","NTNG1",16.3989538524656,0.975305664674349,0.488730601705334,1.
99558951551469,0.045978621677066,0.528365701109583
"1233","HLX",9.46724110999254,0.888695745936645,0.44588802186407,1.993
09176824572,0.0462513992291796,0.528365701109583
"1234","CCNT2",37.5176621938885,0.55948948557399,0.280676135526917,1.9
9336322100792,0.0462216881839527,0.528365701109583
"1235","RBSN",32.4627670497762,0.549486438096308,0.275449772834676,1.9
948698176132,0.0460570801551476,0.528365701109583
"1236","SCAF8",34.9275591017604,0.604816986520514,0.303427524779071,1.
99328319657515,0.0462304453464655,0.528365701109583
"1237","HGF",40.7014926877621,-0.967783370726358,0.485608755896378,-1.
99292817309264,0.0462693128090313,0.528365701109583
"1238","VLDLR",23.7750185330834,1.03744716609551,0.52020922980255,1.99
42882722194,0.0461205601578557,0.528365701109583
"1239","ZNF215",3.66542718600347,-1.55071294913981,0.778025809935268,-
1.99313818299784,0.0462463179027432,0.528365701109583

"1240","NUCB2",256.164045152384,-0.418356949565573,0.209842033886772,-1.99367563217249,0.0461875140526649,0.528365701109583
"1241","PAPPA",22.3305294216429,0.870921059653401,0.437126053258899,1.99237966522571,0.0463294167543962,0.52862573830963
"1242","FMN2",3.09300992856444,1.95752334405471,0.983124374638937,1.99112482057382,0.0464671663350848,0.528715575478388
"1243","COQ10B",88.8242990719702,-0.47009942120988,0.236081687174967,-1.99125746192027,0.0464525894594636,0.528715575478388
"1244","AC106791.1",7.37219461783681,1.08532087557166,0.544916337017522,1.99172019967675,0.0464017661404409,0.528715575478388
"1245","AC096576.6",2.54240257123017,-2.0553143993217,1.03232971416202,-1.99094763148425,0.0464866448778668,0.528715575478388
"1246","PI16",276.518606811266,0.606303257997895,0.304641655839234,1.99021783914493,0.0465669440191485,0.528824973494118
"1247","ZNF91",53.4734164760814,0.512246361969496,0.257386769702674,1.99018140117003,0.0465709563522009,0.528824973494118
"1248","MICOS10",307.436682006155,-0.446315173968014,0.224303530709936,-1.98978220519042,0.0466149324982561,0.52890019565329
"1249","APOC1",269.142267128506,-1.23235291513365,0.619666605638511,-1.98873540048817,0.0467304164013048,0.529785985782607
"1250","AC079848.1",1.55824938766604,-2.45238340934544,1.23359761182987,-1.98799299368591,0.0468124648547984,0.530291601875156
"1251","LINC01503",13.2580529757234,1.0111367547566,0.509170661491452,1.98585038618448,0.0470499390902235,0.532130301531601
"1252","IFNAR2",26.3503205607252,-0.580142171212628,0.292118026509709,-1.98598552148354,0.0470349315901392,0.532130301531601
"1253","ATP23",9.30209390670472,-0.87812834085583,0.44228717147764,-1.98542575386504,0.0470971230056966,0.532238836201647
"1254","LRIG2",20.5842015595749,0.697006691891241,0.351219106488424,1.98453523460067,0.0471962042040117,0.532508566955224
"1255","YIF1A",152.447836681896,-0.472464477923263,0.238032650650306,-1.9848725653077,0.0471586514149385,0.532508566955224
"1256","AP002748.4",3.27209242123377,-1.60477031285604,0.808800289185465,-1.98413667046557,0.0472406061149252,0.53258517721922
"1257","NCKAP5",5.96103062855732,1.35245783027763,0.682218066205845,1.98244212118175,0.0474297792510229,0.533020376344829
"1258","ANKS1A",24.9804441340184,0.671522087308785,0.338589656901816,1.98329179176171,0.0473348457389753,0.533020376344829
"1259","CRNDE",106.277343302723,-0.404847300215209,0.204185623915536,-1.98274145089999,0.0473963169565884,0.533020376344829
"1260","LPAR4",17.0695845904384,0.723505443734796,0.364948090174417,1.98248864212173,0.0474245773367553,0.533020376344829
"1261","AGAP5",6.03122167264949,1.34270608258966,0.677570814939049,1.9816468669927,0.0475187778238949,0.533395777683097
"1262","ZGPAT",28.6570258672908,-0.58998767738853,0.297752423930524,-1.98147061105436,0.0475385219940726,0.533395777683097
"1263","GMPPB",25.5148972492659,-0.710748955851907,0.358989942055807,-1.97985757423092,0.0477195347941331,0.533685921630484
"1264","COL25A1",2.90225097600626,1.75200183616276,0.885049609398002,1.9795521262977,0.047753876905726,0.533685921630484

"1265","EFS",41.0823613815446,0.461426638783402,0.233134841391016,1.97
92264254895,0.0477905189708654,0.533685921630484
"1266","TMC6",2.63012929608669,-1.86606013232227,0.942429886520995,-1.
98005194764237,0.0476976918196782,0.533685921630484
"1267","HM13",178.952490386957,-0.421262213212159,0.212677730253677,-1
.98075375691515,0.0476188949985859,0.533685921630484
"1268","ZNRF3",15.4655417216911,0.776435579017677,0.392102572672403,1.
98018486266444,0.0476827601536814,0.533685921630484
"1269","CHMP2B",118.560536496886,-0.347483552871216,0.175622194174096,
-1.97858564804601,0.047862676839644,0.534070531165768
"1270","PRRT1",1.52151538038848,-2.48671504000969,1.25821735959049,-1.
97637953494699,0.0481118076260156,0.536427713373529
"1271","ZDBF2",12.1286779920843,0.911991586404738,0.461637086790384,1.
9755596170696,0.0482046761740963,0.53685295354846
"1272","NOCT",8.09255710993106,-1.2915686088827,0.653835128260676,-1.9
753735354026,0.0482257737933362,0.53685295354846
"1273","DTD1",85.0218495500309,-0.530210206851097,0.268456742712708,-1
.97503032143434,0.0482647071507181,0.53686429949267
"1274","ACTG1",3217.23990090506,-0.514923729333905,0.260826455361944,-
1.97420054119646,0.0483589444980806,0.537490309335025
"1275","KCTD15",6.36102370004947,1.12965129449291,0.572344279071413,1.
97372689096445,0.0484128057558888,0.537666925100694
"1276","C3orf18",9.76648236973658,0.906432424141345,0.459996000138339,
1.97052240425731,0.0487785298739805,0.539208024671126
"1277","ABHD18",11.5238886702055,1.09684186498573,0.556541357900751,1.
97081824991941,0.0487446684715161,0.539208024671126
"1278","PDZD2",2.88765680626367,1.83487973075359,0.930409920360679,1.9
7211969756544,0.0485959438539546,0.539208024671126
"1279","ZNF395",30.7276073587485,0.554122899194535,0.28120798156663,1.
97050914453948,0.0487800479946125,0.539208024671126
"1280","CD320",46.9472155384917,-0.688922837458569,0.349546972862714,-
1.97090202731965,0.0487350832058838,0.539208024671126
"1281","RLIM",51.3056949272523,0.513572675961616,0.260506844214687,1.9
7143640317709,0.0486739806260536,0.539208024671126
"1282","AZIN2",12.7182189073894,-0.891234331214117,0.452396152638962,-
1.97003074852711,0.0488348466529627,0.53937951016208
"1283","RAD23A",160.197790508508,-0.316686453097351,0.160780469584905,
-1.96968234957241,0.0488747870989631,0.53937951016208
"1284","ROMO1",183.058100022562,-0.442657089034053,0.22477013399846,-1
.96937680802862,0.0489098369384259,0.53937951016208
"1285","VCAM1",86.9264410505493,0.506784644140605,0.25742705727282,1.9
6865337120921,0.0489929092717797,0.539718854436993
"1286","PCAT6",3.9535332314706,-1.40374574865576,0.713364288652221,-1.
96778247942281,0.049093070940314,0.539718854436993
"1287","TRABD2A",15.9966618603457,-1.10616935175897,0.562119200712635,
-1.96785548395537,0.0490846680611498,0.539718854436993
"1288","AC040168.1",1.51110299640017,-2.39508617255131,1.2169333064181
,-1.96813264943908,0.0490527770854874,0.539718854436993
"1289","MAPK14",47.8861083668286,0.46117026141998,0.234488088863599,1.
96671081953438,0.0492165588331801,0.540656689742304

"1290","CAPN2",233.77774128476,-0.451514969918666,0.229637826159519,-1
.96620468617839,0.0492749713861595,0.540878755680634
"1291","AP2M1",307.28884915611,-0.325082994731159,0.165446911605,-1.96
487798761265,0.0494283610241472,0.54130362884913
"1292","MT1H",3.22782954703425,-2.53226643731558,1.28861218729692,-1.9
6511135179269,0.0494013510323078,0.54130362884913
"1293","JUND",2204.29167729861,-0.350289121571921,0.178269692846817,-1
.9649392781134,0.0494212659515151,0.54130362884913
"1294","SEPTIN4",11.0754796537038,0.820662498277294,0.417769157450057,
1.9643922574045,0.0494846200470659,0.541500942709778
"1295","LIN9",5.41775131426166,1.14970062687559,0.585859189366155,1.96
241801399319,0.0497138371881496,0.541915269117935
"1296","MAP4K3-
DT",42.1607948383024,0.577939296004562,0.294439561006471,1.96284525771
27,0.0496641572187276,0.541915269117935
"1297","CNNM3",20.4798430816123,0.828555713723972,0.42218681258035,1.9
6253338340899,0.0497004179077823,0.541915269117935
"1298","MLIP",4.67833167773153,-1.6036145814517,0.816858923589756,-1.9
6314753397622,0.049629033641803,0.541915269117935
"1299","ZFP82",12.6934804904218,0.82565301557252,0.420542635895505,1.9
6330394375917,0.049610867484461,0.541915269117935
"1300","MAP7D1",61.5440128221234,-0.479040686722855,0.244513181320126,
-1.95916099138916,0.0500939358719398,0.541986578217459
"1301","EFCAB14",107.437612236467,0.438355128411568,0.224031758393555,
1.95666512442184,0.0503868527983921,0.541986578217459
"1302","CD58",41.1341990871009,-0.520085089337236,0.265619214755047,-1
.95801004011271,0.0502288345911721,0.541986578217459
"1303","LAMB3",6.73583256227483,-1.34789379198785,0.689283719408035,-1
.95549924368654,0.0505241725456953,0.541986578217459
"1304","WDCP",5.33769810084491,1.21014080521148,0.616793002925121,1.96
198854311321,0.0497638181292807,0.541986578217459
"1305","AL121658.1",2.28737945800598,1.93809655923068,0.99032340430712
,1.95703398586917,0.0503434727481018,0.541986578217459
"1306","TMEM192",23.4823679927807,0.650180487942043,0.332029709325324,
1.95819973237694,0.0502065805331281,0.541986578217459
"1307","IQGAP2",3.94194799016248,-1.58507874698196,0.810036249175669,-
1.9567997711152,0.0503710140087013,0.541986578217459
"1308","GALNT10",22.5073028217993,0.74779661552424,0.381786479143092,1
.95867757601748,0.050150558152759,0.541986578217459
"1309","FGF18",50.1039259551133,-0.589813885327233,0.301216507575816,-
1.95810611468157,0.0502175624130111,0.541986578217459
"1310","LPL",20.6124076573472,0.900693938635993,0.460109815135572,1.95
756297520104,0.0502813154477501,0.541986578217459
"1311","YTHDF3",51.440244124957,0.421075242129754,0.215185181863626,1.
95680408141027,0.0503705070480439,0.541986578217459
"1312","KLHL9",32.5302635360577,0.48903873930824,0.249332293669616,1.9
6139349664931,0.0498331380522576,0.541986578217459
"1313","PRXL2C",70.40325587637,-0.503833083090643,0.257008243591416,-1
.96037713051579,0.0499517268578467,0.541986578217459
"1314","ARFIP2",33.702918063573,-0.488629707496278,0.249578817434462,-

1.95781722391,0.0502514636116421,0.541986578217459
"1315","PTGDR2",2.03523110167085,3.22466576457485,1.64570920015145,1.95943837725286,0.0500614699632122,0.541986578217459
"1316","C11orf95",29.3499232071412,0.672337630280699,0.343474576079647,1.9574596698091 4,0.0502934489806193,0.541986578217459
"1317","LOXL1-AS1",6.44867765535238,-1.20457312863544,0.615857577589573,-1.95592807894003,0.0504736270655882,0.541986578217459
"1318","GPS2",87.0935917364009,-0.37230943087398,0.190323059092296,-1.95619717678787,0.0504419309792509,0.541986578217459
"1319","POLG2",25.8500869944599,0.873994917543662,0.446673797033137,1.95667380390085,0.0503858316861607,0.541986578217459
"1320","ANKRD29",14.1384037761488,-0.804752527910466,0.411518758976397,-1.95556705583043,0.0505161769169108,0.541986578217459
"1321","ABCB5",4.42947489900682,2.06725459202429,1.05750009508838,1.95485050226073,0.0506007182896882,0.542226532800185
"1322","RHOJ",34.6413928441877,0.841123696308754,0.43031697465678,1.95466074044519,0.0506231268617122,0.542226532800185
"1323","SMARCAD1",32.9981369755796,0.520003222634137,0.266136134406327,1.95389935979236,0.0507131203118943,0.542779881796239
"1324","SH3D19",177.491911110177,0.369974574476492,0.189442627471192,1.95296369890537,0.0508238967933722,0.54314443667483
"1325","LGALS3",2478.16097936394,-0.547727906346004,0.280431791103916,-1.95315910578427,0.0508007450942434,0.54314443667483
"1326","BOLA3",58.7027218104386,-0.499172988705947,0.255905085463172,-1.95061769797375,0.0511025403116815,0.54563613082674
"1327","PHLDA2",20.7178561877524,-0.860633020811687,0.441380793946851,-1.94986513372243,0.0511921957894827,0.54563613082674
"1328","SMCO4",25.9498839872408,-0.601545368482773,0.30853134383956,-1.94970585807186,0.0512111877025945,0.54563613082674
"1329","PLEKHA4",85.7675627524853,-0.613510108667173,0.314571144744395,-1.95030637398634,0.0511396134143734,0.54563613082674
"1330","UCP2",14.1028650521783,-0.906842157748198,0.465246751107994,-1.94916386968536,0.0512758582002972,0.545914400087374
"1331","BMP6",5.41597035927643,1.8392529369811,0.94464814238388,1.94702435166985,0.0515318152625111,0.547815693781649
"1332","NUTM2A-AS1",65.3946098160478,-0.504595109625145,0.259135214942344,-1.94722708659035,0.0515075156917875,0.547815693781649
"1333","LIPT1",18.2118436528817,0.630291771612175,0.323868700715908,1.94613363446027,0.0516386892916458,0.548540015281099
"1334","MPV17L2",6.33735078110804,-1.04494491008326,0.537117089496843,-1.9454694898316,0.0517184983314622,0.54897596429798
"1335","ALS2CL",2.47736696796547,2.00018655655326,1.02903514987695,1.94374949853991,0.0519256664135638,0.54919891510391
"1336","NPR3",9.13523066429921,1.09202155966861,0.561487586599842,1.94487213204745,0.051790369858911,0.54919891510391
"1337","TNNT3",273.151090675191,0.445182472324383,0.229021599131805,1.94384492123022,0.0519141548593409,0.54919891510391
"1338","LRP1",1044.22537864443,0.321054132024623,0.165213212016889,1.9

4327153443275,0.0519833589463098,0.54919891510391
"1339","AC002546.1",2.86007713501892,2.45618439571709,1.26354342182235
,1.94388602187857,0.0519091972379968,0.54919891510391
"1340","ZNF626",28.5447955306037,0.606679139523283,0.312231500916113,1
.94304270306883,0.0520109989515779,0.54919891510391
"1341","DDX17",375.360546436566,0.397062632645308,0.204313921283777,1.
9433949001146,0.0519684629924904,0.54919891510391
"1342","CDKN2C",60.4480758537399,-0.515710004816346,0.265581446154974,
-1.94181488309019,0.0521595144789506,0.549946928534579
"1343","APLNR",12.2798012276439,1.23307179733874,0.634941886227595,1.9
4202307972598,0.052134306338284,0.549946928534579
"1344","RASSF1",53.0369981005156,-0.428828139698131,0.220908518096515,
-1.94120237369378,0.0522337353281317,0.550101226793174
"1345","PCSK1",6.48756192876111,-1.38899964610153,0.715699896020478,-1
.94075708802644,0.0522877482622012,0.550101226793174
"1346","DAZAP1",56.8025564547315,-0.415248970110787,0.213965039430221,
-1.94073280016481,0.0522906957107071,0.550101226793174
"1347","AC007364.1",3.63184953336059,1.4779124947483,0.761678907786783
,1.94033532980804,0.0523389503895543,0.550200102090638
"1348","PDAP1",206.679105793773,-0.359041937283066,0.185089274870451,-
1.93983113032545,0.0524002160314915,0.55043550371359
"1349","IPPK",5.6874850671126,1.19650248864121,0.617261229061774,1.938
40538220725,0.0525737843043366,0.55171738641679
"1350","YWHAE",358.67125945844,-0.318947606976068,0.164586725709831,-1
.93786956755174,0.0526391376447093,0.55171738641679
"1351","KCNJ12",17.323367138282,0.656724447917904,0.338839654779335,1.
93815699743168,0.0526040713694447,0.55171738641679
"1352","SLC27A3",104.142498545698,-0.398251127063137,0.205573793512598
,-1.93726603113315,0.0527128323227139,0.55208114326156
"1353","LSM10",73.2924463987774,-0.474803865256342,0.24517375748367,-1
.93660149491312,0.052794075096617,0.552115290522967
"1354","DSEL",67.0835814712712,0.464572622081595,0.23988479612044,1.93
664888144201,0.0527882784007597,0.552115290522967
"1355","MATN4",2.59557256568208,1.7735589353284,0.916816454104435,1.93
447546386027,0.0530546959616937,0.554431361488991
"1356","KPNA3",38.8336680148952,-0.51283457051304,0.265200315089478,-1
.93376305129959,0.0531422676048087,0.55493695374933
"1357","SRSF11",303.84910402524,0.352942388928878,0.182566511314026,1.
93322634249058,0.0532083210044983,0.555217262655635
"1358","LMO4",403.238868029383,-0.545554323961328,0.282935664988735,-1
.92819213506734,0.053831232112361,0.555818639576699
"1359","COQ8A",33.5641597401286,0.499879111903383,0.258852618778971,1.
93113407259063,0.0534664743577632,0.555818639576699
"1360","RPSA",1860.68988562002,-0.423188174039467,0.219040310944867,-1
.93201046973488,0.0533582136407289,0.555818639576699
"1361","SERPINI1",11.3828330745934,1.04490510522599,0.542033642376514,
1.92774954086737,0.0538862867562129,0.555818639576699
"1362","ISOC1",25.6010035780195,-0.642881923292908,0.333350968706062,-
1.92854373811579,0.0537875294052454,0.555818639576699
"1363","AC011379.1",6.20387684588119,-1.0953160495499,0.56762383232074

5,-1.92965127110973,0.0536500614979833,0.555818639576699
"1364","GARS",71.249434639097,-0.387838407724763,0.201226513454167,-1.
92737229834825,0.0539332493487559,0.555818639576699
"1365","NAPEPLD",14.2948737878177,0.688436523795571,0.35695908787774,1
.92861464289533,0.0537787198439316,0.555818639576699
"1366","DENND11",12.5194817247778,0.779311926542968,0.404325805540915,
1.92743553803198,0.0539253743129844,0.555818639576699
"1367","SLC3A2",109.281079380753,-0.491341928612005,0.254538646538616,
-1.93032349033672,0.0535667682431297,0.555818639576699
"1368","EXOSC6",53.7837917545651,-0.440585030229612,0.22825780020187,-
1.93020799219111,0.0535810716851265,0.555818639576699
"1369","ZNF516",20.7556141780163,0.742601293658485,0.384764361170001,1
.93001579304373,0.0536048809492296,0.555818639576699
"1370","MLLT1",32.4805315432463,0.555784279856557,0.287854115913872,1.
9307845506814,0.0535097016896048,0.555818639576699
"1371","CHERP",26.4207365123052,0.527838245456168,0.273353012737776,1.
93097650605562,0.0534859578820205,0.555818639576699
"1372","RABAC1",509.053380266154,-0.398107418078563,0.206515675180628,
-1.92773462707061,0.0538881427134949,0.555818639576699
"1373","ISOC2",95.958447707531,-0.520358916521498,0.269780231831354,-1
.92882522558875,0.0537525631071287,0.555818639576699
"1374","FAM210B",100.527145098957,0.361639603575848,0.187447813655736,
1.92928152387005,0.0536959221736018,0.555818639576699
"1375","TOMM40",47.8778556928949,-0.592547331791779,0.307529961965014,
-1.92679545110206,0.0540051266163932,0.556154613009547
"1376","SLC25A39",43.5528362446198,-0.499816368455039,0.25944929300298
1,-1.92645107130547,0.0540480756940616,0.556192406851681
"1377","SLC35E2B",43.1843933819813,0.555067289695131,0.288717788817044
,1.92252542515442,0.0545396775316971,0.556566225225844
"1378","ZFYVE9",23.3761804154874,0.596087560236421,0.309920013940145,1
.92335936184987,0.0544349342420699,0.556566225225844
"1379","RMDN2",14.3547420215103,0.719057116527842,0.373538253150248,1.
92498923594477,0.0542307048917429,0.556566225225844
"1380","GORASP2",68.8790748095463,-0.365582485765956,0.190043344368164
,-1.92367950049188,0.0543947691076591,0.556566225225844
"1381","KLF7",71.633091166799,0.569520562651436,0.295963999815022,1.92
428999137526,0.0543182444203714,0.556566225225844
"1382","HSP90AB1",881.773366173531,-0.310194685244216,0.16118265234698
7,-1.92449175347011,0.0542929734128205,0.556566225225844
"1383","MTDH",438.336740877218,-0.281649345602034,0.146517959933528,-1
.92228547087205,0.0545698471594752,0.556566225225844
"1384","PTPRD-
AS1",8.01905055432792,0.971752865932042,0.505354802769112,1.9229121017
6946,0.0544910896736082,0.556566225225844
"1385","TPM2",359.338360578208,-0.660036162056419,0.342770200258745,-1
.92559376969813,0.0541551172720981,0.556566225225844
"1386","AL079343.1",6.35795903204903,1.07742598868932,0.56025274272776
9,1.92310703102234,0.0544666095271007,0.556566225225844
"1387","RILP",41.4381741437765,-0.534546750372753,0.277711832430855,-1
.9248252611125,0.0542512225859714,0.556566225225844

"1388","SHKBP1",15.2484185060424,-0.727614089395587,0.378555070525057,
-1.9220825344814,0.0545953733643148,0.556566225225844
"1389","C20orf27",35.6027821419733,-0.585625845199381,0.30439657090393
5,-1.92389107229529,0.0543682385238054,0.556566225225844
"1390","HEXD",25.1136910563857,-0.592508182454781,0.308382245620984,-1
.9213433680712,0.0546884330858091,0.55711382193889
"1391","RBL2",65.3855877996692,0.392098166074203,0.204174990439694,1.9
204025195731,0.0548070755465762,0.557921056606412
"1392","AC113383.1",1.59384040701104,-2.41366552127367,1.2573675251069
8,-1.91961814909153,0.0549061498725639,0.558429167309399
"1393","METTL7A",283.135196329034,0.466626985292944,0.243112996450979,
1.91938313502312,0.054935863704943,0.558429167309399
"1394","DIRC3",4.08715623327912,1.58212765300107,0.824897993532532,1.9
1796763406562,0.0551151152388067,0.55984937717468
"1395","BCKDK",42.4425872367775,-0.453887350282019,0.236794016045077,-
1.91680245076638,0.0552630334938434,0.56094950127084
"1396","PDGFC",7.31775476400476,-1.5526018273529,0.810565106519634,-1.
91545603784918,0.0554343705055079,0.562285591946985
"1397","PDPR",28.4621100175847,0.560314028145655,0.292592938322507,1.9
1499504860932,0.0554931350643331,0.562478734796676
"1398","LRRC75A",35.4008552299958,-0.951362125530315,0.496904286573227
,-1.91457822207802,0.0555463146651963,0.562615032660357
"1399","ZFAND5",332.356492603576,0.578433724405477,0.302476076908576,1
.91232883710109,0.055834029294295,0.563089230091287
"1400","UROS",73.7915118147459,-0.63359677866842,0.331372718613507,-1.
91203663753445,0.0558714949349053,0.563089230091287
"1401","VWCE",5.09943682989068,1.24485292705096,0.651036532049382,1.91
210917631968,0.0558621921050937,0.563089230091287
"1402","RABEP2",15.9584534441197,-0.659635578028116,0.344945940462911,
-1.91228682715586,0.0558394144940601,0.563089230091287
"1403","TRIR",337.824272510832,-0.331556936353635,0.173359030650427,-1
.91254493699961,0.0558063345676021,0.563089230091287
"1404","USF2",71.9620145844002,-0.392484599568393,0.205183476613146,-1
.91284700915945,0.0557676410669764,0.563089230091287
"1405","AL391097.1",1.79435571839243,2.39993549323081,1.25446045350816
,1.91312168232906,0.0557324766032679,0.563089230091287
"1406","STAT1",108.367547110175,0.466463810910508,0.24428667003639,1.9
0949350957636,0.0561984582114735,0.5644819435841
"1407","SNED1",116.21313002193,0.52253557964334,0.273718568385526,1.90
902496211861,0.056258871594307,0.5644819435841
"1408","FAM53C",63.8454360787534,0.694823729826213,0.363733736795565,1
.91025373655877,0.0561005513011256,0.5644819435841
"1409","FAM20C",74.4015336696605,-0.477711552864934,0.250260760572009,
-1.90885519476985,0.0562807743290255,0.5644819435841
"1410","BMT2",13.1723027437072,0.760772409227186,0.398204801823385,1.9
1050536242557,0.0560681766102566,0.5644819435841
"1411","AQP5",4.15765813846469,-1.36606249719234,0.715298717599119,-1.
90977903857775,0.0561616692841594,0.5644819435841
"1412","SDR39U1",27.9087945134138,-0.519050356545493,0.271925882783157
,-1.90879349634916,0.0562887361822563,0.5644819435841

"1413","ZNF292",139.420874625148,0.425962785566397,0.223332377127557,1
.90730422093304,0.0564812039406169,0.56601121571064
"1414","MCM6",31.4820750063214,0.496264198774298,0.260339140457065,1.9
06222006814,0.0566214081953158,0.566349186946192
"1415","TMEM140",47.5367714410744,0.50943185652796 6,0.267244745525814,
1.90623712928627,0.0566194470376759,0.566349186946192
"1416","NR1H3",39.8080245269092,0.593858216549384,0.31155377926087,1.9
0611783929649,0.0566349186946192,0.566349186946192
"1417","PTPRU",8.69763431233514,0.999402086492759,0.52457953426483,1.9
0514883104116,0.0567607274170468,0.566729621030747
"1418","KCTD16",3.3693936016031,1.89743265005299,0.996079767323271,1.9
0490030246462,0.0567930319380388,0.566729621030747
"1419","GPRC5B",62.2219445035455,0.496953660547603,0.260818048789239,1
.90536530295563,0.0567326021819217,0.566729621030747
"1420","SNRPG",161.716122994437,-0.419879516886349,0.220476448675902,-
1.90441890464032,0.0568556490488151,0.566954922909311
"1421","MAN2B1",60.8427278924204,-0.40158424689976,0.210904050774204,-
1.90410874246176,0.0568960233559388,0.566958262294225
"1422","ZBTB33",12.2636765562879,0.818075491871893,0.429753470652196,1
.90359251928874,0.0569632738417209,0.567229224753001
"1423","OGT",85.6171538251872,0.429713442560877,0.225775817004899,1.90
327488683853,0.0570046859630935,0.567242693771893
"1424","PIGO",6.90867194583263,-1.0998527789478,0.577976762300219,-1.9
0293598408806,0.0570488988676658,0.567283994358249
"1425","UBR4",29.3574530837948,0.681246940440576,0.358354884769193,1.9
0103991711833,0.0572967843714103,0.567897289046067
"1426","SPATA6",64.6066204376349,0.462485131042742,0.243262899383787,1
.90117412977594,0.057279208443542,0.567897289046067
"1427","PRKRA",65.9361114564878,-0.409567520152092,0.215586285271573,-
1.89978467153493,0.0574613833184274,0.567897289046067
"1428","MSMO1",19.9502914815475,-0.7806787098042,0.410498224704409,-1.
90178340080849,0.0571994772294187,0.567897289046067
"1429","BMPER",44.7875305844724,0.809837465040168,0.426302126731135,1.
89967962686455,0.0574751755185118,0.567897289046067
"1430","UPP1",43.0727103259208,-0.826038271838219,0.434384565090776,-1
.90162896710107,0.057219678194327,0.567897289046067
"1431","CCDC136",5.97834177645017,1.25839274468982,0.662266219145116,1
.90013125886778,0.0574158964646892,0.567897289046067
"1432","ARL6IP1",157.182889662047,0.323372163709484,0.170116991087123,
1.90088104452702,0.0573175954577912,0.567897289046067
"1433","PSMD8",271.102019715325,-0.467846638144454,0.246194941213882,-
1.90030971326097,0.0573924874130157,0.567897289046067
"1434","HSPA13",25.7905366449656,-0.530122179343378,0.279099506728894,
-1.89940206472065,0.0575116322381398,0.567897289046067
"1435","ZNF518A",42.8684814015349,0.528659037749771,0.278479010689153,
1.89838019189129,0.0576460170388937,0.568827596704345
"1436","TUBB4B",390.464089299393,-0.36450317577436,0.19206038377814,-1
.89785716660558,0.0577149001638426,0.568864803864387
"1437","RAMP2",309.610344589623,-0.647449042617893,0.341168185668339,-
1.89774155333258,0.0577301358158986,0.568864803864387

"1438","AP5S1",14.1275054581648,0.743201110914671,0.391903187170823,1.
89638955549173,0.0579085519486041,0.570226074820747
"1439","HOXB6",1.60254696446277,-2.60791709417128,1.37574427176107,-1.
89564088886442,0.0580075465820116,0.570803933009927
"1440","C1orf21",151.58629938291,0.37234962638778,0.196491595678184,1.
89499008902964,0.05809371471421,0.571006448625169
"1441","RCOR3",38.4189657851003,0.451665798999806,0.23836161435099,1.8
948764054548,0.058108777716728,0.571006448625169
"1442","DDX59",23.4838997227186,-0.566113298719299,0.299149620214816,-
1.89240854898221,0.0584365678663102,0.571704106319424
"1443","SMC4",53.9268590500462,-0.649227154127982,0.343066915622732,-1
.89242134570018,0.0584348642040399,0.571704106319424
"1444","RGS10",116.283280633887,-0.524273619509316,0.276824800510877,-
1.89388240700174,0.0582406201783046,0.571704106319424
"1445","FIBIN",65.5801543021178,0.803055135784626,0.424351506514088,1.
89242909111238,0.0584338330560472,0.571704106319424
"1446","MRPL49",21.7361841343274,-0.610450265878004,0.322490083594054,
-1.89292724624188,0.0583675453213875,0.571704106319424
"1447","IL27RA",6.13245240923797,-1.41086531437113,0.745046822691072,-
1.89365993035867,0.0582701631583628,0.571704106319424
"1448","ERCC1",92.1584873780215,-0.422179108756571,0.223113760198337,-
1.89221457422113,0.0584623973128903,0.571704106319424
"1449","NICN1",11.2844161987601,1.00158490036438,0.529784781283949,1.8
9055053249549,0.0586843689603818,0.572732291462894
"1450","MRPL3",96.5939275472656,-0.401302266400697,0.212274782378396,-
1.89048487957154,0.058693140937198,0.572732291462894
"1451","SOCS2",24.6965226484697,0.721598791191324,0.381755051585534,1.
89021412603272,0.0587293281923814,0.572732291462894
"1452","MYO15A",2.19896748855758,2.21396255838184,1.17088014058215,1.8
9085328348047,0.0586439320722627,0.572732291462894
"1453","FAM114A1",242.10593571995,-0.284990507330616,0.15088545069957,
-1.88878719591105,0.0589203491483386,0.572934329783507
"1454","ANKHD1",104.189467693144,0.479422926540065,0.253744385228477,1
.889393241582,0.0588391558178279,0.572934329783507
"1455","HCG11",52.7727394784392,0.405745318113673,0.21488653190993,1.8
8818403139264,0.0590012488165208,0.572934329783507
"1456","HLA-
DQA1",19.0840101450154,-1.07074094178884,0.566961902471814,-1.88855889
103072,0.0589509597880192,0.572934329783507
"1457","TMEM135",34.9795005872605,0.599071557701475,0.317314020954034,
1.88794543619696,0.0590332759289645,0.572934329783507
"1458","LAYN",12.2831175244927,-0.829726739969449,0.439359614406188,-1
.88849114202464,0.0589600459724726,0.572934329783507
"1459","SLC35A2",19.6666361337865,-0.647180960264759,0.342599082439821
,-1.88903296429125,0.0588874117889589,0.572934329783507
"1460","PTPRO",2.8599258780637,1.73182184679992,0.918136038990394,1.88
623665040344,0.059263071706589,0.573147751966514
"1461","PDIA3",459.375457548979,-0.353265683932069,0.187278419248681,-
1.8863128242394,0.059252812140993,0.573147751966514
"1462","MILR1",9.87529280213577,0.957053545865221,0.507168145350714,1.

88705374073406,0.0591530977780828,0.573147751966514
"1463","KIAA1328",27.7226360676064,0.521605717974157,0.276417003287061
,1.88702471907079,0.0591570009622777,0.573147751966514
"1464","SLC16A2",20.259746811888,0.620563650030056,0.328920219863992,1
.88666920594501,0.0592048320166432,0.573147751966514
"1465","FUNDC2",171.750012551231,-0.356304843144126,0.188923270631317,
-1.88597647051884,0.0592981254682869,0.573147751966514
"1466","TMEM72-
AS1",1.72333849648706,2.35209377012613,1.24781578893355,1.884968751786
96,0.0594340567729858,0.574069743455306
"1467","MTR",71.4975038087681,0.42858535208795,0.227460059996619,1.884
22245247944,0.0595348917360241,0.574260263611785
"1468","NCOA2",50.4207486276459,0.552309146138967,0.293117180457236,1.
88426057209412,0.0595297378315393,0.574260263611785
"1469","IRS2",43.2681864235605,0.620362774514247,0.329348623850706,1.8
8360518183145,0.059618400420475,0.574283367315596
"1470","TVP23B",12.1968714583415,-0.8792896177578,0.46678066854711,-1.
88373186167854,0.0596012543655481,0.574283367315596
"1471","MED19",42.9092619856706,-0.474537520189235,0.25204861596918,-1
.88272218184789,0.0597380277335653,0.574653853741361
"1472","TSIX",14.6141976773873,1.20967063237747,0.642483865794005,1.88
280312826614,0.0597270529693698,0.574653853741361
"1473","NOTCH2NLC",10.2247010481224,1.0578299887988,0.562071692062751,
1.88201968492073,0.0598333429590146,0.574789780393247
"1474","MPDZ",70.1213101452336,0.444068541236449,0.235947123779396,1.8
8206804187043,0.0598267778176233,0.574789780393247
"1475","MRC2",349.8846224186,0.396476817807643,0.210699911077031,1.881
71326594767,0.0598749575687043,0.574799592659561
"1476","ZNF638",124.588597171284,0.476607571446023,0.253435263044367,1
.8805890140181,0.0600278473254146,0.575876909300726
"1477","FAM222B",17.1949836881145,0.670379963446162,0.356570463081334,
1.88007710356325,0.060097570460463,0.576155448693403
"1478","IGF1R",45.6169673191984,0.51066187614548,0.27174640040719,1.87
918542943087,0.0602191784445106,0.576930694705189
"1479","EML1",61.0993567432181,-0.426171835248655,0.22685952159667,-1.
8785715153113,0.0603030235888815,0.577343349573064
"1480","MERTK",1.8924544336976,-2.1282597281591,1.13340048904652,-1.87
776496368862,0.060413325213114,0.578008570957901
"1481","AFDN-
DT",4.22463685895523,1.30011126414036,0.692850678404929,1.876466754905
19,0.0605912156049423,0.579319117465214
"1482","IFT57",143.648919624924,-0.412756910413563,0.220063981677814,-
1.87562229523713,0.0607071627184305,0.579644925214414
"1483","MICOS13",201.514027448934,-0.428516612572537,0.228461489061045
,-1.87566234612976,0.0607016594479349,0.579644925214414
"1484","HRH1",16.7462740155411,-0.788754111075717,0.420933850529992,-1
.87381962767453,0.0609552901552249,0.579720078935269
"1485","ATP6V1A",37.6955971325542,0.489269900797378,0.261055448215884,
1.87419915631398,0.0609029803872372,0.579720078935269
"1486","SSNA1",94.2708351628877,-0.433789513632374,0.231513905718146,-

1.8737082435147,0.0609706491016166,0.579720078935269
"1487","PRC1",3.28271259310972,-1.5870170713771,0.847012796378086,-1.8
736636307779,0.0609768017249478,0.579720078935269
"1488","AC025280.2",12.3097340166782,3.21637939028628,1.7160647730091,
1.87427621665259,0.060892363839102,0.579720078935269
"1489","NCAM2",17.7947238025185,0.929722219873479,0.496253161372535,1.
87348372210276,0.0610016184755333,0.579720078935269
"1490","TAF1",36.0600446216657,0.627735969959994,0.335051499507969,1.8
7355069558512,0.0609923791209091,0.579720078935269
"1491","ZNF559",24.1363866182594,0.62520029896094,0.333844683780045,1.
87272803592959,0.0611059498435898,0.580322099118197
"1492","ENOSF1",35.7806943064961,0.465596630790061,0.248659561312273,1
.87242601222703,0.0611476890880731,0.580329274455171
"1493","ANKRD6",16.5309227967608,0.625752904666524,0.334259646571028,1
.87205638217401,0.061198803564857,0.580425357319742
"1494","AC108863.2",6.96903707989141,1.1054872750212,0.590726739250519
,1.87140212481964,0.0612893646208491,0.580895182751823
"1495","GPBAR1",2.25989164971571,-2.00465880027347,1.0715702929884,-1.
87076742738254,0.0613773242525002,0.581041147048171
"1496","SFRP2",500.39244693387,0.807830877691228,0.431834878341783,1.8
7069391150906,0.061387519211452,0.581041147048171
"1497","IRF7",30.9561126497064,-0.588669632871614,0.314728758037746,-1
.87040306243961,0.0614278670290334,0.581041147048171
"1498","RGL1",58.588921873636,0.485317374223092,0.259980237398677,1.86
674717693585,0.0619369022246084,0.581197173956564
"1499","IL17RE",11.2661296127322,-0.926380976195383,0.496185731558989,
-1.86700446480946,0.0619009643559172,0.581197173956564
"1500","LRRFIP2",79.3642444052124,-0.450340110208632,0.24096118470862,
-1.86893217159934,0.061632251704706,0.581197173956564
"1501","TENT4A",17.0791863766836,-0.636574215813163,0.340804539337632,
-1.86785720944437,0.0617819766844355,0.581197173956564
"1502","DHFR",13.9868635094884,0.84064958771481,0.449997384008004,1.86
812105489897,0.0617451993961638,0.581197173956564
"1503","LSM1",61.6536288904523,-0.450311646182482,0.241210736282159,-1
.86688060872931,0.0619182623695979,0.581197173956564
"1504","C9orf40",9.04824364901469,0.961619858121519,0.515096287036593,
1.86687398516077,0.0619191875444832,0.581197173956564
"1505","AOPEP",17.2175069662415,-0.829540950507169,0.444055810468817,-
1.86810065525631,0.0617480422458169,0.581197173956564
"1506","SH2B3",12.9689160031663,-0.763550257160114,0.408418261350996,-
1.86953015919119,0.0615490917337293,0.581197173956564
"1507","ZNF106",237.776438025569,0.379032120553274,0.202902111299659,1
.8680540982321,0.0617545307368241,0.581197173956564
"1508","UPF1",54.0331601327535,0.511130970887079,0.273580680194706,1.8
6830068016247,0.0617201718881817,0.581197173956564
"1509","HOMER3",79.2113918735197,-0.473647890870888,0.253612280343883,
-1.86760629346753,0.0618169685574744,0.581197173956564
"1510","TCP11L1",10.7318010495139,0.829648850542881,0.44450773058146,1
.86644414363192,0.0619792519395813,0.58119859421189
"1511","G2E3",24.6574287055756,0.542426404603787,0.290664646976465,1.8

6615885435736,0.0620191437750117,0.58119859421189
"1512","SCRN1",41.3717006334432,0.485360369075736,0.260217940438245,1.86520717310389,0.0621523704962732,0.581975513286113
"1513","PIWIL4",4.71750657185974,1.15930119799275,0.621615983501991,1.86497971217151,0.0621842479944837,0.581975513286113
"1514","C2orf68",32.5385147657847,0.511182591497043,0.27420858977022,1.86421071605642,0.062292119091672 1,0.582600004186312
"1515","VPS51",99.3478405309285,-0.33523862446323,0.179925418835521,-1.86320880414173,0.0624328946847606,0.583531213687268
"1516","GPATCH4",33.9792065505928,-0.542679939796626,0.29158738343778,-1.86112284214253,0.062726831178593,0.5856038337506
"1517","AFAP1L2",8.19203123500771,-1.14273524410264,0.614027728436726,-1.86104827384908,0.0627373598728574,0.5856038337506
"1518","FAM3C",263.62940064154,-0.333521953892854,0.179365592515034,-1.85945336123983,0.0629629041700062,0.585631099170149
"1519","SMARCA2",142.148273407356,0.374529263474197,0.201418083030893,1.85946196010987,0.0629616863674456,0.585631099170149
"1520","PC",4.48954084162874,-1.23700503288272,0.664972206915118,-1.86023569108449,0.0628521875097915,0.585631099170149
"1521","SNHG30",24.1399060494071,-0.536161344685481,0.288205539440407,-1.86034364823978,0.0628369218742505,0.585631099170149
"1522","NAT14",16.7332115426561,-0.776695075422012,0.417806823409219,-1.85898130883631,0.0630297877920414,0.585631099170149
"1523","MAVS",51.5544945414329,0.633681601828734,0.340873327171425,1.85899438682117,0.0630279340227721,0.585631099170149
"1524","DYRK1A",35.577662907051,0.473983118517366,0.254893777397051,1.85953193270402,0.0629517773236556,0.585631099170149
"1525","CTNND2",3.06344852128994,1.84353802297143,0.992699883357302,1.85709503333133,0.063297634711116,0.587648925401865
"1526","CD93",1.62653788522664,-2.5588811949366,1.3780631918655,-1.85686782002545,0.0633299618759354,0.587648925401865
"1527","RASGEF1B",11.9353247172792,-1.01769903621105,0.54840935255237,-1.85572881183471,0.0634922219129831,0.588768737582083
"1528","MCUB",111.210726634784,-0.434639712712964,0.23433011958239,-1.85481795292707,0.0636222274548121,0.589296178205325
"1529","MSC",43.1655996033076,-0.54578082110707,0.294261568281922,-1.85474720431101,0.0636323344968885,0.589296178205325
"1530","AUP1",109.864321155093,-0.361908319805319,0.195281398454576,-1.85326571127306,0.0638442832606647,0.589569131280539
"1531","NIT2",61.4515051191301,-0.391929980164341,0.211487200341153,-1.85320898632216,0.0638524101519712,0.589569131280539
"1532","ZDHHC17",43.2327434021772,0.499949857754514,0.269793077645255,1.8530863064318,0.0638699892220584,0.589569131280539
"1533","WDR59",19.2813807451393,0.602052722287916,0.324757182136827,1.85385498890755,0.0637599089368316,0.589569131280539
"1534","ZNF519",14.2602739514063,0.878210219018815,0.47387660433928,1.85324662787118,0.063847017213203,0.589569131280539
"1535","CETN3",43.6911172309639,0.489773465141419,0.26437490181024,1.85257171459098,0.0639437697647324,0.589865654637532
"1536","LTBP2",124.305715844701,1.19767487323624,0.646766803371074,1.8

5178779583881,0.0640563008131293,0.590134820763768
"1537","PTPRA",234.338399444683,0.327339441016895,0.17674791622903,1.8
5201301379264,0.064023954187256,0.590134820763768
"1538","PRDX1",613.887131199281,-0.427159041446828,0.230791118806433,-
1.85084696350509,0.0641915725554893,0.590360007981182
"1539","UFC1",171.428342076936,-0.456604396797694,0.247008846177118,-1
.84853459244243,0.0645250448913262,0.590360007981182
"1540","SH3BP4",20.4376126425215,0.605014667371882,0.327060295122931,1
.84985666677907,0.06433421091149,0.590360007981182
"1541","FJX1",12.4611542876156,-0.819524279305701,0.443361019436032,-1
.84843557141798,0.0645393568047224,0.590360007981182
"1542","OSBP",43.9362347741927,0.505357204430695,0.273267562288507,1.8
4931281341455,0.0644126565970634,0.590360007981182
"1543","ACAT1",83.9952941301554,0.369709063229993,0.199957223747302,1.
84894076993796,0.0644663658038554,0.590360007981182
"1544","SECISBP2L",72.7904587913465,0.501993742283607,0.27155153634854
4,1.84861315473938,0.0645136918250187,0.590360007981182
"1545","PDP2",5.38073793432152,1.23248323652497,0.666690509569885,1.84
865873870036,0.0645071052259709,0.590360007981182
"1546","MIR497HG",26.4594895564407,0.665674710290336,0.359820501787135
,1.85001884824267,0.0643108330461108,0.590360007981182
"1547","PARD6B",1.96712152372978,2.03430162117864,1.09940194070603,1.8
5037113894144,0.0642600757899483,0.590360007981182
"1548","FAM50A",97.5059855957333,-0.377177618285853,0.203941153288287,
-1.84944339190179,0.0643938146907851,0.590360007981182
"1549","AC093525.4",3.04444934699656,1.60382639056293,0.86813267710168
2,1.84744386758648,0.0646828363737018,0.59059730041404
"1550","APPBP2",59.3889493310097,0.413893312790093,0.224042017965851,1
.84739146945721,0.064690424642809,0.59059730041404
"1551","TAFA5",5.74586314725102,1.2949305265756,0.700743847973015,1.84
793706048415,0.0646114484287378,0.59059730041404
"1552","TCAF1",101.764683163623,0.411230920842794,0.222718947177371,1.
84641192882119,0.0648324164732196,0.591131369775138
"1553","ZWILCH",11.1978028192371,0.79092174869242,0.428299851928682,1.
84665426600269,0.0647972639471282,0.591131369775138
"1554","AC096642.1",4.67511705765336,1.33443659829164,0.72306097599902
,1.84553812553348,0.0649592977857531,0.591441898828205
"1555","AOX1",73.7103781320023,0.371775009075046,0.201517718866285,1.8
4487503712631,0.0650557187214173,0.591441898828205
"1556","TRMT112",271.223986612959,-0.351855446361279,0.190688776470356
,-1.84518172948672,0.0650111073428636,0.591441898828205
"1557","ZNF234",12.7901326074007,0.725556555346599,0.393235467520468,1
.84509439070074,0.0650238090456961,0.591441898828205
"1558","C19orf48",28.9634341347793,-0.585605348153042,0.31744594508163
8,-1.84474036359936,0.0650753162693746,0.591441898828205
"1559","CITED4",14.6023318408308,0.913326394245013,0.496018789704668,1
.84131410584025,0.0655755417899827,0.593721081901698
"1560","LINC02580",11.8871259641877,0.940814287580729,0.51256079158892
2,1.83551747035554,0.0664290529464122,0.593721081901698
"1561","ZNF518B",11.194055273617,0.951901533249693,0.517880164168027,1

.83807297346273,0.0660516530555432,0.593721081901698
"1562","CD14",18.8233609127065,-0.76173219776358,0.414899172625983,-1.83594532845756,0.0663657428141718,0.593721081901698
"1563","NPM1",881.627332267485,-0.48468458313955,0.263183645198102,-1.84162121006691,0.0655305763461651,0.593721081901698
"1564","MBOAT1",35.4361919337228,-0.542219811023804,0.294915444574964,-1.8385602415813,0.0659798936295163,0.593721081901698
"1565","KMT2C",105.828748316902,0.486605467105095,0.265106036942164,1.83551258476718,0.066429776153737,0.593721081901698
"1566","ABITRAM",16.0515214435968,0.631300005515755,0.343972585444238,1.83532069772489,0.0664581860744485,0.593721081901698
"1567","ZBTB26",3.59593899893756,1.48504147969108,0.807231062703919,1.83967335785711,0.065816207248641,0.593721081901698
"1568","NCS1",24.1458116559539,-0.629356488153742,0.34172349735879,-1.84171265077787,0.0655171927351278,0.593721081901698
"1569","NDUFS8",115.154607556047,-0.468674261680465,0.25462494131677,-1.84064553635932,0.0656735200549205,0.593721081901698
"1570","STK38L",18.9392945572843,-0.633901736032283,0.344693767488055,-1.83902871424633,0.0659109627775026,0.593721081901698
"1571","FOXO1",69.0783618968324,0.483099572892037,0.262634295029633,1.83943826847719,0.0658507497897431,0.593721081901698
"1572","FNDC3A",102.423295221885,0.325367800037213,0.176961934089118,1.83863157753101,0.0659693934579956,0.593721081901698
"1573","CAB39L",106.985233550325,-0.529984908711459,0.287899259494186,-1.84086930144453,0.0656407140528189,0.593721081901698
"1574","SIPA1L1",23.8234729153206,-0.732230780294628,0.397914946172402,-1.84016907969418,0.0657434179765331,0.593721081901698
"1575","BDKRB1",5.21902542690882,-1.22460013071679,0.665811070342066,-1.83926069310869,0.0658768514917799,0.593721081901698
"1576","USP8",115.105510598951,0.363541132138926,0.197924917818474,1.8367628297936,0.0662449154120516,0.593721081901698
"1577","SMG6",60.7737166957645,0.407212289214671,0.221726415677637,1.83655288870366,0.066275927577863,0.593721081901698
"1578","AC017100.1",1.53922972774523,-2.40747179590635,1.31060366137821,-1.83691825900646,0.0662219633573457,0.593721081901698
"1579","MIDN",124.954495079096,-0.536743964430874,0.291610624116392,-1.8406186882157,0.0656774571455854,0.593721081901698
"1580","UBXN6",110.09989489086,-0.319785883285806,0.174207546674841,-1.83566033383552,0.0664079079176857,0.593721081901698
"1581","ADGRE5",48.2243052514188,-0.417456809059662,0.226859998045106,-1.84015169116179,0.0657459700967123,0.593721081901698
"1582","PPP1R15A",468.643453536541,-0.320467603443212,0.174512205747421,-1.83636211616647,0.0663041185667936,0.593721081901698
"1583","LINC00649",2.27730530718287,2.03722782887932,1.1056401739342,1.84257760970304,0.0653907054991166,0.593721081901698
"1584","SLC35E4",10.4055637122755,-0.941861955385154,0.512522206500231,-1.8376997980569,0.0661066536527617,0.593721081901698
"1585","SLC25A6",749.433173333967,-0.46314274932687,0.251938632153363,-1.8383157254142,0.0660158952148144,0.593721081901698
"1586","HDGF",107.009389181439,-0.317467187449361,0.173019603774989,-1

.83486252726729,0.0665260611809936,0.59395272782022
"1587","TMEM61",1.68196371264538,2.41542193859179,1.31695848370356,1.83409117939628,0.0666404605438691,0.594224761524676
"1588","OSR2",164.540469638514,0.758617709289971,0.413560601593274,1.83435681824463,0.0666010451089565,0.594224761524676
"1589","FAM117A",26.3366163394676,0.551495579818698,0.300763553299122,1.83365163022333,0.0667057229458226,0.594432370618533
"1590","PLA2G2A",2602.31123119069,-0.829297570717439,0.452585658743592,-1.83235494694999,0.0668985556971484,0.595261292358182
"1591","LINC01137",2.79898459191412,-1.56046655722488,0.851831346235164,-1.83189614249542,0.0669668953902955,0.595261292358182
"1592","ETHE1",53.1573022359974,-0.632932137905177,0.345478586876288,-1.83204447959556,0.0669447940461753,0.595261292358182
"1593","NCF4",1.97511477706336,-2.25823848107168,1.23235564281287,-1.83245680274342,0.0668833918968764,0.595261292358182
"1594","PPP1R26",5.06652503117412,-1.1322707903234,0.618636411062844,-1.83026858761532,0.0672097861241382,0.596759008098989
"1595","CXorf40B",13.4364013859357,-0.877359659199079,0.479378483323553,-1.83020241775623,0.0672196764066305,0.596759008098989
"1596","PLOD1",52.3151662314766,0.697503922823502,0.381849260840127,1.82664730393582,0.067752816113558,0.597000545219652
"1597","FCGR1A",2.98922284460856,-1.61325540829441,0.882683761452422,-1.82767088140362,0.0675989607957864,0.597000545219652
"1598","DUSP10",8.81085229491039,-0.913394043316863,0.49988297649348,-1.82721574102009,0.0676673380375343,0.597000545219652
"1599","FN1",1763.94878288507,-0.730565901465298,0.399584908161508,-1.82831204718575,0.0675027327987652,0.597000545219652
"1600","XRCC4",19.4836949139279,-0.624718927349886,0.341439485924743,-1.82966221864445,0.0673004637513432,0.597000545219652
"1601","AL358781.1",2.59663426172157,1.88578581988373,1.03151249492871,1.82817545027804,0.0675232242002034,0.597000545219652
"1602","BBIP1",50.9817734835655,-0.426736308396386,0.233360605512165,-1.82865615839406,0.0674511340983237,0.597000545219652
"1603","TUB",12.2654104129351,0.70382844651945,0.384774184737227,1.82919872080325,0.0673698440172375,0.597000545219652
"1604","GTF2A1",42.9187169624851,0.443069690856302,0.242333983467889,1.82834320022232,0.0674980601344778,0.597000545219652
"1605","RNF24",132.054689439852,-0.474325430891638,0.259498528374122,-1.82785403009222,0.0675714618083839,0.597000545219652
"1606","LINC00654",4.28089970982164,1.29230008139809,0.707334511267425,1.82699990006497,0.067699784420612,0.597000545219652
"1607","CBR3",130.331340209693,-0.468433650250102,0.256443892589084,-1.82665161381208,0.0677521676868381,0.597000545219652
"1608","TOB2",77.1678851016385,0.403044050102186,0.22069777370676,1.82622617044479,0.0678162008416998,0.597187440247805
"1609","GDI2",245.867219990765,-0.318189702198721,0.174270006461227,-1.82584317668866,0.0678738875064677,0.59732395717314
"1610","SUSD5",2.45239952126148,2.64548043689147,1.44979674930994,1.824725043804,0.0680425320543833,0.597830933967222
"1611","KLHL24",56.431372921706,0.53693197345303,0.294285910362769,1.8

2452490773734,0.0680727542911032,0.597830933967222
"1612","CAMSAP1",11.3228009586225,-0.770525350994479,0.422357935961738
,-1.82434206957646,0.0681003740458424,0.597830933967222
"1613","ACACB",40.0683140129931,0.622367189157526,0.341002161015392,1.
82511215560723,0.0679841062140943,0.597830933967222
"1614","GPRASP1",15.8199375763559,0.645512526841258,0.354024801589704,
1.8233539682606,0.0682497973406349,0.59877145622267
"1615","RBBP9",13.9373225587252,0.751582850075371,0.412301393445294,1.
82289670135469,0.0683190376316977,0.599007785055628
"1616","CCNL2",73.6003713136483,0.444566806595962,0.244193380515278,1.
82055224288992,0.068674947982147,0.599380105471049
"1617","TPM3",238.84078620415,-0.325044280528707,0.17855246689057,-1.8
2044127526906,0.0686918315845421,0.599380105471049
"1618","CFAP36",102.143120102301,-0.482494749348821,0.264978940965519,
-1.82087960496305,0.0686251600695579,0.599380105471049
"1619","GNL3",86.9840185872358,-0.395750602819778,0.217399193683646,-1
.82038670941744,0.0687001349703046,0.599380105471049
"1620","VRK3",26.506835750408,0.568524350324427,0.312183211720209,1.82
112403543968,0.0685880044228233,0.599380105471049
"1621","UBE2M",124.068423188134,-0.424971124691781,0.233238141282901,-
1.82204815367792,0.0684476794724993,0.599380105471049
"1622","MN1",18.8616186353575,0.645718964489829,0.354410872859022,1.82
195021072811,0.0684625406652614,0.599380105471049
"1623","SPIN2A",14.5648871077301,0.74460853083968,0.409030259954765,1.
82042407063484,0.0686944495562698,0.599380105471049
"1624","EVA1B",235.645972792373,-0.377431925645026,0.207516423518551,-
1.81880508176398,0.0689411728342101,0.599506458555288
"1625","ZNF462",32.8966136734455,0.55133135926673,0.303212025250194,1.
81830307954245,0.0690178223849786,0.599506458555288
"1626","PLXDC2",160.91854565486,0.590225364283313,0.324418062434499,1.
81933570484375,0.0688602292788142,0.599506458555288
"1627","NEK9",41.0635735797212,0.834598138397731,0.458925745432862,1.8
1859079971757,0.0689738825016007,0.599506458555288
"1628","MCRIP2",16.1055784604534,-0.698383189445461,0.383884519250482,
-1.81925332860263,0.0688727901866464,0.599506458555288
"1629","IGSF6",2.81997756259315,-1.52336086936436,0.837899668382944,-1
.81807073907104,0.0690533216033668,0.599506458555288
"1630","ARHGAP44",7.10603169492471,-1.0958005905582,0.602495878283005,
-1.81876860914139,0.0689467393969775,0.599506458555288
"1631","CHD6",66.6610609010548,0.401673582792387,0.220844971662871,1.8
1880338849444,0.0689414312579918,0.599506458555288
"1632","TRNP1",3.09595172501948,-1.42696862883058,0.786137054938636,-1
.8151652054386,0.0694985250560643,0.599864625539098
"1633","RGS4",2.24995074431678,-1.87712139774544,1.03455217280987,-1.8
1442893561098,0.0696117143020027,0.599864625539098
"1634","PUM2",51.179440339727,0.400166491756359,0.220577897595218,1.81
417311579741,0.0696510777587065,0.599864625539098
"1635","BAZ2B",91.9303606106392,0.607429817446891,0.334635289451863,1.
81519952196874,0.0694932531482712,0.599864625539098
"1636","RELL1",27.4300898223725,0.578794849323575,0.318705331229204,1.

81608147906168,0.0693578743292509,0.599864625539098
"1637","PDE5A",91.5188950283006,-0.522964574017014,0.287883877022432,-1.81658166975522,0.069281192261023,0.599864625539098
"1638","F2R",49.6199841974121,-0.79937149075131,0.440533027361639,-1.81455518905986,0.0695922941884058,0.599864625539098
"1639","SMPDL3A",33.7995142380089,0.645778591647498,0.356015649936126,1.8139050678344,0.0696923423868613,0.599864625539098
"1640","RFC2",22.3503650856097,-0.612208221929178,0.337359385390101,-1.81470635898052,0.0695690473129714,0.599864625539098
"1641","MFHAS1",26.3942622505155,-0.589410776773234,0.3247671131910 4,-1.81487211245592,0.0695435651106196,0.599864625539098
"1642","ACTN1",121.970194281452,-0.537976095669454,0.296317638404884,-1.81553855033891,0.0694411872556944,0.599864625539098
"1643","KIFC3",14.5119830852465,-0.832749747985275,0.459154178632529,-1.81366039282361,0.0697300263868189,0.599864625539098
"1644","MBTD1",23.216424284649,0.66372240155409,0.36531334962985,1.81685778038662,0.0692388927723131,0.599864625539098
"1645","NUP85",24.2835181200658,0.517707121542569,0.285264481449969,1.81483204257017,0.0695497245741786,0.599864625539098
"1646","SNRPD1",135.650231136141,-0.364783301689709,0.201021423543313,-1.81464888298889,0.0695778852063036,0.599864625539098
"1647","EIF5A",243.074501304178,-0.401100193390281,0.221253793674684,-1.81285114586568,0.0698547829820643,0.600572997587146
"1648","ELOA-AS1",6.42048678676305,-1.09106512164375,0.60194200829739,-1.81257514279467,0.0698973745447783,0.60057452885562
"1649","MAD2L2",43.7311612225425,-0.537001191921793,0.296790678924846,-1.8093600306692,0.0703950884084172,0.6008523426949
"1650","HSPG2",682.231331580643,0.302616425182808,0.167170848716404,1.81022246107143,0.0702612957878271,0.6008523426949
"1651","DDR2",341.154538555861,0.391718253073245,0.216802486603868,1.80679778728265,0.0707938138541591,0.6008523426949
"1652","LMOD1",65.3113983999417,0.544707109673554,0.300707485567717,1.81141852403568,0.0700760906740725,0.6008523426949
"1653","PJVK",7.40772086925191,0.943296108526218,0.52100348394492,1.810537045518,0.0702125448818295,0.6008523426949
"1654","CCNYL1",10.3849023028478,-0.77048027746748,0.426539178476111,-1.80635288936449,0.070863235331955,0.6008523426949
"1655","LSAMP",29.4283449968379,1.16128771081325,0.641537085477055,1.81016458300256,0.0702702681312299,0.6008523426949
"1656","NPY1R",78.5049049573135,0.95760056381821,0.530013250846265,1.80674834504462,0.0708015260216169,0.6008523426949
"1657","APC",65.7555076574182,0.428054384562517,0.236450203758333,1.81033628966552,0.0702436526538745,0.6008523426949
"1658","GRAMD2B",29.2221763507065,0.554879704310254,0.306798216788811,1.80861450277664,0.0705109137965673,0.6008523426949
"1659","GAL3ST4",17.2077120436317,0.656785489631062,0.363061020319009,1.80902232096954,0.070447535713484,0.6008523426949
"1660","SRPK2",106.263327085939,0.315441974150911,0.174621154179968,1.80643619973906,0.0708502314085955,0.6008523426949

"1661","FAM160B2",25.1864940721141,0.616817737508419,0.34068062698324,
1.81054538665847,0.0702112526396233,0.6008523426949
"1662","CCDC186",148.759121499416,-0.346899307645272,0.191852291022506
,-1.80815827528782,0.0705818704776418,0.6008523426949
"1663","CTTN",179.505627303513,-0.329826746391705,0.182400174376525,-1
.80825894229054,0.0705662087888038,0.6008523426949
"1664","DCLK1",422.014661423759,0.355529978311676,0.196715250388849,1.
80733307462892,0.0707103620193278,0.6008523426949
"1665","VPS13C",116.953139118727,0.379713845500649,0.209845507022208,1
.80949237793528,0.0703745432291819,0.6008523426949
"1666","MYO9A",89.2208077334379,0.452891718305032,0.250715531089679,1.
8063967411059,0.0708563902660163,0.6008523426949
"1667","RARA-
AS1",23.3288827174653,-0.574539546923443,0.317589705221,-1.80906225069
115,0.0704413328403043,0.6008523426949
"1668","SNHG25",33.096798911081,-0.832442578890302,0.459409013278304,-
1.81198573565212,0.0699884005897273,0.6008523426949
"1669","HOOK2",23.9732838158333,0.548403297939111,0.303366732431287,1.
80772391733271,0.070649480231403,0.6008523426949
"1670","RUVBL2",39.4186323466875,-0.565748608182047,0.312963892560863,
-1.80771207679181,0.0706513239993965,0.6008523426949
"1671","R3HDM4",25.7217068942886,-0.557438205046948,0.308743949407106,
-1.8055032531566,0.0709959669106023,0.601617529296306
"1672","SEC13",97.9711114641686,-0.398751472784195,0.220892326902421,-
1.8051848082543,0.0710457673890386,0.601679465447839
"1673","ACVR2B",10.7037773271874,0.805919192951459,0.446794224698737,1
.80378158087176,0.0712655544490338,0.601922938663635
"1674","CCZ1",60.0230704177704,-0.672528730441933,0.372822293349224,-1
.80388550373508,0.0712492579573697,0.601922938663635
"1675","RASGRP1",2.45395198742587,1.80976970378317,1.00354677105579,1.
80337355067088,0.0713295685789251,0.601922938663635
"1676","TMC3-
AS1",3.1228288910153,1.61415673025642,0.894888491907665,1.803751802434
58,0.0712702246684526,0.601922938663635
"1677","CDC37",149.089927216115,-0.299344251550853,0.165879230720892,-
1.80459151064264,0.0711386275015447,0.601922938663635
"1678","MID1IP1",70.0755266383771,-0.568344119795394,0.315149174153606
,-1.80341300694121,0.0713233763942106,0.601922938663635
"1679","PTPN12",124.271316548784,0.319196208103802,0.177042923202974,1
.80293118939216,0.0713990220016869,0.602150179597312
"1680","PCMT1",151.07082402934,-0.404296293326309,0.224484393248234,-1
.80099955937356,0.0717029494105134,0.604353430745756
"1681","LATS1",38.8473227861427,0.499296155879168,0.277367701465554,1.
80012363819215,0.0718411178884109,0.604927792508627
"1682","MZT1",27.6265162398066,-0.544886180903111,0.302710175545462,-1
.80002598168781,0.0718565358050502,0.604927792508627
"1683","B4GALNT1",2.06218932839835,-1.82203033046703,1.0124091490868,-
1.79969761445805,0.0719083979944317,0.605004703268659
"1684","DOCK7",50.2781927976191,0.509578456876163,0.283274211438928,1.
7988875665303,0.0720364677667185,0.605049215664377

"1685","RNASEH1",56.8490326479023,-0.420786849941495,0.234052190317629
,-1.79783342070181,0.072203409457597,0.605049215664377
"1686","NCOA1",93.6907111317956,0.48679858496563,0.270765271532532,1.7
9786197177485,0.0721988837452125,0.605049215664377
"1687","ATG16L1",8.63632198974947,0.894561810061765,0.49735300303737,1
.79864563921121,0.0720747530415734,0.605049215664377
"1688","PLPP5",52.2578481676781,-0.56805599273274,0.315932980677125,-1
.79802688378798,0.072172747615294,0.605049215664377
"1689","ALDH1A2",9.51092606321749,3.43561547490375,1.91129822786027,1.
79752977574305,0.0722515554648494,0.605049215664377
"1690","AC093525.6",2.52193313166664,1.84924864169533,1.02803381191346
,1.79882083669346,0.0720470261757847,0.605049215664377
"1691","SULT2B1",1.57727623279884,2.2535002976101,1.25368252519741,1.7
9750475285221,0.072255524271784,0.605049215664377
"1692","TRAPPC11",26.1464251629707,0.599826455044336,0.333931281651063
,1.79625715829497,0.0724536280111711,0.60509216569308
"1693","ANXA11",237.964921906075,-0.325415267360103,0.181114221514106,
-1.79674055764173,0.0723768170224197,0.60509216569308
"1694","ATP6V1E1",86.6874312222902,0.306909444386789,0.170834119943398
,1.7965348168649,0.072409500581568,0.60509216569308
"1695","MIR222HG",37.9690120333266,-1.03991065863801,0.578936283507471
,-1.79624371154929,0.072455765620257,0.60509216569308
"1696","LAGE3",35.3529038461927,-0.551548220945473,0.3070763986042,-1.
79612703370401,0.0724743158909226,0.60509216569308
"1697","ZNF451",60.776078952803,0.507018608544377,0.282498348641985,1.
79476662777569,0.0726908899511935,0.606542723458397
"1698","NUMA1",126.275063198151,0.350056076707327,0.195158940105469,1.
79369736543018,0.0728614860585204,0.607608152290134
"1699","BAALC",3.01732867699311,-1.71647496407543,0.957414789654617,-1
.79282269568307,0.0730012792787804,0.608057714463253
"1700","KCNK15-
AS1",4.46608920629285,-1.22703229319714,0.684369908767973,-1.792937236
8316,0.0729829603754417,0.608057714463253
"1701","OLA1",54.2691334441596,-0.436374780862728,0.243705531626331,-1
.79058217493321,0.0733603695816034,0.609075554382899
"1702","KALRN",5.13563130018484,-1.64625772111232,0.919484757267106,-1
.79041328102636,0.0733874968202905,0.609075554382899
"1703","UNC5CL",1.58061754557542,2.19944869888559,1.22894427844424,1.7
8970579664518,0.073501220048102,0.609075554382899
"1704","YWHAG",91.5241380944552,-0.347968279544021,0.194253213377793,-
1.79131286166822,0.0732431033287703,0.609075554382899
"1705","STX17",60.1088925876162,0.370388680301633,0.206947091925251,1.
78977475284077,0.0734901294805306,0.609075554382899
"1706","CAMK2G",31.6773771426514,0.497785141334159,0.278105543206601,1
.78991448927848,0.0734676591717969,0.609075554382899
"1707","MMP14",188.806724068715,0.494725051536671,0.276468905807651,1.
78944192690106,0.0735436721717392,0.609075554382899
"1708","AP4E1",10.9181158017865,0.799966578296305,0.446717634242674,1.
79076561338909,0.0733309155330193,0.609075554382899
"1709","NPIPB11",3.88380114013516,1.68594161062801,0.941569013408694,1

.79056615778436,0.0733629418564866,0.609075554382899
"1710","EID2",25.6542867924086,-0.570721326463522,0.318949179110688,-1
.7893801390392,0.0735536156775959,0.609075554382899
"1711","SPON2",734.494622892394,-0.471825259202094,0.263844496915424,-
1.78827023007169,0.0737324203991899,0.609842916385823
"1712","LUC7L3",203.710409711911,0.460902118733951,0.257719291372714,1
.78838811902286,0.0737134118065731,0.609842916385823
"1713","CAPN3",4.31623869902348,1.31439751658655,0.735200648509916,1.7
8780788516786,0.0738070082763002,0.610103465961711
"1714","CTXN1",7.80234718248886,-1.21556189018096,0.680252539876313,-1
.78692738200136,0.0739492262225422,0.610922429003032
"1715","LINC01750",2.937756623267,1.44496427522478,0.809262589232659,1
.7855320318154,0.0741750604524758,0.611005733570132
"1716","EPAS1",38.3787127087722,0.547388934055677,0.306553801117018,1.
7856210950936,0.0741606289437191,0.611005733570132
"1717","C5orf38",38.6602984807115,-0.551425261970459,0.308651455966808
,-1.78656297033557,0.0740081511180655,0.611005733570132
"1718","NAIP",17.8279320226578,0.647845217368784,0.362751988037738,1.7
8591775850278,0.0741125751720878,0.611005733570132
"1719","ZNF624",14.0663843901688,0.727261405583444,0.407216425226649,1
.78593337726656,0.0741100459378651,0.611005733570132
"1720","SPRTN",17.1666914289099,-0.673291368608254,0.377356634001916,-
1.78423090504045,0.0743861523963188,0.612388324378997
"1721","SMIM1",6.27780946303988,1.12492860710591,0.630886666345125,1.7
830914284857,0.0745714215143131,0.613200539281459
"1722","PYCARD-
AS1",1.97731308135128,2.16306151080862,1.213011094147,1.78321659319176
,0.0745510523937931,0.613200539281459
"1723","UBN1",51.5633179535854,0.408974677697227,0.229405432598952,1.7
8275934036924,0.0746254870826739,0.613288971033466
"1724","METTL14",30.3214954848578,0.474204319266208,0.266066324809591,
1.78227860893546,0.0747038092354879,0.613576530611664
"1725","EIF3I",242.280357134587,-0.364255360723749,0.204904738319527,-
1.77768149097525,0.0754561812662081,0.614096004133073
"1726","MCOLN3",17.3308239742226,0.990703594819908,0.557545350162789,1
.77690226370043,0.0755843218854422,0.614096004133073
"1727","OSBPL10",21.8872659321494,0.597617796784607,0.335592434837709,
1.78078447171675,0.0749476671511871,0.614096004133073
"1728","FAM107A",14.0930006029966,-1.1736869184859,0.660722361414412,-
1.77636929976667,0.0756720677801935,0.614096004133073
"1729","RARRES1",112.307443256254,-0.826603306078539,0.465287490005965
,-1.77654315629234,0.0756434353305092,0.614096004133073
"1730","DCUN1D4",27.4773507773535,0.547117798039246,0.307837862457051,
1.77729209029828,0.0755201943724214,0.614096004133073
"1731","INTU",23.749549382342,0.608882505330013,0.342320849339753,1.77
868951454283,0.0752906791921734,0.614096004133073
"1732","SELENOP",1423.88469687629,0.442198885471318,0.248673894230022,
1.77822801561263,0.0753664134505693,0.614096004133073
"1733","TNFAIP8",20.0034494259663,-0.670806658759602,0.376529704928761
,-1.78155043275143,0.0748225736766805,0.614096004133073

"1734","FLOT1",121.914431554594,-0.379111230942032,0.212898893077502,-1.78071020220863,0.0749598056071011,0.614096004133073
"1735","BAK1",11.7339690797971,-0.733281923021015,0.412294824771817,-1.77853778161501,0.0753155724579579,0.614096004133073
"1736","ZNF316",13.4147659661301,0.805171499946567,0.452695035044223,1.77861791629305,0.0753024247458359,0.614096004133073
"1737","AQP1",48.1971766726532,-1.41529924405889,0.796716952007056,-1.77641412109222,0.0756646853052833,0.614096004133073
"1738","ERLIN1",39.7011544804153,0.502328077529976,0.282476983331059,1.77829737349345,0.0753550275098847,0.614096004133073
"1739","GALNT18",1.59675122752327,-2.40356700567175,1.35310491095021,-1.77633455190392,0.075677791469789,0.614096004133073
"1740","SRSF9",232.814550409505,-0.384897863003298,0.216468822405913,-1.77807528458558,0.0753914910597952,0.614096004133073
"1741","SEPTIN1",49.5819904683528,-0.482920279627377,0.27123942393701,-1.78042068006871,0.0750071398403172,0.614096004133073
"1742","OMG",5.887119931915,1.54164336502757,0.866411493081252,1.77934316123273,0.0751835188651242,0.614096004133073
"1743","LASP1",102.493217375073,0.365697858190222,0.205480570408673,1.77971989012343,0.0751218136629938,0.614096004133073
"1744","RALGAPA2",11.5054746046072,0.741710054872426,0.417518625293333,1.77647177859749,0.0756551894606526,0.614096004133073
"1745","ADARB1",18.7716581342587,0.846245262978216,0.47623985661477,1.77693078650228,0.0755796283021859,0.614096004133073
"1746","PLA2G5",27.103211261848,-0.899112037434318,0.506776618438984,-1.77417821722683,0.0760336760976445,0.615924973422566
"1747","PABPC1",594.498243393326,-0.30137554143509,0.169858712830107,-1.77427190171002,0.0760181859537814,0.615924973422566
"1748","HEXIM1",62.2552841556203,0.581267717323556,0.327584585609914,1.77440497159328,0.0759961881057168,0.615924973422566
"1749","LRIF1",18.4749109604206,-0.698762340722357,0.394074932272069,-1.7731712511973,0.0762003342736429,0.616393322615494
"1750","HBEGF",7.29338526019061,-1.05939564943498,0.597590934240873,-1.77277731092214,0.0762656144931035,0.616393322615494
"1751","TMEM258",403.855500381465,-0.363235812641981,0.20489473073618,-1.77279235701615,0.0762631203529637,0.616393322615494
"1752","TMEM88",3.20139216438173,-1.99591393152536,1.12565341427002,-1.77311586872386,0.0762095090019887,0.616393322615494
"1753","PARL",63.3763980907077,-0.432691212832202,0.244266579769671,-1.77138932898722,0.0764959818637924,0.616445231821891
"1754","ANKRD37",10.977657294725,-0.787812072414173,0.444905209668647,-1.77074139680431,0.0766037151348355,0.616445231821891
"1755","TCF7L2",275.606899711815,0.39637998928491,0.223753832117854,1.77150033826519,0.0764775364819547,0.616445231821891
"1756","ACER3",25.6047435346677,0.49784035691677,0.281104404998697,1.77101585056655,0.0765580659428194,0.616445231821891
"1757","BCL11B",2.72785860136709,1.61736277083814,0.913433145007146,1.77064164977885,0.0766203112998961,0.616445231821891
"1758","IRX3",40.6906075759979,0.59996696779216,0.338714758400845,1.77130447644133,0.0765100834701786,0.616445231821891

"1759","ZNF397",38.0785209425284,0.534853551403177,0.3017923819846,1.7
7225663512762,0.076351966153612,0.616445231821891
"1760","BCL3",42.0550448994083,-0.64430248259324,0.363662847632907,-1.
77170279226219,0.0764439059183896,0.616445231821891
"1761","OSTF1",55.7264442951432,-0.464936779303227,0.262675038259523,-
1.77000746772089,0.0767258967134326,0.616594039422364
"1762","MIR99AHG",170.017682598707,0.320588848654173,0.181106633231887
,1.77016624368305,0.076699450867913,0.616594039422364
"1763","ADRM1",88.5326523174059,-0.398688643178789,0.225288098388967,-
1.769683556432,0.0767798706766134,0.616677804186526
"1764","SPSB1",72.2081612607846,-0.566958542349754,0.320560165593903,-
1.76864939316258,0.076952402495692,0.617013600984711
"1765","HNRNPF",219.331673361799,-0.33276625343976,0.188119947138955,-
1.7689046722619,0.0769097843545442,0.617013600984711
"1766","RAD51B",19.4470796160163,0.644155156273218,0.364129754987549,1
.76902641833059,0.0768894659608354,0.617013600984711
"1767","FRS2",43.909112054499,0.455443377298044,0.257590930796695,1.76
808778123289,0.0770462298127293,0.617416306818476
"1768","MPEG1",2.78455545610189,-1.95550647867141,1.10631781122541,-1.
76758112255772,0.0771309561836166,0.617745667171952
"1769","UBXN10",2.58055347000616,-1.89026123715968,1.07043207556767,-1
.76588620642487,0.0774149417800527,0.619378921460817
"1770","TRAPPC3",68.52927161768,-0.388204383383491,0.219960136652901,-
1.76488517097114,0.0775830664215158,0.619378921460817
"1771","ECHDC2",82.1845513197491,0.419502801624387,0.238035459276916,1
.76235424292968,0.0780094644735773,0.619378921460817
"1772","AL390728.6",17.5551311437759,0.780871088087303,0.4431651681888
5,1.76203173024317,0.0780639365967277,0.619378921460817
"1773","CLHC1",5.98132224208239,1.03782946927489,0.588378989429642,1.7
638792137716,0.0777523171609443,0.619378921460817
"1774","CLASP1",26.7907814228214,0.655223524245709,0.371699620428818,1
.76277695277116,0.0779381160029138,0.619378921460817
"1775","DAB2",490.958694863683,0.33390512462554,0.189187001214164,1.76
494749894339,0.0775725897174615,0.619378921460817
"1776","TUBB2B",4.65348088027331,-1.53404984752304,0.870131017555598,-
1.76301018648036,0.0778987716373134,0.619378921460817
"1777","DCHS1",26.4703196377098,-0.65290289682288,0.369922839880408,-1
.7649704923166,0.0775687250541258,0.619378921460817
"1778","NAV3",47.5816581797276,0.492148032247674,0.279352866517829,1.7
6174326894284,0.0781126837016761,0.619378921460817
"1779","USP44",5.7387582646397,1.38098150351873,0.783255542473732,1.76
313020289294,0.0778785322839012,0.619378921460817
"1780","ANAPC5",133.349377556536,-0.286374140004905,0.162514644702408,
-1.76214359345464,0.0780450394870521,0.619378921460817
"1781","COX5A",124.684761560877,-0.481891414022999,0.273506363192173,-
1.76190202084772,0.078085853135396,0.619378921460817
"1782","HCFC1R1",277.669894793699,-0.450687590141998,0.255303358649101
,-1.76530223702009,0.0775129837637428,0.619378921460817
"1783","PHLPP1",10.2882434023855,0.74061500237416,0.419992196213899,1.
76340181805895,0.0778327433989223,0.619378921460817

"1784","GPX4",1549.18750828083,-0.510400737467439,0.28938195975314,-1.76376142418429,0.0777721547279611,0.619378921460817
"1785","KANK3",1.54973246121585,2.28243750906539,1.29559777707853,1.76168680546219,0.0781222283706934,0.619378921460817
"1786","FKBP1A",674.420841622385,-0.530380811157028,0.301061689632321,-1.76170143668817,0.0781197549958037,0.619378921460817
"1787","ECSIT",42.4060863813085,-0.479180371098395,0.272195987969381,-1.76042407778728,0.0783359298605824,0.620725666942276
"1788","SUCLG2-AS1",13.268476383357,0.745279020250687,0.423431393560283,1.76009391742132,0.0783918839365024,0.620821631174986
"1789","PDE8A",32.1868642867904,0.538015897900152,0.305906640606374,1.75875847884076,0.0786185399641132,0.622268600274926
"1790","PLA2R1",50.5038610929167,0.612737635358042,0.348684088331402,1.75728590969048,0.0788690882585356,0.623206634900036
"1791","HAND2",13.0359217597259,-0.861904099288512,0.490452630798003,-1.75736461620387,0.0788556804331254,0.623206634900036
"1792","BECN1",44.0666682388597,-0.408733142286255,0.232544326846982,-1.75765690708597,0.0788059042761329,0.623206634900036
"1793","AL392172.1",6.98425425914063,-0.94927857561522,0.540475163158884,-1.75637779554388,0.0790239216620224,0.623449803363782
"1794","STARD10",27.0874124123094,-0.623867243946956,0.355151847833545,-1.75662114037304,0.0789824071543117,0.623449803363782
"1795","EIF4A1",777.812966628578,-0.314778038149943,0.179224805693289,-1.75633075417378,0.0790319489433608,0.623449803363782
"1796","VPS45",25.3122641904712,0.503989668122355,0.287058164866578,1.75570574122706,0.0791386660000907,0.623624394361919
"1797","NXF1",26.7376613370996,0.500458348561261,0.28505014970417,1.75568526829628,0.0791421636065232,0.623624394361919
"1798","TSSC4",57.9331340561284,-0.508953380547317,0.289998621170119,-1.75502000145289,0.0792558865850039,0.624173166876337
"1799","CLDN1",6.1684655289927,-1.26751244284356,0.722437107113383,-1.7544952084592,0.0793456902181095,0.624533059193124
"1800","KAZN",26.0665449180318,0.601366718334083,0.343528331610335,1.75055930762711,0.0800218498243065,0.624648849805836
"1801","HS6ST1",38.2010438453909,0.546882287332102,0.31255268199361,1.74972834609457,0.0801652000121295,0.624648849805836
"1802","HNRNPA3",288.089643331904,-0.308932192299224,0.176501837319657,-1.75030581545577,0.0800655579713914,0.624648849805836
"1803","BOK",23.8278067488702,-0.516151914199186,0.294835991284589,-1.75064079507503,0.0800078035473809,0.624648849805836
"1804","PPM1L",10.5144828308818,1.26776336751628,0.723222958916214,1.75293573287011,0.0796130391762864,0.624648849805836
"1805","CD38",8.2048904549243,-1.10234445555335,0.629913288434189,-1.74999396868339,0.0801193544647794,0.624648849805836
"1806","TLR1",2.34097826246779,1.83962515208422,1.04960726678503,1.75267951194643,0.0796570344154786,0.624648849805836
"1807","CARD6",57.4901905679557,0.442020840817123,0.25253095742117,1.75036298650673,0.0800556986133598,0.624648849805836
"1808","PCDHGB7",9.79801698622633,0.739743457110207,0.422819559222264,

1.74954881101266,0.0801961992216125,0.624648849805836
"1809","AC022868.2",2.00867525179131,2.39321793088392,1.36453137065259
,1.75387534677152,0.0794518687621926,0.624648849805836
"1810","SLC35F2",9.62119565086592,-0.779695502394431,0.445101396434027
,-1.75172558127437,0.0798210059498223,0.624648849805836
"1811","IQSEC3",2.17952610358308,1.6379586174664,0.935592200773442,1.7
5071854608485,0.0799944032013156,0.624648849805836
"1812","GALNT8",3.78468522291385,1.45996342469545,0.833445308284154,1.
75172073102329,0.0798218403619103,0.624648849805836
"1813","PAWR",28.6878799716886,0.606712167570927,0.346484608806114,1.7
510508465628,0.0799371519407592,0.624648849805836
"1814","LINC01089",8.7713358340899,1.10084215322675,0.628329640422352,
1.7520137240172,0.0797714480833345,0.624648849805836
"1815","L2HGDH",4.70115130796562,1.08830488252868,0.621413577335387,1.
75133747028076,0.0798877969726132,0.624648849805836
"1816","CEP152",13.6174006804797,0.79104758367151,0.451298243019288,1.
75282664160007,0.0796317686289163,0.624648849805836
"1817","TLCD2",29.4601071438649,0.584058011260779,0.33304150247106,1.7
5370939335566,0.0794803151712361,0.624648849805836
"1818","BORCS8",24.3669613996378,-0.639448843300213,0.365496427222756,
-1.74953514090165,0.0801985599538849,0.624648849805836
"1819","BROX",46.1144532017668,0.380452278788995,0.217530765826147,1.7
4895848568408,0.080298195701669,0.624737610514084
"1820","PIK3IP1",68.5643345086926,0.352350967763005,0.201448490498328,
1.74908715816825,0.0802759546773341,0.624737610514084
"1821","CASZ1",1.72209180559362,-1.99103712701941,1.13958926644214,-1.
74715328202023,0.0806107530407818,0.625000255872506
"1822","TSPAN2",2.57291014599231,-1.88765742490551,1.0811432945587,-1.
74598264115952,0.0808139682505739,0.625000255872506
"1823","LINC02591",7.80092015295707,0.958311947089413,0.54828585994198
7,1.74783268565564,0.0804930036888408,0.625000255872506
"1824","NPR1",54.7813165408257,-0.456236629775187,0.261108462432123,-1
.7473069448823,0.0805841090640844,0.625000255872506
"1825","TAB2",43.9692901197049,0.472342929145193,0.270534414181888,1.7
4596245203623,0.0808174765891638,0.625000255872506
"1826","STAT2",69.8139595513627,0.51982491534633,0.297513027987808,1.7
4723412571913,0.0805967344638201,0.625000255872506
"1827","PPP4R3A",56.0005628736923,0.374712031293086,0.214436162605604,
1.7474292896309,0.080562900525842,0.625000255872506
"1828","DNASE1",15.6390655883028,0.71874859064537,0.411241119388841,1.
74775467908833,0.0805065161227133,0.625000255872506
"1829","TRIM16",15.0834945129161,-0.708546223103855,0.405653871660524,
-1.74667684103064,0.0806934097853338,0.625000255872506
"1830","NR1H2",76.2210384929285,-0.324370922654947,0.185766395743542,-
1.74612271157349,0.0807896311020746,0.625000255872506
"1831","KREMEN1",62.3424377714226,0.546099951739403,0.312674310218603,
1.7465456351614,0.0807161844985475,0.625000255872506
"1832","LINC00886",2.40788254669079,1.96788963478473,1.12764999071164,
1.74512450759905,0.0809631984120346,0.625785420040617
"1833","PNMA8B",7.25279878768313,1.02101002305412,0.585380458174187,1.

74418193979121,0.0811273695074828,0.626712248895776
"1834","ZCWPW1",13.2161129651763,0.716507705138821,0.410955351528989,1.74351715453516,0.0812433204426162,0.627074222575266
"1835","FAM13C",30.052570357584,0.770623165649746,0.442021741853027,1.74340556738039,0.081262796498984,0.627074222575266
"1836","AL445222.1",2.53196585011182,1.95586591663018,1.12308021969138,1.74151933436032,0.081592587215518,0.62893360640813
"1837","THRA",79.4274048771652,0.45000990906567,0.258375353506914,1.74169054036196,0.0815626086605892,0.62893360640813
"1838","CFAP298",82.5597978955706,-0.318796145043699,0.183090565743728,-1.74119372971909,0.0816496259881902,0.629030850920986
"1839","FLYWCH1",24.5840147655092,0.489331611949369,0.281076934307469,1.7409169953949,0.0816981291842333,0.629062267128191
"1840","SLC40A1",88.5516744391607,0.447812249456307,0.257279449020341,1.7405675080597,0.0817594171917785,0.629192036649774
"1841","TNFAIP2",293.007145184554,0.57327690964258,0.329454987730182,1.74007658403425,0.0818455712990162,0.62924521646001
"1842","MPRIP-AS1",1.73327766690201,-2.54920882735404,1.46522117048175,-1.73981162619694,0.0818921003382648,0.62924521646001
"1843","ABCC3",4.22947921334513,-1.28452614807828,0.73833156956123,-1.73976869070035,0.0818996422271045,0.62924521646001
"1844","AC106881.1",1.63478541410717,2.17335053752329,1.25115643750446,1.73707337657809,0.0823742199709352,0.630089130566752
"1845","ARPC1B",504.171029044967,-0.492027786106783,0.283237033738509,-1.73715908408021,0.0823590947651383,0.630089130566752
"1846","ARHGEF10",77.5488669696776,0.597047136086207,0.343562727428149,1.73781114312254,0.0822440965581688,0.630089130566752
"1847","CD44",784.355264593108,-0.348230553315068,0.200336913061393,-1.7382246136954,0.0821712436876609,0.630089130566752
"1848","N4BP2L1",44.9548966342414,0.398405274384521,0.229202963671999,1.73822043136693,0.0821719803453351,0.630089130566752
"1849","WBP4",77.4447200867721,-0.353812137628318,0.203706642520045,-1.73687089066574,0.0824099625571769,0.630089130566752
"1850","SMCR8",12.0793573219563,0.759318884734286,0.436935821532834,1.7378270384664,0.0822412948556392,0.630089130566752
"1851","SCAMP4",35.7375571298468,0.50618745371628,0.291297628269429,1.73769850693083,0.082263951954285,0.630089130566752
"1852","GSTT2B",5.6330308749621,-1.26960746805155,0.730688224844462,-1.73755019566903,0.082290102044534,0.630089130566752
"1853","ACOT7",8.68455663468767,-0.878780936185552,0.506112341143569,-1.7363357198521,0.0825044908443864,0.63013138638431
"1854","ATP5PB",158.67352197091,-0.338309515097,0.194827545725323,-1.73645627900053,0.0824831885751071,0.63013138638431
"1855","UBA7",29.5706142728854,0.465795447279563,0.26832870247624,1.73591361259912,0.0825791104788923,0.630361296162326
"1856","BNIP1",16.6371226364243,-0.604636152990499,0.348426741003721,-1.73533222865934,0.0826819764180375,0.630495118390068
"1857","TMEM215",3.57829156889304,5.62044873631678,3.2388707307809,1.73531122526822,0.0826856945515788,0.630495118390068

"1858","FAM172A",67.4263436790656,0.362371519895656,0.208867370603063,
1.73493599718032,0.0827521422907458,0.630662182366502
"1859","KDM5A",109.945522503496,0.400230649864738,0.23072994991273,1.7
3462807934609,0.0828067026321294,0.630738520317887
"1860","AL136084.3",1.76457155653291,2.38689696096445,1.37635852410356
,1.73421163102766,0.0828805399977978,0.630961530305816
"1861","MRPL48",24.9179156621768,-0.53968271479952,0.311434419177525,-
1.73289360959133,0.0831145803224357,0.632327620797795
"1862","NRK",5.59667610773309,1.48977732003458,0.859801886524695,1.732
69836154494,0.0831492958986931,0.632327620797795
"1863","WDR46",62.1603196839899,-0.398134745789556,0.229823789046981,-
1.73234784545376,0.0832116479842125,0.632462123165029
"1864","DNAJC16",22.4686639760523,0.616820806909074,0.356216022018389,
1.73159198009693,0.0833462351506135,0.632510373016391
"1865","PER2",63.1522988229146,-0.743789577081159,0.429735455122941,-1
.73080803134657,0.0834860089807651,0.632510373016391
"1866","RBMS3-
AS3",5.26728341244104,1.19715528143264,0.691376814623788,1.73155254285
475,0.0833532620672025,0.632510373016391
"1867","AADAC",278.46115783138,-1.35200527707505,0.780924806183062,-1.
73128739972196,0.0834005176545941,0.632510373016391
"1868","CCDC146",88.9398082425555,0.791649648279221,0.457323510316238,
1.73104953150517,0.0834429305873372,0.632510373016391
"1869","APOBR",2.16449485525257,1.81892284745303,1.05089912335958,1.73
082535423398,0.0834829183536065,0.632510373016391
"1870","RHOBTB2",8.80220047663863,0.853532176332576,0.493515501871514,
1.72949415589947,0.0837206915408403,0.633949193699625
"1871","ATP1A1-
AS1",14.9506181457592,1.13580345869524,0.657554620580469,1.72731423846
218,0.084111243410271,0.634059147246676
"1872","WDFY1",48.7972080120654,0.427768325565696,0.247411393012389,1.
72897585821472,0.0838134159334573,0.634059147246676
"1873","NPHP3",55.0470748751528,0.496917421014317,0.287629296151862,1.
72763146057262,0.0840543186941437,0.634059147246676
"1874","RASA2",40.110341070922,0.478605041114134,0.276875813933594,1.7
2859100372315,0.0838823208754805,0.634059147246676
"1875","HIST1H4E",1.77784972771056,2.27832004614185,1.31886650597445,1
.72748343810468,0.0840808770747459,0.634059147246676
"1876","FIGNL1",9.59812154083782,1.04628932630231,0.605329653805413,1.
728462036718,0.0839054215838118,0.634059147246676
"1877","RAB30",55.7319203067136,0.528694556084095,0.306149790355214,1.
72691464354972,0.0841829941259711,0.634059147246676
"1878","ZNF592",22.1359087150346,0.565664951040581,0.327482623949968,1
.72731286997078,0.0841114890502182,0.634059147246676
"1879","TRPV1",4.42938521870009,1.15976063543694,0.67134594978829,1.72
751565091391,0.0840750968331237,0.634059147246676
"1880","CRELD2",84.7757827762835,-0.452179591340967,0.261807753809863,
-1.72714361878434,0.0841418735884241,0.634059147246676
"1881","GPCPD1",38.3916560291286,0.530197217022985,0.307322244392537,1
.72521588234197,0.0844885744903262,0.636022442734194

"1882","AC007877.1",1.96524871508169,2.58866501264931,1.50128445711068
,1.72430014870823,0.0846536724742845,0.636588423917084
"1883","PCOTH",1.95169852317089,-2.12416445762013,1.23180388913804,-1.
72443395929406,0.0846295314404407,0.636588423917084
"1884","PATZ1",16.4586359476897,0.629136115716457,0.364919145533649,1.
72404249932248,0.0847001712026968,0.636600012861033
"1885","NIN",49.2802071968001,0.375709024732885,0.217964085661646,1.72
371986693309,0.0847584267342882,0.636699905866059
"1886","MFAP3",4.11559276311976,1.12153513288788,0.650888314252355,1.7
2308383532148,0.084873365553073,0.637225268415436
"1887","DYNC1I2",237.156064497591,0.280918015404421,0.163089247514981,
1.72248029643166,0.0849825490727145,0.637370784575394
"1888","CAMKK1",9.7411042121675,-0.954545274811084,0.55429984715007,-1
.72207385536704,0.0850561404920478,0.637370784575394
"1889","PAK4",11.5685347454946,0.702691029137572,0.40807108642931,1.72
198191076519,0.0850727953988344,0.637370784575394
"1890","PPP1R1B",2.36624483980072,-2.13671023367742,1.24079285800794,-
1.72205233120687,0.0850600391564439,0.637370784575394
"1891","TMEM108",6.1526323933869,1.29785392618532,0.754024455036351,1.
72123585318297,0.0852080343073172,0.637709178536793
"1892","GLIS2",51.5421683110093,-0.568453931247498,0.33023609049636,-1
.72135616792545,0.0851862129358158,0.637709178536793
"1893","ARPC4",125.621798492211,-0.358405963746789,0.208271626134268,-
1.72085833485419,0.0852765337925013,0.637884690175287
"1894","APLP2",418.056659051773,0.208513500609207,0.121195378919177,1.
72047401863615,0.0853463124580373,0.638069579939708
"1895","EPS15",117.927539103521,0.289208985810051,0.168152254688758,1.
71992332987365,0.0854463791339441,0.638143844164899
"1896","LRCH3",50.6266204148478,0.424774542803212,0.246965905718529,1.
71997240496562,0.0854374577603198,0.638143844164899
"1897","FIGN",14.1707681127471,0.820585065591983,0.477363410666145,1.7
1899447518796,0.0856153779256769,0.639068925370366
"1898","SCARA3",154.78981629506,-0.456177427152753,0.265654314099214,-
1.71718433671807,0.0859454965093711,0.64052012135405
"1899","CNNM2",8.40482769625454,1.02426060533636,0.596400496120129,1.7
1740401290687,0.0859053789710684,0.64052012135405
"1900","RPL41",8106.41074372848,-0.399663859740089,0.232726763620087,-
1.71730940405512,0.0859226547016135,0.64052012135405
"1901","IFT172",12.8516461944563,0.772641391326304,0.450387496643761,1
.71550364316049,0.0862529280251639,0.640743246908101
"1902","LIMS1",275.987812626733,-0.237821144783334,0.13854574543685,-1
.7165532152103,0.0860608369248688,0.640743246908101
"1903","MAATS1",3.44719755254678,-1.45278682443731,0.847116905837383,-
1.71497796163236,0.0863492675562729,0.640743246908101
"1904","HIST1H2BC",4.36146946306906,1.33829543609023,0.780440157177763
,1.71479571339561,0.0863826877364099,0.640743246908101
"1905","TRRAP",25.1742082731264,0.513699625724027,0.299476272797803,1.
71532662980236,0.0862853588483683,0.640743246908101
"1906","SPRY2",153.634343425618,0.448136093619312,0.261263215905579,1.
71526669786255,0.0862963412803888,0.640743246908101

"1907","FANCM",9.09800994192577,0.828131315844671,0.482682206666109,1.
7156864380077,0.0862194483029366,0.640743246908101
"1908","PLSCR3",55.3386263877784,-0.502486555278682,0.292988917939968,
-1.71503604577167,0.0863386184414528,0.640743246908101
"1909","IMPA2",35.0871556718241,-0.531981736199566,0.310094445231092,-
1.7155474545928,0.0862449028019121,0.640743246908101
"1910","SLC39A7",121.982986505153,-0.383285639090677,0.223582777625313
,-1.71428963877083,0.086475545092688,0.64109618770286
"1911","FRG1",122.468483951406,-0.349368966722953,0.203935157047391,-1
.71313750792742,0.0866872450682916,0.641993404899063
"1912","SPECC1L",63.2895937654275,0.508242215048669,0.296659155597543,
1.71321938143101,0.0866721873022624,0.641993404899063
"1913","BORCS7",150.750336666958,-0.315189944257998,0.184052449756482,
-1.71250067399278,0.0868044405100191,0.642525288877089
"1914","ARHGEF10L",31.4220261654058,0.476655098081465,0.27885147193609
5,1.70935119966195,0.0873859146513307,0.642895204304235
"1915","CEBPZOS",58.9455140951858,0.374627604829835,0.218891338828189,
1.71147751590064,0.0869929983291707,0.642895204304235
"1916","LINC00240",1.72845969797654,1.83713820715503,1.07468111728637,
1.70947286372158,0.0873633941168075,0.642895204304235
"1917","CCDC170",12.5829373233969,0.834521223526222,0.487733927218736,
1.71101737433976,0.0870779055328178,0.642895204304235
"1918","WDR86",15.178987783712,0.958574761277467,0.560517211000179,1.7
101611555638,0.0872360765464392,0.642895204304235
"1919","ADAM9",91.4769461166743,0.375182767680505,0.219522156641705,1.
70908838278618,0.0874345791607646,0.642895204304235
"1920","SQLE",27.4879692894263,-0.708712039589819,0.414464557501455,-1
.70994606598498,0.0872758468925601,0.642895204304235
"1921","NACC2",16.9231749317666,0.621207014933827,0.363484284380216,1.
70903403978816,0.0874446443142625,0.642895204304235
"1922","TMEM9B-
AS1",4.59235014927795,1.16525826104067,0.681382183703404,1.71013902169
753,0.0872401684522527,0.642895204304235
"1923","IREB2",58.584068762,0.384277129002917,0.224524866440697,1.7115
1256025539,0.0869865345409839,0.642895204304235
"1924","YIPF2",65.9896852580817,-0.472108516812581,0.276099662745747,-
1.70992065733645,0.0872805459512317,0.642895204304235
"1925","C19orf12",58.8946927084842,0.346085508884526,0.202461811132786
,1.70938660949517,0.0873793596574895,0.642895204304235
"1926","AL354822.1",3.17832070755152,1.40765841561462,0.82324425033041
,1.70989158448377,0.0872859229158504,0.642895204304235
"1927","CCDC167",37.7671224809347,-0.474783212147632,0.277969952554538
,-1.70803789324847,0.087629311558778,0.643702689546045
"1928","AL627171.1",1.64926287332242,-1.98651216850393,1.1630965496898
1,-1.70795121783631,0.0876453944523146,0.643702689546045
"1929","CALCOCO1",96.7466895667049,0.409966368043254,0.240096850333486
,1.70750414873758,0.0877283873396303,0.643978208776135
"1930","AC108734.4",12.9441770995863,0.898377358880058,0.5263325968687
74,1.70686247483935,0.0878476169911602,0.644185529049626
"1931","C8orf48",7.05998849005238,-0.892569861347409,0.52285597873375,

-1.70710462852319,0.0878026069855936,0.644185529049626
"1932","P4HA1",90.4780733612786,0.423131506090113,0.248007948320223,1.70612074716159,0.0879856004820365,0.644670140902266
"1933","SNAPC2",52.8180663223708,-0.530666701192924,0.311055775432217,-1.70601783701188,0.0880047586415311,0.644670140902266
"1934","RGL3",2.40300709207181,2.23706949222157,1.31173753898265,1.7054246186753,0.0881152601025631,0.645145854732313
"1935","CIB2",10.8693119056314,-0.712414386898732,0.417889784235746,-1.70479014748261,0.0882335697087926,0.645678215543413
"1936","IFT52",65.0431838159999,-0.372687497187172,0.218661132651614,-1.70440668932674,0.0883051350774302,0.645868136723353
"1937","AC093227.1",3.28749827510212,1.32287773925543,0.776372041642017,1.70392243447814,0.0883955791388534,0.64619587021485
"1938","NBPF9",13.8427380016237,0.878024093984367,0.515725106387388,1.70250407263446,0.0886609156746242,0.647762244677668
"1939","RALB",52.7540324247998,-0.395206302925669,0.23216200363036,-1.70228675125884,0.0887016271838052,0.647762244677668
"1940","BCS1L",20.1970462812035,-0.522628230392028,0.30705914538859,-1.70204417696347,0.0887470871945393,0.647762244677668
"1941","KCNIP4-IT1",6.82716292009514,-1.15982561243338,0.681644340966657,-1.70151139344691,0.0888470002173903,0.648157404986217
"1942","USP12",29.7059321374895,-0.483332044515898,0.284170126753629,-1.70085451992264,0.0889703086070346,0.64872274452915
"1943","SNRNP40",51.2762975162092,-0.459458382661953,0.270325090371668,-1.69965126814624,0.0891965407818436,0.649035466326262
"1944","EXOC6B",19.0881958893738,0.637074152299621,0.374821386313852,1.69967396621861,0.0891922688671004,0.649035466326262
"1945","ZNF337",12.3621833175964,0.816726848023151,0.480494877728421,1.69976182032219,0.0891757357458986,0.649035466326262
"1946","NIPSNAP1",22.062380198995,-0.525442467979228,0.309121511992573,-1.69979263038753,0.0891699382344036,0.649035466326262
"1947","ACTR1B",33.3840509434106,-0.454943937704829,0.267747259805611,-1.69915441164599,0.089290093481777,0.649146917168528
"1948","AC090578.1",2.15528485845124,1.76537526438052,1.03923515800031,1.69872550095153,0.0893709162653028,0.649146917168528
"1949","IFT74",43.4002308199736,0.414720171789771,0.244144705626415,1.69866543173935,0.0893822402491135,0.649146917168528
"1950","SUCLA2",69.8058816581148,-0.331949260668853,0.195425609539072,-1.69859652197982,0.0893952322371914,0.649146917168528
"1951","CCN1",427.523961480866,0.629681546557642,0.370896130797462,1.69773015750735,0.0895587031434228,0.649813959922209
"1952","AC105345.1",1.64193738985181,2.2290554773745,1.31304478235694,1.69762334638222,0.0895788735711971,0.649813959922209
"1953","NFE2L3",1.94355085787308,-1.82867918795782,1.07752764257701,-1.69710652024143,0.0896765237358117,0.650189235073781
"1954","PPP2R3C",45.0076378569123,-0.5153741448766,0.303764654221078,-1.69662315122915,0.0897679299890963,0.650518878528968
"1955","OSGEPL1",9.49616598687823,0.782094060793836,0.461121600588984,1.69606901909362,0.089872809987178,0.650529296667013

"1956","N4BP2L2",250.990200443245,0.279995597797493,0.165063080673134,
1.69629451150227,0.0898301193829409,0.650529296667013
"1957","PAQR5",3.00996447354478,-1.57269469718701,0.927357928023843,-1
.69588747738249,0.0899071916368181,0.650529296667013
"1958","SOS1",52.7171953412824,0.384998138121116,0.227134555234923,1.6
9502230835338,0.0900711889354881,0.650945867321516
"1959","ZEB1-
AS1",17.4391124848309,0.590037007430435,0.348059537149735,1.6952186176
6024,0.0900339563820934,0.650945867321516
"1960","SERTAD1",265.786511383529,-0.553368321101322,0.326498658288495
,-1.69485634030497,0.0901026765501533,0.650945867321516
"1961","HYOU1",17.4210830117438,-0.622756183919477,0.367502293208842,-
1.69456407600042,0.0901581467454403,0.651014460946168
"1962","CYC1",116.624191512319,-0.305906554537466,0.180549958122532,-1
.69430421207775,0.0902074906003749,0.651038770082216
"1963","ERCC5",47.7619182948171,0.383366985014523,0.226334876042993,1.
69380429440181,0.0903024778052585,0.651392300418982
"1964","ECRG4",7.99274217901372,-1.26134740773676,0.745139496900055,-1
.69276680807318,0.0904998629415757,0.65237435336174
"1965","CPNE3",170.386837295058,0.260228253308271,0.153744270727382,1.
69260455740627,0.0905307630194787,0.65237435336174
"1966","UBAP2L",55.2925861274543,0.373781552809283,0.220900332238573,1
.69208234782372,0.0906302736516242,0.652759244611901
"1967","UBR2",47.1425669901582,0.568676794294986,0.336202508249789,1.6
9147100435216,0.0907468811363431,0.653266820991672
"1968","SYDE2",23.1624778037238,0.744820389096559,0.440494747033721,1.
69087235230876,0.0908611847728609,0.653495787585183
"1969","LANCL1",21.2597410432456,0.575305784851719,0.34030093228275,1.
69057951440964,0.090917139939464,0.653495787585183
"1970","N4BP2",21.95954398618,0.525932724326803,0.311084360470292,1.69
064341110465,0.0909049282612144,0.653495787585183
"1971","BSG",313.241723345563,-0.401549131260259,0.237658559894669,-1.
68960516902159,0.0911035163810027,0.654503192265347
"1972","ADAMTSL2",19.6178087346601,1.21576549667742,0.719976928600784,
1.68861729922396,0.0912927933236136,0.654534659980946
"1973","COLCA1",16.0152523567465,0.719648398025162,0.426139849031333,1
.68876109488706,0.091265222272973,0.654534659980946
"1974","PXN",78.4243123861901,0.359439509459301,0.21279611822032,1.689
12625129352,0.0911952381035869,0.654534659980946
"1975","SLC46A3",7.41917861762639,0.848126325790738,0.502236812277217,
1.68869805051765,0.0912773094315888,0.654534659980946
"1976","PTCD3",47.141560517376,0.3750320772654,0.222197112122402,1.687
83506537568,0.0914428943546773,0.654947589308159
"1977","TLL1",4.42243891605843,-1.29321551463584,0.766118846334609,-1.
68800900907614,0.0914094995635301,0.654947589308159
"1978","CXCL3",37.2092058994544,-1.91693651474235,1.13701070039786,-1.
68594412882094,0.0918065611979708,0.656856290019993
"1979","VIPR2",17.1001486436953,-0.884877093089289,0.524923380614031,-
1.6857261950386,0.0918485490282193,0.656856290019993
"1980","UBE2N",80.1785582075115,-0.349749735953392,0.207462051714979,-

1.68584920982992,0.0918248467027956,0.656856290019993
"1981","DIPK1C",3.06697522797379,2.11476396949808,1.25469766098018,1.6
8547693620948,0.0918965909578735,0.656868111036592
"1982","CCDC14",55.4479944576377,0.433062342149274,0.256992163347091,1
.68511886319422,0.0919656409710479,0.656926363326716
"1983","GJA1",132.025096091174,0.588906200253491,0.349508856008767,1.6
8495358595068,0.0919975267285931,0.656926363326716
"1984","LAMC2",10.9257012469294,0.839444691490669,0.498469436673386,1.
6840444563519,0.0921730774288323,0.657649315421148
"1985","SLC11A1",2.38635636431822,-1.58494860721004,0.941230919236663,
-1.68391047809546,0.0921989710361118,0.657649315421148
"1986","ADGRA3",16.7499582371209,0.569545351027274,0.338268471219617,1
.68370805879068,0.0922381031374576,0.657649315421148
"1987","CENPB",39.7071208593057,-0.453641373401005,0.269501944770833,-
1.68325825547104,0.0923251077654375,0.657938362334472
"1988","ROR1",31.195310166424,0.61589128151861,0.366101892270955,1.682
29472319358,0.0925117038582218,0.65820682051281
"1989","C3orf14",44.1827278584631,-0.50781258335416,0.301876242748444,
-1.68218796792606,0.0925323965311159,0.65820682051281
"1990","DPEP1",13.1799891165772,-1.12960833863515,0.671544964842731,-1
.6821038020882,0.0925487132514834,0.65820682051281
"1991","CNDP2",43.1839992113891,0.406503380159569,0.241586243895767,1.
68264290881958,0.0924442398837541,0.65820682051281
"1992","SPOCK2",3.1186725748009,1.71164119958937,1.01789281800071,1.68
155346940288,0.0926554598586034,0.658635196585253
"1993","ABCD4",52.3623226046898,0.380955899792492,0.226605755934341,1.
68113955544392,0.0927358107942384,0.658875605040851
"1994","YBX1",1164.93494293309,-0.333922854291341,0.198727025368599,-1
.68030922654823,0.0928971668327467,0.659030001178203
"1995","TSTD1",2.40547563954738,-1.63895597709433,0.975140265563716,-1
.68073869470139,0.0928136810909546,0.659030001178203
"1996","TRMT5",23.0492047329403,0.555335308447086,0.330449107108416,1.
68054715991376,0.0928509067311481,0.659030001178203
"1997","DCX",10.9200893053576,-1.16168454938048,0.691504650539739,-1.6
7993743566836,0.0929694891675062,0.659212802509708
"1998","TRMT61B",15.7313303527336,-0.6574713805423,0.391490091239454,-
1.6794074620401,0.0930726599964951,0.659554407336227
"1999","APH1B",37.5034956739041,0.553184583345167,0.329431146550628,1.
67921154128076,0.0931108234650507,0.659554407336227
"2000","KIF5B",174.173317310361,-0.314304649905623,0.187242721978073,-
1.67859474902544,0.0932310506374834,0.659571709119741
"2001","NADSYN1",19.4413677678043,-0.557824520425384,0.332338660485264
,-1.67848218323706,0.0932530057667882,0.659571709119741
"2002","CENPV",13.138438297496,-0.650245839952479,0.38737196726421,-1.
67860840459055,0.093228387502059,0.659571709119741
"2003","CREB3L2",88.2762122067479,-0.317689654935648,0.189303680033019
,-1.67820115742195,0.0933078358938042,0.659630033078516
"2004","APLF",6.67220708374959,0.933885199118319,0.556712140374866,1.6
7750104836852,0.0934445446281862,0.660182275585872
"2005","CCNT1",32.7269829883956,0.432345204919519,0.257758954529415,1.

67732370620777,0.093479199332604,0.660182275585872
"2006","NIPAL3",11.9045854733651,0.859136971090548,0.512497017291751,1.67637457800357,0.093664845228955,0.660834184574989
"2007","OLFML1",140.509044929886,0.307289835458721,0.183294356876628,1.67648279355132,0.0936436637507628,0.660834184574989
"2008","DAP",57.238510751567,-0.395716806900235,0.236098558174542,-1.67606617321098,0.0937252316620737,0.660930916501476
"2009","DBT",27.3141832018383,0.492762059365856,0.294134435186507,1.67529537659676,0.093876292129984,0.661260168675547
"2010","AL137796.1",1.91144078344455,-1.71653540576248,1.0250166915725,-1.67464141791594,0.0940046077851167,0.661260168675547
"2011","ENPP4",2.27626399407353,1.74159363244189,1.03998259331068,1.67463729070475,0.0940054180468839,0.661260168675547
"2012","MED13",75.2730471485774,0.444021134498813,0.264999624751025,1.67555382357988,0.0938256200313821,0.661260168675547
"2013","ERGIC3",175.625817373702,-0.235853346139272,0.140822029025445,-1.67483275004265,0.0939670512537426,0.661260168675547
"2014","PHC3",51.4827752241901,0.434656384779119,0.259883381905454,1.67250549685877,0.0944246848457668,0.66223553116199
"2015","JADE1",50.3894403832489,0.579870704203533,0.346516554198367,1.67342857701274,0.094242956052408,0.66223553116199
"2016","RAB23",25.8115825656609,-0.583139227406622,0.348609409443177,-1.67275814022935,0.0943749185011922,0.66223553116199
"2017","PENK",125.12195576096,-2.27604806473422,1.36066243226972,-1.67274998615016,0.0943765243843936,0.66223553116199
"2018","SLC25A21-AS1",6.10498855892046,0.949901066085765,0.567797169495249,1.67295843853923,0.0943354781660647,0.66223553116199
"2019","HELZ",53.3946822233631,0.431662544120779,0.258065558453308,1.67268560247988,0.094389205022502 5,0.66223553116199
"2020","SERTAD4",11.1938538505866,-0.83546470816563,0.499721578581787,-1.67186037980726,0.0945518571081899,0.6627991567584
"2021","FBLIM1",11.3806470954751,-0.841755136049583,0.503638424990429,-1.67134812254561,0.0946529363967082,0.662910389035786
"2022","TTC14",75.8891968793869,0.470628372618577,0.281593269956777,1.67130547079771,0.0946613564004491,0.662910389035786
"2023","CYP4V2",39.0869607977013,0.498749044729999,0.29858302565065,1.67038646501475,0.0948429258097815,0.663658009940541
"2024","ANKFN1",2.17358529618153,1.83283382661463,1.09731423679648,1.67029075642493,0.0948618511383937,0.663658009940541
"2025","ITPR3",24.5702815575824,0.524488569568105,0.314568832715284,1.66732528788959,0.0954497409381504,0.666319786094635
"2026","COX6A1",329.416980305402,-0.410026466834166,0.245939376378057,-1.66718511233384,0.0954776021176564,0.666319786094635
"2027","CHD8",54.2234368057796,0.492476213501175,0.295343527217108,1.66746912702493,0.0954211583569139,0.666319786094635
"2028","HERC1",61.8372678015163,0.364112180525747,0.218394742554179,1.66722044801705,0.0954705782121622,0.666319786094635
"2029","YWHAH",160.965760091007,-0.340181363348834,0.203942376009531,-1.66802687114391,0.0953103926452065,0.666319786094635

"2030","PITHD1",101.321688617296,-0.356622422926552,0.214001159912034,
-1.66645088780427,0.0956236423934195,0.666739609783817
"2031","SPAG16",57.077458467623,-0.444405029160159,0.266759917828903,-
1.66593629499165,0.0957261035798376,0.666739609783817
"2032","NNMT",1192.67957283552,-0.461746014648536,0.27715685067999,-1.
66600974688399,0.0957114731110049,0.666739609783817
"2033","PLEKHM1",26.5217792865965,0.46896654304142,0.281439224880194,1
.66631550112127,0.0956505908809908,0.666739609783817
"2034","SGSM2",21.4353728326269,0.57673873517039,0.346308986184265,1.6
653877265071,0.0958354264737219,0.667172880466028
"2035","CEP97",19.5237420683974,0.565631291251642,0.339849366240534,1.
6643588231714,0.0960407436544012,0.668273675747578
"2036","UTRN",119.071167603589,0.421871679874795,0.253516832202793,1.6
6407759283349,0.0960969242583963,0.668336172641892
"2037","CRYGS",2.14917095953017,1.75997968527716,1.05816733775613,1.66
323380289666,0.0962656436841948,0.668485821632297
"2038","EXOC3",36.3847099896118,-0.441114624027687,0.265205166867225,-
1.66329573906277,0.0962532512260405,0.668485821632297
"2039","SECTM1",9.76187447601884,0.78963325635988,0.474751933327205,1.
66325443021557,0.0962615163385074,0.668485821632297
"2040","LINC00667",65.3172320535568,-0.317308966041469,0.1908261461784
53,-1.66281703213109,0.0963490661993049,0.668485821632297
"2041","LINC01697",38.597433224867,0.541855547567136,0.325871314769797
,1.66278995114963,0.0963544888383841,0.668485821632297
"2042","B4GALT1",147.701035645425,-0.425167001823013,0.255774863052952
,-1.66227047000703,0.096458555932556,0.668552693100828
"2043","DRAM1",55.0805235596955,0.384944745451984,0.231548005194503,1.
66248353177833,0.0964158626268065,0.668552693100828
"2044","STAMBPL1",12.5734691106295,0.719522599393833,0.432964200019644
,1.6618524103406,0.096542370657697,0.66865835445845
"2045","C21orf62-
AS1",6.72314682189116,0.872335020684643,0.524958012042426,1.6617234153
4649,0.09656824398782,0.66865835445845
"2046","TMEM63A",20.5813936065556,0.634027800645466,0.381877145431557,
1.6602926051753,0.0968556026439284,0.668678001086702
"2047","GMPPA",34.2007651151913,-0.497594618508655,0.299611302484422,-
1.66080055853209,0.0967535089645552,0.668678001086702
"2048","PLK2",61.0188256366527,-0.706201099902315,0.425526337752003,-1
.65959433588313,0.0969960885757123,0.668678001086702
"2049","UBE2H",106.139152762341,0.346850884103795,0.208919917236309,1.
66020975257937,0.0968722633786321,0.668678001086702
"2050","INTS6",165.405261385254,0.347361138678545,0.209211842920436,1.
66033210084884,0.0968476613102723,0.668678001086702
"2051","MED24",18.9079970071137,-0.555685351725724,0.334730147271934,-
1.66009950479389,0.0968944365349075,0.668678001086702
"2052","CANT1",25.8863406452535,0.45441689182711,0.273810471644278,1.6
5960377299765,0.09699418882171,0.668678001086702
"2053","BAIAP2",19.0163811069233,-0.589528640169035,0.355158240272112,
-1.65990415910766,0.096933734641843,0.668678001086702
"2054","UQCR11",335.125451419789,-0.31956282310591,0.192364630684697,-

1.66123482247474,0.0966662944045848,0.668678001086702
"2055","RAD51-
AS1",14.2394927263858,0.817441640214047,0.492738259582396,1.6589774070
0477,0.0971203450313843,0.669208800800195
"2056","LGALSL",22.52595264844,-0.686896300671924,0.414133607359479,-1
.65863452872512,0.0971894596005822,0.669359313202453
"2057","CHL1-
AS2",4.91584130837473,1.36736258567282,0.824908607420453,1.65759282103
827,0.0973996796631747,0.669834736223282
"2058","BCL6",91.3561520948971,0.473052598401571,0.285537900305783,1.6
567068606128 2,0.0975787552859191,0.669834736223282
"2059","BTN3A3",35.320367338212,0.501375668829503,0.302648901964987,1.
65662477403439,0.0975953604271066,0.669834736223282
"2060","UBAC1",56.2639999455777,-0.339043463723008,0.204741781980161,-
1.65595639758503,0.0977306491238122,0.669834736223282
"2061","TTC17",71.3603432317385,0.369410985608774,0.22282388234672,1.6
5786082586049,0.0973455607070097,0.669834736223282
"2062","C3AR1",1.82804445178538,-2.20178781164706,1.32980807402658,-1.
65571848648819,0.0977788417919155,0.669834736223282
"2063","AC073896.2",6.84014306694299,-0.880852961966328,0.531906503521
315,-1.65602968968216,0.0977158064714094,0.669834736223282
"2064","KLF5",24.624230132746,-0.639872540072,0.386455882067675,-1.655
74537680332,0.0977733937801856,0.669834736223282
"2065","DAAM1",122.177163863991,-0.36931711398998,0.222843762505279,-1
.65729168201974,0.0974605181903595,0.669834736223282
"2066","SMAD2",62.7389761563272,0.323047505397041,0.194975285300647,1.
65686386815083,0.0975470008130152,0.669834736223282
"2067","ENTPD6",16.7614447686769,-0.675479431921503,0.407618424934111,
-1.65713665183484,0.09749185047709,0.669834736223282
"2068","FKRP",7.71431650912201,0.847135028423937,0.511741360378002,1.6
5539683522589,0.0978440275488372,0.669957171224146
"2069","ANK2",275.464391046587,-0.362445429655854,0.218999458954082,-1
.65500605063983,0.0979232705604608,0.670175694120892
"2070","LOXL2",71.7293016997981,-0.869716785136903,0.525582944014344,-
1.65476599848181,0.0979719735784506,0.670185094623604
"2071","IER5L",124.651852142026,-0.356658259133135,0.215612855785275,-
1.65416045269733,0.0980949158079225,0.670702080077346
"2072","CMTM8",13.4779480260709,0.865498579852881,0.523392229788843,1.
65363284090415,0.0982021357632155,0.670730041886703
"2073","LINC00377",11.2935409722558,1.17528856836043,0.710726962812513
,1.65364286126073,0.0982000985796305,0.670730041886703
"2074","PDXK",58.5575968561705,-0.358822844139283,0.217033068379432,-1
.65330954779649,0.0982678808225758,0.670730041886703
"2075","CD99L2",37.0197840321346,0.42157603002402,0.255004786257618,1.
65320830330661,0.0982884771832563,0.670730041886703
"2076","PRRX2",218.029806657951,-0.412322773747108,0.249583254879529,-
1.65204502179496,0.0985253732437819,0.671699222499736
"2077","CBX4",21.1882746314258,0.559110677667098,0.338402216408774,1.6
5220749320306,0.0984922594442426,0.671699222499736
"2078","CYBRD1",689.659898074321,0.373995531379532,0.226533138443352,1

.65095285373912,0.0987482021865315,0.672547248692906
"2079","NR2F1-
AS1",8.16381903336337,0.967899149653762,0.586439645997591,1.6504667722
5799,0.0988475039656482,0.672547248692906
"2080","FAM86B1",3.25330064851856,1.28677990129676,0.779778848953065,1
.65018569434705,0.0989049618485932,0.672547248692906
"2081","ZDHHC16",19.9923291681779,-0.510131229992189,0.309206445063221
,-1.64980788122927,0.0989822363189276,0.672547248692906
"2082","USP31",11.7300843605681,0.679618253463858,0.411731827256491,1.
65063327261432,0.0988134805694777,0.672547248692906
"2083","ZADH2",38.0604642074976,0.418404125477644,0.253576438734709,1.
65001183692534,0.0989405150863834,0.672547248692906
"2084","MRPL4",66.1038115335196,-0.350751563282027,0.212560653940979,-
1.65012459633955,0.0989174550161703,0.672547248692906
"2085","PARP15",3.12273536258474,1.38019869916716,0.837371762579132,1.
64825082579344,0.0993012109879329,0.674067664232564
"2086","GJB2",2.29822361596478,2.82296940953055,1.71267153604024,1.648
28418650394,0.099294368204135,0.674067664232564
"2087","KBTBD6",15.2600062834644,0.604584703408658,0.36697225782947,1.
6474943010259,0.099456486644675,0.674798203588212
"2088","MGST3",668.911723229949,-0.385495812073485,0.234072955099897,-
1.64690453841182,0.0995776689194182,0.674973571995673
"2089","GAS1RR",26.9584108258608,0.578260191205018,0.351076602901763,1
.647105464806,0.0995363700735108,0.674973571995673
"2090","SELENOW",511.757190576377,-0.394203523945469,0.239422733805597
,-1.64647490937744,0.0996660216639616,0.675249218546266
"2091","IRS1",39.4546480735779,0.464120276519973,0.282071392812541,1.6
4540002405851,0.0998873446062606,0.675778690695007
"2092","ANXA6",177.05799824839,0.382757614752338,0.232615475496964,1.6
4545206605282,0.0998766199416975,0.675778690695007
"2093","LMO7",8.44351078989977,0.839829716650746,0.510271145063563,1.6
4584990700607,0.0997946643624197,0.675778690695007
"2094","ZNF438",21.067098526106,0.542705509162148,0.329892003725889,1.
64510052693817,0.0999490819640169,0.675873448238051
"2095","UBR3",39.7549713394186,0.473036263991294,0.287609506839146,1.6
4471706512766,0.100028171946143,0.67608540083885
"2096","ADH1C",50.1752547627833,0.504263067408745,0.306676232909276,1.
64428479711345,0.100117388160963,0.676291532157434
"2097","CYP11A1",5.47131891775039,0.93915663760359,0.571251939289915,1
.64403229645223,0.100169531364922,0.676291532157434
"2098","ARL2BP",107.32348172016,-0.314738176335728,0.191488018377567,-
1.64364422903548,0.100249712288026,0.676291532157434
"2099","FSTL3",78.1831450159584,-0.431622728694402,0.262571310338661,-
1.64383050127488,0.100211219086449,0.676291532157434
"2100","TYMS",7.69997952652364,1.39823912315261,0.851041085908447,1.64
297487665952,0.10038813140717,0.676902828916917
"2101","C2orf76",28.1434767045563,-0.499196313421041,0.304145938536032
,-1.64130520967618,0.100734075215463,0.676966212021833
"2102","KANSL1L",22.8209898200111,0.555946795454025,0.33890288155926,1
.6404310075387,0.100915582584535,0.676966212021833

"2103","CMTM7",16.0808035516882,-0.625503831680683,0.381741036460637,-1.63855538686678,0.101305890061742,0.676966212021833
"2104","PTH1R",31.8872363910278,0.546958630942003,0.333758002427081,1.63878806489891,0.101257405676119,0.676966212021833
"2105","ARRDC3",170.71635836819,0.569550092637376,0.347534237799225,1.638831604748,0.10124833509439,0.676966212021833
"2106","EPB41L4A",17.3318903801123,0.630247995926608,0.383888936839013,1.64174565986752,0.100642724770798,0.676966212021833
"2107","SNHG4",1.65163960667812,-1.9716342008326,1.20261477892309,-1.63945615452867,0.101118294926705,0.676966212021833
"2108","HEBP2",148.359411046837,-0.378849741089284,0.230709233243474,-1.64210914215762,0.100567387389976,0.676966212021833
"2109","CCL26",12.8270175883899,-1.02255166572392,0.623949640863833,-1.63883685277587,0.101247241825493,0.676966212021833
"2110","COL15A1",281.97841611517,0.724312454150924,0.441614358474867,1.6401469749588,0.100974611275613,0.676966212021833
"2111","SURF4",146.43657456154,-0.295195816477303,0.179930678004081,-1.64060859299718,0.100878690095125,0.676966212021833
"2112","AL117336.2",4.34305283613774,1.19587663805118,0.729581638809523,1.63912655477806,0.101186905703852,0.676966212021833
"2113","ZNF503",223.018394708297,0.295010224518292,0.179776448681835,1.64098371439295,0.10080079582905,0.676966212021833
"2114","SYT7",3.43000099842715,-1.40958631823865,0.860198243974272,-1.63867611694498,0.101280730571969,0.676966212021833
"2115","ATM",66.9214861920195,0.388058728877905,0.236470281033174,1.64104650775742,0.1007877614194,0.676966212021833
"2116","GNAL",9.41039359976354,0.742447173865949,0.453101319466268,1.638589741342,0.101298730269839,0.676966212021833
"2117","MRPL39",24.9464252696764,-0.49911021534153,0.304031493946949,-1.64163984744495,0.100664664498146,0.676966212021833
"2118","NBDY",94.4423176972378,-0.427037990698697,0.260241528354304,-1.64092946040998,0.100812058747711,0.676966212021833
"2119","PBDC1",96.2250373587997,-0.44714866155978,0.272279835478266,-1.64223935560396,0.100540409557331,0.676966212021833
"2120","ARHGAP29",85.1093218059678,0.480681069560476,0.293645213431505,1.63694501927442,0.101641958127278,0.678891569378425
"2121","TXLNG",52.8026075292848,0.37107160483346,0.226773286665073,1.63631091779123,0.101774532279928,0.679456566281842
"2122","RABGAP1L",66.4922939647689,0.481998077217701,0.294776526362206,1.63513046023701,0.102021701958491,0.67989269119301
"2123","PAX8",1.77824690357856,-2.06411222399627,1.26203249688644,-1.63554601730831,0.101934636255329,0.67989269119301
"2124","WDFY3",39.960797834109,0.428587315904981,0.262290090251639,1.63402023878903,0.102254601037793,0.67989269119301
"2125","SDHAF3",42.4152829537407,-0.450740595509521,0.27562384193595,-1.63534690012146,0.101976347035828,0.67989269119301
"2126","COX6C",487.99265050084,-0.38642017575854,0.236515933118166,-1.63380187822476,0.102300457844248,0.67989269119301
"2127","PRG2",3.91190808252997,-1.25151028005544,0.765736227934827,-1.63438823239529,0.102177357560284,0.67989269119301

"2128","KARS",54.9956572443324,0.413420088752909,0.253056167467328,1.6
3370880421749,0.102320008822903,0.67989269119301
"2129","ZNF407",18.6500230729103,0.608045483194197,0.372137918739846,1
.63392509221634,0.102274580280806,0.67989269119301
"2130","SNRPB",178.289639891942,-0.352180841495967,0.215499766220994,-
1.63425161739993,0.102206028227645,0.67989269119301
"2131","BACH1",43.8669077896258,-0.420331159674884,0.257099964574695,-
1.63489388405873,0.102071294800684,0.67989269119301
"2132","PROCR",301.540897261882,0.589787134675867,0.361083022421453,1.
63338373186506,0.102388316333832,0.680027466832578
"2133","WDR34",31.0670548558081,-0.461853387442808,0.282805616923281,-
1.63311249779066,0.102445338560958,0.680087198323098
"2134","GSK3A",36.2676701746181,-0.400290838417557,0.245164336398921,-
1.63274497546095,0.102522644022366,0.680281461741658
"2135","AC080038.1",13.1912847757777,-0.757572668336089,0.464197584697
871,-1.63200476113887,0.102678483205584,0.68099640383657
"2136","EIF3M",170.828326587663,-0.325671138494338,0.199582865582148,-
1.63175900668834,0.102730264153403,0.681020852252897
"2137","COL16A1",107.120442877173,0.373835296078315,0.229949290096926,
1.62572928979576,0.104007256342947,0.682898144496703
"2138","ZNF326",56.4540272683618,0.355636832335167,0.218767249877745,1
.62564018395765,0.104026221587527,0.682898144496703
"2139","METTL21A",15.5581441054088,-0.630509807653513,0.38781366807496
,-1.6258060495476,0.103990921036124,0.682898144496703
"2140","ANKRD28",154.344527974553,-0.443137116166823,0.272185696416241
,-1.62806907931398,0.103510239060276,0.682898144496703
"2141","LAMB2",335.282312991095,0.278838340242192,0.171370260438423,1.
62711044220175,0.103713643659858,0.682898144496703
"2142","THAP9-
AS1",16.4098641378743,-0.654342959509179,0.402087253312053,-1.62736558
823802,0.103659475518097,0.682898144496703
"2143","FNIP2",42.9640779751882,-0.599336356774494,0.368604000236975,-
1.62596270357669,0.103957589686527,0.682898144496703
"2144","AL590617.2",2.67130933586867,-1.57443870207373,0.9682896832040
83,-1.62599966661205,0.103949726282387,0.682898144496703
"2145","MDFIC",98.8915992072015,-0.388271093516442,0.238184045637096,-
1.63013056763685,0.103073904490673,0.682898144496703
"2146","RNF170",46.8211860662323,0.373674460555284,0.229541248375811,1
.62791856888176,0.103542153517195,0.682898144496703
"2147","AC091563.1",7.75873319257451,0.920255981278461,0.5654406354907
44,1.62750238224349,0.10363044306882,0.682898144496703
"2148","AC139768.1",3.75557628640457,-1.20835784661035,0.7423544219891
54,-1.62773711695894,0.103580639251577,0.682898144496703
"2149","EP400",39.4982838667266,0.526587345495972,0.323511876888726,1.
62772183377087,0.103583881316441,0.682898144496703
"2150","BTBD1",38.0728012838592,-0.407932250336419,0.250579391951004,-
1.62795610269571,0.103534194059292,0.682898144496703
"2151","CDK12",65.2274161364813,0.358903085009039,0.22061714974794,1.6
2681407777724,0.103776590805526,0.682898144496703
"2152","EPB41L3",78.7736892205202,0.320442832601144,0.196830445681337,

1.62801456599826,0.103521797241951,0.682898144496703
"2153","ZNF532",39.980976321486,0.40485026703639,0.248621748043878,1.6
2837833062352,0.103444689488129,0.682898144496703
"2154","MYDGF",280.517240871541,-0.417510725890991,0.256793875578353,-
1.62585935879767,0.103979577449602,0.682898144496703
"2155","DIDO1",48.9692652364327,0.453850320987951,0.279040794870224,1.
62646584059161,0.103850594401981,0.682898144496703
"2156","DGCR2",31.8451200941604,0.430386462980431,0.264697724500495,1.
62595452527068,0.103959329578417,0.682898144496703
"2157","ARSA",45.6261348563497,0.484229701634417,0.297320442637999,1.6
2864583860447,0.103388014541842,0.682898144496703
"2158","ARPC2",527.636550292291,-0.365253458621924,0.22497753401543,-1
.62351081062553,0.104480254272597,0.683241756209676
"2159","MUSK",2.29392835088497,1.76432224589279,1.08669865090865,1.623
56164187518,0.104469397544112,0.683241756209676
"2160","IL17D",37.4635308829446,-0.528336741958887,0.325201401861872,-
1.62464472457377,0.104238281542197,0.683241756209676
"2161","NTRK3",3.32113432364509,-1.67587342236888,1.03226552223117,-1.
62349064874955,0.104484560769676,0.683241756209676
"2162","SGCA",9.01765001519144,1.46473827103648,0.90158438003279,1.624
62693839396,0.104242073602914,0.683241756209676
"2163","RPL28",4514.11606851281,-0.365948370405556,0.225328492798618,-
1.62406611725137,0.104361698367682,0.683241756209676
"2164","CTNNBL1",63.0424066962403,-0.314132847888836,0.193451247385302
,-1.62383469806824,0.104411092512904,0.683241756209676
"2165","PSMA7",494.753781489248,-0.328566585863395,0.202399298216862,-
1.62335832563684,0.104512827962582,0.683241756209676
"2166","MT-
ATP6",18023.5547180776,-0.267170805601869,0.164481539542828,-1.6243209
1980938,0.104307334739977,0.683241756209676
"2167","AL391807.1",2.33069416454194,1.67794164080277,1.03407309788518
,1.62265283202358,0.104663639784209,0.683911924016795
"2168","ZNF142",7.50118235951106,0.80109766791348,0.494417027979895,1.
62028737397381,0.105170559855293,0.684844319972019
"2169","PYURF",191.509772054335,-0.299944438822969,0.185110127247581,-
1.62035672106583,0.105155671027893,0.684844319972019
"2170","AL391840.3",8.23548149721906,0.828666009365715,0.5114590666279
39,1.62020005790322,0.105189309003621,0.684844319972019
"2171","CFL1",775.870173295369,-0.392953057694943,0.24253641077702,-1.
62018171389619,0.105193248300787,0.684844319972019
"2172","CALM1",1100.10473856164,-0.275363562640757,0.169948944886974,-
1.62027226955654,0.105173802997033,0.684844319972019
"2173","CDR2",30.7925061065499,-0.47924509741324,0.295524051485713,-1.
62167882784461,0.104872134420586,0.684844319972019
"2174","VMP1",366.985606800141,-0.509976954059797,0.314651699118857,-1
.62076656661294,0.10506771130526,0.684844319972019
"2175","TBL1X",30.1103933252028,0.457746863628666,0.282520339802444,1.
62022622494632,0.10518368994549,0.684844319972019
"2176","NDUFS5",452.771287383948,-0.351185040188777,0.217114495636233,
-1.61751079383099,0.105768067651065,0.684847563545193

"2177","LINC01341",2.43356242936717,1.45602252808866,0.900334919527965
,1.6172009954385,0.105834901545125,0.684847563545193
"2178","MAPRE3",11.9685360696,-0.741025815731912,0.457853117705663,-1.
61847934867245,0.105559333843599,0.684847563545193
"2179","PXDC1",162.038056338502,-0.448149351894612,0.276849587745425,-
1.61874668315094,0.1055017780266,0.684847563545193
"2180","LINC02544",1.61631682751426,-1.88452099744053,1.16565759284032
,-1.61670203069546,0.105942615376863,0.684847563545193
"2181","WDR86-
AS1",6.4007302851828,1.40924220721394,0.87192707324453,1.6162386172618
8,0.106042732437565,0.684847563545193
"2182","PCMTD1",163.125746454577,0.336647319815914,0.207841988886894,1
.61972718611308,0.105290893597556,0.684847563545193
"2183","PTBP3",58.5410171240363,0.500438991542801,0.309333409413893,1.
61779806614166,0.10570612330678,0.684847563545193
"2184","C9orf78",89.1456650088595,-0.287351349877367,0.177686403043577
,-1.61718254720309,0.105838882504179,0.684847563545193
"2185","FBXW5",101.169126691079,-0.330002098077267,0.204090601202164,-
1.61693922274441,0.105891400793246,0.684847563545193
"2186","RPS13",3485.84348453011,-0.408651758354071,0.2528443392964,-1.
61621873557163,0.106047029408328,0.684847563545193
"2187","APAF1",19.3432405323046,0.65112701018048,0.402717701612555,1.6
168323557004,0.105914473128756,0.684847563545193
"2188","SOS2",39.1803678705884,0.458724609740153,0.283513781519176,1.6
1799757063706,0.105663121215708,0.684847563545193
"2189","TMED10",268.904155109117,-0.264719466695862,0.163588589333917,
-1.61820251506367,0.105618961029062,0.684847563545193
"2190","RPL4",1295.33881905369,-0.288269383226316,0.178162641478409,-1
.61801251280421,0.105659901072877,0.684847563545193
"2191","KDM4B",26.9727919664318,0.458509754631907,0.283406949559856,1.
61784936941029,0.105695063845093,0.684847563545193
"2192","ZC3H4",25.4700166459404,0.519812268527296,0.321012249764667,1.
61929106726727,0.10538465174272,0.684847563545193
"2193","TTC28",61.723842613995,0.412384236623297,0.255166354868677,1.6
1613876106641,0.106064315455834,0.684847563545193
"2194","AC005332.7",1.72696971872134,1.89784061423765,1.17448813560868
,1.61588742933883,0.106118653965639,0.684886116751797
"2195","MAP3K2",111.838921426644,-0.420755116049596,0.260508337795283,
-1.61513109181266,0.106282309094347,0.685005688109217
"2196","STK3",50.6977232783685,0.385010269787537,0.238351286065939,1.6
1530603061662,0.106244438332079,0.685005688109217
"2197","METTL2A",20.7044486399849,-0.505665080872805,0.313052674107818
,-1.61527155873663,0.10625189996008,0.685005688109217
"2198","ZNF507",27.7213599079299,0.610405901631357,0.377999710263215,1
.61483166536374,0.106347153784896,0.685111782344917
"2199","MBD5",39.0166505823975,0.488586259535089,0.303114547826648,1.6
1188653939009,0.106986631837399,0.685726008718908
"2200","SPATS2L",186.625390876712,-0.256988091318167,0.159284879065342
,-1.61338661162398,0.106660540024313,0.685726008718908
"2201","PAIP2",176.145118745758,0.226658226353347,0.14061222097632,1.6

1193831360872,0.106975363800467,0.685726008718908
"2202","PTGDS",1306.40811891595,1.51412880664064,0.939736419579963,1.6
1122712187468,0.107130228420254,0.685726008718908
"2203","ABI1",62.976511423219,0.355297922040828,0.220457404009284,1.61
163977974568,0.107040349033385,0.685726008718908
"2204","PSMC3",163.48833304238,-0.34144681873744,0.211829160686767,-1.
61189714216136,0.106984324194989,0.685726008718908
"2205","SDHAF2",45.910777976274,-0.397761763574736,0.246801558143139,-
1.61166633860571,0.107034566399555,0.685726008718908
"2206","ZBTB44",81.7561879385649,0.394924128528163,0.245134673387705,1
.61104964495802,0.107168902351338,0.685726008718908
"2207","TMEM30B",18.1162645495585,-0.773306890896851,0.479079927265846
,-1.61415005489832,0.106494882360192,0.685726008718908
"2208","AC022467.1",2.97330698632741,1.78000330167988,1.10419430584786
,1.61203810982624,0.106953647005286,0.685726008718908
"2209","STAT3",273.256854369078,-0.261271426731434,0.162141494953048,-
1.61137916488985,0.107097105575628,0.685726008718908
"2210","COX11",70.4354149506229,-0.377706444646891,0.234185383104444,-
1.61285234646109,0.106776590323989,0.685726008718908
"2211","PLEKHF1",70.9312148313841,0.429350136706608,0.266076305652374,
1.61363536544118,0.10660654113285,0.685726008718908
"2212","ZNF181",23.0770376480841,0.516037096006148,0.320145690160044,1
.61188206453186,0.106987605780708,0.685726008718908
"2213","LRCH2",11.1249290117145,0.736052763260463,0.456399421121917,1.
61273816134803,0.1068014059941,0.685726008718908
"2214","PTGER4",66.8216282675456,0.481203241230414,0.298783908137538,1
.61053935009413,0.107280162142895,0.685818102006047
"2215","ADAMTS1",312.767842837141,0.702801698197307,0.436370071668592,
1.61056347313172,0.107274900527844,0.685818102006047
"2216","DAP3",56.8393603608406,-0.342132775697241,0.212528960835802,-1
.60981719550951,0.107437770132082,0.686034104241197
"2217","FAM160B1",44.6968539827762,0.451143833917597,0.280251402829455
,1.60978260719765,0.1074453235231,0.686034104241197
"2218","DBX2",9.55414074916416,0.722384986753868,0.44876475620322,1.60
971862600266,0.107459296836651,0.686034104241197
"2219","RHOBTB1",9.15419759763918,0.808917397146913,0.502713640710018,
1.60910174628328,0.107594095483413,0.686275852272582
"2220","TNFSF13B",132.00685927965,0.420512577612586,0.261324731810217,
1.60915721485549,0.107581969186429,0.686275852272582
"2221","OSTM1",36.7365367096644,0.44976887864702,0.27979123096721,1.60
751599359356,0.107941223820724,0.687122456356498
"2222","ENTPD1",10.4873418422559,1.06645841530555,0.663436269118786,1.
60747680666009,0.107949813227472,0.687122456356498
"2223","HIF1AN",17.1805792095406,0.566488133457989,0.352305367946558,1
.60794635846684,0.107846927489475,0.687122456356498
"2224","KNTC1",5.62208514761597,1.02416704564008,0.636882937205898,1.6
0809308243247,0.10781479402562,0.687122456356498
"2225","CHEK2",20.0813454328672,0.588633269148844,0.366205022098237,1.
60738721106599,0.107969453770707,0.687122456356498
"2226","PVT1",5.53910165455941,-1.00398656472475,0.624904307290393,-1.

60662449116102,0.108136766660302,0.687156921585137
"2227","TRIM66",5.52134131888523,1.00300933845689,0.624185546144171,1.
60690894663111,0.108074343539672,0.687156921585137
"2228","SLC15A3",13.2842216786285,-0.586973757790027,0.36537932789662,
-1.60647774237547,0.108168981512237,0.687156921585137
"2229","C16orf58",32.4047953304185,-0.442280362166758,0.27525307407986
2,-1.6068135247726,0.108095280474368,0.687156921585137
"2230","SLC25A33",16.2291754446171,-0.813931261737708,0.50693545189174
8,-1.60559151801345,0.108363690336002,0.687162615054404
"2231","HSPA4L",2.90155835324741,1.56410474314084,0.974143135737471,1.
60562106918378,0.108357193299109,0.687162615054404
"2232","TMED9",245.221744683436,-0.429796981508247,0.267726720384381,-
1.6053570629453,0.108415247891431,0.687162615054404
"2233","SIM1",10.5389807758329,0.850027498522879,0.529558822147071,1.6
0516162317244,0.108458240668883,0.687162615054404
"2234","NUMBL",100.523185225822,-0.42577990684061,0.265119145848218,-1
.60599456323072,0.108275104518569,0.687162615054404
"2235","MIF",734.062207529962,-0.397868472193378,0.247870139417242,-1.
6051488619355,0.108461048350748,0.687162615054404
"2236","POLR3G",5.69263817963098,0.870343531090658,0.542633157371454,1
.60392618708862,0.108730323750503,0.687330975137109
"2237","SPOCK1",192.624513695084,-0.492004872998165,0.306731389223468,
-1.60402518387095,0.108708501568053,0.687330975137109
"2238","RPP30",51.9668349179081,0.409529508407117,0.255261319359953,1.
60435395944039,0.108636053360952,0.687330975137109
"2239","DLGAP1",2.68392605653868,1.45982818841691,0.909988029155065,1.
60422790371471,0.108663826186054,0.687330975137109
"2240","PSMG1",31.6344427119963,-0.481357932179985,0.30006751163884,-1
.60416544114028,0.108677590134384,0.687330975137109
"2241","KLHL29",22.199325735986,0.655755314902417,0.409630771066641,1.
60084486132448,0.109411284596539,0.687334453128302
"2242","CSRNP1",91.4757774919561,-0.602490904980379,0.375724595529333,
-1.6035439578598,0.108814612303733,0.687334453128302
"2243","TMEM115",66.9251698147353,-0.349985673328259,0.218343112949552
,-1.60291601873939,0.108953196588557,0.687334453128302
"2244","MRAS",38.8932978090142,-0.462000734216197,0.288629972359784,-1
.60066790859926,0.109450492594482,0.687334453128302
"2245","ATP1B3",120.407988925478,-0.446825257576162,0.278870189195687,
-1.60226971145568,0.109095980416114,0.687334453128302
"2246","NCEH1",8.50209671388805,-0.868895322075197,0.542480275671221,-
1.6017085985294,0.10922006286083,0.687334453128302
"2247","DNAJB11",75.6293356103981,-0.3690581384257,0.230570607146413,-
1.60062959886013,0.109458982472057,0.687334453128302
"2248","JMY",30.8492566473084,0.579632427716969,0.362109001758954,1.60
07125614149,0.109440597675424,0.687334453128302
"2249","BTAF1",25.6635127969469,0.472331448924998,0.294806494482013,1.
602174503499,0.109117026496208,0.687334453128302
"2250","SMPD1",31.0897402384446,0.440974323811527,0.27523403514661,1.6
0217948182401,0.109115925939025,0.687334453128302
"2251","RESF1",48.0658049261342,0.418962724601634,0.261454880393134,1.

60242839594986,0.109060909740747,0.687334453128302
"2252","NCKAP5L",40.1606960750626,0.520265430690113,0.324819600508754,
1.60170577722292,0.109220687035991,0.687334453128302
"2253","RILPL2",37.7050469019974,-0.430694377003846,0.268761197890018,
-1.60251695700543,0.109041340841831,0.687334453128302
"2254","DLEU2",22.4973805960433,0.488900853832367,0.305324812796035,1.
60124835369658,0.109321922991023,0.687334453128302
"2255","RFWD3",8.58221194201712,0.745757729713036,0.465612385467812,1.
60167073082421,0.109228440803667,0.687334453128302
"2256","GRB10",11.5509879251542,0.713718091244551,0.44618092700259,1.5
9961586892397,0.109683825868921,0.688441034709185
"2257","INPP5B",12.8618244793592,0.653033650978427,0.408999327375019,1
.59666191915188,0.110341088518498,0.690161425342235
"2258","L1TD1",3.20693677206762,-1.44029901445315,0.902332897449122,-1
.59619472871359,0.110445324140219,0.690161425342235
"2259","PACC1",4.06191037359671,-1.0851018233931,0.679185631832906,-1.
59765132319533,0.110120597522431,0.690161425342235
"2260","CCN2",655.316250821713,-0.591299863171274,0.370328007936989,-1
.59669225793984,0.110334322269917,0.690161425342235
"2261","FRMD3",2.37535084240187,-1.48668441590984,0.931156672099217,-1
.59659964907756,0.110354977206272,0.690161425342235
"2262","PHB2",211.18173283095,-0.398708831404287,0.249731179184809,-1.
59655207133439,0.11036558985409,0.690161425342235
"2263","DCT",1.83101535056809,1.90554255963045,1.19261902495761,1.5977
7977690586,0.110091996863157,0.690161425342235
"2264","SNAI3-
AS1",2.21738918299702,1.63369744975333,1.02287937393148,1.597155531129
88,0.110231042386048,0.690161425342235
"2265","MPRIP",90.1549754378445,0.317399042425309,0.198832504928818,1.
59631365374056,0.110418783206873,0.690161425342235
"2266","ATP7A",20.4016638987311,0.506517331105077,0.317194883222373,1.
5968647601102,0.110295856541879,0.690161425342235
"2267","GLRX2",39.0751110386768,-0.421872071058153,0.264717236090807,-
1.59367057955167,0.111009837071561,0.69113816723191
"2268","TFB2M",15.1830786634628,-0.578746269666653,0.362872872829796,-
1.59490089505288,0.11073439945686,0.69113816723191
"2269","GTDC1",24.3822916231761,0.54276130248633,0.340660312354452,1.5
9326250461955,0.111101314445503,0.69113816723191
"2270","PLCD4",1.64325187813184,1.80041659434633,1.12940513753798,1.59
412821361084,0.110907320866435,0.69113816723191
"2271","USP40",24.2774088220295,0.464588458569033,0.291562930336352,1.
59344145030054,0.111061193212759,0.69113816723191
"2272","MAP4",174.137547260854,-0.331799605493924,0.208026639541346,-1
.59498613362919,0.11071533662724,0.69113816723191
"2273","RBM5",97.3960776875758,0.305340880407693,0.191678181147705,1.5
9298715471637,0.111163072719279,0.69113816723191
"2274","IFT80",40.4130803111941,-0.512073461152841,0.32147668771085,-1
.59287898851758,0.11118734074536,0.69113816723191
"2275","SRD5A1",6.32744516141813,-0.904750965859109,0.567841984990837,
-1.59331467164005,0.111089616948482,0.69113816723191

"2276","HLA-
DPA1",94.3987621782531,-0.508674074209128,0.319255208376941,-1.5933148
8057837,0.111089570099853,0.69113816723191
"2277","TMEM126A",55.2696229675896,-0.393173336287629,0.24657036996134
8,-1.5945684647724,0.110808769220769,0.69113816723191
"2278","RPLP0",3335.37239263055,-0.30105340525523,0.188893433901855,-1
.59377379634938,0.110986708584817,0.69113816723191
"2279","MR1",38.6450904628242,0.400654298188791,0.251842892200513,1.59
088983885159,0.111634369727664,0.691423247971339
"2280","TMEM243",118.139769225223,-0.378940771249065,0.238082643877409
,-1.59163542993997,0.111466644413245,0.691423247971339
"2281","CLU",1083.2408119113,-0.66751278182296,0.419551824909183,-1.59
101389194875,0.11160644941696,0.691423247971339
"2282","AL392086.3",3.19075913566973,-1.88291378189517,1.1835091778339
5,-1.59095832728671,0.111618954530845,0.691423247971339
"2283","NAP1L4",96.3978645517332,0.321511818473169,0.202117201877972,1
.59071971848928,0.111672667239439,0.691423247971339
"2284","HAUS4",42.4385310859548,-0.444726645724726,0.279487672252863,-
1.59122097278898,0.111559854541901,0.691423247971339
"2285","NF1",91.394535235601,0.365211098103957,0.229380374517138,1.592
16366645469,0.11134793474999,0.691423247971339
"2286","DMD",40.6621931841297,0.426733656017681,0.268088782442813,1.59
176244574392,0.111438091258949,0.691423247971339
"2287","AR",104.440280344251,0.448460323633527,0.281794386046023,1.591
44520203566,0.111509418446152,0.691423247971339
"2288","RTN4",835.950419706948,-0.256784219056025,0.161555511571311,-1
.58944883129338,0.111959097550614,0.692354511927673
"2289","LST1",7.817678045331,-0.807534695344484,0.508070922900216,-1.5
8941332586963,0.111967108013414,0.692354511927673
"2290","SLC25A22",6.14903693574909,-0.924157335207117,0.58152914884646
2,-1.58918488787759,0.112018657261744,0.692354511927673
"2291","IDH3A",26.8930800375945,-0.446755429072525,0.281106569614397,-
1.58927423747283,0.111998492430532,0.692354511927673
"2292","RDH13",12.7757622043213,0.687047142705401,0.432580563360134,1.
58825245722706,0.112229263447568,0.69335356475461
"2293","EXO5",5.65937766249057,0.956478609550972,0.603341937516214,1.5
8530105413942,0.112897949667278,0.694581462784304
"2294","FMO2",92.0182556275875,1.21320448443722,0.764873520306932,1.58
615045785658,0.112705183053841,0.694581462784304
"2295","AC137936.1",3.33459387818415,-1.21179184233696,0.7645821932064
63,-1.58490722528471,0.11298741467076,0.694581462784304
"2296","PIGX",36.3808787860555,-0.410770780300745,0.259147177003248,-1
.58508684158113,0.11294660480988,0.694581462784304
"2297","ZCCHC4",7.6844929801276,0.769014117289243,0.485364666733875,1.
58440481970826,0.113101625831608,0.694581462784304
"2298","FKBPL",8.05252289712933,-0.786415099800576,0.496361770916119,-
1.58435871954666,0.113112110272168,0.694581462784304
"2299","CITED2",198.713921215194,0.538582120076333,0.34006092241437,1.
58378127146307,0.113243502680985,0.694581462784304
"2300","DDX21",166.327939512186,-0.308769204314718,0.194983090288842,-

1.58356913852025,0.113291801554592,0.694581462784304
"2301","COX15",25.4339352983934,0.460919229520632,0.291063733605846,1.58356805161031,0.113292049066311,0.694581462784304
"2302","PRH1",24.5899212559358,0.502543357431504,0.317307909245858,1.58377192243929,0.113245630944554,0.694581462784304
"2303","CKB",101.680365459864,-0.434602572416944,0.274276406137911,-1.58454231822776,0.11307035944679,0.694581462784304
"2304","UTP18",30.7211499354112,-0.443929521234186,0.280155499332573,-1.5845825703646,0.113061207627681,0.694581462784304
"2305","P4HB",461.597855953778,-0.324526171006989,0.20485611023815,-1.58416642115023,0.113155852459319,0.694581462784304
"2306","INO80C",11.1992604081981,-0.675349922530668,0.425559369460383,-1.58696992945314,0.112519455320166,0.694581462784304
"2307","ALPK2",1.67335256784142,2.05809647410452,1.29941309399997,1.58386619590626,0.113224171453443,0.694581462784304
"2308","PLIN3",130.056003842465,-0.337320147080556,0.213023895661044,-1.58348501717989,0.113310958971168,0.694581462784304
"2309","GDF15",9.12070245771294,-1.31378290633035,0.828883517167005,-1.58500305425382,0.11296564032926,0.694581462784304
"2310","SCO2",45.5270772511723,-0.463959913922483,0.292868587505598,-1.58419145554016,0.11315015712289,0.694581462784304
"2311","C2orf88",7.32465893862843,0.929736233141917,0.587864067824172,1.58154968815001,0.113752407278356,0.696555693119644
"2312","TPST1",110.603371367694,0.436667023161061,0.27605851852433,1.58179151831744,0.113697171914665,0.696555693119644
"2313","AC025259.3",1.93120152749197,2.06603057988631,1.30661280233216,1.58121103374977,0.113829793353027,0.696555693119644
"2314","ZNF253",11.8310877000818,0.68736976911745,0.434668174032877,1.58136668424558,0.113794220439105,0.696555693119644
"2315","GNB4",79.9545953239846,0.448047356874348,0.283584824200787,1.57994123323439,0.114120325253842,0.696827859247262
"2316","RPLP2",3188.50129037757,-0.24913200457983,0.157650783569515,-1.58027761701531,0.11404330345605,0.696827859247262
"2317","FGD4",26.4145912832806,0.472996344584718,0.299264812231909,1.58052776421365,0.113986053787266,0.696827859247262
"2318","DEF8",38.2089673029904,-0.462287490987228,0.292564671309562,-1.58012069235141,0.114079229403588,0.696827859247262
"2319","LAIR1",2.20391679179321,-1.70789040215279,1.08056189785848,-1.58055767609203,0.11397920955239,0.696827859247262
"2320","COPS9",249.975245525231,-0.328469478400097,0.207979197295622,-1.57933813896401,0.114258518276279,0.697070494955671
"2321","PLEKHG2",30.444542549975,0.635634001891393,0.402464500438559,1.57935420688968,0.114254834764552,0.697070494955671
"2322","CAVIN3",482.397659914574,-0.43778321733145,0.277266817218059,-1.57892394670204,0.114353502565352,0.697327572340794
"2323","NPAT",21.2747748747972,0.50564512680469,0.320287021714229,1.57872499515714,0.114399149049976,0.697327572340794
"2324","TNKS",40.5011161553562,0.467022751700123,0.295939718758144,1.5781009513015,0.114542419709019,0.697600285195574
"2325","PTPDC1",14.1272209727787,0.596187163028953,0.377743825319266,1

.57828433734174,0.11450030251448,0.697600285195574
"2326","GPNMB",703.115046301772,0.25885926992372,0.164124762134868,1.5
7721032802475,0.114747137329083,0.698546631375673
"2327","SNHG18",36.3263607368143,-0.509384164623228,0.323203541315842,
-1.57604759697062,0.115014834441092,0.69902106830902
"2328","HOTAIRM1",16.6456291615055,-0.745536733823495,0.47299329938508
2,-1.5762099268483,0.11497743154045,0.69902106830902
"2329","ZNF596",4.57168941240441,1.02916203381388,0.653015718163416,1.
57601418340797,0.115022534545199,0.69902106830902
"2330","RTN1",1.94355749488126,-1.63588935762573,1.03772031765733,-1.5
7642606566554,0.114927645217276,0.69902106830902
"2331","WNT4",3.69363059764213,1.73563665845842,1.10185759279155,1.575
1914492518,0.115212260313005,0.699271703795128
"2332","IKBIP",119.764155240482,-0.332230664354511,0.21087599782437,-1
.57547880167572,0.115145967751657,0.699271703795128
"2333","IL32",172.104562500106,0.472914072946525,0.300233984861978,1.5
75151704308,0.115221431883467,0.699271703795128
"2334","MRTFB",23.7633621476817,-0.531871849038892,0.337700932027469,-
1.57497891950037,0.115261310498434,0.699271703795128
"2335","SPTBN1",886.072646965603,0.321433101553276,0.204196858983043,1
.57413342768396,0.115456605961851,0.699295384571299
"2336","SCN7A",35.0203830134095,2.69922518621123,1.71407010549536,1.57
474608393054,0.115315065955927,0.699295384571299
"2337","RPL26L1",58.9212341100683,-0.387303718376274,0.246020348018668
,-1.57427514226134,0.115423853959536,0.699295384571299
"2338","AL353150.1",1.89818954403788,1.66402681614832,1.05712445256973
,1.57410682545777,0.1154627548819,0.699295384571299
"2339","GPAM",14.0652510523251,0.659415962103033,0.419222181373128,1.5
7295103027032,0.115730157631181,0.700315825665605
"2340","ZFP14",19.9668696263214,0.613301078706707,0.38987497527609,1.5
7307115767663,0.115702342515674,0.700315825665605
"2341","CAMTA1",101.452310772095,-0.396563440098844,0.252149148959014,
-1.5727336052334,0.115780515067725,0.700321270123446
"2342","CD302",272.604389821809,0.352003697789527,0.223970527558409,1.
57165186699722,0.116031310657408,0.701538581942313
"2343","TTBK2",34.9191956429067,0.4976469727553,0.316691917480148,1.57
139145424037,0.116091749765792,0.701604428802226
"2344","FAM111A",43.0986832641619,0.386261735378673,0.245952428513161,
1.57047335419176,0.116305028678762,0.702580538535436
"2345","DAD1",562.288238382392,-0.420830069079689,0.268071503809349,-1
.56984260952623,0.116451731916855,0.702580538535436
"2346","BTBD6",85.5779277833539,-0.300475912762763,0.191371103731861,-
1.57012164795671,0.116386813183876,0.702580538535436
"2347","AC074212.1",6.0382744294502,0.931562800770502,0.59336002534769
6,1.56997903629357,0.116419988460019,0.702580538535436
"2348","LINC02066",1.74965754383725,2.18528523218439,1.39261547741852,
1.56919499145251,0.116602510914138,0.702891253530947
"2349","AC004951.1",8.09233064219036,0.98978028222119,0.63070256770527
6,1.56932971721103,0.116571131321238,0.702891253530947
"2350","SGIP1",6.83812411285253,-1.04106842236673,0.664376448689093,-1

.56698574192524,0.117118024367812,0.705398224180442
"2351","PPM1G",101.011645670354,-0.343710686137337,0.219338927248884,-1.56703003177967,0.117107672081243,0.705398224180442
"2352","C15orf48",2.1051010628518 5,-1.69861415819644,1.08437911407648,-1.56643939019711,0.117245787432591,0.70586749576764
"2353","EPHB2",30.4817976761007,-0.512771509864114,0.327528306374347,-1.56557921829829,0.117447158310841,0.706726651339492
"2354","NMUR1",6.42564820799169,0.907995421332163,0.580141452418942,1.56512763834787,0.117552984224116,0.706726651339492
"2355","IRF4",8.46079974331727,0.780299787069457,0.498672344946941,1.56475448252997,0.117640488220844,0.706726651339492
"2356","RPL30",4985.68988704152,-0.399983094857157,0.25570969547481,-1.5642077791163,0.117768780938671,0.706726651339492
"2357","APEX1",163.741097272371,-0.410012638703987,0.261959925524864,-1.56517313815265,0.117542318141968,0.706726651339492
"2358","SEL1L",83.8259070678529,0.348973669189937,0.223095413276138,1.56423506904641,0.117762374317014,0.706726651339492
"2359","TMOD3",215.022555997663,-0.356575358535609,0.227985154483467,-1.56402884803391,0.117810793821991,0.706726651339492
"2360","AC002310.1",1.86665651080476,1.63899952517585,1.04801105685225,1.56391434466222,0.117837685297496,0.706726651339492
"2361","LILRB2",2.04907812046349,-1.7997012831532,1.15067329614153,-1.56404193022296,0.117807721735441,0.706726651339492
"2362","SLC25A48",3.67248561269209,1.29732937521155,0.82968715049943,1.56363681711911,0.117902883493574,0.706818302400086
"2363","WASHC4",68.4354860036969,0.317656343084338,0.203200200917431,1.56326785923512,0.117989604795509,0.707038850573172
"2364","IL4I1",1.58972841586828,-1.84059202302164,1.17774702587813,-1.5628076170681,0.118097852046941,0.707388149316701
"2365","LYSMD3",31.6230612177316,0.440375663733348,0.282033211263906,1.56143193831622,0.118421871056979,0.708729372006268
"2366","DPY19L4",39.187682718719,0.374001196588979,0.239523302072092,1.56143971527418,0.118420037362119,0.708729372006268
"2367","AC099778.1",3.48179360078012,-1.17717782046496,0.7544887718531,-1.56023239096546,0.118704973767722,0.70898617122859
"2368","SLC9B2",20.2530633497957,-0.562306207170259,0.360408937750242,-1.56018940784407,0.1187151279649,0.70898617122859
"2369","ADGRA2",63.4088702155734,0.356569846809584,0.22849149696489,1.56053880142583,0.118632608053596,0.70898617122859
"2370","RPS19BP1",181.366777609943,-0.300028523964214,0.192290863728361,-1.56028486297741,0.118692578867969,0.70898617122859
"2371","JPX",151.523658253065,0.26951542161057,0.172658610006197,1.56097296046167,0.118530130876871,0.70898617122859
"2372","TMEM234",18.5148928373294,-0.577239545022649,0.371089861721753,-1.55552496730689,0.11982109304766,0.709250045149294
"2373","MED8",62.1656111221393,-0.377696847439473,0.242497706437211,-1.55752750402721,0.119345295739694,0.709250045149294
"2374","FAM49A",33.7546815708233,0.551028390384371,0.353638061497277,1.55817048665904,0.119192839189529,0.709250045149294
"2375","GULP1",88.4665037625053,0.414028103101092,0.266296287878065,1.

55476483130952,0.120002087831766,0.709250045149294
"2376","TF",2.54719111083077,1.50648980018679,0.966512417929276,1.5586
8644027813,0.119070612687031,0.709250045149294
"2377","HS3ST1",1.50181964308318,2.14560203916102,1.37879381565237,1.5
5614422896569,0.119673799735858,0.709250045149294
"2378","LGI2",23.4527382507017,-1.03080276044375,0.662710433769518,-1.
55543463316326,0.119842591166719,0.709250045149294
"2379","PURPL",2.40421776522766,5.04773838104726,3.24515512184054,1.55
546905818923,0.119834398190534,0.709250045149294
"2380","AC034236.2",3.95819863822826,1.2554544787828,0.806794605455757
,1.55610172687458,0.119683904455071,0.709250045149294
"2381","HOXA6",5.38300672220427,1.09933183975487,0.707090749020571,1.5
5472524747016,0.120011518939104,0.709250045149294
"2382","SEMA3C",198.39867462778,0.52973856593552,0.339812848131287,1.5
5891270400363,0.119017043081657,0.709250045149294
"2383","ZNF3",19.1025210325575,0.551770557634222,0.354815467888353,1.5
5509161119166,0.119924252569736,0.709250045149294
"2384","IFT22",63.3840311041736,-0.444086632422598,0.285489544467128,-
1.55552678208056,0.119820661190747,0.709250045149294
"2385","ZNF862",6.25137608482494,0.881429250858558,0.566248673250671,1
.55661159574737,0.119562729039087,0.709250045149294
"2386","GALNT11",103.475359108496,0.288971891277745,0.185299361782418,
1.55948670571819,0.118881228689435,0.709250045149294
"2387","PDCD1LG2",10.537140002661,0.845208538479008,0.54189923003549,1
.55971533383366,0.118827166896083,0.709250045149294
"2388","CCDC91",49.7397943486393,0.336942477146987,0.216327667464503,1
.55755609578824,0.119338513149806,0.709250045149294
"2389","ITGA5",73.8997188264236,-0.532432116693965,0.341586504447787,-
1.55870360731816,0.119066547601324,0.709250045149294
"2390","ATP5F1B",253.64338073127,-0.310024592228778,0.199326781572895,
-1.55535844095992,0.119860726072784,0.709250045149294
"2391","CNOT2",101.960963819408,0.302770057475928,0.19436695619489,1.5
5772392284799,0.119298706997032,0.709250045149294
"2392","BDKRB2",37.9057965110492,0.786915973186357,0.505098659978654,1
.5579450818967,0.119246267157337,0.709250045149294
"2393","NMT1",104.597224266035,0.416017968058055,0.267307361693529,1.5
5632813635348,0.119630084118061,0.709250045149294
"2394","RPS6KB1",40.3295273586661,0.45616294234549,0.293198667171358,1
.55581519775084,0.119752043164258,0.709250045149294
"2395","PDCD5",211.344449019457,-0.416971404170683,0.267951951004124,-
1.5561424449724,0.119674223860405,0.709250045149294
"2396","MSN",174.107657363292,-0.311306973690348,0.200202484669366,-1.
55496059004698,0.119955455570907,0.709250045149294
"2397","LINC01266",2.84525605872928,1.3027969292926,0.838285591173928,
1.55412062787358,0.120155645647001,0.709805566275148
"2398","TMED2",240.481631758994,-0.274814223096801,0.176876104919792,-
1.55371028337277,0.120253539042783,0.70985407666354
"2399","ADORA2B",4.42316631635922,-1.22779585120316,0.790257295615285,
-1.55366594907195,0.120264119344338,0.70985407666354
"2400","SENP7",41.3410675393321,0.381170813376665,0.245386818634123,1.

55334673434517,0.120340320884726,0.710007893219881
"2401","FAM118A",18.8799093755904,0.650094046906643,0.418603568510959,
1.55300646198295,0.120421590827767,0.710191472770168
"2402","HACL1",21.9638886139345,-0.606145187191776,0.390585653651459,-
1.55188799569344,0.120689025725338,0.710210534473432
"2403","NAALADL2",38.8249594913012,0.467285366784029,0.301030309390227
,1.55228677049355,0.120593621975792,0.710210534473432
"2404","C9orf16",215.99796638886,-0.472371762568893,0.304456425745243,
-1.55152502172562,0.120775915749437,0.710210534473432
"2405","CABCOCO1",1.58403050574134,-2.37981222429195,1.53358223742309,
-1.55179954893765,0.120710193920827,0.710210534473432
"2406","AC007262.2",2.85255187239306,1.60381127479743,1.03358758382265
,1.55169363477244,0.120735546460497,0.710210534473432
"2407","KRTAP13-4",5.66243887566558,1.12294026510094,0.723507916304291
,1.55207737164366,0.12064371164909,0.710210534473432
"2408","RBX1",273.981608888337,-0.400632132818416,0.258173439264811,-1
.55179453765375,0.120711393371604,0.710210534473432
"2409","RAPH1",21.9382347158986,-0.579128745779969,0.373436095723082,-
1.55081084129965,0.1209470217634,0.710921472880875
"2410","BANK1",3.17031890824487,-1.43464491345582,0.925691913705994,-1
.54980819451284,0.121187559617529,0.711179308761652
"2411","TBCK",17.5878400162776,0.51217675931021,0.330594751353604,1.54
92585929242,0.12131956932051,0.711179308761652
"2412","TMEM173",133.533906763516,-0.315214091644225,0.203546624665646
,-1.54860878760337,0.121475792198886,0.711179308761652
"2413","ZNF277",41.2386427927119,-0.396349861210303,0.255764533892674,
-1.5496670127713,0.121221459560631,0.711179308761652
"2414","UBXN8",16.8010307600993,0.589961405175584,0.381031293823086,1.
54832795820048,0.121543356440904,0.711179308761652
"2415","AL583785.1",19.4910913778846,0.990365972337231,0.6394271440150
24,1.54883317295326,0.12142182884423,0.711179308761652
"2416","RET",7.9657060101672,2.53341447702198,1.63619282921672,1.54835
935702932,0.121535800794132,0.711179308761652
"2417","LRCH1",16.4177960403465,0.542824826432141,0.350449128853068,1.
54894043597332,0.121396039344263,0.711179308761652
"2418","TMC5",1.52943786141173,2.17564340565592,1.40481921264609,1.548
69992243196,0.121453872548007,0.711179308761652
"2419","MRPS23",54.1246264755065,-0.445647076716241,0.287650249925498,
-1.54926712850646,0.121317518285568,0.711179308761652
"2420","PRDM15",4.11026688353988,1.12243843726517,0.724291002060106,1.
54970644957981,0.121211989419867,0.711179308761652
"2421","AC010896.1",2.73666965540504,1.3272321773783,0.85738411875929,
1.54800182128277,0.121621858015002,0.711344696196787
"2422","EXOC1",68.4330707306002,0.349716315156878,0.226039109807643,1.
54714958599192,0.121827179313513,0.711346090325491
"2423","CD164",275.066003590275,0.23576610119403,0.152406232534767,1.5
4695839712622,0.121873277904636,0.711346090325491
"2424","WASF1",20.0457605225191,0.552649050309045,0.357189091560039,1.
54721704376617,0.121810917455311,0.711346090325491
"2425","ISLR",559.725699603828,0.34272160281333,0.221511885354363,1.54

719283917909,0.121816752192625,0.711346090325491
"2426","MAPK12",2.2514321038089,-1.51139487733391,0.976571015361262,-1
.54765485925752,0.121705415739597,0.711346090325491
"2427","KCNA2",2.02938228131199,1.85955937748982,1.20563859305181,1.54
2385411521,0.1229799645044,0.711354696643099
"2428","LYST",56.8193976324479,0.354258586951754,0.2295684468335,1.543
15016648908,0.122794345179726,0.711354696643099
"2429","SLC12A8",7.82438847909093,-1.20639876865908,0.781183936335103,
-1.54432101397124,0.122510584260644,0.711354696643099
"2430","PFN2",50.2086973434313,-0.331663024150229,0.21449438364475,-1.
54625505113242,0.122042982870564,0.711354696643099
"2431","ARHGEF26",32.9324723504477,-0.544320316771098,0.352535567454543,-1.54401531936571,0.122584621453399,0.711354696643099
"2432","ADH5",285.241716783451,0.283397361549365,0.183655469669971,1.5
4309241134299,0.122808355713326,0.711354696643099
"2433","USP47",79.0885634129198,0.353210449074733,0.228872428989089,1.
54326342685677,0.122766873535696,0.711354696643099
"2434","MICAL2",8.72285056148242,-1.04835999219074,0.678946459654423,-
1.54409817929435,0.122564549879092,0.711354696643099
"2435","DRAP1",258.463703558569,-0.399214804584834,0.258794057562691,-
1.54259648905627,0.122928710435104,0.711354696643099
"2436","ZNF385A",65.6458371805647,-0.620349350276119,0.40133221289281,
-1.54572528779743,0.122170927326356,0.711354696643099
"2437","TXNRD1",70.1509994562389,0.457953345693497,0.296571287543255,1
.5441594143759,0.12254971824957,0.711354696643099
"2438","NOB1",59.3649657359137,-0.450474254280616,0.291451665803196,-1
.54562250669997,0.122195762382494,0.711354696643099
"2439","SNF8",147.048890985306,-0.370864666474532,0.240233663505751,-1
.54376643582115,0.122644925199398,0.711354696643099
"2440","FASN",17.6095903687136,0.576811339303716,0.373220256324459,1.5
4549848120319,0.122225735988412,0.711354696643099
"2441","SLC44A2",29.0848243153061,0.521976076522174,0.338077601436422,
1.54395344235881,0.122599611904701,0.711354696643099
"2442","POP4",35.3849918637041,-0.38857609642295,0.251917822034,-1.542
47164128986,0.122959024085026,0.711354696643099
"2443","PANK2",38.3891264266477,0.368069475633388,0.238161496765532,1.
54546171665922,0.122234622087726,0.711354696643099
"2444","ARFGEF2",23.4276071717467,0.578772450371425,0.374724127021057,
1.5445294514994,0.122460122119678,0.711354696643099
"2445","SUSD2",16.8972802078997,0.702406843159534,0.454244970403646,1.
54631727135111,0.122027962787031,0.711354696643099
"2446","CA5B",24.6317000753841,-0.498211710455661,0.322495159451582,-1
.5448657006291,0.122378751384002,0.711354696643099
"2447","ZNF41",4.65726839795216,0.980435043647307,0.635435324272295,1.
54293443596342,0.122846684553936,0.711354696643099
"2448","GABRE",33.5701265318562,0.617815515768332,0.399455810692992,1.
54664295581662,0.121949365490932,0.711354696643099
"2449","MGLL",135.458267371521,-0.357827027416695,0.232045631678853,-1
.54205457274852,0.123060332703637,0.711473735207669
"2450","TAF4",8.91954197615203,0.71918398305326,0.466431030955796,1.54

188708581316,0.123101034693417,0.711473735207669
"2451","GHDC",27.4038771199246,0.46943904852344,0.304554046669037,1.54139816448929,0.123219910493806,0.711870229535818
"2452","NSUN6",18.1767308776374,0.567345377024662,0.368260780879177,1.540607652192,0.123412304409451,0.712297914616585
"2453","TPI1",352.490272188767,-0.322865795535291,0.209553810952325,-1.54072977278731,0.12338256754064,0.712297914616585
"2454","TNK1",2.23184254166416,-1.47972049914253,0.960561800157306,-1.54047402145308,0.123444850456857,0.712297914616585
"2455","ANKRD2",1.88988104825723,-1.70509643148947,1.10707441559439,-1.54018231066608,0.123515920511705,0.712318104505712
"2456","CASP1",17.1737869459651,0.607391855996619,0.394398324435418,1.54004674554868,0.12354895936907,0.712318104505712
"2457","DPH2",7.74123166889554,-0.765547488038353,0.498029643068548,-1.53715245406182,0.124255980379762,0.712986955907471
"2458","MBOAT2",18.4824621221087,0.555721944071725,0.3611456082092,1.53877530680039,0.12385916022089,0.712986955907471
"2459","TMUB1",29.6499186815698,-0.581963261340984,0.378345516935486,-1.53817935006805,0.124004768571939,0.712986955907471
"2460","SAR1A",162.665246476371,-0.276221122577002,0.179579599061667,-1.53815424480455,0.124010905397656,0.712986955907471
"2461","AC132217.1",11.1156887153935,1.3293496815377,0.864871030029569,1.53704961246332,0.12428116060139,0.712986955907471
"2462","SLC39A13",69.5296695170257,-0.299643012158131,0.19481789249518,-1.53806720892201,0.124032182613473,0.712986955907471
"2463","STAT6",87.6789205505817,0.366433974605955,0.238204777811501,1.53831496568858,0.123971622273362,0.712986955907471
"2464","DCAF5",68.0719585141658,0.332225870795166,0.216065419406529,1.53761704074487,0.12414227839173,0.712986955907471
"2465","POLG",20.2928446796245,0.56904158577736,0.370254556834002,1.53689286269198,0.124319547608442,0.712986955907471
"2466","LINC01197",10.3580852398849,-1.10043652276879,0.714920007674947,-1.53924426642865,0.12374467453713,0.712986955907471
"2467","UQCRFS1",131.355983428599,-0.351823341845325,0.228738428657856,-1.53810334323656,0.12402334869712,0.712986955907471
"2468","RPS5",2533.91986487649,-0.351496389723236,0.228645573230121,-1.53729803187342,0.124220343228944,0.712986955907471
"2469","MCM3AP-AS1",1.98230949892068,1.87329287886577,1.21822352197239,1.53772509320192,0.124115845495187,0.712986955907471
"2470","TRIML2",5.62232664844975,-3.73984154291395,2.43403782364869,-1.5364763466608,0.124421594642087,0.712994927984188
"2471","JCAD",8.16858854819014,-0.914296475797257,0.595060637002654,-1.53647614872092,0.124421643153173,0.712994927984188
"2472","AGA",18.0879096842543,-0.564870411200788,0.367881232046783,-1.53546949937624,0.124668543564156,0.713291672233459
"2473","NFIB",916.141097371995,0.277514983405851,0.180739774055717,1.53543947288714,0.124675914011262,0.713291672233459
"2474","ARHGAP22",9.05883531954623,1.12560955904126,0.733326296351979,1.53493685504085,0.124799339475403,0.713291672233459

"2475","EIF4EBP2",123.765202846139,0.334767280570477,0.217971471162797
,1.5358307157566,0.124579904275431,0.713291672233459
"2476","MRPL23",26.0226049351282,-0.610572540607581,0.397763821151698,
-1.53501275917883,0.124780693953216,0.713291672233459
"2477","RALGAPA1",53.4688586994203,0.511392020184337,0.333028170794575
,1.53558186673579,0.124640964344277,0.713291672233459
"2478","SPATA33",18.5325587783289,-0.555677480269136,0.362045402316326
,-1.53482816440693,0.124826042640855,0.713291672233459
"2479","ZNF678",13.4346617909867,0.637951035660206,0.416192403693299,1
.53282719722661,0.125318437586047,0.713911870455739
"2480","TEX41",6.70325227516254,-0.912036541285561,0.594737497842825,-
1.53351107773364,0.125149979313803,0.713911870455739
"2481","AC097662.1",2.54313732698068,-1.41430367089741,0.9232166052682
17,-1.53193049477973,0.125539587389463,0.713911870455739
"2482","SLC26A2",37.5410079191186,0.479169608074,0.312647454368552,1.5
3261957319233,0.125369615961,0.713911870455739
"2483","XPO5",11.9759972382959,0.684242132961981,0.446442405912847,1.5
3265488201754,0.125360911347694,0.713911870455739
"2484","DGAT1",26.0349297648252,-0.43176428406616,0.281579504133952,-1
.53336545354794,0.125185835690537,0.713911870455739
"2485","OBI1",23.5768511262554,0.486986105785678,0.317472226448464,1.5
3394868972808,0.12504227629211,0.713911870455739
"2486","COL4A2",533.700620775548,-0.367361087452961,0.239772315766342,
-1.53212470038015,0.12549166552967,0.713911870455739
"2487","AL357093.2",2.03081049486018,1.94329111166436,1.26833867828987
,1.53215473510951,0.125484255481443,0.713911870455739
"2488","FLYWCH2",75.6060929734648,-0.416918460272637,0.271922531375279
,-1.53322513645348,0.125220392901504,0.713911870455739
"2489","RND2",1.84796560027442,-1.62278147258134,1.05881299681864,-1.5
3264219220696,0.125364039686821,0.713911870455739
"2490","WDR13",112.809220095189,-0.298756632371694,0.194952494887059,-
1.53245862559887,0.125409300013581,0.713911870455739
"2491","ZNF486",10.2398998577779,0.707078239122143,0.461662514196964,1
.53159118918733,0.125623348110016,0.713966228480256
"2492","NAP1L2",2.07006517274219,1.79804046130106,1.17405162196264,1.5
314833076039,0.125649988797514,0.713966228480256
"2493","MMADHC",73.9119703727409,-0.328712429145843,0.214666009727326,
-1.53127376599295,0.125701746376654,0.713973818168238
"2494","CXCL14",8568.39946325564,0.511398282141191,0.334029042514808,1
.53099945528994,0.125769527274184,0.71407237618382
"2495","KLF4",754.679942841943,0.616146137782054,0.40256770580176,1.53
054040078781,0.125883021199823,0.714430292661121
"2496","MTA3",30.9127196422576,0.421106666175474,0.275337298529423,1.5
2942107162598,0.126160091984241,0.715297998557298
"2497","GOLGA1",19.8471556635809,0.47462473599105,0.310393453194577,1.
52910678722827,0.126237973050472,0.715297998557298
"2498","C11orf54",34.7509894890539,0.441713173818652,0.28886663214148,
1.5291249478836,0.126233471740596,0.715297998557298
"2499","C1RL-
AS1",19.9190332442549,0.694355915736244,0.45403069978627,1.52931490329

421,0.126186396780702,0.715297998557298
"2500","OMA1",21.8029868188652,0.495616426643559,0.32475130445491,1.52614144991794,0.126974641172276,0.715443567334934
"2501","AL513166.1",2.66769203442469,1.323126605067,0.866925252277909,1.52622916634438,0.126952802179427,0.715443567334934
"2502","RHOC",247.303192878718,-0.40607080311553,0.26669501984688,-1.52260362172744,0.127857903140224,0.715443567334934
"2503","POLR3GL",88.8443990985202,-0.334292292433941,0.219381726801079,-1.52379278487974,0.127560482496023,0.715443567334934
"2504","INPP4A",14.493545407681,0.575224372659525,0.378025591142561,1.52165458142911,0.128095653557741,0.715443567334934
"2505","PAX8-
AS1",37.74401749835,-0.893735779602885,0.587400316966355,-1.52151055045153,0.128131765740989,0.715443567334934
"2506","ACADL",12.2871992342792,0.777189328128129,0.51033598539834,1.52289736637227,0.127784384796213,0.715443567334934
"2507","RRP9",15.7588599246036,-0.579278750785676,0.379544103604461,-1.52624884772118,0.126947902456086,0.715443567334934
"2508","ZMAT3",57.5027858053186,0.479447040792648,0.314075601351221,1.52653386232475,0.126876963921852,0.715443567334934
"2509","WDR1",80.0687643598173,-0.351240830931387,0.230280083321264,-1.5252766364574,0.127190112635088,0.715443567334934
"2510","LIMCH1",40.713307647808,0.482062619244226,0.315489435252938,1.52798339778871,0.126516660066937,0.715443567334934
"2511","AADAT",8.02100418900339,-0.755584661424508,0.496326727278417,-1.52235336099613,0.127920564286071,0.715443567334934
"2512","POLK",75.6642796815553,0.324890601023415,0.213020111692772,1.52516397837585,0.127218202745159,0.715443567334934
"2513","PPP1R11",37.3987528312026,-0.412191601732376,0.270910417070798,-1.52150517572994,0.12813311347185,0.715443567334934
"2514","ARPC1A",134.227441880089,-0.423173010786705,0.277124512211355,-1.52701400323607,0.126757529318977,0.715443567334934
"2515","MKLN1-
AS",9.26101990682243,0.72043991910613,0.472897415109298,1.52345920296396,0.127643860041023,0.715443567334934
"2516","AC087854.1",2.24826550919915,1.44866630194965,0.95142991161349,1.52262009451954,0.127853779463111,0.715443567334934
"2517","TMEM203",64.5383423757387,-0.305161090548984,0.200464915001373,-1.52226682931971,0.12794223594102,0.715443567334934
"2518","BLOC1S2",59.938511881664,-0.478194969859544,0.313447092244565,-1.52560027414909,0.127109443820256,0.715443567334934
"2519","EIF4G2",285.344856479114,0.227718805494608,0.14957791357544,1.52240929192903,0.127906558034308,0.715443567334934
"2520","PTPMT1",78.9475923107623,-0.37970349272486,0.248589920285937,-1.52742915838306,0.126654330431057,0.715443567334934
"2521","SNX15",37.7354084879581,-0.3729256076453,0.244942548208133,-1.52250235973056,0.127883254592814,0.715443567334934
"2522","TBCEL",10.6944631006811,0.670443826771874,0.439924833107477,1.52399632008971,0.127509630450007,0.715443567334934
"2523","TUBA1A",756.358174663328,-0.411771696444044,0.270072786748619,

-1.52466933600132,0.127341593758196,0.715443567334934
"2524","ARL1",201.278327392097,-0.281183648001859,0.184715559589122,-1
.5222520973724,0.12794592580698,0.715443567334934
"2525","NHLRC3",48.9828825979811,0.331981146668739,0.218187131158408,1
.52154320424936,0.12812357792007,0.715443567334934
"2526","AP1G2",6.69458118012037,-0.831872871122365,0.546372829393217,-
1.52253704132069,0.127874571441385,0.715443567334934
"2527","RAB27A",19.2318413556622,-0.519576246027484,0.34074319912216,-
1.52483232934962,0.127300923970901,0.715443567334934
"2528","SELENOS",218.387372864759,-0.289488414422647,0.189792470922517
,-1.52528924364355,0.127186969465842,0.715443567334934
"2529","C18orf54",6.50497323102246,0.833934719254371,0.547039570469632
,1.52445044978819,0.127396225727869,0.715443567334934
"2530","ZBTB7A",185.41841162787,-0.365224361935328,0.239355825888196,-
1.5258636825741,0.127043816938774,0.715443567334934
"2531","CRNKL1",38.7628003029371,0.482971679409295,0.31646481910089,1.
52614651063417,0.126973381112775,0.715443567334934
"2532","POFUT1",31.0229805474401,0.443296302564384,0.290252013519521,1
.52728071440087,0.126691222985737,0.715443567334934
"2533","OSER1-
DT",20.7167680297522,0.520664656590424,0.341807560170009,1.52326840381
019,0.127691568624771,0.715443567334934
"2534","RGS19",11.3172778162642,-0.667708957134504,0.438595618032656,-
1.52237945314992,0.127914030127662,0.715443567334934
"2535","TSPO",532.378719409008,-0.420294014256212,0.275851033475044,-1
.52362675231461,0.127601976414092,0.715443567334934
"2536","MMGT1",33.0205940954353,-0.402778056993263,0.264107409292998,-
1.52505398493545,0.127245633111938,0.715443567334934
"2537","PDS5A",60.0973118465366,0.326569047680712,0.214747548072413,1.
52071141492425,0.128332272894405,0.716273151038542
"2538","AC245407.2",9.87143999420459,-0.76021896137566,0.5004081175258
58,-1.51919790017471,0.128712689721763,0.716382971920982
"2539","RIT1",24.9478947674381,-0.470912673307936,0.311210031843686,-1
.51316675274936,0.130237307087813,0.716382971920982
"2540","ETV3",14.8254736356735,-0.596178948899545,0.392797722977847,-1
.51777597991109,0.12907088238217,0.716382971920982
"2541","FAM71A",5.60782815971113,1.15785231460695,0.76473685296765,1.5
1405324604636,0.130012335382804,0.716382971920982
"2542","TCF7L1",141.570523274212,0.266759982908843,0.176355989497343,1
.51262218918208,0.130375654569421,0.716382971920982
"2543","CNPPD1",39.810228346747,0.394112461680599,0.259823166870695,1.
51684881077882,0.129304860182763,0.716382971920982
"2544","FRYL",31.6318296867397,0.472480306032749,0.311823036660072,1.5
1521937280027,0.129716858614618,0.716382971920982
"2545","INPP4B",1.655171324476,1.78341136741141,1.17829409216679,1.513
55368686595,0.130139074941716,0.716382971920982
"2546","NDUFS6",220.834218786344,-0.418934888326717,0.275836327415033,
-1.51878069235012,0.128817707420327,0.716382971920982
"2547","RREB1",38.4213695483927,0.458486358857214,0.303075053449705,1.
51278158211494,0.130335148671922,0.716382971920982

"2548","NEDD9",14.6169732742368,-0.623838226259729,0.411931023418076,-1.51442399526822,0.129918337220061,0.716382971920982
"2549","TMEM120A",36.1077057141514,-0.37488934637072,0.24705536389552,-1.51743050812392,0.129158026200023,0.716382971920982
"2550","FOXP2",19.0499055551418,0.514224300141906,0.33961438899672,1.51414167597849,0.129989910445813,0.716382971920982
"2551","AC062004.1",26.0147740553083,0.712785891192747,0.470353559464973,1.51542574059297,0.129664622850191,0.716382971920982
"2552","SLC26A7",3.21717834960071,1.7087268472353,1.12793757915169,1.51491259695454,0.129794539833884,0.716382971920982
"2553","MTSS1",21.5584919013556,0.585680022918615,0.38714327781223,1.51282498362965,0.130324120907813,0.716382971920982
"2554","BOP1",16.5529981941721,-0.570299104198592,0.376148082344286,-1.51615581992148,0.129479956436377,0.716382971920982
"2555","NTMT1",48.7802965356223,-0.369020134414386,0.243577785684934,-1.51499913416452,0.129772623383693,0.716382971920982
"2556","BMPR1A",41.3603827026783,0.357305155051892,0.235807727834995,1.51523937884646,0.129711793974137,0.716382971920982
"2557","STIP1",42.5578590818585,-0.465783766465342,0.307945777247626,-1.51255123752126,0.130393688376666,0.716382971920982
"2558","PTS",30.4344008997575,-0.445431281867596,0.294013344113227,-1.51500362410781,0.129771486337172,0.716382971920982
"2559","AVIL",5.51292194034576,-1.03444081381274,0.681715101914673,-1.51740926804672,0.129163385411868,0.716382971920982
"2560","TBX5",7.71597604789382,-0.901542363263619,0.594023292318138,-1.51768857370122,0.129092925916847,0.716382971920982
"2561","HTR2A",2.75159106800974,-1.74030083428882,1.14652775263377,-1.51788810195919,0.129042609882502,0.716382971920982
"2562","NUMB",64.6011964038395,0.307620526952482,0.203332249259428,1.51289590349239,0.130306102645209,0.716382971920982
"2563","TTC8",23.3214958208875,-0.474461316363003,0.313274409055388,-1.51452305917243,0.129893229917196,0.716382971920982
"2564","PYGO1",16.6126979438732,0.665794368970822,0.43959817472842,1.51455216888047,0.129885852908113,0.716382971920982
"2565","ZKSCAN2",3.01761841065196,1.31487019631388,0.8668265506728,1.5168780828106,0.12929746814234,0.716382971920982
"2566","CD68",103.613540129157,-0.324697836429949,0.21438276120453,-1.51457064274013,0.129881171414238,0.716382971920982
"2567","EVI2B",4.19927216367524,-1.06577563858707,0.703333838449956,-1.51531972489179,0.129691455477821,0.716382971920982
"2568","SSTR2",1.88844995117663,-2.07799266797123,1.37204553793678,-1.51452164707012,0.129893587781739,0.716382971920982
"2569","NDUFV2",226.639250162754,-0.390292988675808,0.257501158884296,-1.51569410548238,0.129596718826411,0.716382971920982
"2570","TWSG1",64.0245392516383,0.318564185617393,0.209579083358645,1.52001898525458,0.128506204156477,0.716382971920982
"2571","MYO1F",6.60674003345666,0.973270390742821,0.642391068480908,1.51507459940929,0.129753513369328,0.716382971920982
"2572","IGFLR1",6.14704286929764,-0.839611049986836,0.553658682260584,-1.51647770889948,0.129398602673725,0.716382971920982

"2573","SIPA1L3",7.04314736780042,-0.833605918756021,0.550207987115235
,-1.51507418699364,0.129753617799136,0.716382971920982
"2574","FOSB",2501.10646361295,0.51104520227339,0.336793811160793,1.51
738299617806,0.129170014464285,0.716382971920982
"2575","TEAD2",27.7341920746815,-0.401034357462624,0.265160136448671,-
1.51242325801207,0.130426221865275,0.716382971920982
"2576","EBF4",2.29271929746449,-1.48586289877893,0.98095180295233,-1.5
1471549805708,0.129844467908919,0.716382971920982
"2577","TSR2",60.0039708386039,-0.387059685051288,0.255889609126954,-1
.51260415134425,0.130380239069196,0.716382971920982
"2578","CHRDL1",350.262635567354,0.348265221607227,0.230151482814373,1
.51319999049547,0.130228866661229,0.716382971920982
"2579","RNF146",101.930473713467,0.307685261350669,0.203513860451708,1
.51186391269739,0.130568486161927,0.716608435679412
"2580","GRB2",64.3951422744573,-0.309759170739433,0.204882876025297,-1
.51188413960563,0.130563339538157,0.716608435679412
"2581","LRRC61",4.07551646309109,-1.10170617765874,0.728804246485831,-
1.51166267618634,0.130619698240227,0.716611750128481
"2582","ZBTB41",33.854898142876,0.390269622997468,0.258229141907639,1.
51133067365827,0.130704222630725,0.716797750755642
"2583","SYNJ2",29.9011717117915,-0.771893274255677,0.510873850625146,-
1.51092735185237,0.130806961238968,0.716948415989686
"2584","POLR3D",31.3011923933863,-0.422091563886569,0.279378135747105,
-1.51082532911111,0.130832959528061,0.716948415989686
"2585","KDM4A",24.1796931560902,0.443065142380423,0.293375216338307,1.
51023371336691,0.13098379901819,0.717219873974313
"2586","FCGR3A",2.65691900425512,-1.47985599225327,0.97981295870813,-1
.51034539715053,0.130955313583441,0.717219873974313
"2587","GGNBP2",125.587678188376,0.275603307427081,0.182608877435616,1
.50925470490477,0.131233705105392,0.71831050030628
"2588","EAF1-
AS1",1.51449342083711,-1.76412166783103,1.17029331279331,-1.5074183954
9638,0.131703446506201,0.718562391088779
"2589","RBM5-
AS1",1.92953498019091,1.60913234682918,1.06667946981523,1.508543468177
85,0.13141549030556,0.718562391088779
"2590","TAF3",49.1315623990709,0.36023727415452,0.238864450451562,1.50
812426660187,0.131522725555799,0.718562391088779
"2591","SLC25A28",39.3399238862231,-0.385192309264441,0.25538535920289
1,-1.50827874576171,0.131483200612185,0.718562391088779
"2592","MDK",114.752395338151,0.521194566304094,0.345761744590827,1.50
738065866967,0.131713113510169,0.718562391088779
"2593","SUPT20H",36.7573304892226,0.361319462386434,0.23963570109291,1
.50778644725539,0.131609191899572,0.718562391088779
"2594","AL606834.1",7.22680806028443,0.791395984916169,0.5251112505596
21,1.50710156004611,0.131784626991314,0.718562391088779
"2595","UQCRC2",118.025875966928,-0.322918318203911,0.214152401923302,
-1.50789024686991,0.13158261924497,0.718562391088779
"2596","CAMTA2",26.2514789848914,-0.509674671525111,0.338184273410568,
-1.50709158171393,0.13178718429785,0.718562391088779

"2597","RCN3",193.049931820234,-0.411926375874792,0.273127459884175,-1.50818367384033,0.131507524566258,0.718562391088779
"2598","AC109347.1",1.53758300356937,-1.8152879682304,1.2054815811927,-1.50586122305938,0.132102802779267,0.720006038242657
"2599","SMIM3",38.8508646643188,-0.496994190049584,0.33025118724119,-1.5048975121068,0.132350428046477,0.720301054318786
"2600","C17orf58",132.397760357081,0.508842802210968,0.338133050638523,1.50485970315555,0.132360150370781,0.720301054318786
"2601","TPD52L2",61.6983699378488,-0.3329527679426,0.221251846165683,-1.50485871061736,0.132360405602929,0.720301054318786
"2602","COL6A2",4337.10981893459,-0.257801408742782,0.171303784871521,-1.50493702714236,0.132340267604937,0.720301054318786
"2603","F8A1",32.9217312137059,-0.465572011752885,0.309467880041024,-1.50442757319812,0.132471309057294,0.720627635901377
"2604","FYCO1",23.8144878945588,0.505294372001336,0.336083237272396,1.50347984059614,0.132715351636591,0.720968039762886
"2605","FAT4",40.8171416685654,0.43049987324342,0.286389681292682,1.50319617417871,0.132788463820735,0.720968039762886
"2606","TNKS1BP1",82.9963287421909,0.342712062746616,0.227924435588774,1.50362141672666,0.132678873466601,0.720968039762886
"2607","ATF7IP2",8.47973298208145,-0.909862904387853,0.60504210340003,-1.50380097397336,0.132632620340653,0.720968039762886
"2608","POLR3H",26.6426853063088,-0.463167778946902,0.308119522317847,-1.50320815592175,0.13278537501553,0.720968039762886
"2609","JRK",9.74942704655623,0.944527510107514,0.628438487392198,1.50297527770295,0.1328454192749,0.721000819061933
"2610","MAP2K2",256.165554772944,-0.351914277852141,0.234252837564624,-1.50228394887664,0.13302379218793,0.721692297847162
"2611","LINC02606",2.83648044556812,-1.29195920850001,0.860607008369858,-1.50121855380566,0.133299042597692,0.721802846341611
"2612","FAM149A",19.8236171681958,0.520039139473115,0.346309936168065,1.50165815404367,0.133185416199688,0.721802846341611
"2613","NNT-AS1",46.5472260208409,0.350092115292407,0.233086971575741,1.50198062519614,0.133102112613824,0.721802846341611
"2614","AL928654.1",1.50176907042429,-1.72264479445829,1.14722797492786,-1.50157146801325,0.133207816578562,0.721802846341611
"2615","ZNF765",10.9382402851307,0.719598686979203,0.479293069076645,1.50137511557491,0.133258566436852,0.721802846341611
"2616","KIF13B",31.3507147889356,-0.613642587787551,0.408845400561007,-1.50091596223298,0.133377299039296,0.721950517735636
"2617","MIF4GD",24.0756563854217,-0.530845233426438,0.35381286596412,-1.50035593527645,0.133522227385707,0.72245882299641
"2618","MSRB3",49.4544503374423,-0.433811997884182,0.289221321780746,-1.49993090140514,0.133632302433069,0.722778228591387
"2619","HMCN1",89.1455105341383,0.543212804429223,0.362600018508712,1.49810473442149,0.13410604123365,0.723010157376564
"2620","RIF1",107.379947111469,0.409889692429672,0.273423590987788,1.49910141604416,0.133847324312875,0.723010157376564
"2621","FNDC3B",178.944316896903,0.393392751514575,0.262573702775374,1

.49821839489811,0.134076517917168,0.723010157376564
"2622","NDUFA8",77.2371274532547,-0.390195526666894,0.260323617438047,
-1.49888638805411,0.133903108223987,0.723010157376564
"2623","PLCE1",28.1413884282449,0.497014997595258,0.331614194541563,1.
49877479847433,0.133932064580506,0.723010157376564
"2624","AP001527.2",2.13603685909704,-1.54727776040485,1.0328996102548
3,-1.49799433075894,0.134134723405949,0.723010157376564
"2625","AL096870.8",14.5966191750917,-0.597138144078848,0.398252256392
554,-1.49939676296587,0.133770732866308,0.723010157376564
"2626","DYRK1B",11.8453046522435,0.60410683369581,0.402992385010743,1.
49905272696333,0.133859953965174,0.723010157376564
"2627","CBFA2T2",25.6183673555857,0.463329358079593,0.309204368416307,
1.49845670180111,0.134014634001801,0.723010157376564
"2628","TTL",32.2315654114412,-0.485111983190282,0.323915623643608,-1.
49764922646656,0.134224409908221,0.723010801154824
"2629","FGD5",25.9209164460559,0.486745344291817,0.325045486782402,1.4
9746839776201,0.134271422602234,0.723010801154824
"2630","MAP3K5",18.8376535240055,0.523405088288002,0.349541535462641,1
.49740455764504,0.134288023095847,0.723010801154824
"2631","SLC1A4",6.0896293877346,-0.972497957599544,0.650516831371977,-
1.49496202204098,0.134924353987243,0.723609609755008
"2632","ZDHHC4",45.8880546148701,-0.391692940524971,0.261839754675594,
-1.49592616678952,0.134672896581859,0.723609609755008
"2633","CDK6",32.7118590769518,0.493980452266502,0.330461447256272,1.4
9482021690542,0.13496136859908,0.723609609755008
"2634","CHRAC1",44.6753484407892,-0.345419763070713,0.230829238533263,
-1.49642985119902,0.134541675408808,0.723609609755008
"2635","PAXX",60.6090804933242,-0.326953836079657,0.218579480885444,-1
.49581211719965,0.134702622809917,0.723609609755008
"2636","CD151",298.688220360149,-0.432339143414366,0.289196973970124,-
1.49496427116499,0.134923766974029,0.723609609755008
"2637","MIPOL1",15.927703017943,0.608013708016607,0.406523773856959,1.
49564120751901,0.13474717871783,0.723609609755008
"2638","MAGEL2",1.62837123779818,-1.8552219937889,1.2409885055094,-1.4
94955018159,0.134926181987701,0.723609609755008
"2639","ZCCHC14",35.7921183027291,0.394573620453938,0.263821201339039,
1.4956099754351,0.134755322108109,0.723609609755008
"2640","SARM1",5.84469503181557,-0.829313034127854,0.554353534271887,-
1.49600026491598,0.134653586141053,0.723609609755008
"2641","ASB16-
AS1",8.63468062187156,0.771991234408769,0.516422562097823,1.4948828557
6209,0.134945017356573,0.723609609755008
"2642","DTWD2",5.47668785098452,1.00119204012924,0.670086472823385,1.4
9412364035758,0.135143306093407,0.723988275877554
"2643","CD82",22.3839574169691,-0.563221547994019,0.376998412571062,-1
.49396265133571,0.135185381456233,0.723988275877554
"2644","LINC01303",2.04830413697117,-1.7111757039678,1.14533755982486,
-1.4940361374593,0.135166174199814,0.723988275877554
"2645","TCTE3",2.1802901928224,1.39499927011355,0.934042158575602,1.49
350782221746,0.135304308230723,0.724077477153077

"2646","CRLF1",163.154627648514,0.478932969078738,0.320651174085013,1.49362612017682,0.13527336841094,0.724077477153077
"2647","KCNMB4",24.012407958564,0.499968120263122,0.334935780534056,1.49272830590366,0.135508320782179,0.724895286088272
"2648","ATAD3A",17.716379190402,-0.512207208131429,0.343250283592037,-1.4922266131037,0.135639747860033,0.725324331456976
"2649","CDC14A",9.18150721915244,0.655611406912951,0.439620696284851,1.49131151570751,0.135879726932836,0.725788118827322
"2650","C3orf33",8.78019874176128,0.719911648085444,0.482782559751413,1.49117161244625,0.13591644464063,0.725788118827322
"2651","TSG101",70.2622147936303,-0.327688926292354,0.219714828344627,-1.49142836084949,0.135849066714737,0.725788118827322
"2652","MRPS12",68.2941888324462,-0.396026947168281,0.265591284593543,-1.49111424260158,0.13593150361088,0.725788118827322
"2653","ZEB1",153.613436904079,0.389408115247683,0.261300700228556,1.49026816578399,0.136153739350154,0.726286000440695
"2654","CPNE8",33.6863276600568,0.441245590598635,0.296103503216463,1.49017348935608,0.13617862508263,0.726286000440695
"2655","ZNF594",4.41789308522708,1.14986322657214,0.771515269320499,1.49039594198164,0.136120158898204,0.726286000440695
"2656","TBX15",73.7554982872415,0.301374101176966,0.202304070694436,1.48970853696843,0.136300888949847,0.72643948990897
"2657","ESCO2",2.53920182196762,-2.11125451835609,1.4174450863146,-1.48947887910453,0.136361311029523,0.72643948990897
"2658","ATF7IP",68.3086778219963,0.335870107042322,0.225473502124151,1.48962119219395,0.136323866580339,0.72643948990897
"2659","SEC22A",15.07838015491,-0.556759496022915,0.373982280853504,-1.48873228633259,0.136557879504252,0.727213077766155
"2660","RAB15",1.66614599596572,-1.80213841196365,1.21075447336753,-1.48844249730606,0.136634236190661,0.727346159571339
"2661","IGFBP4",1941.8430339866,-0.420356685954822,0.282663381749144,-1.48712819946334,0.136980954948564,0.728917821146813
"2662","CDC16",49.4880451275797,0.354460077767689,0.238525884789154,1.48604449400121,0.137267352433841,0.730167434433952
"2663","EIF3B",94.1486218953849,-0.286385741171166,0.192769151501649,-1.48564092823076,0.137374123166346,0.730460977857853
"2664","AK8",2.83552891177953,-1.34451939018715,0.905323879662698,-1.485125290949,0.137510637653989,0.730638134776915
"2665","CERS5",39.1357457920126,-0.394815798534523,0.265818298267525,-1.48528450113382,0.137468475748349,0.730638134776915
"2666","NAGK",73.9266695880605,-0.36226304989049,0.244004660901183,-1.48465627071443,0.137634901146968,0.73102408111068
"2667","MRPL41",156.223135392437,-0.336495709260134,0.226723790719401,-1.48416585746218,0.137764925135869,0.731440322431163
"2668","TCTEX1D2",25.0732260836046,0.48046520846588,0.323838842233294,1.48365528098001,0.137900395623918,0.731863961778607
"2669","MOK",25.5512889891561,-0.464246136711132,0.312944903180061,-1.4834756277977,0.137948087145982,0.731863961778607
"2670","SOCS4",19.1207798404257,-0.486955253959163,0.328499862063596,-1.4823606040507,0.138244370603142,0.733161156457111

"2671","KCTD1",8.62945552855141,0.845748857406902,0.570919739804808,1.48137960284234,0.13850544695095,0.734270733367822
"2672","SLC8A1",4.69881186222833,1.00342805900843,0.677698421483294,1.48064098601883,0.1387022674632,0.734527529412552
"2673","TENT5A",263.178674710193,-0.532163306930333,0.359399343727628,-1.48070194400142,0.138686015731479,0.734527529412552
"2674","CRAMP1",14.3198662616667,0.625617072498245,0.422594509893667,1.48041930941238,0.138761380026736,0.734527529412552
"2675","ANAPC11",234.220762547009,-0.310708495647013,0.209869915221012,-1.48048135112557,0.138744833950147,0.734527529412552
"2676","DNLZ",5.24030728567752,-0.912372644863646,0.616487586659083,-1.4799529862524,0.138885793690568,0.734911374685517
"2677","AL645728.1",2.5649260231136,-1.2857865549063,0.869096851205876,-1.47945140190333,0.139019710817791,0.735345201785553
"2678","SRGAP2C",42.7429435839667,0.327639368198765,0.221693355041165,1.47789440120083,0.139436045147775,0.735447718433309
"2679","ITGA4",14.3488021109402,-0.60495361965865,0.409188595781053,-1.47842248267922,0.139294731332392,0.735447718433309
"2680","NECTIN3",64.3080601088488,-0.31446305797381,0.212704558961343,-1.47840299949076,0.13929994304488,0.735447718433309
"2681","ANKRD17",94.5696194285417,0.35995387918867,0.243510281422623,1.47818760294541,0.139357571178372,0.735447718433309
"2682","PURB",48.5043783304018,0.407161071536855,0.275513707597332,1.47782509657171,0.139454599152079,0.735447718433309
"2683","ITGAE",70.8302623283344,-0.373479444132422,0.252656621633366,-1.47820960209934,0.139351684587164,0.735447718433309
"2684","MFSD12",27.2262819510847,-0.429828204676536,0.290754571518181,-1.47831967845657,0.139322232942448,0.735447718433309
"2685","SLC24A3",2.14908140854414,1.43326497332816,0.969559087480166,1.47826469973391,0.139336942257925,0.735447718433309
"2686","PPCS",118.356382562348,-0.364595962560346,0.24703642312907,-1.47587937819944,0.139976276400731,0.736552981729601
"2687","ZNF672",11.9347141605541,-0.670248054932046,0.454088506681551,-1.47602955166202,0.139935959171565,0.736552981729601
"2688","PPP1CB",163.910462225096,-0.390156213136394,0.264349320158583,-1.4759115434923,0.139967640198313,0.736552981729601
"2689","NOTCH4",6.13218946749311,-0.928188218023336,0.628781975623775,-1.47616861488839,0.139898632687912,0.736552981729601
"2690","MSRA",15.7663814410649,-0.574610596239327,0.38908753307767,-1.47681574810217,0.139725033998141,0.736552981729601
"2691","NUDT2",51.2957796199062,-0.433105491586734,0.293332994469492,-1.47649769972187,0.139810332306505,0.736552981729601
"2692","GMDS",11.1637063118545,-0.783809441113303,0.531174285592206,-1.47561631346562,0.140046923209365,0.736650977951193
"2693","RBM8A",185.971371586418,-0.284286989223624,0.192810895731555,-1.47443425406534,0.140364707357444,0.737047740174877
"2694","MARCH7",87.8330060785008,0.261168000111623,0.177049314412665,1.47511443903643,0.140181779151086,0.737047740174877
"2695","CLASP2",29.7903915494152,0.556173790488393,0.37722605068596,1.47437800087515,0.140379844245571,0.737047740174877

"2696","LERFS",7.67665085308748,1.5310200570444,1.03852962694228,1.474
2189508374,0.140422649013911,0.737047740174877
"2697","LIMA1",625.340065090654,-0.230842942048369,0.156607372191317,-
1.47402346912739,0.140475272303966,0.737047740174877
"2698","NEMP1",8.35064200408888,0.702390993235079,0.476622215652307,1.
47368496509082,0.140566432790678,0.737047740174877
"2699","DIS3L",24.2088257313466,0.456423809598463,0.30973502123482,1.4
7359445431401,0.140590815410476,0.737047740174877
"2700","FAM217B",32.3590042617891,0.54276969917634,0.368194975935727,1
.47413662502306,0.140444809112091,0.737047740174877
"2701","PPP6R2",37.7826521294691,0.446770168851053,0.303148675651051,1
.47376586056844,0.140544643180191,0.737047740174877
"2702","ARL4C",29.959593070971,0.87281270836647,0.592612914837059,1.47
282093676013,0.140799325323228,0.737594689817575
"2703","ZNF333",17.9995955412558,0.496162161657838,0.336874048136062,1
.47284174724388,0.140793712524637,0.737594689817575
"2704","SEL1L3",3.30519147998563,1.15738442499402,0.786371344036226,1.
47180391779472,0.141073835353431,0.738219037827926
"2705","ETV2",3.55113517040817,-1.13633599663041,0.771909610243204,-1.
47211018175092,0.14099112645412,0.738219037827926
"2706","EIF3K",508.450932580827,-0.396732178253253,0.269555777920196,-
1.47179994179427,0.141074909347625,0.738219037827926
"2707","GLUD1",123.198833341745,-0.241967718923434,0.164488518188894,-
1.4710310578977,0.141282717823782,0.738487738790975
"2708","MOB2",47.0707471844502,-0.324896805476839,0.220810921259894,-1
.47138014561534,0.141188339736261,0.738487738790975
"2709","ATP5MG",492.741811903902,-0.327886976439507,0.222893120914721,
-1.47105022844091,0.14127753368747,0.738487738790975
"2710","GLB1",41.3840982009996,0.357602606417857,0.243344439493496,1.4
6953268035292,0.141688364503444,0.738596861996728
"2711","ZBED3",20.5904932462998,0.515793820461307,0.350946737297163,1.
46972108768904,0.141637308999151,0.738596861996728
"2712","UBLCP1",49.4523047820524,-0.323184302854471,0.219950289665384,
-1.46935156732978,0.141737456687844,0.738596861996728
"2713","ZNF33A",40.4475124239067,0.389170650434647,0.264882432070756,1
.4692203155651,0.141773041730728,0.738596861996728
"2714","PDCD4",177.619410745551,0.294973373837736,0.200702353478516,1.
46970560496846,0.141641504046338,0.738596861996728
"2715","AC090559.2",2.14877447287836,1.68499503181525,1.14609582688703
,1.47020431650288,0.141506426003385,0.738596861996728
"2716","RERG",55.5438094949163,-0.502397399010484,0.341829508068715,-1
.46973092477871,0.141634643686728,0.738596861996728
"2717","AL161772.1",21.0929158028592,-0.499801708982329,0.339878653313
566,-1.47052986149507,0.141418304230455,0.738596861996728
"2718","NXN",15.0394809514342,0.572947112131312,0.389923866378569,1.46
938200385777,0.141729205701599,0.738596861996728
"2719","WFDC2",3.31328817479251,-1.18245560086055,0.805023348113627,-1
.46884634294314,0.141874471008436,0.738853442250626
"2720","DYNC1H1",210.58731039509,0.285971476773558,0.194745452993926,1
.46843724655526,0.141985490422805,0.739159758965781

"2721","PILRA",6.66602312099271,0.924104179363906,0.629528590518183,1.
46793043760451,0.142123119314512,0.739604325429435
"2722","P4HA3",5.45281868382328,-1.04460258193416,0.711773317175553,-1
.4676057063776,0.142211357070329,0.739791629726618
"2723","EAF1",15.6631786476034,-0.505420819269796,0.344457103900442,-1
.46729683768077,0.142295323602362,0.739956585460688
"2724","MFSD9",6.3427117699306,0.890265593010805,0.606832160332281,1.4
6707055295706,0.142356863684075,0.74000484205819
"2725","PCDH8",1.98463040583864,4.76531367103902,3.24929407912476,1.46
656890850662,0.142493363081603,0.740170954231658
"2726","FERMT1",1.98082874126859,4.76585625872998,3.24928533253515,1.4
667398430693,0.142446839843652,0.740170954231658
"2727","AC114296.1",1.66785155003971,1.54172438670639,1.05144870449912
,1.46628587786489,0.142570421141927,0.740299656534537
"2728","SLC9A6",9.79321045724489,0.611548687342819,0.417150511652786,1
.46601447261759,0.142644344108896,0.740411991415675
"2729","NKTR",181.663578712317,0.405770753122223,0.277022530039669,1.4
6475722773927,0.142987165257764,0.740445039884866
"2730","MAPKAPK3",12.5358328904014,-0.568811793101411,0.38837384389343
7,-1.46459861302473,0.143030460641835,0.740445039884866
"2731","RGS12",27.1395382144095,0.530726811795129,0.362326546795608,1.
46477484603003,0.142982356799593,0.740445039884866
"2732","RIPOR2",7.37275797674949,0.95020193797443,0.648342821639121,1.
46558565354693,0.142761202027618,0.740445039884866
"2733","HOXA3",9.44316303040681,0.734388888618678,0.501565344945996,1.
46419384038136,0.143140992748396,0.740445039884866
"2734","AC007566.1",3.1692563569378,1.21373693995804,0.829013163180009
,1.46407438852029,0.143173624237625,0.740445039884866
"2735","NDUFS3",120.818422433199,-0.429609002321557,0.293238752527116,
-1.46504852656482,0.142907678688774,0.740445039884866
"2736","AP001160.3",4.10240357761476,-0.997099753026148,0.681042858701
808,-1.46407783340802,0.143172683093949,0.740445039884866
"2737","ZMYM2",53.5566806944705,0.341498216665407,0.233212581503832,1.
46432158360974,0.143106102563819,0.740445039884866
"2738","AL031846.2",3.4724220159661,1.13352378302776,0.774004145771767
,1.46449316740741,0.143059248585018,0.740445039884866
"2739","ZNF615",8.19602279885592,0.769411258779762,0.525731093112747,1
.46350723565584,0.143328635187919,0.740976076765583
"2740","RABGGTB",41.6828718545579,-0.410478158310259,0.280586035867994
,-1.46293152843634,0.143486115809814,0.740978628689628
"2741","KLF13",116.203574535597,-0.351895985407075,0.240503958976305,-
1.46316088477257,0.143423361114805,0.740978628689628
"2742","LINC01894",4.90625443156855,1.31705686004172,0.9002326803263,1
.46301827163654,0.143462379334672,0.740978628689628
"2743","TXNDC15",128.68742554479,-0.25924183254339,0.177256987014279,-
1.46251968348366,0.143598854482159,0.741290477385116
"2744","GMEB1",21.6386355643065,-0.493935966883356,0.337909644296007,-
1.46173977340129,0.143812533642401,0.741388563495501
"2745","SLC30A1",30.9806303096583,0.46402829052042,0.317427660256399,1
.46183949484933,0.143785198450293,0.741388563495501

"2746","CCDC74A",18.2914580403701,-0.589964633773666,0.403526875534178
,-1.46202067208705,0.143735545161368,0.741388563495501
"2747","EIF3G",356.490643940582,-0.344290833229844,0.235574416411469,-
1.46149500643773,0.143879644949551,0.741388563495501
"2748","EIF3D",208.442526724067,-0.35017211830252 4,0.239580296941212,-
1.46160649591502,0.143849073285725,0.741388563495501
"2749","DYNLL1",874.377334570911,-0.362892196643275,0.248343511018984,
-1.46125097110161,0.143946579559972,0.741463647351474
"2750","ERCC6L2",37.9605234235552,0.34893489092734,0.238847427492213,1
.46091123773446,0.144039802213119,0.741472171916117
"2751","OLFM2",27.0361532370545,-0.596728449106112,0.408476585289942,-
1.46086329203557,0.144052962213364,0.741472171916117
"2752","PCDHB16",11.646895620067,0.680211590832974,0.465792479646081,1
.46033184423633,0.144198894250111,0.741684105550877
"2753","RANGRF",38.6098933629627,-0.423383736825819,0.289907341061096,
-1.46041054109283,0.144177277470448,0.741684105550877
"2754","PPARGC1B",3.91447402637982,1.09566513520499,0.750481522077731,
1.45994951637397,0.144303949013472,0.741954944818725
"2755","FAM86C1",3.04304125398774,-1.14116581160145,0.781867019868504,
-1.4595395158033,0.144416672768591,0.742265004139112
"2756","EPS8L2",27.4393918316996,-0.438663935437319,0.300599680127518,
-1.45929608192275,0.14448363333347,0.742339712627697
"2757","ALG14",33.680274020623,-0.419388836359865,0.287539049476191,-1
.45854567274902,0.144690195668913,0.74286191830015
"2758","INHBB",18.6185691581306,1.49572717448118,1.02541146857471,1.45
866046979189,0.144658581262597,0.74286191830015
"2759","EIF4EBP3",47.9384236042337,0.446153533901505,0.306056846779806
,1.45774727340928,0.144910216451195,0.743721879285584
"2760","MTHFD2",18.2436778559364,-0.69910682357938,0.479963089764699,-
1.45658455512093,0.145231093605993,0.743751278647688
"2761","POLR1A",13.6365133145729,0.638988617170528,0.438504936631818,1
.45719822923463,0.14506166927057,0.743751278647688
"2762","MKRN1",64.7700759828979,-0.315261166733359,0.216386127832734,-
1.45693797421735,0.145133502481006,0.743751278647688
"2763","VEZT",49.8284146368595,0.332260009435,0.2280867918674,1.456726
21686994,0.145191969908462,0.743751278647688
"2764","NFKBID",3.94851513196323,-1.24992637918417,0.85790440442382,-1
.45695298070376,0.145129359787754,0.743751278647688
"2765","PPP1R12C",31.9114034052755,-0.395580765378442,0.2714163318711,
-1.45746854159944,0.144987088959205,0.743751278647688
"2766","ZNF669",7.90104612878373,-0.74342839015678,0.510838821708566,-
1.45530910839996,0.145583706169513,0.743763414709056
"2767","SATB1",23.2421782757914,0.505964219148378,0.347916154643724,1.
45427055454352,0.145871310727034,0.743763414709056
"2768","RGMB",18.1190540612965,-0.567473384590253,0.390369507985221,-1
.45368265958861,0.146034307916852,0.743763414709056
"2769","GGCT",63.3148305913892,-0.454826092663145,0.312813521273832,-1
.45398476002896,0.14595053146451,0.743763414709056
"2770","URGCP",25.9196866243202,0.438987025643804,0.30171956986719,1.4
5495045560696,0.145682978023059,0.743763414709056

"2771","AGK",12.3974509885448,0.621507983889676,0.427582296637763,1.45
35400290817,0.146073874032902,0.743763414709056
"2772","VDAC2",231.786939009152,-0.315619205320928,0.217121670800068,-
1.4536513290355,0.146042998400047,0.743763414709056
"2773","POLE2",2.02049264453917,1.70830630196462,1.1746678575669,1.454
28879402817,0.145866255955313,0.743763414709056
"2774","UNKL",28.3357221920411,0.482709106628565,0.331821833011986,1.4
5472376620597,0.145745750326179,0.743763414709056
"2775","AC005736.1",5.65939825061193,0.980787214304073,0.6734586134900
47,1.45634370792492,0.145297628561599,0.743763414709056
"2776","ULK2",22.1034547539507,0.602149450901006,0.414212917216474,1.4
5371963517573,0.146024052120725,0.743763414709056
"2777","TUBG1",15.4117262469832,-0.678859907098105,0.46619578518503,-1
.45616912179648,0.145345873237057,0.743763414709056
"2778","ALKBH7",144.399013467894,-0.43584978242347,0.299413302901107,-
1.45567941771587,0.145481262249791,0.743763414709056
"2779","SIRPA",51.1250644303904,0.367859728013135,0.252695275981434,1.
45574438059603,0.145463296339075,0.743763414709056
"2780","NEURL2",4.89459261085975,-1.00131212984637,0.688415582736091,-
1.45451694435312,0.145803039190726,0.743763414709056
"2781","G6PD",46.9138621993173,-0.344213001337042,0.236753890129396,-1
.4538853032105,0.145978108094494,0.743763414709056
"2782","ANKIB1",72.4576717519572,0.304602295376285,0.209588944255357,1
.45333188474468,0.146131628596868,0.743790029091175
"2783","STRN4",22.2383955752548,-0.437938498388672,0.301405040846414,-
1.45298996048255,0.146226541491846,0.744005687216866
"2784","AL512625.1",4.99795564900479,0.909481536574105,0.6262507434608
79,1.45226420259079,0.14642815699413,0.744641384436783
"2785","DPYSL4",2.09287810749206,-1.74404358571665,1.20099821977957,-1
.45216167434183,0.146456656472912,0.744641384436783
"2786","DOK1",16.7566785536664,-0.575797232039144,0.396870498667523,-1
.45084412666691,0.146823269129108,0.744667151694079
"2787","MRPL35",32.4889652437943,-0.388497073207181,0.267649134439177,
-1.45151626969101,0.146636155170469,0.744667151694079
"2788","IL7R",8.72227119705341,0.78040637433985,0.537906912961221,1.45
082049614059,0.146829850814256,0.744667151694079
"2789","LINC00472",14.2730865783522,0.685000415117022,0.47184828508450
4,1.45173869815876,0.146574274798905,0.744667151694079
"2790","MDH2",141.273915215992,-0.411653503525531,0.283635462820675,-1
.45134708978825,0.146683234985346,0.744667151694079
"2791","AC105446.1",5.13544006335645,-0.93207859785709,0.6421428745465
3,-1.45151279380824,0.146637122331674,0.744667151694079
"2792","EMC9",8.12003472185767,-0.814201566945127,0.561142240593221,-1
.45097180009899,0.146787712828214,0.744667151694079
"2793","PFN4",3.27027485954628,1.09130232876807,0.753840938299972,1.44
765596205099,0.147713291806407,0.744881714624646
"2794","TMSB10",5880.24056135843,-0.367700336002689,0.253617104077345,
-1.44982467700819,0.147107416202155,0.744881714624646
"2795","FAM200B",104.223666260284,-0.331564666096865,0.229035845936864
,-1.44765403310827,0.147713831544209,0.744881714624646

"2796","SEPTIN8",70.6853188128872,0.367862015302258,0.254005544459963,
1.44824403768179,0.147548812522999,0.744881714624646
"2797","SMAD5-
AS1",3.43250747232944,1.14493867242435,0.790463748204964,1.44843919158
133,0.147494260739393,0.744881714624646
"2798","PAK1IP1",43.0265745022112,-0.427350314007943,0.295146052586907
,-1.44792827233259,0.147637111742438,0.744881714624646
"2799","SMIM29",22.6484657109391,0.412701992206251,0.284842351299303,1
.44887861767648,0.147371483470539,0.744881714624646
"2800","KPNA5",25.3067809997604,0.50531619692437,0.348776842237236,1.4
4882382007649,0.147386789856392,0.744881714624646
"2801","WDR27",20.0012811486301,0.488355368065902,0.336954209005391,1.
44932265279431,0.147247497801781,0.744881714624646
"2802","SRRT",30.7613077773712,-0.444615917711573,0.307126576228498,-1
.44766344603401,0.14771119772607,0.744881714624646
"2803","MICU3",43.5387330510606,0.460212318348111,0.317283942833403,1.
45047465761529,0.146926201203325,0.744881714624646
"2804","ARAP1",63.2864610720114,0.293984989704461,0.202808122151288,1.
4495720713057,0.147177888926562,0.744881714624646
"2805","VAT1",219.580691365569,0.291086952168037,0.200916326578099,1.4
4879690528728,0.147394308297631,0.744881714624646
"2806","SDCBP2",9.21431841151251,-0.83245485393523,0.57476919001332,-1
.44832894385995,0.147525076623984,0.744881714624646
"2807","SLC25A1",44.6931505196252,-0.341052768243661,0.235271530635936
,-1.44961342038197,0.147166351466191,0.744881714624646
"2808","AL121672.2",1.69744666712469,1.65827936960366,1.14429686715778
,1.44916884525125,0.147290435669258,0.744881714624646
"2809","ALPL",80.1935773159931,1.45609015795016,1.00626586646759,1.447
02330315708,0.147890397034385,0.745241288970422
"2810","EIF3F",431.485938196536,-0.328833604678647,0.227231726223963,-
1.44712892932276,0.147860816985012,0.745241288970422
"2811","FBF1",5.94177412948394,0.905500629284109,0.626095891223349,1.4
4626508810787,0.148102863891705,0.746046443509978
"2812","VSTM4",66.56314422497,0.396774820222976,0.274429163663749,1.44
581871301818,0.148228056031332,0.746146204551601
"2813","MAFB",266.230327354714,0.417224364105593,0.288551711785497,1.4
4592579792335,0.148198015203494,0.746146204551601
"2814","CHCHD5",78.4999626699014,-0.428699291924541,0.296583457501841,
-1.44545921588321,0.148328940789091,0.74638869991952
"2815","RBBP4",88.8368194881079,0.261491331451101,0.181175024085757,1.
44330783324377,0.148933774145638,0.748184212195193
"2816","HMGCS1",31.595121981836,-0.392088429122278,0.27169406129O715,-
1.44312476783488,0.148985327409265,0.748184212195193
"2817","CDO1",101.373631085873,-0.457730034218154,0.317103959508674,-1
.44346994256196,0.148888133721458,0.748184212195193
"2818","IFFO1",26.5129225418584,-0.416256453789437,0.288392374787056,-
1.44336844584325,0.14891670796203,0.748184212195193
"2819","ALDH1A3",73.8206953033174,-0.753309768884425,0.522021481870119
,-1.44306277624002,0.149002788021924,0.748184212195193
"2820","SAMD1",45.9178491787687,-0.354347250701421,0.245463272279884,-

1.4435856224445,0.148855571617347,0.748184212195193
"2821","KCTD10",36.1164158947556,0.459492655206918,0.318550818900695,1
.44244694392125,0.149176328797239,0.748524739818887
"2822","NHS",6.47049211384693,-0.896607690858792,0.621565576537551,-1.
4424989489498,0.149161667884373,0.748524739818887
"2823","SHB",6.02886225671618,-0.98717967593698,0.684472486943408,-1.4
4224887744626,0.149232176456195,0.748539716124591
"2824","PEG10",57.6184203383196,0.518021136638788,0.359637496467224,1.
44039801669012,0.14975482448115,0.750404003094926
"2825","GVQW3",10.0021031785386,0.647508598972671,0.449543336836943,1.
44036969500792,0.149762832821064,0.750404003094926
"2826","CTDNEP1",146.351150654327,-0.253766337590978,0.176139918088994
,-1.44070884297087,0.149666955598551,0.750404003094926
"2827","NBPF14",25.9895007475066,0.717756026993523,0.498837944739623,1
.43885611462089,0.150191293655479,0.750451322154067
"2828","NCSTN",64.0688308093729,0.365951740977115,0.254167259899136,1.
43980676788324,0.149922075892024,0.750451322154067
"2829","GPAT2",9.11120159635007,-1.02343082497256,0.710712111190819,-1
.4400075767075,0.149865255478789,0.750451322154067
"2830","CANX",263.869406157302,-0.232575926313794,0.161625158070895,-1
.43898344224219,0.15015521407586,0.750451322154067
"2831","PSMG4",49.0830428584621,-0.385629285769676,0.26799271812698,-1
.43895434347942,0.150163458924242,0.750451322154067
"2832","C8orf88",9.62396742814765,-0.708370774741191,0.492321223802783
,-1.43883858849229,0.15019626038027,0.750451322154067
"2833","AP1G1",37.9879526431085,0.354467045999993,0.246242707384487,1.
43950271569474,0.150008141089833,0.750451322154067
"2834","GEMIN8",21.1427871328882,-0.551005879053024,0.382821955840392,
-1.43932674353389,0.150057969089701,0.750451322154067
"2835","CPXM2",51.2798671054105,0.384749846073117,0.267560313691595,1.
43799295480196,0.150436053125526,0.751384307674586
"2836","CHST14",36.2619189700101,0.37796859255927,0.263002928691717,1.
43712693405901,0.150681929347019,0.752347009715724
"2837","UQCRH",396.801687841798,-0.364950449488589,0.254046095478901,-
1.43655209028355,0.150845305148742,0.752897257985967
"2838","STRIP1",14.8185255727608,0.534025952302938,0.371890638627169,1
.43597578652232,0.15100923137662,0.753449864796666
"2839","USP33",93.0318409583064,0.32559837104929,0.226832998127809,1.4
3541007585603,0.151170276456071,0.753778869773698
"2840","OSMR-
AS1",11.9567981686254,-0.611077033523566,0.425841262792489,-1.43498783
916894,0.151290562987449,0.753778869773698
"2841","SRSF8",52.8452436356319,-0.287771565333628,0.20049923203752,-1
.43527514998051,0.151208706132982,0.753778869773698
"2842","NT5M",3.42807446595338,-1.14225951353641,0.796105242111954,-1.
4348096873551,0.151341336636061,0.753778869773698
"2843","METTL23",46.7963840290846,-0.357108479029211,0.248831090277662
,-1.43514413183146,0.151246029931285,0.753778869773698
"2844","MNDA",2.92070261951221,-1.34988710796871,0.942187426102148,-1.
43271611419527,0.151938982479441,0.754217365217166

"2845","TIMM17A",41.5995177478511,-0.396519671389026,0.276782517692664,-1.43260374497105,0.151971110915776,0.754217365217166
"2846","CGGBP1",92.8435781590003,0.28080872740798,0.196014474403022,1.43259179335203,0.151974528408687,0.754217365217166
"2847","GZMA",6.0904876514865,0.806507584062142,0.563302500293138,1.43174863176081,0.152215773054189,0.754217365217166
"2848","AC008393.1",3.34785455112867,1.08388577661769,0.755896931316536,1.43390683532728,0.151598851011376,0.754217365217166
"2849","LRRC6",6.87227932820993,0.822886596837592,0.574338663335753,1.43275500914091,0.151927862901173,0.754217365217166
"2850","DNM1",125.343668075908,-0.32609980401402,0.227744560351465,-1.43186648897681,0.152182034336831,0.754217365217166
"2851","DPF2",46.6049550396074,0.341161142950718,0.238097420361422,1.43286366745532,0.151896802132158,0.754217365217166
"2852","VDR",14.6520427326301,0.694608120335171,0.485034991393909,1.43207837096244,0.152121393679732,0.754217365217166
"2853","COX14",121.361459591524,-0.33701194870741,0.235265381683595,-1.43247572717967,0.152007719869573,0.754217365217166
"2854","POSTN",398.946176012237,-1.42507668799571,0.994053798890399,-1.43360116885669,0.151686109768509,0.754217365217166
"2855","PRIMA1",6.79989793537303,1.03927275609557,0.725732921932552,1.4320319840639,0.152134668044205,0.754217365217166
"2856","SERPINA5",36.3974340688917,-0.505427199914639,0.352688331819331,-1.43307037493248,0.151837726646309,0.754217365217166
"2857","JPT2",12.1177122499852,0.627744956542036,0.438459783698597,1.43170475350496,0.152228335437194,0.754217365217166
"2858","DDX3X",356.262555752566,0.351228568808104,0.245160800800027,1.43264570707041,0.151959112571546,0.754217365217166
"2859","ZNF501",6.89974238865993,0.747378573425256,0.522188999843744,1.43124151150043,0.152361010180996,0.754351725970674
"2860","TNPO3",35.5681298275932,0.358490733140756,0.250541048246729,1.43086626183395,0.152468548003395,0.754351725970674
"2861","LINC00511",1.99691363529681,-1.69004533487366,1.18108261796527,-1.43092897073129,0.152450573073663,0.754351725970674
"2862","SMARCA1",127.93106677212,0.342427559058204,0.239257603744489,1.43120867926059,0.15237041683174,0.754351725970674
"2863","SH3RF1",27.3798724434968,0.448038566108506,0.313302777253882,1.43004977496718,0.152702733713307,0.755246492972555
"2864","CDC26",131.52254778519,-0.367449979581789,0.25709247309912,-1.42925218755868,0.152931762830021,0.756115140248989
"2865","NFKB2",37.2704668439167,-0.401969182808802,0.281304449650673,-1.4289471187106,0.153019433133276,0.756284528156084
"2866","LINC01358",6.6379318333025,-0.803799532693145,0.562613282733227,-1.42868922110799,0.153093577239872,0.756346814396665
"2867","PHIP",146.245652235677,0.339492066508909,0.237651048155951,1.42853174493945,0.153138864186104,0.756346814396665
"2868","SKIL",96.0231022647362,-0.372643652026488,0.261189675976568,-1.42671662129524,0.153661592709548,0.758663930532494
"2869","ZNF681",7.25388460182929,0.830249786214262,0.582201230091851,1.42605295781199,0.153853056192537,0.759344466952359

"2870","DANT2",3.76871882921621,1.19518254677761,0.838324535227658,1.4
2568002790595,0.153960724094531,0.759611098668488
"2871","NOP14-
AS1",11.285767642831,0.595719126769484,0.418061706876365,1.42495501733
589,0.154170204432743,0.760114935504053
"2872","CBX6",91.6640223542769,-0.301601126921235,0.211656335248604,-1
.42495676572584,0.154169699002712,0.760114935504053
"2873","SH3GL1",88.1702211531535,-0.350401143803078,0.245939720360935,
-1.42474401161731,0.15423121187221,0.760151047723807
"2874","DENND5A",45.2763001439651,0.305382455773007,0.214391244419927,
1.42441663883837,0.154325900472779,0.76035307957361
"2875","HMGN1",209.837383758935,-0.332413048676701,0.233435903957889,-
1.42400137699755,0.154446073460446,0.760680487026059
"2876","RNF187",111.735938961852,-0.327377648553402,0.229965635677319,
-1.42359377995401,0.154564097452964,0.760997086207916
"2877","PPP2R5A",43.9873346438226,-0.346766070178911,0.243784131497089
,-1.42243085326926,0.154901211667766,0.761840210418851
"2878","C2orf69",28.0868783805307,-0.442141641545955,0.310892450249474
,-1.42216911729815,0.154977161747434,0.761840210418851
"2879","EEF1D",1124.07807107934,-0.304040362440945,0.213768178164841,-
1.42229009505098,0.154942053123747,0.761840210418851
"2880","NBR1",71.6839294989394,0.290097576173149,0.203973203035739,1.4
2223376333566,0.154958400245683,0.761840210418851
"2881","SCRN2",49.3653585738021,0.344705298056471,0.242395930537139,1.
42207543374437,0.155004353546378,0.761840210418851
"2882","HEATR5B",16.8878117852226,0.520255957886277,0.365895307593662,
1.4218710846766,0.155063678755198,0.761867346000557
"2883","SRGAP2B",36.3217567536485,0.454807833803175,0.32091478112992,1
.41722307773368,0.156417716589257,0.762685748249505
"2884","DUSP23",41.6080687453304,-0.430335412658303,0.303840730985096,
-1.41631904077868,0.156682115451147,0.762685748249505
"2885","PELI1",19.2387033017636,-0.577871203588197,0.40791409860129,-1
.41664925426623,0.15658550041729,0.762685748249505
"2886","ORC2",15.5652155777968,0.556442555717955,0.391958358137551,1.4
1964712364338,0.155710439073757,0.762685748249505
"2887","IGFBP5",1210.26696908643,0.519102773795155,0.365331442828868,1
.42090910592198,0.155343185423704,0.762685748249505
"2888","LDB2",75.8769169854857,0.348278974213496,0.246156361566677,1.4
1486887438883,0.157106945493839,0.762685748249505
"2889","CERT1",84.0891146180868,0.321438291242549,0.226839468649946,1.
41702981917396,0.156474209431461,0.762685748249505
"2890","CYSTM1",280.289446518788,-0.36598967517264,0.258677295053321,-
1.41485040307538,0.157112362339438,0.762685748249505
"2891","CARMN",7.94781858245076,1.33072619190621,0.937604026247297,1.4
1928378574947,0.155816297309773,0.762685748249505
"2892","CD83",20.6345911193762,-0.830483245998522,0.585816640990343,-1
.41765048632719,0.15629283255401,0.762685748249505
"2893","GTF2IRD2B",22.2068604924401,0.460965676020834,0.32487465553325
5,1.41890316209554,0.155927250301272,0.762685748249505
"2894","AC078846.1",3.03586696628629,-1.10553756131124,0.7815898613848

05,-1.41447275090349,0.157223142594654,0.762685748249505
"2895","PGM5",29.5362746952113,0.62392022920149,0.440180857841234,1.41741790467982,0.156360780939957,0.762685748249505
"2896","NEK6",34.3980490600828,0.381370018102183,0.269603163483246,1.41456062004214,0.157197361832318,0.762685748249505
"2897","FCN1",5.42490743647716,-1.21475912367404,0.856731356897569,-1.41789968803404,0.156220053500503,0.762685748249505
"2898","TASOR2",59.6790746525898,0.300440695984885,0.212357625305452,1.41478647424473,0.157131111021948,0.762685748249505
"2899","JMJD1C-AS1",4.63748070126669,-1.12991325124023,0.798641915508853,-1.4147933251416,0.157129101748895,0.762685748249505
"2900","PLAAT3",115.513480397448,-0.345265762805495,0.243488030726946,-1.41799891261466,0.15619108224031,0.762685748249505
"2901","ZFPL1",40.9053472409705,-0.468378453973294,0.330832371139398,-1.41575763085149,0.156846478504816,0.762685748249505
"2902","TRIM29",1.57978941645054,-1.76141109161431,1.24291677749146,-1.41715931710996,0.156436353219634,0.762685748249505
"2903","SPA17",11.4104099182714,-0.855069769873068,0.604126354434235,-1.4153823345016,0.156956426275994,0.762685748249505
"2904","AC021054.1",8.07978783270504,0.777209180958499,0.549354890495499,1.41476701928963,0.157136816996679,0.762685748249505
"2905","CCND2-AS1",3.72876394358367,-1.07120478021313,0.754304323345803,-1.42012281655988,0.155571928799476,0.762685748249505
"2906","FAM177A1",94.1698124982023,-0.271864836881172,0.192046896486272,-1.41561692407045,0.156887693476156,0.762685748249505
"2907","PLIN1",3.00403251925907,-1.21073126719513,0.854263663140062,-1.41728054163604,0.15640092187403,0.762685748249505
"2908","FUS",396.525389016375,0.373529202204152,0.262956436632966,1.4204984178635,0.155462628870349,0.762685748249505
"2909","LONP2",75.7207480563789,0.311684982412027,0.22034364285371,1.4145403896175,0.157203297145074,0.762685748249505
"2910","AC129507.1",2.96959639795732,1.808210881891,1.27558805720031,1.4175508085735,0.156321950519939,0.762685748249505
"2911","RNF213",133.518980968955,0.310375499624237,0.218474321650097,1.42064979206722,0.155418595480954,0.762685748249505
"2912","ZNF562",12.7716657795113,0.611579625557892,0.430669510769784,1.42006715187418,0.155588132176881,0.762685748249505
"2913","ZNF254",23.7365111324456,0.442243264996897,0.311503856361235,1.41970398107704,0.155693878640998,0.762685748249505
"2914","MAP3K10",9.50254262147196,0.712287206624044,0.501624933891593,1.41995973186209,0.155619404581193,0.762685748249505
"2915","AC010642.2",47.1892098826056,-0.400413339529793,0.282583631783047,-1.41697286924676,0.156490859838357,0.762685748249505
"2916","TNRC6B",224.574778074528,0.320251745625644,0.225670098867834,1.41911465999402,0.155865590600681,0.762685748249505
"2917","PDHA1",58.4321470645049,-0.346262939117796,0.244039477781063,-1.41888083955188,0.155933759253444,0.762685748249505
"2918","SMS",97.1090609253496,-0.27476501876689,0.193737065836465,-1.4

1823670953508,0.156121667681921,0.762685748249505
"2919","MORC4",60.8457494980739,-0.309717375944361,0.21869029645906,-1
.41623739580206,0.156706010396521,0.762685748249505
"2920","TBC1D15",60.2703760121786,0.285151445492289,0.201656325295025,
1.41404662152357,0.157348214202216,0.763031066131295
"2921","TAOK2",15.7996841020768,0.531309015019797,0.375860213154913,1.
41358142315749,0.157484838870674,0.763432152827369
"2922","KANSL3",18.654672444625,0.505902474623548,0.358025317302659,1.
41303547591267,0.157645293404298,0.763948444423293
"2923","NBEAL1",98.9990437583877,0.285344880652203,0.201994241042126,1
.41263869296498,0.157761986075877,0.763991013281263
"2924","ARHGAP23",22.9041169296181,0.439121352255854,0.310826470275172
,1.41275404204508,0.157728055513806,0.763991013281263
"2925","LINC00623",41.5615076268407,-0.334091959355345,0.2370300934301
48,-1.4094917422534,0.158689813515727,0.764808839200991
"2926","CDC42BPA",73.992920229189,0.418057759125851,0.296528366414183,
1.4098406981473,0.158586726364188,0.764808839200991
"2927","CBWD2",14.8576213468369,-0.744213852896537,0.527871066834037,-
1.40984020465459,0.158586872114023,0.764808839200991
"2928","FAM193A",31.8606653169269,0.485005411088861,0.343916522542549,
1.41024166999379,0.158468335471437,0.764808839200991
"2929","RSPH9",1.61022169939223,-1.74732626813856,1.23931854736223,-1.
409908914748,0.158566580014605,0.764808839200991
"2930","PHF3",150.450065854089,0.338718659106024,0.240124393038052,1.4
105966279417,0.158363586484183,0.764808839200991
"2931","TMEM30A",103.398399174148,0.232074997473175,0.164714506598342,
1.40895299549475,0.158849067520488,0.764808839200991
"2932","PTPA",29.9571296056281,-0.378701253108532,0.268705794157016,-1
.40935276180625,0.158730884683493,0.764808839200991
"2933","NAA16",19.6999167553509,0.484297718118982,0.343321525574984,1.
41062439154637,0.158355395585505,0.764808839200991
"2934","LIN52",8.69225404402878,0.643932460651257,0.456969795768682,1.
40913571665733,0.158795041437993,0.764808839200991
"2935","FAM174B",4.8195920462043,1.07089736582185,0.760053400431422,1.
40897648140773,0.158842122543769,0.764808839200991
"2936","WDR90",14.4238394786391,0.529818787576097,0.375719934351197,1.
41014287275175,0.158497500117264,0.764808839200991
"2937","EMC6",56.9865818549357,-0.406958519024871,0.288384554404969,-1
.41116614190576,0.158195631024718,0.764808839200991
"2938","RPL23",2400.47317079181,-0.211817037132423,0.150087534425661,-
1.41129000448293,0.158159120556748,0.764808839200991
"2939","ICAM3",20.7704976452723,-0.444302087902456,0.314714431274431,-
1.41176267673288,0.15801985151951,0.764808839200991
"2940","TGIF2",6.61530926503093,0.89466566069585,0.633998427029451,1.4
1114807632526,0.158200956675688,0.764808839200991
"2941","MPP1",15.447486247546,-0.573374756993275,0.406561436415295,-1.
41030286110949,0.158450274077536,0.764808839200991
"2942","NUBP2",64.3371312912405,-0.321028286490349,0.22789893925072,-1
.40864317993676,0.158940704030346,0.764989928303773
"2943","CLIC3",15.6763083133321,-0.633110443719708,0.449595545171128,-

1.4081777511357,0.159078442641919,0.76513272683749
"2944","MED13L",148.035909936414,0.297469500960343,0.211230192146009,1
.40827169609695,0.159050633375105,0.76513272683749
"2945","NKIRAS1",17.5570792813846,0.536045215098171,0.380752946846426,
1.40785572255697,0.159173796365348,0.765331394408599
"2946","PDE8B",1.58631940586709,-1.79327650999808,1.27417376470351,-1.
40740341676659,0.159307798620795,0.765483708180537
"2947","AP005205.2",1.92230017489296,-1.48548649174867,1.0556317019863
,-1.40720147846408,0.15936765336979,0.765483708180537
"2948","TFE3",34.28444216847,0.348915453066321,0.247946697577334,1.407
2196019368,0.159362280856469,0.765483708180537
"2949","DCTN3",206.555641493513,-0.298802083068917,0.212413475121488,-
1.40670022416431,0.159516299254595,0.765678236422055
"2950","NSMCE1",74.6856970513691,0.322829340361716,0.229493845763017,1
.40670151431895,0.159515916527439,0.765678236422055
"2951","MRPL20-
AS1",11.845961051672,-0.585823062482233,0.417106267874862,-1.404493549
01084,0.160171931314804,0.766598007978737
"2952","NTRK1",1.94524462473904,1.96665255699984,1.40121676228176,1.40
353199443412,0.160458258190065,0.766598007978737
"2953","ADAM17",68.6237219005174,-0.408344727434479,0.291232864513607,
-1.40212447560292,0.1608780794033,0.766598007978737
"2954","TCF23",3.20015619382539,1.48120361876616,1.05633046101467,1.40
221613730932,0.160850714191646,0.766598007978737
"2955","AC008280.3",2.23483955162565,1.36688522906118,0.97290257229616
2,1.40495592054524,0.16003438624818,0.766598007978737
"2956","TET3",2.84104905522396,-1.18009472471827,0.839958020672459,-1.
40494488495211,0.160037668046755,0.766598007978737
"2957","SETD2",66.9724284974966,0.424530571825362,0.302254510519906,1.
40454668846837,0.160156118985089,0.766598007978737
"2958","SPICE1",14.053754477252,-0.545078883627386,0.388469963637589,-
1.40314293162676,0.160574221219089,0.766598007978737
"2959","FAM162A",119.359426667089,-0.429184703009994,0.306043862336063
,-1.40236337279887,0.160806765025798,0.766598007978737
"2960","ALG3",45.1097854278191,-0.38563529124786,0.274891654835025,-1.
40286285329141,0.160657739824435,0.766598007978737
"2961","DDX60L",27.3174985107803,0.383511663920186,0.273076638090761,1
.4044103757888,0.160196682947044,0.766598007978737
"2962","BRD8",36.5353057480855,0.479114591710794,0.341944176425523,1.4
0114856383626,0.161169651818693,0.766598007978737
"2963","TRIM7",9.55658530980152,-0.746109074658178,0.530985935578061,-
1.40513905296931,0.15997993319349,0.766598007978737
"2964","MRPL2",57.6677983666018,-0.416024298249811,0.296280577744237,-
1.40415649725424,0.160272252888713,0.766598007978737
"2965","WTAP",157.768115909992,-0.330514949807606,0.235761074797039,-1
.4019063583424,0.160943211524781,0.766598007978737
"2966","NDUFB2",343.621024611204,-0.362416886746385,0.258486571037516,
-1.40207239893242,0.160893628238032,0.766598007978737
"2967","RSU1",84.9678150457366,0.321953581785267,0.229705000144755,1.4
0159587985625,0.161035958043105,0.766598007978737

"2968","TRIM34",15.3939400943248,0.499131094792259,0.356102281180475,1
.40165093337129,0.161019509444646,0.766598007978737
"2969","FTH1",8588.77314602461,-0.384125977548168,0.273985831778255,-1
.40199212147237,0.160917599370753,0.766598007978737
"2970","DYNC2H1",53.7584543991116,0.392235640305414,0.2796817663384,1.
40243550890203,0.16078523601838,0.766598007978737
"2971","IGHA1",58.6548807829576,-1.35623603797787,0.967734419229551,-1
.40145479072411,0.161078117703495,0.766598007978737
"2972","MEIS2",77.4476722505829,0.27823965249785,0.198507291985665,1.4
0165960511891,0.161016918661401,0.766598007978737
"2973","CD300A",1.69247666140437,-1.57904748569968,1.12683807669218,-1
.40130824327037,0.161121917223671,0.766598007978737
"2974","VPS4B",115.61155099796,0.268390995280621,0.191034555563684,1.4
0493427740694,0.160040822599075,0.766598007978737
"2975","TNFAIP8L1",2.69018452657152,1.31074597752378,0.934759815063224
,1.40222756306134,0.16084730332862,0.766598007978737
"2976","TTYH1",1.51854973707424,-1.7157546733292,1.22084945398971,-1.4
0537776195267,0.159908975898223,0.766598007978737
"2977","CCDC117",27.7991949647591,0.401040187243385,0.285437225877538,
1.40500310010525,0.160020356419494,0.766598007978737
"2978","MTMR11",14.7350650060768,-0.582002389136404,0.416806740585439,
-1.39633631720767,0.16261324231788,0.766764480652352
"2979","PPP1R12B",69.2569086386817,0.350914290269773,0.251326804598846
,1.39624697345706,0.162640135821722,0.766764480652352
"2980","HNRNPLL",33.3484313135394,-0.377379070664124,0.26966718961977,
-1.39942523670094,0.161685503428908,0.766764480652352
"2981","R3HDM1",20.0272697425691,-0.459635095645385,0.328893215673162,
-1.39752075671317,0.162257029275749,0.766764480652352
"2982","HMCES",30.745821746047,-0.388215600389438,0.277703160716138,-1
.39795168117032,0.162127577530624,0.766764480652352
"2983","ART3",1.69107057484004,1.81657942748795,1.29788177685652,1.399
64938246357,0.161618338388835,0.766764480652352
"2984","PPP3CA",119.119977228479,-0.32930356841741,0.235578445646206,-
1.39785101100447,0.162157812344849,0.766764480652352
"2985","TMEM184C",17.5719355858018,0.455524126207214,0.326095651889754
,1.39690340416197,0.16244262079398,0.766764480652352
"2986","MCC",38.7648855024817,0.378327705434275,0.270910217143274,1.39
650585874431,0.16256221757489,0.766764480652352
"2987","MGAT1",120.582582347084,-0.243074138588903,0.173692724879679,-
1.39944916378787,0.161678332698136,0.766764480652352
"2988","NQO2",98.2481263534729,-0.362459142727181,0.259453712915253,-1
.39700888707487,0.16241089866894,0.766764480652352
"2989","RRP36",52.4798045256734,-0.360217345545002,0.257788888901634,-
1.39733464494838,0.162312962241772,0.766764480652352
"2990","MTHFD1L",13.6602566732577,-0.552202459032364,0.39451446156252,
-1.39970143767431,0.161602743114814,0.766764480652352
"2991","AMBRA1",10.9292192420265,0.628068717991367,0.449244361523003,1
.39805587289315,0.162096289548747,0.766764480652352
"2992","UBXN1",224.796731813803,-0.383598569637946,0.274688237280196,-
1.39648706270103,0.162567873783713,0.766764480652352

"2993","SF3B2",170.922687532805,-0.262291188658794,0.187374012641498,-1.39982692883156,0.161565151761474,0.766764480652352
"2994","KMT2A",156.589542778859,0.292922765582701,0.209833234265666,1.39597889060718,0.162720852002847,0.766764480652352
"2995","PIGB",7.61852248784685,0.691085292563762,0.495004992271561,1.39611782376657,0.16267901730488,0.766764480652352
"2996","SCAMP2",68.4491315880834,-0.348809895606639,0.24903171886309,-1.40066453060304,0.161314414055118,0.766764480652352
"2997","GFER",41.9047903579249,-0.347065740383527,0.248375890960005,-1.39734069616045,0.162311143416279,0.766764480652352
"2998","ATXN1L",21.2961046878106,0.430092151961607,0.308082104133581,1.39603094821478,0.162705175785837,0.766764480652352
"2999","ALDH3A2",101.231574874458,0.24817354793828,0.177385161539648,1.3990659973146,0.161793193079495,0.766764480652352
"3000","GSDMB",2.50237788000645,1.32677539926599,0.95031119122126,1.39614834753333,0.162669827263225,0.766764480652352
"3001","PSMD3",85.8682104623795,-0.275394306352201,0.197178606393632,-1.39667437248454,0.162511514124475,0.766764480652352
"3002","MCRIP1",173.938441879759,-0.287912939395944,0.205900811655726,-1.39830890942453,0.162020323575353,0.766764480652352
"3003","ZNF571",9.49913414543979,0.773001136817978,0.552129354485191,1.40003629681803,0.161502449482554,0.766764480652352
"3004","RASSF2",46.4355826212409,-0.447282168038179,0.319709045283474,-1.3990288189738,0.161804341169438,0.766764480652352
"3005","NANP",7.31667847588707,-0.724082892213126,0.518586415300575,-1.39626274589828,0.162635387890357,0.766764480652352
"3006","ARL6IP5",637.122717017425,-0.233917733891607,0.167587170904801,-1.39579737893233,0.162775519907344,0.766766920122419
"3007","TRIM4",37.2088990985206,0.356485767331867,0.255537753459972,1.39504148606248,0.163003329607774,0.767329503738721
"3008","ERLIN2",40.8005967450441,0.352306216611651,0.252522033353079,1.39515040305038,0.162970489573975,0.767329503738721
"3009","MTOR",11.7926658719461,0.563886609898143,0.40586838025813,1.38933367891215,0.164731307197993,0.767776017455756
"3010","AL031293.1",1.74792593371115,1.51598067186211,1.09100098125342,1.38953190502216,0.164671066073146,0.767776017455756
"3011","CYB5RL",6.52870437372395,0.8376873708602,0.605451242329472,1.38357527789884,0.166488542539889,0.767776017455756
"3012","IGSF8",35.8029397449775,-0.408073196764319,0.293002431879554,-1.39272972632551,0.163701535909385,0.767776017455756
"3013","HSPA6",2.47340330932706,1.3563813274343,0.979354399506162,1.38497496730321,0.166060121884937,0.767776017455756
"3014","PRG4",370.358695447093,-1.86090114245371,1.33599611263104,-1.39289412960114,0.16365180789111,0.767776017455756
"3015","HEATR1",29.178393360164,0.416746797801017,0.301248112817337,1.38340052624234,0.166542089290808,0.767776017455756
"3016","LDAH",8.49385893344601,0.636610157960825,0.456817095419701,1.39357778932494,0.163445139453105,0.767776017455756
"3017","AL133243.3",10.8787194941336,0.589910212140759,0.426448736065071,1.38330861895378,0.166570256374377,0.767776017455756

"3018","ARL5A",140.478357498702,-0.24473817371869,0.175924030155484,-1
.39115829430685,0.164177430925869,0.767776017455756
"3019","PPP1R1C",2.37986289315359,-1.38852259076513,1.00170697131938,-
1.38615646144127,0.165699132821888,0.767776017455756
"3020","COPS7B",17.4443456221881,0.459360342614121,0.331069784423511,1
.3875030710338,0.165288415327545,0.767776017455756
"3021","IQCA1",1.75783070253279,1.45727428313423,1.0539001896792,1.382
74411315725,0.16674334060303,0.767776017455756
"3022","AC007620.2",2.5405790432843,1.33235064496015,0.95712529000019,
1.39203368553754,0.163912197607609,0.767776017455756
"3023","FAM13A",136.855766635853,0.366529631573779,0.265105455497956,1
.38258049380883,0.166793533514423,0.767776017455756
"3024","CISD2",51.9853536228886,-0.294716540048453,0.212585273357593,-
1.38634504353792,0.165641568871388,0.767776017455756
"3025","AC073475.1",1.75452416449488,1.67812213864131,1.20530813004739
,1.39227646176695,0.163838696466781,0.767776017455756
"3026","HSPA9",109.972570947568,-0.233112745984369,0.167173731605541,-
1.39443406416515,0.1631865675491,0.767776017455756
"3027","Z84485.1",1.58308680885556,1.58602889695545,1.14447871263395,1
.38580899709817,0.165805234370262,0.767776017455756
"3028","COL21A1",14.8596401695924,-0.64008435889047,0.461426585535731,
-1.38718569530906,0.165385146213968,0.767776017455756
"3029","TBPL1",28.5115113791995,-0.408808443263886,0.293307523064354,-
1.39378778625529,0.163381697478283,0.767776017455756
"3030","RMND1",26.5431167904491,0.411800626693311,0.295738590353717,1.
39244806097432,0.163786759340188,0.767776017455756
"3031","TMEM184A",1.79332986227689,2.07944013679639,1.50050463644788,1
.38582719858768,0.165799675103737,0.767776017455756
"3032","AKAP9",258.152655714765,0.263825690986309,0.190773820713006,1.
38292397772543,0.166688177306253,0.767776017455756
"3033","ARMC10",66.2155490274569,-0.407446786906404,0.29319726256613,
-1.38966777295381,0.164629785247795,0.767776017455756
"3034","LZTS1",5.04129339242393,-0.900855200378553,0.651760218374678,-
1.38218807313072,0.166913961256521,0.767776017455756
"3035","DOK2",2.51902666314373,-1.26083600915989,0.911819586929691,-1.
3827691653405,0.166735656438434,0.767776017455756
"3036","YTHDF3-
AS1",5.72423616640553,-0.832130998401919,0.598556485214677,-1.39022969
253014,0.1644591396878,0.767776017455756
"3037","PREX2",30.655360248209,-0.503697464019292,0.362535151175837,-1
.38937551954787,0.164718590403004,0.767776017455756
"3038","ZNF623",16.3554530541771,0.529420278014382,0.379755680538051,1
.39410759376734,0.163285116398544,0.767776017455756
"3039","C8orf82",45.8561374220509,-0.364795751415092,0.262115788790075
,-1.39173513010791,0.164002620110116,0.767776017455756
"3040","PLIN2",340.435286959707,-0.447521867229789,0.321516199057522,-
1.39191079187187,0.163949413458361,0.767776017455756
"3041","SLC44A1",207.620410097879,-0.26119507134945,0.188485495475266,
-1.385756875832,0.165821154503017,0.767776017455756
"3042","FKBP15",35.0982023838237,0.367709530811824,0.264871511863877,1

.38825624629952,0.165059030364592,0.767776017455756
"3043","SPTAN1",217.537818374771,-0.230678065216979,0.166017156465035,-1.38948329274367,0.164685837860571,0.767776017455756
"3044","UAP1L1",11.8904199005268,0.671173718168905,0.484530411392104,1.38520452460467,0.165989937592951,0.767776017455756
"3045","PPA1",301.588047881024,-0.31107789438327,0.224723437927828,-1.38426991528661,0.166275822325611,0.767776017455756
"3046","LCOR",42.4475803193276,0.448034174045456,0.32333851481514,1.38565049790498,0.165853650580734,0.767776017455756
"3047","CHST15",6.51719953237752,0.878617553904678,0.635927526492589,1.38163158111841,0.167084851942893,0.767776017455756
"3048","ZRANB1",41.5204809668096,0.405071467515777,0.291094821968504,1.39154473712901,0.164060303429669,0.767776017455756
"3049","SERGEF",25.2461547962086,-0.411323790128517,0.296055346840891,-1.3893476152943,0.164727071374444,0.767776017455756
"3050","ABTB2",2.09553842622284,-1.38487578616651,1.000451228157,-1.38425117306086,0.166281559111249,0.767776017455756
"3051","NDUFV1",115.61341757877,-0.314766766562096,0.226609136030984,-1.38902946313276,0.164823790969184,0.767776017455756
"3052","KCTD21-AS1",4.00885247817093,1.01633200364431,0.731443520802597,1.38948801204652,0.164684403764773,0.767776017455756
"3053","BIRC3",19.9096879564682,-0.533572689781263,0.385473743525573,-1.38419982876438,0.166297275788399,0.767776017455756
"3054","VAMP1",9.42965061124987,0.811232337568937,0.585924840034315,1.38453310414596,0.166195278878795,0.767776017455756
"3055","A2M",197.362739399312,-0.58491534508941,0.423214919954227,-1.38207638131631,0.166948249663995,0.767776017455756
"3056","NABP2",41.0148733387362,-0.34930626197041,0.251779767607925,-1.38734841678921,0.165335546070089,0.767776017455756
"3057","LIN7A",2.5970300974695,-1.30305462326101,0.940357156674741,-1.38570181979454,0.165837972293176,0.767776017455756
"3058","RAN",297.960606584596,-0.321708690247325,0.232714182165004,-1.38241978745937,0.166842843870045,0.767776017455756
"3059","MRPL52",84.1348261617771,-0.384614078823423,0.278199521880272,-1.38251164568481,0.166814657196989,0.767776017455756
"3060","CYP19A1",17.984265264835,-0.598278657771047,0.431330667572555,-1.38705337401127,0.165425488144404,0.767776017455756
"3061","AC104041.1",3.28726312661826,1.34633128284842,0.972706633972082,1.38410825610455,0.166325309287591,0.767776017455756
"3062","WDR73",34.4193440706838,0.38943273901451,0.280188961166915,1.38989322560255,0.164561303045931,0.767776017455756
"3063","LINC01578",236.484541603893,-0.268990737632924,0.194011492700474,-1.38646805861242,0.165604027108503,0.767776017455756
"3064","NSMCE1-DT",2.41780361342086,1.24971850614031,0.902702888344038,1.38441841970047,0.166230372047396,0.767776017455756
"3065","CTU2",14.0700048086542,-0.55507167349308,0.401348589446639,-1.38301638049454,0.166659843310801,0.767776017455756
"3066","VMO1",7.05017336063945,-0.799177351616134,0.574686687701325,-1

.3906313974537,0.164337230317592,0.767776017455756
"3067","SOX9",31.6350486890885,-0.466548535700233,0.336818285159965,-1
.38516391851664,0.166002350775173,0.767776017455756
"3068","ATP5F1A",221.83270432817,-0.278373459091373,0.20151343168106,-
1.38141391751971,0.1671517290586,0.767776017455756
"3069","RNF165",3.94413363008977,-1.12867295920041,0.814126993444479,-
1.38635982873522,0.16563705638365,0.767776017455756
"3070","TRMT1",16.1327073195003,-0.56371170820462,0.406710603613348,-1
.38602658302101,0.165738786455187,0.767776017455756
"3071","AC020910.5",7.46911127952316,-0.913020840270842,0.659716126922
35,-1.3839601656097,0.166370652334238,0.767776017455756
"3072","PRPF31",76.9455082339684,-0.395971297153122,0.285432620517003,
-1.38726714709728,0.165360316989501,0.767776017455756
"3073","ZNF587",17.1981614517659,0.602595298962576,0.435623580660855,1
.38329357205232,0.166574868181467,0.767776017455756
"3074","TRMT6",21.2240660009452,-0.460187656368386,0.333091514163488,-
1.38156523598052,0.16710523434822,0.767776017455756
"3075","RALGAPB",23.6833226704379,0.466951782412377,0.33654669035444,1
.38747994199735,0.165295463237585,0.767776017455756
"3076","PAXBP1-
AS1",3.43733073655115,1.17295214887238,0.846798201174409,1.38516136104
876,0.16600313261025,0.767776017455756
"3077","TOM1",29.1586224745752,-0.392346969717893,0.281628803051014,-1
.39313509650795,0.163578941799039,0.767776017455756
"3078","H1F0",81.6807142162812,-0.245690555643186,0.177658104084854,-1
.3829403218546,0.166683165347825,0.767776017455756
"3079","ATF4",281.184999095157,-0.16361905980099,0.117738202177587,-1.
38968539331185,0.164624432216033,0.767776017455756
"3080","PARVB",31.3313979915546,-0.390387112517509,0.280375016585233,-
1.39237481738617,0.163808926115268,0.767776017455756
"3081","BCLAF3",27.4550500113451,0.467844950091085,0.338681506969712,1
.38137140783694,0.167164792501137,0.767776017455756
"3082","DYNLT3",42.3555831184145,-0.367054107863279,0.264775446805731,
-1.38628453767691,0.16566003641025,0.767776017455756
"3083","PRPS1",28.9080611842389,-0.391187422830507,0.281145717465941,-
1.39140452273792,0.164102793926265,0.767776017455756
"3084","S100A13",1409.47480334774,-0.334030322091078,0.241880204858193
,-1.38097419872333,0.167286893965428,0.767969222795358
"3085","CXCL16",29.0048173595525,-0.468172232088913,0.339038617265689,
-1.38088172923979,0.167315328553932,0.767969222795358
"3086","AL353807.5",13.9147178776836,0.528440003331625,0.3828728122968
24,1.38019725182772,0.167525919933809,0.768002647410921
"3087","ARFIP1",52.7711979491878,0.33219661837631,0.240695512162575,1.
3801529384226,0.167539560582792,0.768002647410921
"3088","TMEM176A",286.986377063973,0.409154380365858,0.296361015254623
,1.38059447533721,0.16740368298045,0.768002647410921
"3089","CUEDC1",43.1080608547519,0.349966634257862,0.253560304204214,1
.38021065780077,0.167521793442321,0.768002647410921
"3090","FCGRT",653.353210898777,0.334036516843604,0.242079100215424,1.
37986516203319,0.167628164840693,0.768160134027252

"3091","SIAE",11.3699029228037,0.585625594300034,0.424537631403187,1.3
79443307215,0.167758114478331,0.768258376783042
"3092","MORF4L1",591.799509479414,-0.179753476612468,0.130298620393807
,-1.3795501139551,0.167725206205018,0.768258376783042
"3093","TBL1XR1",169.123983996548,0.287629318542286,0.208665703858038,
1.37842162475329,0.168073150609176,0.768955028312095
"3094","CCDC152",19.920703559981,0.559485436788045,0.405836358876111,1
.37859860150884,0.168018547982901,0.768955028312095
"3095","UBE2D2",184.208110710435,-0.321406221917127,0.233157100762514,
-1.37849639091413,0.168050081385593,0.768955028312095
"3096","ACSL3",74.2658820332229,0.352844657562485,0.256173229818104,1.
37736740803488,0.16839868410528,0.770195531954384
"3097","CPE",764.710631083988,0.469419756657315,0.340862411560075,1.37
715318773007,0.168464891398889,0.770249551891593
"3098","TAF6",29.472304976515,-0.370036449281842,0.268774838008779,-1.
37675257112332,0.168588759080776,0.770318434522034
"3099","TMED3",128.435727158546,-0.404778617228158,0.293998481607309,-
1.37680512843198,0.168572504856759,0.770318434522034
"3100","AL358472.4",2.75421326294345,-1.24086947816129,0.9015371009471
52,-1.37639313663035,0.168699951719896,0.770577843985075
"3101","GPN1",21.393385895691,-0.427567469894987,0.310782668399997,-1.
37577642954233,0.168890860967519,0.770704025555936
"3102","BBS12",6.03805695540062,-0.884128882832098,0.642608834130164,-
1.37584302591927,0.168870237438286,0.770704025555936
"3103","ITM2B",3396.09265299687,-0.291914825243774,0.212157811204052,-
1.37593248906123,0.168842535516042,0.770704025555936
"3104","ZKSCAN1",109.487223873288,0.341242858261109,0.248105587315119,
1.37539368602649,0.16900942540875,0.770996605601773
"3105","COLGALT2",12.7818678242904,1.98970769402731,1.44773468098507,1
.37435934923757,0.169330149647411,0.771096808369242
"3106","AC023794.3",2.13390936044806,1.53532979400159,1.11705214397685
,1.374447739329,0.169302724072424,0.771096808369242
"3107","BIVM",27.0268890209782,0.460570685786179,0.334970296039799,1.3
7495978369215,0.169143913089375,0.771096808369242
"3108","RPS15",3725.38862807282,-0.291355004671611,0.212007229868765,-
1.37426919285707,0.169358126696917,0.771096808369242
"3109","HMG20B",98.5822894166395,-0.411100798952375,0.299046164891535,
-1.37470680856744,0.169222359562113,0.771096808369242
"3110","SIX5",26.8182881393752,0.389873811670729,0.283563042085077,1.3
749105271404,0.169159185190316,0.771096808369242
"3111","MT-
ND4",10936.7690616811,-0.22356538210566,0.162707091704437,-1.374035881
06518,0.169430543385172,0.7711785581273
"3112","AC015912.3",12.0703302709607,-0.97111972867489,0.7069621538329
46,-1.37365164939843,0.169549854080609,0.771225805904729
"3113","KRBOX4",24.7816517728576,0.38090111539678,0.277267126392522,1.
37376947766085,0.169513259635613,0.771225805904729
"3114","SRP9",241.699863402947,-0.269681793796845,0.196431907473391,-1
.37290217900764,0.169782759102251,0.771260842415052
"3115","TMEM217",1.68164222617047,-2.00872400555979,1.4628352889344,-1

.37317168976901,0.169698978478644,0.771260842415052
"3116","LACTB2",24.695236272768,-0.416215654977117,0.303177882553064,-1.37284306979177,0.169801138050177,0.771260842415052
"3117","UCK1",32.9512222801079,0.378646585566107,0.275830574798499,1.37275059460945,0.169829894537439,0.771260842415052
"3118","CPT1C",7.38690504773317,-0.930136317138442,0.677560958608379,-1.37277141683136,0.169823419249762,0.771260842415052
"3119","USP48",61.7905219046033,0.299630032945522,0.218520677591218,1.37117473846587,0.170320491012214,0.771270815697791
"3120","ADRA1B",2.63515239430849,1.56930117813309,1.14425325268032,1.37146315682925,0.170230621127487,0.771270815697791
"3121","CCDC7",9.29043025163392,0.655644981341121,0.47796917283191,1.37173068601162,0.170147291992654,0.771270815697791
"3122","TRPM7",48.232047662678,0.407510390441102,0.29718879613555,1.37121720515747,0.170307256347626,0.771270815697791
"3123","TRAF7",44.8193675008001,-0.316738577295929,0.230998947853669,-1.37116891760292,0.170322305133262,0.771270815697791
"3124","ZNF75A",15.857294153667,0.511530909972334,0.372882040379019,1.37183037684621,0.170116248429614,0.771270815697791
"3125","BCAR1",23.4676931825097,-0.46278134132728,0.337493380593906,-1.37123086832962,0.170302998409578,0.771270815697791
"3126","AC005332.3",4.46492107018599,1.07306219616326,0.782323657440666,1.37163459900181,0.170177217348571,0.771270815697791
"3127","HTATSF1",78.6700836873517,-0.294404227548146,0.214585347404905,-1.3719679890008,0.170073403203981,0.771270815697791
"3128","WDR31",4.56459900561009,-0.897027122270949,0.654295288560004,-1.37098209012808,0.170380539182759,0.771287862796633
"3129","AC242426.2",4.86363817418501,-0.870467095828349,0.635218174427618,-1.37034349908629,0.170579700406509,0.771705353795015
"3130","NDUFB5",154.928382074827,-0.360857304787835,0.263398966158196,-1.37000273786613,0.170686046994914,0.771705353795015
"3131","HARS2",10.9714590280468,0.64292186111255,0.469207113505441,1.37023042193305,0.170614984626157,0.771705353795015
"3132","AC092828.1",3.05634630283337,1.1271856189975,0.82277064944368,1.36998763842591,0.17069076045805,0.771705353795015
"3133","CD200",17.2969637107531,-0.519544902562039,0.37929136954539,-1.36977781272681,0.170756270044494,0.771755117724235
"3134","GALK1",56.1915476361585,-0.460326042675487,0.336192202448555,-1.36923473930341,0.170925910200956,0.772275331348288
"3135","MMRN2",2.92473794684846,-1.10789936328233,0.809351937856234,-1.36887219448299,0.171039228771859,0.772540822778157
"3136","TEX261",43.1353601931798,0.360696687241928,0.263616960526304,1.36826054940398,0.171230534703273,0.772608168505787
"3137","PDCL3",27.6755327326777,-0.428450455046548,0.313182704794696,-1.36805273243749,0.171295570645562,0.772608168505787
"3138","GTF2E1",7.69943911132333,0.673241859556404,0.492152959863747,1.36795247506546,0.171326952620634,0.772608168505787
"3139","DUSP5",25.9916903942294,-0.930600672238957,0.680251218146607,-1.36802499931488,0.171304251074456,0.772608168505787
"3140","IGHMBP2",8.78951642949149,0.613752831384971,0.448561429911915,

1.36826929481096,0.171227798249484,0.772608168505787
"3141","SLC39A4",17.7841935529476,-0.500425530006021,0.365888387004526
,-1.36769995381086,0.171406014418253,0.772718613232237
"3142","AL021368.2",17.1486636172333,0.605835897828394,0.4430839042408
71,1.36731642027566,0.171526147049636,0.772889203289748
"3143","DIP2A",36.3926101658576,0.47314438450753,0.346060398412275,1.3
6723065302565,0.17155302019348,0.772889203289748
"3144","PELO",24.1330734234658,-0.413675668671421,0.302678504373923,-1
.36671637626561,0.171714222788207,0.77312349592401
"3145","ESRRA",43.0158625975486,-0.336932145449551,0.246519768131062,-
1.36675508014603,0.17170208692177,0.77312349592401
"3146","FAM135A",14.39657240048,0.598595893864102,0.438225169571071,1.
36595507384936,0.171953064892867,0.773163194694421
"3147","GARS-
DT",45.6325077823989,0.384650896608622,0.28158574301731,1.366016945627
02,0.171933644690189,0.773163194694421
"3148","AC016831.5",28.5858936043928,0.728588956308138,0.5335592569527
33,1.36552584706197,0.172087835020998,0.773163194694421
"3149","CRTAC1",3.31911147473521,-1.26647081284167,0.927633222823992,-
1.36527108094097,0.17216786474524,0.773163194694421
"3150","ZNF10",6.72990989913963,0.689557277025181,0.504831405452777,1.
3659159663546,0.171965340729827,0.773163194694421
"3151","MYCBP2",266.647476248403,0.307317079808543,0.225033726858565,1
.36564898114892,0.172049164868295,0.773163194694421
"3152","AL627171.2",38.091418225947,0.628352511063613,0.46029010097746
6,1.36512279914178,0.172214457349308,0.773163194694421
"3153","ZNF429",26.9997453915334,0.427992955163634,0.313397966195322,1
.36565326303647,0.172047820262229,0.773163194694421
"3154","ZNF335",17.2059669594071,0.44439250983368,0.325478362244367,1.
36535192929364,0.172142464823189,0.773163194694421
"3155","GXYLT2",78.0820053812247,-0.351363471874017,0.257499362591557,
-1.36452171507448,0.172403424570541,0.773521068415355
"3156","SMO",21.5779090597247,0.428950356570563,0.314354641413338,1.36
454278086051,0.172396799342924,0.773521068415355
"3157","CRYBG3",76.5583192364903,0.352534369215515,0.258575164107804,1
.36337289171569,0.17276502004125,0.773793088712926
"3158","HNRNPA0",267.163577657653,-0.195271423702256,0.143220591072593
,-1.36343120943609,0.172746650717774,0.773793088712926
"3159","RAC1",707.972692155718,-0.253409540634294,0.185881222631614,-1
.3632874641486,0.172791931250725,0.773793088712926
"3160","LY6K",4.56455955361565,1.03044391042342,0.755566695037408,1.36
380271548682,0.172629665428584,0.773793088712926
"3161","QSER1",59.309817735956,0.275560278779308,0.202106786863257,1.3
634390168488,0.172744191595166,0.773793088712926
"3162","LINC02454",1.53603624152068,-1.71065316732692,1.25414278594847
,-1.364001919473,0.172566961554157,0.773793088712926
"3163","IRX2",23.6162088667155,-0.734318878896594,0.538731412218068,-1
.36305190720781,0.172866152131629,0.773880718995848
"3164","ZNF324B",2.80068399511746,-1.12339052102015,0.824319036317172,
-1.36281035803704,0.172942285836664,0.773976854439683

"3165","RPRD2",55.7120062050433,0.334326477712782,0.24566479075589,1.3
6090514511293,0.173543667368435,0.774651487348915
"3166","TMEM167A",145.699365649742,-0.286499370613955,0.21053399878573
2,-1.36082234824949,0.173569837631236,0.774651487348915
"3167","MOCS1",8.98378015129819,0.761582087973212,0.559356038824685,1.
36153368357914,0.173345096363477,0.774651487348915
"3168","DTX2",16.1696041388072,0.530213739505694,0.389502897195144,1.3
6125749852909,0.173432329196424,0.774651487348915
"3169","RAB11FIP2",57.7153054556166,0.313195192166658,0.23015974039327
3,1.36077313795846,0.17358539331625,0.774651487348915
"3170","SMYD4",50.3847642165019,0.359032681665783,0.263748368469181,1.
36126977296444,0.173428451629107,0.774651487348915
"3171","CCDC124",93.3531150663064,-0.29796409659347,0.218915232882388,
-1.36109348203079,0.173484149203557,0.774651487348915
"3172","ZFP36",3469.48615956706,-0.35386102452807,0.259850833506036,-1
.36178522021115,0.173265677250952,0.774651487348915
"3173","CXorf38",23.5414475968937,0.405579175241807,0.298042343430293,
1.36081058340177,0.173573556479446,0.774651487348915
"3174","SESN1",44.4820323927789,0.417966173029804,0.307402901763485,1.
35966892515343,0.173934715978811,0.775477197185129
"3175","SH2D3C",1.56418048918063,-1.61603149847957,1.18848430183529,-1
.35974155988772,0.173911721532654,0.775477197185129
"3176","CLBA1",5.99496135025543,-0.740997944191314,0.544866530935285,-
1.35996230658426,0.173841852265515,0.775477197185129
"3177","TPRA1",34.2581155025741,0.327483041215509,0.24095562604338,1.3
5910103695421,0.174114574304664,0.775974732312184
"3178","TENM1",19.9621428924936,0.74426878760574,0.547671007474813,1.3
5897058169538,0.174155910966675,0.775974732312184
"3179","AL450326.1",10.2844256723526,-0.60935953640207,0.4485443154459
78,-1.35852694018917,0.174296540129448,0.776357033102542
"3180","RASGRP2",3.35096274584931,-1.04472471011204,0.76912751577726,-
1.35832445034328,0.17436075521789,0.776398834555135
"3181","ZNF813",5.61748050090206,0.88653783881323,0.65286199551755,1.3
5792532709832,0.174487379880755,0.776718421600592
"3182","SLC2A4RG",154.547458215143,-0.417806006767001,0.30781151893759
,-1.35734363746054,0.174672047951724,0.777296102764431
"3183","LRATD1",2.16492395129467,-1.44228142735026,1.06308600320528,-1
.3566930831576,0.174878751166686,0.777482925124103
"3184","DAAM2",13.7765914262354,0.533415042919856,0.393089440369104,1.
35698135879455,0.174787133683006,0.777482925124103
"3185","AKR1C3",203.398587276085,-0.449019516377347,0.330929691354212,
-1.3568426409244,0.174831215426616,0.777482925124103
"3186","OXSM",5.76021389406678,-0.7819114481935,0.576561913430631,-1.3
561621570544,0.175047579692407,0.777538819135928
"3187","CARD19",51.1054965947686,-0.371369553317549,0.27379908241492,-
1.35635791779159,0.174985315830672,0.777538819135928
"3188","ZNF22",116.532589089711,0.26654127819352,0.196588354071122,1.3
5583452770091,0.175151822810857,0.777538819135928
"3189","MDP1",49.2766968707018,-0.441485993182511,0.325598633586221,-1
.35592090273807,0.175124336030616,0.777538819135928

"3190","POLR2A",95.0876257801879,0.408690329227965,0.30147901104754,1.
35561785149786,0.175220788973358,0.777538819135928
"3191","PES1",20.0585984018996,-0.4365196859005,0.321969419369422,-1.3
5577995809486,0.175169189936175,0.777538819135928
"3192","FOXO6",2.64364823065426,1.15261120402892,0.85074740490401,1.35
482188647871,0.175474311697822,0.778330874234364
"3193","URM1",64.2385266879885,-0.407410949788322,0.300736125571532,-1
.35471237123262,0.175509214790277,0.778330874234364
"3194","IL10RA",4.21969853925133,-0.926545121578628,0.684048722031144,
-1.35450164840224,0.175576387848192,0.778384988080903
"3195","CUL4B",37.3694083434264,0.321400272070842,0.237384043522772,1.
35392534098447,0.175760197881022,0.778955994364718
"3196","PSMA1",188.661363307819,-0.24479784133725,0.180841385980665,-1
.35366050204583,0.17584471489603,0.779086721817206
"3197","MRPL20",218.275754644291,-0.247912231278376,0.183594551933189,
-1.35032455303245,0.176911899889614,0.779744141672851
"3198","AC092802.1",5.00122496712828,0.881911681029165,0.6526965151483
15,1.35118184418185,0.176637189019642,0.779744141672851
"3199","PEAR1",67.4835496143355,0.417931906794224,0.309389604829292,1.
35082724264385,0.176750779163921,0.779744141672851
"3200","ACTR3",146.572684537311,-0.219051724856342,0.162116413662258,-
1.35120016479453,0.176631321823363,0.779744141672851
"3201","AC104162.2",2.38502126123594,1.25026031276948,0.92443754127264
1,1.35245514915836,0.176229757355928,0.779744141672851
"3202","AC091607.2",6.53826220442795,-0.8805144588394,0.65154402590141
3,-1.3514274152406,0.176558556692738,0.779744141672851
"3203","ATP6V1H",37.0505600153175,0.334375412999307,0.24740596419117,1
.35152527180361,0.176527230102729,0.779744141672851
"3204","ADPRHL1",1.6362629256869,1.55792845724645,1.15374215265019,1.3
5032637376369,0.176911316116328,0.779744141672851
"3205","SNHG10",7.99893926532211,-0.805066570607998,0.596090936561062,
-1.35057676812291,0.176831046912078,0.779744141672851
"3206","FAH",15.3097433869404,-0.586850394196736,0.434387197501757,-1.
35098455380781,0.176700380681939,0.779744141672851
"3207","ZFHX3",104.530614457486,0.384170604833513,0.284399238196107,1.
35081446515201,0.176754873216505,0.779744141672851
"3208","SENP3",6.2526437193773,-0.873356866147001,0.646474792017268,-1
.35095270060224,0.176710584771266,0.779744141672851
"3209","TOB1",61.9390483170025,0.486939802033066,0.360003496883158,1.3
5259742266089,0.176184276283434,0.779744141672851
"3210","DCAF7",68.6222505884553,0.321280985589576,0.237888498084795,1.
35055283536683,0.176838717889744,0.779744141672851
"3211","RNF213-
AS1",4.14537290150711,1.03993561354845,0.770019891253741,1.35053084389
188,0.176845766862466,0.779744141672851
"3212","APMAP",51.0456730486317,0.300357081367447,0.222442156537818,1.
35027049747372,0.17692923214653,0.779744141672851
"3213","AC011043.1",5.63860522506731,0.803907729960072,0.5948323710841
78,1.35148618172011,0.176539743421751,0.779744141672851
"3214","NUDT16L1",30.3026836958159,-0.481963849658741,0.35698610362978

5,-1.35009134741717,0.17698668347605,0.779754647797406
"3215","CENPQ",15.6192298845374,0.512322573478754,0.379539180011405,1.
34985424551784,0.177062740652365,0.779771423185623
"3216","CRTC1",19.2584898362659,0.445597569716981,0.33013679428391,1.3
4973616219759,0.177100628316735,0.779771423185623
"3217","MIB1",58.10638522754,0.359974964648947,0.266745086337499,1.349
50926216309,0.177173447356801,0.779849553799284
"3218","SLC66A1L",36.0631001101131,0.702923514582265,0.520983120752496
,1.34922512185611,0.177264667970184,0.779875845558826
"3219","VPS33A",13.1466470550856,0.505188989853997,0.37445050671111,1.
3491475663665,0.177289572517928,0.779875845558826
"3220","MSX1",238.371096150884,-0.33056790467831,0.245147358563393,-1.
34844571287855,0.17751506958494,0.780555698460928
"3221","IL13RA1",143.678121035698,0.308864686309509,0.229073139276197,
1.34832345374683,0.177554371803859,0.780555698460928
"3222","AC008982.2",2.77877806670084,-1.33948294451267,0.9936989256679
27,-1.34797664555421,0.177665894290153,0.780601741960127
"3223","OCRL",19.0478805981796,0.451693660349186,0.335097237999296,1.3
47948025612,0.177675099882591,0.780601741960127
"3224","L3HYPDH",36.3645635774374,-0.352920533362815,0.26188177477223,
-1.34763304422297,0.177776436978462,0.780804698391758
"3225","APIP",37.0496516419976,0.353426718250075,0.262349799966777,1.3
4715832943205,0.177929245442805,0.780966527976699
"3226","ZNF426",28.582722635249,0.347169805850277,0.257701540612081,1.
34717784389529,0.177922961902177,0.780966527976699
"3227","PHLDB3",5.60769064629679,-0.813065355620407,0.603609919590462,
-1.34700462870467,0.177978741933673,0.780966527976699
"3228","TACC2",18.1661113910411,-0.489235291909172,0.3632898088709,-1.
34668047372347,0.178083163680898,0.781182651090928
"3229","CDC42",422.135118775498,-0.257807102453242,0.19151356152455,-1
.34615585654071,0.178252257979617,0.781198382231935
"3230","CBWD1",34.2285198335311,-0.420330734523205,0.312214194000731,-
1.34628963897209,0.178209125959878,0.781198382231935
"3231","AC006942.1",2.19164264506559,-1.2931100997408,0.96055130817632
3,-1.34621658284539,0.178232678600272,0.781198382231935
"3232","MYCBP",13.2060629539932,-0.493914874821468,0.367207852159253,-
1.3450553192617,0.178607370701027,0.78208790887214
"3233","DDAH1",11.0279219934591,-0.57765523168573,0.429417182774886,-1
.34520753909504,0.178558222260598,0.78208790887214
"3234","GUCY1A2",8.48664973349151,0.823902134647475,0.612595991273972,
1.34493556337851,0.178646044318298,0.78208790887214
"3235","NDUFS7",134.996518200293,-0.273943302182763,0.203699196605721,
-1.34484233000195,0.17867615714699,0.78208790887214
"3236","OST4",628.54121131494,-0.30160395783669,0.224366980388241,-1.3
4424395833468,0.178869511127362,0.782536860111508
"3237","LHFPL5",1.56558552811878,1.84597871625134,1.37330918853734,1.3
4418289170367,0.178889252555152,0.782536860111508
"3238","ZBED4",6.99659113585777,0.728708575084828,0.542216649726899,1.
34394356103203,0.178966638241305,0.78263360021522
"3239","TAFA2",1.81465749863309,-1.52239266284681,1.13330946037071,-1.

34331593980415,0.179169692870396,0.782947037988894
"3240","RASSF9",12.9874102691152,0.532035627154412,0.39604580300794,1.34336893135501,0.179152541866967,0.782947037988894
"3241","CARS2",50.631140753944,-0.315767790624829,0.235125885268505,-1.34297331943794,0.179280613282203,0.782947037988894
"3242","MED1",49.0693292808198,0.413369556442161,0.307771996661575,1.34310320927833,0.179238556550245,0.782947037988894
"3243","AC011447.3",1.8096896438345,1.48812852805665,1.10817200514995,1.34286782299223,0.179314777132626,0.782947037988894
"3244","MINDY1",13.824935108114,0.546827581030097,0.407371006469151,1.34233308788878,0.179488019599517,0.783369039302703
"3245","ATP1B2",2.61890078504438,1.29006187949779,0.96113466052736,1.34222802743369,0.179522071506869,0.783369039302703
"3246","TRIB2",9.5145051356102,-0.767862881936152,0.57312222294384,-1.33978905731863,0.180313934220213,0.784092791697354
"3247","ZNF718",7.7149563841085,0.648095099550725,0.483320563965294,1.34092183919007,0.179945830758134,0.784092791697354
"3248","RNF130",134.888234181045,-0.250537838321523,0.187024314213868,-1.33960035824553,0.180375307295996,0.784092791697354
"3249","MKLN1",92.7864319911651,0.294849684628126,0.220097355873302,1.33963301584529,0.180364684526038,0.784092791697354
"3250","AC120053.1",3.15108208023973,1.04883453577818,0.782676195234444,1.34006188276112,0.180225227051743,0.784092791697354
"3251","AL158071.1",6.4150773848319,0.874896906748205,0.652651396767662,1.34052713451813,0.180074028688045,0.784092791697354
"3252","ATP5F1C",263.945386263214,-0.335165218891552,0.249907250214709,-1.34115844419717,0.179869015258923,0.784092791697354
"3253","FRMD8",15.3105139389061,-0.461131367858856,0.344199656584016,-1.33972059250529,0.180336200136496,0.784092791697354
"3254","KLRD1",5.09048674752234,0.832680588637806,0.621635144176056,1.33950050353327,0.180407790632061,0.784092791697354
"3255","ACADS",45.5396460501189,-0.376145208939475,0.280654465741896,-1.34024309196418,0.180166326144973,0.784092791697354
"3256","VPS18",14.2612340480269,0.527457690462816,0.393723625483179,1.33966482152428,0.180354339313117,0.784092791697354
"3257","RSL1D1",211.405878699723,-0.251209100738011,0.187302242333862,-1.34119644061835,0.17985668172168,0.784092791697354
"3258","TNFSF13",14.1388750324612,0.641955128704023,0.478730047404089,1.34095432736053,0.17993528179881,0.784092791697354
"3259","MAP1B",494.976547788882,-0.301354765551826,0.225201085865977,-1.33815858122092,0.180844747662368,0.784755472973073
"3260","SEM1",375.955711146126,-0.274285443387783,0.204867981733982,-1.33883997424224,0.180622774215079,0.784755472973073
"3261","WDR11",49.2344590202093,0.379176915036466,0.283374372826496,1.33807765061602,0.180871125407294,0.784755472973073
"3262","ARHGEF25",31.8230295963468,-0.393912135486501,0.294286879133333,-1.33853108452053,0.180723374340334,0.784755472973073
"3263","WDFY2",50.3663376360989,0.381630496200781,0.285222199354913,1.3380119640728,0.180892786990403,0.784755472973073
"3264","PPP1R9B",22.3579420974566,-0.506206032996189,0.378244091531067

,-1.33830519585158,0.180796968771007,0.784755472973073
"3265","CHD2",103.907646206056,0.349401824381626,0.261206892868992,1.3
37643966987,0.181012524761253,0.784794044892635
"3266","CST7",3.43886803398178,1.20055261582708,0.897479461100119,1.33
769369424394,0.180996307390938,0.784794044892635
"3267","PRDM8",37.2756206533689,0.347379636899176,0.259729009626093,1.
3374695317988,0.181069421207198,0.784800429842035
"3268","TTC21B",22.706113044877,0.397717236527552,0.297557014778281,1.
33660850450426,0.181350461544613,0.785063370645706
"3269","MED10",127.560270860389,-0.278766798800642,0.20856345636958,-1
.33660423380528,0.181351856312296,0.785063370645706
"3270","RGL2",32.1938314713926,-0.414544547675396,0.310126845491142,-1
.33669352944564,0.181322694904106,0.785063370645706
"3271","PHKG2",12.0464906618796,-0.518304529242662,0.387647066770016,-
1.33705262769385,0.181205458802906,0.785063370645706
"3272","PDLIM7",86.8813857614299,-0.364954669942543,0.273081950993661,
-1.33642911446394,0.181409055398924,0.785070973242289
"3273","PHLDA3",274.753753005318,-0.341087174903065,0.255343507808004,
-1.3357973258499,0.181615527285082,0.785484381904936
"3274","BZW1",286.163111545452,-0.205649551245985,0.153940809572629,-1
.33590015420154,0.181581910556123,0.785484381904936
"3275","B4GALT2",52.3726834237015,-0.386528973391873,0.28945041456178,
-1.33538925476085,0.18174897974799,0.785574525077954
"3276","COL11A1",1.7559909207842,-1.82506516740106,1.36655220796465,-1
.33552538773423,0.181704451771666,0.785574525077954
"3277","EIF2A",111.15423923621,-0.308043519288555,0.230705372800573,-1
.33522473078611,0.181802804991557,0.785574525077954
"3278","FAM228A",1.80660154548859,-1.52239571598511,1.14090900559531,-
1.33437084685886,0.182082349457387,0.786302552094116
"3279","ELL2",22.4055010997148,0.858440649529326,0.643282166626731,1.3
3446983930994,0.182049924986332,0.786302552094116
"3280","GLRX",95.7155356289719,-0.389046394740839,0.291668271407248,-1
.33386601450943,0.182247771336679,0.786413747088473
"3281","POC1B",12.6714200267745,0.560547215672493,0.420232872952756,1.
33389663624795,0.182237734127371,0.786413747088473
"3282","CCDC120",3.91588179612297,0.895731418126257,0.671571657807296,
1.33378383038208,0.182274711719235,0.786413747088473
"3283","ATP6V0B",123.756323146747,-0.343059532403633,0.257335207641992
,-1.33312318802837,0.182491381003594,0.786869048419881
"3284","VPS8",20.0821891125658,0.442824914652021,0.332158156521798,1.3
3317489261463,0.182474416689746,0.786869048419881
"3285","TTC3",384.740566385769,-0.237384616471763,0.178098377449489,-1
.33288477902663,0.182569618304898,0.786938886793351
"3286","SLC16A8",5.48926883485676,0.810140261541046,0.607877904155551,
1.33273516935358,0.182618727542581,0.786938886793351
"3287","KCNN4",2.90975130275377,-1.28282535824377,0.962734729094556,-1
.33248060911883,0.182702309213024,0.787059537102651
"3288","TMCO4",17.6904953538071,-0.500271439839102,0.376214579850298,-
1.32975027187455,0.183600565246056,0.787405490689838
"3289","RPL5",3242.29164779413,-0.341395121182637,0.256504536373392,-1

.33095159255067,0.183204939496991,0.787405490689838
"3290","TAF13",21.2445841502677,-0.484825386839004,0.36636467703041,-1
.32334096935541,0.18572201300246,0.787405490689838
"3291","LINC02767",4.98846312968297,0.889874417206678,0.67347065287706
8,1.32132619796443,0.186392622324557,0.787405490689838
"3292","RAB3GAP2",51.0294785353274,0.314689613407741,0.237875544546597
,1.32291704894488,0.185862964972191,0.787405490689838
"3293","TTC32",24.4188089464234,-0.76913574284185,0.58114356002044,-1.
32348664900425,0.185673593309139,0.787405490689838
"3294","PTRHD1",67.4638116067795,-0.34410333644462,0.259662572575459,-
1.3251942050471,0.18510674690883,0.787405490689838
"3295","NRBP1",115.986552366352,-0.217659975258911,0.164412470025737,-
1.32386536875724,0.185547761513841,0.787405490689838
"3296","SRSF7",345.949409159569,0.216490915238608,0.16272782741986,1.3
303865643092,0.183390939050695,0.787405490689838
"3297","PLEKHH2",103.012190429484,0.44625665891627,0.335843403667919,1
.3287641026814,0.183925808793627,0.787405490689838
"3298","LRRTM4",4.20381558029514,3.3841805123394,2.54817443550869,1.32
808039558871,0.18415154913914,0.787405490689838
"3299","AC092683.1",25.7308585103192,-0.558741148039225,0.420196129663
807,-1.32971512252211,0.183612150352195,0.787405490689838
"3300","PPIG",243.501758346504,0.190791150908922,0.143985164495741,1.3
2507506295598,0.185146256148982,0.787405490689838
"3301","HOXD4",2.84946153517681,-1.2292996951747,0.925988488734038,-1.
32755397084399,0.184325499122596,0.787405490689838
"3302","EIF4E2",101.53312845648,-0.306547857546074,0.232002504836949,-
1.32131270634994,0.18639711898251,0.787405490689838
"3303","SEPTIN2",308.928140440098,0.224376546057799,0.169325903854854,
1.32511648217826,0.185132520228653,0.787405490689838
"3304","TADA3",76.1460379659601,-0.333961877303321,0.252544003331018,-
1.32239084238158,0.186038036700773,0.787405490689838
"3305","RPL14",3192.50926159179,-0.304129416192956,0.230139429146879,-
1.32150069773075,0.186334470032137,0.787405490689838
"3306","PHLDB2",59.4667322139178,0.355136241036721,0.267201994142434,1
.32909277932789,0.18381736221594,0.787405490689838
"3307","STK32B",9.96889263217756,-0.639464029572705,0.483411353231083,
-1.32281549719215,0.1858967422934,0.787405490689838
"3308","MRFAP1L1",50.5472187994455,0.315536267375326,0.238492629546592
,1.32304410402621,0.185820711330301,0.787405490689838
"3309","PPARGC1A",1.93544976892089,1.32712829133514,1.00325360474506,1
.32282434377336,0.185893799634804,0.787405490689838
"3310","KDM3B",30.3109902939315,0.399829107659628,0.300720747373008,1.
32956941332581,0.183660181379151,0.787405490689838
"3311","RWDD1",327.932508611375,-0.310353291478689,0.234423096340138,-
1.32390236424648,0.185535472933615,0.787405490689838
"3312","SERINC1",270.95946132515,0.201448081369687,0.151919025160789,1
.32602273583889,0.184832166605425,0.787405490689838
"3313","ZFAND2A",24.888268589848,-0.482948757554748,0.365053449793681,
-1.32295355057649,0.185850825203828,0.787405490689838
"3314","PDK4",759.381996706432,0.47697435401883,0.359757865891184,1.32

582050106752,0.184899160657238,0.787405490689838
"3315","ZKSCAN5",7.04600820936751,0.748460856458319,0.564501397524828,
1.32587954563106,0.184879599184045,0.787405490689838
"3316","PILRB",22.6779955298845,0.383134895310924,0.28783115060207б,1.
33110990422508,0.183152850541769,0.787405490689838
"3317","ADCK2",13.0813269949261,-0.526675733420784,0.398340623981154,-
1.32217429434388,0.186110118780036,0.787405490689838
"3318","UBE2W",32.4311631153952,0.341280662481362,0.256992260973416,1.
32798031033574,0.184184611605459,0.787405490689838
"3319","AC087672.2",3.2147636967528,-1.1176234978609,0.84366696009711,
-1.32472118824264,0.185263642901806,0.787405490689838
"3320","TP53INP1",17.7636930324736,0.48671332341609,0.368320971633694,
1.32143798724592,0.186355366845815,0.787405490689838
"3321","LRP12",23.0005480165878,-0.422189599406748,0.318455856206855,-
1.3257397883508,0.184925903270474,0.787405490689838
"3322","DENND4C",39.7591953962095,0.34729228332742,0.261956842337316,1
.3257614507363,0.184918725572867,0.787405490689838
"3323","ENHO",5.75594227532872,-0.985525627791847,0.74543699137808,-1.
32207770635305,0.186142276562293,0.787405490689838
"3324","GALT",28.1386702260867,0.376919560413318,0.283445523299341,1.3
2977778596016,0.183591497075519,0.787405490689838
"3325","CCNY",99.0871142693443,-0.246040416849246,0.186177304219525,-1
.32153818576691,0.186321978841131,0.787405490689838
"3326","TIMM10",71.2856515032255,-0.427704405708797,0.32218428810238,-
1.32751478424946,0.184338452668915,0.787405490689838
"3327","LRRC10B",1.78130107187921,1.4672598818102,1.10608114868457,1.3
2653909123681,0.184661195698165,0.787405490689838
"3328","ROBO4",5.97852812805669,0.937951745868207,0.705614274991721,1.
32926979953631,0.183758974174662,0.787405490689838
"3329","TIGAR",4.92131103949092,-0.948188864370722,0.712833380104759,-
1.33016899998619,0.183462595538935,0.787405490689838
"3330","HEBP1",198.564059366581,-0.337177931457477,0.253536754745714,-
1.3298976386901,0.183551999542457,0.787405490689838
"3331","ZDHHC20",30.5350145095015,0.375062960970003,0.283839177578839,
1.32139250180087,0.186370524898315,0.787405490689838
"3332","DNAJC15",193.867938322394,-0.291891146000058,0.219413289941351
,-1.33032573404319,0.183410971879479,0.787405490689838
"3333","PCCA",20.8004246006565,0.413525329826077,0.31289468540973,1.32
161186849362,0.186297429211444,0.787405490689838
"3334","CCDC88C",1.72580601905723,1.5399649280222,1.16070187387987,1.3
2675320224526,0.18459033554499,0.787405490689838
"3335","SNRNP25",33.6921938016964,-0.558312860846339,0.421677488675558
,-1.32402813960958,0.18549369935133,0.787405490689838
"3336","RPS2",5459.73191018318,-0.342585094683649,0.257978550309043,-1
.32795960855371,0.184191450843487,0.787405490689838
"3337","SNN",10.0303354798339,0.57862733071497,0.436646343331935,1.325
1624330565,0.185117282350407,0.787405490689838
"3338","CCP110",28.5672772128499,0.428231042658907,0.323033822625255,1
.32565388719586,0.184954368116946,0.787405490689838
"3339","CES4A",6.53136316608786,0.944176595767185,0.712971677737069,1.

32428345367652,0.185408923664916,0.787405490689838
"3340","RFLNB",18.4752923052773,0.596055029325782,0.447789256851569,1.33110614023364,0.183154088872529,0.787405490689838
"3341","PSMB6",221.511202831759,-0.292726184384738,0.220457399508709,-1.32781292456992,0.184239916148537,0.787405490689838
"3342","KDM6B",55.222101954174,-0.55365749057071,0.41901611459495,-1.32132744132266,0.186392207926246,0.787405490689838
"3343","SLFN12L",2.070379444586,-1.25605681146774,0.947535462367745,-1.32560401309841,0.184970896256727,0.787405490689838
"3344","AC060780.1",15.7000613110372,0.491591621908577,0.371109343012848,1.32465439408665,0.185285805926967,0.787405490689838
"3345","SLC9A3R1",8.0632591386278,-0.632192397321678,0.478042309512261,-1.32246118124292,0.186014627531191,0.787405490689838
"3346","FDX2",40.9001049426203,0.428780480343891,0.322695948010939,1.32874454416563,0.18393226360242,0.787405490689838
"3347","OXT",7.71045525897887,-0.710329416325443,0.535806615445231,-1.32571975755692,0.184932540533712,0.787405490689838
"3348","BTG3",57.8466770250781,-0.30512962419358,0.230112430628337,-1.32600235180865,0.184838918382745,0.787405490689838
"3349","LCA5L",2.31796530611479,-1.21552960166106,0.915171819365063,-1.32819824205731,0.184112625012269,0.787405490689838
"3350","IFT27",38.5330294193701,-0.373836743672449,0.282002925932845,-1.32564845714215,0.184956167568916,0.787405490689838
"3351","APOBEC3F",11.0251803367347,0.618095965593081,0.466337916931695,1.32542506871388,0.18503020693168,0.787405490689838
"3352","COX7B",239.711611985944,-0.260268944206352,0.196205685706515,-1.32651071384171,0.184670588722952,0.787405490689838
"3353","LRP4",10.1051500011768,0.588802123832645,0.445951689495387,1.32032715135332,0.186725814211607,0.788183579805655
"3354","AP000873.2",11.9983607013061,-0.65105168615302,0.493058752324403,-1.32043429527171,0.186690059609796,0.788183579805655
"3355","CBFA2T3",3.79287068480557,1.08789701244856,0.824002236669222,1.32025978090309,0.186748298746326,0.788183579805655
"3356","AL591895.1",9.28766278884504,0.650953765902137,0.493794400098816,1.3182688296422,0.187413671516409,0.788564239672637
"3357","NSUN3",16.1192633622061,0.467505069630076,0.354541426181376,1.31861902476499,0.187296510177564,0.788564239672637
"3358","AC104596.1",1.55886152679424,1.40489224308198,1.06483701959796,1.31934955042454,0.187052279483788,0.788564239672637
"3359","ZFP37",10.7518742427831,0.528467735485974,0.400429807611203,1.31975124089436,0.186918085841909,0.788564239672637
"3360","MRPL17",68.4594530369132,-0.295025300128472,0.223679846985761,-1.31896236564956,0.187181694505786,0.788564239672637
"3361","CCDC86",12.448686060395,0.534944057930719,0.405761320249684,1.31837124741595,0.18737940101198,0.788564239672637
"3362","FAM216A",11.9580688038539,0.545401718659683,0.413667462558249,1.31845447859676,0.187351554033832,0.788564239672637
"3363","TEKT3",12.6320838283163,0.624746346736604,0.473896382783534,1.31831845406167,0.187397065872618,0.788564239672637
"3364","LSM4",87.3315398281511,-0.389760215812386,0.295583629183216,-1

.31861232264252,0.187298751933062,0.788564239672637
"3365","SMG9",26.5421240984173,-0.397191010292681,0.301322976967778,-1
.318157062862,0.187451075617097,0.788564239672637
"3366","UBQLN2",47.530605859623,0.368725799339625,0.279470539434086,1.
31937269698007,0.187044544930422,0.788564239672637
"3367","VPS50",18.3019490728182,0.436780738277293,0.331621147345349,1.
31710761443821,0.187802555045956,0.789105097759862
"3368","ABCF2",22.8605908892398,-0.429679683017314,0.326184986337341,-
1.31728835174817,0.187741988176441,0.789105097759862
"3369","SLC25A37",70.7176523574119,0.317302433198119,0.240885733146752
,1.3172321542381,0.187760818976144,0.789105097759862
"3370","PCID2",68.6292228227672,-0.286710578233664,0.217601427543906,-
1.31759511630875,0.187639221325566,0.789105097759862
"3371","WDR43",50.6029852005672,-0.36638480953092,0.278592591157045,-1
.31512761344176,0.188467017654851,0.789844629911439
"3372","SLC30A6",17.2174903774028,0.462107383157929,0.35117203561068,1
.31590028902596,0.188207511748205,0.789844629911439
"3373","FGF1",6.75043506420576,-0.911655507769542,0.69269887668311,-1.
31609208338098,0.188143137775693,0.789844629911439
"3374","MXD3",2.63375855476319,-1.07210246491832,0.815318159097769,-1.
31494981800075,0.188526768236055,0.789844629911439
"3375","WAKMAR2",4.12216412048637,0.895067338502258,0.680398195815499,
1.31550516154656,0.188340183794655,0.789844629911439
"3376","ZNF273",15.7255460420984,0.477518284151654,0.362871223307623,1
.31594420687044,0.188192769702196,0.789844629911439
"3377","CAND1",73.882493541992,0.277829477670903,0.21119524576791,1.31
551009427655,0.188338527105588,0.789844629911439
"3378","MYZAP",3.03653334619844,-1.34343395365266,1.02203681111602,-1.
31446728634528,0.188688999989809,0.789844629911439
"3379","MAP3K3",46.5670035760436,0.403154370786932,0.306691375384548,1
.3145279037646,0.188668614181595,0.789844629911439
"3380","ELOF1",61.9264809272915,-0.400354420862402,0.30418313084831,-1
.31616247010769,0.188119517207939,0.789844629911439
"3381","PRMT2",126.069712212699,0.253705320809046,0.193016410047962,1.
3144235805961,0.188703699363729,0.789844629911439
"3382","UFD1",98.0668200571054,-0.331494838751931,0.252127144575122,-1
.31479234142185,0.188579702053816,0.789844629911439
"3383","AC100801.1",1.94036267140927,-4.21835288659644,3.2071272276964
9,-1.31530575094343,0.188407166129308,0.789844629911439
"3384","FNDC10",11.3247275776226,0.651981350699208,0.496140084048386,1
.31410738954852,0.188810067746633,0.790056311847614
"3385","SSBP2",104.371032561448,-0.227153374533551,0.172943536904717,-
1.31345396653187,0.189030022871969,0.790300979226546
"3386","AC087645.2",3.04567627769333,-1.06352934937618,0.8098326153515
41,-1.31327058112436,0.189091787967481,0.790300979226546
"3387","TRPC4AP",47.2370287503997,0.32752113663211,0.249384956429514,1
.31331553162342,0.189076647044871,0.790300979226546
"3388","MTFP1",5.43145144907329,-0.898566084259293,0.683970911607558,-
1.31374897529979,0.188930693698864,0.790300979226546
"3389","PLEKHG4",25.5767511008119,-0.512800264162002,0.390541855916232

,-1.31304815705079,0.189166721439907,0.790380872112445
"3390","SLC22A23",4.11661519907415,-0.981491256765768,0.748279244002528,-1.31166441489917,0.189633388470369,0.791109159708078
"3391","FIS1",344.81353080255,-0.346941120643053,0.264463479437047,-1.31186779127905,0.189564746658986,0.791109159708078
"3392","CCDC6",56.1872361550879,0.324006969748464,0.246944723232868,1.312062738198,0.189498967074006,0.791109159708078
"3393","KMT5B",64.0631273504362,0.276394039878246,0.210767073512646,1.31137200546489,0.189732112031683,0.791109159708078
"3394","ATP6V1D",72.865295421889,-0.264963707310627,0.202018858270467,-1.31157907523608,0.189662197019168,0.791109159708078
"3395","DUS1L",49.5644110677731,-0.375884259875151,0.286628451808102,-1.31139898186662,0.189723002647013,0.791109159708078
"3396","YWHAB",548.228297069207,-0.232318593977596,0.177085034285084,-1.3119041646602,0.189552472164901,0.791109159708078
"3397","SNHG21",7.71555405491011,-0.700028516952893,0.533998285155033,-1.31091903553521,0.189885118945456,0.791179675264737
"3398","METTL9",150.721331964585,-0.273572403695136,0.208702344537474,-1.31082573270284,0.189916646626048,0.791179675264737
"3399","AL021707.2",2.28575957426184,1.33511324088543,1.01829766031557,1.31112276195516,0.189816291762692,0.791179675264737
"3400","EGFLAM",52.32766793377,-0.483384894227111,0.368974447021227,-1.31007688507845,0.19016982717634,0.791681545065247
"3401","ZNF704",96.8012201317445,0.325110349154913,0.248180884848364,1.30997336621454,0.190204845784744,0.791681545065247
"3402","DDA1",38.4841067343359,-0.395752183142562,0.302042997139552,-1.31025114599732,0.190110888502067,0.791681545065247
"3403","RPS7",2526.48984925633,-0.36042590605825,0.275219785759749,-1.30959300423582,0.190333556327737,0.791777748767167
"3404","ABR",33.7641297226678,0.445090619917964,0.339874205207519,1.30957458111951,0.190339792147135,0.791777748767167
"3405","PLEKHG1",9.12881597525815,0.672274657955535,0.513823143844933,1.30837753419379,0.190745288726433,0.792998616666555
"3406","MED14OS",5.44968249642258,-0.785898478857242,0.600662191867178,-1.30838679293972,0.19074214991151,0.792998616666555
"3407","TMEM35B",220.468198764817,0.30721967871913,0.234906323477327,1.30783911719083,0.190927883312475,0.793059204372145
"3408","ROR2",4.47886581254338,1.08147419059627,0.826872543497892,1.307909180321,0.190904115364527,0.793059204372145
"3409","FES",26.3762939588391,0.413783900900265,0.316346671734244,1.30800775817195,0.19087067787973,0.793059204372145
"3410","PPFIA3",2.58464452837912,1.26215198742288,0.965384565154936,1.30740850121248,0.191074011660423,0.793433432584042
"3411","PYGO2",9.39916580535803,0.645665498825894,0.494448818093813,1.30582878388717,0.191610789221948,0.794009393148543
"3412","RGS1",23.6895588310496,-0.607992386625163,0.465425026978105,-1.30631648790508,0.191444952369187,0.794009393148543
"3413","AC078785.1",2.34189056065061,1.47632606704145,1.13115396262648,1.30515041790907,0.191841633657583,0.794009393148543
"3414","MBNL1",336.311153682146,-0.249768116879642,0.191390072777774,-

1.30502127542243,0.191885603344231,0.794009393148543
"3415","ELOC",137.376872576014,-0.290667120368626,0.222594176584556,-1
.30581637322489,0.191615010670418,0.794009393148543
"3416","CPSF7",39.8141596450312,0.394947827035546,0.302535479318643,1.
30545953791942,0.191736416362157,0.794009393148543
"3417","PGM2L1",12.1807204163662,-0.590957120847839,0.452615136307189,
-1.30565037145987,0.191671482236107,0.794009393148543
"3418","TMEM119",139.840634860436,-0.285382569395906,0.218670803569632
,-1.30507852322878,0.191866111027926,0.794009393148543
"3419","RBM26",77.0979270651164,0.297687572394677,0.228090569998248,1.
30512880211121,0.191848992762981,0.794009393148543
"3420","AC116913.1",2.57311347092039,-1.14537859839464,0.8775171278128
72,-1.30524927900768,0.191807979010535,0.794009393148543
"3421","ZNF383",13.4579937918428,0.521874769188273,0.399523004863048,1
.30624460377987,0.191469388911941,0.794009393148543
"3422","PRPS2",27.745963442163,-0.462961987514125,0.354659200810222,-1
.30537142827956,0.191766402517973,0.794009393148543
"3423","NFIC",518.088238608608,-0.234897800644138,0.18007989978511,-1.
30440877035384,0.192094246463789,0.794640528754674
"3424","TSTD2",20.1812198289598,0.424866848700311,0.325794287172807,1.
30409545356747,0.19220103885068,0.794775388941346
"3425","CCNK",46.2855866706436,0.306295497585238,0.234892102176312,1.3
0398380680901,0.19223910361046,0.794775388941346
"3426","ZNF692",7.31658531812964,-0.6837451900746,0.524430858986841,-1
.30378519562244,0.192306831654189,0.794823332230972
"3427","IDO1",3.08687926634375,1.05602277515245,0.810201711487413,1.30
340723819719,0.192435766687632,0.795124148321234
"3428","ZDHHC18",19.2404280723969,0.446996159847082,0.34314766478467,1
.3026350044596,0.192699401251509,0.795749058536416
"3429","FEZ1",91.8170764123855,0.408062824972994,0.313244775660378,1.3
0269634701081,0.192678449683998,0.795749058536416
"3430","PPAT",14.1147762403029,0.459669183489789,0.353050407444728,1.3
019930689692,0.192918754963597,0.795992186511305
"3431","JHY",16.7902297677981,0.451458463792511,0.346750498366886,1.30
196918510218,0.192926919781554,0.795992186511305
"3432","RARG",62.9935346773938,-0.31466846975341,0.241668573764573,-1.
30206615138943,0.192893772954166,0.795992186511305
"3433","MYL3",6.19565106965244,0.705661869436375,0.542327358339001,1.3
0117328323178,0.193199147992875,0.796187408492172
"3434","BIN3",24.771292312776,-0.381130727446723,0.292847649729918,-1.
30146418384517,0.193099616400929,0.796187408492172
"3435","SLCO2B1",5.90442316729306,-0.877841126903061,0.674579822327535
,-1.30131542309434,0.193150510157431,0.796187408492172
"3436","RAB3IP",3.94161331039429,0.945192491906368,0.726217832451456,1
.30152751649699,0.193077952139889,0.796187408492172
"3437","NDUFC1",215.862217363065,-0.300473125051264,0.23105969689007,-
1.3004133957391,0.193459321148975,0.796278927018424
"3438","SLC25A27",7.05911950621438,0.780886220487822,0.600548997830922
,1.30028727598955,0.193502527391977,0.796278927018424
"3439","POM121C",32.6204938793512,0.36041127075959,0.277141245567698,1

.30046060095212,0.193443151357367,0.796278927018424
"3440","EMC10",61.9703200198292,-0.273183859795148,0.210053277038175,-1.30054557418545,0.193414046915268,0.796278927018424
"3441","SPIN3",10.489050740333,0.679897904289001,0.522665487091748,1.30082800774189,0.193317332843661,0.796278927018424
"3442","TNFRSF14",62.8881403058937,0.279391561027903,0.214980662807985,1.29961252039421,0.19373380629066,0.796536207048706
"3443","FRRS1",8.86631921743578,0.61739770544412,0.474952235587444,1.29991535818437,0.193629980664833,0.796536207048706
"3444","LSM8",137.95686479377,-0.257270669603527,0.197944978972471,-1.29970798420356,0.193701072841082,0.796536207048706
"3445","MOGS",20.8310634270904,0.458336924247708,0.352748162085049,1.29933185629809,0.193830066326123,0.79670064997907
"3446","SHE",17.5860756345626,0.558407869821923,0.430271031585522,1.29780493881781,0.194354372091473,0.796868129626583
"3447","LOXL3",10.6274093801565,-0.603423638936759,0.465023073448098,-1.2976208566651,0.194417651590451,0.796868129626583
"3448","TMEM129",28.7550338962281,-0.367348275215312,0.283024263829935,-1.29793915985961,0.194308242219312,0.796868129626583
"3449","HCP5",2.20955056697871,1.23302991204781,0.949955220785382,1.29798740516253,0.194291662954943,0.796868129626583
"3450","RXRA",70.0224409384742,0.304727545210637,0.234843959812799,1.29757454887724,0.194433572588972,0.796868129626583
"3451","TRAFD1",25.7583195324807,0.385281615938052,0.296705862897303,1.29853051158415,0.19410509868638,0.796868129626583
"3452","DIO3",8.53580035737406,0.936149457417359,0.720828542711935,1.29871308077692,0.194042413284027,0.796868129626583
"3453","LINC02185",2.74436352961936,-1.48686739993921,1.14512410851188,-1.29843340899654,0.19413844505805,0.796868129626583
"3454","IST1",65.4595703416658,0.253108070593132,0.195042846279258,1.29770496801886,0.194388735861789,0.796868129626583
"3455","EWSR1",100.746407880295,0.294898126943194,0.227155976849947,1.29821865588857,0.19421220917835,0.796868129626583
"3456","DPM2",55.6900871933108,-0.343001162200983,0.26452040172654,-1.29669076548423,0.194737607978176,0.797376873502963
"3457","ARHGEF12",78.2238111553434,0.318222670264196,0.245436127459895,1.29656001973953,0.194782616169405,0.797376873502963
"3458","ARNTL2",7.92393849233486,-0.700880429505196,0.540476636984828,-1.29678210221115,0.194706170557293,0.797376873502963
"3459","ZNF667",15.6622220431167,0.537010003257058,0.414180909130963,1.29655904320606,0.194782952362058,0.797376873502963
"3460","SLC17A5",36.186339644646,0.34455404783278,0.266039560881589,1.29512335192185,0.195277680425286,0.799171085208685
"3461","TMEM131",45.1113086101925,0.373493799985748,0.288610637900264,1.29410960976018,0.195627562662739,0.7996842623955
"3462","DIP2C",38.2450858050366,0.326615770525762,0.252387278570745,1.29410552059267,0.195628974925001,0.7996842623955
"3463","SPATA41",2.66202853746584,1.15449307023994,0.892080309189602,1.29415822583139,0.195610772863592,0.7996842623955
"3464","BCORL1",14.3673272441713,0.520170825827321,0.401893030407783,1

.29430168345922,0.195561235224055,0.7996842623955
"3465","PIK3AP1",1.50131507452934,-1.55440184650307,1.20154121121481,-1.29367335218699,0.195778273633785,0.800063594416855
"3466","LETM1",20.7087076245563,-0.44048575619436,0.340574218696933,-1.29336201042961,0.195885882803156,0.800272389063096
"3467","ORMDL3",18.9154794524011,0.427878069296811,0.330985974700544,1.29273776535072,0.196101771438557,0.800923300712422
"3468","ANO1",5.63632237090593,-1.28456518300471,0.994012280159625,-1.29230313210862,0.196252187718702,0.800997274699864
"3469","NPIPB5",14.1261324235014,0.661652960844357,0.511982826631387,1.29233428628404,0.196241403184355,0.800997274699864
"3470","ZNF493",29.1741210938665,0.371815271373285,0.287739266210601,1.29219510520732,0.196289586384783,0.800997274699864
"3471","WDR6",27.6023459016155,0.376379794804522,0.291432485805827,1.291481949117,0.19653661040713,0.801387466347348
"3472","TPPP",5.73014487158275,-0.957464560329458,0.741399353891204,-1.29142891115867,0.196554990863301,0.801387466347348
"3473","SLC27A1",42.6222823373665,0.342965093603003,0.265518359492227,1.29168127680091,0.196467544084512,0.801387466347348
"3474","CAPZB",240.191177983869,-0.304553737749682,0.23586004030709,-1.29124771348785,0.196617794932601,0.801412773818548
"3475","NIPA2",40.2324363688717,-0.310930952760802,0.240858437892956,-1.29092821277446,0.196728571369969,0.801633545496048
"3476","ENPP1",24.8178628791656,0.732226295974762,0.567309312867296,1.29070029235717,0.19680762325841,0.801724955506066
"3477","POLR1D",181.627951316247,-0.274740215394177,0.212930266178776,-1.29028259028007,0.196952559367204,0.802037226599651
"3478","ZNF428",146.827283323013,-0.311680852699887,0.241584422227273,-1.29015294043533,0.196997561731185,0.802037226599651
"3479","C1GALT1C1L",8.94656581690507,-0.730863810657669,0.56662129059923,-1.28986295217524,0.197098245926442,0.802216488162809
"3480","TADA1",6.42623165003926,0.670765130267342,0.520310803055018,1.28916241279045,0.197341629275834,0.802442253531683
"3481","IER3-
AS1",1.95948478435528,-1.28217481861867,0.99444002216816,-1.28934354011936,0.197278680438652,0.802442253531683
"3482","TREM1",2.48207484357872,-1.21604029864821,0.94336096667662,-1.28905089526039,0.197380393294552,0.802442253531683
"3483","ZNF419",4.64977533039257,0.835648045541738,0.648192937762193,1.289196467377,0.197329792851831,0.802442253531683
"3484","ZNF605",22.2239582114749,0.434644140487317,0.337256902802284,1.28876277068264,0.197480572555018,0.802619089374011
"3485","MRPS15",128.320707784152,-0.328250616102937,0.254810010599768,-1.28821711254713,0.197670396710266,0.802699402758062
"3486","RPS27A",4962.87193052175,-0.305993093645895,0.23751363119346,-1.28831803087822,0.197635279070341,0.802699402758062
"3487","ACAD10",9.04897026540869,0.605045042783529,0.469606999147344,1.28840720832972,0.19760425083059,0.802699402758062
"3488","HOMER1",8.43538480736317,-0.677116812028029,0.525779878599568,-1.28783325415866,0.197804013766511,0.802781552001663

"3489","SPRYD3",107.957686090901,-0.268125213513853,0.208172597964815,
-1.28799475115919,0.197747790313747,0.802781552001663
"3490","TNIP2",52.0782426381107,-0.326791035349567,0.253844852341706,-
1.28736522460446,0.19796701930793,0.80305339566012
"3491","CROT",19.3453752137028,-0.438771477115872,0.340842282593228,-1
.28731527607892,0.197984421204059,0.80305339566012
"3492","CALCRL",10.8036171333119,1.08495609355719,0.843163639957047,1.
28676812203673,0.198175121080283,0.803366651731121
"3493","ATP6AP2",345.268176327001,-0.242766476266311,0.188655851041167
,-1.28682187658912,0.198156380029981,0.803366651731121
"3494","DKK2",14.7972254399147,0.631079457781496,0.490687025470237,1.2
8611400959036,0.198403275487447,0.804061356869564
"3495","RELA",52.1364542879281,-0.267276548639245,0.207873403422155,-1
.28576597216938,0.19852474919324,0.804093377739211
"3496","IKZF3",1.54150533747856,1.65046767664838,1.28348764568783,1.28
592408520136,0.198469557078252,0.804093377739211
"3497","WARS2-
AS1",9.24867258885116,0.651360373855491,0.507054099902796,1.2845973910
4833,0.198933010848953,0.804123015106572
"3498","CYP1B1-
AS1",8.46539509977729,0.670412565936248,0.521756986951096,1.2849134418
9529,0.198822533167945,0.804123015106572
"3499","WDR55",29.2135311654361,-0.340004052200332,0.264614074510642,-
1.28490539601535,0.198825345102474,0.804123015106572
"3500","HABP4",34.0381876137723,-0.368930156139832,0.2871935984535,-1.
28460438577487,0.19893056531022,0.804123015106572
"3501","SCAI",18.0076213475651,0.562250332849379,0.437786123508791,1.2
843036877072,0.199035717026836,0.804123015106572
"3502","CMTR2",22.0638001975319,0.415486510703181,0.323460117460677,1.
28450615168558,0.198964912521327,0.804123015106572
"3503","C19orf53",278.162780051807,-0.345111322360063,0.26848772257556
2,-1.28538958522745,0.198656178780984,0.804123015106572
"3504","AHCY",39.3895976513222,-0.317279971955765,0.246893361298925,-1
.28508911817851,0.198761143748109,0.804123015106572
"3505","PHKA2",7.45538416743189,-0.704846157837551,0.548824897129612,-
1.28428240322904,0.199043161578286,0.804123015106572
"3506","RPS10",2180.56477325492,-0.429333943619486,0.334662495468108,-
1.28288633902331,0.199531899577009,0.805295115399006
"3507","SFXN4",17.8482367391789,-0.430046587016284,0.335221443196616,-
1.28287314473511,0.199536522847973,0.805295115399006
"3508","LINC00205",14.5175832689554,0.472414463175182,0.36820466315554
5,1.28302140208261,0.199484578059573,0.805295115399006
"3509","SLC25A5",209.317691112073,-0.303153983130117,0.236321369203001
,-1.28280393835103,0.199560774006717,0.805295115399006
"3510","AC018647.2",10.2562822427918,-0.622734411311632,0.485543933085
071,-1.28255008224462,0.199649748175482,0.805424625118184
"3511","ZNF780A",13.9058856314332,0.50708552666823,0.39553107522435,1.
28203713546554,0.199829619602276,0.805920653252129
"3512","FAM214B",13.3230498173724,-0.536398772556407,0.418485153548808
,-1.28176296819057,0.199925808427654,0.806078999810815

"3513","MAN1A2",98.0863792309499,0.239631922022902,0.187119189113479,1
.28063788197359,0.200320887672876,0.806523676271799
"3514","AC098818.2",14.5911461118094,-0.529934810536288,0.413764031084
4,-1.2807657764437,0.200275948245183,0.806523676271799
"3515","FAM182A",10.1657362798117,-0.653649438126826,0.510289109800751
,-1.28093942350064,0.200214944101719,0.806523676271799
"3516","TRABD",27.3459500183467,-0.353155030979818,0.275701940548149,-
1.28093052329511,0.200218070513069,0.806523676271799
"3517","INTS6L",5.52483456126691,0.779932507474766,0.60898318436509,1.
2807127150611,0.200294592005925,0.806523676271799
"3518","ZNF436",40.0447439098759,0.337458683394389,0.263668381344031,1
.27986026111366,0.200594285752355,0.807342665859469
"3519","PHC2",75.1905415141551,-0.34268980327771,0.268336168449946,-1.
27709136363268,0.201569993543512,0.807342665859469
"3520","STON1",10.0600407858274,0.709547915985013,0.55540932700478,1.2
775225072497,0.201417839507684,0.807342665859469
"3521","PARM1",3.30601307005698,-1.09813405567979,0.859463199775975,-1
.27769758608167,0.201356076705711,0.807342665859469
"3522","PPA2",29.7111673611521,-0.358441826416159,0.280211230822404,-1
.27918436874979,0.200832138986596,0.807342665859469
"3523","PCDHB12",5.08339477117916,0.81931500543503,0.641466598747428,1
.27725279388651,0.20151301370054,0.807342665859469
"3524","TNXB",1353.45179511014,0.224574126646405,0.175519758723699,1.2
7948060252252,0.200727866059507,0.807342665859469
"3525","DLL1",4.09591490140061,0.886343864503178,0.693525069857304,1.2
7802714426105,0.201239855485574,0.807342665859469
"3526","ADAM32",6.23639636942727,0.671405617605648,0.525774153798743,1
.27698482847571,0.201607603564907,0.807342665859469
"3527","RFX3",14.3958752611493,0.514513533643869,0.402849907107997,1.2
7718419333267,0.201537226120317,0.807342665859469
"3528","FERMT3",10.2908636969463,-0.579101100070411,0.452766868964651,
-1.27902711034191,0.200887509281892,0.807342665859469
"3529","MON2",47.4947306430274,0.298404076131856,0.233427454838329,1.2
7835895027228,0.201122890992373,0.807342665859469
"3530","RNF31",15.9819869630806,0.447022849070678,0.3497346612827,1.27
8177139867,0.201186974591385,0.807342665859469
"3531","NDUFB10",291.989462602849,-0.344602361143529,0.269535291257705
,-1.27850553274692,0.201071235197373,0.807342665859469
"3532","SULT1A2",2.58842952158476,1.12756608419335,0.881766019808793,1
.27875883041837,0.200981995686791,0.807342665859469
"3533","PRAC2",2.4576793274123,-1.40617408333184,1.09902305356853,-1.2
7947642114147,0.20072933761147,0.807342665859469
"3534","AC015813.1",5.54590078878008,0.744127679510461,0.5824189613952
55,1.27765016050956,0.20137280572617,0.807342665859469
"3535","ZNF43",30.4103528987805,0.366079207740559,0.286192781329756,1.
27913501535441,0.200849514995464,0.807342665859469
"3536","AL022323.4",8.02177953005617,0.671653003422979,0.5258470365921
58,1.27727828947319,0.201504015628098,0.807342665859469
"3537","MMACHC",3.89676719990534,0.934133672258966,0.732608227542288,1
.27507941781209,0.202281133598747,0.807549014363149

"3538","RNF7",212.47995059355,-0.347884928877164,0.272944075011102,-1.27456486777745,0.202463299115402,0.807549014363149
"3539","ELF2",111.798795650196,0.234114637556985,0.183671023971282,1.27464110829801,0.202436300236413,0.807549014363149
"3540","ID4",205.581143118483,-0.357912165877262,0.280789195981455,-1.27466501916584,0.202427833276609,0.807549014363149
"3541","NEIL2",17.9700768663463,-0.438481376364716,0.344087794124133,-1.2743299351285,0.202546511769777,0.807549014363149
"3542","ARID5B",611.057911791968,-0.341932728087866,0.268241900983295,-1.27471780819641,0.202409141326379,0.807549014363149
"3543","LINC02716",1.57423955612638,1.6001720312841,1.2555539210677,1.27447495837005,0.202495141897104,0.807549014363149
"3544","YARS2",18.3493165086942,-0.444535360543034,0.348777516670132,-1.27455280026971,0.202467572794477,0.807549014363149
"3545","ARPC3",370.867469012922,-0.333339712288562,0.261521410718812,-1.2746172918399,0.202444734021789,0.807549014363149
"3546","MEG9",2.51147731411182,1.35069348554558,1.05955968013733,1.27476867123758,0.202391132535211,0.807549014363149
"3547","SMAD6",17.1702730244512,0.477783754869603,0.37495024862016,1.27425906937754,0.202571617162281,0.807549014363149
"3548","MRPS34",105.590035068906,-0.303066200134069,0.237516578805087,-1.27597914073516,0.201962892887503,0.807549014363149
"3549","FOPNL",34.9090685743124,0.323956130609598,0.253927353981835,1.27578272104065,0.202032337274273,0.807549014363149
"3550","TUBD1",11.2064663510748,-0.541242172004058,0.424745501385498,-1.2742740540831,0.202566308387536,0.807549014363149
"3551","RAB5IF",103.213402461442,-0.272985045595593,0.214207922891023,-1.27439285116672,0.202524224597512,0.807549014363149
"3552","TMEM50B",69.9904464665251,0.248484957466873,0.19469662955799,1.27626738085295,0.201861016801033,0.807549014363149
"3553","CCDC36",8.30759388359676,0.656465006783821,0.515324846960846,1.27388580359623,0.202703890309361,0.807817270537668
"3554","DCAKD",19.7827539431231,-0.474359967387046,0.372412948753657,-1.27374724475766,0.202753007026192,0.807817270537668
"3555","NCBP1",13.9054619493815,0.492871727885155,0.387010787001112,1.27353485856129,0.202828311096107,0.807889981749896
"3556","EFNA5",37.6241235827401,0.365374277424269,0.286969748015385,1.27321531259344,0.202941648328694,0.808114100206496
"3557","GNG5",305.584448363162,-0.310627107879746,0.244373747444172,-1.27111488500093,0.203687780650984,0.808810704996618
"3558","TOMM40L",5.53864843323209,-0.854616369588765,0.671702021324815,-1.27231472060064,0.203261320367475,0.808810704996618
"3559","AHCTF1",47.4520386252153,-0.350661326675398,0.275597117270184,-1.27236935621364,0.203241916596275,0.808810704996618
"3560","RAD17",28.1058059896939,0.390608644690339,0.307290541493596,1.27113787099262,0.203679604573697,0.808810704996618
"3561","RGMB-AS1",1.78563318492298,1.26827159091803,0.997568440969778,1.27136298506505,0.203599544515093,0.808810704996618
"3562","RASA4",4.52852644254495,-0.808884261053959,0.636007528231829,-

1.27181554486115,0.203438664486413,0.808810704996618
"3563","LINC02381",66.4227922971866,0.340485157364198,0.26766012296021
3,1.2720802546101,0.203344605993097,0.808810704996618
"3564","ZNF846",17.7157083392828,-0.503010092941157,0.395471708298556,
-1.27192434347647,0.203400001578922,0.808810704996618
"3565","SUGP2",61.4440213773365,0.36316630077958,0.285623524373131,1.2
7148595892665,0.203555819499425,0.808810704996618
"3566","CDK16",33.022094085613,-0.323729913594727,0.254630867294336,-1
.27136948098487,0.20359723463213,0.808810704996618
"3567","FAT1",94.3404218760489,-0.41944194658287,0.33002290643688,-1.2
7094798088839,0.203747155318787,0.808819657783579
"3568","EEPD1",8.93628181987215,-0.637491863798894,0.501727674373613,-
1.27059338433898,0.203873341727682,0.808867054879232
"3569","GDPD3",4.44467853148734,0.843585576664823,0.663913259842213,1.
27062619123665,0.203861664707549,0.808867054879232
"3570","LINC01142",2.2663147017369,-1.15607948921639,0.910349005611248
,-1.26992997420825,0.204109574983499,0.809577473884131
"3571","DIRAS3",5.59396235125126,-1.42247306833642,1.12043663478403,-1
.26957029445097,0.204237736334049,0.809858960092447
"3572","ILKAP",34.9577766483255,-0.314115416588579,0.247539882769849,-
1.26894871676347,0.204459355339098,0.810510770325203
"3573","TFG",155.664531635183,-0.240979023444089,0.189943286139308,-1.
26868934586796,0.204551883852339,0.810557113183504
"3574","WRN",28.2118351889801,0.403250055274161,0.317871377285616,1.26
859504846777,0.20458553125126,0.810557113183504
"3575","TTC7A",25.045648200042,0.358941593374573,0.283001743010751,1.2
6833704116422,0.204677614540788,0.810695111020297
"3576","NDUFAF2",94.42779779736,-0.343291657699846,0.270739769008821,-
1.26797647407559,0.204806352100071,0.810900878072758
"3577","TMEM87A",116.481601891953,-0.206809661763121,0.163115724663838
,-1.26787078431176,0.204844098931233,0.810900878072758
"3578","CHI3L2",108.765929607638,-1.16021331477882,0.915338493516433,-
1.26752378819081,0.204968063284009,0.811164834013851
"3579","PRUNE2",4.8473692078315,-0.980126645747833,0.773507375508773,-
1.26711997426418,0.205112394456837,0.811282543438215
"3580","EID3",18.743489974033,-0.6175262201468,0.487299389175085,-1.26
724193353118,0.205068795990468,0.811282543438215
"3581","RIPOR1",31.6217669008231,-0.339765915596106,0.268234813570714,
-1.26667344582598,0.205272078655283,0.811687415179786
"3582","INTS4",14.5325531864883,0.482061026324176,0.380645655674036,1.
26642986498968,0.205359224314713,0.811766034694597
"3583","URB1",17.1338557640323,0.467608430088574,0.369272194432409,1.2
66297428127,0.205406617394826,0.811766034694597
"3584","AC254633.1",3.44043548775624,1.11472119291136,0.88192954193265
6,1.26395719829111,0.206245390008497,0.812634768016382
"3585","HNRNPR",215.073949584506,-0.226273366462412,0.179225868669113,
-1.26250394623645,0.206767507206976,0.812634768016382
"3586","WDTC1",27.9685205363089,0.391762515539765,0.309661457993821,1.
2651316637138,0.205824134003629,0.812634768016382
"3587","NBPF19",33.1979041526684,0.72346959901215,0.573611601757426,1.

26125342792159,0.207217554724524,0.812634768016382
"3588","C1orf43",124.796780830935,-0.257432134445926,0.204167210272863
,-1.26088872988898,0.207348939235325,0.812634768016382
"3589","COA6-
AS1",5.68749477480657,-0.838549458433729,0.664955307403138,-1.26106138
126565,0.207286733077185,0.812634768016382
"3590","RNF181",118.254878619461,-0.327341958677544,0.259071340103773,
-1.2635205366461,0.206402170912843,0.812634768016382
"3591","PSD4",1.61323813248218,-1.66786027608994,1.31871158143164,-1.2
6476501729002,0.205955575184708,0.812634768016382
"3592","MZT2B",508.587249167599,-0.321935544974922,0.254801265463384,-
1.26347702547492,0.206417798093945,0.812634768016382
"3593","SUMF1",45.1180502728316,0.279097722003422,0.220678786530943,1.
26472383862002,0.205970341370417,0.812634768016382
"3594","WWC2",34.3600354840173,0.433770320751206,0.343706101872579,1.2
6203846364071,0.206934946199808,0.812634768016382
"3595","LYRM7",39.2716874192331,0.366646089138945,0.289987106172573,1.
264353074101,0.206103327793169,0.812634768016382
"3596","KIAA1191",24.1530227032908,-0.387311161550058,0.30721139746008
6,-1.26073174612729,0.207405512119435,0.812634768016382
"3597","HFE",10.9766311388905,0.563130812598353,0.446337479561623,1.26
167045875565,0.207067391075027,0.812634768016382
"3598","RPL7L1",139.046482449155,0.209353485474078,0.165456939757722,1
.26530495354643,0.205762031550588,0.812634768016382
"3599","AL080250.1",2.44229210589262,1.14546221008737,0.90723041328681
2,1.26259238371151,0.206735706436482,0.812634768016382
"3600","TBX18",30.7432469735368,0.418374031083421,0.331795507459481,1.
2609394089958,0.207330678187928,0.812634768016382
"3601","AL133338.1",2.87612969768182,1.0608433322604,0.841237889067909
,1.26105034740626,0.207290708163143,0.812634768016382
"3602","SNHG6",403.635741128489,-0.299537853742646,0.237520035128645,-
1.26110563085936,0.207270792162857,0.812634768016382
"3603","PPP1R16A",14.5438217606714,-0.517095133987725,0.40888927127415
6,-1.26463365589511,0.206002682519003,0.812634768016382
"3604","PROSER2",3.63488622188953,1.21158199350649,0.959270175586581,1
.26302477064465,0.206580277789163,0.812634768016382
"3605","AL157394.3",4.52466112534785,0.757878310268895,0.6011039153243
47,1.26081080317029,0.20737702060798,0.812634768016382
"3606","LPXN",5.45123115283239,-0.740332533512674,0.586427291626415,-1
.26244556500673,0.206788502154665,0.812634768016382
"3607","ZNF410",24.6335018870761,-0.506759533067358,0.401411586262426,
-1.26244371216545,0.206789168495285,0.812634768016382
"3608","CALML4",5.89509124787983,-0.720695300104669,0.571251224685105,
-1.26160832390678,0.207089759462209,0.812634768016382
"3609","ABHD2",77.5607756477994,-0.411289638067057,0.32534631016756,-1
.26415952851973,0.206172773808814,0.812634768016382
"3610","FDXR",9.0051117817076,0.69559748363805,0.551722185949712,1.260
77489967288,0.207389959582692,0.812634768016382
"3611","CBX8",3.93866938171638,-0.868845783373094,0.688663719203126,-1
.26164012876773,0.207078309574289,0.812634768016382

"3612","MEX3D",32.3836254819734,-0.360643776832296,0.285948666804873,-1.26121859864587,0.20723009955663,0.812634768016382
"3613","ZNF606",7.37524817716821,0.63033435940609,0.49931249717843,1.26240453216783,0.206803259232788,0.812634768016382
"3614","CXorf40A",9.27583845153187,-0.593467901347424,0.470730676474166,-1.26073768081693,0.207403373207256,0.812634768016382
"3615","THAP3",28.357351245753,0.35765936250899,0.287025333832343,1.24608987552961,0.212731401360281,0.812647571392276
"3616","FBXO42",27.3499754494839,0.439604155322385,0.353370050918937,1.24403342665627,0.213487260785301,0.812647571392276
"3617","AL604028.1",2.07098076903894,1.19154680482516,0.95734912468694,1.24463142452322,0.213267263710521,0.812647571392276
"3618","MAGOH",103.075154016429,-0.307713117626665,0.24737298198917,-1.2439237104727,0.213527641979649,0.812647571392276
"3619","NEXN",116.375605314587,0.337564539279308,0.2725659217097,1.23846934775227,0.215542080875662,0.812647571392276
"3620","DNTTIP2",56.4038315322978,-0.300781057866513,0.240841766559067,-1.24887415569071,0.211711105639748,0.812647571392276
"3621","PSMA5",112.721459746033,-0.305690864162732,0.247267571000982,-1.23627559782806,0.216356138445652,0.812647571392276
"3622","AL160006.1",5.08833078485971,0.717144459506836,0.574449352422697,1.24840328652532,0.211883406062918,0.812647571392276
"3623","GBA",28.8383760776861,0.430220240383205,0.345050546453116,1.24683251426661,0.212458915803714,0.812647571392276
"3624","CCT3",151.962139818337,-0.267509045076957,0.214990607905182,-1.24428247207402,0.213395619725004,0.812647571392276
"3625","AL645568.1",6.71532466986158,0.655812064011171,0.529407013689038,1.23876723778424,0.215431710097316,0.812647571392276
"3626","BTG2",408.58258755713,0.384578164192232,0.305922834871655,1.2571083958266,0.208714387381704,0.812647571392276
"3627","UTP25",22.9832751173864,0.393385294537117,0.312426049979406,1.25913090333872,0.207983053879142,0.812647571392276
"3628","NENF",433.616449484434,-0.267952121996397,0.21481962968056,-1.24733536872233,0.212274553981751,0.812647571392276
"3629","VASH2",17.4011470918068,0.548603841582891,0.44425739506751,1.23487835582236,0.216875779885015,0.812647571392276
"3630","TBCE",6.87536530103375,-0.68125785981662,0.542605165602665,-1.25553146745287,0.209285892242525,0.812647571392276
"3631","COX20",91.9824503349286,-0.315218093966426,0.252630124011508,-1.24774547453441,0.212124282234394,0.812647571392276
"3632","NOL10",22.2555588708348,-0.389877491543949,0.316103058587384,-1.23338727972533,0.21743130851168,0.812647571392276
"3633","FAM228B",31.1813126816735,-0.316955032109955,0.254546938591677,-1.24517322370311,0.213068082867337,0.812647571392276
"3634","BIRC6",63.3828954599462,0.275313883711402,0.222613296804495,1.23673602459241,0.216185099864909,0.812647571392276
"3635","MCFD2",110.586069515933,0.290088485772489,0.234497000059638,1.23706693773785,0.216062232928353,0.812647571392276
"3636","UGP2",261.59099792352,-0.240076123720243,0.194068122734705,-1.23707139708066,0.216060577532795,0.812647571392276

"3637","DGUOK",118.724520752411,-0.305733533345214,0.243524077423388,-
1.25545505224795,0.209313615139527,0.812647571392276
"3638","VAMP8",10.6327157824443,-0.577013775248808,0.458633269846801,-
1.25811582627128,0.208349870518277,0.812647571392276
"3639","FAM168B",43.9149945271964,0.301953431631918,0.240804396015436,
1.25393654197477,0.209865071651057,0.812647571392276
"3640","ALS2",12.6778116165776,0.479852015008998,0.388795749254989,1.2
3420077490171,0.217128098762355,0.812647571392276
"3641","AC007038.2",2.10640212527278,1.19564084802789,0.96829584831438
8,1.23478877876969,0.216909124652285,0.812647571392276
"3642","AC009950.1",6.0891043502307,0.746501133406963,0.60145160913751
8,1.24116574312179,0.214544527009184,0.812647571392276
"3643","PSMD1",89.8370865637783,-0.266429593163824,0.215791645598303,-
1.23466129759159,0.216956585467075,0.812647571392276
"3644","HDAC4",37.3000212407517,0.393889383750669,0.314537857114045,1.
25227973308107,0.210467951178717,0.812647571392276
"3645","JAGN1",79.3545645706381,-0.2688401583255,0.214749594452005,-1.
25187737379213,0.210614550585399,0.812647571392276
"3646","MYD88",23.2924098418653,-0.385764489750289,0.311094834534981,-
1.24002216342461,0.214967196574851,0.812647571392276
"3647","AC116036.2",1.62301478757086,1.37869097159452,1.10274658224977
,1.25023372893324,0.211214179053972,0.812647571392276
"3648","ADAMTS9",6.05171799104833,1.04814854576971,0.847201516560844,1
.23718917551588,0.216016859172112,0.812647571392276
"3649","ZBTB11",34.7565141290522,0.410367361107321,0.329758591436668,1
.24444782263128,0.213334791485443,0.812647571392276
"3650","NDUFB4",293.241155041958,-0.260695566865007,0.210338083961024,
-1.23941210243845,0.215192921768103,0.812647571392276
"3651","AC010207.1",1.52769552824374,1.99024342361801,1.59262075376474
,1.24966563377586,0.211421716230424,0.812647571392276
"3652","CEP63",58.9661259354093,-0.347477739475261,0.276120553375234,-
1.25842765135653,0.208237136956775,0.812647571392276
"3653","SLC9A9",40.8687945927581,0.360188463177704,0.29146050453835,1.
23580539239172,0.216530910101337,0.812647571392276
"3654","SIAH2",21.6878310659538,-0.367060896795777,0.292428691455999,-
1.25521505762032,0.209400700791247,0.812647571392276
"3655","PSMD2",110.713731684853,-0.261111549362285,0.20907481125473,-1
.24889051816077,0.211705120106676,0.812647571392276
"3656","WDR53",6.39219521933459,0.713016601210296,0.568563402827585,1.
25406700055669,0.209817653541064,0.812647571392276
"3657","TBC1D1",15.361390977872,-0.471923142595798,0.378977296486452,-
1.2452543911497,0.213038254967336,0.812647571392276
"3658","RHOH",1.7752712823209,-1.35649444543905,1.08600650242496,-1.24
906659620372,0.21164071697551,0.812647571392276
"3659","HOPX",3.8936311376147,-0.950767909635054,0.763900086451702,-1.
24462338268784,0.213270221130612,0.812647571392276
"3660","TIGD2",2.17544180787446,1.20826737573286,0.961345798642067,1.2
5684990503893,0.208807991128479,0.812647571392276
"3661","TRMT10A",11.9789387470603,0.472918946485217,0.383457672784188,
1.23330156116443,0.217463275661259,0.812647571392276

"3662","SMARCA5",117.151918451342,0.20960725708345,0.166686068912512,1
.25749715288724,0.2085736693457,0.812647571392276
"3663","LINC01060",5.28457717654137,-1.23772547166581,0.99255709601583
3,-1.24700682372238,0.212394995534152,0.812647571392276
"3664","NNT",59.0551712912499,-0.437265403257159,0.350670012179489,-1.
24694267564957,0.212418517381953,0.812647571392276
"3665","STARD4-
AS1",6.94293157993252,0.767867158550769,0.613861518144943,1.2508801021
9507,0.210978224433772,0.812647571392276
"3666","LOX",137.089133840706,0.380478073908399,0.307534713289851,1.23
718740508425,0.21601751629422б,0.812647571392276
"3667","PURA",174.927669901842,-0.238765835859945,0.192876471744883,-1
.23792100560487,0.21574535276819,0.812647571392276
"3668","TIGD6",6.50827048389942,-0.739480304699457,0.594767521236172,-
1.24330982828806,0.213753683872671,0.812647571392276
"3669","DUSP1",1538.54939065768,-0.385822697329127,0.311491936578318,-
1.23862820196028,0.215483218979039,0.812647571392276
"3670","ARL10",14.6766175000997,0.595225645547514,0.482391260723702,1.
23390636193229,0.217237798610609,0.812647571392276
"3671","CLK4",44.8495819919563,0.325967805990183,0.260193839851045,1.2
5278832956534,0.210282749970864,0.812647571392276
"3672","EEF1E1",30.876651910123,-0.358221485068359,0.285782807737994,-
1.25347458058701,0.210033044297367,0.812647571392276
"3673","HIVEP1",46.2001038227208,0.308688629434555,0.246847256882527,1
.25052485222251,0.211107882664841,0.812647571392276
"3674","HACE1",6.80238052951239,-0.626218468817791,0.503493068369857,-
1.24374794442608,0.213592344405551,0.812647571392276
"3675","PHF10",38.2948451986746,0.346271212896526,0.277743959658538,1.
2467281496319,0.212497193558773,0.812647571392276
"3676","GPR146",8.28638805079509,0.638977959514181,0.511389340143281,1
.24949409257368,0.2114844128301,0.812647571392276
"3677","MTURN",157.985564173908,0.29440701529529,0.235505735599785,1.2
5010549974715,0.211261011010169,0.812647571392276
"3678","PSMA2",230.150496810901,-0.340872400844679,0.27077026660016,-1
.25889893718662,0.208066837776119,0.812647571392276
"3679","ZNF680",29.7410718025698,0.369210873659099,0.298252592048328,1
.23791337779648,0.215748181390365,0.812647571392276
"3680","SRI",391.736442434294,-0.263364640888343,0.211774032099132,-1.
2436115905139,0.21364254830826,0.812647571392276
"3681","DYNC1I1",4.38419612382073,-0.905204795631328,0.729733844146174
,-1.24045883700305,0.214805730290001,0.812647571392276
"3682","AGFG2",4.38381135363371,-0.789196272620886,0.63740145121883,-1
.23814633793475,0.215661804769909,0.812647571392276
"3683","ZC3HAV1L",1.86610670162671,1.31672606690192,1.06122645747219,1
.24075880094276,0.214694865007285,0.812647571392276
"3684","TBXAS1",1.83499716300355,-1.44228909405214,1.16249373277267,-1
.2406854793205,0.214721960529476,0.812647571392276
"3685","PTPRN2",28.8225279983531,0.342879704540207,0.276528974118818,1
.23994133212558,0.214997094692839,0.812647571392276
"3686","CLN8",25.0414645965187,0.388225883421346,0.310353982495571,1.2

5091316792394,0.210966159118668,0.812647571392276
"3687","ASAH1",157.880107399014,0.304784323142742,0.246265682710213,1.23762401560996,0.215855505371407,0.812647571392276
"3688","TACC1",398.572325154127,0.227683589211589,0.183387153702102,1.24154601134954,0.214404111870683,0.812647571392276
"3689","GGH",12.8427341374141,0.568938719871878,0.453704252676143,1.25398586527685,0.209847143073964,0.812647571392276
"3690","ARMC1",40.651887726235,-0.350721085889725,0.280460514591001,-1.25051858512485,0.211110170531327,0.812647571392276
"3691","AZIN1-AS1",14.2804800032763,-0.491297229046761,0.390892972215922,-1.25685869014646,0.208804809397929,0.812647571392276
"3692","EIF3H",317.325783218409,-0.274744325867276,0.222784479151846,-1.23322920390704,0.217490262590676,0.812647571392276
"3693","SMU1",56.6051774600645,-0.296237210808615,0.237359536132186,-1.24805270365729,0.212011757126379,0.812647571392276
"3694","CNTFR",35.5927079984494,0.600482616828187,0.479300256593416,1.252831828416,0.210266915700263,0.812647571392276
"3695","ALDH1A1",261.294365121025,0.432122075737643,0.343602232734551,1.25762301454973,0.208528126053402,0.812647571392276
"3696","BICD2",42.7011898173265,0.354907903513977,0.28338318623763,1.25239576922665,0.210425687186801,0.812647571392276
"3697","NANS",74.7656264433492,-0.278796689320476,0.223294194213905,-1.24856219527769,0.211825246854437,0.812647571392276
"3698","FBXW2",47.0787253841337,-0.316386282388211,0.251371084770736,-1.25864230834971,0.208159558103995,0.812647571392276
"3699","SWI5",79.3394516961038,-0.294342220152989,0.23825936648988,-1.23538572476433,0.216686983046268,0.812647571392276
"3700","CRAT",57.9791551066667,-0.299687722217237,0.239921000556667,-1.24911000505124,0.211624841727993,0.812647571392276
"3701","AL137186.2",9.84629197445079,0.606259970133782,0.490822668270897,1.23519146389378,0.216759255397722,0.812647571392276
"3702","WAC-AS1",54.3370527007087,0.393513164365287,0.318389101410342,1.23595048518361,0.216476969417797,0.812647571392276
"3703","KIF1BP",46.4975677450364,0.310645310255852,0.246450357086578,1.26047823151144,0.207496895868054,0.812647571392276
"3704","HTRA1",800.389098379876,-0.313713941452016,0.252026025922962,-1.24476803656742,0.213217028556909,0.812647571392276
"3705","TAF10",145.455431500991,-0.304857394412229,0.245123638652291,-1.24368827130814,0.213614314296324,0.812647571392276
"3706","PRDM11",8.4335868315835,0.618663288725033,0.496164150160511,1.24689236117703,0.212436968034878,0.812647571392276
"3707","SELENOH",240.129586346919,-0.313072084528846,0.252033311531519,-1.24218533901894,0.214168187666057,0.812647571392276
"3708","DNAJC4",63.0404321440404,-0.339954941905781,0.275474302595853,-1.23407134060169,0.21717632176593,0.812647571392276
"3709","FOSL1",9.04135148706618,-0.990077215884365,0.794308260604969,-1.24646471022509,0.212593837228416,0.812647571392276
"3710","GSTP1",857.528802721366,-0.308411817432227,0.247627721219903,-

1.24546563653245,0.212960639379938,0.812647571392276
"3711","COA4",88.9296855955461,-0.274896078551462,0.21913270940018,-1.
25447305107448,0.209670114978551,0.812647571392276
"3712","AP002433.1",1.72860452599071,-1.35811804235731,1.0874715709865
1,-1.24887682454566,0.211710129341177,0.812647571392276
"3713","ZPR1",31.4130032685915,-0.348116093778276,0.276761192143535,-1
.2578211962526,0.208456428221962,0.812647571392276
"3714","SIK3",32.2227442140472,-0.397197515448289,0.320507183292397,-1
.2392780447792,0.215242546559843,0.812647571392276
"3715","SMIM10L1",61.7140438864236,-0.279212246946496,0.22554866383378
7,-1.23792463320578,0.215744007553369,0.812647571392276
"3716","FMNL3",16.5643058485113,-0.509518455522429,0.413175219984211,-
1.23317767106628,0.217509484150899,0.812647571392276
"3717","BCDIN3D",12.2106651731717,0.610484722931085,0.484493295428257,
1.26004782458643,0.207652110409831,0.812647571392276
"3718","MARS",15.8851050338407,-0.548895879209686,0.442295800107998,-1
.24101535460128,0.214600076694701,0.812647571392276
"3719","C12orf43",28.1470639287952,0.406280684221776,0.327867661605642
,1.23916058763505,0.215286033032975,0.812647571392276
"3720","FRY",6.62918547602486,0.769841272305626,0.623708501785673,1.23
429658262085,0.217092408888453,0.812647571392276
"3721","KBTBD7",20.4437439282492,0.394559990258391,0.318028126715403,1
.24064495280153,0.214736937895165,0.812647571392276
"3722","LINC01232",3.6302005033277,0.922722320563193,0.738479189264527
,1.24948994362612,0.21148592939554,0.812647571392276
"3723","LINC00346",4.14769207643493,-1.11012651559715,0.89068916918065
5,-1.24636804174722,0.212629308358447,0.812647571392276
"3724","ZNF219",10.8157866781026,0.665562470214731,0.535801969343433,1
.24217996255278,0.214170170904506,0.812647571392276
"3725","LINC00645",4.77509494952794,0.92955873619353,0.740466935960913
,1.25536832375538,0.209345082846932,0.812647571392276
"3726","LINC01588",2.28147023772627,-1.11390392684849,0.89817873721211
,-1.24018069087895,0.21490856867647,0.812647571392276
"3727","YLPM1",37.5035796159187,0.375441500813455,0.302632033891973,1.
2405874420666,0.214758193401803,0.812647571392276
"3728","MLH3",40.8186405488227,0.371161011254662,0.295830937785729,1.2
5463893003474,0.209609864431703,0.812647571392276
"3729","MOAP1",22.210305425057,0.384069638149534,0.309768715580824,1.2
3985934935164,0.215027421782803,0.812647571392276
"3730","SERPINA1",6.85859269937973,-1.06437147571372,0.862271768000915
,-1.23438052272238,0.217061143356234,0.812647571392276
"3731","ZBTB42",2.39477971887442,-1.10954147761532,0.883553788377275,-
1.2557128660508,0.209198904817762,0.812647571392276
"3732","GOLGA8R",2.42599512036778,1.1479947678633,0.919856555157437,1.
24801498823565,0.212025568376414,0.812647571392276
"3733","ADAL",11.2072559943773,0.522157577319652,0.41742500794422,1.25
090152094919,0.21097040891272,0.812647571392276
"3734","ZSCAN29",12.163588640344,0.543545547955847,0.438844219065895,1
.23858427282651,0.215499495337945,0.812647571392276
"3735","ADAM10",43.5295966380403,0.280348105630378,0.226807492607247,1

.23606192373832,0.216435546829669,0.812647571392276
"3736","ICE2",40.8365117915117,0.309630299315039,0.250313807214766,1.2
3696851867776,0.216098770339664,0.812647571392276
"3737","RAB8B",88.5402974629823,0.274682966051299,0.219106426363371,1.
25365088833934,0.209968925989049,0.812647571392276
"3738","RPLP1",8979.72951579486,-0.306422654037877,0.245313046347839,-
1.24910867399767,0.21162532850087,0.812647571392276
"3739","SIN3A",34.5594097308055,0.326052292663402,0.259522093353386,1.
25635659165027,0.208986712398275,0.812647571392276
"3740","AXIN1",15.5932595325367,-0.425924894249181,0.339902166691419,-
1.25308084498284,0.21017628633452,0.812647571392276
"3741","AC099518.5",21.3358930087653,-0.569644252363812,0.460496754208
419,-1.23702121059032,0.216079208269769,0.812647571392276
"3742","PPP4C",82.1060077849446,-0.302317111671583,0.241359537279267,-
1.25255921137181,0.210366166865329,0.812647571392276
"3743","FTO",41.4272140742188,0.317341076526976,0.255834197669939,1.24
041695526721,0.214821212868026,0.812647571392276
"3744","AC007336.1",1.64790913878875,1.37945508794669,1.1098228541209,
1.24295069508131,0.213886002862327,0.812647571392276
"3745","CPNE2",18.4758040535638,-0.398882252574189,0.316533232195407,-
1.26015916182837,0.207611951591465,0.812647571392276
"3746","AC010542.2",2.33204611297697,1.33577813680359,1.06998671581895
,1.24840628117632,0.211882309940266,0.812647571392276
"3747","CBFB",37.9479358156908,0.299750793056377,0.238575542601187,1.2
5641878370346,0.208964174892021,0.812647571392276
"3748","GAN",2.79477721260013,1.15175718092751,0.926979992840914,1.242
4833219946,0.214058290210234,0.812647571392276
"3749","TAX1BP3",205.848206561476,-0.30242282310889,0.243332331464654,
-1.242838636726,0.213927301719882,0.812647571392276
"3750","C1QBP",162.270942371406,-0.268091507640507,0.213157033894107,-
1.25771832504336,0.208493642558294,0.812647571392276
"3751","NATD1",13.8195109283973,0.478771351846164,0.388233204682628,1.
2332055735355,0.217499076480779,0.812647571392276
"3752","AC104564.5",2.29806726917306,1.47955016762592,1.18723335508962
,1.24621681262841,0.212684808316825,0.812647571392276
"3753","PSMB3",188.707456835326,-0.352624223813738,0.283422956049645,-
1.24416253619192,0.21343974889928,0.812647571392276
"3754","AC142472.1",6.38457215686919,0.667697789379989,0.5312689913694
11,1.25679796906444,0.208826801664569,0.812647571392276
"3755","HOXB2",49.0204892502178,-0.396398104081744,0.317927193835278,-
1.24682037827542,0.212463366658077,0.812647571392276
"3756","TRIM37",21.3626283943369,0.407314445786373,0.32885590859856,1.
23858028740359,0.215500972036734,0.812647571392276
"3757","AC099850.1",3.18799770379959,-0.959415497629358,0.764381866773
847,-1.255152089987,0.209423553886849,0.812647571392276
"3758","CCDC47",103.385927583448,-0.209297990031743,0.167950067412649,
-1.24619175958712,0.212694003606076,0.812647571392276
"3759","ARHGDIA",172.458004816807,-0.230593535456736,0.185677272462749
,-1.24190501291965,0.214271610327958,0.812647571392276
"3760","IER3IP1",129.187637437126,-0.280859139678115,0.225208422904936

,-1.24710761727003,0.212358040305846,0.812647571392276
"3761","UBL5",378.229086781981,-0.27355076642222,0.2216540160787,-1.23413404034643,0.21715296092167,0.812647571392276
"3762","AC024075.1",3.67859098654292,1.1286162343001,0.913097360152151,1.23603055222067,0.216447207292757,0.812647571392276
"3763","ZNF875",12.2044990188911,0.5065558333122,0.403383321025431,1.2557679182780,0.209200126399082,0.812647571392276
"3764","FBXO27",3.68256771167718,1.16204104571214,0.926432731695473,1.2543177782433,0.209726524529684,0.812647571392276
"3765","ZNF526",6.90169691519785,-0.746086690081341,0.602721861768969,-1.23786233320225,0.215767110957972,0.812647571392276
"3766","LYPD3",2.13590502462817,-1.2972612770139,1.04944442434812,-1.23614099700394,0.216406158134777,0.812647571392276
"3767","ZNF155",4.16848861020233,0.836440668399465,0.66679363037569,1.25442210347479,0.209688622679868,0.812647571392276
"3768","INAFM1",104.030705804899,-0.309995719202782,0.247938806208534,-1.25029124703478,0.211193174690288,0.812647571392276
"3769","CYTH2",53.8817255730425,-0.322474464909784,0.260532419316383,-1.23775177674983,0.215808114225819,0.812647571392276
"3770","AC012313.1",3.68723037372684,0.870789611176823,0.6992265753861,1.24536114877497,0.212999027625192,0.812647571392276
"3771","MCM8",6.80275673456256,0.679973231652111,0.544961437301134,1.24774559282508,0.212124238901193,0.812647571392276
"3772","JAG1",103.311123169951,0.2475606931372,0.197391553987896,1.25416051566411,0.209783668142163,0.812647571392276
"3773","SNTA1",36.3602118369501,-0.370929852227189,0.294729455888998,-1.25854353820302,0.208195251867239,0.812647571392276
"3774","CHMP4B",118.810599309933,-0.225701027043299,0.180547695870993,-1.25009087462722,0.211266352884031,0.812647571392276
"3775","MOCS3",7.36828257881595,0.634301392851325,0.503422976692252,1.25997704161024,0.207677644425325,0.812647571392276
"3776","PPDPF",427.321781906706,-0.360592725080758,0.287733458660818,-1.2532179147988,0.21012641195845,0.812647571392276
"3777","LIME1",5.67524135104816,-0.730055067634534,0.581508102485629,-1.25545123879435,0.209314998703257,0.812647571392276
"3778","AC003681.1",13.4695355993362,0.621699268738808,0.498131700834242,1.24806204402896,0.212008336816728,0.812647571392276
"3779","CDC42EP1",66.442517614327,-0.309114471903489,0.250615801738555,-1.23341971958321,0.217419211549047,0.812647571392276
"3780","CDKL5",19.3228564558735,0.495394650002644,0.395302196537682,1.25320490081168,0.210131146874246,0.812647571392276
"3781","PRRG1",30.2627563254418,0.398686263105042,0.316893714055773,1.25810719942168,0.208352989996479,0.812647571392276
"3782","FAM104B",34.6254765466547,-0.341141178811073,0.275057149119284,-1.24025563379606,0.214880856696185,0.812647571392276
"3783","HEPH",15.476082555203,-0.491030509807972,0.397884668785657,-1.23410261397253,0.217164669621004,0.812647571392276
"3784","MED12",17.7364359375092,0.408571279751998,0.326498184988798,1.25137381626184,0.210798125585639,0.812647571392276
"3785","BRWD3",14.729263140647,0.481901294377426,0.389723598675645,1.2

3652069316566,0.216265078709762,0.812647571392276
"3786","MBNL3",14.7879261554127,0.502490229614997,0.40028766148476,1.2
5532280398338,0.209361600161295,0.812647571392276
"3787","MIR503HG",5.90232263674681,0.72510931096202,0.583859970074549,
1.24192331745133,0.214264856010575,0.812647571392276
"3788","PLXNA3",19.1859449133238,0.602817947465839,0.480390034351177,1
.25485106759139,0.209532830110013,0.812647571392276
"3789","UBL4A",32.0892009890726,-0.326434036750613,0.264110815060745,-
1.23597375849805,0.216468318072234,0.812647571392276
"3790","MT-
CO3",13822.934463904,-0.208226776350473,0.165537170410861,-1.257885318
64872,0.208433233959622,0.812647571392276
"3791","MCMBP",51.7140646247537,0.263435041223775,0.213665540088788,1.
23293181069023,0.217601205984534,0.812775804996308
"3792","LRRC8E",3.31746025095612,-1.06138121172751,0.861004385584863,-
1.23272451278693,0.21767856312722,0.812850330664936
"3793","DENND1B",22.0913541669582,0.404433529043707,0.328122421837522,
1.23256901122098,0.217736604462266,0.81285270740461
"3794","ORC4",47.7718025735477,0.333024377287147,0.270262835323072,1.2
3222409359041,0.21786538550966,0.813048044373422
"3795","KRT10",358.535398259855,-0.289460563748318,0.23492862075364,-1
.23212132612766,0.217903766129741,0.813048044373422
"3796","AL645608.7",3.3458693732947,-0.97490333308468,0.79288584091510
3,-1.22956330252979,0.218860679240153,0.813233781299523
"3797","PLEKHM2",60.7483071350029,-0.249231873178052,0.203100631744525
,-1.2271348987804,0.219771892936451,0.813233781299523
"3798","FCER1A",3.81992618641066,-1.20105661718487,0.981047825127402,-
1.22425898760735,0.220854543084296,0.813233781299523
"3799","CD84",2.35034916790505,-1.31952730202833,1.07284257776258,-1.2
299356209186,0.218721213894363,0.813233781299523
"3800","SLC19A2",37.391519995302,0.657530502076509,0.53387443854232,1.
23162012377258,0.218091020101501,0.813233781299523
"3801","PRDX6",625.185327583744,-0.348021879220978,0.283536278901205,-
1.2274333308234,0.2196597653099,0.813233781299523
"3802","CFH",2102.10184731371,-0.405729438390319,0.329669473536294,-1.
23071582587901,0.218429166931084,0.813233781299523
"3803","CD46",114.084939244936,0.258247774202554,0.210169621335515,1.2
28758811866,0.219162248458651,0.813233781299523
"3804","GNG4",1.73935328611482,1.40888308316865,1.14894260702848,1.226
24322098426,0.220107161030158,0.813233781299523
"3805","HAAO",16.2620337058219,0.558801736091644,0.455427272834081,1.2
2698347117921,0.219828803396527,0.813233781299523
"3806","NCKAP1",114.229330883727,0.235121176012374,0.19088158172002,1.
23176460449308,0.218037028868348,0.813233781299523
"3807","PARP14",100.496233469183,0.316421257184729,0.257240969871037,1
.23005778334361,0.218675467443318,0.813233781299523
"3808","C3orf70",3.03401103380815,0.977597005005647,0.798580679320979,
1.22416811515761,0.220888814653009,0.813233781299523
"3809","EIF4A2",491.217044297328,0.246160436213131,0.200743335214432,1
.22624462700187,0.220106632083197,0.813233781299523

"3810","RFC1",133.080104149812,0.218017441820194,0.177221577599616,1.2
3019693636147,0.218623366859075,0.813233781299523
"3811","NAF1",19.8453137867742,-0.422195484434766,0.344064149911324,-1
.22708362537503,0.219791161641516,0.813233781299523
"3812","ATP6AP1L",4.17638501657347,0.983303109449137,0.80023832663949,
1.22876282816696,0.219160742178114,0.813233781299523
"3813","NHSL1",34.866357855035,0.336940143717987,0.274680225472583,1.2
2666327049312,0.2199491780271,0.813233781299523
"3814","AC018647.1",1.72226030720958,1.3438549747298,1.09804631108638,
1.22386001497534,0.221005039706843,0.813233781299523
"3815","CADPS2",21.6817546618859,-0.37080958658418,0.303069697491412,-
1.22351257698631,0.221136156801281,0.813233781299523
"3816","AC007938.3",2.47749653355174,1.10718694118252,0.90419022402820
3,1.2245066488885,0.220761159645321,0.813233781299523
"3817","ARHGEF5",14.3711529571031,0.52057748132494,0.423779856864633,1
.22841487836744,0.219291265455268,0.813233781299523
"3818","AC100810.1",85.1477852038186,-0.310344934855531,0.253509844358
479,-1.2241928341713,0.22087949176491,0.813233781299523
"3819","AF117829.1",38.5101796349465,0.375717502868506,0.3069362330135
7,1.22408976998129,0.220918364755567,0.813233781299523
"3820","FSBP",3.04491456607561,1.104945144555,0.903158232643015,1.2234
2365337408,0.221169723996078,0.813233781299523
"3821","RPL8",3721.86158720103,-0.281241226447601,0.229661952453606,-1
.2245878058727,0.220730564662148,0.813233781299523
"3822","ZNF517",2.97554547029435,0.979132895178587,0.800082029295805,1
.22379063561817,0.221031217823254,0.813233781299523
"3823","TUT7",35.9379662013049,0.399024107909183,0.325134868176628,1.2
272571999027,0.219725936689287,0.813233781299523
"3824","GAS1",466.927285754648,0.371701626698,0.303073652975749,1.2264
3991996145,0.220033171296446,0.813233781299523
"3825","CKS2",10.8187004174112,-0.6536742891078,0.534155207106706,-1.2
2375347167067,0.221045241383161,0.813233781299523
"3826","NOL8",51.9254036370376,0.282154519060107,0.230214948616618,1.2
2561337026809,0.220344203991337,0.813233781299523
"3827","GRIN3A",3.38008313532373,1.31396799542341,1.06735681566284,1.2
3104848926029,0.218304729339415,0.813233781299523
"3828","GSN",17344.1767226283,-0.299948629067147,0.24506506294373,-1.2
2395508141452,0.220969172988094,0.813233781299523
"3829","AVPI1",12.0491939784172,-0.545184317009581,0.443177571353137,-
1.23017127275866,0.218632974953734,0.813233781299523
"3830","PELI3",12.5235551110043,-0.583894408838852,0.475497920053392,-
1.22796417021822,0.219460418502268,0.813233781299523
"3831","AP001528.1",63.4752075150308,0.39278529610452,0.32052202721771
7,1.22545492275237,0.220403864188456,0.813233781299523
"3832","KLRB1",5.45423050334907,0.878947345280947,0.716918444487712,1.
22600743785999,0.220195876067416,0.813233781299523
"3833","SLC35E3",43.8142062560015,0.27627081074105,0.224600785307777,1
.23005273718196,0.218677356955255,0.813233781299523
"3834","RPL6",3156.91676126306,-0.315190511997677,0.257411560620405,-1
.22446136932628,0.220778230692288,0.813233781299523

"3835","GPC6",41.2612319099924,-0.464406506676676,0.378110961778179,-1
.22822809604002,0.219361354445556,0.813233781299523
"3836","KLHL33",2.92686418773775,1.04035591557029,0.847447029625314,1.
22763533200449,0.219583892231348,0.813233781299523
"3837","CYP46A1",9.43249410302852,0.64859207367923,0.529215421653979,1
.2255728898681,0.220359444962811,0.813233781299523
"3838","EIF3C",9.57389980005021,0.614851141621671,0.501086344513971,1.
22703631490506,0.219808942138574,0.813233781299523
"3839","LDHD",2.80283525607669,1.0402221852269,0.849148060226647,1.225
01862036787,0.220568204873923,0.813233781299523
"3840","AC005332.4",5.95083402785753,0.688705118277308,0.5621066736404
99,1.22522138692446,0.220491818487941,0.813233781299523
"3841","PCYT2",11.7936519725257,0.606390008906319,0.49510622501391,1.2
2476749083347,0.220662836906467,0.813233781299523
"3842","SBNO2",17.756585458965,-0.492223729382057,0.400849029354016,-1
.22795290330451,0.219464648231603,0.813233781299523
"3843","CLPP",65.052138568531,-0.341851644019897,0.278293896443656,-1.
2283835484301,0.21930302071102 1,0.813233781299523
"3844","LYL1",3.10217790358414,-0.941118897382929,0.767589028921126,-1
.22607132452858,0.220171835731671,0.813233781299523
"3845","TM6SF2",2.96791109872594,0.96503546489404,0.786057009817285,1.
22769144329412,0.219562819771151,0.813233781299523
"3846","ZNF222",3.98781659967078,-0.985403648505523,0.803172222486218,
-1.22688960215184,0.219864087145889,0.813233781299523
"3847","ACSS1",9.69904860772048,0.660684349521825,0.538191247485597,1.
22760143835209,0.219596621614431,0.813233781299523
"3848","TCFL5",15.8790127103711,-0.426981930380785,0.346952201483435,-
1.23066499810399,0.218448184289444,0.813233781299523
"3849","USP25",36.5172564017209,0.324411904842768,0.26512602607703,1.2
2361395311871,0.221097893440585,0.813233781299523
"3850","UPF3B",31.9899216842452,0.335229558730172,0.273228127333158,1.
22692184733021,0.219851966280948,0.813233781299523
"3851","TMEM255A",2.43040770430397,1.26181393770957,1.03109246969741,1
.22376408982977,0.221041234628798,0.813233781299523
"3852","SOGA1",26.6971496356282,0.426384523218316,0.348615414158628,1.
22307994971301,0.221299500789429,0.813499722528118
"3853","NCBP2",73.6216794851259,-0.250625618164387,0.204956251637057,-
1.22282495002008,0.221395819697243,0.813556148592704
"3854","EXOC7",78.9774010034595,0.252685099344934,0.206655633208137,1.
22273511455862,0.22142975965228,0.813556148592704
"3855","TMEM200B",18.8086274649848,-0.429188569284202,0.35136632174908
7,-1.22148465210815,0.221902573199235,0.813637129406364
"3856","RAI14",30.0826962151687,-0.482371063969798,0.395306007725545,-
1.22024723769111,0.222371164604705,0.813637129406364
"3857","HINT1",417.425568166985,-0.279952590425631,0.22903400760972,-1
.22231887459559,0.221587064315158,0.813637129406364
"3858","HLA-
C",1005.21473222247,-0.318634934575002,0.261093520819614,-1.2203862186
038,0.222318499199401,0.813637129406364
"3859","RHBDD2",34.1954098391548,-0.502750946010705,0.411249336225047,

-1.2224966747073,0.2215198606212,0.813637129406364
"3860","LINC00710",4.94918404837566,1.14251205844212,0.936272307236706
,1.22027752995718,0.222359684896885,0.813637129406364
"3861","REEP3",102.281142898008,-0.292441188500364,0.239459971538767,-
1.2212529159723,0.221990274538833,0.813637129406364
"3862","PPP1R12A",174.948223084077,0.21411028950279,0.175238094415998,
1.2218250273511 5,0.221773802017938,0.813637129406364
"3863","POC1B-
AS1",5.42209471512098,0.741544552733165,0.607565302092898,1.2205182721
5732,0.222268467121643,0.813637129406364
"3864","KLHL28",31.0171527468226,0.362623811285322,0.297139821491226,1
.22038106324981,0.222320452608917,0.813637129406364
"3865","SAV1",185.919408561396,0.215768794153692,0.176562408076266,1.2
220539836571,0.22168721310749,0.813637129406364
"3866","NDUFA11",354.591215715106,-0.27686814163366,0.226653490877453,
-1.22154810217927,0.221878564629647,0.813637129406364
"3867","PNKP",33.7130254931825,-0.372732562142686,0.305306078505892,-1
.22084880840488,0.222143269830145,0.813637129406364
"3868","ZNF329",8.1072221844306,0.66587607724324,0.545508362324196,1.2
2065237351487,0.222217667429407,0.813637129406364
"3869","MGME1",16.4956827752645,0.419703497046059,0.343884757992844,1.
22047717234037,0.222284038040055,0.813637129406364
"3870","PPM1F",27.099887077755,0.432027576553562,0.353676184497942,1.2
2153426068776,0.221883801886871,0.813637129406364
"3871","C4orf33",16.7571701638674,0.413105252530483,0.338651404586217,
1.2198539469672,0.22252024653468 7,0.813972278721561
"3872","RPL36AL",664.859191146292,-0.322432313365091,0.264370858110556
,-1.21962123839782,0.22260849141198,0.814084772312407
"3873","ANTXR1",136.015653422078,0.344890157066501,0.282899653687759,1
.21912541274144,0.222796595856492,0.814562302434271
"3874","AC072022.2",4.70204276288778,0.892220658720389,0.7320317011109
28,1.21882789688802,0.222909520883313,0.814673189358789
"3875","PPP2R5D",27.5995090207103,0.334060065523607,0.274102283269318,
1.21874236704325,0.222941992144442,0.814673189358789
"3876","AC012358.3",4.86953532637949,0.796882643506755,0.6557863599600
41,1.21515586807159,0.224306648765912,0.81506091216864
"3877","MITD1",61.1487134460882,0.281126042608619,0.231512642132222,1.
21430104213515,0.224632787846226,0.81506091216864
"3878","TMEM42",42.2943812726499,0.328443308625947,0.269993767038748,1
.21648478121649,0.223800304981468,0.81506091216864
"3879","PPM1M",28.2684711371261,0.360111495795277,0.296337913107401,1.
21520561449308,0.224287679595308,0.81506091216864
"3880","CFAP44",13.3598144576992,0.544452385221254,0.448290155243481,1
.21450890422865,0.224553451705112,0.81506091216864
"3881","P2RY14",3.4328771061533,-1.09194565098124,0.898019954874521,-1
.21594809230472,0.224004696233991,0.81506091216864
"3882","THAP9",3.93118901225733,-0.92694306496529,0.762485133841891,-1
.21568673777907,0.224104278139529,0.81506091216864
"3883","PRKAA1",75.9561785836866,0.246948639638214,0.203218385854543,1
.21518847125856,0.22429421647753 5,0.81506091216864

"3884","RPS14",4968.32805442716,-0.319854436839905,0.263137265890914,-1.2155421458719,0.224159384478444,0.81506091216864
"3885","CCND3",30.8325470020527,-0.358819143395948,0.294810465276209,-1.21711806621169,0.223559297891209,0.81506091216864
"3886","FAM120B",14.8322615736787,0.482457611677445,0.397026828176452,1.21517634940031,0.224298838742291,0.81506091216864
"3887","VAV2",15.1510702332399,0.517243821098838,0.425122980138616,1.21669221675616,0.22372134149891,0.81506091216864
"3888","STN1",24.9909147315926,-0.41800139370039,0.343747211706519,-1.21601391797548,0.223979620173133,0.81506091216864
"3889","GANAB",112.22307754887,0.207825381205373,0.170794407345025,1.21681607984704,0.223674200637592,0.81506091216864
"3890","ERGIC2",121.662922581767,-0.216063937286513,0.177590312647642,-1.21664258632849,0.223740232259995,0.81506091216864
"3891","IPO5",99.8355110457574,0.239077539527365,0.196848079182037,1.21452818092411,0.22454609525217,0.81506091216864
"3892","BMP4",56.197399339783,0.501087670275377,0.41213823232739,1.21582428168743,0.224051866897144,0.81506091216864
"3893","AAGAB",13.9639035902886,-0.506860838974861,0.416651471724464,-1.21651037707135,0.22379056045357,0.81506091216864
"3894","PIAS1",62.5712225028576,0.290270003106077,0.238293621172055,1.21811906537143,0.223178729796844,0.81506091216864
"3895","CELF6",4.28665048475394,0.778896414744325,0.6402025183789,1.21664066039075,0.223740965350009,0.81506091216864
"3896","ALDOA",21.450586431225,-0.457277026649469,0.375746114608239,-1.21698404553361,0.223610286187073,0.81506091216864
"3897","RECQL5",6.6848795951918,0.620828490947452,0.511293860505911,1.21423028692963,0.224659798036314,0.81506091216864
"3898","ENGASE",9.41211048913208,0.581600959072318,0.47863945573888,1.21511286229944,0.224323048534552,0.81506091216864
"3899","BAHCC1",24.5759977600438,-0.472656867078844,0.388320046099409,-1.21718379420939,0.223534294650789,0.81506091216864
"3900","DNAJB1",297.54631512101,0.277642556866579,0.228573948885374,1.21467279285537,0.22449091333595,0.81506091216864
"3901","ZNF586",5.37053915851177,0.710628427498008,0.584954718971456,1.21484348181262,0.22442579325801,0.81506091216864
"3902","EIF2S2",234.466063290917,-0.195716876736555,0.160899372012162,-1.2163930429869,0.223835232884548,0.81506091216864
"3903","ZMYND8",47.6078428284532,0.308458933853508,0.25329868157677,1.21776762489788,0.223312290489475,0.81506091216864
"3904","ARF6",110.048733567842,-0.248040334047914,0.204315113990712,-1.21400873975084,0.224744386772487,0.815158943826437
"3905","ACSL4",72.8779600759032,-0.373173434110282,0.307428364227007,-1.21385492535337,0.224803127896744,0.815163198724173
"3906","SLC49A3",31.7603843762477,-0.341243963485346,0.281198818629262,-1.2135327066763,0.224926217521462,0.815184864911212
"3907","SPAG1",6.97880656683211,-0.667918718284256,0.550546178541241,-1.21319290609557,0.22505607569965,0.815184864911212
"3908","TJP2",74.6625512002413,-0.503554076836213,0.415359416000936,-1.21233336103082,0.225384798455325,0.815184864911212

"3909","HERC4",53.4106262692505,0.280846622934548,0.231625059172192,1.
21250534781629,0.225318996737309,0.815184864911212
"3910","AL136295.7",1.89686900861232,1.23829648478848,1.02074681450827
,1.2131279443522,0.225080907573696,0.815184864911212
"3911","DOK4",13.8202369780254,-0.449549893237454,0.370588642548153,-1
.21306980739174,0.225103132307863,0.815184864911212
"3912","AC015802.6",4.77835790894964,0.867288131389065,0.7145979450664
2,1.21367285951047,0.224872672314342,0.815184864911212
"3913","LINC01278",19.7525783355437,0.372757732526565,0.30739936403260
4,1.2126171233296,0.225276239059075,0.815184864911212
"3914","KLHL4",5.11266406241723,-1.12813743902538,0.930204645957073,-1
.21278413726332,0.225212361731788,0.815184864911212
"3915","THOC2",120.544111269856,0.277478171286429,0.228863602284395,1.
21241721495593,0.225352714437266,0.815184864911212
"3916","EBNA1BP2",69.9925286162715,-0.250185531723129,0.20644450677962
,-1.21187788246748,0.225559129715961,0.815245768152091
"3917","HSPA1L",1.58915962502078,1.50077000248853,1.23871456554314,1.2
1155433562734,0.225683023495286,0.815245768152091
"3918","STK17A",31.7197726178782,0.405296479997957,0.334530370368578,1
.21153867002093,0.225689023461378,0.815245768152091
"3919","GZMB",4.77460619500239,0.905036566638294,0.746968517791473,1.2
1161273210573,0.22566065850273,0.815245768152091
"3920","TMX4",133.809834771739,0.190172129590917,0.156967608409798,1.2
1153740900754,0.225689506437585,0.815245768152091
"3921","SARS",131.873302772345,-0.224775421592496,0.185610221564315,-1
.21100777585468,0.225892424569919,0.815354762148881
"3922","FBXO31",15.01452827064,0.434062947271783,0.358419182590296,1.2
1104831536864,0.225876888081709,0.815354762148881
"3923","TPM4",331.890416102688,-0.370902917793882,0.306251030925495,-1
.21110749137075,0.225854210657683,0.815354762148881
"3924","ATF6",49.3065869308651,0.285775374012813,0.236093847897503,1.2
1043126094874,0.226113452477678,0.815643168965155
"3925","SCCPDH",70.0545121935617,-0.276335722662277,0.228257041848774,
-1.21063394331272,0.226035729253989,0.815643168965155
"3926","GLTP",65.7244956988014,-0.268423857019081,0.221773994308176,-1
.21034866083568,0.226145132864209,0.815643168965155
"3927","YTHDF2",99.6625505974095,-0.249415806199655,0.206229754235554,
-1.20940747432,0.226506338536449,0.816322156700463
"3928","AVL9",19.6527171702597,0.437903097322912,0.362058469940093,1.2
0948170994417,0.22647783367409,0.816322156700463
"3929","ZSCAN2",3.07998683445096,0.96383911660829,0.796906946979396,1.
20947510905964,0.226480368166274,0.816322156700463
"3930","RPL19",4741.99037176434,-0.302764360143692,0.250444265340945,-
1.20890913485889,0.226697756292294,0.8168041295417
"3931","LINC00944",1.96065376284723,-1.1780024060682,0.974733394014037
,-1.20853806107646,0.226840364804513,0.816902229306181
"3932","RPL18",3355.76616481078,-0.303387721400572,0.251020983221138,-
1.20861498312793,0.226810797397709,0.816902229306181
"3933","CDH5",1.56129850941762,1.76298455768361,1.45914070322022,1.208
23478763414,0.22695696426213,0.816906612595772

"3934","GPR108",59.5445489396076,-0.27098236434975,0.224253833856995,-
1.20837338514602,0.226903672412745,0.816906612595772
"3935","POU2F1",39.0179659689941,0.413081352430646,0.342206179118394,1
.20711248842684,0.227388826043764,0.816984714181298
"3936","SRGAP2",26.1457255431788,0.357288679881022,0.295965250374233,1
.20719807284554,0.227355872462142,0.816984714181298
"3937","DINOL",2.74266793771089,-1.09331949599429,0.905424931026909,-1
.20752086509732,0.22723161457529,0.816984714181298
"3938","KDELR2",406.294181684079,-0.308577060545861,0.255755882543024,
-1.20652966992441,0.227613326090764,0.816984714181298
"3939","NT5DC3",5.11800425192693,-0.759976641396948,0.629842662885263,
-1.20661347060161,0.22758103658563,0.816984714181298
"3940","COQ6",20.1916241969247,-0.380772457824704,0.315459420670067,-1
.20704101026974,0.227416350733652,0.816984714181298
"3941","SEC11A",489.462708979139,-0.331650984944312,0.274800474917774,
-1.20687922771439,0.227478658461842,0.816984714181298
"3942","KIF1C",18.4603154417638,-0.451142486677041,0.373643629828712,-
1.20741383141968,0.227272811494548,0.816984714181298
"3943","RCVRN",3.19349819352417,-1.01306658260388,0.839187888002451,-1
.20719876572017,0.227355605690314,0.816984714181298
"3944","NAGLU",78.9665675143278,0.393196915945202,0.32560451519285,1.2
0759048968445,0.227204819153109,0.816984714181298
"3945","RCAN1",29.8748017517592,-0.388656051591064,0.322066197870077,-
1.206758281873,0.227525246589983,0.816984714181298
"3946","EVI5",75.9551491249955,0.231286259374787,0.192562070455009,1.2
0109977436509,0.229712500557321,0.817272610582037
"3947","TARS2",14.9372941220711,0.439967519944443,0.366252368793122,1.
20126873552853,0.229646973986152,0.817272610582037
"3948","PFDN2",125.946550937376,-0.297670038683348,0.247020967546983,-
1.20503956258989,0.228188029358733,0.817272610582037
"3949","CCT7",164.329245465624,-0.236843827332381,0.197287350010687,-1
.20050184322285,0.229944497214607,0.817272610582037
"3950","KIAA1143",57.5991560778595,0.272510049992905,0.226933422774488
,1.20083699730607,0.229814437264733,0.817272610582037
"3951","MANF",113.896719828402,-0.250323713204243,0.207914713321858,-1
.2039730580141,0.228599992042272,0.817272610582037
"3952","ZNF654",17.8664993455812,0.512188881365404,0.424865500852342,1
.20553182204222,0.227998060953229,0.817272610582037
"3953","ATP10D",34.2749052864017,0.345464771695817,0.287245746617447,1
.2026801989723,0.229100099278345,0.817272610582037
"3954","SEC24D",77.7718858636839,-0.246884282706302,0.205483452457118,
-1.20148011800524,0.229565014293785,0.817272610582037
"3955","FAM114A2",27.3280535738253,0.337427311198647,0.280977018680742
,1.20090715170569,0.229787219760626,0.817272610582037
"3956","AL662884.5",4.79782508400267,-0.870809050120597,0.724533547752
158,-1.20188920557544,0.229406457178302,0.817272610582037
"3957","TMEM209",12.2310123512772,-0.512694700406374,0.425228925880349
,-1.20569102712132,0.227936646059715,0.817272610582037
"3958","AC019270.1",4.01950785013649,0.930027215500248,0.7739388510828
8,1.2016804870294,0.229487344080879,0.817272610582037

"3959","CIZ1",84.7313052178596,-0.248255184696549,0.206521406394211,-1.20207967314863,0.229332660972942,0.817272610582037
"3960","DPP7",164.990244757221,0.242723415535225,0.201619836194886,1.20386674305502,0.228641087725485,0.817272610582037
"3961","SLC35G1",6.05245160184041,0.716243066717766,0.595969132363412,1.20181235541074,0.229436237374377,0.817272610582037
"3962","PYROXD2",12.7452084827984,0.468926995364275,0.389794194711316,1.20301174754941,0.228971774578697,0.817272610582037
"3963","BSCL2",84.0746271664451,-0.267621144288259,0.222139058901795,-1.20474600734926,0.228301369247835,0.817272610582037
"3964","PRICKLE1",86.5149650236816,0.292349998571947,0.243474153698998,1.2007434634453,0.22985072876556,0.817272610582037
"3965","ZNF641",19.0333552218606,-0.448091773333663,0.372504346260148,-1.20291689971512,0.229008479870252,0.817272610582037
"3966","TUBA1B",900.799987157754,-0.348569476767276,0.28963774373713,-1.20346703530335,0.228795640453466,0.817272610582037
"3967","PCBP2-OT1",4.18552674919858,0.888461581920405,0.737564036818368,1.20458907643188,0.228361975762887,0.817272610582037
"3968","RFXAP",15.3553219697949,-0.449411384420751,0.373767874710569,-1.20238098249818,0.229215953888393,0.817272610582037
"3969","TINF2",47.6821848839269,-0.307975319781087,0.255713097483009,-1.2043783553228,0.228443374001859,0.817272610582037
"3970","NEMF",90.8739475427735,0.228482159820293,0.190273114604956,1.20081158231244,0.229824297977552,0.817272610582037
"3971","AC123768.4",1.7863500884467,1.47441869294923,1.22632360535456,1.20230800949391,0.229244214868888,0.817272610582037
"3972","SYNM",16.4004421822672,0.680048875759637,0.566328463758699,1.2008029249425,0.22982765700201,0.817272610582037
"3973","TARSL2",18.2859886658573,0.433604356285735,0.359648552016827,1.20563353822553,0.227958821595511,0.817272610582037
"3974","DUSP14",35.7013412816121,-0.412899393951078,0.343070037996815,-1.2035425662992 8,0.228766429613888,0.817272610582037
"3975","EZH1",64.9490806919598,0.3495241902617,0.290245957542163,1.20423448175303,0.228498961960082,0.817272610582037
"3976","ATP5MC1",206.096205835227,-0.310222777180047,0.257791452000998,-1.20338659320188,0.2288267535328,0.817272610582037
"3977","SLC16A5",7.38017304309379,0.66220522868491,0.550768189326656,1.20233020264749,0.229235619643385,0.817272610582037
"3978","AC027307.2",4.27310274446407,0.858917824874775,0.715390109572659,1.2006285988324,0.229895302257474,0.817272610582037
"3979","NDUFA7",57.5087535895191,-0.323370147586641,0.269172117716601,-1.20135083206167,0.229615140083677,0.817272610582037
"3980","ZNF302",60.6529727232674,0.307775244712857,0.255642119650681,1.20393010797052,0.228616593601219,0.817272610582037
"3981","PLCG1",12.405754784594,0.513650571731301,0.426907642687949,1.20318898133842,0.228903197859461,0.817272610582037
"3982","TNNC2",9.61512228161882,0.583318775121589,0.483781383852598,1.20574870094488,0.227914400735069,0.817272610582037
"3983","NDUFA1",275.136031595123,-0.274821275375328,0.22824764145381,-

1.20404869739231,0.228570757102133,0.817272610582037
"3984","ZNF449",11.7278059792068,0.494720063094389,0.41183367233549,1.20126181108226,0.229649659165728,0.817272610582037
"3985","HSPE1",230.084461437207,-0.284950593488938,0.23762836221037,-1.19914386834293,0.230472008916399,0.817473183311035
"3986","GMDS-DT",18.0989071546382,0.412443000346693,0.343829402390745,1.1995570986043,0.230311396705672,0.817473183311035
"3987","FAM180B",65.2807323052549,-0.422279398481369,0.351946795597906,-1.19983873631802,0.230201976819964,0.817473183311035
"3988","PYGL",43.9384337860294,-0.330094159800528,0.275248238432174,-1.199259845152,0.230426923611926,0.817473183311035
"3989","MINDY2",75.5749826537703,0.327172478900189,0.272810006682814,1.19926861510099,0.230423514600685,0.817473183311035
"3990","CCR10",5.24920701402709,-0.715997032849508,0.596976397626203,-1.19937243029469,0.230383162795807,0.817473183311035
"3991","NPC1",14.4166130519886,0.465627595225337,0.388236954600144,1.19933867631148,0.230396282040427,0.817473183311035
"3992","CEBPB",483.989011634931,-0.388874741356,0.324068651808423,-1.19997642223626,0.230148497518808,0.817473183311035
"3993","NCAPH2",21.705823204853,-0.385005997363505,0.321100798138442,-1.19901912295313,0.23052050995487,0.817473183311035
"3994","TMEM222",51.3741241949475,-0.295804980538346,0.247664357508095,-1.19437848673351,0.232329948305224,0.817598288770842
"3995","POU3F1",3.02410350400292,1.13071028163799,0.945002671638248,1.19651543384295,0.231495479370102,0.817598288770842
"3996","PLPPR4",7.42891028901247,-0.763155551375158,0.639847173512277,-1.19271535917868,0.232980868304403,0.817598288770842
"3997","AC253572.1",2.67699117827561,-1.13438538905252,0.947467332388767,-1.19728179566096,0.231196737492053,0.817598288770842
"3998","CTSS",91.8030101843083,0.276020637711311,0.231330662359673,1.19318656202243,0.232796316294264,0.817598288770842
"3999","MIR29B2CHG",5.76451143335404,0.798492928573191,0.667422467242686,1.19638305235962,0.231547111855263,0.817598288770842
"4000","XPC",63.9234777065294,0.270276552410884,0.226081103121717,1.19548493296838,0.231897619534725,0.817598288770842
"4001","FILIP1L",391.3898584224,-0.316990259764034,0.265731096428639,-1.19289862580746,0.232909077494818,0.817598288770842
"4002","COX17",102.773418709082,-0.277616373078194,0.231935093789266,-1.19695716824309,0.231323249754572,0.817598288770842
"4003","AC107294.2",2.62920104025369,1.13558363722922,0.949403553182619,1.19610215637226,0.231656696242655,0.817598288770842
"4004","OCIAD2",1.62736550188744,-1.37064232001711,1.14756664096317,-1.19439017403531,0.232325378664346,0.817598288770842
"4005","LAMTOR3",56.0458340989422,0.248300258720529,0.207469884154531,1.19680145256931,0.231383951979159,0.817598288770842
"4006","MTRR",20.7919160165909,0.367524255234337,0.307734432887873,1.194290322943,0.232364421692827,0.817598288770842
"4007","SFXN1",29.4259614135647,0.367284445037404,0.307653694351021,1.19382426338865,0.232546718408691,0.817598288770842

"4008","HOXA9",8.97188059426062,-0.54877008270536,0.4584275361532,-1.1
9707050608314,0.23127907468126,0.817598288770842
"4009","HBP1",125.41692825451,0.231942126277176,0.194012661828835,1.19
549994361607,0.231891758258548,0.817598288770842
"4010","DDHD2",27.4892316938391,0.371687399959232,0.310137076653908,1.
19846167368763,0.230737335020082,0.817598288770842
"4011","KAT6A",62.6671843575691,0.338895722583842,0.283019976787538,1.
19742686163192,0.231140218961165,0.817598288770842
"4012","HDHD3",13.9978476736101,0.429769491911743,0.360184943278215,1.
19319116451733,0.232794514185812,0.817598288770842
"4013","SPTY2D1",55.1612814183856,-0.255090839435477,0.213188378943776
,-1.1965513350179,0.231481478316268,0.817598288770842
"4014","HTATIP2",44.926651358214,0.432173730021313,0.361009327012005,1
.19712621720419,0.231257362657652,0.817598288770842
"4015","METTL15",24.2375727804057,0.360244907491881,0.30083372495896,1
.19748843830932,0.231116231303818,0.817598288770842
"4016","CTNND1",65.991246035187,0.268672500441953,0.224654798168088,1.
19593484151151,0.231721987357084,0.817598288770842
"4017","DPP3",9.82625365163184,-0.535859677889232,0.447743010659656,-1
.19680188217735,0.231383784490639,0.817598288770842
"4018","RSF1",178.567374985577,0.249602240276927,0.20844988611675,1.19
742085220967,0.231142560071488,0.817598288770842
"4019","AP003307.1",6.29465686098225,-0.658312305890594,0.551363618054
229,-1.19397124571583,0.232489216101051,0.817598288770842
"4020","SMUG1",28.0404539041661,0.340284998971942,0.283869314099297,1.
19873822942663,0.230629747988608,0.817598288770842
"4021","KLHDC1",16.3748836078887,0.481383837285734,0.40347935429219,1.
1930817083075,0.232837374483203,0.817598288770842
"4022","SNRPN",87.6127085342663,-0.236101631890072,0.197709535828499,-
1.19418434169445,0.232405866782992,0.817598288770842
"4023","MAP2K5",22.8508627917069,0.408114274917041,0.341423790019312,1
.19533051546864,0.231957921747893,0.817598288770842
"4024","AC012291.3",1.76971403292656,1.39088161585291,1.1652460659859,
1.19363768430843,0.232619726260973,0.817598288770842
"4025","PRMT7",15.0385585916401,0.429752906247839,0.359623996669659,1.
19500620155389,0.232084607150199,0.817598288770842
"4026","HP",13.1728260533603,1.77320409860922,1.48297621739541,1.19570
636252248,0.231811167593177,0.817598288770842
"4027","EMC8",39.7093860223949,-0.339481877332809,0.284302512008996,-1
.1940868018854,0.232444015394375,0.817598288770842
"4028","STX8",87.5445059569553,-0.268279978334837,0.224575753403783,-1
.19460794083355,0.232240245267031,0.817598288770842
"4029","SAP30BP",57.7035867708482,-0.262597476543122,0.219338362984504
,-1.19722547834313,0.231218681686149,0.817598288770842
"4030","COLEC12",280.765328274493,0.29058141760872,0.243231347748974,1
.19467091844022,0.232215629032207,0.817598288770842
"4031","AC068473.5",1.79910309794026,1.3723522618752,1.15056382061812,
1.19276500554131,0.232961418846397,0.817598288770842
"4032","ATP5F1D",362.165665518776,-0.289902753060499,0.24272246630948,
-1.19437956225635,0.23232952778179,0.817598288770842

"4033","TIMM29",26.9356153903743,-0.33458979590733,0.280436426849881,-1.193103904745,0.232828682464379,0.817598288770842
"4034","EXOSC5",17.9286747627492,-0.466025921255965,0.388948307023103,-1.19816930126986,0.230851113914009,0.817598288770842
"4035","ARMCX4",13.7578287236686,0.543486115257905,0.45493068664864,1.19465696909046,0.232221081294233,0.817598288770842
"4036","BTF3",1209.5417002896,-0.287577731975657,0.241275569826985,-1.19190572083976,0.233298214760893,0.817698693320357
"4037","PPP1R18",57.6094780227734,-0.301041115767836,0.252558760205017,-1.19196465615947,0.233275104089704,0.817698693320357
"4038","SGK1",87.6454991517,-0.881307052533272,0.739374573434551,-1.19196288890408,0.233275797070872,0.817698693320357
"4039","SLC2A13",29.5258556558477,0.339320532436491,0.284672438115059,1.19196833625089,0.233273661046563,0.817698693320357
"4040","COMMD4",84.679607353594,-0.293156250269012,0.245900808791612,-1.19217277775384,0.233193505071316,0.817698693320357
"4041","HADHA",206.753717567874,-0.170533828613127,0.143120386190502,-1.19154114345483,0.233441214830008,0.817914926031203
"4042","ADCY7",20.672087111121,0.475219637014394,0.398856914077824,1.19145392806622,0.233475432981506,0.817914926031203
"4043","INKA1",5.40456431561505,-0.725220903466974,0.608791290618397,-1.19124717229498,0.233556565904074,0.817996777937592
"4044","SDHB",77.408182938036,-0.243619656942459,0.204594632089884,-1.1907431512447,0.233754432282754,0.818027982290495
"4045","SEPSECS-AS1",6.45023311049702,0.661484804761597,0.555391666305029,1.19102400142659,0.233644162694569,0.818027982290495
"4046","AL590004.3",1.87574204232106,-1.34228868261565,1.12747695865757,-1.19052426952817,0.233840396902108,0.818027982290495
"4047","ASIC1",12.5731404354485,0.466691287632397,0.391906867912647,1.19082191674278,0.233723503037484,0.818027982290495
"4048","NUDT11",11.5185557535761,-0.636008578156468,0.534241544294404,-1.19048880595101,0.233854327140672,0.818027982290495
"4049","IFFO2",5.82682879952629,0.717157112877518,0.602576943639986,1.19015027117597,0.23398733459407,0.818291098506306
"4050","HAGH",40.4099758902375,-0.313547551169652,0.263537513335244,-1.18976439900907,0.234139005876139,0.81861933906324
"4051","AAK1",83.1018588098738,0.33892646872918,0.285553152836421,1.18691201747415,0.235262326577012,0.818831411775021
"4052","UBA5",44.5263198984276,-0.359353748288148,0.302766083222885,-1.18690225953614,0.235266175970032,0.818831411775021
"4053","DVL3",51.6822386339368,0.27661195964507,0.232643651702853,1.18899423053407,0.234441937273954,0.818831411775021
"4054","AC147067.1",12.8589938407884,-0.467571705146183,0.393486288746958,-1.18827953735096,0.234723296762752,0.818831411775021
"4055","DPYSL3",174.959588963334,-0.293358373210304,0.24720043066016,-1.186722743269,0.235337001003601,0.818831411775021
"4056","CPQ",343.907022306569,0.219024442805187,0.184365734395883,1.18798888265692,0.234837789700551,0.818831411775021
"4057","SLC2A8",11.636378238411,-0.467795154844072,0.393656077880006,-

1.18833464318228,0.234701594265838,0.818831411775021
"4058","ENG",129.468614489454,0.256157623611519,0.215703293574102,1.18
754618609252,0.23501225002906,0.818831411775021
"4059","ALKBH3",24.9585446179853,-0.444553246634412,0.374025909843476,
-1.1885627036385,0.234611791634574,0.818831411775021
"4060","RBMS2",32.036933760978,0.306893713605397,0.258616682881764,1.1
8667407757954,0.235356203808216,0.818831411775021
"4061","TPM1",286.537945381052,0.284122503686718,0.239344381184049,1.1
8708658327866,0.235193469930814,0.818831411775021
"4062","MMP2",1322.78254759198,-0.289930957709108,0.244037480570362,-1
.18805913350475,0.234810113288429,0.818831411775021
"4063","PARD6A",3.98909889581224,-0.843929015029279,0.709959804200318,
-1.18869971234479,0.234557853860686,0.818831411775021
"4064","TMEM92",2.28558155539028,1.12872392216986,0.950119543164462,1.
18798095491284,0.234840913103451,0.818831411775021
"4065","NDC80",4.04906458312674,-0.811754142373235,0.683202369432166,-
1.18816060759261,0.234770140074055,0.818831411775021
"4066","CHMP2A",245.800550609358,-0.255324031919766,0.214972811314165,
-1.18770383267971,0.234950113266463,0.818831411775021
"4067","MZF1",15.9624704421421,0.481772333926226,0.405657020587414,1.1
8763465064303,0.234977380091879,0.818831411775021
"4068","ASXL1",74.7066139095902,0.259887774547965,0.218562649906391,1.
18907679175409,0.234409450079232,0.818831411775021
"4069","MAP7D2",2.07944203189339,1.26876999993791,1.06861882618809,1.1
8729894031887,0.235109725947145,0.818831411775021
"4070","ELK1",20.9944407947536,-0.385407928690807,0.324566508596637,-1
.18745439989245,0.235048433128187,0.818831411775021
"4071","FBXO28",21.3434549367871,0.375024462314874,0.316219027927494,1
.1859642500731,0.235636417940352,0.819604931966441
"4072","CENPF",9.37131224273189,0.654853024981109,0.552663448200241,1.
18490380920549,0.236055481147322,0.819647947756489
"4073","WDR70",44.1638572629057,0.306518291560317,0.258683666381192,1.
18491552191253,0.236050849662497,0.819647947756489
"4074","BTN2A1",22.0466631217488,0.38341458698539,0.323555664715615,1.
18500347481905,0.236016073030769,0.819647947756489
"4075","COQ4",58.2407211444813,-0.245559702888955,0.207305251502514,-1
.1845319938071,0.236202539267917,0.819647947756489
"4076","TBC1D13",19.2644801250209,0.389350292520684,0.32872302440779,1
.18443267922019,0.236241830520062,0.819647947756489
"4077","COX8A",287.595606802259,-0.275541372914851,0.232409798559725,-
1.18558414758077,0.235786565622514,0.819647947756489
"4078","BAZ2A",54.3531264418834,0.306155144844586,0.258279818193249,1.
1853622438882,0.235874253070949,0.819647947756489
"4079","DYRK2",50.0967585268785,-0.381910060221114,0.322210525667196,-
1.18528114322243,0.235906306560608,0.819647947756489
"4080","MIR22HG",91.6779588677577,-0.348588695722243,0.294336013961962
,-1.184322268383,0.236285517142796,0.819647947756489
"4081","RPL36",2387.42369551461,-0.280795362520281,0.237018149695902,-
1.18469983366483,0.236136148267374,0.819647947756489
"4082","NCOA3",51.7015036968087,0.292739728803856,0.24693904140935,1.1

8547365832923,0.23583022366448,0.819647947756489
"4083","PRIMPOL",19.1149799090934,0.402716097723262,0.340712087313684,1.18198359470732,0.237212210343222,0.819722495079582
"4084","LUCAT1",3.74901662599544,0.812595689213052,0.686454339947739,1.18375781450361,0.23650894574759,0.819722495079582
"4085","FAM50B",24.2932795744652,0.385658176062933,0.325928090623217,1.18326154497915,0.236705508208036,0.819722495079582
"4086","TAX1BP1",196.84858782524,-0.163366391012769,0.138062444245517,-1.18327900035041,0.236698592524643,0.819722495079582
"4087","SND1-IT1",8.19298852192688,-0.644631713252127,0.54531757160095,-1.18212166052088,0.23715743090428,0.819722495079582
"4088","SCRIB",17.0582913396967,0.429168661500474,0.362816118941769,1.18288201404126,0.236855910755349,0.819722495079582
"4089","PUF60",102.365809805813,-0.351206946564798,0.296986620841599,-1.18256824354427,0.236980304401273,0.819722495079582
"4090","MARCH8",19.7943698972688,0.38555402935587,0.326144460717526,1.18215722109044,0.237143323225464,0.819722495079582
"4091","FNBP4",95.3982194132935,0.347310727490736,0.293607083146145,1.1829099072445,0.236844854788504,0.819722495079582
"4092","ANKRD13D",33.670797768228,-0.339612447431356,0.287156082167993,-1.18267544558807,0.236937799187404,0.819722495079582
"4093","PRCP",97.3083430832934,0.227693752792654,0.1926456230964,1.18193057871196,0.237233247516676,0.819722495079582
"4094","UBE4A",39.7150308524756,0.302364320276305,0.255683209979299,1.18257401532461,0.236978015774459,0.819722495079582
"4095","VAPA",243.808525819892,-0.230322873499524,0.194660420348916,-1.18320341180136,0.236728541154495,0.819722495079582
"4096","ZNF439",5.38237533034887,1.04219859336186,0.880590745444013,1.18352208304933,0.23660229987988,0.819722495079582
"4097","AC245060.6",2.05526810219063,-1.24709217518455,1.05419490511714,-1.18298065104572,0.236816815861406,0.819722495079582
"4098","ARHGAP4",3.1073623715354,-1.08087444337487,0.914242351653866,-1.18226249464331,0.237101562312674,0.819722495079582
"4099","CD47",233.920309425884,-0.247273426130309,0.209596061279258,-1.17976179810388,0.23809496736952,0.820454189200786
"4100","GSK3B",38.3062847205506,0.387719078530534,0.328570570260379,1.18001766933442,0.237993187425742,0.820454189200786
"4101","RABL3",24.452309964917,0.340240409865996,0.288141332639476,1.18081084289183,0.237677875682933,0.820454189200786
"4102","RAB28",40.5619644701344,-0.318695278789697,0.269952642160767,-1.18055995391926,0.237777580116428,0.820454189200786
"4103","EMB",4.0170385595311,-1.02037465466637,0.864479074770965,-1.18033470611964,0.237867119791729,0.820454189200786
"4104","ATF1",49.8068222769086,0.260996662822507,0.221249641338048,1.17964784595393,0.23814030491633,0.820454189200786
"4105","ZNF213",10.4284007176091,-0.518438802281312,0.439462196164923,-1.17971194520393,0.238114801332546,0.820454189200786
"4106","CES1",40.4963693885181,0.988098107832453,0.837254026417256,1.18016525051624,0.237934496851995,0.820454189200786

"4107","ZNF266",11.1176745473724,0.543759306377003,0.460412136524504,1
.18102730844947,0.237591875006294,0.820454189200786
"4108","RRBP1",404.631010458628,0.290450259493165,0.246059246015269,1.
18040782533789,0.237838051099672,0.820454189200786
"4109","CYB5R3",351.295650552741,-0.246007152402321,0.208380078575882,
-1.1805694387083,0.237773810280393,0.820454189200786
"4110","NDUFB11",236.854090074578,-0.303923766948045,0.257565307343146
,-1.17998720434479,0.238005304116642,0.820454189200786
"4111","SASS6",14.2729500647509,0.425331711535262,0.36097372431655,1.1
7828994988642,0.238681033045079,0.821049911193205
"4112","MFSD10",122.944731393696,-0.312912450465597,0.26545737138758,-
1.17876723042183,0.238490876382042,0.821049911193205
"4113","TAGAP",3.03802939346273,-1.20951760330434,1.0268392761307,-1.1
7790352533261,0.23883506950599,0.821049911193205
"4114","SEMA4D",5.13936685916035,0.828490224364945,0.703167512805844,1
.17822596931281,0.238706532121625,0.821049911193205
"4115","ZCRB1",181.401038962132,-0.211810329935942,0.17973230938273,-1
.17847665043297,0.238606635642856,0.821049911193205
"4116","RPS6KA5",39.4795093752925,-0.455972081002781,0.38675266080531,
-1.17897593788583,0.238407757398345,0.821049911193205
"4117","IFT20",101.362320369038,-0.286491154806171,0.243106863324981,-
1.17845769916909,0.238614186694404,0.821049911193205
"4118","CHMP1B",63.2326876107125,-0.30369088305021,0.257792048388019,-
1.17804596747339,0.238778281094393,0.821049911193205
"4119","RPRD1B",22.2459246117706,0.36304644513152,0.308173560795704,1.
17805837786387,0.238773333807864,0.821049911193205
"4120","RACK1",2751.98618052666,-0.28566596932733 2,0.242610813779567,-
1.17746593763493,0.239009585278529,0.821231616144602
"4121","SEC23IP",27.6322128062467,0.328839905416725,0.279298437075025,
1.17737825123738,0.239044566592719,0.821231616144602
"4122","VKORC1",364.539483613603,-0.276654602346146,0.234983804178195,
-1.17733476702229,0.239061915377687,0.821231616144602
"4123","BGLAP",6.91799479023224,-0.608510563518319,0.517119608439984,-
1.1767307864307,0.239302975805229,0.821302403564832
"4124","IER5",90.8101102269677,0.282732006517177,0.24027497349587,1.17
670185289621,0.239314528044385,0.821302403564832
"4125","PTX3",46.6311440790567,1.03365747590548,0.878382531497364,1.17
677371627988,0.239285836007949,0.821302403564832
"4126","KLHDC4",16.5926875251833,0.392928702493999,0.333887503621526,1
.17682961546054,0.239263519486199,0.821302403564832
"4127","NEK1",41.2726997447176,-0.28837424902516,0.245117960041255,-1.
17647131600077,0.239406588139305,0.821419260492502
"4128","ERBB2",32.3340834108043,0.315998975344349,0.268687058456279,1.
1760855813447,0.239560679067179,0.821549822133993
"4129","APCDD1L-
DT",4.52685173136767,1.31748750998491,1.12013203642957,1.1761894733271
,0.239519170052095,0.821549822133993
"4130","SSX2IP",6.45618620169866,-0.647362089370243,0.550682822204651,
-1.17556252577216,0.239769737807136,0.821830117077517
"4131","KRTCAP2",489.639517291205,-0.257993010982888,0.219515796866293

,-1.17528221051004,0.239881829215305,0.821830117077517
"4132","C2orf42",8.35267570531594,0.540835775764904,0.46011392681709,1
.17543882991376,0.239819196286839,0.821830117077517
"4133","MBTPS1",114.226823322346,0.241802450104163,0.205694780049579,1
.1755400406655,0.23977872770423,0.821830117077517
"4134","ESS2",14.3988712235628,-0.420613488098596,0.35792163192015,-1.
17515525910552,0.23993260621458,0.821830117077517
"4135","NCDN",16.5828552619851,0.385972732061603,0.328622529864118,1.1
7451695177807,0.240188026045811,0.821863776436313
"4136","LGALS8",39.1621564746518,0.32900832033811,0.280266426661701,1.
17391270962056,0.240429991135779,0.821863776436313
"4137","DNAJB14",93.1983120162458,0.236299280492418,0.201356634231642,
1.17353610619344,0.2405808865345,0.821863776436313
"4138","LINC00968",5.48937417765862,0.91478764308662,0.778779449759932
,1.17464276101355,0.240137668096708,0.821863776436313
"4139","COMTD1",24.3841514355038,-0.398552366916041,0.33923418801462,-
1.17485908259596,0.240051097961947,0.821863776436313
"4140","WNK1",115.142303144301,0.243394438359221,0.207243809966859,1.1
7443526249659,0.240220727985179,0.821863776436313
"4141","AC023509.3",2.2503540312538,-1.14242046554582,0.97338986802542
9,-1.17365148649356,0.240534649500526,0.821863776436313
"4142","AC009053.2",2.1760373519424,1.20741167610523,1.02876111822698,
1.17365601665249,0.24053283423073,0.821863776436313
"4143","RPL18A",4168.75395048525,-0.254260809121307,0.21665959497777,-
1.17354972969185,0.240575426782963,0.821863776436313
"4144","C19orf54",5.05416379807079,0.721790704080475,0.614859114590018
,1.17391234341831,0.240430137831295,0.821863776436313
"4145","AC008735.2",1.86651812031035,1.70114267819304,1.44912493063194
,1.17391029733454,0.240430957465084,0.821863776436313
"4146","DBR1",19.5456489067137,0.419043932184827,0.357193522144741,1.1
7315658377204,0.240733018981534,0.822185129951402
"4147","FLVCR1",8.47070562098005,0.615087521615666,0.524490545696178,1
.17273328692557,0.240902778431892,0.822558963497174
"4148","CLDN12",15.5665628806576,0.429150888442451,0.366016682827767,1
.17248996719746,0.241000397804774,0.822558963497174
"4149","TBXA2R",13.2299414857809,-0.508097986479661,0.433364598355825,
-1.17244922268079,0.241016747143346,0.822558963497174
"4150","WDR82",84.6937126334241,0.26163752213802,0.223224382296003,1.1
7208308271217,0.241163701254832,0.822849635330233
"4151","DHTKD1",15.9584458605446,0.415200048479848,0.354325879695531,1
.17180277330187,0.241276249003611,0.822849635330233
"4152","CHAF1A",9.03145938655311,0.555300140252972,0.473831475524466,1
.17193594967141,0.241222772408481,0.822849635330233
"4153","SPEN",69.2164675837066,0.285747492238721,0.243904351566632,1.1
7155553151604,0.241375550374653,0.822990077848564
"4154","PASK",2.31711510748099,-1.10346712802007,0.942085670582443,-1.
17130231620851,0.241477280749674,0.823138732646939
"4155","TCIRG1",43.8780385293056,-0.291800286557078,0.249168897437348,
-1.17109434427083,0.241560856970458,0.823150828417551
"4156","HMOX2",63.2629342550189,-0.339312154284172,0.289761691305757,-

1.1710041888392,0.241597093425377,0.823150828417551
"4157","VANGL1",25.5747079753472,-0.316949416360461,0.271209593567525,
-1.16865119773703,0.242544191757891,0.823381930871905
"4158","MRPL9",62.4801894283665,-0.261491358578413,0.22400017434957,-1
.1673712278917,0.24306048524326,0.823381930871905
"4159","TAF5L",17.9366287450408,0.382617430050598,0.32767746061481,1.
16766478028911,0.242942008621884,0.823381930871905
"4160","CDK7",12.1763880274139,-0.484907405843318,0.414243008132081,-1
.17058682059567,0.241764897403828,0.823381930871905
"4161","TAF8",19.5802850810359,0.413012372143741,0.352965160201346,1.1
7012220670205,0.241951793070934,0.823381930871905
"4162","CUL9",8.52181174592897,0.551334374644797,0.471789575761979,1.1
6860228154543,0.242563908593642,0.823381930871905
"4163","MCM3",32.4974841099373,0.335023206732532,0.286485149613017,1.1
6942608433659,0.24223200559684,0.823381930871905
"4164","FOXO3",154.00236114786,0.397272244420325,0.340033245501659,1.1
68333537017,0.242672252581774,0.823381930871905
"4165","SFRP4",519.12022836122,-0.456068060372619,0.390522760439687,-1
.16783989711415,0.242871351478859,0.823381930871905
"4166","COPS6",167.570120507841,-0.257788255884931,0.220645254736297,-
1.16833809180726,0.242670416040901,0.823381930871905
"4167","ADAMTS8",5.3738869829897,-1.18246643330486,1.01289669657229,-1
.16741069183699,0.243044555383852,0.823381930871905
"4168","ERBB3",2.57466969910979,1.21294407970777,1.03759825592064,1.16
899201862242,0.242406847068121,0.823381930871905
"4169","ALDH2",153.258828908906,-0.299268731622686,0.255842091780607,-
1.1697400124422,0.242105610790474,0.823381930871905
"4170","AHSA1",98.3300617390737,-0.210983029518133,0.1806573171234,-1.
16786318360977,0.242861956798194,0.823381930871905
"4171","RPAP1",11.5814517614489,0.464821811781109,0.397673397699952,1.
16885317064085,0.242462793704154,0.823381930871905
"4172","ELOB",541.062986279296,-0.273664592045243,0.234402477975678,-1
.16749871592074,0.24300902657584,0.823381930871905
"4173","CDK10",22.4151534440326,-0.352211233201686,0.301629267867271,-
1.16769581311543,0.242929486265374,0.823381930871905
"4174","SHPK",12.0953755009277,0.537486128489263,0.459328108043324,1.1
7015727772219,0.24193768185,0.823381930871905
"4175","PLD2",14.9030953628145,-0.441177168314795,0.377727978213927,-1
.16797588148192,0.242816493690801,0.823381930871905
"4176","NDEL1",39.2530373611215,-0.281786397718563,0.241274230992331,-
1.16790921500241,0.24284338669054,0.823381930871905
"4177","SMCHD1",84.6763156626268,0.277100950369254,0.237159424055093,1
.16841635736508,0.242638860045178,0.823381930871905
"4178","LSM7",177.624698839903,-0.316762081177053,0.270747339667909,-1
.1699545471641,0.242019260780684,0.823381930871905
"4179","VASP",35.573491064689,-0.347181167129373,0.29713993525567,-1.1
684096479009,0.242641565130002,0.823381930871905
"4180","COL18A1",192.414328981334,-0.446359902945026,0.381857239272678
,-1.16891826850057,0.242436562402781,0.823381930871905
"4181","CRY2",41.6969838431672,0.311804472718933,0.26728685288411,1.16

655372067299,0.243390642039653,0.824303155054169
"4182","KCNE4",60.0941867040166,0.484664932086637,0.415782151896451,1.16567036337659,0.243747747008777,0.825117881339774
"4183","PPP2R5B",6.49006770694681,-0.652873095234634,0.560015372934471,-1.16581280941198,0.243690137066977,0.825117881339774
"4184","LONRF2",4.34100380796671,-0.961770144005391,0.82519419960O521,-1.16550763986342,0.243813569547253,0.82511890843875
"4185","ENDOG",42.4651175610169,-0.360421220755445,0.309273155016488,-1.16538152409714,0.243864592642385,0.82511890843875
"4186","NR3C1",311.071982265362,0.21621691711403,0.185575549233682,1.16511532907691,0.243972312710413,0.825286179641531
"4187","CDC42EP3",4.6867291925003,1.06076275100623,0.911477930509761,1.16378325299986,0.244511860608078,0.825359211128459
"4188","SLC4A5",2.07342713049962,1.15986802826215,0.996401331027549,1.16405708437385,0.244400878825997,0.825359211128459
"4189","NR2F1",10.6888564270955,0.762166905366739,0.654684248838477,1.16417480139312,0.244353179880728,0.825359211128459
"4190","LSM2",118.116931987001,-0.372988805915986,0.32044806126292,-1.16396025129937,0.244440120495215,0.825359211128459
"4191","UBE3D",11.6200821684783,0.47287826439293,0.406334180871469,1.1637668861102,0.244518495104794,0.825359211128459
"4192","TCAF2",2.84387914209884,1.27936679456159,1.09851159874653,1.16463658282847,0.24416612911807,0.825359211128459
"4193","MAST3",3.72934965983869,0.804997680739792,0.69154332738082,1.16405964581955,0.244399840858433,0.825359211128459
"4194","AL118506.1",2.05611237669313,1.15778467068351,0.994657516355775,1.16400333948654,0.2444226584319,0.825359211128459
"4195","APOBEC3D",7.05541030874169,0.620884294730021,0.53310359685724,1.16465973666332,0.244156752994628,0.825359211128459
"4196","HUS1",15.073427498431,-0.419866471554801,0.360908997230482,-1.16335828360263,0.244684167534657,0.825524854012567
"4197","Z69706.1",3.03965827500892,1.09626552421306,0.942320159564306,1.16336842960033,0.244680052773421,0.825524854012567
"4198","MRPS30-DT",8.61566523721244,0.577315597771755,0.496326996559325,1.16317589366258,0.244758144991609,0.825577735369506
"4199","THUMPD2",25.9392733765326,0.348550840398624,0.299704097472996,1.16298323358769,0.244836305067467,0.825644696298008
"4200","SSSCA1-AS1",2.16508879336428,1.23571837439334,1.0627682845398,1.16273546394775,0.244936848234604,0.825787088333808
"4201","SETD5",99.3176027215824,0.258368259048591,0.222332217396466,1.16208196038393,0.24520217433395,0.826484834222501
"4202","BX322562.1",2.74153373885124,-1.15243265848352,0.991876599588633,-1.16187100185797,0.245287867710092,0.826576917366708
"4203","AGO3",68.6543992182405,0.305980217242043,0.263970855526141,1.15914393894799,0.24639751824604,0.826632979302721
"4204","SNRNP27",68.9008198538832,0.253061199473826,0.218205750129169,1.15973662162443,0.246156055087031,0.826632979302721
"4205","AC108463.3",5.337833317835,0.660956650323554,0.570091036822992

,1.15938790058328,0.246298106438738,0.826632979302721
"4206","AMT",7.67392079510699,0.668540247595796,0.575816864511833,1.16102929385816,0.245629986698265,0.826632979302721
"4207","COMMD8",51.5526909945492,0.332619756576719,0.286545436286656,1.16079237166413,0.245726345954528,0.826632979302721
"4208","TTC37",93.0420620399564,0.243388585353338,0.20985063947749,1.15981817334155,0.246122843320508,0.826632979302721
"4209","SRA1",59.7242656838957,-0.394109393151011,0.3399603908989,-1.15928032706673,0.246341938052941,0.826632979302721
"4210","RNF19A",128.468551498131,0.293879844764727,0.253198238067535,1.16067097072903,0.245775731521872,0.826632979302721
"4211","DCAF10",44.6120667653705,0.279712391648815,0.241292216714533,1.15922674778912,0.246363771359922,0.826632979302721
"4212","LDHB",238.552543932564,-0.283773826215299,0.244713372465731,-1.15961716090949,0.246204710884747,0.826632979302721
"4213","PRKD1",7.64353025647011,0.616069244557305,0.531380404378066,1.15937516604204,0.24630329493523,0.826632979302721
"4214","ALPK3",6.08455504845808,1.05269895645879,0.90748998980312,1.16001164562397,0.246044064703036,0.826632979302721
"4215","KIAA0556",7.77557552451362,-0.541509481585272,0.467179266817656,-1.1591042669207 9,0.246413686838756,0.826632979302721
"4216","ZDHHC1",32.5663696032216,-0.329309517671534,0.283580219038738,-1.16125701146507,0.245537396041963,0.826632979302721
"4217","GGA3",18.894527896529,0.417598019129358,0.359664514381762,1.16107651000038,0.245610786462526,0.826632979302721
"4218","ATP9B",25.3408171418336,0.422804490531498,0.364208937925472,1.1608844443516,0.24568889567818,0.826632979302721
"4219","YPEL1",6.44776149977389,0.620741969307243,0.53468010170563,1.16095954819915,0.245658350388293,0.826632979302721
"4220","SCML1",28.9235119862152,0.425432964359562,0.366468674520109,1.16089858134987,0.245683145853025,0.826632979302721
"4221","CHIC1",11.6704484782098,0.49858819426373,0.429925121446287,1.15970937587098,0.246167151563276,0.826632979302721
"4222","YME1L1",140.166785277868,0.187607007307176,0.161881053485502,1.15891886831573,0.246489257102575,0.826690639642932
"4223","MEF2C",25.028617300658,-0.371267419028808,0.320565311145159,-1.15816467384611,0.246796841462186,0.827330320810738
"4224","RTTN",7.12055710700676,0.72128658799954,0.622778363779951,1.1581754119101,0.246792460253971,0.827330320810738
"4225","KRT24",2.25670729968756,1.48514430457633,1.28254151323759,1.15796977271114,0.246876372012688,0.827401047976252
"4226","MRTO4",50.1429105157499,-0.31448608943382,0.271648215660329,-1.15769613531,0.246988061652339,0.827579496686495
"4227","ZBTB37",20.2479413029765,0.466312774055058,0.403289542033344,1.15627291425401,0.247569543649231,0.827811178963334
"4228","ZNF800",41.533718757557,-0.269497532767156,0.232965217369024,-1.156814462737,0.247348171637613,0.827811178963334
"4229","CTSL",423.726121712865,-0.374729416465738,0.32381245619503,-1.15724212980875,0.247173449586155,0.827811178963334
"4230","CDC14B",44.61439020475,0.284975933923254,0.246381811212596,1.1

5664355465492,0.247418019782601,0.827811178963334
"4231","RFX7",12.641849871826,0.456066329456458,0.394487814952001,1.15
60973803765,0.247641327437386,0.827811178963334
"4232","QPRT",23.7503375150318,0.68627977720297,0.593307257846307,1.15
670214400234,0.247394073442391,0.827811178963334
"4233","CYB5D2",48.0869461907074,0.3678990929107,0.31826468163293,1.15
595324942469,0.247700280033026,0.827811178963334
"4234","ADAP2",2.15563145802471,-1.04969138225196,0.907947510786203,-1
.15611460991068,0.24763428085222,0.827811178963334
"4235","OAZ1",996.688161351861,-0.215996842372305,0.18683105816115,-1.
15610779330918,0.247637068709013,0.827811178963334
"4236","SLC17A7",35.7165909329681,0.324941438904718,0.281074701018755,
1.15606789841622,0.247653385381114,0.827811178963334
"4237","LILRB3",1.74990326261949,-1.26427113327868,1.09316100664304,-1
.15652783587762,0.247465320540866,0.827811178963334
"4238","TESK1",31.5797616527175,-0.339821177439228,0.294116701586273,-
1.15539571743616,0.247928414854419,0.828372063191903
"4239","CPT1A",42.535436141098,0.394021293107754,0.34110972524582,1.15
511597572835,0.248042936897245,0.828372063191903
"4240","PPIB",1019.40654881846,-0.285264092119135,0.246957453088613,-1
.15511432658312,0.248043612142208,0.828372063191903
"4241","NDUFAF5",13.3504294978095,0.445003625758845,0.385351535420182,
1.15479915053048,0.248172685059464,0.828607691686398
"4242","RAP2A",33.1356545727186,-0.334530522904005,0.289878643314612,-
1.15403645842557,0.248485222052345,0.829455620995097
"4243","STXBP3",64.491346432589,-0.249554395473821,0.216302560461201,-
1.15372834672747,0.248611558519562,0.829681750798257
"4244","MANEAL",2.44974521432168,0.951422394902923,0.829630227708596,1
.14680295284167,0.251463068551642,0.829837367408644
"4245","ZYG11B",68.9930135807266,0.283150957883046,0.246021680792368,1
.15091871972866,0.249765675723892,0.829837367408644
"4246","NOTCH2NLA",10.7816369327427,0.569240468989961,0.49524554595056
7,1.14941057752951,0.250386720485436,0.829837367408644
"4247","AC245297.3",105.569628706853,-0.220462399699298,0.192166652282
721,-1.14724587789013,0.251280015371936,0.829837367408644
"4248","NMNAT2",2.35405389426889,-1.08848637980876,0.949556414879838,-
1.14631038530397,0.251666747329896,0.829837367408644
"4249","DISC1",4.5977408591429,0.890712085649859,0.776968968511568,1.1
4639338473992,0.251632418650553,0.829837367408644
"4250","PLD5",5.52774090418248,-0.988857499169834,0.859764420575582,-1
.15014936127251,0.250082358751241,0.829837367408644
"4251","HPCAL1",16.9917813844586,0.476025412261853,0.414411884683547,1
.14867702847218,0.2506891814724,0.829837367408644
"4252","AGAP1",38.6301026477241,0.456935070235383,0.397190562443085,1.
1504177426191,0.249971855888529,0.829837367408644
"4253","NEPRO",35.8934918641154,-0.312584374705718,0.27233320099759,-1
.1478011992687,0.251050642137322,0.829837367408644
"4254","LXN",62.0094109572425,-0.378028612121832,0.328372261418462,-1.
15121968734165,0.249641867825995,0.829837367408644
"4255","DLG1",47.0961045077541,0.354923351427404,0.307932494112027,1.1

5260116491078,0.24907412487452,0.829837367408644
"4256","SLAIN2",68.1065319348043,0.301819628196213,0.262498571873774,1
.1497953152345,0.250228185230288,0.829837367408644
"4257","LINC02104",5.093930074526,-0.761596659311235,0.663669688081804
,-1.14755378012286,0.251152819554167,0.829837367408644
"4258","YTHDC2",28.9760445598137,0.328277668878736,0.285279284908804,1
.15072382133767,0.249845873212514,0.829837367408644
"4259","LYRM2",51.3103484233299,-0.261549798755401,0.227632035951241,-
1.14900258947482,0.250554912901893,0.829837367408644
"4260","FRK",4.14063730603605,0.770573609717451,0.668725193768554,1.15
230234616246,0.249196853217703,0.829837367408644
"4261","RPS12",6905.34394657409,-0.285073768046092,0.248311003163316,-
1.14805129218779,0.250947390009451,0.829837367408644
"4262","PEX7",13.283444065552,-0.445147579920602,0.386800780483465,-1.
15084457524674,0.249796182841244,0.829837367408644
"4263","MPC1",116.470126182218,-0.286348693813456,0.249363498811232,-1
.14831839935893,0.250837146196306,0.829837367408644
"4264","CHST12",86.0780174043479,-0.234773841851093,0.204218519719977,
-1.14962072084849,0.250300120010193,0.829837367408644
"4265","SNAPC3",29.6878788078756,0.33373569792747,0.289462589448126,1.
15294932780002,0.248931183624404,0.829837367408644
"4266","LCNL1",1.60770205614581,1.48388232323129,1.29399037349366,1.14
674912088019,0.251485322698753,0.829837367408644
"4267","ZMYND19",13.8875550932003,-0.443324230341117,0.384934965789186
,-1.15168605021948,0.249450106628537,0.829837367408644
"4268","AL157893.2",3.55402626645732,-0.89631930763204,0.7808149505977
11,-1.14792795264219,0.250998307660707,0.829837367408644
"4269","PPP6R3",46.992018303109,0.304075172464612,0.26408145092877,1.1
5144464480631,0.24954935594235,0.829837367408644
"4270","JRKL",20.5615795065296,-0.361320747808249,0.315038244545275,-1
.1469107451693,0.251418511304497,0.829837367408644
"4271","IRAK3",47.3328064726911,0.284532004572836,0.247424346184294,1.
14997577627588,0.250153848499987,0.829837367408644
"4272","TCP11L2",30.8898161493854,-0.347604939272353,0.301659275203157
,-1.15230980064596,0.249193791060406,0.829837367408644
"4273","ORAI1",16.3338859165344,0.56236216121445,0.489369795731877,1.1
4915584516901,0.250491724241926,0.829837367408644
"4274","ENOX1",3.8746589225192,-0.855210697465105,0.745651946256469,-1
.14693014852127,0.251410491282016,0.829837367408644
"4275","RNASEH2B",64.0128329152475,-0.284793127120805,0.24820851990018
1,-1.14739464719155,0.251218552483741,0.829837367408644
"4276","DDHD1",43.6143834093122,0.330222337939147,0.287572223859951,1.
1483109651785,0.250840214066845,0.829837367408644
"4277","TCF12",78.8232300312394,0.240560048940611,0.209818500771418,1.
14651495485941,0.251582142979797,0.829837367408644
"4278","ORAI3",54.1160420516977,0.327404818226234,0.284837937177948,1.
1494424565422,0.250373581735528,0.829837367408644
"4279","SLC9A5",2.07422887408642,1.02136570929589,0.890920679135612,1.
14641598653523,0.251623071083162,0.829837367408644
"4280","NCBP3",58.8443551211109,0.285905142580389,0.249290278649128,1.

14687642105289,0.251432698990583,0.829837367408644
"4281","RPL23A",3358.84255955261,-0.245610772367771,0.213964954592252,
-1.14790187409815,0.251009074459701,0.829837367408644
"4282","ZNF207",111.645088599324,0.20790147032299,0.180555754514751,1.
15145302835532,0.249545908740756,0.829837367408644
"4283","CEP131",26.4060960698497,0.405714964718952,0.352129107570476,1
.15217673289832,0.249248456667359,0.829837367408644
"4284","AC145207.5",3.64789618914997,-0.797112455148354,0.695199892590
571,-1.14659461781275,0.251549201937721,0.829837367408644
"4285","FN3KRP",37.2877294680425,-0.342725190591909,0.297998381052613,
-1.15009078029655,0.250106483314897,0.829837367408644
"4286","MKNK2",61.071205004963,0.263001641792858,0.229038516369779,1.1
4828565064683,0.250850660835911,0.829837367408644
"4287","MOB3A",28.9522152215925,0.344010954765538,0.299842817058973,1.
14730430476805,0.251255875507447,0.829837367408644
"4288","EPS15L1",26.4795647961732,0.346668043180163,0.301455731319201,
1.14997993789373,0.250152134400236,0.829837367408644
"4289","ZNF841",8.37557785473447,0.594353688867149,0.516343428423532,1
.1510821212188,0.249698452554836,0.829837367408644
"4290","ZNF766",25.767605912121,-0.357348146603695,0.311763240528803,-
1.1462164237117,0.251705613913851,0.829837367408644
"4291","LENG8",89.4746836189328,0.239060662407412,0.207422937640206,1.
15252760917929,0.249104331149536,0.829837367408644
"4292","ACOT8",21.4376147856345,0.438921924997477,0.382343928077475,1.
14797671092749,0.250978178158562,0.829837367408644
"4293","RTEL1",4.95652378927904,0.752444393825859,0.656130983398255,1.
14678991369799,0.251468458811007,0.829837367408644
"4294","Z93241.1",8.48591331977626,0.584576165292589,0.507043876605575
,1.15291041320932,0.248947157494475,0.829837367408644
"4295","HUWE1",78.5244813540115,0.26139346199534,0.226623430505356,1.1
5342646350578,0.248735384660589,0.829837367408644
"4296","USP54",5.44225962772284,0.687166755694882,0.59962777421082,1.1
45988870511,0.251799757117816,0.829954506701183
"4297","GAR1",35.1604129371393,-0.295020163403556,0.257541838571513,-1
.1455232479504,0.251992470790612,0.829999165839061
"4298","ALDH5A1",5.50186542108439,0.64706421821485,0.56485761343174,1.
14553509208041,0.251987567421733,0.829999165839061
"4299","CCDC77",9.18064126175517,0.563664096414909,0.492176394372658,1
.14524813229487,0.252106385047585,0.829999165839061
"4300","ARRDC4",26.1887404248835,0.359570011463357,0.31382029748812,1.
14578315788184,0.251884885582088,0.829999165839061
"4301","CES2",31.0742250780948,0.336806118722614,0.29406561260146,1.14
534343455887,0.252066920174049,0.829999165839061
"4302","PEX13",23.0877756140374,-0.404539455833566,0.353794303426252,-
1.14343123084765,0.252859591877162,0.830340843441775
"4303","KLHL18",12.6288807366936,-0.560094489726859,0.489973906501128,
-1.14311085201753,0.252992569179072,0.830340843441775
"4304","SH3BP2",18.8691216662256,0.367162541761776,0.321235009570559,1
.14297175221535,0.253050319483173,0.830340843441775
"4305","ZNF330",43.4119051498375,-0.294230744167522,0.257144233734203,

-1.14422454625855,0.252530525849514,0.830340843441775
"4306","LRBA",17.9976171808595,0.412508290996716,0.360809007105715,1.14328712108856,0.252919400435702,0.830340843441775
"4307","TCERG1",56.8667276044593,0.270466919743868,0.23665429886558,1.14287769561073,0.253089374329059,0.830340843441775
"4308","COX7A2",306.775941322874,-0.240398205522598,0.210121769412999,-1.14408995409747,0.252586333441537,0.830340843441775
"4309","ZDHHC21",30.0473199980754,0.368379594307548,0.322030536992572,1.14392752236427,0.252653695940057,0.830340843441775
"4310","HPS5",16.2319421193473,0.473886385254154,0.414175667175846,1.14416761488056,0.25255413095351,0.830340843441775
"4311","LINC00900",4.55541641279566,0.720572667141791,0.630489767712838,1.1428776548678,0.253089391247537,0.830340843441775
"4312","CAPRIN2",19.59095676175,0.361333504525882,0.315629375203042,1.14480315494538,0.252290708142524,0.830340843441775
"4313","SIAH1",48.0468692899856,-0.279049393362635,0.244138534943367,-1.14299610025663,0.253040210202043,0.830340843441775
"4314","ZNHIT3",59.5408080868844,-0.361080824228294,0.315940299892994,-1.14287675345813,0.253089765557535,0.830340843441775
"4315","WDR83OS",326.874381255268,-0.278967290347982,0.243981660305799,-1.14339450759673,0.252874831857303,0.830340843441775
"4316","ZNF805",13.895596720288,-0.485018541298528,0.424341300308565,-1.14299159885177,0.253042079159405,0.830340843441775
"4317","MTREX",53.382510856151,0.254350191357477,0.222646440312844,1.14239505019746,0.253289847807324,0.830804782245009
"4318","ADAMTSL4",126.564003401854,0.221023873401244,0.193512817837864,1.14216658033697,0.253384784494402,0.830923702742181
"4319","XAF1",72.2163785044638,0.302842687500891,0.265203084102451,1.14192747239658,0.253484168185451,0.831057147836535
"4320","POGZ",69.2325984286712,0.24113650130482,0.211337087897298,1.14100418295725,0.25386818230094,0.831349564939045
"4321","SFT2D2",124.522368975856,0.361606073669813,0.317034922214384,1.14058751365343,0.254041615748205,0.831349564939045
"4322","INTS7",7.71216527199945,0.59737855649112,0.523721149594826,1.14064241429486,0.254018759319275,0.831349564939045
"4323","ARID5A",17.7847889935194,-0.486035339487113,0.426145202053997,-1.14053927427658,0.254061700117271,0.831349564939045
"4324","GSEC",6.2916212099798,0.812430133614239,0.711796205125973,1.14138025429688,0.253711718032431,0.831349564939045
"4325","TC2N",27.1803968313019,0.591638860810228,0.518725877039882,1.14056168584923,0.254052368965923,0.831349564939045
"4326","ZNF770",37.5358595848436,0.35286533824335,0.309179489392687,1.14129607671089,0.253746734236566,0.831349564939045
"4327","LAT",4.06812446718185,0.770077035581262,0.675243735317524,1.1404430656719,0.254101759679109,0.831349564939045
"4328","BCAP31",157.015887817536,-0.187463694203326,0.164341174421408,-1.1406982751786,0.253995504587912,0.831349564939045
"4329","TRIP6",111.175282513128,-0.235256717723165,0.206346892952147,-1.1401030292117,0.254243380071533,0.83142869787827
"4330","GID4",19.9895699980793,0.355405681593654,0.311724198357127,1.1

4012862481238,0.254232717953526,0.83142869787827
"4331","TPPP3",468.847036461384,-0.344709933945431,0.302434340761888,-1.13978436799552,0.25437614782016,0.831670804233078
"4332","RAB4A",108.146744111751,-0.216116942514871,0.189673431377147,-1.13941600015209,0.254529685555976,0.831980689628951
"4333","P3H4",42.1499254087493,-0.422681983785568,0.371101351846375,-1.13899338194959,0.254705914534255,0.832172531104072
"4334","TRIOBP",211.184837876993,-0.190792917977807,0.167505864750272,-1.13902231579923,0.254693846605896,0.832172531104072
"4335","PTHLH",16.1643405214318,-0.459986589239244,0.40391032181103,-1.13883345980559,0.254772623132536,0.832198464488283
"4336","AC107959.1",7.8724763131937,0.546329274220497,0.479792345369858,1.13867859604835,0.254837233297247,0.832217533092486
"4337","FDPS",106.000765853353,-0.260128231766605,0.228612720515441,-1.13785545782452,0.255180843155637,0.832623199352199
"4338","LPCAT4",18.6854835536475,-0.374549037241655,0.32909395471393,-1.13812190068103,0.255069584321074,0.832623199352199
"4339","ZNF652",70.77192652771,0.226207438313871,0.198808174857053,1.13781758962637,0.255196658558513,0.832623199352199
"4340","RTF2",134.349493121376,-0.223725066201146,0.196586856336117,-1.13804691916243,0.255100891024758,0.832623199352199
"4341","TMEM39A",29.5556269348797,-0.341144224149531,0.29990954611123,-1.13749038192671,0.255333343077524,0.832877249015835
"4342","GSTM5",97.2118664364995,0.386717628010522,0.340122706123033,1.13699444655904,0.255540607235896,0.833065839234355
"4343","CLCN3",51.8015897098609,0.291137136523195,0.256073110474675,1.13692974628817,0.255567655765116,0.833065839234355
"4344","GDF10",211.812756792288,-0.378410649758207,0.332803905021085,-1.13703788942691,0.25552244667141,0.833065839234355
"4345","AC239799.2",6.38928845724955,0.682792431186545,0.600685428728131,1.13668885331922,0.255668380729611,0.83320236389673
"4346","C1QTNF2",36.4295798640761,-0.408488063181599,0.359442517124888,-1.1364489277702,0.255768728603635,0.833337597107104
"4347","ZRANB2-AS2",2.24817587259372,1.06345467189815,0.936382533037178,1.13570537080482,0.256079892070276,0.833835326673687
"4348","FAU",2598.4997758587,-0.257603005329716,0.226796788411427,-1.13583180403068,0.256026963788908,0.833835326673687
"4349","NKG7",12.1719590290117,0.592500522396108,0.521722704307382,1.13566175576485,0.256098152238974,0.833835326673687
"4350","HIST2H2AC",24.837997415469,0.361111736465658,0.318094733056216,1.13523330926023,0.256277576645723,0.833898579345477
"4351","CRYBG1",13.6678065503921,-0.455920438549074,0.401673286231177,-1.13505292529381,0.256353143777603,0.833898579345477
"4352","NUTF2",57.3439456274092,-0.318009076142463,0.28015727356664,-1.13510911958107,0.256329600995399,0.833898579345477
"4353","CENPN",5.85912906283179,-0.65905725697528,0.580493280881686,-1.13534002663091,0.256232877486924,0.833898579345477
"4354","TRIM22",89.745815390185,0.248056242915404,0.218573030080596,1.1348895278797,0.256421608195382,0.833929713377723

"4355","APH1A",120.15530178229,-0.220654632401477,0.194462375935941,-1
.13469061220441,0.256504972097636,0.833966981971016
"4356","SPATA17",3.10844144421415,-0.940243035305405,0.829279250860682
,-1.13380750130859,0.256875303755907,0.833966981971016
"4357","LIMD1",14.9511204750902,0.496480623361122,0.43776667713341,1.1
341215521752,0.256743564340866,0.833966981971016
"4358","ING2",39.9815464434675,0.341784746381288,0.301676431588033,1.1
3295143602078,0.257234647987008,0.833966981971016
"4359","MIER3",12.6714239622555,0.47712855533512,0.420878207178028,1.1
3364994242455,0.256941414911137,0.833966981971016
"4360","LMNB1",2.69083367617207,0.906037021653633,0.79969525146834,1.1
3297786874442,0.257223547300323,0.833966981971016
"4361","NUDCD2",90.2347991108249,-0.277421532803464,0.244739425047635,
-1.13353838577282,0.256988230831895,0.833966981971016
"4362","CREBRF",164.300187995538,0.326648903795888,0.288261631206853,1
.13316816542084,0.257143640119132,0.833966981971016
"4363","MANEA-
DT",4.02923709005525,0.84857179740228,0.748605239095936,1.133537080807
86,0.256988778509179,0.833966981971016
"4364","OTUD6B",13.2522231959836,0.429365402815707,0.378997820914048,1
.13289675856232,0.257257611387669,0.833966981971016
"4365","RMI1",4.76951510513907,0.770142044797992,0.679736868511763,1.1
3300025417801,0.257214146573557,0.833966981971016
"4366","GOT1",15.4908406513491,-0.480088795291597,0.423260804113372,-1
.13426235225647,0.256684516145783,0.833966981971016
"4367","AC103703.1",1.53177123583885,-1.2541560223122 5,1.1061407798497
2,-1.13381230053071,0.256873290204465,0.833966981971016
"4368","AL022322.1",2.40611705559068,1.13477193787135,1.00084487930553
,1.13381400188484,0.256872576390574,0.833966981971016
"4369","WNT9A",5.56427430391472,-0.712005726434608,0.628584517904932,-
1.13271279542124,0.257334882542998,0.834026536234575
"4370","LINC00622",2.41644164039577,1.02069081286487,0.901924085408732
,1.13168151219991,0.257768356888289,0.834116327413388
"4371","SLC39A1",145.787977203933,-0.231404283481416,0.204513470793683
,-1.13148675528988,0.257850274948961,0.834116327413388
"4372","MDM4",90.7840514634989,0.292738395193853,0.258830955724455,1.1
3100225734009,0.258054141329782,0.834116327413388
"4373","EPHA3",22.3827433776714,-0.549564315709574,0.485604648986829,-
1.13171139703088,0.25775578841899,0.834116327413388
"4374","AC096586.1",1.89918680290086,1.45134678889662,1.28307080501582
,1.13115097251295,0.257991553278978,0.834116327413388
"4375","BCLAF1",285.892528516502,0.247498334180788,0.218786706363749,1
.13123113508233,0.257957820543764,0.834116327413388
"4376","CFAP69",10.9386706831071,-0.552777134983705,0.488538539908905,
-1.13149135600802,0.257848339600651,0.834116327413388
"4377","COPG2",9.79164729408704,-0.622527848749003,0.550239271106425,-
1.13137662365905,0.257896606172478,0.834116327413388
"4378","UTP23",50.8393938818588,0.239035711603647,0.21135537530046,1.1
3096584964464,0.258069465423591,0.834116327413388
"4379","VPS36",114.253671355567,0.251130414012922,0.221896095594381,1.

13174778195278,0.257740486822469,0.834116327413388
"4380","PYCARD",100.456362948006,-0.361390686911906,0.319304758376601,-1.1318048899405,0.257716471458112,0.834116327413388
"4381","ZNF823",4.7030923457675,0.719093848190535,0.635453884929506,1.13162239659658,0.257793220005769,0.834116327413388
"4382","GUF1",14.9744566406627,0.421447789407535,0.372819122247302,1.13043501327696,0.258292967506324,0.834353766599387
"4383","DOCK1",78.9592012958968,0.249583366290777,0.220776806940835,1.13047819537341,0.25827478120647,0.834353766599387
"4384","UEVLD",20.1508043825854,0.457155108366695,0.404429064648759,1.13037154924493,0.258319697229641,0.834353766599387
"4385","COBL",1.61356413219894,-1.5590384731245,1.37967743438617,-1.13000215432104,0.258475316712507,0.834666017023739
"4386","RAP2C",28.2874849251501,0.352066784269642,0.311647083155642,1.12969703006594,0.25860390910907,0.834890869353495
"4387","LINC01006",5.33155355842794,-0.755512935029603,0.668867429991213,-1.12954062517221,0.258669841999776,0.834913371943659
"4388","CCDC18",14.5768981536422,-0.409965542918821,0.363079963195501,-1.12913293069294,0.258841761430122,0.835087569343933
"4389","BCAT1",23.0328732198316,-0.437677586989446,0.387583748139153,-1.12924648954142,0.258793867195317,0.835087569343933
"4390","ALG6",18.2091072556695,0.463429959286696,0.41086416654991,1.12793958932508,0.259345432865756,0.835307697216534
"4391","ARHGEF33",2.28814312774126,1.08983700226832,0.966332207781402,1.12780780097403,0.259401098130884,0.835307697216534
"4392","VPS54",23.6175679392466,0.406780762171537,0.360663566396991,1.12786763086511,0.259375825928348,0.835307697216534
"4393","STRADB",16.8102491738957,0.413845395632155,0.366710451923456,1.12853449761649,0.259094256209881,0.835307697216534
"4394","NSD1",56.628904512026,0.294291863073666,0.260820546385976,1.12833082804051,0.259180228708849,0.835307697216534
"4395","RPS25",2762.67161076121,-0.234447056783584,0.207882465156813,-1.12778659136417,0.259410057499552,0.835307697216534
"4396","PA2G4",206.758294851925,-0.217714807453297,0.192983341774778,-1.12815337039495,0.259255152800848,0.835307697216534
"4397","AC027277.1",1.53179896145158,1.45197120613023,1.28639979466448,1.12870914015416,0.259020552267824,0.835307697216534
"4398","GGT5",490.007616170036,0.349950431692155,0.310318544590168,1.1277135633461,0.259440907652424,0.835307697216534
"4399","MPV17L",7.98737729621744,0.552745381904939,0.490355984471104,1.12723286634531,0.259644037996187,0.835771670385544
"4400","ASB8",57.8257270279049,0.23052115923422,0.204564029216289,1.12689000171426,0.259788991150749,0.836048207885139
"4401","TMEM220-AS1",6.73461534691704,0.608047837799538,0.53970351745539,1.1266330830423,0.259897645577026,0.836207830350077
"4402","TSNAX",83.2870728793398,-0.196700292071902,0.17474034311655,-1.1256718887218,0.260304426814971,0.836376374790105
"4403","MZT2A",186.185093222767,-0.238015181837451,0.211320069979769,-1.12632549222721,0.260027771319523,0.836376374790105

"4404","TCF7",18.1700009732928,0.612964059044938,0.544444740490963,1.1
2585174115593,0.260228279065883,0.836376374790105
"4405","SF3B5",267.529788527527,-0.285997739009758,0.254044620836788,-
1.12577758217324,0.260259675380731,0.836376374790105
"4406","RPUSD4",19.5897036357815,-0.37893025643249,0.336481780228291,-
1.12615386240348,0.260100398607029,0.836376374790105
"4407","PCNX1",49.1086754762563,0.330809539421981,0.293799789009378,1.
12596928860089,0.260178518966645,0.836376374790105
"4408","HAUS1",40.5529307057512,-0.301975213943266,0.268332644815809,-
1.12537635571904,0.260429586060229,0.836499247598907
"4409","RPS21",2058.53879300316,-0.283979513318028,0.252358349781884,-
1.12530262447616,0.260460817984716,0.836499247598907
"4410","AURKAIP1",205.792692060823,-0.273515739907579,0.24392745877544
,-1.12129950961928,0.262160394227174,0.836649561845293
"4411","STMN1",10.2882419408596,-0.545689995010439,0.486545642784181,-
1.12155972025114,0.262049686030275,0.836649561845293
"4412","JAK1",272.321261930998,-0.169014958765903,0.150687636171607,-1
.12162459415996,0.262022090061989,0.836649561845293
"4413","ABCD3",31.062209898166,-0.285785093383111,0.254400016977071,-1
.12336900279715,0.261280808865048,0.836649561845293
"4414","SMG7",23.3138890645445,0.393947460561249,0.351094304438113,1.1
2205597066497,0.261838642821201,0.836649561845293
"4415","ZNF124",4.24240121539031,-0.774109967831912,0.689914783935266,
-1.12203707741469,0.26184667550888,0.836649561845293
"4416","NMI",56.7330047905714,-0.236936994253517,0.210969981013181,-1.
12308392462107,0.261401852760064,0.836649561845293
"4417","MYO1B",88.5805656750365,0.233871163756467,0.208623907565694,1.
12101803904148,0.262280183971134,0.836649561845293
"4418","ZFAND2B",35.9640813717425,-0.310124643286026,0.276206097309239
,-1.12280158297451,0.261521772931616,0.836649561845293
"4419","PDCD6IP",109.338636897481,0.218676599374878,0.194492092670503,
1.12434699206691,0.260865850181735,0.836649561845293
"4420","KLF15",3.3929194583441,0.842075402014689,0.749301043853647,1.1
2381453211903,0.26109171519855,0.836649561845293
"4421","C3orf80",5.94422517214006,-1.03864806066494,0.925737909829259,
-1.12196772935064,0.261876161113978,0.836649561845293
"4422","EVC2",23.9182044224036,-0.393262812429022,0.35000030717446,-1.
12360704938752,0.261179764226204,0.836649561845293
"4423","BUD31",166.253698043335,-0.263491191080058,0.234730494829977,-
1.12252645857077,0.261638664269714,0.836649561845293
"4424","LYNX1",6.14934769276166,-0.614094340295861,0.54773318219461,-1
.12115599393734,0.262221467670136,0.836649561845293
"4425","AL158206.1",3.68299511510334,0.835891637117194,0.7443778904759
16,1.12293990433108,0.261463018361286,0.836649561845293
"4426","TNC",181.637878788367,-0.948665872130826,0.84348161610849,-1.1
247024878949,0.260715127106101,0.836649561845293
"4427","RUFY2",51.1103829800025,0.30174035310342,0.269114717510452,1.1
2123318967756,0.262188615616187,0.836649561845293
"4428","CLNS1A",93.3739426878025,-0.300840530679773,0.268262310466945,
-1.12144165968049,0.262099911614968,0.836649561845293

"4429","ETV6",24.0277367141716,-0.339438064533022,0.302762917051351,-1
.12113487291923,0.26223045659968,0.836649561845293
"4430","AC004846.1",10.7563040201998,0.486138047597022,0.4323104583368
85,1.12451142048984,0.260796128350614,0.836649561845293
"4431","TMEM251",28.8269395488778,-0.362496091774561,0.322510137043232
,-1.12398355939419,0.26102000608919,0.836649561845293
"4432","SIVA1",188.821809715481,-0.284812291025242,0.253342216128429,-
1.12421962426057,0.260919866229472,0.836649561845293
"4433","HAGHL",12.0335915159468,-0.475899651793412,0.423071954868109,-
1.12486693177706,0.260645426591832,0.836649561845293
"4434","TANGO6",7.99088732067196,0.568352472235684,0.506980219014307,1
.12105453214861,0.262264650907845,0.836649561845293
"4435","KSR1",10.0599573933667,0.490462767254867,0.436307195032633,1.1
2412257427518,0.260961029833818,0.836649561845293
"4436","KANSL1",79.7100469795091,0.325375779843953,0.289908791918221,1
.1223384351025,0.261718570045281,0.836649561845293
"4437","PYCR1",28.8981150983437,-0.382746129445843,0.341352935206624,-
1.12126215998161,0.262176287509151,0.836649561845293
"4438","ZNF776",5.2619423111854,0.732014342286464,0.651426779179095,1.
12370931881082,0.261136361792058,0.836649561845293
"4439","KDM6A",21.9636535948491,0.403110440080474,0.35913295094322,1.1
2245462027851,0.261669191949134,0.836649561845293
"4440","KDM1B",14.8496674945255,0.404518154263166,0.360900103567127,1.
1208590694902,0.26234785578192,0.836676945466663
"4441","SEPHS2",77.5844610178422,-0.409400236296107,0.365328729808786,
-1.12063520575124,0.262443172872888,0.836792462931795
"4442","ASH1L",160.789070871491,0.245232274572646,0.21893636002544,1.1
2010757164388,0.262667924450112,0.837127211169514
"4443","CARD16",12.8505579426874,-0.479036006286071,0.42772125454391,-
1.11997241473744,0.262725517403766,0.837127211169514
"4444","SMOC1",9.74726878979757,0.766153907642407,0.683935257402583,1.
12021408364305,0.262622543784143,0.837127211169514
"4445","B3GAT2",5.40738302666017,-0.708993912844243,0.633192824578442,
-1.1197124877659,0.262836301785262,0.837136943231241
"4446","IFNGR1",175.962303077072,-0.21674227385137,0.193573840274194,-
1.11968783356448,0.26284681141286,0.837136943231241
"4447","HP1BP3",290.3196530725,0.20685389557376,0.184897713647189,1.11
874771998786,0.263247780820771,0.837583078776815
"4448","PABPC4L",2.49865125164231,-1.00934305575884,0.902211294774542,
-1.11874353779961,0.263249565515481,0.837583078776815
"4449","FKTN",19.0249856573742,0.484857242905927,0.43325720058934,1.11
909794516144,0.263098356388284,0.837583078776815
"4450","DTX3",21.0304483569886,0.35154506763671,0.314211007417381,1.11
88184351853,0.263217605289698,0.837583078776815
"4451","TMEM187",18.5801769180782,0.406899916856461,0.363736727429249,
1.1186660190525,0.263282647149407,0.837583078776815
"4452","LSG1",30.4080468510028,-0.316485804069981,0.282988767179779,-1
.11836878623851,0.263409519792813,0.837798472656387
"4453","WDR92",21.0275230964536,-0.357047917783878,0.319344265196985,-
1.11806585148362,0.263538869690044,0.838021647161693

"4454","IRF9",86.9215949805078,-0.290734871582256,0.260237203871161,-1
.11719180523548,0.263912323693165,0.838882550541662
"4455","AC092329.4",3.33019396775379,0.969524194292011,0.8678511589627
53,1.11715492256849,0.263928090583553,0.838882550541662
"4456","TMEM189",25.3237972968024,0.374926568296306,0.335692496724624,
1.1168750328187,0.264047761210417,0.839074573325742
"4457","ZNF282",11.8658534830904,0.451319012119987,0.404274856415965,1
.11636676127001,0.264265175213799,0.839124773880015
"4458","STK24",83.1585942558774,0.232163152321828,0.207978577063404,1.
11628397308946,0.264300599682547,0.839124773880015
"4459","NOVA1",766.443470046621,0.164532411293266,0.14734637978823,1.1
1663694438734,0.264149588571804,0.839124773880015
"4460","COMP",40.3533016112195,-1.37500104095556,1.23164631278708,-1.1
1639277175611,0.264254046189202,0.839124773880015
"4461","KIAA0355",35.7600119559989,0.287492054332084,0.257638753031789
,1.11587271304877,0.264476623433052,0.839495401885681
"4462","FMC1",56.4807732855485,-0.292579062436177,0.262272329221947,-1
.11555444413117,0.264612901435079,0.839739732030641
"4463","TMEM204",59.1390099417256,0.305983061494826,0.274324652943591,
1.11540489785198,0.264676951632084,0.839754791644702
"4464","DNAJC13",41.0706224118874,0.272393930086581,0.244344099741843,
1.1147964300115,0.264937666660663,0.839851049352611
"4465","GNRH1",2.32694897297085,1.02410299383404,0.918325711397979,1.1
1518492962049,0.264771182744658,0.839851049352611
"4466","THAP1",24.9363124822354,-0.34161147632561,0.306377543632085,-1
.11500168150651,0.264849701157538,0.839851049352611
"4467","NOC4L",23.33052972694,-0.372042132859013,0.333735803095541,-1.
11478040236667,0.264944536543652,0.839851049352611
"4468","SMARCB1",71.9459668286591,-0.300312199825742,0.269440120577872
,-1.11457862764334,0.265031033158737,0.839937204460098
"4469","OSBPL9",75.0682161551443,0.225536046687232,0.202553945797268,1
.11346163018205,0.265510218646651,0.840045048650282
"4470","TNNI3K",3.18708955776258,0.889702681568422,0.798921549486851,1
.11362959497072,0.265438124626816,0.840045048650282
"4471","PCBP1",330.974412687493,-0.14033546294116,0.125953400015805,-1
.11418558707863,0.265199577308682,0.840045048650282
"4472","CSNK1G3",74.0743986667796,0.258904014568818,0.232372491996025,
1.11417669253746,0.265203392331244,0.840045048650282
"4473","FAM185A",6.14955126317338,0.6651963312527,0.597449695195564,1.
1133930381117,0.265539663683521,0.840045048650282
"4474","PLBD1",132.153273704945,0.289025792841454,0.259573196324272,1.
11346547692231,0.265508567393954,0.840045048650282
"4475","SPATA20",38.5184565291441,-0.299886316801205,0.269244239657109
,-1.11380773524856,0.265361677807034,0.840045048650282
"4476","PDE4C",1.55237534128936,3.62646726410355,3.25708878498047,1.11
340755610544,0.265533431246919,0.840045048650282
"4477","PCDHGA5",8.38707188813252,0.570899328198169,0.512879631865931,
1.11312536651369,0.26565459060614,0.840055995980167
"4478","PDZD8",54.37712180803,-0.333716506188569,0.299843084860485,-1.
11297049369621,0.265721102118303,0.840055995980167

"4479","NIPA1",7.31912196158783,0.808540881403717,0.726431954054987,1.11303044544006,0.265695353947335,0.840055995980167
"4480","CDK11A",35.4463730349338,-0.355925780245399,0.321326145774588,-1.10767761953328,0.268001068235823,0.840619238248545
"4481","IPO13",11.309781043883,0.440487837716713,0.398129609512292,1.10639306193857,0.268556427203801,0.840619238248545
"4482","NRDC",79.1926395974529,0.224763935353438,0.203539800720549,1.10427510765832,0.269473817412239,0.840619238248545
"4483","ATG4C",20.9633637023626,0.361568550074992,0.326465493362847,1.1075245544347,0.268067202162326,0.840619238248545
"4484","S100A11",2198.25717258693,-0.310140834577179,0.281406730996269,-1.10210879988258,0.270414374134703,0.840619238248545
"4485","IL10",3.12603308303735,-0.983404442021982,0.891648448184468,-1.10290602089236,0.270067979287763,0.840619238248545
"4486","OPN3",7.90370736513349,-0.574553441522768,0.5202956589589,-1.10428259707651,0.269470569592663,0.840619238248545
"4487","FAIM",47.1973217957172,-0.260315165720802,0.23571744255044,-1.10435257953004,0.26944022268794,0.840619238248545
"4488","GK5",29.9944069843429,0.47106895409069,0.425529260916881,1.10701894641907,0.268285736899376,0.840619238248545
"4489","PCOLCE2",348.433911368876,0.428478437709112,0.389070461429292,1.10128750492919,0.270771547596061,0.840619238248545
"4490","HLTF",52.0928787334211,0.28461503780662,0.257169364492298,1.10672217263711,0.268414065940861,0.840619238248545
"4491","MBNL1-AS1",4.90164970002238,-0.708543633767005,0.638345589801736,-1.10996871457524,0.267012508119797,0.840619238248545
"4492","DCUN1D1",46.0990420804473,0.276901924901699,0.249275298677177,1.11082777303298,0.266642489437332,0.840619238248545
"4493","MASP1",30.6979313813041,0.544888135098297,0.494518408757191,1.10185611991208,0.270524227861693,0.840619238248545
"4494","SNHG8",198.831036418155,-0.273488416006643,0.248398367799318,-1.10100729899963,0.270893480465546,0.840619238248545
"4495","C4orf3",342.823915335581,-0.236052221703679,0.214276982420449,-1.10162192428351,0.270626072754491,0.840619238248545
"4496","FAM198B-AS1",7.48421787893756,0.785123514880135,0.710436759358444,1.10512794353312,0.269104153945161,0.840619238248545
"4497","NKD2",3.25692336559288,1.16159420824093,1.05193019715846,1.10425027380971,0.269484586913567,0.840619238248545
"4498","IPO11",10.2791494512808,0.497924462041789,0.449137284336429,1.10862419889598,0.267592334368625,0.840619238248545
"4499","VDAC1",175.717626666452,-0.202623586902331,0.183310244359095,-1.10535877365043,0.269004160041169,0.840619238248545
"4500","FGFR4",2.90154941183395,3.51273520316681,3.17500836604385,1.10637037707834,0.268566241755432,0.840619238248545
"4501","CFB",213.11186572072,-0.344796119055019,0.311388136710725,-1.10728726757927,0.268169747445874,0.840619238248545
"4502","HOXA10",68.3564564038078,0.216413233041624,0.19466452678773,1.11172403422844,0.266256822757945,0.840619238248545

"4503","MAGI2-
AS3",161.378650856739,0.230753643145223,0.209132277540646,1.1033860763
094,0.269859540670438,0.840619238248545
"4504","TMEM225B",3.9634081248221,-0.787774461780436,0.710438927621434
,-1.10885599191182,0.267492311269692,0.840619238248545
"4505","MAP11",5.02996690821313,0.745510420401023,0.67038777921935,1.1
1205848840077,0.266113003490131,0.840619238248545
"4506","MEPCE",30.1691372226713,0.299829790352078,0.27168501048868,1.1
0359342170838,0.269769546055777,0.840619238248545
"4507","BCAP29",122.954139386176,-0.194975454453514,0.176936108047501,
-1.10195401382498,0.270481664423342,0.840619238248545
"4508","FAM71F2",3.38067816837654,0.982449371563977,0.892486741472588,
1.10079996252152,0.270983728084792,0.840619238248545
"4509","AC005229.4",2.77408916574481,-0.888672940024162,0.805688152844
375,-1.10299864393788,0.270027754045884,0.840619238248545
"4510","NAPRT",37.0775186715572,0.342692667291052,0.308382575107553,1.
11125820637412,0.266457223395128,0.840619238248545
"4511","MLLT3",25.5826683872884,0.357789653038058,0.323624101062825,1.
10557171688706,0.268911937223599,0.840619238248545
"4512","FOCAD",18.0199492461785,0.397387995667423,0.358455105316836,1.
1086130167296,0.267597160336259,0.840619238248545
"4513","TOMM5",203.166954367922,-0.26970072330722,0.242967605490811,-1
.11002749836714,0.266987177171063,0.840619238248545
"4514","RC3H2",36.8846604998313,0.296198386730948,0.268569595611767,1.
10287386052113,0.270081947169388,0.840619238248545
"4515","SKIDA1",1.70389793436692,1.24927831573592,1.13287223250006,1.1
0275305537234,0.270134419646833,0.840619238248545
"4516","ITGB1",621.972519310335,0.170046017959276,0.153542295044417,1.
10748649360806,0.26808364861787,0.840619238248545
"4517","SEC24C",19.8012594608372,0.374274193652821,0.338996578496805,1
.10406481184101,0.269565024096898,0.840619238248545
"4518","FRAT1",4.45949267622998,-0.734195835832982,0.663793252443072,-
1.10606101091084,0.268700112889454,0.840619238248545
"4519","HPS6",8.92993793512401,-0.481395432249852,0.436223920138387,-1
.10355120392558,0.269787868270596,0.840619238248545
"4520","AS3MT",6.8292527156137,0.638061954947446,0.575731585927651,1.1
0826289636231,0.267748294553611,0.840619238248545
"4521","PLEKHA1",55.6651476034841,-0.251733490419651,0.227238131552372
,-1.10779598784738,0.267949933230234,0.840619238248545
"4522","LINC00294",13.3387045280227,0.550514631644172,0.49491065217593
9,1.11235155118154,0.265987026975211,0.840619238248545
"4523","DDB2",36.1570764003351,0.278629498331371,0.252901494789226,1.1
0173132255935,0.270578495343675,0.840619238248545
"4524","DDB1",85.7753719843311,0.197544437074502,0.178278835722784,1.1
0806443329973,0.267833989880365,0.840619238248545
"4525","NINJ2",35.6215120775043,0.407629122359722,0.369836420154605,1.
10218761632323,0.270380114599574,0.840619238248545
"4526","C1R",2223.61683408339,0.204553922873285,0.184750127035297,1.10
719232595821,0.268210784722975,0.840619238248545
"4527","PFDN5",1221.08693700165,-0.294153934862181,0.264958364383627,-

1.11018927651698,0.266917472712552,0.840619238248545
"4528","PHLDA1",153.736415022385,-0.772086618909567,0.6979352033721,-1
.10624398250611,0.268620930580793,0.840619238248545
"4529","SLC41A2",27.8837534939484,-0.303551875495423,0.274600472103455
,-1.10543100370585,0.268972875754231,0.840619238248545
"4530","BRAP",16.8909233715227,0.370265292958182,0.336213675938199,1.1
0127969043782,0.270774947597119,0.840619238248545
"4531","ZNF891",12.3738935132498,0.517086285415896,0.465095949778065,1
.11178410747855,0.266230986606156,0.840619238248545
"4532","ZMYM5",36.3609412792154,0.270284617999966,0.244627726691518,1.
10488137078918,0.269210995619433,0.840619238248545
"4533","AKAP11",56.1738313883242,0.239393872328682,0.217181665893594,1
.10227477694167,0.270342231524384,0.840619238248545
"4534","MCF2L",5.87377253243107,0.648148545041335,0.585143044899797,1.
10767538073075,0.268002035461115,0.840619238248545
"4535","GPR135",4.76339197948527,0.749618206930538,0.678084040204633,1
.10549454416346,0.268945357171672,0.840619238248545
"4536","EXOC3L4",10.7343924911573,0.490260848729888,0.444549072120491,
1.10282729056514,0.270102174295154,0.840619238248545
"4537","SLC30A4",13.3292193100489,0.435785807547664,0.392423957662893,
1.11049745826686,0.266784722779279,0.840619238248545
"4538","HACD3",96.7385660875263,0.241143603290464,0.217259726771642,1.
10993236930619,0.267028170749323,0.840619238248545
"4539","SENP8",2.60429050378498,0.97551939117383,0.880966056922108,1.1
0732914566773,0.268151647559566,0.840619238248545
"4540","ANPEP",111.288089186033,0.203914313129049,0.183612631008186,1.
11056800400599,0.266754341443799,0.840619238248545
"4541","RAB40C",8.9969669913817,-0.531219039861704,0.479713412262682,-
1.10736749543041,0.26813507336676,0.840619238248545
"4542","STUB1",217.528047497572,-0.265640908327833,0.239864371342794,-
1.10746296684555,0.268093815108825,0.840619238248545
"4543","JMJD8",63.957411810526,-0.241965990056097,0.219544638160421,-1
.10212662027889,0.270406627793841,0.840619238248545
"4544","AC092140.1",1.72179013784875,-1.27582908577034,1.1559090999219
2,-1.10374516980316,0.269703695519949,0.840619238248545
"4545","KIAA0513",7.05326566209878,0.589267884227091,0.532293677659598
,1.1070352870205,0.268278672224295,0.840619238248545
"4546","AC005562.1",19.8721106248581,-0.358898337996728,0.325232480469
487,-1.10351320839371,0.269804358788658,0.840619238248545
"4547","RARA",60.7287717348997,-0.24417998185503,0.221306312748671,-1.
1033575085241,0.269871941627922,0.840619238248545
"4548","CHMP6",30.0409545591982,-0.412640415020807,0.372771129891162,-
1.10695378996031,0.268313907831572,0.840619238248545
"4549","SLC16A3",2.89994761803494,-0.913758567520302,0.825847600852842
,-1.10644938191583,0.268532061562529,0.840619238248545
"4550","RAB12",34.677824233135,-0.393508117891871,0.355656763978464,-1
.10642663867824,0.268541900758179,0.840619238248545
"4551","SHFL",66.5090406311008,0.259580316994371,0.234718149569673,1.1
0592349790708,0.268759633205449,0.840619238248545
"4552","PPAN",16.8564409700987,-0.419896456670271,0.379446872375493,-1

.10660144341564,0.268466282969852,0.840619238248545
"4553","ELL",16.4744843492612,0.452721292693588,0.409046248546897,1.10
677287544341,0.268392138368286,0.840619238248545
"4554","ITPKC",43.033114668242,-0.385898302322748,0.350500666243529,-1
.10099163707331,0.270900296933383,0.840619238248545
"4555","PRNP",212.605258087282,0.173154813368903,0.156932517331341,1.1
0337115795646,0.269866016512565,0.840619238248545
"4556","MYL9",369.711577722807,0.361721485931513,0.328613139024086,1.1
0075174415044,0.271004719110495,0.840619238248545
"4557","CD40",69.1532742017431,-0.290186673806393,0.262808576552209,-1
.10417505247872,0.269517209363259,0.840619238248545
"4558","STAU1",134.2650650178,-0.185772098001556,0.167698556932499,-1.
10777398088364,0.26795943971144,0.840619238248545
"4559","ITGB2",9.59579218826354,-0.615494875576897,0.558330612429309,-
1.10238425383639,0.270294654165847,0.840619238248545
"4560","PLCXD1",4.17000470173054,0.83318145689011,0.755149522913897,1.
10333309047871,0.269882541539367,0.840619238248545
"4561","PUDP",9.50565956437246,0.492833233364222,0.444719642156864,1.1
0818859039823,0.26778037727841,0.840619238248545
"4562","EIF2S3",196.407213510377,-0.19858504748922,0.180333093153674,-
1.10121245089493,0.270804204007377,0.840619238248545
"4563","MAGEH1",91.4604689228075,-0.298844228375082,0.270331923118004,
-1.10547146977027,0.268955350181096,0.840619238248545
"4564","MCTS1",97.6833645530189,-0.279501485191933,0.251893870384342,-
1.10960018505201,0.267171351504014,0.840619238248545
"4565","TMEM185A",34.2435071700171,0.352174247093673,0.31890109815831,
1.10433689042628,0.269447025842728,0.840619238248545
"4566","CAMSAP2",43.6209948575191,0.312027395163357,0.283943196850478,
1.0989078048863,0.271808281940203,0.840688567317613
"4567","CEP19",11.0418845552249,-0.49438856638499,0.450195986161643,-1
.09816298141645,0.272133327671529,0.840688567317613
"4568","DDX60",24.0649650635724,0.376444217528555,0.342668592063021,1.
09856644655464,0.271957219888403,0.840688567317613
"4569","STOX2",1.64274747209202,1.21155081922921,1.10123542249135,1.10
01742175059,0.2712562218402,0.840688567317613
"4570","RIOK2",30.4325857883832,-0.322815182830055,0.293943882325467,-
1.09822045036685,0.272108238384742,0.840688567317613
"4571","C5orf24",90.3074086178028,0.246758565877341,0.224229491576502,
1.10047328806948,0.271125961897707,0.840688567317613
"4572","USP42",23.4749963294017,0.344117077869807,0.313197633550605,1.
09872183250135,0.271889416567425,0.840688567317613
"4573","GNAI1",49.2889598232074,-0.292795677862598,0.266321379162319,-
1.09940733554156,0.27159043255389,0.840688567317613
"4574","PTCD1",7.40420740336658,0.557233491759626,0.507204120383346,1.
09863754919512,0.271926192511328,0.840688567317613
"4575","AP1S1",66.1924199106076,-0.321023916479155,0.292164037956866,-
1.09877970856409,0.271864165058975,0.840688567317613
"4576","RRM2B",21.0498348517236,0.390602659139077,0.355318979094296,1.
09930142244222,0.271636612127124,0.840688567317613
"4577","HDAC7",110.72164930505,0.292950888422343,0.266676474040185,1.0

9852543039923,0.271975119393807,0.840688567317613
"4578","PARPBP",6.14945363473336,0.627402125483212,0.570379464550459,1
.09997320113496,0.271343798447051,0.840688567317613
"4579","FGF7",564.76197273851,0.213785937418782,0.194648401120178,1.09
831848701798,0.272065442064025,0.840688567317613
"4580","AC090517.2",2.30032478401532,-0.999233348237025,0.909318104987
534,-1.09888205541747,0.271819514735063,0.840688567317613
"4581","RPL13",9215.13934272327,-0.25482792581659,0.231638382886903,-1
.10011096883287,0.271283775240013,0.840688567317613
"4582","TEN1",34.8145049758675,-0.434935690526075,0.396075501356595,-1
.09811308459216,0.272155112470617,0.840688567317613
"4583","RAB4B",15.8392899904898,-0.427943365393671,0.389336594224639,-
1.09916039679218,0.271698109603544,0.840688567317613
"4584","NF2",33.0322866499581,0.299421401332809,0.272571627936641,1.09
850538590322,0.271983867132212,0.840688567317613
"4585","MFF",93.0317161943103,-0.228369591100191,0.208102330433974,-1.
09739083951608,0.272470576129186,0.841020134087486
"4586","NCBP2AS2",57.7156888703734,-0.292992769309847,0.26703982656052
6,-1.09718753597017,0.272559420573974,0.841020134087486
"4587","HOXC4",15.8062367593951,-0.482004756699386,0.439289078079486,-
1.09723819860636,0.272537278949676,0.841020134087486
"4588","AL161421.1",4.38430036731118,-0.757891063751489,0.690725794127
826,-1.09723868747145,0.272537065301827,0.841020134087486
"4589","CCDC102A",38.3033453895244,-0.353362475211886,0.32205593200781
1,-1.09720840417035,0.272550300176076,0.841020134087486
"4590","UBA52",2206.94904353764,-0.242665191879343,0.221202287374864,-
1.09702840218875,0.272628976487531,0.841051483020356
"4591","POLD2",66.8472039687986,-0.293304675430861,0.267413821837326,-
1.09681942921142,0.272720335099957,0.841150064259505
"4592","PLAC9",2219.14655232257,-0.252021951834513,0.229832768848407,-
1.09654490566026,0.27284038287797,0.841153891041161
"4593","SLPI",50.2303373391836,0.505658721069654,0.461131515900042,1.0
9656075031589,0.272833453106373,0.841153891041161
"4594","TANK",104.010579542941,0.174578984024635,0.159275994620305,1.0
9607844195738,0.273044448009698,0.841416623246425
"4595","TSPYL1",84.085512738073,-0.274244485641477,0.250202704503347,-
1.09608921368717,0.273039734494959,0.841416623246425
"4596","CCDC125",7.61433745318802,0.622998174499343,0.568611667339758,
1.09564789166925,0.273232894621276,0.841538295515109
"4597","PDE9A",7.22536779107807,-0.538272390328134,0.491306059780214,-
1.09559485296992,0.273256115164595,0.841538295515109
"4598","TRAPPC10",24.5681350524541,0.355572388163818,0.324591679954802
,1.09544517041635,0.273321654030649,0.841538295515109
"4599","RPS4X",6273.76084317652,-0.289663881946698,0.264403676168106,-
1.09553651501627,0.273281657299836,0.841538295515109
"4600","LUZP1",74.5959568504613,-0.300669601403858,0.27474950882528,-1
.09434081498235,0.273805530889671,0.841949924033371
"4601","CLK1",124.075819366754,0.252355348351001,0.230562233634432,1.0
9452161515369,0.27372627266402,0.841949924033371
"4602","TTLL3",55.5353938684819,0.258768870930464,0.236372083190425,1.

0947522543176,0.27362518904691,0.841949924033371
"4603","PRSS23",615.741850296838,-0.286501391357798,0.261708323283802,-1.09473549699491,0.273632532520036,0.841949924033371
"4604","ALG11",11.2205888179554,0.476416350433815,0.435259539951835,1.09455694063945,0.273710788695321,0.841949924033371
"4605","REPS2",8.62451547916443,0.555945801511046,0.508025846407538,1.09432582110215,0.273812104532039,0.841949924033371
"4606","SDHAF1",43.1097909981244,-0.272795236307986,0.249325427657394,-1.0941332333052,0.273896548790744,0.842026732713188
"4607","CCNC",50.2603530806213,-0.272433047597019,0.24904024859582,-1.09393180071533,0.273984890279392,0.842115486511003
"4608","AC023481.1",1.70802187699343,1.13401202123862,1.03727449801952,1.09326125669127,0.274279108378597,0.842306718322151
"4609","PIGZ",14.7651287836656,0.392180440310194,0.358596405711343,1.09365413056004,0.274106698889051,0.842306718322151
"4610","MAPK13",10.1983072335853,0.54176320785543,0.495530036863107,1.09330044104893,0.274261909317962,0.842306718322151
"4611","CWF19L2",83.5120176731248,0.27659441358562,0.25300250548596,1.0932477251731,0.274285047894311,0.842306718322151
"4612","SIMC1",9.31034665165558,-0.509053216932319,0.465720308938445,-1.09304491808108,0.274374078433606,0.842397430750187
"4613","TYSND1",20.7590939804278,-0.374529084402585,0.342809258865528,-1.09252908057976,0.274600615534536,0.842850764924493
"4614","POLR3B",8.95722821546366,0.493764127492214,0.451988072068541,1.09242734046605,0.274645311175722,0.842850764924493
"4615","SLC39A9",32.7545393848006,0.320919405104865,0.293800937914153,1.09230218046015,0.274700302268823,0.842850764924493
"4616","PSMD14",74.0763707224778,-0.233729616218126,0.214118012807314,-1.09159249683706,0.275012255546523,0.842905927980496
"4617","AC093297.2",6.85601397887076,0.585830509934731,0.536666647243573,1.09160968534876,0.275004697192744,0.842905927980496
"4618","GSR",14.5217758918654,0.41653084148436,0.381583805381735,1.0915841700034,0.275015917180077,0.842905927980496
"4619","CRACR2A",7.2536007086545,0.588700379399954,0.539252451584851,1.09169717758311,0.274966226178563,0.842905927980496
"4620","TPT1-AS1",53.0088494456133,0.295682726449122,0.270781458192665,1.09196075840148,0.274850349856607,0.842905927980496
"4621","FBXO6",16.8708942758942,0.386292999000428,0.353992887174569,1.09124508710801,0.275165053428821,0.843180514293899
"4622","TAF9",67.2783640023281,-0.257443910796351,0.2360561018366,-1.09060477061744,0.275446829482918,0.843451571737785
"4623","ITK",2.25465419869142,1.11668472543148,1.02398688213557,1.09052639727433,0.275481331778456,0.843451571737785
"4624","RBM4B",13.6917845535809,0.398079055794499,0.365041795447219,1.09050267875985,0.275491773960964,0.843451571737785
"4625","STXBP2",1.82825510827642,-1.37783037184488,1.26336340742917,-1.09060493896102,0.275446755376198,0.843451571737785
"4626","NFIA",881.825823257219,0.220343674355784,0.20217688827477,1.0898558991388,0.275776625699566,0.844141162971434

"4627","TARBP1",21.1922281390783,0.402761397941015,0.369684070547302,1
.08947458121402,0.275944658122778,0.844407077006341
"4628","CLN5",75.79841067398,-0.218050900639801,0.200159057420464,-1.0
8938812687228,0.275982764998965,0.844407077006341
"4629","BRD3OS",17.5816657407031,-0.348682041683575,0.320141930403605,
-1.08914830757717,0.276088490035188,0.844548070619628
"4630","TEX26-
AS1",5.37108777535051,-0.916979666698889,0.842187258393275,-1.08880733
775087,0.276238855127647,0.844643098382096
"4631","ZNF816",3.70065071375733,0.768605981496489,0.705913226097178,1
.08881085249801,0.276237304866174,0.844643098382096
"4632","MRPL43",137.312702791825,-0.223707188972511,0.205512027080544,
-1.0885357521428,0.276358662256047,0.844826998606568
"4633","GTF2H4",16.6827227527966,-0.387456640411585,0.356038606976251,
-1.08824333322209,0.276487699384934,0.844856673131347
"4634","GOLGA8B",14.6044870880472,-0.563935940164725,0.518186063741749
,-1.08828851183805,0.276467760513498,0.844856673131347
"4635","AC012360.3",2.5315471016935,-1.06460008424658,0.97869794657936
1,-1.0877718584855,0.276695835841221,0.845310255773826
"4636","ANKRD9",25.3930768424487,0.381237060778789,0.350797571189928,1
.086772235867,0.27713748076922,0.846476861020741
"4637","UBE2J2",61.1825933160725,-0.263936488127499,0.243135651004023,
-1.08555239446613,0.277677071259774,0.846482780888752
"4638","TMEM53",8.48381657933389,-0.611884998076467,0.564509577439385,
-1.08392314768507,0.278398875336641,0.846482780888752
"4639","FAF1",36.0180531018105,-0.269995632094437,0.24946100837468,-1.
08231596534283,0.27911215423514,0.846482780888752
"4640","SHISA4",33.4874585031831,-0.290896605686554,0.268369404439249,
-1.08394101888915,0.278390950949585,0.846482780888752
"4641","FLVCR1-
DT",6.38590109527513,-0.630154141059892,0.581653629608172,-1.083383837
01722,0.278638086810079,0.846482780888752
"4642","ASB1",24.144535632945,0.324738245578057,0.299107917120334,1.08
568923452271,0.277616505171039,0.846482780888752
"4643","RPL29",3739.73192797715,-0.306182553963411,0.282146548007447,-
1.08518979277156,0.277837604080893,0.846482780888752
"4644","TRIM61",4.0415177191699,0.830233336250295,0.766965190720181,1.
08249154758993,0.279034169174418,0.846482780888752
"4645","SKP1",592.861422745983,-0.204466076503163,0.18875135521755,-1.
08325620373693,0.278694719063335,0.846482780888752
"4646","C7orf26",20.2241099236829,0.347367388733264,0.320641989605599,
1.08334965473654,0.278653253086328,0.846482780888752
"4647","BBS9",18.867259914724,0.353763766575095,0.326448734650609,1.08
367326635136,0.278509692926067,0.846482780888752
"4648","ZSCAN21",14.3789854029766,0.454381948454405,0.419544989169672,
1.08303509798479,0.278792844585815,0.846482780888752
"4649","ERMP1",4.9980965024986,0.691428805349089,0.636967420487014,1.0
8550105250349,0.277699797788686,0.846482780888752
"4650","EDF1",601.431557795608,-0.292855029344671,0.270007553796541,-1
.0846179124505,0.278090918177081,0.846482780888752

"4651","SVIL",86.067119589728,-0.276362469811412,0.254532569831744,-1.
08576466262883,0.277583124163232,0.846482780888752
"4652","ATP5MD",283.966682133415,-0.290765932894672,0.268642404778689,
-1.08235307502629,0.279095670690546,0.846482780888752
"4653","TMEM9B",78.6570600396295,-0.216221115811136,0.199503561134086,
-1.0837957707723,0.278455360809754,0.846482780888752
"4654","BIRC2",92.0153196545255,0.230322412513351,0.212229939601025,1.
08524939010178,0.277811214516476,0.846482780888752
"4655","CACNA2D4",3.5116762066401,0.838841323254214,0.77321548482129,1
.08487393193903,0.277977495044826,0.846482780888752
"4656","DDX23",44.2288448681956,0.262077011314827,0.241215372065543,1.
08648552980121,0.277264239435531,0.846482780888752
"4657","WSB2",34.7340448362172,-0.286308607310881,0.263974488988888,-1
.08460710884427,0.278095705141546,0.846482780888752
"4658","TP53BP1",44.0167049743068,0.279115171127944,0.257697227258354,
1.08311282235147,0.278758348258853,0.846482780888752
"4659","SEC11C",9.35011041259412,-0.489112174936543,0.451898980566453,
-1.08234848045783,0.279097711490533,0.846482780888752
"4660","ZNF763",3.59517762270435,0.81062910199638,0.746968993043895,1.
08522456694363,0.277822205947941,0.846482780888752
"4661","ZNF443",2.08179779521693,1.09071310021241,1.00728171776646,1.0
828282505027,0.278884663642231,0.846482780888752
"4662","ZNF524",41.8388918045811,-0.382207418467127,0.351987869197352,
-1.08585395098611,0.27754361277959,0.846482780888752
"4663","ZNF835",4.75077289293479,0.679206104347729,0.627436543705796,1
.08250963569347,0.279026136164106,0.846482780888752
"4664","DOK5",19.4030184140077,-0.47044682225975,0.434162527932277,-1.
08357306767187,0.27855413755289,0.846482780888752
"4665","PISD",14.7066763057038,0.388746982221655,0.358982508746846,1.0
8291343658696,0.278846847271171,0.846482780888752
"4666","CYTH4",1.67464374658288,-1.1904137052782,1.09667922514131,-1.0
8547119156453,0.277713016321208,0.846482780888752
"4667","APOBEC3C",71.8036372008518,0.250610017969989,0.231128841536684
,1.08428708552244,0.278237529555162,0.846482780888752
"4668","RPGR",19.8170930977449,-0.42459843899473,0.391244811538617,-1.
08525001858797,0.277810936233293,0.846482780888752
"4669","MT-
ND3",12869.8944470408,-0.406887261806863,0.375138126930272,-1.08463318
601176,0.278084150718193,0.846482780888752
"4670","DDX19A",28.343373069251,0.327688967433488,0.302976406239934,1.
08156595921196,0.279445437211976,0.847312075143808
"4671","EEF2",1125.72627828792,-0.142988033898058,0.132231838374718,-1
.08134346202507,0.279544361121159,0.847430561651811
"4672","CFAP97",118.104921536361,-0.201670092157143,0.186533164347992,
-1.0811487215266,0.279630963734075,0.847511653783068
"4673","AL133243.2",4.29031297960496,0.725205217938142,0.6708899130463
55,1.08096008575409,0.279714868914934,0.847584537520964
"4674","C12orf57",585.366903153534,-0.268649964484675,0.24866421642027
3,-1.08037243296247,0.279976366526734,0.848013978613594
"4675","GYS1",6.22699787489274,0.615339767632403,0.569561798854201,1.0

8037401537514,0.279975662151323,0.848013978613594
"4676","RMND5B",28.938476652272,-0.3220994298642,0.298254811354876,-1.07994713782153,0.280165720747475,0.848043310342933
"4677","RBM12B",25.760722565575,0.389937759426902,0.361031079948181,1.08006701108079,0.280112340754157,0.848043310342933
"4678","DCAF13",48.3136617312615,-0.292122680333391,0.270457170978622,-1.08010698801727,0.280094540417261,0.848043310342933
"4679","MARCKSL1",102.499737999625,-0.350579185526031,0.324716272247676,-1.07964772784355,0.280299079275133,0.848246756892705
"4680","SPCS2",383.981663518534,-0.234485507851898,0.217211286172613,-1.07952727495733,0.280352741684877,0.848246756892705
"4681","DPH3",40.5258563892717,-0.268005204110096,0.248354691300691,-1.07912277680961,0.280532998854257,0.848429573638676
"4682","TARS",60.742734573993,0.229253944387332,0.212424996121336,1.07922301317301,0.280488323025588,0.848429573638676
"4683","RPL22",2143.13978499092,-0.26530673516457,0.246150051582015,-1.07782522676487,0.281111759359565,0.848454011279475
"4684","MTFR1L",49.0094464640865,-0.249042751498814,0.230983556988952,-1.07818389648717,0.280951696965486,0.848454011279475
"4685","KCMF1",66.7336088275569,-0.249296536638708,0.231481251767675,-1.07696210701726,0.281497194732609,0.848454011279475
"4686","TBC1D8",4.10212338516292,0.859311108998404,0.798420726675398,1.07626352910019,0.281809414646644,0.848454011279475
"4687","LINC02478",1.71294843187391,-1.34335750551015,1.24871242583154,-1.0757941362004,0.282019335334316,0.848454011279475
"4688","TNS1",146.717365354051,-0.267376152147188,0.248091811474035,-1.07773066171985,0.281153970910553,0.848454011279475
"4689","FARP2",19.1396300742684,0.419222052750034,0.389294038456808,1.07687765888186,0.281534925202725,0.848454011279475
"4690","RPN1",123.020233245685,0.202440636562163,0.187800120761832,1.07795796797648,0.281052514148253,0.848454011279475
"4691","XRN1",70.439431907574,0.301232503239957,0.280052951606443,1.07562695380293,0.282094127824105,0.848454011279475
"4692","ACOX3",14.313098774999,0.445670895517654,0.414366656554786,1.07554719586548,0.28212981391976,0.848454011279475
"4693","PPWD1",37.3211337245547,0.266472558524829,0.247535010190243,1.07650452483482,0.281701678344389,0.848454011279475
"4694","BAG6",60.1824559929227,-0.224921119121125,0.208533146827139,-1.07858689394627,0.280771926404771,0.848454011279475
"4695","MTRF1L",32.558142638505,-0.375262910883035,0.34882787848241,-1.07578245327074,0.282024561496458,0.848454011279475
"4696","TOP1MT",10.2113645765949,-0.511731179415607,0.47457271967915,-1.07829876896755,0.280900446274096,0.848454011279475
"4697","AL162231.1",15.7227365529651,-0.487337732940591,0.452272700416931,-1.07753072978169,0.281243229859939,0.848454011279475
"4698","SEC31B",5.28944994486024,0.618791281509959,0.574678348840834,1.07676108340971,0.281587015440753,0.848454011279475
"4699","PICALM",69.4235670665531,0.200100436499186,0.185996207076971,1.07583073678689,0.282002963102582,0.848454011279475
"4700","MAML2",89.9461095838595,0.250803229884699,0.232819993786972,1.

07724094398087,0.281372637905929,0.848454011279475
"4701","CRY1",17.947200328562,-0.362720237626718,0.337275016454285,-1.
07544353993348,0.282176197270407,0.848454011279475
"4702","ITGBL1",86.8397480367247,-0.411753399456987,0.382002985940784,
-1.07788005489783,0.2810872873762,0.848454011279475
"4703","PPP1R13B",1.93495290167878,1.03710051801655,0.961853012448227,
1.07823181358739,0.28093031784009,0.848454011279475
"4704","MTHFSD",10.0207129464091,0.466718171494358,0.433770186219922,1
.07595723800559,0.281946381343618,0.848454011279475
"4705","WDR81",23.1677446705689,0.364533570161699,0.33869880277696,1.0
7627652407662,0.281803604574917,0.848454011279475
"4706","SLFN5",134.731989669113,0.276697255667642,0.256595258988932,1.
07834126303782,0.280881489029441,0.848454011279475
"4707","DNAJC7",99.3784145442112,-0.182748513196927,0.169532220095613,
-1.07795741183511,0.281052762348232,0.848454011279475
"4708","SMARCD2",81.2623659036235,-0.241168305709176,0.224269943627154
,-1.07534831377189,0.282218813073893,0.848454011279475
"4709","PECAM1",2.39822195526354,-1.10816761194578,1.02912138958854,-1
.076809425163,0.281565413761603,0.848454011279475
"4710","TOMM22",92.6558781883143,-0.241479469451811,0.224114931142169,
-1.07748050619004,0.281265655037781,0.848454011279475
"4711","NBPF12",22.8443765170235,0.456149738052257,0.42488614792662,1.
07358109996807,0.283010472219524,0.848641546816936
"4712","ANKRD36B",26.5934447997959,-0.384342235288892,0.35783602915294
5,-1.07407360907366,0.282789691717557,0.848641546816936
"4713","NPAS2",14.8934396834224,0.564178969715532,0.526023871052241,1.
07253491859023,0.283479838731929,0.848641546816936
"4714","MANBA",27.4597422723128,0.385948228525853,0.35953888504075,1.0
7345337203822,0.283067748779994,0.848641546816936
"4715","BUD23",67.0846409463582,-0.226459048837634,0.211026326382987,-
1.07313174009691,0.283212011783204,0.848641546816936
"4716","PSMD5",32.3587560237385,0.287247653606148,0.267765848486638,1.
07275687033884,0.283380216629705,0.848641546816936
"4717","SGPL1",19.1223519153001,0.399646501298806,0.372377577635847,1.
07322923102966,0.283168278482302,0.848641546816936
"4718","EIF5AL1",6.3500125537998,-0.648943988214698,0.604531430968171,
-1.07346608459282,0.283062047784487,0.848641546816936
"4719","PLCB3",15.0962295380173,-0.410787162794596,0.382933025413874,-
1.07273892699805,0.283388269541017,0.848641546816936
"4720","GAB2",17.2615579359551,0.41393402804994,0.385429596172886,1.07
395496391582,0.28284286693633,0.848641546816936
"4721","CLEC2D",11.7667405281694,0.459522884738717,0.427982635030209,1
.07369516220274,0.28295933035406,0.848641546816936
"4722","RHOT2",33.5517196355902,-0.272599399790329,0.253655498345428,-
1.07468358292436,0.282516416323948,0.848641546816936
"4723","IL4R",38.5981621089735,0.28055789589317,0.261566249973318,1.07
260740222329,0.28344730215222,0.848641546816936
"4724","WWP2",15.8907115490594,0.468238092724122,0.436181302622845,1.0
7349418672583,0.283049445547616,0.848641546816936
"4725","TAOK1",102.318652294433,0.263673481323189,0.245513734002571,1.

07396631962116,0.282837777163454,0.848641546816936
"4726","ZNF460",12.3142701218469,-0.493415488622946,0.459252321916494,
-1.07438866408751,0.282648521038048,0.848641546816936
"4727","SNAP25",2.01085051796125,-1.48711170884231,1.38620881077783,-1
.07279054734031,0.283365102922594,0.848641546816936
"4728","CCN5",1547.59131705169,0.266111482690456,0.248100353107747,1.0
7259614650724,0.283452354471783,0.848641546816936
"4729","BRWD1",63.1851017418027,0.243207976745557,0.226722120356748,1.
07271392999884,0.283399488369017,0.848641546816936
"4730","MCAT",16.6742941137105,-0.38422647791811,0.357717896242685,-1.
07410471199193,0.282775752914539,0.848641546816936
"4731","NOTCH2",147.355602088938,0.211556892718052,0.197803746335986,1
.06952925127467,0.284831255433788,0.848893002995484
"4732","SDCCAG8",67.3317413496164,0.227787643398721,0.212767839839061,
1.07059245218179,0.284352718735889,0.848893002995484
"4733","KIF3C",7.53185775868023,0.611968305674365,0.572461158553978,1.
06901279943635,0.28506390200125,0.848893002995484
"4734","IQSEC1",53.6039340933521,0.311181832634095,0.290879605996666,1
.06979597819471,0.284711153004425,0.848893002995484
"4735","ZNF595",15.2336708889971,0.408524773889807,0.381152940084861,1
.07181325637643,0.283803917693662,0.848893002995484
"4736","TFEC",1.56313634458045,-1.23521574499936,1.15317025579011,-1.0
7114776746738,0.284102992992866,0.848893002995484
"4737","FBP1",3.49925010288662,-0.881785299956859,0.824959853594191,-1
.06888268091482,0.285122536881817,0.848893002995484
"4738","ILK",150.722715747152,-0.216633860273452,0.202604009847053,-1.
06924764439258,0.284958095235311,0.848893002995484
"4739","PEX16",30.8072614774195,-0.355305783192286,0.331424325775804,-
1.07205704457746,0.283694410966343,0.848893002995484
"4740","C11orf24",37.7837551936592,-0.323685946811644,0.30270789103823
9,-1.06930131785284,0.284933916988267,0.848893002995484
"4741","FZD10",4.89323603328936,-0.695098447598213,0.649584227837665,-
1.07006669467954,0.284589289182536,0.848893002995484
"4742","NEDD8",351.037181979444,-0.244196771992595,0.228201585065541,-
1.07009235681891,0.284577739126589,0.848893002995484
"4743","STRN3",50.2907724942275,-0.254384414775181,0.23796376987814,-1
.0690048107132,0.285067501698252,0.848893002995484
"4744","AL161669.3",7.86244238341367,0.535077094092869,0.4996807333491
64,1.07083795387998,0.284242298105126,0.848893002995484
"4745","GOLGA8A",38.9482489560825,0.455706036767251,0.425779360750922,
1.0702868170114,0.284490226485907,0.848893002995484
"4746","RGMA",62.0831149525073,0.360627103833572,0.336959439331872,1.0
7023891228163,0.284511783291893,0.848893002995484
"4747","RPUSD1",11.0388280923567,-0.428685075677913,0.400664471625638,
-1.06993533501632,0.284648416712946,0.848893002995484
"4748","NME3",162.863112776734,-0.195083217940589,0.182503048163179,-1
.06893128582796,0.285100633255747,0.848893002995484
"4749","MINK1",24.1553907916877,0.326972425978329,0.305366739778837,1.
07075323990799,0.284280397088129,0.848893002995484
"4750","DLG4",14.2131190339145,-0.431859686361654,0.403008454258437,-1

.07158964482843,0.283904386473795,0.848893002995484
"4751","SCO1",34.2230065007017,-0.300838608095025,0.281050177003995,-1
.07040889033402,0.284435299308938,0.848893002995484
"4752","ANO8",6.99100467119688,0.595953749158125,0.557170220533538,1.0
696080429199,0.284795773379057,0.848893002995484
"4753","TBC1D20",43.9875737255241,0.256050139998903,0.238934297586413,
1.07163409600625,0.283884412620038,0.848893002995484
"4754","SBF1",18.0612309347408,0.364371089191054,0.340383824497041,1.0
7047122385871,0.28440725496169,0.848893002995484
"4755","CHKB-
DT",4.57372607581481,-0.752541709103513,0.702543649943888,-1.071167192
47782,0.284094260237787,0.848893002995484
"4756","ZMYM3",10.6564515817315,0.445859802888793,0.416252113408253,1.
07112922319628,0.284111329970151,0.848893002995484
"4757","PSPN",2.02078780613468,1.07753908827088,1.00846981289119,1.068
48918479937,0.285299906365057,0.849242521364138
"4758","FMO3",33.4136123597872,0.99293335638851,0.929630946031228,1.06
809412985608,0.285478053528187,0.849300510564007
"4759","NOSTRIN",8.80220202282748,0.575792190933624,0.539211195717314,
1.06784168338279,0.285591931808591,0.849300510564007
"4760","FRZB",25.464247941711,-0.565486648585894,0.529508146201565,-1.
06794702336956,0.285544409342912,0.849300510564007
"4761","MRPL22",80.402035669991,-0.279248549410516,0.261550060720444,-
1.0676676910008,0.285670437421803,0.849300510564007
"4762","RIPK2",34.1813075224554,-0.426658015544115,0.39962418366043,-1
.06764813789812,0.285679260721495,0.849300510564007
"4763","PAF1",47.6065925161571,0.281157130544973,0.263292254336435,1.0
678518866936,0.285587328512788,0.849300510564007
"4764","SLC25A10",3.30737860120849,-0.822931036135693,0.77096014003354
6,-1.06741061360174,0.285786457809316,0.849440856964717
"4765","MAP2K4",22.4690963140615,0.313819870215179,0.294044719710211,1
.06725218709745,0.285857972384588,0.8494751078627
"4766","FBXL4",28.1656517699062,0.401784782916905,0.376530032834945,1.
06707233920177,0.285939171347026,0.849538117136779
"4767","PKIA",5.39862216723843,-0.641063574017881,0.600870459530182,-1
.06689148026868,0.286020842495684,0.849602502567418
"4768","TRAF3IP2",25.900332700294,-0.329318169874284,0.308773145644533
,-1.06653760056389,0.286180690874114,0.849899031622789
"4769","TPRG1L",45.3111733544967,0.248536871055352,0.233460471142586,1
.0645779554842,0.287066959978375,0.850242021560169
"4770","CEP85",2.99420109321897,-0.943720688147881,0.886534394515696,-
1.06450544275095,0.287099790095864,0.850242021560169
"4771","SERBP1",419.306453953045,-0.136123063345973,0.127894204181294,
-1.06434114209752,0.287174186542236,0.850242021560169
"4772","SHC1",80.1662594735257,-0.238758375083189,0.224028296748774,-1
.0657509723021,0.286536229046299,0.850242021560169
"4773","AL445423.1",2.28587757382167,1.09088993033182,1.02480919411359
,1.06448101421982,0.287110850676449,0.850242021560169
"4774","TTN-
AS1",17.3866803761937,0.341974511746938,0.320999122487155,1.0653440704

0512,0.28672025650990l,0.850242021560169
"4775","PCGF3",31.2540733281405,0.312676662160275,0.293836971439316,1.
06411613429268,0.287276092707373,0.850242021560169
"4776","AC139887.2",10.2300767405273,0.519773015930412,0.4884640480862
85,1.0640967702061,0.287284863853807,0.850242021560169
"4777","SEC24B",25.1506325539435,0.333469133948961,0.313307595273241,1
.06435062213585,0.287169893563105,0.850242021560169
"4778","TLR3",30.8977711038883,0.319262209123541,0.300074606045106,1.0
6394277520288,0.287354623782984,0.850242021560169
"4779","C5orf63",3.71014910167415,0.733828707972162,0.688381500969941,
1.06602037814524,0.286414430129175,0.850242021560169
"4780","SUPT3H",12.933963342292,0.400554646091524,0.37648107935477,1.0
6394362972506,0.287354236651828,0.850242021560169
"4781","MSC-
AS1",33.6249810215665,0.290783875476349,0.273320503120365,1.0638933858
1121,0.287376999660097,0.850242021560169
"4782","PTP4A3",4.63053443063096,0.674697220926347,0.633275588815147,1
.06540854067768,0.286691093546189,0.850242021560169
"4783","MOB3B",16.1104418803465,0.473600035035153,0.444495825283859,1.
06547690235945,0.286660172501484,0.850242021560169
"4784","PLPP7",8.61714076993196,0.496579580914596,0.466680545866168,1.
06406745537879,0.287298142626414,0.850242021560169
"4785","CPM",8.41823800250497,0.557372325841922,0.523617354221104,1.06
446495966703,0.287118119901931,0.850242021560169
"4786","YDJC",11.407351129151,-0.490019993612291,0.460370193717239,-1.
06440425618272,0.287145606518848,0.850242021560169
"4787","ULBP3",1.89966759643295,-1.34606637248089,1.26577084081712,-1.
0634360731615,0.287584241125328,0.850444715616907
"4788","TULP4",27.9721977637494,0.351117802605831,0.330146760990864,1.
06352036152657,0.287546036387863,0.850444715616907
"4789","MRPL18",143.850729692337,-0.282802409005065,0.265955550336328,
-1.06334464028832,0.287625688071283,0.850444715616907
"4790","GTF2H2",15.7337323582263,0.380627316694003,0.358044529352983,1
.06307256636997,0.287749044279115,0.850454282402894
"4791","TBC1D25",9.68092723503939,-0.524384012003318,0.493226839051638
,-1.06317006797843,0.287704833702304,0.850454282402894
"4792","IFI16",461.089654388823,-0.255433090655869,0.240344376002825,-
1.06277956199344,0.287881930101966,0.850625396505537
"4793","ACBD5",35.0441142372884,0.319103210832227,0.30028155714486,1.0
6268001893399,0.287927085130723,0.850625396505537
"4794","FMR1",47.025537038032,0.249643039172248,0.235186279176097,1.06
146940224062,0.288476631130517,0.852071150773492
"4795","MFSD6",7.08622638021314,-0.520647881070094,0.49087213823189,-1
.06065885699982,0.288844964073394,0.852154838837665
"4796","LRRC34",11.5486925839745,0.483325021674371,0.455570749972099,1
.06092197908661,0.288725359793613,0.852154838837665
"4797","AL356599.1",16.7632867405079,0.39653225571544,0.37391766862047
5,1.06048012435037,0.288926227490087,0.852154838837665
"4798","POT1-
AS1",1.56980419990286,1.17495362071279,1.10728516137391,1.061112043852

3,0.288638985078454,0.852154838837665
"4799","KTN1",251.477043364349,-0.246550633043869,0.232481328263502,-1
.06051799895267,0.288909005961192,0.852154838837665
"4800","NFATC2IP",52.4870729130853,0.359161765201941,0.338648473516248
,1.06057399719745,0.288883544904729,0.852154838837665
"4801","RBM10",33.3902678519619,0.28598431799437,0.269625534471709,1.0
6067223401045,0.288838882639191,0.852154838837665
"4802","AC005261.1",61.5293418701886,0.352882526803979,0.3328757596114
65,1.06010280597141,0.289097831403137,0.852483401222078
"4803","DTYMK",45.0771234586583,-0.361915576172392,0.341629598517708,-
1.05938003540297,0.289426738243193,0.852742949131007
"4804","C9orf85",34.6015856752655,0.27342486152391,0.258089473709745,1
.05941888134272,0.289409054449642,0.852742949131007
"4805","FKBP11",116.623674577139,-0.24224221588743,0.228659017550967,-
1.05940372910697,0.289415952098348,0.852742949131007
"4806","DOP1B",10.1132542678427,0.471641192150802,0.445045123531339,1.
05976038656132,0.289253622733287,0.852742949131007
"4807","HVCN1",10.2634435898892,0.459706140277174,0.434298741774879,1.
05850212321238,0.289826583304146,0.853435257047159
"4808","SEC23A",92.6456079435849,-0.215527412511466,0.203669290802428,
-1.05822243334927,0.289954046115861,0.853435257047159
"4809","ZZEF1",26.224244761294,0.392732473700004,0.371006608659775,1.0
5855924000576,0.289800558158171,0.853435257047159
"4810","TNFSF12",127.700288658546,-0.247206734556027,0.233610014252987
,-1.05820264318085,0.28996306650098,0.853435257047159
"4811","TSHZ3",28.9126853038371,-0.292945122779113,0.276773933892965,-
1.0584274272461,0.289860620737464,0.853435257047159
"4812","RPL15",3895.63016207276,-0.253339612494372,0.239472645457281,-
1.05790626737602,0.29009817758607,0.853526308997486
"4813","PALLD",225.664102306218,-0.211918853396554,0.200334119749689,-
1.05782706241623,0.290134292525974,0.853526308997486
"4814","FOXN3",191.392548486709,0.219246837956659,0.207330876882989,1.
05747316199504,0.290295696817608,0.853526308997486
"4815","SORD",14.1411487453119,0.415267306354141,0.392795628815629,1.0
5720959168071,0.290415943273297,0.853526308997486
"4816","PAGR1",57.9845878480132,-0.24605907839342,0.232733504568914,-1
.05725679183661,0.29039440708209,0.853526308997486
"4817","SLC7A6",16.466666478868,0.429069260416035,0.405764015907369,1.
057435463952,0.290312893423281,0.853526308997486
"4818","ZNF93",3.97116054033716,0.782075395097395,0.739684714639319,1.
05730912052001,0.29037053213469,0.853526308997486
"4819","THBS4",171.08599570429,-1.22929151548113,1.1629536499323,-1.05
704257048653,0.290492159286489,0.853573142871278
"4820","ATAD5",5.19528376794122,0.713463447525942,0.675133255313398,1.
05677426183776,0.290614623510689,0.853755823425591
"4821","OXSR1",26.2123959421861,0.288802838307024,0.27332784048938,1.0
5661698343622,0.290686426299829,0.853789627962161
"4822","SPR",21.2305529824785,-0.342544930176015,0.324328682516212,-1.
05616600887247,0.290892377261435,0.853855256895078
"4823","MAP2",2.47614328779256,-1.03983385196271,0.984432371564883,-1.

05627758899249,0.290841411742682,0.853855256895078
"4824","HSPB2",111.20011530406,-0.241152208961784,0.228355199702599,-1
.05603992935502,0.290949972776748,0.853855256895078
"4825","TMEM160",88.8110825205956,-0.261668758585567,0.247692735519592
,-1.05642484038402,0.290774162149058,0.853855256895078
"4826","GRASP",34.7545682572436,0.272350288952375,0.257998244200193,1.
05562845901015,0.291137993592154,0.854053032787425
"4827","CBR3-
AS1",7.88999230349905,0.574704710893769,0.54438586228588,1.05569367374
931,0.291108188358384,0.854053032787425
"4828","TRAF3",26.7695054551766,-0.364825374255544,0.345668171760179,-
1.05542078808649,0.29123291955613,0.854154544514252
"4829","AL035071.1",6.36916822804101,-0.708786494344486,0.671983823933
794,-1.0547671969174,0.291531810573076,0.854854097683735
"4830","DBF4B",3.93286966369668,0.982559131567828,0.93172692714022,1.0
5455697688552,0.291627989217524,0.854959073979324
"4831","ZNF593",51.8123013936935,-0.291051103264848,0.276155546625569,
-1.05393900945063,0.291910841557697,0.854961517348921
"4832","BARD1",8.74341474792742,0.528495170277791,0.501859909051026,1.
05307310017477,0.292307490370948,0.854961517348921
"4833","RPL35A",3512.88560320635,-0.253239215076905,0.240306890696776,
-1.05381586995958,0.291967226244147,0.854961517348921
"4834","GSDMD",81.6952319384906,-0.201291380226996,0.191164806720652,-
1.05297300104586,0.292353366313805,0.854961517348921
"4835","RIC1",22.1431400434729,0.339973562760477,0.322717673291758,1.0
5347054375023,0.292125387662852,0.854961517348921
"4836","SEPHS1",46.4313884869415,-0.22751195764294,0.216041426821899,-
1.05309412638946,0.292297854563739,0.854961517348921
"4837","PSAP",908.554774628639,0.14735223738797,0.139938362421043,1.05
29795749976,0.292350353289731,0.854961517348921
"4838","TPCN2",6.29381358334857,-0.613887638531389,0.582888905085199,-
1.05318120344332,0.292257951521007,0.854961517348921
"4839","PEAK1",63.4815315457072,0.305106390136383,0.289447035660537,1.
05410093228321,0.29183670939537,0.854961517348921
"4840","PTPN2",66.662476605433,-0.284424427762614,0.269830741208563,-1
.05408459573096,0.291844188087561,0.854961517348921
"4841","SCAND1",161.080384455972,-0.285084978801058,0.270567284321642,
-1.05365650365237,0.292040209791953,0.854961517348921
"4842","WDR44",13.4117881205481,0.403790100216936,0.383075240700431,1.
05407517196525,0.291848502241422,0.854961517348921
"4843","PPTC7",36.7619474473919,-0.374025567714137,0.355260245487982,-
1.05282134003026,0.292422882546647,0.854988233917102
"4844","ARV1",23.711196964943,-0.390736878261377,0.37125654332015,-1.0
5247135785679,0.292583344794685,0.855280793206594
"4845","MASTL",6.46328877096307,-0.58227840974326,0.553354808518466,-1
.05226953986762,0.292675902576174,0.855374774092596
"4846","ZSWIM6",19.3136336452674,-0.391477137780723,0.372095656488521,
-1.05208736236027,0.292759469720121,0.855442445570969
"4847","S100A8",4.31769539655159,-0.733234122983605,0.699433878350921,
-1.04832514649187,0.294488827700741,0.855865374121091

"4848","S100A2",2.52981646010763,-1.00755204173313,0.958631783367649,-1.05103133363013,0.293244199117442,0.855865374121091
"4849","CYB5R1",49.3364135517243,0.273645204497439,0.260856245182657,1.04902684735735,0.29416576217400З,0.855865374121091
"4850","PLEKHB2",31.9493243999167,-0.277992596864035,0.264923795268358,-1.04933041813944,0.29402607079567З,0.855865374121091
"4851","MST1",2.77603007111576,-0.923372327685773,0.881340119681948,-1.04769124548533,0.294780882326914,0.855865374121091
"4852","HEMK1",17.4689145416924,0.374239211008438,0.357230079839423,1.04761393882805,0.294816512781747,0.855865374121091
"4853","GAP43",9.08818169876862,-0.867143299174345,0.825016435050393,-1.05106184838776,0.293230184938449,0.855865374121091
"4854","TLR6",2.22854218559985,1.11914457996765,1.06742383876365,1.04845380000497,0.294429577376677,0.855865374121091
"4855","UBE2K",114.061491518146,0.183221551240977,0.174236507425038,1.05156808953947,0.292997754682256,0.855865374121091
"4856","MMAA",10.1946385344212,0.441027138028476,0.420744230181503,1.04820721567168,0.294543146796535,0.855865374121091
"4857","ATF6B",37.618850203688,-0.277947464317126,0.26458519260068,-1.05050271931359,0.293487041369071,0.855865374121091
"4858","TBC1D22B",4.73837194020559,-0.65366983350047,0.624431880058965,-1.04682328749574,0.295181087790297,0.855865374121091
"4859","GTF3C6",122.35138427222,-0.279319755392782,0.266837096594418,-1.04678007277727,0.295201023107868,0.855865374121091
"4860","RPS6KA2",7.91231029129143,0.572473033852235,0.545781993525567,1.04890421568189,0.294222205082526,0.855865374121091
"4861","FOXK1",32.1706053232944,0.30464379655593,0.290994201144151,1.04690676088427,0.295142583358788,0.855865374121091
"4862","CHCHD2",818.218142381241,-0.240790618095555,0.229947867667586,-1.04715308099157,0.295028980944436,0.855865374121091
"4863","ADHFE1",2.58890881770976,0.96532783117153,0.921498817136997,1.04756274584346,0.294840109095028,0.855865374121091
"4864","FAM92A",71.9155783465613,-0.205719930136753,0.195983667825165,-1.04967894733083,0.293865746181587,0.855865374121091
"4865","POLR2K",123.117602241821,-0.277337546582386,0.264515091343988,-1.0484753258245,0.294419664616832,0.855865374121091
"4866","GLIPR2",120.24098628938,-0.255779382969841,0.244033588515521,-1.04813187613136,0.294577851806284,0.855865374121091
"4867","ZBTB5",5.94285557848089,0.583477848008302,0.555215046898986,1.05090424199987,0.293302571934861,0.855865374121091
"4868","SNAPC4",11.1969178091262,0.47527738920469,0.452863499111428,1.04949369983945,0.293950953356634,0.855865374121091
"4869","CDK2AP1",293.278114377824,-0.202134474835489,0.19279126600300З,-1.04846282212982,0.294425422610678,0.855865374121091
"4870","IPO4",11.6483587581548,-0.486965926831487,0.465142835154068,-1.04691696835487,0.295137875108806,0.855865374121091
"4871","AL049775.1",5.65771845243801,0.601342473414348,0.574233247276494,1.04720943321626,0.295002995512412,0.855865374121091
"4872","DAPK2",14.0797626059751,0.453045313233803,0.431173265486504,1.05072681795941,0.293384075322436,0.855865374121091

"4873","SRRM2",495.935679463464,0.219012802903264,0.209149841454765,1.
04715739385646,0.295026992119476,0.855865374121091
"4874","RPS15A",3527.89929300124,-0.233270893560003,0.22268611114595,-
1.04753229718541,0.294854144354046,0.855865374121091
"4875","MT1M",554.824488490714,-0.616803462516078,0.589076926293921,-1
.04706776820575,0.295068323739621,0.855865374121091
"4876","CTCF",68.8879903550254,0.20778244246802,0.198050889394226,1.04
913662899247,0.294115239831802,0.855865374121091
"4877","AC105429.1",1.8171241196462,-1.32420436221447,1.26216992094273
,-1.04914904106208,0.29410952806923,0.855865374121091
"4878","RPL27",1867.09384572406,-0.224170993367219,0.213978256332699,-
1.04763445225328,0.294807057911482,0.855865374121091
"4879","AFMID",22.8634113863043,0.361249919799364,0.3442554212962,1.04
936595751834,0.294009719875361,0.855865374121091
"4880","TNPO2",34.9335063702468,0.337403511672652,0.32165732970213,1.0
4895328200698,0.294199620762904,0.855865374121091
"4881","LPIN3",30.1371452190891,-0.398493974208784,0.379108047641611,-
1.05113562396729,0.293196304691899,0.855865374121091
"4882","AC245060.5",18.927956898794,0.54089164751339,0.515973999919155
,1.04829244806548,0.294503887967483,0.855865374121091
"4883","LINC00685",8.98653553329065,0.550057392133273,0.52494044610065
,1.04784722956518,0.294708998420293,0.855865374121091
"4884","IDH3G",114.676283873808,-0.248853052305998,0.237317796302609,-
1.04860678880011,0.294359130039337,0.855865374121091
"4885","AIP",132.664018496486,-0.209001684550849,0.199733935141235,-1.
04640047472684,0.295376173673358,0.856197874967196
"4886","NOA1",42.9501960670336,-0.327651422920129,0.313225666702477,-1
.04605547294231,0.295535421516544,0.856308894756347
"4887","SPATA7",23.4493498774929,-0.336627850268155,0.321776288603066,
-1.04615492872257,0.295489508267403,0.856308894756347
"4888","TMEM43",156.282845033068,-0.234811108258465,0.224705883508484,
-1.04497089525295,0.29603642131894,0.856358677400653
"4889","GPD1L",11.7131575829864,0.433581929865973,0.414647482007402,1.
04566396440394,0.29571620583264,0.856358677400653
"4890","FAM8A1",55.5233986492909,0.240650057055659,0.230112374767846,1
.04579363582009,0.295656320119289,0.856358677400653
"4891","MEA1",63.7658416204153,-0.23599615891435,0.225836216531706,-1.
04498810039716,0.296028469298871,0.856358677400653
"4892","HMGN3-
AS1",11.7095682087105,0.453036045492783,0.433398049223752,1.0453116858
8369,0.29587893843298,0.856358677400653
"4893","STX7",65.3050891558931,0.217094297217896,0.20774723812757,1.04
49924589832,0.296026454833158,0.856358677400653
"4894","TSR3",82.5847724106443,-0.223490466537569,0.213769627440225,-1
.0454734342467,0.295804212465302,0.856358677400653
"4895","ACBD4",11.9513807011502,0.422081853609581,0.403915626437628,1.
04497530172866,0.296034384682853,0.856358677400653
"4896","ZCWPW2",11.8512739401957,0.428940720184351,0.410570106261967,1
.04474415853029,0.296141229785258,0.856374959812755
"4897","TIFA",21.8062800106057,-0.401643316708997,0.384459126445865,-1

.04469705381165,0.296163006935245,0.856374959812755
"4898","PDS5B",70.3596833520647,0.243595993717075,0.233204324050112,1.
04456036443274,0.296226206355117,0.856382826049093
"4899","KIAA1671",16.1123637702743,0.50948322093126,0.487838427040601,
1.04436877599406,0.296314803980089,0.856464099685253
"4900","PHPT1",391.788513190809,-0.257739214420952,0.246927248234035,-
1.04378603926558,0.296584392106287,0.856718684664428
"4901","PAAF1",22.5675783601684,0.358158033296253,0.343111962045269,1.
04385178284457,0.296553969325485,0.856718684664428
"4902","LAG3",3.23036848499415,-0.750892512968205,0.719279509918888,-1
.04395092952513,0.296508093238431,0.856718684664428
"4903","TNFSF4",5.55217435703943,0.585118075782164,0.560704064077399,1
.04354170634546,0.296697475238315,0.856870538318282
"4904","PSMB7",175.454869308063,-0.241250297410403,0.231245562073307,-
1.04326455066811,0.296825784436747,0.857066294376903
"4905","ECM1",319.172460645881,-0.342001893006808,0.328014063751291,-1
.04264399244211,0.297113206368016,0.857197025707234
"4906","CDK2AP2",25.8416860176831,-0.286606536454528,0.274851869774664
,-1.04276727929667,0.297056089189742,0.857197025707234
"4907","HHIPL1",7.13952109841544,-0.50880200762821,0.487953330901644,-
1.04272678431776,0.297074849171103,0.857197025707234
"4908","MFAP1",41.8129715550708,-0.264889200405829,0.254052469536538,-
1.04265548329076,0.29710788249868,0.857197025707234
"4909","RAP1B",420.408296289769,-0.187672601150026,0.180030290671156,-
1.04245013686519,0.297203031956952,0.857281509983793
"4910","PTN",24.6833600035915,-0.583894315110188,0.560262082997971,-1.
04218067370499,0.297327921492342,0.857292479806874
"4911","AC012645.1",3.26293524256721,-0.872214571096551,0.836896330943
919,-1.04220145177694,0.297318290119839,0.857292479806874
"4912","YEATS4",31.9765990165631,0.290406905751199,0.27870298960952,1.
04199422531519,0.297414356259436,0.857367118207168
"4913","GNAI2",357.392709404214,-0.185508525750227,0.178055536278473,-
1.04185766771159,0.297477673036742,0.857375096723034
"4914","SH2D2A",3.16830472747788,0.927195488292728,0.891354717751059,1
.0402093238841,0.298242660624888,0.857410413711119
"4915","FAP",122.756954173565,-0.355573389379228,0.341809560005245,-1.
0402675377885,0.298215621500071,0.857410413711119
"4916","RNF217-
AS1",2.10736897127345,-1.0300076156086,0.9908710747357,-1.039497107010
95,0.298573602831045,0.857410413711119
"4917","NUP205",14.6268139922366,0.369379853827733,0.354907066099399,1
.04077909151654,0.297978086048023,0.857410413711119
"4918","TGS1",58.9128102413992,0.220221548565041,0.211842353316272,1.0
3955391883444,0.298547195373174,0.857410413711119
"4919","FAM120AOS",86.095934085746,-0.223812175434083,0.21531091331820
4,-1.03948365637795,0.298579855226661,0.857410413711119
"4920","PKNOX2",5.13798547971084,0.746968371883853,0.718455234838888,1
.03968672738721,0.298485468950863,0.857410413711119
"4921","CSRP2",15.5361099063454,-0.51479118431903,0.494895542074643,-1
.04020170026382,0.298246201756187,0.857410413711119

"4922","TEP1",7.51735838893044,0.535586901903987,0.51511557689129,1.03
974122688396,0.298460141279245,0.857410413711119
"4923","TGFB3",43.064183615098,-0.30468290712703,0.293073146637288,-1.
03961386644581,0.298519332019156,0.857410413711119
"4924","NOTCH3",97.7725598145511,0.182484209365008,0.175300259139043,1
.0409808306117,0.297884445044886,0.857410413711119
"4925","ZNF829",7.98418978323735,0.514264482465545,0.494219470404872,1
.04055892829202,0.298080301397219,0.857410413711119
"4926","ETFB",233.625082152673,-0.288673722794945,0.277629196555214,-1
.03978157332431,0.298441391913323,0.857410413711119
"4927","KMT5C",8.46234220021167,0.502119310940819,0.48231884394248,1.0
4105265064182,0.297851113170483,0.857410413711119
"4928","SAMHD1",215.898474356996,0.229118048120684,0.22019412018026,1.
04052754875161,0.298094871907364,0.857410413711119
"4929","PLA2G6",7.37260817637137,0.560581303707967,0.538143727967389,1
.04169439236118,0.297553389683533,0.857410413711119
"4930","SRPX",646.670128462535,-0.31949904286965,0.306783388753398,-1.
04144831363889,0.297667529561952,0.857410413711119
"4931","RBMX2",64.1829658196292,-0.206916868278954,0.198845942413473,-
1.04058883861306,0.298066413534822,0.857410413711119
"4932","GALE",4.31848515555332,-0.698347700789814,0.673167188610386,-1
.03740603018903,0.299546669179608,0.857787035505717
"4933","FAM13B",29.6114052898417,0.286788922775062,0.276157817170655,1
.03849648622417,0.299038970301312,0.857787035505717
"4934","SNX13",51.3369059015918,0.242646513351081,0.233748138198453,1.
03806821830202,0.299238296468191,0.857787035505717
"4935","INPP5E",14.5317678882575,0.395480572495834,0.381002422038065,1
.03800015333321,0.299269983700536,0.857787035505717
"4936","SINHCAF",8.43728679809237,-0.516778561827915,0.498031818511149
,-1.03764165786196,0.299436916036957,0.857787035505717
"4937","EEF1AKMT3",13.5730807851486,0.391039292625748,0.37694918066096
2,1.03737934100315,0.299559102441792,0.857787035505717
"4938","USP30",7.79245805000033,0.497890041628752,0.479393809099797,1.
03858254357454,0.298998927839993,0.857787035505717
"4939","CDK8",11.4077998906806,0.562368372048979,0.541976146348435,1.0
3762568858046,0.299444353529721,0.857787035505717
"4940","VPS9D1",6.16928410674326,-0.556052716299897,0.53577642703891,-
1.03784468341216,0.29934237017414,0.857787035505717
"4941","USP22",93.8904063068712,0.228599494819433,0.220190829598757,1.
03818808092961,0.299182500593471,0.857787035505717
"4942","SOCS7",9.3884230577102,0.477600434135812,0.460300675344189,1.0
3758360506138,0.299463953991753,0.857787035505717
"4943","SOCS3",1489.34562534209,-0.338016298221706,0.325468266817511,-
1.038553778305,0.299012311913763,0.857787035505717
"4944","ZNF607",3.4532834694512,0.84344180963199,0.812184369605058,1.0
3848564586651,0.299044014570531,0.857787035505717
"4945","GCNA",2.581530920214,0.910023206725181,0.875934805563727,1.038
91659624088,0.298843527134554,0.857787035505717
"4946","OGFRL1",96.0197995022385,0.269694594729185,0.260084734717212,1
.03694895827862,0.299759645460556,0.857862241511732

"4947","CACNA2D1",41.6568412708743,0.282367819712207,0.272310592141565
,1.03693292828438,0.299767116596049,0.857862241511732
"4948","CHP1",69.6259293133974,-0.249167969527192,0.240279907249766,-1
.03699045159106,0.299740307155809,0.857862241511732
"4949","DDAH2",440.870780212586,-0.211149740437054,0.203679204372559,-
1.03667795191713,0.299885970703205,0.857874080665196
"4950","ZNF34",16.8465346497416,-0.389261324618907,0.375688691656477,-
1.03612733964013,0.300142739053101,0.857874080665196
"4951","NPM3",35.7987090389115,-0.26866787829125,0.259239490183302,-1.
03636941309089,0.300029834308576,0.857874080665196
"4952","ERI2",6.33266127394222,0.543546254858122,0.524615175653831,1.0
360856492203,0.300162186614331,0.857874080665196
"4953","BANP",14.7889409017393,0.368702808781121,0.355885735989657,1.0
3601457292415,0.300195343905088,0.857874080665196
"4954","NUFIP2",131.045817710988,0.288640804439351,0.278508466306281,1
.03638071857366,0.300024562044861,0.857874080665196
"4955","TGIF1",42.5842067195728,0.264081774842213,0.25482924232546,1.0
3630875496202,0.300058123023362,0.857874080665196
"4956","KLHL17",2.24106048394139,0.979135746334716,0.948177383564904,1
.03265039148415,0.301767536991337,0.858210147378456
"4957","S100PBP",26.8702555665253,0.294872477902874,0.28546180199952,1
.03296649792524,0.30161957713416,0.858210147378456
"4958","SLC16A1",51.0615740090234,0.348948386019269,0.337118687768702,
1.03509060363537,0.300626600345372,0.858210147378456
"4959","SEC16B",4.54083473168761,0.62884505670268,0.607438659338344,1.
03524042639573,0.300556643551325,0.858210147378456
"4960","PYCR2",33.4124208139058,-0.270436477250824,0.261670603482424,-
1.03349965052146,0.301370133983704,0.858210147378456
"4961","MAP1LC3C",1.69446250383092,1.34688615147894,1.30274093934115,1
.03388640888196,0.30118926948689,0.858210147378456
"4962","CHROMR",17.4705942580035,0.361549691001412,0.349965643578688,1
.03310052753827,0.30155685651195,0.858210147378456
"4963","VGLL4",145.994644281749,-0.237657941011035,0.229751769948664,-
1.03441179610559,0.300943692123211,0.858210147378456
"4964","GABBR1",38.5221342356171,0.335840438765815,0.324422025602778,1
.03519617122734,0.30057730650354,0.858210147378456
"4965","SNX10",7.22923374678162,0.887107271008015,0.858075888953269,1.
03383311712693,0.301214186660577,0.858210147378456
"4966","SARAF",259.330976545285,-0.192242118990713,0.185835834499645,-
1.03447281579635,0.300915178846831,0.858210147378456
"4967","SLC52A2",43.4281379700342,-0.323115291347999,0.31224690654391,
-1.0348070215471,0.300759043132838,0.858210147378456
"4968","STOML2",121.564852391689,-0.261477155871482,0.253169534621559,
-1.03281445874734,0.301690736041969,0.858210147378456
"4969","AL022344.1",1.55531407632697,1.14357016342787,1.10667581984665
,1.03333798653551,0.301445756300894,0.858210147378456
"4970","AC136475.1",6.00891360931229,-0.57611071790906,0.5573662486583
97,-1.03363043473799,0.301308965684911,0.858210147378456
"4971","HARBI1",2.0668273122084,-1.02231363145319,0.989155778368009,-1
.03352136620977,0.301359976897351,0.858210147378456

"4972","TMBIM4",461.628754851409,-0.231966244046059,0.224551913264015,
-1.03301833715986,0.301595317367926,0.858210147378456
"4973","TDG",51.8637626127517,0.243234847070009,0.235230988780406,1.03
40255267008,0.301124229818454,0.858210147378456
"4974","LMO7DN",1.84036248152968,0.999112540741678,0.967481549051282,1
.03269415496493,0.301747049749707,0.858210147378456
"4975","DCXR",33.5540281272947,0.281514793877562,0.272269239559985,1.0
3395739574738,0.301156080892873,0.858210147378456
"4976","ZNF700",6.69679516261259,0.566716187993209,0.548603098127368,1
.03301674731271,0.3015960613665 5,0.858210147378456
"4977","ZNF566",20.421519069534,0.349666079902069,0.338346167418879,1.
0334566002906,0.301390270556884,0.858210147378456
"4978","KLHL15",19.2147215585685,0.491126008578905,0.47539723252403,1.
03308554400152,0.30156386779058,0.858210147378456
"4979","RPL36A",1721.3995268879,-0.258284665276531,0.249996613587002,-
1.03315265583245,0.301532464852548,0.858210147378456
"4980","RER1",239.078932360526,-0.202029063319972,0.19587894988534,-1.
03139752095992,0.302354442917811,0.85821360017130 3
"4981","DNER",5.12865598858232,0.65692910526414,0.636634640083907,1.03
187772688203,0.302129401251037,0.858213600171303
"4982","B4GALT7",36.9141645862806,-0.3657046822099,0.354366812366802,-
1.03199472819526,0.302074587142411,0.858213600171303
"4983","SLC4A2",26.2693826463759,-0.29344725716027,0.284545161965993,-
1.03128535074282,0.302407025955057,0.858213600171303
"4984","UBE3C",38.2764866628567,-0.281501637242207,0.272700584565932,-
1.03227368467246,0.301943925132578,0.858213600171303
"4985","TTC9C",28.3097259440745,-0.34932004024149,0.338642307910239,-1
.03153100508068,0.302291876297777,0.858213600171303
"4986","MCAM",4.48155616758866,1.15490152416967,1.11886666787041,1.032
20656878432,0.301975358488091,0.858213600171303
"4987","CDK2",7.58848378971031,-0.543739884033,0.526895007462566,-1.03
197008195533,0.302086133143554,0.858213600171303
"4988","CNP",56.2920595068255,0.24342028320842,0.23595241249981,1.0316
4990189967,0.302236154285341,0.858213600171303
"4989","AC036176.1",16.0022888698841,0.426486158275884,0.4135724663423
17,1.03122473806774,0.302435442433249,0.858213600171303
"4990","PTPN1",48.0467849119866,-0.268125885432592,0.259949450638752,-
1.03145394142495,0.302327996490505,0.858213600171303
"4991","MRPL30",28.987899730346,0.327072539296637,0.317406444272516,1.
0304533672789,0.302797232186141,0.858349979647734
"4992","GLB1L",11.57844218408,-0.453131637719851,0.439785529060698,-1.
03034685722302,0.302847210333339,0.858349979647734
"4993","CDKN2AIP",33.006371850935,0.349852055570105,0.339546343993301,
1.03035141375873,0.302845072139229,0.858349979647734
"4994","KHDC1",2.73660925629262,-0.83334403894644,0.808452100892224,-1
.03078962628305,0.302639483997084,0.858349979647734
"4995","COL5A1",287.162704269785,0.299788859538767,0.290821429097669,1
.0308348338323,0.302618280082555,0.858349979647734
"4996","KRT7",3.10784258732316,-0.841244971329573,0.816432739508128,-1
.03039102992905,0.302826482328947,0.858349979647734

"4997","AC005670.3",24.1676704750514,0.445591624988722,0.432746948208456,1.02968172700799,0.303159436169884,0.859062961009718
"4998","C1S",2677.48701049639,0.163685889482307,0.158993271659172,1.02951456859881,0.303237937407874,0.859113484132751
"4999","TGFB2",22.3858422247667,-0.582110969385083,0.566085006201227,-1.02831017074874,0.303803948304618,0.859169943677531
"5000","RHOU",30.7214818303109,-0.349172699897767,0.339479278518332,-1.02855379398042,0.303689400141197,0.859169943677531
"5001","CCL19",62.1268781117738,-1.23650449713629,1.20235419622315,-1.02840286250127,0.303760362584178,0.859169943677531
"5002","IPO8",23.9293059655742,0.332251223891368,0.323028003433489,1.02855238666569,0.303690061758066,0.859169943677531
"5003","HOXC11",7.50586545184898,-0.569665711230883,0.553973199456892,-1.0283272038961,0.303795938628892,0.859169943677531
"5004","BMERB1",92.0826105719691,-0.297256342843538,0.288800050748184,-1.02928078465861,0.303347750110979,0.859169943677531
"5005","RNF40",29.3472469370955,0.285061799654284,0.27716924951588,1.02847556196147,0.303726180590652,0.859169943677531
"5006","SLC46A1",8.6022822469565,0.495114571937639,0.481174656568285,1.02897059348215,0.303493493521198,0.859169943677531
"5007","LINC01524",1.59786966063615,-1.52718908006039,1.48461108059255,-1.02867956465127,0.303630275801026,0.859169943677531
"5008","PHYHIP",12.3340258434217,-0.565023242523183,0.54974198399415,-1.02779714661414,0.304045258777953,0.859680683765138
"5009","BBOX1-AS1",5.39271960504294,-0.870442208315408,0.847046884188143,-1.02761986917606,0.304128674116761,0.85974486434285
"5010","TOR2A",13.9476621549121,-0.398531477732068,0.387881394570219,-1.02745706112986,0.304205294479607,0.859789814337571
"5011","MOB3C",17.909282931011,0.360630689827431,0.351512075431354,1.02594111279075,0.304919342084379,0.860086300817487
"5012","PANK3",33.5310238956166,0.289026532502038,0.281656180508304,1.02616790435925,0.304812447183667,0.860086300817487
"5013","AC021097.1",2.54894994108882,1.05487090424004,1.02805246798778,1.02608664157459,0.304850746353317,0.860086300817487
"5014","RAD21",115.874897855911,0.180840419241117,0.17628934062817,1.02581596026584,0.304978341553997,0.860086300817487
"5015","HNRNPUL2",83.8302463617421,0.194865533642046,0.189728730477042,1.02707446126945,0.304385403235487,0.860086300817487
"5016","PHC1",26.1123229198483,0.336679219529832,0.328072609084999,1.02623385862305,0.304781365271368,0.860086300817487
"5017","AC090517.5",9.40366825406734,0.465576704551885,0.453485082356816,1.02666377057483,0.30457881451828,0.860086300817487
"5018","DHX33",18.1005607440161,0.385949247125207,0.37616333926709,1.02601504941227,0.304884490402516,0.860086300817487
"5019","METRNL",222.394315764271,-0.249859183747465,0.243308950509564,-1.02692146435296,0.304457446296303,0.860086300817487
"5020","KHSRP",94.0158172105462,0.225890972174147,0.220082865127907,1.02639054631929,0.304707532336257,0.860086300817487
"5021","DGCR6",5.59615667213575,0.688569308856343,0.67099566777815,1.0

2619039424857,0.304801848257735,0.860086300817487
"5022","TMEM165",153.144570119845,-0.200141422732734,0.195167891048226
,-1.02548335004183,0.305135177659479,0.860185967680315
"5023","SORBS1",7.504672479577,-0.679856211499552,0.662917669163823,-1
.02555150228096,0.305103037392559,0.860185967680315
"5024","ALMS1",19.7728793162434,0.317994329350489,0.310438244774705,1.
02434005700962,0.305674684062015,0.861093420515457
"5025","MANSC1",17.5242770034438,0.351230732552606,0.342886350964955,1
.02433570646416,0.305676738240142,0.861093420515457
"5026","MT2A",4820.29426940357,-0.629267770986855,0.61434787214673,-1.
02428575000674,0.305700326619435,0.861093420515457
"5027","RBL1",8.77278798629333,0.524921512801935,0.512296094435151,1.0
2464476794559,0.30553083279869,0.861093420515457
"5028","CASP3",29.769679370236,-0.331461535838142,0.323691973012366,-1
.0240029517985,0.30583388069016,0.861298279747944
"5029","MMUT",35.2650538450352,0.268446297634018,0.262211589918718,1.0
2377739182785,0.30594043120328,0.861427024426017
"5030","WDR61",90.8802462792127,-0.251634548801883,0.245887178908334,-
1.02337401209394,0.306131041959842,0.861792356690131
"5031","RPS6KA1",2.46557924901347,-1.1405786099854,1.11653235042782,-1
.02153655426855,0.307000299573168,0.861830654434111
"5032","THEMIS2",2.82647145036304,-0.935661552325113,0.9149242788264,-
1.02266556258111,0.30646599913994,0.861830654434111
"5033","RTCA",45.0620415690848,0.220234912701289,0.215703841934575,1.0
2100598082109,0.30725160512704,0.861830654434111
"5034","DENND4B",10.9284563896833,-0.426534208679167,0.417741595102657
,-1.02104797242982,0.307231710883708,0.861830654434111
"5035","SOX13",18.0403594252682,0.346945451776319,0.339548268668635,1.
02178536541119,0.306882497339278,0.861830654434111
"5036","CMTM6",108.60262142462,-0.218684314786378,0.213931562886413,-1
.0222162257679,0.306678572829784,0.861830654434111
"5037","NUP155",14.6413737020522,0.425912713438917,0.416602493255222,1
.02234797039006,0.306616236545318,0.861830654434111
"5038","SPRY4",21.2481164730409,0.350825500191037,0.343104305656812,1.
02250392783455,0.306542454573683,0.861830654434111
"5039","KCTD20",75.2853547610434,0.192482639181361,0.188486855730767,1
.02119926843228,0.307160038886061,0.861830654434111
"5040","DNAJB9",48.4900405784951,-0.293949027675112,0.287466788853182,
-1.02254952249542,0.306520886399323,0.861830654434111
"5041","POLR2G",89.7859372219388,-0.32075307666395,0.313495972443421,-
1.02314895519699,0.306237423265321,0.861830654434111
"5042","DENND5B",23.9672645312215,0.318414024449534,0.311712925707711,
1.02149766079353,0.307018716801448,0.861830654434111
"5043","FAM219B",33.3994328723301,0.248332512460631,0.243129470695802,
1.02140029240363,0.307064826866659,0.861830654434111
"5044","AC015871.7",1.73247346732804,-1.07343520221611,1.0511405770124
3,-1.02120993679747,0.307154985482117,0.861830654434111
"5045","ZNF689",16.2097177024782,0.351991162324081,0.344784609763163,1
.02090160742925,0.307301057502672,0.861830654434111
"5046","ADAT1",11.0232145144278,-0.517387700539991,0.506175321247436,-

1.02215117731327,0.306709354252072,0.861830654434111
"5047","CPD",42.2570484171979,0.299804567917215,0.293443703581118,1.02
167660869349,0.306933985659104,0.861830654434111
"5048","ADNP2",22.9964124415672,0.415796325050097,0.407154455305349,1.
02122504035543,0.307147831304913,0.861830654434111
"5049","CHCHD10",59.2310230931669,0.326104731933498,0.318821980224599,
1.02284269015508,0.30638222989993,0.861830654434111
"5050","CHKA",6.53605044614478,-0.59395168624507,0.582375585312007,-1.
01987738020793,0.307786618523508,0.863003287684483
"5051","ASIP",3.80478949694524,-0.961203495281386,0.942575765828375,-1
.01976258050369,0.307841073876718,0.863003287684483
"5052","MARK2",31.3534772054914,0.338842633647072,0.332400378986602,1.
01938100877054,0.308022118617555,0.863169048015947
"5053","PTPRS",18.7306571680465,0.414479320007953,0.406558971021156,1.
01948142717624,0.307974466162213,0.863169048015947
"5054","SEC14L5",2.72852682454835,0.880977784470477,0.864531598464548,
1.01902323296816,0.308191936860483,0.8633032296626
"5055","PHF12",20.8593987770121,0.355707144062525,0.349040851338856,1.
01909888970904,0.308156021234451,0.8633032296626
"5056","DENND6A",33.1482775682731,0.339850820718359,0.333653311597931,
1.01857469686348,0.308404921950095,0.863400812956209
"5057","NANOS1",3.88888515916229,1.03246687042555,1.01364884172038,1.0
1856464283354,0.308409697170375,0.863400812956209
"5058","RNF227",3.1124674615535,-0.781705622363058,0.767268990778624,-
1.01881560672716,0.308290515026828,0.863400812956209
"5059","ZBTB10",41.5303296637675,0.309914173806385,0.304405336821101,1
.01809704469315,0.308631839665675,0.863510541328979
"5060","NUDCD1",18.075914690663,-0.32818629080111,0.322311681928025,-1
.01822648449459,0.308570335932361,0.863510541328979
"5061","PHF11",67.6687518402408,0.222690328280726,0.218728568680659,1.
01811267555932,0.308624412179595,0.863510541328979
"5062","TNFRSF14-
AS1",1.8301597254686,1.04924174924583,1.03082570311155,1.0178653346329
,0.308741957679754,0.863647989084417
"5063","RBM12",27.7283701291653,0.312118661821075,0.306708105494195,1.
01764073472451,0.308848721452453,0.863776001534019
"5064","EYA3",18.0838466118376,0.339972486301085,0.33447099063749,1.01
644834923683,0.309415931699034,0.863899548769649
"5065","THUMPD3-
AS1",44.0287047125406,-0.264775924179506,0.260446378859927,-1.01662355
736536,0.309332543187511,0.863899548769649
"5066","LRIG1",15.2715240475013,0.565142294023333,0.555505604893465,1.
01734760017717,0.308988099940277,0.863899548769649
"5067","ATP11B",37.9251920856234,0.32411025805489,0.318905760145878,1.
01631986172539,0.309477093451522,0.863899548769649
"5068","MELTF-
AS1",9.14380646907194,0.470732046579247,0.463250164682269,1.0161508456
2811,0.309557559509942,0.863899548769649
"5069","COX7C",727.282563552402,-0.212777749147113,0.209309535739457,-
1.01656978214301,0.309358135373316,0.863899548769649

"5070","SHARPIN",84.8052945750128,-0.228974620761533,0.225288071403356
,-1.0163637130684,0.309456218735259,0.863899548769649
"5071","NPR2",25.0648429178695,0.344960055283489,0.339346634868112,1.0
1654184788825,0.309371430126896,0.863899548769649
"5072","PLAAT5",7.75522607691398,-0.519584997407126,0.511333451794278,
-1.01613730841175,0.309564004975791,0.863899548769649
"5073","KDELR1",361.270162246585,-0.228978800176086,0.225291434235415,
-1.01636709337479,0.309454609634184,0.863899548769649
"5074","PRR12",26.3341316792102,0.378832620028817,0.372468011819805,1.
0170876639256,0.309111728206088,0.863899548769649
"5075","LPIN1",30.1951108570254,0.297724673905348,0.29315555617578,1.0
1558598373223,0.309826582165915,0.864325721513323
"5076","ATP5PD",288.606397896977,-0.239333408545205,0.235666358786327,
-1.01556034462349,0.309838796779776,0.864325721513323
"5077","SVBP",92.4224298597242,0.265174408897119,0.261377734459352,1.0
1452562302455,0.310332009322453,0.864523275797082
"5078","RXRG",8.08230039190254,-0.585247601012826,0.576666995974165,-1
.01487965341968,0.310163198161918,0.864523275797082
"5079","FAM241A",33.7593144790976,-0.417100290316276,0.411214829874951
,-1.0143123740044,0.310433721423807,0.864523275797082
"5080","TPMT",20.7502026382695,-0.337246780966187,0.332379258489465,-1
.01464448322932,0.310275326835833,0.864523275797082
"5081","AEBP1",663.594108501733,0.178581094714661,0.176029563849569,1.
01449489965943,0.310346661904313,0.864523275797082
"5082","MAPKAPK5-
AS1",33.0268122758122,0.271650490894276,0.267777953108485,1.0144617498
9702,0.310362472195376,0.864523275797082
"5083","ACOT2",25.936656894916,-0.40577149538451,0.400066872871622,-1.
01425917240271,0.310459100100859,0.864523275797082
"5084","ZNF85",10.2154128508994,0.472876640854379,0.46578600994279,1.0
1522293662804,0.309999569456692,0.864523275797082
"5085","RNF215",18.0098791276241,-0.331863857797871,0.327138613739719,
-1.01444416482706,0.310370859351396,0.864523275797082
"5086","PCNX4",56.2252871152681,0.279536603334931,0.275680997001016,1.
01398575301112,0.310589550531409,0.864703318166149
"5087","ACSM5",3.39000115264148,-1.04348972138459,1.02921685660496,-1.
01386769434259,0.310645888383559,0.864703318166149
"5088","CNIH4",115.510951494079,-0.222277782134718,0.219298929250871,-
1.01358352680527,0.310781521399333,0.864910837856635
"5089","SSU72",149.315314001206,-0.24932502234074,0.246348509786136,-1
.01208252713681,0.31149859600683,0.866565838793068
"5090","GSTA4",57.4676096405401,0.266207860627244,0.263000179985911,1.
01219649599291,0.311444111266372,0.866565838793068
"5091","AL590822.3",1.53955101463621,-1.1544706787484,1.14098431828827
,-1.01181993498417,0.311624156552584,0.866619530080659
"5092","ZNRD2",47.8852468755139,-0.268566920336785,0.265438415806595,-
1.01178617842743,0.311640299941435,0.866619530080659
"5093","ARHGAP10",120.357311803479,0.209301058501591,0.206916707057856
,1.0115232427465,0.311766062473647,0.866734105548017
"5094","RRP7A",42.373286849127,-0.32984308021668,0.326111032102288,-1.

01144410261233,0.311803921868757,0.866734105548017
"5095","FHAD1",3.9398768616414,-1.0318191947461,1.02062708360394,-1.01
096591627046,0.312032743170016,0.867108676166264
"5096","AC068587.4",10.1793862389657,0.636791547942682,0.6301616792821
95,1.01052090102978,0.312245790864577,0.867108676166264
"5097","TRIM9",5.30081419339051,0.679055311479934,0.671877812816488,1.
01068274398489,0.312168298680729,0.867108676166264
"5098","FERMT2",218.844765238914,0.188926616944471,0.186970335627455,1
.01046305720344,0.312273490198229,0.867108676166264
"5099","C15orf61",111.124436594785,-0.254756234273785,0.25213529241949
2,-1.01039498211116,0.312306090992087,0.867108676166264
"5100","ASMTL",44.5839716000565,-0.255068286801119,0.252415795099064,-
1.01050842203046,0.31225176647278,0.867108676166264
"5101","TYW3",64.4180509891241,-0.214628648424281,0.212481211184795,-1
.01010648060368,0.31244427769473,0.867152287761148
"5102","MYMX",3.48597671812521,0.983913266522842,0.974020885605571,1.0
1015623079902,0.312420445429145,0.867152287761148
"5103","RPS6KC1",25.0647102220691,0.318167949901554,0.315040409385973,
1.00992742652181,0.312530061429552,0.867220393855078
"5104","SNX9",309.66738414761,-0.161779743321408,0.160230767722776,-1.
00966715457116,0.312654783822589,0.867226589408003
"5105","AL022311.1",9.32699995345725,-0.483407962199275,0.478763514434
881,-1.0097009225314,0.312638600355849,0.867226589408003
"5106","OXR1",90.0425507949473,0.187286925256118,0.185739274190087,1.0
0833238459007,0.313294921093001,0.868039593319679
"5107","PYM1",28.7534905720123,-0.309981431054728,0.307407288382847,-1
.0083737203676,0.313275084088309,0.868039593319679
"5108","POLE",10.4386231421458,0.474356158798245,0.470456506859466,1.0
082890806736,0.313315703492718,0.868039593319679
"5109","TSHZ1",44.8828143230802,0.269997794284888,0.26768516572939,1.0
0863936015728,0.313147623507909,0.868039593319679
"5110","ZNF506",36.6736096115267,-0.254880536775258,0.252719684469509,
-1.00855039175237,0.313190308959126,0.868039593319679
"5111","TMSB15B.1",6.68046710977676,0.543330290528177,0.53882302277352
2,1.00836502444059,0.313279257187654,0.868039593319679
"5112","CPLANE1",34.7693049801888,0.270061145735982,0.267987443297607,
1.00773805821966,0.313580229223931,0.868093068584724
"5113","SYNCRIP",123.182982497863,-0.172629620193293,0.171290735277049
,-1.00781644678026,0.313542588778148,0.868093068584724
"5114","PGAM1",205.880185050859,-0.233623254649508,0.231816721664341,-
1.00779293647238,0.313553877593652,0.868093068584724
"5115","ZNF358",92.2241089136299,-0.266683014417748,0.264559803406153,
-1.00802544825124,0.313442245446781,0.868093068584724
"5116","RNASEH2A",5.94803349071723,-0.561201475912498,0.55707423150585
1,-1.00740878714761,0.313738370357555,0.868361087619816
"5117","IL1R1",227.186556146458,0.169913185978753,0.168773789470067,1.
00675102758706,0.314054433854388,0.868474767455972
"5118","SULT1C4",1.82371756809624,1.27619302692662,1.26729230262226,1.
00702341857987,0.313923520430935,0.868474767455972
"5119","GTF3C3",22.0900256374833,0.309999688684462,0.307970287412571,1

.00658960086358,0.314132033852414,0.868474767455972
"5120","CCM2",63.6236672013353,-0.226491923739212,0.225016366712041,-1
.0065575542292,0.314147440600953,0.868474767455972
"5121","AP4S1",12.7722770398266,-0.425469303654623,0.422683125009133,-
1.00659164863852,0.314131049380479,0.868474767455972
"5122","SNHG29",929.460444963616,-0.273820018985363,0.2718919541507,-1
.00709129051165,0.313890906192233,0.868474767455972
"5123","FPGT",14.2065439258716,0.416854952657617,0.414310075606843,1.0
061424454789,0.314347053307349,0.868724024878274
"5124","ARF4",292.895206175125,-0.229577314786102,0.228182005545622,-1
.00611489603286,0.314360303917816,0.868724024878274
"5125","HELQ",21.4058802917508,0.353474878509024,0.35144047131204,1.00
578876755255,0.314517191657944,0.868987987097852
"5126","LRPAP1",243.227617892794,-0.196547086718464,0.195448350700815,
-1.00562161826237,0.314597620636909,0.869040637576791
"5127","ACAP3",31.8457242771806,-0.309654369381365,0.308616384029981,-
1.00336335141326,0.31568557990615,0.869232954306816
"5128","AL359504.2",4.07505071826664,0.732554269602728,0.7287996619870
03,1.00515176915078,0.314823775285146,0.869232954306816
"5129","BLZF1",32.9037889413618,-0.249095544108681,0.247797707838855,-
1.00523748294988,0.314782510298549,0.869232954306816
"5130","NDUFAF4",24.5067563443266,-0.313860218456275,0.312399536481463
,-1.004675685474,0.315053039812484,0.869232954306816
"5131","AC005076.1",2.13972280280536,-0.949198166450776,0.945644961908
119,-1.00375744035635,0.315495542953474,0.869232954306816
"5132","WDR60",62.1376973789123,-0.239041913003564,0.238137061626131,-
1.0037997083329,0.315475165018881,0.869232954306816
"5133","AP3M2",13.8584706999158,0.377336755414501,0.375975706402954,1.
00362004509432,0.315561788947627,0.869232954306816
"5134","AL157392.3",7.38309054452695,0.563406196778448,0.5613311319161
67,1.00369668586739,0.31552483498773,0.869232954306816
"5135","PDSS1",5.42596856829523,-0.585225105047098,0.582930471488232,-
1.00393637607073,0.315409281663675,0.869232954306816
"5136","SCD",2.78434998538116,0.873164093982563,0.870237089911983,1.00
336345589554,0.315685529512911,0.869232954306816
"5137","ACCS",19.044863633828,0.351269659622723,0.349767462303638,1.00
429484580753,0.315236517265229,0.869232954306816
"5138","AL138995.1",3.14095948791628,-0.859075720912274,0.856433544468
407,-1.00308509219534,0.315819806899308,0.869232954306816
"5139","DHRS7",163.588662765216,-0.181368789208134,0.180529957070831,-
1.00464649829266,0.3150670988624,0.869232954306816
"5140","MARK3",73.5742520168443,0.230990287438191,0.23021311396441,1.0
0337588706568,0.315679533826148,0.869232954306816
"5141","LMF1",23.8026367519639,-0.404212967762431,0.402423373992734,-1
.00444704230756,0.315163185006782,0.869232954306816
"5142","ARMC7",6.34610812838092,-0.576206735186523,0.574008700290437,-
1.00382927104585,0.315460912968938,0.869232954306816
"5143","ACTN4",109.183980806327,-0.212294482697025,0.211647574286337,-
1.00305653590819,0.315833584033091,0.869232954306816
"5144","LINC01637",4.73086952742917,-0.723033315508991,0.7206391432386

54,-1.00332229007097,0.315705384765739,0.869232954306816
"5145","RPA4",4.49632257052466,-0.665555777492095,0.663367961132253,-1
.00329804345104,0.315717079868953,0.869232954306816
"5146","CYTOR",136.426153148899,-0.300066176705029,0.29929115490414,-1
.00258952457562,0.316058952153628,0.869241950324849
"5147","PRKCD",11.6755648396701,-0.401091933311258,0.40051854592509,-1
.00143161257325,0.316618187122106,0.869241950324849
"5148","ETV7",9.82295797735696,-0.509759180915119,0.50871309896863,-1.
00205632988144,0.316316387771083,0.869241950324849
"5149","AMD1",141.943758861519,0.264017253271649,0.263645665724576,1.0
0140942027646,0.316628911638361,0.869241950324849
"5150","HILPDA",81.9902769909232,0.322282867068523,0.321468164958606,1
.00253431660962,0.316085601128927,0.869241950324849
"5151","TMEM65",37.046602029712,0.231805308985595,0.23149457613612,1.0
0134228997785,0.316661354074783,0.869241950324849
"5152","LY6E",619.462022223382,-0.258347162629946,0.258076595801568,-1
.00104839738581,0.316803410871803,0.869241950324849
"5153","NUP188",18.839960230327,0.358784344229553,0.358349431823963,1.
00121365451419,0.316723526551139,0.869241950324849
"5154","PRF1",3.86907816782805,0.815217289830723,0.814389825769517,1.0
01016054026,0.316819047007525,0.869241950324849
"5155","CYB5R2",10.1676643199342,-0.457663458160917,0.456338569251987,
-1.00290330249994,0.315907519052491,0.869241950324849
"5156","TRIM13",37.3607289923964,0.275085938505073,0.27434510007467,1.
00270038878114,0.316005442275675,0.869241950324849
"5157","POLR3E",21.2231661210574,0.343444652032414,0.342673996716724,1
.00224894600429,0.316223373503158,0.869241950324849
"5158","AKTIP",28.1197655427875,-0.312505685975384,0.311963538718198,-
1.00173785455638,0.316470218795405,0.869241950324849
"5159","CRK",86.1863442102833,0.191395966202634,0.190974436588243,1.00
220725675081,0.316243503708675,0.869241950324849
"5160","AARSD1",41.2261151836203,-0.26099278520568,0.26059901621003,-1
.00151101489705,0.316579817583242,0.869241950324849
"5161","KANSL1-
AS1",11.0762016676051,-0.511559437486719,0.510906716817839,-1.00127757
308212,0.316692632237112,0.869241950324849
"5162","TRIP10",133.88781196132,-0.185433499997998,0.185314112233789,-
1.00064424539918,0.316998831241359,0.869566728085555
"5163","RUNX3",2.95030615435209,1.02597984318476,1.02560461892048,1.00
036585664432,0.317133487056364,0.869568157862792
"5164","RPL32",5547.9070553088,-0.252401770847731,0.252399630139097,-1
.0000084814254,0.317306403367023,0.869568157862792
"5165","VEGFA",81.7284706071435,-0.294411915160884,0.294381846030208,-
1.00010214329138,0.317261079015016,0.869568157862792
"5166","ATP6V0E2",7.60043539561911,0.634743017496935,0.634662549422716
,1.00012678875458,0.317249153419075,0.869568157862792
"5167","ZNF844",14.2862104988636,0.519415379621901,0.519218440037027,1
.00037930005887,0.317126983654776,0.869568157862792
"5168","PDE6D",17.6366129936698,0.37431391885737,0.374865295125288,0.9
98529134931701,0.318022843925342,0.869967784877692

"5169","CLEC3B",369.902177045643,0.317443886646613,0.317622641592441,0
.999437209687156,0.317582942062511,0.869967784877692
"5170","TBC1D23",61.2690413309644,0.216870857288816,0.216989565330256,
0.999452932028046,0.317575329147793,0.869967784877692
"5171","SGCE",301.853008458348,0.2276306796877,0.228022609647516,0.998
281179395231,0.318143031261494,0.869967784877692
"5172","ARF5",155.132361457094,-0.454910198582465,0.455638506565235,-0
.998401566214716,0.318084674457811,0.869967784877692
"5173","TSTA3",40.9203764267858,-0.388262750252755,0.388777031529972,-
0.998677181943614,0.317951097761706,0.869967784877692
"5174","DNAJC3-
DT",4.74058334185942,0.627491732589625,0.628163128738015,0.99893117548
9814,0.317828032769016,0.869967784877692
"5175","LRR1",9.83638895620079,0.460945568234343,0.461818684220918,0.9
98109396574008,0.318226314119566,0.869967784877692
"5176","SPIRE2",2.88993141699241,0.798140612490536,0.798781857883926,0
.99919722088445,0.31769916189041,0.869967784877692
"5177","BABAM1",66.390731443707,-0.200920059917538,0.2012915206021,-0.
998154613351567,0.318204390970784,0.869967784877692
"5178","KAT14",8.72652287741767,-0.469859927094059,0.470431020816611,-
0.998786020272302,0.317898359574928,0.869967784877692
"5179","RANBP1",156.411477185134,-0.20525946075009,0.205658719448205,-
0.998058634716845,0.318250926953845,0.869967784877692
"5180","TSPYL2",40.9252037815103,0.308487364422692,0.309048456574517,0
.998184452502871,0.318189924136013,0.869967784877692
"5181","KCNIP3",8.3727658230239,-0.464690926316413,0.465833415232673,-
0.997547430306841,0.318498863470274,0.870179946091116
"5182","DUBR",18.0777495906361,0.341798947937307,0.342623178132953,0.9
97594353656582,0.318476100155957,0.870179946091116
"5183","SLC12A7",14.4341053559994,0.355773174856636,0.356700006286444,
0.997401650088385,0.318569590728947,0.870179946091116
"5184","LETMD1",81.1097783477897,-0.219404587583221,0.219978327413269,
-0.997391834746659,0.318574353145222,0.870179946091116
"5185","AP000766.1",4.13926934778558,0.771868397627341,0.7740475337447
43,0.997184751552841,0.318674841042238,0.870286547571474
"5186","BEND5",21.5817508445339,0.323135686391599,0.324665847210566,0.
995286967101361,0.319596714631386,0.870506748532431
"5187","NES",23.3731202497796,-0.304495998335639,0.305676006428093,-0.
996139677084104,0.31918228398987,0.870506748532431
"5188","PRRT3-
AS1",2.31588522812604,-0.917163052839238,0.92105776057457,-0.995771483
720084,0.319361188667022,0.870506748532431
"5189","NREP",46.0678555685106,0.274719274338664,0.275979850454246,0.9
95432361770226,0.319526025621357,0.870506748532431
"5190","CELF1",78.5243717414075,0.213577241298138,0.21438385561123,0.9
96237523059782,0.319134751791138,0.870506748532431
"5191","PUS1",8.66950622803725,-0.551578596133666,0.553821648269619,-0
.995949865551554,0.319274504974162,0.870506748532431
"5192","PAN3",31.5054789475191,0.28088028648654,0.281882021736593,0.99
6446260588447,0.319033365520528,0.870506748532431

"5193","DMAC2L",27.8572123261431,-0.327362970433696,0.328868424710721,-0.995422320405648,0.319530907274254,0.870506748532431
"5194","RIOX1",3.677764342058,-0.688863809481148,0.692095443161945,-0.995330653145131,0.319575473966058,0.870506748532431
"5195","TCF25",287.538779439501,-0.146306737073317,0.146965082602477,-0.995520394929856,0.319483230008461,0.870506748532431
"5196","RSAD1",26.2774017982489,0.280824299750039,0.282070018300232,0.995583654875128,0.319452479728982,0.870506748532431
"5197","ADGRL1",12.3334505979969,-0.401851197016852,0.403375545534681,-0.996221019011432,0.319142768900854,0.870506748532431
"5198","LTBP4",931.344042038167,0.268728000088682,0.270011361390522,0.995247009995318,0.319616143052268,0.870506748532431
"5199","MORC3",55.1245295925115,0.233437640925271,0.234363339959661,0.996050154283734,0.319225776965809,0.870506748532431
"5200","BAHD1",8.40857215327677,0.521924020206182,0.524522382595957,0.995046231627114,0.319713779601468,0.870605215222459
"5201","NHLRC1",1.69966189651881,1.12022634899263,1.12627505865148,0.994629456088553,0.319916515731114,0.87065498034837
"5202","C10orf143",6.78063257760605,-0.539596006298477,0.542424677168293,-0.994785136095609,0.319840776974286,0.87065498034837
"5203","LINC02256",13.5268655470253,0.389467112845275,0.391534222681195,0.99472048746655,0.319872227288327,0.87065498034837
"5204","GCA",12.7569589199555,-0.381855732201098,0.38452712221681,-0.993052791698253,0.32068422822439,0.871070146107302
"5205","JARID2",24.2068245026579,0.368609997407998,0.370794670242821,0.994108133125559,0.320170226201798,0.871070146107302
"5206","LINC02550",3.76101155613781,0.911034710115569,0.917043477293582,0.993447674699409,0.320491838110114,0.871070146107302
"5207","NUP107",31.8204106898503,0.282714089058279,0.284424810467889,0.993985329877532,0.320230009579666,0.871070146107302
"5208","RCBTB2",21.089630942243,-0.377976436156555,0.380569963439249,-0.993185149823029,0.320619733894087,0.871070146107302
"5209","AC048382.5",2.74123866645922,0.887828638137933,0.893438351892325,0.993721207800728,0.320358614863168,0.871070146107302
"5210","HOXB-AS1",29.7992654260057,0.327586397223662,0.329717080920177,0.993537842532852,0.320447918212482,0.871070146107302
"5211","COX6B1",423.568633217952,-0.263356443054858,0.265087328820293,-0.993470507348888,0.320480716170816,0.871070146107302
"5212","CD37",6.21527761511062,-0.577022686109238,0.581030161056163,-0.993102810808238,0.320659854345544,0.871070146107302
"5213","RP2",17.5155390188219,-0.329446496732429,0.331666583056525,-0.993306270702232,0.32056072258084,0.871070146107302
"5214","CSGALNACT2",51.0466238501559,-0.22915851496321,0.230850912503885,-0.992668872207094,0.320871349175214,0.871411258979867
"5215","DYSF",3.60423908145397,-0.912656695627938,0.919682795193682,-0.992360301179425,0.321021797263642,0.871485553921236
"5216","ESCO1",34.7668848756715,0.278420537761119,0.280556973342983,0.99238502056674З,0.321009743285451,0.871485553921236
"5217","ACKR2",11.2448480313887,0.613550864026656,0.619161862031882,0.

990937752550177,0.321715976436193,0.871634508679716
"5218","ZIC4",5.07378820312973,-0.747332319704675,0.754623001972692,-0
.990338642939643,0.322008624634208,0.871634508679716
"5219","TAPT1",24.9754068382819,0.302085486453053,0.304720687798052,0.
991352076014134,0.321513692667049,0.871634508679716
"5220","CYTH3",57.9963395330796,0.287801701952828,0.290465721316174,0.
990828455243275,0.321769352144398,0.871634508679716
"5221","ACTA2",104.301766347174,0.319230567446537,0.322129706830674,0.
991000086851162,0.321685537858733,0.871634508679716
"5222","NAALAD2",15.7461446891725,0.533739456240996,0.539005375475627,
0.99023030293532,0.322061564224031,0.871634508679716
"5223","SESN3",113.446858284324,0.305928895578397,0.308376026731344,0.
992064457218399,0.321166083358905,0.871634508679716
"5224","UPF3A",110.085221779144,-0.199405274805378,0.201145495652193,-
0.991348447345676,0.321515463919227,0.871634508679716
"5225","ITGA11",63.816317107845,0.36490574117298,0.368096733423099,0.9
91331104135468,0.321523929701799,0.871634508679716
"5226","MESD",139.473381332697,0.177900613810022,0.179449680700849,0.9
91367680985681,0.321506075526864,0.871634508679716
"5227","COX4I1",750.637160515822,-0.23134444610419,0.233302199607769,-
0.991608508162932,0.321388537267067,0.871634508679716
"5228","SSH2",37.906602241948,0.43494237616651,0.439203702592959,0.990
297608145624,0.322028675332765,0.871634508679716
"5229","G6PC3",40.8620959882871,-0.277517668269,0.280248819367924,-0.9
90254549135716,0.32204971599347,0.871634508679716
"5230","KCNK15",12.5085955105087,-0.428900224196365,0.432592129407241,
-0.991465620939671,0.321458271506885,0.871634508679716
"5231","MMP9",2.52106118256465,-0.879517818705257,0.887450228347414,-0
.991061572369046,0.321655515594117,0.871634508679716
"5232","PKNOX1",26.4692505655446,-0.304438386886787,0.307308646812732,
-0.990660009226181,0.321851624644163,0.871634508679716
"5233","WWTR1",94.8358528489334,-0.269189036085462,0.271902756139679,-
0.990019519872674,0.322164578176785,0.871746689658565
"5234","SIRT2",62.8012772750634,-0.22020022613219,0.222523548241967,-0
.989559207876505,0.322389616714635,0.872022344351333
"5235","UQCC1",15.821857722877,0.348086394100381,0.351737281273426,0.9
8962041453261,0.322359687933606,0.872022344351333
"5236","GNL1",66.1292205507316,-0.218294415010897,0.220654106130129,-0
.989305926997612,0.322513485064964,0.872034555240454
"5237","PKIG",102.351327205282,-0.223624313953116,0.226043401570498,-0
.989298127702135,0.322517299844227,0.872034555240454
"5238","HS1BP3",28.8242033868867,0.297349991736871,0.300767090415613,0
.98863872149702,0.322839934025404,0.872141648693504
"5239","STK10",14.6341408624972,-0.380046279903823,0.384273547223291,-
0.988999327822554,0.322663470540993,0.872141648693504
"5240","CD2BP2",75.7728163281698,-0.244938463775691,0.247705011464395,
-0.988831280916164,0.322745696828711,0.872141648693504
"5241","DNAJA2",105.23329703165,-0.196210516231649,0.198441021570976,-
0.988759857605706,0.322780648790609,0.872141648693504
"5242","SP2",18.0555876295392,0.435449078586999,0.440475886283918,0.98

8587780050054,0.322864867404756,0.872141648693504
"5243","AL031432.4",1.55766628867722,1.15491861654065,1.16867336738665
,0.988230457517178,0.32303979483114,0.872303280442415
"5244","ARPC5",246.367327958684,-0.163038518281401,0.165004033157925,-
0.988088079794738,0.323109513129977,0.872303280442415
"5245","AC114760.2",2.69689090871014,1.07235966930601,1.08517502832258
,0.988190514265348,0.323059352909329,0.872303280442415
"5246","DNAJC11",16.4040973855253,0.36097667183665,0.36603336681846,0.
986185152939026,0.324042263607511,0.873099706397128
"5247","ZNF691",3.78619245002049,-0.72137974896676,0.731438175251474,-
0.986248425875151,0.324011221190564,0.873099706397128
"5248","MKNK1",30.2693754814384,0.308173179556308,0.312614302942637,0.
985793601429862,0.32423440628284,0.873099706397128
"5249","MORN2",51.5697321415132,-0.268334007830171,0.271963679762291,-
0.986653835779495,0.323812368481508,0.873099706397128
"5250","TIA1",53.3037098583825,0.290192543837196,0.29433964321915,0.98
5910496674529,0.32417703552633,0.873099706397128
"5251","CWC27",52.6065492149081,-0.21121448444544,0.214230347710742,-0
.985922334078579,0.324171226241844,0.873099706397128
"5252","REEP2",1.57030635485896,-1.06671457591704,1.08373751432184,-0.
984292378754228,0.324971775670134,0.873099706397128
"5253","HIST1H1D",1.52418896332013,-1.59501916231195,1.61933759167257,
-0.984982483278547,0.32463267525203,0.873099706397128
"5254","BRD2",210.064678571891,0.283285112436945,0.287393317211846,0.9
85705287740308,0.324277753945929,0.873099706397128
"5255","SNX3",456.290637088607,-0.216862874964405,0.220312331643747,-0
.984342879703533,0.324946952929751,0.873099706397128
"5256","RIDA",23.889751698446,0.397413424950229,0.402501479898958,0.98
7358916170927,0.323466717452149,0.873099706397128
"5257","FBXO10",4.55321846767799,0.647183021296426,0.656955164087943,0
.985125099358822,0.324562625941657,0.873099706397128
"5258","AL499604.1",3.92656246936365,0.655449389655146,0.6656872207971
01,0.984620658438214,0.324810438359757,0.873099706397128
"5259","DPCD",37.1228124748053,-0.274020098609475,0.278050974631184,-0
.985503104144644,0.3243770074283,0.873099706397128
"5260","GAS2",2.23846093863181,0.857492661224761,0.870713536685178,0.9
84816044653734,0.324714438020676,0.873099706397128
"5261","PYGM",7.10106658419146,0.600192386443785,0.607969415173028,0.9
87208190847841,0.323540587199233,0.873099706397128
"5262","C11orf68",55.4885953299288,-0.240349170253463,0.24378481295438
4,-0.985907068371958,0.324178718001198,0.873099706397128
"5263","KBTBD3",13.9479072804831,0.392772600648135,0.398499107383227,0
.985629812892943,0.324314802815513,0.873099706397128
"5264","MFAP5",2197.287988855,0.36076029501566,0.366515369065433,0.984
297864331181,0.324969079283805,0.873099706397128
"5265","AC026124.1",2.26514412301968,-0.940564663229261,0.955282136154
362,-0.984593585111569,0.324823741924722,0.873099706397128
"5266","GREM1",8.19254364684072,-0.889821635990405,0.903670963802737,-
0.984674368916257,0.324784046611677,0.873099706397128
"5267","MED31",41.7849460567513,-0.267940315069358,0.271695449025368,-

0.986178885331057,0.324045338672199,0.873099706397128
"5268","GRN",269.966099158673,0.28310826879563,0.287647529357555,0.984219365373784,0.325007666131304,0.873099706397128
"5269","LIG1",8.02638993254019,0.590106719291327,0.598362596523366,0.986202551295807,0.32403372758412,0.873099706397128
"5270","TTLL12",10.6507137724976,0.416358623714223,0.422925271395906,0.984473267203898,0.32488286924467,0.873099706397128
"5271","ZNF185",3.77427358289111,0.667843896724089,0.678246965052628,0.984661828412706,0.324790208521127,0.873099706397128
"5272","TCEANC2",8.26174762588605,0.538683204775727,0.547400306778319,0.984075452836525,0.325078415337796,0.873100727490279
"5273","ZNF90",4.85365642311209,0.749936157404175,0.762155213479438,0.983967758982478,0.325131365540695,0.873100727490279
"5274","LRPPRC",53.7894173902173,0.229799645126562,0.233627478703529,0.983615653440215,0.325304525632581,0.873146033008929
"5275","DAGLB",6.76391739369185,0.515712071256006,0.524250090042952,0.983713843928484,0.325256231028952,0.873146033008929
"5276","ZBTB6",11.7914112361468,0.397350903297546,0.404003570024868,0.98353314866274,0.325345108887722,0.873146033008929
"5277","NRIP3",2.8818077666233,0.849138484080837,0.863628512832506,0.983221919452213,0.325498228992507,0.873146033008929
"5278","SDHD",192.069497072474,-0.214067911218715,0.217729842910255,-0.983181305591402,0.325518213859755,0.873146033008929
"5279","SMAD4",59.3509582960936,0.244223740041956,0.248395451109577,0.983205364474324,0.325506375106069,0.873146033008929
"5280","ADD3-AS1",4.59275757058899,-0.677084114837943,0.688772185129989,-0.983030571581749,0.325592392538625,0.873179598171766
"5281","SRSF10",166.22452382073,-0.139661567120274,0.143034750035576,-0.976417039114881,0.3288578410751,0.873286005057144
"5282","PTCH2",56.6683528453876,0.228857709348997,0.234979591966805,0.973947173171223,0.3300827695325,0.873286005057144
"5283","GADD45A",187.742750553957,0.354273329949634,0.362362501847182,0.977676575649211,0.328234310678915,0.873286005057144
"5284","GBP3",57.3482936129184,-0.24691635523038,0.25342636425793,-0.974312029268887,0.329901633659364,0.873286005057144
"5285","NDUFS2",86.7337291189247,-0.216672275542074,0.222300021123423,-0.974684007887591,0.329717028027703,0.873286005057144
"5286","SUCO",40.8613472215101,0.24781234315796,0.252368044207143,0.981948185779639,0.326125375211934,0.873286005057144
"5287","RASAL2",21.7880976180033,-0.348176966709907,0.356181144685902,-0.977527788611456,0.328307927359674,0.873286005057144
"5288","NEK7",59.3095072209769,0.301570809744833,0.309958768583451,0.972938468955237,0.330583884230932,0.873286005057144
"5289","LPGAT1",38.6372895915033,-0.276756587082395,0.283246114785463,-0.977088731797845,0.328525225808738,0.873286005057144
"5290","MAP10",6.10895274710697,-0.584637214657329,0.601582597719131,-0.971831992604091,0.331134137087975,0.873286005057144
"5291","PCBP1-AS1",26.516975003945,0.352724060806766,0.362914469310279,0.97192063319

2498,0.331090034135723,0.873286005057144
"5292","CCDC115",72.7281651954356,-0.304134241597573,0.311482749522322,-0.976407977854254,0.328862329609375,0.873286005057144
"5293","AC019080.1",3.36369648142019,0.805267266857384,0.827850636024685,0.972720478568759,0.330692244447719,0.873286005057144
"5294","PARD3B",54.6936658993377,0.255376452367367,0.262512866856103,0.972814991607067,0.330645260410489,0.873286005057144
"5295","SETMAR",17.7700689853225,0.329383498678121,0.338153783502205,0.974064212047988,0.330024657586,0.873286005057144
"5296","AC093495.1",2.00810973964792,0.929411934666241,0.951224532798134,0.977068928123911,0.328535029261047,0.873286005057144
"5297","ZNF385D",19.1390245431119,0.356870714175136,0.363155150202148,0.982694900448157,0.32575762130555,0.873286005057144
"5298","ZBTB47",29.259593910178,-0.329482378017419,0.335523686034253,-0.981994391846847,0.326102611084116,0.873286005057144
"5299","ACOX2",21.5679621117881,0.333822684329558,0.340620463147344,0.980042952337698,0.327064917012446,0.873286005057144
"5300","RIOX2",31.1754426190469,0.268389058551784,0.274134041320748,0.979043161727432,0.327558653630267,0.873286005057144
"5301","LNP1",5.46387523200827,0.671721645997519,0.68795904344198,0.9763977265809,0.328867407669432,0.873286005057144
"5302","PIK3CA",52.3240037397542,0.285653874721807,0.29358062562396,0.972999747904664,0.330553427391348,0.873286005057144
"5303","UCHL1",18.6860076876899,-0.387897105329479,0.397972785722776,-0.974682488967183,0.329717781701794,0.873286005057144
"5304","AC107214.1",2.05525128249692,0.88387647113888,0.905129249670371,0.976519620220835,0.328807029854345,0.873286005057144
"5305","ZNF622",44.9603692041303,-0.248739882061222,0.25461398653382,-0.976929372370448,0.328604119203208,0.873286005057144
"5306","FST",159.89766981688,0.395683835243653,0.406143782721767,0.974245703312218,0.329934556949579,0.873286005057144
"5307","MZB1",2.08532421491529,0.945928006365983,0.965150440300933,0.980083484260799,0.327044910925396,0.873286005057144
"5308","AC139795.3",2.69751456242031,-0.950655989318814,0.97710636400116,-0.972929892121433,0.330588147230398,0.873286005057144
"5309","DTNBP1",40.9966087199831,0.220171025001372,0.225811133382786,0.975022895032134,0.329548903294038,0.873286005057144
"5310","NELFE",84.8819096832538,-0.223743894828401,0.229012247203052,-0.976995324752305,0.328571466947962,0.873286005057144
"5311","ME1",12.5987967417565,0.558015124660037,0.573594194755502,0.972839561073129,0.330633047218874,0.873286005057144
"5312","AK9",8.91316133671675,-0.500416661726364,0.513769689632395,-0.974009700892271,0.330051722549288,0.873286005057144
"5313","RPA3",66.0246842982796,-0.261485722168069,0.26909171655844,-0.971734565122819,0.331182616324637,0.873286005057144
"5314","THSD7A",2.95321003186379,0.761508212772396,0.783630687903227,0.971769258820089,0.331165352456099,0.873286005057144
"5315","PHKG1",15.682689884194,-0.4300862882325,0.439872429208685,-0.977752320158393,0.328196838010227,0.873286005057144
"5316","GTF2IRD2",29.1938305429521,0.358626094620277,0.368373871933588

,0.973538358564507,0.330285805456504,0.873286005057144
"5317","CTSB",251.850235806372,-0.154343223560724,0.158768857524221,-0
.972125301948326,0.330988216108864,0.873286005057144
"5318","SORBS3",123.336258260133,-0.180671083086297,0.185919053524795,
-0.971772820810979,0.33116358001314,0.873286005057144
"5319","AC090136.3",3.81267873559412,0.724787476764706,0.7404668799175
7,0.978824977081204,0.327666466215359,0.873286005057144
"5320","UBE2R2",162.577392710261,-0.163168213823558,0.166126915690343,
-0.982190111370637,0.326006198311847,0.873286005057144
"5321","DENND1A",15.217261833386,0.399520480435251,0.409559983403751,0
.975487099874689,0.329318697557054,0.873286005057144
"5322","CDNF",2.07247839426672,-0.959905489007579,0.982690450579457,-0
.9768136939171,0.3286613953379,0.873286005057144
"5323","NMT2",45.6529398712879,0.248610218569832,0.254543135390679,0.9
76691900130243,0.328721706366167,0.873286005057144
"5324","ZNF511",63.7940693129223,-0.294654607020679,0.302783091312796,
-0.973154100987364,0.330476718929999,0.873286005057144
"5325","PACSIN3",13.5419385372916,0.436463438483164,0.444812207060523,
0.981230801572352,0.326478938050456,0.873286005057144
"5326","MRPL11",114.243830037934,-0.249581070185299,0.254176864050762,
-0.981918913498969,0.326139797184488,0.873286005057144
"5327","C2CD3",8.61304614988735,0.445466207886621,0.456227144477855,0.
97641320398954,0.328859740816022,0.873286005057144
"5328","YAP1",87.3019853424676,0.233548193711324,0.238921343876487,0.9
77510798834529,0.328316334223282,0.873286005057144
"5329","EXPH5",2.43540244190014,1.18461436368378,1.21503857820922,0.97
4960289268936,0.329579958351698,0.873286005057144
"5330","C11orf71",17.8024912223729,0.359100176446967,0.36626743151752,
0.980431634227324,0.326873100871088,0.873286005057144
"5331","ANO6",124.739979494294,0.23172140243066,0.236290239337623,0.98
0664301158737,0.326758313732917,0.873286005057144
"5332","LMBR1L",24.9877836476491,0.316968702028096,0.323679496255348,0
.979267163027351,0.32744799079193,0.873286005057144
"5333","FARP1",56.8883364665128,0.230591618905141,0.235604325769397,0.
978724045715686,0.32771634768639,0.873286005057144
"5334","ABHD13",29.6860112227826,0.292331534816298,0.298477041875779,0
.979410453075858,0.327377214273571,0.873286005057144
"5335","PSME2",215.097110602959,-0.321756149887709,0.329502013480356,-
0.976492211653482,0.32882060556734,0.873286005057144
"5336","KHNYN",26.9565804962112,0.305639416835497,0.314352788821279,0.
972281550233882,0.330910499793633,0.873286005057144
"5337","JDP2",194.83784623309,-0.205191507602688,0.210208617111681,-0.
976132712455229,0.328998702431385,0.873286005057144
"5338","VRK1",11.8182291820872,-0.370364639574696,0.378892403001237,-0
.9774929152472,0.328325183512048,0.873286005057144
"5339","AC022613.3",3.45458301203554,0.812760664378372,0.8333171506060
79,0.975331737487035,0.32939573235593,0.873286005057144
"5340","EHD4",27.4090931042079,0.305514518550883,0.310866762413113,0.9
82782836541666,0.325714330894959,0.873286005057144
"5341","ZNF280D",90.2026683432371,0.218807380341377,0.223258770977832,

0.980061743523181,0.327055641802466,0.873286005057144
"5342","POLR3K",25.5583252534778,-0.347850271686567,0.357338088302686,
-0.973448627709447,0.330330380696475,0.873286005057144
"5343","C16orf72",36.8781512254919,0.300914014412278,0.309508203456126
,0.97223275846042,0.330934767060077,0.873286005057144
"5344","DCUN1D3",48.4473377340124,0.370745304637358,0.378750496008757,
0.978864208876934,0.327647078729244,0.873286005057144
"5345","ZNF843",2.07257912761036,-0.904385560647656,0.921686972176836,
-0.981228538482738,0.326480053808217,0.873286005057144
"5346","ORC6",2.77870261284088,0.840905664047878,0.863480225453523,0.9
73856307602426,0.33012789052012,0.873286005057144
"5347","PSKH1",16.366438583251,0.334442148824642,0.342486536539852,0.9
76511813292041,0.32881089666026,0.873286005057144
"5348","APRT",393.474628741551,-0.285622640580909,0.290810410590544,-0
.982160989356949,0.326020542840828,0.873286005057144
"5349","HSD17B1",6.13755314693555,-0.616853779258402,0.62834431313207,
-0.981712997741012,0.326241260215447,0.873286005057144
"5350","LRRC37A2",4.36114452568494,0.669094686865734,0.683805220711789
,0.97848724548967,0.327833396493187,0.873286005057144
"5351","NME2",1424.78869085332,-0.273430444071471,0.280578910103613,-0
.974522439945673,0.329797202781316,0.873286005057144
"5352","GNA13",45.138247204098,0.263682149955696,0.270851090486804,0.9
73531801115429,0.330289062842522,0.873286005057144
"5353","SEPTIN9",61.5973610965095,-0.232283939014004,0.238666302932501
,-0.973258210982966,0.330424986154996,0.873286005057144
"5354","ROCK1",190.809189466236,-0.221793700987968,0.227024259406801,-
0.976960354666498,0.328588779967667,0.873286005057144
"5355","THOP1",12.9641528598461,-0.38975515726551,0.397780481851842,-0
.979824740145693,0.327172637675468,0.873286005057144
"5356","TMEM38A",3.4949687537905,-0.707885044567302,0.725627969958156,
-0.975548178783851,0.329288415422012,0.873286005057144
"5357","GMIP",2.48419979978874,-0.845376489283794,0.866608159494613,-0
.975500265052662,0.329312170279148,0.873286005057144
"5358","RPS16",3317.59442060226,-0.19746188218517,0.202680022810744,-0
.974254292291814,0.329930293378642,0.873286005057144
"5359","SNRPD2",299.314600032614,-0.211797529796794,0.217498835144619,
-0.973786961461038,0.330162328322415,0.873286005057144
"5360","ZNF577",10.6245675596271,0.398118040068043,0.405662235669109,0
.981402765804359,0.326394162783748,0.873286005057144
"5361","ZNF550",4.31771436015481,0.646705403412496,0.663167433640973,0
.975176660684171,0.329472637192395,0.873286005057144
"5362","NSFL1C",79.2805553774878,-0.187165184761549,0.191534025555409,
-0.977190262768241,0.328474967708749,0.873286005057144
"5363","NXT1",53.8033911175634,-0.288569733881948,0.294911773215899,-0
.978495130035694,0.327829498776997,0.873286005057144
"5364","LINC00310",14.1048754769847,0.589915754978139,0.60184593525362
1,0.980177351749572,0.326998582073273,0.873286005057144
"5365","CSTB",455.719421686873,-0.235135460292404,0.239951554718519,-0
.979928888430148,0.327121221899159,0.873286005057144
"5366","LSS",26.408241505063,0.333529431404971,0.340143702546466,0.980

554480085981,0.326812491124338,0.873286005057144
"5367","CRKL",25.890084214761,0.29023992687163,0.298224734623141,0.973225534891996,0.330441222502844,0.873286005057144
"5368","HCCS",12.7574563672448,-0.382417629659347,0.390087010161642,-0.980339308147902,0.326918657560131,0.873286005057144
"5369","AL732509.1",2.68343413704305,0.846943892406869,0.866131470885025,0.977846806029863,0.328150097419406,0.873286005057144
"5370","PIN4",84.1739764556952,0.241227238678901,0.246606195528709,0.978188070910887,0.327981315317511,0.873286005057144
"5371","ADAT2",9.80184237166822,0.476079757313443,0.490129357524105,0.97133491394673,0.331381527999003,0.87348518921554
"5372","SLC25A45",16.0355896405482,0.47748259649197,0.491515243887648,0.971450229529635,0.331324125981422,0.87348518921554
"5373","HMGXB3",27.2432166483805,-0.285176616506163,0.293694751150651,-0.970996639840804,0.331549951844999,0.873766483924287
"5374","ID2",258.392350645963,0.218522071744839,0.225146485326559,0.970577317375787,0.331758805793815,0.873824143486762
"5375","HK2",1.91789418713847,-1.00717413961294,1.03783473528924,-0.970457150224645,0.331818673695503,0.873824143486762
"5376","NAPB",8.45497384678221,0.524969011325409,0.540772335829991,0.970776381376229,0.331659646433354,0.873824143486762
"5377","COL6A1",3178.07151425882,-0.22359177942278,0.230340555142898,-0.970700879330906,0.331697253863809,0.873824143486762
"5378","ATAD2B",30.1136175375168,0.29589208055514,0.305146475165599,0.969672287364825,0.332209868191361,0.87399852478661
"5379","COBLL1",43.6580148507309,-0.252449882753946,0.260439838831944,-0.969321298485544,0.332384905854955,0.87399852478661
"5380","NCK1",70.5088304718915,0.185799177407159,0.191721864796911,0.969107918932327,0.33249134705158,0.87399852478661
"5381","TMEM14C",321.716803909074,-0.203060537751113,0.209538132443883,-0.969086320388464,0.332502122388804,0.87399852478661
"5382","DCP1B",14.6172892309,0.364381465948095,0.375933559356794,0.96927091737044,0.332410035735033,0.87399852478661
"5383","AC022364.1",3.0775234628152,0.783727143022245,0.8082917843295,0.969609190909156,0.332241329914351,0.87399852478661
"5384","ISCA2",43.5313671178501,0.275171810539955,0.283646808494283,0.970121299797743,0.331986033156426,0.87399852478661
"5385","CIPC",7.83688277107841,0.542415934359664,0.55971140811372,0.969099300990946,0.332495646445145,0.87399852478661
"5386","AC092279.1",8.85884442179819,0.639456074869291,0.659447437854507,0.969684675627437,0.332203691270348,0.87399852478661
"5387","MYH9",125.006877342475,0.170277111126472,0.175691006019359,0.969185133516223,0.332452827170394,0.87399852478661
"5388","CAMLG",246.843947660605,-0.179879217088926,0.185674445096945,-0.968788230362055,0.332650860106105,0.874227204733193
"5389","PTGES2",35.2716110386994,-0.279199160218043,0.288240941697568,-0.968631168680361,0.332729246315518,0.874270945969148
"5390","RBM17",108.257296800027,-0.177396371047435,0.183180483432147,-0.968423970303289,0.332832672948214,0.874380454350039
"5391","LYPLA1",51.3743581115994,-0.24089492325663,0.248789967171163,-

0.968266228721751,0.332911426286329,0.874425115231759
"5392","AIDA",151.485805219864,0.175514595753186,0.181317429206557,0.967996273282914,0.333046230909123,0.87445478021012
"5393","ZBTB38",122.548626660173,0.257806946285128,0.266296967401726,0.968118220799,0.332985330981473,0.87445478021012
"5394","ZDHHC9",51.2596077645075,0.249031504943164,0.257309488860084,0.967828687727016,0.333129933954784,0.874512396143816
"5395","SEMA4C",17.4039746649528,-0.348367440581814,0.360096105437401,-0.967429070521769,0.33332958337412,0.874524309987485
"5396","CENPC",74.3830905946775,0.193241413742798,0.199806992218902,0.967140396823992,0.333473853258988,0.874524309987485
"5397","TRIB1",21.8178040748221,-0.623046527471998,0.644143239157236,-0.967248415565395,0.333419864228803,0.874524309987485
"5398","TSPAN9",22.9278113174924,0.329420365472475,0.340467920723517,0.967551846800825,0.333268235925484,0.874524309987485
"5399","PHETA1",8.14525390903696,-0.4919766460569,0.508789875935825,-0.966954472417518,0.333566793661187,0.874524309987485
"5400","ZNF563",7.19436983186382,0.504367652426663,0.521277720590199,0.967560347401017,0.333263988712418,0.874524309987485
"5401","AC020911.2",2.98986322544923,0.977025946023238,1.01034056823596,0.967026344125835,0.333530864253233,0.874524309987485
"5402","PEA15",124.371551644981,0.192354952234564,0.199360245386294,0.964861132979867,0.334614371104184,0.874927483702429
"5403","AC079922.2",6.42527041395393,-0.498233505262866,0.516022831297067,-0.965526087306085,0.334281376046033,0.874927483702429
"5404","IL20RB",1.72740611624828,-0.95547056757114,0.989641071428793,-0.965471821204511,0.334308543352537,0.874927483702429
"5405","RSRC1",118.557668444827,-0.18259516807242,0.189272620773352,-0.964720450989433,0.334684849059286,0.874927483702429
"5406","PDCD6",193.810548073337,-0.198941852291542,0.205951680710744,-0.965963723165498,0.334062333884607,0.874927483702429
"5407","PLPP1",243.034389271929,0.342540327121798,0.355035731404231,0.964805220497071,0.334642380633331,0.874927483702429
"5408","PNMA2",1.92190992366073,-1.22656773491484,1.2700727997307,-0.965746006980792,0.334171291956488,0.874927483702429
"5409","EXTL3",7.06589424870586,-0.491087006593141,0.508116030787458,-0.966485953674939,0.333801072205554,0.874927483702429
"5410","PIP5KL1",2.0574692856844,-0.919489004037538,0.953162459647585,-0.964671861266444,0.334709193447462,0.874927483702429
"5411","TRIP11",130.614895554755,0.194203276913717,0.20107880432993,0.965806801770458,0.334140864337821,0.874927483702429
"5412","CATSPER2",1.68283485113275,-1.04709728481785,1.08468930936581,-0.965343048720622,0.334373016579128,0.874927483702429
"5413","KPNB1",155.169301968191,0.169724987109864,0.175803953535791,0.965421901477948,0.334333535984823,0.874927483702429
"5414","SMAD7",45.0500456858375,-0.300643681560184,0.31143961926897,-0.965335374689557,0.334376859031459,0.874927483702429
"5415","NCLN",29.2162193748824,0.29055901819856,0.300881628474172,0.965692121755788,0.334198262851265,0.874927483702429
"5416","ZNF175",5.97759133149369,0.532239239407322,0.551382207501191,0

.9652818537968,0.334403658187164,0.874927483702429
"5417","PYGB",34.7799539603357,-0.261732740760836,0.271250268118421,-0
.964912376221395,0.334588701972725,0.874927483702429
"5418","ARMCX2",39.58034304723,0.300994369805847,0.312087642878819,0.9
64454622519996,0.334818048204151,0.875050491430559
"5419","CD55",161.755830366466,0.283618423554854,0.294203853993278,0.9
64020082351927,0.335035857567022,0.875296631577313
"5420","REXO2",243.185346260588,-0.201162357109234,0.208653733926489,-
0.964096607924136,0.334997493189393,0.875296631577313
"5421","OTUD3",11.0092224541779,0.460695378795069,0.47802617622475,0.9
63745087002239,0.335173743671692,0.875333863960008
"5422","POLR1C",15.700425122339,-0.422135185222548,0.437966785395988,-
0.963852052937928,0.335120105271594,0.875333863960008
"5423","AJAP1",1.98546317374806,1.44626609998627,1.51278444933226,0.95
6029195451242,0.339057484539167,0.875458527392018
"5424","EPHA2",5.44694775827147,0.591530599962347,0.617472639393989,0.
957986738558809,0.338069445748452,0.875458527392018
"5425","MEAF6",119.882811118132,-0.20774624646545,0.217818485531583,-0
.953758566259646,0.34020586704607,0.875458527392018
"5426","YRDC",12.0001479133105,-0.396167788140893,0.414379252364115,-0
.956051216079662,0.339046359683221,0.875458527392018
"5427","PLK3",85.2779931171923,-0.278210730188063,0.291469566442826,-0
.954510392228672,0.339825351430524,0.875458527392018
"5428","WLS",58.2085251740093,0.275918296781924,0.289157575531624,0.95
4214311261398,0.33997517192183,0.875458527392018
"5429","AC239868.1",1.64485015015949,1.0216697248913,1.06236432760637,
0.961694306126822,0.336203183360643,0.875458527392018
"5430","PRUNE1",12.6541883658359,0.363880099138249,0.377892767075356,0
.962918930559175,0.335588208666628,0.875458527392018
"5431","CEP350",78.6869247133218,0.274791167237702,0.286337016206439,0
.959677413972865,0.337217593969033,0.875458527392018
"5432","AC108488.1",12.6658101393207,-0.409363553858735,0.427446691591
787,-0.957694987260958,0.338216585202989,0.875458527392018
"5433","PLEK",6.43260301403584,-0.767983747977657,0.797631170760591,-0
.962830661752269,0.335632510740637,0.875458527392018
"5434","ANKRD23",1.57263643663514,1.18795422438434,1.24544979638065,0.
953835496088728,0.340166918635883,0.875458527392018
"5435","CNOT11",18.5429223942849,0.315832045878329,0.330274835296528,0
.956270390975346,0.338935644949411,0.875458527392018
"5436","IRAK2",2.56932151023896,-0.804442141007115,0.840148863792649,-
0.957499528566468,0.338315184237419,0.875458527392018
"5437","TMA7",625.870850777925,-0.189036320866265,0.197653400883656,-0
.956403077412958,0.3388686305572,0.875458527392018
"5438","CCDC66",67.3253430757207,-0.24991171453152,0.261209549680338,-
0.956748001125361,0.338694463744283,0.875458527392018
"5439","RAP1GDS1",17.7881893579351,-0.313871983096096,0.32753689233347
4,-0.958279786011203,0.337921693995227,0.875458527392018
"5440","BDH2",187.470575267385,0.248162296581108,0.257787000732823,0.9
62664121447729,0.335716107497572,0.875458527392018
"5441","TET2",22.9827415782245,0.310081015811012,0.322707860940481,0.9

60872210882405,0.336616424853748,0.875458527392018
"5442","CBR4",31.2037151581214,0.248111686537445,0.260013643700939,0.954225643723591,0.339969436781999,0.875458527392018
"5443","CCT5",130.008811253682,-0.212969866992236,0.222332376848982,-0.957889579604029,0.33811844153156,0.875458527392018
"5444","SCAMP1-AS1",13.2891393570337,-0.364592402694546,0.379006146609988,-0.961969630191051,0.336064859590332,0.875458527392018
"5445","MBLAC2",10.6092736879456,0.433673530726549,0.454177023013058,0.95485572530621,0.339650662269998,0.875458527392018
"5446","RAD50",108.879904705003,0.192536844182714,0.201517372327531,0.955435464242651,0.339357526762467,0.875458527392018
"5447","DIAPH1",64.0197995722857,-0.299996091867893,0.312706387800674,-0.959353897366234,0.337380491921644,0.875458527392018
"5448","NHP2",140.253522872144,-0.238009128109555,0.249150531752154,-0.955282440843104,0.339434884772789,0.875458527392018
"5449","LINC01011",6.10284901157266,0.584029777476218,0.612095286658038,0.954148463819981,0.340008497264873,0.875458527392018
"5450","HIST1H4J",2.07452366161221,-0.960297755589567,1.00688190754996,-0.953734244690376,0.340218181283718,0.875458527392018
"5451","PSMG3",51.6249495767727,-0.315490096353943,0.329088984572024,-0.958677169836704,0.337721403101396,0.875458527392018
"5452","UMAD1",17.2673040226803,-0.318514560656561,0.331982353210513,-0.959432203477961,0.337341058379013,0.875458527392018
"5453","BLVRA",62.4390433782351,-0.269253472084659,0.281739452771002,-0.955682526662351,0.339232653020039,0.875458527392018
"5454","TECPR1",12.3958120052958,0.454608859089786,0.474053619152077,0.9589819394332,0.337567843671211,0.875458527392018
"5455","GNB2",200.572502032879,-0.190899791093114,0.199600856242462,-0.956407676233719,0.338866308036883,0.875458527392018
"5456","MYOM2",4.34313749151699,-0.751451042216299,0.783304328553049,-0.959334724479831,0.337390147492199,0.875458527392018
"5457","UHRF2",26.1424740013373,0.299060607828363,0.311642764619186,0.959626347153615,0.33724330391033,0.875458527392018
"5458","HAUS6",19.1596962946915,0.374759296501361,0.391236311632619,0.957884749852334,0.338120877220855,0.875458527392018
"5459","RPP25L",31.9542004430364,0.277233091792372,0.290382468118333,0.954717044692233,0.339720807911598,0.875458527392018
"5460","CNTRL",27.4556596113504,0.268986812228068,0.280869758359921,0.957692326147032,0.338217927476575,0.875458527392018
"5461","DAB2IP",13.7844919597556,-0.397919462834057,0.415117278620507,-0.958571187777102,0.337774813118787,0.875458527392018
"5462","PPP6C",42.5367508411736,0.227603185218329,0.236613856549885,0.961918243238405,0.336090673794235,0.875458527392018
"5463","ZNF37A",62.295193516918,0.340755855783342,0.356859867653393,0.954873009464622,0.339641920470589,0.875458527392018
"5464","SHLD2",40.5534955340562,-0.224970594306987,0.234067632463969,-0.961135001618036,0.336484292683027,0.875458527392018
"5465","FGFR2",2.07070975955511,-1.11079484053134,1.15508051110752,-0.961660100616086,0.336220370886663,0.875458527392018

"5466","ARNTL",20.0418285281242,0.948602706210753,0.990578348085262,0.95762511672535,0.338251829238095,0.875458527392018
"5467","LTBP3",246.19684738713,0.160580680000305,0.168380742857149,0.953676039643908,0.340247652202063,0.875458527392018
"5468","KAT5",42.7848507235986,0.234801657584855,0.245295086856053,0.957221200776205,0.338455618392797,0.875458527392018
"5469","GDPD5",18.400813861769,0.454903920256825,0.475211320910112,0.957266589073688,0.338432714542829,0.875458527392018
"5470","SC5D",29.3646732983046,0.267604393066573,0.279316410772704,0.958068995395827,0.338027968441328,0.875458527392018
"5471","VWA5A",21.8646756965991,0.296878675619025,0.308144904161738,0.96343853690081,0.335327494802595,0.875458527392018
"5472","RBP5",52.6875993192162,-0.343370950298514,0.359597873495679,-0.954874807686093,0.339641010993378,0.875458527392018
"5473","AC092747.4",9.92578956001682,0.445246266614682,0.463466254053237,0.960687564026049,0.336709285948097,0.875458527392018
"5474","KLHL42",38.0231205945785,0.276751576868301,0.28793601512223,0.961156515105684,0.336473477098196,0.875458527392018
"5475","MARCH9",17.4324638967782,-0.328884621672473,0.343713808232832,-0.956856005766537,0.338639939371405,0.875458527392018
"5476","RXYLT1",50.874499379161,-0.216981045481751,0.226253205595725,-0.959018657483499,0.3375493461575,0.875458527392018
"5477","C12orf73",13.4877509901574,0.372737945624597,0.39083360536709,0.953699836723363,0.340235602846638,0.875458527392018
"5478","AC004812.2",6.19231237737474,0.531368694555073,0.557229731475029,0.953589990879525,0.340291224206615,0.875458527392018
"5479","P2RX7",5.22863413989459,0.636368776721231,0.665526030123798,0.956189161531153,0.338976674754233,0.875458527392018
"5480","AL162377.1",6.69377493878034,-0.585353025209557,0.61033665030955,-0.959065828527057,0.337525583677746,0.875458527392018
"5481","SLC22A17",28.7687968490786,-0.329159044273222,0.342204429554648,-0.961878385681907,0.336110697091813,0.875458527392018
"5482","AL365295.1",10.1825258770441,-0.457666012370431,0.477104732698468,-0.959256911542058,0.337429336336681,0.875458527392018
"5483","GPATCH2L",52.9027674824515,0.350521102892952,0.367163878642502,0.9546720777352,0.339743554512575,0.875458527392018
"5484","ANGEL1",4.02205541921815,0.741622563636948,0.7725356080768,0.959984958470964,0.337062785219659,0.875458527392018
"5485","NPTN",146.714343008817,-0.19167893539821,0.200482405550697,-0.956088564837874,0.3390274915676,0.875458527392018
"5486","HDGFL3",71.0305714732113,-0.219339460630322,0.228075111978873,-0.961698357735059,0.336201147552977,0.875458527392018
"5487","CHTF18",4.31051405097852,0.664724334628108,0.691842243462996,0.960803334732569,0.33665106156008,0.875458527392018
"5488","AP2B1",136.788582861022,0.250753950175955,0.262878136823766,0.953879060486727,0.340144863906777,0.875458527392018
"5489","CDC6",4.77135759752551,0.689203974254632,0.716341740100601,0.962116173989585,0.335991250411936,0.875458527392018
"5490","MRPL27",112.560277185048,-0.238575358354957,0.249004492772852,-0.958116681744339,0.338003924504305,0.875458527392018

"5491","UNC13D",1.65722857524059,-1.06834239304853,1.11131594479552,-0
.961330932082598,0.336385799822599,0.875458527392018
"5492","TIMM13",154.738404765693,-0.26450287901176,0.276954587121068,-
0.95504061427997,0.33955715855699,0.875458527392018
"5493","GADD45B",699.576810205561,0.300768793520962,0.313363444322878,
0.959808168342258,0.3371517705258,0.875458527392018
"5494","DUS3L",16.6161638068005,-0.377724018256366,0.393271022389045,-
0.960467455653777,0.33682000258286,0.875458527392018
"5495","MISP3",4.94335243223849,-0.613454688717262,0.641220459920469,-
0.956698556988261,0.338719426686651,0.875458527392018
"5496","RAB8A",72.0302265702603,-0.227546009820592,0.237487341302319,-
0.958139531028426,0.337992404054512,0.875458527392018
"5497","POLR3F",13.9418023177529,-0.393443565783915,0.409230039972054,
-0.961423960496113,0.336339041590945,0.875458527392018
"5498","E2F1",3.39275464155948,0.709222169147892,0.737924646232794,0.9
61103783114669,0.336499987717392,0.875458527392018
"5499","CNBD2",6.69520082378315,-0.493401725939467,0.514608409796111,-
0.958790638759585,0.337664226068789,0.875458527392018
"5500","SYS1",65.4444812050592,-0.201711979239525,0.209577950599173,-0
.962467562369232,0.335814789756938,0.875458527392018
"5501","VAPB",49.3654581901614,0.245584679594133,0.256939505014087,0.9
55807397467619,0.339169550287342,0.875458527392018
"5502","AC234772.2",1.97369276713186,-1.16446586891031,1.2153102006404
4,-0.958163494634263,0.33798032204242,0.875458527392018
"5503","SNX12",19.3195644120396,-0.294626275803904,0.308008062335267,-
0.956553778398187,0.338792528081308,0.875458527392018
"5504","HMGN5",12.476102204812,-0.38573237726848,0.40330904825609,-0.9
56418852828591,0.338860663617545,0.875458527392018
"5505","PUSL1",17.6110916048459,-0.359848509156955,0.378006401193188,-
0.951964062039909,0.341115208222232,0.875546096671453
"5506","PEX10",24.0813139606675,-0.30730382179697,0.323210490266982,-0
.950785420185857,0.341713315287175,0.875546096671453
"5507","ACADM",59.638851669723,-0.232218299107754,0.243738785936391,-0
.952734289766901,0.340724715087234,0.875546096671453
"5508","DIPK1A",15.9066914007465,0.359321691143069,0.377443849497779,0
.951987140924876,0.341103503434064,0.875546096671453
"5509","RAP1A",131.210553200492,-0.175793498561762,0.18520733319693,-0
.949171372036557,0.342533459210325,0.875546096671453
"5510","TTC27",16.4230620577794,0.375163078316276,0.394415407101007,0.
951187685779727,0.341509108429874,0.875546096671453
"5511","SOCS5",68.4486529896331,-0.286381904800527,0.301131857812235,-
0.951018291060702,0.341595090759621,0.875546096671453
"5512","TPRKB",63.9929309063345,-0.22145313066543,0.233200884195462,-0
.949623889418081,0.342303395357698,0.875546096671453
"5513","ZNF385B",7.19254132523798,0.684114670061243,0.719578768235817,
0.950715474469153,0.341748830633942,0.875546096671453
"5514","CAPN10-
DT",2.0117798375547,-0.988311174862322,1.04093826815582,-0.94944263756
7031,0.342395533541162,0.875546096671453
"5515","MSH3",30.4694196528235,0.341301018485288,0.359479831923427,0.9

49430227167762,0.342401842865858,0.875546096671453
"5516","KIAA0825",8.11915009990571,0.484074287686458,0.509578623500051
,0.949950145792193,0.342137585042832,0.875546096671453
"5517","GPC2",2.86628756843833,0.831785116159061,0.875425258321648,0.9
5014977949545,0.342036152356964,0.875546096671453
"5518","KLHDC10",45.6889318243659,0.244621795118876,0.257752061865402,
0.949058538459405,0.342590840197393,0.875546096671453
"5519","TTC26",8.95001874337763,0.450222823703088,0.472743095414955,0.
952362558162591,0.340913141302047,0.875546096671453
"5520","RHEB",170.75435676231,-0.230822569597957,0.242931445567756,-0.
950155172618765,0.342033412410271,0.875546096671453
"5521","HSF1",46.9664862671446,-0.215755227421043,0.227297589268389,-0
.949219162928662,0.34250915722861,0.875546096671453
"5522","CYHR1",69.0297942028594,-0.250426278011916,0.263430865697315,-
0.950633773870899,0.34179031758109,0.875546096671453
"5523","CARNMT1",15.0560496033701,0.367328965056702,0.385388052015064,
0.953140511585822,0.340518882498087,0.875546096671453
"5524","AL691447.2",3.98739344731464,0.682071880660231,0.7176364993484
8,0.950442015253493,0.341887703816729,0.875546096671453
"5525","DOLK",18.7070462264663,-0.337805853046339,0.355995440670982,-0
.948904998360768,0.342668932165522,0.875546096671453
"5526","CELF2",309.690804657272,0.216929607643223,0.22804095886045,0.9
51274756636916,0.341464917901901,0.875546096671453
"5527","EDRF1",19.6692478822159,0.440565990263336,0.463485235615483,0.
950550214783625,0.341832751588025,0.875546096671453
"5528","TNS2",131.035602329891,0.218320617388287,0.229364580490356,0.9
51849744723189,0.341173189683877,0.875546096671453
"5529","HMGB1",1201.9936899873,-0.168655934150567,0.177082474612308,-0
.952414599580281,0.340886758125886,0.875546096671453
"5530","AC000403.1",2.67206599361947,0.794640462926109,0.8355769847500
98,0.951008078763404,0.341600274817998,0.875546096671453
"5531","ZC3H7A",49.0286280178085,0.207449405449946,0.217834452359908,0
.952325966818123,0.340931692615129,0.875546096671453
"5532","KRBA2",7.44626274283497,0.514217640343466,0.539363155024389,0.
953379250238579,0.340397950801237,0.875546096671453
"5533","COX10",8.31653063843193,0.459470081830316,0.48289506097755,0.9
51490538959306,0.341355419023233,0.875546096671453
"5534","LINC00672",2.79743047201541,0.870336517792501,0.91649757033520
1,0.9496331970353,0.342298664319915,0.875546096671453
"5535","C17orf80",14.6814325227568,0.452015190010259,0.476002585309996
,0.949606586098446,0.342312190701491,0.875546096671453
"5536","CARM1",44.5978809190005,0.283089066487972,0.297947217847362,0.
950131598922996,0.342045389000437,0.875546096671453
"5537","GNAS",1631.19428595185,-0.185557760863886,0.194665852210001,-0
.953211663767874,0.340482837888999,0.875546096671453
"5538","PRELID3B",25.1357092211762,-0.259477281383939,0.27309885563572
1,-0.950122184803472,0.342050171908445,0.875546096671453
"5539","LSM14B",17.9916800339576,0.31279260092535,0.329434885646787,0.
949482324287659,0.342375357702458,0.875546096671453
"5540","BCL2L13",30.1834629359608,0.258742762491055,0.271672641520516,

0.952406400007398,0.340890914935386,0.875546096671453
"5541","NOL12",18.2364926548539,-0.322733407285431,0.339461554400148,-0.950721526788868,0.341745757439866,0.875546096671453
"5542","NKAP",70.384138397457,-0.225928647406811,0.238097003890007,-0.948893281795278,0.34267489179048,0.875546096671453
"5543","EDEM3",55.9462302447031,0.218027803332672,0.229805772577571,0.948748157573266,0.342748714643663,0.875576727287439
"5544","MGAT2",38.1089358923388,-0.250334425263029,0.263941036165487,-0.948448293224374,0.342901284040293,0.875808474388626
"5545","TRIM45",3.64454895015174,0.639076233590179,0.675391063863675,0.946231402491837,0.344030572639138,0.875946798424478
"5546","KCNK1",16.7043815468617,-0.384788764720274,0.406042822802216,-0.947655624263319,0.343304798801341,0.875946798424478
"5547","ADSS",56.0131639119767,-0.205526042688633,0.217112198169377,-0.946635170301644,0.343824715866526,0.875946798424478
"5548","ADAMTS9-AS2",13.1645867091688,0.548465789667386,0.57953357688254,0.946391739056302,0.343948817310761,0.875946798424478
"5549","NSA2",314.273750272132,-0.246692803433184,0.260648789022522,-0.946456741112533,0.34391567641206,0.875946798424478
"5550","DBN1",175.72966226498,-0.257719278599816,0.272163392916562,-0.94692851907099,0.343675204419825,0.875946798424478
"5551","LEPROTL1",42.6615551583799,-0.234391260040007,0.247576103241232,-0.946744281743628,0.343769100046586,0.875946798424478
"5552","PGM5-AS1",6.60798674220489,0.765325561693159,0.808982389842323,0.946034884445787,0.34413079375956,0.875946798424478
"5553","FUBP3",37.2202723119221,-0.239766091772571,0.252991535816364,-0.947723768697964,0.343270097393482,0.875946798424478
"5554","TM7SF2",1.67384246548848,1.13011099122421,1.19200140092817,0.948078576370994,0.343089453761402,0.875946798424478
"5555","AAAS",22.5944371505447,0.306228741391401,0.323117180751436,0.947732771990769,0.343265512785565,0.875946798424478
"5556","AC027288.3",1.94494275938465,1.23470709098062,1.30378628318879,0.947016475707035,0.343630383539356,0.875946798424478
"5557","LATS2",71.8905149243679,0.205777803725275,0.217279034555105,0.947067001409592,0.343604638375749,0.875946798424478
"5558","LMO7-AS1",3.63315084331602,0.928754327071784,0.980403383442341,0.947318565762993,0.34347647313207,0.875946798424478
"5559","ZNF200",7.99670674454786,0.529260023212238,0.558534308981384,0.947587309681057,0.343339589103414,0.875946798424478
"5560","PLA2G15",14.232028293361,0.374295532338151,0.395292468474541,0.946882529238621,0.343698641372434,0.875946798424478
"5561","PTOV1",75.6879123563736,-0.242519837585354,0.256077998086703,-0.947054566957533,0.343610974185294,0.875946798424478
"5562","PFKL",69.5848865558433,-0.181575377130811,0.191910921545185,-0.946144053026497,0.344075117195505,0.875946798424478
"5563","TIMP3",2210.24889943627,-0.176266777190401,0.186093285084707,-0.947195795432204,0.34353901748455,0.875946798424478

"5564","NR2C1",24.3319667438518,0.269008912777751,0.284430244221591,0.
945781674919824,0.344259954118141,0.876118071587504
"5565","GZMM",2.30454781617588,0.872018710352859,0.922392544307562,0.9
45387856541579,0.344460899513481,0.876341326659644
"5566","ADAMTSL5",2.32427640864937,0.876480061007595,0.927131899830522
,0.945367170699028,0.344471456510422,0.876341326659644
"5567","SFI1",10.64503341381,0.446799529458169,0.472752594070227,0.945
102226962709,0.344606688509717,0.876527880240271
"5568","AC069148.1",1.60955885876392,0.986568939760569,1.0440312101996
,0.944961156450446,0.344678707227212,0.87655360889679
"5569","SHPRH",61.0146027215218,0.214360853628143,0.226899181300415,0.
944740533657229,0.344791357885741,0.876682640987987
"5570","CSF3R",4.17735336648949,0.814606747434548,0.862965336448731,0.
94396230419499,0.345188911682508,0.876833075622269
"5571","INMT",111.732369844796,0.330010912470766,0.349493087338136,0.9
44255907847125,0.345038891718182,0.876833075622269
"5572","CTR9",56.1687536922155,0.228484544256944,0.242034159325856,0.9
44017757218023,0.345160574174773,0.876833075622269
"5573","LUM",4117.46637820765,-0.257425180913771,0.272629639312568,-0.
944230354274266,0.345051946940703,0.876833075622269
"5574","PSMD9",122.207894751813,-0.202408012168422,0.214386424271141,-
0.944127002708113,0.345104752073536,0.876833075622269
"5575","ZNF423",36.6803276681723,0.242914156677174,0.257352279140827,0
.943897436961296,0.345222061906367,0.876833075622269
"5576","VOPP1",33.5310237107695,-0.260448453260667,0.276020593171435,-
0.943583412629302,0.345382571887044,0.876926164231763
"5577","ASXL3",3.5446803712277,0.837434766196103,0.887395407415063,0.9
43699684716092,0.345323135181058,0.876926164231763
"5578","AL078459.1",2.6291595745659,-0.799965577618879,0.8482293501546
28,-0.943100562923281,0.345629467846449,0.877123531567249
"5579","AC015982.2",2.26158380052695,0.860228655111473,0.9122769115803
76,0.942946866452273,0.345708081191865,0.877123531567249
"5580","NIF3L1",20.6420782670703,0.358445598777517,0.380126834716001,0
.942963153457234,0.345699750103364,0.877123531567249
"5581","COIL",37.310664579421,0.311204902384179,0.329928063246389,0.94
3250778130301,0.345552646123285,0.877123531567249
"5582","CHML",10.5297599730811,0.418158178158528,0.443674484977613,0.9
42488676534166,0.345942505795043,0.877561068086315
"5583","AC004908.2",2.05979599182717,0.895372469741685,0.9509655879505
17,0.941540347081703,0.346428023103792,0.878635286969317
"5584","C1QTNF12",3.27193461108169,-0.722795853488417,0.76835742804873
,-0.940702630186034,0.346857270754138,0.878779159297576
"5585","EIF1B",228.367575430416,-0.183108925842211,0.194594010367872,-
0.940979249546535,0.346715493070682,0.878779159297576
"5586","KIAA0408",35.4021574451075,0.371356322202798,0.394676984240759
,0.940912029408496,0.346749942491151,0.878779159297576
"5587","IQCE",15.4536984244538,-0.366421847173661,0.389492417268644,-0
.940767601441981,0.34682396725988,0.878779159297576
"5588","GLG1",268.375046415142,0.169800525347756,0.18053091551825,0.94
0562035373885,0.346929344953515,0.878779159297576

"5589","PLCG1-
AS1",4.25801011445252,-0.700742253530626,0.745054842136864,-0.94052439
3507534,0.346948643210419,0.878779159297576
"5590","TUBA8",2.36218297027482,0.933808029043993,0.992654669236525,0.
940717913272102,0.346849436642615,0.878779159297576
"5591","AKAP17A",29.790307871235,-0.286205017022644,0.304324230225908,
-0.940460826304191,0.346981234437341,0.878779159297576
"5592","DNAJC2",55.0454487396655,-0.217709564807497,0.231531380234962,
-0.940302625875426,0.347062353056497,0.878827417610871
"5593","RAD52",5.91810655167582,0.533707331429006,0.567784891693229,0.
939981565619688,0.347227016537936,0.878980293921016
"5594","LDLR",30.76576454368,-0.449279833488155,0.477986330090077,-0.9
39942850255754,0.347246876002413,0.878980293921016
"5595","ERI1",16.4902768638329,0.320501137063726,0.341103468083751,0.9
39600933594241,0.347422297225343,0.879110030148474
"5596","CCDC159",8.4775563817253,-0.585978998850592,0.623579025896242,
-0.939702867665235,0.347369993790661,0.879110030148474
"5597","HSPB11",63.273594337964,-0.266042298993861,0.283223672868501,-
0.939336377850669,0.34755806690923,0.879139376104804
"5598","RBBP8",13.4314668750497,0.39923608759367,0.425010940856566,0.9
3935484764004,0.347548587140242,0.879139376104804
"5599","AC008014.1",3.34967143966605,0.715779881928469,0.7625569443945
4,0.938657613952738,0.347906562059029,0.879706592634972
"5600","SYNRG",44.2222376659868,0.252601866386751,0.269091103677544,0.
938722473298289,0.347873251975527,0.879706592634972
"5601","CAPS2",8.90797126360219,0.491234415717867,0.523501429385785,0.
938363083925527,0.348057850582823,0.879824164943276
"5602","ZNF441",11.926874861832,0.408192057141222,0.435021962752473,0.
938325169971897,0.348077328531937,0.879824164943276
"5603","PIK3CD",3.41792495126003,0.887603234418879,0.948237647662643,0
.936055678243503,0.349244521360359,0.880474101117453
"5604","ERMAP",10.2456013637441,0.393329629755712,0.420437961936504,0.
935523585796265,0.349518534147977,0.880474101117453
"5605","SZT2",18.958201816697,0.358530808857983,0.38375619766303,0.934
267149407194,0.350166105313125,0.880474101117453
"5606","INTS3",31.6214699062828,0.283141764116523,0.303504345947472,0.
932908434087224,0.350867247337928,0.880474101117453
"5607","SDHC",97.2660283410361,-0.195922070817043,0.209717483707823,-0
.934219061535188,0.35019090506285,0.880474101117453
"5608","FANCL",16.9445634159773,0.358509306581761,0.382933053105599,0.
936219278211269,0.349160299359053,0.880474101117453
"5609","METAP1D",9.10808734454941,0.531091302900859,0.570072391135849,
0.931620810196893,0.351532524559861,0.880474101117453
"5610","PDK1",6.42079298780723,0.493180782856548,0.52804020930479,0.93
3983386427831,0.350312462914114,0.880474101117453
"5611","PMS1",22.3364932223538,0.275351358227341,0.295312161917524,0.9
32407783138448,0.351125824001655,0.880474101117453
"5612","CARF",14.1576086865142,0.358571399776026,0.383253064673267,0.9
35599562867731,0.349479399722713,0.880474101117453
"5613","CYP20A1",48.7777179874624,-0.208870373456832,0.222874274999752

,-0.937166810557496,0.348672759139861,0.880474101117453
"5614","NDUFAF3",148.900076580393,-0.228906134089143,0.245745956694578
,-0.931474670704904,0.351608080978281,0.880474101117453
"5615","AC135507.1",2.15085576140962,0.822721983815339,0.8777169038892
62,0.937343214161383,0.348582040781725,0.880474101117453
"5616","YEATS2",18.2802737605167,0.364074850201504,0.389114256695423,0
.935650246519961,0.349453295030313,0.880474101117453
"5617","ANKRD50",32.0840142417648,0.26046048704824,0.277854277295536,0
.93739959515255,0.348553049123597,0.880474101117453
"5618","SCLT1",24.5941948758538,-0.321959257419911,0.343991017646086,-
0.935952513013458,0.349297637917589,0.880474101117453
"5619","CCDC112",28.65614710453,-0.306560126958633,0.326987596695158,-
0.937528303999954,0.348486871514436,0.880474101117453
"5620","TRERF1",33.1108398861213,0.231501762631763,0.248395758367624,0
.931987583657295,0.351342942209009,0.880474101117453
"5621","SLC2A12",2.73538359882429,0.916452031267935,0.983553723616022,
0.931776281521878,0.351452154729104,0.880474101117453
"5622","SUN1",97.1717330088723,0.186970900603036,0.200263976909416,0.9
33622229461702,0.350498794438748,0.880474101117453
"5623","MRM2",25.8679494128939,-0.268592158335867,0.287819021957131,-0
.933198078811733,0.35071770649666,0.880474101117453
"5624","LSM5",109.36866192008,-0.233917743738029,0.250531310207214,-0.
933686665928324,0.35046554516056,0.880474101117453
"5625","IKZF1",3.73776558138307,0.71899426197995,0.767819464832323,0.9
36410569035214,0.349061838338686,0.880474101117453
"5626","LAMTOR4",290.587785812905,-0.229994876939358,0.246957923280324
,-0.931311997948286,0.351692197452,0.880474101117453
"5627","PUS7",13.8903977192569,-0.340683276907991,0.365453951971759,-0
.93221943577263,0.351223133077566,0.880474101117453
"5628","PARP10",29.0219585192365,0.280484800634876,0.300345236009477,0
.933874644930361,0.350368559246097,0.880474101117453
"5629","MRRF",17.1508639492416,0.328513081507071,0.351138094411403,0.9
35566623888365,0.349496365657958,0.880474101117453
"5630","ZNF79",2.86200780742521,0.799470405544207,0.855608558524046,0.
934388041797198,0.350103763931901,0.880474101117453
"5631","PTRH1",27.6841357939711,-0.27104643126533,0.290884126590995,-0
.931802069923331,0.351438824718254,0.880474101117453
"5632","CERCAM",177.647630774817,-0.286562573007462,0.305961659073146,
-0.936596349606515,0.348966230430752,0.880474101117453
"5633","STKLD1",1.75543811151905,-1.01044143774884,1.0807342090161,-0.
934958317520779,0.349809781021221,0.880474101117453
"5634","MTPAP",17.6493316598383,0.356548146454835,0.38240382134833,0.9
32386463078926,0.351136838085913,0.880474101117453
"5635","PGGHG",9.98576695472638,0.597002564879402,0.637213696295281,0.
93689537489595,0.348812378539958,0.880474101117453
"5636","NUDT22",39.4862536472498,-0.249314548484339,0.267208839138154,
-0.933032564672896,0.350803154937897,0.880474101117453
"5637","AC018653.3",2.38919596635834,-0.835173376755412,0.896522200351
373,-0.931570212570401,0.351558683165315,0.880474101117453
"5638","EMP1",1058.24264484281,-0.177907041775702,0.190444102018911,-0

.934169343601072,0.350216546635313,0.880474101117453
"5639","AC024941.2",2.21782915281361,0.847151822395016,0.905943156801161,0.935104830844206,0.349734277220618,0.880474101117453
"5640","BRCA2",6.31900687568172,0.702520993660459,0.751697152045777,0.934579826128802,0.350004879678997,0.880474101117453
"5641","ATG2B",24.3619075736424,0.275014791678397,0.294405059985214,0.934137448901896,0.35023299666348,0.880474101117453
"5642","CCNDBP1",102.770137686799,-0.228106946217574,0.243890160879983,-0.935285562133949,0.349641153870422,0.880474101117453
"5643","PIGBOS1",52.1117751922759,-0.218183035015767,0.233823016191008,-0.933111883380781,0.350762204161354,0.880474101117453
"5644","TPSB2",8.12686968646192,0.773456573639586,0.827043191109328,0.93520699034126,0.349681636727889,0.880474101117453
"5645","ZNF267",28.7946195286669,-0.294410960321927,0.314194803545237,-0.937033194056431,0.348741483533056,0.880474101117453
"5646","CNTROB",10.4372288200987,-0.426500294524307,0.457383773107314,-0.932477974954828,0.351089563990447,0.880474101117453
"5647","NSRP1",121.2541582602,-0.169564612228943,0.181023449399638,-0.936699708194169,0.348913046395722,0.880474101117453
"5648","MYO1D",43.6721815244783,-0.270132356930899,0.28953405137525,-0.932989938999591,0.35082516303379,0.880474101117453
"5649","PSMD12",93.4475489188194,-0.163153767182835,0.17513589774641,-0.931583811669928,0.351551652408452,0.880474101117453
"5650","HAS1",37.8603924426951,-0.966571855462993,1.03735877822501,-0.931762352381939,0.351459354827281,0.880474101117453
"5651","SRXN1",7.84151421226866,-0.481305271753966,0.516076720739478,-0.932623488740804,0.351014401369568,0.880474101117453
"5652","LINC00653",3.38322692704166,0.761788427109483,0.813854175275239,0.936025703685616,0.349259953796096,0.880474101117453
"5653","N6AMT1",15.2565825999733,0.372023284780758,0.3990549013524,0.932260908260915,0.35120170497954,0.880474101117453
"5654","ADA2",3.77753297377972,-0.708187098378158,0.756395555055024,-0.936265547365148,0.349136482129829,0.880474101117453
"5655","ATRX",238.463401822374,0.180984295403434,0.194313044048855,0.931405795680553,0.351643694016551,0.880474101117453
"5656","AL683813.1",5.17895554859884,0.557131774902164,0.597858501811109,0.931878986774345,0.351399068341743,0.880474101117453
"5657","NEU1",97.3447382950784,0.234132951066867,0.251465882093081,0.931072434630326,0.351816096478656,0.880565681339604
"5658","TRAPPC9",10.7813792709895,0.406382780583311,0.43650100144443,0.931000797795528,0.351853151484426,0.880565681339604
"5659","PCDHGA6",4.22183701482439,0.679609032942445,0.730096094463335,0.930848744564234,0.351931811018827,0.880606899456899
"5660","RSPRY1",42.7465503878725,-0.245107547577571,0.263357711647586,-0.930701994804552,0.352007737542126,0.880641265653092
"5661","S100A9",9.08356775366245,-0.571154671278337,0.614271612398458,-0.929808019368226,0.35247049346581,0.880652327535129
"5662","EIPR1",14.8878187832734,-0.329882250965908,0.354672176595031,-0.930104679010587,0.352316888409708,0.880652327535129
"5663","FBXL2",10.0122903001468,0.390539735433128,0.419907884629273,0.

930060495953598,0.352339762920553,0.880652327535129
"5664","NDRG1",219.317176201385,0.218245010106003,0.234567457632938,0.
930414697368305,0.352156411732727,0.880652327535129
"5665","BAZ1A",49.9774425163604,-0.240869560545368,0.259066037038912,-
0.929761242725878,0.352494717442148,0.880652327535129
"5666","FRMD6-
AS1",9.39254615723622,0.602047217385233,0.647332340168718,0.9300434723
04069,0.352348576677823,0.880652327535129
"5667","ZNF235",1.74463467620684,0.994972881273424,1.06930704046407,0.
930483802707983,0.35212064662597 7,0.880652327535129
"5668","ATP5F1E",987.02652312309,-0.234163708404994,0.25186143009981,-
0.929732306817276,0.352509702857988,0.880652327535129
"5669","EIF2AK3",17.1425728301729,-0.299714204380613,0.322567472433663
,-0.929151975924202,0.352810331496677,0.881247890984819
"5670","MSH6",34.5608545988255,0.255786443099466,0.275376267173004,0.9
28861610789317,0.352960810122445,0.881468266549174
"5671","GLS",116.955315758179,0.221034549233305,0.238060129891336,0.92
8482015590755,0.353157592492234,0.881774251290742
"5672","ESRG",6.73444201430588,0.557397793553306,0.600395064689399,0.9
28385035679237,0.353207878059399,0.881774251290742
"5673","SPPL3",63.2813824830865,0.175092882214358,0.188653265369122,0.
928120071877728,0.353345288925962,0.881806360802189
"5674","ZNF433",6.30611960322282,-0.517014683807556,0.557042277980291,
-0.928142628028404,0.353333589935744,0.881806360802189
"5675","SLC33A1",33.6580206284754,0.290205466049654,0.312766221842474,
0.92786703225202,0.353476547435043,0.881978486639685
"5676","KRBOX1",8.73030383508394,-0.549109687085652,0.591938643853995,
-0.927646290349465,0.35359107741164,0.88210881891276
"5677","AC124016.1",2.60842227573206,-0.803951322632492,0.867233728132
674,-0.927029584473795,0.353911174077058,0.882403717748334
"5678","RNF5",57.6393760131273,-0.232684892568161,0.251057717452643,-0
.926818322611625,0.354020870093805,0.882403717748334
"5679","FAM180A",24.4997708793108,0.356765021546098,0.384887881868403,
0.926932331083575,0.353961669446709,0.882403717748334
"5680","FARSA",22.3612149903372,-0.297571130443487,0.320950683903443,-
0.92715530879819,0.353845902932167,0.882403717748334
"5681","ZMAT1",10.2943863149067,0.60070883490104,0.647823541472916,0.9
27272314827036,0.353785164819735,0.882403717748334
"5682","NCOR2",111.472819784026,0.238834686619129,0.257751707858659,0.
926607581394171,0.354130317171378,0.882464495783963
"5683","SNRPA",51.4138286514697,0.235070425034442,0.25371015988419,0.9
26531381879795,0.354169896153973,0.882464495783963
"5684","TIMMDC1",102.094116833169,-0.249829309532312,0.269818664107631
,-0.925915597271858,0.354489844930215,0.88303805826233
"5685","AFAP1L1",2.91674310120576,0.809752642580569,0.87478433646658,0
.925659741292709,0.354622835995465,0.88303805826233
"5686","AC022506.2",2.17510071605951,0.892426079170303,0.9642757789447
31,0.925488432517658,0.354711897979953,0.88303805826233
"5687","ZNF598",16.8659057434914,-0.365309728538264,0.394703520297467,
-0.925529441092771,0.354690576682678,0.88303805826233

"5688","SGF29",29.8639166191562,0.304992969534332,0.329496417606381,0.925633643454901,0.354636403129,0.88303805826233
"5689","ADGRL2",39.2281391656235,0.241957304348698,0.263120148702958,0.919569654932999,0.357797690287566,0.883420278024748
"5690","GLMN",10.7744192204324,0.383171851901657,0.415214856449647,0.922827894883198,0.35609690148331,0.883420278024748
"5691","TDRD10",4.79194782205893,0.571669764323299,0.621657788497876,0.919589161272533,0.357787492864778,0.883420278024748
"5692","SSR2",555.534255082734,-0.252687172658268,0.274695486864173,-0.919881049167776,0.357634923075943,0.883420278024748
"5693","NTPCR",67.5160669451403,0.283913373109734,0.308048500793999,0.921651533371995,0.356710368857102,0.883420278024748
"5694","SH3BP5L",11.9812067280788,0.391657355451288,0.424511051636915,0.922608148694968,0.356211447606404,0.883420278024748
"5695","FAM171B",33.8805947379592,0.361990217660307,0.392591919541975,0.922052135160169,0.356501382048938,0.883420278024748
"5696","DNAJB2",42.4388764279829,-0.211629761693949,0.229959755771405,-0.920290426401058,0.357421010624572,0.883420278024748
"5697","ULK4",4.66916068487591,0.663936207265539,0.721677136171452,0.919990635684771,0.357577652784403,0.883420278024748
"5698","MFSD1",42.8860577659833,-0.240528530931415,0.261205251665793,-0.920841098704886,0.357133394156032,0.883420278024748
"5699","MAEA",52.3585273964584,0.211747901217341,0.229409743717657,0.9230118031863,0.356001054269201,0.883420278024748
"5700","AC025171.1",8.41530146755774,-0.483385739468646,0.524216781398001,-0.922110387575793,0.356470999229987,0.883420278024748
"5701","KLHL3",3.23340166254392,0.774321701520266,0.838867818303012,0.923055676502981,0.355978191282088,0.883420278024748
"5702","ETF1",60.248905891511,-0.185305965890696,0.201497397843722,-0.919644461286867,0.357758584405083,0.883420278024748
"5703","DNAJC18",27.6419084469183,0.306209435839847,0.332535043871544,0.920833582755066,0.357137318759616,0.883420278024748
"5704","HLA-F-AS1",25.6729646351395,-0.357765801041214,0.388637111234127,-0.920565202600236,0.357277476628843,0.883420278024748
"5705","SMPD2",9.72143755789716,-0.391697672468987,0.425390798697559,-0.920794887120897,0.357157524889161,0.883420278024748
"5706","RNF217",37.5843123500239,0.278346810694961,0.30157178262313,0.922986919644295,0.356014021835735,0.883420278024748
"5707","EPDR1",57.7109552264864,0.251261866092422,0.272945147092781,0.920558100294825,0.357281186181187,0.883420278024748
"5708","ZNF394",29.888202178838,0.270873246557428,0.294319484919817,0.920337457886025,0.357396440357862,0.883420278024748
"5709","KBTBD11",2.25121035908626,0.823827049098407,0.893954544575055,0.92155362271805,0.356761458835461,0.883420278024748
"5710","UBR5",64.0071254981566,0.231169294163618,0.251166474964973,0.920382762850242,0.357372773068648,0.883420278024748
"5711","SLC25A32",20.5707146383125,-0.313910303194434,0.341112384635516,-0.920254782100193,0.357439632688025,0.883420278024748
"5712","MAF1",143.087897273756,-0.181130995174278,0.196733323596626,-0

.920693006466265,0.357210728486602,0.883420278024748
"5713","VCP",170.064589275983,0.154652774071199,0.167356460697036,0.92
4092045368751,0.355438394515815,0.883420278024748
"5714","NTRK2",691.496720064751,0.256618990016024,0.27854756106211,0.9
21275307662103,0.356906709374278,0.883420278024748
"5715","AL360020.1",1.84200942788584,0.905783825238475,0.9812202008225
29,0.923119830267642,0.355944761542129,0.883420278024748
"5716","BMI1",70.2359032891936,0.20651836097732,0.224569909750302,0.91
9617241717496,0.357772813436914,0.883420278024748
"5717","PLAU",108.582400872793,-0.33756091994916,0.366380349318325,-0.
921340133490277,0.356872873933522,0.883420278024748
"5718","DNHD1",3.24830211429057,-0.75059297079926,0.813429143184939,-0
.92275151079583,0.356136715244993,0.883420278024748
"5719","CPSF6",54.4992332644364,-0.214592243030414,0.232501563180115,-
0.922971183914893,0.356022222354097,0.883420278024748
"5720","PLA2G4B",5.04885818094213,0.637731807024595,0.693244652108114,
0.919923154236087,0.357612918139106,0.883420278024748
"5721","AC007950.2",2.08390334284107,0.851587631168843,0.9211188396239
41,0.924514399810251,0.355218557750984,0.883420278024748
"5722","MRPL28",56.1415212160286,-0.269615147799828,0.292228539887386,
-0.922617441485102,0.35620660312449,0.883420278024748
"5723","GSPT1",107.651230116679,-0.179549589905053,0.194129050799531,-
0.924898098278275,0.355018915937655,0.883420278024748
"5724","TK2",73.78854744452,0.223863579416456,0.242523511188855,0.9230
59287402991,0.3559763096338,0.883420278024748
"5725","NUDT7",14.8638580570407,0.403058144553649,0.436740059300117,0.
922878806216119,0.356070366471974,0.883420278024748
"5726","RAB5C",185.051151519193,-0.176925853953697,0.191928960587098,-
0.921829896918591,0.356617310244449,0.883420278024748
"5727","SRSF1",82.827298380818,0.202297836853746,0.218744077651473,0.9
24815149400612,0.355062068994022,0.883420278024748
"5728","RPL17",4047.41165910235,-0.219060322338391,0.237917138775838,-
0.920742084683465,0.35718509848654,0.883420278024748
"5729","ZNF665",8.83377422667803,0.462743490220246,0.50299857605205,0.
919969781728291,0.357588550689138,0.883420278024748
"5730","ATP5PF",401.041051196827,-0.205458171474746,0.222569085345441,
-0.923120886963534,0.355944210927432,0.883420278024748
"5731","HUNK",5.97412125132244,0.605917309530196,0.656766054263711,0.9
22577081438045,0.356227643771847,0.883420278024748
"5732","SPATC1L",37.9446098726052,0.268028275068604,0.291295037198446,
0.920126472618238,0.357506671948523,0.883420278024748
"5733","ARMCX1",91.7762244503248,0.224454579157762,0.244059801216848,0
.919670416998878,0.357745016386535,0.883420278024748
"5734","TCEAL1",51.0638857561284,-0.196951225612519,0.21376153872326,-
0.921359505497834,0.356862763225959,0.883420278024748
"5735","ALG13",58.0231850293476,-0.213476107252723,0.231599186807232,-
0.921748086405873,0.356659991802339,0.883420278024748
"5736","AZI2",118.444240662219,0.200073136923238,0.218017893139001,0.9
1769136029435,0.358780472121709,0.884150972022867
"5737","ZHX1",72.5045548123744,0.19791785938102,0.215498564798953,0.91

8418457058707,0.358399831452069,0.884150972022867
"5738","ZNF485",5.77845843411799,0.581319665464505,0.632834063397056,0
.918597305498977,0.358306241874367,0.884150972022867
"5739","RCN1",249.434395999939,-0.172730332590177,0.18808924531992,-0.
918342419293465,0.358439625902281,0.884150972022867
"5740","AP000866.2",1.85191790622551,0.993091013562046,1.0810787044317
2,0.918611207020372,0.358298967992946,0.884150972022867
"5741","VSIG10",24.056237621139,0.32061025725406,0.349356081040831,0.9
17717694504903,0.358766681528297,0.884150972022867
"5742","MYO18A",10.8449911181725,0.465280409238527,0.506935298699469,0
.917829968503266,0.358707890072073,0.884150972022867
"5743","ICAM4",6.1418216423362,0.771153585541247,0.84016904843315,0.91
7855266126964,0.358694643993146,0.884150972022867
"5744","ZNF611",11.9235950031636,0.425252135694125,0.463263649124236,0
.917948422022819,0.358645869322261,0.884150972022867
"5745","DYNLRB1",284.061458549753,-0.236677844207832,0.257650907446106
,-0.918598915695023,0.358305399344913,0.884150972022867
"5746","UBE2V1",193.011021299547,-0.148663007315697,0.1619880677867 7,-
0.917740481424758,0.358754748834765,0.884150972022867
"5747","RANGAP1",16.0440883593313,0.309282672019234,0.33718706005747,0
.917243597564272,0.35901500571447,0.884267260552601
"5748","ZXDB",7.73887753193282,0.431838086907236,0.470745066639524,0.9
17350212483307,0.358959153158094,0.884267260552601
"5749","HCFC1",33.01235228558,0.236900032628759,0.258239798298428,0.91
7364535558504,0.358951650118194,0.884267260552601
"5750","KATNA1",42.7869669108094,0.228861030084479,0.249577727714899,0
.916993003261551,0.359146306526538,0.884302205628836
"5751","FSCN1",55.0881163493301,-0.278150708253724,0.303334066519101,-
0.916978140456271,0.35915409495561,0.884302205628836
"5752","RIN3",12.1570072594553,-0.456706091771898,0.498197025687852,-0
.91671782090897,0.359290525198312,0.884484324897096
"5753","AC004825.2",3.41431106945595,-0.739213210171673,0.806487074382
834,-0.916584076362733,0.35936063171662,0.884503136643027
"5754","AL133453.1",11.2935361825718,-0.414319113816827,0.452265898640
839,-0.916096294374502,0.359616391213785,0.884671316815011
"5755","AC116667.1",2.01158349852002,0.931436745380212,1.0164933077273
3,0.916323539269252,0.359497225317509,0.884671316815011
"5756","RUNX1",73.7536456892733,-0.272048847025062,0.296942092659139,-
0.916168013058856,0.35957877967484,0.884671316815011
"5757","LINC01128",17.3626354746827,-0.317317562081167,0.3465797709389
85,-0.915568618507255,0.359893197050179,0.884852392970403
"5758","VTI1B",123.89573183678,-0.215887343623759,0.235801799995554,-0
.915545783059459,0.35990517898651,0.884852392970403
"5759","TMEM219",207.737815355012,-0.250049582671727,0.273135095889955
,-0.915479506055386,0.359939956462537,0.884852392970403
"5760","CDKN2D",17.8796678277925,-0.332191013089344,0.362851814229552,
-0.915500488249423,0.35992894626541,0.884852392970403
"5761","TRMT1L",18.662977212494,0.313737687741186,0.342897022804994,0.
914961830740679,0.360211668399484,0.885212985861975
"5762","CHD7",4.23780024428883,-0.781147736134808,0.853665424335758,-0

.915051393515935,0.360164650429153,0.885212985861975
"5763","ATG4D",9.03330804468139,0.395730401480483,0.432571906526844,0.914831489307373,0.360280100917106,0.885227525418397
"5764","ZNF354A",17.1499025366245,-0.328433571009302,0.359075349878665,-0.914664766379209,0.360367646525841,0.885289013672087
"5765","FHL3",62.8284220512491,-0.229894678499952,0.25157204121072,-0.913832385322143,0.360804926538289,0.885441552821867
"5766","POP1",7.65830081401288,0.461297112372722,0.504644712959582,0.914102735105181,0.360662865486185,0.885441552821867
"5767","BET1L",28.662254506096,-0.244002521425218,0.266980286252639,-0.913934601127524,0.360751210939209,0.885441552821867
"5768","COLCA2",10.8562874371567,0.427241198887245,0.467453474500143,0.913975876089278,0.360729521887115,0.885441552821867
"5769","EEA1",297.693866190998,-0.133761114537597,0.146321243892445,-0.914160589257423,0.360632469349229,0.885441552821867
"5770","NUPR1",713.448891595111,0.177651728044356,0.19439876255229,0.913852154776814,0.360794537067666,0.885441552821867
"5771","AC097448.1",15.3786015019438,-0.401658775110959,0.439671250968938,-0.913543412778963,0.360956812127619,0.885507356155073
"5772","CACUL1",47.1428203351977,0.201747731377337,0.220814882018033,0.913650971046695,0.360900274213532,0.885507356155073
"5773","COQ9",37.494674305106,-0.225097274303331,0.246462864882475,-0.913311116507016,0.361078937426084,0.885555967164116
"5774","PCIF1",18.898417051867,-0.34874340619134,0.381863239779563,-0.91326781387142,0.361101705819605,0.885555967164116
"5775","DPY30",85.6970215890966,-0.202578188419151,0.221850675823019,-0.913128561216363,0.361174930543921,0.885582167359639
"5776","ZNF32",69.4689472140106,-0.220988351942056,0.242210105561688,-0.912382872835058,0.361567202332404,0.885886117999557
"5777","AP001107.9",1.79640381430458,-0.892098144779444,0.977857943903763,-0.912298305025828,0.361611706358576,0.885886117999557
"5778","SLIRP",153.066811181549,-0.245457835735982,0.268988941677446,-0.912520173525643,0.361494954805359,0.885886117999557
"5779","ATP5MPL",285.848285478072,-0.20386789430866,0.223372700699366,-0.912680437987106,0.361410635206872,0.885886117999557
"5780","LRRC29",5.52115203955068,-0.564654958919633,0.618758413039501,-0.912561263039483,0.361473335291694,0.885886117999557
"5781","SBNO1-AS1",3.51882081363096,-0.712924681793832,0.781844982250594,-0.911849149100669,0.361848133361286,0.886158693946006
"5782","LTB4R",4.83647136066746,0.590557407136511,0.647643399654042,0.911855825986916,0.361844618068155,0.886158693946006
"5783","SLC25A24",47.3113944257854,-0.245802528968176,0.26966868028401,-0.911498245585292,0.362032909583266,0.886286709902169
"5784","MAP3K20",159.472779405824,0.184448508344968,0.202388272731032,0.911359664549806,0.362105898859207,0.886286709902169
"5785","FBXO3",42.4685644340137,0.229901668119601,0.252220440622854,0.911510849603874,0.362026271627328,0.886286709902169
"5786","C15orf65",5.23779806583106,0.620136381176242,0.680515471148582,0.91127447863833,0.362150770020759,0.886286709902169

"5787","AC018557.1",1.67311934926595,-1.04074163233435,1.1425889440292
6,-0.91086268405876,0.362367729433487,0.886664428681212
"5788","TSC22D3",850.971278109528,0.314588996027685,0.345431303915617,
0.910713628040307,0.36244628162883,0.886703411863205
"5789","AL049775.3",2.92270746993475,0.983041527876453,1.0797486585052
7,0.910435516759339,0.362592874175326,0.886863223694482
"5790","CDK3",4.24730664616462,-0.597307980905959,0.656128550756263,-0
.910352064724959,0.362636869010668,0.886863223694482
"5791","ATG14",31.2027910279777,0.301977623100339,0.33176294793242,0.9
10221062907399,0.362705938219655,0.886878964805788
"5792","B9D2",9.21949788709205,-0.483520524963799,0.531310570322175,-0
.910052522897489,0.362794811125543,0.886943115596976
"5793","STRN",51.3045980536467,0.209743404830801,0.230540690809425,0.9
09789087967052,0.362933750419686,0.886999468390663
"5794","AC097382.3",9.6870124923362,0.461833550099761,0.50763698580730
9,0.909771279500635,0.362943144057592,0.886999468390663
"5795","VPS4A",53.0798942195356,-0.21314620716167,0.23436453723914,-0.
909464416726925,0.363105032421881,0.887241977410497
"5796","NUP62",39.4927170433585,0.226535781775498,0.249155091681809,0.
909215943556961,0.363236149904744,0.887409227510555
"5797","ITGA1",13.8717349531814,0.461041641319879,0.507380743206792,0.
908669963321752,0.363524363764952,0.88765390427862
"5798","MRPS35",43.8050439232165,-0.229666453757611,0.252739834713068,
-0.908706987239855,0.363504814935426,0.88765390427862
"5799","ZNF630",2.17376871426786,0.829096615157299,0.912312692125015,0
.908785575728554,0.363463321966901,0.88765390427862
"5800","ZNF362",23.8096588924353,0.302105100196832,0.334584870054223,0
.902925168576429,0.366565611798036,0.887779915269231
"5801","P3H1",61.2988625038995,-0.231441135100111,0.255311865827656,-0
.906503637619184,0.364669340388284,0.887779915269231
"5802","INKA2",2.83391118654034,-0.901323511238458,0.998461226563048,-
0.902712581379888,0.366678457331984,0.887779915269231
"5803","HHAT",3.79108531965532,0.628009900503233,0.694925535051665,0.9
03708194370126,0.36615015286717,0.887779915269231
"5804","STUM",5.62682974907321,0.634993735447448,0.702129585592794,0.9
04382536325307,0.365792595088853,0.887779915269231
"5805","RSAD2",7.33697008831837,-0.555401014326391,0.612588292869116,-
0.906646471686745,0.364593778421468,0.887779915269231
"5806","THADA",26.1474755115506,0.258584631698562,0.28622374506095,0.9
03435288513526,0.366294918279878,0.887779915269231
"5807","PAPOLG",14.4120070302038,0.343887485304918,0.380809166186572,0
.903044138219182,0.366502469785124,0.887779915269231
"5808","ABCB11",5.67965579954591,0.754059296877703,0.834104006796692,0
.904035097221995,0.365976791246082,0.887779915269231
"5809","ATP5MC3",404.672512732335,-0.27723091874965,0.305876443969697,
-0.906349358426291,0.364750968038145,0.887779915269231
"5810","ASNSD1",43.1867739312972,-0.229629099217693,0.252875193163165,
-0.908072857385924,0.363839729768321,0.887779915269231
"5811","SUMO1",216.1343804751,-0.20605605446637,0.227511698698578,-0.9
05694325369026,0.365097666896093,0.887779915269231

"5812","POPDC2",7.22591081062097,-0.649657862200033,0.719233242055789,
-0.903264510332019,0.366385527289948,0.887779915269231
"5813","LINC02614",7.83662081876384,0.490176369954184,0.54275467801021
1,0.903126937111286,0.366458529059357,0.887779915269231
"5814","TMCC1",22.0592040557126,0.265454628872174,0.294150058093172,0.
90244629082511,0.366819840237467,0.887779915269231
"5815","MARCH6",97.1733793834828,0.177173657474771,0.195973868701113,0
.904067764998734,0.365959469835823,0.887779915269231
"5816","AC116366.1",6.45045434741013,0.509421205357354,0.5643047852391
44,0.902741246720899,0.366663239932601,0.887779915269231
"5817","AGER",2.15098780394212,-0.874105364387747,0.965085498399414,-0
.905728420784939,0.365079615644076,0.887779915269231
"5818","RING1",44.4775167788741,-0.232466679702286,0.256913527468071,-
0.904844061709352,0.365548005149561,0.887779915269231
"5819","UFL1",99.1634998931494,-0.165153283225963,0.182427791439124,-0
.905307694201161,0.365302401349629,0.887779915269231
"5820","C7orf50",54.7879749344481,-0.276986270243857,0.305285895136608
,-0.907301236828884,0.364247520315575,0.887779915269231
"5821","ZNF117",19.6860870986352,0.370466261486129,0.408070472267247,0
.90784873364596,0.363958146562341,0.887779915269231
"5822","UBAP2",29.5120859684191,0.281152346557458,0.31135279499909,0.9
03002481664827,0.366524577835705,0.887779915269231
"5823","ALDH1B1",11.8989158128635,0.431827402568883,0.475402145767465,
0.908341298863435,0.363697929213358,0.887779915269231
"5824","MRPL50",37.708444228035,-0.317830028229826,0.351996122450948,-
0.902936163094001,0.366559776277332,0.887779915269231
"5825","CRB2",2.95799465953023,1.06638176351446,1.17703339600888,0.905
99108498567,0.364940571131815,0.887779915269231
"5826","TRUB2",32.8886901579099,-0.270291719461388,0.298477746294912,-
0.905567409351601,0.365164865374255,0.887779915269231
"5827","TOR1A",45.9842217408858,-0.226047846976509,0.249395638987726,-
0.906382516927789,0.364733423228138,0.887779915269231
"5828","ENTPD2",2.38875507003661,0.942535883132047,1.04451654758894,0.
902365678464079,0.3668626468405,0.887779915269231
"5829","POLR3A",19.8961525281357,0.286211879485336,0.316375208907175,0
.904659630171311,0.365645734216094,0.887779915269231
"5830","ARHGAP19",11.290607332403,0.409975264731299,0.453393738496363,
0.904236715070091,0.365869895733102,0.887779915269231
"5831","HSPA12A",35.6379476277936,0.318901858307995,0.351928893635351,
0.906154237618306,0.36485422094461,0.887779915269231
"5832","TSPAN4",346.74010139489,0.179266768324892,0.198092008581756,0.
904967189783963,0.365482769465411,0.887779915269231
"5833","NFATC4",46.0623114585001,0.254847923144048,0.280807929663539,0
.907552445008955,0.364114728990839,0.887779915269231
"5834","SETD3",32.7913950601186,-0.230657505604128,0.25395209407354,-0
.908271721269344,0.363734679310932,0.887779915269231
"5835","GOLGA8N",5.91327389576162,0.582584343605461,0.645633500735634,
0.902345282488695,0.366873477961204,0.887779915269231
"5836","METTL26",135.827776326984,-0.229758935852931,0.25394263950182,
-0.904767061977728,0.365588804821121,0.887779915269231

"5837","CREBBP",94.6207806872428,0.172101704473944,0.190434200578176,0.903733173723138,0.366136904123718,0.887779915269231
"5838","AC106739.1",8.83211380446417,-0.526333611609068,0.579624853347533,-0.908059080919856,0.363847007931532,0.887779915269231
"5839","FANCA",2.42872229139384,-0.876312560970974,0.971201405286725,-0.902297459827359,0.366898874587255,0.887779915269231
"5840","DDX52",46.1382060720973,0.245928404854408,0.271991016938699,0.904178408619409,0.365900807153984,0.887779915269231
"5841","AC009283.1",3.79047231204744,0.625810968802962,0.692869148353828,0.903216675601464,0.366410909253344,0.887779915269231
"5842","LRRC37A3",3.66611894116263,0.717792309442282,0.792808745365618,0.905378899562036,0.365264690286109,0.887779915269231
"5843","H3F3B",2381.32831693578,-0.185413600320824,0.204464234669969,-0.906826568568849,0.364498517656215,0.887779915269231
"5844","SPIRE1",17.4068196654157,-0.334080592772001,0.370059140301851,-0.902776222469462,0.366644673097984,0.887779915269231
"5845","CDH2",1.6912541624237,2.6629616557511,2.95047537412002,0.902553425495148,0.366762954634149,0.887779915269231
"5846","NFIX",354.740920046988,0.186907555879785,0.206009451612279,0.907276605112057,0.364260542533486,0.887779915269231
"5847","AL117332.1",4.17032565461246,0.590498668779377,0.652796867937136,0.904567251747662,0.365694691068625,0.887779915269231
"5848","ZHX3",48.8246767945177,0.289941833063655,0.32061284916735,0.90433628538797,0.365817111859653,0.887779915269231
"5849","RRP1B",41.0533819846837,0.232624804712207,0.256395188545245,0.907290054981498,0.364253431863109,0.887779915269231
"5850","IGBP1",158.615623617224,-0.227056607351178,0.25054231906357,-0.906260500021822,0.364797987353238,0.887779915269231
"5851","ARMCX6",51.7688295281283,0.251087325263697,0.277067296043848,0.906232272263414,0.364812924829278,0.887779915269231
"5852","ELF4",11.4414035177966,-0.365355919672502,0.402403337082725,-0.907934616847855,0.363912766935516,0.887779915269231
"5853","SYPL1",166.170017249855,-0.191886787803785,0.212838638126587,-0.90155993053131,0.367290684485387,0.888576130584842
"5854","HPF1",92.2123301939213,-0.246295440021387,0.273490960948642,-0.900561536538819,0.367821493028568,0.88970829198574
"5855","FAM161A",12.9642135552301,0.340314216755039,0.378121266872672,0.90001342577125,0.368113105937289,0.890002940505339
"5856","SAP30L",58.1018117572098,0.199500903778269,0.221673046699852,0.899978173929266,0.368131865998571,0.890002940505339
"5857","TMEM14B",178.126334245314,-0.198646819770409,0.220668394414273,-0.900205125875339,0.368011098830436,0.890002940505339
"5858","NMNAT1",12.8603275679184,0.352022173927671,0.391633672115088,0.898855739412067,0.368729505951784,0.89007585871351
"5859","FBXO44",18.5687809126685,0.277150261035346,0.31220844548618,0.887709045166152,0.374697279148315,0.89007585871351
"5860","KIAA2013",48.2189634073385,-0.191894610226624,0.216107866197047,-0.887957544551641,0.374563588967467,0.89007585871351
"5861","DDI2",34.3121230964702,0.319984039934308,0.356213314887689,0.898293316282118,0.369029194876971,0.89007585871351

"5862","NASP",128.138669892693,0.23708285026723,0.268056199385424,0.88
4452032114138,0.376452245573114,0.89007585871351
"5863","FOXD2",2.29271120587404,-0.865158039976194,0.976502357592689,-
0.88597639652301,0.375630246436355,0.89007585871351
"5864","JUN",6471.65117423472,0.17454646060495 6,0.196389828647531,0.88
8775461575569,0.374123766125777,0.89007585871351
"5865","MOV10",15.9403317431773,0.3106316026402,0.346472015688945,0.89
655611008734,0.369955826899515,0.89007585871351
"5866","SLC16A1-
AS1",40.9525178160321,0.256955945096015,0.287910338475531,0.8924859956
63032,0.372132495766626,0.89007585871351
"5867","MLLT11",5.39080431282783,-0.548011751428104,0.611651788774711,
-0.895953811442138,0.370277432379755,0.89007585871351
"5868","SNAPIN",44.2413153970129,-0.291248524182088,0.325072319471235,
-0.895949937096565,0.370279501700748,0.89007585871351
"5869","XCL2",1.77233331265287,1.03953481717831,1.15889585333374,0.897
004518730416,0.369716505804044,0.89007585871351
"5870","SOAT1",37.8634185247743,0.242151254347723,0.272633391566624,0.
888193676336775,0.374436579762653,0.89007585871351
"5871","TOR1AIP2",78.2945714711997,0.188263269843983,0.21005691098600 7
,0.896248873508967,0.370119858319925,0.89007585871351
"5872","RNF2",22.8694135626805,-0.323303625733253,0.361456064079291,-0
.894447922894251,0.371082281420279,0.89007585871351
"5873","TP53BP2",31.149576102576,0.273471092876949,0.308456850535796,0
.886578114254631,0.375306081189021,0.89007585871351
"5874","PDIA6",353.892780893568,-0.188623497169043,0.213258616259779,-
0.884482420814702,0.376435847917777,0.89007585871351
"5875","MEMO1",8.85387829019591,0.396906343888993,0.44754391181501,0.8
86854526250491,0.375157226852329,0.89007585871351
"5876","COX7A2L",207.950269059239,-0.219519770906524,0.247675990934119
,-0.886318330971834,0.375446013830817,0.89007585871351
"5877","PREPL",41.4185209132615,0.204808280560055,0.230305929364768,0.
889287918574042,0.373848362769219,0.89007585871351
"5878","LINC00342",113.812259520017,-0.297654219985,0.335559154128386,
-0.887039487145435,0.375057641451606,0.89007585871351
"5879","EIF5B",209.904165412617,-0.245247843645781,0.276338016434165,-
0.88749223436729,0.374813945279698,0.89007585871351
"5880","ZC3H8",23.1557614530632,0.31546046652961,0.351191808505312,0.8
98256903747908,0.369048602629914,0.89007585871351
"5881","AC008063.1",1.53375442559308,-1.0607757307595,1.19338262048195
,-0.888881497479078,0.37406677028118,0.89007585871351
"5882","AC108047.1",1.59522964014174,0.997260127114765,1.1211910517862
5,0.889464935994588,0.373753259667793,0.89007585871351
"5883","UQCRC1",83.5487381566408,-0.19523152206717,0.220635309217528,-
0.884860735843001,0.376231747115209,0.89007585871351
"5884","IMPDH2",151.330338789149,-0.224077574597449,0.251044900914473,
-0.892579669139697,0.372082310760394,0.89007585871351
"5885","PARP3",14.0528040755636,0.349526892306579,0.392760473284198,0.
88992379855308,0.373506804194773,0.89007585871351
"5886","ST3GAL6",1.87607701940741,0.986558216993939,1.10151919618501,0

.895634157271859,0.370448186547878,0.89007585871351
"5887","ZBTB11-
AS1",3.49542448550181,-0.668350176950805,0.754661070300234,-0.88562959
354046,0.375817159202786,0.89007585871351
"5888","MYLK",21.8016619245764,-0.606801550072696,0.675840226001538,-0
.89784763724809,0.369266783901517,0.89007585871351
"5889","TRPC1",25.9512519498105,0.308197902281802,0.343748672500165,0.
896579178154219,0.369943512846249,0.89007585871351
"5890","CCNL1",605.532975091905,0.244607683876017,0.273230687885982,0.
895242352784657,0.37065754895800 5,0.89007585871351
"5891","TBCCD1",10.01417750724,-0.409329035013547,0.458584555798809,-0
.892592281701541,0.372075553976141,0.89007585871351
"5892","SLC4A4",1.944568937443,0.888335663250648,0.989910756961718,0.8
97389645483974,0.369511035841784,0.89007585871351
"5893","MTHFD2L",7.32821975875435,-0.508420418592814,0.571779161743704
,-0.889190184969891,0.373900876835034,0.89007585871351
"5894","ELMOD2",27.4876549462574,0.281532056620444,0.31752110541318,0.
886656199606309,0.37526402669994,0.89007585871351
"5895","ZNF131",49.1578673742575,0.195108416330905,0.218209162434676,0
.894134848206997,0.371249746003408,0.89007585871351
"5896","SLC30A5",34.0019562827354,-0.251808660030063,0.281393235823297
,-0.894863941179414,0.370859824549957,0.89007585871351
"5897","AC146944.4",3.92813342026596,-0.673661768694297,0.753410539442
144,-0.894149648069834,0.371241828456845,0.89007585871351
"5898","YIPF5",107.057556227019,-0.17937977927985,0.200183760274462,-0
.8960755809258,0.37021239792136,0.89007585871351
"5899","C5orf58",2.45913123321894,0.908064914011443,1.01267275960975,0
.896701234820796,0.369878361547748,0.89007585871351
"5900","HSPA1A",289.827590844452,-0.298394162943966,0.333693917320742,
-0.894215169817294,0.371206777263252,0.89007585871351
"5901","STK19",53.4819854969536,-0.265765839921772,0.296202530722809,-
0.897243650394331,0.36958891770095,0.89007585871351
"5902","DST",337.050196802633,0.24778094164316,0.279589644480803,0.886
230754730879,0.375493194162486,0.89007585871351
"5903","BAG2",28.5389099095496,0.26098322780541,0.291793731483193,0.89
4409987763711,0.371102570538197,0.89007585871351
"5904","MTO1",17.7863307833315,0.289840103779498,0.323972620279943,0.8
94643823694266,0.370977517347506,0.89007585871351
"5905","EEF1A1",11679.2898426802,-0.181594876024048,0.202167020282085,
-0.898241838706766,0.36905663242962,0.89007585871351
"5906","DYNLT1",184.039676180036,-0.251147829702022,0.28327217869882,-
0.886595467495757,0.37529673498872,0.89007585871351
"5907","TNRC18",63.8861250463346,0.231921158111092,0.259162050778456,0
.894888574212394,0.370846655159457,0.89007585871351
"5908","MPP6",21.5577931832125,0.278992510213625,0.315497707678706,0.8
84293303638654,0.376537902164614,0.89007585871351
"5909","UBE2D4",34.0758239296,-0.269103273726597,0.301145407698938,-0.
893599128018669,0.37153641308179,0.89007585871351
"5910","CALD1",1574.56020555173,0.174421733757348,0.194270948507613,0.
89782715891004,0.369277703075203,0.89007585871351

"5911","UBN2",37.8781241567907,0.32295657207894,0.361982057199382,0.89
2189448774399,0.37229139665872,0.89007585871351
"5912","KDM7A",41.1396900526364,0.28380982496176,0.32068336392618,0.88
5015741031991,0.376148141607641,0.89007585871351
"5913","BRAF",41.1080453851845,0.265160549251075,0.297871513943615,0.8
90184313835622,0.373366925992393,0.89007585871351
"5914","ENTPD4",28.2528221347619,0.335076890462936,0.376498026297017,0
.889983126229184,0.373474946594673,0.89007585871351
"5915","AC009812.1",4.29873551587179,0.607617637062978,0.6765156064212
62,0.898157605376244,0.369101531539467,0.89007585871351
"5916","STRBP",4.80797941809061,0.58999775011112,0.665826501285336,0.8
8611334780482,0.375556451055116,0.89007585871351
"5917","RPP38",23.285266977248,-0.292704211023633,0.326170761957689,-0
.897395613471948,0.369507852403833,0.89007585871351
"5918","NRP1",377.14074037631,0.249354820967675,0.277800462185947,0.89
7604053663411,0.369396677149957,0.89007585871351
"5919","PRXL2A",31.2665236959515,0.247166074632786,0.274941751837911,0
.898976139420616,0.36866537011281,0.89007585871351
"5920","INPP5F",8.59279557563129,-0.439066396439492,0.495370142850767,
-0.886340048499376,0.375434314418791,0.89007585871351
"5921","BNIP3",78.3817616720426,-0.189103875078609,0.21382900070696,-0
.884369633928955,0.376496709614275,0.89007585871351
"5922","TMEM80",39.0499459943489,0.285103595549933,0.320475036601343,0
.889628092638583,0.373665616567275,0.89007585871351
"5923","AP006621.3",12.3412008565708,0.359685552764045,0.4062064082701
13,0.885474836046571,0.375900585754181,0.89007585871351
"5924","ARHGAP1",81.4351675207069,0.1787783179251,0.199346005749403,0.
896824178909518,0.36981274377011,0.89007585871351
"5925","BRMS1",41.2914908913012,-0.231033001562655,0.260750842590805,-
0.886029741139568,0.375601500943261,0.89007585871351
"5926","MED17",24.1843237745894,-0.282895908430447,0.319733793256986,-
0.884785763646415,0.376272189157244,0.89007585871351
"5927","POGLUT3",113.65762460131,0.19497100969956,0.220152993698227,0.
88561598197849,0.375824496454618,0.89007585871351
"5928","RBM7",74.4337193234738,-0.202988480826858,0.228316428388543,-0
.889066469108467,0.37396735819136,0.89007585871351
"5929","WNT5B",8.8853026466598,0.544881774120176,0.610273853801471,0.8
92847974275876,0.371938591105115,0.89007585871351
"5930","CCND2",61.8047681570875,-0.267028722580864,0.30082687557375,-0
.887649157248919,0.374729502658725,0.89007585871351
"5931","SLC2A3",382.602665791232,-0.262636086118545,0.292270396402227,
-0.898606527898573,0.368862280399829,0.89007585871351
"5932","LINC00937",1.55881418136791,-1.09871746954944,1.23486872767459
,-0.889744346849283,0.373603175878453,0.89007585871351
"5933","AC092821.3",8.69228062223605,0.417074307436952,0.4711429408169
15,0.885239428004135,0.376027511264373,0.89007585871351
"5934","TMEM106C",39.5941921491208,0.355822006615713,0.396486605950578
,0.897437646758906,0.369485431534442,0.89007585871351
"5935","SP1",59.8445422966105,0.219248657092904,0.246350827529457,0.88
9985470280946,0.37347368792723,0.89007585871351

"5936","ATP5MC2",513.907477398401,-0.237661664529071,0.268659205973835
,-0.884621331577287,0.376360897682211,0.89007585871351
"5937","TSFM",35.7424807902964,-0.257528345616706,0.289744426431704,-0
.888812077554868,0.374104083895684,0.89007585871351
"5938","ACSS3",22.6253435202414,0.294315484284329,0.330102501887555,0.
891588166104187,0.372613715413945,0.89007585871351
"5939","APPL2",63.6391803011769,0.216146859570407,0.242971041293665,0.
889599264256199,0.373681101427705,0.89007585871351
"5940","DTX1",34.5510041988296,-0.303990236141973,0.338867703581576,-0
.897076448800006,0.369678124816386,0.89007585871351
"5941","VPS37B",15.1967804200441,0.493530219729527,0.549055572626651,0
.898871160470161,0.368721290926079,0.89007585871351
"5942","USPL1",15.100186338368,0.382855913255004,0.427876197674011,0.8
94781984453113,0.370903642605517,0.89007585871351
"5943","PCDH17",2.37173917815304,-1.00272920513336,1.12147951619626,-0
.894112813165178,0.371261534379054,0.89007585871351
"5944","DIS3",58.9375581301693,0.207133305065882,0.232763203160373,0.8
89888531578457,0.373525742547656,0.89007585871351
"5945","AL354811.1",1.70710313791756,-1.05595295862499,1.1840695265986
1,-0.89179979292124,0.372500252731136,0.89007585871351
"5946","ANKRD10-
IT1",2.52826739061004,0.780239938557669,0.871023992140693,0.8957731883
36745,0.370373912358387,0.89007585871351
"5947","PPP1R3E",7.95822612876536,0.493876583410411,0.557446724559276,
0.885961943360373,0.375638034959204,0.89007585871351
"5948","SUSD6",15.3001896917711,-0.390911468141989,0.44059321712286,-0
.887238960905253,0.374950260442385,0.89007585871351
"5949","GSTZ1",30.9071277348063,-0.305080909035307,0.341078284788134,-
0.89446007747111,0.371075780846392,0.89007585871351
"5950","GPR65",1.85580307459062,-0.97006781684721,1.08257262823221,-0.
896076430854605,0.370211944017628,0.89007585871351
"5951","OIP5-
AS1",98.5192443663918,0.200710262729522,0.225281390512043,0.8909313915
06979,0.372965978227979,0.89007585871351
"5952","AC018926.2",11.8099274935965,-0.422900187780847,0.471565571462
616,-0.896800388690746,0.369825440532505,0.89007585871351
"5953","ZNF609",26.5645597148305,0.289161077801521,0.3224314515835,0.8
9681411779594,0.369818113320652,0.89007585871351
"5954","BOLA2",31.1265213082596,-0.249971256267332,0.280560729317092,-
0.890970225504412,0.372945143773926,0.89007585871351
"5955","CDH11",226.328450499503,0.16914565458751,0.189896987710313,0.8
90723210657458,0.373077679621057,0.89007585871351
"5956","SPATA2L",11.1624682732999,-0.462284776790553,0.51770466674612,
-0.892950762248499,0.371883541078057,0.89007585871351
"5957","RNASEK",483.63671017009,-0.216842564411896,0.242385897842448,-
0.894617081035156,0.370991817737856,0.89007585871351
"5958","RSKR",1.97039097219384,0.921658832725055,1.03874199946331,0.88
7283688539842,0.374926185204173,0.89007585871351
"5959","LRRC37B",5.02715898302027,0.544313053272287,0.614316510608444,
0.886046596294762,0.375592418589485,0.89007585871351

"5960","DHRS11",5.54156279958799,-0.583029923323572,0.650317641664145,
-0.896530996501362,0.369969233168732,0.89007585871351
"5961","CACNB1",6.9211844137398,-0.518783283940746,0.585809338355394,-
0.885583840976627,0.375841822269472,0.89007585871351
"5962","TOP2A",2.90376866956431,0.738281753804595,0.829325421350834,0.
890219610779634,0.373347976535545,0.89007585871351
"5963","PPM1D",11.2859436860289,-0.386174277485206,0.432978596263785,-
0.891901541594763,0.37244570829952,0.89007585871351
"5964","ABCA5",28.2366940723341,0.288476041136956,0.323626058499412,0.
891386937363947,0.372721623077216,0.89007585871351
"5965","MXRA7",372.50192793853,-0.217563101269374,0.241931732253396,-0
.899274763351427,0.368506326193962,0.89007585871351
"5966","RAB40B",44.2445704779882,-0.192817678347441,0.216813899897963,
-0.889323417171063,0.373829289849045,0.89007585871351
"5967","PSMG2",209.359898199129,-0.200724398311019,0.22527327823457,-0
.891026223278959,0.372915102216245,0.89007585871351
"5968","WDR7",12.7744973424741,0.324516628920062,0.366653481250451,0.8
85077179175596,0.376115006747565,0.89007585871351
"5969","WDR18",34.0893647356357,-0.2595949029603,0.292792803794062,-0.
886616404489531,0.375285458823383,0.89007585871351
"5970","ZNF177",3.89440534259585,0.623345975565642,0.696016711741276,0
.895590529724741,0.370471495480884,0.89007585871351
"5971","HAUS8",7.27864710841268,-0.495865841365447,0.555225851670512,-
0.893088533024051,0.371809763279114,0.89007585871351
"5972","MVB12A",97.9270656754358,-0.202031543998828,0.226283549552682,
-0.892824707753634,0.37195105262884,0.89007585871351
"5973","ZNF100",12.5724216147546,0.349294717160233,0.39191987620298,0.
891240119139376,0.372800365653071,0.89007585871351
"5974","ZNF257",2.88975211201107,0.869914906568429,0.973247533815402,0
.893826982697937,0.371414469903029,0.89007585871351
"5975","GRAMD1A",36.9776868452135,-0.229493733683764,0.258817304884311
,-0.886701659250897,0.375239544804429,0.89007585871351
"5976","ZNF574",6.51138219482244,0.465913722535609,0.526752348363039,0
.884502411775677,0.37642506109312,0.89007585871351
"5977","VPS16",32.8365169963184,-0.261242870366859,0.291481836068078,-
0.896257804228473,0.370115089634918,0.89007585871351
"5978","ITCH",40.5002298593868,0.230752459836712,0.25864728042208,0.89
2151115836799,0.372311939946465,0.89007585871351
"5979","TTI1",10.9481102779333,0.452084195802664,0.511035451077371,0.8
84643511227205,0.376348931355655,0.89007585871351
"5980","MICAL3",9.94102628676496,-0.3909242638911,0.439390430020067,-0
.889696809903772,0.373628707417224,0.89007585871351
"5981","SNAP29",51.8710176221409,-0.229714959629291,0.259556351173674,
-0.885029237737992,0.376140862400845,0.89007585871351
"5982","ASCC2",37.1846265003559,-0.210147063561247,0.236143352928315,-
0.889913101322994,0.373512548540741,0.89007585871351
"5983","Z82217.1",5.7112669569649,-0.570984080600692,0.643160996980433
,-0.887777839889851,0.374660265323407,0.89007585871351
"5984","TRAPPC2",22.271988359059,0.288418050759723,0.323201122396443,0
.892379483775264,0.372189563972739,0.89007585871351

"5985","NLGN3",1.84310219403958,1.03721675264528,1.16392890281874,0.89
1134114921802,0.372857224981218,0.89007585871351
"5986","NAP1L3",11.3984701489273,0.41550063171682,0.46991387499171,0.8
8420592331765,0.376585061409085,0.89007585871351
"5987","SRPX2",118.625482765088,0.174731506638553,0.19700975026605,0.8
86918065743388,0.375123014503893,0.89007585871351
"5988","CAPN6",30.192336527334,0.414304509674233,0.465018659681557,0.8
90941688142037,0.372960454009218,0.89007585871351
"5989","STAG2",124.877131431283,0.192278205020383,0.215736364471289,0.
891264694719427,0.372787184388246,0.89007585871351
"5990","DCAF12L2",7.44888354266168,0.518454752625741,0.581155964624725
,0.892109492432943,0.372334247447905,0.89007585871351
"5991","RENBP",7.68554642686074,0.445152039043749,0.500101251925483,0.
890123824585183,0.373399401532192,0.89007585871351
"5992","AP003555.1",7.93965694106806,-0.543966693004165,0.615392854248
43,-0.883934041886956,0.37673181938892,0.890125573593711
"5993","VPS39",25.5743765283246,0.286201495773821,0.32376343139256,0.8
83983390412009,0.376705179091251,0.890125573593711
"5994","SAMD4A",40.3958663497393,0.265556758310826,0.300510389607686,0
.883685780905971,0.376865858174483,0.890293719010791
"5995","GUK1",584.395123992974,-0.248030853120747,0.280730829343751,-0
.883518399815776,0.376956245631679,0.890358705278494
"5996","MGAT4C",2.83414887370805,0.932501842147054,1.05591725557248,0.
883120185057955,0.377171339302606,0.890718172869397
"5997","CORO1B",80.0672874632871,-0.200707691944539,0.227354964432312,
-0.882794411134547,0.377347360689758,0.890985263859759
"5998","PRDM2",96.2241844892504,0.256006143501766,0.291854575301521,0.
877170225059108,0.380394185948751,0.890994621432525
"5999","RPL11",5491.92995337142,-0.226728764588416,0.257711586959242,-
0.879777146474498,0.378980047373145,0.890994621432525
"6000","TAF12",73.8752025323471,-0.25494070722548,0.291034287974985,-0
.875981689303195,0.381039988171793,0.890994621432525
"6001","BTF3L4",107.88296123616,-0.175606713872768,0.200950654606878,-
0.87387978016946,0.382183727208233,0.890994621432525
"6002","HIST2H2BE",1.83912636466481,0.999686364966163,1.14200417444229
,0.875378906083574,0.381367773294798,0.890994621432525
"6003","BX470102.2",2.55867075835555,-0.716360250469522,0.819277784691
985,-0.874380172213317,0.381911251649828,0.890994621432525
"6004","TOR3A",41.6288272471924,-0.208387868563524,0.23695661826704,-0
.879434683392889,0.379165633528657,0.890994621432525
"6005","TRAF3IP3",2.31752725442165,-0.77182007087703,0.874606829243032
,-0.882476611284908,0.377519122330246,0.890994621432525
"6006","MTIF2",24.3952586397717,-0.26248333347987,0.299455684147379,-0
.876534817588193,0.380739356950326,0.890994621432525
"6007","GYPC",298.932696603488,0.180202126792341,0.205249475833033,0.8
77966319090298,0.379961996982833,0.890994621432525
"6008","TTN",2.68164004483831,0.743582652788638,0.846541151686287,0.87
8377443680607,0.379738921092912,0.890994621432525
"6009","ITPRID2",24.4126705884981,0.28263893820275,0.321979930146705,0
.877815390772866,0.3800439107829,0.890994621432525

"6010","POGLUT1",27.3807784330652,0.280773812202605,0.320780213416008,
0.875284074452808,0.38141935716599,0.890994621432525
"6011","THAP6",21.0127466909534,0.304323379547342,0.345139642567607,0.
881739858346554,0.377917501318203,0.890994621432525
"6012","MGST2",33.3286286186037,-0.271280258709166,0.307790419952783,-
0.881379799770188,0.378112287316323,0.890994621432525
"6013","AC079298.3",2.84999570238812,0.790456983120799,0.8959609396423
28,0.882244915092341,0.377644377797202,0.890994621432525
"6014","RWDD4",56.7999245952876,-0.184636009457719,0.21010426162905,-0
.87878279110637,0.379519058741483,0.890994621432525
"6015","ZFR",100.129458182437,0.165433439236648,0.189318801039298,0.87
3835236270635,0.382207988214191,0.890994621432525
"6016","NIPBL",117.813097121701,0.228009761528363,0.258476663719104,0.
882129002470219,0.377707050023127,0.890994621432525
"6017","HIGD2A",246.920420911171,-0.283527425188207,0.323872859361671,
-0.875428171866632,0.381340976755359,0.890994621432525
"6018","NUP153",32.5296391261678,0.26170234108115,0.298959327971852,0.
875377740699864,0.381368407181807,0.890994621432525
"6019","CDKAL1",14.5212109479127,0.31723497225478,0.361610970541167,0.
877282488913497,0.380333221093792,0.890994621432525
"6020","HIST1H3A",1.63492199923125,0.937813883073572,1.06277176545745,
0.88242265513133,0.377548288857234,0.890994621432525
"6021","HLA-
A",1443.3724793399,0.206975082664136,0.236834345299277,0.8739234269530
94,0.382159955735545,0.890994621432525
"6022","SNHG32",264.720684527579,0.214737622558458,0.245201900890329,0
.875758392486944,0.381161393696226,0.890994621432525
"6023","CUTA",208.359765423549,-0.278857523275872,0.31619104658693 8,-0
.881927323009128,0.377816110375209,0.890994621432525
"6024","L3MBTL3",9.76507701667796,-0.368713077934542,0.418824857488835
,-0.880351467544812,0.378668939208529,0.890994621432525
"6025","NDUFA4",612.665955419425,-0.247096125164189,0.281774720244061,
-0.876927940697324,0.380525778864392,0.890994621432525
"6026","GNG11",288.578571910333,-0.191732429559923,0.219350631321783,-
0.874091076941808,0.382068656472621,0.890994621432525
"6027","PMPCB",71.2194673362393,-0.194012353107538,0.220277105698036,-
0.880764946010765,0.378445056407867,0.890994621432525
"6028","AC007032.1",9.64738953986281,-0.412914057144079,0.469737028995
548,-0.879032376959987,0.379383721149971,0.890994621432525
"6029","METTL2B",31.0939552522239,0.288595049115689,0.329964552407115,
0.874624401349682,0.381778306266784,0.890994621432525
"6030","AC016831.1",18.7137366892306,-0.578905093888935,0.656982107793
715,-0.881158081812945,0.378232263983679,0.890994621432525
"6031","MRPL15",62.8774336348483,-0.278375649080322,0.316732611222993,
-0.878897970137763,0.379456599378533,0.890994621432525
"6032","EIF3E",641.886434690173,-0.198847168886435,0.22696534081343,-0
.876112485605859,0.380968885808945,0.890994621432525
"6033","AC107375.1",4.96734217472879,0.61308135722615,0.69839979264907
1,0.877837255507618,0.380032043394422,0.890994621432525
"6034","APTX",30.6601464778275,0.242440522532874,0.277158505721097,0.8

747359995397,0.381717567585287,0.890994621432525
"6035","GRHPR",142.181866424534,-0.202855324242426,0.231401345286002,-0.876638482769861,0.380683030013729,0.890994621432525
"6036","GKAP1",6.3905231659624,0.475049969675212,0.540103248638146,0.879553994302082,0.379100970716069,0.890994621432525
"6037","AL359091.5",2.49427794123957,-0.771486050413838,0.880143746255938,-0.876545511679971,0.380733546031314,0.890994621432525
"6038","PTGES",147.619277340133,-0.2088014421585,0.237892163379018,-0.87771467202907,0.380098580221256,0.890994621432525
"6039","ZNF33B",8.2997302233895,0.429901542906992,0.489750727884236,0.87779663904575,0.380054088724685,0.890994621432525
"6040","SLC29A3",9.63191818774342,0.426557249217698,0.483709609056421,0.881845721547251,0.377860242761907,0.890994621432525
"6041","FBXL15",45.6521251211835,-0.243586833426516,0.278034290195285,-0.876103567137083,0.380973733731531,0.890994621432525
"6042","CASP7",41.3802824427224,-0.237736566938016,0.270773107496631,-0.877991795920774,0.379948170933233,0.890994621432525
"6043","LINC02709",2.66171593708865,0.821647606850666,0.938663202661181,0.875338038735547,0.381390002652343,0.890994621432525
"6044","PHF21A",36.4801319491539,0.23691501563839,0.270645828862231,0.875369173928737,0.381373066924355,0.890994621432525
"6045","FADS3",31.3867693259427,-0.350155457208259,0.39751481782715,-0.880861395613471,0.378392844356022,0.890994621432525
"6046","UVRAG",50.7917437477683,0.187262641981006,0.212662771423463,0.880561467000355,0.378555222335463,0.890994621432525
"6047","CEP57",84.3121084031747,0.162384658331928,0.18579238122354,0.874011395206521,0.382112048089236,0.890994621432525
"6048","HOXC8",51.5173071784398,0.23742112941736,0.271253284484105,0.875274671305492,0.381424472261701,0.890994621432525
"6049","NACA",2624.23264577761,-0.241132701118452,0.275213995399223,-0.876164385349177,0.380940674773966,0.890994621432525
"6050","GLIPR1",60.8499008031656,0.215443392049829,0.245082537166528,0.879064638960549,0.379366229290632,0.890994621432525
"6051","CEP83",26.9935120824354,0.29232624156665,0.331813137825569,0.880996585856474,0.378319667902378,0.890994621432525
"6052","CKAP4",141.821770644756,-0.153578618255796,0.174783158852778,-0.878680870993741,0.379574333321502,0.890994621432525
"6053","ZNF140",13.6823032469148,0.341644364974777,0.39089265837771,0.874010697444859,0.38211242807682,0.890994621432525
"6054","GOLGA6L4",12.105107324751,0.465527136999764,0.530300866908475,0.877854753875237,0.38002254607644,0.890994621432525
"6055","METRN",70.580628529755,-0.259662097924485,0.296309874108998,-0.876319423054283,0.38085640889247,0.890994621432525
"6056","ZNF263",16.0371651246807,-0.304672033069512,0.346418703725225,-0.879490713963221,0.379135265852187,0.890994621432525
"6057","ASPA",43.9471803103923,0.267145643031867,0.30407310478955,0.878557290414617,0.379641361714692,0.890994621432525
"6058","PEX12",6.22767580476589,0.469553933062418,0.53565336812241,0.87660035576424,0.380703745896387,0.890994621432525
"6059","CWC25",46.6401116449615,-0.212339903855158,0.243023866495986,-

0.873740949466233,0.382259345000183,0.890994621432525
"6060","MYL4",2.41217728812106,-1.11290764718576,1.27265436610847,-0.874477530445927,0.381858251590411,0.890994621432525
"6061","ASPSCR1",26.2464307681753,-0.242517070971265,0.277026940949521,-0.875427747713013,0.381341207455157,0.890994621432525
"6062","CSNK1G2",52.8591011230371,-0.195108954119787,0.222909904050237,-0.875281674679721,0.381420662583314,0.890994621432525
"6063","HDGFL2",66.5837179558562,-0.169121229424434,0.192592754514778,-0.878128722186466,0.379873867562871,0.890994621432525
"6064","HNRNPM",196.32587428615,-0.175556105358907,0.200805716654958,-0.874258503609055,0.381977492178096,0.890994621432525
"6065","NR2C2AP",26.6115588700502,0.281297193470039,0.319168702240364,0.881343288033913,0.37813204304112,0.890994621432525
"6066","RINL",13.1042704188681,0.349572071248196,0.398362044558094,0.877523539261828,0.380202339055282,0.890994621432525
"6067","ARHGAP35",39.5713068068298,0.23417747265373,0.26682579630167,0.877641801877999,0.380138136649212,0.890994621432525
"6068","ZNF132",1.61404852601066,1.13834110620695,1.29374191204414,0.87988268418107,0.378922866091285,0.890994621432525
"6069","ZNF337-AS1",6.50989676585507,0.510390692206617,0.581716143833217,0.877387876573956,0.380275995817635,0.890994621432525
"6070","NFS1",14.3301950882105,-0.372076359831297,0.425765947341025,-0.873898822005311,0.382173356287178,0.890994621432525
"6071","PTGIS",165.996009725442,0.32180739679759,0.36617948545722,0.878824209378563,0.379496597684877,0.890994621432525
"6072","RBM38",7.22136259173438,0.472734144127227,0.540570276151308,0.874510059067522,0.381840544603276,0.890994621432525
"6073","RTL10",9.33009690240022,-0.423341045249915,0.480216273999035,-0.881563304226473,0.378013006580676,0.890994621432525
"6074","ARMCX5-GPRASP2",8.35383653105181,-0.409326822155087,0.466149960305654,-0.8781011627389,0.37988882204589,0.890994621432525
"6075","RPL10",10987.7222998637,-0.220062779375636,0.249892831840491,-0.880628618895738,0.378518863327646,0.890994621432525
"6076","ANKLE2",53.7477961696658,0.195611015575708,0.223950626896746,0.873455985751252,0.382414586716379,0.890998228061535
"6077","AP3D1",55.0363494556952,0.22573945204093,0.25846304196273,0.873391608822284,0.382449663146752,0.890998228061535
"6078","TM9SF4",36.9165415221615,0.214287535477213,0.24534742582286,0.873404458019167,0.382442661972432,0.890998228061535
"6079","AC246817.2",2.01235106428567,0.963925736225427,1.10396360738459,0.873149920683591,0.382581367010326,0.891011867905628
"6080","SCML2",2.11876902120636,0.899658238687695,1.03028608823705,0.873212061153932,0.382547501957288,0.891011867905628
"6081","SCNN1D",1.63761537504013,0.926907341516455,1.06255800789637,0.872335754498269,0.383025237626312,0.89118042190035
"6082","LRRC8B",4.21646697812646,-0.617120769158872,0.710029333704294,-0.869148272986541,0.384766035973053,0.89118042190035
"6083","RWDD3",38.7904605591042,-0.229429124366576,0.265372810746325,-

0.864553997530259,0.387283630885255,0.89118042190035
"6084","IPO9",36.3271479423994,0.217865747198971,0.250410018057065,0.8
7003606680513,0.384280694409344,0.89118042190035
"6085","IAH1",151.851283116786,-0.231307974604211,0.266554409012745,-0
.867770206694092,0.385520143118343,0.89118042190035
"6086","AC092164.1",5.19764699685548,0.509049161753964,0.5871445162810
77,0.866991256221275,0.385946800557226,0.89118042190035
"6087","FAM136A",49.3714863435911,-0.197552969274622,0.228072253874419
,-0.866185894683177,0.386388227288997,0.89118042190035
"6088","WBP1",53.489113894717,0.203558143768685,0.234137567672107,0.86
9395483144992,0.38463085280401,0.89118042190035
"6089","THNSL2",23.780731661813,-0.312594897615233,0.35995212553725,-0
.868434648492953,0.385156433783359,0.89118042190035
"6090","MAP4K4",109.363901392416,0.214140235831415,0.24753713223289,0.
865083286292363,0.386993077552275,0.89118042190035
"6091","PTPN18",40.8653987406199,0.214913041755966,0.24690107918226,0.
870441888985503,0.384058963219719,0.89118042190035
"6092","NDUFS1",78.2915775989847,0.18266345571511,0.210526643770996,0.
867650062924128,0.385585931109527,0.89118042190035
"6093","PIKFYVE",25.6043792358146,0.292901578381921,0.338609311722942,
0.865013359767202,0.387031456128649,0.89118042190035
"6094","P4HTM",57.1184041302639,-0.202421869881104,0.233507252820121,-
0.866876156677828,0.38600986890166,0.89118042190035
"6095","QRICH1",37.4932429800954,0.223540128768756,0.257182810060031,0
.869187675166074,0.384744487535801,0.89118042190035
"6096","CCDC71",10.5277941199054,0.383327177123231,0.440755291483071,0
.869705218588293,0.384461519622139,0.89118042190035
"6097","C3orf62",7.15903972255916,0.4249705490991,0.491278269816782,0.
865030218530913,0.387022203127729,0.89118042190035
"6098","RAB6B",8.45049269062995,0.471793171907645,0.544926567596654,0.
865792200201291,0.386604127283576,0.89118042190035
"6099","PLOD2",35.88793379983,0.298055559152566,0.341879801587005,0.87
1813888299319,0.383309916739912,0.89118042190035
"6100","LNX1",9.68925585328488,-0.394819207466389,0.452724717895484,-0
.872095540313609,0.383156258878493,0.89118042190035
"6101","MAP9",37.6241688989785,-0.212345942298399,0.243751259201718,-0
.871158339833109,0.383667703389466,0.89118042190035
"6102","TMEM144",6.02630554117276,0.564879320371756,0.653228337355467,
0.864750177033985,0.38717592253579,0.89118042190035
"6103","TMEM267",19.6482084647979,0.313206647122223,0.362433298638568,
0.864177348766634,0.38749047340491,0.89118042190035
"6104","BDP1",88.6902186785003,0.234419857552036,0.2692132765062,0.870
758903848629,0.383885808629454,0.89118042190035
"6105","AC011346.1",3.172668260258,0.677162995997138,0.780511343158662
,0.867588923508425,0.385619412293699,0.89118042190035
"6106","SERPINB9P1",2.36017189049165,-1.13660081816682,1.3125968485075
,-0.865917680252853,0.386535306688122,0.89118042190035
"6107","GPSM3",5.46351428897987,-0.515073836463732,0.591131916940686,-
0.871334843717149,0.383571350612392,0.89118042190035
"6108","UHRF1BP1",8.76728217587288,0.408585491574054,0.468963398947198

,0.871252410084264,0.383616348964751,0.89118042190035
"6109","TOMM6",233.463974084828,-0.200733462356288,0.230768822619591,-0.869846541996644,0.384384272895108,0.89118042190035
"6110","MRPS10",80.1850706147326,-0.174033626115372,0.201359936540924,-0.86429122448597,0.387427929682634,0.89118042190035
"6111","YIPF3",157.030323699142,-0.183706850078274,0.211335924256492,-0.869264658739772,0.384702388549874,0.89118042190035
"6112","TMEM63B",17.3357044546869,-0.308355246956102,0.355916696214358,-0.866369153894339,0.386287754003369,0.89118042190035
"6113","AKAP7",12.8774040199125,0.38882064659649,0.445525942280857,0.872722797253811,0.382814188666497,0.89118042190035
"6114","HIVEP2",47.998981128063,0.245283149999373,0.281498132535714,0.871349119761045,0.383563558023786,0.89118042190035
"6115","EZR",9.88893572637618,-0.425763764455176,0.491555266999696,-0.866156448803629,0.386404372708459,0.89118042190035
"6116","DNAJC30",25.6858368053043,-0.276740612696332,0.319994589915508,-0.864829035920272,0.387132631823239,0.89118042190035
"6117","ADAM22",21.9412331606507,0.382169086638805,0.440004276361292,0.868557664482793,0.3850891189778,0.89118042190035
"6118","VPS28",309.041381693551,-0.212143954684333,0.245491443717236,-0.864160279772056,0.387499848703422,0.89118042190035
"6119","CREB3",56.1484901271995,-0.195657115727343,0.225582710577575,-0.867340919995104,0.385755242261145,0.89118042190035
"6120","GPR107",37.4860680698576,0.238646356394523,0.276024753552286,0.864583169890658,0.38726761324023,0.89118042190035
"6121","RABL6",52.6206374649082,-0.178218930553883,0.206091541910358,-0.864756160790925,0.387172637563398,0.89118042190035
"6122","MMS19",19.9225569692421,0.275263551761494,0.316912878667385,0.868577991904192,0.385077996429862,0.89118042190035
"6123","PHRF1",22.7955637225526,0.266264876623687,0.305352379910989,0.871992144620926,0.383212662971111,0.89118042190035
"6124","SPTY2D1OS",4.47350633490373,0.582884527185616,0.669591101127881,0.870508174621476,0.384022753829679,0.89118042190035
"6125","AP001160.1",2.24292998466004,0.857212013525624,0.986040308496311,0.869347841198148,0.384656902828628,0.89118042190035
"6126","CEP126",133.60529938054,0.2121621142694,0.244504725895561,0.867721936630476,0.385546573881226,0.89118042190035
"6127","YAF2",43.9969427306455,-0.203863985698359,0.233759403363904,-0.872110309851341,0.383148202261909,0.89118042190035
"6128","KMT2D",19.5644817605176,0.279339912458076,0.320938109921099,0.870385609632806,0.384089708187471,0.89118042190035
"6129","NAP1L1",414.61333961976,-0.1388289272696,0.160650566531738,-0.86416705690343,0.387496126286619,0.89118042190035
"6130","UBL3",157.662435007251,0.173143275315475,0.199365328749877,0.868472348733714,0.385135803307599,0.89118042190035
"6131","DLEU1",6.07829606251457,-0.524675547460653,0.605097615099744,-0.867092406857638,0.385891380657322,0.89118042190035
"6132","NEK3",16.1094656275167,0.327082423231105,0.37720985829009,0.8671099549566,0.38588176664125,0.89118042190035
"6133","NAXD",19.1430582715037,0.301415627763523,0.34630063083485,0.87

0387174972453,0.384088853034371,0.89118042190035
"6134","ARHGEF40",52.4670761535029,-0.213944646077895,0.245329506378717,-0.872070584724638,0.383169872107177,0.89118042190035
"6135","RIPK3",5.53242744904183,-0.578931881602911,0.666393378537773,-0.868753952617637,0.38498172427641,0.89118042190035
"6136","DCAF4",8.09137753887279,0.43961739742353,0.505876854337651,0.86902058011554,0.384835874282998,0.89118042190035
"6137","POMT2",10.6353223677292,0.365681331090162,0.422467326089919,0.865584883154558,0.386717848452939,0.89118042190035
"6138","FAM98B",24.0584698823312,0.247433200059101,0.286201020275578,0.864543389191457,0.38728945569768,0.89118042190035
"6139","PIGQ",15.0079876763033,-0.315375913319067,0.36449468756004,-0.865241453668972,0.386906277302278,0.89118042190035
"6140","VPS35L",33.9114483084752,0.262897269745228,0.30233044810332,0.86956927889507,0.38453583259445,0.89118042190035
"6141","PSMD7",154.201741499458,-0.170246354263472,0.195481226109172,-0.870908975005061,0.383803855923032,0.89118042190035
"6142","MPDU1",33.0889706465052,-0.29520662535875,0.339522173312031,-0.869476719234613,0.384586436407939,0.89118042190035
"6143","KIAA0100",41.7009479670847,0.200780313205654,0.231094051915429,0.868825101907564,0.384942801041511,0.89118042190035
"6144","SGSH",18.8270210362808,0.290818353783879,0.334721888873326,0.868835781139901,0.384936959022655,0.89118042190035
"6145","CENPX",82.6427541858151,-0.213156777560948,0.245748048414768,-0.867379329911045,0.385734203478901,0.89118042190035
"6146","ZSCAN30",17.424299851035,0.283029180438459,0.327440619634384,0.864367960073145,0.387385787828583,0.89118042190035
"6147","PARD6G-AS1",3.17933909734539,-0.720590828733447,0.82627587961501,-0.872094716197204,0.383156708427772,0.89118042190035
"6148","CNN2",59.8459441210746,0.306989503699555,0.353592389059264,0.868201672881886,0.38528393860839,0.89118042190035
"6149","SLC39A3",47.7986952514643,-0.257201373246458,0.296508167350265,-0.867434362921363,0.38570406072614,0.89118042190035
"6150","NR2F6",53.3131684900847,-0.225101336141504,0.259864203668021,-0.866226794472522,0.386365802279214,0.89118042190035
"6151","ZNF211",2.89718502682931,0.717338546242183,0.828957903946583,0.86534978776004,0.386846831781275,0.89118042190035
"6152","XRN2",117.600518736462,-0.150455752129757,0.174020504829711,-0.864586344448236,0.387265870212766,0.89118042190035
"6153","CTSZ",240.893547989481,-0.136941132927637,0.15710175836743,-0.871671548114433,0.383387586098525,0.89118042190035
"6154","RRP1",26.5785532313246,-0.250315453950838,0.289183710762042,-0.865593201260264,0.386713285267223,0.89118042190035
"6155","AC008079.1",2.11935939824631,0.874568078129017,1.00637451914196,0.869028439705209,0.384831575460068,0.89118042190035
"6156","TSPAN7",4.6946311373123,-0.696539873609393,0.805870044170031,-0.864332752716677,0.387405122751326,0.89118042190035
"6157","NEXMIF",1.80297620592055,-1.03384452823036,1.19223869035277,-0.867145594750379,0.385862241241393,0.89118042190035

"6158","COPB2",135.153518937868,-0.177958120831271,0.2059986046157,-0.
863880224641619,0.387653691245702,0.89138945567378
"6159","EPRS",114.291406716392,-0.162224833679278,0.187826947270874,-0
.863693075122632,0.38775651874528,0.891481134183011
"6160","EEF1B2",1198.36109590842,-0.225170198620637,0.260744743092826,
-0.863565631083407,0.387826551158272,0.891497396818366
"6161","DNAJC25",22.472720211994,0.290227230942254,0.336219584635183,0
.863207392446121,0.38802344995225,0.891669794972109
"6162","AC119403.1",1.7243679202377,-0.866380097941837,1.0036840326794
2,-0.863200040782719,0.388027491286591,0.891669794972109
"6163","BLMH",28.6093238651202,-0.238970243709697,0.276913414168529,-0
.862978214425754,0.388149445067229,0.891805312697056
"6164","SLC2A5",4.6322397329185,0.891538751818124,1.03384350275394,0.8
62353682586631,0.38849292018974,0.892015526169404
"6165","C16orf91",28.4625035503335,-0.281708940671848,0.32661566475151
1,-0.862508970248478,0.388407499013919,0.892015526169404
"6166","JMJD6",17.8243048157441,-0.301205909606669,0.349174890913237,-
0.862621905082838,0.388345382613849,0.892015526169404
"6167","CYBB",2.50032050225839,-0.942558764331856,1.09287870126111,-0.
862455058593605,0.388437153627677,0.892015526169404
"6168","AC015726.1",2.04363173350752,1.06412172557553,1.23466145047094
,0.861873289369841,0.388757248989994,0.892130170109219
"6169","KDSR",179.382648711605,-0.159124113854143,0.184689580624835,-0
.861576020237849,0.388920871474873,0.892130170109219
"6170","ELAVL1",64.9415565764767,-0.177517690461138,0.205938506162113,
-0.861993678449807,0.388690996515656,0.892130170109219
"6171","CA11",9.4908503572481,-0.536078358929227,0.622198957675527,-0.
861586719675588,0.388914981577279,0.892130170109219
"6172","MAPK11",6.90406661346945,-0.567324902493625,0.658461920667705,
-0.861591057412001,0.388912593726778,0.892130170109219
"6173","ZNF182",8.16634992108134,0.418514303098268,0.485681579498456,0
.861705118671479,0.388849808135977,0.892130170109219
"6174","INTS5",14.8519032881542,0.319012543569342,0.370397860001414,0.
861269942456268,0.389089386198707,0.892227645113148
"6175","CLPTM1",69.8643133071931,0.179970396129485,0.208942002009724,0
.861341398083806,0.389050041486725,0.892227645113148
"6176","PFDN4",68.4516133592269,-0.217605905050888,0.252706144763268,-
0.861102547604209,0.389181566197849,0.892294523536519
"6177","NBPF15",11.5036119752861,0.447842408520434,0.520412166350085,0
.860553302705009,0.389484114430068,0.892330582460725
"6178","SRGAP3",2.20009383345028,-0.923593849028752,1.07326818493458,-
0.860543396322744,0.389489572614098,0.892330582460725
"6179","SDHAF4",15.6065035530603,0.378953324120821,0.440754810946372,0
.859782615434523,0.389908884010342,0.892330582460725
"6180","APBA1",4.40935893991882,0.555761079856553,0.645935985746929,0.
860396528634177,0.389570498720579,0.892330582460725
"6181","RPL7A",4713.25002612103,-0.212246070092961,0.246875784845705,-
0.859728183651601,0.389938895107003,0.892330582460725
"6182","HOTAIR",5.93054226678792,-0.74400367383838,0.865202379569223,-
0.859918663433188,0.389833879755668,0.892330582460725

"6183","GLCE",44.3232629450396,0.316491513124054,0.36796755876272,0.86
0107108866465,0.389730002906888,0.892330582460725
"6184","PARP6",17.577029070352,-0.295240789330388,0.343158387841612,-0
.860363027077336,0.3895889599694,0.892330582460725
"6185","MED29",58.9206788892479,0.190813603664478,0.221953274130018,0.
859701684565833,0.389953505951057,0.892330582460725
"6186","ZNF334",2.22116794774595,0.756432968052946,0.878862068209079,0
.860695887802263,0.389405558544046,0.892330582460725
"6187","SMDT1",191.769289752932,-0.214514332787113,0.249311475751656,-
0.860427030646576,0.389553690852853,0.892330582460725
"6188","SHANK3",2.32411045505625,0.900510978015888,1.04710359953814,0.
860001797733378,0.389788051528138,0.892330582460725
"6189","LINC01422",2.16951107494267,0.975966621460115,1.13553904402601
,0.859474296894151,0.39007889474422,0.892473283176307
"6190","MROH6",5.51334105409471,-0.516574806651128,0.601136403463392,-
0.859330434282351,0.390158237820965,0.892510605419202
"6191","SHISA2",1.52417764742131,1.38934520013866,1.6173153881798,0.85
9044074082725,0.390316200332361,0.892693788599446
"6192","NLK",4.32674787640735,-0.577851889405003,0.67273692107676,-0.8
58956705512925,0.3903644024723,0.892693788599446
"6193","TRAC",65.8037909819309,-0.616425063799239,0.717757257509827,-0
.858821081012614,0.390439235072502,0.892720744167065
"6194","AKT2",68.7267082851257,0.190500704954508,0.22184811924542,0.85
8698760225983,0.390506734650035,0.892730927130207
"6195","GNAI3",152.225474829281,-0.136180662065412,0.159119811446179,-
0.855837251362463,0.392087807128177,0.893086430964576
"6196","AL596325.2",6.04250637752655,-0.565634077187948,0.661124255445
295,-0.855563946609354,0.392239019362196,0.893086430964576
"6197","GTF3C2",21.8865231021033,-0.282786472600846,0.330146590614697,
-0.856548214156411,0.391694616032133,0.893086430964576
"6198","CEP68",40.72681339823,0.245531525787284,0.286981077600408,0.85
5566951801477,0.392237356477225,0.893086430964576
"6199","MAPK10",34.1327771322679,0.341016141370831,0.397425931657201,0
.858062129838507,0.390858157217906,0.893086430964576
"6200","CDKN2AIPNL",27.7565198546693,-0.250916519202274,0.292617963761
232,-0.857488432962423,0.391175004720395,0.893086430964576
"6201","CARMIL1",55.1152222708866,0.233632420691958,0.272657417525912,
0.856871684665444,0.391515803021752,0.893086430964576
"6202","RADIL",9.55228154126744,0.38716906169682,0.45156717072649,0.85
7389745746874,0.391229524478006,0.893086430964576
"6203","S1PR3",112.869189236107,-0.397092294224367,0.4638033333331,-0.
856165244373476,0.391906384027127,0.893086430964576
"6204","BX255925.3",4.91638446255762,0.548611284347742,0.6393805606514
21,0.85803560212841,0.390872804789641,0.893086430964576
"6205","TSPAN15",30.3295751903468,-0.30335035392713,0.354489336937492,
-0.855739009099222,0.392142157882222,0.893086430964576
"6206","LDLRAD3",12.1669062259569,0.357014106775504,0.416091245790899,
0.85801878887622,0.390882088587448,0.893086430964576
"6207","PCF11-
AS1",3.11585628010855,0.819348294553208,0.955857294580117,0.8571868407

54431,0.391341633845464,0.893086430964576
"6208","AP003120.1",2.22706343247016,0.886242895779567,1.0350075930615
4,0.856267047431093,0.391850083959171,0.893086430964576
"6209","PRKAB1",9.46038879107857,-0.471162891001004,0.550281956305978,
-0.856220862053887,0.391875625215233,0.893086430964576
"6210","THSD1",24.8141390055335,-0.29655313772355,0.345928018627042,-0
.857268338368611,0.391296602319427,0.893086430964576
"6211","DZIP1",37.2866882479157,0.27984724593848,0.326295442056705,0.8
5764987759114,0.391085824573475,0.893086430964576
"6212","TMEM208",100.78917931221,-0.219142005218005,0.255997182689832,
-0.856032878625541,0.391979593494935,0.893086430964576
"6213","SERPINF2",5.30282491433205,0.571863160049835,0.66661859376278,
0.857856599561541,0.390971651773497,0.893086430964576
"6214","PTPRM",22.1399137084516,0.320814792818715,0.374311171361965,0.
857080465035018,0.391400416507473,0.893086430964576
"6215","AC012615.1",3.28927893453302,-0.681900564227093,0.796174784195
61,-0.856470937993885,0.391737341272387,0.893086430964576
"6216","ZNF69",2.8420842895382,0.706971386855276,0.825914222209523,0.8
55986454578727,0.392005271889193,0.893086430964576
"6217","AC011484.1",2.51654271408626,-0.714348600153081,0.834753027390
882,-0.855760418606521,0.392130313070402,0.893086430964576
"6218","ZNF613",2.45794684758397,-0.827329218638367,0.966731855243759,
-0.855800100256092,0.392108359768105,0.893086430964576
"6219","TIAM1",32.1142673359482,-0.251678243130213,0.293996193086745,-
0.856059530865947,0.391964851881951,0.893086430964576
"6220","CHPT1",123.835117354274,0.200970230729076,0.234975094798461,0.
8552831137336,0.392394433542277,0.893153058826337
"6221","MMP11",13.1080301456333,0.373661738624475,0.436866073907344,0.
855323315180856,0.39237218359032,0.893153058826337
"6222","FCHSD2",12.2909499674657,0.33583779933432,0.392984047361965,0.
854583796947335,0.392781600832516,0.893577600011903
"6223","ARRB2",10.1961834707661,-0.405431379127683,0.474392487544838,-
0.854632798309993,0.392754464348851,0.893577600011903
"6224","BLOC1S3",7.10984143675512,0.415883490993291,0.48670357434347,0
.854490315905917,0.392833372886589,0.893577600011903
"6225","OGFR",54.4356898770713,0.207423519111545,0.242667865544272,0.8
547630261894,0.392682350924263,0.893577600011903
"6226","WDR47",14.9410955861068,0.322963213857995,0.378702111061165,0.
852815984978316,0.393761357992775,0.894012310131176
"6227","MEIS1",24.560544591279,0.288851988092415,0.339080580692762,0.8
51868271259513,0.394287208811384,0.894012310131176
"6228","MITF",60.9002091887829,0.264895237074298,0.310927474373241,0.8
51951850212872,0.394240816905317,0.894012310131176
"6229","AUXG01000058.1",24.4948861953525,0.400096764734661,0.469379241
089799,0.852395525216924,0.393994602931619,0.894012310131176
"6230","GRINA",174.911958871532,0.160198277859904,0.187690458365071,0.
853523824574538,0.393368881475245,0.894012310131176
"6231","DDX50",74.3222313112132,-0.172213187622146,0.202099798794851,-
0.8521195401929,0.394147747719346,0.894012310131176
"6232","ANAPC16",186.250128281335,-0.166245722945283,0.194775636618999

,-0.853524218074957,0.393368663356601,0.894012310131176
"6233","AL158212.2",7.33407943092776,-0.47450390196975,0.556072677046698,-0.853312744099999,0.393485894671801,0.894012310131176
"6234","BCCIP",40.3397268228892,-0.193672160383358,0.227087066764371,-0.852854207608025,0.393740158600021,0.894012310131176
"6235","BICD1",17.8841125324606,0.438238974801004,0.514341753924763,0.852038496693989,0.394192725803601,0.894012310131176
"6236","TESPA1",3.33968562675116,-0.781063673079881,0.915961229131099,-0.852725692135261,0.393811439799132,0.894012310131176
"6237","ZC2HC1C",2.07795772079147,-0.814477392795693,0.955778709449752,-0.852161054376899,0.394124709092021,0.894012310131176
"6238","CILP",650.793876502029,0.309969705457739,0.363807051775539,0.852016759831757,0.394204790006713,0.894012310131176
"6239","FBXO22",31.7859021166982,0.216511971803659,0.254157433813149,0.851881326291773,0.394279962178116,0.894012310131176
"6240","REXO5",6.01363274638465,-0.459549990886765,0.539169671556723,-0.852329081418702,0.394031469471083,0.894012310131176
"6241","SF3B3",47.6343391341243,0.180081364994787,0.211291094542697,0.852290369286701,0.394052949975505,0.894012310131176
"6242","MAFTRR",1.80175120294632,0.874741858193101,1.02517781117928,0.853258672451046,0.393515872870184,0.894012310131176
"6243","WRAP53",7.4379670832206,-0.422508419999786,0.495339023101033,-0.85296816987021,0.393676955885002,0.894012310131176
"6244","BCAS3",13.3834912486993,0.341122416144777,0.39978628844309,0.853261920195487,0.393514072228994,0.894012310131176
"6245","GAPLINC",2.17732289125743,0.828259331714595,0.972105427064138,0.85202623980407 8,0.394199528487937,0.894012310131176
"6246","CHMP3",204.816903123984,-0.168020519722789,0.197281563195154,-0.851678773229206,0.394392405120018,0.894074201278117
"6247","COX5B",406.136695891771,-0.181634199290679,0.213334716616686,-0.851404788546604,0.394544532644523,0.894074201278117
"6248","SP3",98.6460861505847,0.162145729040255,0.190457132617528,0.851350258254033,0.39457481433266,0.894074201278117
"6249","CYP21A2",1.68233759736847,0.986197911985333,1.15854922831056,0.851235224094401,0.394638699557159,0.894074201278117
"6250","TIAL1",95.4053272166088,-0.146780342414556,0.172410578344004,-0.851341859788272,0.394579478282214,0.894074201278117
"6251","RBM23",50.8855475631472,-0.217981241792423,0.25610599219566,-0.851136827856373,0.394693349730898,0.894074201278117
"6252","WDR36",30.4071908015212,0.230180560410989,0.27064872725822,0.850477158133385,0.395059854547256,0.894498348688602
"6253","AARS2",9.50887535694728,0.42281545086801,0.497356239768582,0.850125960146283,0.395255059960515,0.894498348688602
"6254","SLC38A6",27.1666602402532,-0.243982489539221,0.287054294250128,-0.8499524111861,0.395351544722527,0.894498348688602
"6255","INTS14",17.1626591433119,0.30176995362864,0.355012631839302,0.850025961231818,0.395310652744498,0.894498348688602
"6256","KIF7",16.6305668719248,-0.302222125598907,0.35552729898472,-0.850067284458784,0.395287679189884,0.894498348688602
"6257","TBC1D24",14.3529843379957,0.354365093552131,0.41685192622018,0

.850098251351145,0.395270463745046,0.894498348688602
"6258","TIGD7",4.22203845413637,-0.535673769267688,0.629772023860118,-0.850583622283402,0.395000690385443,0.894498348688602
"6259","UBE2L3",180.685751471741,-0.171115644579302,0.201338455978298,-0.849890517675106,0.395385957940816,0.894498348688602
"6260","RC3H1",40.3552632143267,0.280419486216255,0.33008388372438,0.849540071609205,0.395580842508364,0.894498851863638
"6261","GGPS1",51.3756527394699,-0.188129554406854,0.221477042040034,-0.84943140234304,0.395641285742917,0.894498851863638
"6262","ITPRIP",12.8711074222247,0.373119979726898,0.439315000108568,0.849322193948964,0.395702034468491,0.894498851863638
"6263","HOXC-AS1",11.5953033982101,0.377021542807546,0.443781410260453,0.849565876556826,0.395566490286539,0.894498851863638
"6264","PHKB",53.152401027865,0.188685092314184,0.222142297067411,0.84938840916427,0.395665200638793,0.894498851863638
"6265","SYTL1",2.09932887578699,0.85475491842499,1.0069476281448,0.848857373049074,0.39596066074828,0.894512277631723
"6266","B3GALNT1",15.9622854615739,-0.28956582344232,0.34109892319445,-0.84892036811634,0.395925604322938,0.894512277631723
"6267","THAP12",79.5991476611578,0.161774325787667,0.190570991366794,0.848892712513093,0.395940994290603,0.894512277631723
"6268","AATK",2.19033580532233,0.781286786569175,0.920355193788443,0.848897025672422,0.395938594052225,0.894512277631723
"6269","LIMK1",17.1952927244732,-0.297012637711178,0.35004704535996,-0.848493485799187,0.396163198788158,0.894827068885039
"6270","DDOST",197.055602292549,-0.188515969964235,0.222528008645993,-0.847156145023229,0.396908094029808,0.895097667901589
"6271","PTGFR",151.616751041432,-0.301132574483232,0.355314989570427,-0.847508783255385,0.396711593488345,0.895097667901589
"6272","ARHGAP30",1.54871940652301,-1.01468501830358,1.19868090525927,-0.846501361497958,0.39727311465482,0.895097667901589
"6273","SNAP47",28.0614320019602,-0.241375500494461,0.284893363874612,-0.847248588776172,0.396856575934803,0.895097667901589
"6274","MRPL19",68.7450153622289,-0.159259588976475,0.188166108384739,-0.846377651871511,0.397342101499197,0.895097667901589
"6275","LIMS2",43.7506329365558,0.238812844713899,0.28199426429216,0.846871284114053,0.397066869894744,0.895097667901589
"6276","FER",39.7491804871469,0.24817908386928,0.292734525533859,0.847795740583306,0.396551735637308,0.895097667901589
"6277","NEURL1B",1.75715337613698,1.1682220660525,1.3785785049104,0.847410620353774,0.396766286887804,0.895097667901589
"6278","AGPAT4",23.9436984834006,-0.27443486486922,0.323607764342484,-0.848047837871953,0.396411329662633,0.895097667901589
"6279","DDX31",6.27786612266709,0.537324101199862,0.634728785908292,0.846541252152217,0.397250871116513,0.895097667901589
"6280","TWF1",59.5341441227474,-0.192275378722016,0.226965346511727,-0.847157425911633,0.396907380174338,0.895097667901589
"6281","MAP3K12",38.287004795006,0.217525026376134,0.257016506850069,0.846346520859952,0.397359462888506,0.895097667901589

"6282","DHX37",9.20980333247007,0.396589115384969,0.468520897423677,0.84647049377253,0.397290327408258,0.895097667901589
"6283","PEX11A",12.3465429264641,-0.375729421099875,0.444000501968349,-0.84623647818907,0.39742083602382,0.895097667901589
"6284","UNC45A",37.1544750724859,-0.221655204013676,0.261812628345686,-0.846617695312281,0.397208247533005,0.895097667901589
"6285","COL5A3",129.356277738443,0.306759762747092,0.361951321094941,0.84751662687433,0.396707223457433,0.895097667901589
"6286","EVA1C",14.5521281426683,-0.40474341953907,0.477960138550764,-0.846814173178341,0.397098707016529,0.895097667901589
"6287","TRO",5.3836824248059,-0.630982188097811,0.744677801617777,-0.847322408062968,0.396815439989307,0.895097667901589
"6288","ZNHIT6",48.1736166658056,0.304379525784449,0.359944656976872,0.845628681755947,0.397759919964722,0.895543641766952
"6289","OSBPL6",2.76745995326952,-0.845560800814374,1.00002284529764,-0.845541484167505,0.397808580982636,0.895543641766952
"6290","AC007114.1",3.91474801781614,0.620093037127376,0.733220342520361,0.845711720157514,0.397713583335641,0.895543641766952
"6291","MTF2",36.2095243106669,0.226099232822234,0.267631599738723,0.844815160253738,0.398214048806227,0.895921815077561
"6292","EIF2B4",27.8083107664455,-0.231265420161647,0.273935941325728,-0.844231753753908,0.398539913715659,0.895921815077561
"6293","AC007098.1",2.42652574905825,0.79057893814495,0.938243727514166,0.842615745739705,0.399443381997447,0.895921815077561
"6294","AC009404.1",3.83257587323898,0.629175608618732,0.748707654657229,0.840348839370127,0.400712825261657,0.895921815077561
"6295","ELP6",37.9957513066734,0.274958971354133,0.32714262559535,0.840486533522648,0.400635648944085,0.895921815077561
"6296","CCDC51",19.5527050246485,0.35000475858907,0.415312266040341,0.842750834031644,0.3993678104646,0.895921815077561
"6297","ABHD6",7.85167160884738,-0.477199288760742,0.567241112908256,-0.841263578928777,0.400200289500794,0.895921815077561
"6298","ADCY5",1.6585886731306,0.944361361162609,1.12379920820752,0.840329263684823,0.40072379797995,0.895921815077561
"6299","ACAD9",25.8832626657625,-0.280159304168762,0.333299528112028,-0.840563158777099,0.400592705055206,0.895921815077561
"6300","TSC22D2",109.072768749492,-0.353428422838924,0.41938212365988,-0.842736022591071,0.39937609590908,0.895921815077561
"6301","AASDH",31.6344951518468,0.216619664754622,0.257798315599757,0.840267960054997,0.400758161544193,0.895921815077561
"6302","SLC9B1",2.72724908946488,-0.728825637439911,0.864997968892341,-0.842574969711429,0.399466194746426,0.895921815077561
"6303","ADCY2",2.15214848676902,0.772610066805799,0.918478739123911,0.841184486799065,0.400244589873154,0.895921815077561
"6304","EPB41L4A-AS1",188.874160894143,-0.201077500657084,0.23930283125496,-0.840263776247804,0.400760506829186,0.895921815077561
"6305","PCDHB5",5.36693297507566,0.745757264289675,0.883932155902439,0.843681564597346,0.398847371983151,0.895921815077561
"6306","CCDC69",17.3771414894918,0.355307603252534,0.421256622753411,0

.843446925368622,0.39897853716599,0.895921815077561
"6307","AL662844.4",13.4738703465994,-0.467703269577985,0.556406135197924,-0.840578922465717,0.400583870790152,0.895921815077561
"6308","ASF1A",29.3410071397449,-0.240540996979787,0.285963736280549,-0.841159092787207,0.400258813963778,0.895921815077561
"6309","TRMT11",36.4435534097728,0.20306510173857,0.241462930110879,0.840978371484696,0.400360051178164,0.895921815077561
"6310","ULBP2",2.07139130760505,0.905636198965485,1.07768570427914,0.840352799865022,0.400710605315619,0.895921815077561
"6311","SEPTIN7",580.042065805429,-0.139134051306903,0.165477062109297,-0.840805665349589,0.40045681280153,0.895921815077561
"6312","GTF2IRD1",3.5890562687715,0.647527630006823,0.767455252800009,0.843733400278858,0.398818398928915,0.895921815077561
"6313","PARP12",11.1336858715219,0.344351121496536,0.408950822441883,0.842035527500308,0.399768067396896,0.895921815077561
"6314","LMBR1",17.6224334141615,0.330265565134768,0.391360217502425,0.84389151059464,0.398730032520013,0.895921815077561
"6315","NUDT18",14.4768505464762,-0.354215423239393,0.420127397571969,-0.843114315530242,0.399164513176594,0.895921815077561
"6316","TIGD5",13.7036153751395,-0.340791957881316,0.403896576326762,-0.843760452194593,0.398803279024721,0.895921815077561
"6317","IFITM2",598.671159136278,-0.187747815742416,0.222999686523522,-0.841919639750762,0.399832936248745,0.895921815077561
"6318","ELP4",19.5496107548294,-0.27189673381394,0.321747199529242,-0.845063249071818,0.398075526088886,0.895921815077561
"6319","BACE1-AS",6.15991270713512,0.499981065279818,0.593897172772745,0.841864700155991,0.399863691222804,0.895921815077561
"6320","C2CD5",21.2683058566504,0.310152676541836,0.367165918127085,0.844720768539537,0.398266760930248,0.895921815077561
"6321","ISCU",327.397078342174,-0.170151604659413,0.20203306708495,-0.842196810227448,0.399677798972916,0.895921815077561
"6322","PARP2",18.7134326767364,0.287833150976672,0.341935922527493,0.841775116370022,0.399913842932814,0.895921815077561
"6323","PCK2",9.47303898557549,-0.382531060957155,0.454792142957936,-0.841111850502079,0.400285276858943,0.895921815077561
"6324","AHNAK2",122.452256586629,-0.28354996328274,0.336529955559421,-0.842569758199948,0.399469110459693,0.895921815077561
"6325","JMJD7",5.60367619360344,-0.531262560519936,0.630722225745605,-0.842308291723677,0.399615410995237,0.895921815077561
"6326","LYSMD2",33.5936939900388,0.281381531271314,0.334720550104856,0.840646118629904,0.40054621411973,0.895921815077561
"6327","CCPG1",172.060302625435,0.12660307756668,0.149909180146965,0.844531852169184,0.398372272042534,0.895921815077561
"6328","ASB7",8.23260471330144,0.411286780501442,0.487857133943253,0.843047588905982,0.399201829092626,0.895921815077561
"6329","SLX4",6.06415003138183,0.458759553605161,0.544192922554802,0.843009040711959,0.399223387578795,0.895921815077561
"6330","TRADD",68.8392878799261,-0.240170651857656,0.285432364811825,-0.841427537539382,0.400108463866947,0.895921815077561

"6331","HSF4",5.3908680765256,0.546536252550485,0.648594688340839,0.842646821466533,0.399425996751127,0.895921815077561
"6332","TTC39C",42.6208351065533,-0.234683974139258,0.278461853213546,-0.842786799810904,0.399347691821186,0.895921815077561
"6333","EP300",74.805749688425,0.23338079616036,0.276855042347576,0.842971087618349,0.399244613933196,0.895921815077561
"6334","MID2",3.00493096886529,-0.671649303332931,0.795243889390686,-0.844582790629862,0.398343820898737,0.895921815077561
"6335","CACNA1D",1.75511653410522,0.948963350312045,1.13067525517787,0.839289040744736,0.401307131595837,0.89598517045293
"6336","CENPH",6.28115598266725,-0.442933874931847,0.527772035544526,-0.83925226253197,0.401327765316428,0.89598517045293
"6337","DMGDH",6.13855197972024,0.488700889562146,0.582421186214583,0.839085014641094,0.401421604615352,0.89598517045293
"6338","GPANK1",35.6214791455046,0.224893197907375,0.267990400304425,0.839183782896349,0.401366186217619,0.89598517045293
"6339","NRXN2",39.0125380920987,-0.247362228951547,0.294588149820643,-0.839688321143097,0.401083163873326,0.89598517045293
"6340","UBL7-AS1",15.4726876592691,0.293696552678424,0.35000183138807,0.839128616880816,0.401397139039995,0.89598517045293
"6341","FBXL19",5.25162601610295,0.499151033247315,0.594678192564337,0.839363271578707,0.401265487730576,0.89598517045293
"6342","PIGN",17.225084161107,0.30185761103415,0.359482415306411,0.839700631189028,0.401076260011693,0.89598517045293
"6343","LRRC4B",3.97052769154382,0.604374864454258,0.719655495342941,0.839811365806708,0.401014159757284,0.89598517045293
"6344","FIZ1",4.59194952034126,0.56273319953934,0.670168094022498,0.839689630942724,0.401082429293033,0.89598517045293
"6345","SETD7",102.72474850629,0.167677222234645,0.199911035070853,0.838759211942535,0.401604443521149,0.896251996888806
"6346","PAXIP1-AS1",17.7629032248789,-0.309446666050143,0.36930714554376,-0.837911396473309,0.402080467677881,0.89717293134554
"6347","ZKSCAN8",15.2341894756908,0.314041464097964,0.374847316071609,0.837785014413633,0.402151456567331,0.897189951944763
"6348","WDR78",6.3478019773488,-0.485660298978581,0.581092568646342,-0.835770968659828,0.403283761597813,0.897336927547651
"6349","KHDC4",20.6385778959807,0.265494912375095,0.317711313139506,0.835648280042573,0.40335279931199,0.897336927547651
"6350","MARK1",7.40446444534412,0.439518749707937,0.525420507200765,0.836508555879405,0.402868865494857,0.897336927547651
"6351","TBCE.1",6.49989123579749,0.446722861287457,0.53468689856924,0.835484958548331,0.403444712403673,0.897336927547651
"6352","SLC10A7",13.6276165687165,0.365144893253718,0.436291128624631,0.836929447556738,0.402632226724354,0.897336927547651
"6353","UFSP2",60.8934241709257,-0.205075675472695,0.245628185712183,-0.834902862951544,0.403772402685217,0.897336927547651
"6354","MAD2L1BP",17.2643955320277,-0.322136131330561,0.385520686074696,-0.835587149967216,0.403387200260648,0.897336927547651

"6355","HYMAI",156.581975824825,0.190598845553895,0.227894372780107,0.836347309627525,0.402959545376002,0.897336927547651
"6356","FBXO30",17.1670123333601,0.294657505896633,0.35252310865076,0.835853022584531,0.403237593250014,0.897336927547651
"6357","ZNF107",14.5111495963271,0.338564005400627,0.405200971306818,0.835545888028651,0.403410421409089,0.897336927547651
"6358","PEX1",21.8932110916316,0.282568394852979,0.337916892935159,0.836206773797484,0.403038588333953,0.897336927547651
"6359","PAG1",8.11883655506531,0.610187407258025,0.728904639362742,0.837129268091217,0.402519910400555,0.897336927547651
"6360","TRPS1",29.2081623443605,0.339448673040487,0.405795880272773,0.836501033012737,0.402873095849303,0.897336927547651
"6361","AC103706.1",2.21014502967897,-0.787002149942721,0.941758358106617,-0.835673124818313,0.40333881841718,0.897336927547651
"6362","RNF214",24.5451001600799,0.255890209107298,0.306454541715744,0.835002175770174,0.40371648333684,0.897336927547651
"6363","SLC7A1",11.904283820279,-0.437255918822948,0.522329312588161,-0.837126901142745,0.402521240719197,0.897336927547651
"6364","DNAJC17",26.3662344169719,-0.249791113731205,0.29922744514763,-0.834786774348072,0.403837773730917,0.897336927547651
"6365","TLNRD1",42.406615096903,0.240065594916101,0.287532307757037,0.83491694129536,0.40376447541222,0.897336927547651
"6366","INO80E",32.3208191642716,-0.287479985772917,0.343509422627494,-0.836891120988735,0.402653771698534,0.897336927547651
"6367","CHD9",278.088903430578,0.122849953503531,0.146791658962821,0.836900096174032,0.402648726307815,0.897336927547651
"6368","MRM1",2.64745480546935,0.743777796746704,0.888859921366535,0.836777290625522,0.402717764602479,0.897336927547651
"6369","EIF1",3590.44609185041,-0.15749964943116,0.18832598124852,-0.836313972119008,0.402978294880436,0.897336927547651
"6370","CDC34",29.8304935708709,-0.249536727586647,0.298736951315036,-0.835305865204118,0.403545515900505,0.897336927547651
"6371","RASAL3",2.186076668331,0.757322466643582,0.906982480410494,0.834991284838075,0.403722615388606,0.897336927547651
"6372","JOSD2",102.586465718745,-0.187590900838534,0.224138238141046,-0.836942872373641,0.402624680234153,0.897336927547651
"6373","ZNF134",9.9861972605934,0.440628860347094,0.52793574519205,0.83462592627973,0.403928359900334,0.897336927547651
"6374","NUDT10",1.65853664579394,-0.859034762358907,1.02916518441778,-0.834690849792864,0.403891794911588,0.897336927547651
"6375","IP6K1",26.8818651625682,0.227324781874988,0.27264442300844,0.833777487052982,0.404406383858203,0.898117063273551
"6376","FIP1L1",89.2542911258217,0.158088534530615,0.189596800473203,0.833814358343874,0.404385602963295,0.898117063273551
"6377","ZFAS1",681.691414281521,-0.172490511613955,0.20690778687346,-0.833658869105038,0.404473242024447,0.898124683560634
"6378","SLIT2",154.514166343466,0.2021770267562,0.242556409572062,0.833525805864693,0.404548250045943,0.898150395210183
"6379","EIF4G3",64.9006313510079,0.169000496153881,0.20278863138208,0.8333824978357,0.40462904238101,0.898159094538773

"6380","MX1",45.7158317392523,-0.410975524193701,0.493193999843785,-0.
833293844458518,0.404679027059136,0.898159094538773
"6381","TAF1B",44.5399876661967,-0.194105996501949,0.23302954404066,-0
.832967327387812,0.404863156241749,0.898286163018359
"6382","ITGB1-
DT",1.50499162314466,-1.11222182129424,1.33517736715993,-0.83301428607
95,0.404836672259144,0.898286163018359
"6383","SDC3",44.4252658833654,0.266344926865273,0.320151420613043,0.8
31934233979849,0.405446067157542,0.898752663666154
"6384","ARL8A",47.8869767124792,-0.190165167678029,0.228927086312529,-
0.830680068231061,0.406154389110319,0.898752663666154
"6385","DEGS1",116.521134779974,-0.20126962849643,0.242046913211263,-0
.831531482166676,0.405673450970509,0.898752663666154
"6386","AL162595.1",1.62511351797007,1.04092087388976,1.2509794184289,
0.832084731815209,0.405361119314375,0.898752663666154
"6387","EMILIN1",210.853595624612,0.181733156038035,0.218960934964946,
0.829979813829023,0.40655019679045,0.898752663666154
"6388","WDR33",84.9503421806356,-0.146357231511577,0.175885327541628,-
0.832117343483,0.405342713203708,0.898752663666154
"6389","SATB2-
AS1",1.94402393585534,0.872237207053792,1.05029762956649,0.83046670058
0677,0.406274967407282,0.898752663666154
"6390","PAICS",33.6134974672899,-0.220821044288863,0.265735300020584,-
0.830981221808913,0.40598423760875,0.898752663666154
"6391","ZKSCAN3",5.68057733351468,0.461147805353003,0.55566836597846,0
.829897531670677,0.406596720582301,0.898752663666154
"6392","HLA-
DQB1",19.7021144412024,-0.82819116625068,0.996949548266919,-0.83072525
3540056,0.406128856736622,0.898752663666154
"6393","VIRMA",52.5508907340849,0.17242114749223,0.20719734687722,0.83
2159050735349,0.40531917424917,0.898752663666154
"6394","NDUFB6",133.742964534195,-0.227225462798985,0.273777943358675,
-0.82996263326187,0.406559910725115,0.898752663666154
"6395","TGFBR1",40.7309863232507,0.221586302792225,0.266180072095076,0
.832467663894375,0.405145022948786,0.898752663666154
"6396","ANKRD26",42.0996620488882,0.206015039697217,0.248073812111974,
0.830458636255519,0.40627952513667,0.898752663666154
"6397","EXT2",82.6163395474148,0.175214578323202,0.21110080971754,0.83
0004293008846,0.406536356440637,0.898752663666154
"6398","AASDHPPT",54.2059917596139,0.178267428501817,0.214574710988315
,0.830794214661784,0.406089891469139,0.898752663666154
"6399","MIR17HG",3.52250269986536,-0.69093378914054,0.832366993109838,
-0.83008311821582,0.406491791155231,0.898752663666154
"6400","CDAN1",4.06103325074525,-0.563033019948855,0.677511223716894,-
0.831031280721816,0.405955958494427,0.898752663666154
"6401","EMP2",296.811113950195,-0.175429676301499,0.210951738595861,-0
.831610478629828,0.405628845496782,0.898752663666154
"6402","LYRM1",59.2766885550943,0.243043510614092,0.292554180871302,0.
830764099457561,0.406106907260413,0.898752663666154
"6403","PBX4",5.66492002003815,0.513259988208867,0.617518894468122,0.8

31164832050921,0.405880518878358,0.898752663666154
"6404","DMAC2",22.1789872577297,-0.285789322175443,0.344035283803577,-0.830697709304173,0.406144420745951,0.898752663666154
"6405","CSNK2A1",94.4381748723601,0.173280860881187,0.208668552477104,0.830411956301851,0.406305907931099,0.898752663666154
"6406","TCN2",24.123936453217,0.297251879746057,0.357734843727214,0.830927948334614,0.406014333974505,0.898752663666154
"6407","SERPINB6",137.769813577142,-0.139889376623195,0.168629982219759,-0.829564083336561,0.406785290710882,0.898825788929413
"6408","COMMD5",45.756186309619,-0.215454778659138,0.259756068672767,-0.829450413844081,0.406849584469394,0.898825788929413
"6409","SPOP",145.550750135573,0.13754406860026,0.165827511967578,0.829440585390632,0.406855143925724,0.898825788929413
"6410","NNAT",4.07501624802592,-0.70752307414225,0.853064298564739,-0.829390088569691,0.40688370812412,0.898825788929413
"6411","DVL1",28.2730618158331,-0.234678142837631,0.283146585106632,-0.828822084325165,0.407205089685934,0.899114933721008
"6412","SNX27",72.58990087221,0.176863056663583,0.213366881583479,0.828915225038736,0.407152379515584,0.899114933721008
"6413","UCK2",9.07396427929306,-0.410519059644585,0.495261324281035,-0.828893837491813,0.407164482791098,0.899114933721008
"6414","RPARP-AS1",6.34997065199157,-0.439821417250694,0.53073209519766,-0.828707027953326,0.407270207978438,0.899118513404222
"6415","FOSL2",65.2302553722551,0.241269024050508,0.291231702673079,0.828443544559242,0.407419354693957,0.899167403751003
"6416","AC016588.2",1.89183108878304,0.801246845646249,0.96705415268198,0.828543927373777,0.40736252842306,0.899167403751003
"6417","NFATC3",25.9416666050287,0.314934276866434,0.380289513595346,0.828143468614139,0.407589254548512,0.899402188625047
"6418","UBE4B",23.8027696875407,0.28105984424238,0.339655418409123,0.827485236534152,0.407962086548049,0.899477141791094
"6419","HENMT1",5.85622716698356,-0.524343914809468,0.633965428809205,-0.827085975010274,0.408188332922742,0.899477141791094
"6420","UBXN2A",56.5075609731017,0.173905935039451,0.210241124603258,0.827173729058126,0.408138599622096,0.899477141791094
"6421","CEP44",26.7857477393079,0.260834534594436,0.315246100739864,0.827399717180555,0.408010540835621,0.899477141791094
"6422","TMEM161B",16.4812952409707,0.292329632418726,0.353384084319217,0.827229197324744,0.408107165669502,0.899477141791094
"6423","AC004889.1",34.3058583320919,0.273734237536145,0.331025355167031,0.826928310062602,0.40827769625436,0.899477141791094
"6424","TMEM250",32.911502962871,-0.218417393018322,0.264076507943405,-0.827098914323466,0.408180999532663,0.899477141791094
"6425","ASCC1",19.9421676568284,0.282597199385352,0.341397562107788,0.827765721701692,0.407803190925548,0.899477141791094
"6426","AC068888.1",47.8444965008736,-0.248311817101961,0.300296340838347,-0.826889253491205,0.408299835032202,0.899477141791094
"6427","CCBE1",42.1313208943406,0.425405643932283,0.514461777375513,0.826894557847342,0.408296828275677,0.899477141791094

"6428","RPN2",240.27709358264,-0.151973655761949,0.183798291667703,-0.
826850208361613,0.408321968039065,0.899477141791094
"6429","ODF2L",142.178742679955,0.163161918003964,0.197592510895762,0.
825749504696754,0.408946203634866,0.899508825695687
"6430","C1orf162",7.78389368495459,-0.515473973346361,0.62489823852340
6,-0.824892665027178,0.409432531010137,0.899508825695687
"6431","RBM34",4.12688671771216,-0.561237047953847,0.680487276703743,-
0.824757592342446,0.409509227308014,0.899508825695687
"6432","SNRNP200",85.0423388179277,0.152340856256101,0.18452636556462
,0.825577720497786,0.409043677810564,0.899508825695687
"6433","HYAL1",3.07102655179185,-0.648772854452604,0.785465154743016,-
0.825972801638624,0.408819520649432,0.899508825695687
"6434","CEP70",11.2518811697591,0.353720406257725,0.428115329559279,0.
826226910916414,0.408675385332068,0.899508825695687
"6435","MTX3",24.116913654762,0.283452909707333,0.342891062123198,0.82
665587126179,0.408432140022602,0.899508825695687
"6436","JADE2",29.4988177539444,0.282915807253259,0.342720011064543,0.
825501278359781,0.409087057224852,0.899508825695687
"6437","DCLRE1C",26.527391511925,0.264581320427177,0.320783017595937,0
.82479840239064,0.409486053849245,0.899508825695687
"6438","THNSL1",6.699758250249,0.441931981272053,0.535631946975964,0.8
25066510254073,0.409333831657284,0.899508825695687
"6439","CRYL1",77.064364944555,-0.221592159129752,0.268695059557083,-0
.824697556758297,0.409543319156786,0.899508825695687
"6440","TEX30",21.9904256912301,0.25554269272265,0.309665993816768,0.8
25220391729087,0.409246478388899,0.899508825695687
"6441","AC005520.2",6.17467044712324,0.455937721921406,0.5526457009904
19,0.825009081051931,0.40936643509999,0.899508825695687
"6442","FMN1",1.77220177712147,0.87932154668813,1.06461299284519,0.825
95417545876,0.408830086941238,0.899508825695687
"6443","NEDD4",22.6805650095748,0.26725580994641,0.323428578804799,0.8
26320948303425,0.40862205331847,0.899508825695687
"6444","KAT8",44.5135298728947,-0.190211967948681,0.230506855222509,-0
.825190069792366,0.409263690241404,0.899508825695687
"6445","CCL2",703.519800655994,-0.473895664050026,0.573647291377977,-0
.826109825972795,0.408741794242841,0.899508825695687
"6446","ZNF66",7.62769099272341,0.489144909569845,0.591755073809152,0.
826600279776561,0.408463658744204,0.899508825695687
"6447","FAM3A",49.17952829529,-0.207372649585813,0.25132273624284,-0.8
25124907861257,0.409300680020223,0.899508825695687
"6448","NOP10",171.300753010998,-0.209299148626239,0.253856174848057,-
0.824479249919814,0.409667301259025,0.899641592094881
"6449","RPAP2",43.7676378359977,0.196637714623264,0.238718979028528,0.
823720491028763,0.410098393637235,0.900295254353937
"6450","CAPZA1",101.881730953766,-0.150637120081049,0.18298815774878,-
0.823206932810672,0.410390327067984,0.900295254353937
"6451","AIMP2",25.6769929961615,-0.248088569171057,0.301355828082266,-
0.823241318244331,0.410370776727516,0.900295254353937
"6452","IKBKB",18.6203315115746,0.315392176696991,0.383008982389631,0.
823458955790614,0.410247048558981,0.900295254353937

"6453","TIMM23B",10.8268600560309,-0.34297728453067,0.416619802413,-0.
823238075924852,0.410372620171975,0.900295254353937
"6454","MVK",9.11839054089101,-0.384315678383682,0.466650359933772,-0.
823562374275733,0.410188262339874,0.900295254353937
"6455","MAGED1",66.5581323951255,-0.276189683097012,0.335518679663492,
-0.823172299598986,0.410410018845669,0.900295254353937
"6456","WDR54",11.4067578456515,-0.334876734061028,0.407164160354018,-
0.822461224902166,0.410814446315224,0.900345544006124
"6457","APBB3",7.34510586332653,0.523067895042734,0.635870687657154,0.
822601049546664,0.410734901624637,0.900345544006124
"6458","LRRN3",29.0769450479423,0.555252680930134,0.674945429620362,0.
822663072542692,0.410699620358691,0.900345544006124
"6459","RALGPS1",4.36506552603741,0.585342557212055,0.711424331579491,
0.82277556618353,0.410635633879261,0.900345544006124
"6460","ZNF264",39.1738776049338,0.220874064095355,0.268531709127551,0
.822525074647483,0.4107781217678,0.900345544006124
"6461","AP1B1",19.7336961320515,0.286113941159285,0.34770043258515,0.8
22874849571024,0.410579166323055,0.900345544006124
"6462","TRIM52-
AS1",32.9677119169898,-0.210863592294931,0.256554563438818,-0.82190544
369411,0.41113071452911,0.900702309000638
"6463","ARID1B",151.214044489881,0.190462711705811,0.231751618698863,0
.821839833417937,0.411168059701986,0.900702309000638
"6464","CCT2",109.694549881667,-0.153151175432237,0.186343990138953,-0
.821873435886153,0.411148933026051,0.900702309000638
"6465","NXPE3",8.72818457558891,-0.398541970331219,0.48517504749885,-0
.821439545141002,0.411395946210547,0.90092276497701
"6466","DNAJC19",127.593822994148,0.168916040262546,0.205626381181841,
0.821470665834304,0.41137822632689,0.90092276497701
"6467","FGFR1",480.906759304147,0.162417615130118,0.197763967890754,0.
821270006171391,0.411492488353044,0.900994840742091
"6468","POLN",2.64698281161261,-0.739132761428443,0.900434664627677,-0
.820862179638619,0.411724775846368,0.901111262027331
"6469","ZC3HAV1",147.102906505414,0.320569630018286,0.390563684693786,
0.820787089484836,0.411767553743585,0.901111262027331
"6470","SNX1",66.4500209420135,0.166487160869344,0.20285259185065,0.82
0729769092228,0.411800210210371,0.901111262027331
"6471","ZNF17",5.52530355449544,0.448428399972435,0.546280029185863,0.
820876429696214,0.411716658069044,0.901111262027331
"6472","CD81",2740.62015475394,-0.180895443865748,0.220448677147029,-0
.820578495669985,0.411886400798673,0.901123755358949
"6473","DICER1",82.215728638601,0.202469029477821,0.246797666791781,0.
820384698566217,0.411996835606909,0.901123755358949
"6474","UBOX5",7.97221292577241,-0.433927041405477,0.52887622300816,-0
.820469937062725,0.411948260493787,0.901123755358949
"6475","LINC02328",3.73129477388769,0.596370313567943,0.72708805644693
5,0.820217452728119,0.412092154356488,0.901193035627471
"6476","AGO2",71.8521226945933,0.229725973467967,0.280169967777762,0.8
19952171498238,0.412243373430244,0.901384522509613
"6477","OAT",133.795463064117,-0.17110433348491,0.208804291495305,-0.8

19448356447012,0.412530655231914,0.901630836472056
"6478","POMP",278.547939365572,-0.215774786495468,0.26331147796333,-0.819465935038038,0.412520629696465,0.901630836472056
"6479","FBXL20",35.1279511235555,0.218010741142819,0.266055067545843,0.819419615472102,0.412547047281246,0.901630836472056
"6480","SART3",34.2340875123113,0.218874875648761,0.267149624020667,0.819296963082487,0.412617004850012,0.901644566153729
"6481","GPR153",32.0822204931261,-0.25481254446332,0.311673301265406,-0.817562952709683,0.413606789057694,0.901690073994726
"6482","TXNDC12",85.7157803614297,0.180816423655614,0.220822116879846,0.818832942145918,0.412881733309437,0.901690073994726
"6483","COP1",30.7128966652019,0.272646734546025,0.334331712328845,0.815497676385101,0.414787494085979,0.901690073994726
"6484","AL133245.1",1.76215058089278,0.850673960342817,1.03894964507039,0.81878266610812,0.412910422319223,0.901690073994726
"6485","HSPD1",196.079256782007,-0.131473996531048,0.160574485211365,-0.818772648456499,0.412916138831744,0.901690073994726
"6486","ZNF445",19.5951968859966,0.345157057180152,0.423370101932294,0.815260821689648,0.414923029618755,0.901690073994726
"6487","NPY5R",3.98732756244368,0.602683813746346,0.738419388522334,0.816180917124061,0.414396669421055,0.901690073994726
"6488","RWDD2A",10.3689305360794,-0.361908170364113,0.443762331822519,-0.815545043847608,0.414760392109693,0.901690073994726
"6489","SYTL3",1.84975575811497,0.93483793111854,1.14187797045371,0.818684618941457,0.412966374360268,0.901690073994726
"6490","FKBP9",200.964982818168,0.226109441260413,0.277350438924914,0.815248182540743,0.414930262863675,0.901690073994726
"6491","SBDS",224.11751547976,-0.150444739334212,0.184511205525054,-0.81536912029868,0.414861054579799,0.901690073994726
"6492","STEAP2",37.6351544115598,-0.241760797478843,0.296144251354681,-0.816361608820478,0.414293347299303,0.901690073994726
"6493","LINC00535",3.31197301222529,0.779329143407355,0.951902361585134,0.818707017502925,0.412953591899707,0.901690073994726
"6494","CDK9",32.0908470471371,-0.213804329939937,0.262052063366803,-0.815884932150553,0.414565950829179,0.901690073994726
"6495","SLC16A9",1.80137976827586,1.14451092391879,1.40188050983061,0.816411181903855,0.414265003358285,0.901690073994726
"6496","VSIR",128.409176178697,0.212261227174008,0.260185302271407,0.815807908136918,0.41461000954502,0.901690073994726
"6497","SIK2",49.3886371157417,0.205306758367355,0.25176866470079,0.815457946727992,0.41481022678915,0.901690073994726
"6498","KLRK1",2.09356835500254,0.787547887236818,0.965348931219944,0.815816811690636,0.414604916457484,0.901690073994726
"6499","DNM1L",55.5757368838749,-0.180306722379263,0.221040911142489,-0.815716518029698,0.414662289440504,0.901690073994726
"6500","AL136038.5",8.14520487467769,0.417900692836774,0.511485164592911,0.817033849201431,0.413909084367269,0.901690073994726
"6501","GABPB1-
IT1",54.1712631716486,0.228211510565949,0.278992939549716,0.817983103566256,0.413366835328136,0.901690073994726

"6502","UBAP1L",4.11815645757327,0.581375595609002,0.711540864653228,0
.817065645122628,0.413890914555554,0.901690073994726
"6503","UACA",207.926970317423,0.204158473378667,0.250008129130156,0.8
16607340285245,0.414152858954389,0.901690073994726
"6504","SMG1",74.2779947169091,0.222642621994175,0.272213304872777,0.8
17897648677498,0.413415633069958,0.901690073994726
"6505","MLKL",9.64513982072066,-0.349366417262129,0.428297316376308,-0
.815710031101783,0.414666000448869,0.901690073994726
"6506","CFDP1",199.904259037368,0.139876708577264,0.171166281145002,0.
817197801118135,0.413815398934999,0.901690073994726
"6507","GABARAPL2",360.472068892541,-0.200668933830262,0.2454152873252
32,-0.817670879501198,0.413545142765967,0.901690073994726
"6508","CLUH",11.0751174509986,0.365998052372096,0.447585497320212,0.8
17716513522897,0.413519078879788,0.901690073994726
"6509","CRLF3",11.2348574106272,0.347660129852887,0.425868886731429,0.
81635484696053,0.414297213554007,0.901690073994726
"6510","DSG3",1.5069655854982,-1.25621505219715,1.54046057719387,-0.81
5480169239704,0.414797511316494,0.901690073994726
"6511","SMIM7",151.883034419906,0.208211222364326,0.254430790781321,0.
818341293225315,0.413162333569696,0.901690073994726
"6512","TIMM50",36.346102004914,-0.216413138587872,0.264515826445699,-
0.818148167146807,0.413272587885563,0.901690073994726
"6513","ZNF667-
AS1",47.0676751517287,-0.22750956083374,0.27855949174298,-0.8167359848
70541,0.414079322166677,0.901690073994726
"6514","NELFCD",41.8134585482541,0.189597285820817,0.23243734348585,0.
815692018233544,0.414676305260875,0.901690073994726
"6515","ARSD",21.2096054904733,0.304492865298124,0.372178741201887,0.8
18136103945153,0.413279475260412,0.901690073994726
"6516","AC244090.1",10.378577492894,0.437559065861657,0.53663806466058
4,0.815370907649659,0.41486003179496,0.901690073994726
"6517","ATPAF1",29.681847765934,0.224338251064835,0.275359027473508,0.
814711807792171,0.415237292881772,0.901834658608148
"6518","TMED5",80.9588326424251,-0.182718906709004,0.224459570222637,-
0.814039279001419,0.415622449402977,0.901834658608148
"6519","CHD1L",4.28334457031859,-0.55718019395431,0.687184032380413,-0
.810816561066228,0.417471022458975,0.901834658608148
"6520","NBPF11",13.4660476891864,0.36969543300056,0.454894413629332,0.
812706030067461,0.416386623114647,0.901834658608148
"6521","TMEM17",3.66663611151259,-0.620345766418979,0.761808676577389,
-0.81430651224141,0.415469379954586,0.901834658608148
"6522","KDM3A",28.4389614535037,0.311149542360185,0.38354728968489,0.8
11241666225343,0.417226902166753,0.901834658608148
"6523","SCLY",6.29464446647373,0.474205234181965,0.582829898874235,0.8
13625442170892,0.415859558143422,0.901834658608148
"6524","CIP2A",6.22262409814553,0.464851927337114,0.572249712361203,0.
81232356661055,0.416605991317092,0.901834658608148
"6525","MXD4",178.905650105243,0.161904644526202,0.198745916422066,0.8
14631301316269,0.415283387739767,0.901834658608148
"6526","LIAS",23.5359326922797,0.251679581809216,0.309182802457433,0.8

14015462078835,0.41563609319646,0.901834658608148
"6527","AC004854.2",2.37977698334303,0.760997398275063,0.9381682950823
16,0.811152329772871,0.417278197407218,0.901834658608148
"6528","ABHD11",17.2861786052512,-0.278383096288711,0.342819440377274,
-0.812039993946519,0.416768683086425,0.901834658608148
"6529","ATP6V1F",242.585723499216,-0.221674687042975,0.272467396328593
,-0.813582432356925,0.415884205293675,0.901834658608148
"6530","ZNF786",3.48798104090068,-0.62637179408036,0.771736360721026,-
0.811639603834588,0.416998459349723,0.901834658608148
"6531","AC067838.1",2.33546101935459,0.855081790963089,1.0544387345983
5,0.810935489095816,0.417402718536167,0.901834658608148
"6532","GPAA1",110.442355025938,-0.220284025868464,0.271253546339633,-
0.812096390410502,0.416736324230592,0.901834658608148
"6533","AEBP2",38.8155884918626,0.199406365469994,0.245525470099022,0.
812161627832633,0.416698894506637,0.901834658608148
"6534","TRHDE-
AS1",2.1323126287176,0.944206081206921,1.16477481582901,0.810634011291
614,0.417575879066637,0.901834658608148
"6535","NUP37",31.4538986973873,-0.238736239329319,0.293923304336946,-
0.812239913632836,0.416653980942907,0.901834658608148
"6536","SETD1B",15.183836129568,0.283856985113507,0.350156385822174,0.
810657742102874,0.417562247208879,0.901834658608148
"6537","ABCB9",2.47852607109707,-0.790492952989406,0.973208823773747,-
0.812254198358133,0.416645785921596,0.901834658608148
"6538","PSEN1",30.452110564557,0.212248029839421,0.26176967098829,0.81
0819790688875,0.417469167503129,0.901834658608148
"6539","STON2",10.7427264081674,0.371120497280703,0.457846728925506,0.
810578024990294,0.417608040712827,0.901834658608148
"6540","EIF3J",84.7453295087489,-0.171033021522389,0.210431244473267,-
0.812773891778779,0.416347707035821,0.901834658608148
"6541","NME4",114.717652454601,-0.21480288546658,0.264877804920225,-0.
810950866688412,0.417393887205937,0.901834658608148
"6542","PKD1",66.7141107797005,0.207070719097804,0.254623436718665,0.8
13242966815338,0.416078769259109,0.901834658608148
"6543","AC009107.2",23.0999215983132,-0.353411226326999,0.435813833216
478,-0.810922461360816,0.417410200432589,0.901834658608148
"6544","TMEM256",111.773184220301,0.182134990151753,0.224137303456864,
0.81260453901555,0.416444828330928,0.901834658608148
"6545","ZNF286A",17.636792036495,-0.306778774972906,0.376688173124183,
-0.814410424485958,0.41540986869507,0.901834658608148
"6546","NBR2",5.09808141317761,0.590864200555326,0.725574120545484,0.8
14340235992867,0.415450065577436,0.901834658608148
"6547","UBTF",116.140274723499,-0.162406983571395,0.200182003559643,-0
.811296623489965,0.417195348629107,0.901834658608148
"6548","MYO15B",15.2345628620915,0.387982262359852,0.478524636096276,0
.810788480035105,0.417487151195899,0.901834658608148
"6549","PIEZO2",4.41123046705103,0.597863939128006,0.735851564335971,0
.81247899454765,0.41651683484639,0.901834658608148
"6550","CAPNS1",229.984019492864,-0.15477287897155,0.190587355015082,-
0.812083671339589,0.416743621980377,0.901834658608148

"6551","CRLS1",40.8130237335548,-0.199215434014786,0.244894804339468,-0.813473501620872,0.415946632863495,0.901834658608148
"6552","PET117",19.5609169580251,0.266787012071719,0.328454005495465,0.812250749292212,0.416647764611683,0.901834658608148
"6553","RIN2",54.8357460732126,-0.242602489868275,0.298987047526938,-0.811414714700698,0.417127551697699,0.901834658608148
"6554","ZSWIM3",2.24336892315496,0.756881004866327,0.9288216848406,0.814883004154042,0.41513928259372,0.901834658608148
"6555","ZNF512B",12.0992305237349,0.346792037522872,0.426339521447006,0.813417523071406,0.4159787159958,0.901834658608148
"6556","LINC02246",3.23374616693778,0.599642344868224,0.737998010525575,0.812525692909634,0.416490049962169,0.901834658608148
"6557","ACO2",40.9139383021582,-0.199286066770082,0.244616858170564,-0.814686560282473,0.415251748292542,0.901834658608148
"6558","MGA",37.9195501390529,0.202289252256697,0.249641296853097,0.810319665883384,0.417756475465427,0.902005018391824
"6559","SLC25A35",4.925465090196,0.62973790209134,0.777244074495017,0.810218981084529,0.417814330199998,0.902005018391824
"6560","AL034417.2",3.74873804092538,-0.589782797303889,0.728193764873304,-0.809925634843226,0.417982917496596,0.902153214206671
"6561","KIF1B",70.1082495302527,0.20941873124504,0.25929521369072,0.807645957918949,0.419294422156325,0.902153214206671
"6562","EXOSC10",35.7487230943215,0.201688261049011,0.249461513837791,0.808494496590591,0.418805972918858,0.902153214206671
"6563","GON4L",65.5294987048943,0.187473500332536,0.232030278353337,0.807969984189092,0.419107861507689,0.902153214206671
"6564","ANGPTL1",164.224888247751,-0.200914077785903,0.249207045891058,-0.806213472285748,0.420119771921543,0.902153214206671
"6565","ARHGEF3",19.7798949566847,0.259365302568581,0.321747301521467,0.806114927280212,0.420176585289053,0.902153214206671
"6566","AC106707.1",12.4419272456014,-0.390971644899738,0.484867126407084,-0.806348014964179,0.420042212390127,0.902153214206671
"6567","LIN54",10.4511181675209,-0.352123485235603,0.436300214163824,-0.807066954827086,0.419627909273762,0.902153214206671
"6568","ECI2",125.128985831389,-0.200350653049417,0.248381926530671,-0.806623315342861,0.419883536789592,0.902153214206671
"6569","MRPS18B",87.9592918360008,-0.183332447918444,0.227206559066257,-0.80689769112247,0.419725429157528,0.902153214206671
"6570","SMAP1",87.8096436030445,-0.175857262509481,0.217921097991822,-0.806976764205182,0.419679870196902,0.902153214206671
"6571","ZNF92",16.756146036886,-0.304593500087391,0.377627290946634,-0.806598218375153,0.419898000535165,0.902153214206671
"6572","CBLL1",22.5827848903632,0.271281522853422,0.33580144004279,0.807862893080072,0.419169514645137,0.902153214206671
"6573","LINC-PINT",112.589196451148,0.344690781648979,0.426792129389141,0.807631532808086,0.419302728661582,0.902153214206671
"6574","NECAB1",4.15032695067482,0.600484460743227,0.744025696400964,0.807074894923548,0.419623334979933,0.902153214206671
"6575","SPAAR",2.25363051736137,-0.853570639671627,1.05837327190292,-0

.806493004246917,0.419958640146124,0.902153214206671
"6576","CARD9",2.02160243785428,0.869254122191,1.07464548218707,0.8088
7523988091,0.418586912318269,0.902153214206671
"6577","ROM1",12.4478446919924,-0.362253937844662,0.449275126322848,-0
.806307575515202,0.420065523548802,0.902153214206671
"6578","KLC2",10.6544855703174,-0.358091573453817,0.444095161837981,-0
.806339731267912,0.420046987431815,0.902153214206671
"6579","LRTOMT",10.5436463943914,-0.341727804447057,0.42268004480776,-
0.808478679428737,0.418815074779964,0.902153214206671
"6580","KCTD21",5.32845783347313,0.593068011212768,0.734860018704923,0
.807048956422964,0.419638278272559,0.902153214206671
"6581","USP35",3.91709038855092,-0.534630258478499,0.661828823593661,-
0.807807456277762,0.419201432114455,0.902153214206671
"6582","AC023157.3",9.78800403672528,0.496915861866035,0.6150473212553
08,0.807931104962514,0.41913024394702,0.902153214206671
"6583","MTRF1",6.14526000946764,-0.457075619888258,0.564653315797405,-
0.809480095309056,0.418239047531343,0.902153214206671
"6584","TMEM260",24.121449843757,0.264821809831943,0.327956652268235,0
.80749028263450 6,0.41938407077935,0.902153214206671
"6585","AEN",5.1439328320849,-0.488918028958997,0.604721992432245,-0.8
08500492916631,0.418802522410733,0.902153214206671
"6586","SMCR5",4.00015969665343,0.523226815602293,0.647271232884468,0.
808357901633617,0.418884579247686,0.902153214206671
"6587","EVI2A",3.2176654396641,0.682214366497106,0.844778538891863,0.8
07565930109919,0.419340506310054,0.902153214206671
"6588","ENDOV",29.3820229008654,0.230244283918555,0.285086956916793,0.
807628263350383,0.419304611348094,0.902153214206671
"6589","ZNF415",6.64191875116828,0.458613989091366,0.566689521190129,0
.80928616454423,0.418350562556248,0.902153214206671
"6590","AL117335.1",2.48310811989631,0.712866166220345,0.8818991733463
95,0.808330688774037,0.418900240474652,0.902153214206671
"6591","ADORA2A",1.86545441133738,-0.765007044652035,0.944775400507432
,-0.809723712367146,0.418098986455494,0.902153214206671
"6592","RPL3",4865.24887523989,-0.212834178239639,0.263556918327063,-0
.807545404577542,0.419352326453673,0.902153214206671
"6593","MAGED2",235.002088267939,-0.215790965118424,0.267092117448667,
-0.807927119600967,0.41913253832542,0.902153214206671
"6594","RRAGB",27.7847419319089,0.25051368513152,0.309415953118253,0.8
09634030200696,0.418150543592339,0.902153214206671
"6595","HAUS7",15.5526098864086,-0.311053485206757,0.385153165177911,-
0.807609837668281,0.419315221697033,0.902153214206671
"6596","SLC35A3",43.8683728235017,0.219497479214599,0.272576168399134,
0.805270249793768,0.42066374559779,0.902378221127815
"6597","PAK2",107.7557564159,-0.164530592423978,0.204250641501724,-0.8
05532806233999,0.420512282971251,0.902378221127815
"6598","PTDSS1",22.5964231137422,-0.250387618978063,0.310912091986141,
-0.805332521416455,0.42062781966797,0.902378221127815
"6599","VPS26A",90.8906780879185,0.147807423900972,0.183545751384778,0
.805289268674567,0.420652772977405,0.902378221127815
"6600","FGFBP3",4.55294630033462,-0.540131506645835,0.670540705014916,

-0.805516358076755,0.420521770585364,0.902378221127815
"6601","BACE1",83.8907278095106,0.148187696596835,0.183990373911223,0.805410051877699,0.420583093081517,0.902378221127815
"6602","PAQR3",18.3849772504047,-0.345644943866628,0.429312657070305,-0.805112400424813,0.420754820596545,0.902436876650571
"6603","WDR19",22.4867956857811,0.272181128952846,0.338131886046814,0.804955522340659,0.420845346657639,0.902494337221288
"6604","HEYL",16.5401425749929,-0.444184115590129,0.55212340940 7297,-0.804501508217084,0.421107398658184,0.902509575450263
"6605","SLFN12",13.0085066343691,0.389254313138764,0.483704631858438,0.804735550377536,0.420972300119318,0.902509575450263
"6606","NECAB3",22.5085735700715,-0.300479801135881,0.373454204226446,-0.804596113085083,0.421052785863708,0.902509575450263
"6607","PCBP3",3.84226350615287,0.574563848211709,0.714153686671867,0.804538097239712,0.421086276326424,0.902509575450263
"6608","NDUFV2-AS1",6.08132756514681,-0.518372643890609,0.64446511746285,-0.804345541511003,0.421197443033261,0.902565949356989
"6609","PSIP1",169.760724727444,-0.137530724200449,0.171060465052189,-0.803988953020149,0.421403354971876,0.902615890862532
"6610","ZFYVE26",8.38320658471177,0.509742647278989,0.63398509610713,0.804029385562801,0.421380004241874,0.902615890862532
"6611","DCTN5",26.0013203994062,0.292189914549203,0.363432036967773,0.803974016674574,0.421411981249449,0.902615890862532
"6612","AC006001.2",1.82786806445476,0.82579015220954,1.02729396280674,0.803849902858714,0.421483665455281,0.902632895167389
"6613","RPS6",4199.26904338393,-0.195195062386386,0.242904126619073,-0.80358891017317,0.421634429890395,0.902819223839104
"6614","FAM98A",35.5778766658559,-0.200515840170343,0.249564296725565,-0.803463647650055,0.421706799982863,0.902837660682997
"6615","MNS1",39.1753492813806,0.266234263875076,0.331522870020564,0.803064548332855,0.421937427133647,0.903058338605266
"6616","BRD4",155.522344985732,0.154981871893279,0.192983037906901,0.803085460640561,0.421925340722364,0.903058338605266
"6617","RPS17",1215.70075667888,0.16248653481174,0.202449581153763,0.802602474580224,0.422204537608603,0.903493464188881
"6618","AC010680.3",1.81724919983791,1.08570419155947,1.35298024988369,0.802453836005962,0.422290482011577,0.903526742361292
"6619","TRMT44",8.81594614442746,0.384791330429998,0.47970940628942,0.802134219977843,0.422475322116816,0.903526742361292
"6620","FOS",7655.90163161007,-0.146230876496772,0.182290921999029,-0.802184085160044,0.422446481000987,0.903526742361292
"6621","FAM207A",46.4526646062703,0.236573097980208,0.294867319462052,0.802303552702301,0.422377387837027,0.903526742361292
"6622","MOB1A",123.587716432178,0.119085396606178,0.148511615379103,0.801859142816478,0.422634442485703,0.903730550528172
"6623","TSKU",35.1776820391953,-0.269910072300481,0.336708267472107,-0.801614033201133,0.42277625748527,0.903897298201937
"6624","TMEM54",73.4860894342852,-0.176900289290547,0.222666052835251,-0.794464567176007,0.426925028642705,0.903962172579489

"6625","GPBP1L1",45.3119607449759,0.232007048873957,0.290154184478826,
0.799599183071191,0.423943060953647,0.903962172579489
"6626","MRPL37",55.9714864379777,-0.232713247608523,0.293011288384629,
-0.794212567343297,0.42707169351302,0.903962172579489
"6627","LINC01359",4.24447203347103,0.559705682735578,0.70531875146960
9,0.793549982287256,0.427457460619944,0.903962172579489
"6628","PRPF3",26.1397156749368,-0.250302518180011,0.313677326914705,-
0.797961780157841,0.424892669795334,0.903962172579489
"6629","AL451085.2",6.37321385107404,-0.413375165090378,0.519398269751
106,-0.795873203983652,0.426105737463128,0.903962172579489
"6630","EFNA1",3.75144506133178,-0.679982224631295,0.853001945826583,-
0.79716374383223,0.425355939581212,0.903962172579489
"6631","AC082651.1",1.54015254306823,0.948887713312257,1.1843919255322
5,0.801160234933079,0.423038888675833,0.903962172579489
"6632","AC104794.3",15.1527502853707,-0.283874493162994,0.356098838914
874,-0.79717893500589,0.425347118167393,0.903962172579489
"6633","GREB1",2.42890549024409,-0.878575319100986,1.09930833547476,-0
.799207365894802,0.424170181562214,0.903962172579489
"6634","EML4",33.5793304757483,-0.208788794948304,0.26259755226559,-0.
795090407914907,0.42656091393756,0.903962172579489
"6635","ASB3",30.6132535594347,0.203170692299262,0.25471842286748,0.79
7628573591491,0.425086064401012,0.903962172579489
"6636","LINC01886",9.82764713996633,-0.476717762567578,0.5968865580507
99,-0.798673979397953,0.424479478518827,0.903962172579489
"6637","BMPR2",103.824142481788,0.194128533763992,0.243741615479384,0.
796452150291142,0.4257692769225,0.903962172579489
"6638","HES6",2.82182638748982,0.629891828246524,0.792174093645954,0.7
95143180392922,0.426530219142622,0.903962172579489
"6639","THOC7",156.909164847753,-0.155294761432196,0.194067835674205,-
0.800208653292243,0.423589917064326,0.903962172579489
"6640","SLC35A5",28.4950009417374,0.242533550096748,0.30286692896513,0
.80079245008844,0.423251810526601,0.903962172579489
"6641","STAG1",79.7982608433132,0.209576779377868,0.262592374764073,0.
798106874071128,0.424808472696583,0.903962172579489
"6642","MYNN",17.3302424801814,0.296603953051395,0.371785791901152,0.7
97781839738118,0.424997101647769,0.903962172579489
"6643","TRA2B",216.667524841093,0.134656538110906,0.168805329704991,0.
797703119600762,0.425042793094217,0.903962172579489
"6644","IDUA",27.7787914599231,-0.257246943231065,0.324019123759649,-0
.793925186409325,0.427238986148508,0.903962172579489
"6645","RNF4",33.7542971143445,0.216310897202389,0.272565941364003,0.7
93609414734298,0.427422849860439,0.903962172579489
"6646","SOD3",1268.95683983471,-0.281967327053481,0.352949270785873,-0
.798889105014029,0.424354716909766,0.903962172579489
"6647","PDLIM5",75.1641989495375,-0.15550747574842,0.195648177978793,-
0.794832220544758,0.426711105635284,0.903962172579489
"6648","EXOSC9",27.6558991691356,0.234652913402922,0.295400764065054,0
.794354456548547,0.426989109839264,0.903962172579489
"6649","RNF150",7.4920132509451,0.664387450771402,0.836032900191773,0.
794690556578577,0.426793526913346,0.903962172579489

"6650","DCTD",80.4568803726482,-0.164836692242925,0.205911322788874,-0
.800522720219402,0.423408005231146,0.903962172579489
"6651","TNPO1",96.10161251187,0.164306217443236,0.205905404872705,0.79
7969424575396,0.424888233543909,0.903962172579489
"6652","MARCH3",34.974522788704,0.261770208358004,0.329413260524001,0.
79465595265231,0.426813661176697,0.903962172579489
"6653","PCDHB9",2.68877120560697,-0.898046242704921,1.12824351550199,-
0.7959684503973,0.42605037338208,0.903962172579489
"6654","ERGIC1",148.484695366392,0.15548381136729,0.194536524910462,0.
799252538508406,0.424143993192752,0.903962172579489
"6655","BTN3A2",65.8547417199262,0.267626256530836,0.336454643009727,0
.79543041563287,0.426363173074405,0.903962172579489
"6656","SDK1",38.3275939083489,0.296937841601157,0.372745257832977,0.7
96624062576839,0.425669398212556,0.903962172579489
"6657","CDCA7L",7.22534466548644,0.383531890194293,0.482587630236858,0
.794740408091381,0.426764521828868,0.903962172579489
"6658","MTERF1",9.16662106012378,0.409031803837484,0.514276637355784,0
.795353656235622,0.42640780994536,0.903962172579489
"6659","MOSPD3",34.2491304490215,-0.311648320126315,0.390247388040943,
-0.798591687418594,0.424527209232956,0.903962172579489
"6660","IFRD1",129.23823985911,0.258665816476918,0.325634875726945,0.7
94343099459094,0.426995719655332,0.903962172579489
"6661","SSBP1",225.298716738642,-0.182291938384877,0.229525645562285,-
0.794211635646657,0.427072235818514,0.903962172579489
"6662","ZNF783",14.870833168231,0.306051560973222,0.383156693913958,0.
798763445437677,0.424427590289703,0.903962172579489
"6663","FAM66B",5.18330523688689,0.529198223700529,0.662785233765985,0
.798446007454924,0.42461171372204,0.903962172579489
"6664","AC145124.1",18.3950245623455,0.274211980892282,0.3423311196367
64,0.801013887324177,0.423123606251247,0.903962172579489
"6665","TAF2",25.3712282278868,-0.25195396707866,0.317508784678561,-0.
793533846106753,0.427466857880809,0.903962172579489
"6666","AC083843.3",2.90669067243417,0.699817374925632,0.8799516296876
4,0.795290731121265,0.426444403982585,0.903962172579489
"6667","CBWD6",2.32185566799931,-0.87642036933337,1.09988029062992,-0.
796832506955306,0.425548313197515,0.903962172579489
"6668","ZNF510",9.09056229634422,0.364097790609103,0.457683589813226,0
.795522930498091,0.426309377757595,0.903962172579489
"6669","RALGDS",47.7685400365787,-0.218060276159666,0.272894280434763,
-0.799065029183688,0.424252706025305,0.903962172579489
"6670","SUFU",13.8782590163831,0.320623190767517,0.401031758625429,0.7
99495760302079,0.424003004049483,0.903962172579489
"6671","TRIM8",115.146349078401,-0.157175190332759,0.197164613739502,-
0.797177482062895,0.425347961877137,0.903962172579489
"6672","CADM1",1.60523196179047,-0.994589279573176,1.24594654585737,-0
.798259991875312,0.424719629956826,0.903962172579489
"6673","SLC48A1",18.9788889818461,0.270175407280492,0.340433242358482,
0.793622283795637,0.427415355718248,0.903962172579489
"6674","C12orf10",92.7786957623617,-0.257867509903631,0.3242336257045,
-0.795313901645248,0.426430928974551,0.903962172579489

"6675","GATC",35.4890327294002,0.254599216842015,0.318503857761638,0.799359915547875,0.42408174622652,0.903962172579489
"6676","MTIF3",106.96539706734,-0.158358479711281,0.198407884559445,-0.798146102222236,0.424785710517844,0.903962172579489
"6677","PLEKHG3",8.47834886440859,0.391538778269079,0.492514764455806,0.79497876312744,0.426625855702343,0.903962172579489
"6678","GABPB1",11.5371428904493,0.31618498776398,0.394878222553119,0.800715181808861,0.423296551460378,0.903962172579489
"6679","RSL24D1",237.594985034479,-0.185199527292863,0.231428991273887,-0.800243419259808,0.423569777892663,0.903962172579489
"6680","PPCDC",12.606484353817,0.387837944990863,0.486474173634455,0.797242620493747,0.425310137589549,0.903962172579489
"6681","IFT140",10.4823215058977,0.368758257078405,0.463493821028279,0.79560555146194,0.426261338868881,0.903962172579489
"6682","C18orf25",15.2948271845538,-0.307939807017153,0.384965999408854,-0.799914297600357,0.423760453391432,0.903962172579489
"6683","FEM1A",10.9004379498433,0.355442048535913,0.446825679513652,0.79548258936862,0.42633283473179,0.903962172579489
"6684","ZNF490",6.19027005322443,0.462422121419444,0.581033931699609,0.79586078573207,0.426112956155343,0.903962172579489
"6685","ARMC6",13.6110153526947,-0.375713814745876,0.46910871969855,-0.800909893525982,0.423183812126123,0.903962172579489
"6686","ERVK3-1",53.1330862977632,-0.23019625884909,0.287794019055427,-0.799864637926183,0.423789227911795,0.903962172579489
"6687","RWDD2B",23.1134613860975,0.258899502846126,0.325326168341644,0.795815178858408,0.42613946790568,0.903962172579489
"6688","LRRC3",4.27314231557804,0.589813975386095,0.742661459255765,0.794189557079155,0.427085087037832,0.903962172579489
"6689","DNAL4",20.50387209637,-0.279807087297472,0.351375262025193,-0.796319825376352,0.425846165221022,0.903962172579489
"6690","LMF2",101.611067162296,-0.140220383159723,0.17555931680356,-0.798706589389506,0.424460565050222,0.903962172579489
"6691","ZC3H12B",3.00459177538071,0.624983182690819,0.782237161893299,0.798968922900738,0.424308432161944,0.903962172579489
"6692","TCEAL9",247.708799093747,-0.175803649521704,0.221286944580994,-0.79446010633202,0.426927624615782,0.903962172579489
"6693","RPL39",4055.1435367711,-0.180425761356224,0.226109327315256,-0.797958065235685,0.424894825669641,0.903962172579489
"6694","RTL8C",167.436112946442,-0.145021234789819,0.182734532721931,-0.793617017154055,0.427418422674009,0.903962172579489
"6695","IDS",146.868171422731,-0.2167298720005,0.272985231230269,-0.793925264834873,0.427238940489552,0.903962172579489
"6696","LINC02458",4.19859096063232,-0.889373772369439,1.11080966345723,-0.800653614770864,0.423332202828562,0.903962172579489
"6697","MIA2",25.6480700615429,-0.231937185829498,0.292348243262228,-0.793359259632893,0.427568539861044,0.904042186715303
"6698","TFDP1",42.2359147388889,-0.183160483941837,0.230964824706271,-0.793023284713468,0.427764256708935,0.904320972678191
"6699","NBPF3",3.39646782726273,-0.556515448223502,0.70748917444286,-0.78660630908134,0.431512364848547,0.905021886622305

"6700","CAP1",180.437562798544,-0.157828432867941,0.200150060543788,-0
.78855051274597,0.430374764396781,0.905021886622305
"6701","IGFN1",9.02763573992384,-0.546620334201964,0.694057204670106,-
0.787572451555747,0.430946833856564,0.905021886622305
"6702","CSRP1",120.772615336916,-0.187681703169727,0.237578363425184,-
0.789978095917098,0.429540560587942,0.905021886622305
"6703","SLC41A1",19.8450307020288,0.273096748826924,0.347003239123247,
0.787014984404589,0.43127309448091,0.905021886622305
"6704","MARC2",32.0642524295385,0.220847729090588,0.27981828816042,0.7
89254092512981,0.429963512269787,0.905021886622305
"6705","DISP1",6.80659361107871,0.421530499846136,0.532761792465088,0.
791217586936396,0.42881703041022,0.905021886622305
"6706","CCT4",147.962802064841,-0.14963690820289,0.190615846904724,-0.
785018195668085,0.432442900339674,0.905021886622305
"6707","ATF2",48.5415433871495,0.20996433175166,0.267094441605608,0.78
610520866508,0.431805853058007,0.905021886622305
"6708","PAX3",1.5795611900538,0.892517563780128,1.13661702961693,0.785
240358470547,0.432312656925911,0.905021886622305
"6709","SHISA5",110.099343083758,-0.129108668385137,0.164588422321061,
-0.784433476938528,0.43278580150237,0.905021886622305
"6710","ARIH2OS",4.92297766869674,0.5402713215416,0.684855228896543,0.
788883984155453,0.430179817279257,0.905021886622305
"6711","PPP4R2",77.9820331352469,0.160571591834287,0.204117891852876,0
.786661033859902,0.431480320239082,0.905021886622305
"6712","ARHGAP31",6.83067768948303,-0.41759150475322,0.529341632123002
,-0.78888845957271,0.430177201302625,0.905021886622305
"6713","SRPRB",64.816599627634,-0.177690673410674,0.224796096155322,-0
.79045266554762,0.429263455497481,0.905021886622305
"6714","CTBP1-
DT",16.9157769790784,0.33921684939686,0.43299929240202,0.7834120178697
95,0.433385201132213,0.905021886622305
"6715","FBXL5",75.8533719885777,-0.146345055541373,0.186414145098894,-
0.785053384568729,0.43242226925792,0.905021886622305
"6716","SEPSECS",8.2660242848778,0.415329847451711,0.527599098011209,0
.787207273509946,0.431160540078844,0.905021886622305
"6717","GALNT7",14.6155817323701,0.287133740044213,0.366360693444838,0
.7837460327535,0.43318914595126,0.905021886622305
"6718","DCP2",46.0159223492453,0.194767306497423,0.246520799434268,0.7
90064395963294,0.429490161554553,0.905021886622305
"6719","PCDHGA12",6.94455146866577,0.481456834429527,0.610768223897872
,0.78828075134117,0.430532504210049,0.905021886622305
"6720","DCTN4",33.2434426855469,0.212916243494955,0.268753204251051,0.
79223704174356,0.428222473867294,0.905021886622305
"6721","LARP1",91.0918729303104,0.155092173992714,0.19638516476012,0.7
89734673605084,0.429682737186482,0.905021886622305
"6722","THOC3",19.2037856962955,-0.292560869123403,0.369973758278629,-
0.79076113528861,0.429083393222244,0.905021886622305
"6723","TAP2",35.5104854418897,-0.236483062215778,0.298628212759481,-0
.79189792561979,0.42842019661651,0.905021886622305
"6724","SOBP",77.1405603286148,-0.232725334601393,0.296565816240707,-0

.784734186668707,0.4326094342999,0.905021886622305
"6725","AL360178.1",2.62552937367571,0.713372516447878,0.900517325951035,0.792180778636864,0.428255274578244,0.905021886622305
"6726","ZC2HC1B",2.42825430520568,0.793132829225562,1.00223110779751,0.791367203686726,0.428729742336031,0.905021886622305
"6727","MAP3K4",30.423227746049,0.214945247829953,0.272922256559175,0.787569509866445,0.430948555120093,0.905021886622305
"6728","WIPF3",2.2165344546769,-0.840868472953016,1.07007482891638,-0.78580343190067,0.431982655701586,0.905021886622305
"6729","PAXIP1-AS2",22.3685532564026,0.303971151955397,0.388066610435366,0.783296330530412,0.433453117561479,0.905021886622305
"6730","TRIM14",1.88086320999629,-0.947277573390096,1.20416967540246,-0.786664531369716,0.431478272286171,0.905021886622305
"6731","MAPKAP1",48.7587766023524,-0.208887943019568,0.265197759781391,-0.787668580578357,0.430890588309018,0.905021886622305
"6732","SLC27A4",8.75387622147499,-0.368819232499269,0.468484869432753,-0.787259646071045,0.431129887303795,0.905021886622305
"6733","AL954642.1",2.62054299464537,1.10333228363744,1.40861094212785,0.783276808833202,0.433464578748056,0.905021886622305
"6734","FAM107B",44.8116418403036,0.188117382132338,0.239165363611111,0.786557799557556,0.431540771210388,0.905021886622305
"6735","LIPA",67.2019305420462,0.16433406867507,0.209355848404803,0.784950933672124,0.43248233730746,0.905021886622305
"6736","RHOG",64.7496807913889,-0.188051978955585,0.239357304904595,-0.785653811696037,0.432070329582119,0.905021886622305
"6737","DNAJC24",22.0819979852243,-0.252040200960966,0.319739940452644,-0.788266241008748,0.430540989907314,0.905021886622305
"6738","API5",42.7152321163705,0.182463821718004,0.23216334927016,0.785928624356973,0.431909303820933,0.905021886622305
"6739","FKBP4",39.7084141036685,-0.189966306672829,0.241594922168254,-0.786300908015488,0.431691220647632,0.905021886622305
"6740","NDUFA9",65.1736656250627,-0.203967091781803,0.260307236029728,-0.783562896263512,0.433296634380705,0.905021886622305
"6741","CREBL2",65.9024347737455,0.160565000955899,0.202712390812917,0.792082814040136,0.428312390242619,0.905021886622305
"6742","SNRPF",170.617029783028,-0.170564361769657,0.216648713376402,-0.787285366764773,0.431114833879112,0.905021886622305
"6743","SACS",26.6320645289527,0.239114581035776,0.303362020228628,0.788215284350915,0.430570790322396,0.905021886622305
"6744","COG3",17.8099543090256,0.304563381974437,0.387489362482694,0.785991594770653,0.431872411368377,0.905021886622305
"6745","HIF1A",68.9608646379166,-0.201918412441195,0.255407685224273,-0.790572970675846,0.429193224860501,0.905021886622305
"6746","ERH",293.858484877099,-0.194855014294491,0.24710484655431,-0.788551973025201,0.430373910606948,0.905021886622305
"6747","AC004816.2",10.0638759637703,-0.427088479818211,0.543442264894999,-0.785894854719721,0.431929089177738,0.905021886622305
"6748","SPRED1",76.0036164497505,-0.180230532458331,0.229184433109047,-0.786399538630869,0.4316334536854,0.905021886622305

"6749","AC103691.1",2.84687363476269,0.654636081568486,0.828281214362966,0.79035485800794,0.429320557618451,0.905021886622305
"6750","ANP32A",93.071200599288,-0.163645583862002,0.208901207461905,-0.783363513549073,0.433413675757008,0.905021886622305
"6751","CLK3",30.4147412780973,0.223092466395296,0.281923837817912,0.791321755982148,0.428756255938962,0.905021886622305
"6752","LCMT1",44.5037913610956,-0.211412417767194,0.269497311331989,-0.784469487737332,0.432764678862529,0.905021886622305
"6753","GTF3C1",43.9215824526749,0.182192829973621,0.232163181906792,0.784761944065561,0.432593156597144,0.905021886622305
"6754","ZNF319",6.64974617570989,0.453737250456228,0.578824041272678,0.783894963067848,0.433101745550489,0.905021886622305
"6755","MC1R",3.08488060426438,-0.644115095823213,0.820042600792905,-0.785465407773222,0.432180744459122,0.905021886622305
"6756","DERL2",85.5578804339528,-0.201547291714951,0.254999748631638,-0.790382315262975,0.429304527043567,0.905021886622305
"6757","TXNDC17",110.223473707589,-0.166734749757743,0.211824153019911,-0.787137573221268,0.431201336440188,0.905021886622305
"6758","CHRNB1",19.4294147959048,0.25848815856952,0.329109517832796,0.785416843218874,0.432209208560625,0.905021886622305
"6759","CCL8",28.3339699619303,-1.10795854658563,1.41272391203558,-0.784271107147316,0.432881049194455,0.905021886622305
"6760","CAVIN1",904.697889578641,-0.156857651403138,0.19987633930732,-0.784773485179561,0.432586388674081,0.905021886622305
"6761","TMEM101",45.182125803186,0.251860028092627,0.318785623628069,0.790060810227991,0.429492255547506,0.905021886622305
"6762","NAT9",24.4610059240339,-0.276122358373666,0.348827000457538,-0.791573926363184,0.428609155034238,0.905021886622305
"6763","PGS1",17.0913028306925,0.259110930969766,0.32895147999319,0.787687384702237,0.430879586424849,0.905021886622305
"6764","AP005329.3",6.3364806174101,0.535526163896268,0.676890111376382,0.791156725287852,0.428852540728885,0.905021886622305
"6765","AC010618.3",4.11266227170634,-0.667951727003192,0.852403126909786,-0.783610132244253,0.433268908656867,0.905021886622305
"6766","HSPB6",142.09909931761,0.302256296246614,0.383627094496328,0.78789089869539,0.430760525236859,0.905021886622305
"6767","FBXO17",119.069768250037,0.172521970757102,0.218862367844387,0.788266948111273,0.430540576388834,0.905021886622305
"6768","BLVRB",246.279561244359,-0.231715354506803,0.293575332662833,-0.789287548122867,0.429943962687583,0.905021886622305
"6769","RPS19",3315.0487080322,-0.174576497186064,0.220427143307348,-0.791991832614945,0.428365438523206,0.905021886622305
"6770","ZNF224",32.6439580192196,0.206121162003917,0.262785116866299,0.784371521728106,0.432822143597623,0.905021886622305
"6771","NDUFA3",131.831299489142,-0.241887300656287,0.305720397168752,-0.791204325574554,0.428824767734605,0.905021886622305
"6772","AURKC",2.08389963332303,0.743711042722854,0.949137405223828,0.783565202076795,0.433295280932196,0.905021886622305
"6773","TMEM230",277.122925736896,-0.227948200871411,0.288877451871421,-0.789082704083356,0.430063670152215,0.905021886622305

"6774","ZNF133",10.1444178015114,0.418840417113347,0.533714310269107,0
.784765199385719,0.432591247610889,0.905021886622305
"6775","CPNE1",120.982093389112,0.23056893200567,0.292865678358938,0.7
87285602388286,0.431114695978299,0.905021886622305
"6776","TP53RK",24.1297216758146,-0.256657006291648,0.326764070892625,
-0.785450510487689,0.432189475770397,0.905021886622305
"6777","SAMSN1",7.86550278478607,0.480751587875057,0.611332387473004,0
.78639967017335,0.431633376645279,0.905021886622305
"6778","GRK3",5.84244335082471,-0.458020909978307,0.580620119726447,-0
.788847810155285,0.430200962091236,0.905021886622305
"6779","HSD17B10",82.6975121231917,-0.19876380476043,0.25244515926787,
-0.787354391491903,0.431074437621155,0.905021886622305
"6780","NKRF",10.703447733417,0.336643894719021,0.427705193348909,0.78
7093306216642,0.431227247577093,0.905021886622305
"6781","RTL8A",78.2568394948464,-0.17746218079233,0.226431171209622,-0
.783735648428199,0.433195240418083,0.905021886622305
"6782","ZNF75D",10.1499609471458,0.354894554410126,0.450471293199113,0
.787829457210848,0.430796468154837,0.905021886622305
"6783","ATPSCKMT",10.0988707601523,0.334486687722929,0.42716701062221,
0.783034924058666,0.433606603814365,0.905184949139232
"6784","MADD",19.6445904428171,0.29998873506456,0.383174833885984,0.78
2903021114961,0.433684063283312,0.905213198126725
"6785","SLC11A2",21.6823761984388,0.248100142673414,0.317014263599744,
0.782615078123614,0.43385318442874,0.905432732720849
"6786","ATG4B",30.607061250417,-0.205152588250121,0.262492730196483,-0
.781555314299788,0.434475956974282,0.905599348431698
"6787","FGFRL1",13.8863032412978,-0.304804874107843,0.390188463731024,
-0.781173464723344,0.434700478198464,0.905599348431698
"6788","CCDC110",3.68328755851117,-0.703894807175969,0.900847229819931
,-0.781369785992092,0.434585036178297,0.905599348431698
"6789","PHF20L1",82.4468623725336,-0.160484315263425,0.205420443944435
,-0.781248020799891,0.434656635197957,0.905599348431698
"6790","NIPSNAP3A",24.1492228149623,-0.24679641083569,0.31549840458074
7,-0.782242975724861,0.434071792420935,0.905599348431698
"6791","PTER",27.124848828714,0.207779239738868,0.265869478802201,0.78
1508432915874,0.434503518843785,0.905599348431698
"6792","FUT11",31.8621842388725,0.208421092458976,0.266757360666204,0.
781313370092064,0.4346182083841,0.905599348431698
"6793","NDN",253.085437918668,0.174815623192497,0.223625594221071,0.78
1733521162513,0.434371197219946,0.905599348431698
"6794","GRWD1",26.3156599239257,-0.216802874390448,0.277453450701173,-
0.781402695992968,0.434565685973406,0.905599348431698
"6795","MAP3K7CL",7.84802123775181,-0.458221036614288,0.58634854601995
1,-0.781482344800931,0.434518856652997,0.905599348431698
"6796","SYNJ1",11.3643507093604,-0.335584615468421,0.429547805635508,-
0.781250913322511,0.434654934289861,0.905599348431698
"6797","TMSB15B",3.11963825013227,0.582011166491646,0.744603161017733,
0.781639397952791,0.434426526179503,0.905599348431698
"6798","LIG3",11.792844768806,-0.314146848881613,0.402251065983271,-0.
780972073035297,0.43481892015474,0.905712843393811

"6799","BTBD8",16.1439260194372,0.280802565575804,0.359852078576571,0.780327757690173,0.435197978359562,0.905969328663834
"6800","LY75",2.88862109535534,-0.639654124420807,0.819644455587991,-0.780404381509464,0.435152889687951,0.905969328663834
"6801","AC012459.1",1.68159960124767,0.869131065780473,1.11336150956149,0.780636889560505,0.435016088694424,0.905969328663834
"6802","LPIN2",70.0013427736765,0.170560329853866,0.21854971682884,0.780418901148438,0.435144346026755,0.905969328663834
"6803","RAB1A",162.696538687925,-0.143437953044652,0.183861742274934,-0.780140290578585,0.435308303243785,0.906065790670587
"6804","HMGCL",44.1745112394126,0.200988128462616,0.257717992045398,0.779876200599962,0.435463748261897,0.906101627134651
"6805","KIAA1841",12.2539226344217,0.306660735435391,0.393425947356911,0.779462405811259,0.435707374856921,0.906101627134651
"6806","PNO1",25.7567892140064,-0.303055112510233,0.388660939858811,-0.779741624204182,0.435542973088213,0.906101627134651
"6807","ST7-OT4",2.1041144484993,-0.700043731396211,0.897729458422657,-0.779793650334494,0.435512344434655,0.906101627134651
"6808","ABHD3",12.2873174363126,0.375925200957582,0.48232314059645,0.779405276911876,0.435741016349328,0.906101627134651
"6809","PROSER3",6.754197401871,-0.405405745720776,0.520066698572626,-0.779526446960461,0.435669664729134,0.906101627134651
"6810","UBE2G2",79.5506464036604,0.197388195610055,0.253272785577858,0.779350198086628,0.435773452032978,0.906101627134651
"6811","RNH1",539.991185979205,-0.162812086773574,0.209010913714167,-0.778964523336939,0.436000613243838,0.906440857955182
"6812","MUTYH",12.0409294288835,-0.307552909199232,0.395178117333836,-0.77826401743652,0.436413383574081,0.906769197834209
"6813","LINC01004",5.87101114494521,-0.526096996811606,0.676150995945358,-0.778076198905905,0.436524093170241,0.906769197834209
"6814","TMEM132A",3.60614913371221,-0.591766860945756,0.760582265299877,-0.778044516607863,0.436542769889534,0.906769197834209
"6815","ITPK1",17.2823810986704,-0.28280923890602,0.363286834915308,-0.778473678992388,0.436289817724791,0.906769197834209
"6816","SH2B1",24.3008322754601,0.242887923897343,0.31212206471262,0.778182484858856,0.436461440948585,0.906769197834209
"6817","CCDC102B",73.1659541701412,-0.306638723394092,0.393926604585186,-0.778415877031179,0.436323881796632,0.906769197834209
"6818","RBP1",9.16072534680799,0.472527342759106,0.60770601312653,0.777559103501452,0.43682897854512,0.907102798489233
"6819","DERL1",83.7629692212023,-0.163560824748707,0.210382174608594,-0.777446212128016,0.436895556899475,0.907102798489233
"6820","DNAJC1",109.610844606732,0.133584414795719,0.171805228492823,0.777534048105527,0.436843754608305,0.907102798489233
"6821","AL360012.1",16.1002782981557,0.421449465748215,0.542346250861573,0.77708560735637,0.43710826483737,0.907206547113762
"6822","EXOC8",10.6889612303466,-0.327674511426594,0.42186594487448,-0.776726624672416,0.43732007517531,0.907206547113762
"6823","PGRMC2",121.99081577963,0.133614509069155,0.172003979905708,0.

77681056649039,0.437270541743218,0.907206547113762
"6824","ABCC8",1.71771133932511,1.06366961459642,1.36945543499896,0.776709915059943,0.437329935776733,0.907206547113762
"6825","TMEM86A",3.97649582379835,0.625471391619086,0.805153311069894,0.776835148063857,0.437256036953972,0.907206547113762
"6826","MEIS3",30.9448611826381,-0.236874879785463,0.304850640862629,-0.777019458168707,0.437147290363111,0.907206547113762
"6827","CALHM6",7.89040760870681,0.434919698431589,0.560152080365462,0.776431461519938,0.437494274363385,0.907332335051409
"6828","SLF2",35.4493208551768,0.21164713458764,0.272604136481025,0.776390033253849,0.43751872766462,0.907332335051409
"6829","MAD2L1",2.95231305570855,-0.60720645165592,0.782397556836152,-0.776084289055468,0.437699219470503,0.907573722024063
"6830","TMEM201",6.4108161876395,0.4410588016672,0.568533134404303,0.77578381096351,0.437876644236743,0.907587832566443
"6831","GBP4",45.5167225571985,0.260474486083884,0.335772416343845,0.775747123364498,0.437898310176126,0.907587832566443
"6832","SLX4IP",12.0784094362637,0.306444825252484,0.394951869173541,0.775904228263908,0.43780553587827,0.907587832566443
"6833","SPACA9",12.9538017258846,0.353900825457757,0.45627644029062,0.775628093425871,0.437968607804382,0.907600685864195
"6834","ZNF746",9.76236986248911,0.358015093678105,0.461672532621014,0.77547410422183,0.43805956157751,0.907646760733518
"6835","TP53I13",93.227846592433,-0.191227976865411,0.246626945726284,-0.775373413891451,0.438119040226949,0.907646760733518
"6836","ALDH9A1",139.318229537043,0.142624972746012,0.184168484778055,0.774426595939539,0.438678560891875,0.907696858981667
"6837","RPE",30.3397329297648,-0.212733580944857,0.274725257842685,-0.774350282225139,0.438723676243763,0.907696858981667
"6838","CDS1",2.29574991425319,0.892201040193946,1.15253989372346,0.774117273556188,0.438861443447839,0.907696858981667
"6839","ZFP2",1.51948079528733,-0.848219549742037,1.09597129435768,-0.773943217408039,0.438964370980484,0.907696858981667
"6840","NFYA",11.0969320809923,0.329620449515107,0.425908801108337,0.773922606570561,0.438976560049891,0.907696858981667
"6841","TBCC",26.2997371657259,-0.247841112464451,0.319976574033245,-0.774560179017045,0.438599595298465,0.907696858981667
"6842","ATP6V1C1",40.1933793237304,0.193107282879294,0.249253578807518,0.774742267706486,0.438491969513001,0.907696858981667
"6843","ACP2",30.0052381583242,-0.250923984204447,0.324181775231422,-0.774022487924626,0.438917492900767,0.907696858981667
"6844","HCFC2",33.8946861757392,0.233634388230869,0.301707087732939,0.774374874605777,0.438709137361284,0.907696858981667
"6845","ZNF561-AS1",11.0170880203416,0.311471417865933,0.401844240315255,0.775104845652578,0.438277708521003,0.907696858981667
"6846","CLIP3",35.7322418607189,-0.292274738229202,0.377054884092805,-0.775151710161284,0.438250018907788,0.907696858981667
"6847","ZNF787",28.0641549386624,-0.287195981751888,0.370809271360361,-0.774511329499053,0.438628470996454,0.907696858981667

"6848","IFNGR2",161.665619590436,-0.169248529171862,0.218655584266636,
-0.774041649745723,0.438906161636661,0.907696858981667
"6849","ZNF343",6.65240135022544,0.407952570380419,0.527208833975793,0
.773796917065979,0.439050895934028,0.907718015246873
"6850","SETX",99.3434124201446,0.211943814460127,0.27397624423162,0.77
3584640721442,0.439176457835242,0.90784505736453
"6851","PSMD4",148.359972322926,-0.153777478649328,0.199521327240529,-
0.770732035397623,0.440865779364493,0.907913841987828
"6852","HTR2B",4.82888945236122,0.679292039665925,0.88055328193131,0.7
71437746704027,0.440447508625515,0.907913841987828
"6853","SELENOK",211.166020869448,-0.141987479800873,0.184435607010986
,-0.769848523839622,0.441389751994647,0.907913841987828
"6854","PDHB",80.0458843944378,-0.20571767084565,0.266863013527713,-0.
770873670825453,0.440781814664332,0.907913841987828
"6855","HCLS1",6.4784254534617,-0.445222531029373,0.577562458133218,-0
.770864734644301,0.440787111964653,0.907913841987828
"6856","HHIP-
AS1",46.992410692575,-0.406954066997926,0.527840891985416,-0.770978666
444756,0.440719576791851,0.907913841987828
"6857","CMYA5",55.389457430993,0.40617027436268,0.527129538669674,0.77
0532183394121,0.440984271765595,0.907913841987828
"6858","AC109454.2",2.36427886388652,0.758636759813349,0.9828650881467
79,0.771862556684947,0.4401958361275,0.907913841987828
"6859","TMCO6",7.65907347475989,-0.366577305802472,0.475597557419246,-
0.770772053144354,0.440842055005772,0.907913841987828
"6860","BOD1",113.150985434817,-0.186138814285229,0.240969418759462,-0
.772458244882246,0.439843068285074,0.907913841987828
"6861","RAB24",16.2251019893278,-0.314030817156196,0.407787453359287,-
0.770084549118079,0.441249740667973,0.907913841987828
"6862","HIST1H1E",11.0401385820904,0.460634318948401,0.597220409383378
,0.771297014822383,0.440530901374294,0.907913841987828
"6863","CDK14",71.8218205804164,-0.186881046551729,0.242553606205039,-
0.77047317282,0.441019262668442,0.907913841987828
"6864","NAMPT",351.434255945059,-0.289357586982705,0.37584272498694,-0
.769890083658691,0.441365096665058,0.907913841987828
"6865","VDAC3",137.855397813495,-0.187800037024358,0.242858964783467,-
0.773288468851873,0.439351678569899,0.907913841987828
"6866","RB1CC1",160.958105321726,0.170059181564075,0.220021594224318,0
.772920413396763,0.439569482885908,0.907913841987828
"6867","LINC01504",11.811548412321,0.306110244491088,0.396414816117922
,0.772196779849996,0.439997888464035,0.907913841987828
"6868","AL354733.3",4.21510570792633,0.633692049098532,0.8218047074197
7,0.771098100774019,0.440648786262394,0.907913841987828
"6869","PALM2-
AKAP2",57.0384386809039,-0.285120474937983,0.37020443722488,-0.7701703
33654826,0.441198859174314,0.907913841987828
"6870","ASTN2",4.69930288454163,0.545673349120014,0.705807360137516,0.
77311938063907,0.439451732269738,0.907913841987828
"6871","AL359644.1",2.51346302683991,0.710285944271595,0.9203397902815
04,0.771764897890963,0.440253685357415,0.907913841987828

"6872","ACADSB",18.1990877140184,0.314678035276236,0.408724650678747,0
.769902267342249,0.441357868854014,0.907913841987828
"6873","BAD",163.707014943436,-0.193088533882678,0.250519268847675,-0.
770753222978958,0.440853218301746,0.907913841987828
"6874","FGFR1OP2",109.898469176067,-0.172518795619545,0.22371309404526
2,-0.771160920892008,0.440611554446625,0.907913841987828
"6875","AC026124.2",2.59649121058651,-0.662483823465316,0.856567267543
187,-0.773417160061997,0.439275537547852,0.907913841987828
"6876","EMC7",169.648520872557,-0.144368457318879,0.187177456982203,-0
.771291904732984,0.440533929603337,0.907913841987828
"6877","PLEKHO2",16.720389031232,-0.266099634850921,0.345640074973892,
-0.769874948299963,0.441374075613247,0.907913841987828
"6878","ECI1",88.0887604381393,-0.147618941576701,0.191551671375846,-0
.770648152096027,0.440915511613852,0.907913841987828
"6879","EARS2",5.8118037403259,-0.43160685322092,0.559328137165624,-0.
771652317382196,0.440320379034945,0.907913841987828
"6880","E2F4",37.4906154624743,-0.186103160466769,0.241453942767631,-0
.770760495080711,0.440848907081737,0.907913841987828
"6881","LINC02693",14.5392302112059,0.295311890550386,0.38278779390001
7,0.771476769260621,0.440424386838126,0.907913841987828
"6882","TBCB",145.701005810379,-0.183316167729303,0.237295073576479,-0
.772524119301706,0.439804067275662,0.907913841987828
"6883","SNX21",108.329523497751,-0.149981903787066,0.19448764817116,-0
.771164159767484,0.44060963490003,0.907913841987828
"6884","PCDH19",2.13065475105579,0.854812274410827,1.10813250448447,0.
771398971649613,0.440470484451459,0.907913841987828
"6885","MGAT4A",2.4551214698753,0.743446982311618,0.967206236860211,0.
768654040864158,0.442098715158148,0.908107788311373
"6886","RHBDD1",14.0005249946986,0.297148560746637,0.38691782284711,0.
76798881622999,0.442493829229079,0.908107788311373
"6887","HAUS3",20.7938656519644,0.245222951357984,0.319347798463153,0.
767886775916753,0.442554454534121,0.908107788311373
"6888","OSTC",361.759588163438,-0.225170308048116,0.293030137781874,-0
.768420305681076,0.44223752052749,0.908107788311373
"6889","UIMC1",25.6738677934756,-0.220731997859606,0.287055030675576,-
0.768953595204792,0.441920859052264,0.908107788311373
"6890","ASCC3",38.5544634175861,0.17849322516153,0.232383457974031,0.7
68097810049268,0.442429077878516,0.908107788311373
"6891","AL035530.2",5.02965542646718,-0.554658212376825,0.721687043758
99,-0.768557807949306,0.442155860767643,0.908107788311373
"6892","SMIM30",69.1074169468742,0.227011297232093,0.295363025635286,0
.768584005204521,0.442140303736957,0.908107788311373
"6893","LY6E-
DT",4.83725386487993,0.500393039576294,0.651877065783783,0.76761872113
8237,0.442713736796233,0.908107788311373
"6894","C8orf31",3.89902471591357,0.520412884649049,0.678226898133306,
0.767313838600901,0.442894942519657,0.908107788311373
"6895","RAB30-
DT",26.122300979844,0.275304263925117,0.35842179056251,0.7681013576017
57,0.442426970429775,0.908107788311373

"6896","MRE11",40.9561464881267,0.241145012541685,0.313627595424401,0.
768889651484167,0.441958821283367,0.908107788311373
"6897","ALG10B",11.5051031085597,0.412964208628796,0.537721486937042,0
.767989040164852,0.442493696187678,0.908107788311373
"6898","AC008149.2",3.09730037550762,-0.620580045465721,0.808766519044
651,-0.767316686401381,0.442893249744602,0.908107788311373
"6899","TMPO",41.6221706912224,0.21461235760746,0.279544834610883,0.76
7720705360888,0.442653132343108,0.908107788311373
"6900","ZC3H13",160.776423950762,0.194038244986772,0.252444766448182,0
.768636433691333,0.442109170439704,0.908107788311373
"6901","ACYP1",27.2032106897723,-0.268586860730112,0.349777683478668,-
0.767878779626295,0.442559205578241,0.908107788311373
"6902","FHOD1",25.4597001723877,0.279076484304924,0.363524723248848,0.
767696023012675,0.442667799472908,0.908107788311373
"6903","TMEM97",12.6942287530614,-0.332185922848775,0.43274394779927,-
0.767626964023677,0.442708838258736,0.908107788311373
"6904","FAM171A2",4.46726188981219,0.517426271738148,0.672664130134686
,0.769219360090667,0.441763099244655,0.908107788311373
"6905","TMEM205",101.843235156317,-0.163726804773446,0.213331913624312
,-0.767474504830892,0.442799445900343,0.908107788311373
"6906","CBX7",37.8005401302551,0.27528518850488,0.358267171536584,0.76
8379607107735,0.442261692225369,0.908107788311373
"6907","ILRUN",30.6321786337545,0.215800848955861,0.281289831618522,0.
767183256195782,0.442972566621106,0.908135448581852
"6908","PPIA",864.982947382645,-0.166414657891652,0.216960983649028,-0
.767025734732361,0.443066214868858,0.908195947096559
"6909","CTU1",25.5128676498939,0.24447399088708,0.31880004067927,0.766
856837176612,0.443166638899831,0.908270315070431
"6910","GRSF1",54.6113843009566,-0.169647885790735,0.221334163874794,-
0.766478535535541,0.443391618750113,0.908486988150054
"6911","TBCD",30.7744630334886,0.202783714798092,0.264570675119457,0.7
66463307796801,0.443400676207982,0.908486988150054
"6912","GSAP",5.7656767704374,-0.552147490324555,0.720995410700195,-0.
765812766808511,0.443787716654439,0.909148447312913
"6913","DR1",52.0275972253657,-0.170496570277079,0.222706598157063,-0.
765565868671913,0.443934659606433,0.90931791986505
"6914","GSTM3",225.300561132634,0.212620176313627,0.277796228265795,0.
76538179672545,0.444044229237473,0.909410802140962
"6915","ZBTB21",18.6055755051907,-0.304178117322463,0.397540130267996,
-0.765150721054013,0.444181799895516,0.90956099588149
"6916","CDK11B",30.8462566537208,-0.249663597771332,0.326458137118706,
-0.764764511538426,0.444411783627546,0.909881769648998
"6917","SCNM1",29.2213661923322,0.272831042888515,0.356823132590909,0.
76461142221211,0.444502965525202,0.909881769648998
"6918","HOXA5",8.04935050640209,0.442032936634811,0.578154780727639,0.
764558127632343,0.444534710941342,0.909881769648998
"6919","AC006449.2",2.08588324353459,0.708282504137347,0.9265181973184
8,0.764456117739782,0.44459547769784,0.909881769648998
"6920","METTL5",95.5862770814409,-0.173504645431183,0.227183318602798,
-0.76372088627922,0.445033591360499,0.909921960475102

"6921","ZNF35",6.28440922705678,-0.429598988465004,0.562315006396798,-
0.763982791812348,0.444877497466887,0.909921960475102
"6922","AC024560.5",5.83856276371298,-0.428860842579643,0.561265351287
279,-0.764096414638883,0.44480978875784,0.909921960475102
"6923","PTTG1",8.69785386946844,-0.366570186992766,0.479710893216019,-
0.764148140425269,0.444778966914236,0.909921960475102
"6924","TIAM2",9.66323978554666,0.408831042053613,0.535351594628556,0.
763668299778341,0.44506493631713,0.909921960475102
"6925","ARID3A",8.13654838811871,-0.414262831023645,0.5424107163806,-0
.763743817209106,0.445019923436905,0.909921960475102
"6926","CTSA",135.193338294479,0.210852281753023,0.276080660580074,0.7
63734342383856,0.445025570853857,0.909921960475102
"6927","GRIP2",2.4761274631164,-0.686978617880803,0.899715164289538,-0
.763551227263438,0.445134723630749,0.909933259219202
"6928","RNPC3",35.6538136287242,0.238132261767992,0.312197301040248,0.
762762076976741,0.445605301467203,0.910086832184608
"6929","IDH1",36.752215528689,0.207521171303868,0.272028092971531,0.76
2866691586835,0.445542902484497,0.910086832184608
"6930","AC008875.3",26.4668405868633,0.215474009463433,0.2824545643681
98,0.762862550815601,0.445545372216122,0.910086832184608
"6931","TMEM8A",18.6244945888508,0.31790794911779,0.416650830464372,0.
763008077443336,0.445458578642825,0.910086832184608
"6932","RTN4RL1",1.70908935092526,0.900513526789716,1.18040386485216,0
.762885952514651,0.445531414552596,0.910086832184608
"6933","MYBBP1A",13.7241284439565,-0.293768946959926,0.385035754418774
,-0.762965370328741,0.445484048604937,0.910086832184608
"6934","SHC2",34.8745228928072,0.263034359206397,0.344885842148684,0.7
62670794392888,0.445659752427124,0.910086832184608
"6935","TMEM170B",3.2132181565565,0.587300205937953,0.770538471703118,
0.762194526951842,0.445943912128743,0.910535803279452
"6936","RPS8",5575.85238246339,-0.168683564940074,0.221412436881095,-0
.761852257787406,0.446148186955683,0.91063254919154
"6937","DDX58",25.4962957766452,-0.248839973186017,0.326743269238281,-
0.761576432059715,0.446312845437096,0.91063254919154
"6938","AL512598.1",1.96874702777478,0.776408587426621,1.0193590214173
6,0.761663526896609,0.446260849075084,0.91063254919154
"6939","EXOC6",6.57688365091763,-0.473252031336097,0.621272010963806,-
0.76174690471235,0.446211075037969,0.91063254919154
"6940","RHOT1",28.3925602162837,-0.202300714458184,0.265536867810289,-
0.76185546710115,0.446146271309265,0.91063254919154
"6941","AGT",5.60834119813136,-0.772560830075656,1.01471492162442,-0.7
61357513930013,0.446443556661225,0.910768010707816
"6942","DLX5",1.74906562017936,-0.893374524129557,1.1735758961748,-0.7
61241370959864,0.446512911932949,0.91077828190299
"6943","COPA",143.033229497004,0.156752136149334,0.206159287751299,0.7
6034476961539,0.447048527627746,0.911345688339891
"6944","ECPAS",41.8520990547185,0.184642051247563,0.242742535427839,0.
760649759722281,0.446866290280978,0.911345688339891
"6945","ANK3",2.33728639522412,0.680334326915485,0.89475849565484,0.76
0355258116409,0.447042259848582,0.911345688339891

"6946","SAP18",424.382037434905,-0.184434824481174,0.242513943593375,-
0.760512248278873,0.446948450731873,0.911345688339891
"6947","CASC4",149.721960779232,-0.129048870182967,0.169785428303463,-
0.760070351575249,0.447212533710333,0.911417598926068
"6948","ZNF710-
AS1",1.54520999709171,0.871592108186456,1.14657457919224,0.76017044508
3902,0.447152708809702,0.911417598926068
"6949","TBC1D9",29.2554559533053,0.21227329694641,0.279412252079347,0.
759713632336099,0.447425778286694,0.911519433555007
"6950","EID1",1089.64382763219,-0.118918862072722,0.156541452430918,-0
.759663719903203,0.447455620242998,0.911519433555007
"6951","ADCY9",24.3154676162531,0.240378091583141,0.316394401540961,0.
759741924675052,0.447408863189331,0.911519433555007
"6952","BEGAIN",1.72797721014601,-0.900341851696203,1.1857337082717,-0
.759312015349988,0.447665931628449,0.911685544636681
"6953","RAB11FIP3",14.1444820459147,-0.274976429017825,0.3620912182785
4,-0.759412035246594,0.447606116271288,0.911685544636681
"6954","KCTD3",55.6674994025253,0.153180796419129,0.201848032752589,0.
758891698522952,0.44791734528247,0.911806610769165
"6955","RCBTB1",8.51219199178449,0.350570101406919,0.461867479547355,0
.759027463354833,0.447836128408271,0.911806610769165
"6956","SPPL2A",182.300832023796,0.118726478509944,0.156469806001743,0
.758782039447413,0.447982951350359,0.911806610769165
"6957","MAPRE2",39.8678911354483,-0.181379255853287,0.239017484782057,
-0.758853504038306,0.447940195393103,0.911806610769165
"6958","PARD3",46.7602251043327,0.192302103821278,0.253658781613321,0.
758113330822603,0.448383139824205,0.911834735015186
"6959","ADCY6",19.8488699325725,-0.249709020522953,0.329276199273255,-
0.758357333673327,0.448237092874215,0.911834735015186
"6960","CA12",39.9669150817091,-0.376353778350439,0.496383588174231,-0
.758191421547038,0.448336395987164,0.911834735015186
"6961","MPHOSPH6",24.377317879781,-0.257967135481042,0.340242488641178
,-0.758186129284681,0.44833956376188,0.911834735015186
"6962","YJEFN3",3.436298698462,-0.562489016192532,0.741683910118044,-0
.758394524296756,0.448214834945461,0.911834735015186
"6963","NRSN2",20.2955405808472,-0.262105809161999,0.345607302060778,-
0.758391988824085,0.448216352360962,0.911834735015186
"6964","TEFM",19.2208761613436,0.266173295724053,0.35126008477592,0.75
7766985946762,0.448590489584986,0.911994448316354
"6965","CASC3",49.6481957674271,0.203692561757522,0.268784117068648,0.
757829606819732,0.44855299568792,0.911994448316354
"6966","MRPS24",193.910509649119,-0.175459114459648,0.231605929751466,
-0.757576089040256,0.448704799017681,0.91203822710509
"6967","UBAP1",32.542640590508,0.204129354906151,0.269578333306042,0.7
572721550378,0.448919737930423,0.91203822710509
"6968","ZNF503-
AS2",5.10412241362444,0.525464826324191,0.693763357872089,0.7574121930
21979,0.448802953457219,0.91203822710509
"6969","LOXL1",183.622053829855,-0.261636324881662,0.345534395284198,-
0.757193287998055,0.448934070827858,0.91203822710509

"6970","ZNF557",11.9922284535427,0.359405085870987,0.474522740787458,0
.757403291725416,0.448808284635878,0.91203822710509
"6971","ZBTB40",15.9929390260852,0.365696387942198,0.48379799013099,0.
755886538187528,0.449717225778126,0.912204138652595
"6972","BCAS2",69.8263774331553,-0.19359459286938,0.256119979740048,-0
.755874621987209,0.449724370899277,0.912204138652595
"6973","RN7SL832P",8.71554522266858,0.374627738468074,0.49553294354875
1,0.75600975342867,0.449643347961529,0.912204138652595
"6974","TRAF3IP1",24.7536219811197,-0.271220237957586,0.35871176197698
4,-0.756095190363422,0.449592125431462,0.912204138652595
"6975","NFKB1",68.9112453112816,-0.188961372244399,0.249764078097848,-
0.756559444750781,0.44931384599152,0.912204138652595
"6976","ZNF454",2.52886877142817,-0.631877748250993,0.835344197208549,
-0.756428009391249,0.44939261994761,0.912204138652595
"6977","SHOC2",57.1766210300359,0.195040484550426,0.257931395135448,0.
756171944280008,0.449546111506049,0.912204138652595
"6978","GOLGA6L10",4.62242930689672,0.577355058559791,0.76353784450888
1,0.756157750021094,0.449554620754205,0.912204138652595
"6979","NAE1",63.6375778307885,-0.199239535195827,0.263580899110758,-0
.755895195243664,0.449712034925984,0.912204138652595
"6980","PCNA",45.3045752395254,0.193141358732675,0.255391701852258,0.7
56255419936884,0.449496070933084,0.912204138652595
"6981","AP000692.1",2.07657874880919,0.667612429513523,0.8823835499598
03,0.75660117365508,0.449288837990798,0.912204138652595
"6982","ARSB",7.68975680642887,-0.397916697159365,0.526617297179557,-0
.755608863002634,0.449883740443092,0.912212254994739
"6983","KIAA1217",40.0623168919474,-0.379887004671364,0.50279819912498
7,-0.755545674850221,0.449921637632998,0.912212254994739
"6984","TXNRD2",18.5271108022424,0.246933732493221,0.326823563207036,0
.755556698758569,0.449915025897012,0.912212254994739
"6985","PRICKLE2",58.2212546128476,0.1833410436744,0.242698364827,0.75
5427601686105,0.449992457040757,0.912225224294505
"6986","CTBS",56.4799749547745,-0.191914951818281,0.254205524713115,-0
.754959798906289,0.450273103767278,0.912271734059902
"6987","PRKAB2",26.9968518574831,0.237454480311141,0.314400324251909,0
.755261563028428,0.450092056483201,0.912271734059902
"6988","LPAR1",146.841149236965,0.145209020926985,0.192301828411199,0.
755109933830087,0.450183023223227,0.912271734059902
"6989","PRDX5",510.071801598378,-0.199274369518415,0.263946692353096,-
0.754979604941722,0.450261219615454,0.912271734059902
"6990","NUP160",34.4133953405275,0.194083055276058,0.257121310310044,0
.754830686892608,0.450350578788987,0.912298168190566
"6991","EPM2A",4.60431420442293,0.464104167116011,0.615034069855971,0.
754599118752389,0.450489552602937,0.912318659161554
"6992","MYPOP",11.1085246984676,-0.30941794641831,0.410031619806249,-0
.754619720704755,0.450477187517562,0.912318659161554
"6993","KTI12",12.5797866536314,-0.296425344544435,0.39314092037003,-0
.753992599563116,0.450853665467269,0.91232981438595
"6994","AC007389.5",2.66870886312959,0.729169094933975,0.9676407519841
76,0.7535535201869,0.451117362789617,0.91232981438595

"6995","CXCR4",32.3341534690021,0.288420035571647,0.382796641808014,0.
753454978626223,0.451176555755498,0.91232981438595
"6996","CCNH",52.9841414288337,-0.173216924892832,0.229812960630471,-0
.753730009037035,0.4510113586237,0.91232981438595
"6997","CYB5R4",17.1205970028454,0.258032774766842,0.342344405511259,0
.753722773361796,0.45101570429652,0.91232981438595
"6998","TMEM268",6.01224695232924,0.50429534319486,0.668736404201817,0
.754101825511908,0.450788081339682,0.91232981438595
"6999","CASP4",128.862326863722,-0.149211843840631,0.197830844829402,-
0.754239532107863,0.450705403860449,0.91232981438595
"7000","GOLGA3",76.819053307155,-0.189239912200434,0.251155030406101,-
0.753478486552492,0.451162434371542,0.91232981438595
"7001","LPCAT2",11.0262892574665,-0.378258621338907,0.501990244489813,
-0.753517873088036,0.451138775150991,0.91232981438595
"7002","AC005332.5",3.70196452306672,-0.546573066901899,0.724559325634
705,-0.754352400920529,0.45063764510799,0.91232981438595
"7003","ZNF676",4.91401008769297,0.577682416244889,0.766757399342086,0
.753409640051166,0.451203791676893,0.91232981438595
"7004","GZMK",5.77313143170471,0.478249029142001,0.635124904773267,0.7
5299996197241,0.451449936871972,0.912436640894537
"7005","STEAP1",60.2453116186801,-0.224546456431217,0.298148399885044,
-0.753136547161731,0.451367864514486,0.912436640894537
"7006","MRPL46",30.1737487798495,-0.242597030005975,0.322157835082951,
-0.753037808139943,0.451427194730695,0.912436640894537
"7007","TMEM231",8.33796988206762,0.368717554773671,0.48977102014383,0
.752836610596909,0.451548103785835,0.912504802284491
"7008","PIK3C2B",10.9819138006222,-0.313760372237981,0.416845949286604
,-0.752701022463948,0.451629595394852,0.912528192400656
"7009","RAET1G",1.78531440506484,0.814000823272549,1.08204311472862,0.
75228132058008,0.4518818986662,0.912528192400656
"7010","UBR7",22.9078426371371,-0.215937437169779,0.286985398213792,-0
.752433533252152,0.451790386996689,0.912528192400656
"7011","SYDE1",40.9661471671427,0.187043776367303,0.24853644433536,0.7
52580881518195,0.451701809836395,0.912528192400656
"7012","ZBTB46",4.59552396467566,0.463759823501769,0.616427951177507,0
.752334190258391,0.451850111737601,0.912528192400656
"7013","AC017083.1",1.8819412656571,0.772770395292446,1.02798948443546
,0.751729863965317,0.452213527245036,0.912614885561435
"7014","CAMK4",2.32742973549197,-0.747503970788113,0.994481421290061,-
0.751652021631974,0.452260350239833,0.912614885561435
"7015","COMMD6",529.105798013532,-0.191503915750676,0.254759699417536,
-0.751704120347594,0.452229012002802,0.912614885561435
"7016","AC090510.1",2.07694807114761,1.04448544670995,1.38958688860204
,0.751651771672031,0.452260500597821,0.912614885561435
"7017","ITGAX",3.43194795106805,-0.600870766401628,0.799490681989463,-
0.751566941226148,0.452311530146197,0.912614885561435
"7018","RAP2C-
AS1",10.0316568465977,-0.325544740221231,0.432922936064305,-0.75196926
0812912,0.45206954465508,0.912614885561435
"7019","COG8",16.81357896955,0.249193888085605,0.331713204451804,0.751

232946838604,0.452512475278356,0.912890247890229
"7020","COA7",10.1466309634149,0.402653316491905,0.536507756810083,0.750507912291824,0.452948860217272,0.913120139617962
"7021","SLC25A20",12.4311348981443,-0.327930307461907,0.436839128048775,-0.750688952536531,0.452839873175058,0.913120139617962
"7022","ERICH1",76.2639400034335,0.181151990613483,0.24135177205731,0.750572448958313,0.452910007154835,0.913120139617962
"7023","AC009060.1",1.64886677240584,0.908354192469186,1.21020662030593,0.750577775090636,0.452906800743076,0.913120139617962
"7024","CCDC40",4.83494167054695,0.468977372071567,0.624841029744619,0.750554700710425,0.452920691959506,0.913120139617962
"7025","FZD5",2.96282775439706,-0.672676246576631,0.896702604232279,-0.750166491545488,0.453154437341491,0.913136760920082
"7026","NDUFA10",137.474683481016,-0.189195878649006,0.252153063759903,-0.750321554011162,0.453061064209281,0.913136760920082
"7027","C11orf98",2.57573167493227,0.640142174643909,0.853442860965559,0.750070337362331,0.453212343445771,0.913136760920082
"7028","AL136441.1",3.55280022645936,0.600084252164623,0.800270577110254,0.749851699323377,0.453344027490691,0.913136760920082
"7029","LINC02175",3.02195065403631,0.674910951767631,0.899824524617099,0.750047296226812,0.453226219930373,0.913136760920082
"7030","ZNF646",8.78596981302213,0.358301592515263,0.477783823223451,0.749924076746508,0.453300432722806,0.913136760920082
"7031","PTPRG-AS1",3.73404055241183,0.599885933860671,0.800544583130826,0.74934731494228,0.453647896759002,0.91348893886625
"7032","ARGLU1",304.101146123828,0.156163552140776,0.208385190561181,0.749398513974179,0.453617046369113,0.91348893886625
"7033","TBX2-AS1",4.43557333866895,-0.513161645854152,0.684990908091542,-0.749151032214245,0.453766179467577,0.913597199098663
"7034","ZNF248",16.5177618691257,0.26722162331686,0.356875091147682,0.748781940643424,0.453988646299087,0.913785249693685
"7035","FAM104A",34.3858770844443,-0.187078777552223,0.249843872193112,-0.748782733432919,0.453988168386002,0.913785249693685
"7036","PNRC2",205.250943763245,0.111023940862707,0.149143383103761,0.744410771381428,0.45662800448071,0.913995752331737
"7037","IFI6",256.755642980452,0.246053436600164,0.332329355136266,0.740390317007278,0.459063192540962,0.913995752331737
"7038","ZMYM4",53.1469142075425,0.169350778753037,0.229576518855216,0.737665940739518,0.460717473963423,0.913995752331737
"7039","AC119674.1",3.54669030498284,0.627744930113008,0.844418050173896,0.743405390237374,0.457236281868967,0.913995752331737
"7040","ANKRD35",88.2675251654699,-0.184466013797631,0.249062456624394,-0.74064158965484,0.458910783905854,0.913995752331737
"7041","RPS27",6561.19037462348,-0.196722926706733,0.2665080456859,-0.738150047967352,0.460423273622749,0.913995752331737
"7042","HAX1",107.880356841008,-0.185080462586979,0.248005747311102,-0.746274893197582,0.455501374701076,0.913995752331737
"7043","UHMK1",41.7050081198734,0.194600176121249,0.2620530804652,0.74

259831548552,0.457724908690417,0.913995752331737
"7044","RBBP5",23.0925607958374,0.23038240181389,0.308843460597713,0.745952015198975,0.455696402194539,0.913995752331737
"7045","OBSCN",3.55083682642219,-0.634570602246675,0.858460800317927,-0.739195781579851,0.459788122011181,0.913995752331737
"7046","PRKD3",51.3770485764758,0.15908690214949,0.214467630318545,0.74177581909774,0.45822317361076,0.913995752331737
"7047","USP34",93.4243121272882,0.156606562544163,0.20963567477184,0.747041564918043,0.455038471443126,0.913995752331737
"7048","SMYD5",9.7824132411205,0.323733495310502,0.435988806181885,0.742527080329322,0.45776805062268,0.913995752331737
"7049","IMP4",62.1990174002848,-0.180686570542915,0.244465508309163,-0.739108644784399,0.45984102791285,0.913995752331737
"7050","FZD7",65.6144294218433,0.159215351024122,0.214215183387278,0.743249607738016,0.457330574360289,0.913995752331737
"7051","KAT2B",18.7808762883547,0.315615484370375,0.422555211600361,0.746921291480541,0.455111072960095,0.913995752331737
"7052","GOLGA4",239.087489884904,-0.128126131614529,0.171942259711001,-0.74516952277981,0.456169244736256,0.913995752331737
"7053","TCAIM",6.43677791177538,-0.408080089359822,0.549482289392552,-0.742662861456282,0.457685819880027,0.913995752331737
"7054","RPL24",3085.06845554739,-0.176257426007534,0.238114995738242,-0.740219764240689,0.459166656941825,0.913995752331737
"7055","RNF13",155.21249823487,-0.131068507665795,0.177282920872151,-0.739318299929839,0.459713739639388,0.913995752331737
"7056","EHHADH",2.47759583314777,0.730274822184213,0.984204958233926,0.741994658810325,0.458090571718458,0.913995752331737
"7057","UBE2D3",311.379200412785,-0.127178093192402,0.171500874092619,-0.741559446068595,0.458354302026098,0.913995752331737
"7058","SPATA5",11.6342380661091,0.333587630045083,0.449869565281045,0.741520777998579,0.458377738229337,0.913995752331737
"7059","SREK1IP1",91.4999946414476,0.159613920418068,0.214712307822638,0.743385053407914,0.457248590785496,0.913995752331737
"7060","ATG10",24.4857036789517,-0.235935625128638,0.317635357844597,-0.742787662965623,0.457610245843695,0.913995752331737
"7061","ARHGEF37",1.67239337270629,0.976171457576469,1.31794890011954,0.740674738973513,0.458890679408224,0.913995752331737
"7062","FAM193B",17.9173698874764,0.243208383224923,0.326901179677091,0.743981356889445,0.45688775389816,0.913995752331737
"7063","ZNF354B",11.9025660279527,0.290473386787671,0.391605237497943,0.741750515502738,0.458238507246499,0.913995752331737
"7064","AL121944.1",5.17320609458671,0.441273323454321,0.597077547258601,0.739055296050517,0.459873420765881,0.913995752331737
"7065","AL645933.2",3.12965353272344,0.616988188234779,0.828075114726357,0.74508722368582,0.4562189922556,0.913995752331737
"7066","EPHA7",2.23810909585303,2.34719512761316,3.17471202247146,0.739341115351277,0.459699888865254,0.913995752331737
"7067","PREP",18.5560131847687,0.256288905015345,0.343689325059385,0.745699346265881,0.455849054182198,0.913995752331737
"7068","AL355312.2",3.37538621756002,0.585329524286417,0.7886514186001

04,0.742190415792831,0.457971974624374,0.913995752331737
"7069","PRKAR1B",3.16632969687323,0.588286296863718,0.792154750381377,0.742640622404262,0.457699287559286,0.913995752331737
"7070","GNA12",53.1891315451596,0.174231964772009,0.234338402615117,0.743505813932564,0.457175502906423,0.913995752331737
"7071","ITGB8",11.2395161307998,-0.476114086912992,0.645087412058348,-0.738061351086986,0.460477168387994,0.913995752331737
"7072","YAE1",23.0270536926905,-0.22433866339168,0.302590085500757,-0.741394626398356,0.458454201709631,0.913995752331737
"7073","CASD1",31.8990815199863,0.190394252243286,0.257801461616636,0.738530538381555,0.460192116784245,0.913995752331737
"7074","ZNF655",63.4586375421546,0.194365030031183,0.262530332771724,0.740352659363697,0.459086036109823,0.913995752331737
"7075","AC004522.5",1.90937806358324,0.737290860984867,0.987124783357656,0.746907456296466,0.455119424809706,0.913995752331737
"7076","MEST",188.482017521097,-0.345521993004278,0.462039216863357,-0.747819623082909,0.454568964224709,0.913995752331737
"7077","TRBC2",6.27207830784685,0.479055826784713,0.644151306340397,0.743700776617784,0.457057519510897,0.913995752331737
"7078","EPHA1-AS1",2.14446708481911,0.829832604575958,1.11592137292678,0.743629994646951,0.457100351869685,0.913995752331737
"7079","RBPMS",91.6950046202203,0.17841245479657,0.239905696113011,0.74367744362571,0.457071638776632,0.913995752331737
"7080","MATN2",62.9079136726392,-0.246126859693525,0.331456388530482,-0.74256182173689,0.457747010003973,0.913995752331737
"7081","EBAG9",57.3694385845251,-0.15313132859012,0.205487419298194,-0.745210237751357,0.456144634793346,0.913995752331737
"7082","MED30",53.0007322365706,-0.187189968633728,0.250387597890851,-0.747600800560927,0.454700981708318,0.913995752331737
"7083","EXT1",88.8651134006975,-0.198810349542605,0.268031670259428,-0.741742008883415,0.458243662207195,0.913995752331737
"7084","ENPP2",60.9856712558837,-0.294855768818445,0.398650554166756,-0.73963466433589,0.459521702143404,0.913995752331737
"7085","SLA",3.43959574010787,0.562563697354994,0.762054304354472,0.738219959050734,0.460380796132485,0.913995752331737
"7086","FAM219A",15.1025491733262,-0.303214445072409,0.411207148057948,-0.737376396554467,0.460893485196873,0.913995752331737
"7087","FBH1",58.6233904271727,-0.159781712057601,0.216078021841387,-0.739463045320219,0.459625871644151,0.913995752331737
"7088","CDC123",75.4815244259765,-0.174601645188266,0.236286288946675,-0.738941078496817,0.459942776892219,0.913995752331737
"7089","FAM149B1",18.62564313665,0.248514043020326,0.334902921639541,0.742047999473124,0.458058254189815,0.913995752331737
"7090","RPS24",4796.65936121427,-0.188244916804012,0.255312922682333,-0.737310570989905,0.460933505192616,0.913995752331737
"7091","ZDHHC6",28.6712159784545,-0.194312856751774,0.260873098416501,-0.744855862606196,0.456358859952227,0.913995752331737
"7092","ST5",71.9916013125587,0.18285370224396,0.246052161656527,0.743150155694273,0.457390776697197,0.913995752331737

"7093","BDNF",4.35216577888944,-0.596424575485855,0.80449612546118,-0.741364136643856,0.458472683346969,0.913995752331737
"7094","FADD",23.192724118159,-0.218556346573928,0.292241210776035,-0.747862856143939,0.454542883907715,0.913995752331737
"7095","ALG8",27.4581280155471,0.20549801740873,0.278435213365284,0.738046078744838,0.46048644865663,0.913995752331737
"7096","SYTL2",17.8045090052291,-0.261860345608057,0.350884274508945,-0.74628692315871,0.455494109170333,0.913995752331737
"7097","CHD4",114.71121200804,0.193644253385556,0.262020615742536,0.739042051469083,0.45988146294995,0.913995752331737
"7098","METAP2",158.081479206232,-0.148696007874738,0.199201184373435,-0.746461464787194,0.455388701574534,0.913995752331737
"7099","TMEM116",11.552657620346,0.32683111633162,0.443273782174451,0.737312084482802,0.460932585011896,0.913995752331737
"7100","TRIAP1",41.4244320169679,-0.22666223743393,0.306363574015789,-0.739847216373864,0.459392705399879,0.913995752331737
"7101","ARL6IP4",389.271532358405,-0.151375128280791,0.205094161464638,-0.738076243613063,0.460468119016075,0.913995752331737
"7102","UBC",2076.95460099888,-0.128478608087411,0.171750182201562,-0.748055148707975,0.454426893755209,0.913995752331737
"7103","VWA8",9.34748596196382,0.320555765921161,0.42954185888501,0.746273638506963,0.455502132478957,0.913995752331737
"7104","DOCK9",25.9751078341536,0.262077598667374,0.355382515293225,0.737452146319394,0.460847433984738,0.913995752331737
"7105","RASA3",43.1475417748407,0.170094429303762,0.229338453137885,0.741674267775304,0.458284714076477,0.913995752331737
"7106","PRMT5",30.5230009734109,0.198297125976065,0.2674758949754,0.74136447321469,0.458472479329268,0.913995752331737
"7107","AL110292.1",2.46081140596303,0.701492229973411,0.949682770965789,0.738659530761016,0.460113765632933,0.913995752331737
"7108","PNN",170.091998889183,0.162531056112919,0.219838067348875,0.739321711080132,0.459711668785194,0.913995752331737
"7109","CYFIP1",84.2806709304217,0.128163883121943,0.171604385730112,0.746856687704417,0.455150072893124,0.913995752331737
"7110","DPH6",21.3780703691286,-0.235416954194483,0.319209583901949,-0.737499643077121,0.460818560180682,0.913995752331737
"7111","CTDSPL2",42.3690290861947,0.17584403526834,0.237605104376082,0.740068424582383,0.459258476810645,0.913995752331737
"7112","AC103740.1",1.70438460761363,0.737135486675755,0.99263290709583,0.742606336548332,0.457720051061346,0.913995752331737
"7113","ULK3",13.4316990942711,-0.286400522244868,0.386926171921463,-0.740194236080265,0.459182144509537,0.913995752331737
"7114","IMP3",107.482037753971,-0.179923367458952,0.240882899007412,-0.746932921350369,0.455104052453571,0.913995752331737
"7115","ZNF710",5.04163376140221,0.530563411442085,0.718849378706788,0.738073130697518,0.460470010556028,0.913995752331737
"7116","PAM16",42.8695791773096,-0.207357330296503,0.278195659296162,-0.74536508161601,0.45605104708222,0.913995752331737
"7117","LINC01290",1.67676668021025,1.02437016520719,1.38243189350219,0.740991415216915,0.4586986455389,0.913995752331737

"7118","NOMO1",12.6691118189713,-0.355806540140616,0.477332941876304,-0.745405374165061,0.456026696015822,0.913995752331737
"7119","MAPK3",105.826446554699,-0.145912624310803,0.197403900748794,-0.739157756038893,0.459811209146828,0.913995752331737
"7120","MT1X",845.983822276239,-0.268942994354743,0.361961218902846,-0.743016047879236,0.457471964617,0.913995752331737
"7121","FLCN",35.3345921721567,0.197082805927541,0.265376762071577,0.74265283964232,0.457691888932054,0.913995752331737
"7122","PRPSAP2",20.6392715038257,0.259115957827775,0.351336889179098,0.737514237213182,0.460809688447172,0.913995752331737
"7123","AC005899.5",1.85249715646955,0.78152880124038,1.04863301759318,0.745283419583853,0.456100402310303,0.913995752331737
"7124","FKBP10",170.83192021527,-0.156454523872627,0.210040872786424,-0.74487656519936,0.456346343364596,0.913995752331737
"7125","GJC1",3.54669712417722,-0.671269611813548,0.905899119635469,-0.740998194240066,0.458694535201939,0.913995752331737
"7126","EIF4A3",109.367727564356,-0.192214907045179,0.25873031114888,-0.742916074238298,0.457532493213565,0.913995752331737
"7127","FN3K",5.32850270354511,0.439955271573797,0.59400382658848,0.740660668973423,0.458899212563539,0.913995752331737
"7128","DOT1L",10.5852876197257,0.340813707897506,0.457880008352324,0.744329740719451,0.456677012930804,0.913995752331737
"7129","AC011462.5",1.58161705005913,-0.890160204306666,1.20694685254288,-0.737530573472404,0.460799757795251,0.913995752331737
"7130","TMEM91",52.1546244875799,-0.192970085359557,0.260927646257638,-0.739554003292619,0.459570660226328,0.913995752331737
"7131","NOP53",653.539830515286,-0.171228024462893,0.231103606842796,-0.740914548250076,0.458745253865657,0.913995752331737
"7132","STMN3",81.0236891352763,-0.184794614261672,0.250225559582444,-0.738512143084193,0.460203290864304,0.913995752331737
"7133","AP001347.1",9.15931182331332,0.385828724607128,0.523062197480226,0.737634504014628,0.460736582238933,0.913995752331737
"7134","ITSN1",60.2861775131679,0.164228329995876,0.222740578159824,0.737307639912997,0.460935287245828,0.913995752331737
"7135","BID",24.5378393254849,-0.249355483606425,0.33650183647382,-0.741022653009575,0.458679705245095,0.913995752331737
"7136","LZTR1",25.8487190211739,0.239217536433225,0.321350236181122,0.744413756392559,0.456626199161698,0.913995752331737
"7137","MRTFA",30.0189742211656,0.207810031293773,0.280015951124401,0.742136404941624,0.458004694750227,0.913995752331737
"7138","APOO",9.79247640826913,-0.362009247183042,0.483746800911939,-0.748344477938869,0.454252402886415,0.913995752331737
"7139","EDA",12.231788850621,0.389647747963863,0.520740063346878,0.748257672858002,0.454304749987115,0.913995752331737
"7140","FAM122B",14.4757984639572,-0.284632243940439,0.381692891715824,-0.745710098663173,0.455842557448033,0.913995752331737
"7141","MT-ND6",95.777965297579,0.296228184229342,0.398411031420134,0.743524051463731,0.45716446557776,0.913995752331737
"7142","COL8A2",34.2753354220612,-0.223658133347824,0.303627377327043,

-0.736620443508021,0.461353199201605,0.914109970422277
"7143","ST3GAL3",16.8410470471051,0.264954828174287,0.364054080845752,
0.727789749145945,0.466742300504189,0.914109970422277
"7144","HECTD3",29.4664089684186,0.20368642141581,0.276370833609358,0.
737004041836465,0.461119891430136,0.914109970422277
"7145","TOE1",10.2430346140086,-0.323474268230232,0.443828109318812,-0
.728827808420474,0.466106998835873,0.914109970422277
"7146","ST7L",21.1232456210416,-0.240111312291619,0.327618635506564,-0
.732898822804628,0.463620141161239,0.914109970422277
"7147","DUSP12",28.9001604182938,-0.22682238069149,0.309521484930624,-
0.732816272002344,0.463670495381991,0.914109970422277
"7148","Z97200.1",45.718908824715,0.213963060292944,0.293833416941356,
0.728178103498852,0.466504567895439,0.914109970422277
"7149","STX6",17.023525021668,-0.257248067716819,0.351210773945028,-0.
73246063845719,0.463887459053043,0.914109970422277
"7150","ZNF281",24.0023032327912,0.230517879221141,0.314271028272878,0
.733500254503209,0.463253372669688,0.914109970422277
"7151","PROX1",2.74579575145819,-0.582257729777696,0.792425180264383,-
0.734779439471411,0.462473830824638,0.914109970422277
"7152","ITPKB",11.994957673837,0.361791118975861,0.492516880594091,0.7
34576078975113,0.462597710804128,0.914109970422277
"7153","ZNF512",17.012190632234,0.27489179967114,0.376878842893134,0.7
29390372674985,0.465762905148945,0.914109970422277
"7154","SLC4A1AP",36.1093626076755,0.187756684142433,0.257797918283466
,0.728309543353187,0.466424121708204,0.914109970422277
"7155","C2orf49",46.689996344512,0.19523725703508,0.267364370429361,0.
730229150284863,0.465250127101595,0.914109970422277
"7156","ERICH2",2.29077698353539,0.671014468356118,0.921251654393586,0
.728372606069091,0.466385527662489,0.914109970422277
"7157","COL4A4",21.5853063675841,-0.446048624763761,0.613382247824049,
-0.727195197360376,0.467106387854129,0.914109970422277
"7158","SP100",160.377823557183,0.106858385904183,0.145550825294419,0.
734165441439651,0.462847912994863,0.914109970422277
"7159","TTC21A",4.00598448601749,0.532620673550017,0.728676270896997,0
.730942799735147,0.464814092073492,0.914109970422277
"7160","ATXN7",49.014654761015,0.269141329269146,0.369060621277975,0.7
29260489339581,0.465842336050623,0.914109970422277
"7161","ADPRH",4.64614425936242,0.458213257617921,0.625009313698794,0.
733130287141199,0.46347896912167,0.914109970422277
"7162","PCDH7",32.0620044656801,0.3356960292304,0.459841824175357,0.73
0025003341987,0.465374901327968,0.914109970422277
"7163","KIT",2.20349700389801,0.773062180650824,1.05256212025078,0.734
457535358234,0.462669931896754,0.914109970422277
"7164","ARL9",2.41911796967609,-0.770462536894506,1.05614648404107,-0.
729503481322526,0.465693739033818,0.914109970422277
"7165","TMEM131L",9.80283984968829,0.332668530459762,0.452345221535382
,0.735430628250244,0.462077274328969,0.914109970422277
"7166","SPCS3",90.9661904761339,-0.137958631858007,0.188526789178025,-
0.731772033351361,0.464307721520753,0.914109970422277
"7167","MOCS2",67.0056255915577,-0.170047876219628,0.233436359872648,-

0.728454968679251,0.466335124853968,0.914109970422277
"7168","SLC22A4",7.53584221397273,0.431884768112097,0.593537671329761,
0.72764508298943,0.466830875627875,0.914109970422277
"7169","MOXD1",15.8796711178361,-0.808028293162841,1.09877290106427,-0
.735391537578133,0.462101074143563,0.914109970422277
"7170","ELFN1",1.54733348348642,-1.0931934685139,1.48890016619103,-0.7
34228857876052,0.462809268319614,0.914109970422277
"7171","NOD1",10.2922273357575,-0.350481905433221,0.477241877597334,-0
.734390509059508,0.462710769544129,0.914109970422277
"7172","AC073349.1",2.69929477931578,-0.591976712286615,0.812074732020
137,-0.728968269723156,0.466021072145399,0.914109970422277
"7173","CLDN15",5.60436933310559,-0.421717187517679,0.575908539983733,
-0.732264167379061,0.464007345681984,0.914109970422277
"7174","TRBC1",5.01756239766433,0.51949814353739,0.713866439537375,0.7
27724564099208,0.466782210367969,0.914109970422277
"7175","REEP4",7.61770089295461,-0.349431657454133,0.474210839272532,-
0.736869823537103,0.461201516643354,0.914109970422277
"7176","AC026979.2",5.93306185423839,-0.4707184024257,0.64580852227843
4,-0.728882302086987,0.466073661489354,0.914109970422277
"7177","PRKDC",150.671024220254,0.12628828538623,0.173435148843853,0.7
28158543571404,0.466516539972767,0.914109970422277
"7178","ZNF7",18.5820835674972,-0.279232422201309,0.38005135327662,-0.
734722873090443,0.462508287193375,0.914109970422277
"7179","SLC1A1",3.55683792695364,-0.590129866622791,0.809516328482333,
-0.728990689698818,0.466007357621108,0.914109970422277
"7180","CEP78",19.6857318085824,0.315945763404107,0.430583760049234,0.
733761448336976,0.463094140340949,0.914109970422277
"7181","TDRD7",10.1296244305003,0.328463084656758,0.446155434879572,0.
736207740572337,0.461604282270644,0.914109970422277
"7182","HSDL2",50.7368868835884,-0.156437074448551,0.213100894506366,-
0.734098628778293,0.462888629206304,0.914109970422277
"7183","ZNF618",34.6907670070805,0.245869332715085,0.335941492507083,0
.731881408516101,0.464240954629132,0.914109970422277
"7184","C9orf116",3.99788577575554,-0.528374047484231,0.71786463125432
,-0.736035771202443,0.461708928708115,0.914109970422277
"7185","ASB13",13.6842465900513,0.28459998384421,0.389671529628257,0.7
3035867956716,0.465170968692782,0.914109970422277
"7186","C10orf25",1.77444393363203,-0.789264781148068,1.08042627876534
,-0.730512388175155,0.465077043441531,0.914109970422277
"7187","AP001160.2",2.25031281502612,-0.662447989859935,0.902370380355
292,-0.734119829597142,0.462875709028334,0.914109970422277
"7188","TRPT1",23.8788060838313,-0.206724093910307,0.28428255143944,-0
.727178269871216,0.467116756093767,0.914109970422277
"7189","MAP3K11",12.5525488100772,0.290229688520061,0.395823333003819,
0.733230369007227,0.463417935770103,0.914109970422277
"7190","GAL",13.5722920362202,0.448099919616443,0.60876319290145,0.736
082478115565,0.461680505405705,0.914109970422277
"7191","HNRNPA1",806.82173730444,-0.165576791435646,0.227228665940175,
-0.728679151244233,0.466197948904008,0.914109970422277
"7192","AGAP2-

AS1",14.3492911270187,-0.290586756147209,0.397623712634618,-0.730808417389918,0.464896181410422,0.914109970422277
"7193","XPOT",32.5630538359758,0.187326527763259,0.257216028775965,0.728284814343434,0.466439256214098,0.914109970422277
"7194","WASHC3",121.212628204777,-0.192384808474029,0.262247431744581,-0.733600352896514,0.463192345799722,0.914109970422277
"7195","TMEM233",20.6871968142092,-0.392912398944603,0.535303955213052,-0.733998684519757,0.462949539826559,0.914109970422277
"7196","CENPJ",11.705895109699,0.32000340813863,0.434975014166794,0.735682275340816,0.461924078861524,0.914109970422277
"7197","INTS6-
AS1",6.44069961495281,0.394717857748472,0.539199430099067,0.732044278451761,0.464141542335315,0.914109970422277
"7198","ABCC4",4.24897813388932,0.478628976821978,0.65760277982749,0.727839041294104,0.466712122402487,0.914109970422277
"7199","EXD2",13.4175670883149,0.321425723806101,0.442026643393255,0.727163687099603,0.467125688275113,0.914109970422277
"7200","SRSF5",448.991294313313,0.125038142725967,0.171821817422095,0.727719823954605,0.466785112618537,0.914109970422277
"7201","PTPN21",31.3329717898423,0.23906845511684,0.328403952802062,0.727970699125334,0.466631522916966,0.914109970422277
"7202","YY1",231.735524747347,-0.115899132188793,0.157707945990493,-0.734897227028623,0.462402087300235,0.914109970422277
"7203","DISP2",1.86179439070364,0.924968957303766,1.26512171517952,0.731130409197439,0.464699501692863,0.914109970422277
"7204","GALK2",29.9532998100267,0.1879618894752,0.258231665284283,0.727880871109574,0.46668651380938,0.914109970422277
"7205","UBALD1",15.6446057503861,-0.292753801192034,0.399286480764272,-0.733192370129025,0.463441108260802,0.914109970422277
"7206","NAGPA",14.3614769146229,0.350727139914237,0.481412566235663,0.728537567385699,0.466284580600395,0.914109970422277
"7207","AC020765.2",2.13192568904694,-0.714879345785504,0.975362661516487,-0.732936961800459,0.463596878219752,0.914109970422277
"7208","CKLF",98.7911175046091,-0.171816815599403,0.234695925909963,-0.732082650916223,0.464118122336203,0.914109970422277
"7209","ANKRD11",212.488659891778,0.148866337208593,0.204394159765524,0.728329700708519,0.466411785321522,0.914109970422277
"7210","SOX15",5.9574544301338,0.430678908314244,0.584504419818862,0.736827462224685,0.461227280463525,0.914109970422277
"7211","RUNDC1",14.3887946961398,0.274051274339787,0.375246091933624,0.730324126568819,0.465192084115203,0.914109970422277
"7212","GPATCH8",77.7716994839195,0.166473835075253,0.228452265067609,0.72870293068001 1,0.46618339972604,0.914109970422277
"7213","UBE2Z",70.8036026081778,-0.155524144504958,0.212147753313803,-0.733093525977206,0.463501388463823,0.914109970422277
"7214","DYNLL2",46.667152801321,-0.164946684062989,0.226599560982302,-0.727921463518951,0.466661663512965,0.914109970422277
"7215","TLK2",39.1594624381075,0.218702202827182,0.300670189089292,0.727382396936705,0.466991734937284,0.914109970422277
"7216","KPNA2",30.19095052925,0.287645582964017,0.391596186499749,0.73

4546435538899,0.462615770076403,0.914109970422277
"7217","SEC14L1",47.5250706347091,0.165644540580218,0.22502331405345,0
.7361216826665,0.461656648389698,0.914109970422277
"7218","RPTOR",8.37135692601978,0.389773726473808,0.534874571541086,0.
728719866698444,0.466173037769043,0.914109970422277
"7219","RAC3",2.07290026676799,-0.682934438895544,0.935602360531127,-0
.729940910482368,0.46542630413325,0.914109970422277
"7220","AP001033.2",2.19632240038872,0.674441320221904,0.9260614045871
94,0.728290064655645,0.466436042925514,0.914109970422277
"7221","OSBPL1A",199.808682019957,0.128528909923545,0.175200838499703,
0.733608988542387,0.463187081125609,0.914109970422277
"7222","LINC00683",2.14626518861062,0.6851613296425,0.938588698611166,
0.729991028718262,0.465395668358956,0.914109970422277
"7223","SIN3B",24.8474574663888,0.235252076657826,0.321364158875117,0.
732042046883161,0.464142904356548,0.914109970422277
"7224","ZNF284",5.18534715548278,0.578977759878638,0.78990500345738,0.
732971379272795,0.46357588578566,0.914109970422277
"7225","ERCC2",8.12512274809688,-0.343507644913739,0.468822916722743,-
0.732702333143169,0.463740000648781,0.914109970422277
"7226","AP2A1",81.3537784914851,-0.133112923995102,0.181919616870855,-
0.731712864641748,0.464343842649926,0.914109970422277
"7227","CSTF1",18.2320816325052,-0.258554085434793,0.352390640322407,-
0.733714394906285,0.463122823402473,0.914109970422277
"7228","PCMTD2",65.407665786046,0.1876158464429,0.255194221173796,0.73
5188459910803,0.462224726179481,0.914109970422277
"7229","MIS18A",24.072039697501,0.228989115095659,0.311201582591063,0.
735822463334205,0.461838748766661,0.914109970422277
"7230","CHAF1B",2.65871317941341,0.653272922004094,0.89835514170373,0.
727187825479757,0.467110903181745,0.914109970422277
"7231","MED15",35.7090594550306,0.199302950695741,0.272117000146189,0.
732416389232094,0.463914458419965,0.914109970422277
"7232","L3MBTL2",15.849192148265,0.255056483136651,0.350457756813939,0
.727780961264506,0.466747680817179,0.914109970422277
"7233","SREBF2",19.8635007276672,-0.268511390510597,0.367633448987211,
-0.730378019873644,0.465159150016438,0.914109970422277
"7234","GRAMD4",8.80367780836135,0.379675254492209,0.5155134006175,0.7
36499291846577,0.461426898375408,0.914109970422277
"7235","RIBC1",2.05505072148012,0.767525045863709,1.05129897786972,0.7
30073044890585,0.465345536750768,0.914109970422277
"7236","UTP14A",18.0491719308652,0.264945676256411,0.361992716039388,0
.73190886036387,0.464224197783267,0.914109970422277
"7237","EFCAB7",14.4231643777136,0.264384659048787,0.363761579875253,0
.726807540091105,0.467343863428154,0.914157909399456
"7238","NIBAN1",177.350093149928,0.207634086514427,0.285614298138965,0
.726973711986238,0.467242059731015,0.914157909399456
"7239","SNU13",236.54703224846,-0.158508732877941,0.218069188669571,-0
.726873584686561,0.467303400336222,0.914157909399456
"7240","SLC44A3",4.19606424177206,-0.473810884307082,0.652408945205485
,-0.726248295320122,0.467686569958363,0.914229904517363
"7241","C3orf67",2.08763937559049,-0.711836392723405,0.980192940484157

,-0.726220689134733,0.467703490700832,0.914229904517363
"7242","LINC01615",6.37313178418179,-0.542589353997598,0.746983457219774,-0.726373989615623,0.467609532063496,0.914229904517363
"7243","MMAB",31.1931948536412,-0.278401370570247,0.38332097144305,-0.726287866594352,0.467662316009558,0.914229904517363
"7244","SNX29",56.7119497394252,0.191438133987497,0.263529465500087,0.726439199594678,0.467569567710281,0.914229904517363
"7245","AL034417.4",2.5839309886034,0.59455954900791,0.819863945461679,0.725192944877096,0.468333670436145,0.914510355547201
"7246","TUBA4A",6.15578274247821,0.465473556467165,0.641733093605496,0.725338245923956,0.468244547980482,0.914510355547201
"7247","KIF9-AS1",3.21516903664135,0.573496050174582,0.790403950538085,0.725573360032122,0.468100357306556,0.914510355547201
"7248","UBR5-AS1",2.47585166630976,-0.606960553001481,0.836582229857624,-0.725524080406032,0.46813057745613,0.914510355547201
"7249","BDNF-AS",9.66683417759148,0.324862808910317,0.447910074375666,0.725285782783826,0.468276725907414,0.914510355547201
"7250","DLG2",9.45610987218798,0.366594013690019,0.505466393991771,0.725258925316381,0.468293199234835,0.914510355547201
"7251","SIRT6",16.5669061940298,-0.311676950456462,0.429813817303335,-0.725144092416417,0.468363636894654,0.914510355547201
"7252","GPR173",4.7229087999653,0.474107087670523,0.653219641945671,0.725800415704516,0.467961132052122,0.914510355547201
"7253","WAC",161.032187957313,0.176902341101152,0.244044176023411,0.724878355975117,0.468526660173198,0.914576441694581
"7254","DGKE",6.10282915972062,0.411375115769069,0.56747812722631,0.724918011870813,0.468502330187006,0.914576441694581
"7255","AL445426.1",25.7261676811446,0.280145470669986,0.387007814034457,0.723875489100702,0.469142179112801,0.914602345600504
"7256","SUPT7L",24.4791436528635,0.226459287964332,0.312788310209874,0.724001762765313,0.469064652864015,0.914602345600504
"7257","MTX2",59.6428310662942,0.203219382113111,0.281096890572075,0.722951369897861,0.469709761665523,0.914602345600504
"7258","TRNT1",22.0509278318381,-0.275149598452057,0.380558408694838,-0.723015421983997,0.469670409431354,0.914602345600504
"7259","FRAS1",1.60593808053176,1.16478323944169,1.60799938792829,0.724367961944544,0.46883986341584,0.914602345600504
"7260","ADAMTS12",33.790307109432,-0.206934254908944,0.286184876959385,-0.723078931030698,0.469631392628294,0.914602345600504
"7261","AC146507.3",2.54851575011785,0.721903812429624,0.997002562148927,0.724074179783091,0.469020195328331,0.914602345600504
"7262","RPS18",8399.4151593543,-0.188270052206173,0.260112479127918,-0.723802459756594,0.469187019021016,0.914602345600504
"7263","FAM184A",13.7101744621259,-0.322093199724895,0.445170649119208,-0.72352748403825,0.469355874964655,0.914602345600504
"7264","FZD3",3.57407864795748,0.532703240765483,0.736386746547268,0.723401450750159,0.469433280190253,0.914602345600504

"7265","AP000866.1",3.52145127880394,-0.553129604192353,0.764037239445
248,-0.723956340916013,0.469092538983762,0.914602345600504
"7266","TSC22D1-
AS1",1.86510968689899,0.760793961588251,1.05114338142584,0.72377753124
0564,0.469202325635265,0.914602345600504
"7267","PSMC1",156.695738564017,-0.152127311958996,0.210238695407815,-
0.723593302669155,0.469315454275222,0.914602345600504
"7268","AC007906.2",6.10541454901737,-0.430051524257571,0.594598567524
453,-0.72326363995131,0.469517926827551,0.914602345600504
"7269","PIGL",15.8269531969986,0.25952177490063,0.358826120714936,0.72
3252182376108,0.469524964719915,0.914602345600504
"7270","USP36",33.205287895655,0.208297462479233,0.288065945533187,0.7
23089506792241,0.469624895579843,0.914602345600504
"7271","RNF138",15.6597226668571,0.280278580408325,0.386747697821884,0
.72470652569316,0.468632090872929,0.914602345600504
"7272","LIPG",5.59037901055381,0.447094697312537,0.618599704390244,0.7
22752846694681,0.469831741659468,0.914602345600504
"7273","COMMD7",64.9850604501374,-0.180897252779445,0.250009293755967,
-0.723562112678971,0.469334608503972,0.914602345600504
"7274","LINC00894",3.50299558792607,0.643151588208537,0.88982757945487
6,0.722782259235596,0.469813668402598,0.914602345600504
"7275","TUT4",79.8550552297501,0.141984077582372,0.196524660023577,0.7
22474612424406,0.470002728535019,0.914809434509397
"7276","SLC25A25",23.3417103026913,-0.39097166630163,0.541451003449224
,-0.722081340344758,0.470244469701468,0.915154163135348
"7277","AKR7A2",156.737559314526,-0.205306710568608,0.288806837645728,
-0.710878981405742,0.477159231848856,0.915168480995574
"7278","AL139260.1",2.55433760733478,0.640572263507058,0.9025186265781
52,0.709760712569171,0.477852535842232,0.915168480995574
"7279","NSUN4",17.0312371864557,0.248389726585056,0.349866746743096,0.
709955229804809,0.477731899540162,0.915168480995574
"7280","CPT2",19.757665517162,-0.255063587760247,0.353535629321501,-0.
721465014006539,0.470623458622754,0.915168480995574
"7281","GBP2",121.874729444232,0.164659333579995,0.23282551465481,0.70
722203201879,0.479428509030879,0.915168480995574
"7282","PRPF38B",101.318414904711,-0.141928492152039,0.197424239263888
,-0.718901046199954,0.472201891808024,0.915168480995574
"7283","LINC02802",162.583033464207,-0.189951916160657,0.2674374800016
63,-0.710266624406855,0.477538812551516,0.915168480995574
"7284","RO60",89.9809273897553,0.121968633541443,0.169749773724908,0.7
18520153900775,0.472436625779579,0.915168480995574
"7285","EIF2D",45.882046405854,-0.171354301616875,0.238075274993776,-0
.719748414115473,0.471679911650674,0.915168480995574
"7286","SNX17",121.784726048322,-0.159068779828225,0.223466045953795,-
0.71182527595765,0.47657297875936,0.915168480995574
"7287","CYP1B1",136.411167541447,-0.330245744324358,0.462344119997283,
-0.714285593869559,0.475050598498935,0.915168480995574
"7288","PPM1B",40.6349851260593,0.170307235391729,0.240809977259992,0.
707226657838417,0.479425634817349,0.915168480995574
"7289","RAB11FIP5",15.1318810586604,-0.269179923358017,0.3785587964083

66,-0.711065033785774,0.477043936591195,0.915168480995574
"7290","RETSAT",16.3902257165525,-0.262651138610683,0.370024664419311,
-0.709820625127429,0.47781537731101,0.915168480995574
"7291","GCC2",218.790573829365,0.121289197887347,0.170375571686476,0.7
11893123449305,0.476530960708692,0.915168480995574
"7292","CCDC138",4.81808537529313,0.528056268618837,0.738188864064431,
0.715340334059478,0.474398771239007,0.915168480995574
"7293","MARCO",2.07957131634107,-0.715122200807672,1.00382114241327,-0
.712400018880315,0.476217103854212,0.915168480995574
"7294","RAB3GAP1",57.0871876475922,0.1487333576051,0.207555948116619,0
.71659405068715,0.473624616460313,0.915168480995574
"7295","SPOPL",35.0193893840027,0.223179809848319,0.310631598146986,0.
718471047954092,0.472466893167425,0.915168480995574
"7296","AC092620.1",2.00569649084358,0.707159832119268,0.9874385652881
42,0.716155776144832,0.473895166658909,0.915168480995574
"7297","IFIH1",22.7024264972426,0.232373202818067,0.323511554980931,0.
71828408982722,0.472582138150741,0.915168480995574
"7298","ITGAV",173.782345656899,-0.152921584783599,0.215363981944069,-
0.710061094725272,0.477666250905843,0.915168480995574
"7299","UBP1",23.9890591344034,-0.24813617238462,0.347986923627993,-0.
713061771970155,0.475807533585874,0.915168480995574
"7300","CCDC12",79.3091469536802,-0.16612804082526,0.2318156344367,-0.
716638639274449,0.473597096348102,0.915168480995574
"7301","SFMBT1",10.1053446799864,-0.307208944026087,0.430655434594422,
-0.713352066055795,0.475627926593434,0.915168480995574
"7302","SHQ1",11.6830858870058,0.321363808488223,0.452926349339467,0.7
09527738796583,0.477997044159658,0.915168480995574
"7303","CNBP",407.956663928323,-0.111385703142757,0.155785500641416,-0
.714994031435201,0.474612731355195,0.915168480995574
"7304","PIK3R4",14.754443869902,0.249971611952991,0.352858490353356,0.
708418867015689,0.47868517941698,0.915168480995574
"7305","ABCC5",23.1251530048411,0.230893207442047,0.324878852881636,0.
710705561147032,0.477266712808258,0.915168480995574
"7306","ABCF3",23.1669060404826,-0.243120534488549,0.337379053651074,-
0.720615378629848,0.471146189334213,0.915168480995574
"7307","APOD",9809.13777884228,0.222802321172393,0.309372370553362,0.7
20175239869921,0.471417106735831,0.915168480995574
"7308","UBA6-
AS1",9.25759133973994,0.351878692779814,0.492012834928495,0.7151819379
4874,0.474496628346338,0.915168480995574
"7309","RAB33B",23.4008879568141,0.226110355354253,0.313481189556694,0
.721288430970943,0.47073207342309,0.915168480995574
"7310","WDR41",48.1848886305777,-0.165048566916501,0.22897675247857,-0
.720809274871464,0.471026868172394,0.915168480995574
"7311","DMXL1",40.9099123017519,0.168791253430751,0.236564239432989,0.
713511280637003,0.47552943520372,0.915168480995574
"7312","SPATA24",2.33823577360032,-0.667736862082881,0.943713631983221
,-0.707563014300882,0.479216667802514,0.915168480995574
"7313","TTC1",84.2782192728378,-0.144590401399929,0.204006493541946,-0
.70875391704235,0.478477199574982,0.915168480995574

"7314","CASC15",3.78754081637576,-0.511350050017475,0.712205753735662,-0.717980790432177,0.472769131296937,0.915168480995574
"7315","HLA-B",1691.3216129041,0.23229026776685,0.328266024543591,0.707628113783075,0.479176229415749,0.915168480995574
"7316","RCAN2",34.5237372341701,-0.321054936186556,0.450371962457331,-0.712866170520047,0.475928574546471,0.915168480995574
"7317","PLA2G7",2.53693824460321,0.687855312530336,0.954634317090061,0.720543249091519,0.471190581145387,0.915168480995574
"7318","NT5DC1",21.3034420323955,-0.218063552330593,0.308072898138602,-0.707831015477664,0.479050203234027,0.915168480995574
"7319","SAMD5",1.79085338393975,0.836308685176764,1.17036538960737,0.714570588470091,0.474874423972432,0.915168480995574
"7320","SYNE1",172.90003494471,0.191844231627377,0.266230415063166,0.720594720861854,0.471158902832054,0.915168480995574
"7321","CAHM",2.25644847999907,-0.682084354140012,0.953554249834325,-0.715307340152404,0.474419153958571,0.915168480995574
"7322","AC004130.2",7.81239132785815,-0.343731407510195,0.482118059074521,-0.71296107051046,0.475869846973903,0.915168480995574
"7323","ST7",19.2231899979117,0.246615409105764,0.346010911929178,0.712738820087392,0.476007389878525,0.915168480995574
"7324","DNAJB6",150.810973984684,-0.115728395420712,0.162704464957844,-0.711279776192351,0.476910881301681,0.915168480995574
"7325","PRAG1",1.74197922463724,-1.06100420043753,1.49228148833887,-0.710994680781427,0.477087532024609,0.915168480995574
"7326","BMP1",77.8068013375078,-0.209121592033715,0.29532343697321,-0.708110382897533,0.478876712321123,0.915168480995574
"7327","LYN",7.75941433728334,-0.403992987807942,0.570410242420454,-0.708249883616494,0.478790093350537,0.915168480995574
"7328","TERF1",85.2975819982301,0.144755808037896,0.202120924600048,0.716184176993724,0.473877632025713,0.915168480995574
"7329","STAU2",20.8249600166273,0.248027897296038,0.345396291576063,0.718096584547195,0.472697735956135,0.915168480995574
"7330","TSNARE1",2.18836565780381,-0.653958155135879,0.913302330849822,-0.716036884004637,0.473968574324592,0.915168480995574
"7331","DNAJB5",4.94486955019539,-0.513537797362328,0.720292756761974,-0.712957047730011,0.475872336336407,0.915168480995574
"7332","CLTA",386.105647179746,-0.182504377343789,0.257990022865301,-0.707408663780291,0.479312554442896,0.915168480995574
"7333","BX664615.2",4.05600196017506,0.634161941998162,0.893680180698092,0.709607257377903,0.47794771791173,0.915168480995574
"7334","UGCG",95.5262903601571,-0.261489994069569,0.365583947413656,-0.715266619115787,0.474444310947426,0.915168480995574
"7335","ZBTB43",34.6438597929068,0.247720586282809,0.344249754612498,0.71959553482225,0.471774062045464,0.915168480995574
"7336","STXBP1",37.6278354830338,0.181459686258496,0.253743070566329,0.715131592967234,0.474527733794619,0.915168480995574
"7337","ABO",1.67999596553579,-0.783254886363143,1.09526185031776,-0.715130255048966,0.474528560437421,0.915168480995574
"7338","USP6NL",7.68399645704459,-0.34685337101435,0.482176206344696,-

0.719349827822888,0.471925401891377,0.915168480995574
"7339","JMJD1C",202.344412870355,-0.157713740313706,0.22137512850312,-0.712427549472808,0.476200060850363,0.915168480995574
"7340","ABCC2",41.3014300304294,0.56794589682421,0.788565934148385,0.720226264196367,0.471385695470601,0.915168480995574
"7341","PDCD11",29.1033308233223,0.28839924142327,0.405664192904653,0.710930978054194,0.477127008382181,0.915168480995574
"7342","AL133482.1",10.4327081869417,0.450672967422152,0.627798491789001,0.717862456371781,0.472842098828083,0.915168480995574
"7343","AP006623.1",6.0870288653483,0.477883130616371,0.662499565241253,0.721333500713088,0.470704350073377,0.915168480995574
"7344","MTCH2",40.3655050342744,-0.238068295701094,0.33275245815506,-0.715451651419976,0.474330006014506,0.915168480995574
"7345","FAM111A-DT",8.87993502372403,0.315118779305033,0.444015041893332,0.709702937002606,0.477888370480615,0.915168480995574
"7346","AP003068.2",2.74773136921065,0.555848214238418,0.775816378345443,0.716468780182053,0.473701938223071,0.915168480995574
"7347","MALAT1",56101.6332067868,0.116281246226053,0.161319314609424,0.720814159839362,0.471023862243624,0.915168480995574
"7348","PANX1",25.7077050263788,-0.281760794618328,0.396743724188276,-0.710183368860595,0.47759043278011,0.915168480995574
"7349","CEP164",46.4182682355291,-0.206286455226289,0.287322503824265,-0.717961358684455,0.47278111293869,0.915168480995574
"7350","TMEM25",8.49423222800198,-0.358284885536172,0.498526131077015,-0.718688275702085,0.472333008784396,0.915168480995574
"7351","AC022509.1",2.04721630218786,0.989450207820313,1.39688088421415,0.708328261200991,0.478741430791962,0.915168480995574
"7352","KRT8",1.7518576011704,-0.863833335252786,1.21168947741785,-0.712916428963006,0.475897472295452,0.915168480995574
"7353","RAB5B",51.9086510070124,0.174790637139923,0.243093241517131,0.71902713563349,0.472124200342684,0.915168480995574
"7354","LEMD3",30.3467256631785,0.194201701238815,0.269888758835439,0.71956202280076,0.471794701738388,0.915168480995574
"7355","MRPL42",72.0681018055633,-0.182989751658373,0.257223234955856,-0.711404440931541,0.476833647832869,0.915168480995574
"7356","ATXN2",68.2411838000417,0.156504417447621,0.219338150258533,0.713530305891381,0.475517666780332,0.915168480995574
"7357","OAS1",27.8757369885876,-0.30687418815908,0.432413887971202,-0.709676993953342,0.477904461840043,0.915168480995574
"7358","ZNF26",14.1763298282827,0.307028058909296,0.432219126971697,0.710352781147052,0.477485396734116,0.915168480995574
"7359","TDRD3",44.0561762026456,0.189320980762734,0.266655080611508,0.709984525059763,0.477713732561447,0.915168480995574
"7360","TM9SF2",122.471942428522,0.117167899759972,0.164625354142193,0.711724511515823,0.476635386070313,0.915168480995574
"7361","SLC7A8",14.3144696555948,0.32696982201804,0.453415790613727,0.72112579399907,0.470832122351241,0.915168480995574
"7362","DNAAF2",17.8729323082274,-0.26385548948596,0.372272348458949,-0.708770045850063,0.478467188981843,0.915168480995574

"7363","KNSTRN",5.52911168771341,0.422365475308241,0.586174540049134,0
.72054558233259,0.471189145126191,0.915168480995574
"7364","MYO1E",21.9357153727201,0.301366368677547,0.419651488823301,0.
718134873112391,0.472674129627465,0.915168480995574
"7365","THSD4",5.26123742565453,-0.46303812285208,0.647966263069389,-0
.714602208853727,0.474854879475671,0.915168480995574
"7366","PML",77.6129686656329,-0.197851729101684,0.275404543246245,-0.
718404013127628,0.472508213086549,0.915168480995574
"7367","SCAPER",29.9643915078224,0.255152959421444,0.359690964776106,0
.709367163504559,0.478096659258272,0.915168480995574
"7368","HYKK",9.72878958127015,-0.360971678103317,0.501509569050016,-0
.719770270360119,0.471666452374201,0.915168480995574
"7369","SPSB3",74.8069038003777,-0.156874103393653,0.218777082919945,-
0.717049982109215,0.473343256696267,0.915168480995574
"7370","CCNF",3.18464225857888,0.505971735200612,0.715436873507753,0.7
07220656268186,0.479429363843585,0.915168480995574
"7371","CIITA",3.09992448478209,-0.538647745111732,0.760171170358144,-
0.708587442033557,0.47858053154151,0.915168480995574
"7372","GINS3",4.12255520555516,0.519345338263881,0.728594864731553,0.
712804005906947,0.475967046428302,0.915168480995574
"7373","DDX28",4.66514789221985,-0.457247481523443,0.638891715236465,-
0.715688544113,0.474183686069189,0.915168480995574
"7374","AC004943.2",1.91345828304474,0.679451594115788,0.9504495802149
7,0.714873895743224,0.474686968544734,0.915168480995574
"7375","GINS2",3.72978825567439,0.548362763802083,0.76425462870629,0.7
17513173234341,0.473057511075163,0.915168480995574
"7376","CHMP1A",52.2087337218505,-0.182921135207867,0.258380831210194,
-0.707951647771658,0.478975284752868,0.915168480995574
"7377","RPH3AL",3.14896387842777,0.685459337911022,0.961870509916738,0
.71263161812743,0.476073741159575,0.915168480995574
"7378","SCARF1",3.33780094463012,0.570310470429122,0.797534215722183,0
.715092166814052,0.474552093869807,0.915168480995574
"7379","METTL16",29.4973477085193,0.235508617387801,0.330622190432312,
0.712319451637097,0.47626698148424,0.915168480995574
"7380","NTN1",3.03757378760134,0.6740449313932,0.950166702682754,0.709
396497993525,0.478078460355991,0.915168480995574
"7381","CCL4",53.1757786081206,-0.368782128795907,0.514929043151658,-0
.716180479039891,0.473879915115585,0.915168480995574
"7382","TACO1",21.7450603024488,-0.228508195564733,0.321146038044117,-
0.711539824549672,0.476749781461626,0.915168480995574
"7383","SUMO2",912.886076910061,-0.173277619317577,0.240919054722604,-
0.719235842582442,0.471995618616056,0.915168480995574
"7384","MAFG-
DT",3.22451042347943,-0.672388813270783,0.945289490597775,-0.711304653
186805,0.47689546872569,0.915168480995574
"7385","GATA6",23.758587185592,0.378719970389406,0.528160989618797,0.7
17054038130967,0.473340754098038,0.915168480995574
"7386","RIOK3",69.3257979951518,-0.209442742996368,0.291707737754182,-
0.717988300923519,0.472764500362676,0.915168480995574
"7387","ZNF521",64.2144782857854,0.186284708776068,0.260813043867213,0

.71424613590611,0.475074992973958,0.915168480995574
"7388","GAREM1",39.5795586594892,0.186972194200393,0.263792224482571,0
.708785842975999,0.478457384363043,0.915168480995574
"7389","AC120049.1",3.47435168392822,0.551018612230113,0.7776463001502
78,0.708572280384578,0.478589943067277,0.915168480995574
"7390","CFAP53",1.94561697902683,0.70734702078039,0.987533511200691,0.
716276473413407,0.473820650844534,0.915168480995574
"7391","RELCH",15.7424198037455,0.244601269914222,0.342737762618948,0.
713668864630381,0.47543196352527,0.915168480995574
"7392","ZNF57",2.78775760638481,0.578627953661634,0.80194254881377,0.7
21532925915377,0.470581690239472,0.915168480995574
"7393","DNM2",34.1626715239658,0.198671096036743,0.277793414068484,0.7
15175688030403,0.474500489772849,0.915168480995574
"7394","MAP1S",24.9747016946505,0.2297800243571,0.32187035034516,0.713
889999842154,0.475295201490942,0.915168480995574
"7395","NDUFA13",582.333723045697,-0.181719224188516,0.253398356145316
,-0.717128662367115,0.47329471163958,0.915168480995574
"7396","CEBPA",30.8467141609024,-0.20549516541983,0.288434191517882,-0
.712450782406945,0.476185678601739,0.915168480995574
"7397","FXYD1",438.745352217227,0.175983613390701,0.248438643672843,0.
708358453375092,0.478722685994476,0.915168480995574
"7398","ECH1",188.70822212316,-0.183539924329992,0.254883497945345,-0.
720093398786252,0.471467491623947,0.915168480995574
"7399","LIPE-
AS1",5.39633823016158,-0.4112486458852,0.572699920210369,-0.7180874859
10837,0.472703345700291,0.915168480995574
"7400","DBP",31.134709903835,-0.412312251708622,0.577611122050319,-0.7
13823255766019,0.475336477380892,0.915168480995574
"7401","AC022137.4",3.84457513360318,-0.657858129426687,0.922140768734
634,-0.713403150290603,0.475596324265884,0.915168480995574
"7402","TMEM238",4.10911851775984,-0.478158306621121,0.672435235542102
,-0.711084549630479,0.477031843653118,0.915168480995574
"7403","NOP56",89.4599061066709,0.125778354573576,0.177827569755222,0.
707305142541781,0.479376870450206,0.915168480995574
"7404","MAPRE1",64.9290729542015,-0.18484803933548,0.259267007279241,-
0.712963987494139,0.475868041901039,0.915168480995574
"7405","RBM39",389.981277853518,0.109146183906198,0.154174013016914,0.
707941512129054,0.478981579230292,0.915168480995574
"7406","ATP5PO",298.439569760426,-0.197337365093245,0.278317065397349,
-0.709037962912944,0.478300918866309,0.915168480995574
"7407","C21orf58",5.60854991369229,0.509414772513154,0.708009315062036
,0.71950292415082,0.471831101172844,0.915168480995574
"7408","Z95115.1",2.63604486244941,0.585304724327472,0.818391533868663
,0.71518912415998,0.474492188465402,0.915168480995574
"7409","THOC5",23.9611923718133,-0.217417445324221,0.305283357137825,-
0.712182437203953,0.476351811056777,0.915168480995574
"7410","RAC2",4.31398718980936,-0.475721965909148,0.666951056708654,-0
.713278674835294,0.475673330745458,0.915168480995574
"7411","SH3BP1",7.17393743425734,0.391365798034056,0.545682427566753,0
.717204326661554,0.473248029990501,0.915168480995574

"7412","ST13",369.746407102868,-0.12399666639842,0.175009207557018,-0.708515101172732,0.478625437716871,0.915168480995574
"7413","AL035681.1",3.88503795819361,0.552651898347061,0.768565778901601,0.719069094042784,0.472098348744501,0.915168480995574
"7414","NR0B1",2.37887367001751,-0.966700047410238,1.35492552087429,-0.713470986055719,0.475554360690591,0.915168480995574
"7415","IQSEC2",14.0816153587683,-0.297489361852989,0.418769520968259,-0.710389240279826,0.477462793632326,0.915168480995574
"7416","FOXO4",3.47598823511316,0.639614923232781,0.890612889768117,0.7181738896675,0.472650075133566,0.915168480995574
"7417","ZDHHC15",6.70320564104733,0.388630572942062,0.541394097923999,0.717833043308533,0.47286023656817,0.915168480995574
"7418","PAK3",39.6795160006588,0.244648837297732,0.344206777874756,0.710761243018725,0.477232201318899,0.915168480995574
"7419","AC019171.1",1.98391378699088,0.793017412416707,1.12171609296255,0.706968026394519,0.47958634768983,0.915344747713707
"7420","EHMT2",36.4753365679535,0.19434392735303,0.27498851099002,0.706734716491784,0.479731351039024,0.91549810386962
"7421","AC012184.3",1.56278418014693,0.890851377853639,1.26110695663026,0.706404300737535,0.479936747102611,0.91576665395135
"7422","POP5",45.4160949460456,-0.159375798496523,0.225653686454081,-0.706284931573472,0.480010962244567,0.915784859253984
"7423","PGLS",231.58165449568,-0.157412064224421,0.222957972208928,-0.706016755825686,0.480177717413052,0.915979587574945
"7424","PEF1",48.8616819113583,-0.203598942919526,0.288452127058226,-0.705832697425137,0.480292185593958,0.916074535022959
"7425","MUL1",26.3876918209963,0.219477313472066,0.315282799980698,0.696128407529691,0.486348417621674,0.91616862044142
"7426","ZBTB8A",44.0301716022442,0.181637606609015,0.260626634257539,0.696926494586616,0.485848795990628,0.91616862044142
"7427","MACF1",176.257413506525,0.181867387703537,0.258560217490255,0.703385035288313,0.48181582989767,0.91616862044142
"7428","CEPT1",27.8252790143317,0.215234109440644,0.305875817884982,0.703665006697517,0.48164141753321,0.91616862044142
"7429","DENND2C",13.5448244224198,0.276001515623729,0.392617242902777,0.702978589486134,0.48206909250027,0.91616862044142
"7430","MTX1",62.1490188241291,-0.195771171649807,0.27895898765795,-0.701791949036807,0.482808920351796,0.91616862044142
"7431","Z99127.4",5.51842075544356,-0.395880635989547,0.566353156680468,-0.698999610613805,0.484552274182439,0.91616862044142
"7432","AC092803.1",4.97230441785582,-0.451294742527346,0.646497695656929,-0.69806086790266,0.485139129211208,0.91616862044142
"7433","WDR26",49.7345579034859,-0.17496985979729,0.24865947336382,-0.703652498858497,0.481649208744935,0.91616862044142
"7434","ELMOD3",22.6578210132426,0.212714352293592,0.305350429595511,0.696623720409919,0.486038307186973,0.91616862044142
"7435","PLGLB2",1.52364655238636,0.835267567201609,1.18976522583115,0.702044024373047,0.482651708823559,0.91616862044142
"7436","TMEM177",8.50717649210385,0.368108567936624,0.528423809576271,0.696616165406704,0.486043036495467,0.91616862044142

"7437","RBMS1",285.542786474132,0.127922831106519,0.183502056253732,0.697119333254978,0.485728116028629,0.91616862044142
"7438","NPPC",3.16781168324942,1.8257623268078,2.59080947143209,0.704707292041276,0.480992412426499,0.91616862044142
"7439","AC024060.2",2.39008055929154,-0.597677357585774,0.856434964239933,-0.697866601133221,0.485260623137708,0.91616862044142
"7440","PPARG",57.2376198682623,-0.288864680284651,0.410176819920335,-0.704244282601718,0.481280658061832,0.91616862044142
"7441","SNRK",33.555786027329,0.222157633020111,0.316622173983807,0.701649004000183,0.482898083073172,0.91616862044142
"7442","LRRC58",79.0557509176137,0.154804353888239,0.221360462318135,0.699331543976259,0.484344858143526,0.91616862044142
"7443","SLC41A3",37.8522646658224,0.197567607491919,0.283689794782184,0.696421271140934,0.486165045721949,0.91616862044142
"7444","AC092958.1",2.54449207040599,0.766792327560114,1.09694591421763,0.699024735514888,0.484536572638185,0.91616862044142
"7445","IGFBP7",6107.05381148124,-0.237078209249731,0.337242162934963,-0.702991011522635,0.482061351066864,0.91616862044142
"7446","CXCL1",30.7826856520566,1.17141675780848,1.66400433222482,0.703974584154038,0.481448601639057,0.91616862044142
"7447","LINC01091",26.4517865061818,0.240778581383799,0.342396116143304,0.703216450279552,0.481920869012709,0.91616862044142
"7448","SDHA",95.4780204898491,0.121036569825906,0.171962380892443,0.703854931513251,0.481523120595279,0.91616862044142
"7449","DNAJC21",95.876676780477,-0.179104558221833,0.256068692244284,-0.699439500596859,0.484277409391651,0.91616862044142
"7450","LNPEP",65.287471148731,0.163794701990889,0.234603773089632,0.69817590669486,0.485067192019517,0.91616862044142
"7451","SAR1B",70.025748725804,-0.14066752503563,0.200465477353361,-0.701704487439875,0.482863473922692,0.91616862044142
"7452","NRN1",50.5634101640834,-0.217607505760628,0.311504502712187,-0.698569374972038,0.484821188235292,0.91616862044142
"7453","ZKSCAN4",6.693729306574,-0.347820066896115,0.499613130114207,-0.696178795014068,0.486316865635274,0.91616862044142
"7454","ZSCAN31",3.24581393392979,0.573496122330536,0.821291219923633,0.698285953165142,0.484998382082697,0.91616862044142
"7455","PSORS1C1",10.4041216645253,0.306588640172747,0.437614473047553,0.70059072324016,0.48355846942011,0.91616862044142
"7456","TCF19",9.61512656498415,0.296234044839552,0.424610981399829,0.697659876489645,0.485389926276557,0.91616862044142
"7457","DAXX",49.208387011983,0.166540814468926,0.236121427111966,0.705318515587127,0.480612040395269,0.91616862044142
"7458","RPL10A",3185.84959313382,-0.177524849204364,0.253550089384064,-0.700156918246868,0.483829313148862,0.91616862044142
"7459","COQ3",9.96258421974628,-0.357963274126567,0.509404266309474,-0.702709611601675,0.482236736535456,0.91616862044142
"7460","SOD2",856.035520205138,-0.233453289219162,0.335057533702197,-0.696755827692141,0.485955614211137,0.91616862044142
"7461","ERMARD",15.1796195564521,0.307213924149841,0.43981060721811,0.698514131100727,0.484855723665674,0.91616862044142

"7462","DNAAF5",21.3313328157386,0.232013020259527,0.329427570790261,0
.704291446228844,0.481251292135948,0.91616862044142
"7463","TOMM7",924.439995311567,-0.150288855024236,0.213300372303098,-
0.704587870154662,0.481066749294215,0.91616862044142
"7464","SNHG26",14.3703401411436,-0.307819605592387,0.44108571961724,-
0.697867992324719,0.485259753031644,0.91616862044142
"7465","PLEKHA8",11.4423229394548,0.296615570449895,0.420355687998685,
0.705629967473694,0.480418283053282,0.91616862044142
"7466","MSR1",7.4224186774943,-0.419807147899124,0.597265461736795,-0.
702882009413975,0.482129283664732,0.91616862044142
"7467","CCDC171",17.4850344974839,0.248739538822144,0.356536517845522,
0.697655152760303,0.485392881115794,0.91616862044142
"7468","PKN3",1.52052162706446,-0.898628322059958,1.27751807169242,-0.
703417307333649,0.481795723794095,0.91616862044142
"7469","EXD3",9.23490745697242,0.339868368373994,0.482260293388906,0.7
04740516756407,0.480971732059682,0.91616862044142
"7470","PTEN",180.971804521512,0.165439985668293,0.237212268529974,0.6
97434355708242,0.485531007385873,0.91616862044142
"7471","ADRA2A",17.2230900229106,0.793018102869211,1.13704891164182,0.
697435347547318,0.485530386863393,0.91616862044142
"7472","CAT",145.066788449741,0.159882989309498,0.229521418003855,0.69
6592896209857,0.486057602788649,0.91616862044142
"7473","ARL2",223.181818008567,-0.183520192573509,0.260782840281082,-0
.703728022808952,0.481602165443431,0.91616862044142
"7474","SYVN1",35.7846913194404,-0.192277639687223,0.27268012354509,-0
.705139917011327,0.480723167564812,0.91616862044142
"7475","ANAPC15",23.0702211833437,-0.251027481107151,0.357340951103475
,-0.702487303321865,0.482375317039928,0.91616862044142
"7476","PAK1",23.5236760625657,-0.206511673447836,0.295146852555774,-0
.699691261009845,0.484120135207483,0.91616862044142
"7477","TULP3",73.6383434016174,0.159231901064924,0.226158910519305,0.
704070870784161,0.481388639453655,0.91616862044142
"7478","AC022075.1",1.77837011628601,-0.756817239887611,1.080196386301
51,-0.700629301750289,0.483534387127077,0.91616862044142
"7479","GPRC5A",78.171189623383,-0.519721902864339,0.742336877370448,-
0.700115969861729,0.483854883294929,0.91616862044142
"7480","ETNK1",51.6358372019347,-0.160113101165051,0.227525656145026,-
0.703714490391334,0.481610594500873,0.91616862044142
"7481","AC048341.1",11.1481562747192,0.34427710966461,0.49323077921181
4,0.698004107153992,0.485174625530626,0.91616862044142
"7482","ANKS1B",7.09064617006113,-0.378098213645352,0.539131980189012,
-0.701309192440775,0.483110078225752,0.91616862044142
"7483","CHST11",13.1199843961266,-0.321817509359326,0.459721833399241,
-0.700026594298918,0.483910696251853,0.91616862044142
"7484","CLIP1",109.054396622813,0.176556464216539,0.252760747054056,0.
698512194928671,0.484856934078142,0.91616862044142
"7485","PITPNM2",5.31532452728127,-0.406088066461734,0.582303252235678
,-0.697382446178364,0.485563484049909,0.91616862044142
"7486","XPO4",11.5172153863475,0.326532625297895,0.46372700140575,0.70
4148398320646,0.481340362386817,0.91616862044142

"7487","COG6",23.9709129263598,0.237382476157587,0.339512403294276,0.699186462274351,0.48443550979139,0.91616862044142
"7488","PIP4P1",28.7927664678393,-0.187894432064125,0.269636292172108,-0.696843998819685,0.48590042744697,0.91616862044142
"7489","IGHG1",21.3533978298994,0.455089878610944,0.650127244726117,0.700001241761008,0.483926528956965,0.91616862044142
"7490","C15orf41",8.33478867211281,0.36293343011957,0.520251732262372,0.697611190146957,0.485420381561235,0.91616862044142
"7491","CSK",25.0549585896445,-0.199994252769688,0.285666492962778,-0.700096993159596,0.483866733511315,0.91616862044142
"7492","HAPLN3",11.8331135831707,-0.350067137904791,0.502715433448031,-0.6963524781878,0.486208115977049,0.91616862044142
"7493","ERVK13-1",9.11236020811704,0.384565295562596,0.550275668096761,0.698859349701382,0.484639933839789,0.91616862044142
"7494","ZNF205",20.4121632231396,-0.231937107144585,0.332428667320249,-0.697704891140288,0.485361768704736,0.91616862044142
"7495","ZNF174",11.3532083919962,-0.317306272659973,0.452721403893376,-0.700886394880294,0.483373915642952,0.91616862044142
"7496","TMEM159",158.827418064321,0.146950110663287,0.210666868567773,0.697547325131539,0.485460333290573,0.91616862044142
"7497","TMEM265",8.26940812187804,-0.34967713925009,0.501444612521993,-0.697339507730285,0.48559034893727,0.91616862044142
"7498","CIAO2B",168.256865446446,-0.171434434336588,0.24599143516647,-0.696912208429422,0.485857737026002,0.91616862044142
"7499","CCL5",18.0996599198427,0.327659256666406,0.467539702307467,0.700815898733939,0.483417914802341,0.91616862044142
"7500","KAT2A",12.1308362879848,0.324308571805983,0.462773138274593,0.700793855527435,0.483431673199894,0.91616862044142
"7501","CACNA1A",8.05022090410474,0.410336530523586,0.586492936641992,0.699644454156598,0.484149373250984,0.91616862044142
"7502","ZNF714",9.04962214408122,0.368134075370271,0.5242810889034,0.702169281253821,0.48257360035792,0.91616862044142
"7503","ZNF708",29.4620781838927,0.187883095302149,0.269795675554984,0.696390314320879,0.486184427075861,0.91616862044142
"7504","PSENEN",94.9308269796435,-0.224953499609193,0.321227233096943,-0.700293986410872,0.483743726512432,0.91616862044142
"7505","CLASRP",22.1097821337173,0.230222269447367,0.326475790667558,0.705174092622986,0.480701901816309,0.91616862044142
"7506","BBC3",9.74935138374774,0.448183417366691,0.635769011784771,0.704946936794737,0.480843258975509,0.91616862044142
"7507","SPACA6",7.77172926212402,0.328685217717908,0.468588231865129,0.701437200865324,0.483030212839538,0.91616862044142
"7508","ZNF28",7.55500624663649,0.370239163106809,0.531426503610568,0.696689308100678,0.485997251410856,0.91616862044142
"7509","TSEN34",61.4755097694437,-0.203068621503925,0.289405754138005,-0.701674443581002,0.482882214346109,0.91616862044142
"7510","ZNF784",2.92863694933567,0.544264958917991,0.7744901811415,0.702739650121598,0.482218013057362,0.91616862044142
"7511","ZFX",72.8346224645051,0.158785049398305,0.226632501747017,0.700627880706854,0.4835352741893,0.91616862044142

"7512","DLG3",9.55269018744558,-0.339621780536201,0.481729043110985,-0
.705005823072111,0.480806612367216,0.91616862044142
"7513","ENOX2",20.7430315024248,-0.257154269129161,0.369415104872351,-
0.696111950316755,0.486358723153825,0.91616862044142
"7514","SMIM10",56.6122660606112,-0.165801350398189,0.237216538194704,
-0.698945156438043,0.48458630569676,0.91616862044142
"7515","RTL8B",24.7580058658435,-0.194192575045119,0.275362972479351,-
0.70522399325015,0.480670852109267,0.91616862044142
"7516","ZNF275",18.9417515918181,0.217678112251177,0.309547929160545,0
.703212949417859,0.481923050402792,0.91616862044142
"7517","DNASE1L1",3.98549187188775,-0.492723841698213,0.70665147853495
3,-0.697265705464509,0.48563652597323,0.91616862044142
"7518","DHX40",57.0500980857213,0.156448595378485,0.224859094611879,0.
69576280936524,0.486577383575315,0.916458599551272
"7519","CASP9",6.09703216379295,-0.429575612576662,0.617635765637917,-
0.695516089702126,0.486731931501347,0.916459321800935
"7520","BOLA1",33.2542358793109,0.229350494312607,0.329899349146728,0.
695213539844241,0.486921488282422,0.916459321800935
"7521","PPOX",15.8738145295376,0.25510846787484,0.367798536419181,0.69
3609252387271,0.487927289750394,0.916459321800935
"7522","PTPN4",17.165002400181,0.257259711554725,0.370511284919189,0.6
94337047280043,0.487470862858795,0.916459321800935
"7523","DHX16",20.3718079228784,-0.245587919945377,0.353672952555919,-
0.694392709904914,0.487435964274318,0.916459321800935
"7524","KLHDC3",82.9736922677982,-0.15139494547307,0.218258296544159,-
0.693650357719342,0.487901504946004,0.916459321800935
"7525","LONRF1",27.9318379819059,0.211231594749554,0.304049959351831,0
.694726600851567,0.487226654250556,0.916459321800935
"7526","ZNF169",3.41826254152857,-0.522920924363869,0.75275257478264,-
0.694678360303003,0.487256892353105,0.916459321800935
"7527","ADAMTS14",1.76234136428696,0.8499976551362,1.22559702570193,0.
693537628854302,0.487972219955191,0.916459321800935
"7528","NAALADL1",36.5942347365338,-0.170360701339189,0.24531364586693
6,-0.694460761598222,0.487393299995279,0.916459321800935
"7529","NDUFC2",342.378693540464,-0.171833338733963,0.247309148471389,
-0.694811897562466,0.487173191113634,0.916459321800935
"7530","LPCAT3",35.3383404144804,0.191634721879274,0.276036099734021,0
.694237898825284,0.48753302892985,0.916459321800935
"7531","GABARAPL1",107.870251993809,0.155643836523164,0.22400723555054
4,0.694816112259218,0.487170549465434,0.916459321800935
"7532","RFC3",9.26700309724,0.338737763129784,0.487990917786309,0.6941
47679359305,0.487589600244353,0.916459321800935
"7533","KLF12",32.3151574554868,0.199391291486951,0.286824300571766,0.
695168753447589,0.48694955172432,0.916459321800935
"7534","UBE2Q2",66.1754000741339,-0.136507652755884,0.196710817341895,
-0.69395092044494,0.48771298846195,0.916459321800935
"7535","DDX19B",36.7757419294952,-0.196408150336892,0.282525467585089,
-0.695187418025382,0.486937856274596,0.916459321800935
"7536","NT5C3B",57.7204299180827,-0.147358319508337,0.212506167895535,
-0.693430788233762,0.488039246361764,0.916459321800935

"7537","RMC1",10.5863412002702,-0.304366213914759,0.438571600685789,-0
.693994352207998,0.487685750799118,0.916459321800935
"7538","SMARCA4",42.7520828209568,0.207132047709366,0.298724781487613,
0.693387561212277,0.488066366221811,0.916459321800935
"7539","BAX",83.7190322620292,-0.189969886126852,0.273705370285354,-0.
694067076319321,0.487640144660984,0.916459321800935
"7540","ZNF544",6.69754599772689,-0.367312587673139,0.529406144325044,
-0.693820031389013,0.487795078788409,0.916459321800935
"7541","AAR2",20.6849930076944,0.207066737821483,0.298196677614977,0.6
94396528752885,0.487433570035157,0.916459321800935
"7542","SLC2A1",3.1062775944584,-0.527872692341654,0.763567131170441,-
0.691324535581436,0.489361616270399,0.916586459833353
"7543","AC244033.2",3.51439388697618,0.484878891472236,0.7016941216490
84,0.691011762123216,0.489558149415229,0.916586459833353
"7544","ABI2",75.9780679443955,-0.158923038395003,0.229974342835043,-0
.691046820422905,0.489536118200749,0.916586459833353
"7545","GOLIM4",208.683119435024,0.165216405903721,0.238692722631032,0
.692171944257848,0.488829355310586,0.916586459833353
"7546","ARSJ",8.86674906488454,0.399790402598873,0.57787308869405,0.69
1830802334764,0.489043590468813,0.916586459833353
"7547","FCHO2",50.2285841957905,0.168106963730659,0.243186623398593,0.
691267313067318,0.489397569216983,0.916586459833353
"7548","C6orf136",9.49453634872157,0.327405737579348,0.473138474081458
,0.691987135933022,0.488945407593885,0.916586459833353
"7549","AL356417.2",5.11608401650989,-0.528837713125833,0.763912772978
73,-0.692274997659396,0.48876464831651,0.916586459833353
"7550","COG5",25.916298062709,0.204711109471526,0.295664934040341,0.69
2375340809252,0.488701647518851,0.916586459833353
"7551","PIWIL2",1.67688036551321,-0.760029769281661,1.09853637960897,-
0.69185671352295,0.489027316617491,0.916586459833353
"7552","PNPLA2",39.7671950284601,-0.182180053193746,0.26335930517309,-
0.691754760949153,0.489091350912572,0.916586459833353
"7553","OTUB1",54.95453624347,-0.148144717864938,0.21434277424777,-0.6
91157975279773,0.489466270182002,0.916586459833353
"7554","SCN4B",10.8122478387787,0.449396287258401,0.650254627257265,0.
691108172738312,0.489497564676905,0.916586459833353
"7555","LINC02397",2.35081660389667,-0.699243688889623,1.0093018828439
1,-0.69279935049696,0.488435479880108,0.916586459833353
"7556","MTERF2",34.2561221221121,0.209647919601612,0.303158440800132,0
.691545711372194,0.489222664735864,0.916586459833353
"7557","AL121821.2",2.389716949451,0.633226524031114,0.916084674064757
,0.691231435213763,0.489420112023446,0.916586459833353
"7558","IRX6",2.89375554814042,0.582531536436136,0.841804774680079,0.6
92003127040379,0.488935365227382,0.916586459833353
"7559","ZNF569",8.76434492528496,0.318509274976456,0.460774713681225,0
.691247296171744,0.489410146182564,0.916586459833353
"7560","TMEM143",4.56979374749063,-0.43851675927343,0.632767094296164,
-0.693014480724852,0.488300464026539,0.916586459833353
"7561","TMC4",2.71760975341055,0.639828659045926,0.924567113625739,0.6
92030518516718,0.488918163722239,0.916586459833353

"7562","RNF114",64.090497004562,-0.181363471399734,0.262027987979943,-0.692153051274874,0.488841218671878,0.916586459833353
"7563","AP000254.2",2.42239992207712,-0.567417325073078,0.820719756775488,-0.69136550008543,0.489335879116731,0.916586459833353
"7564","INTS11",58.2952196305488,-0.151847562718091,0.220229317128425,-0.689497496055636,0.490510248421499,0.917189708844217
"7565","GBP5",2.17508903041191,0.639453857561844,0.926249679316353,0.690368776196297,0.489962307035797,0.917189708844217
"7566","CPSF3",14.9009010331876,0.263840637373852,0.382458479640344,0.689854327774252,0.490285799871031,0.917189708844217
"7567","TRIM23",35.3385076509479,0.186852808986515,0.270984199351582,0.689533963358827,0.490487307810805,0.917189708844217
"7568","RNF208",3.04164884283813,0.54221672156314,0.7861750949937,0.689689517024811,0.490389459614376,0.917189708844217
"7569","IFT46",17.2563583522257,-0.27578467259841,0.399592282047073,-0.690165163314946,0.490090328117644,0.917189708844217
"7570","LCP1",10.0834523780087,-0.353493828294233,0.512704347741648,-0.689469145037089,0.490528083691897,0.917189708844217
"7571","TMEM102",2.38480298336704,-0.632733930308693,0.917564487823622,-0.689579793796815,0.490458477919017,0.917189708844217
"7572","SON",466.489025176714,0.141233886848204,0.204621605804701,0.690219814729648,0.490055964412918,0.917189708844217
"7573","DOCK11",47.0438910958179,-0.159713431375035,0.231577294751453,-0.689676557222294,0.49039761134095,0.917189708844217
"7574","SH2D4A",15.4047101017231,-0.258694691004264,0.37532746626771,-0.689250625798179,0.490665563104736,0.917325636858075
"7575","BRPF1",11.0015587552035,0.284299973747769,0.412668476214921,0.688930679550388,0.490866891762777,0.917580882819923
"7576","PPIE",59.4548698754049,0.185029913030647,0.268766605278619,0.688440860570584,0.491175200135129,0.918033316795596
"7577","ZRANB3",4.5067636129551,0.436569047158441,0.635315872275205,0.687168487692574,0.491976559763828,0.918033316795596
"7578","NCL",435.184946422548,-0.122643562780478,0.178484221459762,-0.687139522908064,0.491994810384679,0.918033316795596
"7579","EEFSEC",18.4665986593779,-0.244864086538222,0.356637687688553,-0.686590607193648,0.492340749134587,0.918033316795596
"7580","POU5F2",8.10392158266274,0.373418038333971,0.543414746080548,0.68716949811778,0.491975923104598,0.918033316795596
"7581","RANBP9",35.5337412910186,0.190739701780576,0.277252783684504,0.687963162157555,0.491475979574448,0.918033316795596
"7582","ZYX",127.933882836552,-0.167652862672479,0.243816239529056,-0.687619754107885,0.491692265116605,0.918033316795596
"7583","LARP4B",30.3016723145075,0.213584132788747,0.31071645500936,0.6873924098494,0.491835479367316,0.918033316795596
"7584","DNAJC9",28.1649279678942,0.189497244925452,0.275776440351569,0.687140803920284,0.491994003215176,0.918033316795596
"7585","HPSE2",4.26998189186828,-0.544083208993296,0.792159014242605,-0.686835848877516,0.492186176315488,0.918033316795596
"7586","MAPK8IP1",14.7470323242576,-0.267586544853405,0.389376698068246,-0.68721766397666,0.491945574771709,0.918033316795596

"7587","PLBD1-
AS1",2.88414308823214,0.538932964631201,0.783093694646198,0.688210067729751,0.491320504874681,0.918033316795596
"7588","RNPS1",108.058914319049,-0.123229604884987,0.17919711652857,-0.687676271092984,0.491656666030481,0.918033316795596
"7589","KNOP1",53.3993210474483,-0.207778564786942,0.302616441673811,-0.686606992130673,0.49233042109868,0.918033316795596
"7590","ELAC1",16.1237963729904,-0.297753315947252,0.433352004123015,-0.687093432392964,0.492023852624128,0.918033316795596
"7591","MCOLN1",36.7129223067645,-0.184218737652496,0.268251276921047,-0.686739462219656,0.492246924536215,0.918033316795596
"7592","AP1M1",47.1936825457437,0.207311320783995,0.301696829215441,0.687151142168468,0.491987489081118,0.918033316795596
"7593","LINC01684",7.93490436757493,0.33806532846053,0.492110957524034,0.686969723579096,0.492101807721115,0.918033316795596
"7594","LRRC75B",13.4455749248423,-0.255828496375004,0.371988387467702,-0.687732480351196,0.491621262148546,0.918033316795596
"7595","LYPLA2",21.4572929278066,-0.227435700798349,0.332045366243714,-0.684953695849547,0.493373140686317,0.918058191366878
"7596","E2F6",14.0431129935185,-0.301256364550468,0.439216658290218,-0.685894669212225,0.492779532012176,0.918058191366878
"7597","DCAF16",28.115240911693,0.184734250002883,0.269489444340227,0.685497164666896,0.493030249201325,0.918058191366878
"7598","PAQR8",7.47402112413838,0.344140486573344,0.50279695789745,0.684452205145471,0.493689660121908,0.918058191366878
"7599","MYO6",37.3982040742531,0.172589592017461,0.251847977220106,0.685292746531071,0.493159208018331,0.918058191366878
"7600","AP003469.4",2.69355531708169,-0.578100607479127,0.84423389023996,-0.684763563939386,0.493493131006017,0.918058191366878
"7601","OMD",145.659582041961,0.139683521103177,0.203957295679196,0.684866509128876,0.49342816140163,0.918058191366878
"7602","GAPVD1",40.7476640469539,-0.22039673121178,0.321257989635512,-0.686042801493698,0.492686118335827,0.918058191366878
"7603","AP001372.2",7.99194093738288,0.423503946115172,0.619146373482004,0.684012641039045,0.493967183508969,0.918058191366878
"7604","TBRG1",64.4572739017838,-0.145937029565853,0.212792213920169,-0.685819405124489,0.492826997924022,0.918058191366878
"7605","M6PR",69.1292486303715,-0.135448373142658,0.19798064009515,-0.684149586937193,0.493880712335346,0.918058191366878
"7606","INTS13",17.4725610716189,-0.223314330540614,0.326120589410857,-0.684759986923967,0.493495388573895,0.918058191366878
"7607","BBS10",15.3171471812691,0.248724635688918,0.363348427614121,0.684534779253443,0.493637535410407,0.918058191366878
"7608","ULK1",25.0000220798945,0.270930918078884,0.395503123827657,0.685028516227204,0.493325926589187,0.918058191366878
"7609","GGACT",6.71470984255943,0.351977380691694,0.513426276390728,0.68554609858696,0.492999381526585,0.918058191366878
"7610","CCNB1IP1",54.4900708714142,-0.165608521667346,0.241583663551568,-0.68551208816318,0.493020835301954,0.918058191366878
"7611","AC048382.6",2.31662651330756,-0.640142134925416,0.935482345944

642,-0.68429098389773,0.493791439146567,0.918058191366878
"7612","NLGN2",35.1706481573772,-0.212270357306202,0.310282116123557,-0.684120502844818,0.493899076104182,0.918058191366878
"7613","FOXO3B",2.49735755108735,-0.577842324420614,0.842281557685414,-0.686044137079912,0.492685276145178,0.918058191366878
"7614","ZNF396",5.60834746063697,0.444906807580094,0.649557400827925,0.684938401152873,0.493382792436339,0.918058191366878
"7615","ACAA2",165.002305710462,-0.151909551161184,0.222008627020194,-0.684250667193065,0.493816892850164,0.918058191366878
"7616","GTF2F1",63.3075496426398,-0.17926648576044,0.26209214535299,-0.683982671510436,0.49398610812297,0.918058191366878
"7617","PHF20",75.3105929107115,0.123787865372448,0.180579099765989,0.685504942337533,0.49302534295237,0.918058191366878
"7618","HMOX1",18.2532229117624,0.364304816856041,0.532754718361473,0.683813402866685,0.494093002096853,0.918058191366878
"7619","OFD1",44.4501216878459,-0.154684359960171,0.225705180377726,-0.685338102126417,0.493130593518335,0.918058191366878
"7620","KDM5C",40.5253557876883,-0.164944995666442,0.241219949766889,-0.683795000479196,0.494104624040041,0.918058191366878
"7621","FAM120C",11.1960647253966,0.292757025493182,0.427764816177812,0.684387809425377,0.493730311813423,0.918058191366878
"7622","LRRC42",28.1924612590211,-0.188137613801253,0.27692319026983,-0.679385549537887,0.49689360349089,0.918060206867302
"7623","SLC22A15",5.20419276891311,-0.387786933943313,0.567756308944312,-0.683016512250415,0.49459640862775,0.918060206867302
"7624","KIFAP3",99.8483415184784,-0.149239305222794,0.21855421334386,-0.682847989702168,0.49470290170443,0.918060206867302
"7625","AL354953.1",1.96388874577055,0.641789287109968,0.943894366063409,0.67993761821739,0.496543961004788,0.918060206867302
"7626","ACYP2",20.2861547024278,0.229939728220663,0.338176934632314,0.679939122609493,0.496543008405129,0.918060206867302
"7627","CYP27C1",7.85769200464003,0.401655795884123,0.589121832913497,0.681787320455835,0.495373442995567,0.918060206867302
"7628","HAT1",59.8007121937322,-0.179339644457291,0.263025774389178,-0.681832968171161,0.495344575112372,0.918060206867302
"7629","ALPK1",24.8398163946738,0.197138861931993,0.289948303774379,0.679910381836188,0.496561207586259,0.918060206867302
"7630","DIMT1",44.0009364663356,0.173209862013747,0.253670556893174,0.682814214369741,0.494724246548535,0.918060206867302
"7631","TMEM161B-AS1",34.2003913082936,0.197604233914644,0.290600999001222,0.6799847027154,0.496514146981142,0.918060206867302
"7632","PPP2CA",63.0590862366349,-0.169917855121713,0.249082099264902,-0.68217610026244,0.495127605126247,0.918060206867302
"7633","ABT1",33.3227896803486,-0.173946720988506,0.255706762712993,-0.6802585866051,0.496340741933061,0.918060206867302
"7634","ABCC10",7.25555043147577,-0.336060580234499,0.493545681212374,-0.680910791092287,0.495927939535621,0.918060206867302
"7635","MRPL14",90.6276829418001,-0.196952804206328,0.289875672052284,-0.679438887754624,0.496859816992997,0.918060206867302

"7636","AL096865.1",1.54553510171649,-0.929401385802249,1.36289705884969,-0.681930729666905,0.495282753191103,0.918060206867302
"7637","CNKSR3",5.43339330797278,0.46698409462627,0.685401558166202,0.681329199011,0.495663211436455,0.918060206867302
"7638","LRRC17",124.550614657455,-0.314299233122164,0.462001426083654,-0.680299270472932,0.496314986371594,0.918060206867302
"7639","MTPN",234.266973619024,0.105299471703593,0.154459007658235,0.681730857267873,0.495409151890123,0.918060206867302
"7640","CASP2",22.118737336534,0.226408265443386,0.332033582678609,0.68188363242322,0.495312535793901,0.918060206867302
"7641","C9orf72",8.82162435335518,-0.325746255322232,0.477342990363639,-0.682415499752241,0.494976257624527,0.918060206867302
"7642","RGP1",14.1454668154028,0.29130791898445,0.427399863023128,0.681581685412676,0.495503498943694,0.918060206867302
"7643","RPL35",2531.37549459438,-0.142143854180603,0.20871326444764,-0.681048492805609,0.495840806869193,0.918060206867302
"7644","LRRC8A",35.6660489433966,0.180680141268936,0.265354535287768,0.680900897634913,0.495934200072409,0.918060206867302
"7645","FRMD4A",15.0741458364093,0.356612006033817,0.522920916349733,0.681961640630402,0.495263206729569,0.918060206867302
"7646","ZNF488",1.63565335834387,0.693420774033248,1.01771417062698,0.681351202573958,0.495649291796272,0.918060206867302
"7647","AP3M1",21.4064552388366,0.228783832648533,0.335500164371487,0.681918690195362,0.495290366428839,0.918060206867302
"7648","SCAF11",231.019013907411,0.121306195597084,0.178233326468646,0.680603330479971,0.496122518982393,0.918060206867302
"7649","KDM2B",10.9524309683813,0.28998158295063,0.426691100696298,0.679605415902562,0.496754339476004,0.918060206867302
"7650","DNAJC3",138.353956864906,-0.114779220905023,0.168623547050099,-0.680683231452374,0.496071948956243,0.918060206867302
"7651","GMPR2",49.3698709580241,-0.181527636182897,0.267085490658472,-0.679661166674982,0.496719030076363,0.918060206867302
"7652","PRORP",16.6821020476311,-0.235298907996461,0.346301095384855,-0.679463366221714,0.496844311791205,0.918060206867302
"7653","PNMA1",51.9781120708228,0.154852234424543,0.227845430567911,0.679637217382724,0.496734198045455,0.918060206867302
"7654","NPIPB15",15.4500654430403,0.314898226589991,0.461748819533684,0.681968666228543,0.495258764169652,0.918060206867302
"7655","B9D1",11.962708264967,0.271665903328349,0.398803662980786,0.68120212662499,0.495743602585849,0.918060206867302
"7656","TEX2",12.3216999305798,0.274087663113279,0.402110693573321,0.681622417642325,0.495477735988077,0.918060206867302
"7657","UBE2O",18.2647932420188,0.3029489326196,0.445225101508014,0.680439920376204,0.496225951233582,0.918060206867302
"7658","EPG5",18.7389802822213,0.220564360080215,0.324463920662691,0.679780850917815,0.496643233336105,0.918060206867302
"7659","AC027097.1",6.87553291625812,0.420694898752704,0.619084543309625,0.679543534560999,0.496793533199502,0.918060206867302
"7660","RPS9",4174.03231988647,-0.183211335250043,0.269442233003358,-0.679965175495555,0.496526511550466,0.918060206867302

"7661","ZNF471",9.54391039730826,0.366986793153375,0.539991510802155,0
.679615856568221,0.496747726844529,0.918060206867302
"7662","AJ011932.1",1.75712433140867,-0.744511765922814,1.093229594315
74,-0.68102050090293,0.495858518471683,0.918060206867302
"7663","PQBP1",64.0914513039197,-0.188513686475198,0.276501628652993,-
0.68178146867909,0.495377143758511,0.918060206867302
"7664","STARD8",3.80500213042255,0.551312840769837,0.809166111905735,0
.681334564878642,0.495659816923923,0.918060206867302
"7665","HDAC2",77.1524302763646,-0.148439077634996,0.218525585194881,-
0.679275506813622,0.496963312694606,0.918069211709801
"7666","PYCR3",7.58665658958151,-0.42243404659057,0.622193418984121,-0
.678943289500384,0.497173795332684,0.918338239226559
"7667","NCOA7",175.255347944572,-0.19093910488,0.281313765104345,-0.67
8740710783124,0.497302166261441,0.918455546401723
"7668","C2orf27A",19.9225653544125,0.254942636566803,0.37580559185125,
0.678389683641838,0.497524648401109,0.918626812017173
"7669","CCL13",8.43617587716787,0.479605275588903,0.706915852482773,0.
678447475614632,0.49748801601741,0.918626812017173
"7670","SLC39A14",45.5217858578413,-0.204330323017359,0.30130479278877
9,-0.678151585728671,0.497675585790053,0.918785696843176
"7671","TMEM120B",9.13860444852284,-0.319888525514144,0.47195212731461
,-0.677798672789756,0.497899352693661,0.918970302680672
"7672","NPW",1.84705316626409,-1.96100283465046,2.89367115306753,-0.67
7686831335906,0.497970277716723,0.918970302680672
"7673","ZNF461",1.96774961241173,-0.675446247996028,0.996673765030861,
-0.677700438894479,0.497961648103109,0.918970302680672
"7674","MRPS2",44.4504300013513,-0.188780891312708,0.278672059884583,-
0.677430279127714,0.498132992363371,0.919150791225611
"7675","RPS3A",4282.61045954857,-0.174000482467701,0.257157876487132,-
0.676629022002394,0.498641360549015,0.919184359186512
"7676","GINM1",222.249344008482,-0.0994652982467203,0.147009170249397,
-0.676592474319664,0.498664555285546,0.919184359186512
"7677","PTPN20",3.81258902499286,-0.544028884310558,0.804054467701101,
-0.676607003833968,0.498655334160118,0.919184359186512
"7678","WNT10B",9.80309071297724,0.329878528118984,0.487565419107871,0
.676583111088114,0.498670497688615,0.919184359186512
"7679","LIMD2",12.7016844368598,0.29042654694852,0.429165413038113,0.6
76724027904661,0.498581068387354,0.919184359186512
"7680","SLC5A3",193.940866327082,0.306711642997174,0.453308967239086,0
.676606167456217,0.498655864963024,0.919184359186512
"7681","SEPTIN5",6.95694194702398,0.399291277510923,0.590034636978154,
0.676725148808013,0.498580357068515,0.919184359186512
"7682","PORCN",11.5851229254209,0.276353777723305,0.408047036812267,0.
677259611740419,0.498241251429803,0.919184359186512
"7683","AQR",33.0901543001966,0.190860886393676,0.282194643712259,0.67
6344823143729,0.498821740518401,0.919343465539576
"7684","CCDC84",22.6440790012208,0.219702427169559,0.324906822016533,0
.67620133614301,0.498912824363001,0.91939167009111
"7685","POLA2",6.83320217339722,-0.365716104849476,0.54106465128824,-0
.675919419201992,0.499091807629922,0.919403532706915

"7686","MEX3C",20.8604079107952,-0.213137880562245,0.31525662551154,-0
.676077402707729,0.498991502949862,0.919403532706915
"7687","AC008764.8",1.55181983066147,-0.761996745757611,1.127406934100
18,-0.675884388067726,0.499114050559185,0.919403532706915
"7688","RPS6KB2",28.8869926232049,-0.196348806842252,0.290629675263302
,-0.675597929441876,0.499295956492222,0.919499381444903
"7689","PINLYP",10.4596136689333,-0.288202214761678,0.426539175700238,
-0.675675837485606,0.499246480118715,0.919499381444903
"7690","TFDP2",122.224458532755,0.140587210251891,0.20817570216427,0.6
75329583569529,0.49946639246832,0.919574062846367
"7691","SPATA22",2.18591159127134,-0.70741350004092,1.04746995575444,-
0.675354453991382,0.499450595082907,0.919574062846367
"7692","WDR12",18.8972377015831,0.229265210220737,0.339583350325202,0.
675136781591856,0.499588866909018,0.919679973405056
"7693","C1orf167",8.47479446514045,0.380727038438277,0.570512229554167
,0.667342466498572,0.504553406557648,0.920140247238679
"7694","PATJ",6.70098027920734,0.380873919134405,0.573128821328692,0.6
64552025583743,0.506337062604787,0.920140247238679
"7695","PRKACB",30.9857546135825,0.16646561306088,0.248802412033173,0.
669067521092539,0.50345240714913,0.920140247238679
"7696","HIPK1",48.9207330343375,0.174080823454595,0.261371295432535,0.
666028850515182,0.505392659909762,0.920140247238679
"7697","PIAS3",21.4012135220654,-0.224056075914567,0.334763311459085,-
0.669296987588053,0.503306048043237,0.920140247238679
"7698","ZBTB7B",11.7675884625095,-0.288595758950877,0.430655230421605,
-0.670131786553124,0.502773783362029,0.920140247238679
"7699","ARHGEF2",32.3380689928241,0.190030048800604,0.28223076486126,0
.67331443789985,0.500747269409237,0.920140247238679
"7700","RASAL2-
AS1",2.12626764231611,-0.665927019704834,0.99947710639659,-0.666275410
85528,0.505235079468465,0.920140247238679
"7701","DNAJC27",16.4607911306292,0.23047299157889,0.344972665025512,0
.66809059077723,0.504075767630154,0.920140247238679
"7702","MSH2",21.5227037668233,0.220339469909276,0.326905357880862,0.6
74016086299746,0.500301086661145,0.920140247238679
"7703","RTKN",13.6811488983107,0.280296343786431,0.420883254247591,0.6
65971717709497,0.505429178043068,0.920140247238679
"7704","HTRA2",50.268100963548,-0.198570235036982,0.295049947015706,-0
.67300549295273,0.500943796359234,0.920140247238679
"7705","IGKC",224.349865945414,0.514155835897676,0.773506680056477,0.6
64707686635796,0.506237476452253,0.920140247238679
"7706","ANKRD36C",77.87377252433,-0.279534953638294,0.418132458575912,
-0.668532059410893,0.503794024547339,0.920140247238679
"7707","AC068491.3",6.10183428713013,0.429169893922357,0.6409594780916
92,0.66957414406307,0.503129301120273,0.920140247238679
"7708","GTPBP8",24.8990419306825,0.251310416171401,0.377287003139812,0
.666098789727651,0.505347958072069,0.920140247238679
"7709","PLXND1",55.7869035033594,0.168269890211316,0.251165696442505,0
.669955700936395,0.502886030123236,0.920140247238679
"7710","LPP-

AS2",7.87778760268523,0.339646735512635,0.508069119759071,0.668504977578044,0.503811305644787,0.920140247238679
"7711","NUP54",56.7824439092151,0.147514136034779,0.221371900525562,0.666363416877046,0.50517883975752,0.920140247238679
"7712","MIR4453HG",1.68766870789486,0.665558894713066,0.993438200745699,0.669955004965061,0.50288647380033,0.920140247238679
"7713","C5orf51",16.7257164316859,0.237204443039398,0.35254386738069,0.672836673636577,0.501051203456488,0.920140247238679
"7714","SGTB",20.9023086192903,0.215411484413448,0.321580804870221,0.669851810652631,0.502952261777156,0.920140247238679
"7715","RPS23",5718.88773837052,-0.167557333181439,0.251638923311271,-0.665864131735196,0.505497948592639,0.920140247238679
"7716","SLU7",90.4778005460504,0.134325521831833,0.200386221545166,0.670333123685137,0.502645456110838,0.920140247238679
"7717","SERPINB1",123.716361183722,-0.147381128100677,0.218817095854027,-0.673535710386155,0.500606538039684,0.920140247238679
"7718","ZSCAN16",2.39660969303818,0.594592342435335,0.890700029836223,0.667556217040507,0.504416913469431,0.920140247238679
"7719","TRAF3IP2-
AS1",12.8084870215843,0.274901736505795,0.413552428320352,0.664732492618438,0.506221607458797,0.920140247238679
"7720","IL20RA",2.59505838610417,0.577935262402669,0.866235476998262,0.667180319611671,0.504656960472871,0.920140247238679
"7721","MAD1L1",22.9503650801769,-0.261609915815519,0.390423357675804,-0.67006727613042,0.502814904354604,0.920140247238679
"7722","OSBPL3",2.77065532304379,-0.596164676173128,0.887933086377619,-0.671407209979325,0.501961153077441,0.920140247238679
"7723","MAGI2",25.0791221925186,-0.19357316310605,0.290073628611753,-0.667324237754603,0.504565047651752,0.920140247238679
"7724","FAM133B",187.819925655523,-0.166682391112688,0.249609017453144,-0.667773916236727,0.504277918936386,0.920140247238679
"7725","TRIM56",111.606727787469,0.218904691233094,0.328003367487496,0.667385499453567,0.50452592575922,0.920140247238679
"7726","GSTK1",173.986292313533,-0.164577468408702,0.246048698264397,-0.668881687119723,0.503570952878965,0.920140247238679
"7727","AC093673.1",15.8890240701716,-0.301411724375304,0.453135870874523,-0.665168537184221,0.505942701822758,0.920140247238679
"7728","OR2A1-
AS1",6.74988584690741,-0.471667016494135,0.704830986495072,-0.669191658045006,0.50337322690264,0.920140247238679
"7729","HOOK3",142.254141600476,0.154421497327411,0.231153268267558,0.668048081192038,0.504102901422819,0.920140247238679
"7730","UBE2V2",91.2176557711647,-0.168460996235552,0.251841607082104,-0.668916459783515,0.503548769857066,0.920140247238679
"7731","GDF6",2.70085651329842,0.638829450129836,0.951690949371971,0.671257250635204,0.502056662927417,0.920140247238679
"7732","CCN4",1.74558709377753,-0.70251323764041,1.04937960111185,-0.669455778343774,0.503204780737776,0.920140247238679
"7733","MTAP",47.3279992442076,0.161926790335448,0.243040119377153,0.666255393349964,0.505247871993399,0.920140247238679

"7734","UNC13B",9.64714016181998,0.299637644455406,0.445688955133979,0
.672302153786448,0.501391358900103,0.920140247238679
"7735","GCNT1",2.34280388590073,-0.715781991521643,1.06921150734595,-0
.669448454869696,0.503209450977463,0.920140247238679
"7736","NFIL3",147.558408197026,-0.277353480485335,0.417284201403234,-
0.664663266791929,0.50626589356597,0.920140247238679
"7737","ZDHHC12",21.2826954273681,-0.2152116602517б,0.320171438240987,
-0.672176323516322,0.501471452006376,0.920140247238679
"7738","AL441992.1",1.82879304334536,-0.832094312380754,1.251620863604
36,-0.664813392439407,0.506169855684074,0.920140247238679
"7739","NELFB",47.7098849767568,-0.15833930727716,0.237534330801184,-0
.666595463245644,0.505030567755207,0.920140247238679
"7740","MLLT10",46.6127995600651,0.187179284806782,0.27792913420674,0.
673478458244492,0.500642948925894,0.920140247238679
"7741","ERCC6",18.4697285359803,0.218464954243704,0.325393013311926,0.
67138796872163,0.501973407392169,0.920140247238679
"7742","MORN4",9.88590596984885,-0.365388137128437,0.541999749340618,-
0.674148166254612,0.500217119754865,0.920140247238679
"7743","TALDO1",174.360851002046,-0.178334331212141,0.268024597798217,
-0.665365539868845,0.505816720220011,0.920140247238679
"7744","STIM1",40.5924987592529,0.185122176796617,0.276661451618568,0.
669128914467796,0.503413246814249,0.920140247238679
"7745","ZNF214",5.53909314210552,0.392507471595678,0.589460403533744,0
.665875891311177,0.505490431457521,0.920140247238679
"7746","FAR1",46.7102084589741,-0.180088076279858,0.269676282313304,-0
.667793529097365,0.50426539769387,0.920140247238679
"7747","ATG16L2",9.349724097429,0.31485269867853,0.46914678865368,0.67
1117667845642,0.502145572538067,0.920140247238679
"7748","CRYAB",232.852338644301,-0.127372219373076,0.191154932193884,-
0.666329756241318,0.505200349991822,0.920140247238679
"7749","CCDC15",5.76605968268832,0.375693556126803,0.562386380253775,0
.668034592084668,0.50411151165713,0.920140247238679
"7750","ITFG2",22.1023374648345,0.240460793034614,0.3614099487415,0.66
534082382609,0.505832525021033,0.920140247238679
"7751","TSPAN31",73.0048131278067,0.14903967700574,0.223839862165607,0
.665831704700898,0.505518677399923,0.920140247238679
"7752","LRIG3",51.8935820650297,0.1579036399443,0.235577941436511,0.67
028194143065,0.502678076757892,0.920140247238679
"7753","CRADD",10.9178438633738,-0.266450678338115,0.399816408489912,-
0.666432574251983,0.505134647473386,0.920140247238679
"7754","SSH1",20.8540709023656,0.256094307599287,0.380918867309401,0.6
72306702495979,0.501388463696035,0.920140247238679
"7755","ING1",43.0564137399144,-0.170238670610601,0.255228230926308,-0
.667005644292359,0.50476852811422,0.920140247238679
"7756","OSGEP",43.820325129424,-0.206520040726415,0.307294486082476,-0
.672059051105089,0.501546103989098,0.920140247238679
"7757","CDKL1",11.0632581126986,0.325525214648587,0.489331345575666,0.
665244966609748,0.505893823871087,0.920140247238679
"7758","SNAPC1",34.0244787802763,0.172679284039348,0.257572414695735,0
.670410627020484,0.502596062039985,0.920140247238679

"7759","MAN2A2",15.2357070605642,0.283411035668458,0.423694229937758,0
.668904638399469,0.503556311183489,0.920140247238679
"7760","MLST8",25.4723752705799,-0.253436146091544,0.377787747029148,-
0.670842683714647,0.502320752681938,0.920140247238679
"7761","TBC1D10B",23.209664574769,0.214659485531616,0.322101051576701,
0.666435221123456,0.505132956135366,0.920140247238679
"7762","CYBA",403.675392898177,0.193010006280897,0.286466690938243,0.6
73760728162656,0.50046344613838,0.920140247238679
"7763","BORCS6",12.3417606220018,-0.26654244713739,0.396020731627958,-
0.673051751714337,0.500914367500861,0.920140247238679
"7764","LINC00324",5.92983191565408,0.438249550915338,0.65167719598796
9,0.672494838876376,0.501268724695821,0.920140247238679
"7765","ADPRM",10.2380660494409,0.301220989623174,0.449778104340782,0.
669710212027015,0.503042540501372,0.920140247238679
"7766","WIPF2",61.4199930415247,0.151226220300524,0.224987662568794,0.
672153390874418,0.501486049752708,0.920140247238679
"7767","MLX",48.5674923033156,-0.162412607896608,0.244213855797353,-0.
665042560203369,0.506023271724331,0.920140247238679
"7768","MEOX1",12.9481304105306,-0.459476245017653,0.681488551969409,-
0.674224451298307,0.500168626633658,0.920140247238679
"7769","DUSP3",27.1611902163572,0.21113149447598,0.314823729104487,0.6
70633992795084,0.502453721944209,0.920140247238679
"7770","CEP95",41.4091980134904,0.161591925426353,0.242755781091921,0.
665656342763532,0.505630784329669,0.920140247238679
"7771","MYOM1",4.11242684555503,0.477802733530668,0.718573339232415,0.
664932453576778,0.506093697102521,0.920140247238679
"7772","AFG3L2",45.5547194823771,0.15071498030861,0.224902966731664,0.
670133357949121,0.502772781726483,0.920140247238679
"7773","EPOR",10.0739961013904,0.379103222689287,0.566617435278118,0.6
69063814641018,0.503454771395163,0.920140247238679
"7774","ZNF433-
AS1",7.66897402789125,-0.337860859728989,0.502519194731328,-0.67233423
771927,0.501370938015365,0.920140247238679
"7775","ZNF709",3.8645170018319,-0.442067328161594,0.658998489145018,-
0.670816906932716,0.502337175578644,0.920140247238679
"7776","FBXW9",11.286820745461,-0.293310387259952,0.438591614806579,-0
.668755118333267,0.503651701068877,0.920140247238679
"7777","PRDX2",306.439846104296,-0.162681381901992,0.242730352580226,-
0.670214417656002,0.502721114269097,0.920140247238679
"7778","STX10",76.4153147130018,-0.174200571400108,0.259173332537923,-
0.672139257902309,0.50149504619161,0.920140247238679
"7779","EML2-
AS1",1.55055280607546,-0.897590651808404,1.34843085973492,-0.665655673
280022,0.505631212348084,0.920140247238679
"7780","AP2S1",252.391389137403,-0.175327819171984,0.263790483771566,-
0.664648006498263,0.506275656367093,0.920140247238679
"7781","CARD8",17.0117355012457,0.225493063538202,0.337441375354396,0.
668243671367743,0.503978062945489,0.920140247238679
"7782","POLD1",4.84075549270494,0.429690513748316,0.64028924154343,0.6
7108813621878,0.502164384273136,0.920140247238679

"7783","ZNF71",9.18826241860008,-0.291676992780534,0.436617965109174,-0.668037085252784,0.504109920236741,0.920140247238679
"7784","CSE1L",33.7147994279823,0.180760730575162,0.270252894211973,0.668857704936851,0.503586252475939,0.920140247238679
"7785","TSHZ2",540.90535324772,-0.122899911363932,0.183975529804873,-0.668023141416041,0.504118820796164,0.920140247238679
"7786","OSBPL2",23.8534668057823,-0.230393841603836,0.34438186568313,-0.669006891947743,0.503491081591195,0.920140247238679
"7787","AP001062.1",1.69321303634085,-0.706069573281501,1.05056323382066,-0.67208669649868,0.501528505258216,0.920140247238679
"7788","TTC28-AS1",11.4863408363619,0.324701600044919,0.487844621022728,0.665584052898252,0.505677002243434,0.920140247238679
"7789","NAGA",26.9318724912799,0.179096256330791,0.267609806074499,0.669243997288101,0.503339844502054,0.920140247238679
"7790","MBTPS2",15.8136584760232,0.265384472857714,0.396996362200375,0.66848086815407,0.503826690291627,0.920140247238679
"7791","POLA1",8.20433201250807,0.32458083057,0.487335238054938,0.66603193289587,0.505390689753905,0.920140247238679
"7792","CLIC2",50.7312773556882,-0.180320421869099,0.271172928413573,-0.664964688488695,0.506073078773623,0.920140247238679
"7793","C1orf174",39.7765211140215,-0.167875603561933,0.255036671299043,-0.658241039246825,0.510383257894256,0.920211999147571
"7794","AC093157.1",11.4369223131466,0.301968470479727,0.45875565846303,0.658233778502945,0.510387922730314,0.920211999147571
"7795","CSDE1",287.793726138169,-0.106059448834564,0.160845525843558,-0.659387000529435,0.509647287588936,0.920211999147571
"7796","CTSK",700.102984399057,0.140883267387511,0.212104903183716,0.664215042994478,0.506552686824598,0.920211999147571
"7797","THBS3",79.8296051265145,-0.150607517026544,0.228815143615025,-0.6582060725838,0.510405723256004,0.920211999147571
"7798","GAS5",529.880182584888,-0.128209129752438,0.194677931110249,-0.658570434878061,0.510171653357002,0.920211999147571
"7799","MIA3",99.2469372021302,0.143723670511839,0.217364195341983,0.66121133835182,0.508476790926315,0.920211999147571
"7800","TMEM18",77.3440980514405,-0.186451783390257,0.282802007916631,-0.659301483620379,0.50970218989517,0.920211999147571
"7801","ATP6V1E2",13.4725498852147,-0.261196472163857,0.396410771610516,-0.658903568898198,0.509957693952637,0.920211999147571
"7802","ANKRD53",2.20008672665576,0.700811232170648,1.05682039208095,0.663131822040927,0.507246131633779,0.920211999147571
"7803","LINC01943",4.38694299352943,-0.441034748867877,0.668817165830389,-0.659424983986913,0.50962290300196,0.920211999147571
"7804","SLC35F5",51.4035770738523,-0.188839991369066,0.285058188763699,-0.662461205510592,0.507675689521344,0.920211999147571
"7805","TLK1",66.001058290127,0.148779190732649,0.224152536235269,0.663740831272554,0.506856201263593,0.920211999147571
"7806","INO80D",45.4839932775074,0.229242021871395,0.346617109688151,0.6613696077428,0.508375311641287,0.920211999147571
"7807","THRB",39.2049719429918,0.201571669792308,0.303816110872549,0.6

63466032836116,0.507032126965665,0.920211999147571
"7808","ADD1",164.679278710816,0.116944586898527,0.176859368238877,0.661229247073723,0.508465307665876,0.920211999147571
"7809","USP46",30.0534298958664,0.216121431136373,0.326160311637296,0.662623327931785,0.507571825842875,0.920211999147571
"7810","DCK",10.7047260026932,-0.284059552500041,0.431380480967202,-0.658489581779752,0.510223589329835,0.920211999147571
"7811","SAP30",39.2463877083052,-0.19745049522224,0.299355295551604,-0.659585777022615,0.509519683994305,0.920211999147571
"7812","ITGA2",2.71546745009719,0.622193526707516,0.93867767826368,0.662840441522397,0.507432749564714,0.920211999147571
"7813","TICAM2",22.1629914634363,-0.197213315741284,0.298817173178411,-0.659979858733006,0.509266754635662,0.920211999147571
"7814","HSPA4",57.5104720563361,0.145361311670032,0.22000065173898,0.660731277480469,0.50878466166589,0.920211999147571
"7815","NDST1",32.2075109353132,0.207905220700464,0.315517703970269,0.658933613183415,0.509938399950845,0.920211999147571
"7816","CCNG1",94.0705547312466,0.135620916253146,0.204963479805025,0.661683322229687,0.508174195349282,0.920211999147571
"7817","HIST1H1C",25.420628835996,0.242761845281891,0.368497039607119,0.658789133124968,0.510031186435208,0.920211999147571
"7818","AHI1",121.799186672471,-0.180667976051446,0.273607854626777,-0.660317213107391,0.5090502862453,0.920211999147571
"7819","ACTB",2927.51037629891,-0.198111120744915,0.299188784524078,-0.662160919768607,0.50786809695386,0.920211999147571
"7820","FASTK",55.8411474678104,-0.174906070714319,0.263769568761472,-0.663101780601866,0.507265370344483,0.920211999147571
"7821","SMARCD3",26.3245087616007,0.217937217449788,0.328727219853769,0.66297283670861,0.507347951109502,0.920211999147571
"7822","YWHAZ",351.698766259593,-0.130174411176152,0.19680819872225,-0.661427786145553,0.508338011439806,0.920211999147571
"7823","RCL1",17.1118090015692,-0.237499713047889,0.359422824147636,-0.660780832745151,0.508752876558146,0.920211999147571
"7824","SUSD3",3.06908746592768,-0.527171696798096,0.7971371537792,-0.661331233024071,0.508399915796368,0.920211999147571
"7825","PHF2",27.5273718533878,0.211693878095895,0.321051947267027,0.659375779832366,0.509654491159135,0.920211999147571
"7826","ATP6V1G1",300.824502644699,-0.158627452494854,0.240757734818345,-0.658867523465198,0.509980842308023,0.920211999147571
"7827","FANK1",3.31335382757219,-0.489809393196522,0.74173383871633,-0.660357351424336,0.509024534113887,0.920211999147571
"7828","USP28",24.3644476062063,0.242036473764963,0.364519433600911,0.663987846612186,0.506698089657057,0.920211999147571
"7829","ERC1",58.5785962815521,-0.164167153380583,0.247352609582395,-0.663696872483968,0.506884341483379,0.920211999147571
"7830","CD27-AS1",38.5904480647463,0.16582565919214,0.250458182762839,0.662089205323194,0.507914053488264,0.920211999147571
"7831","FAM234B",4.73884510670604,0.444347707270153,0.673719012411341,0.659544556535174,0.50954614390923,0.920211999147571

"7832","TSC22D1",240.444094275354,-0.21855090444913,0.329831324965366,-0.662614154286524,0.50757770263806,0.920211999147571
"7833","HNRNPC",270.871918800436,0.0937983817401924,0.142093737141696,0.660116227688883,0.509179245686826,0.920211999147571
"7834","PTGR2",10.0430815176286,-0.308814664492515,0.466853435616514,-0.661480972255678,0.508303913226957,0.920211999147571
"7835","TDP1",4.9177955768023,-0.415127924602732,0.62664550780088,-0.662460544973126,0.507676112717285,0.920211999147571
"7836","AL049840.4",5.01720957331916,0.469106083253247,0.710960194667028,0.65982046079661 2,0.50936905143204,0.920211999147571
"7837","ALG1",18.1959827709883,0.218281965504601,0.33111667614552,0.659229755642601,0.50974824202197,0.920211999147571
"7838","CHTF8",3.06470689068376,-0.594368802756894,0.896102806837289,-0.663281933972134,0.507150004830327,0.920211999147571
"7839","AC091132.4",6.04362394499545,0.437708202290758,0.659883438127733,0.663311392588749,0.507131141612721,0.920211999147571
"7840","AC018521.5",17.3076013352886,-0.251841204974776,0.382117405661451,-0.65906760917848,0.509852354327802,0.920211999147571
"7841","AC103702.1",3.00870976379282,0.530946927520205,0.806495558357172,0.658338315714648,0.510320762484787,0.920211999147571
"7842","ELP2",51.1896651755801,0.158509438351613,0.24028550126853,0.659671255713723,0.509464816511443,0.920211999147571
"7843","ZNF555",4.80343620041254,0.451265342443521,0.682662765184662,0.661036994337097,0.50858858924348,0.920211999147571
"7844","SAFB",66.8580493872968,0.13896305852003,0.210906936183897,0.65888330196435,0.509970709297705,0.920211999147571
"7845","DHX34",5.297833035385,0.389604365728458,0.589681103190521,0.660703494855898,0.508802482098974,0.920211999147571
"7846","PRMT1",128.994229916263,-0.206818348958655,0.313903903313212,-0.658858799701807,0.509986444786253,0.920211999147571
"7847","ZNF160",27.1968489079925,0.210970676186017,0.317986794463742,0.663457350616718,0.507037685837199,0.920211999147571
"7848","PEG3",14.6216540789473,0.319886894460512,0.482534348941151,0.662930825883082,0.507374858027219,0.920211999147571
"7849","DDRGK1",78.1749303260875,-0.133654809935479,0.201558746290375,-0.663105979747113,0.507262681164626,0.920211999147571
"7850","ZNF74",6.21556838443973,-0.367846955012346,0.55773977595332,-0.659531507114768,0.50955452063534,0.920211999147571
"7851","SLC2A11",9.37710626056229,0.289543052495118,0.438743905415155,0.659936352212442,0.509294674742981,0.920211999147571
"7852","SF3A1",51.598172838615,-0.142958988416894,0.217062142541628,-0.658608575143303,0.510147154924552,0.920211999147571
"7853","LINC00891",11.5721989527105,0.298012975871801,0.452505859638055,0.658583683557539,0.510163143333865,0.920211999147571
"7854","ITM2A",1565.42845479872,0.208333235946205,0.314983973721729,0.66140900276487,0.508350053962858,0.920211999147571
"7855","SYT11",8.10396740863005,0.399960671993049,0.608192652923946,0.657621676404999,0.510781262301075,0.920297583158213
"7856","DHX57",15.7690092040394,0.250037811467904,0.380332051854129,0.657419773718684,0.51091104061597 8,0.920297583158213

"7857","HNMT",168.477151952193,0.145091260428976,0.220868326196356,0.6
56912935085078,0.511236900507578,0.920297583158213
"7858","SCFD2",14.4472834500172,-0.331458634138778,0.504010383388233,-
0.657642471392219,0.510767896749302,0.920297583158213
"7859","SMIM15",84.4766669206753,-0.143282027339803,0.217950720361984,
-0.657405614910714,0.510920142212352,0.920297583158213
"7860","ZNF879",1.99129469852057,-0.673748773352944,1.02528102070845,-
0.657135711814302,0.511093658118899,0.920297583158213
"7861","HEIH",27.9360071619971,0.22576701089169,0.34352582636705,0.657
205349825614,0.511048886131985,0.920297583158213
"7862","GLI4",20.6893065945594,0.261154252971813,0.397376354956775,0.6
57196256682713,0.511054732220465,0.920297583158213
"7863","TMEFF1",3.49645767976627,-0.527630886228827,0.803051400912453,
-0.657032520744396,0.511160005958298,0.920297583158213
"7864","ANKRD16",21.0840772383715,0.222359661438126,0.337935573482106,
0.657994241763067,0.510541831254684,0.920297583158213
"7865","MPHOSPH8",231.043703818451,0.114759475754146,0.174720419729099
,0.656817765960489,0.51129809934362,0.920297583158213
"7866","TEX9",4.65715097014928,0.427431408151366,0.649976934310459,0.6
57610117511039,0.510788691622265,0.920297583158213
"7867","PEX26",26.2612172869614,0.230356583932597,0.350680208272168,0.
656885043691471,0.511254835768778,0.920297583158213
"7868","AP000547.3",4.83588810292118,0.461386355866018,0.7026008249125
86,0.656683481582051,0.51138445788663,0.920336035037453
"7869","BLOC1S6",128.322194048703,-0.109269400833926,0.166465384716337
,-0.656409144880931,0.511560908019484,0.92041962611892
"7870","DRG1",48.3105177117147,0.149326894630904,0.227488138987959,0.6
56416177543253,0.511556384297064,0.92041962611892
"7871","FAM122A",23.0647606428271,0.198191296326162,0.302017105945696,
0.65622540056323,0.511679107969118,0.920449968594848
"7872","TAPBPL",35.9258797239446,-0.230573819800159,0.351387617364785,
-0.656180833944397,0.511707779151034,0.920449968594848
"7873","GUCD1",36.0480983914708,0.189129145117676,0.288286024214658,0.
656046874394611,0.511793964813853,0.920488065764531
"7874","TENT5C",8.52909415927749,0.503686022363953,0.768017635655033,0
.655826115157324,0.511936011387496,0.920554249952169
"7875","LMAN2L",13.7058701906169,0.282268683369845,0.430553852243711,0
.655594374313179,0.512085146155405,0.920554249952169
"7876","WEE2-
AS1",2.05390117742651,0.624330627095084,0.952309841382802,0.6555961095
48259,0.512084029376031,0.920554249952169
"7877","TLCD1",5.32494203582895,-0.395830894899675,0.603782187710478,-
0.655585578634992,0.512090806982573,0.920554249952169
"7878","AL139393.3",11.425813085719,-0.369975137073058,0.5644697374571
82,-0.655438392038019,0.512185539926304,0.920607672677895
"7879","S1PR1",3.54048054601766,0.541494413777948,0.828151742139623,0.
653858932155279,0.513202694499398,0.92073412850485
"7880","TMEM185B",31.5919309891971,-0.194384439846215,0.29781468153439
9,-0.652702676861694,0.513947976430428,0.92073412850485
"7881","FARSB",24.5884831512931,-0.216485349641726,0.331090241849318,-

0.653856025573386,0.513204567275038,0.92073412850485
"7882","C4orf36",5.82736046888112,0.510741693434571,0.781737764264286,
0.653341461526096,0.51353616846583,0.92073412850485
"7883","MYOZ3",3.53452668483804,-0.544241085333065,0.834385177450432,-
0.652266003809011,0.514229587092908,0.92073412850485
"7884","TBC1D7",13.5322220506031,-0.314022919803437,0.481369710171511,
-0.65235288629098,0.514173550144583,0.92073412850485
"7885","MCUR1",61.2729263013232,-0.159571698031848,0.243802028730852,-
0.654513413454853,0.512781088226603,0.92073412850485
"7886","MCM9",7.38858469931208,0.343574015279353,0.526537752118471,0.6
52515444328575,0.514068712955236,0.92073412850485
"7887","MTFR1",18.5313418961783,-0.213422284646592,0.326550200841595,-
0.653566539222924,0.513391107625533,0.92073412850485
"7888","PDP1",47.0711334262129,0.191129153052719,0.292289109338285,0.6
53904462897771,0.513173358489531,0.92073412850485
"7889","ZNF782",4.91333279263612,0.41042214100665,0.62928797906111,0.6
5220082801994,0.514271625871812,0.92073412850485
"7890","PPRC1",18.2738540328704,-0.215646669827999,0.329731248240299,-
0.654007380188733,0.513107050848578,0.92073412850485
"7891","AL391121.1",11.864134764022,0.333489588758844,0.50907382637785
3,0.655090816850041,0.512409284208735,0.92073412850485
"7892","MAP4K2",2.59139017518897,0.594659930393025,0.909030030691231,0
.654169730719285,0.51300246058195,0.92073412850485
"7893","PACS1",51.4342150958358,0.144133339342644,0.220581792823368,0.
653423555488372,0.513483257068368,0.92073412850485
"7894","TMEM126B",101.036395992594,0.159742409888645,0.2448507939477,0
.652407155039758,0.514138549820047,0.92073412850485
"7895","MMP27",6.24734753355874,0.481432332003936,0.738130892520917,0.
65223165278954,0.514251743485899,0.92073412850485
"7896","PUS3",19.7964275036436,-0.217137917392009,0.332179637976276,-0
.653676181703578,0.513320451620513,0.92073412850485
"7897","PAN3-
AS1",1.67318038572004,-0.694910541603461,1.06193979370081,-0.654378473
926222,0.512867999316625,0.92073412850485
"7898","DCUN1D2",5.7267666262239,0.374976520972078,0.572969935238718,0
.654443624194436,0.51282603674131,0.92073412850485
"7899","GMFB",43.1441583171465,-0.150927500794117,0.231296062579546,-0
.652529485849811,0.514059657796634,0.92073412850485
"7900","GOLGA5",40.5601418343606,-0.153734525640434,0.23569046127343,-
0.652273005915514,0.514225070798093,0.92073412850485
"7901","FAM192A",38.5407286860519,-0.16266830601938,0.249202528459771,
-0.652755439620829,0.513913955135227,0.92073412850485
"7902","PRPF8",89.8551987189007,0.140091805091959,0.21435315891944,0.6
53556055801397,0.513397863636146,0.92073412850485
"7903","CTNS",9.82297802929466,0.295054559207284,0.450953791584325,0.6
54290006456484,0.51292498311048,0.92073412850485
"7904","CDRT4",4.18057666549802,0.494974052397359,0.758808054914613,0.
652304689165506,0.514204635648716,0.92073412850485
"7905","WNT3",1.74845009479878,0.733828475644699,1.12051034163749,0.65
4905580409276,0.512528547119905,0.92073412850485

"7906","RBFA",46.1240906197452,-0.152571821499694,0.233415787307022,-0.653648252588031,0.513338449270857,0.92073412850485
"7907","IER2",544.441969729534,-0.14915067462187,0.227737701977416,-0.654923068630333,0.512517286858555,0.92073412850485
"7908","MAMSTR",4.67539051657067,-0.426805066835567,0.652436683934573,-0.654170860322697,0.513001732902127,0.92073412850485
"7909","SNHG11",17.1983440092012,0.293601140065615,0.449793938375819,0.652745879870664,0.513920119151323,0.92073412850485
"7910","PCDHGA3",2.33010059128042,0.58984167635337,0.904895928506757,0.651833716753169,0.51450844824116,0.921041672199093
"7911","NR1D2",65.7001356958017,-0.325261338417,0.499122966332854,-0.651665742425666,0.514616826873064,0.921046432338365
"7912","AMER1",3.0848822556273,0.510510685898222,0.783438876454734,0.651627971550782,0.514641198634261,0.921046432338365
"7913","SLC39A10",51.989870850525,0.147837518379157,0.227110871727836,0.650948663330929,0.515079626526609,0.921248896250699
"7914","PPP1R7",105.962315620141,-0.161353793344375,0.247811059995471,-0.6511161904853,0.514971485898741,0.921248896250699
"7915","PLCXD3",5.45269477242368,-0.538146557242009,0.826581242782639,-0.651051015179547,0.515013555876518,0.921248896250699
"7916","KANK2",169.393864971784,0.189440958905073,0.291000507210329,0.650998724095518,0.515047310514715,0.921248896250699
"7917","AL359555.4",3.16580264496305,-0.506457891450543,0.777942527119448,-0.651022246239509,0.515032126494148,0.921248896250699
"7918","ATRAID",327.533318388049,-0.153485976986472,0.236087106730493,-0.650124350762134,0.515611901139906,0.921377569362109
"7919","PAIP2B",6.96498149254989,0.341963055277133,0.525818714765869,0.650344017194974,0.515470030368807,0.921377569362109
"7920","POLR2D",21.2580256538515,0.197465792493035,0.303732147585722,0.650131354427357,0.515607377535322,0.921377569362109
"7921","ALDH7A1",57.013845212783,0.152698012040145,0.234841152960713,0.650218286339662,0.515551230707004,0.921377569362109
"7922","CNOT6",18.700570735793,0.278841806562249,0.428957069618849,0.650045951707977,0.515662539791907,0.921377569362109
"7923","SP4",9.64383706523546,0.369098016869721,0.568080003104585,0.649728937566156,0.515867328382966,0.921377569362109
"7924","LINC00863",12.308322010233,-0.29749760715358,0.45771537517106,-0.649962014149945,0.515716758673895,0.921377569362109
"7925","CHUK",10.7065089450417,0.281796911323926,0.433608539378217,0.649887826766547,0.515764681960982,0.921377569362109
"7926","TRDC",2.26989083001314,-0.720401206526492,1.10863840545103,-0.649807189597952,0.515816774276722,0.921377569362109
"7927","TM2D3",83.1029661121083,0.142243525911406,0.218760775058242,0.650224090098123,0.515547482338916,0.921377569362109
"7928","ISYNA1",13.881806331869,-0.254542746813372,0.391228987693216,-0.650623432364303,0.515289600377048,0.921377569362109
"7929","GPD2",18.2183844261045,0.250454256068213,0.385673752702978,0.649394091023605,0.516083682373036,0.921647741506141
"7930","SEMA6C",13.6199107908108,0.251906369128983,0.388338846224473,0.648676720287138,0.516547354615774,0.921894446856486

"7931","ELK4",51.1284733182743,0.171627631208318,0.264575826530023,0.648689766783522,0.516538920091684,0.921894446856486
"7932","IRF2BP2",240.012242621171,0.141179399400167,0.217560651771021,0.648919729973764,0.516390261239378,0.921894446856486
"7933","AL049840.2",8.71883309567726,0.303595219198677,0.467887857866127,0.648863213897597,0.516426793791705,0.921894446856486
"7934","PRR34-AS1",8.45065708166484,-0.326804117456356,0.503659210534005,-0.648859607093975,0.516429125310828,0.921894446856486
"7935","FAM76B",23.0828290844481,0.21941802433312,0.338413250565901,0.648373029029463,0.516743710347935,0.921896300683729
"7936","OCEL1",37.4431193715439,-0.250230878356867,0.385910433867212,-0.648416980720893,0.516715290387946,0.921896300683729
"7937","ABHD12",43.8771499440199,-0.197723618616192,0.30491419713154,-0.648456583774268,0.5166896830298,0.921896300683729
"7938","MRI1",27.1265222409845,0.200117560874231,0.308834375561376,0.647976963414361,0.51699985007974,0.922237071948743
"7939","SPAST",18.5474775727141,0.233691493947433,0.361161885698077,0.64705469541931,0.517596545082982,0.923185171731329
"7940","USP1",67.4259761299269,0.146394788019,0.226637529388691,0.645942392744354,0.518316663784098,0.923460944034328
"7941","RBMXL1",20.7596722426341,-0.205398395157094,0.317962543800844,-0.645982991272535,0.518290370683717,0.923460944034328
"7942","SFXN5",15.5043998760295,0.265281380499742,0.410702642302805,0.645920802973929,0.518330646394507,0.923460944034328
"7943","REV1",50.4749441687582,-0.147329559023493,0.22813192736806,-0.64580859296295,0.518403322325485,0.923460944034328
"7944","AC108075.1",5.82705214830297,0.51779811211676,0.801673123520668,0.645896808717713,0.518346186498423,0.923460944034328
"7945","BASP1",410.194692347725,0.151678408268902,0.234800672860255,0.645987962560805,0.518287151141938,0.923460944034328
"7946","CINP",53.5906765854511,-0.154505773159963,0.238956404092589,-0.646585613583708,0.517900171401126,0.923460944034328
"7947","CYB5D1",8.57669966967022,-0.338673525224461,0.524035667217156,-0.646279530977263,0.518098341576086,0.923460944034328
"7948","ZNF331",40.2866098995736,-0.15803076876638,0.244505817312534,-0.64632723467835,0.518067453704492,0.923460944034328
"7949","CHKB",42.6809445754261,-0.154659884544365,0.239331852599787,-0.646215214833893,0.518139987415799,0.923460944034328
"7950","PSMB2",146.155120624692,-0.190173573555991,0.29644261631514,-0.641519009378268,0.521185534383335,0.923660682125824
"7951","KYAT3",37.5240795343857,0.200090807813023,0.312232337940162,0.640839475926959,0.521626982142432,0.923660682125824
"7952","LINC01138",5.84243336836171,0.376830059834408,0.587261372381277,0.64167349932519,0.521085199362417,0.923660682125824
"7953","LYPLAL1",51.5647214924847,0.227227579639801,0.353187232331439,0.643362949843458,0.519988618445259,0.923660682125824
"7954","BPNT1",13.0870100885062,0.263439367999,0.409451203264432,0.643396248194356,0.519967017266913,0.923660682125824
"7955","AL356512.1",1.62735843256544,-0.668734127436191,1.044630123961

31,-0.640163548893562,0.522066277803444,0.923660682125824
"7956","SH3YL1",56.3804436540676,-0.145449625373815,0.226597493317233,
-0.641885412078182,0.520947586711806,0.923660682125824
"7957","DTNB",2.70732947494888,0.553533021403858,0.864030601864599,0.6
40640528482812,0.521756261478771,0.923660682125824
"7958","ORMDL1",105.005776631555,0.131221829593581,0.204091901169435,0
.642954614277627,0.520253549954119,0.923660682125824
"7959","AC018809.2",1.82335974199429,0.69777419077565,1.08782569888859
,0.641439333055425,0.52123728484467,0.923660682125824
"7960","IGF2BP2",17.498960879953,-0.314160402505042,0.49077771662383,-
0.640127682785236,0.522089593067001,0.923660682125824
"7961","AP002026.1",3.55341480546738,0.498892413663679,0.7766398727899
84,0.642372908142701,0.520631085860913,0.923660682125824
"7962","SPEF2",16.2915473087359,0.244164274223593,0.379703239536559,0.
643039744726971,0.520198310870519,0.923660682125824
"7963","DUSP22",42.0660647300679,0.173881351983751,0.269970072163299,0
.644076399248336,0.519525893573284,0.923660682125824
"7964","EXOC2",12.8064706021404,-0.242420805238165,0.377190046388298,-
0.642702021326949,0.52041746918718,0.923660682125824
"7965","CD109",99.6891888632213,-0.119808339456533,0.186524706488443,-
0.642318874062705,0.520666161943271,0.923660682125824
"7966","IRAK1BP1",17.6230023425975,0.24517770591133,0.382725746887597,
0.64060938649977,0.521776499545553,0.923660682125824
"7967","ZNRF2",18.8292424257127,-0.229761386754863,0.35744179976827,-0
.642793839175547,0.520357881265085,0.923660682125824
"7968","RBM48",19.2457586823651,0.223986627449465,0.347825336570983,0.
643962943176094,0.519599464042511,0.923660682125824
"7969","AC009403.1",4.39358538622867,0.433054875972379,0.6739028128930
37,0.642607313231552,0.520478936505102,0.923660682125824
"7970","ZFHX4-
AS1",4.68560182051475,-0.463049816204416,0.719776789501774,-0.64332418
460581,0.520013766664354,0.923660682125824
"7971","EXOSC4",24.8046674448148,-0.211862547241058,0.328944270760708,
-0.644068208730647,0.519531204525726,0.923660682125824
"7972","TLN1",231.068895590975,0.119871756782864,0.187033849753525,0.6
40909423298684,0.521581533100357,0.923660682125824
"7973","RAB18",91.442128263792,-0.123063360604415,0.191678242889734,-0
.64203093031904,0.520853100402823,0.923660682125824
"7974","TCTN3",38.4962696060033,0.15941120958282,0.249038285867715,0.
64010688317602,0.522103114388072,0.923660682125824
"7975","HIKESHI",105.041273015919,-0.164440398617257,0.256226000883911
,-0.64177873459361,0.521016858973488,0.923660682125824
"7976","AMOTL1",43.6813805088869,-0.180897718736364,0.28102082854726,-
0.643716409461591,0.519759347096983,0.923660682125824
"7977","C12orf66",6.06171981320843,0.343395475691901,0.535398644688032
,0.641382788505175,0.521274012624058,0.923660682125824
"7978","VPS29",172.926139275888,-0.165848993639793,0.25777809077291,-0
.643378935512011,0.519978248225977,0.923660682125824
"7979","DDX55",27.1235721052504,0.18062840309265,0.280843291417698,0.6
43164386020536,0.520117439613216,0.923660682125824

"7980","AACS",15.9487539339066,0.219239039123653,0.341365454727194,0.6
42241433887503,0.520716434154033,0.923660682125824
"7981","ANKRD10",82.8783494369874,-0.159000269714947,0.248183967194743
,-0.640654879975319,0.521746935090309,0.923660682125824
"7982","NYNRIN",24.7052688140424,0.193943055036886,0.301532262346639,0
.643191721932332,0.520099704066212,0.923660682125824
"7983","SCFD1",95.0760474746465,-0.114493468182754,0.177599266350338,-
0.644673092043634,0.519139057263091,0.923660682125824
"7984","GSKIP",15.8521655599755,-0.234724115229301,0.366569639711117,-
0.640326120336318,0.521960602684135,0.923660682125824
"7985","CSNK1G1",36.1769625555839,0.170078728879042,0.265370503704949,
0.640910449746681,0.52158086617012,0.923660682125824
"7986","ABHD17C",4.02959379591438,-0.445570553838397,0.694858014646953
,-0.641239712928669,0.521366951458667,0.923660682125824
"7987","CLUAP1",45.6554301712889,0.160466364628007,0.250489268789338,0
.640611733203468,0.521774974492247,0.923660682125824
"7988","TFAP4",9.95345528531339,-0.268760820745738,0.417970555069019,-
0.643013766128473,0.520215167432358,0.923660682125824
"7989","BOLA2B",5.87191969884641,-0.379946865889921,0.592005854441902,
-0.64179579144214,0.521005782592545,0.923660682125824
"7990","RANBP10",9.95109068743277,0.296594238761106,0.463227832480837,
0.640277241487591,0.521992373886312,0.923660682125824
"7991","NLRP1",15.6274479243306,0.261025905104786,0.406025887379836,0.
642879957209717,0.520301995603062,0.923660682125824
"7992","ATXN7L3",17.9981756632004,0.217182461795845,0.338007567070928,
0.642537277132235,0.520524393644175,0.923660682125824
"7993","ATP8B1",12.2431664825438,-0.308990806302101,0.480437611145086,
-0.643144498128789,0.520130343078742,0.923660682125824
"7994","AC090337.1",2.12667506761419,0.660370214682619,1.0312005378916
1,0.640389711231931,0.521919270154747,0.923660682125824
"7995","IZUMO4",2.51296154829023,0.538827101813643,0.836093488318542,0
.644457957563181,0.519278511934373,0.923660682125824
"7996","PRKCSH",118.49561440771,0.109741762645383,0.171352009527284,0.
640446312524329,0.521882482112584,0.923660682125824
"7997","RTN2",36.763945787272,0.179885537377553,0.279293171909092,0.64
4074239795967,0.519527293817688,0.923660682125824
"7998","ZNF547",1.92846619995326,0.622538011745968,0.96770906440751,0.
643311130011087,0.520022235728406,0.923660682125824
"7999","DEPDC5",8.45951647058202,0.293505080809122,0.456243420729154,0
.643308083961082,0.520024211839251,0.923660682125824
"8000","PACSIN2",26.5547631524512,-0.185017786236044,0.288149554423871
,-0.642089440693291,0.520815111576114,0.923660682125824
"8001","ARHGAP6",79.9687683220208,-0.148276032336817,0.231201240237713
,-0.641328879483364,0.521309029770464,0.923660682125824
"8002","HDX",9.2131691042576,-0.287758219128371,0.447510174983352,-0.6
43020505933919,0.520210794192207,0.923660682125824
"8003","DIAPH2",91.4835782033506,-0.130701501176531,0.203012467179413,
-0.643810220093644,0.519698505650511,0.923660682125824
"8004","SOSTDC1",2.21801625349506,-0.995231723465109,1.54871309977434,
-0.642618522184725,0.520471661490532,0.923660682125824

"8005","SRPK1",34.1638670231051,0.162878865373877,0.254829412166986,0.639168234109274,0.522713494685288,0.924021780910822
"8006","C1GALT1",76.8198229303341,0.126216450637044,0.197586876049038,0.638789646159083,0.522959784761252,0.924021780910822
"8007","SLC37A3",43.2701027319681,0.158713204729502,0.248248171402056,0.639332825023931,0.522606438786446,0.924021780910822
"8008","TIMM22",39.392847573269,-0.222706002859679,0.348526037313118,-0.638993874249914,0.522826916945531,0.924021780910822
"8009","WIPI1",55.8394922571239,-0.146454904707648,0.229155396769677,-0.639107377666734,0.522753080774317,0.924021780910822
"8010","ZNF792",2.43767462168573,0.534689687672803,0.836920637667706,0.638877407973656,0.522902686081095,0.924021780910822
"8011","DSTN",1099.51635745103,-0.0855359738631083,0.133864025067624,-0.638976557143702,0.522838182529432,0.924021780910822
"8012","TOP1",141.30726097713,-0.099537619837101,0.155790232155478,-0.638920800488714,0.522874455676429,0.924021780910822
"8013","SMTN",25.7916920674272,-0.189402639646748,0.296458205348824,-0.638884794650529,0.52289788038418,0.924021780910822
"8014","LANCL3",3.10818069295105,-0.513507247035854,0.803555568421899,-0.639043853612178,0.52279440374235,0.924021780910822
"8015","CH25H",16.3566009350933,-0.6999609288229,1.09597298947286,-0.638666222202765,0.523040090990138,0.924048370358123
"8016","DHCR7",7.90495343195401,-0.323491717026124,0.506696395985568,-0.638433033250424,0.523191833475924,0.924201142966453
"8017","RPL26",4672.11796818177,-0.162809267280467,0.255077744664155,-0.638273117456122,0.523295908155407,0.924269684355814
"8018","DROSHA",21.0709378467109,0.201526029085307,0.315963986249771,0.637813288398001,0.523595228350012,0.924391773329807
"8019","IL6",34.9511261605479,0.633238421021369,0.992901221882755,0.63776577877567,0.523626159172202,0.924391773329807
"8020","AC016405.3",1.93323559637248,-0.649117600539468,1.01777536986856,-0.637780810733609,0.523616372615062,0.924391773329807
"8021","XPA",64.4249967411705,-0.13455786077836,0.210899778521928,-0.638018027905942,0.523461944784378,0.924391773329807
"8022","PLEKHA6",26.3133887504016,0.349456313468223,0.548204178028227,0.637456494266685,0.523827539693399,0.92448046501916
"8023","TAMM41",15.814527806626,-0.240828377043638,0.378014853952671,-0.637087073498415,0.524068128016158,0.92448046501916
"8024","USP38",17.1481102240005,-0.230372869452982,0.36154815922178,-0.637184462365544,0.52400469721556,0.92448046501916
"8025","TSBP1-
AS1",1.59198823668846,-0.771233275884985,1.20983155735619,-0.63747161428847,0.523817693861101,0.92448046501916
"8026","AL022345.4",2.29781554273594,-0.640352294014015,1.0048686652038,-0.637249738386601,0.523962184186817,0.92448046501916
"8027","GABPA",49.928995502283,-0.141448192278058,0.222007420745705,-0.637132721973639,0.524038396003236,0.92448046501916
"8028","NDUFB9",175.040769255265,-0.138935399845808,0.218250581338896,-0.636586619808682,0.524394142928269,0.924598993631515
"8029","TNKS2",49.1052777971848,0.174160750507321,0.273542991730938,0.

636685112659107,0.524329972659827,0.924598993631515
"8030","NUDT14",12.3978668353098,0.317267631470754,0.498470095451148,0
.636482778738423,0.524461802037311,0.924598993631515
"8031","HAUS5",8.05661478121,-0.334048630760587,0.524819258218625,-0.6
36502234873079,0.524449124778817,0.924598993631515
"8032","HIF3A",17.8516499234257,-0.285993681819667,0.449294021080668,-
0.636540146098045,0.524424422974856,0.924598993631515
"8033","LIX1L",54.3683401338469,0.143244396836925,0.22509504691412,0.6
36372940234338,0.524533373725487,0.924610055017166
"8034","TEAD3",15.5254384947768,0.235064330958149,0.369503479613271,0.
636162699209685,0.52467038248832,0.924736447104133
"8035","ABL2",134.89001788049,-0.302779132936259,0.476403948482728,-0.
635551266736061,0.525068941713823,0.92476257961077
"8036","CCDC121",10.3667229231196,0.285437470261712,0.448956094709535,
0.635780366109928,0.524919586270999,0.92476257961077
"8037","AC108673.3",3.79842741897798,-0.475526286164596,0.748511347428
477,-0.635296028307752,0.525235363452526,0.92476257961077
"8038","ABCF1",87.1675097444358,0.133542328633956,0.210203962558233,0.
635298816486205,0.52523354534573,0.92476257961077
"8039","GNA14",3.0363482714501,0.543036520492616,0.854401194306942,0.6
35575563460099,0.52505310105864,0.92476257961077
"8040","CAMK1D",58.4242319622367,0.235851548687292,0.371112384396515,0
.635525944710311,0.525085451091898,0.92476257961077
"8041","RPS3",3547.57181096519,-0.158725255579299,0.249617881088516,-0
.635872938617782,0.524859242176572,0.92476257961077
"8042","HIC2",14.2686066557443,-0.284413851043368,0.447630915879237,-0
.63537579946801,0.525183347792671,0.92476257961077
"8043","MT-
ND5",2454.30664723658,-0.137617106930309,0.216638541049854,-0.63523833
8771127,0.525272982190105,0.92476257961077
"8044","LINC01914",15.8092411558292,-0.249442000719396,0.3928025128555
93,-0.635031580898004,0.525407818139694,0.924884970768035
"8045","ARNT",52.8535757296626,0.144910538694006,0.228431403106442,0.6
34372230452404,0.525837927954381,0.924930495067611
"8046","RFX5",10.5971861645502,0.256331491758915,0.404757166283307,0.6
3329698177474,0.526539723914342,0.924930495067611
"8047","GORAB-
AS1",2.3129625207904,-0.565188285712969,0.891350589459315,-0.634080789
755024,0.526028098933529,0.924930495067611
"8048","TMEM87B",34.0953633344971,-0.202503481637268,0.319633244217637
,-0.633549498685387,0.526374867584813,0.924930495067611
"8049","KLF3",92.0507947233081,-0.14062827138388,0.221767725712972,-0.
634124153691736,0.525999800857448,0.924930495067611
"8050","RPL34",5080.82920358264,-0.15239609847328,0.240321699557668,-0
.634133741371579,0.525993544314356,0.924930495067611
"8051","REPS1",17.4894906681856,0.246622471791321,0.389156648854583,0.
633735727032321,0.526253304814373,0.924930495067611
"8052","LINC00957",1.83147697473222,0.702487529562926,1.10937273554522
,0.633229488209548,0.526583791731557,0.924930495067611
"8053","MICU1",80.1274507725302,0.169964262447273,0.268211192997756,0.

633695635695168,0.526279473700294,0.924930495067611
"8054","R3HCC1L",16.9970859663631,0.209849092616755,0.33083181440542,0
.634307474309572,0.525880179616333,0.924930495067611
"8055","AL121929.3",1.82202548536237,-0.635917241385776,1.004260436350
03,-0.6332194502225,0.52659034588285,0.924930495067611
"8056","AC009318.3",1.81764839469836,0.665584902431742,1.0511610774022
9,0.633190209131972,0.526609438646545,0.924930495067611
"8057","AMN1",6.37097868924754,0.398865217142089,0.629310763365945,0.6
33812800227203,0.526202998565986,0.924930495067611
"8058","RPL21",4423.88759239338,-0.130684986710976,0.206269958493866,-
0.633562869092556,0.526366139414403,0.924930495067611
"8059","NLRC5",6.38656762372555,-0.333808744008958,0.526596383538303,-
0.633898664031896,0.526146957514907,0.924930495067611
"8060","TBC1D16",50.0040860893147,0.141363864641586,0.222877190754567,
0.634267975843503,0.525905952167715,0.924930495067611
"8061","GET1",48.2261916992895,0.153360171877849,0.241980102887,0.6337
71826890514,0.526229741862102,0.924930495067611
"8062","PMM1",71.4743154371415,-0.170784503984738,0.269292099765881,-0
.634197973624979,0.525951629851044,0.924930495067611
"8063","DDX20",11.359924315939,0.271663980286392,0.429781359043727,0.6
32098099579865,0.527322776943908,0.924975828849437
"8064","LINC01703",2.75234353552494,0.508431126852409,0.80637914668736
6,0.63051125384512,0.528360142124303,0.924975828849437
"8065","SERTAD2",57.0020829853968,-0.177893623345071,0.281423468304638
,-0.632120783731166,0.527307955219435,0.924975828849437
"8066","CHST10",6.68610753261554,0.342164485069677,0.542008854942815,0
.631289474238898,0.527851267960272,0.924975828849437
"8067","PGM5P4-
AS1",3.38909959359491,-0.495468036073319,0.784790069708017,-0.63133830
969301,0.527819343049724,0.924975828849437
"8068","ZC3H15",156.216132007061,-0.0969481575294064,0.153162977501352
,-0.632973836830447,0.526750728399094,0.924975828849437
"8069","ITIH4",3.978348804149,0.601464733541053,0.951792624891579,0.63
1928340072573,0.527433703803785,0.924975828849437
"8070","RASA1",29.7429331913321,0.164171130505951,0.260064921236371,0.
631269798808188,0.527864130540556,0.924975828849437
"8071","PHAX",97.0112868104519,0.109154525593828,0.172500740384085,0.6
32777142583782,0.526879185297786,0.924975828849437
"8072","PHF1",40.8925590411477,-0.150282111800361,0.238119692217919,-0
.631120048915682,0.527962032997923,0.924975828849437
"8073","MRPS17",40.0773833182266,-0.192148487064682,0.304227842761418,
-0.631594022823772,0.527652193299365,0.924975828849437
"8074","MTBP",2.94099411481503,0.50937145601826,0.806987352626327,0.63
1201287554902,0.527908920209329,0.924975828849437
"8075","FAM171A1",27.5970381349596,0.180786219293302,0.286586126156798
,0.630826836308153,0.528153754320242,0.924975828849437
"8076","CDK17",20.7469581324106,0.209250510009313,0.330796539279554,0.
632565595955273,0.52701735982215,0.924975828849437
"8077","POGLUT2",15.1633326150077,0.26639753782426,0.422550150033712,0
.630451883174117,0.528398974545416,0.924975828849437

"8078","LINC00517",1.76482575756407,-0.664728536814351,1.05162180434916,-0.632098473106256,0.52732253288169,0.924975828849437
"8079","FAHD1",32.3163480363432,0.165808752128771,0.262536587099623,0.631564361983013,0.527671580056132,0.924975828849437
"8080","ABCA3",2.99026597535139,0.570583042884207,0.90282027360728,0.632000697773881,0.527386421269976,0.924975828849437
"8081","AC009093.2",2.21981210841017,-0.568503828141523,0.90006144869639,-0.631627794929912,0.527630119801951,0.924975828849437
"8082","GABARAP",811.694622758852,-0.158747026895497,0.251022909166051,-0.632400554287603,0.527125171846983,0.924975828849437
"8083","C17orf113",1.9071656078634,0.861792624051733,1.36594586695177,0.6309127212888,0.528097593505057,0.924975828849437
"8084","TRAPPC8",23.4550645195355,-0.215663079770473,0.341442662865745,-0.631623119268112,0.527633175793184,0.924975828849437
"8085","FECH",16.8130372586019,-0.264873768747852,0.420115367618561,-0.630478647447005,0.528381468726784,0.924975828849437
"8086","APOE",2829.02741818807,-0.398811976134424,0.632224816211743,-0.630807215895263,0.528166584678291,0.924975828849437
"8087","MZF1-AS1",2.79976821651361,-0.507946321460296,0.805507368275912,-0.630591775401747,0.528307477920835,0.924975828849437
"8088","APOL6",198.307766597398,0.148917842555369,0.236200559403345,0.630472014679149,0.528385807021149,0.924975828849437
"8089","KCNE5",1.62990087927369,-0.722121323916232,1.14359887112103,-0.63144634202757,0.527748723212265,0.924975828849437
"8090","ACVR1",59.3988269398353,0.17146701822633,0.272359059071047,0.629562382874885,0.528980941680467,0.925104103809715
"8091","SP110",66.8536040150149,0.123220498675984,0.195974409439668,0.628758106878839,0.529507430351135,0.925104103809715
"8092","PRRT3",8.06508866450738,-0.317713195958561,0.505315185518672,-0.628742624531359,0.529517567893908,0.925104103809715
"8093","CAND2",2.42874456958183,0.519384391880237,0.8258559611147,0.628904332396169,0.529411689567913,0.925104103809715
"8094","DCAF1",9.34641362169969,0.297353578887011,0.472476639744645,0.629350858590001,0.529119382182011,0.925104103809715
"8095","TASOR",66.3746712040767,0.15304035540332,0.243171314771199,0.629352008674693,0.529118629413394,0.925104103809715
"8096","AC025181.2",10.2387072027854,-0.264572216371958,0.420740094128817,-0.628825776444672,0.529463122781766,0.925104103809715
"8097","AC007036.3",2.78197307067164,0.509487064511423,0.808865692137014,0.629878445166049,0.528774116465794,0.925104103809715
"8098","CTSD",251.17639889311,0.129553807420432,0.205838853908007,0.62939432940261,0.529090929470765,0.925104103809715
"8099","ADAMTS15",18.7525224553963,0.331224826158391,0.526576928172456,0.629015075362196,0.529339186753019,0.925104103809715
"8100","TFCP2",24.3762414174763,0.197854106553223,0.314590743647335,0.62892539131737,0.529397902018396,0.925104103809715
"8101","KMT5A",33.4755585294277,-0.186785537819964,0.296436284689583,-0.630103490925745,0.528626875849503,0.925104103809715
"8102","ZNF785",13.4083536699907,0.317993791323547,0.504563030406845,0

.630236010488399,0.528540182084485,0.925104103809715
"8103","IRX5",11.0137881169801,-0.267119551150226,0.424080650361758,-0
.629879130119147,0.528773668290082,0.925104103809715
"8104","OGFOD3",52.8298993714274,0.157861054207751,0.250650471892723,0
.629805533640945,0.528821824742688,0.925104103809715
"8105","ZNF711",2.61757975038447,-0.637915179749245,1.0139513839689,-0
.629137836226685,0.529258821787803,0.925104103809715
"8106","FXYD6",19.4188933974375,0.434609167525683,0.691387763740443,0.
628604077651628,0.52960829011348,0.925148456452859
"8107","NGF",1.6949524870974,0.703311983014551,1.11931946859943,0.6283
38917301766,0.529781942438952,0.925207747561936
"8108","TTC39B",19.69458793778,0.207330827584381,0.330011707295029,0.6
28252946793273,0.529838250351677,0.925207747561936
"8109","POU2F2",2.32639860769895,-0.631672746416231,1.00517537673517,-
0.628420433922606,0.529728554488424,0.925207747561936
"8110","SMAD1",17.6199841370243,0.201574994748849,0.320947623890985,0.
628061963210909,0.529963349326232,0.925238439124795
"8111","MTMR2",21.9325005143582,-0.197308947876398,0.314172911680232,-
0.628026607453734,0.52998650986873,0.925238439124795
"8112","ZNF487",9.83066426231411,0.30087344413656,0.479173790218667,0.
627900461749501,0.53006914849224,0.925268631983497
"8113","ADAM8",2.12496130757911,-0.59403153778434,0.946476661306376,-0
.627624073650614,0.530250234472303,0.925424619348416
"8114","ZNF101",3.31680246281927,-0.478722104005115,0.76282524862054,-
0.627564576383406,0.530289220437362,0.925424619348416
"8115","FNDC5",25.2818858540754,0.352369926111301,0.56246090703382,0.6
26478963612868,0.531000830672923,0.925474117810924
"8116","NEXN-
AS1",2.02264526420016,-0.668309925336929,1.06589571618103,-0.626993724
800206,0.530663348564345,0.925474117810924
"8117","TOMM20",320.456018773372,-0.11237777809261,0.179407518238172,-
0.626382769218309,0.53106390866431,0.925474117810924
"8118","NIFK-
AS1",17.5199321568595,0.221013413728251,0.353083419975958,0.6259523988
50393,0.531346163935854,0.925474117810924
"8119","ACTR8",13.926009673679,-0.239946475042871,0.383472822171863,-0
.625719636880375,0.53149885086783,0.925474117810924
"8120","LINC00884",2.50410993318622,-0.549520096634694,0.8776878627866
9,-0.626099687524387,0.531249557170527,0.925474117810924
"8121","NRM",13.5099908918816,-0.240185944896994,0.382947043560814,-0.
627204071517661,0.530525474686729,0.925474117810924
"8122","C2",108.083410635673,-0.1863323403893,0.29731275765449,-0.6267
21644436929,0.530841713355735,0.925474117810924
"8123","HLA-
DRB5",17.75361629384,1.08209946967941,1.72919016471629,0.6257839604685
43,0.531456653729781,0.925474117810924
"8124","AKR1B10",2.40715085157429,0.624227516656757,0.996642016062414,
0.626330725171499,0.531098037330231,0.925474117810924
"8125","TCIM",22.1154375746996,-0.362177987829917,0.578070598934383,-0
.626528988842465,0.530968028902138,0.925474117810924

"8126","CAAP1",21.1960223949243,0.192902497338595,0.307796928705531,0.626720020079033,0.530842778309881,0.925474117810924
"8127","CCDC180",6.58294545945262,0.324992632144202,0.519252688492882,0.625885314311006,0.53139016769413,0.925474117810924
"8128","KYAT1",1.78861761497728,-0.623138518791016,0.995753163159275,-0.625796173033439,0.53144864231498,0.925474117810924
"8129","STAM",37.8871107457524,0.157435796641303,0.251074617626048,0.627047839920595,0.530627876525324,0.925474117810924
"8130","PBLD",5.83760375212246,0.346178157405283,0.553459457391133,0.625480607083812,0.531655672507354,0.925474117810924
"8131","CYP2R1",5.3669444059345,0.411083091644622,0.656352740683949,0.626314276095282,0.531108824290569,0.925474117810924
"8132","ING4",49.282308607182,0.174520100551573,0.278382383468742,0.626907846599312,0.530719643525265,0.925474117810924
"8133","ITFG1",78.0330129556406,0.116860157620858,0.186299305253612,0.627271032824168,0.530481588039452,0.925474117810924
"8134","TMEM99",31.2827003905141,0.231237698219324,0.369679251072617,0.625508998810166,0.53163704415854,0.925474117810924
"8135","PMAIP1",6.46934448780085,-0.469142985596857,0.750114950926754,-0.625428122739375,0.531690109349708,0.925474117810924
"8136","ZNF836",10.6502776326282,0.280944462131831,0.44929238019181,0.625304310773957,0.531771351255986,0.925501761772955
"8137","NME7",34.7382669955049,-0.155264380498864,0.248400846480403,-0.62505576248555,0.53193446059263,0.925671864568224
"8138","FAAP20",93.3040125204295,-0.146392166321798,0.235560396355737,-0.621463406355968,0.534294764427799,0.92567361104187
"8139","PARK7",638.168196974207,-0.158588045711292,0.254037390837409,-0.624270487066971,0.532449962565166,0.92567361104187
"8140","PAFAH2",6.33334467010882,0.337282141460997,0.541978646994095,0.622316291115195,0.533733911015292,0.92567361104187
"8141","AHCYL1",201.197640425915,0.115388575293868,0.184889961817667,0.624093239889685,0.53256635323633,0.92567361104187
"8142","ISG20L2",31.9024040864635,-0.175327328521099,0.281556373638012,-0.622707723699097,0.533476606222883,0.92567361104187
"8143","RAB29",34.4971147536662,-0.20179142766326,0.324530773749327,-0.621794430561851,0.534077048987436,0.92567361104187
"8144","THAP4",32.9192947725846,-0.16950582555075,0.272256542237086,-0.622595968339087,0.533550061238248,0.92567361104187
"8145","FAM131A",12.9084059117784,-0.250798706949137,0.403696386581225,-0.621255763701704,0.534431354377967,0.92567361104187
"8146","ZDHHC11B",2.4399163828569,0.710334238423801,1.13710041925892,0.624689100797928,0.532175127886623,0.92567361104187
"8147","RPL37",4919.92551442557,-0.151076594100929,0.242062449183524,-0.624122389121114,0.532547211293031,0.92567361104187
"8148","PWWP2A",43.2714924704608,0.182043011714604,0.292865934939487,0.621591622638591,0.534210430945432,0.92567361104187
"8149","GRK6",14.9069448739658,-0.218344771328849,0.350233852448705,-0.623425662031994,0.5330048387319,0.92567361104187
"8150","AL138724.1",7.70796919354704,-0.347962782510098,0.560541604197291,-0.620761741688003,0.534756399101583,0.92567361104187

"8151","HECA",37.1776292809629,-0.18334479388086,0.29501926402375,-0.621467192956253,0.534292273718118,0.92567361104187
"8152","AC073332.1",11.8320566199461,-0.277771622291248,0.446413425588079,-0.622229544116708,0.533790941883789,0.92567361104187
"8153","AC087623.3",1.91602742187936,0.6647774173057,1.06861840311975,0.622090556708485,0.533882324057495,0.92567361104187
"8154","VCPIP1",33.6863919866787,0.183898039314566,0.295775821527402,0.621748046763618,0.534107553027408,0.92567361104187
"8155","WDYHV1",16.6795097078071,-0.206396060452097,0.331339212388371,-0.622914683005208,0.533340588671206,0.92567361104187
"8156","LDB1",16.2138909670572,0.229248001737964,0.367894950014477,0.623134407604517,0.533196200718214,0.92567361104187
"8157","RIC8A",60.1348169008149,-0.147114706764817,0.236835413806317,-0.621168533879511,0.534488740508761,0.92567361104187
"8158","POLR2L",409.627526763694,-0.185352665930844,0.298006280727613,-0.621975702922391,0.533957844692775,0.92567361104187
"8159","EI24",119.329338370797,-0.0973463052146126,0.155822021344495,-0.624727521660096,0.532149906835497,0.92567361104187
"8160","TNFRSF1A",282.481941415178,-0.152446381596824,0.245355205392256,-0.621329314587412,0.534382969664477,0.92567361104187
"8161","LARP4",33.9686447804626,0.207169744599945,0.33257605949541,0.62292440686881,0.533334198396247,0.92567361104187
"8162","FBXW8",13.6258913521326,0.273447540186839,0.440476192552715,0.620799818038095,0.53473134299394,0.92567361104187
"8163","EXOSC8",57.7295750351043,-0.150543182793776,0.241743554457913,-0.622739179670601,0.533455931636464,0.92567361104187
"8164","ZNF768",14.7246177889444,-0.222847108580338,0.358919564842097,-0.620883146000628,0.534676511170133,0.92567361104187
"8165","PER1",85.3747907055958,0.235173100263568,0.377668711484734,0.62269680572433,0.533483782208241,0.92567361104187
"8166","CYBC1",51.6956505216318,-0.13551474665679,0.217414895296075,-0.623300195105063,0.533087269615693,0.92567361104187
"8167","KLF16",9.24827084167064,-0.306252626430425,0.492285261400308,-0.622103992224523,0.533873490058591,0.92567361104187
"8168","GIPC3",4.27904801713,-0.578918032756972,0.929413773289128,-0.622885144802861,0.533360000661205,0.92567361104187
"8169","ARRDC2",14.249083876957,0.234533730971319,0.37764715984177,0.621039308410489,0.534573760150329,0.92567361104187
"8170","AC006504.5",6.53846496468285,0.313407138750973,0.503483492316973,0.622477486419087,0.533627943095951,0.92567361104187
"8171","ZNF146",35.4978633808416,0.151017701501823,0.24173523410228,0.624723582652937,0.532152492535695,0.92567361104187
"8172","ZNF260",18.0328520065789,0.202046112963313,0.324741907309812,0.622174435806821,0.533827173840394,0.92567361104187
"8173","CCDC97",26.9116910040722,0.170822176087377,0.274513782636236,0.622271765180317,0.533763183721239,0.92567361104187
"8174","MROH8",1.88297845350091,-0.594734071068259,0.957628170545678,-0.621049055740879,0.534567346977365,0.92567361104187
"8175","TOMM34",11.0394594477355,-0.27167643512053,0.436555435769607,-0.622318296510474,0.533732592626435,0.92567361104187

"8176","WFDC3",6.35549081047705,-0.474508936003761,0.760209002591978,-0.624182210926067,0.53250792813704,0.92567361104187
"8177","ZSWIM1",3.58575566324708,0.488004186943292,0.781758283246941,0.624239227650295,0.532470488357301,0.92567361104187
"8178","HLCS",10.3102673782672,0.272440810271443,0.438941028330094,0.620677477582617,0.53481185112525,0.92567361104187
"8179","PRICKLE3",6.24656862368511,-0.410404153147077,0.658970065535663,-0.622796352385853,0.533418355635268,0.92567361104187
"8180","UPRT",9.34341581697208,0.268810510314825,0.430769293843908,0.624024307573395,0.532611621629685,0.92567361104187
"8181","SSR4",510.855923551782,-0.155807495531713,0.250013951365201,-0.623195204431298,0.533156252700331,0.92567361104187
"8182","ARPP19",86.0987286847921,0.108779364683216,0.175327808218,0.620434178632753,0.534971976133462,0.925837592526255
"8183","ZNF496",15.3408967999635,0.228632262476276,0.368660731422922,0.620169828215287,0.535145983375583,0.925844826600661
"8184","TRPV2",35.5545455034896,-0.163432667704114,0.263532843031899,-0.620160530368244,0.535152104152864,0.925844826600661
"8185","REXO1",17.0798748047136,0.253697397966031,0.409103679172388,0.620129837207179,0.535172309726441,0.925844826600661
"8186","HSD17B7",10.5272760785553,-0.346108417351458,0.558359060095132,-0.619867110766483,0.535345280550482,0.925917817588229
"8187","CAPN10",10.3317091291054,-0.290272777207615,0.468265178866775,-0.619889734082064,0.535330384962576,0.925917817588229
"8188","MGP",17516.3712804674,0.217797677768596,0.351524767073124,0.61957989356491,0.535534407553893,0.926018709361721
"8189","DNAJC14",13.3436252497223,0.24451871243511,0.394648573597183,0.61958595265237,0.535530417413755,0.926018709361721
"8190","SYF2",191.936026664726,-0.147867533036422,0.238964236978184,-0.618785199435182,0.536057873729565,0.926374149216423
"8191","AC016065.1",7.13391365231085,0.329188517168895,0.531963469221382,0.618817900504931,0.536036328406635,0.926374149216423
"8192","ZNF484",7.71067921738492,0.293376814105553,0.473932923320956,0.619026025982314,0.535899213733977,0.926374149216423
"8193","AC009041.2",19.739496103993,-0.233080950872078,0.376454402070034,-0.619147895709071,0.53581893321607,0.926374149216423
"8194","EPOP",1.63437941972452,-0.691438890543153,1.11743864615694,-0.618771234484442,0.536067074765492,0.926374149216423
"8195","E4F1",17.377153782066,-0.210211535911391,0.339783202384483,-0.618663708023815,0.536137922993482,0.926383525270006
"8196","AL080317.2",2.49624516512,-0.607702218480841,0.982454725811114,-0.61855493440588,0.53620959775781,0.926394327019349
"8197","ACP1",105.231499550385,-0.148405654976127,0.240427903665852,-0.61725636963662,0.537065640079752,0.92644985402202
"8198","GLT8D1",151.433063879446,-0.127219479021479,0.206102623557901,-0.617262783099596,0.537061410497094,0.92644985402202
"8199","HES1",170.92963648549,0.23039161271728,0.373145407172644,0.61743118979536,0.536950354799246,0.92644985402202
"8200","UNC5C",1.88232516206851,0.728146334512557,1.1791075988088,0.617540193319226,0.536878478644274,0.92644985402202

"8201","RAPGEF2",23.3701926814112,-0.181951205708735,0.2949356290503,-0.616918363829498,0.537288573533109,0.92644985402202
"8202","MDC1",14.4397743761111,0.281853967282834,0.456754477280381,0.617079812684171,0.537182083317108,0.92644985402202
"8203","PSMB9",166.82831041735,0.175381623563777,0.284047904516337,0.617436780117804,0.536946668462356,0.92644985402202
"8204","KBTBD2",29.3774868890044,-0.196691658864792,0.318622777262248,-0.617318261283314,0.537024824165746,0.92644985402202
"8205","LINC00689",1.64557394278845,0.725900722803141,1.17651235322859,0.616993710955199,0.537238873924921,0.92644985402202
"8206","AL132656.3",1.86338983613406,0.644997493400068,1.04316799895131,0.618306441578425,0.536373356517865,0.92644985402202
"8207","SLC35C1",19.3253326179821,-0.22090269767008,0.357395808267419,-0.618089783260107,0.536516156615794,0.92644985402202
"8208","TMEM263",101.897394356586,-0.12413780848105,0.201153511013512,-0.617129712802829,0.537149171792651,0.92644985402202
"8209","ACIN1",68.9879768724508,0.143042896520881,0.231811076475541,0.617066702315122,0.537190730403166,0.92644985402202
"8210","PRR14",19.1148296289784,-0.205012928050603,0.331668236390489,-0.618126505817191,0.536491951330526,0.92644985402202
"8211","ALG12",20.5916281781221,-0.1947742260812,0.315294406595439,-0.617753509123044,0.536737833706241,0.92644985402202
"8212","PIR",20.0436089561659,0.245292947081653,0.397316676094855,0.617373903085537,0.536988131191034,0.92644985402202
"8213","SELENON",77.5195112150262,-0.135175684465086,0.219846200824227,-0.614864773456616,0.538644028258556,0.92650603415345
"8214","STK40",32.1628401527649,-0.156924219005836,0.255309755695999,-0.614642470586542,0.538790860154864,0.92650603415345
"8215","SIKE1",45.3793548457386,-0.149737086189748,0.242979625228612,-0.616253671676647,0.537727110224341,0.92650603415345
"8216","COL5A2",553.908310937146,-0.176418561936993,0.286309112053483,-0.616182141992174,0.537774313293918,0.92650603415345
"8217","ZNF589",5.3576603594382,-0.353080302877684,0.57442837664692,-0.614663754842162,0.538776800953968,0.92650603415345
"8218","MCCC1",14.9528578211693,0.231621091103096,0.375621346871266,0.61663452578609,0.537475816274925,0.92650603415345
"8219","LINC01023",3.25326401634754,0.462613969830718,0.752263685754664,0.614962517254342,0.538579474457697,0.92650603415345
"8220","MALINC1",7.02086333909484,-0.338167191631807,0.54977856287236,-0.615097085388392,0.538490606782993,0.92650603415345
"8221","DDX39B",188.769960173327,-0.0976530469337432,0.158613553753757,-0.615666471261002,0.538114670512392,0.92650603415345
"8222","NMRK1",25.2071760211057,-0.269290745226838,0.43790754329108,-0.614948861586153,0.538588492958253,0.92650603415345
"8223","REXO4",42.7204349782644,-0.160558387838933,0.260474912426049,-0.616406341568567,0.537626368976242,0.92650603415345
"8224","TUBGCP2",60.0166409222872,-0.120963901598361,0.196506982487754,-0.615570500686402,0.538178025726953,0.92650603415345
"8225","YPEL4",10.7873920961863,-0.31266017657339,0.507945707613449,-0.615538574077935,0.538199102987,0.92650603415345

"8226","TMEM179B",145.452563257211,-0.143426122931472,0.23267201709978
,-0.616430478917301,0.537610442495171,0.92650603415345
"8227","TMEM121",15.5729721794483,-0.247155062265934,0.402141422507446
,-0.614597374040368,0.53882064904426,0.92650603415345
"8228","THBS1",158.686452027515,-0.612357497836486,0.995322201528577,-
0.615235445261897,0.538399242744447,0.92650603415345
"8229","GGA2",27.484518862336,0.183247792896933,0.297822933427321,0.61
5291075096646,0.538362510540532,0.92650603415345
"8230","HYDIN",1.92271589203293,0.63298388697865,1.02834351746962,0.61
5537392151012,0.538199883277677,0.92650603415345
"8231","RNF112",2.57031560961878,-0.612032667692204,0.994281865757485,
-0.615552479402742,0.538189922939802,0.92650603415345
"8232","KATNAL2",5.57079503854911,0.463842889235022,0.752851233365173,
0.616114935698104,0.537818665215752,0.92650603415345
"8233","TNFRSF11A",3.22266246270919,-0.579139180585807,0.9424711116888
52,-0.614490113705475,0.538891504045749,0.92650603415345
"8234","PPM1N",3.00938526942036,-0.575755671641523,0.936914798225548,-
0.614522977683738,0.538869793965673,0.92650603415345
"8235","AL021707.6",1.57556875489935,-0.713952220492686,1.161325177301
78,-0.614773738180277,0.538704154980773,0.92650603415345
"8236","AL121601.1",1.7247330148391,-0.704403375180836,1.1450681207714
8,-0.615162855731454,0.538447175272309,0.92650603415345
"8237","STAM2",43.4442313096668,0.160367821011686,0.261421469520372,0.
613445488260438,0.539581816317557,0.926714778402903
"8238","NAA50",84.0047266894955,-0.111070627355632,0.180877119043384,-
0.614066765011836,0.539171209140889,0.926714778402903
"8239","SEC24B-
AS1",3.00545198277514,0.497751453190826,0.811187004236009,0.6136087617
17795,0.539473892311942,0.926714778402903
"8240","TBP",12.6668381970243,0.283356673198398,0.461565183254216,0.61
3903915370353,0.539278822734873,0.926714778402903
"8241","ANKS6",10.0539551616401,-0.281878524214867,0.459204770752389,-
0.613840583043203,0.539320676634529,0.926714778402903
"8242","FAM66C",6.33964699915461,0.332045664562069,0.541306672752441,0
.613415058923402,0.539601931351126,0.926714778402903
"8243","B2M",6664.88846442357,0.156387807319326,0.254928433570498,0.61
3457687433982,0.539573752271449,0.926714778402903
"8244","BFAR",81.4885277314893,-0.136878998057793,0.222983103175946,-0
.613853678185599,0.539312022425033,0.926714778402903
"8245","ZNF585A",26.8016697356465,-0.170285381332688,0.277476531911018
,-0.613692913630965,0.539418271990531,0.926714778402903
"8246","AC118549.1",29.690323659721,0.169748772709426,0.27740609536084
5,0.611914357860882,0.540594421631265,0.926717580814274
"8247","PDGFRA",668.355812743686,0.151990489790915,0.247946405576459,0
.61299735092972,0.539878091328685,0.926717580814274
"8248","TMEM154",4.84850635910939,-0.365704040453397,0.596832746251341
,-0.612741245768359,0.540047445532792,0.926717580814274
"8249","C7",209.689345791256,-0.27479649986361,0.449163242305396,-0.61
1796500651249,0.540672405260083,0.926717580814274
"8250","OARD1",44.2933160770229,-0.144497526749564,0.236287526885021,-

0.611532604596085,0.540847040102342,0.926717580814274
"8251","GATAD1",108.839593957894,0.146275865883023,0.238502587028984,0
.613309346892939,0.539671814235082,0.926717580814274
"8252","DCTN6",85.1527209759906,-0.153627006451252,0.250898161207176,-
0.612308219845409,0.540333852271202,0.926717580814274
"8253","PRPF4",24.2528012205653,-0.182832810813969,0.298281194308877,-
0.61295453519152,0.539906402165108,0.926717580814274
"8254","DCLRE1A",2.48716112901244,0.549632360820107,0.898032418798654,
0.612040667257179,0.540510851612192,0.926717580814274
"8255","STX5",60.8134896647941,-0.171674387762546,0.280140560106885,-0
.612815179983383,0.539998552456869,0.926717580814274
"8256","ARMH4",24.4311993635173,0.200437345636157,0.327741792084775,0.
611570908797345,0.540821690312103,0.926717580814274
"8257","IGHG4",32.3448302681869,0.541400430313167,0.883301161818302,0.
61292847073615,0.539923636998092,0.926717580814274
"8258","LYSMD4",8.26557728466634,0.327528053917236,0.535165540156702,0
.612012600477476,0.540529420862568,0.926717580814274
"8259","THUMPD1",84.0253574523077,0.104367508311767,0.170396902839306,
0.612496510046265,0.540209306322664,0.926717580814274
"8260","STX1B",1.93905611936166,-0.660312474601953,1.07885524086703,-0
.612049188426138,0.540505213988285,0.926717580814274
"8261","SLC7A5",1.83301264805084,0.674165021942948,1.10036355329103,0.
612674801820378,0.540091387146465,0.926717580814274
"8262","FTSJ3",34.2690173534434,-0.174915398710811,0.285970261128947,-
0.611655904429658,0.540765442168484,0.926717580814274
"8263","ZNF790",9.72118123759068,-0.311127827620214,0.508144305277523,
-0.612282425265577,0.540350915405584,0.926717580814274
"8264","ZNF880",9.41009351870344,0.292865389163751,0.478378078114315,0
.612204870085555,0.540402219839956,0.926717580814274
"8265","TNFRSF21",6.07031437490061,-0.373149690195214,0.61056553332101
4,-0.611154200214287,0.541097500717363,0.926872246582317
"8266","NT5C2",26.5537366580205,0.199062590960007,0.325788958975155,0.
611016995745357,0.541188328764763,0.926872246582317
"8267","RAB11B",101.409327362763,-0.123072585181275,0.201397727936373,
-0.611092222550581,0.541138528395475,0.926872246582317
"8268","IFI30",37.2582646119328,-0.232515055217322,0.380547951807959,-
0.6110006744555,0.541199133809505,0.926872246582317
"8269","MESP1",4.05467377611722,-0.425898757479746,0.697301998788873,-
0.610780921637224,0.541344625390728,0.927009299254167
"8270","NUP98",31.5206179397447,0.168434500070621,0.275824425131097,0.
610658392528382,0.54142575662519,0.927036120170821
"8271","RABGGTA",12.2609258770515,-0.291211230528499,0.477285164397976
,-0.610140964460562,0.541768432583511,0.927398574151657
"8272","IFT43",54.3645195013475,-0.211493874077205,0.346582904056228,-
0.610225927482255,0.54171215688207,0.927398574151657
"8273","MAT2A",114.772664959066,-0.13194888271631,0.216583248127009,-0
.609229401892306,0.542372394322117,0.92753728578617
"8274","CREB1",66.3599969446951,0.136245879356416,0.22372454278775,0.6
08989419125438,0.542531452275883,0.92753728578617
"8275","DHX36",159.345719267835,0.103854968849639,0.170552864351618,0.

608931249817813,0.542570009757546,0.92753728578617
"8276","ERCC8",6.97175888089131,-0.315064121241124,0.517226840450089,-0.609141089752722,0.542430923938821,0.92753728578617
"8277","PSMB8-AS1",13.7116188966929,-0.239310428495121,0.392980194855015,-0.608963076583063,0.542548913241008,0.92753728578617
"8278","BHLHE22",21.6771202995662,-0.307879140123732,0.50553361838768,-0.609018132375972,0.542512420222932,0.92753728578617
"8279","MALRD1",1.98148260141433,0.66506738699418,1.09087795573403,0.609662504864416,0.542085397263207,0.92753728578617
"8280","SLC38A1",4.94977363260486,-0.465967390598205,0.764421606900601,-0.609568576282794,0.542147632902646,0.92753728578617
"8281","NRDE2",31.5230399846314,0.188805827898331,0.309622634078527,0.609793364946459,0.541998697318503,0.92753728578617
"8282","EIF5",333.690383851759,-0.130988917690821,0.215019746994971,-0.609194827551743,0.542395308383394,0.92753728578617
"8283","TMEM161A",27.3866542396011,-0.223513780004075,0.366722597683945,-0.609490065285554,0.542199655820942,0.92753728578617
"8284","PRSS12",3.6336468746813,0.542092063186235,0.890456643975729,0.608779851162535,0.542670370645095,0.927596867254292
"8285","EXOC3-AS1",6.59885680681517,0.303104776222597,0.498012252189441,0.608629154985723,0.542770275051867,0.927655654162274
"8286","MSTO1",10.5851083083793,0.3005433082149,0.494273021118574,0.60805120929876,0.543153510538195,0.928131824651191
"8287","ABCB4",4.95580381357896,-0.369760748777015,0.608147815365833,-0.608011308162941,0.543179973932515,0.928131824651191
"8288","PRPF40B",8.75280447625072,0.28072762149115,0.461825866494823,0.607864656048443,0.543277242663039,0.928184982283638
"8289","AC027020.2",2.70277936816557,0.542370185040245,0.892398577647806,0.607766752015484,0.543342183485104,0.928184982283638
"8290","CCDC92",38.8863357095459,0.153780396444141,0.253079207462154,0.607637419076152,0.543427977370578,0.928219560864581
"8291","GAS7",220.8588653995,0.164046375222216,0.270036938180545,0.60749605712288,0.543521758496711,0.928267772321002
"8292","ERCC3",18.5340098369722,0.198879667459583,0.327478266485633,0.607306462178029,0.543647550582167,0.928316124064996
"8293","PSTK",7.04391297464505,-0.328615618150473,0.541274469451779,-0.607114572544509,0.543774879892033,0.928316124064996
"8294","TIMM44",38.9556623004886,-0.163636495060788,0.269506046531977,-0.607171887853629,0.543736846482976,0.928316124064996
"8295","PRRG3",35.691272978426,0.232831115265564,0.383540037741935,0.607058174777112,0.543812305728753,0.928316124064996
"8296","KLHDC8B",30.6294036426173,-0.181152571803836,0.298631210371147,-0.606609642638137,0.544109999481702,0.928374537657415
"8297","FAM13A-AS1",4.89082484140515,0.399881357464151,0.659312404364367,0.606512716607645,0.544174340576027,0.928374537657415
"8298","FARS2",21.2874137148961,-0.184619183169761,0.304388137099708,-0.606525552962943,0.544165819374804,0.928374537657415

"8299","CHAMP1",23.9488933097121,0.199482480344004,0.328705505772379,0
.606872951139859,0.543935230062608,0.928374537657415
"8300","PRKX",8.79339519928441,0.315416545184398,0.519850182815241,0.6
06745088510434,0.544020094619193,0.928374537657415
"8301","TNFRSF1B",33.1544153486016,-0.160985923406941,0.26572909328982
1,-0.605827240871061,0.544629478597376,0.928579009554262
"8302","BNIP3L",203.328802849857,0.113208019026755,0.186892209416134,0
.60573963666236,0.544687659135431,0.928579009554262
"8303","PTAR1",34.9867779661465,0.167238644851313,0.276000543425771,0.
605935925978679,0.544557301904474,0.928579009554262
"8304","AC011603.2",3.12051690971462,0.490083692644221,0.8089345809172
9,0.60583847471138,0.544622018096142,0.928579009554262
"8305","ZNF578",1.99453947092301,0.583957617032831,0.963291977909581,0
.606210401855588,0.54437504638013,0.928579009554262
"8306","FBXO7",77.6905183653679,0.125702807488635,0.207510253291116,0.
605766729571121,0.54466966560609,0.928579009554262
"8307","ANKRD13B",24.5390526251741,-0.220471111462303,0.36408778527398
7,-0.605543828657675,0.544817712167125,0.92858689534124
"8308","ARMCX5",11.1410487345672,0.28505128201095,0.470742708753629,0.
605535203648022,0.544823441136654,0.92858689534124
"8309","COX10-
AS1",10.5263062850385,0.276178716654062,0.456188792101704,0.6054044321
90631,0.544910306814802,0.928623173004887
"8310","ATP6V1B2",55.349220723866,0.151941090248025,0.251126765812337,
0.6050374190761,0.54515413407906,0.928826689727527
"8311","ZNF703",82.6904669936278,0.22682805827575,0.374905550841515,0.
605027206896806,0.545160919373268,0.928826689727527
"8312","SKI",103.442785736727,0.154358699526137,0.257675350929763,0.59
9043327074818,0.549143992333959,0.928950798040326
"8313","PIK3CD-
AS2",3.42313662325736,0.502679680422566,0.839693949865038,0.5986463050
05961,0.549408770655786,0.928950798040326
"8314","MFAP2",82.5685142975902,-0.190665236887577,0.316995824494328,-
0.60147554685216,0.547523294006262,0.928950798040326
"8315","MECR",30.0074260889004,-0.184315981508249,0.307817561606889,-0
.598783190101537,0.549317473391068,0.928950798040326
"8316","UTP11",51.839934261518,-0.140094619329275,0.23306194955851,-0.
601104640181103,0.547770292990909,0.928950798040326
"8317","TM2D1",109.980449966706,0.127280028076538,0.211954717475735,0.
600505757042701,0.548169225271263,0.928950798040326
"8318","SPATA1",2.67903893378522,-0.525786991968821,0.872750816274401,
-0.602448009402388,0.546875960778527,0.928950798040326
"8319","MCL1",699.402941689155,-0.171949898977409,0.284591768726744,-0
.604198426914133,0.545711726672625,0.928950798040326
"8320","MRPL24",63.2349450052033,-0.116746579071681,0.194549596281592,
-0.600086462799445,0.548448613930848,0.928950798040326
"8321","ACBD6",85.7920974946716,0.108522555521253,0.180924465820055,0.
599822445402094,0.548624572923577,0.928950798040326
"8322","DSTYK",39.8429152311731,0.189780034167257,0.31395099624807,0.6
04489351635315,0.545518346623232,0.928950798040326

"8323","NCK2",37.9108015070165,0.158930555918745,0.263650373348092,0.602808006302014,0.546636419967027,0.928950798040326
"8324","AMMECR1L",15.0470707188655,0.238614664626769,0.395912810837042,0.602694982570247,0.546711620008112,0.928950798040326
"8325","HOXD9",3.65785951757146,0.438718849929056,0.726851620049217,0.603587909591987,0.546117653057455,0.928950798040326
"8326","DOCK10",15.3032964147682,0.21117035372786,0.351152100962674,0.601364346529445,0.547597340186425,0.928950798040326
"8327","LIMD1-AS1",2.1438830880044,0.6621592688462,1.10670526476852,0.598315820775187,0.549629222173859,0.928950798040326
"8328","CPOX",17.4754994445847,0.206277443591337,0.344517340717165,0.59874328288364,0.549344089247585,0.928950798040326
"8329","CPA3",3.01597993642104,0.556856582787009,0.92805639021112,0.600024512153116,0.548489899515302,0.928950798040326
"8330","AC007920.2",13.6469835135052,0.263362675585584,0.438295155278792,0.600879732330292,0.547920093371001,0.928950798040326
"8331","G3BP2",74.9307460907921,0.144231011729244,0.239946738295195,0.601095946350408,0.547776083160172,0.928950798040326
"8332","PAPSS1",54.6567010571274,-0.131951881086232,0.220426011694127,-0.598622095786653,0.549424918082787,0.928950798040326
"8333","WWC2-AS2",3.11500120404524,0.488744380473434,0.812232518189662,0.601729639639113,0.547354117039304,0.928950798040326
"8334","AMACR",14.8964212049914,-0.239215523627326,0.397119708490939,-0.602376357840181,0.546923643704372,0.928950798040326
"8335","TENT2",33.3307592797902,0.165306909803899,0.276263249426749,0.59836735485778,0.549594843180967,0.928950798040326
"8336","RGS14",7.35012343960826,-0.400899162532299,0.666651070299926,-0.601362812410898,0.54759836176142,0.928950798040326
"8337","GMPR",2.45522885417396,0.502511552358354,0.837677183993665,0.599886880006217,0.548581626788451,0.928950798040326
"8338","TAPBP",188.327252791028,-0.105605580430765,0.175029713784129,-0.603358013605693,0.546270547201261,0.928950798040326
"8339","FRS3",7.00973644780822,0.329538349596965,0.549092901334551,0.600150445937352,0.548405975450247,0.928950798040326
"8340","PTK7",28.9587693930195,-0.15745637223231,0.263134682992192,-0.598386994986069,0.549581741299274,0.928950798040326
"8341","CYP39A1",2.67412150764333,0.509922307346092,0.848405153847089,0.601036315059912,0.547815798950312,0.928950798040326
"8342","RPF2",63.1450389733742,-0.14202053939776,0.234816852914849,-0.604814082272284,0.545302535666423,0.928950798040326
"8343","LANCL2",12.1741303122543,-0.248698234268495,0.414299471195505,-0.600286149414698,0.548315547822607,0.928950798040326
"8344","KCTD7",11.7994064391091,0.296998378299367,0.495568727498734,0.599308152066811,0.548967412681679,0.928950798040326
"8345","UFSP1",1.99897740071736,-0.692529152837407,1.1563901111846,-0.598871562580195,0.549258536211263,0.928950798040326
"8346","TES",29.9966655227064,-0.363925338729981,0.604339598590599,-0.602186816119123,0.547049790497742,0.928950798040326

"8347","KIAA1549",3.23997227875175,0.59361209844459,0.991890122355179,
0.5984655810818,0.549529318247141,0.928950798040326
"8348","ZFAT",2.92894822453833,0.495500397262791,0.821572888687596,0.6
03111913848956,0.546434241295001,0.928950798040326
"8349","FANCG",11.7106722665984,-0.237545590295707,0.394124439974253,-
0.602717228881378,0.546696818078885,0.928950798040326
"8350","CACFD1",5.18323338619258,-0.3916436535836,0.649528250365735,-0
.602966311878003,0.546531100384175,0.928950798040326
"8351","ARRDC1-
AS1",12.2439858755869,0.251389593774223,0.416699689257419,0.6032872120
02036,0.546317638639712,0.928950798040326
"8352","SRGN",52.0247176894432,-0.212926601373667,0.354741468631869,-0
.600230365496486,0.548352719209033,0.928950798040326
"8353","AP002807.1",2.5767606663691,-0.532542118602129,0.8881362611970
25,-0.599617583324851,0.548761126024564,0.928950798040326
"8354","ARHGEF17",19.1971999148228,0.23473123940274,0.391507543436861,
0.599557386154413,0.548801254308464,0.928950798040326
"8355","RNF169",40.9772721978739,0.147712211404792,0.245505457391796,0
.601665694009652,0.547396690104833,0.928950798040326
"8356","AC079174.2",2.24448738003018,0.631011394055147,1.0514204247909
1,0.600151356371677,0.548405368746955,0.928950798040326
"8357","GZMH",2.90465209458604,0.517653484919891,0.857719031555448,0.6
03523375225966,0.546160570006431,0.928950798040326
"8358","PSMA6",171.238781272122,-0.135442391870525,0.22527894417813,-0
.601220821433848,0.547692918138167,0.928950798040326
"8359","PRPF39",16.5907966369268,0.215254881847595,0.356368868936177,0
.604022687195344,0.545828558776419,0.928950798040326
"8360","RCOR1",38.4237086329839,-0.189043005228312,0.314424941288517,-
0.601234127463335,0.547684056880648,0.928950798040326
"8361","KLC1",91.3688193441597,-0.135010568165485,0.225161996727996,-0
.599615255360267,0.548762677851708,0.928950798040326
"8362","LINGO1",3.5659438360943,0.471579420686404,0.786381882010298,0.
599682458961113,0.548717880630925,0.928950798040326
"8363","EFL1",7.57369899523551,0.293736773409087,0.487409505861374,0.6
02648840198513,0.546742322185763,0.928950798040326
"8364","LINC00924",2.13863870755236,0.663621004322045,1.10084146416593
,0.602830676281662,0.54662133717286,0.928950798040326
"8365","TRAP1",32.6254015473626,-0.16136949472788,0.268880062049886,-0
.600154185838966,0.54840348322359,0.928950798040326
"8366","MT1G",50.6307536734998,-0.319191790947298,0.532484855165006,-0
.599438252283038,0.548880674898875,0.928950798040326
"8367","RRAD",6.58833108476987,0.505219226602837,0.839308251573717,0.6
01947169774088,0.547209304321541,0.928950798040326
"8368","MOCOS",2.12414163002338,-0.526808997919827,0.87234360468606,-0
.603900796761633,0.545909599056089,0.928950798040326
"8369","ICAM1",46.7465638071248,-0.272559211645384,0.451847497336765,-
0.603210625823703,0.546368579769039,0.928950798040326
"8370","FUZ",21.2319220708059,0.206817073249764,0.342745141327898,0.60
341358143982,0.546233589429841,0.928950798040326
"8371","AKT1S1",40.1299065544792,-0.144893138559801,0.240465740677057,

-0.602552106390869,0.546806689627163,0.928950798040326
"8372","LINC01597",2.06686831364207,0.656438213404342,1.09074059604937
,0.601827983465491,0.547288645891831,0.928950798040326
"8373","CBR1",150.385884315416,-0.119099324553195,0.198318675388262,-0
.600545179721607,0.548142960343984,0.928950798040326
"8374","TUBGCP6",14.6577837428612,0.226541767309879,0.377892655114333,
0.599487087785121,0.54884811802742,0.928950798040326
"8375","SMC1A",65.7547166995484,-0.135155909015265,0.225719472023554,-
0.598778243647324,0.549320772361645,0.928950798040326
"8376","SASH3",1.57696164474422,0.752266200765319,1.25071508500125,0.6
0146887951269,0.547527733521261,0.928950798040326
"8377","MECP2",69.0405194032843,0.156789861048298,0.26027219830600 7,0.
602407256974706,0.546903080590601,0.928950798040326
"8378","TMLHE",13.7504206796511,-0.225998181357154,0.37623681947952,-0
.600680660839616,0.548052702276196,0.928950798040326
"8379","HDAC4-
AS1",4.68976875914345,-0.411396402000343,0.688283639483943,-0.59771346
9273798,0.550031136539075,0.928964801215804
"8380","FIG4",11.8878120065358,0.234071224614348,0.391571295392767,0.5
9777421728414,0.549990596336972,0.928964801215804
"8381","FBXL18",10.2050085732894,0.287847218883337,0.48137671741373,0.
597966641240649,0.549862191871839,0.928964801215804
"8382","SULF1",43.6142641640011,0.204788698733727,0.342432714796676,0.
598040694959075,0.549812779778029,0.928964801215804
"8383","AL133467.1",15.5585105408416,0.25494243833828,0.42651081206983
4,0.597739684724656,0.550013641469874,0.928964801215804
"8384","ZNF699",8.85996617543453,0.319996831459963,0.534972501549723,0
.598155663203226,0.549736071940796,0.928964801215804
"8385","LARS",73.3259846880511,-0.133164116385873,0.222896091094457,-0
.597426880534401,0.550222411468185,0.929057165020347
"8386","TMEM168",31.2839114610999,0.159650183390319,0.267270040791189,
0.597336622233127,0.550282658405766,0.929057165020347
"8387","AL138976.2",1.56775084852223,-0.812288353802568,1.359481062794
34,-0.597498836896598,0.550174383305179,0.929057165020347
"8388","PPP1R35",28.9888416431658,-0.166556167176472,0.27901093898141,
-0.596952104403222,0.55053935841083,0.929379746673504
"8389","NGF-
AS1",2.14177330951197,0.632510254139657,1.05997878344037,0.59671973064
0948,0.550694517226456,0.929420067214137
"8390","ZFYVE1",15.7721197879023,0.227198129377745,0.380719312150306,0
.596760190846446,0.550667499903054,0.929420067214137
"8391","H2AFX",47.8106200579765,0.224688542597527,0.376643309706884,0.
596555246852486,0.55080435807812,0.929494662184027
"8392","LINC02593",3.34657041371645,0.482823231984035,0.81087385186342
8,0.595435690612643,0.551552274312555,0.929533305998065
"8393","CTH",3.6960414386717,0.416372674026898,0.701042951617409,0.593
933186356506,0.552556801545646,0.929533305998065
"8394","TLCD4",12.1559873965039,-0.319903288790782,0.538733585968261,-
0.593806098455552,0.552641809688601,0.929533305998065
"8395","IKBKE",10.3295738308321,0.292581060325319,0.492461693848732,0.

594119428942202,0.552432236869585,0.929533305998065
"8396","YIPF4",115.870774910037,0.0983908299890315,0.165345884211879,0
.595060653961912,0.551802927987871,0.929533305998065
"8397","SAP130",10.1435891015624,0.274201433668224,0.46135139779934,0.
594343996736918,0.55228205746286,0.929533305998065
"8398","UGGT1",39.5661137262898,0.14857932257511,0.249940664825403,0.5
94458379467386,0.552205571871803,0.929533305998065
"8399","RCHY1",28.4054946247497,0.159114448993459,0.267972772424701,0.
593770954988232,0.552665318022437,0.929533305998065
"8400","NRG2",2.66774212346578,-0.487030995899385,0.81685005301115,-0.
596230598387115,0.551021187145967,0.929533305998065
"8401","LTC4S",43.6078636885713,-0.244146818746324,0.409823751626474,-
0.595736137247718,0.55135151283881,0.929533305998065
"8402","ACAT2",24.6911472961216,0.187191580834904,0.315193432777119,0.
593894292738237,0.552582816513647,0.929533305998065
"8403","ASAP1",80.8917714221342,0.131105720461865,0.220620897810811,0.
594257940942166,0.552339604789913,0.929533305998065
"8404","RNF121",23.4931822973571,-0.180637668627228,0.304167706140202,
-0.593875237182364,0.552595562517462,0.929533305998065
"8405","RASSF3",6.02122557779438,-0.325824567434214,0.5474911126185,-0
.595123025606543,0.551761238364514,0.929533305998065
"8406","CCDC59",100.798315089233,-0.128725464407415,0.216169016198086,
-0.595485267368093,0.551519144149432,0.929533305998065
"8407","ZNF84",22.3322507729551,0.199227175535771,0.335170125792183,0.
594406124546134,0.552240513114449,0.929533305998065
"8408","PCDH9",50.0190226373334,0.31146867115196,0.522706402640661,0.5
95876900643366,0.551257465676924,0.929533305998065
"8409","HOMEZ",6.50527690039093,0.301711472553392,0.507789901191748,0.
594165956915047,0.552401119705219,0.929533305998065
"8410","ADPGK",44.7933182872595,-0.136171239056692,0.228896465876155,-
0.594903195798434,0.551908180958377,0.929533305998065
"8411","MT1A",179.087651881818,-0.457903117688919,0.768859728895561,-0
.595561323450612,0.551468320812093,0.929533305998065
"8412","TXNL1",164.577520108112,-0.13836924159375,0.232585808747147,-0
.594916957053802,0.551898981848661,0.929533305998065
"8413","ZNF546",8.13575778646537,0.302081125452346,0.507580917004185,0
.59513885438261,0.551750658551179,0.929533305998065
"8414","SNRNP70",134.00902803439,0.0971380756230118,0.163463031237872,
0.594251035768791,0.552344222552708,0.929533305998065
"8415","ZNF543",3.75457956101016,0.479456553010194,0.807130242605774,0
.594026252147727,0.552494554606163,0.929533305998065
"8416","CU638689.4",5.1124420583524,-0.365576064689853,0.6139465616940
34,-0.595452580890976,0.551540987105135,0.929533305998065
"8417","EIF4ENIF1",16.8993754174584,-0.19967571636575,0.33583939719994
,-0.594557154492728,0.552139527045529,0.929533305998065
"8418","GPR174",1.6334653609671,0.618184785983908,1.03775414631248,0.5
95694835988413,0.55137910862724,0.929533305998065
"8419","GDI1",53.7900701462605,-0.136014625330768,0.228140391694816,-0
.596188269513954,0.551049461218975,0.929533305998065
"8420","SLC66A1",10.2524255661058,0.351162560567879,0.591652217228731,

0.593528681786584,0.552827393901387,0.929695474779531
"8421","MHENCR",4.04292535332196,0.419167493290852,0.706826340472655,0
.593027550459643,0.553162714597388,0.930148918026245
"8422","NUP35",16.8888885179052,0.214047937531772,0.362029387593421,0.
591244647167042,0.554356511712764,0.930428454638291
"8423","PFDN6",49.377871493752,0.13498378521255,0.228124959604914,0.59
1709848174116,0.55404490073001,0.930428454638291
"8424","KLC4",9.39148743934651,0.285698053030935,0.483129435010634,0.5
91348885676251,0.554286680970579,0.930428454638291
"8425","AL135925.1",15.9310778654747,0.227506914096037,0.3847392128541
2,0.591327596707175,0.554300942379956,0.930428454638291
"8426","TMX2",70.5421921857346,-0.141284450716963,0.238755914702354,-0
.591752672988628,0.554016219198397,0.930428454638291
"8427","PAFAH1B2",80.7198310797065,0.127406530227137,0.215375060867149
,0.591556560514338,0.554147569708099,0.930428454638291
"8428","C2CD2L",5.34047905928656,-0.375769064928922,0.635594121025582,
-0.591209157697349,0.554380287555315,0.930428454638291
"8429","KRAS",86.3629642651899,-0.111989285751804,0.189116352127511,-0
.592171351086001,0.553735851628641,0.930428454638291
"8430","IKZF4",17.1490889426683,0.249707189173852,0.421926027012462,0.
591826939290657,0.553966481738502,0.930428454638291
"8431","LPAR6",27.4003979467278,0.167442179732463,0.282803917826653,0.
592078713121288,0.553797880606709,0.930428454638291
"8432","CLYBL",5.83982311343745,0.353267666270129,0.596553434196387,0.
592181095639846,0.553729327020761,0.930428454638291
"8433","ACTR10",120.177528317239,-0.157583370669919,0.266375088344759,
-0.591584489559943,0.554128862703846,0.930428454638291
"8434","JAM2",33.2759713464737,-0.196587293818028,0.332094095317272,-0
.591962629236797,0.55387561342044,0.930428454638291
"8435","WAS",3.00250183980655,-0.541472288925029,0.913769264882905,-0.
592570038996022,0.553468935081155,0.930428454638291
"8436","CYSLTR1",4.2103119326561,0.396113504745313,0.669631926366024,0
.591539156286875,0.554159227296668,0.930428454638291
"8437","TSPAN6",31.6289500037419,0.166573834466225,0.281540415035637,0
.591651590927507,0.554083919160077,0.930428454638291
"8438","DUSP16",14.2986364805166,0.238207527633719,0.402992697742314,0
.591096387026935,0.554455840535773,0.930444975348014
"8439","FAM76A",49.9201735671871,-0.154058490227604,0.260878317543527,
-0.590537732986952,0.554830196316408,0.930610518259371
"8440","CAMKMT",10.0897550428709,-0.249246606071083,0.422277921664081,
-0.590243044412245,0.555027717800615,0.930610518259371
"8441","RNF8",41.872764974135,-0.1724787872427,0.292446223031132,-0.58
9779500159038,0.555338488036394,0.930610518259371
"8442","AC004918.1",4.19418893763702,0.405019754552963,0.6862125272849
77,0.590224949922494,0.555039847149214,0.930610518259371
"8443","KIAA1549L",11.2074613752696,-0.275417685407891,0.4668768557261
74,-0.589915053680505,0.555247601189096,0.930610518259371
"8444","RASSF8-
AS1",67.7260611765735,-0.116919673545674,0.198044980791483,-0.59036928
4181843,0.55494309861721,0.930610518259371

"8445","NGDN",32.6298917441754,-0.153217220547725,0.259329527363214,-0
.590820575295042,0.554640647378118,0.930610518259371
"8446","POLR2C",71.2844214572078,-0.121963328763342,0.206710535712537,
-0.590019895903858,0.555177310846041,0.930610518259371
"8447","VAC14",16.8832115222322,0.207183224355332,0.35119236750793,0.5
89942275299174,0.55522935032924,0.930610518259371
"8448","PIP5K1C",26.2004484370964,0.203147734431779,0.344038397207474,
0.590479830393089,0.554869004087763,0.930610518259371
"8449","TBC1D10A",21.0279008594247,-0.212606672490868,0.35994595490560
7,-0.590662763654677,0.554746402151886,0.930610518259371
"8450","PDZD11",58.9676065875887,-0.180292329891389,0.305698063917451,
-0.58977256048332,0.555343141192916,0.930610518259371
"8451","C6orf47",23.3107506836145,-0.221881276770835,0.376416104784396
,-0.589457448686805,0.555554448436119,0.930854453893674
"8452","LRRC40",22.9290284642885,0.173424321087288,0.294387623758951,0
.589101942781707,0.555792890228587,0.930865000788682
"8453","AL592295.5",19.5053904573686,-0.249870251496485,0.424226989576
762,-0.589001307403313,0.555860396574589,0.930865000788682
"8454","INSIG1",47.1958334507758,0.14829748717606,0.251907576834282,0.
588698002020071,0.556063878432453,0.930865000788682
"8455","TTC12",6.30908760864109,0.35641865083168,0.605470383564317,0.5
88664054438955,0.556086655485273,0.930865000788682
"8456","AC008124.1",30.151777005676,-0.173388490995831,0.2943016914388
14,-0.589152206866874,0.555759174511817,0.930865000788682
"8457","CIDEB",6.00843852597877,-0.414650577148443,0.704363373186805,-
0.588688442546931,0.556070292293308,0.930865000788682
"8458","MAPKBP1",8.06839619592532,-0.341838872633808,0.580515149586504
,-0.588854352685364,0.555958981183228,0.930865000788682
"8459","TMEM47",77.4553350026585,0.170837344113875,0.290164376619263,0
.588760571178031,0.556021898989996,0.930865000788682
"8460","LRRK1",28.7106354368535,0.201668045381969,0.34272255773986,0.5
88429447748937,0.556244076755114,0.930908418254628
"8461","ZNF614",6.79701137623374,-0.375501304911156,0.638128077301789,
-0.588441910437316,0.556235713734962,0.930908418254628
"8462","F3",385.347941523471,0.165119612910546,0.280793381488224,0.588
046669887303,0.556500967650099,0.931091041995156
"8463","B4GALT1-
AS1",7.51908786324345,-0.315997189361655,0.537414701161524,-0.58799505
9827513,0.556535608755323,0.931091041995156
"8464","PRR13",89.9171838560068,-0.117040725766338,0.199091188087186,-
0.587874967701148,0.556616219667302,0.931091041995156
"8465","PAXBP1",43.4737955475909,0.130847182472204,0.222575833695709,0
.587876861111029,0.556614948686388,0.931091041995156
"8466","HINT3",16.8816262526125,0.200879679832163,0.341879953073986,0.
587573731732352,0.556818447024746,0.931319301898229
"8467","KIAA0319L",30.7478633650622,0.156977078476168,0.26757582281976
6,0.586663910146712,0.55742945064871,0.931486976086239
"8468","CTNNB1",139.530560606785,-0.144106101794906,0.245646222375456,
-0.586640821916031,0.557444960123926,0.931486976086239
"8469","ASB6",8.96324678619038,0.25646465703445,0.436821126023573,0.58

7115965221448,0.557125825838735,0.931486976086239
"8470","NOLC1",48.9279373989655,-0.159674166617768,0.272129546304702,-0.586757920211212,0.557366301735196,0.931486976086239
"8471","CD4",3.03033278105495,-0.53603945680318,0.913549891506753,-0.586765388280075,0.557361285395745,0.931486976086239
"8472","LAMP1",367.159414202758,0.116718425639448,0.198920900756761,0.586757978650874,0.557366262480895,0.931486976086239
"8473","MVP",121.094015183649,-0.121491900737296,0.207016651034103,-0.586870187158432,0.557290893780235,0.931486976086239
"8474","TNFSF14",8.75090237191434,0.287790651677002,0.490541727794975,0.586679247391744,0.557419147999544,0.931486976086239
"8475","RAB11FIP1",6.43559562048413,-0.458131829742924,0.781375287501028,-0.586314715951803,0.557664043622661,0.931633182833516
"8476","SUV39H2",2.85857281252127,-0.463253139001194,0.790045650915312,-0.586362494957715,0.557631942259244,0.931633182833516
"8477","ATAD3B",7.70480348060406,-0.34051833803489,0.590339709849243,-0.576817605784725,0.564062674998404,0.931645499788421
"8478","PDIK1L",7.92999627113474,-0.307753437713512,0.532336153609589,-0.578118611006863,0.563184042794361,0.931645499788421
"8479","AL020997.2",1.93564442002055,-0.681495627301808,1.16579135450166,-0.584577698805399,0.558831706595768,0.931645499788421
"8480","ADGRB2",2.4909164138931,-0.493188714190045,0.849912109233192,-0.580282018378359,0.561724451470945,0.931645499788421
"8481","TMEM39B",27.1830791284231,-0.172094390749517,0.297408221419711,-0.578647052620152,0.562827349029383,0.931645499788421
"8482","TXLNA",79.1600767863569,-0.139047961802447,0.240061086856229,-0.5792190797075,0.562441358344593,0.931645499788421
"8483","HDAC1",20.5729793162219,-0.17723257289605,0.307989977529513,-0.575449156877407,0.564987566977802,0.931645499788421
"8484","SETDB1",15.1925058128465,0.207521939916456,0.359499133561585,0.577252962644223,0.563768583821758,0.931645499788421
"8485","CERS2",101.465441224755,-0.114511538184981,0.196757083839671,-0.581994487569713,0.560570393926047,0.931645499788421
"8486","PIGM",24.4537072556778,0.194546457694737,0.334777913228301,0.581120946178031,0.561158942494043,0.931645499788421
"8487","DCAF8",70.259161213035,0.156979073387696,0.271513683624274,0.578162659399983,0.563154306324812,0.931645499788421
"8488","GORAB",13.5522024199081,0.236172505286499,0.406614773320389,0.580826179427593,0.561357609060051,0.931645499788421
"8489","SWT1",8.75492100393635,0.267217829007192,0.461052243156546,0.579582537496647,0.562196171840527,0.931645499788421
"8490","TRAF5",19.6293511170962,0.226837213051284,0.391425631201451,0.579515481280631,0.562241403688174,0.931645499788421
"8491","ATF3",522.521362620581,-0.187182961954245,0.321827373625909,-0.581625359724141,0.560819057356944,0.931645499788421
"8492","H3F3A",1529.21707925797,-0.120763915429461,0.208086752007072,-0.580353695103842,0.561676124475088,0.931645499788421
"8493","HIST3H2A",15.1494514308457,0.248556408525036,0.431374095195753,0.576196881762787,0.564482112658501,0.931645499788421
"8494","GAREM2",9.24721305256845,-0.276401345488301,0.476722166188376,

-0.579795455491956,0.562052562590065,0.931645499788421
"8495","BABAM2",30.3311913051112,0.174428506672082,0.298721087673471,0
.58391762038155,0.559275737703973,0.931645499788421
"8496","AC016727.1",3.56989930728344,0.41680353604451,0.72075726833906
3,0.578285581503751,0.563071327294233,0.931645499788421
"8497","ETAA1",23.506053230841,0.183700283913568,0.314170646628726,0.5
84714981761671,0.558739378624275,0.931645499788421
"8498","ANKRD39",22.6432255748326,0.186547892588148,0.318644312446034,
0.585442404906387,0.55825028279282,0.931645499788421
"8499","CERS6",10.0482425289473,-0.252361745213052,0.434489019487463,-
0.58082421855159,0.561358930762983,0.931645499788421
"8500","TMEM237",44.0007717123854,-0.165997371326445,0.284153816803765
,-0.584181388776074,0.559098281621045,0.931645499788421
"8501","LMCD1",94.8427240267877,-0.224829483476249,0.387978620186013,-
0.579489363018139,0.562259021878343,0.931645499788421
"8502","NGLY1",34.6004963030656,-0.140063157407788,0.242056999226511,-
0.578637089013568,0.562834073373806,0.931645499788421
"8503","XYLB",4.33604498227527,0.536072384899887,0.917535303836754,0.5
84252597865448,0.55905037880132,0.931645499788421
"8504","TCTA",24.8874705330531,0.204387000064583,0.351213066544303,0.5
81945888504694,0.560603129693625,0.931645499788421
"8505","ARL13B",40.9356872305752,-0.1626757444405,0.282457773469785,-0
.575929429883089,0.564662882373211,0.931645499788421
"8506","AC124016.2",4.43993105408585,0.39843154488133,0.68394348432186
5,0.582550391976288,0.560196009034269,0.931645499788421
"8507","ABRAXAS1",58.0280206080634,-0.129973621268499,0.22481263068565
1,-0.578141988161853,0.563168261101497,0.931645499788421
"8508","HHIP",61.3268001632858,-0.550804714514644,0.953593784112409,-0
.577609380106566,0.563527872602212,0.931645499788421
"8509","CKMT2-
AS1",11.0486174425517,0.264625366845301,0.455985890347659,0.5803367438
48677,0.561687553432316,0.931645499788421
"8510","FBXL17",29.0855729616401,-0.165418252838518,0.286874047216594,
-0.576623275766819,0.564193972171364,0.931645499788421
"8511","PCBD2",10.577700893971,0.279292111404946,0.478577084989747,0.5
83588559011198,0.559497159415219,0.931645499788421
"8512","EGR1",1789.15822704393,0.170274394516338,0.293795547950199,0.5
79567647312345,0.562206215657591,0.931645499788421
"8513","CYFIP2",1.75593932426147,0.55492208601527,0.954922218249942,0.
581117577337617,0.561161212829147,0.931645499788421
"8514","FOXF2",13.5511005127365,0.220707026253195,0.383655639564218,0.
575273770258895,0.565106158028443,0.931645499788421
"8515","SLC29A1",89.7026125444619,0.179987508259657,0.307945001843009,
0.584479394640135,0.558897824411358,0.931645499788421
"8516","LMBRD1",103.493860765244,0.130464441247205,0.222799400272554,0
.585569086306364,0.558165127596499,0.931645499788421
"8517","MANEA",20.2964347674838,0.223850309496301,0.384444111678762,0.
582270095174062,0.560384765350112,0.931645499788421
"8518","LINC00242",1.611704775832,-0.609303414813387,1.05208401740183,
-0.579139502867929,0.56249504730556,0.931645499788421

"8519","ARL4A",83.8874115354202,-0.167097538374429,0.28955289143091,-0
.577088135948732,0.563879918447942,0.931645499788421
"8520","SKAP2",71.5865252402007,-0.214503036198506,0.372704658136298,-
0.575530870129513,0.564932319070514,0.931645499788421
"8521","THAP5",39.0941802810844,0.173857146458454,0.299509523814088,0.
580472848557466,0.561595791430333,0.931645499788421
"8522","CEP41",11.1266171501576,-0.242779843775218,0.421610027384104,-
0.575839823548682,0.564723453184156,0.931645499788421
"8523","DOCK5",7.61299793162874,0.376919767430832,0.649116227632093,0.
580666067779256,0.561465535187782,0.931645499788421
"8524","MRPS28",44.7032831187271,-0.176969252475992,0.302601380153049,
-0.584826322954923,0.558664502910523,0.931645499788421
"8525","FABP5",138.703227609133,-0.486868992270391,0.839619720422649,-
0.579868457621874,0.56200332808422,0.931645499788421
"8526","TMEM67",13.1719641081481,-0.215452750498042,0.373651293430525,
-0.576614491334826,0.564199907634394,0.931645499788421
"8527","ZNF707",2.30391959561748,-0.555530781113291,0.950630645355345,
-0.584381309215667,0.558963798891728,0.931645499788421
"8528","DMAC1",98.1886592128295,-0.126362036500655,0.218966262009388,-
0.577084503069416,0.56388237244923,0.931645499788421
"8529","GNAQ",92.9811833238216,0.133328876384901,0.227594033606875,0.5
85818855933634,0.557997251038778,0.931645499788421
"8530","SPTLC1",88.3091151157039,-0.110258638107823,0.190032644556465,
-0.580208933918515,0.56177372968104,0.931645499788421
"8531","SLC35D2",38.528117865334,-0.153000868949255,0.265150843926987,
-0.577033309354226,0.563916954228442,0.931645499788421
"8532","INIP",30.2714596249386,0.195172043091174,0.334514665098965,0.5
83448390920119,0.559591489870062,0.931645499788421
"8533","C5",5.56967738939311,0.360530736027305,0.626645349973233,0.575
334574879883,0.565065042453959,0.931645499788421
"8534","GLE1",14.5720516590159,-0.218885582142123,0.380155569578004,-0
.575778969607362,0.56476459014488,0.931645499788421
"8535","IL15RA",68.6751506584428,0.149442383085796,0.257209485704515,0
.581014275878912,0.561230832095093,0.931645499788421
"8536","WASHC2A",44.2929478683752,0.148133768624659,0.253686419980336,
0.583924707661298,0.559270969220725,0.931645499788421
"8537","CDH23",4.74746237705002,-0.376899295879186,0.644402084919548,-
0.584882179464459,0.558626941865665,0.931645499788421
"8538","PSMD13",143.284283853931,-0.130591655650899,0.226854768541763,
-0.575661937769044,0.564843707135166,0.931645499788421
"8539","TOLLIP-
AS1",3.39846732890114,-0.421840854417254,0.722183578170437,-0.58411859
1405714,0.559140527475989,0.931645499788421
"8540","SBF2-
AS1",8.86959466402697,0.304427463734048,0.525418158797265,0.5794003473
93612,0.562319069766981,0.931645499788421
"8541","CCDC89",9.02160211913465,0.271268264121329,0.467750061775443,0
.579942764928077,0.561953215474426,0.931645499788421
"8542","PHLDB1",180.349904197809,-0.136881973504126,0.235817662324244,
-0.580456833279587,0.56160658857877,0.931645499788421

"8543","TRAPPC4",63.7443829484417,-0.141729199262916,0.244022069845862
,-0.580804840121389,0.561371992622428,0.931645499788421
"8544","AP000866.6",1.81262057310117,-0.57263593398552,0.9837530393618
73,-0.58209317895167,0.560503919402994,0.931645499788421
"8545","VSIG10L2",2.88173979717518,-0.446216371084784,0.77363224039062
8,-0.576780992037608,0.564087411592903,0.931645499788421
"8546","IGSF9B",1.92201456598194,0.598156253356684,1.02664181130129,0.
58263383272741,0.560139824660295,0.931645499788421
"8547","C12orf4",14.1489529922477,0.252295056155081,0.431382838692399,
0.5848518613303,0.558647329324749,0.931645499788421
"8548","SSPN",631.445475980135,0.139074598726757,0.237866588649247,0.5
84674794036891,0.558766405619418,0.931645499788421
"8549","PCBP2",502.683966207103,-0.097611707549196,0.169017310484743,-
0.577524913094669,0.563584914030114,0.931645499788421
"8550","METTL1",16.7793944164037,-0.225507717130988,0.385735179915682,
-0.584617968162203,0.558804623164827,0.931645499788421
"8551","CTDSP2",166.185966329937,-0.117481941097601,0.201016358340152,
-0.584439704647337,0.558924520344849,0.931645499788421
"8552","LINC01048",7.6003129800702,-1.35171098290703,2.32155999572029,
-0.582242537517387,0.560403324777122,0.931645499788421
"8553","EBPL",113.527645960351,-0.179300599714207,0.31156639605548,-0.
575481187907951,0.564965909870066,0.931645499788421
"8554","PCCA-
DT",8.65429391666958,0.27017625602108,0.464515326562882,0.581630444834
215,0.560815631403486,0.931645499788421
"8555","ANG",91.8726001864291,0.138899957728411,0.240150036630429,0.57
8388242939001,0.563002029824573,0.931645499788421
"8556","NUBPL",20.696462783687,-0.184674078866651,0.315419479876717,-0
.585487234139223,0.558220147874841,0.931645499788421
"8557","KIAA0586",30.472406382346,-0.15755204501786,0.271056824757877,
-0.581250979969217,0.56107131328018,0.931645499788421
"8558","EIF2S1",68.0417366608398,-0.115130043831775,0.199224695822569,
-0.577890423455886,0.563338101220004,0.931645499788421
"8559","PGF",20.110291610488,0.345834053960961,0.596045654858872,0.580
214034179723,0.561770290691397,0.931645499788421
"8560","FLVCR2",2.98371538355613,-0.547792598692685,0.951853189425053,
-0.575501143221013,0.564952417706594,0.931645499788421
"8561","VASH1",7.57377842349638,-0.290330485610143,0.501121923866118,-
0.579360973413945,0.562345631534116,0.931645499788421
"8562","SLC9A3R2",54.899063575871,0.191168863280274,0.328646926614757,
0.58168462200276,0.560779131651001,0.931645499788421
"8563","NTHL1",42.0321011601318,0.165162969597621,0.285597808494319,0.
578306151816662,0.56305744180287,0.931645499788421
"8564","AF001548.2",2.52220957174949,-0.680919509678975,1.174076353987
63,-0.579961863098853,0.56194033607793,0.931645499788421
"8565","TRPV3",3.03077194702331,0.474614036119128,0.820839202119501,0.
57820585919096,0.563125143470549,0.931645499788421
"8566","CLEC10A",4.80704873706071,-0.477067317528337,0.822858296155527
,-0.579768496905532,0.562070744677993,0.931645499788421
"8567","SPAG5",1.71756571200621,0.60002297927908,1.03473666388399,0.57

9879886566337,0.561995620300083,0.931645499788421
"8568","VPS25",68.243638546309,-0.149630293823877,0.259013980165133,-0
.577691959825803,0.563472108372487,0.931645499788421
"8569","ARL4D",56.8534383403044,-0.155940006945028,0.269357237050885,-
0.578933793100831,0.562633847188418,0.931645499788421
"8570","CD79B",1.66461151847698,-0.657056711476893,1.13021873135415,-0
.581353585150418,0.561002172763618,0.931645499788421
"8571","NPLOC4",39.4746351891525,0.156801594705445,0.268778229266821,0
.583386515839365,0.559633133075093,0.931645499788421
"8572","GATA6-
AS1",4.40608587304549,-0.385941245251049,0.664186958335591,-0.58107320
5981328,0.561191116069658,0.931645499788421
"8573","DYM",42.1213484668402,0.140895649918039,0.242329298863618,0.58
1422265399836,0.5609558948715,0.931645499788421
"8574","NEDD4L",2.20450692070536,0.529640947189205,0.912985177594445,0
.580119984625287,0.561833707794235,0.931645499788421
"8575","AC104532.2",4.49540019922767,0.421883307844905,0.7310494038497
7,0.57709274588452,0.563876804454751,0.931645499788421
"8576","DOCK6",16.3578027265577,0.244342996454212,0.423983596533845,0.
576302947688937,0.564410430813301,0.931645499788421
"8577","PEPD",107.662953024311,-0.162398747609215,0.281233781179097,-0
.577451069101104,0.563634783905961,0.931645499788421
"8578","THAP8",7.55254503695602,0.307381601779205,0.533284834249341,0.
576392918077034,0.56434963014551,0.931645499788421
"8579","IRF2BP1",25.0025169831972,-0.180187731429523,0.311902843541498
,-0.577704676826872,0.563463521103648,0.931645499788421
"8580","CCDC9",13.2133285572739,-0.236529753382965,0.407531758644043,-
0.580395879256029,0.561647683360061,0.931645499788421
"8581","RRAS",326.521438099393,0.143202958901398,0.248726203169031,0.5
75745366096709,0.564787306568164,0.931645499788421
"8582","STK35",15.3897617357448,-0.234007076118536,0.399830336563313,-
0.585265935871481,0.558368915747905,0.931645499788421
"8583","ATRN",30.6399936175813,0.147363940966645,0.252238259545986,0.5
84225173579499,0.559068827061722,0.931645499788421
"8584","AL118522.1",3.18095604064869,-0.458819685861733,0.791414673791
786,-0.579746245622992,0.562085752161719,0.931645499788421
"8585","PCNT",23.4526127098351,0.187471546620762,0.320502633377827,0.5
84929816784873,0.558594908833347,0.931645499788421
"8586","CCDC157",1.72501420420519,-0.645744287619007,1.12222950652087,
-0.575411966863124,0.565012712724718,0.931645499788421
"8587","ZNF81",2.66249027020306,0.477669622868717,0.828934122266208,0.
57624557855433,0.564449201678521,0.931645499788421
"8588","AL136169.1",4.26305778741151,0.461069866129301,0.7875974881273
12,0.585413073403264,0.558270000320276,0.931645499788421
"8589","PDZD4",2.64410872872932,-0.459876320020354,0.794777389083568,-
0.578622802229719,0.562843715457575,0.931645499788421
"8590","EMX2OS",93.0325511129425,0.135570477513771,0.235793843908357,0
.57495342230589,0.565322798372609,0.93167723754145
"8591","FEN1",7.81288295723704,-0.295876118086353,0.514551337076624,-0
.575017683886209,0.565279337267613,0.93167723754145

"8592","MCM5",17.0749400092724,0.20240245488837,0.351991418483051,0.57
5020992729446,0.565277099489006,0.93167723754145
"8593","DDX18",139.824570954611,-0.151890643911931,0.264564577345812,-
0.574115572975573,0.56588959580105,0.931743799598008
"8594","LAP3",116.284459929859,-0.132759570863635,0.231033439704706,-0
.574633572669482,0.565539141534886,0.931743799598008
"8595","SNRNP48",28.1407880544198,0.192547354984005,0.335327563327143,
0.574206763898372,0.565827892743495,0.931743799598008
"8596","TMED4",103.835122530024,0.106221869455345,0.184901436469664,0.
574478335503746,0.565644156768694,0.931743799598008
"8597","AL391834.2",2.31942000458351,-0.524758313130777,0.913387193718
066,-0.574519017498677,0.565616635203335,0.931743799598008
"8598","PGR",23.4404950372255,-0.205769117828047,0.358133140257312,-0.
574560393043228,0.565588645108106,0.931743799598008
"8599","LIG4",9.97237911840444,0.256205760895109,0.446075363946379,0.5
74355325585537,0.565727377483036,0.931743799598008
"8600","CTF1",8.66139890450283,0.297143589764072,0.517550131556115,0.5
74134893697452,0.565876522436133,0.931743799598008
"8601","LRRC47",39.3175634699243,0.140030878007105,0.244256480161187,0
.573294423610367,0.56644536046574,0.931933353655951
"8602","AGL",55.8688216467553,-0.159958573630296,0.279121765170657,-0.
573078109951389,0.566591807947382,0.931933353655951
"8603","ADAR",110.775521796469,0.121144931661228,0.211887005939462,0.5
71743090729403,0.567496036922118,0.931933353655951
"8604","EFCAB2",16.4013159072987,0.190453486066458,0.333470321550219,0
.57112574570681,0.56791440769296,0.931933353655951
"8605","ATL2",19.1298239511408,0.200076764731672,0.349613653217198,0.5
72279608906962,0.56713256189611,0.931933353655951
"8606","ST6GAL2",3.15639625915537,0.540842380834334,0.944658803789558,
0.572526692880764,0.56696520741305,0.931933353655951
"8607","FHIT",13.3698507176417,0.261156613672098,0.455278046883546,0.5
73620044848986,0.566224944413745,0.931933353655951
"8608","MRPL1",57.5965832853413,0.158977426099113,0.277087216692274,0.
573745075636848,0.566140320845115,0.931933353655951
"8609","MAP3K1",18.8410170279858,0.248227415478647,0.434542828733993,0
.571238089929681,0.567838261747647,0.931933353655951
"8610","PCDHB4",11.5868649712829,-0.26638666721308,0.466133218676222,-
0.57148183510627,0.56767307017221,0.931933353655951
"8611","B3GALT4",28.8261304089942,-0.158086695126,0.276938878592035,-0
.570836048480146,0.56811078451682,0.931933353655951
"8612","NUDT3",62.9766089318318,0.17350956002501,0.303681813995494,0.5
71353146710276,0.567760282319043,0.931933353655951
"8613","TMEM106B",99.0997087431853,0.107179264469154,0.187463637818043
,0.571733621072613,0.56750245333319,0.931933353655951
"8614","KCTD9",49.1117164682764,0.154760341468949,0.270836406702306,0.
57141631493825,0.56771747239933,0.931933353655951
"8615","NAA35",28.2284128415181,-0.184598192932209,0.322726487245363,-
0.571995792808486,0.567324824921523,0.931933353655951
"8616","PRPF18",53.4853696653739,-0.120120333215103,0.210051321091577,
-0.571861831627011,0.567415583902413,0.931933353655951

"8617","OTUD1",36.8524746609122,0.20372838484246,0.356723277080093,0.5
71110431901304,0.56792478763666,0.931933353655951
"8618","HPS1",47.6964866739565,-0.131116684078938,0.229096456968992,-0
.572320872237167,0.567104611843506,0.931933353655951
"8619","CFAP300",9.13511780223088,-0.283659567978335,0.496857472512799
,-0.570907319847197,0.568062468840478,0.931933353655951
"8620","AREL1",8.11645176492889,0.289268581969274,0.50625275724839,0.5
71391617779073,0.567734209797464,0.931933353655951
"8621","THAP10",3.53900211681896,0.444429982784668,0.777568766300609,0
.57156357359762,0.567617679313903,0.931933353655951
"8622","ZNF213-
AS1",6.37335210243721,0.326332518542761,0.570964476778967,0.5715460975
50113,0.567629521904569,0.931933353655951
"8623","TNRC6A",156.098724107896,0.13316380732335,0.232642492036174,0.
572396754169243,0.567053214322149,0.931933353655951
"8624","AC092376.2",4.3050763709969,0.373548108010365,0.65239546350180
3,0.57257925431502,0.56692960964804,0.931933353655951
"8625","AC005899.6",2.46033110047132,0.586657235798614,1.0234058214602
2,0.573240080813257,0.566482149618985,0.931933353655951
"8626","AC067852.3",3.64440427146279,0.482305683529518,0.8446575204421
05,0.571007386848427,0.567994635591589,0.931933353655951
"8627","AC002558.3",14.3197165648965,0.241379073659802,0.4212030840272
16,0.573070527765189,0.56659694152668,0.931933353655951
"8628","VEZF1",63.464659646332,0.145290665085233,0.254000765108791,0.5
7200876943423,0.567316033599845,0.931933353655951
"8629","CD7",1.61789698055459,-0.618556984893399,1.08036781737544,-0.5
7254295707926,0.566954192205029,0.931933353655951
"8630","AP001269.4",1.71985219068964,-0.642212349530173,1.120449871858
26,-0.57317365610035З,0.566527119581164,0.931933353655951
"8631","ZNF221",2.24465080572691,0.503255907749366,0.878315297940309,0
.572978643238395,0.566659154447256,0.931933353655951
"8632","PIGA",3.5525271535699,-0.434052500697038,0.758442586225421,-0.
5722944736756,0.567122493048013,0.931933353655951
"8633","EMC1-
AS1",8.02966934753952,-0.310328255496,0.54383283623514,-0.570631699336
783,0.56824932603996,0.931944690378253
"8634","TCEAL4",241.409638053159,-0.105690730528642,0.185185579155268,
-0.570728730664419,0.568183540202113,0.931944690378253
"8635","AC234582.1",1.69761862174372,0.595439493092061,1.0448380615793
7,0.569886870499335,0.568754430494889,0.931950128083756
"8636","SMN2",3.99746759987056,-0.428938091173238,0.752484735262401,-0
.570028960153805,0.568658056070231,0.931950128083756
"8637","LRRC24",3.13171868797392,-0.426681443656654,0.748885159033058,
-0.569755507249702,0.568843536518478,0.931950128083756
"8638","AP2A2",64.858895628235,0.137412655077712,0.240910895846971,0.5
70387879695561,0.568414648308616,0.931950128083756
"8639","BEST1",6.04548732877861,0.320082317369116,0.561466400115997,0.
57008276417429,0.568621564729257,0.931950128083756
"8640","NELL2",3.20297182758126,0.471458892314566,0.826996820744345,0.
570085495480172,0.568619712313634,0.931950128083756

"8641","NGFR",43.0544145096069,0.19929601200328,0.34954976943524,0.570150603518572,0.568575555847643,0.931950128083756
"8642","KRI1",15.5966607872132,0.244794189732174,0.42964938941248,0.569753375111076,0.568844982840954,0.931950128083756
"8643","RADX",1.85451365635221,-0.68754442446254,1.20530906754566,-0.570429977650934,0.568386102085195,0.931950128083756
"8644","THUMPD3",43.5067275200526,-0.133034609309514,0.233558612007403,-0.569598389740802,0.568950120872006,0.93201454321467
"8645","MDH1",161.417900084301,-0.16079445756933,0.282518692624957,-0.569146260997266,0.569256886242305,0.932122249426755
"8646","KCTD18",21.1979888410515,-0.225599391102831,0.396404985465169,-0.569113405165925,0.569279181712047,0.932122249426755
"8647","ARIH1",64.8814951180952,0.161989785696724,0.284497888757997,0.56938835786763,0.569092615826344,0.932122249426755
"8648","GNAO1",7.15647864439951,0.290815970041683,0.510920267989822,0.56920030044194,0.569220216800042,0.932122249426755
"8649","USP24",22.6463985895212,0.173116690745237,0.304529651262436,0.568472363947409,0.569714265975459,0.93261896025578
"8650","BANF1",183.880280870135,-0.152882859895124,0.268895837140471,-0.568557927563818,0.569656183495855,0.93261896025578
"8651","AC007686.3",2.97580510071889,0.443658180206817,0.780850976165244,0.568172665142357,0.569917730444782,0.932844187157336
"8652","EFHC1",57.9478758606687,-0.131537344410555,0.231554729976517,-0.568061574142295,0.56999315853855,0.932859815638682
"8653","SYNC",46.3513807819986,0.141847321810628,0.249754285145887,0.567947499790733,0.570070617209223,0.932878763397965
"8654","ZNF280C",5.62066923642598,0.339483021016396,0.597880144729641,0.567811164175571,0.570163198287577,0.932922450630008
"8655","WWOX",21.327478374899,0.186088657203192,0.327816361380756,0.567661285786318,0.570264984093471,0.932981187147724
"8656","UTP3",33.6007822988719,-0.161827875814368,0.28536094456767,-0.567098893156322,0.570646994859415,0.933158648588799
"8657","TBCA",300.901539613653,-0.132419363294931,0.233537046887162,-0.567016518620758,0.570702958812076,0.933158648588799
"8658","PPP1R32",1.90684835371167,0.615263988539356,1.08456543582788,0.567290795201957,0.570516629793582,0.933158648588799
"8659","LYRM9",33.8784633311539,-0.163595873129856,0.288437997663863,-0.567178646554417,0.570592814156777,0.933158648588799
"8660","PTTG1IP",152.573415257499,0.101678240482376,0.179252501072752,0.567234710109334,0.570554728684829,0.933158648588799
"8661","LIPT2",2.00052661502558,0.533650869418673,0.941396863442978,0.566871305972857,0.570801620346888,0.93321220922664
"8662","NOL9",26.1336340929822,0.162277725904808,0.287342284109352,0.564754075119174,0.572241047974503,0.933256410467606
"8663","ZSWIM5",3.57223816083527,0.46316655954137,0.820369112888742,0.564583127600252,0.57235734412211,0.933256410467606
"8664","GLMP",52.90615133663,0.158702364173293,0.280424986827938,0.565935175636359,0.571437848367549,0.933256410467606
"8665","RCSD1",2.03021516477781,-0.581707932244036,1.02681788621084,-0.566515192280741,0.571043608328996,0.933256410467606

"8666","C1orf220",3.46233561715514,-0.412879710536036,0.731053916179361,-0.564773269656813,0.572227990569773,0.933256410467606
"8667","DARS",84.1190570814617,0.119089499573356,0.210918762846291,0.564622596711057,0.572330492162218,0.933256410467606
"8668","ZNF502",8.13512896394748,0.259830256703747,0.460206551745777,0.564594866626931,0.572349357665494,0.933256410467606
"8669","CXCL10",32.8677426804998,1.25925649517639,2.22901627650936,0.564938223397986,0.572115783868499,0.933256410467606
"8670","ZSCAN12",2.5963310921517,0.533034617984165,0.942863460822238,0.565335958102909,0.57184527526879,0.933256410467606
"8671","CBX3",180.525570668445,-0.103033335150692,0.182076025870079,-0.565880843775728,0.571474784619865,0.933256410467606
"8672","CNPY4",66.7890431823678,0.179270760204689,0.316973649834348,0.565569915033556,0.571686184139947,0.933256410467606
"8673","AC016831.7",6.27173109248913,-0.487422841676199,0.863599847429513,-0.564408207258261,0.572476354613109,0.933256410467606
"8674","CNOT4",83.4871303972643,0.114146803495482,0.201683043116723,0.565971247416274,0.571413326433893,0.933256410467606
"8675","CCL27",1.65068698793162,0.626976738951429,1.11062212099048,0.564527508593359,0.572395184331854,0.933256410467606
"8676","GTF3C4",25.5854109643407,0.179857432288695,0.318231002570185,0.565178850696761,0.571952120394973,0.933256410467606
"8677","TMEM273",1.94210399450261,0.666841972360857,1.18142300886705,0.564439635385413,0.572454971003404,0.933256410467606
"8678","SGMS1-AS1",7.65891835009567,0.292227415310962,0.517423303940693,0.564774359958974,0.572227248877771,0.933256410467606
"8679","IFIT5",34.8660139866672,-0.148185813479391,0.261783883928303,-0.566061635482404,0.571351881979384,0.933256410467606
"8680","TUT1",7.47726168931767,-0.300622669389523,0.531208273263512,-0.565922415971853,0.571446522626783,0.933256410467606
"8681","PPHLN1",77.7258178022962,0.122146380902653,0.215693206216028,0.566296839133249,0.571192008792402,0.933256410467606
"8682","LINC01852",4.32763449396348,-0.403712874514788,0.71371297573128,-0.565651582978631,0.571630654748111,0.933256410467606
"8683","ZFP90",35.8833079946698,0.157321921965765,0.278427286982642,0.565037729134545,0.57204810200582,0.933256410467606
"8684","PIK3R5",2.22019741477818,-0.599645613366448,1.06143382734096,-0.564939234006368,0.572115096453248,0.933256410467606
"8685","AC022966.1",2.10514800977863,0.539151933402117,0.952956586445124,0.565767571231498,0.571551793977385,0.933256410467606
"8686","AL353194.1",3.4888289959086,0.429696526901792,0.761064944922051,0.564599026362726,0.572346527669339,0.933256410467606
"8687","AC017033.1",2.68938202355318,-0.447830450148376,0.793669753941501,-0.564252887204499,0.572582039511933,0.933258623287009
"8688","PPIP5K2",49.1302408805581,0.13343686087957,0.23679132025458,0.563520912574449,0.573080223901741,0.933258623287009
"8689","SRFBP1",51.5808285384302,-0.133428710299214,0.236626102050879,-0.563879931853519,0.572835848481626,0.933258623287009
"8690","SNHG5",445.806746500373,0.224856346463199,0.398943189367634,0.

563629991577546,0.573005971329455,0.933258623287009
"8691","AHCYL2",9.1323254283192,0.261101172707222,0.463455523813523,0.
563379136273451,0.573176741094148,0.933258623287009
"8692","TTC7B",14.0292232371176,-0.215172872115789,0.381935836864298,-
0.563374397863168,0.573179966999541,0.933258623287009
"8693","NPRL3",35.6639439569542,0.166680309627146,0.295873365363524,0.
563350166455013,0.573196463854697,0.933258623287009
"8694","NTAN1",194.975432661352,-0.108546262396342,0.192476644691199,-
0.563945109135132,0.57279148924523,0.933258623287009
"8695","PGPEP1",18.3567133014014,0.234910287300205,0.416522612325691,0
.563979674449276,0.572767964977551,0.933258623287009
"8696","FBL",127.561340754046,0.109720864714895,0.194685490116369,0.56
3580083186023,0.573039944546615,0.933258623287009
"8697","PTGIR",33.1793911143661,-0.345738178431142,0.613728053401897,-
0.56334100505055,0.57320270104005,0.933258623287009
"8698","LSMEM1",6.9005833961996,-0.32382076294477,0.574948588070116,-0
.563216902630743,0.573287194509593,0.933288879541946
"8699","AKT3",76.4829226701777,0.176792368634564,0.313955626546865,0.5
63112598360056,0.573358213242522,0.933297195024038
"8700","UBE2D1",44.2156906184777,-0.14217333387263,0.252554519707187,-
0.562941158358464,0.573474952441024,0.933379922593667
"8701","RAB42",16.094380000135,-0.227215485414847,0.405319016821179,-0
.560584319968118,0.575080943193133,0.933408175195541
"8702","CSMD2",3.4980426474786,-0.500488745947888,0.891469675525172,-0
.561419821322633,0.574511374762378,0.933408175195541
"8703","FNBP1L",17.6548548515496,-0.20736846588883,0.370160189470235,-
0.56021277216659,0.5753343162424,0.933408175195541
"8704","RBM15",8.71717516360564,-0.282112071846112,0.501820939211384,-
0.562176764264667,0.573995590879084,0.933408175195541
"8705","NUCKS1",888.771473495995,0.0807953690325543,0.144226962328949,
0.560196011396803,0.575345747313593,0.933408175195541
"8706","TTC30A",5.36039455007642,-0.319823882769267,0.56881816790171,-
0.562260315891548,0.573938671917448,0.933408175195541
"8707","EIF4E3",26.4323127490127,0.189837045839798,0.338010252478304,0
.56163102878657l,0.574367435109225,0.933408175195541
"8708","EVC",11.3196758722577,-0.247930602038771,0.442891037581338,-0.
559800449773694,0.575615556831142,0.933408175195541
"8709","CPEB2",15.3969547096435,0.201428217456745,0.358810833428729,0.
561377190125323,0.574540430350374,0.933408175195541
"8710","ADAMTS3",13.6270036704441,-0.290212525757222,0.516091482522947
,-0.562327679461981,0.573892782903032,0.933408175195541
"8711","ENC1",20.4179628225518,-0.216476846977578,0.386433244960499,-0
.560192089579938,0.575348422061118,0.933408175195541
"8712","ZFYVE16",79.6200862363084,0.122044669492151,0.217475071978218,
0.561189235998391,0.574668540078938,0.933408175195541
"8713","FZD1",35.7608241698286,0.151196082365513,0.269268176731035,0.5
61507431739842,0.57445166545839,0.933408175195541
"8714","ZNHIT1",231.675506860057,-0.137782811293296,0.24528885760117,-
0.561716551826929,0.574309155298053,0.933408175195541
"8715","AC026979.4",3.23600565560613,-0.488425692018561,0.872229270316

467,-0.559973975467881,0.575497188941564,0.933408175195541
"8716","SMC5-
AS1",1.51687937484266,0.616487626182183,1.09961329573834,0.56064038928
1796,0.575042711906171,0.933408175195541
"8717","TRAF6",12.8392429596778,0.213957592238882,0.381332891875067,0.
561078251568657,0.574744193527625,0.933408175195541
"8718","USP15",60.4970965283919,0.127459893768813,0.227719136790787,0.
559724121411518,0.575667626693691,0.933408175195541
"8719","COA8",80.0809844065685,-0.147315823142256,0.262292173377368,-0
.561647803841663,0.574356003500687,0.933408175195541
"8720","NPIPA5",1.61255310142106,0.812681377196748,1.44740308543718,0.
561475504214005,0.574473424749192,0.933408175195541
"8721","AC008915.2",6.5266538878394,-0.334664477191302,0.5955734621305
29,-0.561919726903404,0.57417071254728,0.933408175195541
"8722","CLN3",50.6940919296148,0.116864720741505,0.208480591509839,0.5
60554437682463,0.57510131914723,0.933408175195541
"8723","ZNF688",43.5420411897765,-0.163888491015654,0.29224437460357,-
0.560792628559467,0.574938912461232,0.933408175195541
"8724","RNFT1",37.7215809295791,-0.167159717556119,0.298503421128288,-
0.559992635676624,0.575484460851359,0.933408175195541
"8725","CYGB",492.730954262353,-0.157703343341926,0.281641323732895,-0
.559943907561981,0.575517698478471,0.933408175195541
"8726","PRKACA",65.9021240652881,0.127112052572365,0.225900771089083,0
.562689768430399,0.573646152576427,0.933408175195541
"8727","CERS1",2.27055440519032,-0.539984608053484,0.963415375262327,-
0.56048992150084,0.575145312218947,0.933408175195541
"8728","SCN1B",52.1405873324374,0.141247198989127,0.252346965076984,0.
559734090505254,0.575660825827452,0.933408175195541
"8729","HNRNPL",81.0542317951371,-0.130446201519888,0.232740705153663,
-0.560478672751992,0.575152982814294,0.933408175195541
"8730","AL050403.2",8.84927677817863,0.351248557817247,0.6253052410470
47,0.56172335486761,0.574304519475423,0.933408175195541
"8731","SMIM26",179.835176319857,-0.144987079646653,0.257807942741434,
-0.562384068174607,0.573854371453095,0.933408175195541
"8732","CR381653.1",2.07481178397233,0.546704792823426,0.9758396933733
35,0.560240371995474,0.575315493020517,0.933408175195541
"8733","STK26",3.6474315289226,-0.461044096877326,0.820330749012432,-0
.562022205594952,0.574100889939346,0.933408175195541
"8734","STK17B",71.2162084503456,0.200649407834608,0.358561558671389,0
.559595425059209,0.575755426113068,0.933443649388716
"8735","ZNF318",17.457268646368,0.260917757748179,0.466470070237476,0.
559345120717709,0.575926207226119,0.933506764459918
"8736","AIG1",77.1582696410643,0.128905335091834,0.230457429378251,0.5
59345539172275,0.575925921697185,0.933506764459918
"8737","HLA-
DMB",7.26107451557624,-0.277438118451138,0.496207594956149,-0.55911703
3417548,0.576081850611339,0.933652169469676
"8738","OPA1",37.2651146655047,0.152019484386236,0.272055805092068,0.5
58780520543534,0.576311518242851,0.93368407122608
"8739","ARFGEF1",49.6372212173944,0.140602972495918,0.251682850409018,

0.55865138314915,0.576399665059894,0.93368407122608
"8740","C1QTNF4",62.6019621761644,-0.260473887757675,0.466377228269911
,-0.558504729581111,0.576499775807539,0.93368407122608
"8741","TKFC",14.6178895184213,0.265454580647322,0.475291384545362,0.5
58509136245425,0.576496767548069,0.93368407122608
"8742","MSANTD4",18.8623834100769,0.205988951583871,0.368777448106305,
0.558572528340973,0.576453493033038,0.93368407122608
"8743","IQCK",28.3947837737527,-0.153315762262723,0.274436579897861,-0
.558656438291806,0.576396214392623,0.93368407122608
"8744","RALY",126.088319302624,-0.120273214970273,0.215384378654844,-0
.558411968971122,0.576563101610229,0.93368407122608
"8745","KLHL21",21.5938716638171,0.180328196630926,0.324471776894668,0
.555759266204115,0.578375435188788,0.934120018503207
"8746","PHACTR4",51.9953539335463,-0.139442921825541,0.251561744294812
,-0.554308932053374,0.579367438473782,0.934120018503207
"8747","IPP",19.902076558713,-0.192044094593983,0.345291700562654,-0.5
56179295016492,0.578088291992929,0.934120018503207
"8748","AL356488.3",4.1071558703318,-0.444905693261293,0.8008873077863
61,-0.555515974514573,0.578541786711325,0.934120018503207
"8749","DRAM2",72.1481309519309,0.133694633322794,0.241257464989081,0.
554157498624324,0.579471062325718,0.934120018503207
"8750","ARHGAP15",18.3360132610313,0.282562869858998,0.507009326397161
,0.557312962794801,0.577313620576003,0.934120018503207
"8751","CAB39",46.3266091555431,-0.128434093488689,0.231443370598631,-
0.554926646447004,0.578944834970628,0.934120018503207
"8752","PLCL2",23.8286825215554,0.16693416603267,0.300666743229373,0.5
55213271144256,0.578748792563184,0.934120018503207
"8753","ZNF141",25.0488947643474,0.177544945832608,0.319078694765389,0
.556429961464999,0.577916961501303,0.934120018503207
"8754","NADK2",32.6102885574193,-0.170835571555941,0.308241280204684,-
0.554226777939995,0.579423654345639,0.934120018503207
"8755","TTC33",24.3346810052615,-0.161501652563702,0.290579573378488,-
0.555791484879572,0.578353407245853,0.934120018503207
"8756","PAIP1",106.853033436052,-0.113681308760927,0.204957221865243,-
0.554658712322278,0.579128121806297,0.934120018503207
"8757","PCDHB15",3.05465569155629,-0.447807982314673,0.807414540677559
,-0.554619665307099,0.579154835138385,0.934120018503207
"8758","LSM11",4.8066767666623,0.334737050849918,0.603406892234277,0.5
54745156473874,0.579068984611014,0.934120018503207
"8759","SENP6",109.471051196731,0.125642773559883,0.226167241156999,0.
555530380603019,0.578531935872879,0.934120018503207
"8760","ARMC2",5.72228492529122,0.366720896998031,0.659132310654464,0.
556369170605379,0.577958509853041,0.934120018503207
"8761","ANKMY2",33.1044177886544,0.161453562064342,0.289882708952659,0
.55696168511627,0.57755360755377,0.934120018503207
"8762","AC005072.1",2.11757689079624,0.557655738975929,1.0036778719778
1,0.555612268184247,0.578475942897404,0.934120018503207
"8763","CLEC2L",2.53729572284478,0.450067308769498,0.81079366139649,0.
555094754927306,0.57882985019949,0.934120018503207
"8764","PURG",2.23255282348212,-0.505628071003384,0.910689786044435,-0

.555214386667902,0.578748029640526,0.934120018503207
"8765","LAPTM4B",72.7368359912639,0.150543762992093,0.270691595014201,0.556144947848106,0.578111770139979,0.934120018503207
"8766","INVS",9.63945324389028,0.262175092224955,0.472164425608734,0.555262273067159,0.57871527988918,0.934120018503207
"8767","AC004803.1",1.86123996633705,0.575831881173338,1.03522272311264,0.556239607494282,0.578047066237976,0.934120018503207
"8768","AMIGO2",13.1581075821753,0.237383302355831,0.427462608390822,0.555331151067126,0.578668175402682,0.934120018503207
"8769","CACNB3",11.5571984378505,-0.262190440228931,0.470357131150981,-0.557428436531496,0.57723474112781,0.934120018503207
"8770","ZBTB39",4.49927425491355,0.351468479964249,0.632396141873946,0.555772650545844,0.578366284252111,0.934120018503207
"8771","RNF34",23.2642967678976,0.172657235369546,0.309873101357795,0.557186908489313,0.577399733345589,0.934120018503207
"8772","REC8",3.24050677621818,0.530124985032213,0.951166893209767,0.557341712392108,0.577293981416101,0.934120018503207
"8773","SERF2",2204.09094823582,-0.163437772855473,0.294168113762945,-0.555593095270614,0.57848905269701,0.934120018503207
"8774","EDC4",17.0800912607894,0.191845233174275,0.345804302660833,0.554779774855601,0.579045302670466,0.934120018503207
"8775","KLHL36",42.5421227274465,0.134506233496726,0.241696432943418,0.556508972261972,0.577862962582344,0.934120018503207
"8776","HDAC5",37.9408160645921,0.147606632368336,0.265539603974839,0.555874265679491,0.578296811732991,0.934120018503207
"8777","RNFT1-DT",5.19476925609204,0.407324898353987,0.734598461659075,0.554486457042194,0.579245971594675,0.934120018503207
"8778","ILF3",96.6664223059585,0.132635720991692,0.238284107278449,0.556628482304529,0.577781289483607,0.934120018503207
"8779","GET3",112.889574630471,-0.144857582442508,0.259852608914976,-0.557460566000727,0.57721279457423,0.934120018503207
"8780","SLC1A5",38.9461027096055,-0.176719719503652,0.317225124649791,-0.557079833127172,0.577472885586116,0.934120018503207
"8781","ZSCAN22",2.92855742707319,0.437145044199554,0.78834023561557,0.55451317140779,0.579227694016298,0.934120018503207
"8782","GID8",56.1496255463899,0.110898207809908,0.199854957860494,0.554893453718165,0.57896753978313,0.934120018503207
"8783","APOL4",11.7109205582329,-0.280695114855827,0.506344704496668,-0.554355782460197,0.579335381134295,0.934120018503207
"8784","CDR1",2.48942350434447,-0.494916263259552,0.887851059268111,-0.557431630106439,0.577232559687204,0.934120018503207
"8785","MAN2C1",35.0816064166187,-0.157968920188796,0.285121203802493,-0.554041292201555,0.579550586709849,0.934141867707622
"8786","TRMT9B",3.76366024919916,0.381619040195635,0.689478902718731,0.553489075141889,0.579928559380159,0.934148250270743
"8787","GOLGA7",75.6848045982073,0.117676465203644,0.2126819974687,0.553297724321791,0.580059559077992,0.934148250270743
"8788","TSPAN18",28.8305694708569,-0.203939920277502,0.368504097514459,-0.553426465684007,0.579971420591248,0.934148250270743

"8789","ATP2B1",169.303151759789,-0.120044566445864,0.21697500732091,-0.553264488514643,0.580082313886345,0.934148250270743
"8790","LINC00345",1.93286330001817,0.574667556238686,1.03844823933379,0.553390659709107,0.579995933329346,0.934148250270743
"8791","RMDN3",23.4897385935305,-0.170419331680709,0.307794048471765,-0.553679749582104,0.579798036535313,0.934148250270743
"8792","LACTB",45.5531335422207,-0.216890407949107,0.391974277710887,-0.553328165347321,0.580038718076697,0.934148250270743
"8793","XYLT1",31.3081132524059,-0.194118762695414,0.350650058015111,-0.553596836099905,0.579854791812862,0.934148250270743
"8794","AC010913.1",2.10582962025269,-0.591697949381317,1.07015387429385,-0.552909225107233,0.580325570185164,0.934226634678863
"8795","RPUSD2",10.2774933643784,-0.236057643434223,0.426794514324445,-0.553094370971164,0.580198790968753,0.934226634678863
"8796","MRPL54",121.389007947851,-0.150115492242152,0.271550871275401,-0.552807993349827,0.580394894439969,0.934226634678863
"8797","GADD45GIP1",198.41242868415,-0.137480073663657,0.248665059105587,-0.552872503109821,0.580350717231646,0.934226634678863
"8798","LOXL4",11.1047828729519,0.310210683877618,0.561685810244108,0.552285064390003,0.580753061823509,0.934590675692793
"8799","KCNJ15",11.9252092746853,0.290663402025348,0.526220235791434,0.55236074604427,0.580701219123479,0.934590675692793
"8800","DCBLD2",59.6244955729118,0.14683156178369,0.265960457100865,0.552080423474399,0.58089325377517,0.934603848819045
"8801","SLC2A6",3.03954314151719,-0.432923216322102,0.784157797872245,-0.552086859936619,0.58088884415076,0.934603848819045
"8802","TMEM240",13.1015879970066,0.226866878667552,0.411348179218777,0.55152031813636,0.581277042299631,0.934779608516841
"8803","TMEM254-AS1",4.9942677881263,0.339536292660111,0.615527556219595,0.55161834629379,0.581209864082773,0.934779608516841
"8804","PIP4K2C",8.18073235039835,-0.287083242001533,0.520275864433145,-0.551790428168945,0.581091946000784,0.934779608516841
"8805","PSMB5",211.704531545621,-0.166556788052228,0.302040117450675,-0.551439290442694,0.581332572923679,0.934779608516841
"8806","CDC42EP5",114.856484281313,0.155354330760617,0.281662670633298,0.551561662080794,0.581248709053797,0.934779608516841
"8807","CEP120",16.7316158336396,0.205419314172307,0.372792863662671,0.551028021711768,0.581614465543606,0.93491438666108
"8808","DPYSL2",445.977775393085,0.110648333768569,0.20079242116316,0.551058317478318,0.581593697979639,0.93491438666108
"8809","MEGF8",21.385794779988,0.179796939607687,0.326287025800158,0.551039193687731,0.581606807181585,0.93491438666108
"8810","PNRC1",1073.02681012752,0.127691313133538,0.231784228596945,0.550905960713935,0.581698141128424,0.934942755775084
"8811","LINC02035",4.19967255862059,0.36320825106527,0.659611174171419,0.550639930443143,0.581880530412949,0.935023639428887
"8812","BTG1",778.770280021037,-0.143195196131183,0.260028053543848,-0.550691335721728,0.581845285077565,0.935023639428887
"8813","CPSF1",21.0555295298157,0.175403737091472,0.318803000089015,0.

550194750496378,0.582185803759192,0.935195800479882
"8814","C9orf92",1.75901592896149,0.562745821614488,1.02277101167979,0
.550216827802188,0.582170662920585,0.935195800479882
"8815","ZMYND15",1.53723441907632,-0.61178912211395,1.11191204819503,-
0.550213592079582,0.582172882000224,0.935195800479882
"8816","AC013643.2",1.85999276283244,0.729901894662365,1.3277376535876
3,0.549733520541594,0.582502161954772,0.935279579689258
"8817","SLC31A2",38.7971802977996,0.188599925731972,0.343066745880917,
0.549747033183559,0.582492892476,0.935279579689258
"8818","SFXN3",63.8766421874366,-0.150469247595699,0.273685331321709,-
0.549789230095153,0.582463946442534,0.935279579689258
"8819","NAA30",22.4317364320717,0.168458038797616,0.306370174626412,0.
549851299993655,0.582421369253953,0.935279579689258
"8820","AC096921.2",3.08165034664206,0.448426687161411,0.8159841594966
27,0.54955317691221,0.582625881692026,0.935372163804886
"8821","CCDC130",31.545018477603,0.143295233748436,0.260850303747463,0
.549338956826227,0.582772857364769,0.935502058755824
"8822","RPL9",4249.16464605975,-0.111324115166569,0.202769399398557,-0
.549018320795801,0.582992876973708,0.935749165489425
"8823","AL450998.2",5.81823904354433,-0.30196745226534,0.5548262729560
79,-0.544255863473221,0.586265414574712,0.935760070216027
"8824","AL031280.1",1.79855133076555,0.637371627514934,1.1792810515621
3,0.540474746601452,0.588869672058452,0.935760070216027
"8825","SMAP2",36.1841199231218,0.184151230088984,0.340950917598494,0.
540110674539498,0.589120709565224,0.935760070216027
"8826","LRP8",1.93132776608982,0.544763894716949,1.00370137440347,0.54
2754955417608,0.587298530919157,0.935760070216027
"8827","ROR1-
AS1",15.1802076495156,-0.77637282918033,1.43406824465798,-0.5413778821
701,0.588247149007025,0.935760070216027
"8828","MIER1",130.780217102311,-0.0894510467379009,0.16310749559748,-
0.548417756095341,0.583405087116248,0.935760070216027
"8829","PRRC2C",368.209498672401,0.0815205222006386,0.149567949881205,
0.545040045446815,0.585725975939305,0.935760070216027
"8830","NPL",4.5734836776967,-0.347341797403955,0.642712870919845,-0.5
40430747725407,0.588900007846841,0.935760070216027
"8831","TMEM183A",52.7418943565867,-0.126274608788381,0.23159088164898
9,-0.545248620711111,0.585582535853128,0.935760070216027
"8832","AL591846.2",3.87319329391082,-0.419882900435806,0.777591422312
45,-0.539978822280642,0.589211637432634,0.935760070216027
"8833","NVL",15.8368272629621,0.202173372502104,0.369957788298784,0.54
6476865460191,0.584738185995469,0.935760070216027
"8834","CEP170",77.515158305405,0.133574302973023,0.244148576665626,0.
54710252583598,0.584308297078158,0.935760070216027
"8835","ARHGAP25",1.65947024281033,-0.574254182561402,1.0592787431873,
-0.542118102770105,0.587737148644047,0.935760070216027
"8836","ARHGEF4",8.80355365598169,0.280752860258249,0.512827465800301,
0.547460654861993,0.584062294158751,0.935760070216027
"8837","IKZF2",7.67835236840673,-0.318747694578624,0.58618688695547,-0
.543764628093495,0.586603452626327,0.935760070216027

"8838","PECR",8.02033544979673,0.299903947906224,0.555325450204678,0.5
4005078966881,0.589162006527546,0.935760070216027
"8839","CRBN",96.7898387510706,0.107798805032005,0.197117372861556,0.5
46876226418239,0.584463769588986,0.935760070216027
"8840","AC022007.1",1.87457648848092,-0.531720004592491,0.971772511024
426,-0.547165101461823,0.584265309689702,0.935760070216027
"8841","ZNF621",25.2409699952493,0.18066082165937,0.329382169789042,0.
54848391391397,0.583359671656641,0.935760070216027
"8842","WDR5B",5.65077698930312,0.306809575297156,0.564279893458225,0.
543718780084213,0.586635007022104,0.935760070216027
"8843","IQCG",10.0730524062348,0.254601330953664,0.463877015847409,0.5
48855240194558,0.583104797319147,0.935760070216027
"8844","SDAD1",47.4365862935628,0.137765854379888,0.254076849139111,0.
54222120136754,0.587666131560017,0.935760070216027
"8845","NUDT6",12.5799840074923,0.231123787539517,0.421309203590951,0.
548584710634319,0.583290480744949,0.935760070216027
"8846","MRPL36",96.8943389321959,-0.153692727731289,0.281848571077388,
-0.54530249042522,0.585545491558041,0.935760070216027
"8847","ERBIN",61.1031377554341,0.111609327033239,0.205687654823442,0.
542615584435742,0.58739450654942,0.935760070216027
"8848","SMN1",6.58850266205983,-0.29714851756721,0.544756945618619,-0.
545469901682064,0.58543037570994,0.935760070216027
"8849","GPR150",2.06506116846419,-0.495465341226019,0.910018339221434,
-0.544456435515261,0.586127419177432,0.935760070216027
"8850","UBE2B",252.349225717474,-0.103688257195413,0.191837571644979,-
0.54050025918438,0.588852082295971,0.935760070216027
"8851","GPLD1",1.75699695591033,-0.607562786202682,1.10817809764799,-0
.548253739621981,0.583517686863435,0.935760070216027
"8852","VARS",27.3961370216648,0.181441780898624,0.335977346489455,0.5
40041710533359,0.589168267669846,0.935760070216027
"8853","AGPAT1",47.1088984726121,-0.127367831830556,0.235815162414543,
-0.540117227944208,0.589116190379515,0.935760070216027
"8854","HSD17B8",12.0716124504756,-0.2328599500957,0.427728515886832,-
0.54441062834658,0.586158933600375,0.935760070216027
"8855","UBE2J1",82.4716622327641,0.110927012775815,0.203831459567964,0
.544209480768735,0.586297328443968,0.935760070216027
"8856","TCF21",8.25729386685646,-0.353484661579761,0.651757510803499,-
0.542356099807701,0.587573215885449,0.935760070216027
"8857","CDK13",76.0513139559599,0.145421467506406,0.267936501705393,0.
542746011016828,0.587304690121806,0.935760070216027
"8858","LINC02604",3.14001659401745,0.463975663679218,0.85286318306851
1,0.544021213355562,0.586426875120601,0.935760070216027
"8859","PON2",39.2024695307654,0.130393268276286,0.240845480444927,0.5
41398028459589,0.588233265823141,0.935760070216027
"8860","LMTK2",8.11604985594137,0.313652760739864,0.579537957249065,0.
541211765021746,0.588361629204011,0.935760070216027
"8861","WASL",104.252374998712,-0.099858019863774,0.1830703711817,-0.5
45462486470099,0.585435474358539,0.935760070216027
"8862","EFR3A",64.6280156316141,-0.134579234811564,0.24861790723767,-0
.541309499009301,0.588294274247973,0.935760070216027

"8863","PTK2",59.7087770021877,0.116048400402639,0.214713860400342,0.540479315989486,0.58886652165595,0.935760070216027
"8864","KDM4C",12.4361893154624,0.22866802171823,0.422791174304185,0.540853347032524,0.588608669237701,0.935760070216027
"8865","ACO1",58.4779327165912,0.114972825656065,0.211056787395825,0.544748297719703,0.585926642232673,0.935760070216027
"8866","TLR4",19.3218490978018,-0.198884581614109,0.363832996963292,-0.546637010040556,0.584628137246727,0.935760070216027
"8867","FPGS",21.3057839167252,0.174470839665243,0.321360498929089,0.542913146595972,0.587189603853661,0.935760070216027
"8868","FNBP1",78.0801113501032,0.136832294960853,0.252841654263459,0.541177818818867,0.588385024611669,0.935760070216027
"8869","BEND7",13.1297832730693,0.228096563379541,0.41994148921008,0.543162724427626,0.587017768914005,0.935760070216027
"8870","LRMDA",31.6771779738162,0.158539847338902,0.291041343903103,0.544733078856607,0.585937110757192,0.935760070216027
"8871","UBE2L6",135.58084465071,-0.176707332693711,0.324988117171809,-0.543734750154859,0.586624015704684,0.935760070216027
"8872","SERPING1",1614.66131128681,0.0684338240160416,0.125871404096065,0.543680469027047,0.5866613747955,0.935760070216027
"8873","PRPF19",37.1946995899832,-0.134160347121047,0.247245659504469,-0.542619625314886,0.5873917237595,0.935760070216027
"8874","GRK2",26.9966312224784,-0.170609252634011,0.315221228709951,-0.541236557360788,0.588344542825857,0.935760070216027
"8875","TENM4",6.91633785960852,0.32612277058648,0.599722741017456,0.543789235060852,0.586586517471978,0.935760070216027
"8876","TIMM8B",139.483288424486,-0.140561807578449,0.259607401009976,-0.541439908999542,0.588204405646434,0.935760070216027
"8877","ENO2",8.45665290241889,-0.288151781406971,0.528611979305389,-0.545110199329213,0.585677728326455,0.935760070216027
"8878","PCED1B",4.82904807374366,0.40122423574215,0.733851780602415,0.546737428929841,0.584559135934725,0.935760070216027
"8879","SPRYD4",10.6539093679229,0.229677227163817,0.419131789387239,0.547983314507355,0.583703359834242,0.935760070216027
"8880","SLC26A10",1.85409602750506,0.598117143613759,1.09896851441451,0.544253211778695,0.586267239065679,0.935760070216027
"8881","AC025159.1",12.6990563012185,0.207378005120495,0.383816734639591,0.540304750691044,0.588986882678363,0.935760070216027
"8882","RAB21",89.4119861251439,0.105408056486631,0.19447393854089,0.54201636104813,0.587807234967157,0.935760070216027
"8883","C12orf29",34.1842305526791,0.185516589787178,0.342336147669958,0.54191352870522,0.587878076508417,0.935760070216027
"8884","RFC5",11.2695525784543,-0.220638148855782,0.402595703699539,-0.548039004957804,0.583665120708797,0.935760070216027
"8885","HSPB8",43.6485621809761,0.186616864812416,0.344983941340198,0.540943627948142,0.588546438493704,0.935760070216027
"8886","OGFOD2",9.1465360047013,0.262308027252681,0.48391122157758,0.542058161820553,0.58777843940541,0.935760070216027
"8887","PGAM5",9.80832366066219,-0.247146035890583,0.453898705066022,-0.544496014489916,0.5861001902613,0.935760070216027

"8888","NUP58",27.9527580828524,0.174108593508528,0.321224674009927,0.
542015005681499,0.587808168658038,0.935760070216027
"8889","ATP7B",2.17767776313657,0.493773338718016,0.906337490850256,0.
544800743324427,0.585890567397338,0.935760070216027
"8890","COX16",148.54478717331,-0.137025890358658,0.252736969761407,-0
.542167972054168,0.587702796845588,0.935760070216027
"8891","TBL3",18.255693867729,-0.181359418525641,0.332724944103904,-0.
545073105396647,0.585703239068142,0.935760070216027
"8892","CRYM",2.05691413598848,-0.635224781075464,1.15782008196224,-0.
548638593311407,0.583253495083417,0.935760070216027
"8893","RBBP6",152.205878986601,0.124311535445538,0.229843837367576,0.
540852158009932,0.588609488852682,0.935760070216027
"8894","AC026471.1",4.42465052887062,0.3722512461881,0.683776017230601
,0.544405239153861,0.586162641309681,0.935760070216027
"8895","DYNLRB2",4.60099154569409,-0.343400248866931,0.63519856760039,
-0.540618739371856,0.588770398788618,0.935760070216027
"8896","TP53",24.9946457217221,-0.183983168423093,0.336592076824408,-0
.546605761368153,0.584649610069315,0.935760070216027
"8897","EPN2",104.111694543133,0.16187232759549,0.29905486925538,0.541
279692246903,0.588314815682066,0.935760070216027
"8898","MAPK7",22.56948556564,-0.175628248604398,0.32406569595413,-0.5
41952606514875,0.587851155210962,0.935760070216027
"8899","MIEN1",60.4421066584511,-0.14155249608622,0.261244114496115,-0
.541839942917929,0.58792877242408,0.935760070216027
"8900","TUBG2",20.7810701253644,-0.182168316940237,0.332444765141765,-
0.547965665401754,0.5837154786072,0.935760070216027
"8901","MAP3K14",10.8323152593533,-0.345147331055355,0.638251715806723
,-0.540769922755481,0.588666176393779,0.935760070216027
"8902","AC005921.2",16.4577440886262,-0.328680711894471,0.599886460973
261,-0.547904867466447,0.583757226457025,0.935760070216027
"8903","HEATR6",14.5251789770278,-0.212196920993508,0.390754542403551,
-0.543044028837833,0.587099488209976,0.935760070216027
"8904","DDX42",93.6834530795001,0.104340285048152,0.192744916027561,0.
541338714393027,0.58827414068618,0.935760070216027
"8905","CASKIN2",7.22495313905135,0.355268036655637,0.656661053918939,
0.541021938997913,0.588492461054046,0.935760070216027
"8906","RPRD1A",23.1161688935049,0.19276528122619,0.353884139561677,0.
54471297149669,0.585950942041058,0.935760070216027
"8907","USE1",64.910379031753,-0.137171370740138,0.251636505724742,-0.
545117133720597,0.585672959370123,0.935760070216027
"8908","CEP89",40.2156885869278,0.135322972347807,0.250207994240218,0.
540841921373173,0.588616545175097,0.935760070216027
"8909","LRFN3",11.7045065209865,0.239875738703518,0.441801249574354,0.
542949434694045,0.587164617963677,0.935760070216027
"8910","HNRNPUL1",146.420835195055,-0.0985088832323362,0.1808623032092
39,-0.544662328657685,0.585985778488116,0.935760070216027
"8911","FBXO46",7.41398294415113,0.29471830997945,0.539339355411338,0.
546443175381995,0.584761338495069,0.935760070216027
"8912","BCAT2",17.6632802107526,-0.187414900720875,0.34504116746742,-0
.543166782376979,0.587014975198497,0.935760070216027

"8913","ZNF808",7.37604578056047,0.270071209822223,0.495457122584661,0
.545095019349681,0.585688168045217,0.935760070216027
"8914","ZNF845",4.51464207138156,0.35997875811838,0.666225729504423,0.
540325511574212,0.588972567662796,0.935760070216027
"8915","SNRPB2",157.722510240483,-0.0962634577796294,0.176815083516728
,-0.544430123635476,0.586145521132461,0.935760070216027
"8916","CCDC22",19.8753804321105,-0.189071317094354,0.346303688943282,
-0.545969688256254,0.585086773456501,0.935760070216027
"8917","MTCL1",2.02779128268093,0.490992139727206,0.909526656705566,0.
539832599855455,0.589312482815475,0.935815269335777
"8918","ZBTB34",6.84652863029087,0.270591470556233,0.501398063767751,0
.539673943937629,0.589421912223722,0.935884085791422
"8919","C12orf65",34.5501412881112,0.157623102791409,0.292212839911448
,0.539411967109916,0.589602625452044,0.935961118430599
"8920","AL121832.2",2.95697653291633,-0.414890899759156,0.769037757198
804,-0.539493537053867,0.589546355264212,0.935961118430599
"8921","KCNMB2",2.11044597953649,0.609727077436595,1.13104289796217,0.
539083953875804,0.589828927005726,0.936001869256253
"8922","COPS2",110.618938851117,0.11224351680528,0.208192816152803,0.5
39132516094596,0.589795420633373,0.936001869256253
"8923","AC040169.1",2.49409884356454,0.506127129237173,0.9387249594162
81,0.539164452974483,0.589773385689426,0.936001869256253
"8924","LINC01534",5.94341910978967,0.332414977341853,0.61673507448300
2,0.538991523419533,0.589892703477599,0.936001869256253
"8925","IBA57",12.0776776100937,0.241809986926243,0.448775676254238,0.
538821508653365,0.59001002096451,0.936083125698316
"8926","KPNA6",54.3235534114443,-0.121927591008649,0.226357481156801,-
0.538650591027685,0.59012797229667,0.936099670884318
"8927","AGO4",17.1353685999406,0.180956258251276,0.337633083584749,0.5
35955352271794,0.591989407831545,0.936099670884318
"8928","PPT1",70.5318895685623,0.120772839591079,0.226858035760738,0.5
32371882644947,0.594468455248799,0.936099670884318
"8929","SCMH1",19.4581373069669,-0.168280289025663,0.315258636505988,-
0.533784865945986,0.59349038560712,0.936099670884318
"8930","SLC6A9",2.64127751146221,-0.493693214549558,0.918374931531333,
-0.537572616149654,0.590872139697736,0.936099670884318
"8931","HADHB",160.078296286074,-0.127964175497095,0.239309229590122,-
0.534723109995656,0.592841338657631,0.936099670884318
"8932","GMCL1",24.8508066483598,0.15014156601037 8,0.281252278434593,0.
533832354518311,0.593457526730322,0.936099670884318
"8933","ZNF2",3.882855189673,0.347909194903747,0.650863666005078,0.534
534669970397,0.59297166927196,0.936099670884318
"8934","UXS1",57.3865727686786,0.116429904167084,0.217904483216716,0.5
34316240071522,0.593122758185749,0.936099670884318
"8935","AC012510.1",1.90344721601941,-0.509038906284103,0.955269640995
541,-0.532874577437219,0.594120404569844,0.936099670884318
"8936","RPUSD3",22.0510990530074,-0.18542832992008,0.346460631992393,-
0.535207503530015,0.592506378241341,0.936099670884318
"8937","ZNF660",7.90649283489472,-0.255338018754433,0.478666550335634,
-0.533436101969928,0.593731732183445,0.936099670884318

"8938","EOGT",5.94228868290109,-0.359572965986554,0.667893778291932,-0
.538368491627702,0.590322674625415,0.936099670884318
"8939","PARP9",41.0279469676476,0.147549446601585,0.275393540134459,0.
535776716220523,0.592112875946715,0.936099670884318
"8940","PCCB",13.9668232208089,-0.204339648148234,0.381630758484693,-0
.535438099799887,0.592346950334274,0.936099670884318
"8941","SELENOT",70.3930571254257,-0.117090715165476,0.219785236808331
,-0.532750592650533,0.594206239230182,0.936099670884318
"8942","POLR2H",67.9807453833317,-0.133586938713005,0.251456052708579,
-0.531253621752442,0.595243039310904,0.936099670884318
"8943","SCRG1",2.47333545451097,0.480910293611965,0.900494275065029,0.
534051472539608,0.59330592268499,0.936099670884318
"8944","TMED7",102.886711721328,-0.100701978875651,0.188846734810102,-
0.533247127502168,0.593862522306191,0.936099670884318
"8945","CDKL3",7.74582391196809,0.256822017214594,0.481366970009329,0.
533526463624243,0.593669197114215,0.936099670884318
"8946","ZMAT2",97.6772094574377,0.122971274689735,0.22995344535566,0.5
34765958820663,0.592811704993633,0.936099670884318
"8947","AL512329.2",1.80337183690245,0.517936468471815,0.9722528618028
85,0.532717864683254,0.59422889774585,0.936099670884318
"8948","C6orf62",99.7970984389505,0.109692150292871,0.204871868118806,
0.535418314383996,0.592360628653759,0.936099670884318
"8949","BTN3A1",27.5856212773877,0.196278896729582,0.367789024378751,0
.533672523428685,0.593568122338729,0.936099670884318
"8950","HLA-
DRB1",129.833606344814,0.15636358376897,0.294271132775151,0.5313588944
12678,0.595170100620876,0.936099670884318
"8951","HMGCLL1",21.992387076741,-0.231413877800295,0.431157803632845,
-0.536726636629212,0.591456453936023,0.936099670884318
"8952","ECHDC1",82.0892406562549,0.111589613308199,0.2076480428257,0.5
37397857401754,0.590992823014269,0.936099670884318
"8953","HDAC9",5.79920495022839,-0.416593934486925,0.78111019588605,-0
.53333567617097,0.593801235712545,0.936099670884318
"8954","STX1A",1.63488046795874,-0.566966082948314,1.06216213354983,-0
.533784876187848,0.593490378520355,0.936099670884318
"8955","STYXL1",15.4887189350798,0.217258135343804,0.407732984764053,0
.532844149142182,0.594141469551616,0.936099670884318
"8956","AGAP3",34.7665538158841,-0.151425584115961,0.284438342462969,-
0.532366989642668,0.594471843473568,0.936099670884318
"8957","PLEKHF2",18.0126842864382,0.189917529368569,0.356342305163769,
0.532963744737764,0.594058677558668,0.936099670884318
"8958","ST6GALNAC6",138.711320677006,-0.120595698335899,0.225248786471
699,-0.535388892543718,0.592380969223543,0.936099670884318
"8959","GOLGA2",138.46519512594,-0.122681373160268,0.228296854152959,-
0.537376538171968,0.591007546231031,0.936099670884318
"8960","HMCN2",90.4919981071815,0.264035072813817,0.491414750223952,0.
537295782622496,0.591063318136499,0.936099670884318
"8961","ACSL5",31.108707595009,-0.162281318723234,0.305308703822405,-0
.531531910788993,0.59505023422087,0.936099670884318
"8962","COMMD9",47.1617397539705,0.166597785891768,0.313515181159285,0

.531386662922476,0.595150861749755,0.936099670884318
"8963","TMEM223",47.5954544529945,-0.140835800720633,0.263294764783355
,-0.534897839068376,0.592720502671084,0.936099670884318
"8964","RAB6A",84.7187843197135,-0.121346711758917,0.225724054706285,-
0.537588747095719,0.590861000706293,0.936099670884318
"8965","CD3E",3.32434817630502,0.43020191740065,0.80439230613522,0.534
816051967972,0.592777062115888,0.936099670884318
"8966","NFRKB",19.7151281698937,0.16935155784723,0.315584985224234,0.5
36627424549047,0.591524996663678,0.936099670884318
"8967","EIF4B",284.779457658714,-0.0944221571330305,0.176505528636851,
-0.534952972081107,0.592682377118403,0.936099670884318
"8968","TCHP",21.8482668688706,0.186387132880797,0.346519161548592,0.5
37884058266313,0.590657094922834,0.936099670884318
"8969","ANKRD13A",23.6094432544159,0.167171361210513,0.314376421456682
,0.531755404670346,0.594895413130644,0.936099670884318
"8970","TCTN2",9.88780136850022,0.293249635173558,0.551978346418865,0.
531270179484592,0.595231566932871,0.936099670884318
"8971","N4BP2L2-
IT2",2.06390651018645,0.517684722779925,0.964265850985078,0.5368692899
89962,0.591357905293713,0.936099670884318
"8972","WDHD1",5.23884754322395,0.30942642224797,0.581300772040534,0.5
32300036626124,0.594518206870579,0.936099670884318
"8973","LRRC57",21.7249328508081,0.164115135728655,0.307179045170538,0
.534265400940817,0.593157926364267,0.936099670884318
"8974","DNAJA4",11.3609096061681,-0.255441631493159,0.479254078542785,
-0.532998346659568,0.594034724800668,0.936099670884318
"8975","AC133552.5",3.80776084071061,-0.380892435072178,0.715183520289
034,-0.532579994178618,0.594324353809792,0.936099670884318
"8976","MT1E",1139.91987966612,-0.237577915229412,0.441481424170603,-0
.538137965092738,0.590481803809653,0.936099670884318
"8977","ZFP1",12.8915531090134,0.20952857294056,0.393616522654993,0.53
2316508278828,0.59450680048332,0.936099670884318
"8978","ZNF469",13.7929527479229,0.200372051042741,0.372837901049619,0
.537424039988024,0.590974741358569,0.936099670884318
"8979","SPG7",68.2001594498801,0.14726152528539,0.275860945832038,0.53
3825202553508,0.593462475352458,0.936099670884318
"8980","AC015908.2",4.86920187642366,-0.403063910782857,0.754066296778
606,-0.534520522273383,0.592981454761483,0.936099670884318
"8981","CNTNAP1",16.1725928048181,0.196437593266264,0.368211655842161,
0.533490969526693,0.593693760557405,0.936099670884318
"8982","HOXB3",13.1842747109888,0.210396209261934,0.395798954195456,0.
531573434016793,0.595021468411644,0.936099670884318
"8983","SUPT4H1",99.8186212806809,-0.116977040835108,0.217725264925422
,-0.537269025141277,0.591081798090232,0.936099670884318
"8984","TSEN54",6.70616987323921,-0.264018438114255,0.492709317944045,
-0.535850304629796,0.592062012317274,0.936099670884318
"8985","AC132872.1",6.25652776531198,-0.325028829568068,0.604727080092
993,-0.537480196054864,0.590935960919181,0.936099670884318
"8986","POLR2E",153.674040506508,-0.124541833744541,0.234139519698445,
-0.53191291203186,0.594786313985469,0.936099670884318

"8987","TRAPPC5",238.066684819982,-0.128746937471409,0.242212992777217
,-0.531544307327181,0.595041646275473,0.936099670884318
"8988","ZNF20",3.99488252597932,-0.366014694095326,0.68691622800118,-0
.532837454081369,0.594146104472131,0.936099670884318
"8989","LPAR2",2.85891066596011,-0.425919921668375,0.795617222140441,-
0.535332707507923,0.592419813219032,0.936099670884318
"8990","URI1",121.932023992223,0.0924656146052193,0.173369564223027,0.
533343986988795,0.593795483750752,0.936099670884318
"8991","SPHK2",11.2968129761946,0.245525372510194,0.458151362395291,0.
535904490661224,0.592024560714487,0.936099670884318
"8992","ZNF888",3.40611583837667,0.442015900395051,0.82727110948775,0.
534305979413145,0.593129855961591,0.936099670884318
"8993","SPTLC3",14.7503279170198,0.218616574971514,0.408946191058402,0
.534585184436388,0.592936730706734,0.936099670884318
"8994","MACROD2",3.10402168164598,0.471502709234297,0.88416797833505,0
.533272772581257,0.593844772452644,0.936099670884318
"8995","NOL4L",6.50248137122989,0.293250745147337,0.551350373408361,0.
531877294894183,0.594810983774139,0.936099670884318
"8996","HELZ2",35.4784805626717,-0.224482173857998,0.422370499883248,-
0.531481658686034,0.595085047932174,0.936099670884318
"8997","SUMO3",204.45946739095,-0.0930759660360086,0.173392775650277,-
0.536792641371271,0.591410855209863,0.936099670884318
"8998","TMEM191B",5.66243054292567,0.448023369853654,0.840266214288076
,0.533192174379219,0.593900558096283,0.936099670884318
"8999","DGCR11",1.77800869643511,0.561589820309728,1.05361327051957,0.
533013237421347,0.59402441698854‌8,0.936099670884318
"9000","EMID1",6.93535704677357,0.308704908037139,0.575655969147798,0.
536266319784968,0.59177450420528,0.936099670884318
"9001","HMGXB4",61.011098383721,0.111345025094325,0.208334156221518,0.
534454009432492,0.593027460463866,0.936099670884318
"9002","YIPF6",67.7401476652762,0.10726159585145,0.199754105018781,0.5
36968168145358,0.591289602127371,0.936099670884318
"9003","CXorf56",15.8212001547558,0.188565865948777,0.351761540590337,
0.536061633208451,0.591915955104574,0.936099670884318
"9004","AIFM1",23.7225009926216,-0.176357604493823,0.329177367171807,-
0.535752521532797,0.592129599529691,0.936099670884318
"9005","AC008267.5",6.08334841697369,-0.368706834343035,0.694239386779
068,-0.531094664700104,0.595353181230903,0.936168911297011
"9006","SUPT6H",50.5420134605257,0.140744095975112,0.265082509923941,0
.530944482212329,0.595457251766152,0.936228590385156
"9007","CCDC74B",6.81960705381669,-0.294567489569432,0.555123286829031
,-0.53063435917462,0.595672181048453,0.936372398708696
"9008","AC073111.4",8.97319495861634,0.236761373521057,0.4461961969008
7,0.530621675320235,0.595680972285871,0.936372398708696
"9009","EPB41L5",7.85349944099899,0.319314744841894,0.602054600103584,
0.530375060313392,0.595851914031612,0.936537140935468
"9010","NRAS",25.8180459871044,-0.152785631621834,0.288592833862173,-0
.529415888735483,0.596516978502767,0.936862869563264
"9011","EEF1AKMT4",2.59584624197806,-0.431723703345187,0.8147388147315
81,-0.529892151372977,0.596186708202276,0.936862869563264

"9012","KIAA0232",40.5901808705728,-0.148207111158209,0.279913552985785,-0.529474580910112,0.59647627317751,0.936862869563264
"9013","INTS1",35.1999904059982,-0.143861714455936,0.271649209140296,-0.529586354811129,0.596398757091304,0.936862869563264
"9014","CDKN2A",9.76610512451858,-0.416097710021401,0.78559285265737,-0.529660763350756,0.596347156723255,0.936862869563264
"9015","AC084824.5",1.70062263993314,0.600316562052957,1.13303669968123,0.529829759461324,0.596229969913533,0.936862869563264
"9016","STARD3",30.9515600060427,-0.151042814658059,0.285304999368475,-0.529408229762514,0.596522290394236,0.936862869563264
"9017","POLDIP3",41.0637133073025,0.141766590193165,0.267909709005052,0.52915809105855,0.596695786281296,0.937031422174021
"9018","RCC2",24.4606021117695,-0.16594329817053,0.313688869165611,-0.529006013544334,0.596801278278108,0.937093158174541
"9019","HMGCR",14.7297979271173,0.236939155227661,0.448128082993462,0.528730879004336,0.596992153121437,0.937288933163272
"9020","ETFDH",31.9213093138692,0.13958164570975,0.264234150495231,0.528249832385951,0.597325946374164,0.937489725646076
"9021","LINC02202",11.6893476242928,0.222871641279449,0.421882350852905,0.528279130020198,0.597305614623494,0.937489725646076
"9022","CLDN23",10.1039255440152,-0.331922254554502,0.628386698227494,-0.52821336844138,0.597351251786214,0.937489725646076
"9023","SH3GLB2",44.5092416764387,-0.123793886239861,0.234384906500101,-0.528164923622366,0.597384872493258,0.937489725646076
"9024","POMZP3",5.48612238193291,0.321166887394896,0.60843063231823,0.527861140342643,0.597595717714385,0.937612782585673
"9025","DHX58",13.4951247964111,0.200861819772083,0.380496115067685,0.527894535102815,0.597572537938994,0.937612782585673
"9026","EIF4H",169.503831297272,-0.0847096018001556,0.16062104407098,-0.527387941537234,0.597924215606146,0.937920338205719
"9027","EFEMP2",325.397151191195,0.136003834743322,0.257872421547048,0.527407444066324,0.59791067519492,0.937920338205719
"9028","ANAPC13",68.4596576771959,0.157270512694661,0.298428057648964,0.526996402193709,0.598196086922961,0.938112056804502
"9029","NDUFA12",175.214470820092,-0.126499616783269,0.240071072111892,-0.526925696088493,0.598245188767278,0.938112056804502
"9030","MRPL12",42.8133450693481,-0.160542287313044,0.304589626763923,-0.527077330304077,0.598139888654157,0.938112056804502
"9031","DHX30",40.0815432930345,-0.132010179503888,0.25063013047343,-0.526713126049632,0.598392819016065,0.938135774719607
"9032","PCF11",87.4165776456551,0.107794927438901,0.204636571776829,0.526762770226915,0.598358339571988,0.938135774719607
"9033","LAMTOR5",213.832601135555,-0.122616519119887,0.232843120241493,-0.526605720592973,0.598467418572449,0.938148859402843
"9034","TMEM141",66.3881783277236,-0.143258018837375,0.272147447572045,-0.526398539157531,0.598611330472732,0.938234965790205
"9035","MALT1",25.7396352998111,0.17814841721176,0.338469081192655,0.526335866732712,0.598654866943115,0.938234965790205
"9036","ZER1",22.0985344382669,-0.164628774656886,0.313083445859403,-0.525830339592008,0.599006092507895,0.938621939385642

"9037","ARL14EP",55.313147806324,0.14158445346055,0.269524109318802,0.525312758915675,0.599365789260238,0.938621939385642
"9038","USP7",70.8657002186694,-0.119339773430673,0.227059717572846,-0.525587606231326,0.59917476977772,0.938621939385642
"9039","PIN1",98.9219603370984,-0.108164309517494,0.205868876392836,-0.525403895006919,0.599302446421423,0.938621939385642
"9040","ERF",24.7554726110126,-0.166507294984696,0.316823949620772,-0.525551477986435,0.599199877411684,0.938621939385642
"9041","SLC25A5-AS1",1.79481369862325,-0.511480360591875,0.973428618688789,-0.525442082523565,0.59927590563496,0.938621939385642
"9042","LAMP2",216.351962345399,0.117901710609426,0.224239332896372,0.525785146996993,0.599037495557273,0.938621939385642
"9043","SECISBP2",56.177693996487,-0.125093689953529,0.238212950925475,-0.525133874827251,0.599490128909182,0.938691718807808
"9044","TFAM",66.3846505689424,0.0984416475154517,0.187487217591536,0.525057914774324,0.599542931136852,0.938691718807808
"9045","ALKBH4",25.1087723936556,0.146485814460859,0.279179871660113,0.524700486427609,0.599791419115614,0.93897694800189
"9046","FAM110B",42.9130622380397,0.168842029888253,0.321865943954244,0.524572521758486,0.599880392842221,0.939012421252028
"9047","PABPC4",119.004526343327,-0.106099206475201,0.202611594300516,-0.523658119573522,0.600516349675855,0.93914010957289
"9048","CEBPZ",80.2831886992765,-0.108783717301406,0.207712837011134,-0.523721686472248,0.6004721297294,0.93914010957289
"9049","MEIS1-AS2",1.80664703557292,-0.50871378039823,0.973003061450009,-0.522828550652373,0.601093569416157,0.93914010957289
"9050","USP19",23.6171148344079,0.155240505995919,0.296508029296515,0.52356257051196,0.600582820598565,0.93914010957289
"9051","KCTD6",19.3750761635298,-0.16363920618526,0.312097722844565,-0.524320410587415,0.600055703001298,0.93914010957289
"9052","RNF168",45.0281459883251,0.168183644914315,0.321718177079701,0.522766995763034,0.601136409713132,0.93914010957289
"9053","CDC5L",87.8745349839773,-0.107320746624592,0.205223286496076,-0.52294624287992,0.601011663108206,0.93914010957289
"9054","AC104051.2",1.59756645732637,0.548678120818368,1.04979072952397,0.522654759074854,0.601214526521138,0.93914010957289
"9055","PWWP2B",1.97560465491734,-0.496876192764649,0.949417795012308,-0.523348303955275,0.600731892191565,0.93914010957289
"9056","KBTBD4",12.3226705817409,-0.2145690991252,0.410617415664208,-0.522552358813415,0.601285801167476,0.93914010957289
"9057","ME3",22.9129338291761,-0.206356269850957,0.394584277324593,-0.522971344043707,0.600994194946225,0.93914010957289
"9058","PARP11",12.2041867959829,0.197131060879938,0.376690765727837,0.523323316670755,0.600749277674405,0.93914010957289
"9059","LINC00539",2.63239817124394,-0.44009439525302,0.842224804656554,-0.522537917216157,0.601295853399257,0.93914010957289
"9060","LCMT2",3.72036618683561,0.397493946810872,0.758495745301726,0.524055605154055,0.600239865304978,0.93914010957289

"9061","MTHFS",36.5628694457553,-0.160480218495727,0.306960680497389,-0.522803826977744,0.601110776162484,0.93914010957289
"9062","ST20-AS1",3.09155147153037,-0.394582087968237,0.753981284726061,-0.523331408831451,0.600743647340995,0.93914010957289
"9063","WDR24",8.4624668821185,0.295098395460303,0.563593929689549,0.52360108921481,0.600556023768679,0.93914010957289
"9064","PTRH2",21.8374341460742,-0.162679736983107,0.31043015016609,-0.524046188477725,0.600246414722851,0.93914010957289
"9065","SPPL2B",33.7415861460272,-0.142533057710555,0.272692814007847,-0.522687252427759,0.601191910655043,0.93914010957289
"9066","CCDC106",13.7085396552383,-0.23680439191508,0.453254663621171,-0.522453293747022,0.601354758015353,0.93914010957289
"9067","MIRLET7BHG",15.6225616159969,-0.256830894759818,0.49025545252906,-0.52387157233013,0.600367868325026,0.93914010957289
"9068","MOB4",78.6294528926472,-0.122894699007048,0.235384457308073,-0.52210201307473,0.601599304939689,0.93918245181888
"9069","MRPS18C",100.676480828649,-0.116775053114129,0.223733574287458,-0.521937994715509,0.601713503029723,0.93918245181888
"9070","CTSH",129.338204709353,0.215664154087933,0.412999254322301,0.522190177901945,0.601537924054308,0.93918245181888
"9071","GSE1",45.5506527962848,-0.139801710014056,0.267659962119913,-0.522310878723893,0.601453895995401,0.93918245181888
"9072","ZNF283",7.58444954397487,0.277110760402548,0.530842145703365,0.522021023849523,0.601655692617512,0.93918245181888
"9073","AUH",24.9499770830848,0.157071049866235,0.301075084420423,0.521700592291123,0.601878812228535,0.939233412073623
"9074","AL161717.1",2.78314397009032,0.454890354982851,0.871898942070831,0.521723714794801,0.601862710555364,0.939233412073623
"9075","NDUFB8",284.084866294509,-0.127054340519278,0.243618875021983,-0.521529132370443,0.60199821676031,0.939316225821045
"9076","SF3B4",46.8586979898959,-0.134094677708188,0.257210557100461,-0.521342044509526,0.602128516757274,0.939416019973887
"9077","HDAC11",10.0989533312584,-0.240106200047378,0.460674698575816,-0.521205529172041,0.602223602823069,0.939460858871286
"9078","DXO",11.3342235985168,-0.226116224177466,0.434242185548635,-0.520714549858356,0.602565637099174,0.939890881397258
"9079","PEX14",12.3054026048922,-0.247864157885982,0.476433614119534,-0.520249097755294,0.602889968934315,0.940166688911066
"9080","CHPF2",25.7533798418813,0.157498826713089,0.302690219839642,0.520330081350259,0.602833533082812,0.940166688911066
"9081","ZNF529",14.5623287234951,0.213273304372966,0.410003030422932,0.520174946397266,0.602941645621567,0.940166688911066
"9082","TMEM41A",27.9343531986907,0.144638233505165,0.278163089248142,0.519976370323301,0.603080044725933,0.940249731107545
"9083","C15orf40",58.4095304517033,0.12026470194161,0.231319202557986,0.519907991259234,0.603127705342502,0.940249731107545
"9084","TUBE1",27.4755953542149,0.157856534102011,0.304016681600406,0.519236422393081,0.603595882872218,0.940461894968157
"9085","MTA1",43.8031411988855,-0.118441917778252,0.228019293044649,-0

.519438141381567,0.603455239329627,0.940461894968157
"9086","GAMT",61.5858923359271,-0.164102742406343,0.316035634687746,-0
.519253920743724,0.603583681999289,0.940461894968157
"9087","ZNF540",4.08070986186122,-0.362686446385026,0.698131241808692,
-0.519510408165364,0.603404856698872,0.940461894968157
"9088","BFSP1",2.08783773737415,-0.516179672049064,0.993812575385936,-
0.519393379429327,0.603486447214384,0.940461894968157
"9089","AC010883.3",2.99072218029498,0.534254508136139,1.0299513770990
2,0.51871818419325,0.603957279085543,0.940622134522218
"9090","AC092902.4",1.88520462747584,0.568811817404114,1.0965936633134
6,0.518707919290171,0.603964438352825,0.940622134522218
"9091","VASH1-
AS1",1.83927435139912,-0.534034565557256,1.02900481865025,-0.518981598
412483,0.60377357360763,0.940622134522218
"9092","ZNF599",13.1735825304636,0.196803961458645,0.379373080807818,0
.51876100707931З,0.603927412630549,0.940622134522218
"9093","SLC49A4",14.8695155193447,0.194828621352751,0.37577738668031,0
.518468189568044,0.604131648935383,0.940706909733583
"9094","CHRD",18.0856915717841,-0.175886536397902,0.339323857118951,-0
.518344150309019,0.604218174013202,0.940706909733583
"9095","ICK",61.2490762062192,0.126102462213675,0.243269494799876,0.51
8365289973627,0.604203427392458,0.940706909733583
"9096","SRSF3",489.230358992651,0.0830632728815862,0.160378005056746,0
.517921848773441,0.60451279723845,0.94075186381981
"9097","CCDC82",87.8723916977758,0.094257106467167,0.181975062938582,0
.517967159593617,0.604481182568476,0.94075186381981
"9098","PLEKHA5",161.24392760917,0.0971587669495855,0.187535758078343,
0.518081287244417,0.604401555693218,0.94075186381981
"9099","LGMN",170.912368543912,0.108748878143115,0.209870212529724,0.5
18172049440855,0.604338234259004,0.94075186381981
"9100","CPEB4",19.9037206438786,0.182336774132068,0.352151559793668,0.
517779260267659,0.604612290204474,0.940803299922567
"9101","TEX264",90.9892837568309,-0.153492594820215,0.296658746871283,
-0.517404581658309,0.604873762036691,0.940825916777896
"9102","LINC01252",3.28769603191449,0.453518383158876,0.87657135789735
5,0.517377597468776,0.60489259508093,0.940825916777896
"9103","CISD3",65.9911470741832,-0.137652133932576,0.266004193974504,-
0.517481066278863,0.604820382642697,0.940825916777896
"9104","MKKS",26.3373610828799,-0.15359036509763,0.296817745537365,-0.
51745681451615,0.604837307965634,0.940825916777896
"9105","SLC30A9",31.0870343485021,-0.139844055167621,0.270352913278068
,-0.517264835329465,0.604971297875569,0.940844983846025
"9106","AC021546.1",1.60413944866313,0.625547858525278,1.2097180924728
5,0.517102176463743,0.605084834380728,0.94091821379652
"9107","ZNF644",83.1495413575725,0.103543684085739,0.200332615766128,0
.516858843427763,0.605254699591575,0.940975685794544
"9108","GYG1",40.5382254377175,0.141038801755175,0.272861039347174,0.5
16888750745116,0.605233820827609,0.940975685794544
"9109","FAM225A",3.72698883770293,-0.352040574309602,0.681941983257575
,-0.51623244051926,0.605692075366792,0.941229338384674

"9110","ATG13",30.4679664721513,-0.163996564297624,0.317632654965794,-
0.51630889246978,0.605638686438328,0.941229338384674
"9111","LDLRAD4",23.5868297848973,-0.187843327136101,0.363932227406235
,-0.516149197543924,0.60575020908895,0.941229338384674
"9112","CNFN",5.04470831238769,0.379665985963438,0.735224840882069,0.5
16394393731233,0.605578980572709,0.941229338384674
"9113","MAGT1",84.3300182313241,0.103906782513194,0.201247320587675,0.
516313867979803,0.605635211947968,0.941229338384674
"9114","AFAP1",49.1543212069955,0.128016962002902,0.248286818524761,0.
515601121169206,0.606133027094651,0.941266194972417
"9115","TXLNB",3.43075848179016,-0.565091756306177,1.09593290136653,-0
.515626235512737,0.606115482971107,0.941266194972417
"9116","THBS2",133.609819579457,0.183747601722436,0.356095287500807,0.
5160068334856,0.6058496365114,0.941266194972417
"9117","AC073611.1",8.39744415721924,-0.345603057146348,0.669898726035
468,-0.515903439900034,0.605921851434848,0.941266194972417
"9118","DACT1",44.3857558285766,-0.196147514785802,0.380466899822417,-
0.51554422967505,0.606172770618183,0.941266194972417
"9119","CD99",1128.84474201394,-0.117373023192213,0.22757819243851,-0.
515748112481938,0.606030346619915,0.941266194972417
"9120","CZIB",164.624026434074,-0.146856566966197,0.284983495436998,-0
.515316042218531,0.606332190582011,0.941410506429965
"9121","OSR1",114.914885318327,-0.179111816662163,0.347800549033888,-0
.514984283836514,0.606564002359328,0.941614704173812
"9122","ITM2C",95.1342097559914,0.176564454348865,0.342936163077917,0.
514860995598031,0.606650158479439,0.941614704173812
"9123","TRAM1",269.800144768544,-0.08285331979644,0.160929501857246,-0
.514842330587313,0.606663202413679,0.941614704173812
"9124","PLAC8",2.30340977641605,0.50043285908926,0.972530167203546,0.5
14567954769183,0.606854962821858,0.941705892992603
"9125","GTF2H3",30.8706342824657,-0.14247966194271,0.276874345630758,-
0.514600446704881,0.606832252895267,0.941705892992603
"9126","GTPBP10",17.1615122073,0.190085514667103,0.369495156320266,0.5
14446566932378,0.606939809044168,0.941734351968598
"9127","GREM2",4.95592268121234,-0.394986151137308,0.768106737195414,-
0.514233415761356,0.607088807727573,0.941759149586156
"9128","MRPS36",101.591811058775,-0.116212709449516,0.22596544968256,-
0.514294152547539,0.607046349336,0.941759149586156
"9129","DNAJA3",18.9246143715613,0.18744466180061,0.364726883458674,0.
513931575383443,0.607299830608102,0.94198330610261
"9130","KLRF1",1.70189569270398,0.537084764051874,1.04538683651706,0.5
13766526696749,0.607415233420442,0.942059113388111
"9131","SH3GLB1",211.677159341885,-0.0818058749104109,0.15927005536326
3,-0.513629977234754,0.607510716839279,0.942104013847792
"9132","AP000915.1",1.78552281047217,-0.590908296075805,1.150690628203
22,-0.513524905472202,0.607584193757749,0.942114781385208
"9133","PTPN14",28.6397524428521,0.225927519376127,0.440242034928229,0
.513189340070536,0.607818881940575,0.942169170035965
"9134","EIF2B2",23.3981975239897,0.165929414978218,0.323320444681126,0
.513204214913986,0.607808477896248,0.942169170035965

"9135","RBMX",252.70387264839,0.0991246625044658,0.193090021131035,0.5
13359840782232,0.607699631870815,0.942169170035965
"9136","CDC42-
IT1",1.97970313524909,0.67623347911158,1.31825470295409,0.512976344856
726,0.607967867791235,0.942193828162843
"9137","AZIN1",77.9236717478588,-0.104627277515476,0.203929991291723,-
0.51305488149512,0.607912931103806,0.942193828162843
"9138","GPSM2",42.3521976416087,-0.19869836008962,0.388202419418824,-0
.511842147679271,0.608761489838564,0.942355026461374
"9139","AC092040.1",30.6733503445212,-0.243465005283887,0.475772336559
298,-0.511725854101949,0.608842889006258,0.942355026461374
"9140","NAAA",46.2686121853114,0.1834936770955,0.358526318114459,0.511
799741956237,0.608791170972943,0.942355026461374
"9141","AFF4",132.391933028877,0.141897762172018,0.277036868354449,0.5
12198116499317,0.608512361176407,0.942355026461374
"9142","CAP2",4.16508018433283,0.414335278282209,0.810337633580672,0.5
11311903966954,0.609132670850883,0.942355026461374
"9143","FGL2",442.326550698452,0.107391301740437,0.209569033298628,0.5
12438789500971,0.608343949283973,0.942355026461374
"9144","HECTD2",18.3115103402667,-0.178464739274734,0.349036943102354,
-0.511306160569943,0.609136691892723,0.942355026461374
"9145","EMSY",20.833664531317,0.163790587056409,0.320302155705916,0.51
136273714871,0.609097082262156,0.942355026461374
"9146","NCKAP1L",2.61301715705697,-0.483448676244375,0.945328764997284
,-0.511407982222739,0.609065406716359,0.942355026461374
"9147","SLC15A4",42.2620708749278,0.141235737550824,0.27615577379465,0
.511435033966907,0.609046468457571,0.942355026461374
"9148","IGHM",5.76402705016347,-0.337647973113178,0.658572773583211,-0
.512696525967933,0.608163620193785,0.942355026461374
"9149","ZSCAN32",8.25853687877395,0.238232485856931,0.465365691661128,
0.511925331251982,0.608703268842271,0.942355026461374
"9150","C16orf87",24.7604689935054,-0.150885227547955,0.29446385553493
7,-0.512406615317351,0.608366462094735,0.942355026461374
"9151","STXBP4",15.6924715603832,0.18003645975129,0.351675344934918,0.
51193938484544,0.60869343283953,0.942355026461374
"9152","ALKBH6",8.76530767244215,0.241166210300395,0.470749159566539,0
.512303007662209,0.608438960614094,0.942355026461374
"9153","ZNF628",6.66980191522581,-0.287330675026822,0.561802544566952,
-0.511444239271471,0.609040024110668,0.942355026461374
"9154","SNX24",20.4468216982017,-0.15797945657875,0.309151202955424,-0
.511010324619468,0.60934382722308,0.942572492186893
"9155","RBM25",273.525067090752,0.125870969968421,0.246422953500603,0.
510792392430738,0.609496436742794,0.94260261514613
"9156","WDR4",5.62035441994465,-0.28827084425696,0.564343427488537,-0.
510807480366757,0.609485870694756,0.94260261514613
"9157","NPIPA1",21.063398238968,0.243788289058093,0.477459033898559,0.
510595196131294,0.609634540359194,0.94271323484615
"9158","RFNG",78.5169167035717,-0.112158354362933,0.219719121364744,-0
.510462419776133,0.609727536220274,0.942754085267425
"9159","PPFIA1",65.6445211694052,0.146455778687074,0.287189825129456,0

.509961585933821,0.610078374329468,0.943193556120238
"9160","GPBP1",140.404247034941,0.0959029679084048,0.188096848899372,0
.509859513700364,0.610149887740316,0.943201136506863
"9161","C4orf46",13.0114018164093,0.185935491484627,0.364864693940264,
0.509601215389366,0.610330872220761,0.943377922786375
"9162","Z99289.3",3.11941995059138,-0.443281353371212,0.87051172657056
1,-0.509219278547285,0.610598531413687,0.943676952707418
"9163","PUS7L",49.4993408138374,0.106913034776391,0.209989572611502,0.
509134970116773,0.610657621303536,0.943676952707418
"9164","PPIH",36.406367720725,-0.176108165181201,0.346289404779526,-0.
508557763392516,0.611062240674821,0.943850203880631
"9165","PDE1A",26.911851263913,-0.225967827674411,0.44431012832684,-0.
508581311268661,0.611045731392434,0.943850203880631
"9166","NR3C2",11.142431394255,0.23646758398822,0.465203965533233,0.50
8309476075021,0.611236325535691,0.943850203880631
"9167","HAND2-
AS1",21.5990282167481,-0.173676862830379,0.341652964301459,-0.50834291
2187158,0.611212880766563,0.943850203880631
"9168","UQCRB",673.647703072045,-0.121779455545619,0.239378266685981,-
0.508732297344982,0.610939880609188,0.943850203880631
"9169","NECAP1",34.8075660767232,0.135251529802886,0.265956485923279,0
.508547589404915,0.611069373660979,0.943850203880631
"9170","CDADC1",33.1429218829417,0.141642461791366,0.278606464626195,0
.508396178033441,0.611175532586152,0.943850203880631
"9171","AL031595.3",3.36255487146105,-0.396650766293688,0.780606075417
928,-0.508131795004703,0.611360918824449,0.943939658767223
"9172","CPS1",2.64543982487938,0.438272988745788,0.863049508926803,0.5
07819058133498,0.611580243089541,0.944175342580452
"9173","AGRN",6.26637660624702,-0.273814455480218,0.53994160597701,-0.
50711864477411,0.612071573575444,0.944321171431438
"9174","C1orf35",49.2747322945978,-0.120413100934545,0.237397859610134
,-0.507220668005569,0.611999994795281,0.944321171431438
"9175","C1orf198",22.6417034057202,0.1934802138261,0.381491528209998,0
.50716778622563 7,0.612037095817046,0.944321171431438
"9176","WAPL",52.4288245102105,-0.113041751715259,0.22291191647713,-0.
507113991489354,0.612074838375547,0.944321171431438
"9177","GPR180",18.4239522551251,0.172593137927387,0.340340247537663,0
.507119387660101,0.612071052358482,0.944321171431438
"9178","ZNF738",9.93941272641199,0.258814095753692,0.509986927222084,0
.50749162760595,0.611809909432624,0.944321171431438
"9179","UBQLN4",9.29602835744708,-0.230552177489354,0.454894693951904,
-0.506825383005522,0.612277344586065,0.944347568304321
"9180","RDH14",115.974982677247,0.124234544107186,0.245091968031243,0.
506889495829373,0.612232356359696,0.944347568304321
"9181","NARF",36.4033794628337,-0.128987483724875,0.254552444661623,-0
.506722628008301,0.612349451229907,0.944347568304321
"9182","MAPK1",81.5780260293432,-0.0921954742118467,0.181949394829842,
-0.506709430377976,0.61235871272389,0.944347568304321
"9183","TMTC1",20.6423483044511,0.358774244638616,0.708338845506844,0.
506500874425288,0.612505075984693,0.944367582310894

"9184","CLEC16A",8.95283841378885,0.239752978700194,0.473284880976134,
0.506572232363966,0.612454995690119,0.944367582310894
"9185","LDLRAD2",4.6333565848397,0.315837627293666,0.631778889223337,0
.499917981878017,0.617132830109925,0.944513813946432
"9186","TINAGL1",2.41663969447524,0.626814840900802,1.24855001214378,0
.502034227547322,0.61564344465741,0.944513813946432
"9187","COL24A1",4.13486474093221,0.388568477474975,0.776815080932568,
0.500207175443218,0.616929206428143,0.944513813946432
"9188","CREB3L4",6.02953433622446,-0.360550579718526,0.717797309444029
,-0.502301380870027,0.615455538038921,0.944513813946432
"9189","VAMP4",33.0958598390407,0.13223891028767,0.262984247085736,0.5
02839663413598,0.615077004882072,0.944513813946432
"9190","FAM72A",2.44858457448314,0.448292069601911,0.891073253011981,0
.503092274497755,0.614899398040597,0.944513813946432
"9191","PFKFB2",2.49972026021469,-0.419557225453168,0.829228150898615,
-0.505961145914431,0.612883925843603,0.944513813946432
"9192","GPATCH11",34.5609392905866,-0.135675008711453,0.27147903596127
6,-0.499762378450488,0.617242404007251,0.944513813946432
"9193","GGCX",47.3574632078201,0.125376281794884,0.250567067620784,0.5
00370152332357,0.616814465971468,0.944513813946432
"9194","AC159540.2",5.61317600834721,-0.344859349597326,0.690418180109
129,-0.499493436778877,0.617431809303563,0.944513813946432
"9195","RND3",521.10141631482,0.182906500305439,0.366466132615236,0.49
9108878084181,0.617702683433857,0.944513813946432
"9196","AC064836.3",2.78032295361444,-0.408093368722895,0.810410153023
877,-0.50356398818571,0.614567804068536,0.944513813946432
"9197","RPL37A",4222.5160194155,-0.126089960243642,0.249230071663716,-
0.505917923154049,0.61291426952966,0.944513813946432
"9198","SLC26A6",3.54629470501191,-0.377978441514116,0.755921475219884
,-0.5000234202953,0.617058586600695,0.944513813946432
"9199","USP4",36.6578404430634,0.140795112533995,0.280456251817696,0.5
02021658000033,0.615652286273066,0.944513813946432
"9200","ARL6",9.9657140550766,-0.209732203143668,0.417714047106183,-0.
502095164375342,0.615600581532842,0.944513813946432
"9201","ZXDC",16.3917304197752,0.184097713116831,0.365126730580089,0.5
04202233630904,0.61411927097781,0.944513813946432
"9202","ACTL6A",47.1955604768424,-0.143391921882591,0.287074118702324,
-0.49949442510099,0.617431113219646,0.944513813946432
"9203","OCIAD1",232.842397625545,-0.0959634696858598,0.191469690241763
,-0.501194050947123,0.616234560707908,0.944513813946432
"9204","COX18",11.4161914942982,0.235306779293068,0.46784609594642,0.5
0295766349628,0.614994038089418,0.944513813946432
"9205","HSD17B11",135.283913597203,-0.102618052707894,0.20411749336679
9,-0.502740118033342,0.615146999855626,0.944513813946432
"9206","NUDT9",52.8488859575722,0.113975051319144,0.225576353774527,0.
505261519711714,0.613375166144142,0.944513813946432
"9207","HERC3",21.3755154683623,-0.187380138580318,0.374019206150103,-
0.500990685770071,0.616377678087511,0.944513813946432
"9208","EIF4E",55.7407265151005,-0.116853568214592,0.232955915396565,-
0.501612367368608,0.615940218168792,0.944513813946432

"9209","ZGRF1",2.16162782277713,0.48379431300694,0.96642815165107,0.50
0600393500969,0.616652385457715,0.944513813946432
"9210","ANAPC10",35.1131747169808,-0.167452407765271,0.332123881045649
,-0.504186592177801,0.614130261434082,0.944513813946432
"9211","PPIC",447.884930217898,-0.161549208726525,0.320589570900024,-0
.503912863643664,0.614322610629228,0.944513813946432
"9212","TCOF1",29.542843099899,0.140209225455842,0.280797335462537,0.4
99325341620089,0.617550205194612,0.944513813946432
"9213","RBM22",65.6897243891439,0.104200036940223,0.208528269574673,0.
499692617949384,0.617291531205822,0.944513813946432
"9214","AL359643.3",2.51672168158777,-0.470671511576064,0.942622750139
036,-0.499321188149385,0.617553130769735,0.944513813946432
"9215","PPP1R10",98.6885841405314,0.106648783388502,0.213698279034204,
0.499062434524482,0.617735400708226,0.944513813946432
"9216","BMP5",19.8857955077912,0.503382738357606,1.00204282745083,0.50
235651068747,0.615416764726584,0.944513813946432
"9217","AC019205.1",10.7740506918192,-0.229315189485906,0.458849170879
02,-0.499761586245445,0.617242961889479,0.944513813946432
"9218","AFDN",43.8113562770682,0.152718846529837,0.304390131708381,0.5
01720754456481,0.615863963172066,0.944513813946432
"9219","PSMB1",446.616254834631,-0.134758073385071,0.266821633134763,-
0.505049278807949,0.613524224760252,0.944513813946432
"9220","CCZ1B",16.6560345736058,-0.255789985906419,0.508623002597269,-
0.502906837874485,0.615029773383007,0.944513813946432
"9221","C7orf25",13.3315279728232,0.193524329979997,0.384445625971811,
0.503385438423967,0.614693307436244,0.944513813946432
"9222","PCOLCE",1008.77601056964,0.159813324394174,0.319720342502238,0
.499853475519953,0.61717825360891,0.944513813946432
"9223","GIGYF1",39.0066930656378,0.160198892671067,0.319288687953325,0
.501736825372547,0.615852656941618,0.944513813946432
"9224","ST7-
AS1",6.15974087064284,-0.287828398147885,0.577031025937224,-0.49880922
3785479,0.61791378894258,0.944513813946432
"9225","CEBPD",1743.64109179896,-0.136890014228443,0.271808859822051,-
0.503626019836374,0.614524204405031,0.944513813946432
"9226","MYBL1",3.88376354724243,0.400059421428599,0.797366998865694,0.
501725581818296,0.615860567011022,0.944513813946432
"9227","MFSD3",7.71200008047481,0.251958295775242,0.504032702997192,0.
499884817546542,0.617156183283493,0.944513813946432
"9228","SMIM27",19.1484148981918,0.186891523728634,0.373339170015504,0
.500594469422729,0.616656555536199,0.944513813946432
"9229","GARNL3",10.1976602234546,0.230328720876525,0.455709400373933,0
.505428943724945,0.613257594093706,0.944513813946432
"9230","TMEM254",14.2881809216221,-0.183526060755419,0.36612517238729,
-0.501265891003212,0.616184007059857,0.944513813946432
"9231","VTI1A",23.688921248928,0.1509503835824,0.299697994006925,0.503
674988157952,0.614489787415546,0.944513813946432
"9232","STK32C",15.5762384356559,-0.201791280569755,0.400708763395127,
-0.503585893305694,0.614552407645908,0.944513813946432
"9233","IFITM1",143.284004095452,-0.344865985579126,0.687399399622694,

-0.501696663931361,0.615880911538398,0.944513813946432
"9234","OSBPL5",20.2772552616308,-0.168350831906805,0.337519000460755,
-0.498789199058378,0.617927897424704,0.944513813946432
"9235","ZBED5",39.2809457981546,0.140937752463382,0.281528530396192,0.
500616233335364,0.616641235538673,0.944513813946432
"9236","DDX6",108.830753516149,0.12629875963467,0.250868535182103,0.50
3445996298384,0.614650739790944,0.944513813946432
"9237","HYLS1",6.05923280724812,-0.281050998322747,0.560937284118942,-
0.501038184267231,0.616344249916529,0.944513813946432
"9238","A2M-
AS1",4.96111126437763,0.304667175545021,0.605009819045574,0.5035739354
20825,0.614560812447535,0.944513813946432
"9239","PWP1",75.4403312812651,-0.108248489767756,0.215936439301847,-0
.501297928768942,0.616161462759985,0.944513813946432
"9240","GIT2",35.5406418933998,0.149414349657763,0.295597544743203,0.5
05465462467103,0.613231950447687,0.944513813946432
"9241","NDRG2",87.7805388067955,-0.128759940553767,0.257086867093455,-
0.500842155064112,0.61648221515565,0.944513813946432
"9242","ABHD4",14.7745084613769,0.19164951732774,0.382364719263905,0.5
01221759415164,0.616215062120211,0.944513813946432
"9243","BTBD7",77.1670576105348,-0.132834351869294,0.265821720070018,-
0.499712182414233,0.617277753217002,0.944513813946432
"9244","IGHG3",21.8314659249482,0.728886456654076,1.44289078194875,0.5
05157054000755,0.613448531316209,0.944513813946432
"9245","GCHFR",15.8992522674741,0.187438996754603,0.370590677801506,0.
505784435449286,0.613007986127668,0.944513813946432
"9246","ANKDD1A",8.84266586087295,-0.245410161700209,0.491933635251722
,-0.498868432882482,0.617872073818342,0.944513813946432
"9247","CPPED1",15.4216006644324,0.204104360640549,0.409209256668856,0
.498777477083605,0.61793615624292,0.944513813946432
"9248","ABCC1",18.6758356939929,0.17601487475889,0.34786755563876,0.50
5982440459813,0.612868976672194,0.944513813946432
"9249","AC079414.3",2.43030465220155,0.426186564931924,0.8499091648392
04,0.501449546096559,0.616054777756578,0.944513813946432
"9250","KCTD11",20.3667315676988,-0.183260596952258,0.366321557481027,
-0.500272487954111,0.616883223402917,0.944513813946432
"9251","MRPL45",40.1330047001055,-0.127236725771168,0.254730483751954,
-0.499495482036873,0.61743036881083,0.944513813946432
"9252","KLHL11",3.13008264604239,0.412290281304166,0.819044284689555,0
.503379718302335,0.614697328319954,0.944513813946432
"9253","DLGAP1-
AS2",3.39482514927629,-0.36535342178898,0.722388534272315,-0.505757503
691572,0.613026894649006,0.944513813946432
"9254","PEX11G",11.5276330349352,0.208798714418484,0.417354855381649,0
.500290608162564,0.616870466205212,0.944513813946432
"9255","ZNF568",15.2188999007489,0.185829047443685,0.370029825233281,0
.502200187043115,0.615526711390881,0.944513813946432
"9256","ZNF112",3.42885187043572,0.358810366504366,0.717519301223253,0
.500070682269665,0.617025308778729,0.944513813946432
"9257","U2AF2",39.4094116435903,-0.123734974639951,0.247438061673313,-

0.50006443553262,0.61702970714918,0.944513813946432
"9258","IL17RA",31.3867213188815,0.139860764767591,0.279249668916952,0
.500844872296644,0.616480302676384,0.944513813946432
"9259","BCR",15.7737914724841,-0.191703427678968,0.383929874609886,-0.
499318860960636,0.617554769971505,0.944513813946432
"9260","SUV39H1",2.18833416723224,-0.46289035904026,0.918914183766394,
-0.503736221747053,0.614446751076217,0.944513813946432
"9261","PHKA1",2.07829010722393,-0.539118740230245,1.06626392665015,-0
.505614723292739,0.61312714366007,0.944513813946432
"9262","TCEAL7",37.4079662531987,-0.142233270366973,0.282468907212931,
-0.50353602373572,0.614587459651208,0.944513813946432
"9263","PSMD10",28.883884449591,-0.154636294229248,0.307416665119309,-
0.50301857958557,0.614951209431595,0.944513813946432
"9264","ABCD1",8.49024494727823,0.246656639678226,0.491471380655295,0.
501873861605839,0.615756252747045,0.944513813946432
"9265","TRIP12",73.8767591767959,0.121294856072783,0.24349408903621,0.
498142918182895,0.618383312214471,0.944545439983491
"9266","NDNF",30.3902075929285,0.158735309296517,0.318663560842235,0.4
98128210445449,0.618393678023941,0.944545439983491
"9267","KLHL35",6.40172275803081,-0.278618675243354,0.558886965894436,
-0.498524195849613,0.618114619508533,0.944545439983491
"9268","DCTN2",162.631465137654,-0.0824667246348143,0.165550703394454,
-0.49813575505217,0.618388360680223,0.944545439983491
"9269","FAM214A",34.4036689882396,0.146718641555032,0.294621156996996,
0.497990853917282,0.618490488667156,0.944545439983491
"9270","ANTKMT",66.7307497046114,0.153069398829898,0.307202289434877,0
.498269069255574,0.618294405806639,0.944545439983491
"9271","ACSF2",10.8914429171407,-0.246793093542206,0.495510410386152,-
0.498058342204918,0.618442921228591,0.944545439983491
"9272","PDCD2L",9.38945076156157,0.231435007390601,0.464630712377492,0
.498105272047903,0.618409844840138,0.944545439983491
"9273","HYAL2",40.0870334906302,0.163345367725639,0.32849825081639,0.4
97248820411341,0.619013597887895,0.944904580876449
"9274","ABHD10",17.8380260615027,0.17435249768818,0.350736455644048,0.
49710400753187,0.619115708726471,0.944904580876449
"9275","COPS4",65.2368372206356,-0.133636756508452,0.268889686091054,-
0.496994728400252,0.619192768781961,0.944904580876449
"9276","AC016596.1",1.79742162870584,0.539564121528904,1.0846430061873
6,0.497457798050559,0.618866255986537,0.944904580876449
"9277","ATMIN",49.2269247828652,0.117196439184337,0.235799051565535,0.
497018280634452,0.619176160165892,0.944904580876449
"9278","NUP88",17.3602916298578,0.179670352166299,0.36146164423768,0.4
97066161875136,0.619142395767169,0.944904580876449
"9279","XYLT2",16.5096643459573,-0.190852237514364,0.383895794614872,-
0.497145944789076,0.619086137061108,0.944904580876449
"9280","CNOT3",26.3877769322822,0.141202964195967,0.284205843720111,0.
496833430121251,0.619306518679944,0.944976325916811
"9281","HIVEP3",8.42827103602704,-0.238284913060724,0.481599215777083,
-0.49477844908082,0.620756518411965,0.945131522017987
"9282","GNPAT",34.1644709679261,-0.127320364307114,0.257187061273714,-

0.495049648596481,0.620565074771943,0.945131522017987
"9283","KLF11",35.1354796110491,0.130975804697158,0.263853875805861,0.
496395227461156,0.619615591602133,0.945131522017987
"9284","ICA1L",19.5263565384915,0.168983588280089,0.341431803439621,0.
494926326656539,0.620652126318014,0.945131522017987
"9285","ZNF148",100.009622350705,0.0882069165882613,0.1782843855829,0.
494754020661256,0.620773764046215,0.945131522017987
"9286","STX18-
AS1",4.91332522895574,0.362975775952826,0.731403248870079,0.4962731250
01257,0.619701724839333,0.945131522017987
"9287","DDR1",2.03020933691379,-0.495649147188429,0.999959258155281,-0
.49566934167178,0.620127720990453,0.945131522017987
"9288","AC018645.3",5.03509084742432,-0.305703055458387,0.617008495237
886,-0.495460042799774,0.620275420475634,0.945131522017987
"9289","FBXO25",33.3253007942216,-0.137765011830725,0.277668036228591,
-0.496150056383549,0.619788544902973,0.945131522017987
"9290","COPS5",95.8303228021615,-0.134610129886819,0.27141605225102,-0
.495954932548812,0.619926207950454,0.945131522017987
"9291","TGDS",18.9128749337321,0.164143436689714,0.331031771104584,0.4
95854026766076,0.619997403852259,0.945131522017987
"9292","TRMT61A",17.9120109924086,-0.176408570893128,0.355362323069175
,-0.496418892609468,0.61959889839141,0.945131522017987
"9293","AC127502.2",14.3505792899031,-0.249266077395706,0.502749933046
195,-0.49580529207709,0.620031790770134,0.945131522017987
"9294","BMF",4.48137649032823,0.380955524800039,0.769743988899234,0.49
4911984106328,0.620662250904998,0.945131522017987
"9295","UTP4",16.3501249987395,-0.171297806664833,0.346320038957481,-0
.494622855727571,0.620866365596978,0.945131522017987
"9296","SLC38A10",76.662135924166,0.101193862416838,0.204199840421005,
0.495562886867118,0.620202842843795,0.945131522017987
"9297","DNASE2",83.0815798079825,-0.113767622373544,0.229957347169218,
-0.494733583310238,0.620788192281805,0.945131522017987
"9298","AC024075.2",11.3667023680895,0.227667875058989,0.4586091493974
41,0.49643116662204,0.619590240475956,0.945131522017987
"9299","PLCB1",22.5569987282396,0.162463114542195,0.328468244332931,0.
494608283586539,0.620876653800234,0.945131522017987
"9300","IGLC2",62.5645278748093,0.441335358814049,0.892176083829022,0.
494672931513626,0.620831011723658,0.945131522017987
"9301","PITPNB",93.2990786175271,-0.107909143698264,0.217804631708573,
-0.495440077888923,0.620289510253232,0.945131522017987
"9302","C1GALT1C1",61.4453398120777,-0.127434465680787,0.2576126576844
84,-0.494674705917847,0.620829758997291,0.945131522017987
"9303","LAPTM4A",2026.44640995428,-0.129797868040963,0.262606741135694
,-0.494267083470998,0.62111756863456,0.945396621720452
"9304","PPP1R37",22.759906109855,0.147736027926142,0.298968029380414,0
.494153265258202,0.621197942522756,0.945417332988201
"9305","ERI3",68.5560901470149,-0.149800963955569,0.303491649480258,-0
.493591715660414,0.621594552637801,0.945628305181333
"9306","PMVK",89.7420802092233,-0.136846132279602,0.27741226503003 4,-0
.49329517663823,0.621804035984703,0.945628305181333

"9307","TMEM198",3.57182623602946,-0.366661552063035,0.743159314626628
,-0.493382165635979,0.621742581379659,0.945628305181333
"9308","EIF4G1",101.139711926521,-0.087998838661175,0.178222635384792,
-0.493757925143351,0.6214771510807,0.945628305181333
"9309","TEX10",10.0950301928624,0.213511138667269,0.432788161334589,0.
493338676383533,0.621773304646101,0.945628305181333
"9310","NUDT4",81.1957718404232,0.177384100538956,0.359198662167458,0.
493832854133127,0.621424228369721,0.945628305181333
"9311","ZSCAN5A",4.42154498119298,-0.346286879619943,0.701920815760296
,-0.493341801303979,0.6217710970036б,0.945628305181333
"9312","METAP1",19.3587415879067,-0.18855306089313,0.38236495622493,-0
.493123278751025,0.621925483427152,0.945711430984586
"9313","CEBPA-
DT",3.75398555234642,0.466634665534033,0.946622231265419,0.49294708081
2003,0.622049979580515,0.945799174364876
"9314","MED18",7.13098066035045,-0.279651198690485,0.570044760446156,-
0.490577614416823,0.623725222442193,0.94584951768863
"9315","DUSP11",45.4312866425934,-0.135480059545929,0.275484558205434,
-0.491788216473821,0.62286906750991,0.94584951768863
"9316","CCDC142",6.44585642337453,0.34019673958346,0.692132477093749,0
.491519688560115,0.623058930310805,0.94584951768863
"9317","RFT1",23.9818461889969,-0.148182092706617,0.301864146768818,-0
.49089000562926,0.623504246221626,0.94584951768863
"9318","TMEM45A",63.2494892362798,0.209595880928039,0.427421704506919,
0.490372572843095,0.62387028114313,0.94584951768863
"9319","PCNP",158.2059872714,-0.0908075209086823,0.184531201038156,-0.
492098465721826,0.622649736835653,0.94584951768863
"9320","KPNA4",93.8041854274828,-0.103870146516208,0.211472958284921,-
0.491174603876594,0.623302959410917,0.94584951768863
"9321","LCORL",16.0177474439159,0.192859319787549,0.391459930722927,0.
49266682143275,0.622248024689556,0.94584951768863
"9322","ANXA2R",12.3442405734158,-0.232879950528993,0.474267144152845,
-0.491031169669096,0.623404402107132,0.94584951768863
"9323","LEAP2",4.71400139532363,-0.39338841920763,0.801160308512801,-0
.491023350792152,0.623409932150695,0.94584951768863
"9324","LINC00271",3.132563417511,-0.382871525138442,0.779670745730536
,-0.49106821980309,0.62337819801166,0.94584951768863
"9325","MPLKIP",75.6169835159679,0.113773013644104,0.232036081210388,0
.490324664382459,0.6239041765625,0.94584951768863
"9326","TMEM74",2.54998500958795,-0.415568462606142,0.844727937865536,
-0.491955390579602,0.622750879633668,0.94584951768863
"9327","GADD45G",53.4341867495795,0.371268693648393,0.754172517727207,
0.492286161218467,0.622517061778365,0.94584951768863
"9328","PAPSS2",60.6621913535994,0.125935296036396,0.255779602494765,0
.492358635356678,0.622465835750523,0.94584951768863
"9329","ACTR1A",49.758192134158,-0.141931393675065,0.289315432769696,-
0.490576642650263,0.623725909893705,0.94584951768863
"9330","DUSP8",4.55356113360021,0.331618918684804,0.673461778054805,0.
492409412814246,0.622429946416781,0.94584951768863
"9331","ALOX5AP",6.52422578209003,-0.27541153635029,0.56157833839325,-

0.49042407358211,0.623833845061146,0.94584951768863
"9332","PACS2",36.3813292169542,-0.12232500702745,0.248760111412177,-0.49173883358159,0.622903981828439,0.94584951768863
"9333","DNAAF4",11.1031777802801,-0.237160810135942,0.483119552062465,-0.490894663905631,0.623500951352305,0.94584951768863
"9334","TLE3",42.9551282162816,-0.153817474146445,0.312689475278119,-0.491917657316842,0.62277755524947,0.94584951768863
"9335","AC087392.1",4.8052640009338,-0.368591839584795,0.751708792596034,-0.490338603479493,0.623894314515834,0.94584951768863
"9336","GOSR2",39.8956070756987,-0.114383081605235,0.232982851350621,-0.490950646977436,0.62346135426842,0.94584951768863
"9337","HLF",22.7658789068109,0.25205566667751,0.51267364513005,0.491649354461298,0.622967246838413,0.94584951768863
"9338","AC132938.3",2.60541056378425,0.419030457762037,0.854194882685404,0.490556038505751,0.623740485848301,0.94584951768863
"9339","NDUFB7",331.098373008234,-0.104721859149416,0.212625170107155,-0.492518637829382,0.622352749590003,0.94584951768863
"9340","ZNF530",2.29547541386533,0.442612975563349,0.902821816584744,0.490255072964115,0.623953414175812,0.94584951768863
"9341","MORC2",14.2703675711735,0.176701259350948,0.358991348756268,0.49221592654846,0.622566706653531,0.94584951768863
"9342","AC022306.2",2.14149836837524,-0.485145473114534,0.98978040037802,-0.490154657466693,0.62402446353208,0.945855962707584
"9343","LARGE1",17.6398741463194,0.164128724174762,0.334983905283388,0.489960029679376,0.624162183099532,0.945963449929292
"9344","DHCR24",13.4110312838653,-0.258493613111486,0.528829367509311,-0.488803438297958,0.624980863567094,0.946011399527136
"9345","DPP4",46.0369000787646,0.282812114688469,0.5777786727782,0.489481747965169,0.624500673348312,0.946011399527136
"9346","TATDN2",16.3731372063355,-0.1879664023137,0.384147684563676,-0.489307653974792,0.624623903084567,0.946011399527136
"9347","CAST",518.702709318656,-0.0894123340857069,0.183016852174233,-0.488547000035745,0.62516244329925,0.946011399527136
"9348","RGS17",6.16630753012919,-0.281921301132849,0.576080152160443,-0.489378604827079,0.624573680354515,0.946011399527136
"9349","MCM4",20.079321424911,0.166461835775669,0.340181987185994,0.489331716686856,0.624606870041025,0.946011399527136
"9350","AL137026.3",7.03281094136718,0.24722044807553,0.505631080976378,0.488934437333529,0.624888114078399,0.946011399527136
"9351","RNLS",8.43192202658429,-0.229063659955856,0.469070037965012,-0.488335731162054,0.625312056420007,0.946011399527136
"9352","FANCF",10.2046623954568,-0.268198582601838,0.548445250972338,-0.489016145415334,0.624830266410102,0.946011399527136
"9353","CD248",676.762609050334,-0.107149492008679,0.218978281945129,-0.489315611835554,0.624618270007024,0.946011399527136
"9354","ALG5",82.7420972532749,-0.140230850409953,0.286841237827509,-0.48887967250469,0.624926887762492,0.946011399527136
"9355","ARHGAP5",70.344153195539,0.142147587691496,0.291100521751189,0.488311002798521,0.625329569178954,0.946011399527136
"9356","SLTM",133.739221938151,-0.113936712002922,0.233239275113326,-0

.488497110735584,0.625197771739139,0.946011399527136
"9357","NGRN",125.825847872807,0.0916090411186675,0.187327890071312,0.489030443271386,0.624820144054985,0.946011399527136
"9358","ZNF720",34.0710773527944,-0.120378030300736,0.246004181145544,-0.489333269622425,0.624605770786157,0.946011399527136
"9359","ELAC2",26.9042611451821,0.135236230326164,0.276789424397977,0.48858886361105,0.625132798833374,0.946011399527136
"9360","TSKS",8.77186171960873,0.237841609354262,0.486608260412294,0.488774294856284,0.625001498414563,0.946011399527136
"9361","CCDC17",1.79308682841249,0.511194360974121,1.04814414270712,0.487713798269979,0.625752576815884,0.946547010950835
"9362","DUT",224.127557960527,-0.116220804098756,0.238341796424872,-0.487622422261092,0.625817310488822,0.946547010950835
"9363","ADAMTS2",96.4421153295972,0.094305402077585,0.193508293099757,0.487345532157471,0.626013485862049,0.946742599573493
"9364","SLC31A1",21.3172492841754,-0.181311896281363,0.372129119463918,-0.487228455925614,0.626096441801479,0.946766938905269
"9365","AC010359.3",3.3219962170051,-0.354722504981984,0.728233907762655,-0.487099682122457,0.626187691677438,0.946803813577418
"9366","CPSF2",30.5189811194995,0.140411548995571,0.288449175401387,0.486780899269978,0.626413607706754,0.946943171253084
"9367","DDTL",1.79166963974247,-0.549600619546484,1.12886978671862,-0.486859180759948,0.626358127687212,0.946943171253084
"9368","MKS1",6.45678014594366,0.288292186757477,0.592374977185958,0.486671783685044,0.62649094413587,0.946958984731417
"9369","MROH1",13.1595066768553,-0.189461024748248,0.389512329414408,-0.486405719256906,0.62667953636327,0.947069613019486
"9370","NAIF1",5.64715684389308,0.304788169593273,0.626646422312592,0.486379812827264,0.626697900705691,0.947069613019486
"9371","AL022069.3",3.41886744556509,-0.39150801703237,0.805142427836498,-0.486259329401375,0.626783311072382,0.947097608023149
"9372","TPR",229.362773977491,0.0804986218639574,0.16561527243347,0.486057962415845,0.626926070740454,0.947212245164834
"9373","E2F5",2.03975306727709,-0.460577844139728,0.948366136640953,-0.485654038398147,0.627212475885898,0.947299870568915
"9374","TRAF1",5.13333565612136,0.495864882605356,1.02109012048755,0.48562303430043,0.627234461880322,0.947299870568915
"9375","LINC02762",37.7623228825245,0.157873786472586,0.325111583584961,0.485598774216943,0.627251665710039,0.947299870568915
"9376","TTYH2",3.73564200795028,0.350856176456684,0.722203474602392,0.4858134705733,0.62709942267463,0.947299870568915
"9377","RRAGC",85.1095165407514,0.105295676964332,0.217059313693039,0.485100939336973,0.627604745778286,0.947428912603469
"9378","HACD2",25.6514645037758,0.167021811591526,0.34417807546677,0.485277312812599,0.627479646434784,0.947428912603469
"9379","AC144652.1",3.88801297744636,0.337934217915983,0.696530285116132,0.48516801801323,0.627557166510342,0.947428912603469
"9380","ISCA1",86.446720367023,-0.123535188300722,0.25457505601663,-0.48526037952694,0.627491656522881,0.947428912603469
"9381","AC092944.1",18.4581654086221,0.184206578772137,0.3802167064776

88,0.484477866526748,0.628046768653532,0.947861159619174
"9382","MED23",21.355303857123,0.172817519837442,0.35681208570669,0.48
4337629694256,0.628146274446925,0.947861159619174
"9383","SAP25",2.77973556308953,-0.441148103972566,0.910538053002031,-
0.484491672278942,0.628036973073837,0.947861159619174
"9384","VPS37A",38.1685748899365,-0.11771474499056,0.243051624635358,-
0.484319926547143,0.628158836289995,0.947861159619174
"9385","SLC1A7",11.1674336242051,0.887892866357148,1.85369253129882,0.
478986051551403,0.631948551698315,0.948310649553928
"9386","ANKRD13C",30.564610783021,0.133504612008356,0.278996114184168,
0.478517818783054,0.632281694913495,0.948310649553928
"9387","GBP1",78.935889261299,-0.120939568166209,0.252980953892147,-0.
478057997274248,0.63260892624904,0.948310649553928
"9388","SLC25A44",13.0160175756437,0.188886108565364,0.391385615296706
,0.482608714227197,0.629373590497131,0.948310649553928
"9389","SCYL3",13.8083369865144,0.210467882109051,0.438035342530751,0.
480481508393985,0.630885048815566,0.948310649553928
"9390","PPP1R15B",89.0767706198515,-0.220377220325635,0.45999524390642
2,-0.479085867180111,0.631877543474892,0.948310649553928
"9391","B3GNT2",5.66483476025462,-0.303425008728281,0.634335030969243,
-0.478335570187031,0.632411383263229,0.948310649553928
"9392","ZC3H6",71.0714186980473,0.105395155985224,0.220219389650743,0.
478591626978782,0.632229176118026,0.948310649553928
"9393","POLR1B",5.75775547355015,0.277726583753699,0.577932424206207,0
.480552002485685,0.630834935368744,0.948310649553928
"9394","SF3B1",237.71715162901,0.0823669253510332,0.170982999626989,0.
481725817951038,0.630000732949974,0.948310649553928
"9395","DYNC1LI1",71.4339645889304,-0.105011829845922,0.21717484526473
9,-0.483535879663738,0.628715290195977,0.948310649553928
"9396","RAP2B",79.1791804353259,-0.109967477029987,0.227671341372713,-
0.483009747151105,0.629088815383433,0.948310649553928
"9397","FAM184B",2.83994569864166,0.566258677201577,1.17396495270802,0
.482347173904441,0.629559341044696,0.948310649553928
"9398","SLC22A5",7.66487464916306,0.266676925718309,0.557392305361945,
0.478436683020124,0.632339429836655,0.948310649553928
"9399","AL513548.1",2.3352257425285,0.490258298031102,1.02341171162193
,0.479043079597094,0.631907981882116,0.948310649553928
"9400","MICB",1.51140982385019,0.524631224259026,1.09599867254634,0.47
8678704090166,0.632167218107867,0.948310649553928
"9401","NFKBIL1",46.2705389774178,-0.139711229894126,0.290532282592323
,-0.480880226622422,0.630601626924951,0.948310649553928
"9402","HSPA1B",116.826121682224,0.175037791959543,0.364868622327516,0
.479728267240323,0.631420625301854,0.948310649553928
"9403","PPARD",14.6188090219898,0.190470654510949,0.397684457413757,0.
478949204476403,0.631974765338488,0.948310649553928
"9404","TRAM2",99.7576304069226,0.145009998280651,0.301819587333466,0.
48045257619558,0.630905616877159,0.948310649553928
"9405","BCKDHB",16.6556999280628,0.16067923323017,0.335392159936924,0.
479078679896358,0.631882656350842,0.948310649553928
"9406","SMIM8",33.802550117608,-0.134962242354137,0.281148304342114,-0

.480039325401403,0.631199430377577,0.948310649553928
"9407","AC092428.1",1.5060596614151,0.510280631011259,1.05624974761398
,0.483106038286832,0.629020446865862,0.948310649553928
"9408","NCF1",4.47830025328167,-0.330446625904012,0.685339200761882,-0
.48216507320267,0.629688686012277,0.948310649553928
"9409","AC105046.1",1.79994041908563,-0.553796426850963,1.145311696719
74,-0.483533372126625,0.628717070184331,0.948310649553928
"9410","ELP3",21.491096633647,0.152512154845937,0.31646313895879,0.481
927074817384,0.629857751869368,0.948310649553928
"9411","LETM2",2.67259236375573,0.389544046950155,0.811347544565317,0.
480119832197009,0.631142186995177,0.948310649553928
"9412","HINT2",69.1114377946065,-0.129811772740011,0.270994161288709,-
0.479020552039544,0.631924007879608,0.948310649553928
"9413","NINJ1",31.4076056141172,-0.149979335777765,0.311018200913216,-
0.482220446705027,0.62964935336471,0.948310649553928
"9414","LINC00476",15.4329599341132,0.182070595457659,0.38081148220620
6,0.478112147256813,0.632570386751306,0.948310649553928
"9415","FSD1L",8.84795498744375,0.289330601974342,0.598672609319742,0.
483286854067204,0.628892072854316,0.948310649553928
"9416","RAPGEF1",33.7869568279727,0.14406670560391,0.300674198660279,0
.4791455543769,0.631835083993026,0.948310649553928
"9417","NDOR1",1.58246363493821,0.541027083250558,1.12112716291602,0.4
82574235239616,0.629398076740493,0.948310649553928
"9418","MAP3K8",49.8009444881093,-0.181415626195962,0.378596772019252,
-0.479179009446855,0.631811285702668,0.948310649553928
"9419","ZSWIM8",31.5657867629124,-0.135519559647418,0.282227750098619,
-0.480178010844304,0.631100821149517,0.948310649553928
"9420","ABLIM1",204.757862926929,0.135615495783614,0.282569834584093,0
.479936211107681,0.631272751779177,0.948310649553928
"9421","ZNF195",18.7874853268175,0.165009840151173,0.345104981201902,0
.478143895740047,0.632547791259768,0.948310649553928
"9422","RRP8",23.2815302778359,-0.141975484582355,0.296553477965886,-0
.478751709661916,0.632115274415847,0.948310649553928
"9423","MUS81",23.272264139641,0.1697350077714,0.352534661597579,0.481
470409185335,0.630182205706064,0.948310649553928
"9424","SRPRA",80.2359297787492,-0.0924026590117782,0.191501800021419,
-0.482515877142894,0.629439522347459,0.948310649553928
"9425","PDE3A",14.9303977938196,0.177499726983358,0.370523014821615,0.
479051826426527,0.631901759475794,0.948310649553928
"9426","PROSER1",10.3725202942861,0.209465237732087,0.436193109122249,
0.480212166014254,0.631076536876257,0.948310649553928
"9427","NDFIP2",40.0288868619536,-0.143097985508369,0.298463572260392,
-0.479448746205866,0.631619422186672,0.948310649553928
"9428","PPIP5K1",1.70998666748305,-0.576907021215025,1.19349635724287,
-0.483375602878046,0.628829067851174,0.948310649553928
"9429","NPTN-
IT1",3.27605002687625,-0.372012047755951,0.769254449700024,-0.48360077
4621478,0.62866922492101,0.948310649553928
"9430","AC022819.1",1.85396201499852,0.492794389918204,1.0244806227755
4,0.481018751319196,0.630503171779625,0.948310649553928

"9431","CMIP",16.7758865747066,0.18100313331043,0.374587537382717,0.48
3206501142878,0.628949119738416,0.948310649553928
"9432","GAS8",10.7733921375,-0.207787553211211,0.431255791843031,-0.48
1819739331968,0.629934005647528,0.948310649553928
"9433","MPP3",3.75738304586872,0.364937349302637,0.756249042491725,0.4
8256239518694,0.629406485387958,0.948310649553928
"9434","PNPO",14.1935839637696,0.184771801181057,0.384963714985615,0.4
79971992134275,0.631247308580951,0.948310649553928
"9435","KAT7",23.6491961355206,-0.170723056742117,0.354719348089864,-0
.481290512235793,0.630310039285426,0.948310649553928
"9436","AC027601.6",2.99835424424816,-0.413467651863283,0.858404515173
561,-0.481669940633623,0.630040432932631,0.948310649553928
"9437","ARL16",72.1037748358134,-0.110057813709322,0.228801248988784,-
0.481019287244874,0.630502790888151,0.948310649553928
"9438","CAPS",12.0966464358702,-0.249779020536694,0.522159400777864,-0
.478357796804189,0.632395566166692,0.948310649553928
"9439","RPS28",3806.81957472291,-0.122985209457681,0.255232866865749,-
0.481854907512247,0.629909020869798,0.948310649553928
"9440","ZNF625",4.46532511348725,-0.389050078769451,0.812369554437333,
-0.478907754044175,0.63200425443875,0.948310649553928
"9441","AC011477.2",1.97974961939769,0.47250690571375,0.98296820778093
2,0.480693985800866,0.630734006200385,0.948310649553928
"9442","APLP1",1.67010441980558,0.509422302939995,1.05918886730287,0.4
80955114489821,0.630548400252229,0.948310649553928
"9443","BCAM",6.2473836685392,0.259903810003027,0.539340773514557,0.48
1891640250729,0.629882925024037,0.948310649553928
"9444","LENG9",16.2014101663466,-0.184799161546272,0.38235656948909,-0
.48331629764647,0.628871169820533,0.948310649553928
"9445","UBE2S",66.6174817963266,-0.13331076768787,0.276061072967012,-0
.482903171588412,0.629164489745018,0.948310649553928
"9446","DONSON",8.29981990657352,-0.223723210636445,0.467570142807799,
-0.478480531911143,0.632308227382331,0.948310649553928
"9447","AAMDC",120.102967329667,-0.101249757554537,0.212009572789464,-
0.477571631423845,0.632955126116762,0.948644361178943
"9448","PLCB2",2.44034854495056,-0.419302747778594,0.878016099651712,-
0.477557015121843,0.632965531385498,0.948644361178943
"9449","GNG12",207.020238409169,0.0661367646536372,0.138601181282001,0
.477173167226287,0.633238816683267,0.948692601920265
"9450","UBE2T",1.69582389671687,0.558377054504478,1.16995909221429,0.4
77262032681572,0.63317554335944,0.948692601920265
"9451","LINC01960",1.90258588683314,0.489309330963802,1.02551463486511
,0.477135395564747,0.633265711394799,0.948692601920265
"9452","RFPL4B",4.09325782864668,0.380444274781396,0.797177089058596,0
.477239348700639,0.633191694384617,0.948692601920265
"9453","MFN2",18.3412919832882,0.169797930746567,0.362270759142225,0.4
68704488180637,0.639280878413534,0.94907377854194
"9454","EMC1",35.2169707934893,0.116677613791219,0.248551368435781,0.4
6943058300387,0.638761892329091,0.94907377854194
"9455","ZNF436-
AS1",4.03426334193967,-0.323299991536266,0.680371498441847,-0.47518156

2244556,0.634657567695009,0.94907377854194
"9456","AHDC1",56.0302924449974,0.118119110554418,0.250850302308376,0.470874898166204,0.637730074578675,0.94907377854194
"9457","XKR8",11.6217156405035,0.189016399594737,0.402701680881878,0.469370773871144,0.638804635033309,0.94907377854194
"9458","LCK",1.71589936588097,-0.484738878281822,1.01809691504997,-0.476122529315426,0.633987087389336,0.94907377854194
"9459","BTBD19",6.21494445669868,-0.260746949783458,0.554906957326562,-0.469893098907407,0.638431395038823,0.94907377854194
"9460","RNF115",141.245407284933,-0.0775507501415071,0.163380547768474,-0.474663301113448,0.635026979568533,0.94907377854194
"9461","POGK",23.6989668465786,0.163986194893832,0.349050184172377,0.469806928429661,0.63849296393205,0.94907377854194
"9462","DNAH14",7.90436688642499,-0.263229183869974,0.553399401078605,-0.475658599118334,0.634317620497238,0.94907377854194
"9463","AC093690.1",3.66326152876375,0.345849406278236,0.735240310783329,0.47038961439664,0.638076682797986,0.94907377854194
"9464","LBX2",3.09500371069273,0.42024464030379,0.886540203566899,0.474027730060048,0.635480132964993,0.94907377854194
"9465","PLEKHM3",16.8699566864821,0.181415461060985,0.383389284902472,0.473188657599375,0.636078589160723,0.94907377854194
"9466","XRCC5",230.406299346295,-0.0816572457056794,0.171857675574781,-0.475144595273824,0.634683914407463,0.94907377854194
"9467","NCKIPSD",11.1042793268081,0.20842842850038,0.440341372722017,0.473333739257697,0.635975094780854,0.94907377854194
"9468","MAGI1",4.63149002265925,0.317787891914913,0.670766164025844,0.473768518685551,0.635664986309008,0.94907377854194
"9469","P2RY12",1.78820600426302,0.518098293039649,1.09880409249531,0.471511069696766,0.637275816519288,0.94907377854194
"9470","ACTRT3",3.43310071543749,-0.347557502105701,0.734046287201267,-0.473481724743612,0.635869536269718,0.94907377854194
"9471","FBXO45",15.7694915967741,0.169270326447274,0.36087260356298,0.469058401153284,0.63902789225405,0.94907377854194
"9472","TADA2B",15.6443872494169,-0.195812729536917,0.417291130456107,-0.469247283839678,0.638892891202615,0.94907377854194
"9473","SCD5",29.1216708549258,-0.194266648059224,0.413415013566477,-0.469907095011648,0.638421395043562,0.94907377854194
"9474","CENPU",1.8824991026026,0.491896501851396,1.03708813095055,0.474305401027532,0.635282140589677,0.94907377854194
"9475","C5orf49",2.71594982396319,0.444614107177409,0.936240551047872,0.474893024746452,0.634863223409791,0.94907377854194
"9476","CHD1",75.127907682402,-0.118544714292574,0.25051536343932,-0.473203370304624,0.636068093490246,0.94907377854194
"9477","MRS2",18.3858146276826,-0.153989527457237,0.325683493614417,-0.472819564013731,0.636341915061118,0.94907377854194
"9478","MED20",8.51461989400239,0.221459745097119,0.472061011807967,0.469133733897957,0.638974047876785,0.94907377854194
"9479","TRA2A",110.233753682734,0.0989857017215064,0.211323403693955,0.468408609700705,0.639492412225676,0.94907377854194
"9480","MLXIPL",5.95659310886368,0.32508599817828,0.686931038845012,0.

473244008197491,0.636039103828629,0.94907377854194
"9481","IQUB",2.25396234810232,0.465033791057721,0.991798228987773,0.468879432797872,0.639155818222942,0.94907377854194
"9482","PPP2R2A",66.5263375287747,0.0987286508476248,0.208531369804214,0.473447476704917,0.635893964845409,0.94907377854194
"9483","NSD3",173.945621506463,0.0835715555014946,0.178390211339185,0.468476128113299,0.639444138382705,0.94907377854194
"9484","RRS1",16.2378528092558,-0.165650765416321,0.353520264512931,-0.468575021136482,0.639373435295941,0.94907377854194
"9485","PEX2",67.8617052709516,0.138990430101108,0.294308420089574,0.472261140400963,0.636740403817133,0.94907377854194
"9486","CA13",1.92380777233817,-0.511284529505066,1.07449279516256,-0.475838024979699,0.634189777553037,0.94907377854194
"9487","NCALD",100.982682272551,-0.125686499179746,0.266851862637473,-0.470997271435556,0.637642683412201,0.94907377854194
"9488","C8orf33",61.6817799860486,0.115037774598508,0.244625771908327,0.470260241597185,0.638169099159229,0.94907377854194
"9489","RNF20",41.8445032775034,0.124257733151459,0.264651517405136,0.469514531296799,0.638701900554954,0.94907377854194
"9490","ABCA2",23.6809074114786,0.13988220823277,0.297292912255443,0.470519822257248,0.637983675596351,0.94907377854194
"9491","MAN1B1",65.9899804630858,-0.103502813813639,0.221006519033662,-0.468324709452911,0.639552400766045,0.94907377854194
"9492","CREM",38.0779476488242,-0.135963600348444,0.286253308608227,-0.474976519955362,0.634803709124062,0.94907377854194
"9493","LINC00857",2.38249387079065,0.446955904405399,0.947250304278636,0.471845617136826,0.637036987688252,0.94907377854194
"9494","ADIRF-AS1",5.19791758740232,-0.32329718270963,0.688425392128567,-0.469618329605792,0.638627726679134,0.94907377854194
"9495","IDE",15.1376594511302,0.207932549651723,0.438775137377334,0.473893190244521,0.635576075514465,0.94907377854194
"9496","EXOSC1",40.2634143354832,-0.129109977029237,0.274562828838952,-0.470238369757501,0.638184723677942,0.94907377854194
"9497","GSTO1",220.581423219993,-0.124047541658963,0.264729906434095,-0.468581518914431,0.639368789855843,0.94907377854194
"9498","NAT10",19.1434072260561,0.156386820529763,0.333003835535115,0.469624682485892,0.638623187052647,0.94907377854194
"9499","B4GAT1",43.0231002324167,0.136342208810661,0.288895621236874,0.471942801441319,0.636967616210705,0.94907377854194
"9500","FDX1",78.4157787494831,-0.134517514854167,0.286976741303216,-0.468740129403163,0.639255399243677,0.94907377854194
"9501","DIXDC1",94.6170943039482,0.121639430870708,0.259364690834233,0.468989940301669,0.639076826568797,0.94907377854194
"9502","HIST4H4",2.07688394585534,0.46213279727311,0.980623256810094,0.471264365865031,0.637451959139769,0.94907377854194
"9503","ETFBKMT",13.5470743109247,0.187659205243304,0.397161432566476,0.472501078542903,0.636569172078215,0.94907377854194
"9504","SARNP",85.0951326096335,-0.114479130244716,0.2430617713838,-0.470987805251985,0.637649443375668,0.94907377854194

"9505","MYL6",1565.62853930801,-0.119960552328086,0.25218906068965 6,-0.475677065452531,0.634304462522679,0.94907377854194
"9506","KITLG",70.799762411478,0.135573751842661,0.288721690434645,0.469565523943028,0.638665460952627,0.94907377854194
"9507","RBM19",17.4852905925722,-0.170375082596051,0.360831679937995,-0.472173293169071,0.636803100804734,0.94907377854194
"9508","RAB35",25.4088917311275,0.143892728841549,0.306249447490487,0.46985465613295,0.638458862131954,0.94907377854194
"9509","ESD",296.641349187736,-0.10794070825181,0.228524783150783,-0.472336990166136,0.636686271571088,0.94907377854194
"9510","SETDB2",19.6315803969028,0.152863068811136,0.324850889742439,0.4705637990782,0.637952264332559,0.94907377854194
"9511","DCAF11",20.1096757972655,-0.164570679250873,0.349325351343573,-0.471110037155627,0.63756215780247,0.94907377854194
"9512","C14orf28",17.0607634512172,0.162854622113439,0.347199167497095,0.469052455647958,0.639032141908511,0.94907377854194
"9513","ARID3B",3.25712109982867,0.42542970614768,0.892942022616085,0.476435978341889,0.633763807870953,0.94907377854194
"9514","AC087477.2",17.6409110119187,-0.181422848282559,0.385058391820092,-0.47115671840058,0.637528824139231,0.94907377854194
"9515","PDXDC1",47.1579459319176,-0.121354381021535,0.257087057089087,-0.472036135912836,0.636900995791378,0.94907377854194
"9516","DYNC1LI2",141.349620707273,-0.109553779115186,0.232911665887975,-0.470366216726466,0.638093396308545,0.94907377854194
"9517","B3GNT9",21.8652047107115,0.166927710710454,0.351171097166659,0.47534581307309,0.634540510160511,0.94907377854194
"9518","DUS2",4.92457042175149,0.295345776343744,0.627283065141284,0.470833333077823,0.637759758853592,0.94907377854194
"9519","SNTB2",187.282706882267,0.111376242729992,0.235198315798852,0.473541837881374,0.63582665949233,0.94907377854194
"9520","RPA1",36.7089206658591,0.116921277759156,0.24546312949412,0.476329288232093,0.633839802821861,0.94907377854194
"9521","ACACA",10.5046081522325,0.238511772187055,0.50661275985941,0.47079700924478,0.637785700490178,0.94907377854194
"9522","NKIRAS2",28.8714773294674,-0.123733346550506,0.260431361029045,-0.475109242072835,0.634709111403751,0.94907377854194
"9523","RAD51C",29.9364744960571,0.143217636596374,0.300976082330037,0.475843912538299,0.634185582786247,0.94907377854194
"9524","CDR2L",11.7379488075305,0.189853651405133,0.400230046922243,0.474361315111402,0.635242274388526,0.94907377854194
"9525","SLC26A11",13.1813100836762,0.227826349204071,0.480710320689296,0.473936879236103,0.635544919507123,0.94907377854194
"9526","PSTPIP2",8.31633095264213,-0.266148413371727,0.560665174415718,-0.474701168391787,0.634999985034397,0.94907377854194
"9527","AC090409.1",3.15247960986785,-0.366384673564028,0.7710488591665,-0.475176986786659,0.634660828642376,0.94907377854194
"9528","GIPC1",60.6921705082133,-0.112905150399121,0.240461304357654,-0.469535631525935,0.638686822118915,0.94907377854194
"9529","CYP4F12",8.88007639046887,-0.271077774515977,0.575706994200247,-0.470860658715028,0.637740243811572,0.94907377854194

"9530","FAM32A",89.5523054879043,-0.0917299480775228,0.193926014615732
,-0.473015176737826,0.636202351513836,0.94907377854194
"9531","PIK3R2",27.1965488587278,0.133123837763386,0.281944093277134,0
.472163953555619,0.636809766684268,0.94907377854194
"9532","UBA2",97.6488998837678,-0.117699980650431,0.249466515848102,-0
.471806728250846,0.637064747994874,0.94907377854194
"9533","AC010632.2",1.86971664322584,0.514399547175882,1.0970221413889
1,0.46890534636303,0.639137294630371,0.94907377854194
"9534","ZNF226",27.8164699066659,-0.165590614928609,0.351048917985945,
-0.471702393725194,0.63713922830478,0.94907377854194
"9535","PLA2G4C",17.9170924534958,0.263210186233499,0.554051989963886,
0.475064057166649,0.634741316286592,0.94907377854194
"9536","ZNF444",15.8123613848003,-0.192028143347348,0.406671169975569,
-0.47219512354142,0.636787520121213,0.94907377854194
"9537","NCOA6",25.4543027053486,0.14773873888681,0.310020777972494,0.4
76544636307952,0.633686415194003,0.94907377854194
"9538","TPST2",13.379107644051,-0.185265203347326,0.394590010330228,-0
.469513161755615,0.638702879248026,0.94907377854194
"9539","EDA2R",4.92456338426933,-0.31973436556201,0.673270490165712,-0
.474897341012695,0.634860146774189,0.94907377854194
"9540","UBE2A",90.5642019168381,-0.0941589093912502,0.199980568566615,
-0.470840292465141,0.637754788671704,0.94907377854194
"9541","FAM122C",4.88306524925647,-0.299025512443396,0.629811437662981
,-0.474785776442835,0.634939672051059,0.94907377854194
"9542","LINC00632",92.5868365298299,0.122212788369081,0.26060444148300
6,0.468958961994706,0.63909896969931,0.94907377854194
"9543","BAG5",46.6770267612472,0.128328865369096,0.274104193336445,0.4
68175491250442,0.639659097361395,0.949132643679908
"9544","AL161729.1",3.55963066145778,0.413184144691704,0.8829668908332
26,0.467949760043433,0.639820517783179,0.949272687742017
"9545","GPATCH2",50.8709385528811,-0.100703898055469,0.215374737119686
,-0.467575256978756,0.640088362593524,0.949380515724655
"9546","AC138331.1",1.92888656882496,-0.489285193945276,1.046449580941
49,-0.467566907050663,0.640094335001644,0.949380515724655
"9547","ZNF726",2.08708165327702,0.411469121042137,0.879726542047463,0
.467723890749607,0.639982054018396,0.949380515724655
"9548","AL391001.1",1.62614447645325,0.497726337090813,1.0653423221109
8,0.467198502078248,0.640357865247991,0.949537781011854
"9549","PSMG3-
AS1",8.87657399603803,-0.247669226802578,0.529977536024807,-0.46732023
5231604,0.640270781143526,0.949537781011854
"9550","TUSC3",78.6948768836771,-0.115237849510197,0.246699443979989,-
0.467118399827219,0.640415170604112,0.949537781011854
"9551","LBHD2",10.797011955774,-0.331867618722979,0.710570774755624,-0
.467043721066509,0.640468597912727,0.949537781011854
"9552","AK4",18.8145818163653,-0.187597198158786,0.401912191804025,-0.
466761650888809,0.640670415747555,0.949574358374706
"9553","CLCC1",34.8673189514914,-0.12280385947299,0.263589779995221,-0
.465890064004822,0.64129419358465,0.949574358374706
"9554","MATN3",1.75219151010115,0.505162768270665,1.0848326649103,0.46

565962162693,0.641459159088959,0.949574358374706
"9555","PARTICL",4.34230518976177,-0.329026735046608,0.706178334816158,-0.465925841710033,0.641268583185726,0.949574358374706
"9556","MRPS9",36.8319386024059,0.125939495575723,0.2706487184413,0.465324559085388,0.641699050029625,0.949574358374706
"9557","NME5",2.6987076385616,-0.43450934972342,0.934446074024851,-0.464991358839895,0.641937644736996,0.949574358374706
"9558","AL365272.1",1.52493477895098,0.52760481496919,1.13004848524282,0.466886883048934,0.640580810355764,0.949574358374706
"9559","HLA-F",64.0207720991957,0.149830926023613,0.322276893814271,0.464913646927357,0.64199329722542,0.949574358374706
"9560","TAF11",54.0956667718116,-0.131839792718886,0.282608947828026,-0.466509619501198,0.640850763666993,0.949574358374706
"9561","COL12A1",320.160438401282,0.142461817205182,0.30540771066262,0.466464376083016,0.640883141071662,0.949574358374706
"9562","HIP1",23.2805186818368,0.152643416464402,0.32723042995286,0.466470726718145,0.640878596345446,0.949574358374706
"9563","SEC61A2",4.75467297418005,0.315278609850916,0.676998263408868,0.465700766001088,0.641429703996083,0.949574358374706
"9564","DGKA",11.8511600580702,-0.19905186999072,0.427954867902678,-0.46512350932292,0.641843011312848,0.949574358374706
"9565","AC007541.1",11.9201294750354,0.217244569738457,0.467368541438125,0.464824973178513,0.642056802362434,0.949574358374706
"9566","GAS6-DT",2.08055629869789,-0.501927096547432,1.07853258247977,-0.46537963219748,0.641659617386695,0.949574358374706
"9567","CDKN3",4.56999285969877,0.431595848204021,0.92864068959281 5,0.464760862883647,0.642102717615665,0.949574358374706
"9568","SNW1",124.465441142214,0.0855596158336131,0.183818457843982,0.465457151785228,0.641604114659571,0.949574358374706
"9569","NEIL1",9.98208786269512,0.295766679897701,0.634296756506213,0.466290702047448,0.641007433219489,0.949574358374706
"9570","MAPK8IP3",10.8135740925702,0.197072923671716,0.423977773809262,0.464818997234452,0.642061082225416,0.949574358374706
"9571","RAB3D",8.99246331240575,-0.218415612080775,0.469576855336808,-0.465132830969947,0.641836336268336,0.949574358374706
"9572","MED26",9.27565882278543,-0.227662666403004,0.488133700664476,-0.466394076239965,0.640933450882367,0.949574358374706
"9573","ZNF256",3.33766680354472,0.329251870316246,0.707254564701538,0.465535164775064,0.641548260686588,0.949574358374706
"9574","CDC25B",18.9561130569781,-0.16051543051228,0.344377251752189,-0.466103465590652,0.641141442771176,0.949574358374706
"9575","SCARF2",9.7179378069369,0.222061086987111,0.476918247644818,0.465616671376536,0.641489907603451,0.949574358374706
"9576","NBEAL2",4.04607465016147,0.334197361996176,0.719277501609918,0.464629244273818,0.642196986042872,0.949614590890462
"9577","SLC25A34",1.73398519379533,0.494377345305081,1.06564956754326,0.463921124131654,0.642704257703186,0.949670025531849
"9578","NECAP2",56.3586304193731,-0.107391195244236,0.231423321703277,

-0.464046555264337,0.642614391223521,0.949670025531849
"9579","ANKAR",19.474554881134,0.170349179450436,0.367467873498106,0.463575707527297,0.642951762158781,0.949670025531849
"9580","FGD5-AS1",125.996572978036,-0.0971945697961072,0.210032623557145,-0.462759394945437,0.64353683940475,0.949670025531849
"9581","DTX3L",35.8338387655591,0.122896925876639,0.265411040886595,0.463043758338413,0.643333001991369,0.949670025531849
"9582","RTP4",17.2430753228828,0.206909376667124,0.447209633474263,0.462667530347446,0.64360269553413,0.949670025531849
"9583","MYL5",16.4175950390537,-0.214148714321372,0.463722198591039,-0.461803888129651,0.644221962385617,0.949670025531849
"9584","NSUN7",3.0027432337584,0.414504365937838,0.893804787255713,0.463752680504776,0.642824949172119,0.949670025531849
"9585","FBXW11",50.3543520800656,0.111187240610741,0.239836178555897,0.463596615323937,0.642936779780681,0.949670025531849
"9586","TPK1",10.6390416561432,-0.199818507350207,0.431139652864335,-0.463465853865878,0.643030484895648,0.949670025531849
"9587","GIMAP2",5.20637331348498,0.265707009574334,0.572755378807383,0.463910107885152,0.64271215066092,0.949670025531849
"9588","SNX30",16.3491357692578,-0.196295075819279,0.424566213385125,-0.46234266795323,0.643835606171823,0.949670025531849
"9589","ARRDC1",18.6048525811638,0.153841457265585,0.332391688055818,0.462831842051811,0.643484905311185,0.949670025531849
"9590","UPF2",58.3155010342238,0.113619121616369,0.245991383086471,0.461882526903103,0.644165564935223,0.949670025531849
"9591","CREB3L1",49.5013280232173,0.222519887200918,0.479128951804083,0.464425884436862,0.642342648604162,0.949670025531849
"9592","NPFF",2.80700639741606,0.388286838997066,0.840589001817517,0.461922340356006,0.644137012661127,0.949670025531849
"9593","ELK3",69.2725243182427,-0.109467893034884,0.237061667287164,-0.461769691775095,0.64424648765953,0.949670025531849
"9594","LINC01550",4.85060951466488,-0.339420678213238,0.733417614111727,-0.462793191331142,0.643512612069208,0.949670025531849
"9595","MTFMT",14.7547263609194,-0.175505136553387,0.378862672837482,-0.463242090435951,0.643190849431264,0.949670025531849
"9596","RCN2",115.353941771986,-0.104201111055161,0.225052921159872,-0.463007147466169,0.643359243897784,0.949670025531849
"9597","SUZ12",54.7294827697863,0.121165465255738,0.262182211123712,0.462142205363299,0.643979345617609,0.949670025531849
"9598","AC144831.1",3.36177592119217,0.370367059155938,0.800667011699024,0.462573146819195,0.64367036035602,0.949670025531849
"9599","RNMT",95.2534538650999,-0.0922652637889625,0.199071483227842,-0.463478054681307,0.643021741415392,0.949670025531849
"9600","PLD3",236.771401801084,0.0982896921297309,0.211687579031388,0.464314876571747,0.642422167110405,0.949670025531849
"9601","TBC1D17",36.2755434330796,0.116061424789605,0.24998683329156,0.464270150797272,0.642454206780446,0.949670025531849
"9602","ZNF347",13.8927528742353,0.196731985437805,0.425062418422102,0.46283081474976,0.643485641725953,0.949670025531849

"9603","ZNF677",51.4722079621321,-0.0980727465390775,0.212032345407489
,-0.462536724529453,0.643696472775827,0.949670025531849
"9604","ZNF551",3.1200486950254,-0.425332643716341,0.920355321910046,-
0.462139603684405,0.643981211209751,0.949670025531849
"9605","SCAF4",21.786551263412,0.148797337285981,0.322076609474474,0.4
61993615521383,0.644085898886884,0.949670025531849
"9606","SYTL4",21.4348519958715,0.167930693679254,0.363345842032576,0.
462178658051626,0.643953206636622,0.949670025531849
"9607","MKRN2",24.8012581703302,-0.129811858931624,0.281221357307143,-
0.4616002858909 7,0.644367989528929,0.949750258325141
"9608","HNRNPH2",74.4834111727267,-0.100689757321413,0.218327010903549
,-0.461187815949604,0.644663862360923,0.950087457434499
"9609","KLHL7",28.3066535249329,0.158281886174634,0.343340087359248,0.
461006133574546,0.644794204574866,0.950180657381632
"9610","WNK3",3.01945754230511,0.336514302284382,0.730170270832437,0.4
608709991722,0.644891159513644,0.950224642946223
"9611","H2AFJ",332.811121791297,0.120305855610109,0.261161204988011,0.
460657453374946,0.645044384620456,0.950351522861894
"9612","RERE",89.200196904754,0.0885132687759975,0.193350029531615,0.4
57787717904251,0.647104960588853,0.950405928414926
"9613","MAST2",20.2150937137331,-0.147531177496915,0.32255661326553,-0
.457380724590714,0.647397416445777,0.950405928414926
"9614","LRRC8C-
DT",2.96686000995926,-0.446344304464322,0.972061987206241,-0.459172676
577077,0.646110170133183,0.950405928414926
"9615","PRMT6",13.6140905419981,0.183779109198636,0.400940588595627,0.
458369929176686,0.64668669194078,0.950405928414926
"9616","UBXN4",312.124279618029,0.0605175907798492,0.132263537304754,0
.457553094473863,0.647273548838624,0.950405928414926
"9617","SLC4A7",51.9519626910974,-0.191812554836121,0.419621563600194,
-0.457108431679348,0.647593110151245,0.950405928414926
"9618","SCAP",29.0180574361155,-0.128541545949216,0.280563504142617,-0
.458154906291288,0.646841154370009,0.950405928414926
"9619","RBM6",81.5259763736259,0.103675896368814,0.226404163815986,0.4
57923982586639,0.647007056154295,0.950405928414926
"9620","GMPS",46.2978051046957,-0.103731212811515,0.226063116067092,-0
.458859519483618,0.646335049860869,0.950405928414926
"9621","E2F3",16.9595945314,-0.181361831764658,0.396656223252694,-0.45
7226739763314,0.647508080532698,0.950405928414926
"9622","HIST1H1A",1.60991874021771,-0.54457105200326,1.18887481308116,
-0.458055840708675,0.646912323592876,0.950405928414926
"9623","PEX6",12.4440692869352,-0.19442977554031,0.423594900022584,-0.
458999330563102,0.646234646807666,0.950405928414926
"9624","MAP3K7",36.2615142293432,0.110175490317747,0.240646196747546,0
.457831836974048,0.647073260931816,0.950405928414926
"9625","CD24",3.14119718496607,-0.605104640677355,1.32211059155218,-0.
457680805632873,0.647181779966719,0.950405928414926
"9626","TBC1D32",10.2103513294027,0.223315070962578,0.488020393947213,
0.457593727090703,0.64724435104783,0.950405928414926
"9627","HOXA10-

AS",5.61758994238604,0.305338676404199,0.665028149448612,0.45913646912
1436,0.646136169241474,0.950405928414926
"9628","IL11RA",163.354865495725,-0.137524710239457,0.300861609002219,
-0.457102887588568,0.647597094877004,0.950405928414926
"9629","PARG",15.3371209060566,0.190087711097552,0.413938116739002,0.4
59217702865974,0.646077839180683,0.950405928414926
"9630","FZD4-
DT",1.91940461022763,0.442357982954919,0.964706789745157,0.45854138030
0407,0.646563540344592,0.950405928414926
"9631","FUT4",18.0336169540012,0.16689546204517,0.36419523952848,0.458
258219578178,0.646766937015432,0.950405928414926
"9632","TAGLN",519.807690033936,0.285586374154142,0.621904847317538,0.
459212330288085,0.646081696904323,0.950405928414926
"9633","AP003396.5",5.20229993886281,-0.324681045671326,0.705982322912
575,-0.459899681810492,0.645588228610475,0.950405928414926
"9634","TIRAP",5.18477941618506,-0.283241844322744,0.618783464925706,-
0.457739840150304,0.647139361593821,0.950405928414926
"9635","AC004241.1",2.53088607341883,0.393010231978156,0.8557917028484
11,0.459235852217384,0.646064807298173,0.950405928414926
"9636","C12orf75",44.6530828476273,0.170510258180498,0.37251943372187,
0.457721779712046,0.647152338530946,0.950405928414926
"9637","WDR25",11.9105874302058,-0.204797825832119,0.448081799749751,-
0.457054551080844,0.647631836389521,0.950405928414926
"9638","MYO5A",21.3261205512856,0.213282369727167,0.463867984645499,0.
459791097439423,0.645666173905916,0.950405928414926
"9639","TELO2",21.2914281120567,-0.146142133068009,0.318478464034936,-
0.458876029532654,0.646323193102185,0.950405928414926
"9640","FAM106A",9.48705217369911,-0.260929359741861,0.569019918701214
,-0.458559272120791,0.646550689390035,0.950405928414926
"9641","MRPL10",47.8238748986354,-0.10529512235172,0.22974783677855,-0
.458307350476653,0.64673164399491,0.950405928414926
"9642","CXXC1",24.7256794263416,-0.139533436677141,0.303549934865204,-
0.459672102183462,0.645751596932911,0.950405928414926
"9643","RFX1",31.9056885169546,-0.154680269992638,0.336418418219008,-0
.459785379205787,0.645670278742725,0.950405928414926
"9644","LSR",5.91004437573124,-0.254546756786682,0.55279913901988,-0.4
60468801087492,0.64517976047542,0.950405928414926
"9645","ZNF420",12.6944704563574,0.171595549252608,0.373427782043499,0
.459514683973405,0.645864609793778,0.950405928414926
"9646","ZNF793",2.56790356166234,0.539340645212941,1.17998096567749,0.
45707571638944,0.647616623883815,0.950405928414926
"9647","PVR",6.06242370211084,0.305916251319659,0.666830743510157,0.45
8761468778921,0.646405467222865,0.950405928414926
"9648","YBEY",47.3711581182324,0.153235974880486,0.333801454805502,0.4
59063232572706,0.646188758763907,0.950405928414926
"9649","SEPTIN6",33.4675026501821,-0.143853289337601,0.313701823591449
,-0.458566952817428,0.646545172695669,0.950405928414926
"9650","PIGV",21.1733537699732,0.151893796071326,0.337735108191312,0.4
49742393927508,0.652896199210056,0.950545977875221
"9651","HS2ST1",25.7723985408887,0.124520511924937,0.276362564511511,0

.450569389327513,0.652299932782076,0.950545977875221
"9652","SELENBP1",158.473057653823,0.108687757621952,0.241228800381658
,0.450558795011179,0.652307569915368,0.950545977875221
"9653","TSEN15",20.5611703901389,0.157790989712521,0.347854080081699,0
.453612588575822,0.650107691240013,0.950545977875221
"9654","RABL2A",13.2500585634401,-0.189747593903734,0.421819762380551,
-0.449830972434502,0.652832323200066,0.950545977875221
"9655","FSIP2",3.26916370101574,-0.407421050839186,0.905746887894043,-
0.449817776118981,0.652841839207056,0.950545977875221
"9656","ADAM23",8.72513128479406,0.268014279004943,0.593646203551277,0
.451471393907084,0.651649837772555,0.950545977875221
"9657","TRAIP",3.88622607209443,0.295340568293915,0.655702566765799,0.
450418502630939,0.652408706033168,0.950545977875221
"9658","EBLN2",5.05276854892643,0.301101611960782,0.665794793960439,0.
452243866566901,0.65109330971636,0.950545977875221
"9659","SEC61A1",102.616408830146,0.0810162981138467,0.180137101270231
,0.449747983855423,0.652892168108524,0.950545977875221
"9660","AC026304.1",4.23910682549239,0.2848078827233,0.633187494604491
,0.44980023318559,0.652854489695037,0.950545977875221
"9661","ACAP2",99.6119508536268,0.0833829504988669,0.182914375154734,0
.455857831995599,0.648492214080505,0.950545977875221
"9662","BMP2K",20.3356717902703,-0.146855285091865,0.321649867861301,-
0.456568771715432,0.647981029373745,0.950545977875221
"9663","AC098487.1",12.2955841885791,0.213407541050766,0.4727503994420
35,0.451416944972741,0.651689072843198,0.950545977875221
"9664","HADH",44.2627430325333,0.140796408830695,0.308215304898788,0.4
56811866876402,0.64780627549145,0.950545977875221
"9665","OTULIN",28.3974856836401,-0.132877668178348,0.293864028716088,
-0.45217398250102,0.65114364970977,0.950545977875221
"9666","AC008966.1",3.32952796198907,-0.333195320775798,0.740105627309
441,-0.450199685667959,0.65256646291107З,0.950545977875221
"9667","PTCD2",11.4110000692094,0.188191846395466,0.416568820706116,0.
451766519818902,0.651437191330868,0.950545977875221
"9668","FAM174A",33.7251177532513,-0.118559455349657,0.261236660367032
,-0.453839270426609,0.649944516356258,0.950545977875221
"9669","AC008467.1",3.76499260260773,0.386551643386456,0.8574117943930
64,0.450835463092836,0.652108139930782,0.950545977875221
"9670","FNIP1",85.9244104272509,-0.107162743488,0.236765640309688,-0.4
52611043341558,0.65082884528988,0.950545977875221
"9671","ZNF346",17.3594982198109,0.179084728643485,0.395521946791055,0
.452780762474582,0.65070661745915,0.950545977875221
"9672","BLOC1S5",37.0087693695539,0.10574841082994,0.233528912991004,0
.452827915291217,0.650672660748145,0.950545977875221
"9673","C6orf89",95.8941021097957,0.083461075075002,0.182767800913207,
0.456650868796282,0.647922010054039,0.950545977875221
"9674","MDGA1",15.1804005938011,-0.183650840422538,0.402481973362086,-
0.456295815905572,0.648177272305391,0.950545977875221
"9675","AKIRIN2",76.5087749963546,-0.128197463192167,0.282874112482829
,-0.45319616583843,0.650407493108147,0.950545977875221
"9676","TPD52L1",3.9528967061411,-0.38474221475549,0.855073426405614,-

0.449952253074677,0.6527448690536,0.950545977875221
"9677","COX19",36.8200765922623,-0.130874436531256,0.287747967878252,-0.454823147827164,0.649236475719985,0.950545977875221
"9678","LRCH4",9.88350378291969,-0.19463176161412,0.427863055941124,-0.45489265528198,0.649186467129406,0.950545977875221
"9679","POLR2J3",77.1265213026976,0.117400563960325,0.2582154929153,0.454661192614166,0.649353003914862,0.950545977875221
"9680","CUL1",45.7294828566734,-0.115225101161591,0.255743928754562,-0.450548725526824,0.652314828746402,0.950545977875221
"9681","ABCB8",27.5868632069302,0.123304470349079,0.273861405451645,0.450244057375404,0.652534471712285,0.950545977875221
"9682","NCAPG2",7.10283193356935,-0.238860925179908,0.529466450788961,-0.451135147135348,0.651892147470776,0.950545977875221
"9683","DMRT3",4.52215155015493,0.288561075657036,0.635326296642274,0.454193502113943,0.649689559488003,0.950545977875221
"9684","TRMT10B",22.6849351971578,0.141640218420399,0.314442982248689,0.450448018930115,0.652387427340851,0.950545977875221
"9685","UBQLN1",56.9722481310034,0.0910262864289386,0.201881657131715,0.450889336466807,0.652069309409749,0.950545977875221
"9686","SPOUT1",14.7319992413371,-0.183721949172899,0.407998954109345,-0.45030004935665,0.652494103418293,0.950545977875221
"9687","TPRN",8.39356194044246,-0.216698806881139,0.479037455065955,-0.45236297201709,0.651007517464984,0.950545977875221
"9688","DPH7",23.2339272388053,0.134222124159323,0.294647942251089,0.455533892868471,0.648725189954537,0.950545977875221
"9689","ITGA8",12.1934514723898,0.358028624560138,0.794244510926372,0.450778846608016,0.652148948630858,0.950545977875221
"9690","ECD",20.3887080592145,0.138480492783666,0.304843318652904,0.454267764160319,0.649636114871166,0.950545977875221
"9691","AL450306.1",2.65579871496786,-0.382437956041044,0.844872113769401,-0.452657804427697,0.650795168089874,0.950545977875221
"9692","ZMIZ1-AS1",3.62779722124889,-0.350242652819667,0.769903181122555,-0.454917788895218,0.64916838461548,0.950545977875221
"9693","MXI1",62.0881523656835,0.162996749117333,0.360834587386119,0.451721522313255,0.651469611455972,0.950545977875221
"9694","DENND10",59.0891396980128,-0.0987205091388702,0.217355586148657,-0.454188966973923,0.649692823378106,0.950545977875221
"9695","MAP6",3.4192784846468,0.347192114158098,0.7636611755877,0.454641567827387,0.649367124703843,0.950545977875221
"9696","AP001267.3",2.59695142303233,-0.390666578840098,0.868026606680754,-0.450062908018416,0.652665081144317,0.950545977875221
"9697","ARHGAP32",37.5087696988574,-0.143335373095298,0.317999905240334,-0.450740301280814,0.652176732383966,0.950545977875221
"9698","CACNA1C",10.8570276192031,0.236285219605945,0.517562046951243,0.456535058932178,0.648005266022819,0.950545977875221
"9699","GOLT1B",47.5602544997042,-0.113329636482083,0.251740113418822,-0.450185053716631,0.65257701242354,0.950545977875221
"9700","HELLPAR",146.677465668731,0.0870671935575637,0.192980737889454,0.451170383685851,0.651866753211166,0.950545977875221

"9701","NRAV",5.29410160449113,-0.285333555795581,0.629939615699649,-0
.452953820785938,0.650581994500025,0.950545977875221
"9702","AC156455.1",2.43269699375517,-0.370093793638915,0.820075685032
28,-0.451292240940355,0.65177893629818,0.950545977875221
"9703","FKBP3",114.851903848173,-0.0846648253209782,0.187100994161719,
-0.452508687622466,0.650902564072524,0.950545977875221
"9704","BBOF1",22.4508049789468,-0.13568528318276,0.297605891583624,-0
.45592270522855,0.648445561614764,0.950545977875221
"9705","ZFYVE21",94.8657806674307,-0.0849890810370587,0.18687041332358
9,-0.454802231800545,0.649251524501793,0.950545977875221
"9706","OAZ2",82.5443586207039,0.101555945406911,0.224002152998337,0.4
53370398666059,0.650282048000458,0.950545977875221
"9707","MFGE8",234.150868248678,0.109687739617477,0.240775922503062,0.
45555942004991,0.648706829647789,0.950545977875221
"9708","EME2",3.53182480306077,-0.317570220853066,0.70199682566069,-0.
452381277585098,0.65099433228258,0.950545977875221
"9709","SNHG19",43.7296180305554,-0.126981196079505,0.281156317813807,
-0.451639134652479,0.651528972415626,0.950545977875221
"9710","CORO7",11.2765735964839,-0.19495121394699,0.430150207480765,-0
.453216598659219,0.650392781260889,0.950545977875221
"9711","LITAF",128.954613642665,-0.0850680056427813,0.188763260946192,
-0.450659758770699,0.652234789571086,0.950545977875221
"9712","CENPT",30.8009282879888,0.123514973194559,0.273038013446522,0.
452372809322213,0.651000431811548,0.950545977875221
"9713","TAF1C",16.2482382691474,0.173771243824545,0.382904961255588,0.
453823432464102,0.649955916625217,0.950545977875221
"9714","HIC1",211.984460589619,-0.0839287032893837,0.185246614434865,-
0.453064708067277,0.650502147352424,0.950545977875221
"9715","AC002553.2",1.65962394285096,-0.532894098167637,1.172558461978
19,-0.454471239982873,0.64948968743148,0.950545977875221
"9716","COPRS",179.878100318143,-0.126468613106322,0.279692295026311,-
0.452170529382745,0.651146137155676,0.950545977875221
"9717","LGALS3BP",649.062435696141,0.119209892607046,0.264026250198539
,0.451507728937573,0.651623655840976,0.950545977875221
"9718","DEDD2",15.2588966446781,-0.158169414958116,0.351291580414014,-
0.450251084218145,0.652529405544339,0.950545977875221
"9719","CCDC8",37.8640263742315,0.131838548674911,0.289007919119979,0.
456176249690165,0.648263242727643,0.950545977875221
"9720","ZNF350",11.6680183344544,0.180940291220001,0.401913829765497,0
.450196728302614,0.652568595140732,0.950545977875221
"9721","AC022150.4",2.70785037598841,0.381882374529935,0.8394626795254
83,0.454912867294825,0.649171925471212,0.950545977875221
"9722","FAM110A",2.91553238197529,-0.383070425179485,0.847068807951064
,-0.452230588098358,0.651102874549991,0.950545977875221
"9723","DSN1",14.4771533357023,-0.166336009808628,0.367598958300167,-0
.45249314790714,0.650913756407121,0.950545977875221
"9724","DHX35",6.36940529899098,0.294097574372899,0.653248824154554,0.
450207583233732,0.652560768862194,0.950545977875221
"9725","NCOA5",13.3273968504756,-0.196005832214255,0.430624835550007,-
0.45516611220045,0.648989738184916,0.950545977875221

"9726","LINC00630",5.65358383529233,0.259518373069245,0.57466244096559
1,0.451601417752625,0.65155614847665,0.950545977875221
"9727","SNHG20",6.92779583964758,-0.266584560013404,0.593138590871645,
-0.449447336787926,0.653108990091048,0.950660289852923
"9728","TEF",18.2926590282405,-0.253222794783224,0.563310709071787,-0.
449525973330917,0.653052275810783,0.950660289852923
"9729","CCDC107",132.522937051883,-0.101243527735161,0.225392410342267
,-0.449187830155501,0.653296165793395,0.95083499924293
"9730","SMARCC2",92.3764737506957,-0.0913492340688035,0.20349758418649
7,-0.448895914091471,0.653506743692581,0.951043729772553
"9731","STX12",85.2177574127997,-0.0949786430549229,0.212136902028036,
-0.447723343496222,0.654352872453439,0.951196309460509
"9732","LEPROT",439.082942402976,0.0598291188975724,0.133600710236547,
0.447820365562742,0.654282844305634,0.951196309460509
"9733","SLC29A4",1.53270600356997,-0.488091299247407,1.08834590751398,
-0.448470744344798,0.653813495436738,0.951196309460509
"9734","IDNK",13.321164673262,-0.191586549886394,0.427639007229503,-0.
448009995925311,0.654145982546535,0.951196309460509
"9735","AL589843.1",2.86161607912901,-0.475801642404186,1.061639536699
51,-0.448176265065813,0.654025990828577,0.951196309460509
"9736","ATP6V0A1",34.6876676356717,-0.12711540411562,0.28339957127587
2,-0.448524109755363,0.653774990114561,0.951196309460509
"9737","ACTL10",2.69663997837215,-0.440198384230435,0.983263929799104,
-0.447690971762154,0.6543762382539,0.951196309460509
"9738","ACSS2",17.016852417374,-0.177406243595261,0.396079496060107,-0
.447905648638622,0.654221291585945,0.951196309460509
"9739","CASTOR1",10.9522239771887,-0.210197524129247,0.468943981102887
,-0.448235892984262,0.653982961230885,0.951196309460509
"9740","GGA1",30.2818942053214,0.137832379357757,0.307321724793577,0.4
48495398268173,0.653795706510081,0.951196309460509
"9741","MAMLD1",2.34297284464161,-0.448330669122745,1.00155734646354,-
0.447633548598873,0.654417686918381,0.951196309460509
"9742","IRAK1",29.5182531094309,-0.16020973484794,0.357433072843623,-0
.448223029764322,0.65399224368435,0.951196309460509
"9743","CBWD3",5.43540793961168,-0.27232142262085,0.608690223127654,-0
.447389184635776,0.65459408340031,0.951355046797535
"9744","C1orf50",27.1504739495663,0.130777763626088,0.292933373889879,
0.446442007919693,0.655277994217114,0.951363930984203
"9745","CC2D1B",6.39668049904594,-0.248019351840523,0.556961830526473,
-0.445307628363115,0.656097455835706,0.951363930984203
"9746","ADAMTS4",32.1525071749379,-0.412721630045628,0.926765226387097
,-0.445335688364768,0.656077180643271,0.951363930984203
"9747","YOD1",4.45002702604604,0.31236643573431,0.701183603606793,0.44
5484512369571,0.655969649769968,0.951363930984203
"9748","COL3A1",6901.3725818814,-0.142715588468975,0.320791661568931,-
0.444885592633485,0.65640243489704,0.951363930984203
"9749","AC007563.2",3.37734056019546,0.415999414581867,0.9336578606707
54,0.445558734205918,0.655916024399483,0.951363930984203
"9750","ITPR1",7.4493537249482,0.273967086754766,0.616481372022307,0.4
4440448517697,0.656750171283233,0.951363930984203

"9751","SPCS1",297.679324356954,-0.115908530434125,0.260773207765296,-0.444480211089962,0.656695432933255,0.951363930984203
"9752","IQCB1",19.3017695865885,0.140936316327404,0.316607987927567,0.445144537413399,0.656215304763695,0.951363930984203
"9753","GTF2H2C",15.7255124153783,0.192149497527063,0.431984678660353,0.444806279062828,0.656459756291966,0.951363930984203
"9754","EPB41L4A-DT",2.15705523663002,-0.403316736180171,0.905589765923826,-0.445363619771843,0.656056998620477,0.951363930984203
"9755","AC011374.2",1.89269303864916,-0.453342761314874,1.01756177967481,-0.445518660753699,0.655944977299071,0.951363930984203
"9756","NKAPL",3.64571623980824,0.341715918285772,0.76515837686157,0.446595016952411,0.655167494140685,0.951363930984203
"9757","RNF216",62.1728019913224,0.0935752484860077,0.209764537315672,0.446096607574746,0.655527463217433,0.951363930984203
"9758","CRCP",41.6201345196582,-0.104517102858891,0.233779202113218,-0.447076138142833,0.654820087205588,0.951363930984203
"9759","RGS22",6.05953834526268,0.257548481939775,0.578882050716245,0.444906663837845,0.656387206685179,0.951363930984203
"9760","NSMCE2",38.2024880464165,-0.119409091617362,0.268531389121019,-0.444674613303953,0.65655491805346,0.951363930984203
"9761","LINC00963",62.7581363159056,-0.107933464136251,0.241632221713606,-0.446684897282361,0.655102587876491,0.951363930984203
"9762","CUBN",4.65445237919444,0.28653445386539,0.641934066589584,0.44636119000144,0.655336362354886,0.951363930984203
"9763","CSTF2T",37.9113489444765,-0.116889942490987,0.262214622937522,-0.445779648676718,0.655756423910585,0.951363930984203
"9764","PCED1B-AS1",5.19369291851594,0.264263044979501,0.594407596907769,0.444582213205639,0.656621703786253,0.951363930984203
"9765","SLC16A7",33.478977282318,-0.155694258469864,0.349513364053792,-0.44546010105039,0.655987287366405,0.951363930984203
"9766","OSBPL8",214.655116487138,0.0737313257775948,0.164896199958637,0.447137810307878,0.654775560505138,0.951363930984203
"9767","GSC",55.3560761075421,-0.145140546003787,0.326568495608335,-0.44444135902766,0.656723516851602,0.951363930984203
"9768","ZFP3",3.2668656063342,0.340639805265657,0.762279597889744,0.446869896831382,0.65496900033395,0.951363930984203
"9769","RABEP1",68.9623339864187,0.104205477950026,0.234201327716946,0.444939740375714,0.656363302476835,0.951363930984203
"9770","NAA38",199.71864533497,-0.129709311197075,0.291278424792381,-0.445310397738967,0.656095454768663,0.951363930984203
"9771","DRG2",43.9133774363214,0.104758822032435,0.234915575210353,0.445942428204812,0.655638833285865,0.951363930984203
"9772","KEAP1",44.8645596975464,-0.112829752183152,0.253259920324488,-0.445509704175022,0.655951448459928,0.951363930984203
"9773","RFXANK",45.8489036639851,-0.125459784482975,0.282001445701155,-0.44489057200057,0.656398836283167,0.951363930984203
"9774","ZNF567",19.0719169235266,-0.151291709037658,0.339390329040419,-0.445774956126226,0.655759813897954,0.951363930984203

"9775","EMD",57.6158239760277,-0.0929151836537188,0.209053120693206,-0
.444457290786277,0.65671200064215,0.951363930984203
"9776","ANKRD52",11.8951826464391,0.201694322372725,0.454349150618513,
0.443919224011215,0.657100985222388,0.951774749462869
"9777","WBP11",69.8623184103319,-0.0893479723815772,0.201465673544416,
-0.443489805531954,0.657411491373823,0.951828955304309
"9778","AC011939.2",11.3360971030718,0.218118841079041,0.4915904243328
71,0.443700345414675,0.657259245705948,0.951828955304309
"9779","CCDC189",3.19864698503529,-0.322724759160884,0.727536685525391
,-0.443585547755338,0.657342256463587,0.951828955304309
"9780","SLC16A11",1.89016938989886,0.499217789241145,1.12567404125173,
0.443483433877559,0.657416099068256,0.951828955304309
"9781","PPP4R1",30.7881033638839,0.139207249148476,0.313952213661798,0
.443402667956456,0.657474506485272,0.951828955304309
"9782","TMEM98",211.535433047903,-0.102155167093936,0.230463850566137,
-0.443258961624526,0.657578435633158,0.951882094517022
"9783","MXRA8",660.15101902999,0.0847018929235797,0.192566977027252,0.
439856792847679,0.660040831199212,0.952084566017316
"9784","ERO1B",17.1304568719186,0.183968896321028,0.416521045535545,0.
441679714129423,0.658720991191062,0.952084566017316
"9785","ACTR2",193.424975955755,-0.0825983064732366,0.186771571549154,
-0.442242391538151,0.658313813235035,0.952084566017316
"9786","RNF149",67.8125581307309,-0.0985331118456421,0.223451800538672
,-0.440959131267279,0.659242584211145,0.952084566017316
"9787","AC012306.2",13.7395625842407,-0.204930368561119,0.466636830398
584,-0.439164581985684,0.66054228647981,0.952084566017316
"9788","STK36",7.11501404047933,-0.227370917186684,0.51680436141585,-0
.439955492178458,0.659969343306263,0.952084566017316
"9789","EXOSC7",61.5623294993201,0.116317911520335,0.264080655781432,0
.440463581764982,0.659601383318292,0.952084566017316
"9790","PPP2R3A",26.9952030568488,0.149631283795145,0.340155767150507,
0.439890480319089,0.660016431025802,0.952084566017316
"9791","MRPS30",37.8234813966562,-0.103890893661463,0.2357551241515,-0
.440672897504874,0.659449820180662,0.952084566017316
"9792","LINC01184",38.4000868674412,0.109568493334489,0.24895379861952
2,0.440115772251959,0.659853259115298,0.952084566017316
"9793","TNIP1",50.6269781990375,-0.108387390584465,0.245563386666372,-
0.441382536932194,0.658936082442106,0.952084566017316
"9794","TXNDC5",143.023345282,-0.0882858812388826,0.200752215222169,-0
.439775377527855,0.660099802624667,0.952084566017316
"9795","FAM162B",6.81100799751041,0.26932074035589,0.612404244118872,0
.439776084085422,0.660099290835897,0.952084566017316
"9796","RP9",31.2044774986886,-0.144782479021236,0.328375379016603,-0.
440905403611017,0.659281481552325,0.952084566017316
"9797","AC044849.1",5.56470399608739,-0.27873114877682,0.6339818276510
61,-0.439651637665286,0.660189434960146,0.952084566017316
"9798","AGAP9",5.65106224303093,0.267469759689203,0.604427778249688,0.
442517318551682,0.658114900873417,0.952084566017316
"9799","H2AFY2",8.33325236464147,0.312426104694572,0.708380876002277,0
.441042545442133,0.659182196475348,0.952084566017316

"9800","PCGF5",38.4783815237217,0.116736295877252,0.263961968425836,0.442246648535851,0.658310733071517,0.952084566017316
"9801","PPP2R2D",42.9158047964421,-0.100017369658404,0.226174971693387,-0.442212367308226,0.658335537521782,0.952084566017316
"9802","PIDD1",6.95903020714448,0.249044350902093,0.566626718615272,0.439521015723915,0.660284057696063,0.952084566017316
"9803","CD3D",3.10232841638632,0.364856428213568,0.828109204825199,0.440589750829522,0.659510024065733,0.952084566017316
"9804","THYN1",78.5308589046984,-0.131814058237733,0.299690249885025,-0.439834323233083,0.660057106367429,0.952084566017316
"9805","AC005906.2",4.18651666149801,-0.310400847758913,0.706600397544005,-0.439287677784786,0.660453101578542,0.952084566017316
"9806","ACVR1B",20.5396031243683,0.147131480465636,0.334852175260737,0.439392338876313,0.660377276714867,0.952084566017316
"9807","AC009779.2",14.541877458161,-0.17520974068969,0.398526434929932,-0.439643961687246,0.66019499530022,0.952084566017316
"9808","UGGT2",41.5921816362206,0.123379491885805,0.280357697617721,0.440078845468471,0.659880002922878,0.952084566017316
"9809","CTSG",5.2732473001341,0.42488618263559,0.962086053188732,0.441630123654064,0.658756881879965,0.952084566017316
"9810","GEMIN2",21.6399533945609,0.139732338401422,0.315999197111841,0.442192067823409,0.658350225549984,0.952084566017316
"9811","PARN",31.7314925318987,-0.114227551930437,0.257955334423858,-0.442819111244836,0.657896578793076,0.952084566017316
"9812","MT1F",47.2404462659298,-0.207064323376674,0.470748322959102,-0.439862052136643,0.66003702181965,0.952084566017316
"9813","NUP93",16.3813596850914,-0.159566167829764,0.361076668569975,-0.441917691502243,0.658548768010775,0.952084566017316
"9814","TRAPPC1",148.772972754041,-0.125607915372142,0.284267860311531,-0.441864638635152,0.658587160569401,0.952084566017316
"9815","TMEM100",24.9975248542465,-0.217667262403164,0.495607233126023,-0.439193070347734,0.660521645771004,0.952084566017316
"9816","C17orf67",6.01851268309373,0.236446655575136,0.537984073445009,0.439504935640636,0.660295706508782,0.952084566017316
"9817","REEP6",1.56199661103934,0.496518214725764,1.12270190873708,0.442252935406776,0.658306184197712,0.952084566017316
"9818","TCF3",34.5039481718639,-0.109599492008138,0.249378815829705,-0.439489984919093,0.660306537257593,0.952084566017316
"9819","PNPLA6",13.1539858013546,-0.176444524014115,0.398665562325838,-0.442587824703812,0.658063892886426,0.952084566017316
"9820","EVI5L",5.70565358428922,0.240776370280749,0.545615937115515,0.441292773729542,0.659001056904431,0.952084566017316
"9821","ZNF781",5.75785077370166,0.252361127936666,0.571444678854442,0.441619525520068,0.658764552292136,0.952084566017316
"9822","BMP7",4.51862746691809,-0.326905523450918,0.741839910966961,-0.440668557485413,0.659452962597302,0.952084566017316
"9823","MRGBP",19.4825217708594,-0.150590428329652,0.341654909945281,-0.440767639937534,0.659381222862247,0.952084566017316
"9824","SH3KBP1",142.432266591093,0.0931721932473251,0.211700559748797,0.440113117121101,0.659855182049454,0.952084566017316

"9825","DHX15",55.2599954239801,-0.0972132377870436,0.221549599811884,
-0.438787693002319,0.660815378630948,0.952209413700977
"9826","DHX29",89.0840052956431,-0.0762243236083172,0.173733802396859,
-0.438742044188952,0.660848458633751,0.952209413700977
"9827","CLN6",15.9665860823798,-0.168317633631098,0.383574411343888,-0
.438813509590959,0.660796670599529,0.952209413700977
"9828","ZNF575",6.51837769809248,-0.247539696941669,0.564291001831178,
-0.438673833427041,0.660897889678898,0.952209413700977
"9829","TENT4B",34.8432103611778,-0.134187457860661,0.305978562708595,
-0.438551827529359,0.660986308727164,0.952239915716415
"9830","EIF2B3",50.0205760567557,-0.111660900677302,0.255113065699388,
-0.437691814690813,0.661609703869483,0.952241147688144
"9831","TRAPPC12",49.0046429280754,-0.0953503581407594,0.2181085554089
75,-0.437169270879668,0.661988593415645,0.952241147688144
"9832","MPHOSPH10",87.8501609217015,-0.085595331001049,0.1957701091213
48,-0.437223697658527,0.661949125244413,0.952241147688144
"9833","XXYLT1",19.9641396052041,-0.151528450991354,0.34618817451608,-
0.437705450809141,0.661599817661699,0.952241147688144
"9834","DEK",383.17728270194,-0.0801622986253591,0.183522356885296,-0.
436798545887582,0.662257453658457,0.952241147688144
"9835","PTPRK",25.1374152678005,0.15230421363115,0.348679077665727,0.4
36803420069734,0.66225391848126,0.952241147688144
"9836","AKR1B1",91.3693424528539,0.0913059932907057,0.209037244682881,
0.436792942947661,0.662261517402928,0.952241147688144
"9837","AC021097.2",3.32242390755768,0.335596791028666,0.7683282609774
,0.436788294890713,0.66226488858989,0.952241147688144
"9838","CCDC183",2.88466217972131,0.455499022407254,1.03929628267979,0
.438276389513073,0.661185939155044,0.952241147688144
"9839","SLK",112.718798692413,-0.11542111062849,0.264028085739915,-0.4
37154669758076,0.661999181737694,0.952241147688144
"9840","SPRYD7",23.3870002311039,-0.127396910138693,0.290977915900281,
-0.437823295780124,0.661514382326267,0.952241147688144
"9841","PAPLN",40.9414468142981,-0.179043418543548,0.409743716329741,-
0.436964403377118,0.66213716373498,0.952241147688144
"9842","COA3",100.527998113912,-0.107973506932801,0.246430286679413,-0
.438150311748271,0.661277325134954,0.952241147688144
"9843","COPZ2",270.675434451446,-0.106326724757909,0.242966494964353,-
0.437618877341539,0.661662584567198,0.952241147688144
"9844","HOXB5",2.32907192838964,-0.427397210989149,0.978095558164608,-
0.436968767950606,0.662133998398947,0.952241147688144
"9845","SIRT7",21.2438213733676,-0.143958509383363,0.329311345802691,-
0.437150165696436,0.662002447969951,0.952241147688144
"9846","TYK2",20.1388307795541,0.152570121748682,0.348762216728751,0.4
37461727304433,0.661776526492089,0.952241147688144
"9847","MRPL34",127.852723736799,-0.12295939067881,0.28101569867681,-0
.437553457894973,0.661710016105977,0.952241147688144
"9848","ZXDA",3.76865310845465,-0.30035910590344,0.686481899668332,-0.
437533904460636,0.661724193338739,0.952241147688144
"9849","SOWAHC",32.8195581357659,0.130500463487467,0.299134283840203,0
.436260470756271,0.66264775840347,0.952383453822388

"9850","LRTM1",1.76145370167006,0.511406387505892,1.17286665490831,0.436031142471069,0.662814134621901,0.952383453822388
"9851","H2AFZ",217.923533814393,-0.123351360481198,0.282685711832498,-0.436355129806803,0.662579088714527,0.952383453822388
"9852","PSMC2",87.58328027996,-0.0982216485919669,0.225081469655216,-0.436382651767934,0.662559123649143,0.952383453822388
"9853","NTNG2",3.58190312022178,0.353836677907764,0.811310866632767,0.436129592811093,0.662742707485245,0.952383453822388
"9854","MSS51",2.15376036537099,0.377275296430875,0.864969528132216,0.436171777340584,0.662712102941346,0.952383453822388
"9855","NKAPD1",53.3372542286999,0.1041084832564,0.238779370818377,0.436002837680596,0.662834670721725,0.952383453822388
"9856","GNG12-AS1",2.35677068436132,0.359264618876538,0.827575251024662,0.434117161468657,0.664203363920789,0.95239097757155
"9857","IFI44L",40.9573916453586,0.164421597288862,0.378474964296853,0.434431898538737,0.66397483813492,0.95239097757155
"9858","RNPEP",39.2391047117047,0.11328505904945,0.260469351693775,0.434926636522809,0.663615679596001,0.95239097757155
"9859","KDM5B",103.998704171706,0.0994035294886629,0.229575907871173,0.43298763537698,0.665023752576986,0.95239097757155
"9860","CCDC150",3.3513003642981,-0.312555729394409,0.719683514113216,-0.43429608052025,0.664073449695239,0.95239097757155
"9861","COLQ",4.14639692992394,0.396523503922362,0.915397466516506,0.433170855750027,0.664890650080407,0.95239097757155
"9862","ABHD14A",105.043292741245,0.118032315036883,0.272453925005388,0.433219360060783,0.664855415347653,0.95239097757155
"9863","SLC38A9",12.4607584694277,0.190626419817271,0.439742287933896,0.433495765697038,0.664654641580129,0.95239097757155
"9864","GPER1",7.61584173011266,0.224072264323464,0.517561440191056,0.432938482126389,0.665059462304201,0.95239097757155
"9865","IMMP2L",29.7255318517657,-0.128905289964697,0.296920833864357,-0.43414026657215,0.664186586595313,0.95239097757155
"9866","LUC7L2",103.518443267055,-0.0772611222022582,0.178059104483903,-0.433907170465651,0.664355852553357,0.95239097757155
"9867","PNPLA7",8.83399654222975,0.24046887714934,0.553273366863079,0.434629410254714,0.663831443845417,0.95239097757155
"9868","NUTM2B-AS1",46.3234980779627,0.124603618913083,0.287798014139077,0.432955103202586,0.665047387044276,0.95239097757155
"9869","CUTC",127.686714897139,-0.103928306917594,0.239510335728615,-0.433919925006301,0.664346590224611,0.95239097757155
"9870","CSTF3",24.0369704811767,0.147228383530899,0.338589335479501,0.434828767782653,0.663686721971274,0.95239097757155
"9871","PRKAG1",59.849837479654,0.128221074965381,0.294836492775498,0.434888753961044,0.663643177978647,0.95239097757155
"9872","PEBP1",698.859804917826,-0.0868101405524392,0.199822636026808,-0.434435969210179,0.663971882687102,0.95239097757155
"9873","GCN1",21.8531813201423,0.13669019058669,0.314382088570388,0.43479000730694,0.663714858821293,0.95239097757155

"9874","MAB21L1",5.11071392984221,0.404734574839227,0.930946434084251,
0.434756028940971,0.66373952465131,0.95239097757155
"9875","EPSTI1",91.343329928546,0.148965390751956,0.342758680635034,0.
434607200832859,0.663847567359657,0.95239097757155
"9876","FGF14",14.7594917066522,0.164368173203664,0.377840574766841,0.
435019910990482,0.663547974991925,0.95239097757155
"9877","RORA",101.158182610178,0.109366371914372,0.252174345226312,0.4
33693490177291,0.664511034488052,0.95239097757155
"9878","DECR2",10.6223284478757,0.212664496133156,0.489846148156226,0.
434145490239378,0.664182793553383,0.95239097757155
"9879","ACSM3",1.84072554432888,-0.529571112085567,1.22021615473676,-0
.433997788039296,0.664290047315768,0.95239097757155
"9880","UBFD1",29.7851280635688,0.119935626047542,0.276790186191099,0.
433308809455898,0.664790439033153,0.95239097757155
"9881","SLC7A6OS",28.4262598502516,0.134368205750093,0.308928015122011
,0.434949888559069,0.663598801516476,0.95239097757155
"9882","OSGIN1",4.72144157196403,-0.280762797912821,0.644875676830892,
-0.435375077708888,0.663290197308294,0.95239097757155
"9883","TANC2",22.8126566231062,-0.171653949545762,0.394501114655387,-
0.435116513411397,0.663477857641394,0.95239097757155
"9884","PLEKHJ1",91.6519713770294,0.100466948739112,0.231714481633727,
0.43358079318461,0.664592884692202,0.95239097757155
"9885","COX7A1",199.346277404202,-0.107412479208685,0.247900377381483,
-0.433288889445266,0.664804908771916,0.95239097757155
"9886","ZFP30",6.54366546342512,0.235948474023787,0.544478052123054,0.
43334799833302,0.664761972906152,0.95239097757155
"9887","B3GNT8",11.2780409747094,-0.231350050609568,0.532089118523158,
-0.43479568094099,0.663710740209855,0.95239097757155
"9888","ZSWIM9",9.41414690918323,-0.206771498996978,0.477397331682203,
-0.433122443873697,0.664925818404702,0.95239097757155
"9889","ASH2L",34.2266936013166,0.150189124919754,0.347003234635057,0.
432817651044978,0.665147249046069,0.952420370764722
"9890","ROBO3",59.6597948763579,-0.12523490287751,0.289434824251286,-0
.432687750001991,0.665241630458323,0.952459199928196
"9891","FAM117B",9.8667525692842,-0.185186825138481,0.428127404873046,
-0.43255073847327,0.665341183841538,0.9525054254571
"9892","PRKAR1A",195.20701103533,0.0582662846339065,0.134747150519157,
0.432411998394154,0.665441999211912,0.952553448123805
"9893","PNKD",107.785327446532,-0.101476507330764,0.235092142536256,-0
.431645678311492,0.665998954026499,0.952783862378141
"9894","AC012467.2",1.70240761910783,-0.437295290119612,1.014087467296
08,-0.431220485630888,0.666308059890889,0.952783862378141
"9895","LINC02018",1.80889733272124,-0.464629773029746,1.0778279609804
1,-0.431079717589728,0.666410407697253,0.952783862378141
"9896","GOLGB1",217.29101223122,0.121253659381811,0.281262770488279,0.
431104547435522,0.666392354282986,0.952783862378141
"9897","TMEM33",35.8499725920101,-0.106966185026681,0.24776800620493,-
0.431719117674172,0.66594557094505,0.952783862378141
"9898","PPID",43.8595770141453,-0.1071237699026,0.24804544980391,-0.43
1871538007595,0.66583478198844,0.952783862378141

"9899","CDK5",9.30343696352916,-0.192915373184645,0.446971040572007,-0
.431605978180964,0.666027812760776,0.952783862378141
"9900","PLAA",20.0974488354492,-0.154253522982739,0.357040550676347,-0
.432033623885394,0.665716975489965,0.952783862378141
"9901","COQ7",25.8580707557206,-0.131846229319855,0.305729986525911,-0
.431250564650388,0.66628619123201,0.952783862378141
"9902","YES1",63.8133146287406,0.0914269351837051,0.211756504109968,0.
431755027161886,0.66591946895027,0.952783862378141
"9903","ZMAT5",31.6323838749301,0.139382187694829,0.323129848226353,0.
431350395080776,0.666213612520743,0.952783862378141
"9904","SUN2",59.8763956017858,0.102592552270489,0.237953724069496,0.4
31144974392273,0.666362960853093,0.952783862378141
"9905","TXNDC9",53.4038374002735,0.130851511626598,0.303737000838382,0
.430805306121475,0.666609941090983,0.952972919318357
"9906","C1orf56",31.9235642539588,-0.179481103818188,0.417009966219457
,-0.430400034429234,0.666904670299984,0.953201789789823
"9907","BUB3",77.4262858851455,-0.0834852626279425,0.193931192439313,-
0.430489090371924,0.666839900973909,0.953201789789823
"9908","AC104695.4",11.6326528900417,0.192454655360118,0.4473813670556
91,0.430180310428889,0.667064483565693,0.953237772458393
"9909","PRDM5",23.2518748983179,0.151502794080987,0.352158306366034,0.
430212183958868,0.667041299849921,0.953237772458393
"9910","TMED8",14.6356570372871,0.174860261802334,0.406697866710408,0.
429951263862527,0.667231093554436,0.953379645280607
"9911","EPHX1",567.291352013629,0.119838489420304,0.27885510022964,0.4
29751829253313,0.667376176973256,0.953422812785694
"9912","RHBDF1",40.4134446697866,-0.123488441602708,0.28736645439833,-
0.429724624125874,0.667395968949986,0.953422812785694
"9913","AL050343.2",1.55976683029301,-0.487774422358782,1.141064113510
12,-0.427473282687244,0.669034640981663,0.953469883495222
"9914","CHTOP",54.0855158597983,0.0942367722018138,0.220370328811403,0
.427629130972814,0.668921153602386,0.953469883495222
"9915","SLC25A26",39.4576579213868,-0.131346686090949,0.30727310777369
6,-0.427459099960301,0.669044969096647,0.953469883495222
"9916","CCDC191",13.8920978019078,0.175326261035484,0.408795556081891,
0.428884948544701,0.668006954267089,0.953469883495222
"9917","CHCHD6",23.441964682129,-0.129747977365302,0.302458758724935,-
0.428977417986756,0.667939658596749,0.953469883495222
"9918","USP46-
AS1",5.09082938162866,0.273451150750086,0.637470790078227,0.4289626364
15905,0.667950415870969,0.953469883495222
"9919","IRF2",69.1175302478317,-0.0920530026046035,0.215250688964639,-
0.427654856982715,0.668902420870756,0.953469883495222
"9920","HMGN4",74.166410775665,-0.084854802064611,0.198211205067567,-0
.42810295228105,0.668576167467079,0.953469883495222
"9921","PPP1R3B",22.2793263782893,-0.158464656636802,0.370367879887749
,-0.427857449962534,0.668754907337375,0.953469883495222
"9922","CCDC25",109.607648959661,-0.0973771003935018,0.227785894862716
,-0.427493987071455,0.669019563791842,0.953469883495222
"9923","CHCHD7",28.5549996228994,0.125016887283701,0.291535881490552,0

.428821614150959,0.668053048120314,0.953469883495222
"9924","GDAP1",5.51814398634014,-0.2693961170336,0.627696713378131,-0.
429181978002986,0.667790797242757,0.953469883495222
"9925","ZNF239",3.97061002666441,0.307730932097586,0.71886287834104,0.
428080154601603,0.668592764703662,0.953469883495222
"9926","RNF141",22.2069348836602,-0.139704712318673,0.326284060252908,
-0.428168977088201,0.668528100798194,0.953469883495222
"9927","TMEM216",28.7390870709942,-0.123160973957898,0.287149109927921
,-0.428909474902474,0.667989104675908,0.953469883495222
"9928","MACROD1",27.4632977941744,-0.133444543381772,0.310963053545736
,-0.429133113597192,0.667826355406863,0.953469883495222
"9929","BLOC1S1",268.727212898672,-0.117718653119198,0.275347553691767
,-0.42752750674871,0.668995154629319,0.953469883495222
"9930","RNASE4",357.973781877238,0.0881148639548335,0.205825021853844,
0.428105694639031,0.668574170978121,0.953469883495222
"9931","PSMB10",103.932038544247,-0.113630802529639,0.265552730931572,
-0.427902971025817,0.668721763955393,0.953469883495222
"9932","AC100793.2",4.80401364676284,-0.256582301474187,0.599828187941
856,-0.427759659569482,0.668826109622256,0.953469883495222
"9933","BACE2",78.0649510109616,0.087707771225884,0.204309932818178,0.
429287847223454,0.667713759774434,0.953469883495222
"9934","LINC00899",11.8521091920009,0.220212772908167,0.51491038043732
,0.427672040173549,0.66888990881971,0.953469883495222
"9935","SYAP1",105.173439470872,-0.0764693931940565,0.178453945007468,
-0.428510522369546,0.668279474380709,0.953469883495222
"9936","GLA",34.2833426918581,0.125527512452948,0.292768489159862,0.42
8760324627713,0.668097654938359,0.953469883495222
"9937","H1FX",259.33075973588,-0.0741636973579724,0.173577797806881,-0
.427264882346794,0.669186408129336,0.953494712562604
"9938","GFM1",30.9764707191861,0.11756667708587,0.27517052963515,0.427
250248207003,0.669197065921409,0.953494712562604
"9939","NUDT1",78.0765465165998,-0.1063794377435,0.249040803300926,-0.
427156660007067,0.669265226171925,0.953495885158916
"9940","GEMIN6",32.1685429113551,-0.120117910411584,0.282171416782227,
-0.425691275825744,0.670332819962032,0.953513880915195
"9941","ZFP36L2",1857.88372579059,0.0674731149962599,0.158485077986468
,0.425737967596047,0.670298792765527,0.953513880915195
"9942","TMEM150A",17.8315109648129,0.16765811041899,0.393734406823134,
0.425815238682714,0.670242482005613,0.953513880915195
"9943","FOXP1",283.05183711024,-0.123662200095574,0.29057988050252,-0.
425570414172229,0.67042090250568,0.953513880915195
"9944","AL080276.2",5.00425047781917,-0.247527018362428,0.581674398263
767,-0.425542226202956,0.670441446215896,0.953513880915195
"9945","AUTS2",46.6914995197454,0.12561560318782,0.295183765509317,0.4
25550514172348,0.670435405824665,0.953513880915195
"9946","CD59",679.542859688326,0.0957178021948871,0.224623548775243,0.
426125411680062,0.67001646426762,0.953513880915195
"9947","IGF1",717.681743369318,0.086401399722704,0.203013914047897,0.4
25593487657793,0.670404086472352,0.953513880915195
"9948","SLC24A1",4.30064635433796,0.355608505682419,0.833740934146361,

0.426521586164549,0.669727822168083,0.953513880915195
"9949","PSTPIP1",1.5442001040467,0.475624753919527,1.11650047444214,0.425996015950796,0.670110749083582,0.953513880915195
"9950","DCTPP1",54.792666545326,-0.136543508916756,0.320319439055954,-0.426272939660415,0.669908973642773,0.953513880915195
"9951","AC012146.1",11.2239679896546,-0.172739190998019,0.404562293536547,-0.426977980295669,0.669395366104972,0.953513880915195
"9952","ACAP1",7.73813278842339,0.26132381501521,0.612806134801999,0.426437987765324,0.669788725660186,0.953513880915195
"9953","PEMT",38.8302410844634,-0.115360495044819,0.270213865598435,-0.426922929322421,0.66943546404027,0.953513880915195
"9954","LTN1",58.6613675993939,0.0893323269903935,0.209958718015664,0.425475673668997,0.67048995143168,0.953513880915195
"9955","SELENOO",19.8478372455927,-0.138266275747503,0.324696711884525,-0.42583207863428,0.670230210255453,0.953513880915195
"9956","TAF9B",13.2255316452891,0.157800262388418,0.369825932896075,0.426687931678285,0.669606641825628,0.953513880915195
"9957","SLC25A53",6.12756422191861,-0.252873338535561,0.593849925779835,-0.425820274715858,0.670238812096255,0.953513880915195
"9958","RPP14",22.4471231545688,0.131928661523555,0.310251306102505,0.425231607179655,0.670667845183305,0.953671087346415
"9959","MMP16",10.4753003132617,0.211494557269319,0.497577540573882,0.425048439737436,0.670801363322901,0.953756912511575
"9960","TMEM218",37.003150727914,-0.125510941642684,0.295344875067504,-0.424964007294853,0.670862913037803,0.953756912511575
"9961","BX284668.5",34.7336547386313,-0.141968708208053,0.334673018407919,-0.424201236428966,0.671419059180027,0.953781488562317
"9962","NHEJ1",15.6023464136973,0.156460827794846,0.368703621042477,0.424353922406475,0.671307719435075,0.953781488562317
"9963","PXYLP1",21.5422374102458,-0.177979750665921,0.419509539533246,-0.424256742442483,0.671378582949711,0.953781488562317
"9964","VPS41",33.2172405291823,-0.115111205781933,0.271184545185057,-0.424475538247877,0.671219041424484,0.953781488562317
"9965","ZNF736",12.0602148076039,0.179943360718416,0.423901616018197,0.42449321710227,0.671206151004324,0.953781488562317
"9966","MED22",15.1707916284553,0.156432891524763,0.368608999117566,0.424387065696324,0.671283552055618,0.953781488562317
"9967","LTBR",95.4351233528602,0.0772950591143381,0.181997827651783,0.424703196250381,0.671053053491305,0.953781488562317
"9968","ZNF579",11.4318130672494,-0.178662601226728,0.420850515574963,-0.42452746192467,0.671181181892958,0.953781488562317
"9969","NPHP1",20.6802846583194,-0.156316683214317,0.368778597703377,-0.423876776439311,0.671655681748679,0.953830553962622
"9970","PARP8",12.6133336652172,0.166202188207258,0.392042078108972,0.423939667417691,0.671609813997459,0.953830553962622
"9971","CUX1",62.0324622647125,-0.101782155426378,0.240119744054774,-0.423880825906429,0.671652728347864,0.953830553962622
"9972","AC009951.6",16.8976139736143,0.154033372428622,0.364103152516187,0.423048719474555,0.672259715589665,0.953903584416826
"9973","GASK1A",10.23844945662,0.207612467487474,0.490179627227598,0.4

23543647992282,0.671898659921133,0.953903584416826
"9974","GOLPH3",75.272851757499,0.0798021650752056,0.188655712668951,0
.423004233194045,0.672292172513378,0.953903584416826
"9975","TDP2",28.1795058306484,0.119922519508196,0.283521439708216,0.4
22975135960135,0.672313402011294,0.953903584416826
"9976","PIP4P2",107.243462372057,-0.094124938992394,0.222518498377986,
-0.422998266114965,0.67229652610498,0.953903584416826
"9977","CEMIP2",31.0314089433549,-0.241693508255011,0.570554734242917,
-0.423611432434647,0.671849216333007,0.953903584416826
"9978","MICU2",49.3913335524694,-0.0999386370884672,0.236257479594662,
-0.423007293821674,0.672289939478232,0.953903584416826
"9979","ARHGEF7",41.0131066898584,0.113096402254664,0.267173327195926,
0.423307234452064,0.672071116701059,0.953903584416826
"9980","LRFN5",7.98857397789261,-0.207071702202041,0.489376728023858,-
0.423133529537077,0.672197840368525,0.953903584416826
"9981","ZNF230",7.29598423224687,0.204466473483831,0.483575546037492,0
.422822194296768,0.672424993364819,0.953966326625171
"9982","SCARB1",5.77355770198694,-0.254596654939312,0.602359158028115,
-0.422665865615392,0.672539063457983,0.954032572486981
"9983","RPF1",39.8629110929775,0.127601062527599,0.30205174143235,0.42
2447696949227,0.67269826962189,0.954076691918573
"9984","PHACTR1",17.1857682851864,0.145369565897386,0.344195228077038,
0.422346255959276,0.672772300056988,0.954076691918573
"9985","AC002456.1",2.44777631740634,-0.370382586135391,0.876932707390
274,-0.422361468575666,0.672761197867234,0.954076691918573
"9986","AIMP1",96.1071276808937,0.0763552141470047,0.180873499769641,0
.422147048872554,0.67291768826314,0.954099332950229
"9987","CDC27",73.3156063649136,-0.0786850079120496,0.186395643676175,
-0.422139736531338,0.67292302529477,0.954099332950229
"9988","TMEM9",103.386929290286,-0.0742125901086873,0.175899703589197,
-0.421902871888892,0.673095913730083,0.954153382562616
"9989","SRC",16.3341154273264,0.162331907182002,0.384726893362952,0.42
1940628488525,0.673068353881534,0.954153382562616
"9990","FBXO2",3.1409780883567,-0.315986122930666,0.749172634603494,-0
.421780118941349,0.673185518387807,0.954184878916052
"9991","ITPR2",27.6638663006154,-0.126913107926217,0.301106509819286,-
0.421489087042275,0.67339797837078,0.954390488812956
"9992","PTGFRN",57.1267382282216,0.12445652723149,0.295350377620655,0.
421386044040682,0.67347320838138,0.954392839816706
"9993","AC121247.1",4.06925159745638,-0.294449001627288,0.698951951870
484,-0.421272164473259,0.673556353791468,0.954392839816706
"9994","CDON",103.397733101986,0.0927126307087154,0.22011030061846,0.4
21209868180698,0.673601839062723,0.954392839816706
"9995","EMC3",98.9383004795574,-0.102818181800479,0.244439874225478,-0
.420627698841131,0.674026964253229,0.954517633145887
"9996","CBWD5",13.0927786695655,-0.174929321288477,0.415868469780586,-
0.420636172251217,0.674020775856335,0.954517633145887
"9997","GDE1",52.3516783283723,-0.102813042497006,0.244268409100922,-0
.420901920454755,0.673826702811692,0.954517633145887
"9998","ANKRD27",15.9585154823577,0.139897536492441,0.3325488775996,0.

420682630181307,0.673986846566266,0.954517633145887
"9999","PHF5A",60.2834839037375,-0.0948205775076843,0.225253887024077,
-0.420949794742272,0.673791743043373,0.954517633145887
"10000","GATB",11.8666384689385,0.185247187804524,0.440584290046179,0.
420457996323718,0.674150907963072,0.954592331284216
"10001","CPVL",57.5311048089295,0.149149047251404,0.354881391626443,0.
42027857974701,0.674281956038469,0.954592331284216
"10002","PLXDC1",83.9010772343677,0.111661688998129,0.265630182693532,
0.420365215525819,0.67421867496553,0.954592331284216
"10003","UCHL5",32.9430989993476,-0.107815235218134,0.25690391603861,-
0.419671435455777,0.674725494981405,0.954756872244283
"10004","NOP58",64.0358572890739,-0.0785762744582315,0.187091604550992
,-0.419988243977112,0.67449404179038,0.954756872244283
"10005","P2RX4",21.9001464989535,0.134013424927472,0.319339617766825,0
.419657998793391,0.674735312185631,0.954756872244283
"10006","IGHA2",5.27833474369142,0.399551654881587,0.951865428466983,0
.419756451839081,0.674663380899031,0.954756872244283
"10007","IRF3",60.5485667663904,-0.0945205108081437,0.225223681863991,
-0.419673943813879,0.674723662310414,0.954756872244283
"10008","UNC50",100.007238910468,-0.0883325773225357,0.210567586742606
,-0.419497505238125,0.674852577579434,0.954773818611714
"10009","NDFIP1",487.184339483108,-0.0928612361907279,0.22143309110779
7,-0.419364764887475,0.674949570925371,0.954773818611714
"10010","C10orf55",2.23422046966398,-0.401345314985456,0.9568900550398
47,-0.419426780403464,0.674904255528941,0.954773818611714
"10011","WDR77",12.6826793687188,-0.169494839368721,0.404460342789692,
-0.419064173757211,0.675169232755664,0.954962947704533
"10012","DUSP7",13.4863975769912,-0.173438474047853,0.415121478456323,
-0.417801735272297,0.676092082064683,0.954962947704533
"10013","RNF14",25.2716185447646,-0.129908129367153,0.310758286888795,
-0.418035929685893,0.675920847871903,0.954962947704533
"10014","ZSCAN16-
AS1",54.6844846122925,0.122431289943948,0.293039502562637,0.4177979039
45658,0.676094883526691,0.954962947704533
"10015","CUL7",14.5207566966159,0.174514401670829,0.41760793045122,0.4
17890535465331,0.676027152713463,0.954962947704533
"10016","PODXL",2.80985066941803,0.442742988964187,1.05912956705034,0.
418025332063216,0.675928596095032,0.954962947704533
"10017","MRPL13",72.7137456384781,-0.133531377771907,0.319189709328714
,-0.418344870994544,0.675694987189912,0.954962947704533
"10018","AP004609.3",3.64533446839814,-0.302554419240712,0.72362183164
4133,-0.418111237126831,0.675865789450229,0.954962947704533
"10019","PXN-
AS1",9.2762333414759,0.186363269390078,0.445102462693508,0.41869745735
0186,0.675437254172358,0.954962947704533
"10020","CAMKK2",71.6465292180545,-0.122336993059879,0.292277080069511
,-0.418565126731059,0.675533980540859,0.954962947704533
"10021","AC139530.1",9.24854928710728,0.191891448605568,0.459074245881
432,0.417996544844575,0.675949643421984,0.954962947704533
"10022","KLHL26",7.06005990425285,0.217104641126859,0.518153794014573,

0.418996528896888,0.675218669146359,0.954962947704533
"10023","RABL2B",26.2034889950667,-0.125927041254087,0.300920687527245
,-0.418472529385957,0.675601667268428,0.954962947704533
"10024","SAT1",819.397446709387,-0.142530441623224,0.340514618023147,-
0.41857363554811,0.675527760904698,0.954962947704533
"10025","LAS1L",26.0310282681594,0.114520364316844,0.273952839395919,0
.418029484817051,0.67592555989459,0.954962947704533
"10026","AC009318.1",5.12000528540261,-0.269746120188939,0.64621668572
7205,-0.417423638458649,0.676368567666858,0.955158962617205
"10027","JUP",24.9813960702572,0.183106175662051,0.438608114107053,0.4
17471017459014,0.676333919091426,0.955158962617205
"10028","NIT1",38.3732413156282,0.107963443247592,0.258744765130051,0.
417258463928059,0.676489366248726,0.955234286605701
"10029","PDK2",30.4943299922066,-0.112504935746048,0.269764630794626,-
0.417048504152123,0.676642930046357,0.955355856960456
"10030","SDR42E2",6.18074891469228,0.235752357949346,0.565549932438849
,0.41685507225277,0.676784417317778,0.955460353860393
"10031","AKAP1",16.9576925680489,0.168328607641473,0.403984627822098,0
.416670823711638,0.67691919795966,0.955555362686551
"10032","BCL2L11",18.0365385458428,-0.164744227259677,0.39625383843462
1,-0.415754274862018,0.677589821234804,0.955789112279105
"10033","SLC19A3",3.14699838520518,0.453717260928985,1.09164635193803,
0.415626599331816,0.677683259551333,0.955789112279105
"10034","WIPI2",94.3981652507556,-0.0793778724857916,0.190973737943463
,-0.415648106072528,0.677667519667693,0.955789112279105
"10035","GUSB",111.396735442576,-0.0902899846459042,0.216925821827919,
-0.416225158835763,0.677245251449115,0.955789112279105
"10036","GCC1",8.16329657123712,0.222995027407588,0.536995782164102,0.
415264020340932,0.67794863711925,0.955789112279105
"10037","PRKG1",40.2860420304577,0.141086005471172,0.33931406895464,0.
415797688276913,0.677558050603913,0.955789112279105
"10038","IKZF5",18.2463637269258,0.152394740085541,0.366039311899902,0
.41633435298l232,0.677165358174372,0.955789112279105
"10039","TMEM41B",26.5801990399944,-0.129299484579442,0.31147064193742
7,-0.415125752382845,0.678049848253959,0.955789112279105
"10040","PATL1",17.7430127037073,-0.139021003362592,0.335015591000769,
-0.414968756968307,0.67816477479295,0.955789112279105
"10041","AP000821.2",2.95197637018117,-0.35173425400889,0.845800825217
739,-0.415859435840987,0.677512863737558,0.955789112279105
"10042","KCNA1",12.0301039623673,-0.227610481573928,0.547935453792544,
-0.415396521613118,0.677851652609103,0.955789112279105
"10043","GPR162",2.23633075423313,-0.395205110029621,0.951066807887012
,-0.415538747385842,0.677747556175809,0.955789112279105
"10044","FAM161B",9.72410782595873,0.212285638425353,0.510560241949338
,0.415789599313176,0.677563970193389,0.955789112279105
"10045","LINC00663",3.35517785981379,0.304440171434131,0.7331306640643
74,0.415260452681596,0.677951248546778,0.955789112279105
"10046","NFKBIB",20.3271866373747,-0.132619794368099,0.319507309424605
,-0.415075932400207,0.678086317540946,0.955789112279105
"10047","SLC25A14",6.05424071987662,-0.220419216041749,0.5310672721480

86,-0.415049519339022,0.678105652769462,0.955789112279105
"10048","SUSD4",2.43676898218308,0.488204098247678,1.17991742154559,0.413761242382683,0.679048970905845,0.956199675563442
"10049","LINC01118",1.59568059111124,-0.545852265052818,1.31792449749238,-0.414175672499762,0.678745456806819,0.956199675563442
"10050","SMPD4",22.8961924987235,0.129955746548834,0.313995928390346,0.413877171003565,0.678964063599873,0.956199675563442
"10051","CASTOR2",14.8830705939882,0.189904927669852,0.458994764719793,0.413740944922946,0.679063837391665,0.956199675563442
"10052","KIN",48.0882320064867,0.116328392195859,0.28079731991305,0.414278855054174,0.67866989761654,0.956199675563442
"10053","INCENP",3.68487180643135,0.306024488625569,0.739328368500221,0.41392228631286,0.678931021787677,0.956199675563442
"10054","CSNK2A2",79.6948625101117,-0.0929436413303652,0.224512006675841,-0.413980716249891,0.678888229448156,0.956199675563442
"10055","PHLPP2",12.1864480342933,0.18175693492563,0.438825367108308,0.414189672131625,0.67873520487623,0.956199675563442
"10056","RAB3A",2.54887436224023,-0.343591102427764,0.830084253517729,-0.413923166198726,0.678930377377891,0.956199675563442
"10057","AC093702.1",2.2432349822921,0.393527728318949,0.952022960327126,0.413359493119503,0.679343247677146,0.956397382157233
"10058","TPRG1",8.21102824742232,0.223138925292812,0.539794834626719,0.413377288886282,0.679330211445355,0.956397382157233
"10059","HGH1",10.1142158696848,-0.178328513381479,0.431503307589034,-0.413272645296429,0.679406869146865,0.956397382157233
"10060","GATD1",20.6557452837567,0.126883959023161,0.307130068682298,0.413127765599572,0.679513007680608,0.956451708623997
"10061","ADIPOR1",37.3221459677082,-0.104826647709612,0.25391881938181,-0.412835283201231,0.679727298971723,0.956537864216542
"10062","SATB2",5.79407937842747,-0.25296069128766,0.61383367312847,-0.412099730531917,0.68026632618292,0.956537864216542
"10063","ATIC",41.7225847659535,-0.0979029419467074,0.237485293156684,-0.412248441347122,0.680157334836269,0.956537864216542
"10064","CD200R1",1.94586646823091,-0.394014145319111,0.955705936346311,-0.412275502677568,0.680137502089107,0.956537864216542
"10065","DOCK8",5.67277514804598,-0.301068096218127,0.73069425140252,-0.412030196816584,0.680317290291299,0.956537864216542
"10066","AL352979.4",1.58123998774718,-0.489361807439936,1.18537825632502,-0.41283177317347,0.679729870799474,0.956537864216542
"10067","FBRS",28.5416838478692,0.129828919363047,0.314863984962271,0.412333342533933,0.680095113073054,0.956537864216542
"10068","WIZ",29.0726811731519,-0.108514520357262,0.26290893730707,-0.412745650523549,0.679792974778644,0.956537864216542
"10069","ZNF865",12.7203548552334,-0.176621258404548,0.428342410354803,-0.412336612333692,0.680092716769587,0.956537864216542
"10070","A1BG-AS1",2.8012423183177,-0.352776563408841,0.855848800958691,-0.412194961322225,0.680196530005273,0.956537864216542
"10071","COX4I2",7.69688736160589,-0.351604045095639,0.852155019471287,-0.412605731424066,0.679895501394163,0.956537864216542

"10072","ELMO1",9.02311307510627,0.26582424840224,0.645935831464575,0.
411533523073829,0.680681365275167,0.956670736034913
"10073","RNF41",33.6698047251853,-0.103355953982974,0.251261413443303,
-0.411348294855853,0.680817161512981,0.956670736034913
"10074","INAFM2",2.5054389110858,0.348555054046343,0.847279399453181,0
.411381480856604,0.680792831121803,0.956670736034913
"10075","PMM2",29.556439697591,-0.119591323244403,0.290629021533893,-0
.411491332191187,0.680712295738134,0.956670736034913
"10076","MYL12B",869.855432223209,-0.108380477982743,0.263427062853944
,-0.411424994867875,0.680760929229762,0.956670736034913
"10077","ZNF529-
AS1",3.7290899464606,-0.280378905194619,0.681129204883202,-0.411638354
638894,0.68060451476762,0.956670736034913
"10078","MARS2",1.75152711995612,0.423367554456601,1.03036236311157,0.
410891905230392,0.681151798277418,0.956677141489681
"10079","GNPDA1",40.1064426508758,0.124550887908483,0.303069543972723,
0.410964712177391,0.681098410118078,0.956677141489681
"10080","BRAT1",26.1816597094083,0.111398650252706,0.2711820133643,0.4
10789229236438,0.681227091641275,0.956677141489681
"10081","DDX51",17.7175148495523,0.160025766961023,0.389536730594508,0
.410810468930087,0.68121151609462,0.956677141489681
"10082","MPI",15.8781093949521,0.136704328410598,0.332654507470919,0.4
10949875442615,0.681109289526681,0.956677141489681
"10083","RFFL",12.4425596417112,0.163353148668598,0.39741829171795,0.4
11035808046124,0.681046278206352,0.956677141489681
"10084","GNG2",41.2065992352137,0.10068219434193,0.245259977557329,0.4
10512124092469,0.681430311546736,0.956677891285126
"10085","DZANK1",3.5236025656152,0.329796474752615,0.803061359094975,0
.41067406745144,0.681311544742838,0.956677891285126
"10086","MAGEE1",5.73450651437424,-0.224405118086245,0.546638666190371
,-0.410518194130259,0.681425859730614,0.956677891285126
"10087","NDUFAF7",16.9892365125032,-0.150417901042122,0.36671626650448
2,-0.410175153875492,0.681677465699426,0.95683514217921
"10088","NISCH",38.7298608811341,0.109597578351633,0.267144587010784,0
.41025565809877,0.681618415970408,0.95683514217921
"10089","IARS2",77.4512805669607,0.0936144641434936,0.228729324659213,
0.409280551512016,0.68233378636954,0.956975596174734
"10090","D2HGDH",26.3095619635196,0.148471897121836,0.362654651571487,
0.409402985673739,0.682243948936697,0.956975596174734
"10091","ORC3",27.3265933971365,0.124314294859296,0.30365761943383,0.4
09389677397457,0.682253713814864,0.956975596174734
"10092","TMEM248",117.669240499551,0.071496833110983,0.174665678435786
,0.409335329935859,0.682293591533004,0.956975596174734
"10093","OAF",429.576854148603,0.141866417640629,0.346137492702349,0.4
09855680565131,0.681911818120966,0.956975596174734
"10094","PIBF1",41.4404931641409,-0.114134681107998,0.278914896408677,
-0.409209700082721,0.682385776453128,0.956975596174734
"10095","ZNF223",3.90438856937642,-0.281425858386171,0.686852459704201
,-0.409732620754346,0.682002097902096,0.956975596174734
"10096","ARFGAP1",21.8267151008291,-0.141532917278816,0.34566874673221

8,-0.40944667001804,0.682211896151334,0.956975596174734
"10097","PICK1",5.77578880683799,-0.222103925063837,0.542705102544901,
-0.409253430679622,0.682353687192148,0.956975596174734
"10098","KDM4A-
AS1",3.30138281567085,-0.297278519449381,0.729205121690051,-0.40767475
5164075,0.683512473669619,0.957094671243766
"10099","AL359258.2",3.00627548928257,0.356034354478592,0.874342238441
928,0.407202510441498,0.683859257927408,0.957094671243766
"10100","PI4KB",44.6868650971476,-0.113154200757362,0.278833384026728,
-0.405812959421372,0.684880035842813,0.957094671243766
"10101","MEF2D",44.7540474128632,0.11710137828105,0.286897614899021,0.
408164349230531,0.68315301972103,0.957094671243766
"10102","XPR1",14.9094574989321,-0.161148351796656,0.395923134295711,-
0.407019286921223,0.68399382272292,0.957094671243766
"10103","ODR4",47.4894515775649,0.0962757146176246,0.23704621611487,0.
406147443294224,0.684634268003617,0.957094671243766
"10104","CENPO",2.38518353708149,0.365726884219436,0.902110581167335,0
.405412475869847,0.685174341977264,0.957094671243766
"10105","TGOLN2",217.660774157356,0.0672798028871005,0.165901923711845
,0.405539618720508,0.685080902446524,0.957094671243766
"10106","AC106897.1",3.84514008209326,0.311351788652632,0.763376476846
611,0.407861387003668,0.683375442399299,0.957094671243766
"10107","OTULINL",5.63899118407942,0.254525216806893,0.624191575350078
,0.407767786138642,0.683444165947132,0.957094671243766
"10108","FOXD1",5.43882934129482,-0.37321002641415,0.915910972338752,-
0.407474129784873,0.6836597910879,0.957094671243766
"10109","SLC36A1",9.26329418083435,0.178204669631495,0.438719686173052
,0.406192553577827,0.68460112499305,0.957094671243766
"10110","LINC00847",10.535533343483,-0.206284863557348,0.5076590688163
24,-0.406345274276945,0.684488923961318,0.957094671243766
"10111","HCG18",43.662503790266,0.124297136918479,0.3062586413279,0.40
585675029296,0.684847857840311,0.957094671243766
"10112","LRRC1",1.82593991678403,0.447573782447828,1.09533859313363,0.
40861682885415,0.682820878602278,0.957094671243766
"10113","ADAP1",9.95264563743295,0.253847720502993,0.624285446102687,0
.406621237268502,0.684286196789426,0.957094671243766
"10114","AC002467.1",4.40421967243561,-0.320764375344455,0.78817035390
422,-0.40697340842058,0.684027518827701,0.957094671243766
"10115","IMPAD1",85.5318863729805,-0.0773625427724895,0.19077895837145
8,-0.405508780595499,0.685103565487767,0.957094671243766
"10116","GNE",18.9405535681665,0.191390346462427,0.468787428106243,0.4
08266807059193,0.68307780552771,0.957094671243766
"10117","FAM120A",132.465499849477,0.0788247300191119,0.19387873641910
3,0.406567174281136,0.684325910600878,0.957094671243766
"10118","NIPSNAP3B",3.25535697161226,-0.373914200572574,0.915862035414
498,-0.408264767087271,0.683079303038438,0.957094671243766
"10119","FAM24B",6.62933006004191,0.230452801219679,0.566553555661838,
0.406762606847411,0.684182353069583,0.957094671243766
"10120","DTX4",15.6614561588981,0.144366253862752,0.353933742506206,0.
407890620545231,0.683353979110804,0.957094671243766

"10121","LAMTOR1",225.817410974363,-0.0959998325512001,0.236767429318873,-0.405460467376659,0.685139071603731,0.957094671243766
"10122","THY1",1626.88757907251,-0.141871262654862,0.349174402847272,-0.406304876583168,0.684518602711643,0.957094671243766
"10123","MRPL51",261.106869435998,-0.113806072262066,0.278679694201439,-0.40837590477548,0.682997720457892,0.957094671243766
"10124","ALKBH2",38.1794616202569,0.123403843504914,0.304035416865085,0.405886408818201,0.684826064762427,0.957094671243766
"10125","OAS2",10.9368930253568,0.233927933739046,0.574058664905899,0.407498306427256,0.683642037757711,0.957094671243766
"10126","ACOT4",4.0828573783222,0.291887749688483,0.715358081197201,0.408030268142059,0.683251453270399,0.957094671243766
"10127","FOXN3-AS1",6.48924424820543,0.247815676652248,0.609593217767539,0.406526302178693,0.684355935177307,0.957094671243766
"10128","ARHGAP28",25.5862996382206,0.17160494267409,0.423163342672681,0.405528847537314,0.685088818192882,0.957094671243766
"10129","AC027575.2",2.03774371646438,0.501531296213232,1.22683422253437,0.408801194979039,0.682685562883221,0.957094671243766
"10130","CACTIN",12.6958685228795,-0.158669401587711,0.388937366600982,-0.407956178071449,0.683305847655615,0.957094671243766
"10131","ABHD8",17.3479614345103,-0.179056712920565,0.441334434552599,-0.405716615115434,0.684950832685945,0.957094671243766
"10132","GRIN2D",4.78612179898599,-0.250847692641217,0.615470420378239,-0.407570671693787,0.683588899725598,0.957094671243766
"10133","LENG1",29.9388435234656,-0.114470348510504,0.281041159472133,-0.407308127839738,0.683781693934294,0.957094671243766
"10134","DDX27",74.5849128079038,0.0998811187618748,0.245365184940388,0.407071275356938,0.683955639851589,0.957094671243766
"10135","U2AF1L5",2.07132039495823,0.547573708049247,1.34956077576624,0.405742162844315,0.684932059137109,0.957094671243766
"10136","CABIN1",53.1468071787575,-0.0858190459577813,0.2113963690202,-0.405962724693635,0.684769989079854,0.957094671243766
"10137","MPST",64.6271277228304,-0.0995792395902082,0.243827219682236,-0.408400832851981,0.682979422071951,0.957094671243766
"10138","PCBP4",20.5240848986784,0.136082272522028,0.336081004914244,0.404909145510177,0.685544295628349,0.957419868467036
"10139","ADAMTS6",8.56065076915557,0.3041301001836,0.751260550474645,0.404826394772694,0.685605125599731,0.957419868467036
"10140","LNX2",6.03821780764743,0.232330679194071,0.573911386031852,0.404819776795954,0.685609990554784,0.957419868467036
"10141","EMC4",131.912232663912,-0.0855735202330653,0.211522167943448,-0.404560529352857,0.685800576751682,0.957591575466306
"10142","IQGAP1",228.34492384591,-0.0731588796271304,0.180915508573183,-0.404381471793704,0.685932222891604,0.957680958010758
"10143","NBPF26",24.1673784840349,0.156699316953633,0.388313724833554,0.403537930627613,0.686552536771071,0.957697164878177
"10144","RNF103",44.027714947437,0.103417163308008,0.255875038672291,0.404170582033439,0.686087284827973,0.957697164878177
"10145","COA5",63.191741912392,0.105062433873057,0.260311532876062,0.4

03602686028813,0.686504910178932,0.957697164878177
"10146","LINC00607",1.69438355227662,0.439058492756389,1.0872264390434
6,0.403833531810236,0.686335136820063,0.957697164878177
"10147","QDPR",53.1946555030723,-0.0903616572931912,0.223726796498867,
-0.403892867136497,0.686291501768818,0.957697164878177
"10148","CREBZF",66.4978373253985,0.0915589137517639,0.226657771737116
,0.403952236228443,0.686247842932972,0.957697164878177
"10149","C5AR2",5.11848722290072,-0.364956589367383,0.904242718285598,
-0.403604676031369,0.686503446582663,0.957697164878177
"10150","ZDHHC8",27.9485258827017,-0.12944818791069,0.320670735527869,
-0.403679455493811,0.686448449041972,0.957697164878177
"10151","GCAT",9.09228751438361,-0.177719515548644,0.440226264152551,-
0.40370039232157,0.686433051073673,0.957697164878177
"10152","PSEN2",9.17016048839196,-0.193267431124922,0.480303182227761,
-0.402386322381837,0.687399734473531,0.957746751957611
"10153","SUCLG1",117.972355648197,-0.100943174972491,0.250522908948298
,-0.40292991725289,0.686999781848211,0.957746751957611
"10154","SEPTIN10",142.977455371396,0.0612514239620369,0.1520824422689
03,0.402751448807849,0.687131081239517,0.957746751957611
"10155","PTPN23",32.2709092990999,0.103928685779967,0.258256549654949,
0.402424201511341,0.687371861878822,0.957746751957611
"10156","HNRNPA2B1",670.76015368257,0.0570053131382759,0.1414840772251
56,0.40290974261053,0.687014623877007,0.957746751957611
"10157","MRPL21",96.395988518342,-0.102853917968874,0.255069268109326,
-0.403239162174526,0.686772292444427,0.957746751957611
"10158","CTSC",114.121064290147,0.164226320538515,0.407814623816128,0.
402698458926671,0.687170067758809,0.957746751957611
"10159","AC002070.1",1.99400381526511,0.425062166184701,1.055665723958
27,0.402648448782548,0.687206862743833,0.957746751957611
"10160","SMAD3",84.9766775365568,0.10375082271078,0.257276279124714,0.
403266181646255,0.686752417495917,0.957746751957611
"10161","AC018529.1",1.67895167253476,0.417350640814982,1.036954149551
1,0.402477429687371,0.687332695715303,0.957746751957611
"10162","SNAI1",4.3137895682601,0.270494785857773,0.671461430548958,0.
40284485981068,0.687062357505553,0.957746751957611
"10163","LINC01436",10.669581363551,-0.498212944502071,1.2376407967498
3,-0.402550518543367,0.687278917105335,0.957746751957611
"10164","TM4SF1",3.78269460946547,-0.374015508844091,0.930012078127921
,-0.402161990838839,0.687564813029488,0.957882502213454
"10165","SNCAIP",16.864095512773,-0.132721564975489,0.330235623086625,
-0.401899600457926,0.687757916789855,0.958057265297033
"10166","PRKAR2B",9.69264399236838,-0.185077064341505,0.46103204813418
6,-0.401440778554374,0.688095631435246,0.958074073839373
"10167","ZC3H3",9.11210899554883,-0.182759126107033,0.455079831557044,
-0.401597947071677,0.687979940956818,0.958074073839373
"10168","MFSD14C",29.7695200982569,0.113707070897502,0.283259565311422
,0.401423587487645,0.688108286083787,0.958074073839373
"10169","APBB1",17.6204646436918,0.129997261887705,0.323738575349329,0
.401550114154398,0.688015149608708,0.958074073839373
"10170","HEATR3",7.04788226405621,-0.231604109436939,0.576944160120021

,-0.401432452992952,0.688101760017485,0.958074073839373
"10171","PKN2",88.7778544067763,0.0805078998141326,0.200634155260229,0.401267170635584,0.688223431309265,0.958117403697663
"10172","JMJD4",17.2713099367609,-0.149248971701625,0.372008743963023,-0.401197482918467,0.688274733786203,0.958117403697663
"10173","AL136419.3",3.36200164269185,-0.337817690561385,0.842375525767855,-0.401029802300408,0.688398182235742,0.958195051652228
"10174","ARAP2",3.26516581444401,-0.371677178010945,0.927292028588191,-0.40081998610171,0.68855266308932,0.958315874714446
"10175","TRIM62",1.79871298883004,-0.408824801785348,1.020500460323,-0.400612069940615,0.688705757823139,0.958368215103706
"10176","CDC7",3.7626554856807,0.275782690016186,0.692594265268628,0.398187949057334,0.690491648365449,0.958368215103706
"10177","AL355488.1",2.09979413782549,0.427277002116935,1.06817077762881,0.400008136400653,0.689150524002283,0.958368215103706
"10178","TTC13",4.79534397253763,-0.237860406652033,0.596414233478667,-0.398817454883128,0.690027714718467,0.958368215103706
"10179","AL445524.1",7.30018280993999,0.226992256813515,0.567585346183622,0.399926210815315,0.689210866288174,0.958368215103706
"10180","RBM45",9.26819867884862,-0.187717422299999,0.471527803622183,-0.398104673484768,0.690553029569429,0.958368215103706
"10181","SLCO2A1",3.40926222053579,-0.45157811619469,1.13372192929362,-0.398314705331712,0.690398222080392,0.958368215103706
"10182","ZIC1",11.0441296752562,-0.263870179560443,0.65948756068406,-0.400113960127984,0.689072582467938,0.958368215103706
"10183","HULC",3.89601593585105,-0.292967822954664,0.734164279304664,-0.399049410619838,0.689856797170087,0.958368215103706
"10184","PDSS2",17.2928700358945,-0.149332932313451,0.373135779711732,-0.400210701929519,0.68900133286181,0.958368215103706
"10185","HSPB1",513.935135731498,-0.0906592552917374,0.226734107614679,-0.399848334445594,0.689268227957437,0.958368215103706
"10186","DUS4L",7.33241266577234,0.225037079019414,0.562416315028394,0.400125446232926,0.689064122894106,0.958368215103706
"10187","ZNF251",13.2474044639679,-0.151284397748369,0.378930299756784,-0.399240699003142,0.689715857432183,0.958368215103706
"10188","PFKFB3",25.9763323974978,-0.208169779346674,0.520539786112351,-0.399911370658886,0.689221797016183,0.958368215103706
"10189","DNMBP",36.5004627890514,0.102552144101669,0.257136449114314,0.398823832462888,0.69002301516089,0.958368215103706
"10190","RAB3IL1",70.7806896217029,0.0939459358883906,0.235750469594769,0.398497343610276,0.690263616006338,0.958368215103706
"10191","NRIP2",2.64883055804038,-0.412411858028648,1.03344977118426,-0.399063282539656,0.689846576089586,0.958368215103706
"10192","RNF6",27.9172162279632,-0.115175325035373,0.289214717717287,-0.398234660892877,0.690457218647136,0.958368215103706
"10193","LLGL1",8.74937178335546,0.226115844070064,0.566739522585537,0.398976663985767,0.689910399127328,0.958368215103706
"10194","C1QTNF1",34.5385792989981,-0.1564745077766,0.391973840841927,-0.39919630208104,0.689748567767304,0.958368215103706
"10195","SETBP1",116.102050989149,-0.109139166229272,0.273306961869968

,-0.399328160111772,0.689651420378139,0.958368215103706
"10196","TMX3",74.0286956799483,0.0845634836745053,0.211642404717873,0.399558320022074,0.689481860602378,0.958368215103706
"10197","SLC35E1",48.3212567507879,0.102424437900273,0.256398118839857,0.399474217532174,0.689543817453651,0.958368215103706
"10198","ZNF850",3.44390241452722,0.291995569859483,0.730616968819448,0.399656156811274,0.689409788549826,0.958368215103706
"10199","ZNF649",8.60695244519964,0.212632311280299,0.533367211510745,0.3986602601199,0.69014355332217,0.958368215103706
"10200","PMEPA1",30.6604274712045,-0.125787977288614,0.315712068979265,-0.398426254958517,0.690316007825639,0.958368215103706
"10201","LIF",4.79433181699736,0.301736523409078,0.755503996126503,0.399384417496258,0.689609973886519,0.958368215103706
"10202","SAMM50",54.9742059306951,-0.0831839376974428,0.208780196536115,-0.398428294817002,0.690314504444091,0.958368215103706
"10203","HDAC6",15.5836984965621,0.149038205402046,0.374348459432735,0.398126936672557,0.690536619500708,0.958368215103706
"10204","DLST",42.4327236945931,-0.0955506813263252,0.240197014612938,-0.397801286083005,0.690776669185538,0.958493655404738
"10205","F8A3",2.89486122626436,0.357550678048511,0.898823819522139,0.397798400846346,0.690778796144446,0.958493655404738
"10206","APOL2",53.3491471683701,-0.0962770169007062,0.242113114966134,-0.3976530429348,0.690885955263973,0.958548415298634
"10207","NFE2L2",301.08303051334,-0.0732964999675937,0.18445607846847,-0.397365598228971,0.691097880240699,0.958654583092506
"10208","LINC00519",7.85806931865104,0.282694276921806,0.711363761964605,0.397397635410998,0.691074258919632,0.958654583092506
"10209","MBD1",26.5864748600139,0.127891143510309,0.32201259770642 8,0.397161926027826,0.691248056736621,0.958768976725493
"10210","VRK2",20.4755843403474,-0.139500670167588,0.351496468656543,-0.396876448576493,0.691458572297406,0.958873115633265
"10211","OLFML2A",75.0382048344972,0.139677137796151,0.351866462861454,0.396960644274725,0.691396482585159,0.958873115633265
"10212","GCLM",29.8515319616042,-0.121111925592492,0.307739412528929,-0.393553508785968,0.693910711260042,0.958905394731979
"10213","PTBP2",20.5984938893867,0.125097250213282,0.319346248063687,0.391729199800507,0.6952583125513,0.958905394731979
"10214","PMF1",79.0004835796352,-0.0992464434890752,0.251562008030305,-0.394520795354437,0.69319657714898,0.958905394731979
"10215","RRNAD1",20.4235348325075,0.149180299990147,0.37967791916239,0.392912762267698,0.694383915179453,0.958905394731979
"10216","PIGC",54.3244487611936,-0.107740341155961,0.274339991899561,-0.392725611785415,0.694522151985531,0.958905394731979
"10217","PPP3R1",31.0373405457881,0.115412976625818,0.292615837226657,0.394418079758343,0.693272397712659,0.958905394731979
"10218","SLC20A1",72.980511761002,-0.14679818512076,0.37520338432404,-0.391249629544865,0.69561272702591,0.958905394731979
"10219","MLPH",1.82293302107614,0.504112186310144,1.27990397775489,0.393867192439248,0.693679093198581,0.958905394731979
"10220","LINC01985",3.32436437436561,-0.290744677408366,0.741206608922

352,-0.392258614411281,0.694867139161439,0.958905394731979
"10221","CISH",10.0473955680717,-0.278900921719501,0.705540822975428,-0.395300899164575,0.692620835900682,0.958905394731979
"10222","CTBP1",74.0270283157167,-0.0947940888657099,0.239776525494314,-0.395343491904749,0.692589406221341,0.958905394731979
"10223","MRFAP1",398.544579826259,0.0758073761297324,0.19299017675622,0.392804325090029,0.694464009962936,0.958905394731979
"10224","CLOCK",44.6585221195673,0.0958201657189136,0.243198555109186,0.393999732752912,0.693581236542633,0.958905394731979
"10225","UBE2D3-AS1",9.05490953610329,-0.185145183568584,0.471214927808899,-0.392910267994883,0.694385757481952,0.958905394731979
"10226","CASP6",18.6348482977487,0.136040949133831,0.34321898402873,0.396367786935828,0.691833726492553,0.958905394731979
"10227","LINC02511",83.1323737525313,-0.0840179986979392,0.214321394658234,-0.392018719511964,0.695044382429747,0.958905394731979
"10228","PDE4D",8.63196676720652,0.205072125251828,0.52175936680153 8,0.393039662151061,0.694290187649961,0.958905394731979
"10229","SERINC5",22.8051850697002,0.160286811947262,0.409639062138594,0.391287908702982,0.695584435322497,0.958905394731979
"10230","GRPEL2",7.72250159297476,-0.199203535220854,0.505341660657051,-0.394195750577633,0.693436522781744,0.958905394731979
"10231","PPT2",7.79485569870912,-0.205460845125848,0.520544249712591,-0.394703899311709,0.693061424718259,0.958905394731979
"10232","RXRB",30.386043224233,-0.101889453451967,0.257328447411838,-0.395950989782716,0.69214118408618,0.958905394731979
"10233","SEC63",192.158053832594,0.062211734776554,0.15682112629557 1,0.396705062934566,0.691584966223521,0.958905394731979
"10234","OSGIN2",25.3694335780202,0.127505463915092,0.324637127658851,0.392763036177066,0.69449450802522 4,0.958905394731979
"10235","MYC",514.939895299329,-0.143211352129766,0.363738059537735,-0.393721108843461,0.693786954996827,0.958905394731979
"10236","ZNF696",6.36409096414773,0.210643900367213,0.537464142756496,0.391921774142703,0.695116014001724,0.958905394731979
"10237","SPIN1",68.4226758126375,-0.0822564935570337,0.20925211497591,-0.39309754917652,0.694247434175886,0.958905394731979
"10238","AL158152.1",19.3756961843024,0.135688294898636,0.346162104840013,0.391979055481383,0.695073689295942,0.958905394731979
"10239","FAM78A",2.7895567980584,0.425679830252009,1.08519453841681,0.392261309085679,0.69486514833054,0.958905394731979
"10240","AGAP4",5.42240249279282,0.237073101828903,0.601093609032432,0.394402965306044,0.693283554858879,0.958905394731979
"10241","AHNAK",1718.72477180055,0.0660015144365821,0.166719231643118,0.395884228748524,0.692190436228246,0.958905394731979
"10242","TMEM134",50.8844290485746,-0.107508733853734,0.27432039299089,-0.391909375316858,0.695125175516346,0.958905394731979
"10243","INPPL1",23.9680877586801,0.122807464537543,0.312132489859742,0.393446592479774,0.693989662768365,0.958905394731979
"10244","SNX19",22.0664992716959,0.134655262991111,0.342423640282499,0.393241725016475,0.694140954819428,0.958905394731979

"10245","MMP17",9.08593951643497,0.1834453149267,0.468785011622881,0.391320776855968,0.695560143171412,0.958905394731979
"10246","NBEA",7.12385314883959,0.248804256401978,0.634465325372754,0.392147918021845,0.694948923696625,0.958905394731979
"10247","UFM1",120.140292046446,-0.0668629134867673,0.168922946897099,-0.395819009287694,0.692238552350283,0.958905394731979
"10248","ELF1",122.425618496697,-0.0725842100849933,0.185367224204928,-0.391569817136332,0.69537609296399,0.958905394731979
"10249","MBIP",53.410520801644,0.109619631964384,0.276741967109149,0.396107728471663,0.69202557138912,0.958905394731979
"10250","FAN1",14.3736265866602,0.161730144595716,0.41111290509144,0.393395932340639,0.694027073510777,0.958905394731979
"10251","RPS27L",429.615082535023,0.0960273502560749,0.245058821596224,0.391854288821711,0.695165879564512,0.958905394731979
"10252","CIB1",176.230004820732,-0.104957465668559,0.267205380623857,-0.392796976705747,0.694469437812003,0.958905394731979
"10253","CRTC3",45.449598643997,0.101674511654209,0.259468295946827,0.391857168072067,0.695163752031372,0.958905394731979
"10254","RASD1",295.829539413559,-0.130664438856796,0.332557420272184,-0.392907903693302,0.694387503787667,0.958905394731979
"10255","ZNF830",33.3176635264557,-0.141953737079638,0.358364543299244,-0.396115463245209,0.692019851342022,0.958905394731979
"10256","NDUFAF8",131.412797095901,-0.0852283644939008,0.217148308521496,-0.392489193557147,0.69469679443699,0.958905394731979
"10257","CEP192",19.4857791101053,0.142999039850101,0.361611792260645,0.395449050364566,0.69251151568488,0.958905394731979
"10258","REX1BD",231.689054540571,-0.101157578875165,0.258400238988469,-0.39147633636546,0.695445176693718,0.958905394731979
"10259","AC008736.1",2.85253643214873,0.324996860474177,0.824185725730469,0.394324786668848,0.693341265619137,0.958905394731979
"10260","NUDT19",12.0882221478749,0.160839501067103,0.408709866594475,0.39352977310599,0.69392823840003,0.958905394731979
"10261","ZNF780B",9.4829034220196,0.169934055854242,0.430120113727212,0.395085117925957,0.692780071521782,0.958905394731979
"10262","CALM3",249.782937877156,0.0834370156816496,0.211009485379458,0.395418317482767,0.692534192836886,0.958905394731979
"10263","ZSCAN18",53.3706577320604,0.0981703045637718,0.248113848171143,0.39566636561155,0.692351171165588,0.958905394731979
"10264","ZNF8",7.64527953845662,0.219270274924063,0.55779813730724,0.393099690118339,0.694245852964553,0.958905394731979
"10265","BTBD3",9.01721466882644,0.198622665437678,0.506774139484353,0.391935282332635,0.695106032826664,0.958905394731979
"10266","PLAGL2",10.5789152823935,0.166931084964114,0.423169739534614,0.394477840376061,0.693228284430139,0.958905394731979
"10267","B4GALT5",50.1882192527551,-0.119271110860689,0.304670938235785,-0.391475181555993,0.695446030131514,0.958905394731979
"10268","RAB22A",94.726616004667,-0.06903511627359,0.175108587809894,-0.394241751001601,0.693402563743232,0.958905394731979
"10269","MIAT",2.39181496140869,0.327471480503796,0.834540071622745,0.392397551224874,0.694764495105498,0.958905394731979

"10270","CERK",25.048888238047,0.138773558384174,0.350752117187657,0.3
9564567563231,0.692366436526856,0.958905394731979
"10271","STS",20.0134933449015,0.133028226199271,0.338476805661472,0.3
93020212830535,0.694304552503785,0.958905394731979
"10272","SLC6A8",12.8616034062929,0.150180778709101,0.383369136518162,
0.391739356154421,0.695250807470088,0.958905394731979
"10273","AVPR1A",2.08139896881837,-0.403671011542623,1.03226095413497,
-0.391055197743964,0.695756435965926,0.95891679319423
"10274","METTL3",22.745388871417,-0.140356452697821,0.358841526601437,
-0.391137709247665,0.695695448513104,0.95891679319423
"10275","LINC01915",11.6305256630043,-0.205433614510765,0.525460459740
649,-0.390959225765837,0.69582737505309,0.958921229270244
"10276","ORMDL2",27.7151500338241,-0.128572152231893,0.329073386165216
,-0.390709664279387,0.696011854523715,0.959080675691848
"10277","NEO1",24.185925333349,0.115340058805207,0.295274737388955,0.3
90619461133493,0.696078538424091,0.959080675691848
"10278","BOD1L1",227.472455241153,0.0793418583933335,0.203228836397642
,0.390406498407006,0.69623598333254,0.959204273592991
"10279","EML2",8.66083468212289,0.182164737121215,0.466755884307548,0.
390278394436235,0.696330697845451,0.959241432191028
"10280","CD34",394.519114256567,0.0961871665983404,0.247059533455877,0
.389327888921634,0.697033608178381,0.959264352728709
"10281","AC104794.5",1.51814837023509,-0.538558893914511,1.38332090016
092,-0.389323181520543,0.69703709000634,0.959264352728709
"10282","ZEB2-
AS1",2.18733302944589,-0.351314519451567,0.90236773486523,-0.389325222
83062,0.697035580151104,0.959264352728709
"10283","CCDC96",3.15229357919878,-0.312985006218567,0.804454153619436
,-0.389065063323212,0.697228016827957,0.959264352728709
"10284","AC097534.2",12.4409950996721,-0.160395807227489,0.41173497021
9017,-0.389560806900052,0.696861338428249,0.959264352728709
"10285","AL359715.1",1.85674736879688,0.359910317009552,0.924670820260
88,0.389230750147398,0.697105458139474,0.959264352728709
"10286","AC067750.1",2.10275498259902,0.396166964171555,1.016663407345
95,0.389673673025932,0.696777866512269,0.959264352728709
"10287","SAAL1",9.45638631142152,0.217351376171522,0.557296786661009,0
.390010101213328,0.696529077546327,0.959264352728709
"10288","SNHG1",22.073245268605,0.114391388534794,0.293562532551015,0.
389666172793748,0.696783413312913,0.959264352728709
"10289","KIF21A",10.5920002595784,-0.167507533052401,0.430514344841393
,-0.389086995728594,0.697211792954788,0.959264352728709
"10290","LLPH",77.1059961214604,-0.0880090864894634,0.225619449038395,
-0.390077570282899,0.696479188033615,0.959264352728709
"10291","IFT88",23.9832238525384,0.11810931555137,0.303162609073898,0.
389590642171114,0.696839272928067,0.959264352728709
"10292","ZNF136",22.474521278206,0.127819024931975,0.32832014677976,0.
389312158226212,0.697045243408014,0.959264352728709
"10293","IP6K2",125.049617005522,-0.0995772216056754,0.256537005304221
,-0.388159289095892,0.697898158395131,0.959460998710593
"10294","SNHG7",249.118487129163,-0.102559355641744,0.264017921221701,

-0.388456038011235,0.697678581256018,0.959460998710593
"10295","ADCY4",2.5798161718167,0.329988460815942,0.849847556311967,0.388291356920496,0.697800432672185,0.959460998710593
"10296","LEO1",41.9832812299943,-0.0960345713692704,0.247423007511927,-0.388139212820138,0.697913014598807,0.959460998710593
"10297","LUC7L",49.1417321954122,0.0926519808422256,0.238616965929961,0.38828748191116,0.697803299976665,0.959460998710593
"10298","RNF166",17.9751584382411,-0.155293631507179,0.399885580661137,-0.38834516426031,0.697760618497485,0.959460998710593
"10299","MARK4",17.1615313791652,0.14383412262294,0.37055161031718,0.388162184748902,0.697896015656127,0.959460998710593
"10300","GGT7",19.5527641060037,-0.123286733492492,0.317625839652282,-0.388150830636006,0.697904417540057,0.959460998710593
"10301","TRAK2",30.1100669173605,0.119229073490344,0.308074847761431,0.387013332495982,0.698746337695302,0.959486694690923
"10302","AC008269.1",1.79098474020768,0.401103537564794,1.03544874367959,0.387371697549628,0.698481053472331,0.959486694690923
"10303","ABTB1",49.144581036049,0.0847918208611985,0.2192198348213,0.386789000777771,0.698912420789488,0.959486694690923
"10304","SNX2",145.42042915562,0.0722477769798945,0.186545257507753,0.387293560528559,0.698538892243385,0.959486694690923
"10305","TONSL",1.66084388052396,0.400287426092382,1.03515370396573,0.386693709889515,0.698982973380533,0.959486694690923
"10306","EDRF1-DT",8.196873869938,0.23269339290929,0.600775017039405,0.387322019573972,0.698517826018619,0.959486694690923
"10307","PTPRJ",3.05597477239959,0.291269359947409,0.751671107288765,0.387495750632217,0.698389230201451,0.959486694690923
"10308","SIX1",59.9509256098286,-0.108857276368998,0.281509374271757,-0.386691479282363,0.698984624934892,0.959486694690923
"10309","AC018362.1",2.21020628551007,-0.352445084724144,0.91153690237548,-0.386649277506667,0.699015871640647,0.959486694690923
"10310","PIK3C3",30.4084633386664,0.110249789357961,0.28512007682842,0.386678449951134,0.698994271955098,0.959486694690923
"10311","IRGQ",13.2566118804555,0.143635628487963,0.371309635905911,0.386835176354972,0.698878233706659,0.959486694690923
"10312","OSCAR",2.53064985886972,-0.353169732261644,0.910593615104455,-0.387845605771276,0.69813029352326,0.959486694690923
"10313","ESF1",78.549685442747,-0.0835853884373872,0.216126896365177,-0.38674218638738,0.698947081454739,0.959486694690923
"10314","AJ009632.2",2.06807160428478,-0.450094864190442,1.16326755855278,-0.386922905982527,0.698813282870196,0.959486694690923
"10315","BRD1",39.5854935060965,-0.103226712885475,0.266223672697042,-0.387744304778432,0.698205265349705,0.959486694690923
"10316","XIAP",82.1039273586441,0.0962044725273652,0.248209633383725,0.387593628884806,0.698316784481613,0.959486694690923
"10317","FUCA1",33.6877385335794,0.111348571897472,0.292215083602945,0.381050048904285,0.703166111334907,0.95952569335573
"10318","HHLA3",14.446332370333,-0.144582702968738,0.379552479752632,-0.380929412088066,0.70325562726261,0.95952569335573

"10319","FMO4",10.609527549979,0.173616153892402,0.455115424462386,0.381477191412463,0.702849192605408,0.95952569335573
"10320","LYPLAL1-AS1",15.4821257207167,-0.280264758797844,0.736114860267922,-0.380735091661968,0.703399827161178,0.95952569335573
"10321","TRIM11",12.678145429412,-0.156029115737622,0.40522315270819,-0.385044918324255,0.700204136305688,0.95952569335573
"10322","WDR35",12.8368599722698,0.141502541317478,0.366361134183428,0.386237862356412,0.699320515106256,0.95952569335573
"10323","EXOG",28.1391386924944,0.121686555001453,0.318892475591576,0.381591176699022,0.702764629856037,0.95952569335573
"10324","PRKAR2A",75.2398490852133,-0.0817828785167833,0.21406431732427,-0.382048159819633,0.702425642785924,0.95952569335573
"10325","CFI",17.1157108398978,0.221836542550128,0.581745285532207,0.381329334447776,0.702958889359043,0.95952569335573
"10326","KIF2A",46.9890038846883,-0.0973167591366104,0.252794539827788,-0.384963849309821,0.700264199381963,0.95952569335573
"10327","SCAMP1",68.1488059859534,0.0861301853370845,0.225531471734169,0.38189874200176,0.702536473431026,0.95952569335573
"10328","SIL1",93.4182344181207,0.094344082121099,0.245753948767626,0.383896505403893,0.70105515656547,0.95952569335573
"10329","SPARC",1892.00466377337,-0.103627107677393,0.270922345721641,-0.382497454764638,0.702092416446932,0.95952569335573
"10330","ZFP62",13.7199684094033,-0.155395709830076,0.403503931972283,-0.385115726309081,0.700151677039084,0.95952569335573
"10331","GFOD1",5.33005867241626,0.240693404099904,0.628989958260552,0.38266652899441,0.701967034832259,0.95952569335573
"10332","APOM",7.6039644685364,0.217629351178147,0.564085226150237,0.385809344207474,0.699637874422415,0.95952569335573
"10333","AL121933.2",6.2303849453922,-0.237983495148588,0.622440439020534,-0.382339385794207,0.702209644145119,0.95952569335573
"10334","ETV1",18.6933784966582,-0.12561260617189l,0.329324873179554,-0.38142459438041,0.702888214229202,0.95952569335573
"10335","VPS37D",8.76150250333186,0.187851689639234,0.493079502988785,0.380976472355021,0.703220706724833,0.95952569335573
"10336","KMT2E-AS1",34.1629325429498,0.142782237701273,0.369543738186084,0.386374393467261,0.699219411586358,0.95952569335573
"10337","ADCK5",7.44301643270293,-0.208125176713644,0.542431933999746,-0.38368901915303,0.701208952293448,0.95952569335573
"10338","BNC2-AS1",5.8371207980884,-0.221174804788321,0.577936342143322,-0.382697519882686,0.701944053571917,0.95952569335573
"10339","CDK20",5.07648924544411,0.253674675976448,0.659905622547755,0.384410538884431,0.700674190587585,0.95952569335573
"10340","TRIM32",11.0177543165865,0.186185643605286,0.488021105614214,0.381511458138591,0.702823770616947,0.95952569335573
"10341","LMX1B",6.50723893595591,0.218910261876461,0.571733974840449,0.382888321334326,0.701802570963164,0.95952569335573
"10342","SURF2",34.0352202341662,-0.106350178725145,0.279149537646432,

-0.380979240094059,0.703218652974939,0.95952569335573
"10343","OLFM1",37.6475583854629,0.133279667186028,0.349027402704331,0
.3818601810441,0.70256507704266,0.95952569335573
"10344","ARMH3",13.6941993634288,-0.141276082675797,0.371052600334759,
-0.380744084661688,0.703393153465399,0.95952569335573
"10345","RRM1",29.6058536798243,-0.100381590584594,0.26296380195065,-0
.381731591344396,0.70266046490206,0.95952569335573
"10346","FKBP2",33.5874234804506,-0.15229696577627,0.399442743022355,-
0.381273582851766,0.703000253704295,0.95952569335573
"10347","MEN1",12.3508560201334,0.147561518676725,0.38694172817332,0.3
81353335483706,0.702941082298584,0.95952569335573
"10348","UNC93B1",23.8618801835673,-0.117465288245592,0.30546276172664
4,-0.384548635590188,0.700571855707231,0.95952569335573
"10349","ZW10",9.81760025271871,-0.165494529272016,0.429133869082757,-
0.385647792437706,0.699757532823934,0.95952569335573
"10350","NCAPD2",10.6250085507624,0.183050226094448,0.478032990911027,
0.382923834912719,0.701776238165327,0.95952569335573
"10351","SPSB2",16.3912372574684,-0.137309191459511,0.357694406612282,
-0.383872906372688,0.701072648337037,0.95952569335573
"10352","CD69",12.4633288130042,0.244512306422287,0.634210280098695,0.
38553822619248,0.699838690753822,0.95952569335573
"10353","SLC8B1",24.0917540755975,0.154441070800024,0.403374956872424,
0.382872233808182,0.701814499745839,0.95952569335573
"10354","ATP6V0A2",18.5048507142541,0.133905440943914,0.34727495470071
3,0.385589110678354,0.699800999161398,0.95952569335573
"10355","GPALPP1",32.3851647055238,-0.116471444054059,0.30514369804506
1,-0.381693755434726,0.702688532477404,0.95952569335573
"10356","LINC00562",1.60539883300257,-0.437444479960449,1.141459812148
07,-0.383232484669993,0.701547393977805,0.95952569335573
"10357","IFI27L1",46.8848837850486,-0.137459624902846,0.35568614427704
8,-0.386463254514005,0.699153611393425,0.95952569335573
"10358","TYRO3",19.678992040392,0.124516685434919,0.325110942330795,0.
382997522452583,0.701721601076989,0.95952569335573
"10359","LINC00926",3.59208824823121,0.264067276762411,0.6883891370175
24,0.383601748723831,0.70127364370409,0.95952569335573
"10360","CHSY1",26.9474430199355,0.12740620925492,0.330938410606802,0.
38498465325107,0.700248785808544,0.95952569335573
"10361","NPIPB4",4.81111241490243,0.271807023617928,0.711736604735189,
0.381892713975359,0.702540944851486,0.95952569335573
"10362","ARHGAP17",28.6155673304792,0.108011483667673,0.28087683889677
2,0.384551051243388,0.700570065665231,0.95952569335573
"10363","HNRNPA1P48",28.9590983010837,-0.13427627272691,0.352574147949
623,-0.380845485999999,0.703317905218817,0.95952569335573
"10364","MVD",20.9654484716957,0.12021261690628 5,0.313147385428273,0.3
83885104906359,0.701063606676407,0.95952569335573
"10365","AC007952.4",55.8554683444019,0.148779989651644,0.386333322098
931,0.385107836008887,0.700157522627659,0.95952569335573
"10366","HEXIM2",14.7992164036464,-0.173069296937951,0.450220752667908
,-0.384409860967939,0.700674692962738,0.95952569335573
"10367","TBKBP1",5.75465832861427,0.21253017102467,0.551287724001116,0

.385515878137421,0.699855244830371,0.95952569335573
"10368","CLTC",156.993397658534,0.0643377954410735,0.167891859470189,0.383209737768719,0.7015642584364,0.95952569335573
"10369","THOC1",30.9209026500144,0.101658683608455,0.266782047426873,0.381055189391334,0.703162297039606,0.95952569335573
"10370","ZBTB14",14.5194789489623,-0.145487882692592,0.376886159810999,-0.38602606889452,0.699477362177444,0.95952569335573
"10371","SS18",59.8189164867402,0.0742777818395962,0.19512433328954,0.380668984679513,0.703448885785722,0.95952569335573
"10372","AC093462.1",2.29902809318876,0.340617295417736,0.889999120018038,0.382716440675618,0.701930023011977,0.95952569335573
"10373","GCDH",19.3215983789536,-0.119841216001702,0.313760804175238,-0.381950882350398,0.702497797616338,0.95952569335573
"10374","ZNF527",5.3859866288641,0.22678172582834,0.594121737586044,0.381709187665426,0.702677084433634,0.95952569335573
"10375","ZNF576",25.2346941062277,-0.109306955836261,0.285937299556513,-0.382275960519299,0.702256683825483,0.95952569335573
"10376","KPTN",8.51349368909592,0.189507287748494,0.491686234205722,0.38542321213167,0.699923887678961,0.95952569335573
"10377","RPL13A",5378.40775994364,0.0691565180360607,0.180484768495996,0.383170937981921,0.701593024777201,0.95952569335573
"10378","MED14",13.65932515398,-0.145459351737066,0.377961011531174,-0.384852795127702,0.700346481157856,0.95952569335573
"10379","TIMM17B",26.5573895260298,-0.113286680973975,0.293343025840977,-0.386191833431855,0.699354601479286,0.95952569335573
"10380","NHSL2",33.6392023718838,0.119817156997058,0.310520697870012,0.38585884232173,0.699601213579074,0.95952569335573
"10381","AC007325.4",12.715241110473,0.222263369342691,0.579416128071355,0.383598865434618,0.701275781052029,0.95952569335573
"10382","PPM1J",2.14259464006793,0.339396122201006,0.892038329123304,0.380472577377437,0.703594648807013,0.959632077355741
"10383","L3MBTL1",3.69788712663616,0.281474094541998,0.740718828761785,0.380001268514425,0.703944473523801,0.96001673361235
"10384","RNASEH1-
AS1",13.2573322620937,-0.217089753354529,0.571940393190923,-0.379567094646628,0.704266790577288,0.960178871035471
"10385","GDF11",20.4669056715944,0.174615214575677,0.460004690677996,0.379594421783643,0.70424650220132,0.960178871035471
"10386","XPNPEP2",30.350208104022,-0.137038530414716,0.360992894210952,-0.379615589703645,0.704230786732612,0.960178871035471
"10387","PRKCA",18.6922784908528,0.127833109759202,0.336902112480643,0.379436949260882,0.704363416777859,0.960218155538122
"10388","GNLY",9.90194760964335,0.251574346602763,0.663583700463803,0.379114716692,0.704602678281999,0.960263980639864
"10389","OBSL1",46.5064864094441,0.0931904468904312,0.246153454381216,0.378586793042148,0.704994731017811,0.960263980639864
"10390","TMEM150C",34.4329410320629,0.113806272598601,0.300574138333751,0.378629622726334,0.704962921422688,0.960263980639864
"10391","MRAP2",8.90283488052665,-0.276676331169069,0.730071779823688,-0.378971409134436,0.704709095235086,0.960263980639864

"10392","RAB32",100.665009616328,0.0994297370207625,0.263019712304643,
0.37803150246624,0.705407191857689,0.960263980639864
"10393","PAXIP1",11.299914244051,0.16814439411143,0.444385640564638,0.
378374949059526,0.705152075071679,0.960263980639864
"10394","SMC2",41.2026079538262,-0.1035712693354,0.274008992132046,-0.
377984928631423,0.705441790084779,0.960263980639864
"10395","MVB12B",5.90293042343512,0.22128716789945,0.584321149646436,0
.378708126572772,0.704904617975991,0.960263980639864
"10396","PITRM1",19.8775968325051,-0.124919259861321,0.329784796850861
,-0.378790232461242,0.704843641209548,0.960263980639864
"10397","GALNT4",16.6078161585555,0.149665828835705,0.395654421287991,
0.378274122019137,0.705226967327933,0.960263980639864
"10398","SUDS3",56.6065177266269,-0.0873577666876314,0.230729070789666
,-0.378616211596791,0.704972881809583,0.960263980639864
"10399","TRIM16L",3.14162692923319,0.310900605860396,0.821919088563871
,0.378261814558449,0.705236109252769,0.960263980639864
"10400","AC100793.4",9.04086391086433,-0.188746385729763,0.49910863912
6873,-0.378166937883406,0.705306584636412,0.960263980639864
"10401","DUSP15",2.53956998214686,0.31560282394900 7,0.832440840575685,
0.379129433066677,0.7045917505621,0.960263980639864
"10402","KIF3B",37.9742688614399,-0.113646902271105,0.300461705563423,
-0.378240887829599,0.705251653627805,0.960263980639864
"10403","PABPC5",15.0032460300965,0.138932072050534,0.367612559819307,
0.377930699970707,0.705482075607098,0.960263980639864
"10404","PFDN1",99.4081652060866,0.0970706645291746,0.257020583074895,
0.377676617832933,0.705670839772635,0.960336289397455
"10405","DLG5",24.2530521736962,0.128036992416809,0.338981626650209,0.
377710714536539,0.705645507400579,0.960336289397455
"10406","FAM87B",1.87862205969227,-0.383513639808229,1.01881256496001,
-0.376431988570224,0.706595768424854,0.960353659417337
"10407","GPATCH3",8.41962636337647,0.180777653900854,0.479696586457626
,0.376858328794515,0.706278890840785,0.960353659417337
"10408","C1orf131",47.8616244633594,0.0796775152219414,0.2115374570167
39,0.376659133307236,0.706426936652709,0.960353659417337
"10409","UQCRQ",268.832253456595,-0.0776721195920159,0.206401167335547
,-0.376316280545759,0.706681777248358,0.960353659417337
"10410","SEC24A",24.3844360828359,-0.113006319995691,0.29946793548,-0.
377356994212284,0.705908322119652,0.960353659417337
"10411","AL139339.1",4.4862460594777,0.255802718005517,0.6797476294097
22,0.376320132558094,0.70667891388582,0.960353659417337
"10412","HOXC9",82.6838318946851,0.0837992613687488,0.222698306869407,
0.376290518534969,0.706700927339594,0.960353659417337
"10413","LINC00641",6.54484104252284,-0.24673672298839,0.6544138583979
97,-0.377034685042276,0.706147828853593,0.960353659417337
"10414","GNPTG",87.6387071728374,0.07493720173174,0.198909259084583,0.
376740640815891,0.706366357404756,0.960353659417337
"10415","ZNF771",22.1960762343414,0.145639283903494,0.386555316742395,
0.376761818026034,0.706350618061571,0.960353659417337
"10416","CMC2",51.3814410822478,-0.0952415492028343,0.252514523324219,
-0.377172560013698,0.706045370916359,0.960353659417337

"10417","LRRC45",10.6862772086168,-0.16687280912592,0.443176963285449,
-0.376537642861272,0.706517236078322,0.960353659417337
"10418","BICRA",7.60862076687339,0.187174540735008,0.496620832638875,0
.37689627263606,0.706250691527954,0.960353659417337
"10419","MANBAL",73.2993299337882,-0.0946688442722997,0.25079677395625
1,-0.377472336581226,0.70582261871445,0.960353659417337
"10420","USP11",53.5950371325905,0.0876301031786909,0.232785112935427,
0.37644204164808,0.706588295884047,0.960353659417337
"10421","KCNAB2",7.08272732636128,-0.195955590895443,0.521130419770807
,-0.376020250327402,0.706901841355591,0.960405569192667
"10422","NBPF1",23.7983628875348,0.141799829287388,0.387372918386674,0
.366055092023351,0.714323966636288,0.960405569192667
"10423","TTC4",30.9266880282859,0.0987106297987182,0.265357869159892,0
.371990588073496,0.709899856955464,0.960405569192667
"10424","AC093425.1",4.46603251762538,0.28052982778735,0.7490537837671
3,0.374512263160215,0.708023234671216,0.960405569192667
"10425","LYSMD1",7.33321968692616,0.218048612852682,0.589528339958418,
0.369869602652286,0.711479651367953,0.960405569192667
"10426","PSMB4",167.243405020795,0.0749007435011746,0.203016685497746,
0.36893885503813,0.71217330103008,0.960405569192667
"10427","C1orf112",3.23269845004795,-0.289909090550769,0.7832662830782
87,-0.370128392877333,0.711286827570103,0.960405569192667
"10428","KLHL12",13.5620314533571,0.13463517126312,0.368437106698465,0
.365422398600334,0.714796123939222,0.960405569192667
"10429","RASSF5",15.6641819507847,-0.143147320005727,0.390401833473336
,-0.366666618166649,0.713867709656593,0.960405569192667
"10430","GPR137B",35.1511228288394,-0.106380669046492,0.29020302932591
3,-0.366573254916031,0.713937360956508,0.960405569192667
"10431","MRPL33",148.183811286967,-0.0833481430741057,0.22546407954409
8,-0.369673711407337,0.711625621604159,0.960405569192667
"10432","PPP1R21",20.1020241460078,0.115240432653819,0.314302318538272
,0.366654732901014,0.71387657622702,0.960405569192667
"10433","RMND5A",33.1597260514843,0.0961881186235581,0.259745056502563
,0.370317417850873,0.71114599731682,0.960405569192667
"10434","GIGYF2",69.1616669630375,0.0899597136686591,0.243512776592271
,0.369425025362362,0.71181094761865,0.960405569192667
"10435","ENTPD3-
AS1",13.7951552844381,0.153132596543498,0.417744695678027,0.3665698167
51243,0.713939925958203,0.960405569192667
"10436","QARS",70.1185500197004,-0.0694735048087968,0.186319697254166,
-0.372872572426012,0.709243286312493,0.960405569192667
"10437","NAA80",11.6647908861033,-0.159286038313501,0.425349626222061,
-0.374482610289972,0.708045291966679,0.960405569192667
"10438","PROS1",151.593991731692,0.0748762030957296,0.201210174979831,
0.372129307592099,0.709796576476193,0.960405569192667
"10439","TOMM70",52.1555021634509,0.0798316671737806,0.216737818076343
,0.368332891243101,0.712625030133191,0.960405569192667
"10440","COPG1",58.2370964625883,-0.0819217156428683,0.222288578474878
,-0.368537673887399,0.712472359097658,0.960405569192667
"10441","NEK11",24.7381448816683,0.112449935719191,0.305615376293949,0

.367945936107067,0.712913547155427,0.960405569192667
"10442","U2SURP",166.162140662164,0.0642218796921421,0.172440920816437
,0.372428304071201,0.709573983543984,0.960405569192667
"10443","PLSCR1",116.023614317214,-0.0698633508107761,0.18670531039388
5,-0.374190485869888,0.708262601884285,0.960405569192667
"10444","COMMD2",66.5503215617071,-0.0765822746833578,0.20619916587542
2,-0.371399536745101,0.710339970561363,0.960405569192667
"10445","CCDC39",2.86696820861046,0.290722172469895,0.790552400535271,
0.367745607088223,0.713062930308009,0.960405569192667
"10446","AC108471.2",4.1049418145512,-0.282303039789849,0.759317377367
413,-0.371785301119548,0.710052708491852,0.960405569192667
"10447","RBM47",14.6159945228523,0.130743657002778,0.357757846145291,0
.365452940897014,0.714773328766258,0.960405569192667
"10448","IL15",4.69196485416962,-0.238222728843804,0.651944544493256,-
0.365403362687803,0.714810331477508,0.960405569192667
"10449","AC099343.3",14.2921546847601,0.170449837109036,0.464563967098
05,0.366902836166502,0.713691495820713,0.960405569192667
"10450","POC5",10.1408535955652,0.165461920847794,0.441884582037468,0.
37444601503151,0.708072513697141,0.960405569192667
"10451","LRRTM2",1.97270122167072,0.376178132865284,1.00038916809363,0
.376031793289144,0.70689326004423,0.960405569192667
"10452","ARHGAP26",35.1092707682592,0.117031056526903,0.31626410097905
3,0.370042177296164,0.711351064482525,0.960405569192667
"10453","PDGFRB",428.906863732791,-0.0631220038648545,0.16947559837103
4,-0.372454822237365,0.70955424284778,0.960405569192667
"10454","DDX41",28.4536712290163,0.108029100442647,0.295278283746226,0
.365855216550541,0.714473115008199,0.960405569192667
"10455","WRNIP1",25.0901083679478,0.111894091522525,0.299566026183261,
0.373520632323217,0.708760992302986,0.960405569192667
"10456","FANCE",3.15843295368977,-0.307370309549282,0.834516535107071,
-0.368321413199855,0.712633587667745,0.960405569192667
"10457","MTCH1",315.134137143546,-0.0662419244480649,0.177218743519745
,-0.373786221098473,0.708563371638332,0.960405569192667
"10458","IBTK",54.6449635288,-0.0887884955618326,0.239162635490101,-0.
371247353834699,0.710453305925916,0.960405569192667
"10459","SLC35A1",38.5772003217149,0.104269810355096,0.28427194228927,
0.366795996521502,0.713771194137429,0.960405569192667
"10460","REV3L",159.221966519018,0.0940886350661525,0.255819954769896,
0.36779239973982,0.713028036555542,0.960405569192667
"10461","TCP1",135.736219122196,-0.0707712854654881,0.189897970781617,
-0.372680577755489,0.709386193455896,0.960405569192667
"10462","RNASET2",96.0393978489584,-0.0817574214675707,0.2215679344731
83,-0.368994826178091,0.712131581198945,0.960405569192667
"10463","FGFR1OP",12.200606706187,0.158510814123032,0.423228695364334,
0.374527568331772,0.708011850011102,0.960405569192667
"10464","DPY19L1",22.9622757586189,0.113488772657695,0.307560567812216
,0.368996498689607,0.712130334553692,0.960405569192667
"10465","NIPSNAP2",86.8251452901164,0.0788763996975757,0.2099960190968
65,0.375609023622454,0.707207581327775,0.960405569192667
"10466","FAM200A",13.0062215240877,0.171075743789539,0.45891280247335,

0.372784857749689,0.709308573594445,0.960405569192667
"10467","LINC00513",10.07136027462,0.190776816134418,0.508147606534273
,0.375435825498768,0.707336365286706,0.960405569192667
"10468","BPGM",26.1225390076494,-0.103202736943183,0.281972163303464,-
0.366003281083154,0.714362627247835,0.960405569192667
"10469","EZH2",4.77989392174644,0.234707653859169,0.635944851520328,0.
369069194125973,0.712076150067472,0.960405569192667
"10470","TOX",2.61225753660368,-0.397768914107225,1.08557649308466,-0.
366412608085283,0.714057213054957,0.960405569192667
"10471","TSPYL5",20.0791838246,0.130414656677249,0.356312728849073,0.3
66011781556337,0.71435628426129,0.960405569192667
"10472","FAM49B",18.5708490567842,-0.129275661152393,0.350927673327716
,-0.368382635448837,0.712587943414618,0.960405569192667
"10473","FBXL6",10.7001096854445,-0.166264597181942,0.445339268413843,
-0.373343670712272,0.708892677694752,0.960405569192667
"10474","AL162253.2",6.5786542138433,-0.235874285726652,0.636027362567
064,-0.37085556315477,0.710745114134578,0.960405569192667
"10475","DNAJA1",451.73255932222,-0.0655026844572313,0.178126076030045
,-0.367732147460448,0.71307296739946,0.960405569192667
"10476","VPS13A",21.2805535957149,-0.132412865907905,0.36096019136916,
-0.366835094489641,0.713742028175995,0.960405569192667
"10477","MFSD14B",40.5756264894855,0.09010644286526,0.243164613517093,
0.370557383173378,0.710967228912988,0.960405569192667
"10478","RAD23B",120.436644023717,-0.0686842380293395,0.18331690991269
9,-0.374674862575683,0.707902289407177,0.960405569192667
"10479","SEC16A",35.6803808159525,0.107991722939455,0.291356631452706,
0.370651329956032,0.710897245144777,0.960405569192667
"10480","GBF1",33.7214112390342,-0.102017675720499,0.275714167127603,-
0.370012454504319,0.711373210610983,0.960405569192667
"10481","LGR4",43.65948896882,-0.126086896310608,0.337902206143437,-0.
373146117480765,0.709039696549937,0.960405569192667
"10482","IMMP1L",17.9403474394815,0.131162142827375,0.349579672201319,
0.375199570391039,0.707512049676302,0.960405569192667
"10483","HSD17B12",101.912378759504,-0.0765243503517983,0.208792171753
112,-0.366509671839062,0.713984796873043,0.960405569192667
"10484","CKAP5",34.8995340155749,0.0970484893574028,0.259928587292746,
0.373365970893004,0.708876082610548,0.960405569192667
"10485","RBM14",23.0354949768205,0.10767426733698,0.289034065563922,0.
372531407766421,0.709497232018911,0.960405569192667
"10486","AP000487.1",2.93187287447268,-0.313371942655131,0.85404104599
6475,-0.366928432917994,0.713672402080296,0.960405569192667
"10487","POLD3",32.4039042562405,0.0917520807116002,0.246706275211894,
0.371908175553278,0.709961217856757,0.960405569192667
"10488","RPAP3",36.7713749572645,0.101328466609117,0.270211566417422,0
.374996777349587,0.707662863373944,0.960405569192667
"10489","MBD6",24.640563503289,0.119538389046906,0.317962135721691,0.3
75951648379719,0.706952842438007,0.960405569192667
"10490","WSCD2",4.73494453920605,0.272754166837207,0.725825420680056,0
.375784808668801,0.707076882391636,0.960405569192667
"10491","C12orf49",28.3317982166964,0.102420611997053,0.28015084264210

3,0.365590947473597,0.714670331047372,0.960405569192667
"10492","RILPL1",34.0496020016932,-0.102598563080305,0.27627750943416,
-0.371360532713776,0.710369017470811,0.960405569192667
"10493","TPP2",45.9235447605135,0.0894953279854733,0.243262520808306,0
.367896080695459,0.712950722760234,0.960405569192667
"10494","ZFHX2-
AS1",5.59829414193234,0.204590972159117,0.555775990732914,0.3681176869
28718,0.712785483217503,0.960405569192667
"10495","MAPK1IP1L",65.0611336555811,0.0711807794799434,0.192571993511
841,0.369632043485942,0.711656672218199,0.960405569192667
"10496","FBXO34",22.6552907354208,0.114931834999357,0.310408125426414,
0.370260394573992,0.711188480625465,0.960405569192667
"10497","PRKCH",3.13365905638158,0.337711417286157,0.92357864196263,0.
365655291214304,0.71462231149955,0.960405569192667
"10498","ARG2",1.74650377909344,-0.433077495601489,1.18463098867488,-0
.36558008336919,0.714678439008578,0.960405569192667
"10499","WDR20",29.3224038021723,0.10074941374857,0.274629776453632,0.
366855390007501,0.713726888467437,0.960405569192667
"10500","AL049840.5",5.25815550831242,-0.231734208868184,0.63312247649
6159,-0.366017978307535,0.714351660306492,0.960405569192667
"10501","AC091057.6",1.90749387135629,0.370301529910021,0.993433147936
701,0.372749319548189,0.709335025791323,0.960405569192667
"10502","KATNBL1",68.7361401851538,-0.0768034044233885,0.2094010639936
77,-0.366776571993481,0.713785684433032,0.960405569192667
"10503","NDUFAF1",22.2653124172064,0.123429065958517,0.330230787823611
,0.373766076664072,0.708578360125503,0.960405569192667
"10504","SHF",10.545077471679,0.158132565812285,0.428660058832715,0.36
8899696983423,0.712202489217066,0.960405569192667
"10505","RGS11",12.1037464753223,0.158456400387476,0.433083739780586,0
.365879357344969,0.714455100411882,0.960405569192667
"10506","CLCN7",26.656933771894,-0.109457741149824,0.295230744226502,-
0.37075319318996,0.710821366928828,0.960405569192667
"10507","MSRB1",20.4419335913202,-0.128376363022251,0.349119691036784,
-0.367714472480801,0.713086148031616,0.960405569192667
"10508","TXNDC11",23.5355970923617,0.11994737100204,0.322925411511017,
0.371439864211329,0.710309938511767,0.960405569192667
"10509","ZNF764",7.12938184926918,0.193568222866733,0.516264826781416,
0.374939784438751,0.707705250086509,0.960405569192667
"10510","SETD6",11.2114049799263,-0.179874254177327,0.483550104132805,
-0.371986796487021,0.709902679970231,0.960405569192667
"10511","BEAN1",2.2522977405967,-0.329236313568193,0.898708123752204,-
0.36634398295366,0.71410841363891,0.960405569192667
"10512","SLC12A4",42.0538493750872,0.0974357154809448,0.26080531760568
2,0.373595586069531,0.70870521834687,0.960405569192667
"10513","NQO1",27.9504702819477,-0.132271107206936,0.356996652936598,-
0.370510776834726,0.711001948272747,0.960405569192667
"10514","USP10",35.0261790090225,0.111850494916249,0.299322920353063,0
.373678349737858,0.70864363475114,0.960405569192667
"10515","PIEZO1",25.5063912886796,-0.120510731801074,0.320760693582801
,-0.375702928108197,0.707137760797433,0.960405569192667

"10516","MNT",23.9944836793028,0.118084078470825,0.315061215972624,0.3
7479725362669,0.707811256929089,0.960405569192667
"10517","CHRNE",1.72245268462916,-0.468745224891404,1.25292344408653,-
0.374121201980663,0.70831414531411,0.960405569192667
"10518","VAMP2",186.411964982536,0.0830842597996155,0.226995520324835,
0.366017178139553,0.714352257383382,0.960405569192667
"10519","CTC1",4.17736787661505,0.244941011336694,0.658103577242346,0.
372192189507723,0.709749760924529,0.960405569192667
"10520","CCDC137",21.0823978189319,0.116067615456426,0.313118171890176
,0.370683102663029,0.710873577256476,0.960405569192667
"10521","L3MBTL4",4.30450569755649,0.238148222125157,0.640604215579112
,0.371755627474085,0.710074803711995,0.960405569192667
"10522","ZBTB7C",7.05120857035401,-0.192163556986591,0.51441859949626,
-0.373554838753432,0.708735538705915,0.960405569192667
"10523","AC005523.2",5.03623310907429,0.238477079252673,0.643944984261
855,0.370337660951015,0.711130916079557,0.960405569192667
"10524","TICAM1",11.7696255938718,0.149794825342707,0.405478247196965,
0.369427525097154,0.711809084679546,0.960405569192667
"10525","LONP1",29.8974034119637,0.097716186473028,0.266416420580738,0
.366779893897024,0.713783206354471,0.960405569192667
"10526","CCDC151",1.97768817661689,0.497937564406788,1.34329510431854,
0.370683673904547,0.710873151734049,0.960405569192667
"10527","ILVBL",43.8992563768947,0.118086279988292,0.314979159990113,0
.374901882372151,0.707733439076333,0.960405569192667
"10528","JAK3",5.12429689947031,0.217955123782056,0.591165248730363,0.
368687307398659,0.712360810526856,0.960405569192667
"10529","ZNF430",35.8843864783707,-0.0967817678082677,0.25796110730419
,-0.375179688208354,0.707526835206401,0.960405569192667
"10530","ZNF675",12.1895606131981,0.149417294513559,0.403140138350607,
0.370633634063032,0.71091042715847,0.960405569192667
"10531","PRX",3.63672570028643,0.251193891023261,0.683187884891054,0.3
67679077130179,0.713112543402814,0.960405569192667
"10532","XRCC1",26.0551801089441,0.112136128891255,0.303363203511005,0
.369643145883997,0.711648398749956,0.960405569192667
"10533","TRAPPC6A",41.4000593146815,-0.130459981620074,0.3563008889250
15,-0.366151153912864,0.714252288514551,0.960405569192667
"10534","PTPRH",2.29873953162703,0.364847256255754,0.993952703609024,0
.367067019316916,0.713569027516755,0.960405569192667
"10535","CEP250",11.0455242181939,0.213657784175519,0.575694753686257,
0.371130330452791,0.710540461247182,0.960405569192667
"10536","Z95114.1",2.31124210212057,-0.365267163752098,0.9782486531750
13,-0.37338887466554,0.708859038496382,0.960405569192667
"10537","SERHL2",5.85567714983387,0.209717651103309,0.560057828180311,
0.374457137372233,0.708064240198935,0.960405569192667
"10538","AL118516.1",21.7461755545648,-0.150451531872914,0.40944513008
4116,-0.367452244069932,0.713281707751125,0.960405569192667
"10539","MT-
ND1",10330.1181911006,-0.0586463168585053,0.159062157748206,-0.3687006
24263767,0.71235088338793,0.960405569192667
"10540","NOM1",23.6736958402672,-0.109022157554214,0.298544650251007,-

0.365178734445756,0.714977991230947,0.960539692204004
"10541","SH3PXD2A",103.887821175836,0.100731046787568,0.276143317030733,0.364778144445759,0.715277020802879,0.960759117299257
"10542","GART",27.6267366827401,0.102795224647839,0.281791918246971,0.36479124485659,0.715267241010043,0.960759117299257
"10543","RAB7B",33.4704198501076,0.107263548209025,0.295657412172274,0.362796749863064,0.716756719943199,0.961148400657165
"10544","ENOPH1",33.8119231158502,-0.112783693150495,0.310836616063134,-0.362839148678635,0.716725045484326,0.961148400657165
"10545","CCDC127",53.7274742898482,-0.0848793899122242,0.233574227157348,-0.363393645545683,0.716310847934283,0.961148400657165
"10546","GM2A",25.1985185767026,0.103540680123731,0.28436766707175,0.364108483886131,0.715777001815587,0.961148400657165
"10547","GEMIN5",20.6980753873792,0.109537993440493,0.301726971645835,0.363036797283961,0.716577396549606,0.961148400657165
"10548","SERPINB9",16.0995605985512,-0.164522288423348,0.452243884979708,-0.363791073550349,0.71601402887147,0.961148400657165
"10549","ORAI2",33.86040194386,-0.119613468456312,0.329198638900018,-0.36334739674498,0.716345391631629,0.961148400657165
"10550","UBXN2B",11.427980851681,0.175422075301768,0.482849827581114,0.363305660023868,0.716376565709365,0.961148400657165
"10551","TMTC2",8.42958824147035,0.260309999466828,0.716626404234,0.363243662149279,0.716422874162204,0.961148400657165
"10552","RIC8B",14.4605574843319,-0.151566333265983,0.416070699610539,-0.364280237488137,0.715648755752888,0.961148400657165
"10553","SNX6",205.452591720456,-0.0546256825484561,0.150011354980886,-0.364143651361701,0.715750742090059,0.961148400657165
"10554","LINC00921",7.85704960444347,-0.18942425656763,0.520685812713122,-0.363797614497316,0.716009144124747,0.961148400657165
"10555","AC092338.3",7.68656779012389,0.181008753894966,0.498126686410442,0.363378953252507,0.716321821693844,0.961148400657165
"10556","DDX5",1425.19860239609,0.0695213702624311,0.191648790346196,0.362754025928612,0.716788637778225,0.961148400657165
"10557","MRPL58",32.4932431604192,0.109369222388546,0.301334538700072,0.362949507415759,0.71664260317183,0.961148400657165
"10558","CEBPB-
AS1",3.92307559285805,-0.244879197510095,0.674243991339762,-0.363190774638579,0.716462378579335,0.961148400657165
"10559","C2CD2",27.9881649594657,0.119261252624045,0.328553915174635,0.362988377602057,0.716613566401815,0.961148400657165
"10560","TRMT2A",16.1244624058679,0.140797107627897,0.387772508137057,0.363092031212622,0.716536137182147,0.961148400657165
"10561","TTC38",11.545845359748,0.154574406733548,0.426247523350119,0.362640011415577,0.716873817196135,0.961171598475265
"10562","AC010969.2",7.89985142696747,0.171835606095906,0.474457146911111,0.362173079728314,0.717222695218273,0.961275282942806
"10563","TOM1L1",1.77162486826148,0.529652066550925,1.46204654593009,0.362267581716412,0.717152081266978,0.961275282942806
"10564","ZNF227",12.1463765012939,-0.138491635438683,0.382380318621581,-0.362182959462773,0.717215312751579,0.961275282942806

"10565","VSIG10L",2.02341464624305,-0.376728049743031,1.03983189323278
,-0.36229707147355,0.717130046370668,0.961275282942806
"10566","C5orf22",8.54661116403414,0.210389606666959,0.58126201500279,
0.361953131697327,0.71738705455278,0.961282806897318
"10567","TSLP",2.52475581359519,-0.408987695819088,1.13013422410077,-0
.361893027480442,0.717431970571388,0.961282806897318
"10568","ZDHHC13",8.72529224128739,0.184939690219092,0.510933654446483
,0.361964197522759,0.717378785142657,0.961282806897318
"10569","KIAA1522",10.8099133370307,-0.210732474438623,0.5839543873487
99,-0.36087146360072,0.718195536898532,0.961410733534083
"10570","SF3A3",63.6021869191887,-0.0814808708671671,0.225445155982727
,-0.361422140617693,0.717783899199488,0.961410733534083
"10571","DZIP1L",12.3812257280401,-0.143478362197147,0.397251935593088
,-0.361177251365529,0.717966946755205,0.961410733534083
"10572","FBXO8",36.7560252481874,-0.0998656043630952,0.276290651095818
,-0.361451261441566,0.717762133312695,0.961410733534083
"10573","AL139274.2",4.18863169097163,0.25229450177743,0.6990089993102
38,0.360931693334974,0.718150510460575,0.961410733534083
"10574","PHYH",85.8643939447921,0.0904871115084518,0.250419197666329,0
.361342550218619,0.717843388919116,0.961410733534083
"10575","COPB1",141.686447794243,-0.0704973941310696,0.195361069341295
,-0.360856921846137,0.718206408144317,0.961410733534083
"10576","ZNF384",22.9495934049679,-0.106669968618116,0.295573186054013
,-0.360891899709142,0.718180259199123,0.961410733534083
"10577","LRRC28",17.6210561073748,0.129215601204331,0.357746848596887,
0.361192842679467,0.717955292221207,0.961410733534083
"10578","RPS6KA6",3.1888396406746,0.279561893619301,0.774715293297198,
0.360857589927627,0.718205908693153,0.961410733534083
"10579","SRP14",1173.90820368275,-0.0747551170089302,0.207323190340123
,-0.360572866384561,0.718418776126152,0.961513220221768
"10580","CHADL",7.95801663180007,0.201995483980457,0.560072639225184,0
.360659439211139,0.718354049501399,0.961513220221768
"10581","UBXN7",53.7856266111106,0.0958117583469466,0.265830195904269,
0.360424661393434,0.71852958704974,0.961570641019215
"10582","ELOVL1",75.5652407663016,0.0776955835920062,0.217332148159239
,0.357496966049766,0.720719797082786,0.962034234195174
"10583","BCAR3",4.50960350062447,-0.242018824997918,0.676705366855862,
-0.357642833723037,0.720610618993764,0.962034234195174
"10584","TMEM79",4.53214896761757,0.229404665674109,0.638930731677766,
0.359044657425877,0.719561681763646,0.962034234195174
"10585","ZBTB18",3.6536564042994,0.335306045728563,0.93783759837273,0.
357531033422378,0.720694298047267,0.962034234195174
"10586","PIGF",50.8605400332016,0.110289061892129,0.308204403331912,0.
357843887692143,0.720460144760068,0.962034234195174
"10587","AC064836.4",7.65671322962695,-0.211173066527189,0.59008021277
9218,-0.357871797687614,0.720439257019424,0.962034234195174
"10588","NUDT16",37.1450813199569,0.102395566811037,0.286436992121246,
0.35748024741055,0.720732310905033,0.962034234195174
"10589","MBLAC1",4.98113270249113,-0.22653939543153,0.631571620678444,
-0.358691537134265,0.719825860064059,0.962034234195174

"10590","AC009275.1",4.08471013674571,0.26111238016373,0.731107257959745,0.357146475186692,0.720982153480169,0.962034234195174
"10591","ARHGEF35",13.4731364699466,-0.170304310643658,0.476815815409754,-0.357170012276767,0.720964533999963,0.962034234195174
"10592","ENY2",234.542798263067,-0.0766044430902701,0.212924551463261,-0.359772710867905,0.719017112150584,0.962034234195174
"10593","AL135791.1",2.34991544232526,-0.350200061423872,0.976355147854696,-0.358681021135958,0.719833727863973,0.962034234195174
"10594","MTG1",48.8350692583821,-0.092323282416209,0.258436955843662,-0.357237153312001,0.72091427413437,0.962034234195174
"10595","AP002360.1",10.6399504153264,0.184223302216015,0.513463714846831,0.358785434859734,0.719755609512512,0.962034234195174
"10596","NLRX1",7.97936133668522,0.167368137992862,0.466291989757479,0.358934190741537,0.719644321110389,0.962034234195174
"10597","AC010198.2",2.23805155267474,0.373753134820265,1.03992217489003,0.359404909179659,0.719292202748005,0.962034234195174
"10598","TSPAN8",141.4337174475,0.158824995542899,0.442410804842599,0.358998907360335,0.719595906667143,0.962034234195174
"10599","AC011611.4",2.17693921797959,0.344535046168167,0.962554467521461,0.357938233932186,0.720389537211346,0.962034234195174
"10600","ANAPC7",27.2263829013817,0.105051397557116,0.292061626995447,0.359689147245466,0.719079608851095,0.962034234195174
"10601","TVP23A",1.99878181469795,0.477932441583399,1.33526108845449,0.35793182750243,0.72039433162798,0.962034234195174
"10602","MOSMO",55.432561707613,-0.0725362273291393,0.20302288295616,-0.357281042771926,0.720881420386983,0.962034234195174
"10603","CNEP1R1",16.8865916736642,-0.135154368223951,0.376202460539183,-0.359259660423923,0.719400848937039,0.962034234195174
"10604","PMP22",793.25201336558,0.0442302812264931,0.123828632170788,0.357189451672934,0.720949982098002,0.962034234195174
"10605","RAPGEFL1",2.52542137084324,0.297059395164424,0.82653496225288,0.359403302619809,0.719293404425082,0.962034234195174
"10606","STAT5A",29.0408867380386,0.101078589527857,0.281986886305667,0.358451383509693,0.720005544237919,0.962034234195174
"10607","COASY",27.0315069636896,-0.0950847196170833,0.265181099277193,-0.358565221564648,0.719920368092334,0.962034234195174
"10608","TPGS2",70.7344795727838,-0.0701940611749886,0.195770638905197,-0.358552546835076,0.719929851432802,0.962034234195174
"10609","YJU2",22.5974191849006,-0.109654202908427,0.306249058691089,-0.358055640651076,0.720301674817897,0.962034234195174
"10610","LAMA5",3.99189306615434,0.24867800663312,0.694639103405293,0.357995980091012,0.720346321882974,0.962034234195174
"10611","GRIA3",9.80430495079155,-0.168795931440934,0.470771728970758,-0.358551546436253,0.719930599941545,0.962034234195174
"10612","VBP1",75.6270297892776,-0.0755793644981042,0.211343214542043,-0.357614341496017,0.720631944223319,0.962034234195174
"10613","STPG1",4.95530172629791,-0.228938506546708,0.644196046804241,-0.355386388479775,0.722300144631783,0.962105211115006
"10614","SLC30A7",48.0587942939672,0.0835408167270898,0.234189665384563,0.356722900602413,0.721299259786134,0.962105211115006

"10615","VPS72",38.4238953590054,-0.0946824290623763,0.267393022700385
,-0.354094613637202,0.72326797862091,0.962105211115006
"10616","GJC2",4.98721794843304,0.228381105851204,0.646733656845748,0.
353130076707412,0.723990925602816,0.962105211115006
"10617","RAB10",118.512299018344,0.0608069770787608,0.171964474921674,
0.353601969863003,0.723637197884204,0.962105211115006
"10618","SELENOI",7.93553323471849,0.170632165669353,0.48057672327082,
0.355057074982793,0.722546833420992,0.962105211115006
"10619","STAMBP",39.4890036696499,0.0855800124039148,0.24162721400723,
0.354182010315088,0.723202484593981,0.962105211115006
"10620","PLCD1",16.3897497155765,0.134274751753254,0.378206704959467,0
.355030066872147,0.722567066480864,0.962105211115006
"10621","QTRT2",13.6141666276314,-0.128195920290135,0.361722292841006,
-0.354404256600471,0.723035945014809,0.962105211115006
"10622","UMPS",24.3021329198028,-0.108131798141032,0.306483895752694,-
0.352813963929395,0.724227914445447,0.962105211115006
"10623","MRPL47",66.3550614282746,-0.0968715819401992,0.27203332516823
7,-0.356101892590879,0.721764259931394,0.962105211115006
"10624","NDUFS4",219.992788668016,0.0880394139066865,0.24882570649968,
0.35381960789007,0.723474077865489,0.962105211115006
"10625","MRPS27",32.1906990378837,-0.0978009313772633,0.27608760663073
5,-0.354238759829851,0.723159958270736,0.962105211115006
"10626","GFM2",12.3061183936818,0.1388776744367,0.392243446750781,0.35
4059897206998,0.723293995261701,0.962105211115006
"10627","AP3B1",55.2366223537287,-0.080103681746376,0.225717246888108,
-0.354885073474624,0.722675691245338,0.962105211115006
"10628","SHROOM1",10.8720962456679,-0.173811171789932,0.48836073655649
6,-0.355907342214898,0.721909957078756,0.962105211115006
"10629","SH3PXD2B",71.1646883172222,-0.12136054377291,0.34261931336002
7,-0.354213960044288,0.723178542354388,0.962105211115006
"10630","MSX2",4.5730991585151,-0.235342360229172,0.66679422599898,-0.
352946008008072,0.724128918162773,0.962105211115006
"10631","NUP43",12.1790245204872,0.167960882388334,0.475183582610447,0
.353465246980193,0.723739678305094,0.962105211115006
"10632","MAFK",24.923854133364,0.142664376816449,0.401490663042228,0.3
55336723737069,0.722337346649837,0.962105211115006
"10633","NUDCD3",36.9590242716762,0.118047544062378,0.334896802749416,
0.352489313404123,0.724471331525606,0.962105211115006
"10634","POR",60.6704853741115,-0.0826253174256976,0.233729219479313,-
0.353508720945396,0.723707091926021,0.962105211115006
"10635","APTR",14.374858903875,0.143401552999706,0.404229387315803,0.3
54752913814438,0.722774706222795,0.962105211115006
"10636","ZC3HC1",11.7421307128139,0.148250895862717,0.417089766062467,
0.355441221352128,0.722259072122023,0.962105211115006
"10637","PCAT1",1.54102966591969,-0.428379470045579,1.21363216391837,-
0.352973069420391,0.724108630210154,0.962105211115006
"10638","MIGA2",8.04912733677233,0.170550634616926,0.483838467670592,0
.352494987506947,0.724467076946892,0.962105211115006
"10639","ZNF143",20.592341917971,0.115581925523207,0.325158494202454,0
.355463343520229,0.72224250176012,0.962105211115006

"10640","EHBP1L1",30.5627809213081,-0.100772330199713,0.28254977084131
7,-0.356653377915171,0.721351312064527,0.962105211115006
"10641","HINFP",7.84855014515235,-0.184691807904461,0.523605012509187,
-0.352731168518407,0.724289990296562,0.962105211115006
"10642","NRGN",2.67160992590427,-0.303069839991282,0.858276349772237,-
0.353114518501772,0.724002588926171,0.962105211115006
"10643","MAGOHB",39.1638245595535,-0.0995266991572842,0.28212782402279
7,-0.352771654132355,0.724259635985022,0.962105211115006
"10644","KATNAL1",34.5453623010817,0.0956201511459783,0.26855395267529
8,0.356055646150143,0.721798892589272,0.962105211115006
"10645","TECPR2",20.0838585807714,0.124850985093651,0.354184709427685,
0.352502470519953,0.724461466016846,0.962105211115006
"10646","SNAP23",60.4876852330121,0.0801874923405703,0.227426995176876
,0.352585638649477,0.724399105694847,0.962105211115006
"10647","MEF2A",81.6747822760537,0.0838830355447557,0.236881129590199,
0.354114469522635,0.723253098679488,0.962105211115006
"10648","AC087190.1",6.53735312173774,0.219630443925331,0.622155158082
219,0.353015547765188,0.724076784567375,0.962105211115006
"10649","PRRT2",25.5364624330965,0.133009371867433,0.374863470003988,0
.354820841481349,0.722723813741366,0.962105211115006
"10650","N4BP1",50.7327251787344,0.0775091320989599,0.219894852593509,
0.352482703368418,0.724476287900027,0.962105211115006
"10651","MLYCD",24.9663267715891,0.10791861086807,0.303040084762627,0.
3561199204145,0.721750759556167,0.962105211115006
"10652","ZNF287",10.593986018151,-0.164487784949461,0.462994364672165,
-0.355269518379409,0.722387688737732,0.962105211115006
"10653","MIEF2",9.52924011443617,-0.163200997979733,0.4572748427678,-0
.356899139676934,0.721167313746887,0.962105211115006
"10654","ACE",55.5852022877633,0.124610896714369,0.350312199620838,0.3
55713837112274,0.722054881437201,0.962105211115006
"10655","AC023043.1",2.68579941878793,0.334808088840924,0.942029188431
219,0.355411586979048,0.722281269620986,0.962105211115006
"10656","ARHGEF18",14.5177414851628,0.154594207171548,0.43655974723136
5,0.354119242903119,0.723249521537965,0.962105211115006
"10657","ADAMTS10",16.309723049472,-0.119645725448235,0.33814431360852
8,-0.353830363643938,0.723466016737933,0.962105211115006
"10658","AC022098.1",3.34250074195699,0.268787090730439,0.757239727529
2,0.354956404106617,0.722622251747196,0.962105211115006
"10659","BISPR",3.30000452995314,-0.25758590653243,0.72428825460107,-0
.355640043720307,0.722110151134664,0.962105211115006
"10660","SUGP1",13.1131072364608,0.139234813266703,0.3949328806047,0.3
52553104855474,0.724423499651682,0.962105211115006
"10661","TP53INP2",15.4260892579122,-0.163698429672842,0.4589699574765
76,-0.35666480345001,0.721342757572377,0.962105211115006
"10662","ANKRD54",18.2557768235748,-0.119267486887762,0.33506567485068
8,-0.355952566436146,0.721876088137994,0.962105211115006
"10663","SPIN2B",20.8491839792446,0.107296306651921,0.304429574054855,
0.352450339245249,0.724500555516901,0.962105211115006
"10664","AC073263.2",2.18995180801799,0.41076444199924,1.1664066142459
8,0.352162305136427,0.724716544559682,0.962211558458987

"10665","PALB2",7.79672160746945,0.198695107083225,0.564158142116788,0
.35219753514094,0.724690125349084,0.962211558458987
"10666","NAP1L5",14.422074220845,-0.131198659424611,0.372920299352603,
-0.351814207090292,0.724977603174138,0.962310544112265
"10667","TRAPPC2L",134.620486312769,0.119024492928129,0.33819618664697
1,0.351939192775031,0.72488386558726,0.962310544112265
"10668","U2AF1",12.875856106062,-0.185040691681574,0.525996031747431,-
0.351791041211552,0.724994977725258,0.962310544112265
"10669","PGAP1",17.2882754657623,0.119162669843687,0.339034109600741,0
.351476935415192,0.725230572971216,0.96244282223734
"10670","AC012485.3",3.37718674832608,-0.290046251587125,0.82512992476
0921,-0.351515855725588,0.725201379356193,0.96244282223734
"10671","MRPS5",66.5818326011891,-0.0932455419552206,0.265453538714199
,-0.351268784763173,0.725386710827079,0.962559818696602
"10672","RAD51D",17.3462310187684,0.134813001924872,0.383978922015318,
0.351094797644891,0.725517230711294,0.962642802368059
"10673","HYI",101.232778208255,0.0783461530930714,0.223884325310851,0.
349940322906894,0.726383484622706,0.962807652663906
"10674","CWC22",53.7405580224237,0.0732777687320052,0.20930120429824,0
.35010677065952,0.726258569827463,0.962807652663906
"10675","SPRY1",525.848949647143,0.080604142014936,0.229909991941443,0
.350589991040779,0.725895966501679,0.962807652663906
"10676","GPX8",178.071606542281,-0.0834757972215335,0.23807276173635,-
0.350631448187162,0.725864860369206,0.962807652663906
"10677","ZSCAN9",6.89713266882004,-0.179621422247989,0.513011210268276
,-0.350131573448575,0.726239956589635,0.962807652663906
"10678","TNS3",16.4340187491734,0.165677861412663,0.473083153157102,0.
350208753592299,0.726182037832258,0.962807652663906
"10679","GNPTAB",43.7864101020127,0.105131697225087,0.299952384087364,
0.35049462115449,0.72596752616574,0.962807652663906
"10680","SDSL",6.94424662661256,-0.23634449063129,0.675203436787481,-0
.350034489983912,0.72631281373794,0.962807652663906
"10681","KIAA0753",8.37373493141817,0.159458212545303,0.45459652176134
9,0.350768659486168,0.725761911191208,0.962807652663906
"10682","SAMD4B",122.42755957751,0.0679016745065401,0.193766911484341,
0.350429668235836,0.72601626418794,0.962807652663906
"10683","SULF2",44.1011090213251,0.109891923416312,0.314037562461001,0
.349932417495308,0.726389417613595,0.962807652663906
"10684","BCL9",11.8309558725246,0.157223763323301,0.449760717590305,0.
349572021686693,0.726659911157545,0.962939716302291
"10685","RRP15",51.9091826684411,-0.099854018031866,0.285682579296356,
-0.349527851077967,0.726693065565416,0.962939716302291
"10686","HOXB4",13.9830531277948,-0.133120484099068,0.380764621066209,
-0.349613584703082,0.726628714472956,0.962939716302291
"10687","DCLRE1B",4.16378669056023,0.232462048625875,0.665704495178334
,0.349197054112728,0.726941377652531,0.963178619590141
"10688","BPTF",221.98742260817,0.0849218005982702,0.24325680157048,0.3
49103499059472,0.727011609772591,0.963181548875365
"10689","TNFRSF11B",4.15005083882334,-0.247353586108756,0.709163756946
637,-0.348796147132162,0.727242356130246,0.963243546106393

"10690","RORB",2.40273391051377,0.399943958914017,1.14678199469394,0.348753259786536,0.727274556037077,0.963243546106393
"10691","DTD2",14.001063911237,-0.134786321882088,0.386764053219761,-0.348497542002699,0.727466559467639,0.963243546106393
"10692","ZNF304",4.46616833922988,-0.234827915605272,0.673791652203156,-0.348517104415637,0.727451870598757,0.963243546106393
"10693","AP000550.1",2.11425824272981,0.364547992460063,1.04586035572711,0.348562779403392,0.727417574917482,0.963243546106393
"10694","ZRSR2",24.988699610151,0.0994449376174689,0.285235307202505,0.348641753339699,0.727358277559302,0.963243546106393
"10695","VAMP3",82.5752950666393,-0.0760826101638637,0.21870922087901,-0.347871067612428,0.727937014497018,0.963352292554777
"10696","RBKS",4.89713217912516,-0.22123863402934,0.636192881264273,-0.3477540232605,0.728024921089595,0.963352292554777
"10697","RAB7A",215.111267887512,-0.0575717191615371,0.165374200961119,-0.348129991419112,0.727742561534403,0.963352292554777
"10698","SRD5A3-AS1",1.75347642873633,0.374223662474145,1.07464736757084,0.34822926456336,0.727668011596808,0.963352292554777
"10699","MAK16",21.865331325492,0.104737171584442,0.300891668171033,0.348089304769007,0.727773116234256,0.963352292554777
"10700","ALKBH8",9.68582183533162,-0.159047196275997,0.457250906981924,-0.347833528260851,0.727965208175765,0.963352292554777
"10701","AC091271.1",13.6149332666026,0.151637528786384,0.435801957983849,0.347950544985857,0.727877324762605,0.963352292554777
"10702","ROCK2",154.489708003693,0.0651650678335337,0.187496542167905,0.347553437946486,0.728175579749907,0.963461615516603
"10703","DPM3",88.264615516359,-0.0819513288441534,0.236067973595423,-0.347151405571867,0.728477575941971,0.963571080178514
"10704","IPO9-AS1",2.82663132905575,0.269708448485047,0.77647210690859,0.347351110342974,0.728327557676075,0.963571080178514
"10705","MGAT5",13.7057999453896,-0.167788898610489,0.483563042831154,-0.34698453717249,0.728602935494936,0.963571080178514
"10706","TMF1",125.983131716857,-0.0665017009003368,0.19175298127684,-0.346809214946839,0.72873465378755,0.963571080178514
"10707","TYW1B",6.95968917935437,0.210099579326494,0.60540406697443,0.347040250945933,0.728561079826184,0.963571080178514
"10708","SYBU",9.15837315938477,-0.178136006287426,0.513632211895768,-0.34681626689639,0.728729355555341,0.963571080178514
"10709","PRMT3",13.4728311168033,0.136021961026524,0.391701176381907,0.347259516254051,0.728396361883321,0.963571080178514
"10710","CHDH",4.80468632691628,-0.221615789549169,0.639374112529683,-0.346613641694603,0.728881595976897,0.963675387398026
"10711","ARHGAP27",4.10513390144792,0.233311625809861,0.673615120362886,0.346357465497765,0.729074086707942,0.963839890559655
"10712","ZNF425",2.93188288335772,0.306271114528293,0.884630574173034,0.346213576005554,0.729182212728664,0.963892842815337
"10713","SLC35E2A",18.580687263433,0.15919198121994,0.465358339626825,0.342084728400049,0.732287129901417,0.963917147058281

"10714","C1orf122",210.052456167771,0.130758688375646,0.37793105561253
3,0.345985561212265,0.729353565890294,0.963917147058281
"10715","PTPRF",15.8401975777466,-0.142672053917825,0.418525885023504,
-0.340891827777422,0.733185017230023,0.963917147058281
"10716","TESK2",7.02235384552325,0.175215179507343,0.519564236024998,0
.337234873685402,0.735939850059821,0.963917147058281
"10717","LRRC8C",7.33130193556306,-0.203950287788354,0.593376675943574
,-0.343711332205697,0.73106338798793,0.963917147058281
"10718","MRPS21",208.340885571433,-0.0859172533988919,0.24983036239029
7,-0.343902368698756,0.730919710439207,0.963917147058281
"10719","MYOC",1222.87040920687,0.2020122085905,0.597965237805878,0.3
37832696314081,0.735489268903272,0.963917147058281
"10720","KIAA1614",12.2951874472347,0.132465584766086,0.39037624986133
5,0.339327981180052,0.734362665828785,0.963917147058281
"10721","PTPRC",16.6827427257848,0.218796441002741,0.642488917969754,0
.340545081608771,0.733446079019362,0.963917147058281
"10722","SNRPE",203.785605295877,-0.0689281397544415,0.200780729813106
,-0.343300573808066,0.731372349178644,0.963917147058281
"10723","TAF1A",6.4987949385092,0.187670088600144,0.544912025735119,0.
344404380407948,0.73054219505905,0.963917147058281
"10724","COG2",18.897194713193,-0.11675272577433,0.340116854763121,-0.
343272390471928,0.731393549508252,0.963917147058281
"10725","COLEC11",3.59115804405073,0.286377723260114,0.830537090421473
,0.344810275859909,0.730237007310891,0.963917147058281
"10726","C2orf74",45.2540424466943,-0.11262627623242,0.329378992798683
,-0.341935213522429,0.732399648568797,0.963917147058281
"10727","AC007878.1",2.79871671579622,0.308077002952512,0.893770351419
998,0.34469369280716,0.730324660290015,0.963917147058281
"10728","AGPS",58.372848422464,-0.090316767909054,0.264647271616242,-0
.341272242700541,0.732898642062825,0.963917147058281
"10729","C2CD6",2.41792350688899,-0.330167030316257,0.960031961245015,
-0.343912540045105,0.730912060887356,0.963917147058281
"10730","CCL20",4.56496629947353,-0.295334961346875,0.873917216951157,
-0.337943864268074,0.735405491228708,0.963917147058281
"10731","MTERF4",32.6250818557249,-0.094514033298237,0.276608170165247
,-0.341689232251433,0.732584776340797,0.963917147058281
"10732","CCDC174",52.5273744289827,0.06921126393351,0.204243212515778,
0.33886689834632,0.734710001898634,0.963917147058281
"10733","CAPN7",38.9259959785505,0.0876681054441123,0.259482817786946,
0.337857073511874,0.735470897647757,0.963917147058281
"10734","TOP2B",62.0831712718156,0.0772577335999282,0.225700640430897,
0.34230179166719,0.732123787373093,0.963917147058281
"10735","MRPS22",61.0763704768252,-0.0774170530198444,0.22820837607756
9,-0.33923843791575,0.734430114980591,0.963917147058281
"10736","UVSSA",14.1226827238686,0.164085508600395,0.484392535181297,0
.338744915916141,0.734801900971876,0.963917147058281
"10737","EDNRA",8.44075804116619,0.179815989880257,0.531021520480788,0
.338622792005588,0.734893910437586,0.963917147058281
"10738","CCL28",6.35226082177672,-0.256832467172198,0.753058371634127,
-0.341052535695042,0.733064032278155,0.963917147058281

"10739","COMMD10",38.2647132891957,-0.10224208520703,0.297076572614126
,-0.344160713540452,0.730725425647236,0.963917147058281
"10740","HSD17B4",73.7570808576907,0.0605563167219459,0.17646413727008
6,0.343165005982274,0.731474329141059,0.963917147058281
"10741","CD74",233.224833348411,0.0839166234174894,0.24274195328267,0.
345703007999486,0.729565923439416,0.963917147058281
"10742","LMAN2",200.445613760932,-0.070482397396022,0.205679107922796,
-0.342681364713417,0.731838183594719,0.963917147058281
"10743","ZNF354C",12.9884027570554,0.139485641054714,0.413485743714772
,0.337340871299624,0.735859952630465,0.963917147058281
"10744","AC008443.5",4.34546426907096,-0.236465924526773,0.68505320102
6475,-0.345178920662593,0.729959864872498,0.963917147058281
"10745","FKBP5",141.516482958288,0.253881078711895,0.750655920442812,0
.338212317784866,0.735203194004275,0.963917147058281
"10746","GTF2H5",161.26264511865,0.0994734141113394,0.287801903023958,
0.345631537061306,0.729619641895091,0.963917147058281
"10747","AL009176.1",2.76668611470727,-0.321808121454774,0.93462654746
4172,-0.344317334370507,0.730607649450851,0.963917147058281
"10748","VKORC1L1",55.54342448043,0.0786005735102984,0.230149724357699
,0.341519303269454,0.732712675547376,0.963917147058281
"10749","AC002074.1",5.66053364345018,-0.21498240432874,0.630864868290
578,-0.340774094634984,0.733273653899772,0.963917147058281
"10750","RNF139",53.5539333473979,0.0844786087201379,0.250245030173658
,0.337583562245027,0.735677031134762,0.963917147058281
"10751","SMC5",83.6825244540392,-0.0920136982785113,0.269832128592988,
-0.341003492646732,0.733100952412313,0.963917147058281
"10752","SHC3",22.5793293375112,-0.153299068703328,0.454242150151248,-
0.337483143412129,0.735752717197612,0.963917147058281
"10753","PTPN3",1.63184113304933,-0.400803382702331,1.16579426012986,-
0.343802844472476,0.730994560912509,0.963917147058281
"10754","WHRN",3.02908808738645,-0.281226027822726,0.817844288092384,-
0.343862556622709,0.730949652115258,0.963917147058281
"10755","IDI1",42.4648331550946,-0.0994158205410144,0.289658101443844,
-0.343217814538801,0.731434603704249,0.963917147058281
"10756","MACORIS",1.93440031341143,-0.337499708907209,0.98600310403138
7,-0.342290716456472,0.732132121298869,0.963917147058281
"10757","TIMM23",26.31632707294,-0.104893771701705,0.309221457387347,-
0.339218929332933,0.734444810242322,0.963917147058281
"10758","NDST2",18.2008016490728,0.114403838826625,0.333192728291826,0
.343356349381144,0.731330393766193,0.963917147058281
"10759","TWNK",3.08018752283801,-0.262970800566393,0.761370292655065,-
0.345391464709446,0.72980009300658,0.963917147058281
"10760","POLL",17.2797459765145,0.121880320784302,0.355079783221004,0.
343247705286682,0.731412118594961,0.963917147058281
"10761","CHID1",129.371811038289,0.0900888973783321,0.264564473938603,
0.34051774237549,0.733466663771477,0.963917147058281
"10762","TAF6L",7.81538173819199,-0.187940836045138,0.55642841004754,-
0.337762832830698,0.735541920579896,0.963917147058281
"10763","FDXACB1",2.54998662468938,0.293423120409599,0.851464067780021
,0.344610103365403,0.730387509187886,0.963917147058281

"10764","NECTIN1",12.8061903547943,0.140623397432747,0.415243160090842
,0.338653133749351,0.734871050286702,0.963917147058281
"10765","FBXL14",5.64667431949045,0.192733136240673,0.557386434552193,
0.345780098497581,0.729507982739749,0.963917147058281
"10766","TEAD4",1.88663263738447,-0.333133971000383,0.97338823245637,-
0.342241625584183,0.732169061805825,0.963917147058281
"10767","LRRC23",14.3771993307009,-0.12942828663594,0.378588033323917
,-0.34187104515586,0.732447940772276,0.963917147058281
"10768","EMG1",42.3243206384467,-0.0890808240036271,0.260329385095733,
-0.342185051337438,0.732211634264741,0.963917147058281
"10769","AC079630.1",4.42360045372287,0.227239772109648,0.672276975518
058,0.33801510446574,0.735351805306315,0.963917147058281
"10770","CDK4",135.096772746566,-0.0914077338632441,0.268982206309296,
-0.339828180895121,0.73398592426304,0.963917147058281
"10771","NFKBIA",949.127944731876,-0.0926099331607752,0.26889300889104
8,-0.34441182960729,0.730536593714139,0.963917147058281
"10772","GNPNAT1",37.398276990945,-0.0857426270886705,0.25184014353870
4,-0.340464494198056,0.733506756906525,0.963917147058281
"10773","ZFP36L1",1579.33503688492,0.0872878322374259,0.25265485734674
3,0.34548250191617,0.729731662852242,0.963917147058281
"10774","CEP170B",11.5241275342268,-0.144802376647456,0.42823711451265
2,-0.338135980605618,0.735260717176758,0.963917147058281
"10775","TMEM62",7.1512846680334,0.186965171510574,0.541855735352375,0
.345046032204473,0.730059764649317,0.963917147058281
"10776","SQOR",66.3216635087416,0.0820620006680563,0.23935651115304,0.
342844237964295,0.73171564356207,0.963917147058281
"10777","PCLAF",3.39278019668986,0.300138910420576,0.883817875827573,0
.339593618356652,0.734162584644139,0.963917147058281
"10778","PIF1",2.80940532600619,-0.272352853207392,0.801786764517989,-
0.339682400932526,0.734095716540859,0.963917147058281
"10779","FURIN",27.4987904348185,-0.0927634034727435,0.26838020023034,
-0.345641755215655,0.729611961720515,0.963917147058281
"10780","DHODH",5.86207043179742,0.205242521897037,0.598367249671373,0
.343004270387054,0.731595247531781,0.963917147058281
"10781","C16orf95",10.7299715570218,0.15219958054599,0.451218134039509
,0.337308208744695,0.735884572259111,0.963917147058281
"10782","SAT2",221.721517062008,-0.0894951115177752,0.264908829050468,
-0.337833630681767,0.735488564738026,0.963917147058281
"10783","MED9",9.55523207145009,-0.161380325941309,0.47475473390047,-0
.33992357404306,0.733914083101499,0.963917147058281
"10784","LGALS9",11.3136004744679,-0.177039304900969,0.512735301466899
,-0.345284017688021,0.729880860733895,0.963917147058281
"10785","SDF2",108.085369472223,-0.0862719120045423,0.253124406521158,
-0.340828105792836,0.733232990573802,0.963917147058281
"10786","GJD3",3.14019407946587,-0.349538264534484,1.0282644559766,-0.
339930318998051,0.733909003522928,0.963917147058281
"10787","TTC25",1.71395274435033,-0.33825159730922,0.99237403275914,-0
.340850915222725,0.733215818286115,0.963917147058281
"10788","GPS1",48.1599784101822,-0.0804799925706314,0.235013038425597,
-0.342449053506922,0.732012978106259,0.963917147058281

"10789","SEH1L",22.7403986347229,0.121670612728484,0.357305397786351,0
.340522739041396,0.733462901576711,0.963917147058281
"10790","ZNF24",70.4237827018094,0.0634513635510793,0.185319888343384,
0.342388310927041,0.732058684047884,0.963917147058281
"10791","POLRMT",12.0206372653024,0.130922276911219,0.387964148705987,
0.337459730101083,0.735770364269497,0.963917147058281
"10792","SF3A2",34.7789534698014,-0.133456924462514,0.392819689574892,
-0.339740924409722,0.734051639693841,0.963917147058281
"10793","CC2D1A",17.9757132396458,-0.125148170626897,0.369536996629795
,-0.338662087337013,0.734864304497645,0.963917147058281
"10794","NAPA-
AS1",5.20246778785861,0.197293842921632,0.580285258081003,0.3399945805
5178,0.733860609179007,0.963917147058281
"10795","BCL2L12",13.3524478127117,-0.149135747062907,0.43532671436243
8,-0.342583494517045,0.73191182093442,0.963917147058281
"10796","PPP6R1",27.8155175727153,0.0902114387441163,0.266086658832263
,0.339030296144928,0.734586907284472,0.963917147058281
"10797","ZNF587B",3.2283632932447,0.297407920379375,0.880464221243104,
0.337785355956284,0.735524946192857,0.963917147058281
"10798","CDS2",31.6383530440737,0.0990914569220737,0.292557707313413,0
.33870738813221,0.734830174402254,0.963917147058281
"10799","MAP1LC3A",136.110292218837,0.0666894075518867,0.1958142783661
77,0.340574794179084,0.733423707508547,0.963917147058281
"10800","KIAA1755",19.8372088336047,-0.146993953566844,0.4320218223676
42,-0.340246593936534,0.733670832152506,0.963917147058281
"10801","SLC13A3",2.86541794102281,-0.256334176425007,0.75521453306095
4,-0.339419019634144,0.734294092514781,0.963917147058281
"10802","DPM1",60.5306293713078,0.0880323647711591,0.259361954898954,0
.33941895913553,0.734294138083727,0.963917147058281
"10803","CBLN4",4.05295472466137,1.04596802192705,3.10134158562953,0.3
37263082136349,0.735918587201224,0.963917147058281
"10804","LINC01426",7.98896455396612,0.223733485228341,0.6572353887234
56,0.340416065639583,0.733543221747885,0.963917147058281
"10805","LRP5L",3.1731583750508,-0.29986961490806,0.873983813520205,-0
.343106600224384,0.73151826593222,0.963917147058281
"10806","TFIP11",16.9521937304009,0.122804342395435,0.35839976487244,0
.342646269422478,0.731864588937341,0.963917147058281
"10807","LINC01638",4.82918889130812,-0.219766971277526,0.641230731853
006,-0.342726822593876,0.731803982016409,0.963917147058281
"10808","CBY1",72.0922138958675,0.0895358321216224,0.25959901614815,0.
344900506366039,0.730169169915184,0.963917147058281
"10809","TCF20",15.7491125696391,0.139579806270455,0.410151986920811,0
.340312398138897,0.733621281408225,0.963917147058281
"10810","ATG4A",10.1214546683218,0.159746610661169,0.469426023975152,0
.340301991160219,0.733629117817758,0.963917147058281
"10811","DKC1",43.7809474546197,0.0879311425443812,0.255031860978856,0
.34478493081957,0.730256062682141,0.963917147058281
"10812","ARRB1",34.0507533337438,0.0959994797629527,0.284790687771055,
0.337087846917688,0.736050678610487,0.963973141798418
"10813","ZFP69",4.05378202361455,0.235748205878421,0.700910526660253,0

.336345648854399,0.736610230173196,0.964228861510347
"10814","CAVIN2",9.19483445042654,0.216256578992208,0.642684217109293,
0.33648963711121,0.736501664857737,0.964228861510347
"10815","OTUD4",32.1659995231918,0.0931469314931117,0.277342223786696,
0.335855573022127,0.736979780512362,0.964228861510347
"10816","DENND3",4.57632227369517,-0.252277942512,0.749695923956705,-0
.336507021647579,0.73648855749411,0.964228861510347
"10817","CDK5RAP2",37.8706020754548,0.112641524081013,0.33511432514971
1,0.336128645144282,0.736773858145075,0.964228861510347
"10818","FAM102A",45.9694916251946,0.0996599139961784,0.29675224843812
3,0.335835413280648,0.736994983624752,0.964228861510347
"10819","USP5",31.3246380690719,-0.0987005613310646,0.293482083007444,
-0.336308643851902,0.736638132324088,0.964228861510347
"10820","CKAP2",12.775831104155,-0.139202797665724,0.414157228724466,-
0.336110993630232,0.736787168495266,0.964228861510347
"10821","SCG5",8.25965982054327,0.187313859745635,0.5570633077258,0.33
625237409791,0.736680560960375,0.964228861510347
"10822","CU638689.5",1.90930771993552,0.349340436313322,1.040064362702
,0.335883478793335,0.73695873603855,0.964228861510347
"10823","CASK",36.319159820354,0.0838375235026751,0.249439664030975,0.
336103417346907,0.73679288151294,0.964228861510347
"10824","ALX4",2.32192757190361,-0.2895261990898,0.862425970376297,-0.
335711364261761,0.737088535262906,0.964262163647704
"10825","PACRGL",17.9851517475906,-0.118324436726559,0.353226613245944
,-0.334981658486111,0.737638922111554,0.964358520644409
"10826","RANBP3L",2.40349343140497,0.284999784699534,0.850688629713818
,0.335022445045976,0.737608154945873,0.964358520644409
"10827","AL590428.1",2.31015928299479,-0.302314477407338,0.90182208199
6987,-0.335226297340042,0.737454386148795,0.964358520644409
"10828","STARD3NL",35.1932809913386,0.0865334490536349,0.2581241355595
97,0.335239666240569,0.737444302156707,0.964358520644409
"10829","CCSER2",103.730014733607,0.0548514893218378,0.16359793673859,
0.335282280542962,0.737412159030596,0.964358520644409
"10830","ACAD8",19.3142029650446,-0.115017409778978,0.343156300570332,
-0.335174990486309,0.737493086677273,0.964358520644409
"10831","TSR1",26.8369667170813,0.0976539963400665,0.291386216075678,0
.335135949995328,0.737522535189508,0.964358520644409
"10832","ALDH4A1",2.23563479262938,-0.312557689412924,0.93409987436932
,-0.334608426774444,0.737920487362937,0.964459488744617
"10833","PUM1",122.898884762374,0.065641674275213,0.196171751538546,0.
334613285350185,0.737916821837202,0.964459488744617
"10834","TAS2R14",3.47316696145699,0.252091149216748,0.753088666256636
,0.334742986466405,0.737818971746885,0.964459488744617
"10835","CYB561A3",40.1344982422196,-0.0844276972766153,0.252454803631
281,-0.334426978858064,0.738057384011326,0.964549382335061
"10836","C2orf92",1.6770670598505,0.34191724413218,1.02320130392183,0.
334164198991581,0.7382556577405,0.96463044325971
"10837","TAB1",14.6595465039432,0.117926962633859,0.352869853369174,0.
334193928747106,0.738233225051223,0.96463044325971
"10838","ZCCHC7",59.3968756661485,-0.0721777095048779,0.21611556987756

6,-0.333977369357368,0.738396635783698,0.964725628593574
"10839","TANC1",13.0275366962774,-0.143492728963684,0.430011667127189,
-0.333694966748987,0.738609748048766,0.964737019866296
"10840","SLC25A15",3.15230604687426,-0.265822974331405,0.7964670268265
19,-0.333752641826948,0.738566222503456,0.964737019866296
"10841","POFUT2",27.0100895707045,-0.0984338028606812,0.29488134870055
6,-0.333808168249522,0.73852431927155,0.964737019866296
"10842","STARD7-
AS1",2.40824123141012,-0.28974446240206,0.869755775829112,-0.333133128
234596,0.739033793497456,0.964845889353123
"10843","LTB",4.79258601911689,-0.252647428439948,0.758374319499563,-0
.33314343846277,0.739026011177267,0.964845889353123
"10844","SMARCD1",36.9046868471196,0.0881563283556779,0.26444626531583
2,0.333361971477993,0.738861065355766,0.964845889353123
"10845","IGHG2",10.8728096346101,0.268863038529464,0.806734997156077,0
.333273056799651,0.738928175526217,0.964845889353123
"10846","HCST",9.00450630919404,-0.18903917157881,0.567131280973567,-0
.33332524218078,0.738888787302295,0.964845889353123
"10847","ZNF468",6.91058184121405,0.193466013944761,0.580953132436219,
0.33301483913765,0.739123081855455,0.964873498577785
"10848","RNF207",14.1360958489647,-0.159695153508234,0.483070664613862
,-0.330583422274017,0.740959169918126,0.965100233273692
"10849","SH3D21",3.87943299355481,0.23355521641667,0.705461800939475,0
.331067133763502,0.740593776515248,0.965100233273692
"10850","YIPF1",13.708756443166,-0.136370833416904,0.412450760186813,-
0.330635427499606,0.74091988260848,0.965100233273692
"10851","SEC22B",101.949774865286,0.0579484723087178,0.175418059384232
,0.330344962839823,0.741139322570926,0.965100233273692
"10852","RUSC1",42.9453050042316,-0.0835576830608345,0.252279399624379
,-0.33121088438154,0.740485199243231,0.965100233273692
"10853","ZNF670",5.30893376168954,-0.196008069099202,0.592419409920192
,-0.33086030912729,0.740750003724856,0.965100233273692
"10854","DYNC2LI1",53.2891993102342,0.0719768547341234,0.2185358774813
52,0.329359442319787,0.741884019494673,0.965100233273692
"10855","ZAP70",1.59134724552456,-0.417907311577322,1.26812499618945,-
0.329547412781138,0.741741963160907,0.965100233273692
"10856","LNPK",76.5501957180353,0.0791279540001754,0.239376034490596,0
.330559214787577,0.740977457676439,0.965100233273692
"10857","STBD1",11.3626783871374,-0.135386795096239,0.409162370475932,
-0.330887698540701,0.740729314223081,0.965100233273692
"10858","PCDHGA9",2.13621382497494,-0.331041219945968,1.00621914604892
,-0.328995151052188,0.742159353115624,0.965100233273692
"10859","DOCK2",3.12417004124311,-0.333890565814031,1.01199115973393,-
0.329934271265587,0.741449627377944,0.965100233273692
"10860","HLA-
DPB1",118.582385399676,-0.0845039330282756,0.256792035655505,-0.329075
365645842,0.742098723589113,0.965100233273692
"10861","QKI",246.325195516064,0.0494708310689406,0.148984377928907,0.
332053815015069,0.739848623581362,0.965100233273692
"10862","AHR",121.361079801885,0.070392403022901,0.213720983146155,0.3

29365895602129,0.741879142361147,0.965100233273692
"10863","EPHB4",10.8783318746634,0.141436603482035,0.427717050568569,0.330677964074664,0.740887748886185,0.965100233273692
"10864","AK3",149.373117545442,0.0847436014315002,0.256033590591504,0.330986263309124,0.740654861608,0.965100233273692
"10865","AL590399.1",2.85648043706729,0.308851162172268,0.937552862143149,0.329422664729823,0.741836238964326,0.965100233273692
"10866","VPS37C",12.9960464037222,-0.134957061862172,0.407163975081951,-0.331456293094222,0.740299849840491,0.965100233273692
"10867","MTA2",13.1634675638658,0.146074583996074,0.442645717413515,0.330003382501072,0.741397406297733,0.965100233273692
"10868","DLAT",17.5606508967973,-0.109833475982547,0.333804902192727,-0.329034940053495,0.742129278733347,0.965100233273692
"10869","KANSL2",15.7853774263833,0.115059920534273,0.349720664760345,0.329005209380811,0.742151750524717,0.965100233273692
"10870","HOXC5",12.9925969424367,0.128494865328708,0.389725774616122,0.329705843692977,0.741622237807237,0.965100233273692
"10871","SCYL2",20.1268486867383,0.112874381048122,0.342361918408135,0.329693155047584,0.741631826315231,0.965100233273692
"10872","SPATA5L1",7.50103698961722,-0.179564247368516,0.539819554782375,-0.332637537446946,0.739407904621783,0.965100233273692
"10873","ARPIN",36.2032777454501,0.0945878407976514,0.287168361001141,0.329381135400482,0.741867624770252,0.965100233273692
"10874","SLCO3A1",94.7934899771673,0.0936884019689258,0.282064814444595,0.332152034465571,0.739774460480263,0.965100233273692
"10875","AC009061.2",4.63985691096734,0.264541954707141,0.796409898773654,0.332168089716733,0.73976233778345,0.965100233273692
"10876","GCSH",179.106857510946,-0.0975975696475935,0.296568524375347,-0.329089440132463,0.742088085671707,0.965100233273692
"10877","ZC3H18",38.8555633484781,0.0937542154725296,0.284369713096859,0.329691282702093,0.741633241205651,0.965100233273692
"10878","ZNF232",3.80706788748416,-0.218586943558174,0.662735734929226,-0.329825195832419,0.741532048193246,0.965100233273692
"10879","SP2-AS1",3.75953041588176,0.292706585915776,0.88795688716887,0.329640537897094,0.741671588274341,0.965100233273692
"10880","CEP112",31.5390959301737,-0.0904468086986467,0.272324041185826,-0.332129357014529,0.739791583453244,0.965100233273692
"10881","RNF125",5.7894966900103,-0.213823390346057,0.643618908722364,-0.332220491735573,0.739722771503388,0.965100233273692
"10882","STAP2",6.36889010966554,0.226941546934274,0.685659600554591,0.330982818224545,0.740657463871699,0.965100233273692
"10883","MAP2K7",35.0832870836705,-0.0893746067803603,0.270963817212732,-0.329839635784998,0.741521136742276,0.965100233273692
"10884","TASP1",38.6645298868111,0.0871550797417785,0.262162705567394,0.332446522296717,0.739552114437206,0.965100233273692
"10885","GMEB2",8.02620055204496,0.175672407729406,0.531177510921977,0.330722600481499,0.74085402935831,0.965100233273692
"10886","SOD1",497.826624554509,0.0669857328335292,0.202507047401771,0.330782230509887,0.74080898402695,0.965100233273692

"10887","URB1-
AS1",8.74148509028241,-0.18620827703008,0.564685657505971,-0.329755634
050456,0.741584612804059,0.965100233273692
"10888","PIGP",76.5683034629743,0.0864934263235882,0.260772410873656,0
.331681660777735,0.740129650069668,0.965100233273692
"10889","TBX1",6.58261333885633,0.237801059055287,0.717842627526254,0.
331271855329586,0.740439148416573,0.965100233273692
"10890","G3BP1",116.689680547349,0.0583357994764737,0.177376179667779,
0.328881812573341,0.742245021779868,0.965123003526439
"10891","ARF4-
AS1",4.22498218615803,0.223519776669138,0.680114980725796,0.3286499827
2881,0.742420263943835,0.965150412752032
"10892","NDUFB1",215.59075213443,-0.0872612870015224,0.265455090104299
,-0.328723351913262,0.74236480210467,0.965150412752032
"10893","DLGAP1-
AS1",14.52734841481,0.141006131755913,0.429133436187911,0.328583419200
568,0.742470582352252,0.965150412752032
"10894","SAMD11",31.1760960326487,-0.139474032862782,0.436376428316053
,-0.319618622392147,0.749257455699264,0.965198383399543
"10895","B3GALT6",25.4146345696377,-0.0961083886226266,0.3025228510524
56,-0.317689682905843,0.750720337953652,0.965198383399543
"10896","MTHFR",29.0815112665918,-0.104531837713011,0.326765420784577,
-0.319898713462477,0.749045113176769,0.965198383399543
"10897","ZSCAN20",2.33213019762411,0.30817149280691,0.948420063293881,
0.324931435693826,0.745232964458001,0.965198383399543
"10898","ZMPSTE24",60.7645061189235,0.0677660835444862,0.2121209417530
9,0.31946908675979,0.749370829373753,0.965198383399543
"10899","ITGB3BP",26.6067342099774,-0.138970049070082,0.43553765781827
,-0.319076999601417,0.749668124447952,0.965198383399543
"10900","PEX11B",10.986308378801,-0.15904864942997,0.494937085192668,-
0.32135124683182,0.74794422469691,0.965198383399543
"10901","HIST2H2AA4",8.08786457692119,0.237719169138511,0.727551374626
887,0.326738670874509,0.743865548991003,0.965198383399543
"10902","LMX1A",4.3707595450276,0.255774156159267,0.794716596949229,0.
321843229575344,0.747571462793099,0.965198383399543
"10903","C1orf74",1.55669127502036,0.344745452545999,1.06984744563068,
0.322237954536377,0.747272433125485,0.965198383399543
"10904","DDX1",111.601417337625,-0.0726174953657002,0.227174365258025,
-0.319655324152535,0.749229630293749,0.965198383399543
"10905","AC079807.1",2.03500700589157,0.310370025848101,0.975351272698
938,0.318213585746664,0.750322928044392,0.965198383399543
"10906","PSME4",22.5670352397073,0.125295326410413,0.389164707393455,0
.321959633106545,0.747483275643811,0.965198383399543
"10907","POLE4",139.855349704555,-0.0701910637473376,0.216667650473945
,-0.323957284780629,0.745970373519267,0.965198383399543
"10908","MALL",7.53224565161262,0.179918590631653,0.550716693988808,0.
326698995319197,0.743895560211016,0.965198383399543
"10909","BBS5",18.1176705841227,-0.122531508459646,0.373582365312625,-
0.32799061154053,0.742918761236426,0.965198383399543
"10910","PHOSPHO2",7.46817327722058,-0.183949841783977,0.5743602788825

55,-0.320269086403154,0.748764355415921,0.965198383399543
"10911","NEMP2",6.15601778089503,-0.175347109961635,0.541257076604711,
-0.323962711141961,0.745966265247637,0.965198383399543
"10912","CASP10",3.86831418471416,-0.233360019536462,0.710708771590163
,-0.328348303643889,0.742648325738189,0.965198383399543
"10913","FANCD2",2.23891085827964,0.298814679543092,0.939663783610638,
0.318001698857545,0.750483648250036,0.965198383399543
"10914","TRANK1",23.5636658193133,0.108086117566427,0.334688891260431,
0.322945040569995,0.746736864872498,0.965198383399543
"10915","NME6",27.6737691611717,-0.0896176152847352,0.278697513261017,
-0.321558718756141,0.747787021688261,0.965198383399543
"10916","COL8A1",42.4753641666674,-0.338952925121451,1.04573137769738,
-0.32413001307066,0.745839605319943,0.965198383399543
"10917","IFT122",18.2546921806426,0.114553643317996,0.350575653585155,
0.326758695723777,0.743850402024788,0.965198383399543
"10918","CDV3",141.188265991354,-0.0626921420036973,0.193020960893722,
-0.324794476793719,0.745336625001733,0.965198383399543
"10919","SLC35G2",21.1301221865768,-0.100423781424335,0.30727296932709
3,-0.326822699843259,0.743801989429392,0.965198383399543
"10920","CP",123.425237739744,0.12029519454495,0.374961181881596,0.320
820395170764,0.748346502623375,0.965198383399543
"10921","EIF2B5",27.1927119995149,-0.0901061427262746,0.27813544791795
4,-0.323964972464979,0.74596455321317,0.965198383399543
"10922","FYTTD1",65.0212888114389,0.066721256097725,0.205596069720236,
0.324525931787098,0.74553989294837,0.965198383399543
"10923","ATP5ME",361.007248747632,-0.0804914721509192,0.24993743859068
7,-0.322046479330121,0.747417483221282,0.965198383399543
"10924","HTT",40.4906613843434,0.0763271887782933,0.232651876040385,0.
328074675680002,0.742855201130255,0.965198383399543
"10925","KLHL5",22.1660877865165,-0.0948916251893333,0.298228097053752
,-0.318184725472833,0.750344818469632,0.965198383399543
"10926","REST",87.391469238951,-0.0647385337183895,0.200477339642638,-
0.322921951347666,0.746754351414712,0.965198383399543
"10927","SEC31A",271.168681166064,0.0475783561981483,0.149299845622874
,0.318676526420056,0.7499718165141,0.965198383399543
"10928","SORBS2",5.96580213909071,-0.187421374437246,0.588302046536575
,-0.318580184346842,0.750044881681959,0.965198383399543
"10929","LHFPL2",45.4043174026208,0.0937551893220239,0.287305068202315
,0.326326263259663,0.744177519646871,0.965198383399543
"10930","AC010226.1",29.914598174742,-0.10621989701381,0.3265499459629
62,-0.325279174983719,0.744969790580448,0.965198383399543
"10931","PCDHB3",2.35886227578906,0.316892185470202,0.975355717429235,
0.324899090462545,0.745257445286063,0.965198383399543
"10932","PCDHGB6",7.086334363594,0.165381569000197,0.51507923077712,0.
321079863287594,0.748149869832557,0.965198383399543
"10933","LCP2",6.27328036585467,-0.217672883714084,0.670609433121095,-
0.324589653773596,0.745491658694649,0.965198383399543
"10934","RNF44",16.3203439726752,0.11372802155739,0.35735049342195,0.3
18253433676116,0.750292703847979,0.965198383399543
"10935","BPHL",26.4863466967828,0.0922946842555653,0.28451492053611,0.

324393125259143,0.745640423838625,0.965198383399543
"10936","LYRM4",60.1276818890212,-0.0725318246488217,0.223815081714758
,-0.324070317751241,0.745884798301714,0.965198383399543
"10937","ZNF184",6.54168185388108,-0.173581977962162,0.543870256271528
,-0.319160637965649,0.749604703605421,0.965198383399543
"10938","AL035587.2",2.29417893992313,-0.313395715687316,0.96059171944
0647,-0.326252776642512,0.744233113883148,0.965198383399543
"10939","CEP57L1",37.4041659522682,-0.0797802453958048,0.2445600844843
64,-0.326219405607482,0.744258360242539,0.965198383399543
"10940","MFSD4B",11.8310945549907,0.171972512932225,0.526428447511818,
0.326677849088625,0.743911555714505,0.965198383399543
"10941","LTV1",52.0176073340788,0.0796237228660485,0.249431026790796,0
.319221405173586,0.749558626440312,0.965198383399543
"10942","PDGFA",8.86585011808775,-0.185073445701364,0.575261803721335,
-0.321720379319702,0.747664537555022,0.965198383399543
"10943","ZMIZ2",18.1693705419936,0.111386491679795,0.34484883146235,0.
323000925383608,0.746694541243044,0.965198383399543
"10944","TP53TG1",47.7131240890176,-0.0921732844065839,0.2895895871066
14,-0.318289360220158,0.750265454305891,0.965198383399543
"10945","ORC5",9.63189253102324,0.156002994731677,0.487840673069006,0.
319782673614036,0.749133082949641,0.965198383399543
"10946","CYREN",22.8027568870576,-0.0950656280394954,0.289589127848677
,-0.328277614376364,0.742701768312817,0.965198383399543
"10947","MCPH1",28.9385295156607,0.0984755616534007,0.306083162934435,
0.3217281235247,0.747658670221814,0.965198383399543
"10948","PCM1",152.418858975373,0.0729231029795433,0.229575230118068,0
.317643601803381,0.750755296240295,0.965198383399543
"10949","BAG4",32.9509041073333,-0.0964876354725905,0.295592360317488,
-0.326421276141764,0.744105642295614,0.965198383399543
"10950","GPAT4",33.5375981538152,-0.0953055263729332,0.294112343112131
,-0.324044633300541,0.745904243259591,0.965198383399543
"10951","RMDN1",38.9906469335798,-0.0824650478392823,0.259015866606606
,-0.318378363919035,0.750197948179677,0.965198383399543
"10952","C8orf37",4.66238034778082,0.198274305790437,0.604822096176144
,0.327822523422975,0.743045856338184,0.965198383399543
"10953","RANBP6",15.6121155531325,0.11503732709343,0.362122088299645,0
.317675532121202,0.750731073041365,0.965198383399543
"10954","AL162231.2",15.685567121905,0.1157358337886,0.354876851441198
,0.32612956668935,0.74432632790347,0.965198383399543
"10955","SURF1",76.1152061682992,-0.0703948856781479,0.215738689998786
,-0.326296992340799,0.744199663578702,0.965198383399543
"10956","MAN1B1-
DT",4.55821890030494,-0.227881717454968,0.709276876726254,-0.321287391
331268,0.747992610590747,0.965198383399543
"10957","SUPV3L1",14.1197839162687,-0.123409902083144,0.38770070259601
,-0.318312299298923,0.75024805565113,0.965198383399543
"10958","AIFM2",16.1236635976585,-0.122035324452461,0.380540997747824,
-0.320689032652748,0.748446059356895,0.965198383399543
"10959","CHCHD1",81.9842789154853,0.0885970800869768,0.271673050129414
,0.326116558284941,0.744336169580862,0.965198383399543

"10960","ZFYVE27",13.925855923377,0.139098556862218,0.435507831208554,
0.319393927948927,0.74942781469748,0.965198383399543
"10961","ENTPD7",5.87779405758554,-0.194417736391011,0.605547283974948
,-0.321061197921341,0.748164014465278,0.965198383399543
"10962","LRRC27",7.92414501887946,-0.1570210166975З,0.490032550785102,
-0.320429768279598,0.748642562370396,0.965198383399543
"10963","TRIM6",4.34611326124356,0.237911897834678,0.741016877344042,0
.321061375399982,0.748163879971392,0.965198383399543
"10964","PPFIBP2",12.7868742510203,0.119879591704941,0.377069763885912
,0.317924169971932,0.75054245808003б,0.965198383399543
"10965","LRRC4C",11.1983596207225,0.229085008300315,0.70867799197001,0
.323256840054389,0.746500737390323,0.965198383399543
"10966","TMEM109",151.99677892571,-0.0560056565191131,0.17233124476293
2,-0.324988405881692,0.745189846598567,0.965198383399543
"10967","EHD1",44.1020552892863,0.0858508447592861,0.267104529648019,0
.32141291228725,0.747897499231158,0.965198383399543
"10968","XRRA1",41.2350864361805,0.15063062923477З,0.467812953479924,0
.321989008885453,0.747461021117808,0.965198383399543
"10969","ANKRD49",28.1854523476655,-0.131338787269327,0.41093538812297
5,-0.319609337782375,0.749264494869263,0.965198383399543
"10970","ALG9",17.0939951668402,0.109890989326862,0.345141233680826,0.
318394264733044,0.750185888181644,0.965198383399543
"10971","PCSK7",66.8460737008866,-0.0891780012955557,0.273846153441891
,-0.325650005211699,0.744689173961081,0.965198383399543
"10972","APOLD1",14.407155843706,0.188322686616947,0.592785334291897,0
.317691204088078,0.750719183955165,0.965198383399543
"10973","PFKM",25.2527354773829,0.10958207724578,0.338023545033465,0.3
2418474646472,0.745798169585403,0.965198383399543
"10974","HOXC-
AS2",6.67517282957753,0.175196200582295,0.54457662019108,0.32171083753
2508,0.747671766831694,0.965198383399543
"10975","RPS26",1178.20834866092,-0.207367631661424,0.648955362363669,
-0.319540670572681,0.749316555881539,0.965198383399543
"10976","PAN2",10.8429109629611,0.139607585135824,0.436524159728919,0.
319816399675382,0.749107514896508,0.965198383399543
"10977","ACTR6",34.8647363463716,-0.0906218751252416,0.28356872566191,
-0.319576409259204,0.74928945995037,0.965198383399543
"10978","RHOF",4.11247756834758,0.232361033586168,0.714454414928849,0.
325228634228971,0.745008038670723,0.965198383399543
"10979","LINC00545",1.95593221966387,-0.299992203782814,0.927976320830
873,-0.323275709787738,0.7464864479999,0.965198383399543
"10980","DNAL1",18.4958887897831,-0.109375302922373,0.342183272871674,
-0.319639537036607,0.749241599241005,0.965198383399543
"10981","DMXL2",20.3499856888619,0.11101799862341,0.347643266720697,0.
319344596173652,0.74946521873624,0.965198383399543
"10982","SNAPC5",40.5727256337538,-0.100371245847291,0.308719579694657
,-0.325121088680428,0.745089428780724,0.965198383399543
"10983","TPSAB1",2.06269400875203,0.441661946020241,1.38407089193574,0
.319103557912802,0.749647985773075,0.965198383399543
"10984","MMP25-

AS1",6.27945688070026,0.190488423111201,0.585161845310843,0.3255311750
71764,0.74477909204782,0.965198383399543
"10985","KATNB1",12.7226598370961,0.121351337096335,0.376163087799475,
0.322602990650227,0.746995928526812,0.965198383399543
"10986","NIP7",29.9549615151154,0.0963966379916155,0.298768477105982,0
.322646615618089,0.746962886016977,0.965198383399543
"10987","COG4",32.3371703231266,-0.0881922471317967,0.269756194500082,
-0.326933167541292,0.743718434253074,0.965198383399543
"10988","LINC00311",1.59535861795569,0.344063793078992,1.0607863123929
5,0.324347881434143,0.745674673159372,0.965198383399543
"10989","TMEM107",33.7755381908623,0.0869838599812361,0.27139897976750
9,0.320501794279956,0.748587970408243,0.965198383399543
"10990","NCOR1",152.045350653008,-0.0551504930860607,0.170558279109713
,-0.323352776387856,0.746428089058646,0.965198383399543
"10991","DRC3",7.61732058257195,0.179374735813271,0.558736482729186,0.
321036376463378,0.748182824321601,0.965198383399543
"10992","PROCA1",3.41419571560909,0.258487252039986,0.805219214031607,
0.321014759130971,0.748199206193622,0.965198383399543
"10993","TADA2A",11.7223800690222,-0.170112512551947,0.532612334618972
,-0.319392739324456,0.749428715922172,0.965198383399543
"10994","ACLY",56.7016538786188,0.0761739897797459,0.237481301746482,0
.320757841647102,0.748393910025847,0.965198383399543
"10995","PHB",166.131114527834,-0.0781468042670534,0.240460506052218,-
0.324988105323554,0.745190074073755,0.965198383399543
"10996","AC025048.2",1.88430300624421,0.315834243790779,0.968935836428
753,0.32595991593712,0.744454682662461,0.965198383399543
"10997","TRIM65",21.2200511783108,-0.103576327389086,0.315772541030008
,-0.328009291280468,0.742904637509074,0.965198383399543
"10998","AP000915.2",2.7896405030747,-0.341900506301598,1.043580339864
38,-0.327622601960889,0.743197030446332,0.965198383399543
"10999","DOHH",20.2986614202717,-0.100497954167501,0.316152129077681,-
0.317878467118621,0.750577126831767,0.965198383399543
"11000","ZNF653",4.08750119888243,-0.217044916164837,0.667585097686363
,-0.32511947453148,0.745090650384907,0.965198383399543
"11001","AC005253.1",2.36120496446745,-0.286476018533833,0.87923830433
7345,-0.325822950525047,0.74455831344981,0.965198383399543
"11002","BCKDHA",29.3111709193418,0.0933734548724631,0.292630885872094
,0.319082705826465,0.749663797508952,0.965198383399543
"11003","AC011468.1",2.12664919734347,-0.278895989237834,0.86647446338
8703,-0.32187444757125,0.747547811759647,0.965198383399543
"11004","ZNF600",5.84276188944637,-0.215777389034828,0.666487722950694
,-0.323752983895235,0.746125053974127,0.965198383399543
"11005","ZNF837",3.41644261203499,0.23943084103877,0.74293148464863,0.
322278495374319,0.747241722973734,0.965198383399543
"11006","ZCCHC3",25.2222713071746,0.100427396042474,0.315901593146877,
0.317907216111382,0.750555318688374,0.965198383399543
"11007","NAA20",96.3712079367116,-0.0772905547693002,0.239493351210068
,-0.322725263055449,0.746903317893766,0.965198383399543
"11008","CCT8",146.405790760489,-0.0777432491531715,0.242848253919563,
-0.32013097849541,0.748869042938326,0.965198383399543

"11009","GP1BB",16.7468690984293,0.165803611598538,0.507699289957548,0
.326578379915407,0.743986798006291,0.965198383399543
"11010","TMEM191C",2.64445690882697,-0.292927065552173,0.9003584469776
79,-0.325344940712746,0.744920021518404,0.965198383399543
"11011","TOP3B",11.4784541449021,-0.13626419485131,0.421433307323373,-
0.323335133895223,0.746441448766985,0.965198383399543
"11012","ASPHD2",4.94357662366019,-0.225666142878922,0.703892665743454
,-0.320597377784258,0.748515525029267,0.965198383399543
"11013","NONO",121.424224707382,0.0559246090047884,0.174690627620713,0
.320135142717625,0.748865886338088,0.965198383399543
"11014","CSTF2",4.94423105392206,-0.212809143893625,0.661871421352272,
-0.321526412877645,0.747811499401673,0.965198383399543
"11015","OXNAD1",7.66297344963938,0.155342394891107,0.489772857607862,
0.317172322798424,0.751112849746133,0.965570399673649
"11016","FGF2",44.6376420010791,-0.0913278031105955,0.288194677027232,
-0.316896217697893,0.751322352078539,0.965576738558006
"11017","INO80",39.2567864085897,-0.0890268823559254,0.280854808954074
,-0.316985429900484,0.75125465786293,0.965576738558006
"11018","XG",96.2193974516243,0.0674302286856679,0.212728461921007,0.3
1697793551813,0.751260344526303,0.965576738558006
"11019","PIFO",7.85484970320806,0.155843408232459,0.49271004405566,0.3
16298419552522,0.751776010641119,0.965888833605325
"11020","SRP72",165.980536795322,-0.0507475008786308,0.160425099348731
,-0.316331428714383,0.75175095832616,0.965888833605325
"11021","BX322234.1",8.43796956602029,0.238829907261547,0.754676963823
631,0.316466407098868,0.751648519156084,0.965888833605325
"11022","PABPC1L",5.02417357135416,0.193090870313473,0.610628345926711
,0.316216683358241,0.751838045480077,0.965888833605325
"11023","NARS2",13.273233788062,0.119069444524919,0.376676301821933,0.
316105483538507,0.751922444728132,0.965909626902871
"11024","NEAT1",2525.82627247233,0.10668237401715,0.337600823985461,0.
316001521435103,0.752001353325013,0.965923363849981
"11025","ARMC9",9.20085197873007,-0.16844835923549,0.533214063459558,-
0.315911321135412,0.752069818633159,0.965923685428166
"11026","FNTA",122.490638171142,-0.0719615979677589,0.228137128663512,
-0.315431330223753,0.75243418199461,0.96615221424925
"11027","IFIT2",66.6033509809845,-0.165202576212374,0.524081563095552,
-0.315223026043092,0.752592323850061,0.96615221424925
"11028","BBS4",31.472947403236,0.0927270643957133,0.294086957438356,0.
315304919345666,0.752530150268689,0.96615221424925
"11029","SRR",9.05016143383914,-0.147360903577289,0.467215030258034,-0
.315402746131486,0.752455882054966,0.96615221424925
"11030","SYNGR2",3.47554728969869,-0.237745665732789,0.75350085382091,
-0.315521428445914,0.752365783801574,0.96615221424925
"11031","CTPS2",12.1373940992496,-0.12231847232412,0.388142989864537,-
0.315137656786768,0.752657138092053,0.96615221424925
"11032","CKS1B",170.464723965999,-0.0781756716691404,0.248723632335069
,-0.314307373751385,0.753287598361976,0.966173223986013
"11033","MRPL53",7.54910559531932,-0.147584599613515,0.469025164837662
,-0.314662433229136,0.753017970266919,0.966173223986013

"11034","SMIM14",267.988601882674,-0.0614192295539349,0.195049685149129,-0.314890175326229,0.752845041385457,0.966173223986013
"11035","LFNG",13.1117641667325,-0.186925177220695,0.594396784904491,-0.314478782469744,0.753157428788355,0.966173223986013
"11036","KIAA0895L",6.54889306151856,-0.16263356335391,0.516584819953344,-0.314824511042733,0.752894900245834,0.966173223986013
"11037","RPAIN",53.3336411935199,-0.0817384088768745,0.259788375879844,-0.314634589019024,0.75303911375263,0.966173223986013
"11038","TRIM25",61.4670576343383,0.119419327217903,0.379738286224341,0.314477974831731,0.753158042100661,0.966173223986013
"11039","ADNP-AS1",3.25884623046651,0.255627405063585,0.813234624140095,0.314334138605919,0.753267272389363,0.966173223986013
"11040","DENND6B",7.67640408831476,0.157734902216046,0.50073514814015,0.31500665132438,0.752756603749723,0.966173223986013
"11041","METTL17",28.0778215671915,0.0918777340021729,0.292438353033348,0.314178126942521,0.753385754378399,0.966211600579489
"11042","CDC73",62.2403254725277,0.0717223223206876,0.228356500670935,0.314080493044691,0.753459904727919,0.966219185921692
"11043","ZMYM6",16.872932704104,0.121611249765175,0.387467471592633,0.313861830169403,0.753625981615929,0.966257144121835
"11044","TMEM60",42.9987694097064,0.0801829576260534,0.255402865405506,0.313946977449709,0.753561309953188,0.966257144121835
"11045","SCAMP3",29.2615385603153,-0.0958609654955516,0.305881135433398,-0.313392865368202,0.753982204031049,0.966397132691161
"11046","EGLN1",37.6787492786546,-0.0759017723207023,0.242145300614786,-0.313455483662059,0.753934636578367,0.966397132691161
"11047","STARD4",2.19339713334081,0.35884742060351,1.14680887313523,0.31290952573681,0.754349400256737,0.966397132691161
"11048","IRF1",588.941613213412,-0.120708226310561,0.385550338157392,-0.313080327947434,0.754219634369676,0.966397132691161
"11049","NCOA4",80.4235366184241,-0.0571031409524267,0.182279369292684,-0.313272649417262,0.754073527668703,0.966397132691161
"11050","AP002387.2",4.8176850474719,0.189947376329221,0.607017266816539,0.312919230988908,0.754342026567649,0.966397132691161
"11051","GOLGA6L9",3.96946992811051,-0.311877664668375,0.996422638495437,-0.312997369408728,0.754282660722394,0.966397132691161
"11052","ALDOC",5.98002542925797,0.198828590461241,0.634054045128981,0.313583032848241,0.753837747815268,0.966397132691161
"11053","CCDC57",12.9516090815071,0.136411566898216,0.435794750714847,0.313017921107255,0.754267046763028,0.966397132691161
"11054","ERLEC1",159.598256851283,0.0485920728985421,0.156357413221018,0.310775625520582,0.75597120079082,0.966854802738808
"11055","ANKRD44",12.3407816541161,0.133687608710499,0.430160437154799,0.310785458548316,0.755963725049624,0.966854802738808
"11056","CHCHD4",10.9493392173114,-0.144497881422318,0.46502337970329,-0.310732508792387,0.756003981350571,0.966854802738808
"11057","C5orf15",122.230718249829,0.0588934368422667,0.188956257129816,0.311677621777859,0.755285536605704,0.966854802738808
"11058","PCYOX1L",6.36467080132674,-0.159318636532173,0.51141182708587

5,-0.311527086575221,0.755399954512468,0.966854802738808
"11059","ZFAND3",67.3909998764303,0.0692521731588568,0.221845748474582
,0.312163625559818,0.754916174379116,0.966854802738808
"11060","BACH2",10.5450995235504,-0.144695718918382,0.463724302175365,
-0.312029622427816,0.755018010989316,0.966854802738808
"11061","PNPLA8",74.9040590100514,-0.0707481428028572,0.22767164507043
9,-0.310746394356524,0.75599342445882,0.966854802738808
"11062","PPP3CC",19.1486552741947,-0.122425803843751,0.392779921875975
,-0.311690585554951,0.755275683425683,0.966854802738808
"11063","NOC3L",50.8966178338388,-0.0744744818607522,0.239347817556392
,-0.311155884440874,0.75568211856056,0.966854802738808
"11064","RND1",3.00244582813403,-0.248655878997828,0.800122250523829,-
0.31077235864274,0.755973684500176,0.966854802738808
"11065","HSP90B1",763.239752451943,-0.0595753213626312,0.1917128640632
64,-0.310752862901113,0.755988506580916,0.966854802738808
"11066","RB1",21.979281813729,0.107228817830633,0.343839722443189,0.31
1856981121052,0.755149217225236,0.966854802738808
"11067","AL359232.1",1.97189417729831,0.351644805750004,1.129415813988
12,0.311351055470261,0.755533757999764,0.966854802738808
"11068","FEM1B",45.9391645144723,0.0721081500877191,0.231061826116233,
0.312072968952673,0.754985069030902,0.966854802738808
"11069","INCA1",1.91798683169494,-0.368997600362998,1.18521042629408,-
0.311335094744975,0.755545890313226,0.966854802738808
"11070","TTC19",45.0659670658651,-0.0896031541669381,0.287593638612075
,-0.311561669442211,0.755373668496558,0.966854802738808
"11071","ARSG",17.0621683603614,0.106227229153735,0.341368266409238,0.
311180738242341,0.755663225276735,0.966854802738808
"11072","FHL1",627.351456421276,0.0770945637541936,0.24797722184768,0.
310893731205478,0.755881410270396,0.966854802738808
"11073","ATXN10",118.378775669144,-0.0650651391330994,0.20947407230721
1,-0.310611897770699,0.756095681197355,0.966884750813198
"11074","TMEM59",758.497603847142,0.0675460334796491,0.21828985711878,
0.309432762342661,0.756992351674241,0.967161843954621
"11075","LINC01133",80.8824619992066,-0.103834163971841,0.335222659190
134,-0.309746853696271,0.756753469700155,0.967161843954621
"11076","MXD1",18.0089873116194,0.129725729005571,0.420124256246283,0.
308779431505911,0.757489316367543,0.967161843954621
"11077","FASTKD2",32.071299667169,0.0937697368809882,0.303356185146152
,0.309107713877044,0.757239591519637,0.967161843954621
"11078","RAB43",11.2338224425225,0.130055933755272,0.420578321871995,0
.309231187133928,0.757145671800898,0.967161843954621
"11079","TBC1D14",18.8993923834928,0.103587956986532,0.334048207521588
,0.310098826020008,0.756485804992577,0.967161843954621
"11080","HEXB",165.167885786161,-0.0572950902578611,0.185045309924588,
-0.309627357111675,0.756844350011909,0.967161843954621
"11081","CHSY3",5.7549901770586,0.192819029548389,0.624576237168891,0.
308719765616458,0.757534707010479,0.967161843954621
"11082","RALA",108.222299411889,0.0592947473424644,0.191926374494541,0
.308945279139584,0.757363152680613,0.967161843954621
"11083","PTK2B",7.47767237868179,0.167183509952106,0.539745411446207,0

.309745125028762,0.756754784373521,0.967161843954621
"11084","HAS2-
AS1",17.4694124049226,-0.112413895696919,0.363664117754959,-0.30911462
0355986,0.757234338023747,0.967161843954621
"11085","PGR-
AS1",1.7056623611635,-0.306302864787754,0.987585712336525,-0.310153195
779911,0.756444460976744,0.967161843954621
"11086","FXYD2",1.79428403942627,-0.310954349799315,1.00504565022243,-
0.309393259630044,0.757022397080669,0.967161843954621
"11087","ZFHX2",3.75675519213139,0.223629128182445,0.722055945370885,0
.309711636080466,0.756780253278493,0.967161843954621
"11088","SMG8",8.51393131752847,0.140745041406781,0.455773578071998,0.
308804740288274,0.757470063041339,0.967161843954621
"11089","TMEM94",12.9242889400753,0.11260419512512,0.364756690999024,0
.308710430552243,0.757541808707677,0.967161843954621
"11090","PRPF6",87.288205417697,-0.0622992525228936,0.20164109403448,-
0.30896109159297,0.757351124161408,0.967161843954621
"11091","Z95331.1",8.68236653010993,0.181896474500264,0.58747385280942
2,0.309624800542861,0.756846294386825,0.967161843954621
"11092","TMEM123",126.409329637545,0.0567728766875035,0.18401753477885
4,0.3085188417274,0.75768756539226,0.967260721777354
"11093","NUS1",30.6086558812267,-0.0906481183254889,0.294137321361733,
-0.308182987136165,0.757943097176484,0.967348287378543
"11094","NACAD",7.25837416910747,0.180121384309017,0.584507339676166,0
.308159319964758,0.75796110511758,0.967348287378543
"11095","GTF2F2",55.386025389025,-0.0725766869408418,0.235400843193692
,-0.30831107465968,0.757845639875991,0.967348287378543
"11096","C16orf46",5.36044484150472,-0.182558774568389,0.5927733420129
23,-0.307973995504692,0.758102119831694,0.967441061356956
"11097","USF1",8.49699282278561,-0.161122895781998,0.523398671472765,-
0.307839711034463,0.758204302879722,0.967484268611053
"11098","SSBP3",68.7620830718724,0.0702057714067958,0.228968079752697,
0.306618160411806,0.759134029061354,0.967521090007851
"11099","YPEL5",99.7877341899889,-0.0642500560023241,0.209585355475805
,-0.306557945599121,0.759179867770409,0.967521090007851
"11100","THORLNC",2.85691524265917,-0.23809386996668,0.774647292546546
,-0.307357777220106,0.758571062605285,0.967521090007851
"11101","WDR75",26.0966120510991,0.0894781700428491,0.291504750904221,
0.306952698936453,0.758879376009699,0.967521090007851
"11102","ACAA1",76.6155441082115,-0.0702760407558872,0.228878708355462
,-0.307044902782064,0.758809194396652,0.967521090007851
"11103","LEMD2",22.6095073990481,0.0980108164087521,0.318628561154097,
0.3076021058933,0.758385117587254,0.967521090007851
"11104","AL021368.3",1.49973052370805,0.400284706432502,1.304537574473
33,0.306840304384568,0.758964928608807,0.967521090007851
"11105","TMEM242",59.6467437805717,-0.078259027655662,0.25569594916252
3,-0.306062837178229,0.759556802627952,0.967521090007851
"11106","DLC1",241.556555739026,0.0539257028305902,0.17556132489061,0.
307161630639268,0.758720349007611,0.967521090007851
"11107","AC027031.2",4.38782729241877,-0.198554051847552,0.64697366038

5641,-0.306896654384972,0.758922035689419,0.967521090007851
"11108","ELP1",14.0867625511741,-0.117187826274847,0.382543885320457,-0.306338254960417,0.759347115110566,0.967521090007851
"11109","COL27A1",23.1370445494601,-0.127680258631559,0.416342767671144,-0.30667101375569,0.759093794990217,0.967521090007851
"11110","AC022387.2",1.51626925846345,-0.334891745005098,1.09279271982977,-0.306454956121291,0.75925827078443,0.967521090007851
"11111","CLP1",7.4586664179341,0.17419788302136,0.568326797560992,0.306510063873358,0.759216318483536,0.967521090007851
"11112","BUD13",15.467703852454,-0.105779465489526,0.346020139412272,-0.30570320464351,0.759830633101294,0.967521090007851
"11113","LACC1",11.1505590823282,0.143581514684008,0.469329271819516,0.30592917021213,0.759658575465656,0.967521090007851
"11114","RCCD1",12.154502587857,0.11910114612717,0.389228169868927,0.305993130372033,0.759609876250091,0.967521090007851
"11115","MPP2",3.93389637235088,0.240722916108226,0.787583378053774,0.305647532459465,0.759873025563369,0.967521090007851
"11116","STRADA",21.5673840229243,0.0965906881978521,0.316007400034308,0.305659576919293,0.759863854058429,0.967521090007851
"11117","METTL4",14.9722611021563,-0.120169794297924,0.391594546173493,-0.306873002885703,0.758940038826145,0.967521090007851
"11118","MAU2",31.8073785945762,0.103382109110286,0.337798889261803,0.306046326369541,0.759569373585315,0.967521090007851
"11119","FP565260.6",3.64477266693876,0.222254013009843,0.722563846790575,0.307590829512205,0.758393699108709,0.967521090007851
"11120","PPARA",27.4451983163406,-0.0843749106390296,0.275918860210775,-0.305796097354764,0.759759900088401,0.967521090007851
"11121","PRAF2",62.6335777469139,-0.0807673092267827,0.26372451497453,-0.306256357072391,0.75940946568159,0.967521090007851
"11122","SLC16A4",25.3637178481619,-0.102729044716332,0.336518136289013,-0.305270455403049,0.760160175815036,0.967538704677834
"11123","ANGPTL2",404.654171093554,0.0978162607593581,0.320239922304659,0.305446803931901,0.760025879453074,0.967538704677834
"11124","AC040977.1",1.65033379001176,0.312550658626001,1.02363302513444,0.305334676540895,0.760111268047692,0.967538704677834
"11125","KANTR",10.0068403809932,-0.172446656822899,0.564526116942337,-0.305471530275567,0.760007049945025,0.967538704677834
"11126","MAPK8",24.0287823409707,0.0948034782636817,0.310847861336154,0.304983530709128,0.76037869573868,0.967671383703284
"11127","ANKS3",25.3089183437533,0.0902477996562913,0.295938939353315,0.304954122811619,0.760401093677008,0.967671383703284
"11128","AFTPH",29.1758391884387,0.0915801624345576,0.300602825517734,0.304655028697176,0.760628904148337,0.967825237704035
"11129","AL353135.2",2.59311923738421,-0.263136433565476,0.86408473121984,-0.304526192927865,0.760727040652959,0.967825237704035
"11130","NHLRC4",6.20595496744159,0.172665741708248,0.566936789939992,0.304559070379829,0.760701996945113,0.967825237704035
"11131","IRAK4",19.5356677341545,-0.109336164998405,0.359515703917678,-0.304120692940413,0.761035942353417,0.968131249997699
"11132","AC092718.4",1.7998181698843,-0.334376702800571,1.100490730946

,-0.30384327045911,0.761247299146809,0.968313129349517
"11133","DBNL",79.8383433340365,0.072611472111148,0.239370600572428,0.303343317589987,0.761628237677766,0.968704819092434
"11134","SLC25A17",18.7110537847753,-0.0965342208642698,0.318322088615583,-0.3032595736102,0.761692051961522,0.968704819092434
"11135","TRDMT1",14.4170186703306,0.125904397550163,0.415304005868288,0.303162010891108,0.761766398394722,0.968712366526203
"11136","DNAJB12",62.3888093539536,0.0719712131406863,0.237594838187066,0.302915726999174,0.761954085692784,0.968777036312276
"11137","PPIL2",22.1000091563761,0.100193069843235,0.330739818572676,0.302936218189944,0.761938469292689,0.968777036312276
"11138","TVP23C",6.49510302654415,0.156523522460988,0.51691201778245,0.30280495921235,0.762038503845039,0.968780005943949
"11139","SGSM3",39.3992338062501,-0.0779909344730361,0.257622734673377,-0.302733120863404,0.762093254675823,0.968780005943949
"11140","PLBD2",78.5193903113109,0.0576562072740594,0.190591756213351,0.30251154834587,0.762262131303675,0.96890770011311
"11141","SPATA13",27.6481621212628,-0.0954615184354599,0.315725015994102,-0.302356524188895,0.762380293287753,0.968970914007233
"11142","ZNF513",6.45591276074963,0.166412601068881,0.550765805177005,0.302147663316534,0.762539499266783,0.968990573242562
"11143","RTN3",187.561375920461,0.055914122195878,0.185054973508283,0.302148713627388,0.762538698633248,0.968990573242562
"11144","PDXP",4.36867547429188,-0.214431011519025,0.709879180088159,-0.302066911572748,0.762601055664909,0.968990573242562
"11145","PTPN13",57.0692123558033,0.0592967426235526,0.196617481954528,0.301584284541221,0.762968989621625,0.969000027039904
"11146","PACRG",1.77716120881322,0.326449912746059,1.08207776436649,0.301688033426304,0.762889891440996,0.969000027039904
"11147","MINDY4",22.3035295235279,-0.0993903034647798,0.329506318891756,-0.301633983223945,0.762931099022367,0.969000027039904
"11148","COA1",42.23359002625,0.0851947070460703,0.282233602629283,0.301858836979007,0.762759676155285,0.969000027039904
"11149","SPINDOC",6.32541308135145,-0.152896753070406,0.506830870348635,-0.301672139594087,0.762902008742809,0.969000027039904
"11150","MCM3AP",26.4087306614308,0.0991755515429968,0.328920206877379,0.301518573408807,0.763019089088625,0.969000027039904
"11151","EGFL8",12.5361892506544,0.124359313521147,0.412584258375956,0.301415555723476,0.763097633807295,0.969012868326723
"11152","AC007383.2",1.60199978880255,-0.340649310416029,1.13093103760352,-0.301211390517565,0.763253304563571,0.969036742815401
"11153","ZNF408",5.34527227438121,-0.179282314510072,0.595127385492272,-0.301250318638546,0.763223622126476,0.969036742815401
"11154","POLM",12.0185416229076,-0.125173932700164,0.415991622792825,-0.300904936161428,0.763486986152958,0.969159634596673
"11155","WBP2",54.9749810257463,0.0676125573427183,0.224631869753364,0.300992719407775,0.763420046319026,0.969159634596673
"11156","ATP13A2",7.97765141487146,0.174702968735681,0.588380275744862,0.296921864884942,0.766526162785248,0.969216967490217
"11157","SESN2",15.961093642432,0.137253267136418,0.463548555493184,0.

296092535528216,0.76715941433208,0.969216967490217
"11158","DNALI1",17.4957545206619,-0.111272702775197,0.37572734407137,
-0.296152794123124,0.76711339739403,0.969216967490217
"11159","CD53",10.3375163895612,0.173696208118562,0.57990206690289,0.2
99526796043735,0.764538129676465,0.969216967490217
"11160","ATP1A1",194.977988411386,-0.0625326617813163,0.21032070727079
5,-0.297320518710521,0.766221817892105,0.969216967490217
"11161","ANGEL2",36.9936424781263,-0.0739251497530019,0.24876885381344
,-0.297164008354682,0.766341298531414,0.969216967490217
"11162","CNST",25.591212931827,0.113417412706488,0.383031952843056,0.2
96104311571521,0.767150421401761,0.969216967490217
"11163","CRIPT",61.130485503024,-0.0754436476186942,0.254837675282145,
-0.296045894843321,0.767195032412426,0.969216967490217
"11164","COL7A1",2.15473394414316,-0.299633090328684,1.00651303257567,
-0.297694198317455,0.765936571856072,0.969216967490217
"11165","FRMD4B",15.9586435502324,-0.111924773543113,0.376672101882093
,-0.297141128806371,0.766358765336852,0.969216967490217
"11166","ATR",30.2058858281036,0.0892583645101862,0.300336466175589,0.
297194561975042,0.766317973337315,0.969216967490217
"11167","MFN1",36.447998606852,0.0737647055784357,0.248540997528292,0.
296790896922504,0.7666261557192,0.969216967490217
"11168","NSD2",10.6392288997069,0.140481513008414,0.471372506204444,0.
298026531372375,0.765682914113486,0.969216967490217
"11169","PRMT9",11.7515738991529,0.120959794954299,0.406820532237924,0
.297329621710334,0.766214868796255,0.969216967490217
"11170","AL451165.2",8.20350308043888,-0.150192522536116,0.50703323131
4425,-0.29621830140553,0.76706337318714,0.969216967490217
"11171","SEMA3A",18.7138175626221,0.167701860639847,0.559627373809142,
0.299667007884859,0.764431166575455,0.969216967490217
"11172","CDHR3",5.73889469709555,0.207203940622681,0.692525747009008,0
.299200342395336,0.764787188060638,0.969216967490217
"11173","NDUFA5",159.970540571253,-0.0682817330890967,0.22742372937159
7,-0.300240143268113,0.763993986789102,0.969216967490217
"11174","MTMR9",28.828723016194,0.0840962053418802,0.282775446299823,0
.297395712542574,0.766164416593337,0.969216967490217
"11175","CSGALNACT1",16.7351495728857,-0.178009308380892,0.59933468164
8663,-0.297011525999497,0.766457709523133,0.969216967490217
"11176","SPIDR",25.8654041609895,0.0983594776788498,0.330415971991575,
0.297683786549391,0.76594451918581,0.969216967490217
"11177","EFCAB1",1.82541471812552,-0.296815383744102,0.991056482715195
,-0.29949391272929,0.764563215949123,0.969216967490217
"11178","ZNF16",4.62025711400071,0.188780907514475,0.637118146914732,0
.296304395705339,0.766997629315568,0.969216967490217
"11179","FAM225B",4.21410530248547,0.230582128605469,0.769173691282835
,0.29977901118914,0.764345726088119,0.969216967490217
"11180","MARVELD1",93.8301624244142,-0.0644247828871427,0.217350920151
323,-0.296409064393604,0.766917703811403,0.969216967490217
"11181","NAV2",12.9911555708219,-0.162331996077628,0.545370311850334,-
0.297654625765872,0.765966777819082,0.969216967490217
"11182","PPP2R1B",20.5312895271494,0.105791879263824,0.353754924611183

,0.299054152758725,0.76489872711035,0.969216967490217
"11183","PSMC6",119.743606317032,-0.0558751709236693,0.185989283842523
,-0.30042145315737,0.763855701755685,0.969216967490217
"11184","PGBD4",3.19524503154633,0.224809613491866,0.747923724682117,0
.30057826229194,0.763736109519259,0.969216967490217
"11185","WDR76",1.91226035558779,0.275791550281142,0.919335358117537,0
.299990148149922,0.764184670384359,0.969216967490217
"11186","GLDN",6.74682850110331,0.217500189073108,0.73476025932210з,0.
296015178166789,0.767218490077837,0.969216967490217
"11187","GNB5",22.322811827829,0.0992689453484311,0.33492841008027,0.2
96388548599506,0.766933369573211,0.969216967490217
"11188","DPP8",38.8600447057271,0.083287653218689,0.278978181599604,0.
298545401440122,0.765286929688784,0.969216967490217
"11189","IQCH-
AS1",9.47357252107284,0.172791820802544,0.582155676103956,0.2968137697
44449,0.766608692232518,0.969216967490217
"11190","HMG20A",26.8799248819355,0.0880520377318919,0.29608880124724,
0.297383884027302,0.76617344613371,0.969216967490217
"11191","SOX8",3.20249741860647,-0.244629382701881,0.826387289754637,-
0.296022682989854,0.767212758786137,0.969216967490217
"11192","UBE2I",227.124811706195,-0.0527941533061687,0.177347834520606
,-0.297687047878978,0.765942029801774,0.969216967490217
"11193","ACD",20.2067097553995,-0.0946688581337105,0.316462222027158,-
0.29914742280229,0.764827563828131,0.969216967490217
"11194","CNTNAP4",3.07755418506908,0.240912734190617,0.813888024672986
,0.296002308533061,0.767228318403802,0.969216967490217
"11195","AC067852.2",5.9848547216645,0.160991629319914,0.5415481676083
44,0.297280350944416,0.766252481597142,0.969216967490217
"11196","CCDC43",35.6906816437971,0.0789634707996933,0.263927395629903
,0.29918633725474,0.764797873422641,0.969216967490217
"11197","SNX11",11.7779986328842,-0.120368426259712,0.404757343439725,
-0.297384169084597,0.766173228528988,0.969216967490217
"11198","ELANE",24.113238842898,-0.128519115761218,0.43247991804495,-0
.297167823056838,0.766338386306261,0.969216967490217
"11199","PWWP3A",81.1637135741809,0.0670907950760704,0.224371277666503
,0.299016860686561,0.764927180812821,0.969216967490217
"11200","DPP9",22.5201177137228,-0.0957809023517037,0.319645471474226,
-0.299647299584618,0.764446201132004,0.969216967490217
"11201","PODNL1",3.90754132748526,-0.20659952911375,0.690482945814929,
-0.29921018377929,0.76477967950517,0.969216967490217
"11202","ZNF417",3.84228923173738,-0.221448942560885,0.742268732371638
,-0.298340658717132,0.765443175222785,0.969216967490217
"11203","SLC35C2",51.7994608278943,0.0689191875093953,0.23203025144266
6,0.29702673285438,0.766446099778837,0.969216967490217
"11204","PREX1",3.32275388571446,-0.25485499086303,0.849753619858448,-
0.299916334461139,0.764240974457124,0.969216967490217
"11205","TMEM184B",23.8883758551525,-0.0979671673590967,0.328603733607
951,-0.298131631930814,0.765602699904486,0.969216967490217
"11206","MOSPD2",33.3300928068071,0.0746890574041289,0.251609279588781
,0.296845400639426,0.766584542119061,0.969216967490217

"11207","RPS6KA3",67.5323640500793,-0.089871816376107,0.300435254047953,-0.29913871679574,0.764834206262743,0.969216967490217
"11208","FGD1",15.957008512605,0.125502623093752,0.418680074400391,0.299757812151652,0.764361897320361,0.969216967490217
"11209","SLITRK4",16.5245998802805,-0.141697603991312,0.472981630963856,-0.299583735847323,0.764494691591026,0.969216967490217
"11210","RAVER2",6.48791328181999,-0.153735678628607,0.51967632257387,-0.295829676955802,0.767360157896893,0.96929704155397
"11211","HOXD3",2.38246295092725,0.288171106340115,0.975297469770096,0.295469962008663,0.767634895408572,0.96955758799263
"11212","VCAN",1027.55041906704,-0.0621059966674298,0.210322101638372,-0.295289920477377,0.767772415708895,0.969644791869243
"11213","ANKRD40",35.2693921140478,-0.0874569314969698,0.296263178955263,-0.295200138624639,0.767840996103631,0.969644921504273
"11214","PDPN",71.2090984932709,0.13716238695467,0.4653749077969,0.294735243900453,0.768196137727375,0.969661018917778
"11215","DPT",1178.34460107196,-0.0695526598735417,0.235826733648163,-0.294931192904149,0.768046442745043,0.969661018917778
"11216","ASAP2",70.384118710871,-0.0991276213460768,0.336114832925051,-0.294921888699214,0.768053550484514,0.969661018917778
"11217","CRIM1-DT",10.1396742175317,0.164714907407659,0.558813054609706,0.29475851726961,0.768178357615224,0.969661018917778
"11218","C11orf53",2.57130077535251,0.312066700267064,1.05829831910812,0.294875929246546,0.768088660467586,0.969661018917778
"11219","PIAS4",16.2167626714436,0.128752439377257,0.437166574828277,0.294515744777222,0.768363834058785,0.969786245678973
"11220","WNT11",180.334774426399,-0.125023805984651,0.424846895159139,-0.294279674417345,0.76854420283922,0.969927443155379
"11221","RPA2",67.4686844560452,-0.0777096455870401,0.264354754989323,-0.293959704224646,0.768788695011032,0.970027198292809
"11222","AC083862.2",4.33133548794738,0.204658177976522,0.696335874396435,0.293907273058298,0.768828760341821,0.970027198292809
"11223","RNF185",23.2250351706654,0.0994888743356646,0.338303976660105,0.29408130320508,0.768695777399463,0.970027198292809
"11224","ZFPM1",44.3285209485236,0.0920551603721587,0.313480689646687,0.293654963168261,0.769021571841618,0.970184021496553
"11225","WARS2-IT1",4.74855728666767,0.18028979112192,0.616530107628802,0.292426580455772,0.769960487691247,0.970498844077561
"11226","AC009506.1",6.50068332244417,0.171788206970278,0.587627231375656,0.292342147875135,0.770025036243743,0.970498844077561
"11227","ANKZF1",18.5489089499709,0.117208637042037,0.399713402364447,0.293231691378639,0.769345061986276,0.970498844077561
"11228","CDHR4",2.64963337177698,-0.296665940309356,1.01295955362726,-0.29287046975078,0.769621161365903,0.970498844077561
"11229","GMNN",18.6644383578752,-0.0952996826983469,0.325445640184208,-0.29282826663281,0.769653421182094,0.970498844077561
"11230","ZBTB22",15.4409894980085,0.105361706336286,0.359969517598959,0.292696190052596,0.769754382322364,0.970498844077561

"11231","C6orf132",19.9438675255413,-0.0992423013824736,0.339413544536
007,-0.292393462135231,0.769985806396407,0.970498844077561
"11232","RABGEF1",35.9594704135342,-0.0753382657972484,0.2575302563763
83,-0.292541415743946,0.769872698882701,0.970498844077561
"11233","CSAD",77.7706736204364,0.0742669985321037,0.253720406196577,0
.292711964502229,0.769742323912529,0.970498844077561
"11234","BRMS1L",28.5026928399169,0.0852623507579591,0.290916064462213
,0.293082305081966,0.769459241704167,0.970498844077561
"11235","TSPAN10",6.82890475344196,0.150867884743171,0.515899503298859
,0.292436576849686,0.769952845572004,0.970498844077561
"11236","DHX8",27.8137586635171,-0.100118010946753,0.342589542509701,-
0.29223895806428,0.770103926827713,0.970511890697795
"11237","SLC66A3",157.196763786797,-0.0641016997732807,0.2195983917397
84,-0.291904231471962,0.770359848080566,0.970730977910154
"11238","LINC01088",15.3631224795202,-0.185085027751417,0.635588053411
647,-0.291202810936951,0.770896212874453,0.970730977910154
"11239","AC093849.2",9.65990824019527,0.14275018699891,0.4902530589626
79,0.291176535034689,0.770916307757138,0.970730977910154
"11240","RAD1",31.7664282402225,-0.0776102713404758,0.266678660609013,
-0.291025427993516,0.771031872073129,0.970730977910154
"11241","TNFRSF10D",7.9984809305255,0.158237128331446,0.54227545973587
6,0.29180211918223,0.770437924852366,0.970730977910154
"11242","IFNG",2.08616206143002,-0.338647236706244,1.16350137775702,-0
.291058732873254,0.771006400582149,0.970730977910154
"11243","CBLN3",9.22846516319988,0.138705924276429,0.475709187594394,0
.291577139760216,0.770609956105704,0.970730977910154
"11244","CYB5B",45.5926332222647,-0.0657292863362917,0.225507025346955
,-0.291473342061799,0.770689329161352,0.970730977910154
"11245","OSBPL7",2.90547745735985,-0.222760797482492,0.764544350158204
,-0.29136412745238,0.770772847066991,0.970730977910154
"11246","ZNF627",3.96497768455903,-0.237405756066013,0.814846160887002
,-0.29135040141514,0.770783343743428,0.970730977910154
"11247","SYP",3.56593778894877,0.212405509661346,0.728352304570478,0.2
91624682627463,0.770573601355539,0.970730977910154
"11248","ZNF480",21.3037180117213,-0.091272616250323,0.313811971676015
,-0.290851288313993,0.771165057695901,0.970763659452426
"11249","GK",4.29891230103447,-0.196207258476437,0.674687132750955,-0.
290812213472081,0.771194943868668,0.970763659452426
"11250","MAML3",8.00029748650493,0.138760794917277,0.477669610983412,0
.290495337628033,0.771437317113721,0.970841145428418
"11251","SARDH",4.77449539014061,0.196089454271944,0.67468331607631,0.
290639251926849,0.771327236610514,0.970841145428418
"11252","PLAAT4",203.062604270425,0.0847915571467167,0.291928479635127
,0.290453186522587,0.771469559499304,0.970841145428418
"11253","LINC02126",3.40356004182769,0.303652090124473,1.0463764075743
9,0.290193937789911,0.771667873714466,0.970841145428418
"11254","TAF4B",11.9216405923222,0.134328214796752,0.462823818788846,0
.290236174854337,0.77163556314524,0.970841145428418
"11255","AC010331.1",1.64488446064404,0.35976382802061,1.2397042419670
6,0.290201336610551,0.771662213725586,0.970841145428418

"11256","AC004540.1",8.05769494630449,0.183783958006379,0.633608483639502,0.29005918126397,0.771770962599233,0.970884579815667
"11257","ARHGEF26-AS1",1.85287238031402,-0.272405019785297,0.940569930285891,-0.289616976913666,0.772109277692588,0.970964075943733
"11258","TMEM44",23.7438333434645,0.0863955203996593,0.298628937732051,0.289307262235848,0.772346255373629,0.970964075943733
"11259","PRR7",1.96925846616218,-0.273269067643606,0.944123270639488,-0.289442148225529,0.772243044966999,0.970964075943733
"11260","ATAD2",13.9282566170112,-0.117161251650264,0.405038414834145,-0.289259604421075,0.772382722558631,0.970964075943733
"11261","CCDC32",53.8709607387732,-0.0876544943348112,0.302942994124294,-0.289343196690159,0.772318759095123,0.970964075943733
"11262","XPO6",28.4313391599394,0.0813772262724852,0.281226410814334,0.289365518824654,0.77230167881755,0.970964075943733
"11263","U2AF1L4",16.9903716985647,0.107818274871007,0.372228359722244,0.289656260880984,0.772079221147581,0.970964075943733
"11264","FUNDC1",28.7535081340972,-0.0996100037584263,0.343946965070412,-0.289608613752514,0.772115676472578,0.970964075943733
"11265","AJUBA",1.61522770529383,-0.291942177038284,1.01061710278997,-0.288875159773499,0.772676913343531,0.971247678024358
"11266","WRAP73",26.5290571855018,-0.0901839124504327,0.315433260611571,-0.285904892450408,0.774950966459719,0.971357199053179
"11267","LINC00339",11.3004541290628,0.122206150353666,0.427580887399062,0.285808262144353,0.77502497970555,0.971357199053179
"11268","AC099063.4",1.62316709761525,0.295939190709846,1.03786362384395,0.285142656425102,0.775534850828439,0.971357199053179
"11269","TMEM167B",46.4618068571988,0.0666569794217342,0.232285955367999,0.28696086819426,0.774142283295623,0.971357199053179
"11270","ENSA",151.971997458248,-0.0585408670103553,0.203880074823441,-0.287133831302796,0.774009848703126,0.971357199053179
"11271","Z99572.1",3.37575917156861,0.216685159245753,0.760063648428902,0.285088176093745,0.775576588453054,0.971357199053179
"11272","PLXNA2",10.3012229640329,-0.184955746157793,0.64247994020344,-0.287877853585945,0.773440239904884,0.971357199053179
"11273","KRCC1",89.9850912216143,-0.0550109731523107,0.192568120268783,-0.285670198553776,0.775130732006178,0.971357199053179
"11274","RGPD5",9.46637126312824,0.153460710161538,0.53304642621082,0.287893704217134,0.773428106300092,0.971357199053179
"11275","BIN1",148.247449358659,0.0853797663030034,0.297010790003832,0.287463517072568,0.773757432741743,0.971357199053179
"11276","GALNT15",79.8557772811048,0.217266213775715,0.759941837759478,0.285898476673263,0.774955880511917,0.971357199053179
"11277","ZNF852",2.11482800945817,-0.294532133588897,1.02673725318803,-0.286862225632089,0.774217814990102,0.971357199053179
"11278","DHFR2",15.9185226241023,0.101318303793182,0.354543187693617,0.285771401933515,0.775053213041493,0.971357199053179
"11279","GATA2",1.65328955432999,-0.34761839348673,1.21335183545985,-0.286494307197373,0.774499553028336,0.971357199053179
"11280","CXCL9",94.7315258400514,0.229615419746153,0.803196835373632,0

.285876897957821,0.774972408417422,0.971357199053179
"11281","SEMA5A",49.8260402212978,0.162586259398651,0.56468630101925,0
.28792315150055,0.773405564650798,0.971357199053179
"11282","PPP1R3G",10.1217393210941,0.142251885322959,0.49650556946934,
0.28650122126678,0.774490505140085,0.971357199053179
"11283","PPIL1",11.4914378568431,-0.142053017296619,0.492373742641944,
-0.288506483985926,0.772959067924496,0.971357199053179
"11284","AC079781.5",3.75373516122775,0.197402637389078,0.691185289400
39,0.285600171786537,0.775184371867645,0.971357199053179
"11285","IFIT3",187.623546518927,0.125883466152465,0.440965750053208,0
.285472207619924,0.775282394016838,0.971357199053179
"11286","RASSF8",82.9151862399249,-0.0527744524226587,0.18453668204441
3,-0.285983533669242,0.774890733327184,0.971357199053179
"11287","TSPAN11",17.640301659486,-0.11894745417863,0.413623751422516,
-0.287574042277678,0.773672817135713,0.971357199053179
"11288","KRR1",114.206904101432,-0.0510435016859054,0.178728107909307,
-0.285593028891721,0.775189843319862,0.971357199053179
"11289","TBX5-
AS1",1.85699829383893,-0.322481809010108,1.12345149299365,-0.287045601
008364,0.774077404155199,0.971357199053179
"11290","ZCCHC8",26.8600036603061,-0.0878632714409857,0.30754825652391
1,-0.2856893823235,0.775116037601743,0.971357199053179
"11291","TMEM132C",21.1678703771747,-0.102051610122656,0.3576427618735
95,-0.285345101318519,0.775379762575481,0.971357199053179
"11292","GCH1",6.69588882449041,0.189499342968153,0.660875433009125,0.
286739880926299,0.77431149864588,0.971357199053179
"11293","MPP5",18.8335358086324,-0.132720551984234,0.465223414798178,-
0.285283474052592,0.775426972820623,0.971357199053179
"11294","TUBGCP5",16.7845201653742,-0.0985244665391475,0.3416087254980
38,-0.288413202547759,0.773030462739424,0.971357199053179
"11295","INSYN1",7.66242653993476,-0.134861872981039,0.472583870563066
,-0.28537129889845,0.775359693882582,0.971357199053179
"11296","AC108134.2",11.1999497905518,0.175006466189154,0.613625335328
6,0.285200848324551,0.775490270471111,0.971357199053179
"11297","AC138150.1",5.34355972719146,0.168342065739705,0.583333854997
446,0.288586140333723,0.772898102865952,0.971357199053179
"11298","BAIAP2-
DT",6.19027465534137,-0.149511884843733,0.52207748704245,-0.2863787245
27488,0.774588068002611,0.971357199053179
"11299","ANKRD12",314.21111551953,0.0542544559000516,0.188739516657884
,0.287456791565251,0.77376258172535,0.971357199053179
"11300","DAPK3",36.6963296409409,-0.0760434407573417,0.26471772571053,
-0.287262368068603,0.773911434783749,0.971357199053179
"11301","KCNJ14",2.16556338522167,-0.2922190223068,1.01556303420847,-0
.2877409008241,0.773545079088362,0.971357199053179
"11302","C20orf96",11.0669333410584,0.123840506174334,0.43189812644219
3,0.286735455868919,0.774314887129961,0.971357199053179
"11303","THAP7",46.684117604778,0.0684088797854254,0.239926474480152,0
.285124348755787,0.775548876343153,0.971357199053179
"11304","ACOT9",68.7879542236379,-0.0623100413030103,0.217430912544653

,-0.286573976872833,0.774438542480189,0.971357199053179
"11305","TMEM164",5.1292810532478,-0.182049615171675,0.631817368101291
,-0.288136452656821,0.773242290105891,0.971357199053179
"11306","TAZ",19.1969914356303,0.0952343166238291,0.332423314241051,0.
286485070522976,0.77450662650695,0.971357199053179
"11307","INO80B",14.9258179679203,-0.101658816071371,0.356747008973141
,-0.284960528089599,0.775674382696447,0.971385355651952
"11308","SGO2",8.8544505247542,0.143103140475163,0.502398087094513,0.2
84840138032297,0.775766619709751,0.971385355651952
"11309","FCSK",4.19133827062915,-0.18913345938202,0.66411502848505,-0.
284790211438918,0.775804871968073,0.971385355651952
"11310","ADAM15",39.5639221254256,-0.066681174715442,0.234243730840674
,-0.284665781560646,0.775900208775333,0.971418829023758
"11311","ATXN1",50.9072455662505,-0.0674635292629991,0.237117849532194
,-0.284514765109826,0.776015920458979,0.971477803350645
"11312","SACM1L",25.2622627027224,-0.0916417030756196,0.32242404508380
3,-0.284227260568611,0.776236225660225,0.971536092212902
"11313","SUCLG2",97.1747266260396,0.0532279803259165,0.187318943818662
,0.284156953059937,0.776290102714199,0.971536092212902
"11314","SIGMAR1",59.78029802317,-0.0717054163834728,0.252287768482951
,-0.2842207405244,0.776241221962845,0.971536092212902
"11315","FANCB",1.97542782386752,0.262142397060057,0.9227251878066,0.2
84095850556754,0.776336926793008,0.971536092212902
"11316","MED6",49.2471915088667,-0.0652278667390197,0.229673670923693,
-0.284002369434376,0.776408564832792,0.971539879642306
"11317","ZBTB8OS",51.1785956343187,-0.0701118622542809,0.2480410887482
09,-0.282662290381464,0.777435725724879,0.971581477278878
"11318","TRIM33",43.013464824393,0.0756217994476067,0.269161387281373,
0.280953372292416,0.778746163396973,0.971581477278878
"11319","FAM78B",5.2782979753408,0.172994767169681,0.611544742178392,0
.282881619672591,0.777267584732261,0.971581477278878
"11320","TOR1AIP1",83.9958227929856,0.0579544594337077,0.2061301901315
09,0.28115464016568,0.778591793802348,0.971581477278878
"11321","GALM",35.473604941592,0.084193760805067,0.298343964619702,0.2
82203666872861,0.777787346790061,0.971581477278878
"11322","RHOQ",127.602475687141,-0.0775315589060457,0.274015796675775,
-0.282945581410344,0.777218552707486,0.971581477278878
"11323","CALM2",779.48588250532,-0.054435002117145,0.193269038573844,-
0.2816540223867 6,0.77820881248217,0.971581477278878
"11324","ANKMY1",5.1179226195393,0.164846138967964,0.58705615682589,0.
280801311852784,0.7788627973795,0.971581477278878
"11325","RBM15B",37.8647810623371,-0.0736324354302853,0.26030200766518
1,-0.282873098408816,0.77727411706104,0.971581477278878
"11326","SCOC",83.5624113526928,-0.0759986960018971,0.270927878001881,
-0.280512646252555,0.779084224475971,0.971581477278878
"11327","SLC9A3-
AS1",1.53227671003428,0.310710663463489,1.10755453433577,0.28053757519
9247,0.779065101488872,0.971581477278878
"11328","ABLIM3",15.8537455418055,-0.119199531679464,0.425249742878806
,-0.280304770727246,0.779243690931247,0.971581477278878

"11329","RARS2",67.8752535434943,-0.0617559666980656,0.218053957093944
,-0.283214152685426,0.777012680036785,0.971581477278878
"11330","AL606970.4",1.95561672886645,-0.290072249041208,1.02394198041
037,-0.283289731831245,0.776954747845436,0.971581477278878
"11331","GALNT17",6.02154398298685,0.213852463771596,0.761740098841927
,0.280742032744129,0.778908267218449,0.971581477278878
"11332","BET1",43.4823490673146,0.0663667790352101,0.23417702905462,0.
283404308710957,0.776866925856205,0.971581477278878
"11333","NUB1",101.248166535223,0.0470456383533593,0.167781512362599,0
.280398225590476,0.77917199824487,0.971581477278878
"11334","TNFSF15",3.86028863497643,-0.328302485345561,1.16848867277014
,-0.280963344357678,0.77873851475924,0.971581477278878
"11335","ATAD1",59.8715401129706,-0.0670035908113713,0.238296736155262
,-0.281177123499145,0.778574549947822,0.971581477278878
"11336","BTRC",16.4427483377529,0.106864228099334,0.377309843594647,0.
283226716486467,0.777003049668332,0.971581477278878
"11337","PTDSS2",24.5193519720709,-0.0950265880790923,0.33765898170897
7,-0.28142769251432,0.778382380501677,0.971581477278878
"11338","LSP1",459.078460673885,0.0703931312553284,0.248280221108965,0
.283522911897337,0.776776020748457,0.971581477278878
"11339","PGAP2",16.0921032227439,0.0998169933689178,0.355721173162371,
0.280604588367743,0.779013696371565,0.971581477278878
"11340","OASL",8.1543823348308,-0.242578195147204,0.865298525389389,-0
.280340469825766,0.779216304609059,0.971581477278878
"11341","TOGARAM1",33.9344107213557,0.0768863126681032,0.2743618491224
44,0.280236894867223,0.779295762194429,0.971581477278878
"11342","ZNF839",6.9985142595127,0.137164344253401,0.489322687850172,0
.280314703689767,0.779236070898654,0.971581477278878
"11343","GPR176",13.7828518161934,-0.120600016726272,0.426206390867791
,-0.282961540019897,0.777206319236041,0.971581477278878
"11344","IVD",25.2963182303805,0.0861242722629563,0.305068347816269,0.
282311399656662,0.777704745282932,0.971581477278878
"11345","FANCI",3.2313160224984,0.213841243468248,0.761413319100818,0.
280847784119118,0.778827151514703,0.971581477278878
"11346","RAI1",11.8298471409321,-0.13121998193792,0.464037470929372,-0
.282778849033708,0.77734636888541,0.971581477278878
"11347","PRR11",1.7421975551891,0.317579125484393,1.1243125590178,0.28
2465158764951,0.777586858556,0.971581477278878
"11348","HDHD2",28.8593529306044,0.0801776021990497,0.283448891955154,
0.28286440509965,0.777280781294588,0.971581477278878
"11349","HSD11B1L",11.6774836622041,0.126620353330837,0.45189182209483
2,0.280200585936396,0.77932361715632,0.971581477278878
"11350","ARHGEF1",31.7260809699137,-0.0734532936475336,0.2611374816992
64,-0.281282078580069,0.778494054868141,0.971581477278878
"11351","CCDC61",12.9395128676827,0.119801666326607,0.423882596402696,
0.282629358561336,0.777460972632652,0.971581477278878
"11352","TRAPPC2B",22.0026769692644,-0.110563520336124,0.3915338166954
26,-0.282385621935005,0.777647838607172,0.971581477278878
"11353","AC020915.2",14.7795942952702,0.109319094492226,0.389741597005
041,0.280491216057732,0.779100663678513,0.971581477278878

"11354","KIZ",45.960774706265,0.0628945354694357,0.223693539244603,0.281163844435677,0.778584734467068,0.971581477278878
"11355","VPS26C",24.9376798283967,0.079276156483817,0.281941583873121,0.28117936841659,0.778572828188221,0.971581477278878
"11356","SLC19A1",4.34384538383294,-0.204654728291435,0.72099735463085,-0.283849485683922,0.776525729400946,0.971581477278878
"11357","Z93930.2",5.83088147472889,0.161439903375687,0.575534371113415,0.280504365123094,0.779090576957344,0.971581477278878
"11358","GRIN2A",6.38776802866697,-0.86983228729851,3.08644277114792,-0.281823559286343,0.778078805147045,0.971581477278878
"11359","PRELID3A",3.70616639497908,-0.195464940618963,0.697833222604292,-0.280102658181699,0.779398745371611,0.97158959718831
"11360","SNIP1",26.1478330688004,0.0974099724836741,0.347922066455929,0.279976413901913,0.779495600499424,0.97162479780562
"11361","CRTC2",17.9835017704222,0.0933618947614216,0.334129682387961,0.279418141166573,0.7799239506491,0.971651808885836
"11362","CNOT9",26.5165537940487,0.0828869811074463,0.296477323794336,0.279572751287193,0.779805315075044,0.971651808885836
"11363","STK25",72.6440027438398,-0.0647778271456007,0.232131238863654,-0.279056913936728,0.780201147816824,0.971651808885836
"11364","ZBTB20-AS2",4.55302954126313,0.196918637231209,0.705588538954013,0.279084234450757,0.780180181728216,0.971651808885836
"11365","GUCY1B1",27.3635517025749,0.131969265951427,0.472916768697049,0.279053894229678,0.780203465185873,0.971651808885836
"11366","OSMR",83.9866682183261,-0.0596040852097646,0.213077960456946,-0.279729001919971,0.77968542591155,0.971651808885836
"11367","ZNF775",11.4880911465634,0.117715124079422,0.420989359771155,0.279615437652416,0.779772561838856,0.971651808885836
"11368","LRRC32",113.258697839513,-0.0797936555297991,0.285778788859954,-0.279214758548445,0.780080018183994,0.971651808885836
"11369","IRF2BPL",52.9624438857176,0.0625044904168864,0.223572298118898,0.279571713234552,0.779806111577415,0.971651808885836
"11370","FLNA",354.797872240318,0.0562225859741939,0.201289270687005,0.279312383527969,0.780005103645704,0.971651808885836
"11371","AP4B1",9.55067796113379,0.122643597484962,0.439972372435951,0.27875295170452,0.78043442284142,0.971653906961377
"11372","CENPK",3.62606230923187,0.211924498470276,0.759944039159308,0.278868558143727,0.780345698649972,0.971653906961377
"11373","BTBD10",24.3446448833866,-0.0804427803707081,0.288641899969383,-0.278694050930378,0.780479628374202,0.971653906961377
"11374","CHURC1",243.148108332162,0.0547294891147888,0.196362908995546,0.278716023279275,0.780462764812312,0.971653906961377
"11375","HGS",45.9811702454315,0.0610101659657548,0.219017801228707,0.278562589997174,0.780580525505699,0.971694087135006
"11376","TP53I3",112.166288696961,-0.0932164457342564,0.334890291308107,-0.278349203167837,0.780744309167428,0.97174117412761
"11377","TMEM175",26.0711532238633,0.093107592616999,0.334731947274427,0.278155680612899,0.780892854547887,0.97174117412761
"11378","POLE3",58.8745076831643,-0.0641493511084556,0.230589628904239

,-0.27819703519752,0.780861110637976,0.97174117412761
"11379","NDRG3",33.5864378490484,-0.0798179314078201,0.286770801931768
,-0.278333536294993,0.780756334556389,0.97174117412761
"11380","TMEM59L",2.82322716747144,0.27077711664903,0.973905392835888,
0.278032259232657,0.780987595360508,0.97177366874383
"11381","CPTP",14.3539992841708,-0.101108131344709,0.366850129945105,-
0.27561154567354,0.782846440858322,0.972029990744741
"11382","NAXE",72.4382946283579,-0.0761966892403671,0.277176504539709,
-0.27490313209232,0.783390660764316,0.972029990744741
"11383","PRCC",35.2085194520919,-0.0714865072142998,0.26139736774411,-
0.273478297930988,0.784485572467834,0.972029990744741
"11384","RABIF",19.0799722837056,-0.0917397792806017,0.330778118742297
,-0.27734536863992,0.781514926192569,0.972029990744741
"11385","MRPL55",87.3231216268029,-0.0708844424629797,0.25586219198219
4,-0.277041488286448,0.781748249387525,0.972029990744741
"11386","TSN",59.4370469554987,-0.0580240459382184,0.211486132979971,-
0.274363359529174,0.783805397852804,0.972029990744741
"11387","SFT2D3",17.5746243632244,0.0978275930335041,0.356806634040867
,0.274175375960917,0.783949850443128,0.972029990744741
"11388","GALNT5",7.87329532876785,-0.273070277669183,0.995358466256312
,-0.274343652991911,0.78382054063727,0.972029990744741
"11389","TTC30B",5.10091144855752,0.175172533518783,0.63438349833684,0
.276130343834656,0.782447955340495,0.972029990744741
"11390","RHOA",625.688999404168,-0.0489566328360379,0.17696734071928,-
0.276642190796645,0.782054864932624,0.972029990744741
"11391","MSL2",18.4562258449543,0.0904910865192711,0.326345148816515,0
.277286446106018,0.781560166126346,0.972029990744741
"11392","GRK4",16.032671977509,0.116846951017339,0.426197001439917,0.2
74161832726578,0.783960257785428,0.972029990744741
"11393","SUB1",447.855321322372,-0.0700371495680961,0.254513985406761,
-0.275179964889409,0.783177978694422,0.972029990744741
"11394","FGF10-
AS1",4.67895222330474,-0.17536758623453,0.638755805426736,-0.274545584
939728,0.78366537712636,0.972029990744741
"11395","ARL15",10.101951706864,0.12755023575533,0.463991770071657,0.2
74897625308379,0.783394891620221,0.972029990744741
"11396","TRIM38",84.2571114240814,0.0725789632748304,0.262809569905285
,0.27616560272515,0.782420875284771,0.972029990744741
"11397","PBX2",24.5957331312857,0.0920323787377271,0.336530210727697,0
.273474344364866,0.784488611174499,0.972029990744741
"11398","KIAA1586",25.1147869041979,-0.0804994471344944,0.293942155486
373,-0.273861525582458,0.784191039708434,0.972029990744741
"11399","CCDC28A",33.1033170870897,0.0818141698258876,0.29883497366560
5,0.273777091156128,0.784255929826857,0.972029990744741
"11400","PDCD2",77.2384852314893,-0.0657170317930588,0.239349138650333
,-0.27456556628376,0.78365002402794,0.972029990744741
"11401","AC090114.2",13.1436081132127,-0.10771159039225,0.391129007816
071,-0.275386351407875,0.783019428860881,0.972029990744741
"11402","TRIM35",8.68972750609323,-0.130031637950847,0.474893831542921
,-0.273812017158397,0.784229088076598,0.972029990744741

"11403","DSCC1",11.2416682940387,0.117141847252801,0.425297022045111,0
.275435380876888,0.782981764865801,0.972029990744741
"11404","ASAP1-
IT2",4.7215725028775,0.173294476689487,0.628445393462422,0.27575104932
303,0.782739283400645,0.972029990744741
"11405","C9orf64",23.1889573972494,0.0900007626581802,0.32833394945137
7,0.27411348356929,0.783997412167181,0.972029990744741
"11406","PTGR1",93.4918711619644,-0.079938351676039,0.289179093417816,
-0.276431987981031,0.782216290655732,0.972029990744741
"11407","EIF3A",211.367354874,-0.0611263806746353,0.222921100840006,-0
.274206346749143,0.783926050985825,0.972029990744741
"11408","IFITM3",2114.73897150644,-0.0633037421244835,0.22953861478485
1,-0.275786896177877,0.782711748888183,0.972029990744741
"11409","TP53I11",43.8183728042152,0.0809452042782379,0.29268081402162
5,0.276564777738581,0.782114313372405,0.972029990744741
"11410","STT3A",62.1180357191796,0.0660589045806522,0.239439600319848,
0.275889637689043,0.782632833084672,0.972029990744741
"11411","KLRG1",2.029290408735,-0.301152627982795,1.08774449941831,-0.
276859711213287,0.781887829524133,0.972029990744741
"11412","AC048341.2",4.09933233659897,-0.174764552570028,0.63385473551
6067,-0.275717041741022,0.78276540539135,0.972029990744741
"11413","MEDAG",327.554211295605,0.0725542782199618,0.263287397943203,
0.275570645563573,0.782877858393212,0.972029990744741
"11414","TM9SF1",94.6753442529613,-0.0580795843472762,0.21056858418426
7,-0.275822647391935,0.782684288109979,0.972029990744741
"11415","HSP90AA1",1240.57308135188,0.038929674555921,0.14230239360259
3,0.273570061404868,0.784415044083812,0.972029990744741
"11416","IDH2",26.3027043321124,0.0881613985649833,0.318940336798863,0
.276419719906991,0.78222571224044,0.972029990744741
"11417","NUDT21",62.2992033200327,0.0650813735149669,0.23778825505183,
0.273694651154159,0.784319288624037,0.972029990744741
"11418","ST3GAL2",37.9123122088463,-0.0761191329749382,0.2761236246833
68,-0.275670482966549,0.782801168633259,0.972029990744741
"11419","HSBP1",263.914691419596,-0.0695650307714482,0.253401240742297
,-0.274525217665347,0.783681026849401,0.972029990744741
"11420","PIGS",21.7564701930939,0.0865090763167273,0.314956126305861,0
.274670244809642,0.783569593392185,0.972029990744741
"11421","CDC42EP4",111.598165512397,-0.0754574407354834,0.273335123070
833,-0.276061999964524,0.782500446563499,0.972029990744741
"11422","FAAP100",16.8852852210606,-0.0955894542606438,0.3474116057785
48,-0.275147556013358,0.783202876593791,0.972029990744741
"11423","SWSAP1",3.94456482801478,0.195111192003844,0.705635007993774,
0.276504410628058,0.782160672462215,0.972029990744741
"11424","ZNF345",8.35664845866229,0.133067112066355,0.47942119499745,0
.277557841528184,0.781351798490129,0.972029990744741
"11425","PXMP4",23.9483684107671,0.0828939421688498,0.298552076466543,
0.277653209282365,0.781278582287199,0.972029990744741
"11426","HDHD5",22.5465711938682,0.0977741401254141,0.355148012684104,
0.275305328013709,0.783081671414724,0.972029990744741
"11427","ADSL",46.7778225076745,0.066212273672529,0.241865220561497,0.

273756902785838,0.784271445352445,0.972029990744741
"11428","DHRSX",37.0739954467056,-0.0798792622906458,0.290617070730096
,-0.274860874792975,0.783423127163843,0.972029990744741
"11429","SERPINH1",323.638193735713,-0.0629133554286078,0.230180187229
002,-0.273322201124185,0.784605550800986,0.972089824074019
"11430","STYX",13.8377792804206,0.111180736078775,0.407067987359532,0.
273125717401545,0.784756578396422,0.972191876648585
"11431","RNF135",32.9058226338851,-0.0707394850886043,0.25927556694329
3,-0.272835138006181,0.784979947668829,0.9723835236629
"11432","TRNAU1AP",44.2820790523153,0.0603383810439573,0.2217523986335
51,0.272097985932804,0.785546678136295,0.972540265214202
"11433","ACY1",15.9107727995493,0.108753024036343,0.399695067038242,0.
272089983101887,0.785552831423371,0.972540265214202
"11434","TMEM128",42.8743695205471,0.075193203860326,0.276319321298657
,0.27212430714917,0.785526440142287,0.972540265214202
"11435","RSPH3",10.9411165646829,-0.136983042463176,0.503530431829694,
-0.272045210783817,0.785587256604523,0.972540265214202
"11436","INTS8",16.4580841024544,0.100833552008808,0.370628713600023,0
.27206082073184,0.785575254156506,0.972540265214202
"11437","AC004687.1",2.14355090274775,-0.317111456144329,1.16547988109
558,-0.27208659822273,0.785555434023082,0.972540265214202
"11438","ATP13A1",21.6223315287239,0.0833676492020749,0.30616981722188
4,0.272292187252598,0.785397363024778,0.972540265214202
"11439","THG1L",28.8954989277175,-0.0857722910693823,0.315643714376527
,-0.271737681324665,0.785823725587532,0.972568686131718
"11440","VARS2",6.95936212723247,0.158929929840786,0.58454608546082,0.
271886056196057,0.785709633057439,0.972568686131718
"11441","PSD3",33.7435334467124,-0.0967781738938728,0.356249961405085,
-0.271658061413313,0.785884951039485,0.972568686131718
"11442","SLC25A23",16.5050723617969,0.0924213102592867,0.3400839910699
38,0.271760249485781,0.785806371553125,0.972568686131718
"11443","CMBL",142.918858343486,-0.0619255258284831,0.228194797074524,
-0.271371331083677,0.786105449493831,0.972653787583152
"11444","NUDT8",24.1778158807769,0.0951069536690898,0.350739435849865,
0.271161277997267,0.786266993280833,0.972653787583152
"11445","CLMP",188.933030139093,-0.0643958933865447,0.237594526928818,
-0.27103273050493,0.786365858774854,0.972653787583152
"11446","BNIP2",81.9080597754716,0.0505382534084833,0.186338564138006,
0.271217359875403,0.786223861954751,0.972653787583152
"11447","MSI2",19.6257317980606,0.120998192841758,0.44618861587715,0.2
71181712253888,0.786251277672244,0.972653787583152
"11448","ZNF324",14.0756087822484,-0.101659931359728,0.375054018821168
,-0.271054104897357,0.786349419554411,0.972653787583152
"11449","EHD3",2.13053274376125,0.308970760079532,1.14298735102869,0.2
70318617088334,0.786915143956928,0.972883825763607
"11450","ATRIP",7.66122221670891,-0.129392801004218,0.480590617405227,
-0.269237051906728,0.787747269294318,0.972883825763607
"11451","TOPBP1",32.9147988937065,0.0665031820449115,0.246257374025733
,0.270055596540074,0.787117482098839,0.972883825763607
"11452","MCCC2",17.4096852559538,-0.0913644545999436,0.337565416961113

,-0.270657034190409,0.786654825432404,0.972883825763607
"11453","GOPC",117.796630747792,-0.0432610532146054,0.160649063830515,-0.269289170961157,0.787707164818457,0.972883825763607
"11454","KIAA1324L",16.5937786169328,0.118759247275552,0.439725742913171,0.270075721491254,0.787101999738787,0.972883825763607
"11455","ATP5MF",253.122103674018,-0.0661728070029461,0.244866384173001,-0.270240471048873,0.786975259144104,0.972883825763607
"11456","HSPA14",9.85057948116343,-0.116520571048284,0.430885199356769,-0.270421381895286,0.786836092291757,0.972883825763607
"11457","MGST1",814.12673294159,-0.0594532549229547,0.220791147926654,-0.269273725333884,0.78771904983315,0.972883825763607
"11458","AC007529.1",1.81881006961512,-0.299869043561559,1.11356656527469,-0.269287039421473,0.787708804980725,0.972883825763607
"11459","TMBIM6",623.745208411299,0.033631037821945,0.124859889964677,0.269350211917207,0.787660195849503,0.972883825763607
"11460","WDR66",6.67555133656076,0.155186229265556,0.576324108963298,0.26926902215614,0.787722668817814,0.972883825763607
"11461","CHFR",14.9355708641211,0.113111235009338,0.420201379135914,0.26918339783162,0.787788555522989,0.972883825763607
"11462","PATL2",2.57521216465199,0.223139091878541,0.8283807694045,0.269367783656965,0.787646675128086,0.972883825763607
"11463","SPESP1",5.47824220655881,0.174454213566514,0.648041616012243,0.269202176613327,0.787774105386374,0.972883825763607
"11464","CHST9",1.67415862660945,0.853319447302786,3.15558779984864,0.270415371533543,0.786840715692543,0.972883825763607
"11465","AKAP8",24.7987173212342,-0.0819618634730822,0.303682513295192,-0.269893259851323,0.787242372686404,0.972883825763607
"11466","NUCB1",284.038191488656,0.0546513519362096,0.202603714437469,0.269745064091987,0.787356389001657,0.972883825763607
"11467","SCOC-AS1",2.74633169115346,0.218767472931077,0.81327803267896,0.268994690795288,0.787933768276006,0.972978299362366
"11468","AC245014.3",2.88013665505867,0.269186636213346,1.01261428265355,0.265833339332271,0.790367565660658,0.97298870105768
"11469","CMSS1",47.3980358095118,0.0594624967240304,0.224187956343634,0.26523501839183,0.790828419864857,0.97298870105768
"11470","BBX",169.586005844447,0.0465356652412188,0.17350948580716,0.268202427231782,0.788543506758974,0.97298870105768
"11471","SNX4",43.3547748978988,-0.0623991024731194,0.234677150000599,-0.265893387886124,0.79032131755756,0.97298870105768
"11472","LINC02447",2.2904537069728,0.235947125426772,0.881884499378333,0.267548783988264,0.78904665843337,0.97298870105768
"11473","SMIM20",102.384298916988,-0.083563375654594,0.314877960767324,-0.265383374088676,0.790714142683913,0.97298870105768
"11474","SKP2",8.51539370283886,-0.123652220647735,0.462353289776786,-0.267440988053598,0.789129644426478,0.97298870105768
"11475","RBM27",89.4725414826056,0.062280498067781,0.234822307255179,0.265223942289697,0.790836951876243,0.97298870105768
"11476","CLINT1",119.726490362937,0.0514477332729516,0.192644137664651,0.267060985590489,0.789422205906108,0.97298870105768

"11477","SSR1",143.026614210967,0.0477001789809783,0.179898782287529,0
.265150093705136,0.790893838681021,0.97298870105768
"11478","RNGTT",16.2152004895833,0.0991221203840867,0.370463087971738,
0.267562744042258,0.789035911552412,0.97298870105768
"11479","IGFBP3",768.175482273642,0.0945878861707102,0.354637601430488
,0.266717025462542,0.789687044223217,0.97298870105768
"11480","COL1A2",7766.76131465794,0.0642849376735248,0.240857504656499
,0.266900289302612,0.789545933891369,0.97298870105768
"11481","MYORG",4.06940576284277,-0.17992207630301,0.675769931337456,-
0.266247531829236,0.790048578543046,0.97298870105768
"11482","DOLPP1",4.09972641117635,-0.17388624711326,0.655671799185922,
-0.265203181422711,0.790852944208131,0.97298870105768
"11483","SURF6",31.2359974259802,0.096758686000571,0.3621524374516,0.2
67176680299169,0.789333130140065,0.97298870105768
"11484","CPEB3",6.47572466154092,0.151943061585779,0.570208969246462,0
.266469083758141,0.789877966491377,0.97298870105768
"11485","SCYL1",50.3439590670583,0.0662513395889765,0.24832445728167,0
.266793453670288,0.789628194842677,0.97298870105768
"11486","ZDHHC24",38.7463020578995,0.0815841859072907,0.30595995080986
9,0.266649885683859,0.789738742544448,0.97298870105768
"11487","RHNO1",11.0511414610542,0.116172668939475,0.438100562241144,0
.265173521680007,0.790875791597917,0.97298870105768
"11488","AC087239.1",6.37584540150961,0.151970901518395,0.573159032415
778,0.265146133836295,0.790896889065953,0.97298870105768
"11489","HOXC-
AS3",5.09964690303698,0.157876283778635,0.588504229119397,0.2682670335
51945,0.788493779862997,0.97298870105768
"11490","CD63",4342.40283835517,-0.0725777140631779,0.272000530792756,
-0.266829310412179,0.789600585724187,0.97298870105768
"11491","NAB2",27.7477894596288,0.0804950773925164,0.302248198134711,0
.266321115855387,0.78999191205803,0.97298870105768
"11492","GLIPR1L2",3.18693530963904,0.26423673972282,0.990682636710933
,0.266721884417078,0.78968330281409,0.97298870105768
"11493","TPT1",10920.228134577,-0.0709752112512575,0.264396187672256,-
0.268442642369859,0.788358619711532,0.97298870105768
"11494","POLR2M",46.8675959882796,0.0649386980825034,0.243521126691104
,0.266665561895479,0.78972667161718,0.97298870105768
"11495","HEXA",157.767218833439,0.0942665455949543,0.354007177672716,0
.266284277665423,0.790020280722208,0.97298870105768
"11496","IL16",32.1330686228742,0.118473777586729,0.44242666756066,0.2
67781728076972,0.788867336130264,0.97298870105768
"11497","ROGDI",11.2761038457027,-0.114121907015143,0.42748765489805,-
0.266959538380962,0.78950031449056,0.97298870105768
"11498","C16orf70",7.27848832710088,0.144863884667966,0.54300962606999
3,0.26677958863531,0.789638870768945,0.97298870105768
"11499","TRAF4",8.09868419470963,0.153084219008964,0.575681089363143,0
.265918443105915,0.790302020785134,0.97298870105768
"11500","EFTUD2",21.9526613303474,-0.0801913072547309,0.30048407066300
6,-0.266873738357551,0.78956637728235,0.97298870105768
"11501","RGS9",36.882416437247,0.083058890793314,0.309215565270678,0.2

68611609899414,0.788228577148973,0.97298870105768
"11502","UNK",28.4744985871417,-0.0786482529919031,0.296456602562109,-
0.265294320693788,0.790782739246641,0.97298870105768
"11503","TLE2",48.3847322006273,-0.0749075599569009,0.280718978303647,
-0.2668418088779,0.789590962167313,0.97298870105768
"11504","ZNF208",22.6568949672884,0.18170937479798,0.680682881555633,0
.266951586005367,0.789506437457127,0.97298870105768
"11505","ZNF45",5.02632794451095,-0.151791151743651,0.570962072023343,
-0.265851549833673,0.790353540246619,0.97298870105768
"11506","PPP1R3D",9.96649144801306,-0.129386691778108,0.48154025224117
5,-0.268693408652588,0.788165624454507,0.97298870105768
"11507","IL10RB",90.9585729810858,0.0545457978344767,0.203867568992097
,0.267555051076276,0.789041833830388,0.97298870105768
"11508","SETD4",19.3314524722053,-0.0989247411673567,0.371146063212114
,-0.266538570586481,0.789824458355407,0.97298870105768
"11509","XPNPEP3",19.3651444628119,-0.112027745191138,0.41999168410277
4,-0.266738007992854,0.789670887648584,0.97298870105768
"11510","NDUFA6",128.700141336467,-0.0670676652109851,0.25284269523470
9,-0.265254509918617,0.790813405444412,0.97298870105768
"11511","MAP3K6",23.1765864550875,-0.100967356920358,0.3811252776219,-
0.264919077397234,0.791071801613575,0.973034808100089
"11512","APOL3",106.089871027532,0.0584578783260183,0.220640551207568,
0.264946212317172,0.791050897719705,0.973034808100089
"11513","KHDRBS1",195.335461954573,-0.0356587410873786,0.1362354750925
92,-0.261743434029523,0.793519252010774,0.973058799823659
"11514","GPR89A",11.0465612376802,0.106740408636909,0.40562510323712,0
.263150401158754,0.792434657934405,0.973058799823659
"11515","AC245297.2",3.540568783643,-0.198890043215455,0.7527585410178
75,-0.264214927334491,0.791614309308507,0.973058799823659
"11516","SEMA4A",24.3308820755531,-0.150628649806774,0.570753287577607
,-0.263912014324215,0.791847717672574,0.973058799823659
"11517","NUP133",23.0348235146624,0.0852288578428381,0.322132849927136
,0.264576735536646,0.791335543981076,0.973058799823659
"11518","POMC",3.2168024401542,-0.242875542435738,0.926936017960319,-0
.26201974864476,0.793306216730465,0.973058799823659
"11519","C2orf81",9.29638687268842,-0.150361995898586,0.57307193630191
9,-0.262378920295564,0.793029322717855,0.973058799823659
"11520","STIMATE",21.2805416206857,0.0986788099117057,0.37759575937574
4,0.261334528954577,0.793834541225618,0.973058799823659
"11521","CSTA",9.47051713888636,-0.15593641337239,0.59603795930742,-0.
261621614760214,0.793613178112596,0.973058799823659
"11522","AC092903.2",5.22279735965361,-0.151280930585325,0.57755838795
0908,-0.261931838825937,0.793373992482524,0.973058799823659
"11523","KLF3-
AS1",15.4317402827715,0.111414973882859,0.423357848705898,0.2631697374
29996,0.792419754902029,0.973058799823659
"11524","ZNF300",5.27307445309216,-0.147278876920616,0.557468742213004
,-0.264192170373458,0.791631843941752,0.973058799823659
"11525","MDN1",26.3036047275482,0.0889395338381614,0.337619069264923,0
.263431606608607,0.792217932102262,0.973058799823659

"11526","FYN",292.396306270695,0.0425387951820667,0.162463084947167,0.261836682443279,0.793447356870135,0.973058799823659
"11527","AC018638.7",1.57750970615744,0.28638907407967,1.08754427957454,0.263335552821544,0.792291959221958,0.973058799823659
"11528","TMEM68",17.6408157674161,0.0893846525538314,0.34002433467281,0.262877222125417,0.79264521314291,0.973058799823659
"11529","ZC2HC1A",86.2039367711815,0.0493633841792363,0.186977268796316,0.264007408478142,0.791774210088957,0.973058799823659
"11530","RBIS",250.200644592052,-0.0614677918650151,0.23410472887171,-0.262565357655375,0.792885603985139,0.973058799823659
"11531","AL365203.2",1.51454213815399,0.336089453730726,1.2773836972988,0.263107674257495,0.792467589081495,0.973058799823659
"11532","SAMD8",46.9571805004235,0.0933881392256069,0.354445427793216,0.263476777813282,0.792183120023734,0.973058799823659
"11533","DHX32",22.6392925279993,0.0820255723852161,0.312525577581781,0.262460349709303,0.792966550463227,0.973058799823659
"11534","SLC1A2",9.52741901244002,0.17773855488941,0.678849133581133,0.261823350870002,0.793457635493335,0.973058799823659
"11535","FBRSL1",27.5195553374415,0.0830052958106949,0.315465390941882,0.263120133599654,0.792457986183765,0.973058799823659
"11536","MED4",115.741771437619,-0.0445119271016619,0.169267062890637,-0.262968626864052,0.792574760430623,0.973058799823659
"11537","GRTP1",7.32514863569564,0.129791949998674,0.493619619081764,0.262939204564264,0.792597438291333,0.973058799823659
"11538","PIGH",34.5554953521156,-0.0762240418405085,0.290750287644871,-0.262163255135315,0.79319558101812,0.973058799823659
"11539","CDT1",3.69696539448541,-0.18346332403375,0.694499345476466,-0.264166302284825,0.791651775880807,0.973058799823659
"11540","C17orf97",12.0452535495923,0.171436664449313,0.649472807492246,0.26396280563503,0.791808579335985,0.973058799823659
"11541","CCDC144A",19.1913601933714,-0.15213141299278,0.578128818745042,-0.263144489716865,0.792439214071493,0.973058799823659
"11542","BTBD2",53.4214492290128,0.0577388267776363,0.219280624128371,0.263310208127804,0.792311492284911,0.973058799823659
"11543","ZNF77",6.07138188802161,0.14815297465973,0.567220250382976,0.261191264874094,0.793945014003848,0.973058799823659
"11544","APBA3",14.1762175255608,0.105835777000687,0.403392746882346,0.262364105003493,0.79304074366499,0.973058799823659
"11545","PET100",152.040204002258,-0.0731571540806825,0.279258725519831,-0.261969089576352,0.793345273115211,0.973058799823659
"11546","WDR83",15.7714278030337,0.105396827552926,0.399817554892313,0.263612305821122,0.792078675181894,0.973058799823659
"11547","ZNF585B",7.14749865248776,-0.134610041909656,0.515452748944885,-0.261149139635395,0.793977498104701,0.973058799823659
"11548","NOSIP",70.6815403785607,0.0586421833872953,0.224480450185514,0.261235146930757,0.793911175545307,0.973058799823659
"11549","ZNF581",42.7867864118704,-0.07871943859295,0.30037649542929,-0.262069235745115,0.793268064393008,0.973058799823659
"11550","FAM227A",2.46452542473983,-0.217890448824198,0.832155876955828,-0.261838502686875,0.793445953467809,0.973058799823659

"11551","AL050309.1",15.7525164557203,0.0945303696797043,0.36112473005
8895,0.261766536078235,0.793501440015651,0.973058799823659
"11552","EFNB1",44.7653691166491,0.0647719724870191,0.247936661924987,
0.261244028955329,0.793904326460014,0.973058799823659
"11553","RBM41",22.7260242621197,0.10370465612150 2,0.396805231719046,0
.261349014155461,0.793823371730792,0.973058799823659
"11554","SYT12",1.77714477659496,-0.303537739273732,1.15865743694064,-
0.261973668485833,0.793341742913501,0.973058799823659
"11555","FAM102B",58.4500676069518,-0.0723762807562421,0.2773514909688
7,-0.26095508087377,0.794127147553636,0.973157975712634
"11556","C19orf47",5.60616148551489,-0.149618719464955,0.5738501005646
88,-0.260727878792258,0.794302365280644,0.973288464206812
"11557","PEX19",37.465863028201,0.0663316533630386,0.255043466741639,0
.260079798202528,0.79480222055587,0.973311085465389
"11558","FAM20B",31.7216633821135,0.0838001041449519,0.322932650835306
,0.259497154989414,0.795251676852486,0.973311085465389
"11559","RNASEL",21.5925989724822,0.0783331976066018,0.301196730926161
,0.260073199884116,0.794807310177975,0.973311085465389
"11560","B4GALT4",29.5349484675543,-0.0733491087659122,0.2817866016869
54,-0.260300199962659,0.794632218370689,0.973311085465389
"11561","GSTCD",7.60967727079587,0.128978065833705,0.494907891893822,0
.260610242726491,0.794393090025185,0.973311085465389
"11562","FAM153CP",7.36960298922893,0.145760285909881,0.56014530234599
7,0.260218706288187,0.794695075638076,0.973311085465389
"11563","LINC00174",7.46083410702537,-0.12732701966079 6,0.489977938507
573,-0.25986276045126,0.794969637507957,0.973311085465389
"11564","SLC22A18",14.623985350635,-0.114599588858265,0.44169117128474
8,-0.259456372027832,0.795283139748203,0.973311085465389
"11565","GTF2H1",34.4116659929843,0.0692566232350068,0.266908636726908
,0.25947689098524,0.795267309914141,0.973311085465389
"11566","NAA40",6.29507705595795,0.138761220074252,0.534567399634228,0
.259576659873382,0.79519034205153,0.973311085465389
"11567","DCPS",32.7889341154169,0.0904731362836423,0.348153334198411,0
.259865775785109,0.794967311497658,0.973311085465389
"11568","FAM155A",4.77875757146344,-0.256206645949714,0.98489025535770
7,-0.26013725342085,0.794757902762297,0.973311085465389
"11569","AC009093.1",5.51084669316664,-0.237775533007329,0.91514217774
7792,-0.259823597675834,0.794999847601675,0.973311085465389
"11570","ARMCX3",140.674076836543,0.0417927086085544,0.160955000708113
,0.259654614175947,0.795130204690384,0.973311085465389
"11571","AC008549.2",2.95026282334471,0.237999037231676,0.917803147176
375,0.259313816872258,0.795393119614397,0.97331354128161
"11572","TMSB15B-
AS1",4.28935880509253,-0.167995179665686,0.64794061968766,-0.259275579
522499,0.795422620036073,0.97331354128161
"11573","CHMP5",174.761254047543,0.0520351226633114,0.200865691598251,
0.259054307628533,0.795593338816911,0.973438320024839
"11574","NSMCE4A",50.6141807518434,-0.06543767324076,0.252795891837826
,-0.258855762113175,0.795746531755141,0.973470838116587
"11575","RNASEH2C",198.985728314083,0.0639529478056319,0.2470736258380

09,0.258841661422664,0.795757411807874,0.973470838116587
"11576","MAFF",168.319869976114,-0.0958513398850834,0.3713846157763,-0
.258091842831795,0.796336026851904,0.97409451798747
"11577","LRRC2",16.2790368345359,0.121815314893995,0.473018271997615,0
.25752771532388,0.79677142286753,0.974374587425876
"11578","ALAS1",19.1574806915307,0.0888553491699446,0.344894716343324,
0.257630357785748,0.796692198266283,0.974374587425876
"11579","TRMO",8.64069958812751,0.132255571420349,0.513415524371412,0.
257599478672314,0.796716032094829,0.974374587425876
"11580","SLC37A4",6.62294552637767,0.136813476089833,0.532170714206667
,0.257085691560062,0.797112622901392,0.974707663236935
"11581","YTHDC1",103.872789791272,0.0528561934071394,0.205669175042316
,0.256996185239058,0.797181717936237,0.974707980828695
"11582","CNTLN",67.7599235905382,0.0755012479813557,0.293912818714153,
0.256883140761496,0.797268985688059,0.97473051608901
"11583","PSRC1",1.52999591347987,-0.27275666243002,1.06302822044394,-0
.256584592190893,0.797499470539528,0.974780936140943
"11584","PYHIN1",10.7821624223591,-0.110887646265164,0.433251567690439
,-0.255942862148843,0.797994957383499,0.974780936140943
"11585","AC107223.1",2.49345195987626,0.241224244099164,0.942510081278
77,0.25593810495043,0.797998630773009,0.974780936140943
"11586","FCHSD1",9.66459961622774,0.121142237817492,0.472574619460814,
0.256345205241259,0.797684294314212,0.974780936140943
"11587","DELE1",26.150449164714,0.0745157934192932,0.290998931626005,0
.256068958751579,0.797897590374637,0.974780936140943
"11588","WDR91",10.8883015898245,0.102852102531612,0.401453583348633,0
.256199238959819,0.797796996246534,0.974780936140943
"11589","ENDOD1",27.5338437562486,-0.0852244884446976,0.33298160343237
4,-0.255943534316021,0.797994438353193,0.974780936140943
"11590","FNTB",7.7393132580953,-0.127587476999587,0.49839076272694,-0.
255998879877895,0.797951702232061,0.974780936140943
"11591","SOCS6",22.5939007899919,-0.0800668286180736,0.312820333309401
,-0.255951484262635,0.797988299614497,0.974780936140943
"11592","VAMP7",29.9518966774937,0.0678219110324707,0.264828223956174,
0.256097745245214,0.797875362977976,0.974780936140943
"11593","SMIM4",35.9011178956039,0.0879952651656428,0.344736518693895,
0.255253680402155,0.798527172845036,0.974986076794954
"11594","NIPBL-
DT",21.7111921338551,0.0907864787954079,0.355349765769074,0.2554848420
93314,0.798348649506126,0.974986076794954
"11595","MLEC",148.206021082344,-0.0503641518189907,0.197062296034739,
-0.255574774233383,0.798279198865578,0.974986076794954
"11596","MTMR10",12.3652707232646,-0.105844078347987,0.41493782160307,
-0.255084190539847,0.798658074480791,0.974986076794954
"11597","CIAO3",15.58945359881,0.0889583462388665,0.348480960762814,0.
25527462402577,0.798510997907289,0.974986076794954
"11598","AC110285.2",2.08461336238916,0.264192736298355,1.036020006437
16,0.255007369217614,0.798717407543889,0.974986076794954
"11599","STK11",32.6835702445168,-0.0661246614009842,0.258975832995907
,-0.255331397667632,0.798467151578212,0.974986076794954

"11600","CLTCL1",1.65780762430882,-0.260498330365593,1.02121477599111,
-0.255086722685513,0.798656118794091,0.974986076794954
"11601","MAML1",27.4965280698209,-0.0765118657851542,0.300189182965238
,-0.25487882351182,0.79881669261155,0.975023219324158
"11602","CYP4X1",7.96342823807542,0.156903472762518,0.616688024824494,
0.254429251820111,0.799163954644565,0.975027820578826
"11603","NAB1",35.1931968361125,0.0672265041822122,0.2642850322576,0.2
54371212807414,0.799208788534503,0.975027820578826
"11604","CXCL11",3.93295481697944,-0.301006539609594,1.18235518311584,
-0.254582162710495,0.799045837438308,0.975027820578826
"11605","GASK1B",88.3917960466699,-0.172619907536155,0.67869989403345,
-0.254339081313673,0.799233609707517,0.975027820578826
"11606","BRD3",60.4200049401974,-0.0573837769332313,0.225485747619595,
-0.254489596522262,0.799117340361104,0.975027820578826
"11607","CCAR1",80.5232343504936,0.0476612970561774,0.187093317925324,
0.25474612126554З,0.798919191485975,0.975027820578826
"11608","TNFRSF10B",22.2705638045059,0.103808999581831,0.4083110013224
47,0.254240025974348,0.799310129982293,0.975037167518028
"11609","TIPIN",10.0595169707785,-0.10639623391182,0.41865535858146,-0
.254137996160673,0.79938895005402,0.975049318008866
"11610","SLC35B2",44.4264346285129,-0.0774138564477733,0.3048291152898
67,-0.253958209911016,0.799527843500922,0.975134734192339
"11611","ALKAL2",12.9471263658421,0.308202624654618,1.2167360930601,0.
253302771580882,0.800034254522831,0.975164418011818
"11612","ABHD14B",91.1280886227186,0.0594032251749505,0.23442191011521
8,0.253403042171927,0.799956777048423,0.975164418011818
"11613","CWC15",146.543680653711,-0.0560597346445871,0.221017698201605
,-0.253643645286049,0.799770874919099,0.975164418011818
"11614","AC026356.2",4.49208523663904,-0.177138953240291,0.69911411266
2433,-0.253376308719751,0.799977433364899,0.975164418011818
"11615","PRSS36",8.71467690043555,-0.127458081599354,0.502180206940843
,-0.253809448954185,0.799642773225179,0.975164418011818
"11616","FAM215B",7.00903407275332,-0.156244479669738,0.61603320930461
2,-0.253629962329643,0.799781446758796,0.975164418011818
"11617","GEMIN7",13.5893696082717,0.0991589220705392,0.391216495540991
,0.253463039520913,0.799910419002302,0.975164418011818
"11618","TEPSIN",9.71777767206259,0.109631307280948,0.432963303118935,
0.253211545854343,0.800104744885402,0.975166395900955
"11619","SAMD13",2.29821167287785,0.240784068345998,0.961027072000013,
0.250548684174836,0.802163060402931,0.975174537005954
"11620","KLHDC9",3.84437283693708,-0.175356183512471,0.69610773770819,
-0.251909545050885,0.80111098158065,0.975174537005954
"11621","USP21",7.90435598898865,0.125323014677135,0.501659407516481,0
.249816933161007,0.802728924092824,0.975174537005954
"11622","DCAF6",67.6538016609528,0.049744962289789,0.197168286749948,0
.252296974882561,0.800811526217813,0.975174537005954
"11623","VAMP5",269.630219174086,-0.0580055497805562,0.231359812513158
,-0.250715753745078,0.802033879895896,0.975174537005954
"11624","ZNF662",10.760858824602,0.110198271404888,0.440866208412608,0
.249958534589598,0.802619415504656,0.975174537005954

"11625","ATG3",68.8505897618928,-0.0496337936367653,0.198389444966702,-0.250183640793471,0.802445335815335,0.975174537005954
"11626","TRAM1L1",5.69546312930507,-0.152330438657705,0.607656626321726,-0.250685061364004,0.802057611264683,0.975174537005954
"11627","AC097376.3",17.7915247089863,0.0946323221169391,0.379075927577524,0.249639492335176,0.802866154831597,0.975174537005954
"11628","SNX14",48.7246096542245,0.0570100021422062,0.225694503735984,0.252598096978454,0.800578800733205,0.975174537005954
"11629","RRAGD",7.45445264810486,-0.135049529841469,0.536618101676205,-0.251667861034918,0.801297800728184,0.975174537005954
"11630","USP45",10.9400787970119,0.110154922807801,0.439562260252744,0.250601411377907,0.802122290414639,0.975174537005954
"11631","ZUP1",8.87785625177927,-0.126696847320783,0.502757842483615,-0.252003721503184,0.801038187273822,0.975174537005954
"11632","SLC22A3",4.17412712076455,0.19476174011792,0.773874799288932,0.251670864973087,0.801295478646493,0.975174537005954
"11633","LINC01176",2.95490773092926,-0.188790883070347,0.752045361048783,-0.251036563548593,0.801785840483384,0.975174537005954
"11634","RAB14",182.984554736618,0.0414271699931689,0.164908944740806,0.251212389105283,0.801649906514735,0.975174537005954
"11635","STOM",207.457513370855,0.0586806553275763,0.234931263582056,0.249777975195202,0.802759053224342,0.975174537005954
"11636","AL356481.1",3.4018825554532,-0.18036029538184,0.722395426745038,-0.249669763545577,0.802842742987794,0.975174537005954
"11637","BMS1",36.5496761609183,-0.0683862150229969,0.271735928420187,-0.251664236748447,0.801300602349191,0.975174537005954
"11638","VCL",149.454099509469,0.0577344660306318,0.230981154113948,0.249953145537363,0.80262358310002,0.975174537005954
"11639","IPO7",54.8348165354986,-0.0660654138623144,0.261468383251822,-0.252670755219709,0.800522648671861,0.975174537005954
"11640","CSKMT",33.5545278630683,-0.0676973096040039,0.270635192983433,-0.25014229988983,0.802477304931093,0.975174537005954
"11641","NOP2",8.97057913229262,-0.120909163468227,0.479710489481137,-0.252046111393154,0.801005422293965,0.975174537005954
"11642","RAPGEF3",1.53209132200282,0.315945083839256,1.2529214778272,0.252166707515593,0.800912210248412,0.975174537005954
"11643","DAZAP2",135.943024238933,0.0449638018455523,0.179837208808033,0.250025020648251,0.802567999320791,0.975174537005954
"11644","ESYT1",29.2251105747609,0.0767919899989661,0.303981452921258,0.252620642677361,0.800561376756794,0.975174537005954
"11645","SIRT4",3.15227982422361,0.194204751979886,0.773781955019384,0.250981236665077,0.801828615954914,0.975174537005954
"11646","RRN3",23.5127955269277,-0.0694567260614876,0.277525668003373,-0.250271358902354,0.802377504083205,0.975174537005954
"11647","PRKCB",1.63131621761974,0.296611899384793,1.18501525333158,0.250302178432633,0.802353671933358,0.975174537005954
"11648","TERF2IP",185.227689276034,-0.0434613976130785,0.173258737857912,-0.250846786432907,0.801932567391645,0.975174537005954
"11649","PITPNA-AS1",35.7682353958154,-0.080907660471903,0.322296023811199,-0.25103524

2430725,0.801786861886599,0.975174537005954
"11650","FLOT2",22.5223190497474,-0.0846746095924152,0.336667552462698
,-0.251508079626405,0.801421316280435,0.975174537005954
"11651","MMD",14.1919908467397,0.10377919058208,0.413750539913827,0.25
0825510955693,0.801949017044361,0.975174537005954
"11652","CBARP",2.3375067521152,-0.213437203728369,0.854806203387893,-
0.249690751988513,0.802826510567719,0.975174537005954
"11653","SGTA",29.9162552695838,-0.0699066726972343,0.27920170314887,-
0.250380538187334,0.802293078670561,0.975174537005954
"11654","DLGAP4",102.753561245865,-0.0467328815806346,0.18694174441410
9,-0.249986335192813,0.802597916142725,0.975174537005954
"11655","SNHG17",25.4296145242766,0.0776843985810705,0.309272444285766
,0.251184352231816,0.801671581934525,0.975174537005954
"11656","FITM2",9.5025074436053,-0.112592331675091,0.445276955713308,-
0.25285910315014,0.800377093596844,0.975174537005954
"11657","HIRA",11.8434483511396,0.107977766974598,0.429054386173681,0.
251664521921211,0.801300381906809,0.975174537005954
"11658","KIAA0930",99.5677804642395,-0.0629000272795714,0.250982550661
563,-0.25061514082861,0.802111674549374,0.975174537005954
"11659","TMEM44-
AS1",11.6115682365073,0.114971875447501,0.460938433345874,0.2494300043
7811,0.803028178285812,0.97528767514599
"11660","AGTRAP",119.137225206054,-0.0686994235539799,0.27688360834474
6,-0.248116614647852,0.804044180852791,0.975309027355228
"11661","B4GALT3",17.2833534686835,-0.0904696107371286,0.3636607156832
92,-0.248774769546232,0.80353500863181,0.975309027355228
"11662","SRD5A3",37.6190647621566,-0.079251457046169,0.319227326613075
,-0.248260253553503,0.80393304954583,0.975309027355228
"11663","AC012640.2",1.60657673615852,0.275814945483574,1.111171522141
54,0.248219955234276,0.803964227362387,0.975309027355228
"11664","TFEB",19.3916686955315,-0.0885215033446582,0.357085228504255,
-0.247900210589651,0.804211616965056,0.975309027355228
"11665","DOP1A",14.4885111079045,-0.095093504306183,0.383355091281027,
-0.24805593161269,0.804091131614066,0.975309027355228
"11666","SLC25A40",9.92785550920103,0.10719184216508,0.431024507669552
,0.248690829077541,0.803599943353651,0.975309027355228
"11667","AL162390.1",11.0620310677291,0.158515722106162,0.639450474343
511,0.247893665680523,0.804216681031048,0.975309027355228
"11668","ECHS1",155.444394975353,-0.0576191447338118,0.23231784378976,
-0.24801859294956,0.804120021071689,0.975309027355228
"11669","CUL5",66.9569972136745,-0.0554800816222376,0.223388858179082,
-0.248356529839825,0.803858564218394,0.975309027355228
"11670","UHRF1BP1L",37.1959388979313,0.0718273914404793,0.288453518442
886,0.249008546778053,0.803354170238532,0.975309027355228
"11671","NAA25",20.8982678892579,-0.102247789462953,0.411092231305846,
-0.248722261518201,0.803575627667174,0.975309027355228
"11672","CNIH1",125.425551114056,-0.0490941712740926,0.197662296686986
,-0.248373979747069,0.803845064074684,0.975309027355228
"11673","PRSS53",4.67238506275969,0.155850986713194,0.62642677501687,0
.248793622700749,0.803520424382858,0.975309027355228

"11674","ME2",27.2087737569306,0.0737138116920323,0.29679599455724,0.248365251027052,0.803851817053102,0.975309027355228
"11675","ELMO2",13.9152671261876,0.0912192864600942,0.367263505298919,0.248375580867612,0.803843825368599,0.975309027355228
"11676","AMMECR1",15.5372820457009,0.0964808703701344,0.387709214804967,0.248848535670394,0.803477945705348,0.975309027355228
"11677","ANKRD65",56.6533428711228,-0.0782714204517299,0.316117730615933,-0.247602120574583,0.804442269829454,0.97549906146999
"11678","CENPS",14.6371449254295,-0.094910463197555,0.384318099598064,-0.246958088356537,0.80494066020619l,0.975572592711579
"11679","DHDDS",23.7418406571724,-0.0789586539563747,0.320608526944192,-0.246277460892732,0.805467456312928,0.975572592711579
"11680","TRMT13",29.4959190492778,0.0703717059879358,0.285702329919518,0.246311277922583,0.805441280319576,0.975572592711579
"11681","ESPNL",1.96799189786871,0.253672806854852,1.02926944029938,0.246459087312519,0.805326871364415,0.975572592711579
"11682","GRM7",1.65179589737264,0.259922913187086,1.05079474400458,0.247358406263549,0.804630861100745,0.975572592711579
"11683","IL17RC",35.6973674347369,-0.078775389056023,0.318771826417132,-0.247121553813042,0.804814153413672,0.975572592711579
"11684","SEC62",530.84234803121,0.0448306240607255,0.181666435584622,0.246774391298292,0.80508283043784,0.975572592711579
"11685","C5orf34",2.10155272494419,0.213253154633719,0.863600931626556,0.24693483624672,0.804958655553513,0.975572592711579
"11686","ASPN",280.103287994952,0.0825773245493121,0.334133321601988,0.247138849107891,0.804800768791222,0.975572592711579
"11687","QSOX2",9.21017778509806,0.10960288063404,0.444874964216428,0.24636783242475,0.805397504925855,0.975572592711579
"11688","PTPN6",2.61560090589808,-0.232659714189173,0.943676229596812,-0.246546121320209,0.805259506349585,0.975572592711579
"11689","SRP54",62.2908703891243,-0.0634106863501946,0.257028231859764,-0.246707086966197,0.805134921467344,0.975572592711579
"11690","USP14",70.0078033127646,0.0548307164073296,0.221727169204217,0.247289119344816,0.804684478855081,0.975572592711579
"11691","AC006115.2",9.68064590055704,0.110241268912119,0.447593961617602,0.246297489165644,0.805451953448509,0.975572592711579
"11692","C1orf109",14.7634173210729,0.0890959193502144,0.36250200453749,0.245780487376588,0.805852162636337,0.975586469881448
"11693","AC104463.2",1.77538929998643,-0.297788884907145,1.21365559386276,-0.245365230806013,0.8061736480579,0.975586469881448
"11694","C5orf30",8.93880920466081,-0.113021065034763,0.460747512340649,-0.245299349443263,0.806224655435003,0.975586469881448
"11695","ARHGAP18",56.0760486110238,0.0736376650831052,0.299323826947529,0.246013375660915,0.805671878369217,0.975586469881448
"11696","HOXA11-AS",5.23485893502284,0.146317212951253,0.596262122029934,0.245390756087483,0.806153885821606,0.975586469881448
"11697","SRGAP1",109.450912994007,0.0652279571099449,0.265928643072571,0.245283683458439,0.806236784643553,0.975586469881448
"11698","DHRS4L2",79.7214975680879,-0.0661969424066091,0.2692300977544

53,-0.245874970735935,0.805779019613726,0.975586469881448
"11699","UBR1",31.9924501119794,0.06482539072334,0.263810325566736,0.245727268574789,0.805893362004243,0.975586469881448
"11700","NPEPPS",96.4378107975198,0.0570892766986135,0.232213279332959,0.245848458204476,0.805799543762102,0.975586469881448
"11701","DACT3",16.0430936688837,-0.111544205112674,0.454126412072877,-0.245623690116429,0.80597354887568,0.975586469881448
"11702","ZBTB45",8.50496951484329,0.115757166331229,0.470775247689383,0.24588626292350 4,0.805770278032168,0.975586469881448
"11703","ZNF12",42.6572026596316,0.0665673361413348,0.271523839796448,0.245162031412188,0.806330973927046,0.975617071759973
"11704","NSUN2",15.1577138198765,0.0879920921761499,0.359256436353424,0.244928366682306,0.806511897081502,0.975752602757525
"11705","ECE1",67.3819570335215,0.0548321312616768,0.224149249756891,0.244623309340304,0.806748114064895,0.97583842160095
"11706","ACOT11",3.17767037621212,-0.213028054904436,0.871031846392051,-0.244569766061747,0.806789576389994,0.97583842160095
"11707","ZNF189",12.9012644302405,0.104357028918669,0.426663130310588,0.244588813762049,0.806774826354222,0.97583842160095
"11708","ARHGEF19",6.62412063366531,-0.144793420913827,0.60251992459515,-0.240313083440549,0.810087553039909,0.975852226629248
"11709","LDLRAP1",11.5838114912355,0.101596697325782,0.421641971729248,0.240954895711903,0.809590074648439,0.975852226629248
"11710","PDE4B",7.15843117815209,-0.144391893375073,0.599583743919733,-0.24082022709809,0.80969445195547,0.975852226629248
"11711","FUBP1",46.1000187893863,-0.0577966242302325,0.240007227719874,-0.240812015451843,0.809700816647627,0.975852226629248
"11712","EFNA4",16.166136754129,0.0904662067866624,0.378562323496116,0.238973086257462,0.811126452233172,0.975852226629248
"11713","HCN3",2.05444369294039,-0.246289914947345,1.02038476814106,-0.241369650583904,0.809268632785887,0.975852226629248
"11714","METTL18",10.9998638516989,0.122617426738568,0.50350755380363,0.24352648895192,0.807597567426887,0.975852226629248
"11715","EEF1AKNMT",21.5126580356694,0.0775034645477859,0.323749990373202,0.239392947806559,0.810800897891981,0.975852226629248
"11716","CACYBP",128.328784682334,0.0586883637421484,0.240476658702848,0.244050146316563,0.807191982677728,0.975852226629248
"11717","MRPS14",60.0598875740734,0.066734166970765,0.279122249748881,0.239085802120053,0.811039050835781,0.975852226629248
"11718","LBH",33.3087163187663,0.111419702578145,0.461654552389841,0.24134864911731,0.8092849084957,0.975852226629248
"11719","CSRNP3",1.86473899642657,-0.227185338583987,0.944692456498368,-0.240486030158514,0.809953491864169,0.975852226629248
"11720","DCAF17",8.59102379996637,0.12717870133408,0.522236482513235,0.243527033427537,0.807597145691039,0.975852226629248
"11721","CDK15",2.05159861795148,0.236994061368525,0.977545418589953,0.242437902998281,0.808440867851327,0.975852226629248
"11722","TMBIM1",91.7744504038155,0.0446573713991638,0.183869199185715,0.242875759490626,0.808101644520115,0.975852226629248
"11723","RAD18",11.958273899909,0.106282192356209,0.437659149457157,0.

242842386565058,0.808127498468777,0.97585226629248
"11724","VILL",9.48561030654914,-0.102571146559707,0.427267981195476,-0.240062796825351,0.810281574785522,0.97585226629248
"11725","ABHD5",122.527490090499,-0.0771763933144207,0.322258456684295,-0.239486014140593,0.810728740082234,0.97585226629248
"11726","ZNF639",30.7592043161915,-0.071687242453578,0.29362986754539,-0.244141520931949,0.807121216231296,0.97585226629248
"11727","LMLN",10.653783603557,0.106293280824685,0.442374280846763,0.240279070069863,0.810113919460358,0.97585226629248
"11728","GPRIN3",3.08471581482617,0.213260836004831,0.878307237934999,0.242808924706384,0.808153421524134,0.97585226629248
"11729","AC124852.1",18.1491927255719,0.103336578466909,0.431176506474774,0.239661894642107,0.810592377742982,0.97585226629248
"11730","SLC35B3",40.5025523125709,-0.0554199682075351,0.228727805206425,-0.242296594231379,0.808550352575837,0.97585226629248
"11731","CCN6",1.85927227107805,0.25171942181824,1.04486550918291,0.240910834558111,0.809624224657536,0.97585226629248
"11732","TTYH3",17.7132445874491,-0.0850713204031759,0.356150725085372,-0.238863252020008,0.811211621444412,0.97585226629248
"11733","FAM221A",6.16170129716575,-0.132735152561708,0.553068192875301,-0.23999780546345,0.810331957882515,0.97585226629248
"11734","YKT6",27.423169582059,0.0900841802898166,0.371410356441592,0.242546226101219,0.808356942653421,0.97585226629248
"11735","TFPI2",1.70936990744032,-0.305275345885323,1.25879748254686,-0.242513470290452,0.808382320551935,0.97585226629248
"11736","ZNF789",4.86182653995034,0.183235756341949,0.766807105657328,0.238959387556632,0.811137074550696,0.97585226629248
"11737","PLOD3",54.7184555020481,0.0544615468681061,0.225208342966659,0.241827394805568,0.808913910824169,0.97585226629248
"11738","RBM28",47.351105709743,0.0802342764214801,0.332742182987116,0.241130462333316,0.809454003698606,0.97585226629248
"11739","ACTR3B",6.3569952880731,-0.142472920681907,0.591714582172367,-0.240779803260627,0.809725783831123,0.97585226629248
"11740","POLR1E",31.480775500402,0.0730483730593095,0.304167168553157,0.240158638444713,0.810207277152115,0.97585226629248
"11741","VIM-AS1",7.49877315171457,-0.145952427619515,0.605010577686158,-0.241239464238302,0.809369525887227,0.97585226629248
"11742","IPMK",19.5141547974638,-0.07676635655607,0.32092165205066,-0.239205912301461,0.810945918376218,0.97585226629248
"11743","SIPA1",19.3740077105258,-0.0882289003279726,0.366537227362649,-0.240709247905887,0.809780470898825,0.97585226629248
"11744","ANKRD42",13.3935793028696,-0.0928609271365362,0.383693394540272,-0.242018571228726,0.808765773166,0.97585226629248
"11745","ANGPTL5",47.8836610046235,-0.10973413666008,0.451135416647076,-0.243239906712812,0.807819553910658,0.97585226629248
"11746","PIH1D2",2.74594219706646,0.205933262248406,0.84365496204044,0.244096545998309,0.807156047548452,0.97585226629248
"11747","PDZRN4",3.06595948394102,0.227460738759542,0.942388485320475,0.241366211814642,0.809271297756533,0.97585226629248

"11748","CNPY2",177.814695264523,0.0532999367382836,0.221042509494965,
0.241129802860376,0.80945451480497,0.975852226629248
"11749","OS9",212.789784441433,0.0329644190847406,0.137804601140675,0.
23921130943291,0.810941733547117,0.975852226629248
"11750","AL121603.2",12.5916436895825,0.0985776186535308,0.40388962686
5634,0.244070686881804,0.807176074590239,0.975852226629248
"11751","CLMN",10.8823916435445,0.18062262985621,0.74888484539933,0.24
1188790193633,0.809408798616238,0.975852226629248
"11752","PAPOLA",147.558027034081,-0.0411950481943186,0.16901849028164
8,-0.243731015024878,0.80743915108781,0.975852226629248
"11753","STOML1",23.8122198299022,-0.0786363585771654,0.32898870500658
8,-0.239024493487066,0.811086590083144,0.975852226629248
"11754","NR2F2",306.324006016894,-0.0517035288703549,0.215256487169144
,-0.240194985760069,0.810179100701552,0.975852226629248
"11755","AC005632.3",2.67693702204061,-0.216241756273276,0.89588932214
8115,-0.241371061053371,0.809267539703671,0.975852226629248
"11756","ATP2A3",2.93745308207442,0.221200772104741,0.919524579167441,
0.240559934031368,0.809896206324621,0.975852226629248
"11757","PSME3",26.3132258798629,-0.0754114861752084,0.312191879405828
,-0.241554925511623,0.809125052057318,0.975852226629248
"11758","SPAG9",171.970239504305,0.0560948659912388,0.2312144786408,0.
242609659745332,0.808307797363911,0.975852226629248
"11759","PRCD",14.8429305359862,-0.0888335318879969,0.369834015814314,
-0.240198381137009,0.810176468616819,0.975852226629248
"11760","VMAC",10.8568015150239,-0.110623278741824,0.461287578606309,-
0.23981412869614,0.810474353428873,0.975852226629248
"11761","FXYD7",2.10847270054873,-0.26682858024958,1.09225113852166,-0
.244292334280125,0.807004420016767,0.975852226629248
"11762","ZNF565",9.46250971081565,-0.108839765981054,0.453686247605698
,-0.239900959210137,0.810407037228261,0.975852226629248
"11763","ZNF583",9.22466629944163,0.112391047533731,0.468056903301899,
0.240122614880521,0.810235202873351,0.975852226629248
"11764","SERINC3",205.774259563974,0.0397005580344034,0.16431806388842
7,0.241607995462753,0.809083926131457,0.975852226629248
"11765","AP001627.1",1.93828135103817,-0.260225042218655,1.08307524544
128,-0.240264970798619,0.810124848969989,0.975852226629248
"11766","USP18",20.797434100766,-0.104834137184466,0.437552079586416,-
0.239592364144532,0.810646284915341,0.975852226629248
"11767","ZNF70",5.88622975228344,-0.142643384875206,0.588690693226184,
-0.242306166067416,0.808542936287496,0.975852226629248
"11768","UQCR10",294.418619511758,0.0638120995395298,0.265478688087597
,0.240366185320588,0.810046390055717,0.975852226629248
"11769","C1QTNF6",23.8592176641701,0.117235672409179,0.489619160887925
,0.23944257450336,0.810762420257653,0.975852226629248
"11770","WWC3",39.9640893701284,-0.0696478994215798,0.290747669655198,
-0.23954757575246,0.81068100994568,0.975852226629248
"11771","GRIPAP1",23.4420919624116,0.0726603107978928,0.30416034860078
4,0.238888175701234,0.811192294576478,0.975852226629248
"11772","KDM1A",43.4484107045446,-0.055869872879798,0.235004897526023,
-0.23773918530192,0.812083389370528,0.975960488531882

"11773","GPR161",21.7404337766369,-0.0726583705529776,0.305429194159247,-0.237889409206556,0.811966869979566,0.975960488531882
"11774","AL592148.3",5.80411252627161,0.136082368146165,0.571021310732687,0.238313992119761,0.811637569799333,0.975960488531882
"11775","EDEM1",23.9191109758692,-0.0748398741510227,0.314743767209641,-0.23778032148028,0.812051482167534,0.975960488531882
"11776","CNOT6L",37.2242016028492,0.0796673122137483,0.335309226853813,0.237593557926401,0.8121963474906,0.975960488531882
"11777","ZNF658",5.21095405690277,-0.139339387292187,0.585128117087034,-0.238134834445942,0.811776517761202,0.975960488531882
"11778","TBK1",33.6541398194188,0.0658581971542054,0.276427982897215,0.238247215292577,0.811689358704795,0.975960488531882
"11779","CD3EAP",8.67181958667277,-0.108028000812794,0.454678787184246,-0.237591908524685,0.812197626896872,0.975960488531882
"11780","NORAD",294.351543152301,0.0405794356861787,0.170729882825972,0.237682091819521,0.812127674344871,0.975960488531882
"11781","SMIM11A",3.03969174620241,0.19642512897149,0.824555339865906,0.238219461417149,0.81171088352228,0.975960488531882
"11782","TBC1D22A",26.5344233972327,0.0776509660762214,0.326284983319318,0.237985105187107,0.811892646697541,0.975960488531882
"11783","PGRMC1",264.938570218596,0.0523814401157862,0.219716892945047,0.238404245634801,0.811567574810141,0.975960488531882
"11784","ATP6AP1",77.4269522979972,0.0512587480257817,0.215254018433216,0.238131433730632,0.811779155285595,0.975960488531882
"11785","WDPCP",13.6202884443135,0.0956599784414869,0.403610107837677,0.237010859202662,0.812648365828147,0.97597974865925
"11786","KLHL2",8.19911383317518,0.120144913823746,0.506327509140381,0.23728695687051,0.812434180102776,0.97597974865925
"11787","KRBA1",3.6862141257366,0.163532109244218,0.689390929501792,0.237212446880319,0.812491980644587,0.97597974865925
"11788","AMPD3",2.05047313148654,0.270847169931597,1.14127958442127,0.237318860013554,0.812409431801654,0.97597974865925
"11789","IFI27",771.260001717583,-0.140496467940905,0.593383940927986,-0.236771604774448,0.812833981351591,0.97597974865925
"11790","DMPK",10.7377708263121,-0.100427379597668,0.423769713026868,-0.236985741336594,0.812667851977792,0.97597974865925
"11791","ZIK1",1.56254813500315,-0.290390182546058,1.2256419436821,-0.236929050970353,0.812711832133791,0.97597974865925
"11792","CNKSR2",22.4584702696653,-0.137050527447879,0.578792890182084,-0.236786819210518,0.812822177555665,0.97597974865925
"11793","RTL3",1.93146788051519,0.22821285425589,0.963808273383747,0.236782418825559,0.81282559149617,0.97597974865925
"11794","EPS8",245.042637979788,-0.0364834426706766,0.154252621623453,-0.236517488563252,0.813031138281641,0.976034232219447
"11795","PPFIBP1",134.972776092224,-0.0517664439806871,0.218804405994377,-0.236587758575654,0.812976617866733,0.976034232219447
"11796","NOL3",11.5018153972936,0.102712661344364,0.434401103733065,0.236446593854606,0.813086144298065,0.976034232219447
"11797","AL355001.2",11.4344575955178,0.13024901931145,0.551339234697825,0.236241158100849,0.813245543663137,0.976096676993416

"11798","BCL2L1",29.5562300255115,0.0682418272010387,0.288913157313294
,0.236201867148051,0.813276030732226,0.976096676993416
"11799","ARIH2",60.330139381779,0.0462672337837426,0.196332511031172,0
.23565752580018I,0.813698431163177,0.976385295031007
"11800","C11orf52",2.36643408814823,-0.196710665639291,0.8348449287547
27,-0.235625394446258,0.813723366289612,0.976385295031007
"11801","ZC3H10",7.63707831234757,0.11301708178614,0.479294452737261,0
.235798852126698,0.813588758916118,0.976385295031007
"11802","CD70",5.71525132246623,0.247322968085114,1.05103112404963,0.2
35314599564071,0.81396456442532,0.976530085088983
"11803","SLC9A8",7.59775916357308,0.11312975066756,0.480805396210336,0
.235292181741799,0.813981962874666,0.976530085088983
"11804","ELOA",36.9244563596333,0.0688897374731886,0.29297862151169,0.
235135714400376,0.814103399576311,0.976593031006486
"11805","AP5Z1",20.8272858193878,0.0780396731581812,0.332060655416608,
0.235016319715058,0.814196066626921,0.976621457300907
"11806","GORASP1",15.7025760192487,0.0879383888982915,0.37435868117106
2,0.234904099520824,0.814283167635031,0.976643202923263
"11807","ALKBH1",4.59565664186375,-0.163779513354212,0.697568896397329
,-0.234786146859571,0.814374720439058,0.97667028385001
"11808","MIR34AHG",3.36851789029989,0.19322018460289,0.834815949098,0.
231452435487919,0.816963333195345,0.976811665639351
"11809","SLC9A1",19.921409939162,0.0775616110055979,0.331565445204004,
0.233925495335849,0.815042819849768,0.976811665639351
"11810","TRIT1",11.9982058026929,0.0967202026843755,0.413288822459956,
0.234025692029808,0.814965033065291,0.976811665639351
"11811","ZNF687",10.7611971742731,-0.101821623083707,0.440926458514891
,-0.230926543684084,0.817371869115327,0.976811665639351
"11812","ARID4B",177.631882541433,0.0593390782094214,0.25546345542888,
0.232280104838483,0.816320463934782,0.976811665639351
"11813","SMC6",37.2673882861878,-0.0642513993734416,0.27773434254432,-
0.231341211838751,0.817049732483207,0.976811665639351
"11814","AC006059.1",2.27742962861055,0.209798343260194,0.907233708313
107,0.231250604268539,0.817120118703847,0.976811665639351
"11815","TIPARP",102.650939369884,-0.0899377165237537,0.38581325318161
,-0.233112045224165,0.815674401805291,0.976811665639351
"11816","SGCB",94.6307088734611,0.0533022987225281,0.230223721726821,0
.231523920831128,0.816907804068147,0.976811665639351
"11817","DCLK2",2.47374885871015,-0.1927377056309,0.831581455988141,-0
.231772491128817,0.816714724206764,0.976811665639351
"11818","SEMA6A",36.5369094092994,0.0901655931264696,0.39013959633768,
0.231111104776015,0.817228488272356,0.976811665639351
"11819","ZCCHC10",40.698236570342,-0.0641752021467048,0.27741114557598
4,-0.231336062628122,0.81705373247893,0.976811665639351
"11820","TSTD3",25.3795299013621,0.0718207386462274,0.309332519698472,
0.232179722701758,0.816398426404302,0.976811665639351
"11821","Z97989.1",5.23890238589337,0.154746424206809,0.66318577362791
5,0.233337973099571,0.815498974015183,0.976811665639351
"11822","HIPK2",22.6182168467013,-0.0831966788457714,0.358393212070693
,-0.232137987115004,0.816430841166569,0.976811665639351

"11823","THEM6",7.93818834210207,-0.140513155122996,0.606966549134963,
-0.231500657364483,0.816925874804472,0.976811665639351
"11824","MINDY3",24.4027845163305,-0.0706058874782077,0.30138452518297
6,-0.234271774356502,0.814773997050068,0.976811665639351
"11825","CHST3",17.1891268083396,-0.0818061733383582,0.352508341013306
,-0.232068759290068,0.816484609007461,0.976811665639351
"11826","PPP1R3C",2.06991224275205,-0.218417712037439,0.94555001253045
9,-0.230995409172397,0.817318368544791,0.976811665639351
"11827","MARCH5",47.4156172006881,-0.0531716498349017,0.22728018847809
1,-0.233947578937472,0.815025675291507,0.976811665639351
"11828","SAC3D1",22.8833295022798,-0.0823245677262529,0.35486132206451
4,-0.231990816151235,0.816545146888831,0.976811665639351
"11829","CDC42EP2",39.4509815621324,-0.075244029168318,0.3220252766056
7,-0.233658767291291,0.815249900605983,0.976811665639351
"11830","EED",23.9621558807441,0.0697258757710763,0.300891265783002,0.
231731139119743,0.816746844088358,0.976811665639351
"11831","POU6F1",6.40654588678585,-0.137701393935294,0.589925372152403
,-0.233421718128305,0.815433950278599,0.976811665639351
"11832","SMAD9-
IT1",1.96869860739702,-0.233559429732494,1.00565984048091,-0.232244960
304676,0.816347758969331,0.976811665639351
"11833","MAX",67.6615377085253,0.0537420549559479,0.231725890876067,0.
231920804156884,0.816599525639969,0.976811665639351
"11834","RDH11",78.4844887474022,0.0469618467798654,0.200945412049773,
0.23370449865376,0.815214395041669,0.976811665639351
"11835","SPG21",75.5689220300167,-0.0470678512807416,0.200729344440377
,-0.234484157819397,0.814609130632071,0.976811665639351
"11836","RAMAC",27.6673479223211,-0.0796585255131661,0.34414988030522,
-0.231464632335535,0.816953858736388,0.976811665639351
"11837","ISG20",10.2140099851053,0.105033221328572,0.454879699923594,0
.23090329453307,0.817389931226036,0.976811665639351
"11838","AC138969.1",3.24552413561556,-0.179142189990832,0.77052733395
1466,-0.232492972146938,0.816155145106752,0.976811665639351
"11839","MYLPF",1.99331077509256,-0.243651707186707,1.04233595877066,-
0.233755446251775,0.815174840069224,0.976811665639351
"11840","MYO1C",134.474719717201,-0.0453395610083261,0.196181691181134
,-0.231110052805407,0.817229305504381,0.976811665639351
"11841","MYL12A",543.954543982297,-0.0687579082569927,0.2955415178404,
-0.232650589194455,0.816032740479845,0.976811665639351
"11842","ATP8B3",3.03456853684011,-0.174923214386369,0.754825393065494
,-0.231739970585742,0.816739984283539,0.976811665639351
"11843","FKBP8",299.685767529769,-0.0441208192820212,0.188735125145121
,-0.233771107779202,0.815162680782282,0.976811665639351
"11844","DMWD",38.4693045446615,-0.067615677309789,0.291094500714214,-
0.232280847435766,0.816319887198321,0.976811665639351
"11845","TRPM4",5.05876281693052,-0.150829201425957,0.648537994500291,
-0.232568026399399,0.816096857782393,0.976811665639351
"11846","ZNFX1",58.0028914249664,-0.0522767859055008,0.223027055672844
,-0.234396610526863,0.814677089771488,0.976811665639351
"11847","TCEA2",72.4929484015155,-0.0473699226626905,0.204589994262708

,-0.231535871699884,0.816898520837875,0.976811665639351
"11848","COMT",247.607770075734,0.062584602599798,0.269574500313327,0.232160692228144,0.81641320675604,0.976811665639351
"11849","MORC2-AS1",5.34433730547428,-0.135236121411761,0.585239685559448,-0.231078180015226,0.817254066237971,0.976811665639351
"11850","RCAN3",11.8990269730018,-0.105072559923678,0.457021284535722,-0.229907366416027,0.818163752073368,0.9768949661166
"11851","AC239809.3",5.82572438719584,-0.133208366780299,0.578901979664548,-0.230105218948273,0.818010009567214,0.9768949661166
"11852","CLK2",12.6473803069525,0.10216896917823,0.444638488196106,0.229779859122696,0.818262836092579,0.9768949661166
"11853","MTMR12",16.4782700587022,-0.0821727620815216,0.357802853367152,-0.229659325822094,0.818356503395135,0.9768949661166
"11854","PRKAG2-AS1",22.5475465468793,0.0852578025742625,0.371123437418362,0.229728963407268,0.818302387206008,0.9768949661166
"11855","BRF2",18.6073377770955,-0.0845558619541015,0.368096113011591,-0.229711368757211,0.81831606013445,0.9768949661166
"11856","LINC01239",11.5023970498499,0.117474101356421,0.509427760602048,0.230600117311214,0.817625476465344,0.9768949661166
"11857","ZFAND4",4.04639536981505,0.171766133630854,0.746263963526485,0.230168066563431,0.817961174912453,0.9768949661166
"11858","TBC1D10C",1.96694925782247,-0.248139420755261,1.08032617988283,-0.229689352508493,0.818333169196684,0.9768949661166
"11859","AL162171.3",1.56347494756377,-0.26562222962999,1.1557095940496,-0.229834753468863,0.818220178157521,0.9768949661166
"11860","APOBEC3G",22.4670952745017,-0.0820965366610071,0.357208724551041,-0.229827915777227,0.818225491640884,0.9768949661166
"11861","NUP50-DT",3.24406886258661,0.215409505889225,0.935389882157043,0.23028847114797,0.81786761858116,0.9768949661166
"11862","CHST7",43.0792434939988,0.0961843305632595,0.418039592645877,0.230084260570835,0.818026295069746,0.9768949661166
"11863","JAZF1",50.6737353281706,-0.0560637670308769,0.244600872156545,-0.229205098643295,0.818709509918278,0.976953372231213
"11864","RARRES2",477.034208513879,0.0556429975589109,0.242897874731717,0.229079804096142,0.81880689011373,0.976953372231213
"11865","NAT1",3.64419572041598,0.185593354295574,0.809988312443916,0.229130904044278,0.818767174377097,0.976953372231213
"11866","RHEBL1",1.63521638160215,0.245528640958771,1.07127147474864,0.22919367008851,0.818718392191323,0.976953372231213
"11867","BCL7C",215.008944882747,-0.064947935260103,0.28353654785321,-0.229063715954274,0.818819394183618,0.976953372231213
"11868","LONRF3",6.30934889895366,-0.152103390444776,0.663828424515721,-0.229130577762981,0.818767427966918,0.976953372231213
"11869","CROCC",39.6774236869613,0.0629192833877583,0.281694171212895,0.22336026023132,0.82325513077455,0.976994722442298
"11870","PAQR7",14.3019608066542,-0.0894085951170099,0.398888114848904,-0.224144545271491,0.822644832031455,0.976994722442298

"11871","SNHG12",30.7438584089015,0.0626034147617415,0.276981462111021
,0.226020233573063,0.821185683375321,0.976994722442298
"11872","DMAP1",42.5289077321609,-0.0615328378377438,0.273454012696725
,-0.225020789532121,0.821963101258984,0.976994722442298
"11873","HIST2H3PS2",2.25085199789496,-0.207249452773028,0.90765364742
0257,-0.228335393530423,0.819385511156825,0.976994722442298
"11874","FLAD1",16.0916739886681,-0.0784506184335326,0.348163362938123
,-0.225327035479822,0.82172486913053,0.976994722442298
"11875","OLFML2B",198.423112381002,-0.0540415021575414,0.2382003936816
05,-0.226874109325684,0.820521634899693,0.976994722442298
"11876","KLHL20",41.5363782720789,0.0606943454613927,0.271952371519414
,0.22318005583952,0.823395373627118,0.976994722442298
"11877","GCFC2",19.900485559374,0.0746669394322338,0.333780915731857,0
.223700445151326,0.822990399544359,0.976994722442298
"11878","INSIG2",39.3876401264122,0.0667884505194356,0.294658975292402
,0.226663553869889,0.820685369309139,0.976994722442298
"11879","SSB",238.32133881789,-0.0447077148230826,0.199075849211788,-0
.224576285873431,0.822308914801107,0.976994722442298
"11880","PLCL1",7.4024307515354,-0.126273996173534,0.558476146760951,-
0.22610454699972,0.821120108169165,0.976994722442298
"11881","LINC01963",1.80890780782632,0.22370533115913,1.00430648179034
,0.222746079225077,0.82373313614004,0.976994722442298
"11882","ZDHHC3",63.6530555578379,0.0456250855310186,0.200801700505156
,0.227214637207951,0.820256846483519,0.976994722442298
"11883","POC1A",2.32227047398186,-0.242062790000985,1.07801829653841,-
0.224544231557355,0.822333853651236,0.976994722442298
"11884","OSBPL11",24.9872976301807,-0.0700487935724873,0.3148715435202
34,-0.222467844471902,0.823949702477217,0.976994722442298
"11885","PDCD10",81.365481070249,-0.0561818691919358,0.251261930940442
,-0.223598811732657,0.82306948836033,0.976994722442298
"11886","ANAPC4",23.447083139107,0.068647808950025,0.30427386962315,0.
225611910201316,0.821503276674197,0.976994722442298
"11887","PGM2",18.9902710535183,-0.0756653097415431,0.337245399205,-0.
224362763494807,0.822475042540507,0.976994722442298
"11888","ELOVL6",10.0413833548068,0.116228706382572,0.511346725105922,
0.227299209471803,0.820191087772131,0.976994722442298
"11889","AP1AR",20.2449158307234,-0.0753409644183034,0.336011959990346
,-0.224221079572489,0.822585281888295,0.976994722442298
"11890","LARP1B",37.4129614872438,-0.0698377302885282,0.30791910509964
2,-0.226805447053793,0.820575027938667,0.976994722442298
"11891","LPCAT1",21.7696063883558,-0.0831001694518645,0.37358670145048
4,-0.222438778278832,0.823972327164281,0.976994722442298
"11892","ADAMTS16",13.1374756254047,-0.16949654767189,0.75526383448226
3,-0.224420315038759,0.822430264680159,0.976994722442298
"11893","CTNNA1",156.65762002362,0.0389926667257536,0.175139218005802,
0.222638122801609,0.823817163328638,0.976994722442298
"11894","NDUFA2",194.966357459981,0.0526105735872241,0.230257679031535
,0.228485641862215,0.819268717070932,0.976994722442298
"11895","FAF2",58.2849049121174,0.046063432751694,0.206865501511665,0.
222673342897131,0.823789749771562,0.976994722442298

"11896","TUBB",770.618452125657,-0.0570858674793857,0.249762314569698,
-0.228560772179486,0.819210316749815,0.976994722442298
"11897","VPS52",16.325149335603,0.0778502134336033,0.348035977127883,0
.223684384804269,0.823002897221738,0.976994722442298
"11898","LCA5",44.3717863409815,0.0578540018934765,0.25853026914382,0.
223780380088849,0.822928197256349,0.976994722442298
"11899","PPIL6",8.52667834855026,-0.113275413910987,0.50173010243083,-
0.225769618689768,0.821380607780448,0.976994722442298
"11900","MICAL1",13.5725021864617,-0.105199387856685,0.462866375169426
,-0.227278094716165,0.820207505319052,0.976994722442298
"11901","VTA1",38.3058104220056,-0.0571616481697513,0.252219135373519,
-0.226634858949536,0.820707683968477,0.976994722442298
"11902","CCDC126",20.7450027063207,-0.0778448720349854,0.3470378853951
43,-0.224312316640444,0.822514293087277,0.976994722442298
"11903","PHTF2",30.9949583256601,0.0608799754521269,0.269544402355324,
0.225862510666694,0.821308356541198,0.976994722442298
"11904","AC058791.1",27.7901688440446,-0.127432562750622,0.55700952583
5762,-0.228779862533619,0.819040019096286,0.976994722442298
"11905","TBC1D31",3.93286088933638,-0.157593334092987,0.69101448844131
9,-0.228060824670205,0.819598954951724,0.976994722442298
"11906","TTLL11",5.11846659802116,-0.156930145261868,0.688631199871268
,-0.22788706827574,0.819734036258893,0.976994722442298
"11907","EPC1",122.092016435761,0.0429386902664173,0.189984846933527,0
.226011131726948,0.821192762458223,0.976994722442298
"11908","ZNF25",24.9534170974722,-0.0739055609152241,0.324919589704478
,-0.227458002709049,0.820067622355151,0.976994722442298
"11909","MINPP1",18.0240950190455,-0.079245671501359,0.35406722597749,
-0.223815325698621,0.822901004279005,0.976994722442298
"11910","FBXW4",23.9695117778894,-0.0775977910825244,0.34146673823995,
-0.227248461980493,0.8202305460609,0.976994722442298
"11911","SSRP1",62.0419430353913,-0.0481452922493028,0.214098032365691
,-0.224874987020283,0.82207652842453,0.976994722442298
"11912","ZC3H12C",11.3540678615373,0.0876654692410721,0.39302710172293
5,0.22305196984322,0.823495059119489,0.976994722442298
"11913","TLCD5",15.0373284824073,0.087782029350217,0.385885302205752,0
.227482178897325,0.820048825200323,0.976994722442298
"11914","SOX5",26.400899149548,0.0916681443530872,0.404747277746772,0.
226482423460397,0.820826228148938,0.976994722442298
"11915","TM7SF3",64.0146562506038,0.0436407992953558,0.195909332711064
,0.222760185497233,0.823722156762978,0.976994722442298
"11916","ZNF740",13.6894485126541,0.0934199173775152,0.417992939257292
,0.223496400545683,0.823149184235919,0.976994722442298
"11917","SNRNP35",37.9611934267263,0.0596660358242445,0.26455343622454
8,0.22553491149365,0.821563169432623,0.976994722442298
"11918","DHRS12",45.9687345165336,-0.0562437896593692,0.24628606629280
7,-0.228367729063899,0.819360375104512,0.976994722442298
"11919","RPS29",3810.44018985507,-0.0599061670194093,0.26775799548747,
-0.223732504832756,0.822965451802108,0.976994722442298
"11920","TXNDC16",9.42262642875185,-0.109865871149153,0.48489490516559
4,-0.226576666363681,0.820752937988984,0.976994722442298

"11921","NSMCE3",51.6707510427264,0.0557841921524894,0.24774969867369,
0.225163511605164,0.821852074127148,0.976994722442298
"11922","WHAMM",22.7416112206575,0.0662970500547756,0.292086223542999,
0.226977668616458,0.820441106873621,0.976994722442298
"11923","KLHL25",2.35721791186595,-0.197367703474065,0.865653764849774
,-0.227998434811077,0.819647457320591,0.976994722442298
"11924","EEF2KMT",13.3044193983723,-0.0875253632984843,0.3875083259789
24,-0.225867052217208,0.821304824168936,0.976994722442298
"11925","NUBP1",35.1766661940187,0.056304355519083,0.248280962955954,0
.226776772768807,0.820597325833484,0.976994722442298
"11926","TXNL4B",14.6231955858061,0.0983637120801679,0.435475825387262
,0.22587640081443,0.82129755293555,0.976994722442298
"11927","CENPBD1",6.2740371227391,-0.132666921438578,0.583356528130591
,-0.227419965391866,0.820097196843528,0.976994722442298
"11928","GIT1",23.1748491163073,0.0643640164229643,0.289447084376099,0
.22236885391919,0.824026755832247,0.976994722442298
"11929","LINC00910",6.78978021289966,-0.12840360296284 7,0.567135146770
748,-0.226407415752618,0.82088456073501,0.976994722442298
"11930","NACA2",1.70944131418296,-0.257201294627543,1.13643684209184,-
0.226322559337404,0.820950553735857,0.976994722442298
"11931","TMEM241",7.45117306855189,0.114357428172664,0.507597210500486
,0.225291679715711,0.821752371933656,0.976994722442298
"11932","XAB2",34.4080396782701,-0.0597270633226042,0.267837728443126,
-0.222997199348212,0.823537686223751,0.976994722442298
"11933","ZNF121",13.6773281326435,-0.0932414102764189,0.41746712872162
3,-0.223350304398684,0.823262878686881,0.976994722442298
"11934","TMEM147-
AS1",1.67737494414623,0.249467780850642,1.11973441582853,0.22279192040
9138,0.823697456560051,0.976994722442298
"11935","TMEM147",140.260412671828,-0.0544591097556663,0.2423178321054
5,-0.224742476781351,0.822179618083095,0.976994722442298
"11936","ZNF180",8.47371631352928,-0.103831812596534,0.461444154284076
,-0.225014905124602,0.82196767896586,0.976994722442298
"11937","SAE1",35.0693287573692,0.062345651305677,0.280373760195386,0.
22236621309437,0.824028811449744,0.976994722442298
"11938","ZNF582",6.11704029965753,0.129160952638683,0.577180800457969,
0.223779017833232,0.822929257301856,0.976994722442298
"11939","AGPAT3",22.9753254438456,0.0648191449270477,0.291267743637593
,0.22541446291074,0.82389241258363,0.976994722442298
"11940","NUP50",29.7186363660997,0.0654788830888165,0.291533541599287,
0.224601542346085,0.822289264925208,0.976994722442298
"11941","EIF1AX",401.587913028584,-0.0283973485699272,0.12473835956709
4,-0.227655299207722,0.819914225973991,0.976994722442298
"11942","INE1",2.99173431810294,-0.195473813613147,0.875430741863599,-
0.223288724356454,0.823310802411315,0.976994722442298
"11943","SLC10A3",28.4155538056636,0.0705078644300721,0.31516873309031
,0.223714655126873,0.822979341807684,0.976994722442298
"11944","NFIA-
AS2",3.06954558804143,-0.195560522735334,0.883297540394047,-0.22139824
2146234,0.824782361035649,0.977180737754725

"11945","CNN3",291.287644002659,-0.0439393025051224,0.198334478747754,
-0.221541422260753,0.824670887458893,0.977180737754725
"11946","WDR3",17.7067316046618,0.0744389279483031,0.336490368009543,0
.221221571329471,0.824919913832075,0.977180737754725
"11947","AGFG1",54.0297567374175,-0.0479660972810212,0.216585551922245
,-0.221464898536913,0.824730464937706,0.977180737754725
"11948","PRDM1",10.2645630249501,0.141407705434576,0.640074850323199,0
.220923701912633,0.825151841902065,0.977180737754725
"11949","CEP85L",28.9067919994788,0.0728950786527078,0.328940539366422
,0.221605639709572,0.824620891799245,0.977180737754725
"11950","NSUN5",24.1899055575855,0.0619127877555312,0.280048038772451,
0.221079169227239,0.825030789522221,0.977180737754725
"11951","AC005070.3",2.37273732286452,-0.239059808572748,1.08165979171
963,-0.221012013576551,0.825083078789743,0.977180737754725
"11952","XPO7",22.0528251188445,-0.0764228651472204,0.345680988526662,
-0.22107916745131,0.825030790904999,0.977180737754725
"11953","TOPORS",89.6803564690809,-0.0557133148865877,0.25154150065344
6,-0.22148756663158,0.824712816607005,0.977180737754725
"11954","CUEDC2",93.6684243709721,-0.0520481610562759,0.23511694841601
9,-0.221371370319851,0.824803282621096,0.977180737754725
"11955","IL18",5.86934097899581,-0.14762959336178,0.666933709669684,-0
.221355722797244,0.824815465362875,0.977180737754725
"11956","COPS7A",65.6707970132861,0.0438579774437672,0.198324580578282
,0.221142418735411,0.824981542396386,0.977180737754725
"11957","DDT",157.160495601606,-0.0496207328807567,0.224117682220113,-
0.221404810139089,0.824777247415416,0.977180737754725
"11958","ID3",326.501276962,0.0895607183035178,0.406137769927264,0.220
518072770128,0.825467698746132,0.97729833009654
"11959","GNPDA2",42.2792125585707,0.0508003258445013,0.230633399081064
,0.220264393825483,0.825665248795544,0.97729833009654
"11960","IRX1",23.5395501934141,-0.0778268942793704,0.353100909297316,
-0.220409781538792,0.825552028154644,0.97729833009654
"11961","DDX46",134.365750069448,-0.0426695483184104,0.193652252859139
,-0.220341089186542,0.825605521852666,0.97729833009654
"11962","ZNF747",11.3346708561711,-0.101675801268211,0.461318662274548
,-0.220402532095483,0.82555767357066,0.97729833009654
"11963","PHETA2",29.5900346298019,-0.0613293405102766,0.27825767728434
1,-0.220404846000373,0.825555871644323,0.97729833009654
"11964","AC046143.1",4.74961972381658,-0.139382146347992,0.63378805493
9028,-0.2199191752855,0.825934102158415,0.977534845082176
"11965","ECT2",4.21962807396433,0.153899193563639,0.700425980009749,0.
21972228037786,0.826087451437933,0.977552925987057
"11966","SARS2",9.12574343925193,-0.100005402110688,0.455140191070797,
-0.219724392775352,0.826085806186812,0.977552925987057
"11967","SZRD1",78.0511487174512,-0.0420119845122218,0.192212319422428
,-0.218570717207212,0.826984465535231,0.977639757529074
"11968","FBXO48",8.47017977459434,-0.103522489878139,0.472133008215271
,-0.219265520683394,0.826443218882861,0.977639757529074
"11969","DAG1",43.9238984616548,-0.0564488286366863,0.258022537888971,
-0.218774798118514,0.826825479511472,0.977639757529074

"11970","SPSB4",5.73909941308516,0.182724020706667,0.835924955698524,0
.218589024602068,0.82697020315759,0.977639757529074
"11971","LYAR",43.9895852301448,-0.0543944913089001,0.248313706065216,
-0.219055533304368,0.826606788780315,0.977639757529074
"11972","RNF180",6.31513521884519,-0.131771265894669,0.60073106375093,
-0.219351509928414,0.82637623963988,0.977639757529074
"11973","GCLC",15.0760556404626,-0.0819564633697926,0.374468492715862,
-0.218860771904725,0.826758505110333,0.977639757529074
"11974","SAMD9",50.1187201637641,-0.062870684443867,0.287676367851967,
-0.218546573405776,0.827003274853214,0.977639757529074
"11975","POLR2J2",5.23123001235374,-0.139076678064778,0.63398223176941
9,-0.219369993503794,0.826361842470764,0.977639757529074
"11976","ING3",28.6806483787045,0.0644200955639466,0.29378856583505,0.
219273664993878,0.826436875015011,0.977639757529074
"11977","LINC01003",25.8130473186596,-0.0668974055436207,0.30620047132
0397,-0.218475841187135,0.827058379621524,0.977639757529074
"11978","RAB1B",89.6424243535212,0.04308184882439,0.196969044250917,0.
218723957301171,0.826865085586848,0.977639757529074
"11979","TFPT",12.7306312329998,-0.0923678049959947,0.421837510809937,
-0.218965366116082,0.826677026919249,0.977639757529074
"11980","HTRA3",317.993450821543,-0.059905112726695,0.274431148782563,
-0.218288313817318,0.827204479149392,0.977729421039914
"11981","BRIX1",50.8126410663462,0.0480556264989398,0.220308673030854,
0.218128618532461,0.827328899867258,0.977729421039914
"11982","EML3",25.6425056072087,0.0676936527343667,0.310361034109186,0
.218112602081845,0.827341378735893,0.977729421039914
"11983","CS",25.3851319004645,-0.082907330545083,0.38037352861492,-0.2
17962934610563,0.827457990992765,0.977785625674501
"11984","AKNAD1",4.65360138323032,-0.133489506303532,0.612954623733029
,-0.217780405163683,0.827600212553223,0.977794166821486
"11985","PRKN",6.38367993345949,-0.120858895151557,0.554967799974017,-
0.217776410013726,0.827603325519457,0.977794166821486
"11986","SLC45A1",5.59438634050664,-0.129828808247329,0.61070161734970
4,-0.212589592951717,0.83164708952364,0.977878951303815
"11987","CTNNBIP1",29.9713285972531,0.060434565472544,0.28654809883491
5,0.210905483994718,0.832961024770559,0.977878951303815
"11988","PGD",49.9883621808863,0.0564761722838253,0.261994374951106,0.
215562537532972,0.829328759942376,0.977878951303815
"11989","NDC1",10.9848998322052,0.0919890228573135,0.431021632899381,0
.213420895463007,0.830998683704595,0.977878951303815
"11990","GIPC2",2.48860362561293,0.188360778449611,0.880471265108442,0
.2139317725791,0.83060026282495,0.977878951303815
"11991","EFNA3",1.55950714709351,0.224717098634446,1.06650105342533,0.
210704994535835,0.83311747702696,0.977878951303815
"11992","TNN",2.0504180922063,0.247783515458068,1.14654939587517,0.216
112377146152,0.828900152111093,0.977878951303815
"11993","KCNT2",16.4057058610429,-0.119176568063554,0.552532511386804,
-0.215691503409333,0.829228224634722,0.977878951303815
"11994","PKP4",12.1548118890498,-0.111816189577604,0.522595538028923,-
0.213963153989684,0.830575790629663,0.977878951303815

"11995","MREG",3.33233375678919,-0.160687187076501,0.741973547365622,-0.216567272036881,0.828545593537115,0.977878951303815
"11996","DUSP28",4.67860005963424,-0.138445544047257,0.63873830753362,-0.216748459289754,0.828404380539287,0.977878951303815
"11997","APEH",25.1919220754433,-0.0609942303856343,0.280866657337874,-0.217164368899296,0.828080251550673,0.977878951303815
"11998","PSMD6-AS1",2.35451966877148,0.194691430687436,0.922981371490191,0.21093755161396,0.832936001368268,0.977878951303815
"11999","RYBP",58.5521103061218,-0.0549622716199619,0.252828585018415,-0.217389468109228,0.827904838189279,0.977878951303815
"12000","AC016949.1",4.33918322407454,-0.141242668715039,0.663601852662288,-0.212842487024999,0.831449823082833,0.977878951303815
"12001","PI4K2B",14.167005826504,-0.0853793753552035,0.392961553870339,-0.217271574061862,0.827996708570748,0.977878951303815
"12002","AC110772.2",2.56638762347339,0.203659132037518,0.964984280963886,0.211049170494353,0.832848902859531,0.977878951303815
"12003","LIFR-AS1",11.7996437342134,0.0894902309700656,0.412631906636963,0.216876663027418,0.828304464971985,0.977878951303815
"12004","ARSK",20.1870362023474,-0.0674966644001643,0.320934114968068,-0.210313149186025,0.833423273222598,0.977878951303815
"12005","ERAP1",37.6304292787659,0.0599311970367048,0.27889519749631,0.21488787750638,0.829854736468171,0.977878951303815
"12006","AP3S1",257.553531305578,-0.0444724607976083,0.206908582096787,-0.21493772924704,0.829815868584211,0.977878951303815
"12007","TGFBI",26.9763968935723,-0.0756800093982252,0.358570395513164,-0.211060395239592,0.832840144073414,0.977878951303815
"12008","TAF7",151.943511248915,-0.0358979766189388,0.168494806950419,-0.213050937703393,0.831287232065235,0.977878951303815
"12009","TBC1D9B",69.5591067297374,0.0483500402005797,0.225473926826246,0.21443738919684,0.830205987354133,0.977878951303815
"12010","TRIM26",17.6750741390597,0.0729466154774417,0.345269328166898,0.211274531290484,0.832673055448191,0.977878951303815
"12011","PSMB8",177.388804382871,-0.0568429665764936,0.263511609369523,-0.21571332933868,0.829211210515127,0.977878951303815
"12012","ZNF76",14.5222820792262,-0.0790047981690592,0.36990915029208,-0.21357892365376,0.830875436641935,0.977878951303815
"12013","CALHM5",12.4715815452258,0.0802245585511489,0.372742026276094,0.215228101195452,0.829589482699953,0.977878951303815
"12014","FUCA2",124.077564872061,-0.0489852909920264,0.232089589807402,-0.211061991331349,0.832838898628007,0.977878951303815
"12015","MICALL2",32.9757624423338,-0.0866509374124521,0.407152787355139,-0.212821673100498,0.831466058289092,0.977878951303815
"12016","ZSCAN25",6.61704370448318,0.117606072981699,0.542872934719516,0.216636464005085,0.828491666322923,0.977878951303815
"12017","CASTOR3",9.8221349436792,-0.127154227206124,0.604322392412366,-0.210407935900809,0.833349299337407,0.977878951303815
"12018","C8orf58",10.3310257969312,-0.103028562628265,0.476383712693117,-0.216272218976209,0.828775562670588,0.977878951303815

"12019","TRIQK",47.8842520756627,-0.0537461291272152,0.250831896688612
,-0.214271509472086,0.830335334223736,0.977878951303815
"12020","TBC1D2",2.70471893318397,-0.170894994618833,0.799938603299927
,-0.213635138889226,0.830831595070833,0.977878951303815
"12021","SFTA1P",3.44616769919362,-0.154826667786857,0.736104746332669
,-0.210332386196687,0.833408260066706,0.977878951303815
"12022","TSPAN14",68.4613201779492,0.0513691969896969,0.23816997317171
8,0.215682927220469,0.829234910113223,0.977878951303815
"12023","PDLIM1",210.076482268117,0.0551514022625991,0.25514061888922,
0.216160807725192,0.828862402221669,0.977878951303815
"12024","FRAT2",12.8575342346389,0.0857364011455167,0.404276069221702,
0.212073896212985,0.832049384313894,0.977878951303815
"12025","XPNPEP1",38.4259018313922,-0.050746630091537,0.23440990786057
7,-0.216486711482009,0.828608382280635,0.977878951303815
"12026","C10orf88",8.20424250847404,-0.101538819591041,0.4830933441540
72,-0.210184679254569,0.833523536672845,0.977878951303815
"12027","AP000662.1",2.48491242203747,0.184954712594241,0.869421231960
437,0.212733144527873,0.831535112847148,0.977878951303815
"12028","ZFP91",106.931691810872,-0.0607232033174005,0.28652207011242,
-0.211932027761684,0.83216006354939,0.977878951303815
"12029","RBM4",155.584345791494,-0.0436429255901432,0.207235718643698,
-0.210595576263467,0.833202864531654,0.977878951303815
"12030","C11orf1",18.0779853093296,-0.0670445805283198,0.3180247895822
41,-0.210815580182884,0.833031180530103,0.977878951303815
"12031","CBL",15.8975972523808,-0.0861246203306618,0.403986263258357,-
0.213187002043145,0.831181106108556,0.977878951303815
"12032","TBX3",39.6437085411017,-0.049112347847261,0.227876690889908,-
0.21552159483915,0.829360677390822,0.977878951303815
"12033","B3GLCT",13.7990057000936,0.11663435688435,0.537734435517278,0
.216899549630206,0.828286628593082,0.977878951303815
"12034","TOX4",86.147959553184,0.037406846230956,0.17563984651713,0.21
2974714865216,0.831346684854891,0.977878951303815
"12035","NOP9",7.66115463620667,0.105862479569447,0.494341552332209,0.
214148454788007,0.830431290682219,0.977878951303815
"12036","ARID4A",77.4398859476528,0.0419037872909439,0.195423362807829
,0.214425679145386,0.830215118267936,0.977878951303815
"12037","AC037198.1",15.2812509884906,-0.239865261422087,1.11009350841
928,-0.216076627421814,0.828928017985165,0.977878951303815
"12038","CIAO2A",33.1031094576816,0.0707188749938005,0.327450246076463
,0.215968306150813,0.829012452590778,0.977878951303815
"12039","AC015712.1",2.34908976469689,-0.208876793645203,0.98235397548
3759,-0.212628847501066,0.831616468872612,0.977878951303815
"12040","NAA60",37.174768622232,-0.0597383993327803,0.283127779311645,
-0.210994482696186,0.832891576617246,0.977878951303815
"12041","YPEL3",214.193705598249,0.0506295499737338,0.235362251358062,
0.215113297402606,0.829678986756964,0.977878951303815
"12042","ZNF629",10.9847107251682,0.102489769230365,0.483905101599641,
0.211797248864633,0.832265214923515,0.977878951303815
"12043","AC026471.2",1.66565719771771,0.214842818188021,1.008778194044
81,0.212973297258325,0.831347790578362,0.977878951303815

"12044","THAP11",34.8692721183529,0.0563406993844597,0.267558742459889
,0.210573195502689,0.833220330205349,0.977878951303815
"12045","FAM157C",2.28234955273206,-0.201670978028352,0.93917344401457
,-0.214732411051034,0.829975951602247,0.977878951303815
"12046","MED11",42.7107889972205,0.0560107925903799,0.261217905560111,
0.214421719944044,0.830218205460768,0.977878951303815
"12047","AC233723.1",2.66623788675387,-0.230972644549844,1.07564097781
272,-0.214730239284412,0.829977644928555,0.977878951303815
"12048","NEURL4",4.60175733285098,0.137626232247605,0.649574033812799,
0.211871511303772,0.832207276716925,0.977878951303815
"12049","FGF11",2.57968187820049,0.184173948621563,0.863076734562741,0
.21339232219586,0.831020968597988,0.977878951303815
"12050","UTP6",41.8607615848559,0.0604441392869049,0.284073852437695,0
.21277614524611,0.831501571021176,0.977878951303815
"12051","AC116407.2",6.38262317349591,0.131691026248304,0.618009217465
272,0.213089097260438,0.831257468486088,0.977878951303815
"12052","FMNL1",36.3071270256887,-0.0656077410676263,0.301639958567811
,-0.217503481233496,0.827815994304215,0.977878951303815
"12053","FAM20A",28.9915499098274,0.0938918646750216,0.44380033469867,
0.211563302985727,0.832447741206215,0.977878951303815
"12054","JPT1",53.5417442547869,-0.0545643258914452,0.252771584860397,
-0.215864160212393,0.829093634457547,0.977878951303815
"12055","ACOX1",32.3754839043831,-0.0533812597700259,0.253440303880336
,-0.210626561571795,0.833178684109511,0.977878951303815
"12056","LAMA3",3.75303597339015,0.197204968051814,0.91245884485886,0.
216124781038555,0.828890483686744,0.977878951303815
"12057","IMPACT",33.1115897969862,-0.0604398524904574,0.27783504024472
4,-0.217538624491787,0.827788609623894,0.977878951303815
"12058","MPND",14.355270256425,-0.0754466602896767,0.359001379441008,-
0.210157020586252,0.83354512303934,0.977878951303815
"12059","DCAF15",13.2290767741654,-0.0899210893121369,0.42520400227188
4,-0.21147752333394,0.832514669380863,0.977878951303815
"12060","PPP1R13L",4.57973189123846,0.130767646176099,0.61864719889909
5,0.211376769197055,0.832593282703851,0.977878951303815
"12061","PIGU",21.3270367290567,0.0653573365356493,0.306199181098272,0
.213447130398018,0.830978222645398,0.977878951303815
"12062","TTPAL",20.3546894061692,0.0869518217563712,0.403879425634725,
0.215291535635222,0.829540028490471,0.977878951303815
"12063","C22orf39",45.5271796688344,0.056208200109256,0.26500905749648
2,0.212099166120019,0.83202967024493,0.977878951303815
"12064","RAB36",3.7499863785272,-0.140705420381534,0.663516705923151,-
0.212060102067468,0.832060145725169,0.977878951303815
"12065","SRRD",27.6173149420947,-0.0618759201110723,0.291152430141025,
-0.212520706356809,0.831700825374996,0.977878951303815
"12066","LINC01315",10.0717891229373,0.0995329957588775,0.472196644982
796,0.21078717270959,0.833053348363496,0.977878951303815
"12067","PHF8",13.7499080747709,0.0862298730301892,0.406270886230206,0
.21224723688748,0.831914156418659,0.977878951303815
"12068","KLF8",21.0142526352096,-0.0894911586204187,0.412802533879748,
-0.216789266721139,0.828372576971314,0.977878951303815

"12069","PHF6",26.8800405057788,0.0595402777195547,0.279274080442659,0
.213196576013002,0.831173638825488,0.977878951303815
"12070","CETN2",82.0237029904437,0.0516787588361083,0.244446467424108,
0.211411354725969,0.832566297187849,0.977878951303815
"12071","POLR3C",21.1607922945586,0.0651431333737082,0.310653047729863
,0.20969739022279,0.833903862551382,0.97787984534529
"12072","FAM189B",10.776032754033,0.0897810773017427,0.428452271180431
,0.209547441665758,0.834020904235983,0.97787984534529
"12073","MLH1",28.1980982874583,0.0559366350065117,0.267075932017088,0
.209440942821208,0.834104033672934,0.97787984534529
"12074","ANAPC2",12.2194022134201,-0.0859240424959118,0.40974369460076
3,-0.209701927395448,0.833900321138632,0.97787984534529
"12075","ZCCHC24",188.590843309242,-0.0376371500114505,0.1793154479192
28,-0.20989351697353,0.833750782236534,0.97787984534529
"12076","LRP10",201.444753146029,-0.0311172958014575,0.148441747699551
,-0.209626309873686,0.833959343545633,0.97787984534529
"12077","CCDC85C",2.70421708405315,0.179757070915804,0.857197137747314
,0.209703302776068,0.833899247609408,0.97787984534529
"12078","DNMT1",58.2343603885661,0.05855903704997,0.279469201372043,0.
209536638608035,0.834029336657077,0.97787984534529
"12079","FAM199X",19.4136387570617,0.0677327883850724,0.32352346396824
9,0.209359740262054,0.83416741892131,0.97787984534529
"12080","HES4",66.8026872946614,-0.0935432761606293,0.448550894706405,
-0.208545512370134,0.834803049989292,0.978170872498475
"12081","SPAG17",27.4900113618579,-0.129314889289094,0.623287636754175
,-0.207472251435168,0.835641061343582,0.978170872498475
"12082","BOLA3-
AS1",9.73510447639576,0.103401072077135,0.496492865045183,0.2082629567
45058,0.835023653697386,0.978170872498475
"12083","DBI",449.787646252453,-0.046529483602811,0.224073883084085,-0
.207652417865007,0.835500372788432,0.978170872498475
"12084","TMCC1-
AS1",2.76850664789089,0.168899404881189,0.813699716888198,0.2075696984
71944,0.835564966143633,0.978170872498475
"12085","TBC1D19",10.9863419530986,0.0999395248609252,0.48175435927143
9,0.207449134476052,0.835659113320201,0.978170872498475
"12086","FEM1C",50.6550958164066,-0.0898608724061742,0.433086911416475
,-0.207489236080284,0.835627798131083,0.978170872498475
"12087","SRSF12",1.72353261252628,0.220853349221594,1.06299313387069,0
.20776554634685,0.835412035587853,0.978170872498475
"12088","ARMT1",26.7440930437957,0.0569676169233857,0.27264233345586,0
.208946337134429,0.834490130621825,0.978170872498475
"12089","SMIM19",172.230723940212,-0.0624026601683736,0.30014696447504
1,-0.207907017409009,0.835301569798436,0.978170872498475
"12090","DDX10",25.4365857487017,0.0702973755246355,0.337294437884394,
0.208415460289118,0.83490458580329,0.978170872498475
"12091","RNF26",14.0237910743268,0.0785454787078651,0.377395663728461,
0.208125016413488,0.835131354548881,0.978170872498475
"12092","RNF111",18.6379291718383,-0.0667631103422012,0.32147468575179
2,-0.207677659552169,0.83548066245292,0.978170872498475

"12093","CCDC113",7.55848023356541,-0.109729742656461,0.525814013406855,-0.208685466455143,0.834693786440798,0.978170872498475
"12094","TMEM220",34.4816318119134,-0.0542826123525927,0.260266523232025,-0.208565480026028,0.834787460846314,0.978170872498475
"12095","MATK",2.17629661876911,0.243056988746193,1.16445824157048,0.208729673653551,0.834659274103668,0.978170872498475
"12096","WTIP",49.5835940793803,0.0474421408459933,0.227687764823769,0.208364910967938,0.834944052015913,0.978170872498475
"12097","EIF3L",235.644602306898,-0.0385400237237603,0.185274200461293,-0.208016138392739,0.835216366355156,0.978170872498475
"12098","C1orf159",7.23090335071873,-0.102137730760374,0.502179277413586,-0.203388979502344,0.838831012880284,0.978259376096817
"12099","UBIAD1",14.6770671124972,0.0777965956632178,0.380368709207827,0.204529430996678,0.837939795449859,0.978259376096817
"12100","TMEM50A",401.180734556217,-0.0446406221958118,0.2194534704853 5,-0.203417253311526,0.838808915520403,0.978259376096817
"12101","DNAJC8",238.814063904852,0.0380113443780611,0.183691342083979,0.206930516957534,0.836064123206584,0.978259376096817
"12102","EPB41",3.54269647707429,0.169127995498282,0.846031002260462,0.199907562543688,0.841552875773843,0.978259376096817
"12103","ADAMTSL4-AS1",8.33284380576813,-0.109804319323083,0.548987739248984,-0.200012334470885,0.841470934524245,0.978259376096817
"12104","CDC42SE1",35.0413174286969,-0.0621366475163958,0.300940320547672,-0.206474982824884,0.836419904527147,0.978259376096817
"12105","CADM3",24.5090597195148,-0.117019805786594,0.581378054454925,-0.201280053297345,0.840479598552088,0.978259376096817
"12106","TBX19",3.96143194170041,-0.132663398197937,0.654361935714986,-0.202737034288193,0.839340575051406,0.978259376096817
"12107","ATP2B4",155.314841840945,0.0502174120649184,0.248635380675866,0.201972108428062,0.839938529065788,0.978259376096817
"12108","LINC01139",4.65192410976868,0.13344170804466,0.65386220278373,0.204082308897119,0.838289178856589,0.978259376096817
"12109","DNAJC27-AS1",3.46365233216324,0.192977750436903,0.954621309346819,0.202151102795877,0.839798598192217,0.978259376096817
"12110","FBXO11",59.2718931905078,0.0453029133425803,0.221811816151505,0.204240306619359,0.838165715013673,0.978259376096817
"12111","FAHD2B",20.7080758653088,-0.0773210676928215,0.383761321799323,-0.201482179940099,0.840321562068693,0.978259376096817
"12112","NIFK",91.2529920712996,-0.0493549681337203,0.241539323141822,-0.204335126437119,0.838091622066572,0.978259376096817
"12113","CYTIP",5.37835874657053,0.128919672549036,0.625627474056719,0.206064595777884,0.836740453722145,0.978259376096817
"12114","EPHA4",6.29584390328208,0.118497090676683,0.586327395932148,0.202100552522018,0.839838115928272,0.978259376096817
"12115","ATG7",23.284346787627,0.0644984999691963,0.316674926159328,0.203674161233546,0.838608135284164,0.978259376096817
"12116","LSM3",187.878167882235,-0.054085815615569,0.265000344021519,-0.204097152459459,0.838277579511982,0.978259376096817

"12117","SLC25A38",20.2438249289677,0.0659209131985119,0.325357146941878,0.202610927155345,0.839439148513855,0.978259376096817
"12118","SMARCC1",45.3108870633377,0.0498665446015609,0.247549251381979,0.201440902459506,0.840353835113579,0.978259376096817
"12119","RNF123",7.91049208627541,-0.0938093363286876,0.464099261425846,-0.202132052614086,0.839813490646831,0.978259376096817
"12120","HYAL3",1.69305366883096,0.2238368033826,1.11171557055387,0.201343589413866,0.840429920961848,0.978259376096817
"12121","CBLB",78.3957077446084,0.0567693011647109,0.28363145696501,0.200151639638879,0.841361987774801,0.978259376096817
"12122","EAF2",3.32010421710066,-0.146715771722595,0.71920940766504,-0.20399590183187,0.83835670144027,0.978259376096817
"12123","CCDC58",19.2353673993883,-0.0767736584402052,0.377616643664741,-0.203311108575943,0.838891873461422,0.978259376096817
"12124","PDIA5",31.3120025140553,-0.0722846319338427,0.356572946885404,-0.202720460330025,0.839353530181466,0.978259376096817
"12125","CRIPAK",6.11464274205751,0.12592901497623,0.628925103723679,0.200228952908131,0.841301524498965,0.978259376096817
"12126","TMA16",71.9295093041705,-0.050169186779607,0.245344254043541,-0.204484865460528,0.837974617746465,0.978259376096817
"12127","ICE1",45.383240281235,-0.0553123836558861,0.276695861284593,-0.1999031839475,0.841556300274735,0.978259376096817
"12128","CDC42SE2",23.6823553982289,0.0579761459152532,0.289430801073392,0.200310905751015,0.841237433840479,0.978259376096817
"12129","ADAM19",12.5902207790058,0.0839813882927072,0.418354797770059,0.200742022657204,0.84090029915662,0.978259376096817
"12130","KCNIP1",2.21811689537305,0.191901876877079,0.931114684901622,0.206099076718304,0.836713519962491,0.978259376096817
"12131","PHYKPL",81.4965363219237,-0.039282116863759,0.190395660887071,-0.206318340873631,0.83654225277769,0.978259376096817
"12132","BVES",6.31207792549158,0.122045789589641,0.590653340288797,0.206628459139785,0.836300032696445,0.978259376096817
"12133","SLC18B1",17.3637870476853,0.0756799832170877,0.372550088823047,0.203140424569954,0.839025276140843,0.978259376096817
"12134","EIF4EBP1",49.5650785353095,0.0682363190904808,0.333902882196569,0.204359778632608,0.838072358883338,0.978259376096817
"12135","ANK1",12.7445201076328,0.11629494297199,0.563007405990149,0.206560236570005,0.836353317089773,0.978259376096817
"12136","RRAGA",180.329815212291,-0.040709785355729,0.203598714759726,-0.199951092047767,0.841518831503649,0.978259376096817
"12137","GLIDR",6.56124593743273,-0.111002995533603,0.55426542743613,-0.200270466168295,0.841269059207218,0.978259376096817
"12138","AKNA",20.129130425877,-0.0857661900151701,0.419823920765118,-0.204290860460889,0.83812621166795,0.978259376096817
"12139","ZWINT",3.42318173646869,0.150922156119412,0.740154859721911,0.203906188194341,0.838426809195932,0.978259376096817
"12140","ADK",75.0055097802887,0.041179069310782,0.200272091160447,0.205615615596642,0.837091178676953,0.978259376096817
"12141","SMC3",144.479905535683,0.0377137953385908,0.18695279920479,0.201728968482995,0.84012861457288,0.978259376096817

"12142","AP002495.1",5.60484990305302,0.129122454791454,0.644346522667
112,0.200392879062937,0.841173328226825,0.978259376096817
"12143","AP000787.1",4.89288087986551,0.149215318157363,0.727553656501
348,0.20509184006429,0.837500371616158,0.978259376096817
"12144","ARHGAP42",35.4659678524753,0.0521389798067214,0.2563718680525
54,0.203372469073063,0.838843916644218,0.978259376096817
"12145","ARHGDIB",62.5949053714098,-0.0576254525013399,0.2801225075838
38,-0.205715181541037,0.837013399036877,0.978259376096817
"12146","GXYLT1",21.8072422139563,-0.0692050418003215,0.33760779188837
9,-0.204986506422821,0.837582667489592,0.978259376096817
"12147","COPZ1",119.327075335739,-0.0582328529136655,0.284306092348129
,-0.204824498950097,0.837709245395537,0.978259376096817
"12148","R3HDM2",80.9482261169993,-0.0418192622205173,0.20579788904801
8,-0.203205496489616,0.838974416884624,0.978259376096817
"12149","GIHCG",27.7809991391304,0.0753304616555499,0.36888798837973,0
.204209581305221,0.838189724322262,0.978259376096817
"12150","UNG",18.9364023435596,-0.0805549245882987,0.396899995280375,-
0.202960255848312,0.839166096873243,0.978259376096817
"12151","ERP29",236.872081696397,-0.0480761759851466,0.240634865737285
,-0.199788903564931,0.841645680043039,0.978259376096817
"12152","RASL11A",25.1807916336323,0.102543552112584,0.509210362144837
,0.201377583285347,0.840403342109841,0.978259376096817
"12153","EDNRB",26.8772154468248,-0.095352318351813,0.47088175485316,-
0.202497373000888,0.839527911914479,0.978259376096817
"12154","COL4A1",691.159850413098,0.0649648813701417,0.324148692684535
,0.200416916175461,0.841154530676171,0.978259376096817
"12155","SALL2",5.80528654838493,-0.116591045660231,0.58277747815059,-
0.200061001036323,0.841432873393688,0.978259376096817
"12156","C14orf93",10.3200023391431,0.0874353459395519,0.4357047208041
09,0.20067569104642,0.840952168772983,0.978259376096817
"12157","HEATR5A",28.0522369457428,0.0617626245353806,0.30526813193518
,0.202322542296997,0.839664578168273,0.978259376096817
"12158","CDC42BPB",77.5846794184537,-0.0498535068567362,0.241668982088
379,-0.206288396739738,0.836565641677656,0.978259376096817
"12159","CDCA4",12.9070978969703,-0.0781821351533022,0.382813969129876
,-0.204230099886396,0.838173690720182,0.978259376096817
"12160","AKAP13",311.162569797579,-0.0444492789081834,0.22252458028910
2,-0.199749973016173,0.841676128459571,0.978259376096817
"12161","MESP2",4.8777383634235,0.137726657441001,0.688318281573437,0.
200091529061482,0.841408998235529,0.978259376096817
"12162","SNRPA1",63.8346913389829,-0.0516482674453173,0.25324289955861
8,-0.203947544177294,0.838394490927211,0.978259376096817
"12163","AARS",35.4008926533622,-0.05329690585932,0.259268141145846,-0
.205566737292797,0.837129362364438,0.978259376096817
"12164","MTSS2",8.46593424626768,-0.103923671489624,0.507558300649748,
-0.204752185820991,0.837765745654373,0.978259376096817
"12165","AC015922.3",18.6117553210612,-0.101851484021616,0.49172059403
9232,-0.207132841813596,0.835906114175278,0.978259376096817
"12166","AC008105.3",12.3671502753285,-0.113805318940441,0.56858568398
5052,-0.200155090333637,0.841359289119662,0.978259376096817

"12167","NSF",28.7400375923217,0.0692277948072469,0.340525882874118,0.
203296719247736,0.83890311964959,0.978259376096817
"12168","KCTD2",16.527947395421,0.0814162179115792,0.403415131643472,0
.20181746177913,0.84005942990808,0.978259376096817
"12169","MRPS7",78.4484484149201,0.0477923978109863,0.235661905146499,
0.202800693566812,0.83929081581359,0.978259376096817
"12170","B4GALT6",6.09450257263859,-0.12141492280303,0.592998209741756
,-0.204747536853282,0.837769378050757,0.978259376096817
"12171","SAFB2",59.6891159085603,0.0570379828233281,0.276614107142706,
0.206200556481026,0.836634253178948,0.978259376096817
"12172","ZNF791",63.4873449565819,0.0654723984448044,0.317922234460693
,0.205938406780099,0.836839024130945,0.978259376096817
"12173","DDX49",43.9754586329056,-0.0562950817273394,0.279387690485944
,-0.201494495442603,0.840311933171444,0.978259376096817
"12174","GPATCH1",13.5693249542045,-0.0824727516142603,0.4068287366089
79,-0.20272105727263,0.839353063577078,0.978259376096817
"12175","PIH1D1",203.292063181368,-0.0468150956609052,0.22970615559372
4,-0.203804271330482,0.838506454859425,0.978259376096817
"12176","AC010332.3",2.50104846970107,0.173843523382547,0.848283077551
065,0.204935743719445,0.837622328397506,0.978259376096817
"12177","ZNF525",5.17659068719038,-0.134374268808165,0.669291743275965
,-0.200770844938929,0.840877761087654,0.978259376096817
"12178","EDEM2",29.3207882534566,0.0631123611947019,0.31395487414296,0
.201023670573637,0.840680065413129,0.978259376096817
"12179","SRSF6",115.315849163701,0.0405385974741292,0.197990887191625,
0.204749814747251,0.837767598254393,0.978259376096817
"12180","MRPS6",303.287682552373,0.0756519498917262,0.374695978569957,
0.201902219982331,0.839993166522968,0.978259376096817
"12181","ICOSLG",11.547651595816,0.0962110387799605,0.475181896415986,
0.202472020726427,0.839547729646022,0.978259376096817
"12182","TRMT2B",7.23686917336248,0.103263706193904,0.511416474458953,
0.201917050683888,0.839981572098206,0.978259376096817
"12183","HMGB3",4.45542488697143,-0.142706154472776,0.694659029500723,
-0.205433383015757,0.837233540558063,0.978259376096817
"12184","SLC5A6",7.04347865162443,-0.113746430914998,0.569764264641594
,-0.199637706282877,0.841763936003549,0.978281133766436
"12185","CLEC4A",1.72018810152976,0.209439017378826,1.05015669787151,0
.199435967797304,0.841921727324065,0.97830392736819
"12186","CLCN5",8.83390286947029,0.111389650919608,0.558370564092907,0
.199490549972964,0.841879034825475,0.97830392736819
"12187","KCNMB1",13.4704517529655,-0.107452263527657,0.53924583530382,
-0.199263965510493,0.842056265260386,0.978379971780346
"12188","CHN1",2.12132042724786,0.191621265695034,0.963652825733349,0.
19884885985698,0.842380974452203,0.978676944391467
"12189","RAVER1",12.19957282378,-0.0787625507771424,0.396480996611957,
-0.198654037520564,0.842533380088268,0.978773702686838
"12190","AC084036.1",2.32000547137842,-0.176597381153397,0.88970228684
6099,-0.19849042063207,0.842661378880789,0.978840990292937
"12191","CCDC149",23.9430150755695,0.0600958399623518,0.30289742054820
9,0.198403274130183,0.842729555978898,0.978840990292937

"12192","CTDSPL",111.275553320234,0.0322858551972087,0.163086211342957
,0.1979680252018,0.843070080786891,0.978915321356489
"12193","ATG12",106.809277466103,-0.0410945304455242,0.207340220403366
,-0.198198547129821,0.842889724161779,0.978915321356489
"12194","EPHX2",23.3477390602854,0.0638611985308208,0.322570894942803,
0.19797569939515,0.843064076489738,0.978915321356489
"12195","MTMR4",17.7740036021314,0.065277303267152,0.329693136626372,0
.197994122459177,0.84304966230181,0.978915321356489
"12196","MED16",31.4835249047577,0.0548387163461012,0.2773173785807,0.
19774713228130l,0.843242911554903,0.979035718892868
"12197","PLPBP",58.1608619965034,-0.0444241096769822,0.224826740406661
,-0.197592642212527,0.843363791972032,0.979082154083465
"12198","MIR100HG",86.3095020428862,-0.0601182576823475,0.304366527576
973,-0.197519280983169,0.843421194598171,0.979082154083465
"12199","AC008771.1",8.86279476324437,0.0968122176566727,0.49129212358
7152,0.197056319465905,0.843783465157922,0.979181598642532
"12200","SLC45A4",2.25859263865443,0.205662342270274,1.04293927691194,
0.197194934377411,0.843674994569934,0.979181598642532
"12201","PI4K2A",20.6576873416412,0.061816794955618,0.313429191774547,
0.19722730549005,0.843649663569119,0.979181598642532
"12202","RTL6",14.2227048604072,0.0851036956537012,0.431740031584711,0
.197117916866143,0.843735262862304,0.979181598642532
"12203","LZIC",92.9153250003429,0.0349705754186618,0.183011476513025,0
.191084057049138,0.84845973812491,0.979188885872077
"12204","RCC1",7.5023082510389,0.0978691429357368,0.499929818196044,0.
195765764260451,0.844793507698711,0.979188885872077
"12205","SNHG3",33.9250562665009,-0.053708877214889,0.278953093395175,
-0.192537306402274,0.847321348246978,0.979188885872077
"12206","AL050341.2",10.9253158755733,0.092700095680339,0.489692030706
843,0.189302847233457,0.849855463902265,0.979188885872077
"12207","RNF11",71.0125014652535,-0.0426920511293087,0.224083514615039
,-0.190518482373194,0.848902861580513,0.979188885872077
"12208","SCP2",305.563683280095,0.0387364550200619,0.204315066821237,0
.189591769333164,0.849629037353734,0.979188885872077
"12209","ZRANB2",165.391531392084,0.046100989347352,0.242258256716593,
0.190296875624279,0.849076501810177,0.979188885872077
"12210","IFI44",38.5000174406046,0.0679054037008481,0.359064879340574,
0.189117364598724,0.8500008320796,0.979188885872077
"12211","QSOX1",50.0822403942339,-0.039707300147776,0.205743116080743,
-0.192994550214711,0.846963236155362,0.979188885872077
"12212","LINC01686",5.7533847378553,0.103070824756126,0.53107133902071
4,0.194080940135438,0.846112505436273,0.979188885872077
"12213","NCF2",2.65492399252534,-0.176917693368775,0.932109562918129,-
0.189803538561394,0.849463082932249,0.979188885872077
"12214","PUS10",4.10852689769397,0.141594918287523,0.741124644875706,0
.191054121957136,0.848483190842728,0.979188885872077
"12215","COMMD1",106.641608666427,0.0582972902236367,0.303832291774947
,0.191873253112998,0.84784148907398,0.979188885872077
"12216","CNNM4",4.23172203592106,-0.137341781518034,0.707491191958501,
-0.194125076155139,0.846077947166414,0.979188885872077

"12217","DNPEP",83.4031473947654,-0.035664833466772,0.187683824948551,
-0.190026143577097,0.849288644162063,0.979188885872077
"12218","STK11IP",10.3195965951449,-0.0888054680610492,0.4547422683604
71,-0.195287472134993,0.845167904048822,0.979188885872077
"12219","FBXO36",7.08327864582425,-0.1005365847937,0.523427607202619,-
0.192073523463928,0.847684613971485,0.979188885872077
"12220","HDLBP",316.116295412342,0.0324129986768418,0.169538233784212,
0.191184005833735,0.848381433987763,0.979188885872077
"12221","ZNF197",19.2997472984019,0.0647268043947843,0.341546869937264
,0.189510752672608,0.849692528383108,0.979188885872077
"12222","PDE12",22.2803917129855,0.0598829309936326,0.311247875714299,
0.192396272122995,0.847431812270655,0.979188885872077
"12223","HEG1",90.6061470567534,0.0531857955869639,0.2740619926481,0.1
94064835744135,0.846125115163156,0.979188885872077
"12224","RYK",79.0212103528745,-0.0433483708435152,0.223625532354395,-
0.193843566909068,0.846298372492085,0.979188885872077
"12225","HNRNPD",208.004134069982,0.0297902990748926,0.156060937619706
,0.190888889489351,0.848612645018861,0.979188885872077
"12226","GPAT3",2.44821722304312,0.200701657059892,1.02425816056047,0.
195948311458967,0.844650623083492,0.979188885872077
"12227","WDR17",1.98398176917723,-0.192767228516194,1.01006181374522,-
0.190846961931399,0.848645494524086,0.979188885872077
"12228","MIR4458HG",28.3062192939917,0.0731686397985356,0.374796640038
014,0.195222240495844,0.845218968625033,0.979188885872077
"12229","SLC12A2",17.0519805320516,-0.0745593020430897,0.3910845489083
83,-0.190647526861401,0.848801751996328,0.979188885872077
"12230","IGIP",15.6995593546638,-0.072933290288846,0.385310297597893,-
0.189284560375177,0.849869795622362,0.979188885872077
"12231","PCDHB10",3.07971201098987,0.172204902084105,0.899367757348671
,0.191473288515216,0.848154806042533,0.979188885872077
"12232","PCDHB11",8.45271759003084,0.14166727978212,0.721867055503887,
0.196251205401293,0.844413551104237,0.979188885872077
"12233","PCDHGC3",17.5024193062516,0.0780796961527632,0.40123916790210
9,0.194596396361315,0.84570892441583,0.979188885872077
"12234","TSPAN17",17.0186732304615,0.0701484838916209,0.36994074334122
8,0.189620865379829,0.849606235640362,0.979188885872077
"12235","TRIM52",17.5893471420109,0.0707126830915539,0.365345260817265
,0.193550295228607,0.846528020746534,0.979188885872077
"12236","ELOVL5",57.0474885741605,-0.053516927071366,0.274261320303536
,-0.195131150875146,0.845290276412348,0.979188885872077
"12237","CGAS",14.4732237932409,0.0886345340867168,0.465320047222183,0
.190480798357684,0.84893238841825,0.979188885872077
"12238","ATG5",45.2197384762617,-0.0503430939698602,0.26004232263002,-
0.193595771106408,0.846492409716764,0.979188885872077
"12239","SFT2D1",120.211089495468,-0.0429990698332491,0.22415679390287
7,-0.191825860303301,0.847878613533703,0.979188885872077
"12240","LINC00997",1.9600513138735,-0.21511577481013,1.12162936732838
,-0.191788643446913,0.847907767046808,0.979188885872077
"12241","POP7",56.3265211368597,-0.0509441885323263,0.26464378477928,-
0.192500982310297,0.847349798528299,0.979188885872077

"12242","CHCHD3",63.8349885427422,-0.0442544817139644,0.231348018309641,-0.191289651138196,0.848298668580831,0.979188885872077
"12243","GINS4",2.23187021450193,0.172641174416359,0.896700419898736,0.192529378357886,0.847327557747735,0.979188885872077
"12244","C8orf76",26.4197420883735,-0.055389357607773,0.285283620608554,-0.194155407484029,0.846054198068061,0.979188885872077
"12245","WASHC5",38.6294974204518,-0.0487871836860961,0.257734789126711,-0.189292193930834,0.849863813069664,0.979188885872077
"12246","SPAG8",1.499351256432,-0.235450623838519,1.21233740788546,-0.194212124699829,0.846009789484509,0.979188885872077
"12247","FAM27C",1.50440167204896,0.229540472390443,1.21038531292883,0.189642479909982,0.849589297052197,0.979188885872077
"12248","KIF27",10.8705809793623,0.081009787046155,0.426302357081719,0.190028944715936,0.849286449167705,0.979188885872077
"12249","AGTPBP1",5.91255112528021,-0.107880574514074,0.559051415509817,-0.192970756394014,0.846981870631252,0.979188885872077
"12250","PHYHD1",65.1406753549116,-0.0560089068049187,0.295790539636254,-0.189353273007971,0.84981594462703,0.979188885872077
"12251","NPDC1",128.170193730159,-0.0491461642614737,0.25437649667066,-0.193202457399604,0.846800414268752,0.979188885872077
"12252","PIP4K2A",49.5722170645852,0.0470862704082142,0.245226543923602,0.192011311886708,0.84773334469058,0.979188885872077
"12253","PCGF6",18.1628755779621,0.0696178100911567,0.364658608658528,0.190912290120505,0.848594311149681,0.979188885872077
"12254","TAF5",3.95274109441722,-0.136821799901225,0.697987420560669,-0.196023303387504,0.844591926359437,0.979188885872077
"12255","HBB",5.5018124206926,-0.183425356988089,0.96649167661298,-0.189784724924785,0.84947782609893,0.979188885872077
"12256","FAM168A",33.7824983623065,-0.0626675520396532,0.319677353147577,-0.196033755355586,0.844583745597038,0.979188885872077
"12257","SORL1",2.54720169134835,0.168160665207887,0.888704867061828,0.189219921529008,0.849920454576811,0.979188885872077
"12258","MED21",67.9612372799699,-0.0481504390305137,0.252117991185751,-0.190983748537955,0.848538325590792,0.979188885872077
"12259","FAIM2",26.6768807643817,0.0664642828982858,0.338145421591246,0.196555323994978,0.844175534780464,0.979188885872077
"12260","MYL6B",105.821842674843,-0.0580819759737508,0.307434262353053,-0.188924863251092,0.85015170642029,0.979188885872077
"12261","MPHOSPH9",7.45494584698403,0.1144668688971,0.584435544849464,0.19585884175916,0.844720652792029,0.979188885872077
"12262","BRI3BP",12.1494813554822,-0.0813984103038222,0.428494345439084,-0.189963791051693,0.849337504392318,0.979188885872077
"12263","CPB2-AS1",3.05348564101171,-0.16440777841496,0.860088587471428,-0.19115214503462,0.848406394933104,0.979188885872077
"12264","NEK5",2.89664445809419,0.197326852731849,1.01484344781268,0.194440682606911,0.845830837617446,0.979188885872077
"12265","HNRNPA1L2",6.47610591962916,0.105162913586409,0.540788822628792,0.194462069454781,0.845814092962058,0.979188885872077
"12266","TIMM9",77.1194581925541,0.051152843132501,0.269468338420292,0

.18982876961492,0.849443310887082,0.979188885872077
"12267","SYNJ2BP",50.1747460318847,-0.0506463452094971,0.2616930803768
26,-0.193533375573282,0.846541270188151,0.979188885872077
"12268","ADSSL1",3.06968411412693,0.17640081746413,0.899942449706904,0
.196013442327986,0.844599644632674,0.979188885872077
"12269","RPP25",18.5302677953617,-0.0791015175706583,0.402792478256179
,-0.196382807129653,0.84431055217263,0.979188885872077
"12270","MRPS11",59.2923270539528,0.0519337836253434,0.271806776615606
,0.191068759476844,0.848471723026931,0.979188885872077
"12271","LINS1",12.710283761382,-0.085289769378125,0.445271098583221,-
0.191545711476678,0.848098070883959,0.979188885872077
"12272","CDIPT",134.545167131052,-0.0331797800823482,0.173856628406388
,-0.190845643254917,0.848646527688212,0.979188885872077
"12273","AC009113.1",7.08118242174494,0.110770104872091,0.565759482967
023,0.195790098455226,0.844774460373141,0.979188885872077
"12274","GLOD4",67.4362805179013,-0.0380678253306729,0.196230671117358
,-0.19399528684232,0.84617957235614,0.979188885872077
"12275","TOM1L2",20.4084082116232,0.069590740364455,0.353805696246284,
0.19669197274883,0.844068592205254,0.979188885872077
"12276","TNFAIP1",17.4095065077338,-0.0695476198832178,0.3604951537900
08,-0.192922482180523,0.847019677544809,0.979188885872077
"12277","TMUB2",36.5203143663499,-0.0454598554317433,0.240458752709313
,-0.189054691998253,0.850049951586357,0.979188885872077
"12278","RHBDF2",2.35016125314318,0.203794474982866,1.03959784728252,0
.196032028649903,0.844585097089573,0.979188885872077
"12279","CSNK1D",49.1888964091998,-0.0424345441926965,0.22451329157091
,-0.189006824031592,0.850087468387374,0.979188885872077
"12280","PARD6G",2.42039292776061,0.164054492746773,0.838507817859993,
0.195650522574097,0.844883713104069,0.979188885872077
"12281","ADAT3",1.88931317493672,0.193435845900165,1.01351073563473,0.
190857224397354,0.848637454037967,0.979188885872077
"12282","ZNF14",5.77458192213814,-0.113572547014394,0.581645023262305,
-0.195260928009653,0.845188683219934,0.979188885872077
"12283","CLEC11A",178.05804294442,0.0516300774016589,0.272923003704095
,0.189174516991747,0.849956039530279,0.979188885872077
"12284","ZNF814",4.30646441537653,0.125931527232303,0.645672643167115,
0.195039279679856,0.845362197324379,0.979188885872077
"12285","SDCBP2-
AS1",5.01923987790833,-0.113140969795372,0.587065214197201,-0.19272300
0885158,0.84717590903445,0.979188885872077
"12286","CST3",4760.90044323983,0.0538687547090039,0.280038142070994,0
.192362205771767,0.847458494929985,0.979188885872077
"12287","HAR1A",2.127337138192,0.199912756886367,1.02462990853638,0.19
5107282366889,0.845308961632346,0.979188885872077
"12288","AC245452.1",13.7594986831498,-0.0866229555872254,0.4536870249
37373,-0.190931084262731,0.848579586420471,0.979188885872077
"12289","DUSP18",18.9426825068592,0.0723164501703935,0.382588874100488
,0.189018696219063,0.85007816345881,0.979188885872077
"12290","LIMK2",8.53419303374437,0.0934862786478623,0.485829356970134,
0.19242616220412,0.847408400842174,0.979188885872077

"12291","NDUFA6-
DT",6.14322516718619,0.112948222547623,0.57555905251684,0.196240893186747,0.844421622137101,0.979188885872077
"12292","TCEANC",4.51035640261319,0.125050626339524,0.641435245456673,0.194954404556447,0.845428642541319,0.979188885872077
"12293","PPP1R3F",7.18754660966572,-0.0978269084303041,0.509290800133556,-0.192084578014466,0.847675954933553,0.979188885872077
"12294","MT-
ND2",7024.25327430469,-0.0404172313116798,0.212165625622533,-0.190498490003214,0.848918526324878,0.979188885872077
"12295","MBD3",45.087956920252,-0.0410328607932,0.217486616026757,-0.188668441041686,0.850352687726183,0.979340712338573
"12296","BNC2",101.501139588068,0.0521848498725043,0.276842848245652,0.188499902392996,0.850484792023776,0.979413195759326
"12297","HACD4",33.000339132474,-0.0753794340218445,0.400311486262573,-0.188301951376937,0.850639955740683,0.979512220321059
"12298","FGGY",8.86249690330188,0.101199621193234,0.537986811716637,0.188107996310023,0.850791992846481,0.979607628777538
"12299","TTC31",13.7491202726716,0.0755494799117647,0.402075014428902,0.187898967109592,0.85095585244396,0.979638658565677
"12300","NMD3",50.1386324969186,-0.0423748029670984,0.22552131469981,-0.187897108632518,0.850957309347304,0.979638658565677
"12301","CCDC28B",47.5703119968959,-0.0632046784626548,0.337072713117517,-0.187510516286197,0.851260379119389,0.979779916115054
"12302","SF3B6",234.072525014328,0.0470684046710769,0.25134396873037,0.187266895278357,0.851451377538792,0.979779916115054
"12303","TMEM14A",61.6133301075137,-0.0428806196027047,0.229049636558076,-0.187211035333087,0.851495172861007,0.979779916115054
"12304","PUM3",63.9377373682695,0.0389878527903914,0.208209454324328,0.187253037653419,0.851462242153138,0.979779916115054
"12305","TMCO3",68.2568251833845,0.0374632910341008,0.199856224453584,0.187451209670988,0.851306874592944,0.979779916115054
"12306","SULT1A3",2.25189296806645,0.169488920684917,0.905161252897022,0.187247211634896,0.851466809859463,0.979779916115054
"12307","LRRC41",62.2030679829534,-0.040681101705126,0.217986846771346,-0.186621818277861,0.851957158769108,0.979821031890888
"12308","PTGS2",24.6226589267082,-0.105513038571419,0.565609337026796,-0.186547554405773,0.852015390261469,0.979821031890888
"12309","NEB",2.30043551017857,0.174431660072695,0.934479913075226,0.186661754449775,0.851925844510307,0.979821031890888
"12310","SLC25A4",45.7519761507646,0.0426370580049193,0.228607236075397,0.186507910846957,0.852046475741608,0.979821031890888
"12311","HLA-
DQA2",2.94570229062307,-0.485802965379203,2.59861931657012,-0.186946569003576,0.85170252600514,0.979821031890888
"12312","CFAP70",3.38094651397016,-0.148717431995801,0.7955773336486,-0.186930202389964,0.851715358481482,0.979821031890888
"12313","C18orf32",177.227120787221,0.0438114157090735,0.2349648464158 8,0.186459448625472,0.852084476462174,0.979821031890888
"12314","AC136469.1",1.91481127934381,0.204609207810752,1.096381038471

17,0.186622351747405,0.851956740470007,0.979821031890888
"12315","MACO1",47.1214737940933,0.0490530803159085,0.263914790820912,
0.18586711325776,0.852548972590769,0.979971926515304
"12316","CNIH3",9.65896967533251,0.0830067086321459,0.446630796810386,
0.185850839720275,0.852561734656923,0.979971926515304
"12317","CMC1",69.0686884224467,-0.0429492338425172,0.230833092939987,
-0.18606185662332,0.852396253554535,0.979971926515304
"12318","RTN4IP1",3.8907803306206,-0.135421259972586,0.728347655102391
,-0.18592942398304,0.852500107505909,0.979971926515304
"12319","ZMYND11",76.2595635817156,-0.042726105338639,0.22979970538830
1,-0.185927589708799,0.852501545965535,0.979971926515304
"12320","ZFP69B",2.84390984852327,0.16988959238919,0.917328978676609,0
.185200289468978,0.853071942056048,0.980043779649734
"12321","COQ2",11.9607397886045,-0.0727735808892677,0.391995022216822,
-0.185649247476961,0.852719830929284,0.980043779649734
"12322","MCM7",18.7317916304127,-0.0655482718565756,0.354246512650979,
-0.185035757631175,0.853200989278035,0.980043779649734
"12323","ALG2",34.7984713787364,-0.0460982867883492,0.248387227528231,
-0.185590407554711,0.85276597654064 8,0.980043779649734
"12324","UNC5B",10.6775844573734,0.10143155971407,0.548347174326768,0.
184976898693063,0.853247155050983,0.980043779649734
"12325","AL138762.1",4.35699061653111,0.157409765021351,0.848976887356
57,0.185411131169274,0.852906578377838,0.980043779649734
"12326","SPATS2",45.797567241827,-0.0436361869060447,0.235588362946914
,-0.185222166155454,0.853054783815216,0.980043779649734
"12327","AC006449.6",4.45664992701207,-0.116167393108788,0.62759336905
1713,-0.18509977771804,0.853150775949544,0.980043779649734
"12328","SLC25A43",35.2408955968481,-0.0492941404737718,0.265898120408
533,-0.18538732202407,0.852925251626931,0.980043779649734
"12329","IMPA1",25.2870426920595,0.0538739773346193,0.291683875129144,
0.184699882058157,0.853464438688891,0.980134343214493
"12330","MCTP2",2.32523285643956,-0.17542539738014,0.949538865173147,-
0.184747990645072,0.853426702941033,0.980134343214493
"12331","COL6A3",3265.35031722134,-0.0420560992544263,0.22789966184679
9,-0.184537786996357,0.853591586408066,0.980200864774813
"12332","FAAH",3.75672385276395,-0.136248803279894,0.74260582336271,-0
.183473922494876,0.854426178287368,0.980255602223134
"12333","AL592295.4",2.0155104502547,-0.176460428439917,0.960702660863
946,-0.18367850494057,0.854265672566783,0.980255602223134
"12334","SP5",8.74508015271665,-0.107594704964476,0.584540278960714,-0
.184067221433867,0.85396072059481,0.980255602223134
"12335","OXCT1",21.7941082290243,0.0629028622845432,0.342801054748463,
0.183496699946561,0.854408307876821,0.980255602223134
"12336","ZNF250",6.04575828622159,0.123798665837432,0.67367388926 2923,
0.183766460019524,0.854196669021135,0.980255602223134
"12337","PXMP2",19.4669992404645,0.0580413448186948,0.315526118831335,
0.183950999155544,0.854051895848621,0.980255602223134
"12338","PARP4",54.022406044443,-0.0479908292847112,0.261405020937929,
-0.183588016452471,0.854336664815571,0.980255602223134
"12339","MEAK7",15.8549249475212,-0.066407466966193,0.362055155171481,

-0.183418095330642,0.854469978689275,0.980255602223134
"12340","IRF8",16.0373410810231,0.0802977378660465,0.435591868674803,0.184341682296172,0.853745416445385,0.980255602223134
"12341","RALBP1",162.767050295099,0.0326720326933533,0.177349689943608,0.184223793702386,0.853837894247731,0.980255602223134
"12342","NACC1",11.4149258437353,0.0794439710232458,0.432006947508554,0.183895123634957,0.854095730348283,0.980255602223134
"12343","PPP2R1A",114.333822351631,0.0391889900674597,0.213605913533188,0.183463975408016,0.85443398245537,0.980255602223134
"12344","SRSF2",258.556257595204,-0.0424446391421027,0.231570618479668,-0.183290261177194,0.854570275411726,0.98029124269524
"12345","CNOT10",19.8497943102219,0.0624983406912818,0.341277719762494,0.183130445007592,0.854695668040441,0.980321957545233
"12346","C4orf47",2.03056525290043,-0.166305166865953,0.908375985045924,-0.183079660409059,0.854735514678916,0.980321957545233
"12347","ATP2C1",34.5278031712963,0.0438182854390844,0.239694396895942,0.182808968447048,0.854947911396677,0.980327348398814
"12348","RUFY1",44.8205217722239,-0.0419096441871342,0.229188338838278,-0.182861154278478,0.854906963299309,0.980327348398814
"12349","H2AFV",133.620357926331,0.0427825659204992,0.233833480483486,0.18296167782321,0.854828087667594,0.980327348398814
"12350","CNOT7",53.3232225230423,-0.038700319024206,0.212311613318143,-0.182280744888951,0.855362408888452,0.980656332159662
"12351","BCO2",5.16267071570465,-0.112142150788252,0.615561683862563,-0.182178575645866,0.855442585793513,0.980656332159662
"12352","AMZ2",30.6709994939428,-0.0483724565565898,0.265482944198402,-0.182205514944267,0.855421445141888,0.980656332159662
"12353","ADAMTS13",2.04286120843582,0.18225500609933,1.00131263115361,0.182016086114238,0.855570101877592,0.980663435431912
"12354","TMTC4",7.96771376526488,0.0876635502092212,0.481683263468491,0.181994179282826,0.855587293878944,0.980663435431912
"12355","AK6",75.1869954945647,-0.0446223987386412,0.245342082013222,-0.181878291618298,0.855678241119346,0.980688295770938
"12356","SMNDC1",45.4325456366965,-0.0418750845462665,0.230498425092962,-0.181671890076376,0.855840227305014,0.980759628112867
"12357","HOXC6",62.2340791096782,0.0366925790779664,0.202048710744658,0.181602639000934,0.855894577667011,0.980759628112867
"12358","GPN3",41.0617216004429,-0.0460622560969266,0.253794308075429,-0.181494441093756,0.855979496061861,0.980759628112867
"12359","KLHL22",19.1716305282854,0.0733633407164713,0.404326074971949,0.181445979514334,0.856017531345122,0.980759628112867
"12360","SRSF4",135.847749028064,-0.0336304924739081,0.185453128088399,-0.181342276728153,0.856098924055397,0.980773524645989
"12361","GDAP2",16.6031036593498,-0.0652933617768529,0.360724410256551,-0.181006219485994,0.856362694210083,0.980996069096456
"12362","LBX2-AS1",7.06456415117912,0.0957556439646686,0.529534068461769,0.180829996911864,0.856501017107308,0.980996069096456
"12363","SLF1",11.7531089028183,0.0778575940458804,0.430448454006988,0.180875534157724,0.856465273002063,0.980996069096456

"12364","TCHH",3.67576214418834,0.150135037968645,0.831181180736904,0.180628533763889,0.856659157561424,0.981097838164814
"12365","CEP162",32.7438150547629,0.0475605664180328,0.264004010332239,0.180150924064296,0.85703408491444,0.981130458597177
"12366","AL353796.1",1.51891596781663,0.217241519013341,1.20361800625367,0.180490419622018,0.85676757492346,0.981130458597177
"12367","HSPA2",8.26603124629465,0.108221763482837,0.6006281241216,0.180180979106012,0.857010490519372,0.981130458597177
"12368","CBX1",91.4559276795862,0.0322953589246527,0.179197069811115,0.180222583765984,0.856977829428565,0.981130458597177
"12369","SH3BGRL",279.542787997391,-0.0422823799947439,0.234523706622268,-0.180290430352295,0.856924568047232,0.981130458597177
"12370","AMY2B",3.89518167037101,0.132338396727287,0.735141155840049,0.180017668274964,0.857138697511904,0.981170893837394
"12371","AC093827.4",10.7475609720122,0.082197664238431,0.45713536093656,0.179810339042746,0.857301466532591,0.981277889103669
"12372","NSMAF",26.2337944974168,-0.0509623857459288,0.283892086926832,-0.179513230881505,0.857534729333084,0.981325837193299
"12373","DKK1",4.97716072970114,0.214734886016798,1.19579007875571,0.179575738109688,0.857485653204491,0.981325837193299
"12374","HSD17B14",5.02886297298805,0.134763579371163,0.750804780165295,0.179492170177039,0.857551264790246,0.981325837193299
"12375","NPHP4",7.89925551588466,-0.0885017033073436,0.499020091707428,-0.177350982010623,0.859232707576013,0.98143607788478
"12376","THRAP3",211.025001320903,-0.0258309722046365,0.14437649609971,-0.17891397078093,0.858005252691646,0.98143607788478
"12377","NFYC",36.5338392940261,0.0438768551439473,0.246206275982592,0.178211765597111,0.858556670020153,0.98143607788478
"12378","PRPF38A",52.7565515160078,0.0377053775722133,0.212979140143831,0.177037889939596,0.859478627756386,0.98143607788478
"12379","PIGK",52.5071716822931,0.040998691201907,0.229883322437144,0.178345652773994,0.858451527740282,0.98143607788478
"12380","MCEE",44.0296871533641,0.0480983855618839,0.269371220026293,0.178557997239605,0.858284777702321,0.98143607788478
"12381","AC079305.3",2.29079467020889,0.168511293088634,0.954342847059894,0.176573118987351,0.859843710219296,0.98143607788478
"12382","CTDSP1",116.194546283511,-0.0303320840864317,0.169911659166626,-0.178516790638165,0.858317135962281,0.98143607788478
"12383","CCDC13",2.56398107269696,0.149958348740226,0.848426326745006,0.176748816029251,0.859705694821612,0.98143607788478
"12384","C4orf48",23.4132048175799,0.0582124051597584,0.327161732233172,0.177931583753413,0.858776706360846,0.98143607788478
"12385","NOP14",25.8184418879118,-0.0650437460456146,0.365116577163198,-0.178145146273492,0.858608987448104,0.98143607788478
"12386","SHLD3",1.7656041480569,-0.190403484943613,1.06608339935794,-0.178600928462338,0.858251065404245,0.98143607788478
"12387","SERF1A",1.77634346824965,-0.182752795296638,1.02342022510838,-0.178570630922684,0.858274856912396,0.98143607788478
"12388","MED7",24.7059272811789,0.0629341436657078,0.355014846666135,0.177271864139509,0.859294849928222,0.98143607788478

"12389","MRNIP",38.5218146373702,0.0458388570484174,0.258698041000187,
0.177190584324465,0.859358691265609,0.98143607788478
"12390","ZNF391",4.16178325106906,0.121369829958378,0.687471179476242,
0.176545335399871,0.859865535468826,0.98143607788478
"12391","TDRD6",2.04525856581331,0.170705754382107,0.965496962377141,0
.176806101970341,0.859660695889905,0.98143607788478
"12392","ELOVL4",10.2958166046701,0.0786840488306858,0.443543402149301
,0.177398758383965,0.859195182516603,0.98143607788478
"12393","AL358852.1",2.09248788911729,-0.173029407738435,0.97066679393
4566,-0.178258295039708,0.858520129928085,0.98143607788478
"12394","ZNF853",10.3876514708541,0.0836642821212018,0.473620822262203
,0.176648234597431,0.859784704086985,0.98143607788478
"12395","UPK3BL1",46.7996378648753,-0.0706791937561974,0.3975480848893
94,-0.177787785786622,0.858889640060617,0.98143607788478
"12396","FBXO33",31.1433449056093,-0.0503509323483111,0.28505815965937
8,-0.176633892565912,0.859795970230823,0.98143607788478
"12397","DICER1-
AS1",3.47140962869802,-0.138286817788364,0.778485465514857,-0.17763570
9225357,0.85900907860909,0.98143607788478
"12398","VPS33B",5.74146677912535,0.114532088612988,0.642149341288734,
0.178357402630256,0.8584423006383,0.98143607788478
"12399","VPS53",26.6647315893384,-0.0572311591087947,0.321598824775697
,-0.17795823460708,0.858755776065769,0.98143607788478
"12400","MRM3",14.7120021928432,0.0661847515070438,0.374704329550392,0
.176631936936674,0.859797506444989,0.98143607788478
"12401","MFAP4",719.734489542285,0.0399369919914791,0.224181977325436,
0.178145417700122,0.858608774290325,0.98143607788478
"12402","PITPNC1",14.7582894345323,0.0801861547039936,0.45296228988441
9,0.177026115627538,0.859487876231603,0.98143607788478
"12403","SLC25A19",9.38103214079021,-0.0817369225932755,0.460418184277
368,-0.177527572507942,0.85909400945568,0.98143607788478
"12404","TMED1",76.6265469298076,0.0492425085945222,0.27666365027765,0
.177986911345614,0.858733254849301,0.98143607788478
"12405","CLIC6",13.1878294816113,-0.0796940775134791,0.448530607933308
,-0.177678125202392,0.858975765440723,0.98143607788478
"12406","MT-
ATP8",128.611842406035,-0.0615604166035123,0.347699103152212,-0.177050
835177343,0.859468459565843,0.98143607788478
"12407","PDE4DIP",151.32172162655,0.0407384298403053,0.23238319319019,
0.175307126479511,0.860838312365323,0.981577613187819
"12408","RNF25",7.66757439888137,0.0867155336002373,0.493130676987226,
0.17584696642688,0.860414170627016,0.981577613187819
"12409","DNPH1",104.51693418075,-0.0592653468145611,0.33819445312213,-
0.175240446043504,0.860890704673695,0.981577613187819
"12410","FAM229B",56.1795660936594,-0.0645125829238769,0.3680376187926
17,-0.175288013044745,0.860853330155454,0.981577613187819
"12411","PPP2CB",91.8047513729967,0.0334019441151866,0.190321602026321
,0.175502642682501,0.86068469444775,0.981577613187819
"12412","MRPS16",87.6446088467206,0.043516970070696,0.247252939486673,
0.176001831003678,0.860292503969914,0.981577613187819

"12413","ZBTB1",44.6762351084811,0.048984473821096,0.27900085292997,0.
175571054018932,0.86063094461245,0.981577613187819
"12414","DEGS2",1.98647998847722,0.167092409285565,0.952422545940472,0
.175439367744669,0.860734409260615,0.981577613187819
"12415","ATP10A",12.2897018193481,-0.0716990854361031,0.40890764002086
8,-0.175342983154934,0.860810139227518,0.981577613187819
"12416","CFAP20",52.3084397574728,0.0401293261980832,0.228567102057578
,0.17556912537647,0.86063245991116,0.981577613187819
"12417","ZNF18",6.48390339215488,0.0963807559825575,0.548027536769821,
0.175868454623,0.860397288602009,0.981577613187819
"12418","TMEM11",57.8440530803919,-0.0411194835344283,0.23412831425889
6,-0.175627982734967,0.860586217004383,0.981577613187819
"12419","GSPT2",28.6226856852826,0.0544256617138157,0.310081427849098,
0.175520546623328,0.860670627508165,0.981577613187819
"12420","CMAS",51.9070876264378,0.0397372067307384,0.226952346997795,0
.175090530044726,0.861008499258579,0.981632878381761
"12421","UNC119",18.3259198064891,0.0553868472127026,0.3164981556873,0
.174998957236973,0.861080452946223,0.981635875832745
"12422","PRKAA2",7.75877734456846,-0.0997355481377102,0.57963157912030
9,-0.172067140111786,0.863384745820999,0.981654729470479
"12423","ANP32E",65.274471074233,0.0416515725195052,0.239245599716062,
0.174095459097001,0.861790441821847,0.981654729470479
"12424","ABCB10",14.0132284347005,-0.0654985083470212,0.38049999339206
2,-0.17213800127332,0.863329037947579,0.981654729470479
"12425","AL391832.2",1.99663533271882,-0.18446114574743,1.070762443306
76,-0.172270840185403,0.863224607771961,0.981654729470479
"12426","UBE2F",41.1626631944869,0.0487284042349527,0.281099707143301,
0.173349181790899,0.862376967329038,0.981654729470479
"12427","ROBO1",84.0495613551049,-0.0457275543410718,0.262361665302771
,-0.174292057066726,0.861635941250582,0.981654729470479
"12428","ETV5",16.0668641000607,0.0710133512658856,0.40929409729186,0.
173502016607993,0.86225684289708,0.981654729470479
"12429","CENPE",6.70150094223714,0.101157446389526,0.584830389922416,0
.172968860942649,0.862675904031084,0.981654729470479
"12430","BRD9",52.681396876141,-0.0402263523016981,0.232837855573379,-
0.172765516168486,0.86283574350867,0.981654729470479
"12431","GIN1",9.2214268120671,0.0847482601651536,0.492466118052182,0.
172089524656747,0.863367148020287,0.981654729470479
"12432","ATOX1",175.86334098375,-0.0480335322554561,0.275142624588144,
-0.174576848379478,0.861412141511275,0.981654729470479
"12433","AC011365.1",6.25159741819283,0.110196377199364,0.635227619107
536,0.173475418707683,0.862277747967988,0.981654729470479
"12434","CPSF4",23.6258261872852,-0.0531235088569663,0.304874893493861
,-0.174246912391413,0.861671418657188,0.981654729470479
"12435","PITPNM1",8.44386876803407,0.0776958155898413,0.45128828843494
9,0.172164484612015,0.863308218106525,0.981654729470479
"12436","ALDH3B1",3.20922800905786,-0.143105809321151,0.82915539831511
9,-0.172592266313345,0.862971931258661,0.981654729470479
"12437","AP002784.1",3.35199739405745,0.137282447232932,0.788741607889
23,0.174052498130936,0.861824204287769,0.981654729470479

"12438","TMEM19",22.4552448169826,-0.0575425812248916,0.333817854198401,-0.172377182649709,0.863141009274573,0.981654729470479
"12439","SUGT1",108.574577108182,-0.0290192984128311,0.167445666118495,-0.173305759925104,0.862411096435276,0.981654729470479
"12440","F7",1.81914869146717,0.174780078822416,1.00887442513196,0.173242649896249,0.862460700677807,0.981654729470479
"12441","MTHFD1",20.0308270208721,-0.054307286169389,0.312171597682667,-0.173966134563575,0.861892077058827,0.981654729470479
"12442","ENTPD5",16.4805812411004,0.0630167059447531,0.361085361124491,0.174520245707294,0.861456621137444,0.981654729470479
"12443","HBA2",2.67919624313999,0.216596429248185,1.25149616333505,0.173069990619058,0.862596412983664,0.981654729470479
"12444","CMTM3",138.863634986933,0.0570336700164743,0.329558873544424,0.17306064134482,0.862603761743464,0.981654729470479
"12445","ZNF778",3.86238411754092,0.145600826952822,0.839054923168064,0.17352955442186,0.862235199186816,0.981654729470479
"12446","USP32",17.6059849314184,0.0704559117871399,0.407572118488286,0.172867349337992,0.862755696684275,0.981654729470479
"12447","FHOD3",1.75839498096729,0.190483461337416,1.10103632625306,0.173003793603842,0.862648445723814,0.981654729470479
"12448","AC027097.2",6.97172797326216,-0.091750821738977,0.531068236091528,-0.172766540161073,0.862834938583623,0.981654729470479
"12449","PTBP1",102.114617215272,-0.0352217330886648,0.201576923442102,-0.174730978562541,0.861291024847863,0.981654729470479
"12450","NECTIN2",47.1497431147534,-0.0584562956214476,0.336804738514592,-0.173561381230137,0.86221018461332,0.981654729470479
"12451","EHD2",164.0486739912,-0.0339709626037712,0.19658177862920,-0.172808298107056,0.862802114232333,0.981654729470479
"12452","ADNP",60.6094697365211,-0.0494243074037537,0.286813145265388,-0.172322322807141,0.863184135791265,0.981654729470479
"12453","XRCC6",184.358031208734,0.0344697642287027,0.199890368261155,0.172443347463687,0.863088996216276,0.981654729470479
"12454","SLC38A5",2.71418446144194,0.144607987311602,0.829486115550169,0.174334427786882,0.861602644039061,0.981654729470479
"12455","NEDD1",25.8413797286531,-0.0543615716563683,0.316122059852896,-0.171963866367519,0.863465936232428,0.981668218149432
"12456","CCAR2",17.2028240502613,-0.0676787424026534,0.393920726641759,-0.171808025892992,0.86358845560268,0.981700583317395
"12457","ERAL1",23.4072327963212,-0.0519979956297161,0.302751706435227,-0.171751288347704,0.863633062597796,0.981700583317395
"12458","SMG7-AS1",1.58404188266486,0.203380113082832,1.1883091508716,0.171150843140151,0.86410515856792,0.981839152797621
"12459","RAMP3",3.16895184019299,-0.403070773658654,2.35525258906841,-0.171136962349369,0.864116072819895,0.981839152797621
"12460","ADIPOR2",27.2120209833278,0.0483975489172053,0.282915576324437,0.171067106116861,0.864171000092991,0.981839152797621
"12461","AC009118.3",2.37854224670899,0.175712960082262,1.02485018782034,0.171452337298167,0.863868104756954,0.981839152797621
"12462","LINC00665",7.03492926356608,0.109241053535047,0.6380457549359

62,0.17121194317171,0.864057116862566,0.981839152797621
"12463","ZNF570",13.4182878484019,-0.0660616913180737,0.38598461431007
2,-0.171151099989194,0.864104956611718,0.981839152797621
"12464","PDHX",18.0063339216346,0.0563020428549767,0.329392006306648,0
.170927168167409,0.864281033909599,0.981885385121945
"12465","FGR",2.83031362602748,-0.1413183979726,0.83373354442455,-0.16
9500674307328,0.865402842014543,0.982056759330496
"12466","MTMR14",20.7272764694292,0.0544682948853241,0.320933151246864
,0.16971850578137,0.865231519702539,0.982056759330496
"12467","MICA",32.137901302734,0.063320932920613,0.373133381388576,0.1
69700530906592,0.865245656525125,0.982056759330496
"12468","TSC22D4",55.1337470896092,-0.0377555337125162,0.2218426613055
02,-0.170190591342225,0.864860250799461,0.982056759330496
"12469","FUT10",18.7955869849852,0.0605800345083606,0.356654822848814,
0.169856204451329,0.865123224319669,0.982056759330496
"12470","CSPP1",30.7254376298272,-0.0497486208510993,0.293227099110341
,-0.169659015152549,0.865278307868686,0.982056759330496
"12471","ST3GAL1",36.3622980809929,-0.0427543807209286,0.2511979887594
66,-0.170201922921715,0.864851339511908,0.982056759330496
"12472","AC016876.1",21.676239301436,0.0642848269931036,0.377188867883
635,0.170431400464703,0.864670879369554,0.982056759330496
"12473","PGAP3",9.00807823774995,-0.0769412518867344,0.453772840031878
,-0.169558962324253,0.865356998442286,0.982056759330496
"12474","MAP2K6",14.2133577916589,0.0743339105946884,0.436636329935084
,0.170242156912916,0.864819699166285,0.982056759330496
"12475","NAPG",35.2264472673336,0.0440336558821498,0.259348430717137,0
.169785703967401,0.865178670268038,0.982056759330496
"12476","LSM14A",100.504699472779,0.030461708748885,0.178787533981429,
0.170379377524437,0.864711789365351,0.982056759330496
"12477","SLC6A16",1.82511559198723,-0.156746915169747,0.91966794823188
6,-0.170438597399313,0.864665219845554,0.982056759330496
"12478","GPR82",4.63575779212794,0.106342383243708,0.626752594234125,0
.169672027243311,0.865268074084353,0.982056759330496
"12479","MFSD8",15.6320685247078,-0.0628479244777429,0.371151664218279
,-0.169332190952487,0.865535356855049,0.982128428004447
"12480","RPP40",11.8379386480567,-0.0655106845928602,0.387743071292851
,-0.16895384970885,0.865832942037098,0.982244842014788
"12481","MED27",28.0255520814594,-0.0474229723035238,0.28093012739458,
-0.168807001026686,0.865948451335345,0.982244842014788
"12482","COQ5",32.8816416799312,-0.050061267860358,0.296429490772244,-
0.168880861785852,0.865890353047265,0.982244842014788
"12483","GPM6B",4.81103928406177,-0.105028116495632,0.621657170915565,
-0.168948612530197,0.865837061485686,0.982244842014788
"12484","RBBP7",144.893141984305,-0.0297126585012512,0.176063739445237
,-0.168760805574581,0.865984788680269,0.982244842014788
"12485","HIBADH",86.6601946624324,-0.044911350429154,0.266646938256674
,-0.16843002482152,0.866244989109153,0.982303919739377
"12486","SMAD9",34.7485573521865,-0.0735323673429551,0.436233613519163
,-0.168561901385265,0.866141249960348,0.982303919739377
"12487","CRYZL1",34.3624746666551,-0.0425679094657439,0.25269756689296

2,-0.168453974405598,0.866226149279051,0.982303919739377
"12488","JAM3",73.3384277629859,0.0353481595839877,0.210043082472352,0.168290043965817,0.866355105924967,0.982350120107105
"12489","NFX1",40.708703464054,0.0517886447347348,0.308168824588275,0.168052835337664,0.866541713497643,0.982400960167219
"12490","AKAP8L",42.3138355503913,-0.03931746607811,0.234076457954733,-0.167968476717609,0.866608078633385,0.982400960167219
"12491","FP565260.1",7.4945033227843,0.0903539605333418,0.537378019455044,0.168138549144548,0.866474283196687,0.982400960167219
"12492","PELI2",24.9936305633761,-0.0616557850575777,0.367518056270258,-0.167762601063167,0.866770045468602,0.982505911290058
"12493","TRAPPC13",15.0915332539086,-0.0818013159116232,0.488027079166697,-0.167616346312788,0.866885110646657,0.982548969410372
"12494","ZBTB16",238.00891636908,0.0749389792002478,0.447295615740039,0.167537924726276,0.866946809591327,0.982548969410372
"12495","TTLL5",14.3344410874619,-0.0628028571783966,0.37514463540117,-0.167409716818253,0.867047680153672,0.982584645936454
"12496","UROD",138.751070762091,0.0585106799601933,0.352868173849057,0.16581455709639,0.868302889806286,0.982612183545667
"12497","FHL2",42.1360755319647,-0.069856490696322,0.420323106116462,-0.166197122356072,0.868001823982572,0.982612183545667
"12498","IWS1",74.5745573782081,0.0393795427918721,0.236269008119005,0.166672485339412,0.867627756186507,0.982612183545667
"12499","EPC2",46.1479826160935,-0.0442894360078938,0.265621612325005,-0.16673882678531,0.8675755538065,0.982612183545667
"12500","CACNB4",45.5748982553564,-0.0541553064054929,0.32759218525533,-0.165313181580579,0.86869748416317,0.982612183545667
"12501","AC104825.1",6.03138767647947,-0.0953785343498792,0.577133780624057,-0.165262435767918,0.868737424138998,0.982612183545667
"12502","RIOK1",37.3757186595148,-0.0390088135463764,0.234868206003699,-0.166088097704302,0.868087620720229,0.982612183545667
"12503","UQCC2",72.5363552510233,0.0470576071899602,0.284477146204775,0.165417882658619,0.868615079240062,0.982612183545667
"12504","AC007036.2",2.11887962781505,0.23334855919909,1.41116269748511,0.165359075615412,0.868661363113884,0.982612183545667
"12505","SLC35B4",31.3771697195894,0.0484213601826604,0.290012334600655,0.166963106066976,0.867399078476855,0.982612183545667
"12506","EPHB6",16.6431085468575,0.0604309987060577,0.363367777245825,0.166308083683422,0.867914504778095,0.982612183545667
"12507","FXN",17.9160205951137,-0.0574751465244007,0.345713108405609,-0.166250989988404,0.867959433527825,0.982612183545667
"12508","MAMDC2",46.9790928827848,-0.0906370427300261,0.544285184139288,-0.166524912621599,0.867743879437968,0.982612183545667
"12509","AL158212.3",2.29695264433537,0.149894611389789,0.902150943716566,0.166152474188269,0.86803695958348,0.982612183545667
"12510","ABRAXAS2",23.5681040748729,-0.0576226938873155,0.348599622472542,-0.165297637096134,0.868709718562311,0.982612183545667
"12511","FUT8",36.1091010938768,0.0452489733774901,0.27256631100752,0.166010880839348,0.868148387321801,0.982612183545667
"12512","SOCS1",108.402972060958,-0.0709976302647515,0.427684108500886

,-0.166004835937467,0.86815314445204,0.982612183545667
"12513","B3GNTL1",6.66843878664792,0.0894945466059988,0.53976064620764
1,0.165804134174634,0.868311092558007,0.982612183545667
"12514","AC020916.1",165.883808833239,-0.0623083455932557,0.3767633316
8987,-0.165377945124831,0.868646511884683,0.982612183545667
"12515","PAFAH1B3",6.76612690067709,-0.0985604905220264,0.589394086981
454,-0.167223412482466,0.867194263294259,0.982612183545667
"12516","AL008721.2",1.7224555932506,0.202368844589195,1.2156184096403
5,0.166473988041253,0.867783952059056,0.982612183545667
"12517","GTPBP1",20.7426705119649,0.0528288455092546,0.316577145946266
,0.166875108281573,0.867468319208784,0.982612183545667
"12518","TTLL1",10.1424918881276,-0.0721716854993853,0.433901790426947
,-0.166331845343091,0.867895806137506,0.982612183545667
"12519","GAB3",7.151517894997,-0.106286677035509,0.642476937286103,-0.
165432672936831,0.868603438675145,0.982612183545667
"12520","FAM229A",6.73142291839597,-0.0940700566088363,0.5767436743117
05,-0.163105484808483,0.87043538137717,0.98261920203461
"12521","SFPQ",291.565308064602,0.0398779043738202,0.24221244673257,0.
164640194638098,0.869227192158594,0.98261920203461
"12522","SELENOF",310.356562645498,-0.0354383428018544,0.2174843575271
68,-0.162946628460061,0.870560457210946,0.98261920203461
"12523","NUDT17",2.40046317245919,-0.142287800248718,0.865615037463829
,-0.164377689955119,0.869433825509339,0.98261920203461
"12524","DEDD",23.0496255213219,-0.0472929859921363,0.288837897138798,
-0.163735390890934,0.869939455614092,0.98261920203461
"12525","ZNF514",13.3451866218927,-0.0771841617766654,0.47308729600056
8,-0.163149935390725,0.870400383710837,0.98261920203461
"12526","MUSTN1",43.9304118392245,-0.146297596962081,0.886276966156768
,-0.165069839958137,0.868889011555076,0.98261920203461
"12527","EIF5A2",16.2644073037872,-0.0578345116458763,0.35388054513743
4,-0.163429474834271,0.870180297291423,0.98261920203461
"12528","ARHGAP24",80.9923555050221,-0.0476000479922082,0.288746144956
554,-0.16485085194599,0.869061377478167,0.98261920203461
"12529","LINC02615",1.97723245676206,-0.209724863738997,1.283952705956
04,-0.163343137769887,0.870248270938931,0.98261920203461
"12530","LINC00603",1.61809196077032,-0.202575465638755,1.248480825594
25,-0.162257570549658,0.871103025645513,0.98261920203461
"12531","SYNGAP1",19.3525786944587,-0.0561030018421037,0.3414926919300
26,-0.164287562129147,0.869504772642214,0.98261920203461
"12532","BTBD9",8.1549043704856,0.0822200282970163,0.502859988683892,0
.16350481276549,0.870120984105777,0.98261920203461
"12533","SUMO4",2.96692062804637,-0.131167925849428,0.797517162103708,
-0.164470348830401,0.869360887072109,0.98261920203461
"12534","ZNF138",19.0839797894801,0.0614270962051752,0.378001610467339
,0.162504853165123,0.870908306801882,0.98261920203461
"12535","ELN",224.95700969503,0.0405990283606888,0.250098327456043,0.1
62332266567534,0.871044206605671,0.98261920203461
"12536","PDLIM2",402.572731366047,0.0438092700907943,0.268201268255056
,0.163344753646475,0.870246998741567,0.98261920203461
"12537","ZFPM2",49.2036949734976,0.0457282394022521,0.281296092724822,

0.162562654032261,0.870862793537214,0.98261920203461
"12538","AL161457.2",2.96565337232236,-0.125567292977744,0.773790601185118,-0.162275546879774,0.871088870200902,0.98261920203461
"12539","FUOM",49.40676988104,-0.0503203445978448,0.308817345767232,-0.162945330913417,0.870561478849982,0.98261920203461
"12540","IL18BP",4.64428483517496,-0.107840842855423,0.663949790322518,-0.162423189866569,0.870972610429503,0.98261920203461
"12541","PEX5",13.765335989194,0.0632280554480711,0.384936398655042,0.164255850236528,0.869529735969081,0.98261920203461
"12542","LOH12CR2",5.76807262140373,0.0942854280783327,0.575063740249844,0.163956482523779,0.869765402040901,0.98261920203461
"12543","ATXN3",81.4436025208862,-0.0351503308781125,0.214923698286159,-0.163547952870752,0.870087020440349,0.98261920203461
"12544","MAP2K1",30.6535585734549,-0.0461647331951741,0.279714895471145,-0.165042098017037,0.868910846950536,0.98261920203461
"12545","SNUPN",59.6723143733976,0.0421913522102357,0.25988260365492,0.162347735542386,0.871032025720851,0.98261920203461
"12546","ABAT",3.2633487239259,-0.138504753910131,0.845963944497938,-0.163724180931057,0.8699482807816,0.98261920203461
"12547","AOC3",5.74517083493318,0.107321505109306,0.652011376679306,0.164600663344089,0.869258309065942,0.98261920203461
"12548","DOK6",14.4422147242934,-0.0669551940608669,0.411628797056183,-0.162659159270939,0.87078680482641,0.98261920203461
"12549","PALM",108.488719235533,-0.0334205569773427,0.203989718066871,-0.163834517220063,0.869861417997077,0.98261920203461
"12550","ZNF554",4.17192474849577,-0.108796581566423,0.668406613174636,-0.162770055564962,0.870699485999115,0.98261920203461
"12551","DHPS",66.7019794279912,-0.0457338379246494,0.279260458740184,-0.163767681722527,0.869914034382792,0.98261920203461
"12552","SHLD1",4.67836926073939,-0.112964815350874,0.694238337493568,-0.162717627722367,0.870740767053605,0.98261920203461
"12553","AL162458.1",4.26851665919539,0.118998763553656,0.724317810174382,0.164290815277629,0.869502211799294,0.98261920203461
"12554","MTMR6",28.0394020039529,-0.0553560356727005,0.341908898446868,-0.161902880925758,0.871382334102201,0.982826746839067
"12555","TAF15",63.138417141625,0.0374970311605784,0.231681045401212,0.161847643149413,0.871425833796927,0.982826746839067
"12556","HOXA13",4.15943690039707,-0.139083595533069,0.861269445835901,-0.161486740538069,0.871710053814752,0.982912435261737
"12557","ZNF761",9.95689823531629,-0.0849710251263064,0.525632615358424,-0.161654780627274,0.871577715892547,0.982912435261737
"12558","SMOX",9.62368809239055,-0.0933587219219263,0.577990203095418,-0.161523017902285,0.871681483715057,0.982912435261737
"12559","PPP2R5C",84.752313037034,-0.033174473894207,0.205782450019509,-0.161211385572783,0.87192691402041,0.983000406252309
"12560","AC068870.2",2.05441449002463,0.149799653777854,0.928947319060745,0.161257426233079,0.871890653291552,0.983000406252309
"12561","SNX8",13.2670276361042,-0.0774849130625187,0.481874491581968,-0.160798951627715,0.87225175097169,0.983210061595219
"12562","MDM1",18.244309066386,0.0589379891028792,0.366370559377337,0.

16086988322164,0.872195883029036,0.983210061595219
"12563","AC006333.2",10.1552694463125,-0.0800394679495111,0.4980651266
7138,-0.160700807310929,0.872329053557047,0.983218928469934
"12564","SLC25A46",33.5578873497927,0.0415899297523081,0.2590545600272
64,0.160545059496081,0.872451729578778,0.983278931139406
"12565","TMEM127",32.1364015512597,-0.0446736879176807,0.2807837547179
46,-0.159103534898437,0.87358730325162,0.98328927491189
"12566","WDSUB1",11.9032327979565,-0.0787358041577549,0.49784412677092
9,-0.15815352622204,0.874335823475679,0.98328927491189
"12567","CNTN3",15.3387133593463,-0.0865807516307459,0.546021308348643
,-0.158566616919395,0.874010331809128,0.98328927491189
"12568","ZNF717",7.79870532233048,0.0805878105857527,0.503169087921533
,0.160160495786101,0.872754647416341,0.98328927491189
"12569","MELTF",19.8601767748743,-0.0838692841837852,0.526739886490038
,-0.159223340276456,0.873492915555858,0.98328927491189
"12570","NELFA",24.2947949982783,0.0528700843429587,0.334194379914555,
0.158201596198225,0.874297946009956,0.98328927491189
"12571","SGMS2",47.2822156426244,-0.0462177935383289,0.291450384504955
,-0.158578598607212,0.874000891247429,0.98328927491189
"12572","CRPPA",5.84653702261278,0.0972018881968937,0.612867670044597,
0.15860175523669,0.873982645822947,0.98328927491189
"12573","AL359091.4",1.49903790303954,0.180452226370011,1.128723277547
99,0.159872866945758,0.872981222610986,0.98328927491189
"12574","BBS1",18.4689616943121,-0.0557466497524869,0.352099934416658,
-0.158326214530103,0.874199752449617,0.98328927491189
"12575","CLPB",12.2881740064615,-0.0746479168147432,0.469417928465327,
-0.15902296099321,0.873651307604591,0.98328927491189
"12576","CHEK1",6.63861776948381,-0.0850914461905922,0.537179501686483
,-0.158404119895577,0.874138367559783,0.98328927491189
"12577","DYRK4",7.2392789372061,0.127524595826226,0.801484166973261,0.
159110561482221,0.873581767364874,0.98328927491189
"12578","AC074032.1",10.3794481205726,-0.0746722799828171,0.4656387009
22233,-0.160365278562376,0.872593339294128,0.98328927491189
"12579","FAM124A",3.08042177939047,0.130490352812613,0.815727494555065
,0.159968069831689,0.872906226847535,0.98328927491189
"12580","AL136418.1",1.58259672982577,0.170537657224035,1.074356109205
4,0.158734758208027,0.873877852264202,0.98328927491189
"12581","FCF1",31.5152132521347,-0.0543457023406836,0.340677594955689,
-0.1595223846398,0.873257323737614,0.98328927491189
"12582","BRD7",99.0284029132333,-0.0297289589617387,0.187129083839102,
-0.158868725009632,0.873772301536347,0.98328927491189
"12583","TMEM170A",34.0034111144846,0.0427795967247217,0.2676796977537
48,0.159816366664,0.873025731063776,0.98328927491189
"12584","SPAG7",100.435667925427,0.0456383443000144,0.288534947407626,
0.158172674437039,0.874320735315172,0.98328927491189
"12585","AC073508.3",4.76894213769812,-0.0994439756243119,0.6247337721
26797,-0.159178165261936,0.873528506113408,0.98328927491189
"12586","AC124283.1",2.73414816955484,0.151344231960434,0.955660135863
405,0.158366166256061,0.874168272722233,0.98328927491189
"12587","AC139099.2",1.53073390960068,-0.178472461160019,1.12614202248

041,-0.158481308393874,0.874077548266619,0.98328927491189
"12588","ZNF236-
DT",1.85437889363369,-0.177691446380949,1.1149796160443,-0.15936743938
9931,0.873379390622865,0.98328927491189
"12589","ZNF573",8.67731186527774,0.0810294173176951,0.505356006278162
,0.160341257076292,0.872612260830151,0.98328927491189
"12590","CARD8-
AS1",4.24387993062121,-0.11410281419865,0.712688500495848,-0.160101943
723329,0.872800770057636,0.98328927491189
"12591","GNB1L",4.51481489084461,0.105499910627178,0.660118656624812,0
.159819616622562,0.873023170877556,0.98328927491189
"12592","AC026748.3",1.54767904109034,-0.186017036682743,1.17780493607
819,-0.157935351588978,0.874507741100096,0.983326420549302
"12593","ZFYVE19",23.6499856392288,-0.0507527908065472,0.3211968060347
61,-0.158011505260904,0.874447732724671,0.983326420549302
"12594","MAPKAPK5",23.0946114194065,-0.0480102935451903,0.304523415077
547,-0.157657149395111,0.87472696793756,0.983416742040163
"12595","C17orf100",2.18358673490945,-0.153966354836947,0.976316583582
375,-0.157701259433699,0.874692208028977,0.983416742040163
"12596","ZNF682",7.38073176307955,-0.0864556295561603,0.54888493415364
1,-0.157511391143339,0.87484183111802,0.983467793635373
"12597","SMAGP",4.76238774761022,0.104129019725369,0.663621711167588,0
.156910206482187,0.875315615893719,0.983922292693106
"12598","LINC02609",33.1699588694409,-0.0515729560426318,0.32934685830
8725,-0.15659161380033,0.875566712222833,0.984048308998755
"12599","ADRB1",1.69916227214517,-0.184600041953463,1.17820871259256,-
0.156678557865409,0.875498186694396,0.984048308998755
"12600","ZCCHC2",24.6465586181255,0.0535485018111031,0.342400011592484
,0.156391647190816,0.875724320741859,0.984069231148697
"12601","DNAJC5",16.3546676967551,0.0549667269333167,0.35133771391056,
0.156449833755421,0.875678459085131,0.984069231148697
"12602","MTERF3",17.2712038422257,-0.0520250085303468,0.33295896712808
6,-0.156250510322893,0.875835564144899,0.984116139366114
"12603","C1orf216",28.412195503699,-0.0493707578188965,0.3174174644998
25,-0.155538882829567,0.876396502850026,0.984165824181402
"12604","RLF",42.8060015218579,-0.0430420443759344,0.275847770781355,-
0.156035498325817,0.876005040285501,0.984165824181402
"12605","AC105942.1",4.97286685225821,-0.0893561687054733,0.5728754127
15062,-0.155978362349298,0.876050076797834,0.984165824181402
"12606","AC253572.2",10.6743557987656,-0.0789398216352553,0.5086899221
78457,-0.155182593940915,0.876677370075211,0.984165824181402
"12607","CXCL2",115.364490130253,-0.138184939012267,0.889021767207258,
-0.155434820731506,0.876478534765453,0.984165824181402
"12608","BTN2A2",8.43425588293621,0.0778091230501163,0.502123823313549
,0.154960030648713,0.876852827533373,0.984165824181402
"12609","ABHD16A",48.4782075500062,0.0332472415274702,0.21340985705356
1,0.155790561816111,0.876198110367342,0.984165824181402
"12610","LRSAM1",9.6650128331307,0.078744693167951,0.507714680348085,0
.155096348827188,0.87674536057056,0.984165824181402
"12611","COMMD3",99.2906732743945,0.036645231601542,0.236323712093066,

0.155063710183728,0.876771091164463,0.984165824181402
"12612","TMEM117",6.86504694223506,0.0806189505884909,0.518739488624807,0.155413174351184,0.87649559872461,0.984165824181402
"12613","AC009133.1",4.42171694219937,0.1056068818549,0.681074190843251,0.155059292034171,0.876774574211754,0.984165824181402
"12614","MON1B",29.1968222417412,-0.0414857001515911,0.266842594582617,-0.155468808180647,0.876451742390575,0.984165824181402
"12615","FZD2",9.08595858186425,0.0900880435557319,0.580205660176699,0.155269156678506,0.876609130099178,0.984165824181402
"12616","ZNF432",8.56962656463816,0.0728057699865622,0.469614012576032,0.155033214590833,0.876795132407428,0.984165824181402
"12617","NOTCH1",6.98708733523831,-0.0797177824308626,0.51557548742391,-0.154619031306502,0.87712166562041,0.984389536750813
"12618","RPS20",2520.58461670033,-0.027666222340030 5,0.179300799596163,-0.154300607706953,0.877372718174415,0.984515230156883
"12619","AL442125.1",2.51208941108404,0.129603381988019,0.839902808974095,0.154307594406458,0.87736720956597,0.984515230156883
"12620","TRIM68",5.70950082829064,-0.0844176419902986,0.548084665980568,-0.154022995405771,0.877591604219859,0.984682814243519
"12621","ZNF398",13.7863969479285,0.0604673008617929,0.393459739433426,0.153681037223439,0.87786123724729,0.984907306823677
"12622","AC012063.1",3.51417204532424,0.113719682988007,0.740514051249193,0.153568568747845,0.877949921409803,0.984911238981566
"12623","RAB2B",25.6702978786905,-0.0453143139517632,0.295206924061073,-0.153500173127336,0.878003853789852,0.984911238981566
"12624","LENG8-
AS1",5.62233602161588,-0.091892671527922,0.599224333576613,-0.153352703451542,0.878120140801934,0.984963656032588
"12625","PHGDH",80.5228032555187,-0.0454503872510739,0.297071081733582,-0.152994990242216,0.878402225987714,0.985202021385033
"12626","BRI3",534.87600026757,-0.036846448761783,0.241145227571095,-0.152797752345814,0.878557770289771,0.985220402890881
"12627","DCUN1D5",53.4133106547387,-0.0419333511861852,0.274350558089938,-0.152845875285002,0.878519819498139,0.985220402890881
"12628","ZCCHC18",2.48913054478537,-0.127065659691836,0.832419776359222,-0.152646132757185,0.87867734260396,0.985276462723477
"12629","MAGEF1",44.6072350280345,-0.0372629729948705,0.244483441842938,-0.152415119461584,0.878859532789108,0.985402722645796
"12630","SMG5",15.2456559467243,-0.0589563655843372,0.387768016983597,-0.15204029987556,0.879155150413611,0.985481269044967
"12631","FAM89A",18.037115203066,-0.0691856356320338,0.456571689683026,-0.151532907526671,0.879555354093944,0.985481269044967
"12632","PGBD2",5.84129832167675,-0.101635503805213,0.668326987956155,-0.152074516870896,0.879128163005799,0.985481269044967
"12633","FYB1",10.0117455695286,0.090342811386438,0.596746692474725,0.151392228102319,0.879666319878608,0.985481269044967
"12634","ZNF777",10.9129972332484,0.0595712349859894,0.393235605234727,0.151489931717731,0.879589252505846,0.985481269044967
"12635","LHPP",13.3487719582703,-0.0693445243375955,0.45673242498409,-0.151827460772051,0.879323022649798,0.985481269044967

"12636","FAR2",9.87868340830586,0.0798522691669138,0.527580240146697,0.151355685998987,0.879695144119237,0.985481269044967
"12637","AP5M1",26.5387489295673,-0.0412585397898904,0.271458580685176,-0.151988342699471,0.879196130010781,0.985481269044967
"12638","UBB",1066.82269484737,-0.0386662591215401,0.255038916817253,-0.151609250870706,0.87949513661838,0.985481269044967
"12639","DHRS7B",51.1594014314606,-0.0410493685908691,0.270799717920528,-0.151585713995891,0.879513701766785,0.985481269044967
"12640","CDK5RAP1",18.7788153603891,0.0529737096739041,0.34919144327613,0.151703916845448,0.879420467736732,0.985481269044967
"12641","NMRAL1",25.0217811166406,0.0475764085568433,0.315484267727962,0.150804377344952,0.880130033138312,0.985890457182066
"12642","WDR5",17.697216043895,-0.0530356747225551,0.352340612610844,-0.15052387611397,0.880351314919661,0.985982331666724
"12643","ZNF821",7.94396430593765,-0.0729696402311883,0.484630791268989,-0.150567486725554,0.880316910771201,0.985982331666724
"12644","SLFN13",2.55708043869513,0.153772230817613,1.0231847407401,0.15028784607009,0.880537521515022,0.986034899537581
"12645","LINC01560",5.70815542014721,-0.0867202309916206,0.576951522942438,-0.150307655917692,0.880521893062884,0.986034899537581
"12646","AC123768.3",2.35307461526199,-0.154280864327202,1.02769952394487,-0.150122541397109,0.88066793605162,0.986102955439739
"12647","PRXL2B",3.04469838768398,-0.113589473673648,0.761256200257809,-0.149213200017523,0.881385404488809,0.98635750264889
"12648","C3orf52",1.75835085611818,0.154133866082845,1.03568075942231,0.14882372263903,0.881692731134851,0.98635750264889
"12649","TXNRD3",12.340952516391,-0.0632885966454381,0.424436116801188,-0.149112184708549,0.881465111374079,0.98635750264889
"12650","SUGCT",2.43655273010679,-0.140950381298033,0.942706298520707,-0.149516749298496,0.881145893890276,0.98635750264889
"12651","CTTNBP2",2.06385034101795,-0.141457894864431,0.949748796420373,-0.148942431301402,0.881599059273588,0.98635750264889
"12652","ENTPD1-AS1",17.8117926830833,0.0523835163704459,0.351244714057762,0.149136810530995,0.881445680073948,0.98635750264889
"12653","ZBTB3",4.66582911336848,-0.0963401033355155,0.647555643794626,-0.148775019195215,0.881731163031755,0.98635750264889
"12654","MAP4K5",72.7521176254016,-0.0303546484417595,0.202847580865113,-0.149642644552633,0.88104656148562,0.98635750264889
"12655","LINC01569",1.82994042542324,-0.144597756935159,0.971106247746102,-0.148900037736102,0.881632511437291,0.98635750264889
"12656","ERCC4",24.9721267606461,-0.041512271353651,0.278586104594948,-0.14901055964010 8,0.881545300606112,0.98635750264889
"12657","CRISPLD2",194.427058287645,-0.0386676519221718,0.258737179870663,-0.149447605255267,0.881200449912157,0.98635750264889
"12658","RAB9B",8.14339185180618,-0.0766245026667302,0.512997181892982,-0.149366322801194,0.881264584101886,0.98635750264889
"12659","PSMD6-AS2",2.20578370186295,0.132932647125554,0.895149144953258,0.148503350391398,0.881945542043639,0.986363547534786

"12660","AC005162.3",1.84522017752331,0.18170447759935,1.2234695582681
8,0.148515732468696,0.881935770923081,0.986363547534786
"12661","Z68871.1",2.21770054265311,-0.142846027134205,0.9608223742287
35,-0.148670587785666,0.881813570815775,0.986363547534786
"12662","TARDBP",64.1863045599803,0.037660017181777,0.253897782001621,
0.148327476060963,0.882084332430539,0.986440858254339
"12663","CCDC24",3.57173681775226,0.10548624902892,0.717493561794275,0
.147020481640456,0.883115853976622,0.986456632972464
"12664","MAGI3",38.6479474368306,-0.0396925097861067,0.270103100064004
,-0.146953181124915,0.883168975064247,0.986456632972464
"12665","AL357568.2",4.18656421099893,0.105734532288278,0.717354736528
221,0.147395043071718,0.88282021784771,0.986456632972464
"12666","NRP2",8.1674459489466,-0.0926663036618316,0.627958104349622,-
0.147567653032851,0.882683984697004,0.986456632972464
"12667","PKD2",87.2992487419579,-0.0438381247785629,0.299263061537138,
-0.146486922085847,0.883537013217175,0.986456632972464
"12668","RANBP17",2.46402123798152,-0.148346182477463,1.00655324728971
,-0.147380362516247,0.882831804698945,0.986456632972464
"12669","STC2",1.88618003048706,0.162134755953475,1.09783805583337,0.1
47685494314914,0.882590979953623,0.986456632972464
"12670","GFPT2",117.117583836806,-0.0417218907562515,0.28247432084459,
-0.147701534891753,0.882578320256094,0.986456632972464
"12671","HSF2",16.4374960019429,-0.0506011973489766,0.344570350816939,
-0.146853022115822,0.88324803271956,0.986456632972464
"12672","SOGA3",2.60359887437747,0.140926847273347,0.96224684992329,0.
146456023508502,0.883561403671593,0.986456632972464
"12673","C14orf119",81.430766176419,0.0462109558547618,0.3140511535580
87,0.147144677964746,0.883017825738814,0.986456632972464
"12674","PPP2R5E",88.2957658162946,0.02554001049087,0.174359988378426,
0.14647862005725,0.883543566590579,0.986456632972464
"12675","ADAMTS7",14.7276455387183,-0.0560074264985202,0.3818267965954
94,-0.146682807487328,0.883382389445558,0.986456632972464
"12676","GOSR1",76.7589129534793,-0.0290989829782108,0.19814510387589,
-0.146856936704514,0.883244942828857,0.986456632972464
"12677","HSBP1L1",11.9561726058743,0.0715802498866582,0.48804119208128
4,0.146668459646612,0.883393714882297,0.986456632972464
"12678","COQ8B",19.1005826680783,-0.0466616888420709,0.318545980836866
,-0.146483370217021,0.883539816955361,0.986456632972464
"12679","OPA3",9.01532955294772,0.0700767350000152,0.476810523979092,0
.146969774104835,0.88315587798144,0.986456632972464
"12680","BPI",1.86465305643742,-0.17921604722503,1.21942464194816,-0.1
46967710065884,0.883157507155966,0.986456632972464
"12681","IFNAR1",106.9928765786,0.0278453061025146,0.189821057074378,0
.146692398260136,0.883374819003276,0.986456632972464
"12682","SNRPD3",159.39797035525,-0.0289364612977092,0.195325162426823
,-0.148145077358121,0.882228275319308,0.986456632972464
"12683","DESI1",28.0950902440276,0.0431296390829458,0.293269667059063,
0.147064780055347,0.883080889004117,0.986456632972464
"12684","ZNF317",8.89017756312295,-0.0656976536620629,0.449084577214003,-0.146292384542869,0.88369057743673,0.986523066580266

"12685","AL034417.3",12.2991365590086,0.0852492055404011,0.58416752097
1178,0.14593280605309,0.883974433326947,0.986660326775945
"12686","ZC3H12A",9.61582235794557,-0.0697022949735632,0.4824578565537
09,-0.144473333839893,0.885126712035208,0.986660326775945
"12687","DENND2D",2.61517547213127,0.138983959496404,0.958253838696924
,0.145038771444319,0.884680260241624,0.986660326775945
"12688","THEM4",25.6269297988481,0.0428648461965994,0.294694281143084,
0.14545530381632,0.88435140275953,0.986660326775945
"12689","MUC1",4.62125441079138,0.094139787370881,0.649603799261909,0.
144918775841281,0.884775001949577,0.986660326775945
"12690","AL136454.1",4.72026079435427,0.107743790081518,0.745754866375
389,0.144476147511028,0.885124490358341,0.986660326775945
"12691","NSL1",60.8853021468267,0.033508937205507,0.232800604894901,0.
143938359699,0.885549144413879,0.986660326775945
"12692","SCRN3",10.3814843666623,0.0630395140398776,0.436091017279304,
0.144555864583431,0.885061546072697,0.986660326775945
"12693","EFHB",1.54546321268836,-0.186562760725939,1.28909369634803,-0
.144723972551,0.884928811063288,0.986660326775945
"12694","USF3",34.4075474123356,-0.0397727172037853,0.273591443554373,
-0.145372664755435,0.884416645742158,0.986660326775945
"12695","DIPK2A",30.9043168603281,0.0481588424787358,0.334488987045217
,0.143977363512496,0.885518344677291,0.986660326775945
"12696","PIGG",20.4910477315959,0.0475682398098689,0.330948650941736,0
.1437329920352,0.885711317816533,0.986660326775945
"12697","P4HA2",114.350250222676,0.0322422751837066,0.224472433392033,
0.143635789466392,0.885788077779147,0.986660326775945
"12698","SIT1",1.67439937476309,0.175305370063198,1.21947848582044,0.1
43754377056727,0.88569443040825,0.986660326775945
"12699","ERP44",138.488173756621,-0.0313406676748776,0.217678331606981
,-0.143976974848665,0.885518651588571,0.986660326775945
"12700","CUL2",38.4151768193997,0.0353774915820438,0.243533765212967,0
.145267296102069,0.884499834721899,0.986660326775945
"12701","TOLLIP",35.6721005121638,-0.047192394268948,0.324562980294651
,-0.145402886755923,0.884392785585156,0.986660326775945
"12702","KDM2A",63.0081832445712,0.0334250131644767,0.232790970570429,
0.143583804314112,0.885829130409272,0.986660326775945
"12703","BCL9L",60.7090076589637,0.0376668203549411,0.261421015029899,
0.144084898265096,0.885433429721216,0.986660326775945
"12704","PPFIA2",7.24820585903003,-0.0718532087850725,0.49696168073020
2,-0.144585008404463,0.885038534407711,0.986660326775945
"12705","TMTC3",40.0410097735622,-0.038966579377508,0.271304312588627,
-0.143626833667742,0.885795150143799,0.986660326775945
"12706","C12orf45",48.4539421021438,0.0378271365338556,0.2634617388179
55,0.143577343350008,0.885834232648488,0.986660326775945
"12707","UNC119B",13.6024425872108,0.0586280827574717,0.40749747861407
7,0.143873485933874,0.885600372989631,0.986660326775945
"12708","AC137932.3",2.96300198105886,-0.110464921117164,0.75795803154
0705,-0.145740155154265,0.884126520557727,0.986660326775945
"12709","ALOX12-
AS1",15.5648524632662,0.061904596224571,0.429839068172021,0.1440180774

81493,0.885486194684459,0.986660326775945
"12710","INTS2",10.4564979315412,-0.0664362921191617,0.459650701003716
,-0.144536475140989,0.885076855833158,0.986660326775945
"12711","PSMF1",101.623519755805,-0.0249017353089256,0.170814902073898
,-0.145781983928736,0.884093498690352,0.986660326775945
"12712","SEC23B",16.3707738984616,-0.0524406107788691,0.36167462739436
,-0.144993889000926,0.884715696674982,0.986660326775945
"12713","PPP2R3B",11.2003219063897,0.0607553555153117,0.41973836218553
,0.144745777343213,0.884911594630416,0.986660326775945
"12714","CTPS1",8.91711591444526,-0.077605284183058,0.54176690554038,-
0.143244785514633,0.886096860774513,0.986766452242729
"12715","NBAS",35.5444157701967,0.034009041285461,0.238475360781038,0.
142610293885612,0.886597967239842,0.986766452242729
"12716","TSEN2",7.01022602062386,-0.075696912619457,0.530096280010494,
-0.142798422614772,0.886449382885888,0.986766452242729
"12717","METTL6",16.5680186567007,0.050878150724299,0.356462190782133,
0.142730847871031,0.886502753061274,0.986766452242729
"12718","NCK1-
DT",4.78106889045288,0.102967110936798,0.724441096174558,0.14213317201
4288,0.886974816676939,0.986766452242729
"12719","PINX1",17.5097077481167,0.0488233369918172,0.34251573108814,0
.142543341985228,0.886650846913784,0.986766452242729
"12720","OTUD6B-
AS1",99.0968969125075,0.0278529776551886,0.195486567905115,0.142480263
24094,0.886700667969264,0.986766452242729
"12721","TATDN1",69.7517383531066,0.0339893647675539,0.239004568835487
,0.14221219675072З,0.886912398094042,0.986766452242729
"12722","KIFC2",3.6996016098648,-0.0998022151418225,0.699283046169988,
-0.142720770492642,0.886510712165566,0.986766452242729
"12723","MLXIP",56.2229464126995,0.0363055050062506,0.253339838972593,
0.143307523812622,0.886047314023273,0.986766452242729
"12724","IFI27L2",350.931391696668,-0.0432020667630736,0.3034487278375
92,-0.142370235231949,0.886787571714679,0.986766452242729
"12725","SRP14-
AS1",8.39837464765184,0.0814723984925828,0.571593293772733,0.142535609
462515,0.886656954215507,0.986766452242729
"12726","HAUS2",19.1843235414416,-0.046190603655863,0.324762759887378,
-0.142228756991353,0.886899317889352,0.986766452242729
"12727","AC026401.3",2.85756978562409,0.131204588512731,0.917120501160
329,0.143061449773212,0.886241650332345,0.986766452242729
"12728","TMEM106A",10.7843323151217,0.0646657740140127,0.4547351522761
25,0.142205355557703,0.886917801660084,0.986766452242729
"12729","CDPF1",7.83610250821186,0.077074665683853,0.542977749481351,0
.141948110686809,0.887120992204637,0.986851539760991
"12730","PLEKHA3",75.9337918685594,0.0298450511660062,0.21059136467657
5,0.141720204016162,0.88730101552265,0.986906058662348
"12731","DENR",89.3470210623543,-0.0336190860806379,0.237239305192703,
-0.141709595942924,0.887309394973895,0.986906058662348
"12732","NBPF10",10.1201637932902,0.0739315995200217,0.522825703845072
,0.141407736796984,0.887547842607357,0.986946536792486

"12733","SESTD1",181.623324360263,0.0367768663784505,0.260918023745379
,0.140951804902293,0.88790801623696,0.986946536792486
"12734","TIMP4",32.1202831529391,-0.0459516210606124,0.328226317096144
,-0.139999807045187,0.888660142842459,0.986946536792486
"12735","UBA3",33.8286045409788,0.035998729857107,0.256433310173841,0.
140382424704118,0.888357843393817,0.986946536792486
"12736","ZFYVE28",8.39562616718944,-0.0639575435188553,0.4568812738825
67,-0.13998722901323,0.888670080798319,0.986946536792486
"12737","TRIM73",11.7038324519049,-0.0609997712832178,0.43466962581569
,-0.140335941736778,0.888394567899017,0.986946536792486
"12738","FSCN3",2.54990496212317,0.119012152162438,0.846828071408965,0
.140538742373553,0.888234344235865,0.986946536792486
"12739","C11orf58",355.073153676227,-0.0202185395359341,0.143345919767
909,-0.141047192474469,0.887832660766964,0.986946536792486
"12740","MFSD5",21.7390511943707,0.0451046379371738,0.321433686582467,
0.140323307170239,0.888404550052825,0.986946536792486
"12741","ZNF597",4.00928191102536,-0.105811518613119,0.752125619948067
,-0.140683305829185,0.888120133938021,0.986946536792486
"12742","METTL22",9.01990719172678,-0.0639473866414855,0.4539105975104
57,-0.140881017081811,0.887963938741706,0.986946536792486
"12743","ARMC5",6.16567970804641,-0.0812847352405578,0.580466804565671
,-0.140033391403628,0.888633607785595,0.986946536792486
"12744","CHD3",92.0610864038861,0.0362972393694148,0.257232771342707,0
.141106590656976,0.887785737158342,0.986946536792486
"12745","EFCAB13",8.71878789680109,0.0881995413528346,0.62620467331048
9,0.140847785256152,0.887990192135773,0.986946536792486
"12746","CDK5RAP3",61.5731240882242,-0.0313270448957012,0.222706036136
763,-0.140665450470608,0.888134240183916,0.986946536792486
"12747","MPPE1",34.0618673725572,-0.0433905389534676,0.309767688342307
,-0.140074451230431,0.88860116651843,0.986946536792486
"12748","FZR1",18.3720949824174,-0.0472480022228667,0.336933681199067,
-0.140229382989324,0.888478757001322,0.986946536792486
"12749","YTHDF1",32.0740925558195,0.0396807393268837,0.281628521029313
,0.140897445975486,0.887950959836621,0.986946536792486
"12750","CCDC134",2.06217887645963,-0.17440008265966,1.24157823961482,
-0.140466445927536,0.888291461883229,0.986946536792486
"12751","HDAC8",33.6394012037293,-0.0372712979098494,0.266567686468854
,-0.139819264681221,0.888802791798678,0.987016511008492
"12752","PPIL3",51.0817503539519,0.0387840911977431,0.277973423145169,
0.139524457982044,0.889035730285561,0.987042962274075
"12753","SART1",40.6549307872499,0.0386408235191264,0.276781746577743,
0.139607557206714,0.888970069315658,0.987042962274075
"12754","PIGT",261.132386615057,-0.0266208152281897,0.190754474887309,
-0.139555390477294,0.889011288841718,0.987042962274075
"12755","SH3BGRL2",8.27811596581361,0.0630988190075666,0.4536735470366
99,0.139084192630835,0.889383619266289,0.987274384510085
"12756","MTMR1",7.60743126088598,0.0690005345033692,0.495892825094881,
0.139144046881838,0.889336322367192,0.987274384510085
"12757","DNM3OS",29.5824054640187,-0.0476252106838222,0.342726630552853,-0.138959761040448,0.889481946514366,0.987279122081507

"12758","ITPA",93.6629629608652,-0.0396329342461164,0.285329522762733,
-0.13890232550199,0.889527333299143,0.987279122081507
"12759","CHI3L1",329.937119912729,-0.197636650980604,1.42563003703722,
-0.138631093513811,0.889741671478301,0.987439616594775
"12760","TACC3",2.75907791619125,0.111199407721608,0.804304255851287,0
.138255401376576,0.890038571483493,0.987541852233103
"12761","MOB1B",13.9511933737904,-0.0545492610708074,0.394695281925072
,-0.138206012508563,0.890077603406034,0.987541852233103
"12762","MYO19",15.8316224092552,0.0611699918814114,0.442742578992219,
0.138161529484352,0.890112758478184,0.987541852233103
"12763","UCKL1",29.9635273946513,-0.0383978621206109,0.277521125692341
,-0.138360141141755,0.889955796740875,0.987541852233103
"12764","UBL7",26.2505239417795,0.0405556889625174,0.293732846584738,0
.138069982414505,0.890185109091532,0.987544746532129
"12765","HELB",10.6730819726555,-0.081213307125726,0.589177817348756,-
0.137841759710469,0.890365479878249,0.987667465340854
"12766","GTF2B",75.2934773031709,-0.0345901132026858,0.252780804704723
,-0.13683836968195,0.891158554274801,0.987668769093806
"12767","KCND3",10.9836892212083,0.07669647119584,0.557927275900329,0.
137466789147519,0.890661841918551,0.987668769093806
"12768","NAV1",257.530666661475,-0.0315732532286096,0.229260563678106,
-0.137717768472994,0.890463476000852,0.987668769093806
"12769","LCLAT1",6.51976551128404,0.0803656966412292,0.586939652545461
,0.136923270207927,0.891091445100785,0.987668769093806
"12770","ANO10",23.7015012854399,-0.0462535916502678,0.339112974517274
,-0.136395818284775,0.891508379311264,0.987668769093806
"12771","CLDND1",72.272351662029,0.0285949455287549,0.209843470593096,
0.136267978450485,0.891609437213215,0.987668769093806
"12772","ASTE1",12.6573121579678,-0.0552404096160539,0.405007195726049
,-0.13639364978942,0.891510093500856,0.987668769093806
"12773","HNRNPH1",205.346087990056,0.0329680707693987,0.2404275541579,
0.137122680821131,0.890933825099729,0.987668769093806
"12774","SOX4",244.047041594487,0.0420754245229344,0.308806203168507,0
.136251876067318,0.891622166336591,0.987668769093806
"12775","CENPW",11.0266225506782,0.0756953862121628,0.553547633594507,
0.136745930464247,0.89123162325458,0.987668769093806
"12776","TMEM181",34.5203977313416,0.0474942814622614,0.34756644753793
3,0.136648061971166,0.891308984835185,0.987668769093806
"12777","RNF139-
AS1",1.85792341661082,-0.129793187330619,0.948394462965456,-0.13685569
9183206,0.891144856198376,0.987668769093806
"12778","ZBED5-
AS1",11.6530947185793,-0.0594130137036159,0.435842875324823,-0.1363175
05842758,0.891570285401906,0.987668769093806
"12779","SLC36A4",20.8704816141865,0.0523282465695742,0.38400123981663
9,0.136271035464784,0.891607020610594,0.987668769093806
"12780","ALG10",4.0184268778461,-0.100010982703398,0.729501617937114,-
0.137094942964224,0.890955749657976,0.987668769093806
"12781","CD276",127.357182283795,0.034864612543128,0.254608046576224,0
.13693444889885,0.891082609021479,0.987668769093806

"12782","GMFG",7.65110930548392,-0.074958716361865,0.546049449374675,-0.13727459380777,0.890813751508629,0.987668769093806
"12783","RNF113A",15.4964443069496,-0.0512946702995173,0.373517232193489,-0.137328792029991,0.890770913280808,0.987668769093806
"12784","ABCB6",33.1495462023984,0.0373502367939865,0.274715384919659,0.135959756330755,0.891853095271522,0.987717107905702
"12785","HOXA11",7.65105286639473,-0.0668848648645082,0.492046631093068,-0.135931963838316,0.891875066502988,0.98771710790570
"12786","PDIA4",96.5070166446683,-0.0325831792911104,0.239541576947345,-0.136023064164233,0.8918030478549,0.98771710790570
"12787","AGBL5",6.19502039483616,0.0712641781832282,0.52460827482711,0.13584264984519,0.891945673845487,0.987718052838985
"12788","CBX5",115.50119090838,-0.0301619027377355,0.222650388389973,-0.135467550520985,0.892242218657586,0.987851918096464
"12789","SMURF2",58.3313978612228,0.0330417597800677,0.243873617278943,0.13548722550941,0.892226663693959,0.987851918096464
"12790","HSPBP1",19.2598504576024,-0.0423671963299311,0.312846170532541,-0.135425011780748,0.89227584974956,0.987851918096464
"12791","AP002840.2",1.66003974653471,-0.145576145842651,1.07598582962153,-0.135295597613825,0.892378165671275,0.98788795449185
"12792","PHF13",17.5089959242903,0.049263864214892,0.3718790656564,0.132472808405972,0.894610329202077,0.988067571698826
"12793","AL109811.2",4.26726417976173,0.0845874548051522,0.635000721203829,0.133208438952309,0.894028537009137,0.988067571698826
"12794","SRRM1",246.25391488899,0.0209724501068444,0.156448787156695,0.134053133220131,0.893360559320109,0.988067571698826
"12795","HMGN2",427.237924835694,-0.0250342918577242,0.188622453781409,-0.132721695407144,0.894413484203041,0.988067571698826
"12796","PPP1R8",29.7220355812099,0.0332711498278518,0.252781249168751,0.131620323648455,0.895284609434126,0.988067571698826
"12797","LINC01116",63.6599626293492,-0.0498411076381171,0.379096883067143,-0.13147327204294,0.895400928845056,0.988067571698826
"12798","ST6GAL1",1.57731402157144,0.161868250642028,1.22941614468898,0.131662701308496,0.895251088664319,0.988067571698826
"12799","RNF212",21.0036177274198,0.0467084795924472,0.35051619980213,0.133256264956697,0.893990714563214,0.988067571698826
"12800","AC096733.2",2.8608150798118,-0.122903595647245,0.928528336542278,-0.132363860972647,0.894696497888233,0.988067571698826
"12801","UTP15",9.7662283016086,-0.0593388235942105,0.443360162714242,-0.133838870932696,0.893529989137858,0.988067571698826
"12802","CDC23",15.6009075759338,0.0507891357615119,0.385808256594406,0.131643465098015,0.895266304502114,0.988067571698826
"12803","SMIM13",19.0742006314813,0.0446494842203279,0.339838216795776,0.131384529501459,0.895471126243717,0.988067571698826
"12804","HLA-DRA",218.510705874575,-0.0458491002976259,0.340679580122067,-0.134581298594996,0.892942928630908,0.988067571698826
"12805","FBXO9",71.8189644073299,-0.028285681615041,0.211674497954791,-0.133628197484055,0.893696585786665,0.988067571698826
"12806","ADCYAP1R1",5.29000038232676,-0.0985800571192293,0.73054514872

9487,-0.134940403465375,0.892658994334002,0.988067571698826
"12807","OGDH",43.4795114137612,-0.0325502505892921,0.245397923473245,-0.132642730340141,0.894475937056482,0.988067571698826
"12808","ASL",24.0623213967469,0.038020820811047,0.287527387964279,0.132233736341564,0.894799417668501,0.988067571698826
"12809","LRWD1",11.6846286662964,0.0773582549762796,0.580528148875959,0.133254959515854,0.893991746947443,0.988067571698826
"12810","DCAF12",22.0517199103243,0.0402011543845104,0.300405620978304,0.133822910016101,0.893542610569776,0.988067571698826
"12811","GTF3C5",25.9376951023804,-0.0397319646261555,0.295868632306108,-0.134289209087391,0.893173885854315,0.988067571698826
"12812","OPTN",204.906105616116,0.0221149496460756,0.165661811134877,0.133494554324715,0.893802270745681,0.988067571698826
"12813","BAMBI",2.80341595890816,0.122625804168988,0.921549463994163,0.133064809823127,0.894142125302732,0.988067571698826
"12814","CLEC2B",252.398922677158,-0.0425077295665666,0.319058170295153,-0.133228776204802,0.894012453582327,0.988067571698826
"12815","ATF7",71.491044427879,-0.0273689774907576,0.204897457146719,-0.133574022205458,0.893739427348783,0.988067571698826
"12816","SUOX",8.77267489741357,0.0687610974843988,0.522946866286506,0.131487732152748,0.895389490642016,0.988067571698826
"12817","AC025164.1",5.66605074120615,0.0884972786021372,0.661892402562073,0.133703421069013,0.893637099834633,0.988067571698826
"12818","FICD",6.2766772447278,-0.0772841459392621,0.573884539977657,-0.134668457774226,0.892874013014102,0.988067571698826
"12819","TJP1",84.6685019486975,-0.0405688906054241,0.305373820988919,-0.132849929552069,0.89431206621616,0.988067571698826
"12820","CYLD",45.8776216355394,0.0334342800409515,0.254321279391413,0.131464736733628,0.895407680436144,0.988067571698826
"12821","FBXL8",18.7015549742153,-0.047720362956586,0.3584813381884,-0.133118123241067,0.894099962430822,0.988067571698826
"12822","UBE2G1",31.5372332517448,-0.0391355315955878,0.296771946211467,-0.131870724626045,0.895086544733817,0.988067571698826
"12823","IFI35",72.3720871926043,0.036782981064243,0.277235748608787,0.132677626348066,0.894448337996254,0.988067571698826
"12824","COG1",17.6596044497056,-0.0545532650473642,0.412054193219535,-0.1323934228678,0.894673116676066,0.988067571698826
"12825","PRPSAP1",43.4304740898741,0.0389201701713575,0.291729068916902,0.133412039862383,0.893867524092348,0.988067571698826
"12826","FAM210A",24.3207235919631,-0.039722685625061,0.300065397993775,-0.132380094108302,0.894683658700428,0.988067571698826
"12827","ZNF274",8.5175798074011,-0.0717713167377565,0.537246841677772,-0.133590951439791,0.893726039754523,0.988067571698826
"12828","ZNF584",10.4776259260389,-0.058060083043029,0.441825083530732,-0.131409657819915,0.895451249080718,0.988067571698826
"12829","PCED1A",28.616808260495,-0.0401537472087698,0.304672605952045,-0.131793099951657,0.895147944385739,0.988067571698826
"12830","ACTR5",5.89587272031909,-0.0769482009145399,0.571188250662844,-0.134716007945269,0.892836416060174,0.988067571698826
"12831","ZNF217",49.8823569129855,0.0374281145762048,0.283405036432052

,0.13206580605415,0.894932241782596,0.988067571698826
"12832","SLC37A1",5.98685260611113,-0.0769290138521888,0.572626953937153,-0.134344032049584,0.893130536262191,0.988067571698826
"12833","AL157700.1",1.72630650005406,0.141443601011405,1.06882120282511,0.132336073271695,0.894718475931055,0.988067571698826
"12834","LINC00115",2.10325431098682,0.150625418333299,1.1626822172634 8,0.129549945889613,0.896922506784665,0.988167147389908
"12835","CEP104",20.4720203452723,0.0549927181058567,0.426951693608039,0.128803138456086,0.897513422090406,0.988167147389908
"12836","ERRFI1",284.637902732519,0.0414255259935869,0.31958357438989,0.129623451620351,0.896864348074054,0.988167147389908
"12837","LINC01715",1.53481521888977,0.182653141822319,1.42925784858647,0.127795794161958,0.898310578545351,0.988167147389908
"12838","KCNS3",13.6609421042959,0.0729650625838215,0.571187556217155,0.127742738422126,0.89835256676987,0.988167147389908
"12839","ASXL2",35.0524769677102,0.0465171569892072,0.358479013964614,0.12976256678110 3,0.896754279827902,0.988167147389908
"12840","AC016747.1",17.0574230648009,-0.0501219100958277,0.386477464948346,-0.129689088347044,0.896812415892296,0.988167147389908
"12841","DCTN1",70.4844001562076,-0.0280970068770562,0.219352224002 15,-0.128090823833972,0.898077097748558,0.988167147389908
"12842","RFX8",3.81535930772363,-0.0890036012426545,0.696315054975031,-0.12782087735536,0.898290727846794,0.988167147389908
"12843","AC083855.2",2.34799847132663,-0.163703323168423,1.24936256652532,-0.131029476594379,0.895751989471162,0.988167147389908
"12844","NAA15",40.9859766256043,0.0323808157249309,0.252131717514816,0.128428172560353,0.897810137281242,0.988167147389908
"12845","SNHG15",37.9279248168965,-0.0412929174439406,0.322131840741849,-0.12818638899168,0.898001471215329,0.988167147389908
"12846","SUMF2",116.278074845546,0.0254710826906099,0.195397823071437,0.130354997257558,0.896285569698497,0.988167147389908
"12847","AC037459.2",2.30248956669247,-0.116346489206009,0.898411502483757,-0.129502448359528,0.896960087756003,0.988167147389908
"12848","GTF2E2",37.6143823386578,0.0313088998011593,0.245263257746166,0.127654260523451,0.89842258866509,0.988167147389908
"12849","PLAG1",2.0590037474646,-0.120477488990192,0.92177964255335,-0.130700965207332,0.896011868770784,0.988167147389908
"12850","AC016394.1",3.15134435492549,-0.0954080928984209,0.73319003114117,-0.130127373322198,0.896465653466951,0.988167147389908
"12851","HELLS",9.97644586852001,-0.0622348743487391,0.477391261553052,-0.130364502580706,0.896278049716168,0.988167147389908
"12852","WEE1",26.8350060898574,-0.0429653178757571,0.335350626429921,-0.12812058332247,0.898053547152419,0.988167147389908
"12853","RELA-DT",3.76823598275652,-0.0928721106336388,0.713717024543987,-0.130124555586967,0.896467882740382,0.988167147389908
"12854","AP5B1",14.2839064585674,0.0527015584628016,0.412836723475848,0.127657147404632,0.898420303958975,0.988167147389908
"12855","CEP295",14.4238132403815,-0.0501781359037983,0.392985060238325,-0.127684589010503,0.898398586444662,0.988167147389908

"12856","TIMELESS",1.58193494604318,0.140595672949242,1.07619729638234
,0.130641169069888,0.896059173593523,0.988167147389908
"12857","AC130895.1",2.47991807802113,-0.109911265620478,0.85884190394
3073,-0.127976132878308,0.898167860913419,0.988167147389908
"12858","EFCAB11",13.2315908258674,0.0496375498178376,0.38644700356896
4,0.128445943064427,0.897796074936816,0.988167147389908
"12859","OTUD7A",3.88466804694581,0.11936786683126,0.931960975573704,0
.12808247336513,0.898083706027437,0.988167147389908
"12860","IL34",64.0990963839693,0.0471888587893079,0.364430226347444,0
.129486676399664,0.896972566889108,0.988167147389908
"12861","NEK8",6.07343361605323,0.0739843553614474,0.567253126286391,0
.130425645859014,0.896229677425436,0.988167147389908
"12862","QTRT1",45.880687254547,0.0289894407067541,0.223513251254113,0
.129698980011686,0.896804589584733,0.988167147389908
"12863","RBM42",73.9677118421061,0.0282028465619528,0.218406544707874,
0.129130043239661,0.897254749948367,0.988167147389908
"12864","ZNF616",9.20270276402435,-0.0648418738778456,0.50380937620796
4,-0.128703190015821,0.897592511138915,0.988167147389908
"12865","ZNF772",1.65586030121119,0.128728679017469,0.998096649936776,
0.1289741619968 6,0.897378093785219,0.988167147389908
"12866","OSER1",58.5152729307945,-0.037898379393555,0.293871239690575,
-0.128962532820358,0.897387295679712,0.988167147389908
"12867","ZFP64",10.7058065086391,-0.0541211847266178,0.421373807793966
,-0.128439840648759,0.897800903961716,0.988167147389908
"12868","NPEPL1",63.359769188822,-0.0296432303012063,0.230901986502583
,-0.128380144104454,0.897848143830729,0.988167147389908
"12869","CABLES2",2.31823501630503,0.116888959793067,0.909684624082842
,0.128493938117198,0.897758095141248,0.988167147389908
"12870","PRDX4",306.501338168295,-0.0385328906663302,0.299596011384118
,-0.128616167112206,0.897661373058527,0.988167147389908
"12871","MAOB",17.7482158120683,0.0524146101764255,0.405170435501977,0
.129364350366501,0.897069354891868,0.988167147389908
"12872","KCND1",2.69514599990491,0.110500689679161,0.862548422975883,0
.128109549256286,0.898062279107768,0.988167147389908
"12873","TBC1D8B",32.1846165346014,-0.038783983710166,0.30370955647688
7,-0.12770090003117,0.898385677803783,0.988167147389908
"12874","AMOT",21.3458852776727,-0.0438715761543078,0.340038775072003,
-0.129019333589289,0.897342350694997,0.988167147389908
"12875","SS18L2",67.9645867495415,-0.0394121546046265,0.30929824645664
6,-0.127424435980923,0.898604476764892,0.988213683674346
"12876","SPARCL1",676.25236207429,0.0906954170901962,0.711474658028112
,0.127475260104925,0.898564252997919,0.988213683674346
"12877","ZNF713",3.69725016592774,0.0884186614502695,0.695359865991756
,0.127155255536876,0.898817518746365,0.988219724660232
"12878","ZNF202",5.18203365453128,-0.0782014252301724,0.61630069474262
3,-0.126888426213491,0.899028707137931,0.988219724660232
"12879","AC027682.6",3.65163086203166,0.0854524051158012,0.67238598182
9917,0.127088320436485,0.898870495445172,0.988219724660232
"12880","CLDN7",2.84243206488708,-0.105031335550268,0.827156182887926,
-0.126978843564419,0.898957143381035,0.988219724660232

"12881","TST",20.9311987470061,-0.0487412584377643,0.384011509078059,-0.126926556328437,0.898998527716575,0.988219724660232
"12882","BX537318.1",2.0228018387622,-0.134863697373823,1.05935508027843,-0.127307358868168,0.898697136165566,0.988219724660232
"12883","GPR89B",6.93266554609978,-0.0684724911123431,0.541083871492419,-0.126546908381287,0.899299019950624,0.988221930477442
"12884","AC012020.1",1.72126935144125,0.122874557659427,0.97498202424242,0.12602751087119,0.899710147667156,0.988221930477442
"12885","ACAD11",19.4873046780584,-0.0444023238443783,0.351394726063291,-0.12636024547614,0.899446769371436,0.988221930477442
"12886","FAM126A",96.8961508196249,-0.0338291664112358,0.267973014622202,-0.126240944294072,0.899541201745888,0.988221930477442
"12887","TSPAN12",3.48789464654445,-0.0921769144295432,0.730688695558245,-0.126150732849535,0.899612609199111,0.988221930477442
"12888","ABL1",76.9595808466666,0.0258451013361573,0.204738107675034,0.126234933152647,0.899545959878535,0.988221930477442
"12889","MTRNR2L8",1.77939880013583,0.137450179183285,1.09160198749824,0.125916021368097,0.899798400398705,0.988221930477442
"12890","RAB11A",108.604909831211,-0.0303631108625866,0.240231497465621,-0.126391048563196,0.899422387542008,0.988221930477442
"12891","ZNF564",7.42416629963469,0.0650939577610895,0.516909201413996,0.125929191399623,0.899787975215871,0.988221930477442
"12892","ZNF473",4.12391141349768,0.0822016772171353,0.65090793450141,0.126287717294614,0.899504178594446,0.988221930477442
"12893","LINC01547",1.90261598840717,-0.124373883910788,0.985743816275857,-0.126172624019772,0.899595281026826,0.988221930477442
"12894","PER3",60.4585126683321,-0.0997542510788568,0.795598399639687,-0.125382669351816,0.90022060769048,0.988271688896768
"12895","PIK3R3",1.50374535736309,-0.147255660143735,1.19220113935676,-0.123515785451426,0.901698675021224,0.988271688896768
"12896","SLC35D1",13.2646667613803,0.0507868320919607,0.408895661786055,0.124204868963696,0.901153067217657,0.988271688896768
"12897","RSBN1",58.1043742734119,0.0266360938471755,0.212177140095748,0.125537057550854,0.900098389393994,0.988271688896768
"12898","USP39",21.3706918166948,-0.039936322181834,0.321702908365952,-0.124140382767086,0.901204124614159,0.988271688896768
"12899","ARL8B",62.6268690718052,0.0253927071463544,0.20452570805777,0.124154109463745,0.90119325637197,0.988271688896768
"12900","ZBTB49",2.71434362777602,0.0968887237026624,0.771728408476704,0.125547696104526,0.900089967685264,0.988271688896768
"12901","ACSL1",26.3603786171619,0.0396319308068015,0.31768736406851,0.124751360265795,0.900720395351239,0.988271688896768
"12902","NUDT12",15.1511151545966,0.0443996212872062,0.354872383810653,0.125114332116912,0.900433037065355,0.988271688896768
"12903","CDC40",82.4463992555283,0.0227655341351787,0.183548506185213,0.124030070352121,0.901291466186586,0.988271688896768
"12904","NUP42",33.2418892074826,-0.0382605076532207,0.304676662479049,-0.125577414895871,0.900066441708129,0.988271688896768
"12905","NRF1",9.46312177423894,-0.0609464504745259,0.488285194557471,-0.12481732224087,0.900668173473438,0.988271688896768

"12906","GIMAP7",1.70640911544174,-0.136299938721734,1.08801942229887,
-0.125273442668649,0.900307076215132,0.988271688896768
"12907","ESYT2",101.913735258084,-0.0266468595726512,0.215778910489983
,-0.12349149188001,0.901717911219627,0.988271688896768
"12908","AP001330.4",2.28954612862799,0.121475462214603,0.968440698536
693,0.125434073968753,0.900179914001866,0.988271688896768
"12909","FANCC",4.15899628609287,-0.0905922057243749,0.730294087424349
,-0.124048937660007,0.901276527617004,0.988271688896768
"12910","EXOSC2",15.653122312888,0.0477860655100059,0.384176988062975,
0.124385548835041,0.901010014846474,0.988271688896768
"12911","SFMBT2",14.3897939593546,-0.0681568268153133,0.54856092154607
5,-0.124246595297417,0.90112003031944,0.988271688896768
"12912","DDX54",48.2420232296489,-0.0312210780717128,0.252161151916724
,-0.123813988928887,0.901462555480491,0.988271688896768
"12913","AL356019.2",2.4375758781092,0.111460718067255,0.9001050343845
87,0.123830790640407,0.901449252030324,0.988271688896768
"12914","MNAT1",58.7352670684161,0.025645692982846,0.207201868042844,
0.123771533647475,0.901496171329193,0.988271688896768
"12915","ZNF500",12.3977497515897,0.0494222203414924,0.397494102712168
,0.124334474409246,0.901050452438145,0.988271688896768
"12916","MFSD11",36.0923984387798,-0.0338843163798691,0.27416139080148
3,-0.123592590046366,0.901637859788443,0.988271688896768
"12917","ZNF440",2.76621809458395,-0.107201435968684,0.857407215245146
,-0.125029780555361,0.90049997385136,0.988271688896768
"12918","LINC01260",1.73740697040948,0.123811254460655,0.9982667707416
2,0.124026220334545,0.901294514519545,0.988271688896768
"12919","RAE1",19.4045008083093,-0.0433083885478163,0.350735999114699,
-0.123478595459639,0.901728122919932,0.988271688896768
"12920","ABCB7",31.7807791151831,-0.033256788838247,0.265966915759573,
-0.125041074162435,0.900491033020357,0.988271688896768
"12921","JADE3",1.51922482160587,-0.128156419541394,1.04053877408151,-
0.123163521373356,0.901977611316477,0.988468615141345
"12922","ERICH6-
AS1",4.76725160448098,0.0753101708845214,0.614094143170837,0.122636197
921807,0.902395188988816,0.988506018423468
"12923","CC2D2A",36.3371893306559,0.0343014658146412,0.279756995966623
,0.122611646211463,0.902414631692228,0.988506018423468
"12924","SLC7A7",11.0045793448379,-0.0546435733532537,0.44541153585873
8,-0.122681091426837,0.902359637600574,0.988506018423468
"12925","TSPAN3",171.147644818902,0.0249975806704847,0.203909605733579
,0.122591481556517,0.902430600293797,0.988506018423468
"12926","GTPBP3",14.9833773899681,-0.0460326595477842,0.37415460974071
2,-0.123031116948378,0.902082457382425,0.988506018423468
"12927","SUPT5H",61.0717782878277,0.0322494603661915,0.262768225701924
,0.12272968042481,0.902321159953873,0.988506018423468
"12928","AC099791.2",2.67003634428741,0.10507270026567,0.8587366567597
01,0.122357301785793,0.902616052597883,0.988632681372681
"12929","DCP1A",55.5131549835454,0.0306242508710743,0.250874480727633,
0.122070011992659,0.902843571197931,0.988788658842441
"12930","MYOF",183.722798857818,0.0323603892686071,0.265630569851898,0

.121824793308427,0.903037777976727,0.988788658842441
"12931","MMP19",63.9831938208913,0.0503589951239291,0.413092911998235,0.12190718761145,0.902972523197803,0.988788658842441
"12932","AC135050.6",5.77936087124184,-0.0793289707614086,0.650708183071025,-0.121911746041082,0.902968913024154,0.988788658842441
"12933","CTIF",19.6292445690798,-0.0417070938867861,0.342736929609118,-0.121688357115038,0.903145834383143,0.988830512245056
"12934","FMNL2",48.6136544724276,-0.0302160371085556,0.248874259286748,-0.121410857013305,0.903365617843492,0.988923632132212
"12935","AC092053.4",2.22176302755978,0.127048305629795,1.04648664522329,0.121404612480923,0.903370563674447,0.988923632132212
"12936","TTLL7",14.9096119146075,0.0638130010284979,0.527783154097232,0.120907612403146,0.903764212583401,0.988955834935721
"12937","AGMO",3.62539732226482,-0.0839595845887231,0.695824238192827,-0.12066205800301,0.903958712681711,0.988955834935721
"12938","AC132192.2",1.52972130834702,-0.134454833842683,1.1121235600295,-0.120899186632748,0.903770886418989,0.988955834935721
"12939","EIF1AD",17.0876457419902,-0.0458889148869218,0.378948528629106,-0.121095376865377,0.903615490956616,0.988955834935721
"12940","RCE1",14.684562369859,-0.0434075051132915,0.359646683574902,-0.120694857191005,0.90393273258429,0.988955834935721
"12941","ELL3",1.81851142869001,0.128694513830132,1.06166420679499,0.121219603153658,0.903517097541133,0.988955834935721
"12942","AC087741.1",3.26924523091856,-0.0967357621650209,0.800954886158043,-0.120775544087177,0.903868821274497,0.988955834935721
"12943","WDR45B",71.719780073295,-0.0245705652138518,0.203443885290559,-0.120773181158825,0.903870692918332,0.988955834935721
"12944","AJM1",2.39715614640483,-0.107349684267093,0.891287983140493,-0.120443320562723,0.904131976306563,0.989068972844634
"12945","CD6",1.58857783383767,0.144599622299676,1.20179313882939,0.120319893355794,0.904229745970654,0.989099513553067
"12946","FOXRED1",19.2340466092745,0.0462597395589853,0.385227117520713,0.120084328062648,0.904416346943077,0.989150805029271
"12947","PFAS",6.47073434598587,0.0650121897949888,0.541054869998626,0.120158219433741,0.904357813975524,0.989150805029271
"12948","SOCS2-AS1",1.86103354753667,0.123117094755611,1.02610907206088,0.11998441306862,0.904495495320543,0.989160968005784
"12949","PPP1R2",83.1643649354999,-0.0214711064683485,0.179479415328724,-0.11962991092334,0.904776324381907,0.989315270521066
"12950","GPI",81.1545926181544,-0.0306121973905845,0.255837479624414,-0.119654858371515,0.90475656115396,0.989315270521066
"12951","FAM91A1",28.7597643941736,0.0365255803847335,0.306883991594917,0.119020807162033,0.905258871222238,0.989690056864337
"12952","ZNF749",13.4960574954367,0.0555869553115068,0.466820621604327,0.119075620782284,0.905215445101673,0.989690056864337
"12953","SYNE3",144.61990102021,0.036665628973516,0.308320739326659,0.118920410782583,0.905338411043242,0.989700602205845
"12954","ABCE1",73.8591580665895,0.0228710933748876,0.19285297024725,0.118593420394642,0.90559747832841,0.989797698794812

"12955","SETD9",12.3326514392871,-0.0536967066467823,0.453531397459784,-0.118396889272796,0.905753190444791,0.989797698794812
"12956","MMS22L",9.6357938776763,-0.0591711541018498,0.499894972340512,-0.11836717185774,0.90577673594514,0.989797698794812
"12957","MSRB2",105.927987199592,-0.0285239348396653,0.24092230150332,-0.118394746611999,0.905754888100462,0.989797698794812
"12958","AC090617.10",1.84835160212655,-0.121298924657587,1.02205478454979,-0.118681431261063,0.905527748267375,0.989797698794812
"12959","TDRP",3.28518363504623,0.0880776249798315,0.745415314103622,0.118159129968703,0.905941572599202,0.989825051543573
"12960","RBM18",25.9651968862572,0.0373200956683525,0.315666153022845,0.118226472211139,0.905888215257007,0.989825051543573
"12961","HSD11B1-AS1",3.17336517020592,0.130353164707509,1.11141631234352,0.117285632089246,0.906633710378229,0.989885167013596
"12962","FKBP7",82.1279410947032,-0.0307870828829958,0.262032820427197,-0.11749323169824,0.906469207219165,0.989885167013596
"12963","MON1A",8.9727765480519,-0.0543693889347994,0.461630352672689,-0.117776893611996,0.906244438327861,0.989885167013596
"12964","PHF7",9.06216856002327,0.0554026564422452,0.473922774490109,0.11690228751267,0.906937485411725,0.989885167013596
"12965","P2RY1",10.6273434296769,0.0729679433603075,0.624506896035766,0.116840892908456,0.906986137810536,0.989885167013596
"12966","AC093323.1",17.9682405333791,0.0450586838938065,0.383561217137034,0.117474556552229,0.906484005351277,0.989885167013596
"12967","C6orf226",31.1944669759517,0.0409063385582376,0.347851838913485,0.117597016839148,0.906386968763092,0.989885167013596
"12968","AL513550.1",3.00173427018885,0.0967452459113099,0.828536955083879,0.116766361859521,0.907045200706314,0.989885167013596
"12969","CWF19L1",7.51976863824365,0.0556717536301353,0.473867179243952,0.117483877484317,0.906476619467378,0.989885167013596
"12970","SBNO1",55.1441612493575,0.0295698925861109,0.252421184245304,0.117145051333627,0.906745109676147,0.989885167013596
"12971","HOXB7",8.47568829094347,-0.0853653491445369,0.727856787439746,-0.117283166987852,0.906635663763243,0.989885167013596
"12972","C19orf44",6.31810579652412,0.0667283041265845,0.568304396608121,0.117416484061793,0.906530022033481,0.989885167013596
"12973","EGLN2",66.6898134512513,-0.0257141138058158,0.220030443167999,-0.116866163770721,0.906966111772739,0.989885167013596
"12974","TANGO2",12.0761455262492,-0.0469289586230848,0.40143375196984,-0.116903370463504,0.906936627226154,0.989885167013596
"12975","FGF13",4.59043327131707,0.0941416669719786,0.805003134360194,0.116945714809919,0.906903071487773,0.989885167013596
"12976","AC009237.14",2.07411358939056,-0.115558300140322,0.99122829849955,-0.116580913110779,0.907192163703294,0.989892967406846
"12977","RAMP2-AS1",23.9397386397359,0.0504983754683109,0.433118007451864,0.116592648191667,0.907182863882498,0.989892967406846
"12978","TENM3",6.62220663872283,-0.0794989966703425,0.684168298346746,-0.116198012773242,0.907495611662757,0.990147777865976

"12979","AC027807.2",2.5244248413287,0.127340109851488,1.0981475885603
4,0.115959012411463,0.907685025947722,0.990278139103147
"12980","PEBP4",6.15856308325598,0.0660197170179753,0.569876956985168,
0.115849072696746,0.907772157924254,0.990296899553732
"12981","BST1",65.2917667039242,-0.0318287783475574,0.275640653525628,
-0.115472002915557,0.90807101045031,0.990394015865084
"12982","CCNJ",4.13281059851312,0.0722871770513564,0.625596861611699,0
.115549136332184,0.90800987609995,0.990394015865084
"12983","COLGALT1",51.9615020471602,0.0269220986553211,0.2331162448675
12,0.115487870313893,0.90805843423414,0.990394015865084
"12984","ETFRF1",70.5575981048431,0.028823469500271,0.24985045935188,0
.115362883762712,0.908157496963448,0.99041205768657
"12985","LINC01355",3.91132833139312,0.107204829333093,0.9370604755488
66,0.114405454216068,0.908916390546924,0.990507068722767
"12986","BAP1",25.5050858487423,0.0404059832899266,0.353042839026222,0
.11445065250828,0.908880562856829,0.990507068722767
"12987","MCM2",3.32211737319473,0.089981347071937,0.785142405233753,0.
114605129556272,0.908758113705076,0.990507068722767
"12988","SREK1",120.442516552117,-0.0211326944715154,0.183978201365428
,-0.114865208566424,0.908551962060825,0.990507068722767
"12989","RELN",19.1762265782884,-0.171951227323902,1.49558764708994,-0
.114972350606451,0.908467037706174,0.990507068722767
"12990","ATL3",127.620155422707,-0.0235216687063326,0.205050899204908,
-0.114711365800095,0.908673904796389,0.990507068722767
"12991","MCRS1",38.500455743993,0.0318315350536162,0.278319544908651,0
.11437046242679,0.908944127894325,0.990507068722767
"12992","ZBTB25",20.8329710714995,0.0364749917020076,0.317635942873661
,0.114832696111206,0.908577732724027,0.990507068722767
"12993","CIAPIN1",29.324018738792,-0.0297712633443336,0.25956725959963
7,-0.114695757046762,0.908686277120627,0.990507068722767
"12994","ARHGAP45",3.0067661231948,-0.102679156027202,0.89712173656236
1,-0.114453983046553,0.908877922819873,0.990507068722767
"12995","TATDN3",18.4929640781198,-0.0408703584868365,0.35818025902551
,-0.114105558463862,0.909154115989446,0.990626279416214
"12996","ATPAF2",16.0219893188218,0.0416171982060512,0.364884061334769
,0.114055949864768,0.909193441193017,0.990626279416214
"12997","ART5",2.18476422449589,-0.123575479123297,1.08459378066222,-0
.11393710836867,0.909287648870682,0.990652697392387
"12998","IVNS1ABP",38.2220327304774,-0.0330383873188989,0.291532317062
022,-0.113326672157139,0.909771572126247,0.990846644386093
"12999","SIX2",8.70471409664932,0.0583809841284527,0.514559844527603,0
.113458103560432,0.909667377058118,0.990846644386093
"13000","VPS13B-
DT",8.96844931633254,-0.0562317015261403,0.496453946204222,-0.11326670
269433,0.909819114721768,0.990846644386093
"13001","SLC25A25-
AS1",1.63253368207416,0.13597881069251,1.20140725047638,0.113182944949
427,0.909885516733924,0.990846644386093
"13002","USP2",2.03900657523981,-0.129066515696884,1.14030771593516,-0
.113185690049495,0.909883340445579,0.990846644386093

"13003","PLEKHH3",45.5493927049409,0.03408081280255,0.300117019748923,
0.113558414084819,0.909587854780575,0.990846644386093
"13004","ISG15",103.807493750334,0.0442769281735281,0.392752000731764,
0.112735079874915,0.910240588804277,0.990972607478855
"13005","MORN1",3.91677227545004,0.0822692492739787,0.739793850515068,
0.111205640891311,0.911453278596306,0.990972607478855
"13006","CLCN6",15.158614847279,0.0470409404159428,0.427826483735277,0
.109953315664885,0.912446399046265,0.990972607478855
"13007","WASF2",434.014760049763,0.0139198315709867,0.130361361240566,
0.106778814201697,0.914964455186464,0.990972607478855
"13008","ZCCHC17",76.6023332640435,0.028551955275907,0.267308873085446
,0.106812598273834,0.914937652679174,0.990972607478855
"13009","LURAP1",8.84998226080023,-0.0556236222903133,0.52059110675454
4,-0.106847046690983,0.914910323215056,0.990972607478855
"13010","LRIG2-
DT",3.38996959358641,0.0798475952319822,0.724679818802705,0.1101832742
68496,0.912264026703089,0.990972607478855
"13011","AL353593.1",2.70870891327562,-0.0911551205069665,0.8213386976
48853,-0.110983593939875,0.911629356492789,0.990972607478855
"13012","LTBP1",61.9881734637103,0.0506287602399413,0.483627190207161,
0.10468551244659,0.916625358182849,0.990972607478855
"13013","SEMA4F",8.70749103395074,-0.0499872508311587,0.46501260624994
6,-0.107496549898457,0.914395062246552,0.990972607478855
"13014","ARL6IP6",27.5593903492607,-0.0324410331639348,0.3004814038625
89,-0.107963530344694,0.914024621633444,0.990972607478855
"13015","FASTKD1",5.45871332390521,-0.0636620681515278,0.5974699389756
4,-0.106552755207528,0.915143800998299,0.990972607478855
"13016","CIR1",160.582307600587,0.0186196746341282,0.169578328004419,0
.109799847971393,0.912568111617771,0.990972607478855
"13017","ATG9A",26.1037597970367,-0.0314928886947102,0.298818073867517
,-0.105391512257297,0.916065150794421,0.990972607478855
"13018","RAF1",47.1191631547434,0.0266165032152913,0.238225518112812,0
.111728178518168,0.911038935985254,0.990972607478855
"13019","ITGA9-
AS1",15.8029938733558,0.0494158786638847,0.472476352448941,0.104589104
635083,0.91670186052788,0.990972607478855
"13020","ZKSCAN7",5.15716651646203,-0.0623864686364024,0.5878979693737
77,-0.10611785018216,0.915488848714751,0.990972607478855
"13021","TUSC2",32.1291911119362,0.0307896516728314,0.276297345648573,
0.111436653872141,0.911270095468797,0.990972607478855
"13022","ADAMTS9-
AS1",3.92607523177834,0.087868794116138,0.781905128470406,0.1123778204
24366,0.910523840655329,0.990972607478855
"13023","MTRNR2L12",14.8115906055735,-0.0517598956732033,0.46307859291
473,-0.111773458037466,0.911003033039657,0.990972607478855
"13024","TIPARP-
AS1",3.84433744857342,0.0804108093703117,0.742573439814224,0.108286675
84775,0.913768291596816,0.990972607478855
"13025","SENP2",23.3783364049881,0.0356147977312435,0.341288680031706,
0.104353879325663,0.916888521748068,0.990972607478855

"13026","STX18",37.9385367024703,-0.0301319066498232,0.27019498229083,
-0.111519119986433,0.911204704610942,0.990972607478855
"13027","HERC6",6.70462599169976,-0.0567165559192323,0.542864761599947
,-0.104476399890233,0.916791295931769,0.990972607478855
"13028","INTS12",14.5756402194899,0.0430625173003715,0.382719408660017
,0.112517202749509,0.910413330532835,0.990972607478855
"13029","LARP7",133.676649999777,-0.0187157913262883,0.179283046295631
,-0.10439242144194,0.916857936636433,0.990972607478855
"13030","SLC35A4",24.8970018678849,-0.0323666983230894,0.2927778892081
16,-0.110550350679256,0.911972920472173,0.990972607478855
"13031","SQSTM1",494.348436262369,-0.0148996648770093,0.13977727177552
2,-0.106595762585334,0.915109680398934,0.990972607478855
"13032","FOXC1",23.0150353750628,0.0394498636775793,0.378127375855954,
0.104329562461004,0.916907818468941,0.990972607478855
"13033","AL391422.4",14.4533717651201,-0.0448823581133514,0.4235600083
00711,-0.105964579360114,0.915610455493243,0.990972607478855
"13034","TRIM27",29.9933016457951,-0.0294630487247477,0.27061795341384
1,-0.10887322275951,0.913303045511499,0.990972607478855
"13035","RPP21",82.141177318595,0.02550906740213,0.226242800678413,0.1
12750846991102,0.910228088166357,0.990972607478855
"13036","PRR3",8.89256166468527,0.0506985338339335,0.450458994958884,0
.112548610198274,0.910388429185636,0.990972607478855
"13037","GLO1",123.669697272677,-0.0200779682609387,0.189476423444589,
-0.105965522759671,0.915609706983475,0.990972607478855
"13038","TNFAIP3",76.0724228480195,0.0412366627070438,0.37255457823911
2,0.1106862326104,0.911865163676197,0.990972607478855
"13039","AL135910.1",2.78862042109495,-0.102734372694267,0.93573565796
1994,-0.109789951702834,0.912575960247503,0.990972607478855
"13040","HOXA4",5.4537605349237,0.0682378165573569,0.619282894471852,0
.110188440802249,0.912259929354022,0.990972607478855
"13041","FKBP14",63.0666048740446,0.0311197376328416,0.295326615645559
,0.105373968969294,0.916079070803584,0.990972607478855
"13042","TYW1",17.544507550622,0.0352680407281108,0.332189351501487,0.
106168486643838,0.915448673616208,0.990972607478855
"13043","SRRM3",21.3891702893792,0.0404080768901832,0.383658150588688,
0.105323129009981,0.916119410747202,0.990972607478855
"13044","SMURF1",12.8040664855632,-0.044776129112066,0.408103117327012
,-0.109717684602215,0.912633274806926,0.990972607478855
"13045","AGPAT5",9.86210541694021,-0.0513741279117396,0.48260316506034
8,-0.106452115591317,0.915223645677859,0.990972607478855
"13046","FDFT1",44.4734785599457,0.0264630446625703,0.238815963285774,
0.110809362567208,0.911767520823028,0.990972607478855
"13047","AC124067.2",2.18986745820219,0.11351229011775,1.0727612449992
2,0.10581319063016,0.915730570937739,0.990972607478855
"13048","SGK3",27.2446230524081,0.0346071396814626,0.322845897830056,0
.10719398918824,0.914635083888102,0.990972607478855
"13049","AC104248.1",2.04589253952975,-0.0973673571887258,0.9201936444
65369,-0.105811812300982,0.915731664545966,0.990972607478855
"13050","EXOSC3",30.3845704346813,-0.0384899595061575,0.35915998312512
8,-0.107166614641331,0.914656800521338,0.990972607478855

"13051","GOLM1",71.889733968172,0.0207734764098408,0.198851474970142,0
.104467298585339,0.916798518201437,0.990972607478855
"13052","CENPP",8.07830227290278,0.0502659703904418,0.474833588112302,
0.105860182701636,0.915693286096586,0.990972607478855
"13053","TTF1",38.6484588994949,-0.0294421831911917,0.270446826527057,
-0.10886496088445,0.913309598583676,0.990972607478855
"13054","EHMT1",48.8651608486527,-0.0252316816719047,0.23842285454184,
-0.105827445612924,0.915719260610868,0.990972607478855
"13055","PCBD1",129.300624425757,-0.033633769494948,0.306778432935417,
-0.109635378123301,0.912698552084375,0.990972607478855
"13056","ATE1",30.3758522339896,-0.0365621809555945,0.332219272317068,
-0.110054364698926,0.912366259927489,0.990972607478855
"13057","AL133330.1",19.9326037163399,0.11787366415195,1.0541650398261
1,0.111817087172037,0.910968438885697,0.990972607478855
"13058","TRIM44",99.9968084247627,-0.0219486798472872,0.21043369402491
5,-0.104302117343853,0.916929597682765,0.990972607478855
"13059","EEF1G",1530.82326769927,-0.0226328673970283,0.208569083181542
,-0.108514968046958,0.913587207841639,0.990972607478855
"13060","ZNHIT2",8.85397005090223,0.0520683124402514,0.489733542900813
,0.106319677700322,0.915328719529875,0.990972607478855
"13061","RHOD",24.7507007670544,-0.0481625621592441,0.443024516607025,
-0.108713085515233,0.913430062633387,0.990972607478855
"13062","AP003071.4",2.227599391863,-0.102341388926231,0.9224811882719
08,-0.110941437318574,0.911662786170794,0.990972607478855
"13063","CHORDC1",48.6529636005889,-0.0255483258786719,0.2276280044845
12,-0.112237182487844,0.910635348051366,0.990972607478855
"13064","PYROXD1",26.5871574556199,-0.0382877455588536,0.3553851004246
79,-0.107735933535482,0.914205164555667,0.990972607478855
"13065","CSRNP2",14.266090930677,-0.0393850449639726,0.370699008838645
,-0.106245347370529,0.915387692531317,0.990972607478855
"13066","PTPN11",47.4944331057991,-0.0248261053629993,0.23334053922752
8,-0.106394308700862,0.915269508447903,0.990972607478855
"13067","MRPS31",53.630860516066,0.0257458753739196,0.239516014880249,
0.107491248077052,0.914399268118371,0.990972607478855
"13068","RBM26-
AS1",6.26043765418748,0.0613696443927417,0.566012852005805,0.108424471
591525,0.913658990038477,0.990972607478855
"13069","BCL2L2",18.5198854534394,-0.0395458447667305,0.36037657915831
3,-0.109734780376385,0.91261971622048,0.990972607478855
"13070","COCH",13.8023948548257,0.0542988336415709,0.496831376215765,0
.109290266760427,0.912972266352054,0.990972607478855
"13071","EAPP",121.717068010803,0.0242219102627662,0.22647665468035,0.
106951024585528,0.914827833528912,0.990972607478855
"13072","PSMA3",167.019145327166,0.0263261426412227,0.235367436262914,
0.111851252914254,0.910941348506356,0.990972607478855
"13073","ERG28",34.5107392763336,0.03560952368862,0.335143849671667,0.
106251461047266,0.915382841978724,0.990972607478855
"13074","EIF3J-
DT",39.3845709723569,-0.0242539549635562,0.231187053011443,-0.10491052
4389771,0.916446807800663,0.990972607478855

"13075","LRRC49",11.5514830819341,0.0480947126648874,0.460121304874647
,0.104526158983206,0.916751810112217,0.990972607478855
"13076","TMEM202-
AS1",3.19547653409852,0.0920321877872352,0.85116039858399,0.1081255518
23537,0.913896099549797,0.990972607478855
"13077","UBE2Q2L",2.52678431417027,0.0879050626778828,0.83272736898037
5,0.105562835992189,0.915929212524887,0.990972607478855
"13078","AC018553.1",1.71403188698197,-0.114520949685081,1.02301650133
828,-0.111944381674458,0.910867506250512,0.990972607478855
"13079","SLC38A7",12.3159857873201,0.04204469594644,0.385723006066592,
0.109002302909517,0.913200663763155,0.990972607478855
"13080","GFOD2",18.1958038702079,0.0353379137816193,0.32036950190002,0
.110303613708671,0.912168591429327,0.990972607478855
"13081","CCDC144NL-
AS1",4.11475151184181,0.0781373388592529,0.717124809427202,0.108959190
69048,0.913234858669884,0.990972607478855
"13082","C17orf75",11.1717688435728,0.0455305370325336,0.4169003069169
7,0.109212049684582,0.913034303212036,0.990972607478855
"13083","AC138150.2",2.12329826862029,-0.0961960557394748,0.9159221201
29948,-0.105026457627016,0.916354814667091,0.990972607478855
"13084","NOL11",37.5649791653424,-0.0272385499700847,0.255754962313502
,-0.106502527746445,0.915183649965538,0.990972607478855
"13085","UBALD2",50.3970034328454,0.0342919921008963,0.320166068741328
,0.107106890607455,0.914704180702495,0.990972607478855
"13086","GAA",75.9439169556665,0.0254110668003157,0.236030266380759,0.
107660204726978,0.914265237998422,0.990972607478855
"13087","AP001020.2",1.71505966402423,-0.130356438739773,1.17954874662
314,-0.110513820741163,0.912001889641054,0.990972607478855
"13088","GALNT1",116.887684132541,0.0194205442622638,0.18250072270083,
0.10641351976506,0.915254266762657,0.990972607478855
"13089","ZNF414",28.5007560049743,-0.0407600666019789,0.36873848168423
,-0.11053922665138,0.911981742096476,0.990972607478855
"13090","ZNF558",7.55443470422209,0.0554980140510425,0.504929367815814
,0.109912430507086,0.912478824156705,0.990972607478855
"13091","PLPPR2",26.3775849906142,0.0328308131953364,0.294430743719125
,0.111506063465491,0.911215057636654,0.990972607478855
"13092","ZNF416",2.8950672992947,0.0987549707678208,0.882100223468295,
0.11195436543427,0.910859590123518,0.990972607478855
"13093","SNPH",2.25614429833356,0.110398791438419,1.04439715955112,0.1
05705756118552,0.915815813219996,0.990972607478855
"13094","RALY-
AS1",3.01183671056033,-0.0843636755501006,0.755934936405405,-0.1116017
68204105,0.911139169959002,0.990972607478855
"13095","AP001505.1",2.40669949913212,0.0875888204439649,0.81862816658
1899,0.106994633240734,0.914793237368721,0.990972607478855
"13096","PI4KA",28.922502343413,-0.0302979834214035,0.281671511260829,
-0.107564954956866,0.914340797542884,0.990972607478855
"13097","ZNF674",3.76132236594928,0.0761886576254531,0.681323996883544
,0.111824415364715,0.910962628275285,0.990972607478855
"13098","OTUD5",36.5175388289949,0.0325606413035869,0.288879436242345,

0.112713600272577,0.910257618506606,0.990972607478855
"13099","GPRASP2",10.484320408171,-0.0473909041013708,0.447970761072239,-0.105790172528088,0.915748834219853,0.990972607478855
"13100","MTM1",6.88021919250566,0.057413290878965,0.522569841955767,0.109867210599239,0.912514687230818,0.990972607478855
"13101","MT-CO1",10514.5846639623,-0.0244351851731816,0.223866113850128,-0.109150888238228,0.913082812989756,0.990972607478855
"13102","HR",1.6497827384753,-0.134582654379278,1.28413348110244,-0.104804256224,0.916531132673749,0.990972607478855
"13103","HIBCH",53.0399037996345,0.0279558120598913,0.273623151963214,0.102169030139853,0.918622509457268,0.991069577988254
"13104","MAIP1",23.2681010202215,0.0332799588741904,0.323768388058506,0.102789401626747,0.918130116984357,0.991069577988254
"13105","COPS8",111.981438399603,-0.0272430202190758,0.264256347237216,-0.103093153689211,0.917889038636654,0.991069577988254
"13106","AC083798.2",3.47169527068642,0.0838378918164167,0.821013110608281,0.102115168117476,0.918665261534246,0.991069577988254
"13107","H1FX-AS1",5.7039860027388,0.0566532512062867,0.553039258328924,0.102439836509024,0.918407565010765,0.991069577988254
"13108","RNF145",70.9999381412494,-0.0297205782489715,0.287639184269779,-0.103325902291172,0.917704318583775,0.991069577988254
"13109","ADTRP",1.50094427864443,0.110026565807355,1.07979089567971,0.101896178461567,0.918839083321314,0.991069577988254
"13110","DEF6",1.62632968313089,-0.107957099527793,1.0533584520112,-0.102488473246375,0.918368961688042,0.991069577988254
"13111","DLD",42.7226658671134,-0.0240479306147592,0.234137456880156,-0.102708600901342,0.918194247217699,0.991069577988254
"13112","HGSNAT",98.0967739232057,-0.021504973770979,0.208732124528713,-0.103026660699852,0.917941811362107,0.991069577988254
"13113","VLDLR-AS1",3.42765450263889,0.109707278055645,1.07601042206272,0.101957449301779,0.918790449551127,0.991069577988254
"13114","BAG3",80.8430316489277,0.029916010479029,0.289588559913133,0.103305222029499,0.917720731216027,0.991069577988254
"13115","HSPH1",69.7942305758461,-0.0219389971227544,0.211172556837529,-0.103891326843354,0.917255590037716,0.991069577988254
"13116","AL132639.2",1.72005408902323,0.110626400620784,1.07099087884805,0.103293503993025,0.917730031105374,0.991069577988254
"13117","NUSAP1",2.45292191638307,0.104079626120346,1.00739875967765,0.103315221624503,0.917712795157546,0.991069577988254
"13118","C16orf86",13.5372829563911,0.0400591618766755,0.385948749504636,0.103793993187156,0.917332833465014,0.991069577988254
"13119","RNF167",55.4595619347964,0.0259569114497172,0.253975470947515,0.102202434561412,0.918595995376051,0.991069577988254
"13120","TNFSF9",22.9964367539861,-0.0432159499969217,0.419134734920297,-0.103107536542252,0.917877623610824,0.991069577988254
"13121","KMT2B",32.9893862448768,0.0285201733963739,0.278435735341389,0.102430003682557,0.918415369418111,0.991069577988254

"13122","ALDH16A1",50.2557761616896,-0.0248141768415391,0.240349270278586,-0.103242155937387,0.917770783051476,0.991069577988254
"13123","FLT3LG",32.9593174691045,0.0295974626785477,0.2858226744042,0.103551835907504,0.917525011467592,0.991069577988254
"13124","GZF1",9.34532006902704,-0.05186996766472,0.508986992937882,-0.101908238097256,0.918829510953456,0.991069577988254
"13125","AL390198.1",1.63396390216848,0.118485022962998,1.1571549510037,0.102393394126021,0.918444426838423,0.991069577988254
"13126","TOX2",1.61938821614394,0.142477968860584,1.36964076467022,0.104025794599425,0.91714887848835,0.991069577988254
"13127","ARFRP1",57.6879085337505,0.0239063584931552,0.233652726337214,0.102315769509374,0.918506038737494,0.991069577988254
"13128","ZC4H2",7.46139413187083,-0.0531053505426298,0.513597454483956,-0.103398780657876,0.917646479854829,0.991069577988254
"13129","POLR2B",52.1097736723318,-0.0226753640173277,0.222951740493851,-0.101705256783824,0.918990629476398,0.991082049762818
"13130","TRIM28",81.9776032850339,-0.0214465848007017,0.210858355707755,-0.101710860490756,0.918986181431221,0.991082049762818
"13131","MATN1-AS1",1.90853769525502,0.108593055252783,1.07065395166147,0.10142684766097,0.919211625014002,0.991093932094591
"13132","PPIL4",97.3418036570293,0.0198161602474515,0.195121041486426,0.101558294771761,0.919107284174118,0.991093932094591
"13133","GNB3",1.71656886668384,0.118932505952704,1.17179550204739,0.10149595705471,0.919156766790381,0.991093932094591
"13134","IMMT",49.6763302401739,0.0240248223247254,0.237518101491571,0.101149437343317,0.919431833927285,0.991174654452656
"13135","APPL1",166.784819538056,-0.0156524621103211,0.154926574269759,-0.101031486587104,0.919525465367159,0.991174654452656
"13136","UTP20",23.8270441271868,0.031770692594916,0.314624198796694,0.100979812476045,0.919566485561056,0.991174654452656
"13137","ADCK1",3.06503638379721,0.0746614860745665,0.739135035285062,0.101011970087133,0.919540958024825,0.991174654452656
"13138","SFR1",15.4322062719067,0.0365215492275333,0.362442594893993,0.100765058362456,0.919736965006907,0.991282952085386
"13139","HOXC10",55.116800223219,0.0243393928286909,0.242095491701151,0.100536332410255,0.919918539851498,0.991403190828618
"13140","RAB5A",73.8786942893651,-0.0211931929388148,0.21162709769053,-0.100144041902452,0.92022997066969,0.991587884079051
"13141","TOP3A",14.3087514520194,-0.0386504482262975,0.385930315211964,-0.100148774799071,0.920226213254003,0.991587884079051
"13142","CCDC30",9.60904388553147,-0.0438860991106077,0.440929995403081,-0.0995307635410213,0.920716863388143,0.991671413791002
"13143","ADGRL4",4.15923822359397,0.0894616015778501,0.905426930015601,0.0988059871118575,0.921292314730478,0.991671413791002
"13144","CGREF1",19.2766216823905,0.0422978859694268,0.428380303784873,0.0987391007376199,0.921345422642119,0.991671413791002
"13145","PIK3CB",8.12011469418407,0.0477658893414858,0.484702241593042,0.0985468711357647,0.921498055272741,0.991671413791002
"13146","NT5C3A",56.3216710129037,0.0249667796293533,0.251931772492169

,0.0991013534433388,0.921057797487291,0.991671413791002
"13147","RCC1L",52.1753840604832,0.0282445160466335,0.286285404190853,
0.0986585960484532,0.921409343987491,0.991671413791002
"13148","PTCH1",34.0053815209988,-0.0456152292926249,0.462631877162054
,-0.0985994081783657,0.921456339937638,0.991671413791002
"13149","GHITM",174.053655650219,0.0151427381987388,0.152375439536293,
0.0993778147240852,0.920838296841553,0.991671413791002
"13150","FLI1",8.76080269999058,-0.0504583844229584,0.506460472940252,
-0.0996294619598301,0.920638502927339,0.991671413791002
"13151","KTN1-
AS1",5.12023262796717,0.0620960975691328,0.628483345430301,0.098803091
6342227,0.921294613738103,0.991671413791002
"13152","SGPP1",21.1062949029966,0.0296879285109997,0.298812809749048,
0.0993529311408453,0.920858053294848,0.991671413791002
"13153","FAM227B",1.90527692501453,0.0993872660626267,1.00261633245619
,0.0991279144826515,0.92103670868407,0.991671413791002
"13154","SAXO2",1.89372950921743,0.109529492424611,1.09682756454598,0.
0998602660664798,0.920455261702417,0.991671413791002
"13155","VPS35",119.420342689297,0.0176778463830481,0.177549188437658,
0.0995659092480464,0.920688959774842,0.991671413791002
"13156","S1PR5",1.51087730880673,0.12180991873956,1.23011927061578,0.0
990228522138209,0.921120125831422,0.991671413791002
"13157","RPS11",3323.28543497663,-0.0167644834360342,0.169128509207929
,-0.099122752956001,0.921040806803241,0.991671413791002
"13158","NSDHL",16.0603442711855,-0.0356748120817644,0.356982393552433
,-0.0999343741486918,0.920396426314577,0.991671413791002
"13159","AL020997.5",2.03233365438369,0.10096861984206,1.0466847734629
6,0.0964651654461402,0.92315113881519,0.991677843661997
"13160","C1orf53",2.63921814591793,-0.0966539860835601,1.0022647642080
4,-0.0964355822285471,0.923174633272574,0.991677843661997
"13161","EIF2AK2",145.647432462562,0.018841769045917,0.193787960355225
,0.0972287907431347,0.922544704649486,0.991677843661997
"13162","AC009948.3",1.52803112684231,0.107174165312615,1.099053476854
63,0.0975149686249449,0.922317447638422,0.991677843661997
"13163","GRPEL1",36.6043719109389,-0.0257994227699729,0.26313906229366
2,-0.0980448229354134,0.921896701243586,0.991677843661997
"13164","PJA2",184.409418412074,-0.0179499000736235,0.184937870578292,
-0.0970590826935285,0.922679474670728,0.991677843661997
"13165","ACOT13",78.1522688757416,0.0314739960784298,0.326370485701531
,0.0964364042011232,0.923173980475887,0.991677843661997
"13166","MEOX2",112.645951335084,-0.0346965773020805,0.353420973580721
,-0.0981735094851575,0.921794517199317,0.991677843661997
"13167","C7orf31",3.71722914915885,-0.0823128900277181,0.8427790130745
5,-0.0976684145555922,0.922195597143854,0.991677843661997
"13168","AC083862.3",10.8007573442825,0.0427226360810555,0.44256536660
3794,0.0965340700039523,0.923096416319207,0.991677843661997
"13169","NDUFAF6",13.685648796411,-0.0399771088344625,0.41498205893159
2,-0.0963345474196814,0.92325487379916,0.991677843661997
"13170","GBGT1",6.63612091750936,0.0533571473516355,0.547891131171163,
0.0973864045537298,0.92241954100133,0.991677843661997

"13171","PMPCA",30.4310560956484,-0.0276774562168404,0.285401732707161
,-0.0969771835451297,0.92274451392378,0.991677843661997
"13172","RACGAP1",3.44624666961664,-0.0699456061339314,0.7197578019823
96,-0.0971793649770568,0.922583954836136,0.991677843661997
"13173","AC121761.2",2.56827000952157,0.103553458815147,1.053637514373
72,0.0982818639261329,0.921708478944388,0.991677843661997
"13174","GPHN",13.7575333951664,0.0384917941460098,0.398389021125091,0
.0966186117210386,0.923029275629906,0.991677843661997
"13175","HERC2",63.236377836318,0.0300308855057857,0.310101113893736,0
.0968422368069164,0.922851681427901,0.991677843661997
"13176","RBPMS2",2.96429799547898,0.0767580642236986,0.792257639369496
,0.096885230775162,0.922817537758646,0.991677843661997
"13177","EXOC3L1",2.80878945566403,-0.0824454492607463,0.8440186161554
25,-0.0976820270106033,0.922184787662996,0.991677843661997
"13178","CALCOCO2",139.724467375933,0.0170378004489108,0.1767386194914
68,0.09640111763877,0.923202004512157,0.991677843661997
"13179","AC009831.1",1.93534567976583,0.101185296484943,1.033001801363
23,0.0979526815455806,0.921969867256787,0.991677843661997
"13180","LIN37",19.0712480994144,0.034174998764533,0.347350620468512,0
.0983876139862289,0.921624509567404,0.991677843661997
"13181","HSPA12B",26.1745873555576,0.0273126232993488,0.28088602411651
8,0.0972373879592488,0.922537877413164,0.991677843661997
"13182","NXT2",9.86314426165521,0.0434186371520582,0.442308325794629,0
.0981637347071287,0.921802278853704,0.991677843661997
"13183","IKBKG",23.7820882446387,0.029622653417103,0.306622772596828,0
.0966094369515509,0.923036561951106,0.991677843661997
"13184","FEZ2",129.689554504302,0.017706343292559,0.184553668826135,0.
0959414321328925,0.923567088253048,0.991801806001427
"13185","INHBA",9.833700505462,0.0525079081523308,0.546525331339563,0.
0960758909813588,0.923460298923821,0.991801806001427
"13186","RPL38",1820.0748751578,0.0198037627298299,0.206451220490092,0
.0959246580515147,0.923580410588617,0.991801806001427
"13187","ADPRHL2",34.9844912014803,0.0272774647084538,0.28532056124357
3,0.0956028706433374,0.923835985202598,0.991804715859083
"13188","SRBD1",26.6741417404875,0.0273059795232508,0.285985468697627,
0.0954803041133585,0.923933333820421,0.991804715859083
"13189","LINC02482",7.83668445856068,0.0566111951893239,0.591091115509
11,0.095774058692397,0.923700020792724,0.991804715859083
"13190","BLOC1S4",65.2829382696404,0.0237374672802841,0.24860111531311
7,0.0954841544068956,0.923930275702684,0.991804715859083
"13191","TRAPPC6B",36.469341169255,-0.0225579260484989,0.2359436249258
92,-0.0956072708282929,0.923832490371464,0.991804715859083
"13192","PSMD11",97.9055347852986,-0.0165909129796969,0.17402885480089
2,-0.0953342651060866,0.924049326969607,0.991854038044999
"13193","PREB",40.2396800547402,0.0274107116096325,0.287932511236932,0
.0951983903862699,0.924157248467705,0.991894689479474
"13194","TMCO1",212.517514380185,0.0169016433387936,0.179554422717346,
0.0941310332711776,0.92500506883522,0.992072981490622
"13195","PCGF1",18.127832959833,0.0322137394080853,0.343589821919417,0
.0937563843658919,0.925302679240458,0.992072981490622

"13196","DNAJC10",95.84469738467,0.0173898229718338,0.184821222246795,
0.0940899684594276,0.925037689039092,0.992072981490622
"13197","RUNX2",9.80063016270493,0.0461202188807258,0.486611395799167,
0.0947783370444542,0.924490893934645,0.992072981490622
"13198","PDGFRL",319.158037333061,0.0248937474378826,0.265362797497562
,0.0938102389356641,0.925259898061855,0.992072981490622
"13199","TLE1",17.6919828120589,0.0507001735182124,0.535662287135904,0
.0946495109620235,0.924593222266105,0.992072981490622
"13200","PDCL",50.6500397730331,-0.0253229988497608,0.267859372764178,
-0.0945384086748197,0.924681473345033,0.992072981490622
"13201","MGMT",177.64432243181,0.0317195730591125,0.338559580448233,0.
0936897813292349,0.925355587888841,0.992072981490622
"13202","NUFIP1",18.2703033584723,0.032386396082572,0.345668789028857,
0.0936919881414816,0.92535383481854,0.992072981490622
"13203","BRF1",15.7759437892403,0.0324652723087227,0.346605912334202,0
.0936662392458535,0.925374289514699,0.992072981490622
"13204","ZNF276",20.6092247634972,-0.0312125354111688,0.33110801847464
,-0.0942669270136065,0.924897121313682,0.992072981490622
"13205","KLF2",719.982758527212,0.0218576921231204,0.233323388020778,0
.0936798162778863,0.925363504033748,0.992072981490622
"13206","ZNF30",2.84288393963278,-0.072758866825841,0.773790208424365,
-0.0940291903848159,0.925085968882556,0.992072981490622
"13207","SCAF1",35.953633049851,-0.0260143636525963,0.277606627172549,
-0.0937094474925009,0.925339965291764,0.992072981490622
"13208","ARAF",25.1471104856673,-0.02772085299082,0.293848776731871,-0
.0943371393242673,0.924841348537314,0.992072981490622
"13209","GON7",24.6079231510742,0.0279292771190015,0.298987952227159,0
.0934127175056939,0.925575687586658,0.992138662848378
"13210","HDAC10",15.623271433964,0.0340488180458218,0.36440471791296,0
.09343682003029,0.925556540297569,0.992138662848378
"13211","WARS2",31.7675998927771,-0.0271322677036336,0.291331855177749
,-0.0931318262023885,0.925798833652155,0.992152537993984
"13212","MRPL44",28.8122643759058,-0.0267520708294391,0.28703240953489
2,-0.093202265461201,0.925742874647705,0.992152537993984
"13213","ARHGAP9",2.05918313542342,-0.0861380526506947,0.9240981902259
19,-0.0932130952768516,0.925734271158433,0.992152537993984
"13214","OXER1",3.32488929191444,0.0718877505215849,0.77494620555203,0
.092764826779655,0.926090394644831,0.992314792899796
"13215","MRPL32",84.1038876372996,-0.0210651663555536,0.22707968752938
6,-0.0927655246699581,0.926089840199677,0.992314792899796
"13216","AC114980.1",4.64469163363832,-0.0656524983863629,0.7097196275
96118,-0.092504836887115,0.92629694842062,0.992332483632918
"13217","GTF2A2",154.943971294989,-0.0232998559904711,0.25194640024770
8,-0.0924794161280461,0.926317144679373,0.992332483632918
"13218","LINC00893",4.94042882144712,-0.0593154537855554,0.64029338591
8439,-0.0926379298772128,0.926191209613055,0.992332483632918
"13219","LINC02193",2.63381873844615,-0.0785095441436949,0.85007111256
4675,-0.0923564428707978,0.926414845017593,0.992362070160309
"13220","CREB5",100.971534609329,0.039101677793845,0.425465082124354,0
.0919033768849131,0.926774808452939,0.992400725777674

"13221","AC022081.1",3.56112557177937,0.258832097450418,2.82110460714297,0.0917484934075331,0.926897867675108,0.992400725777674
"13222","ZFAND6",78.048195107583,-0.019177774609994,0.208710776241242,-0.0918868443468726,0.92678794392988,0.992400725777674
"13223","ZKSCAN2-DT",3.88337594506953,-0.0658395993552731,0.718731034441062,-0.091605338019771,0.927011610159696,0.992400725777674
"13224","RPGRIP1L",17.141884070725,-0.0320937086715176,0.348081641212221,-0.092201670159187,0.926537811104145,0.992400725777674
"13225","ACSF3",20.8581615706764,-0.0333668309612831,0.363227378174016,-0.0918621033717828,0.926807601233751,0.992400725777674
"13226","FXR2",18.970250339738,0.0324306947446183,0.353738272189511,0.0916799150509899,0.926952355641776,0.992400725777674
"13227","MT-ND4L",628.152280270471,0.0308328986408251,0.334884166117431,0.092070338822807,0.926642154520571,0.992400725777674
"13228","AC007390.2",4.19811986402948,0.0640415943411604,0.70825324051327,0.0904218868024516,0.927951964780005,0.992416167857506
"13229","NFU1",63.2700215201776,0.0246658374285389,0.27063075125987,0.0911420350928775,0.927379732442456,0.992416167857506
"13230","RANBP2",57.2672198477195,0.0231723486256578,0.258201656753296,0.0897451585595297,0.928489729363107,0.992416167857506
"13231","LINC01117",4.1271237243413,-0.0720522403904513,0.801377565641685,-0.0899104785055432,0.928358354236773,0.992416167857506
"13232","PLXNB1",6.82779405460055,-0.0470829239829652,0.522698656132803,-0.0900766118882134,0.928226334662321,0.992416167857506
"13233","AC083799.1",4.86727817068408,-0.0565758486907379,0.627896978148342,-0.0901037123280624,0.928204799210759,0.992416167857506
"13234","CEP135",13.6556813963952,0.0342040239675683,0.377955612262672,0.0904974628179277,0.927891910059923,0.992416167857506
"13235","PLRG1",56.9802769411953,-0.018473807504585,0.20314320717354,-0.0909398239873377,0.927540406310922,0.992416167857506
"13236","FASTKD3",7.52046439952659,0.0490206214856307,0.538929088287179,0.0909593164500134,0.92752491776781,0.992416167857506
"13237","FBXO4",22.7088429770552,-0.0363197936707665,0.398642811440254,-0.0911086130953848,0.927406288849161,0.992416167857506
"13238","AC011379.2",3.52557466782927,0.0732950390894713,0.819210142767017,0.089470375503293,0.928708096064959,0.992416167857506
"13239","PCDHB7",4.31952495505551,-0.0720590284812544,0.789260786737433,-0.0912993901282297,0.927254702573414,0.992416167857506
"13240","CNOT8",33.4798111430617,0.0240678506254521,0.268955435577686,0.0894863886047035,0.928695370503522,0.992416167857506
"13241","PSPH",6.82078239450251,0.0484550231055041,0.535629301863804,0.0904637273145763,0.927918717141028,0.992416167857506
"13242","RSBN1L",98.8773313639149,0.019662751057853,0.219165390443149,0.0897164968341727,0.92851250623107,0.992416167857506
"13243","REPIN1",31.732814604909,0.0263488657653244,0.289016666479928,0.0911672883305997,0.927359666811772,0.992416167857506
"13244","RPL7",2676.86793525512,-0.0164339255041225,0.181631163999841,-0.0904796574674646,0.927906058629903,0.992416167857506

"13245","EBLN3P",76.4065252227568,-0.0175075952796647,0.19346034324605
,-0.0904970754517783,0.927892217870362,0.992416167857506
"13246","SLC25A16",15.9022209686822,0.0357234536830906,0.3914276031599
68,0.0912645234896508,0.927282406465362,0.992416167857506
"13247","LINC01001",1.84915063760022,-0.0853411873741276,0.94891431874
9962,-0.0899356092408327,0.928338383716877,0.992416167857506
"13248","C11orf65",1.6255502515009,-0.0926625245683526,1.0301131112429
,-0.0899537376594974,0.928323977722097,0.992416167857506
"13249","DGKH",31.1062040782514,-0.0381596448041111,0.422531685369508,
-0.0903119129888213,0.928039353642061,0.992416167857506
"13250","MBOAT7",30.955391082007,-0.0285637269826134,0.318787998276503
,-0.0896010111329174,0.928604280872691,0.992416167857506
"13251","PDK3",13.4407208746398,-0.0434029993809837,0.480406133103335,
-0.0903464722663058,0.928011891593385,0.992416167857506
"13252","DGKQ",7.49812029860992,0.0442006040908156,0.49455031424236,0.
0893753432520409,0.928783618271844,0.992421976662339
"13253","LINC01719",3.48925870990161,-0.0659036072277761,0.74281065118
3557,-0.0887219470032754,0.929302890129823,0.992445290321551
"13254","CD1C",2.18642316249198,0.0901916920665832,1.02323126887194,0.
0881439952143122,0.929762229310758,0.992445290321551
"13255","TGFBRAP1",20.0989084822781,-0.0293919432623796,0.333298195260
624,-0.0881851257532208,0.929729539185999,0.992445290321551
"13256","TYW5",12.1352553695072,0.0440503793700887,0.499880440510354,0
.0881218303423022,0.929779845769766,0.992445290321551
"13257","HCG25",3.61514630857599,0.0731878793769178,0.824713933224775,
0.0887433526080254,0.929285878032509,0.992445290321551
"13258","DECR1",152.780532799191,-0.0272807175392325,0.309468652982206
,-0.0881534115857642,0.929754745266152,0.992445290321551
"13259","PHF19",31.5969438042654,-0.0235501389578339,0.264026028387383
,-0.0891962777369845,0.928925923523912,0.992445290321551
"13260","PRIM1",9.12381609071609,-0.0422573582889746,0.477227615149495
,-0.0885475964665983,0.929441456363941,0.992445290321551
"13261","TSC2",38.9086074895729,0.0261250291889018,0.29515822791331,0.
0885119461978033,0.929469789912777,0.992445290321551
"13262","HIRIP3",29.3114865490099,0.0242269562104334,0.273822452737504
,0.0884768797015278,0.929497659587475,0.992445290321551
"13263","DVL2",30.5572827127922,0.0265758449204646,0.301483870502543,0
.0881501384341571,0.929757346735956,0.992445290321551
"13264","TMC8",2.61004474126787,0.0888045273091616,0.998646997296877,0
.0889248428619286,0.92914164001242,0.992445290321551
"13265","CIRBP",743.81648281307,0.0148107092813957,0.166863175418988,0
.0887596034547855,0.929272962698986,0.992445290321551
"13266","CILP2",3.86764607599788,-0.0640138647836476,0.726495360860648
,-0.0881132464601194,0.929786668178369,0.992445290321551
"13267","THRB-
IT1",3.02982026768722,-0.0772388899490652,0.878106282555349,-0.0879607
531383271,0.92990786964728,0.992499844290757
"13268","STT3B",66.7488407914525,0.0204383576568451,0.232746703769364,
0.0878137362456403,0.930024719997597,0.992549746394782
"13269","MTMR3",33.641083876586,0.0222842284140055,0.254425575417605,0

.0875864322107911,0.930205386259926,0.992667742063498
"13270","NOC2L",38.5755841309537,-0.0229769607181668,0.265239306042049
,-0.086627284096891,0.930967777845941,0.992858630751707
"13271","OSCP1",10.8967628056061,0.0340068728426605,0.418333679148038,
0.0812912623050516,0.935210322663807,0.992858630751707
"13272","PARS2",2.69225423420017,0.0690106358492454,0.812862141764788,
0.0848983269160719,0.932342221914273,0.992858630751707
"13273","CD48",7.32707494328136,-0.0520441247399803,0.604581009921061,
-0.0860829630536619,0.931400466859813,0.992858630751707
"13274","CENPL",12.6122579004394,0.0374135118476721,0.457226366393498,
0.0818271092780186,0.934784198270297,0.992858630751707
"13275","FMOD",80.2523751664281,0.024402234497349,0.299066477321966,0.
0815946832819991,0.934969029324535,0.992858630751707
"13276","HNRNPU",357.922556846643,0.0117595529038228,0.143848898454228
,0.081749342749153,0.934846039798026,0.992858630751707
"13277","SMYD3",19.9292975876785,0.0332892261526931,0.389140886945652,
0.0855454342358592,0.931827776528649,0.992858630751707
"13278","FOXN2",28.38098755443,-0.0272395298358917,0.312701858924411,-
0.0871102267494876,0.930583896518272,0.992858630751707
"13279","PNPT1",32.7509497013268,-0.0246335124938294,0.30152779026627
,-0.0816956621878198,0.934888727903162,0.992858630751707
"13280","MTLN",98.9928455542398,0.0246905251978293,0.303167234281305,0
.0814419317323696,0.93509050314523,0.992858630751707
"13281","SLC25A12",27.6711191428686,-0.0230666626650845,0.274362535039
562,-0.0840736606467002,0.93299786624305,0.992858630751707
"13282","HOXD8",32.9163664939822,-0.0341932182617009,0.406122704361755
,-0.0841943035798442,0.932901947193639,0.992858630751707
"13283","CUL3",70.9820558724014,0.017624316823236,0.218176213150454,0.
0807801939943028,0.935616759488346,0.992858630751707
"13284","SP140L",20.0413825174502,0.0293746714949758,0.345510369044389
,0.0850182053181795,0.932246917359217,0.992858630751707
"13285","LMCD1-
AS1",3.12792276848067,0.0692314084462538,0.81226038410669,0.0852330235
48715,0.932076137100823,0.992858630751707
"13286","EPM2AIP1",49.4482049293508,-0.0192887206284847,0.235959497603
002,-0.0817458963272487,0.934848780472023,0.992858630751707
"13287","POMGNT2",24.5726486080599,0.0303663908819285,0.35374087852334
4,0.0858436011373352,0.931590745686033,0.992858630751707
"13288","TRIM59",1.99211843345472,0.0850375861904714,1.02291894495865,
0.0831322819951377,0.93374635735826,0.992858630751707
"13289","AC069224.1",1.76742978080489,-0.0797560027873326,0.9794770722
39267,-0.0814271258080554,0.935102277457689,0.992858630751707
"13290","USP13",14.9767770397482,-0.0310333704511285,0.357310857437208
,-0.0868525817371282,0.930788691313446,0.992858630751707
"13291","AC108519.1",4.93789797267671,-0.0640384077300786,0.7503890597
50205,-0.0853402736860212,0.931990874528714,0.992858630751707
"13292","ZNF827",33.6765717011543,0.0295014264909464,0.343681348303112
,0.0858394749572718,0.931594025793931,0.992858630751707
"13293","SNX25",5.42531065986972,0.0543226018428484,0.648969263041674,
0.083705970276993,0.933290210213075,0.992858630751707

"13294","CSNK1A1",229.667432048648,0.0122442112234523,0.144041880805638,0.0850045219832552,0.932257795701269,0.992858630751707
"13295","GUCA1B",2.72173748834231,0.0696137851448927,0.80065671703522,0.0869458578985862,0.930714548178513,0.992858630751707
"13296","CASP8AP2",42.6605853657346,0.0238314731495155,0.283819240626812,0.083967080938149,0.933082604915409,0.992858630751707
"13297","MTRES1",32.6550936872698,-0.0231040428390021,0.276791017786911,-0.0834710715099465,0.933476978691679,0.992858630751707
"13298","SERAC1",3.77104059802511,0.0713099453134821,0.832715042113257,0.0856354715684159,0.931756199787657,0.992858630751707
"13299","GSDME",9.48332792423454,-0.04633358806455,0.543357617855836,-0.0852727311478372,0.932044569945114,0.992858630751707
"13300","METTL27",9.88636417127921,-0.050838608364569,0.632230319113078,-0.0804115317276903,0.935909955613281,0.992858630751707
"13301","KRIT1",56.5989919489529,0.0190333332044138,0.227839292404059,0.0835384143076574,0.933423433923893,0.992858630751707
"13302","SLC7A2",22.8632445477155,-0.0368860117339979,0.456187831995736,-0.0808570705900431,0.935555620834114,0.992858630751707
"13303","AC021321.1",2.38557960422926,-0.0703891245050855,0.830023192520402,-0.0848038044471334,0.932417368927262,0.992858630751707
"13304","WWP1",43.0104781767847,0.0210281786137832,0.261518806528269,0.0804079021808708,0.935912842225533,0.992858630751707
"13305","FBXO32",21.6802980677956,-0.0403871886091467,0.471943655117396,-0.0855762932104689,0.9318032445888,0.992858630751707
"13306","OPLAH",4.42003555858437,-0.0513874671375501,0.616901772800973,-0.0832992696782684,0.933613580990245,0.992858630751707
"13307","TMEM8B",21.2135802639801,0.0268535046335364,0.312870645599411,0.0858294154828406,0.931602022580573,0.992858630751707
"13308","KLF9",465.74376391181,0.017668334182034,0.213633694681661,0.0827038740698739,0.934087004366274,0.992858630751707
"13309","RPL12",4922.0061106877,-0.0211928652122299,0.257491530980055,-0.0823050961387599,0.934404102039569,0.992858630751707
"13310","TOR1B",14.195953056593,-0.0302877594767684,0.35875945033069,-0.0844235864695698,0.932719654935415,0.992858630751707
"13311","NUP214",33.7667703048971,0.0273905776039421,0.321598617095468,0.085170072717729,0.932126182619079,0.992858630751707
"13312","EGFL7",14.1013049816821,0.034901448274366,0.413047690091962,0.0844973815652993,0.932660984606077,0.992858630751707
"13313","ZMIZ1",112.122947285584,0.0248567225521144,0.287039743686048,0.0865967974779887,0.930992011581344,0.992858630751707
"13314","KAZALD1",68.5245906617671,0.0255853599075459,0.308769440407907,0.0828623450356544,0.933960995342701,0.992858630751707
"13315","ADAM12",50.1496329573054,-0.0642717434023311,0.802251261743571,-0.0801142316219564,0.93614640393335,0.992858630751707
"13316","TIMM10B",28.8330079948741,-0.0258231162135558,0.30915021826676,-0.0835293481542153,0.933430642473522,0.992858630751707
"13317","AKIP1",29.8284471989354,-0.0225577912134572,0.264319569399625,-0.0853428721327555,0.931988808804421,0.992858630751707
"13318","MRVI1",1.93428567351507,-0.106501269908066,1.30029570911253,-0.0819054228678146,0.934721922106258,0.992858630751707

"13319","SVIP",3.7719985093842,-0.059429874364036,0.708232574640475,-0
.0839129355130337,0.933125654783604,0.992858630751707
"13320","CTSF",271.351005238504,-0.0161605468074566,0.196036890247055,
-0.0824362536412933,0.934299807908259,0.992858630751707
"13321","YBX3",848.210129427297,-0.0163342742766791,0.195785709628764,
-0.0834293488919649,0.933510152811812,0.992858630751707
"13322","TAS2R30",1.56976962424965,-0.087925390279458,1.08383472183351
,-0.0811243527340732,0.935343058832866,0.992858630751707
"13323","ARF3",93.6242395790599,-0.0151474637655135,0.18858333283662,-
0.0803223887162741,0.935980852120782,0.992858630751707
"13324","ACVRL1",162.444467347419,-0.0188742601035158,0.21834544765790
9,-0.0864421965558308,0.931114904443403,0.992858630751707
"13325","TARBP2",10.6962259758706,0.0343881528524731,0.417752724066567
,0.0823170044655255,0.93439463270179,0.992858630751707
"13326","LINC02384",23.2846463963515,-0.0481744432310944,0.57367814403
2411,-0.0839746881282141,0.933076556617144,0.992858630751707
"13327","THAP2",20.1854351430572,0.0319331206159266,0.378859795718106,
0.0842874355548844,0.9328279018277,0.992858630751707
"13328","NUDT15",25.4557784749776,-0.0251730034531493,0.30141612911809
4,-0.0835157810791427,0.933441429741469,0.992858630751707
"13329","TMX1",62.1238756050822,-0.017308276448965,0.212851141819915,-
0.0813163429661509,0.93519037722336,0.992858630751707
"13330","PSMA3-
AS1",69.6866181017204,-0.0236489238092501,0.289868117077394,-0.0815851
15492146,0.934976637949175,0.992858630751707
"13331","DDX24",253.52499837278,0.0103804046462559,0.129266287693947,0
.0803024890049658,0.935996678669491,0.992858630751707
"13332","AC078909.1",2.35162635262566,-0.0704969039421429,0.8489848278
26411,-0.0830367064658041,0.933822352844608,0.992858630751707
"13333","USP3",33.4637381552931,0.0214574024067601,0.264106634913599,0
.0812452228387968,0.935246935737244,0.992858630751707
"13334","DENND4A",22.6970609443939,0.0301954101471218,0.36098297884201
4,0.0836477394141537,0.933336509346422,0.992858630751707
"13335","AMDHD2",27.4746823114762,-0.0231777047478307,0.28927344532473
7,-0.0801238590075613,0.936138747005639,0.992858630751707
"13336","COG7",19.1623483882603,0.0272079254199137,0.339541618444755,0
.0801313416144317,0.936132795884226,0.992858630751707
"13337","SPNS1",47.6573460667159,-0.0215792580804654,0.254007738569021
,-0.084955120669305,0.932297070180808,0.992858630751707
"13338","GOT2",18.7586957335067,-0.029711084051432,0.365744513772928,-
0.0812345310253324,0.935255438465135,0.992858630751707
"13339","OXLD1",34.5550149947283,0.0234204885719074,0.289846751263241,
0.0808030052772153,0.935598618021858,0.992858630751707
"13340","AC008760.2",18.4683728185428,0.0398426728165879,0.47434030841
5059,0.0839959668401713,0.933059638433242,0.992858630751707
"13341","FBXL12",33.7531307108502,-0.0206470680238571,0.25467598409342
7,-0.081071908281241,0.935384766074,0.992858630751707
"13342","GATAD2A",37.0177804873235,-0.0222773050206106,0.2641263057953
15,-0.0843433786480716,0.932783424076804,0.992858630751707
"13343","LINC00662",30.7238322927964,0.0274649729333529,0.340546146366

867,0.0806497833740426,0.935720473713498,0.992858630751707
"13344","ATF5",41.1857363411582,-0.0410540703974274,0.492120923025338,
-0.0834227290013305,0.933515416371501,0.992858630751707
"13345","ZNF580",50.0214991177653,0.0255499524651167,0.311276994421203
,0.0820810818757261,0.934582236830729,0.992858630751707
"13346","SLC27A5",12.1647164515169,0.0353816577590583,0.44134866186135
6,0.0801671350035111,0.936104328474606,0.992858630751707
"13347","SNX5",107.551322572009,0.0171773265454571,0.204646116323608,0
.0839367335869425,0.933106733420483,0.992858630751707
"13348","DNTTIP1",37.5648478450208,0.0213820380385702,0.26024995425786
5,0.0821596226579316,0.934519781300984,0.992858630751707
"13349","SS18L1",10.323229825252,0.0370366159564641,0.440504712864033,
0.0840776837906291,0.932994667563907,0.992858630751707
"13350","MTG2",23.9872479571256,0.0236302263090453,0.295223457275056,0
.0800418317946509,0.936203985720113,0.992858630751707
"13351","AP000692.2",11.9568392686052,-0.0449728184041869,0.5439754829
51057,-0.0826743480426916,0.934110482325001,0.992858630751707
"13352","GPKOW",18.460962545945,-0.0262150597402109,0.315100192064603,
-0.0831959497340966,0.933695733220488,0.992858630751707
"13353","SLC16A12",8.22236098558318,-0.068813188361146,0.8627221990193
39,-0.0797628581243954,0.936425865090441,0.992945203660375
"13354","DTWD1",140.423305602412,-0.0131435241903347,0.164748186088403
,-0.079779477409736,0.936412646942674,0.992945203660375
"13355","EIF1B-
AS1",7.20490585889896,0.0409449590030372,0.513966605633964,0.079664629
0910919,0.93650399190491,0.992953689657321
"13356","AL118558.3",3.44549680763149,-0.0546027705230398,0.6908839507
20674,-0.0790332015472101,0.937006214633801,0.993411799881299
"13357","ARID1A",77.7843852849666,-0.0150669550383938,0.19360481398710
7,-0.0778232458589443,0.93796865500308,0.99367026651782
"13358","ACP6",2.24880677118216,-0.0855772953631245,1.10525721385853,-
0.0774274931573335,0.938283470051077,0.99367026651782
"13359","DARS2",6.19577445867697,-0.0456228502352136,0.589394549954399
,-0.077406298104968,0.938300330654378,0.99367026651782
"13360","CCDC85A",6.174005283696,0.0594333220312609,0.761178254311158,
0.0780806883205645,0.93776386877706,0.99367026651782
"13361","HPSE",2.78461429562567,0.0664062159076804,0.847125612514918,0
.0783900462064131,0.937517791143341,0.99367026651782
"13362","HIST1H4C",262.303709159593,0.026255238554685,0.33903914722453
4,0.0774401386082331,0.938273410644372,0.99367026651782
"13363","PRRC2A",67.5051124879396,0.0163670737791897,0.210557040944488
,0.0777322558569994,0.938041035261575,0.99367026651782
"13364","MRPS18A",49.5264138212372,0.0232904275177479,0.29881606240454
7,0.077942354672409,0.937873907711784,0.99367026651782
"13365","PRDX3",140.852759983275,-0.0163359867342445,0.208275006593152
,-0.0784346955568963,0.937482275468009,0.99367026651782
"13366","METTL25",13.6586460820263,0.0326698594097001,0.42207257254441
6,0.0774034171724393,0.938302622430069,0.99367026651782
"13367","HECTD1",97.3348755643749,0.0143417432377681,0.18481010957092,
0.0776025904159997,0.938144181743735,0.99367026651782

"13368","GOLGA8O",2.13561971465462,0.0827018486405797,1.06123800041388
,0.077929596007989,0.937884056783476,0.99367026651782
"13369","ATP6V0C",264.841715353802,-0.0181136806613557,0.2323611839393
3,-0.0779548475105257,0.937863970105407,0.99367026651782
"13370","TMEM259",89.2994356274363,0.0148620845795583,0.19094336325345
8,0.0778350413773242,0.937959272002336,0.99367026651782
"13371","RBCK1",121.370411277113,-0.0173506702070136,0.221681919001996
,-0.0782683147327741,0.937614621301756,0.99367026651782
"13372","STK39",14.54397512996,-0.033764804770836,0.441224908457271,-0
.0765251555921755,0.939001301382628,0.993900618230249
"13373","TNFSF10",231.595778452084,0.0188890466287052,0.24615759391577
8,0.07673558360815,0.938833896359136,0.993900618230249
"13374","PRKRIP1",31.5676785082862,0.0194149034466024,0.25323431481751
3,0.0766677433135129,0.938887866093201,0.993900618230249
"13375","CDC37L1",40.3029712125516,-0.022335022370284,0.29195965343589
8,-0.0765003729365906,0.939021017285899,0.993900618230249
"13376","MSANTD2",16.7743518993469,-0.0267306643453633,0.3497672890419
22,-0.0764241402293039,0.939081664640007,0.993900618230249
"13377","ATN1",92.552837855499,-0.0155633493128133,0.203121147568142,-
0.0766210190280272,0.938925037340041,0.993900618230249
"13378","CAPN15",16.4600333263788,-0.0283268766790437,0.36885552715162
5,-0.076796671308654,0.938785298830773,0.993900618230249
"13379","CORO1A",7.5514623543457,-0.0406587676176307,0.53063978563072,
-0.0766221619988474,0.93892412805434,0.993900618230249
"13380","COL13A1",2.64583545678564,0.105775494151874,1.38637162969465,
0.0762966378467882,0.9391831006588,0.993933685002138
"13381","AL096803.2",5.17367842594721,0.0469877765716392,0.61773099722
0556,0.0760651105142171,0.939367297470756,0.994054325699567
"13382","LINC00954",2.16341854030174,0.0665233539111078,0.901997436928
934,0.0737511562533952,0.941208392784236,0.994093969992099
"13383","TSGA10",5.45392971001841,0.0427804578368824,0.568806813996377
,0.075210874385121,0.940046932353967,0.994093969992099
"13384","TMEM182",2.43850887611772,0.0623720302323715,0.83647975105342
,0.0745649014860471,0.940560900907245,0.994093969992099
"13385","PRPF40A",150.060067425556,-0.0138321881897697,0.1840524175497
05,-0.075153526228658,0.940092560426977,0.994093969992099
"13386","GPR155",8.86802589173331,-0.0398992645134817,0.52968322087088
5,-0.0753266536324878,0.939954815189698,0.994093969992099
"13387","SLMAP",40.8109347690849,-0.0188543254946371,0.254620249067312
,-0.0740488062662004,0.940971550042791,0.994093969992099
"13388","ISY1",48.7204189523116,0.0167481124626863,0.22351231609891,0.
0749314970870547,0.940269215942778,0.994093969992099
"13389","C6",12.4909359183737,-0.0478988903075189,0.648732187502574,-0
.0738346134664836,0.941141984617989,0.994093969992099
"13390","NLN",15.6385111473469,-0.0270135256608898,0.363931861386931,-
0.0742268774103544,0.940829859756525,0.994093969992099
"13391","ATAT1",8.51336555713638,-0.0348049736193454,0.465730319458289
,-0.0747320330354024,0.940427920251465,0.994093969992099
"13392","PHF14",114.290086626704,-0.0130252949356776,0.172448695822078
,-0.0755314203658364,0.939791899093511,0.994093969992099

"13393","SLC12A9",15.6933750054964,-0.0255770877813352,0.343450491651375,-0.0744709598706809,0.940635647651946,0.994093969992099
"13394","RINT1",12.6592934399337,-0.0317157303079512,0.429590497068226,-0.0738278209699648,0.941147389494874,0.994093969992099
"13395","MINCR",10.373710530194,-0.0319532801832984,0.429448491258571,-0.0744053846589469,0.940687824343909,0.994093969992099
"13396","NOL6",14.5633611416248,0.027176322728939,0.36218034057949,0.0750353337385922,0.940186598866258,0.994093969992099
"13397","GPSM1",14.1033798718287,-0.0276678461525015,0.365156768113491,-0.075769774980324,0.939602263048962,0.994093969992099
"13398","ZDHHC5",32.6615125880421,-0.0188097862370544,0.254873466686318,-0.0738004880680823,0.941169138664013,0.994093969992099
"13399","CCS",87.5018563132054,0.0158991847229614,0.209661352080546,0.0758326919348179,0.939552206596995,0.994093969992099
"13400","CD3G",2.64365857093127,-0.0629925522502745,0.836347451615209,-0.0753186395541938,0.939961191385681,0.994093969992099
"13401","ZFC3H1",57.4773610414228,-0.0156744974778936,0.213048663255365,-0.0735723812503146,0.941350648132097,0.994093969992099
"13402","AL139089.1",1.90865750426971,-0.0748511944209175,1.00996279133746,-0.0741128238217513,0.940920611390376,0.994093969992099
"13403","CGNL1",8.01607324869934,-0.0497842827620143,0.676333726872983,-0.0736090494735358,0.941321470237789,0.994093969992099
"13404","NPIPB2",4.5361993204951,-0.0463575260741852,0.629913873577232,-0.0735934355770329,0.941333894626782,0.994093969992099
"13405","TNRC6C",86.3689695286421,-0.0195028247091343,0.265173348587155,-0.0735474541957003,0.941370483306783,0.994093969992099
"13406","LINC00526",6.09086628886315,0.0395180168837311,0.525178688328223,0.0752468022065537,0.94001834710187,0.994093969992099
"13407","CTDP1",9.27036191823505,0.0349518449239779,0.461580314837617,0.0757221306898106,0.939640168795496,0.994093969992099
"13408","LIN7B",6.8101899170909,0.042013957633317,0.564599742592078,0.0744137031314093,0.940681205512057,0.994093969992099
"13409","CPXM1",182.348768314649,-0.031895910489786,0.427698444845964,-0.0745756990097855,0.940552309649827,0.994093969992099
"13410","ITGB3",14.4682991725239,-0.0296533234430636,0.404488175026053,-0.073310730137299,0.941558853263026,0.994218744385119
"13411","ZNF684",11.2585353202512,-0.0303394953851633,0.418190732414182,-0.0725494207153177,0.942164677011579,0.99428549256264
"13412","CYS1",48.5240276976468,-0.0233245984510882,0.321526591651493,-0.072543295194601,0.942169551625609,0.99428549256264
"13413","CCDC88A",99.789197718431,0.0157832438568028,0.215865027791887,0.0731162616670787,0.941713601354864,0.99428549256264
"13414","KLHL23",5.34367399955029,0.0502704500014745,0.697979338222356,0.0720228339846069,0.942583736146095,0.99428549256264
"13415","PCDHB13",3.61409053390645,0.0493717485838745,0.683292460303091,0.072255661304933,0.942398449572332,0.99428549256264
"13416","TBRG4",24.7458967710787,0.0259125068314923,0.357298924783109,0.0725233272034668,0.942185441922348,0.99428549256264
"13417","MRPS33",76.3552321968107,-0.0170698451606418,0.235044733400112,-0.072623814682902,0.942105475380336,0.99428549256264

"13418","SLC20A2",19.2508139747234,-0.0235578747240369,0.3254581308553
48,-0.0723837338527189,0.94229652934113,0.99428549256264
"13419","NUDT5",64.6165095464916,-0.0164139435521681,0.22798420566684,
-0.0719959678968038,0.942605116678028,0.99428549256264
"13420","TRPV4",2.60723336879794,0.062585149165639,0.864957579204749,0
.0723563220559099,0.942318343590521,0.99428549256264
"13421","BORA",2.63162432334271,0.0570723762194114,0.785289443339458,0
.0726768667317238,0.942063257531233,0.99428549256264
"13422","AL135999.1",3.04173791429651,0.0620127983495119,0.85670470062
6891,0.072385266830139,0.942295309402249,0.99428549256264
"13423","LINC01579",1.76194083833353,-0.106135040090749,1.473370258591
56,-0.0720355521443784,0.94257361481263,0.99428549256264
"13424","ZNF470",5.98098999882072,0.0424473101633922,0.588437831417809
,0.0721355900267624,0.942494003243728,0.99428549256264
"13425","C1orf52",55.9377806180918,0.0151551088521329,0.21321085368779
5,0.071080381650385,0.943333781846478,0.994473958143831
"13426","BCL10",48.0072155065657,0.0170610109452767,0.252255745062746,
0.0676337854705071,0.946077160049389,0.994473958143831
"13427","LAMTOR2",134.254304716633,0.0178009483599214,0.26841271756922
5,0.0663193179560521,0.94712360357674,0.994473958143831
"13428","TIPRL",83.3480604575653,-0.0150605256217228,0.223923021025391
,-0.0672576028706536,0.946376628431798,0.994473958143831
"13429","DHX9",68.4977482511622,-0.0128028771127862,0.190595628336508,
-0.0671729841052912,0.946443992095211,0.994473958143831
"13430","LBR",30.1653941410844,0.0187963255853475,0.270808438340015,0.
0694081975457046,0.944664703959907,0.994473958143831
"13431","CAD",17.2358123377916,-0.0241703482441664,0.350244842616598,-
0.0690098619685456,0.944981769500318,0.994473958143831
"13432","AC233266.2",2.62503598472165,-0.0649135154888017,0.9311497965
61444,-0.0697132896645789,0.9444218638956,0.994473958143831
"13433","DUSP19",2.83281243799341,-0.0534374064553297,0.79814232760085
6,-0.066952227199825,0.946619734984323,0.994473958143831
"13434","CASP8",13.891381822086,-0.0246840068353437,0.369799796898225,
-0.0667496495195132,0.946781007816595,0.994473958143831
"13435","USP37",17.1068459143205,-0.0261569590683296,0.369976410590955
,-0.0706989913939369,0.943637323581517,0.994473958143831
"13436","CRELD1",29.4820242467844,-0.0237273580983193,0.35007538895748
2,-0.0677778525619265,0.945962474307192,0.994473958143831
"13437","MRPS25",52.9677751964209,-0.0155350048912655,0.23301573859941
3,-0.0666693373788471,0.946844945209564,0.994473958143831
"13438","KPNA1",29.0608026048222,-0.0196637948101758,0.293686365747173
,-0.0669550823721378,0.946617461986817,0.994473958143831
"13439","RUVBL1",30.1311196590193,-0.0194367992389737,0.27754559783938
9,-0.0700310125265306,0.94416897579926,0.994473958143831
"13440","KCNMB3",2.49747168982951,0.058044964448599,0.856125530952863,
0.0677995952112247,0.945945165997263,0.994473958143831
"13441","IL1RAP",9.41134049895377,0.0325991735398429,0.455977482404635
,0.0714929460286689,0.943005438447689,0.994473958143831
"13442","PCYT1A",33.7722451561996,-0.0169968043920841,0.25627998512559
3,-0.0663212321623735,0.947122079616236,0.994473958143831

"13443","RARS",55.9661298932731,-0.0148527434361465,0.214226801495305,
-0.0693318638586499,0.944725463061734,0.994473958143831
"13444","ATP6V0E1",515.808894026185,-0.0180330806535419,0.252205448021
426,-0.0715015507992117,0.942998590359733,0.994473958143831
"13445","BRPF3",14.2649645506811,-0.0320504772512567,0.463070304032473
,-0.0692129833680915,0.944820088658643,0.994473958143831
"13446","CHMP7",21.3653995834962,-0.022280208754349,0.313109066285196,
-0.0711579802485024,0.943272023505702,0.994473958143831
"13447","TM2D2",70.9094512754215,0.0189006292746071,0.268115109799555,
0.0704944577302538,0.943800111668219,0.994473958143831
"13448","HEY1",2.12292588531987,0.0650609441912109,0.945431577505419,0
.0688161319541265,0.945135977086469,0.994473958143831
"13449","PLPP6",5.15563640018215,0.0406164978549155,0.582955346904423,
0.069673428797926,0.94445359111987,0.994473958143831
"13450","WNK2",1.62283235468658,-0.0833567691782465,1.18004815497008,-
0.0706384471067286,0.943685510458898,0.994473958143831
"13451","AL596244.1",2.19201617924223,-0.0670345407367111,0.9469047351
08831,-0.0707933314210396,0.943562239264989,0.994473958143831
"13452","RABEPK",26.8445562838606,0.0233384883373183,0.33136645033237,
0.0704310539401593,0.943850575142223,0.994473958143831
"13453","WDR37",10.7757456660142,0.0284011982030806,0.417404813723836,
0.0680423350888124,0.945751933826444,0.994473958143831
"13454","PPP3CB",73.0302668648615,0.0131154913611675,0.183685297143823
,0.0714019661078168,0.943077844877842,0.994473958143831
"13455","FAS",44.5091472179896,-0.0166824077576803,0.237587226089923,-
0.0702159288284558,0.944021796245429,0.994473958143831
"13456","DGKZ",25.4752069789207,0.0221257530337954,0.329204392839037,0
.0672097745810265,0.946414703731132,0.994473958143831
"13457","LRP5",28.3655954522163,-0.0223921715035599,0.333576963476417,
-0.0671274516986931,0.946480239883863,0.994473958143831
"13458","TAF1D",148.992238910103,-0.0119098848716558,0.177576643192049
,-0.0670689830462403,0.946526786221137,0.994473958143831
"13459","RAD51AP1",1.97483317845785,0.0674659004873222,1.0160132234857
9,0.0664025811158805,0.947057315307192,0.994473958143831
"13460","CLSTN3",89.8259307419694,-0.0211731282793982,0.31454287736371
5,-0.0673139651320577,0.946331759537858,0.994473958143831
"13461","AC008147.2",11.9136103217498,-0.0353649765724853,0.5264087974
98845,-0.0671815834775498,0.946437146253208,0.994473958143831
"13462","AC079866.2",6.32645987929146,0.0434906724952453,0.63349303553
407,0.0686521714616487,0.945266489970972,0.994473958143831
"13463","RITA1",10.8925562309199,0.0279888672431368,0.410246386111238,
0.0682245308933632,0.945606899634435,0.994473958143831
"13464","FBXO21",46.9761783390155,0.0167817337089896,0.241080109623845
,0.0696106109092699,0.944503591145891,0.994473958143831
"13465","RNF10",72.2197041228112,-0.0127059017226471,0.190123049700103
,-0.0668298859222441,0.946717131061714,0.994473958143831
"13466","ZNF664",24.7885877550917,-0.0232084778583541,0.32818289136483
2,-0.070718122330618,0.943622097413259,0.994473958143831
"13467","CUL4A",36.804036163464,0.0215579388439211,0.311695115869965,0
.0691635439450221,0.944859441307312,0.994473958143831

"13468","VCPKMT",9.07282860991012,0.0346023091599925,0.500202342083708
,0.0691766236356444,0.944849030159989,0.994473958143831
"13469","EDC3",11.0647641573482,-0.0266039582416314,0.400328159026374,
-0.066455375775549,0.947015284103579,0.994473958143831
"13470","PTPN9",31.5233452940759,-0.0182555914940874,0.275343792493859
,-0.0663010824712692,0.947138121435573,0.994473958143831
"13471","KCTD13",13.3335574617316,0.025336112726511,0.372545237296382,
0.0680081509305531,0.945779145804836,0.994473958143831
"13472","ENKD1",19.5593776465239,-0.0229972372042213,0.331615768242514
,-0.0693490461147287,0.944711786523515,0.994473958143831
"13473","ZNF23",12.6395802295857,0.0299932628482854,0.419244497678602,
0.0715412200144808,0.942967019755374,0.994473958143831
"13474","SERPINF1",1927.87839698007,0.0194728887807379,0.2808775866328
14,0.0693287385945622,0.944727950675912,0.994473958143831
"13475","PAFAH1B1",122.032729261277,-0.0127667417367365,0.191943893540
465,-0.0665128830162292,0.946969501259284,0.994473958143831
"13476","PHF23",17.8545090329992,-0.0246669632712757,0.358337089121406
,-0.0688373155336943,0.945119115021521,0.994473958143831
"13477","ARL17A",11.558830539475,-0.0386630627087558,0.55015622365334,
-0.0702765160993941,0.943973573722056,0.994473958143831
"13478","SLC39A11",12.6040571468031,0.0282029167990473,0.3988052450892
82,0.0707185202459748,0.943621780715645,0.994473958143831
"13479","NPB",10.379464291687,0.0431349276319155,0.650067028263566,0.0
663545846143525,0.947095526700014,0.994473958143831
"13480","MYO9B",34.8549965759704,-0.023688118939971,0.338794674517508,
-0.0699188054644195,0.944258285162702,0.994473958143831
"13481","FAAP24",8.46231055784615,-0.0342597791820884,0.51140855095877
4,-0.0669910174905351,0.946588854141321,0.994473958143831
"13482","FTL",7734.31410907052,-0.0190693145235552,0.279158672375175,-
0.0683099484651762,0.945538904899306,0.994473958143831
"13483","C19orf18",2.06652014435584,0.0674296640886774,1.0084567770275
9,0.0668642083872207,0.94668980681465,0.994473958143831
"13484","SPATA2",7.66312855849954,-0.0336665925955688,0.50609182736892
3,-0.0665226956352864,0.946961689223798,0.994473958143831
"13485","USP27X",8.39225735727228,0.0341704060957359,0.51141678966292,
0.0668151824234357,0.946728836593139,0.994473958143831
"13486","SHROOM4",2.72640808764704,-0.0564694064842994,0.8397907674007
98,-0.0672422330374928,0.946388864084888,0.994473958143831
"13487","ATP8B2",40.4098435865277,-0.018622237137354,0.287671149102803
,-0.0647344622338166,0.948385423462531,0.994560994800749
"13488","GFPT1",58.3315701437018,-0.0149497389957998,0.22988300247207,
-0.0650319459683243,0.948148564876849,0.994560994800749
"13489","AC005041.4",2.62594660402331,0.0600457379351753,0.91757127600
0378,0.0654398622817728,0.947823786569568,0.994560994800749
"13490","EIF2AK3-
DT",2.29068910650864,0.0632776585309595,0.969888774956975,0.0652421805
106123,0.947981177457409,0.994560994800749
"13491","TRAK1",17.2445610100792,0.0217365558558101,0.330736829605093,
0.0657216067583523,0.947599469895627,0.994560994800749
"13492","HPS3",18.7940865364639,0.0227428162124362,0.344163436670845,0

.0660814420974861,0.947312985613764,0.994560994800749
"13493","ZNRD1",59.7395245138257,-0.0144839430743374,0.223983004935357
,-0.0646653663679417,0.948440438717428,0.994560994800749
"13494","BYSL",8.90300239192995,-0.0368868168783867,0.574954221348422,
-0.0641560936658177,0.948845937511314,0.994560994800749
"13495","SNX16",27.7611072234009,-0.0235141589455217,0.36229185721009,
-0.0649039123501086,0.948250505559604,0.994560994800749
"13496","NSMF",16.0719379194604,0.0218752072996269,0.332205452750121,0
.0658484293937253,0.947498498794256,0.994560994800749
"13497","NCAPD3",10.1817099275645,0.0335963852110787,0.515280281065669
,0.0652002151947225,0.948014589794639,0.994560994800749
"13498","EIF2B1",60.72516722207,-0.0133806240792257,0.208072961035428,
-0.0643073660923557,0.948725488303832,0.994560994800749
"13499","UCHL3",44.0227641723232,-0.02340202561922 8,0.358063957115188,
-0.0653571105222958,0.947889671875582,0.994560994800749
"13500","FBXL3",94.3089664300629,0.0140350499285811,0.214164271802758,
0.0655340398771425,0.947748804674237,0.994560994800749
"13501","ERO1A",34.8639483678552,-0.0164917718248794,0.256310295389608
,-0.0643429941033419,0.948697120014831,0.994560994800749
"13502","INPP5K",16.5131653266223,-0.0235019381233139,0.36334645267657
6,-0.064681897814573,0.948427276087723,0.994560994800749
"13503","RETREG3",36.4352242048245,0.0178722674942078,0.27102746080385
4,0.0659426444877562,0.947423489056735,0.994560994800749
"13504","CEP76",5.66845167443957,0.0420122430610992,0.656468289202106,
0.0639973685738306,0.948972322088352,0.994560994800749
"13505","PIAS2",33.5938293385041,-0.0185969646718473,0.290044417266971
,-0.0641176439356519,0.948876552923747,0.994560994800749
"13506","C19orf25",38.3111478390232,-0.0163832143901849,0.256021497861
374,-0.0639915574552875,0.948976949205715,0.994560994800749
"13507","BST2",183.637462886476,0.0257450331986866,0.401563637452469,0
.0641119633291845,0.948881076085684,0.994560994800749
"13508","ZNF549",2.80874202288548,-0.049429672237492,0.762534129642284
,-0.064822898170709,0.948315009686851,0.994560994800749
"13509","ZNF552",5.59003892214211,0.0455465406007472,0.699834381632494
,0.0650818847946588,0.948108803690098,0.994560994800749
"13510","ZNF135",6.1468365507516,-0.0374432836630888,0.56899404613951,
-0.0658061080201675,0.947532193282693,0.994560994800749
"13511","SOX12",7.9361590309393,0.030548857908453,0.476073506258991,0.
064168363722878 5,0.948836167553377,0.994560994800749
"13512","ZBTB9",1.94884708335468,-0.059234693151832,0.927012609659517,
-0.0638984761745456,0.949051065637732,0.99456505990455
"13513","EXTL2",52.9761881919606,0.0203267010230233,0.319320852573088,
0.0636560401841304,0.949244108566702,0.994693745082846
"13514","HNRNPK",456.055157208687,0.0081004716772228,0.127513314595495
,0.0635264772382364,0.949347276033,0.994728239501796
"13515","TMEM138",26.2920036456425,-0.0170876395793462,0.2707537536604
47,-0.0631113672417478,0.949677822595416,0.994853737364141
"13516","BIN2",2.6511985402935,-0.0599762588958666,0.948945222716635,-
0.0632030779649924,0.949604793820503,0.994853737364141
"13517","MGRN1",29.5209092495383,0.0199199267463921,0.315554963824704,

0.0631266467969679,0.949665655535205,0.994853737364141
"13518","PAOX",9.14691169783399,0.0344119743522979,0.547855942571609,0
.0628120855836111,0.949916141970108,0.994956177993693
"13519","APBA2",1.73153687443351,-0.0717883043005868,1.14240243778283,
-0.0628397681292709,0.949894098042043,0.994956177993693
"13520","AFG1L",3.99117420463396,0.0401865731815056,0.642123451983742,
0.0625838739534513,0.950097870963084,0.995072918109265
"13521","ATP1B1",22.3492548268046,-0.0238424230331362,0.38659342407689
,-0.0616731210316557,0.950823145519615,0.995124028377365
"13522","CCSAP",5.1477831671314,0.042836843104616,0.69099293399873,0.0
619931709818247,0.950568270309821,0.995124028377365
"13523","C1D",89.7188501565428,0.0144895593435025,0.234191350006886,0.
0618706000160827,0.95066588041668,0.995124028377365
"13524","ADO",17.3728181256299,-0.0227649000341468,0.369849138878462,-
0.0615518535562353,0.950919719489698,0.995124028377365
"13525","FRA10AC1",77.2414358070202,0.0117803665950073,0.1896590438444
83,0.0621133923076557,0.950472532072969,0.995124028377365
"13526","PCNX3",14.2906750799774,-0.0215165950985825,0.347515044236659
,-0.0619155787797481,0.950630061228595,0.995124028377365
"13527","RDX",185.550207127328,-0.00841184873963586,0.13639765970524,-
0.0616715034393857,0.950824433719365,0.995124028377365
"13528","SIDT2",42.1965470671914,-0.0156358324494114,0.25309848475332,
-0.0617776612319538,0.950739893255571,0.995124028377365
"13529","SENP1",14.0201157760822,-0.0221774789753406,0.356948036546565
,-0.0621308333557607,0.950458642955976,0.995124028377365
"13530","TTC5",31.5425084904126,-0.0168204564735152,0.27310932925061,-
0.0615887290253656,0.950890352838026,0.995124028377365
"13531","GNA11",92.3708438268553,0.021765489685156,0.35314550572936,0.
0616332059506272,0.950854932675784,0.995124028377365
"13532","HIPK3",101.087820057925,0.0129576379798748,0.211745485095562,
0.0611944003152034,0.95120438927799,0.995348370689946
"13533","GSS",29.1194087687758,0.0181637423290438,0.297780347162959,0.
060997115834188,0.951361506011256,0.995365666109014
"13534","GNL3L",30.1224428375315,-0.0188823962804043,0.309203751569737
,-0.0610678110616186,0.951305204339273,0.995365666109014
"13535","CYB561D2",28.8207612477156,0.0169284205306409,0.2786126741139
36,0.0607596929482045,0.951550591348906,0.995489942630256
"13536","REL",36.6753105960538,-0.0200886122469956,0.332535142214577,-
0.0604104941005992,0.951828700840102,0.995539039646478
"13537","LY86",1.54265825504633,-0.0664119952288256,1.09853959922783,-
0.0604548031545761,0.951793411828492,0.995539039646478
"13538","TCEA1",206.424634292738,-0.0111613070419867,0.184654721654017
,-0.0604442006248794,0.951801855981073,0.995539039646478
"13539","TSPAN32",1.62452404367292,-0.0675197724775856,1.1188466439762
,-0.0603476560805788,0.951878747017915,0.995539039646478
"13540","EIF2AK4",76.0171400706736,-0.0121067758017706,0.2021017041549
71,-0.0599043726642064,0.952231797269284,0.99576118819386
"13541","ZNF548",10.1122968730144,0.0252891232671428,0.422009530273402
,0.0599254790543688,0.952214987006261,0.99576118819386
"13542","AC093772.1",2.50331641269642,-0.0586482457012927,0.9829185184

55873,-0.0596674542193251,0.952420493303307,0.995884964198407
"13543","MIR3936HG",3.09045889374267,-0.0507227490504004,0.85523132891
4939,-0.0593088060919775,0.952706147216509,0.995942535180697
"13544","EMX2",60.461168573838,0.015067024846546,0.253748375076109,0.0
593778180531277,0.952651180509635,0.995942535180697
"13545","FGF14-
AS2",13.6752814255914,-0.025130845007895,0.424184392302945,-0.05924509
59156155,0.952756891352946,0.995942535180697
"13546","EID2B",13.9753012918514,-0.0237643915532637,0.399661988602181
,-0.0594612253128694,0.952584748523451,0.995942535180697
"13547","NADK",37.6620915890617,0.0155929707080202,0.266804460692342,0
.0584434408163843,0.953395413117083,0.996095259332833
"13548","CRYZ",23.9108702490702,-0.018388793416937,0.314383338994771,-
0.0584916283278067,0.953357030706024,0.996095259332833
"13549","CCNB1",6.21314296313731,0.0344645411809406,0.586633008244792,
0.0587497476217008,0.953151434864042,0.996095259332833
"13550","UQCC3",68.3243707300566,-0.0185853154754867,0.317009261482669
,-0.0586270425935261,0.953249170849095,0.996095259332833
"13551","ATG2A",13.8390271054601,-0.0230535522228038,0.392546952345961
,-0.0587281396149681,0.953168645842173,0.996095259332833
"13552","AC027279.1",9.3640232370313,0.0299059727712921,0.510797570212
335,0.0585476018589132,0.953312446695124,0.996095259332833
"13553","TMEM104",6.45447070145208,-0.0304507014278956,0.5206655436412
57,-0.0584841877857706,0.953362957254092,0.996095259332833
"13554","DPAGT1",20.6088559834983,0.0205421822502271,0.352870500289296
,0.0582145071163099,0.953577765311794,0.996212273632507
"13555","YY2",3.5625210619273,-0.0429140711258421,0.738841040805267,-0
.0580829552715017,0.953682551192862,0.996248242337951
"13556","CDKN2B",10.0512747998931,-0.0406588500958061,0.70320735663877
1,-0.0578191478117486,0.953892685944674,0.996320751860779
"13557","HNRNPH3",168.910146995321,0.010440359772468,0.180294835808548
,0.0579071481756384,0.953822589258178,0.996320751860779
"13558","GNL2",32.9389071965344,-0.0152937259932922,0.268286187952911,
-0.0570052678074376,0.954540998863572,0.996348558702178
"13559","PRKCE",12.0097098807355,0.0258168497557414,0.453104934257589,
0.0569776398441615,0.95456300701835,0.996348558702178
"13560","RPL31",1286.74602532487,0.0101015724712077,0.180203181599699,
0.056056571152263,0.955296740704981,0.996348558702178
"13561","NDUFB3",131.553035315884,-0.0157666870545992,0.28209698156153
6,-0.0558910165125603,0.955428627429028,0.996348558702178
"13562","NR2C2",26.3325884751818,-0.0176577601706834,0.315247784025365
,-0.0560123213086968,0.955331991587111,0.996348558702178
"13563","RFC4",15.5190383763412,-0.0223344846870298,0.39739874110928,-
0.0562016996447609,0.955181127184135,0.996348558702178
"13564","BBS7",27.346594290801,0.0151394126162855,0.264876378029131,0.
057156522332922,0.954420511660449,0.996348558702178
"13565","AL355312.3",3.60015144219338,-0.0465314387278466,0.8231287224
95535,-0.0565299660383301,0.954919624239607,0.996348558702178
"13566","AP4M1",24.4082305758103,0.0170076116001853,0.297568393725016,
0.0571553026424645,0.954421483244353,0.996348558702178

"13567","TRIM24",17.6223268483279,-0.0202590278329602,0.35535648539908
3,-0.0570104350570899,0.954536882688858,0.996348558702178
"13568","ZFP41",4.83720260595648,0.0358889993281258,0.626809379575064,
0.057256640531538,0.954340759500555,0.996348558702178
"13569","CD72",2.15645897003602,0.0565243402932228,1.0054457659618,0.0
562181891920865,0.955167991197265,0.996348558702178
"13570","PTPRE",23.3220313577796,-0.0343524624126984,0.598580328928044
,-0.0573898953114913,0.954234612110283,0.996348558702178
"13571","AL355075.4",14.3128448149377,0.0410486024490576,0.72325251807
7054,0.0567555610565941,0.954739913980719,0.996348558702178
"13572","AL136295.2",2.40321489747257,-0.0518914664067601,0.9061179398
26435,-0.0572678943060103,0.954331794996865,0.996348558702178
"13573","ETFA",83.2487457774896,-0.0117105540911303,0.20646506964852,-
0.0567192993520213,0.954768800103328,0.996348558702178
"13574","LCAT",17.1872421131961,0.0203458396656134,0.360972558787238,0
.0563639511379186,0.955051874106265,0.996348558702178
"13575","MIER2",8.70572688494232,0.0281471575597406,0.504045783196932,
0.0558424621295631,0.955467307811926,0.996348558702178
"13576","ZNF790-
AS1",7.06728085641515,0.0294298298001603,0.518049980260831,0.056808861
9274588,0.954697454543261,0.996348558702178
"13577","PPP5C",23.2200479666164,0.0168010780940333,0.297570591529528,
0.0564608149201669,0.954974710868157,0.996348558702178
"13578","HSCB",35.0279432910144,0.0151150977862211,0.264710416936928,0
.0571005023569682,0.954465136255,0.996348558702178
"13579","ARHGEF9",19.3777432541895,-0.017695791610829,0.31687279310055
3,-0.0558450961904249,0.955465209409947,0.996348558702178
"13580","ZNF322",56.8997238751787,-0.0118100353321818,0.21189723788156
1,-0.0557347299580327,0.955553131986093,0.996364679596692
"13581","AL031963.3",3.31869438803642,-0.0430885922957044,0.7833678055
75006,-0.0550042929886257,0.956135043662277,0.996513155155227
"13582","TSPYL4",22.3391459556614,0.0162559842277484,0.295898897256932
,0.0549376303137527,0.956188152478394,0.996513155155227
"13583","HBS1L",40.5468354547832,-0.0136206380165103,0.247671457940175
,-0.054994782724622,0.956142620286972,0.996513155155227
"13584","LINC02823",2.24405113054655,-0.057277559580133,1.037175118983
44,-0.0552245792747824,0.95595954738231,0.996513155155227
"13585","PLCD3",26.0521487817677,-0.0150732673508498,0.272641445913418
,-0.0552860453785759,0.955910579337557,0.996513155155227
"13586","AC008555.4",2.16409818721803,0.0596902441285931,1.08632320697
34,0.0549470394680199,0.956180656382101,0.996513155155227
"13587","USP9X",66.9282501399005,0.0112708301999924,0.204478868919425,
0.0551197796601354,0.956043038208268,0.996513155155227
"13588","KLHDC2",89.5124924363008,-0.0105004471633573,0.19180405517248
2,-0.0547456994791619,0.956341061010362,0.996525824115588
"13589","OGFOD1",22.6771214007966,0.0169158230031481,0.308804282681873
,0.0547784598589087,0.956314961173721,0.996525824115588
"13590","KLHL8",25.9428746814437,0.0169915268049688,0.310894490472032,
0.0546536761689489,0.956414375226591,0.996528885445808
"13591","HECW2",22.6662135994451,0.0179715218666877,0.333455848962723,

0.053894756749931,0.957019014103774,0.996646966232228
"13592","UBA6",34.5267159521568,0.0158437555614107,0.294467977701212,0
.053804680852146,0.957090780143068,0.996646966232228
"13593","KIF13A",48.8062816746132,-0.0138876262679201,0.25636054023284
9,-0.054172246069173l,0.956797932633414,0.996646966232228
"13594","CD2AP",39.4423514292597,-0.013364362300914,0.246229810546033,
-0.0542759719924957,0.956715292900927,0.996646966232228
"13595","ARHGAP39",2.5160791689954,0.0473797133786891,0.88010365379652
,0.0538342423353276,0.957067227627147,0.996646966232228
"13596","RTF1",184.5001692217,-0.00907833197911528,0.168318966221724,-
0.0539352883569671,0.956986721528929,0.996646966232228
"13597","MED25",24.6348355151172,0.0153013382319156,0.281982378673414,
0.0542634554112946,0.956725264992093,0.996646966232228
"13598","SH3BGR",5.1057060626562,0.0348080912826643,0.645504981013281,
0.0539238151625481,0.956995862511149,0.996646966232228
"13599","CLIC4",114.786969472099,0.00930670020295404,0.179095542774454
,0.0519650018017172,0.958556580234431,0.9966663213342
"13600","POMGNT1",23.329681258725,-0.0162441832631009,0.30521551273776
,-0.053222010629118 2,0.957555018520794,0.9966663213342
"13601","AL049597.2",6.46016056974033,0.0264460983830108,0.53648257497
5295,0.0492953538784156,0.960683922086264,0.9966663213342
"13602","WNT2B",16.2196976957884,0.0341560872964217,0.651436003417388,
0.0524319919642778,0.958184483265669,0.9966663213342
"13603","ZC3H11A",2.98332518101432,0.0418466031354937,0.79844218324901
,0.0524103109948575,0.958201758424248,0.9966663213342
"13604","LINC00467",26.4846349991514,0.0177616051314785,0.344725442416
906,0.0515239171409863,0.958908044037049,0.9966663213342
"13605","PARP1",62.2217874776017,0.0115559276299377,0.229433603899489,
0.0503671974529074,0.959829775835239,0.9966663213342
"13606","KHK",6.96158460146338,-0.0259869157104414,0.501177470531422,-
0.0518517236676387,0.958646841423291,0.9966663213342
"13607","CIAO1",83.1250010253431,0.0100139823643181,0.191922212435802,
0.0521772974437121,0.958387422302994,0.9966663213342
"13608","ANAPC1",19.3464695455761,-0.0176526251723635,0.34869259485218
1,-0.0506251794072277,0.959624198282148,0.9966663213342
"13609","CNTN4",41.6291716386993,0.0225040328093562,0.44999106465499,0
.0500099548123477,0.960114455456438,0.9966663213342
"13610","BTD",49.0512518424318,-0.0157831806253884,0.323817309789645,-
0.0487410034863218,0.961125698634463,0.9966663213342
"13611","GLYCTK",3.98469379110298,-0.0357606346051833,0.68092170882742
8,-0.0525179828188536,0.958115966889603,0.9966663213342
"13612","DNAH1",2.19663924401771,-0.0514022496504849,0.982877460970107
,-0.0522977193919479,0.958291470493722,0.9966663213342
"13613","ABI3BP",842.861312164693,0.00859714094312192,0.17719342339948
,0.0485183974562068,0.961303102657847,0.9966663213342
"13614","AC084198.2",2.76287706291705,0.0378944829907557,0.78222045031
5332,0.0484447612888151,0.961361786810889,0.9966663213342
"13615","ARMC8",34.8124733829397,-0.0125634682626399,0.250146683971453
,-0.0502244045900431,0.959943564041587,0.9966663213342
"13616","B3GNT5",3.04811309288579,0.0402484443014704,0.822934606730439

,0.0489084357035118,0.960992266190045,0.9966663213342
"13617","ATP13A3",54.2313316875715,0.0152443611098083,0.31116271967975
1,0.0489916051816806,0.960925986002217,0.9966663213342
"13618","QRSL1",15.7097954141053,0.0203402119716655,0.38606739469826,0
.0526856508759639,0.957982372088704,0.9966663213342
"13619","PEX3",23.4603145607312,-0.0178018756340366,0.336482213657281,
-0.0529058443848935,0.957806927774312,0.9966663213342
"13620","TFB1M",11.3179434236011,0.022376885926329,0.440192192446543,0
.0508343544258714,0.959457515382613,0.9966663213342
"13621","MALSU1",76.8930609089532,0.013361947691848,0.258602799269551,
0.0516697720581142,0.95879182345688,0.9966663213342
"13622","DDX56",38.4507776429654,0.0129422674755809,0.255278337371713,
0.0506986515535612,0.959565651173557,0.9966663213342
"13623","ADCY1",18.5817120527284,-0.0221418004748623,0.449950086215514
,-0.0492094593449128,0.960752372933994,0.9966663213342
"13624","BAZ1B",91.174376375833,-0.0131644183180867,0.253874180636176,
-0.0518541046005482,0.958644944265864,0.9966663213342
"13625","FLNC",49.5441688312629,-0.0165749044311364,0.321830942905456,
-0.0515018981130276,0.958925589385061,0.9966663213342
"13626","INTS10",66.3644951374775,0.0113525360813859,0.233798350995175
,0.0485569553124015,0.96127237415775,0.9966663213342
"13627","RNF122",7.92675442217704,-0.0334482510790801,0.68667549211856
7,-0.0487104192052694,0.96115007240889,0.9966663213342
"13628","AC064807.1",5.07786497448529,0.0291008090658756,0.58731052810
7597,0.0495492719322482,0.960481572069448,0.9966663213342
"13629","SDCBP",582.497791285837,-0.0120887087340662,0.235113395227259
,-0.0514165036083176,0.958993634189257,0.9966663213342
"13630","PLGRKT",46.3069941945987,-0.0164202387462601,0.31152525237584
8,-0.0527091740429746,0.957963629359443,0.9966663213342
"13631","ABHD17B",10.3597192232271,-0.0213297013719554,0.4395867512089
98,-0.0485221661328331,0.9613000992264,0.9966663213342
"13632","CCDC3",3.03263067066365,0.0487537017429288,0.957559775758163,
0.0509145256277367,0.959393630745343,0.9966663213342
"13633","ARFGAP2",55.7052345397256,0.0137583457230948,0.28031913674308
4,0.0490810077504786,0.960854738783497,0.9966663213342
"13634","CCDC90B",106.153513085859,-0.0122557491978594,0.2383334128490
52,-0.0514227067508221,0.958988691336337,0.9966663213342
"13635","VPS26B",43.0803565340672,0.012473706829956,0.246984880159761,
0.0505039289121159,0.959720818583,0.9966663213342
"13636","BORCS5",22.0777077833942,0.0165908253051455,0.31985023471866,
0.0518706053779786,0.958631796244348,0.9966663213342
"13637","SLC38A2",271.854367253729,-0.0149213013980219,0.3083349659713
98,-0.0483931536957312,0.961402915473434,0.9966663213342
"13638","ATP2A2",69.5444483878304,0.011430617047856,0.231292350623988,
0.0494206445523085,0.960584076288892,0.9966663213342
"13639","CCDC122",19.7631694375433,0.0192693995018479,0.39750948565620
7,0.0484753199537817,0.961337433136496,0.9966663213342
"13640","AL109628.2",2.04974787429276,0.0474724594294152,0.98046483478
6913,0.0484183192962066,0.96138285973957,0.9966663213342
"13641","LINC00648",2.5400219583125,0.0459608458026952,0.8643893722421

78,0.0531714610089146,0.957595294253967,0.9966663213342
"13642","LINC00640",4.2698133600045,0.036730295451693,0.70956508920074
6,0.051764518873196,0.95871632746668,0.9966663213342
"13643","CGRRF1",34.6835203072862,0.016024201183741,0.30916912948272,0
.0518298874488263,0.958664240809275,0.9966663213342
"13644","WDR89",10.0247059042732,0.0216012587550189,0.428681919335743,
0.0503899459732072,0.959811648160737,0.9966663213342
"13645","CEP128",4.98242146832578,-0.0395077290563565,0.76299102521146
6,-0.0517800704738391,0.958703935703024,0.9966663213342
"13646","MAPK6",41.893482895721,0.0145540721270633,0.297569116628056,0
.0489098878673455,0.960991108915931,0.9966663213342
"13647","HDDC3",27.3300310489239,0.0168313686821463,0.323726969478192,
0.0519924821502404,0.958534683688539,0.9966663213342
"13648","AC024337.2",3.28272875051016,-0.0417898638590479,0.7838387384
16958,-0.0533143640533087,0.957481435619346,0.9966663213342
"13649","PIGW",3.73827518821971,-0.0359839616065037,0.72049947323425,-
0.0499430782995236,0.96016774859805,0.9966663213342
"13650","C18orf21",21.9137824379409,-0.0167189135967668,0.328635818259
998,-0.0508736804323007,0.959426178316337,0.9966663213342
"13651","ZNF701",11.9100360510504,0.0200261553103614,0.409761482948959
,0.0488727128919922,0.961020734825361,0.9966663213342
"13652","ZNF446",9.05021464783155,-0.0230195315078522,0.467674515401,-
0.0492212655378806,0.960742964356308,0.9966663213342
"13653","PDRG1",20.8011428688496,0.015530554291059,0.296743633251208,0
.0523366049033698,0.958260486776091,0.9966663213342
"13654","MMP24OS",98.3911803954058,-0.0113668350481968,0.2290912815638
47,-0.0496170564440659,0.960427554295928,0.9966663213342
"13655","MRPL40",118.015525549471,-0.0162813879511041,0.32041926737251
7,-0.0508127619309968,0.959474721464598,0.9966663213342
"13656","RBFOX2",174.295782905703,-0.00903279373902076,0.1823797181878
13,-0.0495274026562475,0.960498999829729,0.9966663213342
"13657","RAI2",39.8783060970593,-0.0161593485339644,0.329207090456933,
-0.0490856637125753,0.960851028335475,0.9966663213342
"13658","BCOR",7.9160921926392,-0.0264234513448782,0.540621706382541,-
0.0488760459169967,0.961018078630497,0.9966663213342
"13659","TNMD",6.20413174893737,0.0449772306184752,0.849596985307348,0
.0529394894241583,0.95778012048428,0.9966663213342
"13660","DIS3L2",21.0198152757627,0.0158953522584802,0.331974391205928
,0.0478812603608935,0.961810874324486,0.996870295742551
"13661","RUSC2",18.9658504137928,-0.0158973834255203,0.33155320605192,
-0.0479482120375902,0.961757515901228,0.996870295742551
"13662","DAPK1",4.59335045465825,-0.0306109579800151,0.638953411557172
,-0.0479079654734359,0.961789591152169,0.996870295742551
"13663","POLH",8.44288425672507,-0.0228992261838101,0.48075601731286,-
0.0476316995714441,0.962009768088834,0.996906493699693
"13664","KMT2E",192.63728572949,0.0117641897045514,0.247417224667513,0
.047547981836594,0.962076489578942,0.996906493699693
"13665","ALDH1L2",52.7613130024478,0.0131396537638645,0.27676654968635
7,0.0474755846714672,0.962134188998008,0.996906493699693
"13666","NLE1",16.6352958486931,0.0192049905664506,0.405204887543618,0

.0473957525114582,0.962197814222719,0.996906493699693
"13667","NRIP1",93.7647418783246,0.0156062449031193,0.326971747063888,
0.0477296434424653,0.961931708968418,0.996906493699693
"13668","RIPK1",26.9809478324637,0.0134140937218268,0.28421266228868,
0.0471973824593446,0.962355913693923,0.996993181069424
"13669","MRPL16",68.2987335855517,0.0132490918607478,0.28121297264040
,0.047114084874348,0.962422301697595,0.996993181069424
"13670","OTUD7B",20.341111941558,-0.0184927001638121,0.39653018278056,
-0.0466362989927702,0.962803101080236,0.997095875908443
"13671","SDE2",21.6653117169298,-0.0149646671881489,0.31954827780987,-
0.0468306926600082,0.962648166656996,0.997095875908443
"13672","C11orf74",44.6875550374337,0.0139059625371163,0.2972951876453
59,0.0467749331809051,0.962692607583821,0.997095875908443
"13673","FAM98C",31.2549939154741,0.0139058554535408,0.298148786354852
,0.0466406575842648,0.962799627207524,0.997095875908443
"13674","SDF4",241.874392218691,-0.00879041308718461,0.195192045807794
,-0.0450346890458874,0.964079659132811,0.997118184999501
"13675","ZBTB17",16.0813598618106,0.0156584515889179,0.35146004084856,
0.0445525800062856,0.964463940770234,0.997118184999501
"13676","ATXN7L2",2.22467939273601,0.0402126348940089,0.92029352409934
1,0.0436954448129618,0.965147170263307,0.997118184999501
"13677","CYB561D1",9.10252630080458,0.0192969298483899,0.4434461526676
64,0.0435158355356209,0.965290341551101,0.997118184999501
"13678","AL162258.2",1.66046130322814,-0.0481967596623508,1.1078814974
2533,-0.0435035333421111,0.965300147994719,0.997118184999501
"13679","AC019069.1",1.59400366446841,0.0489575627952284,1.11789011824
547,0.0437946109337358,0.965068122816497,0.997118184999501
"13680","TIGD1",3.29098993368002,0.035681753865801,0.773001951298995,0
.0461599790347741,0.963182740555608,0.997118184999501
"13681","RAD54L2",19.6467212432672,0.01747708488907,0.396589386930176,
0.0440684634159083,0.964849830900897,0.997118184999501
"13682","SORCS2",13.64428226329,-0.025065087702092,0.54506518732817,-0
.0459854862956071,0.963321817930084,0.997118184999501
"13683","USO1",108.426358421364,-0.00902486183952922,0.200126577747903
,-0.0450957685935035,0.96403097416644,0.997118184999501
"13684","AC008443.1",10.0301674702234,-0.0196508339487868,0.4515675172
77746,-0.0435169342278005,0.965289465751207,0.997118184999501
"13685","AL590062.1",4.35890025123452,0.0289660470262156,0.65862596230
7881,0.0439795098946846,0.96492073679702,0.997118184999501
"13686","RPS10-
NUDT3",2.53845435879954,-0.0590662469280476,1.28161308221501,-0.046087
4250955393,0.963240568679154,0.997118184999501
"13687","RBPMS-
AS1",2.18245787281111,0.0398942208255821,0.911686151171143,0.043758721
9838037,0.965096730630455,0.997118184999501
"13688","POLB",7.56378850300907,-0.0248426783391519,0.565004513824493,
-0.0439689909218473,0.964929121612118,0.997118184999501
"13689","RFK",38.5908154055931,-0.0110367531970332,0.252057121884888,-
0.0437867143546675,0.965074417336869,0.997118184999501
"13690","AL354920.1",2.53505738406803,-0.04262342811785,0.968456698789

261,-0.0440117025068201,0.964895075655872,0.997118184999501
"13691","KIF20B",14.903598601563,-0.020092474900765,0.462446902686722,
-0.0434481770426655,0.965344274209091,0.997118184999501
"13692","ALDH18A1",20.4119445875012,0.0146842097975757,0.3381211682109
63,0.0434288390616639,0.965359689135463,0.997118184999501
"13693","C11orf49",28.4639520183232,-0.0141399221961385,0.314585962723
944,-0.0449477213595402,0.96414897911245,0.997118184999501
"13694","ITGB7",2.31815221693031,0.0440379151715106,0.999867109259939,
0.0440437681804592,0.964869515735159,0.997118184999501
"13695","PDCD7",111.02161092344,-0.00896036098239046,0.195902723863982
,-0.0457388279532639,0.96351841594799,0.997118184999501
"13696","CLPX",39.0396322069176,-0.0131659502043083,0.295384080164957,
-0.0445723080165857,0.96444821571665,0.997118184999501
"13697","TMEM186",14.224515667557,-0.0210822818383255,0.45689060319467
3,-0.0461429534573786,0.963196310541504,0.997118184999501
"13698","RMI2",7.05583777794635,0.029908091103659,0.661117067889787,0.
0452387217881431,0.963917030306212,0.997118184999501
"13699","AMFR",44.9464277501629,0.0118899925204186,0.273691724584606,0
.0434430107028795,0.965348392463364,0.997118184999501
"13700","TLCD3A",17.0424853104692,0.0150445862950394,0.334348795657462
,0.0449966815805506,0.964109953992109,0.997118184999501
"13701","SLC25A11",75.5385591138204,-0.0102795984632479,0.224520779868
575,-0.0457846194426419,0.963481917861583,0.997118184999501
"13702","SPECC1",15.1766630889322,-0.0201469691989542,0.46195682183685
3,-0.0436122344050357,0.9652134993327,0.997118184999501
"13703","AC104984.3",2.09594253144923,-0.0443391234882936,0.9728321105
21654,-0.0455773642838721,0.96364711110392,0.997118184999501
"13704","PSMC3IP",4.97665197195237,-0.025797749641359,0.58454667360470
1,-0.0441329166793012,0.964798454621565,0.997118184999501
"13705","RANBP3",31.7514545071454,-0.0120953024156106,0.26190399102132
8,-0.0461821997001398,0.963165029917346,0.997118184999501
"13706","MEF2B",6.24955945499452,0.0225745107918714,0.515140383857246,
0.0438220560827302,0.965046245758819,0.997118184999501
"13707","AL031651.2",2.25605239198775,-0.0468178942542643,1.0603721626
186,-0.0441523230284045,0.964782985673749,0.997118184999501
"13708","C21orf91",17.0079738150349,-0.016162794926787,0.3666179847408
59,-0.0440862030765116,0.964835690442383,0.997118184999501
"13709","F8",9.42373271280036,-0.0219903581350292,0.474021480787969,-0
.0463910582669681,0.96299856331069,0.997118184999501
"13710","DHX38",40.5351902793876,0.0114616933674927,0.264667293020432,
0.0433060437377426,0.965457573537895,0.997146552975681
"13711","RPIA",37.8773868278272,-0.0109737056496058,0.256163867467965,
-0.042838616382844,0.965830180808642,0.997167725865868
"13712","FAHD2A",42.5945742280268,-0.0106548525280428,0.24802371290741
9,-0.0429590074398248,0.965734210990732,0.997167725865868
"13713","SSR3",233.382759144404,-0.00935113149451993,0.217295726444728
,-0.0430341251874482,0.965674331075405,0.997167725865868
"13714","LZTS2",58.243584887301,-0.00939125168890299,0.218110113051055
,-0.0430573876540274,0.965655787500476,0.997167725865868
"13715","PPME1",27.80499053405,0.0128963884012669,0.300995435974937,0.

0428457938556275,0.965824459267148,0.997167725865868
"13716","ZNF860",1.55674823522733,0.0492387417876571,1.16505170477605,
0.0422631386965972,0.966288930074744,0.997205105302702
"13717","AC112220.2",2.58066899294423,-0.0453191518589933,1.0642460481
2273,-0.0425833405150375,0.966033675786498,0.997205105302702
"13718","PODXL2",12.1023152106899,0.0181880818941338,0.426854880678566
,0.042609520746771,0.966012805926112,0.997205105302702
"13719","SNRPC",87.1530959797082,0.0105810381336373,0.248911476415149,
0.0425092417835715,0.966092744533359,0.997205105302702
"13720","AC091729.3",6.74361139210767,0.0216751473915715,0.51205539347
7124,0.0423296925834253,0.966235875234428,0.997205105302702
"13721","ZNF225",6.08561599492769,-0.0244849121003006,0.57790666066033
3,-0.0423682815358512,0.966205113302232,0.997205105302702
"13722","LINC01409",3.19236419132076,-0.0283205701421064,0.77322712225
2911,-0.0366264572556512,0.970782847840282,0.99723300451239
"13723","GNB1",179.860992923181,0.00729505422019467,0.179541347848943,
0.0406316110890088,0.967589582953332,0.99723300451239
"13724","GPR157",6.00052066471961,0.0212585701646822,0.540377900974716
,0.0393401916072747,0.96861916313513,0.99723300451239
"13725","AGO1",26.4529091008218,0.0135144435086005,0.372989547962402,0
.0362327673320292,0.971096758582847,0.99723300451239
"13726","RNF220",29.7222053172038,0.010836875385,0.281138696676035,0.0
385463670178699,0.969252063398765,0.99723300451239
"13727","GOLPH3L",27.0898452853961,0.0113684504045067,0.27679799146355
9,0.0410712893702603,0.967239063046875,0.99723300451239
"13728","PIP5K1A",20.5762909619985,0.0128712563660455,0.3112002785663,
0.0413600412742026,0.967008867964443,0.99723300451239
"13729","DNM3",3.4335885227814,0.0350772809950426,0.858042913968103,0.
0408805671884455,0.967391109632185,0.99723300451239
"13730","AC074117.1",3.20628569797405,-0.0270506604929822,0.7459549260
51625,-0.0362631300474984,0.971072548551123,0.99723300451239
"13731","POU3F3",1.51412053462014,0.0414806024192165,1.05704478057301,
0.0392420483801361,0.968697409679264,0.99723300451239
"13732","STEAP3",3.46346724620052,0.0285206979345107,0.740241584166299
,0.0385289053527469,0.96926598544977,0.99723300451239
"13733","ZEB2",306.656409130103,0.00805262905006955,0.20114878173852,0
.0400331982151273,0.968066659150821,0.99723300451239
"13734","CRTAP",256.985307087123,-0.0061397477628233,0.163804923796377
,-0.0374820708714197,0.970100635472937,0.99723300451239
"13735","LARS2",6.73516321376436,-0.0209117538482512,0.530686316532875
,-0.0394051122042748,0.968567404127222,0.99723300451239
"13736","ALG1L",2.12998679154881,0.0393712732358601,0.958849916320955,
0.0410609341104447,0.967247318384863,0.99723300451239
"13737","LSM6",112.045149880312,-0.0104741656361471,0.252877014749493,
-0.0414199987552174,0.966961069775929,0.99723300451239
"13738","OCLN",6.31753480903407,-0.0391945468827804,1.04160981161226,-
0.0376288188204689,0.969983630092131,0.99723300451239
"13739","AGGF1",53.6334658673795,-0.00903336602446072,0.21599170312104
4,-0.0418227454755442,0.966640002607036,0.99723300451239
"13740","KIF3A",28.4071486013671,0.0117441844952158,0.286596393936855,

0.0409781307220613,0.967313330370699,0.99723300451239
"13741","SIRT5",10.4058507931426,0.0162357813308689,0.430137099639162,
0.037745596333097,0.96989052126372,0.99723300451239
"13742","SRF",15.2828080853594,0.0139109939048115,0.355401047659798,0.
0391416795094189,0.968777430971516,0.99723300451239
"13743","NFKBIE",9.58248724547124,-0.0188330257137484,0.48124185160214
5,-0.0391342225349888,0.968783376221135,0.99723300451239
"13744","FAXC",1.62589196190536,-0.0575666477253246,1.41031666946781,-
0.0408182424356138,0.967440796117648,0.99723300451239
"13745","ZBTB24",17.0344654007269,0.014046302567988,0.360603104450149,
0.0389522508116116,0.968928458027877,0.99723300451239
"13746","MAN1A1",204.346241357311,-0.00773168701130519,0.2076423995272
64,-0.0372355888243818,0.970297162499789,0.99723300451239
"13747","STX11",8.52969063382054,0.019172949501252,0.5121972179156,0.0
374327482278737,0.970139961650592,0.99723300451239
"13748","RBAK",11.5763027506301,-0.0168789961565258,0.449041215940785,
-0.0375889685786697,0.970015403506239,0.99723300451239
"13749","MIOS",12.2774343085344,-0.0151518509649014,0.376790022761731,
-0.0402129834910275,0.967923326674343,0.99723300451239
"13750","ERV3-1",14.9684253381687,0.0162800361632525,0.439413951096198
,0.0370494294107845,0.970445593800826,0.99723300451239
"13751","POM121",32.6887427622131,0.0124844864935304,0.340666865586052
,0.0366471992280588,0.970766309243988,0.99723300451239
"13752","DBF4",15.4040045888296,0.0161217109880704,0.420936608882134,0
.0382996172057457,0.969448795989379,0.99723300451239
"13753","CYP3A5",11.4169018989878,0.020141689212854,0.523902736111647,
0.0384454743686654,0.969332504459494,0.99723300451239
"13754","AGBL3",2.87484093569923,-0.0284194613222409,0.77686666073189,
-0.0365821610821435,0.970818167403388,0.99723300451239
"13755","AP003117.1",2.29142260383466,-0.03688217835247,0.988513369422
008,-0.0373107531909612,0.970237231657288,0.99723300451239
"13756","JAK2",24.7056734701727,-0.0179205345742259,0.437154738146622,
-0.0409935727797495,0.967301019735575,0.99723300451239
"13757","ALAD",18.7325971423556,0.0144108684998968,0.362028954952182,0
.0398058450926951,0.968247916216369,0.99723300451239
"13758","POMT1",12.7532695937514,0.0158292707709546,0.405401438019561,
0.0390459166802236,0.968853780288816,0.99723300451239
"13759","WDFY4",1.91415740413164,-0.0445149330265798,1.06513914833395,
-0.0417925987381164,0.966664035211136,0.99723300451239
"13760","TRIM21",13.922213672888,-0.0188023972425299,0.47186570442741,
-0.0398469248053233,0.968215165331965,0.99723300451239
"13761","TRIM5",22.4862827124913,-0.0119611646878879,0.313765669055684
,-0.0381213302394953,0.969590944597719,0.99723300451239
"13762","CTSW",2.21948354821812,0.0380291447374621,1.04987598670406,0.
0362225112480659,0.971104936385689,0.99723300451239
"13763","SSH3",18.605902662871,0.0145505282565365,0.388323546189952,0.
037470115833303,0.970110167517247,0.99723300451239
"13764","AP003486.1",4.40055437715407,0.0259622393738413,0.65092014590
6446,0.0398854445312755,0.968184455450843,0.99723300451239
"13765","DDX47",4.61865849202235,0.026521233001359,0.66339529965391,0

.0399780241342861,0.968110646484247,0.99723300451239
"13766","C12orf81",10.739041657037,-0.0177806282063287,0.487878259241211,-0.0364448053782569,0.970927688365871,0.99723300451239
"13767","IFT81",19.024429825264,-0.0137869048632685,0.334533239231373,-0.041212361722098,0.967126598817048,0.99723300451239
"13768","EEF1AKMT1",42.012847261412,0.00982530141448105,0.252817963990727,0.0388631458753517,0.968999499688679,0.99723300451239
"13769","UBAC2-AS1",1.69001920329747,-0.0383736658410263,1.05700630204241,-0.0363041031703203,0.971039878241343,0.99723300451239
"13770","SUPT16H",109.006256012368,0.00906449167303261,0.229086009760756,0.0395680717582842,0.968437482540958,0.99723300451239
"13771","WARS",44.1668810089438,0.00961755830744652,0.265008221977565,0.0362915468647638,0.971049890126024,0.99723300451239
"13772","MIR193BHG",14.6851376941987,-0.0134395390924131,0.368444719651056,-0.0364764057553635,0.970902491666371,0.99723300451239
"13773","CMTM4",4.09797405141993,-0.0263435618416769,0.724754782682089,-0.0363482414620124,0.971004684208387,0.99723300451239
"13774","ZNF19",3.18839990304479,0.0311754060251609,0.790428305559042,0.0394411558972596,0.968538667760548,0.99723300451239
"13775","TBX2",8.16338553302799,-0.0258603698874093,0.632096287150924,-0.0409120737031552,0.967365992083982,0.99723300451239
"13776","LINC00909",19.3800551517737,0.0123991344435917,0.337590185761864,0.036728361683885,0.97070159454102,0.99723300451239
"13777","TLE5",347.562466053463,-0.00730727215394417,0.193467711825379,-0.0377699828307247,0.969871077518706,0.99723300451239
"13778","ZNF724",2.26275206257573,-0.0338167468938166,0.930800359231312,-0.0363308270763278,0.971018569706504,0.99723300451239
"13779","PRKD2",16.6051076015666,-0.0145902438979253,0.346530270309209,-0.0421038077998394,0.966415944679127,0.99723300451239
"13780","CLDND2",4.20912565178199,-0.0244165079183776,0.651730148973162,-0.0374641375066769,0.970114934184898,0.99723300451239
"13781","MRPS26",104.803350973056,-0.0096422447948981,0.252755481890576,-0.0381485090759473,0.969569274786365,0.99723300451239
"13782","FASTKD5",9.69306540454193,0.0173262718788727,0.435605328222433,0.0397751605784431,0.96827237954268,0.99723300451239
"13783","LZTS3",6.52640618121268,-0.0224463030817928,0.567123023040738,-0.0395792485401892,0.968428571739459,0.99723300451239
"13784","ZNF341",2.90215400481819,-0.0303693901384857,0.80118787943292,-0.0379054537869209,0.969763064682691,0.99723300451239
"13785","MICALL1",5.25989568632707,0.0215939579020267,0.584842661392459,0.0369226790853687,0.970546656779367,0.99723300451239
"13786","TRMU",16.7974854724578,-0.0137880123358901,0.353879241591439,-0.0389624784824442,0.968920303717377,0.99723300451239
"13787","MSL3",30.3995846310141,-0.0103525051703755,0.278401546133792,-0.0371855160797146,0.970337087119784,0.99723300451239
"13788","UBA1",87.5022421499028,-0.00760302847314519,0.190328637502052,-0.0399468444314546,0.968135504430797,0.99723300451239
"13789","APLN",1.77332494482764,-0.0396677260997253,1.06861819031754,-0.0371205791358822,0.970388863557575,0.99723300451239

"13790","TWIST2",167.923301431193,-0.00831318435343727,0.230642887938839,-0.0360435321796774,0.971247647828766,0.997295359827292
"13791","KLF14",3.25028239185708,0.0297961971361982,0.830408524836837,0.0358813719332334,0.971376949345904,0.997295359827292
"13792","TRIP4",18.8873808333345,0.0122183595629909,0.3399408426616,0.0359426053878258,0.971328123612606,0.997295359827292
"13793","DFFA",56.3065047529498,0.00854236096102817,0.242096999902802,0.0352848691411203,0.971852588494557,0.997307412431196
"13794","MFSD14A",40.4726479951636,-0.00880859532871099,0.247030427277587,-0.0356579366590044,0.971555110888704,0.997307412431196
"13795","WDR48",34.5085084428691,-0.010564714845714,0.300640757270915,-0.0351406607062058,0.971967578865597,0.997307412431196
"13796","AC027644.3",30.1121623483214,0.0119452174915157,0.342318461389616,0.0348950431800407,0.972163433177104,0.997307412431196
"13797","PON3",9.40656021376562,-0.0196804117844737,0.558411712543254,-0.0352435512049709,0.971885534946003,0.997307412431196
"13798","TTI2",16.1201483402632,-0.0123776174948707,0.348279598252877,-0.0355393125436064,0.971649699285727,0.997307412431196
"13799","AC007406.5",4.42025154577777,-0.0264210180587456,0.738749606902126,-0.0357645104808103,0.971470131485774,0.997307412431196
"13800","TRHDE",4.43670860278543,0.0260198679581715,0.740422086468793,0.0351419392177575,0.971966559390644,0.997307412431196
"13801","NFYB",80.8940038054491,-0.00727069639719792,0.207916573090965,-0.0349692970074922,0.972104223331236,0.997307412431196
"13802","CCT6B",5.9301762981012,-0.022564494837336,0.644780727389373,-0.0349956099474257,0.972083241485142,0.997307412431196
"13803","COL1A1",7091.68086663374,0.0103343858833571,0.293915737003184,0.0351610498598282,0.971951320721958,0.997307412431196
"13804","METTL8",32.2959042988218,0.00983567087643261,0.283737954217174,0.034664628859995,0.9723471660466,0.997409508792764
"13805","TK1",3.98633685844026,-0.0290341814507961,0.839294278244251,-0.0345935653362653,0.972403832548313,0.997409508792764
"13806","COA6",64.9738552227799,-0.0125378880073749,0.365686766676093,-0.0342858674415209,0.972649194392684,0.997470936000484
"13807","AL445250.1",5.17049934928391,-0.0228945262551981,0.675561634348511,-0.0338896187870065,0.97296517144236,0.997470936000484
"13808","AL391988.1",3.19503221307956,0.0263015448433145,0.779884684120868,0.0337249152071285,0.973096510808319,0.997470936000484
"13809","KDM4D",2.4688870864694,0.0274931207263898,0.802915090384232,0.0342416291033129,0.972684470766344,0.997470936000484
"13810","PTGES3",355.371886532991,-0.00650735323843842,0.192962451963132,-0.0337234170287271,0.973097705502168,0.997470936000484
"13811","PELP1",25.0723732641131,0.010332530906778,0.306110231041056,0.0337542814940812,0.973073093233967,0.997470936000484
"13812","CERS4",30.0271844277146,-0.010474739353682,0.307239727990884,-0.034093049821971,0.972802950703526,0.997470936000484
"13813","ADA",67.5347263592966,0.0130936249173326,0.385491600756353,0.0339660446340264,0.972904227525291,0.997470936000484
"13814","RGN",7.38903073089133,0.0166267521335206,0.485697061073955,0.0342327624893511,0.972691541155612,0.997470936000484

"13815","URB2",4.86357842131597,-0.0200274360388141,0.60001259440169,-
0.0333783594305794,0.973372866718086,0.997568895151756
"13816","ZBTB2",14.8140638037121,-0.0136572828134555,0.409654496470873
,-0.0333385399918991,0.973404620361004,0.997568895151756
"13817","SLC25A18",3.18882128720162,-0.0341119724818573,1.018161982303
61,-0.0335034828197751,0.973273088504066,0.997568895151756
"13818","ICMT",47.0210541806063,-0.00745617997803425,0.252857480529984
,-0.0294876780485444,0.97647564616227,0.997575798032597
"13819","ATP5IF1",275.177904380022,0.0078171584654015 6,0.2712458424358
21,0.0288194591120827,0.977008581197981,0.997575798032597
"13820","AK2",58.7254695944587,-0.00673525355878624,0.222672753973085,
-0.030247317817789,0.975869811616085,0.997575798032597
"13821","SMIM12",36.0245181496203,0.00789475544222768,0.25601664156013
6,0.0308368838608223,0.975399625330299,0.997575798032597
"13822","LINC01473",9.81259456290285,0.0174297474382545,0.565372189938
141,0.0308288022446974,0.975406070462725,0.997575798032597
"13823","AAMP",86.9430582757506,-0.0064001210293841 5,0.219463407025957
,-0.0291625884976222,0.976734918588313,0.997575798032597
"13824","UBE2E1",76.3569255627818,-0.00805178238852174,0.2427676727647
99,-0.0331666168597438,0.973541719355162,0.997575798032597
"13825","ITGA9",19.6577400102363,-0.01141810159175,0.358769884221715,-
0.0318256968990567,0.974611053851185,0.997575798032597
"13826","SEC22C",58.762339221921,-0.00589903153414475,0.19950740972896
9,-0.0295679821724847,0.976411600668114,0.997575798032597
"13827","FLNB",74.1770814143082,0.00965782983103474,0.336881892189618,
0.02868296085201,0.977129142028579,0.997575798032597
"13828","CCDC80",4134.86593773201,0.00712461002658304,0.21916239718719
5,0.0325083596366106,0.974066649528487,0.997575798032597
"13829","HSPBAP1",7.12891508350559,0.0151613561572402,0.49674952303128
2,0.0305211287667114,0.975651442910418,0.997575798032597
"13830","FXR1",143.144669941419,0.00511802485073859,0.167301433260743,
0.0305916378060076,0.975595211094321,0.997575798032597
"13831","SLBP",69.5514814395643,0.00724719546699346,0.233651282980498,
0.0310171439015738,0.975255867394299,0.997575798032597
"13832","STIM2",18.0672743173238,-0.0101687969399291,0.324121299969994
,-0.0313734300734646,0.97497173044702,0.997575798032597
"13833","FBXW7",38.6941979000168,0.00896507135225286,0.272435195593531
,0.0329071702087589,0.973748614894287,0.997575798032597
"13834","C1QTNF3",16.3381244789084,0.0155281521632204,0.53677762218915
1,0.0289284640814415,0.976921644063467,0.997575798032597
"13835","IK",122.119542056813,-0.00550763231717496,0.191952976107266,-
0.028692612268211,0.977109748499471,0.997575798032597
"13836","STK32A",4.02103343989022,0.0248425327143078,0.840251646275015
,0.0295655864816679,0.976413511317507,0.997575798032597
"13837","CCHCR1",16.2209761100667,0.0113315054131335,0.352050395743472
,0.032187168513767,0.974322788921016,0.997575798032597
"13838","TRAM2-
AS1",15.3138643639786,0.0105500767009235,0.357798432452539,0.029486089
775765,0.976476912869793,0.997575798032597
"13839","HDDC2",118.448877989267,-0.00864052388994313,0.27348936135303

4,-0.031593638038408,0.974796116968726,0.997575798032597
"13840","GFRA2",1.95217142269812,0.0326834045301279,1.08672649943879,0
.0300750966752042,0.976007161718832,0.997575798032597
"13841","LRRCC1",30.1079877557125,0.00903458514434541,0.30348802464427
9,0.0297691652081987,0.976251150461917,0.997575798032597
"13842","EMC2",84.6507092008891,-0.00650336337882611,0.227072195544636
,-0.0286400691340817,0.977151654633054,0.997575798032597
"13843","LRRC14",11.0410850678354,-0.0140993799264745,0.42939583224429
9,-0.0328353907227793,0.973805855704614,0.997575798032597
"13844","RMRP",2.51378585778461,-0.0262096398953629,0.917710766414883,
-0.0285598043027795,0.977215670516253,0.997575798032597
"13845","TLE4",38.2192245545166,0.0111167677322186,0.349630360092238,0
.0317957734828458,0.974634917205993,0.997575798032597
"13846","PHYHIPL",1.9203657024556,0.0424886220679608,1.35974507667308,
0.0312474910164182,0.975072166033346,0.997575798032597
"13847","DEPDC7",1.72857489356442,0.0310006633319359,1.0209258802662,0
.0303652438743669,0.975775763436445,0.997575798032597
"13848","WDR74",82.0612287863313,0.00862098853411299,0.278255442931006
,0.0309822817598956,0.975283669996787,0.997575798032597
"13849","NEU3",3.44246617810457,0.0217937825547958,0.720540748448,0.03
02464261760883,0.975870522717874,0.997575798032597
"13850","AP003119.2",1.53085700767609,-0.0302385628281931,1.0446327954
9618,-0.0289465953572999,0.976907183454177,0.997575798032597
"13851","GLB1L2",1.76308525286059,0.0346726484381481,1.05150696518272,
0.0329742451417077,0.973695125868963,0.997575798032597
"13852","CEP290",59.8448463232306,-0.00725661609041615,0.2237365175134
41,-0.0324337581145206,0.974126141559597,0.997575798032597
"13853","PRDM4",17.3278264912316,-0.0124345021817725,0.377416954896685
,-0.0329463263916597,0.973717389711796,0.997575798032597
"13854","IQCD",2.7572483455905,0.022506823169763,0.783543258559297,0.0
287244168383841,0.977084382579149,0.997575798032597
"13855","PABPN1",111.877767191277,-0.00524750072479677,0.1792711149436
98,-0.0292713119257657,0.976648206861415,0.997575798032597
"13856","AL049838.1",3.51440078698834,-0.0233558912221907,0.8011719353
92288,-0.0291521584698978,0.976743237009688,0.997575798032597
"13857","PPM1A",54.0323260841861,-0.00692392050837178,0.23436665141093
1,-0.0295431131805164,0.97643143458819,0.997575798032597
"13858","DGLUCY",32.2782983987166,-0.00842348881294219,0.2913377131993
58,-0.0289131424848458,0.976933863817389,0.997575798032597
"13859","C14orf132",53.6567872254035,-0.00890878058025373,0.2931781734
06112,-0.0303869161771236,0.975758479416555,0.997575798032597
"13860","INF2",35.6426361379753,0.00744680365744324,0.251955723773792,
0.0295560011334731,0.976421155978005,0.997575798032597
"13861","PDPK1",43.5228117185039,0.00723799866666862,0.238120221296633
,0.0303964049220837,0.975750911989096,0.997575798032597
"13862","SRCAP",26.5998144062606,0.0100026013584226,0.320661597437217,
0.0311936366511147,0.975115114663325,0.997575798032597
"13863","SETD1A",15.2038403663371,-0.0106428920227877,0.35958282514302
,-0.0295978875480346,0.976387750069389,0.997575798032597
"13864","AC087500.1",3.25410889428548,-0.0256955596603472,0.8032760342

65595,-0.0319884554801136,0.974481257379579,0.997575798032597
"13865","MIS12",13.3403371935724,0.0140458172274674,0.433158744006141,
0.0324264889531314,0.974131938462099,0.997575798032597
"13866","FOXK2",25.6032821537626,-0.00876620043121583,0.28875305326122
7,-0.030358814676447,0.975780890830308,0.997575798032597
"13867","TMEM86B",1.84100912409741,-0.0298457138332647,0.9660867635380
41,-0.0308934093289536,0.975354546009677,0.997575798032597
"13868","NDUFV3",72.7325329904974,0.00709973130756249,0.21898198586547
9,0.0324215312940118,0.974135892023,0.997575798032597
"13869","ZBED1",41.5051283749745,0.00799084725348922,0.250242224003666
,0.0319324497906163,0.974525920637613,0.997575798032597
"13870","TCEAL8",193.073797598064,-0.00749605204105353,0.2348766040452
87,-0.0319148519347981,0.974539954542138,0.997575798032597
"13871","MOSPD1",12.7423992221627,0.0123476514083856,0.409234868054646
,0.0301725301831729,0.975929456291816,0.997575798032597
"13872","TSNAXIP1",1.51523387202071,-0.0314050384335421,1.104870319813
38,-0.0284241850562576,0.977323835106537,0.997614295350963
"13873","NARS",115.984174595899,-0.00662608549154611,0.235034155898395
,-0.0281920109280225,0.977509009049169,0.997731389615529
"13874","PRTFDC1",47.0605939852261,-0.007130573228091,0.25555172012854
1,-0.0279026618349678,0.977739785434065,0.997761505492243
"13875","OAS3",14.4932702396104,0.0157448067168493,0.561260892699196,0
.0280525632939255,0.977620228175122,0.997761505492243
"13876","AC004148.1",16.3510076863675,0.0145591937413242,0.52202248662
2118,0.0278899743103659,0.977749904675874,0.997761505492243
"13877","PHTF1",13.4909101060641,0.0104771347824771,0.382127778016669,
0.0274178831930403,0.978126434884842,0.997933387788344
"13878","POT1",11.0731367319442,-0.011797016747998,0.430332788583473,-
0.0274137064638514,0.978129766180397,0.997933387788344
"13879","DHRS1",28.5365128728558,0.00790704445034573,0.287741669548082
,0.0274796641819876,0.978077159354073,0.997933387788344
"13880","TRUB1",14.4186803411981,0.0105369042874246,0.389372723400123,
0.0270612286228297,0.978410898503802,0.998148294150852
"13881","ANKRD36",22.0866174077796,0.0104492701180995,0.38982049650639
1,0.0268053378715251,0.97861499574329,0.998149169200853
"13882","CYP27A1",65.187484999606,0.00621052679236711,0.23328493132048
9,0.0266220658025916,0.978761173530175,0.998149169200853
"13883","SLC25A36",141.021157402506,0.00479088537344787,0.179845010330
141,0.026638967434533,0.97874769276002,0.998149169200853
"13884","GBA2",13.7356313230024,0.0113756099529483,0.427361160829385,0
.026618258736642,0.978764210053237,0.998149169200853
"13885","SLC25A51",7.98682822517848,-0.0125170305751354,0.468159317893
701,-0.0267366900469928,0.978669749158555,0.998149169200853
"13886","HMGA2-
AS1",4.25627911536062,-0.0228246947900947,0.861863802139163,-0.0264829
486207025,0.978872133859269,0.998187340879104
"13887","ETNK2",15.2145474805567,0.00912257438233106,0.356045026461218
,0.0256219683027217,0.979558863649129,0.998212031119848
"13888","SLC35F6",70.186027456205,0.00580608739849238,0.23101026393528
1,0.0251334607371341,0.979948510797556,0.998212031119848

"13889","SPDL1",11.6529279998004,0.0105674833503422,0.401291274286854,
0.0263336983071011,0.978991176862569,0.998212031119848
"13890","ACHE",1.5401282232386,-0.0278147498846841,1.10536715656501,-0
.0251633583642199,0.97992466348472,0.998212031119848
"13891","ANKRD46",14.5958039091484,0.00967475661472678,0.3769676813353
64,0.025664684517397,0.979524792245128,0.998212031119848
"13892","TRMT12",8.75973699027398,-0.0116195875338457,0.45993187460285
5,-0.0252637144226611,0.979844616383053,0.998212031119848
"13893","IFIT1",96.9690739150018,-0.00728631695936118,0.28341884958818
8,-0.0257086533586185,0.979489721757314,0.998212031119848
"13894","SIRT3",14.3541389084858,-0.0098648716047297,0.392488085792563
,-0.0251341937801991,0.979947926098521,0.998212031119848
"13895","CARS",23.8866319824453,-0.00703527831660448,0.279202593657986
,-0.02519775416278,0.979897228307253,0.998212031119848
"13896","LTO1",9.65426767492768,0.0120733358719426,0.466000448548664,0
.025908421138959,0.979330383204895,0.998212031119848
"13897","PSME1",329.959968183393,-0.00465797330483042,0.18155433582696
7,-0.0256560840787067,0.979531652143535,0.998212031119848
"13898","RAB11B-
AS1",22.6226812813715,-0.007922815625634 5,0.307671650970413,-0.0257508
795517738,0.979456041280171,0.998212031119848
"13899","KIF16B",23.4846285731291,0.00934819263455048,0.36729863442006
7,0.0254512044383461,0.979695069081695,0.998212031119848
"13900","FGF16",6.23090643420054,0.0179275393339272,0.713481034206499,
0.0251268617866842,0.979953774335947,0.998212031119848
"13901","CHM",46.2905131652202,0.00611288150944956,0.237466798650509,0
.025742047074320 3,0.979463086242194,0.998212031119848
"13902","CDKN1A",329.049347047505,-0.0100089560864465,0.39993886220845
1,-0.0250262153349572,0.980034053340842,0.998221996497362
"13903","MPV17",60.9565596018487,0.00643063323379379,0.258809747810102
,0.0248469514313355,0.980177040777581,0.998295828052258
"13904","AL691432.2",4.99045739997256,-0.0132537573774982,0.6001916855
85732,-0.0220825407878881,0.982382113513953,0.998408600359838
"13905","GPN2",17.0286974456493,-0.00773508340202614,0.335110908478359
,-0.0230821593876155,0.981584736641818,0.998408600359838
"13906","AC104506.1",5.71536836360105,-0.0136541799350922,0.5770792653
05001,-0.0236608396038552,0.981123142722822,0.998408600359838
"13907","CTTNBP2NL",30.1688025474838,-0.00540416844986812,0.2673859968
66297,-0.0202111124486837,0.983874963244886,0.998408600359838
"13908","OAZ3",1.64272188293098,0.0232408588670744,1.08223395898578,0.
0214748933667306,0.982866831034825,0.998408600359838
"13909","UBE2Q1",68.6022326267674,-0.00800616796927631,0.3671608102113
08,-0.0218056169030366,0.982603013612681,0.998408600359838
"13910","CD247",3.93001541670292,-0.0216451087746446,0.906250558619347
,-0.0238842432358006,0.980944942774951,0.998408600359838
"13911","TTLL4",6.05039156785022,0.0129684237418943,0.536215890678153,
0.0241850791208241,0.980704979786973,0.998408600359838
"13912","NEK4",13.2368954863616,0.00817162448468157,0.407991705458261,
0.0200288985667077,0.984020319463739,0.998408600359838
"13913","C3orf38",33.8964981282428,-0.0053100404998076,0.2497543765706

95,-0.0212610508481102,0.983037413730908,0.998408600359838
"13914","MBD4",60.1199770362413,0.00456455146539439,0.208357810825733,
0.0219072731053606,0.982521923069331,0.998408600359838
"13915","MED28",73.007199759272,0.00515451276542974,0.218647195584474,
0.0235745661024877,0.981191959821924,0.998408600359838
"13916","GUCY1A1",11.7465548957071,0.0172370872157811,0.78828704187926
4,0.0218665109281617,0.982554438792146,0.998408600359838
"13917","FGF10",22.8972460361102,-0.00998910186300236,0.40902035722489
6,-0.024422016377805 7,0.980515987031893,0.998408600359838
"13918","ZCCHC9",56.9285149457763,0.0052274319679171 8,0.23039672453418
,0.0226888293593848,0.981898486423289,0.998408600359838
"13919","RAPGEF6",21.1601253546288,-0.00762434495722079,0.332038056826
917,-0.022962262308369,0.981680375321507,0.998408600359838
"13920","MAPK9",34.9430984291583,-0.00648352102607605,0.30815817769225
3,-0.0210395877683016,0.983214076184557,0.998408600359838
"13921","GTPBP2",18.0490380077845,0.00674431365238386,0.33598440746952
9,0.0200732935887672,0.983984904481014,0.998408600359838
"13922","DCBLD1",4.50874339692624,-0.0147801771377656,0.66298589288645
6,-0.0222933508787296,0.982213952795791,0.998408600359838
"13923","AC004982.2",4.45822438252897,0.0138655390809535,0.67409222164
9526,0.0205692020107042,0.98358930850136,0.998408600359838
"13924","HOXA7",16.2809250544023,-0.0129636567322102,0.583197547668589
,-0.0222285858094469,0.982265615042425,0.998408600359838
"13925","ZNF212",6.99241259622869,0.0118438625851574,0.534351475781019
,0.022164929118697,0.982316393222512,0.998408600359838
"13926","NBN",78.3543229977338,-0.00494337850576919,0.2180998345208,-0
.0226656683010814,0.98191696152303,0.998408600359838
"13927","ENTR1",30.0144701480354,0.0056376819098177,0.268314436731132,
0.0210114743675422,0.983236502475397,0.998408600359838
"13928","ITIH5",493.353923177166,0.00584383267068813,0.27447142500261,
0.0212912242891316,0.983013344256601,0.998408600359838
"13929","ENO4",1.90317735465396,0.0249107824009342,1.02144958084505,0.
0243876769525182,0.980543377771026,0.998408600359838
"13930","RPL27A",3154.36872496777,0.00482466403862368,0.2000060099412,
0.0241225953162213,0.980754820109219,0.998408600359838
"13931","DAGLA",4.29051902252825,-0.0143862542528251,0.651195241366949
,-0.0220920752163766,0.982374507996065,0.998408600359838
"13932","LTA4H",35.5618074831489,-0.00553237808855292,0.27046927283811
1,-0.0204547379097821,0.98368061842927,0.998408600359838
"13933","EXOC5",50.5092115446257,0.00505600340413602,0.231552530449541
,0.0218352327841989,0.982579389183178,0.998408600359838
"13934","ZC3H14",58.5397989055635,0.0042688656983134,0.203570833302819
,0.0209699279069282,0.983269644452735,0.998408600359838
"13935","LIPC",4.2228448520191,-0.0169934563536585,0.804600340869853,-
0.0211203693193653,0.983149636151237,0.998408600359838
"13936","AC092868.2",2.13952570650104,-0.0196517884503208,0.9264925554
06184,-0.0212109512760254,0.98307737839357,0.998408600359838
"13937","ATXN2L",43.4027213202436,0.00481903492443847,0.24036392127625
7,0.0200489112461259,0.984004354861487,0.998408600359838
"13938","ZNF48",4.73564497244567,0.0148926948958759,0.647500806242961,

0.0230002723584064,0.981650055696969,0.998408600359838
"13939","MIR762HG",6.15973027106628,0.0127044551372544,0.607045993030006,0.0209283238553992,0.983302832399787,0.998408600359838
"13940","FBXL19-
AS1",2.22355245156439,0.0191728085205859,0.952007448872475,0.0201393471692827,0.98393221200086,0.998408600359838
"13941","CCL22",1.64494592045422,-0.0237650056776027,1.17053377686378,
-0.0203027081724001,0.983801895424074,0.998408600359838
"13942","GEMIN4",14.0007511860135,0.0102904932529701,0.428436226128278
,0.0240187281686331,0.980837670097633,0.998408600359838
"13943","MAFG",56.1522206893046,-0.00584947923412997,0.251748034971441
,-0.0232354514099526,0.981462460098723,0.998408600359838
"13944","ZNF236",19.7591412883386,0.00699510588838868,0.341974179351174,0.0204550703262464,0.983680353254786,0.998408600359838
"13945","LMNB2",17.2883947873365,0.0076650444572233,0.33407364127088,0
.0229441761045978,0.98169480222335,0.998408600359838
"13946","ZNF442",1.79473912234553,-0.0268175005433734,1.11612430112356
,-0.0240273422201964,0.980830799061829,0.998408600359838
"13947","ZSWIM4",4.24377334574672,0.0166292905209971,0.793230990889419
,0.0209639949926204,0.983274377193059,0.998408600359838
"13948","ARHGAP33",8.92462661191021,0.0128887636778017,0.612590785306677,0.021039760941473,0.983213938042936,0.998408600359838
"13949","BLCAP",61.8562923928465,-0.00444095254639031,0.2202173550409,
-0.0201662241632387,0.983910771616627,0.998408600359838
"13950","STK4",46.2944162768195,-0.0058041479442769,0.278971227638448,
-0.0208055432576695,0.983400775817,0.998408600359838
"13951","DGCR6L",95.1031300677153,-0.0062483134399015,0.295555220998397,-0.0211409340660033,0.983133231520177,0.998408600359838
"13952","TXN2",137.80369105787,0.0048144092130047,0.215054581512022,0
.0223869176799455,0.982139315916995,0.998408600359838
"13953","APEX2",9.81721580038331,-0.00924608823224504,0.46176535702473
,-0.0200233475543075,0.98402474764279,0.998408600359838
"13954","PJA1",30.9063066504273,-0.00667496259917326,0.285795389328586
,-0.0233557392750619,0.981366510307136,0.998408600359838
"13955","IL2RG",3.34529132670184,0.0156168522124121,0.738069971970525,
0.0211590402068759,0.983118788140701,0.998408600359838
"13956","TIMM8A",13.0863242984444,-0.00943968350095943,0.38704306949898,-0.0243892327362402,0.980542136804331,0.998408600359838
"13957","EPB41L1",29.6944522647993,0.00537799494885205,0.26992992349352,0.0199236708522283,0.984104262282008,0.998417736899995
"13958","DET1",9.7424506147797,-0.00917300425617617,0.46686242624705,-
0.0196481955721193,0.984324016734902,0.998569141493496
"13959","NPRL2",18.4382632285162,0.00690337014052413,0.353716737667876
,0.0195166623610729,0.984428944932736,0.998601661008621
"13960","VIPAS39",17.4996781862765,-0.00663103460299129,0.341257008270496,-0.0194312041724729,0.984497117774036,0.998601661008621
"13961","TCTN1",50.9223493980877,0.0057015653574667,0.295199659531529,
0.0193142680669581,0.984590401780651,0.998624746738344
"13962","GPX7",47.8801607046771,0.0055748232744523,0.296927264400079,0
.0187750467634417,0.985020560112306,0.998631316043503

"13963","CD2",2.95379342403518,-0.0150721479129601,0.841014913125656,-0.0179213800822438,0.98570157291314,0.998631316043503
"13964","C1orf54",56.6993320943909,-0.00701501210935876,0.379794194985622,-0.018470561693615,0.985263461923678,0.998631316043503
"13965","B3GALNT2",24.0682122916119,0.00587469787852773,0.309385258594448,0.0189882928010751,0.984850444718804,0.998631316043503
"13966","AC007405.3",9.21918762009509,0.0103325207546556,0.590317231574612,0.0175033358370628,0.986035071641689,0.998631316043503
"13967","SLC4A3",5.9395038991434,-0.0101953516785377,0.550519654773381,-0.0185195053258081,0.985224417233712,0.998631316043503
"13968","PBRM1",79.090379972425,-0.00397363484831039,0.226332319328438,-0.0175566391052801,0.985992548321163,0.998631316043503
"13969","HNRNPDL",313.566873897556,0.00339385620278573,0.197495868326149,0.0171844415356631,0.986289474213869,0.998631316043503
"13970","AP001816.1",18.5755751655585,0.0065796852239476,0.380043389638276,0.017312984262692,0.986186927212569,0.998631316043503
"13971","CDYL",49.3911227334364,0.00433950173125153,0.235991147841734,0.018388408933719,0.985328999211726,0.998631316043503
"13972","U91328.1",1.73095162692012,0.0184862614449312,0.99850438255852,0.0185139512333064,0.985228847998716,0.998631316043503
"13973","TAP1",46.640744344043,0.00532614035397655,0.308490399785704,0.0172651737547632,0.986225068777769,0.998631316043503
"13974","GALNT12",24.2646945835331,0.00597876576653997,0.31907381493566,0.0187378765874146,0.985050212405509,0.998631316043503
"13975","ARCN1",79.3476163430483,0.00348102561387954,0.199849638442735,0.0174182232252424,0.986102971329193,0.998631316043503
"13976","CHMP4A",91.0581405939192,0.0042949789633155,0.248815289346799,0.0172617164105585,0.9862278269283,0.998631316043503
"13977","MIS18BP1",65.964971411149,0.00427952400825889,0.246770256480879,0.017342138672982 6,0.986163668850578,0.998631316043503
"13978","AC015712.2",5.7840211886807,-0.0106661202551778,0.610182035047276,-0.0174802266250782,0.986053507304618,0.998631316043503
"13979","MAP1LC3B",200.593722034478,-0.00351681447521818,0.194734143698342,-0.0180595678211726,0.985591332891274,0.998631316043503
"13980","PCTP",12.5493559857615,-0.00746752802610967,0.409057873806391,-0.0182554315765209,0.985435081984789,0.998631316043503
"13981","PDE4A",44.2708124780278,0.00448087276858323,0.258957382490369,0.0173035142906184,0.986194482025375,0.998631316043503
"13982","ATP9A",28.462095945786,-0.00575552235277477,0.334766402620805,-0.0171926522724986,0.98628292396141,0.998631316043503
"13983","THAP7-AS1",11.5357533732608,-0.00810588562459251,0.427881981319554,-0.0189442088671147,0.984885612283304,0.998631316043503
"13984","IGLC3",11.1259701879931,-0.0245080059172472,1.3632520170250 3,-0.0179776047357187,0.985656719356339,0.998631316043503
"13985","MIATNB",5.86134952566508,0.00979296856541809,0.547895869635129,0.0178737769495136,0.985739548635046,0.998631316043503
"13986","PRELID2",14.4613751809827,0.0066403542060048,0.388672928824372,0.0170846840969605,0.986369057450595,0.998640487165767
"13987","FAM126B",28.5508468969482,-0.00487270418938192,0.286789277147

046,-0.0169905382720552,0.986444164048953,0.998645124968412
"13988","ARHGEF11",27.9934246082511,0.00538767167064785,0.332079618611
277,0.016224035950109,0.987055660070763,0.998778238174975
"13989","ADI1",149.300943096539,-0.00427626388267992,0.25684107341243,
-0.0166494549561907,0.986716270665435,0.998778238174975
"13990","TRMT10C",50.6314488815988,-0.00378618158611816,0.233769618413
748,-0.0161962089505447,0.987077859887195,0.998778238174975
"13991","C1QTNF7",89.1991027467984,-0.00745457495712754,0.465039328244
137,-0.0160299882276065,0.987210467619841,0.998778238174975
"13992","LY6G5C",1.83200554690489,0.0162087359053821,1.00893280318898,
0.0160652283820592,0.987182353664951,0.998778238174975
"13993","KIAA2026",70.3368674366897,0.0042622063091337,0.2632386527120
3,0.0161914151482774,0.987081684286524,0.998778238174975
"13994","P3H3",60.3477495734049,-0.00412576915492277,0.249465676853086
,-0.0165384240708693,0.986804848298403,0.998778238174975
"13995","NPC2",371.466608316669,0.00314992981238759,0.195929497955696,
0.0160768533847816,0.987173079452501,0.998778238174975
"13996","BRCC3",16.9168493175201,-0.00559558108116815,0.34568614427523
1,-0.0161868827369402,0.987085300153683,0.998778238174975
"13997","LZTFL1",18.9540856138392,0.00498456510483782,0.31486990381046
3,0.0158305542845349,0.987369572694044,0.998797747445286
"13998","ARL5B",39.974053226552,0.00490787800228827,0.311797979797297,
0.0157405702419205,0.987441360627582,0.998797747445286
"13999","INPP5A",13.0742094626201,0.00684537560932144,0.43366025400157
6,0.0157851118384867,0.98740582599024,0.998797747445286
"14000","MIB2",31.6973466016609,0.00432253942976959,0.282337701997311,
0.015309820116802,0.987785008081864,0.999002622272638
"14001","ZSWIM7",63.2467418799997,0.0045928484240099,0.298658737099188
,0.0153782489962266,0.98773041616239,0.999002622272638
"14002","AC023632.2",3.16135846780064,-0.011360292861566,0.75221310449
5671,-0.015102492622995,0.987950412363816,0.999098545855709
"14003","PGGT1B",43.0073765507771,-0.00392371775647284,0.2626056906352
86,-0.0149414803121011,0.988078867106268,0.999157091924927
"14004","PANK4",12.4140888408569,0.00592877866875778,0.406212714805382
,0.0145952562602534,0.988355083806713,0.999214805883681
"14005","FMO5",4.31678166878002,-0.00973379263740338,0.716293862315626
,-0.0135891051836408,0.989157796473588,0.999214805883681
"14006","TMEM214",52.2025539383581,-0.00318480466495178,0.239066575567
376,-0.0133218316169598,0.989371030621463,0.999214805883681
"14007","PPP4R3B",71.3287028970999,-0.00357382284868675,0.256998202713
859,-0.0139060227306953,0.988904956750719,0.999214805883681
"14008","PRKCI",36.4782918076006,0.00369422797650276,0.270689575604583
,0.0136474704216139,0.989111232069393,0.999214805883681
"14009","RBM24",2.00805064298928,0.0124473429900769,0.895431321223823,
0.0139009466109188,0.988909006516875,0.999214805883681
"14010","FNDC1",69.7045192253788,0.00499498172148909,0.369460313174186
,0.0135196705664409,0.989213192193701,0.999214805883681
"14011","AC006480.2",1.98058056590025,0.0138340797959856,1.01998362025
587,0.0135630411324794,0.989178590661227,0.999214805883681
"14012","PRR5L",3.20389419322162,-0.0123112547801206,0.864466811463856

,-0.01424144295288,0.988637356638395,0.999214805883681
"14013","RAD9A",15.1343334107379,0.0051854203174129,0.370160583716344,
0.0140085696465902,0.988823144118993,0.999214805883681
"14014","RSRC2",213.102379356099,0.00213748009127605,0.154700012768271
,0.0138169354548007,0.988976031284216,0.999214805883681
"14015","SLC25A30",5.51047833031501,0.00834985830529442,0.586885244994
788,0.0142274122181562,0.988648550410915,0.999214805883681
"14016","HYPK",3.83562793882786,-0.00956049162345286,0.694402141926828
,-0.0137679466208506,0.98901511500082,0.999214805883681
"14017","AC090825.1",3.21876111591522,0.00972789323326168,0.7164287909
46579,0.0135783114193509,0.989166407856957,0.999214805883681
"14018","SRP68",45.362381178349,0.00317309777155494,0.21687848528423,0
.0146307632469696,0.988326756354897,0.999214805883681
"14019","CYTH1",22.3282244219833,-0.00485689941791522,0.36578993321777
1,-0.0132778378431309,0.989406129469992,0.999214805883681
"14020","ZNF582-
AS1",6.59091120965889,-0.00935506496271351,0.70021148588663,-0.0133603
420556117,0.989340306471369,0.999214805883681
"14021","RHBDD3",17.9409044205873,0.00462478478861311,0.32222840715434
7,0.014352504887621,0.988548751091521,0.999214805883681
"14022","CYP51A1",51.9310245871842,-0.00315721476481162,0.240042543732
428,-0.0131527299941085,0.989505942374868,0.999244340609622
"14023","AC240274.1",25.1388085031657,-0.00526924538356071,0.405199402
934655,-0.0130040798318019,0.989624537901223,0.999292837244621
"14024","DDIT3",75.854819734216,0.00339371940981961,0.267172748313993,
0.0127023412052159,0.989865270605914,0.99946464858669
"14025","LRP3",24.8759223180751,0.00409392101714692,0.32693729267004,0
.0125220374332722,0.990009120760223,0.999538620318343
"14026","DFFB",5.98085708946979,0.00769795164669169,0.627618875951929,
0.0122653284368096,0.990213929174322,0.999587002817927
"14027","DPH5",34.1306643512876,0.00362843866650755,0.299148338021522,
0.0121292288986292,0.990322512816994,0.999587002817927
"14028","INTS9",9.4417431067007,-0.00525652962592096,0.432924867555796
,-0.0121418980979269,0.990312405002803,0.999587002817927
"14029","PSMA4",234.86363412883,-0.00233551906001187,0.192889802662955
,-0.0121080483663143,0.990339411195812,0.999587002817927
"14030","ZBTB48",12.6181927929803,-0.00407118936132779,0.4256847146666
99,-0.00956386081307955,0.99236925944313,0.999642093163714
"14031","TMEM69",14.698465367528,0.00191453493687455,0.369987020588849
,0.00517460027064596,0.995871284761149,0.999642093163714
"14032","DLEU2L",2.75810165112277,-0.00570518167713613,0.8838018704326
76,-0.00645527223691334,0.994849473717281,0.999642093163714
"14033","DNAJB4",55.224229682168,-0.00184252104588983,0.26889168230130
7,-0.00685227981066812,0.994532714517716,0.999642093163714
"14034","ZNF697",8.36854017066893,0.00545698931681772,0.52152418964951
3,0.0104635401868609,0.99165145517485,0.999642093163714
"14035","S100A14",1.74469890595833,0.00713133382514865,0.9848636745811
85,0.00724093497324008,0.994222620264885,0.999642093163714
"14036","ASH1L-
AS1",4.12481001430803,-0.00690418752958609,0.78920630924178,-0.0087482

6702313011,0.99301998184124,0.999642093163714
"14037","SLAMF8",1.74739767712992,-0.00576643746235877,1.3517840017813
1,-0.0042657979786416,0.996596395975923,0.999642093163714
"14038","PKDCC",231.767388486791,0.00256055192808676,0.271387575363812
,0.00943503741707475,0.992472041004216,0.999642093163714
"14039","LINC01121",2.09881072758176,-0.00855262656806013,0.9614469212
54302,-0.00889557850671817,0.992902448856631,0.999642093163714
"14040","IL18R1",3.80116559927412,0.00563845244139722,0.70716683502533
4,0.00797329874950262,0.993638295435138,0.999642093163714
"14041","HOXD-
AS2",39.4672730985535,-0.00434796303657488,0.381528729217324,-0.011396
1615564164,0.99090737545579,0.999642093163714
"14042","RETREG2",70.2395843488615,-0.00117155974528124,0.195868193694
581,-0.00598136799641936,0.995227587280107,0.999642093163714
"14043","DGKD",10.6201505486557,-0.00610274141771119,0.51134467071481,
-0.0119346925219347,0.99047771915336,0.999642093163714
"14044","ING5",29.9865066662127,-0.00262409173931135,0.301040964087936
,-0.00871672646698286,0.993045146604521,0.999642093163714
"14045","OGG1",26.4905419016004,0.00139655280633764,0.265246607959815,
0.00526511089841805,0.995799068712524,0.999642093163714
"14046","SH3BP5-
AS1",4.37092137553317,-0.00500170971920116,0.652665711143182,-0.007663
50925719749,0.993885464132648,0.999642093163714
"14047","ITGB5",160.70264825203,0.00215002908092514,0.204169021161386,
0.0105306332405132,0.991597925612475,0.999642093163714
"14048","PLXNA1",54.4699529268,-0.0015402878408566,0.242463926235344,-
0.00635264744233145,0.99493135477738,0.999642093163714
"14049","CLCN2",4.72729409694731,-0.00654302040913061,0.67980837439106
6,-0.00962480112868789,0.992320638344049,0.999642093163714
"14050","MB21D2",8.65773684041748,-0.00500127544414425,0.5330898470015
83,-0.00938167453811852,0.992514616537076,0.999642093163714
"14051","GAK",27.7097946753268,-0.00350077535253533,0.316151315593506,
-0.0110731006953534,0.991165124461253,0.999642093163714
"14052","NFXL1",16.4081604997324,0.00445143351053994,0.407902832751817
,0.0109129752311584,0.991292878377225,0.999642093163714
"14053","PLA2G12A",60.8459969339247,-0.0022672734285002,0.200728666010
799,-0.0112952149464204,0.990987914013522,0.999642093163714
"14054","LRP2BP",2.01401326608641,-0.00625752094532586,0.9517785697343
63,-0.00657455541058494,0.994754301534471,0.999642093163714
"14055","RETREG1",16.2869977764381,0.00354788639694728,0.6924005801023
8,0.00512403729705525,0.995911627642262,0.999642093163714
"14056","LMBRD2",15.3538169287121,0.00203117114319281,0.41344777070730
4,0.00491276356314122,0.996080197569667,0.999642093163714
"14057","ARHGEF28",2.08148355887474,-0.00509615179801514,0.95882700456
8894,-0.005314985679097,0.995759274951861,0.999642093163714
"14058","AL357054.4",2.84366928754947,0.00833920280746228,0.9719337807
4079,0.0085800112854461,0.993154225457698,0.999642093163714
"14059","PRPF4B",150.256069216582,-0.00116238242661101,0.1712129287058
64,-0.00678910427732905,0.994583120127885,0.999642093163714
"14060","HLA-

E",1235.17084682839,0.00180681827198489,0.207889579634723,0.0086912402
01455,0.993065480932026,0.999642093163714
"14061","LY6G5B",5.70445067211137,-0.00546997573215877,0.5793324997531
3,-0.00944185892296684,0.992466598472411,0.999642093163714
"14062","AL662884.3",2.79557957161628,0.00812568033925372,0.9747969591
61718,0.00833576701577059,0.993349097218766,0.999642093163714
"14063","AL354740.1",10.8467458937634,-0.00343368763482415,0.413963650
514802,-0.00829465976192364,0.993381894928063,0.999642093163714
"14064","CMTR1",18.588543795306,0.00192463469865147,0.344633154484663,
0.0055845895080217,0.995544165414301,0.999642093163714
"14065","PRIM2",12.1685590736316,-0.00431864327903873,0.39942554098720
7,-0.0108121360200575,0.991373331680163,0.999642093163714
"14066","HMGN3",217.367601805508,0.00107329417017782,0.196057182520817
,0.00547439352324602,0.995632087745036,0.999642093163714
"14067","FBXO5",13.5342935722493,-0.00408026170683339,0.4035338733823,
-0.0101113239209235,0.991932468223677,0.999642093163714
"14068","GLCCI1",7.99297150627878,-0.00284817597088875,0.4937031175170
15,-0.00576900544038107,0.995397025160206,0.999642093163714
"14069","HERPUD2",40.4839471690548,-0.0014319587973336,0.2534838349650
14,-0.00564911288142393,0.995492684022961,0.999642093163714
"14070","CLIP2",10.6654114891095,-0.00384860691636182,0.42403501087561
7,-0.00907615366102572,0.992758376545874,0.999642093163714
"14071","AC106845.1",2.88346136488776,-0.00436207862422962,0.993572819
97418,-0.00439029584599846,0.996497061980136,0.999642093163714
"14072","ZDHHC2",18.2665658768625,0.0036157035359813,0.331489328806849
,0.0109074507737415,0.991297285994171,0.999642093163714
"14073","LINC00824",3.46931109852135,0.00335692995778057,0.74311034847
4355,0.00451740439986137,0.996395645033429,0.999642093163714
"14074","DOCK8-
AS1",1.62376390500065,-0.010675995698835,1.56134038042292,-0.006837711
8997865,0.994544337756591,0.999642093163714
"14075","LINC01235",2.15690389617464,-0.0112975703504869,1.27589023397
38,-0.0088546569678649,0.992935098234801,0.999642093163714
"14076","ODF2",19.6225524452557,0.00389142612287111,0.34535270551558,0
.011267976363647,0.991009645875176,0.999642093163714
"14077","SIRT1",31.1203180715467,0.00171276137161614,0.297550236264387
,0.00575620907957968,0.9954072350094,0.999642093163714
"14078","MCU",17.1628239751408,-0.00274219813511361,0.343002828455872,
-0.00799468082364925,0.993621235552041,0.999642093163714
"14079","PPIF",26.8529259415904,0.00318711679113818,0.32203646452426,0
.00989675748628797,0.992103658902144,0.999642093163714
"14080","TRIM3",13.4218508667589,0.00463939077824557,0.407542446071538
,0.0113838222814985,0.990917220134134,0.999642093163714
"14081","PIK3C2A",43.6575485582754,-0.00173555026748592,0.263643526576
767,-0.00658294284718789,0.994747609473119,0.999642093163714
"14082","CAPRIN1",109.842410123979,-0.00151744111779972,0.201880069866
367,-0.00751654741750475,0.994002719337962,0.999642093163714
"14083","B3GAT3",58.9496685021759,0.00172215215528454,0.26919051623205
8,0.00639752164894229,0.994895551068319,0.999642093163714
"14084","SF1",202.216891111307,0.00126708251519179,0.135099365937001,0

.00937889313102066,0.992516835681224,0.999642093163714
"14085","LRFN4",19.2594725681804,-0.00341932841650726,0.32985746179906
6,-0.0103660787233916,0.9917292139545,0.999642093163714
"14086","PRR4",5.10840638716766,-0.00410751504926038,0.719549901160481
,-0.00570845057811256,0.995445340154299,0.999642093163714
"14087","ATXN7L3B",98.2633605187401,0.00198581618826948,0.171087116269
041,0.0116070469336025,0.99073912439862,0.999642093163714
"14088","STX2",62.3486061629127,0.00214479641526558,0.250297745184826,
0.00856898017072353,0.993163026690276,0.999642093163714
"14089","C15orf39",8.92748398255606,-0.00425417403619791,0.43858357365
212,-0.00969980248182363,0.992260798715738,0.999642093163714
"14090","AC015871.1",7.95221408555178,0.00417700482229713,0.5246270903
51745,0.00796185499970385,0.993647425936603,0.999642093163714
"14091","TTC23",17.6706452033661,0.00195157489654127,0.339485328953813
,0.00574862808521184,0.995413283667684,0.999642093163714
"14092","KCTD5",12.5403420892807,0.00475491253914986,0.432898155893759
,0.0109839057395218,0.991236287411558,0.999642093163714
"14093","PLD6",2.0422503599049,0.00436502032723665,0.989097091057953,0
.00441313635102067,0.996478838070365,0.999642093163714
"14094","FLII",67.7734660458668,-0.00110593979704778,0.222911686542707
,-0.00496133609771912,0.996041442766569,0.999642093163714
"14095","ABHD15",10.5401852141686,0.0020733321238671,0.452567450899943
,0.00458126654876356,0.996344690938096,0.999642093163714
"14096","SLFN11",45.6358053312422,-0.00196224275784689,0.2375231674622
12,-0.0082612689061 5443,0.993408536063549,0.999642093163714
"14097","AC006141.1",5.44451165459966,-0.00465905388914917,0.708892224
129901,-0.00657230215053878,0.994756099337032,0.999642093163714
"14098","TIMM21",21.6031800464311,0.00221932267890482,0.32472126709162
8,0.00683454674460415,0.994546863126034,0.999642093163714
"14099","CYB5A",310.427710801038,0.00129831270918957,0.296479088347455
,0.00437910382289098,0.996505991836737,0.999642093163714
"14100","TECR",95.6407173965531,0.000871465999324541,0.204897106611713
,0.00425318841117655,0.996606456863853,0.999642093163714
"14101","SLC25A42",7.58602069816437,-0.00339901221568271,0.50353820213
4464,-0.00675025688472996,0.994614115152428,0.999642093163714
"14102","KCNK6",10.3294966252172,-0.0033555222097949,0.533652613235382
,-0.00628783992914667,0.994983062658807,0.999642093163714
"14103","SYMPK",31.138023086095,0.00274905730722123,0.279148006708117,
0.00984802771705156,0.992142537737973,0.999642093163714
"14104","NAPA",70.4712293656423,-0.00217695359083467,0.210914444327436
,-0.0103215007287743,0.991764780145419,0.999642093163714
"14105","EPN1",86.3945751515433,-0.00169979496525731,0.196146644918545
,-0.00866593953703971,0.993085667181333,0.999642093163714
"14106","TRIB3",1.9765122123031,0.00704851491320159,1.10286621236884,0
.00639108790726493,0.994900684346528,0.999642093163714
"14107","IDH3B",70.1294274715146,-0.0029206130001932,0.246871859813432
,-0.0118304816207096,0.990560861552588,0.999642093163714
"14108","IL10RB-
DT",2.47603906221404,0.00616990532792141,0.937394820971654,0.006581970
78742766,0.994748385047791,0.999642093163714

"14109","DGCR8",19.1028940610868,0.00327226153062242,0.369418452305557
,0.00885787244843914,0.992932532753107,0.999642093163714
"14110","POLR2F",131.191752502174,-0.00206847243129431,0.2333266187296
51,-0.0088651369593239,0.992926736739604,0.999642093163714
"14111","JOSD1",51.9411738684508,0.00101635916380022,0.221147708147004
,0.00459583855657512,0.996333064280443,0.999642093163714
"14112","RAB9A",77.0005938240476,0.00220008246836714,0.28235147245444,
0.00779199927396215,0.99378294699324,0.999642093163714
"14113","FTSJ1",23.8872448355275,-0.00195751792158597,0.29031749699076
7,-0.00674267979669248,0.994620160656407,0.999642093163714
"14114","PIM2",1.97103079544628,-0.00430713333219767,0.917966058699003
,-0.00469203985417718,0.996256307578065,0.999642093163714
"14115","APOOL",36.4839703449867,0.00216876004513179,0.264964781939385
,0.0081850879549265,0.993469317613267,0.999642093163714
"14116","BEX4",69.746938555455,0.00268632106900718,0.251498899018819,0
.0106812438523088,0.991477762489264,0.999642093163714
"14117","BEX3",236.225139666267,-0.00314327664815358,0.305731183212211
,-0.0102811777821558,0.991796951494952,0.999642093163714
"14118","GSTM4",22.408811165023,-0.000122080771977435,0.33300222632367
8,-0.000366606473852136,0.999707490361175,0.999846150946599
"14119","NBPF20",4.71866552947274,0.000705424190332917,0.6467425412105
63,0.00109073417222952,0.9991297202166,0.999846150946599
"14120","GLI2",34.824743085496,-0.0006787356784217,0.294201774897869,-
0.00230704141284437,0.99815924890844,0.999846150946599
"14121","ZNF619",3.52592769715223,0.000604974726974175,0.6790822863056
33,0.000890871016921056,0.999289187863955,0.999846150946599
"14122","CCDC50",155.027476498382,-0.000483105112534161,0.151275960526
531,-0.00319353525076069,0.997451931860189,0.999846150946599
"14123","ERAP2",78.3674106087072,-0.00353489059848168,0.99123696063459
6,-0.00356614083096601,0.997154637320242,0.999846150946599
"14124","PRRC1",63.9480860676715,-0.000476737270418471,0.1945700341353
07,-0.00245020911127014,0.99804501793551,0.999846150946599
"14125","AC008608.2",5.32668866277174,-0.00213897661195248,0.646468971
166908,-0.00330870731211047,0.997360038336302,0.999846150946599
"14126","TRIM41",10.8184459917699,0.000237084317467943,0.4554716055329
32,0.000520524912174357,0.999584681227818,0.999846150946599
"14127","TRIM39",8.71206347568954,0.000254365290228625,0.4871659117101
12,0.000522132776769458,0.999583398337656,0.999846150946599
"14128","SKIV2L",12.0960860214671,-0.000481035964943976,0.440832055933
917,-0.00109120005786531,0.999129348493865,0.999846150946599
"14129","CNPY3",55.6892654019202,-0.000778166438090913,0.2547906211606
9,-0.00305414082569445,0.997563151977057,0.999846150946599
"14130","TBL2",36.8364454354644,0.000426918089929509,0.339869971943415
,0.00125612182649836,0.998997760051712,0.999846150946599
"14131","LY96",77.5613449793436,0.000311695140697582,0.375884855096898
,0.000829230378588228,0.999338369959401,0.999846150946599
"14132","WASHC1",29.7421987048911,7.53499420255673e-05,0.3304453060447
74,0.000228025457306262,0.999818062009722,0.999846150946599
"14133","TMEM38B",17.6963782050134,-0.000434516517917117,0.37836241091
848,-0.00114841354579151,0.999083698763807,0.999846150946599

"14134","TRAF2",15.4055150295302,-9.01699557853624e-05,0.367465447258414,-0.000245383495123426,0.99980421229973,0.999846150946599
"14135","HSPA14.1",24.5685699060801,0.000814155291933688,0.329055419803724,0.00247421936529511,0.998025860582594,0.999846150946599
"14136","FAM53B",14.302044964111,0.00103176232118438,0.364883488547146,0.00282764869765015,0.997743865767294,0.999846150946599
"14137","FAM160A2",17.3391887578648,0.00102909125892404,0.382215864045223,0.00269243470962335,0.997851750509739,0.999846150946599
"14138","LIN7C",42.5960195461891,-0.000943990290176166,0.27087489414543,-0.00348496782307683,0.997219403607464,0.999846150946599
"14139","CAPN5",30.5899743905931,0.000703236005204159,0.28237225189763,0.00249045719074092,0.998012904712287,0.999846150946599
"14140","FAM118B",10.5892152515501,0.00180335421553456,0.493781504555261,0.00365212993783315,0.997086028386343,0.999846150946599
"14141","FOXJ2",24.3868950537203,0.000229211852171984,0.296461859606757,0.000773157978824063,0.999383109247095,0.999846150946599
"14142","KCNJ8",27.667880428798,-0.000909824637524797,0.399837929252094,-0.00227548356712092,0.998184428360219,0.999846150946599
"14143","COQ10A",12.4435210424491,-0.00118050041674888,0.433150752326791,-0.00272538004472459,0.99782546403196,0.999846150946599
"14144","TAOK3",74.5055462823741,-0.000590605179996006,0.224699250120987,-0.00262842523808157,0.997902822498077,0.999846150946599
"14145","SFSWAP",62.1367212203552,-0.000230371910850514,0.239125264981251,-0.000963394273158785,0.999231322702386,0.999846150946599
"14146","PSPC1",33.4458627592183,0.000762698656176881,0.270731932946288,0.00281717286866266,0.997752224236213,0.999846150946599
"14147","JKAMP",52.6236736843361,0.000169640134882439,0.241802809162487,0.00070156395399213,0.999440232998613,0.999846150946599
"14148","ELMSAN1",78.722621702547,-0.000551444277768056,0.279234851933281,-0.00197484043968772,0.998424306327324,0.999846150946599
"14149","SEMA4B",4.51725485553767,-0.000795453773682221,0.692347127699564,-0.00114892333896906,0.99908329200797,0.999846150946599
"14150","TMEM199",16.8745796513438,-0.000160573040116096,0.392189896119523,-0.000409426764189664,0.9996733247152,0.999846150946599
"14151","AC005726.1",2.64109668978468,-0.00143567592710848,0.803040958684529,-0.00178779912977326,0.998573543436417,0.999846150946599
"14152","MAPT",3.66505219351341,0.001381085357282,0.715514275399476,0.00193019958478248,0.998459924508336,0.999846150946599
"14153","YPEL2",63.4398720041783,-0.000381048023416585,0.355535780966849,-0.00107175717273901,0.999144861662653,0.999846150946599
"14154","NT5C",120.716434034873,4.83497353351878e-05,0.250749070280531,0.000192821194834723,0.999846150946599,0.999846150946599
"14155","ILF3-DT",52.6215282390612,-0.00052673031503177,0.224886621749581,-0.00234220386670356,0.998131193405192,0.999846150946599
"14156","ZNF799",5.49943226472536,-0.00124225255391814,0.605419495875141,-0.00205188726557681,0.9983628319791,0.999846150946599
"14157","NRSN2-AS1",4.61596534944182,0.000771415337160232,0.627509252121173,0.00122932902511415,0.999019137597769,0.999846150946599

"14158","USP16",125.690619022639,0.000538648959014127,0.163933906919166,0.00328576905862269,0.997378340315123,0.999846150946599
"14159","CSNK1E",108.712897343189,4.48660144389212e-05,0.159638412576637,0.000281047736035226,0.999775756353521,0.999846150946599
"14160","LINC00629",1.51288220870664,-0.00104840464367359,1.54120524341257,-0.000680249855205651,0.999457239184903,0.999846150946599
"14161","AL627309.1",1.31930751786183,1.76866309511719,1.41014423834103,1.25424268456249,0.209753809489001,NA
"14162","AL627309.5",1.03995205973715,0.214918583661529,1.39003022458818,0.154614324105939,0.877125376796604,NA
"14163","AL627309.4",0,NA,NA,NA,NA,NA
"14164","FAM41C",0.973681251642668,-2.3499649328482,1.49704633204585,-1.56973427110753,0.116476944686768,NA
"14165","PLEKHN1",0.358139358300896,-0.359409442181463,2.29893240859354,-0.156337542086044,0.875766965836093,NA
"14166","AL390719.2",0,NA,NA,NA,NA,NA
"14167","TTLL10",0,NA,NA,NA,NA,NA
"14168","TNFRSF18",1.41400865286429,-0.87493635119345,1.22935117083764,-0.711705794038736,0.476646979019782,NA
"14169","TNFRSF4",0.751985814484387,0.00459416429628531,1.63623498762973,0.00280776559052833,0.99775973012851,NA
"14170","AL162741.1",0.330989799689647,1.28767123567888,2.3893126005633,0.538929579735739,0.589935446033768,NA
"14171","LINC01786",0,NA,NA,NA,NA,NA
"14172","TAS1R3",0,NA,NA,NA,NA,NA
"14173","AL391244.2",0.27268524254951,-0.0202790885325299,2.50354399079405,-0.00810015266641987,0.993537083922046,NA
"14174","ATAD3C",1.28642224623787,-0.576542904963561,1.18820366222276,-0.485222292519302,0.627518670442843,NA
"14175","AL691432.4",0.120322481366256,1.41832010653068,3.35247519753738,0.42306654724024,0.672246708730081,NA
"14176","FO704657.1",0.167194048217763,-0.631571198727006,3.33432221989104,-0.189415166584483,0.849767438415713,NA
"14177","AL109917.1",0.2292884040636,0.696980734286753,3.06852794721869,0.227138467133238,0.820316073199257,NA
"14178","CALML6",0.16538586715322,1.41832010653068,3.35247519753738,0.42306654724024,0.672246708730081,NA
"14179","TMEM52",0.50436729447256,-0.207236086690067,1.91351899719312,-0.108301034373871,0.913756902132009,NA
"14180","CFAP74",0,NA,NA,NA,NA,NA
"14181","PRKCZ",0.847971363211281,0.45955375014985,1.3845229604419,0.331922086725939,0.739948092182947,NA
"14182","AL590822.2",0.439761757551905,2.57992481053724,2.54830735454105,1.01240723805935,0.311343379010575,NA
"14183","PRKCZ-AS1",0.839198803482186,1.91074272439296,1.53937071935334,1.24124923280054,0.214513692525929,NA
"14184","AL590822.1",0.225789575149882,0.696980732170056,3.08390169185347,0.226006144752027,0.821196641151035,NA
"14185","AL513477.2",0.900174461046488,-1.51221574977974,1.41616623155

39,-1.06782361850307,0.285600082029793,NA
"14186","PLCH2",0.278383754487592,1.24654657160755,2.93909347309959,0.424126208647913,0.671473772635966,NA
"14187","AL139246.1",0,NA,NA,NA,NA,NA
"14188","HES5",0,NA,NA,NA,NA,NA
"14189","AL139246.5",0.613161497540933,-1.52098113215724,1.74786596567168,-0.870193231076932,0.384194814452414,NA
"14190","AL139246.3",0,NA,NA,NA,NA,NA
"14191","MMEL1",0.527132829425758,-2.37961911772718,1.98644584726065,-1.19792800846231,0.230945044825114,NA
"14192","TTC34",0.0973092156216871,0.119912045671111,3.35247519753738,0.0357682126206911,0.971467179494267,NA
"14193","PRDM16-DT",0.981536408780311,-0.0559239277648951,1.36550377901617,-0.0409547953101876,0.967331933718456,NA
"14194","PRDM16",1.10412790529006,-1.80169924898205,1.52726680686978,-1.17968860508056,0.238124087595351,NA
"14195","AL354743.1",0.724458357684361,-0.868425052940428,1.62327364568979,-0.534983768908168,0.592661081001826,NA
"14196","AL513320.1",0.227597756214425,-1.13126253111685,2.5754042190421,-0.439256301108964,0.660475834030736,NA
"14197","TP73",0.173429405397192,1.41832010653068,3.35247519753738,0.42306654724024,0.672246708730081,NA
"14198","AL365330.1",0.167194048217763,-0.631571198727006,3.33432221989104,-0.189415166584483,0.849767438415713,NA
"14199","LINC01134",0.06390253691038,0.119912045671111,3.35247519753738,0.0357682126206912,0.971467179494267,NA
"14200","AL365255.1",0,NA,NA,NA,NA,NA
"14201","CHD5",0.16538586715322,1.41832010653068,3.35247519753738,0.42306654724024,0.672246708730081,NA
"14202","AL031847.1",0.346321965553037,1.26405017323688,2.3604933630023,0.535502532245692,0.592302407034682,NA
"14203","LINC00337",0.0604037079966622,0.119912045671111,3.35247519753738,0.0357682126206911,0.971467179494267,NA
"14204","AL031848.2",1.3986920107454,0.0148832876362637,1.12220702286082,0.0132625151447743,0.989418354138026,NA
"14205","HES2",0.0973092156216871,0.119912045671111,3.35247519753738,0.0357682126206911,0.971467179494267,NA
"14206","ESPN",0.338472392144151,-0.374118255054918,2.33551135129715,-0.160186870788332,0.872733871436184,NA
"14207","TNFRSF25",1.37356339608372,2.64872591479517,1.50557322045308,1.75928070372963,0.0785298424355025,NA
"14208","PLEKHG5",0.172892560155845,0.696980693484986,3.35247519753738,0.207900328091007,0.835306792989891,NA
"14209","CAMTA1-DT",0,NA,NA,NA,NA,NA
"14210","Z98884.2",0,NA,NA,NA,NA,NA
"14211","Z98884.1",0,NA,NA,NA,NA,NA
"14212","UTS2",0.451464810381797,0.571519235142493,2.00792341380662,0.284631989055304,0.775926100805242,NA
"14213","TNFRSF9",0.643657355740006,-1.65310896315217,1.70442776253351

,-0.969890892116743,0.332100880408206,NA
"14214","RERE-
AS1",0.33658777945989,-0.555619674618605,2.07497246909751,-0.267772070
66283,0.788874770266061,NA
"14215","ENO1-
AS1",0.244628726273298,0.06022438509446,2.57649611869115,0.02337452971
79232,0.98135152179478,NA
"14216","CA6",0,NA,NA,NA,NA,NA
"14217","LNCTAM34A",1.08498390498086,0.296966550172592,1.5328394023206
5,0.19373624511674,0.846382409990527,NA
"14218","BX323043.1",0.237331942307572,0.696980738944435,3.03442443415
691,0.229691249219751,0.818331695238684,NA
"14219","PIK3CD-AS1",0,NA,NA,NA,NA,NA
"14220","RBP7",1.14759111093242,-2.75794925788269,1.50719433599486,-1.
82985643723391,0.0672714089622535,NA
"14221","AL139424.3",0.466620346371172,0.624927545713488,2.24009382046
61,0.278973826901347,0.780264910781832,NA
"14222","AL139424.2",0.607149770604754,-0.354178997060217,1.6862573826
593,-0.210038515295726,0.833637612594052,NA
"14223","CORT",1.03946906490552,-0.262251530493591,1.33755971765686,-0
.196067156502742,0.844557602609226,NA
"14224","AL354956.1",0,NA,NA,NA,NA,NA
"14225","AL139423.2",0,NA,NA,NA,NA,NA
"14226","AL139423.1",0,NA,NA,NA,NA,NA
"14227","C1orf127",0,NA,NA,NA,NA,NA
"14228","MASP2",0,NA,NA,NA,NA,NA
"14229","AL109811.1",0.409517243898923,2.51477181352879,2.589856856302
77,0.971008033671338,0.331544278051701,NA
"14230","ANGPTL7",0.8269293357661,3.48175715344508,2.66175546401277,1.
30806800268425,0.190850245138321,NA
"14231","DISP3",0.150263610326729,1.41832010653068,3.35247519753738,0.
42306654724024,0.672246708730081,NA
"14232","DRAXIN",0.326970069736394,2.31602366272664,3.34194918242485,0
.693015822893567,0.488299621743865,NA
"14233","AL096840.2",0.0604037079966622,0.119912045671111,3.3524751975
3738,0.0357682126206911,0.971467179494267,NA
"14234","TNFRSF8",0.27268524254951,-0.0202790885325299,2.5035439907940
5,-0.00810015266641987,0.993537083922046,NA
"14235","AADACL4",0,NA,NA,NA,NA,NA
"14236","AL357873.1",0.522503445585701,1.09521359033865,1.952228233033
83,0.561006941609816,0.574792805033169,NA
"14237","KAZN-
AS1",0.2292884040636,0.696980734286753,3.06852794721869,0.227138467133
238,0.820316073199257,NA
"14238","TMEM51-
AS1",0.527024054367834,0.179618334086769,1.92520144268284,0.0932984622
307699,0.925666453717934,NA
"14239","TMEM51",1.13771084329423,-0.701091264844919,1.66022167038795,
-0.422287744672728,0.672815002344518,NA
"14240","AL031283.1",0.244628726273298,0.06022438509446,2.576496118691

15,0.0233745297179232,0.98135152179478,NA
"14241","CTRC",0.227597756214425,-1.13126253111685,2.5754042190421,-0.439256301108964,0.660475834030736,NA
"14242","CELA2A",0,NA,NA,NA,NA,NA
"14243","AL121992.3",0,NA,NA,NA,NA,NA
"14244","AGMAT",0.69893895950438,1.21450576357116,2.08040972601747,0.583782006199368,0.55936698581608,NA
"14245","AL121992.1",0.108990023245465,1.41832010653068,3.35247519753738,0.42306654724024,0.672246708730081,NA
"14246","AL121992.2",0.120322481366256,1.41832010653068,3.35247519753738,0.42306654724024,0.672246708730081,NA
"14247","AL450998.3",0,NA,NA,NA,NA,NA
"14248","UQCRHL",0.579408324668645,0.655720377797965,1.72990784776878,0.379049311004462,0.704651246350133,NA
"14249","HSPB7",0.800392624339056,-1.32184986794745,1.68077129243455,-0.786454334326945,0.43160136233178,NA
"14250","CLCNKB",0.735297724831775,0.698094038502734,1.82695561597867,0.382107825935758,0.702381387220268,NA
"14251","FAM131C",0.236795097066225,0.0434742300939003,3.08117651838172,0.014109620086529,0.988742525500995,NA
"14252","AL451042.2",0,NA,NA,NA,NA,NA
"14253","AL109627.1",0.441569938616448,0.529937983934942,2.01849251177153,0.262541466388618,0.792904020622947,NA
"14254","CPLANE2",1.28375482631913,1.48057739296197,1.22698738747126,1.20667694556613,0.227556581018441,NA
"14255","SPATA21",0.662508288274333,3.12214482472418,2.01957358536407,1.54594259270892,0.122118432802639,NA
"14256","LINC01772",1.33595867160847,1.25595423937717,1.18047715356647,1.06393777768817,0.287356887854894,NA
"14257","AL137802.2",0.293215041522101,1.263698751305,2.46803799965885,0.512025646071769,0.608633060816255,NA
"14258","AL137798.1",0,NA,NA,NA,NA,NA
"14259","BX284668.2",0.253555121634112,0.696980747544927,2.97042254430159,0.234640269911095,0.814487951095881,NA
"14260","BX284668.6",0.81054440551054,0.995352214189858,1.51898597741599,0.655274129576291,0.512291274111461,NA
"14261","PADI2",0,NA,NA,NA,NA,NA
"14262","PADI4",0,NA,NA,NA,NA,NA
"14263","PADI6",0,NA,NA,NA,NA,NA
"14264","IGSF21",0.455587014388671,0.22088830814934,2.24216786549866,0.0985155088288694,0.921522957598525,NA
"14265","PAX7",0,NA,NA,NA,NA,NA
"14266","AL137127.1",0.614623184444495,-0.737346369727235,1.54785229137329,-0.476367398773589,0.633812656387717,NA
"14267","AKR7A3",0.501682676821128,0.0150348304000036,1.94109891601604,0.00774552511257977,0.993820026890109,NA
"14268","HTR6",0,NA,NA,NA,NA,NA
"14269","PLA2G2D",0,NA,NA,NA,NA,NA
"14270","Z98257.1",0,NA,NA,NA,NA,NA
"14271","PLA2G2C",0,NA,NA,NA,NA,NA

"14272","UBXN10-
AS1",1.04926459617683,-1.71707685181094,1.37887933284264,-1.2452698440
7772,0.213032576574294,NA
"14273","VWA5B1",0,NA,NA,NA,NA,NA
"14274","PINK1-
AS",1.42141023045086,-0.21471643735746,1.2123986194857,-0.177100529402
238,0.859429426096137,NA
"14275","KIF17",0.577232742192402,0.450512574114315,1.73266190294101,0
.260011819587894,0.794854656373526,NA
"14276","AL031005.1",0,NA,NA,NA,NA,NA
"14277","AL031728.1",0,NA,NA,NA,NA,NA
"14278","LINC02596",0,NA,NA,NA,NA,NA
"14279","RAP1GAP",0.173429405397192,1.41832010653068,3.35247519753738,
0.42306654724024,0.672246708730081,NA
"14280","LINC01635",0.0604037079966622,0.119912045671111,3.35247519753
738,0.0357682126206911,0.971467179494267,NA
"14281","CDC42-
AS1",0.429039553038712,-0.773023508969754,2.60162473255293,-0.29713105
7872131,0.766366453771418,NA
"14282","AL445253.1",0.120322481366256,1.41832010653068,3.352475197537
38,0.42306654724024,0.672246708730081,NA
"14283","TEX46",0,NA,NA,NA,NA,NA
"14284","AL109936.6",0,NA,NA,NA,NA,NA
"14285","AL109936.2",0.108990023245465,1.41832010653068,3.352475197537
38,0.42306654724024,0.672246708730081,NA
"14286","E2F2",0.173429405397192,1.41832010653068,3.35247519753738,0.4
2306654724024,0.672246708730081,NA
"14287","MDS2",0.120322481366256,1.41832010653068,3.35247519753738,0.4
2306654724024,0.672246708730081,NA
"14288","CNR2",0,NA,NA,NA,NA,NA
"14289","MYOM3",0.233833113393854,0.696980736952966,3.04905264581788,0
.228589276052335,0.819188160373076,NA
"14290","IL22RA1",1.35437710599982,1.26415458647925,1.3488876723437,0.
937182993364279,0.348664436249298,NA
"14291","IFNLR1",0.108990023245465,1.41832010653068,3.35247519753738,0
.42306654724024,0.672246708730081,NA
"14292","RCAN3AS",0,NA,NA,NA,NA,NA
"14293","NCMAP-
DT",0.590395962587735,1.11309376180813,1.77007647058049,0.628839363896
577,0.529454226439957,NA
"14294","AL031432.1",0,NA,NA,NA,NA,NA
"14295","AL031432.5",0,NA,NA,NA,NA,NA
"14296","AL031432.3",0.487833472765475,-0.117324090158972,1.9657783779
657,-0.0596832743070387,0.952407893155059,NA
"14297","RHD",0.274375890398685,1.98403204198911,2.89460999865626,0.68
5422921536976,0.493077083984588,NA
"14298","RHCE",0.947095396905698,-0.383133410935716,1.6936665644646,-0
.226215371416293,0.821033915804264,NA
"14299","AL606491.1",0.442215558915719,-2.09596810376261,2.13684131216
193,-0.980872136753119,0.326655799376317,NA

"14300","AL020996.1",0.722399251577769,2.49734181424362,1.65207852771654,1.51163626446703,0.130626420836376,NA
"14301","AUNIP",0,NA,NA,NA,NA,NA
"14302","SLC30A2",0,NA,NA,NA,NA,NA
"14303","TRIM63",0.342286291397972,0.6094932892186,2.40258431168592,0.253682372874112,0.799740953086473,NA
"14304","FAM110D",0.124306244907042,-0.509577950443694,3.33980312193904,-0.152577242381833,0.878731672788745,NA
"14305","CNKSR1",0.398543694839639,-0.549167447531507,2.2514769759509,-0.243914307540084,0.807297187979757,NA
"14306","CRYBG2",0.0973092156216871,0.119912045671111,3.35247519753738,0.0357682126206911,0.971467179494267,NA
"14307","ZNF683",0,NA,NA,NA,NA,NA
"14308","AL513365.2",0,NA,NA,NA,NA,NA
"14309","AL512408.1",0,NA,NA,NA,NA,NA
"14310","SFN",1.32952066308018,0.488320381970493,1.4873143433963,0.328323588176665,0.742667011010782,NA
"14311","TENT5B",0.06390253691038,0.119912045671111,3.35247519753738,0.0357682126206912,0.971467179494267,NA
"14312","FCN3",0,NA,NA,NA,NA,NA
"14313","GPR3",0,NA,NA,NA,NA,NA
"14314","BX293535.1",0.16538586715322,1.41832010653068,3.35247519753738,0.42306654724024,0.672246708730081,NA
"14315","LINC02574",0.227597756214425,-1.13126253111685,2.5754042190421,-0.439256301108964,0.660475834030736,NA
"14316","AL445490.1",0,NA,NA,NA,NA,NA
"14317","AL020997.3",0.221615460528729,-1.10387780491251,2.59193339108378,-0.425889727224409,0.670188200759635,NA
"14318","SMPDL3B",0.276720916704575,0.696980758280457,2.88854265454755,0.241291489043159,0.809329206809347,NA
"14319","PTAFR",0.768153951727415,0.0477735547449352,1.58947635511972,0.0300561594332977,0.976022264624155,NA
"14320","AL353622.1",1.32178838183766,-1.22992172292254,1.40591471320652,-0.874819582844693,0.381672080231235,NA
"14321","MATN1",0.173429405397192,1.41832010653068,3.35247519753738,0.42306654724024,0.672246708730081,NA
"14322","AL137857.1",0.108990023245465,1.41832010653068,3.35247519753738,0.42306654724024,0.672246708730081,NA
"14323","LINC01778",0.36093092865012,1.2894502130469,2.72024374951432,0.474020099587444,0.635485574216522,NA
"14324","NKAIN1",0.718755765647095,-0.067693321129491,1.64140908906471,-0.0412409810451721,0.967103783298334,NA
"14325","SERINC2",1.21061339632538,-0.238385513081697,1.47012908104803,-0.162152777028093,0.871185546029426,NA
"14326","LINC01226",0,NA,NA,NA,NA,NA
"14327","AC114488.2",0.0973092156216871,0.119912045671111,3.35247519753738,0.0357682126206911,0.971467179494267,NA
"14328","AC114488.3",0,NA,NA,NA,NA,NA
"14329","SPOCD1",0.358901658948903,-1.79961066720244,2.68572597578764,-0.670064885035291,0.502816428548756,NA

"14330","AL354919.1",0,NA,NA,NA,NA,NA
"14331","AL136115.2",0.797762675386714,0.330665279941364,1.61397001798539,0.204876965653991,0.837668252224497,NA
"14332","AL136115.1",0,NA,NA,NA,NA,NA
"14333","AL445248.1",0.331142329015541,-0.0194489660721624,2.37337051585543,-0.0081946606912964,0.993461679930862,NA
"14334","AL049795.2",0.373877603000368,1.28717078660063,2.31487324831634,0.556043743447646,0.578180951428236,NA
"14335","AL049795.1",0.233833113393854,0.696980736952966,3.04905264581788,0.228589276052335,0.819188160373076,NA
"14336","IQCC",1.27917152691671,-1.3239867573379,1.30188124004349,-1.01697967265714,0.309163099538534,NA
"14337","DCDC2B",0,NA,NA,NA,NA,NA
"14338","FAM167B",0.370278241918811,-1.79826140597758,2.68786093599126,-0.669030671155014,0.503475912996726,NA
"14339","TSSK3",0.926749732462606,-0.342456018910421,1.43098182055255,-0.239315422454624,0.81086100742443,NA
"14340","HPCA",0.954547251803894,-2.32541324064528,1.44083295280606,-1.61393674132485,0.106541148198902,NA
"14341","AL031602.2",0,NA,NA,NA,NA,NA
"14342","AL020995.1",0,NA,NA,NA,NA,NA
"14343","AL662907.3",0,NA,NA,NA,NA,NA
"14344","AL513327.1",0.173429405397192,1.41832010653068,3.35247519753738,0.42306654724024,0.672246708730081,NA
"14345","GJA4",0.520288216191576,2.79382521304909,2.95368862825364,0.945876686636712,0.344211485698358,NA
"14346","DLGAP3",0,NA,NA,NA,NA,NA
"14347","AL590434.1",0,NA,NA,NA,NA,NA
"14348","AC004865.2",0.43228277885212,-0.901439642708117,1.92008933655091,-0.469477969357087,0.638728028438615,NA
"14349","TFAP2E",0.89285811912423,0.633020580486512,1.44704126576264,0.437458554544324,0.66177882698835,NA
"14350","CLSPN",1.0657747798282,1.1030800951253,1.2713547234436,0.867641481000272,0.385590630637952,NA
"14351","AL139286.3",0.434433840322924,-0.690115991133408,1.97463024943621,-0.349491248465604,0.726720539831794,NA
"14352","AL139286.2",0,NA,NA,NA,NA,NA
"14353","TEKT2",0.103291511307383,0.119912045671111,3.35247519753738,0.0357682126206912,0.971467179494267,NA
"14354","AL591845.1",0.428080949928127,1.28886520766923,2.59768840815572,0.496158509089351,0.619782581682311,NA
"14355","CDCA8",0.318924676150416,-1.60743045140455,2.32803343644947,-0.690467081029591,0.489900504562551,NA
"14356","AL929472.2",0.337124624701237,-0.0193835125736199,2.36388267352518,-0.0081998623665674,0.993457529733912,NA
"14357","GJA9",0.861294617587992,1.31251255848404,2.00950898229277,0.653150879169755,0.513659014004721,NA
"14358","RHBDL2",1.00096334545741,2.87536081482739,1.70489015249737,1.68653728840857,0.0916923592570828,NA
"14359","AL356055.1",0.974086209640185,-1.56588136939743,1.49013223399

043,-1.05083383452766,0.293334913238911,NA
"14360","AL442071.2",0.233833113393854,0.696980736952966,3.04905264581788,0.228589276052335,0.819188160373076,NA
"14361","BMP8A",0.381811850919056,-1.87544131362572,2.21462297199633,-0.846844513644296,0.397081793197015,NA
"14362","PABPC4-AS1",0,NA,NA,NA,NA,NA
"14363","HPCAL4",0,NA,NA,NA,NA,NA
"14364","BMP8B",0.628393044692707,1.16282891151693,1.90280256902687,0.611113801528881,0.541124243470044,NA
"14365","OXCT2",0.06390253691038,0.119912045671111,3.35247519753738,0.0357682126206912,0.971467179494267,NA
"14366","AL033527.4",0.103291511307383,0.119912045671111,3.35247519753738,0.0357682126206912,0.971467179494267,NA
"14367","MYCL",0.0604037079966622,0.119912045671111,3.35247519753738,0.0357682126206911,0.971467179494267,NA
"14368","MFSD2A",0.554195375899712,-1.31868331127517,1.65162101283196,-0.798417615802846,0.424628183995202,NA
"14369","COL9A2",1.29554736830687,1.73192664007689,1.5458181691996,1.12039480100926,0.262545559451873,NA
"14370","AL031985.3",1.40564768402746,0.00305562993180167,1.15575351745922,0.00264384220825829,0.99789052157834,NA
"14371","AL603839.3",0.29319094097398,0.0421599161580697,2.88101411017646,0.0146337069329686,0.988324407884707,NA
"14372","AL603839.2",0,NA,NA,NA,NA,NA
"14373","RIMS3",0,NA,NA,NA,NA,NA
"14374","NFYC-AS1",1.20454802015529,-0.534816214925199,1.26829892015942,-0.421679941868891,0.673258647013206,NA
"14375","KCNQ4",0.330605483774194,-0.533746182735224,2.08108005383055,-0.256475565057087,0.797583645844787,NA
"14376","SLFNL1-AS1",0.439761757551905,2.57992481053724,2.54830735454105,1.01240723805935,0.311343379010575,NA
"14377","SLFNL1",0,NA,NA,NA,NA,NA
"14378","AC093151.2",0.108990023245465,1.41832010653068,3.35247519753738,0.42306654724024,0.672246708730081,NA
"14379","EDN2",0,NA,NA,NA,NA,NA
"14380","RIMKLA",0.0604037079966622,0.119912045671111,3.35247519753738,0.0357682126206911,0.971467179494267,NA
"14381","ZMYND12",1.3301076303445,1.22783541293532,1.24533574895732,0.985947294906889,0.32415897677214,NA
"14382","AC098484.4",0,NA,NA,NA,NA,NA
"14383","AC098484.2",0.225789575149882,0.696980732170056,3.08390169185347,0.226006144752027,0.821196641151035,NA
"14384","TMEM269",0,NA,NA,NA,NA,NA
"14385","AL512353.1",0.993810555507935,-0.820303046052864,1.50545151769502,-0.544888384920439,0.585830285215198,NA
"14386","SLC2A1-AS1",0.65971067077958,-0.0322764502188599,1.65714325522493,-0.0194771635566769,0.984460454429516,NA

"14387","CFAP57",1.49457207117226,-1.33084948619987,1.25730358552483,-
1.05849494228901,0.289829855388021,NA
"14388","TIE1",1.21054890140524,-1.20526951171634,1.3488954325646,-0.8
93523309975785,0.371576994858581,NA
"14389","AL139289.2",0.528124976867883,0.0311370878341213,1.9224860438
528,0.0161962620918279,0.987077817492146,NA
"14390","CDC20",0.322423505064134,-1.62476410664247,2.74149049892963,-
0.592657208652313,0.553410584490248,NA
"14391","AL139289.1",0,NA,NA,NA,NA,NA
"14392","SZT2-
AS1",0.0604037079966622,0.119912045671111,3.35247519753738,0.035768212
6206911,0.971467179494267,NA
"14393","HYI-
AS1",0.180726189362918,0.69698069859353,3.31825525196168,0.21004432922
4369,0.833633074956668,NA
"14394","AL451062.1",0.06390253691038,0.119912045671111,3.352475197537
38,0.0357682126206912,0.971467179494267,NA
"14395","ARTN",0.281534233898323,-0.047105237591176,2.4839065807098,-0
.0189641744005184,0.984869684953748,NA
"14396","AL357079.1",0.523525225454117,0.194674212323013,1.92739927403
674,0.101003572505913,0.91954762423212,NA
"14397","AL139220.2",1.35306131141397,-0.0126146601499735,1.1829386401
7535,-0.0106638330354173,0.991491653520114,NA
"14398","ERI3-IT1",0,NA,NA,NA,NA,NA
"14399","ARMH1",1.2644704072796,-2.11881002720604,1.2668713373909,-1.6
724745162912,0.0944307889257151,NA
"14400","KIF2C",0.338302527857186,2.35457786137086,2.72355548167741,0.
864523552837888,0.3873003475594,NA
"14401","AL592166.1",0.06390253691038,0.119912045671111,3.352475197537
38,0.0357682126206912,0.971467179494267,NA
"14402","BEST4",0.330605483774194,-0.533746182735224,2.08108005383055,
-0.256475565057087,0.797583645844787,NA
"14403","TCTEX1D4",0.120322481366256,1.41832010653068,3.35247519753738
,0.42306654724024,0.672246708730081,NA
"14404","LINC01144",0,NA,NA,NA,NA,NA
"14405","HPDL",0.270485559525146,-1.34694009274034,2.93176047727036,-0
.459430469570426,0.645925071915764,NA
"14406","CCDC163",0.697183745899832,-1.84754197329274,2.06399990804194
,-0.895126964925814,0.370719220956807,NA
"14407","TSPAN1",0.667193263234084,-0.985339859100817,1.58705032515763
,-0.620862390739218,0.534690168371109,NA
"14408","RAD54L",0.217631696987943,0.696980727003399,3.12110949465653,
0.223311847340396,0.823292807226044,NA
"14409","TMEM275",0,NA,NA,NA,NA,NA
"14410","MKNK1-AS1",0,NA,NA,NA,NA,NA
"14411","TEX38",0.150263610326729,1.41832010653068,3.35247519753738,0.
42306654724024,0.672246708730081,NA
"14412","CYP4Z1",0.184225018276636,0.696980701717498,3.29715406410566,
0.21138857577362,0.832584070546688,NA
"14413","CYP4A22-AS1",0,NA,NA,NA,NA,NA

"14414","CYP4A22",0.0604037079966622,0.119912045671111,3.3524751975373
8,0.0357682126206911,0.971467179494267,NA
"14415","LINC00853",0.960792620671638,0.514108465181144,1.332878382563
14,0.385712959191751,0.699709264119822,NA
"14416","PDZK1IP1",0,NA,NA,NA,NA,NA
"14417","TAL1",0.0604037079966622,0.119912045671111,3.35247519753738,0
.0357682126206911,0.971467179494267,NA
"14418","STIL",0.587451862912617,0.655205800998712,1.72596641637959,0.
379616772829844,0.704229908360817,NA
"14419","FOXD2-
AS1",1.03825510879333,1.66190585989605,1.4364540978214,1.1569502028756
7,0.247292706060591,NA
"14420","SLC5A9",0,NA,NA,NA,NA,NA
"14421","AL109659.2",0.398706235853848,1.64861659608777,2.286342034409
84,0.72107172561051 8,0.470865386048721,NA
"14422","AGBL4",0.161211752532067,-0.631571180295204,3.33432222068988,
-0.189415161011203,0.849767442783486,NA
"14423","DMRTA2",0,NA,NA,NA,NA,NA
"14424","LINC01562",0.150263610326729,1.41832010653068,3.3524751975373
8,0.42306654724024,0.672246708730081,NA
"14425","TTC39A",0.0604037079966622,0.119912045671111,3.35247519753738
,0.0357682126206911,0.971467179494267,NA
"14426","TTC39A-AS1",0,NA,NA,NA,NA,NA
"14427","AC104170.2",0.103291511307383,0.119912045671111,3.35247519753
738,0.0357682126206912,0.971467179494267,NA
"14428","AC104170.1",0,NA,NA,NA,NA,NA
"14429","AL050343.3",0.2292884040636,0.696980734286753,3.0685279472186
9,0.227138467133238,0.820316073199257,NA
"14430","RAB3B",0.167194048217763,-0.631571198727006,3.33432221989104,
-0.189415166584483,0.849767438415713,NA
"14431","AL513218.1",0.16538586715322,1.41832010653068,3.3524751975373
8,0.42306654724024,0.672246708730081,NA
"14432","AL513218.2",0.353594648970642,-0.375823671540819,2.3079274993
5522,-0.1628403282364,0.870644154692966,NA
"14433","ORC1",0.124306244907042,-0.509577950443694,3.33980312193904,-
0.152577242381833,0.878731672788745,NA
"14434","SHISAL2A",0.49379166790305,-1.05420061449473,1.88641610978359
,-0.558837792482416,0.576272427503585,NA
"14435","AL445183.1",0.563222303428365,1.91564300391452,2.135573842059
97,0.897015577820852,0.369710604650265,NA
"14436","AL445183.2",0.562238914143684,-1.31864080556137,1.64723198583
289,-0.800519184245094,0.423410053061009,NA
"14437","AL606760.2",0.259253633572194,1.98393725043859,2.947861372725
44,0.673009005374071,0.500941561797964,NA
"14438","AL606760.3",0.566521803822761,-2.46890287839069,1.75279274522
619,-1.40855379799743,0.158967148642468,NA
"14439","AL606760.1",0.835000588312913,-0.0210631279847206,1.459761715
80426,-0.0144291549481525,0.988487599524103,NA
"14440","LINC01771",0,NA,NA,NA,NA,NA
"14441","LINC02812",0.0973092156216871,0.119912045671111,3.35247519753

738,0.0357682126206911,0.971467179494267,NA
"14442","GLIS1",0.150263610326729,1.41832010653068,3.35247519753738,0.42306654724024,0.672246708730081,NA
"14443","DIO1",0,NA,NA,NA,NA,NA
"14444","SSBP3-AS1",0.31557304586023,-0.25535901340068,2.86839237951369,-0.0890251331109634,0.92906193608637,NA
"14445","FAM151A",1.29676986039622,-0.176652361782303,1.45089220206996,-0.121754298169275,0.903093609355764,NA
"14446","MROH7",0.519351110832963,-1.20403504087968,1.80499318500161,-0.667057942868968,0.504735122896889,NA
"14447","TTC22",0.06390253691038,0.119912045671111,3.35247519753738,0.0357682126206912,0.971467179494267,NA
"14448","LEXM",0,NA,NA,NA,NA,NA
"14449","MIR4422HG",0.06390253691038,0.119912045671111,3.35247519753738,0.0357682126206912,0.971467179494267,NA
"14450","AL603840.1",1.16023332844201,-1.37348929910179,1.39270279621381,-0.986204165623669,0.324032935566151,NA
"14451","LINC01767",0,NA,NA,NA,NA,NA
"14452","DAB1",1.26997418606491,-2.01244655281232,1.43181688809297,-1.40551949732391,0.159866855697235,NA
"14453","TACSTD2",1.07945622972203,-2.53401191713624,1.59282907920086,-1.59088752850216,0.111634889764322,NA
"14454","AL136985.3",0.225789575149882,0.696980732170056,3.08390169185347,0.226006144752027,0.821196641151035,NA
"14455","LINC01135",0.966057646104525,-0.441767261090739,1.41143596086805,-0.312991360103257,0.754287226254597,NA
"14456","AL136985.2",0,NA,NA,NA,NA,NA
"14457","LINC02777",1.35124415571374,-0.219528877386304,1.20769974901613,-0.181774383546197,0.855759788550193,NA
"14458","AC093424.1",0.308531263183678,-0.370615225138078,2.39555983459126,-0.154709233218261,0.877050550585805,NA
"14459","HOOK1",0,NA,NA,NA,NA,NA
"14460","AC099792.1",0.120322481366256,1.41832010653068,3.35247519753738,0.42306654724024,0.672246708730081,NA
"14461","NFIA-AS1",0.531568763698089,0.218234717963708,2.1431526441655,0.101828826125767,0.918892544633,NA
"14462","AC099791.4",0.33077173430644,2.01280089522968,3.31423402991417,0.60732008574597,0.543638511160742,NA
"14463","ANGPTL3",0,NA,NA,NA,NA,NA
"14464","AC103923.1",0.337124624701237,-0.0193835125736199,2.36388267352518,-0.0081998623665674,0.993457529733912,NA
"14465","AL357078.2",0.61265431770775,2.20025820833221,2.06142571590479,1.06734780271551,0.285814809534079,NA
"14466","DNAJC6",0.0604037079966622,0.119912045671111,3.35247519753738,0.0357682126206911,0.971467179494267,NA
"14467","AC119800.1",0.362545144879577,1.26414546505463,2.33385265849746,0.541656072611067,0.588055456177385,NA
"14468","AL139147.1",0.224098927300707,-1.11019835680208,3.08249114251

047,-0.360162707847362,0.718725460748659,NA
"14469","TCTEX1D1",0,NA,NA,NA,NA,NA
"14470","IL23R",0.150263610326729,1.41832010653068,3.35247519753738,0.
42306654724024,0.672246708730081,NA
"14471","IL12RB2",0.169393731242127,0.696980693484986,3.35247519753738
,0.207900328091007,0.835306792989891,NA
"14472","DEPDC1",0.167194048217763,-0.631571198727006,3.33432221989104
,-0.189415166584483,0.849767438415713,NA
"14473","LRRC7",0.909116294561296,2.71232857165163,1.99199032256405,1.
36161734368286,0.17331867885702,NA
"14474","AL353771.1",0.120322481366256,1.41832010653068,3.352475197537
38,0.42306654724024,0.672246708730081,NA
"14475","AL354872.2",0,NA,NA,NA,NA,NA
"14476","AL031429.1",0.44731775837266,-2.12691282787499,3.083038421604
73,-0.689875550356561,0.4902724525239,NA
"14477","AL031429.2",0.167194048217763,-0.631571198727006,3.3343222198
9104,-0.189415166584483,0.849767438415713,NA
"14478","ZRANB2-
AS1",0.172892560155845,0.696980693484986,3.35247519753738,0.2079003280
91007,0.835306792989891,NA
"14479","NEGR1-IT1",0,NA,NA,NA,NA,NA
"14480","LRRIQ3",0.206583022614766,-0.859518418402433,3.32508269885954
,-0.25849535071631,0.796024636623985,NA
"14481","AC105271.1",0,NA,NA,NA,NA,NA
"14482","ERICH3",0.931808810473687,-2.30217768508092,1.66003986562075,
-1.3868207220554,0.165496436700118,NA
"14483","AC135803.1",0.667577579149537,0.127824099997876,1.54279628930
419,0.0828522215693977,0.933969045020804,NA
"14484","LHX8",0,NA,NA,NA,NA,NA
"14485","SLC44A5",0,NA,NA,NA,NA,NA
"14486","ST6GALNAC3",0,NA,NA,NA,NA,NA
"14487","AC093433.2",0,NA,NA,NA,NA,NA
"14488","AK5",0.568103676614012,1.99271755336713,2.14593356907841,0.92
8601696753793,0.353095542220556,NA
"14489","AC093575.1",0,NA,NA,NA,NA,NA
"14490","AC096531.2",0.274569855233771,0.0589952543162062,2.4952667603
5574,0.0236428646642155,0.981137480638694,NA
"14491","LINC01781",0.06390253691038,0.119912045671111,3.3524751975373
8,0.0357682126206912,0.971467179494267,NA
"14492","LINC01725",0.434433840322924,-0.690115991133408,1.97463024943
621,-0.349491248465604,0.726720539831794,NA
"14493","TTLL7-
IT1",0.311475362858796,-0.0461613448588672,2.41663261754422,-0.0191015
15275324,0.984760122636486,NA
"14494","DNASE2B",0.324906971836112,-1.62214160027477,2.02889523613539
,-0.799519645659284,0.423989159827425,NA
"14495","AL359762.1",0.150263610326729,1.41832010653068,3.352475197537
38,0.42306654724024,0.672246708730081,NA
"14496","LPAR3",0,NA,NA,NA,NA,NA
"14497","MCOLN2",0.967831692336276,1.56979910132529,1.71552579743687,0

.915054208844128,0.360163172522483,NA
"14498","WDR63",0.06390253691038,0.119912045671111,3.35247519753738,0.0357682126206912,0.971467179494267,NA
"14499","AL360219.1",0.210667318323391,0.69698072231542,3.15449020715825,0.220948767168088,0.825132324921599,NA
"14500","AC092807.2",0.167194048217763,-0.631571198727006,3.3343222198 9104,-0.189415166584483,0.849767438415713,NA
"14501","LINC02795",0.214166147237109,0.696980724704133,3.13752578908925,0.222143424964947,0.824202234028988,NA
"14502","CLCA2",0.06390253691038,0.119912045671111,3.35247519753738,0.0357682126206912,0.971467179494267,NA
"14503","CLCA4-AS1",0.287516529584019,-0.0469351117677705,2.47135887102577,-0.0189916212971083,0.984847789442023,NA
"14504","AL121989.1",0.150263610326729,1.41832010653068,3.35247519753738,0.42306654724024,0.672246708730081,NA
"14505","LINC02801",0.395377271227095,-0.745201301761531,2.23611053975441,-0.333257810163256,0.738939683436358,NA
"14506","PKN2-AS1",0.50738118875921,1.07651084907669,1.96428608477402,0.548041783435299,0.58366321293383,NA
"14507","GBP7",0.167194048217763,-0.631571198727006,3.33432221989104,-0.189415166584483,0.849767438415713,NA
"14508","GBP6",0,NA,NA,NA,NA,NA
"14509","AC099568.1",0.0604037079966622,0.119912045671111,3.35247519753738,0.0357682126206911,0.971467179494267,NA
"14510","AC099568.2",0.103291511307383,0.119912045671111,3.35247519753738,0.0357682126206912,0.971467179494267,NA
"14511","LINC01763",0.180726189362918,0.69698069859353,3.31825525196168,0.210044329224369,0.833633074956668,NA
"14512","HFM1",0.648392416150627,0.85784981050032,2.09669222648671,0.409144365426375,0.682433719718753,NA
"14513","BRDT",0,NA,NA,NA,NA,NA
"14514","EPHX4",0.0604037079966622,0.119912045671111,3.35247519753738,0.0357682126206911,0.971467179494267,NA
"14515","AC104836.1",0.206583022614766,-0.859518418402433,3.32508269885954,-0.25849535071631,0.796024636623985,NA
"14516","GFI1",0.654835344739462,1.24307002258131,1.89416283239042,0.656263548901213,0.511654566545899,NA
"14517","AC093577.1",0.06390253691038,0.119912045671111,3.35247519753738,0.0357682126206912,0.971467179494267,NA
"14518","AL049796.1",0.161211752532067,-0.631571180295204,3.33432222068988,-0.189415161011203,0.849767442783486,NA
"14519","ABCA4",0,NA,NA,NA,NA,NA
"14520","ARHGAP29-AS1",0,NA,NA,NA,NA,NA
"14521","AC118469.2",0,NA,NA,NA,NA,NA
"14522","AC092802.4",0,NA,NA,NA,NA,NA
"14523","DPYD-AS1",0.16538586715322,1.41832010653068,3.35247519753738,0.42306654724024,0.672246708730081,NA

"14524","DPYD-
IT1",0.412958693101636,1.28682861931889,2.26752427558922,0.56750378956
1198,0.570371952675838,NA
"14525","AC118553.1",0.741364493851466,-0.822362496815916,1.6163457787
4526,-0.508778819253824,0.610907267448866,NA
"14526","LRRC39",0.838842297632891,1.67207584500456,1.80126784477556,0
.928277185347133,0.353263805423213,NA
"14527","AL445928.2",0,NA,NA,NA,NA,NA
"14528","GPR88",0.721644340953215,2.47792318928127,1.99319210401144,1.
24319336018554,0.213796588892083,NA
"14529","AC093157.2",0.373877603000368,1.28717078660063,2.314873248316
34,0.556043743447646,0.578180951428236,NA
"14530","AL109741.1",0.108990023245465,1.41832010653068,3.352475197537
38,0.42306654724024,0.672246708730081,NA
"14531","VAV3",1.41009635789432,-0.79900252951967,1.22899685687703,-0.
65012577131401,0.515610983616518,NA
"14532","VAV3-AS1",0,NA,NA,NA,NA,NA
"14533","LINC02785",0.06390253691038,0.119912045671111,3.3524751975373
8,0.0357682126206912,0.971467179494267,NA
"14534","AL160171.1",0,NA,NA,NA,NA,NA
"14535","FNDC7",0.06390253691038,0.119912045671111,3.35247519753738,0.
0357682126206912,0.971467179494267,NA
"14536","SPATA42",0.240644962732512,2.01289191439962,3.35072301158191,
0.600733605088207,0.548017432599981,NA
"14537","AL356488.2",0.792971121113594,0.416577360754519,1.68075877184
876,0.24785077295554,0.804249869103885,NA
"14538","KIAA1324",0.441763903451534,-0.932217476493597,1.915151529018
22,-0.486759121859922,0.6264290422694,NA
"14539","CELSR2",0.471339347947949,-0.860593687670558,2.19901301212838
,-0.391354522653601,0.695535202696949,NA
"14540","SYPL2",1.09487020975809,1.43859362344869,1.45919192546432,0.9
85883760966485,0.324190156501725,NA
"14541","AMIGO1",1.23452745315572,0.373894885185213,1.23546944408056,0
.302633858713896,0.762168908222718,NA
"14542","GNAT2",0.108990023245465,1.41832010653068,3.35247519753738,0.
42306654724024,0.672246708730081,NA
"14543","AC000032.1",0.489078882877141,2.6851779958048,2.1683690527531
2,1.23833993682741,0.215590041407738,NA
"14544","ALX3",0.816716166428097,0.150127671513995,1.72108152048765,0.
087228681341868,0.930489741826163,NA
"14545","UBL4B",0.46718129216064,2.64731687490042,2.51702392494676,1.0
5176468473831,0.29290752537043,NA
"14546","RBM15-
AS1",0.167194048217763,-0.631571198727006,3.33432221989104,-0.18941516
6584483,0.849767438415713,NA
"14547","LAMTOR5-
AS1",0.929072794672059,1.02213393493199,1.77280406842299,0.57656339645
0933,0.564234432010035,NA
"14548","AL365361.1",0.937248556831251,0.464993095962457,1.55125411313
952,0.299753014044474,0.76436555746804,NA

"14549","KCNA3",0.412958693101636,1.28682861931889,2.26752427558922,0.567503789561198,0.570371952675838,NA
"14550","AL360270.2",0.500536060257668,0.0484056021036175,1.93637187866207,0.0249980918629448,0.980056485606958,NA
"14551","AL360270.1",0,NA,NA,NA,NA,NA
"14552","AL360270.3",0.30503243426996,-0.35285406173419,2.4020524594433,-0.146896900751271,0.883213398227782,NA
"14553","AL355816.1",0,NA,NA,NA,NA,NA
"14554","AL355816.2",0.832963446302757,0.569241966270217,1.38444887330021,0.411168644251392,0.680948878523307,NA
"14555","OVGP1",0.276720916704575,0.696980758280457,2.88854265454755,0.241291489043159,0.809329206809347,NA
"14556","AL390195.2",0.474963010753435,0.241898409472134,2.22249720666001,0.108840815973695,0.91332874963847,NA
"14557","AL390195.1",0,NA,NA,NA,NA,NA
"14558","TMIGD3",0.0604037079966622,0.119912045671111,3.35247519753738,0.0357682126206911,0.971467179494267,NA
"14559","ADORA3",0,NA,NA,NA,NA,NA
"14560","LINC01160",0.150263610326729,1.41832010653068,3.35247519753738,0.42306654724024,0.672246708730081,NA
"14561","INKA2-AS1",0.633865588760046,-1.69541850933005,1.8174729470807,-0.932843876467767,0.350900583336015,NA
"14562","AL049557.1",0.2292884040636,0.696980734286753,3.06852794721869,0.227138467133238,0.820316073199257,NA
"14563","AL354760.1",0.167194048217763,-0.631571198727006,3.33432221989104,-0.189415166584483,0.849767438415713,NA
"14564","AL603832.1",0,NA,NA,NA,NA,NA
"14565","TAFA3",0.644021999066895,-0.543291480504819,1.68874944604981,-0.32171230716057,0.747670653375541,NA
"14566","AL390242.1",0,NA,NA,NA,NA,NA
"14567","AL137856.1",0.16538586715322,1.41832010653068,3.35247519753738,0.42306654724024,0.672246708730081,NA
"14568","PTPN22",0.715102467612043,3.28591189770423,1.93408709731187,1.6989472202525,0.0893291287201204,NA
"14569","AP4B1-AS1",0.108990023245465,1.41832010653068,3.35247519753738,0.42306654724024,0.672246708730081,NA
"14570","BCL2L15",0.229797439238789,0.0884527683768807,3.10949972347481,0.0284459804608171,0.977306451918786,NA
"14571","HIPK1-AS1",1.32352723078308,0.195637905714681,1.19804168678117,0.163298078750757,0.870283746534219,NA
"14572","AMPD1",0,NA,NA,NA,NA,NA
"14573","LINC01765",0,NA,NA,NA,NA,NA
"14574","CASQ2",0.693717621588768,3.2145857150237,2.76299910980282,1.16344073496756,0.244650730368457,NA
"14575","MAB21L3",0.274375890398685,1.98403204198911,2.89460999865626,0.685422921536976,0.493077083984588,NA
"14576","LINC01779",1.01310416887077,-0.16569506360647,1.3718764551791

5,-0.120779872692568,0.903865392645645,NA
"14577","LINC01762",0,NA,NA,NA,NA,NA
"14578","AL390066.1",0.120807415993324,-0.48654549181451,3.34088239674
268,-0.145633827844071,0.884210461918222,NA
"14579","AL157904.1",0.289016826352827,-1.48815745083166,2.85197758914
948,-0.521798437860609,0.60181067744985,NA
"14580","CD101",0,NA,NA,NA,NA,NA
"14581","AL445231.1",0.103291511307383,0.119912045671111,3.35247519753
738,0.0357682126206912,0.971467179494267,NA
"14582","AL358072.1",0.420465386104261,0.529345106475082,2.03806251319
317,0.259729573086412,0.795072379253792,NA
"14583","AL157902.1",0,NA,NA,NA,NA,NA
"14584","AL139420.1",1.02848310094571,-1.68376624910135,1.341800010308
58,-1.25485633936918,0.209530916010305,NA
"14585","AL139420.2",0,NA,NA,NA,NA,NA
"14586","AL359915.1",0.214166147237109,0.696980724704133,3.13752578908
925,0.222143424964947,0.824202234028988,NA
"14587","HAO2",0.225114289442447,-1.12060253516837,3.07913523483178,-0
.363934172975546,0.71590716558595,NA
"14588","HSD3B2",0.103291511307383,0.119912045671111,3.35247519753738,
0.0357682126206912,0.971467179494267,NA
"14589","ADAM30",0,NA,NA,NA,NA,NA
"14590","AC245008.1",0,NA,NA,NA,NA,NA
"14591","FCGR1B",0.0604037079966622,0.119912045671111,3.35247519753738
,0.0357682126206911,0.971467179494267,NA
"14592","AC244453.2",0.575400460579738,1.22025914870432,1.930507530874
04,0.632092405333352,0.527326497572851,NA
"14593","FAM72B",1.15667055088648,0.0378281142991579,1.36297187564909,
0.027754141501374,0.977858241638791,NA
"14594","LINC02798",0.286193283146544,0.0861184834588285,2.90156683357
524,0.0296799930514492,0.97632226812405,NA
"14595","AL592494.1",0,NA,NA,NA,NA,NA
"14596","AL592494.2",0,NA,NA,NA,NA,NA
"14597","AC239800.2",0.648738428026121,0.578243868876243,1.70340229207
664,0.339464066454495,0.734260162474437,NA
"14598","FAM72C",0.173429405397192,1.41832010653068,3.35247519753738,0
.42306654724024,0.672246708730081,NA
"14599","AC246785.3",0.402717809460792,1.28699235086224,2.289845126861
23,0.562043404492757,0.574086446830557,NA
"14600","FP700111.1",0.12780507382076,-0.532254130061651,3.33875455416
813,-0.15941696864095,0.873340370825791,NA
"14601","FAM72D",0.47041830142318,0.198599833371119,1.95868195906918,0
.10139463043071,0.919237198803748,NA
"14602","PDZK1",0.326905503981022,-0.253160972816323,2.83819636466522,
-0.0891978356283266,0.928924685441435,NA
"14603","CD160",0.544771631011303,-0.354548196765232,1.71217933062122,
-0.207074218467871,0.835951896391316,NA
"14604","ITGA10",0.50197364661311,0.0122743156034616,1.78232072442968,
0.00688670419146317,0.994505248483833,NA
"14605","LIX1L-

AS1",1.10275195719984,-0.933681041546647,1.4401983704969,-0.6483003040
92491,0.516790737388543,NA
"14606","ANKRD34A",0.172892560155845,0.696980693484986,3.3524751975373
8,0.207900328091007,0.835306792989891,NA
"14607","AC239799.1",0.563929561992859,0.0338345453134477,1.9058380729
6123,0.0177531059923033,0.985835814853936,NA
"14608","LINC00624",0.297198805062887,-0.369239549556464,2.42049461405
861,-0.152547147765528,0.878755406995513,NA
"14609","AC239803.1",0.237331942307572,0.696980738944435,3.03442443415
691,0.229691249219751,0.818331695238684,NA
"14610","LINC02804",0.217631696987943,0.696980727003399,3.121109494656
53,0.223311847340396,0.823292807226044,NA
"14611","LINC02805",0,NA,NA,NA,NA,NA
"14612","LINC01731",0,NA,NA,NA,NA,NA
"14613","AC245100.6",0.120322481366256,1.41832010653068,3.352475197537
38,0.42306654724024,0.672246708730081,NA
"14614","PPIAL4G",0.376053185476611,1.28722100944184,2.32205489746435,
0.554345640513266,0.579342320679753,NA
"14615","NUDT4B",0.391061102385135,1.28701110851324,2.29244108252202,0
.561415130066219,0.57451457208665,NA
"14616","AC239802.2",0.16538586715322,1.41832010653068,3.3524751975373
8,0.42306654724024,0.672246708730081,NA
"14617","AC239804.1",0.225789575149882,0.696980732170056,3.08390169185
347,0.226006144752027,0.821196641151035,NA
"14618","HIST2H2BF",1.06939832800165,-0.0606397391181139,1.31934731409
751,-0.0459619225886657,0.963340599189695,NA
"14619","AC243772.2",0.16538586715322,1.41832010653068,3.3524751975373
8,0.42306654724024,0.672246708730081,NA
"14620","HIST2H3D",0,NA,NA,NA,NA,NA
"14621","HIST2H4A",0.194618431243374,-0.859518418402434,3.325082698859
54,-0.25849535071631,0.796024636623985,NA
"14622","HIST2H4B",0.862574507589548,1.93714798311419,1.77784215026313
,1.08960628637783,0.275886612733052,NA
"14623","HIST2H2AB",1.14259424122722,1.37720026386492,1.54442136556931
,0.891725726260757,0.372539960773383,NA
"14624","SV2A",1.03525843483572,-1.6798043790723,1.40867068136698,-1.1
9247486392079,0.233075101024649,NA
"14625","AC242988.2",0,NA,NA,NA,NA,NA
"14626","CIART",0.464820207020906,-1.16027832598351,2.20696916668511,-
0.525733817897539,0.599073163594171,NA
"14627","FALEC",1.12823973038313,0.820783023042596,1.21375508518403,0.
676234466954387,0.498891792532488,NA
"14628","AL356356.1",0.286193283146544,0.0861184834588285,2.9015668335
7524,0.0296799930514492,0.97632226812405,NA
"14629","HORMAD1",0.2292884040636,0.696980734286753,3.06852794721869,0
.227138467133238,0.820316073199257,NA
"14630","ANXA9",0.240644962732512,2.01289191439962,3.35072301158191,0.
600733605088207,0.548017432599981,NA
"14631","BNIPL",0,NA,NA,NA,NA,NA
"14632","AL592424.1",0.206583022614766,-0.859518418402433,3.3250826988

5954,-0.25849535071631,0.796024636623985,NA
"14633","TNFAIP8L2",0.412735062858386,-1.97634162010436,2.555846672057
43,-0.773262982365615,0.439366758720276,NA
"14634","TMOD4",0.0604037079966622,0.119912045671111,3.35247519753738,
0.0357682126206911,0.971467179494267,NA
"14635","ZNF687-
AS1",1.25258313337644,0.725549222854697,1.27214346719713,0.57033600498
9495,0.568449824996493,NA
"14636","AL391069.2",1.15146334133047,-1.93903916671642,1.361967695940
73,-1.42370422771085,0.154532109382824,NA
"14637","CGN",0,NA,NA,NA,NA,NA
"14638","AL365436.2",1.10560262420771,1.58458648578253,1.4012410563406
1,1.13084503099041,0.258120322831125,NA
"14639","AL391335.1",0.150263610326729,1.41832010653068,3.352475197537
38,0.42306654724024,0.672246708730081,NA
"14640","AL589765.5",0.108990023245465,1.41832010653068,3.352475197537
38,0.42306654724024,0.672246708730081,NA
"14641","TDRKH",1.24200086699546,0.0386780442824463,1.29071504240436,0
.0299663698118807,0.97609387412104,NA
"14642","TDRKH-
AS1",0.108990023245465,1.41832010653068,3.35247519753738,0.42306654724
024,0.672246708730081,NA
"14643","LINGO4",0,NA,NA,NA,NA,NA
"14644","RORC",0.0604037079966622,0.119912045671111,3.35247519753738,0
.0357682126206911,0.971467179494267,NA
"14645","C2CD4D",0.108990023245465,1.41832010653068,3.35247519753738,0
.42306654724024,0.672246708730081,NA
"14646","C2CD4D-AS1",0,NA,NA,NA,NA,NA
"14647","AL450992.2",0,NA,NA,NA,NA,NA
"14648","AL450992.1",0.0973092156216871,0.119912045671111,3.3524751975
3738,0.0357682126206911,0.971467179494267,NA
"14649","AL589986.2",0.266702946863814,-0.0203591384131145,2.517155763
2184,-0.0080881519970316,0.993546658757216,NA
"14650","HRNR",0.338278427309065,1.26399624841041,2.376014849266,0.531
981628313846,0.594738719787115,NA
"14651","FLG",0.0604037079966622,0.119912045671111,3.35247519753738,0.
0357682126206911,0.971467179494267,NA
"14652","FLG2",0.270485559525146,-1.34694009274034,2.93176047727036,-0
.459430469570426,0.645925071915764,NA
"14653","SPRR2D",0,NA,NA,NA,NA,NA
"14654","SPRR2A",0,NA,NA,NA,NA,NA
"14655","SPRR2B",0,NA,NA,NA,NA,NA
"14656","SPRR2E",0,NA,NA,NA,NA,NA
"14657","SPRR2F",0.578947490589698,3.05708790317944,2.32147237327606,1
.31687455701456,0.18788067619118,NA
"14658","LOR",0.0604037079966622,0.119912045671111,3.35247519753738,0.
0357682126206911,0.971467179494267,NA
"14659","S100A12",0.289692112060262,0.0584007698147231,2.4588995720125
4,0.0237507747284382,0.981051405035345,NA
"14660","S100A7",1.29442908066716,-2.08746002583487,1.65485376218928,-

1.26141661186622,0.207158786360049,NA
"14661","BX470102.1",0.06390253691038,0.119912045671111,3.352475197537
38,0.0357682126206912,0.971467179494267,NA
"14662","S100A5",1.17510582813792,0.517186972014574,1.26640643351965,0
.408389406690857,0.682987809390949,NA
"14663","S100A1",1.26929784351175,0.822103732201243,1.16896292480468,0
.703276138837854,0.481883677844451,NA
"14664","AL358472.5",0.108990023245465,1.41832010653068,3.352475197537
38,0.42306654724024,0.672246708730081,NA
"14665","AL358472.3",0.315425847236699,-1.59539646267932,2.75600970027
68,-0.578879117340947,0.562670741698805,NA
"14666","AL358472.2",1.23193150180778,2.45994969442834,1.3967880717186
5,1.76114740971516,0.0782134564818015,NA
"14667","NUP210L",0,NA,NA,NA,NA,NA
"14668","C1orf189",0.0604037079966622,0.119912045671111,3.352475197537
38,0.0357682126206911,0.971467179494267,NA
"14669","IL6R-
AS1",0.936090276292188,-0.92568995744466,2.293525072513,-0.40361013207
9518,0.686499433804041,NA
"14670","AL162591.2",0.180726189362918,0.69698069859353,3.318255251961
68,0.210044329224369,0.833633074956668,NA
"14671","UBE2Q1-AS1",0,NA,NA,NA,NA,NA
"14672","CHRNB2",0.0604037079966622,0.119912045671111,3.35247519753738
,0.0357682126206911,0.971467179494267,NA
"14673","KCNN3",0.880254325180243,2.70556239341602,1.78629436522625,1.
51462292334631,0.129867923602017,NA
"14674","AL451085.1",0.313958829630774,-0.0196902921484575,2.409281375
11917,-0.00817268267285035,0.993479215265255,NA
"14675","DCST2",0.663209499693298,-0.0266327854732443,1.55352413408066
,-0.0171434642623077,0.986322164531962,NA
"14676","DCST1-
AS1",0.515367347292177,0.0450275103008753,1.77484670564396,0.025369802
4498055,0.979759997506232,NA
"14677","TRIM46",0.375377899769176,-0.548801873903334,2.28777018398007
,-0.239885053903699,0.810419367866906,NA
"14678","AC234582.2",0,NA,NA,NA,NA,NA
"14679","RUSC1-
AS1",0.908162474921462,-0.0479999709040645,1.48891131024336,-0.0322383
009477032,0.974282012303054,NA
"14680","ASH1L-
IT1",0.262695082774907,0.696980751978295,2.93688603919854,0.2373196449
14958,0.812408822930581,NA
"14681","AL355388.2",0.184709952903704,-0.898539523146346,3.2645796836
2944,-0.275238961895205,0.783132655214614,NA
"14682","MEX3A",1.31877386591788,-0.296606655313136,1.16025539400763,-
0.255639109152192,0.798229516813758,NA
"14683","PAQR6",0.221615460528729,-1.10387780491251,2.59193339108378,-
0.425889727224409,0.670188200759635,NA
"14684","TSACC",0.06390253691038,0.119912045671111,3.35247519753738,0.
0357682126206912,0.971467179494267,NA

"14685","C1orf61",0.507696259099313,2.00293909214649,2.1561772241097,0
.928930641577256,0.352925031976884,NA
"14686","IQGAP3",0.06390253691038,0.119912045671111,3.35247519753738,0
.0357682126206912,0.971467179494267,NA
"14687","TTC24",0,NA,NA,NA,NA,NA
"14688","HAPLN2",0.214166147237109,0.696980724704133,3.13752578908925,
0.222143424964947,0.824202234028988,NA
"14689","BCAN",0.90240195413701,-0.267888966095015,1.4659040594949,-0.
182746588605069,0.854996858831668,NA
"14690","INSRR",0.26450326383945,-1.3088493347846,2.4903960717771,-0.5
25558705146299,0.599194854796744,NA
"14691","LRRC71",0,NA,NA,NA,NA,NA
"14692","FCRL5",0,NA,NA,NA,NA,NA
"14693","FCRL3",0.120322481366256,1.41832010653068,3.35247519753738,0.
42306654724024,0.672246708730081,NA
"14694","AL356276.1",0,NA,NA,NA,NA,NA
"14695","FCRL2",0,NA,NA,NA,NA,NA
"14696","FCRL1",0,NA,NA,NA,NA,NA
"14697","KIRREL1-
IT1",0.492007587386628,1.76291425050661,1.95740790726491,0.90063713545
0183,0.367781283220468,NA
"14698","CD1D",1.24431537502656,-0.496094858393284,1.39221167099153,-0
.356335799167641,0.721589102618085,NA
"14699","AL138899.1",0,NA,NA,NA,NA,NA
"14700","CD1A",0,NA,NA,NA,NA,NA
"14701","CD1B",0.289016826352827,-1.48815745083166,2.85197758914948,-0
.521798437860609,0.60181067744985,NA
"14702","CD1E",0.513115753653534,-2.31852094094521,1.73690804016138,-1
.33485532183373,0.181923702967788,NA
"14703","SPTA1",0.108990023245465,1.41832010653068,3.35247519753738,0.
42306654724024,0.672246708730081,NA
"14704","AIM2",0.634938531788598,-2.63193084373106,1.85920787935781,-1
.41561945436685,0.156886952244689,NA
"14705","CADM3-
AS1",0.492354081547608,-1.02462635973414,1.88289442343254,-0.544176214
54645,0.586320217943186,NA
"14706","ACKR1",1.43162032784497,-2.26620202966825,1.54024882580622,-1
.47132203037521,0.141204048198658,NA
"14707","FCRL6",0.55349045496271,1.19482807972211,1.94150950433361,0.6
15411913799627,0.538282725458963,NA
"14708","AL590560.2",0.467129381546361,0.179580446571156,1.95926421710
716,0.0916570848398923,0.926970495140261,NA
"14709","VSIG8",0.544937881543549,1.94757450655489,2.15845306804476,0.
902301067087414,0.366896958883285,NA
"14710","AL590560.3",1.1166491621292,-0.0205032844373424,1.44918610761
749,-0.0141481375853447,0.988711796050711,NA
"14711","CFAP45",0.229312504611721,1.98374348132209,3.06703062452501,0
.646796111346072,0.51776390945149,NA
"14712","IGSF9",0.06390253691038,0.119912045671111,3.35247519753738,0.
0357682126206912,0.971467179494267,NA

"14713","SLAMF9",0.425715016371517,-2.0084786650818,2.13831601131646,-0.939280562111714,0.347586715788837,NA
"14714","KCNJ10",0.447712919974346,-1.05347391249752,2.17553602141504,-0.484236483389646,0.628218047543021,NA
"14715","AL121987.1",0.702688994569825,-0.564433751907539,2.05416127956234,-0.27477577224501,0.783488512830329,NA
"14716","KCNJ9",0,NA,NA,NA,NA,NA
"14717","ATP1A2",0.497122421121113,2.72060421588454,2.48713251227995,1.09387183933781,0.274011191062069,NA
"14718","ATP1A4",0,NA,NA,NA,NA,NA
"14719","AL121987.2",0.421020115342861,0.173462977827213,2.29005344881502,0.0757462573272998,0.939620973651438,NA
"14720","AL139011.1",0.618097912810092,-1.55330373748073,1.60994188943026,-0.964819753854863,0.334635099957992,NA
"14721","NHLH1",0,NA,NA,NA,NA,NA
"14722","SLAMF6",0.409517243898923,2.51477181352879,2.58985685630277,0.971008033671338,0.331544278051701,NA
"14723","SLAMF1",0.859628070286938,0.580621934150755,1.38106634663871,0.420415670517129,0.674181822362205,NA
"14724","AL121985.1",0,NA,NA,NA,NA,NA
"14725","SLAMF7",1.0075502680625,-0.909257713738127,1.46933230921681,-0.618823739214435,0.536032481578911,NA
"14726","LY9",0.962320727506604,0.349110472562561,1.45074275888573,0.240642574587588,0.80983214987495,NA
"14727","CD244",0.482445603207939,-1.07281169701047,2.08647454360467,-0.514174352281836,0.607130097656544,NA
"14728","ITLN1",0,NA,NA,NA,NA,NA
"14729","F11R",0.4585768081272,0.631563903537034,2.587657350072,0.24406782587326,0.80717829035575,NA
"14730","NECTIN4",0.06390253691038,0.119912045671111,3.35247519753738,0.0357682126206912,0.971467179494267,NA
"14731","AL590714.1",0.06390253691038,0.119912045671111,3.35247519753738,0.0357682126206912,0.971467179494267,NA
"14732","NR1I3",0,NA,NA,NA,NA,NA
"14733","MPZ",1.01148995264131,-0.0322867323494314,1.33833948832178,-0.0241244711309517,0.980753323861037,NA
"14734","CFAP126",0.301234479217952,0.0419815600776809,2.85689911516166,0.0146947996360401,0.988275668200936,NA
"14735","FCGR3B",0.888036043773038,0.939316694780189,1.98515135113304,0.473171324818165,0.636090954020634,NA
"14736","FCRLA",0,NA,NA,NA,NA,NA
"14737","FCRLB",1.39009807346071,-0.0791253149910829,1.23016824588706,-0.0643207262548275,0.94871485045987,NA
"14738","NOS1AP",0.311475362858796,-0.0461613448588672,2.41663261754422,-0.019101515275324,0.984760122636486,NA
"14739","C1orf226",0.161211752532067,-0.631571180295204,3.33432222068988,-0.189415161011203,0.849767442783486,NA
"14740","SH2D1B",0.120807415993324,-0.48654549181451,3.34088239674268,-0.145633827844071,0.884210461918222,NA
"14741","AL596325.1",0.694190292519439,-1.10586414367313,1.71803673696

593,-0.643678985366809,0.519783619742733,NA
"14742","RGS5",12.8682404179971,3.72813365107524,1.79905248941944,2.07227619705433,NA,NA
"14743","AL499616.1",0,NA,NA,NA,NA,NA
"14744","AL592435.1",1.23889481616879,-1.28589126380214,1.46620923210509,-0.877017574057928,0.380477092664628,NA
"14745","NUF2",0.695419557253485,1.59770724600483,1.86830777748935,0.855162765608053,0.392461046165537,NA
"14746","AL357568.1",0.308531263183678,-0.370615225138078,2.39555983459126,-0.154709233218261,0.877050550585805,NA
"14747","AL356441.1",0.405873757968249,-0.77132395679941,2.6447535303111,-0.291643038929484,0.770559564920624,NA
"14748","AL451074.5",0,NA,NA,NA,NA,NA
"14749","TMCO1-
AS1",0.464820207020906,-1.16027832598351,2.20696916668511,-0.525733817897539,0.599073163594171,NA
"14750","AL358115.1",0.533115125111454,-2.39171219947894,1.98313525734918,-1.20602575674838,0.2278075593249,NA
"14751","AL596087.2",0.465228824660288,-1.18096956821875,2.17799777699842,-0.542227168774379,0.587662021171385,NA
"14752","LINC01675",0.06390253691038,0.119912045671111,3.35247519753738,0.0357682126206912,0.971467179494267,NA
"14753","ILDR2",0,NA,NA,NA,NA,NA
"14754","GPA33",0,NA,NA,NA,NA,NA
"14755","DUSP27",0,NA,NA,NA,NA,NA
"14756","AL451050.2",0,NA,NA,NA,NA,NA
"14757","AL136984.1",0.463121517457454,0.635608040746632,2.00882048040095,0.316408582522898,0.751692403436473,NA
"14758","AL359962.3",0.103291511307383,0.119912045671111,3.35247519753738,0.0357682126206912,0.971467179494267,NA
"14759","AL359962.2",0,NA,NA,NA,NA,NA
"14760","AL031733.2",0,NA,NA,NA,NA,NA
"14761","ADCY10",0.282419428642657,1.98408346788866,2.86695637949831,0.692052199355701,0.488904548657237,NA
"14762","Z99943.1",0.157712923618349,-0.60066841133552,3.33567379711352,-0.180074086337609,0.857094406146103,NA
"14763","AL023755.1",0.157712923618349,-0.60066841133552,3.33567379711352,-0.180074086337609,0.857094406146103,NA
"14764","XCL1",1.04129631883783,-1.28941409013433,1.65903866685034,-0.777205568440583,0.437037497439917,NA
"14765","CCDC181",0.785849713519922,1.76271422809025,2.08985180469097,0.843463744239465,0.398969134420529,NA
"14766","F5",0.487809372217354,-1.04197833925346,2.12436612378093,-0.490489058166187,0.623787870652889,NA
"14767","SELP",0.210667318323391,0.69698072231542,3.15449020715825,0.220948767168088,0.825132324921599,NA
"14768","SELL",1.40146735285544,-1.44520893471057,1.2335676538191,-1.17156844234382,0.241370364200723,NA
"14769","SELE",0.0604037079966622,0.119912045671111,3.35247519753738,0.0357682126206911,0.971467179494267,NA

"14770","LINC01681",0.284017700670301,-0.0201117199416187,2.4757236911
7806,-0.00812357211480602,0.993518398520538,NA
"14771","BX284613.2",1.05066086165511,0.1841481951989,1.54594194802662
,0.119117147596624,0.905182545649045,NA
"14772","MROH9",0.16538586715322,1.41832010653068,3.35247519753738,0.4
2306654724024,0.672246708730081,NA
"14773","AL021026.1",0.06390253691038,0.119912045671111,3.352475197537
38,0.0357682126206912,0.971467179494267,NA
"14774","MYOCOS",0,NA,NA,NA,NA,NA
"14775","FASLG",0.229797439238789,0.0884527683768807,3.10949972347481,
0.0284459804608171,0.977306451918786,NA
"14776","ANKRD45",0.977851400796669,2.14609077496204,1.58805476043672,
1.35139595209668,0.176568629794754,NA
"14777","GAS5-
AS1",0.248127555187016,0.0432008440948624,3.03591950680245,0.014229904
316654,0.988646562205271,NA
"14778","SERPINC1",0.120807415993324,-0.48654549181451,3.3408823967426
8,-0.145633827844071,0.884210461918222,NA
"14779","RC3H1-
IT1",0.361638187214614,-0.376712300772108,2.29395212926016,-0.16421977
4234611,0.869558134843047,NA
"14780","RABGAP1L-
DT",0.06390253691038,0.119912045671111,3.35247519753738,0.035768212620
6912,0.971467179494267,NA
"14781","GPR52",0.163695219304045,-0.600668372859899,3.33567379881164,
-0.18007407471135,0.857094415273326,NA
"14782","RABGAP1L-
IT1",0.549139710467542,-0.184794359146368,2.06961576015358,-0.08928921
14102644,0.928852067870296,NA
"14783","Z99127.1",0,NA,NA,NA,NA,NA
"14784","TNR",0,NA,NA,NA,NA,NA
"14785","LINC02803",0.739864197082658,0.329700077308349,1.690650425178
18,0.195013748790558,0.845382184283977,NA
"14786","AL590723.1",0.266702946863814,-0.0203591384131145,2.517155763
2184,-0.0080881519970316,0.993546658757216,NA
"14787","AL591043.2",0.2292884040636,0.696980734286753,3.0685279472186
9,0.227138467133238,0.820316073199257,NA
"14788","PAPPA2",0.939753786523857,0.184707955778829,1.32670546246147,
0.139223031038204,0.889273909597411,NA
"14789","ASTN1",0,NA,NA,NA,NA,NA
"14790","CLEC20A",0,NA,NA,NA,NA,NA
"14791","TEX35",1.00033412439453,-0.143392972071311,1.28239740651414,-
0.111816330369138,0.910969038963913,NA
"14792","AL449106.1",0.150263610326729,1.41832010653068,3.352475197537
38,0.42306654724024,0.672246708730081,NA
"14793","AXDND1",0,NA,NA,NA,NA,NA
"14794","AL160286.2",0.124306244907042,-0.509577950443694,3.3398031219
3904,-0.152577242381833,0.878731672788745,NA
"14795","AL353708.3",0.985818927878241,0.179505437312684,1.39421647485
226,0.128750047464262,0.897555432784626,NA

"14796","AL353708.1",0.892896308532157,-0.224115577789939,1.5605466390
5079,-0.143613508357724,0.885805673114003,NA
"14797","LHX4",0.589292702460194,-0.104400032627378,2.11748548193045,-
0.0493037772954173,0.960677209334343,NA
"14798","AL162431.1",0.288001464211087,-1.48571521254876,2.37685272244
263,-0.625076681664117,0.531920731445105,NA
"14799","KIAA1614-
AS1",0.266702946863814,-0.0203591384131145,2.5171557632184,-0.00808815
19970316,0.993546658757216,NA
"14800","LINC01732",0,NA,NA,NA,NA,NA
"14801","TEDDM1",0,NA,NA,NA,NA,NA
"14802","RGSL1",0,NA,NA,NA,NA,NA
"14803","AL355999.1",0.750083317802873,0.555943918050764,1.58281531355
4,0.351237389030856,0.725410262368042,NA
"14804","SHCBP1L",0.834269778236479,0.577305234079893,1.54806796631155
,0.372919824350729,0.709208116917367,NA
"14805","AL445228.2",0.58017684186752,0.926405767622686,1.790284497315
19,0.517462877554923,0.604833076546527,NA
"14806","AL078645.1",0.342823136639319,1.26402829119266,2.366751974340
89,0.534077209989303,0.593288116543864,NA
"14807","AL713852.1",0.860994469181247,1.81807040153509,1.641016744919
67,1.10789265689308,0.267908177262914,NA
"14808","AL713852.2",0.473419780809303,1.79503962060784,2.205292797180
63,0.81396884028403,0.415662801784622,NA
"14809","AL078644.2",0.103291511307383,0.119912045671111,3.35247519753
738,0.0357682126206912,0.971467179494267,NA
"14810","OCLM",0.173429405397192,1.41832010653068,3.35247519753738,0.4
2306654724024,0.672246708730081,NA
"14811","AL096803.4",0,NA,NA,NA,NA,NA
"14812","PACERR",0.163695219304045,-0.600668372859899,3.33567379881164
,-0.18007407471135,0.857094415273326,NA
"14813","ERVMER61-1",0.237331942307572,0.696980738944435,3.03442443415
691,0.229691249219751,0.818331695238684,NA
"14814","RGS18",0.979083089701983,1.51842442885152,1.58307803544825,0.
959159558057782,0.33747837048456,NA
"14815","AL390957.1",0.849084700611856,0.224708402818883,1.77801155148
209,0.126381857660921,0.899429662485105,NA
"14816","AL136987.1",0,NA,NA,NA,NA,NA
"14817","RGS13",0.808051961299607,-0.370920380287877,1.62368662517158,
-0.228443330466358,0.819301607019352,NA
"14818","B3GALT2",0.173429405397192,1.41832010653068,3.35247519753738,
0.42306654724024,0.672246708730081,NA
"14819","LINC01031",0,NA,NA,NA,NA,NA
"14820","AL138778.1",0.103291511307383,0.119912045671111,3.35247519753
738,0.0357682126206912,0.971467179494267,NA
"14821","AL136456.1",0,NA,NA,NA,NA,NA
"14822","CFHR3",0.21798004649093,1.96674997191025,3.35148506485532,0.5
86829400654111,0.557318288863578,NA
"14823","CFHR1",0.373877603000368,1.28717078660063,2.31487324831634,0.
556043743447646,0.578180951428236,NA

"14824","ASPM",0.564722380915662,-1.32311033345141,1.79345456513984,-0
.737743993725449,0.460670032706907,NA
"14825","CRB1",0.0604037079966622,0.119912045671111,3.35247519753738,0
.0357682126206911,0.971467179494267,NA
"14826","LHX9",0.88426218926915,1.97328586608528,1.62357960520219,1.21
539212476098,0.224216570214231,NA
"14827","AL450352.1",0.16538586715322,1.41832010653068,3.3524751975373
8,0.42306654724024,0.672246708730081,NA
"14828","AL157402.2",0,NA,NA,NA,NA,NA
"14829","MIR181A1HG",0.735345925928017,2.4523376866506,1.7592911920052
8,1.39393506759696,0.163337213528013,NA
"14830","NR5A2",0,NA,NA,NA,NA,NA
"14831","LINC00862",0.173429405397192,1.41832010653068,3.3524751975373
8,0.42306654724024,0.672246708730081,NA
"14832","AL359834.1",0,NA,NA,NA,NA,NA
"14833","KIF14",0,NA,NA,NA,NA,NA
"14834","DDX59-
AS1",0.120322481366256,1.41832010653068,3.35247519753738,0.42306654724
024,0.672246708730081,NA
"14835","INAVA",0,NA,NA,NA,NA,NA
"14836","KIF21B",0.67396546565486,0.650343382259148,1.84069690576953,0
.353313671697222,0.723853297107913,NA
"14837","AL358473.1",0,NA,NA,NA,NA,NA
"14838","ASCL5",0,NA,NA,NA,NA,NA
"14839","PKP1",0,NA,NA,NA,NA,NA
"14840","LAD1",0,NA,NA,NA,NA,NA
"14841","TNNI1",0,NA,NA,NA,NA,NA
"14842","AC096677.1",0.628673539379602,-0.777245688697867,1.7931200709
0987,-0.433459923463728,0.664680675076373,NA
"14843","AL645504.1",0.860763988897513,2.61781060486262,1.629876862232
92,1.60614011126966,0.108243128420143,NA
"14844","AL513217.1",0.638380866839818,-0.569751214790215,1.6937095140
7565,-0.336392521890722,0.736574887922277,NA
"14845","ELF3-
AS1",0.907798579048715,0.738704615800161,1.74091528238278,0.4243196801
56465,0.671332688509819,NA
"14846","ELF3",0,NA,NA,NA,NA,NA
"14847","GPR37L1",0,NA,NA,NA,NA,NA
"14848","PTPN7",1.28442825668455,0.561767649229128,1.10473642665518,0.
508508306302524,0.611096915403736,NA
"14849","LGR6",0.150263610326729,1.41832010653068,3.35247519753738,0.4
2306654724024,0.672246708730081,NA
"14850","SYT2",0,NA,NA,NA,NA,NA
"14851","AC098934.4",0,NA,NA,NA,NA,NA
"14852","PPFIA4",0.124306244907042,-0.509577950443694,3.33980312193904
,-0.152577242381833,0.878731672788745,NA
"14853","ADORA1",0.253555121634112,0.696980747544927,2.97042254430159,
0.234640269911095,0.814487951095881,NA
"14854","CHIT1",0.345921705435771,-1.76403744204294,2.21812050446305,-
0.795284764057472,0.426447874217624,NA

"14855","LINC01353",0.225789575149882,0.696980732170056,3.083901691853
47,0.226006144752027,0.821196641151035,NA
"14856","LINC01136",0.330889267521808,-1.64733224819506,2.310813272260
67,-0.712879862674264,0.475920101097177,NA
"14857","LAX1",0.859103488741988,3.51250204937672,2.23824248104923,1.5
693125651561,0.116575125916299,NA
"14858","ZBED6",1.18392886476258,1.85014564433773,1.3807714187829,1.33
993622635133,0.180266079272785,NA
"14859","LINC00303",0,NA,NA,NA,NA,NA
"14860","AL592146.2",0.450790830980187,2.62265483135,3.05731026738038,
0.857830773452114,0.390985914459059,NA
"14861","LINC00628",0,NA,NA,NA,NA,NA
"14862","AL512306.3",0.498336377233304,-1.03551003078545,1.72208293461
171,-0.601312521001745,0.547631851511455,NA
"14863","LRRN2",1.29306103799217,3.36727447396651,1.59353200564198,2.1
1308869984695,0.03459317668929,NA
"14864","AL512306.2",0.338302527857186,2.35457786137086,2.723555481677
41,0.864523552837888,0.3873003475594,NA
"14865","NFASC",1.23231881079811,1.05876774019467,1.36008810005033,0.7
78455263416755,0.436300670295561,NA
"14866","CNTN2",0.323693015723921,2.01097763960685,2.76273133237213,0.
727894752574506,0.466678015622723,NA
"14867","TMEM81",1.15900633674944,0.515057925784447,1.33293753382958,0
.386408149453685,0.699194415548738,NA
"14868","TMCC2",1.12319240360437,0.684006514263304,1.28386975223901,0.
532769397417789,0.594193220326165,NA
"14869","NUAK2",0.863320864035742,-0.343523521176883,1.47216983740277,
-0.233345034281461,0.815493491319118,NA
"14870","CDK18",0.715779578482886,-0.348304844937186,1.62468500498949,
-0.214382999699956,0.830248397763868,NA
"14871","MFSD4A",0.367794775146833,-1.77720183208484,2.26623873065255,
-0.784207686527846,0.432918255638315,NA
"14872","SLC45A3",1.19596803241168,0.88207609275943,1.23437462973975,0
.714593504684556,0.474860259461418,NA
"14873","PM20D1",0.394892336600027,0.637794923225675,2.06182977467526,
0.309334422782855,0.757067148536003,NA
"14874","RHEX",0.248612489814084,-1.30424658337368,2.93265112742767,-0
.444732948687857,0.656512755319132,NA
"14875","C1orf147",0,NA,NA,NA,NA,NA
"14876","AC244034.2",0.163695219304045,-0.600668372859899,3.3356737988
1164,-0.18007407471135,0.857094415273326,NA
"14877","IL19",0,NA,NA,NA,NA,NA
"14878","IL20",0,NA,NA,NA,NA,NA
"14879","IL24",0,NA,NA,NA,NA,NA
"14880","FCMR",1.12686552476461,-0.107949097357172,1.27927524072236,-0
.0843830115059657,0.932751913962228,NA
"14881","PIGR",0,NA,NA,NA,NA,NA
"14882","C4BPB",0.0604037079966622,0.119912045671111,3.35247519753738,
0.0357682126206911,0.971467179494267,NA
"14883","AL445493.3",0.293751886763448,2.01128355462242,2.837038982231

16,0.70893758147823,0.478363212179869,NA
"14884","CR2",0.173429405397192,1.41832010653068,3.35247519753738,0.42
306654724024,0.672246708730081,NA
"14885","CR1",0.531031918456741,-0.227092738529779,1.85948057726919,-0
.122126975299352,0.902798458581789,NA
"14886","CR1L",0.866940249418295,1.06820181778284,1.51392813963583,0.7
05582907019478,0.480447557103346,NA
"14887","AL356275.1",0.447805295795877,2.58917019540731,2.198097825213
92,1.1779139971422,0.238830894311389,NA
"14888","IRF6",0.06390253691038,0.119912045671111,3.35247519753738,0.0
357682126206912,0.971467179494267,NA
"14889","KCNH1",0.33077173430644,2.01280089522968,3.31423402991417,0.6
0732008574597,0.543638511160742,NA
"14890","AL590101.1",0,NA,NA,NA,NA,NA
"14891","RD3",0,NA,NA,NA,NA,NA
"14892","NEK2",0,NA,NA,NA,NA,NA
"14893","DTL",0.71400736383081,1.5113311098546,1.68596892093478,0.8964
16945228532,0.370030120429253,NA
"14894","AL360091.2",0.0604037079966622,0.119912045671111,3.3524751975
3738,0.0357682126206911,0.971467179494267,NA
"14895","AL360091.3",0.103291511307383,0.119912045671111,3.35247519753
738,0.0357682126206912,0.971467179494267,NA
"14896","LINC01740",0.06390253691038,0.119912045671111,3.3524751975373
8,0.0357682126206912,0.971467179494267,NA
"14897","AC092803.4",0.342823136639319,1.26402829119266,2.366751974340
89,0.534077209989303,0.593288116543864,NA
"14898","AL590648.2",0,NA,NA,NA,NA,NA
"14899","LINC02773",0,NA,NA,NA,NA,NA
"14900","PROX1-
AS1",0.334388096435525,-1.66805575603244,2.7170708962683,-0.6139169052
68611,0.539270238411727,NA
"14901","AC011700.1",0.35709347788436,-0.373613001150393,2.72627308047
933,-0.137041664617363,0.890997862165426,NA
"14902","KCNK2",0.389484794453927,-0.536595401468397,2.26391910918727,
-0.237020571667435,0.81264083104154,NA
"14903","ESRRG",0.755411235031854,3.34938853458542,1.96118736270891,1.
70783709821537,0.0876665733358514,NA
"14904","TGFB2-
AS1",0.16538586715322,1.41832010653068,3.35247519753738,0.423066547240
24,0.672246708730081,NA
"14905","LYPLAL1-
DT",0.120322481366256,1.41832010653068,3.35247519753738,0.423066547240
24,0.672246708730081,NA
"14906","AL360093.1",0.271071026320053,0.0867249233658518,2.9512515673
7628,0.0293858118787725,0.976556888399647,NA
"14907","AC096642.2",0,NA,NA,NA,NA,NA
"14908","ZC3H11B",0.846196461309622,1.88597098274201,1.53608301289565,
1.22777933673441,0.219529814518219,NA
"14909","SLC30A10",0.16538586715322,1.41832010653068,3.35247519753738,
0.42306654724024,0.672246708730081,NA

"14910","C1orf115",0.221615460528729,-1.10387780491251,2.5919333910837
8,-0.425889727224409,0.670188200759635,NA
"14911","MARC1",0.81799371880216,-0.187554662339561,1.40051076648596,-
0.133918757947258,0.893466817199006,NA
"14912","LINC01352",0,NA,NA,NA,NA,NA
"14913","HLX-
AS1",1.15197331223034,0.583764062047132,1.28616675345161,0.45387898612
7201,0.649915929095699,NA
"14914","LINC02817",0.270485559525146,-1.34694009274034,2.931760477270
36,-0.459430469570426,0.645925071915764,NA
"14915","LINC01655",0,NA,NA,NA,NA,NA
"14916","LINC02257",0,NA,NA,NA,NA,NA
"14917","AL356108.1",0.108990023245465,1.41832010653068,3.352475197537
38,0.42306654724024,0.672246708730081,NA
"14918","AL513314.2",0.639706871362103,-0.531613180219892,1.8394558677
2065,-0.289005672573509,0.772577036618986,NA
"14919","HHIPL2",0.276720916704575,0.696980758280457,2.88854265454755,
0.241291489043159,0.809329206809347,NA
"14920","TAF1A-
AS1",1.46628372764986,1.09402694446688,1.1308198475704,0.9674635149156
92,0.333312371857407,NA
"14921","FAM177B",0.0604037079966622,0.119912045671111,3.3524751975373
8,0.0357682126206911,0.971467179494267,NA
"14922","AL392172.2",0.108990023245465,1.41832010653068,3.352475197537
38,0.42306654724024,0.672246708730081,NA
"14923","AL929091.1",0.520288216191576,2.79382521304909,2.953688628253
64,0.945876686636712,0.344211485698358,NA
"14924","CAPN8",0.0604037079966622,0.119912045671111,3.35247519753738,
0.0357682126206911,0.971467179494267,NA
"14925","AC138393.3",0,NA,NA,NA,NA,NA
"14926","AC092809.2",0.0604037079966622,0.119912045671111,3.3524751975
3738,0.0357682126206911,0.971467179494267,NA
"14927","AC092809.4",0.124306244907042,-0.509577950443694,3.3398031219
3904,-0.152577242381833,0.878731672788745,NA
"14928","AC096537.1",0.925365881884851,-2.35786569830844,1.43641720084
397,-1.64149085441407,0.100695563991283,NA
"14929","AC099066.2",0.0973092156216871,0.119912045671111,3.3524751975
3738,0.0357682126206911,0.971467179494267,NA
"14930","LEFTY1",0.0973092156216871,0.119912045671111,3.35247519753738
,0.0357682126206911,0.971467179494267,NA
"14931","AL117348.1",0,NA,NA,NA,NA,NA
"14932","LEFTY2",1.24978155250487,1.9232692827471,1.5864061023733,1.21
234359844547,0.225380881261781,NA
"14933","AL512343.2",1.25852280187915,0.830536695855232,1.235413023961
21,0.672274518518683,0.501408948637305,NA
"14934","ACBD3-AS1",0,NA,NA,NA,NA,NA
"14935","MIXL1",0,NA,NA,NA,NA,NA
"14936","ITPKB-IT1",0,NA,NA,NA,NA,NA
"14937","ITPKB-AS1",0,NA,NA,NA,NA,NA
"14938","AL359732.1",0.675487525344297,-0.818638479524725,1.6630802989

3023,-0.492242304867245,0.622548061125924,NA
"14939","AL353689.3",0,NA,NA,NA,NA,NA
"14940","AL627308.3",0,NA,NA,NA,NA,NA
"14941","AL731702.1",0.43228277885212,-0.901439642708117,1.92008933655091,-0.469477969357087,0.638728028438615,NA
"14942","PRSS38",0,NA,NA,NA,NA,NA
"14943","LINC02809",0.108990023245465,1.41832010653068,3.35247519753738,0.42306654724024,0.672246708730081,NA
"14944","IBA57-DT",0.0604037079966622,0.119912045671111,3.35247519753738,0.0357682126206911,0.971467179494267,NA
"14945","OBSCN-AS1",0.124306244907042,-0.509577950443694,3.33980312193904,-0.152577242381833,0.878731672788745,NA
"14946","AL353593.3",0,NA,NA,NA,NA,NA
"14947","AL670729.3",0,NA,NA,NA,NA,NA
"14948","TRIM17",0.903887741588693,0.784336678131975,1.51434769523341,0.517936984089432,0.604502236808128,NA
"14949","HIST3H2BB",0.330989799689647,1.28767123567888,2.3893126005633,0.538929579735739,0.589935446033768,NA
"14950","AL117350.1",0,NA,NA,NA,NA,NA
"14951","ACTA1",0.856935666870343,-0.498205715252172,1.78598284142037,-0.278953248428722,0.780280703419024,NA
"14952","LINC01736",0.357602513059549,-0.758784488563382,2.26392616525718,-0.335163089772048,0.737502063427483,NA
"14953","AL136988.2",0,NA,NA,NA,NA,NA
"14954","AL136988.1",0.323693015723921,2.01097763960685,2.76273133237213,0.727894752574506,0.466678015622723,NA
"14955","PGBD5",0.30503243426996,-0.35285406173419,2.4020524594433,-0.146896900751271,0.883213398227782,NA
"14956","AL158214.2",0.167194048217763,-0.631571198727006,3.33432221989104,-0.189415166584483,0.849767438415713,NA
"14957","AL118511.1",0,NA,NA,NA,NA,NA
"14958","TRIM67",0,NA,NA,NA,NA,NA
"14959","AL136171.2",0.499773963588746,-1.07918527743271,2.11767604006399,-0.50960829561074,0.610325910933087,NA
"14960","DISC1-IT1",0.120322481366256,1.41832010653068,3.35247519753738,0.42306654724024,0.672246708730081,NA
"14961","SLC35F3",0.259253633572194,1.98393725043859,2.94786137272544,0.673009005374071,0.500941561797964,NA
"14962","AL160408.2",0.796978985202501,-0.001336692088463,1.52549567584762,-0.000876234596810827,0.999300866033028,NA
"14963","LINC01132",0.06390253691038,0.119912045671111,3.35247519753738,0.0357682126206912,0.971467179494267,NA
"14964","AL391832.4",0,NA,NA,NA,NA,NA
"14965","AL391832.3",0,NA,NA,NA,NA,NA
"14966","LINC01348",0.161211752532067,-0.631571180295204,3.33432222068988,-0.189415161011203,0.849767442783486,NA
"14967","AL732292.2",0.231096585128143,-1.15074146808831,3.05677932246

771,-0.376455526125231,0.706578272798499,NA
"14968","LINC02768",0,NA,NA,NA,NA,NA
"14969","EDARADD",0.150263610326729,1.41832010653068,3.35247519753738,0.42306654724024,0.672246708730081,NA
"14970","LGALS8-AS1",0.801307568944988,-1.16512438918571,1.52454979912014,-0.764241607494967,0.444723275782302,NA
"14971","AL359921.2",1.11014499591047,0.790782928354536,1.31934637674339,0.59937476791081,0.548922999069567,NA
"14972","AL359921.1",0.27107102632005 3,0.0867249233658518,2.95125156737628,0.0293858118787725,0.976556888399647,NA
"14973","ACTN2",0.229312504611721,1.98374348132209,3.06703062452501,0.646796111346072,0.51776390945149,NA
"14974","RYR2",0,NA,NA,NA,NA,NA
"14975","AC093426.1",0,NA,NA,NA,NA,NA
"14976","CHRM3",0.210667318323391,0.69698072231542,3.15449020715825,0.220948767168088,0.825132324921599,NA
"14977","CHRM3-AS2",0.224098927300707,-1.11019835680208,3.08249114251047,-0.360162707847362,0.718725460748659,NA
"14978","AL358176.4",0,NA,NA,NA,NA,NA
"14979","RGS7",0,NA,NA,NA,NA,NA
"14980","AL359764.2",0.223613992673639,0.696980730824599,3.093634143729,0.225295137835683,0.821749681896065,NA
"14981","KMO",0.2292884040636,0.696980734286753,3.06852794721869,0.227138467133238,0.820316073199257,NA
"14982","AL133390.1",0.161211752532067,-0.631571180295204,3.33432222068988,-0.189415161011203,0.849767442783486,NA
"14983","EXO1",0.172892560155845,0.696980693484986,3.35247519753738,0.207900328091007,0.835306792989891,NA
"14984","AL591686.2",0.371685106020372,1.26420130968869,2.31868787890972,0.545222718929784,0.585600347978445,NA
"14985","AL591686.1",1.24425261872604,-1.25297825565493,1.59857831074055,-0.783807866800396,0.433152857134078,NA
"14986","AL591721.1",0,NA,NA,NA,NA,NA
"14987","AKT3-IT1",0.902883275009359,1.97955803396098,1.64699810737113,1.20191882741181,0.229394979159254,NA
"14988","C1orf100",0,NA,NA,NA,NA,NA
"14989","CATSPERE",0.967663104176537,-0.683150727841201,1.32623267847008,-0.515106239599879,0.606478782779322,NA
"14990","AL451007.3",0.206583022614766,-0.859518418402433,3.32508269885954,-0.25849535071631,0.796024636623985,NA
"14991","AL451007.2",0.30503243426996,-0.35285406173419,2.4020524594433,-0.146896900751271,0.883213398227782,NA
"14992","BX323046.2",0.284017700670301,-0.0201117199416187,2.47572369117806,-0.00812357211480602,0.993518398520538,NA
"14993","BX323046.1",0,NA,NA,NA,NA,NA
"14994","KIF26B",0.0973092156216871,0.119912045671111,3.35247519753738,0.0357682126206911,0.971467179494267,NA

"14995","SMYD3-
IT1",0.108990023245465,1.41832010653068,3.35247519753738,0.42306654724
024,0.672246708730081,NA
"14996","AL591623.1",0.375829555233361,-1.85678094543068,2.60946262480
254,-0.711556826981257,0.476739249500974,NA
"14997","AL591848.3",0.212281534552848,0.696980723426082,3.14661376999
226,0.221501834789148,0.82470170812449,NA
"14998","AL591848.4",0.59021933271372,1.25394427494946,1.9252632277298
2,0.651310562051328,0.514846031688698,NA
"14999","ZNF695",0.150263610326729,1.41832010653068,3.35247519753738,0
.42306654724024,0.672246708730081,NA
"15000","C1orf229",1.04602867103982,0.719171029439961,1.35186726635034
,0.531983462682336,0.594737449292598,NA
"15001","AL390728.5",0.942105998874116,0.207584274870814,1.32481573755
899,0.156689167395682,0.875489824788238,NA
"15002","AC104335.1",0.62980282098199,0.0508612574923491,1.66075292736
592,0.0306254209486892,0.975568268670255,NA
"15003","AC104335.2",0.229312504611721,1.98374348132209,3.067030624525
01,0.646796111346072,0.51776390945149,NA
"15004","OR2B11",0.0604037079966622,0.119912045671111,3.35247519753738
,0.0357682126206911,0.971467179494267,NA
"15005","GCSAML",0.421002231345608,1.28676736105846,2.25932589960192,0
.569535967026793,0.568992469434524,NA
"15006","OR2C3",0.815536207920309,3.55529601927911,1.91625380613609,1.
85533670325642,0.0635481600588788,NA
"15007","AL390860.1",0,NA,NA,NA,NA,NA
"15008","TRIM58",0.576588395217082,-1.52164829755172,2.07780094091898,
-0.732335936318671,0.463963550283659,NA
"15009","OR2L2",0.163695219304045,-0.600668372859899,3.33567379881164,
-0.18007407471135,0.857094415273326,NA
"15010","FAM110C",0.173429405397192,1.41832010653068,3.35247519753738,
0.42306654724024,0.672246708730081,NA
"15011","AC079779.2",0.518507267144388,2.86142186343831,2.401072026647
09,1.19172679190056,0.233368389218479,NA
"15012","AC093326.1",0.285708348519476,2.01137472954577,2.860309475841
75,0.703201785168317,0.481930006892637,NA
"15013","AC116609.3",0.225789575149882,0.696980732170056,3.08390169185
347,0.226006144752027,0.821196641151035,NA
"15014","LINC01115",0,NA,NA,NA,NA,NA
"15015","TPO",0,NA,NA,NA,NA,NA
"15016","AC144450.1",0.605147624705125,3.03408526384619,2.372188119020
34,1.27902388496036,0.200888645047297,NA
"15017","MYT1L",0,NA,NA,NA,NA,NA
"15018","EIPR1-
IT1",0.16538586715322,1.41832010653068,3.35247519753738,0.423066547240
24,0.672246708730081,NA
"15019","TRAPPC12-
AS1",0.108990023245465,1.41832010653068,3.35247519753738,0.42306654724
024,0.672246708730081,NA
"15020","AC114810.1",0,NA,NA,NA,NA,NA

"15021","AC231981.1",1.28408109885536,0.122848868103205,1.34488095392134,0.0913455334057696,0.927218038768138,NA
"15022","AC108488.3",1.22097528770957,0.190810325651831,1.32967486163107,0.14350149134787,0.885894133514726,NA
"15023","SOX11",0,NA,NA,NA,NA,NA
"15024","SILC1",0.447405035678612,-0.902104519400194,1.90505207920899,-0.473532733957994,0.635833152951106,NA
"15025","LINC00487",0,NA,NA,NA,NA,NA
"15026","NRIR",0.16538586715322,1.41832010653068,3.35247519753738,0.42306654724024,0.672246708730081,NA
"15027","AC017076.1",0,NA,NA,NA,NA,NA
"15028","RNF144A-AS1",0.951138944535676,1.66508050752383,1.98706680630421,0.837958996769091,0.402053732507922,NA
"15029","AC010904.2",0.270586091692985,2.01155232872955,2.90724650940082,0.691909792384316,0.488993980674386,NA
"15030","AC013460.1",0,NA,NA,NA,NA,NA
"15031","AC092580.4",0,NA,NA,NA,NA,NA
"15032","LINC00298",0,NA,NA,NA,NA,NA
"15033","LINC00299",0,NA,NA,NA,NA,NA
"15034","U91324.1",0.253555121634112,0.696980747544927,2.97042254430159,0.234640269911095,0.814487951095881,NA
"15035","LINC01814",0,NA,NA,NA,NA,NA
"15036","AC092617.1",0,NA,NA,NA,NA,NA
"15037","ID2-AS1",1.00853255221716,-2.49229301097638,1.57125236746144,-1.58618250167094,0.112697916001445,NA
"15038","AC073195.1",0.9747505809165,-0.581278286493146,1.53799885074646,-0.377944551916294,0.705471785160798,NA
"15039","AC082651.4",0,NA,NA,NA,NA,NA
"15040","AC082651.3",0.311475362858796,-0.0461613448588672,2.41663261754422,-0.019101515275324,0.984760122636486,NA
"15041","AC010969.1",0.06390253691038,0.119912045671111,3.35247519753738,0.0357682126206912,0.971467179494267,NA
"15042","RRM2",0.282419428642657,1.98408346788866,2.86695637949831,0.692052199355701,0.488904548657237,NA
"15043","AC007240.3",0.120322481366256,1.41832010653068,3.35247519753738,0.42306654724024,0.672246708730081,NA
"15044","ODC1-DT",1.13824882743442,-0.58711116955613,1.42128319717433,-0.413085281471964,0.679544132659778,NA
"15045","ATP6V1C2",0.221615460528729,-1.10387780491251,2.59193339108378,-0.425889727224409,0.670188200759635,NA
"15046","AC092687.3",0.403989145283987,-0.940043913464617,2.2528578608457,-0.417267298484483,0.676482904981981,NA
"15047","C2orf50",0.435895527226486,0.364488370732983,2.30637108761314,0.158035440476403,0.874428872149669,NA
"15048","LINC00570",0.571820570910937,1.19000690882526,1.93224774417711,0.615866631187117,0.537982546482594,NA
"15049","AC099344.3",0.217631696987943,0.696980727003399,3.12110949465

653,0.223311847340396,0.823292807226044,NA
"15050","MIR3681HG",0.103291511307383,0.119912045671111,3.35247519753738,0.0357682126206912,0.971467179494267,NA
"15051","AC009486.1",0,NA,NA,NA,NA,NA
"15052","LINC00276",0,NA,NA,NA,NA,NA
"15053","MYCN",0.262695082774907,0.696980751978295,2.93688603919854,0.237319644914958,0.812408822930581,NA
"15054","AC079148.1",0.976435663905632,-1.65730020121893,1.35787803566655,-1.22050740765198,0.222272583130362,NA
"15055","NT5C1B",0,NA,NA,NA,NA,NA
"15056","AC130814.1",0,NA,NA,NA,NA,NA
"15057","LINC01376",1.34264822585866,0.4985907643711,1.12527678633509,0.443082777878107,0.657705861645473,NA
"15058","AC010096.1",0,NA,NA,NA,NA,NA
"15059","AC013400.1",1.05029346024341,-0.909632721057968,1.70900253377588,-0.532259433839587,0.594546324154888,NA
"15060","AC079145.1",0.373493287084915,-0.711154961173275,2.04479856801751,-0.347787294209014,0.727999932456857,NA
"15061","LAPTM4A-DT",0.184709952903704,-0.898539523146346,3.26457968362944,-0.275238961895205,0.783132655214614,NA
"15062","SDC1",0.672742955364309,0.845024458814882,2.08632110095783,0.405030873927762,0.685454816913829,NA
"15063","AC012065.3",0.0604037079966622,0.119912045671111,3.35247519753738,0.0357682126206911,0.971467179494267,NA
"15064","GDF7",0.817429159257973,2.56456179712937,1.95665613102608,1.31068599968279,0.189963870604419,NA
"15065","AC012065.4",0.634380188795279,0.971295148781894,2.12526947480837,0.457022114275403,0.647655150537299,NA
"15066","APOB",0.108990023245465,1.41832010653068,3.35247519753738,0.42306654724024,0.672246708730081,NA
"15067","TDRD15",0,NA,NA,NA,NA,NA
"15068","AC018742.1",0,NA,NA,NA,NA,NA
"15069","AC011752.1",0,NA,NA,NA,NA,NA
"15070","AC018467.1",0.229312504611721,1.98374348132209,3.06703062452501,0.646796111346072,0.51776390945149,NA
"15071","AC009242.1",0.163695219304045,-0.600668372859899,3.33567379881164,-0.18007407471135,0.857094415273326,NA
"15072","MFSD2B",0.124306244907042,-0.509577950443694,3.33980312193904,-0.152577242381833,0.878731672788745,NA
"15073","AC009228.1",0.334104312687912,-0.548108789156736,2.36098106779811,-0.232152979383232,0.816419197091169,NA
"15074","AC012073.1",0,NA,NA,NA,NA,NA
"15075","EFR3B",0.959881385583743,0.458004319352563,1.37608293527128,0.332831915586738,0.739261165271894,NA
"15076","AC012074.1",0,NA,NA,NA,NA,NA
"15077","ADGRF3",0.103291511307383,0.119912045671111,3.35247519753738,0.0357682126206912,0.971467179494267,NA
"15078","OTOF",0.06390253691038,0.119912045671111,3.35247519753738,0.0357682126206912,0.971467179494267,NA

"15079","KCNK3",0.301234479217952,0.0419815600776809,2.85689911516166,
0.0146947996360401,0.988275668200936,NA
"15080","CENPA",0.797049200271351,0.11096350068226,1.7722774533195,0.0
626106823592568,0.950076522816486,NA
"15081","DPYSL5",0.16538586715322,1.41832010653068,3.35247519753738,0.
42306654724024,0.672246708730081,NA
"15082","AC013472.1",0.108990023245465,1.41832010653068,3.352475197537
38,0.42306654724024,0.672246708730081,NA
"15083","AGBL5-
AS1",0.120322481366256,1.41832010653068,3.35247519753738,0.42306654724
024,0.672246708730081,NA
"15084","AC013472.3",0,NA,NA,NA,NA,NA
"15085","AC013403.2",0,NA,NA,NA,NA,NA
"15086","ABHD1",1.41288978358555,1.23815161110064,1.18203562431698,1.0
4747402331135,0.294881006849042,NA
"15087","SLC30A3",0.06390253691038,0.119912045671111,3.35247519753738,
0.0357682126206912,0.971467179494267,NA
"15088","TRIM54",0,NA,NA,NA,NA,NA
"15089","UCN",1.13620309467381,1.85582921918583,1.67982046885724,1.104
77831029665,0.269255661069839,NA
"15090","GTF3C2-
AS1",0.06390253691038,0.119912045671111,3.35247519753738,0.03576821262
06912,0.971467179494267,NA
"15091","KRTCAP3",0.697248311655205,-0.264500716103852,2.0819838042906
6,-0.127042638640491,0.898906651184876,NA
"15092","GCKR",0.103291511307383,0.119912045671111,3.35247519753738,0.
0357682126206912,0.971467179494267,NA
"15093","LINC01460",0.172892560155845,0.696980693484986,3.352475197537
38,0.207900328091007,0.835306792989891,NA
"15094","AC104695.2",0.120322481366256,1.41832010653068,3.352475197537
38,0.42306654724024,0.672246708730081,NA
"15095","AC104695.3",0.653603655834148,2.22146083120939,1.840026548247
23,1.20729824975922,0.227317304484175,NA
"15096","PLB1",0.120322481366256,1.41832010653068,3.35247519753738,0.4
2306654724024,0.672246708730081,NA
"15097","SPDYA",1.01316687928413,-0.8962234103448,1.28427214320325,-0.
697845402228708,0.485273881873686,NA
"15098","AC097724.1",0.120322481366256,1.41832010653068,3.352475197537
38,0.42306654724024,0.672246708730081,NA
"15099","TOGARAM2",0.461321378107188,-1.14718650057539,2.2090897510114
1,-0.519302803360599,0.603549598768801,NA
"15100","PCARE",0.38336591364415,2.43735836912973,2.62401805200281,0.9
28864939503518,0.352959084821909,NA
"15101","ALK",0,NA,NA,NA,NA,NA
"15102","AC016907.2",0.426984527031304,1.28670729871888,2.251366204678
44,0.571522880660216,0.567645254942652,NA
"15103","LINC01936",0.33077173430644,2.01280089522968,3.31423402991417
,0.60732008574597,0.543638511160742,NA
"15104","GALNT14",0,NA,NA,NA,NA,NA
"15105","CAPN14",0.0604037079966622,0.119912045671111,3.35247519753738

,0.0357682126206911,0.971467179494267,NA
"15106","AL121652.1",0.223613992673639,0.696980730824599,3.09363414372
9,0.225295137835683,0.821749681896065,NA
"15107","AL121655.1",0.341937941894985,-0.534050001383225,2.0649395232
6993,-0.258627429697085,0.795922717282238,NA
"15108","NLRC4",0,NA,NA,NA,NA,NA
"15109","AL133243.1",0,NA,NA,NA,NA,NA
"15110","LINC00486",0,NA,NA,NA,NA,NA
"15111","AC019127.1",0,NA,NA,NA,NA,NA
"15112","RASGRP3",0.19170761073114,-0.935110177380914,3.32149227750043
,-0.281533148132028,0.778301507243725,NA
"15113","AC020594.1",0.16538586715322,1.41832010653068,3.3524751975373
8,0.42306654724024,0.672246708730081,NA
"15114","AC017050.1",0.172892560155845,0.696980693484986,3.35247519753
738,0.207900328091007,0.835306792989891,NA
"15115","AC007378.1",0,NA,NA,NA,NA,NA
"15116","AC006369.1",0,NA,NA,NA,NA,NA
"15117","LINC00211",0,NA,NA,NA,NA,NA
"15118","RMDN2-
AS1",1.31316330184177,0.313143723896661,1.35739477579231,0.23069465823
8889,0.817552023712866,NA
"15119","AC009229.3",0.194618431243374,-0.859518418402434,3.3250826988
5954,-0.25849535071631,0.796024636623985,NA
"15120","AC009229.1",0.16538586715322,1.41832010653068,3.3524751975373
8,0.42306654724024,0.672246708730081,NA
"15121","LINC02613",0.848295612166147,0.568909524575636,1.450602460900
54,0.392188445773389,0.694918980633917,NA
"15122","AC074366.1",0,NA,NA,NA,NA,NA
"15123","SOS1-
IT1",0.281534233898323,-0.047105237591176,2.4839065807098,-0.018964174
4005184,0.984869684953748,NA
"15124","CDKL4",0.150263610326729,1.41832010653068,3.35247519753738,0.
42306654724024,0.672246708730081,NA
"15125","AC007684.2",0.511901797541343,0.180545511790643,1.93290575556
538,0.0934062673623901,0.925580811652988,NA
"15126","TMEM178A",1.06958813004583,1.57261117733445,1.42674945748556,
1.10223359054641,0.27036013212908,NA
"15127","SLC8A1-
AS1",0.16538586715322,1.41832010653068,3.35247519753738,0.423066547240
24,0.672246708730081,NA
"15128","AC009413.2",0,NA,NA,NA,NA,NA
"15129","LINC01913",0.225789575149882,0.696980732170056,3.083901691853
47,0.226006144752027,0.821196641151035,NA
"15130","C2orf91",1.01437751256678,0.14435424029754,1.70941939506989,0
.0844463568822664,0.932701551422202,NA
"15131","AC083949.1",0.0604037079966622,0.119912045671111,3.3524751975
3738,0.0357682126206911,0.971467179494267,NA
"15132","LINC01819",0.229797439238789,0.0884527683768807,3.10949972347
481,0.0284459804608171,0.977306451918786,NA
"15133","AC010883.2",0.173429405397192,1.41832010653068,3.352475197537

38,0.42306654724024,0.672246708730081,NA
"15134","LINC01126",0.800724659059084,-0.081030149428161,1.63174486503992,-0.0496585901167697,0.960394456020498,NA
"15135","AC010883.1",0.612908436047199,-2.55797597990941,1.63620685880398,-1.56335732621193,0.117968571533295,NA
"15136","ABCG5",0,NA,NA,NA,NA,NA
"15137","ABCG8",0,NA,NA,NA,NA,NA
"15138","AC019129.2",0.225789575149882,0.696980732170056,3.08390169185347,0.226006144752027,0.821196641151035,NA
"15139","SLC3A1",0.518335748691224,-1.18282059391381,2.11748355776135,-0.558597297994756,0.576436584483888,NA
"15140","LINC01833",0,NA,NA,NA,NA,NA
"15141","SIX3",0.247572825948416,0.696980744488543,2.99332389411541,0.23284508096793,0.815881705308138,NA
"15142","AC093833.1",0.173429405397192,1.41832010653068,3.35247519753738,0.42306654724024,0.672246708730081,NA
"15143","AC009236.1",1.18548942966778,0.437791211886906,1.4322445061545,0.305667928908558,0.759857494281973,NA
"15144","U51244.1",0,NA,NA,NA,NA,NA
"15145","AC018682.1",0.124306244907042,-0.509577950443694,3.33980312193904,-0.152577242381833,0.878731672788745,NA
"15146","AC016722.2",0,NA,NA,NA,NA,NA
"15147","STPG4",0,NA,NA,NA,NA,NA
"15148","AC073283.1",0.108990023245465,1.41832010653068,3.35247519753738,0.42306654724024,0.672246708730081,NA
"15149","EPCAM-DT",0,NA,NA,NA,NA,NA
"15150","KCNK12",0.44347865184883,1.74299313978226,1.99921543305451,0.871838577756088,0.38329644566944,NA
"15151","AC092650.1",0.184225018276636,0.696980701717498,3.29715406410566,0.21138857577362,0.832584070546688,NA
"15152","AC093635.1",0.676774920001337,0.988178496742983,1.64560472256979,0.600495661679819,0.548175951296021,NA
"15153","STON1-GTF2A1L",0,NA,NA,NA,NA,NA
"15154","GTF2A1L",0,NA,NA,NA,NA,NA
"15155","LHCGR",0.709142136022783,0.20640165949466,1.65869393655974,0.12443625369654,0.900969870107214,NA
"15156","NRXN1",0.0604037079966622,0.119912045671111,3.35247519753738,0.0357682126206911,0.971467179494267,NA
"15157","CHAC2",1.33575292536248,-0.583022741929509,1.17757418935361,-0.495104892074394,0.620526080756582,NA
"15158","GPR75",0,NA,NA,NA,NA,NA
"15159","TSPYL6",0.120807415993324,-0.48654549181451,3.34088239674268,-0.145633827844071,0.884210461918222,NA
"15160","C2orf73",1.47424406868706,-0.498442327568329,1.2303844047272,-0.405111057693261,0.685395878770685,NA
"15161","AC093110.1",1.04310439448989,2.02376482555491,1.47702946232717,1.37015873899111,0.170637355165021,NA
"15162","EML6",0.662031596537349,-1.62516611701831,1.57514895572133,-1.03175392467823,0.302187408724762,NA
"15163","AC012358.1",0,NA,NA,NA,NA,NA

"15164","AC011306.1",0.180726189362918,0.69698069859353,3.318255251961
68,0.210044329224369,0.833633074956668,NA
"15165","AC007743.1",0.428647364814321,1.71061425986059,2.247037164447
41,0.761275463942432,0.44649255252664,NA
"15166","AC132153.1",0.206299238867152,0.696980719233408,3.17624445737
489,0.219435477522864,0.826310836331857,NA
"15167","AC068193.1",0.379728644264344,1.26425268242219,2.305017521615
96,0.548478556265319,0.583363349468637,NA
"15168","AC007381.1",0,NA,NA,NA,NA,NA
"15169","BCL11A",0.439247157516672,-1.02551403556625,1.93204059095195,
-0.530793214370806,0.595562082797643,NA
"15170","LINC01185",0.16538586715322,1.41832010653068,3.35247519753738
,0.42306654724024,0.672246708730081,NA
"15171","AC016747.3",0.206299238867152,0.696980719233408,3.17624445737
489,0.219435477522864,0.826310836331857,NA
"15172","AC016727.3",0,NA,NA,NA,NA,NA
"15173","AC107081.2",0.108990023245465,1.41832010653068,3.352475197537
38,0.42306654724024,0.672246708730081,NA
"15174","AC107081.4",0,NA,NA,NA,NA,NA
"15175","AC092155.1",0.120322481366256,1.41832010653068,3.352475197537
38,0.42306654724024,0.672246708730081,NA
"15176","AC092567.2",0.0973092156216871,0.119912045671111,3.3524751975
3738,0.0357682126206911,0.971467179494267,NA
"15177","OTX1",0,NA,NA,NA,NA,NA
"15178","AC009501.3",0,NA,NA,NA,NA,NA
"15179","AC012368.2",0,NA,NA,NA,NA,NA
"15180","AC012368.1",0.867894522330993,0.141912316455413,1.40089328529
564,0.101301303921565,0.919311281140784,NA
"15181","LINC00309",0.952863904631119,-0.59917005805974,1.522355791939
36,-0.393580831256566,0.693890535728686,NA
"15182","LGALSL-
DT",0.270738621018879,0.696980755662315,2.90872380220041,0.23961737279
2514,0.810626895543258,NA
"15183","AC008074.2",1.01572677767583,0.100403479773473,1.290465743935
14,0.0778040643429268,0.937983913374115,NA
"15184","AC007365.1",1.42232506042476,-1.42519297734381,1.250578094039
76,-1.1396273324603,0.254441593210124,NA
"15185","LINC01800",0,NA,NA,NA,NA,NA
"15186","LINC02245",1.25543269525429,0.0693375529560519,1.163548460534
5,0.059591461213571,0.952481019248393,NA
"15187","LINC02576",0.06390253691038,0.119912045671111,3.3524751975373
8,0.0357682126206912,0.971467179494267,NA
"15188","AC012370.1",0.108990023245465,1.41832010653068,3.352475197537
38,0.42306654724024,0.672246708730081,NA
"15189","AC007389.1",1.20706379369787,0.327302144637873,1.450885171209
21,0.225587903944934,0.821521949615134,NA
"15190","LINC01873",1.33557435569302,0.423867031337647,1.1643006198600
8,0.364052912201134,0.715818498149289,NA
"15191","LINC01829",0.16538586715322,1.41832010653068,3.35247519753738
,0.42306654724024,0.672246708730081,NA

"15192","LINC02831",0.06390253691038,0.119912045671111,3.3524751975373
8,0.0357682126206912,0.971467179494267,NA
"15193","AC017083.2",0.716333758685305,-0.8789406682509,1.718927229932
13,-0.511330935333199,0.609119346778894,NA
"15194","PROKR1",0,NA,NA,NA,NA,NA
"15195","LINC01888",0,NA,NA,NA,NA,NA
"15196","AC114802.1",0.167194048217763,-0.631571198727006,3.3343222198
9104,-0.189415166584483,0.849767438415713,NA
"15197","ASPRV1",1.37199028144991,-0.102485386183019,1.2539006651425,-
0.0817332576910088,0.93485883101297,NA
"15198","LINC01816",0.326621720233408,1.26392102233239,2.3982324286093
7,0.527021904655539,0.598178377191062,NA
"15199","TGFA",0.0604037079966622,0.119912045671111,3.35247519753738,0
.0357682126206911,0.971467179494267,NA
"15200","ADD2",0.864371592869155,-0.411322196584166,1.43827729542297,-
0.285982541678935,0.77489149310712,NA
"15201","CLEC4F",0,NA,NA,NA,NA,NA
"15202","VAX2",0.381326916291988,-0.693919196636814,2.03324742984739,-
0.341286154577309,0.73288816994981,NA
"15203","ATP6V1B1",0.324906971836112,-1.62214160027477,2.0288952361353
9,-0.799519645659284,0.423989159827425,NA
"15204","AC007881.3",0.297198805062887,-0.369239549556464,2.4204946140
5861,-0.152547147765528,0.878755406995513,NA
"15205","AC007881.4",0.06390253691038,0.119912045671111,3.352475197537
38,0.0357682126206912,0.971467179494267,NA
"15206","FBXO41",0.864227821700704,0.430367859839434,1.5514204197928,0
.277402472178955,0.781471083564147,NA
"15207","EGR4",0,NA,NA,NA,NA,NA
"15208","ALMS1-
IT1",0.878165754841295,-0.0498386045584647,1.75221086056537,-0.0284432
688326015,0.977308614609984,NA
"15209","AC074008.1",0.161211752532067,-0.631571180295204,3.3343222206
8988,-0.189415161011203,0.849767442783486,NA
"15210","AC092653.1",0,NA,NA,NA,NA,NA
"15211","AC073046.4",0.2292884040636,0.696980734286753,3.0685279472186
9,0.227138467133238,0.820316073199257,NA
"15212","ACTG2",0.103291511307383,0.119912045671111,3.35247519753738,0
.0357682126206912,0.971467179494267,NA
"15213","DGUOK-
AS1",0.0604037079966622,0.119912045671111,3.35247519753738,0.035768212
6206911,0.971467179494267,NA
"15214","AC073046.1",0,NA,NA,NA,NA,NA
"15215","DCTN1-AS1",0,NA,NA,NA,NA,NA
"15216","AC005041.5",0.06390253691038,0.119912045671111,3.352475197537
38,0.0357682126206912,0.971467179494267,NA
"15217","AC005041.3",0,NA,NA,NA,NA,NA
"15218","TLX2",0,NA,NA,NA,NA,NA
"15219","M1AP",0,NA,NA,NA,NA,NA
"15220","TACR1",0.12780507382076,-0.532254130061651,3.33875455416813,-
0.15941696864095,0.873340370825791,NA

"15221","AC007681.1",0,NA,NA,NA,NA,NA
"15222","AC005034.3",0,NA,NA,NA,NA,NA
"15223","AC005034.5",0.225789575149882,0.696980732170056,3.08390169185
347,0.226006144752027,0.821196641151035,NA
"15224","AC005034.6",0.0604037079966622,0.119912045671111,3.3524751975
3738,0.0357682126206911,0.971467179494267,NA
"15225","AC005034.4",0.12780507382076,-0.532254130061651,3.33875455416
813,-0.15941696864095,0.873340370825791,NA
"15226","AC079117.2",0.108990023245465,1.41832010653068,3.352475197537
38,0.42306654724024,0.672246708730081,NA
"15227","AC013716.1",0.33077173430644,2.01280089522968,3.3142340299141
7,0.60732008574597,0.543638511160742,NA
"15228","CTNNA2",0.120322481366256,1.41832010653068,3.35247519753738,0
.42306654724024,0.672246708730081,NA
"15229","DNAH6",0.576430282162431,-2.45386830255078,1.96871845486565,-
1.24642926797688,0.212606841763635,NA
"15230","AC022210.2",0.457447106067493,0.571522678248025,2.00384067085
849,0.285213633279123,0.775480476116909,NA
"15231","TCF7L1-
IT1",0.16538586715322,1.41832010653068,3.35247519753738,0.423066547240
24,0.672246708730081,NA
"15232","AC062037.3",0,NA,NA,NA,NA,NA
"15233","SH2D6",0.19170761073114,-0.935110177380914,3.32149227750043,-
0.281533148132028,0.778301507243725,NA
"15234","AC105053.1",0,NA,NA,NA,NA,NA
"15235","ST3GAL5-
AS1",0.609553893569291,2.00490002131528,2.11333637144909,0.94868949799
0582,0.342778556915,NA
"15236","AC012511.1",1.12584787111499,1.78621478449053,1.5255074765685
5,1.17089874151808,0.241639481051502,NA
"15237","AC009309.1",0.701975519454463,-0.72963429965789,1.78711126403
606,-0.40827581043279,0.683071196310195,NA
"15238","REEP1",0.06390253691038,0.119912045671111,3.35247519753738,0.
0357682126206912,0.971467179494267,NA
"15239","AC015971.1",0.16538586715322,1.41832010653068,3.3524751975373
8,0.42306654724024,0.672246708730081,NA
"15240","CD8A",1.07634039470939,-0.105224075494755,1.32744779627197,-0
.0792679574972884,0.936819492293507,NA
"15241","CD8B",1.11846783716755,-0.398830420355849,1.44588519583043,-0
.275838234948374,0.782672315274628,NA
"15242","RGPD1",0,NA,NA,NA,NA,NA
"15243","PLGLB1",1.16512299280204,2.47462643888807,1.50971919656989,1.
63913027304049,0.101186131489003,NA
"15244","AC068279.2",0.167194048217763,-0.631571198727006,3.3343222198
9104,-0.189415166584483,0.849767438415713,NA
"15245","AC133644.2",0.225789575149882,0.696980732170056,3.08390169185
347,0.226006144752027,0.821196641151035,NA
"15246","RGPD2",1.20964351521475,3.18008288309009,1.86750424731454,1.7
0285175397219,0.0885958147349904,NA
"15247","AC108479.3",0,NA,NA,NA,NA,NA

"15248","AC062029.1",0.06390253691038,0.119912045671111,3.352475197537
38,0.0357682126206912,0.971467179494267,NA
"15249","AC233263.6",1.44411645186105,0.734690672080129,1.286549996641
,0.571054894095295,0.567962432816534,NA
"15250","TEKT4",0.998465759647031,-1.74060399260266,1.51784079453919,-
1.14676321710743,0.251479495181915,NA
"15251","MAL",0.413166045229531,-1.95881013422226,3.18080764425016,-0.
615821625606043,0.53801225290182,NA
"15252","AC092835.1",1.40336003743257,0.956282905357762,1.128910696566
57,0.84708463500804,0.396947948703568,NA
"15253","PROM2",0,NA,NA,NA,NA,NA
"15254","AC009237.15",0.16538586715322,1.41832010653068,3.352475197537
38,0.42306654724024,0.672246708730081,NA
"15255","ADRA2B",0.214166147237109,0.696980724704133,3.13752578908925,
0.222143424964947,0.824202234028988,NA
"15256","ASTL",0,NA,NA,NA,NA,NA
"15257","AC021188.1",0,NA,NA,NA,NA,NA
"15258","ITPRIPL1",0.476885330560137,1.74411557875733,1.96969134549772
,0.885476591418237,0.37589963940566,NA
"15259","NCAPH",0.530546983829673,1.09523903454535,1.94644716175597,0.
562686239865507,0.573648555751943,NA
"15260","NEURL3",0.0604037079966622,0.119912045671111,3.35247519753738
,0.0357682126206911,0.971467179494267,NA
"15261","AC013270.1",0,NA,NA,NA,NA,NA
"15262","FER1L5",0,NA,NA,NA,NA,NA
"15263","CNNM3-
DT",0.338815272550412,2.01083448867207,2.72981071828631,0.736620482585
846,0.461353175430781,NA
"15264","FAM178B",1.14047923271266,-1.37018213347113,1.37861823811517,
-0.99388075363375,0.320280925396193,NA
"15265","LINC02611",0.103291511307383,0.119912045671111,3.352475197537
38,0.0357682126206912,0.971467179494267,NA
"15266","KIAA1211L",0,NA,NA,NA,NA,NA
"15267","C2orf15",0.334488628603365,1.28762870382305,2.38272719387433,
0.540401228950328,0.588920360482733,NA
"15268","AC079447.1",0.338815272550412,2.01083448867207,2.729810718286
31,0.736620482585846,0.461353175430781,NA
"15269","LYG2",0.615367601095249,-1.51834169736763,1.79716745385115,-0
.844852656392134,0.398193110623172,NA
"15270","LYG1",0.667193263234084,-0.985339859100817,1.58705032515763,-
0.620862390739218,0.534690168371109,NA
"15271","AC018690.1",0.331142329015541,-0.0194489660721624,2.373370515
85543,-0.0081946606912964,0.993461679930862,NA
"15272","AC092667.1",0.169393731242127,0.696980693484986,3.35247519753
738,0.207900328091007,0.835306792989891,NA
"15273","AC092168.2",0.346112056516138,1.28750669156114,2.364111450606
41,0.54460490482836,0.586025280638922,NA
"15274","AC016738.2",0,NA,NA,NA,NA,NA
"15275","AC016738.1",0,NA,NA,NA,NA,NA
"15276","TBC1D8-

AS1",1.08014477029135,-0.932388457809409,1.36689577899186,-0.682121103993078,0.495162377062876,NA
"15277","LINC01127",0.496860601469936,0.24027005616695,2.20415162975388,0.109007952503603,0.913196182740516,NA
"15278","IL1R2",1.03048163309062,-0.888384902000703,1.33784433976736,-0.664042053020297,0.506663396243677,NA
"15279","AC007271.1",0,NA,NA,NA,NA,NA
"15280","IL1RL2",0.692627986904261,0.327413345834552,1.63171404953308,0.200656080597114,0.840967503773028,NA
"15281","IL1RL1",1.36176489829168,-2.26819992737637,1.68797125946825,-1.34374321520788,0.179031437626585,NA
"15282","IL18RAP",0.289692112060262,0.0584007698147231,2.45889957201254,0.0237507747284382,0.981051405035345,NA
"15283","AC007278.2",0,NA,NA,NA,NA,NA
"15284","AC007278.1",0,NA,NA,NA,NA,NA
"15285","PANTR1",1.41929114183711,-1.59144830963915,1.21252146400078,-1.31251145393177,0.189347623962649,NA
"15286","AC010884.1",0,NA,NA,NA,NA,NA
"15287","LINC01918",0.0973092156216871,0.119912045671111,3.35247519753738,0.0357682126206911,0.971467179494267,NA
"15288","AC012360.2",0,NA,NA,NA,NA,NA
"15289","AC108058.1",0,NA,NA,NA,NA,NA
"15290","AC010978.1",0.06390253691038,0.119912045671111,3.35247519753738,0.0357682126206912,0.971467179494267,NA
"15291","RGPD3",0.210667318323391,0.69698072231542,3.15449020715825,0.220948767168088,0.825132324921599,NA
"15292","LINC01885",0.268677378460603,0.696980754736642,2.91582564400198,0.239033755728972,0.811079408015517,NA
"15293","SULT1C2",0.356846632941494,-0.247554755442067,2.76470448062684,-0.0895411271536475,0.928651870088189,NA
"15294","GCC2-AS1",0.78797637750348,-0.377064360647933,1.75001921032275,-0.215462983733986,0.829406368986108,NA
"15295","SH3RF3-AS1",0.940553791491029,-0.124850518529076,1.6732623999372,-0.0746150266292736,0.940521017931536,NA
"15296","AC011753.2",0.217631696987943,0.696980727003399,3.12110949465653,0.223311847340396,0.823292807226044,NA
"15297","LINC01123",0.811251664075034,-0.0768081806651913,1.58764530421235,-0.0483786778201675,0.961414452038459,NA
"15298","LINC01106",1.12366527119128,1.5664639327367,1.5424241368734,1.0155857233355,0.309826706218764,NA
"15299","RGPD6",0.16538586715322,1.41832010653068,3.35247519753738,0.42306654724024,0.672246708730081,NA
"15300","BUB1",0.172892560155845,0.696980693484986,3.35247519753738,0.207900328091007,0.835306792989891,NA
"15301","ACOXL",0.06390253691038,0.119912045671111,3.35247519753738,0.0357682126206912,0.971467179494267,NA
"15302","ACOXL-AS1",0.36893497118034,-0.76015972323402,2.24421488580268,-0.3387196689

7775,0.734820921974328,NA
"15303","AC017002.3",1.01874067196248,0.936444413005327,1.41154484934464,0.663418107784604,0.507062811824051,NA
"15304","AC017002.1",0,NA,NA,NA,NA,NA
"15305","AC093675.1",0.481423823339523,-0.232479392320112,1.93644357237276,-0.120054824027353,0.904439718676981,NA
"15306","AC092645.2",1.4884484687199,0.921566261424999,1.32569164410998,0.695158836913167,0.486955765606212,NA
"15307","RGPD8",1.35000902654358,1.12325735358885,1.20884959145907,0.929195295696866,0.352787884967521,NA
"15308","AC012442.1",0.16538586715322,1.41832010653068,3.35247519753738,0.42306654724024,0.672246708730081,NA
"15309","CKAP2L",0.253555121634112,0.696980747544927,2.97042254430159,0.234640269911095,0.814487951095881,NA
"15310","IL1A",0.727923907435195,-0.941146208850516,1.62984068131364,-0.577446752704662,0.563637699011202,NA
"15311","AC079753.2",0.0604037079966622,0.119912045671111,3.35247519753738,0.0357682126206911,0.971467179494267,NA
"15312","AC016745.2",1.05283963742875,-1.66238816031389,1.38018379756785,-1.20446868253586,0.228408479495049,NA
"15313","AC104653.1",0.0604037079966622,0.119912045671111,3.35247519753738,0.0357682126206911,0.971467179494267,NA
"15314","AC110769.2",1.16762248822927,-0.108990681762637,1.27707851181389,-0.0853437598036414,0.931988103120131,NA
"15315","LINC01191",0.184709952903704,-0.898539523146346,3.26457968362944,-0.275238961895205,0.783132655214614,NA
"15316","AC009303.2",0,NA,NA,NA,NA,NA
"15317","LINC01956",0.233833113393854,0.696980736952966,3.04905264581788,0.228589276052335,0.819188160373076,NA
"15318","SCTR",0,NA,NA,NA,NA,NA
"15319","AC013275.1",0.274375890398685,1.98403204198911,2.89460999865626,0.685422921536976,0.493077083984588,NA
"15320","CFAP221",0,NA,NA,NA,NA,NA
"15321","AC073257.2",0.108990023245465,1.41832010653068,3.35247519753738,0.42306654724024,0.672246708730081,NA
"15322","AC073257.1",0,NA,NA,NA,NA,NA
"15323","TFCP2L1",0.342286291397972,0.6094932892186,2.40258431168592,0.253682372874112,0.799740953086473,NA
"15324","AC012447.1",0,NA,NA,NA,NA,NA
"15325","AC011246.1",0.739390062254677,-1.04307591651286,1.59875236601763,-0.652431194901735,0.51412304584221,NA
"15326","AC012508.2",0,NA,NA,NA,NA,NA
"15327","AC012508.1",0.12780507382076,-0.532254130061651,3.33875455416813,-0.15941696864095,0.873340370825791,NA
"15328","AC110926.2",0,NA,NA,NA,NA,NA
"15329","AC068282.1",0.06390253691038,0.119912045671111,3.35247519753738,0.0357682126206912,0.971467179494267,NA
"15330","AC068282.2",0.06390253691038,0.119912045671111,3.35247519753738,0.0357682126206912,0.971467179494267,NA
"15331","PROC",0.0973092156216871,0.119912045671111,3.35247519753738,0

.0357682126206911,0.971467179494267,NA
"15332","AC010976.1",0,NA,NA,NA,NA,NA
"15333","MYO7B",0.247572825948416,0.696980744488543,2.99332389411541,0
.23284508096793,0.815881705308138,NA
"15334","AC010976.2",0.0604037079966622,0.119912045671111,3.3524751975
3738,0.0357682126206911,0.971467179494267,NA
"15335","AC012306.3",1.26037144815094,0.623459448380632,1.182223693974
29,0.52736165884541,0.597942463636975,NA
"15336","LINC02572",0,NA,NA,NA,NA,NA
"15337","RAB6C",0.338815272550412,2.01083448867207,2.72981071828631,0.
736620482585846,0.461353175430781,NA
"15338","AC073869.3",0,NA,NA,NA,NA,NA
"15339","AC093787.2",0.120322481366256,1.41832010653068,3.352475197537
38,0.42306654724024,0.672246708730081,NA
"15340","AC097532.2",0.0604037079966622,0.119912045671111,3.3524751975
3738,0.0357682126206911,0.971467179494267,NA
"15341","AC097532.3",0.167194048217763,-0.631571198727006,3.3343222198
9104,-0.189415166584483,0.849767438415713,NA
"15342","GPR39",0.173429405397192,1.41832010653068,3.35247519753738,0.
42306654724024,0.672246708730081,NA
"15343","LYPD1",0.237331942307572,0.696980738944435,3.03442443415691,0
.229691249219751,0.818331695238684,NA
"15344","AC092623.1",0.377667401706068,1.26423683394566,2.309206732520
38,0.547476679390162,0.584051287854355,NA
"15345","TMEM163",0.503664294462285,1.85193819171971,2.18213676097746,
0.848681083989511,0.396058774651061,NA
"15346","AC110620.2",0,NA,NA,NA,NA,NA
"15347","CCNT2-
AS1",1.11882432414813,0.56530051408845,1.21586238509894,0.464937908283
47,0.641975922519617,NA
"15348","MAP3K19",0.358901658948903,-1.79961066720244,2.68572597578764
,-0.670064885035291,0.502816428548756,NA
"15349","AC011893.1",0,NA,NA,NA,NA,NA
"15350","DARS-
AS1",0.315425847236699,-1.59539646267932,2.7560097002768,-0.5788791173
40947,0.562670741698805,NA
"15351","AC068492.1",0,NA,NA,NA,NA,NA
"15352","THSD7B",0,NA,NA,NA,NA,NA
"15353","AC114763.1",0.27268524254951,-0.0202790885325299,2.5035439907
9405,-0.0081001526641987,0.993537083922046,NA
"15354","LINC02631",0.434063245613823,1.28881134843813,2.5871731669192
5,0.49815426540343,0.618375314902635,NA
"15355","KYNU",1.12839419950447,-0.740597301546524,1.41060008465871,-0
.5250228676441,0.599567294212052,NA
"15356","AC079793.1",0.06390253691038,0.119912045671111,3.352475197537
38,0.0357682126206912,0.971467179494267,NA
"15357","AC092652.3",0,NA,NA,NA,NA,NA
"15358","AC092652.1",0.06390253691038,0.119912045671111,3.352475197537
38,0.0357682126206912,0.971467179494267,NA
"15359","AC092652.2",0.16538586715322,1.41832010653068,3.3524751975373

8,0.42306654724024,0.672246708730081,NA
"15360","AC016910.1",0.439879290767273,-1.05989946046786,1.93703501608
1,-0.547176200568769,0.584257685123445,NA
"15361","AC009951.5",0,NA,NA,NA,NA,NA
"15362","LINC01412",0.968079392949049,-1.55023922182317,1.371293181125
16,-1.13049437068679,0.258267969141883,NA
"15363","AC018678.1",0,NA,NA,NA,NA,NA
"15364","AC105402.3",0,NA,NA,NA,NA,NA
"15365","LYPD6B",0,NA,NA,NA,NA,NA
"15366","LYPD6",0,NA,NA,NA,NA,NA
"15367","LINC02612",0,NA,NA,NA,NA,NA
"15368","AC079790.2",0.06390253691038,0.119912045671111,3.352475197537
38,0.0357682126206912,0.971467179494267,NA
"15369","AC012066.2",0.276720916704575,0.696980758280457,2.88854265454
755,0.241291489043159,0.809329206809347,NA
"15370","AC012443.2",0,NA,NA,NA,NA,NA
"15371","RPRM",0,NA,NA,NA,NA,NA
"15372","AC073225.1",0.680867554363365,0.907455922825451,2.08447623245
533,0.435340019087935,0.663315640853762,NA
"15373","LINC01876",0.0973092156216871,0.119912045671111,3.35247519753
738,0.0357682126206911,0.971467179494267,NA
"15374","AC074099.1",0.217631696987943,0.696980727003399,3.12110949465
653,0.223311847340396,0.823292807226044,NA
"15375","ERMN",0.225789575149882,0.696980732170056,3.08390169185347,0.
226006144752027,0.821196641151035,NA
"15376","ACVR1C",0.951235580673142,2.09281902012134,2.04733674128668,1
.02221533855055,0.306678992654,NA
"15377","AC019186.1",0,NA,NA,NA,NA,NA
"15378","UPP2",0,NA,NA,NA,NA,NA
"15379","AC013731.1",0.0973092156216871,0.119912045671111,3.3524751975
3738,0.0357682126206911,0.971467179494267,NA
"15380","AC091488.1",0.747708626088819,-1.9540412486009,1.680770233562
1,-1.16258677693241,0.244997198282165,NA
"15381","CCDC148",0.445577802705355,-0.000823893182234822,1.9906878052
7208,-0.000413873627021198,0.999669776632304,NA
"15382","PKP4-
AS1",0.163695219304045,-0.600668372859899,3.33567379881164,-0.18007407
471135,0.857094415273326,NA
"15383","DAPL1",1.24703781443246,1.37075553833359,1.19962042904113,1.1
4265771501512,0.253180732614121,NA
"15384","AC008277.1",0.282419428642657,1.98408346788866,2.866956379498
31,0.692052199355701,0.488904548657237,NA
"15385","AC009961.1",1.00439431955504,0.0852452251204014,1.29103191788
099,0.0660287510631939,0.94735493535746,NA
"15386","AC009961.4",1.43500762361596,-0.896027427390292,1.21390887250
156,-0.73813401292949,0.460433016709801,NA
"15387","LINC01806",0,NA,NA,NA,NA,NA
"15388","TBR1",0,NA,NA,NA,NA,NA
"15389","SLC4A10",0.236795097066225,0.0434742300939003,3.0811765183817
2,0.01410962008652‌9,0.988742525500995,NA

"15390","AC007750.1",0,NA,NA,NA,NA,NA
"15391","GRB14",0.16538586715322,1.41832010653068,3.35247519753738,0.42306654724024,0.672246708730081,NA
"15392","AC019197.1",0,NA,NA,NA,NA,NA
"15393","SLC38A11",0,NA,NA,NA,NA,NA
"15394","SCN3A",0,NA,NA,NA,NA,NA
"15395","SCN2A",0,NA,NA,NA,NA,NA
"15396","GALNT3",0.403989145283987,-0.940043913464617,2.2528578608457,-0.417267298484483,0.676482904981981,NA
"15397","AC009495.3",0.150263610326729,1.41832010653068,3.35247519753738,0.42306654724024,0.672246708730081,NA
"15398","TTC21B-AS1",0.06390253691038,0.119912045671111,3.35247519753738,0.0357682126206912,0.971467179494267,NA
"15399","SCN9A",1.28581926312808,-1.34492054239534,1.36794387128571,-0.983169390664591,0.325524076990588,NA
"15400","B3GALT1",0.06390253691038,0.119912045671111,3.35247519753738,0.0357682126206912,0.971467179494267,NA
"15401","CERS6-AS1",0.16538586715322,1.41832010653068,3.35247519753738,0.42306654724024,0.672246708730081,NA
"15402","SPC25",0.0973092156216871,0.119912045671111,3.35247519753738,0.0357682126206911,0.971467179494267,NA
"15403","KLHL41",1.08508885389037,-1.68426777904063,1.42720897795328,-1.18011293724903,0.237955299796118,NA
"15404","CCDC173",0.768636128269672,-1.13205322046998,1.74202409716151,-0.649849346122462,0.515789540452901,NA
"15405","MYO3B",0.349610885429856,1.28746559520527,2.35793151790902,0.546014837762114,0.585055737879021,NA
"15406","AC007405.1",0.466244186802027,-1.20150006501784,1.85075129910945,-0.649195851217818,0.516211792992446,NA
"15407","LINC01124",0.525641510095905,0.0392265931951817,2.14896205039185,0.0182537393752621,0.985436431941105,NA
"15408","AC007405.2",0,NA,NA,NA,NA,NA
"15409","AC007405.4",0,NA,NA,NA,NA,NA
"15410","AC010092.1",0,NA,NA,NA,NA,NA
"15411","DLX1",0,NA,NA,NA,NA,NA
"15412","DLX2",0,NA,NA,NA,NA,NA
"15413","ITGA6",0.942466214241448,0.706422521570863,1.44339534940352,0.489417207740618,0.624546355922547,NA
"15414","ITGA6-AS1",0.556193908044622,-0.134033944818542,1.91816082635857,-0.0698762809544975,0.944292132029299,NA
"15415","AC078883.1",0.32840580074983,-1.64140837171611,2.31460259717979,-0.709153430362549,0.478229269166669,NA
"15416","RAPGEF4",1.34196548829961,1.12745069060179,1.27108832026442,0.886996342132421,0.375080869838285,NA
"15417","MAP3K20-AS1",0.2292884040636,0.696980734286753,3.06852794721869,0.227138467133238,0.820316073199257,NA

"15418","CDCA7",1.15525539111988,1.76047613014544,1.49624091150022,1.17659938089801,0.239355445014053,NA
"15419","AC016737.1",0,NA,NA,NA,NA,NA
"15420","AC016737.2",0.0604037079966622,0.119912045671111,3.35247519753738,0.0357682126206911,0.971467179494267,NA
"15421","LINC01305",0.953490367609186,1.79949184691374,1.61708400850501,1.11280047137277,0.26579413292355,NA
"15422","SP9",0.150263610326729,1.41832010653068,3.35247519753738,0.42306654724024,0.672246708730081,NA
"15423","AC010894.2",0.727981287146199,-0.163308794209652,1.50540767410042,-0.108481441286155,0.913613801337436,NA
"15424","AC010894.3",0,NA,NA,NA,NA,NA
"15425","CHRNA1",0,NA,NA,NA,NA,NA
"15426","EVX2",0,NA,NA,NA,NA,NA
"15427","HOXD13",0.16538586715322,1.41832010653068,3.35247519753738,0.42306654724024,0.672246708730081,NA
"15428","HOXD11",0,NA,NA,NA,NA,NA
"15429","HOXD10",0.225789575149882,0.696980732170056,3.08390169185347,0.226006144752027,0.821196641151035,NA
"15430","HAGLR",0.390872606646774,1.60848886152635,2.69475792663702,0.596895493144979,0.55057715646071,NA
"15431","HAGLROS",0.06390253691038,0.119912045671111,3.35247519753738,0.0357682126206912,0.971467179494267,NA
"15432","HOXD1",0.379552014390329,1.28718260850839,2.31655820713756,0.555644405801004,0.578453968578949,NA
"15433","AC017048.3",0.927182336018076,-1.62429407937416,1.66764273053235,-0.974006032368599,0.330053544034643,NA
"15434","AC092162.2",0,NA,NA,NA,NA,NA
"15435","AC074286.1",0,NA,NA,NA,NA,NA
"15436","AC019080.4",0,NA,NA,NA,NA,NA
"15437","AC019080.5",0,NA,NA,NA,NA,NA
"15438","AC019080.6",0.206299238867152,0.696980719233408,3.17624445737489,0.219435477522864,0.826310836331857,NA
"15439","PDE11A",0.740786327732958,1.66466813855645,1.85778400726625,0.896050419233626,0.370225835657559,NA
"15440","AC009948.2",0.947456161309212,0.231515769261418,1.32400072663195,0.174860756950155,0.861189046538833,NA
"15441","AC010680.2",1.02232056163128,0.965152299375942,1.50666150238935,0.640590005017949,0.521789095084849,NA
"15442","AC010680.4",0.454963639295515,0.549611487626288,2.00740622426376,0.273791861847925,0.784244578046276,NA
"15443","AC010680.5",0.0604037079966622,0.119912045671111,3.35247519753738,0.0357682126206911,0.971467179494267,NA
"15444","AC010680.1",0.173429405397192,1.41832010653068,3.35247519753738,0.42306654724024,0.672246708730081,NA
"15445","CCDC141",0.330989799689647,1.28767123567888,2.3893126005633,0.538929579735739,0.589935446033768,NA
"15446","SCHLAP1",0.821913684905266,-1.24664453366001,1.7350049674586,-0.718525051536923,0.472433607086591,NA
"15447","AC104076.1",0.120322481366256,1.41832010653068,3.352475197537

38,0.42306654724024,0.672246708730081,NA
"15448","AC068196.1",0.0604037079966622,0.119912045671111,3.3524751975
3738,0.0357682126206911,0.971467179494267,NA
"15449","LINC01934",0,NA,NA,NA,NA,NA
"15450","CERKL",0,NA,NA,NA,NA,NA
"15451","AC009962.1",0.338472392144151,-0.374118255054918,2.3355113512
9715,-0.160186870788332,0.872733871436184,NA
"15452","AC012500.1",0,NA,NA,NA,NA,NA
"15453","AC021851.1",0.660070886146912,0.599681955654783,1.70079745051
413,0.352588696245697,0.724396813112487,NA
"15454","AC021851.2",0.233833113393854,0.696980736952966,3.04905264581
788,0.228589276052335,0.819188160373076,NA
"15455","AC096667.1",0.20060072692907,-0.855152612088657,3.23855166872
834,-0.264054027714322,0.791738287519029,NA
"15456","ZNF804A",0,NA,NA,NA,NA,NA
"15457","AC009315.1",1.16075436430962,2.32594021870578,1.5208556442716
8,1.52936291321695,0.1261745010733,NA
"15458","FSIP2-
AS1",0.266702946863814,-0.0203591384131145,2.5171557632184,-0.00808815
19970316,0.993546658757216,NA
"15459","FSIP2-
AS2",0.428080949928127,1.28886520766923,2.59768840815572,0.49615850908
9351,0.619782581682311,NA
"15460","AC097500.1",0.686198664889746,0.131528140295228,1.53642943469
207,0.0856063658540812,0.931779337820343,NA
"15461","AC017071.1",1.31248477296077,0.496193779051895,1.142126039171
12,0.434447479554883,0.66396352580827,NA
"15462","AC007319.1",0.906752698632178,0.659478381956812,1.44521733889
515,0.456317789863338,0.648161473114766,NA
"15463","DIRC1",0.814714876198376,-1.12469614218645,1.56322464822555,-
0.719471858035962,0.471850235716408,NA
"15464","AC133106.1",0.902744834543271,1.15218838191045,1.741974553437
55,0.661426643481536,0.50833874402771,NA
"15465","OSGEPL1-AS1",0,NA,NA,NA,NA,NA
"15466","AC013468.1",0,NA,NA,NA,NA,NA
"15467","MSTN",0.49615760145966,2.73730884111826,2.98685308631898,0.91
6452454141876,0.359429634140874,NA
"15468","AC093388.1",0.552504728050537,-2.39822977844623,1.69089911493
139,-1.41831630123217,0.156098439545882,NA
"15469","AC006460.2",1.16467411113122,0.178649252334326,1.307994124544
99,0.136582610718127,0.891360722312017,NA
"15470","AC006460.1",0,NA,NA,NA,NA,NA
"15471","AC005540.1",0.06390253691038,0.119912045671111,3.352475197537
38,0.0357682126206912,0.971467179494267,NA
"15472","AC067945.3",0,NA,NA,NA,NA,NA
"15473","STAT4",1.31916969807559,0.662511835113836,1.23907502699797,0.
534682582312202,0.592869367676542,NA
"15474","AC067945.4",0,NA,NA,NA,NA,NA
"15475","AC092614.1",0.233296268152507,0.0607094481758273,2.6110486817
2048,0.0232509828716883,0.981450071132202,NA

"15476","AC098617.1",0.16538586715322,1.41832010653068,3.3524751975373
8,0.42306654724024,0.672246708730081,NA
"15477","AC096647.1",0.16538586715322,1.41832010653068,3.3524751975373
8,0.42306654724024,0.672246708730081,NA
"15478","LINC01821",0,NA,NA,NA,NA,NA
"15479","LINC01825",0.06390253691038,0.119912045671111,3.3524751975373
8,0.0357682126206912,0.971467179494267,NA
"15480","LINC01827",0,NA,NA,NA,NA,NA
"15481","DNAH7",1.29005404652096,0.31463040791645,1.21919295139583,0.2
58064490576521,0.796357135977981,NA
"15482","HECW2-AS1",0,NA,NA,NA,NA,NA
"15483","C2orf66",0.646639277169596,2.27040972287053,1.85342733760566,
1.22497908431822,0.220583101138149,NA
"15484","AC013264.1",0,NA,NA,NA,NA,NA
"15485","ANKRD44-
IT1",0.0604037079966622,0.119912045671111,3.35247519753738,0.035768212
6206911,0.971467179494267,NA
"15486","AC011997.1",0.184709952903704,-0.898539523146346,3.2645796836
2944,-0.275238961895205,0.783132655214614,NA
"15487","AC011997.2",0.150263610326729,1.41832010653068,3.352475197537
38,0.42306654724024,0.672246708730081,NA
"15488","FTCDNL1",1.12323059301229,0.00731387931692279,1.3625904607370
2,0.00536762844572297,0.995717272700295,NA
"15489","AC097717.1",0.2292884040636,0.696980734286753,3.0685279472186
9,0.227138467133238,0.820316073199257,NA
"15490","LINC01792",0,NA,NA,NA,NA,NA
"15491","AC005037.1",0.06390253691038,0.119912045671111,3.352475197537
38,0.0357682126206912,0.971467179494267,NA
"15492","AC007272.1",0,NA,NA,NA,NA,NA
"15493","CFLAR-
AS1",0.991627564139525,0.909585283763417,2.00680938659724,0.4532494664
60647,0.650369116381779,NA
"15494","FLACC1",0,NA,NA,NA,NA,NA
"15495","AC007279.2",0.167194048217763,-0.631571198727006,3.3343222198
9104,-0.189415166584483,0.849767438415713,NA
"15496","MPP4",0.449888502450589,-0.897435466406063,2.18256630102967,-
0.411183598859142,0.680937913667748,NA
"15497","KIAA2012",0.828517299166304,-2.10950337642237,1.9172835823466
4,-1.10025631880729,0.271220458416162,NA
"15498","KIAA2012-
AS1",0.188208781817422,-0.917509588944765,3.24540722344342,-0.28271015
800947,0.777399028822487,NA
"15499","AC079354.3",0.16538586715322,1.41832010653068,3.3524751975373
8,0.42306654724024,0.672246708730081,NA
"15500","AC079354.2",0.212281534552848,0.696980723426082,3.14661376999
226,0.221501834789148,0.82470170812449,NA
"15501","AC064836.2",0,NA,NA,NA,NA,NA
"15502","CD28",0.806306694627514,-2.07843448327863,1.50092080839923,-1
.38477291516487,0.166121915281501,NA
"15503","CTLA4",0.402689999394634,0.192098974823665,2.27974953657425,0

.0842631928384244,0.932847176148917,NA
"15504","ICOS",0.278520339611674,-1.43972823613246,2.87864192827664,-0
.500141480602412,0.616975460146789,NA
"15505","AC009498.1",0.565551934568624,-0.449433049626299,1.7139236189
9745,-0.262224666633156,0.793148237359286,NA
"15506","AC007679.1",0,NA,NA,NA,NA,NA
"15507","AC007383.3",0.642423727039251,1.01008116911186,1.913223331110
91,0.527947340327151,0.597535885919806,NA
"15508","GPR1-
AS",0.150263610326729,1.41832010653068,3.35247519753738,0.423066547240
24,0.672246708730081,NA
"15509","MDH1B",0.820955702474531,-0.508992903727278,1.45057781455213,
-0.350889761735692,0.725671052695372,NA
"15510","CPO",0.384096723720583,1.28715287945805,2.31232740411653,0.55
6648196603381,0.57776781726439,NA
"15511","KLF7-
IT1",1.11487383977022,1.57569958119974,1.34107834169824,1.174949689519
55,0.240014844346283,NA
"15512","MYOSLID",0.974276560720552,0.199613492440312,1.47774810940188
,0.135079511298516,0.89254900930983,NA
"15513","AC007879.3",0.124306244907042,-0.509577950443694,3.3398031219
3904,-0.152577242381833,0.878731672788745,NA
"15514","AC007879.2",0.16538586715322,1.41832010653068,3.3524751975373
8,0.42306654724024,0.672246708730081,NA
"15515","AC009226.1",0,NA,NA,NA,NA,NA
"15516","LINC01857",0.150263610326729,1.41832010653068,3.3524751975373
8,0.42306654724024,0.672246708730081,NA
"15517","AC096772.1",0,NA,NA,NA,NA,NA
"15518","AC083900.1",0.214166147237109,0.696980724704133,3.13752578908
925,0.222143424964947,0.824202234028988,NA
"15519","IDH1-
AS1",1.0411597127547,0.612494644343322,1.47682728849991,0.414736813920
514,0.678334579515613,NA
"15520","PTH2R",1.07129286675482,1.32238751261263,1.54319929238439,0.8
56912985340614,0.391492975765806,NA
"15521","AC019185.2",0,NA,NA,NA,NA,NA
"15522","AC007038.1",1.05798342012823,-0.815149200377341,1.39687692950
945,-0.583551194208356,0.559522304461438,NA
"15523","AC006994.2",1.42146782944337,0.962939512315567,1.226950825809
88,0.784823231754174,0.432557217062658,NA
"15524","MYL1",0,NA,NA,NA,NA,NA
"15525","LINC01878",0,NA,NA,NA,NA,NA
"15526","AC108066.2",0.374030132326262,-0.206321295990841,2.3389876189
2708,-0.088209657170175,0.929710041927262,NA
"15527","AC079610.1",0.803382418077584,-1.13933012172351,1.56623267188
784,-0.727433504723296,0.466960435993598,NA
"15528","SNHG31",0.385310679832774,-1.88656928783623,1.90991483766999,
-0.987776654030168,0.323262043612321,NA
"15529","AC093382.1",0,NA,NA,NA,NA,NA
"15530","TMEM169",0.276720916704575,0.696980758280457,2.88854265454755

,0.241291489043159,0.809329206809347,NA
"15531","AC098820.1",0.28188258340131,1.24658559047362,2.9277466218199
4,0.425783290528988,0.670265763772875,NA
"15532","AC098820.3",0,NA,NA,NA,NA,NA
"15533","AC007563.4",0.282419428642657,1.98408346788866,2.866956379498
31,0.692052199355701,0.488904548657237,NA
"15534","LINC01921",0,NA,NA,NA,NA,NA
"15535","AC007749.1",0,NA,NA,NA,NA,NA
"15536","AC007749.2",0,NA,NA,NA,NA,NA
"15537","DIRC3-AS1",0,NA,NA,NA,NA,NA
"15538","AC010136.1",0.311475362858796,-0.0461613448588672,2.416632617
54422,-0.019101515275324,0.984760122636486,NA
"15539","RUFY4",0.120322481366256,1.41832010653068,3.35247519753738,0.
42306654724024,0.672246708730081,NA
"15540","CXCR2",0.439761757551905,2.57992481053724,2.54830735454105,1.
01240723805935,0.311343379010575,NA
"15541","CXCR1",0,NA,NA,NA,NA,NA
"15542","AC021016.2",1.33873299145006,-0.268684875046406,1.27816181530
726,-0.210211940169575,0.833502260858002,NA
"15543","CATIP",0,NA,NA,NA,NA,NA
"15544","CATIP-
AS1",0.103291511307383,0.119912045671111,3.35247519753738,0.0357682126
206912,0.971467179494267,NA
"15545","AC021016.3",0.518058385473562,-1.17615074862677,1.88681145747
097,-0.623353618068043,0.533052170318652,NA
"15546","VIL1",0.508402968627625,0.195671594603177,1.93519860566839,0.
10111189313078,0.919461637079921,NA
"15547","AC009974.2",0,NA,NA,NA,NA,NA
"15548","AC009974.1",0.463121517457454,0.635608040746632,2.00882048040
095,0.316408582522898,0.751692403436473,NA
"15549","PRKAG3",0.103291511307383,0.119912045671111,3.35247519753738,
0.0357682126206912,0.971467179494267,NA
"15550","WNT10A",0,NA,NA,NA,NA,NA
"15551","AC097468.4",0,NA,NA,NA,NA,NA
"15552","AC097468.3",1.07907458534761,0.0920119249792258,1.35819800443
143,0.067745589876451,0.945988157174705,NA
"15553","SLC23A3",0.963963124383365,-0.557019651127166,1.4434023881557
4,-0.385907391935855,0.699565255923793,NA
"15554","PTPRN",0.120807415993324,-0.48654549181451,3.34088239674268,-
0.145633827844071,0.884210461918222,NA
"15555","AC053503.2",0.231096585128143,-1.15074146808831,3.05677932246
771,-0.376455526125231,0.706578272798499,NA
"15556","DES",0.172892560155845,0.696980693484986,3.35247519753738,0.2
07900328091007,0.835306792989891,NA
"15557","SPEG",0.0973092156216871,0.119912045671111,3.35247519753738,0
.0357682126206911,0.971467179494267,NA
"15558","SPEGNB",1.23079857627208,-0.687950248102695,1.18683705235061,
-0.579650126982608,0.562150582061887,NA
"15559","AC053503.5",0.523149065884972,-1.42608740353589,1.84213588377
732,-0.774148864964124,0.438842763459179,NA

"15560","ASIC4",0.0604037079966622,0.119912045671111,3.35247519753738,
0.0357682126206911,0.971467179494267,NA
"15561","AC009955.4",0.188208781817422,-0.917509588944765,3.2454072234
4342,-0.28271015800947,0.777399028822487,NA
"15562","INHA",0.0973092156216871,0.119912045671111,3.35247519753738,0
.0357682126206911,0.971467179494267,NA
"15563","AC093843.1",0,NA,NA,NA,NA,NA
"15564","AC093843.2",0.194618431243374,-0.859518418402434,3.3250826988
5954,-0.25849535071631,0.796024636623985,NA
"15565","AC079834.2",1.33456056276558,0.428890806997251,1.326968886779
13,0.323210899117817,0.746535527222455,NA
"15566","AC010980.1",0,NA,NA,NA,NA,NA
"15567","SGPP2",0.297735650304234,0.0580911751384808,2.4406441175237,0
.0238015754617354,0.981010883369774,NA
"15568","MOGAT1",0,NA,NA,NA,NA,NA
"15569","SCG2",1.21212753102291,3.21178056514132,1.64798368923792,1.94
891526300636,0.051305545017562,NA
"15570","AP1S3",0.644654132317495,-0.610139056166979,1.87092024234482,
-0.326117085249072,0.744335770898489,NA
"15571","FAM124B",0.124306244907042,-0.509577950443694,3.3398031219390
4,-0.152577242381833,0.878731672788745,NA
"15572","AC073052.2",0,NA,NA,NA,NA,NA
"15573","NYAP2",0,NA,NA,NA,NA,NA
"15574","AC010735.2",0.06390253691038,0.119912045671111,3.352475197537
38,0.0357682126206912,0.971467179494267,NA
"15575","AC010735.1",0,NA,NA,NA,NA,NA
"15576","COL4A3",1.24720756408739,-1.24472167482554,1.25001023320158,-
0.995769187934967,0.319362304391835,NA
"15577","AC064853.1",0,NA,NA,NA,NA,NA
"15578","SCYGR4",0,NA,NA,NA,NA,NA
"15579","DAW1",0.756604283893325,-2.83424066146994,1.58108754108213,-1
.7925893334977,0.0730386132218726,NA
"15580","LINC01807",1.24969288620138,0.382411824878073,1.3169197777377
2,0.290383538422516,0.77152283585667,NA
"15581","AC008273.1",0.377861366541154,-0.555867632328218,2.0208560581
1447,-0.275065425910078,0.783265973483172,NA
"15582","SLC16A14",0.855244046628886,-0.450616643446922,1.417400743882
98,-0.317917600503336,0.75054744144196,NA
"15583","SP140",0.470628210460079,0.164263600031717,1.95667258102286,0
.0839504788000081,0.933095804899174,NA
"15584","LINC01907",0,NA,NA,NA,NA,NA
"15585","GPR55",0,NA,NA,NA,NA,NA
"15586","AC017104.1",0.612790902831832,-0.318628135563967,1.8285958039
7061,-0.174247438866533,0.861671004919702,NA
"15587","B3GNT7",0.490292838989332,-1.03572415044845,1.72725257331099,
-0.599636767923904,0.54874833760427,NA
"15588","AC017104.3",0,NA,NA,NA,NA,NA
"15589","LINC00471",0.259253633572194,1.98393725043859,2.9478613727254
4,0.673009005374071,0.500941561797964,NA
"15590","AC017104.6",0.439955722386991,0.636248500103901,2.02388140664

003,0.314370445825765,0.753239699953286,NA
"15591","AC073476.3",0.06390253691038,0.119912045671111,3.35247519753738,0.0357682126206912,0.971467179494267,NA
"15592","CHRNG",0,NA,NA,NA,NA,NA
"15593","KCNJ13",0.225789575149882,0.696980732170056,3.08390169185347,0.22600614475202,0.821196641151035,NA
"15594","SNORC",0.0604037079966622,0.119912045671111,3.35247519753738,0.0357682126206911,0.971467179494267,NA
"15595","INPP5D",1.38137282897935,-0.300404847997284,1.16522695215681,-0.257808015375239,0.79655507817786,NA
"15596","SAG",0,NA,NA,NA,NA,NA
"15597","HJURP",0,NA,NA,NA,NA,NA
"15598","AC097713.3",0.16538586715322,1.41832010653068,3.35247519753738,0.42306654724024,0.672246708730081,NA
"15599","AC010148.1",0.44337811968099,-1.08110063041399,2.18587927650445,-0.494583869308112,0.62089389090218,NA
"15600","AGAP1-IT1",0.167194048217763,-0.631571198727006,3.33432221989104,-0.189415166584483,0.849767438415713,NA
"15601","AC084030.1",0.150263610326729,1.41832010653068,3.35247519753738,0.42306654724024,0.672246708730081,NA
"15602","AC105760.1",0.124306244907042,-0.509577950443694,3.33980312193904,-0.152577242381833,0.878731672788745,NA
"15603","AC107079.1",0.291927646865061,-1.45883157994448,3.28423784711834,-0.444191817966075,0.656903907272504,NA
"15604","AC012076.1",0.674915514587085,-2.72535067085968,1.96929037362986,-1.38392524909174,0.16638134459247,NA
"15605","RBM44",0.730018429156355,-0.870809150240382,1.58005704873296,-0.551125132436628,0.581547897957027,NA
"15606","RAMP1",0.974663099631465,0.0564071046304807,1.45006193738984,0.0388997898476085,0.968970284120907,NA
"15607","ERFE",0,NA,NA,NA,NA,NA
"15608","LINC02610",1.16600399457688,0.497342892284768,1.33426053108199,0.372747960910947,0.709336037074546,NA
"15609","AC012485.1",0.103291511307383,0.119912045671111,3.35247519753738,0.0357682126206912,0.971467179494267,NA
"15610","LINC01940",0,NA,NA,NA,NA,NA
"15611","AC017028.1",0,NA,NA,NA,NA,NA
"15612","AC017028.2",0,NA,NA,NA,NA,NA
"15613","AC093802.1",0.06390253691038,0.119912045671111,3.35247519753738,0.0357682126206912,0.971467179494267,NA
"15614","AC110619.1",0.06390253691038,0.119912045671111,3.35247519753738,0.0357682126206912,0.971467179494267,NA
"15615","GPR35",0.460894024366932,-1.21586362240499,1.85852041842661,-0.654210527013913,0.512976180336212,NA
"15616","CROCC2",0.543247233694374,0.0282688721095388,1.76375373022377,0.0160276753070012,0.987212312826428,NA
"15617","AC104809.2",0.270586091692985,2.01155232872955,2.90724650940082,0.691909792384316,0.488993980674386,NA
"15618","AC104809.1",0.108990023245465,1.41832010653068,3.352475197537

38,0.42306654724024,0.672246708730081,NA
"15619","AC005237.1",0.173429405397192,1.41832010653068,3.352475197537
38,0.42306654724024,0.672246708730081,NA
"15620","ANO7",0.994809904702975,-1.66352076320841,1.5622733335541,-1.
0648077564148,0.286962934419861,NA
"15621","AC104841.1",0,NA,NA,NA,NA,NA
"15622","BOK-
AS1",0.16538586715322,1.41832010653068,3.35247519753738,0.423066547240
24,0.672246708730081,NA
"15623","AC133528.1",0.124306244907042,-0.509577950443694,3.3398031219
3904,-0.152577242381833,0.878731672788745,NA
"15624","AC114730.2",1.07782989167911,0.981460058930904,1.410221565730
42,0.695961601198859,0.486452877469532,NA
"15625","GAL3ST2",0,NA,NA,NA,NA,NA
"15626","AC131097.1",0,NA,NA,NA,NA,NA
"15627","NEU4",0.383502498768232,-0.512182144388483,2.2700102808854,-0
.225629878728439,0.821489300165254,NA
"15628","AC131097.3",0,NA,NA,NA,NA,NA
"15629","PDCD1",0.252111318727802,-1.32095011480748,2.95582255360433,-
0.446897637071183,0.654948970153549,NA
"15630","RTP5",0.16538586715322,1.41832010653068,3.35247519753738,0.42
306654724024,0.672246708730081,NA
"15631","LINC01237",0.564604847700294,1.99299626277964,2.1491089275789
9,0.927359352150093,0.353739987808923,NA
"15632","FAM240C",0,NA,NA,NA,NA,NA
"15633","LINC01238",0.418940988787332,1.28676752537837,2.2593477821011
5,0.569530523619388,0.5689961624001,NA
"15634","LINC01238.1",0.184225018276636,0.696980701717498,3.2971540641
0566,0.21138857577362,0.832584070546688,NA
"15635","CHL1-
AS1",0.173429405397192,1.41832010653068,3.35247519753738,0.42306654724
024,0.672246708730081,NA
"15636","AC087430.1",0,NA,NA,NA,NA,NA
"15637","CNTN6",0,NA,NA,NA,NA,NA
"15638","AC087427.1",0.210667318323391,0.69698072231542,3.154490207158
25,0.220948767168088,0.825132324921599,NA
"15639","IL5RA",0,NA,NA,NA,NA,NA
"15640","LRRN1",0.06390253691038,0.119912045671111,3.35247519753738,0.
0357682126206912,0.971467179494267,NA
"15641","AC023480.1",0,NA,NA,NA,NA,NA
"15642","AC023483.1",0.06390253691038,0.119912045671111,3.352475197537
38,0.0357682126206912,0.971467179494267,NA
"15643","ITPR1-
DT",0.613409228332304,2.21207819714346,1.72635374251541,1.281358589879
92,0.200067742190347,NA
"15644","EGOT",0.412307709118129,-2.01591698350677,2.10923764184198,-0
.955756214243498,0.33919541452015,NA
"15645","AC018816.1",1.45556816014485,0.233405542168912,1.169186864509
62,0.199630657214753,0.841769449379723,NA
"15646","AC026202.2",0.808045543572931,0.156379436154744,1.52908389646

007,0.102270017045352,0.918542353428777,NA
"15647","AC026202.3",0.173429405397192,1.41832010653068,3.352475197537
38,0.42306654724024,0.672246708730081,NA
"15648","AC069277.1",0.37947558277061,-0.748608550273953,2.30880372407
626,-0.324240879580817,0.745755674959597,NA
"15649","AC066585.1",0,NA,NA,NA,NA,NA
"15650","GRM7-
AS1",0.16538586715322,1.41832010653068,3.35247519753738,0.423066547240
24,0.672246708730081,NA
"15651","AC077690.1",0.188208781817422,-0.917509588944765,3.2454072234
4342,-0.28271015800947,0.777399028822487,NA
"15652","CAV3",0.321271557798312,1.2470125876493,2.81246093920152,0.44
3388411290554,0.657484816682473,NA
"15653","OXTR",0.646197581543139,-0.424652555048317,2.03288813821317,-
0.208891255286467,0.834533130846442,NA
"15654","AC026191.1",0.06390253691038,0.119912045671111,3.352475197537
38,0.0357682126206912,0.971467179494267,NA
"15655","LHFPL4",0,NA,NA,NA,NA,NA
"15656","AC022382.2",0,NA,NA,NA,NA,NA
"15657","CPNE9",0.06390253691038,0.119912045671111,3.35247519753738,0.
0357682126206912,0.971467179494267,NA
"15658","AC022382.1",0.430398166167859,-1.02974134467151,1.94183928579
181,-0.53029174566917,0.595909668817743,NA
"15659","CIDEC",0.608529510904727,-2.56891847623975,2.36878977059594,-
1.0844856340263,0.278149533218814,NA
"15660","AC018809.1",0.163695219304045,-0.600668372859899,3.3356737988
1164,-0.18007407471135,0.857094415273326,NA
"15661","AC034193.1",0,NA,NA,NA,NA,NA
"15662","LINC00852",0.928249608774095,-0.599028905312839,1.35910530412
444,-0.440752385775395,0.659392267293543,NA
"15663","GHRL",0.443769621640812,1.72577947152038,2.22622500978239,0.7
75204421806884,0.438218875750837,NA
"15664","GHRLOS",1.05517313178384,0.604169829838207,1.26432579626924,0
.477859291980744,0.632750356858315,NA
"15665","SLC6A1",1.30788824877955,2.30017818524649,1.95669266766774,1.
17554392841271,0.239777173305136,NA
"15666","AC022001.2",0,NA,NA,NA,NA,NA
"15667","AC022001.3",0.173429405397192,1.41832010653068,3.352475197537
38,0.42306654724024,0.672246708730081,NA
"15668","SYN2",0.346112056516138,1.28750669156114,2.36411145060641,0.5
4460490482836,0.586025280638922,NA
"15669","AC034198.2",0.06390253691038,0.119912045671111,3.352475197537
38,0.0357682126206912,0.971467179494267,NA
"15670","NUP210",1.0335424103112,0.113530192939037,1.3553652441649,0.0
837635415455759,0.933244435742418,NA
"15671","WNT7A",0,NA,NA,NA,NA,NA
"15672","AC090004.2",0,NA,NA,NA,NA,NA
"15673","AC090946.1",0.240644962732512,2.01289191439962,3.350723011581
91,0.600733605088207,0.548017432599981,NA
"15674","AC090948.1",0.373877603000368,1.28717078660063,2.314873248316

34,0.556043743447646,0.578180951428236,NA
"15675","AC090948.2",0.16538586715322,1.41832010653068,3.3524751975373
8,0.42306654724024,0.672246708730081,NA
"15676","AC090948.3",0.338278427309065,1.26399624841041,2.376014849266
,0.531981628313846,0.594738719787115,NA
"15677","AC090948.4",0.20060072692907,-0.855152612088657,3.23855166872
834,-0.264054027714322,0.791738287519029,NA
"15678","AC010727.1",0,NA,NA,NA,NA,NA
"15679","AC090644.1",0,NA,NA,NA,NA,NA
"15680","AC144521.1",0.270738621018879,0.696980755662315,2.90872380220
041,0.239617372792514,0.810626895543258,NA
"15681","SATB1-
AS1",0.150263610326729,1.41832010653068,3.35247519753738,0.42306654724
024,0.672246708730081,NA
"15682","KCNH8",0,NA,NA,NA,NA,NA
"15683","AC061958.1",0.680701303831118,-0.883954898189208,1.5934389672
9262,-0.554746630610594,0.579067976165865,NA
"15684","PP2D1",0,NA,NA,NA,NA,NA
"15685","SGO1",0.391175442303102,1.28920775753492,2.66750436700999,0.4
83301086018463,0.628881969053795,NA
"15686","SGO1-
AS1",0.124306244907042,-0.509577950443694,3.33980312193904,-0.15257724
2381833,0.878731672788745,NA
"15687","AC116096.1",0.294236821390516,0.0858012426627812,2.8766327541
4391,0.0298269713223494,0.976205048328083,NA
"15688","UBE2E2-
AS1",0.601842760626493,1.32190155730516,2.13698035610648,0.61858385994
4145,0.536190537232101,NA
"15689","UBE2E1-
AS1",0.108990023245465,1.41832010653068,3.35247519753738,0.42306654724
024,0.672246708730081,NA
"15690","LINC00691",0.229312504611721,1.98374348132209,3.0670306245250
1,0.646796111346072,0.51776390945149,NA
"15691","THRB-
AS1",1.0166794294042,0.924340577867225,1.50742515834266,0.613191688324
674,0.539749599925718,NA
"15692","AC012087.2",0,NA,NA,NA,NA,NA
"15693","RARB",0.909695124348017,2.81537042745677,1.72024869884885,1.6
3660663097188,0.101712689213249,NA
"15694","NEK10",1.14300909511505,-1.79429063603792,1.55371526402266,-1
.1548387774684,0.248156454199522,NA
"15695","LINC02084",0.311475362858796,-0.0461613448588672,2.4166326175
4422,-0.019101515275324,0.984760122636486,NA
"15696","EOMES",0.346321965553037,1.26405017323688,2.3604933630023,0.5
35502532245692,0.592302407034682,NA
"15697","RBMS3-
AS2",1.16835135851026,0.60437462932698,1.28491775393401,0.470360556134
101,0.638097439831516,NA
"15698","AC025614.2",0.0604037079966622,0.119912045671111,3.3524751975
3738,0.0357682126206911,0.971467179494267,NA

"15699","GADL1",0,NA,NA,NA,NA,NA
"15700","OSBPL10-
AS1",0.682699835976028,0.142394311221419,1.53672982505733,0.0926606023
385446,0.926173197081108,NA
"15701","AC097639.1",0.278068684147489,0.0425010519860944,2.9289971106
5163,0.014510445173037,0.988422746098443,NA
"15702","TRIM71",0,NA,NA,NA,NA,NA
"15703","CCR4",0,NA,NA,NA,NA,NA
"15704","TMPPE",0.390872606646774,1.60848886152635,2.69475792663702,0.
596895493144979,0.55057715646071,NA
"15705","ARPP21",0,NA,NA,NA,NA,NA
"15706","DCLK3",0.255022139240036,-1.27475032494481,2.97713781476924,-
0.428179817078309,0.668520209315217,NA
"15707","LINC02033",0.371685106020372,1.26420130968869,2.3186878789097
2,0.545222718929784,0.585600347978445,NA
"15708","AC011816.2",0.443421272137825,0.571513944330224,2.01424046811
779,0.283736700446831,0.776612167180248,NA
"15709","AC126118.1",0,NA,NA,NA,NA,NA
"15710","AC097359.3",0.108990023245465,1.41832010653068,3.352475197537
38,0.42306654724024,0.672246708730081,NA
"15711","AC097359.2",1.49005220984427,-0.104230468425783,1.03960240877
442,-0.100259933553501,0.920137965557797,NA
"15712","C3orf35",1.43839513586517,1.30950847261967,1.24920501652395,1
.04827346616293,0.294512630903802,NA
"15713","DLEC1",0.289692112060262,0.0584007698147231,2.45889957201254,
0.0237507747284382,0.981051405035345,NA
"15714","SLC22A13",0,NA,NA,NA,NA,NA
"15715","SLC22A14",0.16538586715322,1.41832010653068,3.35247519753738,
0.42306654724024,0.672246708730081,NA
"15716","ACVR2B-
AS1",0.510554030098429,0.571380524635716,2.19380043801853,0.2604523705
68306,0.794514850518496,NA
"15717","SCN11A",0.701000202062656,1.27061258108924,1.87091673495671,0
.679139032405224,0.497049772921386,NA
"15718","AC092053.3",1.11682094358634,-0.0453128136074335,1.4486050289
6086,-0.0312803094712007,0.975045993489027,NA
"15719","AC092053.2",0.541657118013038,1.19650752800675,2.168341776313
92,0.551807626028749,0.581080161884934,NA
"15720","AC092053.5",0,NA,NA,NA,NA,NA
"15721","CX3CR1",0.412958693101636,1.28682861931889,2.26752427558922,0
.567503789561198,0.570371952675838,NA
"15722","AC104850.2",0.270738621018879,0.696980755662315,2.90872380220
041,0.239617372792514,0.810626895543258,NA
"15723","CCR8",0,NA,NA,NA,NA,NA
"15724","AC099332.2",0.06390253691038,0.119912045671111,3.352475197537
38,0.0357682126206912,0.971467179494267,NA
"15725","AC099332.1",0.371879070855458,-0.534817155388632,2.0257485742
2451,-0.264009641765819,0.791772489213426,NA
"15726","MYRIP",0,NA,NA,NA,NA,NA
"15727","ZNF620",1.23853531724463,0.120656309202835,1.21978144578138,0

.0989163342499806,0.921204699654781,NA
"15728","AC099560.1",0,NA,NA,NA,NA,NA
"15729","AC099541.2",0,NA,NA,NA,NA,NA
"15730","VIPR1",0.473138770855048,-2.18094959299659,2.44689583677332,-0.891312805481971,0.372761380718116,NA
"15731","VIPR1-AS1",0,NA,NA,NA,NA,NA
"15732","AC006059.5",0.423906835306974,-0.698615875509431,2.23556524231357,-0.312500777112834,0.754659972561857,NA
"15733","LINC02158",0.929801068703039,-1.66220317819935,1.74351599618264,-0.953362734749021,0.340406315741761,NA
"15734","AC099329.1",0.173429405397192,1.41832010653068,3.35247519753738,0.42306654724024,0.672246708730081,NA
"15735","AC099329.2",0.304547499642892,1.29002452429831,2.85797012172814,0.45137789037426,0.651717215584432,NA
"15736","CYP8B1",0,NA,NA,NA,NA,NA
"15737","AC092042.4",0,NA,NA,NA,NA,NA
"15738","SNRK-AS1",0.439280436679556,-0.920802326455234,2.20003525840209,-0.418539804277511,0.675552490465555,NA
"15739","AC104187.1",0.06390253691038,0.119912045671111,3.35247519753738,0.0357682126206912,0.971467179494267,NA
"15740","ZKSCAN7-AS1",0,NA,NA,NA,NA,NA
"15741","AC124045.1",0.82895558221385,1.02102869186103,1.51725486083132,0.672944749243771,0.500982441630157,NA
"15742","KIF15",0.675597016845388,-0.643924805496207,1.61564041610552,-0.398557005059566,0.690219647097617,NA
"15743","TGM4",0,NA,NA,NA,NA,NA
"15744","CDCP1",0.304547499642892,1.29002452429831,2.85797012172814,0.45137789037426,0.651717215584432,NA
"15745","SLC6A20",0.33077173430644,2.01280089522968,3.31423402991417,0.60732008574597,0.543638511160742,NA
"15746","CCR9",0,NA,NA,NA,NA,NA
"15747","CXCR6",1.02503803798828,1.51099177837377,1.34276826194015,1.12528112348334,0.260469926109591,NA
"15748","XCR1",0.06390253691038,0.119912045671111,3.35247519753738,0.0357682126206912,0.971467179494267,NA
"15749","CCR3",0.184225018276636,0.696980701717498,3.29715406410566,0.21138857577362,0.832584070546688,NA
"15750","CCR1",1.29549073880786,-0.0517586036389318,1.19360197968327,-0.0433633694648081,0.965411877152037,NA
"15751","CCR2",0.103291511307383,0.119912045671111,3.35247519753738,0.0357682126206912,0.971467179494267,NA
"15752","CCR5AS",0,NA,NA,NA,NA,NA
"15753","CCR5",0.340086608373608,-0.573225375189377,2.3529983306217,-0.243614866925095,0.807529113010069,NA
"15754","CCRL2",1.21171727808605,-1.26059524745599,1.34828400215382,-0.93496269735623,0.349807523778062,NA
"15755","LINC02009",0.06390253691038,0.119912045671111,3.35247519753738,0.0357682126206912,0.971467179494267,NA
"15756","LTF",0.0604037079966622,0.119912045671111,3.35247519753738,0.

0357682126206911,0.971467179494267,NA
"15757","LRRC2-AS1",0,NA,NA,NA,NA,NA
"15758","TMIE",0.424639500725414,2.54525832651505,2.21853738738168,1.1
4726861985362,0.251270619026034,NA
"15759","PRSS45P",0,NA,NA,NA,NA,NA
"15760","CSPG5",0.157712923618349,-0.60066841133552,3.33567379711352,-
0.180074086337609,0.857094406146103,NA
"15761","AC112512.1",0,NA,NA,NA,NA,NA
"15762","CDC25A",0,NA,NA,NA,NA,NA
"15763","CAMP",0.396482452281363,-0.573129016870408,2.25790316759688,-
0.253832416330058,0.799625028809032,NA
"15764","AC134772.1",0,NA,NA,NA,NA,NA
"15765","PFKFB4",0.394791804432188,-1.91531134019006,2.16478486500133,
-0.884758282984798,0.376287013645482,NA
"15766","CELSR3",0.163695219304045,-0.600668372859899,3.33567379881164
,-0.18007407471135,0.857094415273326,NA
"15767","PRKAR2A-
AS1",0.503149694427052,-1.30578459993053,1.8372420824815,-0.7107308353
0008,0.477251047780074,NA
"15768","AC137630.2",0.233296268152507,0.0607094481758273,2.6110486817
2048,0.0232509828716883,0.981450071132202,NA
"15769","AC137630.1",0.287516529584019,-0.0469351117677705,2.471358871
02577,-0.0189916212971083,0.984847789442023,NA
"15770","AC137630.4",1.26656634012312,0.840908368648973,1.326270991870
07,0.634039629761694,0.526054959498409,NA
"15771","AC137630.3",0.682372899771579,-0.27669025198348,1.80572109452
148,-0.153229783283228,0.878217071343013,NA
"15772","RHOA-
IT1",0.813639360552273,2.55788132483926,1.63224440048131,1.56709456260
656,0.117092589966739,NA
"15773","BSN",0.718484820156109,-0.745264858068454,1.61397141160819,-0
.461758400866504,0.644254585468135,NA
"15774","AMIGO3",0.389484794453927,-0.536595401468397,2.26391910918727
,-0.237020571667435,0.81264083104154,NA
"15775","MST1R",0.911433510801028,0.546149760638797,1.44678972010645,0
.377490766660003,0.705808924868482,NA
"15776","SEMA3F-
AS1",0.615536189254987,2.00440787042355,2.1084739113051,0.950643903951
778,0.341785173427479,NA
"15777","SEMA3F",0.0604037079966622,0.119912045671111,3.35247519753738
,0.0357682126206911,0.971467179494267,NA
"15778","U73169.1",0.391175442303102,1.28920775753492,2.66750436700999
,0.483301086018463,0.628881969053795,NA
"15779","U73166.1",0.708671290255521,-0.566925814352213,1.815158865804
65,-0.312328482664739,0.754790895922201,NA
"15780","SEMA3B-
AS1",0.5949863659814,1.24793071475445,2.13653829303868,0.5840900295681
5,0.559159742475536,NA
"15781","LSMEM2",0,NA,NA,NA,NA,NA
"15782","RASSF1-

AS1",0.797788715730274,-0.548018873725501,1.92723970349428,-0.284354288017152,0.776138886515498,NA
"15783","ZMYND10",0.0604037079966622,0.119912045671111,3.35247519753738,0.0357682126206911,0.971467179494267,NA
"15784","CACNA2D2",0.724264392849274,0.239514249070438,1.80085898464803,0.133000002283493,0.894193378693933,NA
"15785","CYB561D2.1",1.06052329421893,0.634514624131954,1.32752046334091,0.477969750112252,0.632671735448565,NA
"15786","LINC02019",1.1882962805506,0.133854101093111,1.33831564229063,0.100016839722511,0.9203309563157,NA
"15787","DOCK3",0.124306244907042,-0.509577950443694,3.33980312193904,-0.152577242381833,0.878731672788745,NA
"15788","AC099050.1",0,NA,NA,NA,NA,NA
"15789","GRM2",0.12780507382076,-0.532254130061651,3.33875455416813,-0.15941696864095,0.873340370825791,NA
"15790","GPR62",0.0973092156216871,0.119912045671111,3.35247519753738,0.0357682126206911,0.971467179494267,NA
"15791","TLR9",0.227597756214425,-1.13126253111685,2.5754042190421,-0.439256301108964,0.660475834030736,NA
"15792","AC006252.1",0.210667318323391,0.69698072231542,3.15449020715825,0.220948767168088,0.825132324921599,NA
"15793","TNNC1",0.567727517044867,-0.327503301906498,1.69898972536073,-0.192763556493528,0.847144145854997,NA
"15794","ITIH3",0,NA,NA,NA,NA,NA
"15795","IL17RB",1.14424663291778,1.86401027766534,1.6796220430218,1.10977959917209,0.267094012417634,NA
"15796","AC115282.2",0,NA,NA,NA,NA,NA
"15797","CACNA2D3-
AS1",0.772906232467075,-0.964333796131469,1.46452907712044,-0.658459986351069,0.51024260063935,NA
"15798","WNT5A-
AS1",0.858713125681006,0.305108445144988,1.58492896740686,0.192506068990702,0.847345814452834,NA
"15799","ERC2",0.297909942550757,-1.47362501367338,2.86206139143832,-0.514882391440531,0.606635206273485,NA
"15800","ERC2-
IT1",0.33077173430644,2.01280089522968,3.31423402991417,0.60732008574597,0.543638511160742,NA
"15801","SPATA12",0.402717809460792,1.28699235086224,2.28984512686123,0.562043404492757,0.574086446830557,NA
"15802","AC097358.2",0.108990023245465,1.41832010653068,3.35247519753738,0.42306654724024,0.672246708730081,NA
"15803","HESX1",0.67892598147214,1.42136041947504,1.69251610449273,0.839791370789377,0.401025372586487,NA
"15804","ASB14",0,NA,NA,NA,NA,NA
"15805","AC093928.1",0,NA,NA,NA,NA,NA
"15806","DNAH12",0,NA,NA,NA,NA,NA
"15807","AC092418.2",0.330989799689647,1.28767123567888,2.3893126005633,0.538929579735739,0.589935446033768,NA
"15808","DENND6A-

AS1",0.673303901153777,2.29363661331253,1.71334291840491,1.33869092326
705,0.180671312387445,NA
"15809","DENND6A-DT",0,NA,NA,NA,NA,NA
"15810","FLNB-AS1",0,NA,NA,NA,NA,NA
"15811","HTD2",0.258520968153754,-1.2931292497918,2.96669614377236,-0.
435881933006964,0.662922394077564,NA
"15812","FAM3D-AS1",0,NA,NA,NA,NA,NA
"15813","AC126121.3",0.16538586715322,1.41832010653068,3.3524751975373
8,0.42306654724024,0.672246708730081,NA
"15814","AC093418.1",0.694108611084506,-0.836334232042864,1.8001715990
2813,-0.464585838646927,0.642228075465349,NA
"15815","CADPS",0.439761757551905,2.57992481053724,2.54830735454105,1.
01240723805935,0.311343379010575,NA
"15816","C3orf49",0,NA,NA,NA,NA,NA
"15817","SCAANT1",1.12232930842314,-2.6215396566459,1.8295465448753,-1
.43289038695902,0.151889164908557,NA
"15818","AC012557.1",0,NA,NA,NA,NA,NA
"15819","AC012557.2",0.626279891520152,-0.657647199018728,1.6309381958
7203,-0.403232446626892,0.686777232291979,NA
"15820","LINC00994",0,NA,NA,NA,NA,NA
"15821","PRICKLE2-
AS3",0.391175442303102,1.28920775753492,2.66750436700999,0.48330108601
8463,0.628881969053795,NA
"15822","MAGI1-
IT1",0.06390253691038,0.119912045671111,3.35247519753738,0.03576821262
06912,0.971467179494267,NA
"15823","MAGI1-AS1",0,NA,NA,NA,NA,NA
"15824","KBTBD8",0.631663333118131,-0.531289894302682,1.8422887852308,
-0.288385783250655,0.773051449012333,NA
"15825","TAFA1",0.124306244907042,-0.509577950443694,3.33980312193904,
-0.152577242381833,0.878731672788745,NA
"15826","AC104167.1",0,NA,NA,NA,NA,NA
"15827","AC109587.1",0.450903864592329,-0.915568657947089,1.9036296488
5454,-0.48095944423749,0.6305453229357,NA
"15828","LMOD3",0.448728282116086,-1.05553832077033,1.92747400988709,-
0.547627784009479,0.583947507741277,NA
"15829","SAMMSON",0,NA,NA,NA,NA,NA
"15830","FOXP1-
IT1",1.07791567131893,2.17550193053014,1.78864796067944,1.216282901026
4,0.223877172870908,NA
"15831","AC097634.3",0,NA,NA,NA,NA,NA
"15832","AC097634.1",1.19224288600513,-0.75852585467617,1.358046273851
83,-0.558541979961228,0.576474346645489,NA
"15833","GPR27",0.696954630851309,0.885703950193214,1.8413797119931,0.
481000167659354,0.63051637956278,NA
"15834","PROK2",0.120807415993324,-0.48654549181451,3.34088239674268,-
0.145633827844071,0.884210461918222,NA
"15835","LINC00877",0.840873392497517,-0.261034787103001,1.49504511563
161,-0.174599939743438,0.861393995935234,NA
"15836","PDZRN3-

AS1",0.06390253691038,0.119912045671111,3.35247519753738,0.03576821262
06912,0.971467179494267,NA
"15837","LINC00960",1.23695880813761,0.711069577777227,1.2168740053522
1,0.584341168148644,0.558990799774055,NA
"15838","AC117461.1",0,NA,NA,NA,NA,NA
"15839","CADM2",0.35415559476011,1.2874229447024,2.35156498325213,0.54
7474959812482,0.584052468926338,NA
"15840","AC107204.1",1.25153917108335,0.462450538466255,1.466113804397
03,0.31542608566909,0.75243816346565,NA
"15841","HTR1F",0.06390253691038,0.119912045671111,3.35247519753738,0.
0357682126206912,0.971467179494267,NA
"15842","STX19",0.705574661254509,0.154925410661129,1.63072745067621,0
.0950038650523028,0.924311756941265,NA
"15843","AC109782.1",0,NA,NA,NA,NA,NA
"15844","EPHA6",0.326905503981022,-0.253160972816323,2.83819636466522,
-0.0891978356283266,0.928924685441435,NA
"15845","AC110491.3",0,NA,NA,NA,NA,NA
"15846","AC026100.1",0,NA,NA,NA,NA,NA
"15847","ST3GAL6-
AS1",0.227597756214425,-1.13126253111685,2.5754042190421,-0.4392563011
08964,0.660475834030736,NA
"15848","AC107029.1",0.16538586715322,1.41832010653068,3.3524751975373
8,0.42306654724024,0.672246708730081,NA
"15849","AC107029.2",0.387595552634301,1.28711517028576,2.306991504066
7,0.557919336944617,0.57689946654674,NA
"15850","AC069222.1",0,NA,NA,NA,NA,NA
"15851","AC129803.1",0.262695082774907,0.696980751978295,2.93688603919
854,0.237319644914958,0.812408822930581,NA
"15852","IMPG2",0.493815768451171,-0.12195031211058,1.79877352124078,-
0.0677963682867977,0.945947734799817,NA
"15853","AC020651.1",0,NA,NA,NA,NA,NA
"15854","AC020651.2",0,NA,NA,NA,NA,NA
"15855","AC106712.1",0,NA,NA,NA,NA,NA
"15856","AC117435.1",0,NA,NA,NA,NA,NA
"15857","AC063944.3",0.285708348519476,2.01137472954577,2.860309475841
75,0.703201785168317,0.481930006892637,NA
"15858","LINC01990",0.684414584373324,2.44140602528465,1.7829084645721
,1.36933896147641,0.170893344477491,NA
"15859","LINC00636",0.06390253691038,0.119912045671111,3.3524751975373
8,0.0357682126206912,0.971467179494267,NA
"15860","LINC01215",0,NA,NA,NA,NA,NA
"15861","HHLA2",0,NA,NA,NA,NA,NA
"15862","MYH15",0.0973092156216871,0.119912045671111,3.35247519753738,
0.0357682126206911,0.971467179494267,NA
"15863","TRAT1",0.919300419170985,0.698477094757418,1.54102005491872,0
.453256330135338,0.650364174567533,NA
"15864","MORC1",0,NA,NA,NA,NA,NA
"15865","DPPA4",0,NA,NA,NA,NA,NA
"15866","LINC01205",0,NA,NA,NA,NA,NA
"15867","NECTIN3-

AS1",1.45677484575924,0.872394275866773,1.19855903376885,0.727869259074828,0.466693622724382,NA
"15868","CD96",1.01974824833906,-0.117494390859849,1.28028118332383,-0.0917723328205238,0.926878926485793,NA
"15869","ZBED2",0,NA,NA,NA,NA,NA
"15870","PLCXD2",0.391393507686309,0.665967175556164,2.32225230553716,0.286776408389492,0.774283527934951,NA
"15871","GCSAM",0,NA,NA,NA,NA,NA
"15872","TBILA",0.387058707392954,0.60359886830084,2.32983482814959,0.259073673810702,0.795578396758788,NA
"15873","SLC9C1",0.0604037079966622,0.119912045671111,3.35247519753738,0.0357682126206911,0.971467179494267,NA
"15874","AC112487.1",0,NA,NA,NA,NA,NA
"15875","BTLA",0.184709952903704,-0.898539523146346,3.26457968362944,-0.275238961895205,0.783132655214614,NA
"15876","AC074044.1",0.06390253691038,0.119912045671111,3.35247519753738,0.0357682126206912,0.971467179494267,NA
"15877","SIDT1",0.443963586475898,0.181935264172628,1.98169087894865,0.091808094847341,0.926850512469563,NA
"15878","SIDT1-AS1",0,NA,NA,NA,NA,NA
"15879","GRAMD1C",0.496048730638418,1.04925854869369,2.19867023317897,0.47722415706543,0.633202510947239,NA
"15880","AC128687.2",0,NA,NA,NA,NA,NA
"15881","ZDHHC23",0.847895352048881,-1.35061551405317,1.41208427108917,-0.956469483943338,0.338835094577996,NA
"15882","AC128687.3",0,NA,NA,NA,NA,NA
"15883","AC093010.2",0,NA,NA,NA,NA,NA
"15884","ZNF80",0.36838024194174,-0.511720676674028,2.29466715960387,-0.223004314387088,0.823532148659855,NA
"15885","TIGIT",0.552904988167802,-0.153034403279081,1.91830730602533,-0.0797757495884031,0.93641561186366,NA
"15886","ZBTB20-AS1",0.51954146191333,1.88442863704037,1.95576638409787,0.96352440269066,0.335284423976889,NA
"15887","ZBTB20-AS5",0,NA,NA,NA,NA,NA
"15888","ZBTB20-AS4",0.651561971232403,-0.759076069786867,1.66427662501753,-0.456099700239961,0.648318285725608,NA
"15889","AC026341.1",0.150263610326729,1.41832010653068,3.35247519753738,0.42306654724024,0.672246708730081,NA
"15890","LSAMP-AS1",0.108990023245465,1.41832010653068,3.35247519753738,0.42306654724024,0.672246708730081,NA
"15891","AC092691.1",0.777482432368902,-1.28052598601582,1.728267520327,-0.740930423649653,0.45873562759106,NA
"15892","UPK1B",0,NA,NA,NA,NA,NA
"15893","ARHGAP31-AS1",0.120322481366256,1.41832010653068,3.35247519753738,0.42306654724024,0.672246708730081,NA
"15894","AC073352.1",0.0604037079966622,0.119912045671111,3.3524751975

3738,0.0357682126206911,0.971467179494267,NA
"15895","CD80",0.0973092156216871,0.119912045671111,3.35247519753738,0
.0357682126206911,0.971467179494267,NA
"15896","AC073352.2",0.0973092156216871,0.119912045671111,3.3524751975
3738,0.0357682126206911,0.971467179494267,NA
"15897","PLA1A",1.38682641690129,-0.163547776044732,1.11016393450387,-
0.147318581483033,0.882880566591873,NA
"15898","AC092910.3",0.390524257143788,0.528407461279756,2.07024121086
178,0.255239562669027,0.798538076138587,NA
"15899","GPR156",0,NA,NA,NA,NA,NA
"15900","AC126182.3",0,NA,NA,NA,NA,NA
"15901","HGD",0,NA,NA,NA,NA,NA
"15902","AC072026.2",0.389484794453927,-0.536595401468397,2.2639191091
8727,-0.237020571667435,0.81264083104154,NA
"15903","POLQ",0.229797439238789,0.0884527683768807,3.10949972347481,0
.0284459804608171,0.977306451918786,NA
"15904","FBXO40",0,NA,NA,NA,NA,NA
"15905","SLC15A2",1.09544496329768,0.729471776393222,1.54178123692087,
0.473135720506023,0.636116353743862,NA
"15906","ILDR1",0,NA,NA,NA,NA,NA
"15907","CD86",0.797871450444637,-2.04425081135985,1.61567257319822,-1
.26526305222429,0.205777046618502,NA
"15908","AC096861.2",0.763285965834576,-1.16539088401793,1.79334956274
026,-0.649840336892935,0.51579536050401,NA
"15909","AC112503.2",0.150263610326729,1.41832010653068,3.352475197537
38,0.42306654724024,0.672246708730081,NA
"15910","AC112503.1",0.184225018276636,0.696980701717498,3.29715406410
566,0.21138857577362,0.832584070546688,NA
"15911","AC020634.2",0.16538586715322,1.41832010653068,3.3524751975373
8,0.42306654724024,0.672246708730081,NA
"15912","AC117488.1",0.274569855233771,0.0589952543162062,2.4952667603
5574,0.0236428646642155,0.981137480638694,NA
"15913","AC092902.6",0.16538586715322,1.41832010653068,3.3524751975373
8,0.42306654724024,0.672246708730081,NA
"15914","AC079848.2",0.60580200316184,-0.116240048298391,1.89465715410
386,-0.0613514946736476,0.951079281186783,NA
"15915","AC016924.1",0.108990023245465,1.41832010653068,3.352475197537
38,0.42306654724024,0.672246708730081,NA
"15916","AC063919.1",0,NA,NA,NA,NA,NA
"15917","CHST13",0.294236821390516,0.0858012426627812,2.87663275414391
,0.0298269713223494,0.976205048328083,NA
"15918","KBTBD12",0.240644962732512,2.01289191439962,3.35072301158191,
0.600733605088207,0.548017432599981,NA
"15919","AL449214.1",0,NA,NA,NA,NA,NA
"15920","GATA2-
AS1",0.108990023245465,1.41832010653068,3.35247519753738,0.42306654724
024,0.672246708730081,NA
"15921","AC112484.3",0,NA,NA,NA,NA,NA
"15922","KIAA1257",0.453387331364307,-0.919205841191184,1.901873005903
74,-0.483316098571151,0.628871311150114,NA

"15923","EFCC1",1.28557495979179,-2.12459705051309,1.29564915459951,-1
.63979349113982,0.101048111994287,NA
"15924","AC108673.2",0.920686376021826,-0.344249181539833,1.5262616025
7402,-0.225550574658539,0.821550985895149,NA
"15925","AC137695.3",0.227597756214425,-1.13126253111685,2.57540421904
21,-0.439256301108964,0.660475834030736,NA
"15926","EFCAB12",0.0604037079966622,0.119912045671111,3.3524751975373
8,0.0357682126206911,0.971467179494267,NA
"15927","TRH",0,NA,NA,NA,NA,NA
"15928","ALG1L2",0,NA,NA,NA,NA,NA
"15929","LINC02021",0.21798004649093,1.96674997191025,3.35148506485532
,0.586829400654111,0.557318288863578,NA
"15930","AC130888.1",0.173429405397192,1.41832010653068,3.352475197537
38,0.42306654724024,0.672246708730081,NA
"15931","AC010210.1",0.653567689421682,1.37233823959467,1.868399741739
54,0.734499266370574,0.46264450712562,NA
"15932","AC107027.3",1.05308394076504,-0.775758852921442,1.52996230327
383,-0.507044422768759,0.612123649538495,NA
"15933","ACPP",0.221615460528729,-1.10387780491251,2.59193339108378,-0
.425889727224409,0.670188200759635,NA
"15934","NPHP3-
AS1",0.0973092156216871,0.119912045671111,3.35247519753738,0.035768212
6206911,0.971467179494267,NA
"15935","TMEM108-
AS1",0.262695082774907,0.696980751978295,2.93688603919854,0.2373196449
14958,0.812408822930581,NA
"15936","BFSP2",0,NA,NA,NA,NA,NA
"15937","AC022296.4",0,NA,NA,NA,NA,NA
"15938","AC080128.2",0,NA,NA,NA,NA,NA
"15939","LINC02004",0.16538586715322,1.41832010653068,3.35247519753738
,0.42306654724024,0.672246708730081,NA
"15940","EPHB1",0,NA,NA,NA,NA,NA
"15941","AC092969.1",0,NA,NA,NA,NA,NA
"15942","AC072039.2",0,NA,NA,NA,NA,NA
"15943","AC117382.2",0,NA,NA,NA,NA,NA
"15944","AC096992.2",1.25724087631883,-0.652810321609965,1.14374128005
006,-0.570767474250288,0.568157273628508,NA
"15945","CLDN18",0.270586091692985,2.01155232872955,2.90724650940082,0
.691909792384316,0.488993980674386,NA
"15946","AC016252.1",0.557447359870564,-1.31969746242781,2.13517402705
848,-0.618074894928302,0.536525970258832,NA
"15947","NME9",0,NA,NA,NA,NA,NA
"15948","ESYT3",0.492201552221714,0.0455172212701957,1.78682792015951,
0.0254737575771328,0.979677080112871,NA
"15949","FOXL2",0.0604037079966622,0.119912045671111,3.35247519753738,
0.0357682126206911,0.971467179494267,NA
"15950","AC024933.1",0.490292838989332,-1.03572415044845,1.72725257331
099,-0.599636767923904,0.54874833760427,NA
"15951","RASA2-
IT1",0.406725673549699,0.61738663533866,2.04856467601613,0.30137522264

5789,0.763128385990047,NA
"15952","ATP1B3-
AS1",0.0604037079966622,0.119912045671111,3.35247519753738,0.035768212
6206911,0.971467179494267,NA
"15953","AC109992.2",0.236795097066225,0.0434742300939003,3.0811765183
8172,0.014109620086529,0.988742525500995,NA
"15954","PLS1",0.0604037079966622,0.119912045671111,3.35247519753738,0
.0357682126206911,0.971467179494267,NA
"15955","AC072028.1",0,NA,NA,NA,NA,NA
"15956","AC021074.3",0,NA,NA,NA,NA,NA
"15957","PAQR9",0.173429405397192,1.41832010653068,3.35247519753738,0.
42306654724024,0.672246708730081,NA
"15958","PAQR9-
AS1",0.120322481366256,1.41832010653068,3.35247519753738,0.42306654724
024,0.672246708730081,NA
"15959","AC018450.1",0,NA,NA,NA,NA,NA
"15960","AC131210.1",0,NA,NA,NA,NA,NA
"15961","AC107021.2",0.19170761073114,-0.935110177380914,3.32149227750
043,-0.281533148132028,0.778301507243725,NA
"15962","LNCSRLR",0,NA,NA,NA,NA,NA
"15963","PLSCR2",0.237331942307572,0.696980738944435,3.03442443415691,
0.229691249219751,0.818331695238684,NA
"15964","AC093001.2",0.124306244907042,-0.509577950443694,3.3398031219
3904,-0.152577242381833,0.878731672788745,NA
"15965","TM4SF18",0.150263610326729,1.41832010653068,3.35247519753738,
0.42306654724024,0.672246708730081,NA
"15966","TM4SF1-AS1",0,NA,NA,NA,NA,NA
"15967","WWTR1-
AS1",0.124306244907042,-0.509577950443694,3.33980312193904,-0.15257724
2381833,0.878731672788745,NA
"15968","LINC01213",0.103291511307383,0.119912045671111,3.352475197537
38,0.0357682126206912,0.971467179494267,NA
"15969","AC018545.1",0,NA,NA,NA,NA,NA
"15970","ERICH6",0,NA,NA,NA,NA,NA
"15971","AC011317.1",0.173429405397192,1.41832010653068,3.352475197537
38,0.42306654724024,0.672246708730081,NA
"15972","SIAH2-
AS1",0.466244186802027,-1.20150006501784,1.85075129910945,-0.649195851
217818,0.516211792992446,NA
"15973","MED12L",1.27655692035504,0.143262962910793,1.2969095410641,0.
110464884692919,0.912040697353704,NA
"15974","GPR171",0.774773510190265,0.142366522019091,1.62167958260069,
0.0877895507513132,0.930043942990863,NA
"15975","P2RY13",0.124306244907042,-0.509577950443694,3.33980312193904
,-0.152577242381833,0.878731672788745,NA
"15976","AADACL2",0,NA,NA,NA,NA,NA
"15977","AC108718.1",0.259253633572194,1.98393725043859,2.947861372725
44,0.673009005374071,0.500941561797964,NA
"15978","AC026347.1",0.384096723720583,1.28715287945805,2.312327404116
53,0.556648196603381,0.57776781726439,NA

"15979","AC078788.1",0,NA,NA,NA,NA,NA
"15980","LINC02006",0.978793459984648,-0.33762499464539,1.48453643956371,-0.227427893076585,0.820091032948707,NA
"15981","MME-AS1",0,NA,NA,NA,NA,NA
"15982","STRIT1",0.2292884040636,0.696980734286753,3.06852794721869,0.227138467133238,0.820316073199257,NA
"15983","PLCH1",0,NA,NA,NA,NA,NA
"15984","AC104472.4",0,NA,NA,NA,NA,NA
"15985","AC104472.5",0.282419428642657,1.98408346788866,2.86695637949831,0.692052199355701,0.488904548657237,NA
"15986","LEKR1",1.03352626493358,0.852324388819578,1.38677173217042,0.614610443122905,0.538812016070807,NA
"15987","LINC00880",0.173429405397192,1.41832010653068,3.35247519753738,0.42306654724024,0.672246708730081,NA
"15988","LINC02029",0.523040290827048,1.88426308834456,1.95313107903045,0.964739698515229,0.334675205993253,NA
"15989","LINC00881",0.253555121634112,0.696980747544927,2.97042254430159,0.234640269911095,0.814487951095881,NA
"15990","AC084212.1",0.16538586715322,1.41832010653068,3.35247519753738,0.42306654724024,0.672246708730081,NA
"15991","AC080013.4",0.169393731242127,0.696980693484986,3.35247519753738,0.207900328091007,0.835306792989891,NA
"15992","AC080013.6",0.20060072692907,-0.855152612088657,3.23855166872834,-0.264054027714322,0.791738287519029,NA
"15993","AC080013.1",1.03706300214554,-0.10603584143679,1.23247905356298,-0.0860345992333507,0.931438912973267,NA
"15994","AC080013.3",0.414766874166179,-0.707921488907536,1.99682887245498,-0.354522863061965,0.72294707299051,NA
"15995","AC080013.5",0.96444397891125,-0.0877214208019188,1.30116757008491,-0.0674174662962082,0.946249364728842,NA
"15996","IQCJ-SCHIP1",1.31036867742189,-2.88607992499333,1.45504049271594,-1.98350488487522,0.0473110619695404,NA
"15997","IQCJ",0,NA,NA,NA,NA,NA
"15998","SCHIP1",0.51909804933923,-2.32698510445798,1.95533674342196,-1.19006872462572,0.234019381555282,NA
"15999","IL12A",0.06390253691038,0.119912045671111,3.35247519753738,0.0357682126206912,0.971467179494267,NA
"16000","SPTSSB",0.120807415993324,-0.48654549181451,3.34088239674268,-0.145633827844071,0.884210461918222,NA
"16001","WDR49",0,NA,NA,NA,NA,NA
"16002","AC069243.1",1.11351001349347,-0.868980114088782,1.42502429584948,-0.609800209455914,0.541994162752508,NA
"16003","MECOM",0.323098790771569,-0.0195554982229674,2.38904070136595,-0.00818550232810448,0.993468987002381,NA
"16004","TERC",0.630339666223338,0.516822160033407,1.60618212380366,0.321770584029103,0.747626500629779,NA
"16005","AC078802.1",1.0520307596721,-3.31367577106567,1.67861954010062,-1.97404813413946,0.048376270010956,NA
"16006","AC078795.1",0.108990023245465,1.41832010653068,3.352475197537

38,0.42306654724024,0.672246708730081,NA
"16007","AC078795.3",0.108990023245465,1.41832010653068,3.352475197537
38,0.42306654724024,0.672246708730081,NA
"16008","AC078795.2",0.276720916704575,0.696980758280457,2.88854265454
755,0.241291489043159,0.809329206809347,NA
"16009","AC008040.1",0,NA,NA,NA,NA,NA
"16010","GPR160",0.469920951895585,1.79501935009981,2.20928314515949,0
.812489496438099,0.416510811164956,NA
"16011","SLC7A14-
AS1",1.02670458528933,2.19397598026274,1.58665781963421,1.382765680862
78,0.166736725203613,NA
"16012","SLC7A14",0,NA,NA,NA,NA,NA
"16013","AC026316.5",0.477976905040084,1.78680061260803,2.198055595176
65,0.812900554712506,0.416275076461693,NA
"16014","GHSR",0,NA,NA,NA,NA,NA
"16015","LINC02068",0,NA,NA,NA,NA,NA
"16016","TBL1XR1-AS1",0,NA,NA,NA,NA,NA
"16017","LINC00578",0.459137753916668,2.6179166044583,2.18961049702244
,1.19560835501031,0.231849429489171,NA
"16018","LINC02015",0.466434537882394,1.78668345555563,2.2108943039274
1,0.808127033654113,0.419017456625707,NA
"16019","AC117453.1",1.28299623518916,3.2972875774526,1.43691880190799
,2.29469304255351,0.0217507218083973,NA
"16020","LINC01014",0,NA,NA,NA,NA,NA
"16021","KCNMB2-
AS1",0.16538586715322,1.41832010653068,3.35247519753738,0.423066547240
24,0.672246708730081,NA
"16022","AC007823.1",0.172892560155845,0.696980693484986,3.35247519753
738,0.207900328091007,0.835306792989891,NA
"16023","AC007620.3",0.06390253691038,0.119912045671111,3.352475197537
38,0.0357682126206912,0.971467179494267,NA
"16024","AC090425.3",0.270586091692985,2.01155232872955,2.907246509400
82,0.691909792384316,0.488993980674386,NA
"16025","PEX5L",0.38336591364415,2.43735836912973,2.62401805200281,0.9
28864939503518,0.352959084821909,NA
"16026","AC007687.1",0.06390253691038,0.119912045671111,3.352475197537
38,0.0357682126206912,0.971467179494267,NA
"16027","SOX2-
OT",0.486657424951532,1.72744950224589,2.17560367520823,0.794009277485
04,0.427190030384709,NA
"16028","LINC01206",0,NA,NA,NA,NA,NA
"16029","AC092953.2",0.120807415993324,-0.48654549181451,3.34088239674
268,-0.145633827844071,0.884210461918222,NA
"16030","MCCC1-AS1",0,NA,NA,NA,NA,NA
"16031","LAMP3",0.365137016128332,-0.374264546092033,2.70796269688093,
-0.138208900190212,0.890075321269731,NA
"16032","MCF2L2",0.663351649677423,1.23926496579513,2.13050525397566,0
.58167655934318,0.560784563480315,NA
"16033","KLHL6",1.06106986711122,0.746655127285527,1.53732606553323,0.
485684295625694,0.627191019840111,NA

"16034","KLHL6-AS1",0,NA,NA,NA,NA,NA
"16035","YEATS2-
AS1",0.180726189362918,0.69698069859353,3.31825525196168,0.21004432922
4369,0.833633074956668,NA
"16036","MAP6D1",0.688911092607337,0.872991762880273,1.8435427086649,0
.473540297589577,0.635827758117939,NA
"16037","AC131235.3",0.225114289442447,-1.12060253516837,3.07913523483
178,-0.363934172975546,0.71590716558595,NA
"16038","AC131235.2",0.0604037079966622,0.119912045671111,3.3524751975
3738,0.0357682126206911,0.971467179494267,NA
"16039","CAMK2N2",0.461738973185525,0.546592215320623,2.23506032219117
,0.244553674857762,0.806802037046688,NA
"16040","THPO",0.721105640369861,-0.972441985187536,1.5925477006604,-0
.610620319117776,0.541450967730643,NA
"16041","LINC02054",0.450150322101767,1.28870059956372,2.5659218790753
4,0.502236880270148,0.615500903272508,NA
"16042","AC107294.3",0.447805295795877,2.58917019540731,2.198097825213
92,1.1779139971422,0.238830894311389,NA
"16043","AC107294.1",0.289692112060262,0.0584007698147231,2.4588995720
1254,0.0237507747284382,0.981051405035345,NA
"16044","AC099661.1",0.796384760250149,-1.12279920272943,1.56543066020
091,-0.717246206603189,0.47322219293818,NA
"16045","LIPH",0.163695219304045,-0.600668372859899,3.33567379881164,-
0.18007407471135,0.857094415273326,NA
"16046","DGKG",0,NA,NA,NA,NA,NA
"16047","AC068631.1",0,NA,NA,NA,NA,NA
"16048","AC112907.3",0,NA,NA,NA,NA,NA
"16049","LINC02043",0.342286291397972,0.6094932892186,2.40258431168592
,0.253682372874112,0.799740953086473,NA
"16050","AC072022.1",0.172892560155845,0.696980693484986,3.35247519753
738,0.207900328091007,0.835306792989891,NA
"16051","LPP-AS1",0,NA,NA,NA,NA,NA
"16052","TPRG1-
AS1",0.315289262112617,1.24694911083339,2.82863815654472,0.44083019524
7237,0.659335931880918,NA
"16053","TP63",0.878443120862233,-0.32351481673444,1.46993007449641,-0
.220088575876832,0.825802171817026,NA
"16054","P3H2",0.840464670208656,0.89495318292087,1.51436328662207,0.5
90976544945929,0.554536136670098,NA
"16055","OSTN",0.410224502463417,0.603203662969439,2.29442219746615,0.
262900029312647,0.79262763371854,NA
"16056","OSTN-
AS1",1.04143108160178,0.00320753975768019,1.33638779845998,0.002400156
42269144,0.998084954085507,NA
"16057","UTS2B",1.28948059928718,3.31493501645409,1.53571921949767,2.1
5855540151305,0.0308846759182368,NA
"16058","AC073365.1",0.150263610326729,1.41832010653068,3.352475197537
38,0.42306654724024,0.672246708730081,NA
"16059","PLAAT1",0.393007723915766,0.551289372322731,2.06602446574534,
0.26683583929576,0.789595558616548,NA

"16060","ATP13A4",0.270738621018879,0.696980755662315,2.90872380220041
,0.239617372792514,0.810626895543258,NA
"16061","OPA1-AS1",0,NA,NA,NA,NA,NA
"16062","LINC02028",0.108990023245465,1.41832010653068,3.3524751975373
8,0.42306654724024,0.672246708730081,NA
"16063","LINC00887",1.01819418561396,-2.47123155292653,1.3672244675387
3,-1.80748049175512,0.0706873937154991,NA
"16064","LRRC15",0.687307459698732,-2.72574434264542,1.91108743217492,
-1.42627924644105,0.153787752728799,NA
"16065","GP5",0.0604037079966622,0.119912045671111,3.35247519753738,0.
0357682126206911,0.971467179494267,NA
"16066","AC108676.1",0.473917130336898,0.183270215314198,1.95611257687
757,0.0936910367432643,0.925354590600036,NA
"16067","AC046143.2",0.268677378460603,0.696980754736642,2.91582564400
198,0.239033755728972,0.811079408015517,NA
"16068","XXYLT1-
AS2",0.173429405397192,1.41832010653068,3.35247519753738,0.42306654724
024,0.672246708730081,NA
"16069","AC090018.2",0.120807415993324,-0.48654549181451,3.34088239674
268,-0.145633827844071,0.884210461918222,NA
"16070","ACAP2-
IT1",0.172892560155845,0.696980693484986,3.35247519753738,0.2079003280
91007,0.835306792989891,NA
"16071","AC069213.1",0,NA,NA,NA,NA,NA
"16072","AC233280.1",0.399191170480916,0.22947666828153,2.687118703114
21,0.0853987834685458,0.931944360282684,NA
"16073","MUC4",0.995990565943735,-0.29976039850532,1.42892337778329,-0
.20978059647281,0.833838917867534,NA
"16074","AC124944.3",0.16538586715322,1.41832010653068,3.3524751975373
8,0.42306654724024,0.672246708730081,NA
"16075","TNK2-AS1",0,NA,NA,NA,NA,NA
"16076","ZDHHC19",0.161211752532067,-0.631571180295204,3.3343222206898
8,-0.189415161011203,0.849767442783486,NA
"16077","SLC51A",0.358313650547419,-1.74570516060008,2.27695697101209,
-0.766683421261196,0.443269763016177,NA
"16078","TM4SF19-
AS1",0.424869981009148,1.68896784705048,2.63938212440917,0.63991031515
7021,0.522230907375244,NA
"16079","TM4SF19",0.225114289442447,-1.12060253516837,3.07913523483178
,-0.363934172975546,0.71590716558595,NA
"16080","AC117490.2",0.237331942307572,0.696980738944435,3.03442443415
691,0.229691249219751,0.818331695238684,NA
"16081","LINC01063",0.286193283146544,0.0861184834588285,2.90156683357
524,0.0296799930514492,0.97632226812405,NA
"16082","NRROS",0.43283750809072,-1.08074021637208,2.15188804304916,-0
.502228831031888,0.615506564649727,NA
"16083","NCBP2-AS1",0,NA,NA,NA,NA,NA
"16084","AC011322.1",0.0604037079966622,0.119912045671111,3.3524751975
3738,0.0357682126206911,0.971467179494267,NA
"16085","DLG1-AS1",0,NA,NA,NA,NA,NA

"16086","LINC02012",0,NA,NA,NA,NA,NA
"16087","BDH1",0.285708348519476,2.01137472954577,2.86030947584175,0.703201785168317,0.481930006892637,NA
"16088","AC024560.3",0.541439052629831,1.94778698656201,2.16180728302594,0.900999363752551,0.36758865797471,NA
"16089","AC024560.4",0.737497407856139,1.63582905652525,1.69042954502308,0.967700228229808,0.333194104113304,NA
"16090","AC024560.1",0.595645592854991,-1.39947149532372,1.6435685528351,-0.85148349480749,0.394500828069415,NA
"16091","AC055764.2",0.214166147237109,0.696980724704133,3.13752578908925,0.222143424964947,0.824202234028988,NA
"16092","AC135893.1",0.0973092156216871,0.119912045671111,3.35247519753738,0.0357682126206911,0.971467179494267,NA
"16093","AC253576.2",0.917411177351359,2.00071495019768,1.77542483952299,1.1268936345034,0.259787455019971,NA
"16094","ZNF732",0.12780507382076,-0.532254130061651,3.33875455416813,-0.15941696864095,0.873340370825791,NA
"16095","AC079140.6",0.958568837099153,-0.62882138093346,1.42096042692489,-0.44253264835411,0.658103810324777,NA
"16096","AC092574.1",0.432372597764648,-0.727293372373792,2.22855968213929,-0.326351310311615,0.744158571307804,NA
"16097","PDE6B",0.0604037079966622,0.119912045671111,3.35247519753738,0.0357682126206911,0.971467179494267,NA
"16098","AC107464.3",0.347920237580681,-0.555689180979515,2.05920330485248,-0.26985639527192,0.787270734499137,NA
"16099","AC139887.4",0.568474271323113,0.0498501963250608,1.90323933301899,0.0261922898818966,0.979103965557294,NA
"16100","CPLX1",0.646562845549878,0.366954521437634,1.7103126701636,0.214554056599799,0.830115017187268,NA
"16101","SLC26A1",1.20641882508942,0.791342339158494,1.21453540576606,0.651559711970159,0.514685244838513,NA
"16102","AC092535.2",1.39097272145629,0.0779956383700248,1.11627838326008,0.0698711356769622,0.944296227357211,NA
"16103","AC092535.5",0.346112056516138,1.28750669156114,2.36411145060641,0.54460490482836,0.586025280638922,NA
"16104","CTBP1-AS",0.976912556818424,-0.427986367682493,1.50144744718373,-0.285049182697183,0.775606461865955,NA
"16105","FAM53A",0.688935193155457,1.58748735983874,1.70121966302857,0.933146609070246,0.350744276886362,NA
"16106","AC016773.1",0.773890170787938,1.70564326799017,1.65045854099242,1.03343599710452,0.301399907931699,NA
"16107","FGFR3",0.681854136945446,0.452294519246553,1.69842224367732,0.266302752999321,0.790006053046393,NA
"16108","NAT8L",0.108990023245465,1.41832010653068,3.35247519753738,0.42306654724024,0.672246708730081,NA
"16109","AL121750.1",0,NA,NA,NA,NA,NA
"16110","HTT-AS",0,NA,NA,NA,NA,NA
"16111","MSANTD1",0.375377899769176,-0.548801873903334,2.28777018398007,-0.239885053903699,0.810419367866906,NA

"16112","HGFAC",0,NA,NA,NA,NA,NA
"16113","DOK7",0,NA,NA,NA,NA,NA
"16114","ADRA2C",0.229312504611721,1.98374348132209,3.06703062452501,0.646796111346072,0.51776390945149,NA
"16115","NSG1",0.0604037079966622,0.119912045671111,3.35247519753738,0.0357682126206911,0.971467179494267,NA
"16116","CYTL1",0.479344696783994,0.571534704324282,1.989747854919,0.287239764029069,0.773928741258432,NA
"16117","CRMP1",0.563553402423714,-1.53024260612534,1.74853499878862,-0.875156978376465,0.381488498067226,NA
"16118","C4orf50",0,NA,NA,NA,NA,NA
"16119","JAKMIP1",0.36838024194174,-0.511720676674028,2.29466715960387,-0.223004314387088,0.823532148659855,NA
"16120","PPP2R2C",0.629608307110723,1.19066586223081,1.90167427180124,0.626114513871523,0.531239833050434,NA
"16121","LINC002481",0.418940988787332,1.28676752537837,2.25934778210115,0.569530523619388,0.5689961624001,NA
"16122","S100P",0.285708348519476,2.01137472954577,2.86030947584175,0.703201785168317,0.481930006892637,NA
"16123","AC097382.1",0,NA,NA,NA,NA,NA
"16124","AC097382.2",0,NA,NA,NA,NA,NA
"16125","AFAP1-AS1",0,NA,NA,NA,NA,NA
"16126","AC097381.1",0.0604037079966622,0.119912045671111,3.35247519753738,0.0357682126206911,0.971467179494267,NA
"16127","ABLIM2",0,NA,NA,NA,NA,NA
"16128","SH3TC1",0.814263217930915,0.262694452828896,2.02175287360537,0.129934007394502,0.896618638340039,NA
"16129","LINC02517",0.270586091692985,2.01155232872955,2.90724650940082,0.691909792384316,0.488993980674386,NA
"16130","GPR78",0.391061102385135,1.28701110851324,2.29244108252202,0.561415130066219,0.57451457208665,NA
"16131","CPZ",1.12804576554805,1.721816199136,1.31620017570274,1.30817198699788,0.190814981239496,NA
"16132","HMX1",0.334488628603365,1.28762870382305,2.38272719387433,0.540401228950328,0.588920360482733,NA
"16133","AC073648.7",0.346858810794384,2.01267811119372,3.26678991323492,0.616102707749784,0.537826735089075,NA
"16134","AC105916.1",0.16538586715322,1.41832010653068,3.35247519753738,0.42306654724024,0.672246708730081,NA
"16135","SLC2A9",0.445714387829436,-2.10907072936444,1.83814578720707,-1.14739034522883,0.251220329659127,NA
"16136","AC005674.2",1.0149959675994,0.581537023167797,1.32819797444201,0.437839113112718,0.661502915400127,NA
"16137","CLNK",0,NA,NA,NA,NA,NA
"16138","AC006230.1",0.4585768081272,0.631563903537034,2.587657350072,0.24406782587326,0.80717829035575,NA
"16139","AC025539.1",0.150263610326729,1.41832010653068,3.35247519753738,0.42306654724024,0.672246708730081,NA
"16140","AC005699.1",0.173429405397192,1.41832010653068,3.35247519753738,0.42306654724024,0.672246708730081,NA

"16141","AC007370.2",1.023628261699,2.90432962860406,1.70512543984131,1.70329382269632,0.0885130960464785,NA
"16142","AC007370.1",0,NA,NA,NA,NA,NA
"16143","NKX3-2",1.00790130481507,-0.153234668426557,1.43629745839663,-0.106687279526078,0.915037074473221,NA
"16144","LINC01096",0,NA,NA,NA,NA,NA
"16145","LINC00504",1.26367992598429,-0.688492287830633,1.36432996034169,-0.504637666725581,0.613813349012492,NA
"16146","CPEB2-DT",0.268677378460603,0.696980754736642,2.91582564400198,0.239033755728972,0.811079408015517,NA
"16147","AC098829.1",0.206583022614766,-0.859518418402433,3.32508269885954,-0.25849535071631,0.796024636623985,NA
"16148","AC116651.1",0.169393731242127,0.696980693484986,3.35247519753738,0.207900328091007,0.835306792989891,NA
"16149","TAPT1-AS1",0.942351874323481,0.565262406773781,1.37467946436998,0.411195788854564,0.680928975901182,NA
"16150","AC006427.2",0.627124840338745,1.16996738533566,1.90394127651513,0.614497621206629,0.538886544520772,NA
"16151","AC097515.1",0,NA,NA,NA,NA,NA
"16152","AC104656.1",0.0604037079966622,0.119912045671111,3.35247519753738,0.0357682126206911,0.971467179494267,NA
"16153","AC006160.1",1.06919212848279,0.0409213897255973,1.38570208990595,0.0295311597086317,0.976440967919306,NA
"16154","NCAPG",0.682751746590307,2.25371552620619,1.83057065291967,1.23115462525941,0.218265038341557,NA
"16155","AC024230.1",0.624168874799281,0.894210179864734,2.09439975061639,0.426952963302046,0.669413587823878,NA
"16156","SLIT2-IT1",0.817786147392754,0.777256537714557,1.76414805251232,0.440584641752525,0.659513723459168,NA
"16157","STIM2-AS1",1.42518849720576,2.24928612764966,1.40819202450811,1.59728651242386,0.110201855903629,NA
"16158","AC098595.1",0,NA,NA,NA,NA,NA
"16159","LINC02506",0.308531263183678,-0.370615225138078,2.39555983459126,-0.154709233218261,0.877050550585805,NA
"16160","AC104078.1",0,NA,NA,NA,NA,NA
"16161","DTHD1",0.305517368897029,-1.60012505214604,2.79208049228924,-0.573094169944251,0.566580934475511,NA
"16162","C4orf19",0.103291511307383,0.119912045671111,3.35247519753738,0.0357682126206912,0.971467179494267,NA
"16163","AC027607.1",0.233833113393854,0.696980736952966,3.04905264581788,0.228589276052335,0.819188160373076,NA
"16164","PTTG2",0,NA,NA,NA,NA,NA
"16165","LINC02278",0.289716212608383,1.26367466627299,2.47600349358036,0.510368692754016,0.60979318592903,NA
"16166","TLR10",0.124306244907042,-0.509577950443694,3.33980312193904,-0.152577242381833,0.878731672788745,NA

"16167","TMEM156",0.377861366541154,-0.555867632328218,2.0208560581144
7,-0.275065425910078,0.783265973483172,NA
"16168","AC079921.1",0.84645776569344,-1.33044000157554,1.503153671275
64,-0.885099126589282,0.376103170490737,NA
"16169","KLB",0.173429405397192,1.41832010653068,3.35247519753738,0.42
306654724024,0.672246708730081,NA
"16170","UGDH-
AS1",1.49186888325901,0.940680587745419,1.23008892890615,0.76472567603
8652,0.444434913521444,NA
"16171","AC021148.2",0.334973563230433,-0.356620301362942,2.3410823910
3228,-0.152331375746965,0.878925579416111,NA
"16172","AC108471.3",1.03806281791752,1.27780036994307,1.5711385036910
7,0.813295815067314,0.416048475863176,NA
"16173","AC095057.3",1.07424587298823,-0.214830719793577,1.22821111644
347,-0.174913511950341,0.861147592950397,NA
"16174","LINC02265",0.334388096435525,-1.66805575603244,2.717070896268
3,-0.613916905268611,0.539270238411727,NA
"16175","AC108050.1",0,NA,NA,NA,NA,NA
"16176","DCAF4L1",0.582409804054767,2.14874201457901,2.07596635246188,
1.03505628211692,0.300642627631436,NA
"16177","AC096734.1",0,NA,NA,NA,NA,NA
"16178","KCTD8",0.672742955364309,0.845024458814882,2.08632110095783,0
.405030873927762,0.685454816913829,NA
"16179","YIPF7",0,NA,NA,NA,NA,NA
"16180","AC096586.2",0.103291511307383,0.119912045671111,3.35247519753
738,0.0357682126206912,0.971467179494267,NA
"16181","GABRA2",0.06390253691038,0.119912045671111,3.35247519753738,0
.0357682126206912,0.971467179494267,NA
"16182","AC107398.3",0,NA,NA,NA,NA,NA
"16183","CORIN",0.403226844635981,0.642312752056105,2.30408337482781,0
.278771488511828,0.780420196250701,NA
"16184","AC107068.2",0,NA,NA,NA,NA,NA
"16185","AC107068.1",1.49728892684941,-0.795134124765443,1.18521932635
516,-0.670875092132252,0.502300105042183,NA
"16186","NIPAL1",0,NA,NA,NA,NA,NA
"16187","TXK",0.471339347947949,-0.860593687670558,2.19901301212838,-0
.391354522653601,0.695535202696949,NA
"16188","TEC",1.47267494680022,-0.578282163086318,1.17201607478906,-0.
493408047488082,0.62172429728909,NA
"16189","SLC10A4",0,NA,NA,NA,NA,NA
"16190","AC027271.1",0.575400460579738,1.22025914870432,1.930507530874
04,0.632092405333352,0.527326497572851,NA
"16191","LRRC66",0.06390253691038,0.119912045671111,3.35247519753738,0
.0357682126206912,0.971467179494267,NA
"16192","RASL11B",1.11079315781632,-1.74847561657376,1.38562439560686,
-1.26186838375344,0.206996150269043,NA
"16193","AC023154.1",0,NA,NA,NA,NA,NA
"16194","AC124017.1",0.590950691826335,0.640554652913891,1.72353704975
437,0.371651223282483,0.710152545797586,NA
"16195","AC110792.3",0.06390253691038,0.119912045671111,3.352475197537

38,0.0357682126206912,0.971467179494267,NA
"16196","AC110792.2",0,NA,NA,NA,NA,NA
"16197","AC110792.4",0.454775143557154,0.953366486241213,2.62047743100
901,0.363814042036654,0.715996876171179,NA
"16198","KDR",1.17704925637115,2.47418984987427,1.73630724587518,1.424
97236923477,0.154165188347338,NA
"16199","AC069200.1",0.587427762364497,-0.212314281990188,1.8334719275
0138,-0.115799036137698,0.907811814427887,NA
"16200","AC024243.1",0.167194048217763,-0.631571198727006,3.3343222198
9104,-0.189415166584483,0.849767438415713,NA
"16201","NMU",0,NA,NA,NA,NA,NA
"16202","EXOC1L",0,NA,NA,NA,NA,NA
"16203","AC110611.1",0.0604037079966622,0.119912045671111,3.3524751975
3738,0.0357682126206911,0.971467179494267,NA
"16204","AC110611.2",0,NA,NA,NA,NA,NA
"16205","KIAA1211",0,NA,NA,NA,NA,NA
"16206","AC068620.1",0.645863459294321,-1.64996882241802,1.75488247280
983,-0.940216138677463,0.347106705206997,NA
"16207","AC068620.2",0.0604037079966622,0.119912045671111,3.3524751975
3738,0.0357682126206911,0.971467179494267,NA
"16208","SPINK2",0.120807415993324,-0.48654549181451,3.34088239674268,
-0.145633827844071,0.884210461918222,NA
"16209","AC069307.1",0.379552014390329,1.28718260850839,2.316558207137
56,0.555644405801004,0.578453968578949,NA
"16210","IGFBP7-
AS1",0.308531263183678,-0.370615225138078,2.39555983459126,-0.15470923
3218261,0.877050550585805,NA
"16211","LINC02271",0,NA,NA,NA,NA,NA
"16212","ADGRL3",0.704801866632702,1.12333173925166,1.89744973531427,0
.592021869325357,0.553835944035897,NA
"16213","AC020741.1",0.206299238867152,0.696980719233408,3.17624445737
489,0.219435477522864,0.826310836331857,NA
"16214","EPHA5",0,NA,NA,NA,NA,NA
"16215","AC104806.2",0.289692112060262,0.0584007698147231,2.4588995720
1254,0.0237507747284382,0.981051405035345,NA
"16216","STAP1",0.169393731242127,0.696980693484986,3.35247519753738,0
.207900328091007,0.835306792989891,NA
"16217","TMPRSS11F",0,NA,NA,NA,NA,NA
"16218","SULT1B1",0.06390253691038,0.119912045671111,3.35247519753738,
0.0357682126206912,0.971467179494267,NA
"16219","AC009570.2",0,NA,NA,NA,NA,NA
"16220","JCHAIN",35.8213589855715,-2.46387037728726,0.976216852355852,
-2.5238965823437,NA,NA
"16221","AC009570.1",1.47791994793212,-0.95279096771022,1.109178666947
78,-0.859005853702624,0.390337286464649,NA
"16222","AC053527.2",0.188208781817422,-0.917509588944765,3.2454072234
4342,-0.28271015800947,0.777399028822487,NA
"16223","ALB",0,NA,NA,NA,NA,NA
"16224","AFP",0,NA,NA,NA,NA,NA
"16225","CXCL6",0.2292884040636,0.696980734286753,3.06852794721869,0.2

27138467133238,0.820316073199257,NA
"16226","PF4V1",0.120322481366256,1.41832010653068,3.35247519753738,0.42306654724024,0.672246708730081,NA
"16227","CXCL5",0.587427762364497,-0.212314281990188,1.83347192750138,-0.115799036137698,0.907811814427887,NA
"16228","AC093677.2",1.07489064042087,0.639216460175281,1.37823964951231,0.463791954034604,0.64279680846959,NA
"16229","EPGN",0.103291511307383,0.119912045671111,3.35247519753738,0.0357682126206912,0.971467179494267,NA
"16230","BTC",0,NA,NA,NA,NA,NA
"16231","LINC02562",0,NA,NA,NA,NA,NA
"16232","CDKL2",0.29319094097398,0.0421599161580697,2.88101411017646,0.0146337069329686,0.988324407884707,NA
"16233","AC110615.1",0.108990023245465,1.41832010653068,3.35247519753738,0.42306654724024,0.672246708730081,NA
"16234","PPEF2",0,NA,NA,NA,NA,NA
"16235","FAM47E",0.657393454539848,2.22237510665255,1.83824270560858,1.20896718364336,0.226675453170051,NA
"16236","CCDC158",0.163695219304045,-0.600668372859899,3.33567379881164,-0.18007407471135,0.857094415273326,NA
"16237","SHROOM3",0.103291511307383,0.119912045671111,3.35247519753738,0.0357682126206912,0.971467179494267,NA
"16238","AC107072.2",0.108990023245465,1.41832010653068,3.35247519753738,0.42306654724024,0.672246708730081,NA
"16239","CXCL13",0,NA,NA,NA,NA,NA
"16240","ANXA3",1.01734099390529,-0.0228266082396298,1.22810920399853,-0.0185867902995189,0.985170740831046,NA
"16241","LINC01094",0.630742919415473,-0.244263387911908,2.13789747405294,-0.114254023346051,0.909036427809868,NA
"16242","AC112253.1",0.161211752532067,-0.631571180295204,3.33432222068988,-0.189415161011203,0.849767442783486,NA
"16243","LINC00989",0.72544600552326,2.4217361056407,2.3811062652778,1.01706343011875,0.309123255711428,NA
"16244","PCAT4",0.16538586715322,1.41832010653068,3.35247519753738,0.42306654724024,0.672246708730081,NA
"16245","FGF5",0,NA,NA,NA,NA,NA
"16246","AC073840.1",0.21798004649093,1.96674997191025,3.35148506485532,0.586829400654111,0.557318288863578,NA
"16247","WDFY3-AS1",0.12780507382076,-0.532254130061651,3.33875455416813,-0.15941696864095,0.873340370825791,NA
"16248","AC108021.1",0.173429405397192,1.41832010653068,3.35247519753738,0.42306654724024,0.672246708730081,NA
"16249","SLC10A6",0.801092916140693,-2.05253602954721,1.67927097099502,-1.22227803910111,0.221602501104125,NA
"16250","HSD17B13",0.733849663371247,-0.962916517102734,1.92703200152648,-0.499688908300417,0.617294143687156,NA
"16251","ABCG2",0.79795302646708,3.40589884073438,1.91598599452025,1.7776219922668,0.0754659593193196,NA
"16252","PPM1K-DT",0,NA,NA,NA,NA,NA

"16253","PIGY-DT",0,NA,NA,NA,NA,NA
"16254","AC104785.1",0.0604037079966622,0.119912045671111,3.3524751975
3738,0.0357682126206911,0.971467179494267,NA
"16255","AC093866.1",0,NA,NA,NA,NA,NA
"16256","SNCA",0.418717358544081,-1.98658953121294,2.14373883043113,-0
.926693822499551,0.354085525831691,NA
"16257","MMRN1",0.06390253691038,0.119912045671111,3.35247519753738,0.
0357682126206912,0.971467179494267,NA
"16258","CCSER1",0,NA,NA,NA,NA,NA
"16259","HPGDS",1.04493173287563,-2.63774836026099,1.44790595822206,-1
.82176773655935,0.0684902351202604,NA
"16260","AC109925.2",0,NA,NA,NA,NA,NA
"16261","LINC02267",0.0604037079966622,0.119912045671111,3.35247519753
738,0.0357682126206911,0.971467179494267,NA
"16262","AC114811.2",0,NA,NA,NA,NA,NA
"16263","AC019131.2",0.720175468005284,0.655462709256299,1.68781498632
506,0.388349857399632,0.69775714589789,NA
"16264","ADH4",0.49615760145966,2.73730884111826,2.98685308631898,0.91
6452454141876,0.359429634140874,NA
"16265","ADH1A",0.555934224845129,2.03484245418561,2.07671494364988,0.
979837151173632,0.327166510341538,NA
"16266","MTTP",0.68989145863855,-0.894140882179205,1.63627348361757,-0
.54644953373099,0.584756968877074,NA
"16267","DAPP1",1.10632445684519,0.0218923672239635,1.70471366654769,0
.0128422547748438,0.989753644834169,NA
"16268","AC097460.1",0.46439285328065,-1.2258797863104,1.8914775548853
9,-0.648106969677827,0.516915766434374,NA
"16269","AF213884.3",0.774310539659633,-1.03627580283005,1.47189295685
286,-0.704042911548252,0.481406050557998,NA
"16270","AC018797.3",0.811924612154977,0.167977371452379,1.61736858688
697,0.103858435742031,0.917281692138385,NA
"16271","AC105460.1",0,NA,NA,NA,NA,NA
"16272","LINC02503",0.300527220653458,2.01289191439962,3.3507230115819
1,0.600733605088207,0.548017432599982,NA
"16273","CXXC4",0.0604037079966622,0.119912045671111,3.35247519753738,
0.0357682126206911,0.971467179494267,NA
"16274","CXXC4-
AS1",0.103291511307383,0.119912045671111,3.35247519753738,0.0357682126
206912,0.971467179494267,NA
"16275","AC004069.1",0.245113660900367,-1.28517145886886,2.97823191585
762,-0.431521619262071,0.666089136408018,NA
"16276","AC109361.1",0.233833113393854,0.696980736952966,3.04905264581
788,0.228589276052335,0.819188160373076,NA
"16277","AC092445.1",0.655736085853557,0.54928109046968,1.702001559823
83,0.322726549396661,0.746902343622434,NA
"16278","AC096564.1",0.473419780809303,1.79503962060784,2.205292797180
63,0.81396884028403,0.415662801784622,NA
"16279","AC096564.2",0.274375890398685,1.98403204198911,2.894609998656
26,0.685422921536976,0.493077083984588,NA
"16280","LEF1",0.640580549864182,0.400901325360293,1.60785131440967,0.

249339800121684,0.803097947220368,NA
"16281","LEF1-AS1",0,NA,NA,NA,NA,NA
"16282","RPL34-
AS1",0.12780507382076,-0.532254130061651,3.33875455416813,-0.159416968
64095,0.873340370825791,NA
"16283","AC107071.1",0.278520339611674,-1.43972823613246,2.87864192827
664,-0.500141480602412,0.616975460146789,NA
"16284","AC004067.1",1.2997999182617,1.7346321790159,1.37298515633253,
1.26340199019296,0.206444749282043,NA
"16285","AC126283.1",0,NA,NA,NA,NA,NA
"16286","RRH",0,NA,NA,NA,NA,NA
"16287","LRIT3",0.108990023245465,1.41832010653068,3.35247519753738,0.
42306654724024,0.672246708730081,NA
"16288","AC109347.2",0.909367639107387,2.00575328478296,1.958181019160
87,1.02429411027714,0.30569637899303,NA
"16289","AC017007.5",0.402689999394634,0.192098974823665,2.27974953657
425,0.0842631928384244,0.932847176148917,NA
"16290","AC093879.1",0.374030132326262,-0.206321295990841,2.3389876189
2708,-0.088209657170175,0.929710041927262,NA
"16291","AC104779.1",0.16538586715322,1.41832010653068,3.3524751975373
8,0.42306654724024,0.672246708730081,NA
"16292","UGT8",0.120322481366256,1.41832010653068,3.35247519753738,0.4
2306654724024,0.672246708730081,NA
"16293","AC092661.1",0.16538586715322,1.41832010653068,3.3524751975373
8,0.42306654724024,0.672246708730081,NA
"16294","METTL14-
DT",0.2292884040636,0.696980734286753,3.06852794721869,0.2271384671332
38,0.820316073199257,NA
"16295","MYOZ2",0.704483086774562,0.0417093651929482,1.46701946278464,
0.0284313645803834,0.977318108989298,NA
"16296","AC093752.3",0.161211752532067,-0.631571180295204,3.3343222206
8988,-0.189415161011203,0.849767442783486,NA
"16297","AC093752.2",0.173429405397192,1.41832010653068,3.352475197537
38,0.42306654724024,0.672246708730081,NA
"16298","TNIP3",0.161211752532067,-0.631571180295204,3.33432222068988,
-0.189415161011203,0.849767442783486,NA
"16299","QRFPR",0,NA,NA,NA,NA,NA
"16300","AC079341.1",0,NA,NA,NA,NA,NA
"16301","CCNA2",1.03890192076639,-0.0764076652970994,1.48959984072442,
-0.0512940879880471,0.95909117900705,NA
"16302","TRPC3",0,NA,NA,NA,NA,NA
"16303","IL2",0,NA,NA,NA,NA,NA
"16304","IL21",0,NA,NA,NA,NA,NA
"16305","IL21-AS1",0,NA,NA,NA,NA,NA
"16306","AC053545.1",0.06390253691038,0.119912045671111,3.352475197537
38,0.0357682126206912,0.971467179494267,NA
"16307","AC021205.3",1.18055127858226,0.219490326991243,1.173711382164
86,0.187005366333249,0.851656425445893,NA
"16308","LINC02516",0.346858810794384,2.01267811119372,3.2667899132349
2,0.616102707749784,0.537826735089075,NA

"16309","AC096773.1",0,NA,NA,NA,NA,NA
"16310","AC093772.2",0.46439285328065,-1.2258797863104,1.8914775548853
9,-0.648106969677827,0.516915766434374,NA
"16311","AC093591.2",0.286193283146544,0.0861184834588285,2.9015668335
7524,0.0296799930514492,0.97632226812405,NA
"16312","PLK4",1.08800338508661,3.07806588047848,1.88158092631118,1.63
589343271723,0.101861892697192,NA
"16313","AC110609.1",0.855804992418354,0.468613863057402,1.57948221208
626,0.296688281432706,0.766704504495406,NA
"16314","AC078850.1",0.364600170886985,-0.768165167619747,2.7308631793
993,-0.281290243105009,0.778487793202432,NA
"16315","PCDH10",1.4911215391181,0.984035239945727,1.14262086276148,0.
861208885655663,0.38912300705413,NA
"16316","AC015631.1",0,NA,NA,NA,NA,NA
"16317","LINC02510",0.120807415993324,-0.48654549181451,3.340882396742
68,-0.145633827844071,0.884210461918222,NA
"16318","LINC02172",0.225789575149882,0.696980732170056,3.083901691853
47,0.226006144752027,0.821196641151035,NA
"16319","AC116563.1",0.364429757563838,1.2894149286128,2.7123850644557
,0.475380485429547,0.634515801191117,NA
"16320","SLC7A11",0.289692112060262,0.0584007698147231,2.4588995720125
4,0.0237507747284382,0.981051405035345,NA
"16321","LINC00499",0,NA,NA,NA,NA,NA
"16322","AC109927.1",0,NA,NA,NA,NA,NA
"16323","MGARP",0.69170266295656,2.35318894548603,2.03585224854853,1.1
5587412945303,0.2477326459443,NA
"16324","AC112236.2",0.849686111608577,2.70529768385357,1.715509144786
92,1.57696488653203,0.114803604791766,NA
"16325","AC108053.1",0,NA,NA,NA,NA,NA
"16326","CLGN",0.06390253691038,0.119912045671111,3.35247519753738,0.0
357682126206912,0.971467179494267,NA
"16327","LINC02432",0.227597756214425,-1.13126253111685,2.575404219042
1,-0.439256301108964,0.660475834030736,NA
"16328","AC139720.1",0,NA,NA,NA,NA,NA
"16329","AC097658.3",0.47395662605065,2.66909678443823,2.5106815498775
,1.06309650643207,0.287738188608552,NA
"16330","SMARCA5-
AS1",0.39467427121682,1.28917448930526,2.66049112848182,0.484562596546
191,0.627986651291867,NA
"16331","GYPE",1.45054675907762,-1.6897762478414,1.1956435469177,-1.41
327760451479,0.157574116246826,NA
"16332","SMAD1-AS1",0,NA,NA,NA,NA,NA
"16333","REELD1",0.420465386104261,0.529345106475082,2.03806251319317,
0.259729573086412,0.795072379253792,NA
"16334","AC002460.2",0.360967444098768,2.39992452792507,3.252122552828
31,0.737956362018982,0.460540967313044,NA
"16335","IQCM",0,NA,NA,NA,NA,NA
"16336","AC110813.1",0.377667401706068,1.26423683394566,2.309206732520
38,0.547476679390162,0.584051287854355,NA
"16337","MAB21L2",0,NA,NA,NA,NA,NA

"16338","AC095055.1",1.43809016375716,-0.325832158994489,1.05591791035
83,-0.308577168545162,0.75764319068304,NA
"16339","PRSS48",0,NA,NA,NA,NA,NA
"16340","FAM160A1",0.150263610326729,1.41832010653068,3.35247519753738
,0.42306654724024,0.672246708730081,NA
"16341","AC092611.1",0.253555121634112,0.696980747544927,2.97042254430
159,0.23464026991l095,0.814487951095881,NA
"16342","LINC02273",0.533629523970879,2.89584459607367,2.0756640744252
1,1.39514126189981,0.16297324557014,NA
"16343","AC023424.3",0.0604037079966622,0.119912045671111,3.3524751975
3738,0.0357682126206911,0.971467179494267,NA
"16344","TIGD4",0.556452438635081,0.616803330889821,1.89013312313248,0
.326327983643611,0.744176218154683,NA
"16345","AC093599.2",0.402205064767566,1.64886010787136,2.281241465434
45,0.722790696581234,0.469808483939629,NA
"16346","MND1",0.969691784015097,1.96038709651388,1.63376411231715,1.1
9992052814374,0.230170206567653,NA
"16347","AC106865.2",0,NA,NA,NA,NA,NA
"16348","TLR2",1.34252383129293,-0.864351500767183,1.3863756187781,-0.
623461267682268,0.532981447245871,NA
"16349","RNF175",1.14147060893831,-0.293676211075253,1.31281796325107,
-0.223699110840921,0.822991437862737,NA
"16350","DCHS2",0.802230470811763,0.501186167818907,2.03128472805965,0
.246733587318238,0.805114411085233,NA
"16351","AC079298.1",0.0604037079966622,0.119912045671111,3.3524751975
3738,0.0357682126206911,0.971467179494267,NA
"16352","LRAT",0.800729201650673,3.48100447783256,1.88058287935664,1.8
5102423086157,0.0641660672512578,NA
"16353","RBM46",0,NA,NA,NA,NA,NA
"16354","AC104083.1",0.888836533828971,-1.23108496169744,1.43549497337
516,-0.85760311567159,0.391111654047148,NA
"16355","TDO2",0.787030809973271,0.3385318147398,1.56359708555122,0.21
6508343401303,0.828591522296246,NA
"16356","GLRB",0.589600586755929,-0.260424845717386,1.91072787180001,-
0.13629614638533,0.891587170196426,NA
"16357","AC084740.1",0,NA,NA,NA,NA,NA
"16358","RXFP1",0.16538586715322,1.41832010653068,3.35247519753738,0.4
2306654724024,0.672246708730081,NA
"16359","C4orf45",0,NA,NA,NA,NA,NA
"16360","MARCH1",1.07181978592726,0.322486471393666,1.23565172056219,0
.260984924819223,0.794104132742686,NA
"16361","AC093788.1",0.629608307110723,1.19066586223081,1.901674271801
24,0.626114513871523,0.531239833050434,NA
"16362","FAM218A",0.747087106337669,0.758015165331852,1.6270104890883,
0.465894455146755,0.641291050294206,NA
"16363","GK3P",0.173429405397192,1.41832010653068,3.35247519753738,0.4
2306654724024,0.672246708730081,NA
"16364","AC068989.1",0.357654423673828,1.28738258176336,2.345583563684
82,0.548853855260194,0.583105747826613,NA
"16365","AC080188.1",0.173429405397192,1.41832010653068,3.352475197537

38,0.42306654724024,0.672246708730081,NA
"16366","AC096741.1",0,NA,NA,NA,NA,NA
"16367","AC084724.1",0.233833113393854,0.696980736952966,3.04905264581
788,0.228589276052335,0.819188160373076,NA
"16368","MFAP3L",1.03619554019433,0.314310657891943,1.38717576896221,0
.226583151843179,0.820747894464328,NA
"16369","GALNTL6",0,NA,NA,NA,NA,NA
"16370","AC105285.1",0.741751147394411,1.64519852134625,1.690237407015
36,0.97335351502565,0.330377633697155,NA
"16371","AC093849.4",0.644616224019247,1.02839689554847,1.769197760522
96,0.581278655498909,0.56105266371425,NA
"16372","AC093849.3",0.0973092156216871,0.119912045671111,3.3524751975
3738,0.0357682126206911,0.971467179494267,NA
"16373","LINC02269",0.184225018276636,0.696980701717498,3.297154064105
66,0.21138857577362,0.832584070546688,NA
"16374","AC106895.1",0,NA,NA,NA,NA,NA
"16375","LINC02268",0.120807415993324,-0.48654549181451,3.340882396742
68,-0.145633827844071,0.884210461918222,NA
"16376","AC096751.2",0.342823136639319,1.26402829119266,2.366751974340
89,0.534077209989303,0.593288116543864,NA
"16377","GLRA3",0.307976533945078,-0.019760906174343,2.42007072380183,
-0.00816542507621408,0.993485005796345,NA
"16378","GPM6A",0.06390253691038,0.119912045671111,3.35247519753738,0.
0357682126206912,0.971467179494267,NA
"16379","SPATA4",0,NA,NA,NA,NA,NA
"16380","AC093801.1",0.12780507382076,-0.532254130061651,3.33875455416
813,-0.15941696864095,0.873340370825791,NA
"16381","AC097518.2",1.25761889122998,2.47555285965173,1.4296465234633
9,1.73158386987476,0.0833476801884984,NA
"16382","NEIL3",0.293751886763448,2.01128355462242,2.83703898223116,0.
70893758147823,0.478363212179869,NA
"16383","AC078881.1",0.108990023245465,1.41832010653068,3.352475197537
38,0.42306654724024,0.672246708730081,NA
"16384","TENM3-
AS1",0.77515160955485,0.0370684340286619,1.63176700887136,0.0227167443
802538,0.981876219198311,NA
"16385","AC079766.1",0.120807415993324,-0.48654549181451,3.34088239674
268,-0.145633827844071,0.884210461918222,NA
"16386","AC019193.2",0,NA,NA,NA,NA,NA
"16387","CLDN22",0,NA,NA,NA,NA,NA
"16388","AC107214.2",0,NA,NA,NA,NA,NA
"16389","AC074194.1",0,NA,NA,NA,NA,NA
"16390","ENPP6",0.663351649677423,1.23926496579513,2.13050525397566,0.
58167655934318,0.560784563480315,NA
"16391","LINC02363",0.16538586715322,1.41832010653068,3.35247519753738
,0.42306654724024,0.672246708730081,NA
"16392","LINC02362",0.556989283876428,1.16789989159151,1.7885599638596
8,0.652983358227032,0.513767007250983,NA
"16393","AC099343.2",0.620096444955001,-0.481428711736102,1.8384255880
6311,-0.261870110415138,0.793421584111249,NA

"16394","AC099343.4",0.497965782524203,0.546927088678929,2.20549455359
785,0.247983876354046,0.804146882038116,NA
"16395","LINC02427",1.14909785840977,0.424780282887439,1.3353605534939
2,0.318101565735201,0.750407895988269,NA
"16396","LINC02365",0,NA,NA,NA,NA,NA
"16397","AC084871.1",0.16538586715322,1.41832010653068,3.3524751975373
8,0.42306654724024,0.672246708730081,NA
"16398","MIR3945HG",1.46168525236332,-1.0890305493349,1.16998069647586
,-0.930810698514268,0.351951494581972,NA
"16399","LINC01093",0,NA,NA,NA,NA,NA
"16400","AC093797.1",0.173429405397192,1.41832010653068,3.352475197537
38,0.42306654724024,0.672246708730081,NA
"16401","KLKB1",0.876388094854825,0.920569614861249,1.51099145491132,0
.609248723326019,0.542359589298494,NA
"16402","AC107050.1",0,NA,NA,NA,NA,NA
"16403","AC108865.1",0,NA,NA,NA,NA,NA
"16404","AC108865.2",0.73752150840426,2.51359263155185,1.6495908387170
5,1.52376733221116,0.1275668428015,NA
"16405","AC097652.1",0,NA,NA,NA,NA,NA
"16406","FRG1-
DT",0.485459497494588,0.191298648061405,2.21302621356049,0.08644210669
04709,0.931114975878197,NA
"16407","AC021087.1",0.223613992673639,0.696980730824599,3.09363414372
9,0.225295137835683,0.821749681896065,NA
"16408","AHRR",0.0604037079966622,0.119912045671111,3.35247519753738,0
.0357682126206911,0.971467179494267,NA
"16409","SLC9A3",0.223613992673639,0.696980730824599,3.093634143729,0.
225295137835683,0.821749681896065,NA
"16410","CEP72",1.28005574926876,0.134590089550075,1.28977127829794,0.
104351904725067,0.916890088696466,NA
"16411","AC026740.1",0.16538586715322,1.41832010653068,3.3524751975373
8,0.42306654724024,0.672246708730081,NA
"16412","TRIP13",0.681210339006308,-1.07356468651796,1.78907508663477,
-0.600066869489156,0.548461671276243,NA
"16413","AC122719.3",0,NA,NA,NA,NA,NA
"16414","AC116351.1",0.78952967191088,1.42055211193397,1.8259599864578
7,0.777975488219574,0.436583463728727,NA
"16415","AC116351.2",1.13138798888875,1.46016852573332,1.5122239404744
6,0.965576914008643,0.334255931901111,NA
"16416","SLC6A3",0,NA,NA,NA,NA,NA
"16417","AC026412.3",0.163695219304045,-0.600668372859899,3.3356737988
1164,-0.18007407471135,0.857094415273326,NA
"16418","AC126768.1",0,NA,NA,NA,NA,NA
"16419","AC091891.1",0,NA,NA,NA,NA,NA
"16420","AC094105.2",0.120322481366256,1.41832010653068,3.352475197537
38,0.42306654724024,0.672246708730081,NA
"16421","AC022424.1",0.791151158638139,-2.12491929219212,1.45413343617
018,-1.46129594391875,0.143934242511177,NA
"16422","AC091978.1",0.941763118467855,-0.871658311694898,1.3654750997
7403,-0.638355332762309,0.523242400345994,NA

"16423","LINC02145",0.169393731242127,0.696980693484986,3.352475197537
38,0.207900328091007,0.835306792989891,NA
"16424","UBE2QL1",1.39622393913905,0.477207220696907,1.14699412313819,
0.416050275298064,0.677373214761397,NA
"16425","LINC01018",0,NA,NA,NA,NA,NA
"16426","LINC02102",0.206299238867152,0.696980719233408,3.176244457374
89,0.219435477522864,0.826310836331857,NA
"16427","AC091965.4",0,NA,NA,NA,NA,NA
"16428","AC091932.1",0,NA,NA,NA,NA,NA
"16429","AC034229.1",0,NA,NA,NA,NA,NA
"16430","AC034229.4",0,NA,NA,NA,NA,NA
"16431","AC012640.1",0.258520968153754,-1.2931292497918,2.966696143772
36,-0.435881933006964,0.662922394077564,NA
"16432","AC012640.4",0.270485559525146,-1.34694009274034,2.93176047727
036,-0.459430469570426,0.645925071915764,NA
"16433","ROPN1L",1.03113808617085,0.457524011435976,1.35871919918277,0
.336731836652609,0.736319061348026,NA
"16434","LINC01513",0.317457658544492,-0.0460044947122103,2.4059816517
4391,-0.019120883436025,0.984744671901947,NA
"16435","DNAH5",1.45008612617448,-1.78058869423887,1.31175193303397,-1
.35741267033663,0.174650124568871,NA
"16436","AC016549.1",0,NA,NA,NA,NA,NA
"16437","AC010491.1",0.349420534349489,-1.77550867513901,2.25175243349
119,-0.788500835496469,0.430403810064774,NA
"16438","AC010491.2",0.233833113393854,0.696980736952966,3.04905264581
788,0.228589276052335,0.819188160373076,NA
"16439","AC016575.1",1.08498044941359,-0.916357007616185,1.30410302246
796,-0.702672251983605,0.482260023919693,NA
"16440","LINC02149",0,NA,NA,NA,NA,NA
"16441","AC114964.2",0.394698371764941,2.47726606480199,2.246784016635
38,1.1025830905241,0.270208256786214,NA
"16442","AC010638.1",0.16538586715322,1.41832010653068,3.3524751975373
8,0.42306654724024,0.672246708730081,NA
"16443","AC024588.1",0,NA,NA,NA,NA,NA
"16444","AC022113.1",0,NA,NA,NA,NA,NA
"16445","MYO10",1.41074664031083,0.838879800353832,1.19715083085822,0.
700730249464434,0.483471374491296,NA
"16446","BASP1-
AS1",0.773915378057947,-2.00139213769084,1.9966292280896,-1.0023854752
471,0.316157454446392,NA
"16447","AC026790.1",0,NA,NA,NA,NA,NA
"16448","AC026785.2",0.344882041570103,-0.2488455020926,2.781197344065
66,-0.0894742340465592,0.928705029691067,NA
"16449","LINC02217",0,NA,NA,NA,NA,NA
"16450","H3.Y",0,NA,NA,NA,NA,NA
"16451","LINC02223",0.938927688438171,1.16297882195865,1.5466788584221
1,0.751920035388021,0.452099148626635,NA
"16452","CDH18",1.46377393942464,1.68239030525249,1.84112191493556,0.9
13785389009057,0.360829625333359,NA
"16453","LINC02241",0.375692970109279,0.533119432585828,2.346714748450

35,0.227176921667992,0.82028617240732,NA
"16454","AC091946.2",0.16538586715322,1.41832010653068,3.3524751975373
8,0.42306654724024,0.672246708730081,NA
"16455","CDH12",1.1633606796969,-3.46775060237718,1.6637893590383,-2.0
8424857602263,0.0371375622818468,NA
"16456","AC091938.1",0.607514148762987,-2.57688024958168,1.93606879266
294,-1.33098589231292,0.183193652987375,NA
"16457","C5orf17",0.72979665425511,-0.476119872374077,1.77990709074364
,-0.267497036699345,0.789086495445621,NA
"16458","CDH10",0,NA,NA,NA,NA,NA
"16459","LINC02211",0.559140345347232,1.88105884735457,2.1335706306212
9,0.881648266224403,0.377967045344915,NA
"16460","AC113370.1",0.54862149667759,1.05379504136515,1.934056834902,
0.544862499564833,0.585848089540312,NA
"16461","CDH6",0.520288216191576,2.79382521304909,2.95368862825364,0.9
45876686636712,0.344211485698358,NA
"16462","AC010343.3",0.237331942307572,0.696980738944435,3.03442443415
691,0.229691249219751,0.818331695238684,NA
"16463","AC034223.1",0.173429405397192,1.41832010653068,3.352475197537
38,0.42306654724024,0.672246708730081,NA
"16464","AC034231.1",0.2292884040636,0.696980734286753,3.0685279472186
9,0.227138467133238,0.820316073199257,NA
"16465","SLC45A2",0.12780507382076,-0.532254130061651,3.33875455416813
,-0.15941696864095,0.873340370825791,NA
"16466","AC139792.1",0.0604037079966622,0.119912045671111,3.3524751975
3738,0.0357682126206911,0.971467179494267,NA
"16467","AC026801.2",0.985442768309097,-0.926457383860793,1.4644538917
5614,-0.632629944224331,0.526975327928081,NA
"16468","PRLR",0.289692112060262,0.0584007698147231,2.45889957201254,0
.0237507747284382,0.981051405035345,NA
"16469","UGT3A2",0.270586091692985,2.01155232872955,2.90724650940082,0
.691909792384316,0.488993980674386,NA
"16470","AC008957.2",0,NA,NA,NA,NA,NA
"16471","AC008957.1",0.210667318323391,0.69698072231542,3.154490207158
25,0.220948767168088,0.825132324921599,NA
"16472","AC026741.1",0.417521286429143,0.169256933264983,2.00882650450
746,0.0842566209103675,0.93285240120763,NA
"16473","AC025449.2",1.25965700354208,0.812305443105684,1.302360342271
02,0.623717888775091,0.532812872825768,NA
"16474","GDNF",0.309874533922148,-1.53623631476011,3.27958300176136,-0
.468424282579536,0.639481206404182,NA
"16475","AC010457.1",1.00979114844583,-0.691115612967617,1.41136461165
204,-0.489679000920001,0.624361063586577,NA
"16476","AC008964.1",0,NA,NA,NA,NA,NA
"16477","C9",0,NA,NA,NA,NA,NA
"16478","AC108105.1",0,NA,NA,NA,NA,NA
"16479","AC114977.1",0,NA,NA,NA,NA,NA
"16480","OXCT1-
AS1",0.762978081538842,-1.04863519873753,1.47454728818649,-0.711157388
534634,0.476986710713829,NA

"16481","AC008945.1",0,NA,NA,NA,NA,NA
"16482","AC008945.2",0,NA,NA,NA,NA,NA
"16483","AC008875.2",1.00094118470473,0.22199498409167,1.4904845265807
5,0.148941488578173,0.881599803160879,NA
"16484","AC008875.1",0.225789575149882,0.696980732170056,3.08390169185
347,0.226006144752027,0.821196641151035,NA
"16485","AC025171.3",0.882531844530416,1.09332425967124,1.551238926668
96,0.704807132463456,0.480930269287588,NA
"16486","AC025171.5",0,NA,NA,NA,NA,NA
"16487","AC025171.4",1.03999349524634,0.022475537809937,1.292960588515
59,0.0173830030161558,0.986131068736242,NA
"16488","NIM1K",1.31289230357583,-0.234341654333983,1.16381968731979,-
0.201355636863008,0.840420501379395,NA
"16489","AC114947.2",0.06390253691038,0.119912045671111,3.352475197537
38,0.0357682126206912,0.971467179494267,NA
"16490","AC114956.3",0.435871426678366,-0.744192748745376,2.2262461306
8578,-0.33428143388446,0.738167199042397,NA
"16491","AC114956.1",0.06390253691038,0.119912045671111,3.352475197537
38,0.0357682126206912,0.971467179494267,NA
"16492","LINC02224",0.06390253691038,0.119912045671111,3.3524751975373
8,0.0357682126206912,0.971467179494267,NA
"16493","HCN1",0,NA,NA,NA,NA,NA
"16494","AC010478.1",0,NA,NA,NA,NA,NA
"16495","ISL1",0.2292884040636,0.696980734286753,3.06852794721869,0.22
7138467133238,0.820316073199257,NA
"16496","AC022126.1",0.309590750174535,-0.21960280990608,2.45825916411
348,-0.0893326517854253,0.928817545423718,NA
"16497","AC008966.2",0.2292884040636,0.696980734286753,3.0685279472186
9,0.227138467133238,0.820316073199257,NA
"16498","AC027329.1",0.541362621010113,-0.137835767784526,1.9257822262
0038,-0.0715739121014113,0.942941001941214,NA
"16499","AC025175.1",0,NA,NA,NA,NA,NA
"16500","AC025175.2",0.426390302078952,-0.690667740944349,1.9827741568
325,-0.348334044280513,0.72758932925112,NA
"16501","AC016583.2",0,NA,NA,NA,NA,NA
"16502","ESM1",0.303608454488839,-0.25693568090626,2.89056459085803,-0
.0888877147803126,0.929171147087986,NA
"16503","AC034238.1",0,NA,NA,NA,NA,NA
"16504","CDC20B",0,NA,NA,NA,NA,NA
"16505","CCNO",0.43228277885212,-0.901439642708117,1.92008933655091,-0
.469477969357087,0.638728028438615,NA
"16506","IL31RA",0.515367347292177,0.0450275103008753,1.77484670564396
,0.0253698024498055,0.979759997506232,NA
"16507","AC008914.1",1.40749644493128,1.26764781334931,1.2514271709330
8,1.0129617150666,0.31107844810367,NA
"16508","AC008892.1",0.06390253691038,0.119912045671111,3.352475197537
38,0.0357682126206912,0.971467179494267,NA
"16509","ANKRD55",0,NA,NA,NA,NA,NA
"16510","LINC01948",1.14312118941229,2.24543542478055,1.61533115275265
,1.39007745932105,0.164505357200143,NA

"16511","AC022431.1",0.157712923618349,-0.60066841133552,3.33567379711352,-0.180074086337609,0.857094406146103,NA
"16512","AC008937.3",0.432088814017034,0.551948044127556,2.02352372132837,0.272765788861236,0.785033259187494,NA
"16513","AC008780.1",0,NA,NA,NA,NA,NA
"16514","GAPT",0.556256618457988,-1.30590000738442,1.79607261685312,-0.727086419073899,0.46717301775983,NA
"16515","RAB3C",0.0604037079966622,0.119912045671111,3.35247519753738,0.0357682126206911,0.971467179494267,NA
"16516","AC092343.1",0.06390253691038,0.119912045671111,3.35247519753738,0.0357682126206912,0.971467179494267,NA
"16517","PART1",0,NA,NA,NA,NA,NA
"16518","DEPDC1B",0.258520968153754,-1.2931292497918,2.96669614377236,-0.435881933006964,0.662922394077564,NA
"16519","ELOVL7",0.317457658544492,-0.0460044947122103,2.40598165174391,-0.019120883436025,0.984744671901947,NA
"16520","AC104113.1",0.762860548323474,1.58221666497184,1.84230968658984,0.858822312279408,0.390438555665661,NA
"16521","C5orf64",0,NA,NA,NA,NA,NA
"16522","LRRC70",0.157712923618349,-0.60066841133552,3.33567379711352,-0.180074086337609,0.857094406146103,NA
"16523","RGS7BP",0.615023444561761,2.14154412200814,1.84794136409035,1.15888099245092,0.24650469766885,NA
"16524","AC092354.2",0.161211752532067,-0.631571180295204,3.33432222068988,-0.189415161011203,0.849767442783486,NA
"16525","AC092354.1",0.549122375506471,1.02373115955517,1.92643411613407,0.531412494713068,0.595132964951686,NA
"16526","AC025176.1",0,NA,NA,NA,NA,NA
"16527","AC010359.2",0.301234479217952,0.0419815600776809,2.85689911516166,0.0146947996360401,0.988275668200936,NA
"16528","AC025442.2",0.108990023245465,1.41832010653068,3.35247519753738,0.42306654724024,0.672246708730081,NA
"16529","AC092353.2",0.0604037079966622,0.119912045671111,3.35247519753738,0.0357682126206911,0.971467179494267,NA
"16530","MAST4-IT1",0,NA,NA,NA,NA,NA
"16531","MAST4-AS1",0.338278427309065,1.26399624841041,2.376014849266,0.531981628313846,0.594738719787115,NA
"16532","CD180",0.329081086457265,-0.0194879299683752,2.3790710924939,-0.00819140295128663,0.99346427914408,NA
"16533","AC112206.2",1.41911071799163,0.725514569513251,1.30463131476429,0.556106971603952,0.578137729478639,NA
"16534","LINC02242",0.0604037079966622,0.119912045671111,3.35247519753738,0.0357682126206911,0.971467179494267,NA
"16535","AC010280.2",0.0604037079966622,0.119912045671111,3.35247519753738,0.0357682126206911,0.971467179494267,NA
"16536","AC093523.1",0.12780507382076,-0.532254130061651,3.33875455416813,-0.15941696864095,0.873340370825791,NA
"16537","LINC02198",0.289716212608383,1.26367466627299,2.47600349358036,0.510368692754016,0.60979318592903,NA

"16538","AC010273.3",1.49595938021607,0.374440785595744,1.24679113128863,0.300323587647546,0.763930342834416,NA
"16539","AC010273.2",1.08460522556912,1.01096612356879,1.53583730007393,0.658250794872691,0.51037699019756,NA
"16540","MARVELD2",0.181211123989987,-0.880500955048307,3.32429392960711,-0.264868562676217,0.791110716975408,NA
"16541","SERF1B",0.616850677535017,1.1619113583576,1.76288179575791,0.659097712140175,0.509833024734898,NA
"16542","LINC02197",0.0604037079966622,0.119912045671111,3.35247519753738,0.0357682126206911,0.971467179494267,NA
"16543","AC025774.1",0.421334636646782,0.674478329035459,2.63942954319792,0.255539433046682,0.798306491120967,NA
"16544","ZNF366",0,NA,NA,NA,NA,NA
"16545","AC035140.1",0,NA,NA,NA,NA,NA
"16546","AC008972.1",0.16538586715322,1.41832010653068,3.35247519753738,0.42306654724024,0.672246708730081,NA
"16547","AC008972.2",0.120807415993324,-0.48654549181451,3.34088239674268,-0.145633827844071,0.884210461918222,NA
"16548","TMEM171",0,NA,NA,NA,NA,NA
"16549","AC099522.2",1.44344032619226,-0.310275058645831,1.07041110808545,-0.289865320251386,0.771919273546501,NA
"16550","FAM169A",0.225789575149882,0.696980732170056,3.08390169185347,0.226006144752027,0.821196641151035,NA
"16551","AC010501.2",0,NA,NA,NA,NA,NA
"16552","GCNT4",1.1687753213436,0.702057495630542,1.42106078421472,0.494037625574547,0.621279607290858,NA
"16553","ANKRD31",1.26764413156855,0.000895838914859662,1.18989189773753,0.000752874203583551,0.999399293353483,NA
"16554","AC008897.3",0,NA,NA,NA,NA,NA
"16555","AC010245.1",0.291927646865061,-1.45883157994448,3.28423784711834,-0.444191817966075,0.656903907272504,NA
"16556","AC010245.2",1.28215877904866,-0.406223932164459,1.30160658844479,-0.312094250114263,0.754968896198753,NA
"16557","ANKDD1B",0.657227204007602,-0.030217581817872,1.65784437627714,-0.0182270315900994,0.985457738125836,NA
"16558","SV2C",0,NA,NA,NA,NA,NA
"16559","AC026725.1",0,NA,NA,NA,NA,NA
"16560","F2RL2",0.880117709877564,-2.19537013220428,1.65278790831528,-1.32828303084699,0.184084623447579,NA
"16561","F2RL1",0.6386718366318,-0.573787764301094,1.69239496388739,-0.339038922086555,0.7345804091798,NA
"16562","S100Z",0.284502635297369,-1.46425231901376,2.86278195697283,-0.511478813622987,0.609015819955434,NA
"16563","CRHBP",0.248612489814084,-1.30424658337368,2.93265112742767,-0.444732948687857,0.656512755319132,NA
"16564","ZBED3-AS1",1.26934923885867,-1.28288708428646,1.27117835174994,-1.0092109282072,0.31287348671584,NA
"16565","BHMT",0.712180130857553,2.42450583932661,1.7647358709665,1.37386329547366,0.169484126457801,NA

"16566","AC012636.1",0.225789575149882,0.696980732170056,3.08390169185
347,0.226006144752027,0.821196641151035,NA
"16567","AC010260.1",0,NA,NA,NA,NA,NA
"16568","FAM151B",1.49425042501354,0.857229150264121,1.07385400889261,
0.798273455390945,0.424711818611175,NA
"16569","ANKRD34B",0,NA,NA,NA,NA,NA
"16570","RASGRF2-AS1",0,NA,NA,NA,NA,NA
"16571","RASGRF2",0.157712923618349,-0.60066841133552,3.33567379711352
,-0.180074086337609,0.857094406146103,NA
"16572","CKMT2",0.558740085229966,-1.30860931231682,1.64750393983474,-
0.794298138338948,0.427021887573003,NA
"16573","AC010623.1",0.745656141687986,-1.89664544570814,1.54769398193
368,-1.22546541360746,0.2203999137117,NA
"16574","AC026726.1",0.163695219304045,-0.600668372859899,3.3356737988
1164,-0.18007407471135,0.857094415273326,NA
"16575","LINC01338",0,NA,NA,NA,NA,NA
"16576","AC104118.1",0.420408006393256,-0.681411085097132,2.2380593467
1369,-0.304465154642882,0.760773535948184,NA
"16577","VCAN-
AS1",0.417521286429143,0.169256933264983,2.00882650450746,0.0842566209
103675,0.93285240120763,NA
"16578","LINC01949",0.150263610326729,1.41832010653068,3.3524751975373
8,0.42306654724024,0.672246708730081,NA
"16579","AC018754.1",0.327106654860476,-0.510402172210371,2.3699393182
4898,-0.215365080565641,0.829482692873131,NA
"16580","AC114971.1",0.669129786532624,0.595608874864662,1.69919867136
216,0.350523387819738,0.725945941187383,NA
"16581","LINC02060",0,NA,NA,NA,NA,NA
"16582","MEF2C-
AS1",1.03574851386551,0.150793158281,1.41865570301063,0.10629299128815
5,0.915349892230748,NA
"16583","AC093510.2",0,NA,NA,NA,NA,NA
"16584","AC093510.1",0.212281534552848,0.696980723426082,3.14661376999
226,0.221501834789148,0.82470170812449,NA
"16585","ADGRV1",0.150263610326729,1.41832010653068,3.35247519753738,0
.42306654724024,0.672246708730081,NA
"16586","AC123595.1",0.351904001121467,-1.78185622650998,2.21049788227
116,-0.806088185291192,0.420192003413668,NA
"16587","AC123595.2",0.413537522888357,1.67494477837355,2.267223716791
15,0.738764668862999,0.460049909309912,NA
"16588","ARRDC3-
AS1",0.612908436047199,-2.55797597990941,1.63620685880398,-1.563357326
21193,0.117968571533295,NA
"16589","AC106818.2",0,NA,NA,NA,NA,NA
"16590","AC108102.1",0.173429405397192,1.41832010653068,3.352475197537
38,0.42306654724024,0.672246708730081,NA
"16591","AC099501.1",0,NA,NA,NA,NA,NA
"16592","MCTP1",0.509616924739816,-2.31028742590548,1.73775062702291,-
1.32947005742903,0.183692938016381,NA
"16593","SPATA9",1.02644587239178,2.29741299111392,1.73935298544761,1.

32084344600283,0.186553569963133,NA
"16594","AC008840.1",0.323693015723921,2.01097763960685,2.762731332372
13,0.727894752574506,0.466678015622723,NA
"16595","LINC01554",1.36002699638434,1.46101692623244,1.247505442535,1
.17115074324932,0.241538190297646,NA
"16596","AC008592.5",0.315649477479949,2.01105601173365,2.781238966705
07,0.723079187300523,0.469631235192475,NA
"16597","AC020900.1",0.0604037079966622,0.119912045671111,3.3524751975
3738,0.0357682126206911,0.971467179494267,NA
"16598","AC008906.1",1.47375761472187,0.607124517973091,1.262954989060
13,0.48071746280119,0.630717318183954,NA
"16599","AC008906.2",0.317457658544492,-0.0460044947122103,2.405981651
74391,-0.019120883436025,0.984744671901947,NA
"16600","AC009126.1",1.17162841252274,0.730101972135941,1.336845472987
56,0.546137894684509,0.584971152960719,NA
"16601","LIX1-AS1",0,NA,NA,NA,NA,NA
"16602","LIX1",0,NA,NA,NA,NA,NA
"16603","AC008883.3",0.707750243730752,0.349680432030999,1.62820419341
916,0.214764483130759,0.829950945095505,NA
"16604","LINC01340",0,NA,NA,NA,NA,NA
"16605","LINC02234",0.2292884040636,0.696980734286753,3.06852794721869
,0.227138467133238,0.820316073199257,NA
"16606","AC008522.1",1.33009071584075,1.36093970211679,1.3899849109720
6,0.979103939455744,0.327528625351903,NA
"16607","LINC02062",1.03017463464339,1.99256638031694,1.56206207659307
,1.27559999706466,0.202096955151689,NA
"16608","AC113385.1",0,NA,NA,NA,NA,NA
"16609","AC021086.1",0.2292884040636,0.696980734286753,3.0685279472186
9,0.227138467133238,0.820316073199257,NA
"16610","SLCO4C1",1.4409373224228,0.325272692972938,1.28587913220567,0
.252957439642868,0.80030110201669,NA
"16611","SLCO6A1",0.223613992673639,0.696980730824599,3.093634143729,0
.225295137835683,0.821749681896065,NA
"16612","AC024587.2",0,NA,NA,NA,NA,NA
"16613","AC012603.1",0.06390253691038,0.119912045671111,3.352475197537
38,0.0357682126206912,0.971467179494267,NA
"16614","TMEM232",1.34406986801229,-0.122794061991664,1.22890970045561
,-0.0999211430637576,0.920406930616175,NA
"16615","AC104126.1",0,NA,NA,NA,NA,NA
"16616","AC079465.1",0,NA,NA,NA,NA,NA
"16617","TRIM36",0.270485559525146,-1.34694009274034,2.93176047727036,
-0.459430469570426,0.645925071915764,NA
"16618","AC008494.3",1.13715990919308,0.0952447431218509,1.26028047968
287,0.0755742429223514,0.939757829015642,NA
"16619","LINCADL",0.35415559476011,1.2874229447024,2.35156498325213,0.
547474959812482,0.584052468926338,NA
"16620","ARL14EPL",0.173429405397192,1.41832010653068,3.35247519753738
,0.42306654724024,0.672246708730081,NA
"16621","SEMA6A-
AS1",0.590085521382997,-1.41156570157857,1.79287271978221,-0.787320642

454774,0.431094188744183,NA
"16622","SEMA6A-
AS2",0.695125060250559,0.421071627357254,1.69547068621607,0.2483508743
50412,0.803862939607777,NA
"16623","LINC02147",0.06390253691038,0.119912045671111,3.3524751975373
8,0.0357682126206912,0.971467179494267,NA
"16624","AC008629.1",0.0973092156216871,0.119912045671111,3.3524751975
3738,0.0357682126206911,0.971467179494267,NA
"16625","AC114284.1",1.32902626580098,-0.130183640889217,1.17846479745
678,-0.110468841470839,0.912037559505685,NA
"16626","AC010255.1",0.27107102632005З,0.0867249233658518,2.9512515673
7628,0.0293858118787725,0.976556888399647,NA
"16627","ZNF474",0.26450326383945,-1.3088493347846,2.4903960717771,-0.
525558705146299,0.599194854796744,NA
"16628","AC010255.2",0.167194048217763,-0.631571198727006,3.3343222198
9104,-0.189415166584483,0.849767438415713,NA
"16629","AC113349.2",0.75596039941041,-0.439462326918956,1.55167608803
888,-0.283217825103163,0.777009865062213,NA
"16630","LINC02201",0,NA,NA,NA,NA,NA
"16631","AC106786.1",0.568496034243742,-0.242539431395719,1.9207064721
1677,-0.126276156672925,0.899513329379965,NA
"16632","LINC01170",0.206583022614766,-0.859518418402433,3.32508269885
954,-0.25849535071631,0.796024636623985,NA
"16633","AC093535.1",0,NA,NA,NA,NA,NA
"16634","AC011416.3",0.749489092850521,-0.809206318415561,1.7485846659
7659,-0.462777887831709,0.643523582515351,NA
"16635","MEGF10",0.214166147237109,0.696980724704133,3.13752578908925,
0.222143424964947,0.824202234028988,NA
"16636","FBN2",0,NA,NA,NA,NA,NA
"16637","MEIKIN",0,NA,NA,NA,NA,NA
"16638","ACSL6",0.38702542823007,0.551208964081818,2.07138664475715,0.
266106265325681,0.7901573698877,NA
"16639","CSF2",0.924993567424831,-2.36640393125552,1.65451438736037,-1
.43027099028792,0.152639257463404,NA
"16640","AC116366.2",0,NA,NA,NA,NA,NA
"16641","IL13",0,NA,NA,NA,NA,NA
"16642","IL4",0.184225018276636,0.696980701717498,3.29715406410566,0.2
1138857577362,0.832584070546688,NA
"16643","CCNI2",0.223613992673639,0.696980730824599,3.093634143729,0.2
25295137835683,0.821749681896065,NA
"16644","GDF9",0.237331942307572,0.696980738944435,3.03442443415691,0.
229691249219751,0.818331695238684,NA
"16645","AC010240.3",0.108990023245465,1.41832010653068,3.352475197537
38,0.42306654724024,0.672246708730081,NA
"16646","AC113410.4",0.618122013358213,-0.746771089252538,1.5477559447
3476,-0.482486332417554,0.629460505297764,NA
"16647","AC104109.2",0,NA,NA,NA,NA,NA
"16648","LINC01843",0,NA,NA,NA,NA,NA
"16649","CATSPER3",0.441763903451534,-0.932217476493597,1.915151529018
22,-0.486759121859922,0.6264290422694,NA

"16650","PITX1",1.00254741399322,0.433053725522466,1.70456996385438,0.254054532641912,0.799453428811401,NA
"16651","C5orf66",0.459622688543737,0.663395808617261,2.24812082679364,0.295089036456916,0.7679258646623,NA
"16652","AC026691.1",0.258520968153754,-1.2931292497918,2.96669614377236,-0.435881933006964,0.662922394077564,NA
"16653","DCANP1",0,NA,NA,NA,NA,NA
"16654","TIFAB",0,NA,NA,NA,NA,NA
"16655","AC034206.1",0.621206891126911,1.479111273123,2.10018566576887,0.70427643480821,0.481260638729958,NA
"16656","SLC25A48-AS1",0.0639025369103 8,0.119912045671111,3.35247519753738,0.0357682126206912,0.971467179494267,NA
"16657","AC002428.2",0,NA,NA,NA,NA,NA
"16658","MYOT",0.103291511307383,0.119912045671111,3.35247519753738,0.0357682126206912,0.971467179494267,NA
"16659","PKD2L2",0.762722107857386,0.592183634934507,1.82402817485155,0.3246570656633 1,0.745440632460684,NA
"16660","KIF20A",0.12780507382076,-0.532254130061651,3.33875455416813,-0.15941696864095,0.873340370825791,NA
"16661","CDC25C",0.0639025369103 8,0.119912045671111,3.35247519753738,0.0357682126206912,0.971467179494267,NA
"16662","AC104116.1",0,NA,NA,NA,NA,NA
"16663","AC034243.1",0.627970539369327,0.736517917312543,1.85954049367465,0.396075223861948,0.692049535368908,NA
"16664","AC011405.1",0.108990023245465,1.41832010653068,3.35247519753738,0.42306654724024,0.672246708730081,NA
"16665","AC135457.1",0,NA,NA,NA,NA,NA
"16666","SLC23A1",0.315649477479949,2.01105601173365,2.78123896670507,0.723079187300523,0.469631235192475,NA
"16667","PROB1",0.231096585128143,-1.15074146808831,3.05677932246771,-0.376455526125231,0.706578272798499,NA
"16668","ECSCR",0.0604037079966622,0.119912045671111,3.35247519753738,0.0357682126206911,0.971467179494267,NA
"16669","SMIM33",1.41157806909887,-0.800904984560716,1.10739737709681,-0.723231787545313,0.469537492525733,NA
"16670","AC113361.1",0.0973092156216871,0.119912045671111,3.35247519753738,0.0357682126206911,0.971467179494267,NA
"16671","PSD2",0.0639025369103 8,0.119912045671111,3.35247519753738,0.0357682126206912,0.971467179494267,NA
"16672","SLC4A9",0.16538586715322,1.41832010653068,3.35247519753738,0.42306654724024,0.672246708730081,NA
"16673","AC008438.1",0,NA,NA,NA,NA,NA
"16674","DND1",0.661971443032986,2.27477626447939,1.71504230280731,1.32636743755876,0.18471801911246,NA
"16675","PCDHA3",0.0604037079966622,0.119912045671111,3.35247519753738,0.0357682126206911,0.971467179494267,NA
"16676","PCDHA4",0.167194048217763,-0.631571198727006,3.33432221989104,-0.189415166584483,0.849767438415713,NA
"16677","PCDHA5",0.259253633572194,1.98393725043859,2.94786137272544,0

.673009005374071,0.500941561797964,NA
"16678","PCDHA7",0.497989883072324,1.76305823507314,1.95263839461026,0
.902910769315812,0.366573254529626,NA
"16679","PCDHA9",0.548654775840474,1.15877662187486,2.16167700794404,0
.536054469569885,0.591920905896563,NA
"16680","PCDHA10",1.21012776421585,-0.454672999507418,1.30375963591572
,-0.348739895746251,0.727284589884284,NA
"16681","PCDHA11",0,NA,NA,NA,NA,NA
"16682","PCDHA12",0.391175442303102,1.28920775753492,2.66750436700999,
0.483301086018463,0.628881969053795,NA
"16683","PCDHA13",0.120322481366256,1.41832010653068,3.35247519753738,
0.42306654724024,0.672246708730081,NA
"16684","PCDHAC1",0,NA,NA,NA,NA,NA
"16685","AC244517.5",0.319657341568856,1.26387583376937,2.411907269400
06,0.524015101991771,0.600268035997735,NA
"16686","AC244517.1",1.09991965583611,-0.146719719584843,1.53087608433
161,-0.0958403629702674,0.923647359876415,NA
"16687","PCDHB6",1.43577841661416,0.176854817732938,1.23004922597523,0
.143778650478578,0.885675262139495,NA
"16688","PCDHB8",0.214166147237109,0.696980724704133,3.13752578908925,
0.222143424964947,0.824202234028988,NA
"16689","SLC25A2",0,NA,NA,NA,NA,NA
"16690","AC005618.4",0.248612489814084,-1.30424658337368,2.93265112742
767,-0.444732948687857,0.656512755319132,NA
"16691","AC005618.1",0.350095820056924,-0.358454129690765,2.3130996548
7499,-0.154967006689618,0.876847327889436,NA
"16692","PCDHGA1",1.23934077034947,0.118976940233253,1.14317100968291,
0.104076239884927,0.917108846268198,NA
"16693","PCDHGA2",1.49237499094405,0.487842596649856,1.04100048724343,
0.46862859588247,0.639335133659587,NA
"16694","PCDHGB1",0.436124488172099,1.28665564971416,2.24458275408073,
0.573227094155905,0.566490941535727,NA
"16695","PCDHGA4",0.828098017318922,1.60672152468172,1.78774542832635,
0.898741789084532,0.368790212505913,NA
"16696","PCDHGB2",1.08279388402272,1.87659998145336,1.67177456459914,1
.12251975905814,0.261641511123255,NA
"16697","PCDHGB3",0.33077173430644,2.01280089522968,3.31423402991417,0
.60732008574597,0.543638511160742,NA
"16698","PCDHGA7",1.18019236952076,1.61945742096888,1.36980736847887,1
.18225194157572,0.237105748369365,NA
"16699","PCDHGB4",0,NA,NA,NA,NA,NA
"16700","PCDHGB5",0.350095820056924,-0.358454129690765,2.3130996548749
9,-0.154967006689618,0.876847327889436,NA
"16701","PCDHGA11",1.33846968327795,-0.486073130428688,1.4203307411932
7,-0.342225311563926,0.732181338119035,NA
"16702","AC008781.3",0.332604015919104,1.26395938665104,2.386817901297
59,0.529558365539279,0.596418167442558,NA
"16703","PCDHGC4",0.16538586715322,1.41832010653068,3.35247519753738,0
.42306654724024,0.672246708730081,NA
"16704","PCDHGC5",0,NA,NA,NA,NA,NA

"16705","AC008781.2",0.06390253691038,0.119912045671111,3.352475197537
38,0.0357682126206912,0.971467179494267,NA
"16706","RELL2",1.2702758691122,-0.649280671349483,1.26217234941012,-0
.514415223604703,0.606961717890239,NA
"16707","ARAP3",0.688796752689369,0.644099927795106,1.69410416682889,0
.380200899334758,0.70379629171001,NA
"16708","AC005753.3",0,NA,NA,NA,NA,NA
"16709","PCDH1",0.180726189362918,0.69698069859353,3.31825525196168,0.
210044329224369,0.833633074956668,NA
"16710","PCDH12",0,NA,NA,NA,NA,NA
"16711","AC005740.4",0.173429405397192,1.41832010653068,3.352475197537
38,0.42306654724024,0.672246708730081,NA
"16712","ARHGAP26-AS1",0,NA,NA,NA,NA,NA
"16713","ARHGAP26-IT1",0,NA,NA,NA,NA,NA
"16714","HMHB1",0,NA,NA,NA,NA,NA
"16715","AC132803.1",0,NA,NA,NA,NA,NA
"16716","AC137770.1",0,NA,NA,NA,NA,NA
"16717","AC008700.1",0.16538586715322,1.41832010653068,3.3524751975373
8,0.42306654724024,0.672246708730081,NA
"16718","SH3RF2",0.285708348519476,2.01137472954577,2.86030947584175,0
.703201785168317,0.481930006892637,NA
"16719","PLAC8L1",0.383502498768232,-0.512182144388483,2.2700102808854
,-0.225629878728439,0.821489300165254,NA
"16720","POU4F3",0,NA,NA,NA,NA,NA
"16721","PPP2R2B",0.227597756214425,-1.13126253111685,2.5754042190421,
-0.439256301108964,0.660475834030736,NA
"16722","JAKMIP2",0.120807415993324,-0.48654549181451,3.34088239674268
,-0.145633827844071,0.884210461918222,NA
"16723","AC011370.1",0,NA,NA,NA,NA,NA
"16724","SCGB3A2",0,NA,NA,NA,NA,NA
"16725","SPINK6",0.06390253691038,0.119912045671111,3.35247519753738,0
.0357682126206912,0.971467179494267,NA
"16726","SPINK9",0,NA,NA,NA,NA,NA
"16727","AC114939.1",0.509165269275632,-1.23074418149883,2.13593309645
929,-0.576209144162343,0.564473825216766,NA
"16728","ADRB2",0.726034665615569,0.222337727841819,1.52802391532093,0
.14550670680774,0.884310820839198,NA
"16729","SH3TC2",0,NA,NA,NA,NA,NA
"16730","AC012613.2",0.439761757551905,2.57992481053724,2.548307354541
05,1.01240723805935,0.311343379010575,NA
"16731","PDE6A",0.387595552634301,1.28711517028576,2.3069915040667,0.5
57919336944617,0.57689946654674,NA
"16732","CDX1",1.47322761952101,-1.08890173914248,1.19058002940587,-0.
914597685370105,0.360402874288646,NA
"16733","SLC6A7",0.268677378460603,0.696980754736642,2.91582564400198,
0.239033755728972,0.811079408015517,NA
"16734","CAMK2A",1.1016111593552,-1.84510550777678,1.31607379203249,-1
.40197724394106,0.160922042150417,NA
"16735","NDST1-
AS1",0.658352057650433,0.245926164623193,1.86011881945409,0.1322099223

18493,0.89481825314149,NA
"16736","IRGM",0.124306244907042,-0.509577950443694,3.33980312193904,-0.152577242381833,0.878731672788745,NA
"16737","AC011337.1",0.480675129265837,1.72722230882804,2.18227203339441,0.791478918483615,0.428664573421544,NA
"16738","AC011374.1",0,NA,NA,NA,NA,NA
"16739","CLMAT3",0.108990023245465,1.41832010653068,3.35247519753738,0.42306654724024,0.672246708730081,NA
"16740","AC091982.3",0.163695219304045,-0.600668372859899,3.33567379881164,-0.18007407471135,0.857094415273326,NA
"16741","LINC01933",0.120322481366256,1.41832010653068,3.35247519753738,0.42306654724024,0.672246708730081,NA
"16742","NMUR2",0.120322481366256,1.41832010653068,3.35247519753738,0.42306654724024,0.672246708730081,NA
"16743","SAP30L-AS1",1.04893949155205,0.876696147878071,1.38518032929167,0.632911202490422,0.526791631841302,NA
"16744","AC008625.1",0,NA,NA,NA,NA,NA
"16745","PPP1R2B",0,NA,NA,NA,NA,NA
"16746","TIMD4",0.16538586715322,1.41832010653068,3.35247519753738,0.42306654724024,0.672246708730081,NA
"16747","HAVCR1",0,NA,NA,NA,NA,NA
"16748","HAVCR2",1.08752032350429,0.477831380743667,1.41302287559946,0.338162523052538,0.735240716193981,NA
"16749","AC010609.1",0,NA,NA,NA,NA,NA
"16750","AC016571.1",0.103291511307383,0.119912045671111,3.35247519753738,0.0357682126206912,0.971467179494267,NA
"16751","AC008676.1",0.150263610326729,1.41832010653068,3.35247519753738,0.42306654724024,0.672246708730081,NA
"16752","NIPAL4",0,NA,NA,NA,NA,NA
"16753","AC106801.1",0,NA,NA,NA,NA,NA
"16754","SOX30",0,NA,NA,NA,NA,NA
"16755","AC025437.4",0.16538586715322,1.41832010653068,3.35247519753738,0.42306654724024,0.672246708730081,NA
"16756","AC025437.2",0,NA,NA,NA,NA,NA
"16757","LINC02227",0.503882359845492,1.09423428306638,1.8112646333271,0.604127228530038,0.545759058000958,NA
"16758","AC091979.2",0.524662663402813,0.986327235214139,2.20497052408041,0.447319918539724,0.654644087307782,NA
"16759","AC136424.2",0.323693015723921,2.01097763960685,2.76273133237213,0.727894752574506,0.466678015622723,NA
"16760","AC136424.1",0,NA,NA,NA,NA,NA
"16761","AC008691.1",0.0973092156216871,0.119912045671111,3.35247519753738,0.0357682126206911,0.971467179494267,NA
"16762","AC008703.1",0,NA,NA,NA,NA,NA
"16763","CCNJL",1.27022082983196,-0.669098860661577,1.15469972587809,-0.579457018709139,0.562280840245468,NA
"16764","MIR3142HG",0.344421408666963,-0.536068076123864,2.34017467331309,-0.229071821961447,0.818813094004659,NA
"16765","GABRB2",0.124306244907042,-0.509577950443694,3.33980312193904

,-0.152577242381833,0.878731672788745,NA
"16766","GABRG2",0.167194048217763,-0.631571198727006,3.3343222198910 4,-0.189415166584483,0.849767438415713,NA
"16767","HMMR",0.474663884615144,0.631265969926065,2.56835380623206,0.245786218547581,0.805847725878953,NA
"16768","AC109466.1",0.332579915370983,-0.0455547330855546,2.3762169463749,-0.0191711169954629,0.984704598665924,NA
"16769","TENM2",0.733050207440257,-0.382969572814403,2.05737424336175,-0.186144827101865,0.852331189236785,NA
"16770","WWC1",0.620333447614891,-2.54761282400384,1.96767966737025,-1.29472945533287,0.195413575063523,NA
"16771","AC011365.2",0.424639500725414,2.54525832651505,2.21853738738168,1.14726861985362,0.251270619026034,NA
"16772","AC011389.1",1.02395251065386,2.94105353935938,1.71469864450632,1.71520141383569,0.0863083057585803,NA
"16773","INSYN2B",0,NA,NA,NA,NA,NA
"16774","LINC01366",0.62070601229803,1.4916776671094,1.85940703391386,0.802232991433603,0.422418195621089,NA
"16775","AC034199.1",0,NA,NA,NA,NA,NA
"16776","AC027306.1",0.422041895211276,-0.766294883910789,2.16210813595914,-0.35442023974941,0.723023968596177,NA
"16777","AC008619.1",0,NA,NA,NA,NA,NA
"16778","GABRP",0.06390253691038,0.119912045671111,3.35247519753738,0.035768212620691 2,0.971467179494267,NA
"16779","AC022217.3",0,NA,NA,NA,NA,NA
"16780","AC008429.1",0.309590750174535,-0.21960280990608,2.45825916411348,-0.0893326517854253,0.928817545423718,NA
"16781","AC008378.1",0,NA,NA,NA,NA,NA
"16782","LINC01484",0,NA,NA,NA,NA,NA
"16783","LINC01485",0,NA,NA,NA,NA,NA
"16784","FAM153B",0.0973092156216871,0.119912045671111,3.35247519753738,0.0357682126206911,0.971467179494267,NA
"16785","AC138956.2",0,NA,NA,NA,NA,NA
"16786","GPRIN1",0.73631206470506,0.704446869022252,2.05541845783167,0.342726740794863,0.731804043559833,NA
"16787","HK3",0.322423505064134,-1.62476410664247,2.74149049892963,-0.592657208652313,0.553410584490248,NA
"16788","ZNF346-IT1",0,NA,NA,NA,NA,NA
"16789","F12",0.206299238867152,0.696980719233408,3.17624445737489,0.219435477522864,0.826310836331857,NA
"16790","PRR7-AS1",0.108990023245465,1.41832010653068,3.35247519753738,0.42306654724024,0.672246708730081,NA
"16791","DOK3",0.503858259297371,0.181053061553326,1.93716394924829,0.0934629521799552,0.925535780702537,NA
"16792","AC139795.2",0.653413304753781,-0.721199937753683,1.63027453291458,-0.442379441739995,0.658214653060356,NA
"16793","FAM153A",0,NA,NA,NA,NA,NA
"16794","AC106795.2",0.108990023245465,1.41832010653068,3.35247519753738,0.42306654724024,0.672246708730081,NA

"16795","N4BP3",0.956748790170264,0.479814110041497,1.52472560408799,0
.314688825815644,0.752997929241809,NA
"16796","COL23A1",0.416233891772103,-1.98245561943386,2.18308789627801
,-0.908097022943412,0.363826963225674,NA
"16797","AC104117.3",0.169393731242127,0.696980693484986,3.35247519753
738,0.207900328091007,0.835306792989891,NA
"16798","GRM6",0.06390253691038,0.119912045671111,3.35247519753738,0.0
357682126206912,0.971467179494267,NA
"16799","AC136604.2",0.39467427121682,1.28917448930526,2.6604911284818
2,0.484562596546191,0.627986651291867,NA
"16800","AC136604.3",0.320923208295326,-0.217085582671771,2.4345717842
2353,-0.089167870949021,0.928948498906702,NA
"16801","AC010285.3",0,NA,NA,NA,NA,NA
"16802","AC008610.1",0.120322481366256,1.41832010653068,3.352475197537
38,0.42306654724024,0.672246708730081,NA
"16803","AC034213.1",0.446920101051543,0.549513071531492,2.01365993017
52,0.272892688232457,0.784935707226451,NA
"16804","SCGB3A1",0,NA,NA,NA,NA,NA
"16805","FLT4",0.0604037079966622,0.119912045671111,3.35247519753738,0
.0357682126206911,0.971467179494267,NA
"16806","AC022413.2",0,NA,NA,NA,NA,NA
"16807","BTNL8",0,NA,NA,NA,NA,NA
"16808","BTNL9",1.30346875986323,-0.803174672488961,1.3475697908052,-0
.59601712502707,0.551163786483845,NA
"16809","LINC01962",0.103291511307383,0.119912045671111,3.352475197537
38,0.0357682126206912,0.971467179494267,NA
"16810","AC008443.6",0.793610323686189,-1.11681963559096,1.59515219506
026,-0.700133591671969,0.483843879297097,NA
"16811","AC008443.8",0.39129297551847,-1.9037979345125,2.1679417627521
1,-0.878159167936184,0.379857347316932,NA
"16812","AC138035.1",0.340623453614955,-0.0453206414787626,2.361162331
91558,-0.0191942082364132,0.984686177910777,NA
"16813","AL512308.1",1.33900776318146,1.9048496787923,1.41560449751851
,1.34560866550751,0.178428755549285,NA
"16814","AL031770.1",0,NA,NA,NA,NA,NA
"16815","HUS1B",0.593837411790448,-0.0781268439787596,1.89762903336486
,-0.0411707676290268,0.967159757958488,NA
"16816","AL357054.2",0,NA,NA,NA,NA,NA
"16817","LINC01394",0,NA,NA,NA,NA,NA
"16818","AL034346.1",0,NA,NA,NA,NA,NA
"16819","AL031768.2",0,NA,NA,NA,NA,NA
"16820","AL031768.1",0.266986730611428,-1.32916053734901,2.94158530001
421,-0.451851774396135,0.651375768309789,NA
"16821","LINC01600",0.957312729034602,-0.810160638537893,1.52422698247
419,-0.531522304652294,0.595056889099724,NA
"16822","MYLK4",0.928617010185796,0.588454704856258,1.54174192930197,0
.381681715773719,0.702697463868561,NA
"16823","AL133351.1",1.15961844569905,0.825952387415719,1.234352140245
97,0.669138376712442,0.50340721137665,NA
"16824","AL031963.2",0,NA,NA,NA,NA,NA

"16825","AL031963.1",0.0973092156216871,0.119912045671111,3.3524751975
3738,0.0357682126206911,0.971467179494267,NA
"16826","AL445309.1",0.06390253691038,0.119912045671111,3.352475197537
38,0.0357682126206912,0.971467179494267,NA
"16827","AL033523.1",0,NA,NA,NA,NA,NA
"16828","AL138831.2",0.16538586715322,1.41832010653068,3.3524751975373
8,0.42306654724024,0.672246708730081,NA
"16829","AL136309.2",0.06390253691038,0.119912045671111,3.352475197537
38,0.0357682126206912,0.971467179494267,NA
"16830","AL159166.1",0,NA,NA,NA,NA,NA
"16831","AL034376.1",1.23533524388805,1.06346801292562,1.4140703809689
8,0.752061585645327,0.452014023829037,NA
"16832","AL359643.2",1.25934966828253,1.0185860099379,1.30644356299674
,0.779663231377137,0.435589126604042,NA
"16833","AL022097.1",0,NA,NA,NA,NA,NA
"16834","LY86-
AS1",0.16538586715322,1.41832010653068,3.35247519753738,0.423066547240
24,0.672246708730081,NA
"16835","AL031123.2",0,NA,NA,NA,NA,NA
"16836","AL031123.1",0.285708348519476,2.01137472954577,2.860309475841
75,0.703201785168317,0.481930006892637,NA
"16837","AL031123.5",0.103291511307383,0.119912045671111,3.35247519753
738,0.0357682126206912,0.971467179494267,NA
"16838","AL355336.1",0.879886923768543,0.907537950219989,1.51072062159
792,0.600731821122601,0.548018620999555,NA
"16839","CAGE1",0.16538586715322,1.41832010653068,3.35247519753738,0.4
2306654724024,0.672246708730081,NA
"16840","DSP",0,NA,NA,NA,NA,NA
"16841","AL355499.1",0.150263610326729,1.41832010653068,3.352475197537
38,0.42306654724024,0.672246708730081,NA
"16842","TFAP2A",0.579016822708824,-1.56397622123729,1.77958327957306,
-0.878844075008672,0.379485824878498,NA
"16843","TFAP2A-
AS2",0.455102079761603,1.76200479400924,1.98843275354502,0.88612742415
7493,0.375548866603951,NA
"16844","TFAP2A-AS1",0,NA,NA,NA,NA,NA
"16845","GCNT2",0.294236821390516,0.0858012426627812,2.87663275414391,
0.0298269713223494,0.976205048328083,NA
"16846","AL358777.1",0,NA,NA,NA,NA,NA
"16847","C6orf52",0.719799308436139,-0.963929404747151,1.4748598913896
5,-0.653573543069851,0.513386594042478,NA
"16848","AL024498.1",0,NA,NA,NA,NA,NA
"16849","MAK",0.551711909127734,-1.31665242067448,2.05823938296876,-0.
639698390561051,0.522368702003177,NA
"16850","GCM2",0,NA,NA,NA,NA,NA
"16851","AL357497.1",1.22535498116973,1.26352299881313,1.2415719820892
4,1.01768001939522,0.30883004568715,NA
"16852","SYCP2L",0.752821671231993,-2.03359599613776,1.49756202403301,
-1.35793774381456,0.174483439555512,NA
"16853","ELOVL2-

AS1",0.403818731960841,1.28903049121967,2.63072424934197,0.48999072842
4731,0.624140459645733,NA
"16854","ERVFRD-1",0.124306244907042,-0.509577950443694,3.339803121939
04,-0.152577242381833,0.878731672788745,NA
"16855","AL022098.1",0.206299238867152,0.696980719233408,3.17624445737
489,0.219435477522864,0.826310836331857,NA
"16856","AL022724.3",0.0604037079966622,0.119912045671111,3.3524751975
3738,0.0357682126206911,0.971467179494267,NA
"16857","AL157373.2",0.0973092156216871,0.119912045671111,3.3524751975
3738,0.0357682126206911,0.971467179494267,NA
"16858","EDN1",0.718201036408495,0.189828399637752,1.52967018528209,0.
124097600557433,0.901237997952966,NA
"16859","AL008729.1",0.173429405397192,1.41832010653068,3.352475197537
38,0.42306654724024,0.672246708730081,NA
"16860","AL008729.2",0.792747490870343,-1.95055802760526,1.66407406457
396,-1.17215818041407,0.241133554863735,NA
"16861","AL441883.1",1.22294084651688,-1.18390143477413,1.279068449918
47,-0.925596620610561,0.354655650261676,NA
"16862","Z93020.1",0,NA,NA,NA,NA,NA
"16863","AL023583.1",0.402341649891647,-0.900074484860014,1.9522052572
897,-0.461055250977866,0.644758965786572,NA
"16864","AL133259.1",0.16538586715322,1.41832010653068,3.3524751975373
8,0.42306654724024,0.672246708730081,NA
"16865","AL138720.1",0,NA,NA,NA,NA,NA
[truncated: 432,680 more chars]
